# Supplementary material for: Bacteroides Fragilis in the gut microbiomes of Alzheimer’s disease activates microglia and triggers pathogenesis in neuronal C/EBPβ transgenic mice
Source: Nat Commun. 2023 Sep 6;14:5471. doi: 10.1038/s41467-023-41283-w (PMC10482867; doi:10.1038/s41467-023-41283-w)
Supplement: Supplementary file 1 — Supplementary Information [file 41467_2023_41283_MOESM1_ESM.pdf]

Bacteroides Fragilis **in the gut microbiomes of Alzheimer's Disease activates microglia and triggers pathogenesis in neuronal C/EBPβ transgenic mice**

Supplementary Information files

By

Yiyuan Xia † [1,2], Yifan Xiao † [2], Zhihao Wang[1], Xia Liu [1], Ashfaqul M. Alam[3], John P. Haran[4,5,6], Beth A. McCormick[5,6], Xiji Shu[2,*], Xiaochuan Wang [7,8,*], and Keqiang Ye [1,9,*]

This file contains Supplementary figures 1-16.

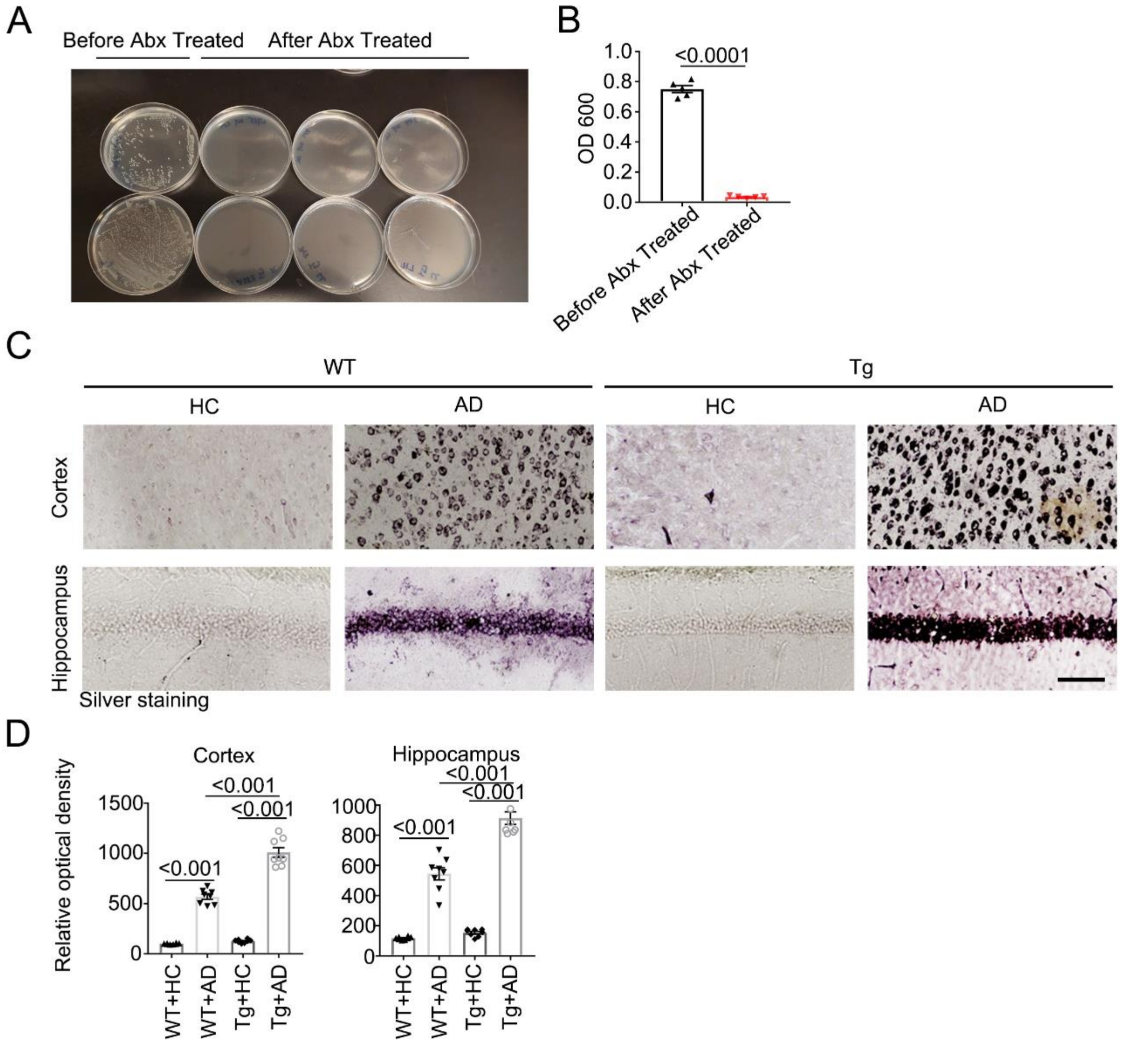
A
Before Abx Treated
After Abx Treated
B
<0.0001
OD 600
1.0
0.8
0.6
0.4
0.2
0.0
Before Abx Treated
After Abx Treated
C
WT
Tg
HC
AD
HC
AD
Cortex
Hippocampus
Silver staining
D
Cortex
Hippocampus
Relative optical density
1500
1000
500
0
<0.001
<0.001
<0.001
1000
800
600
400
200
0
<0.001
<0.001
<0.001
WT+HC
WT+AD
Tg+HC
Tg+AD

1

Figure S1. AD humanized Abx-treated mice display increased aggregated proteins and apoptotic neurons compared with HC humanized Abx-treated mice in WT and C/EBPβ Tg mice.

(A-B) In vitro cultures of bacteria from fecal pallets with/without Abx treated 3-month-old mice, showed that antibiotics treatment was effective in killing the bacteria. (n = 5 biologically independent samples in each group, data are shown as mean ± SEM, two-tailed Student's t test). (C) Silver staining in the cortex and the hippocampus. Scale bar: 50 μm. (D) Quantitative analysis of Silver staining. Aggregated proteins were significantly increased in AD humanized Abx-treated mice. (n = 8 biologically independent samples in each group, data are shown as mean ± SEM, one-way ANOVA and Bonferroni's multiple comparison test).

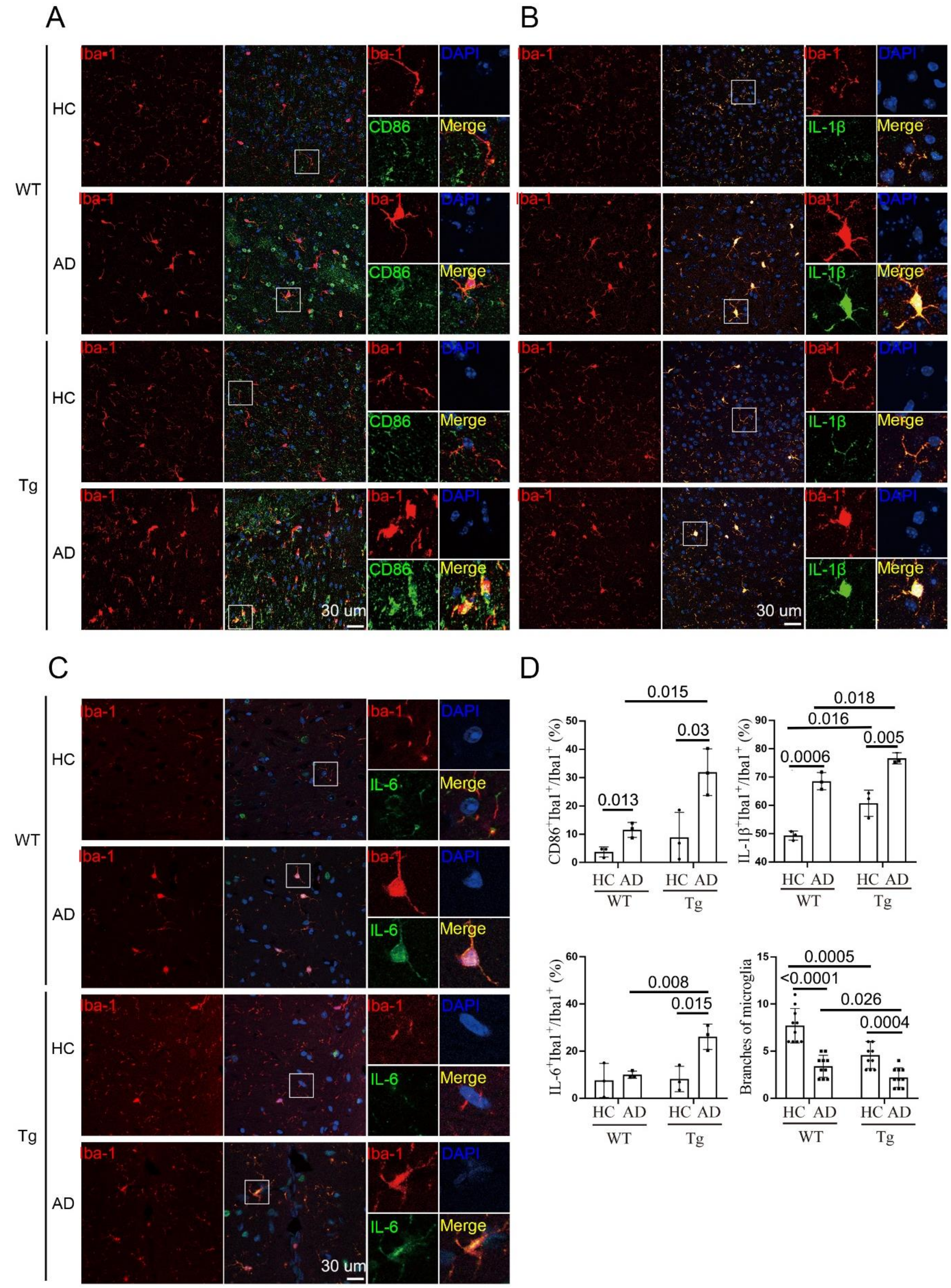
A
B
C
D
WT
Tg
HC
AD
Iba-1
DAPI
CD86
IL-1β
IL-6
Merge
30 um
0.015
0.03
0.013
0.018
0.016
0.005
0.0006
0.008
0.015
0.0005
<0.0001
0.026
0.0004
CD86+Iba1+/Iba1+ (%)
IL-1β+Iba1+/Iba1+ (%)
IL-6+Iba1+/Iba1+ (%)
Branches of microglia

1

Figure S2. AD humanized Abx-treated mice display increased cytokines in microglia compared with HC humanized Abx-treated mice in WT and C/EBPβ Tg mice.

(A) Immunofluorescent staining of Iba-1 (red) and CD86 (green) in the cortex region of the brains from AD or HC humanized Abx-treated 6-month-old mice. Scale bar: 30 μm. (B) Immunofluorescent staining of Iba-1 (red) and IL-1β (green) in the cortex region of the brains from AD or HC humanized Abx-treated 6-month-old mice. Scale bar: 30 μm. (C) Immunofluorescent staining of Iba-1 (red) and IL-6 (green) in the cortex region of the brains from AD or HC humanized Abx-treated 6-month-old mice. Scale bar: 30 μm. (D) Quantitative analysis of Iba-1^+ and $CD86^+$/IL-$1\beta^+$/IL-6^+ percentage in Iba-1^+ microglia, and branches of Iba-1^+ microglia (n = 3 biologically independent samples in each group, data are shown as mean ± SEM, one-way ANOVA and Bonferroni's multiple comparison test).

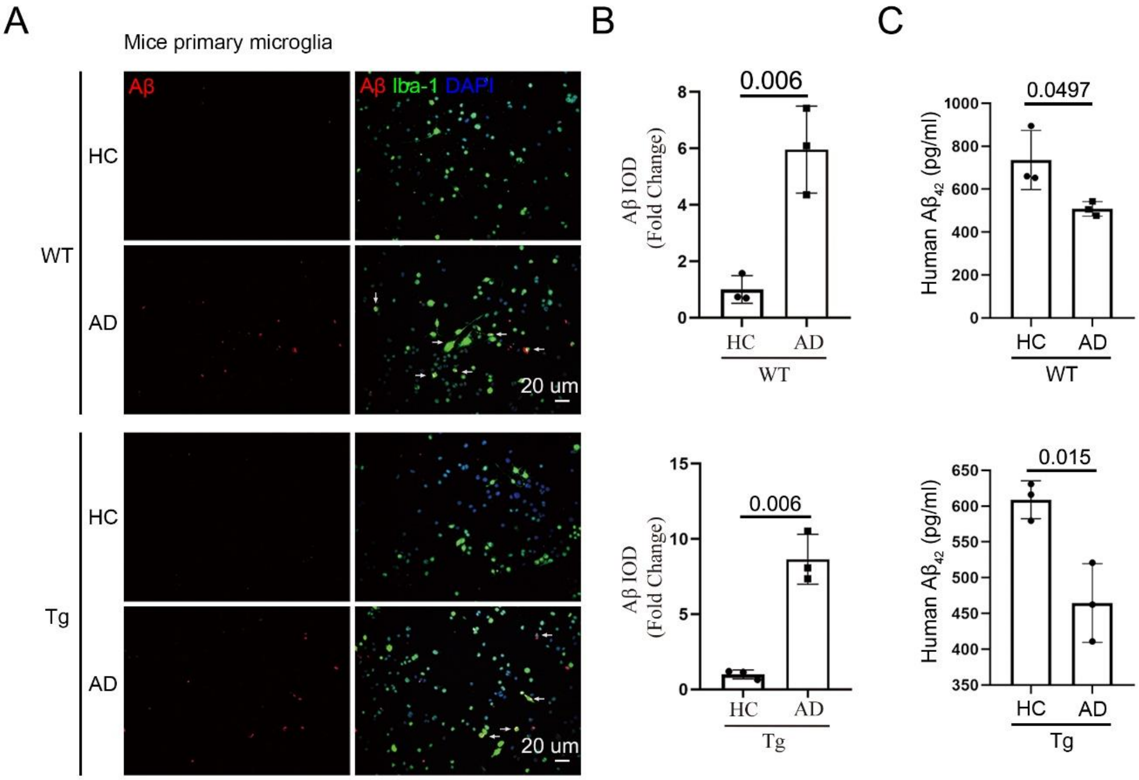
A
Mice primary microglia
Aβ
Aβ Iba-1 DAPI
WT
HC
AD
Tg
20 um
B
0.006
Aβ IOD (Fold Change)
HC
AD
WT
Tg
C
0.0497
0.015
Human Aβ42 (pg/ml)

Figure S3. AD humanized Abx-treated mouse primary microglia display better ability to engulf Aβ than HC.

The primary cultures of microglia cells from AD or HC humanized Abx-treated 6-month-old mice, then treated with human $A\beta_{42}$ fibrils for 24 hours. (A) Immunofluorescent staining of human Aβ (red) and Iba-1 (green) in the primary cultured microglia cells. Phagocytozed microglia cells with engulfed $A\beta_{42}$ was designed with white arrows. Scale bar: 20 μm. (B) Quantitative analysis Aβ IOD (Integrated optical density) in Iba-1^{+} microglia (n = 3 biologically independent samples in each group, data are shown as mean ± SEM, two-tailed Student's t test). (C) Human $A\beta_{42}$ concentration in the medium were detected with ELISA (n = 3 biologically independent samples in each group, data are shown as mean ± SEM, two-tailed Student's t test).

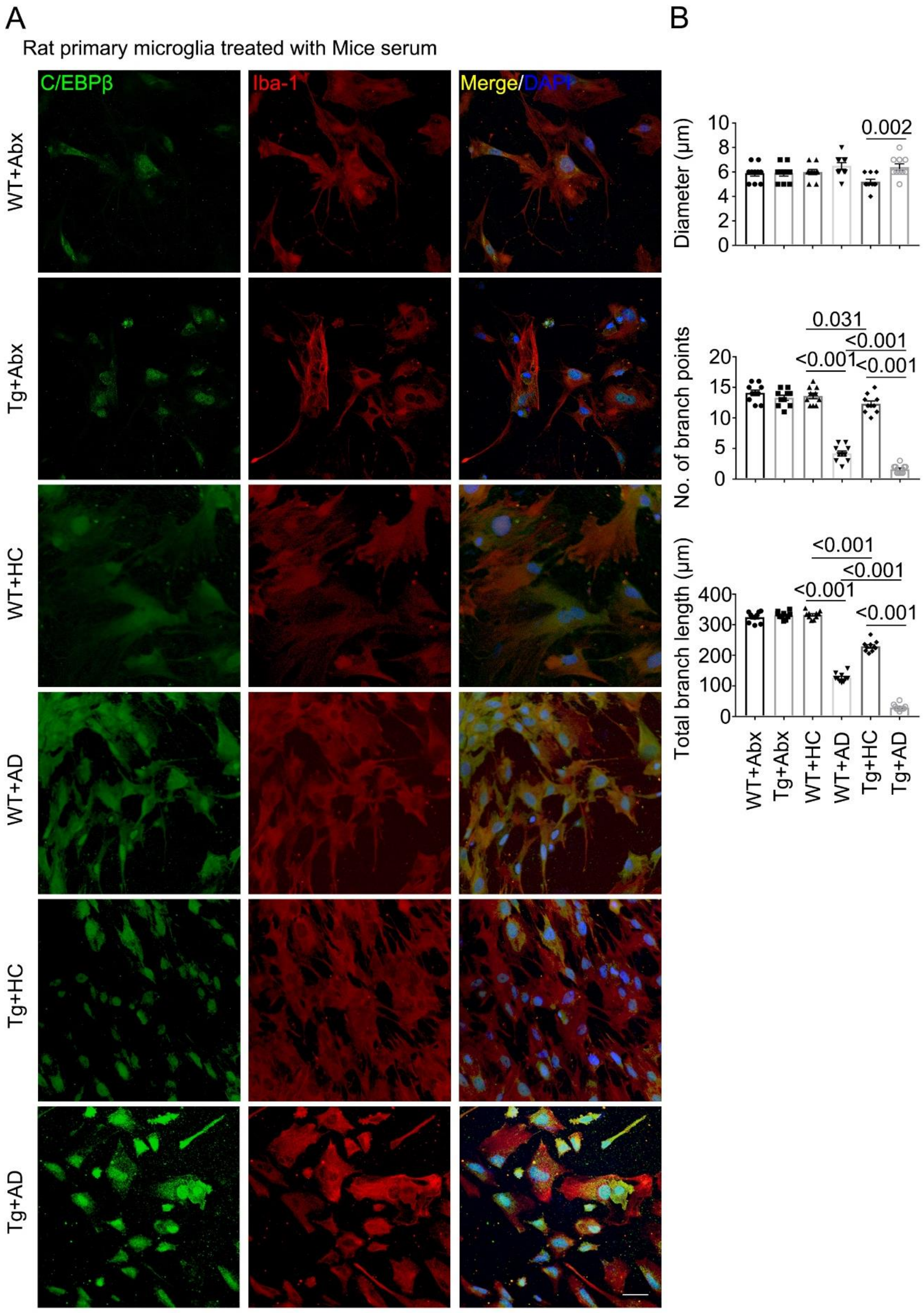
A
Rat primary microglia treated with Mice serum
C/EBPβ
Iba-1
Merge/DAPI
WT+Abx
Tg+Abx
WT+HC
WT+AD
Tg+HC
Tg+AD
B
Diameter (μm)
0.002
No. of branch points
0.031
<0.001
<0.001
<0.001
Total branch length (μm)
<0.001
<0.001
<0.001
<0.001
WT+Abx
Tg+Abx
WT+HC
WT+AD
Tg+HC
Tg+AD

1

Figure S4. AD humanized Abx-treated mouse serum strongly activated rat primary microglia compared with HC humanized Abx-treated mouse serum.

(A) Rat primary microglia treated with HC or AD humanized Abx-treated 6-month-old mice serum (20 μg/ml), 24 h later, the microglia were fixed and staining with C/EBPβ (green) and Iba-1 (red). Scale bar: 10 μm. (B) Quantitative analysis of diameter, number of branch points, and total branch length in Iba-1 (red) positive microglia. (n = 10 biologically independent samples in each group, data are shown as mean ± SEM, one-way ANOVA and Bonferroni's multiple comparison test).

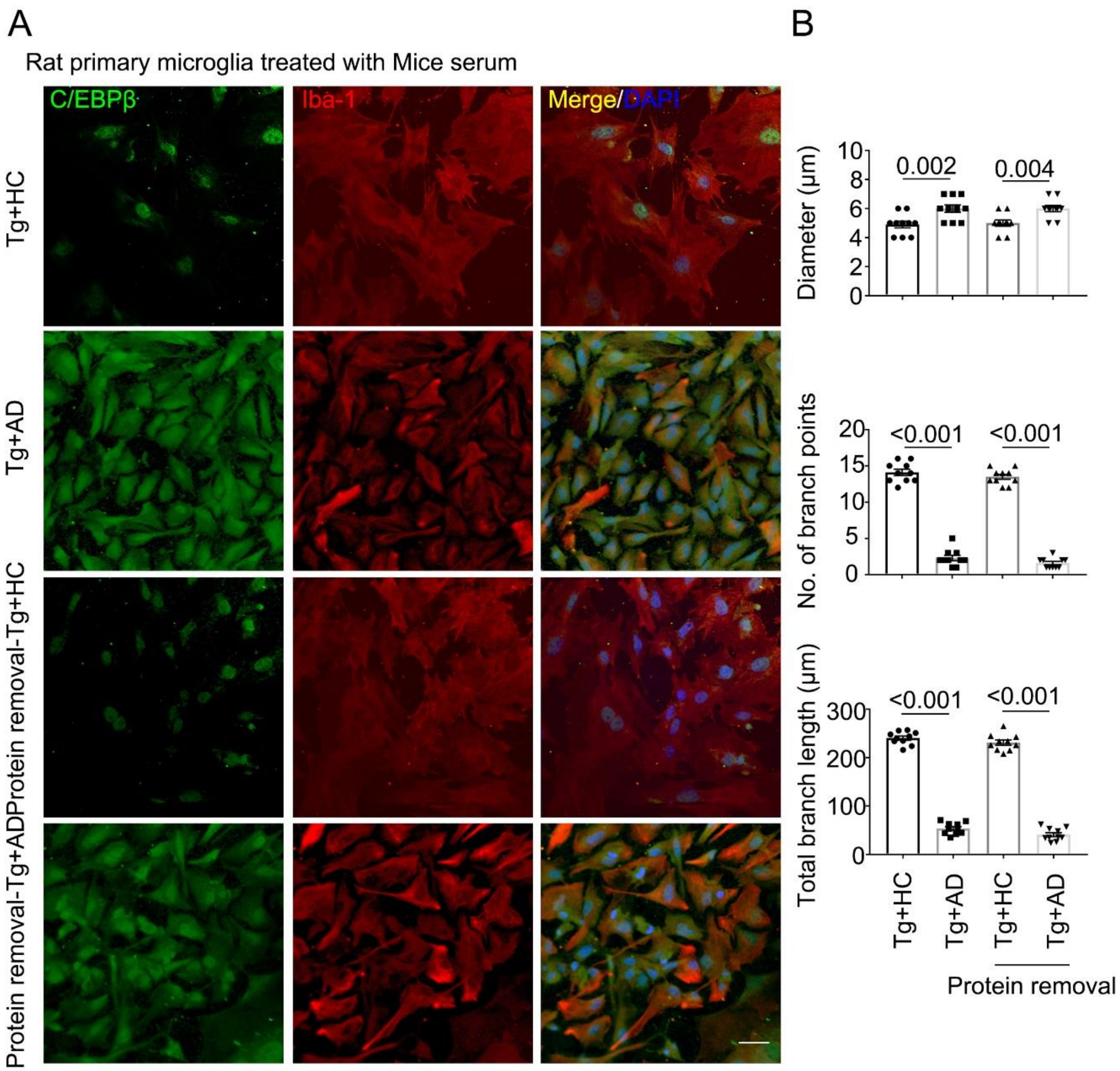
A
Rat primary microglia treated with Mice serum
C/EBPβ
Iba-1
Merge/DAPI
Tg+HC
Tg+AD
Protein removal-Tg+HC
Protein removal-Tg+AD
B
Diameter (μm)
0.002
0.004
No. of branch points
<0.001
<0.001
Total branch length (μm)
<0.001
<0.001
Tg+HC
Tg+AD
Tg+HC
Tg+AD
Protein removal

1

Figure S5. Protein depletion from AD humanized Abx-treated mouse serum did not affect rat primary microglia activation.

(A) Rat primary microglia treated with HC or AD humanized Abx-treated 6-month-old C/EBPβ Tg mice serum (20 μg/ml) or TCA treated protein removal serum, 24 h later, the microglia were fixed and staining with C/EBPβ (green) and Iba-1 (red). Scale bar: 10 μm. (B) Quantitative analysis of diameter, number of branch points, and total branch length in Iba-1 (red) positive microglia. (n = 10 biologically independent samples in each group, data are shown as mean ± SEM, one-way ANOVA and Bonferroni's multiple comparison test).

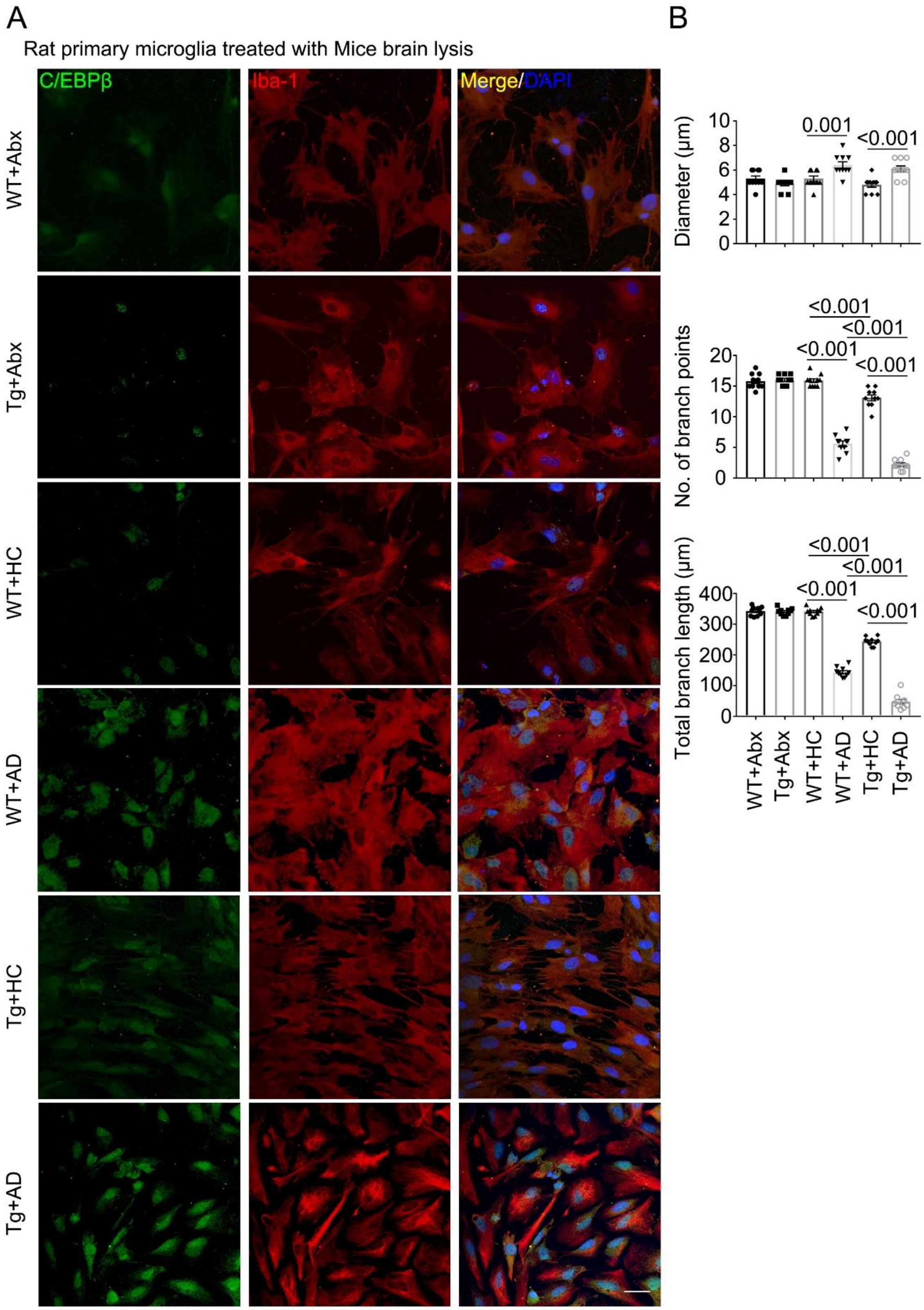
A
Rat primary microglia treated with Mice brain lysis
C/EBPβ
Iba-1
Merge/DAPI
WT+Abx
Tg+Abx
WT+HC
WT+AD
Tg+HC
Tg+AD
B
Diameter (μm)
0.001
<0.001
No. of branch points
<0.001
<0.001
<0.001
<0.001
Total branch length (μm)
<0.001
<0.001
<0.001
<0.001
WT+Abx
Tg+Abx
WT+HC
WT+AD
Tg+HC
Tg+AD

1

Figure S6. AD humanized Abx-treated mouse brain lysates activated rat primary microglia more robust than HC.

(A) Rat primary microglia treated with HC or AD humanized Abx-treated 6-month-old mice brain lysis (20 μg/ml), 24 h later, the microglia were fixed and staining with C/EBPβ (green) and Iba-1 (red). Scale bar: 10 μm. (B) Quantitative analysis of diameter, number of branch points, and total branch length in Iba-1 (red) positive microglia. n=10 biologically independent samples in each group, data are shown as mean ± SEM, one-way ANOVA and Bonferroni's multiple comparison test.

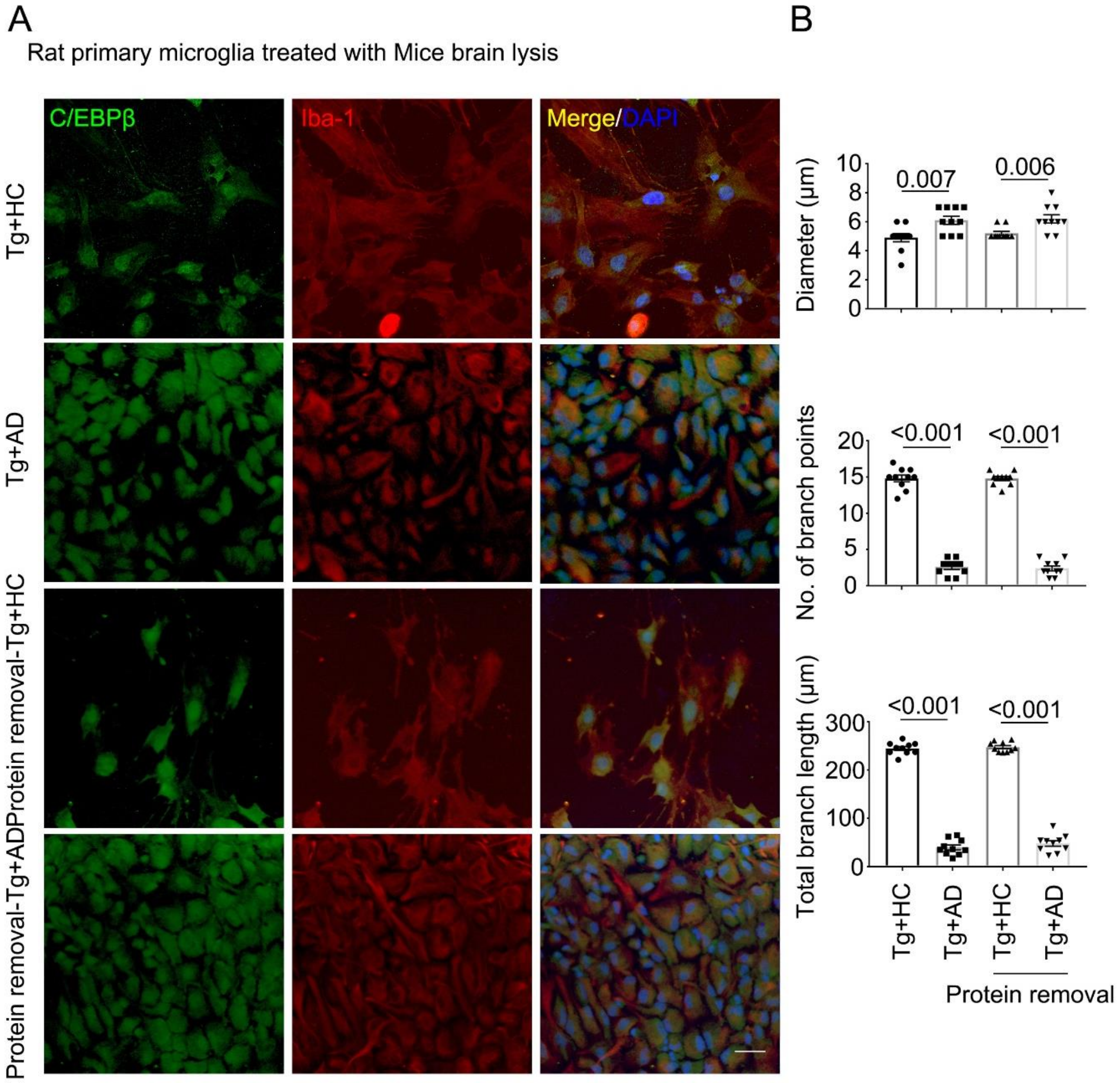
A
Rat primary microglia treated with Mice brain lysis
C/EBPβ
Iba-1
Merge/DAPI
Tg+HC
Tg+AD
Protein removal-Tg+HC
Protein removal-Tg+AD
B
Diameter (μm)
0.007
0.006
No. of branch points
<0.001
<0.001
Total branch length (μm)
<0.001
<0.001
Tg+HC
Tg+AD
Tg+HC
Tg+AD
Protein removal

1

Figure S7. Protein depletion from AD humanized Abx-treated mice brain lysates did not affect rat primary microglia.

(A) Rat primary microglia treated with HC or AD humanized Abx-treated 6-month-old C/EBPβ Tg mice brain lysis (20 μg/ml) or TCA treated protein removal brain lysis, 24 h later, the microglia were fixed and staining with C/EBPβ (green) and Iba-1 (red). Scale bar: 10 μm. (B) Quantitative analysis of diameter, number of branch points, and total branch length in Iba-1 (red) positive microglia. (n = 10 biologically independent samples in each group, data are shown as mean ± SEM, one-way ANOVA and Bonferroni's multiple comparison test).

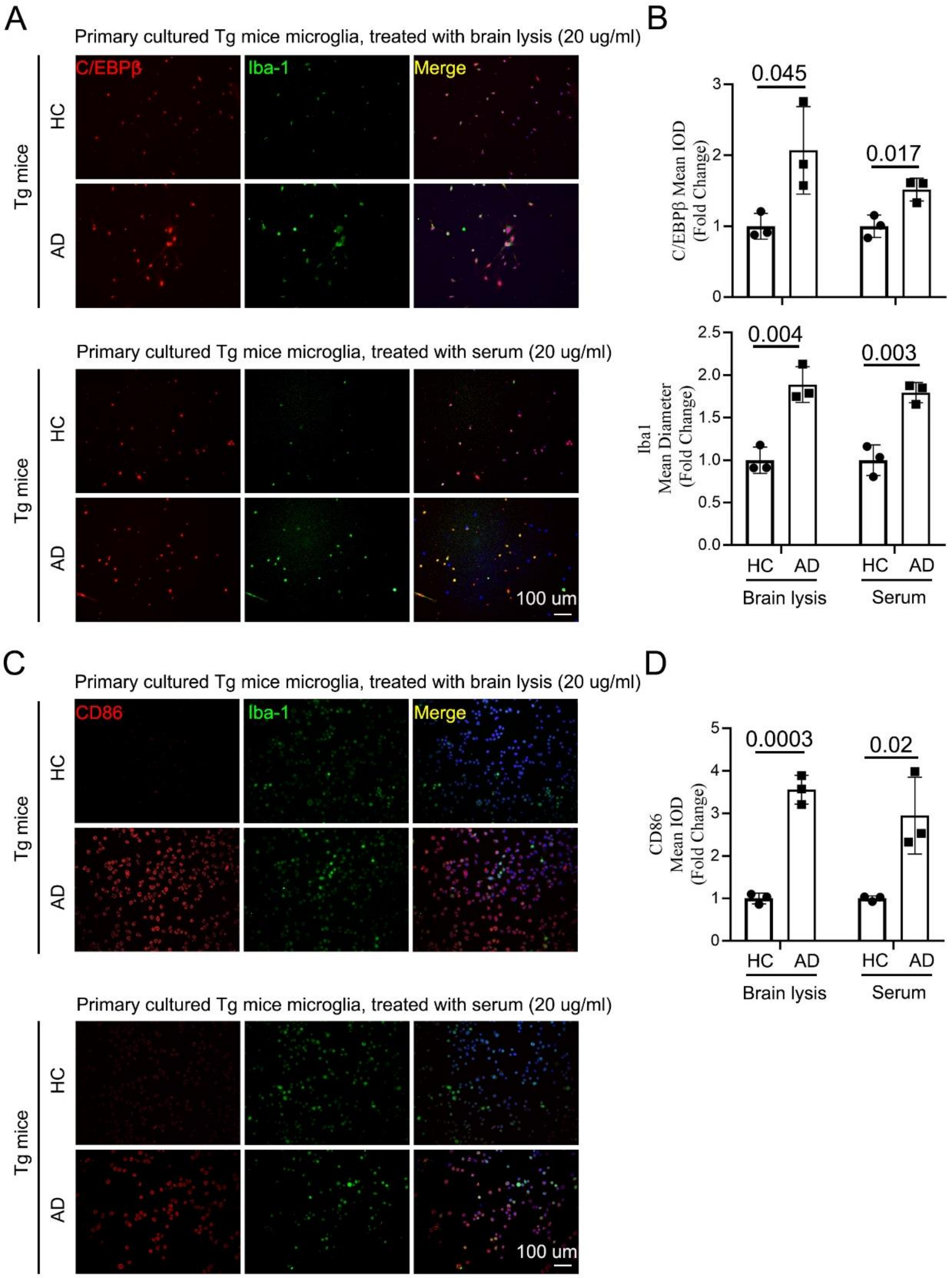
A
Primary cultured Tg mice microglia, treated with brain lysis (20 ug/ml)
C/EBPβ
Iba-1
Merge
Tg mice
HC
AD
Primary cultured Tg mice microglia, treated with serum (20 ug/ml)
100 um
B
C/EBPβ Mean IOD (Fold Change)
0.045
0.017
Iba1 Mean Diameter (Fold Change)
0.004
0.003
Brain lysis
Serum
C
Primary cultured Tg mice microglia, treated with brain lysis (20 ug/ml)
CD86
D
CD86 Mean IOD (Fold Change)
0.0003
0.02
Primary cultured Tg mice microglia, treated with serum (20 ug/ml)

1

Figure S8. AD humanized Abx-treated mice brain lysates/serum activated Tg mice primary microglia more robust than HC.

The primary cultured microglia from C/EBPβ Tg mice, treated with HC/AD humanized Abx-treated 6-month-old mice brain lysis or serum for 24 h. (A) The microglia were fixed and staining with C/EBPβ (red) and Iba-1 (green). Scale bar: 100 µm. (B) Quantitative analysis of C/EBPβ mean IOD and Iba-1$^+$ microglia soma mean diameter (n = 3 biologically independent samples in each group, data were shown as mean ± SEM, two-tailed Student's t test). (C) The microglia were fixed and staining with CD86 (red) and Iba-1 (green). Scale bar: 100 µm. (D) Quantitative analysis of CD86 mean IOD (n = 3 biologically independent samples in each group, data were shown as mean ± SEM, two-tailed Student's t test).

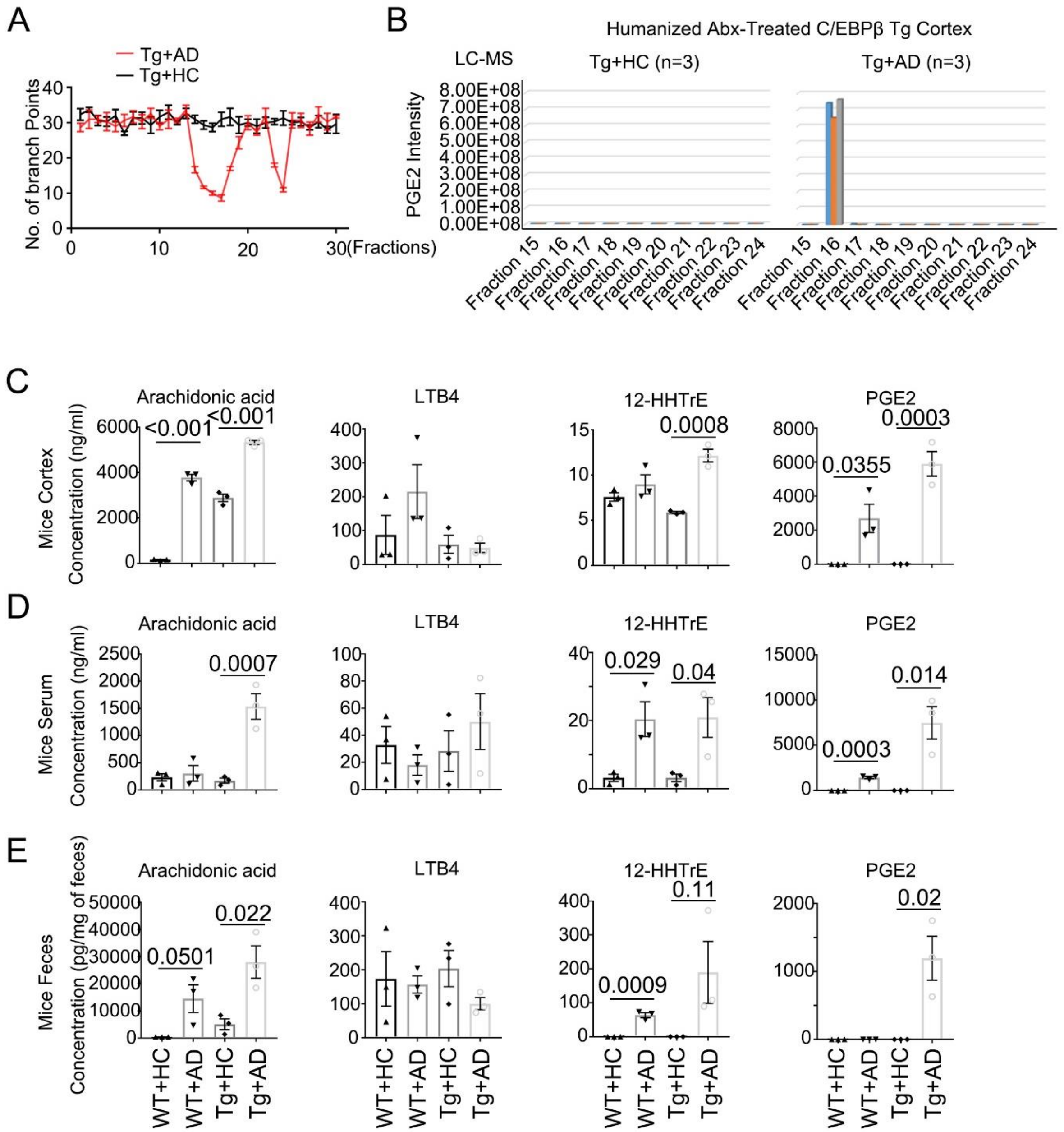

A
Tg+AD
Tg+HC
No. of branch Points
(Fractions)
B
Humanized Abx-Treated C/EBPβ Tg Cortex
LC-MS
Tg+HC (n=3)
Tg+AD (n=3)
PGE2 Intensity
Fraction 15
Fraction 16
Fraction 17
Fraction 18
Fraction 19
Fraction 20
Fraction 21
Fraction 22
Fraction 23
Fraction 24
C
Mice Cortex
Concentration (ng/ml)
Arachidonic acid
<0.001
<0.001
LTB4
12-HHTrE
0.0008
PGE2
0.0355
0.0003
D
Mice Serum
Concentration (ng/ml)
Arachidonic acid
0.0007
LTB4
12-HHTrE
0.029
0.04
PGE2
0.0003
0.014
E
Mice Feces
Concentration (pg/mg of feces)
Arachidonic acid
0.0501
0.022
LTB4
12-HHTrE
0.0009
0.11
PGE2
0.02
WT+HC
WT+AD
Tg+HC
Tg+AD

1

Figure S9. AD humanized Abx-treated mice display increased arachidonic acid, 12-HHTrE, and PGE2 compared with HC.

(A) TCA treated protein removal from HC or AD humanized Abx-treated 6-month-old C/EBPβ Tg mice brain lysates were separated into 30 fractions with HPLC. 96-well planted rat primary microglia were treated with each fraction for 10 ul/well. 24 h later, the number of branch points was counted for the activity of microglia. Only AD humanized Abx-treated C/EBPβ Tg mice showed changes in the activity, including fractions 12-20, 22-25. (B) After LC-MS analysis of fractions 15-24, PGE2 was found increased in AD humanized Abx-treated mice compared with HC humanized Abx-treated 6-month-old mice. Source data are provided as a Source Data file. (C-E) HPLC analysis the concentrations of arachidonic acid (AA) and its metabolites in HC or AD humanized Abx-treated 6-month-old WT or C/EBPβ Tg mice brain lysis (C), serum (D), and feces samples (E). (n=3 biologically independent samples in each group, data are shown as mean ± SEM, one-way ANOVA and Bonferroni's multiple comparison test).

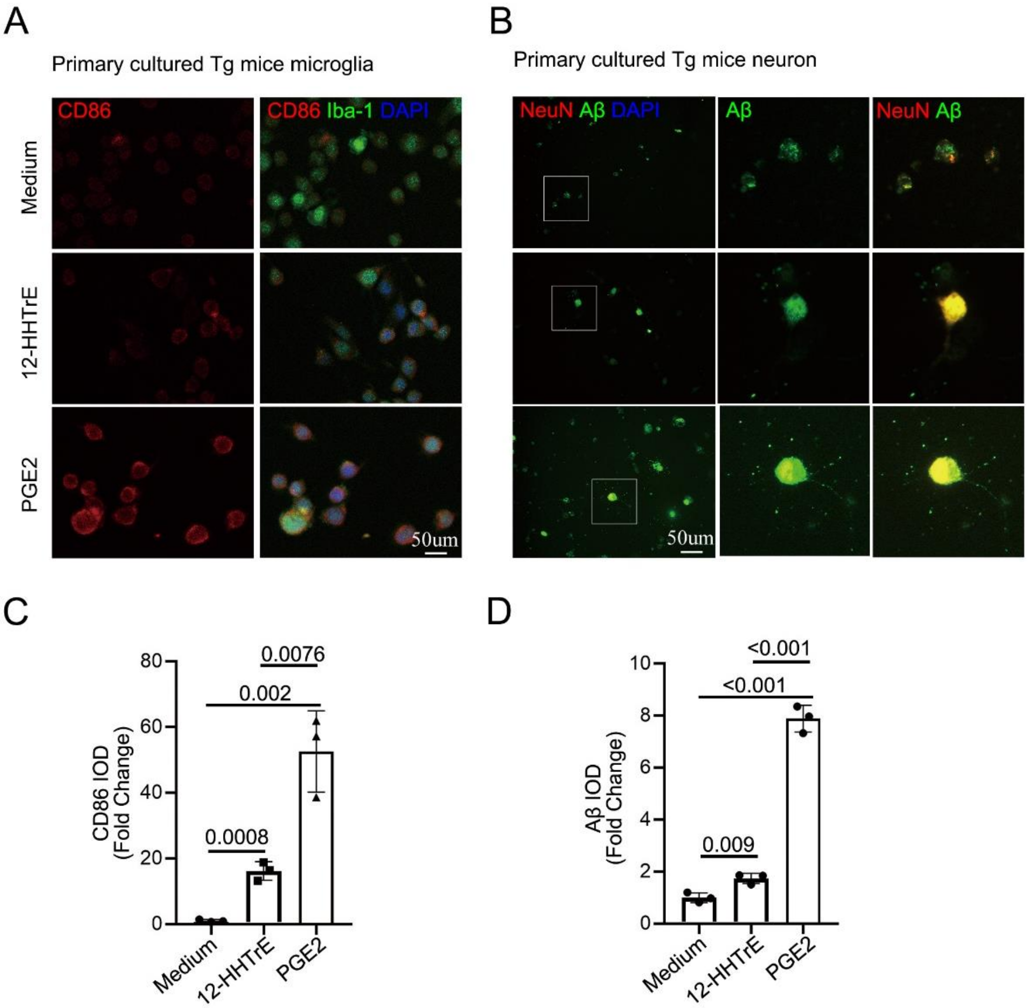
A
Primary cultured Tg mice microglia
CD86
CD86 Iba-1 DAPI
Medium
12-HHTrE
PGE2
50um
B
Primary cultured Tg mice neuron
NeuN Aβ DAPI
Aβ
NeuN Aβ
50um
C
CD86 IOD
(Fold Change)
0.0076
0.002
0.0008
Medium
12-HHTrE
PGE2
D
Aβ IOD
(Fold Change)
<0.001
<0.001
0.009
Medium
12-HHTrE
PGE2

1

Figure S10. 12-HHTrE and PGE2 enhanced the activation of microglia and neuronal Aβ_{42} deposition.

In vitro cultured microglia and neurons from Tg mice were employed for the analysis of microglia activation and Aβ_{42} deposition. (A) The primary cultured microglia from C/EBPβ Tg mice, treated with 12-HHTrE or PGE2 (20 ug/ml) for 24 h. Then fixed for immunofluorescent staining of CD86 (red) and Iba-1 (green). (B) The primary cultured neuron from C/EBPβ Tg mice, treated with 12-HHTrE or PGE2 (20 ug/ml) for 24 h. Then fixed for immunofluorescent staining of NeuN (red) and Aβ (4G8, green). (C-D) Quantitative analysis of CD86/Aβ mean IOD ($n = 3$ biologically independent samples in each group, data were shown as mean ± SEM, one-way ANOVA and Bonferroni's multiple comparison test).

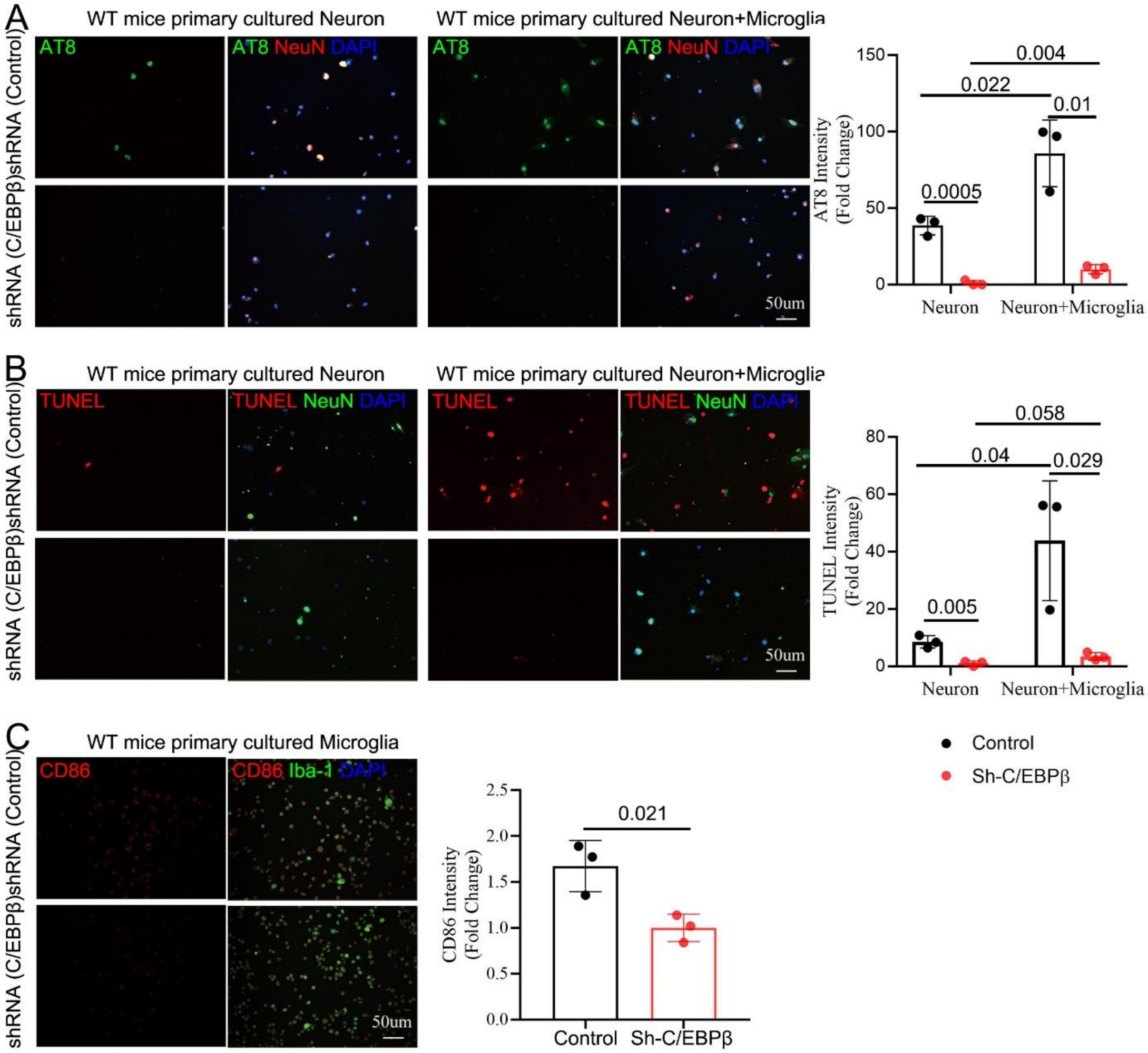
A
WT mice primary cultured Neuron
WT mice primary cultured Neuron+Microglia
shRNA (Control)
shRNA (C/EBPβ)
AT8
AT8 NeuN DAPI
50um
AT8 Intensity (Fold Change)
150
100
50
0
0.004
0.022
0.01
0.0005
Neuron
Neuron+Microglia
B
WT mice primary cultured Neuron
WT mice primary cultured Neuron+Microglia
shRNA (Control)
shRNA (C/EBPβ)
TUNEL
TUNEL NeuN DAPI
50um
TUNEL Intensity (Fold Change)
80
60
40
20
0
0.058
0.04
0.029
0.005
Neuron
Neuron+Microglia
Control
Sh-C/EBPβ
C
WT mice primary cultured Microglia
shRNA (Control)
shRNA (C/EBPβ)
CD86
CD86 Iba-1 DAPI
50um
CD86 Intensity (Fold Change)
2.5
2.0
1.5
1.0
0.5
0.0
0.021
Control
Sh-C/EBPβ

1

Figure S11. Deletion of C/EBPβ from primary neuronal cultures or microglia alleviate the detrimental effect of *B. Fragilis* metabolites.

The primary cultured neurons and neurons + microglia mixture from WT mice, followed w/wo AAV-sh-C/EBPβ knockdown, then treated with PGE2 (20 ug/ml) for 24 h. (A) Immunofluorescent staining of AT8 (green) and NeuN (red) in the primary cultured neurons or neurons + microglia mixture. Scale bar: 50 μm. Quantitative analysis showed AT8 intensity was significantly decreased after C/EBPβ knockdown (n=3 biologically independent samples in each group, data were shown as mean ± SEM, one-way ANOVA and Bonferroni's multiple comparison test). (B) Immunofluorescent staining of NeuN (green) and TUNEL (red) in the primary cultured neuron or neurons + microglia mixture. Scale bar: 50 μm. Quantitative analysis showed TUNEL intensity was significantly decreased after C/EBPβ knockdown (n = 3 biologically independent samples in each group, data were shown as mean ± SEM, one-way ANOVA and Bonferroni's multiple comparison test). (C) Immunofluorescent staining of Iba-1 (green) and CD86 (red) in the primary cultured microglia. Scale bar: 50 μm. Quantitative analysis showed CD86 intensity was significantly decreased after C/EBPβ knockdown (n = 3 biologically independent samples in each group, data were shown as mean ± SEM, two-tailed Student's t test).

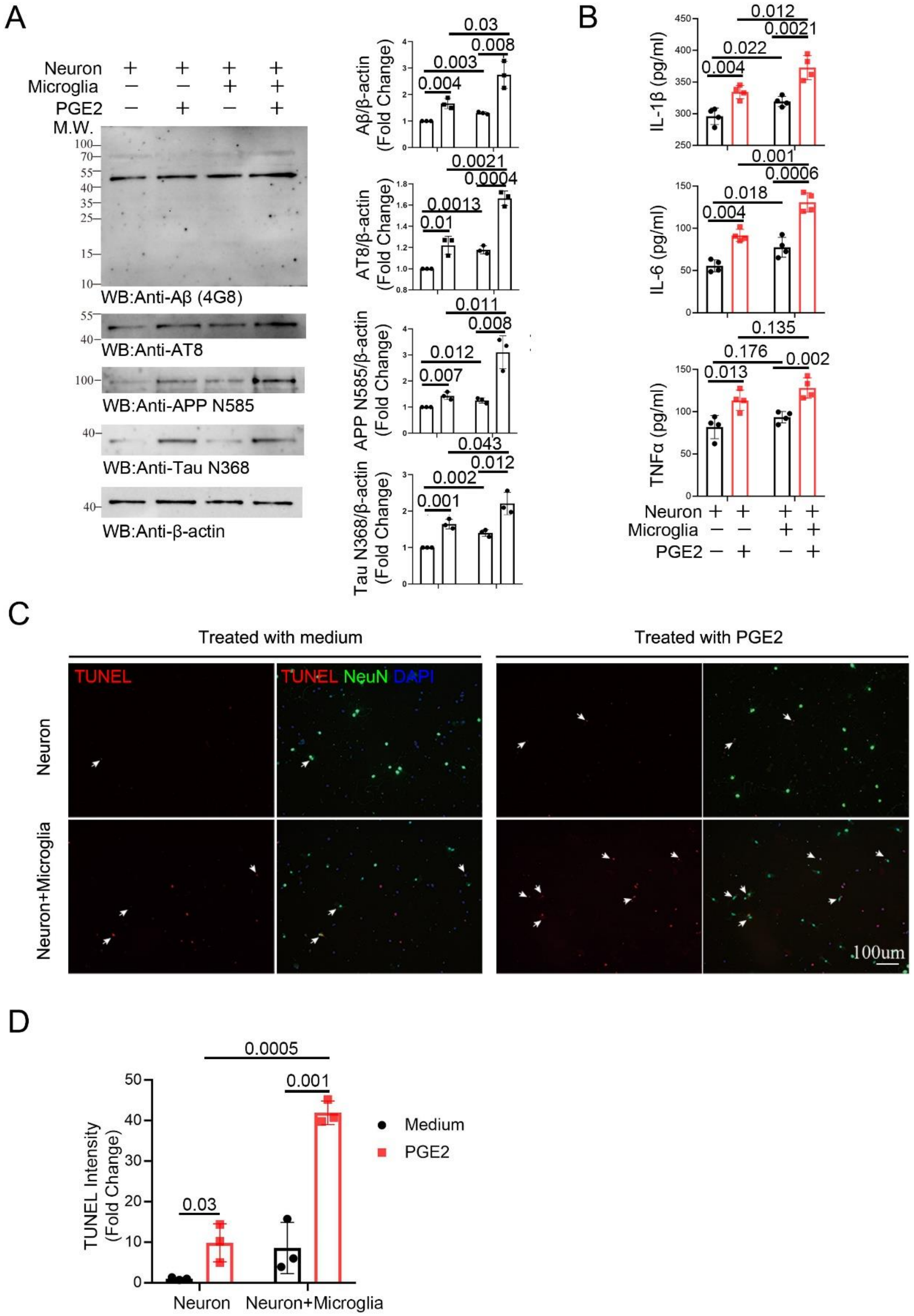
A
Neuron
Microglia
PGE2
M.W.
WB:Anti-Aβ (4G8)
WB:Anti-AT8
WB:Anti-APP N585
WB:Anti-Tau N368
WB:Anti-β-actin
Aβ/β-actin (Fold Change)
AT8/β-actin (Fold Change)
APP N585/β-actin (Fold Change)
Tau N368/β-actin (Fold Change)
B
IL-1β (pg/ml)
IL-6 (pg/ml)
TNFα (pg/ml)
C
Treated with medium
Treated with PGE2
TUNEL
TUNEL NeuN DAPI
Neuron
Neuron+Microglia
100um
D
TUNEL Intensity (Fold Change)
Medium
PGE2
Neuron
Neuron+Microglia

1

Figure S12. Mixed cultures of neurons and microglia showed higher inflammatory cytokines, Aβ_{42}, AT8 levels, and apoptosis than neurons alone.

The primary cultured neurons, mixed cultures of neurons + microglia from Thy1- C/EBPβ Tg mice. Treated w/wo PGE2. (A) WB detecting the protein levels of Aβ, AT8, APP N585, and Tau N368. Quantitative analysis the protein fold changes between neurons, and mixed cultured neurons + microglia (n = 3 biologically independent samples in each group, data were shown as mean ± SEM, one-way ANOVA and Bonferroni's multiple comparison test). (B) inflammatory cytokines including IL-1β, IL-6, and TNF-α were tested by ELISA (n = 3 biologically independent samples in each group, data were shown as mean ± SEM, one-way ANOVA and Bonferroni's multiple comparison test). (C) Immunofluorescent staining of NeuN (green) and TUNEL (red) in the primary cultured neurons or neurons + microglia mixture. Scale bar: 100 μm. Arrows showed the NeuN positive TUNEL deposited. (D) Quantitative analysis showed TUNEL intensity was significantly increased after PGE2 treatment or microglia addition (n = 3 biologically independent samples in each group, data were shown as mean ± SEM, one-way ANOVA and Bonferroni's multiple comparison test).

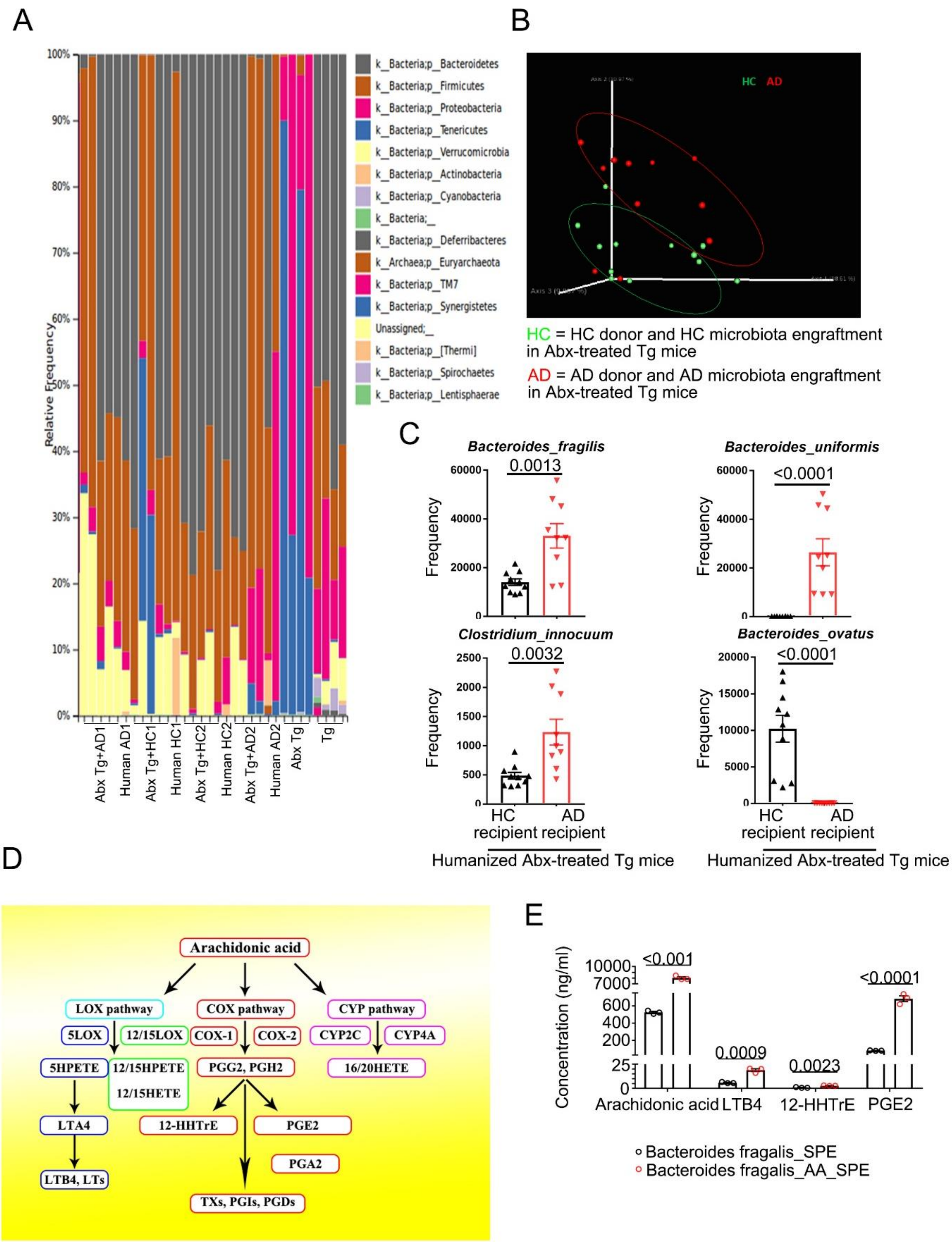

A
B
C
D
E
100%
90%
80%
70%
60%
50%
40%
30%
20%
10%
0%
Relative Frequency
Abx Tg+AD1
Human AD1
Abx Tg+HC1
Human HC1
Abx Tg+HC2
Human HC2
Abx Tg+AD2
Human AD2
Abx Tg
Tg
k__Bacteria;p__Bacteroidetes
k__Bacteria;p__Firmicutes
k__Bacteria;p__Proteobacteria
k__Bacteria;p__Tenericutes
k__Bacteria;p__Verrucomicrobia
k__Bacteria;p__Actinobacteria
k__Bacteria;p__Cyanobacteria
k__Bacteria;__
k__Bacteria;p__Deferribacteres
k__Archaea;p__Euryarchaeota
k__Bacteria;p__TM7
k__Bacteria;p__Synergistetes
Unassigned;__
k__Bacteria;p__[Thermi]
k__Bacteria;p__Spirochaetes
k__Bacteria;p__Lentisphaerae
HC AD
HC = HC donor and HC microbiota engraftment in Abx-treated Tg mice
AD = AD donor and AD microbiota engraftment in Abx-treated Tg mice
Bacteroides_fragilis
0.0013
Bacteroides_uniformis
<0.0001
Clostridium_innocuum
0.0032
Bacteroides_ovatus
<0.0001
Frequency
60000
40000
20000
0
2500
2000
1500
1000
500
15000
10000
5000
HC recipient
AD recipient
Humanized Abx-treated Tg mice
Arachidonic acid
LOX pathway
COX pathway
CYP pathway
5LOX
12/15LOX
COX-1
COX-2
CYP2C
CYP4A
5HPETE
12/15HPETE
12/15HETE
PGG2, PGH2
16/20HETE
LTA4
12-HHTrE
PGE2
PGA2
LTB4, LTs
TXs, PGIs, PGDs
Concentration (ng/ml)
10000
7000
600
400
200
25
0
<0.001
0.0009
0.0023
<0.0001
Arachidonic acid
LTB4
12-HHTrE
PGE2
Bacteroides fragalis_SPE
Bacteroides fragalis_AA_SPE

Figure S13. Microbiome analysis of humanized Abx-treated WT and C/EBPβ Tg mice fecal samples, revealing Bacteroides elevation in the feces from human AD fecal inoculated Abx-treated mice.

(A) Relative abundance of bacterial phyla determined by high throughput sequencing analysis. (B) Principal coordinate plot (PcoA) of microbial community structure. (C) Mean frequency of bacterial species. (n = 9-10 biologically independent samples in each group, data represent the means ± SEM, two-tailed Student's t test). (D) KEGG arachidonic acid metabolism pathway of *Bacteroides fragilis* (1.11.1.9). (E) Concentrations of arachidonic acid (AA) and its metabolites in culture medium from *in vitro* cultures of *Bacteroides fragilis* (n = 3 biologically independent samples in each group, data were shown as mean ± SEM, two-tailed Student's t test).

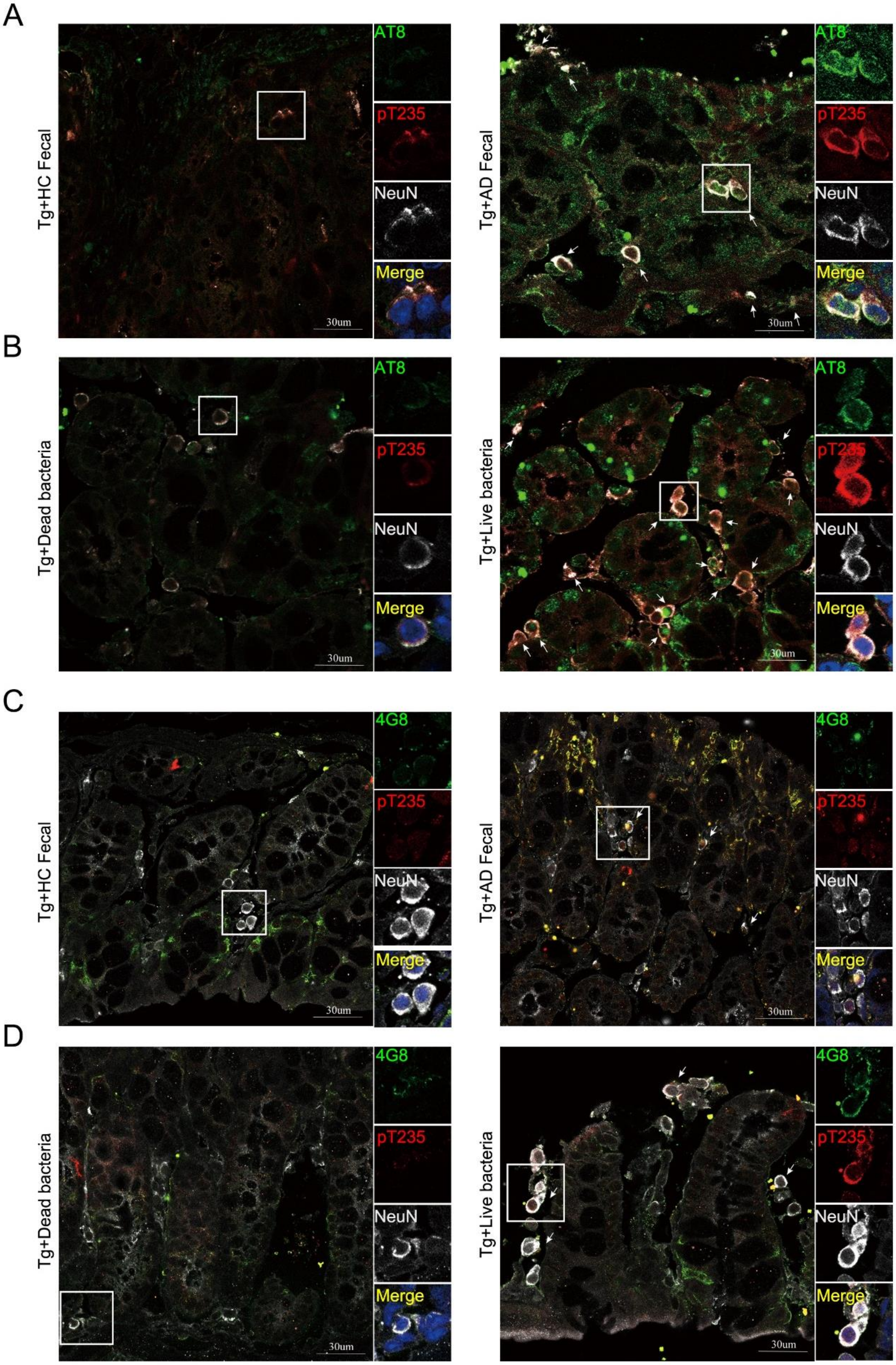
A
B
C
D
Tg+HC Fecal
Tg+AD Fecal
Tg+Dead bacteria
Tg+Live bacteria
AT8
pT235
NeuN
Merge
4G8
30um

1

Figure S14. The enteric neuronal Aβ_{42} and AT8 signals were increased after human AD fecal or live *B. fragilis* treatment.

(A-B) Immunofluorescent staining of AT8 (green) and active C/EBPβ pT235 (red) in the intestine region of gut from human AD fecal or *B. Fragilis* treated C/EBPβ Tg mice (representative of 3 mice). Scale bar: 30 μm. Arrows showed the AT8 and pT235 positive neurons. (C-D) Immunofluorescent staining of Aβ 4G8 (green) and C/EBPβ pT235 (red) in the intestine region of gut from human AD fecal or *B. Fragilis* treated C/EBPβ Tg mice (representative of 3 mice). Scale bar: 30 μm. Arrows showed the 4G8 and pT235 positive neurons.

Lipid Metabolism

A

Feces		Welch's Two-Sample t-Test AD-C/EBPβ Tg / HC-C/EBPβ Tg	
		Colored by:	
Sub Pathway	Biochemical Name	Statistics	Fold of Change
Long Chain Saturated Fatty Acid	myristate (14:0)	1.60	1.60
	palmitate (16:0)	1.74	1.74
	stearate (18:0)	1.59	1.59
	arachidate (20:0)	1.64	1.64
Long Chain Monounsaturated Fatty Acid	palmitoleate (16:1n7)	1.79	1.79
	oleate/vaccenate (18:1)	2.04	2.04
	eicosenoate (20:1)	2.74	2.74
	erucate (22:1n9)	2.02	2.02
Long Chain Polyunsaturated Fatty Acid (n3 and n6)	hexadecatrienoate (16:3n3)	1.58	1.58
	stearidonate (18:4n3)	1.52	1.52
	eicosapentaenoate (EPA; 20:5n3)	2.17	2.17
	heneicosapentaenoate (21:5n3)	1.77	1.77
	docosapentaenoate (n3 DPA; 22:5n3)	2.31	2.31
	docosahexaenoate (DHA; 22:6n3)	2.23	2.23
	nisinate (24:6n3)	3.51	3.51
	hexadecadienoate (16:2n6)	1.57	1.57
	linoleate (18:2n6)	1.76	1.76
	linolenate [alpha or gamma; (18:3n3 or	1.75	1.75
	dihomo-linoleate (20:2n6)	1.45	1.45
	dihomo-linolenate (20:3n3 or n6)	1.61	1.61
	arachidonate (20:4n6)	2.27	2.27
	docosatrienoate (22:3n6)*	0.65	0.65
	adrenate (22:4n6)	1.72	1.72
	docosapentaenoate (n6 DPA; 22:5n6)	1.44	1.44
	docosadienoate (22:2n6)	1.72	1.72

Feces		Welch's Two-Sample t-Test AD-C/EBPβ Tg / HC-C/EBPβ Tg	
		Colored by:	
Sub Pathway	Biochemical Name	Statistics	Fold of Change
Mono-acylglycerol	1-myristoylglycerol (14:0)	1.68	1.68
	1-palmitoylglycerol (16:0)	2.58	2.58
	1-palmitoleoylglycerol (16:1)*	1.89	1.89
	1-oleoylglycerol (18:1)	2.43	2.43
	1-linoleoylglycerol (18:2)	2.07	2.07
	1-linolenoylglycerol (18:3)	1.92	1.92
	1-dihomo-linolenylglycerol (20:3)	2.58	2.58
	1-docosahexaenoylglycerol (22:6)	1.91	1.91
	2-myristoylglycerol (14:0)	1.35	1.35
	2-palmitoylglycerol (16:0)	1.39	1.39
	2-oleoylglycerol (18:1)	2.72	2.72
	2-linoleoylglycerol (18:2)	1.47	1.47
	2-docosahexaenoylglycerol (22:6)*	1.61	1.61
	1-heptadecenoylglycerol (17:1)*	1.62	1.62
Di-acylglycerol	diacylglycerol (16:1/18:2 [2], 16:0/18:3	0.68	0.68
	palmitoyl-oleoyl-glycerol (16:0/18:1) [2	0.62	0.62
	palmitoyl-linoleoyl-glycerol (16:0/18:2)	0.74	0.74
	palmitoyl-linoleoyl-glycerol (16:0/18:2)	0.64	0.64
	oleoyl-linoleoyl-glycerol (18:1/18:2) [2]	0.72	0.72
	linoleoyl-linoleoyl-glycerol (18:2/18:2)	0.78	0.78
	linoleoyl-linoleoyl-glycerol (18:2/18:2)	0.67	0.67
	linoleoyl-linolenoyl-glycerol (18:2/18:3)	0.72	0.72
	linoleoyl-linolenoyl-glycerol (18:2/18:3)	0.62	0.62
	linolenoyl-linolenoyl-glycerol (18:3/18:	0.51	0.51
	linoleoyl-docosahexaenoyl-glycerol (1	0.86	0.86

B

Brain		Welch's Two-Sample t-Test C/EBPβ Tg / WT		AD-C/EBPβ Tg / HC-C/EBPβ Tg	
		Colored by:		Colored by:	
Sub Pathway	Biochemical Name	Statistics	Fold of Change	Statistics	Fold of Change
Long Chain Saturated Fatty Acid	myristate (14:0)	0.98	0.98	1.61	1.61
	palmitate (16:0)	1.14	1.14	1.77	1.77
	margarate (17:0)	1.19	1.19	2.00	2.00
	stearate (18:0)	1.16	1.16	1.86	1.86
	nonadecanoate (19:0)	1.17	1.17	2.25	2.25
	arachidate (20:0)	1.26	1.26	2.10	2.10
Long Chain Monounsaturated Fatty Acid	palmitoleate (16:1n7)	0.92	0.92	2.00	2.00
	10-heptadecenoate (17:1n7)	1.02	1.02	2.36	2.36
	oleate/vaccenate (18:1)	1.15	1.15	2.12	2.12
	10-nonadecenoate (19:1n9)	1.09	1.09	2.74	2.74
	eicosenoate (20:1)	1.20	1.20	2.52	2.52
	erucate (22:1n9)	1.27	1.27	3.13	3.13
Long Chain Polyunsaturated Fatty Acid (n3 and n6)	tetradecadienoate (14:2)*	0.93	0.93	1.50	1.50
	hexadecatrienoate (16:3n3)	1.11	1.11	1.62	1.62
	stearidonate (18:4n3)	1.11	1.11	1.58	1.58
	eicosapentaenoate (EPA; 20:5n3)	1.13	1.13	2.10	2.10
	docosapentaenoate (n3 DPA; 22:5n3)	1.03	1.03	2.68	2.68
	docosahexaenoate (DHA; 22:6n3)	1.18	1.18	2.30	2.30
	docosatrienoate (22:3n3)	1.76	1.76	1.04	1.04
	nisinate (24:6n3)	1.27	1.27	2.67	2.67
	hexadecadienoate (16:2n6)	1.17	1.17	1.59	1.59
	linoleate (18:2n6)	1.34	1.34	1.99	1.99
	linolenate [alpha or gamma; (18:3n3 or	1.06	1.06	2.00	2.00
	dihomo-linoleate (20:2n6)	1.50	1.50	2.37	2.37
	dihomo-linolenate (20:3n3 or n6)	1.32	1.32	2.10	2.10
	arachidonate (20:4n6)	0.99	0.99	1.01	1.01
	docosatrienoate (22:3n6)*	0.82	0.82	3.32	3.32
	adrenate (22:4n6)	1.12	1.12	2.28	2.28
	docosapentaenoate (n6 DPA; 22:5n6)	1.21	1.21	2.35	2.35
	docosadienoate (22:2n6)	1.46	1.46	3.01	3.01
	mead acid (20:3n9)	0.84	0.84	2.21	2.21

Brain		Welch's Two-Sample t-Test C/EBPβ Tg / WT		AD-C/EBPβ Tg / HC-C/EBPβ Tg	
		Colored by:		Colored by:	
Sub Pathway	Biochemical Name	Statistics	Fold of Change	Statistics	Fold of Change
Monoacylglycerol	1-myristoylglycerol (14:0)	0.92	0.92	1.42	1.42
	1-palmitoylglycerol (16:0)	1.06	1.06	2.06	2.06
	1-palmitoleoylglycerol (16:1)*	0.88	0.88	2.37	2.37
	1-oleoylglycerol (18:1)	1.04	1.04	1.87	1.87
	1-linoleoylglycerol (18:2)	1.34	1.34	2.53	2.53
	1-dihomo-linolenylglycerol (20:3)	0.85	0.85	2.78	2.78
	1-arachidonylglycerol (20:4)	0.96	0.96	2.10	2.10
	1-docosahexaenoylglycerol (22:6)	1.00	1.00	1.97	1.97
	2-palmitoylglycerol (16:0)	1.03	1.03	2.23	2.23
	2-palmitoleoylglycerol (16:1)*	0.83	0.83	2.46	2.46
	2-oleoylglycerol (18:1)	0.90	0.90	2.62	2.62
	2-linoleoylglycerol (18:2)	1.32	1.32	2.47	2.47
	2-arachidonoylglycerol (20:4)	0.97	0.97	1.93	1.93
	2-docosahexaenoylglycerol (22:6)*	0.93	0.93	1.84	1.84

1

Figure S15. Metabolomics analysis of the brains, serum and feces samples from HC or AD humanized Abx-treated C/EBPβ Tg mice and the brains from WT and C/EBPβ Tg mice. (A-B) The differences in microbiomes related metabolites from feces samples (A) and brain lysates (B). Red and green shaded cells indicate $p \leq 0.05$ (red indicates the fold change values are significantly higher for that comparison; green values significantly lower). Light red and light green shaded cells indicate $0.05 < p < 0.10$ (light red indicates the fold change values trend higher for that comparison; light green values trend lower). Source data are provided as Supplementary Data 1.

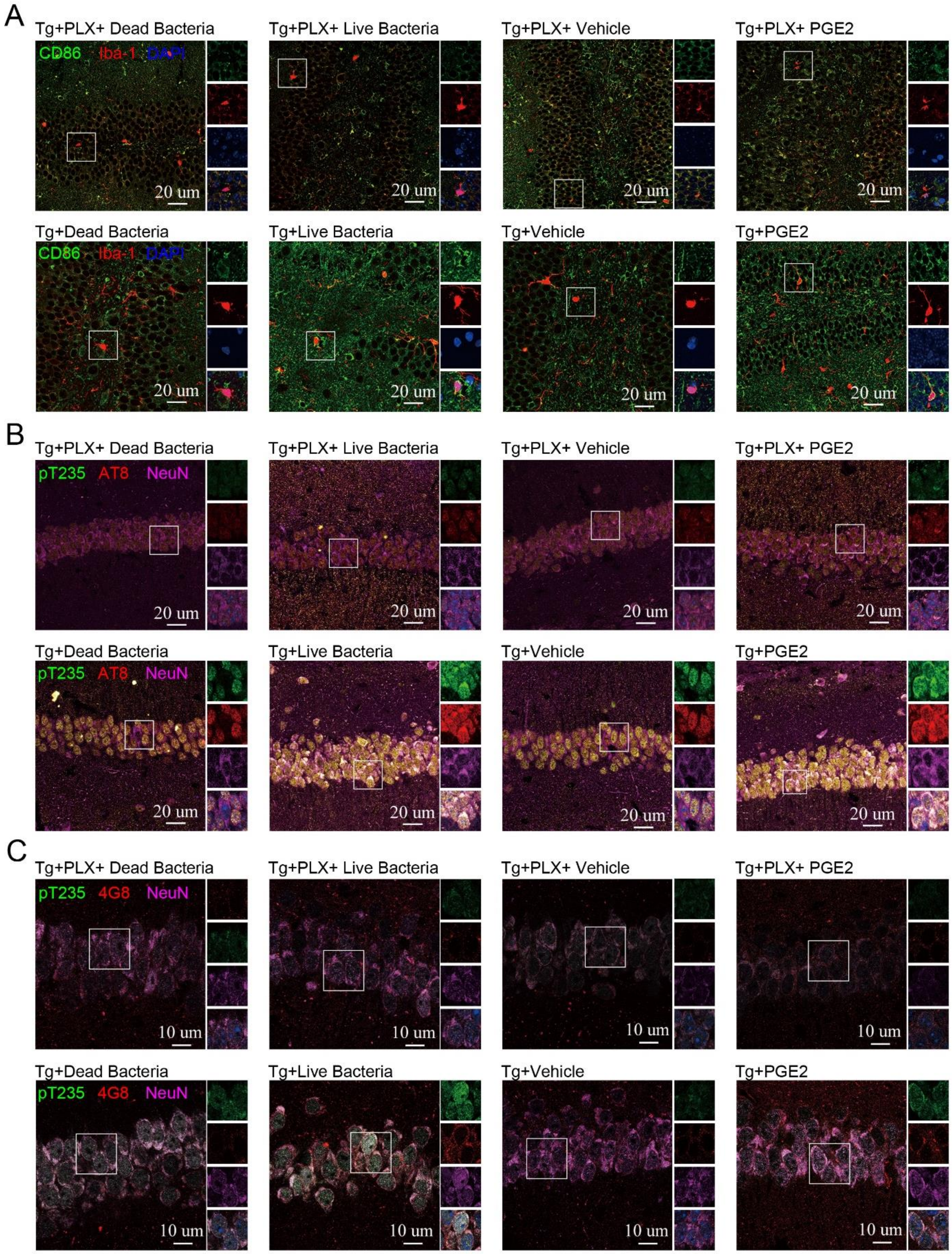
A
Tg+PLX+ Dead Bacteria
Tg+PLX+ Live Bacteria
Tg+PLX+ Vehicle
Tg+PLX+ PGE2
CD86 Iba-1 DAPI
20 um
Tg+Dead Bacteria
Tg+Live Bacteria
Tg+Vehicle
Tg+PGE2
B
pT235 AT8 NeuN
C
pT235 4G8 NeuN
10 um

Figure S16. Deletion of microglia attenuated PGE2-induced AD pathologies in Thy1-C/EBPβ Tg mice.

3-month-old Thy1-C/EBPβ Tg mice feed w/wo PLX3397 (600 ppm in chow, 7 days), followed by bacteria/PGE2 treatment (Tg + B.F (Dead), Tg + B.F (Live), Tg + Vehicle, Tg + PGE2) for 8 weeks, n = 3/group. (A) Immunofluorescent staining of CD86 (green) and Iba-1 (red) in the hippocampus region of the brain from *B. fragilis* or PGE2 treated C/EBPβ Tg mice. Scale bar: 20 μm. (B) Immunofluorescent staining of pT235 (green) and AT8 (red) in the hippocampus region of the brain from *B. fragilis* or PGE2 treated C/EBPβ Tg mice. Scale bar: 20 μm. (C) Immunofluorescent staining of pT235 (green) and 4G8 (red) in the hippocampus region of the brain from *B. fragilis* or PGE2 treated C/EBPβ Tg mice. Scale bar: 20 μm.
