# Supplementary material for: Bacteroides Fragilis in the gut microbiomes of Alzheimer’s disease activates microglia and triggers pathogenesis in neuronal C/EBPβ transgenic mice
Source: Nat Commun. 2023 Sep 6;14:5471. doi: 10.1038/s41467-023-41283-w (PMC10482867; doi:10.1038/s41467-023-41283-w)
Supplement: Supplementary file 3 — Description of Additional Supplementary Files [file 41467_2023_41283_MOESM3_ESM.docx]

**Supplementary data legends**

File Name: Source Data.xlsx

Description: Source Data.xlsx contains all the raw data for the graphs.

File Name: Supplementary Data 1.Zip

Description: Supplementary Data 1.Zip contains the metabolomic data of brain, serum, and fecal samples from the Alzheimer’s disease mouse model. We use proteomics only to quantify certain specific metabolites.

It contains:

EMOR-0201-20VW+ BOX PLOTS (BRAIN).PDF

EMOR-0201-20VW+ BOX PLOTS (FECES).PDF

EMOR-0201-20VW+ BOX PLOTS (SERUM).PDF

EMOR-0201-20VW+ DATA TABLE (BRAIN).XLSX

EMOR-0201-20VW+ DATA TABLE (FECES).XLSX

EMOR-0201-20VW+ DATA TABLE (SERUM).XLSX

EMOR-0201-20VW+ HEATMAP (BRAIN).XLSX

EMOR-0201-20VW+ HEATMAP (FECES).XLSX

EMOR-0201-20VW+ HEATMAP (SERUM).XLSX
