# Supplementary material for: Bacteroides Fragilis in the gut microbiomes of Alzheimer’s disease activates microglia and triggers pathogenesis in neuronal C/EBPβ transgenic mice
Source: Nat Commun. 2023 Sep 6;14:5471. doi: 10.1038/s41467-023-41283-w (PMC10482867; doi:10.1038/s41467-023-41283-w)
Supplement: Supplementary file 4 — Supplementary Data 1 [file 41467_2023_41283_MOESM4_ESM.zip › EMOR-0201-20VW+/EMOR-0201-20VW+ BOX PLOTS (BRAIN).PDF]

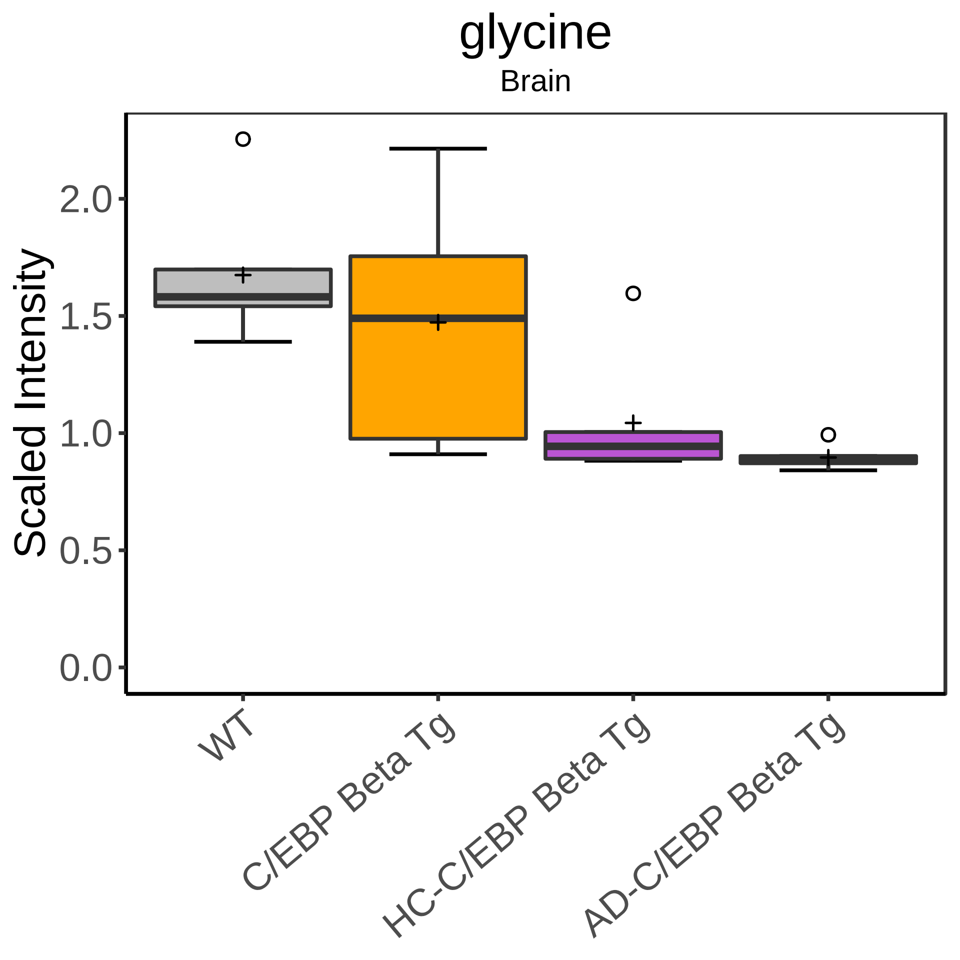
glycine
Brain
Scaled Intensity
2.0
1.5
1.0
0.5
0.0
WT
C/EBP Beta Tg
HC-C/EBP Beta Tg
AD-C/EBP Beta Tg

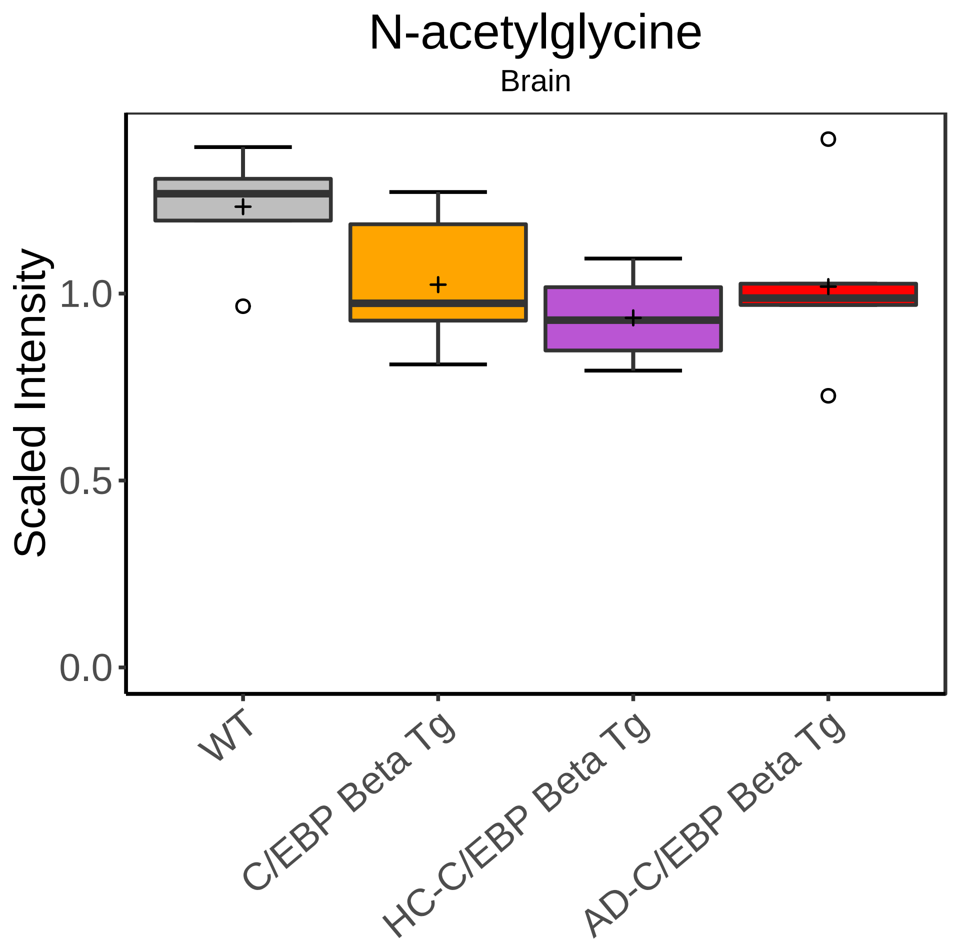
N-acetylglycine
Brain
Scaled Intensity
1.0
0.5
0.0
WT
C/EBP Beta Tg
HC-C/EBP Beta Tg
AD-C/EBP Beta Tg

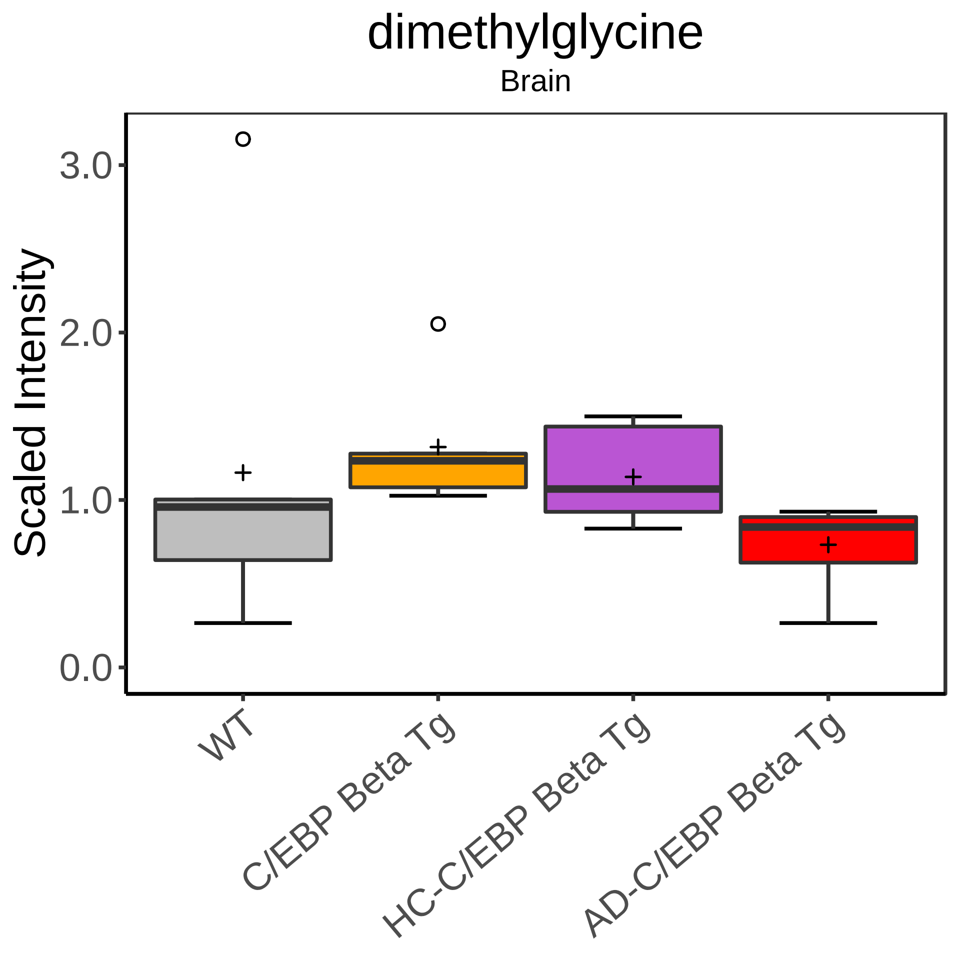
dimethylglycine
Brain
Scaled Intensity
3.0
2.0
1.0
0.0
WT
C/EBP Beta Tg
HC-C/EBP Beta Tg
AD-C/EBP Beta Tg

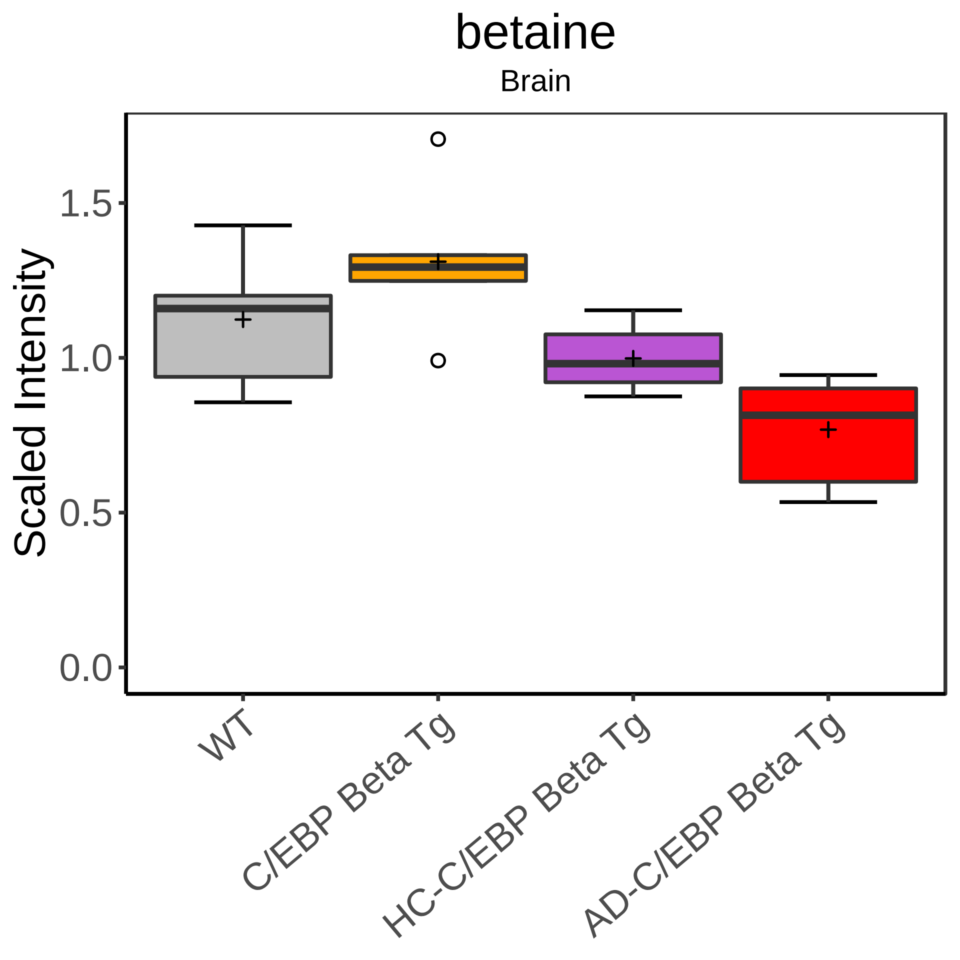

betaine
Brain
Scaled Intensity
1.5
1.0
0.5
0.0
WT
C/EBP Beta Tg
HC-C/EBP Beta Tg
AD-C/EBP Beta Tg

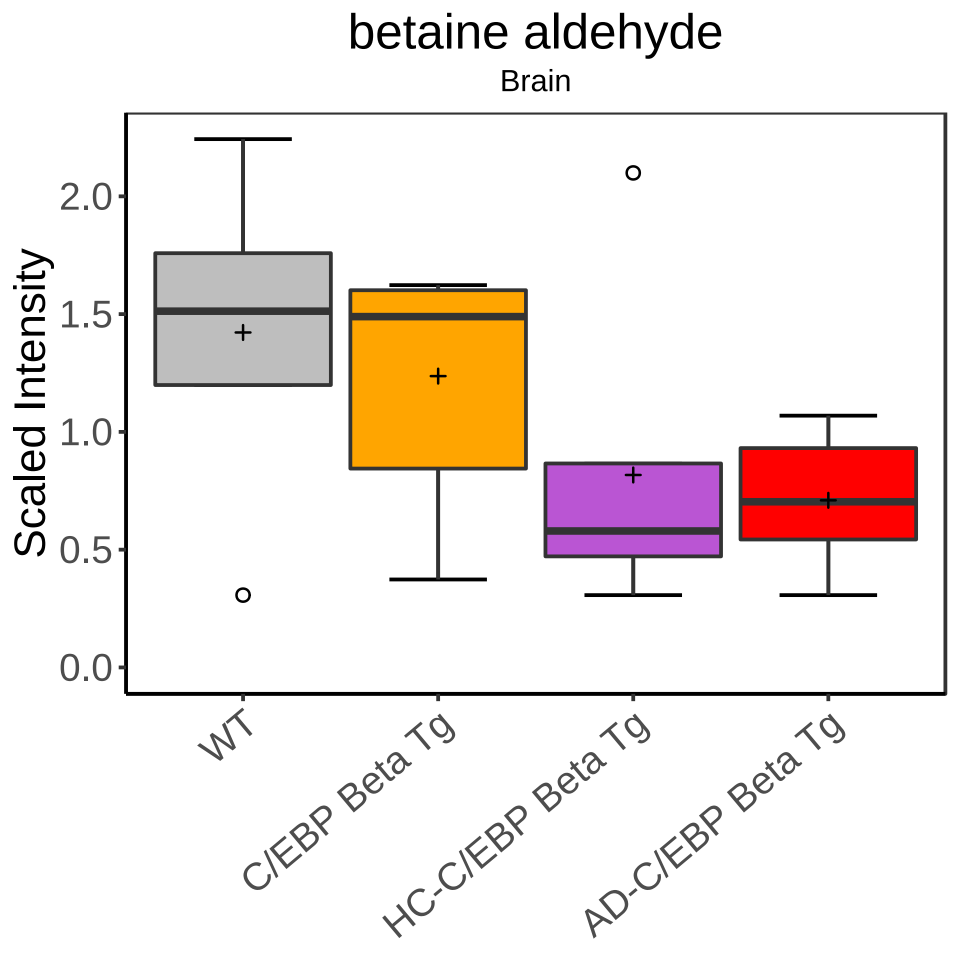
betaine aldehyde
Brain
Scaled Intensity
2.0
1.5
1.0
0.5
0.0
WT
C/EBP Beta Tg
HC-C/EBP Beta Tg
AD-C/EBP Beta Tg

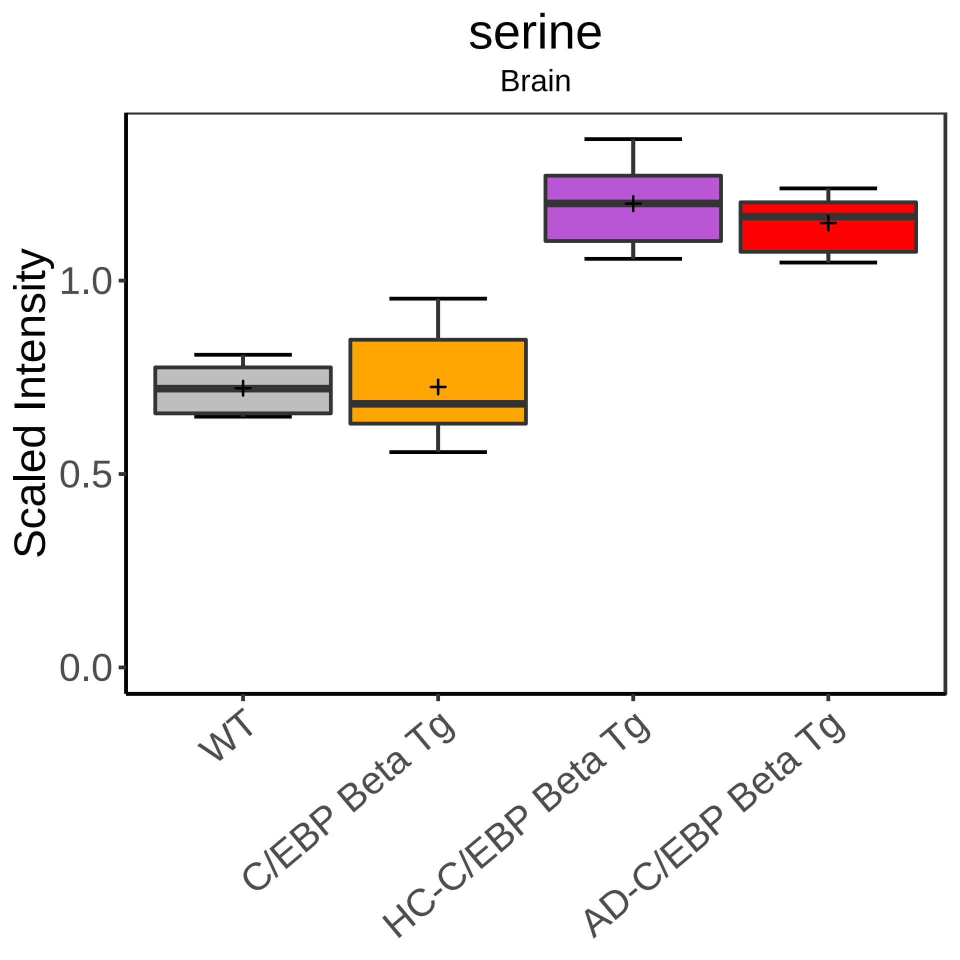

serine
Brain
Scaled Intensity
1.0
0.5
0.0
WT
C/EBP Beta Tg
HC-C/EBP Beta Tg
AD-C/EBP Beta Tg

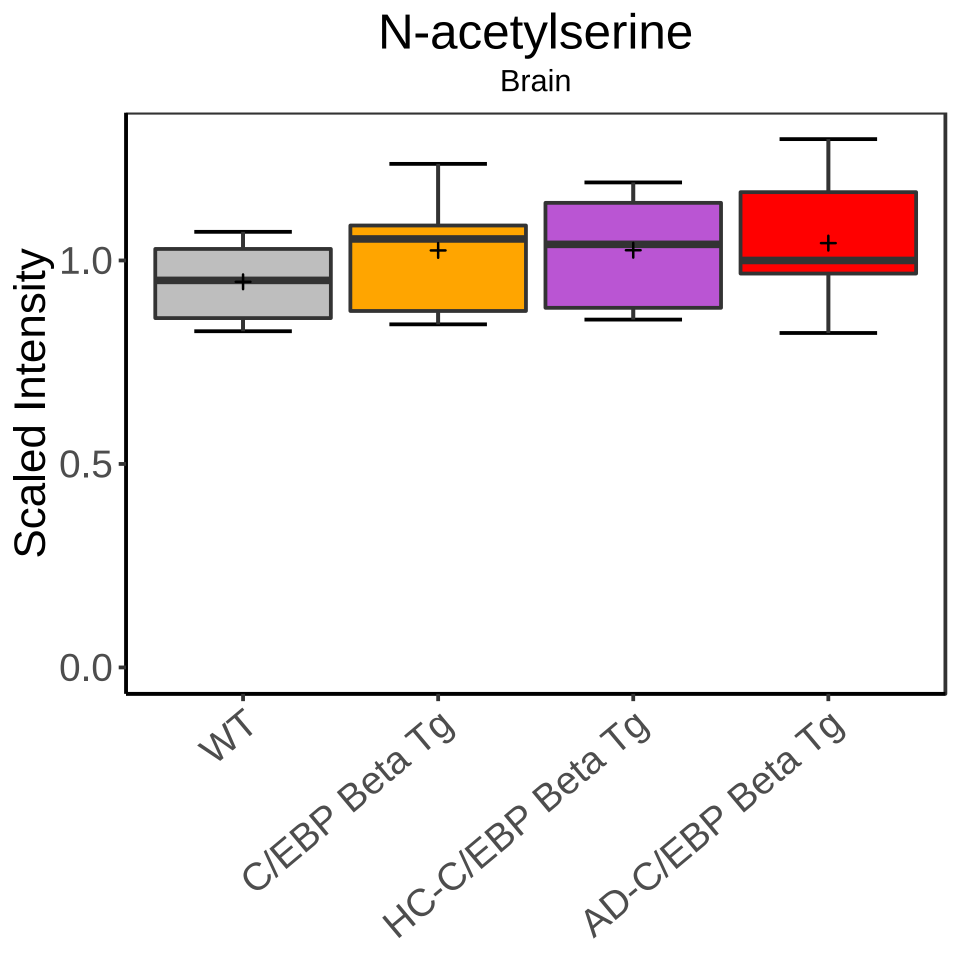
N-acetylserine
Brain
Scaled Intensity
1.0
0.5
0.0
WT
C/EBP Beta Tg
HC-C/EBP Beta Tg
AD-C/EBP Beta Tg

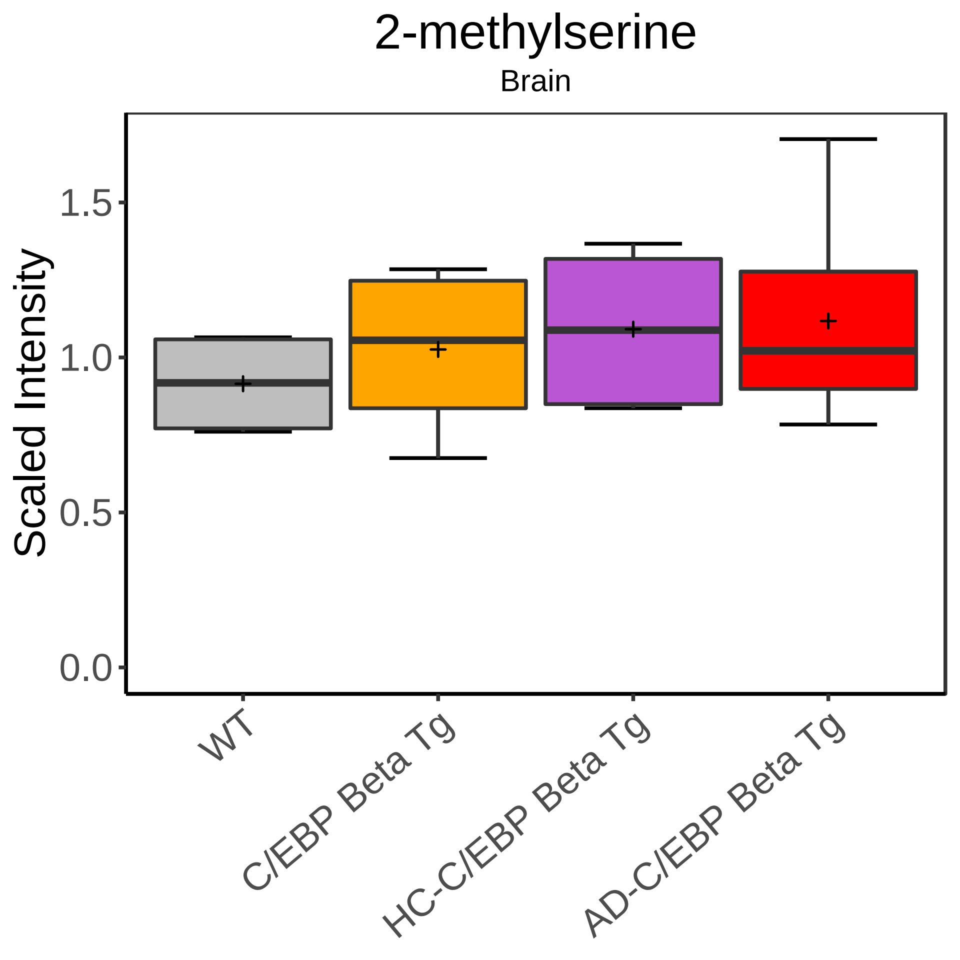

2-methylserine
Brain
Scaled Intensity
0.0
0.5
1.0
1.5
WT
C/EBP Beta Tg
HC-C/EBP Beta Tg
AD-C/EBP Beta Tg

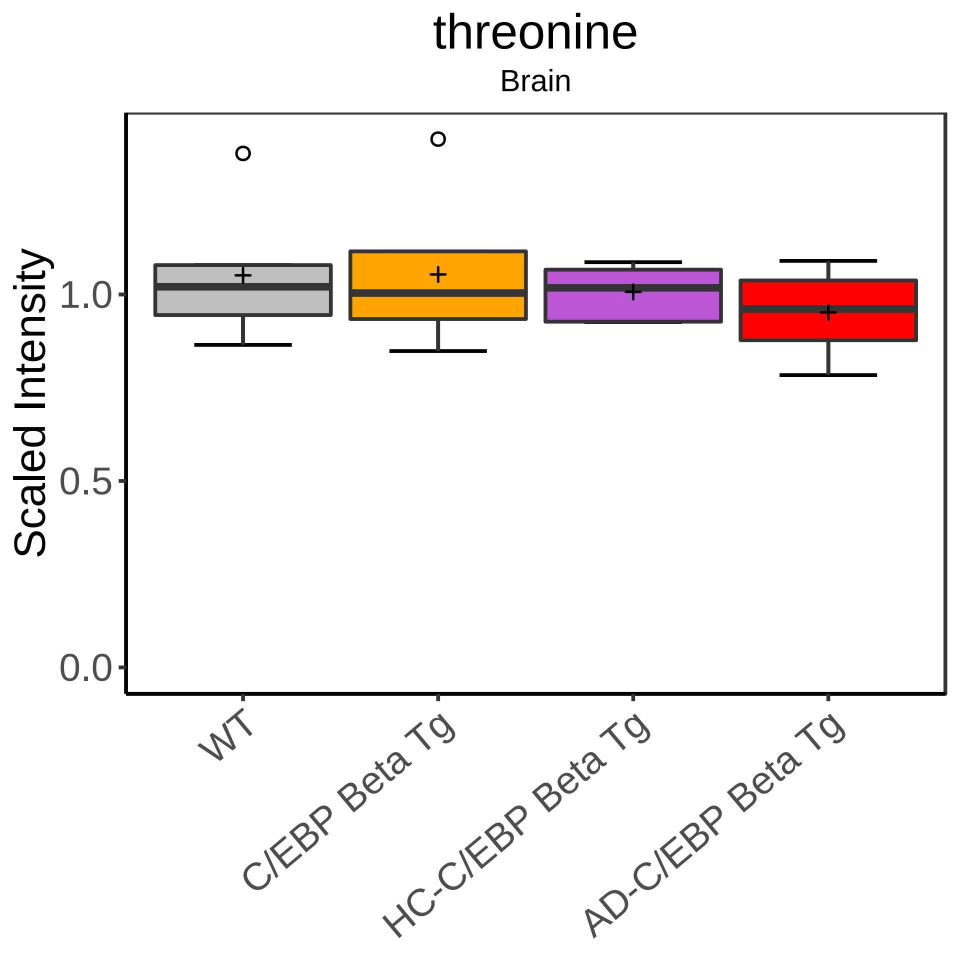
threonine
Brain
Scaled Intensity
1.0
0.5
0.0
WT
C/EBP Beta Tg
HC-C/EBP Beta Tg
AD-C/EBP Beta Tg

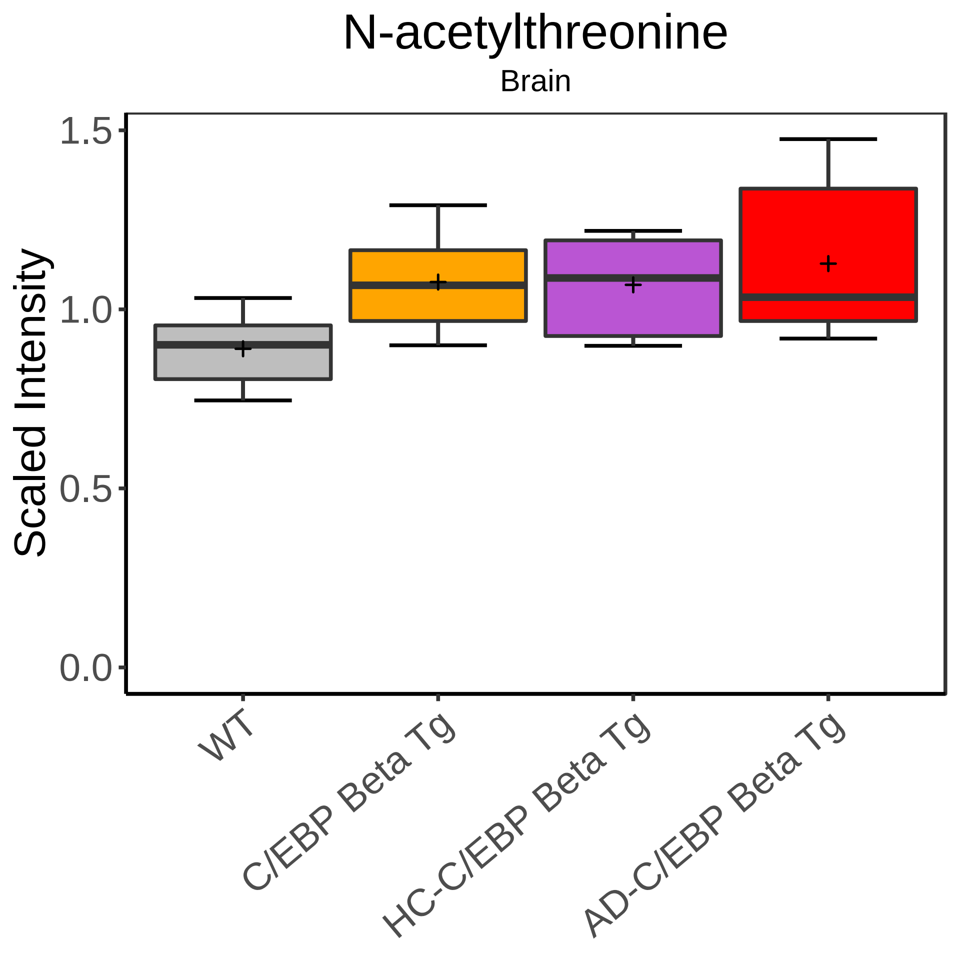

N-acetylthreonine
Brain
Scaled Intensity
1.5
1.0
0.5
0.0
WT
C/EBP Beta Tg
HC-C/EBP Beta Tg
AD-C/EBP Beta Tg

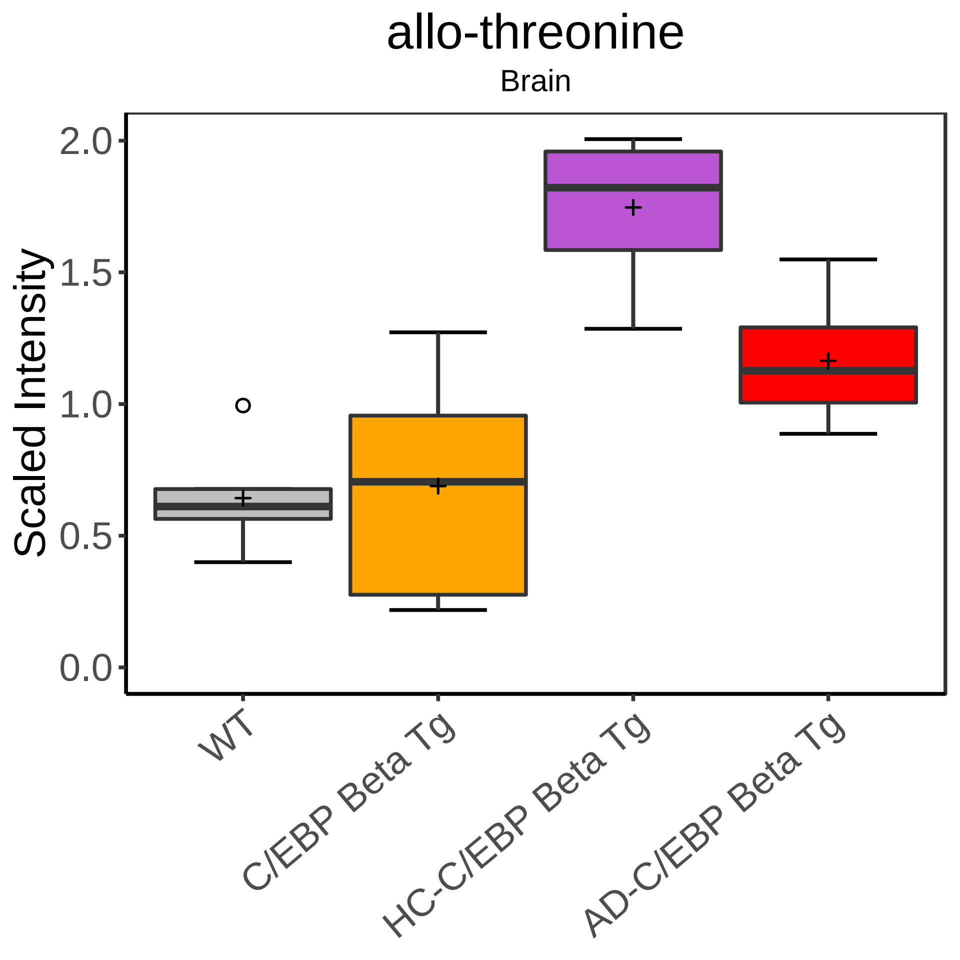
allo-threonine
Brain
Scaled Intensity
2.0
1.5
1.0
0.5
0.0
WT
C/EBP Beta Tg
HC-C/EBP Beta Tg
AD-C/EBP Beta Tg

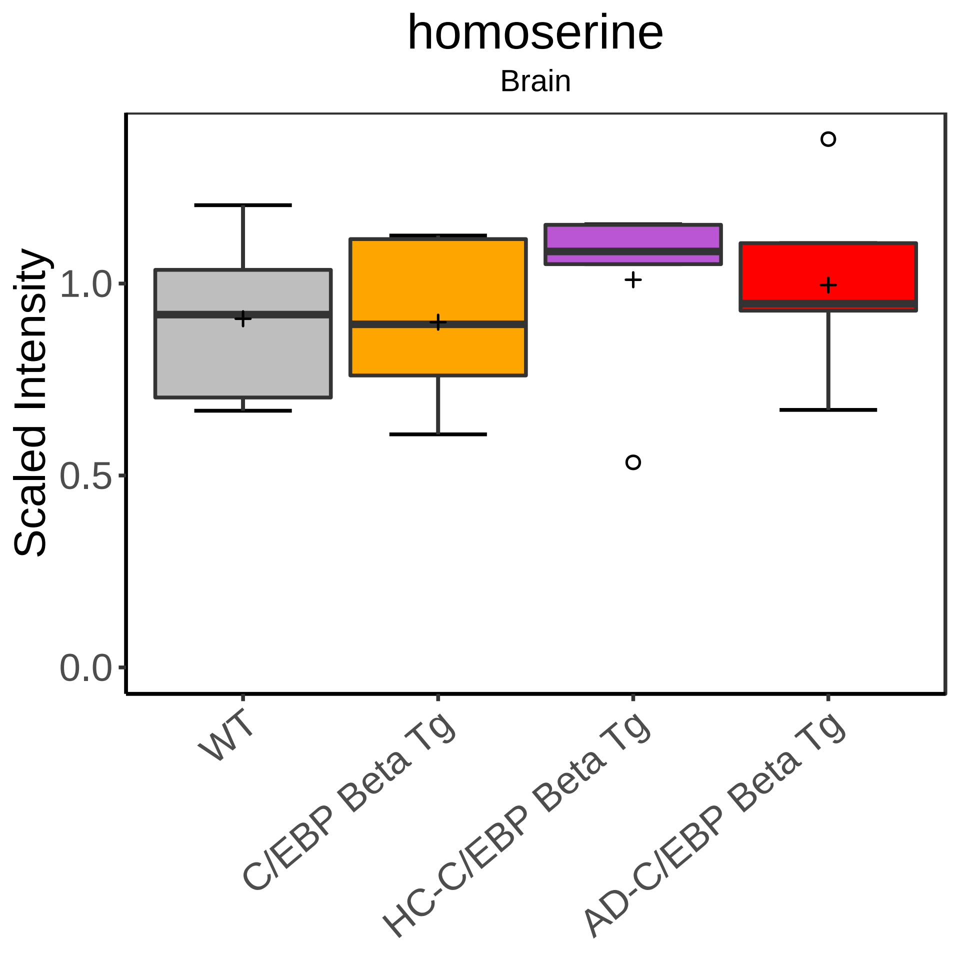
homoserine
Brain
Scaled Intensity
0.0
0.5
1.0
WT
C/EBP Beta Tg
HC-C/EBP Beta Tg
AD-C/EBP Beta Tg

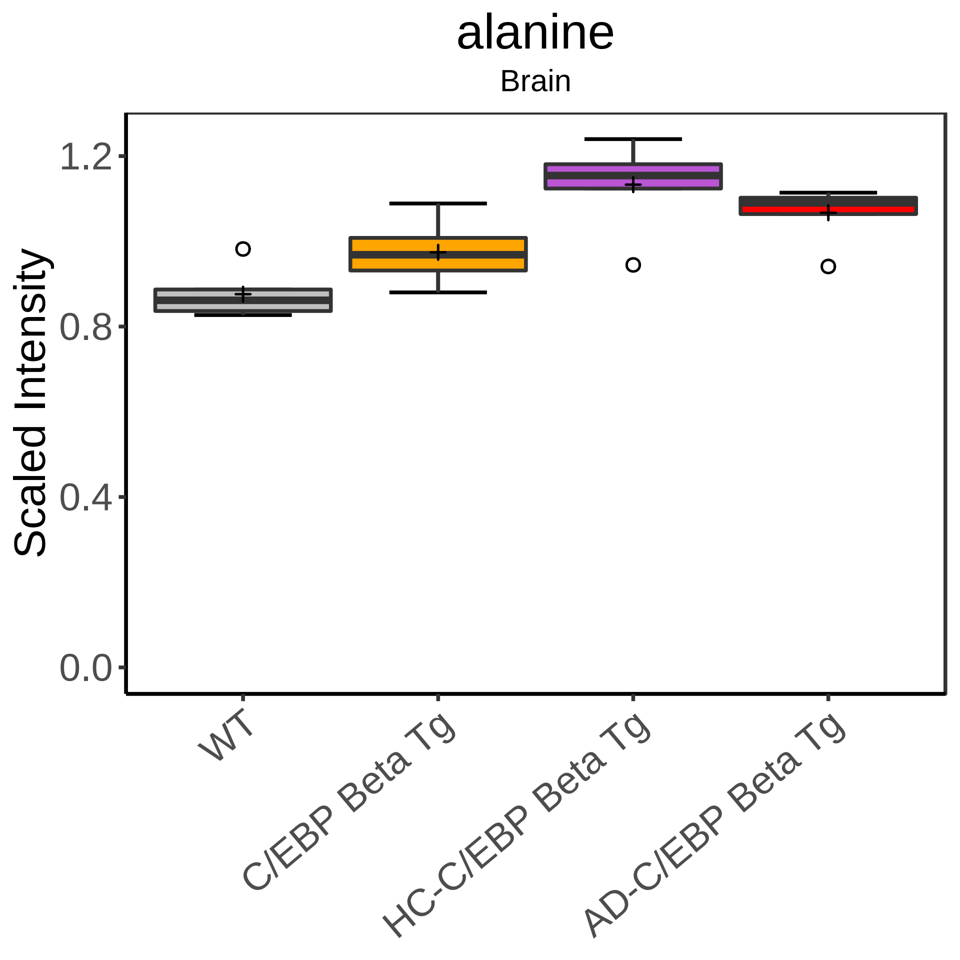
alanine
Brain
Scaled Intensity
1.2
0.8
0.4
0.0
WT
C/EBP Beta Tg
HC-C/EBP Beta Tg
AD-C/EBP Beta Tg

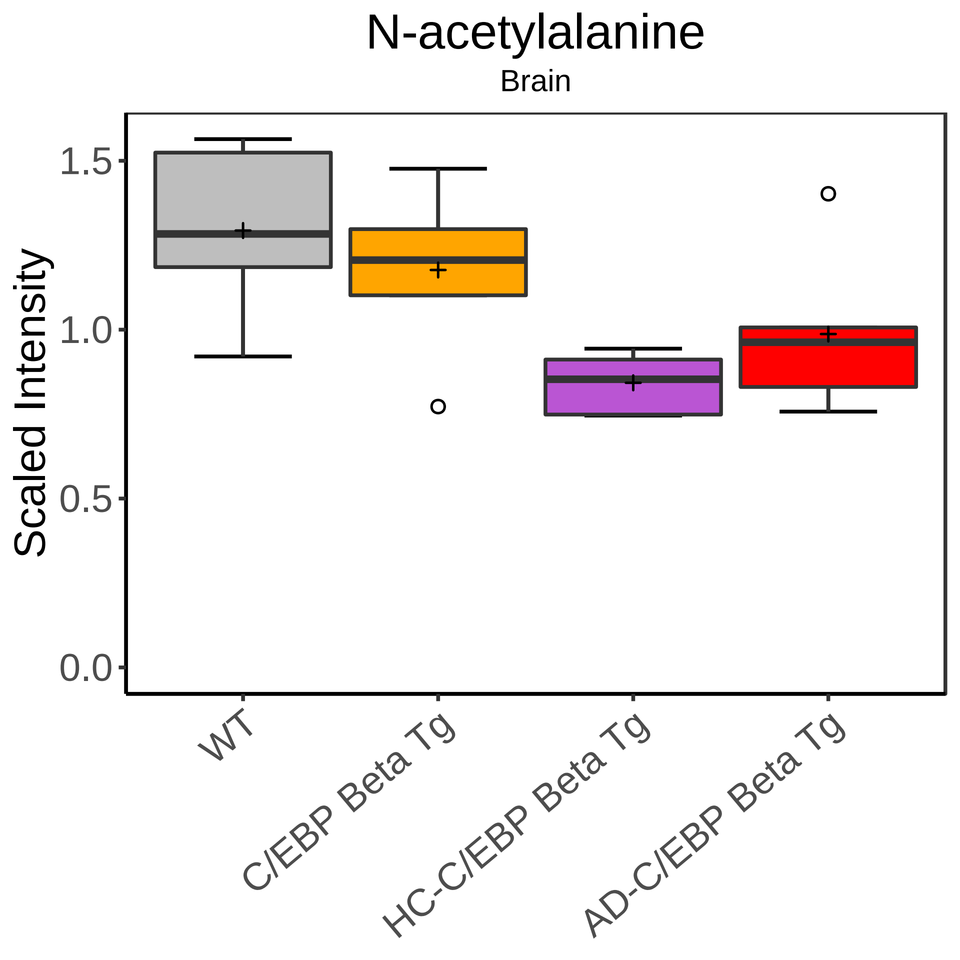

N-acetylalanine
Brain
Scaled Intensity
1.5
1.0
0.5
0.0
WT
C/EBP Beta Tg
HC-C/EBP Beta Tg
AD-C/EBP Beta Tg

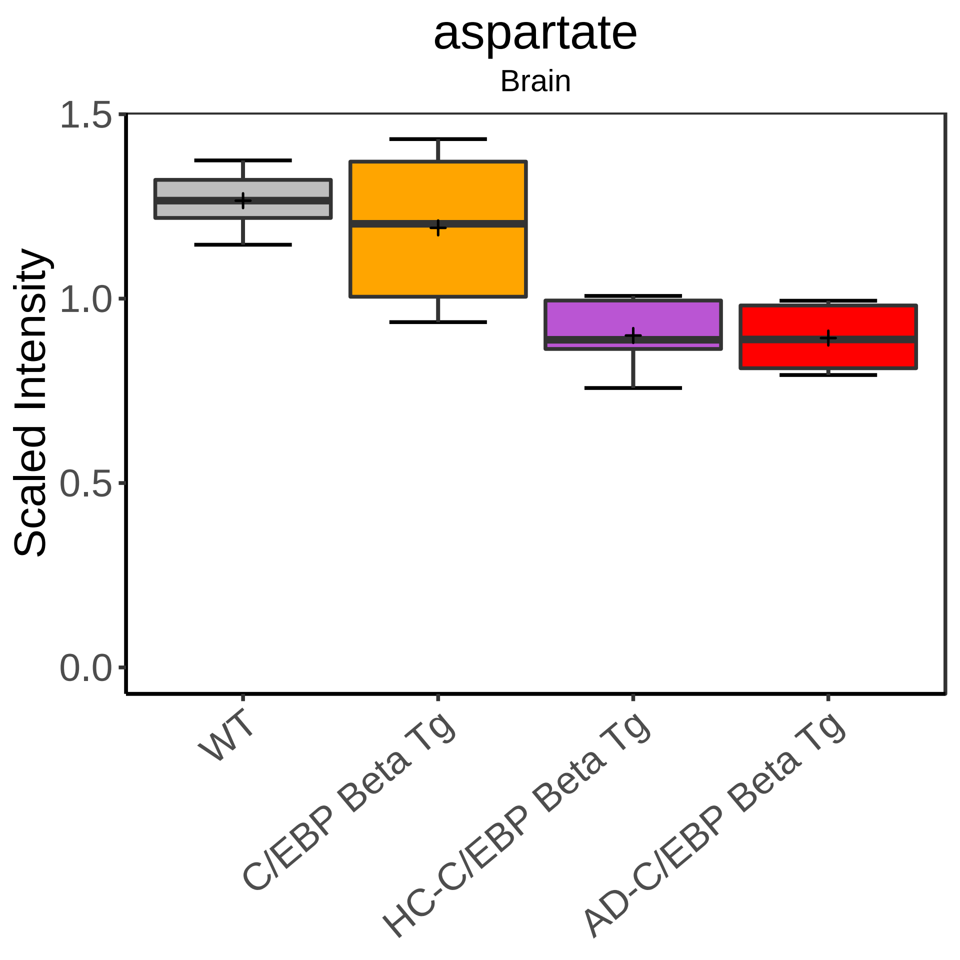
aspartate
Brain
Scaled Intensity
1.5
1.0
0.5
0.0
WT
C/EBP Beta Tg
HC-C/EBP Beta Tg
AD-C/EBP Beta Tg

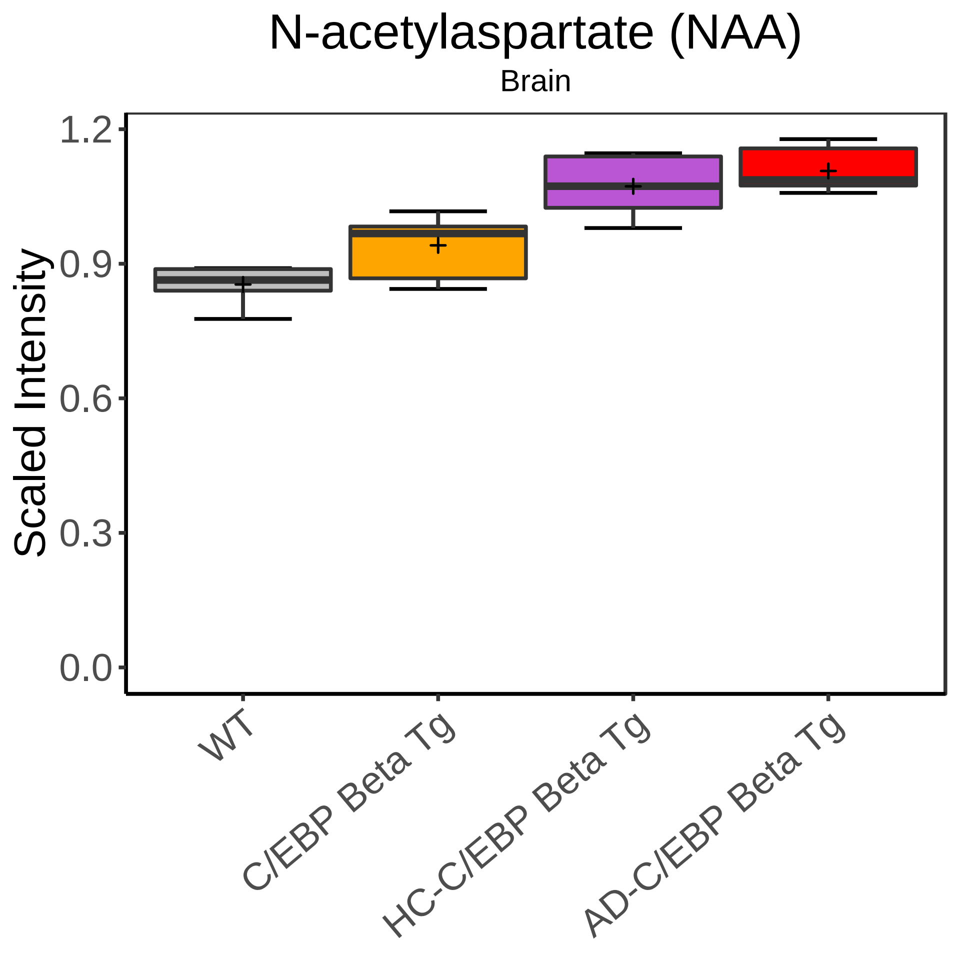
N-acetylaspartate (NAA)
Brain
Scaled Intensity
1.2
0.9
0.6
0.3
0.0
WT
C/EBP Beta Tg
HC-C/EBP Beta Tg
AD-C/EBP Beta Tg

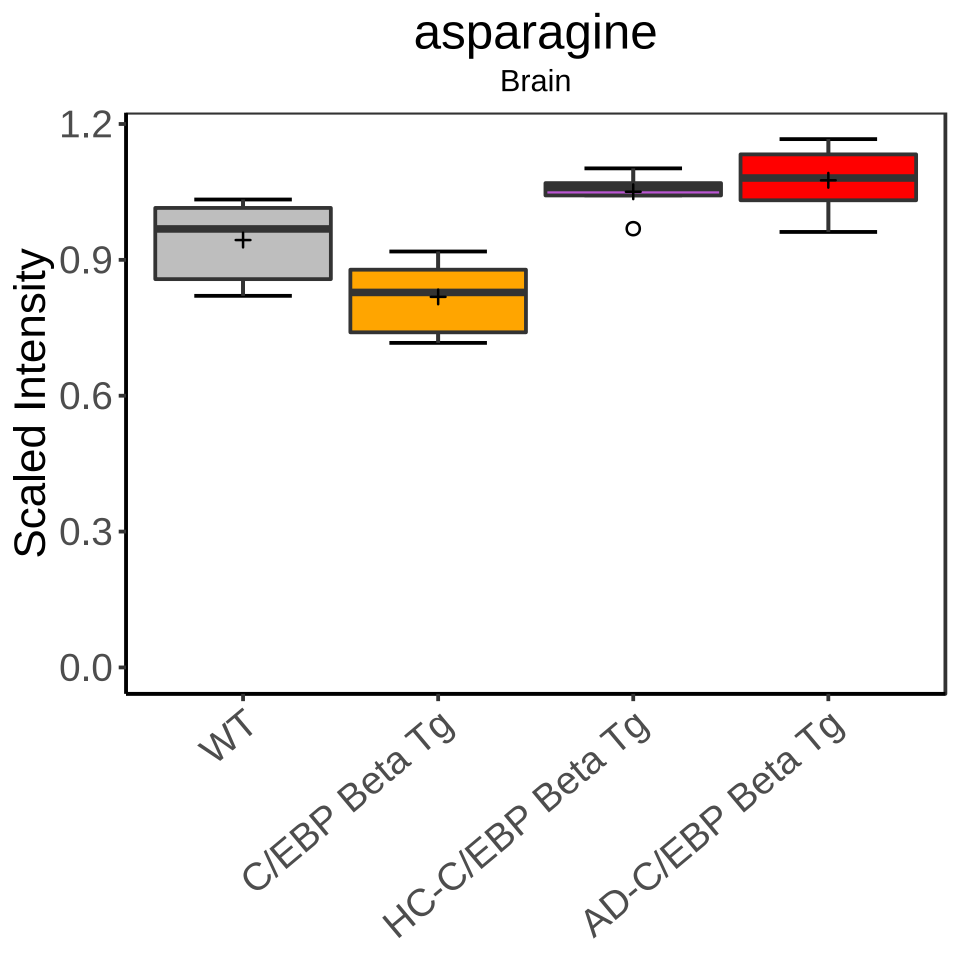

asparagine
Brain
Scaled Intensity
1.2
0.9
0.6
0.3
0.0
WT
C/EBP Beta Tg
HC-C/EBP Beta Tg
AD-C/EBP Beta Tg

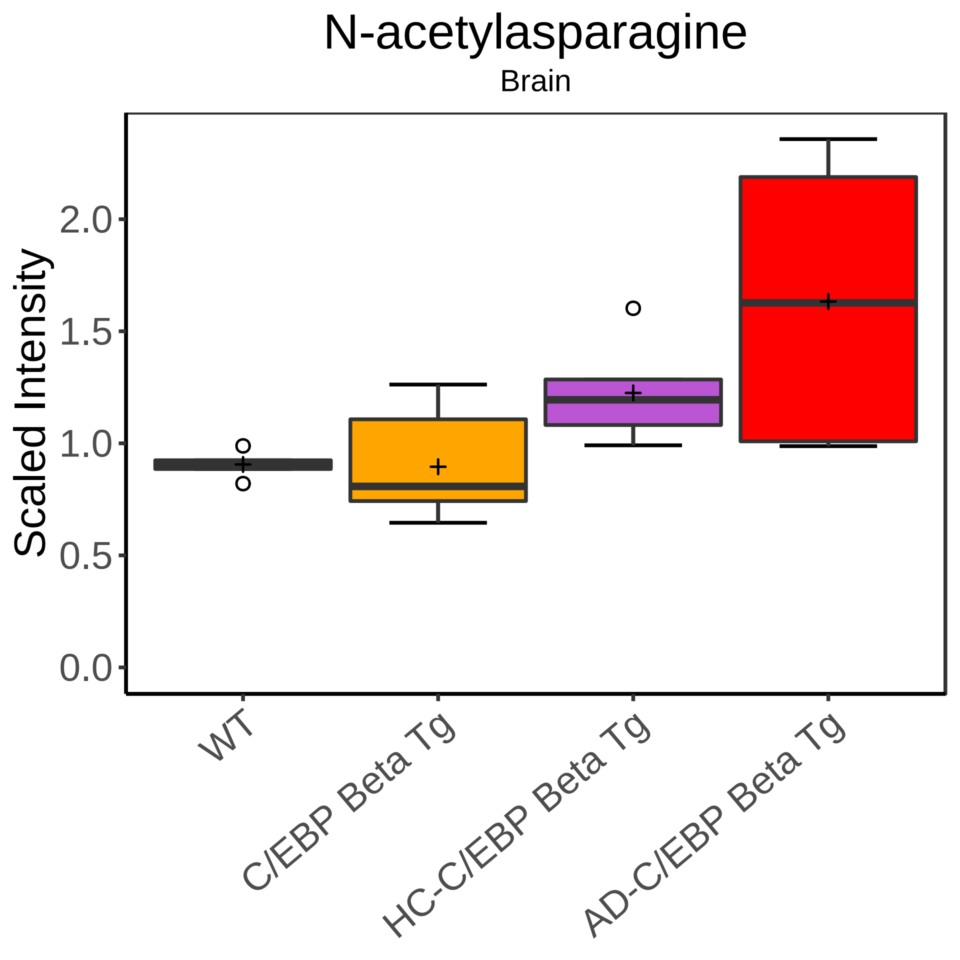
N-acetylasparagine
Brain
Scaled Intensity
2.0
1.5
1.0
0.5
0.0
WT
C/EBP Beta Tg
HC-C/EBP Beta Tg
AD-C/EBP Beta Tg

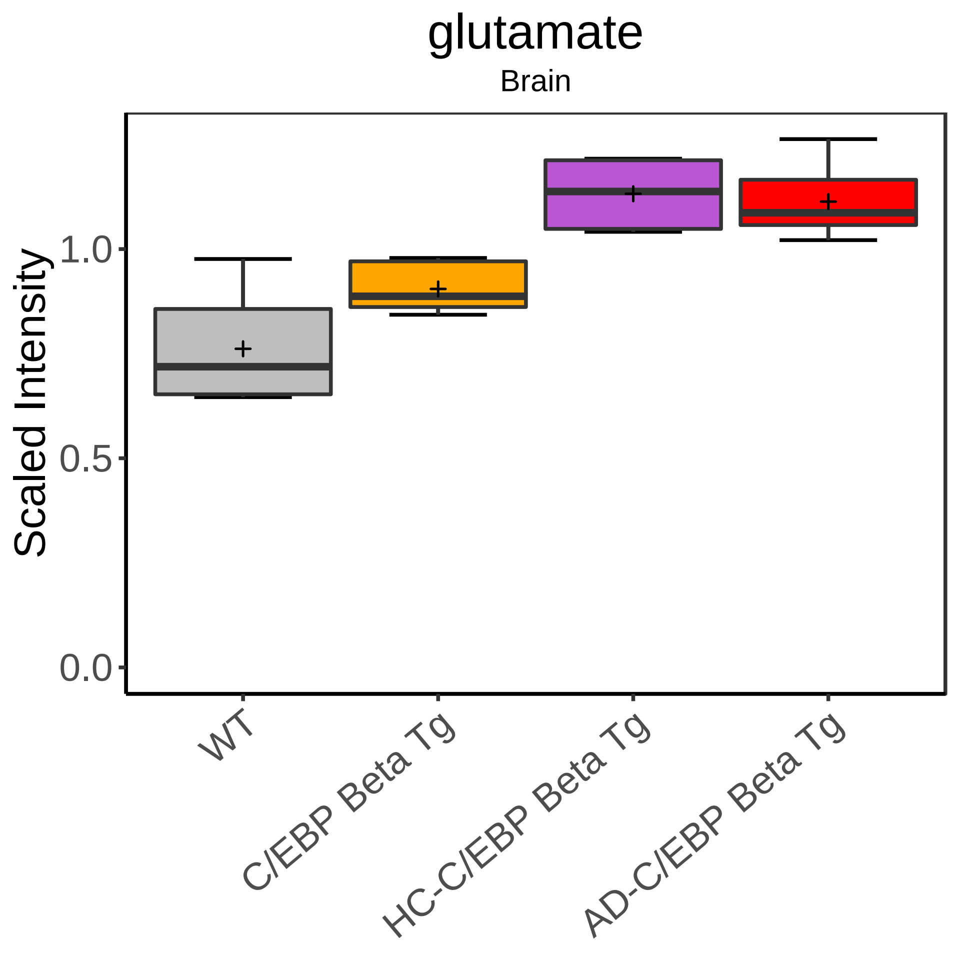
glutamate
Brain
Scaled Intensity
1.0
0.5
0.0
WT
C/EBP Beta Tg
HC-C/EBP Beta Tg
AD-C/EBP Beta Tg

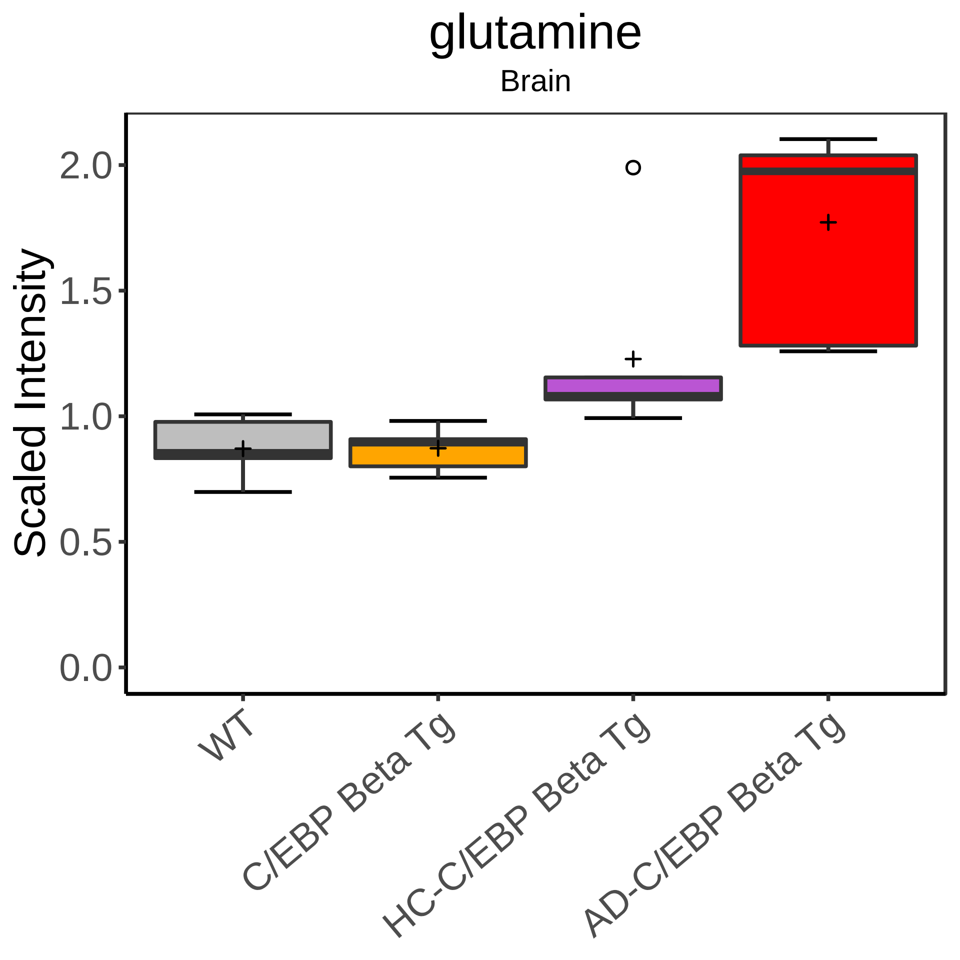

glutamine
Brain
Scaled Intensity
2.0
1.5
1.0
0.5
0.0
WT
C/EBP Beta Tg
HC-C/EBP Beta Tg
AD-C/EBP Beta Tg

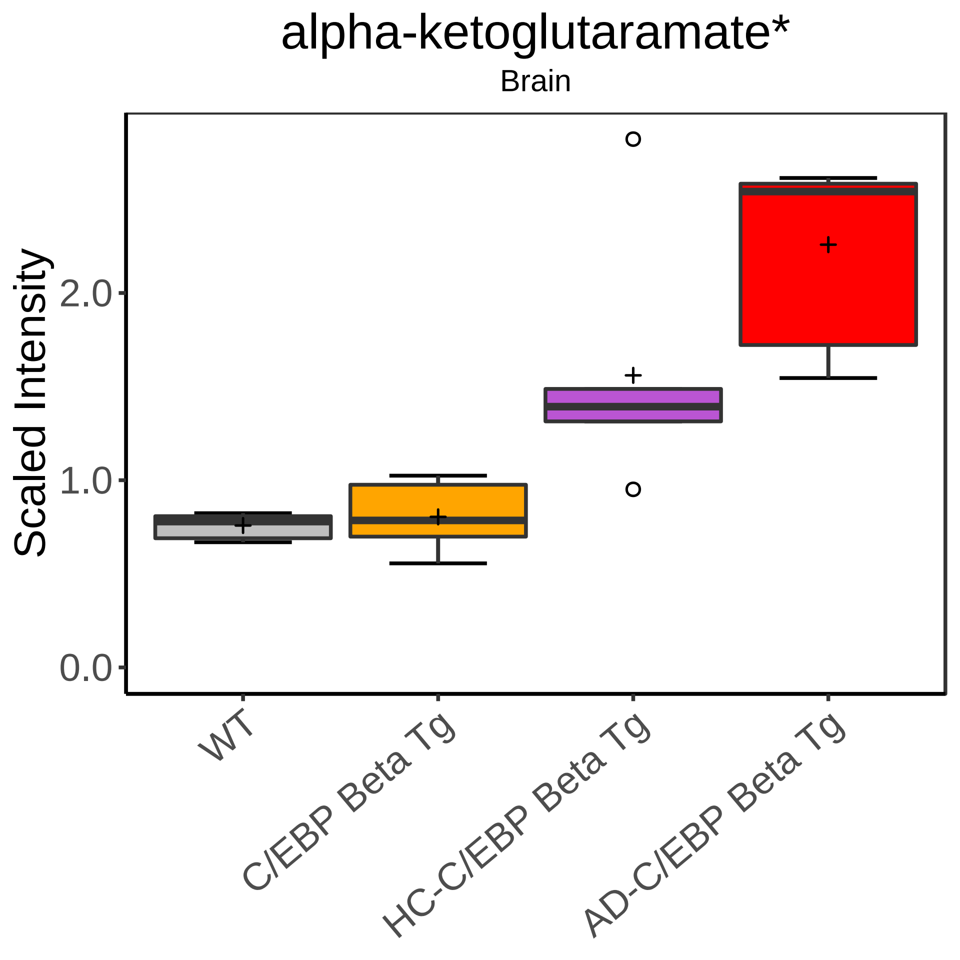

alpha-ketoglutaramate*
Brain
Scaled Intensity
2.0
1.0
0.0
WT
C/EBP Beta Tg
HC-C/EBP Beta Tg
AD-C/EBP Beta Tg

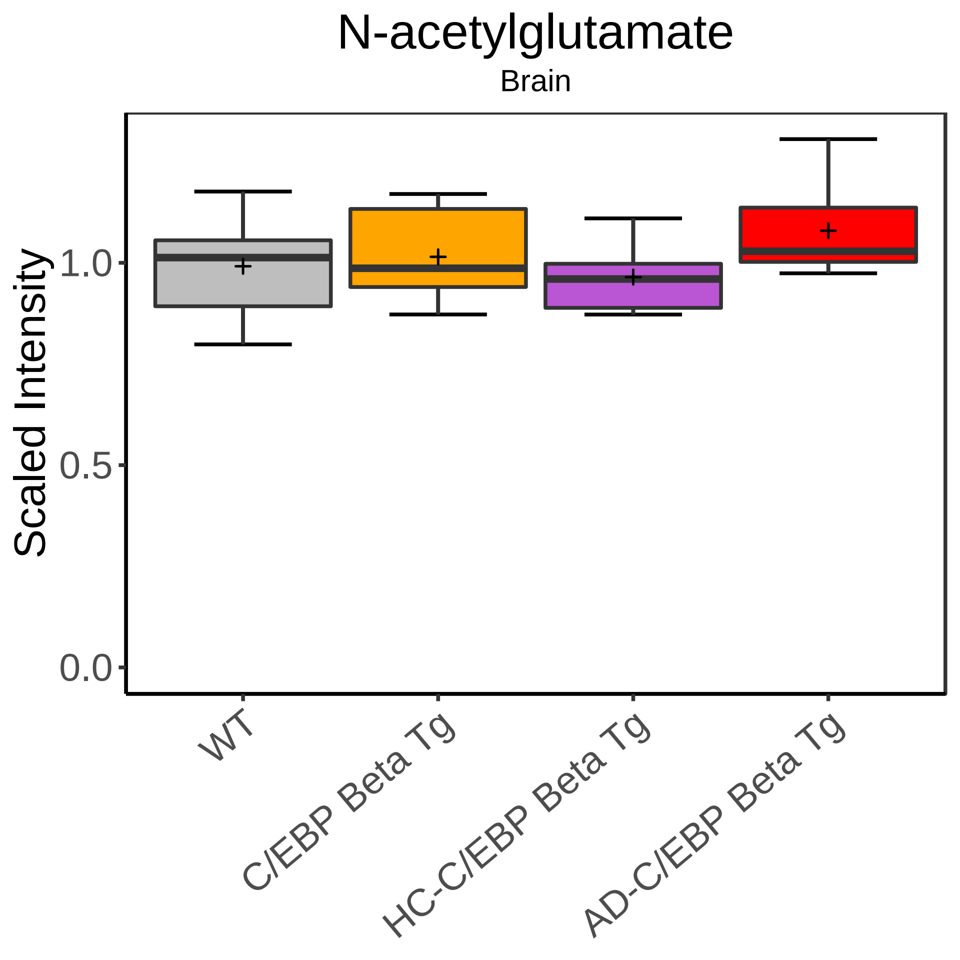
N-acetylglutamate
Brain
Scaled Intensity
1.0
0.5
0.0
WT
C/EBP Beta Tg
HC-C/EBP Beta Tg
AD-C/EBP Beta Tg

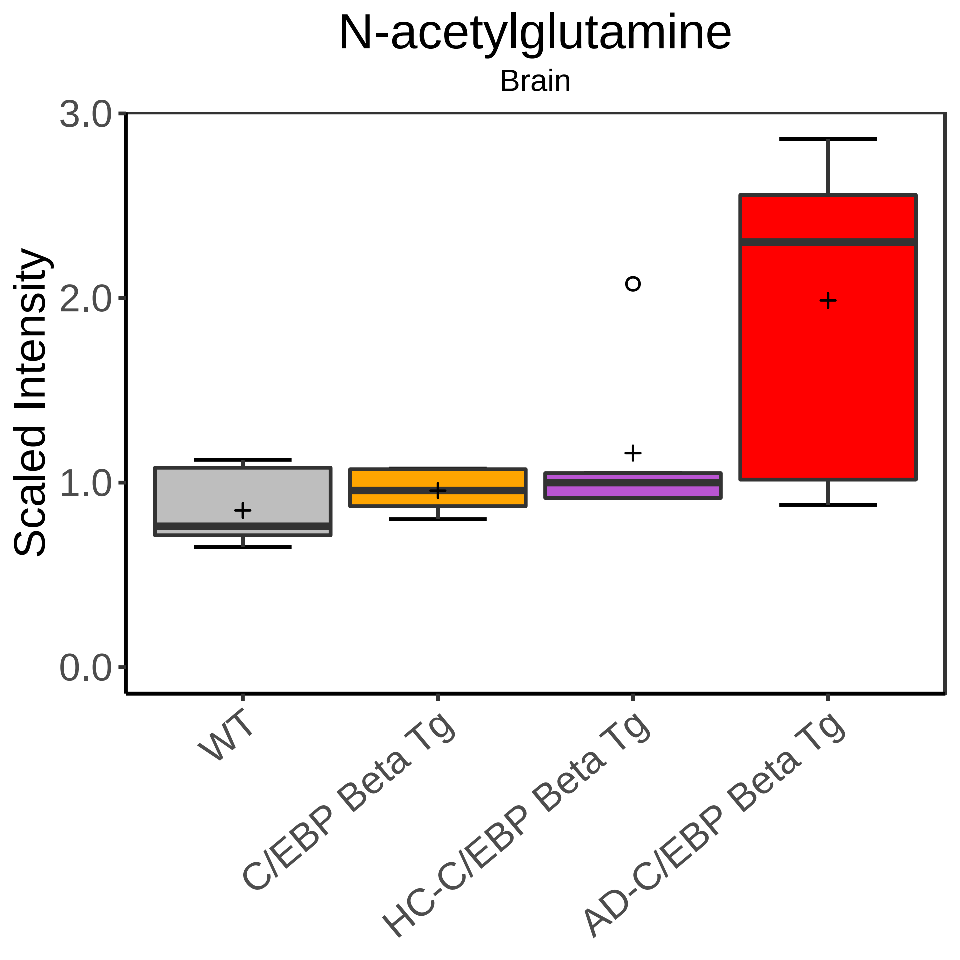
N-acetylglutamine
Brain
Scaled Intensity
3.0
2.0
1.0
0.0
WT
C/EBP Beta Tg
HC-C/EBP Beta Tg
AD-C/EBP Beta Tg

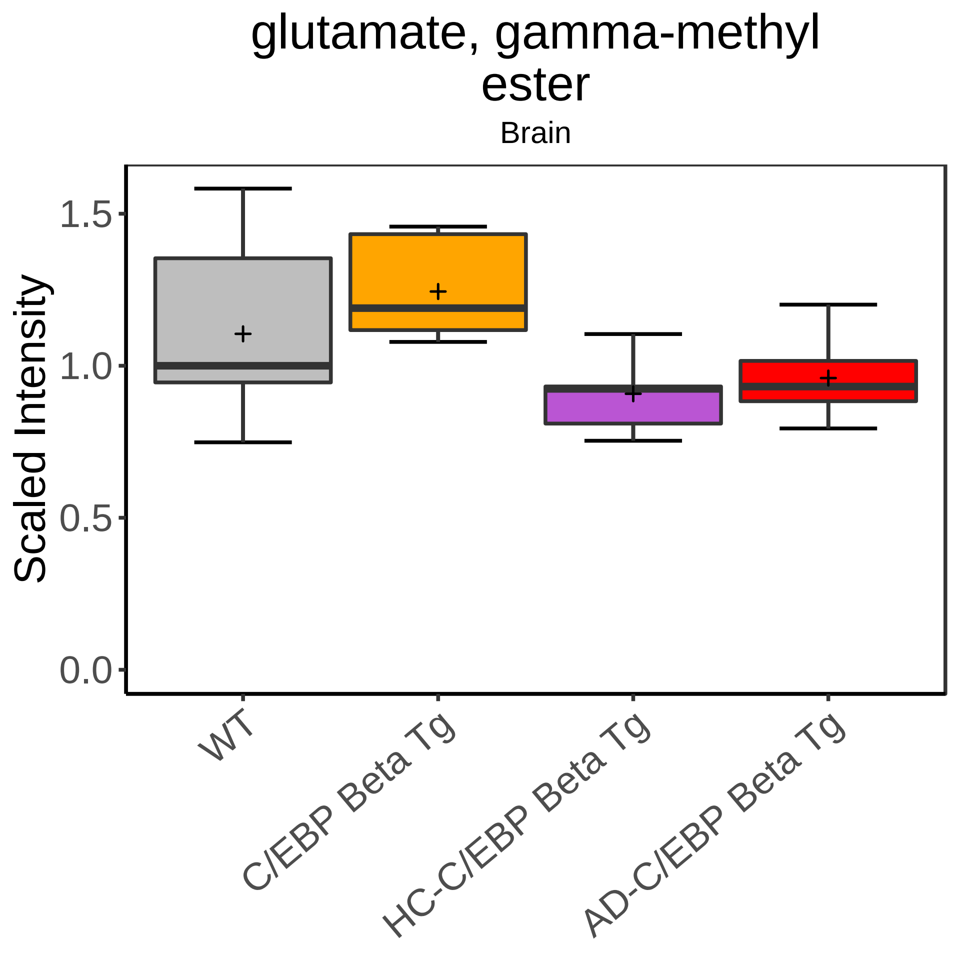

glutamate, gamma-methyl ester
Brain
Scaled Intensity
1.5
1.0
0.5
0.0
WT
C/EBP Beta Tg
HC-C/EBP Beta Tg
AD-C/EBP Beta Tg

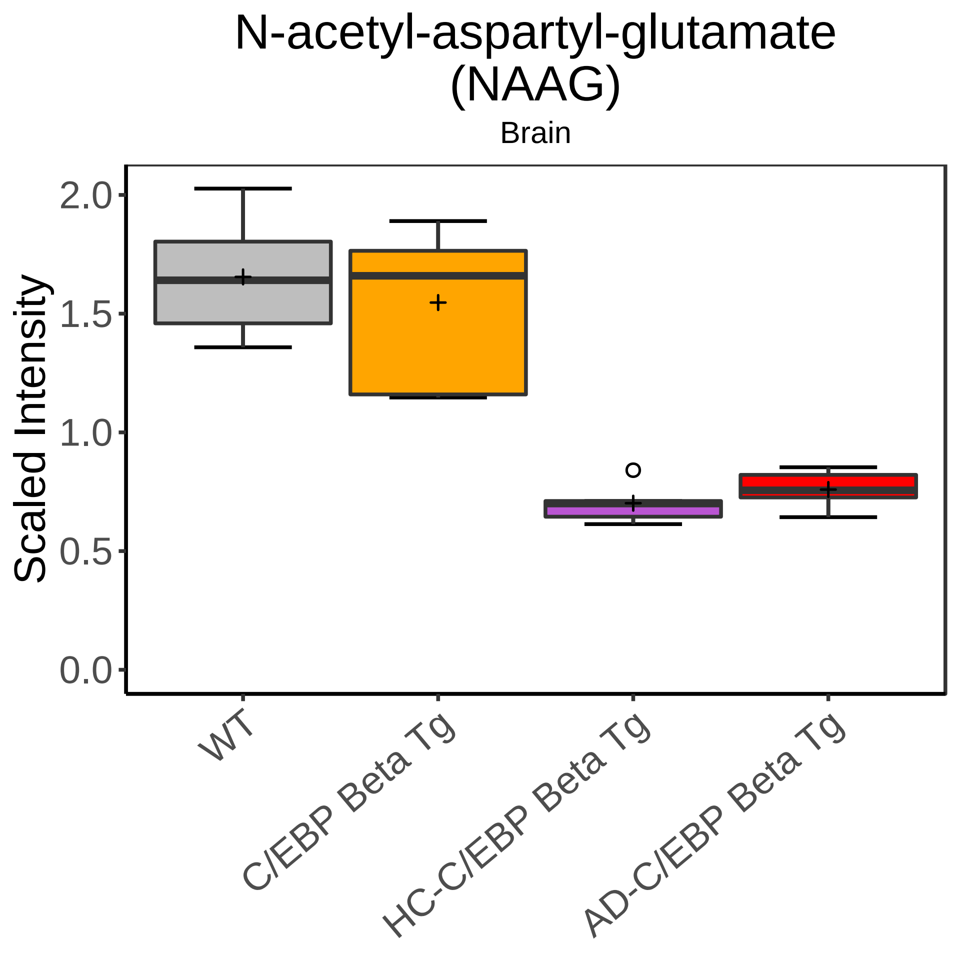

N-acetyl-aspartyl-glutamate
(NAAG)
Brain
Scaled Intensity
2.0
1.5
1.0
0.5
0.0
WT
C/EBP Beta Tg
HC-C/EBP Beta Tg
AD-C/EBP Beta Tg

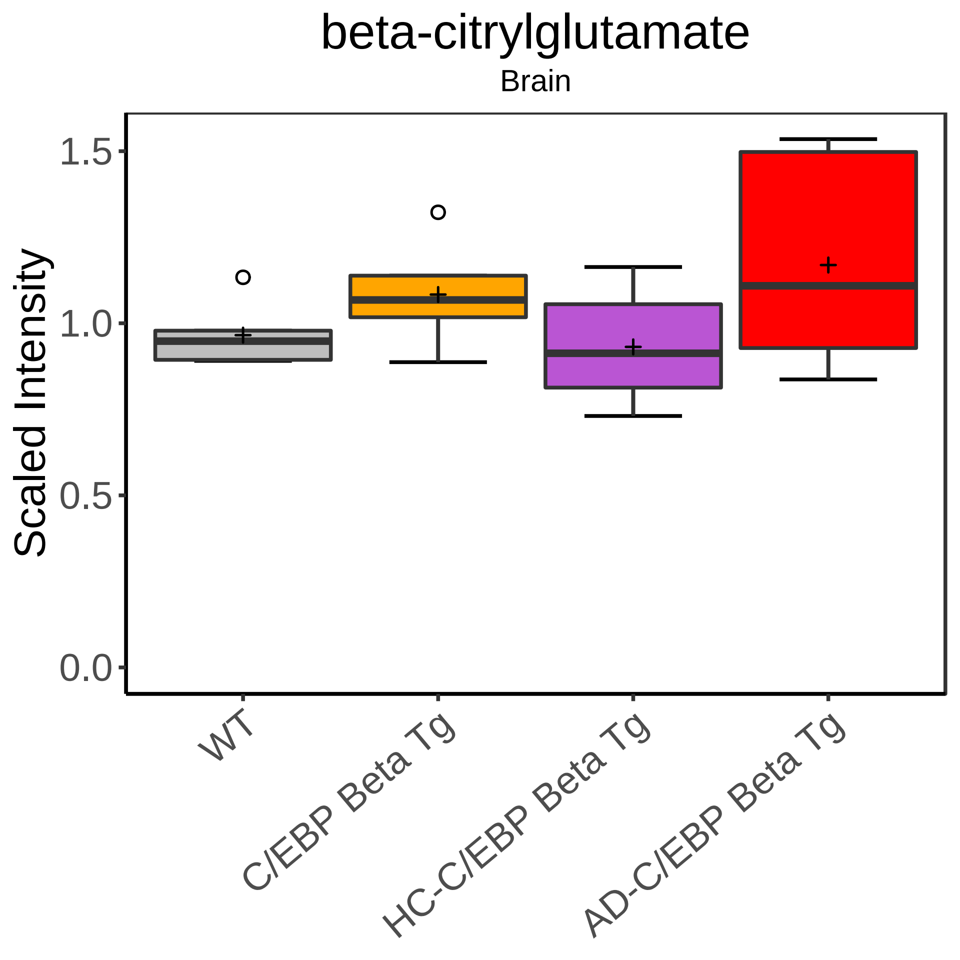
beta-citrylglutamate
Brain
Scaled Intensity
1.5
1.0
0.5
0.0
WT
C/EBP Beta Tg
HC-C/EBP Beta Tg
AD-C/EBP Beta Tg

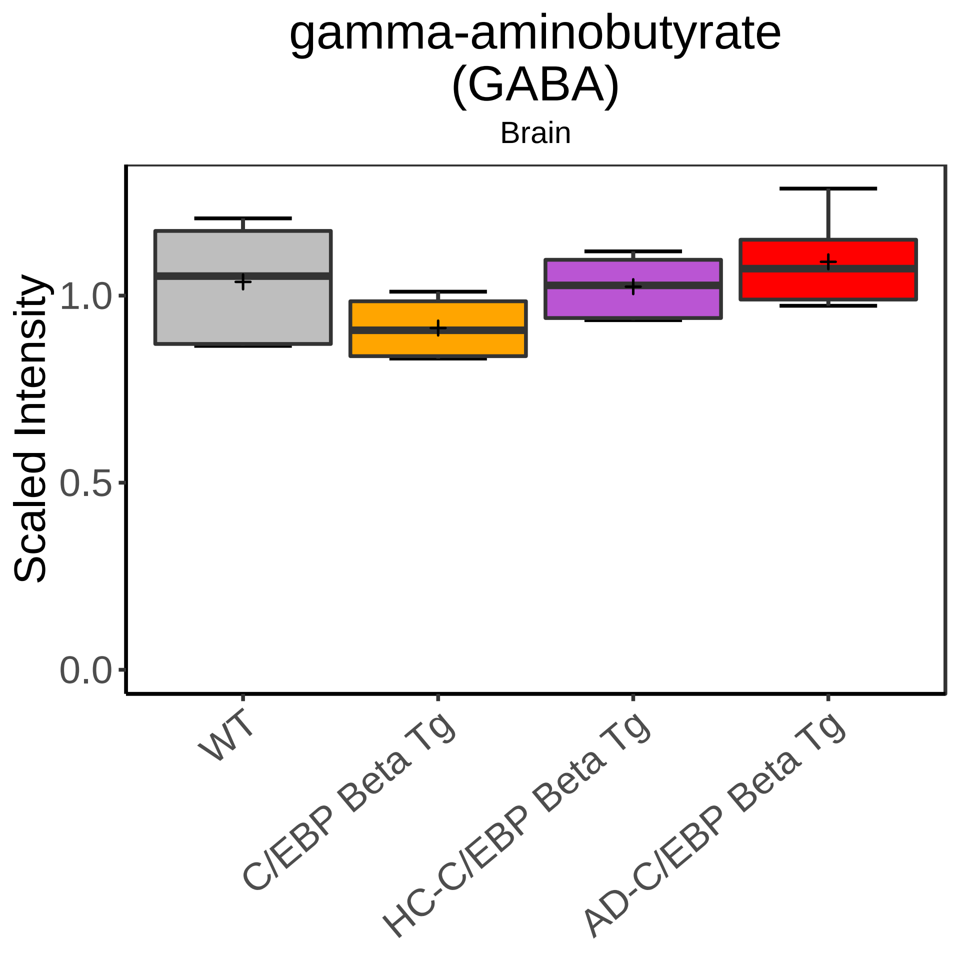
gamma-aminobutyrate
(GABA)
Brain
Scaled Intensity
1.0
0.5
0.0
WT
C/EBP Beta Tg
HC-C/EBP Beta Tg
AD-C/EBP Beta Tg

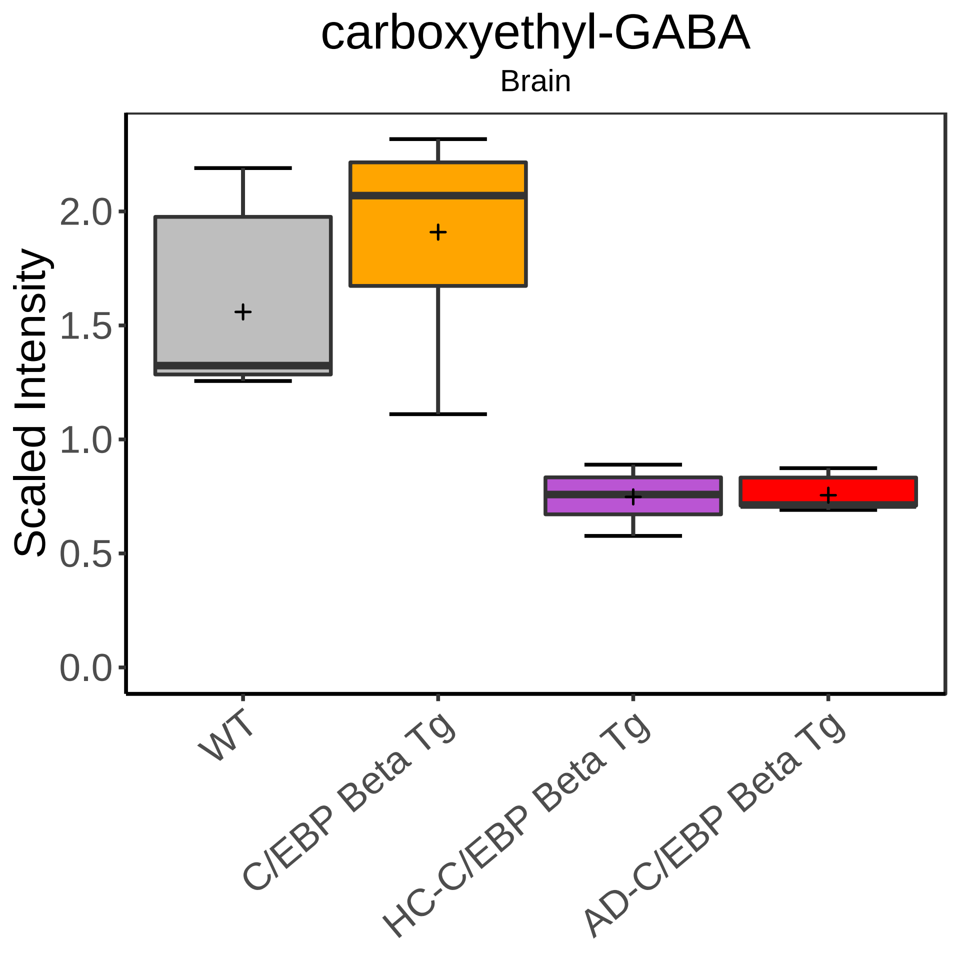
carboxyethyl-GABA
Brain
Scaled Intensity
2.0
1.5
1.0
0.5
0.0
WT
C/EBP Beta Tg
HC-C/EBP Beta Tg
AD-C/EBP Beta Tg

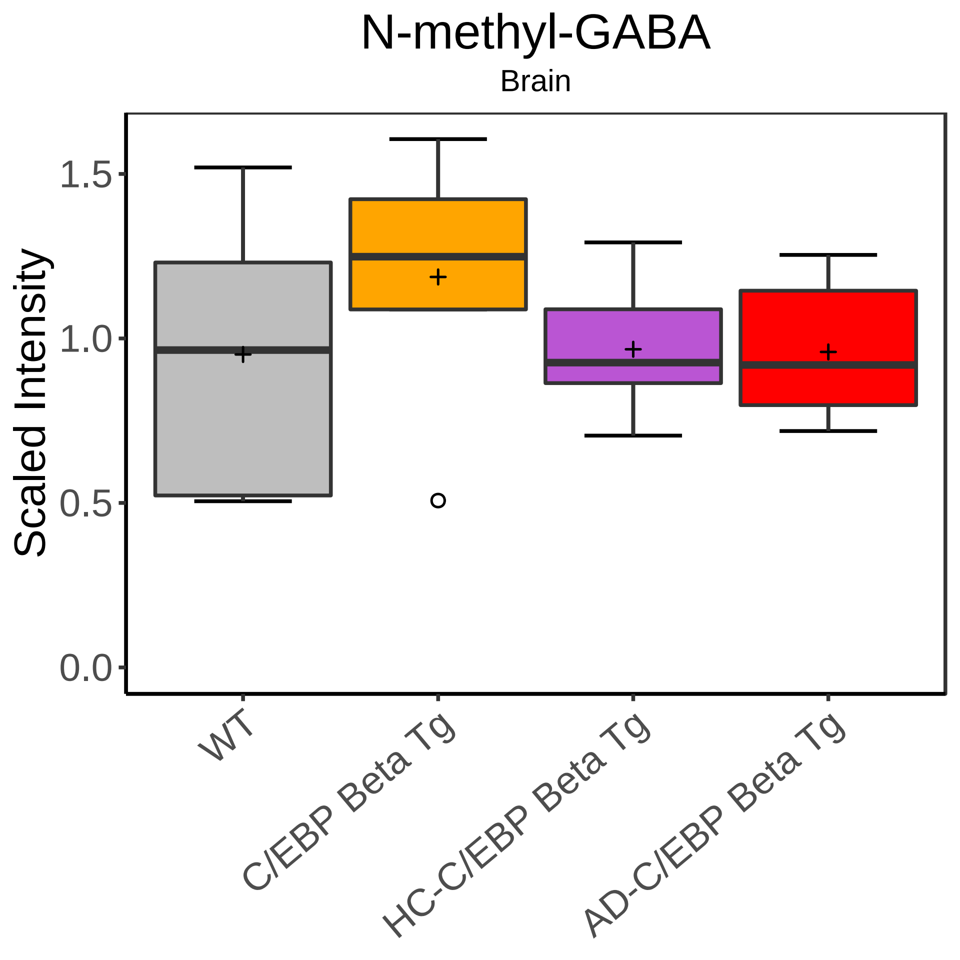

N-methyl-GABA
Brain
Scaled Intensity
1.5
1.0
0.5
0.0
WT
C/EBP Beta Tg
HC-C/EBP Beta Tg
AD-C/EBP Beta Tg

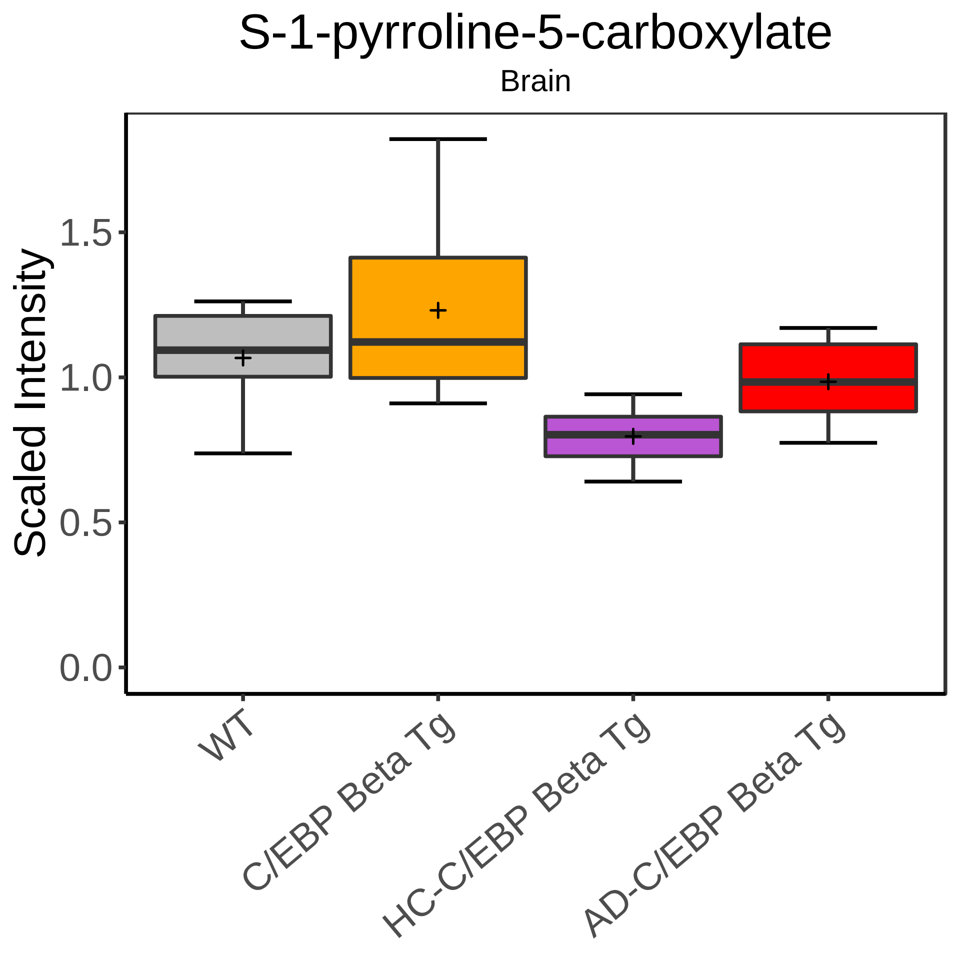

S-1-pyrroline-5-carboxylate
Brain
Scaled Intensity
0.0
0.5
1.0
1.5
WT
C/EBP Beta Tg
HC-C/EBP Beta Tg
AD-C/EBP Beta Tg

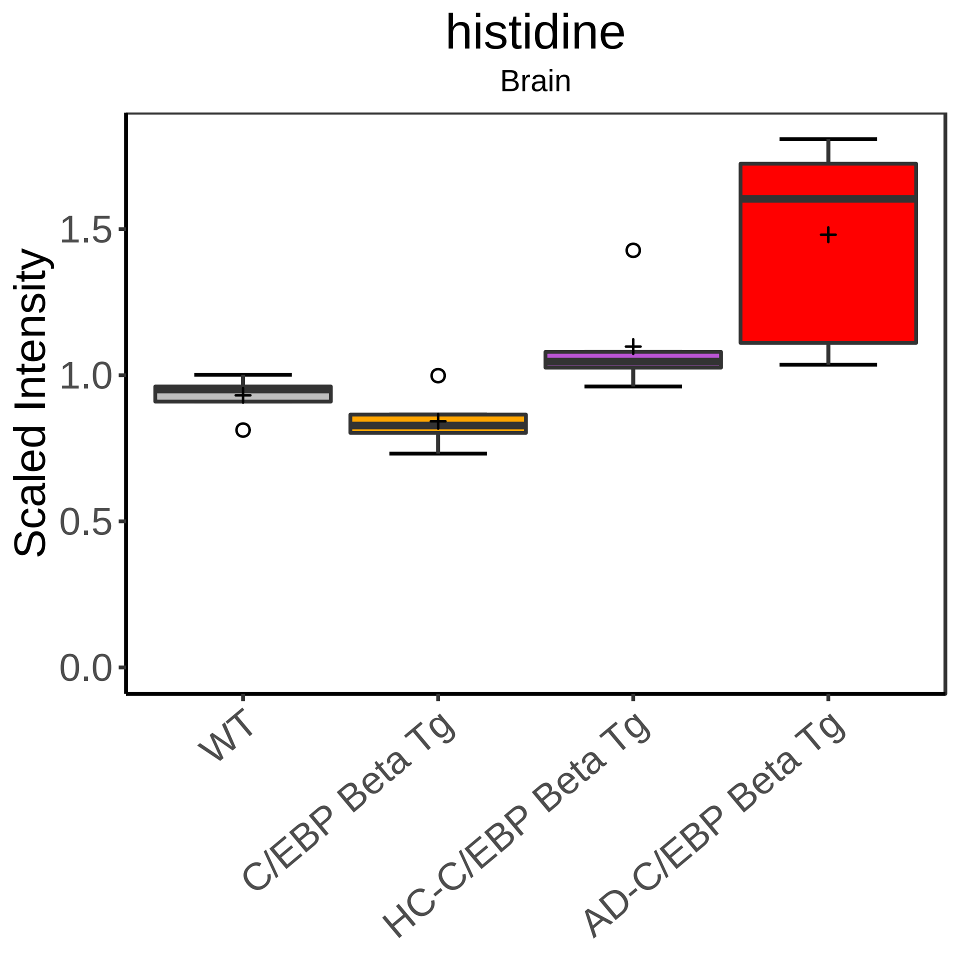

histidine
Brain
Scaled Intensity
0.0
0.5
1.0
1.5
WT
C/EBP Beta Tg
HC-C/EBP Beta Tg
AD-C/EBP Beta Tg

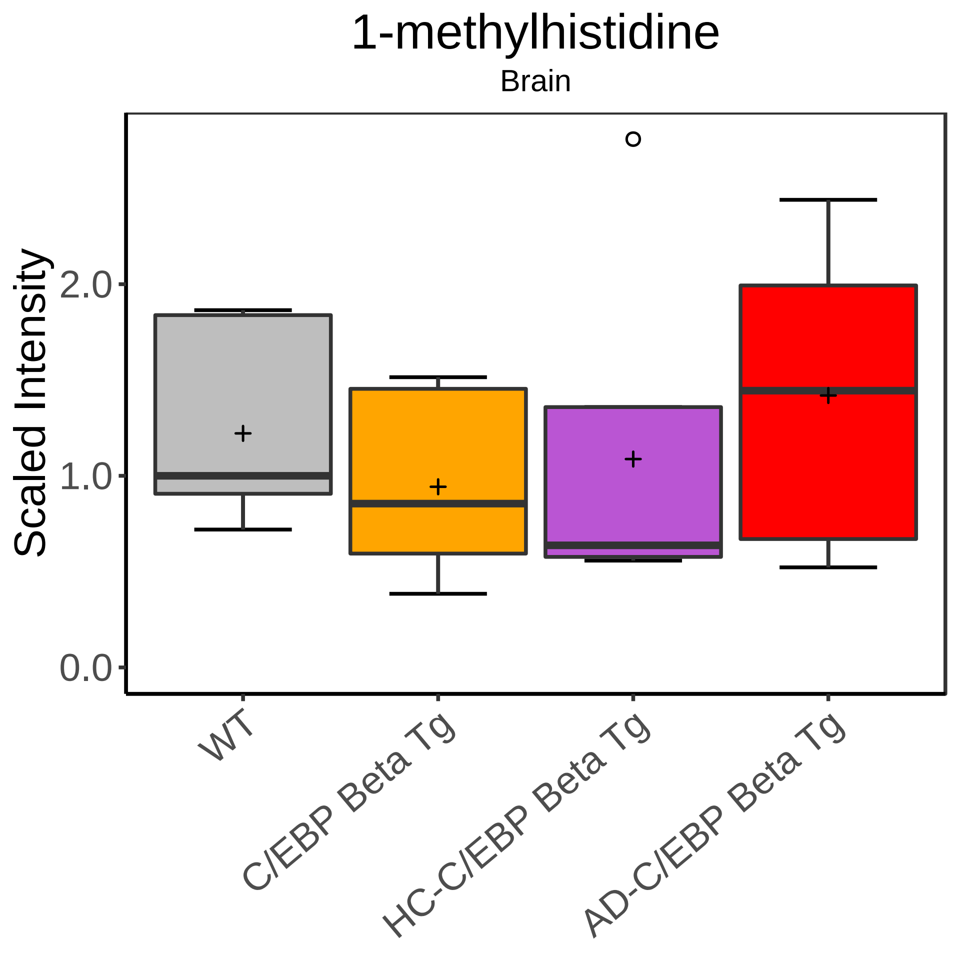
1-methylhistidine
Brain
Scaled Intensity
2.0
1.0
0.0
WT
C/EBP Beta Tg
HC-C/EBP Beta Tg
AD-C/EBP Beta Tg

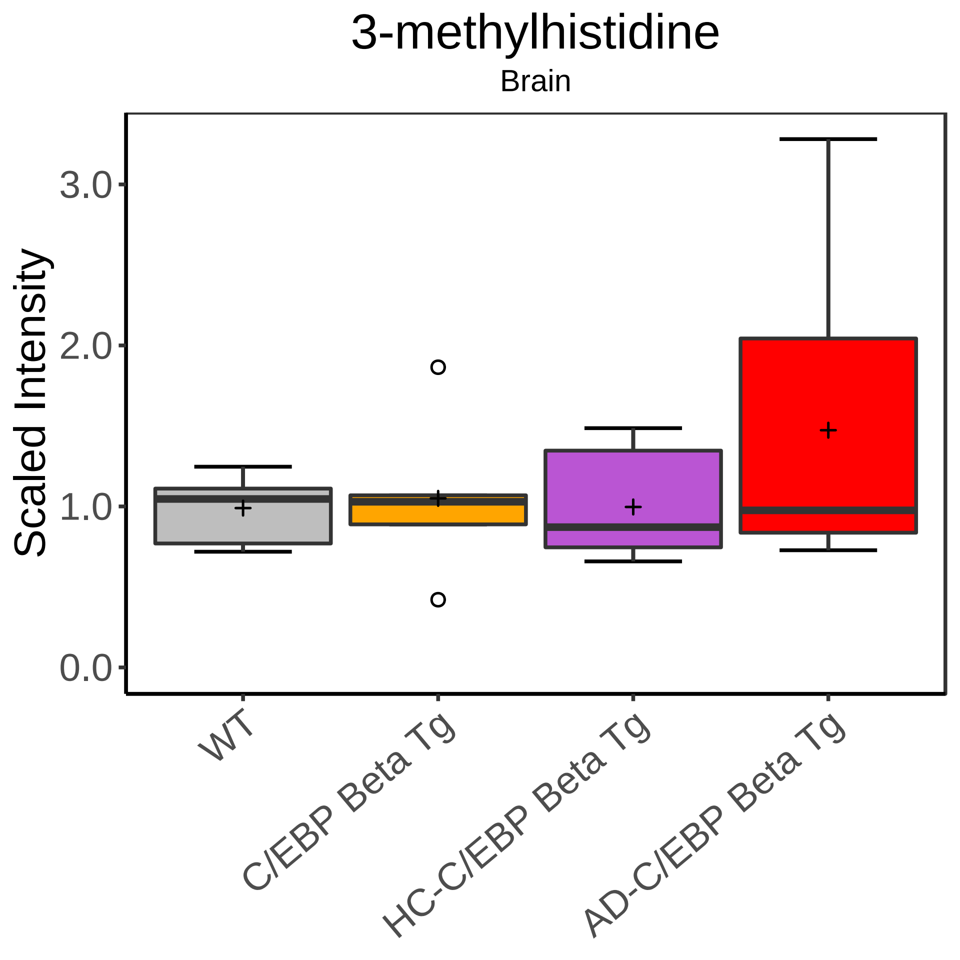

3-methylhistidine
Brain
Scaled Intensity
3.0
2.0
1.0
0.0
WT
C/EBP Beta Tg
HC-C/EBP Beta Tg
AD-C/EBP Beta Tg

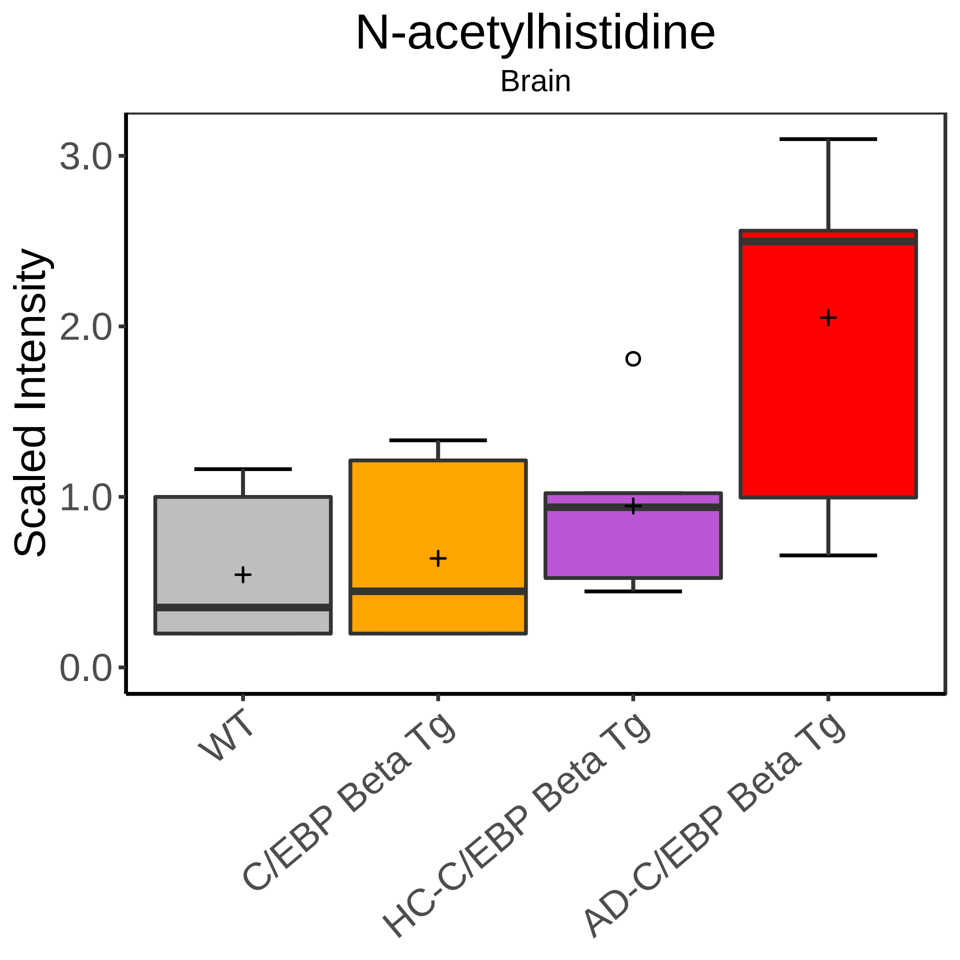

N-acetylhistidine
Brain
Scaled Intensity
3.0
2.0
1.0
0.0
WT
C/EBP Beta Tg
HC-C/EBP Beta Tg
AD-C/EBP Beta Tg

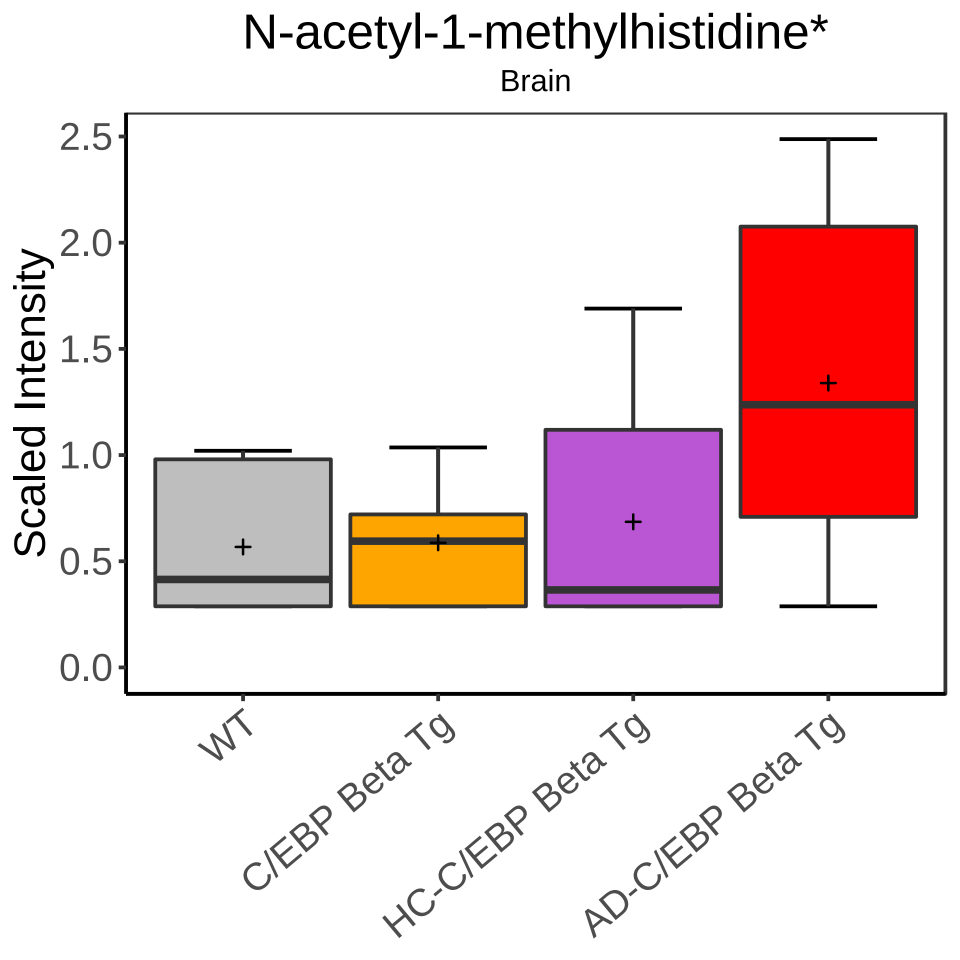

N-acetyl-1-methylhistidine*
Brain
Scaled Intensity
0.0
0.5
1.0
1.5
2.0
2.5
WT
C/EBP Beta Tg
HC-C/EBP Beta Tg
AD-C/EBP Beta Tg

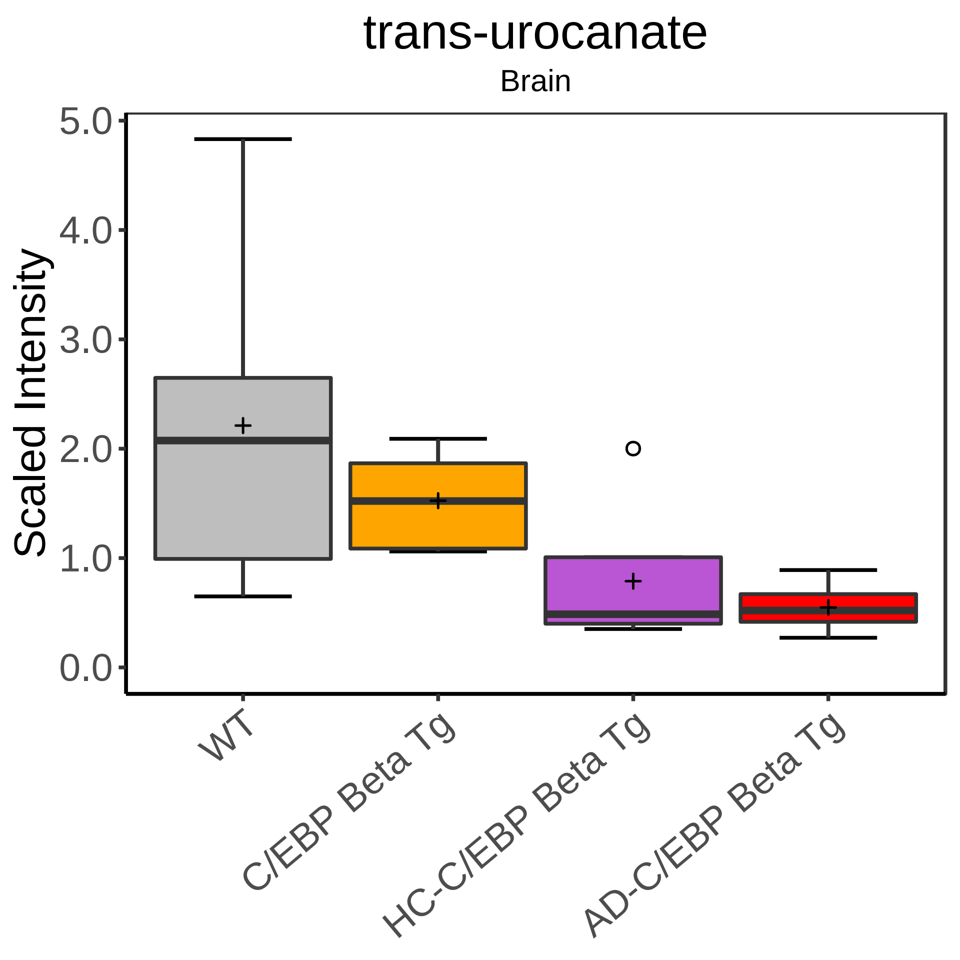
trans-urocanate
Brain
Scaled Intensity
5.0
4.0
3.0
2.0
1.0
0.0
WT
C/EBP Beta Tg
HC-C/EBP Beta Tg
AD-C/EBP Beta Tg

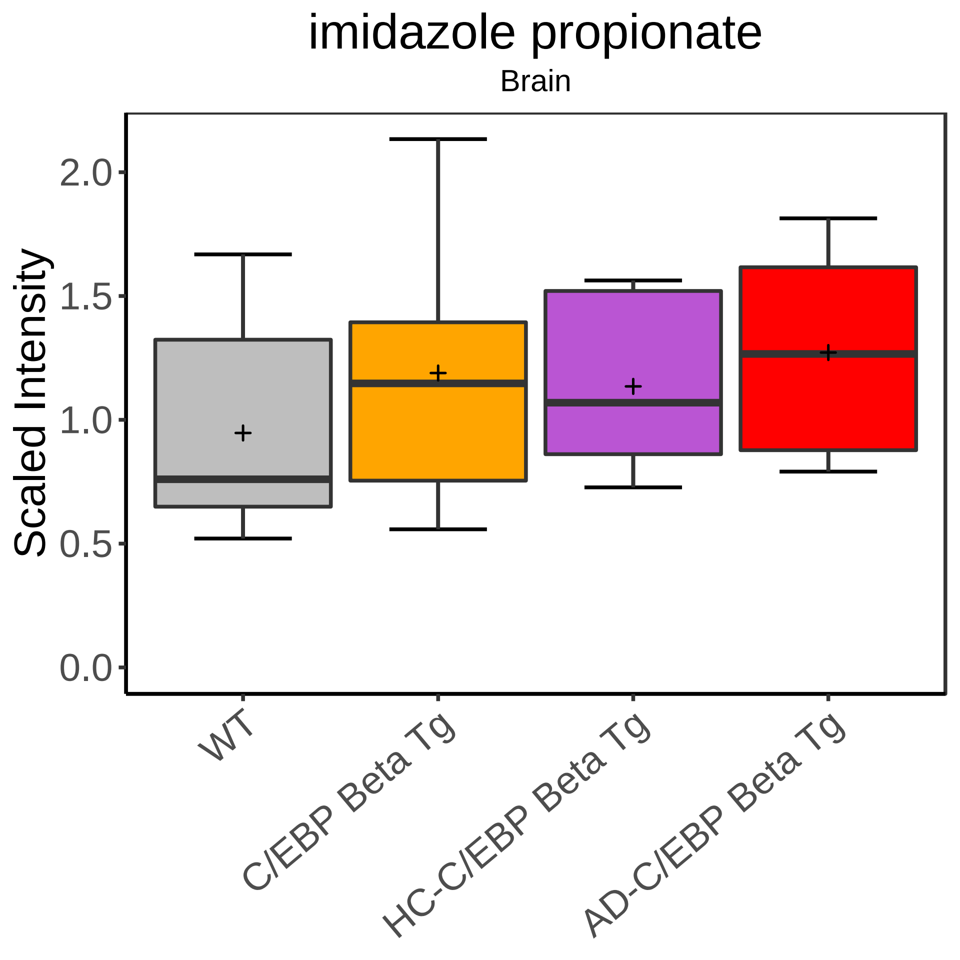

imidazole propionate
Brain
Scaled Intensity
0.0
0.5
1.0
1.5
2.0
WT
C/EBP Beta Tg
HC-C/EBP Beta Tg
AD-C/EBP Beta Tg

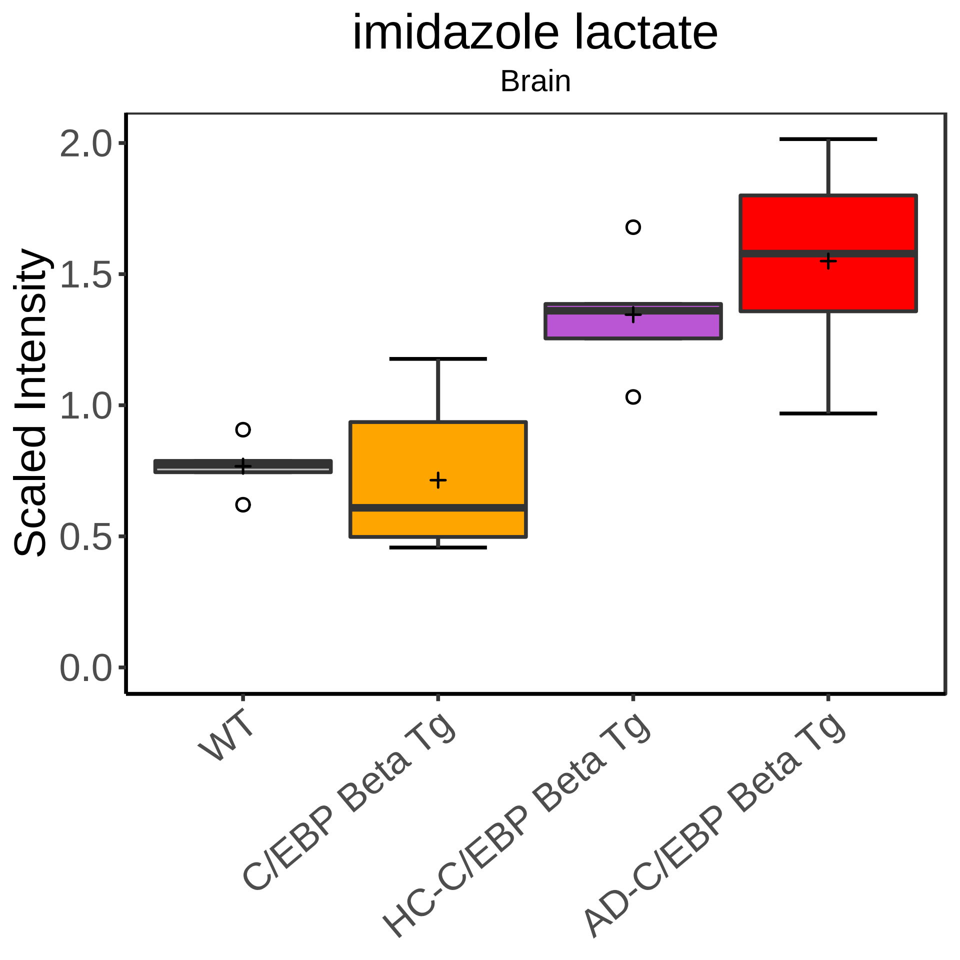

imidazole lactate
Brain
Scaled Intensity
2.0
1.5
1.0
0.5
0.0
WT
C/EBP Beta Tg
HC-C/EBP Beta Tg
AD-C/EBP Beta Tg

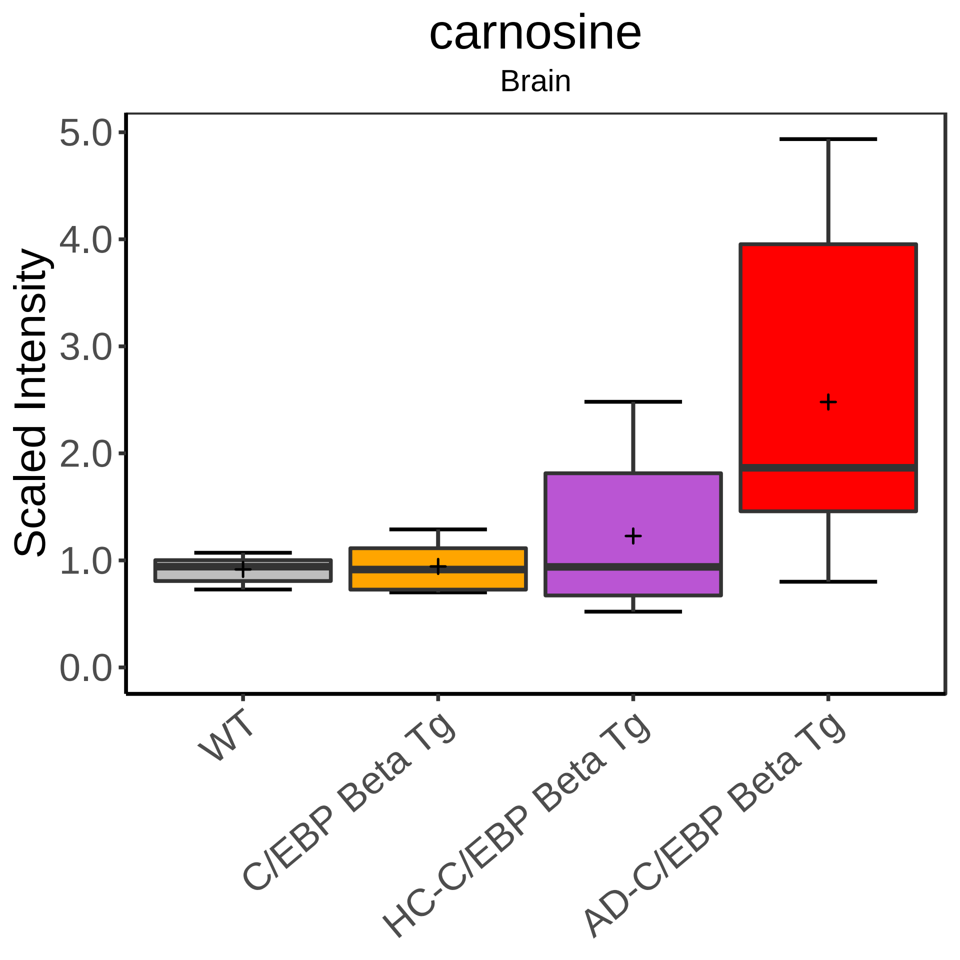
carnosine
Brain
Scaled Intensity
5.0
4.0
3.0
2.0
1.0
0.0
WT
C/EBP Beta Tg
HC-C/EBP Beta Tg
AD-C/EBP Beta Tg

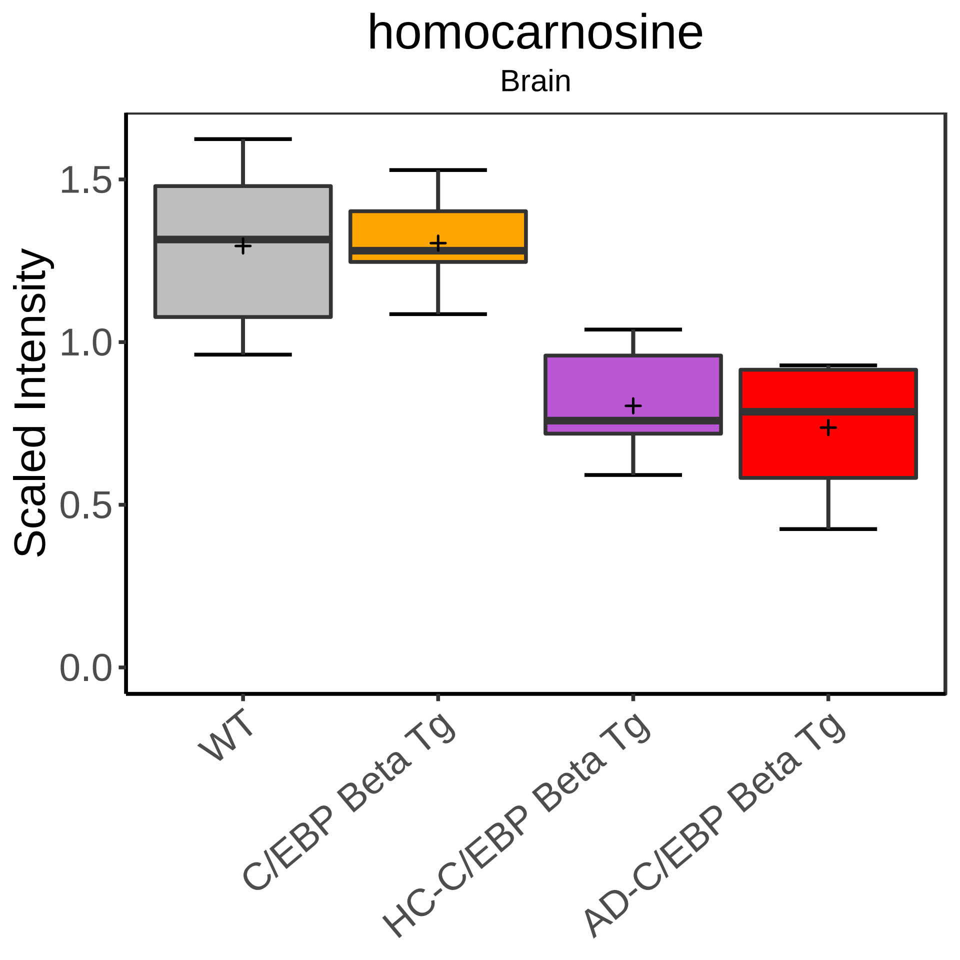

homocarnosine
Brain
Scaled Intensity
1.5
1.0
0.5
0.0
WT
C/EBP Beta Tg
HC-C/EBP Beta Tg
AD-C/EBP Beta Tg

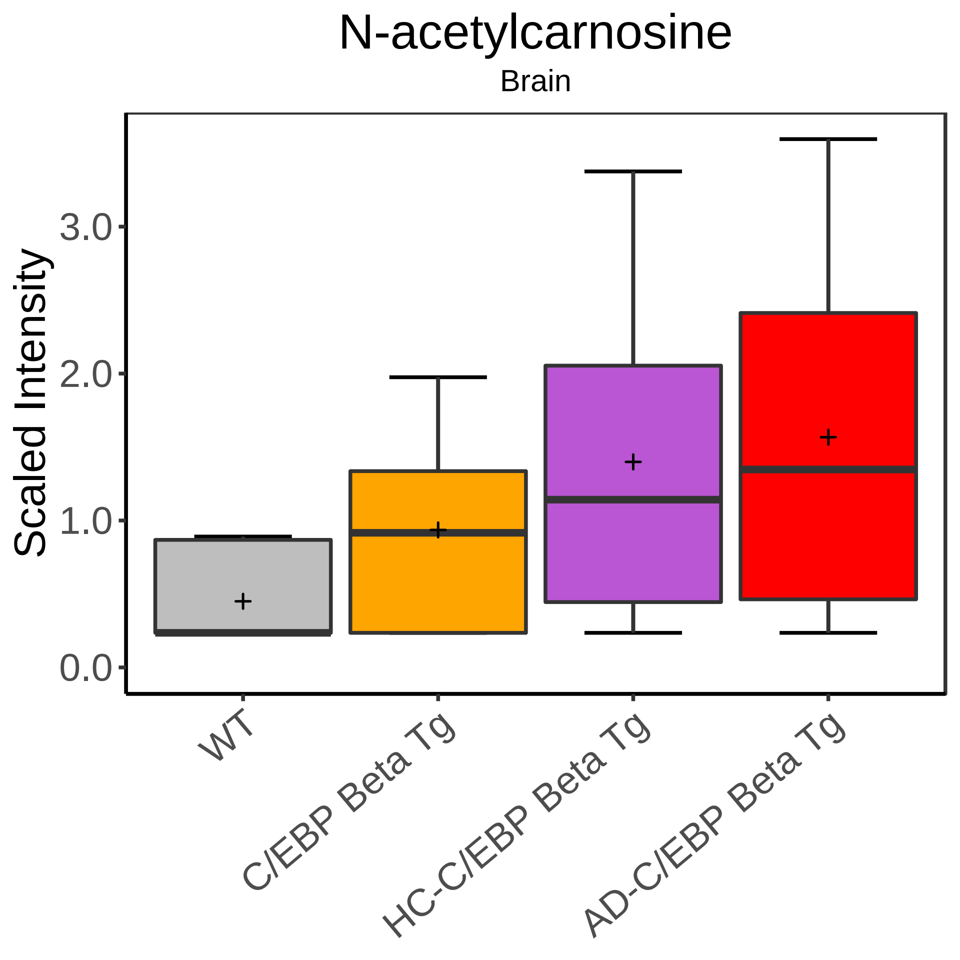

N-acetylcarnosine
Brain
Scaled Intensity
0.0
1.0
2.0
3.0
WT
C/EBP Beta Tg
HC-C/EBP Beta Tg
AD-C/EBP Beta Tg

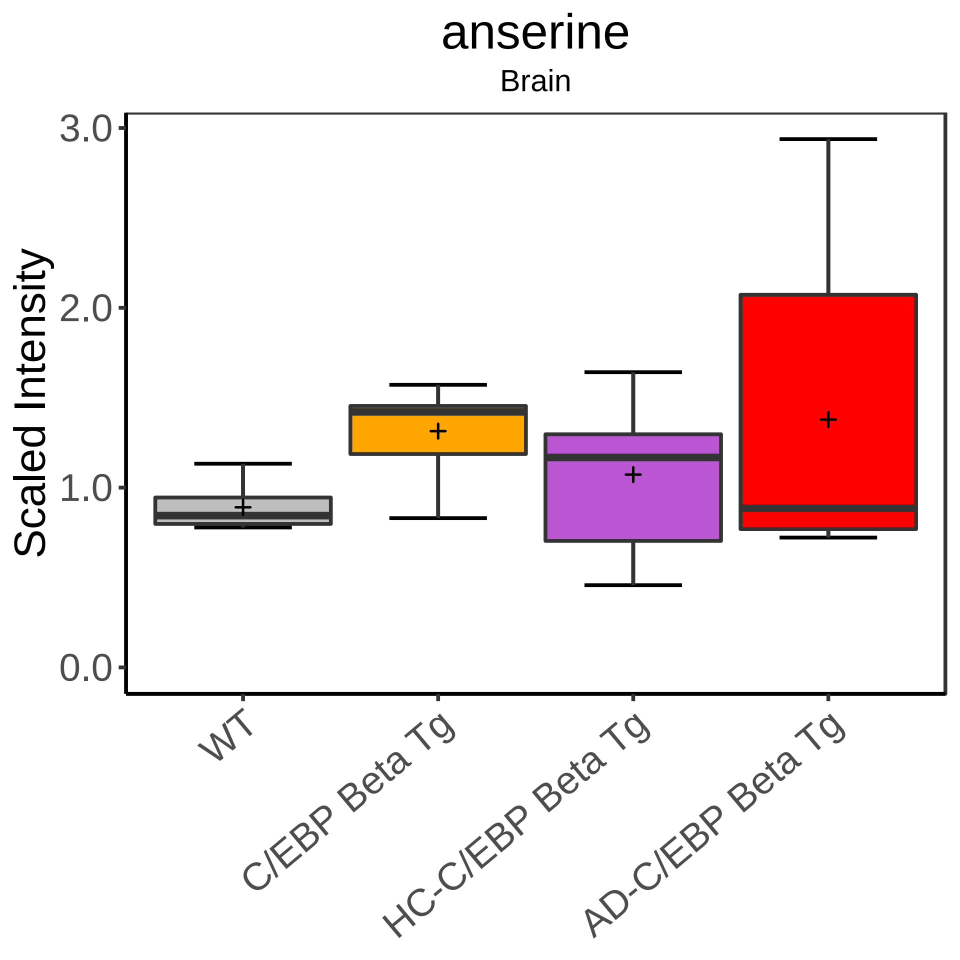
anserine
Brain
Scaled Intensity
3.0
2.0
1.0
0.0
WT
C/EBP Beta Tg
HC-C/EBP Beta Tg
AD-C/EBP Beta Tg

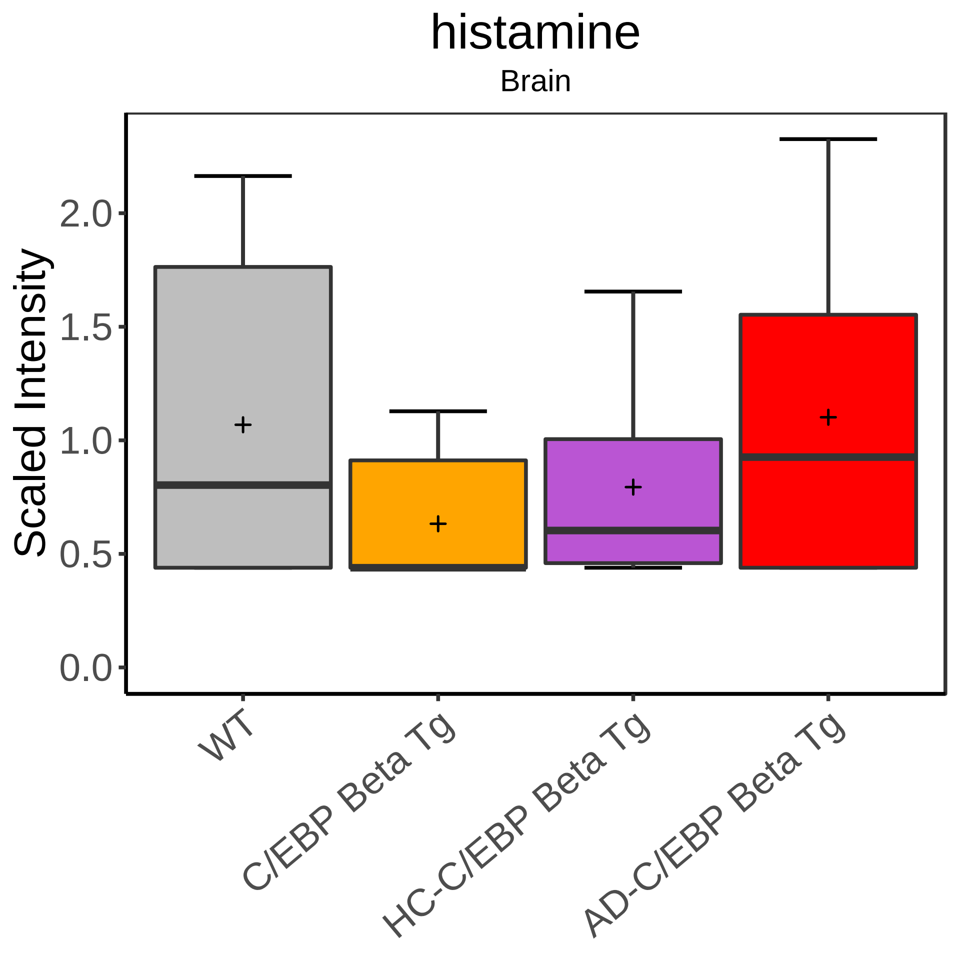
histamine
Brain
Scaled Intensity
0.0
0.5
1.0
1.5
2.0
WT
C/EBP Beta Tg
HC-C/EBP Beta Tg
AD-C/EBP Beta Tg

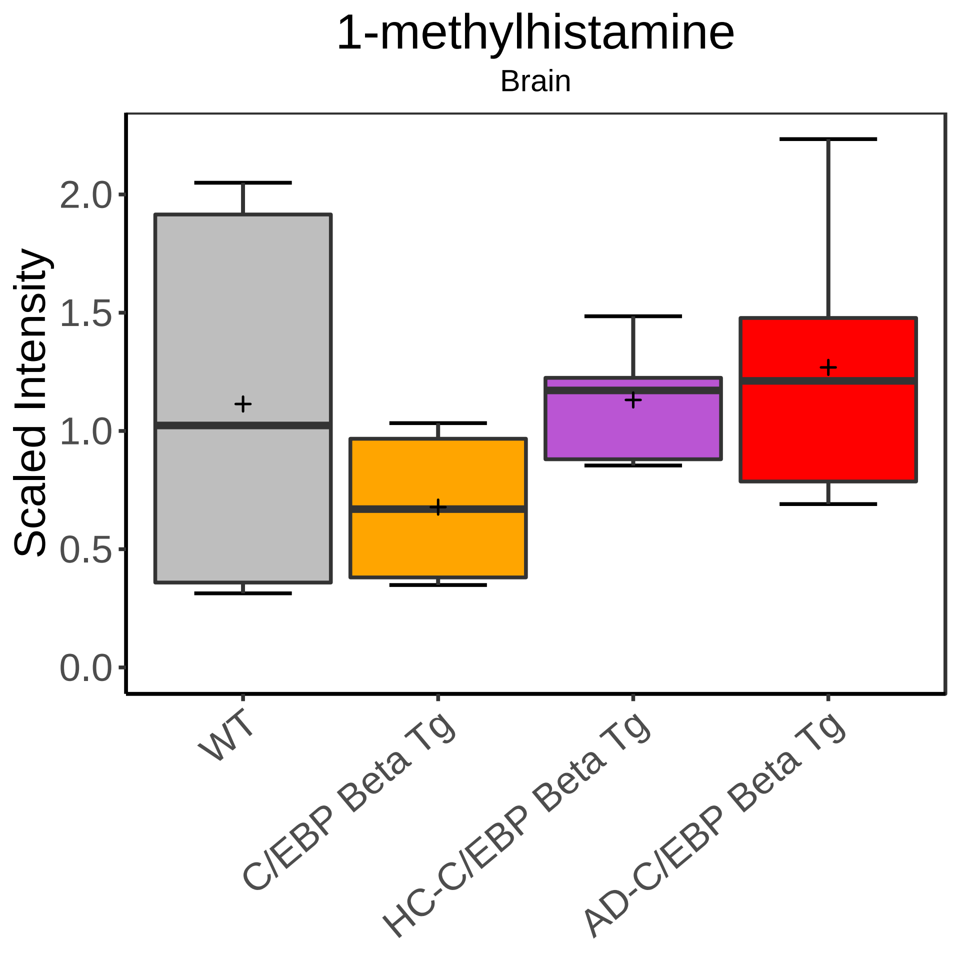

1-methylhistamine
Brain
Scaled Intensity
2.0
1.5
1.0
0.5
0.0
WT
C/EBP Beta Tg
HC-C/EBP Beta Tg
AD-C/EBP Beta Tg

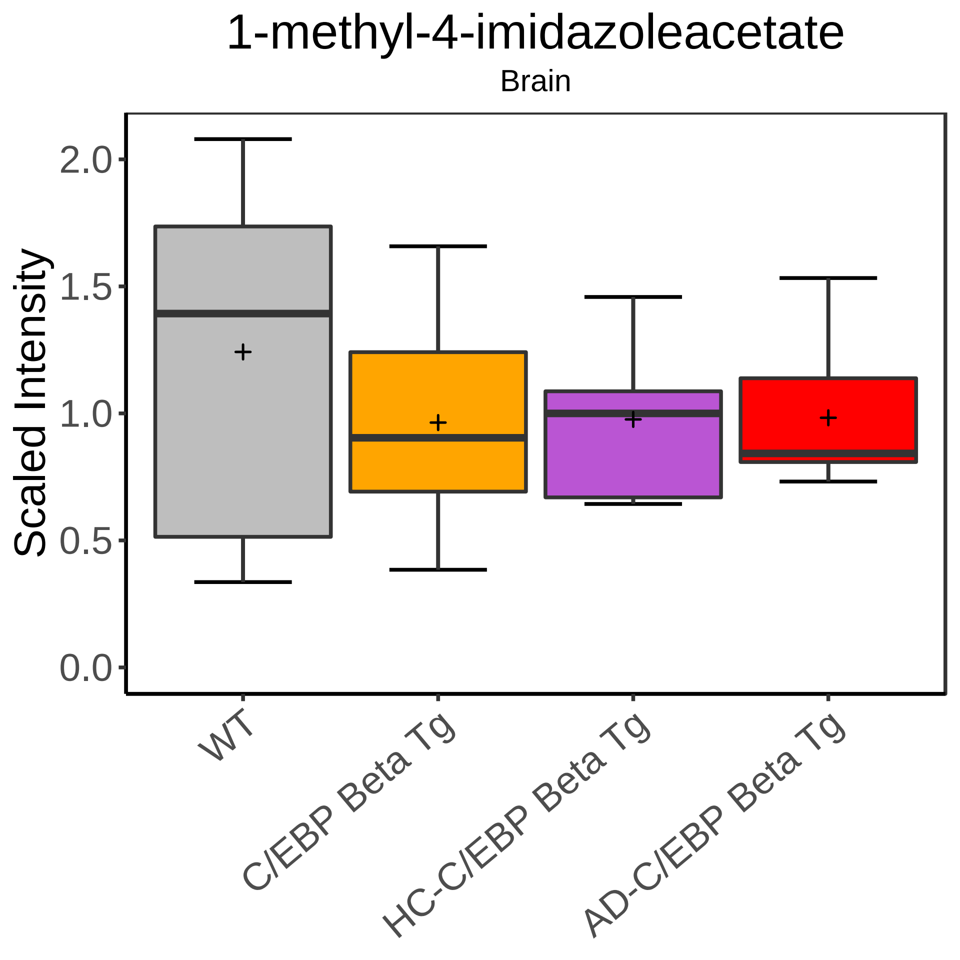

1-methyl-4-imidazoleacetate
Brain
Scaled Intensity
2.0
1.5
1.0
0.5
0.0
WT
C/EBP Beta Tg
HC-C/EBP Beta Tg
AD-C/EBP Beta Tg

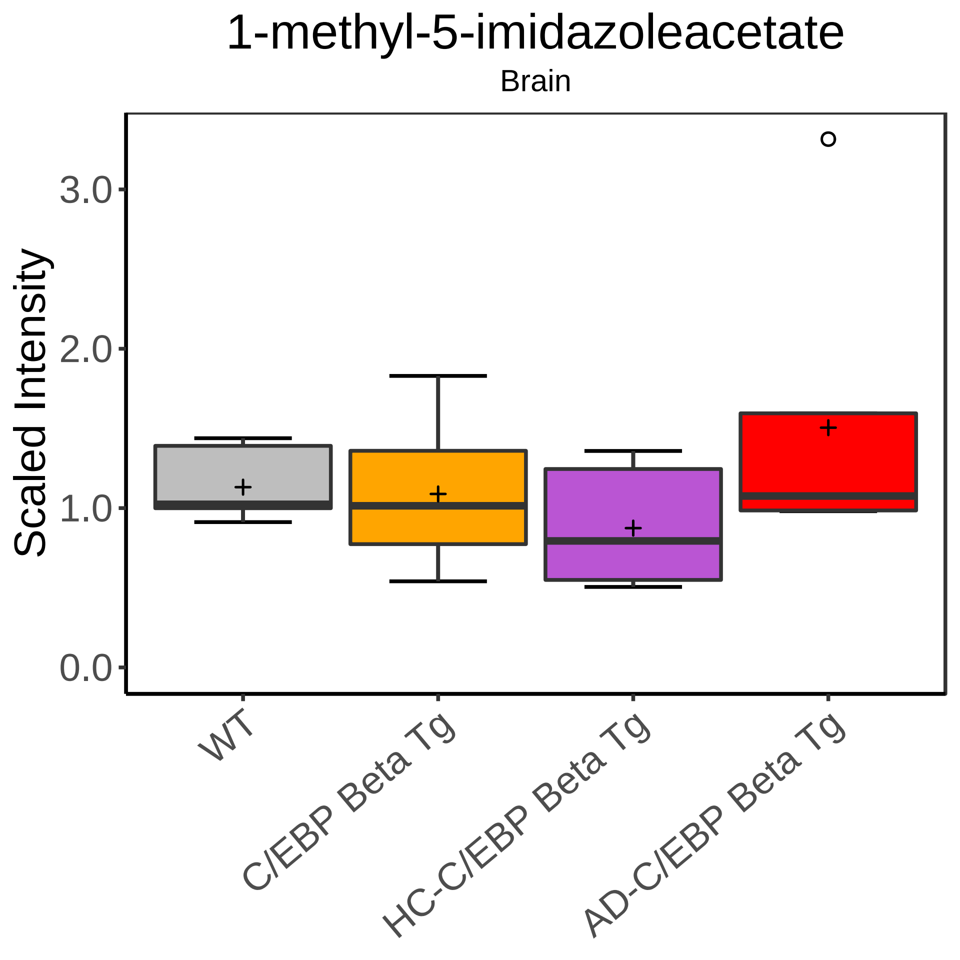

1-methyl-5-imidazoleacetate
Brain
Scaled Intensity
3.0
2.0
1.0
0.0
WT
C/EBP Beta Tg
HC-C/EBP Beta Tg
AD-C/EBP Beta Tg

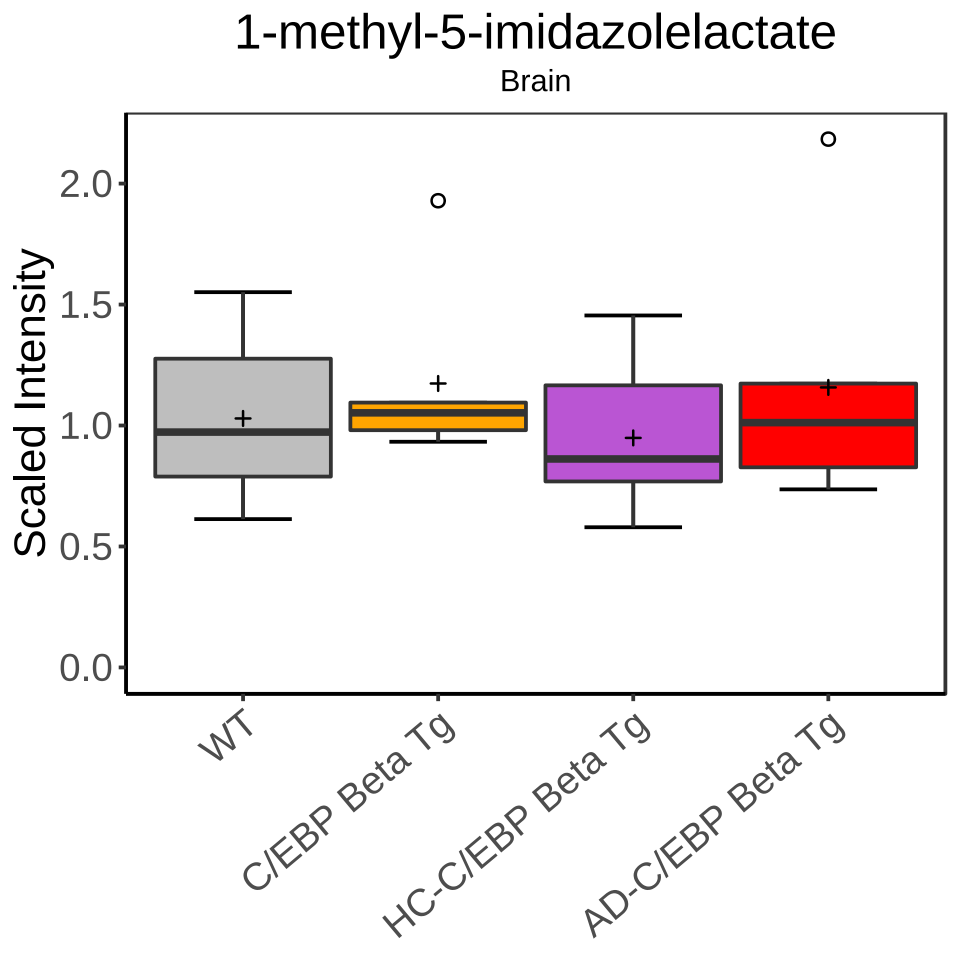
1-methyl-5-imidazolelactate
Brain
Scaled Intensity
2.0
1.5
1.0
0.5
0.0
WT
C/EBP Beta Tg
HC-C/EBP Beta Tg
AD-C/EBP Beta Tg

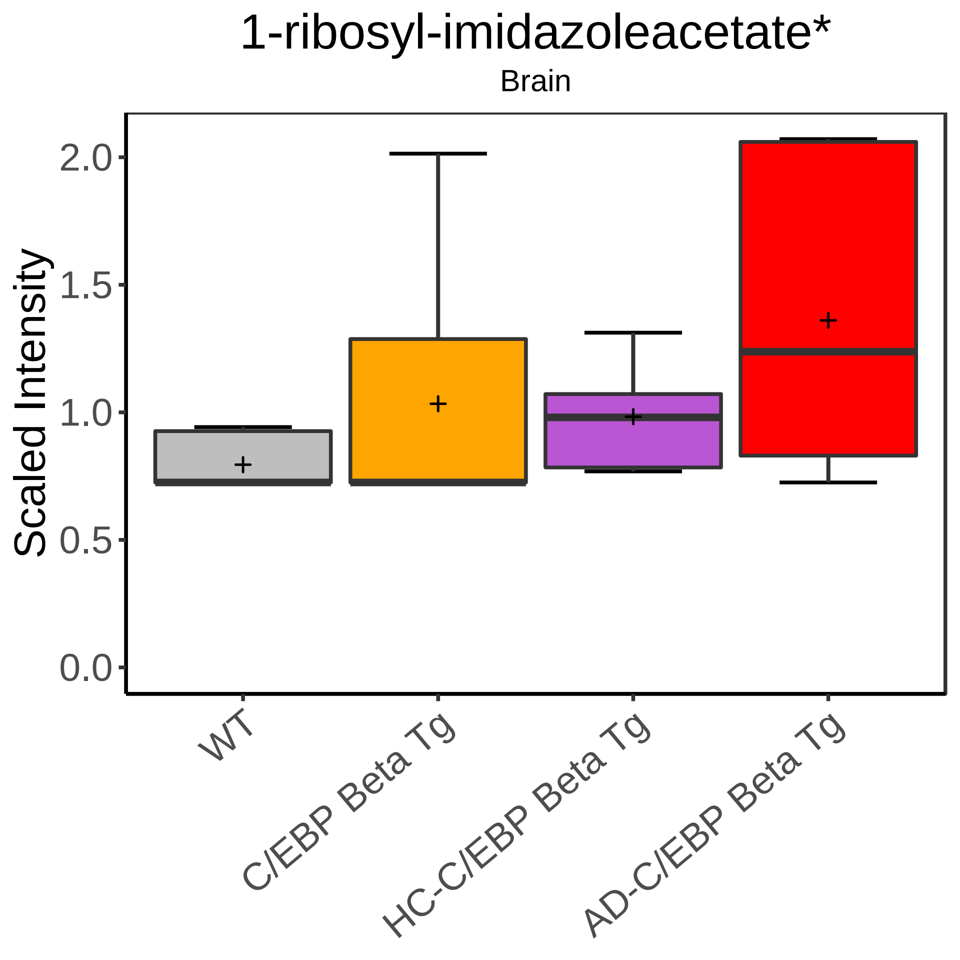
1-ribosyl-imidazoleacetate*
Brain
Scaled Intensity
2.0
1.5
1.0
0.5
0.0
WT
C/EBP Beta Tg
HC-C/EBP Beta Tg
AD-C/EBP Beta Tg

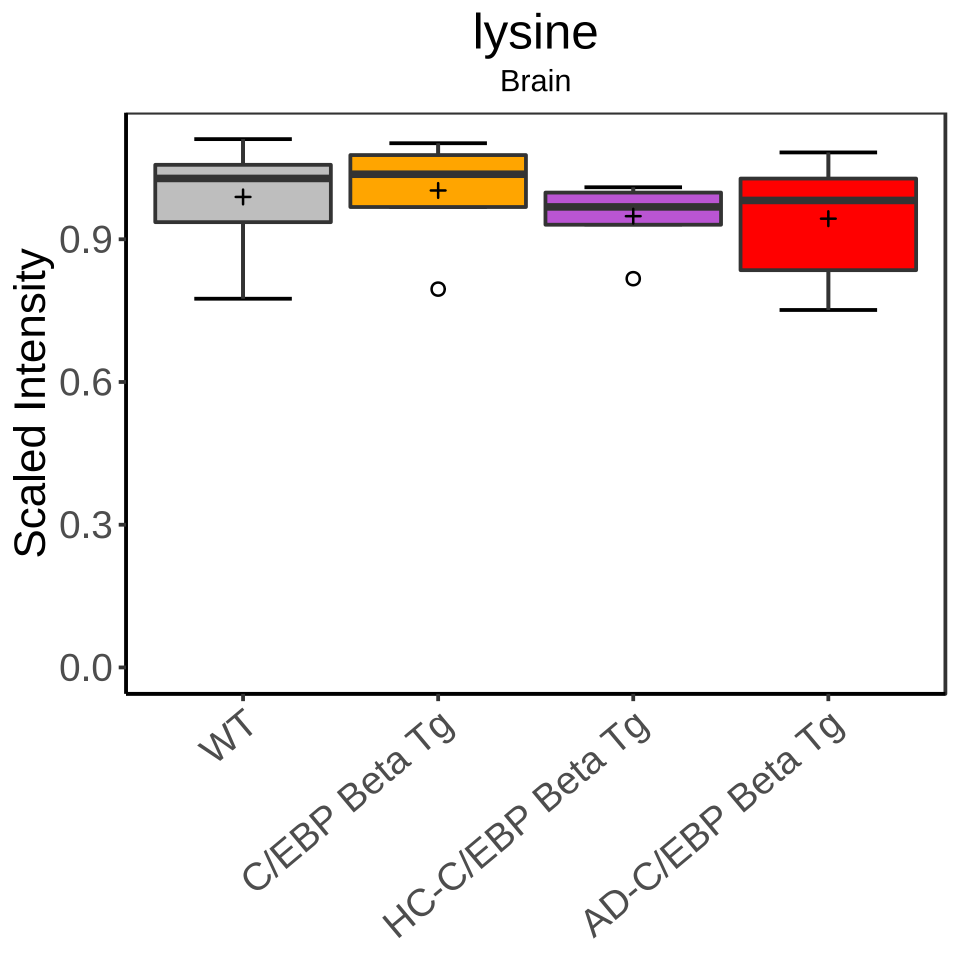

lysine
Brain
Scaled Intensity
0.0
0.3
0.6
0.9
WT
C/EBP Beta Tg
HC-C/EBP Beta Tg
AD-C/EBP Beta Tg

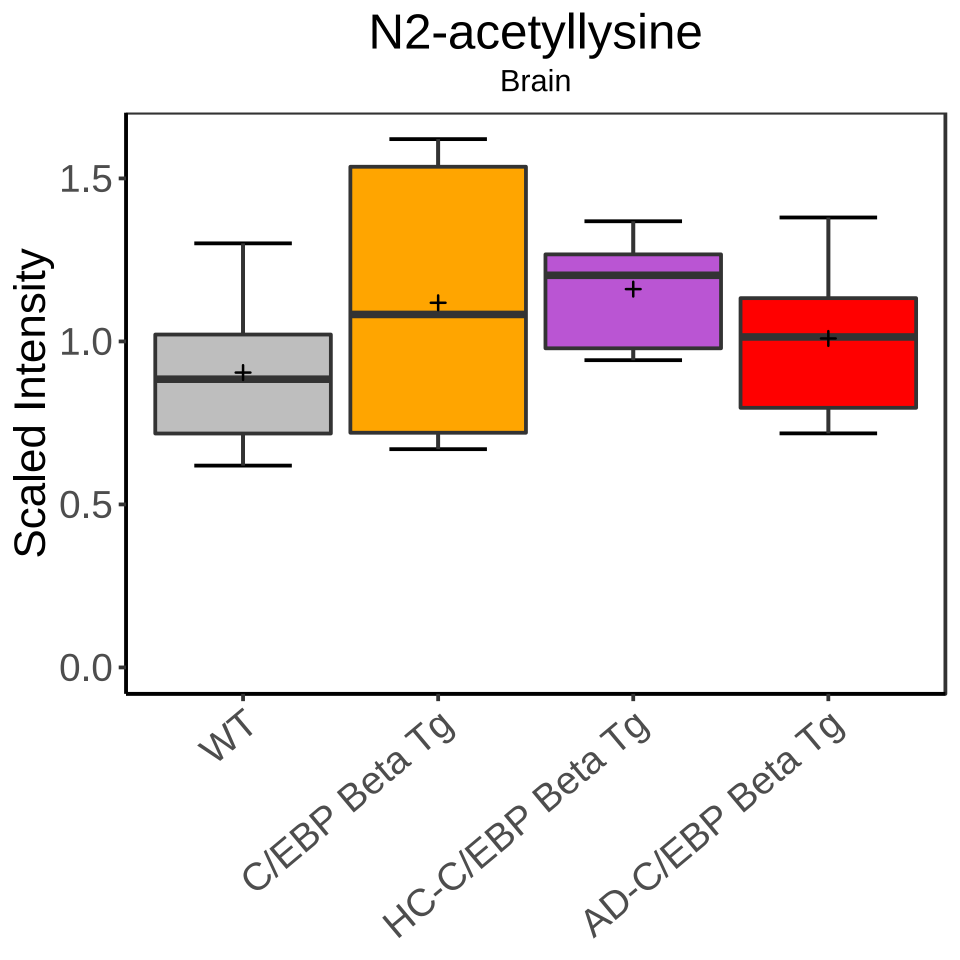

N2-acetyllysine
Brain
Scaled Intensity
1.5
1.0
0.5
0.0
WT
C/EBP Beta Tg
HC-C/EBP Beta Tg
AD-C/EBP Beta Tg

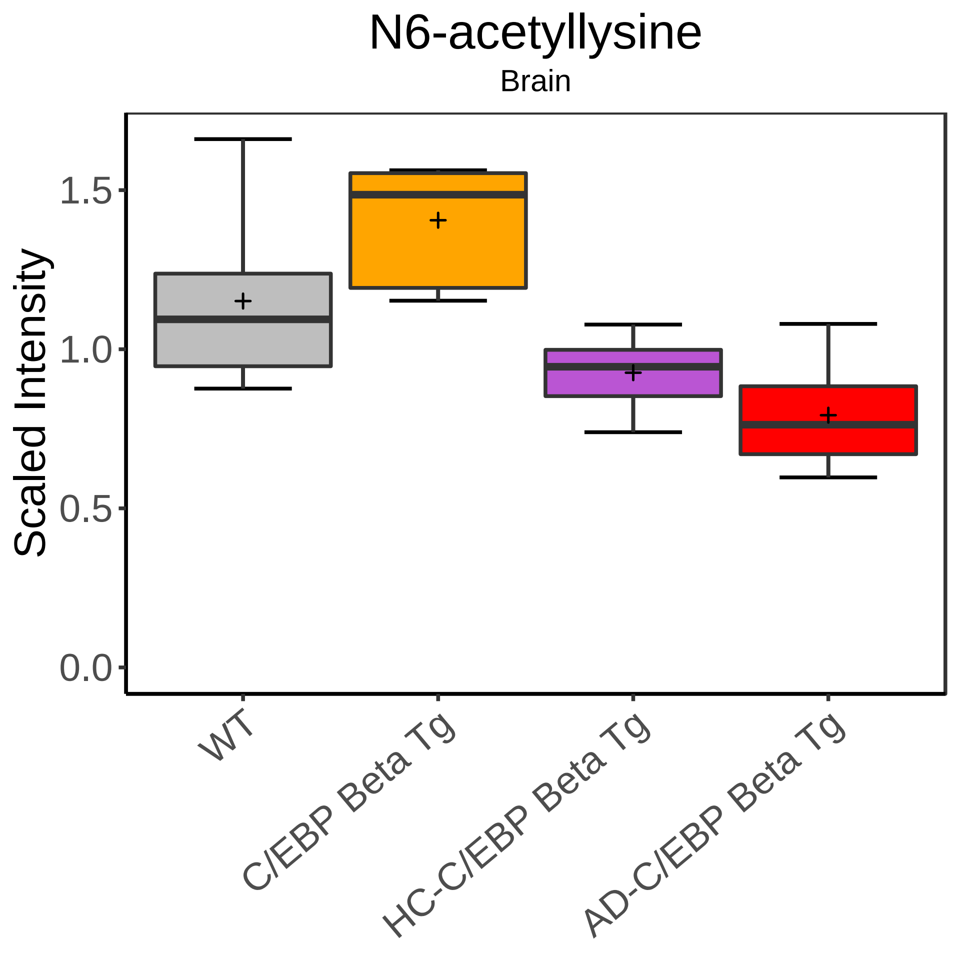
N6-acetyllysine
Brain
Scaled Intensity
1.5
1.0
0.5
0.0
WT
C/EBP Beta Tg
HC-C/EBP Beta Tg
AD-C/EBP Beta Tg

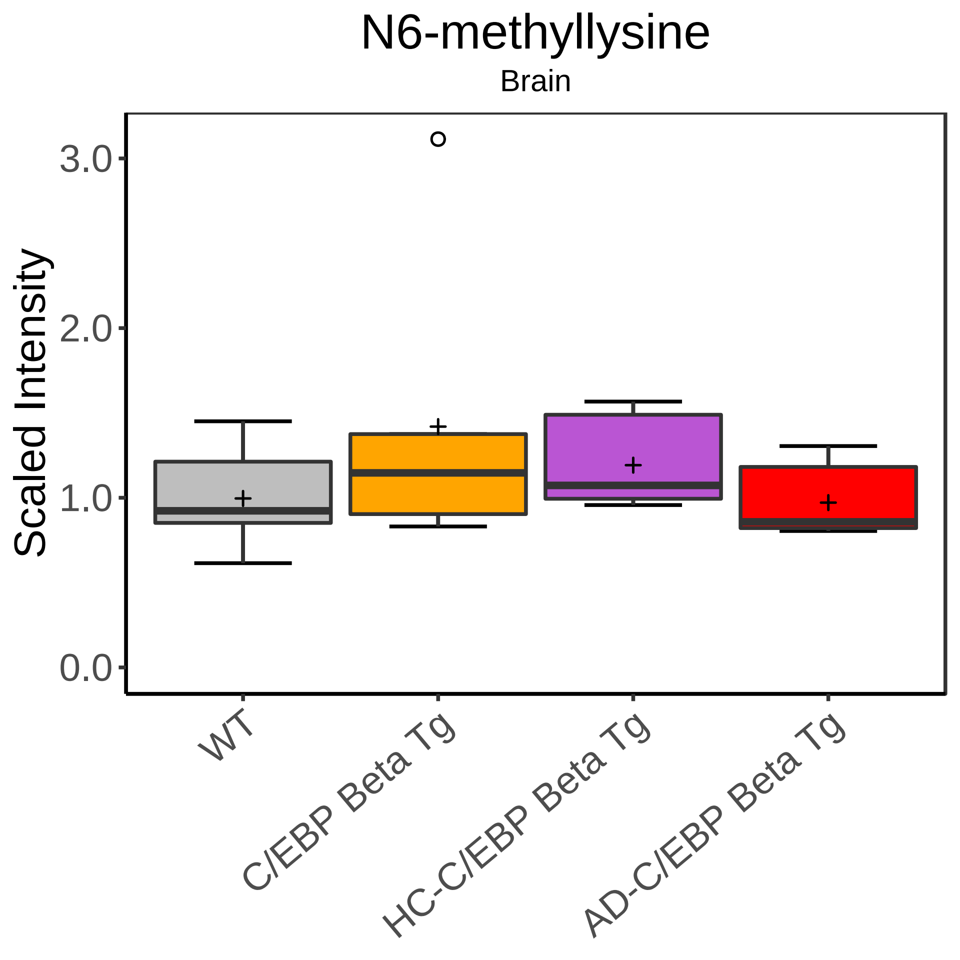

N6-methyllysine
Brain
Scaled Intensity
3.0
2.0
1.0
0.0
WT
C/EBP Beta Tg
HC-C/EBP Beta Tg
AD-C/EBP Beta Tg

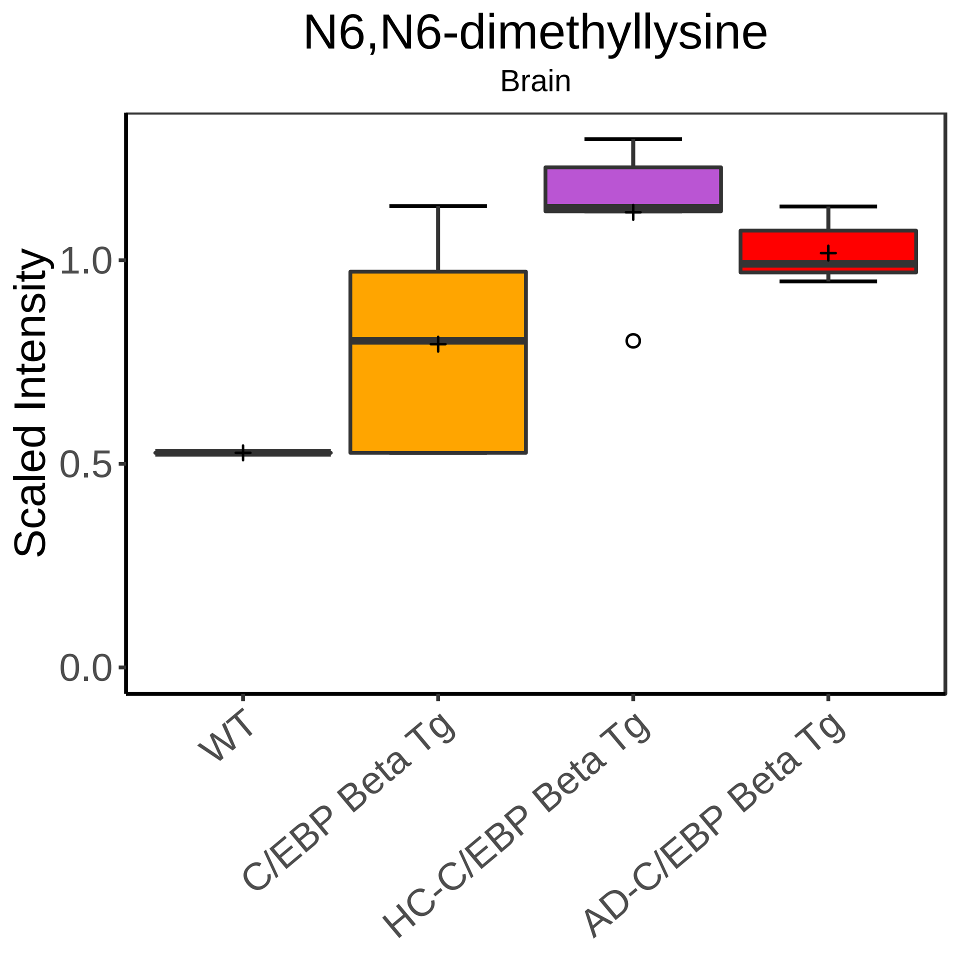

N6,N6-dimethyllysine
Brain
Scaled Intensity
0.0
0.5
1.0
WT
C/EBP Beta Tg
HC-C/EBP Beta Tg
AD-C/EBP Beta Tg

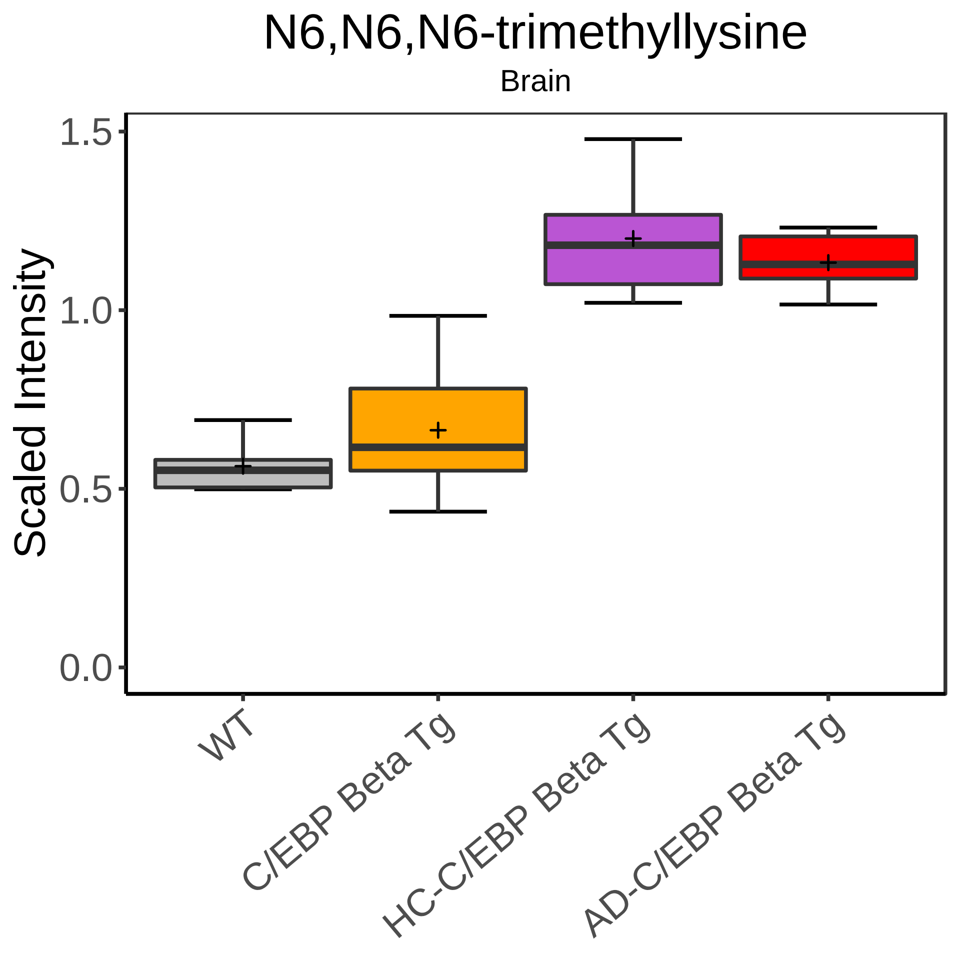
N6,N6,N6-trimethyllysine
Brain
Scaled Intensity
1.5
1.0
0.5
0.0
WT
C/EBP Beta Tg
HC-C/EBP Beta Tg
AD-C/EBP Beta Tg

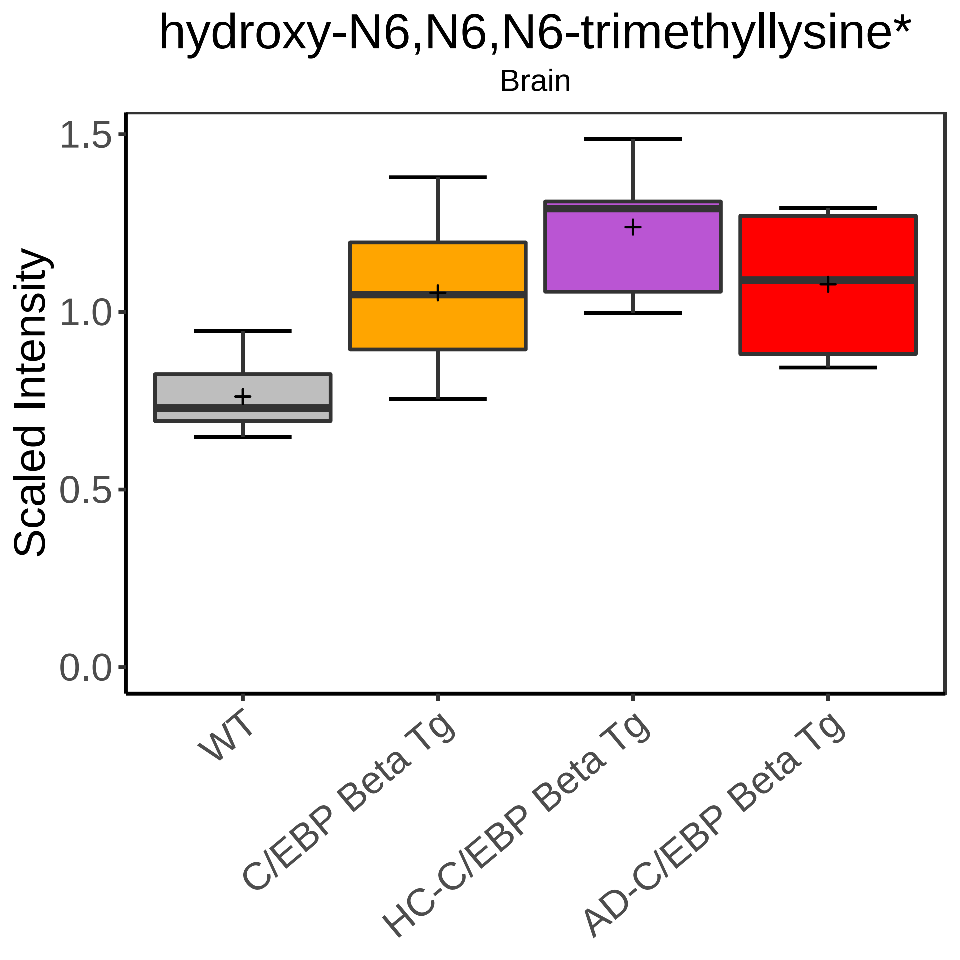
hydroxy-N6,N6,N6-trimethyllysine*
Brain
Scaled Intensity
1.5
1.0
0.5
0.0
WT
C/EBP Beta Tg
HC-C/EBP Beta Tg
AD-C/EBP Beta Tg

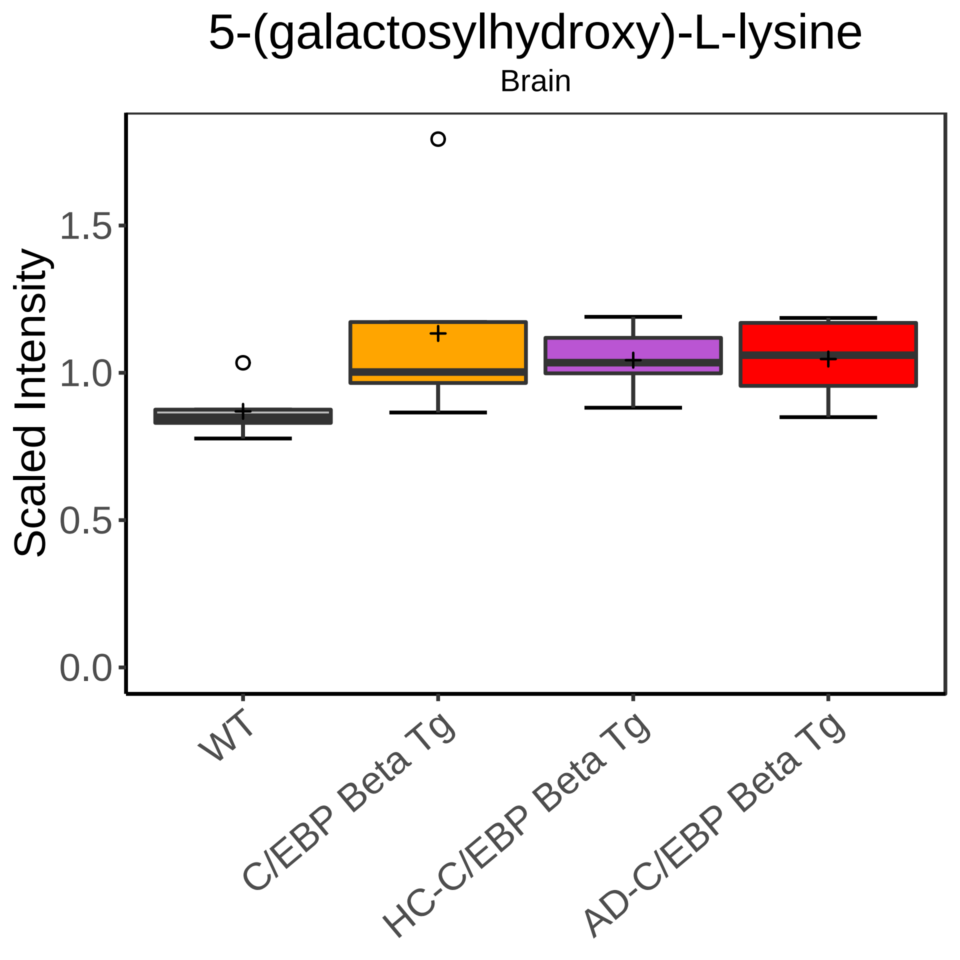
5-(galactosylhydroxy)-L-lysine
Brain
Scaled Intensity
1.5
1.0
0.5
0.0
WT
C/EBP Beta Tg
HC-C/EBP Beta Tg
AD-C/EBP Beta Tg

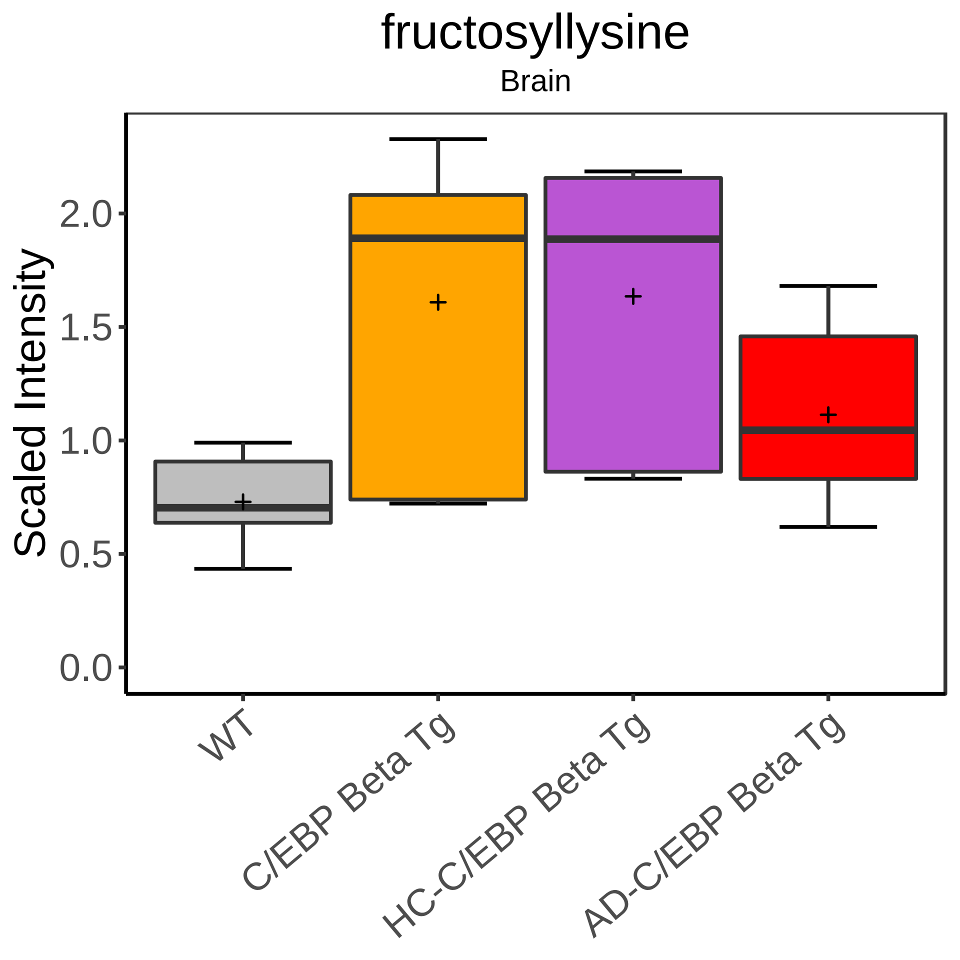
fructosyllysine
Brain
Scaled Intensity
2.0
1.5
1.0
0.5
0.0
WT
C/EBP Beta Tg
HC-C/EBP Beta Tg
AD-C/EBP Beta Tg

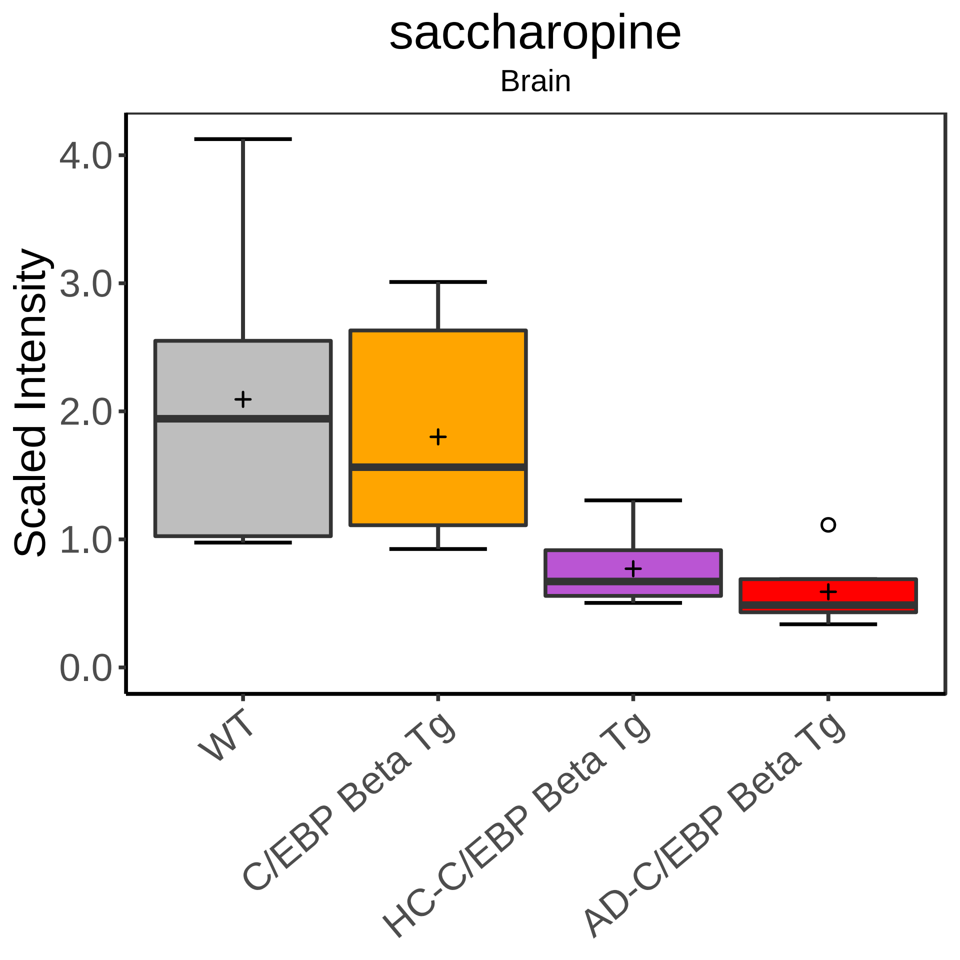
saccharopine
Brain
Scaled Intensity
4.0
3.0
2.0
1.0
0.0
WT
C/EBP Beta Tg
HC-C/EBP Beta Tg
AD-C/EBP Beta Tg

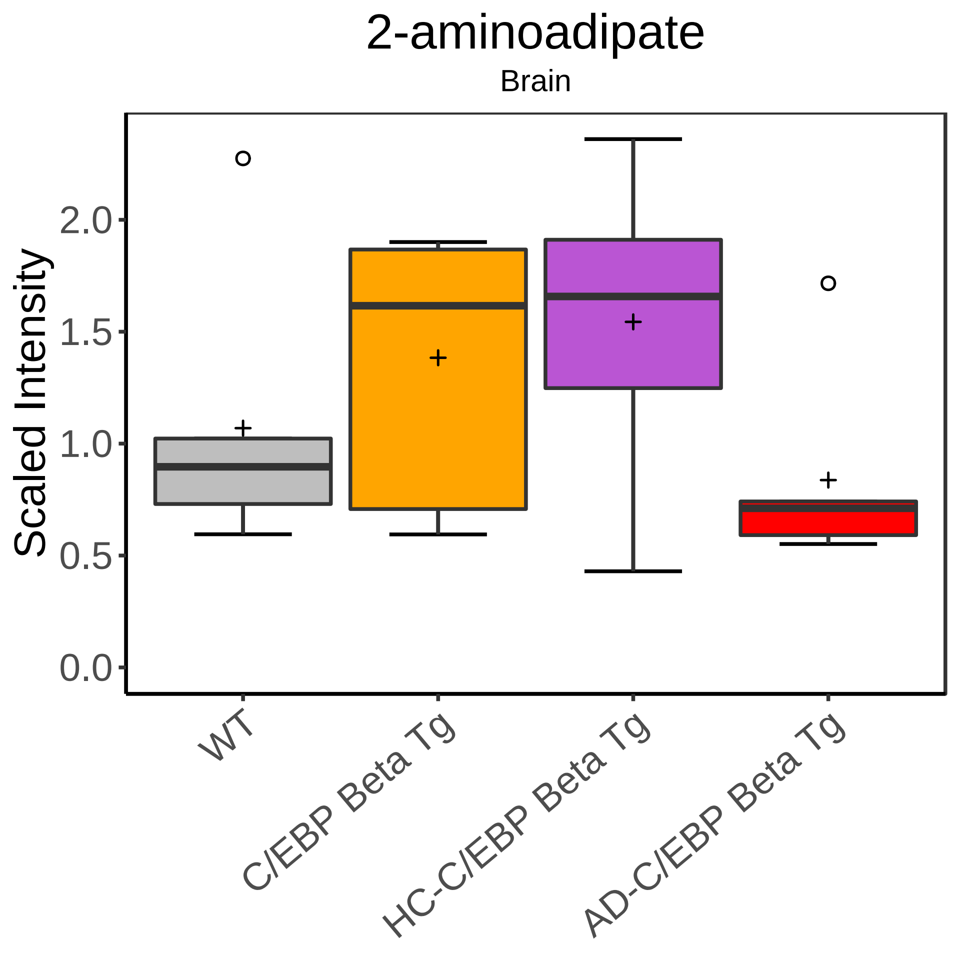

2-aminoadipate
Brain
Scaled Intensity
2.0
1.5
1.0
0.5
0.0
WT
C/EBP Beta Tg
HC-C/EBP Beta Tg
AD-C/EBP Beta Tg

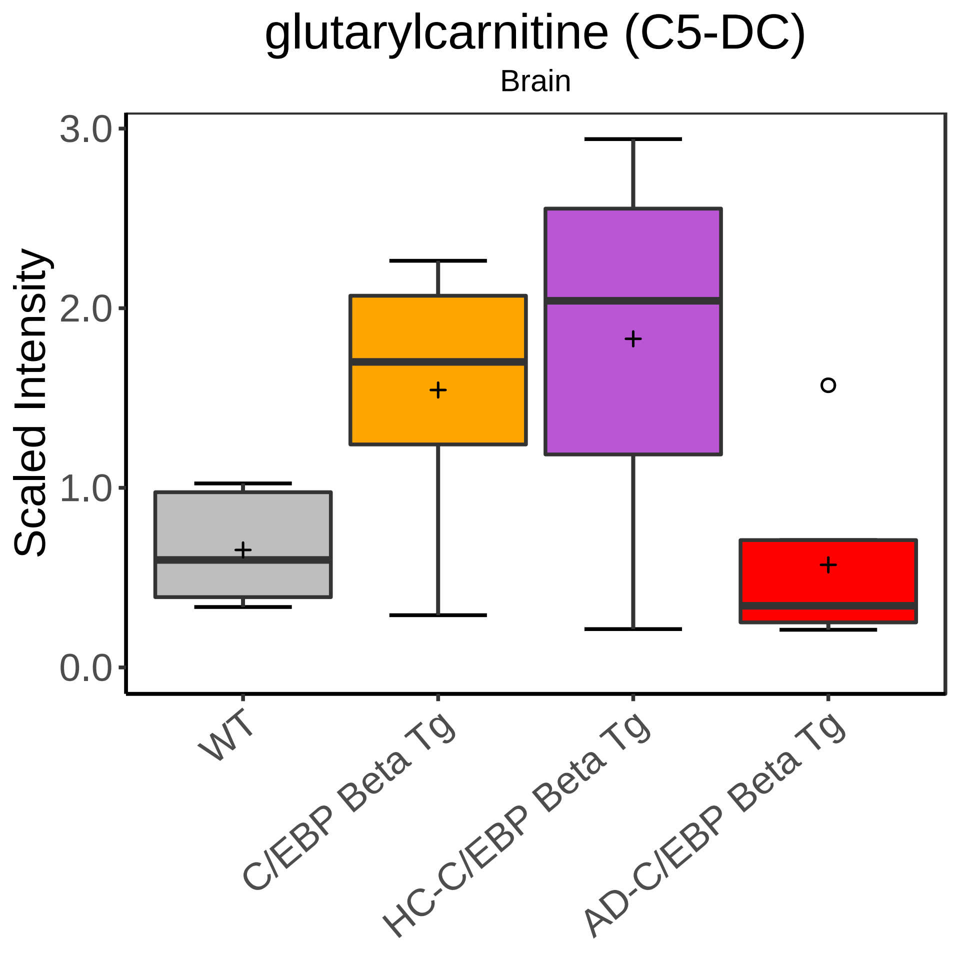
glutarylcarnitine (C5-DC)
Brain
Scaled Intensity
3.0
2.0
1.0
0.0
WT
C/EBP Beta Tg
HC-C/EBP Beta Tg
AD-C/EBP Beta Tg

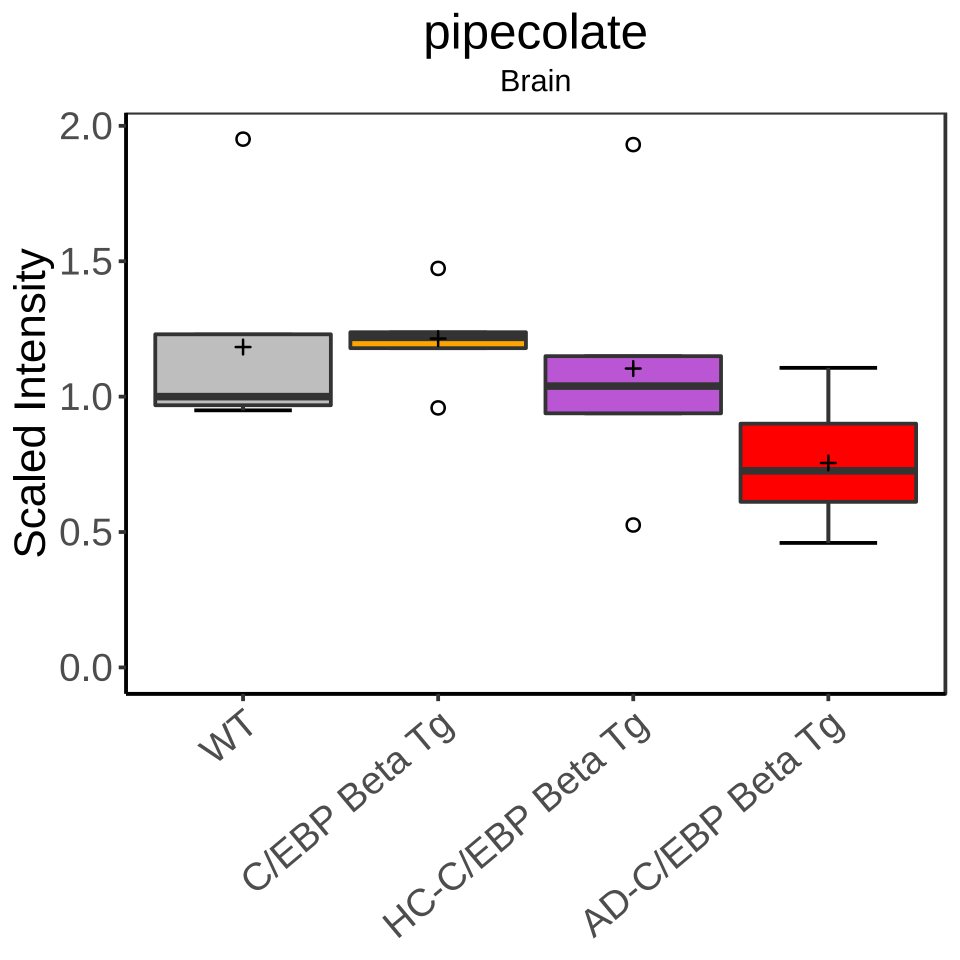

pipecolate
Brain
Scaled Intensity
2.0
1.5
1.0
0.5
0.0
WT
C/EBP Beta Tg
HC-C/EBP Beta Tg
AD-C/EBP Beta Tg

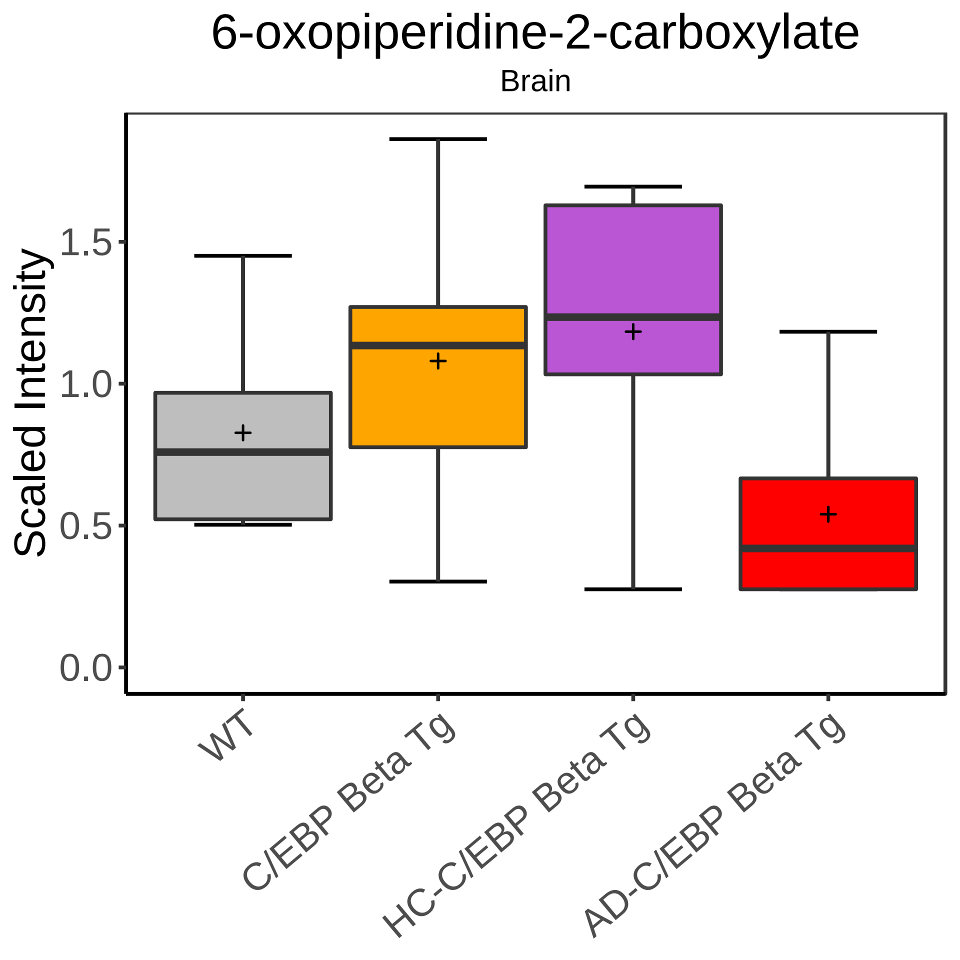

6-oxopiperidine-2-carboxylate
Brain
Scaled Intensity
0.0
0.5
1.0
1.5
WT
C/EBP Beta Tg
HC-C/EBP Beta Tg
AD-C/EBP Beta Tg

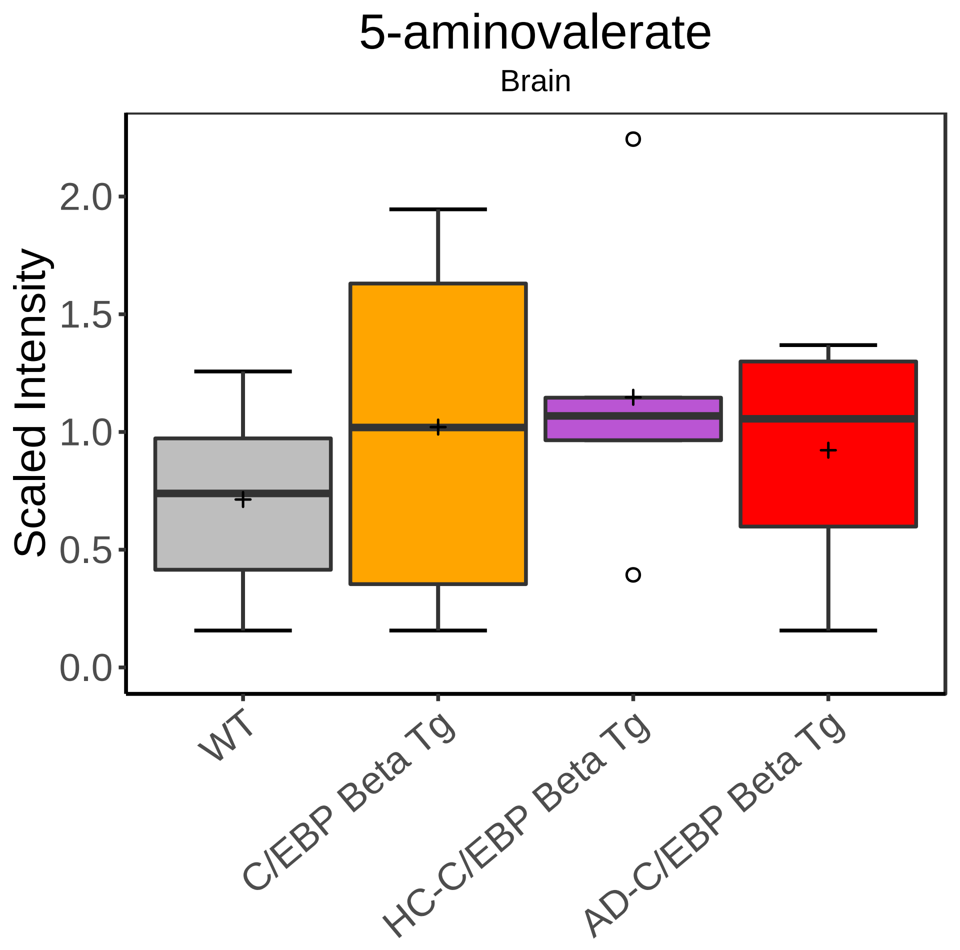

5-aminovalerate
Brain
Scaled Intensity
0.0
0.5
1.0
1.5
2.0
WT
C/EBP Beta Tg
HC-C/EBP Beta Tg
AD-C/EBP Beta Tg

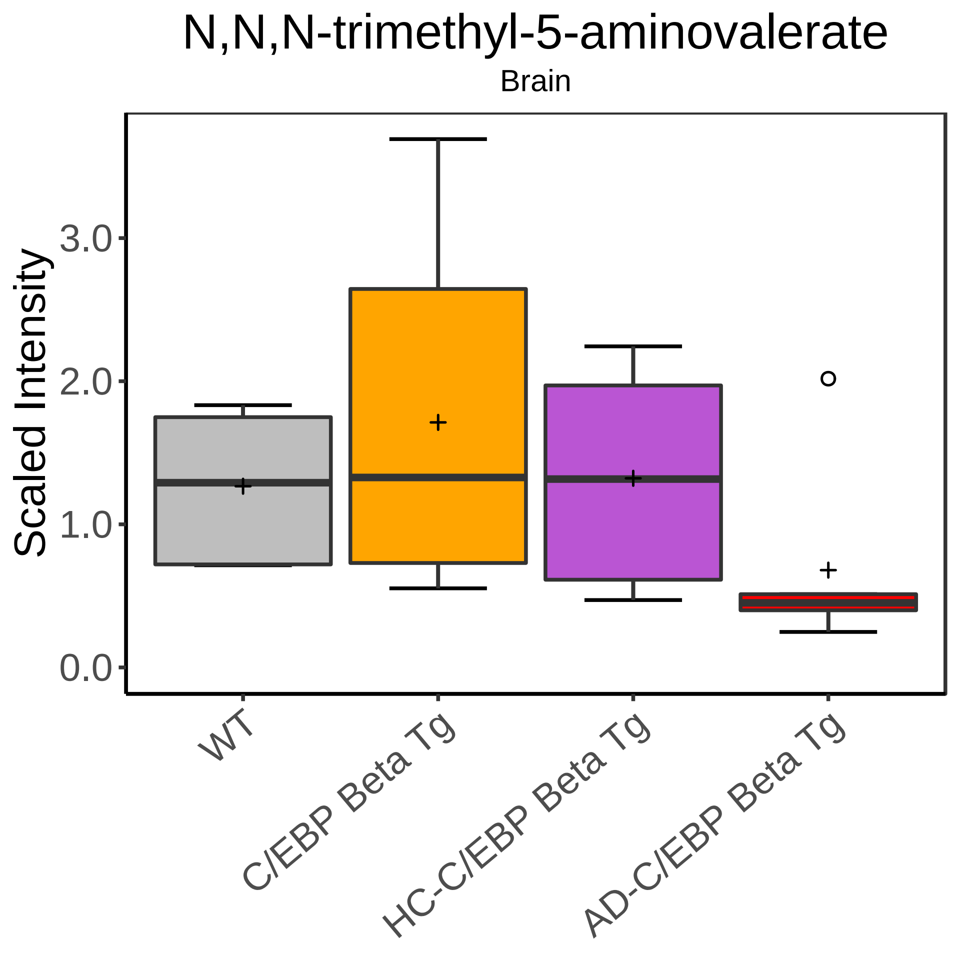
N,N,N-trimethyl-5-aminovalerate
Brain
Scaled Intensity
3.0
2.0
1.0
0.0
WT
C/EBP Beta Tg
HC-C/EBP Beta Tg
AD-C/EBP Beta Tg

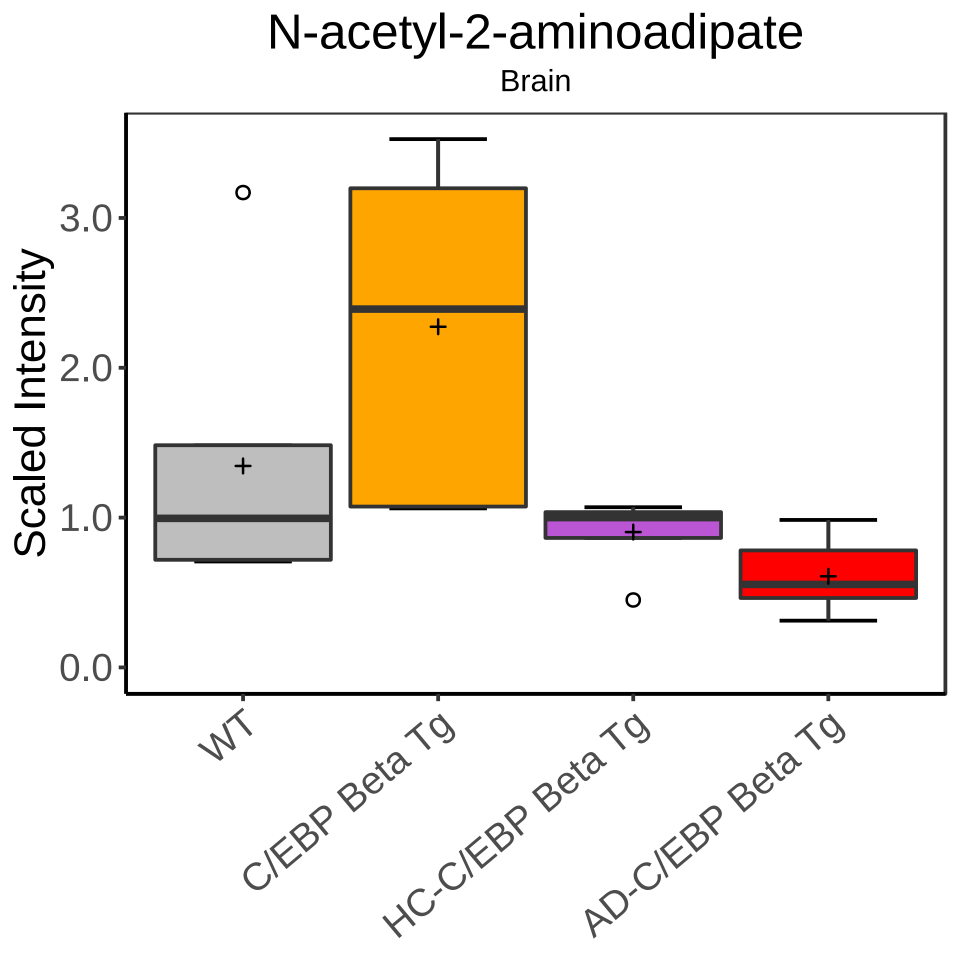
N-acetyl-2-aminoadipate
Brain
Scaled Intensity
3.0
2.0
1.0
0.0
WT
C/EBP Beta Tg
HC-C/EBP Beta Tg
AD-C/EBP Beta Tg

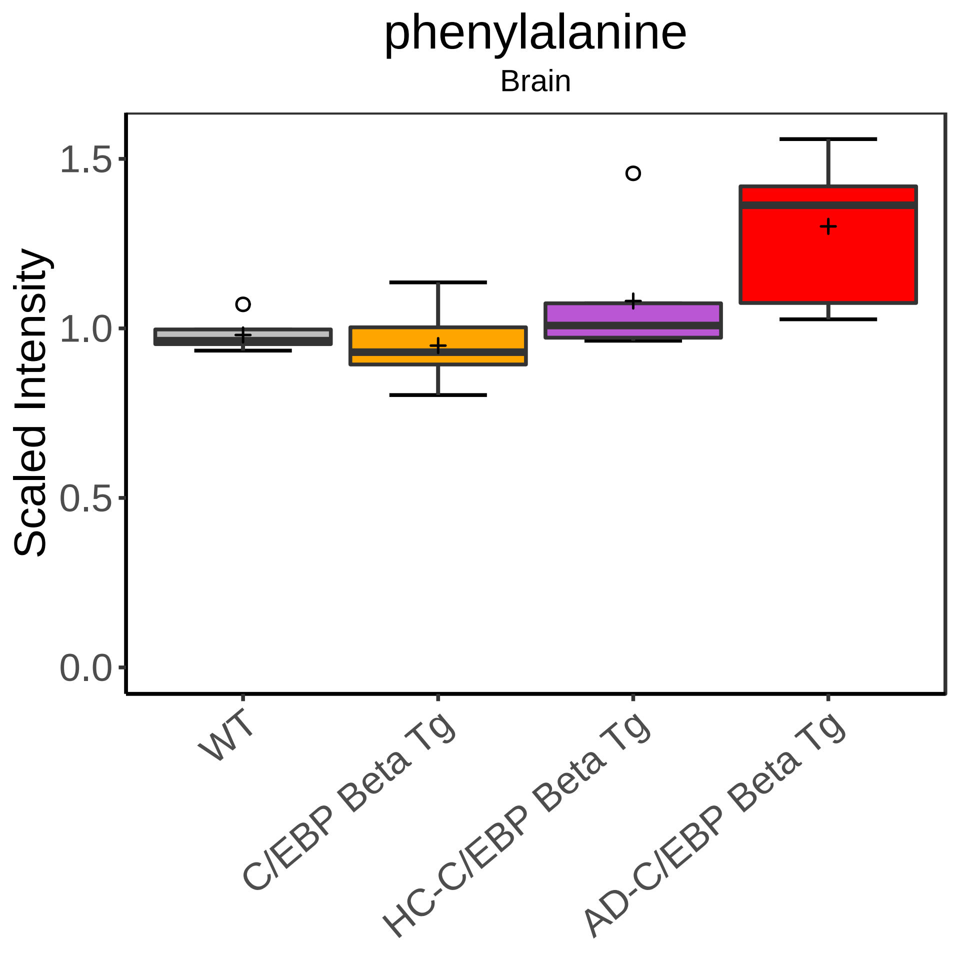
phenylalanine
Brain
Scaled Intensity
1.5
1.0
0.5
0.0
WT
C/EBP Beta Tg
HC-C/EBP Beta Tg
AD-C/EBP Beta Tg

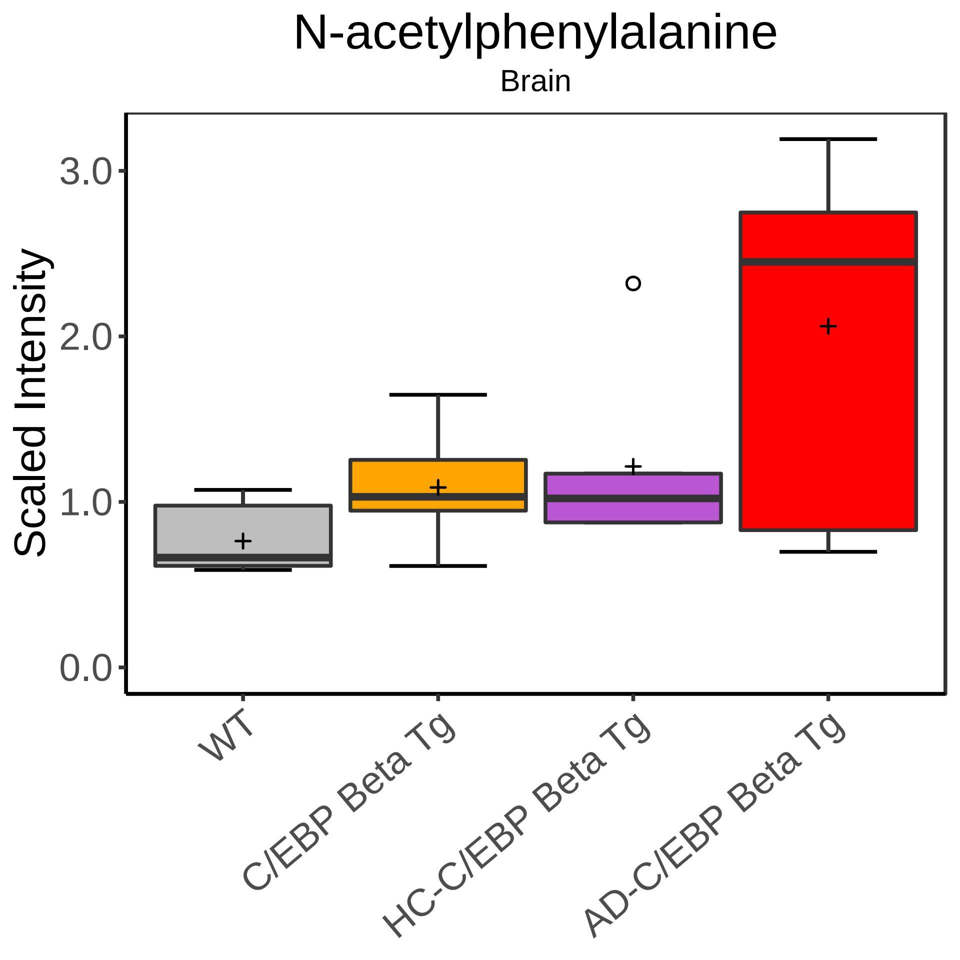

N-acetylphenylalanine
Brain
Scaled Intensity
3.0
2.0
1.0
0.0
WT
C/EBP Beta Tg
HC-C/EBP Beta Tg
AD-C/EBP Beta Tg

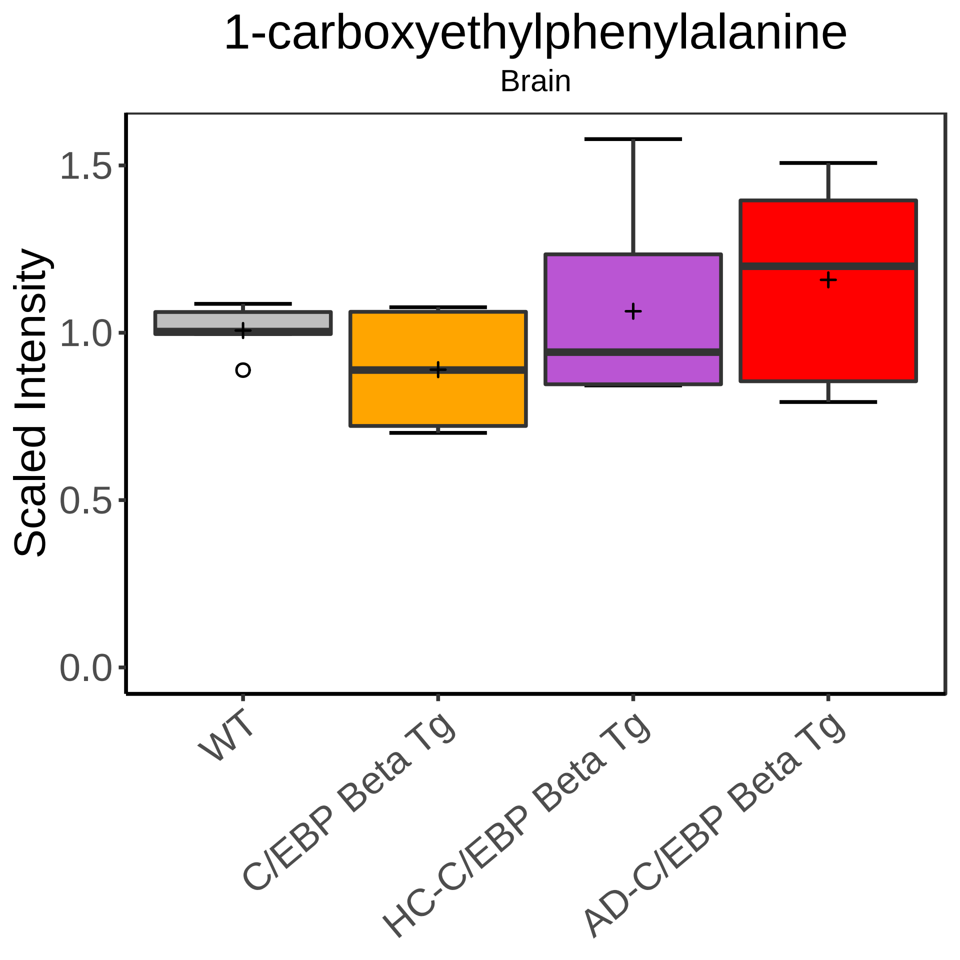
1-carboxyethylphenylalanine
Brain
Scaled Intensity
1.5
1.0
0.5
0.0
WT
C/EBP Beta Tg
HC-C/EBP Beta Tg
AD-C/EBP Beta Tg

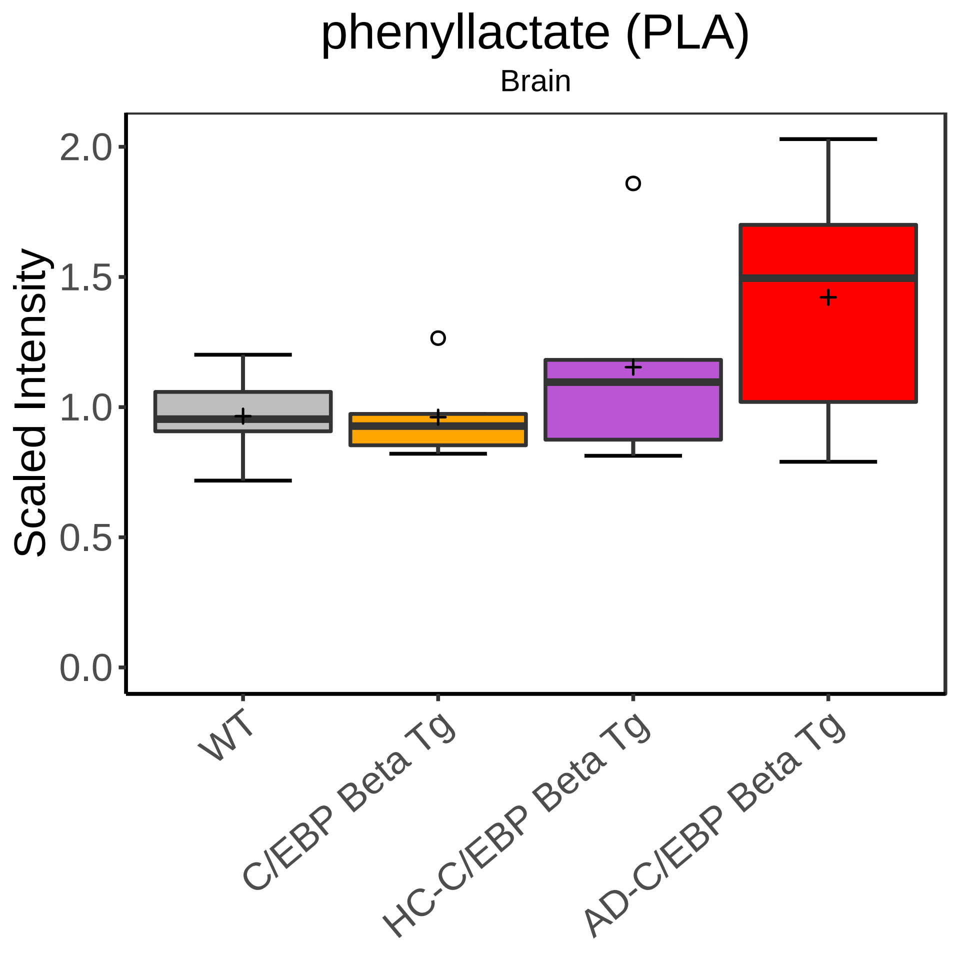

phenyllactate (PLA)
Brain
Scaled Intensity
2.0
1.5
1.0
0.5
0.0
WT
C/EBP Beta Tg
HC-C/EBP Beta Tg
AD-C/EBP Beta Tg

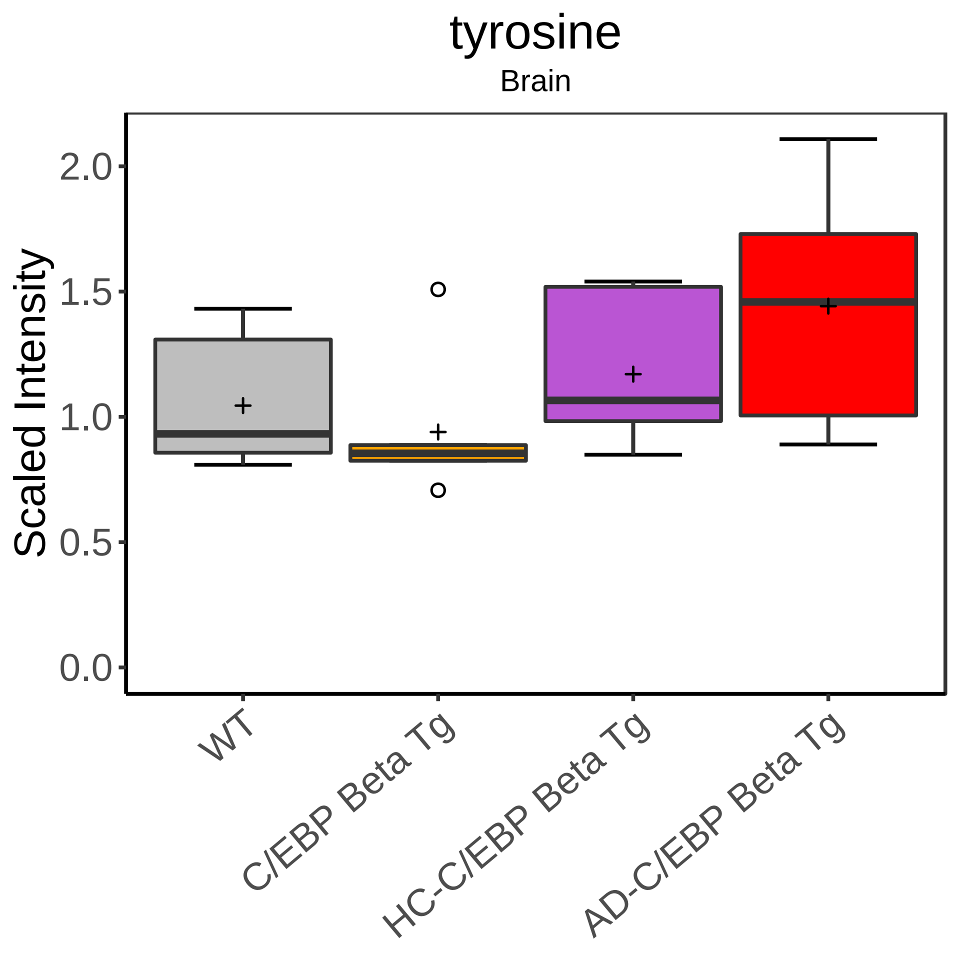

tyrosine
Brain
Scaled Intensity
2.0
1.5
1.0
0.5
0.0
WT
C/EBP Beta Tg
HC-C/EBP Beta Tg
AD-C/EBP Beta Tg

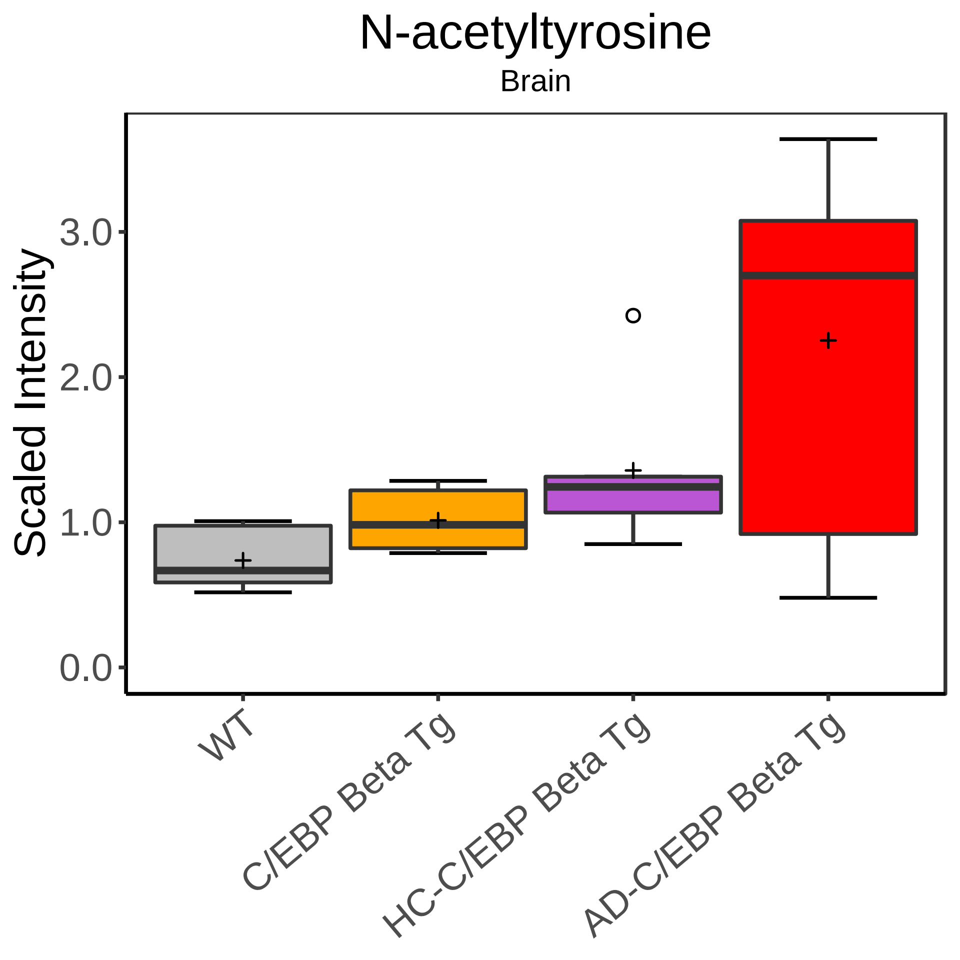

N-acetyltyrosine
Brain
Scaled Intensity
0.0
1.0
2.0
3.0
WT
C/EBP Beta Tg
HC-C/EBP Beta Tg
AD-C/EBP Beta Tg

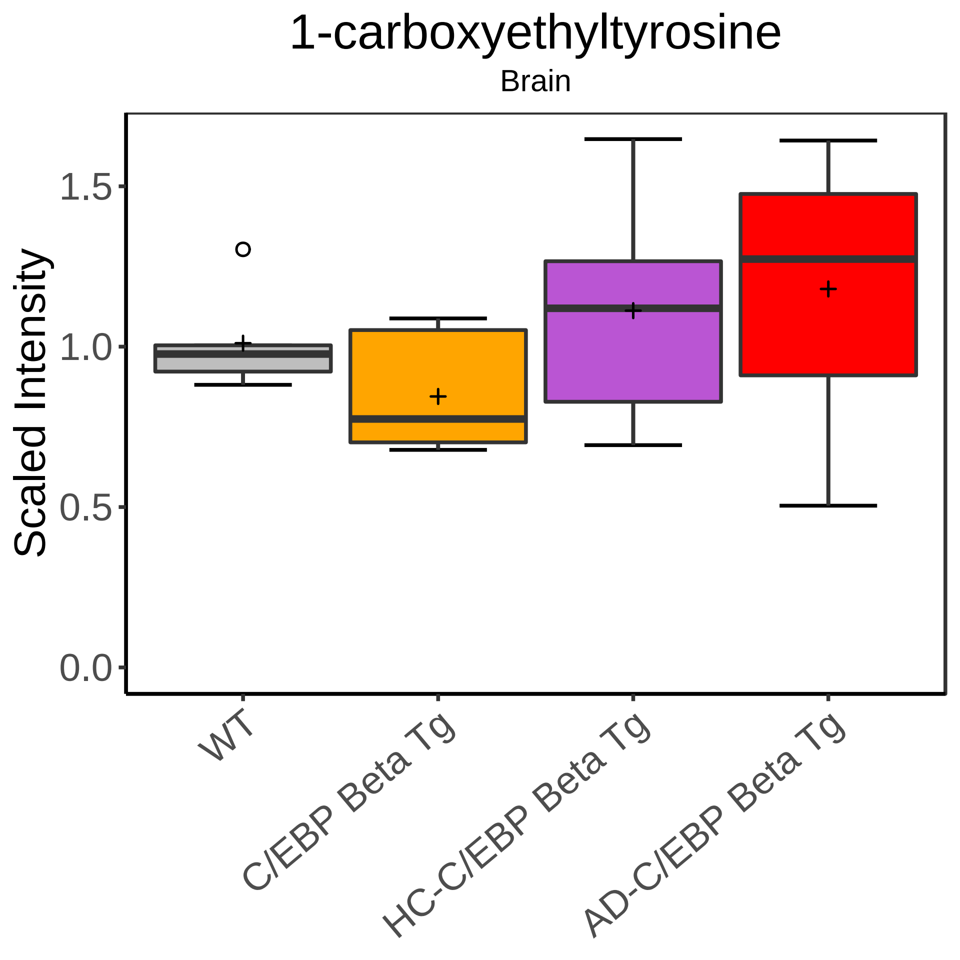
1-carboxyethyltyrosine
Brain
Scaled Intensity
0.0
0.5
1.0
1.5
WT
C/EBP Beta Tg
HC-C/EBP Beta Tg
AD-C/EBP Beta Tg

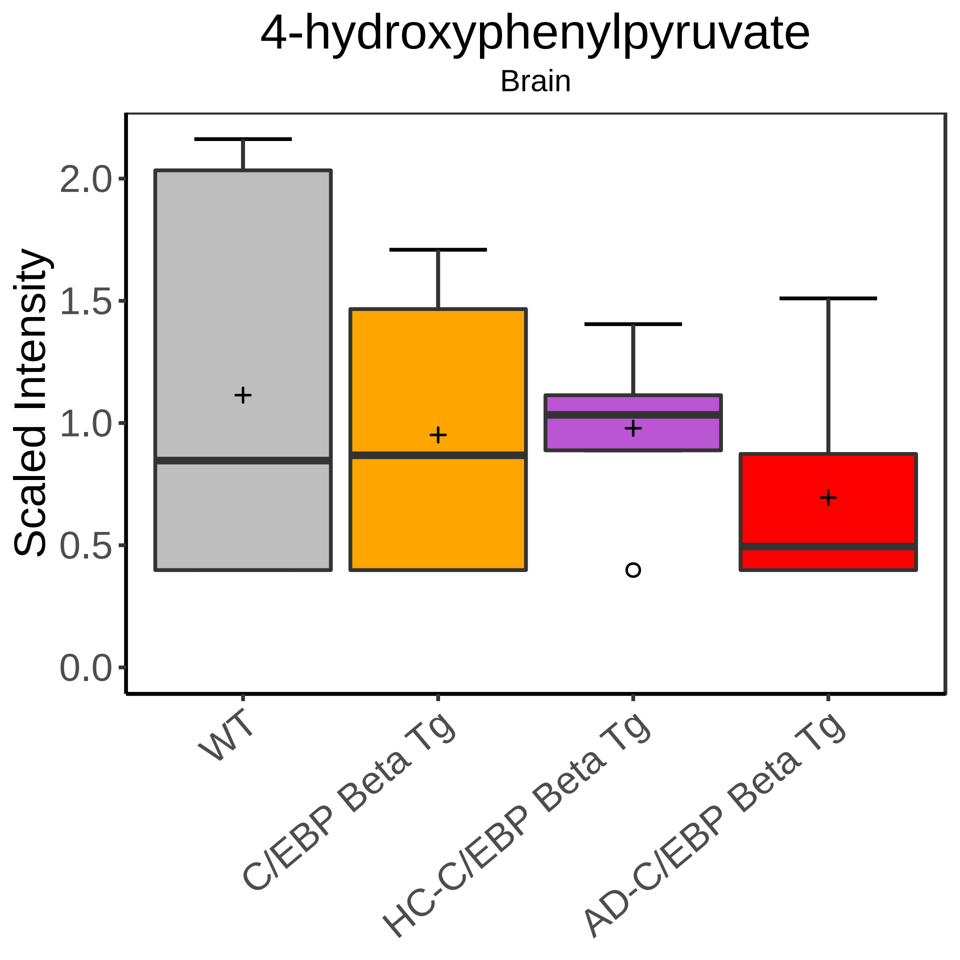
4-hydroxyphenylpyruvate
Brain
Scaled Intensity
2.0
1.5
1.0
0.5
0.0
WT
C/EBP Beta Tg
HC-C/EBP Beta Tg
AD-C/EBP Beta Tg

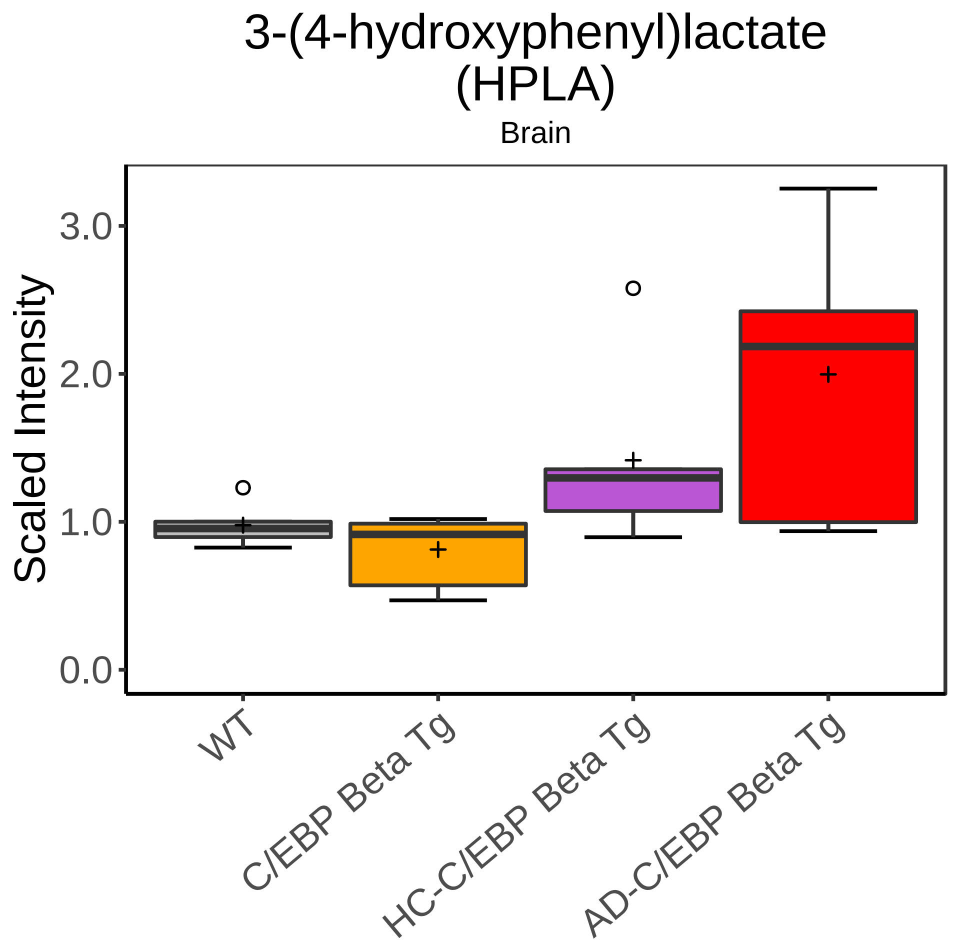

3-(4-hydroxyphenyl)lactate
(HPLA)
Brain
Scaled Intensity
3.0
2.0
1.0
0.0
WT
C/EBP Beta Tg
HC-C/EBP Beta Tg
AD-C/EBP Beta Tg

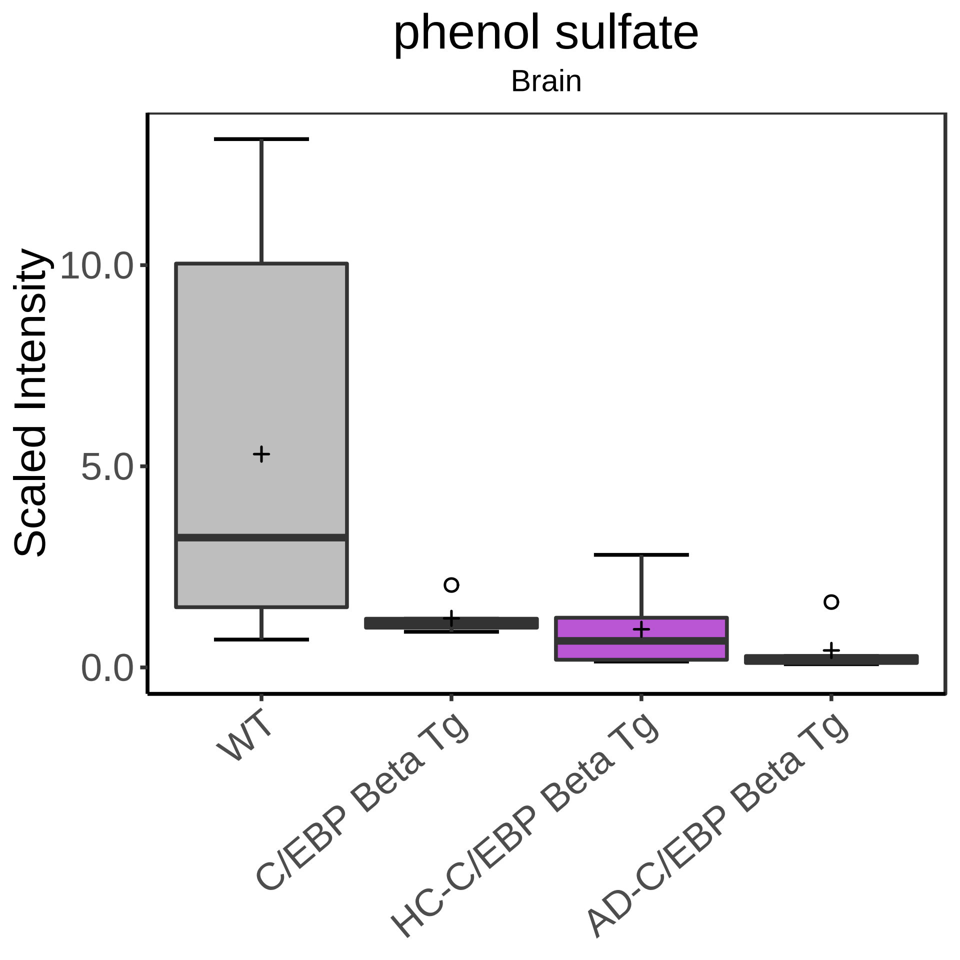

phenol sulfate
Brain
Scaled Intensity
10.0
5.0
0.0
WT
C/EBP Beta Tg
HC-C/EBP Beta Tg
AD-C/EBP Beta Tg

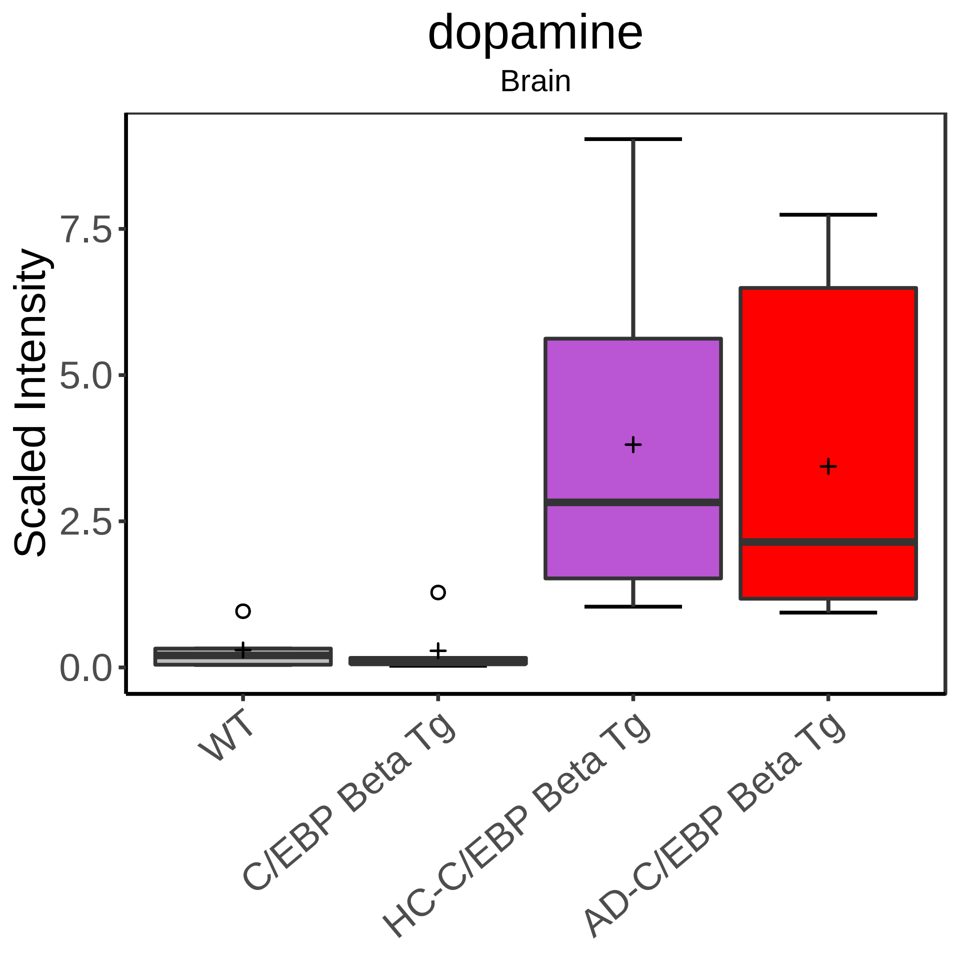

dopamine
Brain
Scaled Intensity
7.5
5.0
2.5
0.0
WT
C/EBP Beta Tg
HC-C/EBP Beta Tg
AD-C/EBP Beta Tg

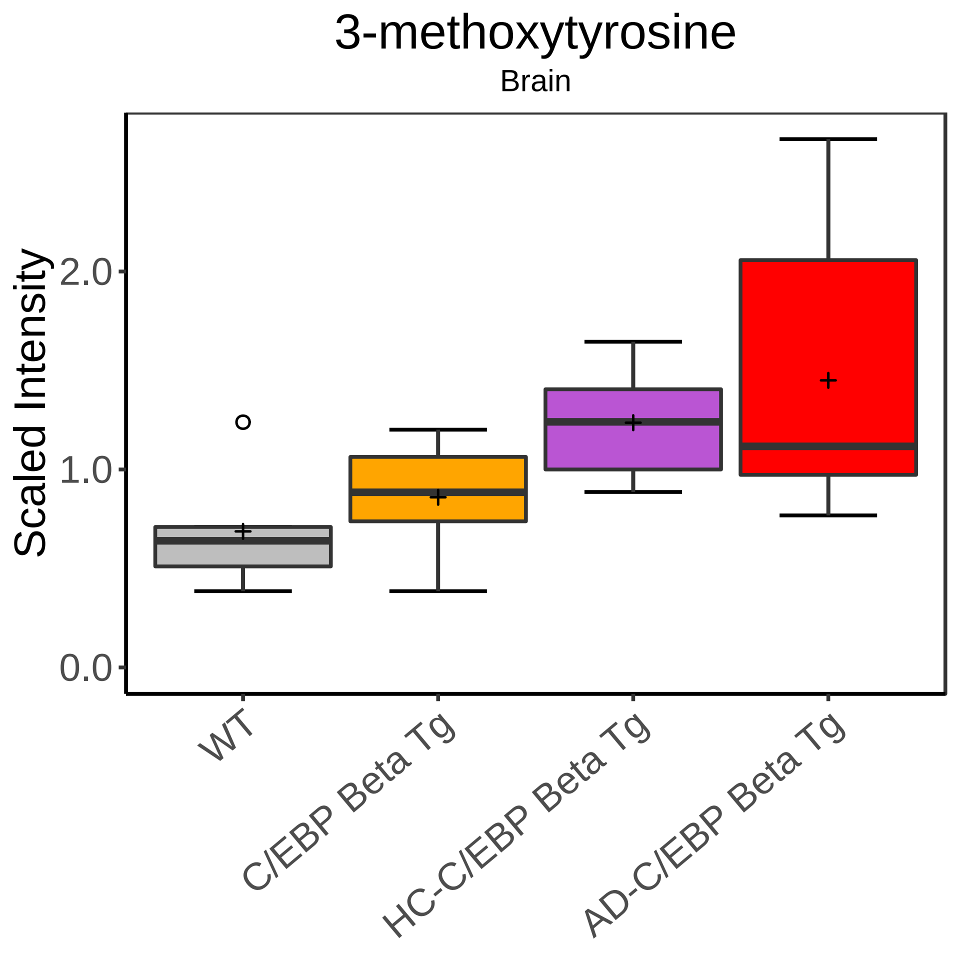
3-methoxytyrosine
Brain
Scaled Intensity
2.0
1.0
0.0
WT
C/EBP Beta Tg
HC-C/EBP Beta Tg
AD-C/EBP Beta Tg

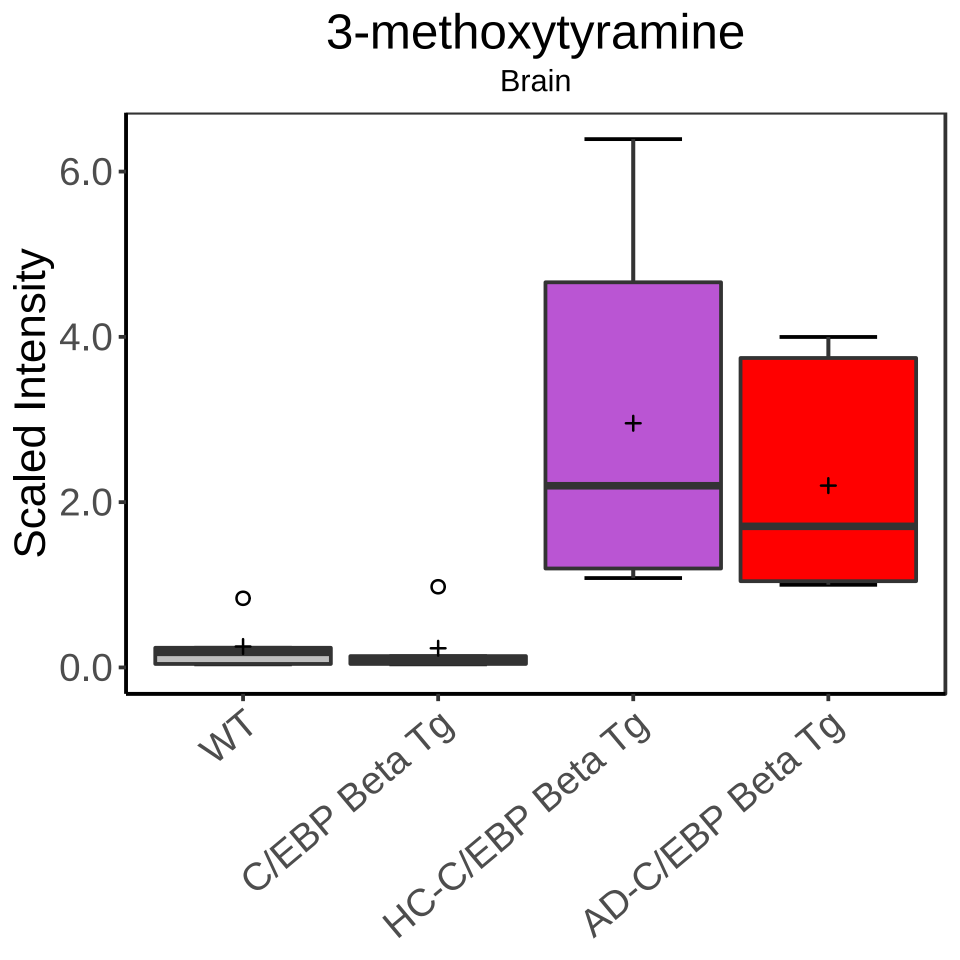

3-methoxytyramine
Brain
Scaled Intensity
6.0
4.0
2.0
0.0
WT
C/EBP Beta Tg
HC-C/EBP Beta Tg
AD-C/EBP Beta Tg

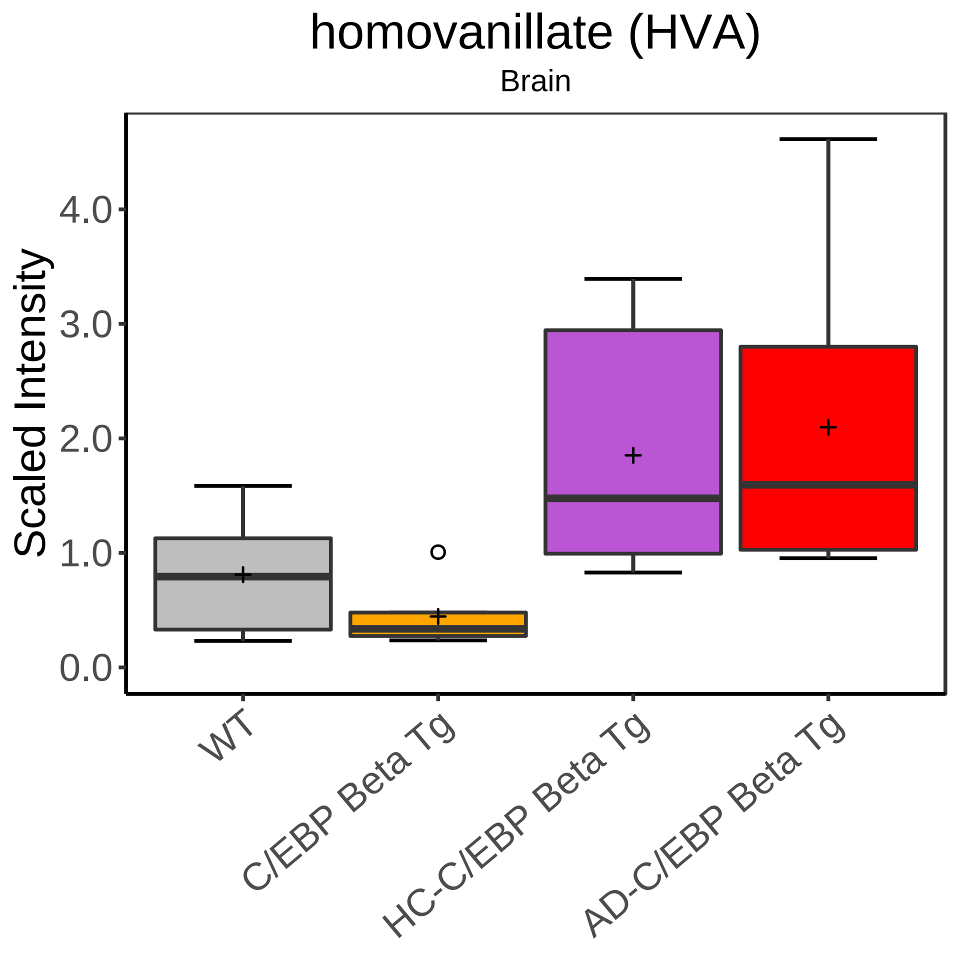
homovanillate (HVA)
Brain
Scaled Intensity
4.0
3.0
2.0
1.0
0.0
WT
C/EBP Beta Tg
HC-C/EBP Beta Tg
AD-C/EBP Beta Tg

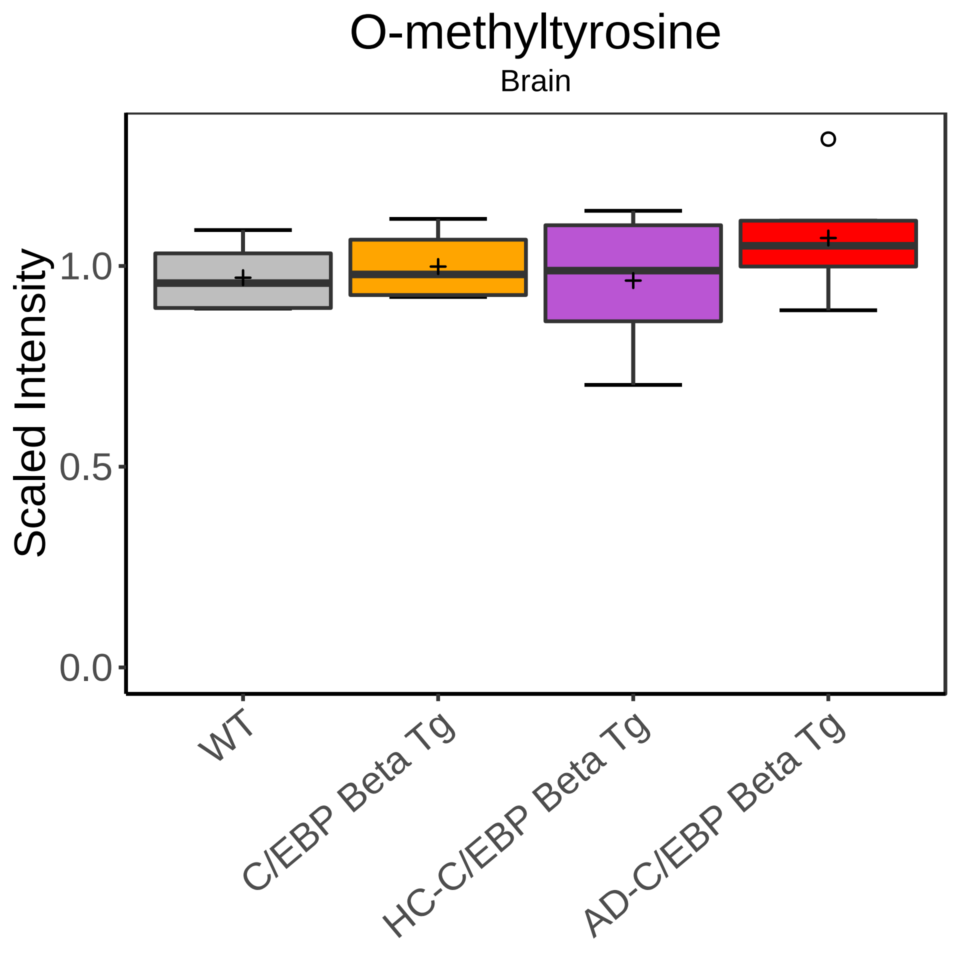
O-methyltyrosine
Brain
Scaled Intensity
0.0
0.5
1.0
WT
C/EBP Beta Tg
HC-C/EBP Beta Tg
AD-C/EBP Beta Tg

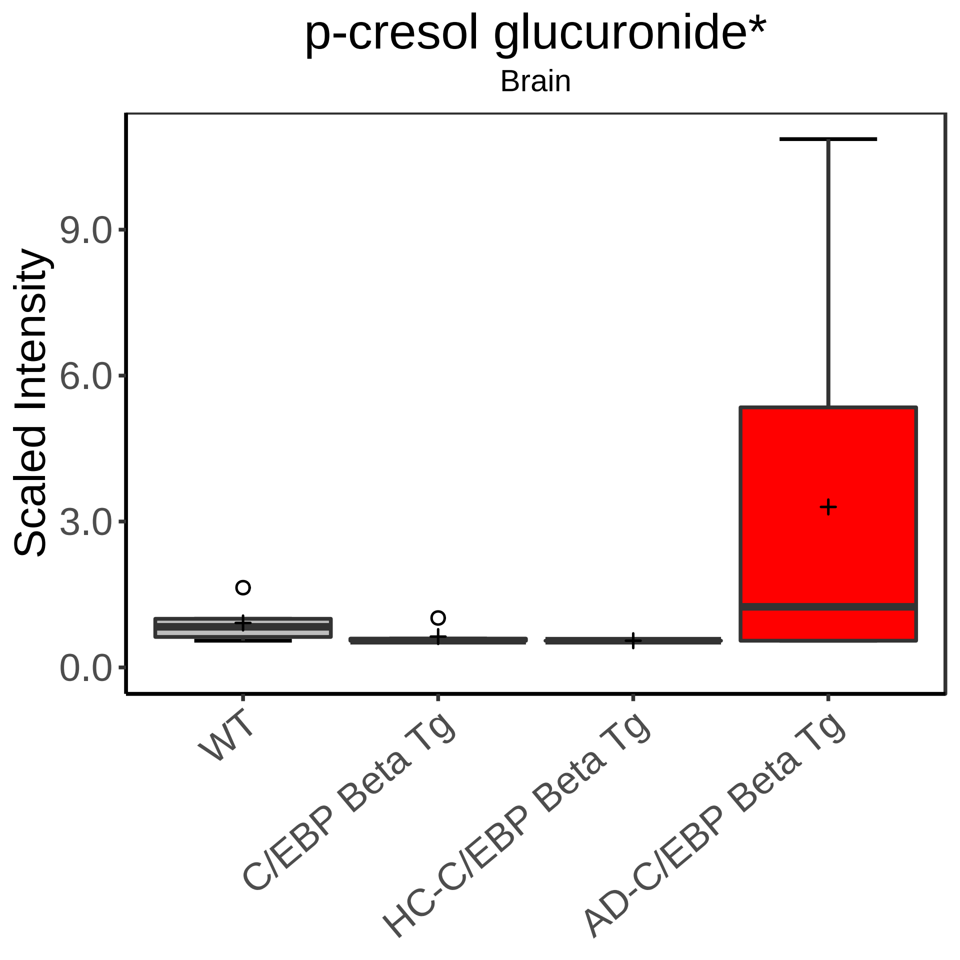
p-cresol glucuronide*
Brain
Scaled Intensity
9.0
6.0
3.0
0.0
WT
C/EBP Beta Tg
HC-C/EBP Beta Tg
AD-C/EBP Beta Tg

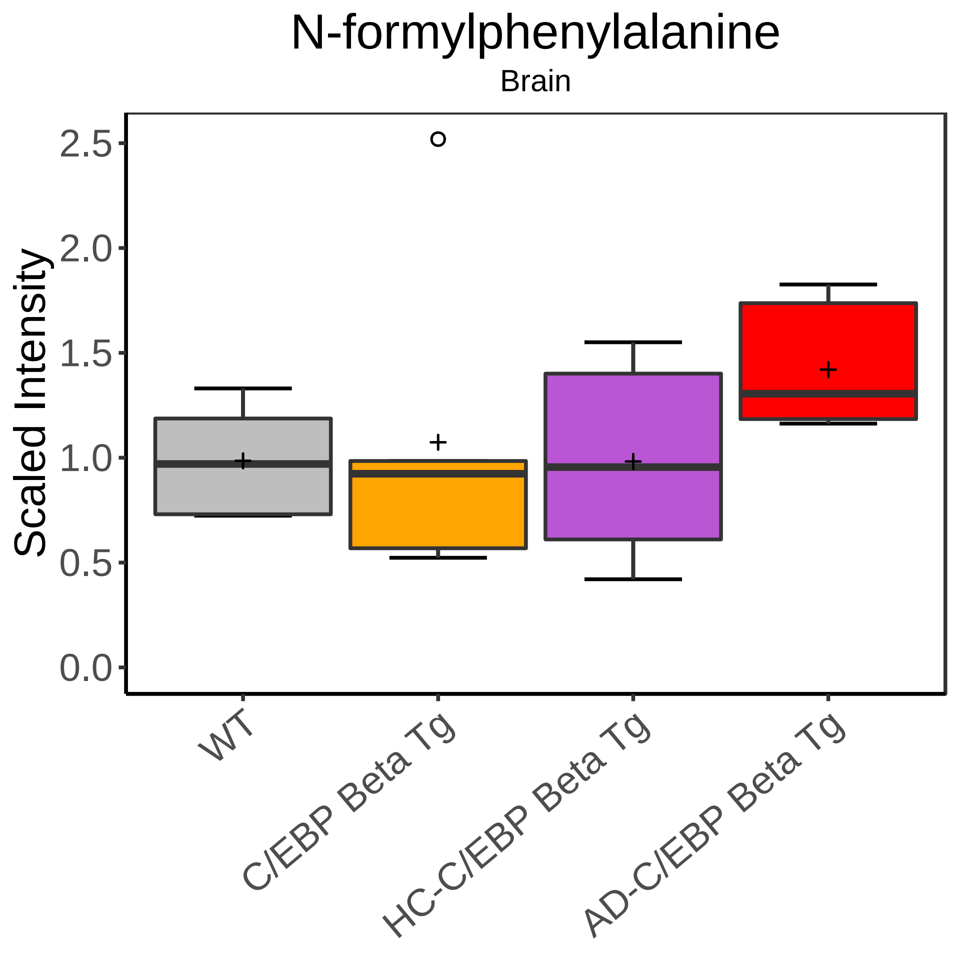

N-formylphenylalanine
Brain
Scaled Intensity
0.0
0.5
1.0
1.5
2.0
2.5
WT
C/EBP Beta Tg
HC-C/EBP Beta Tg
AD-C/EBP Beta Tg

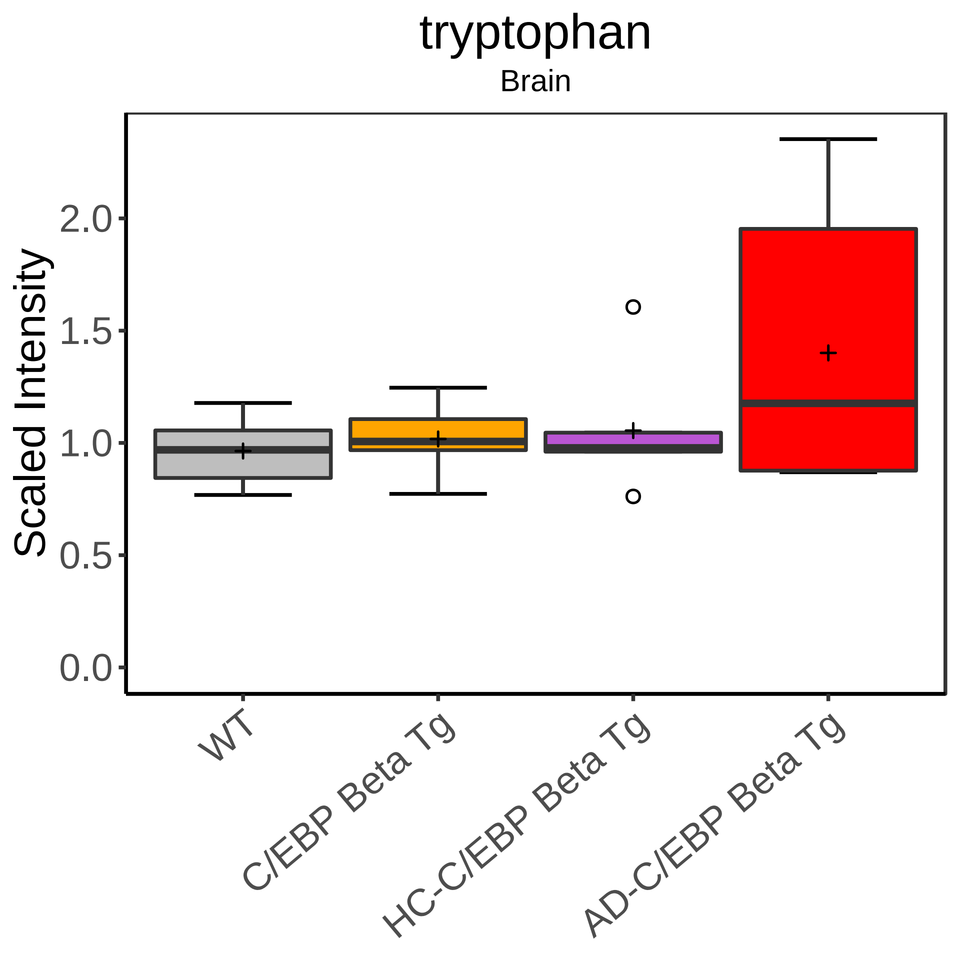
tryptophan
Brain
Scaled Intensity
2.0
1.5
1.0
0.5
0.0
WT
C/EBP Beta Tg
HC-C/EBP Beta Tg
AD-C/EBP Beta Tg

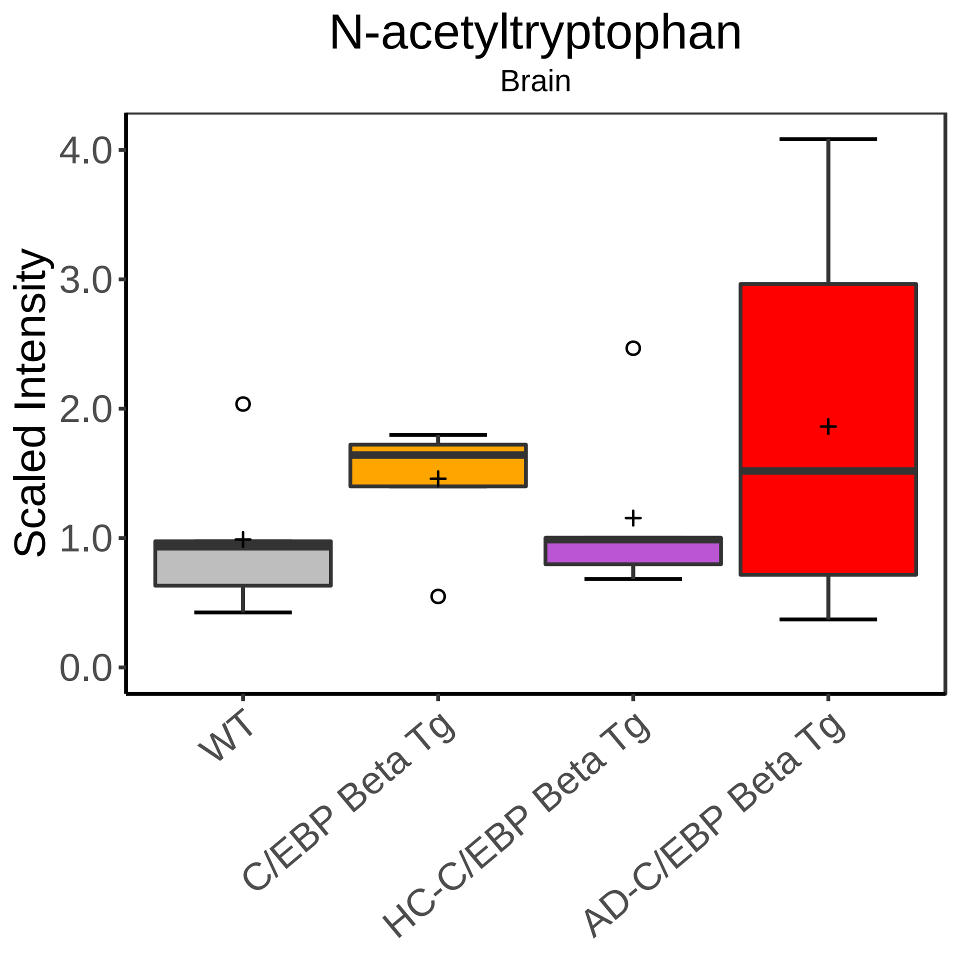
N-acetyltryptophan
Brain
Scaled Intensity
4.0
3.0
2.0
1.0
0.0
WT
C/EBP Beta Tg
HC-C/EBP Beta Tg
AD-C/EBP Beta Tg

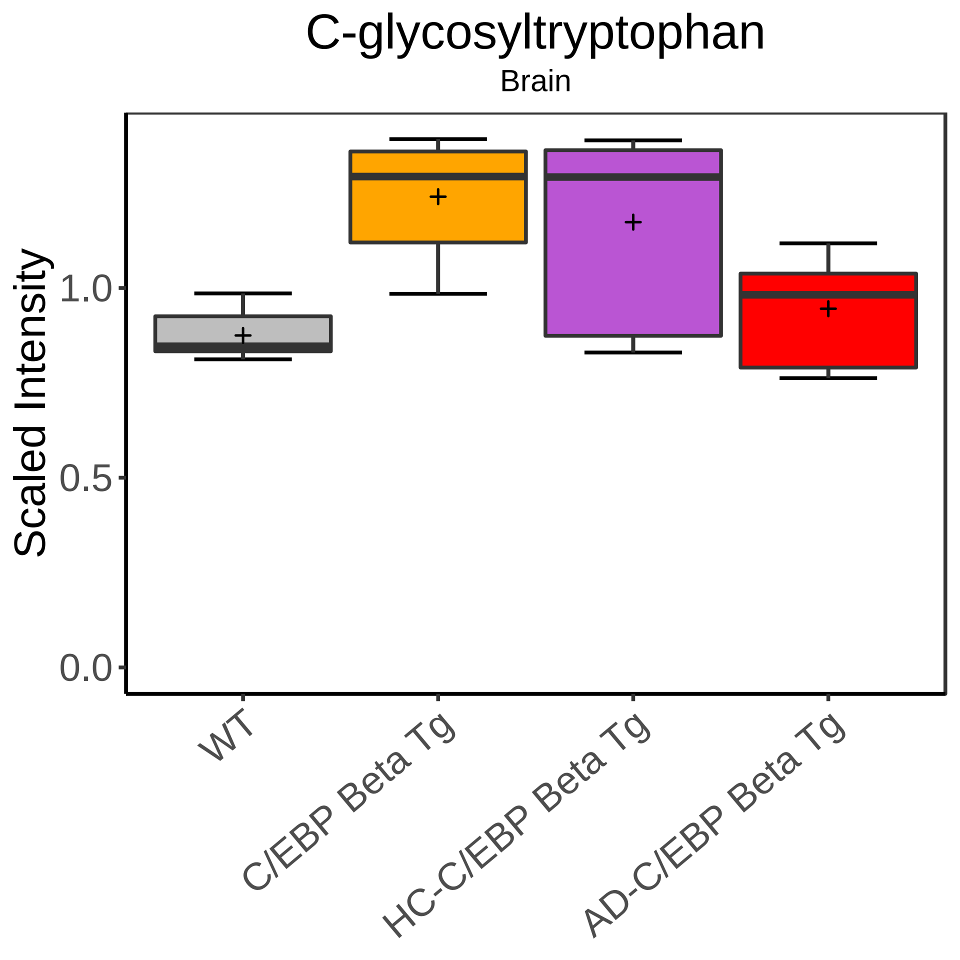
C-glycosyltryptophan
Brain
Scaled Intensity
1.0
0.5
0.0
WT
C/EBP Beta Tg
HC-C/EBP Beta Tg
AD-C/EBP Beta Tg

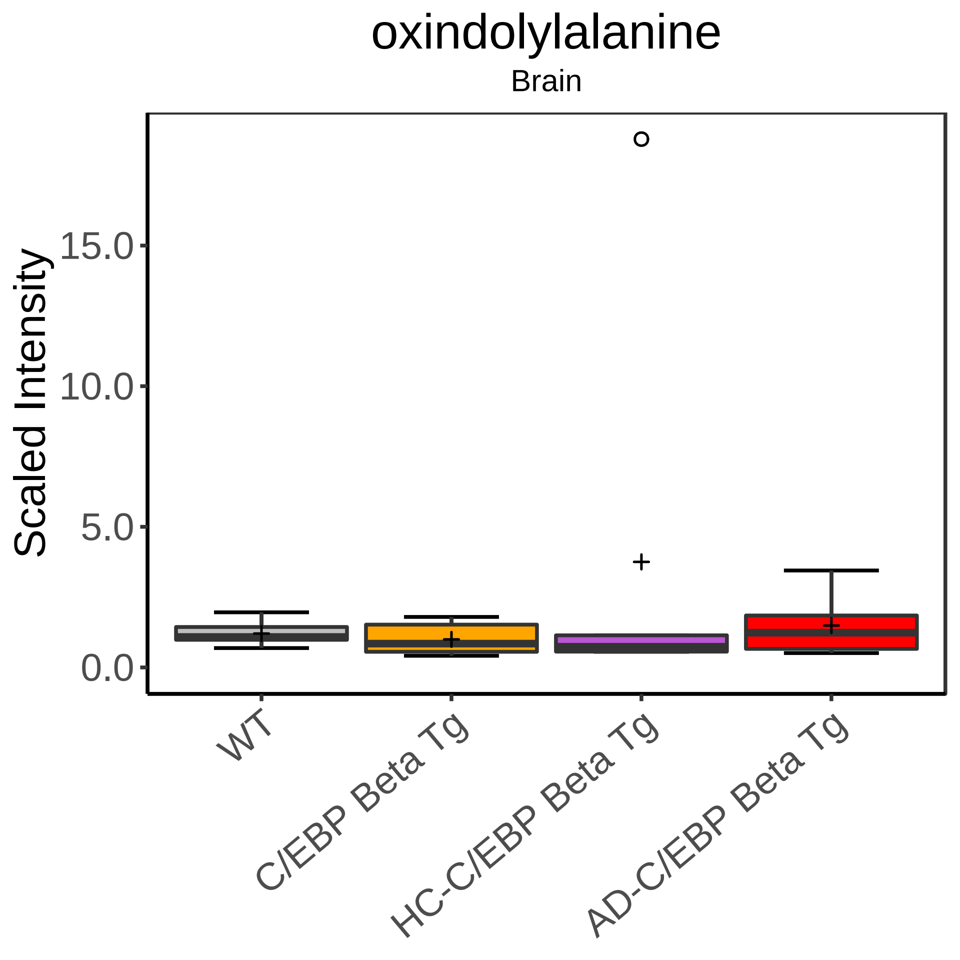
oxindolylalanine
Brain
Scaled Intensity
15.0
10.0
5.0
0.0
WT
C/EBP Beta Tg
HC-C/EBP Beta Tg
AD-C/EBP Beta Tg

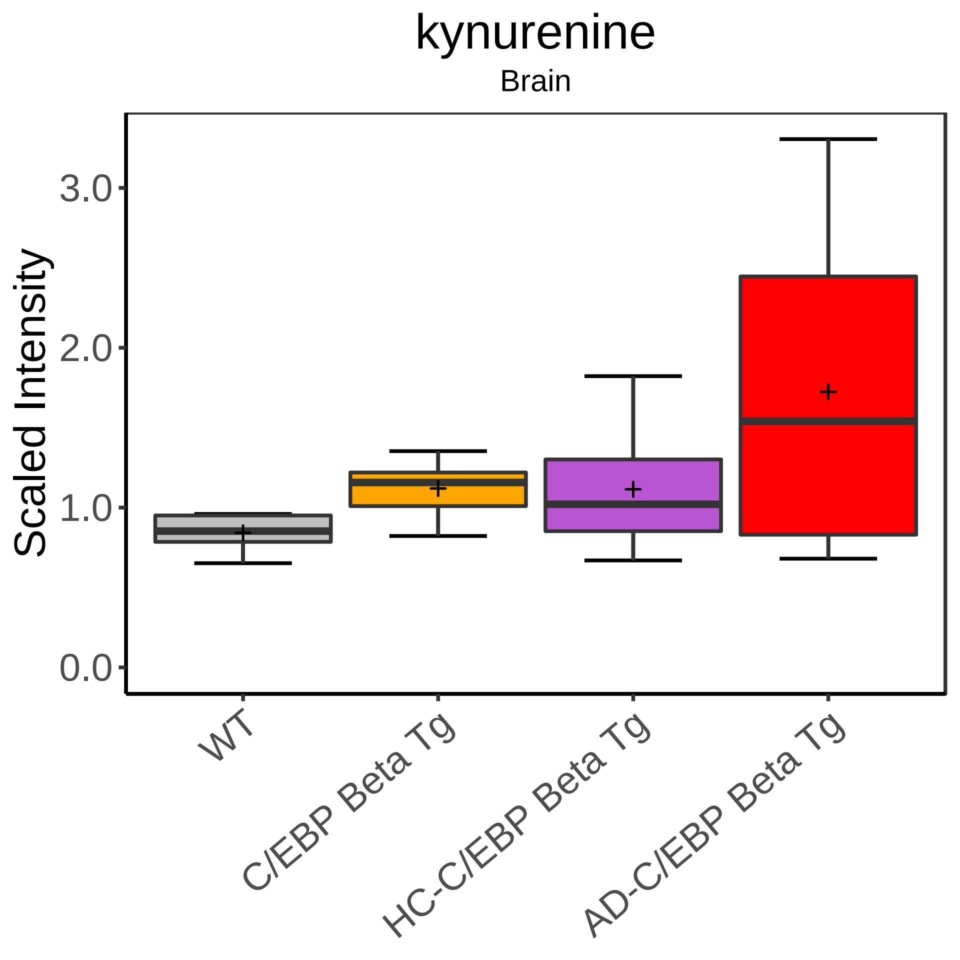

kynurenine
Brain
Scaled Intensity
0.0
1.0
2.0
3.0
WT
C/EBP Beta Tg
HC-C/EBP Beta Tg
AD-C/EBP Beta Tg

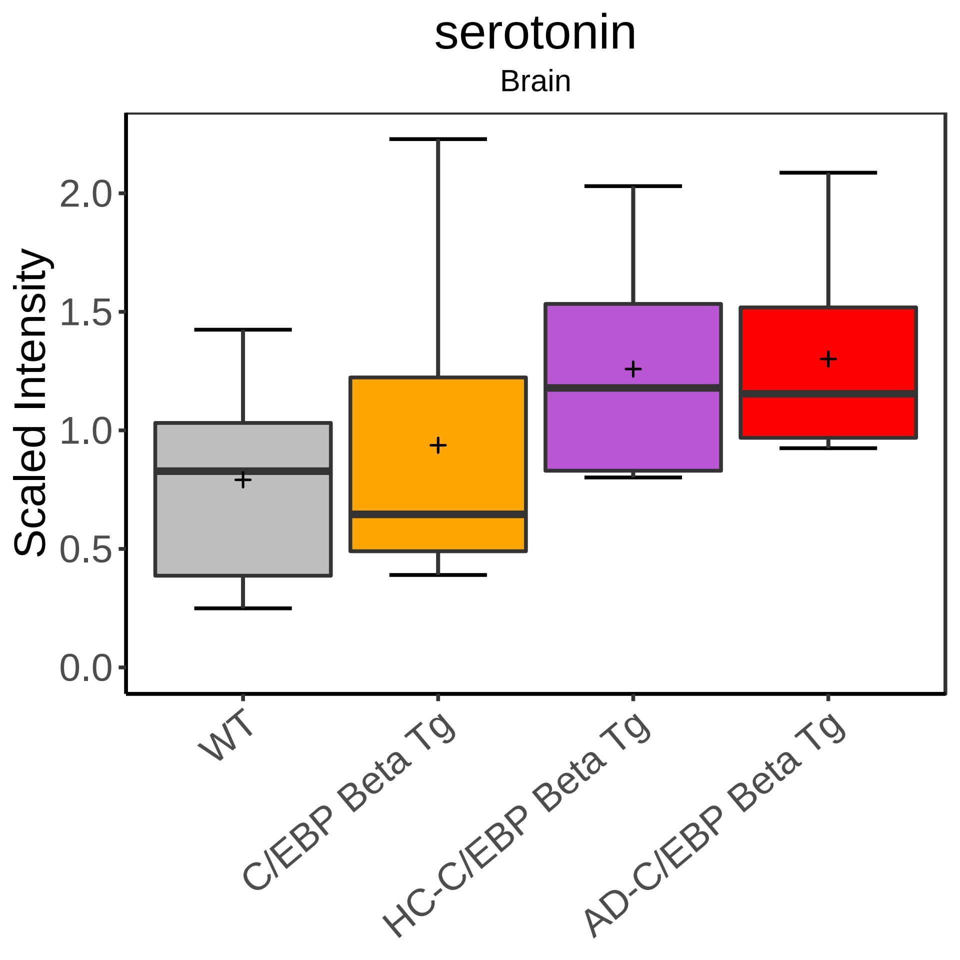

serotonin
Brain
Scaled Intensity
2.0
1.5
1.0
0.5
0.0
WT
C/EBP Beta Tg
HC-C/EBP Beta Tg
AD-C/EBP Beta Tg

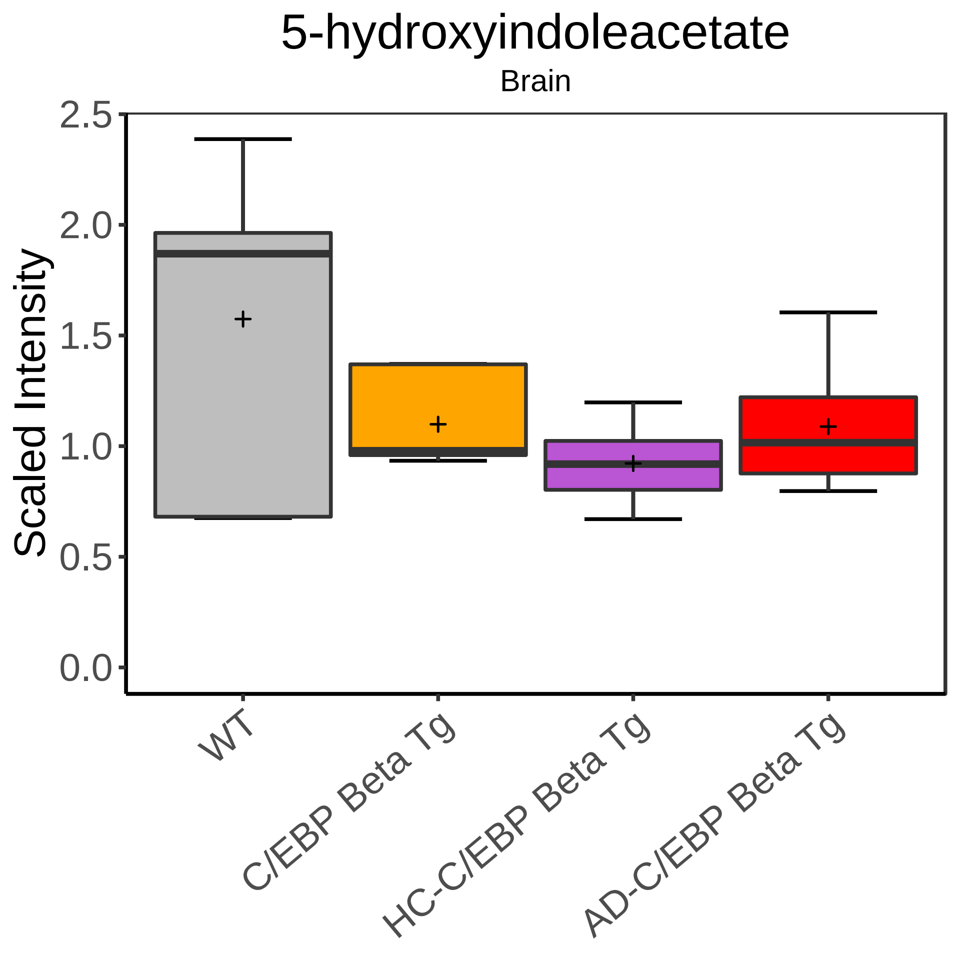

5-hydroxyindoleacetate
Brain
Scaled Intensity
2.5
2.0
1.5
1.0
0.5
0.0
WT
C/EBP Beta Tg
HC-C/EBP Beta Tg
AD-C/EBP Beta Tg

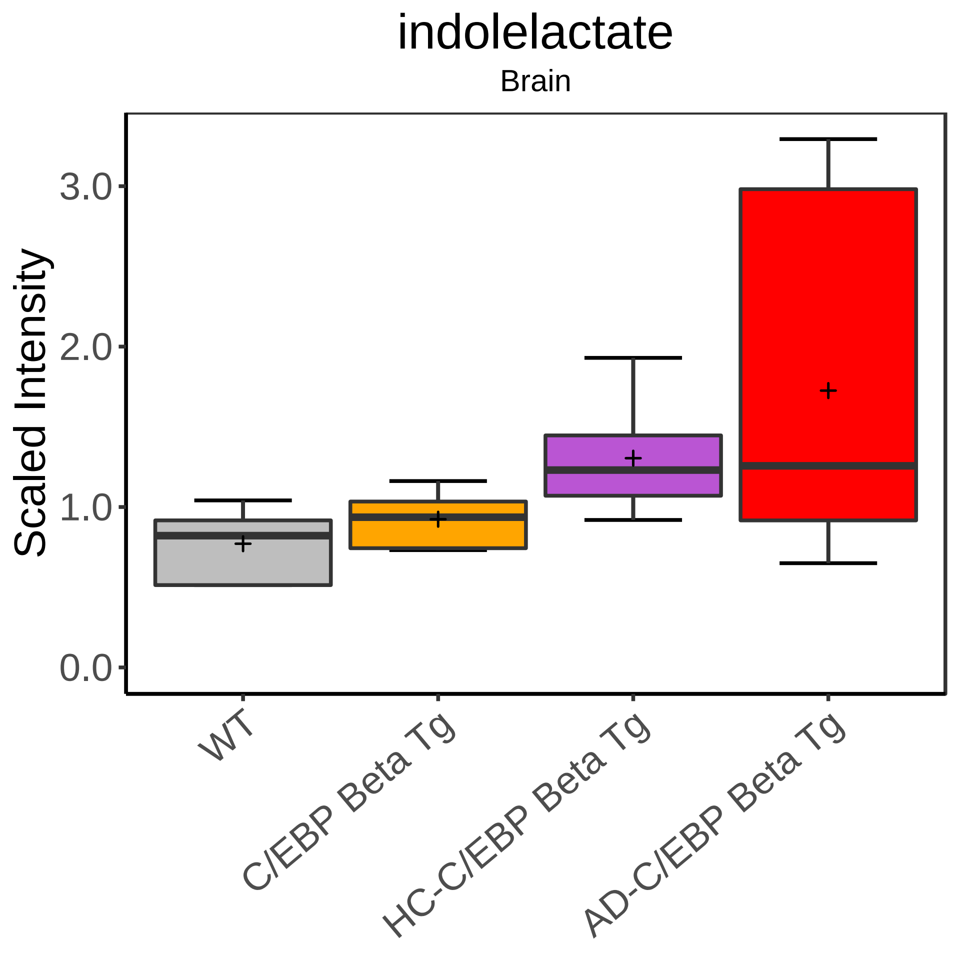

indolelactate
Brain
Scaled Intensity
3.0
2.0
1.0
0.0
WT
C/EBP Beta Tg
HC-C/EBP Beta Tg
AD-C/EBP Beta Tg

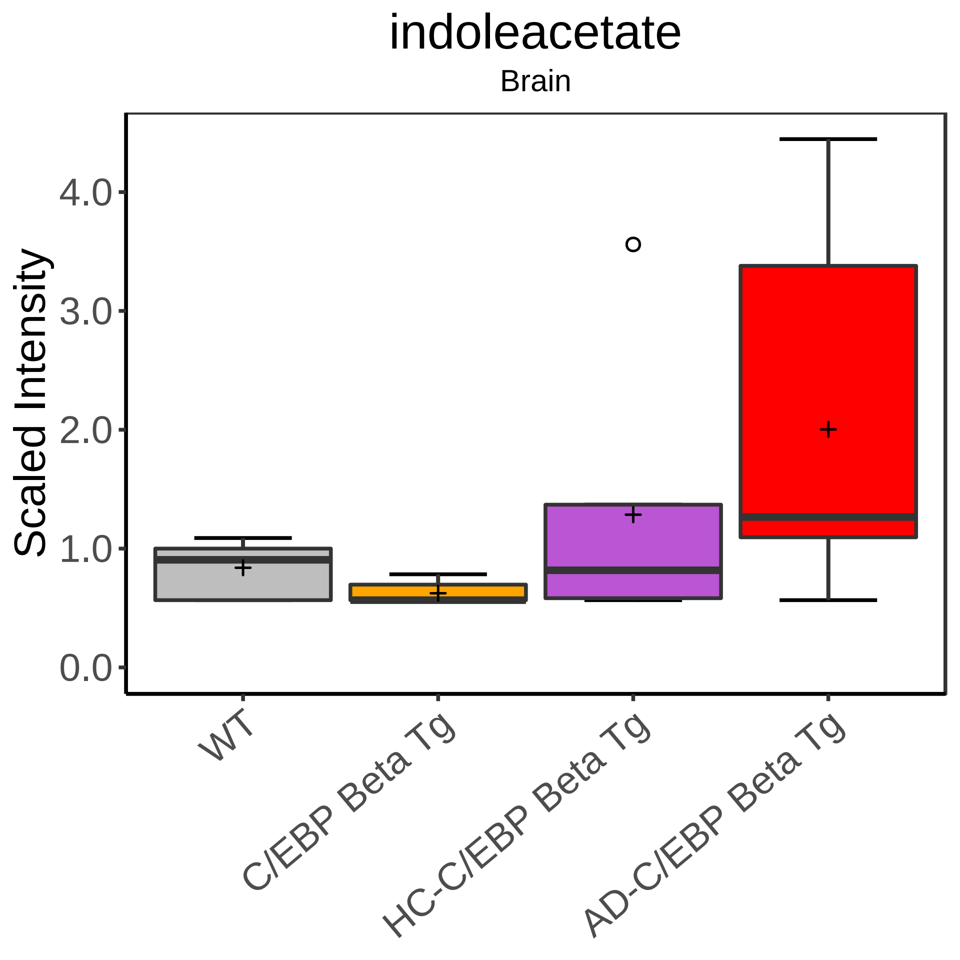
indoleacetate
Brain
Scaled Intensity
4.0
3.0
2.0
1.0
0.0
WT
C/EBP Beta Tg
HC-C/EBP Beta Tg
AD-C/EBP Beta Tg

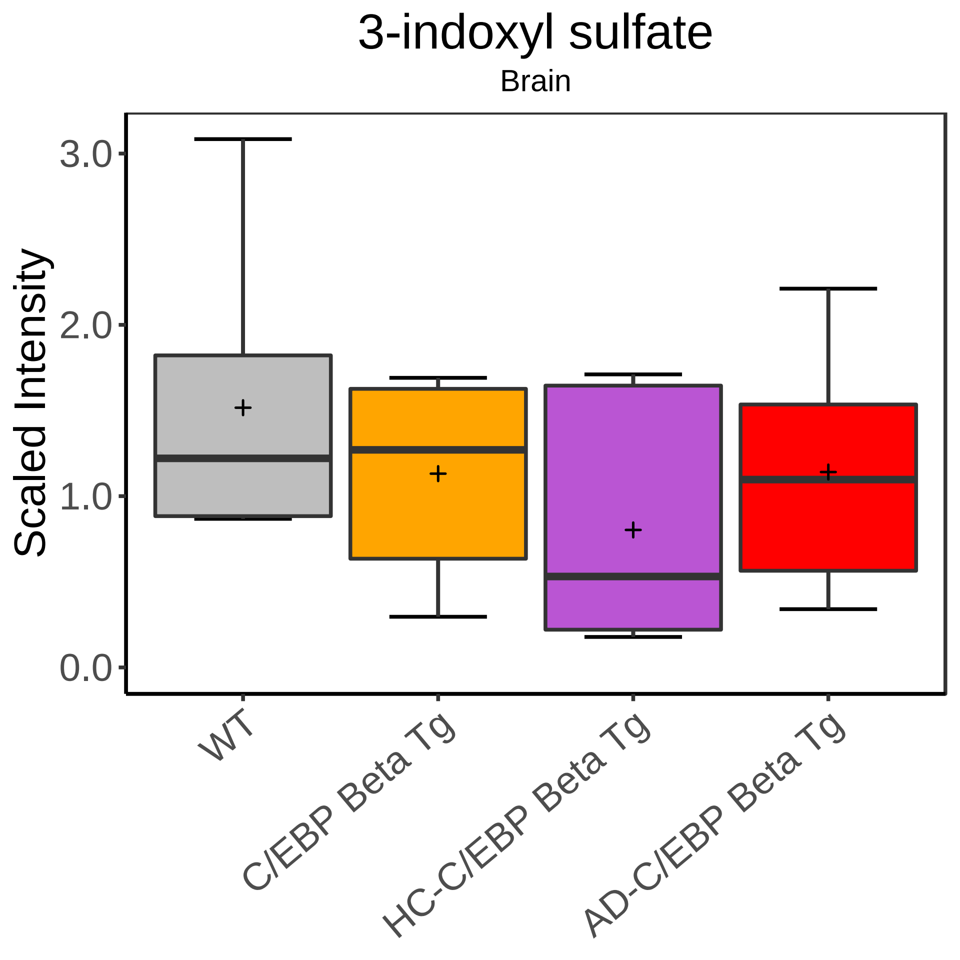

3-indoxyl sulfate
Brain
Scaled Intensity
3.0
2.0
1.0
0.0
WT
C/EBP Beta Tg
HC-C/EBP Beta Tg
AD-C/EBP Beta Tg

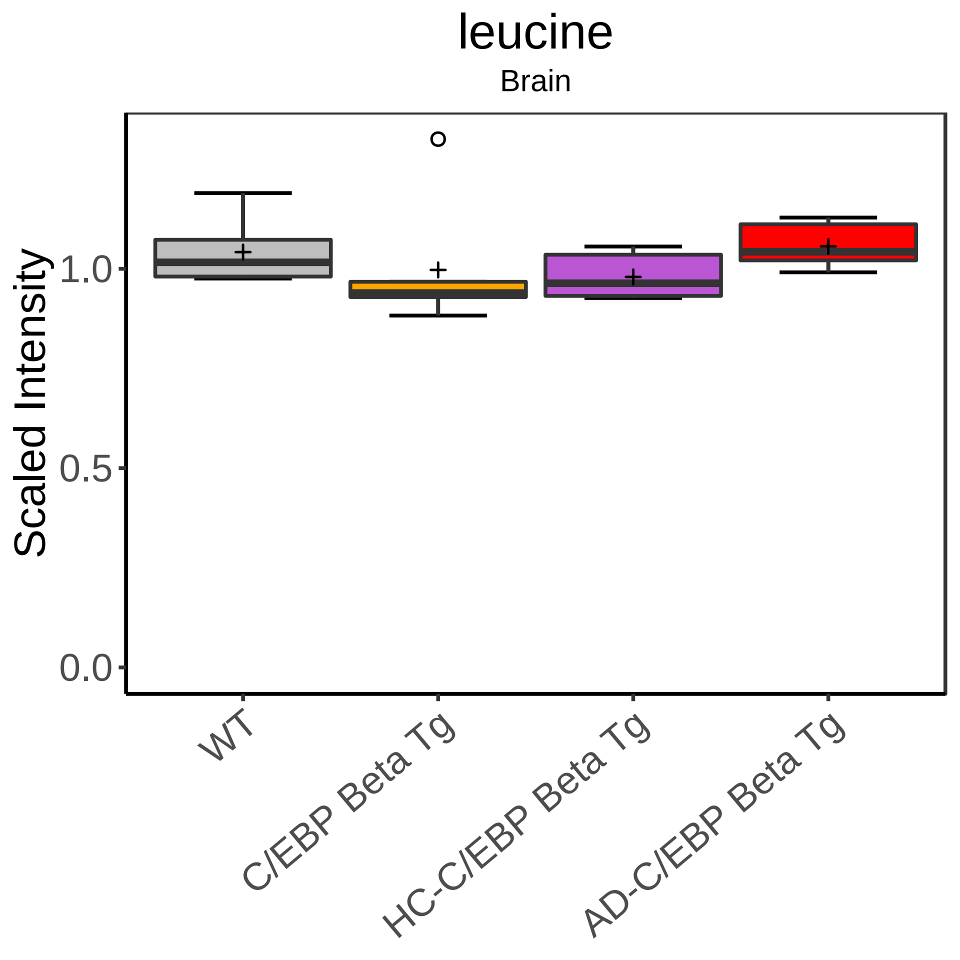
leucine
Brain
Scaled Intensity
0.0
0.5
1.0
WT
C/EBP Beta Tg
HC-C/EBP Beta Tg
AD-C/EBP Beta Tg

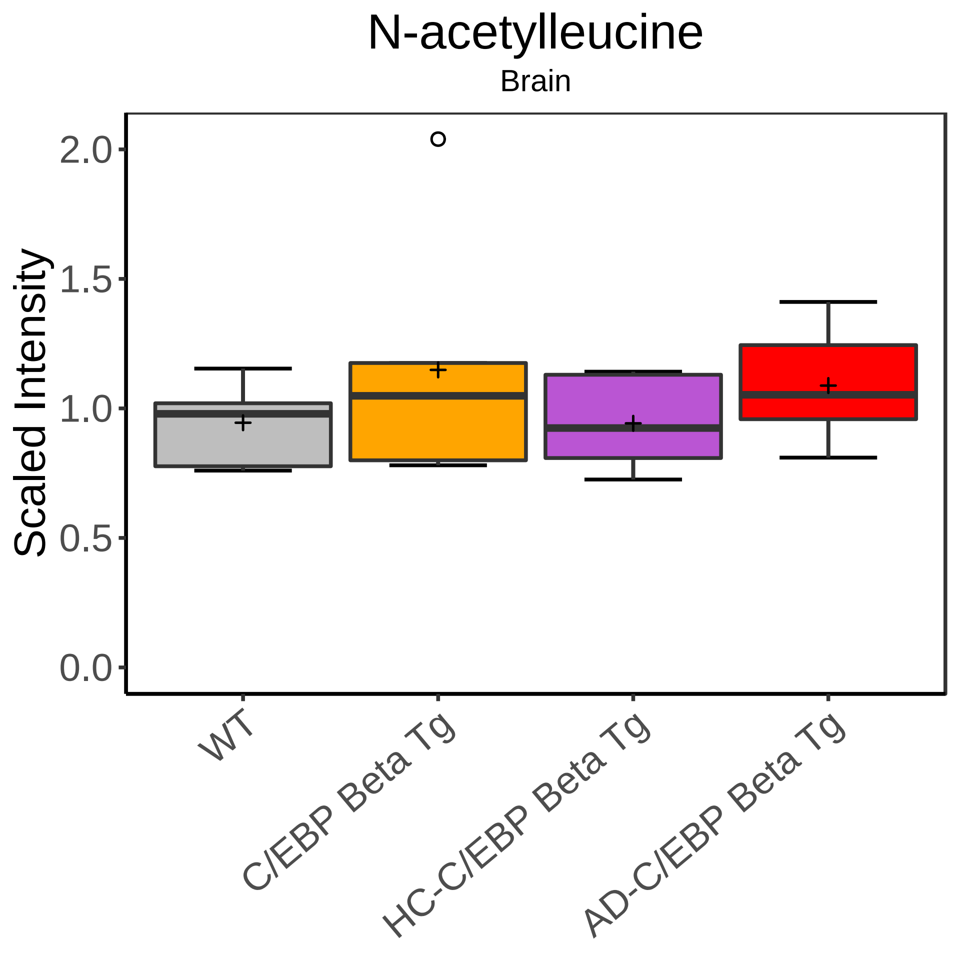
N-acetylleucine
Brain
Scaled Intensity
2.0
1.5
1.0
0.5
0.0
WT
C/EBP Beta Tg
HC-C/EBP Beta Tg
AD-C/EBP Beta Tg

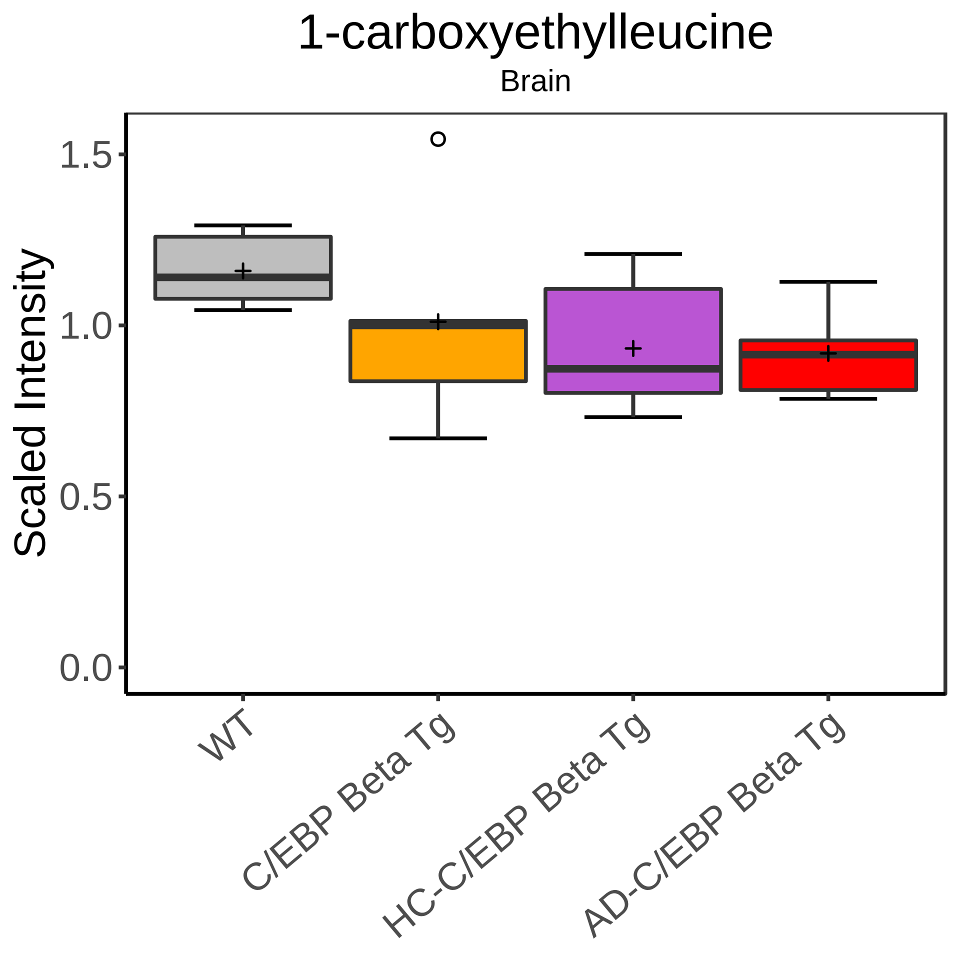
1-carboxyethylleucine
Brain
Scaled Intensity
1.5
1.0
0.5
0.0
WT
C/EBP Beta Tg
HC-C/EBP Beta Tg
AD-C/EBP Beta Tg

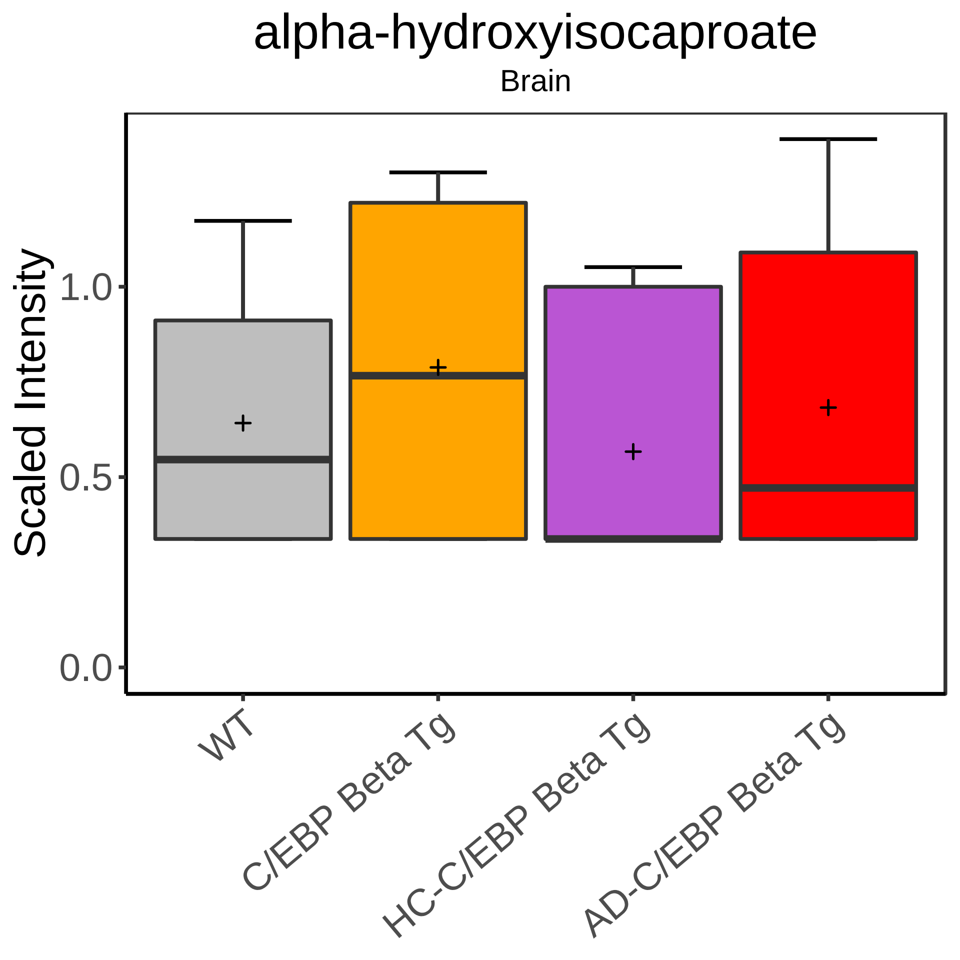

alpha-hydroxyisocaproate
Brain
Scaled Intensity
0.0
0.5
1.0
WT
C/EBP Beta Tg
HC-C/EBP Beta Tg
AD-C/EBP Beta Tg

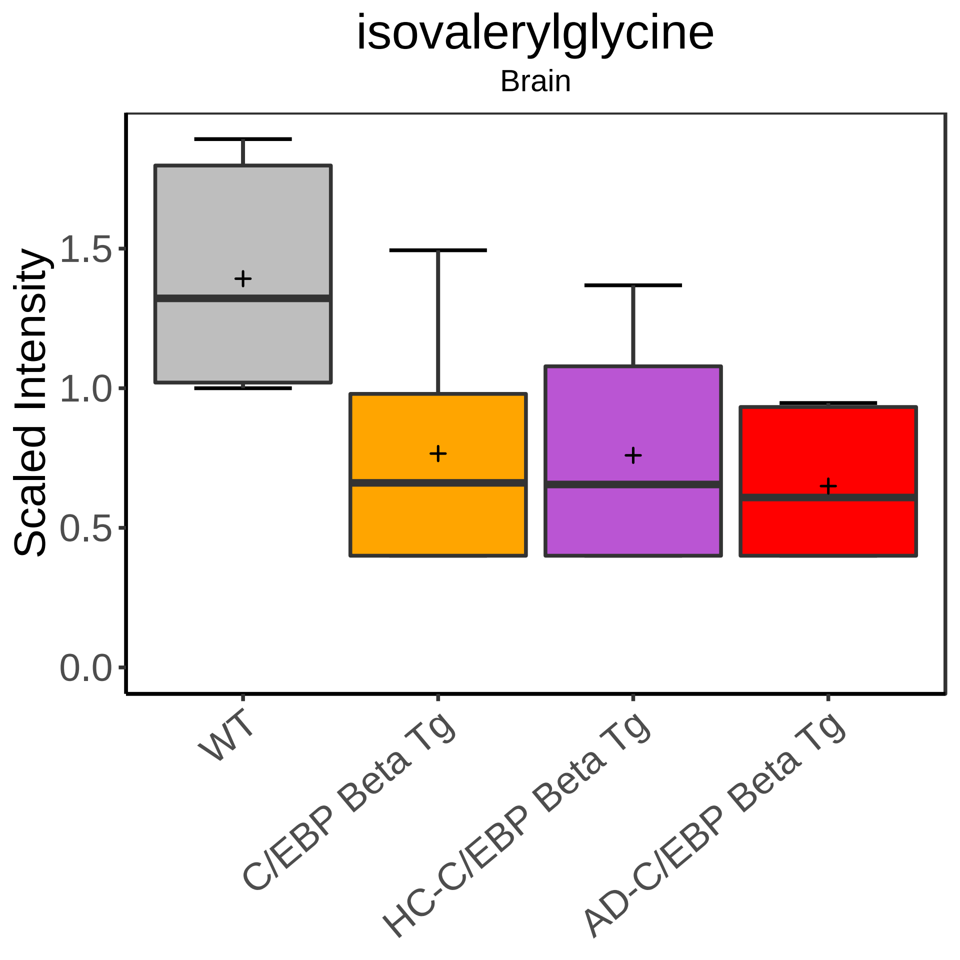

isovalerylglycine
Brain
Scaled Intensity
0.0
0.5
1.0
1.5
WT
C/EBP Beta Tg
HC-C/EBP Beta Tg
AD-C/EBP Beta Tg

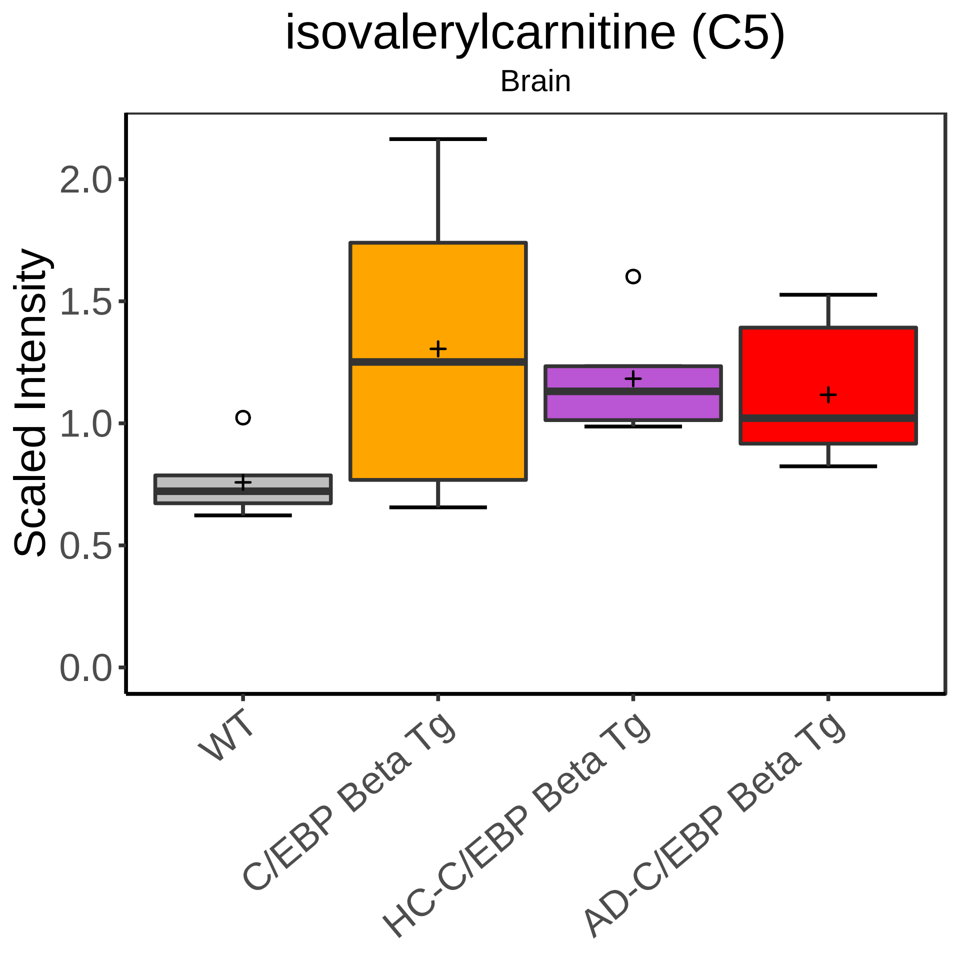
isovalerylcarnitine (C5)
Brain
Scaled Intensity
2.0
1.5
1.0
0.5
0.0
WT
C/EBP Beta Tg
HC-C/EBP Beta Tg
AD-C/EBP Beta Tg

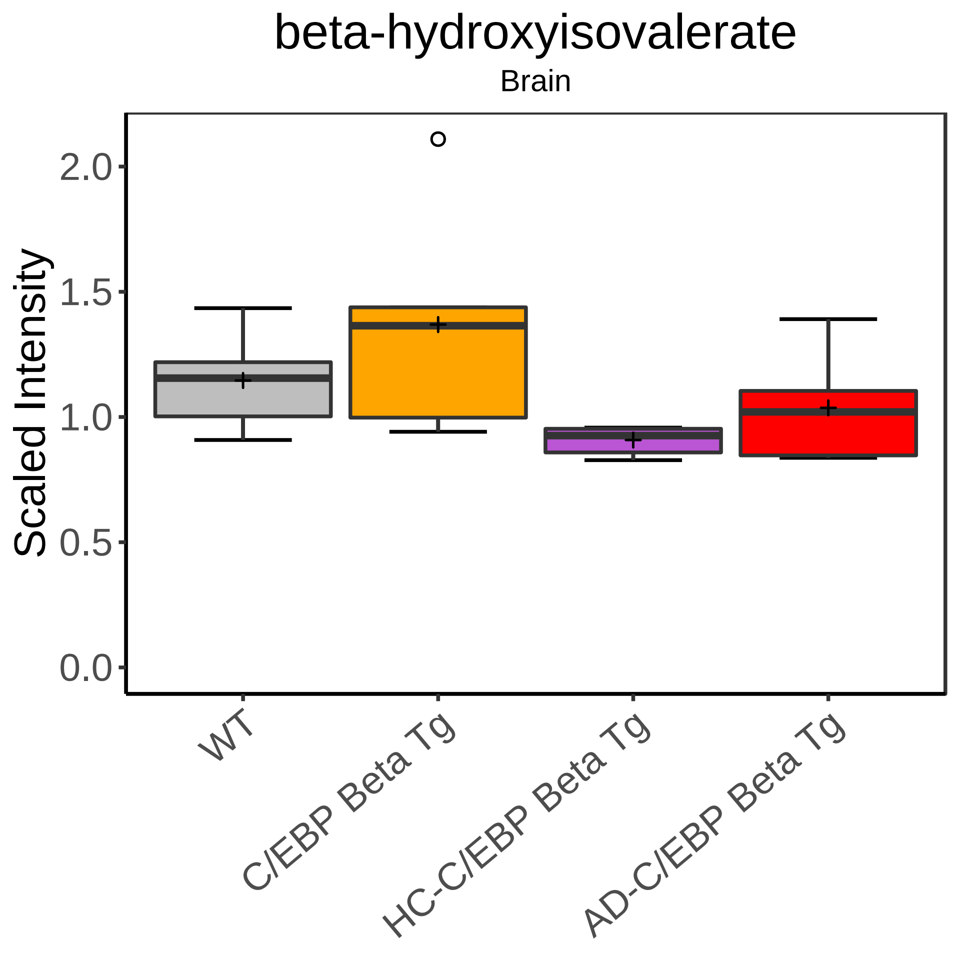
beta-hydroxyisovalerate
Brain
Scaled Intensity
2.0
1.5
1.0
0.5
0.0
WT
C/EBP Beta Tg
HC-C/EBP Beta Tg
AD-C/EBP Beta Tg

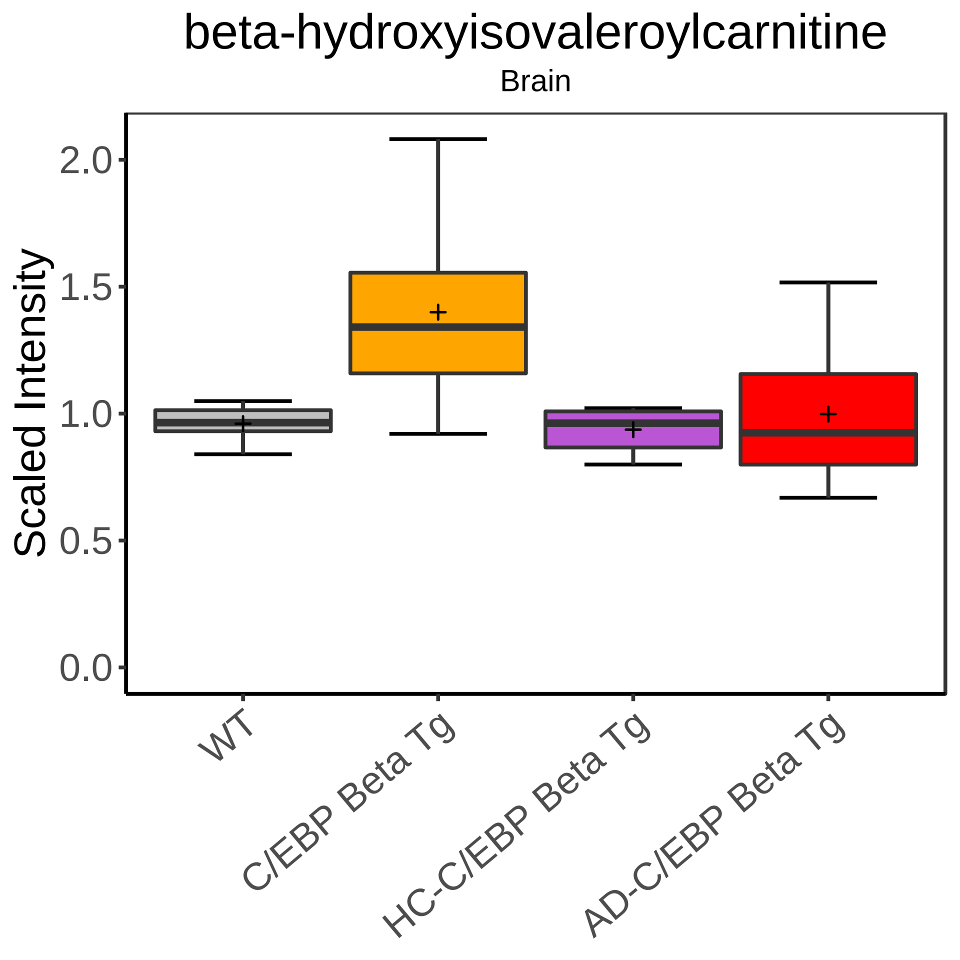

beta-hydroxyisovaleroylcarnitine
Brain
Scaled Intensity
2.0
1.5
1.0
0.5
0.0
WT
C/EBP Beta Tg
HC-C/EBP Beta Tg
AD-C/EBP Beta Tg

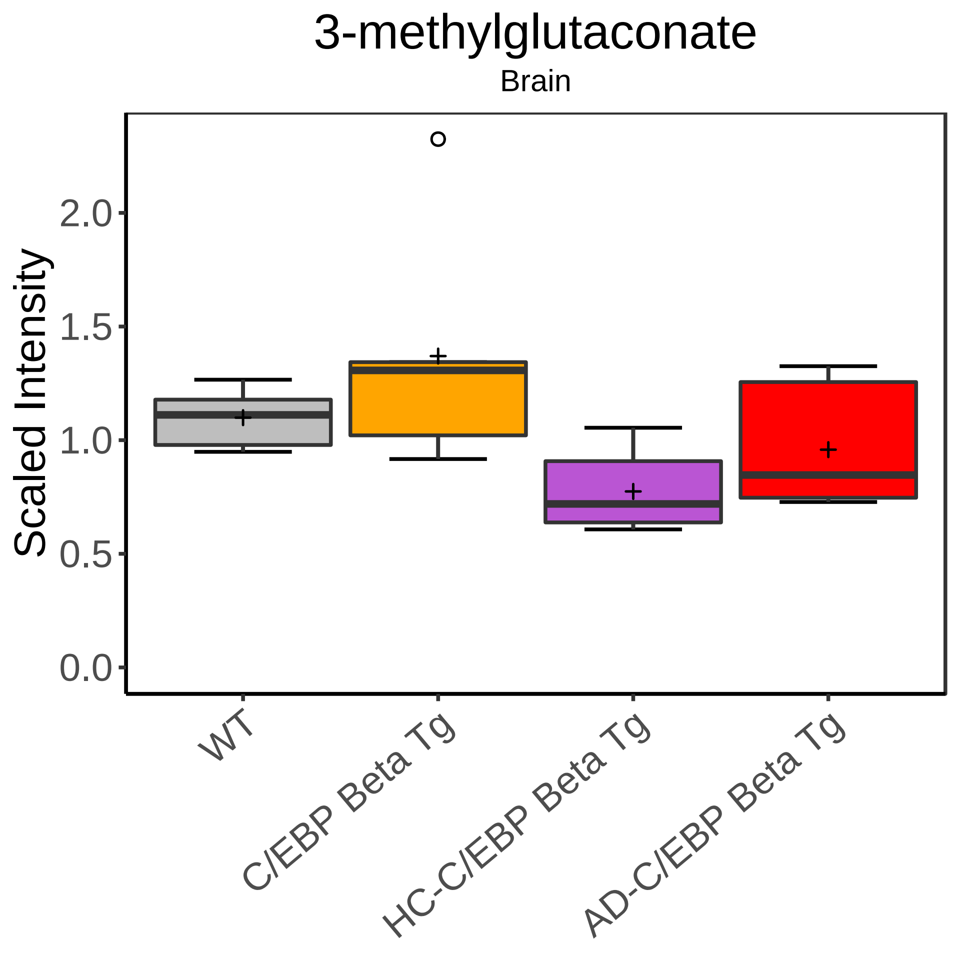
3-methylglutaconate
Brain
Scaled Intensity
2.0
1.5
1.0
0.5
0.0
WT
C/EBP Beta Tg
HC-C/EBP Beta Tg
AD-C/EBP Beta Tg

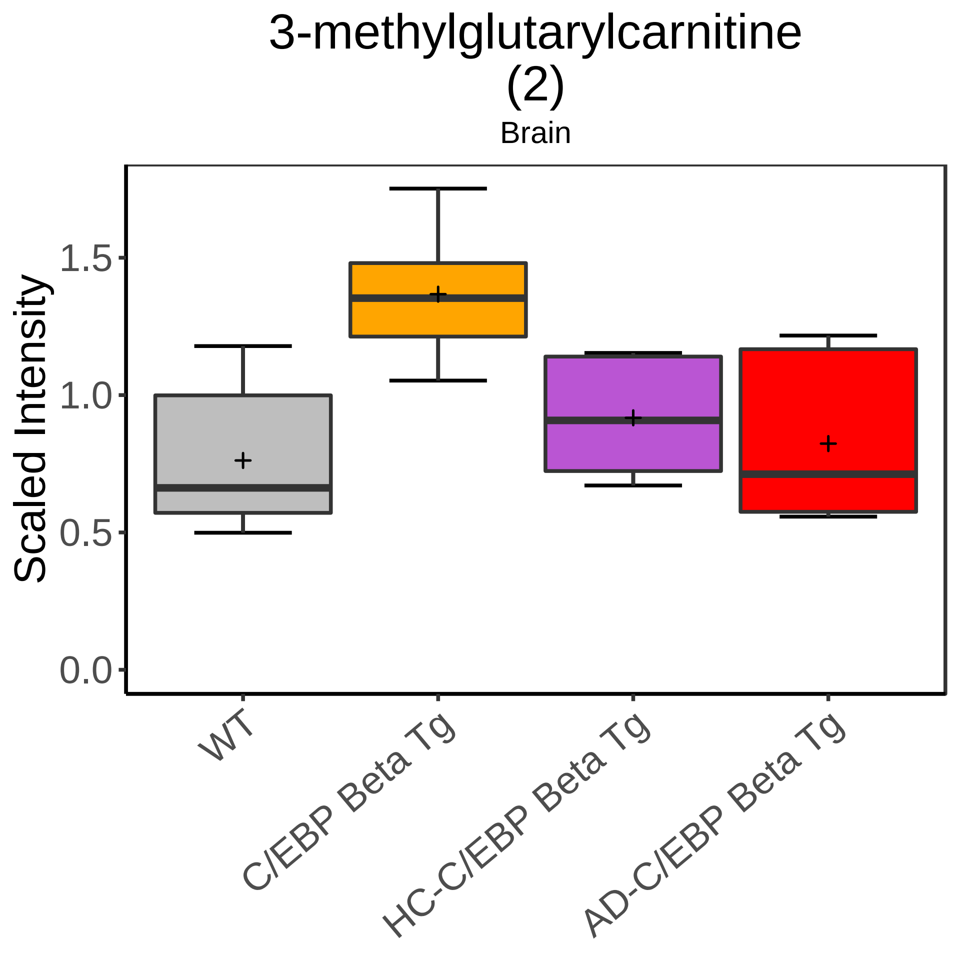
3-methylglutarylcarnitine
(2)
Brain
Scaled Intensity
0.0
0.5
1.0
1.5
WT
C/EBP Beta Tg
HC-C/EBP Beta Tg
AD-C/EBP Beta Tg

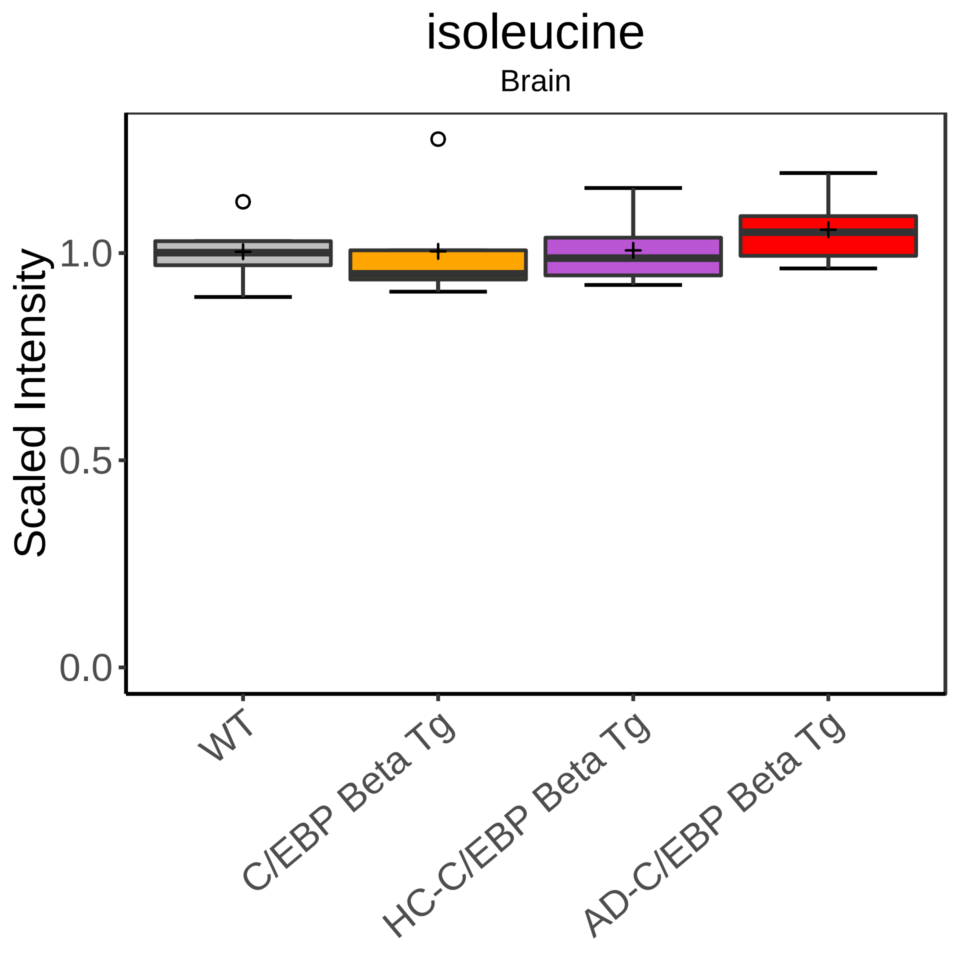
isoleucine
Brain
Scaled Intensity
1.0
0.5
0.0
WT
C/EBP Beta Tg
HC-C/EBP Beta Tg
AD-C/EBP Beta Tg

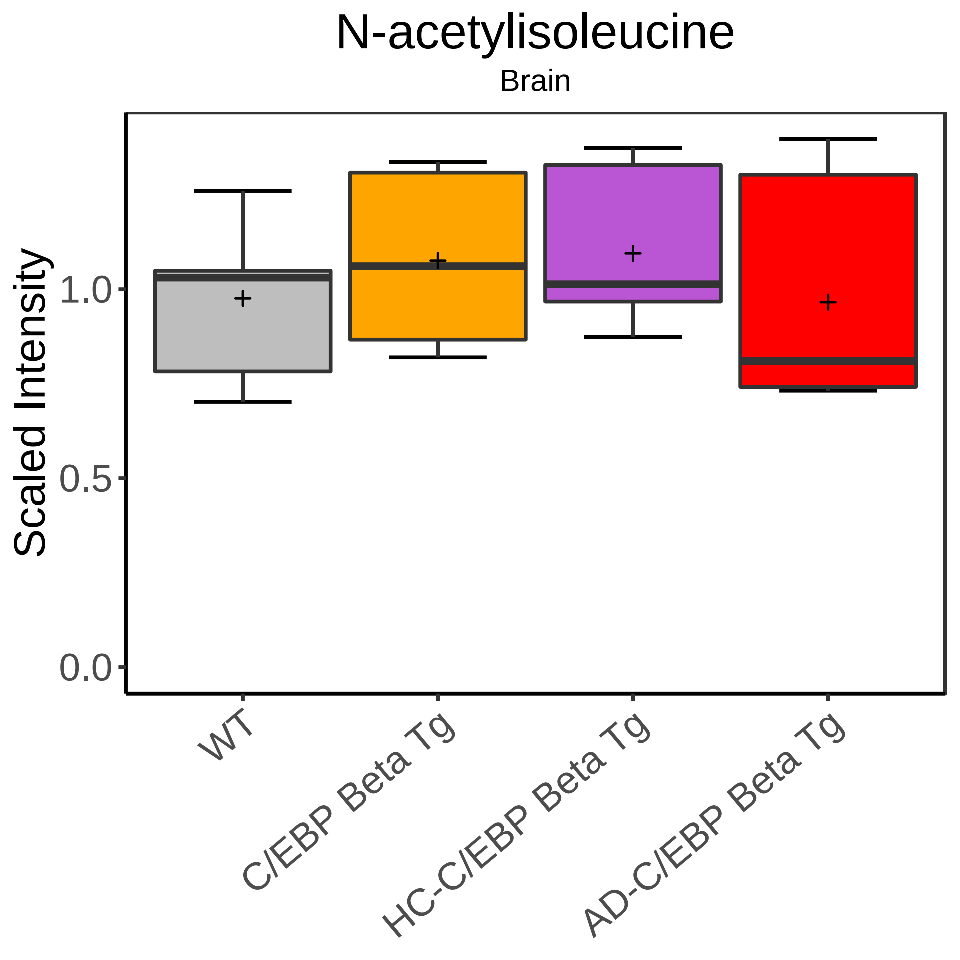
N-acetylisoleucine
Brain
Scaled Intensity
1.0
0.5
0.0
WT
C/EBP Beta Tg
HC-C/EBP Beta Tg
AD-C/EBP Beta Tg

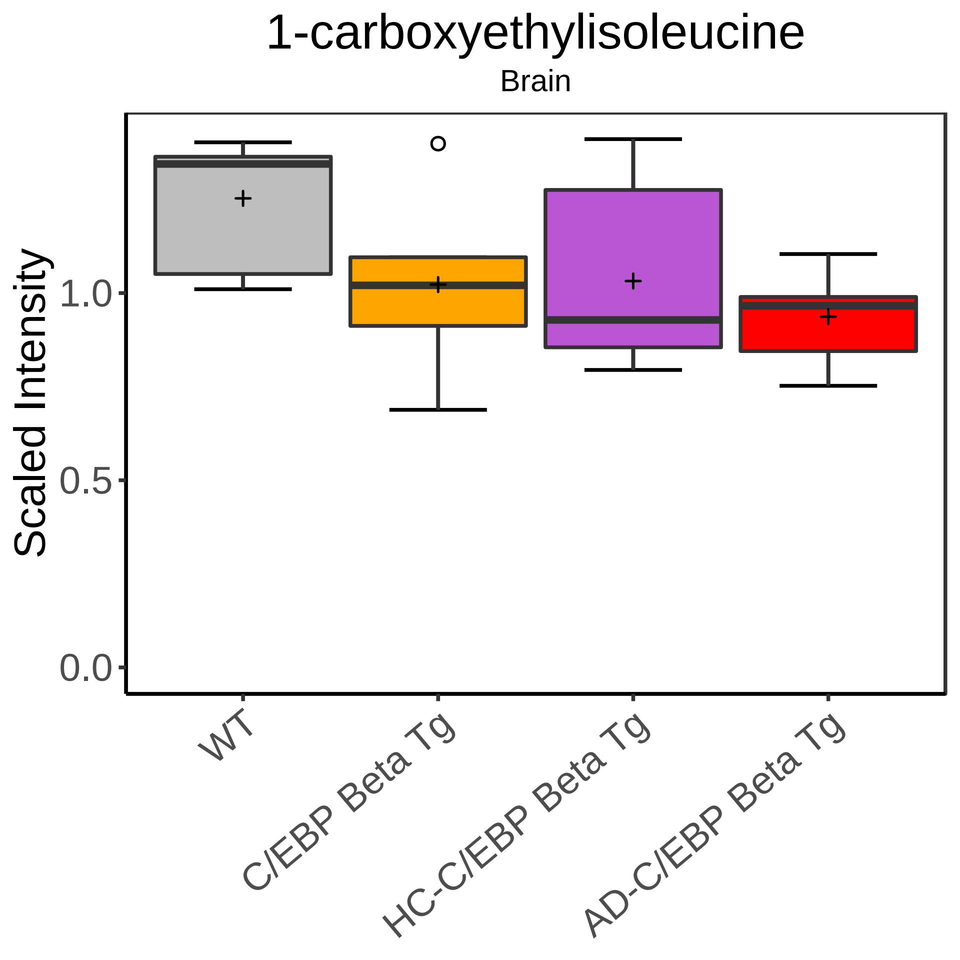

1-carboxyethylisoleucine
Brain
Scaled Intensity
0.0
0.5
1.0
WT
C/EBP Beta Tg
HC-C/EBP Beta Tg
AD-C/EBP Beta Tg

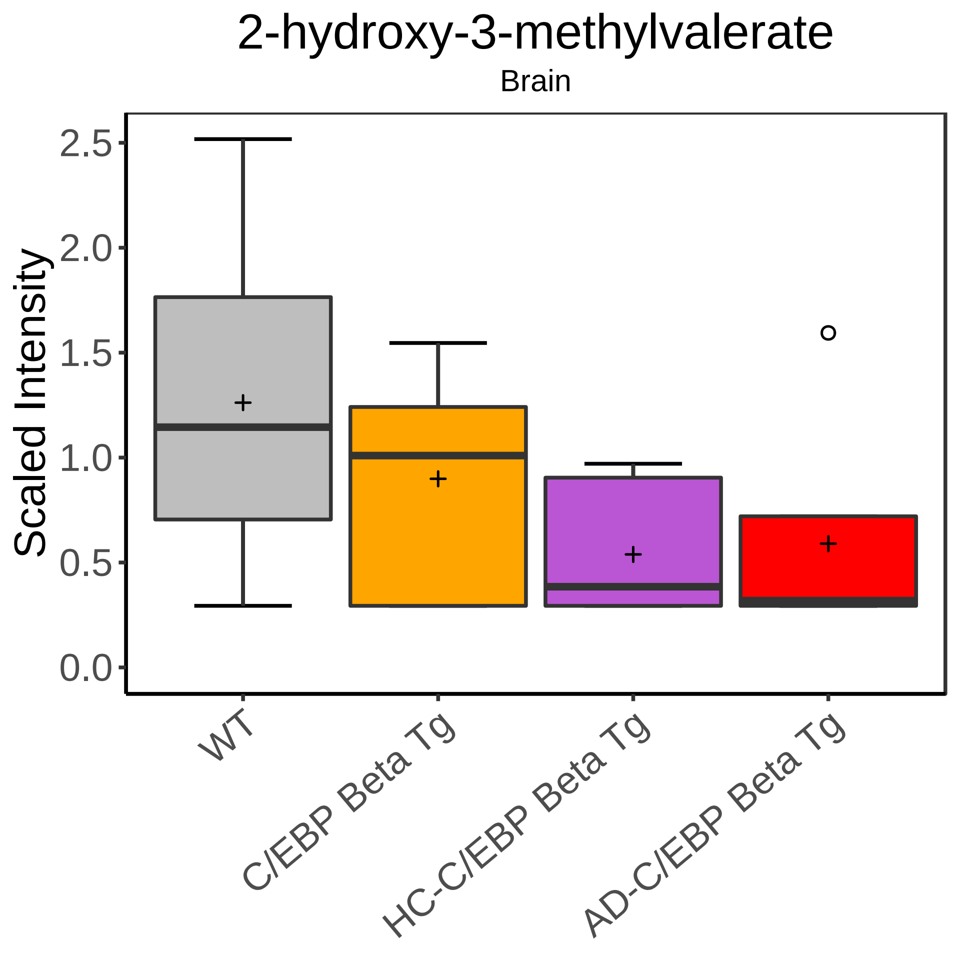
2-hydroxy-3-methylvalerate
Brain
Scaled Intensity
0.0
0.5
1.0
1.5
2.0
2.5
WT
C/EBP Beta Tg
HC-C/EBP Beta Tg
AD-C/EBP Beta Tg

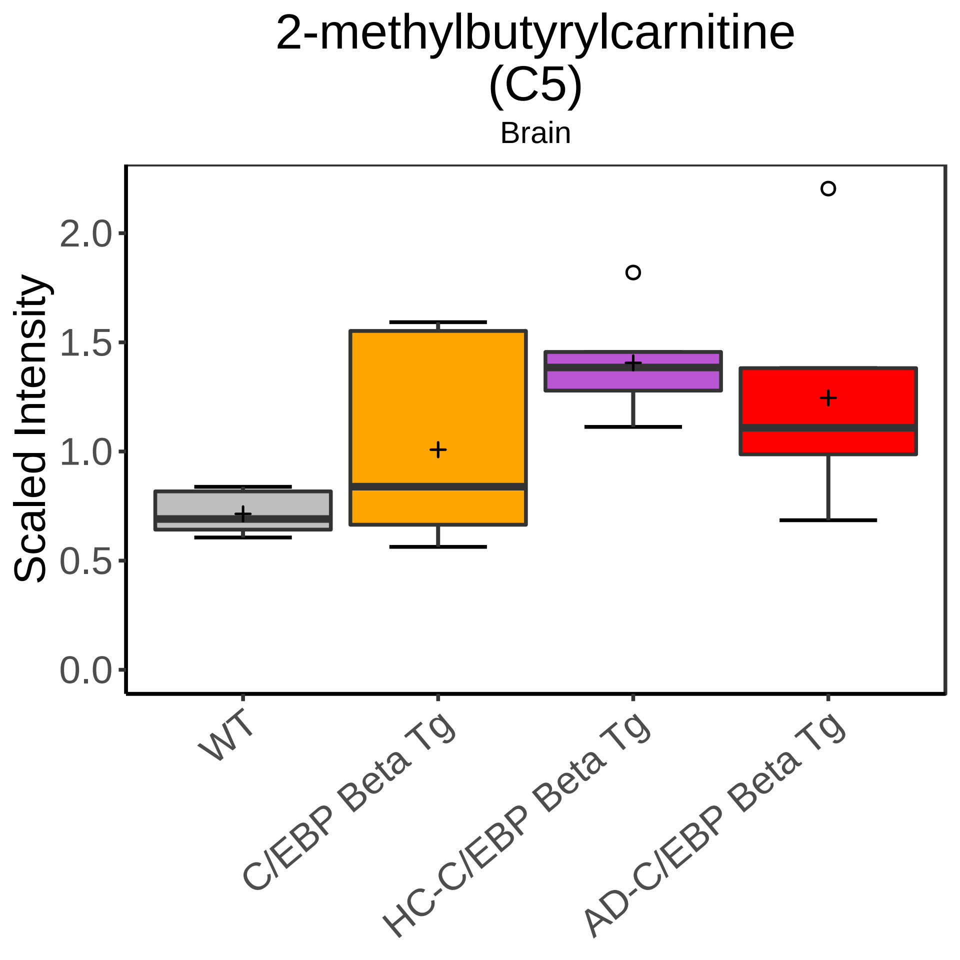
2-methylbutyrylcarnitine
(C5)
Brain
Scaled Intensity
0.0
0.5
1.0
1.5
2.0
WT
C/EBP Beta Tg
HC-C/EBP Beta Tg
AD-C/EBP Beta Tg

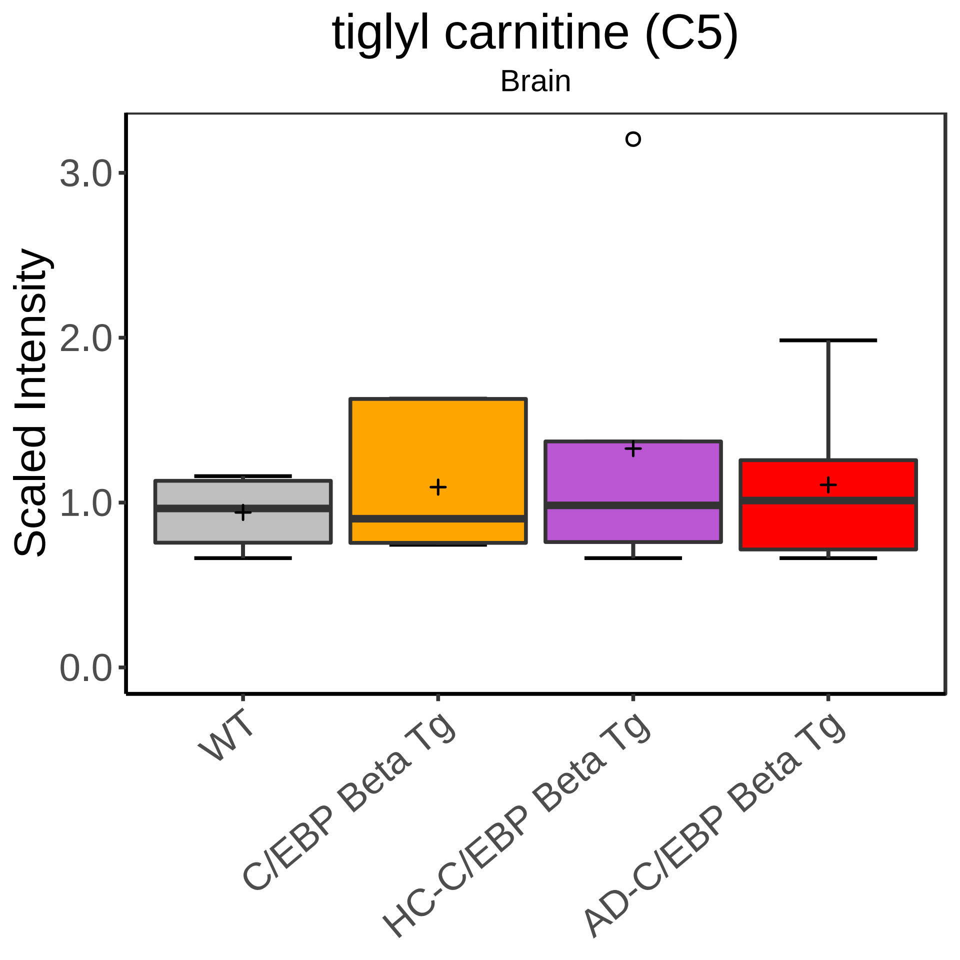
tiglyl carnitine (C5)
Brain
Scaled Intensity
0.0
1.0
2.0
3.0
WT
C/EBP Beta Tg
HC-C/EBP Beta Tg
AD-C/EBP Beta Tg

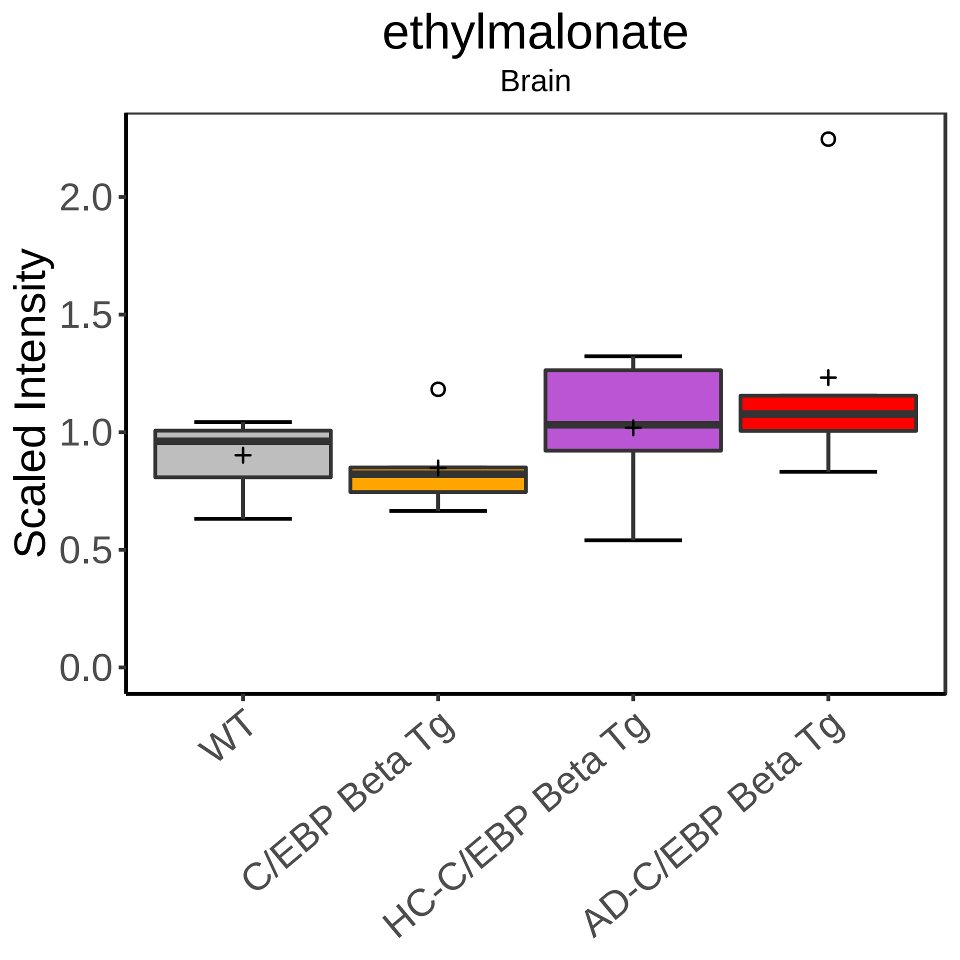
ethylmalonate
Brain
Scaled Intensity
2.0
1.5
1.0
0.5
0.0
WT
C/EBP Beta Tg
HC-C/EBP Beta Tg
AD-C/EBP Beta Tg

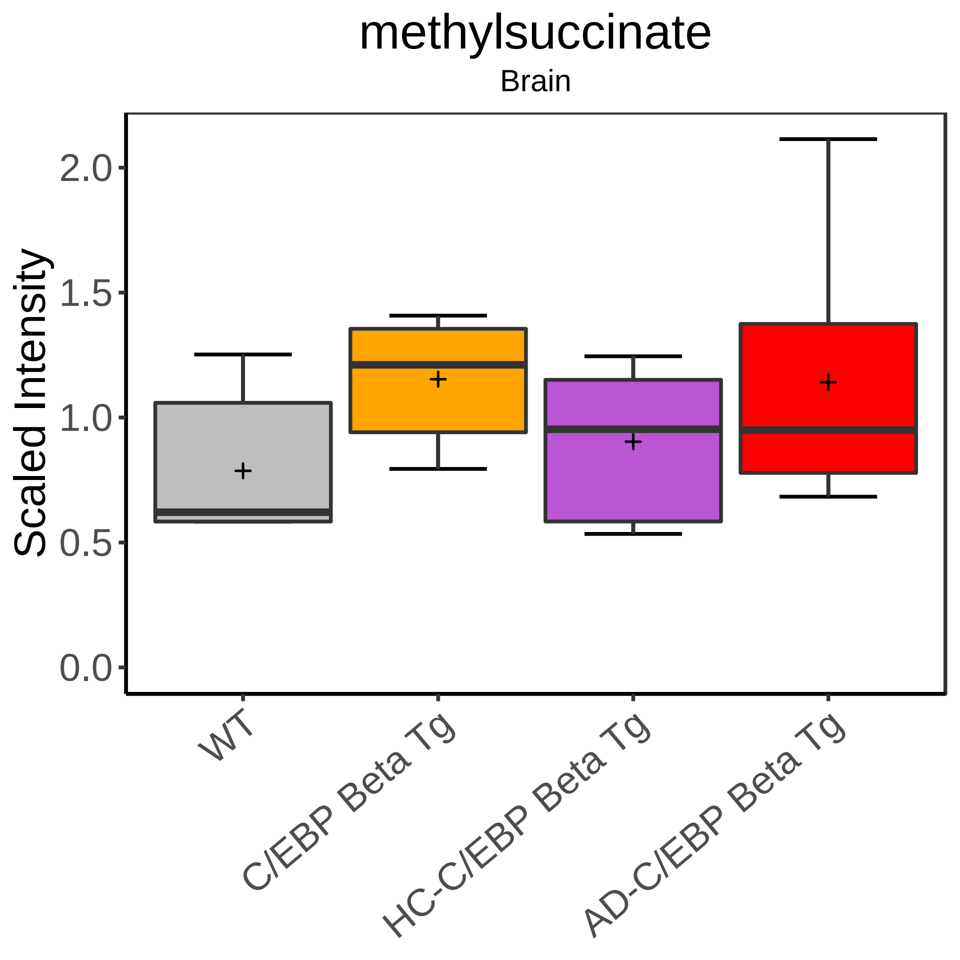
methylsuccinate
Brain
Scaled Intensity
2.0
1.5
1.0
0.5
0.0
WT
C/EBP Beta Tg
HC-C/EBP Beta Tg
AD-C/EBP Beta Tg

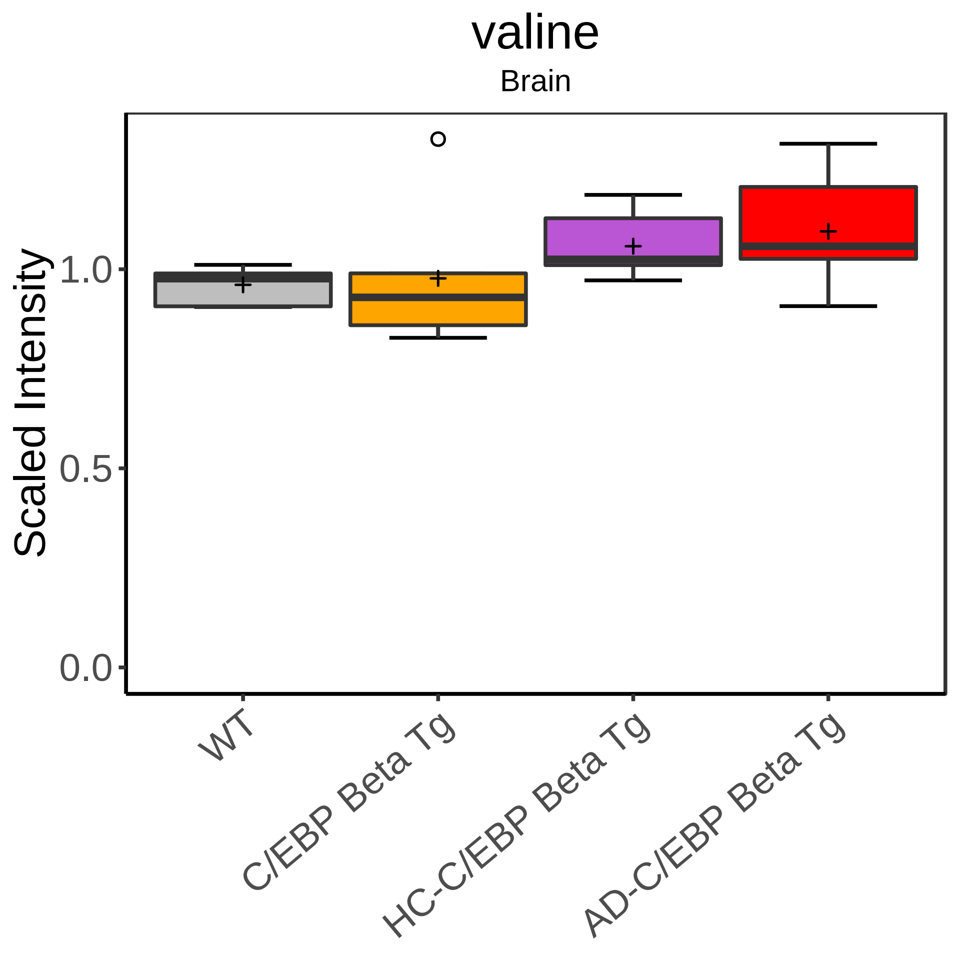

valine
Brain
Scaled Intensity
1.0
0.5
0.0
WT
C/EBP Beta Tg
HC-C/EBP Beta Tg
AD-C/EBP Beta Tg

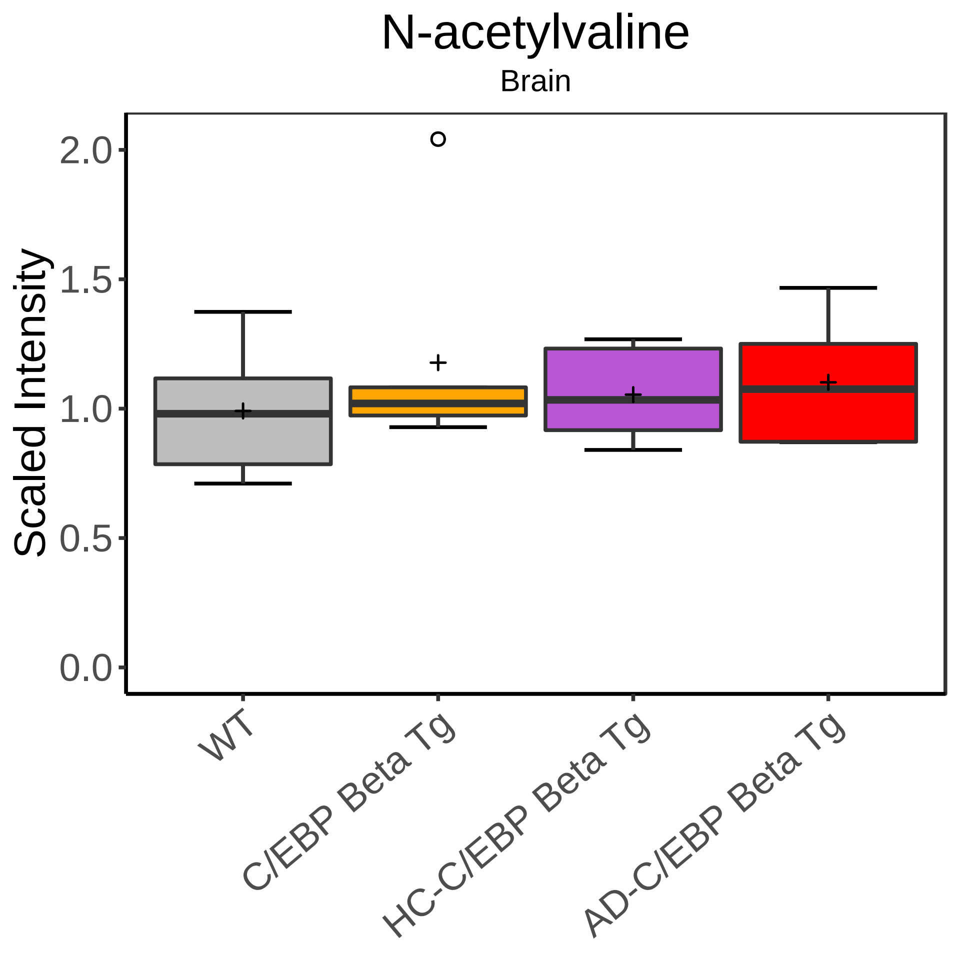
N-acetylvaline
Brain
Scaled Intensity
2.0
1.5
1.0
0.5
0.0
WT
C/EBP Beta Tg
HC-C/EBP Beta Tg
AD-C/EBP Beta Tg

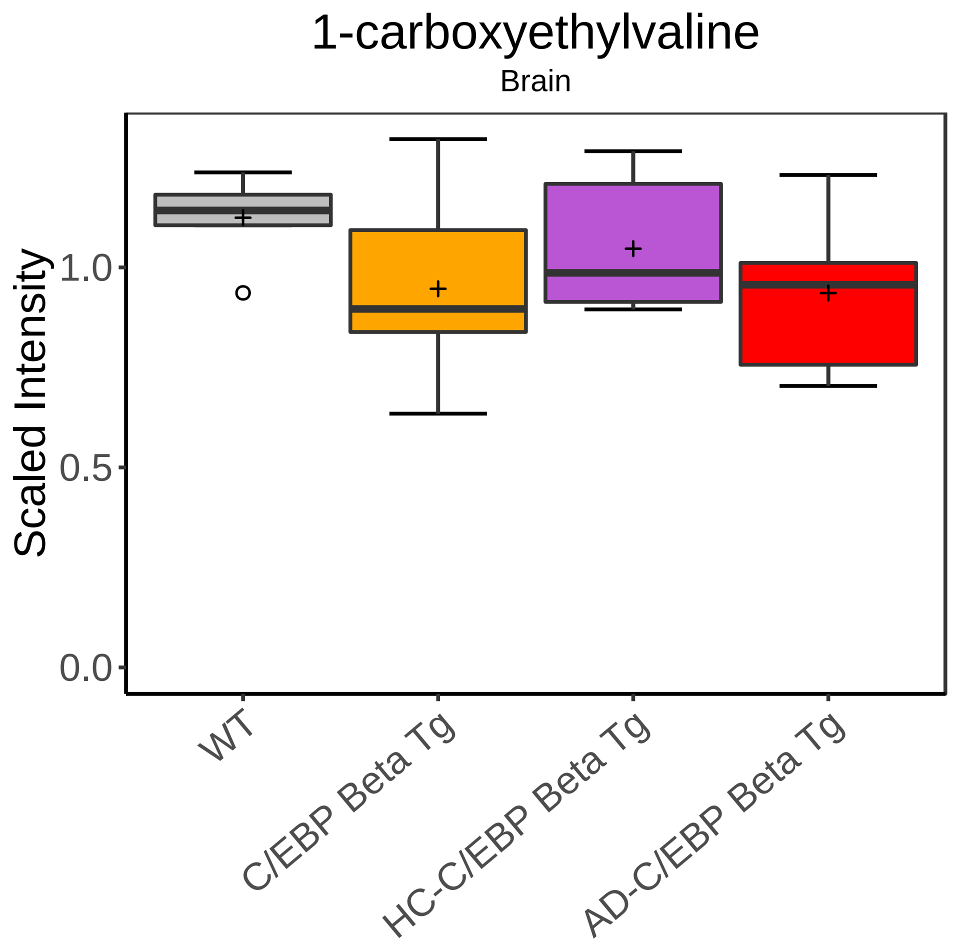
1-carboxyethylvaline
Brain
Scaled Intensity
1.0
0.5
0.0
WT
C/EBP Beta Tg
HC-C/EBP Beta Tg
AD-C/EBP Beta Tg

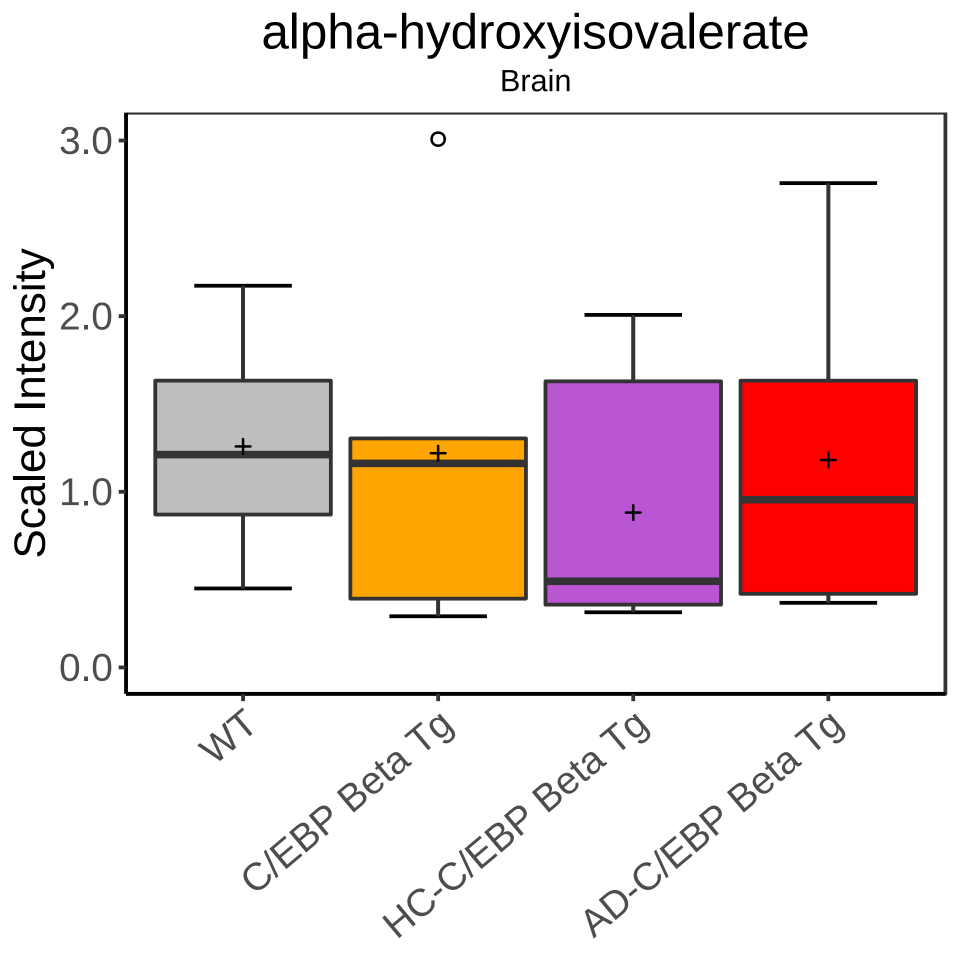
alpha-hydroxyisovalerate
Brain
Scaled Intensity
3.0
2.0
1.0
0.0
WT
C/EBP Beta Tg
HC-C/EBP Beta Tg
AD-C/EBP Beta Tg

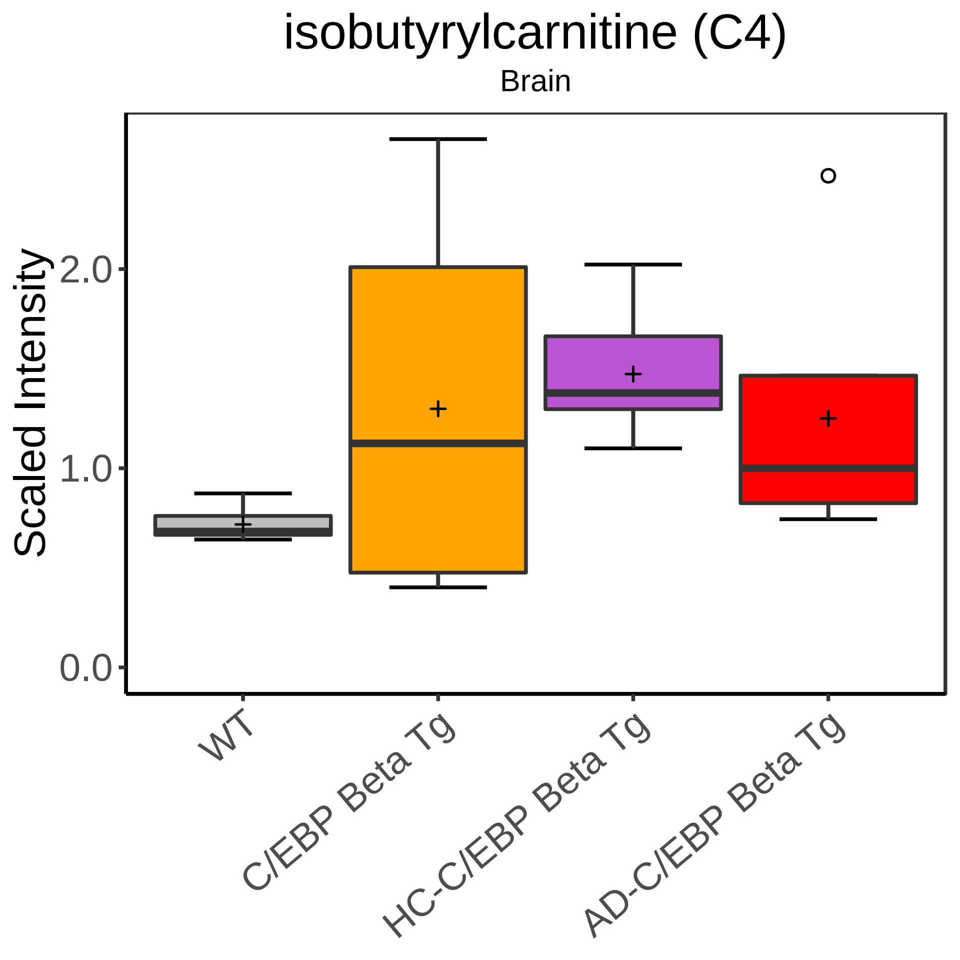
isobutyrylcarnitine (C4)
Brain
Scaled Intensity
2.0
1.0
0.0
WT
C/EBP Beta Tg
HC-C/EBP Beta Tg
AD-C/EBP Beta Tg

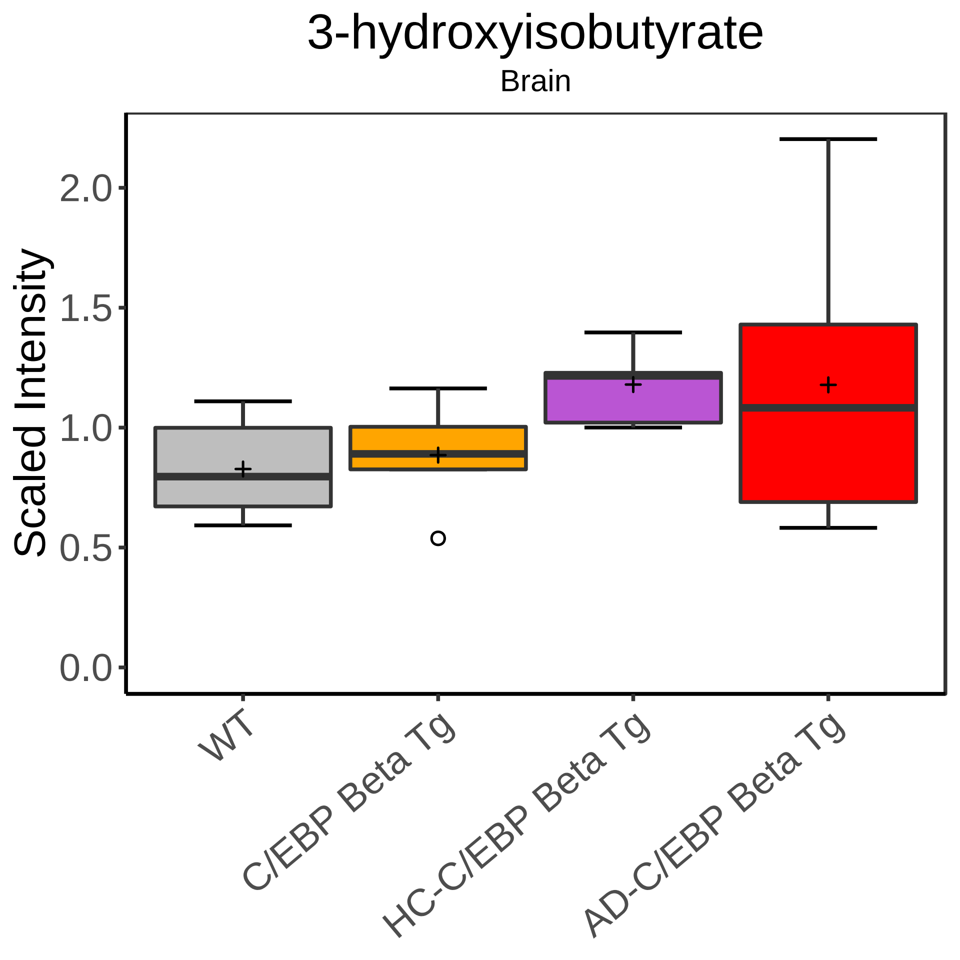
3-hydroxyisobutyrate
Brain
Scaled Intensity
2.0
1.5
1.0
0.5
0.0
WT
C/EBP Beta Tg
HC-C/EBP Beta Tg
AD-C/EBP Beta Tg

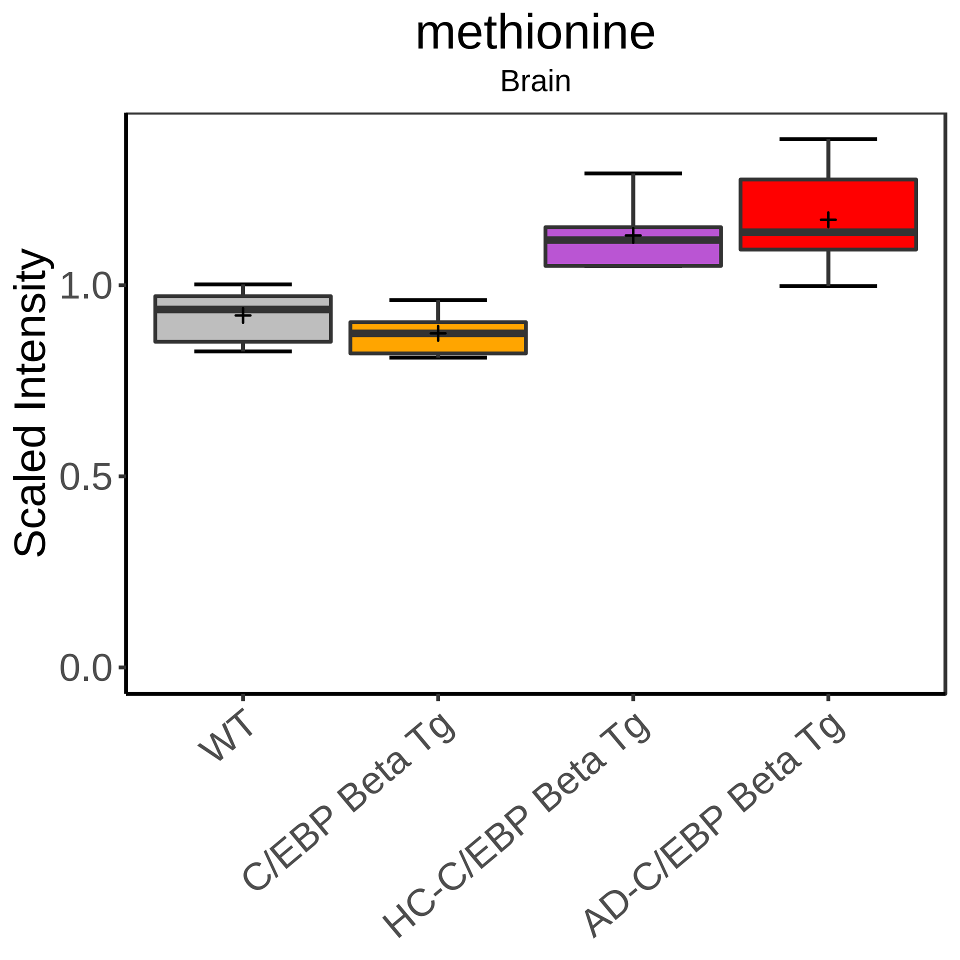
methionine
Brain
Scaled Intensity
0.0
0.5
1.0
WT
C/EBP Beta Tg
HC-C/EBP Beta Tg
AD-C/EBP Beta Tg

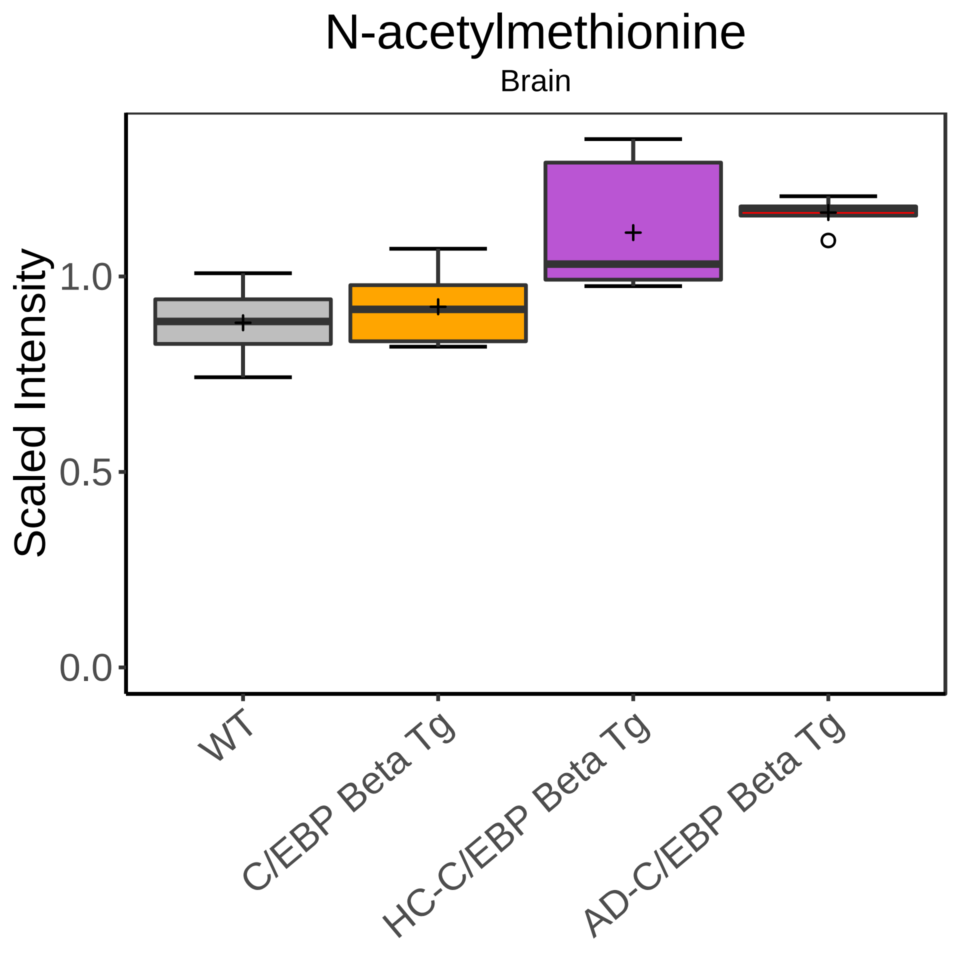

N-acetylmethionine
Brain
Scaled Intensity
1.0
0.5
0.0
WT
C/EBP Beta Tg
HC-C/EBP Beta Tg
AD-C/EBP Beta Tg

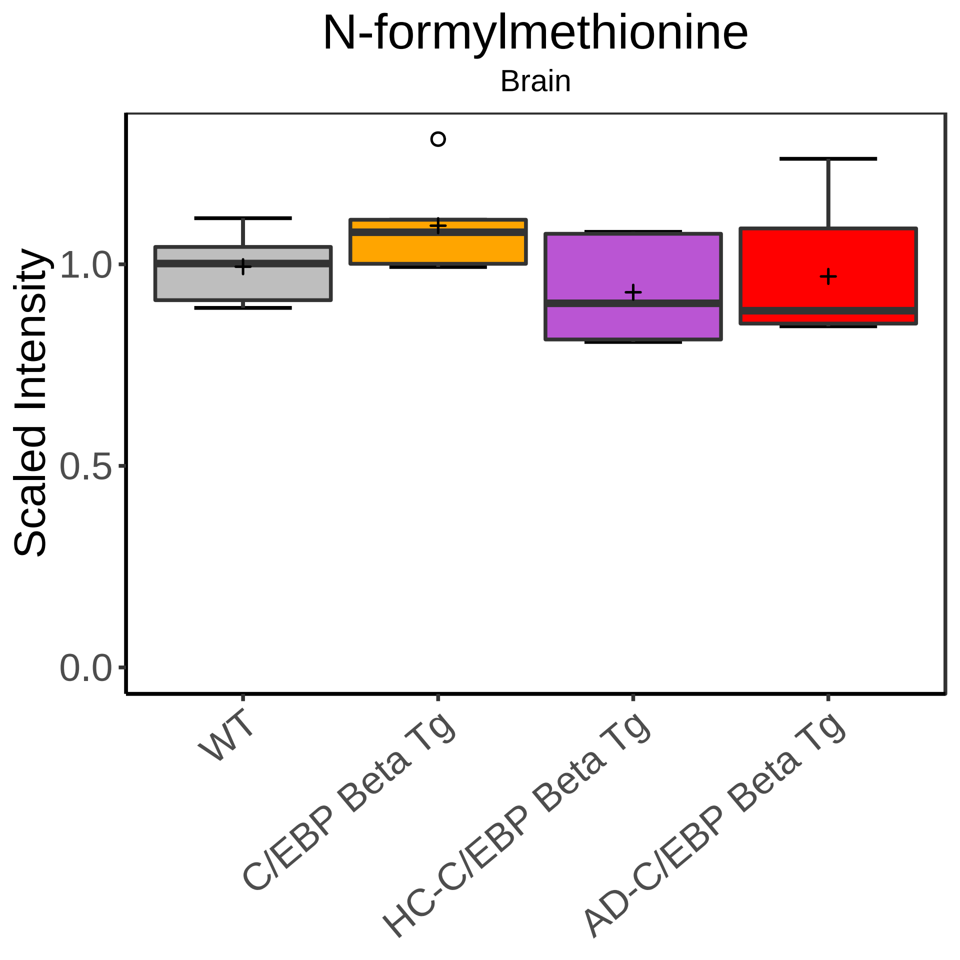
N-formylmethionine
Brain
Scaled Intensity
1.0
0.5
0.0
WT
C/EBP Beta Tg
HC-C/EBP Beta Tg
AD-C/EBP Beta Tg

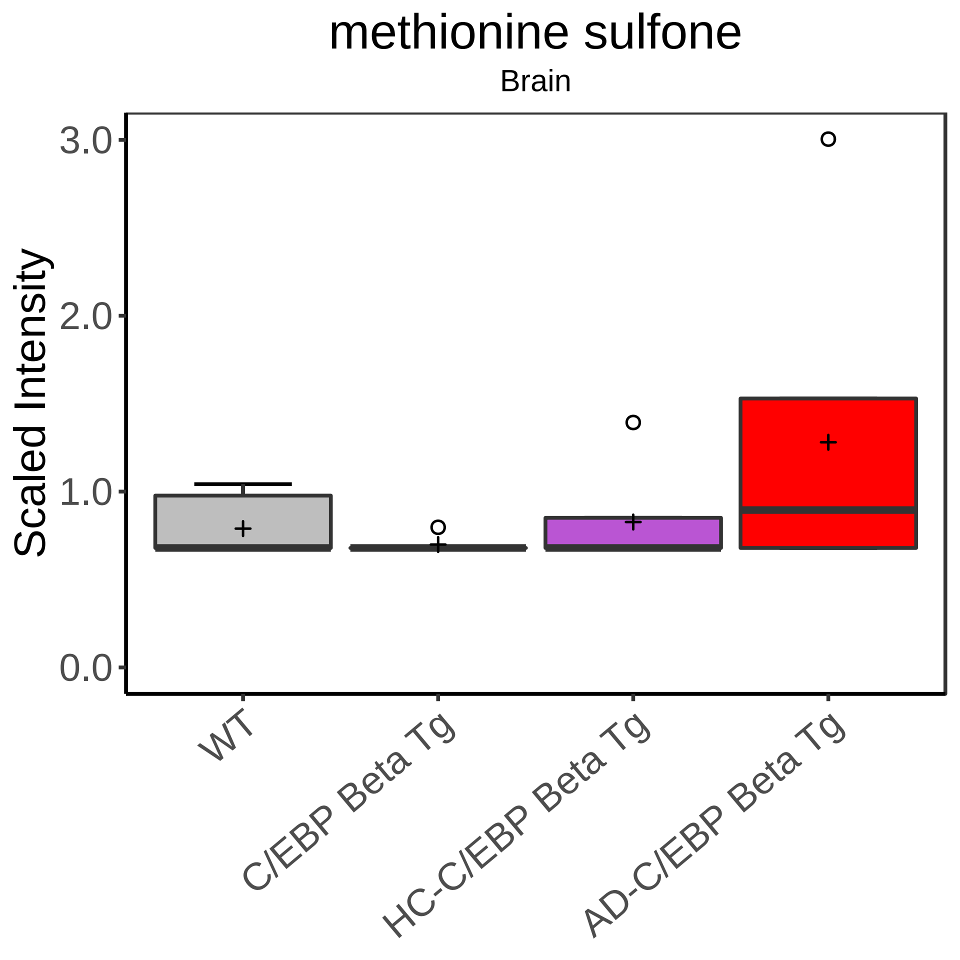
methionine sulfone
Brain
Scaled Intensity
3.0
2.0
1.0
0.0
WT
C/EBP Beta Tg
HC-C/EBP Beta Tg
AD-C/EBP Beta Tg

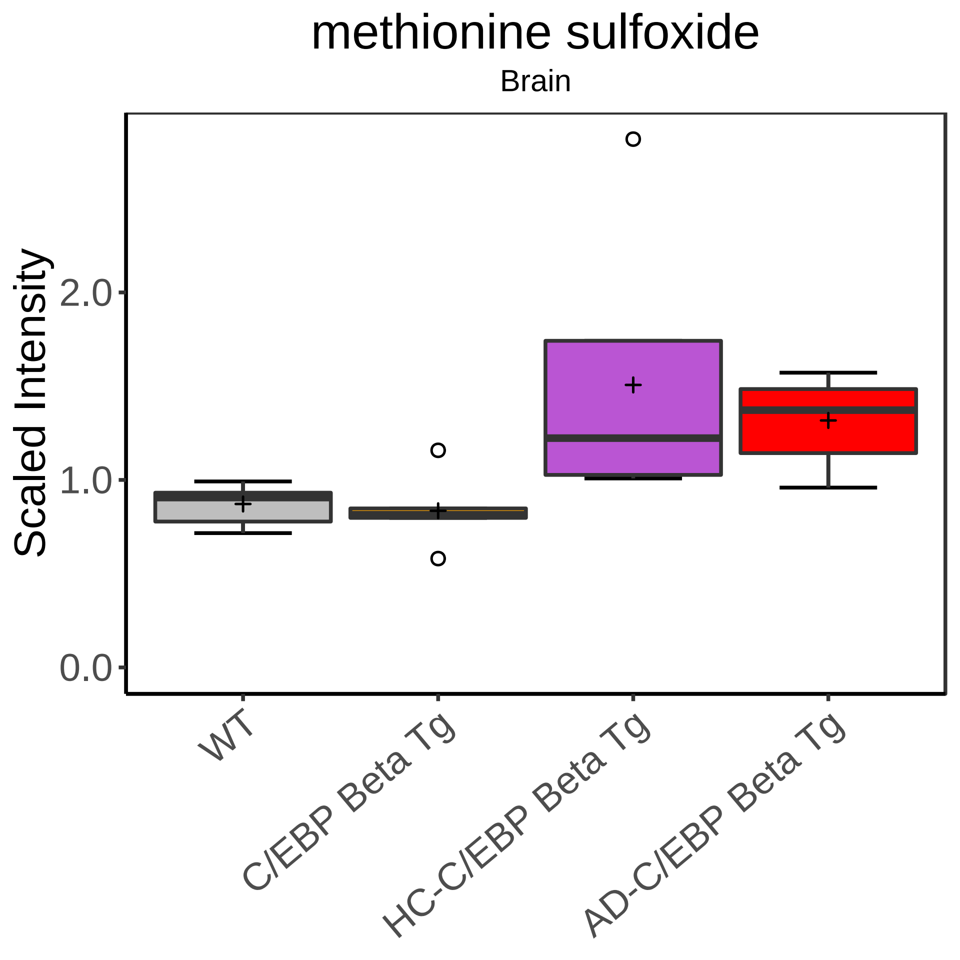

methionine sulfoxide
Brain
Scaled Intensity
2.0
1.0
0.0
WT
C/EBP Beta Tg
HC-C/EBP Beta Tg
AD-C/EBP Beta Tg

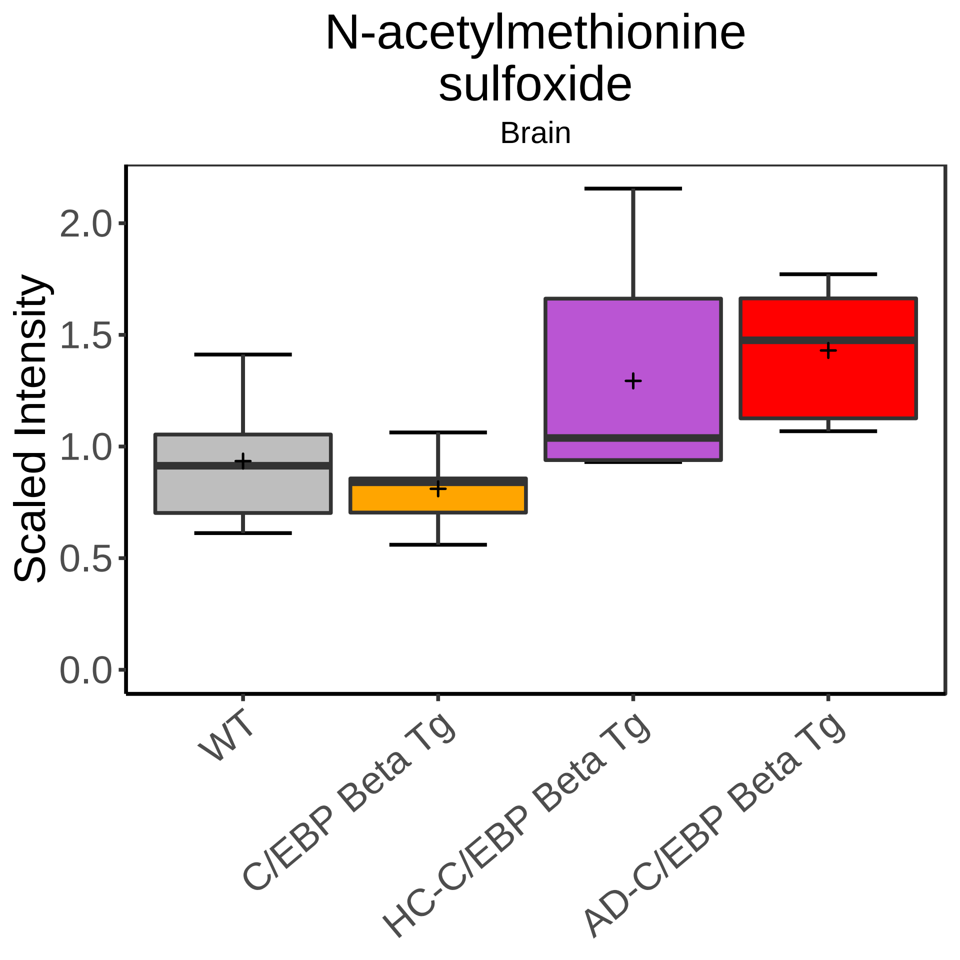
N-acetylmethionine
sulfoxide
Brain
Scaled Intensity
0.0
0.5
1.0
1.5
2.0
WT
C/EBP Beta Tg
HC-C/EBP Beta Tg
AD-C/EBP Beta Tg

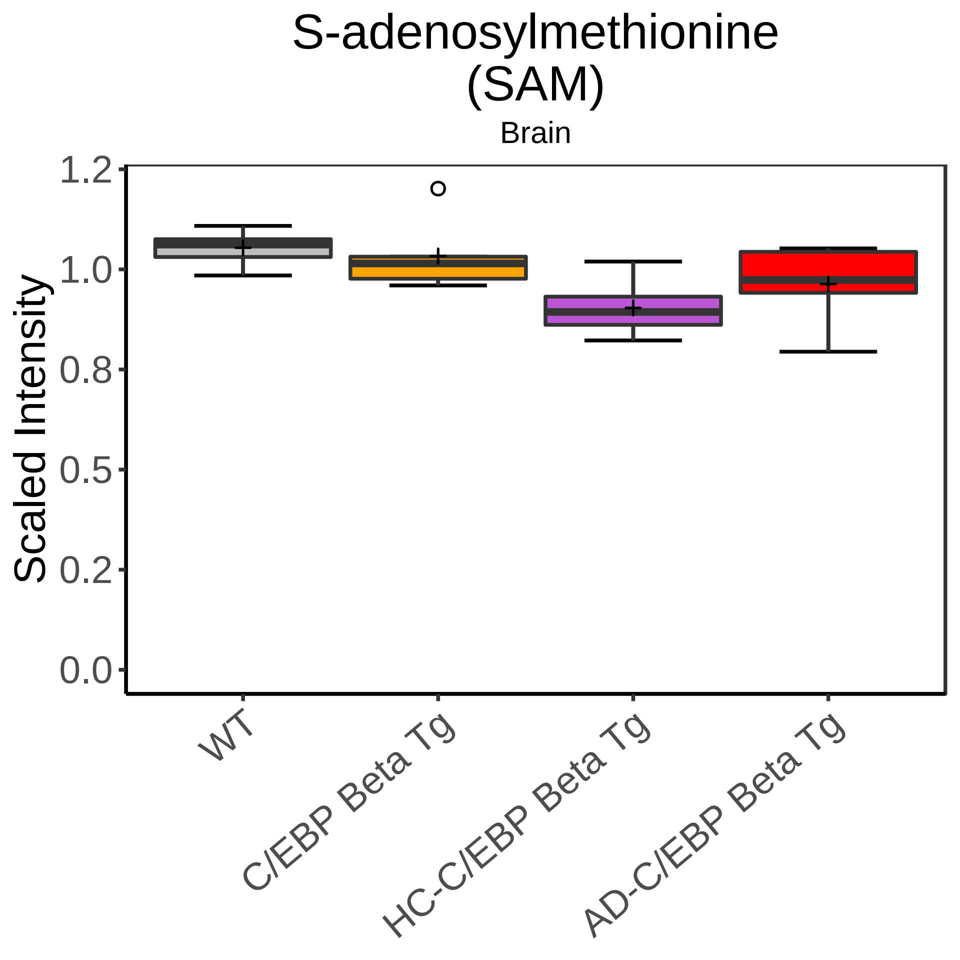
S-adenosylmethionine
(SAM)
Brain
Scaled Intensity
1.2
1.0
0.8
0.5
0.2
0.0
WT
C/EBP Beta Tg
HC-C/EBP Beta Tg
AD-C/EBP Beta Tg

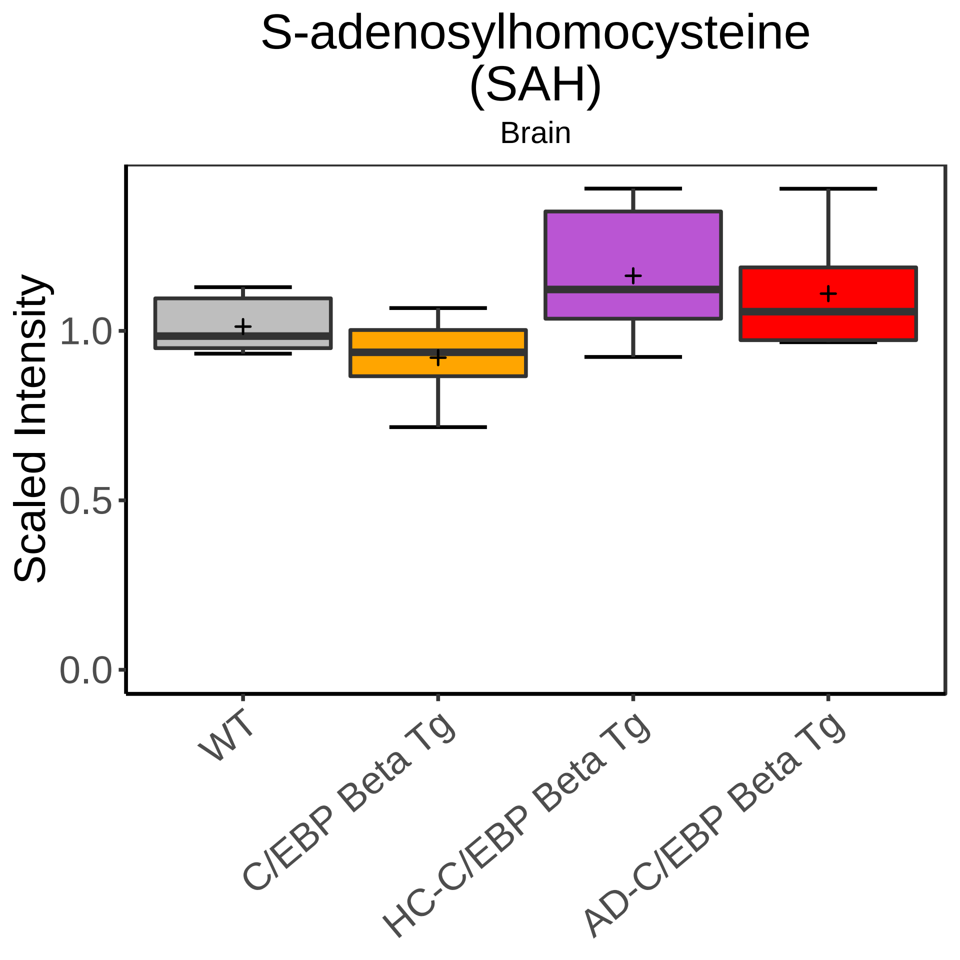

S-adenosylhomocysteine
(SAH)
Brain
Scaled Intensity
1.0
0.5
0.0
WT
C/EBP Beta Tg
HC-C/EBP Beta Tg
AD-C/EBP Beta Tg

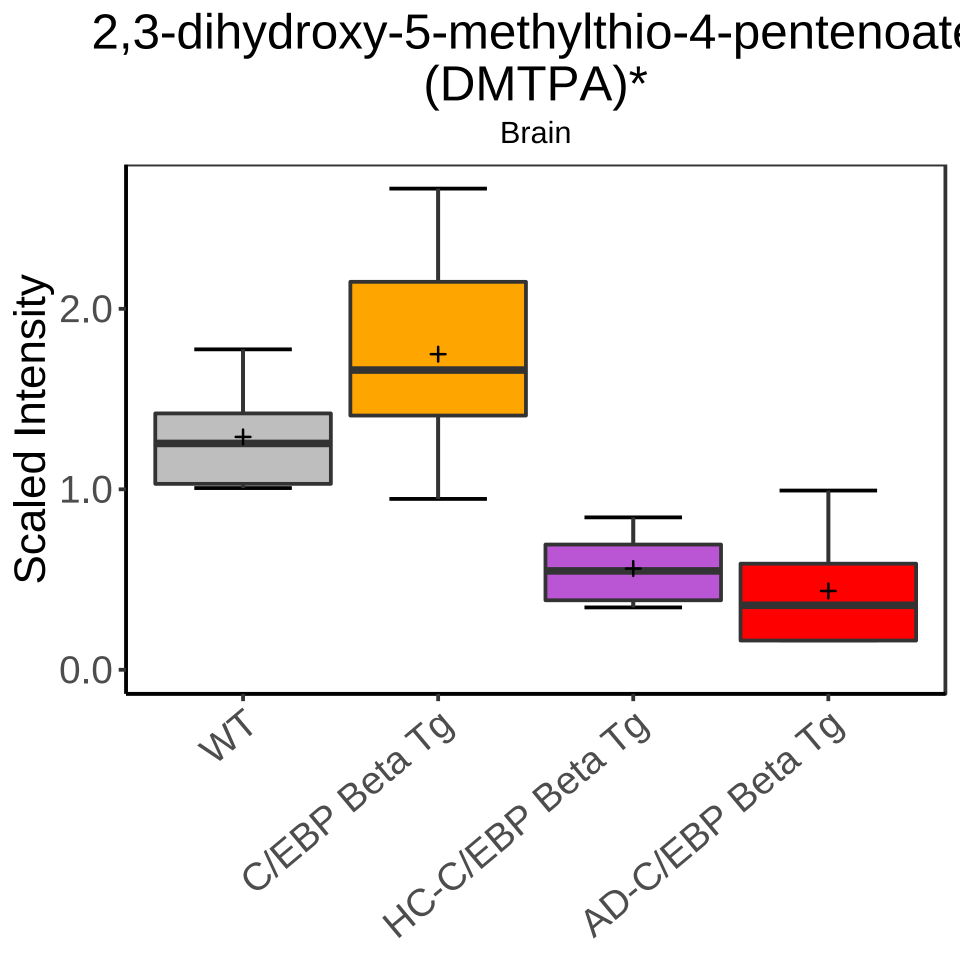
2,3-dihydroxy-5-methylthio-4-pentenoate
(DMTPA)*
Brain
Scaled Intensity
2.0
1.0
0.0
WT
C/EBP Beta Tg
HC-C/EBP Beta Tg
AD-C/EBP Beta Tg

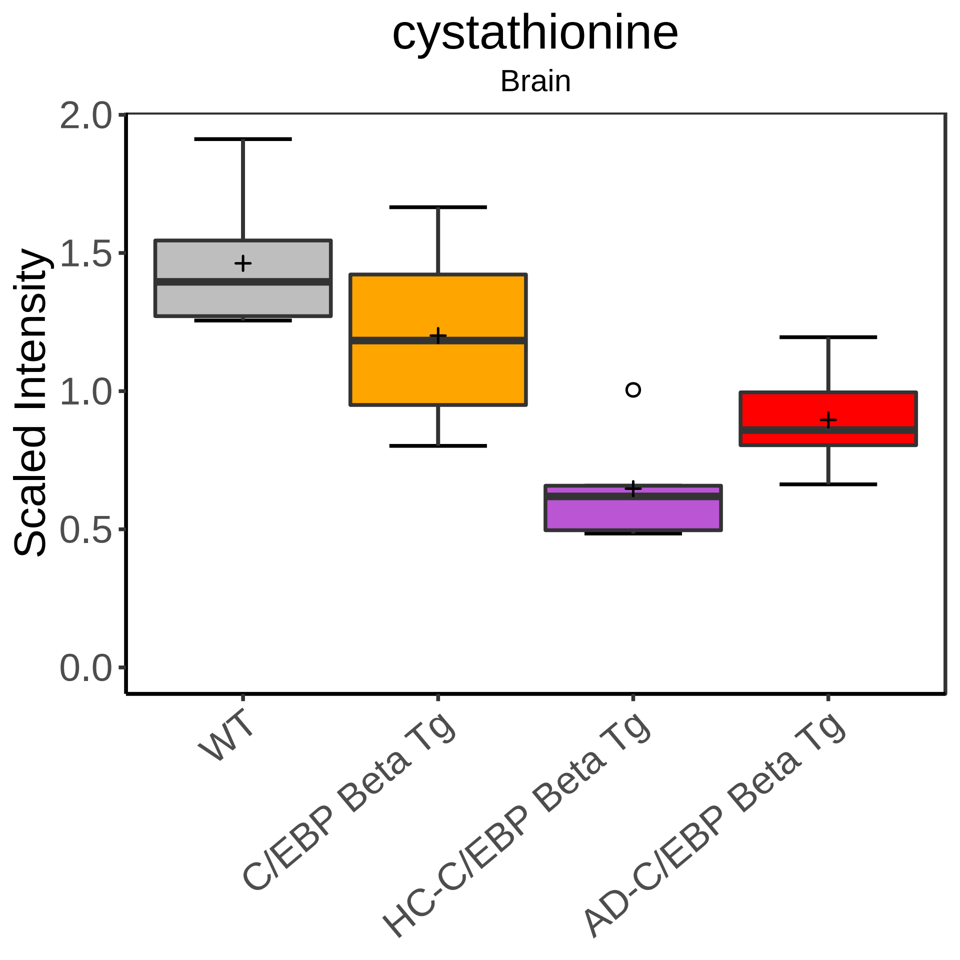
cystathionine
Brain
Scaled Intensity
2.0
1.5
1.0
0.5
0.0
WT
C/EBP Beta Tg
HC-C/EBP Beta Tg
AD-C/EBP Beta Tg

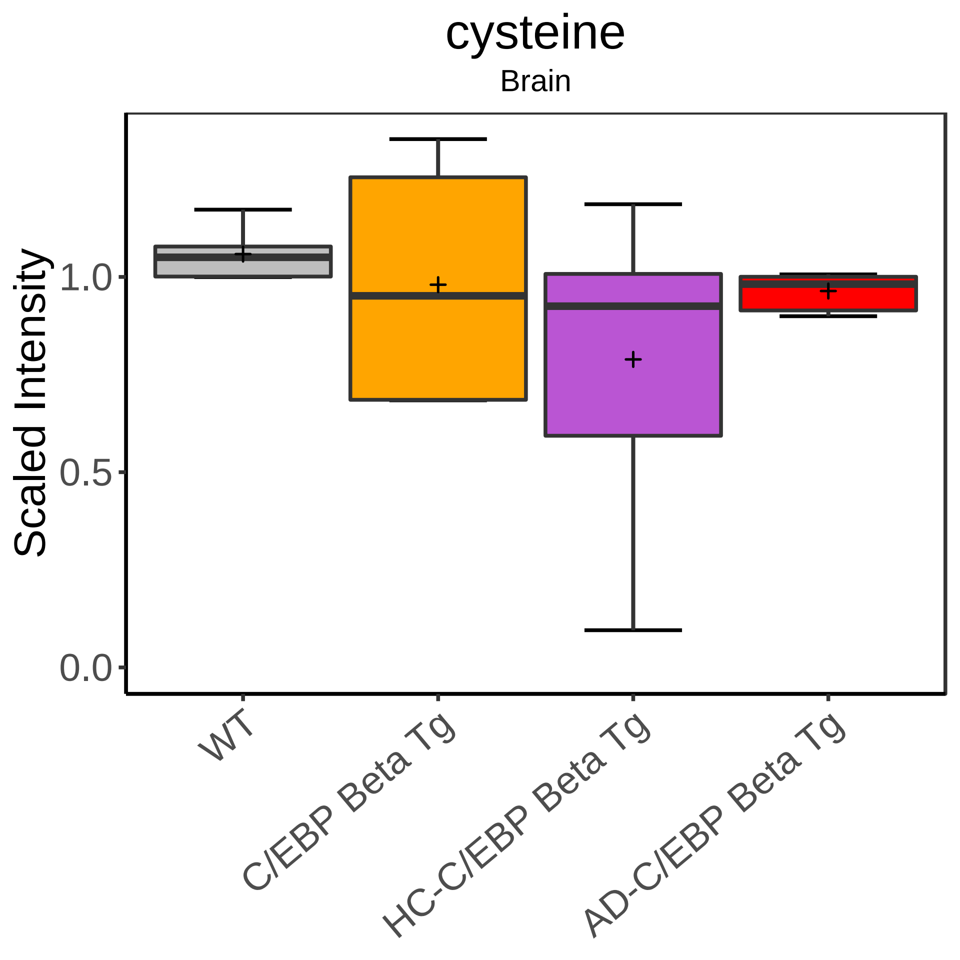

cysteine
Brain
Scaled Intensity
1.0
0.5
0.0
WT
C/EBP Beta Tg
HC-C/EBP Beta Tg
AD-C/EBP Beta Tg

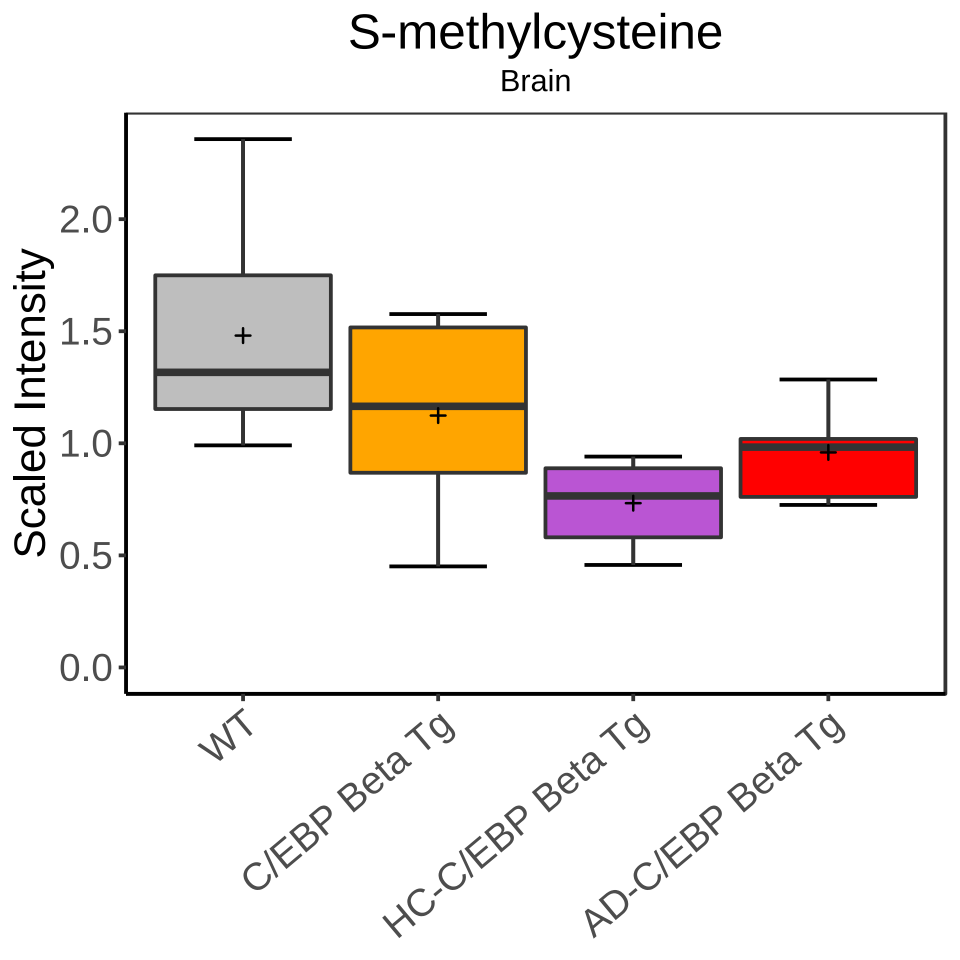

S-methylcysteine
Brain
Scaled Intensity
0.0
0.5
1.0
1.5
2.0
WT
C/EBP Beta Tg
HC-C/EBP Beta Tg
AD-C/EBP Beta Tg

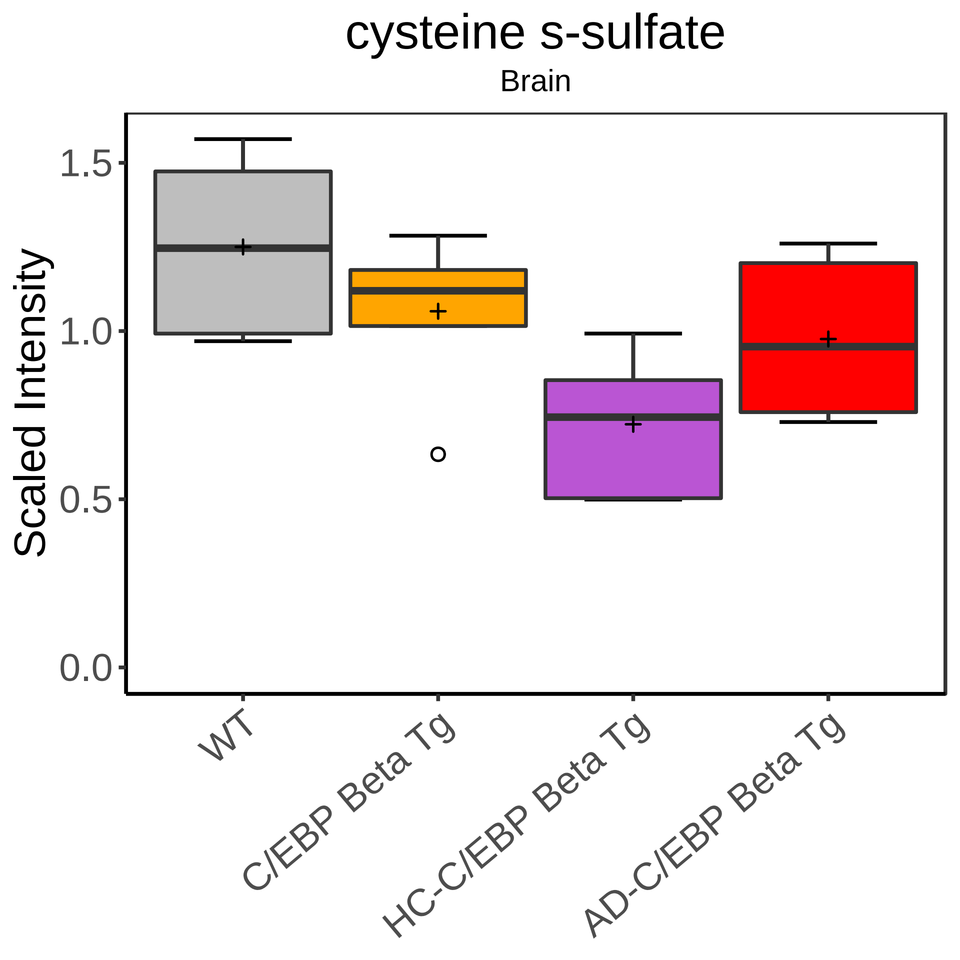
cysteine s-sulfate
Brain
Scaled Intensity
1.5
1.0
0.5
0.0
WT
C/EBP Beta Tg
HC-C/EBP Beta Tg
AD-C/EBP Beta Tg

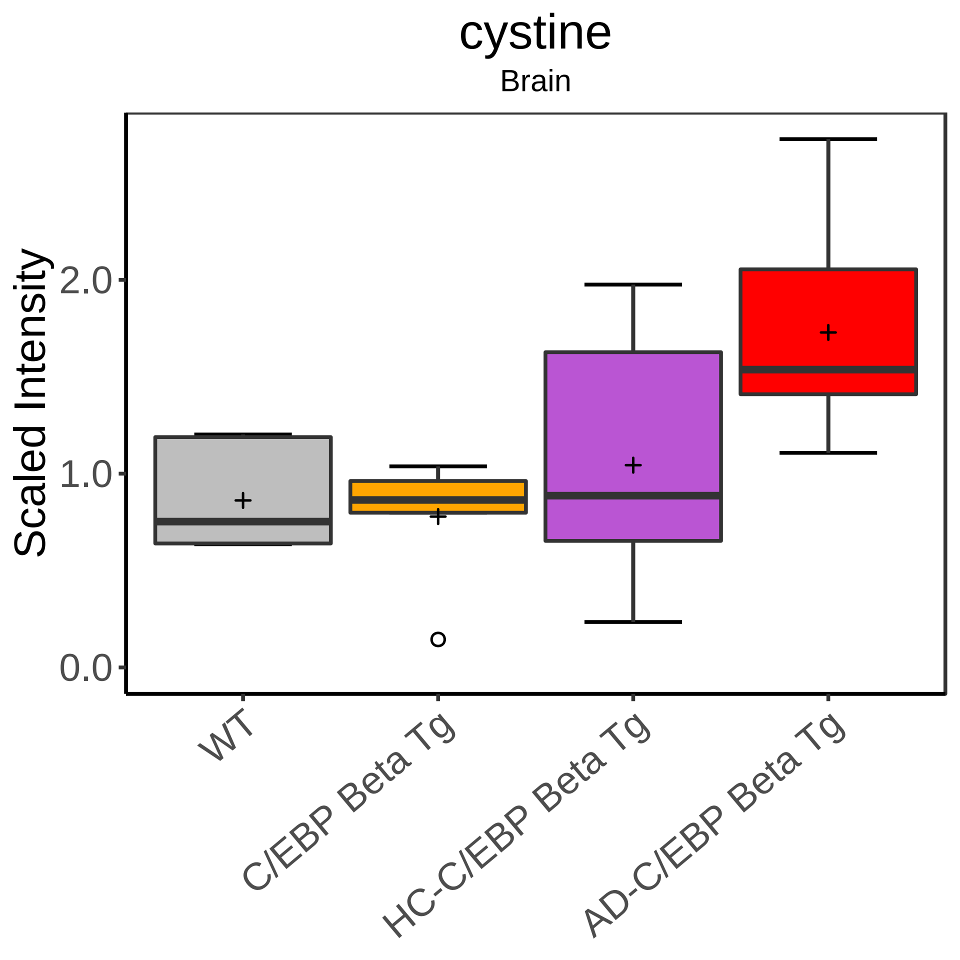

cystine
Brain
Scaled Intensity
2.0
1.0
0.0
WT
C/EBP Beta Tg
HC-C/EBP Beta Tg
AD-C/EBP Beta Tg

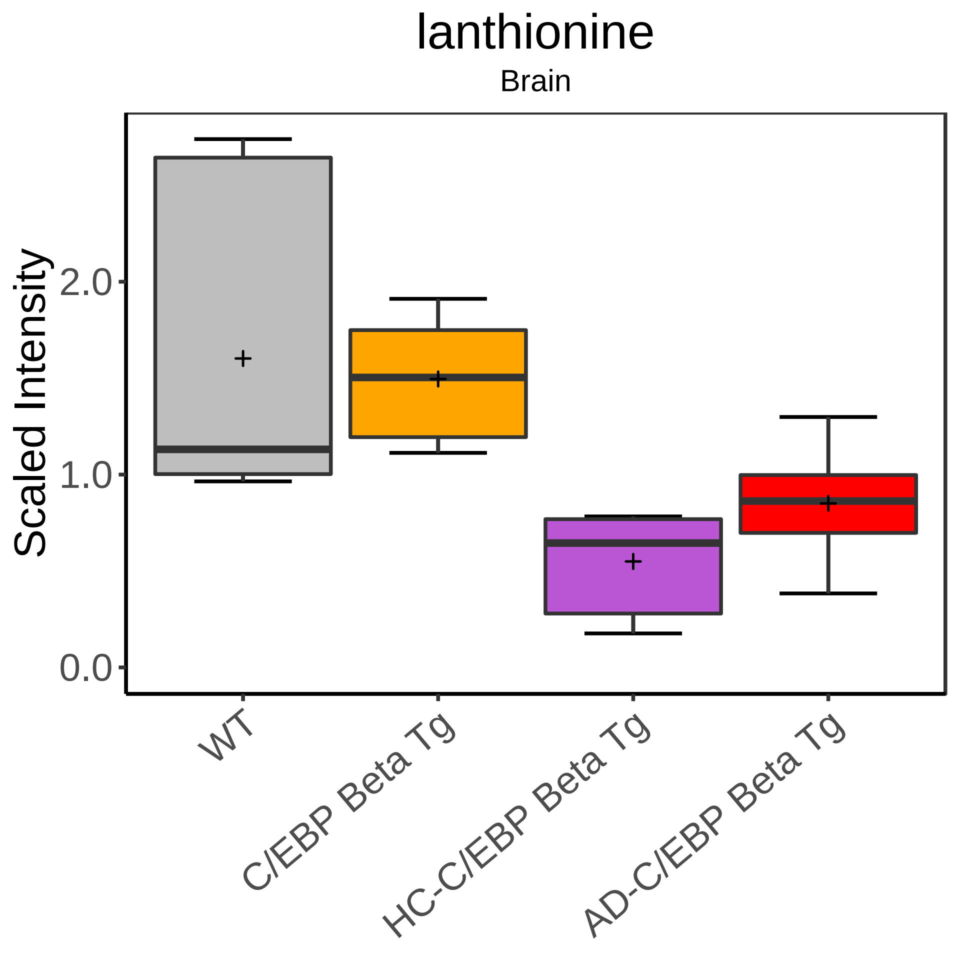

lanthionine
Brain
Scaled Intensity
2.0
1.0
0.0
WT
C/EBP Beta Tg
HC-C/EBP Beta Tg
AD-C/EBP Beta Tg

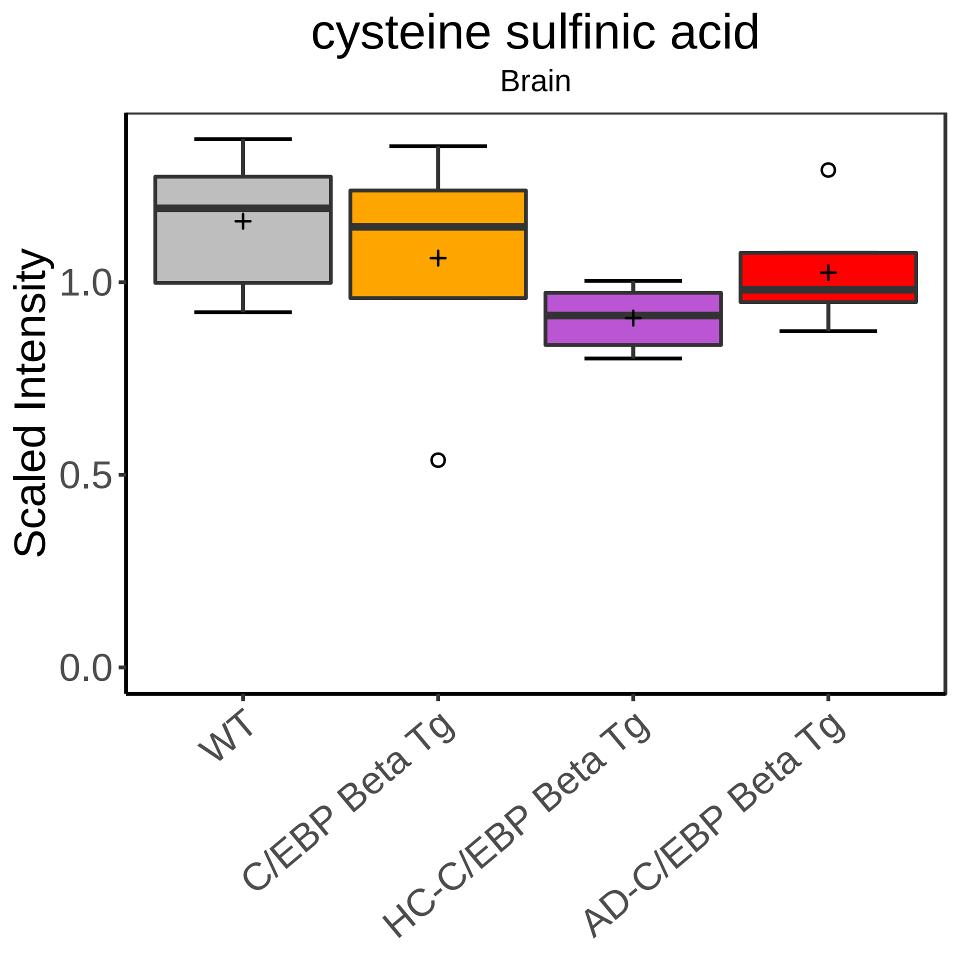
cysteine sulfinic acid
Brain
Scaled Intensity
1.0
0.5
0.0
WT
C/EBP Beta Tg
HC-C/EBP Beta Tg
AD-C/EBP Beta Tg

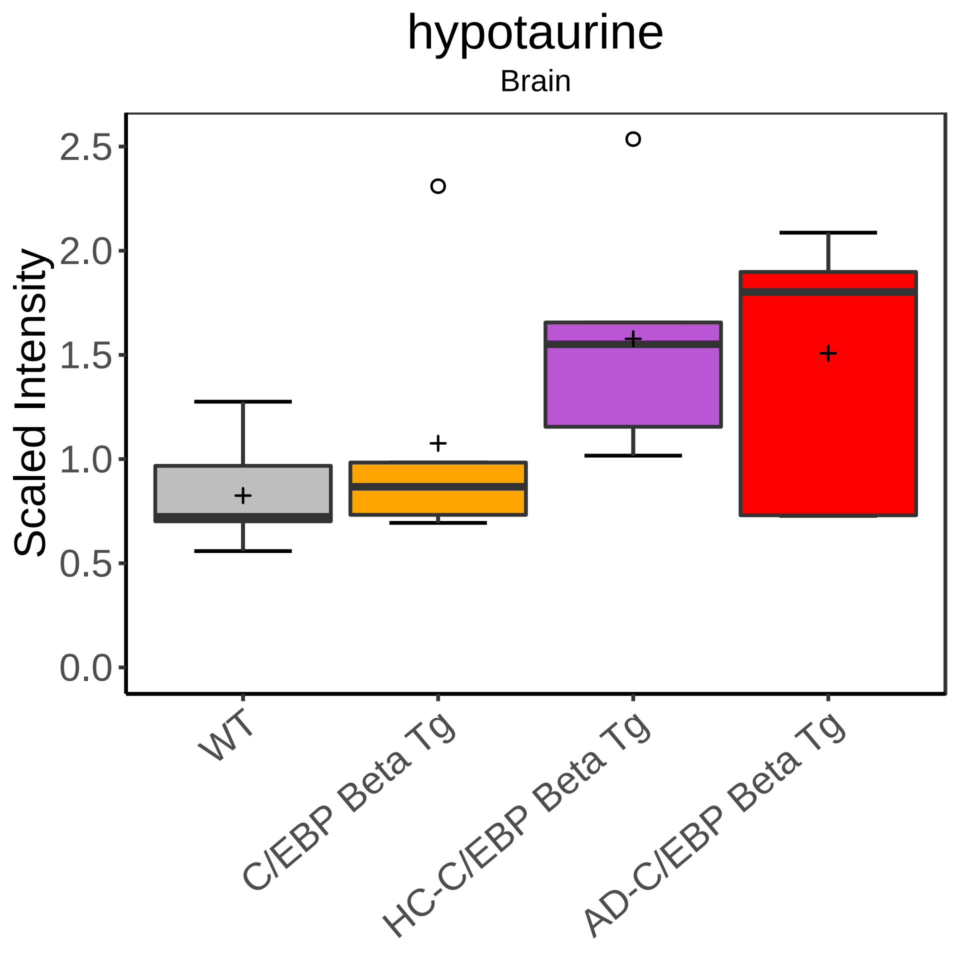
hypotaurine
Brain
Scaled Intensity
0.0
0.5
1.0
1.5
2.0
2.5
WT
C/EBP Beta Tg
HC-C/EBP Beta Tg
AD-C/EBP Beta Tg

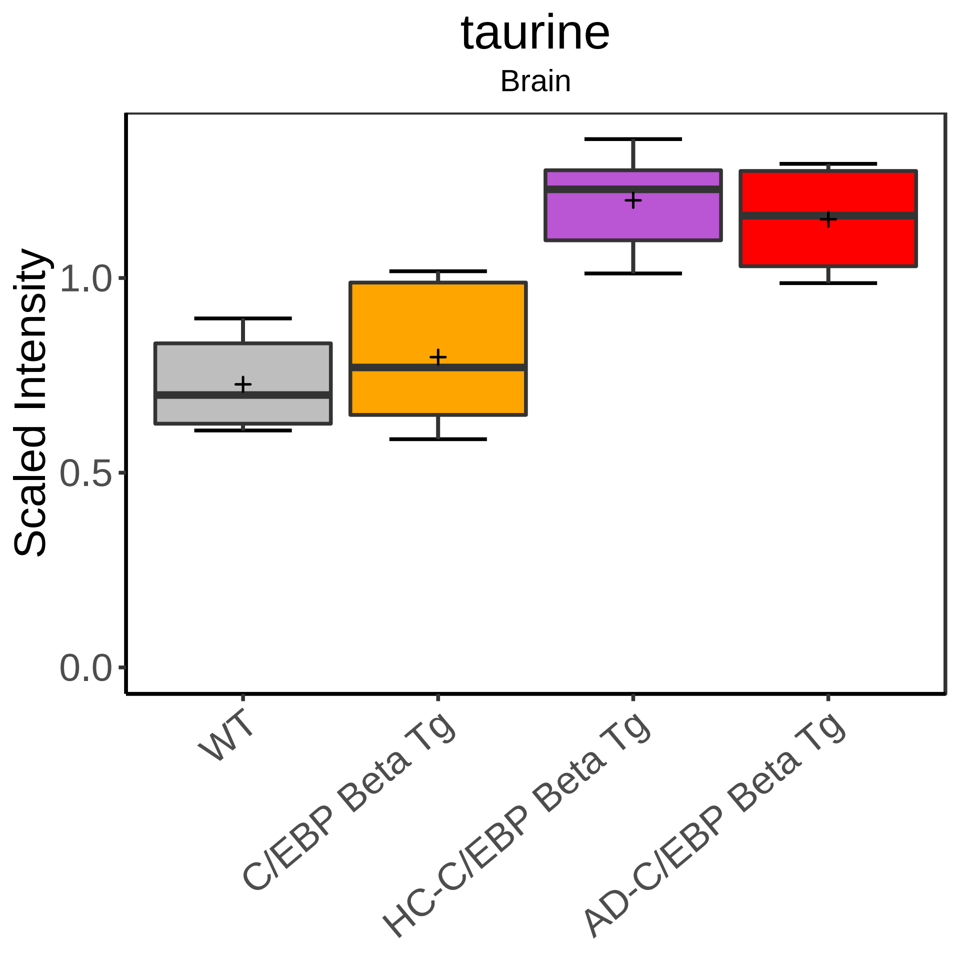
taurine
Brain
Scaled Intensity
1.0
0.5
0.0
WT
C/EBP Beta Tg
HC-C/EBP Beta Tg
AD-C/EBP Beta Tg

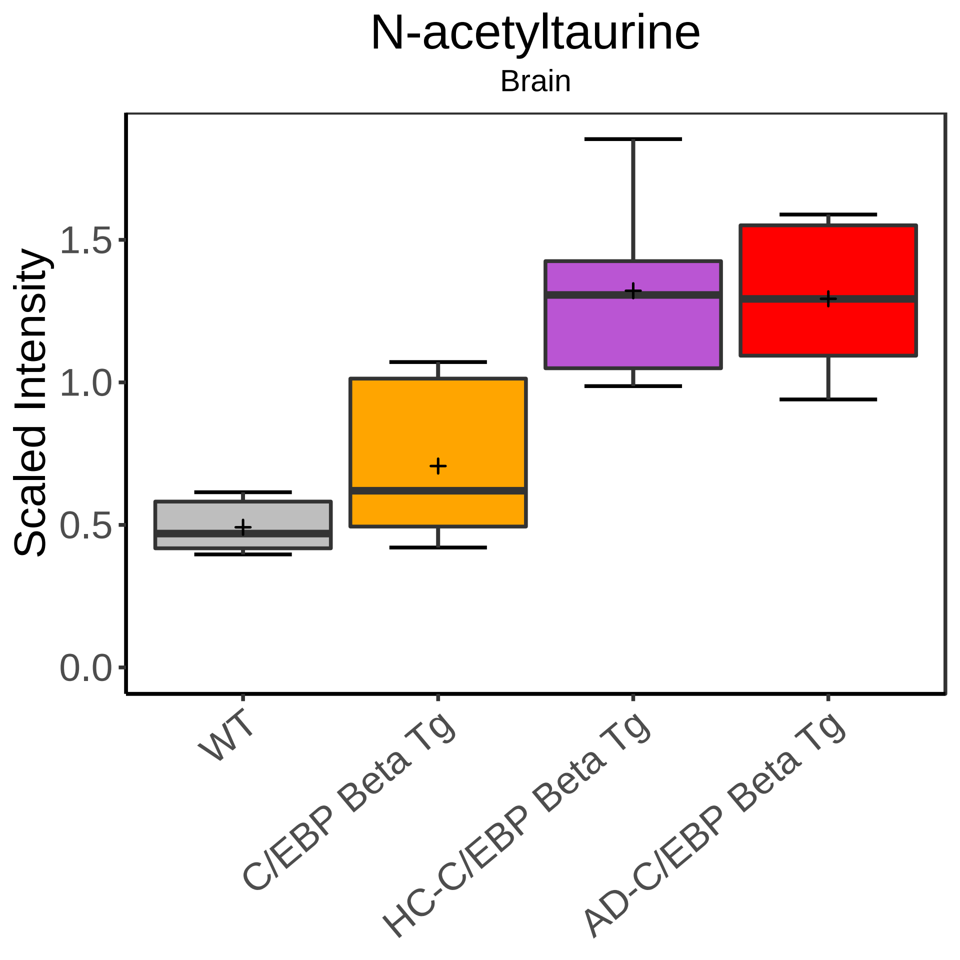
N-acetyltaurine
Brain
Scaled Intensity
0.0
0.5
1.0
1.5
WT
C/EBP Beta Tg
HC-C/EBP Beta Tg
AD-C/EBP Beta Tg

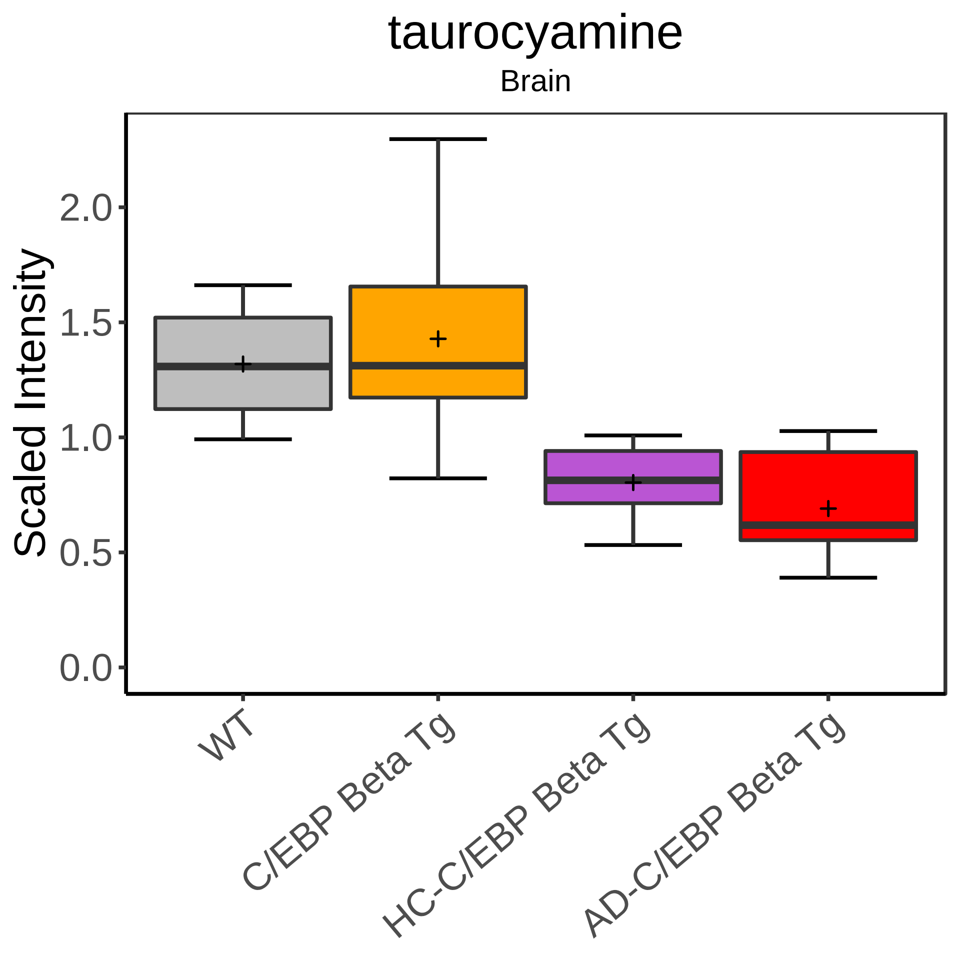

taurocyamine
Brain
Scaled Intensity
2.0
1.5
1.0
0.5
0.0
WT
C/EBP Beta Tg
HC-C/EBP Beta Tg
AD-C/EBP Beta Tg

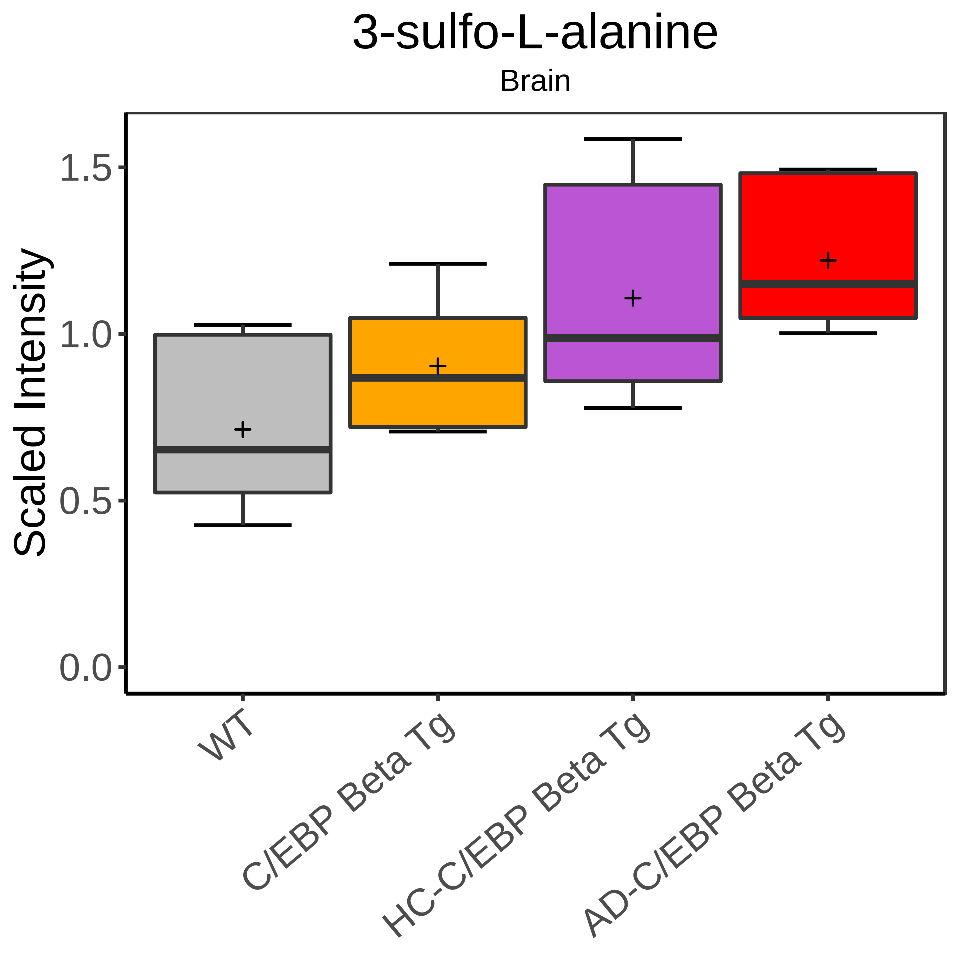

3-sulfo-L-alanine
Brain
Scaled Intensity
0.0
0.5
1.0
1.5
WT
C/EBP Beta Tg
HC-C/EBP Beta Tg
AD-C/EBP Beta Tg

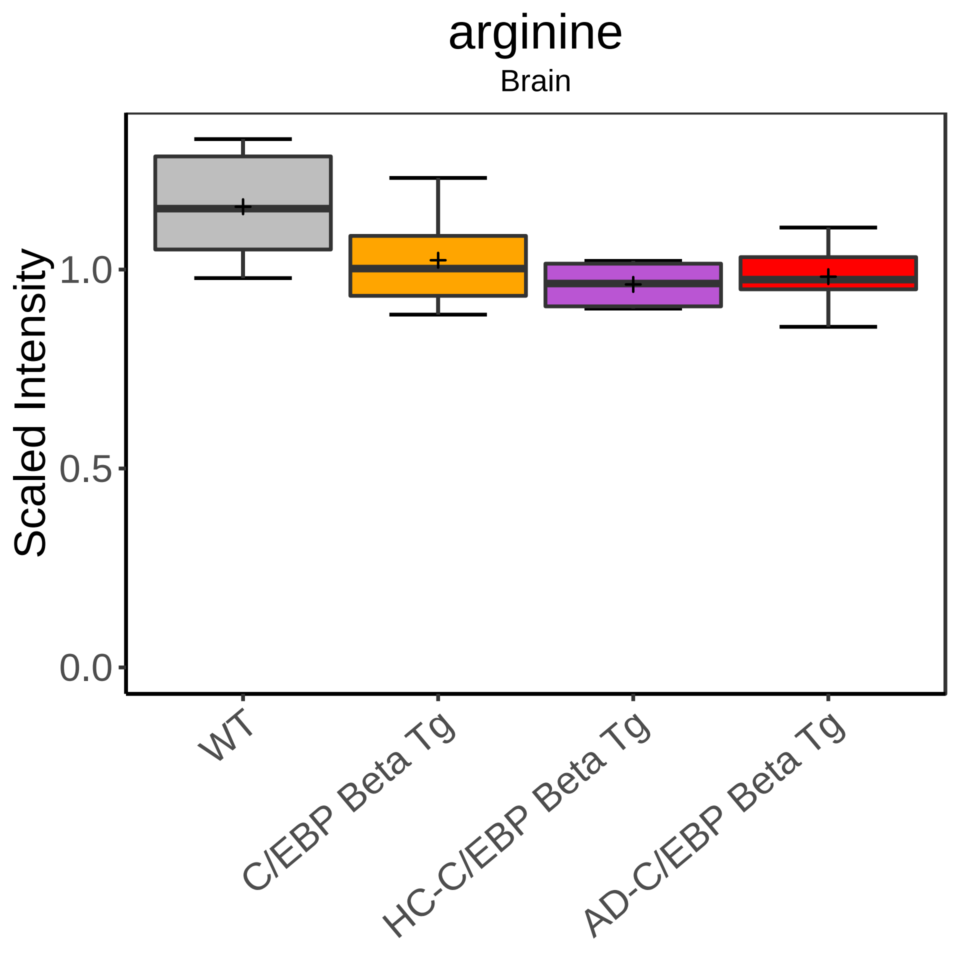

arginine
Brain
Scaled Intensity
0.0
0.5
1.0
WT
C/EBP Beta Tg
HC-C/EBP Beta Tg
AD-C/EBP Beta Tg

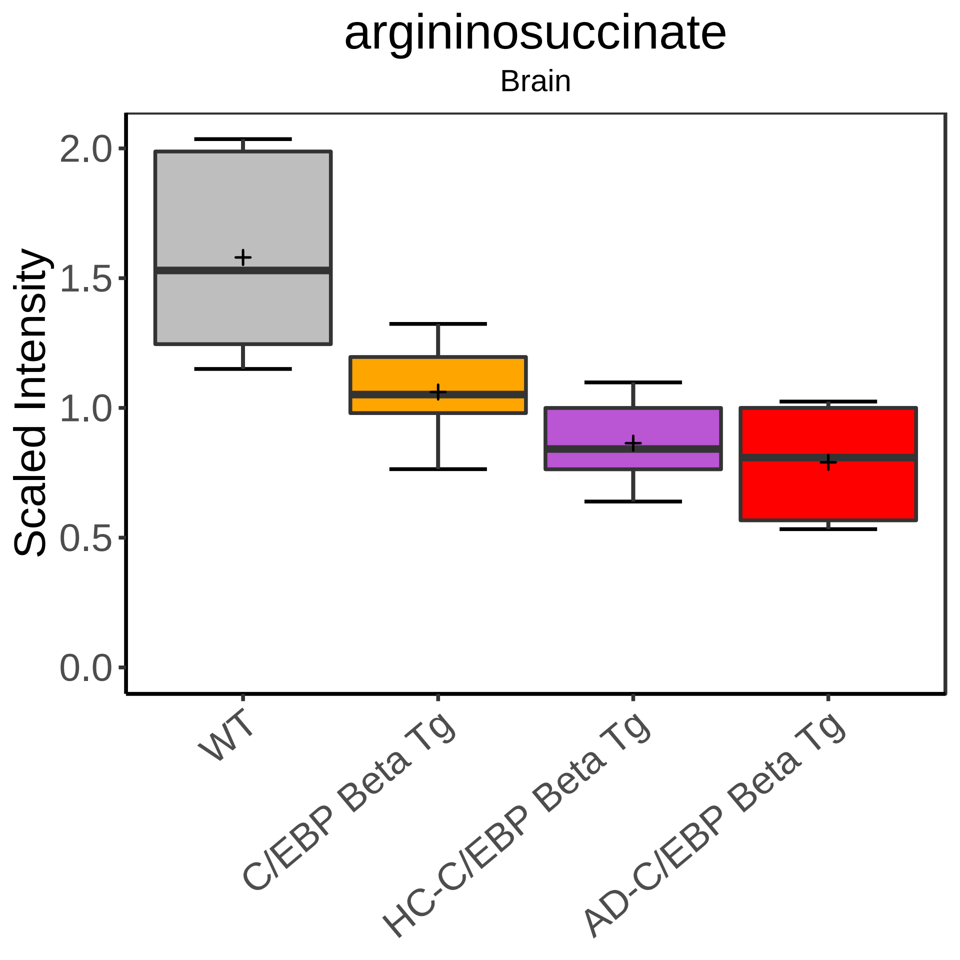
argininosuccinate
Brain
Scaled Intensity
2.0
1.5
1.0
0.5
0.0
WT
C/EBP Beta Tg
HC-C/EBP Beta Tg
AD-C/EBP Beta Tg

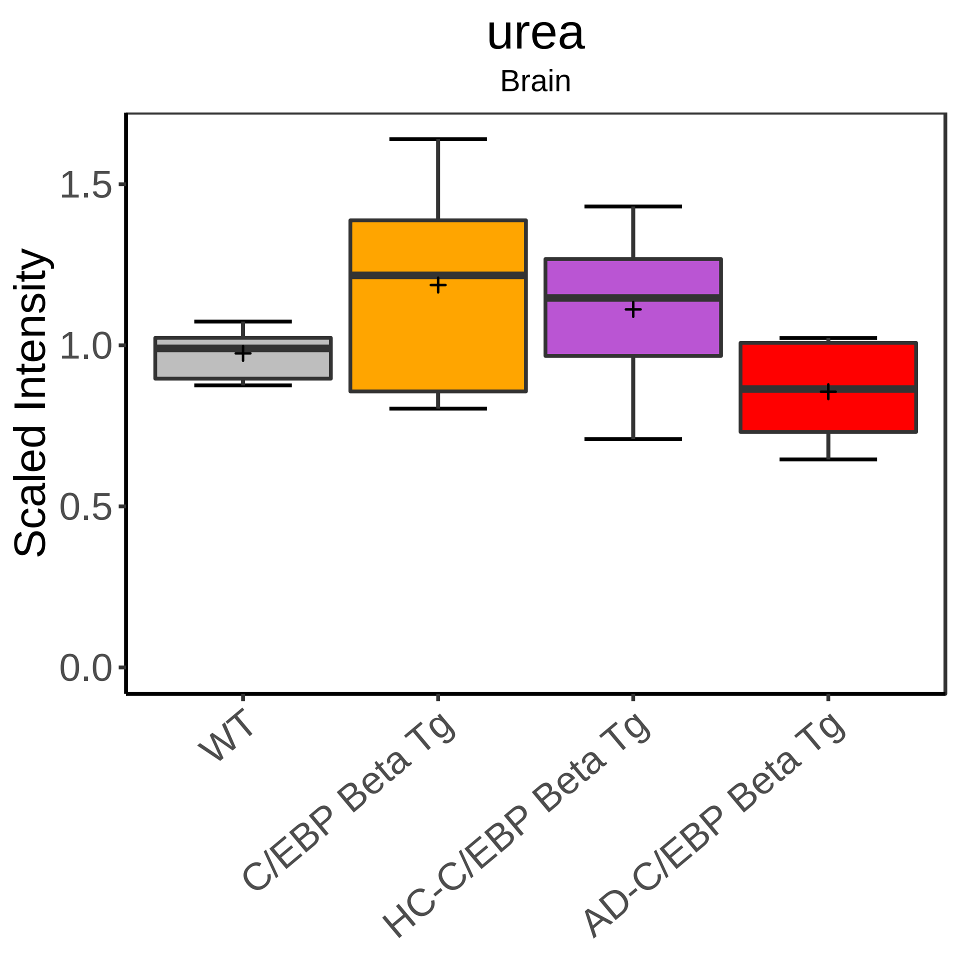
urea
Brain
Scaled Intensity
0.0
0.5
1.0
1.5
WT
C/EBP Beta Tg
HC-C/EBP Beta Tg
AD-C/EBP Beta Tg

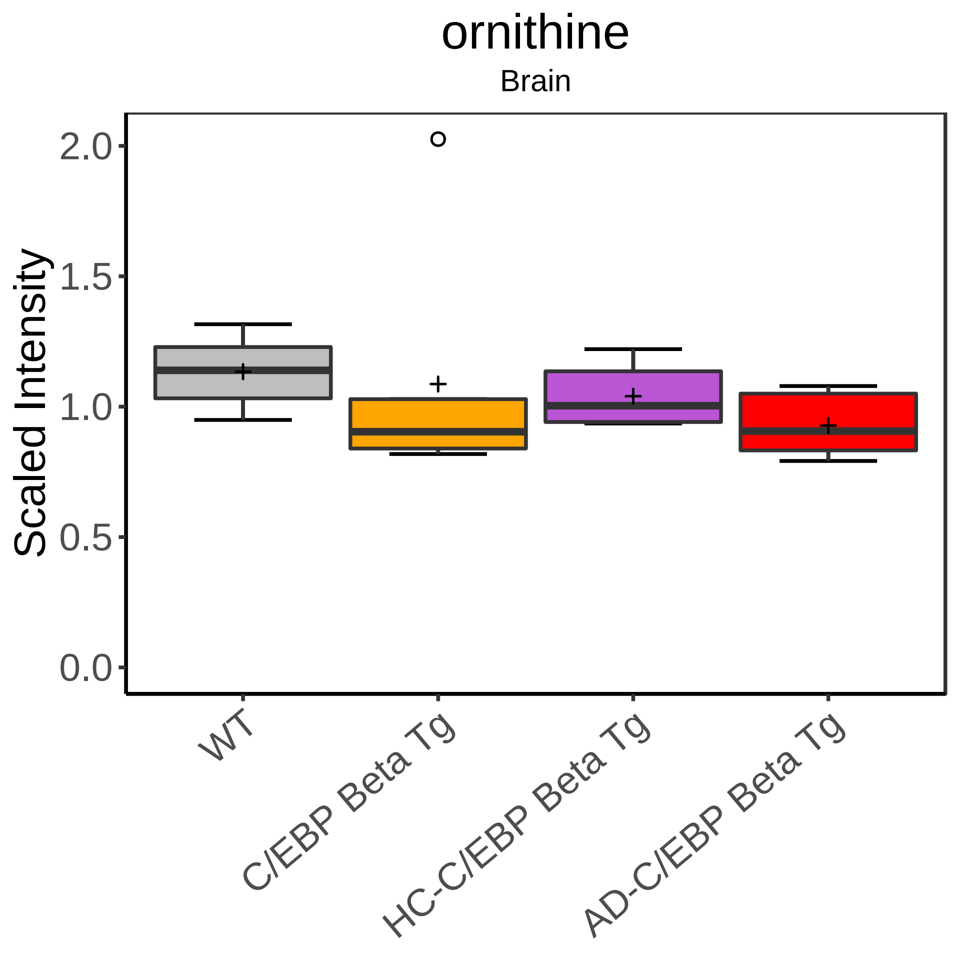
ornithine
Brain
Scaled Intensity
2.0
1.5
1.0
0.5
0.0
WT
C/EBP Beta Tg
HC-C/EBP Beta Tg
AD-C/EBP Beta Tg

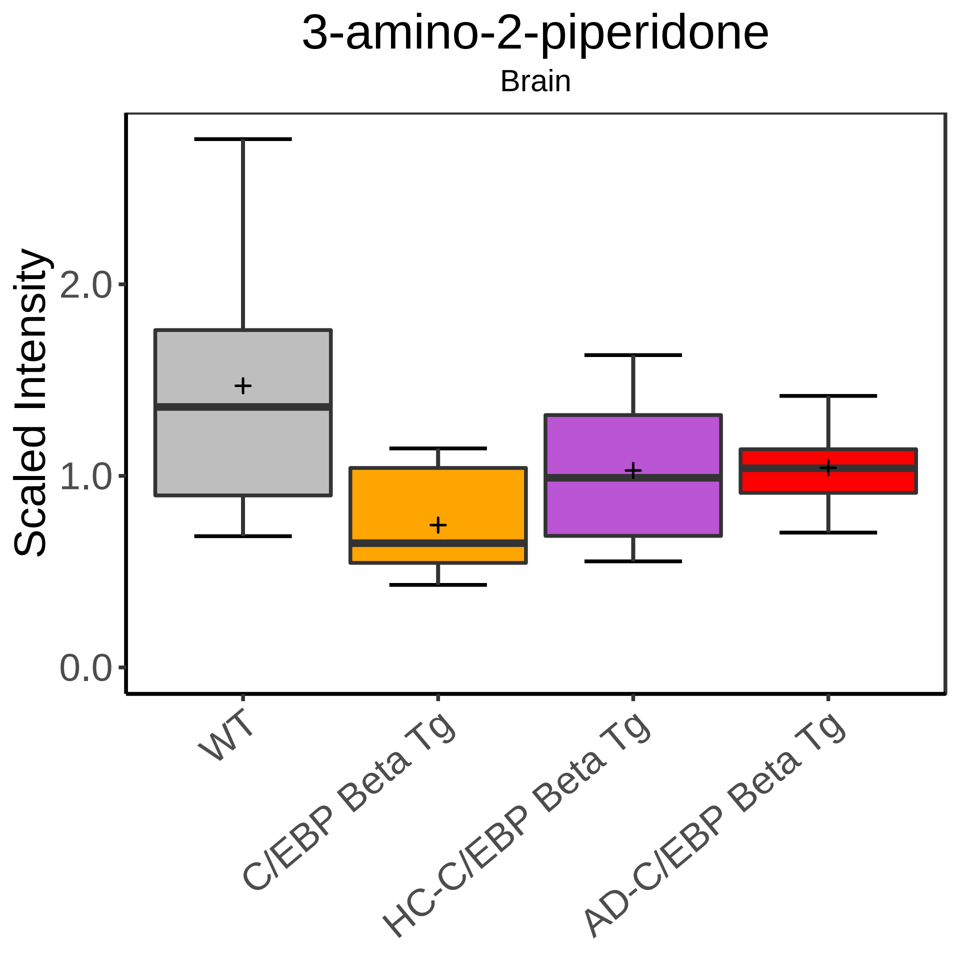

3-amino-2-piperidone
Brain
Scaled Intensity
2.0
1.0
0.0
WT
C/EBP Beta Tg
HC-C/EBP Beta Tg
AD-C/EBP Beta Tg

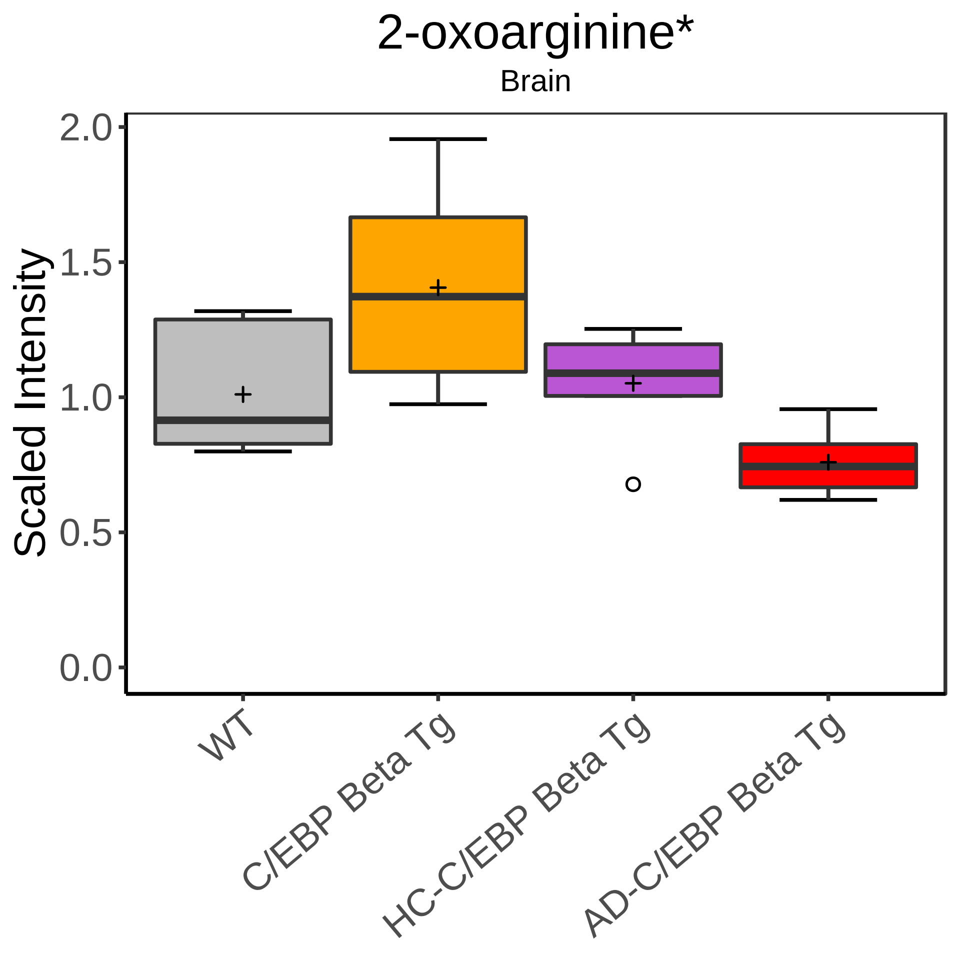

2-oxoarginine*
Brain
Scaled Intensity
2.0
1.5
1.0
0.5
0.0
WT
C/EBP Beta Tg
HC-C/EBP Beta Tg
AD-C/EBP Beta Tg

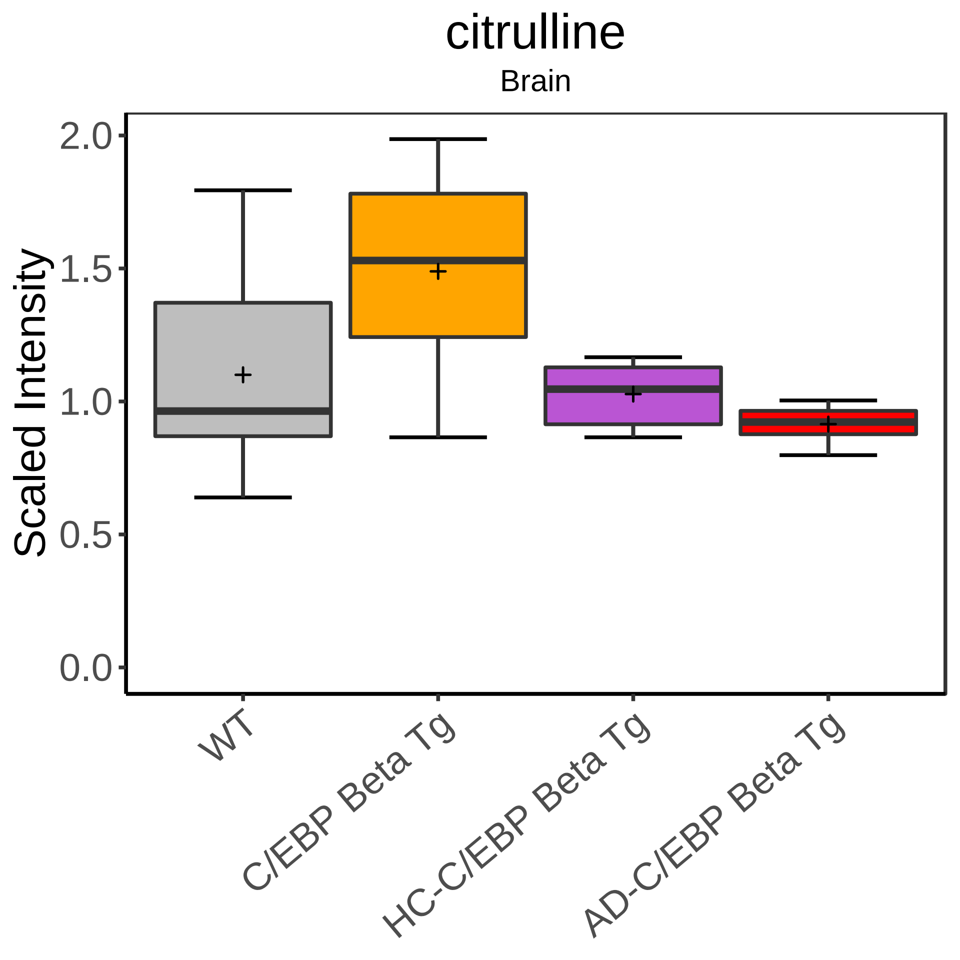

citrulline
Brain
Scaled Intensity
2.0
1.5
1.0
0.5
0.0
WT
C/EBP Beta Tg
HC-C/EBP Beta Tg
AD-C/EBP Beta Tg

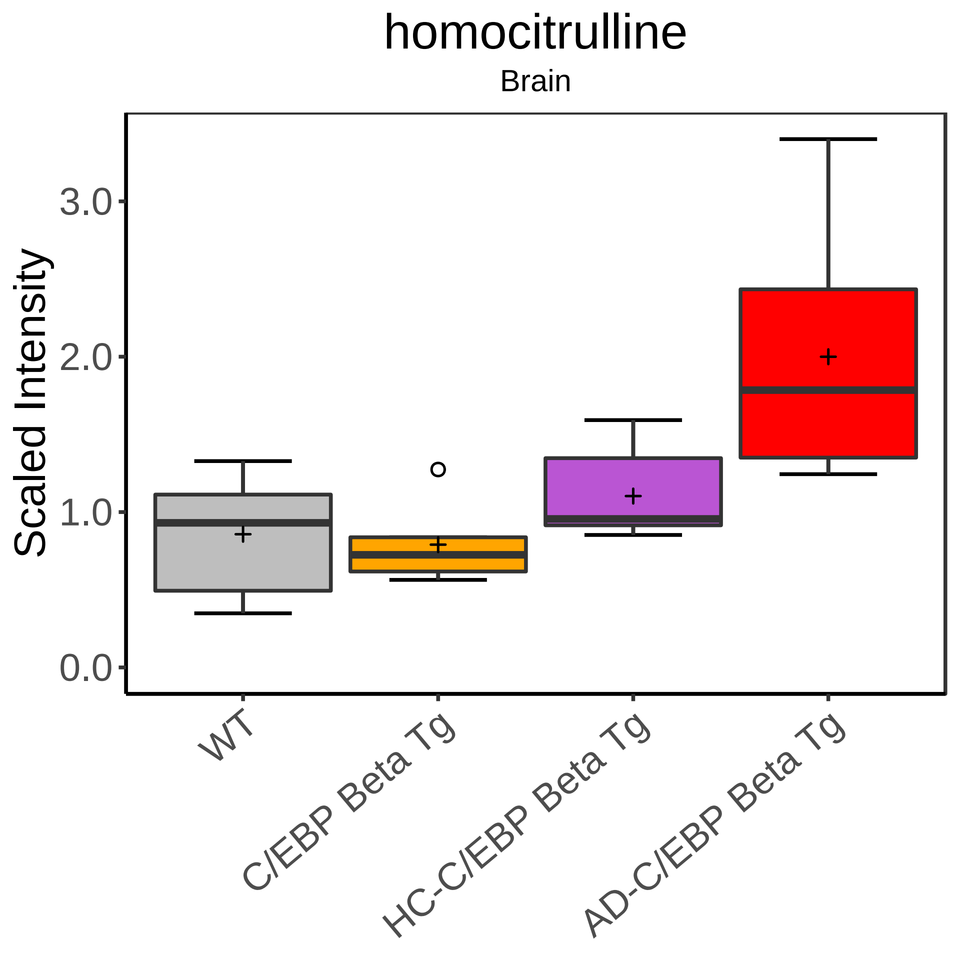
homocitrulline
Brain
Scaled Intensity
0.0
1.0
2.0
3.0
WT
C/EBP Beta Tg
HC-C/EBP Beta Tg
AD-C/EBP Beta Tg

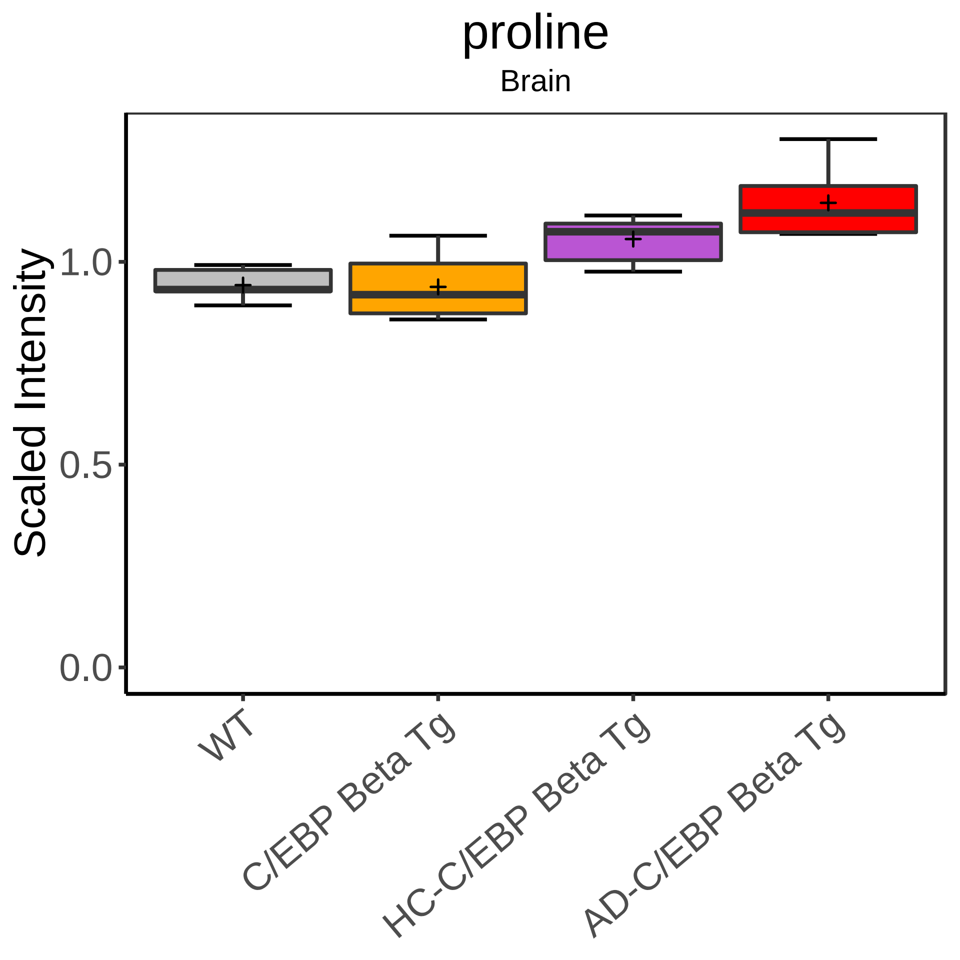
proline
Brain
Scaled Intensity
1.0
0.5
0.0
WT
C/EBP Beta Tg
HC-C/EBP Beta Tg
AD-C/EBP Beta Tg

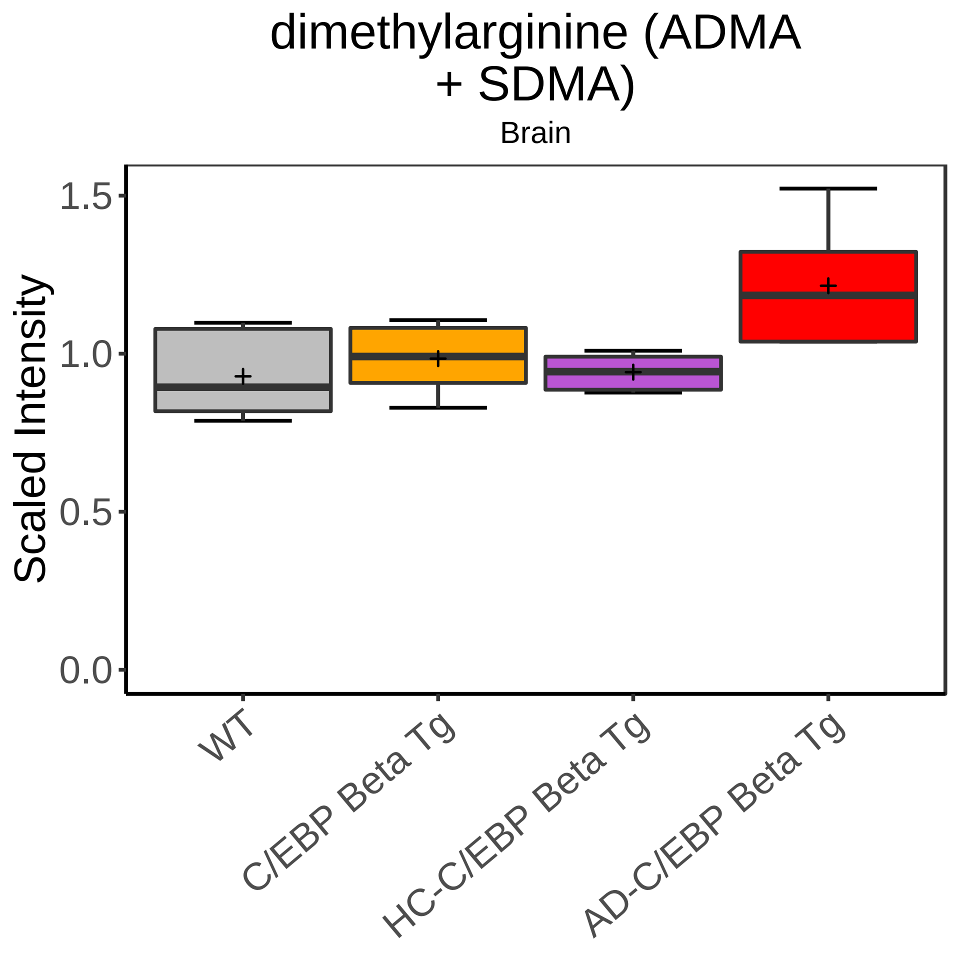
dimethylarginine (ADMA
+ SDMA)
Brain
Scaled Intensity
1.5
1.0
0.5
0.0
WT
C/EBP Beta Tg
HC-C/EBP Beta Tg
AD-C/EBP Beta Tg

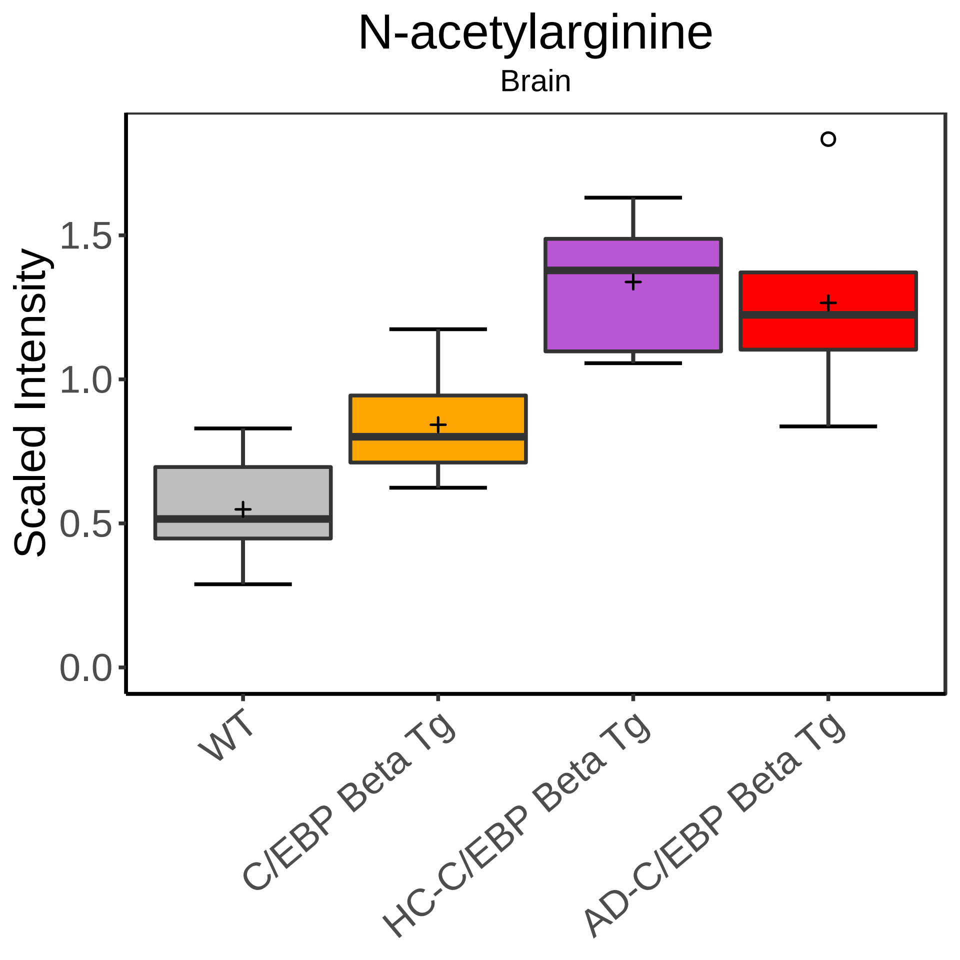
N-acetylarginine
Brain
Scaled Intensity
0.0
0.5
1.0
1.5
WT
C/EBP Beta Tg
HC-C/EBP Beta Tg
AD-C/EBP Beta Tg

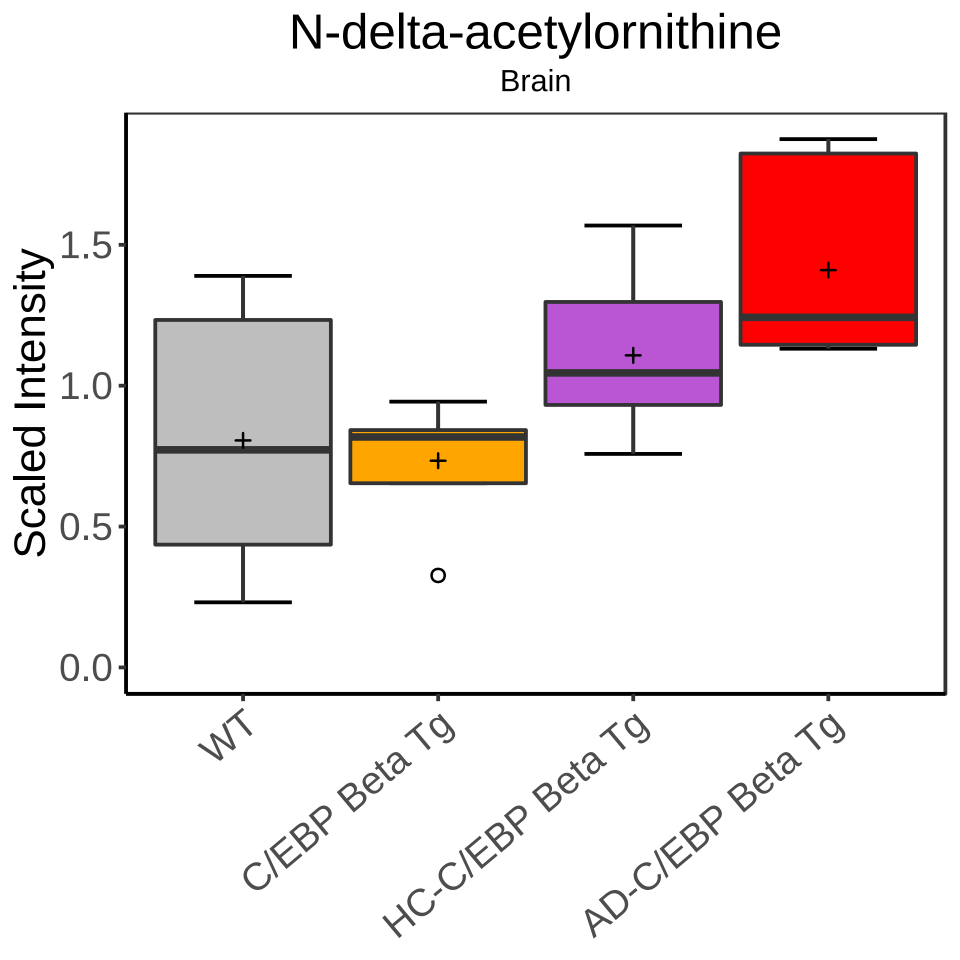
N-delta-acetylornithine
Brain
Scaled Intensity
1.5
1.0
0.5
0.0
WT
C/EBP Beta Tg
HC-C/EBP Beta Tg
AD-C/EBP Beta Tg

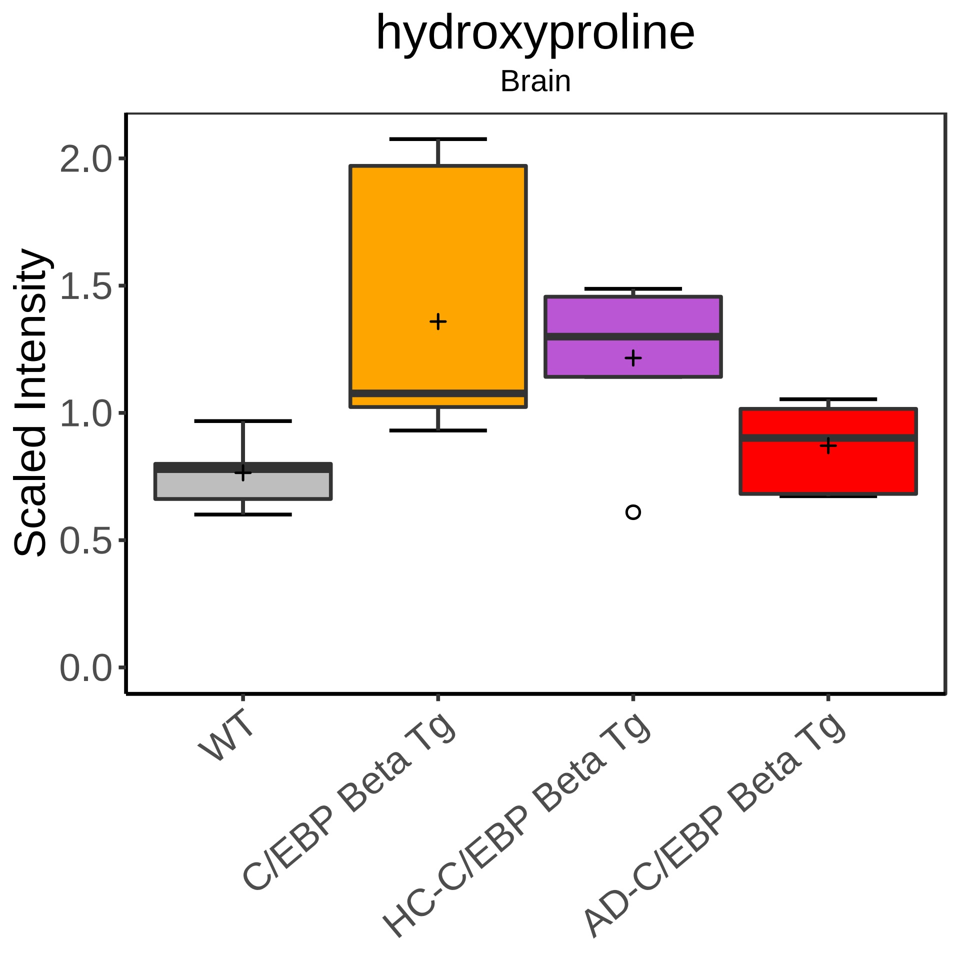
hydroxyproline
Brain
Scaled Intensity
0.0
0.5
1.0
1.5
2.0
WT
C/EBP Beta Tg
HC-C/EBP Beta Tg
AD-C/EBP Beta Tg

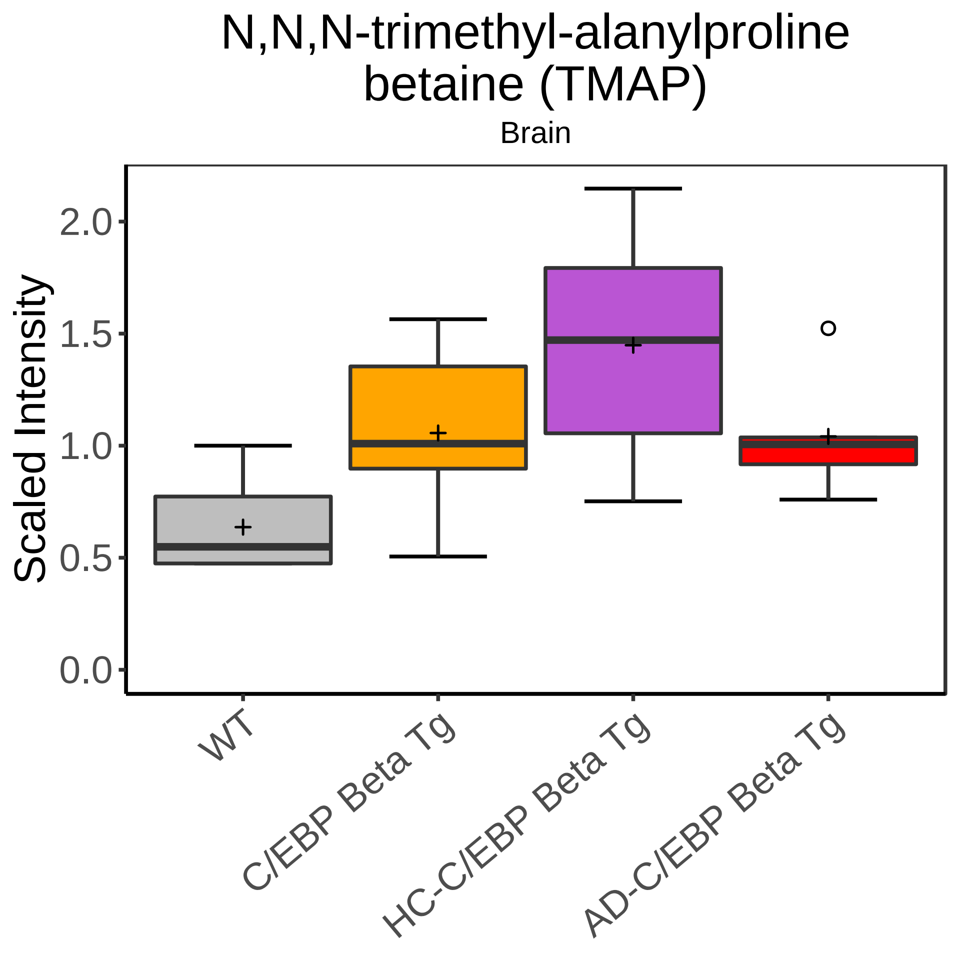
N,N,N-trimethyl-alanylproline betaine (TMAP)
Brain
Scaled Intensity
0.0
0.5
1.0
1.5
2.0
WT
C/EBP Beta Tg
HC-C/EBP Beta Tg
AD-C/EBP Beta Tg

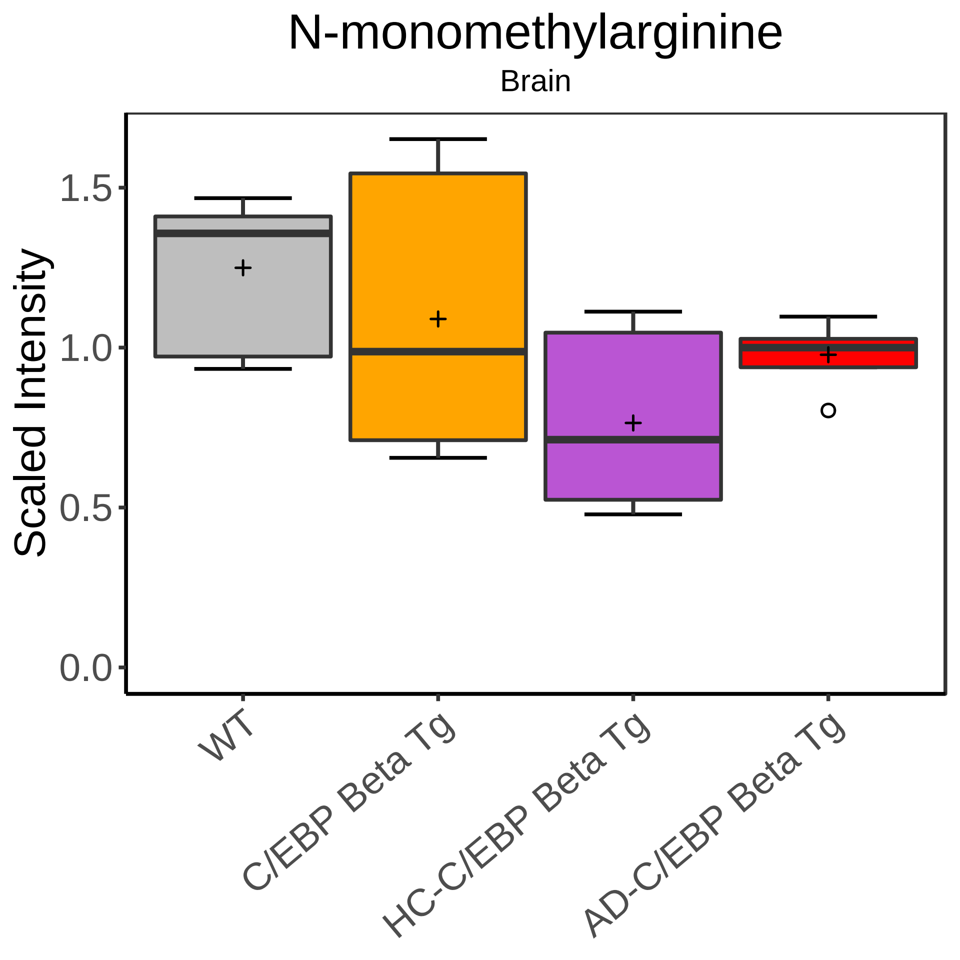

N-monomethylarginine
Brain
Scaled Intensity
1.5
1.0
0.5
0.0
WT
C/EBP Beta Tg
HC-C/EBP Beta Tg
AD-C/EBP Beta Tg

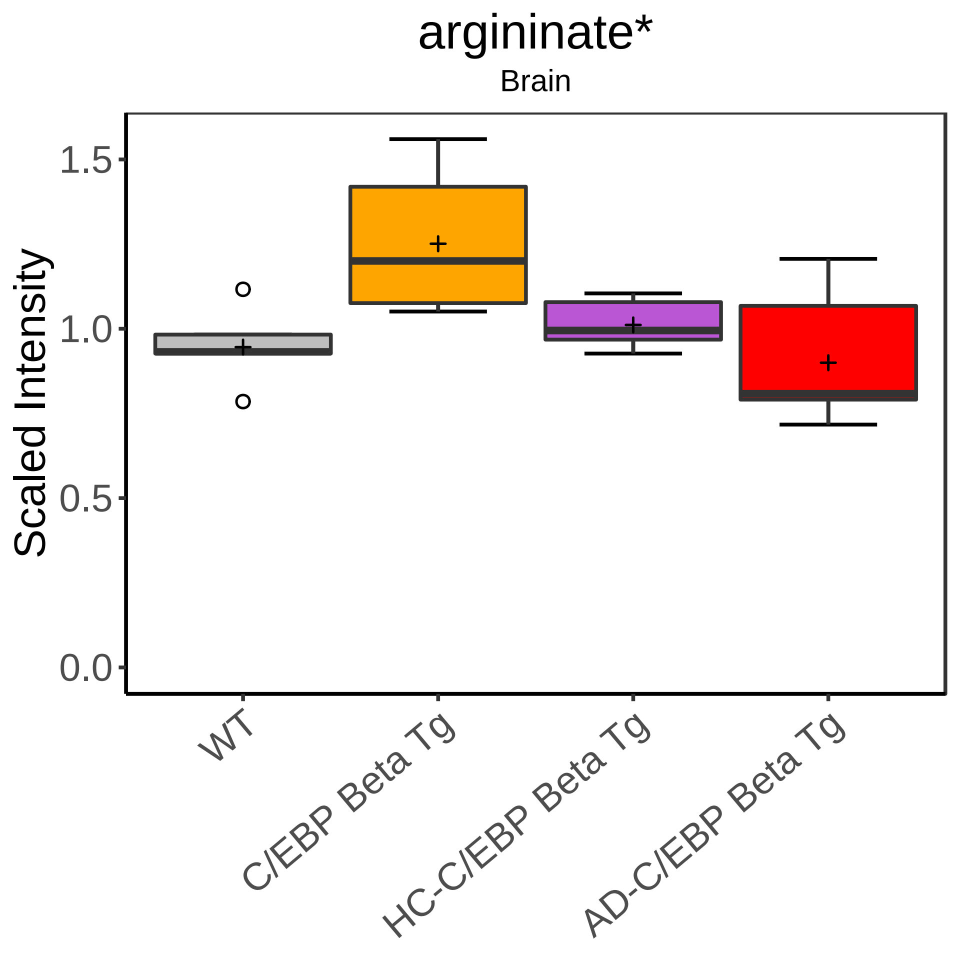
arginate*
Brain
Scaled Intensity
1.5
1.0
0.5
0.0
WT
C/EBP Beta Tg
HC-C/EBP Beta Tg
AD-C/EBP Beta Tg

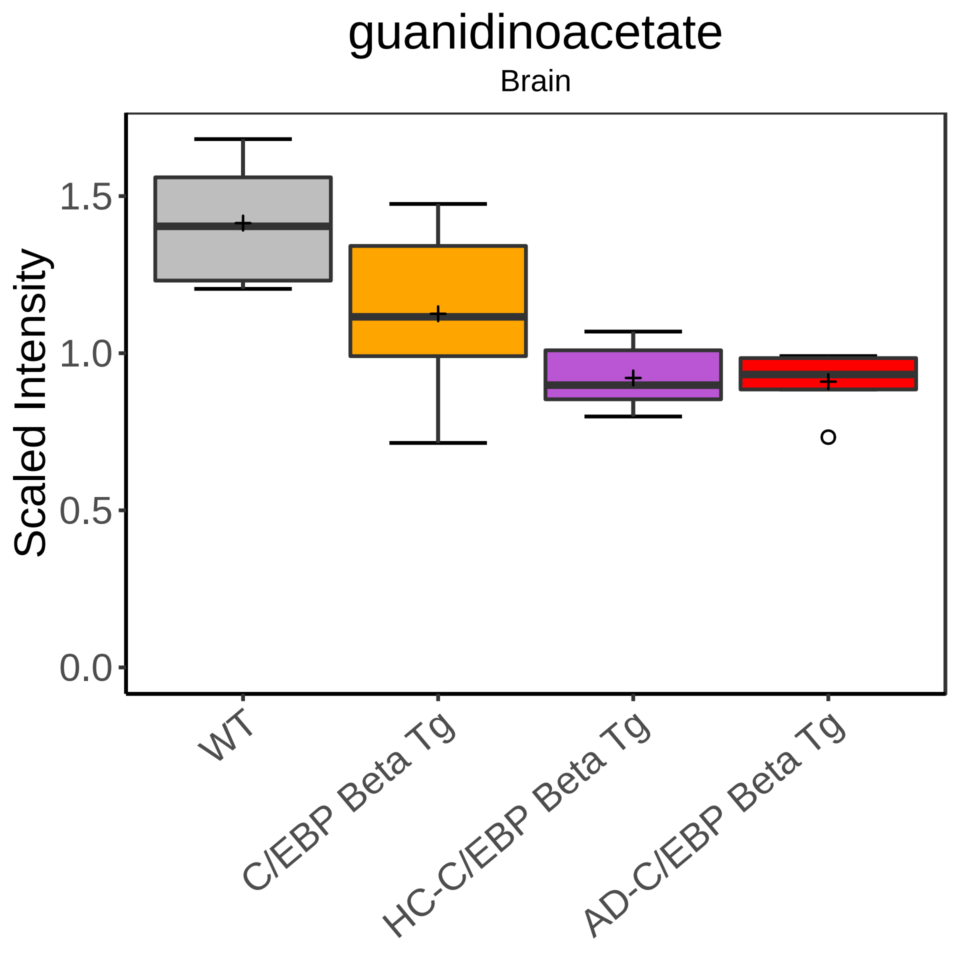
guanidinoacetate
Brain
Scaled Intensity
1.5
1.0
0.5
0.0
WT
C/EBP Beta Tg
HC-C/EBP Beta Tg
AD-C/EBP Beta Tg

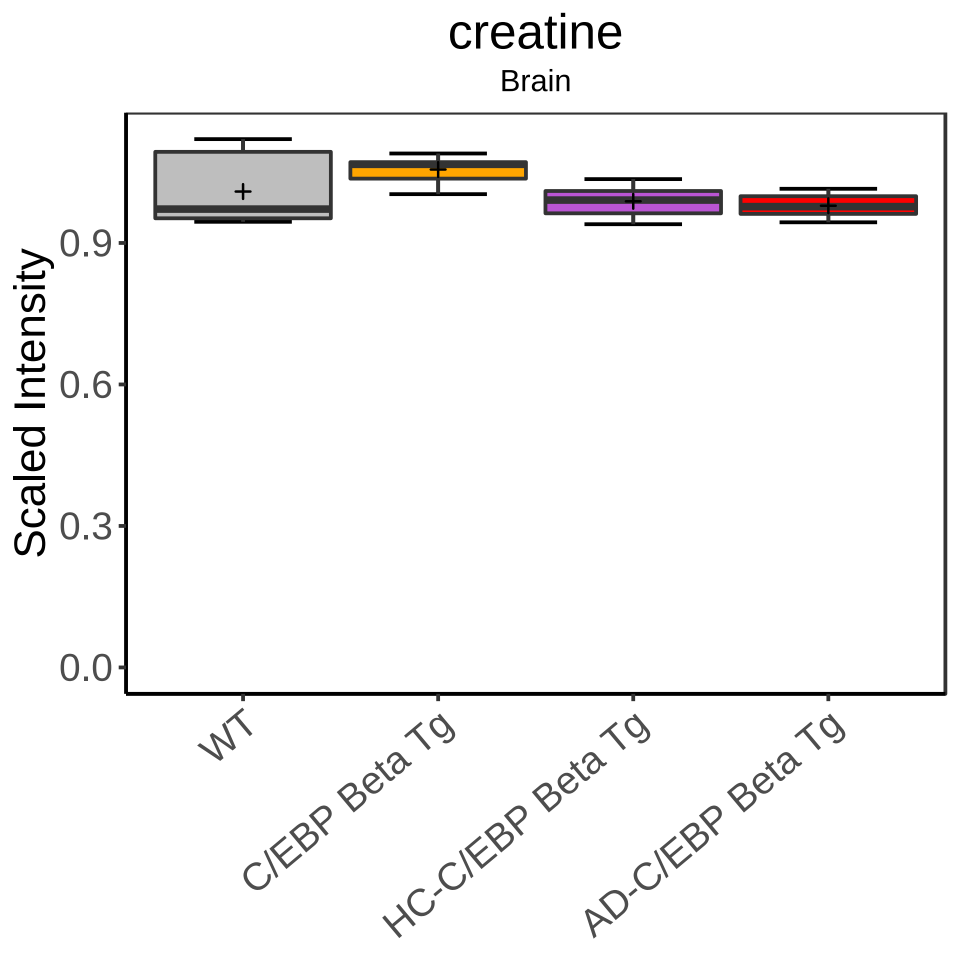
creatine
Brain
Scaled Intensity
0.9
0.6
0.3
0.0
WT
C/EBP Beta Tg
HC-C/EBP Beta Tg
AD-C/EBP Beta Tg

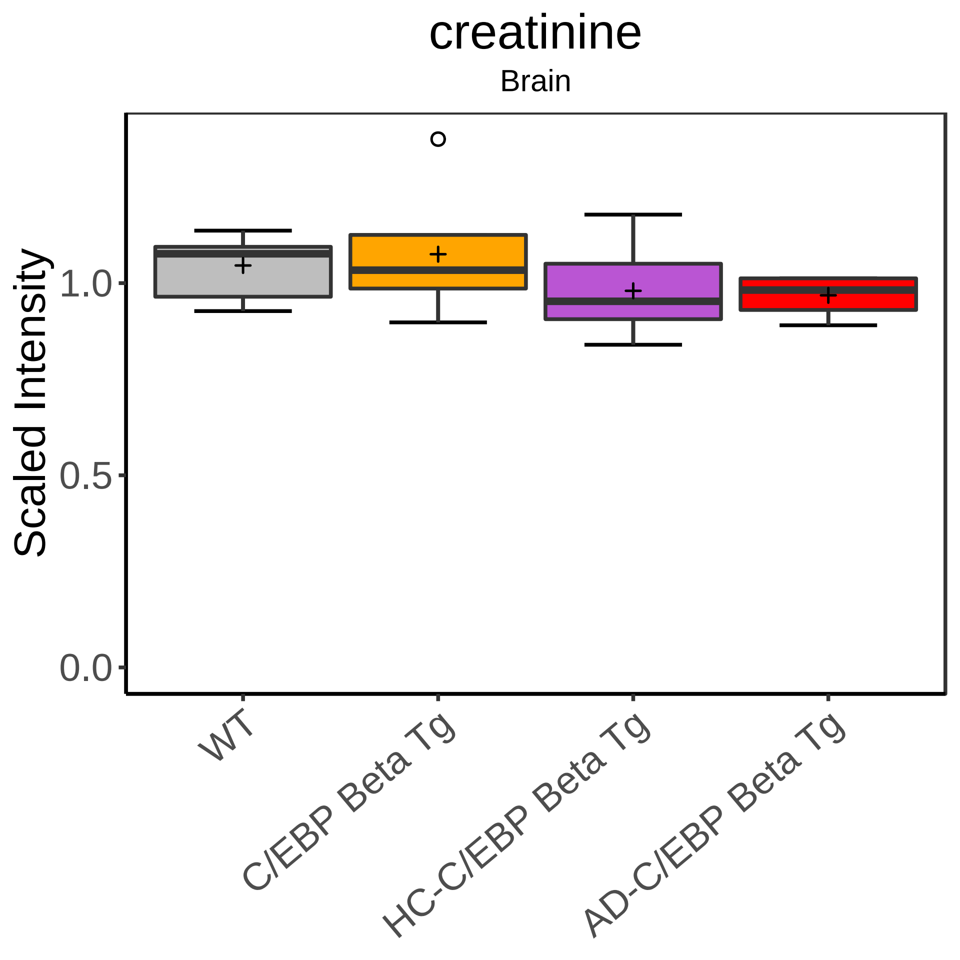

creatinine
Brain
Scaled Intensity
0.0
0.5
1.0
WT
C/EBP Beta Tg
HC-C/EBP Beta Tg
AD-C/EBP Beta Tg

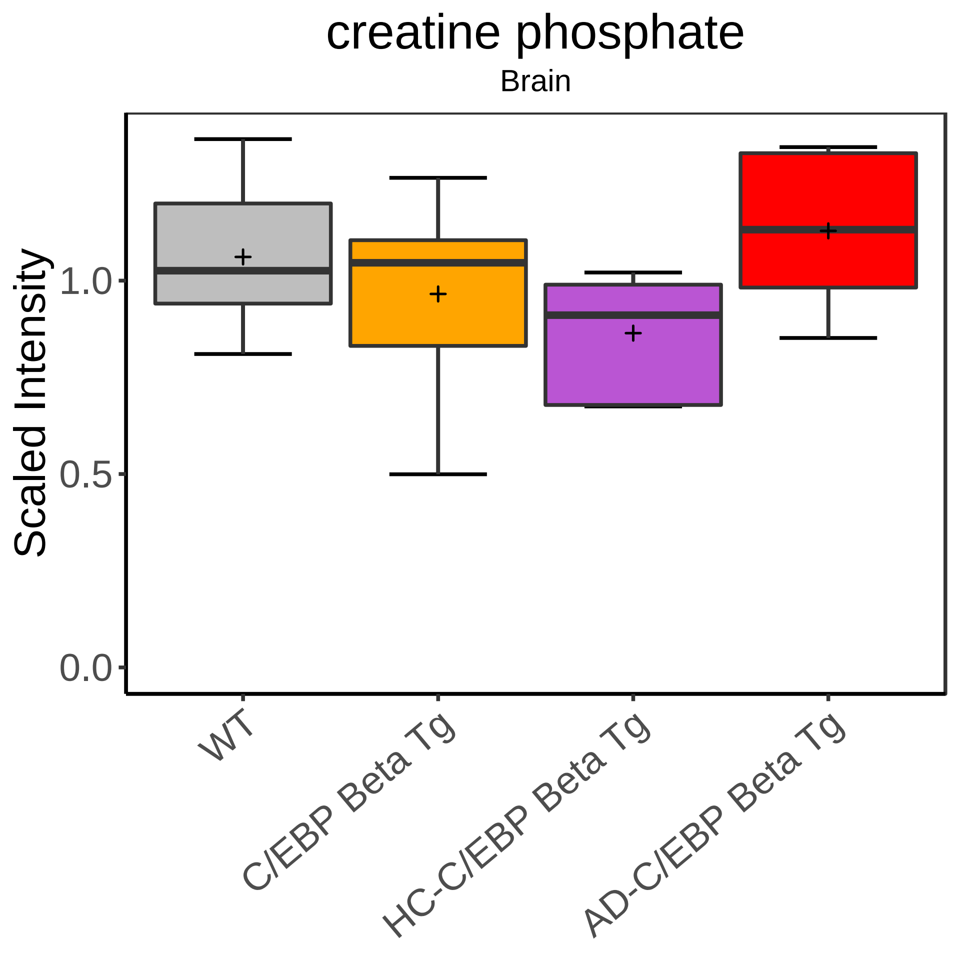
creatine phosphate
Brain
Scaled Intensity
1.0
0.5
0.0
WT
C/EBP Beta Tg
HC-C/EBP Beta Tg
AD-C/EBP Beta Tg

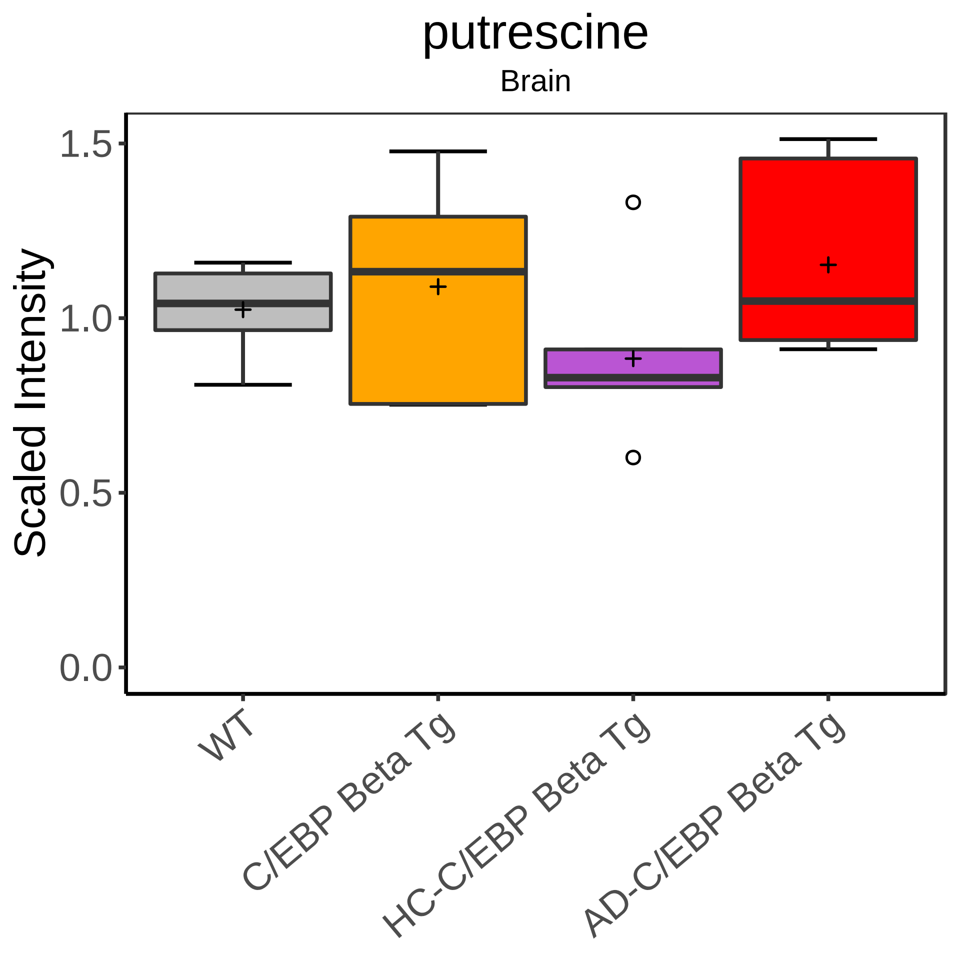
putrescine
Brain
Scaled Intensity
1.5
1.0
0.5
0.0
WT
C/EBP Beta Tg
HC-C/EBP Beta Tg
AD-C/EBP Beta Tg

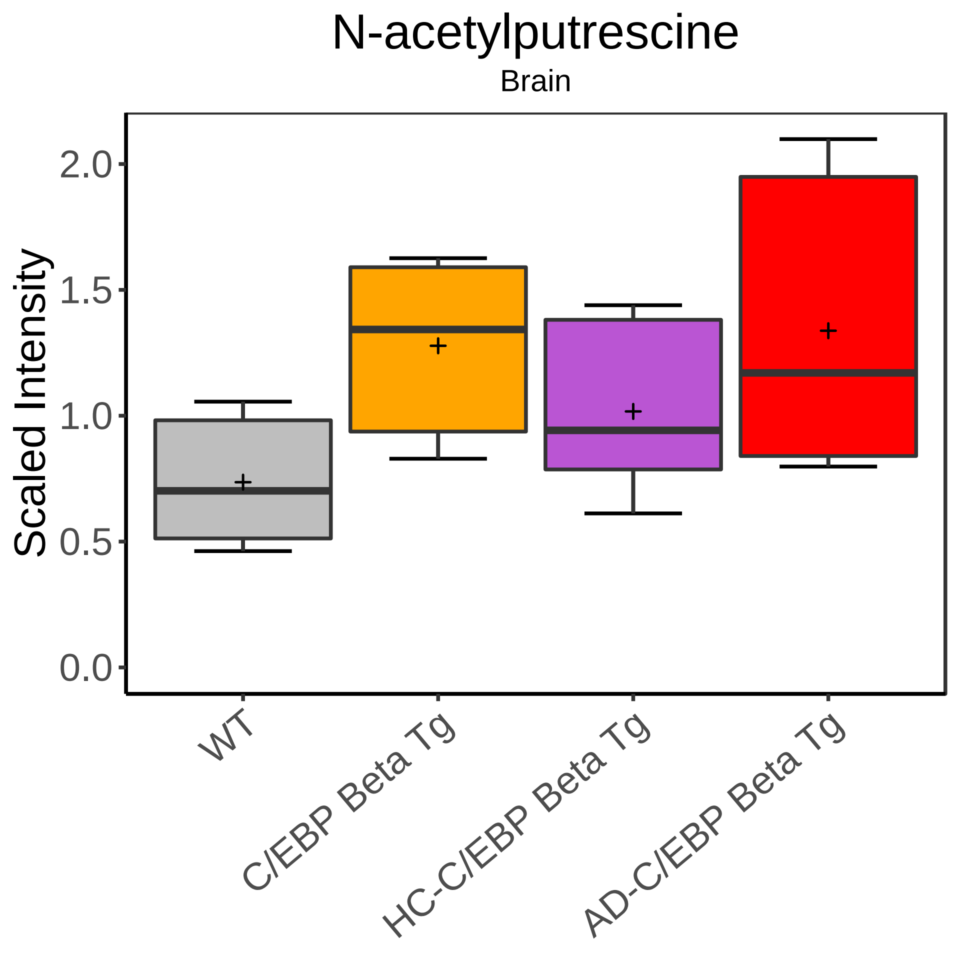

N-acetylputrescine
Brain
Scaled Intensity
2.0
1.5
1.0
0.5
0.0
WT
C/EBP Beta Tg
HC-C/EBP Beta Tg
AD-C/EBP Beta Tg

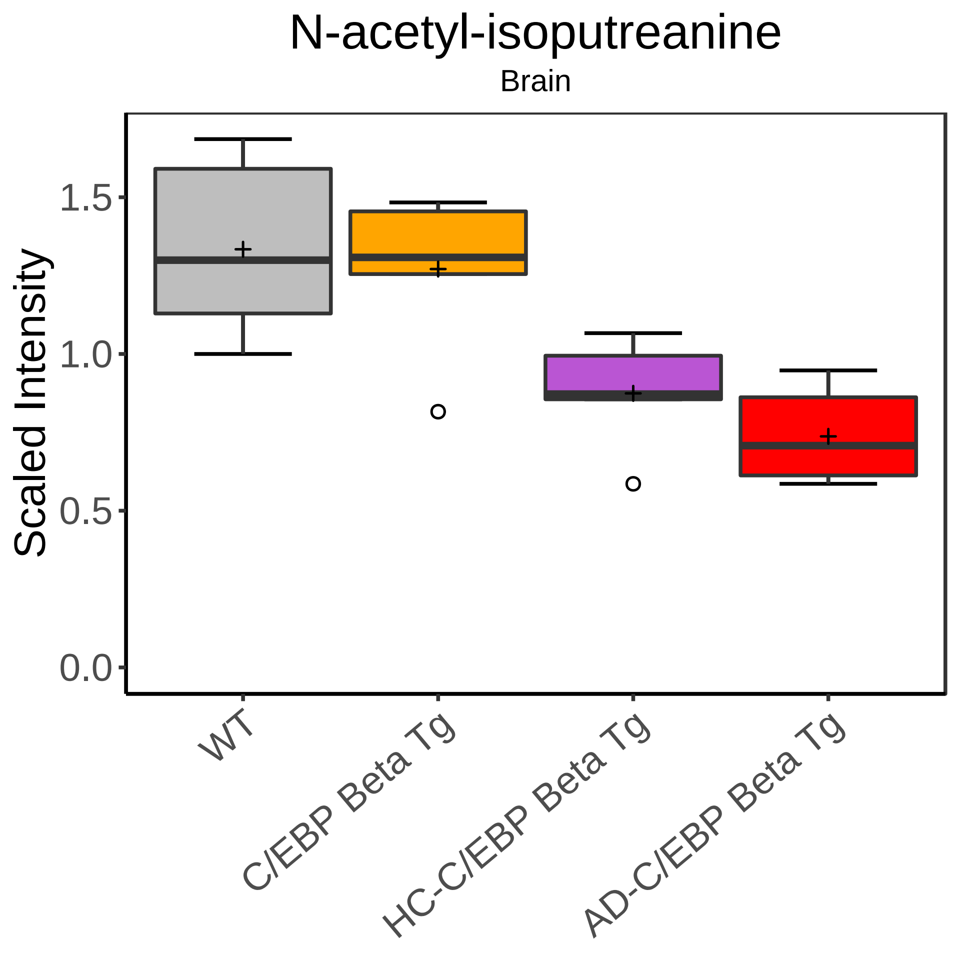
N-acetyl-isoputreanine
Brain
Scaled Intensity
1.5
1.0
0.5
0.0
WT
C/EBP Beta Tg
HC-C/EBP Beta Tg
AD-C/EBP Beta Tg

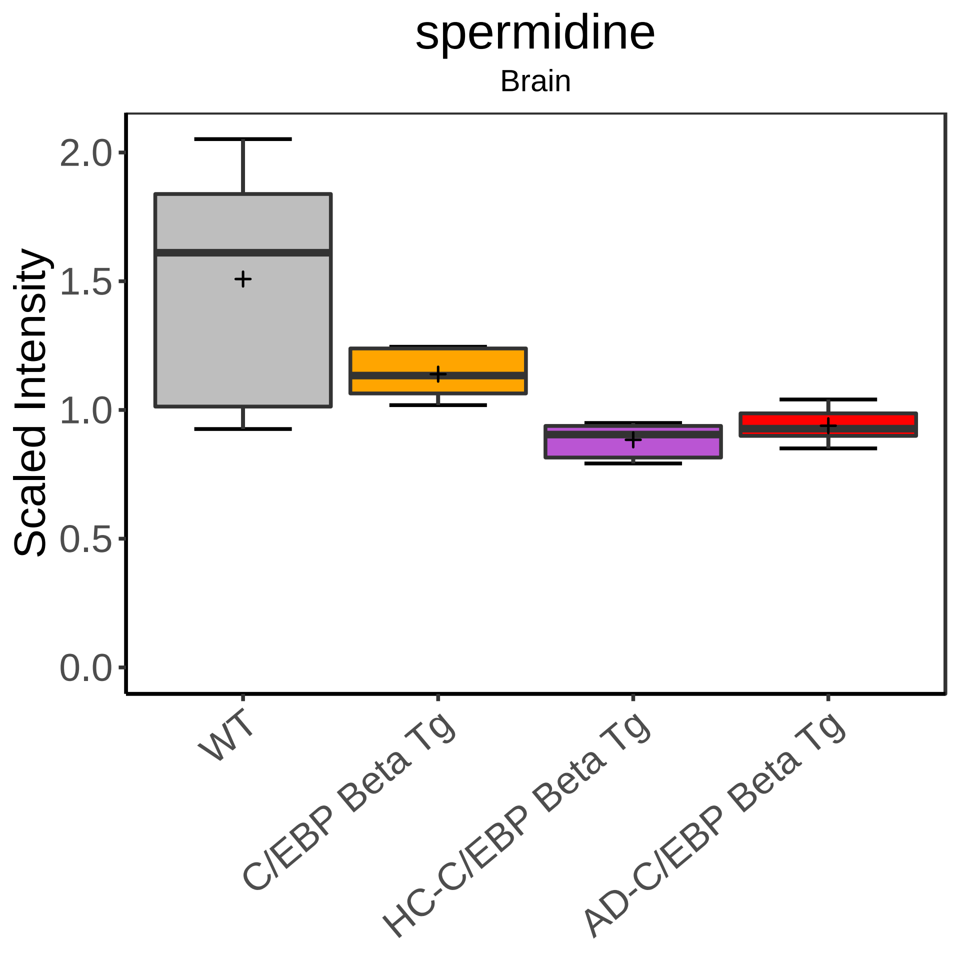
spermidine
Brain
Scaled Intensity
2.0
1.5
1.0
0.5
0.0
WT
C/EBP Beta Tg
HC-C/EBP Beta Tg
AD-C/EBP Beta Tg

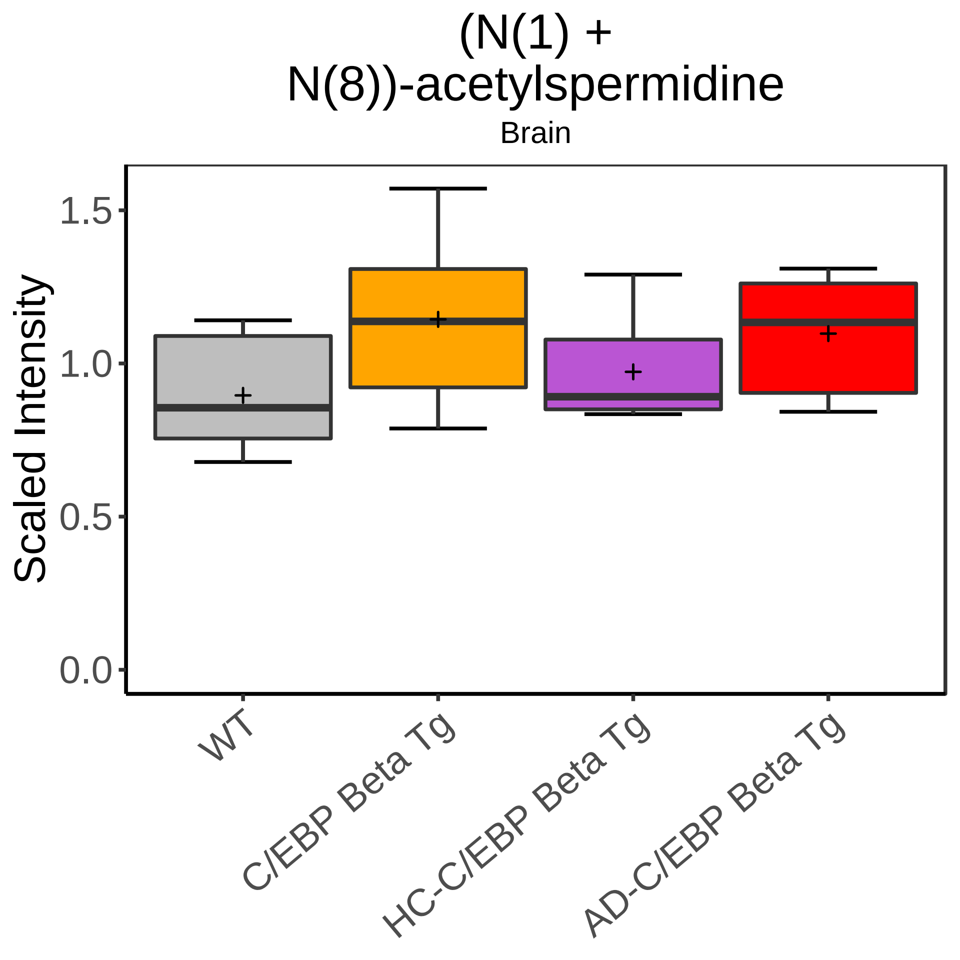
(N(1) +
N(8))-acetylspermidine
Brain
Scaled Intensity
1.5
1.0
0.5
0.0
WT
C/EBP Beta Tg
HC-C/EBP Beta Tg
AD-C/EBP Beta Tg

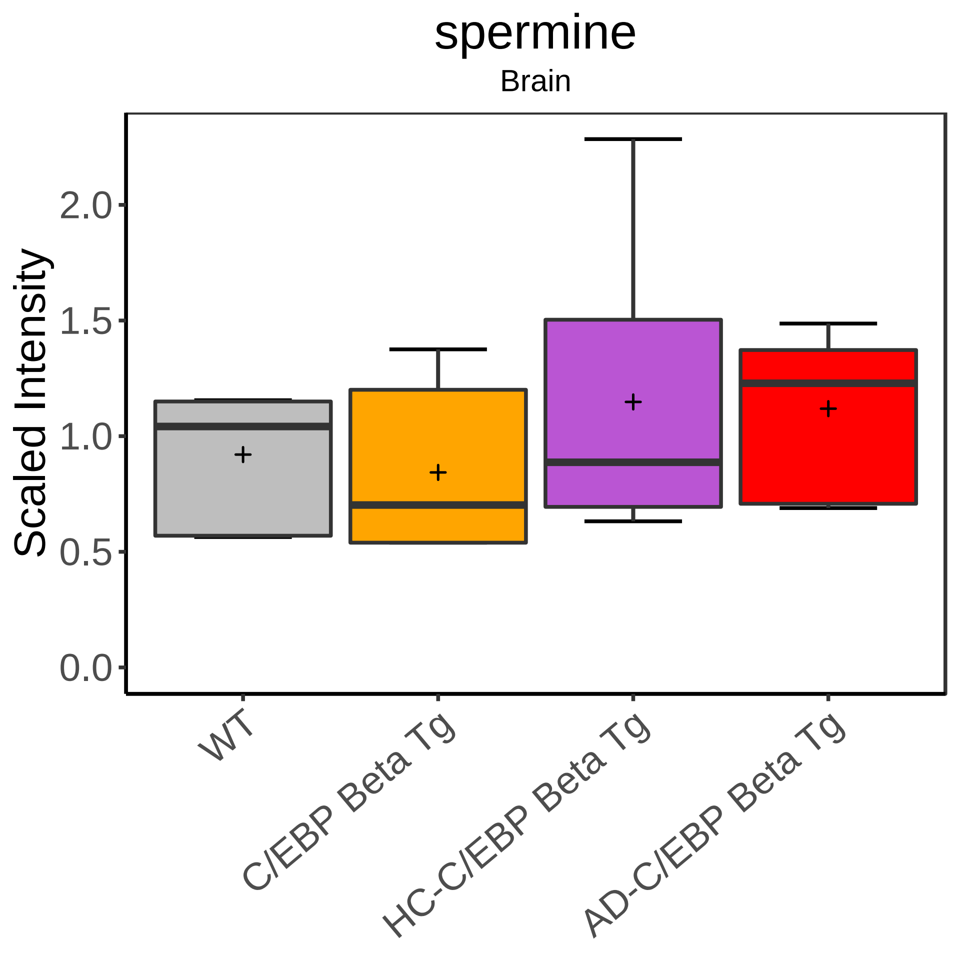
spermine
Brain
Scaled Intensity
2.0
1.5
1.0
0.5
0.0
WT
C/EBP Beta Tg
HC-C/EBP Beta Tg
AD-C/EBP Beta Tg

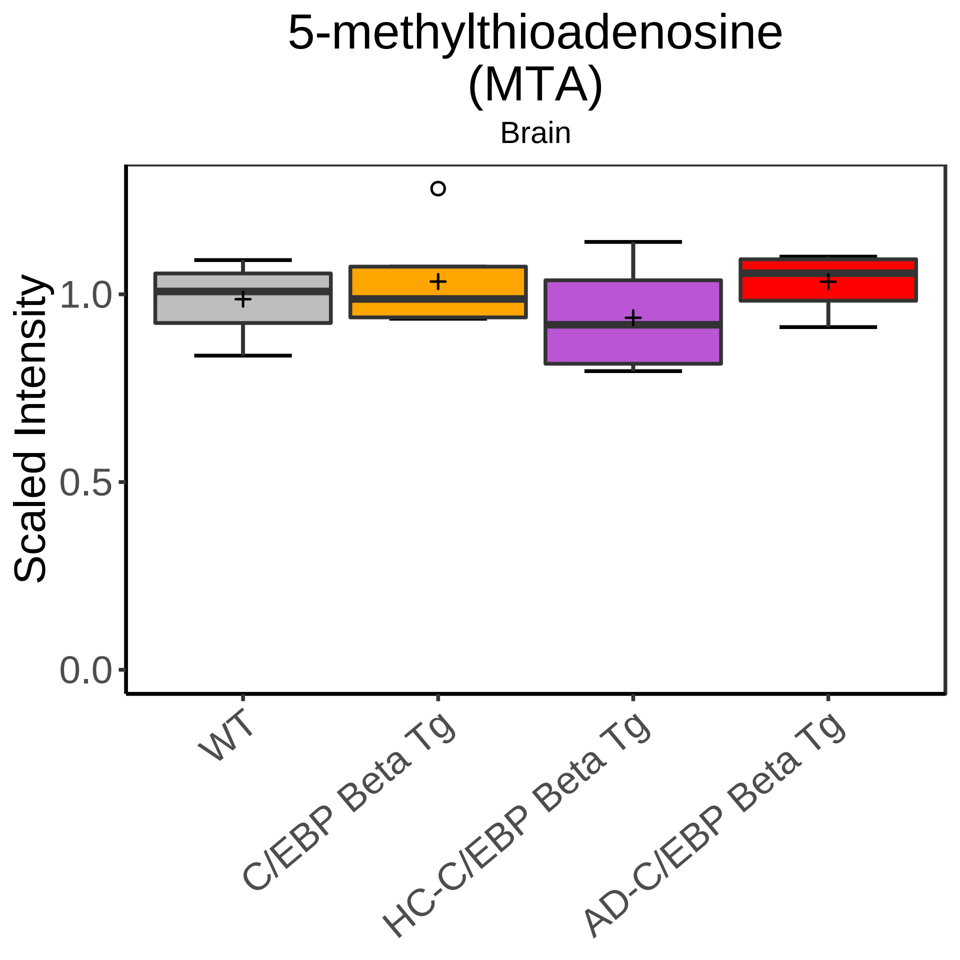
5-methylthioadenosine
(MTA)
Brain
Scaled Intensity
1.0
0.5
0.0
WT
C/EBP Beta Tg
HC-C/EBP Beta Tg
AD-C/EBP Beta Tg

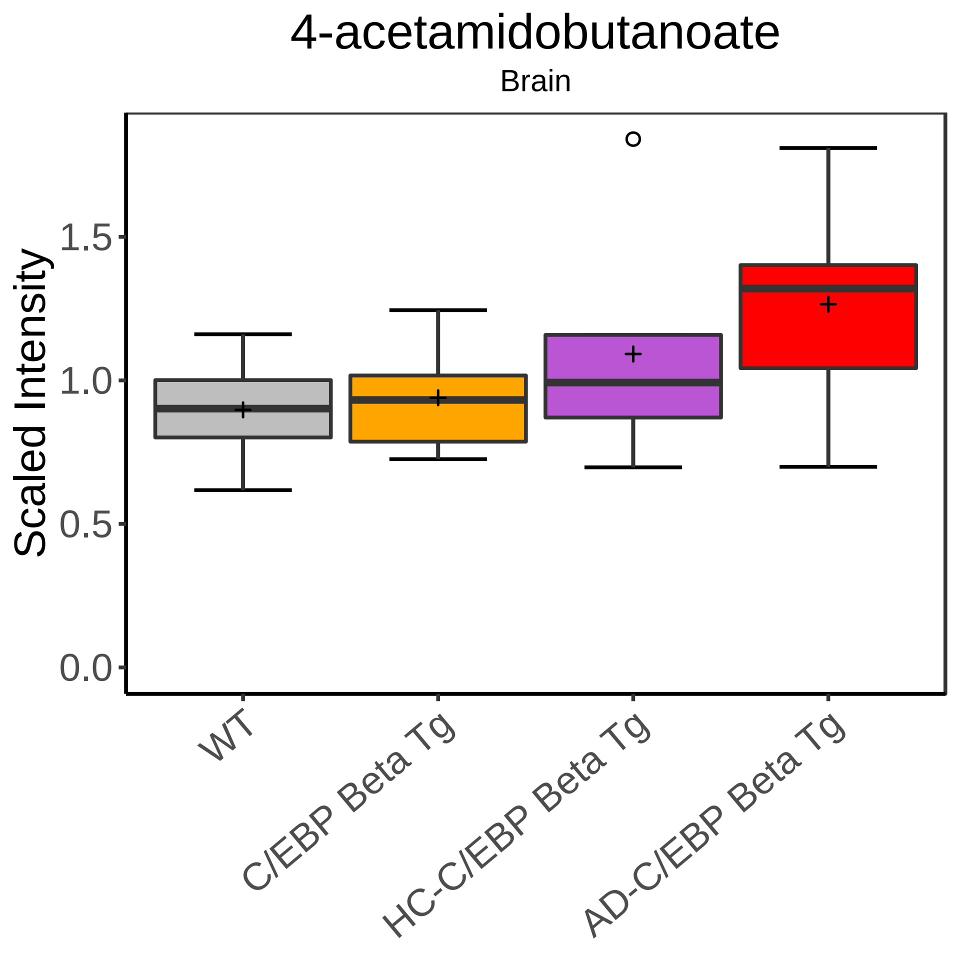
4-acetamidobutanoate
Brain
Scaled Intensity
0.0
0.5
1.0
1.5
WT
C/EBP Beta Tg
HC-C/EBP Beta Tg
AD-C/EBP Beta Tg

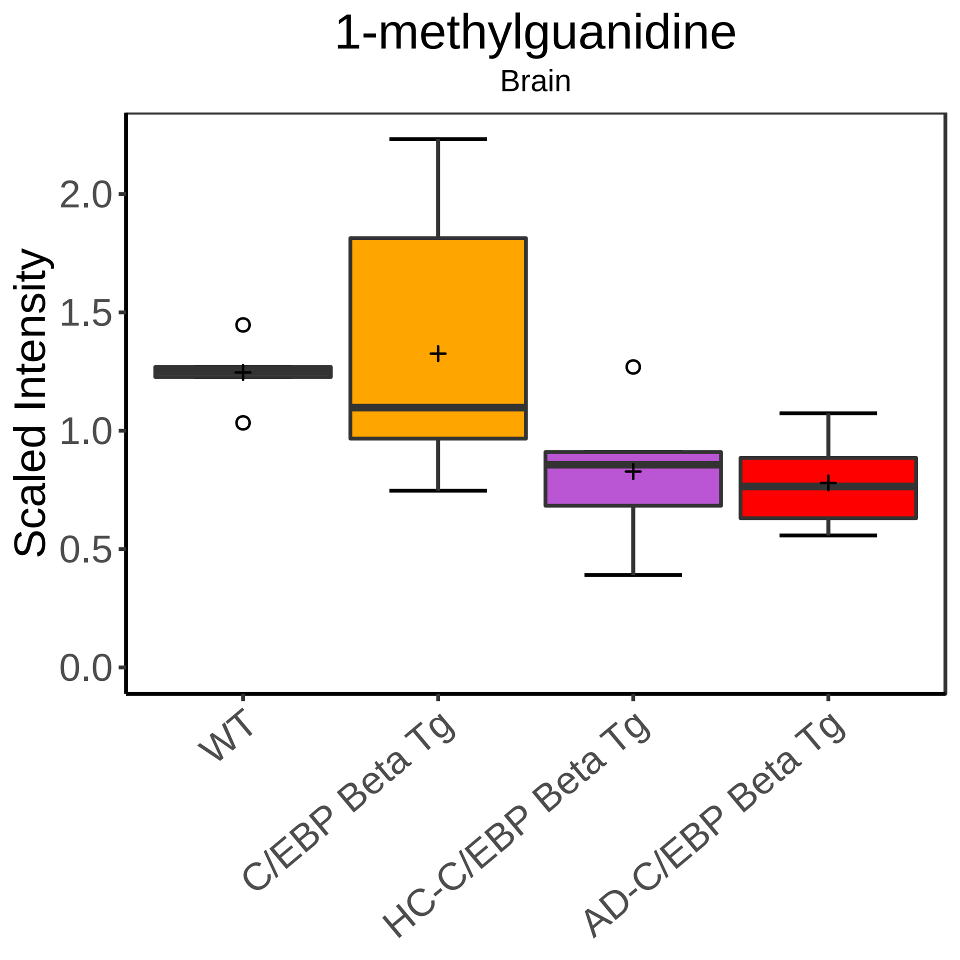
1-methylguanidine
Brain
Scaled Intensity
2.0
1.5
1.0
0.5
0.0
WT
C/EBP Beta Tg
HC-C/EBP Beta Tg
AD-C/EBP Beta Tg

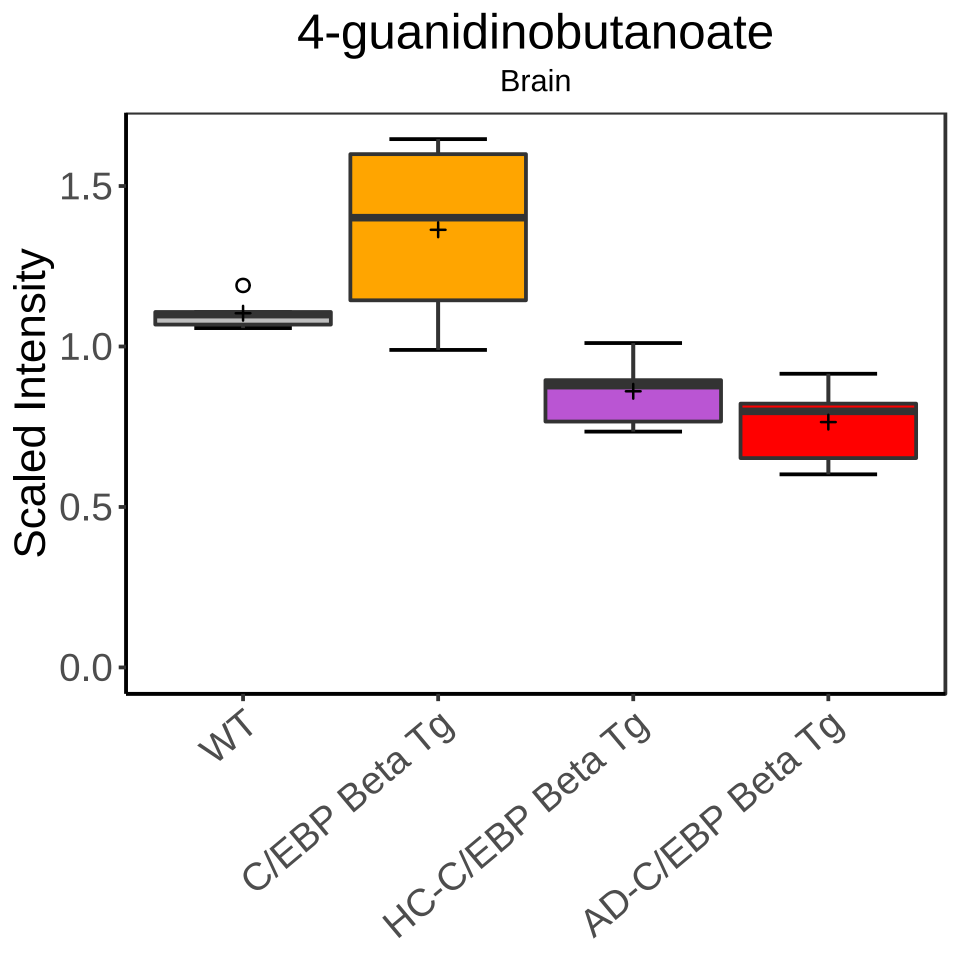
4-guanidinobutanoate
Brain
Scaled Intensity
0.0
0.5
1.0
1.5
WT
C/EBP Beta Tg
HC-C/EBP Beta Tg
AD-C/EBP Beta Tg

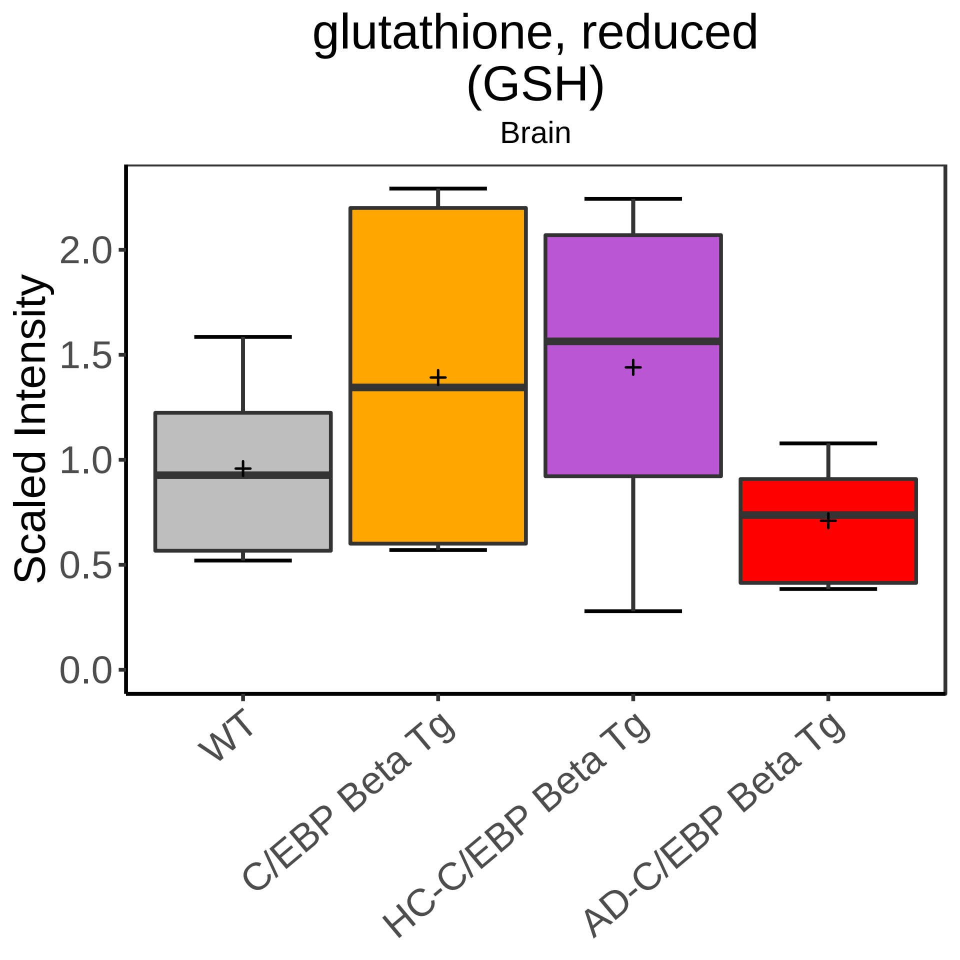

glutathione, reduced
(GSH)
Brain
Scaled Intensity
0.0
0.5
1.0
1.5
2.0
WT
C/EBP Beta Tg
HC-C/EBP Beta Tg
AD-C/EBP Beta Tg

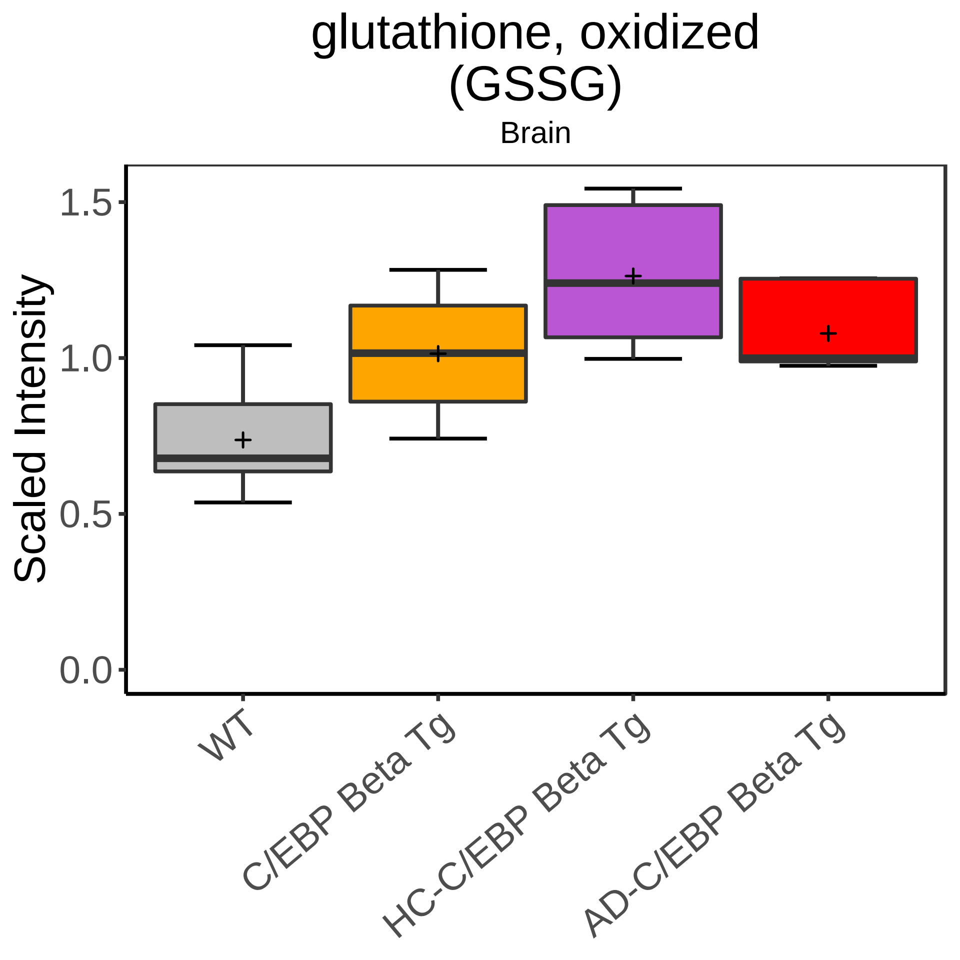
glutathione, oxidized
(GSSG)
Brain
Scaled Intensity
1.5
1.0
0.5
0.0
WT
C/EBP Beta Tg
HC-C/EBP Beta Tg
AD-C/EBP Beta Tg

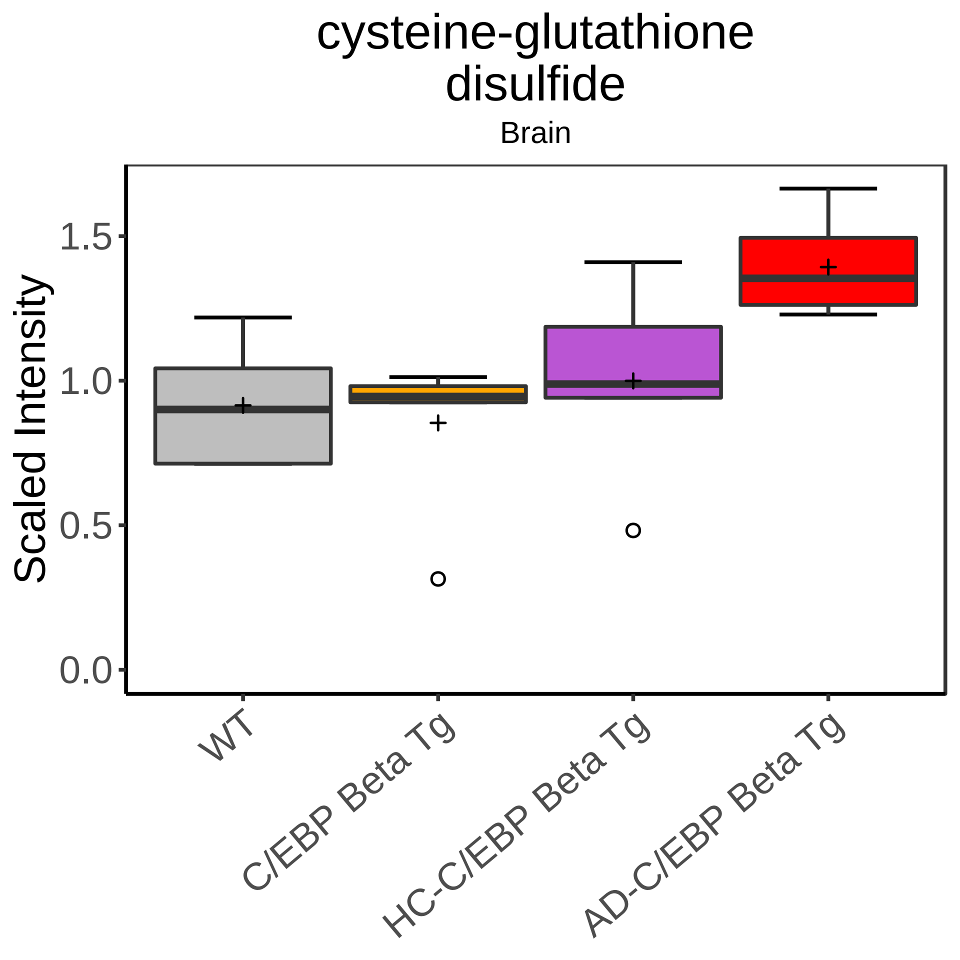
cysteine-glutathione
disulfide
Brain
Scaled Intensity
1.5
1.0
0.5
0.0
WT
C/EBP Beta Tg
HC-C/EBP Beta Tg
AD-C/EBP Beta Tg

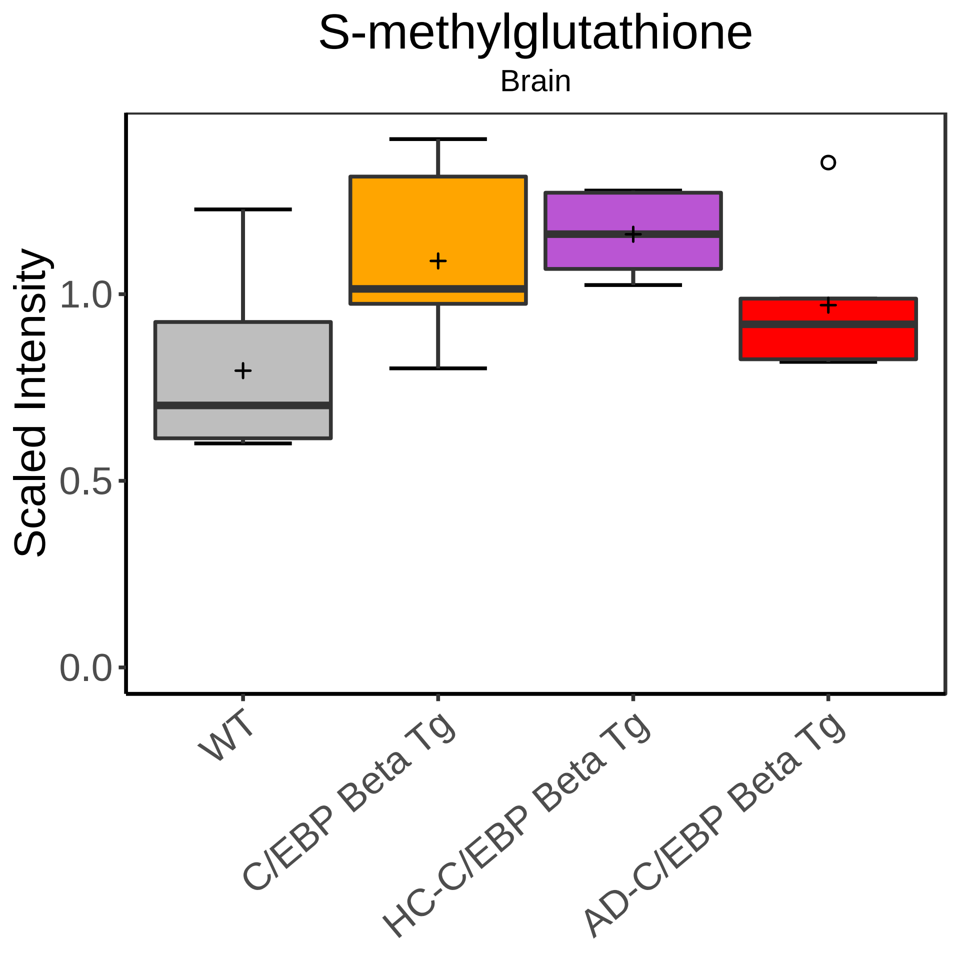

S-methylglutathione
Brain
Scaled Intensity
0.0
0.5
1.0
WT
C/EBP Beta Tg
HC-C/EBP Beta Tg
AD-C/EBP Beta Tg

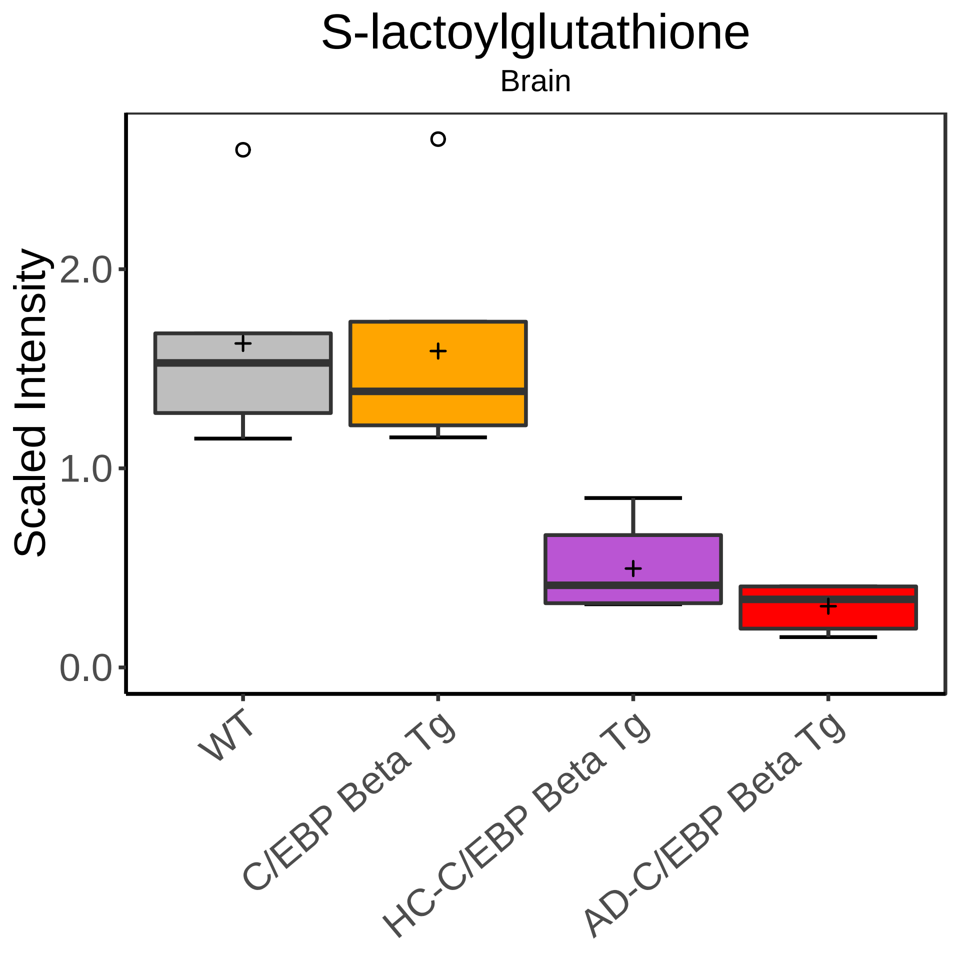

S-lactoylglutathione
Brain
Scaled Intensity
2.0
1.0
0.0
WT
C/EBP Beta Tg
HC-C/EBP Beta Tg
AD-C/EBP Beta Tg

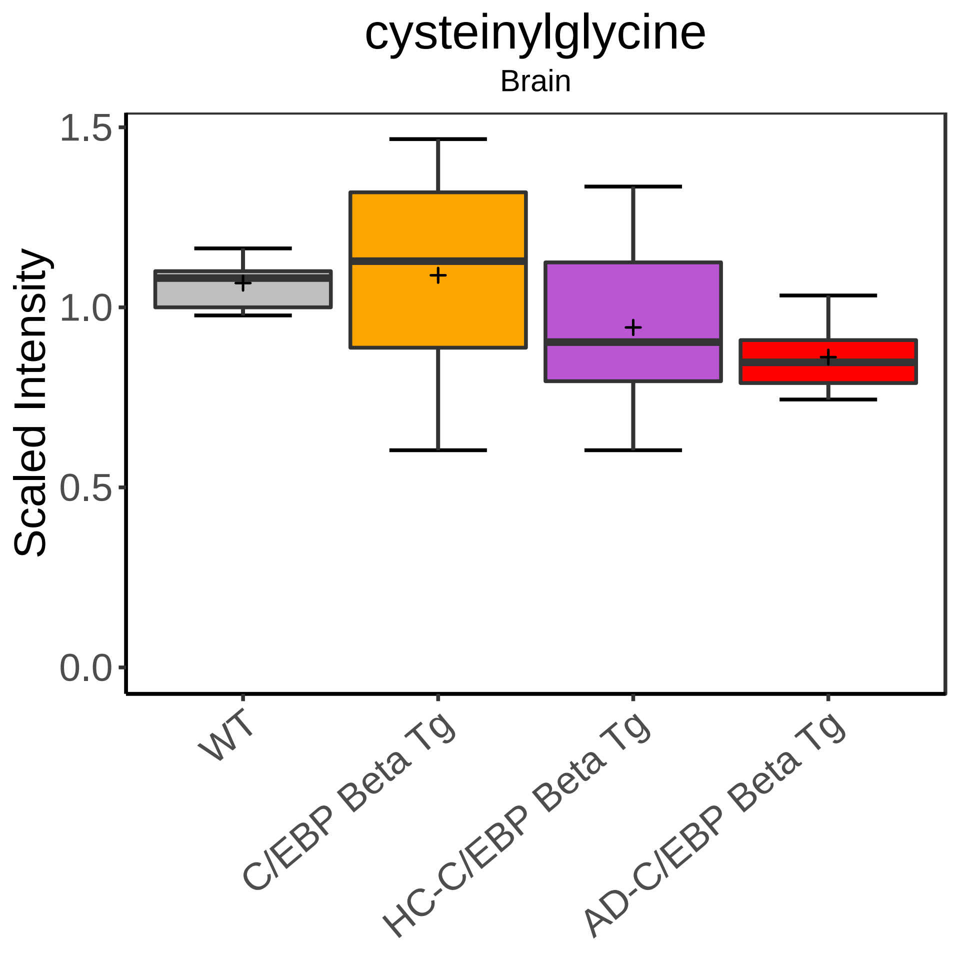

cysteinylglycine
Brain
Scaled Intensity
1.5
1.0
0.5
0.0
WT
C/EBP Beta Tg
HC-C/EBP Beta Tg
AD-C/EBP Beta Tg

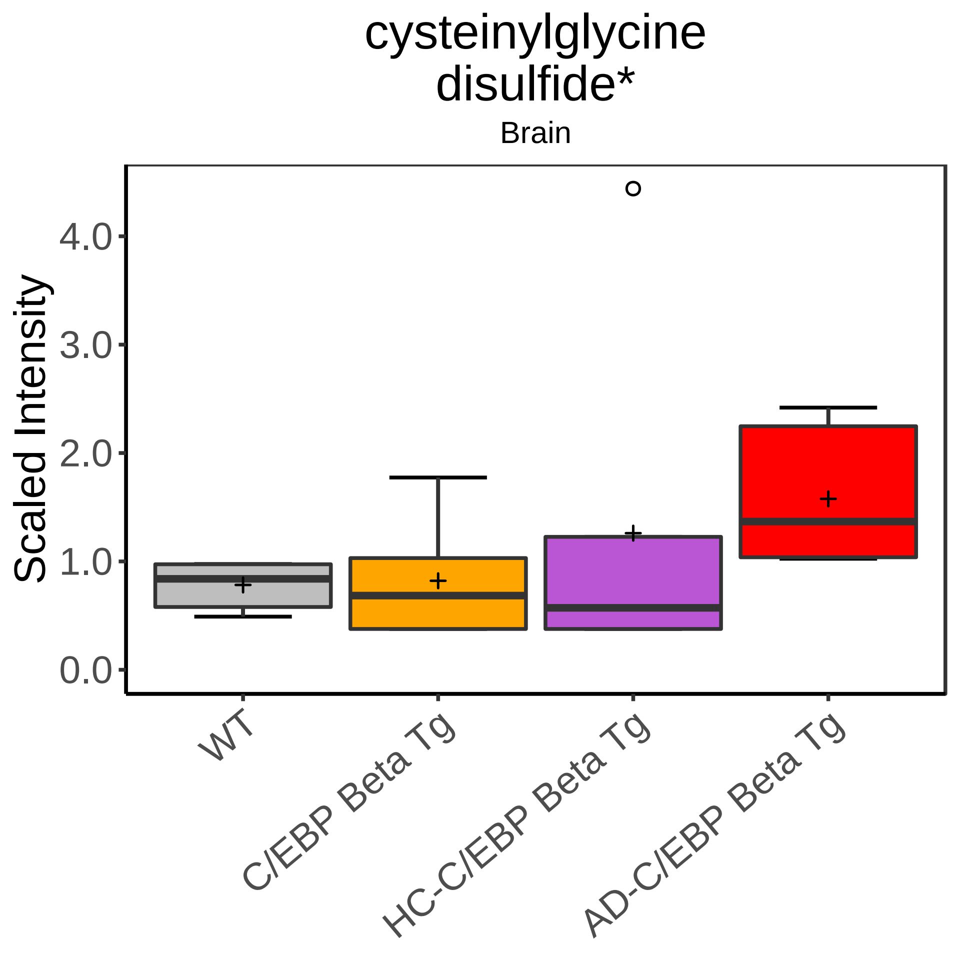
cysteinylglycine
disulfide*
Brain
Scaled Intensity
4.0
3.0
2.0
1.0
0.0
WT
C/EBP Beta Tg
HC-C/EBP Beta Tg
AD-C/EBP Beta Tg

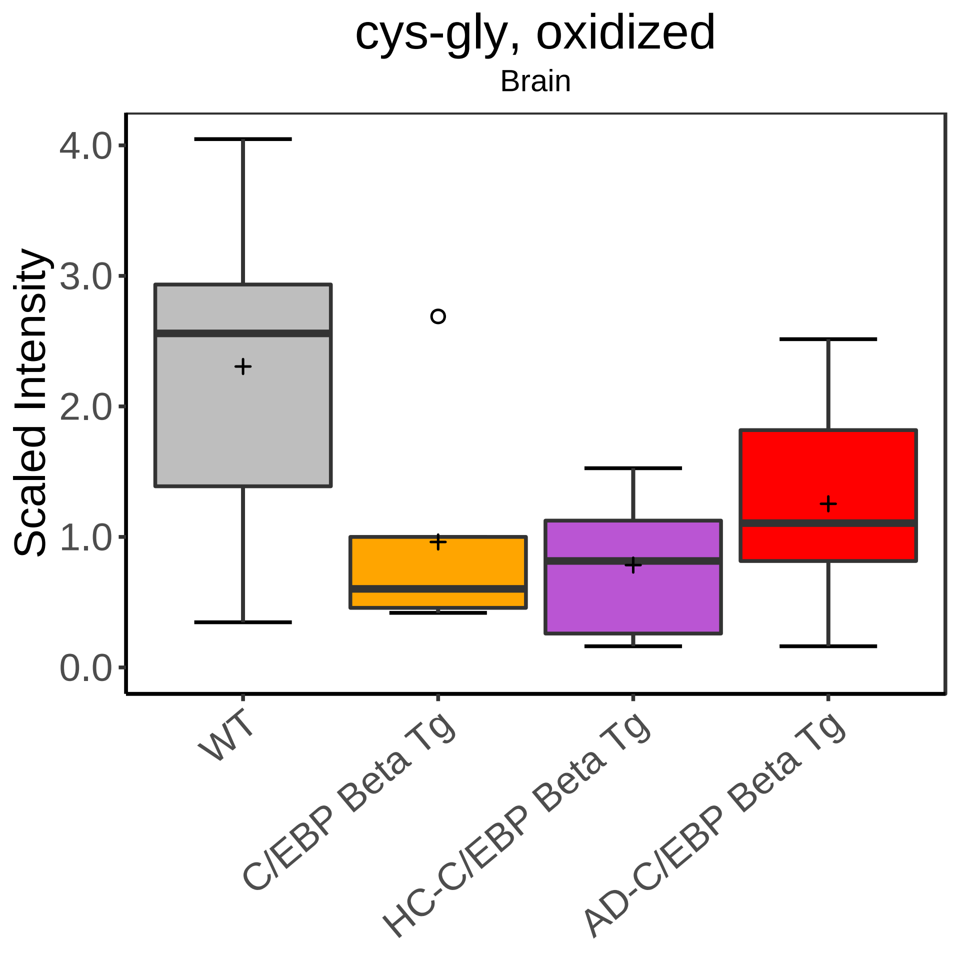
cys-gly, oxidized
Brain
Scaled Intensity
4.0
3.0
2.0
1.0
0.0
WT
C/EBP Beta Tg
HC-C/EBP Beta Tg
AD-C/EBP Beta Tg

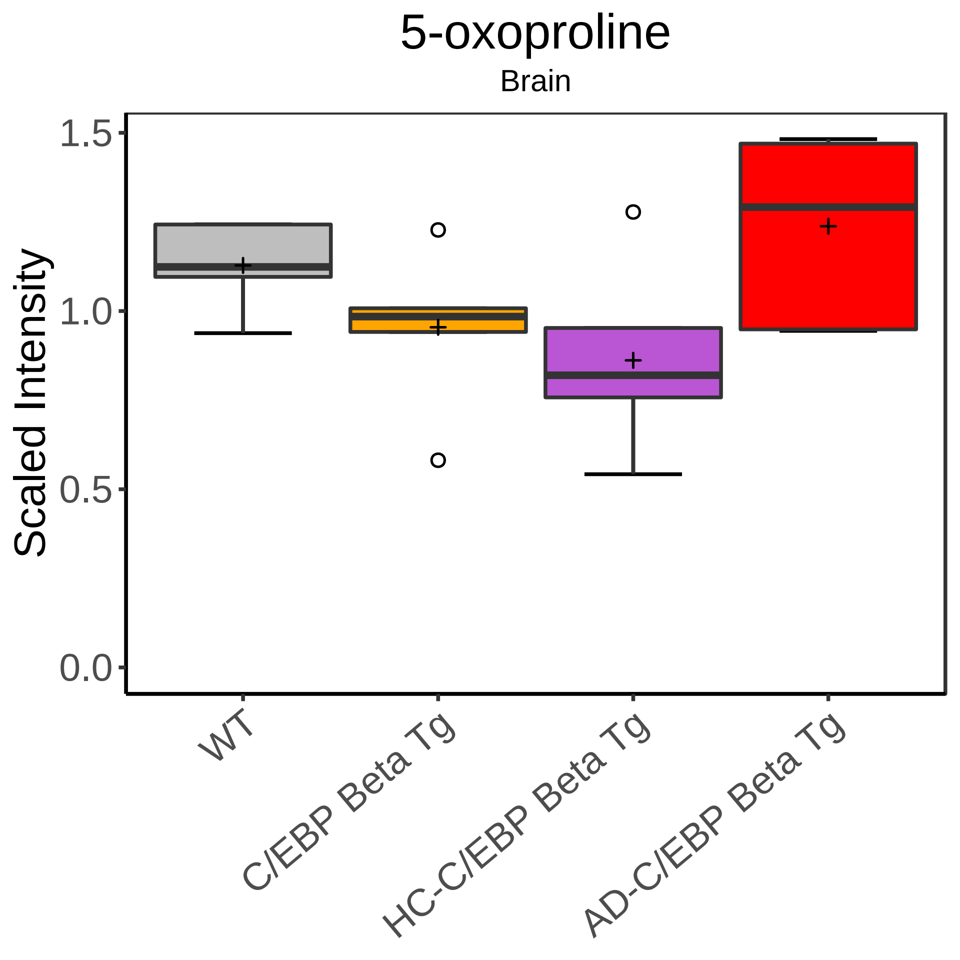
5-oxoproline
Brain
Scaled Intensity
1.5
1.0
0.5
0.0
WT
C/EBP Beta Tg
HC-C/EBP Beta Tg
AD-C/EBP Beta Tg

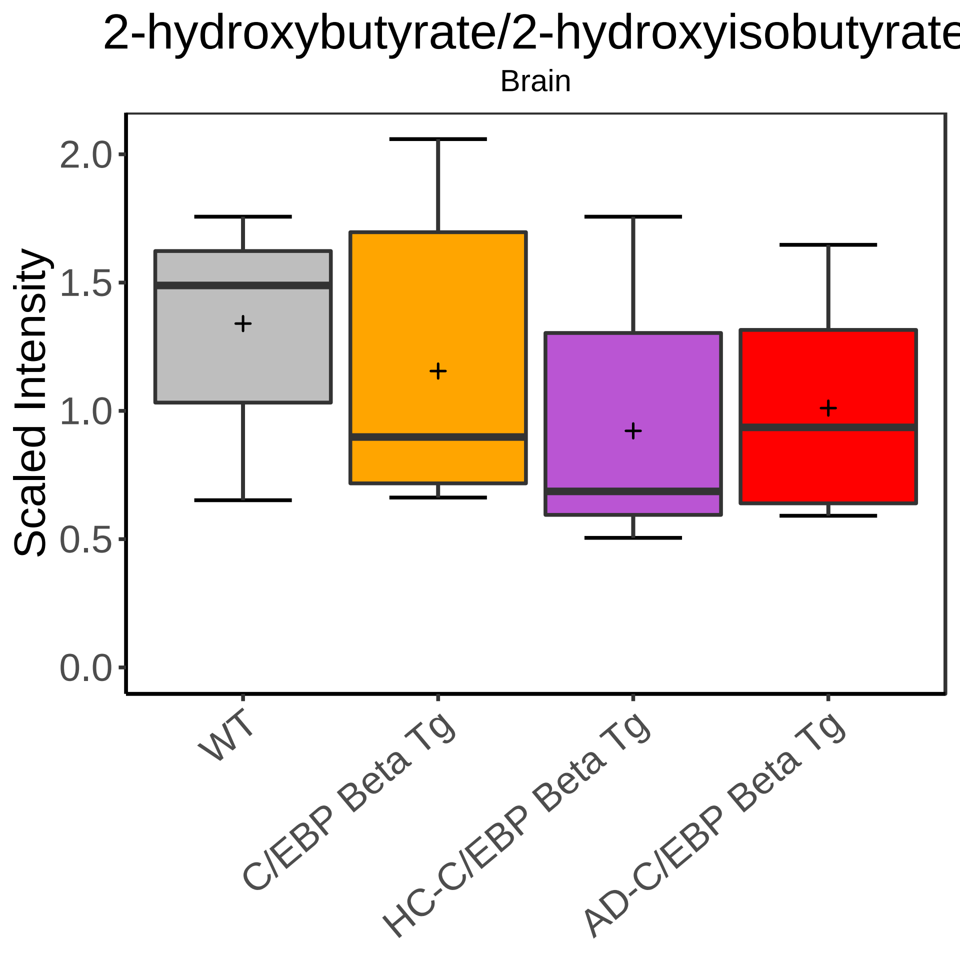
2-hydroxybutyrate/2-hydroxyisobutyrate
Brain
Scaled Intensity
0.0
0.5
1.0
1.5
2.0
WT
C/EBP Beta Tg
HC-C/EBP Beta Tg
AD-C/EBP Beta Tg

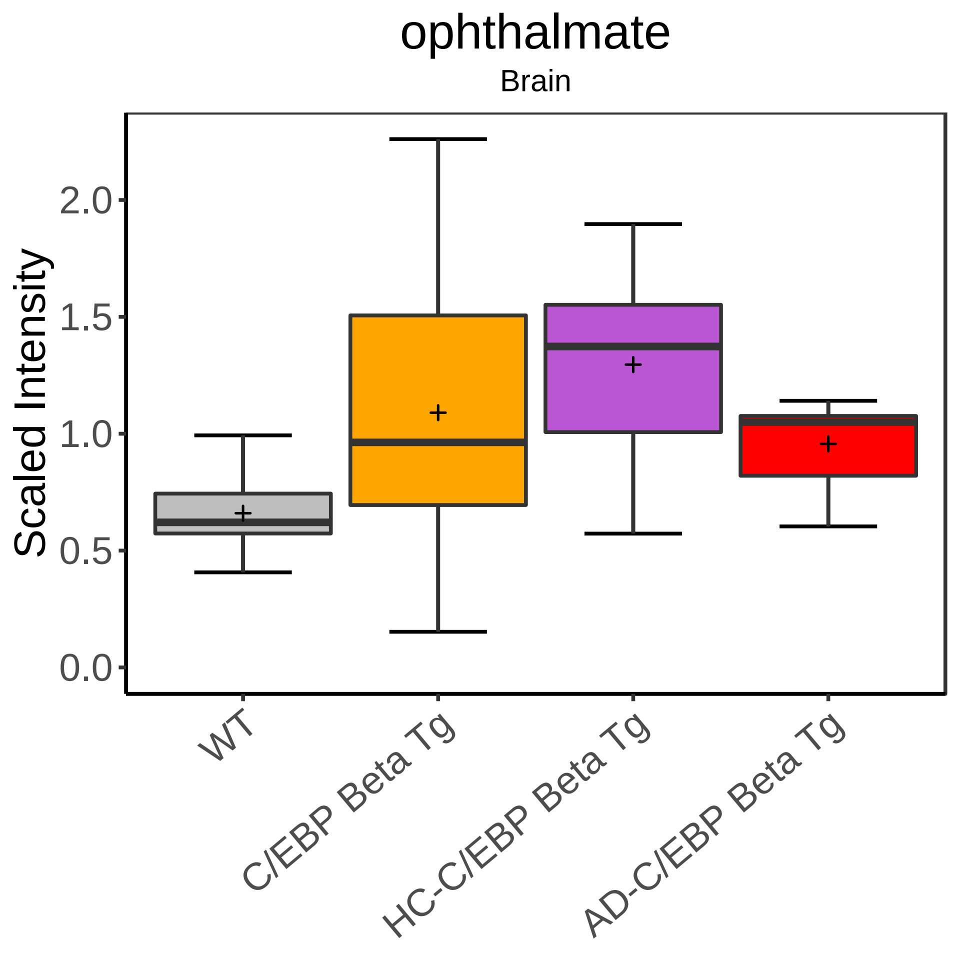
ophthalmate
Brain
Scaled Intensity
2.0
1.5
1.0
0.5
0.0
WT
C/EBP Beta Tg
HC-C/EBP Beta Tg
AD-C/EBP Beta Tg

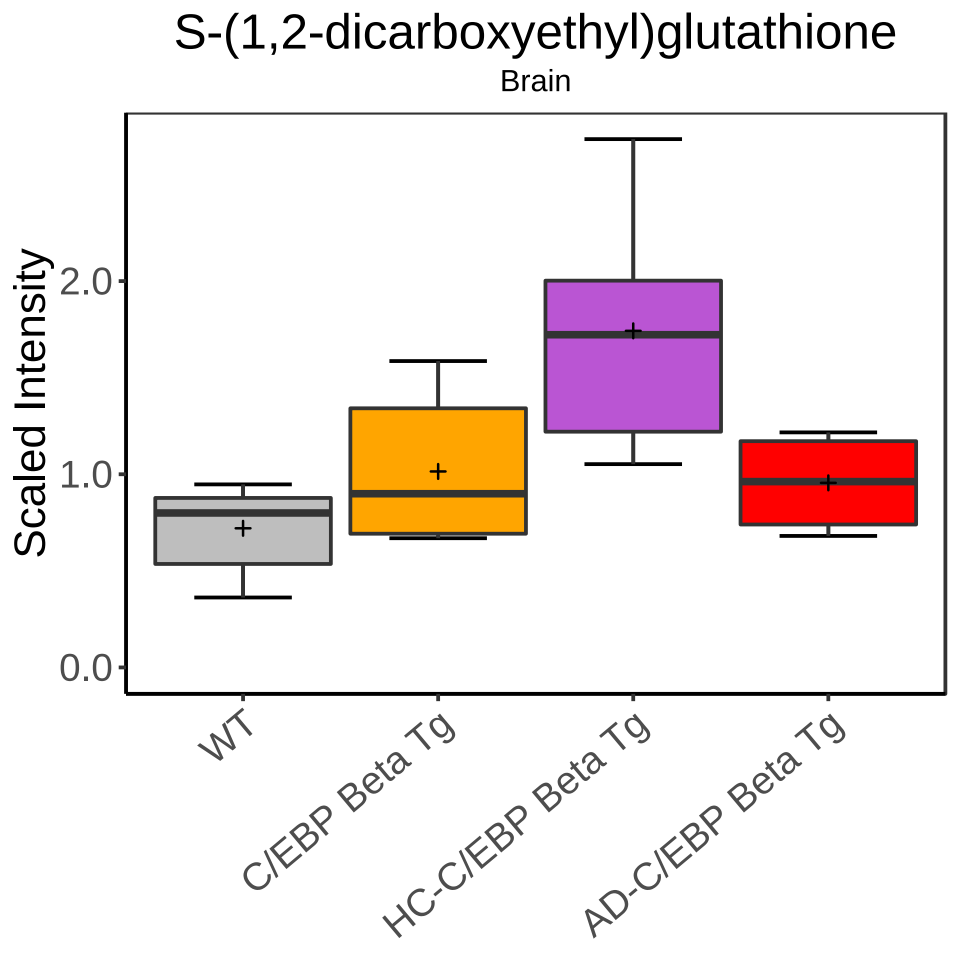
S-(1,2-dicarboxyethyl)glutathione
Brain
Scaled Intensity
2.0
1.0
0.0
WT
C/EBP Beta Tg
HC-C/EBP Beta Tg
AD-C/EBP Beta Tg

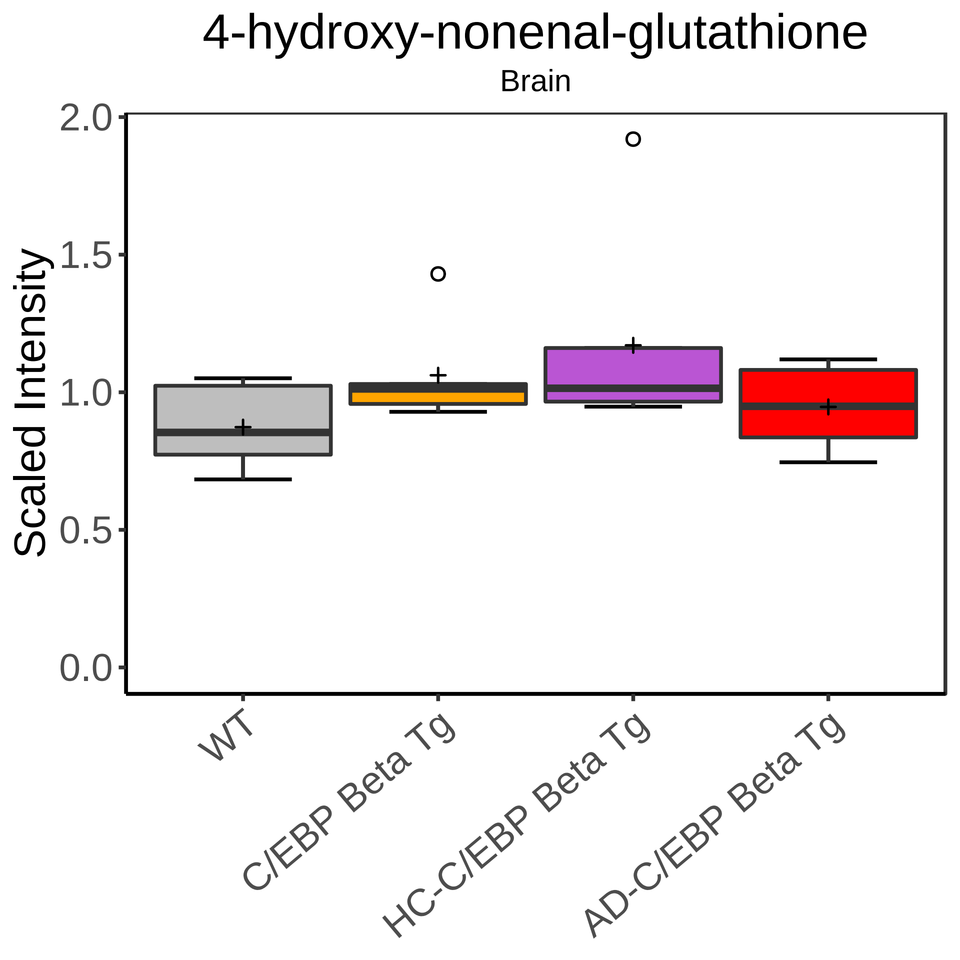

4-hydroxy-nonenal-glutathione
Brain
Scaled Intensity
2.0
1.5
1.0
0.5
0.0
WT
C/EBP Beta Tg
HC-C/EBP Beta Tg
AD-C/EBP Beta Tg

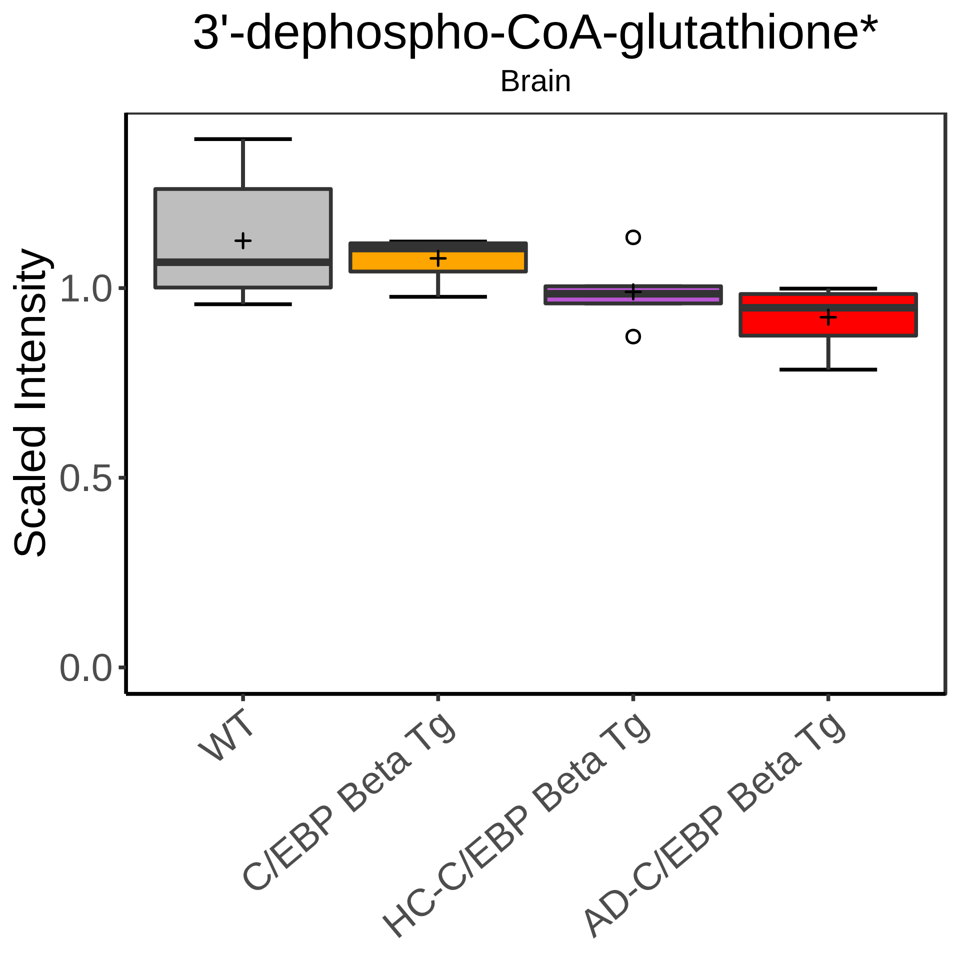

3'-dephospho-CoA-glutathione*
Brain
Scaled Intensity
0.0
0.5
1.0
WT
C/EBP Beta Tg
HC-C/EBP Beta Tg
AD-C/EBP Beta Tg

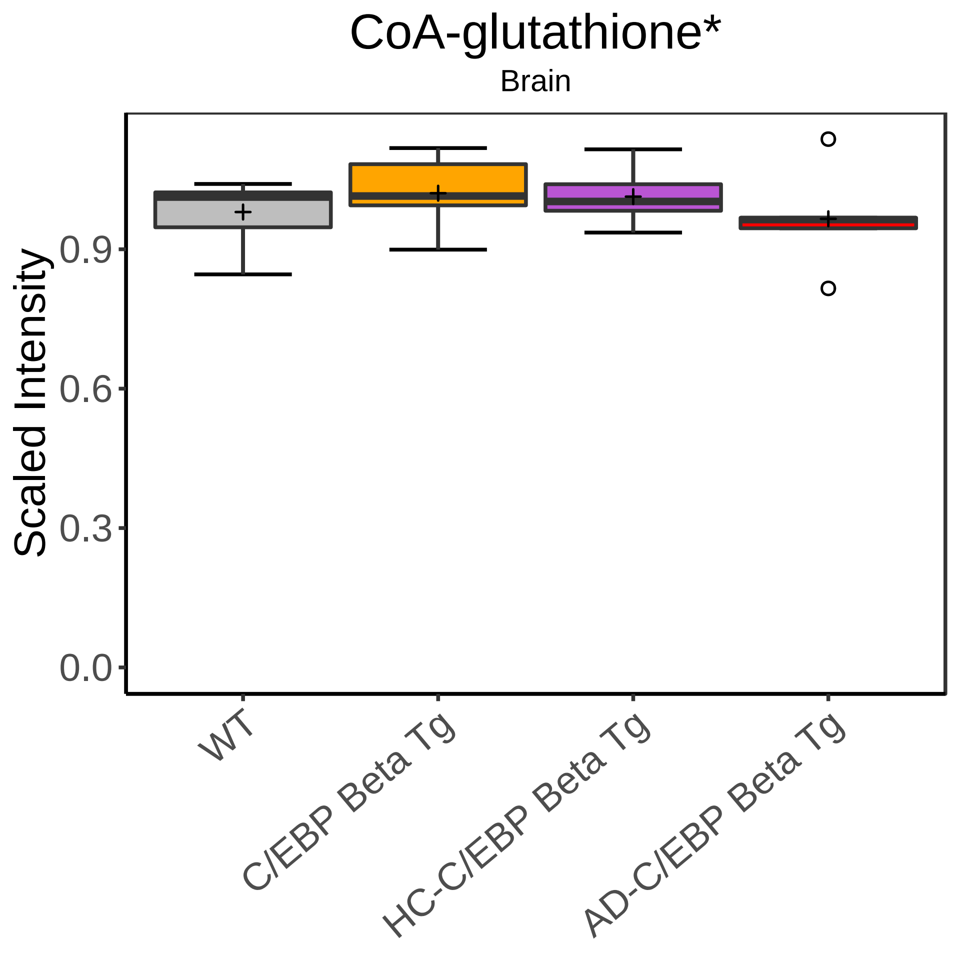
CoA-glutathione*
Brain
Scaled Intensity
0.0
0.3
0.6
0.9
WT
C/EBP Beta Tg
HC-C/EBP Beta Tg
AD-C/EBP Beta Tg

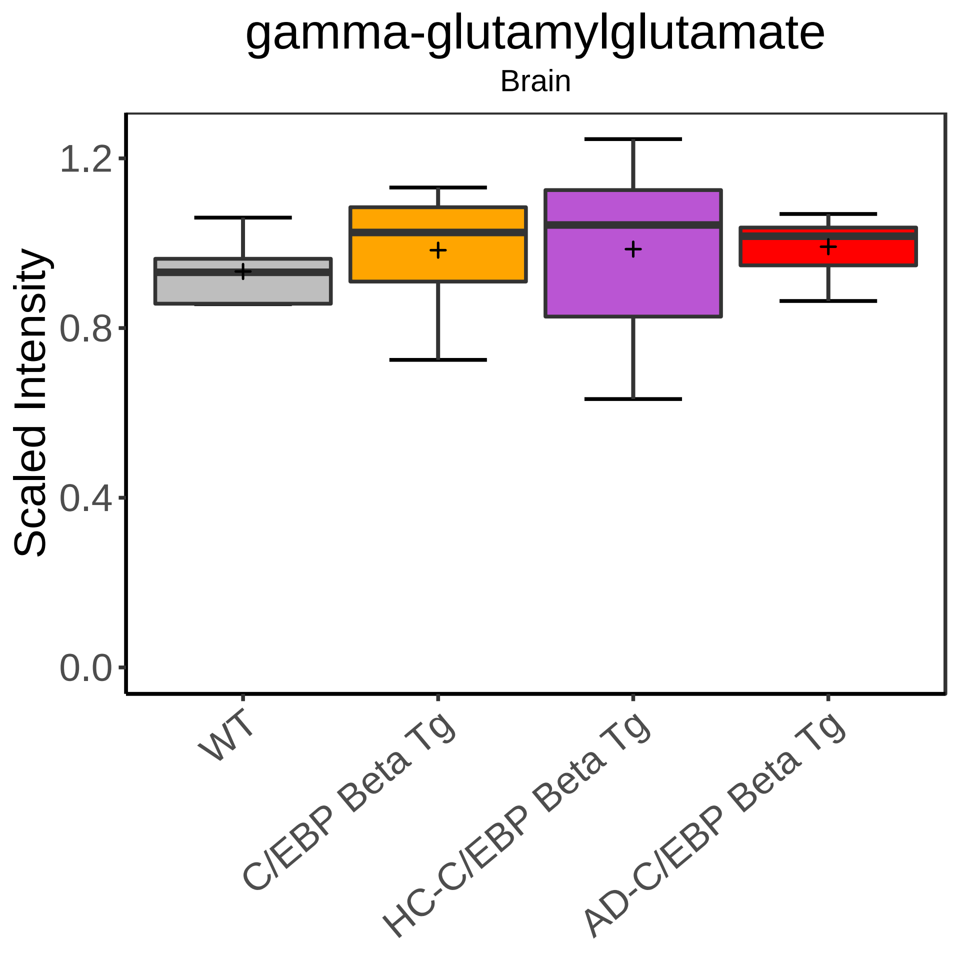
gamma-glutamylglutamate
Brain
Scaled Intensity
1.2
0.8
0.4
0.0
WT
C/EBP Beta Tg
HC-C/EBP Beta Tg
AD-C/EBP Beta Tg

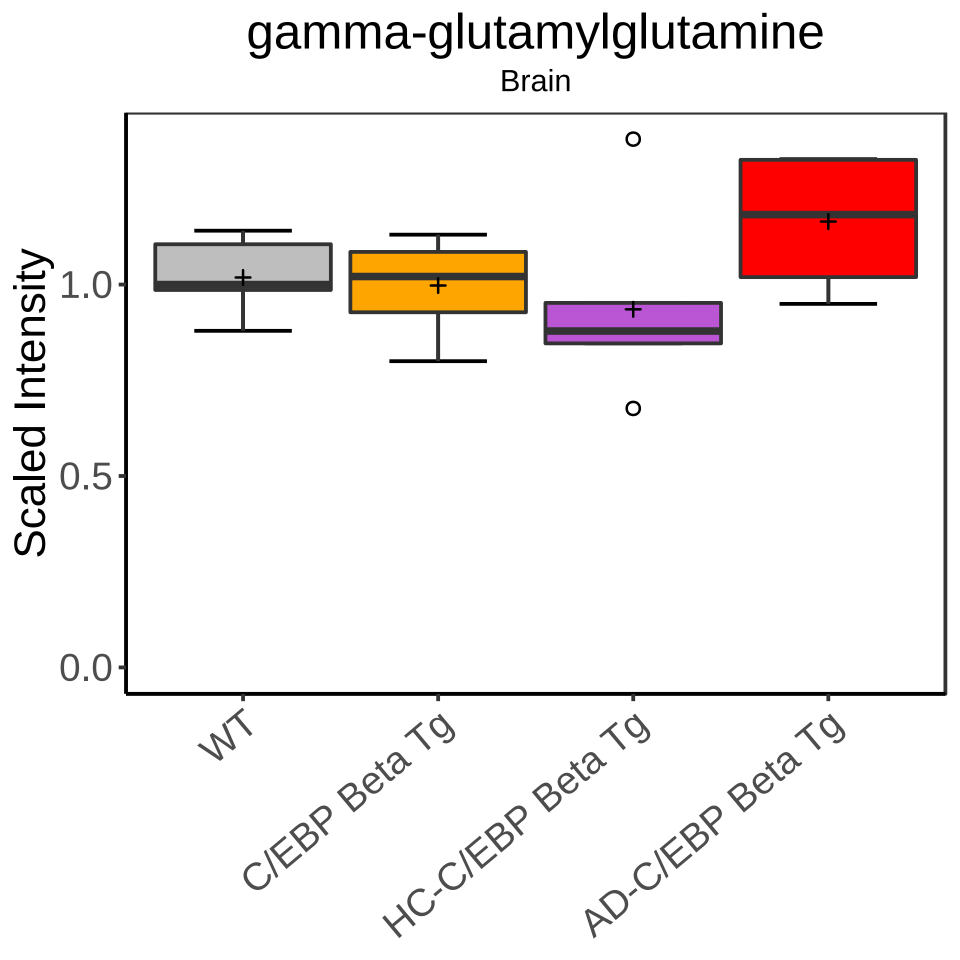
gamma-glutamylglutamine
Brain
Scaled Intensity
1.0
0.5
0.0
WT
C/EBP Beta Tg
HC-C/EBP Beta Tg
AD-C/EBP Beta Tg

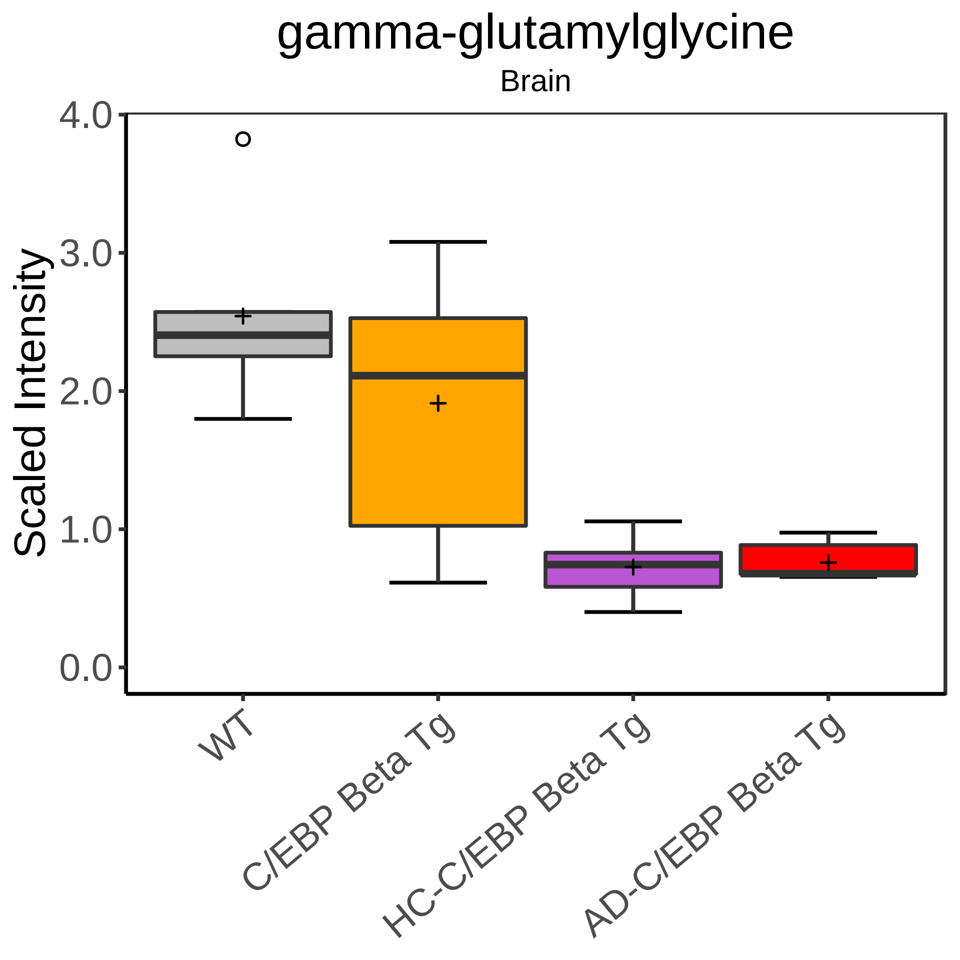
gamma-glutamylglycine
Brain
Scaled Intensity
4.0
3.0
2.0
1.0
0.0
WT
C/EBP Beta Tg
HC-C/EBP Beta Tg
AD-C/EBP Beta Tg

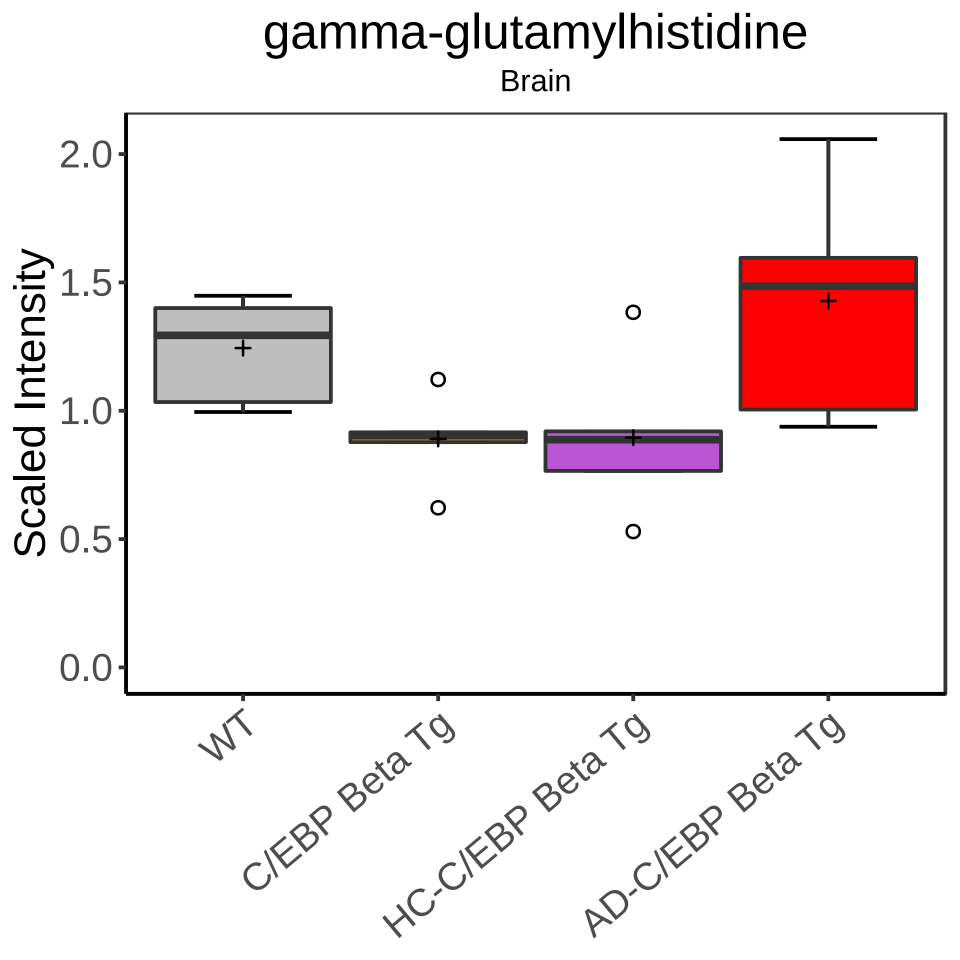
gamma-glutamylhistidine
Brain
Scaled Intensity
2.0
1.5
1.0
0.5
0.0
WT
C/EBP Beta Tg
HC-C/EBP Beta Tg
AD-C/EBP Beta Tg

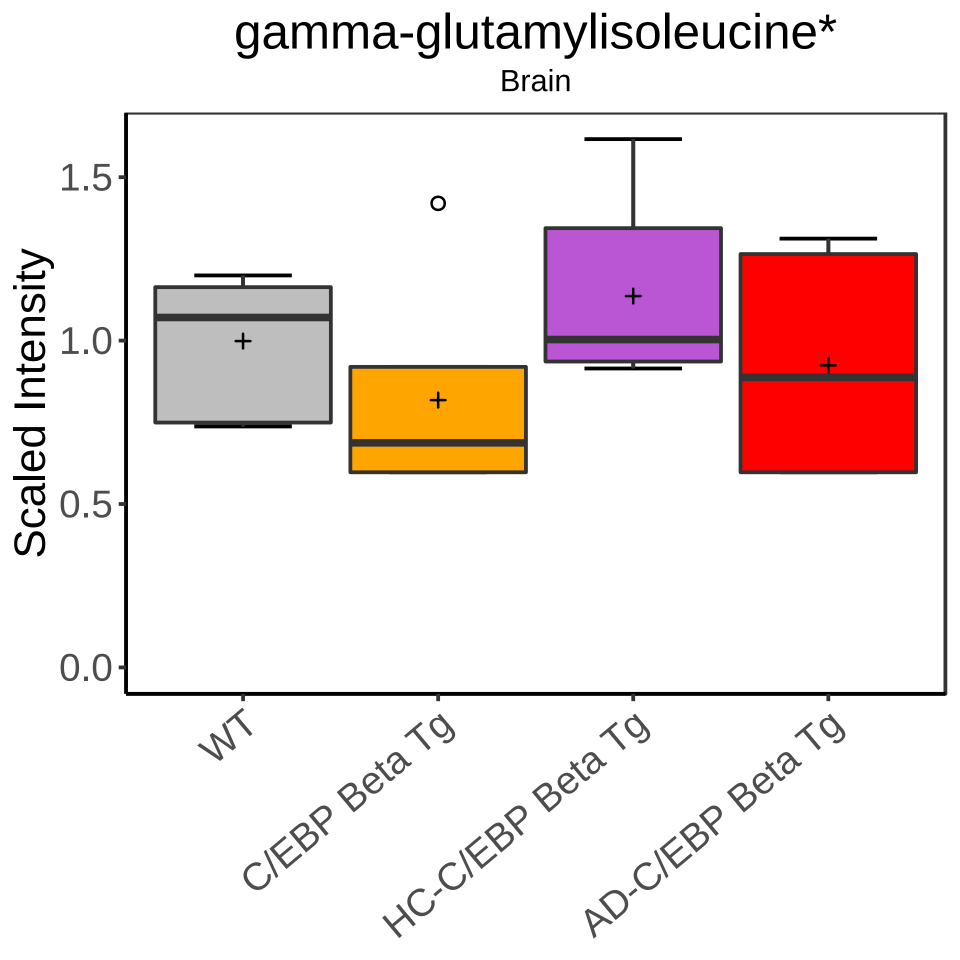

gamma-glutamylisoleucine*
Brain
Scaled Intensity
1.5
1.0
0.5
0.0
WT
C/EBP Beta Tg
HC-C/EBP Beta Tg
AD-C/EBP Beta Tg

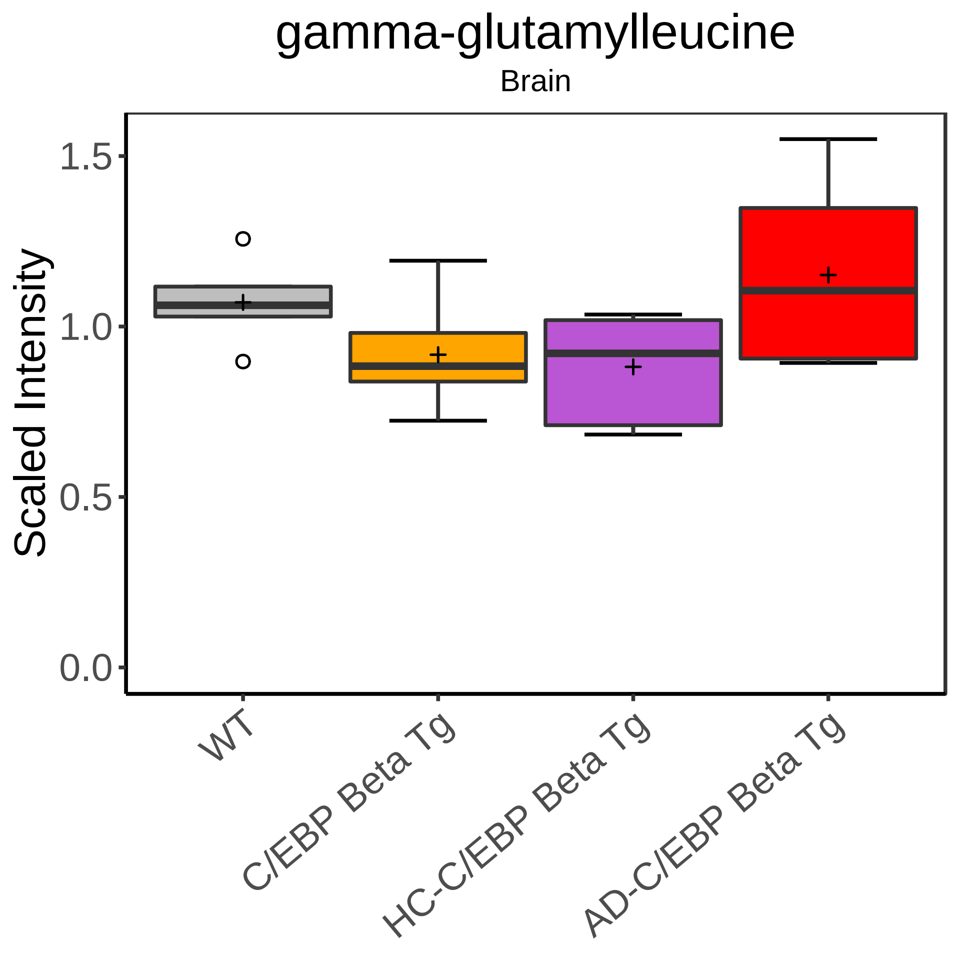
gamma-glutamylleucine
Brain
Scaled Intensity
1.5
1.0
0.5
0.0
WT
C/EBP Beta Tg
HC-C/EBP Beta Tg
AD-C/EBP Beta Tg

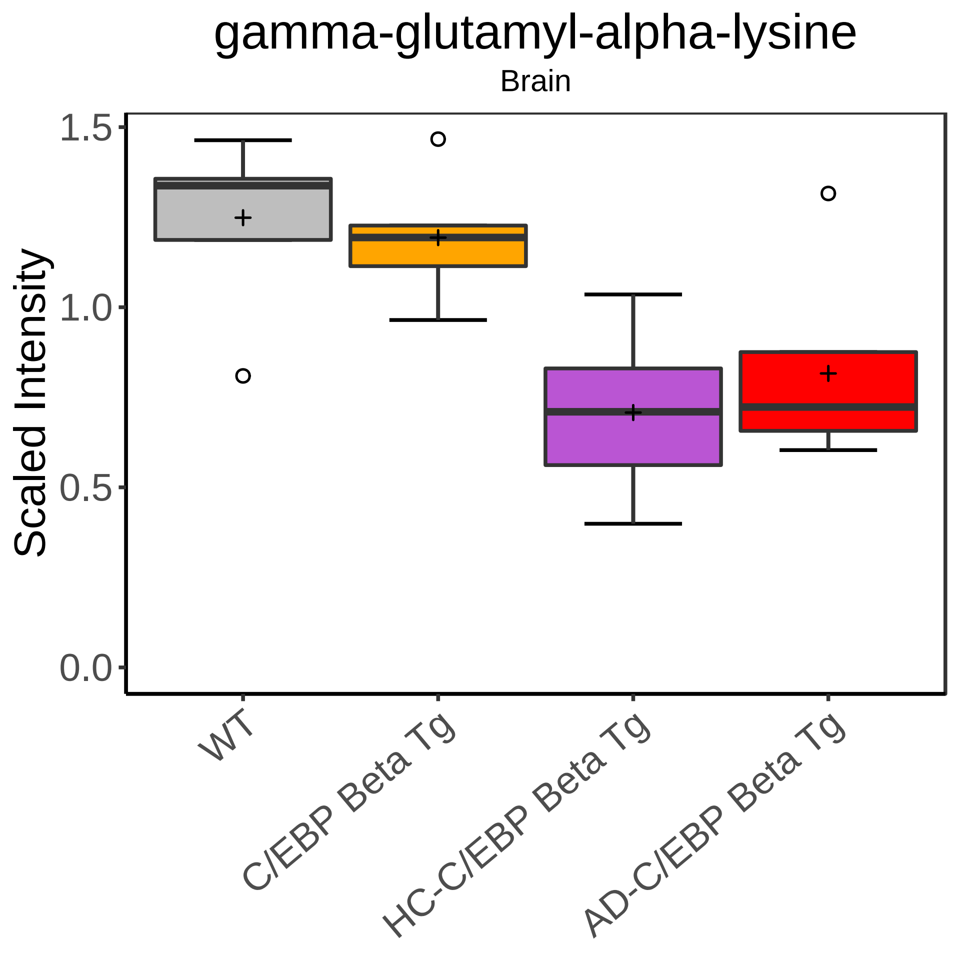
gamma-glutamyl-alpha-lysine
Brain
Scaled Intensity
1.5
1.0
0.5
0.0
WT
C/EBP Beta Tg
HC-C/EBP Beta Tg
AD-C/EBP Beta Tg

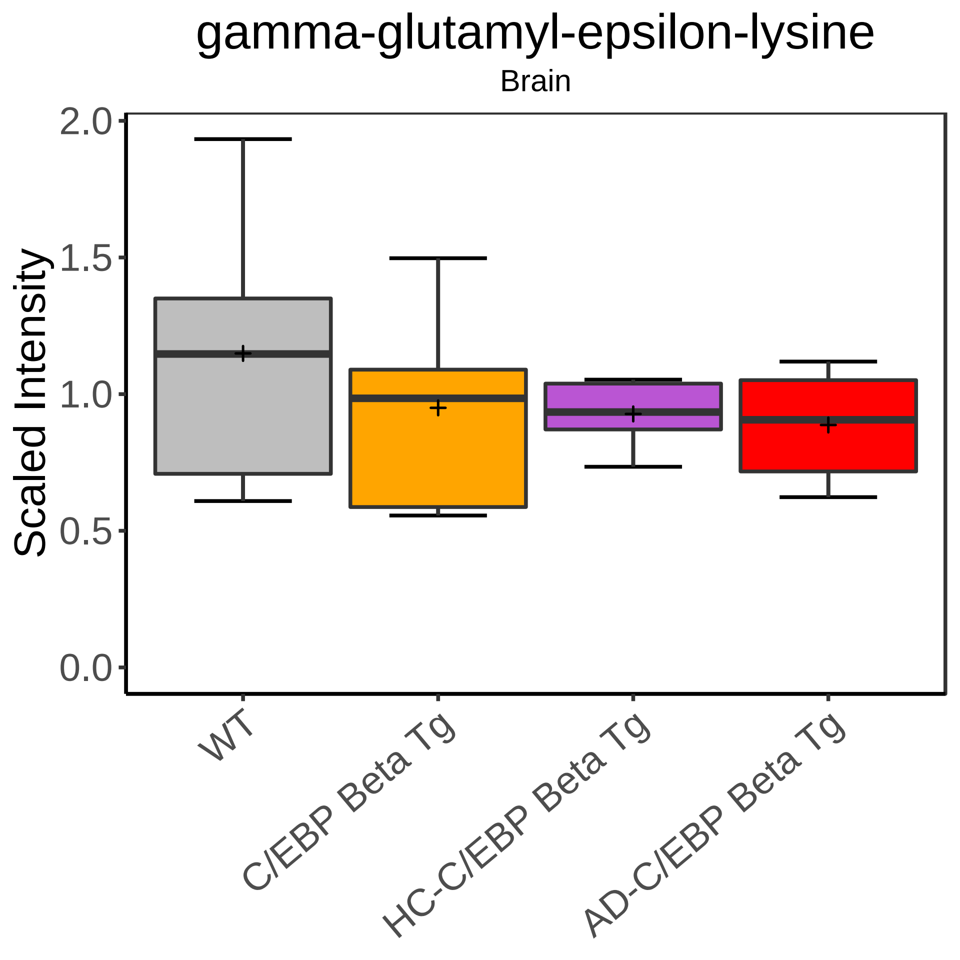

gamma-glutamyl-epsilon-lysine
Brain
Scaled Intensity
2.0
1.5
1.0
0.5
0.0
WT
C/EBP Beta Tg
HC-C/EBP Beta Tg
AD-C/EBP Beta Tg

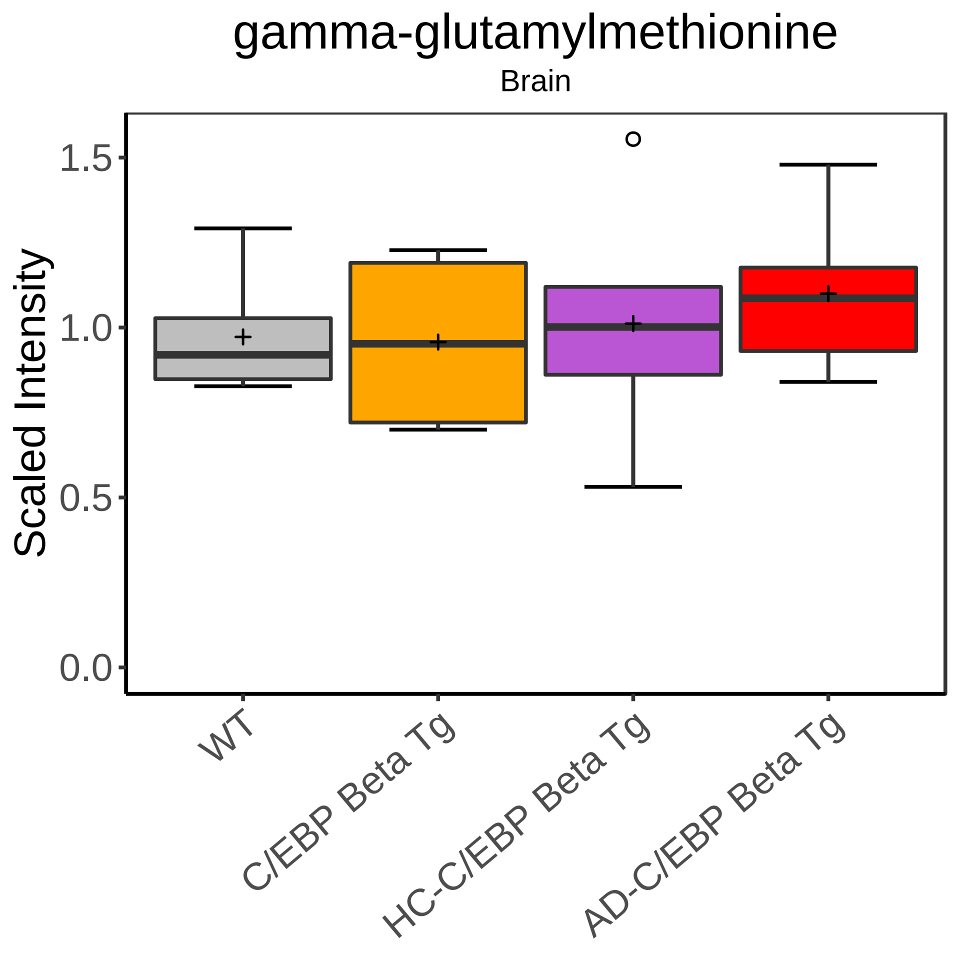
gamma-glutamylmethionine
Brain
Scaled Intensity
1.5
1.0
0.5
0.0
WT
C/EBP Beta Tg
HC-C/EBP Beta Tg
AD-C/EBP Beta Tg

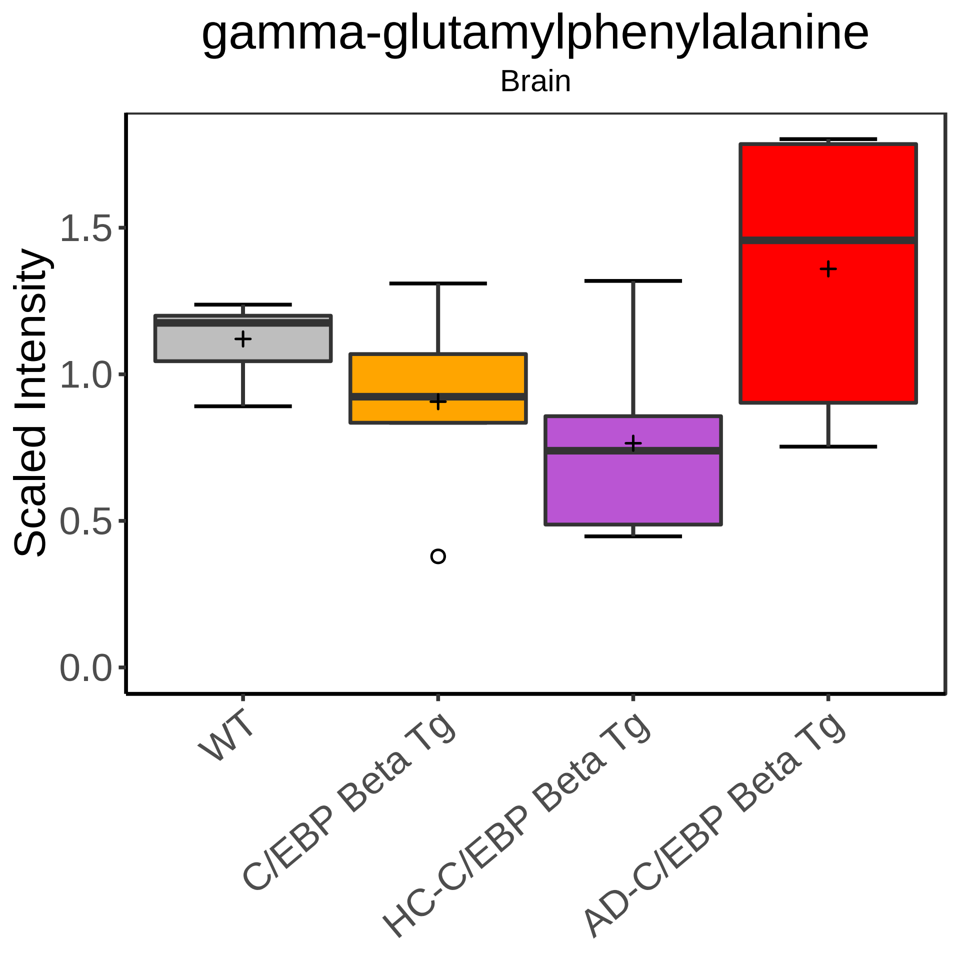

gamma-glutamylphenylalanine
Brain
Scaled Intensity
1.5
1.0
0.5
0.0
WT
C/EBP Beta Tg
HC-C/EBP Beta Tg
AD-C/EBP Beta Tg

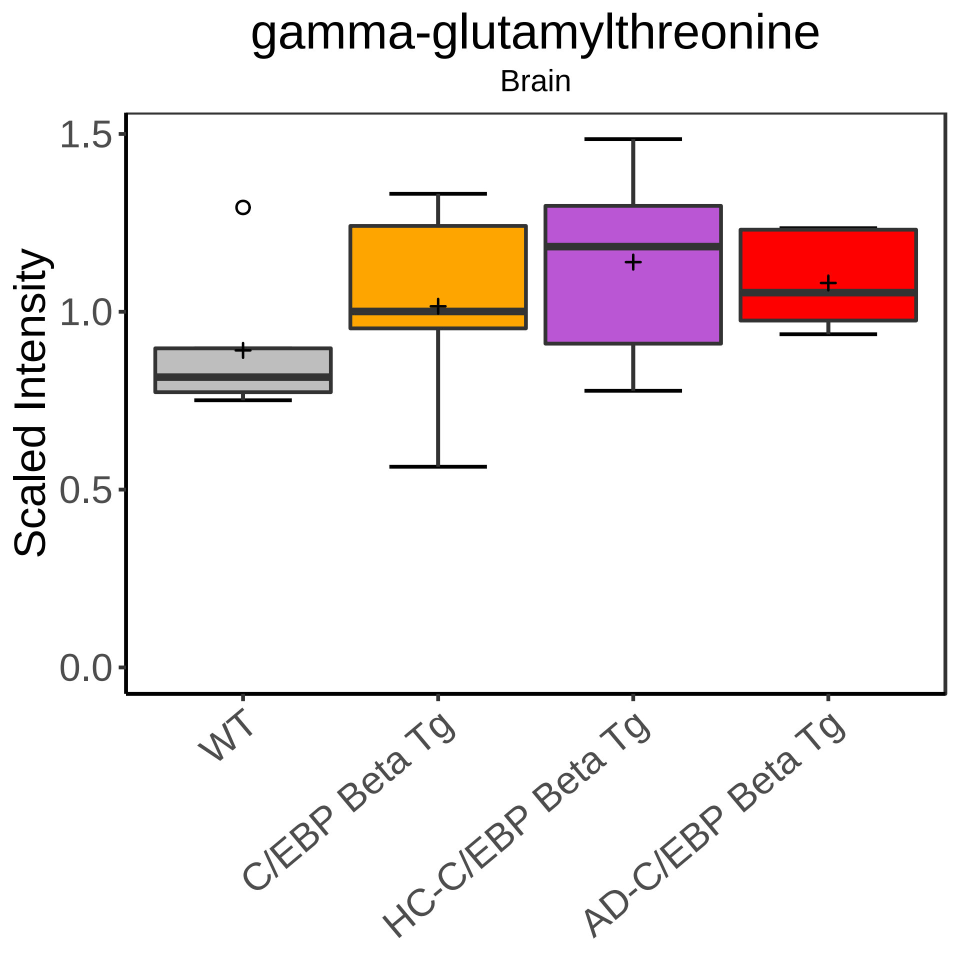
gamma-glutamylthreonine
Brain
Scaled Intensity
1.5
1.0
0.5
0.0
WT
C/EBP Beta Tg
HC-C/EBP Beta Tg
AD-C/EBP Beta Tg

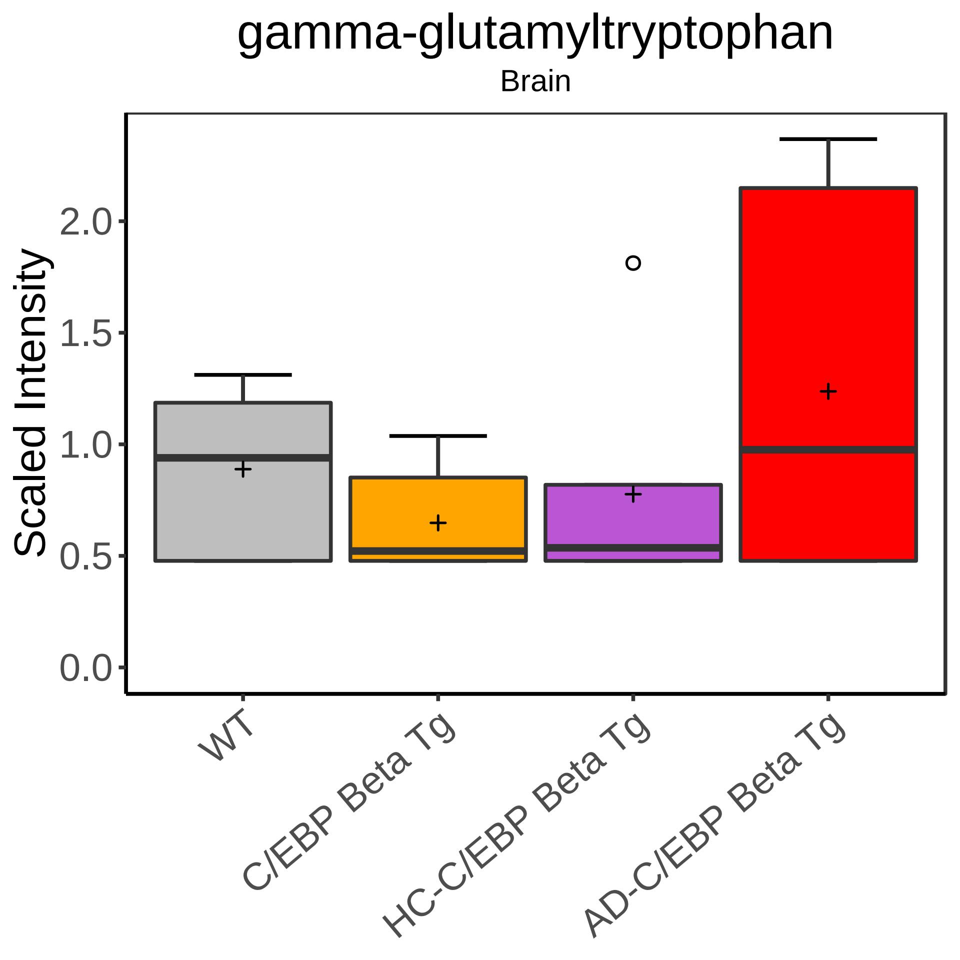
gamma-glutamyltryptophan
Brain
Scaled Intensity
0.0
0.5
1.0
1.5
2.0
WT
C/EBP Beta Tg
HC-C/EBP Beta Tg
AD-C/EBP Beta Tg

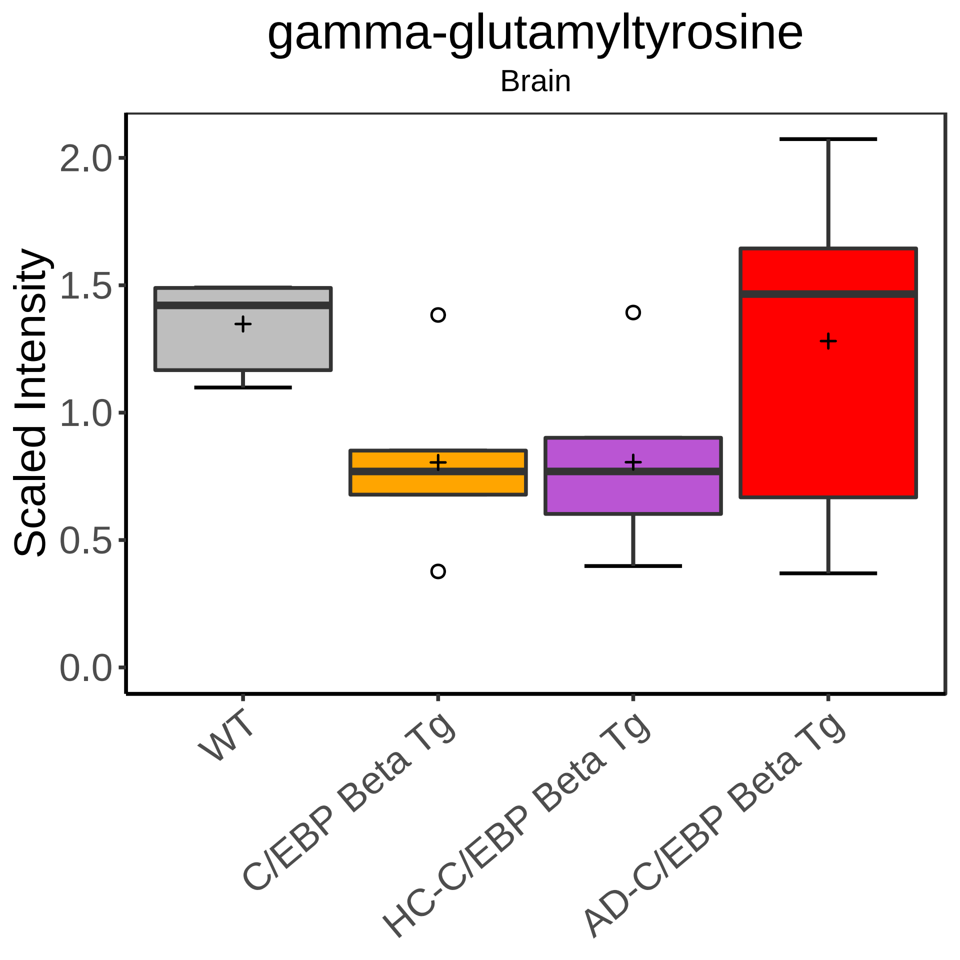

gamma-glutamyltyrosine
Brain
Scaled Intensity
2.0
1.5
1.0
0.5
0.0
WT
C/EBP Beta Tg
HC-C/EBP Beta Tg
AD-C/EBP Beta Tg

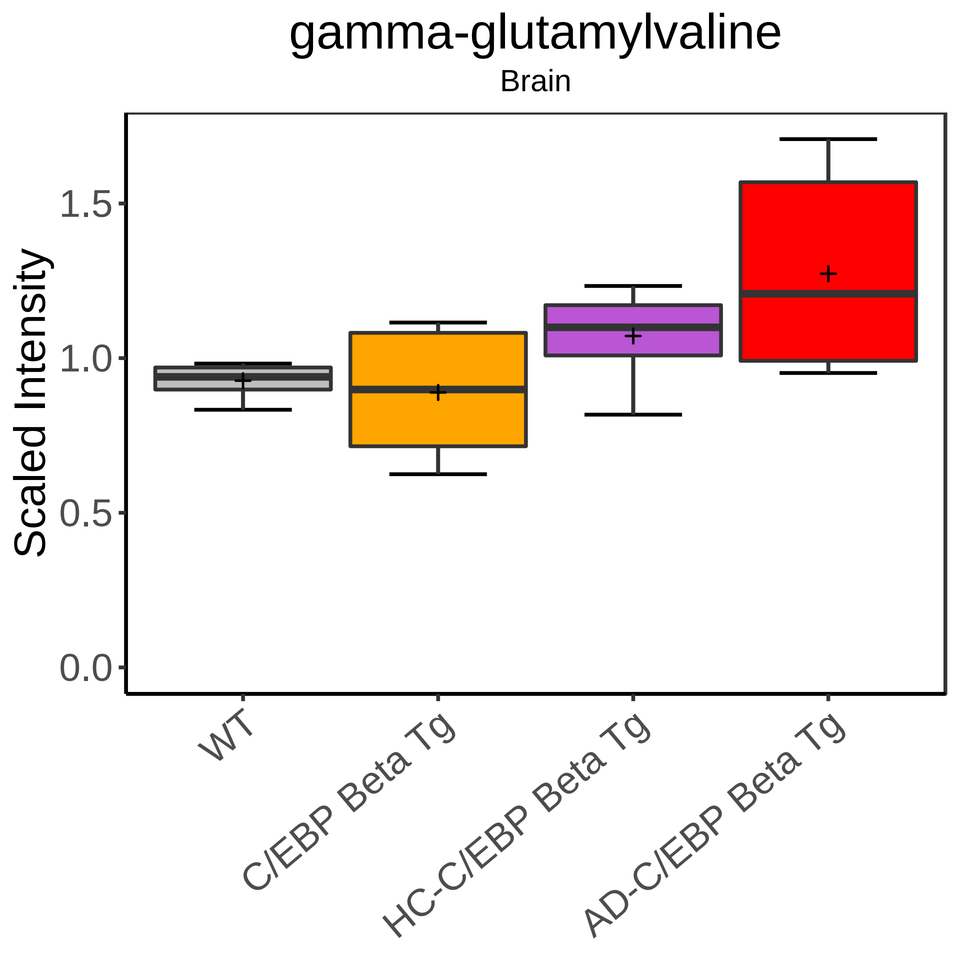
gamma-glutamylvaline
Brain
Scaled Intensity
1.5
1.0
0.5
0.0
WT
C/EBP Beta Tg
HC-C/EBP Beta Tg
AD-C/EBP Beta Tg

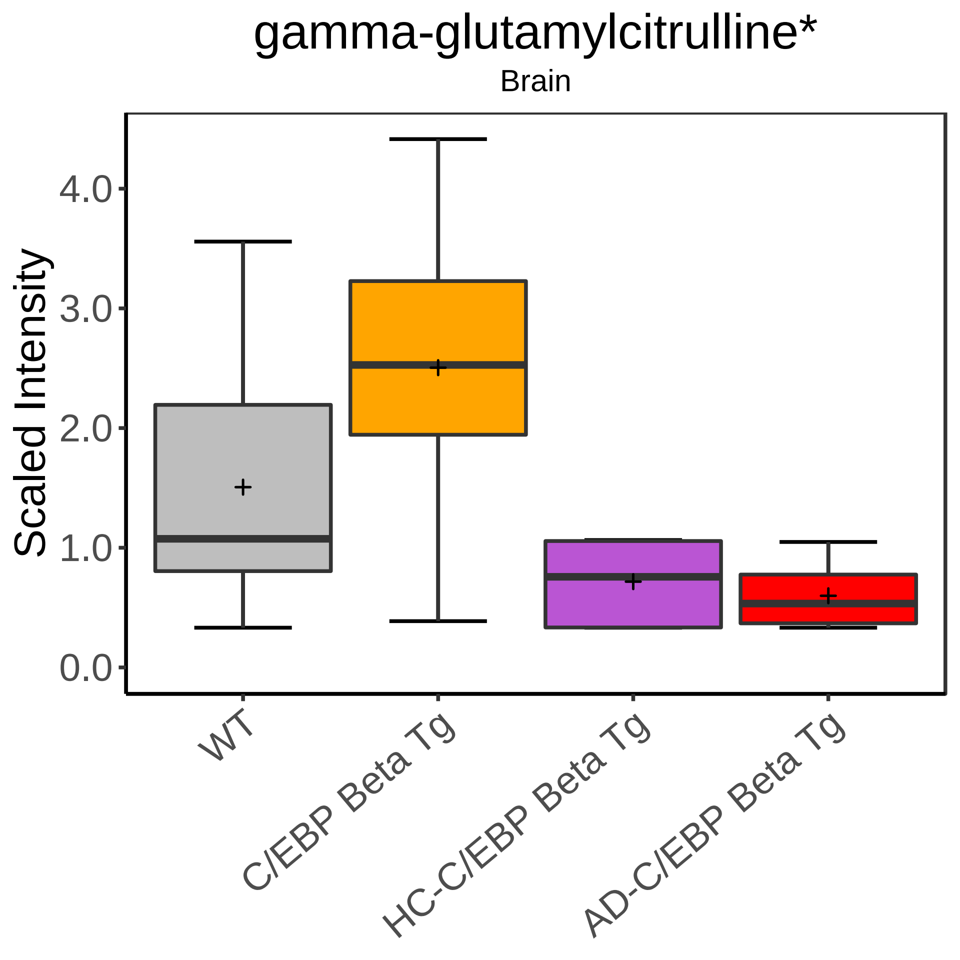
gamma-glutamylcitrulline*
Brain
Scaled Intensity
4.0
3.0
2.0
1.0
0.0
WT
C/EBP Beta Tg
HC-C/EBP Beta Tg
AD-C/EBP Beta Tg

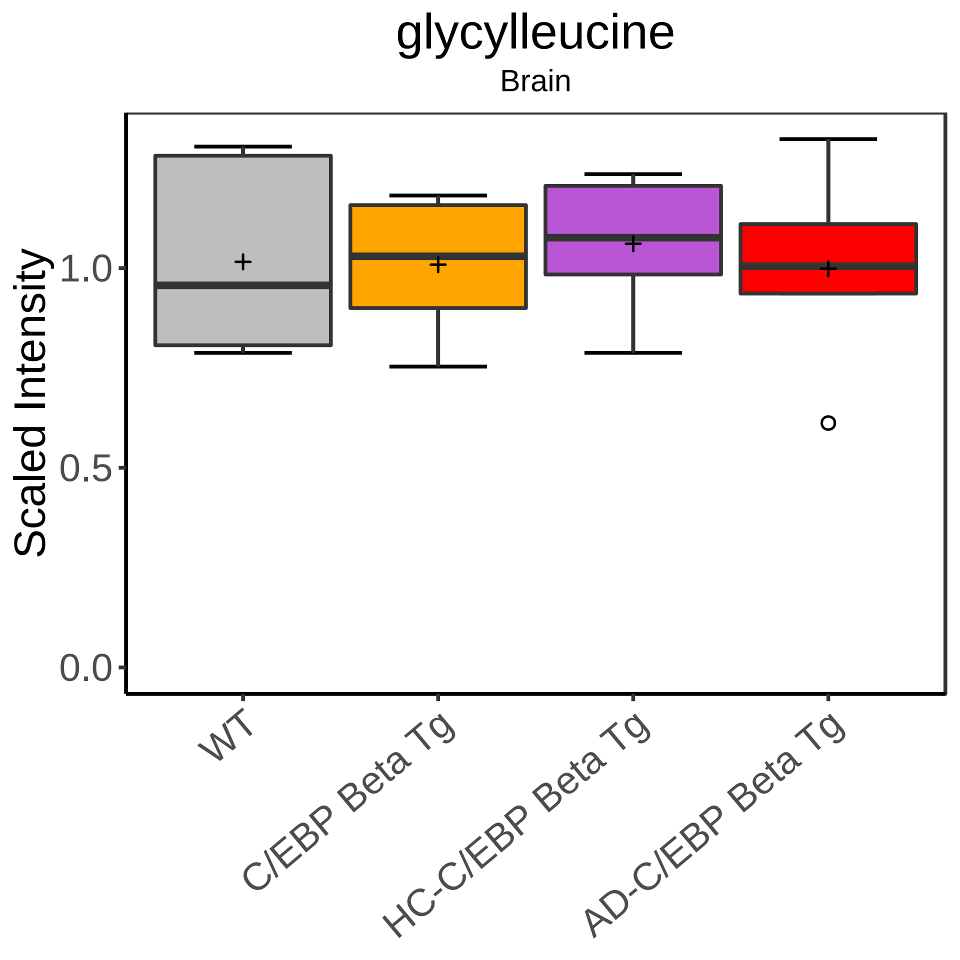
glycylleucine
Brain
Scaled Intensity
1.0
0.5
0.0
WT
C/EBP Beta Tg
HC-C/EBP Beta Tg
AD-C/EBP Beta Tg

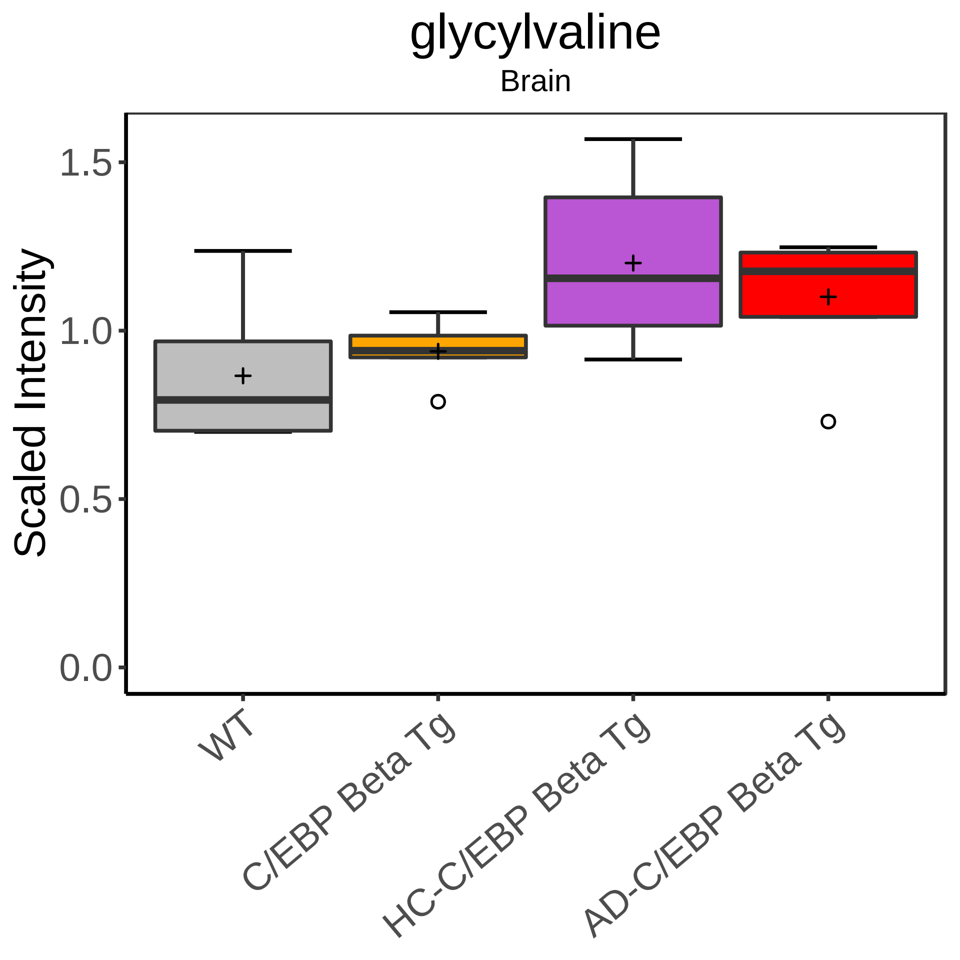

glycylvaline
Brain
Scaled Intensity
0.0
0.5
1.0
1.5
WT
C/EBP Beta Tg
HC-C/EBP Beta Tg
AD-C/EBP Beta Tg

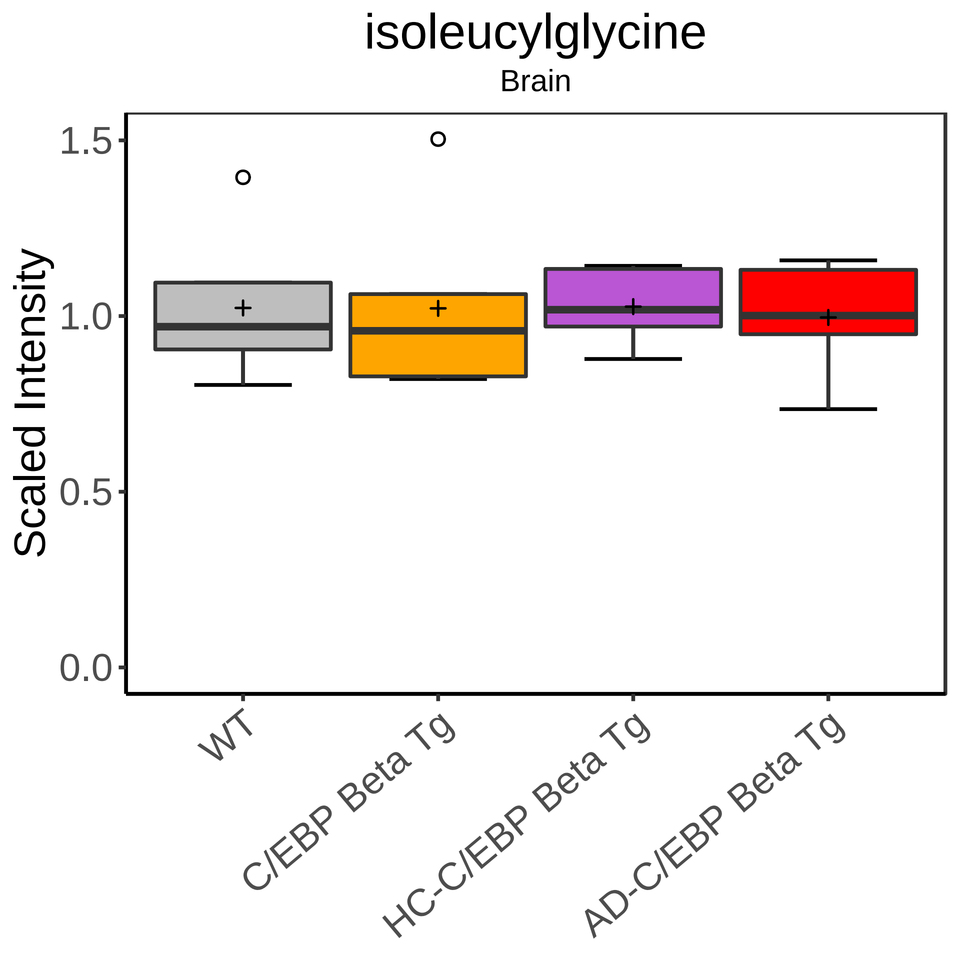
isoleucylglycine
Brain
Scaled Intensity
1.5
1.0
0.5
0.0
WT
C/EBP Beta Tg
HC-C/EBP Beta Tg
AD-C/EBP Beta Tg

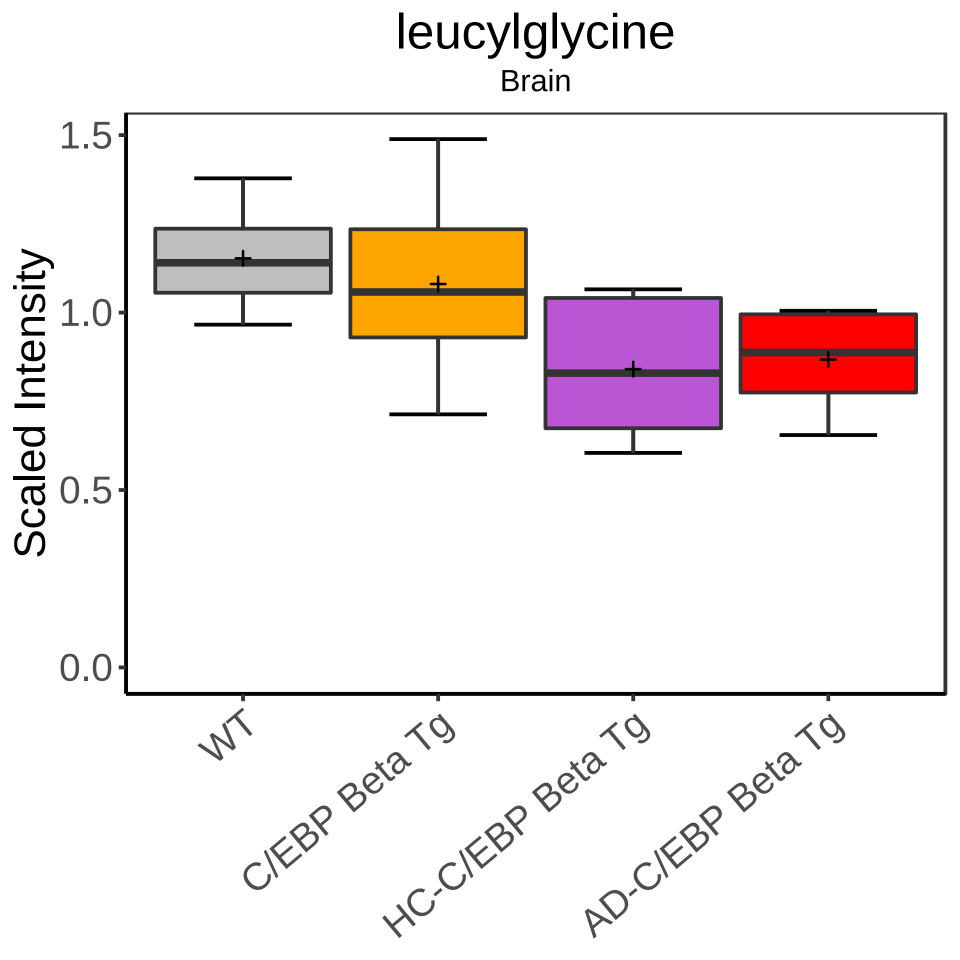
leucylglycine
Brain
Scaled Intensity
1.5
1.0
0.5
0.0
WT
C/EBP Beta Tg
HC-C/EBP Beta Tg
AD-C/EBP Beta Tg

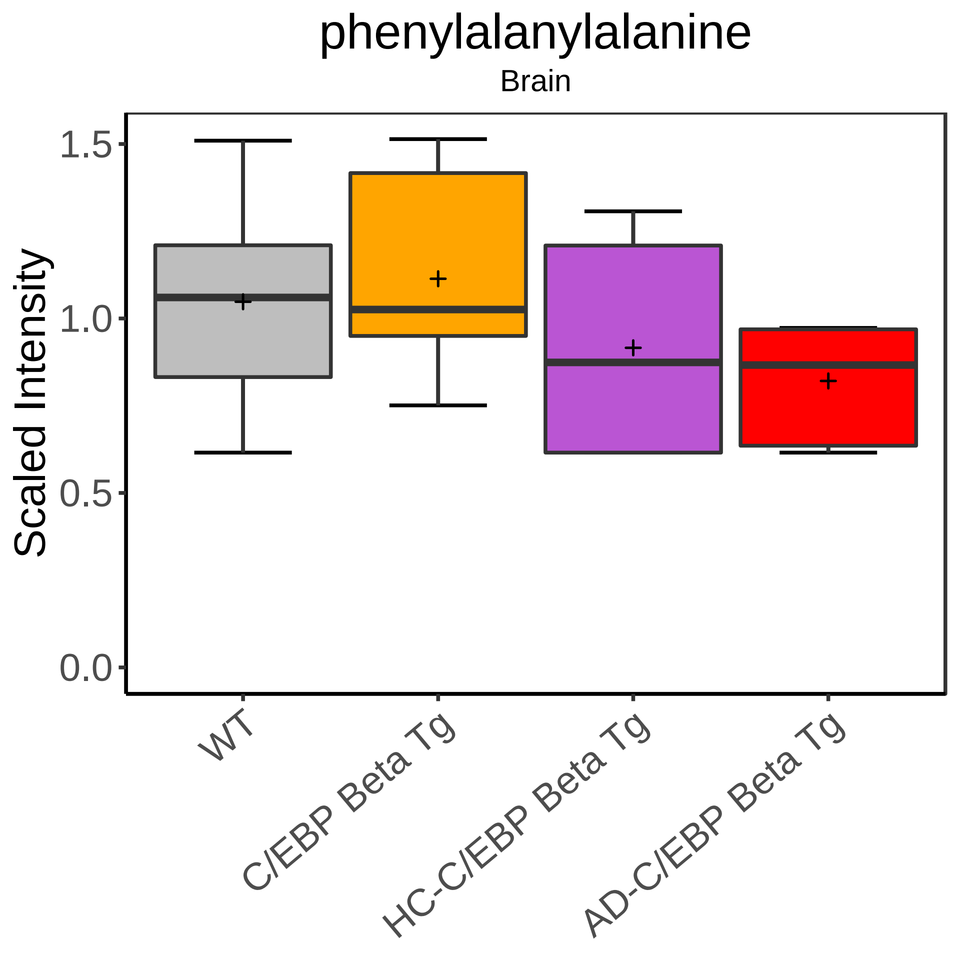
phenylalanylalanine
Brain
Scaled Intensity
1.5
1.0
0.5
0.0
WT
C/EBP Beta Tg
HC-C/EBP Beta Tg
AD-C/EBP Beta Tg

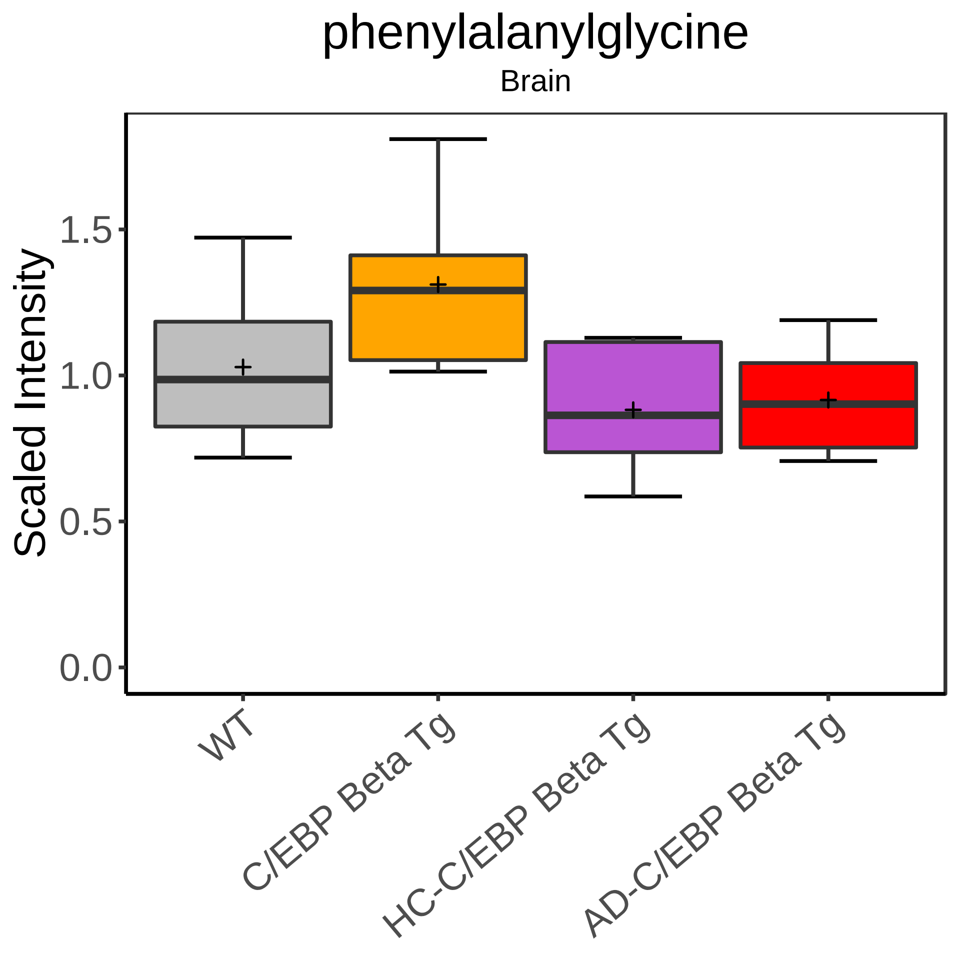

phenylalanylglycine
Brain
Scaled Intensity
1.5
1.0
0.5
0.0
WT
C/EBP Beta Tg
HC-C/EBP Beta Tg
AD-C/EBP Beta Tg

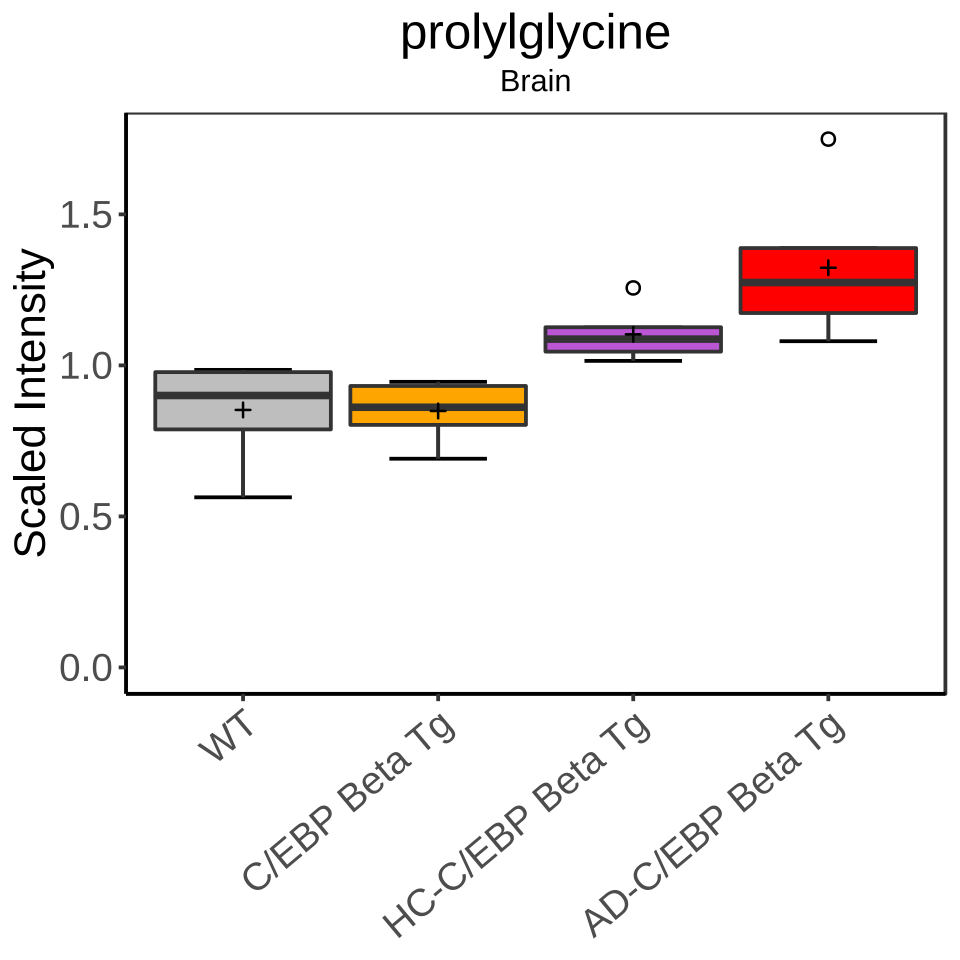
prolylglycine
Brain
Scaled Intensity
1.5
1.0
0.5
0.0
WT
C/EBP Beta Tg
HC-C/EBP Beta Tg
AD-C/EBP Beta Tg

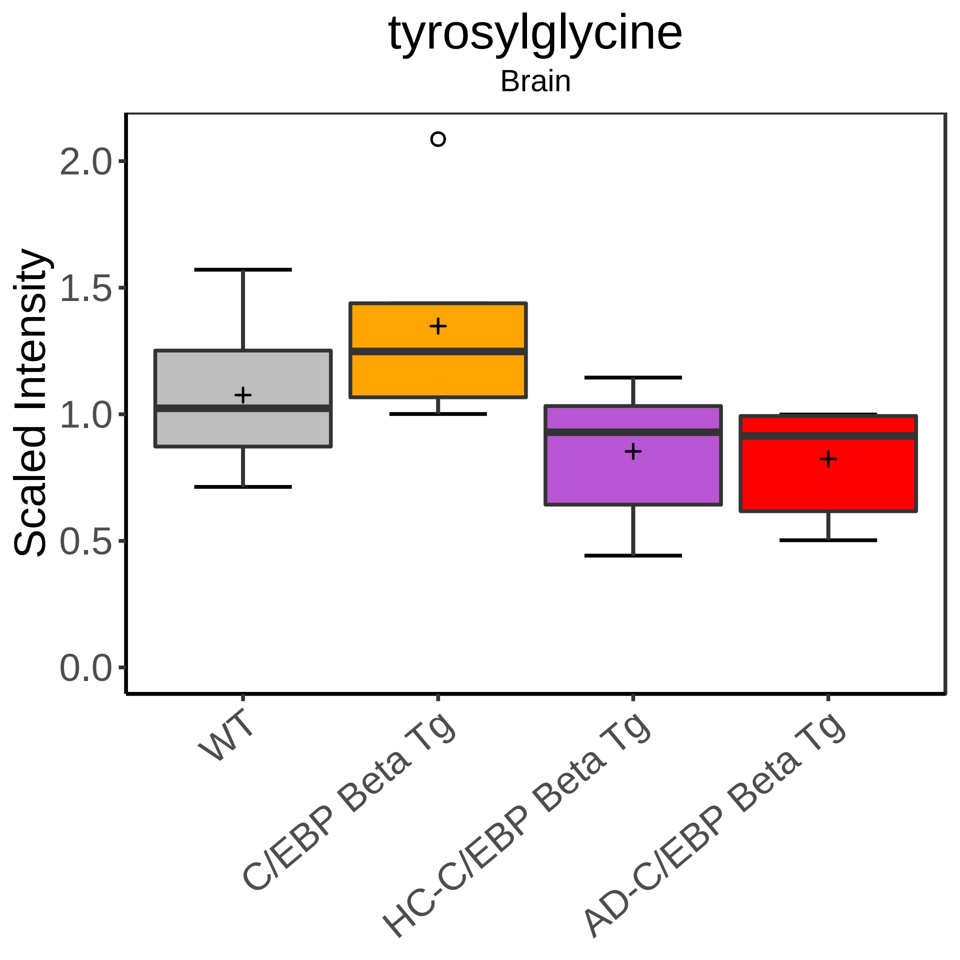

tyrosylglycine
Brain
Scaled Intensity
0.0
0.5
1.0
1.5
2.0
WT
C/EBP Beta Tg
HC-C/EBP Beta Tg
AD-C/EBP Beta Tg

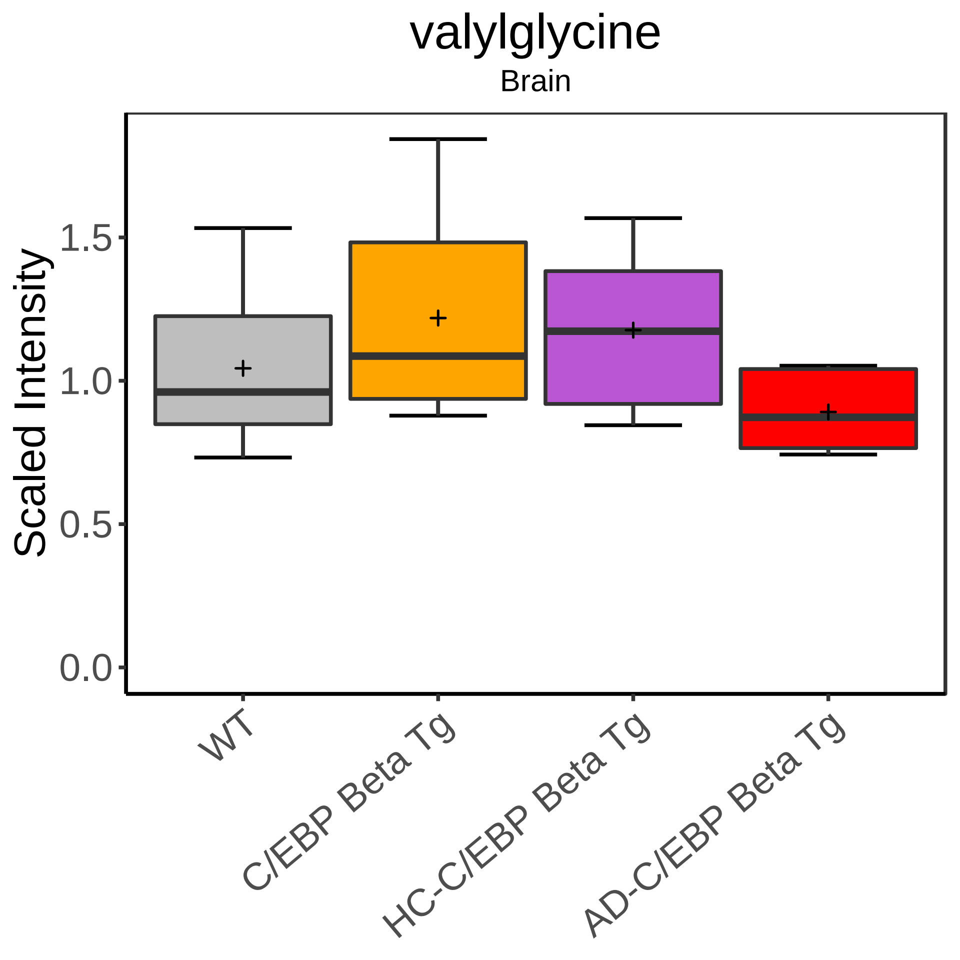
valylglycine
Brain
Scaled Intensity
0.0
0.5
1.0
1.5
WT
C/EBP Beta Tg
HC-C/EBP Beta Tg
AD-C/EBP Beta Tg

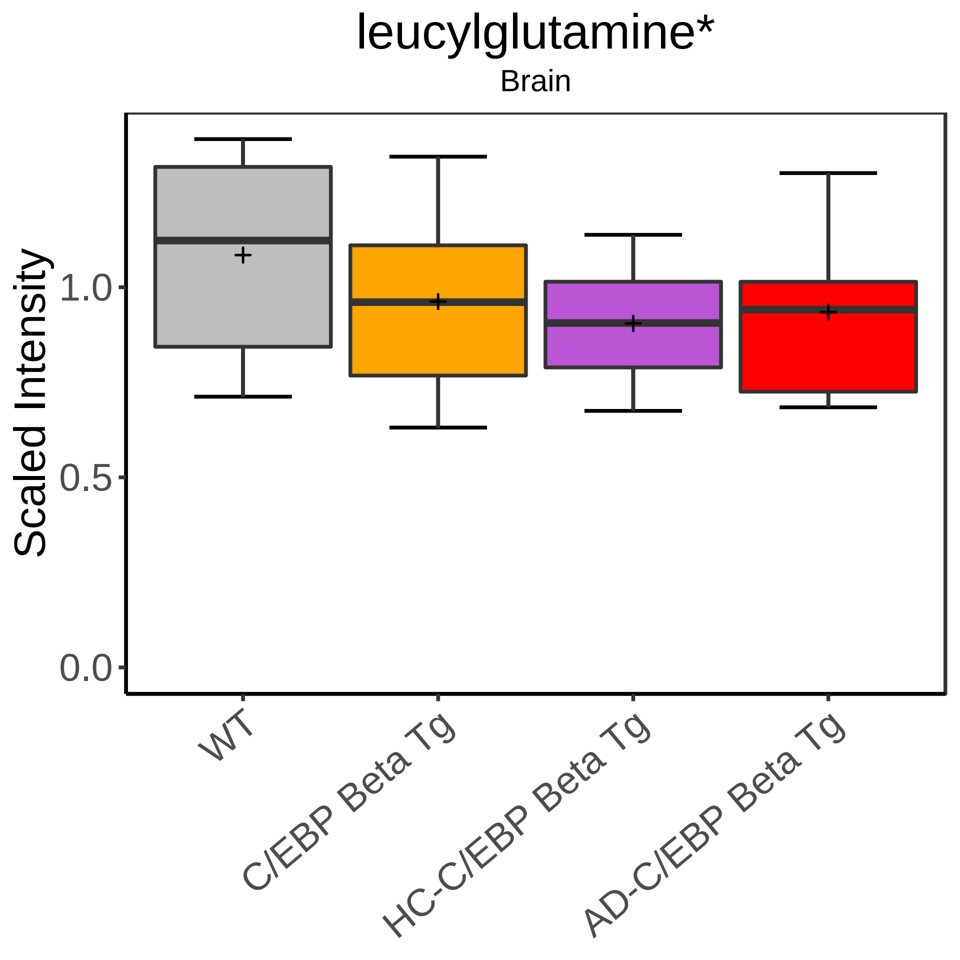
leucylglutamine*
Brain
Scaled Intensity
0.0
0.5
1.0
WT
C/EBP Beta Tg
HC-C/EBP Beta Tg
AD-C/EBP Beta Tg

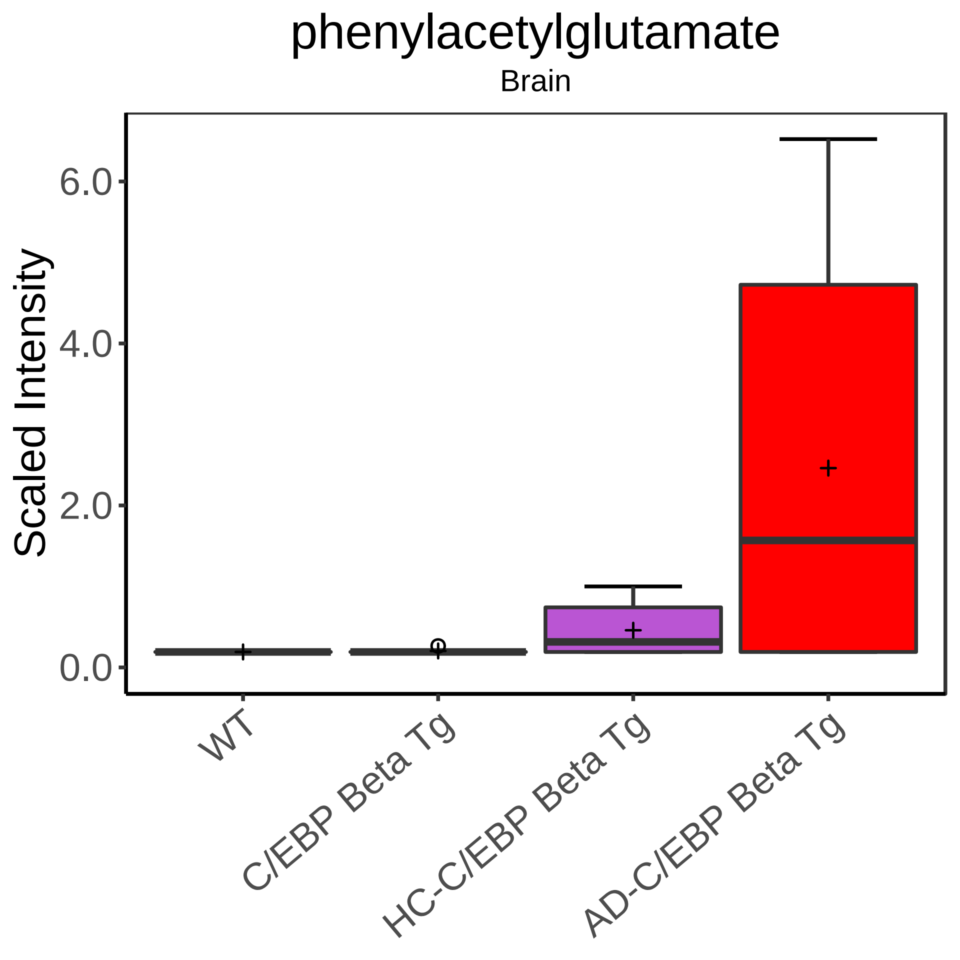

phenylacetylglutamate
Brain
Scaled Intensity
6.0
4.0
2.0
0.0
WT
C/EBP Beta Tg
HC-C/EBP Beta Tg
AD-C/EBP Beta Tg

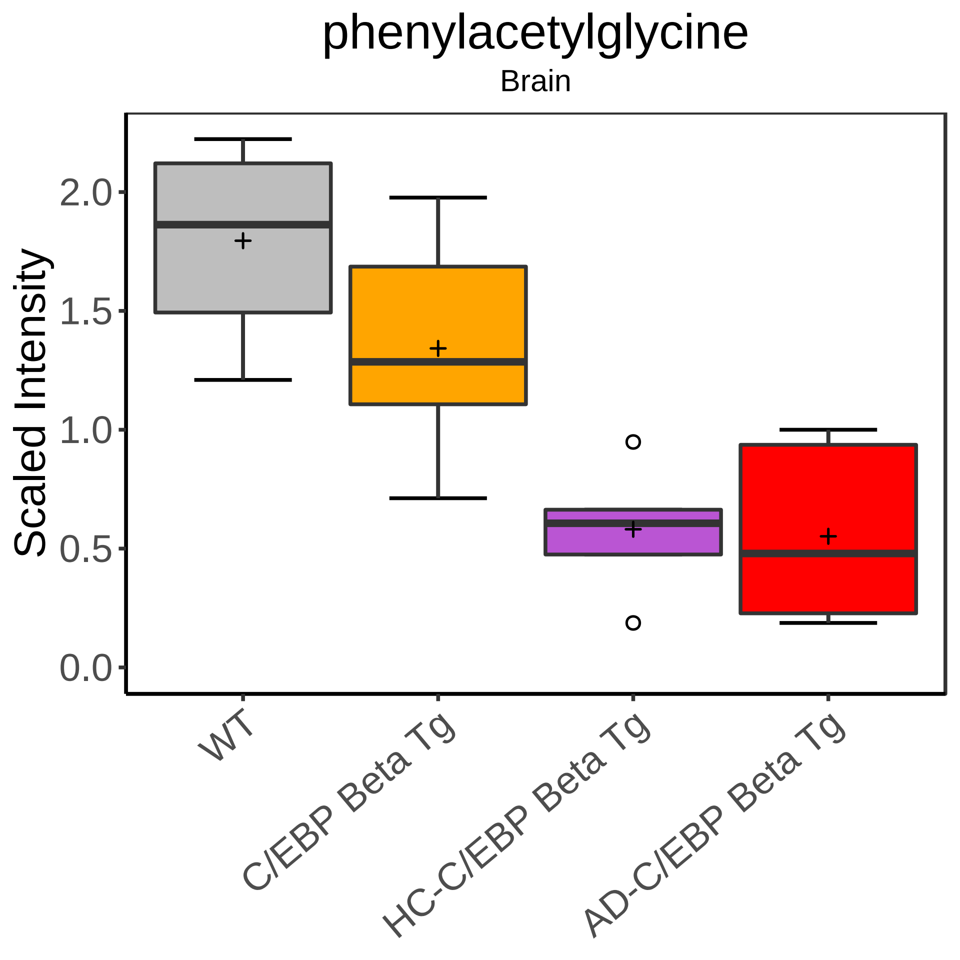
phenylacetylglycine
Brain
Scaled Intensity
2.0
1.5
1.0
0.5
0.0
WT
C/EBP Beta Tg
HC-C/EBP Beta Tg
AD-C/EBP Beta Tg

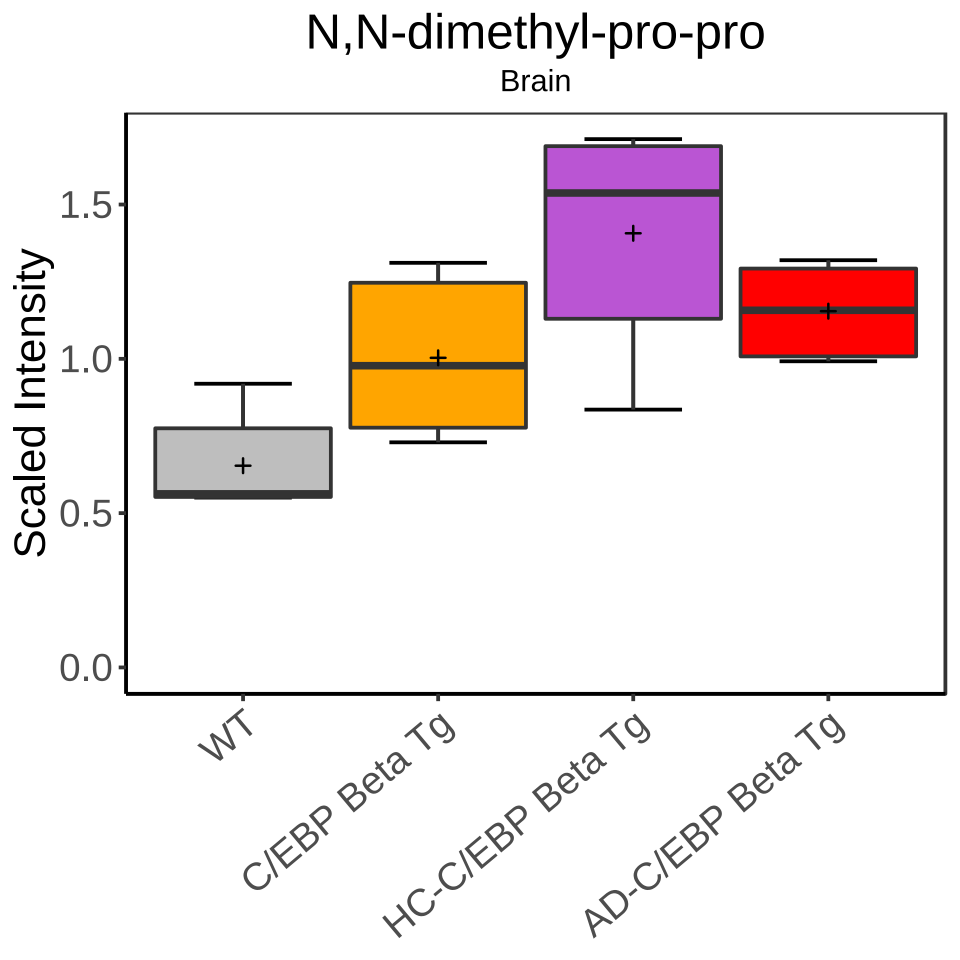
N,N-dimethyl-pro-pro
Brain
Scaled Intensity
1.5
1.0
0.5
0.0
WT
C/EBP Beta Tg
HC-C/EBP Beta Tg
AD-C/EBP Beta Tg

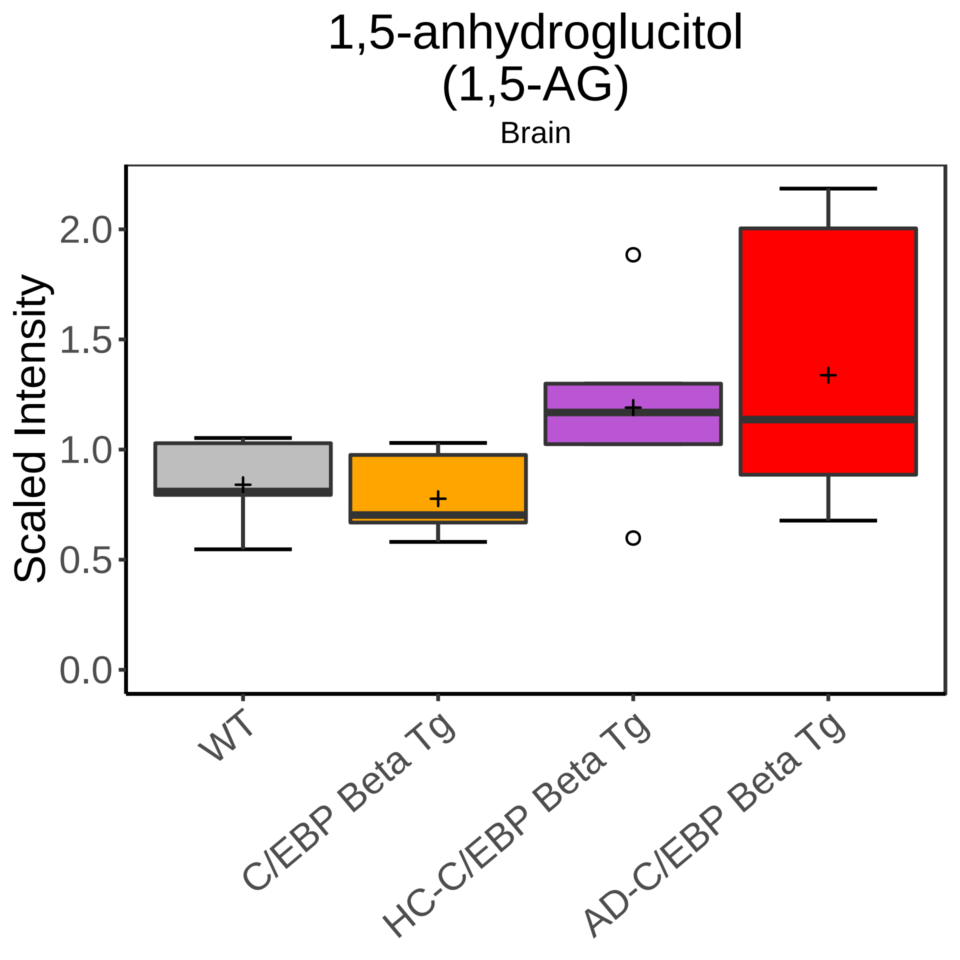

1,5-anhydroglucitol
(1,5-AG)
Brain
Scaled Intensity
2.0
1.5
1.0
0.5
0.0
WT
C/EBP Beta Tg
HC-C/EBP Beta Tg
AD-C/EBP Beta Tg

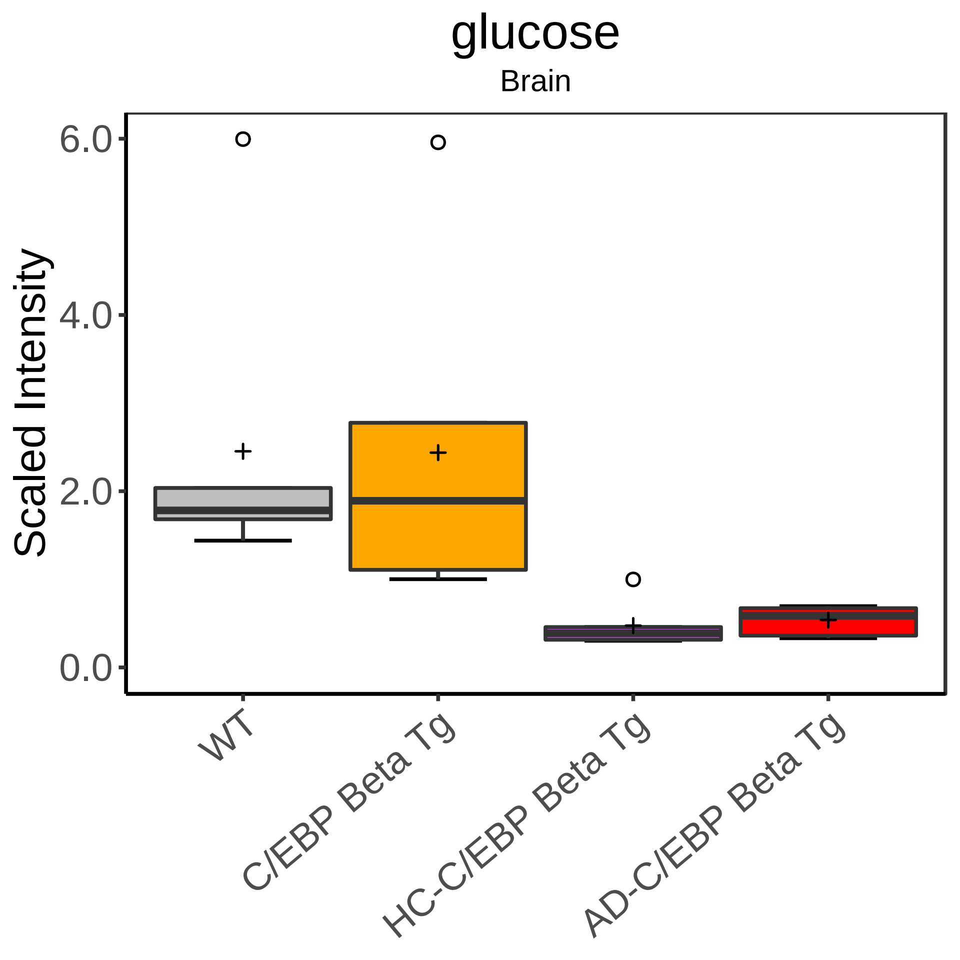
glucose
Brain
Scaled Intensity
6.0
4.0
2.0
0.0
WT
C/EBP Beta Tg
HC-C/EBP Beta Tg
AD-C/EBP Beta Tg

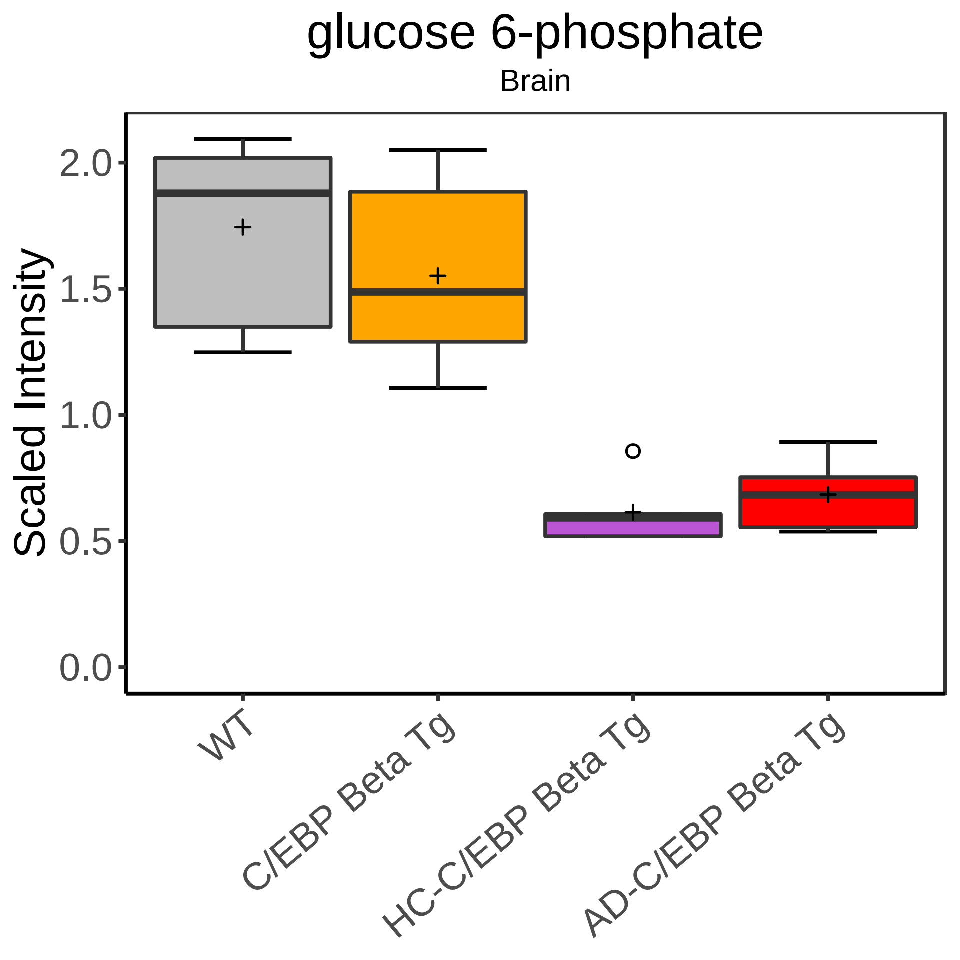
glucose 6-phosphate
Brain
Scaled Intensity
2.0
1.5
1.0
0.5
0.0
WT
C/EBP Beta Tg
HC-C/EBP Beta Tg
AD-C/EBP Beta Tg

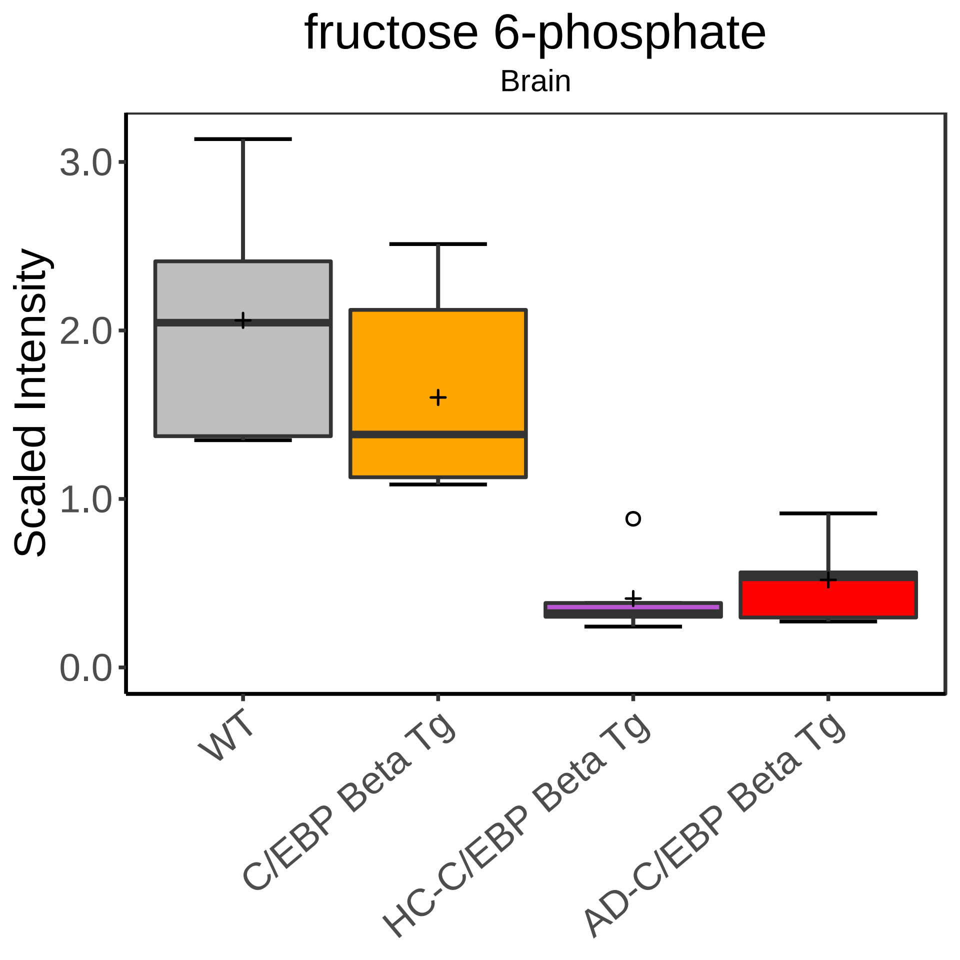

fructose 6-phosphate
Brain
Scaled Intensity
3.0
2.0
1.0
0.0
WT
C/EBP Beta Tg
HC-C/EBP Beta Tg
AD-C/EBP Beta Tg

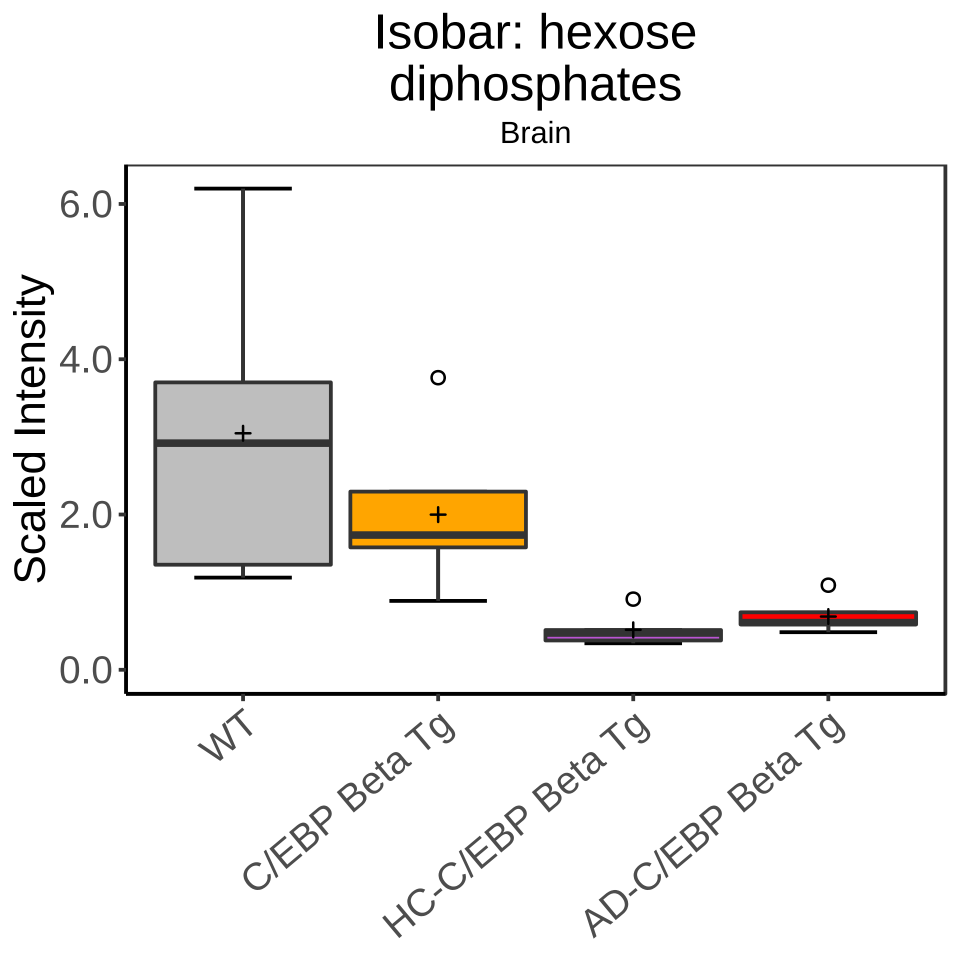

Isobar: hexose diphosphates
Brain
Scaled Intensity
6.0
4.0
2.0
0.0
WT
C/EBP Beta Tg
HC-C/EBP Beta Tg
AD-C/EBP Beta Tg

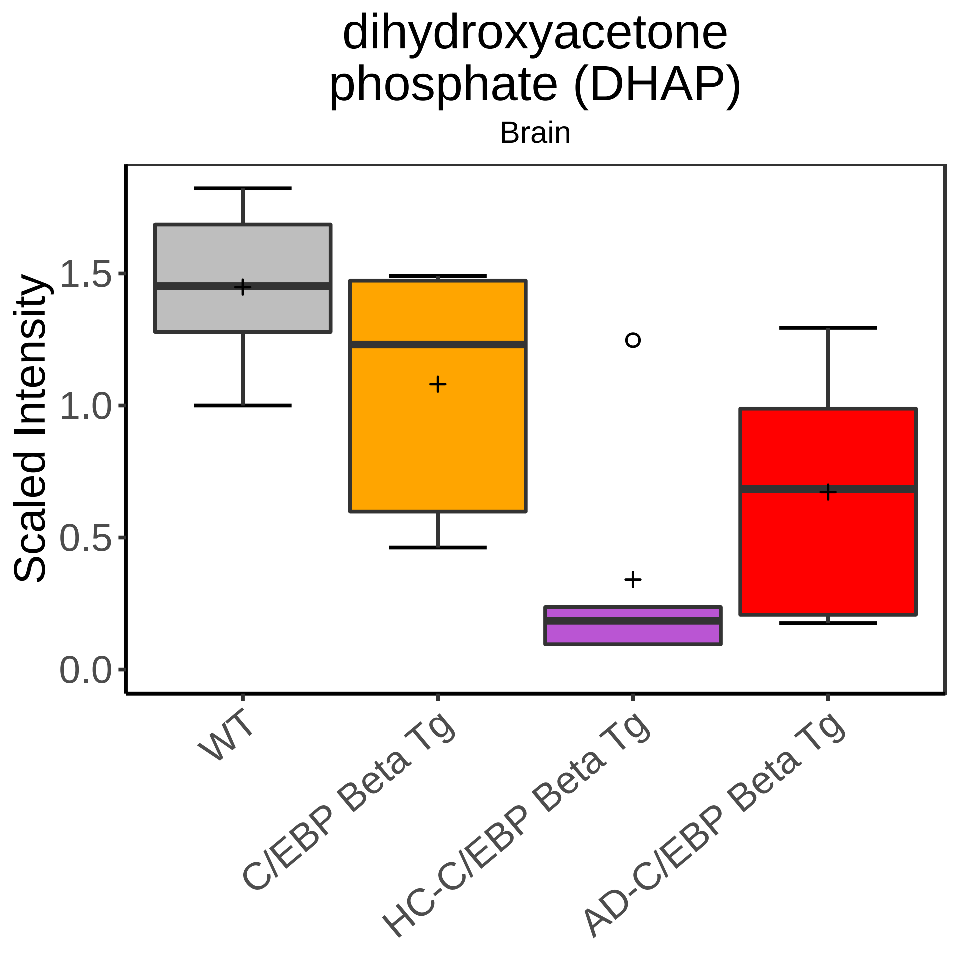
dihydroxyacetone
phosphate (DHAP)
Brain
Scaled Intensity
1.5
1.0
0.5
0.0
WT
C/EBP Beta Tg
HC-C/EBP Beta Tg
AD-C/EBP Beta Tg

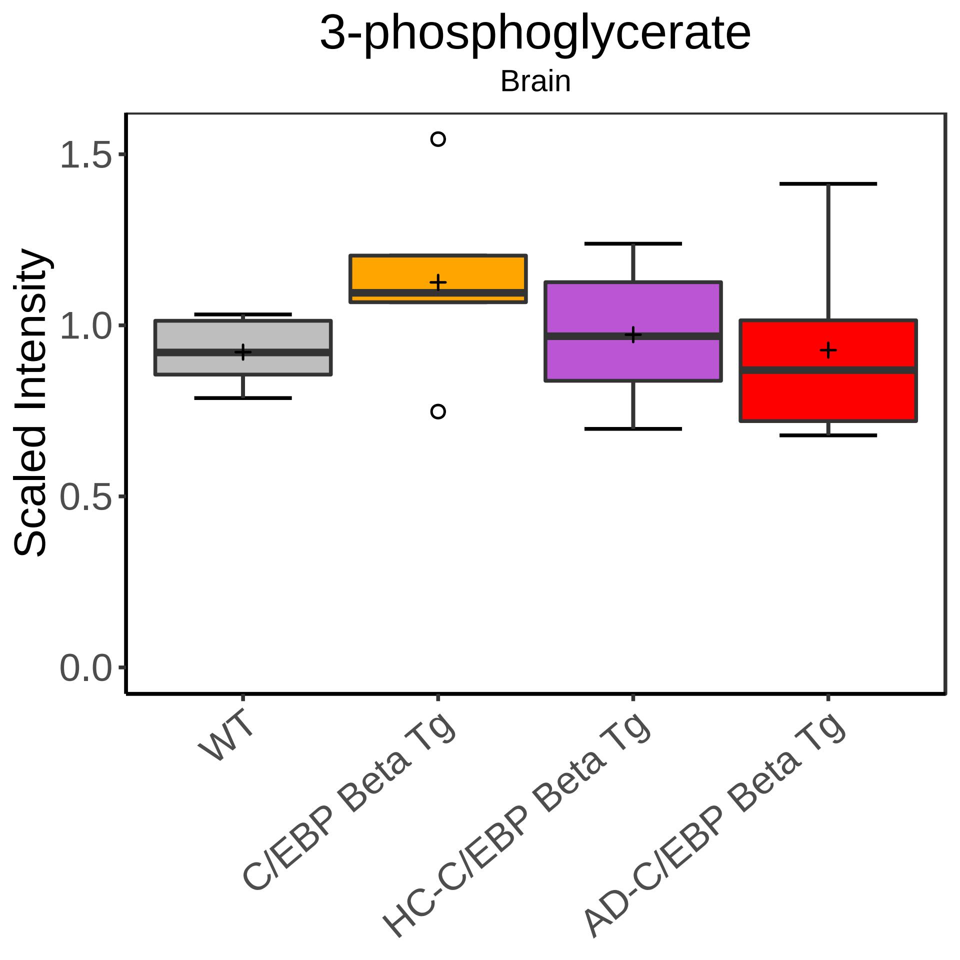
3-phosphoglycerate
Brain
Scaled Intensity
1.5
1.0
0.5
0.0
WT
C/EBP Beta Tg
HC-C/EBP Beta Tg
AD-C/EBP Beta Tg

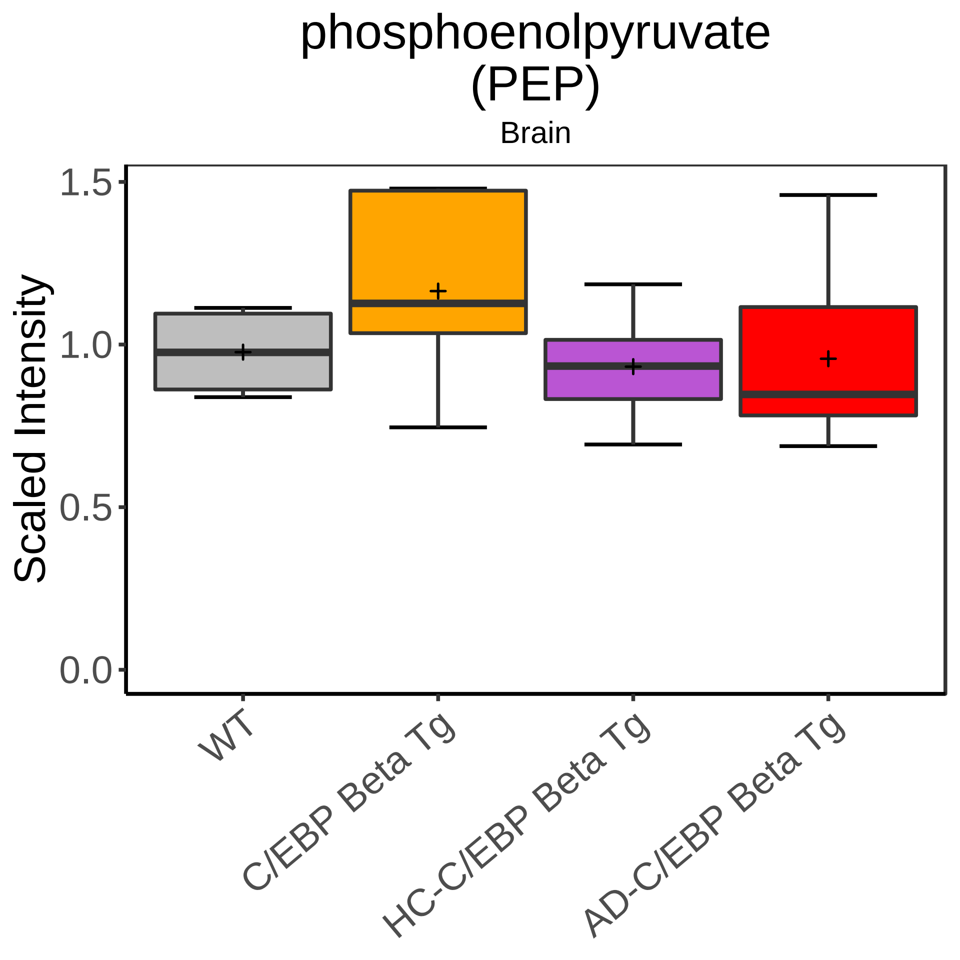
phosphoenolpyruvate
(PEP)
Brain
Scaled Intensity
1.5
1.0
0.5
0.0
WT
C/EBP Beta Tg
HC-C/EBP Beta Tg
AD-C/EBP Beta Tg

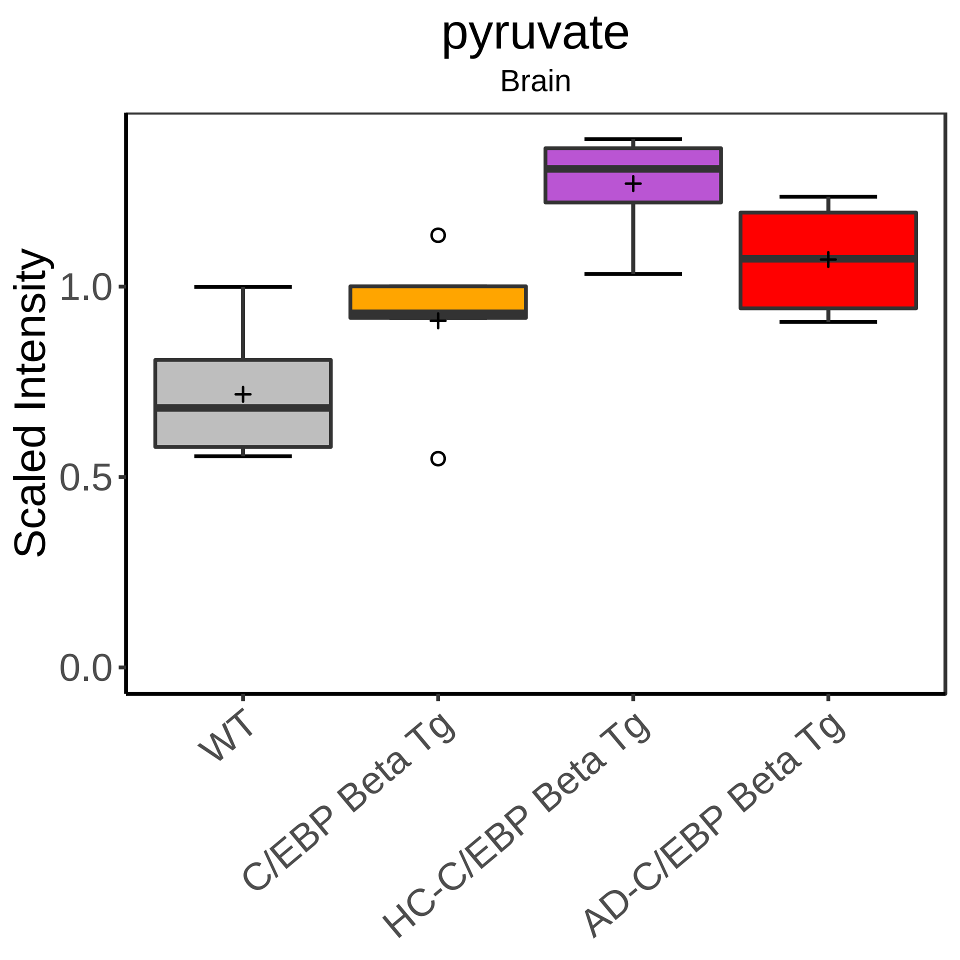
pyruvate
Brain
Scaled Intensity
0.0
0.5
1.0
WT
C/EBP Beta Tg
HC-C/EBP Beta Tg
AD-C/EBP Beta Tg

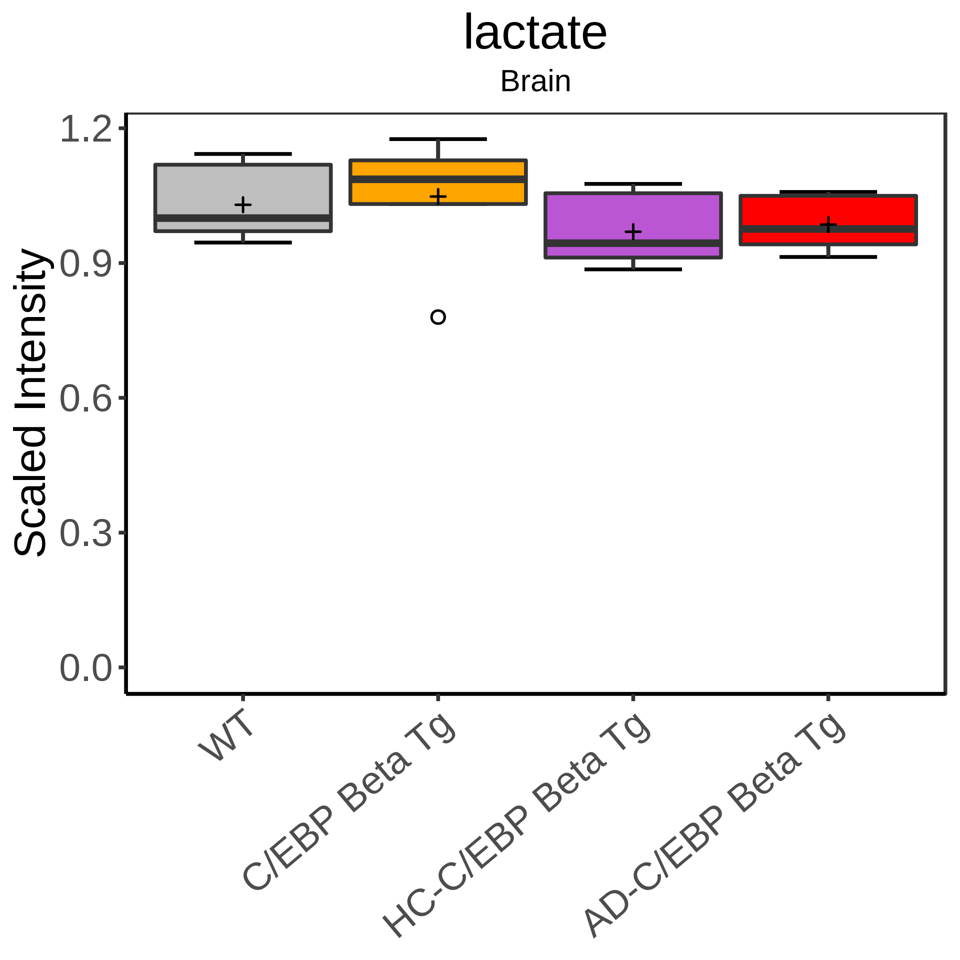
lactate
Brain
Scaled Intensity
1.2
0.9
0.6
0.3
0.0
WT
C/EBP Beta Tg
HC-C/EBP Beta Tg
AD-C/EBP Beta Tg

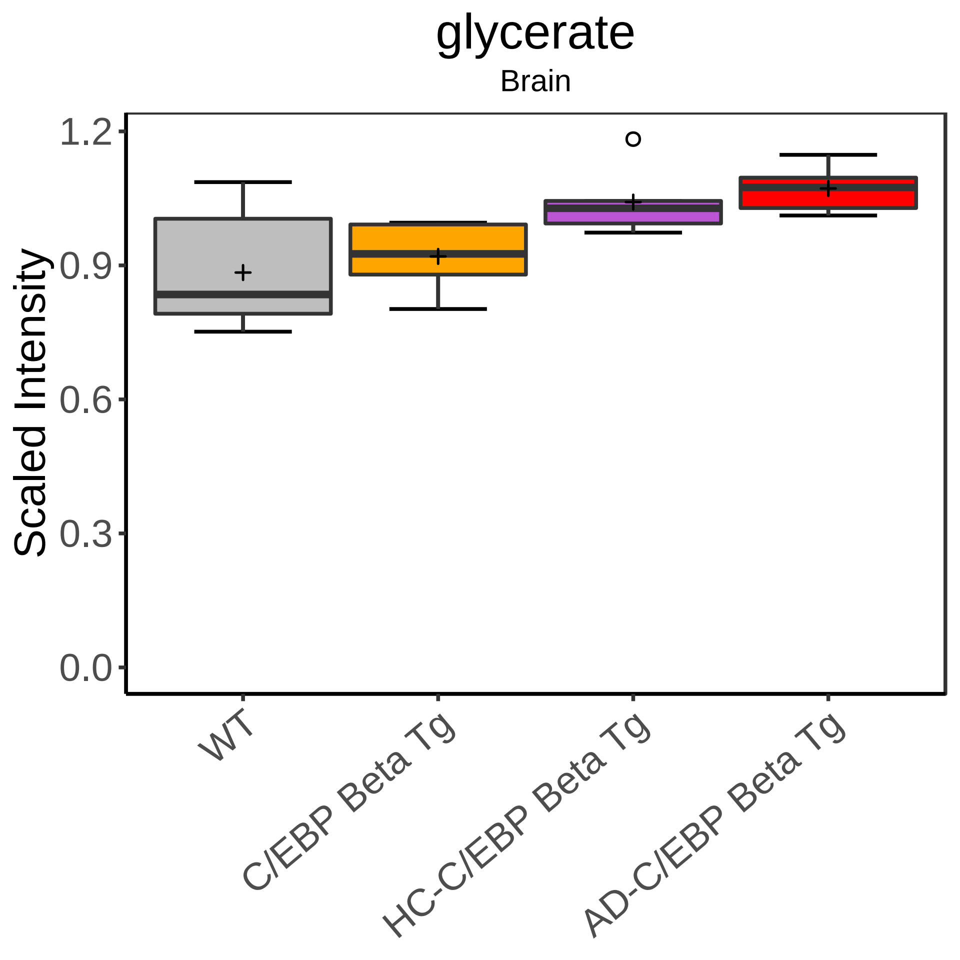

glycerate
Brain
Scaled Intensity
1.2
0.9
0.6
0.3
0.0
WT
C/EBP Beta Tg
HC-C/EBP Beta Tg
AD-C/EBP Beta Tg

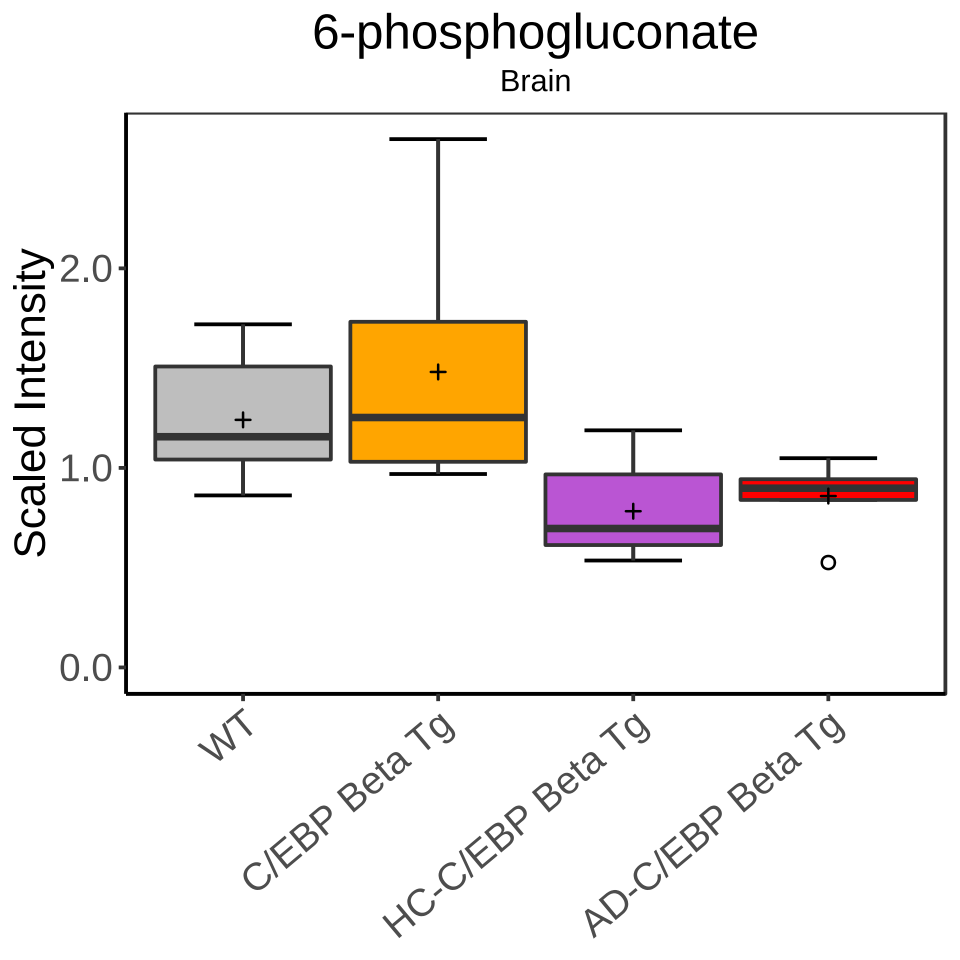
6-phosphogluconate
Brain
Scaled Intensity
2.0
1.0
0.0
WT
C/EBP Beta Tg
HC-C/EBP Beta Tg
AD-C/EBP Beta Tg

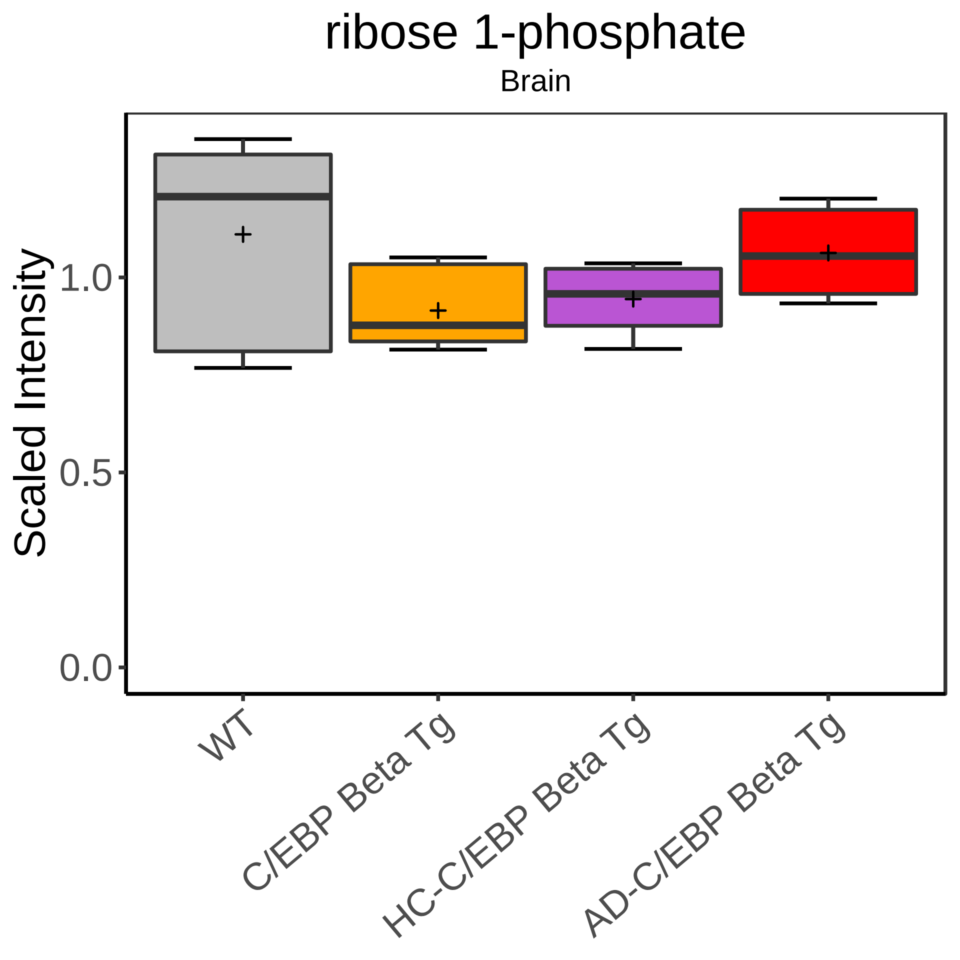

ribose 1-phosphate
Brain
Scaled Intensity
0.0
0.5
1.0
WT
C/EBP Beta Tg
HC-C/EBP Beta Tg
AD-C/EBP Beta Tg

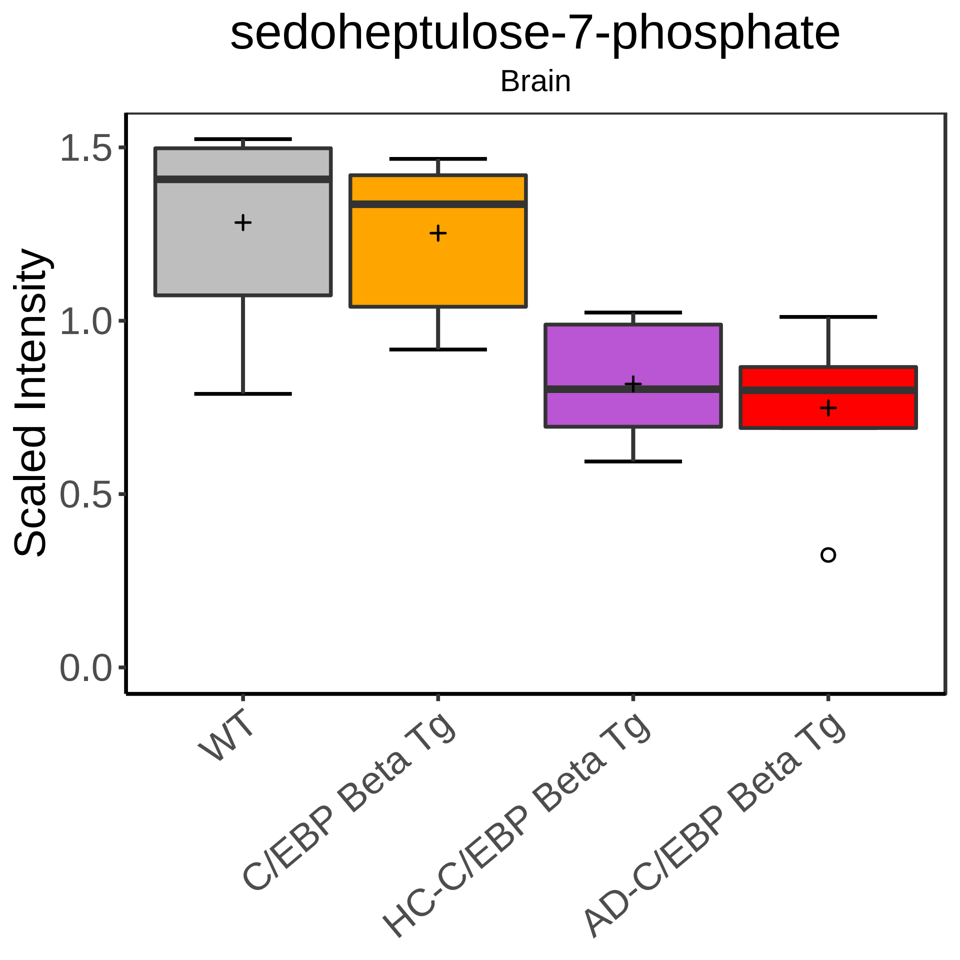
sedoheptulose-7-phosphate
Brain
Scaled Intensity
0.0
0.5
1.0
1.5
WT
C/EBP Beta Tg
HC-C/EBP Beta Tg
AD-C/EBP Beta Tg

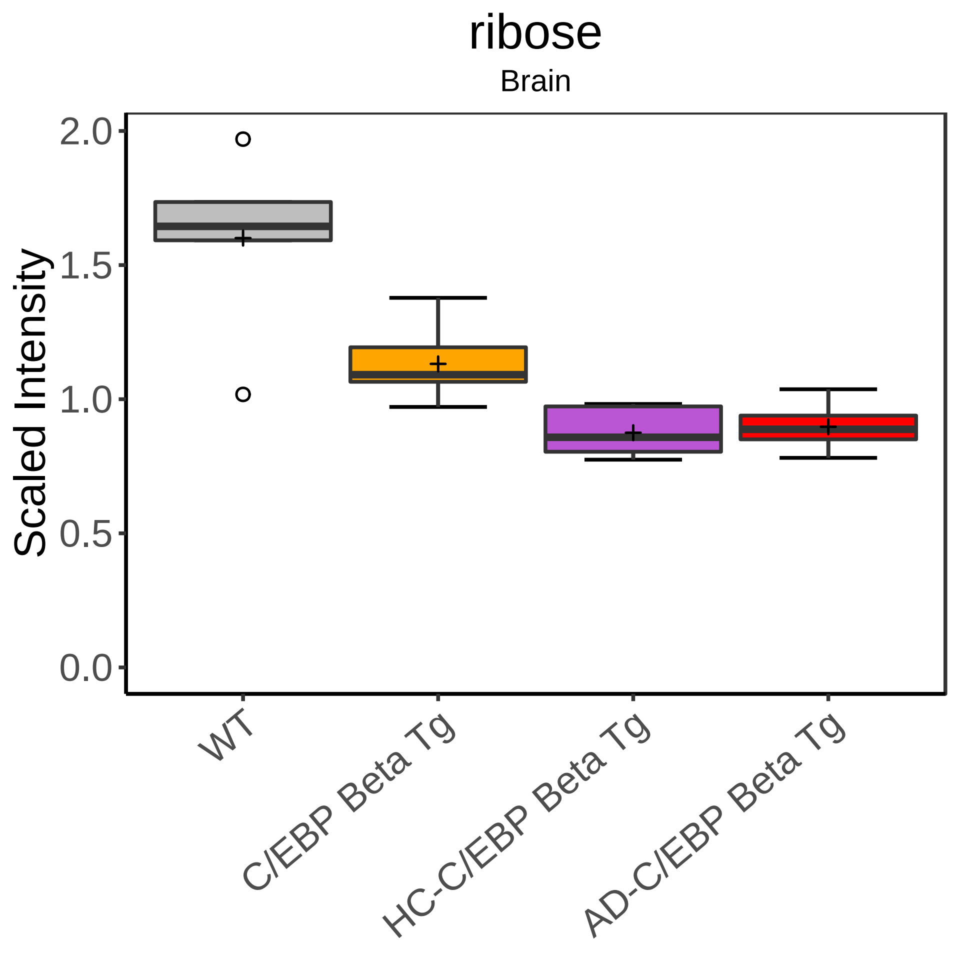
ribose
Brain
Scaled Intensity
2.0
1.5
1.0
0.5
0.0
WT
C/EBP Beta Tg
HC-C/EBP Beta Tg
AD-C/EBP Beta Tg

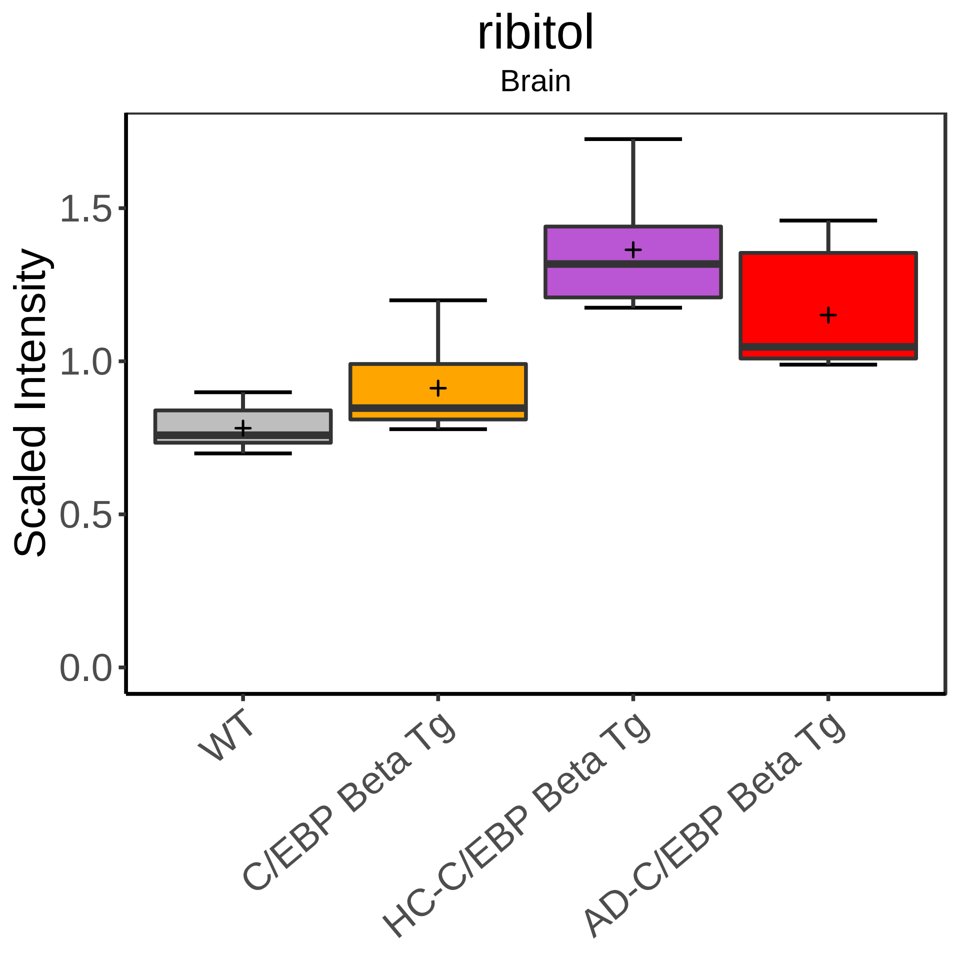
ribitol
Brain
Scaled Intensity
1.5
1.0
0.5
0.0
WT
C/EBP Beta Tg
HC-C/EBP Beta Tg
AD-C/EBP Beta Tg

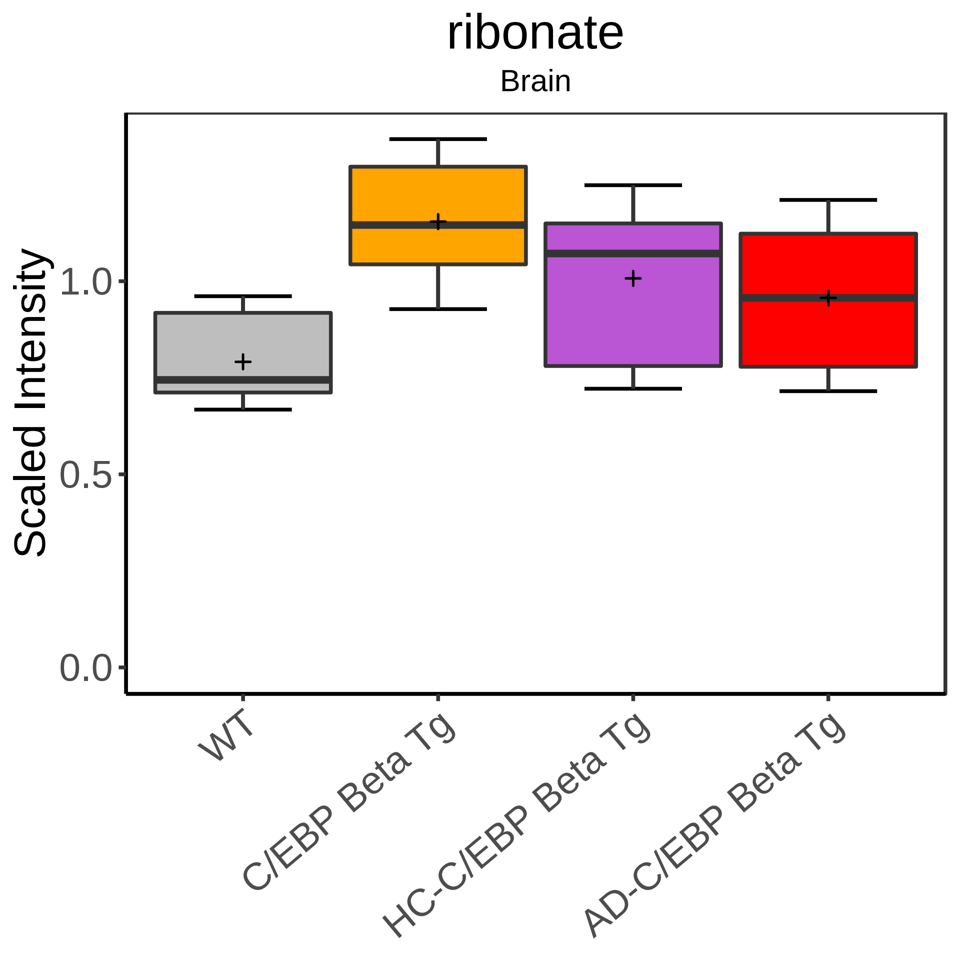

ribonate
Brain
Scaled Intensity
0.0
0.5
1.0
WT
C/EBP Beta Tg
HC-C/EBP Beta Tg
AD-C/EBP Beta Tg

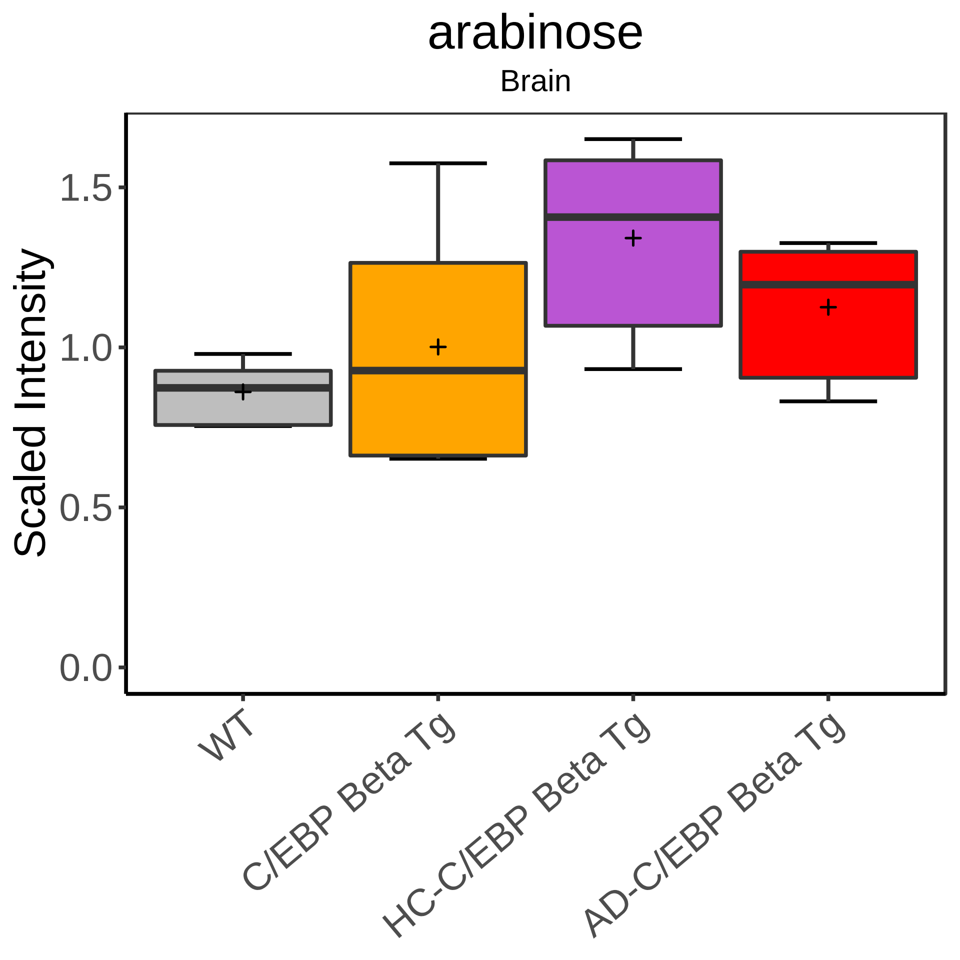
arabinose
Brain
Scaled Intensity
1.5
1.0
0.5
0.0
WT
C/EBP Beta Tg
HC-C/EBP Beta Tg
AD-C/EBP Beta Tg

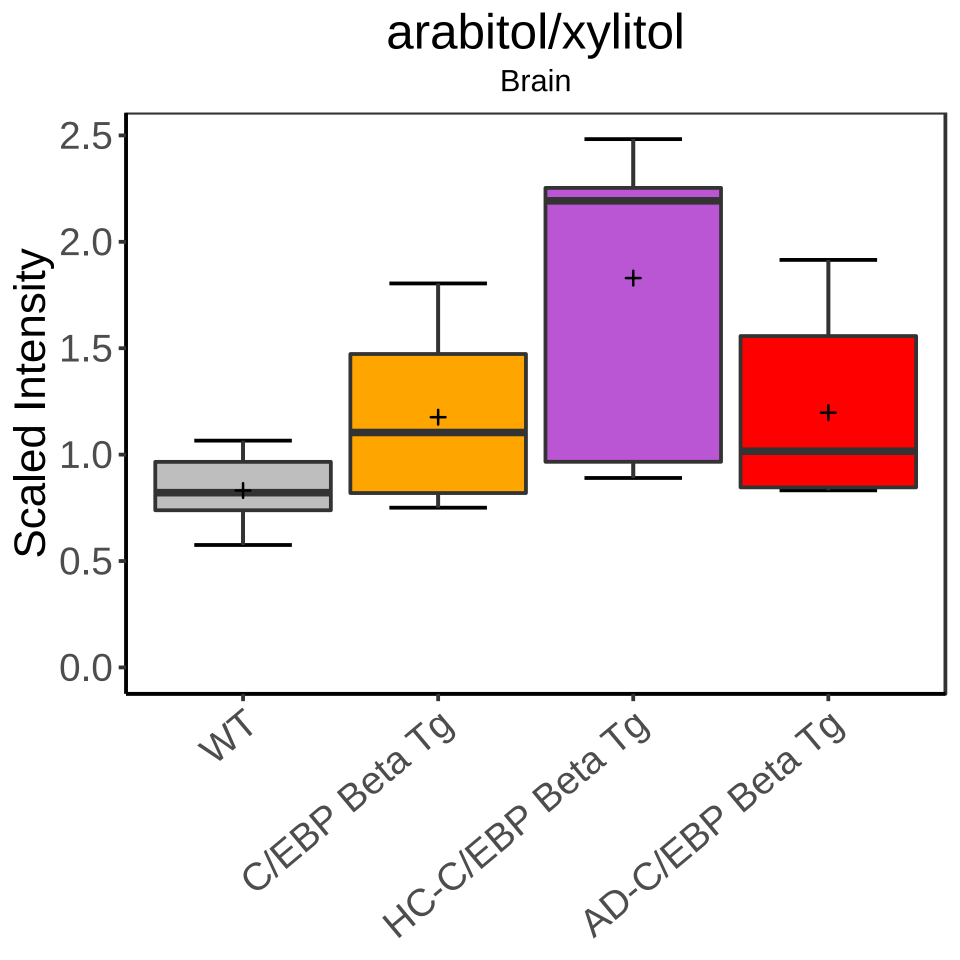
arabitol/xylitol
Brain
Scaled Intensity
2.5
2.0
1.5
1.0
0.5
0.0
WT
C/EBP Beta Tg
HC-C/EBP Beta Tg
AD-C/EBP Beta Tg

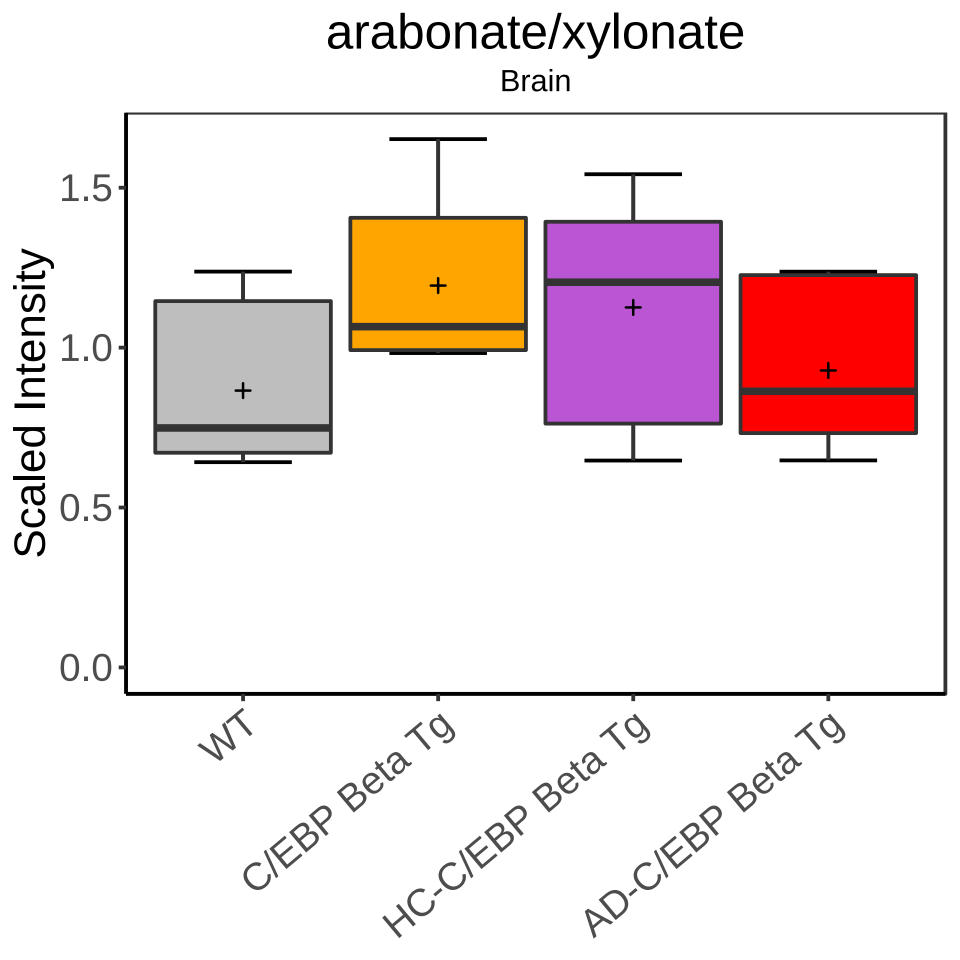

arabonate/xylonate
Brain
Scaled Intensity
0.0
0.5
1.0
1.5
WT
C/EBP Beta Tg
HC-C/EBP Beta Tg
AD-C/EBP Beta Tg

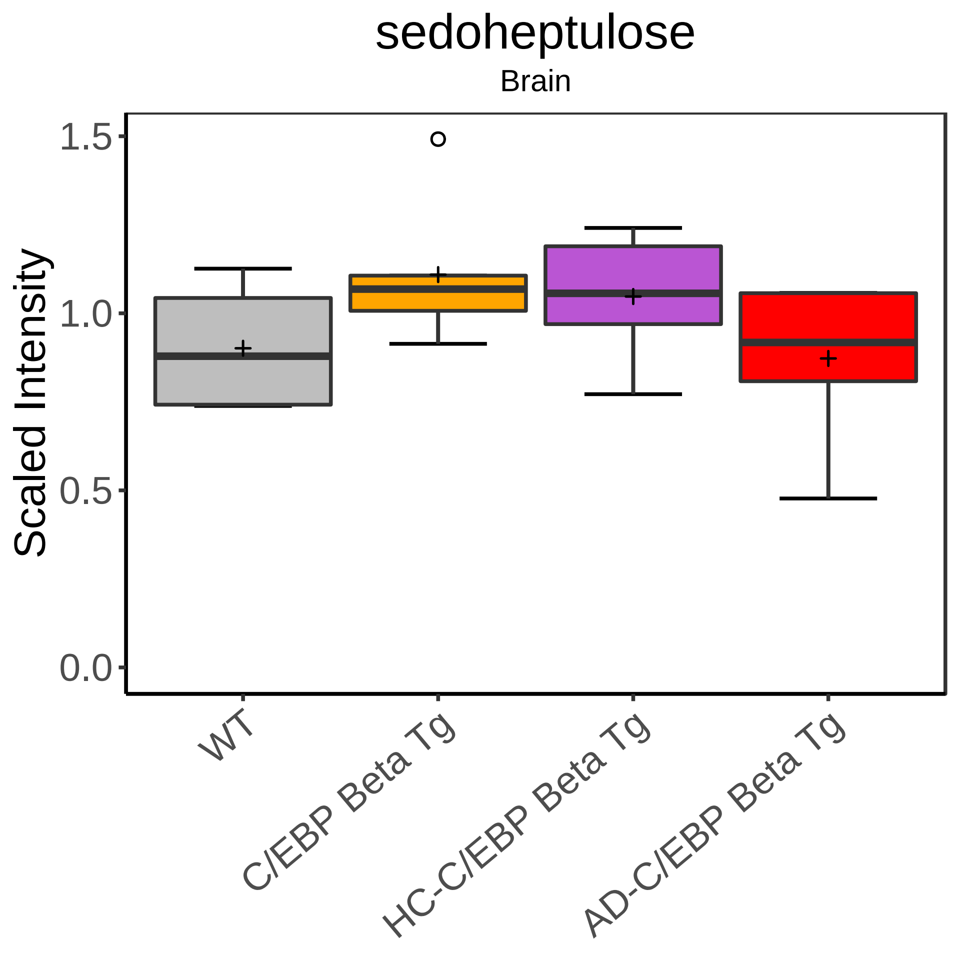
sedoheptulose
Brain
Scaled Intensity
1.5
1.0
0.5
0.0
WT
C/EBP Beta Tg
HC-C/EBP Beta Tg
AD-C/EBP Beta Tg

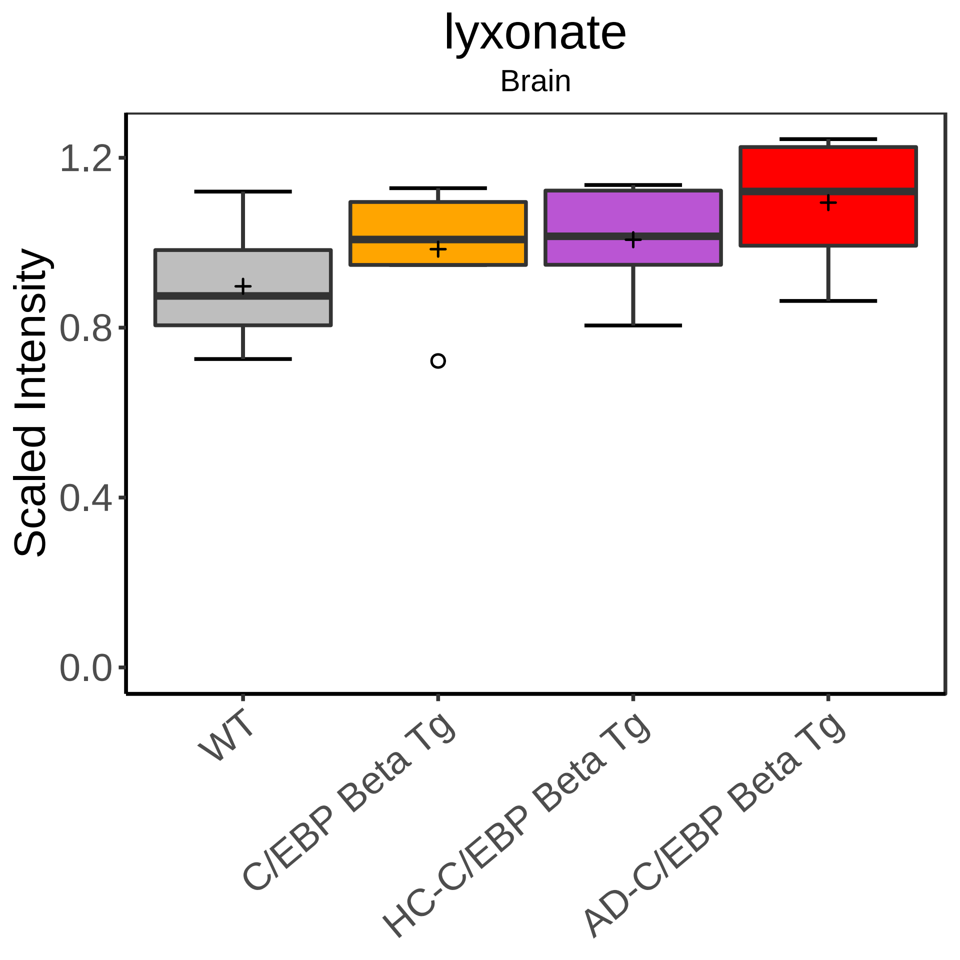

lyxonate
Brain
Scaled Intensity
1.2
0.8
0.4
0.0
WT
C/EBP Beta Tg
HC-C/EBP Beta Tg
AD-C/EBP Beta Tg

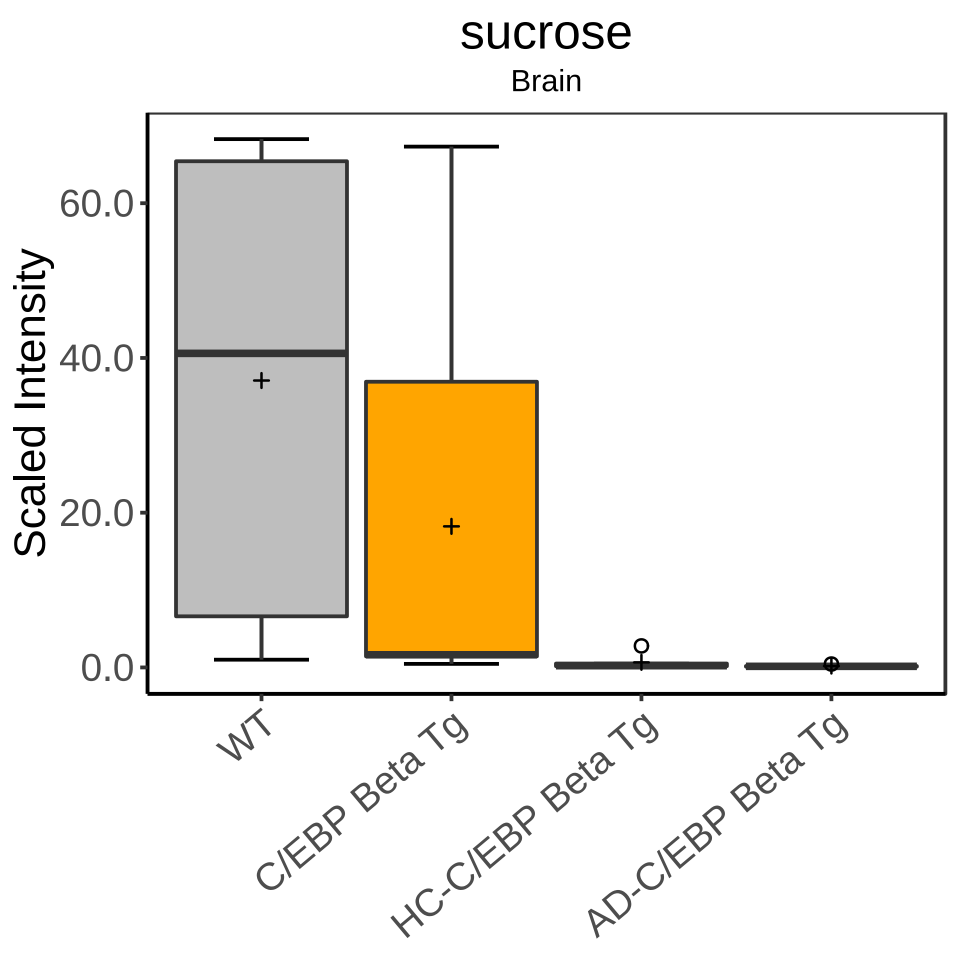
sucrose
Brain
Scaled Intensity
60.0
40.0
20.0
0.0
WT
C/EBP Beta Tg
HC-C/EBP Beta Tg
AD-C/EBP Beta Tg

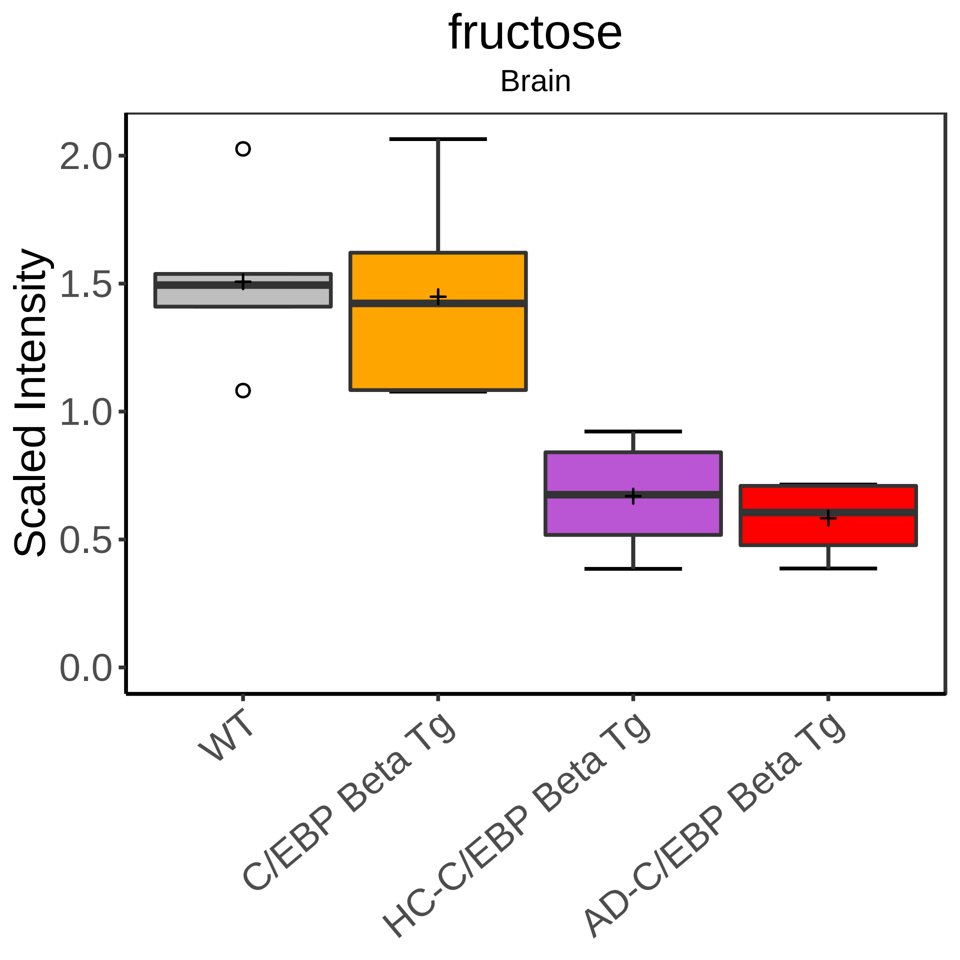
fructose
Brain
Scaled Intensity
2.0
1.5
1.0
0.5
0.0
WT
C/EBP Beta Tg
HC-C/EBP Beta Tg
AD-C/EBP Beta Tg

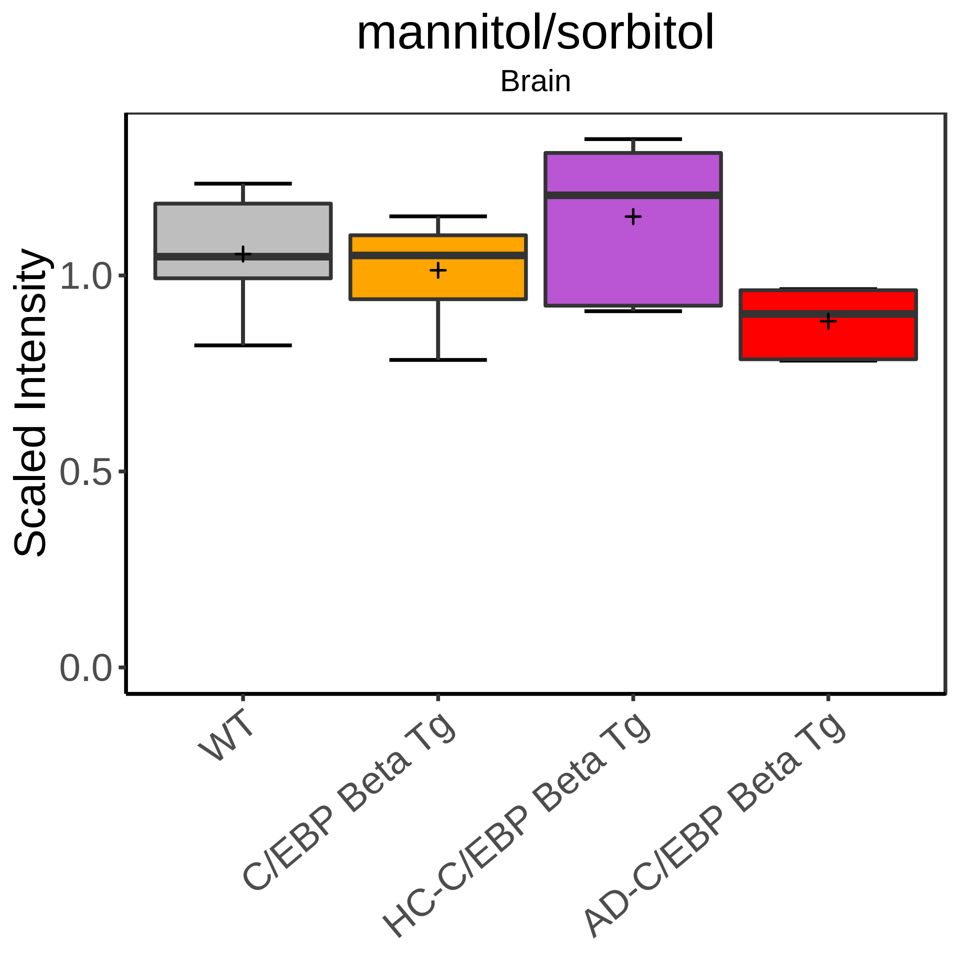
mannitol/sorbitol
Brain
Scaled Intensity
1.0
0.5
0.0
WT
C/EBP Beta Tg
HC-C/EBP Beta Tg
AD-C/EBP Beta Tg

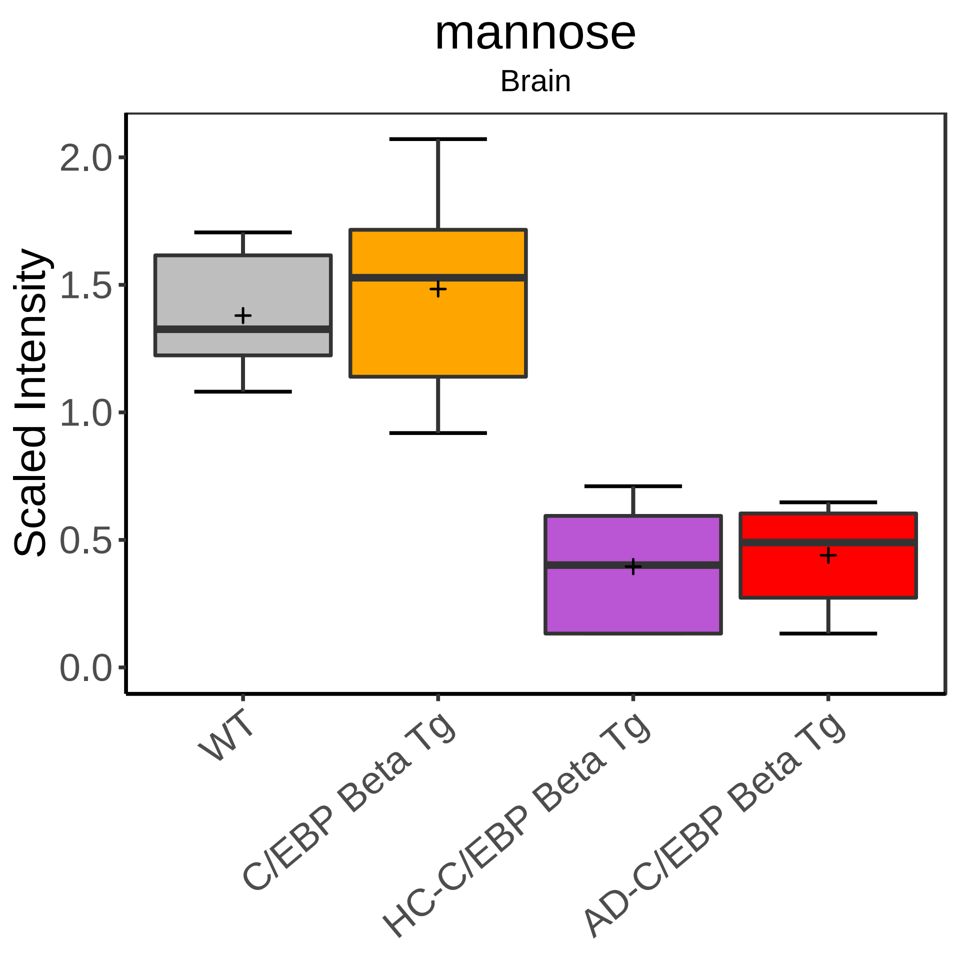

mannose
Brain
Scaled Intensity
2.0
1.5
1.0
0.5
0.0
WT
C/EBP Beta Tg
HC-C/EBP Beta Tg
AD-C/EBP Beta Tg

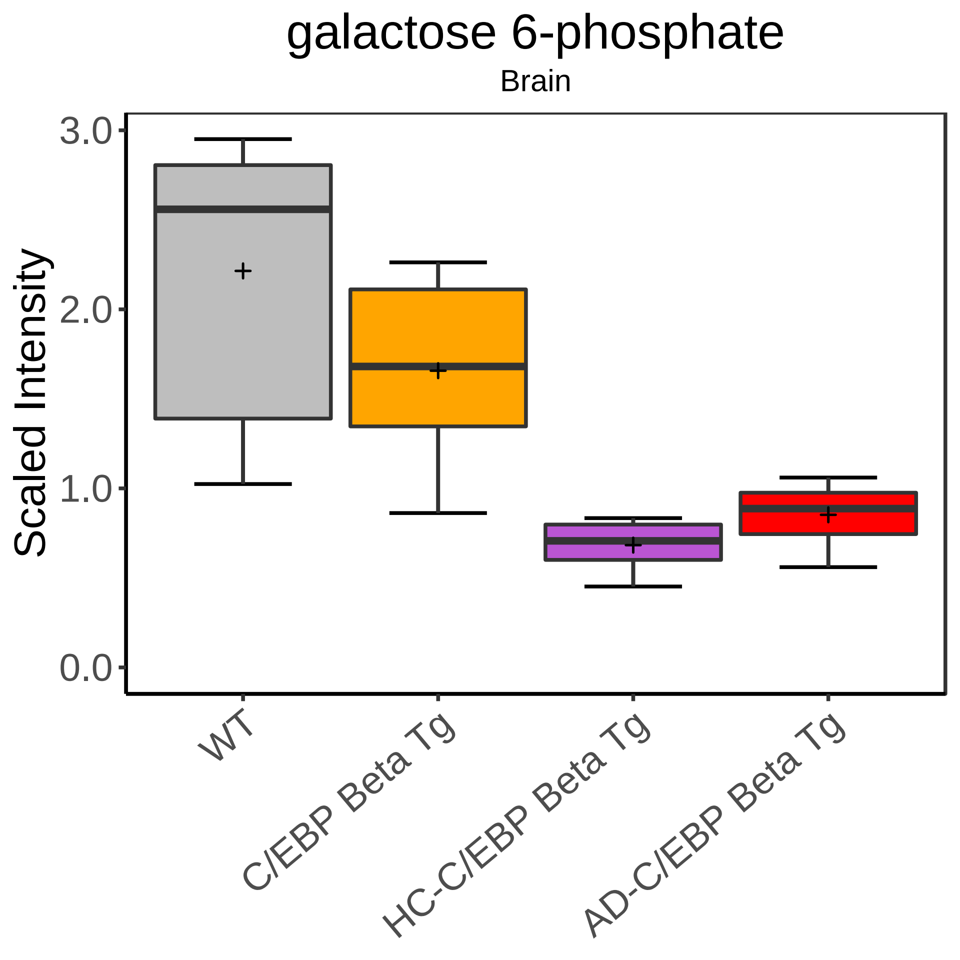

galactose 6-phosphate
Brain
Scaled Intensity
3.0
2.0
1.0
0.0
WT
C/EBP Beta Tg
HC-C/EBP Beta Tg
AD-C/EBP Beta Tg

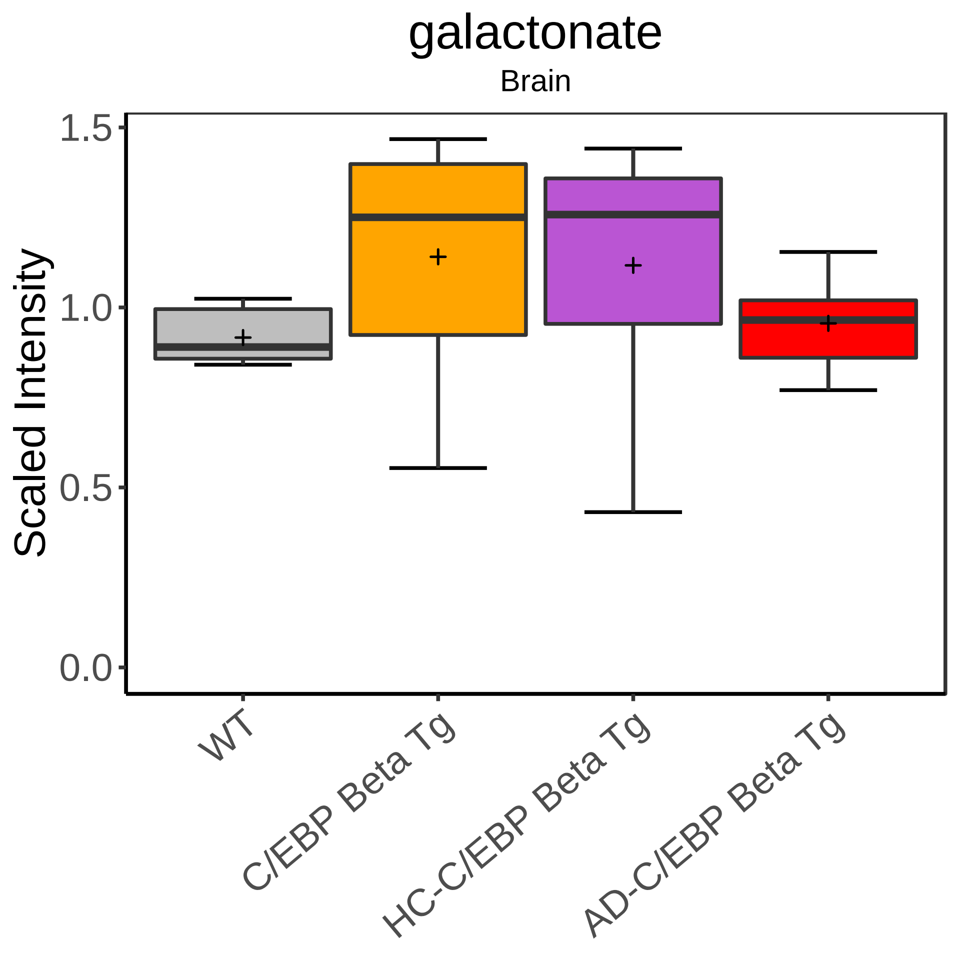

galactonate
Brain
Scaled Intensity
1.5
1.0
0.5
0.0
WT
C/EBP Beta Tg
HC-C/EBP Beta Tg
AD-C/EBP Beta Tg

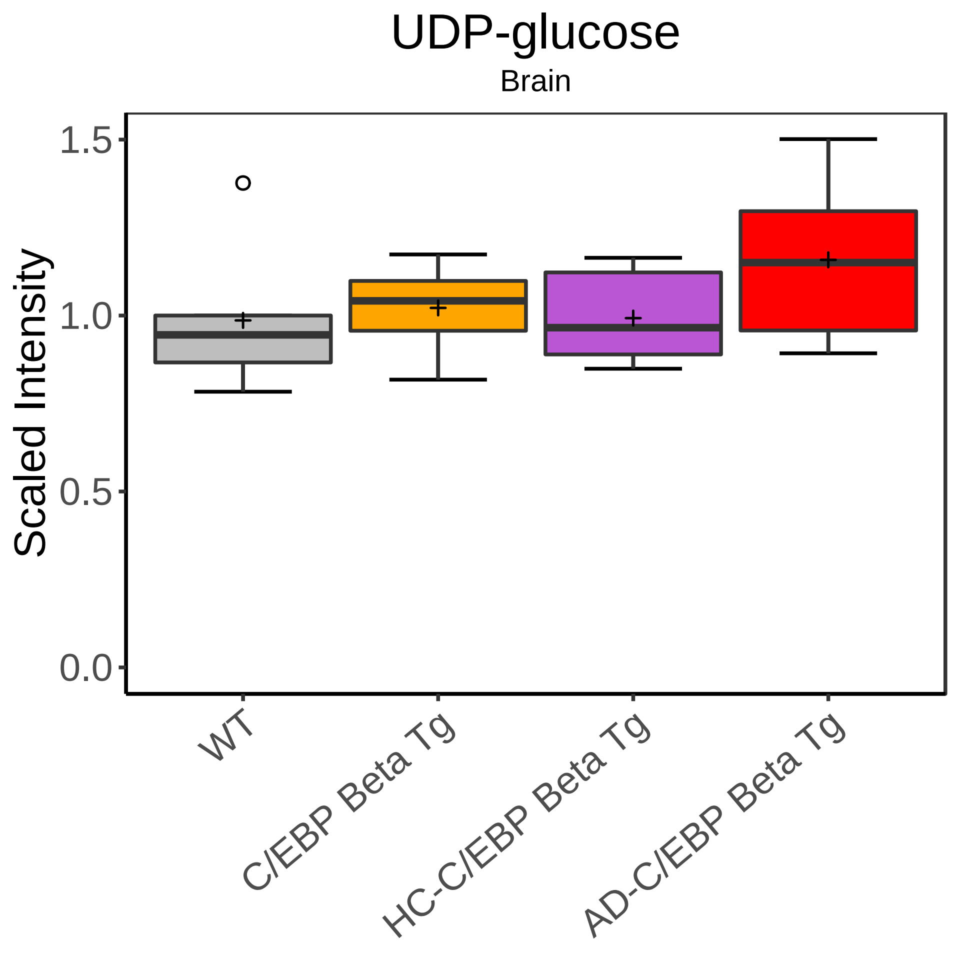
UDP-glucose
Brain
Scaled Intensity
1.5
1.0
0.5
0.0
WT
C/EBP Beta Tg
HC-C/EBP Beta Tg
AD-C/EBP Beta Tg

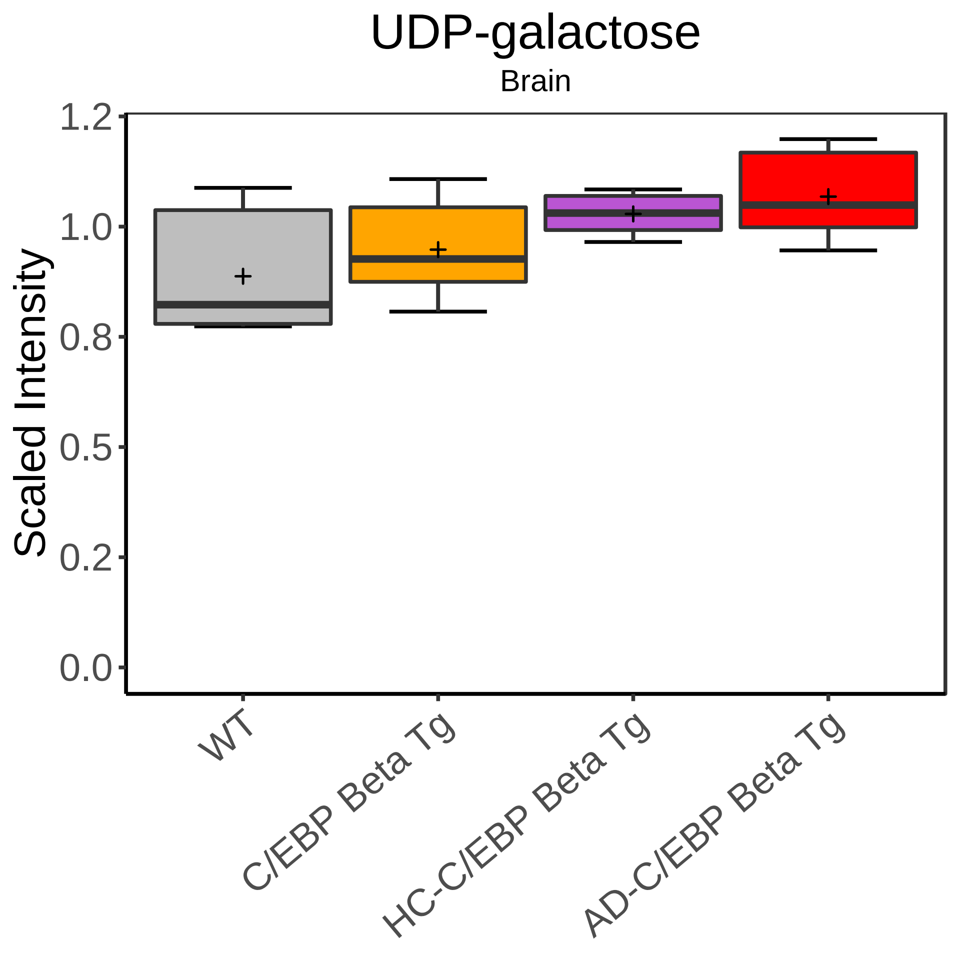
UDP-galactose
Brain
Scaled Intensity
1.2
1.0
0.8
0.5
0.2
0.0
WT
C/EBP Beta Tg
HC-C/EBP Beta Tg
AD-C/EBP Beta Tg

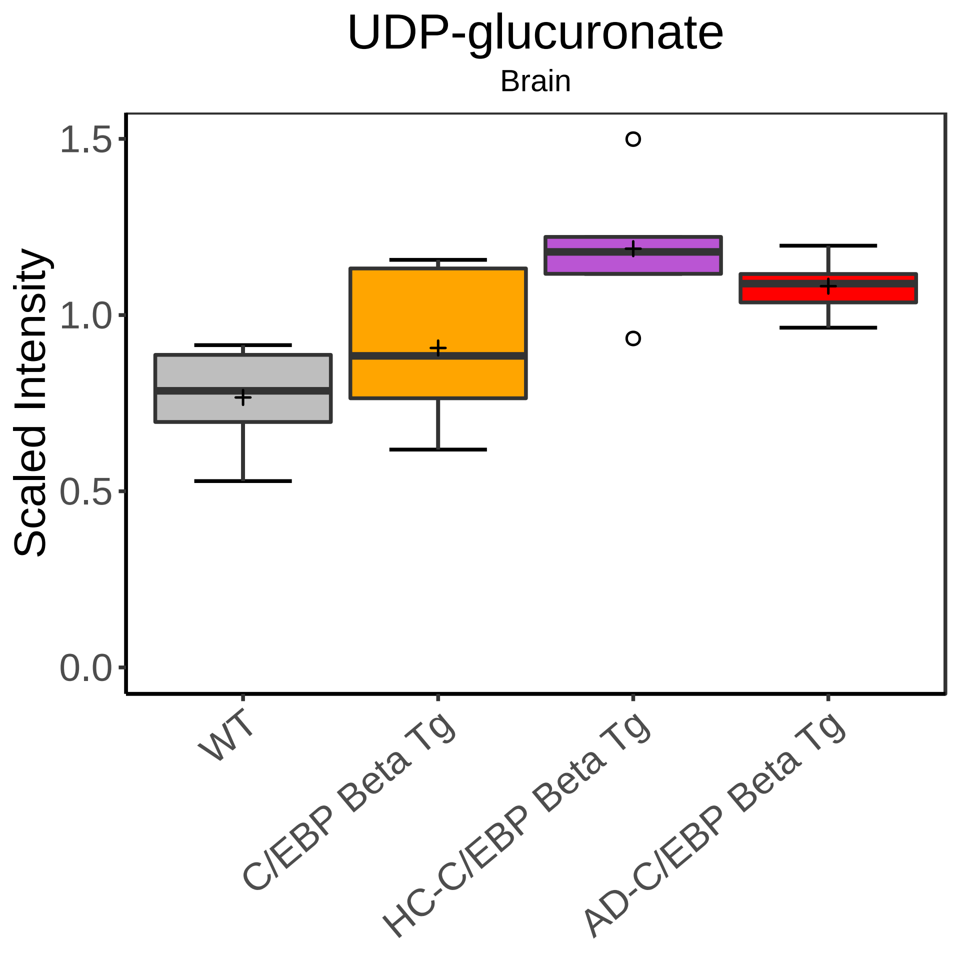

UDP-glucuronate
Brain
Scaled Intensity
1.5
1.0
0.5
0.0
WT
C/EBP Beta Tg
HC-C/EBP Beta Tg
AD-C/EBP Beta Tg

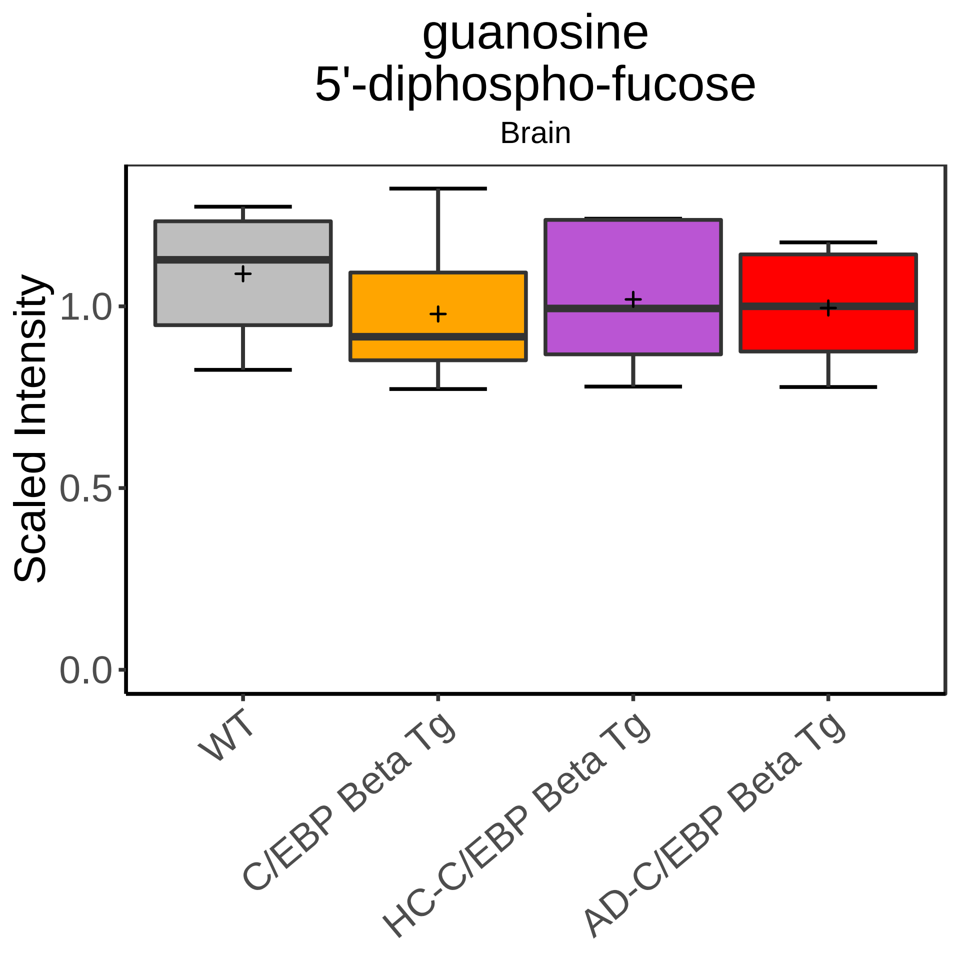

guanosine
5'-diphospho-fucose
Brain
Scaled Intensity
1.0
0.5
0.0
WT
C/EBP Beta Tg
HC-C/EBP Beta Tg
AD-C/EBP Beta Tg

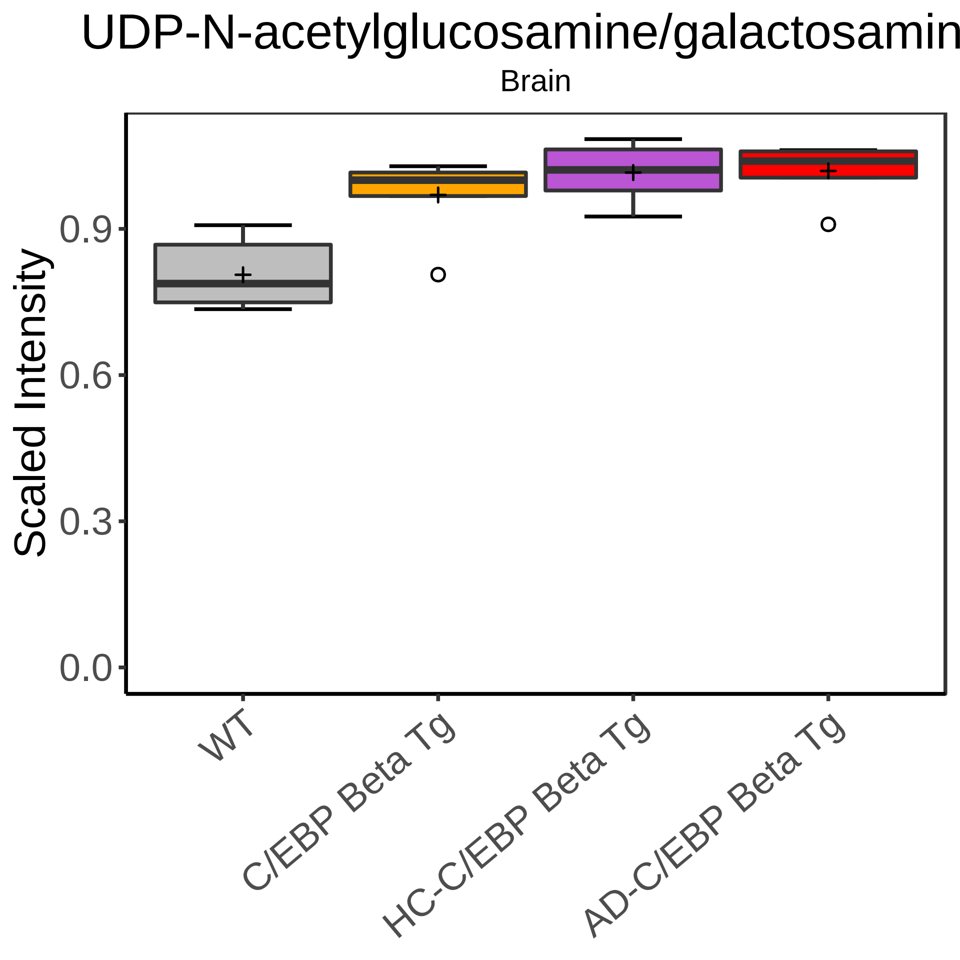
UDP-N-acetylglucosamine/galactosamin
Brain
Scaled Intensity
0.0
0.3
0.6
0.9
WT
C/EBP Beta Tg
HC-C/EBP Beta Tg
AD-C/EBP Beta Tg

cytidine 5'-monophospho-N-acetylneuraminic acid

Brain

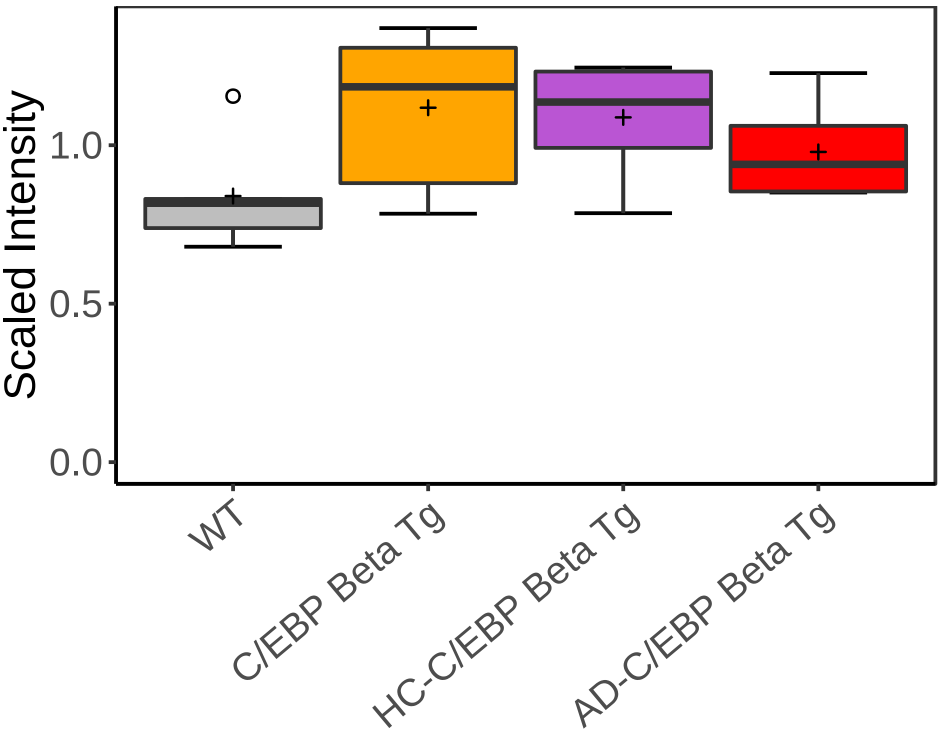

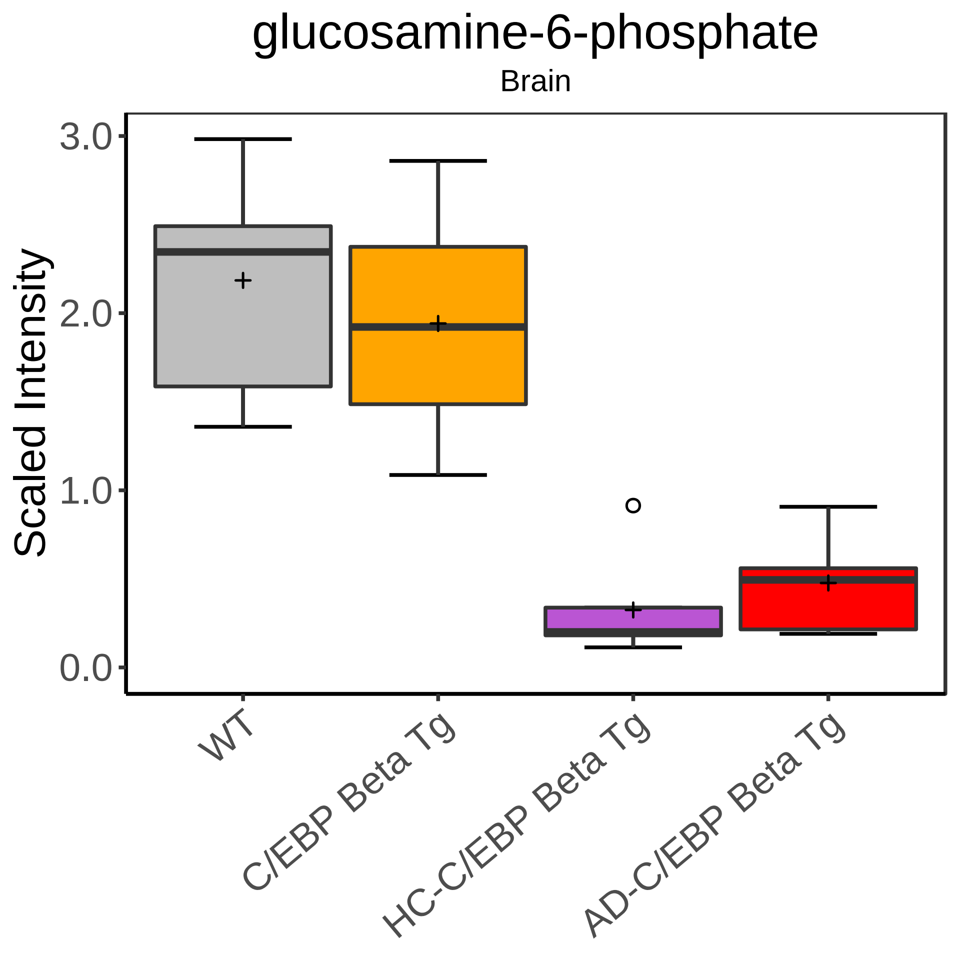
glucosamine-6-phosphate
Brain
Scaled Intensity
3.0
2.0
1.0
0.0
WT
C/EBP Beta Tg
HC-C/EBP Beta Tg
AD-C/EBP Beta Tg

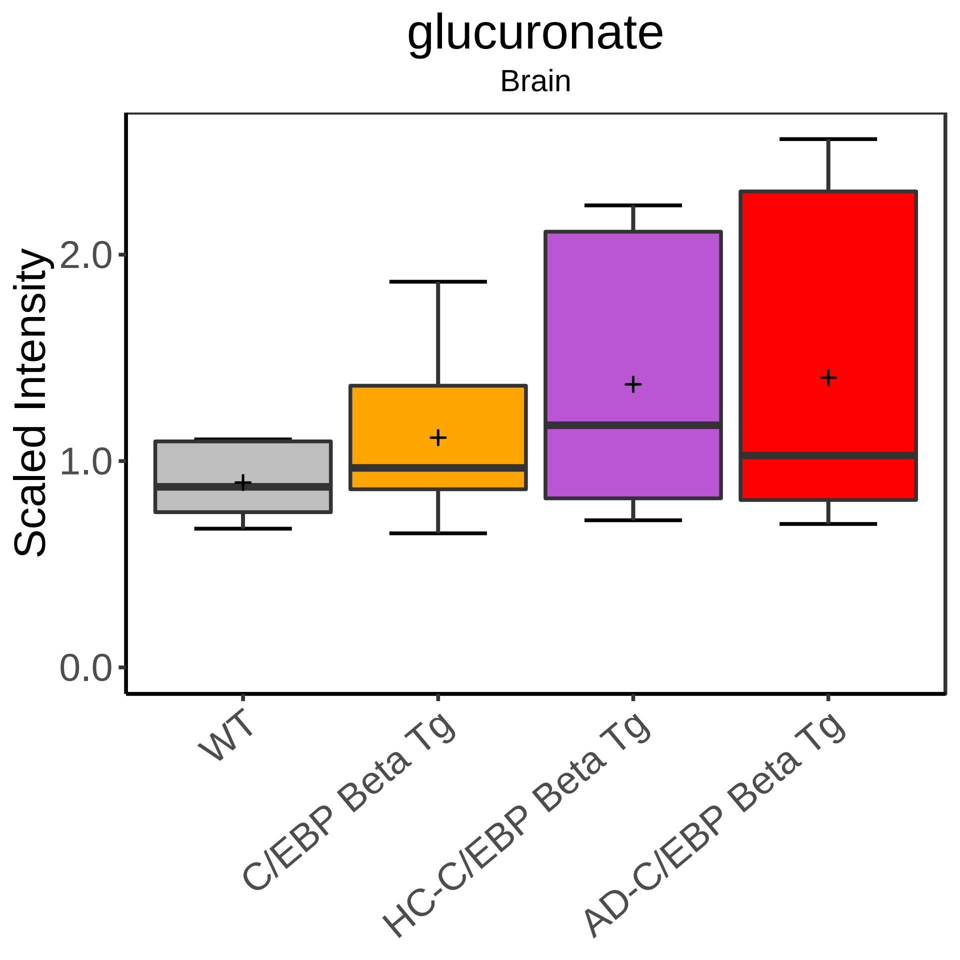
glucuronate
Brain
Scaled Intensity
2.0
1.0
0.0
WT
C/EBP Beta Tg
HC-C/EBP Beta Tg
AD-C/EBP Beta Tg

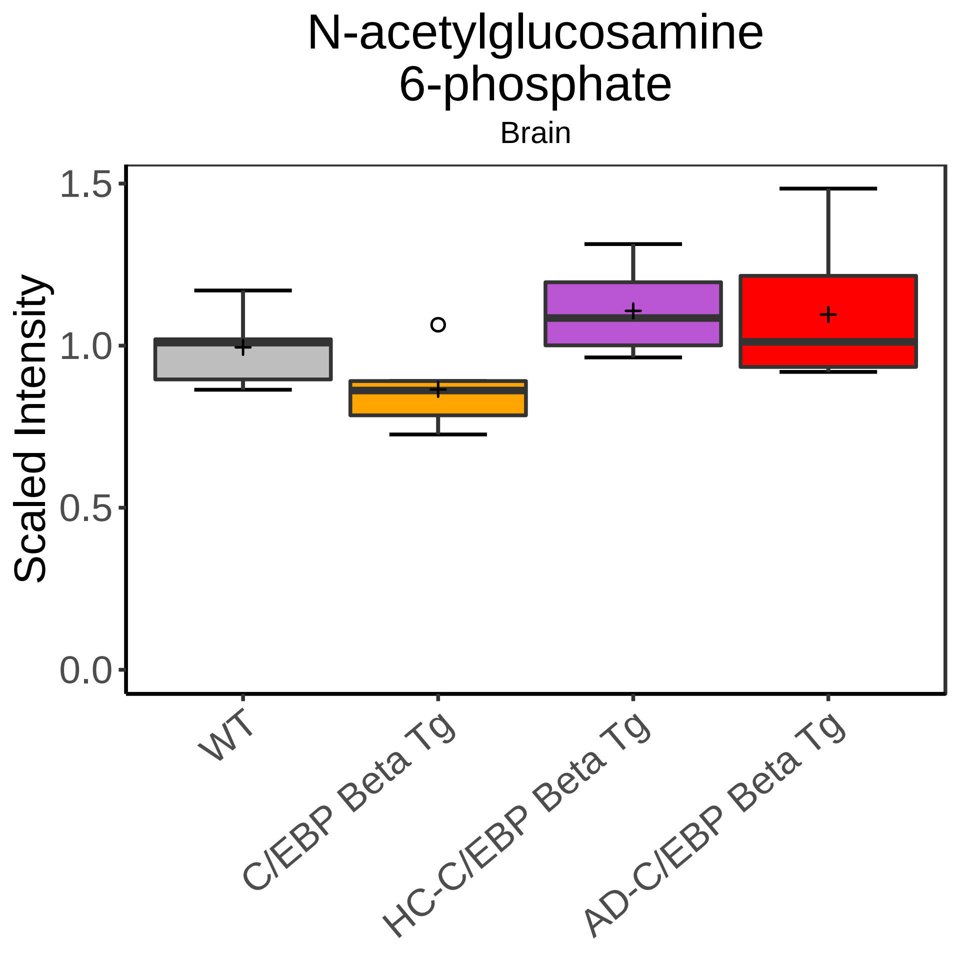
N-acetylglucosamine
6-phosphate
Brain
Scaled Intensity
1.5
1.0
0.5
0.0
WT
C/EBP Beta Tg
HC-C/EBP Beta Tg
AD-C/EBP Beta Tg

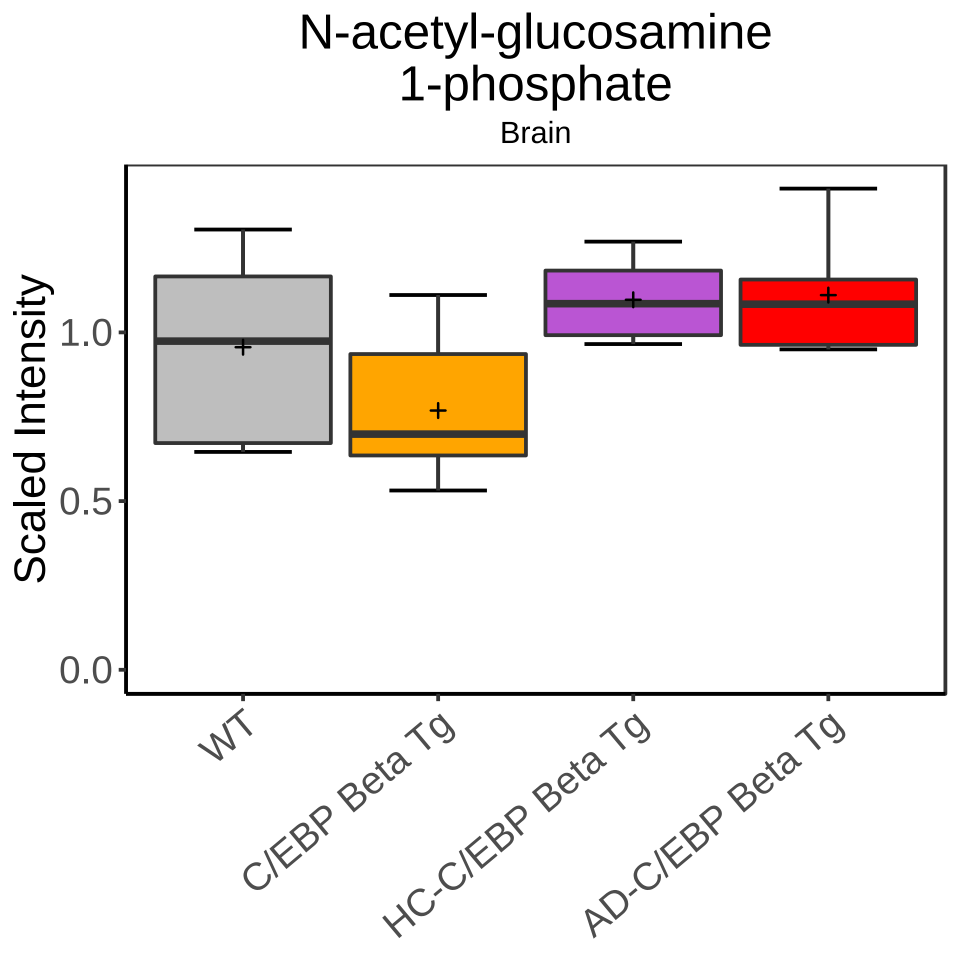
N-acetyl-glucosamine
1-phosphate
Brain
Scaled Intensity
1.0
0.5
0.0
WT
C/EBP Beta Tg
HC-C/EBP Beta Tg
AD-C/EBP Beta Tg

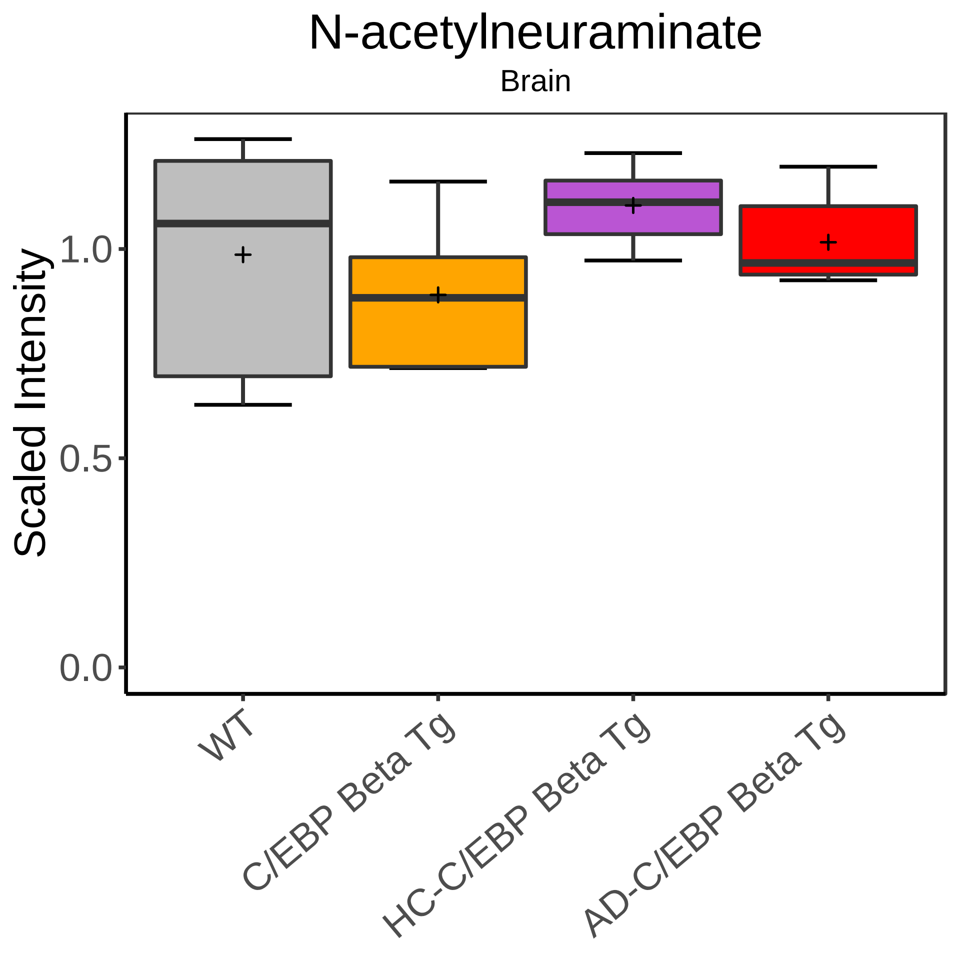

N-acetylneuraminate
Brain
Scaled Intensity
0.0
0.5
1.0
WT
C/EBP Beta Tg
HC-C/EBP Beta Tg
AD-C/EBP Beta Tg

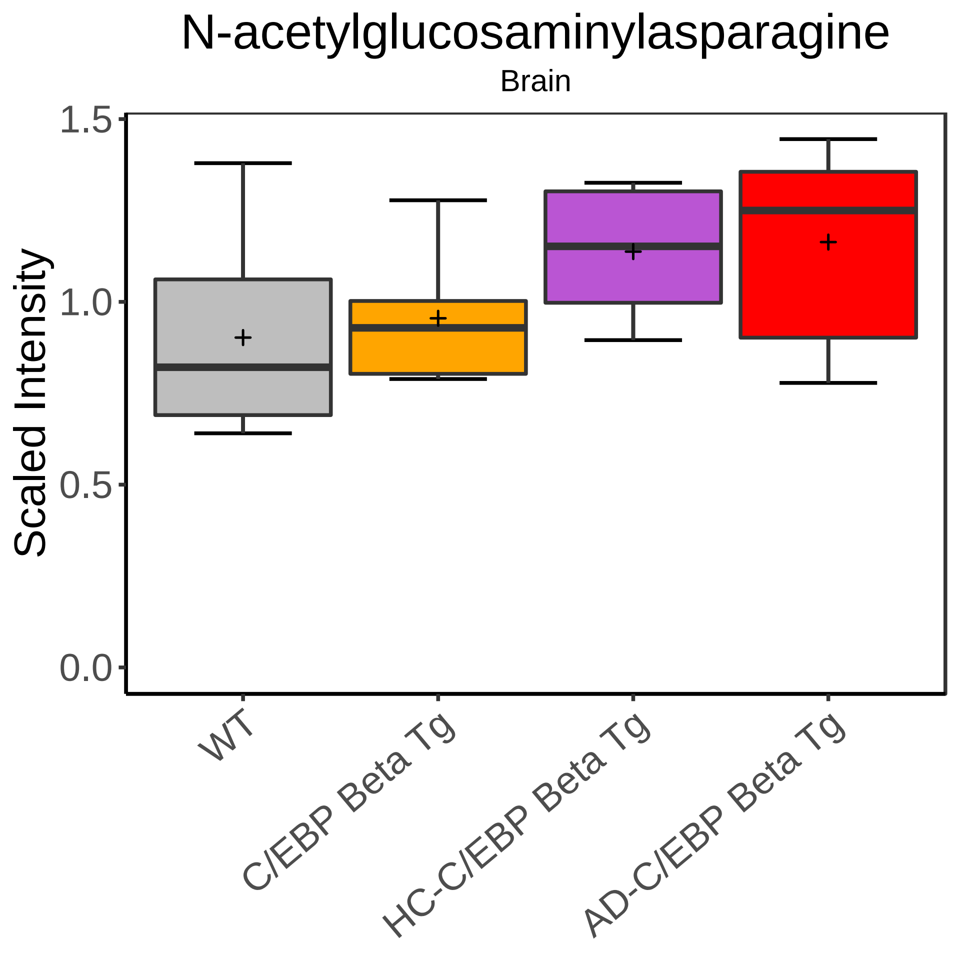
N-acetylglucosaminylasparagine
Brain
Scaled Intensity
1.5
1.0
0.5
0.0
WT
C/EBP Beta Tg
HC-C/EBP Beta Tg
AD-C/EBP Beta Tg

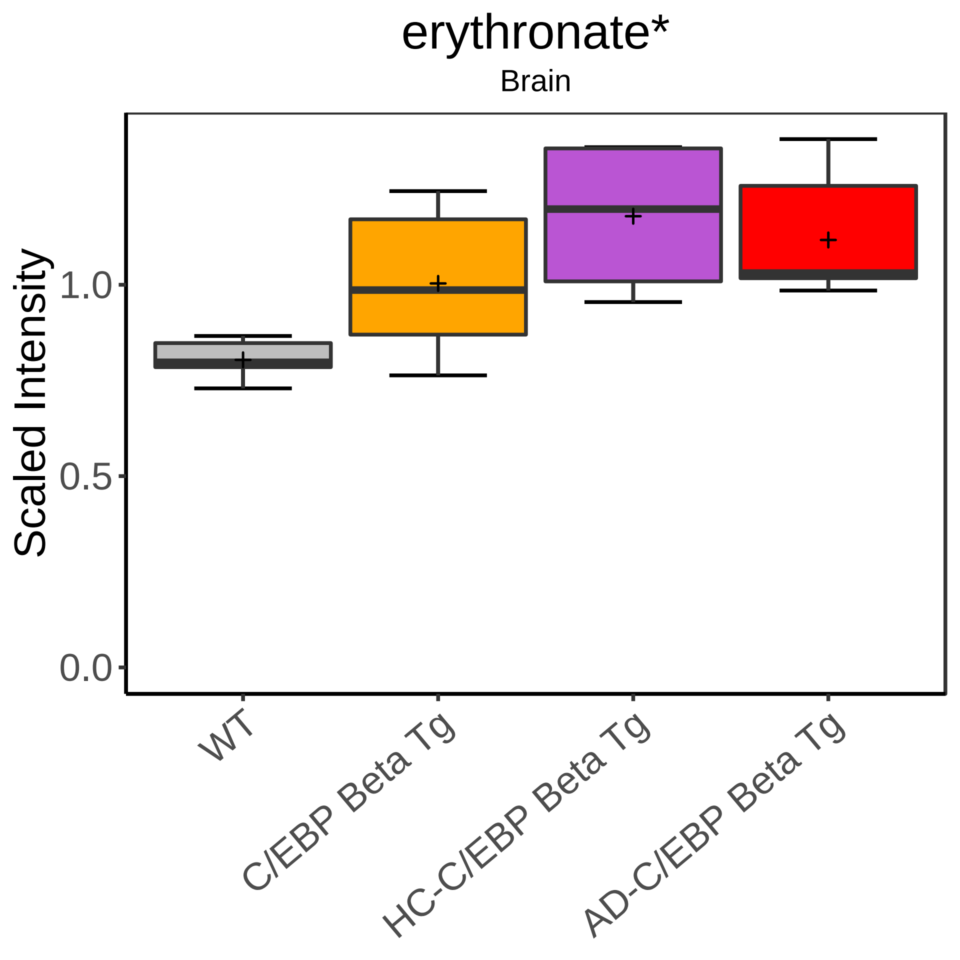
erythronate*
Brain
Scaled Intensity
1.0
0.5
0.0
WT
C/EBP Beta Tg
HC-C/EBP Beta Tg
AD-C/EBP Beta Tg

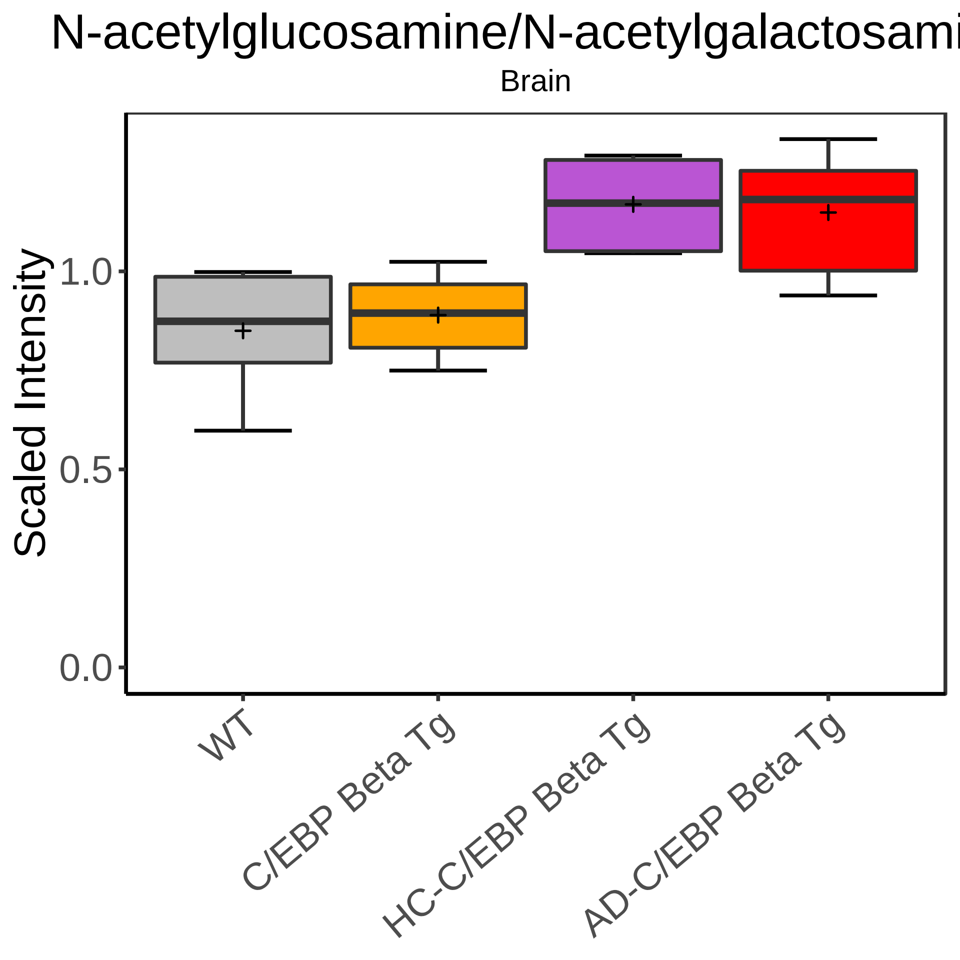
N-acetylglucosamine/N-acetylgalactosami
Brain
Scaled Intensity
1.0
0.5
0.0
WT
C/EBP Beta Tg
HC-C/EBP Beta Tg
AD-C/EBP Beta Tg

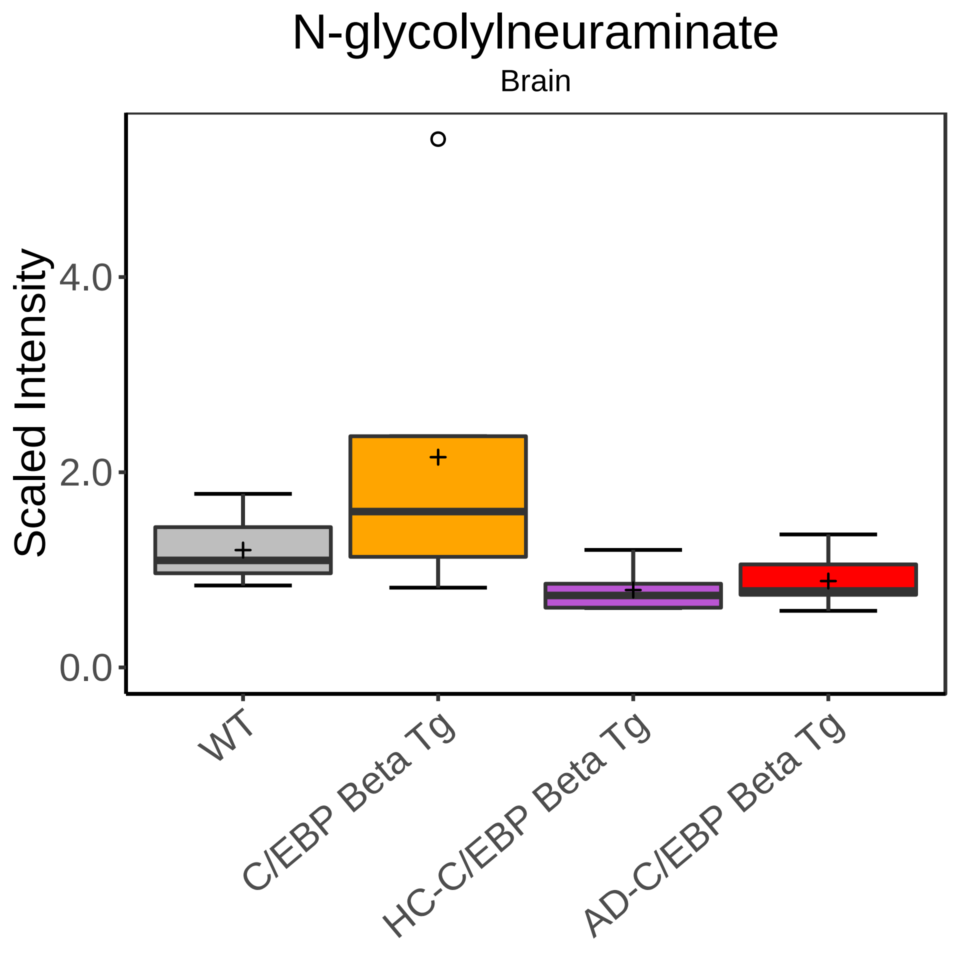

N-glycolylneuraminate
Brain
Scaled Intensity
0.0
2.0
4.0
WT
C/EBP Beta Tg
HC-C/EBP Beta Tg
AD-C/EBP Beta Tg

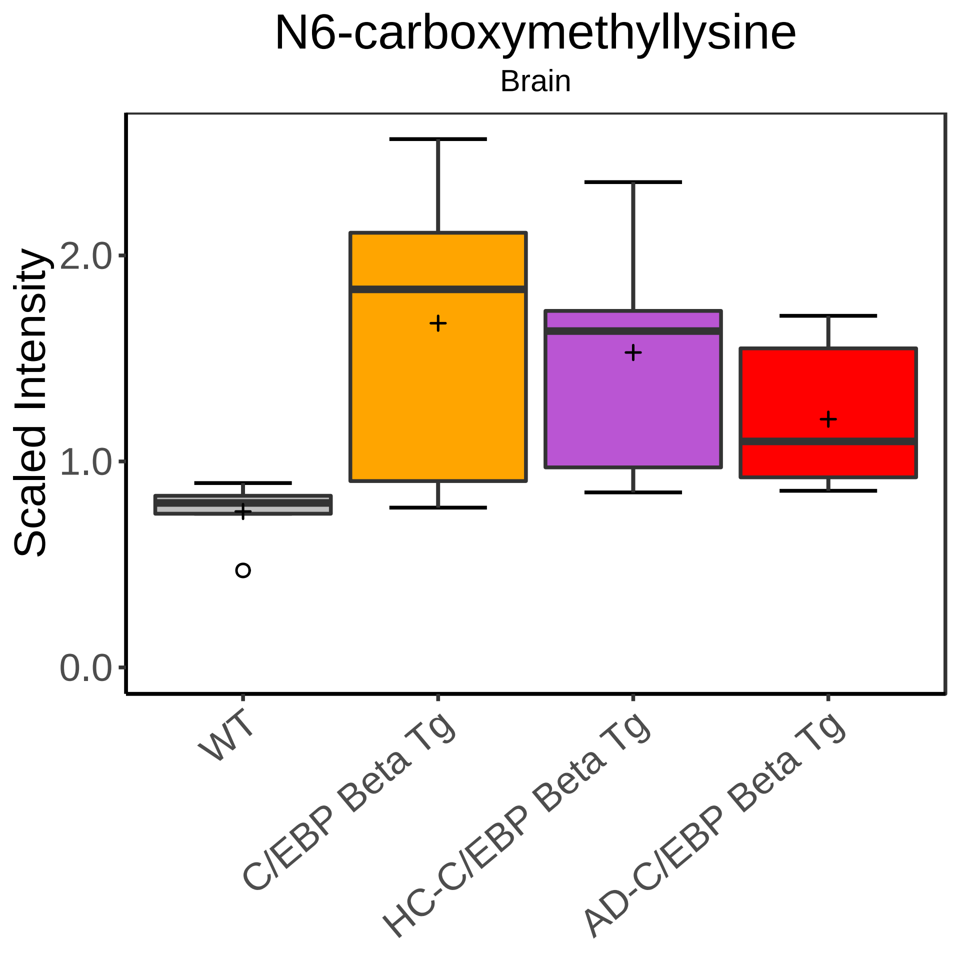

N6-carboxymethyllysine
Brain
Scaled Intensity
2.0
1.0
0.0
WT
C/EBP Beta Tg
HC-C/EBP Beta Tg
AD-C/EBP Beta Tg

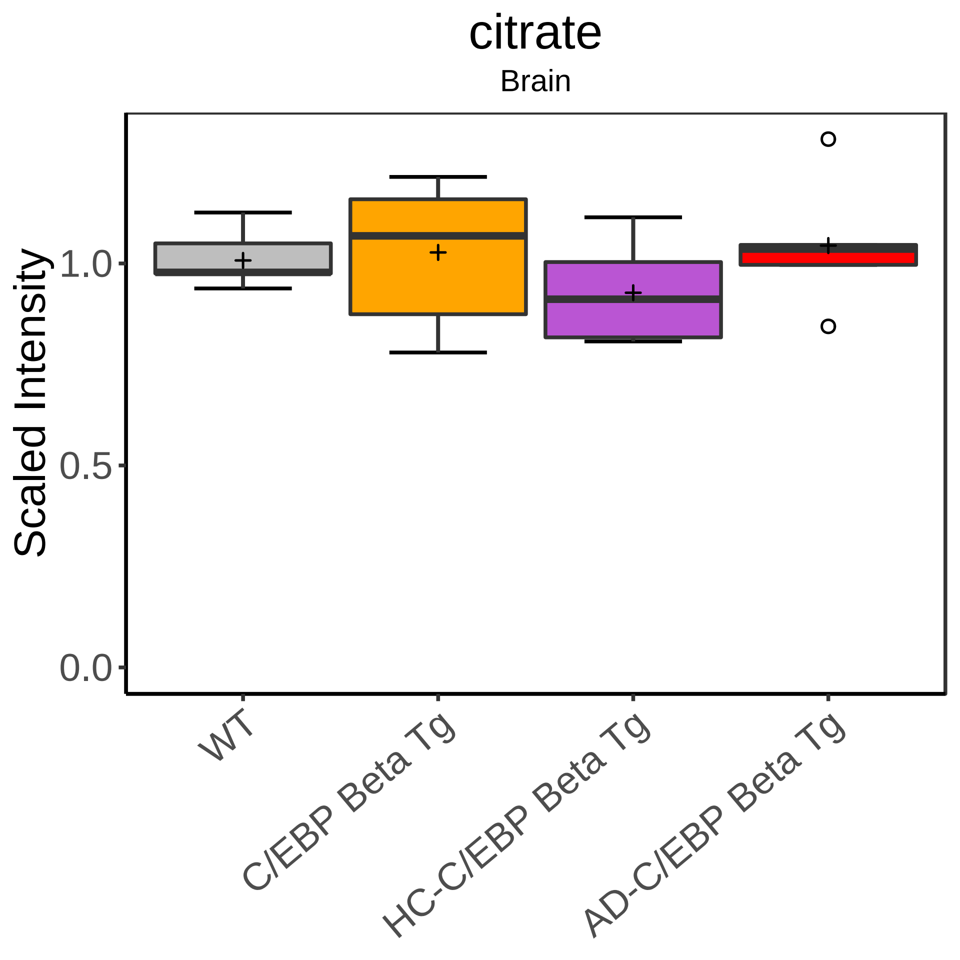

citrate
Brain
Scaled Intensity
0.0
0.5
1.0
WT
C/EBP Beta Tg
HC-C/EBP Beta Tg
AD-C/EBP Beta Tg

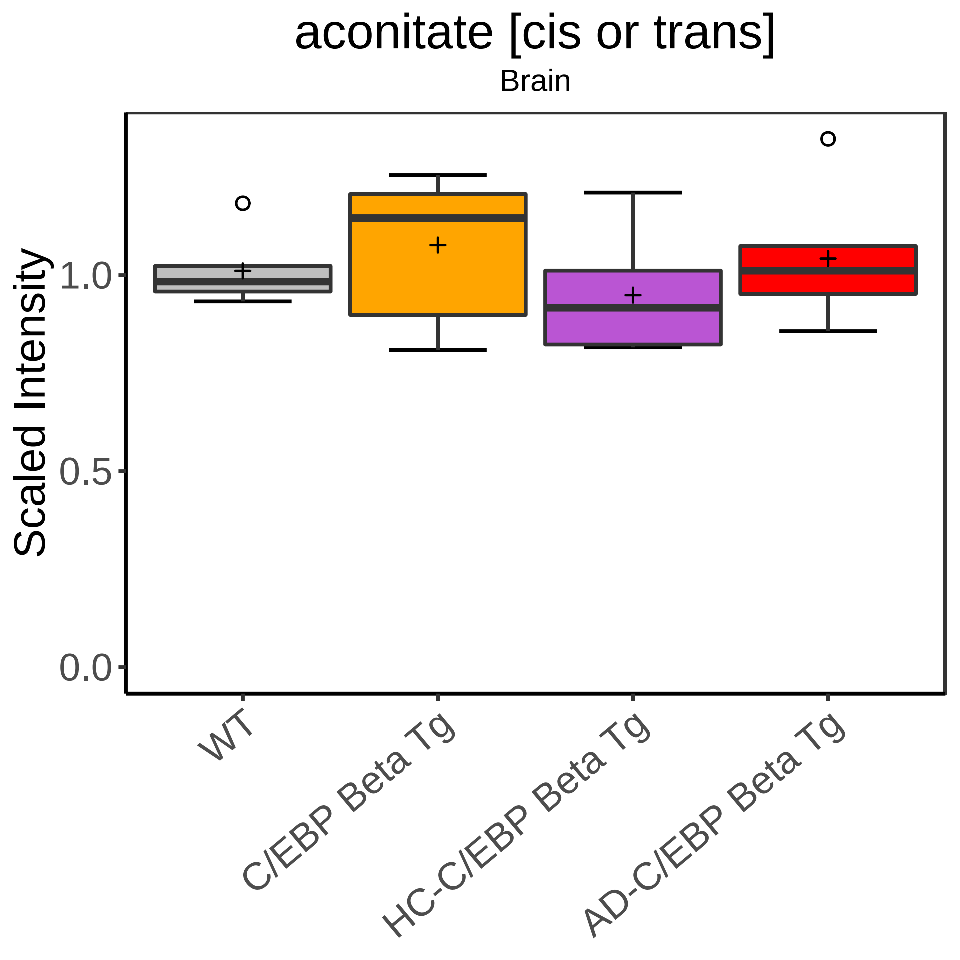

aconitate [cis or trans]
Brain
Scaled Intensity
1.0
0.5
0.0
WT
C/EBP Beta Tg
HC-C/EBP Beta Tg
AD-C/EBP Beta Tg

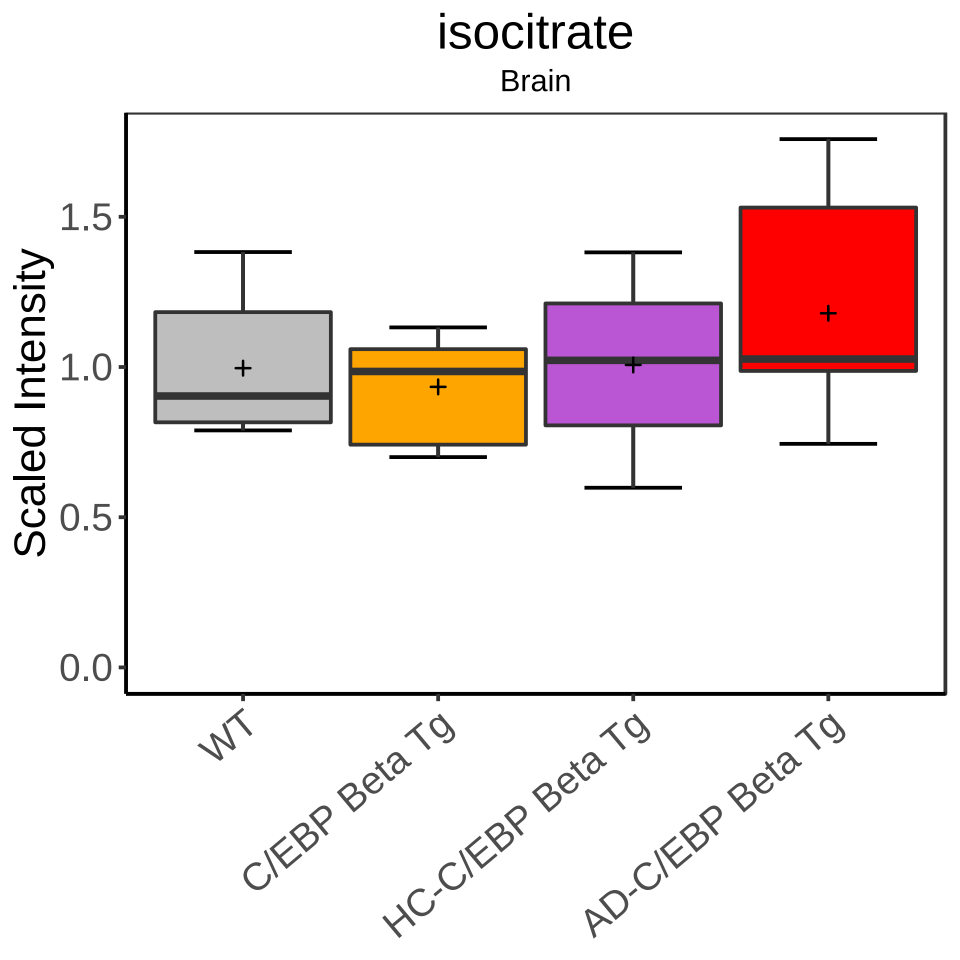
isocitrate
Brain
Scaled Intensity
1.5
1.0
0.5
0.0
WT
C/EBP Beta Tg
HC-C/EBP Beta Tg
AD-C/EBP Beta Tg

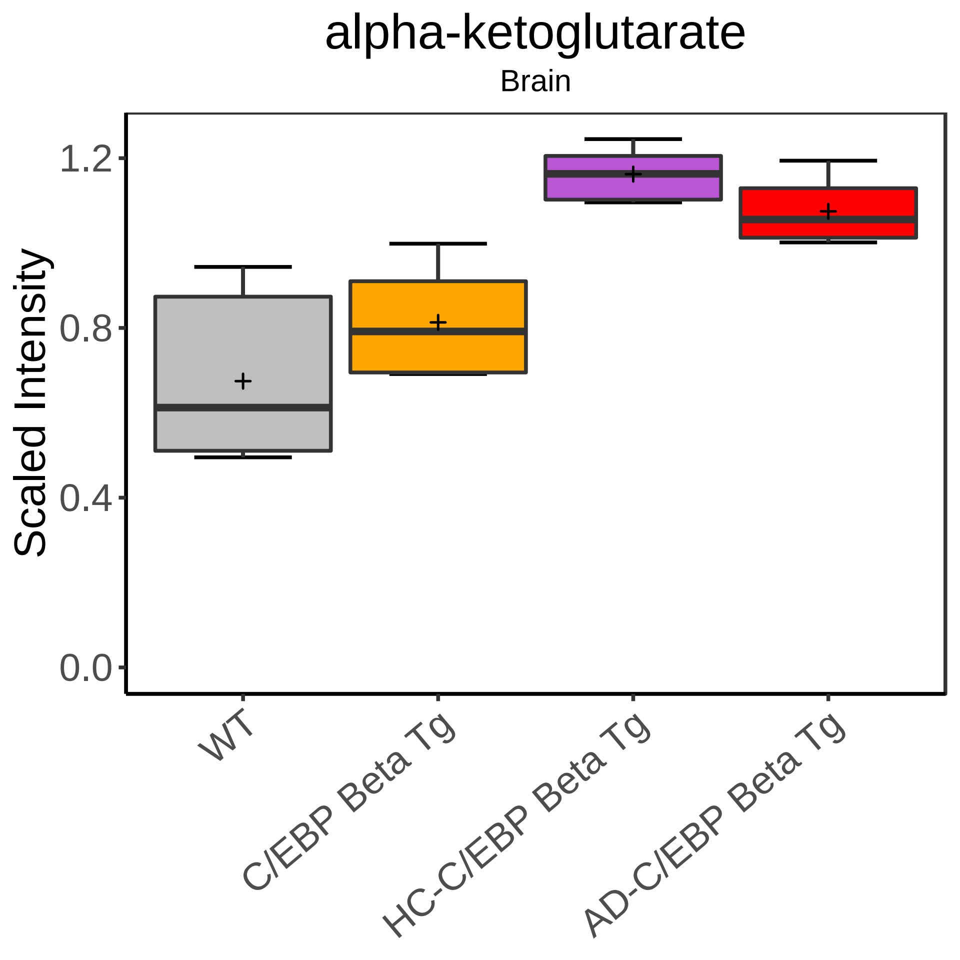

alpha-ketoglutarate
Brain
Scaled Intensity
1.2
0.8
0.4
0.0
WT
C/EBP Beta Tg
HC-C/EBP Beta Tg
AD-C/EBP Beta Tg

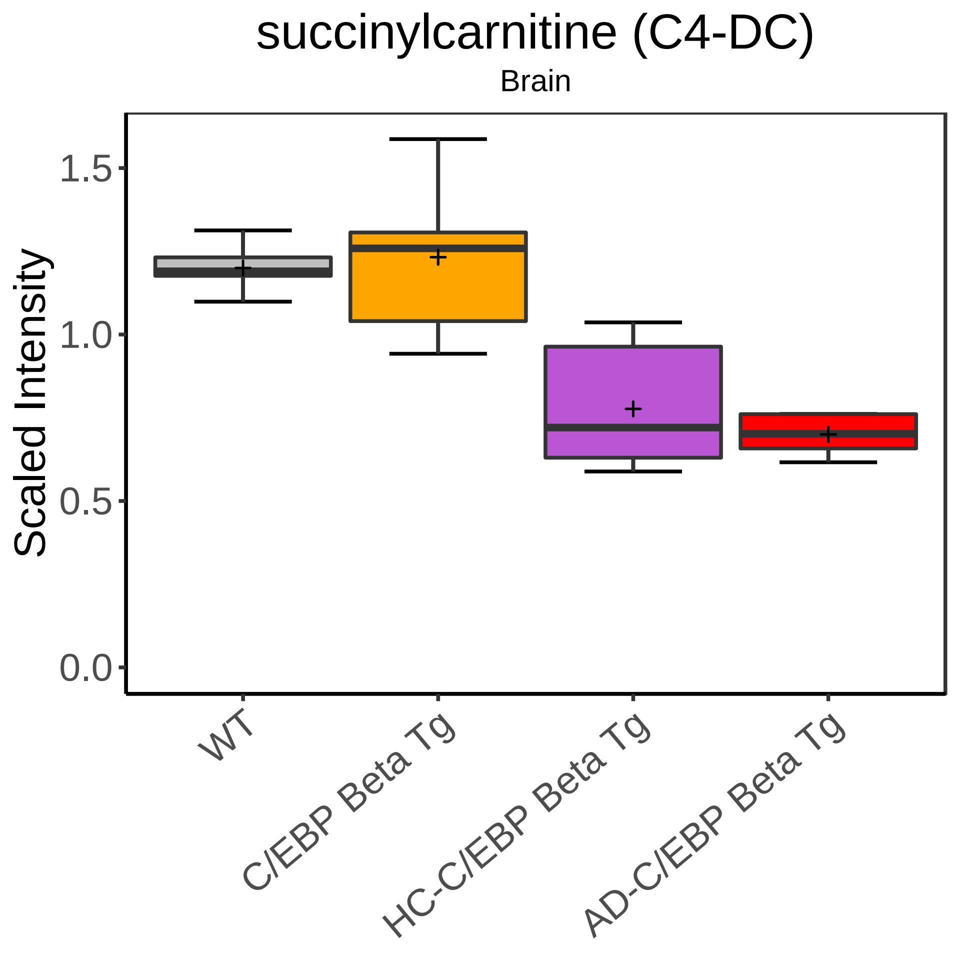

succinylcarnitine (C4-DC)
Brain
Scaled Intensity
0.0
0.5
1.0
1.5
WT
C/EBP Beta Tg
HC-C/EBP Beta Tg
AD-C/EBP Beta Tg

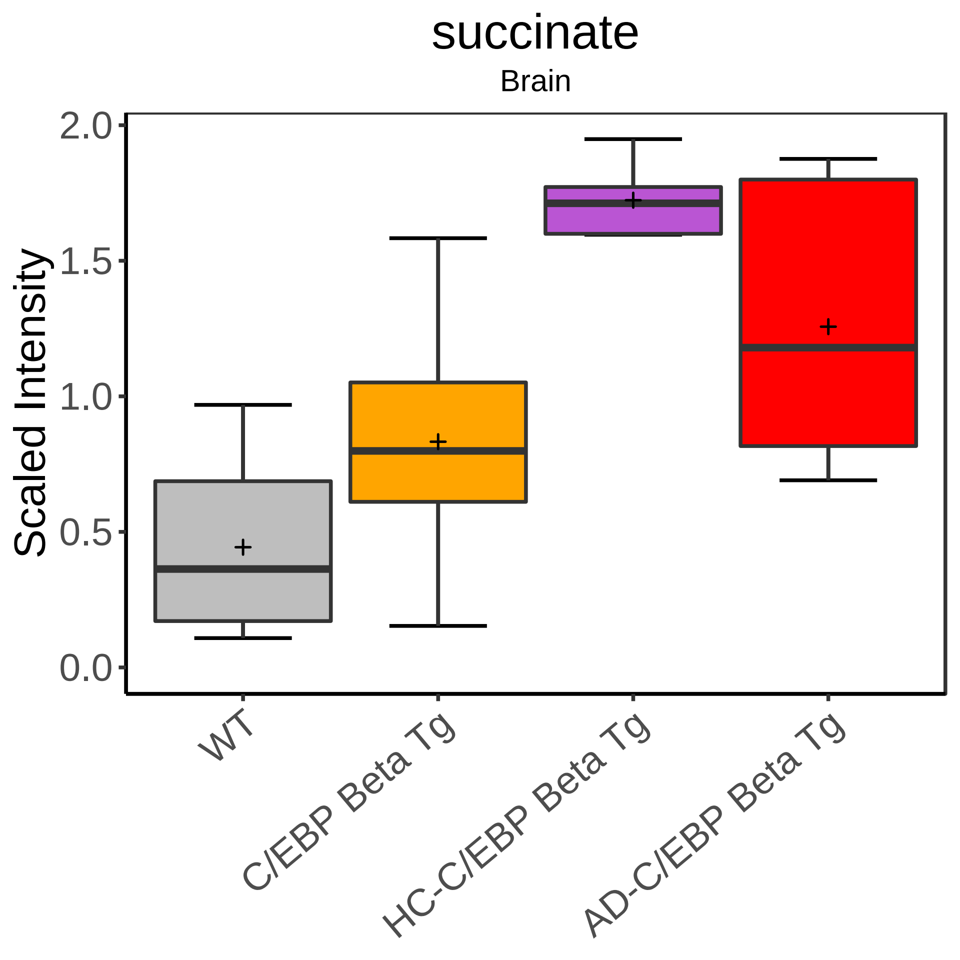
succinate
Brain
Scaled Intensity
2.0
1.5
1.0
0.5
0.0
WT
C/EBP Beta Tg
HC-C/EBP Beta Tg
AD-C/EBP Beta Tg

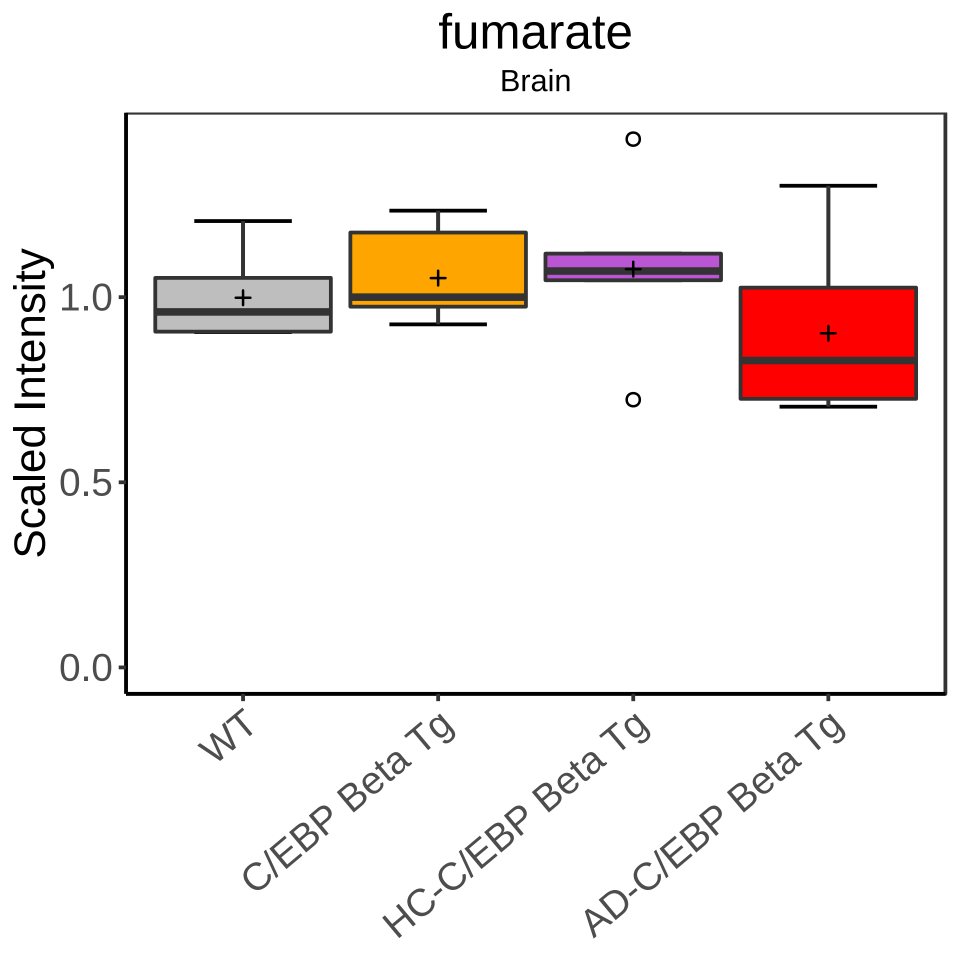
fumarate
Brain
Scaled Intensity
0.0
0.5
1.0
WT
C/EBP Beta Tg
HC-C/EBP Beta Tg
AD-C/EBP Beta Tg

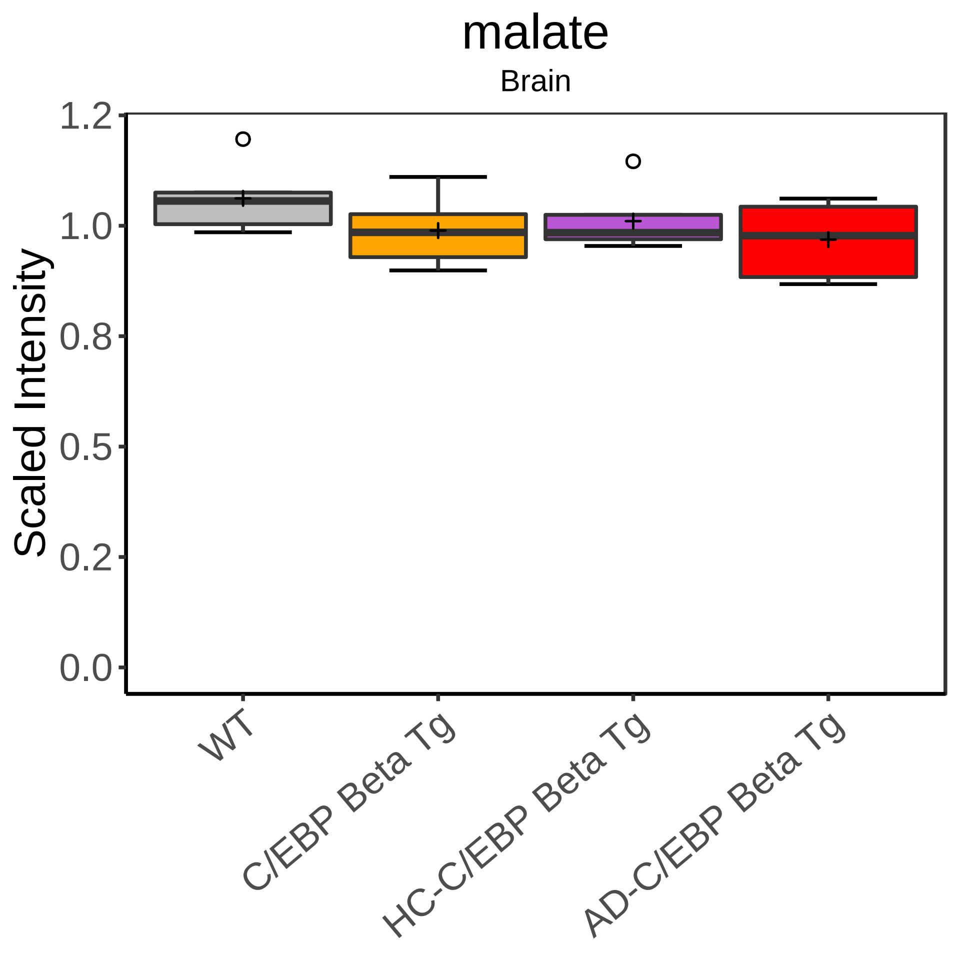
malate
Brain
Scaled Intensity
1.2
1.0
0.8
0.5
0.2
0.0
WT
C/EBP Beta Tg
HC-C/EBP Beta Tg
AD-C/EBP Beta Tg

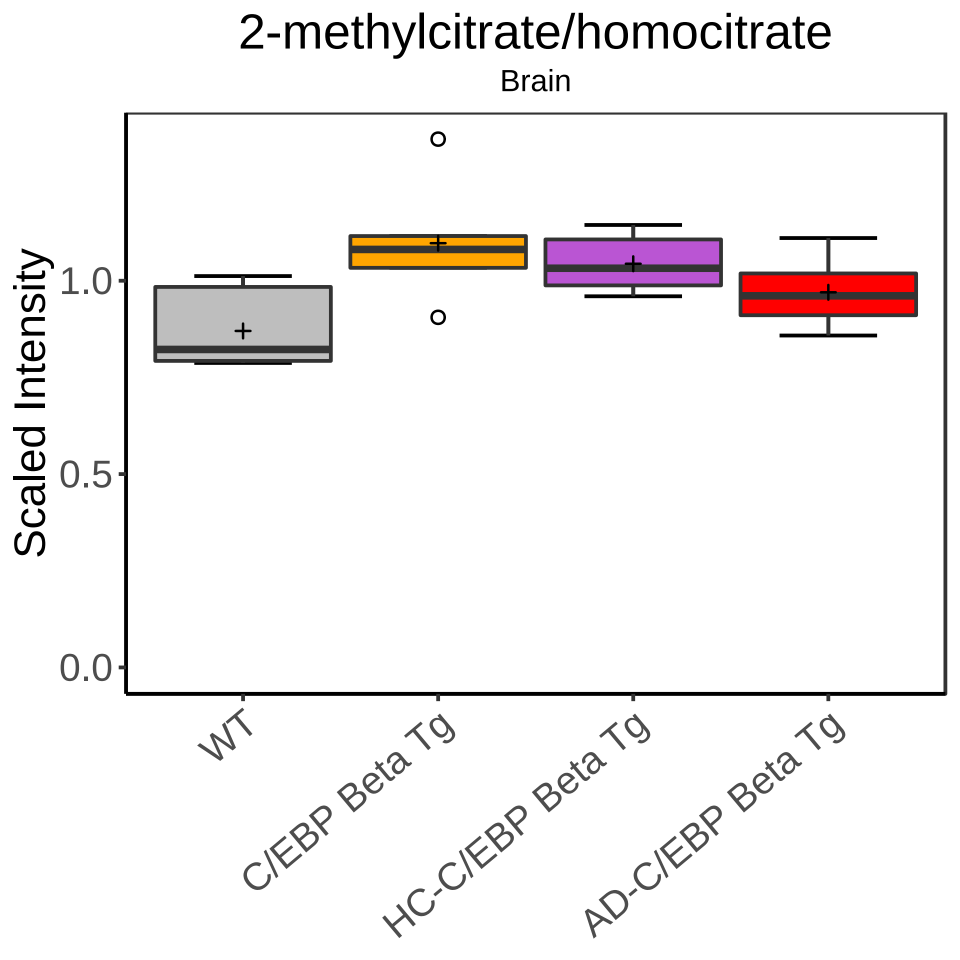
2-methylcitrate/homocitrate
Brain
Scaled Intensity
1.0
0.5
0.0
WT
C/EBP Beta Tg
HC-C/EBP Beta Tg
AD-C/EBP Beta Tg

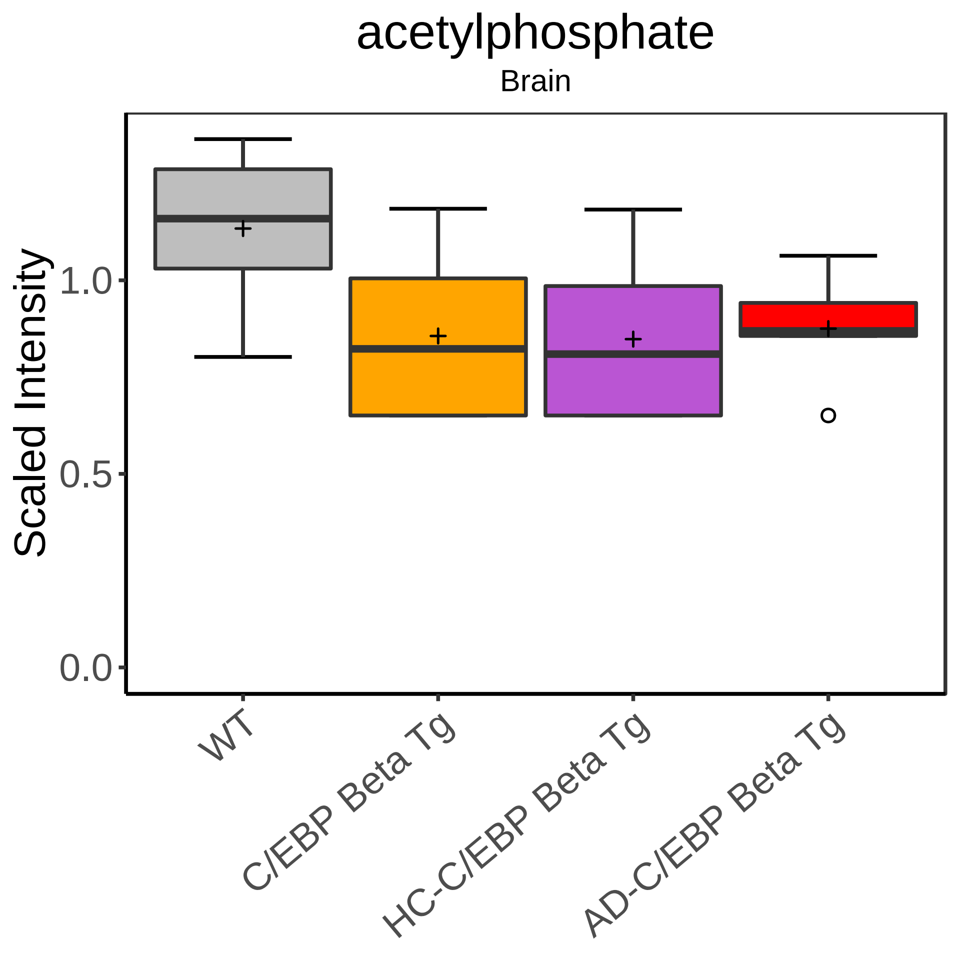
acetylphosphate
Brain
Scaled Intensity
1.0
0.5
0.0
WT
C/EBP Beta Tg
HC-C/EBP Beta Tg
AD-C/EBP Beta Tg

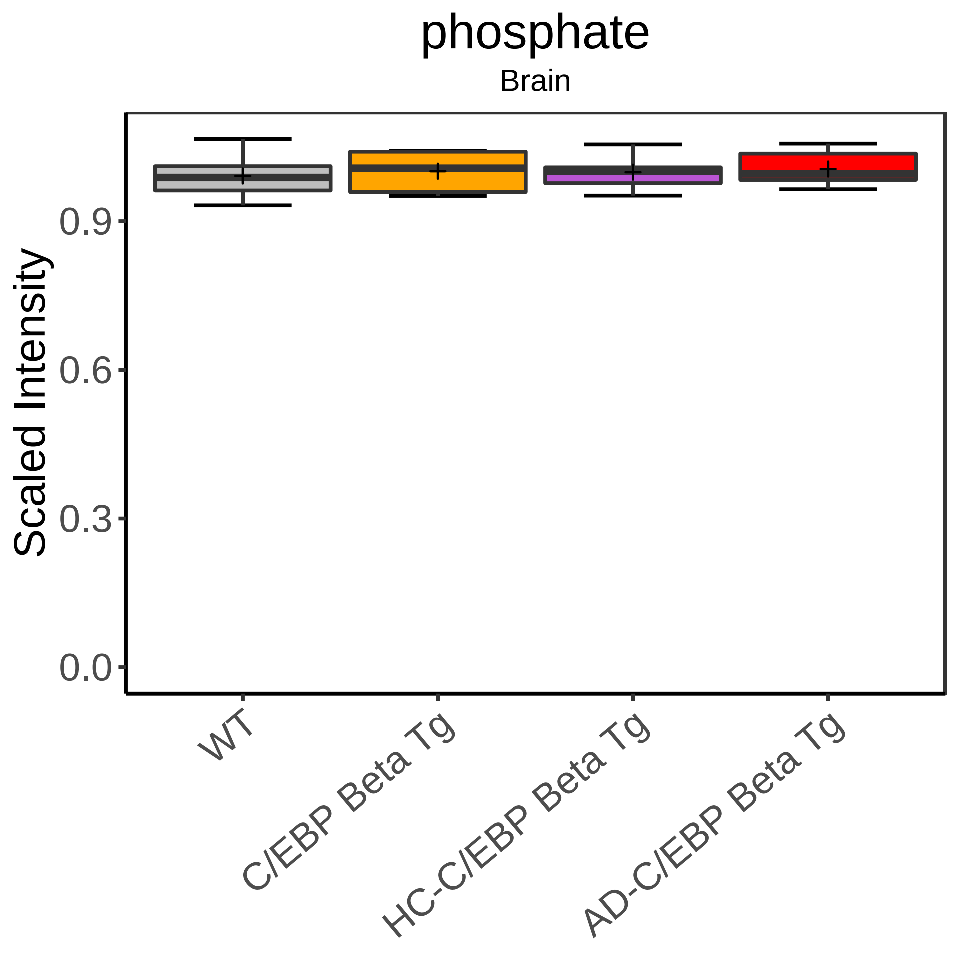
phosphate
Brain
Scaled Intensity
0.0
0.3
0.6
0.9
WT
C/EBP Beta Tg
HC-C/EBP Beta Tg
AD-C/EBP Beta Tg

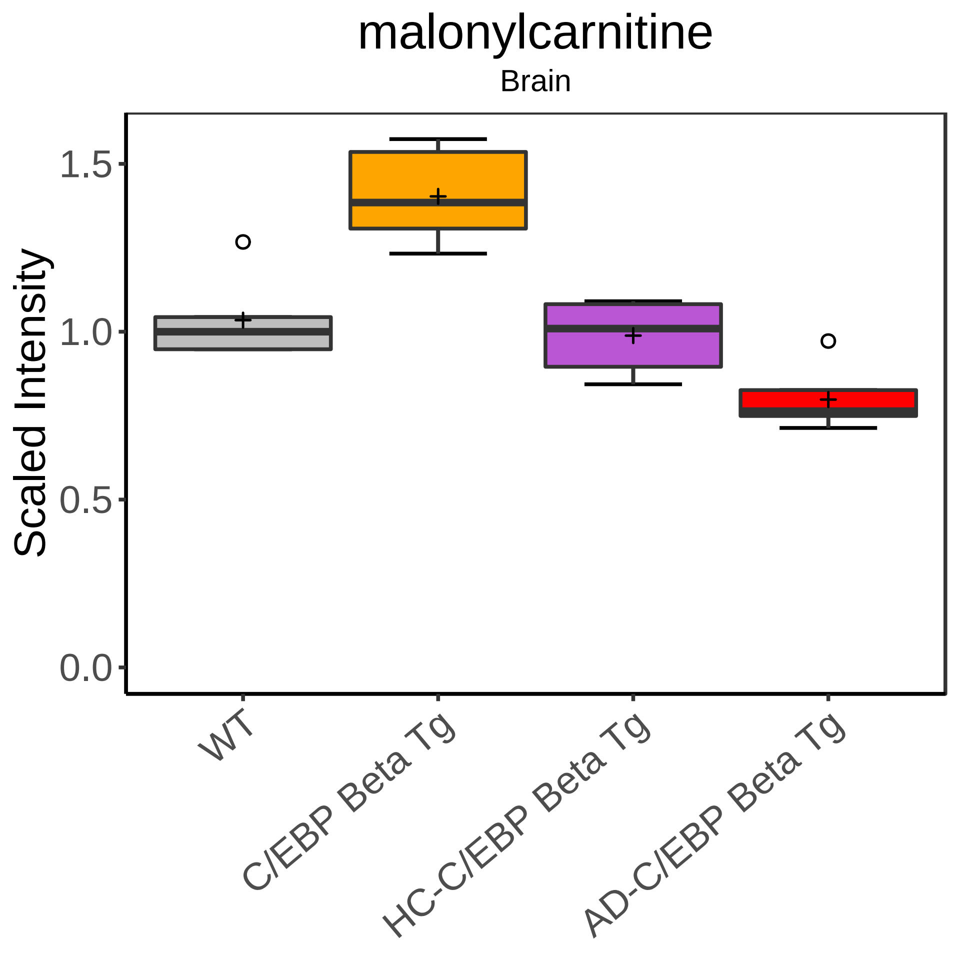
malonylcarnitine
Brain
Scaled Intensity
1.5
1.0
0.5
0.0
WT
C/EBP Beta Tg
HC-C/EBP Beta Tg
AD-C/EBP Beta Tg

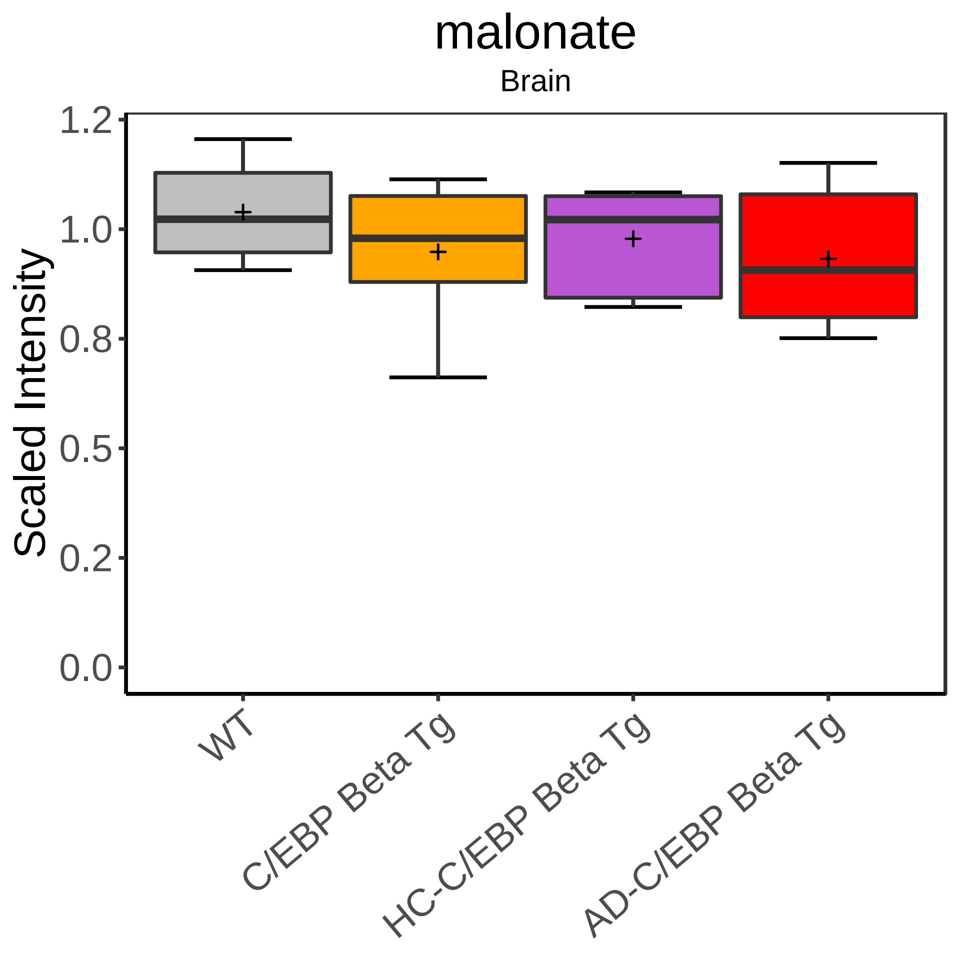

malonate
Brain
Scaled Intensity
1.2
1.0
0.8
0.5
0.2
0.0
WT
C/EBP Beta Tg
HC-C/EBP Beta Tg
AD-C/EBP Beta Tg

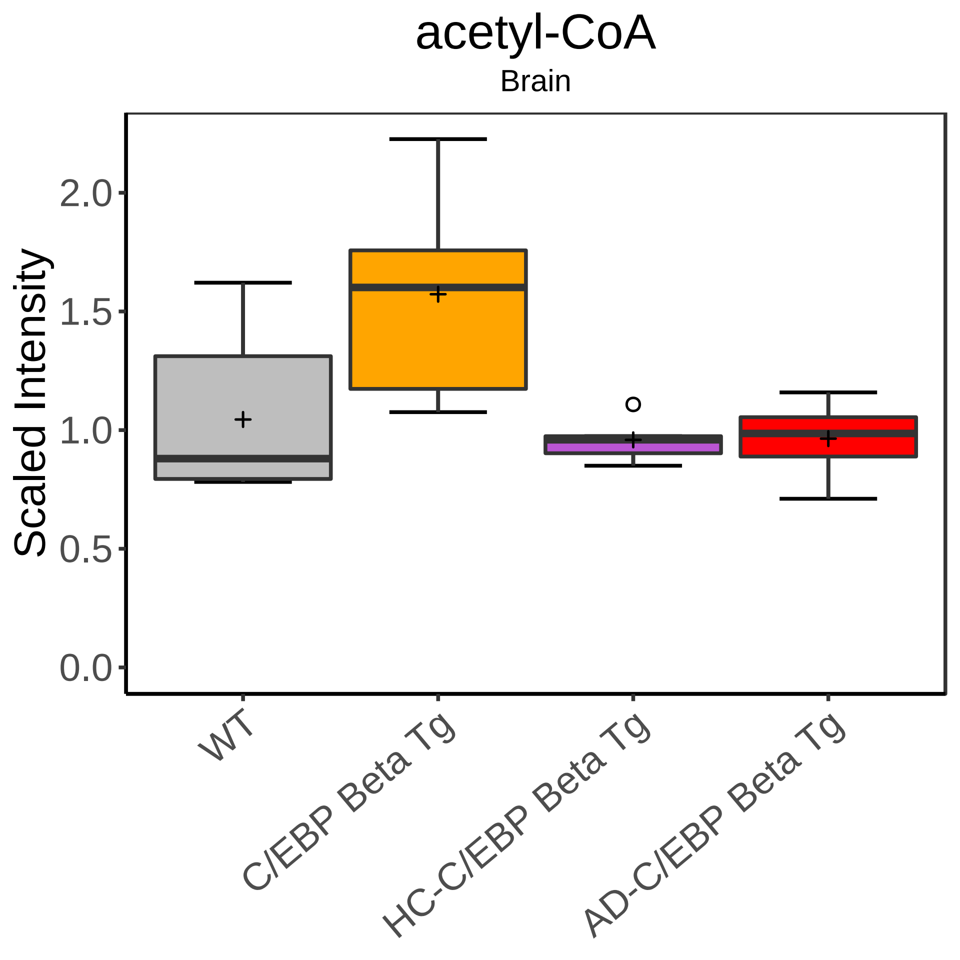
acetyl-CoA
Brain
Scaled Intensity
2.0
1.5
1.0
0.5
0.0
WT
C/EBP Beta Tg
HC-C/EBP Beta Tg
AD-C/EBP Beta Tg

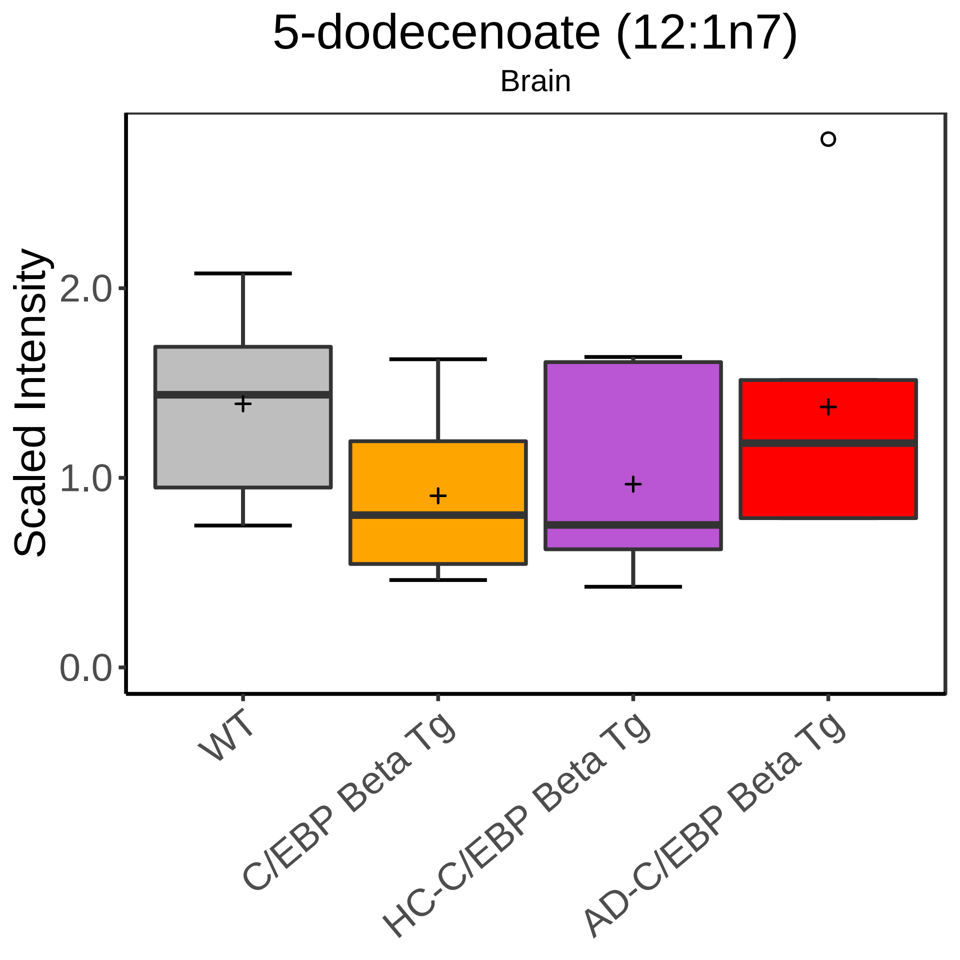
5-dodecenoate (12:1n7)
Brain
Scaled Intensity
2.0
1.0
0.0
WT
C/EBP Beta Tg
HC-C/EBP Beta Tg
AD-C/EBP Beta Tg

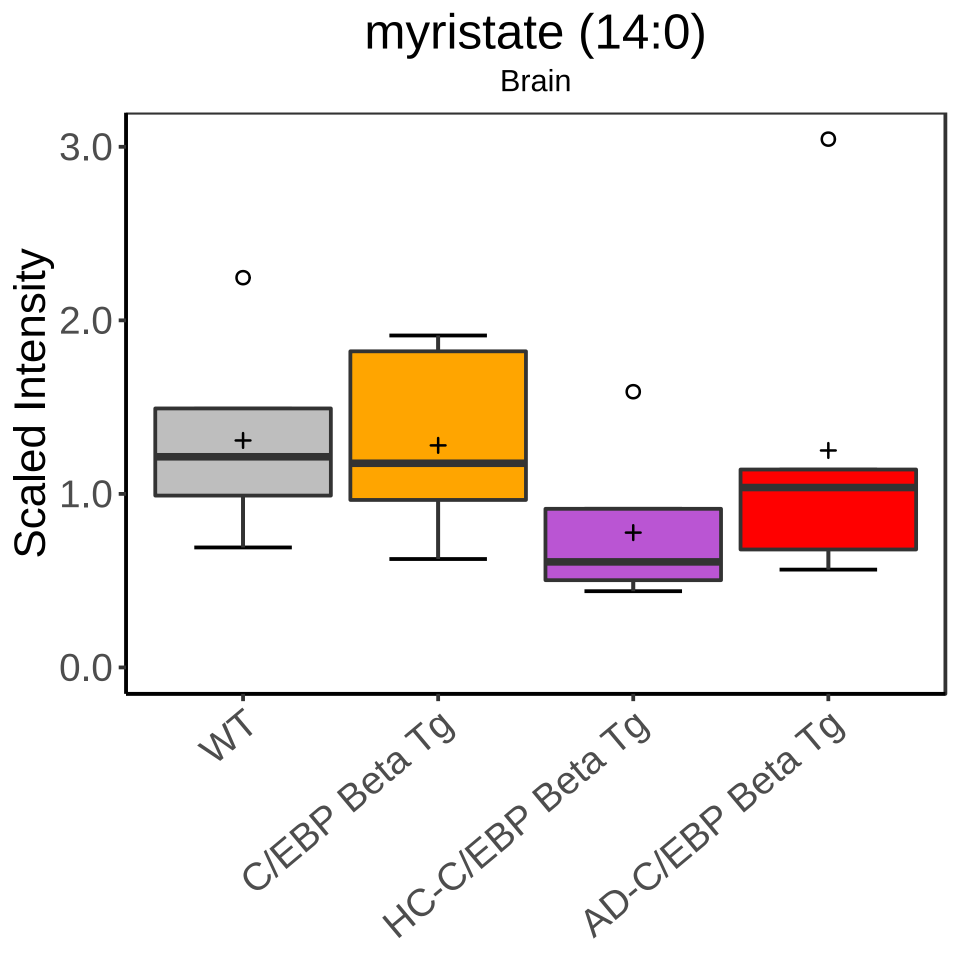

myristate (14:0)
Brain
Scaled Intensity
3.0
2.0
1.0
0.0
WT
C/EBP Beta Tg
HC-C/EBP Beta Tg
AD-C/EBP Beta Tg

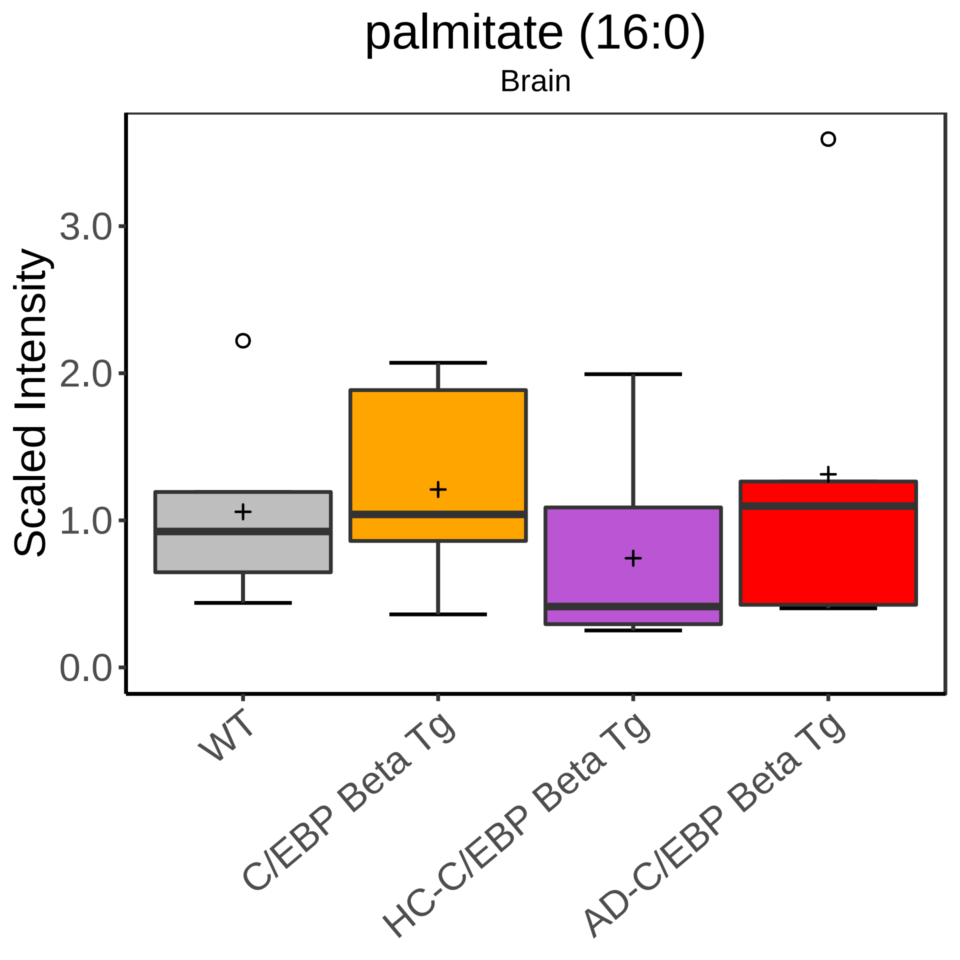

palmitate (16:0)
Brain
Scaled Intensity
3.0
2.0
1.0
0.0
WT
C/EBP Beta Tg
HC-C/EBP Beta Tg
AD-C/EBP Beta Tg

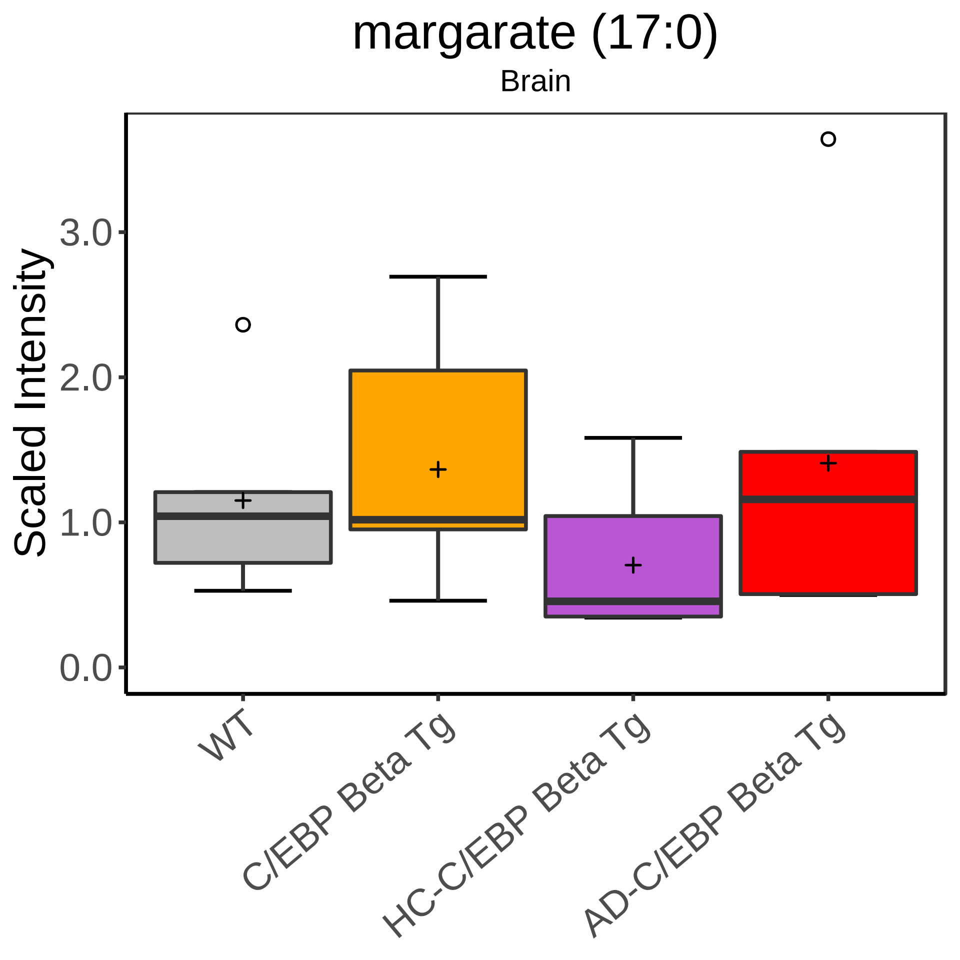

margarate (17:0)
Brain
Scaled Intensity
3.0
2.0
1.0
0.0
WT
C/EBP Beta Tg
HC-C/EBP Beta Tg
AD-C/EBP Beta Tg

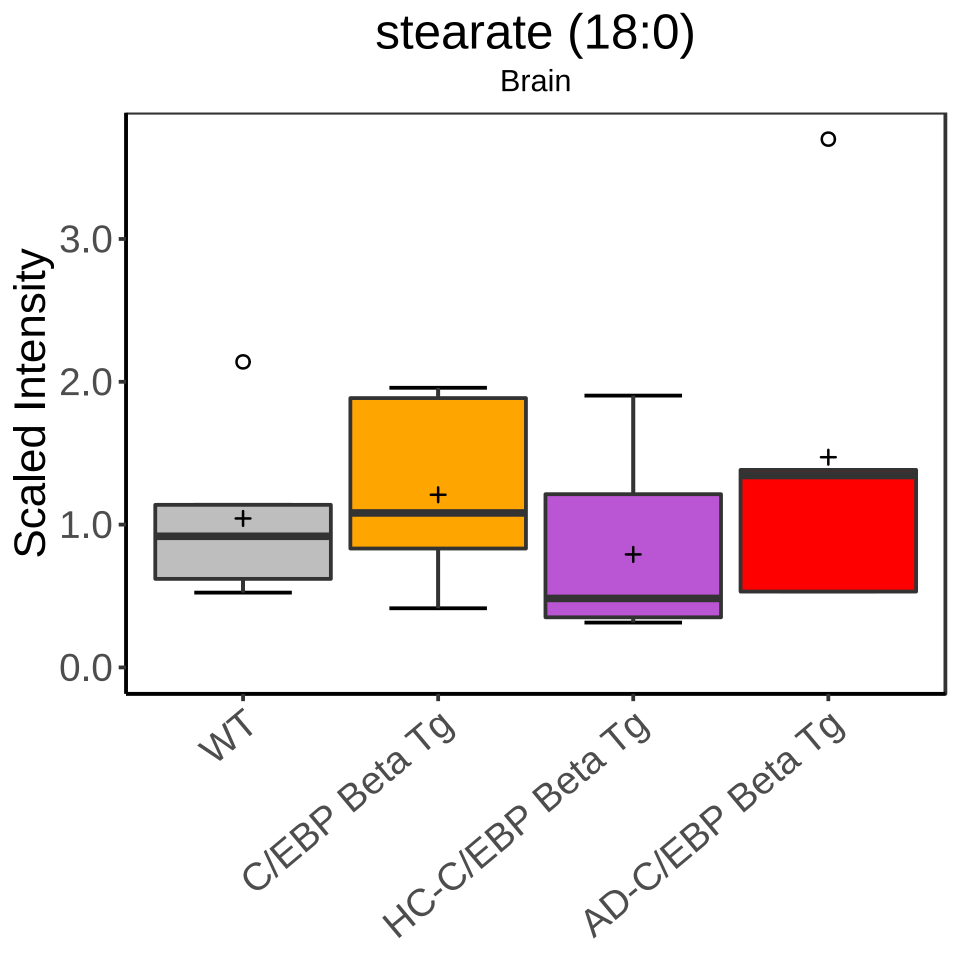
stearate (18:0)
Brain
Scaled Intensity
3.0
2.0
1.0
0.0
WT
C/EBP Beta Tg
HC-C/EBP Beta Tg
AD-C/EBP Beta Tg

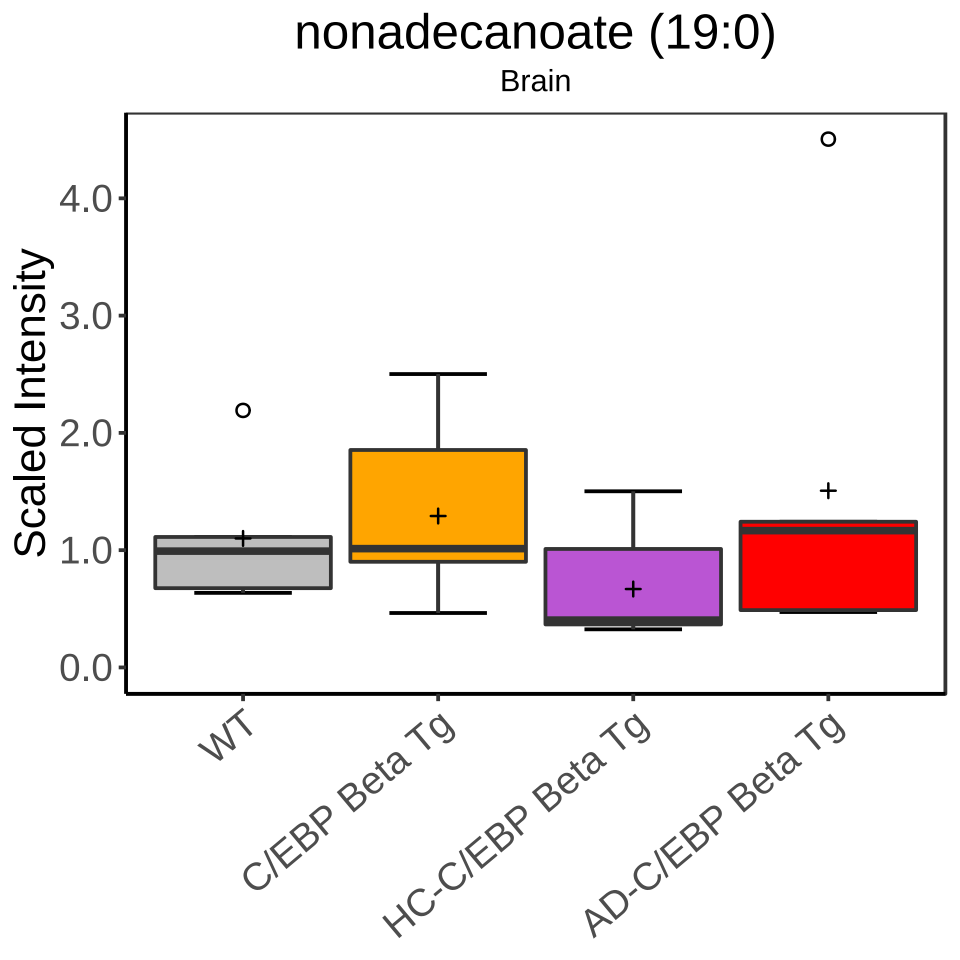
nonadecanoate (19:0)
Brain
Scaled Intensity
4.0
3.0
2.0
1.0
0.0
WT
C/EBP Beta Tg
HC-C/EBP Beta Tg
AD-C/EBP Beta Tg

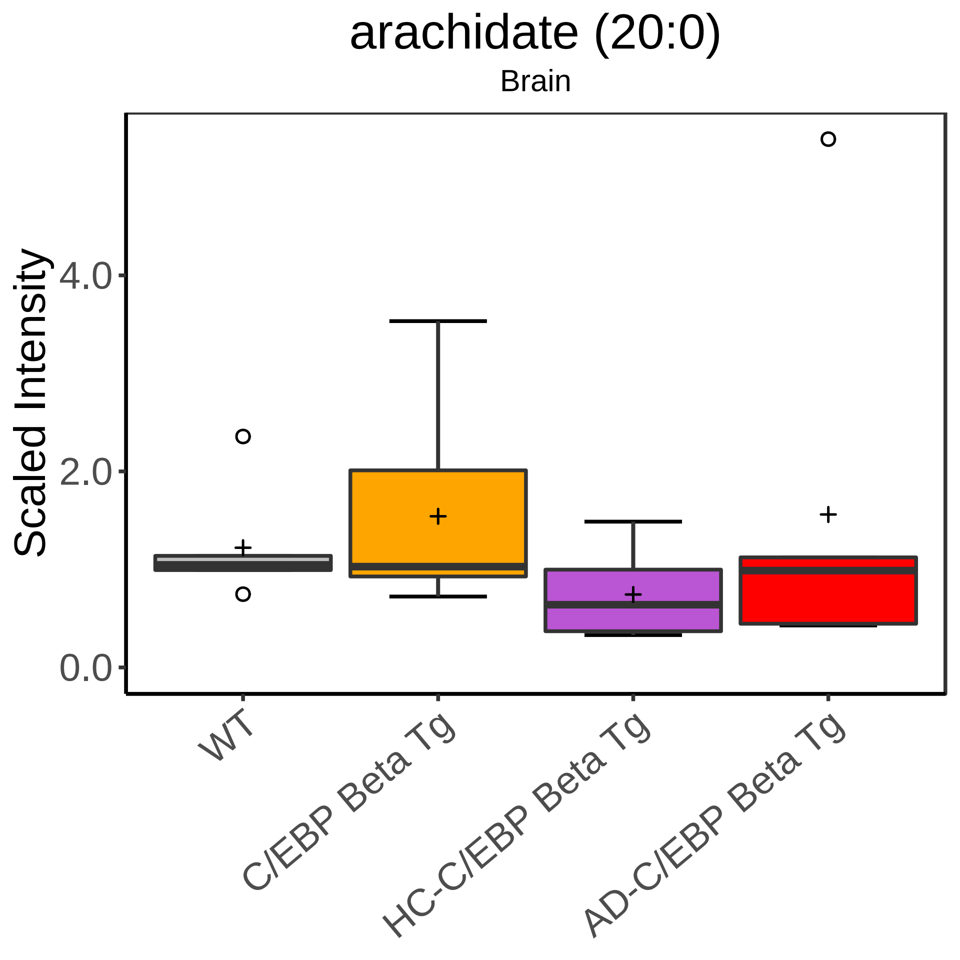
arachidate (20:0)
Brain
Scaled Intensity
4.0
2.0
0.0
WT
C/EBP Beta Tg
HC-C/EBP Beta Tg
AD-C/EBP Beta Tg

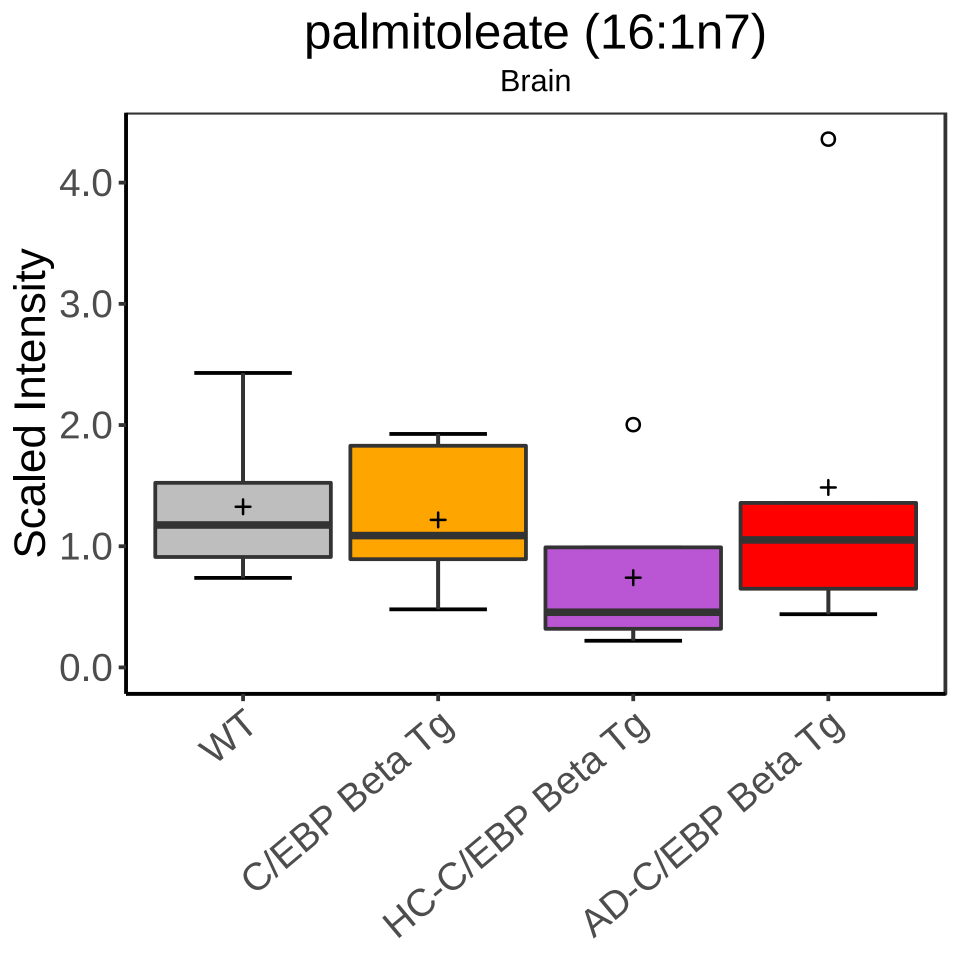
palmitoleate (16:1n7)
Brain
Scaled Intensity
0.0
1.0
2.0
3.0
4.0
WT
C/EBP Beta Tg
HC-C/EBP Beta Tg
AD-C/EBP Beta Tg

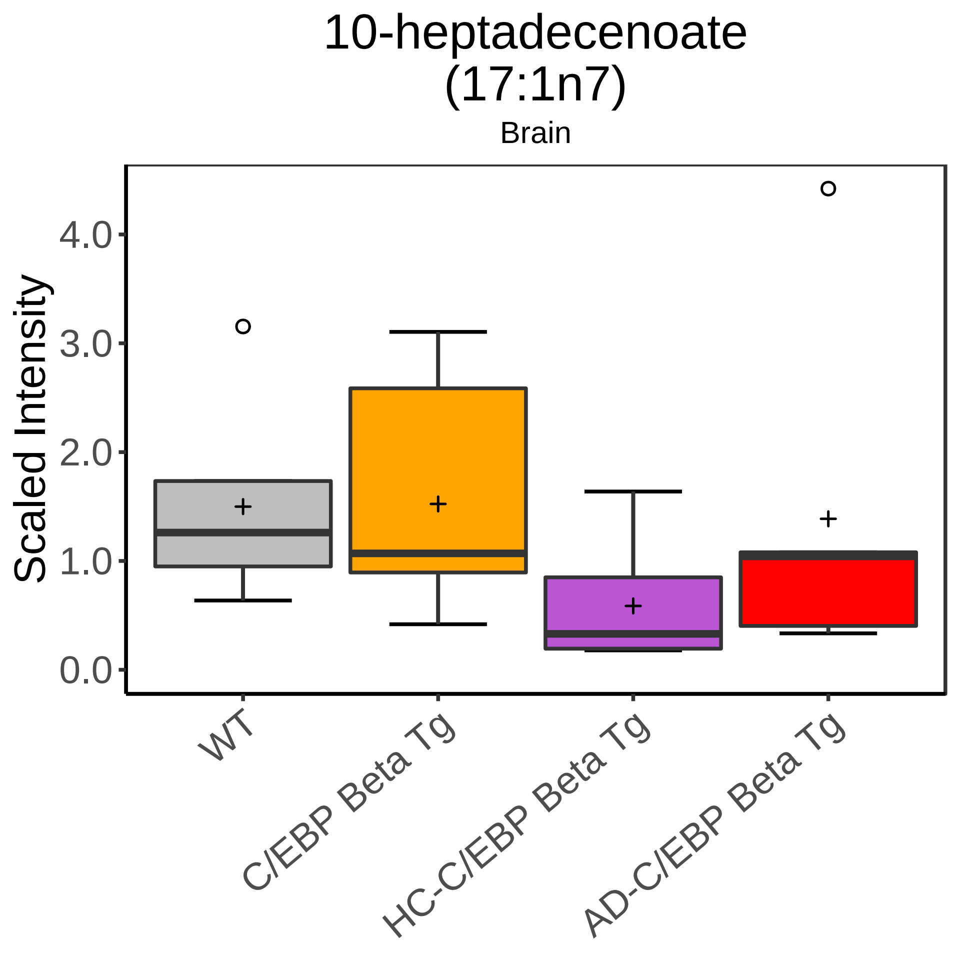

10-heptadecenoate
(17:1n7)
Brain
Scaled Intensity
4.0
3.0
2.0
1.0
0.0
WT
C/EBP Beta Tg
HC-C/EBP Beta Tg
AD-C/EBP Beta Tg

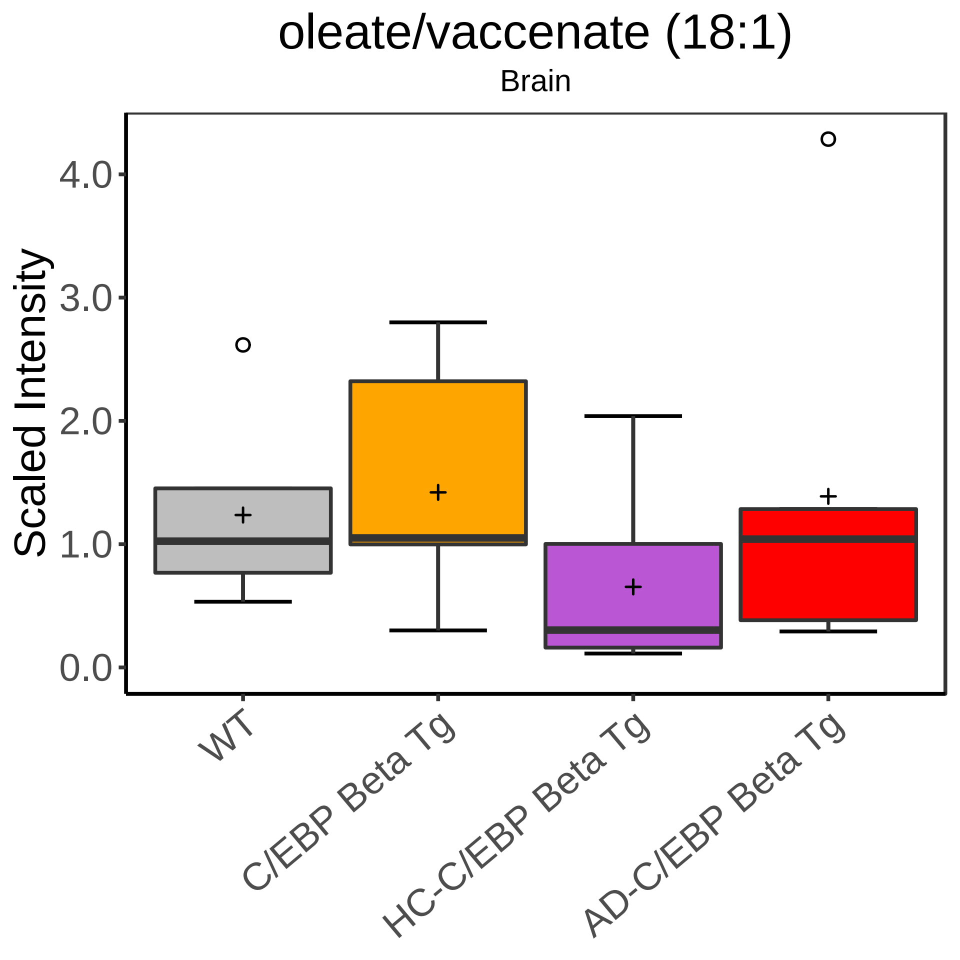

oleate/vaccenate (18:1)
Brain
Scaled Intensity
0.0
1.0
2.0
3.0
4.0
WT
C/EBP Beta Tg
HC-C/EBP Beta Tg
AD-C/EBP Beta Tg

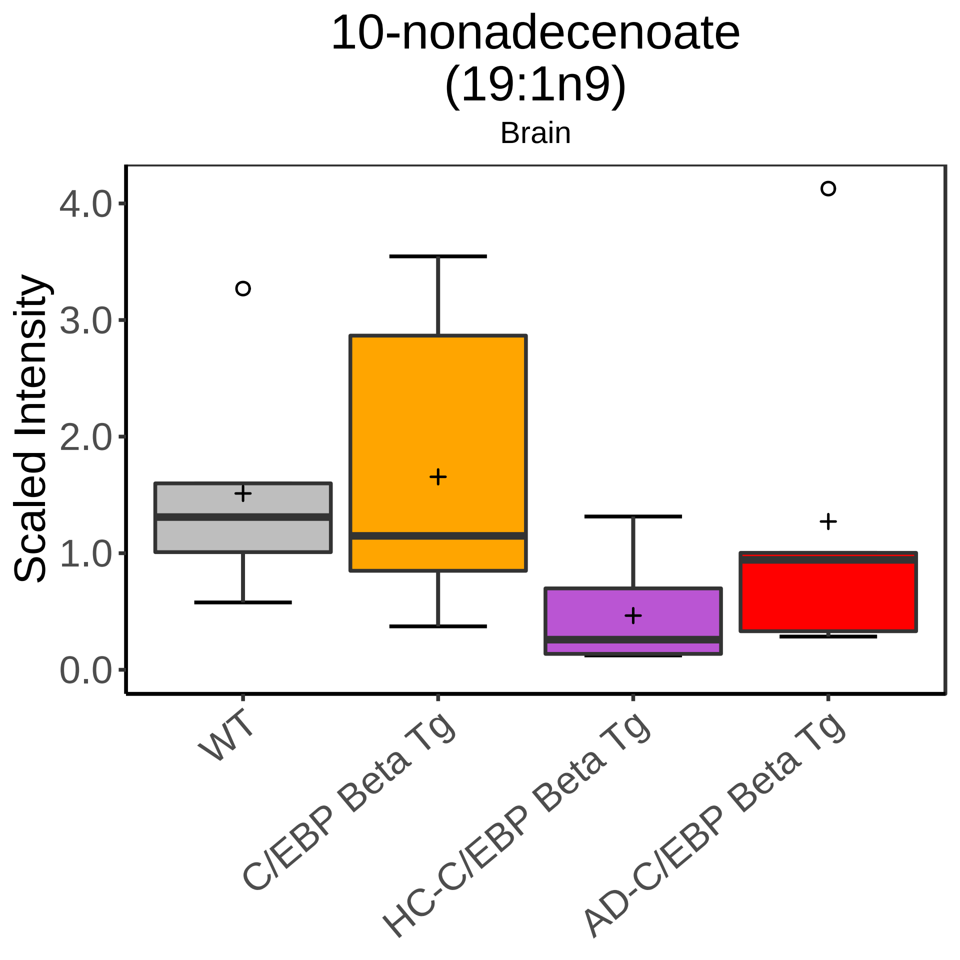
10-nonadecenoate
(19:1n9)
Brain
Scaled Intensity
4.0
3.0
2.0
1.0
0.0
WT
C/EBP Beta Tg
HC-C/EBP Beta Tg
AD-C/EBP Beta Tg

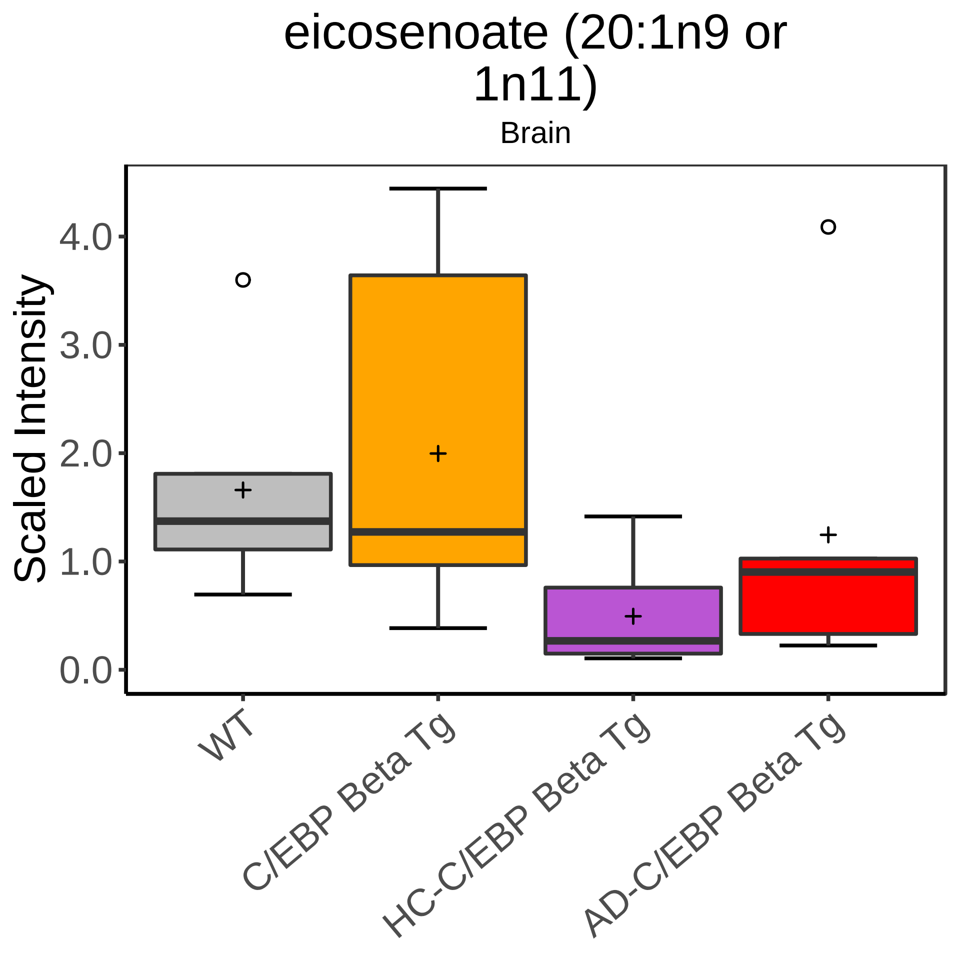
eicosenoate (20:1n9 or 1n11)
Brain
Scaled Intensity
4.0
3.0
2.0
1.0
0.0
WT
C/EBP Beta Tg
HC-C/EBP Beta Tg
AD-C/EBP Beta Tg

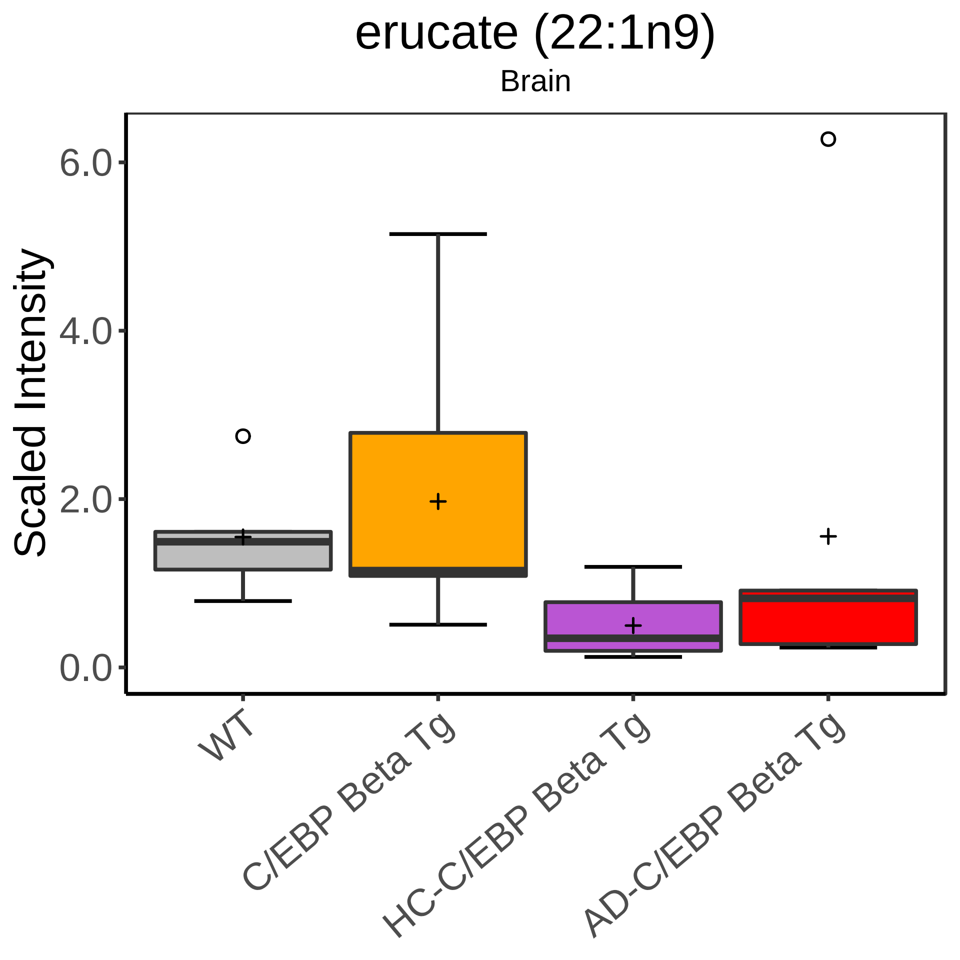
erucate (22:1n9)
Brain
Scaled Intensity
6.0
4.0
2.0
0.0
WT
C/EBP Beta Tg
HC-C/EBP Beta Tg
AD-C/EBP Beta Tg

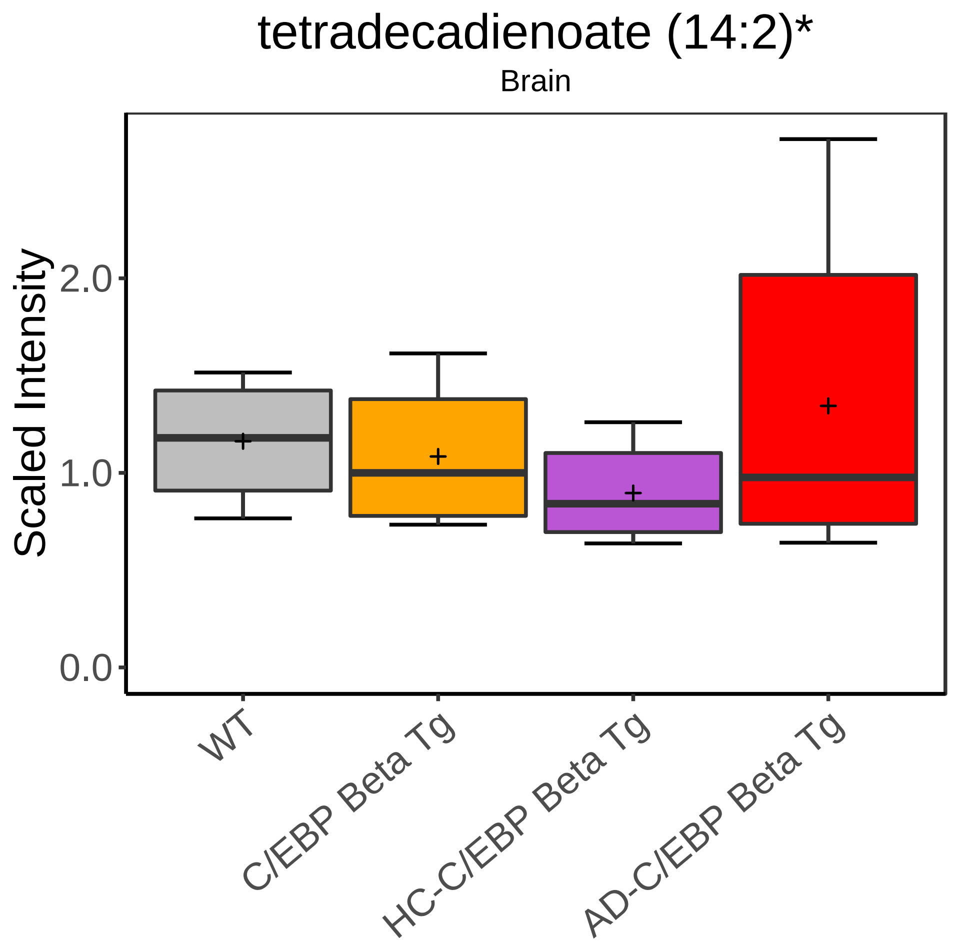
tetradecadienoate (14:2)*
Brain
Scaled Intensity
2.0
1.0
0.0
WT
C/EBP Beta Tg
HC-C/EBP Beta Tg
AD-C/EBP Beta Tg

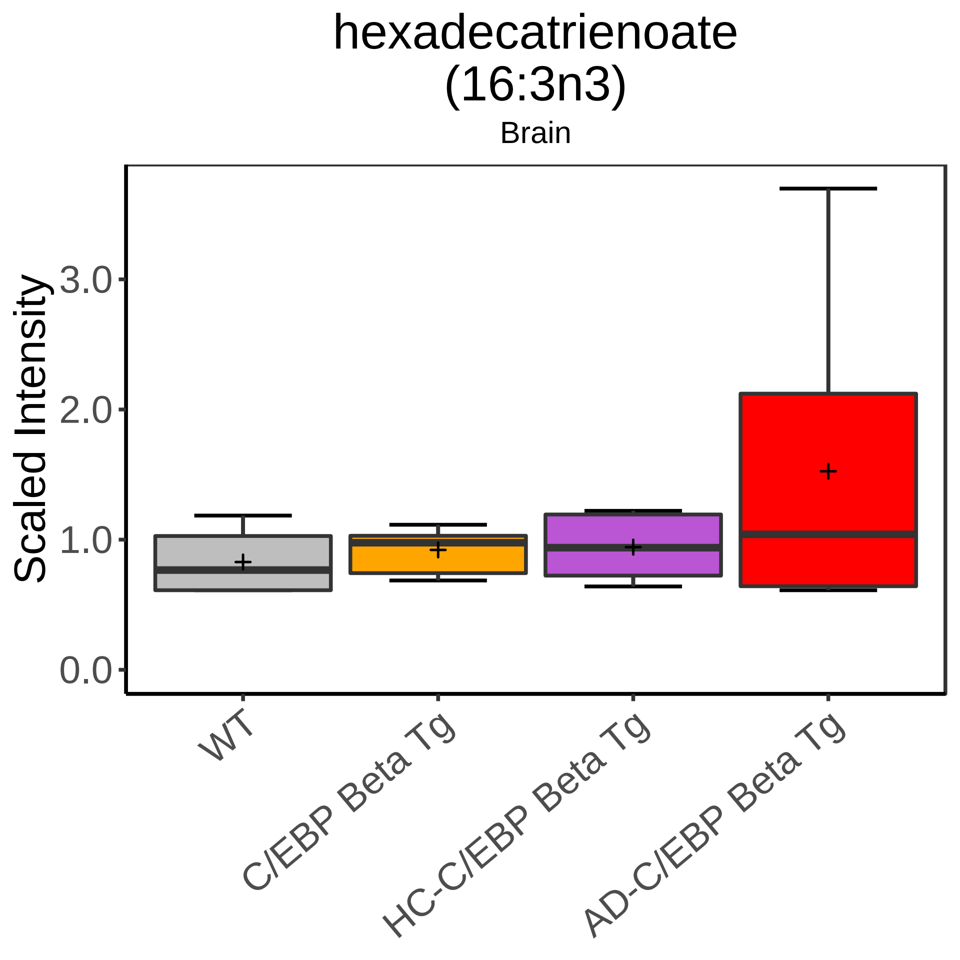
hexadecatrienoate
(16:3n3)
Brain
Scaled Intensity
0.0
1.0
2.0
3.0
WT
C/EBP Beta Tg
HC-C/EBP Beta Tg
AD-C/EBP Beta Tg

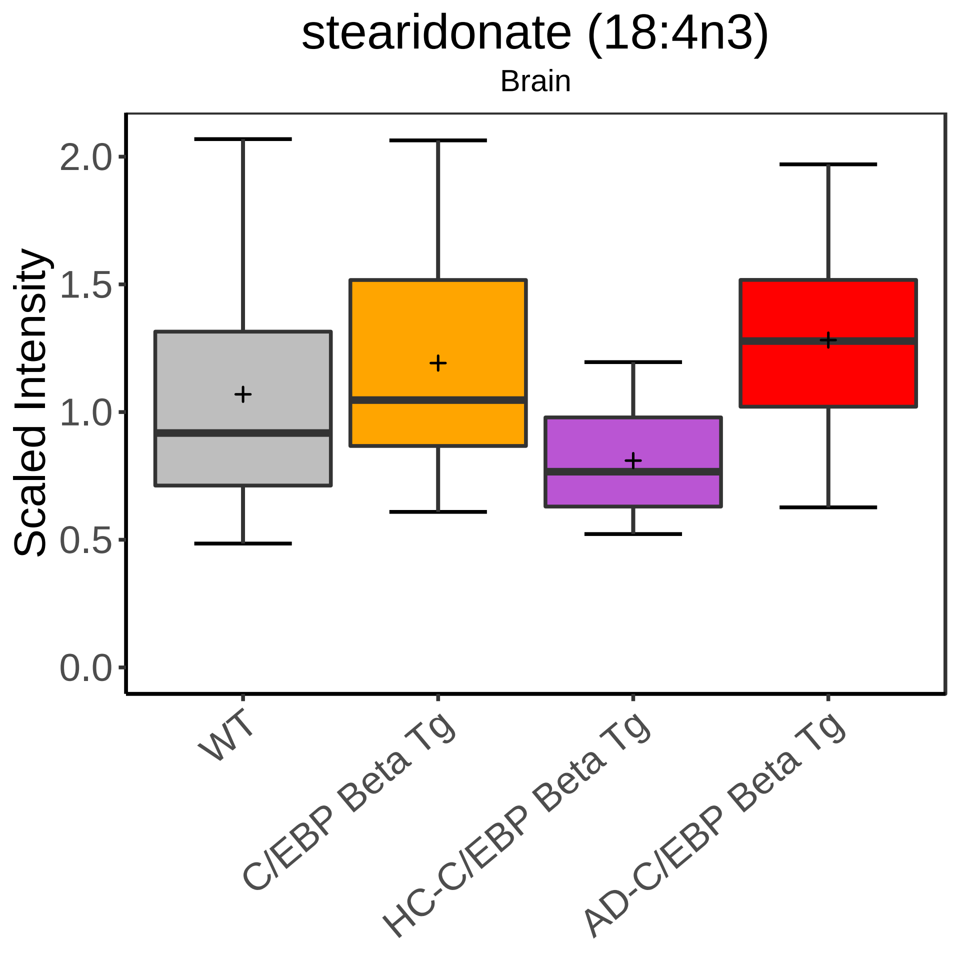
stearidonate (18:4n3)
Brain
Scaled Intensity
2.0
1.5
1.0
0.5
0.0
WT
C/EBP Beta Tg
HC-C/EBP Beta Tg
AD-C/EBP Beta Tg

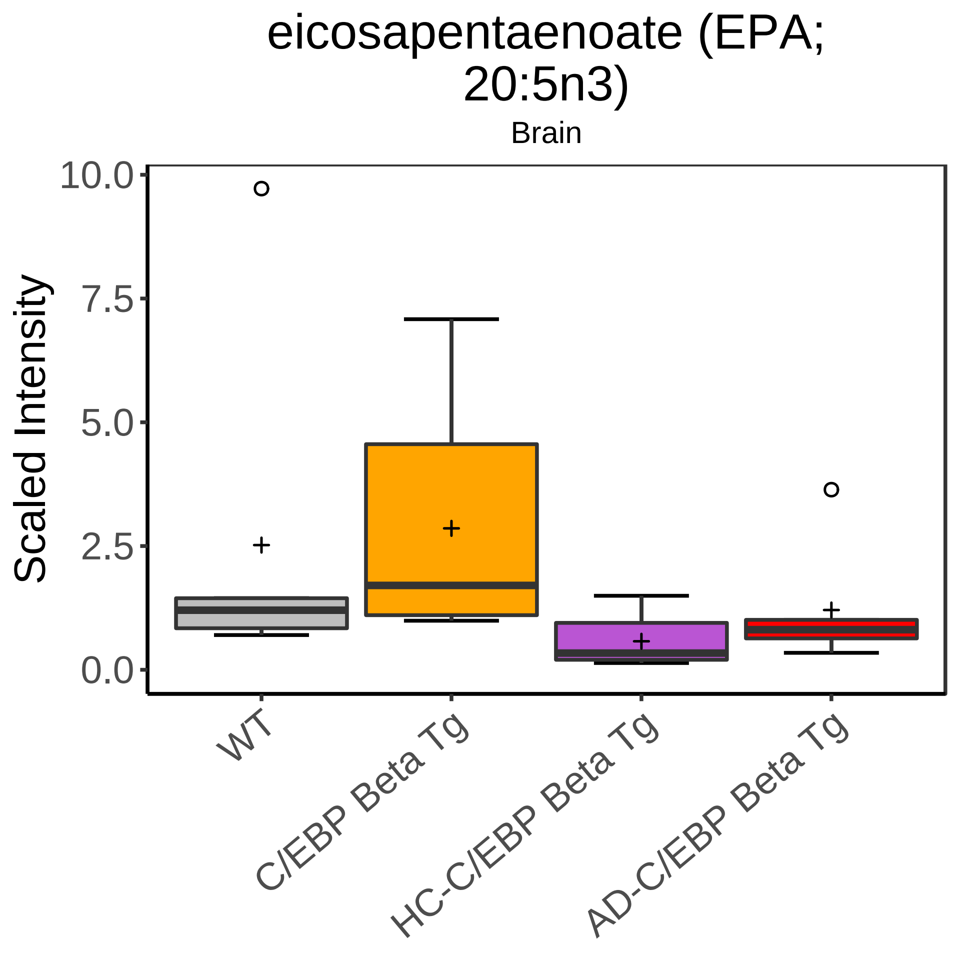

eicosapentaenoate (EPA; 20:5n3)
Brain
Scaled Intensity
10.0
7.5
5.0
2.5
0.0
WT
C/EBP Beta Tg
HC-C/EBP Beta Tg
AD-C/EBP Beta Tg

docosapentaenoate (DPA; 22:5n3)

Brain

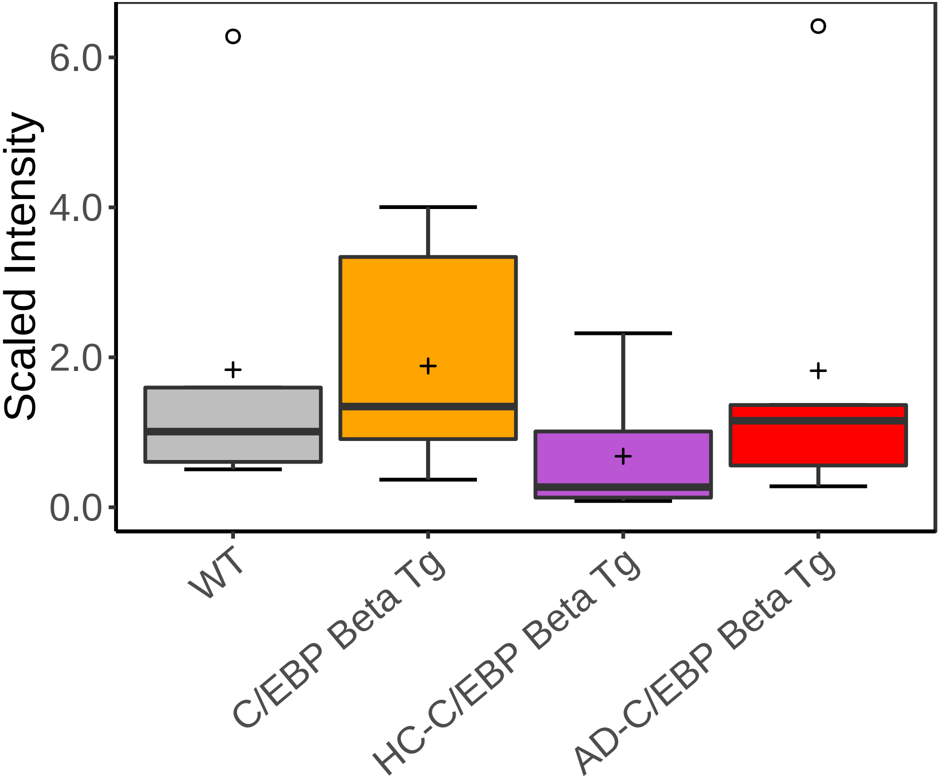

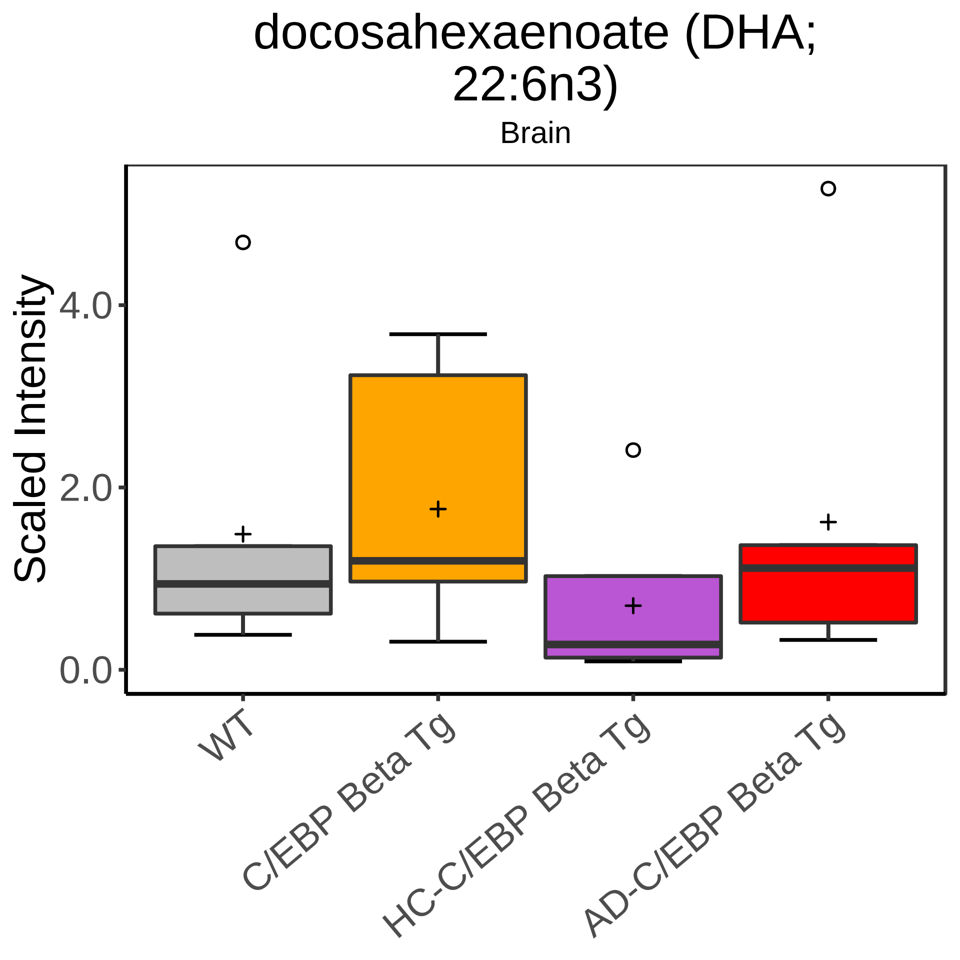
docosahexaenoate (DHA; 22:6n3)
Brain
Scaled Intensity
4.0
2.0
0.0
WT
C/EBP Beta Tg
HC-C/EBP Beta Tg
AD-C/EBP Beta Tg

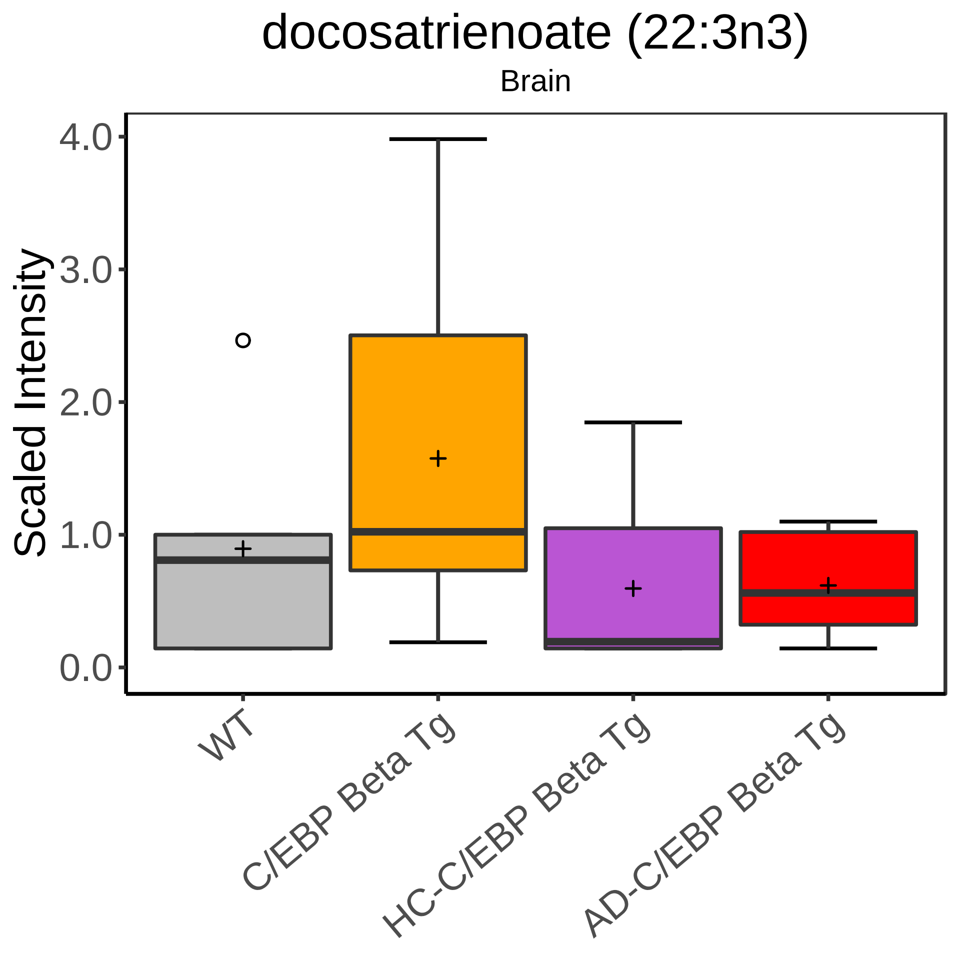

docosatrienoate (22:3n3)
Brain
Scaled Intensity
4.0
3.0
2.0
1.0
0.0
WT
C/EBP Beta Tg
HC-C/EBP Beta Tg
AD-C/EBP Beta Tg

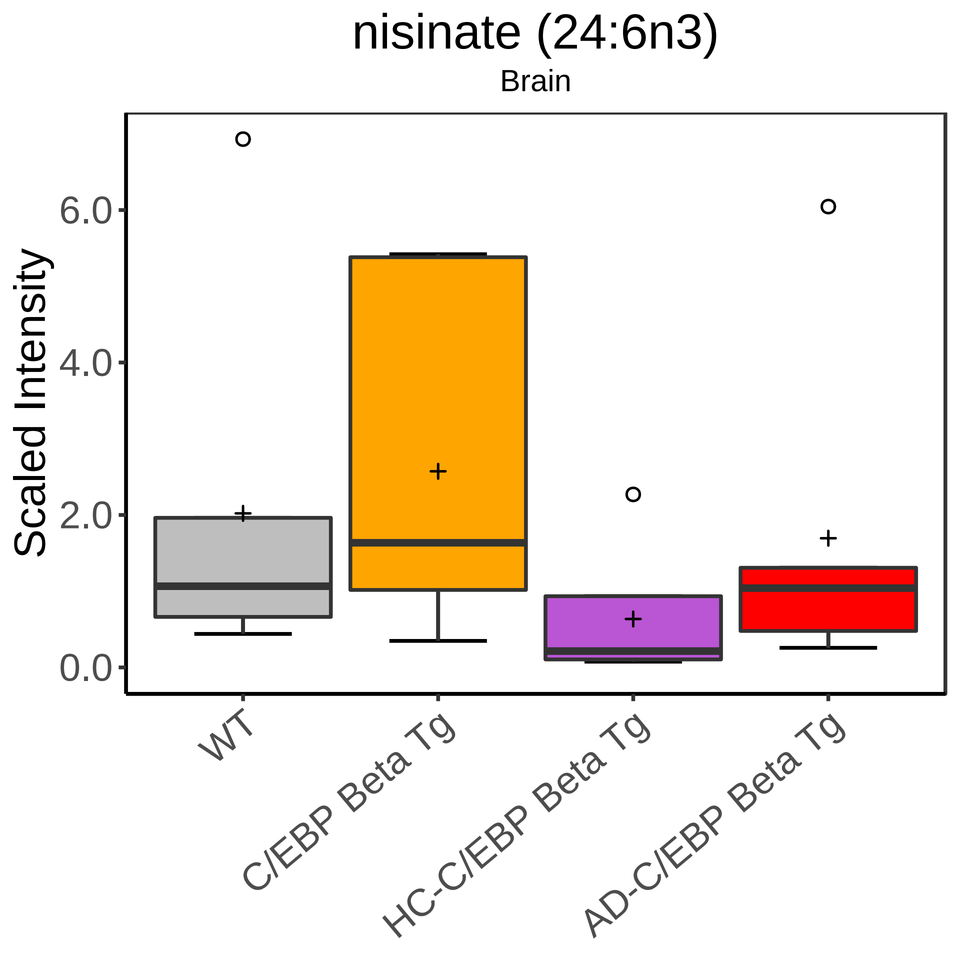

nisinate (24:6n3)
Brain
Scaled Intensity
6.0
4.0
2.0
0.0
WT
C/EBP Beta Tg
HC-C/EBP Beta Tg
AD-C/EBP Beta Tg

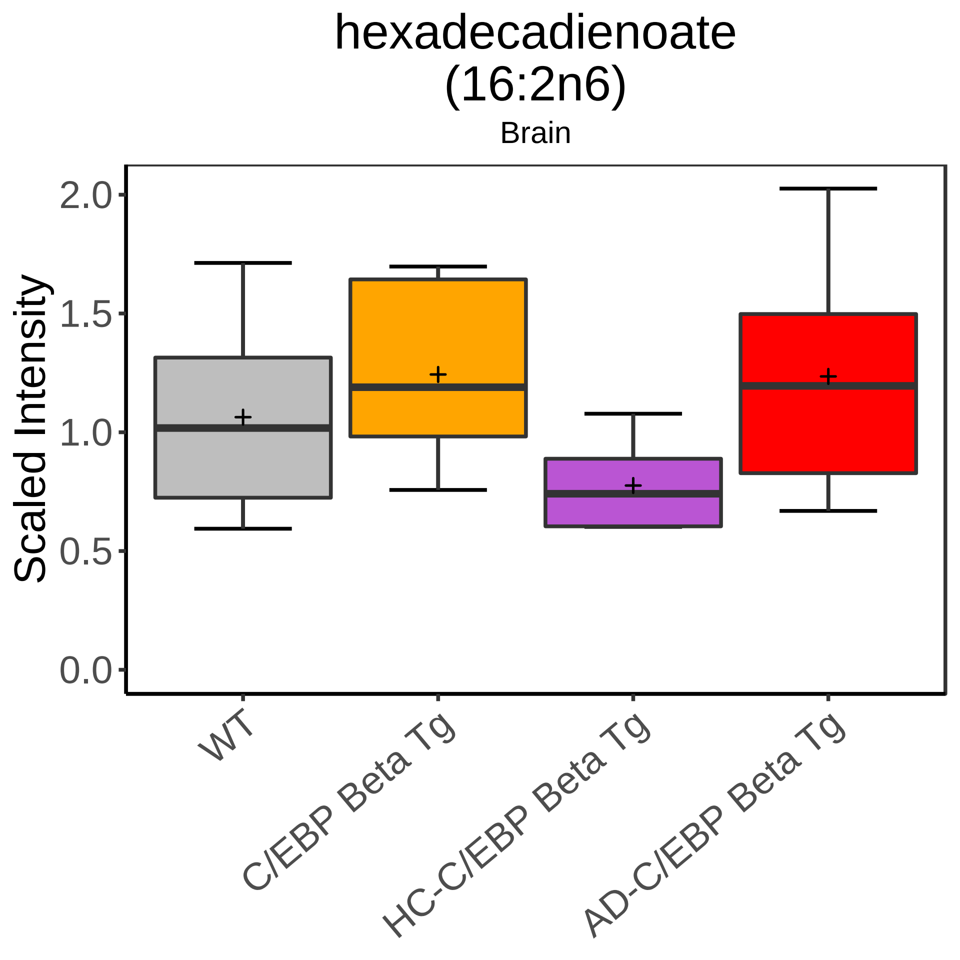
hexadecadienoate
(16:2n6)
Brain
Scaled Intensity
2.0
1.5
1.0
0.5
0.0
WT
C/EBP Beta Tg
HC-C/EBP Beta Tg
AD-C/EBP Beta Tg

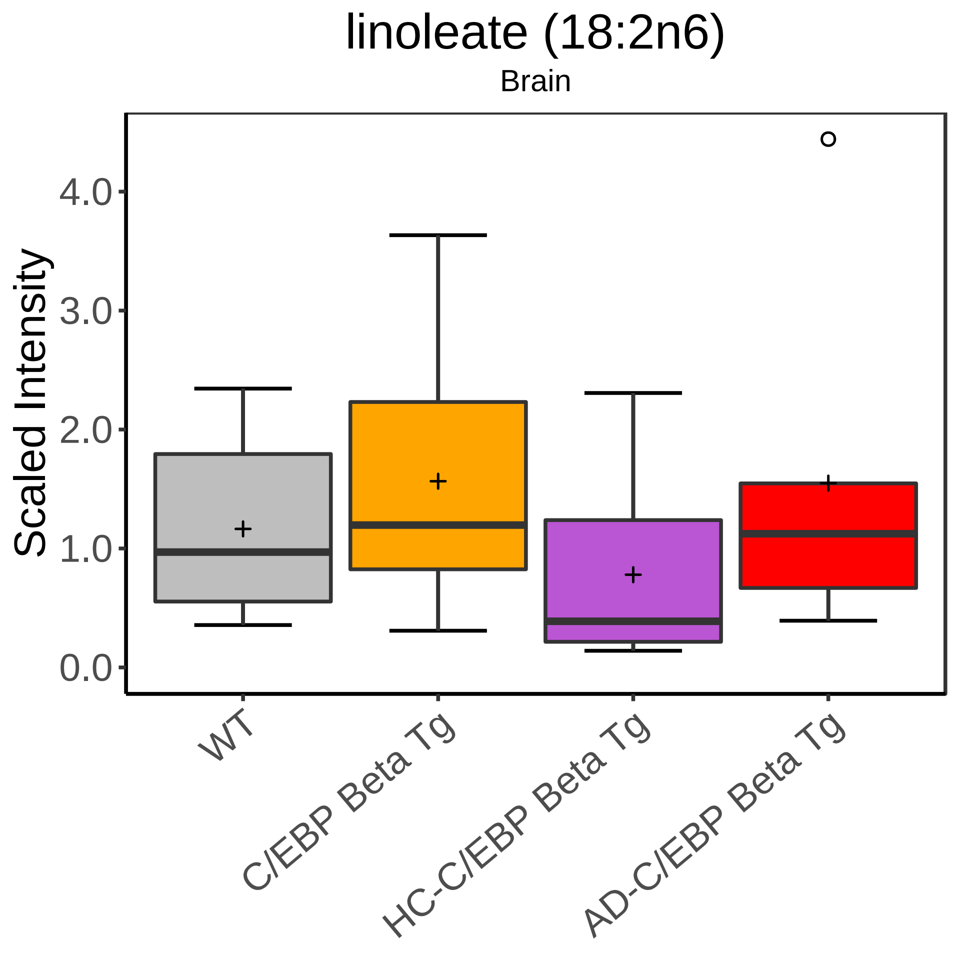

linoleate (18:2n6)
Brain
Scaled Intensity
4.0
3.0
2.0
1.0
0.0
WT
C/EBP Beta Tg
HC-C/EBP Beta Tg
AD-C/EBP Beta Tg

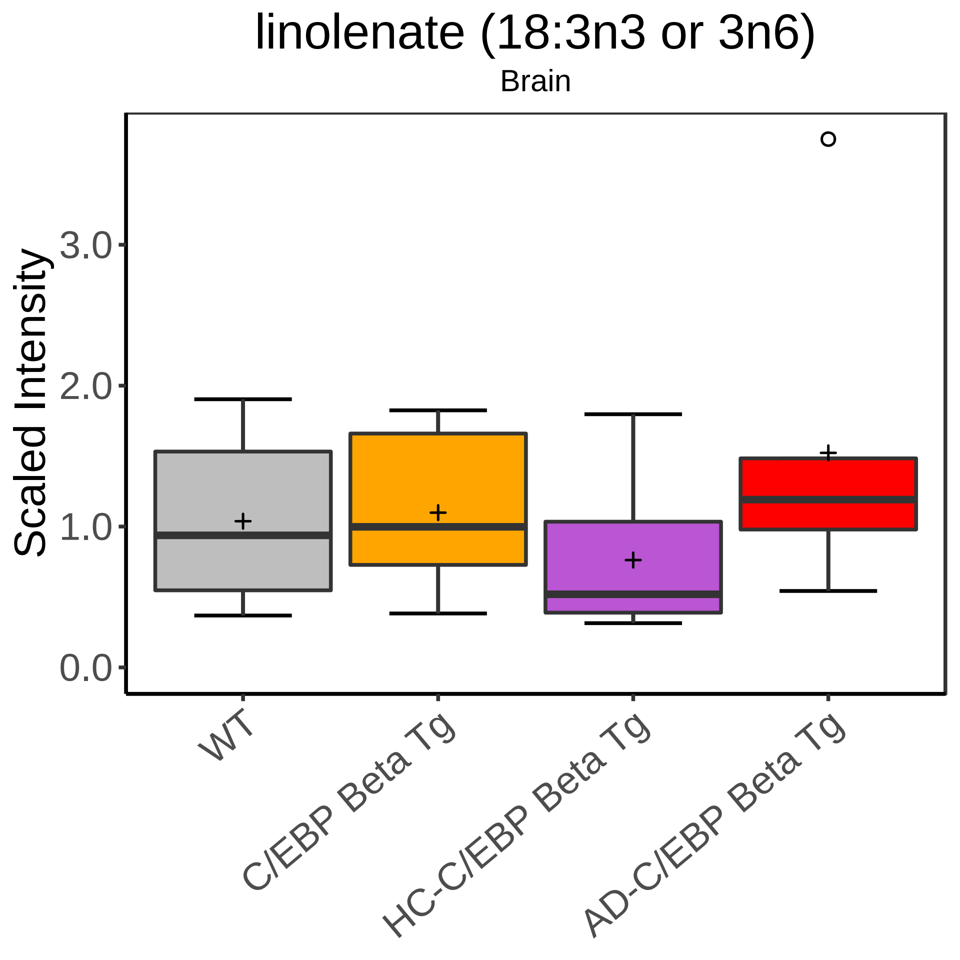

linolenate (18:3n3 or 3n6)
Brain
Scaled Intensity
3.0
2.0
1.0
0.0
WT
C/EBP Beta Tg
HC-C/EBP Beta Tg
AD-C/EBP Beta Tg

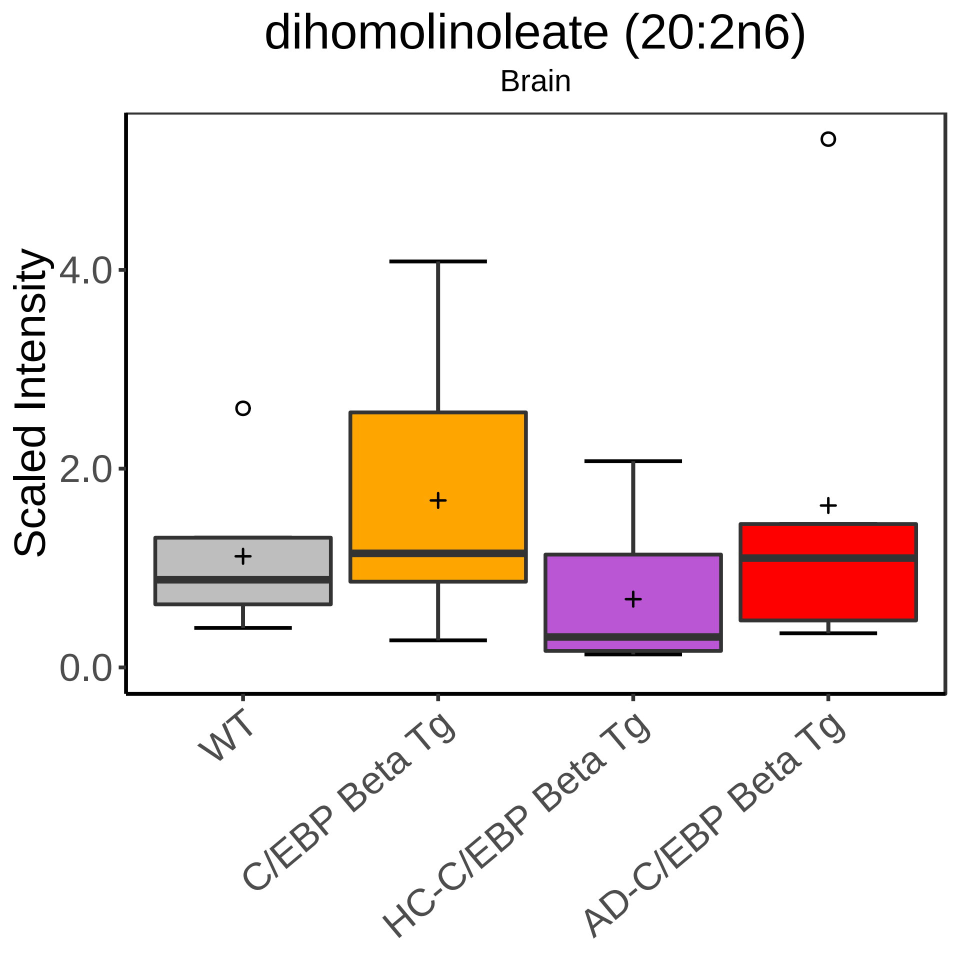

dihomolinoleate (20:2n6)
Brain
Scaled Intensity
4.0
2.0
0.0
WT
C/EBP Beta Tg
HC-C/EBP Beta Tg
AD-C/EBP Beta Tg

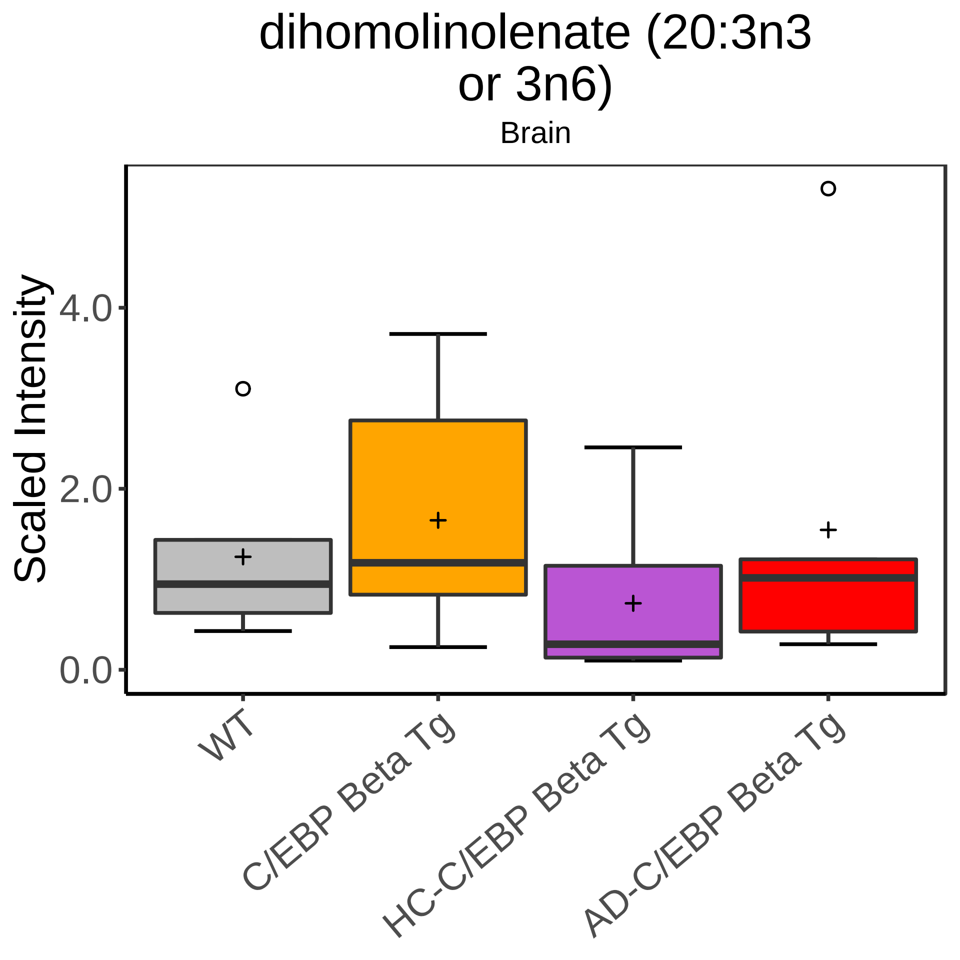
dihomolinolenate (20:3n3
or 3n6)
Brain
Scaled Intensity
0.0
2.0
4.0
WT
C/EBP Beta Tg
HC-C/EBP Beta Tg
AD-C/EBP Beta Tg

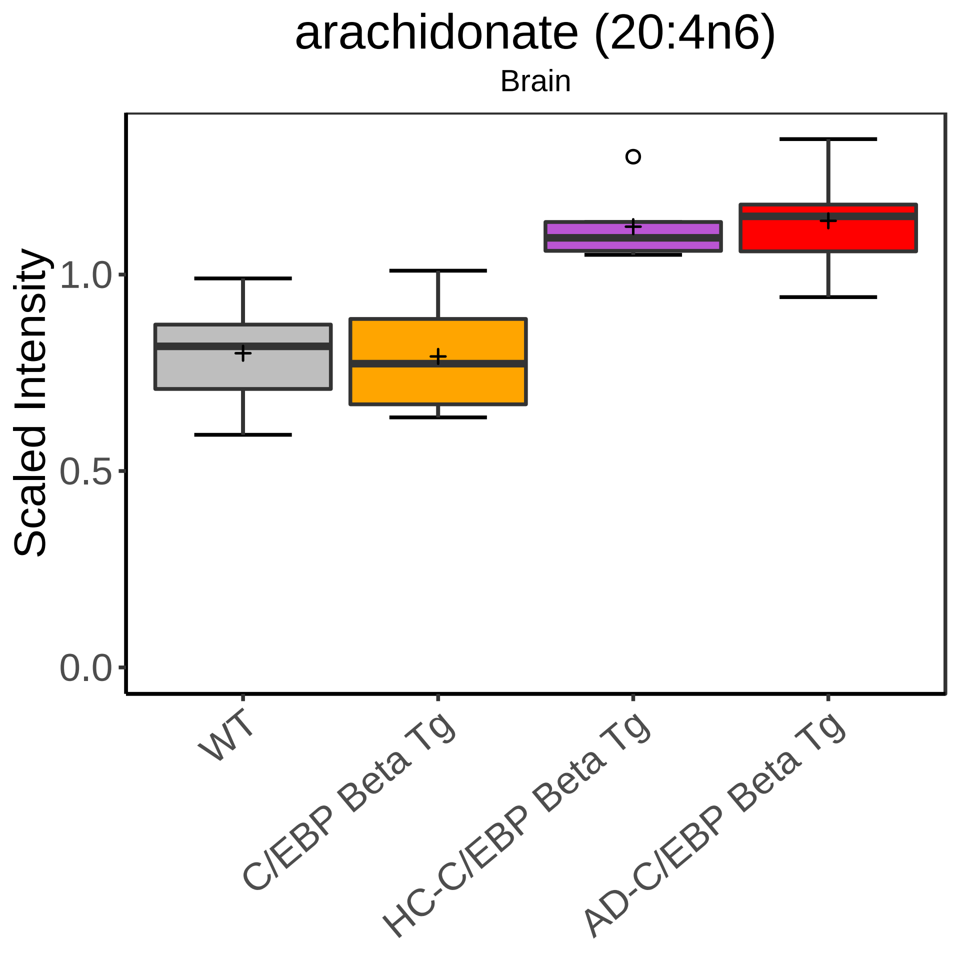
arachidonate (20:4n6)
Brain
Scaled Intensity
1.0
0.5
0.0
WT
C/EBP Beta Tg
HC-C/EBP Beta Tg
AD-C/EBP Beta Tg

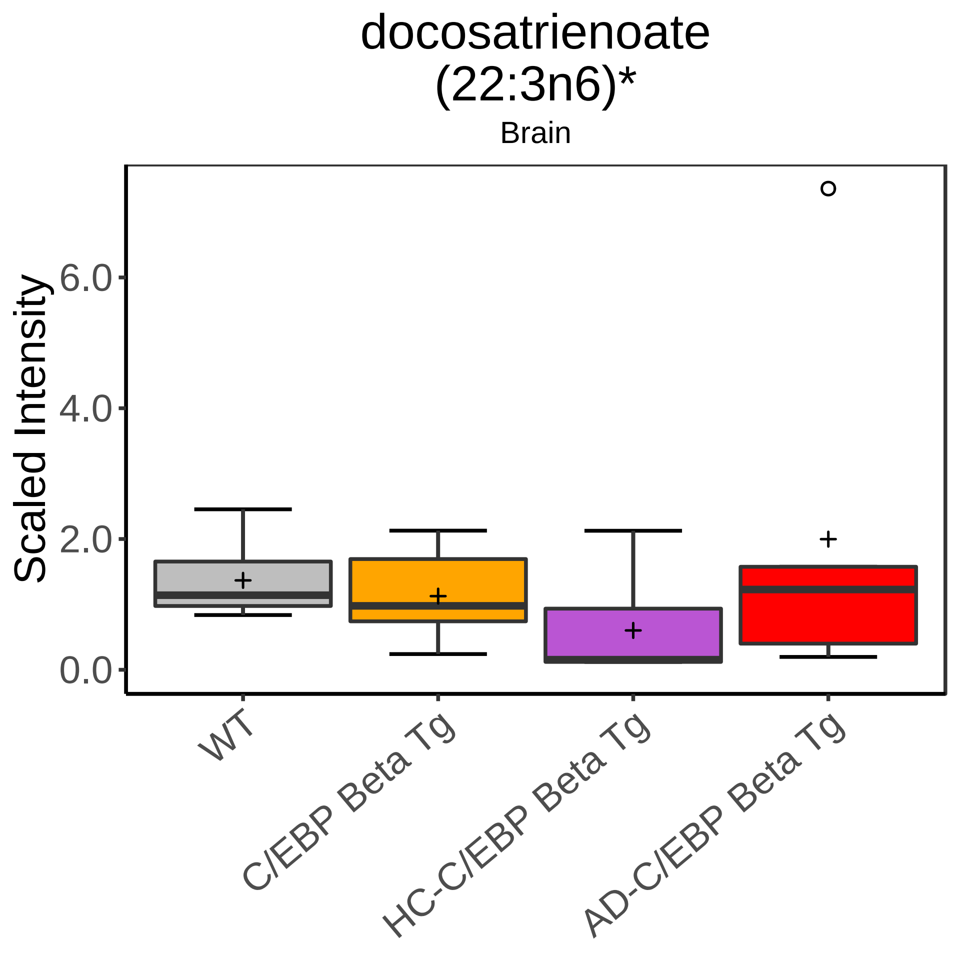

docosatrienoate
(22:3n6)*
Brain
Scaled Intensity
0.0
2.0
4.0
6.0
WT
C/EBP Beta Tg
HC-C/EBP Beta Tg
AD-C/EBP Beta Tg

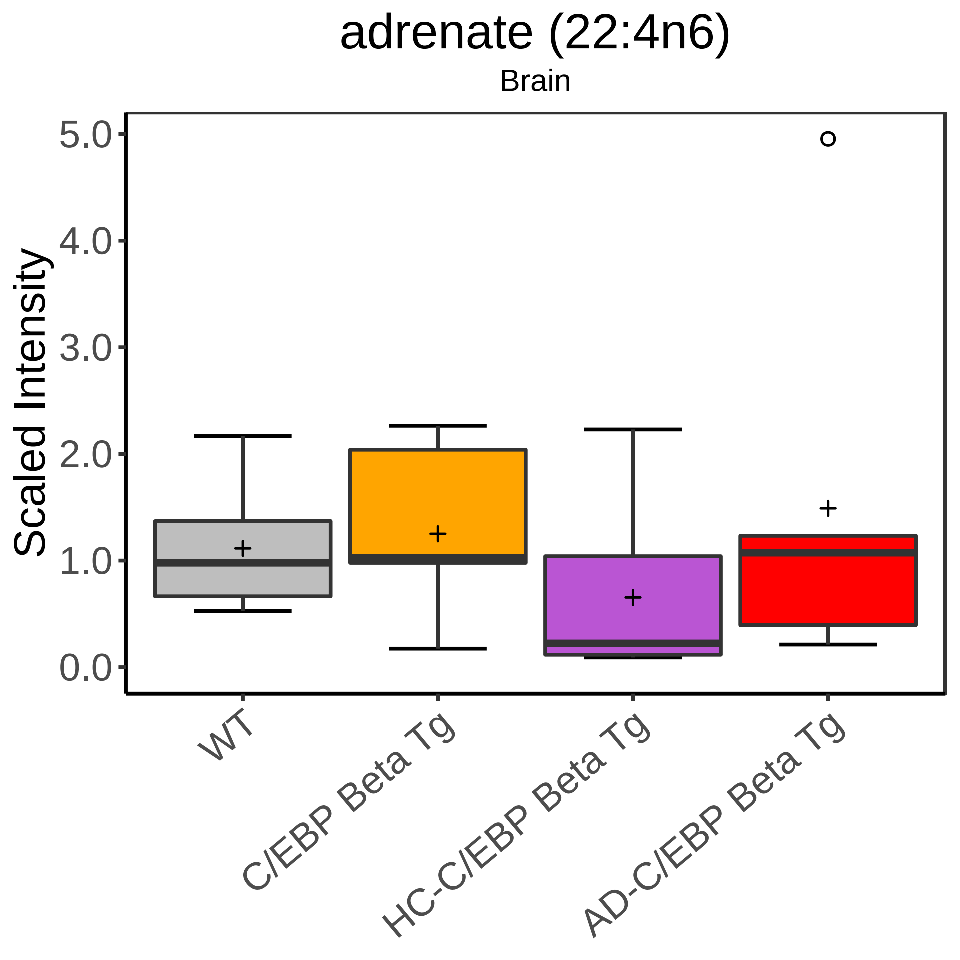

adrenate (22:4n6)
Brain
Scaled Intensity
5.0
4.0
3.0
2.0
1.0
0.0
WT
C/EBP Beta Tg
HC-C/EBP Beta Tg
AD-C/EBP Beta Tg

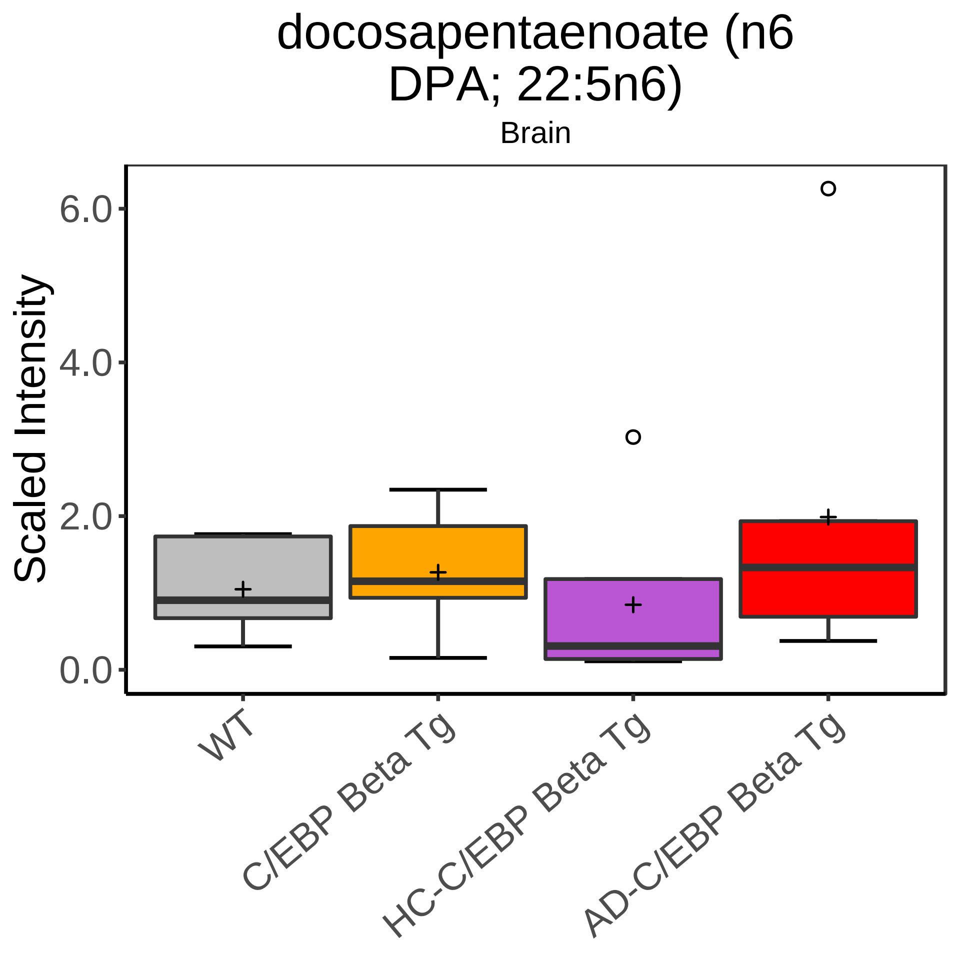
docosapentaenoate (n6
DPA; 22:5n6)
Brain
Scaled Intensity
6.0
4.0
2.0
0.0
WT
C/EBP Beta Tg
HC-C/EBP Beta Tg
AD-C/EBP Beta Tg

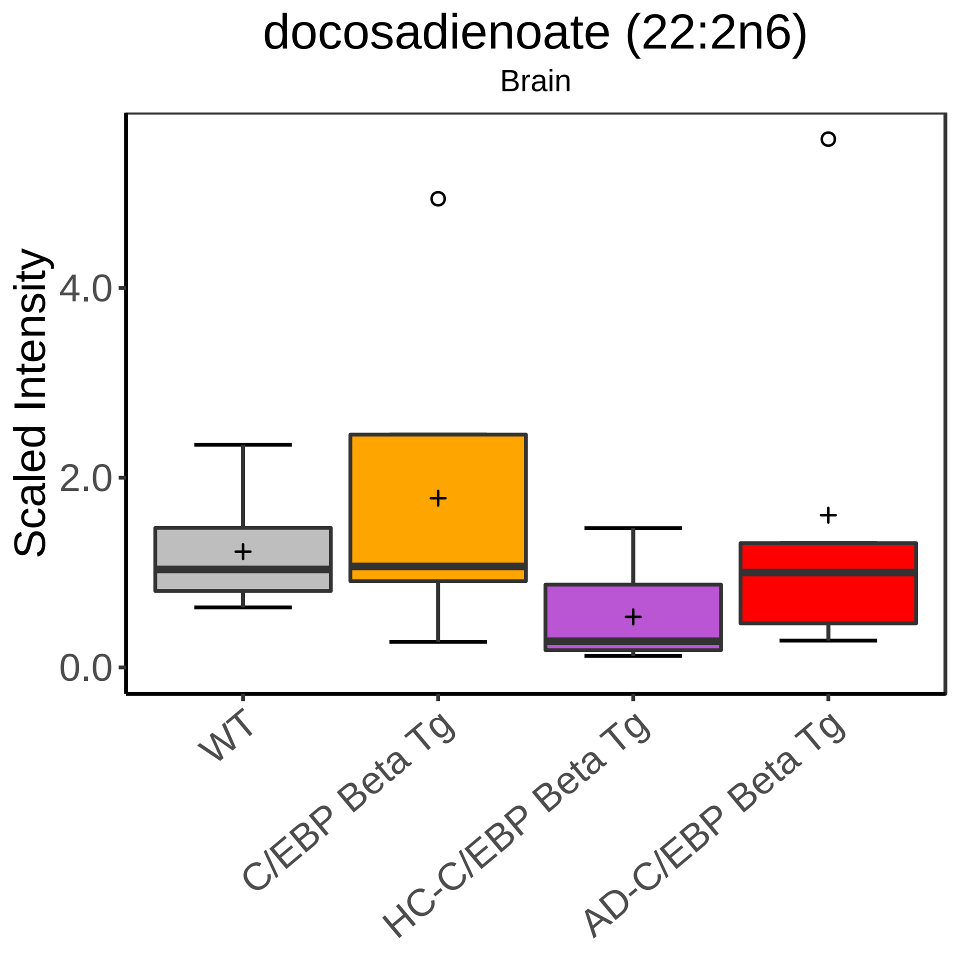
docosadienoate (22:2n6)
Brain
Scaled Intensity
0.0
2.0
4.0
WT
C/EBP Beta Tg
HC-C/EBP Beta Tg
AD-C/EBP Beta Tg

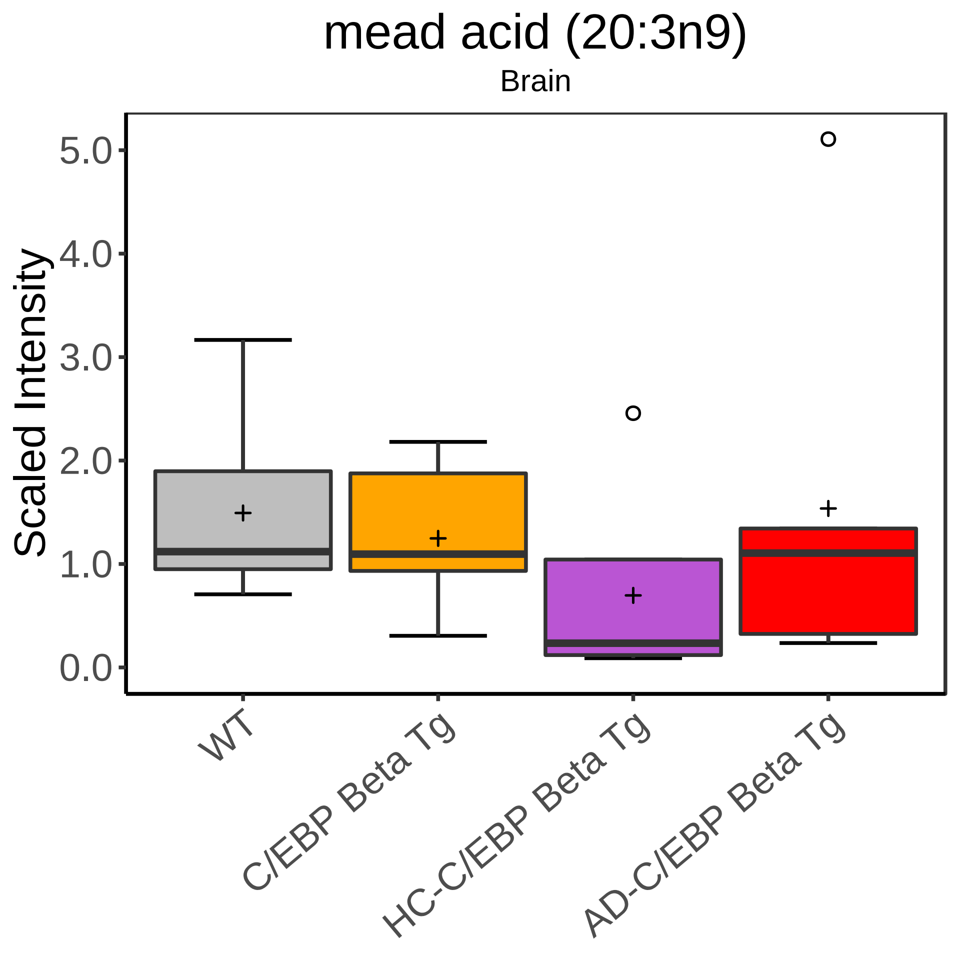

mead acid (20:3n9)
Brain
Scaled Intensity
5.0
4.0
3.0
2.0
1.0
0.0
WT
C/EBP Beta Tg
HC-C/EBP Beta Tg
AD-C/EBP Beta Tg

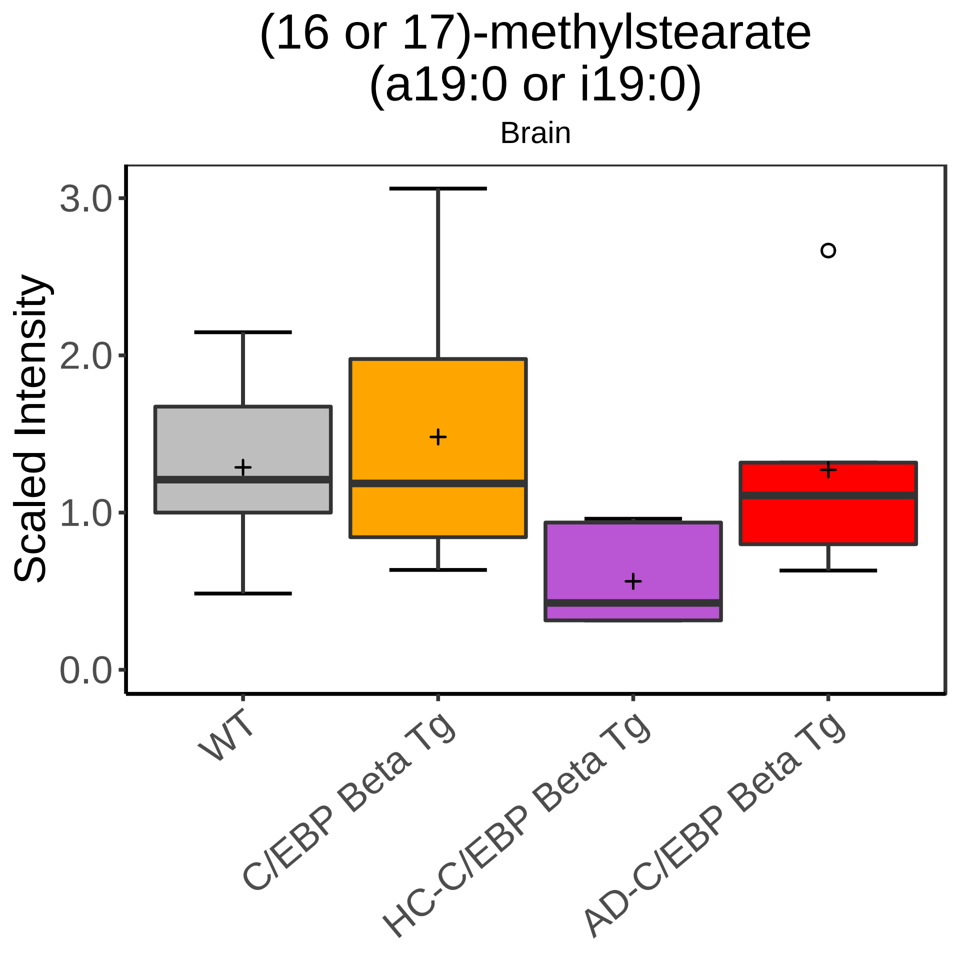

(16 or 17)-methylstearate
(a19:0 or i19:0)
Brain
Scaled Intensity
3.0
2.0
1.0
0.0
WT
C/EBP Beta Tg
HC-C/EBP Beta Tg
AD-C/EBP Beta Tg

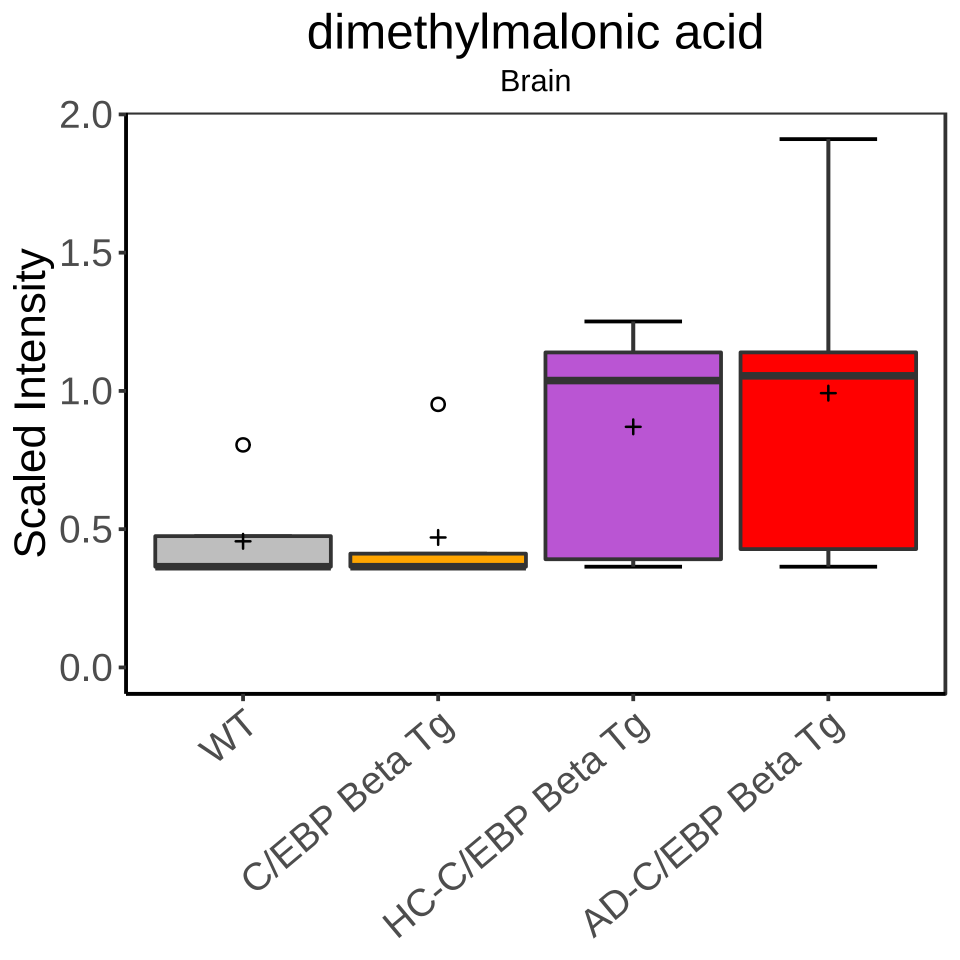

dimethylmalonic acid
Brain
Scaled Intensity
2.0
1.5
1.0
0.5
0.0
WT
C/EBP Beta Tg
HC-C/EBP Beta Tg
AD-C/EBP Beta Tg

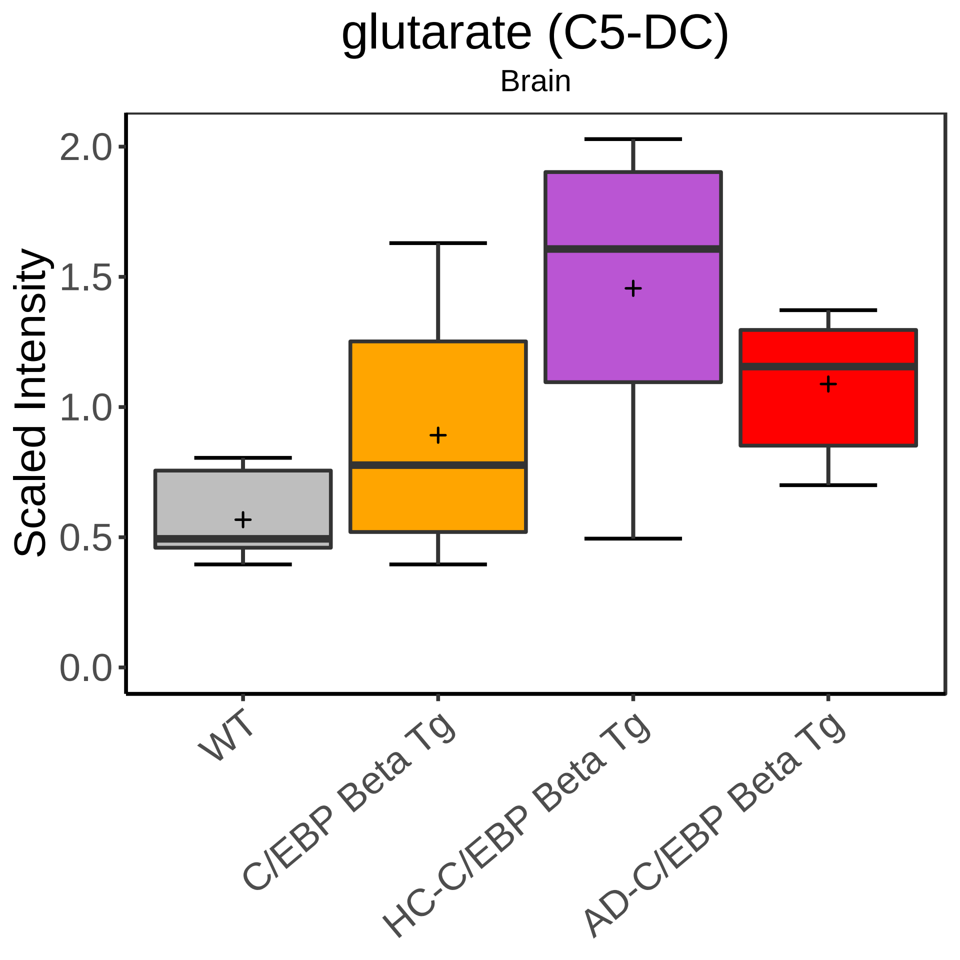
glutarate (C5-DC)
Brain
Scaled Intensity
2.0
1.5
1.0
0.5
0.0
WT
C/EBP Beta Tg
HC-C/EBP Beta Tg
AD-C/EBP Beta Tg

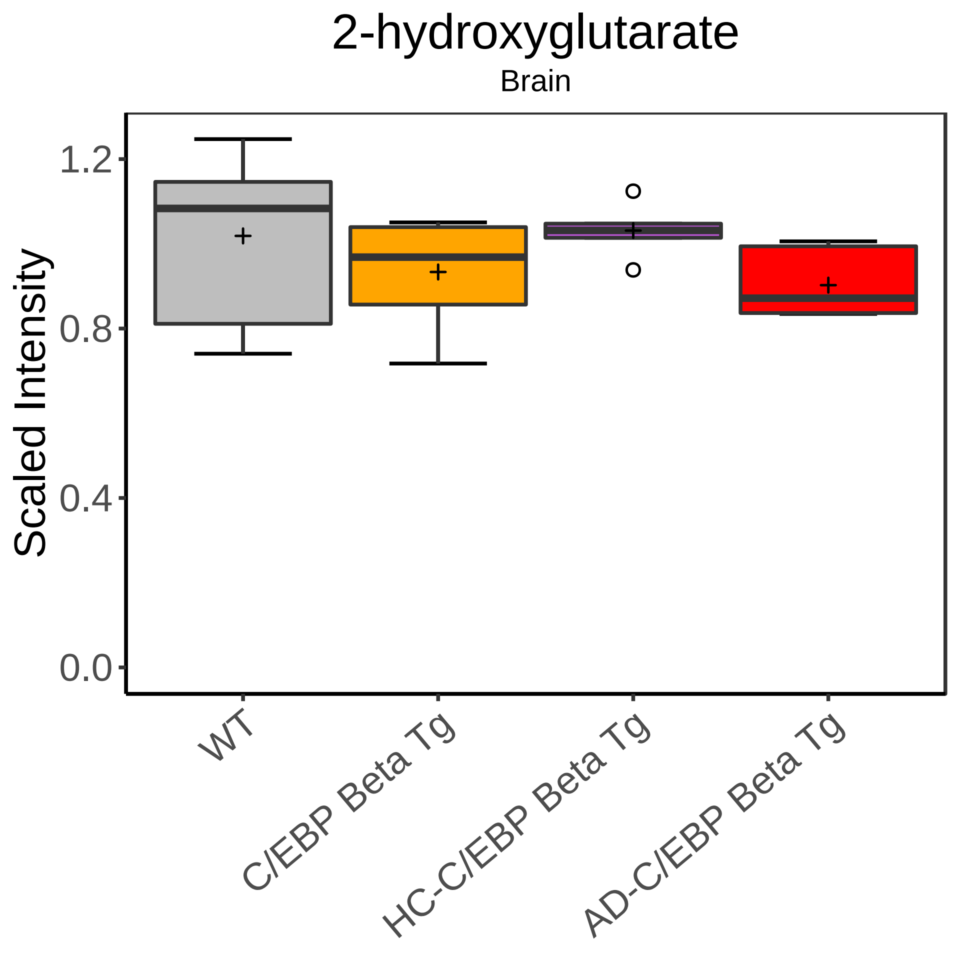

2-hydroxyglutarate
Brain
Scaled Intensity
1.2
0.8
0.4
0.0
WT
C/EBP Beta Tg
HC-C/EBP Beta Tg
AD-C/EBP Beta Tg

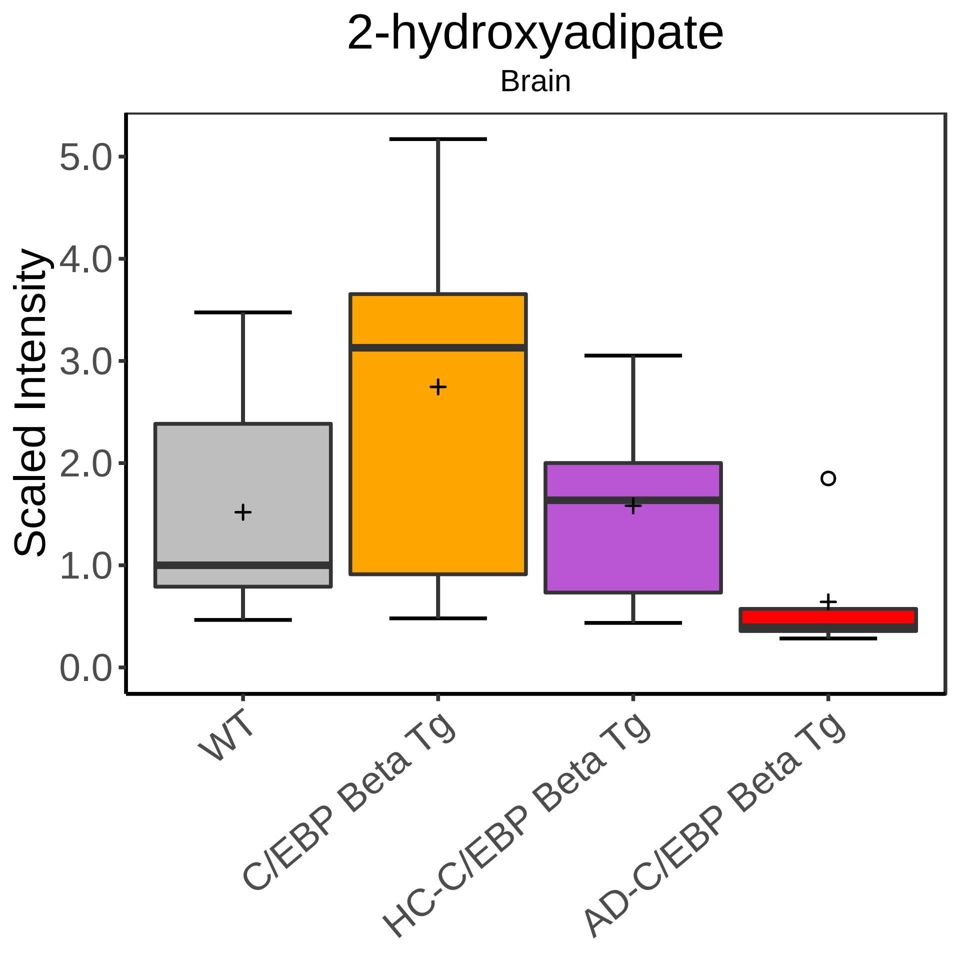
2-hydroxyadipate
Brain
Scaled Intensity
5.0
4.0
3.0
2.0
1.0
0.0
WT
C/EBP Beta Tg
HC-C/EBP Beta Tg
AD-C/EBP Beta Tg

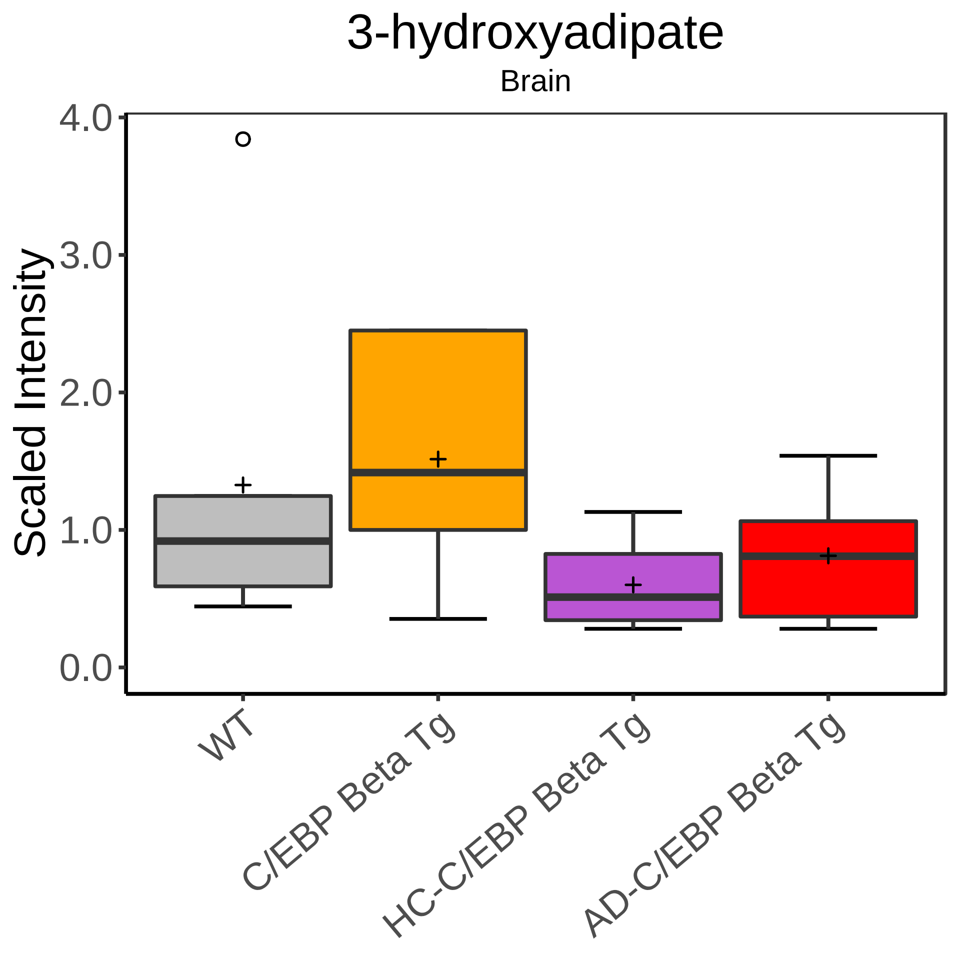
3-hydroxyadipate
Brain
Scaled Intensity
4.0
3.0
2.0
1.0
0.0
WT
C/EBP Beta Tg
HC-C/EBP Beta Tg
AD-C/EBP Beta Tg

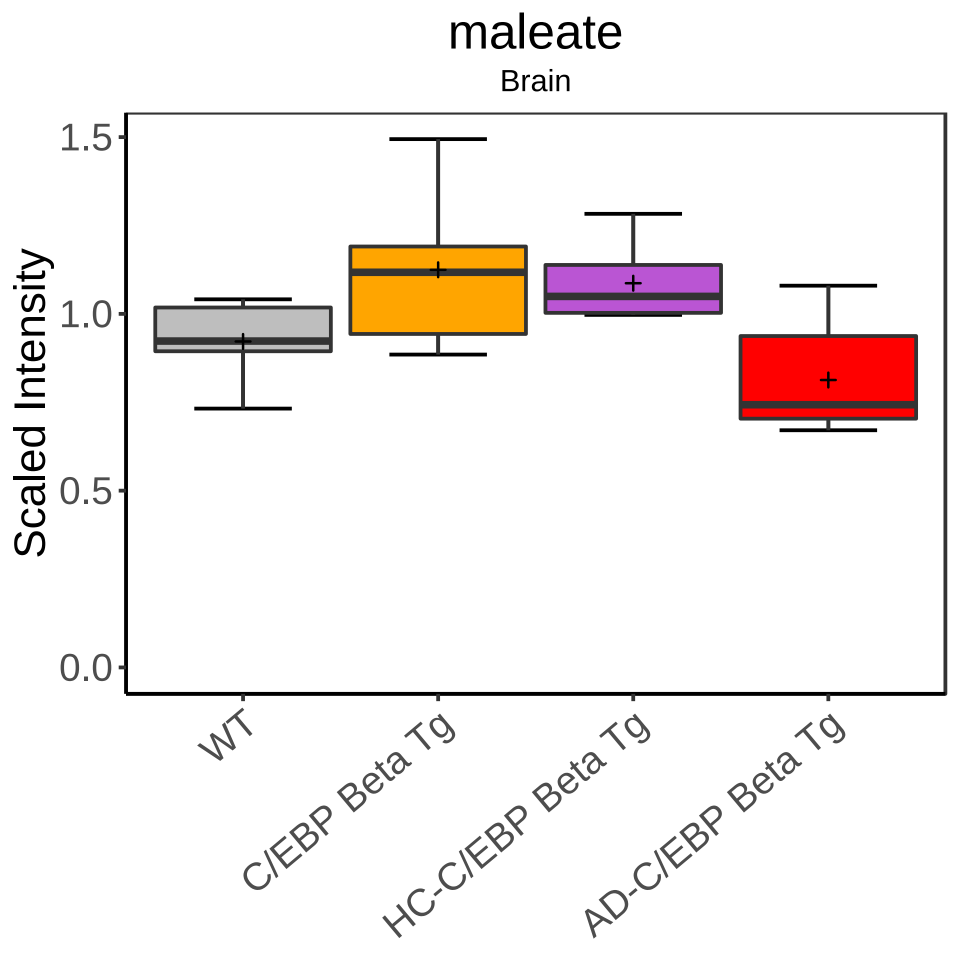
maleate
Brain
Scaled Intensity
1.5
1.0
0.5
0.0
WT
C/EBP Beta Tg
HC-C/EBP Beta Tg
AD-C/EBP Beta Tg

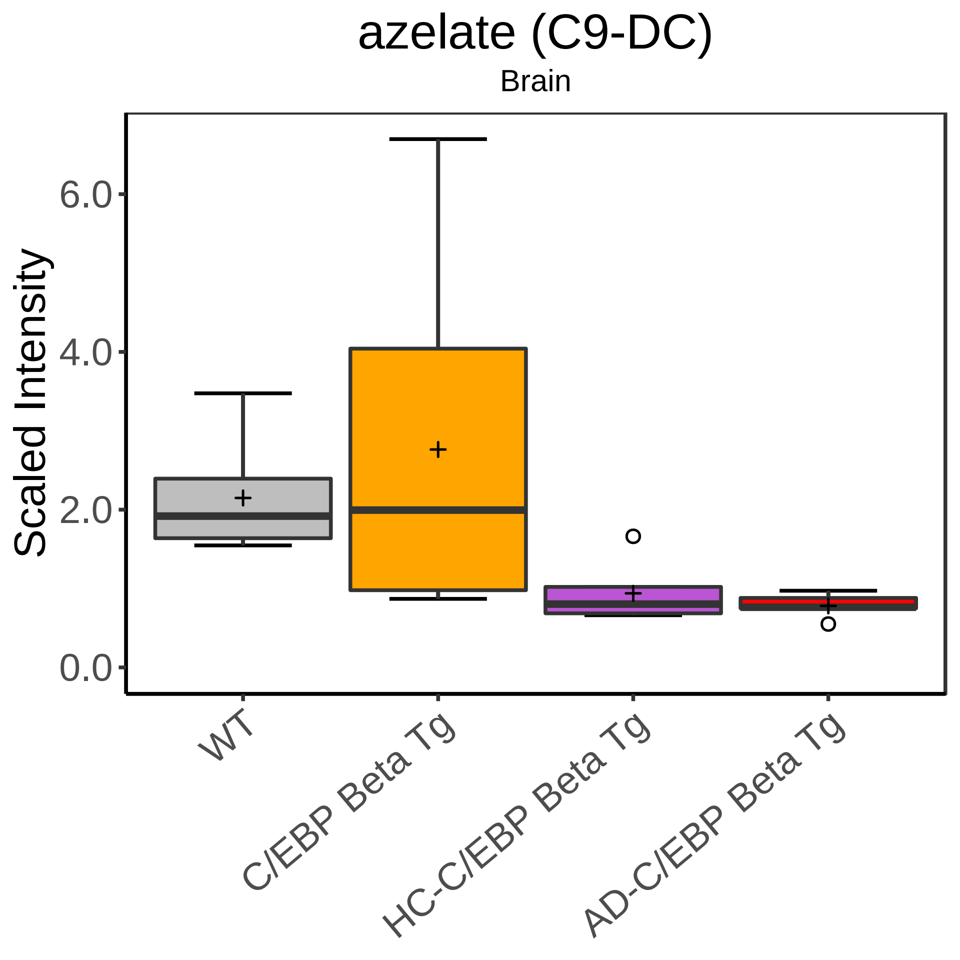

azelate (C9-DC)
Brain
Scaled Intensity
6.0
4.0
2.0
0.0
WT
C/EBP Beta Tg
HC-C/EBP Beta Tg
AD-C/EBP Beta Tg

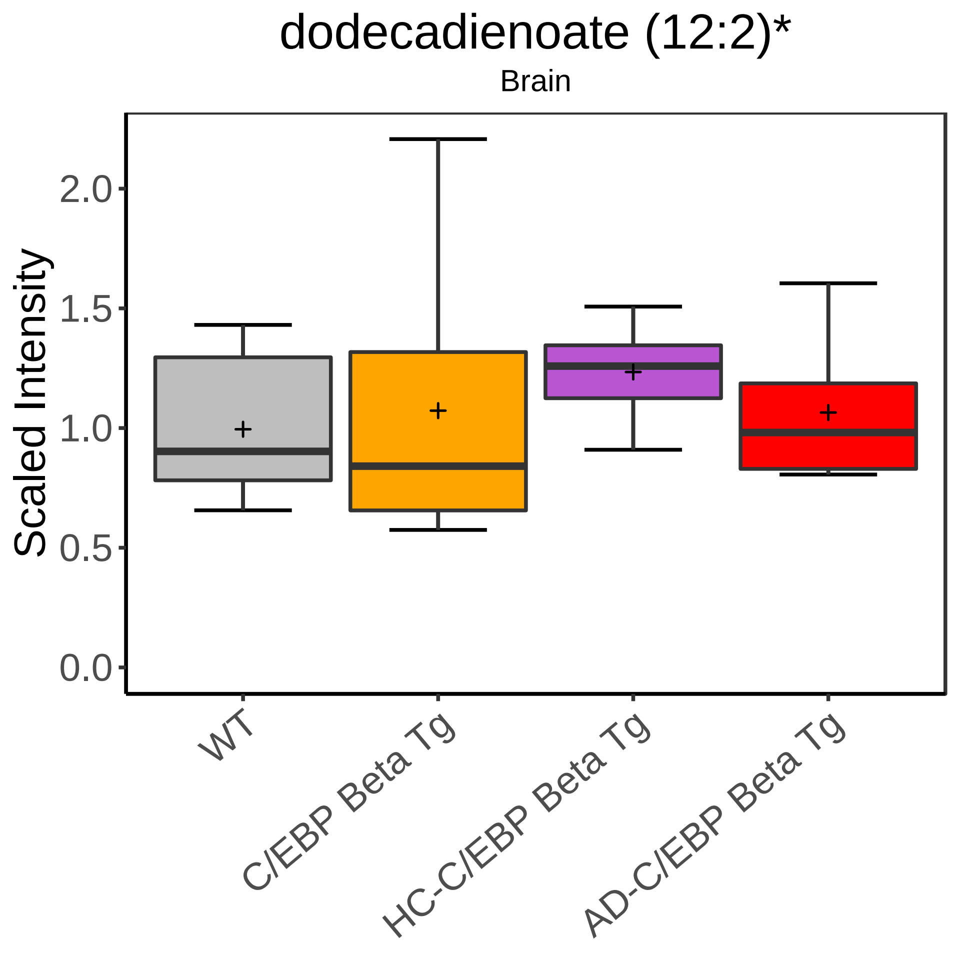
dodecadienoate (12:2)*
Brain
Scaled Intensity
0.0
0.5
1.0
1.5
2.0
WT
C/EBP Beta Tg
HC-C/EBP Beta Tg
AD-C/EBP Beta Tg

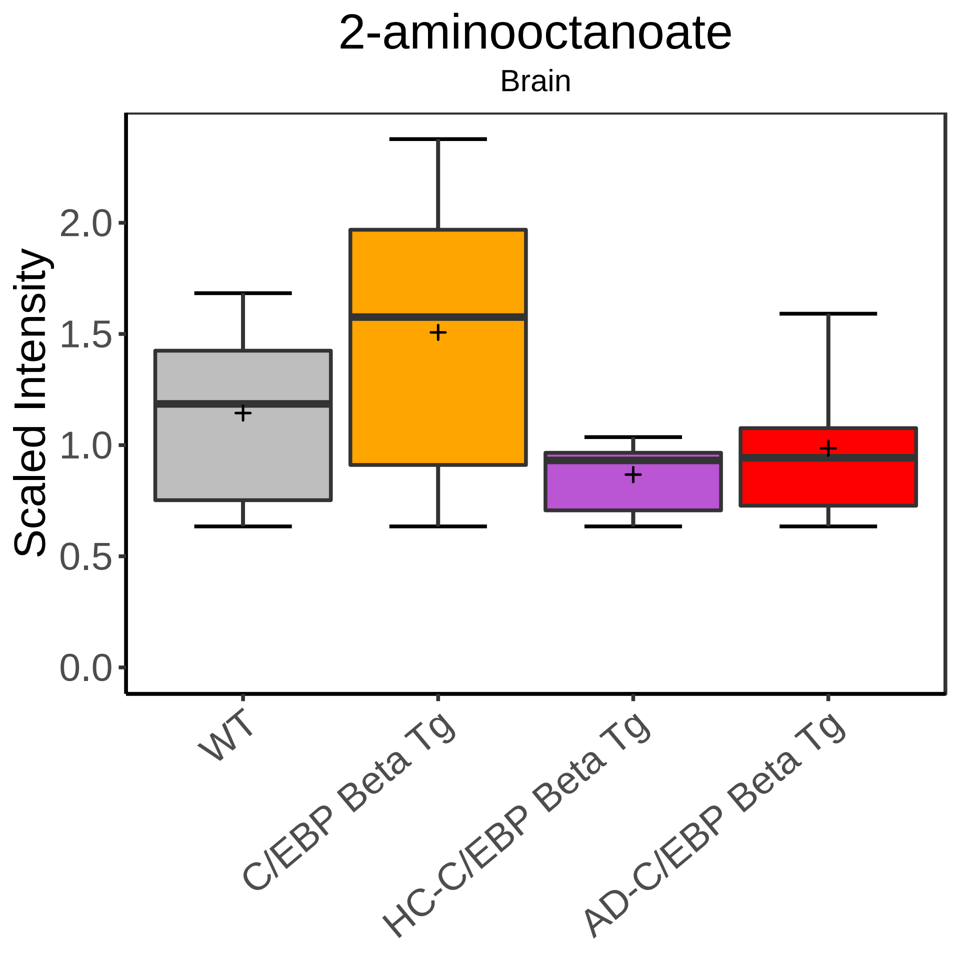
2-aminooctanoate
Brain
Scaled Intensity
2.0
1.5
1.0
0.5
0.0
WT
C/EBP Beta Tg
HC-C/EBP Beta Tg
AD-C/EBP Beta Tg

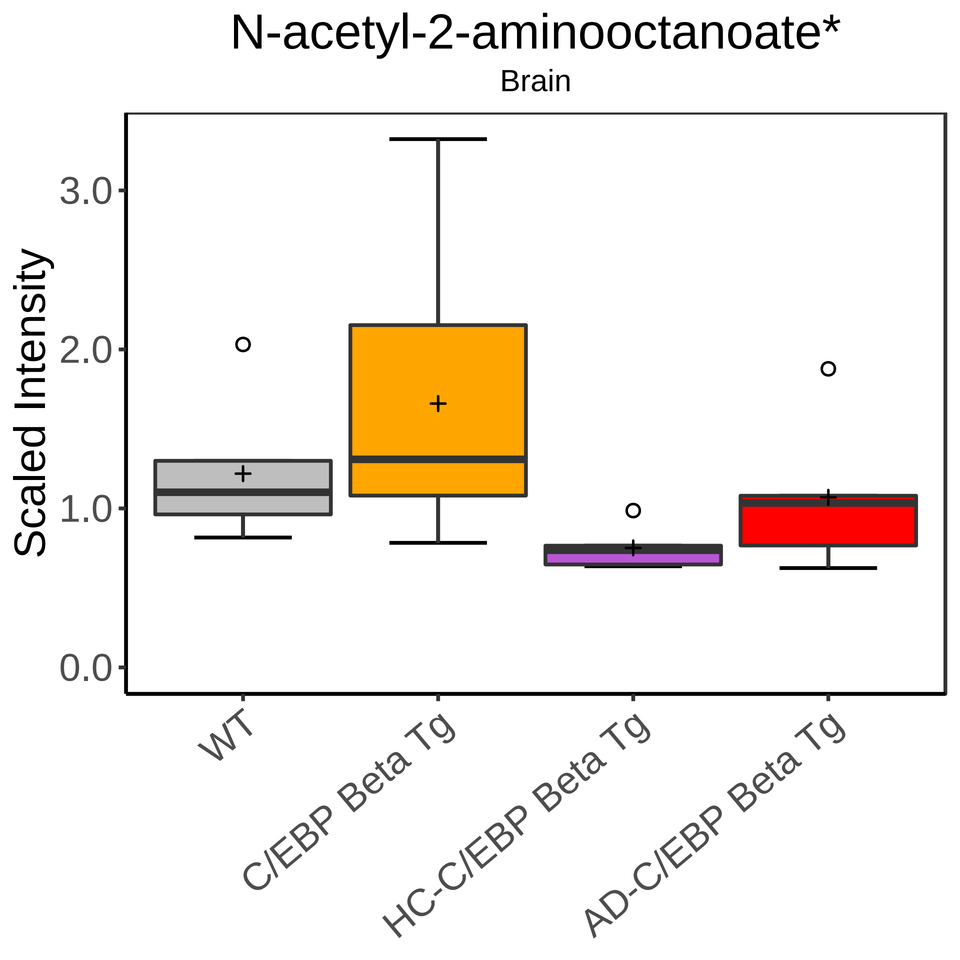
N-acetyl-2-aminooctanoate*
Brain
Scaled Intensity
3.0
2.0
1.0
0.0
WT
C/EBP Beta Tg
HC-C/EBP Beta Tg
AD-C/EBP Beta Tg

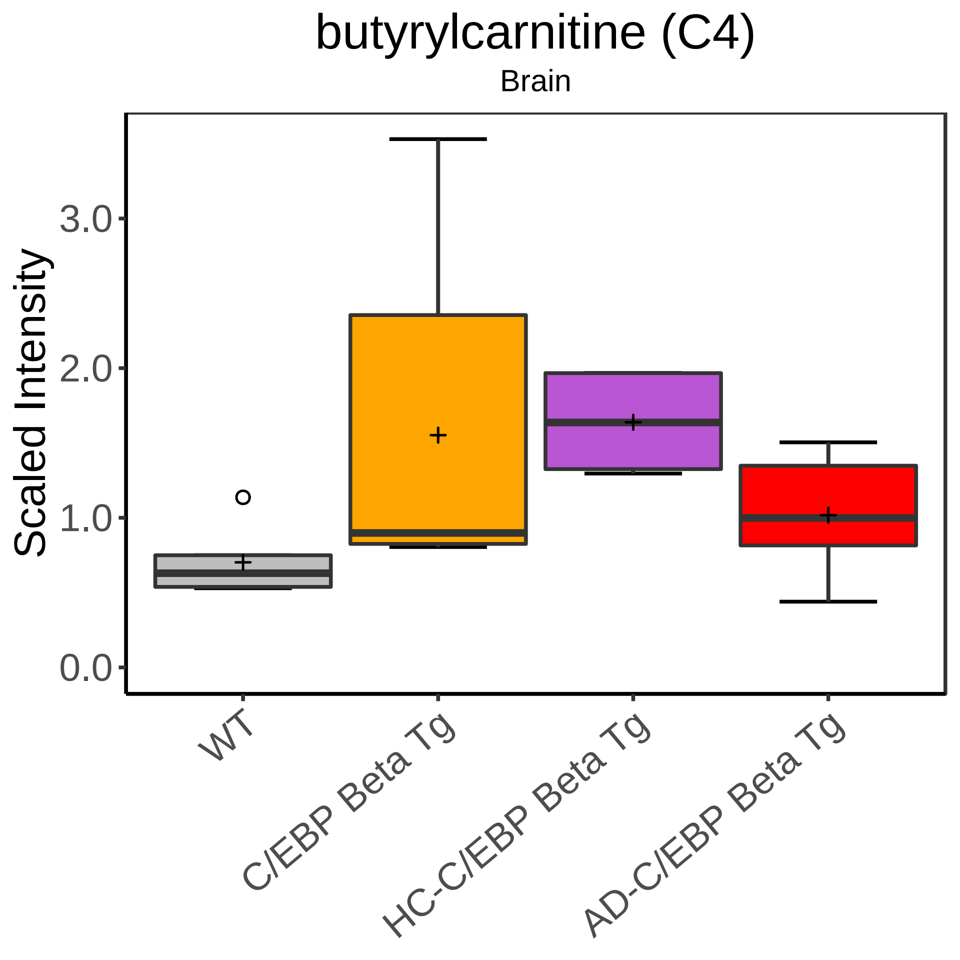

butyrylcarnitine (C4)
Brain
Scaled Intensity
0.0
1.0
2.0
3.0
WT
C/EBP Beta Tg
HC-C/EBP Beta Tg
AD-C/EBP Beta Tg

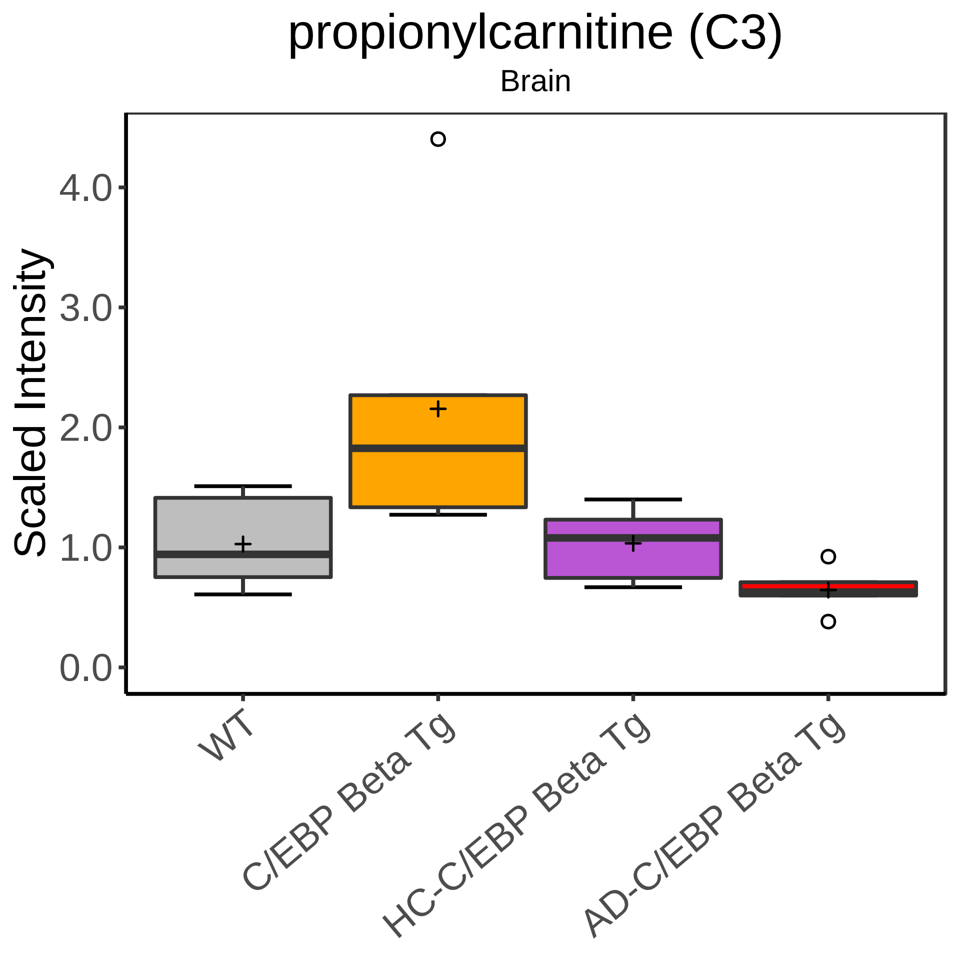
propionylcarnitine (C3)
Brain
Scaled Intensity
4.0
3.0
2.0
1.0
0.0
WT
C/EBP Beta Tg
HC-C/EBP Beta Tg
AD-C/EBP Beta Tg

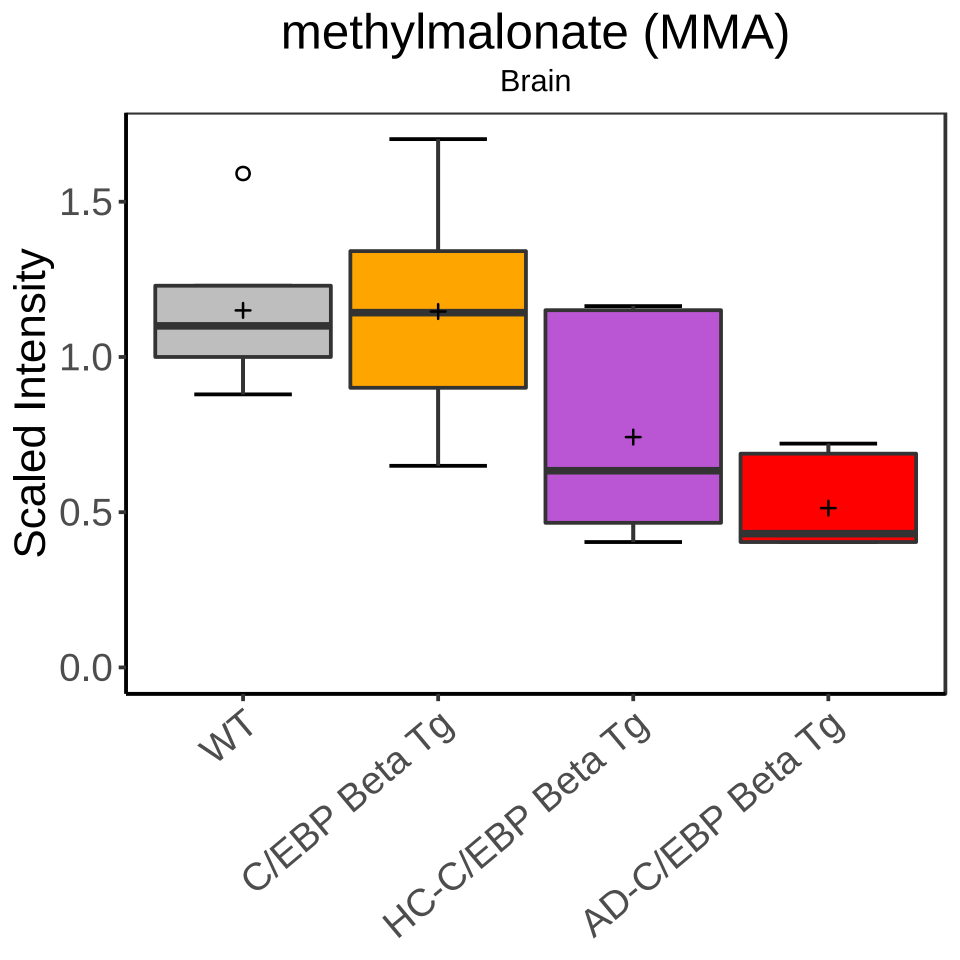
methylmalonate (MMA)
Brain
Scaled Intensity
0.0
0.5
1.0
1.5
WT
C/EBP Beta Tg
HC-C/EBP Beta Tg
AD-C/EBP Beta Tg

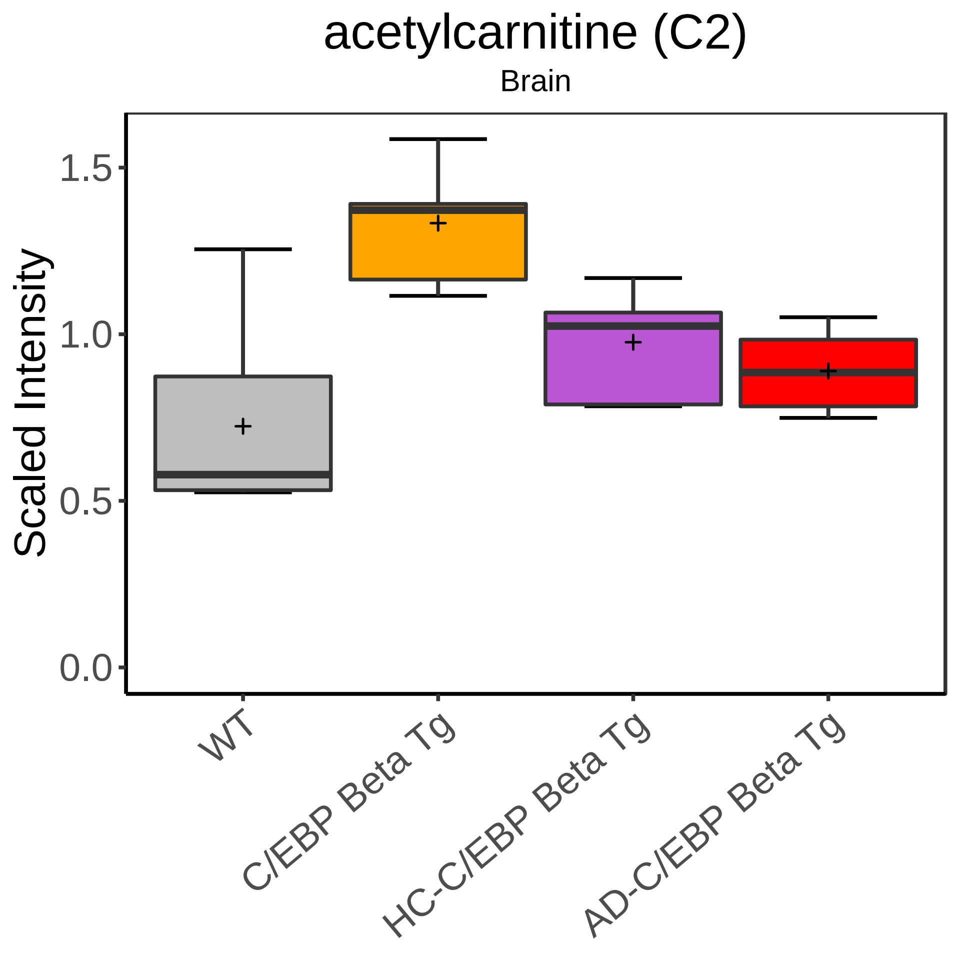
acetylcarnitine (C2)
Brain
Scaled Intensity
1.5
1.0
0.5
0.0
WT
C/EBP Beta Tg
HC-C/EBP Beta Tg
AD-C/EBP Beta Tg

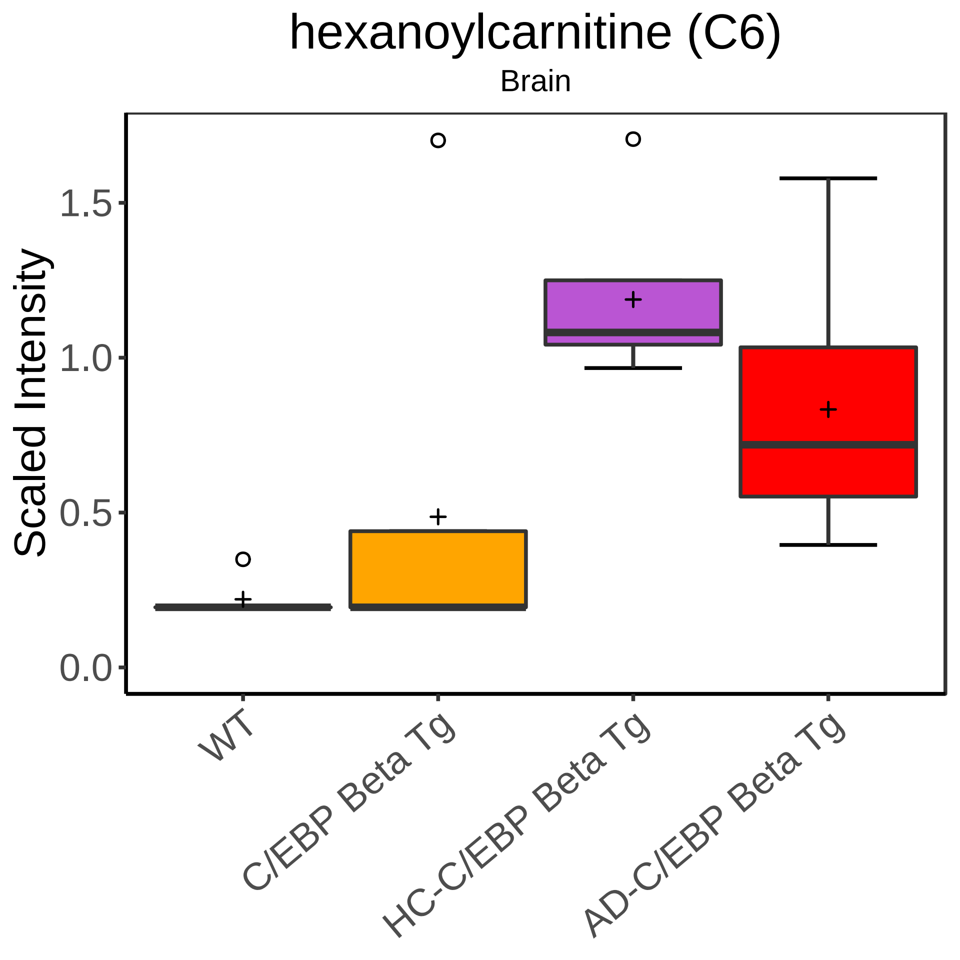
hexanoylcarnitine (C6)
Brain
Scaled Intensity
0.0
0.5
1.0
1.5
WT
C/EBP Beta Tg
HC-C/EBP Beta Tg
AD-C/EBP Beta Tg

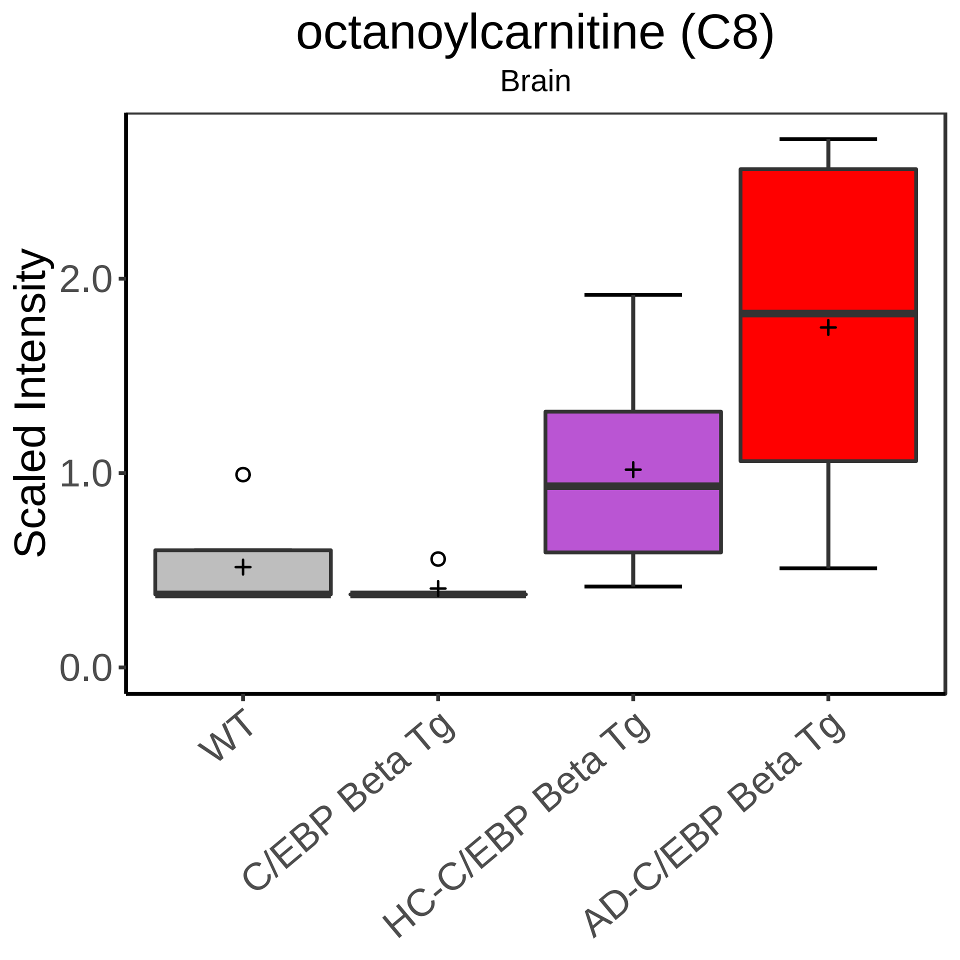

octanoylcarnitine (C8)
Brain
Scaled Intensity
2.0
1.0
0.0
WT
C/EBP Beta Tg
HC-C/EBP Beta Tg
AD-C/EBP Beta Tg

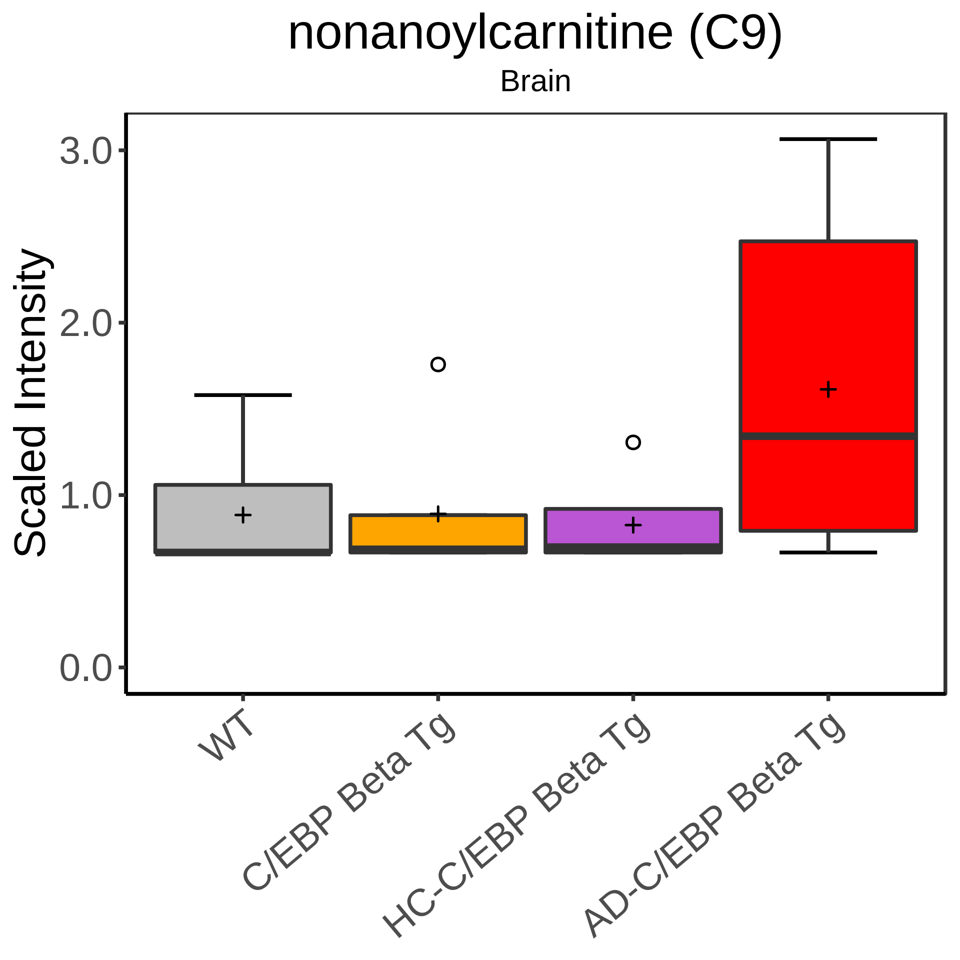

nonanoylcarnitine (C9)
Brain
Scaled Intensity
3.0
2.0
1.0
0.0
WT
C/EBP Beta Tg
HC-C/EBP Beta Tg
AD-C/EBP Beta Tg

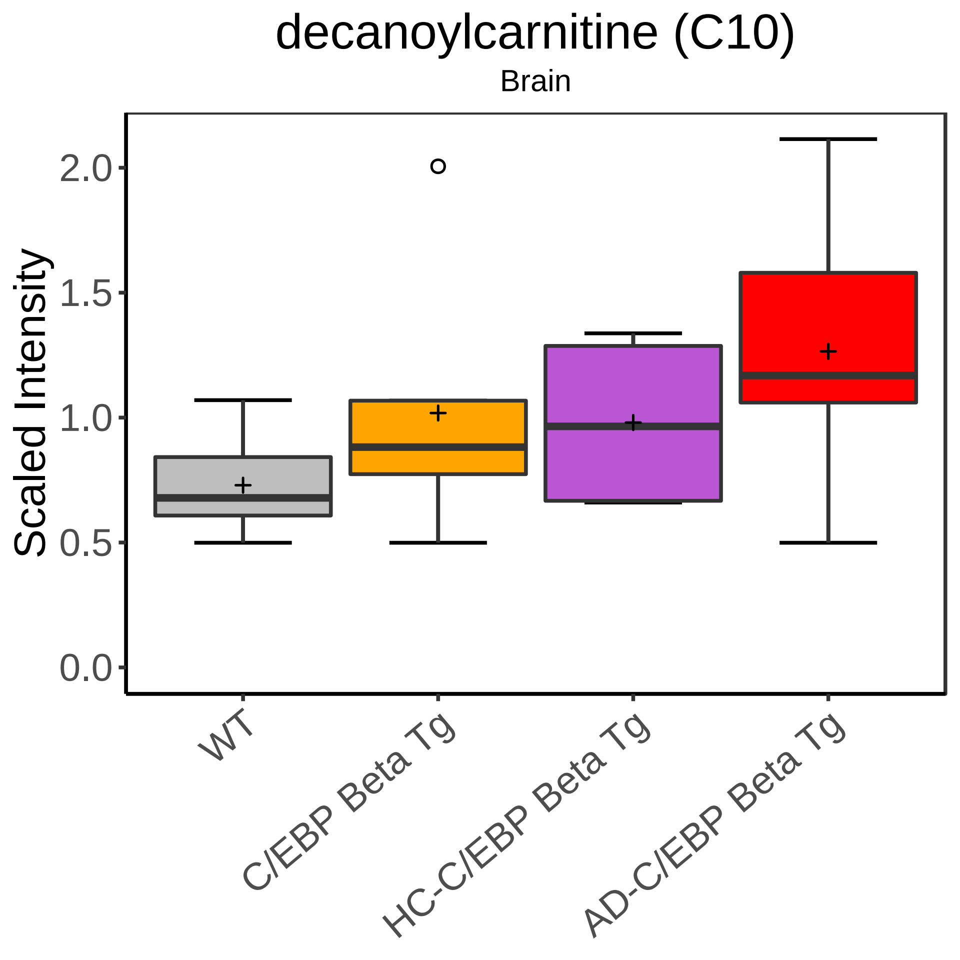

decanoylcarnitine (C10)
Brain
Scaled Intensity
2.0
1.5
1.0
0.5
0.0
WT
C/EBP Beta Tg
HC-C/EBP Beta Tg
AD-C/EBP Beta Tg

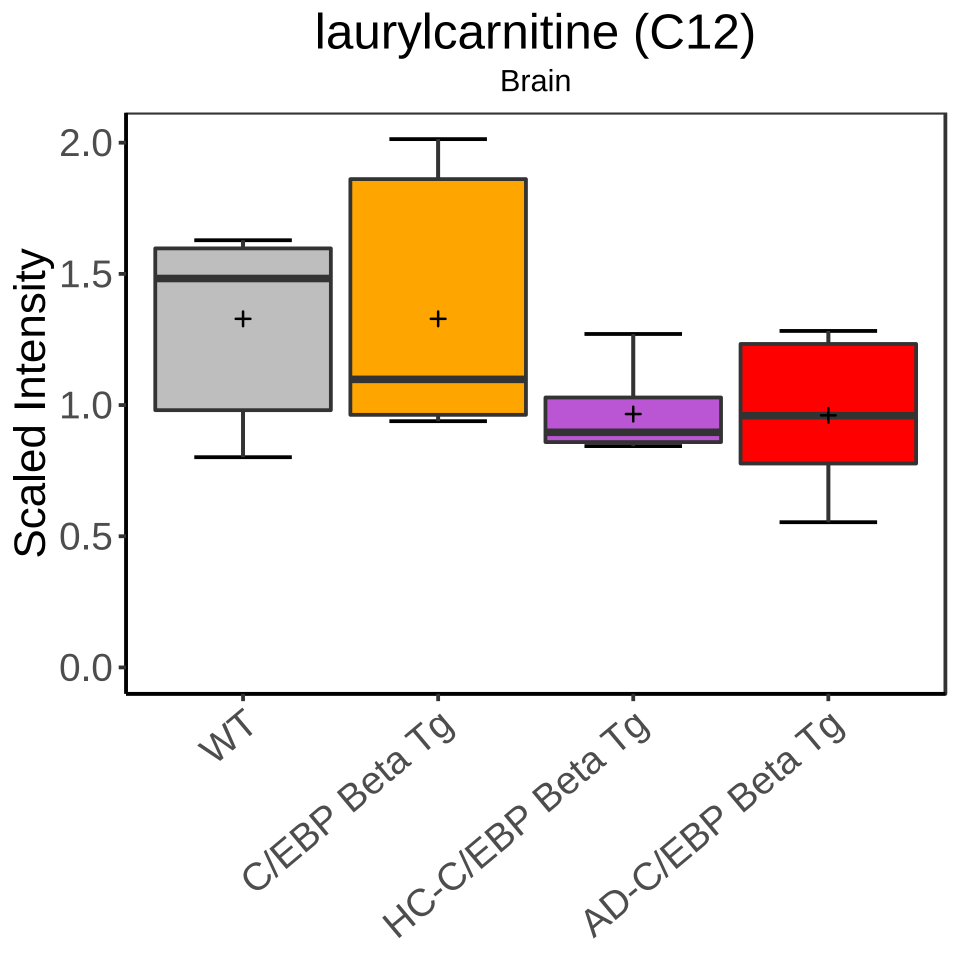

laurylcarnitine (C12)
Brain
Scaled Intensity
2.0
1.5
1.0
0.5
0.0
WT
C/EBP Beta Tg
HC-C/EBP Beta Tg
AD-C/EBP Beta Tg

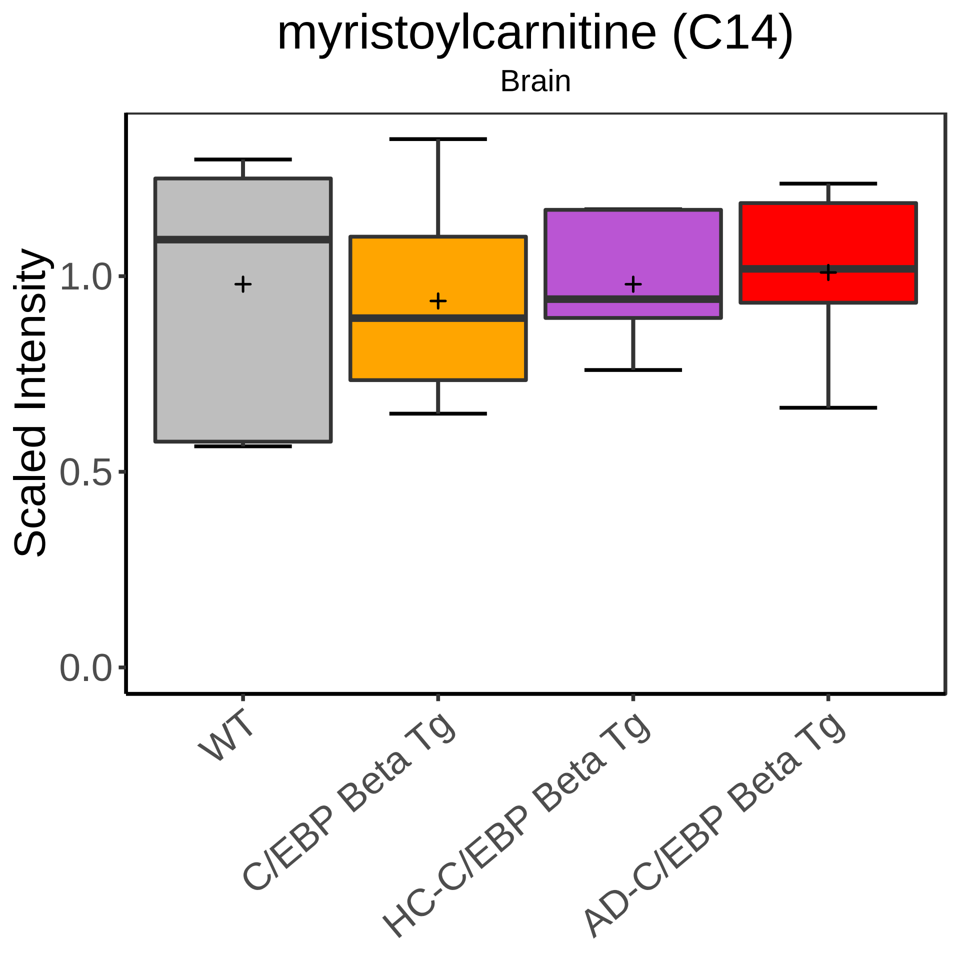
myristoylcarnitine (C14)
Brain
Scaled Intensity
0.0
0.5
1.0
WT
C/EBP Beta Tg
HC-C/EBP Beta Tg
AD-C/EBP Beta Tg

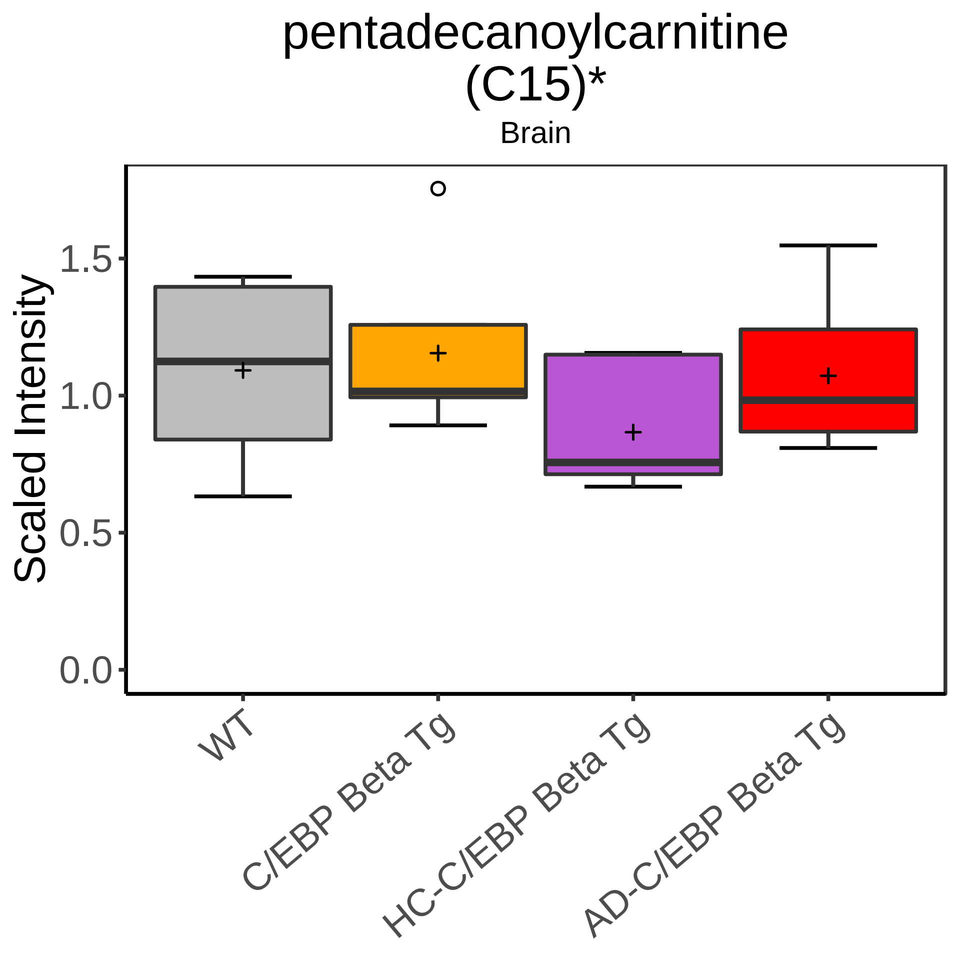
pentadecanoylcarnitine
(C15)*
Brain
Scaled Intensity
0.0
0.5
1.0
1.5
WT
C/EBP Beta Tg
HC-C/EBP Beta Tg
AD-C/EBP Beta Tg

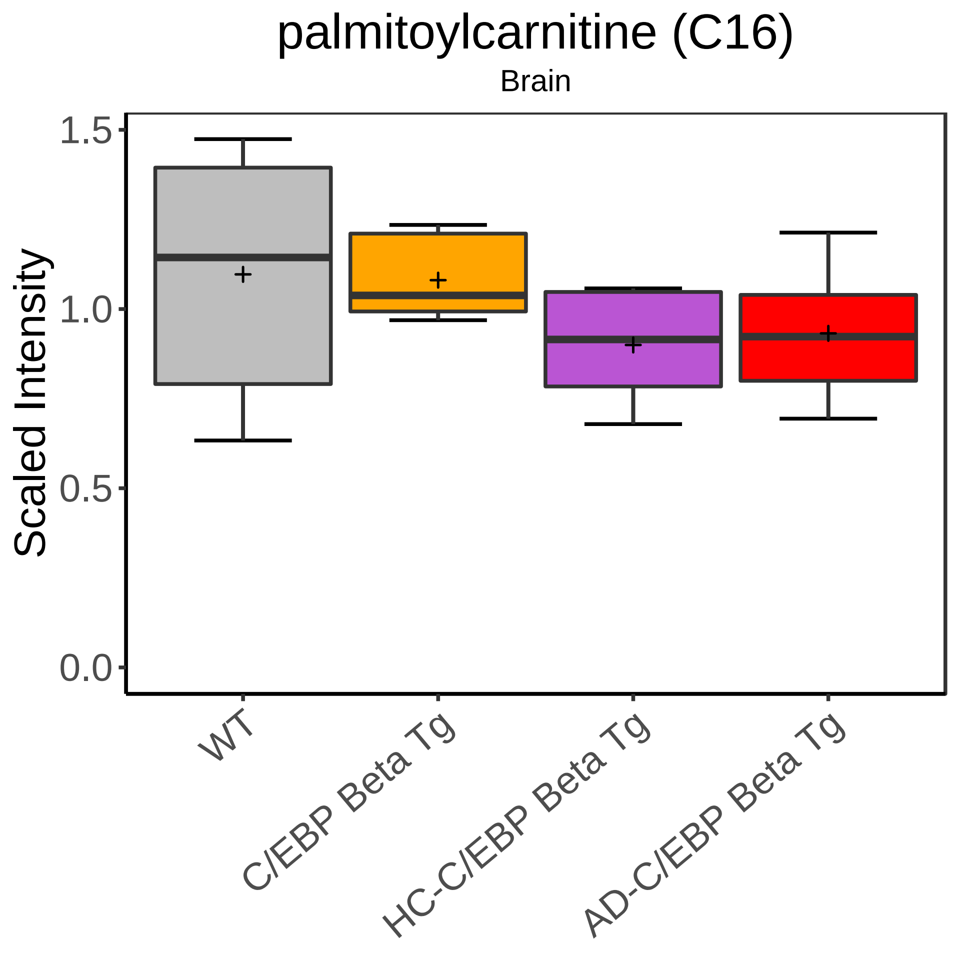

palmitoylcarnitine (C16)
Brain
Scaled Intensity
1.5
1.0
0.5
0.0
WT
C/EBP Beta Tg
HC-C/EBP Beta Tg
AD-C/EBP Beta Tg

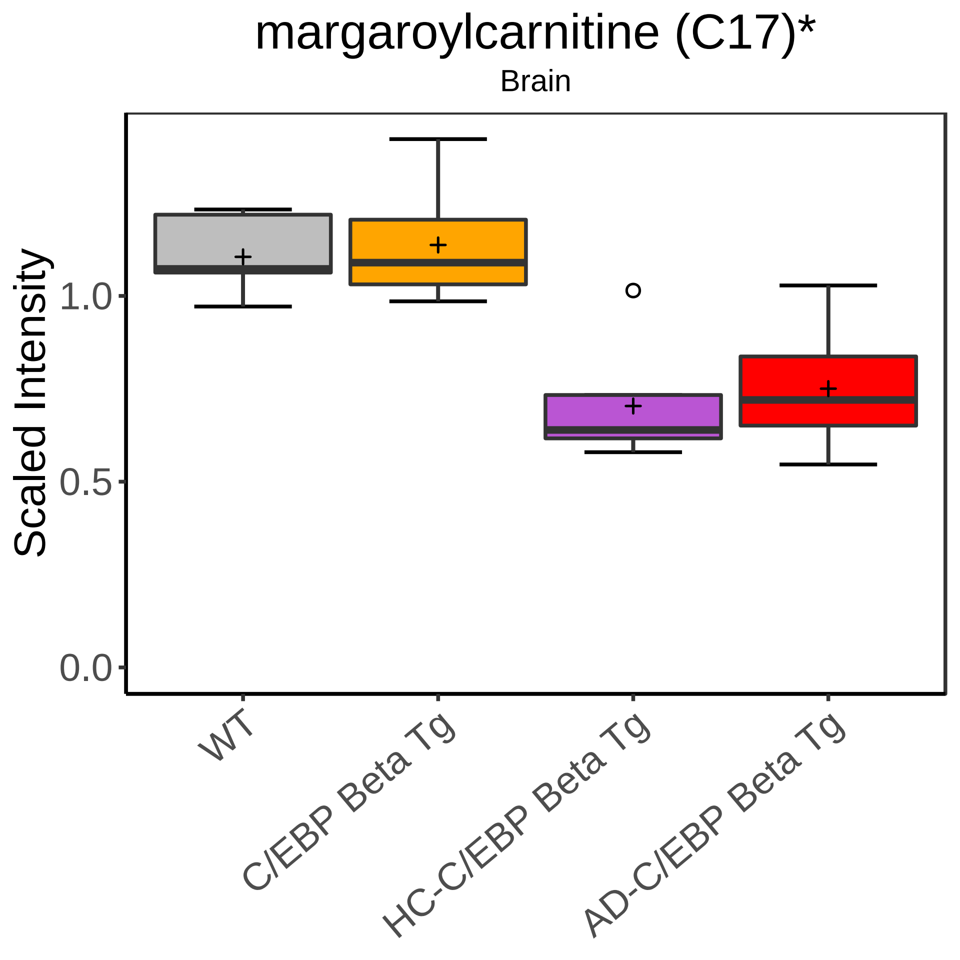

margaroylcarnitine (C17)*
Brain
Scaled Intensity
0.0
0.5
1.0
WT
C/EBP Beta Tg
HC-C/EBP Beta Tg
AD-C/EBP Beta Tg

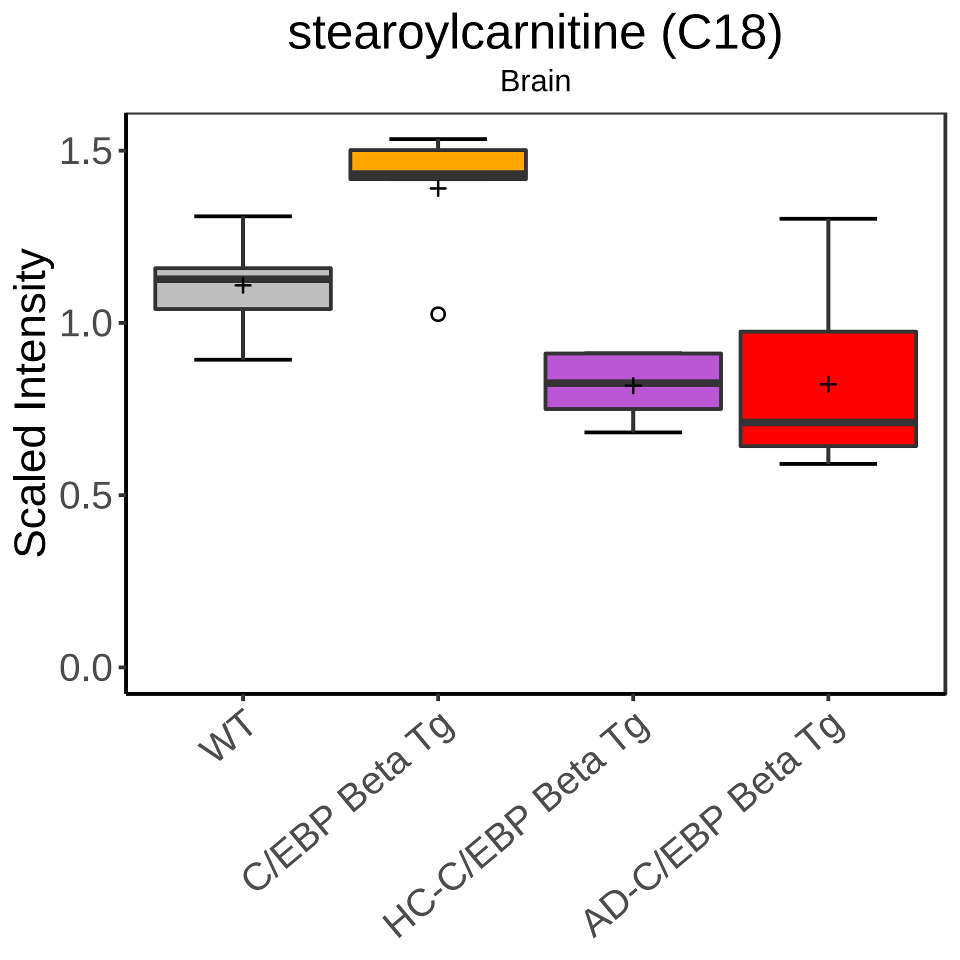
stearoylcarnitine (C18)
Brain
Scaled Intensity
1.5
1.0
0.5
0.0
WT
C/EBP Beta Tg
HC-C/EBP Beta Tg
AD-C/EBP Beta Tg

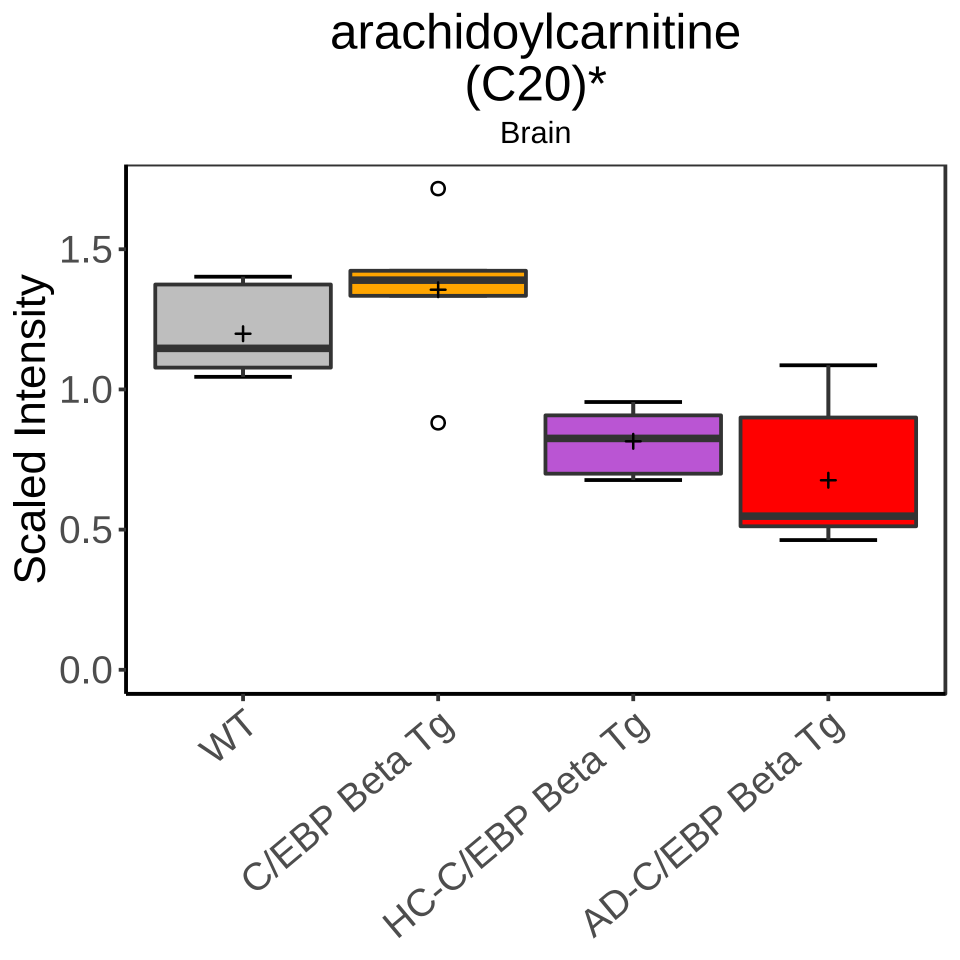

arachidoylcarnitine
(C20)*
Brain
Scaled Intensity
1.5
1.0
0.5
0.0
WT
C/EBP Beta Tg
HC-C/EBP Beta Tg
AD-C/EBP Beta Tg

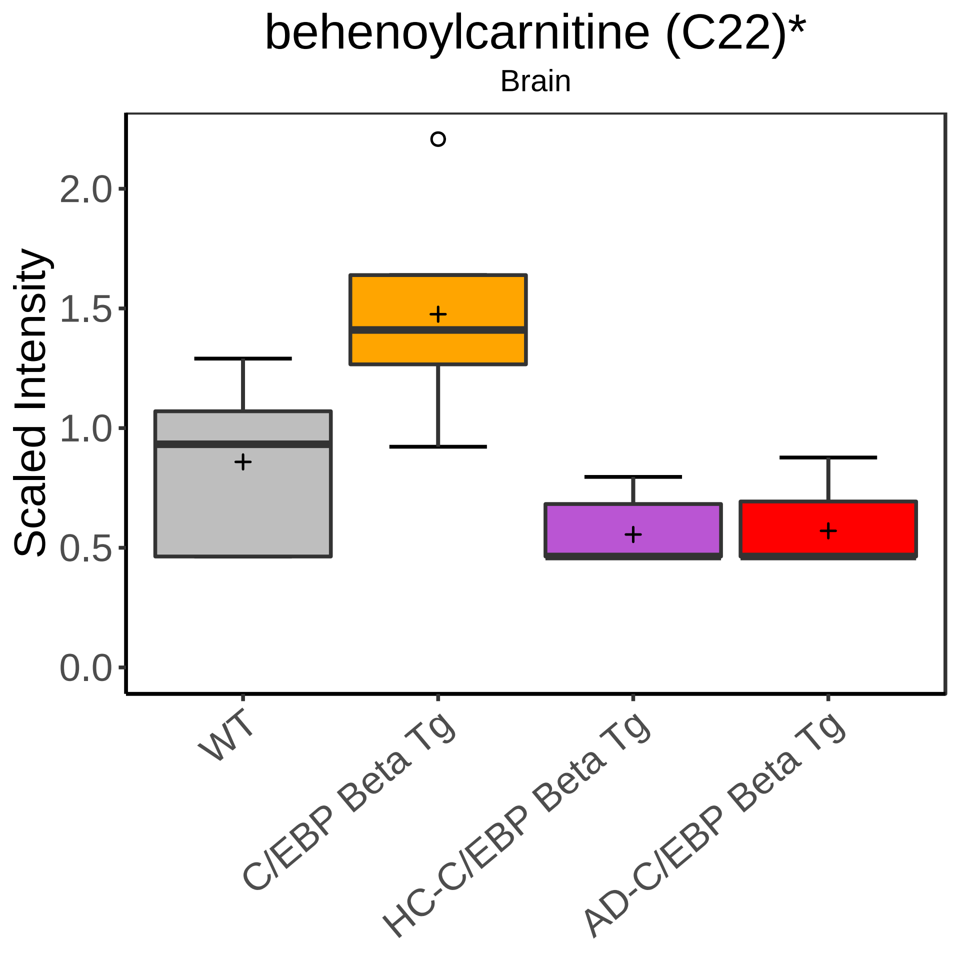

behenoylcarnitine (C22)*
Brain
Scaled Intensity
0.0
0.5
1.0
1.5
2.0
WT
C/EBP Beta Tg
HC-C/EBP Beta Tg
AD-C/EBP Beta Tg

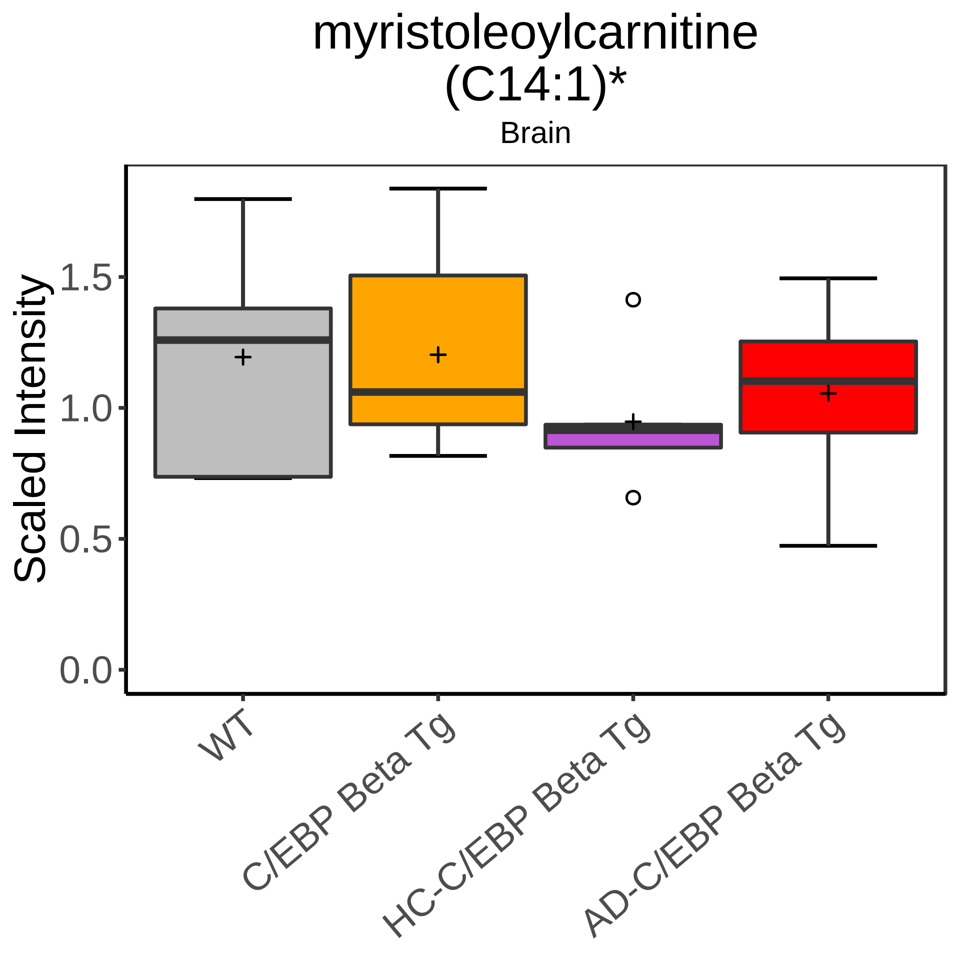

myristoleoylcarnitine
(C14:1)*
Brain
Scaled Intensity
0.0
0.5
1.0
1.5
WT
C/EBP Beta Tg
HC-C/EBP Beta Tg
AD-C/EBP Beta Tg

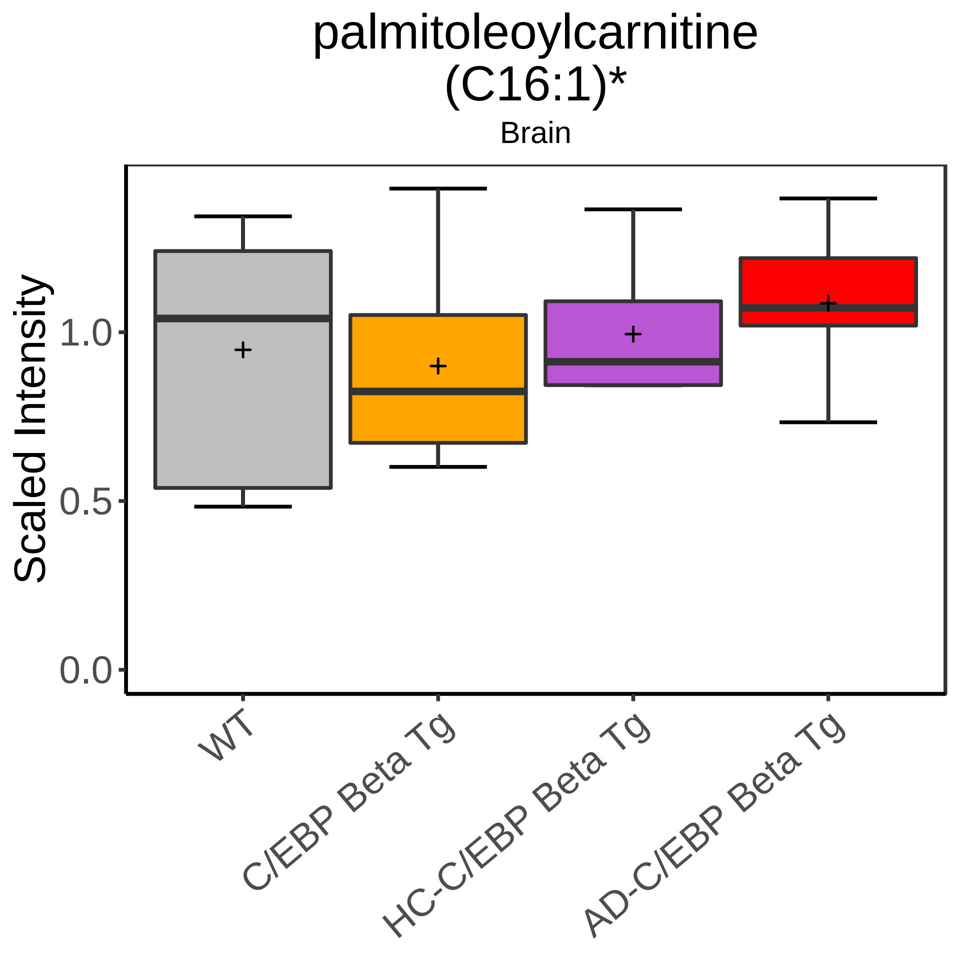
palmitoleoylcarnitine
(C16:1)*
Brain
Scaled Intensity
1.0
0.5
0.0
WT
C/EBP Beta Tg
HC-C/EBP Beta Tg
AD-C/EBP Beta Tg

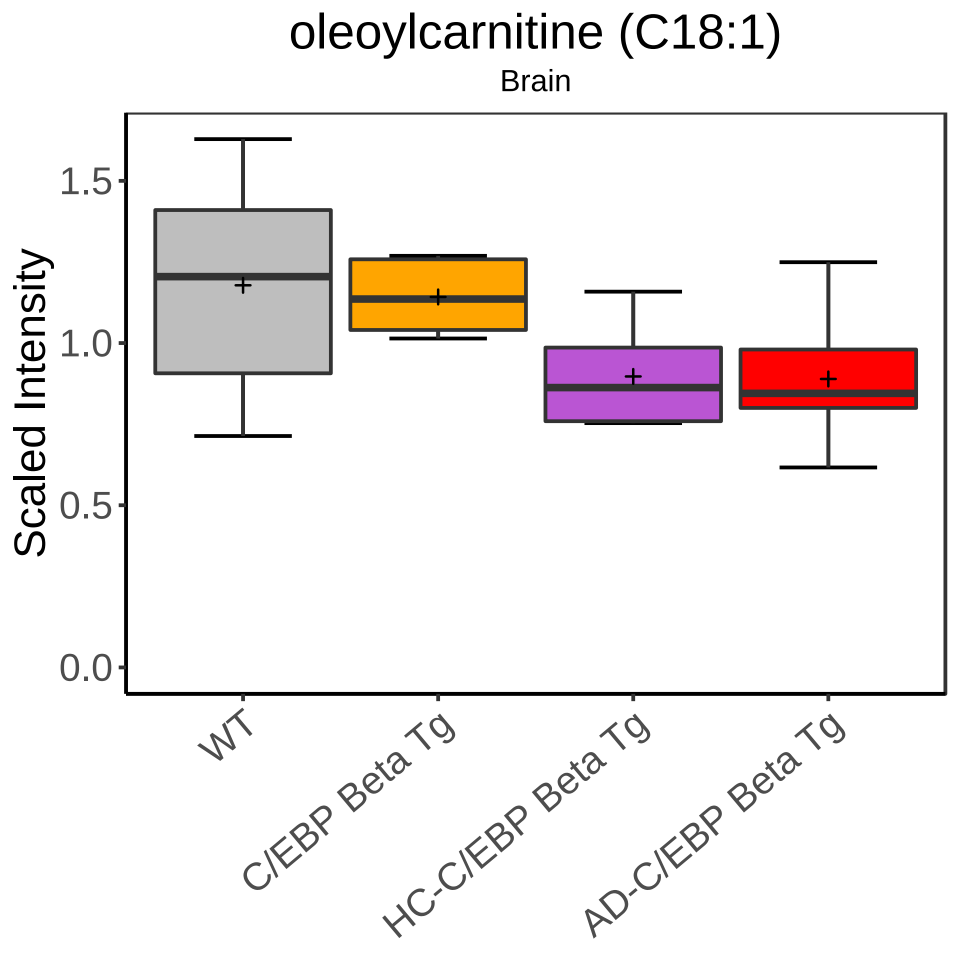
oleoylcarnitine (C18:1)
Brain
Scaled Intensity
1.5
1.0
0.5
0.0
WT
C/EBP Beta Tg
HC-C/EBP Beta Tg
AD-C/EBP Beta Tg

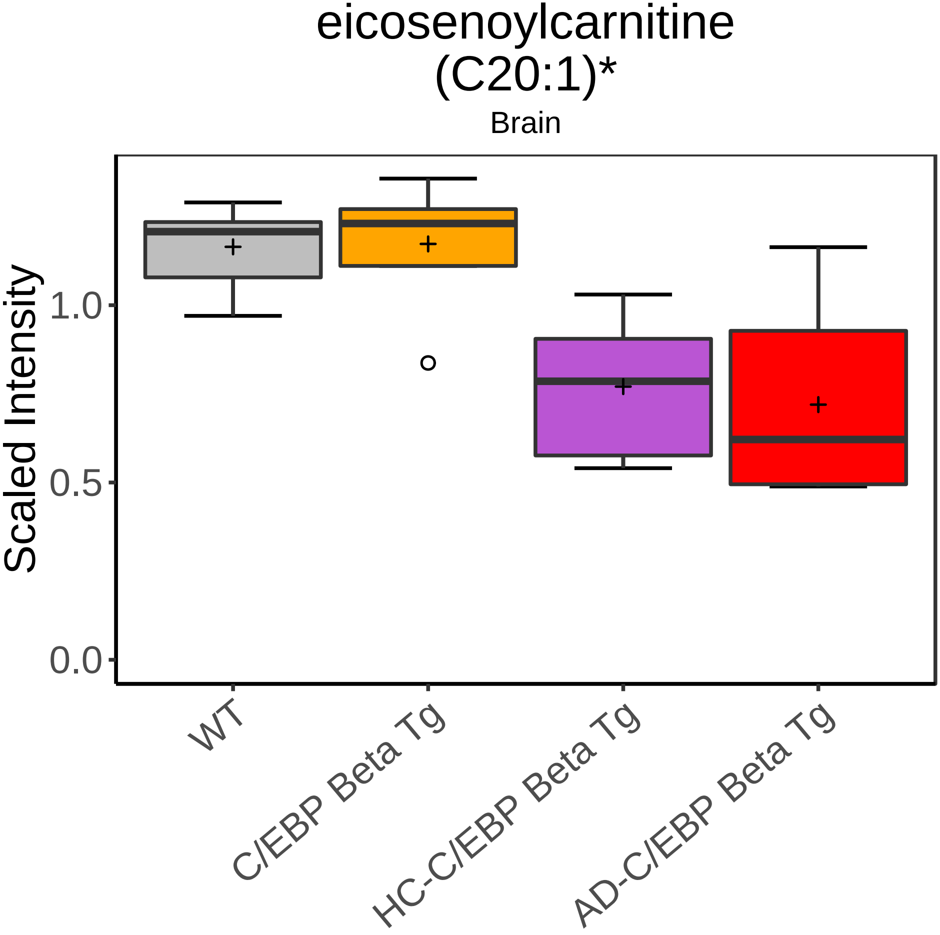

eicosenoylcarnitine
(C20:1)*
Brain
Scaled Intensity
1.0
0.5
0.0
WT
C/EBP Beta Tg
HC-C/EBP Beta Tg
AD-C/EBP Beta Tg

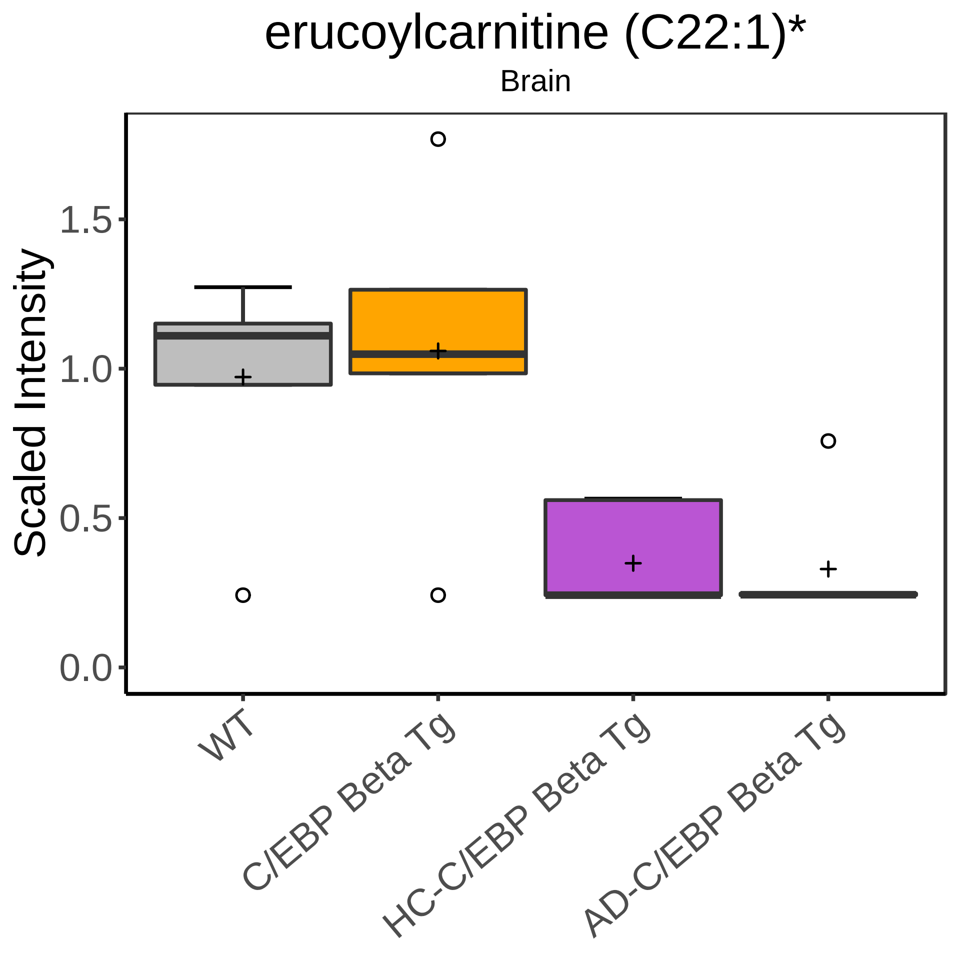

erucoylcarnitine (C22:1)*
Brain
Scaled Intensity
0.0
0.5
1.0
1.5
WT
C/EBP Beta Tg
HC-C/EBP Beta Tg
AD-C/EBP Beta Tg

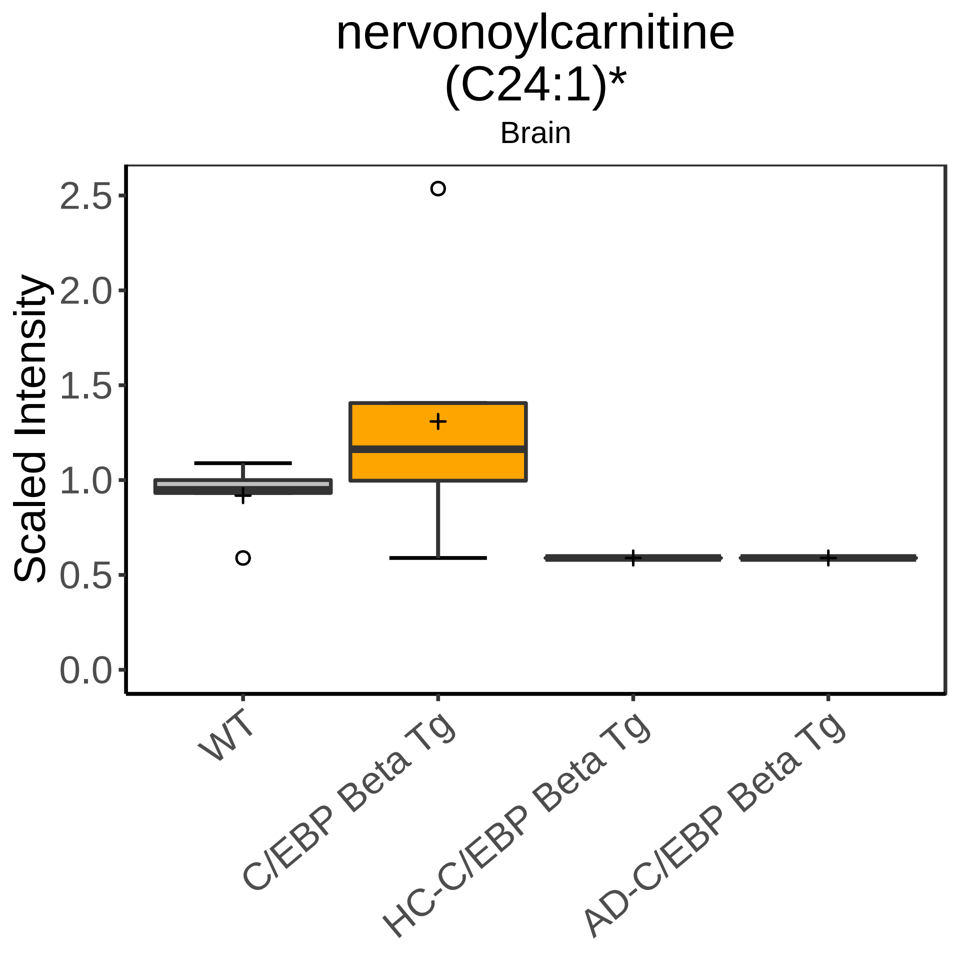

nervonoylcarnitine
(C24:1)*
Brain
Scaled Intensity
2.5
2.0
1.5
1.0
0.5
0.0
WT
C/EBP Beta Tg
HC-C/EBP Beta Tg
AD-C/EBP Beta Tg

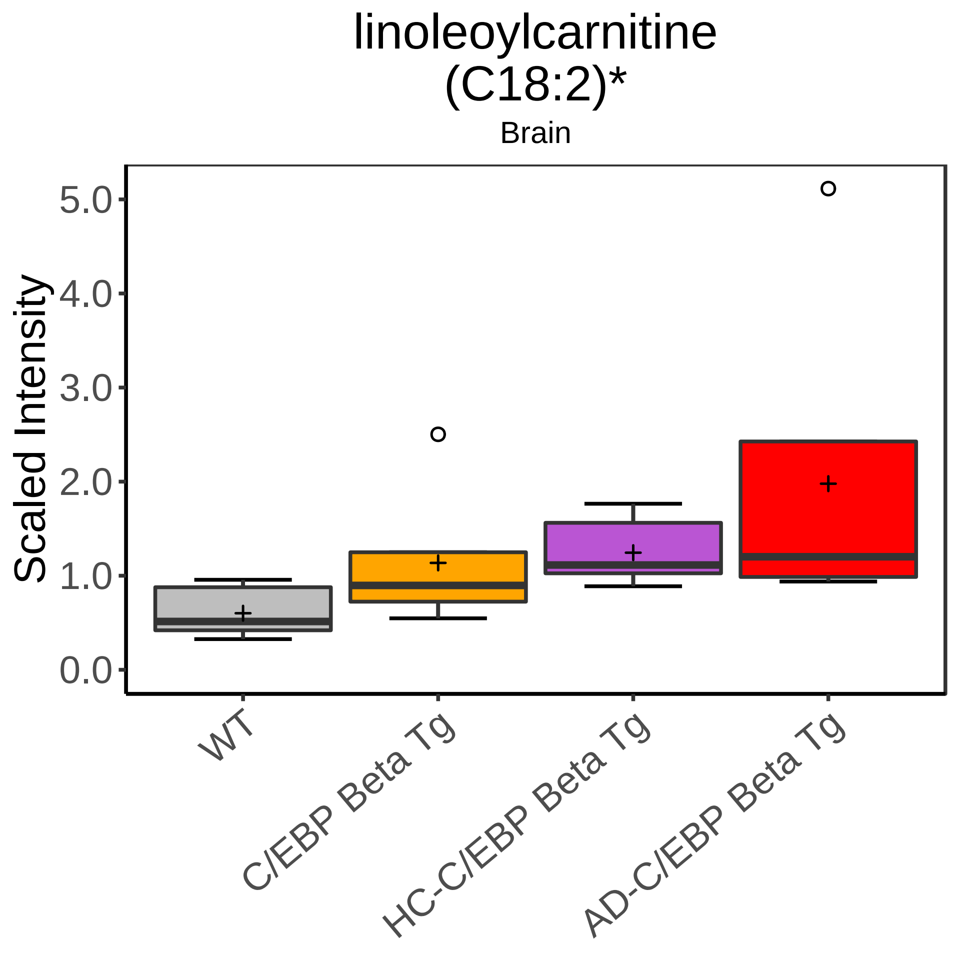
linoleoylcarnitine (C18:2)*
Brain
Scaled Intensity
5.0
4.0
3.0
2.0
1.0
0.0
WT
C/EBP Beta Tg
HC-C/EBP Beta Tg
AD-C/EBP Beta Tg

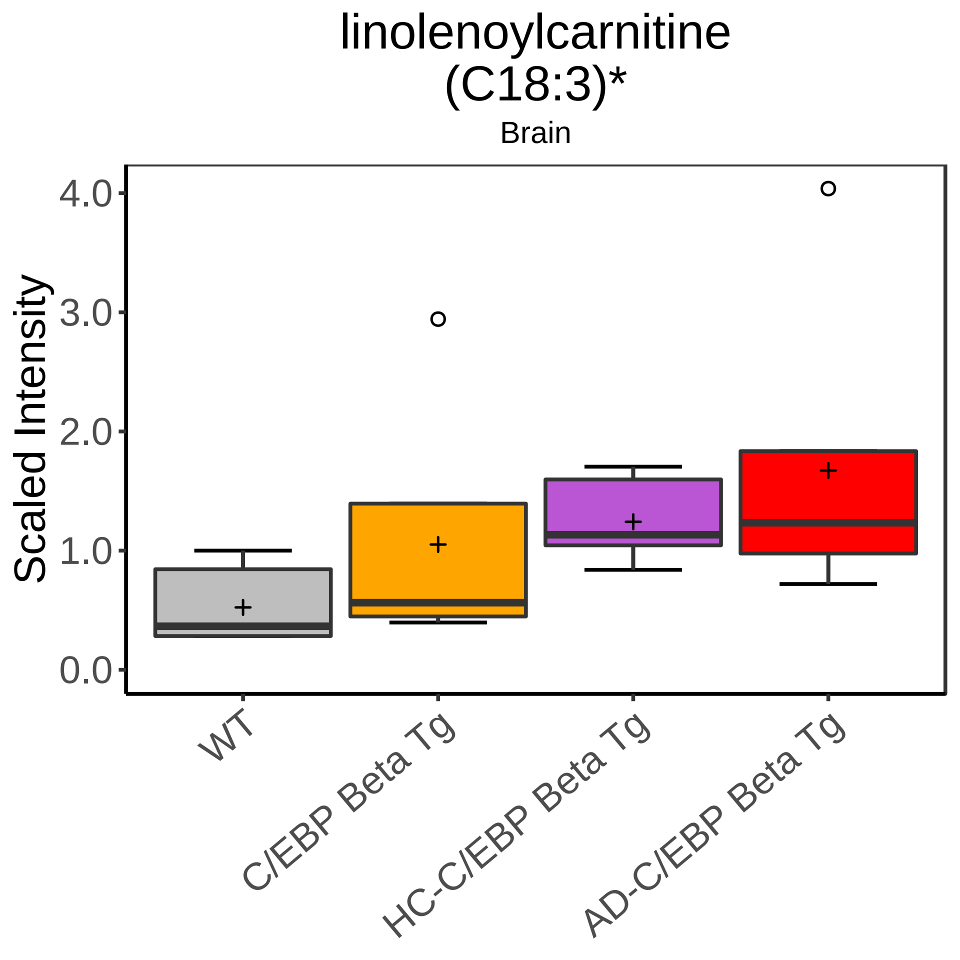

linolenoylcarnitine
(C18:3)*
Brain
Scaled Intensity
4.0
3.0
2.0
1.0
0.0
WT
C/EBP Beta Tg
HC-C/EBP Beta Tg
AD-C/EBP Beta Tg

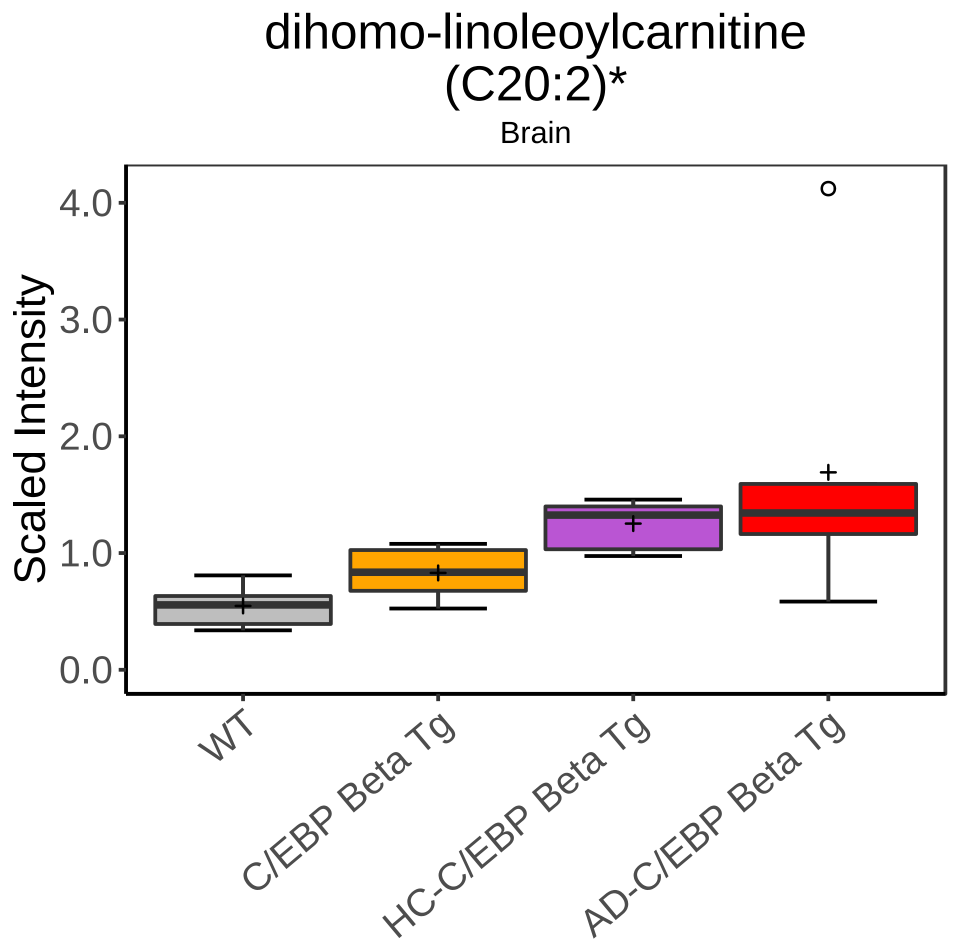
dihomo-linoleoylcarnitine
(C20:2)*
Brain
Scaled Intensity
4.0
3.0
2.0
1.0
0.0
WT
C/EBP Beta Tg
HC-C/EBP Beta Tg
AD-C/EBP Beta Tg

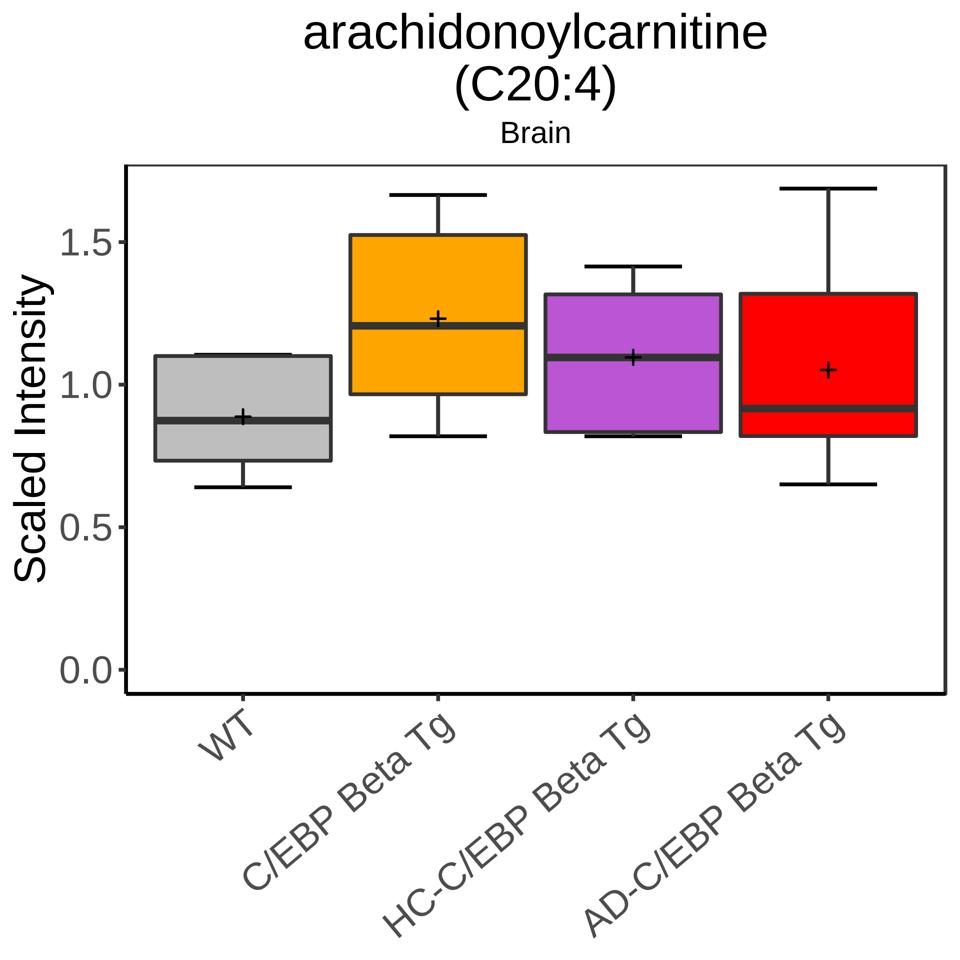
arachidonoylcarnitine
(C20:4)
Brain
Scaled Intensity
1.5
1.0
0.5
0.0
WT
C/EBP Beta Tg
HC-C/EBP Beta Tg
AD-C/EBP Beta Tg

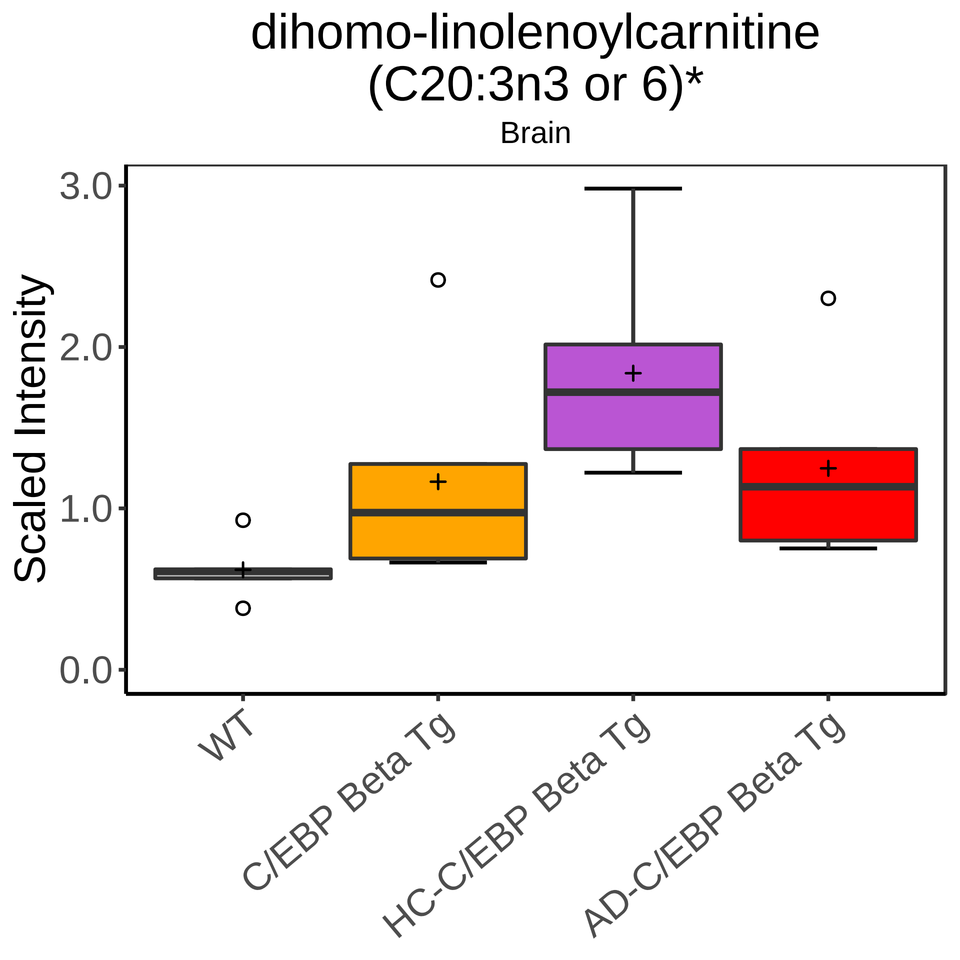
dihomo-linolenoylcarnitine
(C20:3n3 or 6)*
Brain
Scaled Intensity
3.0
2.0
1.0
0.0
WT
C/EBP Beta Tg
HC-C/EBP Beta Tg
AD-C/EBP Beta Tg

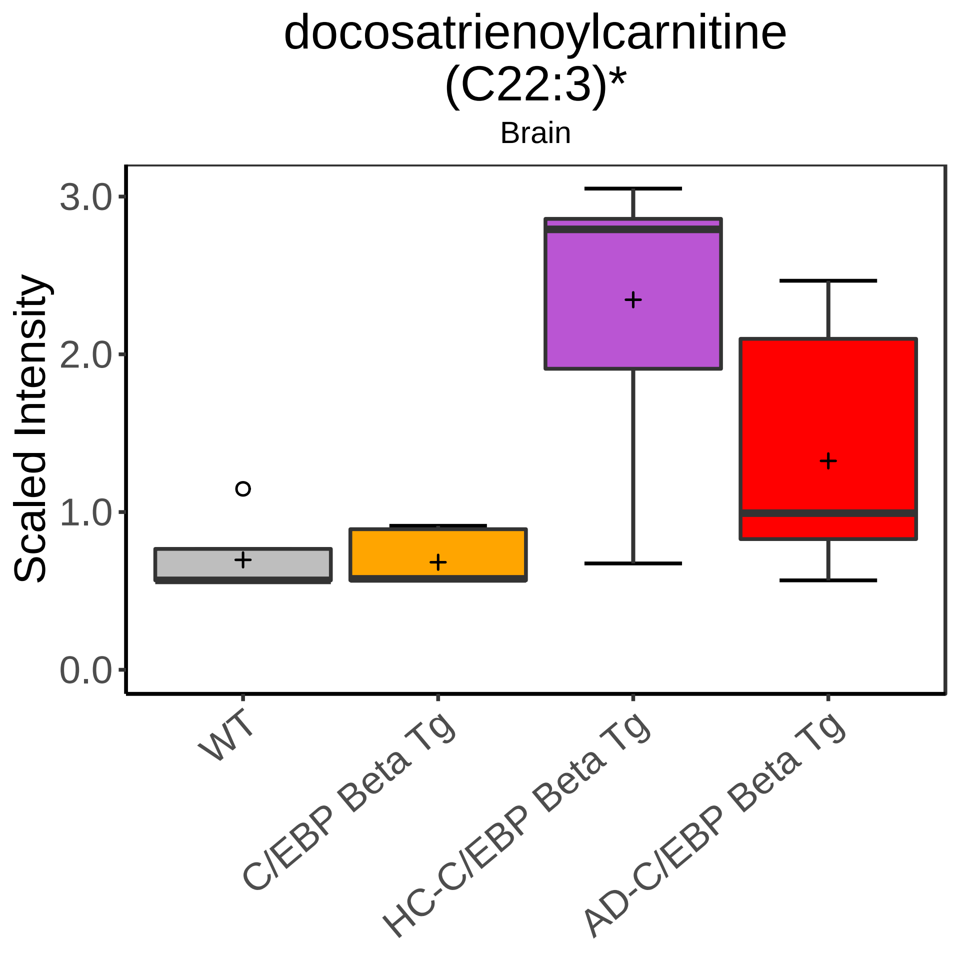

docosatrienoylcarnitine (C22:3)*
Brain
Scaled Intensity
3.0
2.0
1.0
0.0
WT
C/EBP Beta Tg
HC-C/EBP Beta Tg
AD-C/EBP Beta Tg

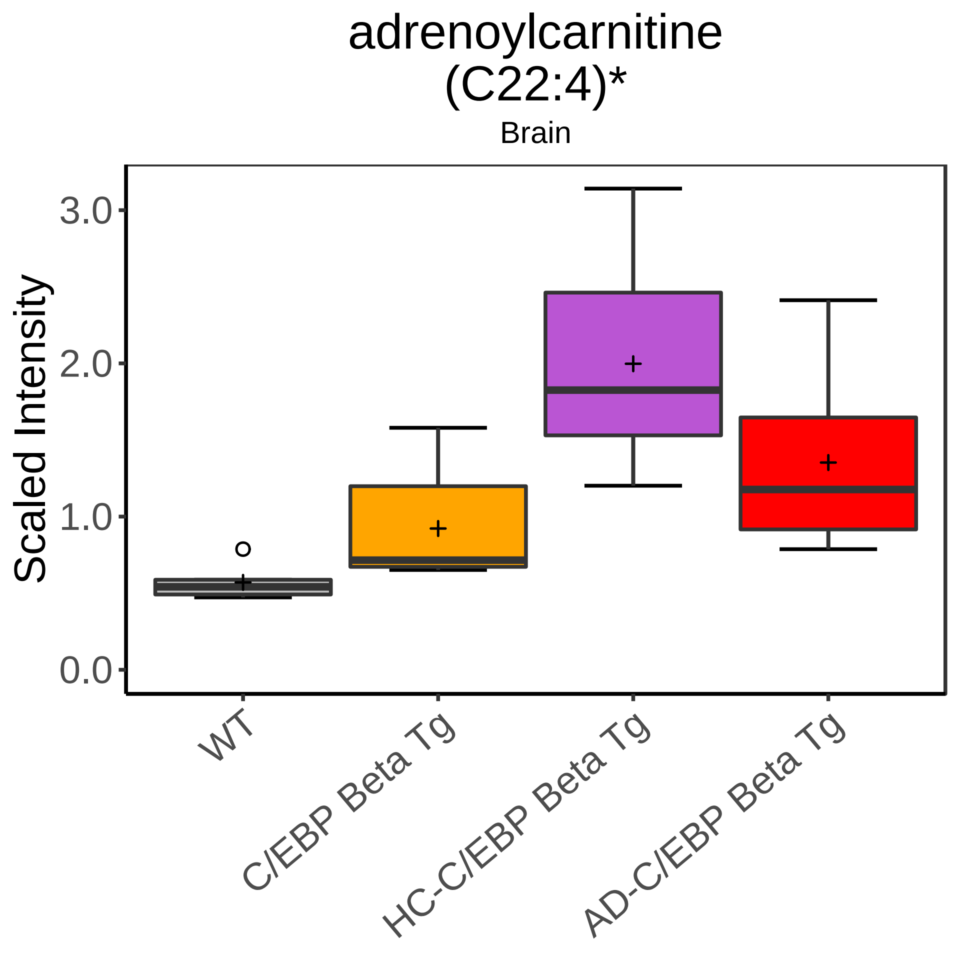

adrenoylcarnitine
(C22:4)*
Brain
Scaled Intensity
3.0
2.0
1.0
0.0
WT
C/EBP Beta Tg
HC-C/EBP Beta Tg
AD-C/EBP Beta Tg

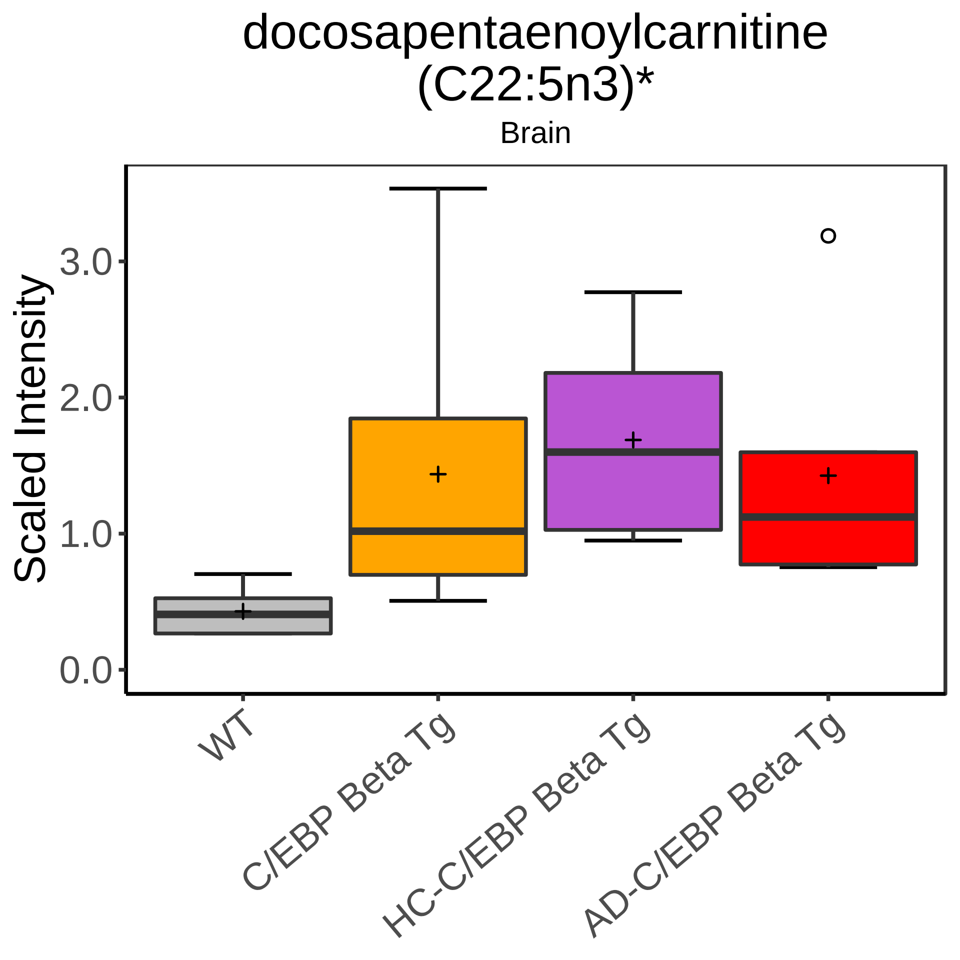
docosapentaenoylcarnitine
(C22:5n3)*
Brain
Scaled Intensity
0.0
1.0
2.0
3.0
WT
C/EBP Beta Tg
HC-C/EBP Beta Tg
AD-C/EBP Beta Tg

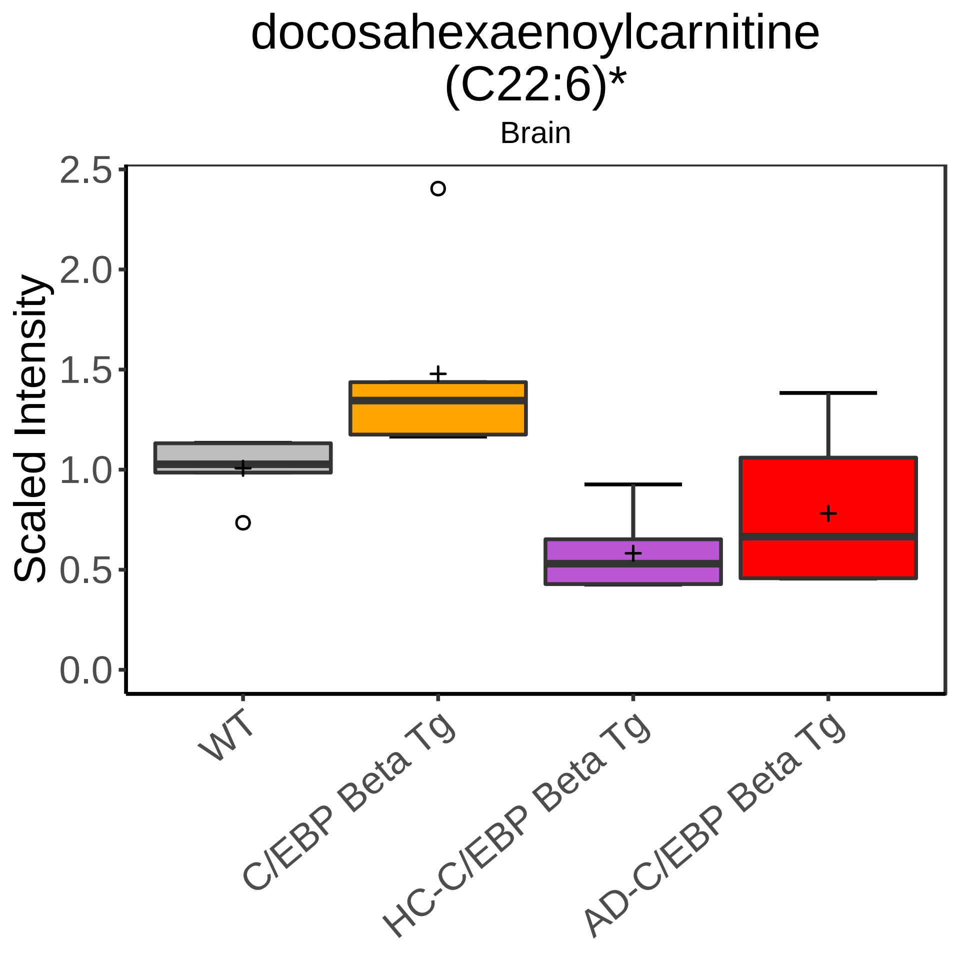

docosahexaenoylcarnitine
(C22:6)*
Brain
Scaled Intensity
2.5
2.0
1.5
1.0
0.5
0.0
WT
C/EBP Beta Tg
HC-C/EBP Beta Tg
AD-C/EBP Beta Tg

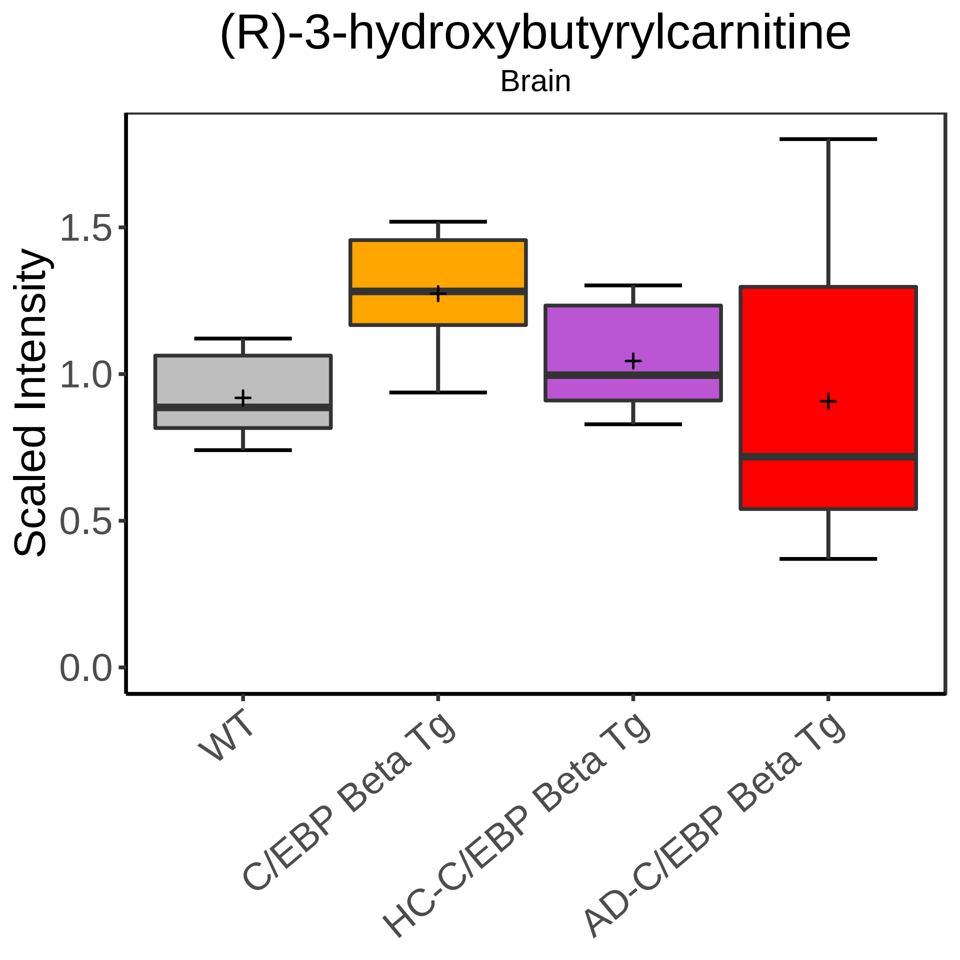

(R)-3-hydroxybutyrylcarnitine
Brain
Scaled Intensity
0.0
0.5
1.0
1.5
WT
C/EBP Beta Tg
HC-C/EBP Beta Tg
AD-C/EBP Beta Tg

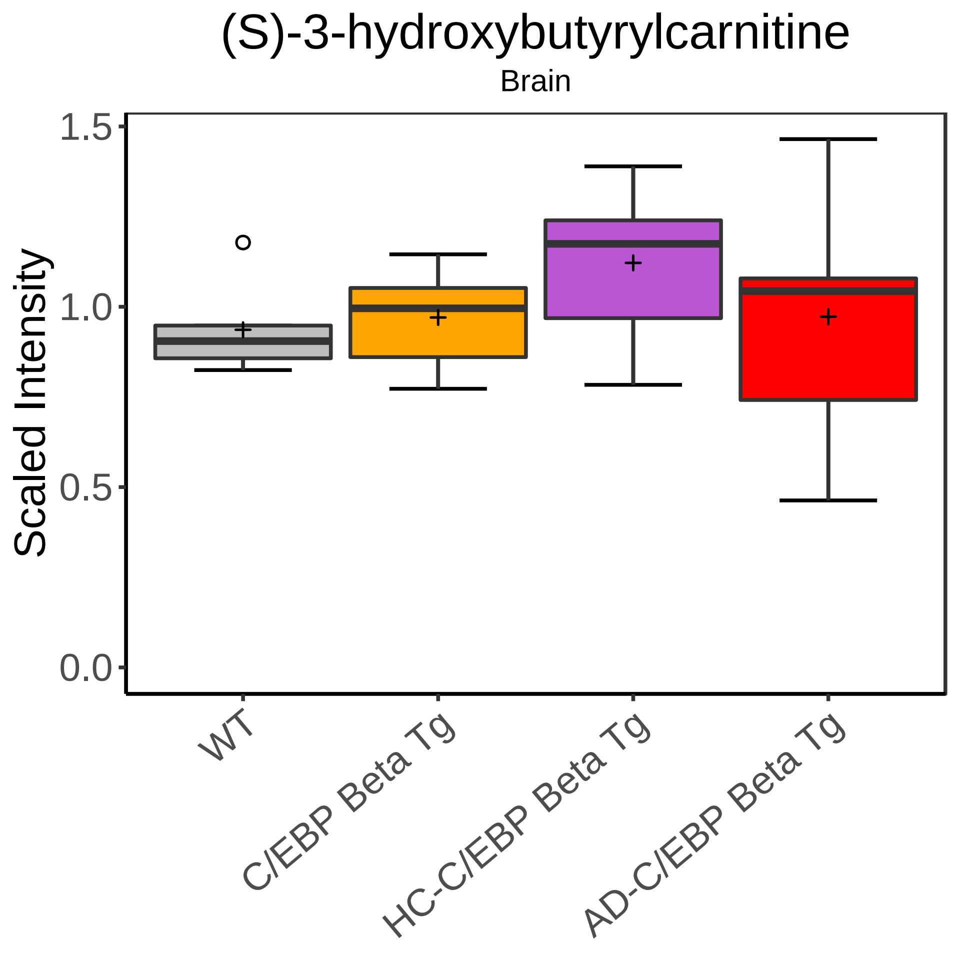

(S)-3-hydroxybutyrylcarnitine
Brain
Scaled Intensity
1.5
1.0
0.5
0.0
WT
C/EBP Beta Tg
HC-C/EBP Beta Tg
AD-C/EBP Beta Tg

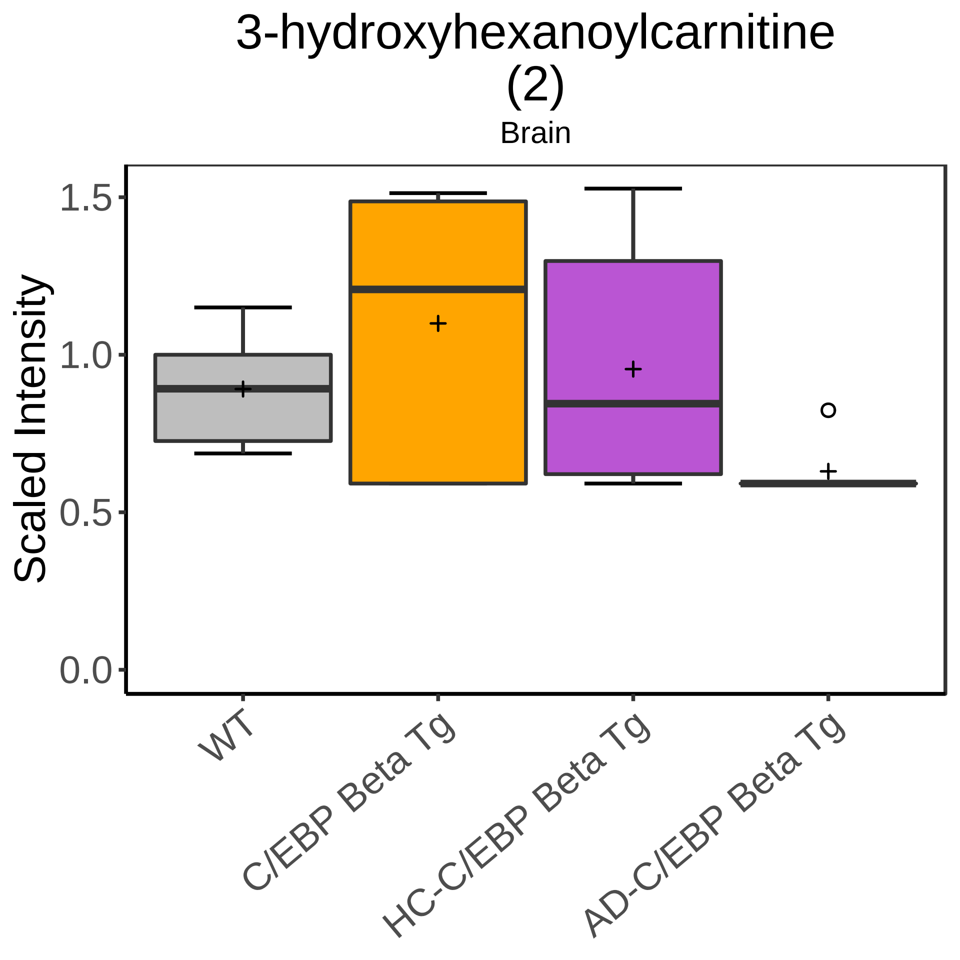

3-hydroxyhexanoylcarnitine (2)
Brain
Scaled Intensity
1.5
1.0
0.5
0.0
WT
C/EBP Beta Tg
HC-C/EBP Beta Tg
AD-C/EBP Beta Tg

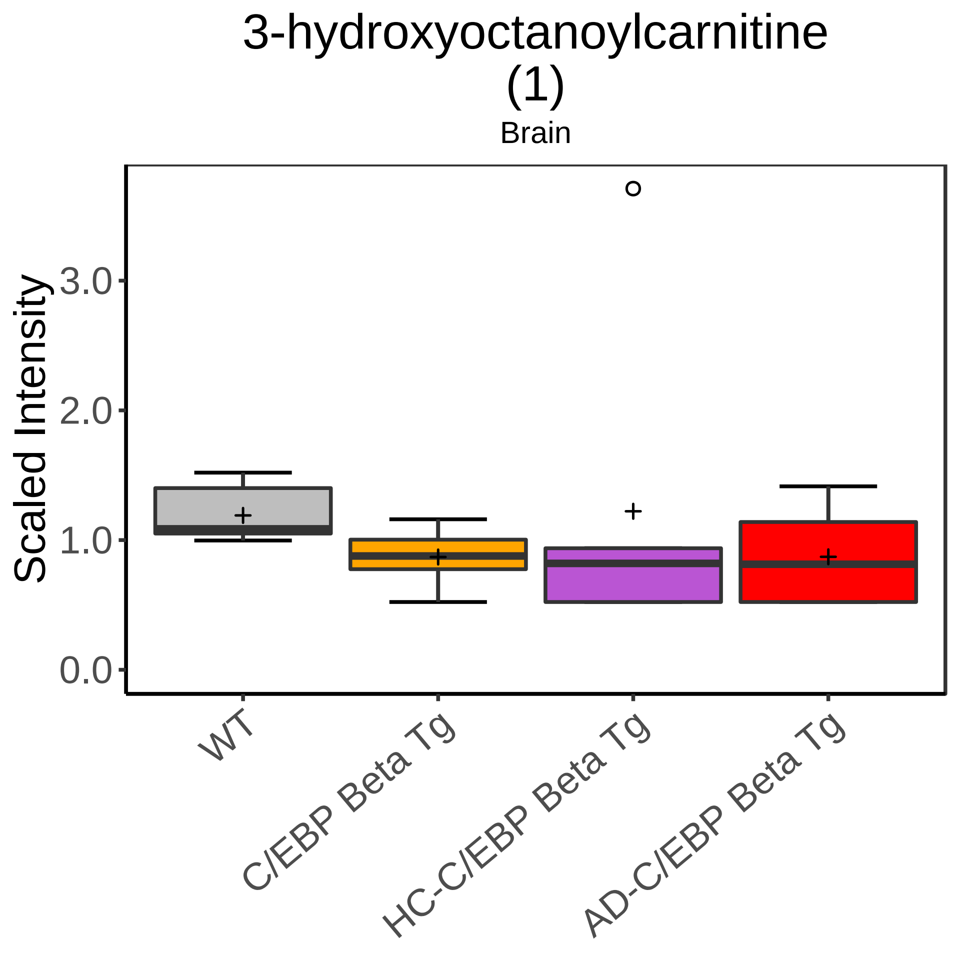

3-hydroxyoctanoylcarnitine
(1)
Brain
Scaled Intensity
0.0
1.0
2.0
3.0
WT
C/EBP Beta Tg
HC-C/EBP Beta Tg
AD-C/EBP Beta Tg

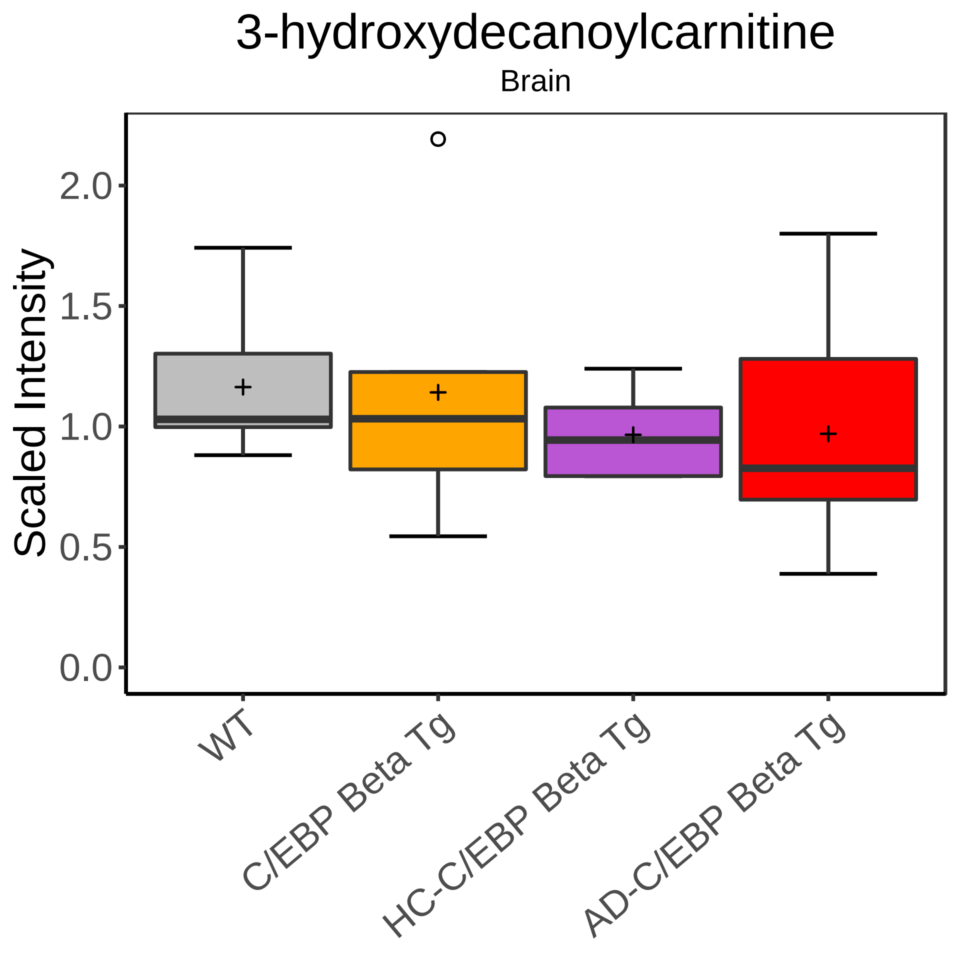
3-hydroxydecanoylcarnitine
Brain
Scaled Intensity
2.0
1.5
1.0
0.5
0.0
WT
C/EBP Beta Tg
HC-C/EBP Beta Tg
AD-C/EBP Beta Tg

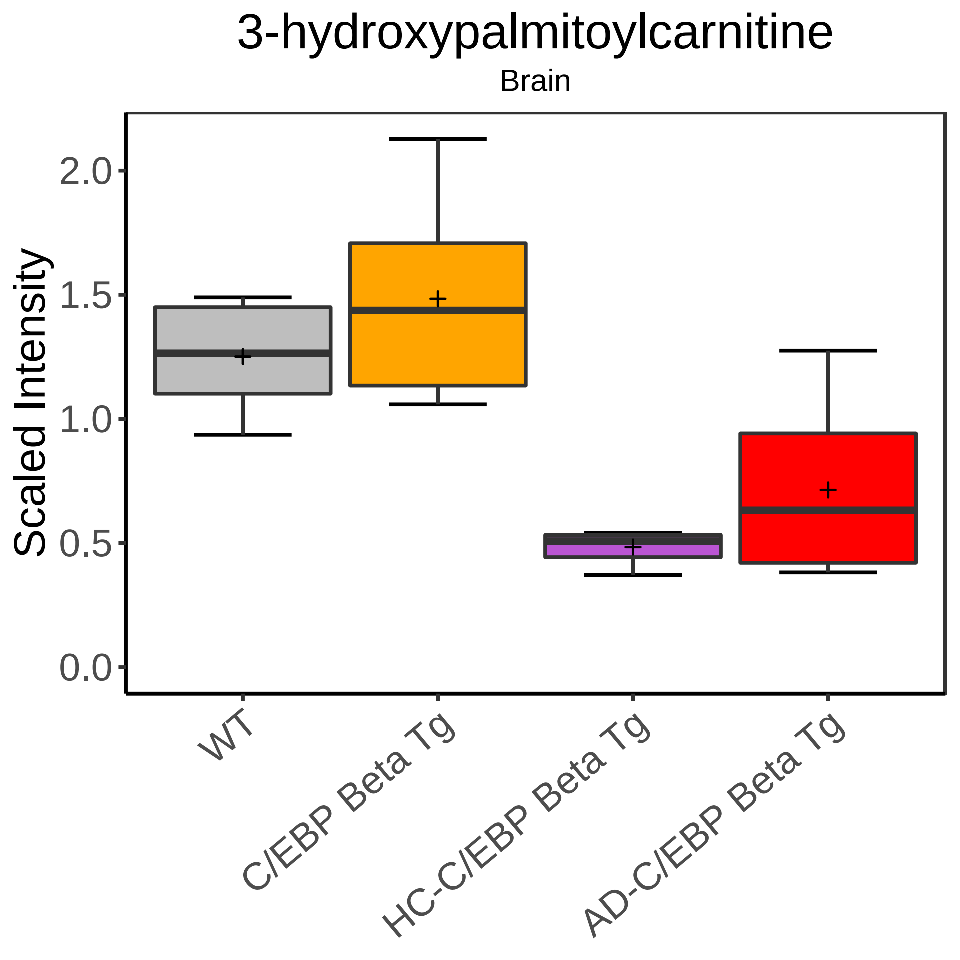
3-hydroxypalmitoylcarnitine
Brain
Scaled Intensity
0.0
0.5
1.0
1.5
2.0
WT
C/EBP Beta Tg
HC-C/EBP Beta Tg
AD-C/EBP Beta Tg

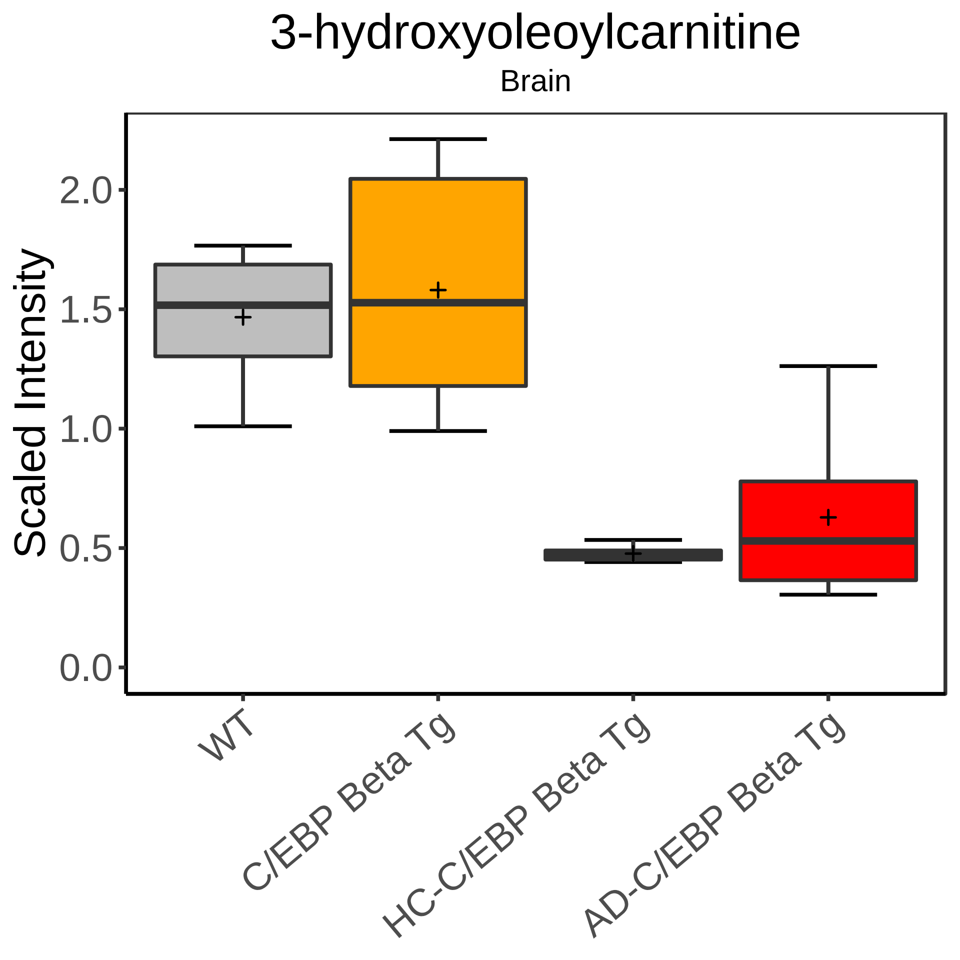
3-hydroxyoleoylcarnitine
Brain
Scaled Intensity
2.0
1.5
1.0
0.5
0.0
WT
C/EBP Beta Tg
HC-C/EBP Beta Tg
AD-C/EBP Beta Tg

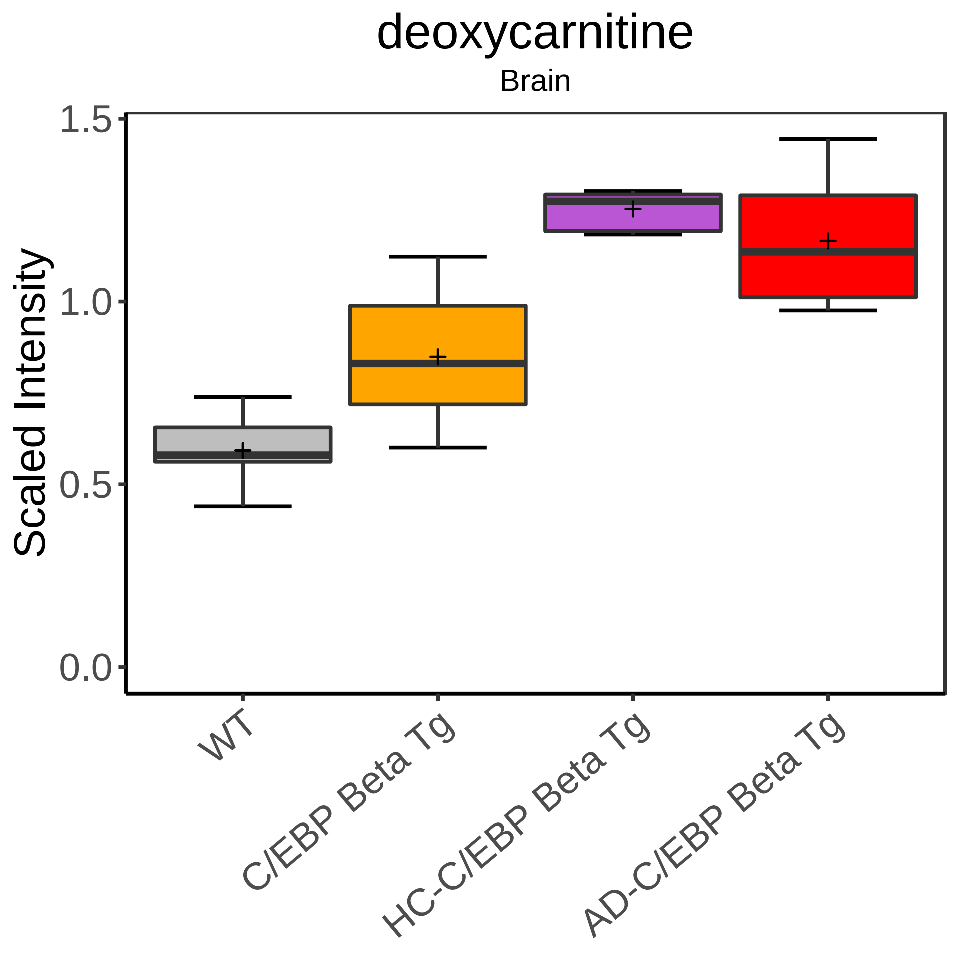

deoxycarnitine
Brain
Scaled Intensity
1.5
1.0
0.5
0.0
WT
C/EBP Beta Tg
HC-C/EBP Beta Tg
AD-C/EBP Beta Tg

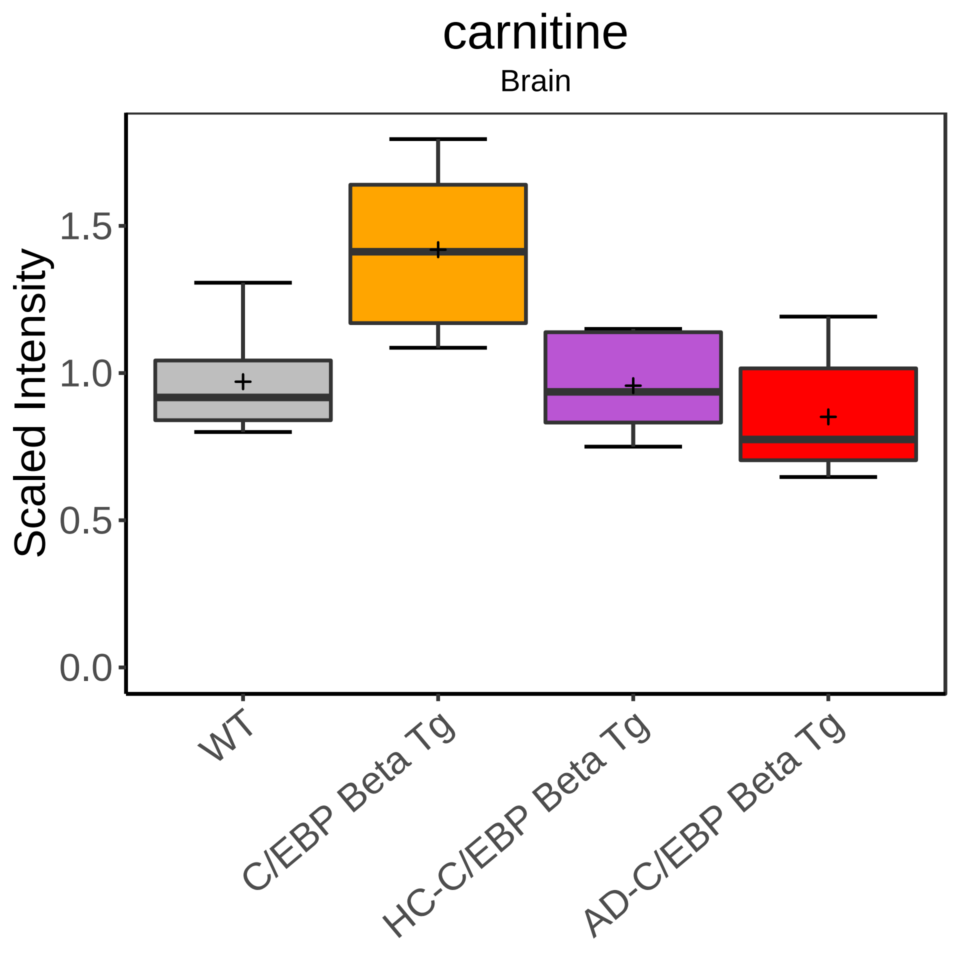

carnitine
Brain
Scaled Intensity
0.0
0.5
1.0
1.5
WT
C/EBP Beta Tg
HC-C/EBP Beta Tg
AD-C/EBP Beta Tg

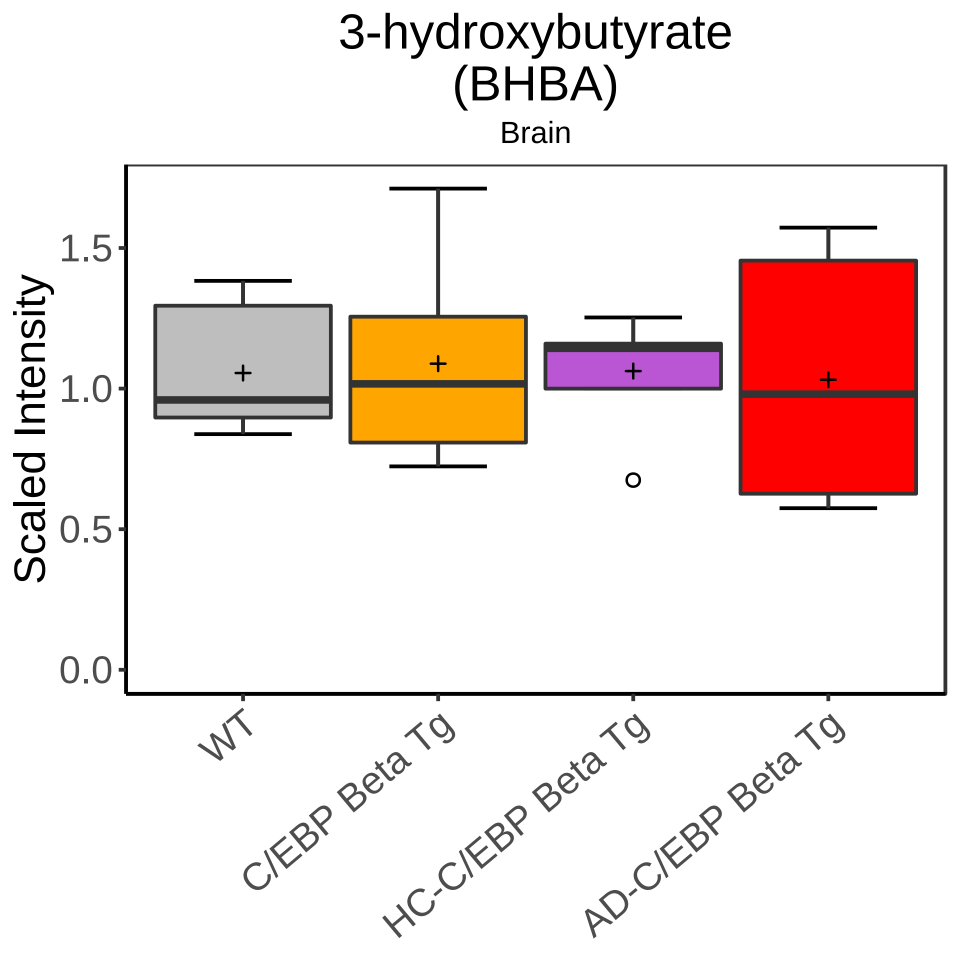

3-hydroxybutyrate
(BHBA)
Brain
Scaled Intensity
0.0
0.5
1.0
1.5
WT
C/EBP Beta Tg
HC-C/EBP Beta Tg
AD-C/EBP Beta Tg

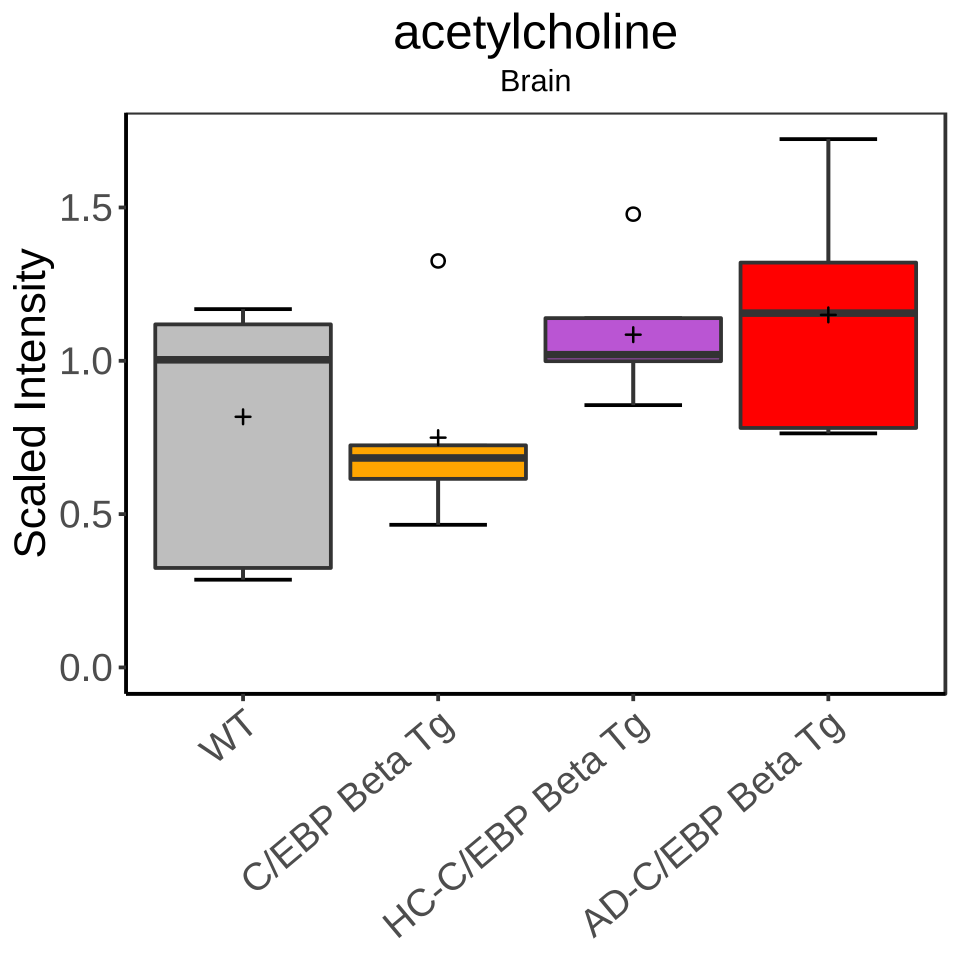
acetylcholine
Brain
Scaled Intensity
0.0
0.5
1.0
1.5
WT
C/EBP Beta Tg
HC-C/EBP Beta Tg
AD-C/EBP Beta Tg

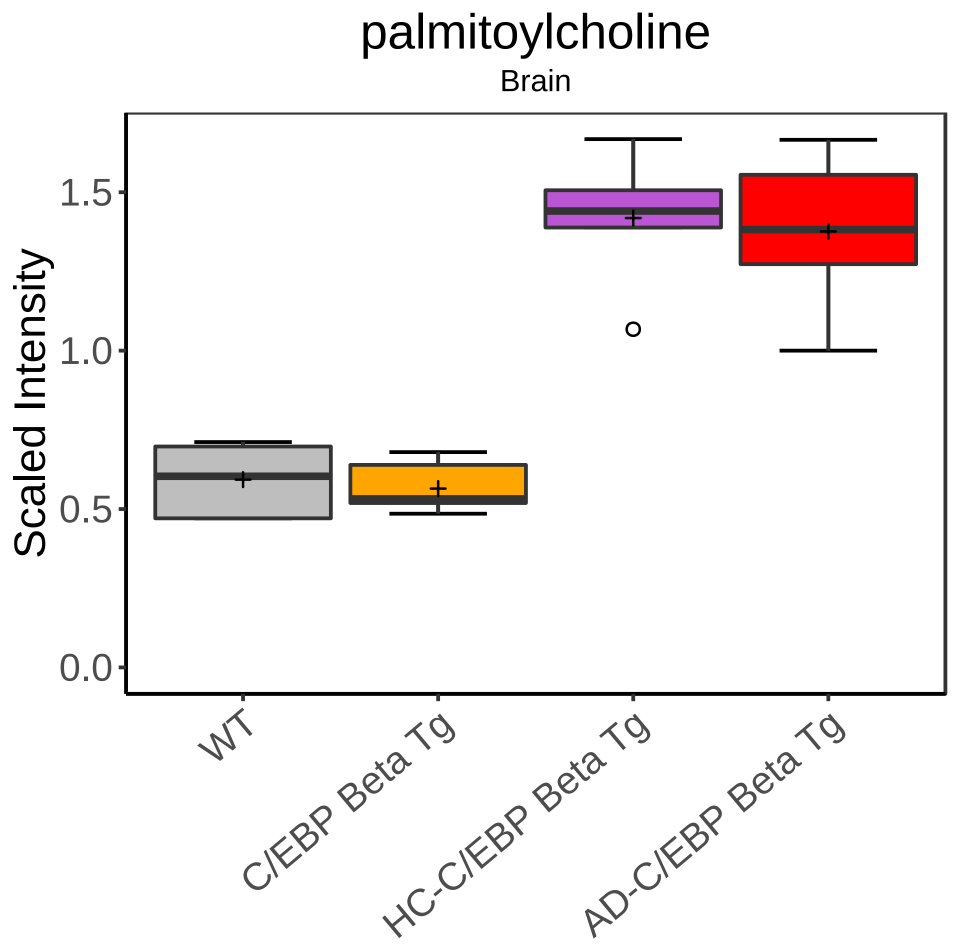
palmitoylcholine
Brain
Scaled Intensity
1.5
1.0
0.5
0.0
WT
C/EBP Beta Tg
HC-C/EBP Beta Tg
AD-C/EBP Beta Tg

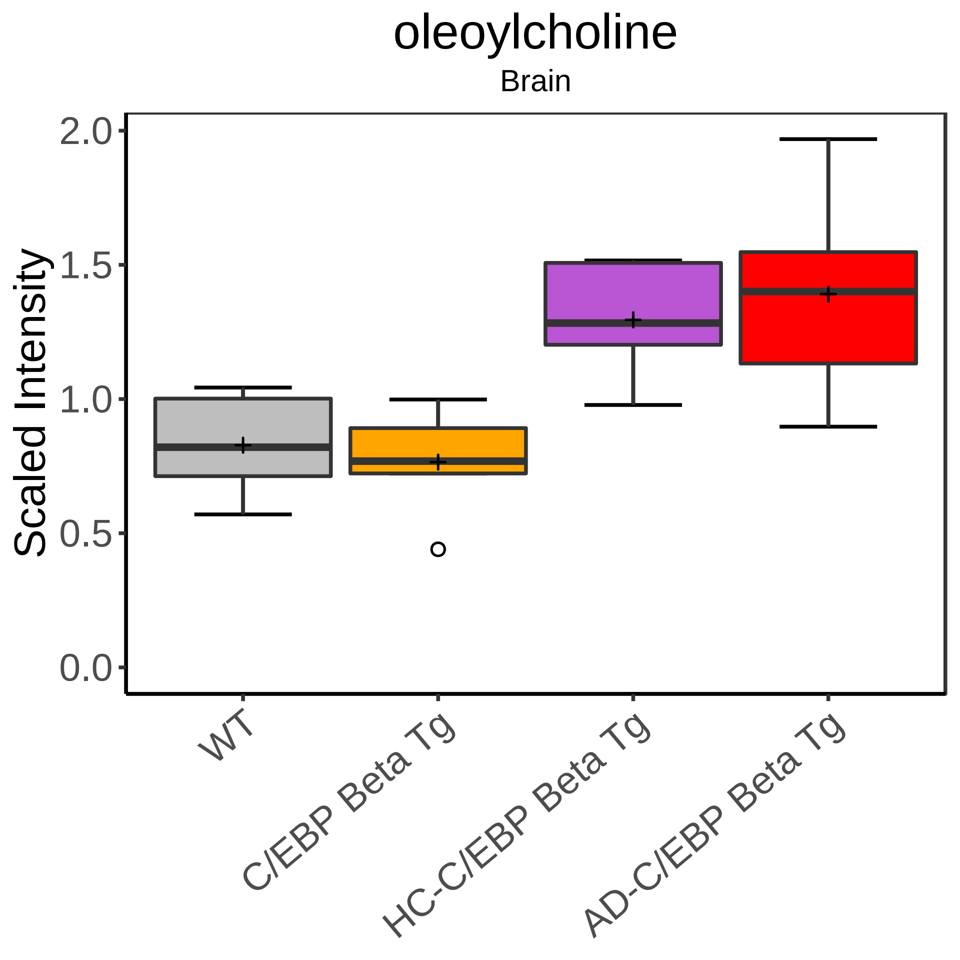

oleoylcholine
Brain
Scaled Intensity
2.0
1.5
1.0
0.5
0.0
WT
C/EBP Beta Tg
HC-C/EBP Beta Tg
AD-C/EBP Beta Tg

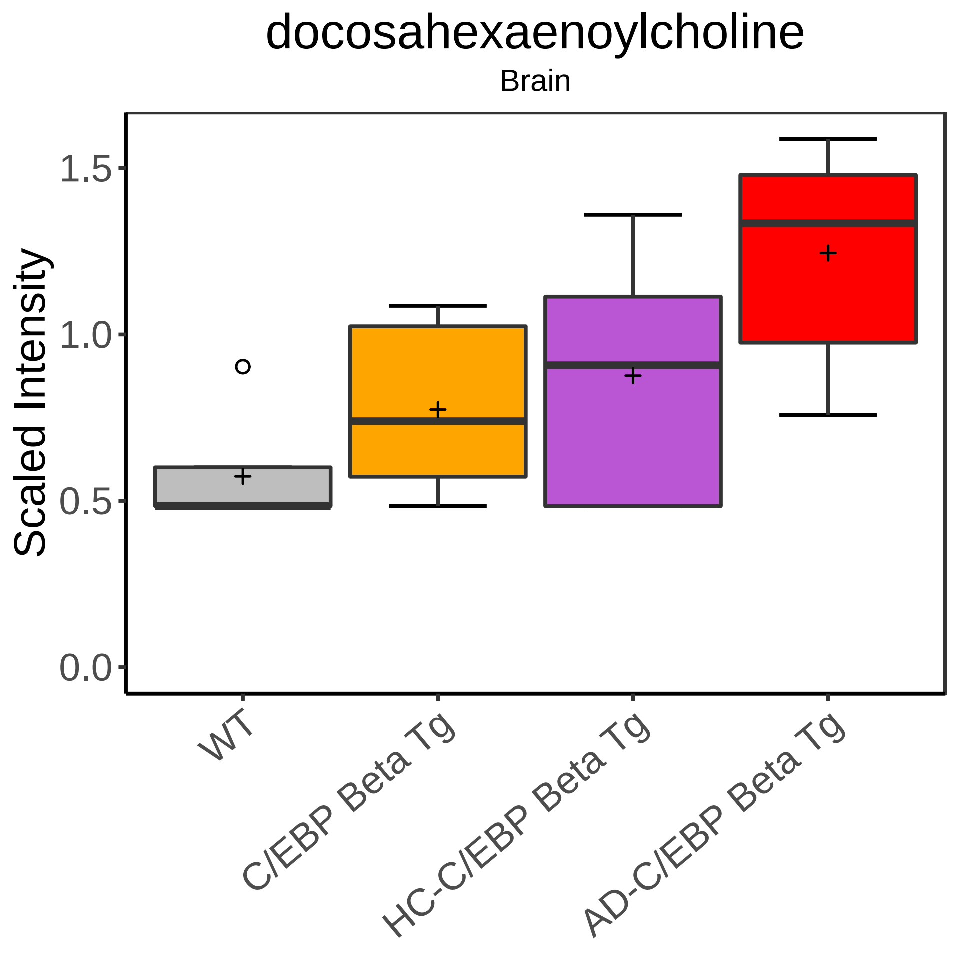

docosahexaenoylcholine
Brain
Scaled Intensity
1.5
1.0
0.5
0.0
WT
C/EBP Beta Tg
HC-C/EBP Beta Tg
AD-C/EBP Beta Tg

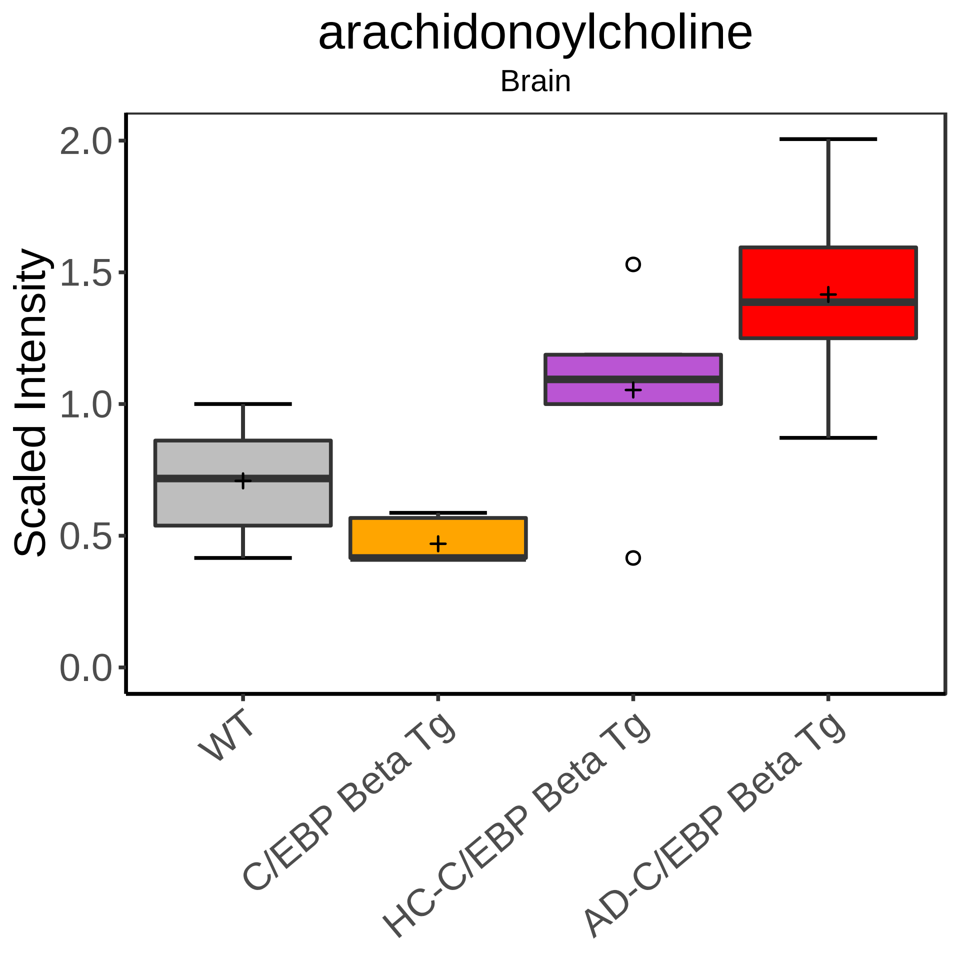
arachidonoylcholine
Brain
Scaled Intensity
2.0
1.5
1.0
0.5
0.0
WT
C/EBP Beta Tg
HC-C/EBP Beta Tg
AD-C/EBP Beta Tg

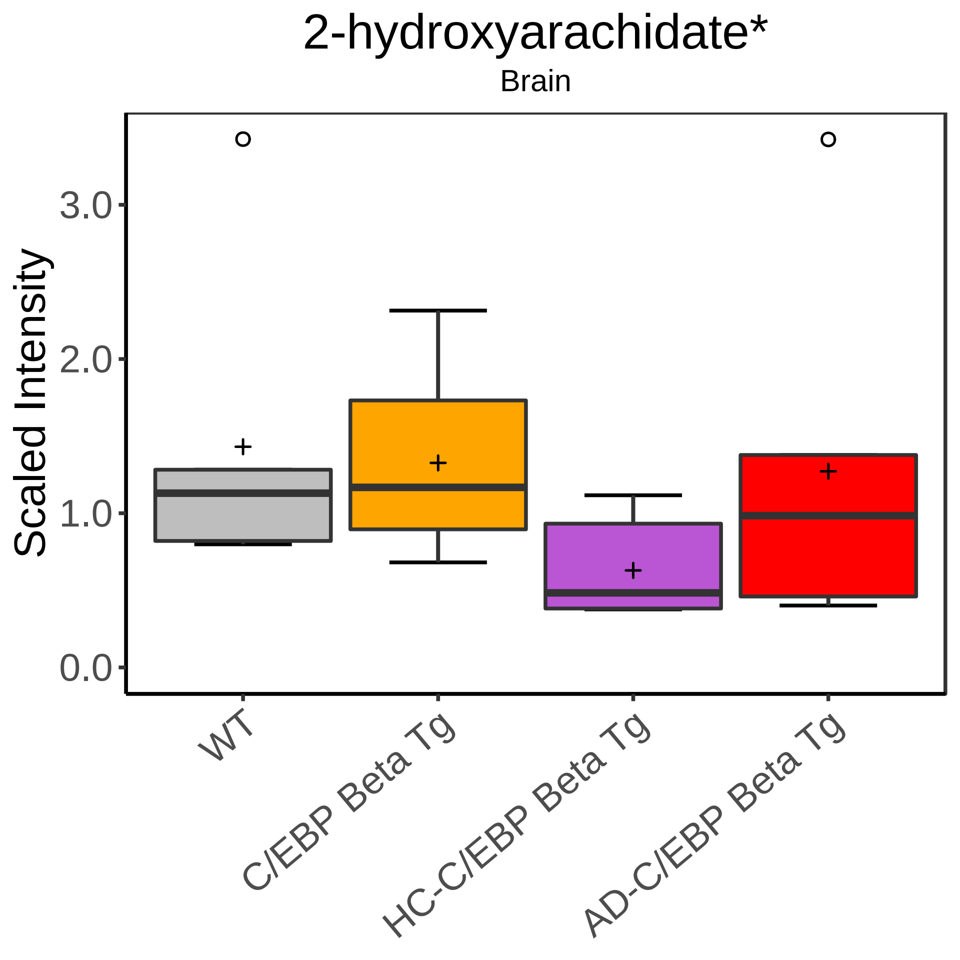
2-hydroxyarachidate*
Brain
Scaled Intensity
0.0
1.0
2.0
3.0
WT
C/EBP Beta Tg
HC-C/EBP Beta Tg
AD-C/EBP Beta Tg

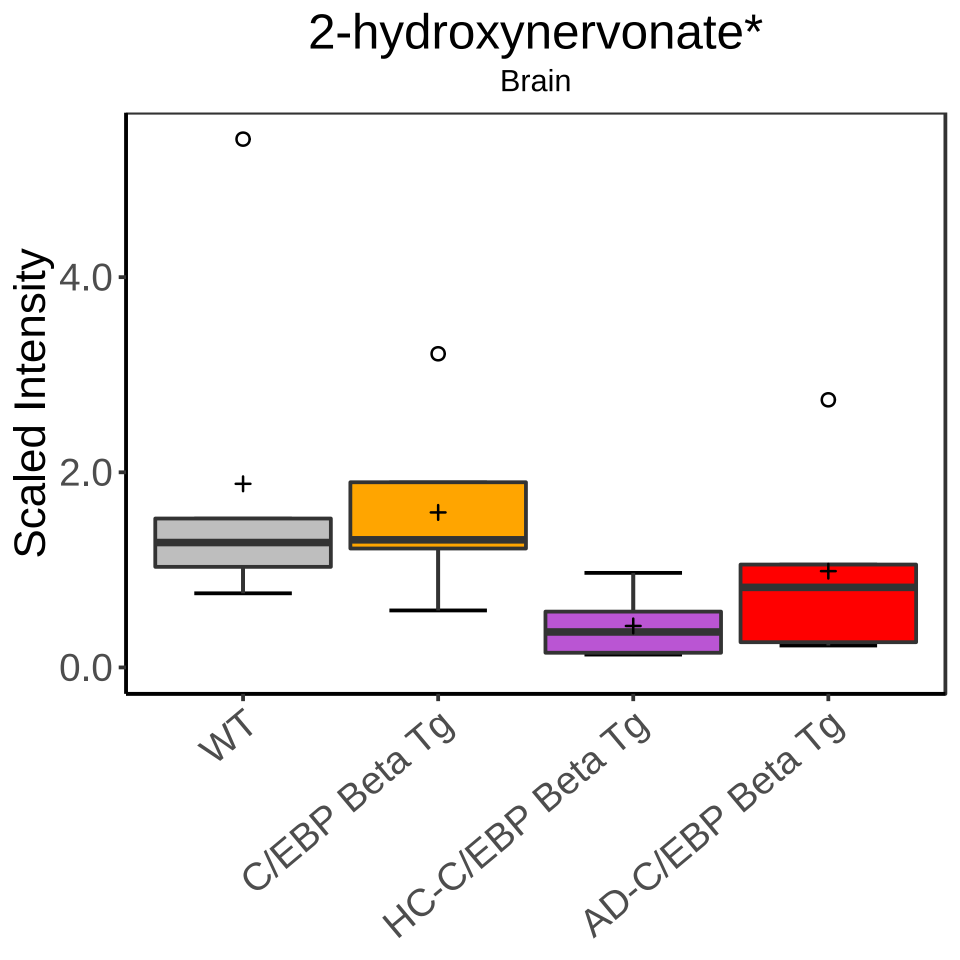
2-hydroxynervonate*
Brain
Scaled Intensity
4.0
2.0
0.0
WT
C/EBP Beta Tg
HC-C/EBP Beta Tg
AD-C/EBP Beta Tg

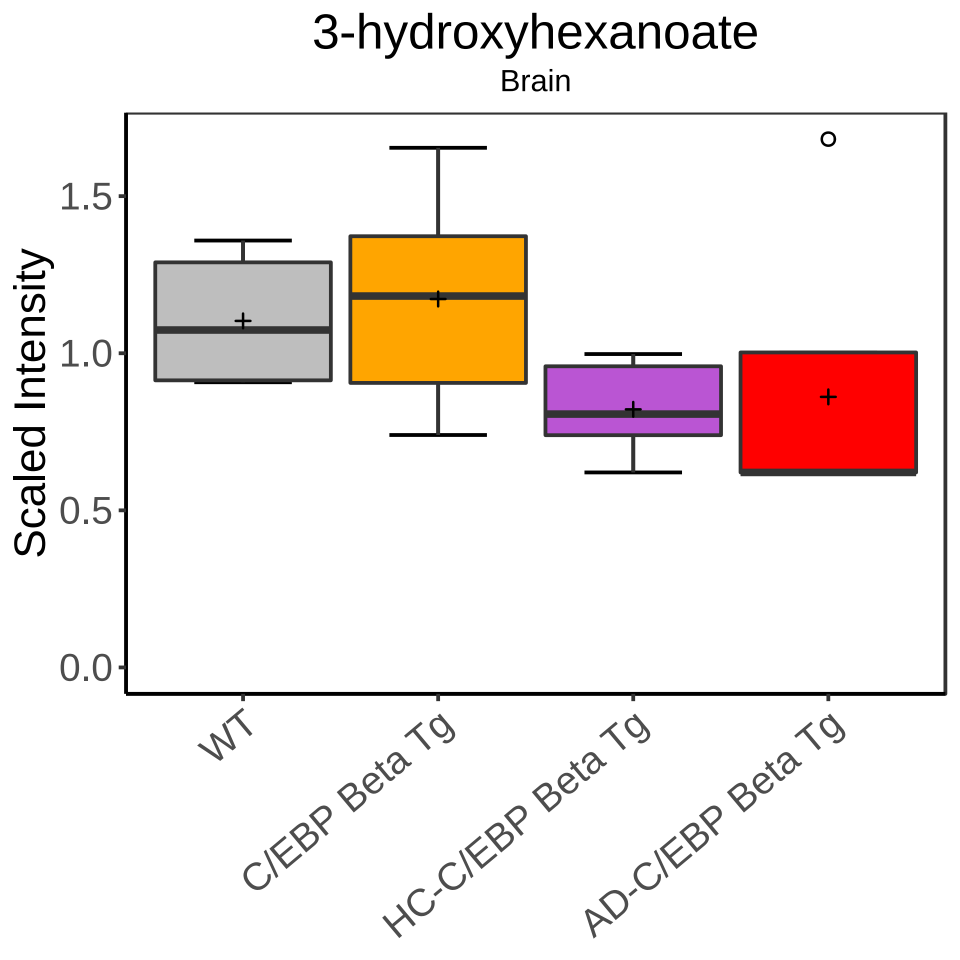
3-hydroxyhexanoate
Brain
Scaled Intensity
1.5
1.0
0.5
0.0
WT
C/EBP Beta Tg
HC-C/EBP Beta Tg
AD-C/EBP Beta Tg

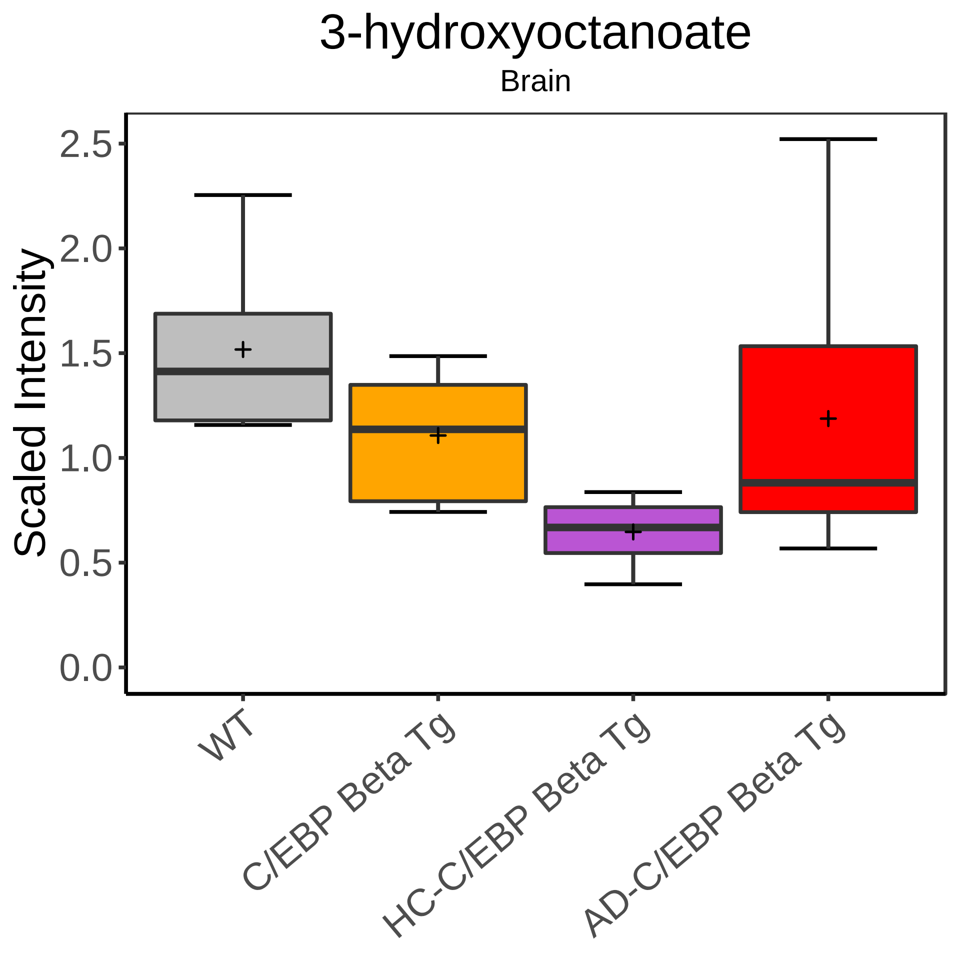
3-hydroxyoctanoate
Brain
Scaled Intensity
0.0
0.5
1.0
1.5
2.0
2.5
WT
C/EBP Beta Tg
HC-C/EBP Beta Tg
AD-C/EBP Beta Tg

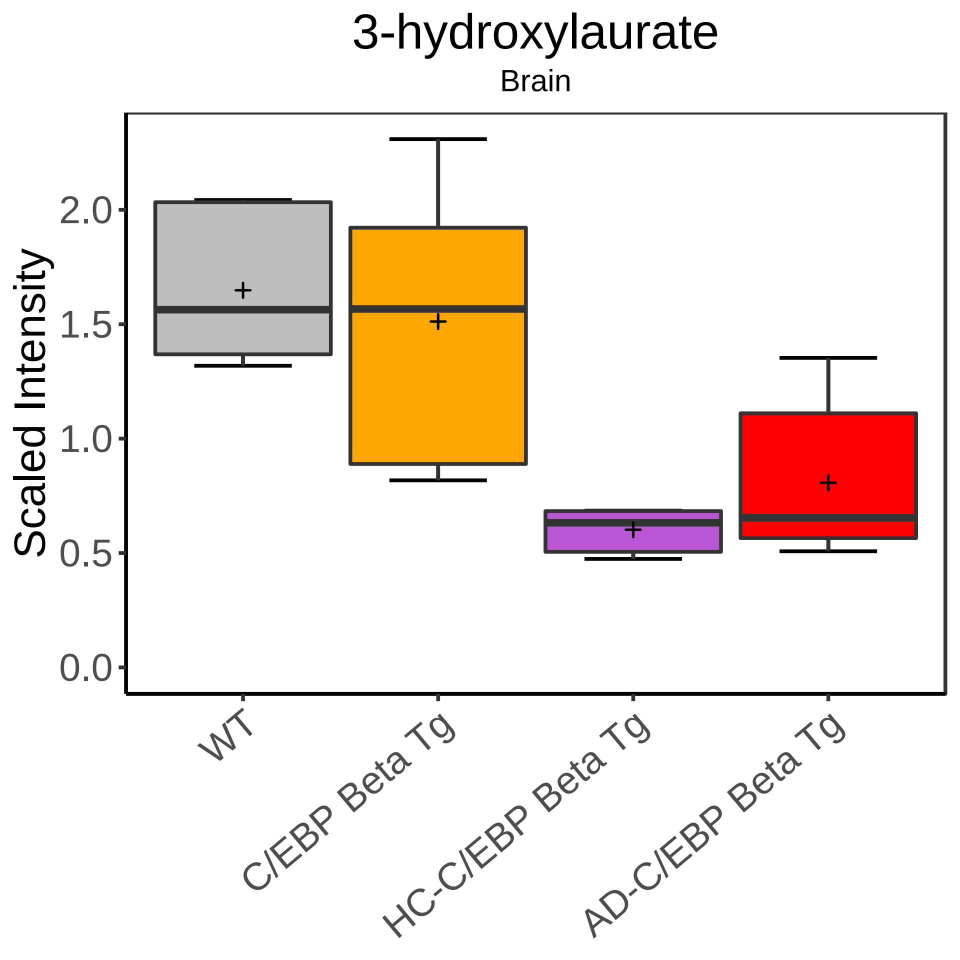

3-hydroxylaurate
Brain
Scaled Intensity
0.0
0.5
1.0
1.5
2.0
WT
C/EBP Beta Tg
HC-C/EBP Beta Tg
AD-C/EBP Beta Tg

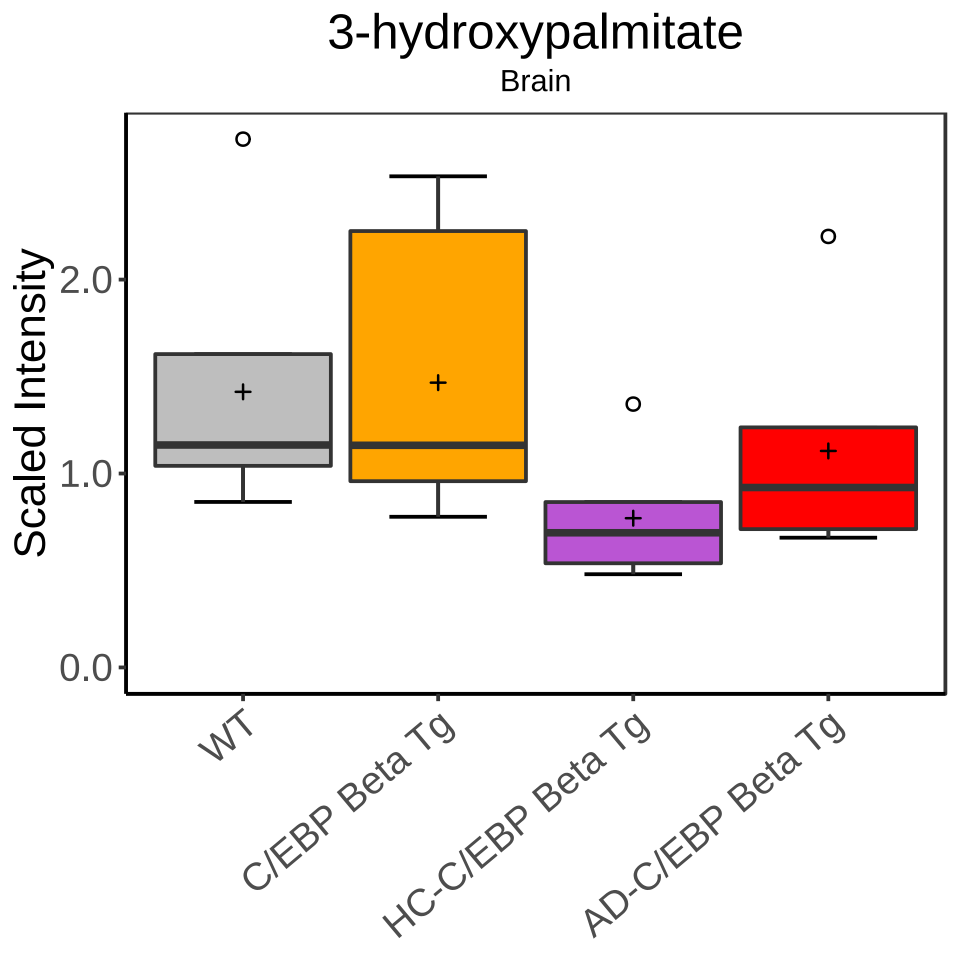
3-hydroxypalmitate
Brain
Scaled Intensity
2.0
1.0
0.0
WT
C/EBP Beta Tg
HC-C/EBP Beta Tg
AD-C/EBP Beta Tg

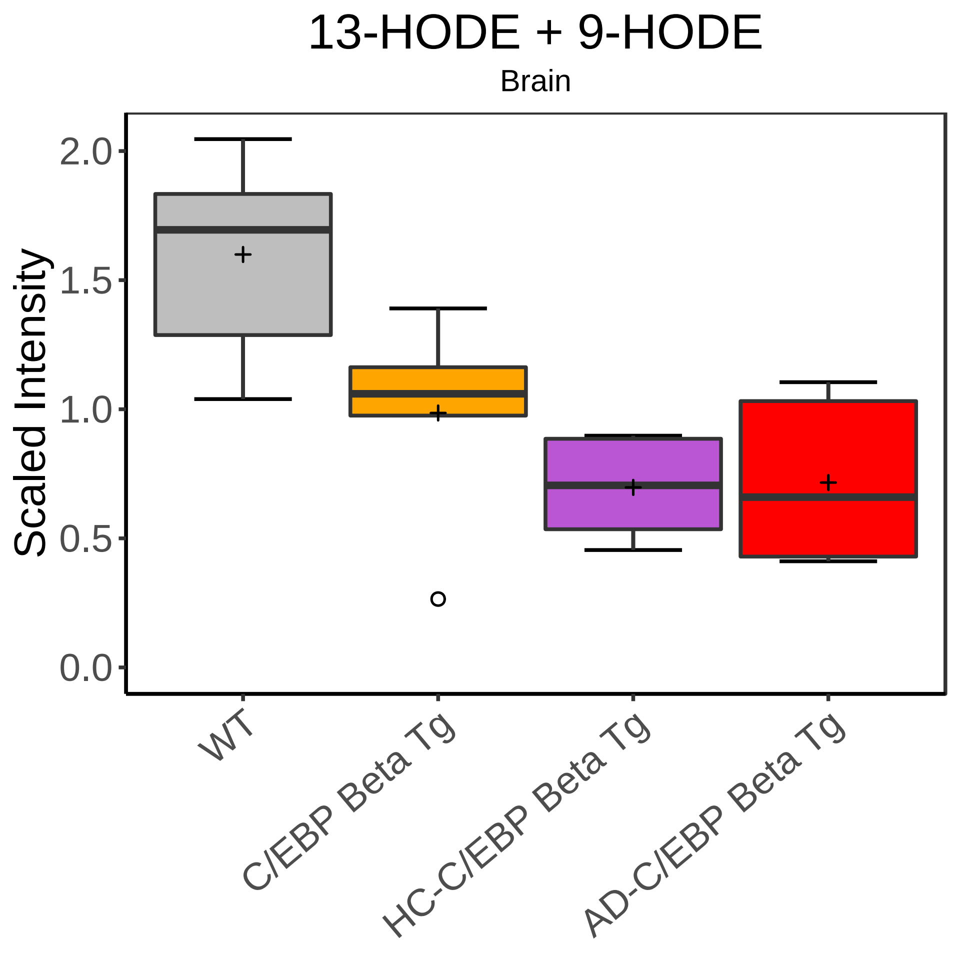
13-HODE + 9-HODE
Brain
Scaled Intensity
2.0
1.5
1.0
0.5
0.0
WT
C/EBP Beta Tg
HC-C/EBP Beta Tg
AD-C/EBP Beta Tg

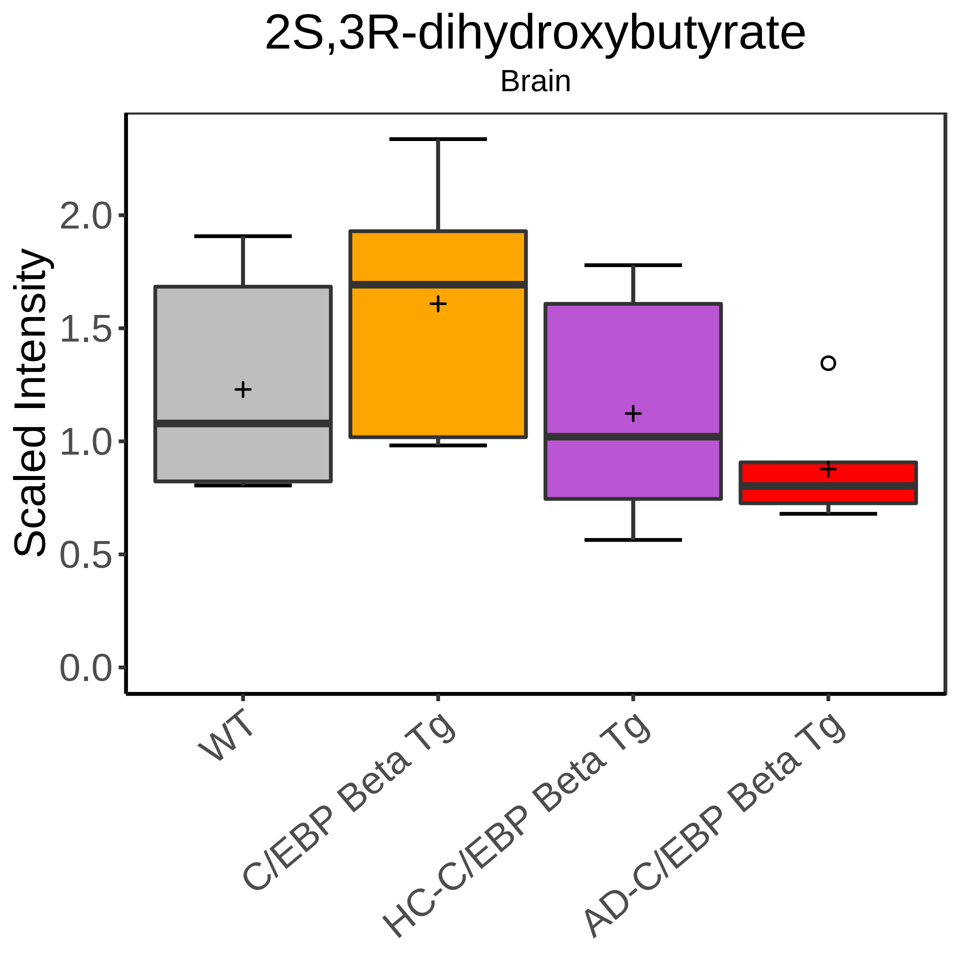
2S,3R-dihydroxybutyrate
Brain
Scaled Intensity
0.0
0.5
1.0
1.5
2.0
WT
C/EBP Beta Tg
HC-C/EBP Beta Tg
AD-C/EBP Beta Tg

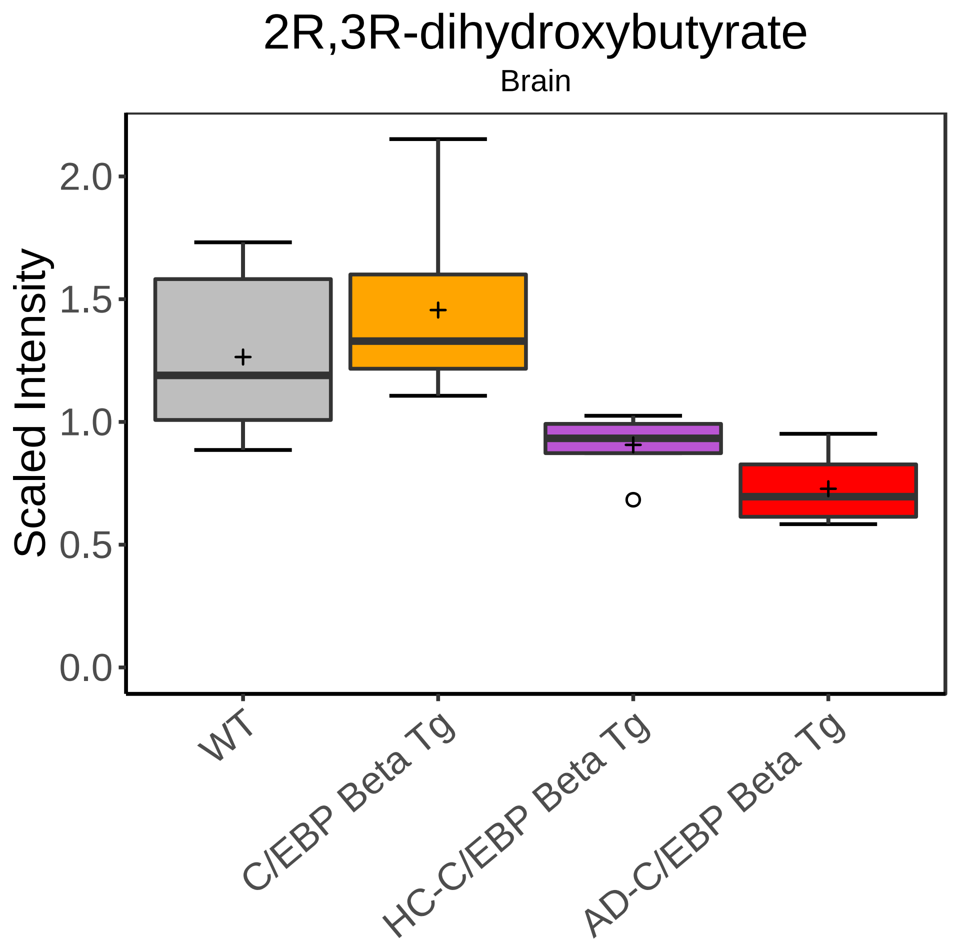

2R,3R-dihydroxybutyrate
Brain
Scaled Intensity
2.0
1.5
1.0
0.5
0.0
WT
C/EBP Beta Tg
HC-C/EBP Beta Tg
AD-C/EBP Beta Tg

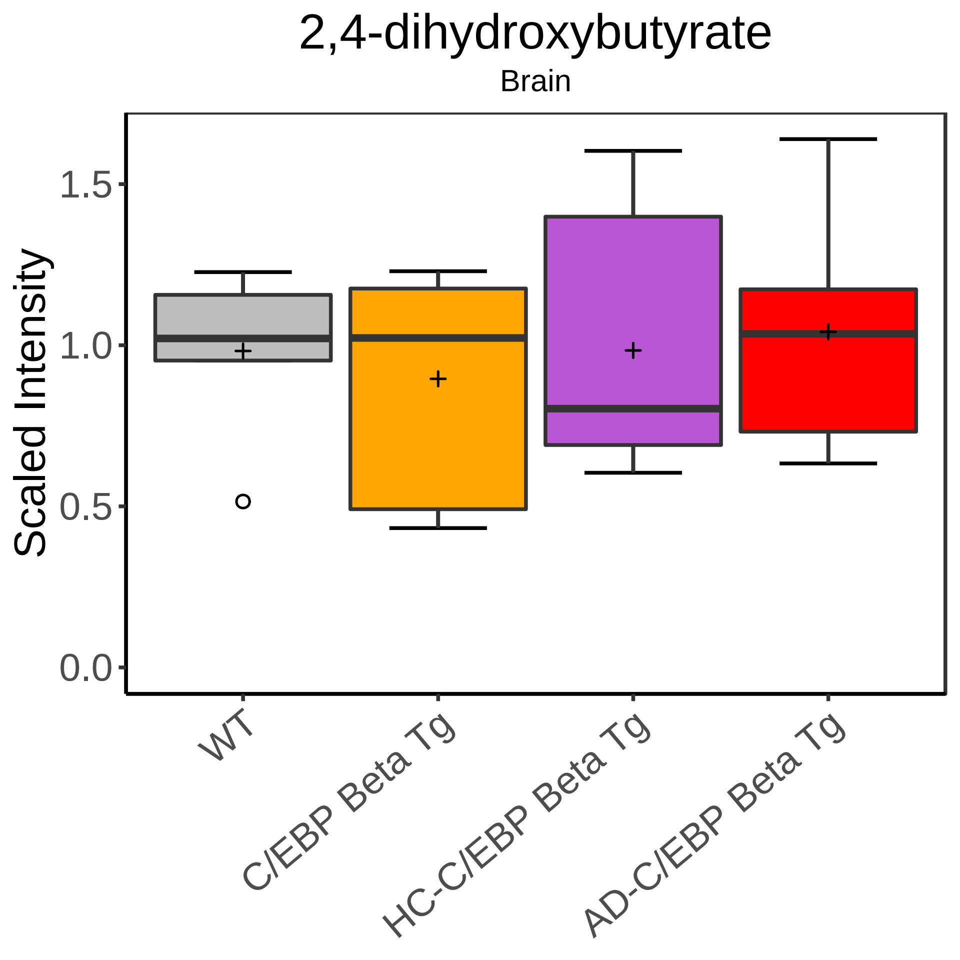
2,4-dihydroxybutyrate
Brain
Scaled Intensity
1.5
1.0
0.5
0.0
WT
C/EBP Beta Tg
HC-C/EBP Beta Tg
AD-C/EBP Beta Tg

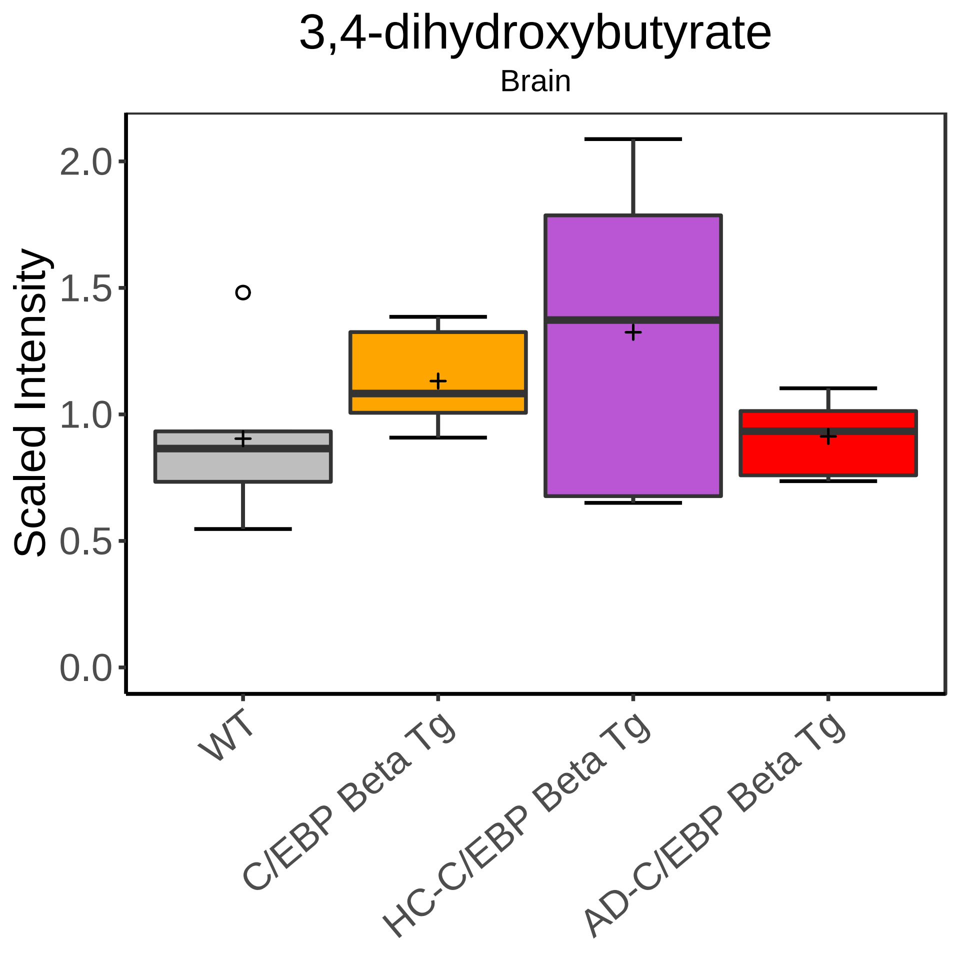
3,4-dihydroxybutyrate
Brain
Scaled Intensity
2.0
1.5
1.0
0.5
0.0
WT
C/EBP Beta Tg
HC-C/EBP Beta Tg
AD-C/EBP Beta Tg

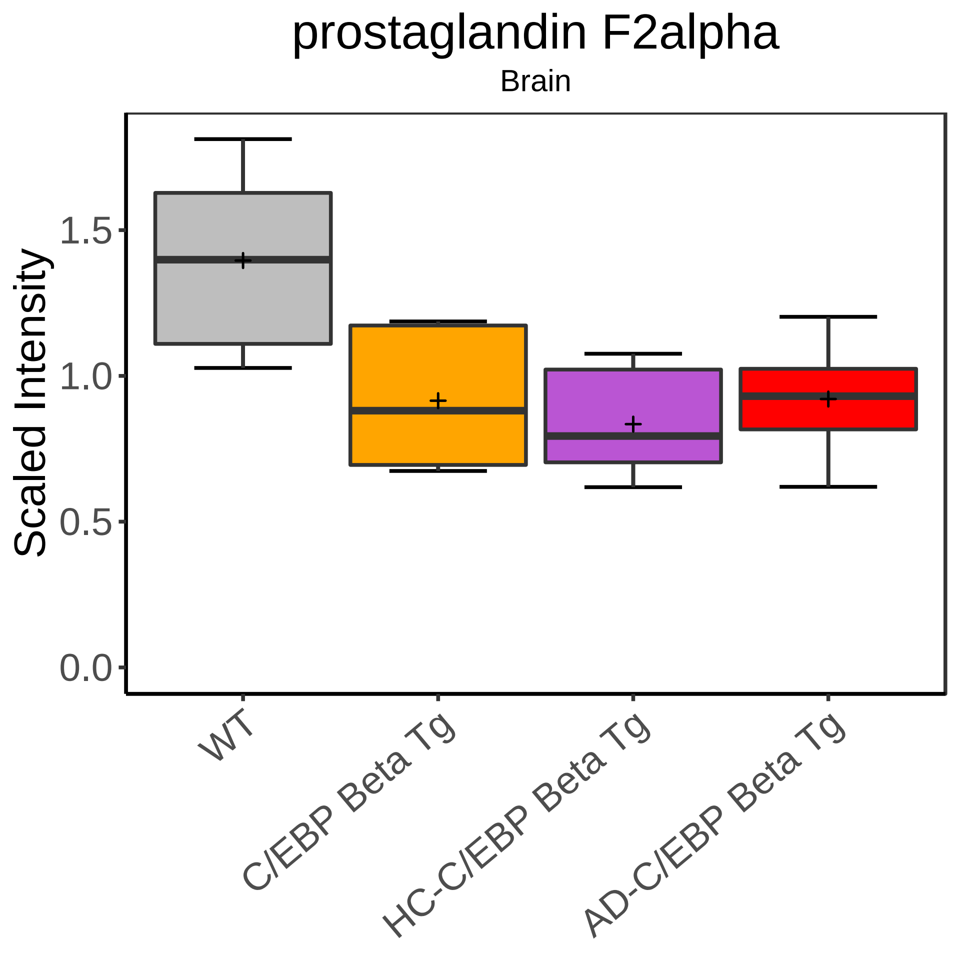
prostaglandin F2alpha
Brain
Scaled Intensity
1.5
1.0
0.5
0.0
WT
C/EBP Beta Tg
HC-C/EBP Beta Tg
AD-C/EBP Beta Tg

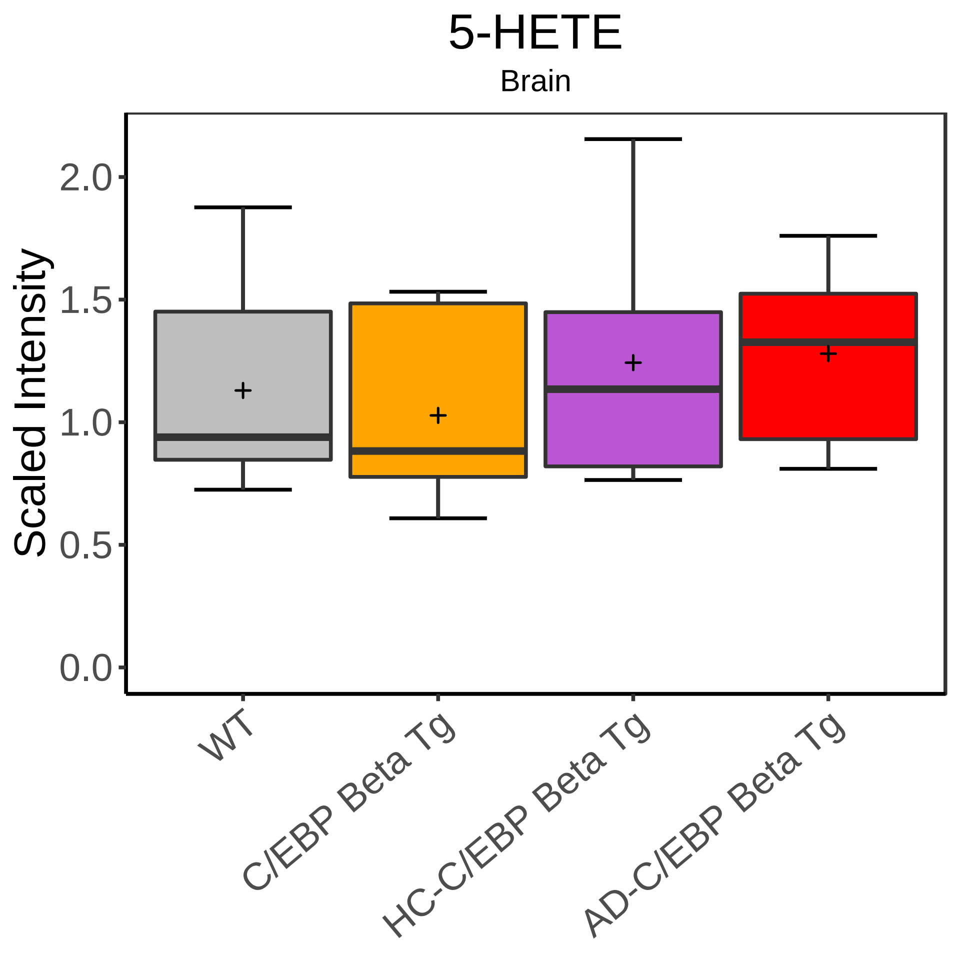

5-HETE
Brain
Scaled Intensity
2.0
1.5
1.0
0.5
0.0
WT
C/EBP Beta Tg
HC-C/EBP Beta Tg
AD-C/EBP Beta Tg

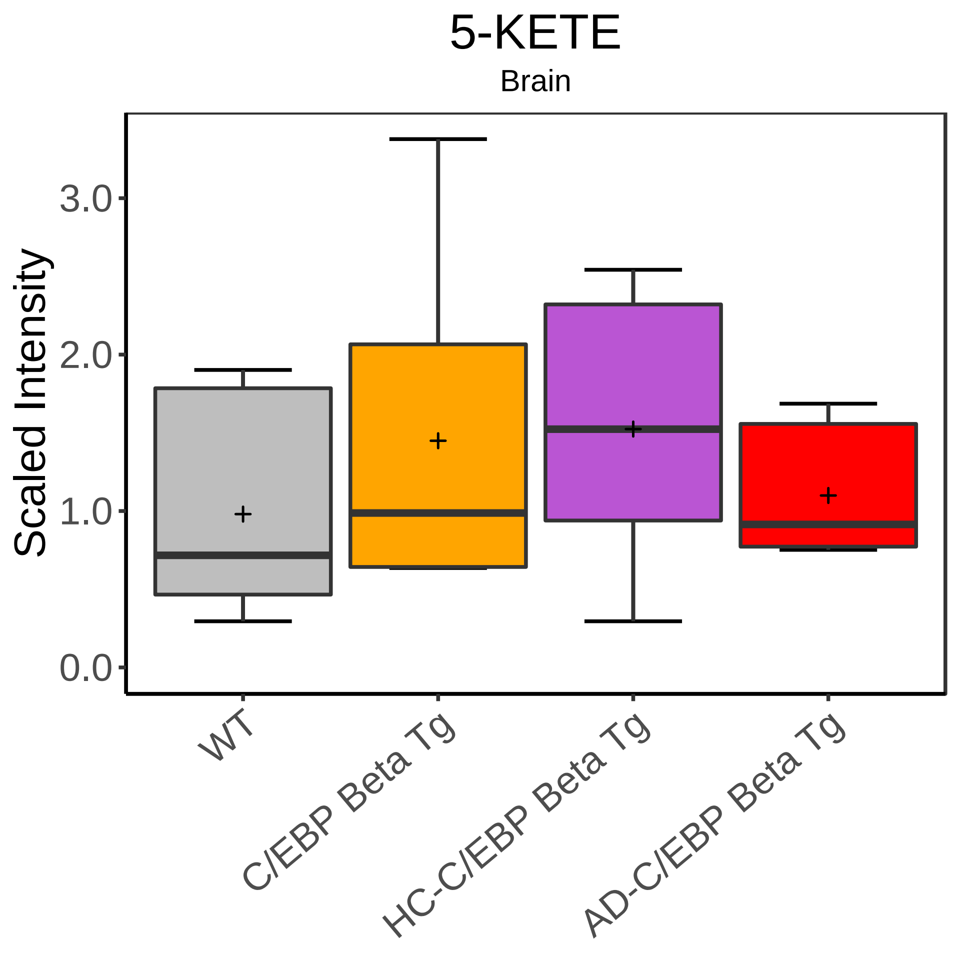
5-KETE
Brain
Scaled Intensity
3.0
2.0
1.0
0.0
WT
C/EBP Beta Tg
HC-C/EBP Beta Tg
AD-C/EBP Beta Tg

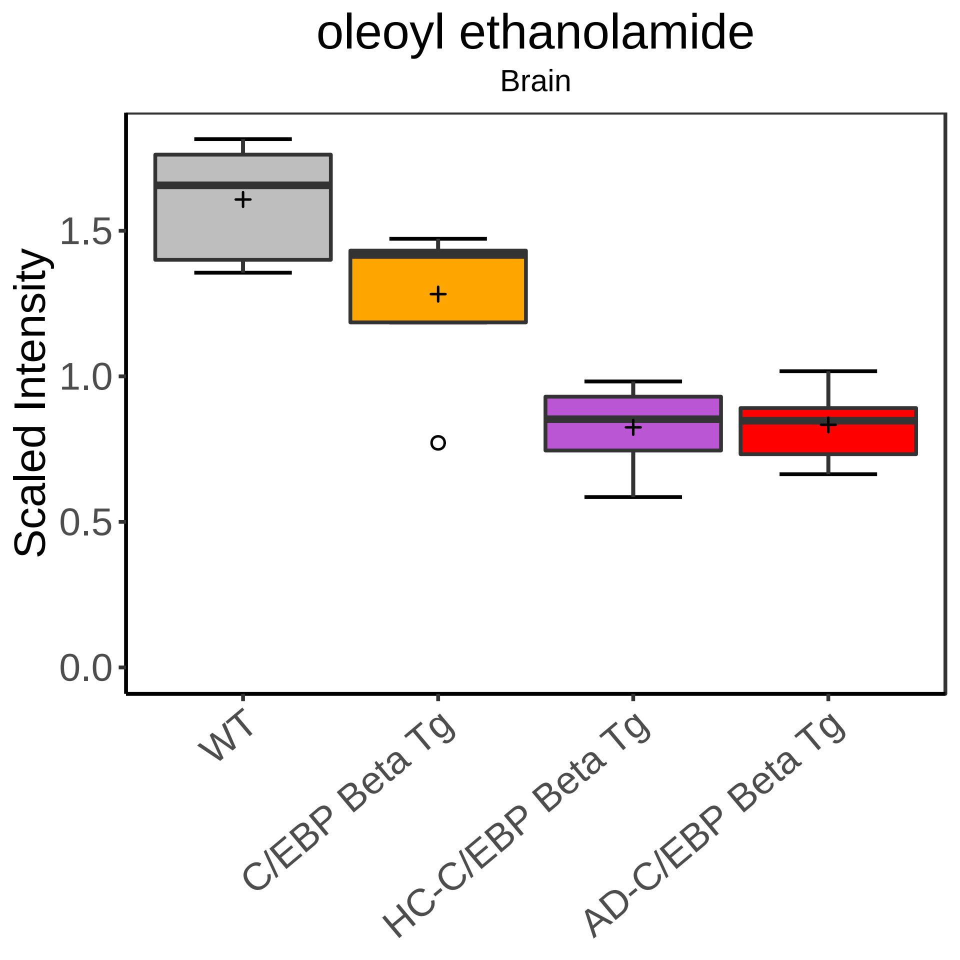

oleoyl ethanolamide
Brain
Scaled Intensity
0.0
0.5
1.0
1.5
WT
C/EBP Beta Tg
HC-C/EBP Beta Tg
AD-C/EBP Beta Tg

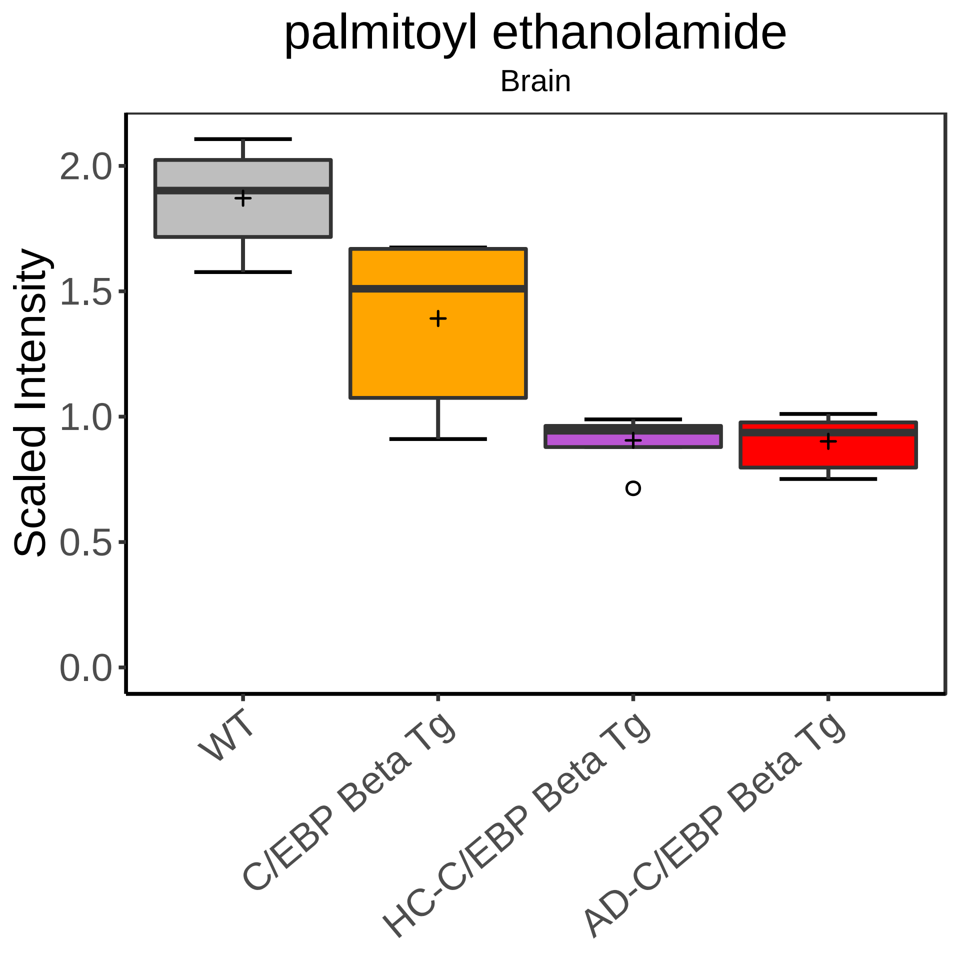
palmitoyl ethanolamide
Brain
Scaled Intensity
2.0
1.5
1.0
0.5
0.0
WT
C/EBP Beta Tg
HC-C/EBP Beta Tg
AD-C/EBP Beta Tg

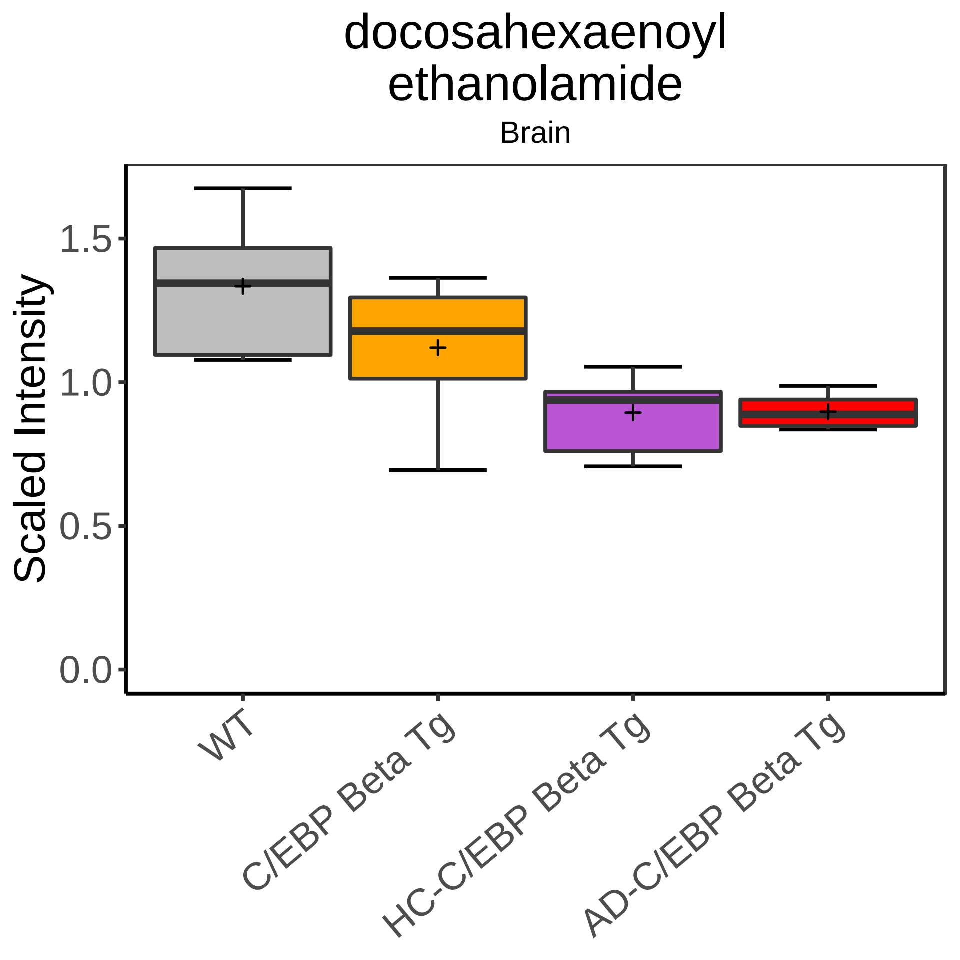

docosahexaenoyl
ethanolamide
Brain
Scaled Intensity
0.0
0.5
1.0
1.5
WT
C/EBP Beta Tg
HC-C/EBP Beta Tg
AD-C/EBP Beta Tg

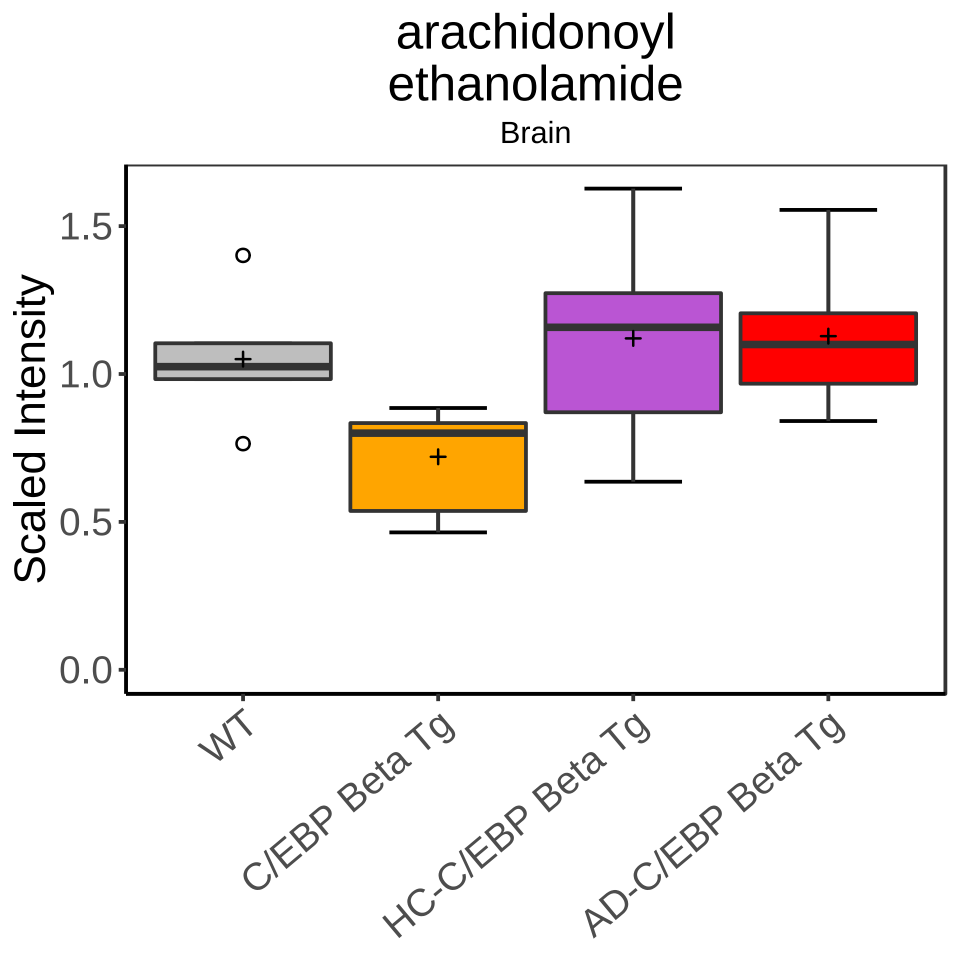
arachidonoyl
ethanolamide
Brain
Scaled Intensity
1.5
1.0
0.5
0.0
WT
C/EBP Beta Tg
HC-C/EBP Beta Tg
AD-C/EBP Beta Tg

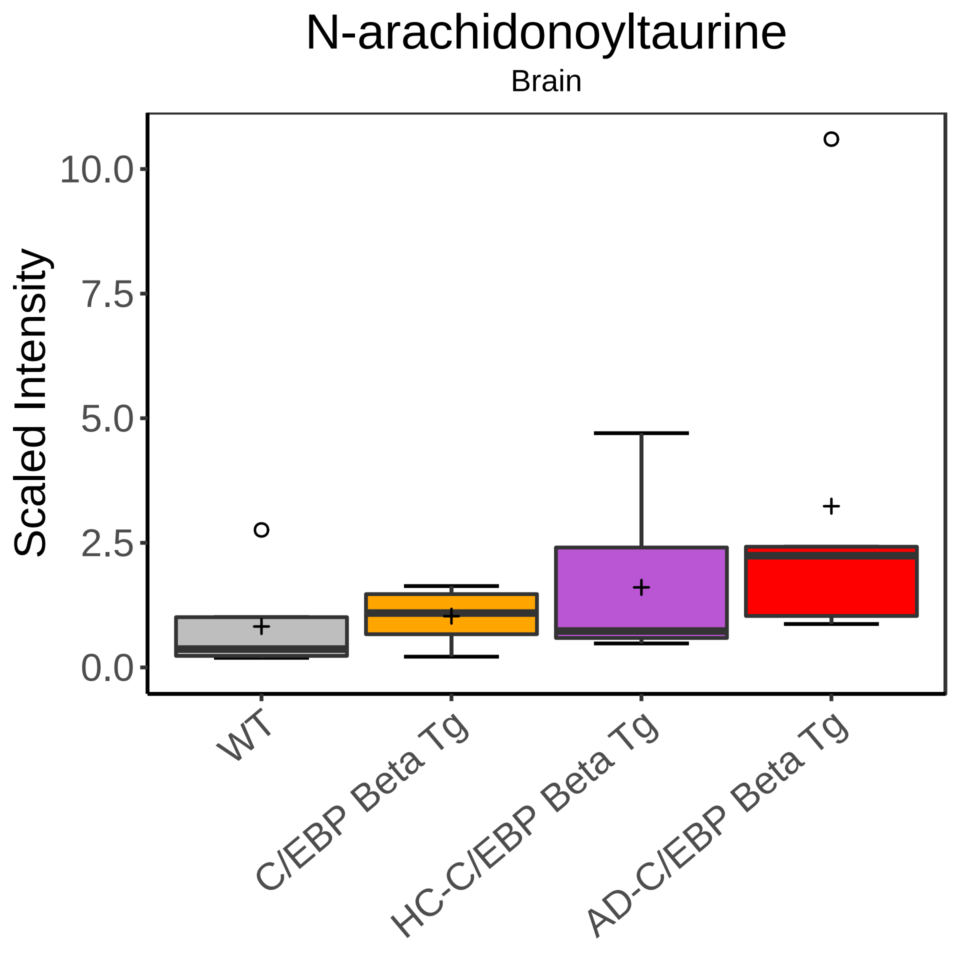

N-arachidonoyltaurine
Brain
Scaled Intensity
10.0
7.5
5.0
2.5
0.0
WT
C/EBP Beta Tg
HC-C/EBP Beta Tg
AD-C/EBP Beta Tg

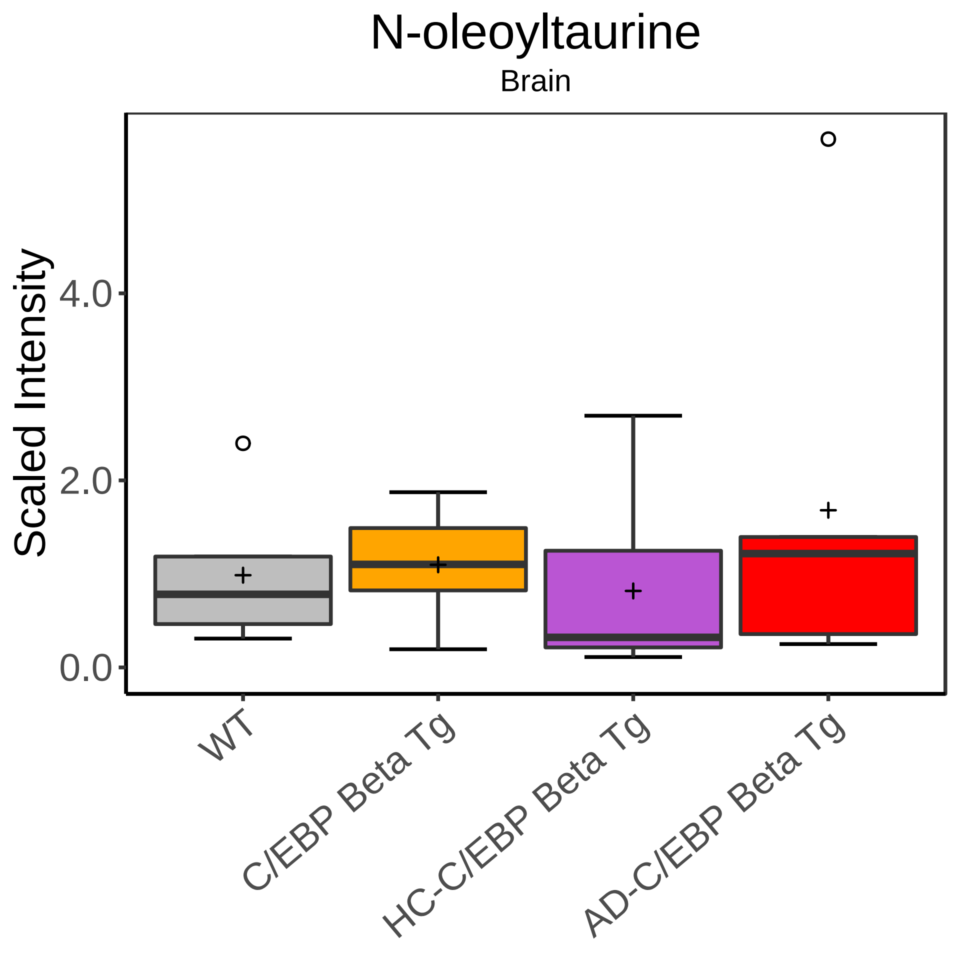

N-oleoyltaurine
Brain
Scaled Intensity
4.0
2.0
0.0
WT
C/EBP Beta Tg
HC-C/EBP Beta Tg
AD-C/EBP Beta Tg

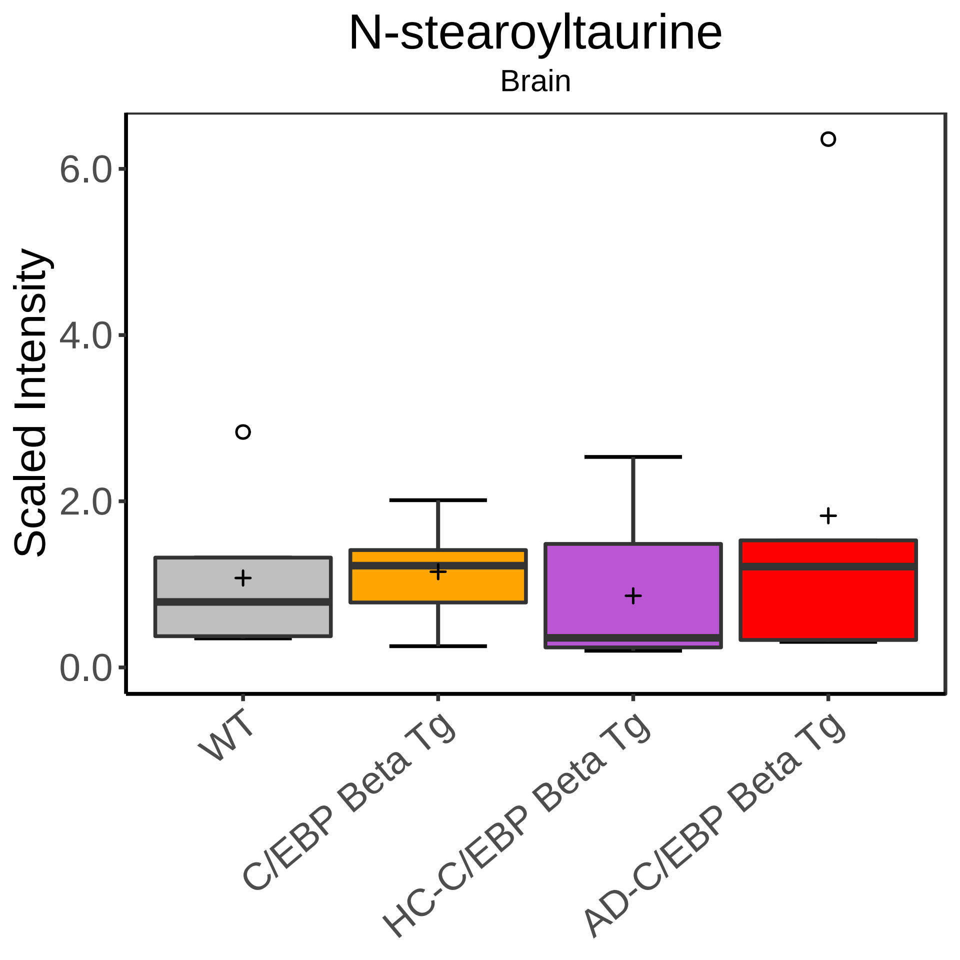

N-stearoyltaurine
Brain
Scaled Intensity
6.0
4.0
2.0
0.0
WT
C/EBP Beta Tg
HC-C/EBP Beta Tg
AD-C/EBP Beta Tg

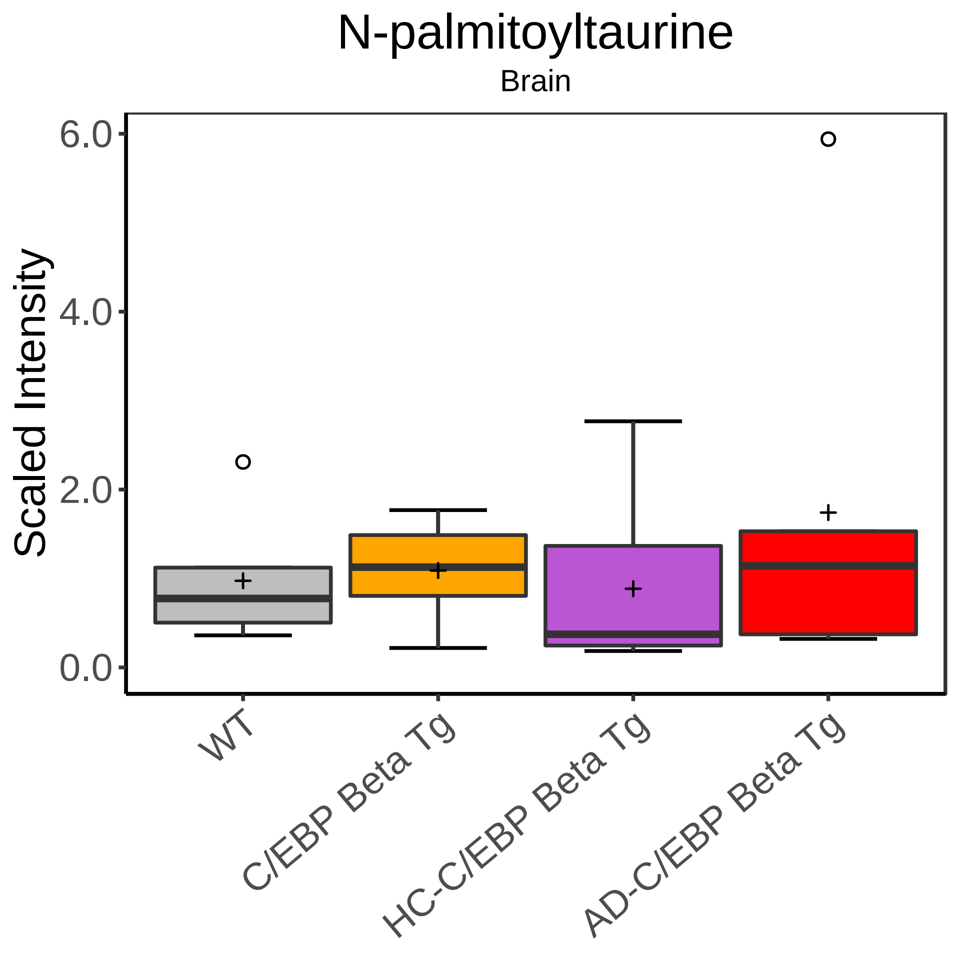
N-palmitoyltaurine
Brain
Scaled Intensity
6.0
4.0
2.0
0.0
WT
C/EBP Beta Tg
HC-C/EBP Beta Tg
AD-C/EBP Beta Tg

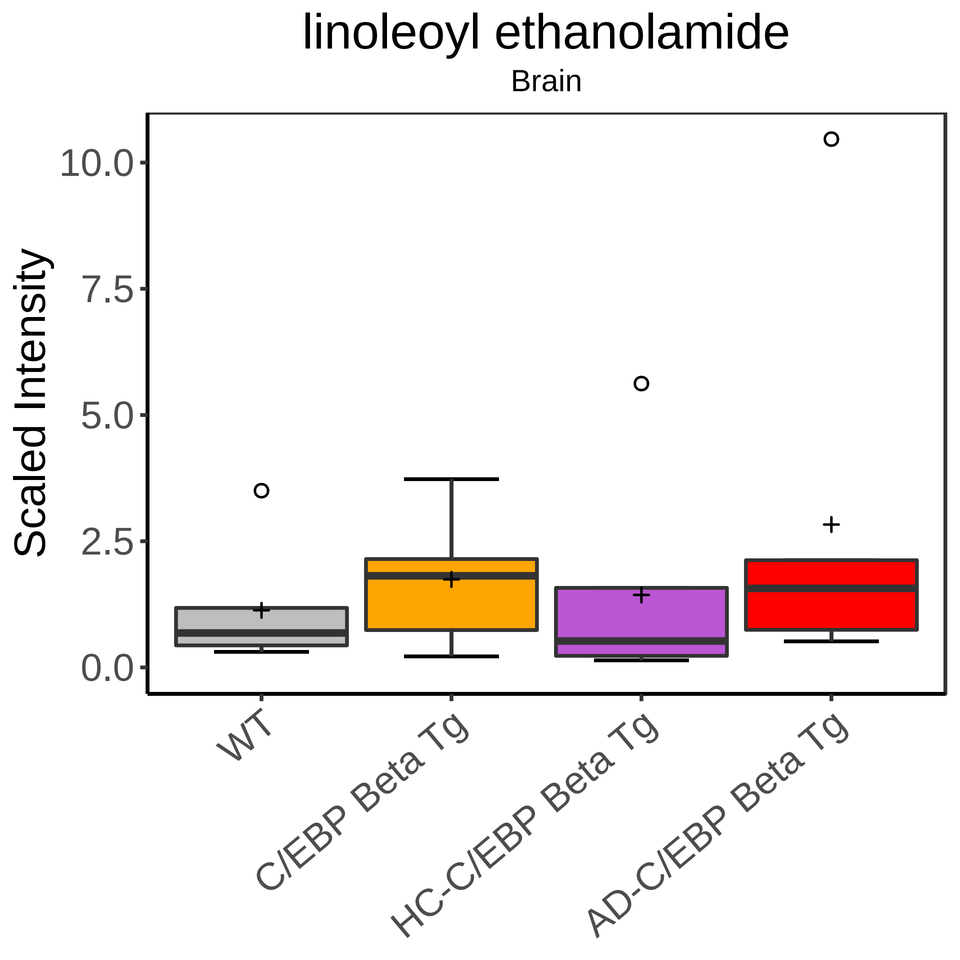

linoleoyl ethanolamide
Brain
Scaled Intensity
10.0
7.5
5.0
2.5
0.0
WT
C/EBP Beta Tg
HC-C/EBP Beta Tg
AD-C/EBP Beta Tg

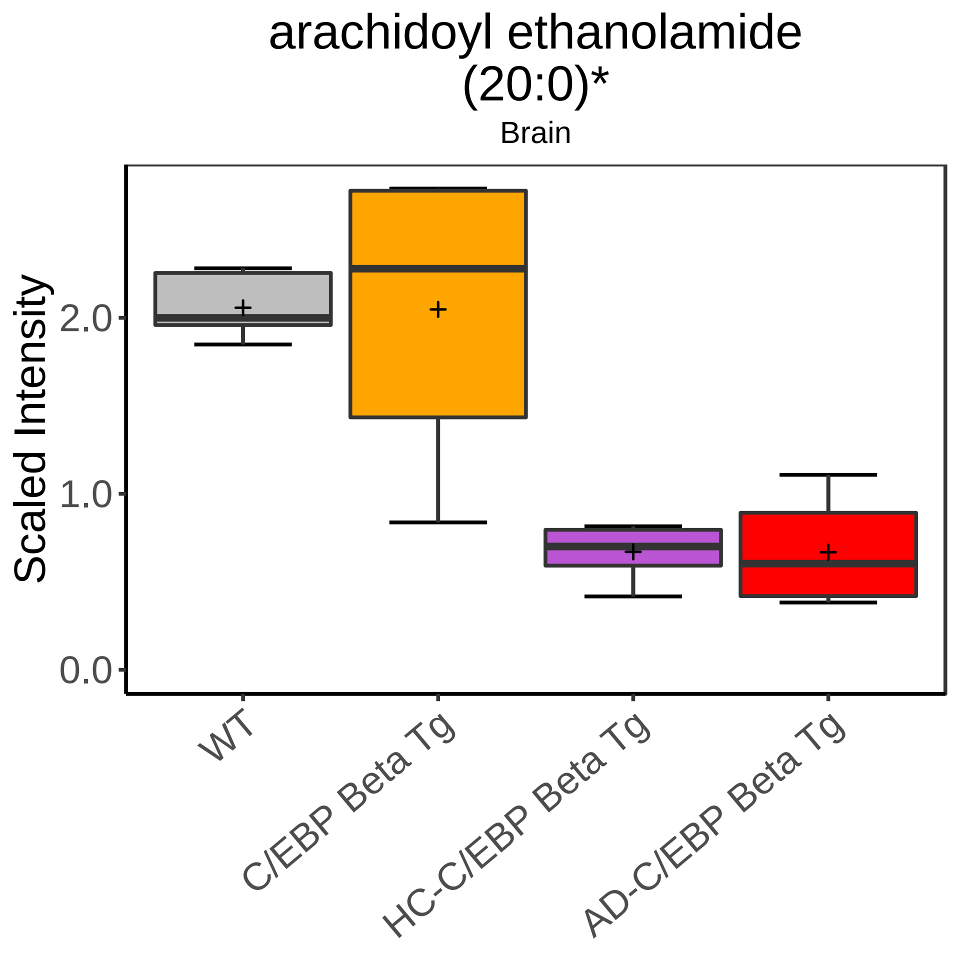

arachidoyl ethanolamide (20:0)*
Brain
Scaled Intensity
2.0
1.0
0.0
WT
C/EBP Beta Tg
HC-C/EBP Beta Tg
AD-C/EBP Beta Tg

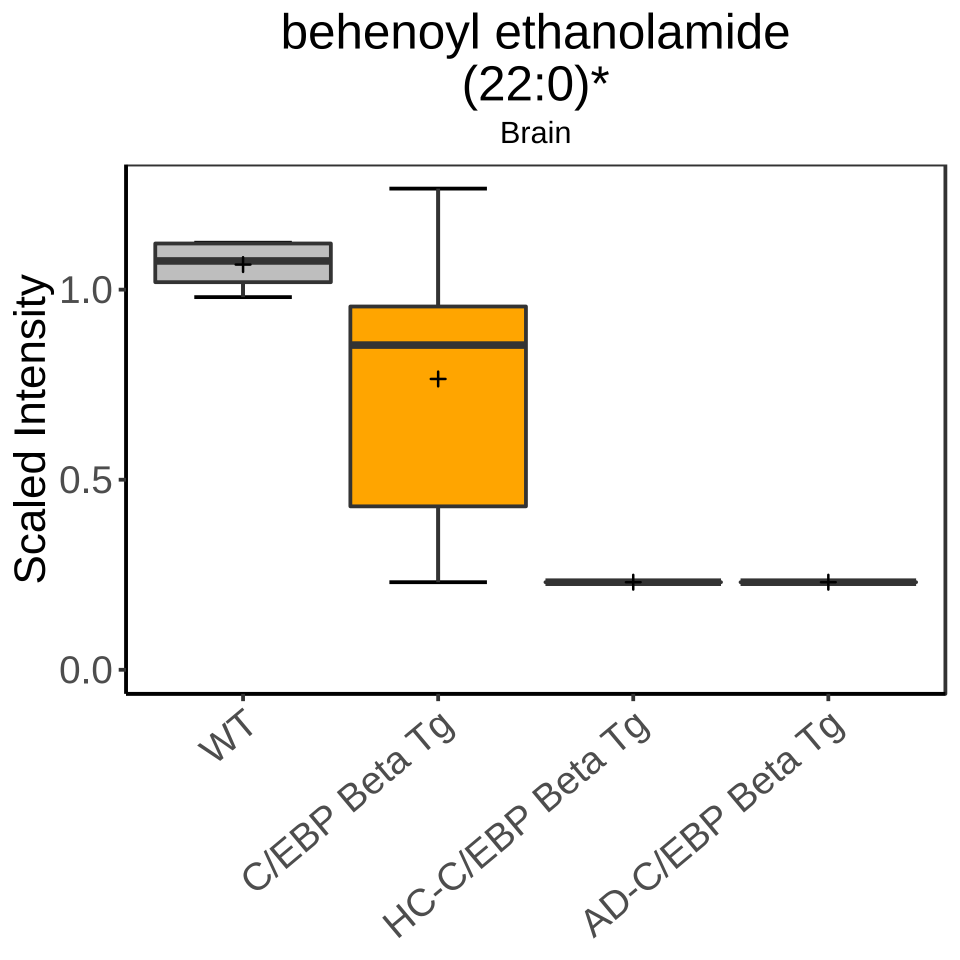

behenoyl ethanolamide
(22:0)*
Brain
Scaled Intensity
1.0
0.5
0.0
WT
C/EBP Beta Tg
HC-C/EBP Beta Tg
AD-C/EBP Beta Tg

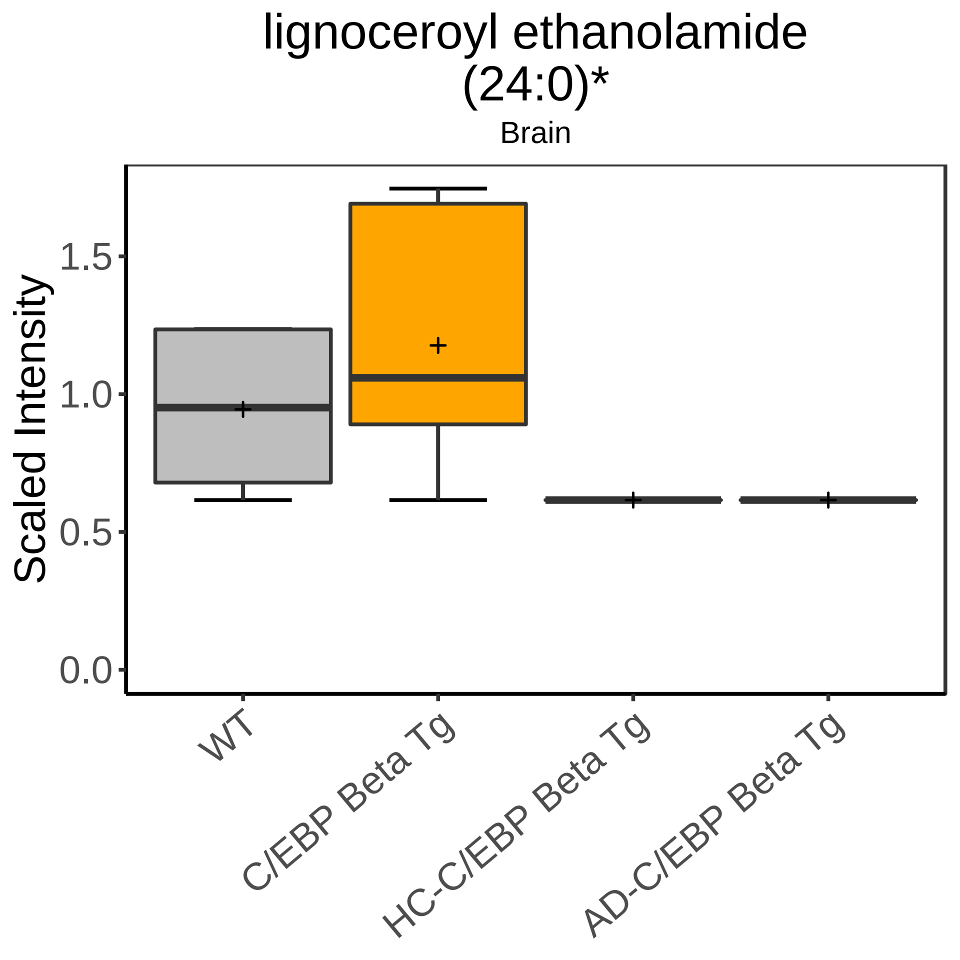
lignoceroyl ethanolamide
(24:0)*
Brain
Scaled Intensity
0.0
0.5
1.0
1.5
WT
C/EBP Beta Tg
HC-C/EBP Beta Tg
AD-C/EBP Beta Tg

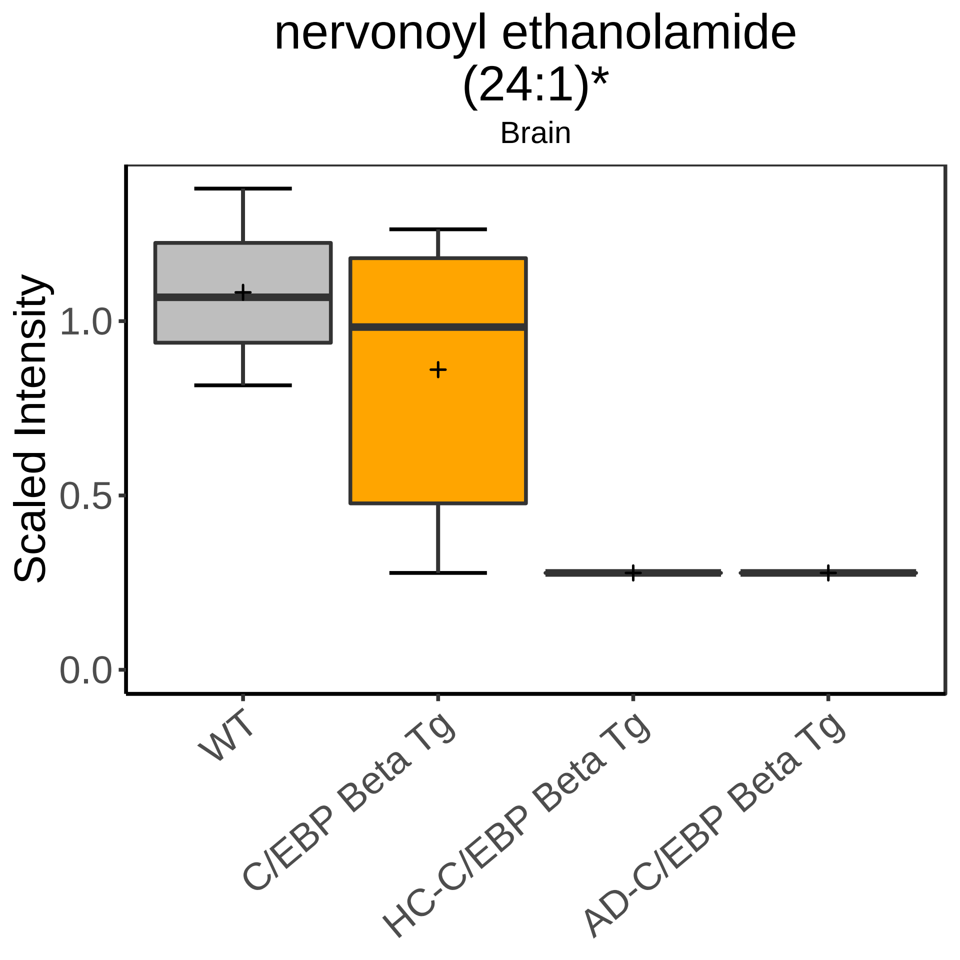
nervonoyl ethanolamide
(24:1)*
Brain
Scaled Intensity
1.0
0.5
0.0
WT
C/EBP Beta Tg
HC-C/EBP Beta Tg
AD-C/EBP Beta Tg

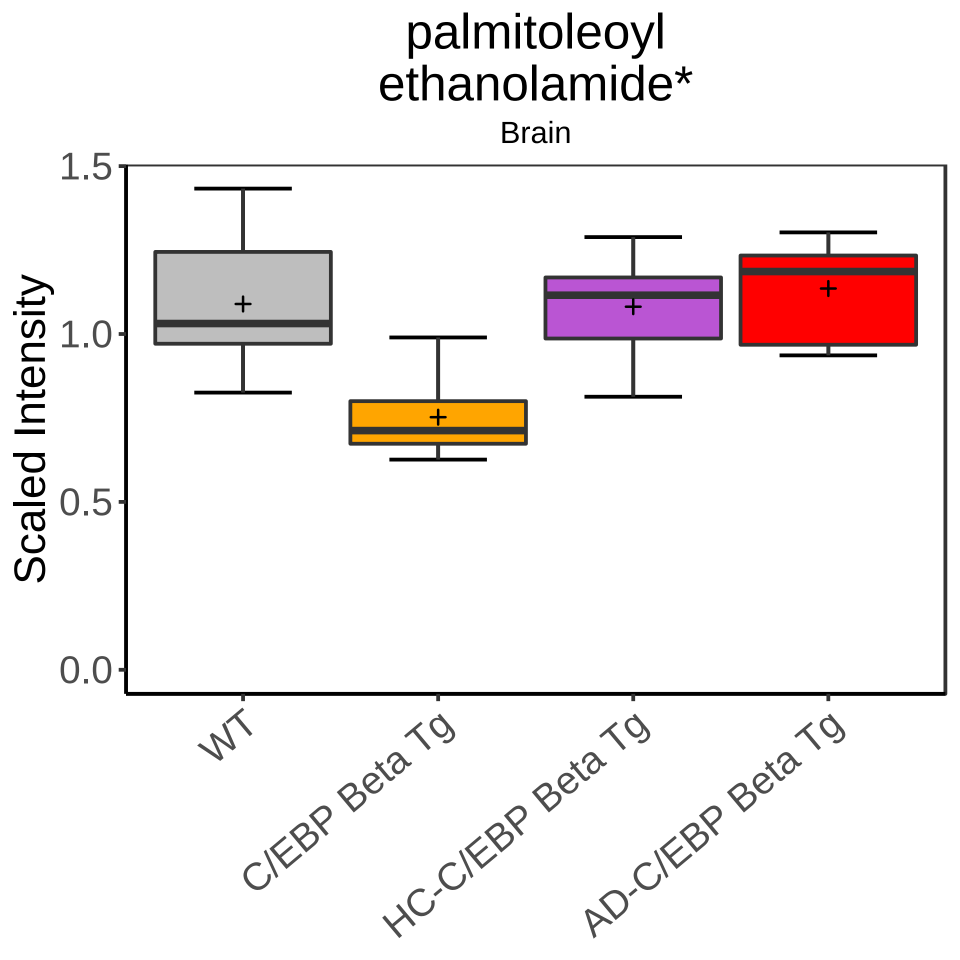

palmitoleoyl ethanolamide*
Brain
Scaled Intensity
1.5
1.0
0.5
0.0
WT
C/EBP Beta Tg
HC-C/EBP Beta Tg
AD-C/EBP Beta Tg

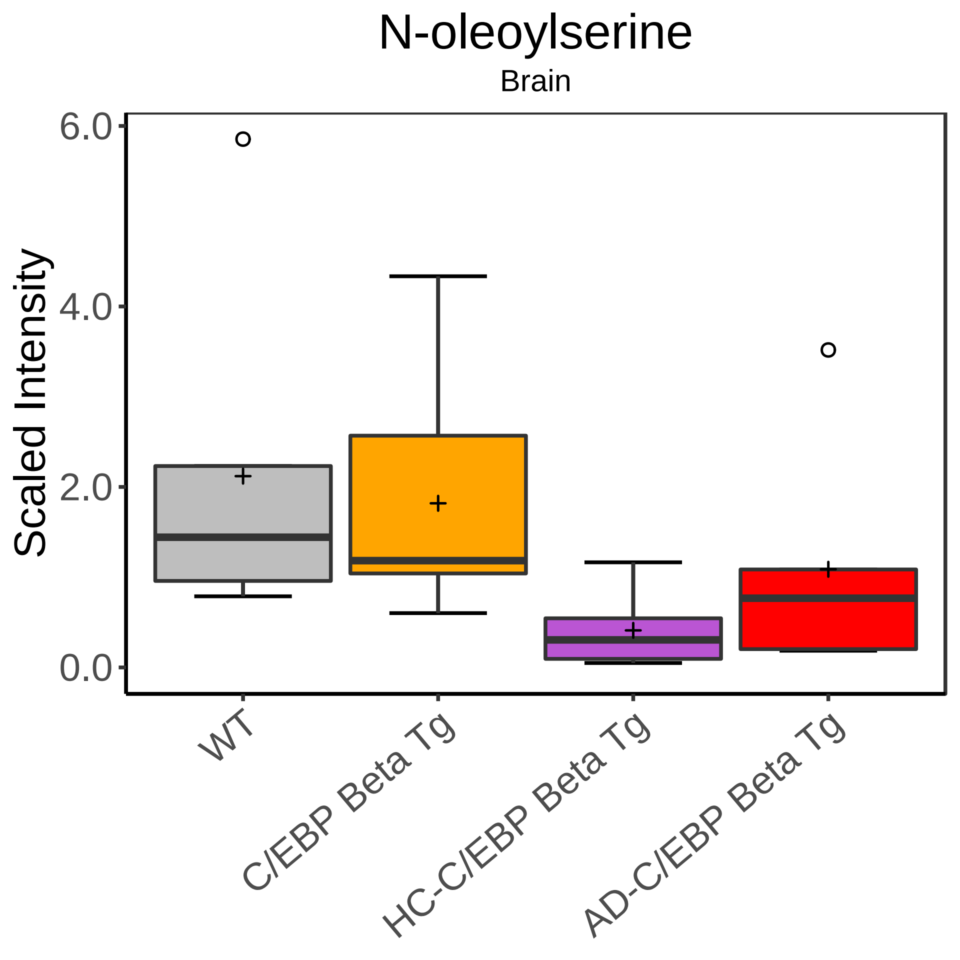
N-oleoylserine
Brain
Scaled Intensity
6.0
4.0
2.0
0.0
WT
C/EBP Beta Tg
HC-C/EBP Beta Tg
AD-C/EBP Beta Tg

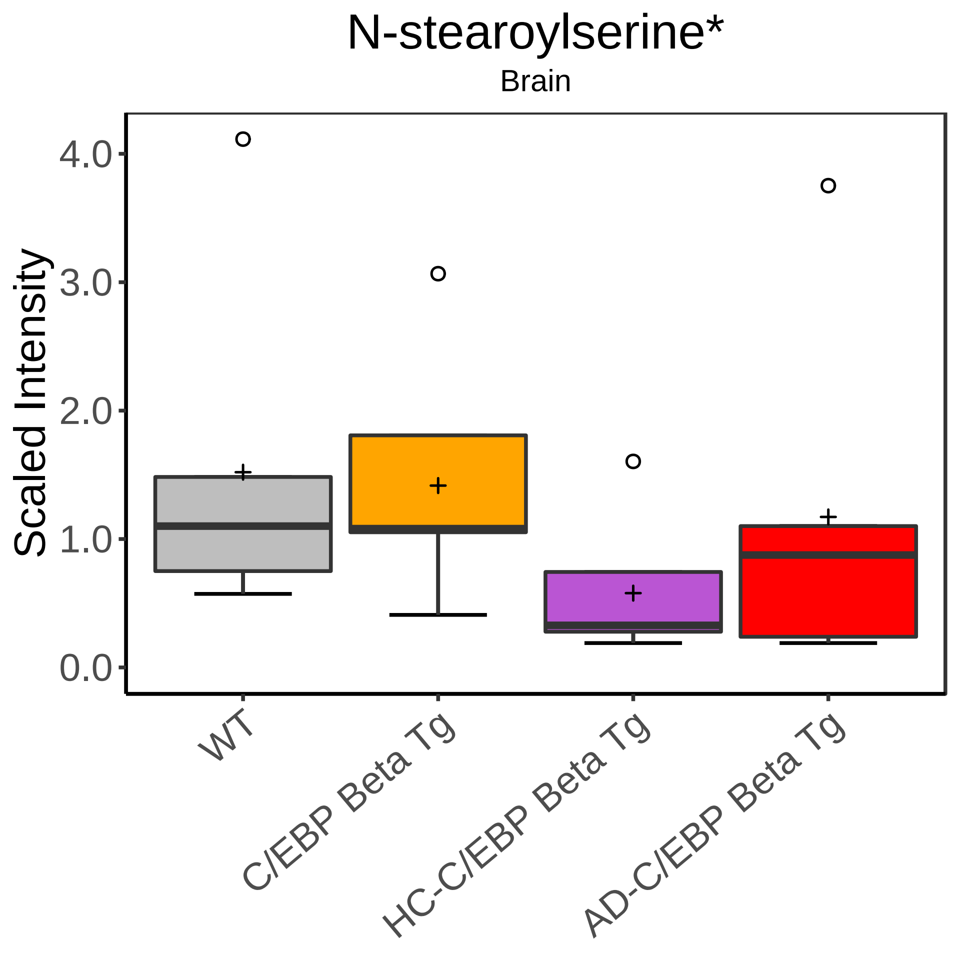

N-stearoylserine*
Brain
Scaled Intensity
4.0
3.0
2.0
1.0
0.0
WT
C/EBP Beta Tg
HC-C/EBP Beta Tg
AD-C/EBP Beta Tg

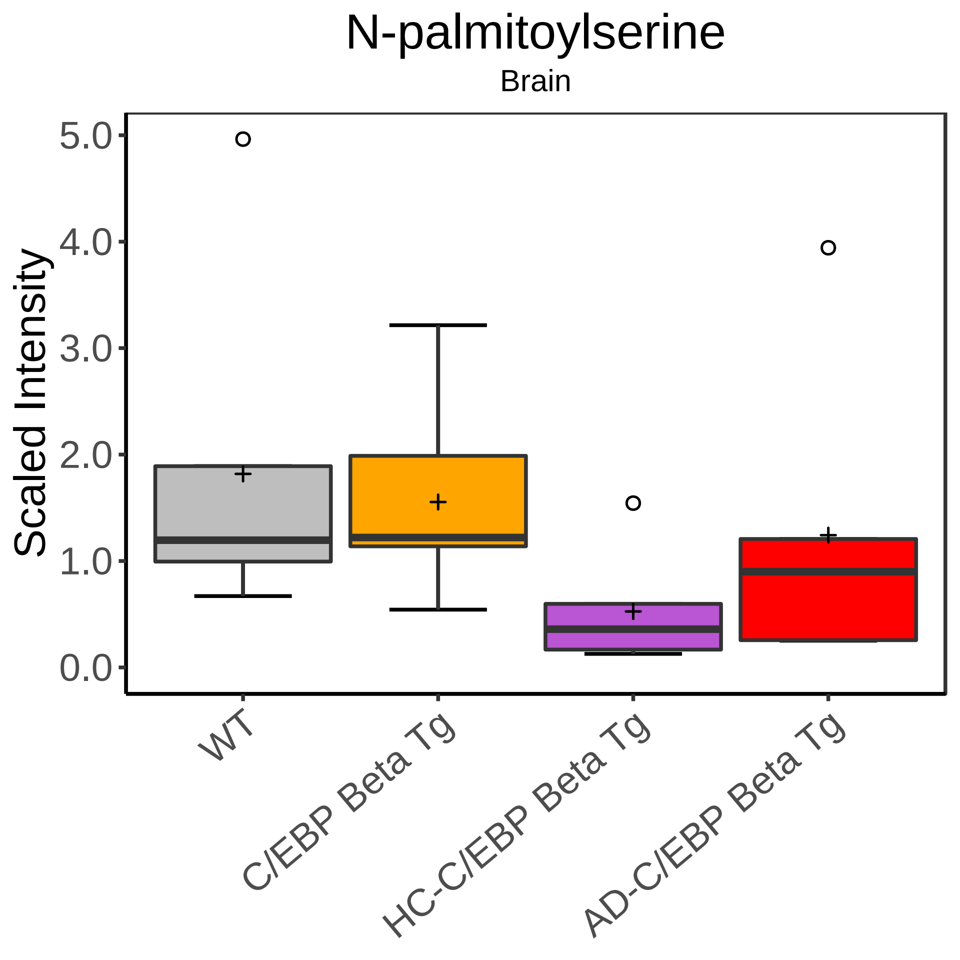
N-palmitoylserine
Brain
Scaled Intensity
5.0
4.0
3.0
2.0
1.0
0.0
WT
C/EBP Beta Tg
HC-C/EBP Beta Tg
AD-C/EBP Beta Tg

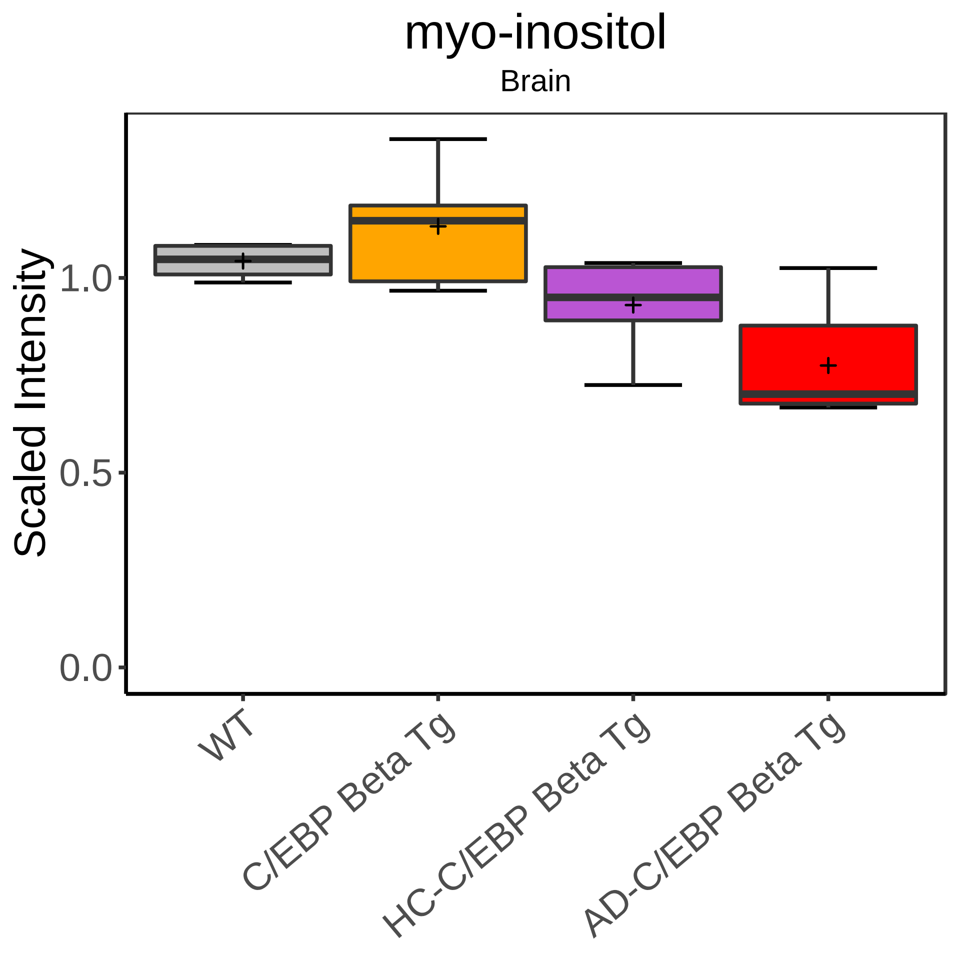

myo-inositol
Brain
Scaled Intensity
1.0
0.5
0.0
WT
C/EBP Beta Tg
HC-C/EBP Beta Tg
AD-C/EBP Beta Tg

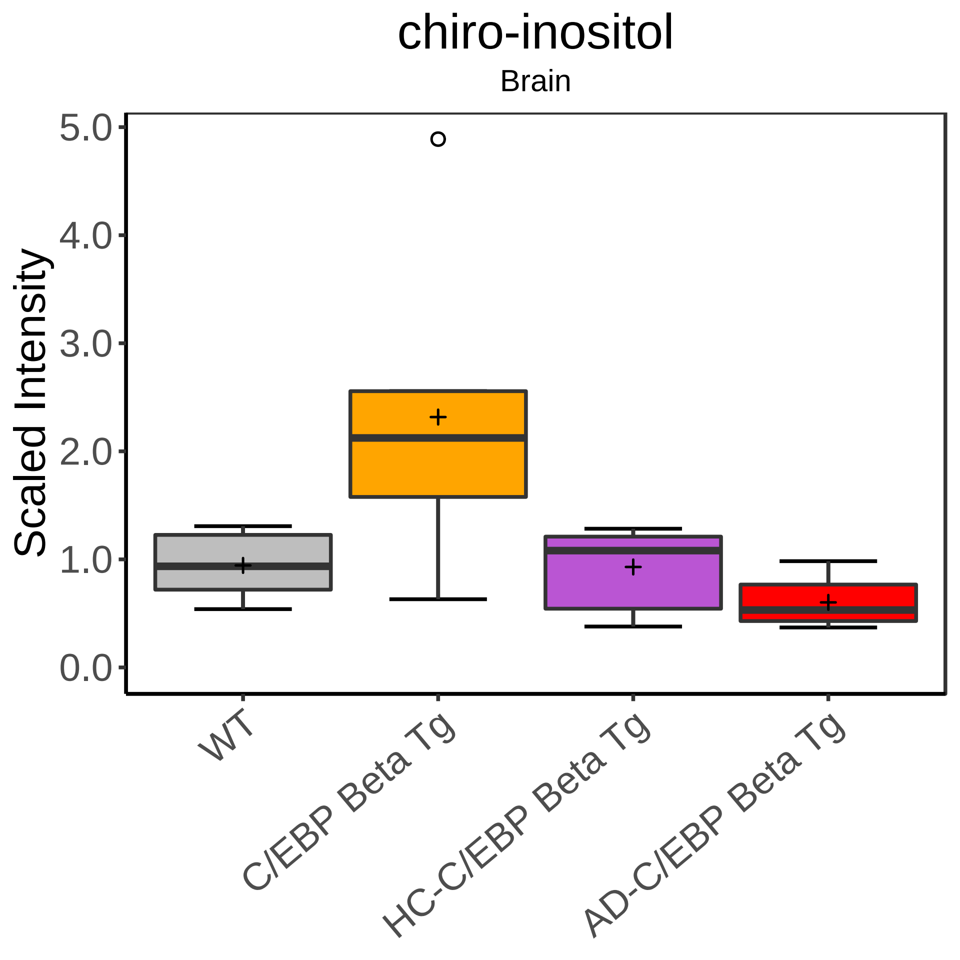
chiro-inositol
Brain
Scaled Intensity
5.0
4.0
3.0
2.0
1.0
0.0
WT
C/EBP Beta Tg
HC-C/EBP Beta Tg
AD-C/EBP Beta Tg

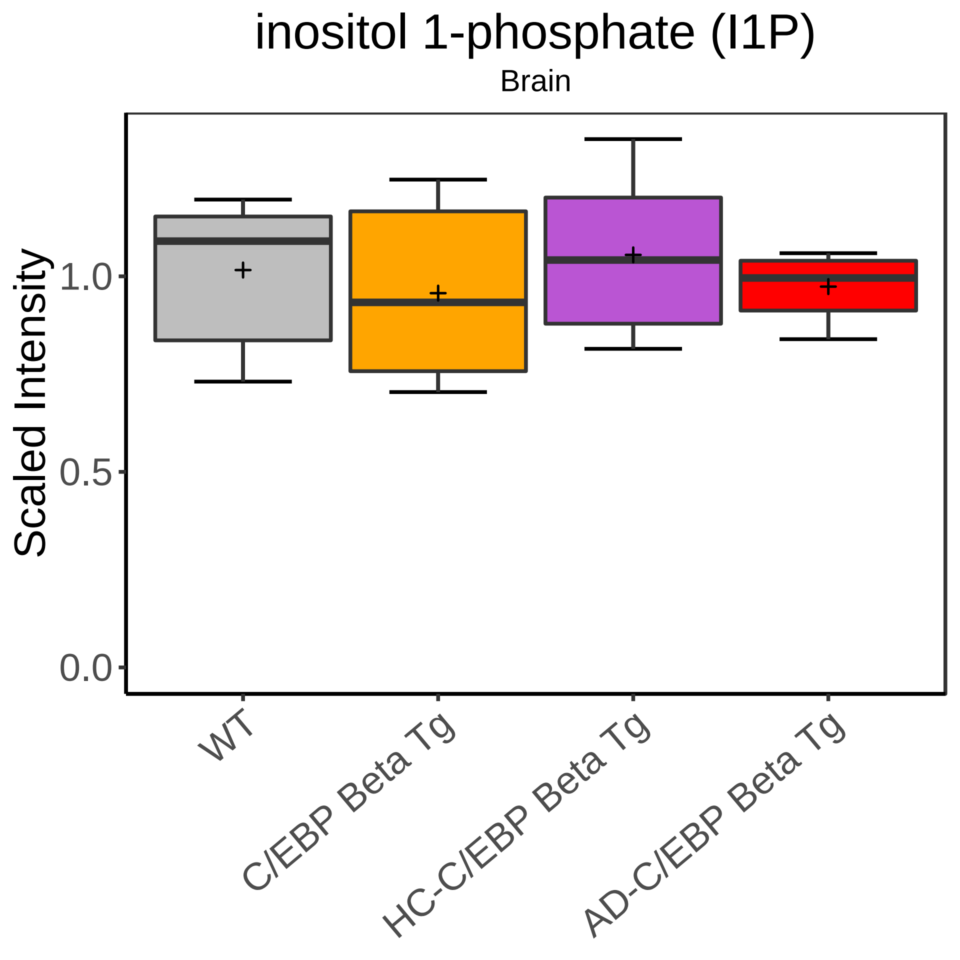

inositol 1-phosphate (I1P)
Brain
Scaled Intensity
1.0
0.5
0.0
WT
C/EBP Beta Tg
HC-C/EBP Beta Tg
AD-C/EBP Beta Tg

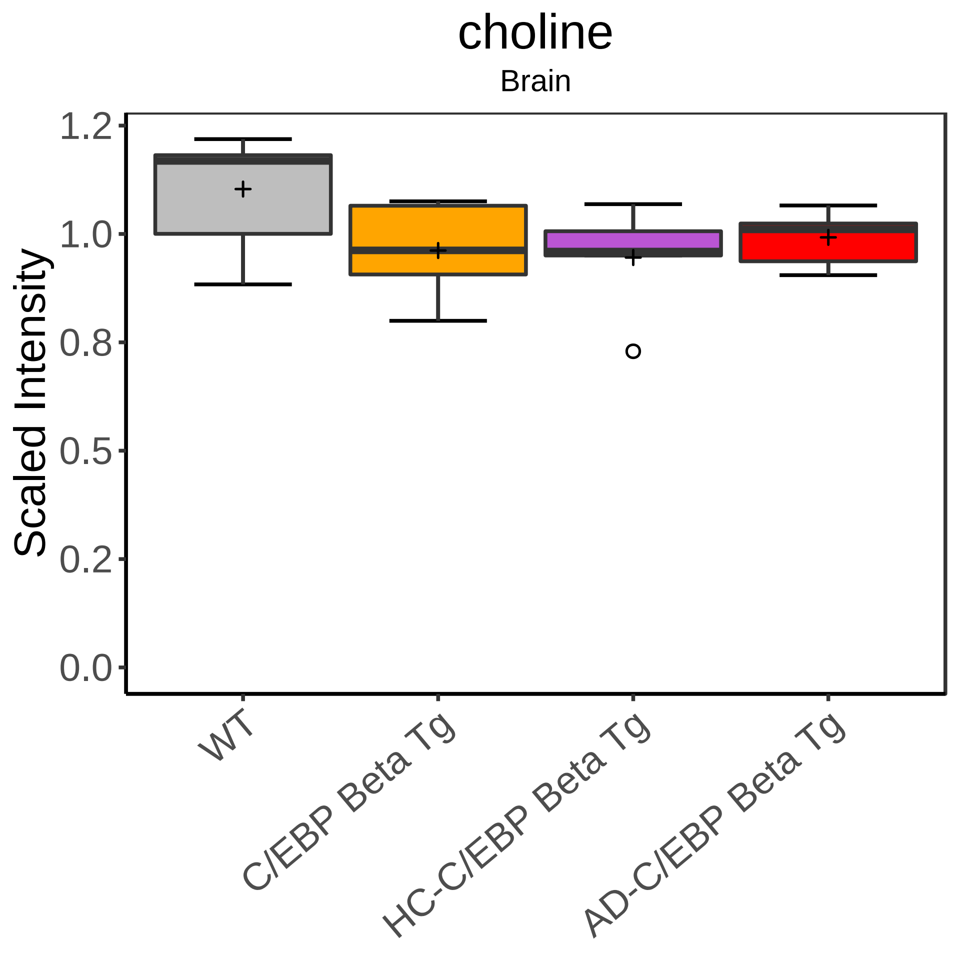

choline
Brain
Scaled Intensity
1.2
1.0
0.8
0.5
0.2
0.0
WT
C/EBP Beta Tg
HC-C/EBP Beta Tg
AD-C/EBP Beta Tg

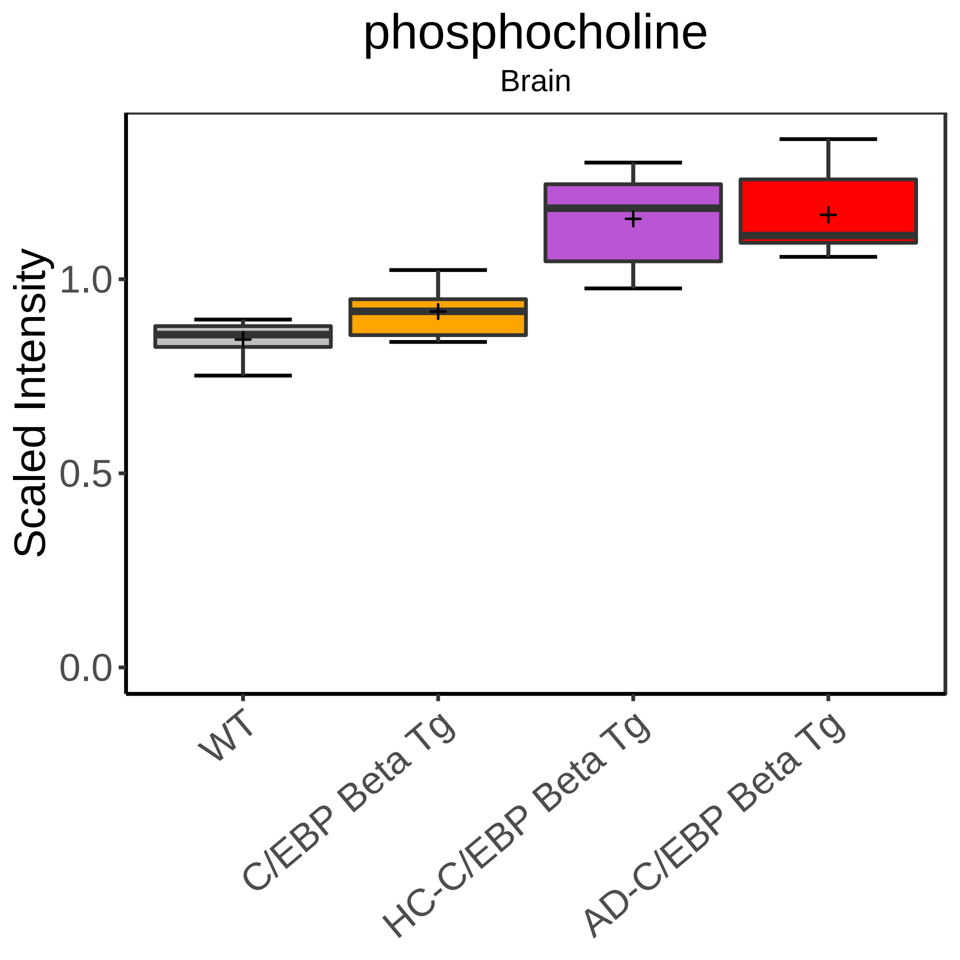

phosphocholine
Brain
Scaled Intensity
1.0
0.5
0.0
WT
C/EBP Beta Tg
HC-C/EBP Beta Tg
AD-C/EBP Beta Tg

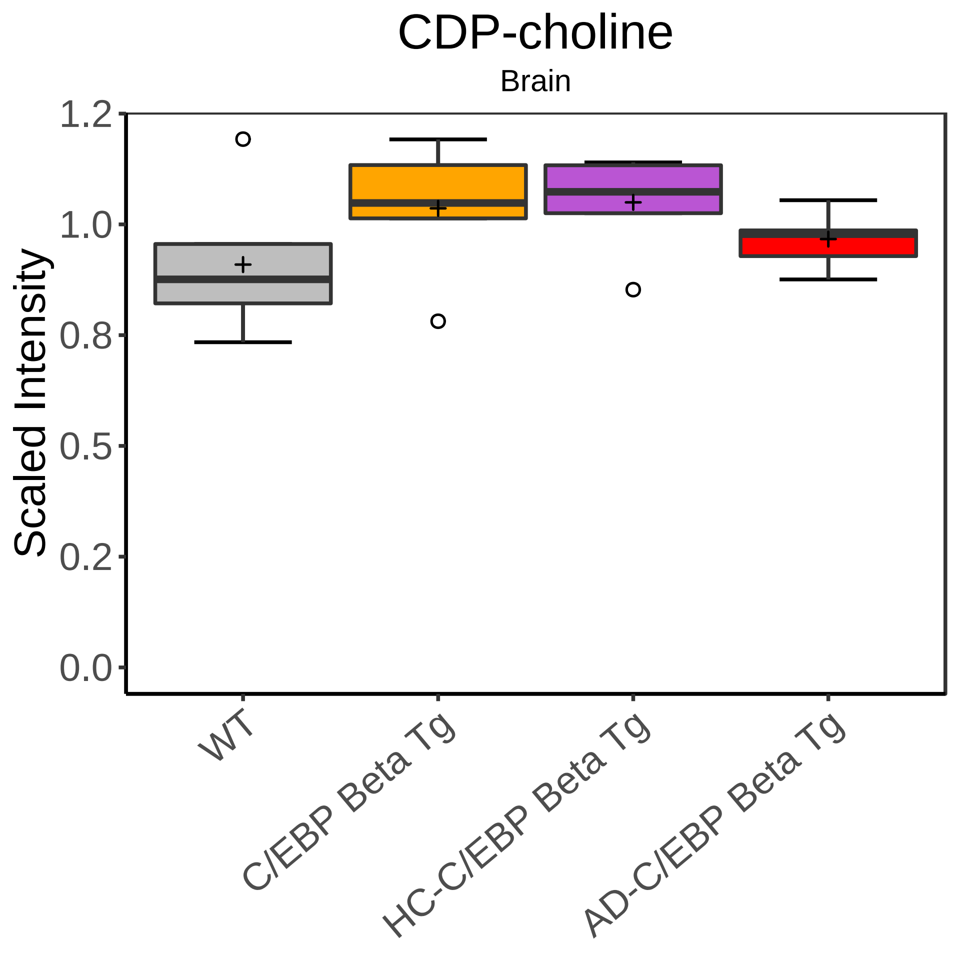

CDP-choline
Brain
Scaled Intensity
1.2
1.0
0.8
0.5
0.2
0.0
WT
C/EBP Beta Tg
HC-C/EBP Beta Tg
AD-C/EBP Beta Tg

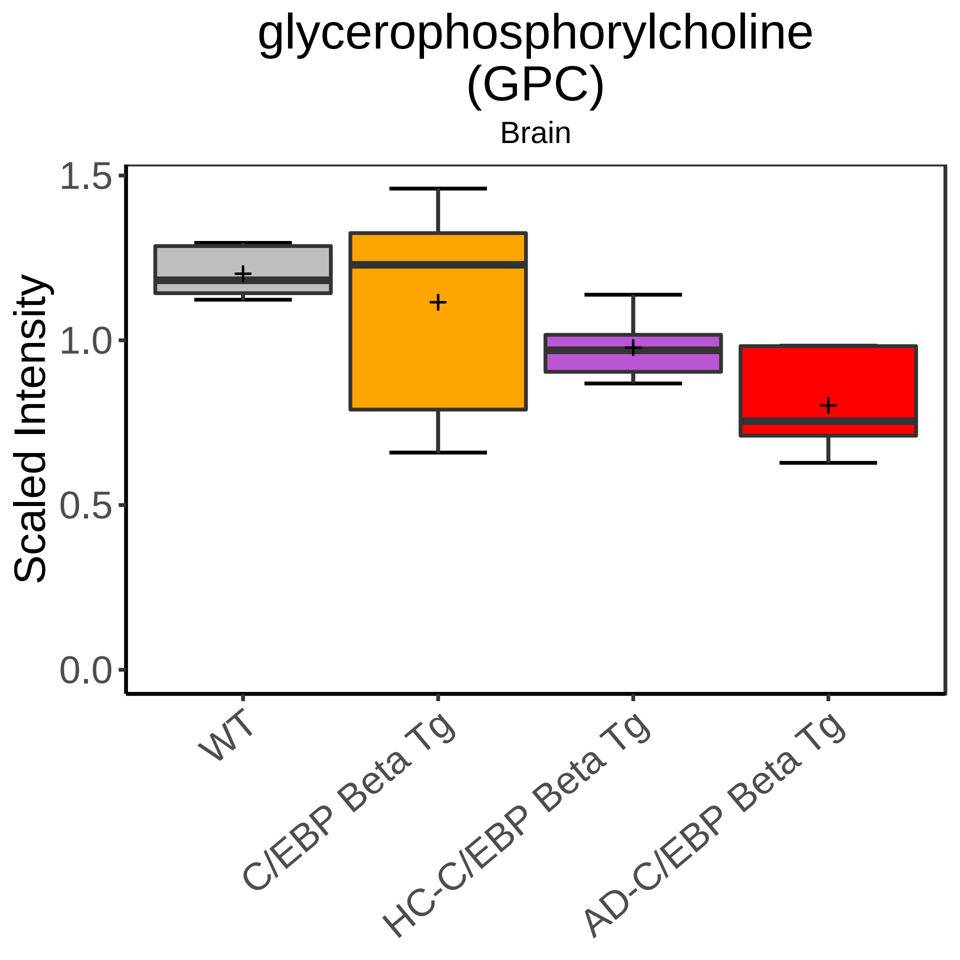
glycerophosphorylcholine
(GPC)
Brain
Scaled Intensity
1.5
1.0
0.5
0.0
WT
C/EBP Beta Tg
HC-C/EBP Beta Tg
AD-C/EBP Beta Tg

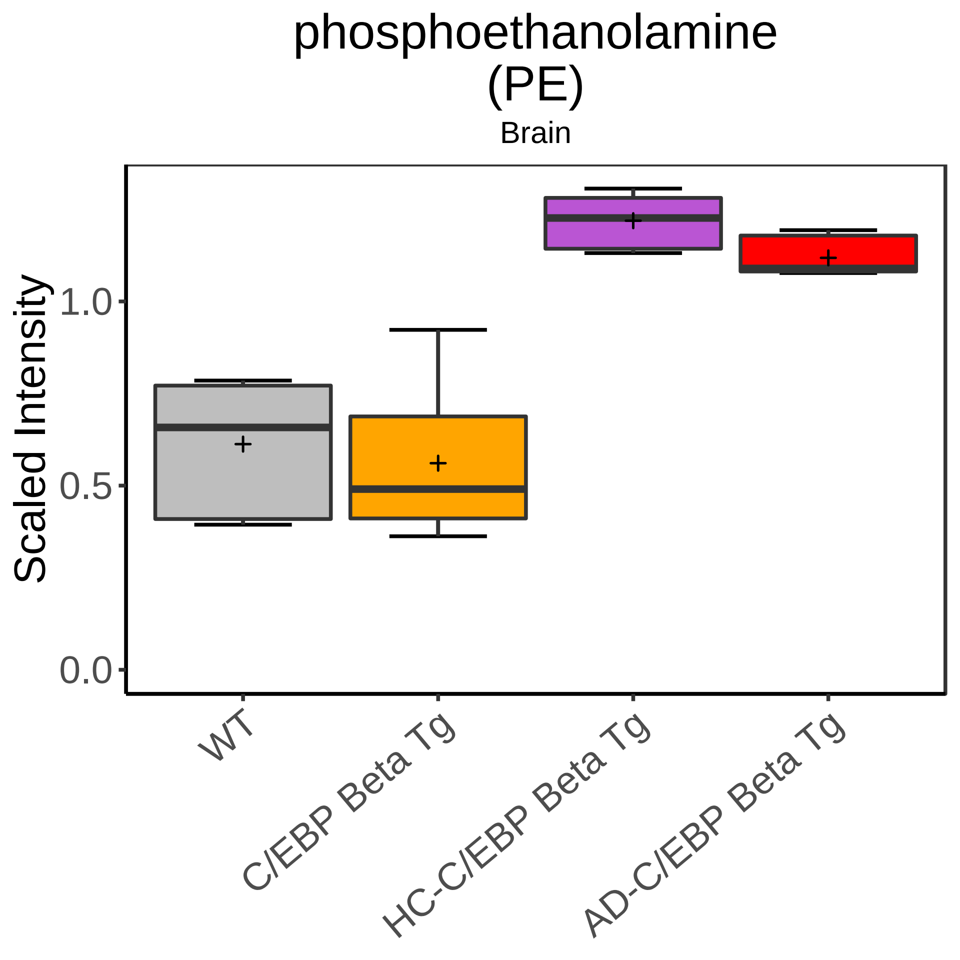

phosphoethanolamine
(PE)
Brain
Scaled Intensity
1.0
0.5
0.0
WT
C/EBP Beta Tg
HC-C/EBP Beta Tg
AD-C/EBP Beta Tg

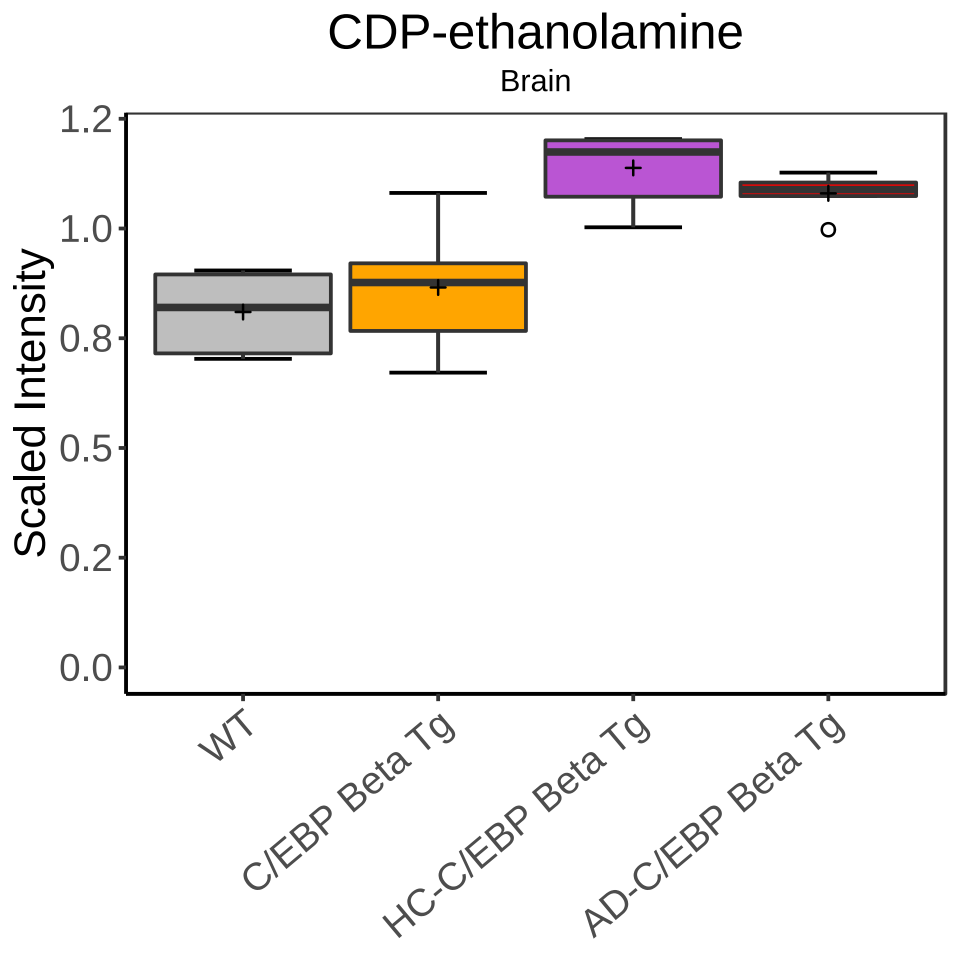

CDP-ethanolamine
Brain
Scaled Intensity
1.2
1.0
0.8
0.5
0.2
0.0
WT
C/EBP Beta Tg
HC-C/EBP Beta Tg
AD-C/EBP Beta Tg

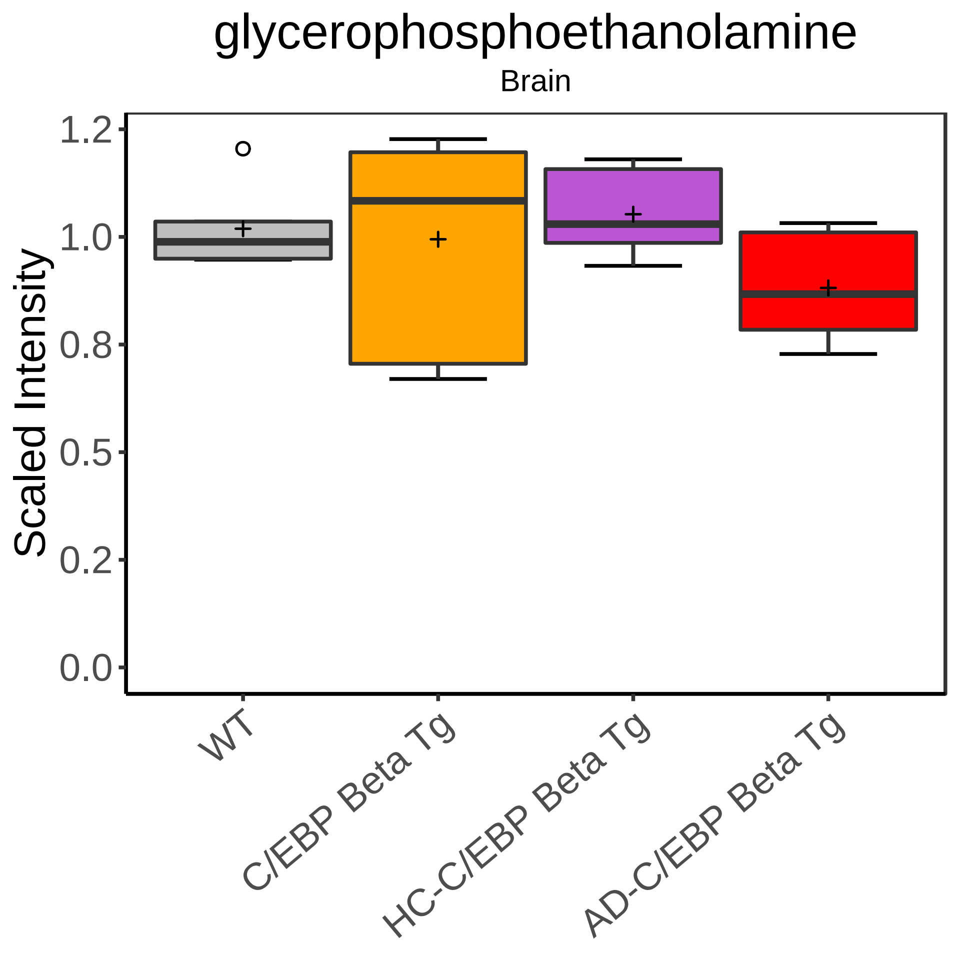
glycerophosphoethanolamine
Brain
Scaled Intensity
1.2
1.0
0.8
0.5
0.2
0.0
WT
C/EBP Beta Tg
HC-C/EBP Beta Tg
AD-C/EBP Beta Tg

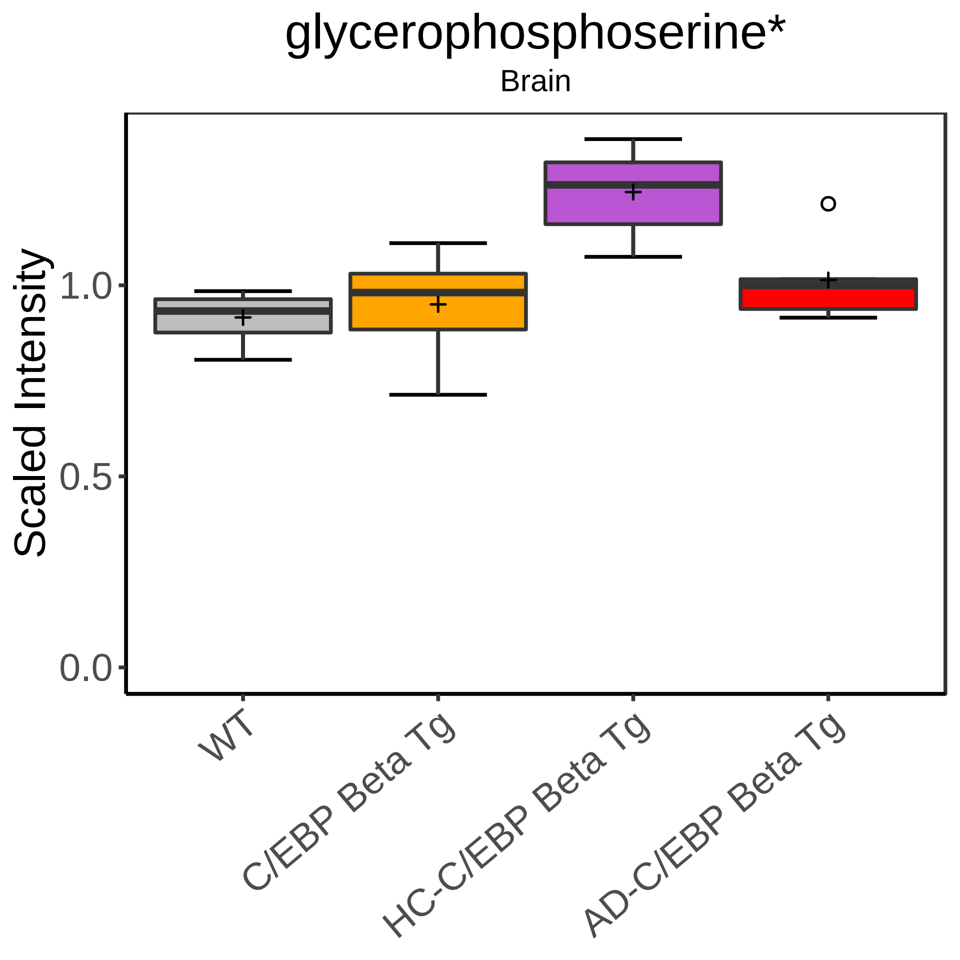

glycerophosphoserine*
Brain
Scaled Intensity
1.0
0.5
0.0
WT
C/EBP Beta Tg
HC-C/EBP Beta Tg
AD-C/EBP Beta Tg

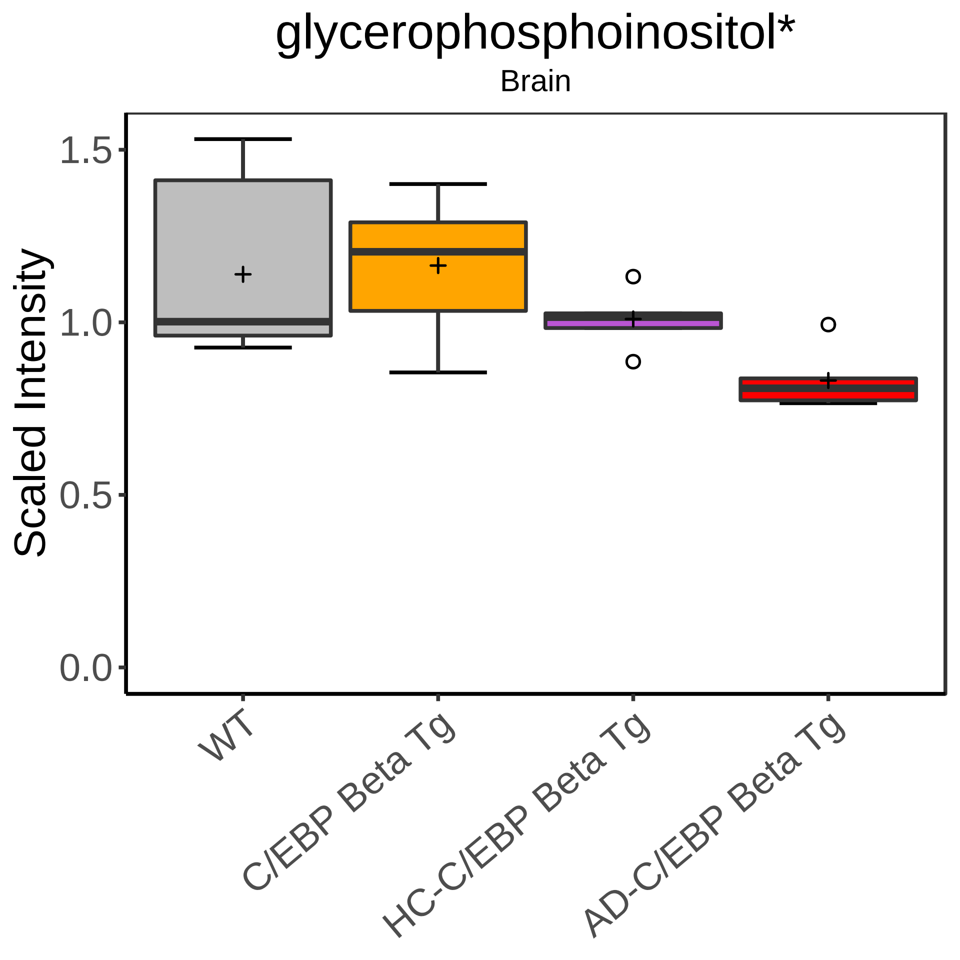
glycerophosphoinositol*
Brain
Scaled Intensity
0.0
0.5
1.0
1.5
WT
C/EBP Beta Tg
HC-C/EBP Beta Tg
AD-C/EBP Beta Tg

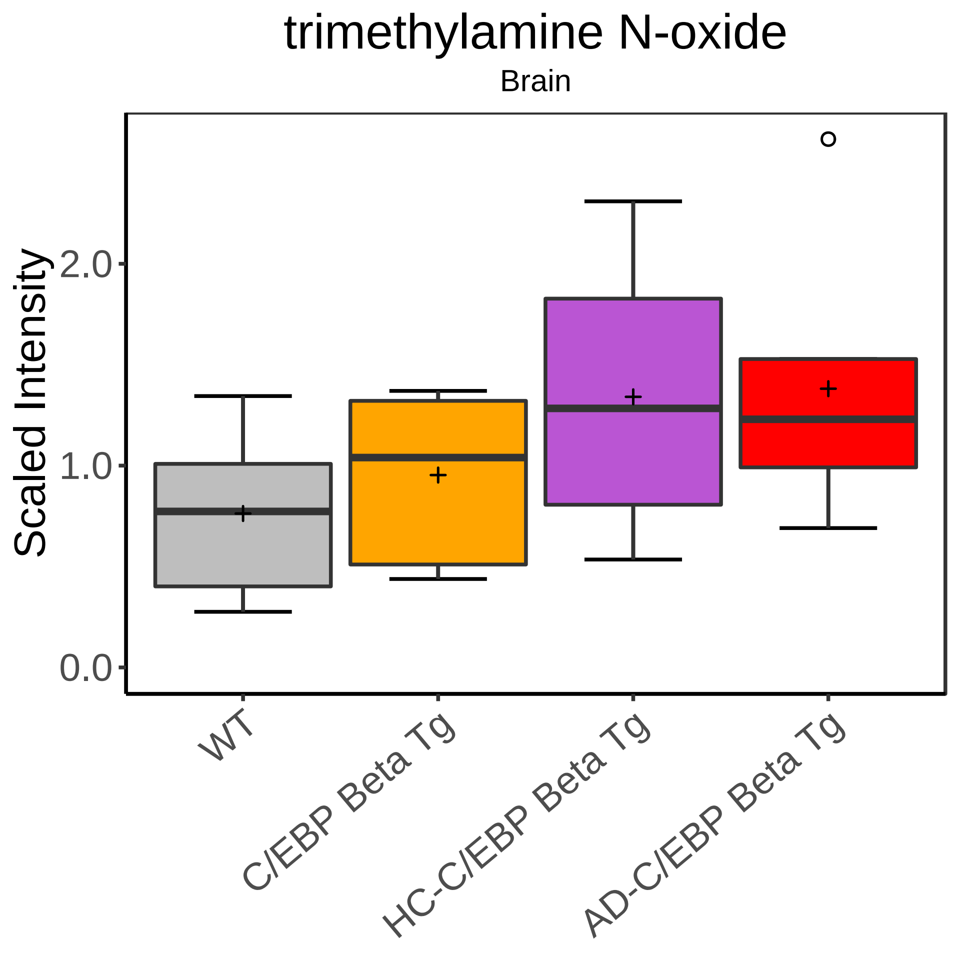

trimethylamine N-oxide
Brain
Scaled Intensity
2.0
1.0
0.0
WT
C/EBP Beta Tg
HC-C/EBP Beta Tg
AD-C/EBP Beta Tg

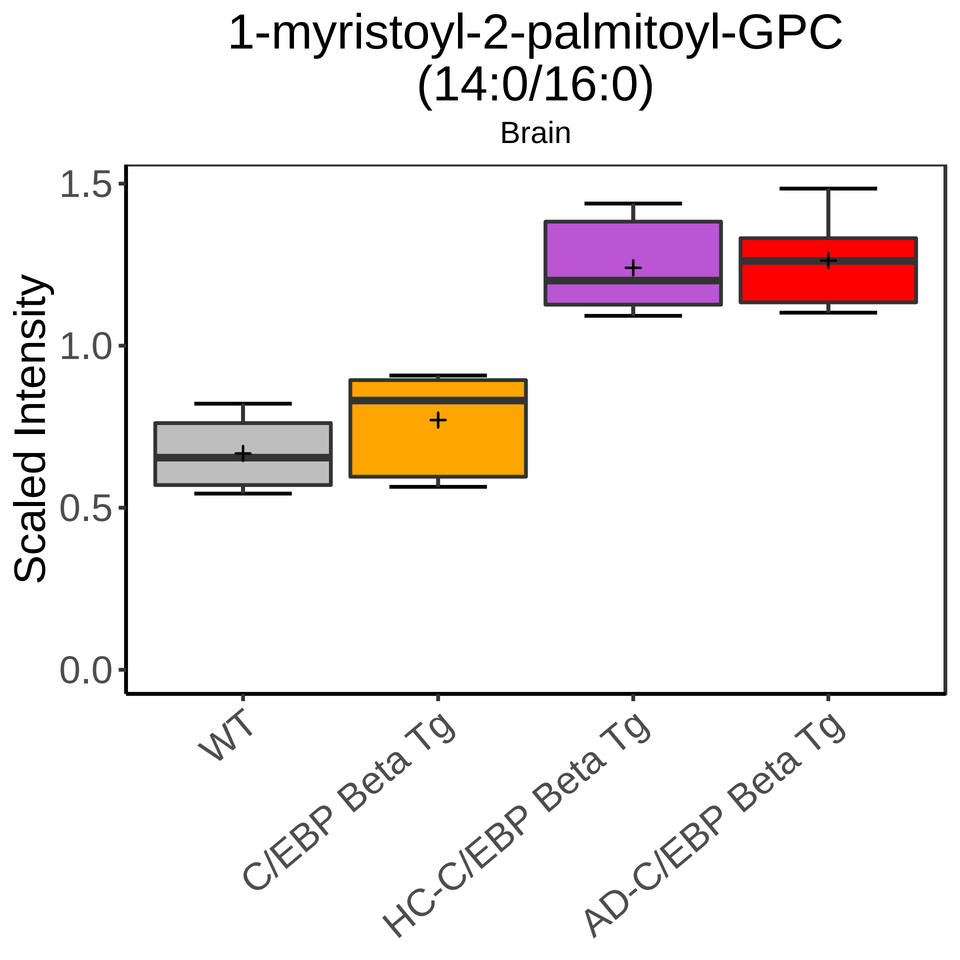

1-myristoyl-2-palmitoyl-GPC
(14:0/16:0)
Brain
Scaled Intensity
1.5
1.0
0.5
0.0
WT
C/EBP Beta Tg
HC-C/EBP Beta Tg
AD-C/EBP Beta Tg

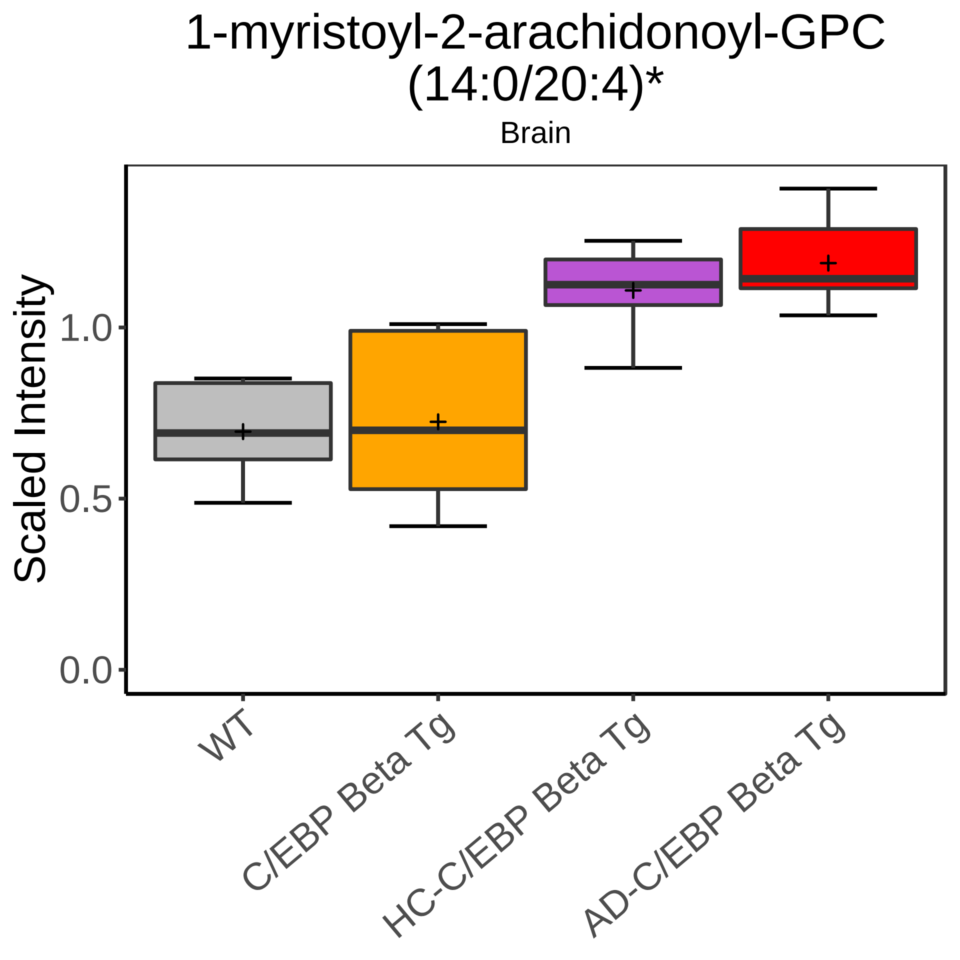

1-myristoyl-2-arachidonoyl-GPC
(14:0/20:4)*
Brain
Scaled Intensity
0.0
0.5
1.0
WT
C/EBP Beta Tg
HC-C/EBP Beta Tg
AD-C/EBP Beta Tg

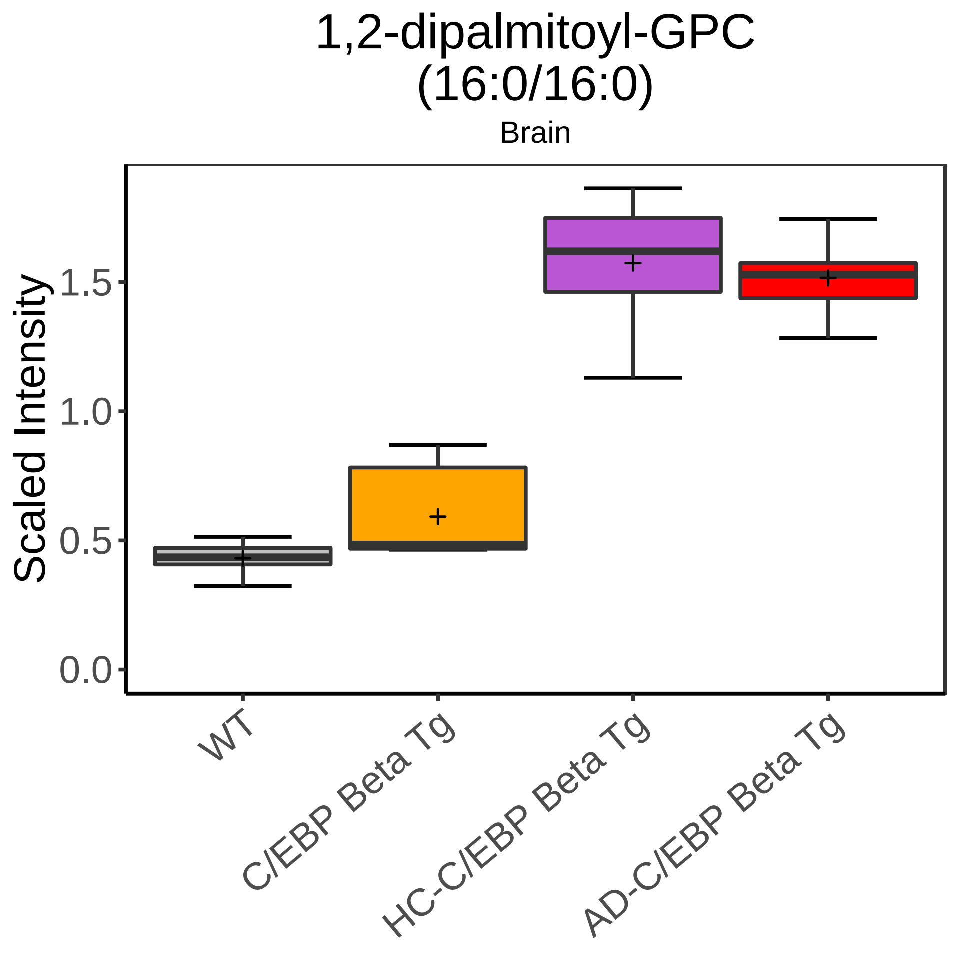

1,2-dipalmitoyl-GPC
(16:0/16:0)
Brain
Scaled Intensity
0.0
0.5
1.0
1.5
WT
C/EBP Beta Tg
HC-C/EBP Beta Tg
AD-C/EBP Beta Tg

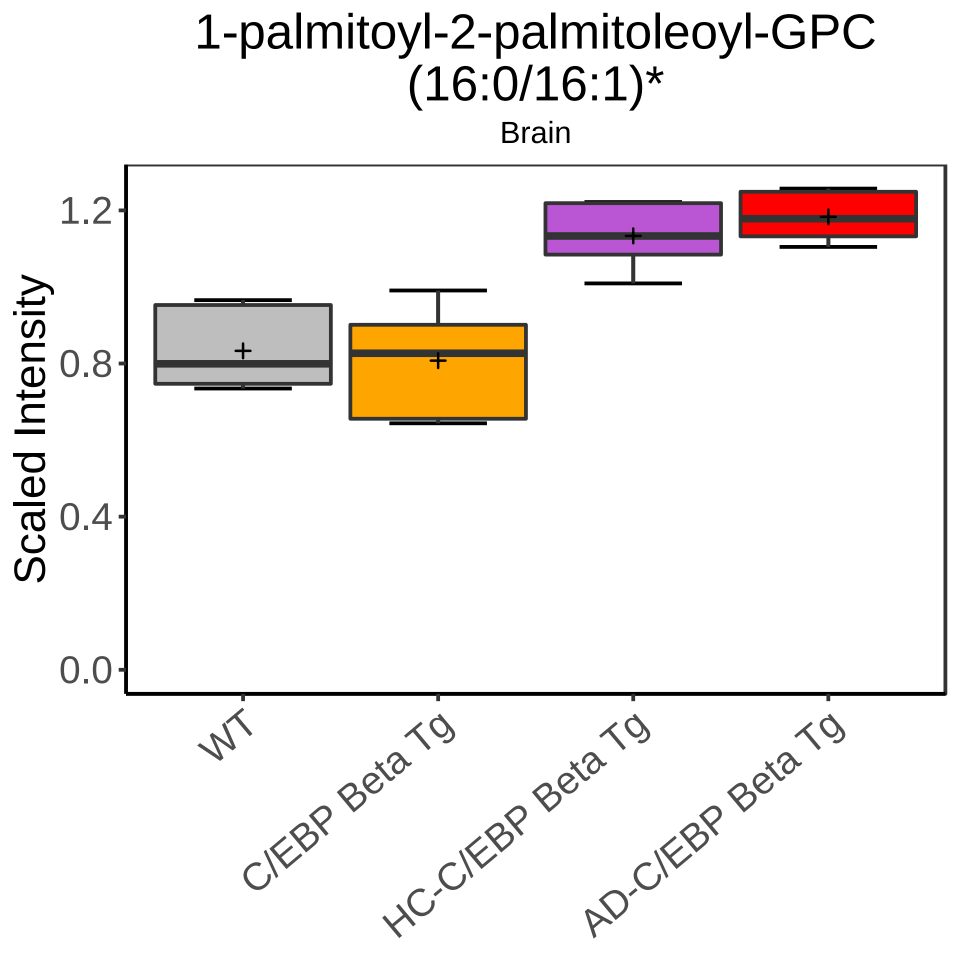

1-palmitoyl-2-palmitoleoyl-GPC
(16:0/16:1)*
Brain
Scaled Intensity
1.2
0.8
0.4
0.0
WT
C/EBP Beta Tg
HC-C/EBP Beta Tg
AD-C/EBP Beta Tg

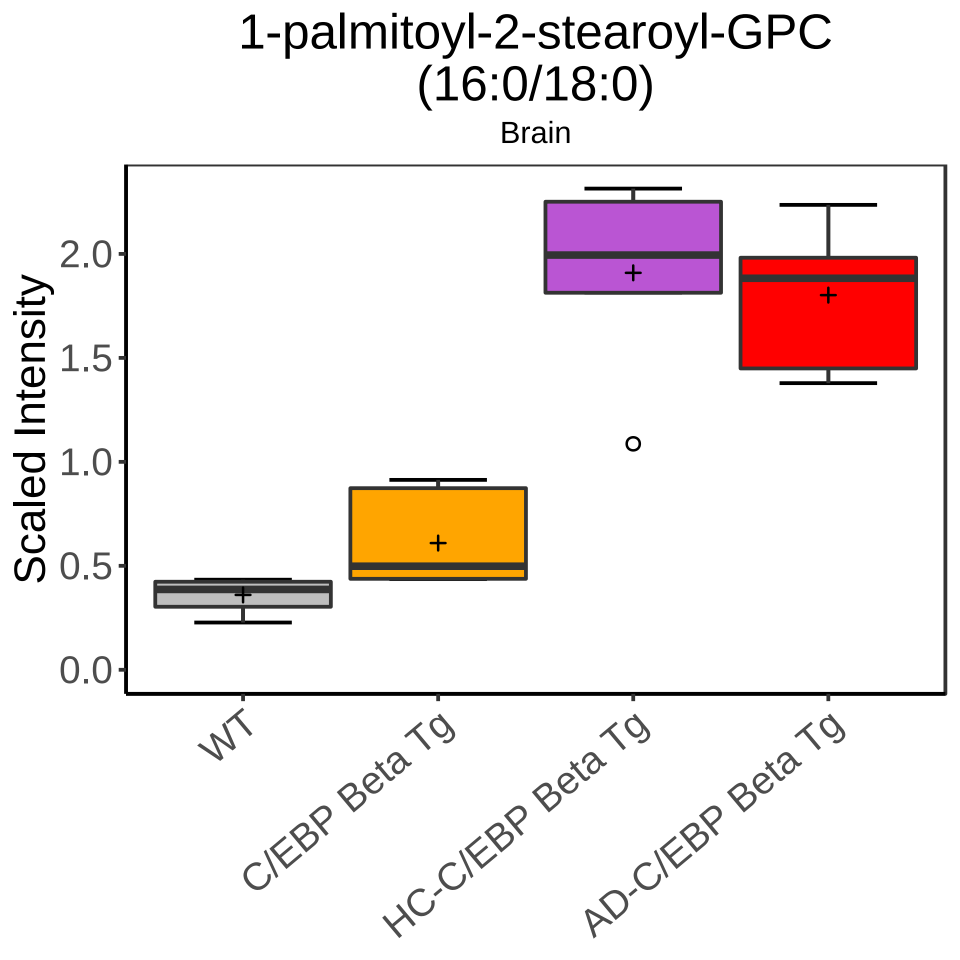

1-palmitoyl-2-stearoyl-GPC
(16:0/18:0)
Brain
Scaled Intensity
2.0
1.5
1.0
0.5
0.0
WT
C/EBP Beta Tg
HC-C/EBP Beta Tg
AD-C/EBP Beta Tg

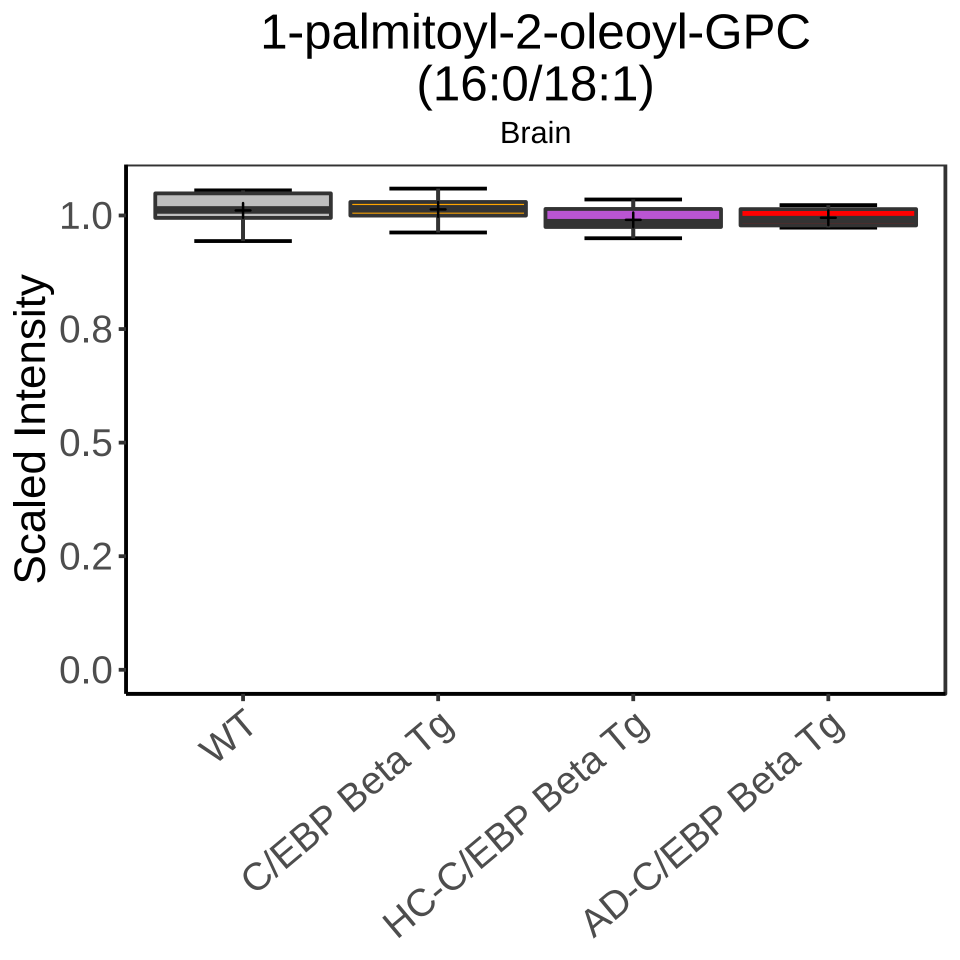

1-palmitoyl-2-oleoyl-GPC
(16:0/18:1)
Brain
Scaled Intensity
1.0
0.8
0.5
0.2
0.0
WT
C/EBP Beta Tg
HC-C/EBP Beta Tg
AD-C/EBP Beta Tg

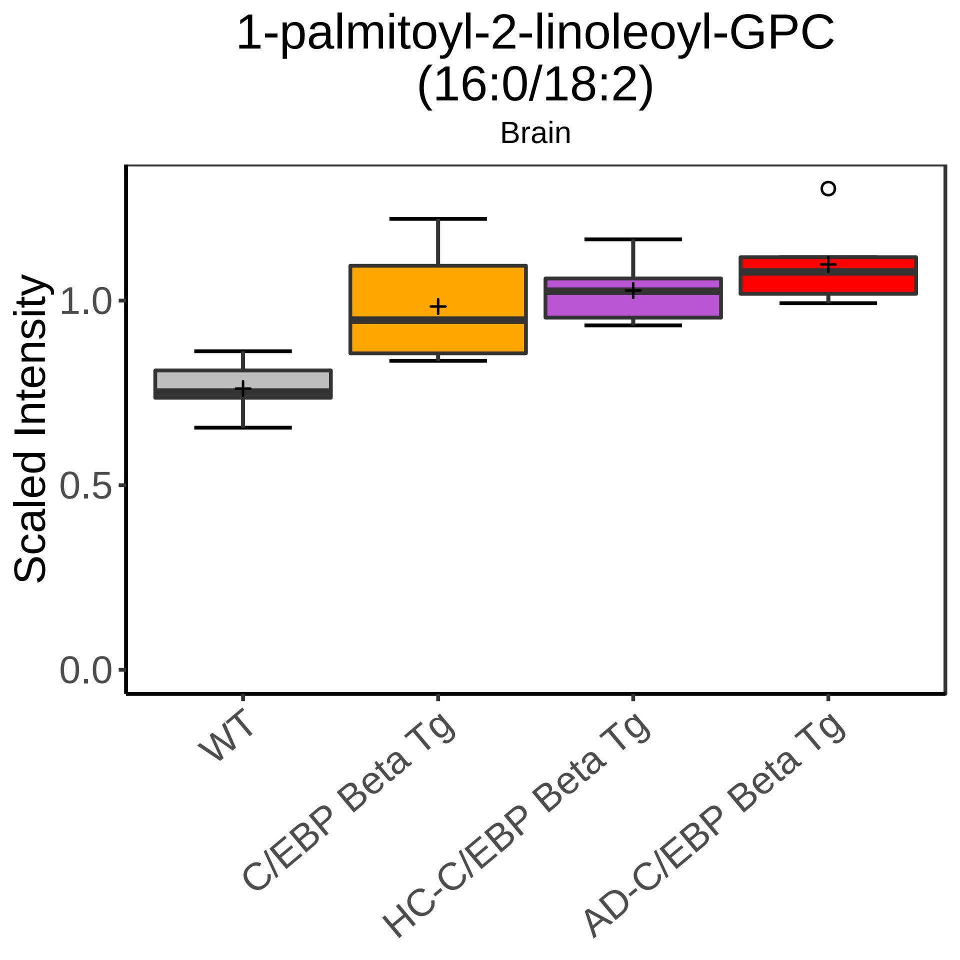

1-palmitoyl-2-linoleoyl-GPC
(16:0/18:2)
Brain
Scaled Intensity
0.0
0.5
1.0
WT
C/EBP Beta Tg
HC-C/EBP Beta Tg
AD-C/EBP Beta Tg

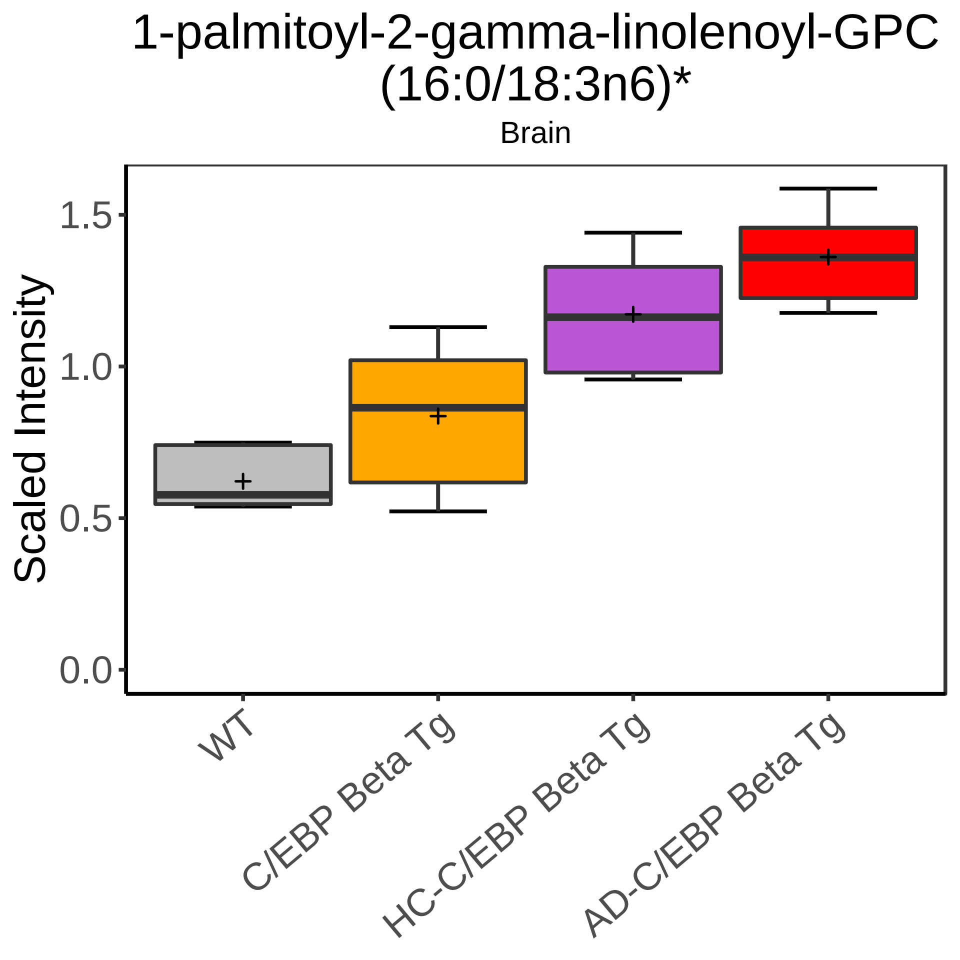

1-palmitoyl-2-gamma-linolenoyl-GPC
(16:0/18:3n6)*
Brain
Scaled Intensity
0.0
0.5
1.0
1.5
WT
C/EBP Beta Tg
HC-C/EBP Beta Tg
AD-C/EBP Beta Tg

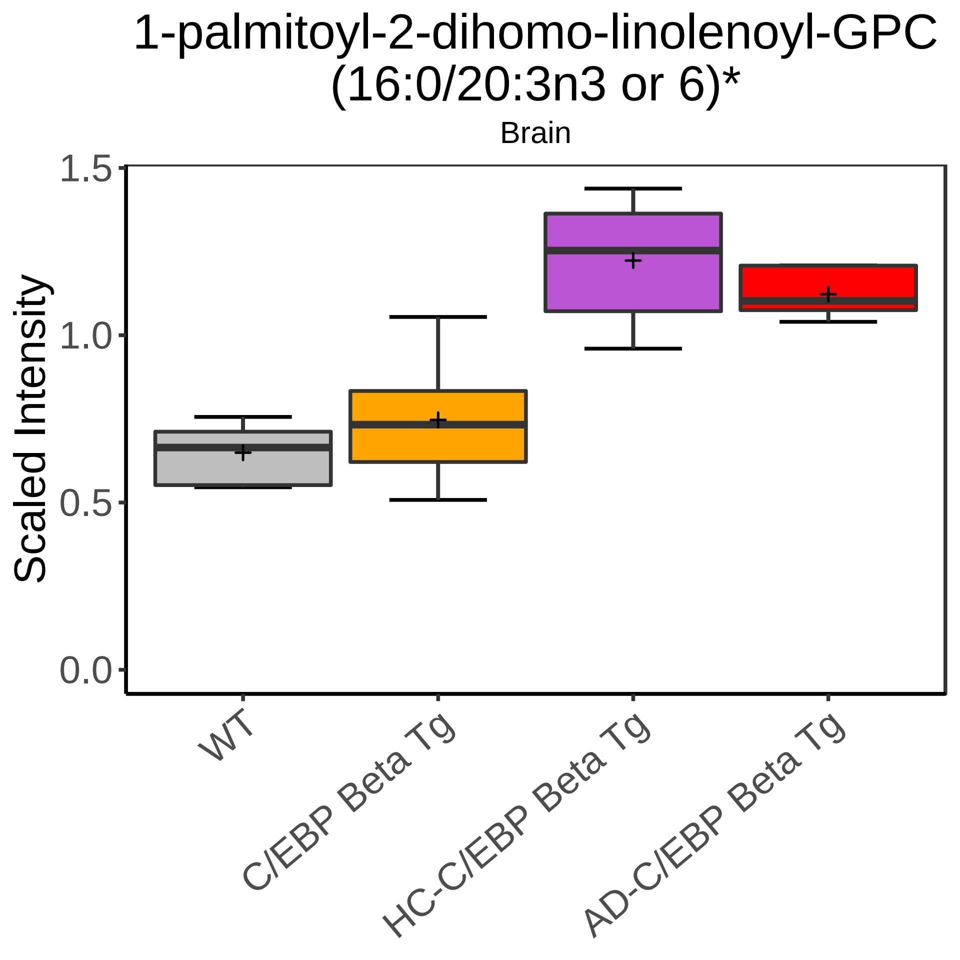
1-palmitoyl-2-dihomo-linolenoyl-GPC (16:0/20:3n3 or 6)*
Brain
Scaled Intensity
1.5
1.0
0.5
0.0
WT
C/EBP Beta Tg
HC-C/EBP Beta Tg
AD-C/EBP Beta Tg

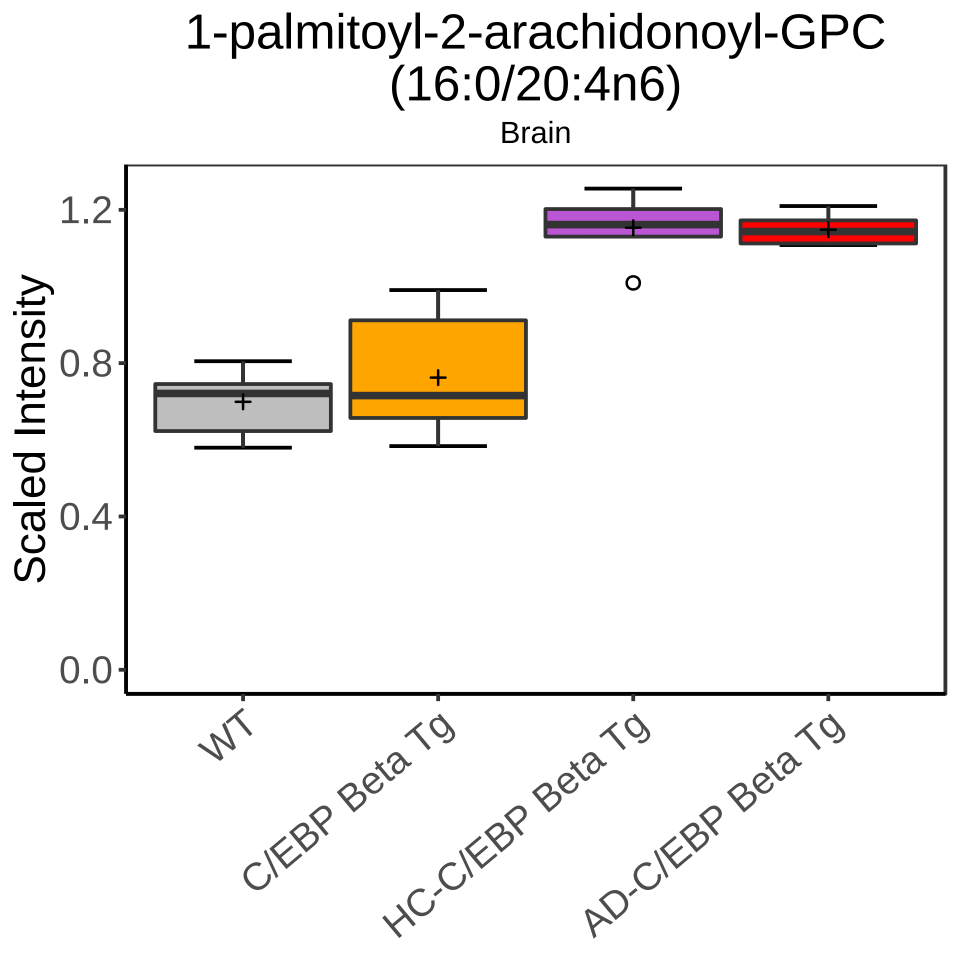

1-palmitoyl-2-arachidonoyl-GPC
(16:0/20:4n6)
Brain
Scaled Intensity
1.2
0.8
0.4
0.0
WT
C/EBP Beta Tg
HC-C/EBP Beta Tg
AD-C/EBP Beta Tg

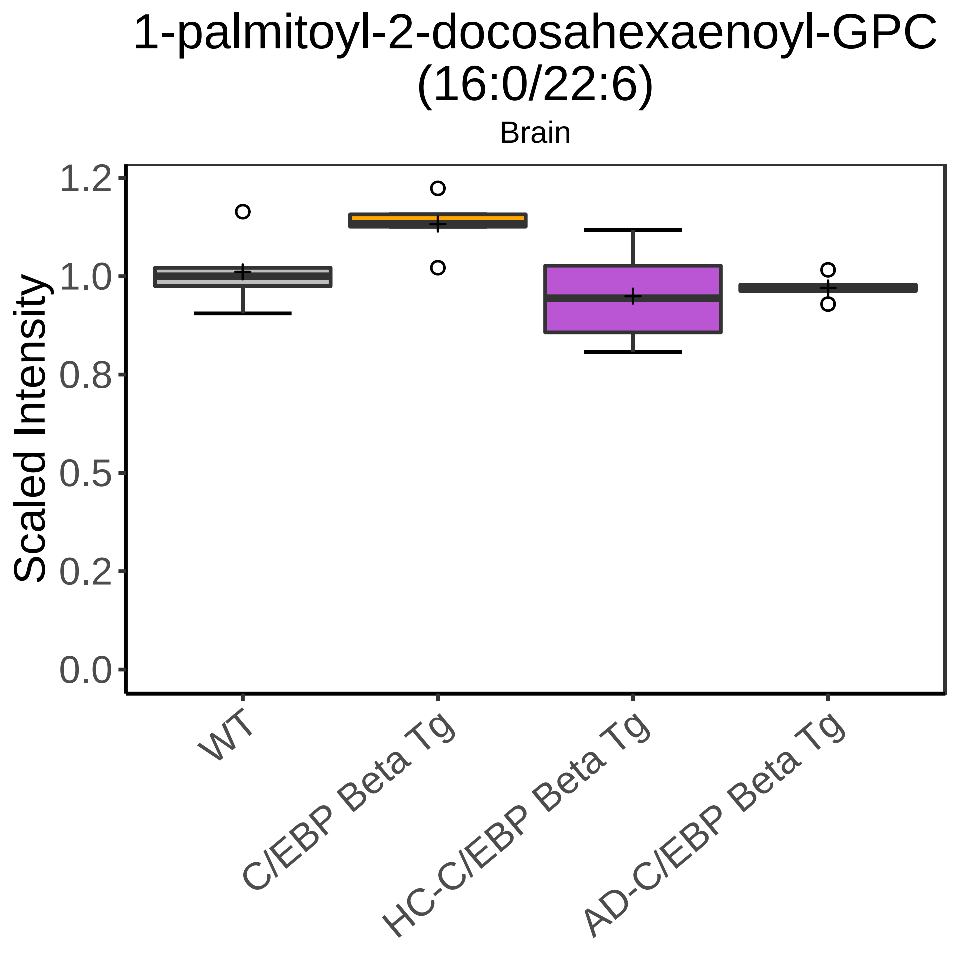
1-palmitoyl-2-docosahexaenoyl-GPC
(16:0/22:6)
Brain
Scaled Intensity
1.2
1.0
0.8
0.5
0.2
0.0
WT
C/EBP Beta Tg
HC-C/EBP Beta Tg
AD-C/EBP Beta Tg

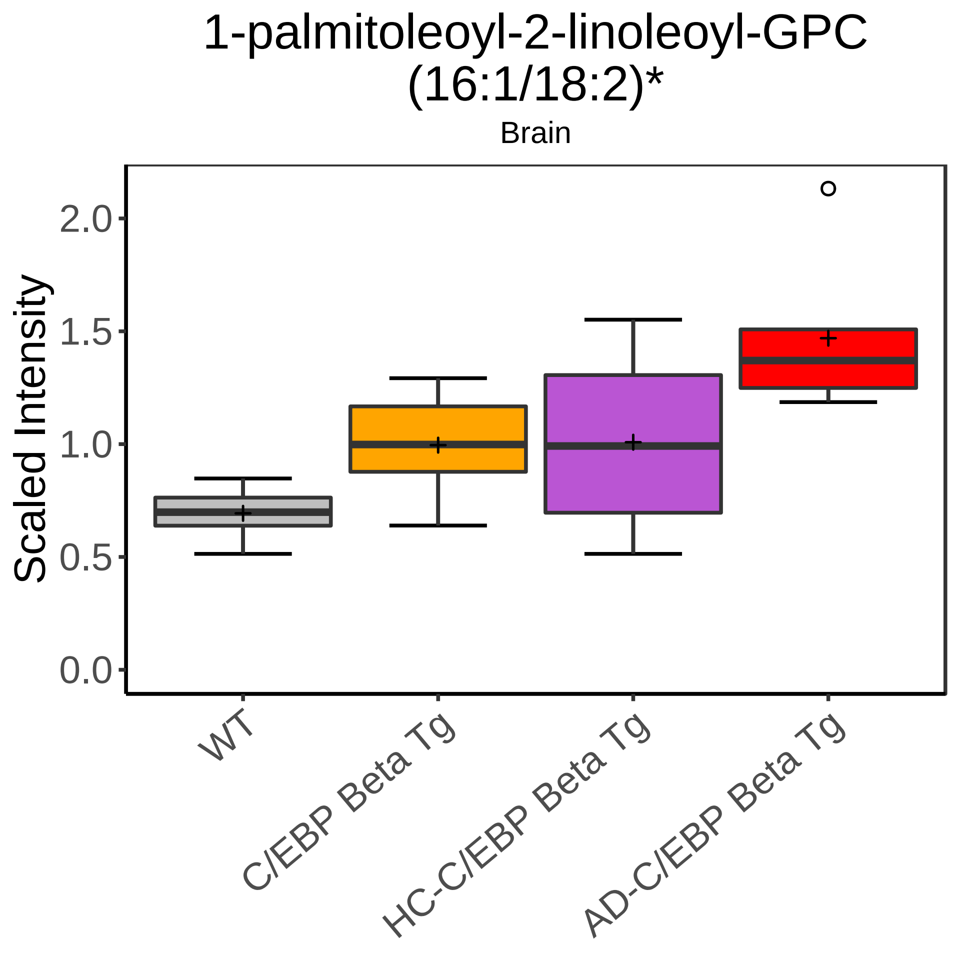

1-palmitoleoyl-2-linoleoyl-GPC (16:1/18:2)*
Brain
Scaled Intensity
2.0
1.5
1.0
0.5
0.0
WT
C/EBP Beta Tg
HC-C/EBP Beta Tg
AD-C/EBP Beta Tg

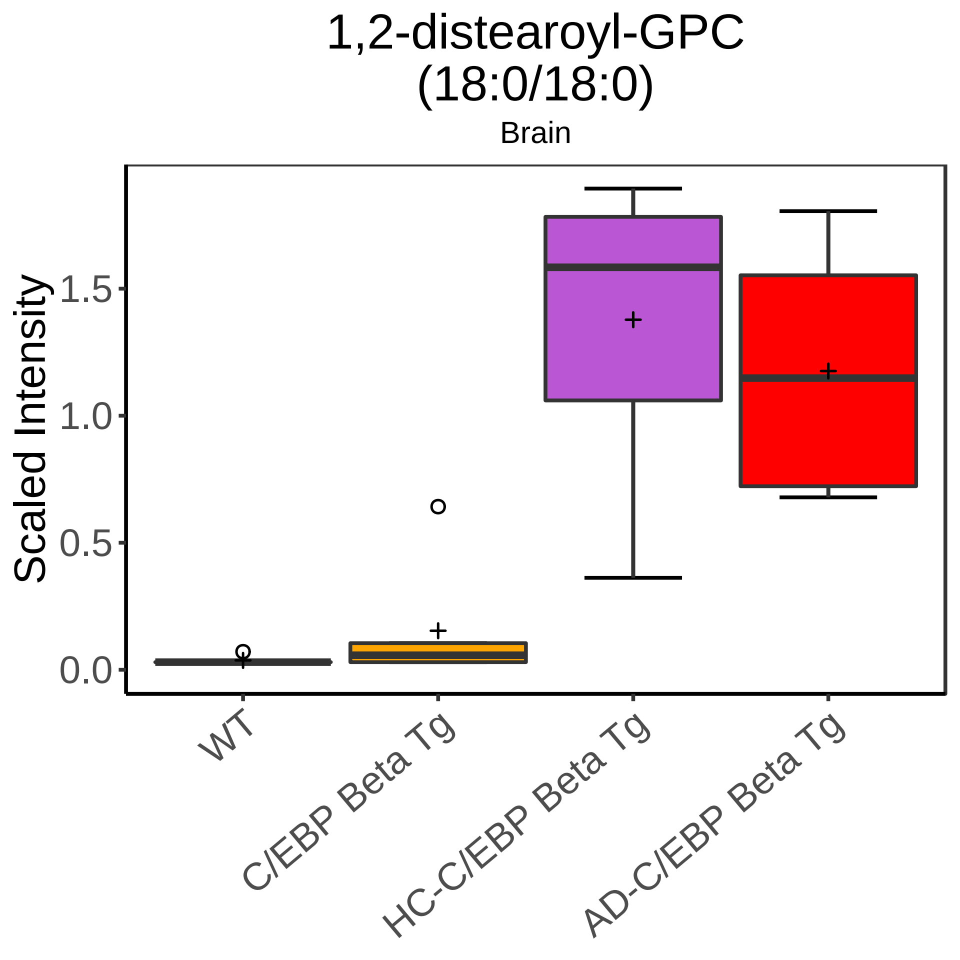
1,2-distearoyl-GPC
(18:0/18:0)
Brain
Scaled Intensity
0.0
0.5
1.0
1.5
WT
C/EBP Beta Tg
HC-C/EBP Beta Tg
AD-C/EBP Beta Tg

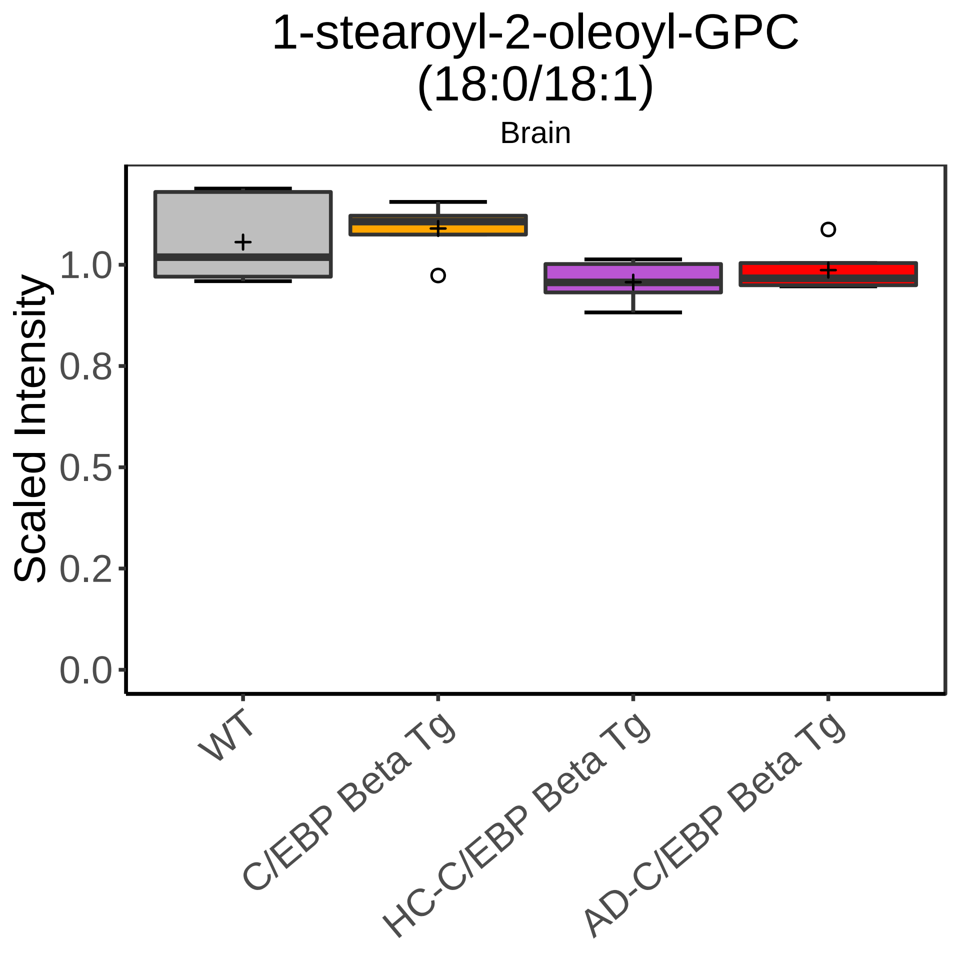

1-stearoyl-2-oleoyl-GPC
(18:0/18:1)
Brain
Scaled Intensity
0.0
0.2
0.5
0.8
1.0
WT
C/EBP Beta Tg
HC-C/EBP Beta Tg
AD-C/EBP Beta Tg

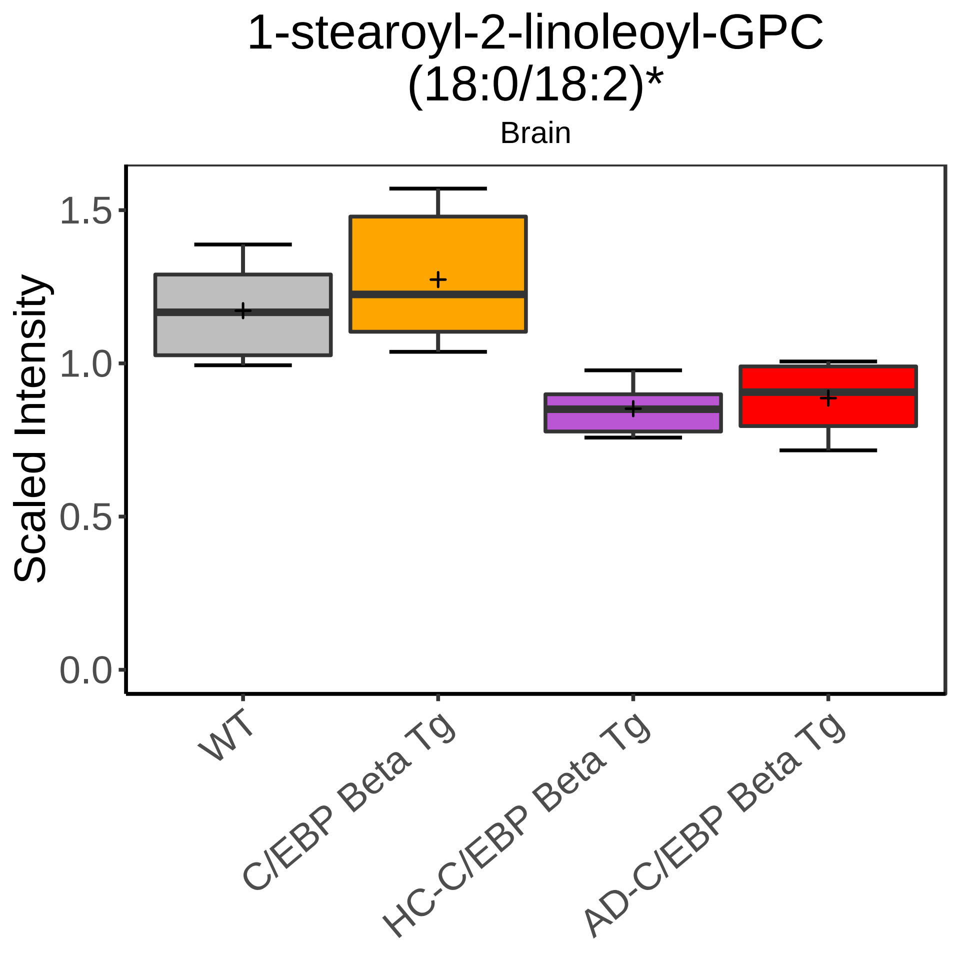

1-stearoyl-2-linoleoyl-GPC (18:0/18:2)*
Brain
Scaled Intensity
1.5
1.0
0.5
0.0
WT
C/EBP Beta Tg
HC-C/EBP Beta Tg
AD-C/EBP Beta Tg

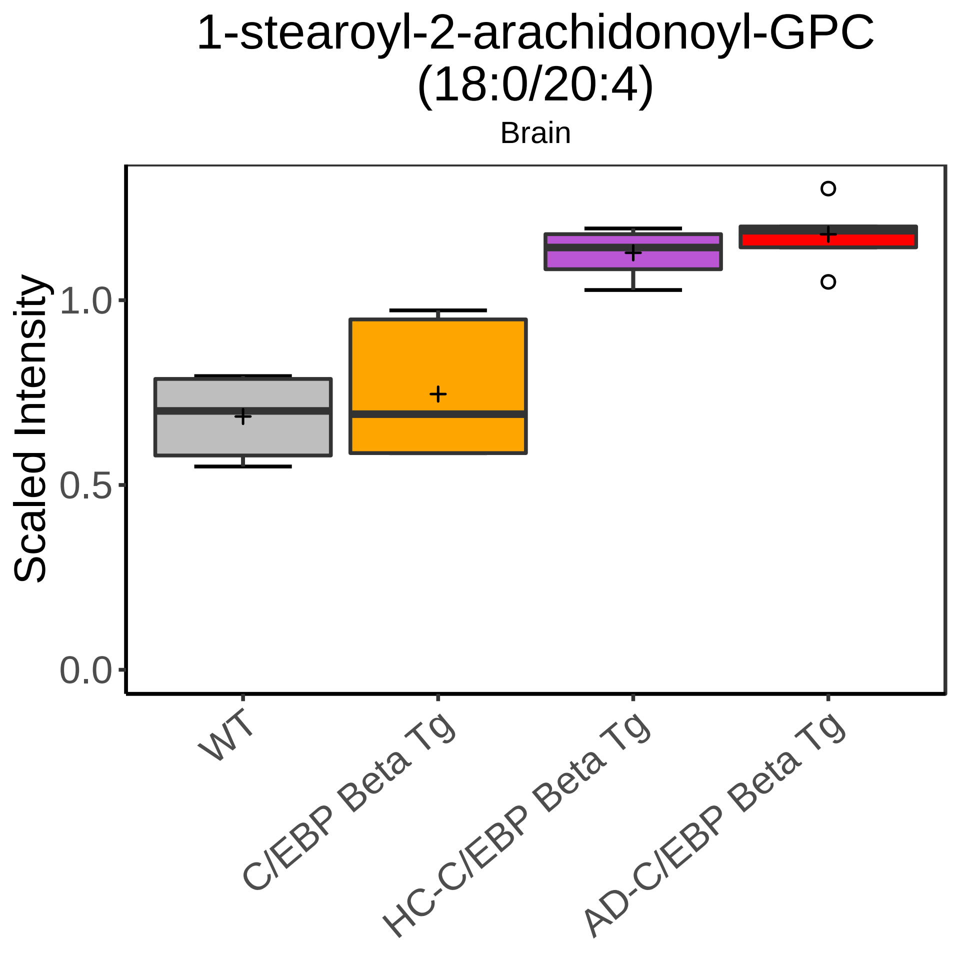
1-stearoyl-2-arachidonoyl-GPC
(18:0/20:4)
Brain
Scaled Intensity
1.0
0.5
0.0
WT
C/EBP Beta Tg
HC-C/EBP Beta Tg
AD-C/EBP Beta Tg

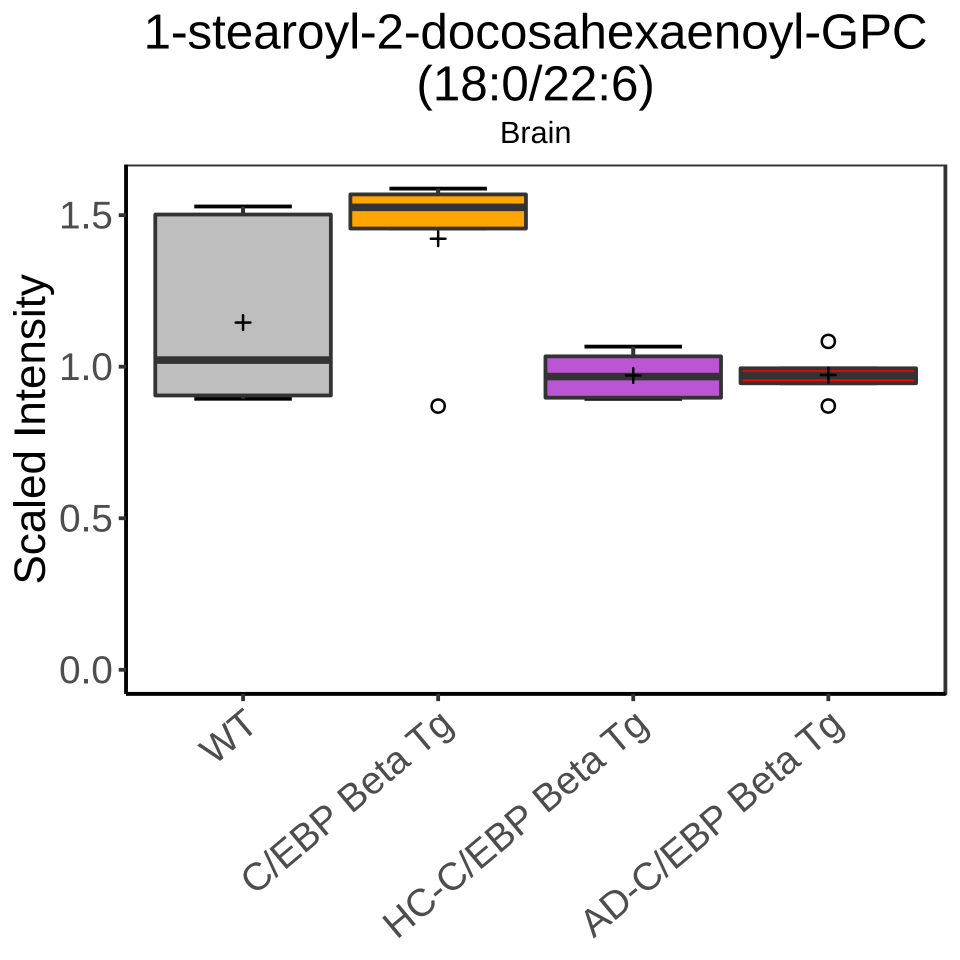
1-stearoyl-2-docosahexaenoyl-GPC
(18:0/22:6)
Brain
Scaled Intensity
1.5
1.0
0.5
0.0
WT
C/EBP Beta Tg
HC-C/EBP Beta Tg
AD-C/EBP Beta Tg

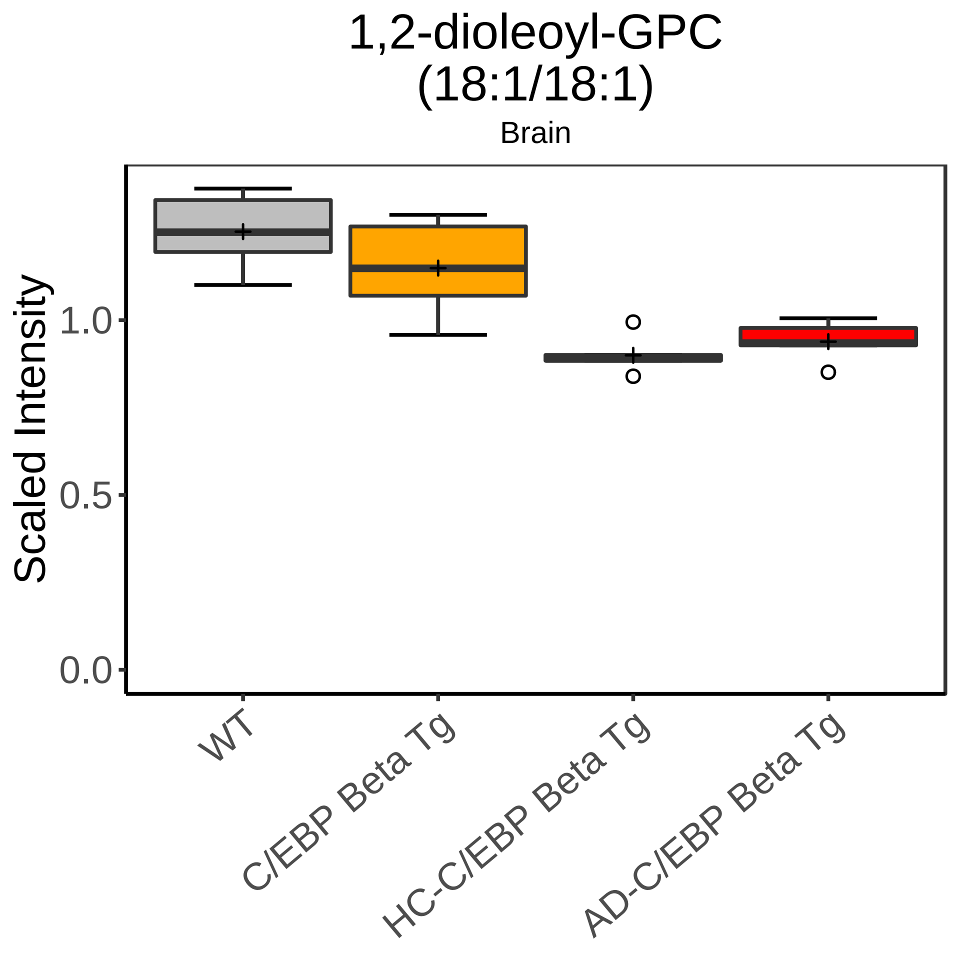
1,2-dioleoyl-GPC
(18:1/18:1)
Brain
Scaled Intensity
1.0
0.5
0.0
WT
C/EBP Beta Tg
HC-C/EBP Beta Tg
AD-C/EBP Beta Tg

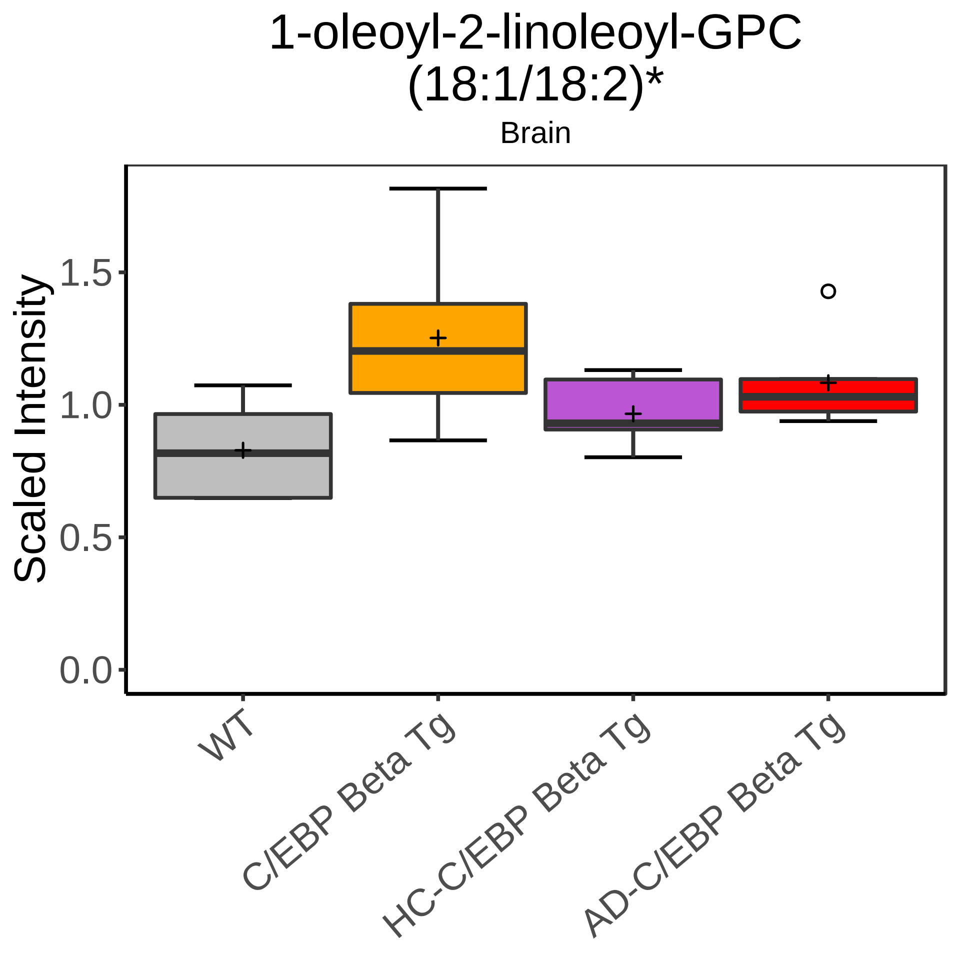

1-oleoyl-2-linoleoyl-GPC
(18:1/18:2)*
Brain
Scaled Intensity
1.5
1.0
0.5
0.0
WT
C/EBP Beta Tg
HC-C/EBP Beta Tg
AD-C/EBP Beta Tg

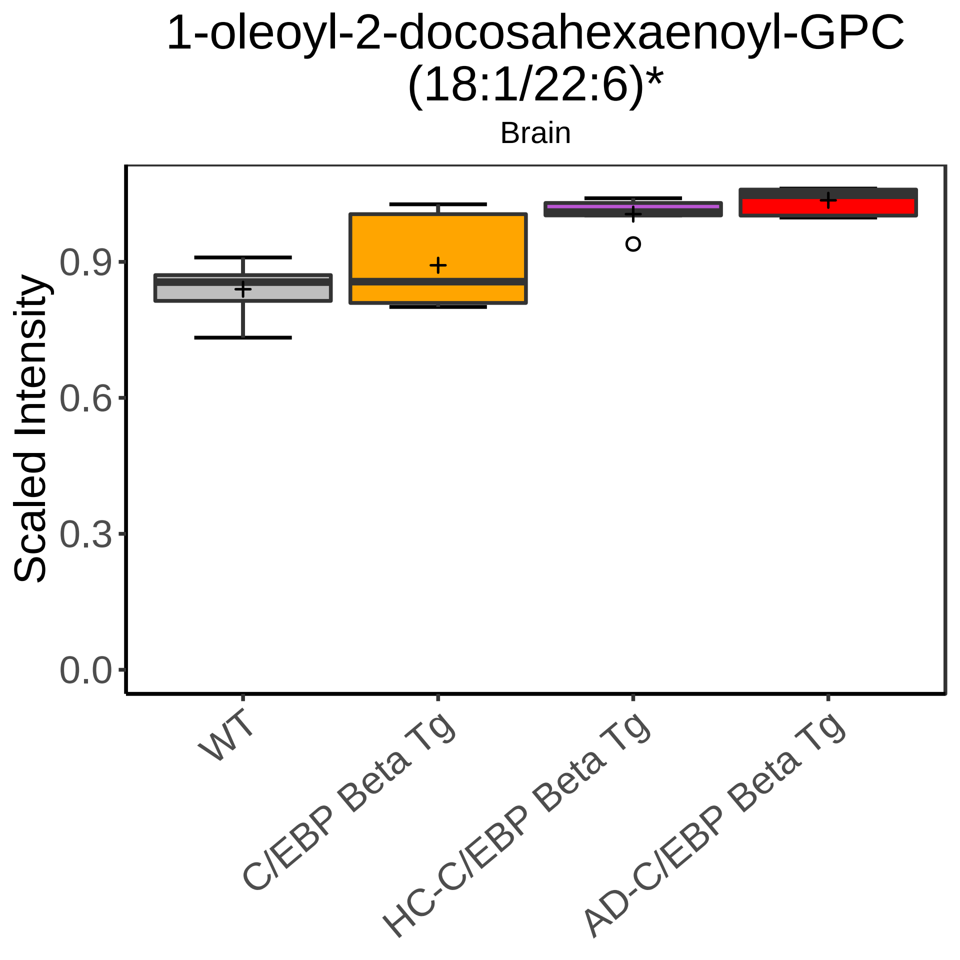
1-oleoyl-2-docosahexaenoyl-GPC (18:1/22:6)*
Brain
Scaled Intensity
0.0
0.3
0.6
0.9
WT
C/EBP Beta Tg
HC-C/EBP Beta Tg
AD-C/EBP Beta Tg

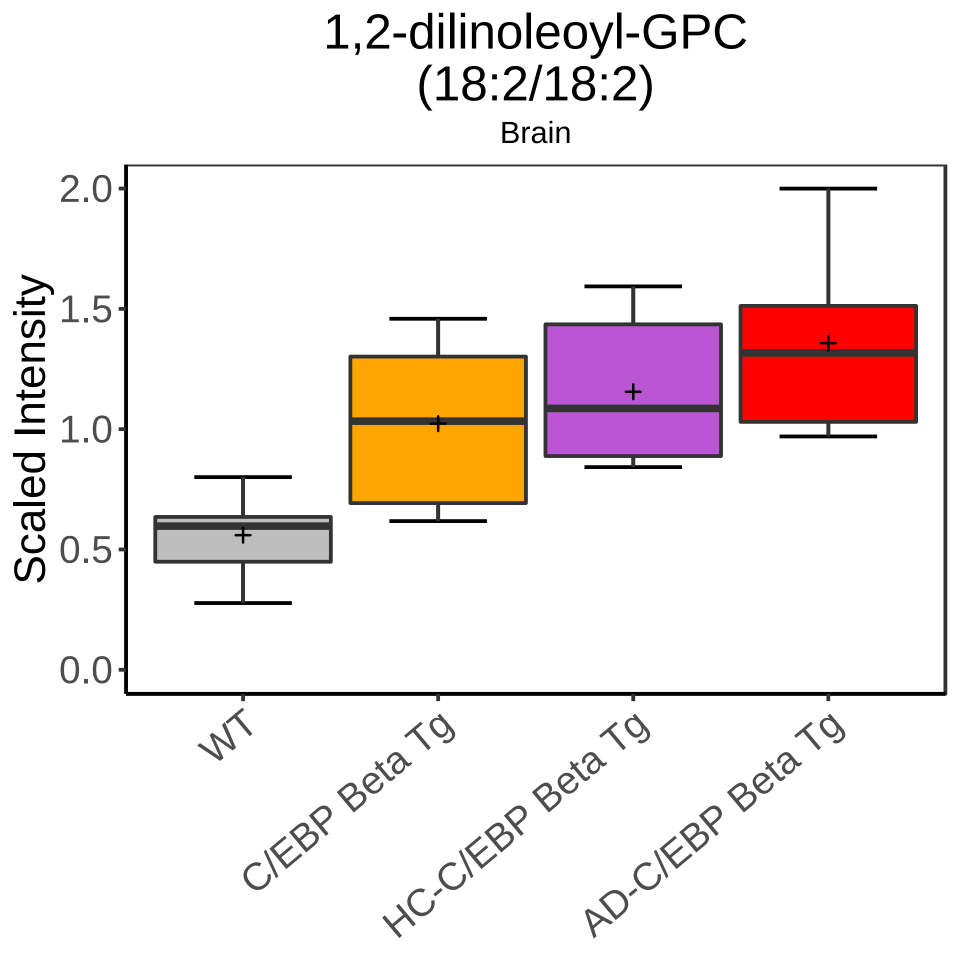

1,2-dilinoleoyl-GPC
(18:2/18:2)
Brain
Scaled Intensity
2.0
1.5
1.0
0.5
0.0
WT
C/EBP Beta Tg
HC-C/EBP Beta Tg
AD-C/EBP Beta Tg

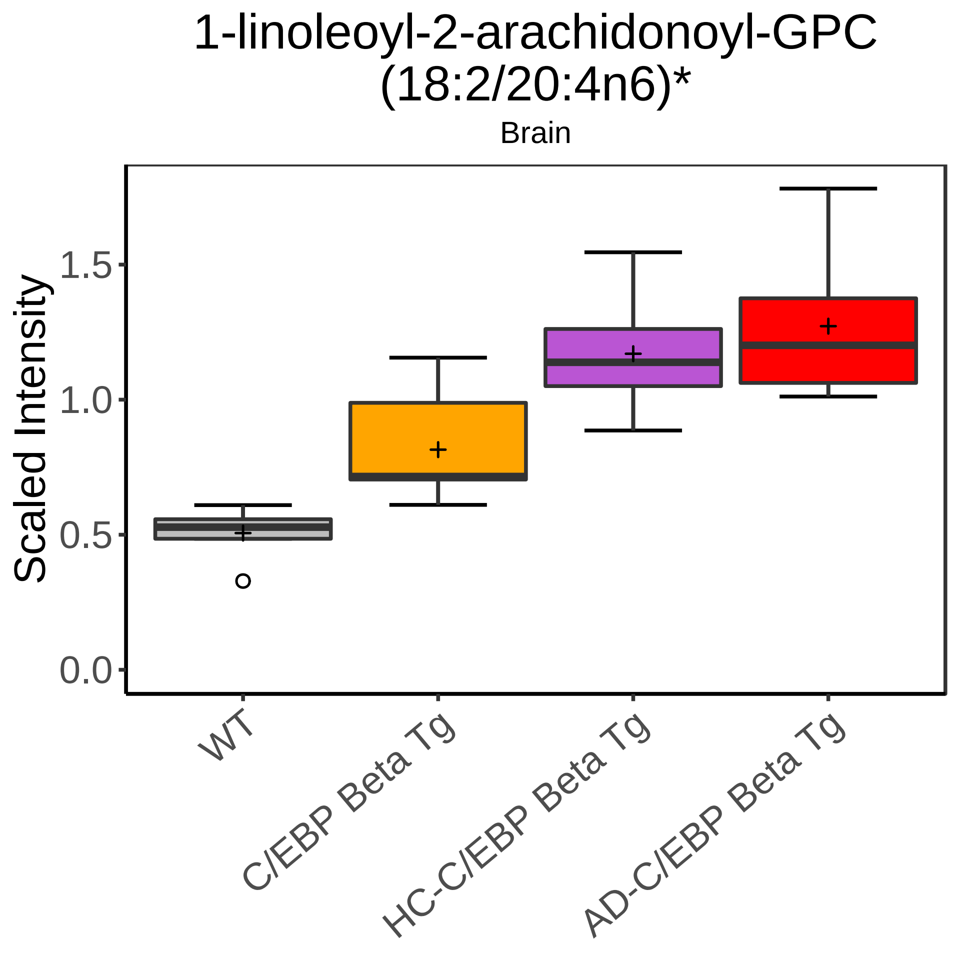

1-linoleoyl-2-arachidonoyl-GPC
(18:2/20:4n6)*
Brain
Scaled Intensity
0.0
0.5
1.0
1.5
WT
C/EBP Beta Tg
HC-C/EBP Beta Tg
AD-C/EBP Beta Tg

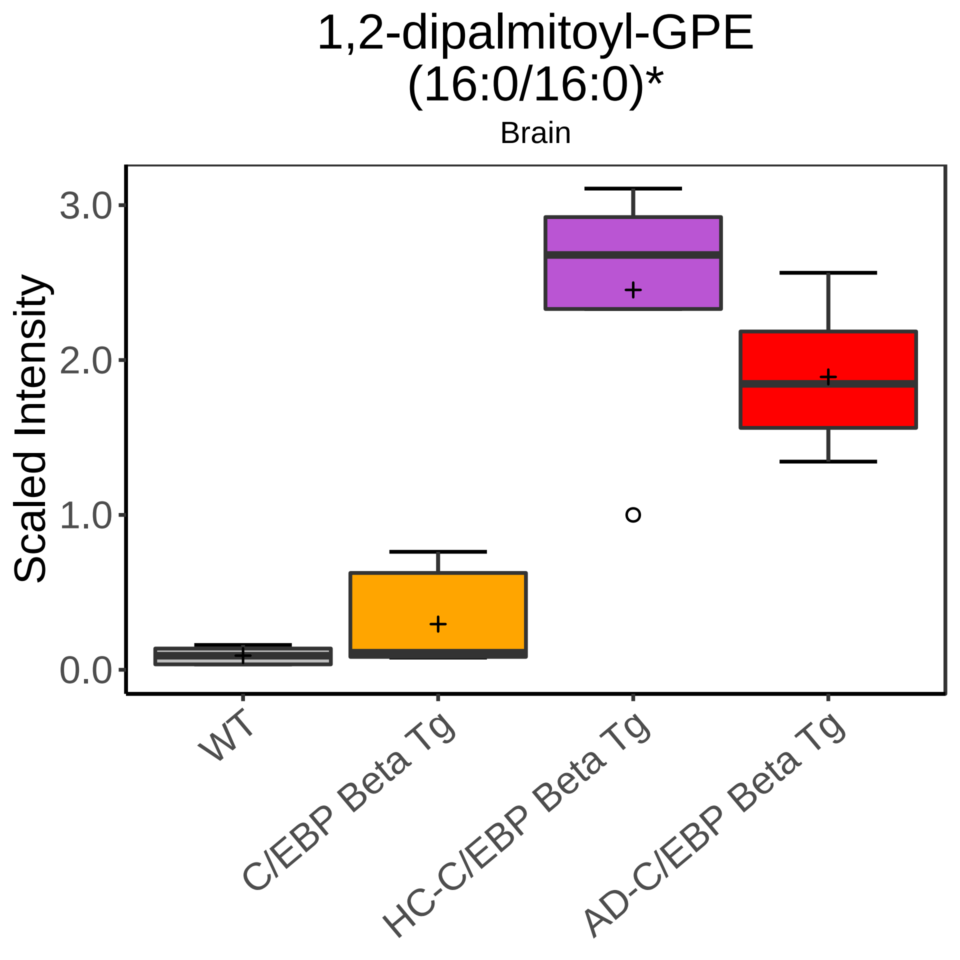

1,2-dipalmitoyl-GPE
(16:0/16:0)*
Brain
Scaled Intensity
3.0
2.0
1.0
0.0
WT
C/EBP Beta Tg
HC-C/EBP Beta Tg
AD-C/EBP Beta Tg

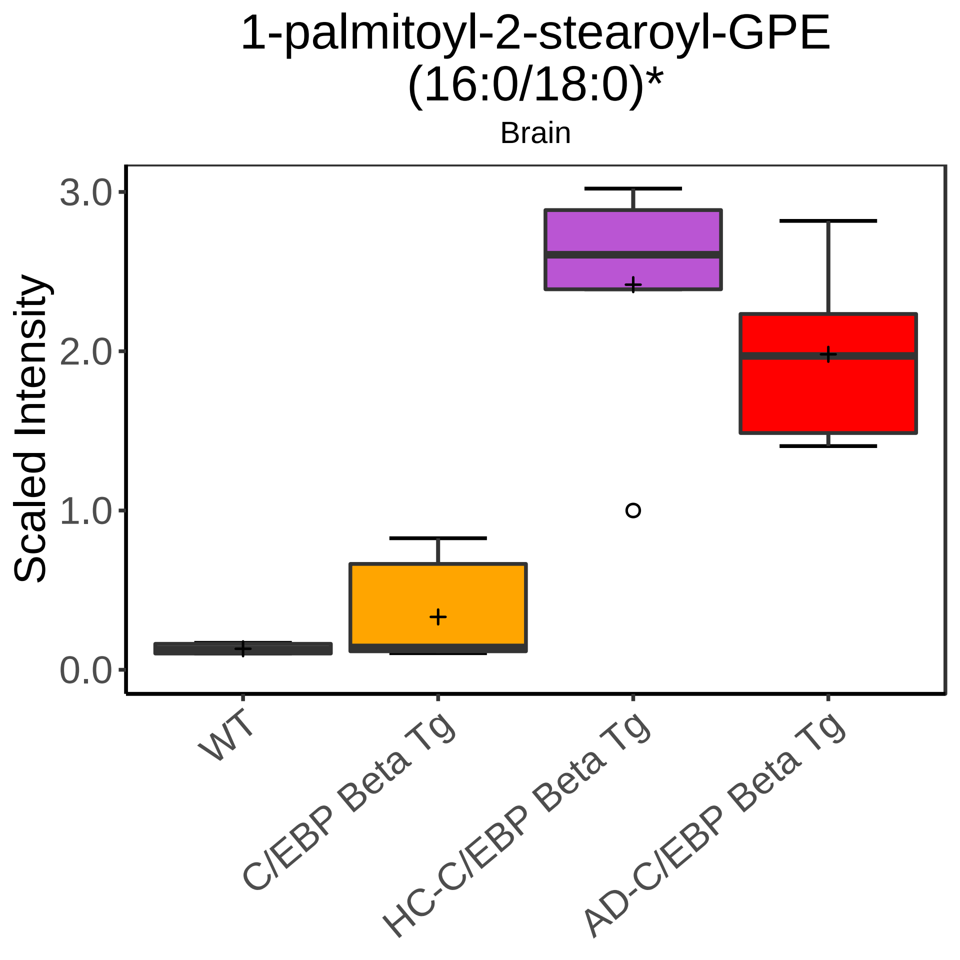
1-palmitoyl-2-stearoyl-GPE
(16:0/18:0)*
Brain
Scaled Intensity
3.0
2.0
1.0
0.0
WT
C/EBP Beta Tg
HC-C/EBP Beta Tg
AD-C/EBP Beta Tg

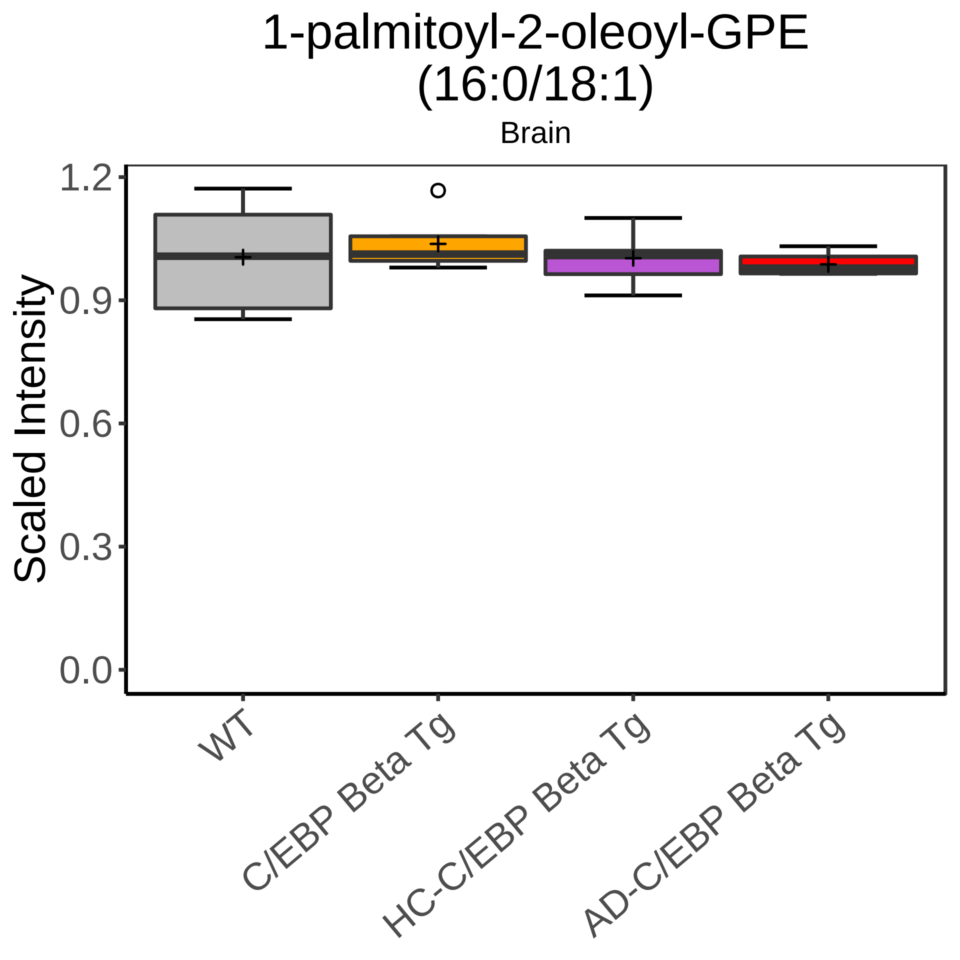
1-palmitoyl-2-oleoyl-GPE
(16:0/18:1)
Brain
Scaled Intensity
1.2
0.9
0.6
0.3
0.0
WT
C/EBP Beta Tg
HC-C/EBP Beta Tg
AD-C/EBP Beta Tg

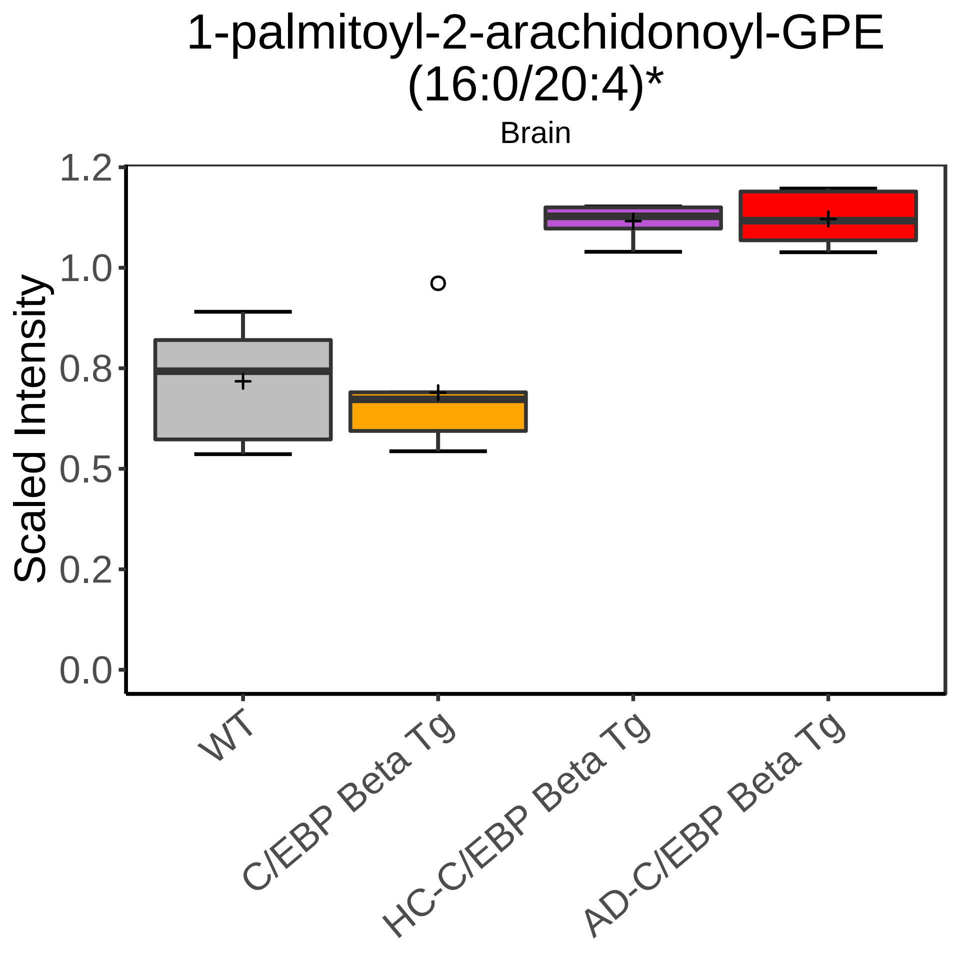

1-palmitoyl-2-arachidonoyl-GPE
(16:0/20:4)*
Brain
Scaled Intensity
1.2
1.0
0.8
0.5
0.2
0.0
WT
C/EBP Beta Tg
HC-C/EBP Beta Tg
AD-C/EBP Beta Tg

1-palmitoyl-2-docosahexaenoyl-GPE (16:0/22:6)*

Brain

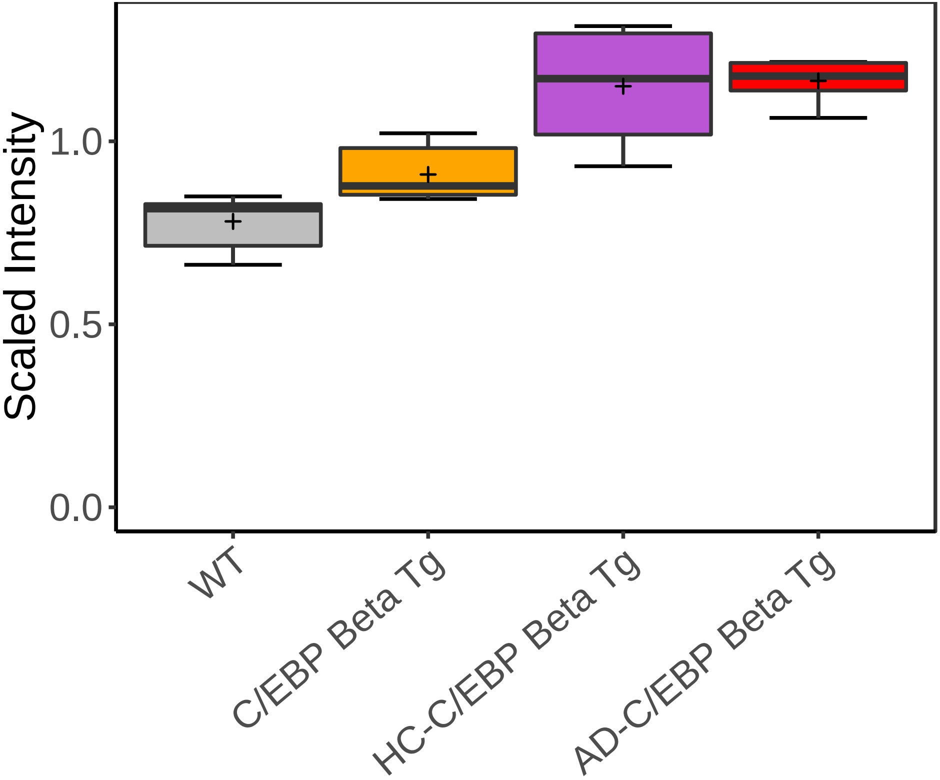

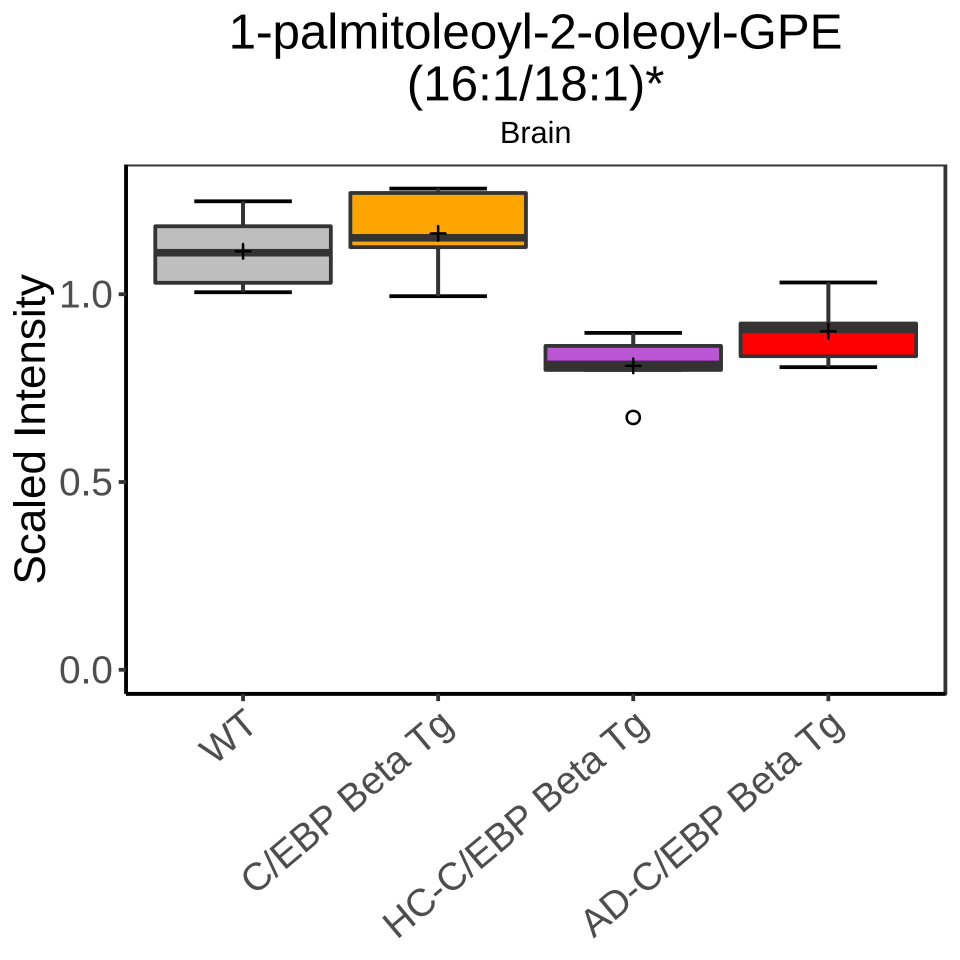

1-palmitoleoyl-2-oleoyl-GPE
(16:1/18:1)*
Brain
Scaled Intensity
1.0
0.5
0.0
WT
C/EBP Beta Tg
HC-C/EBP Beta Tg
AD-C/EBP Beta Tg

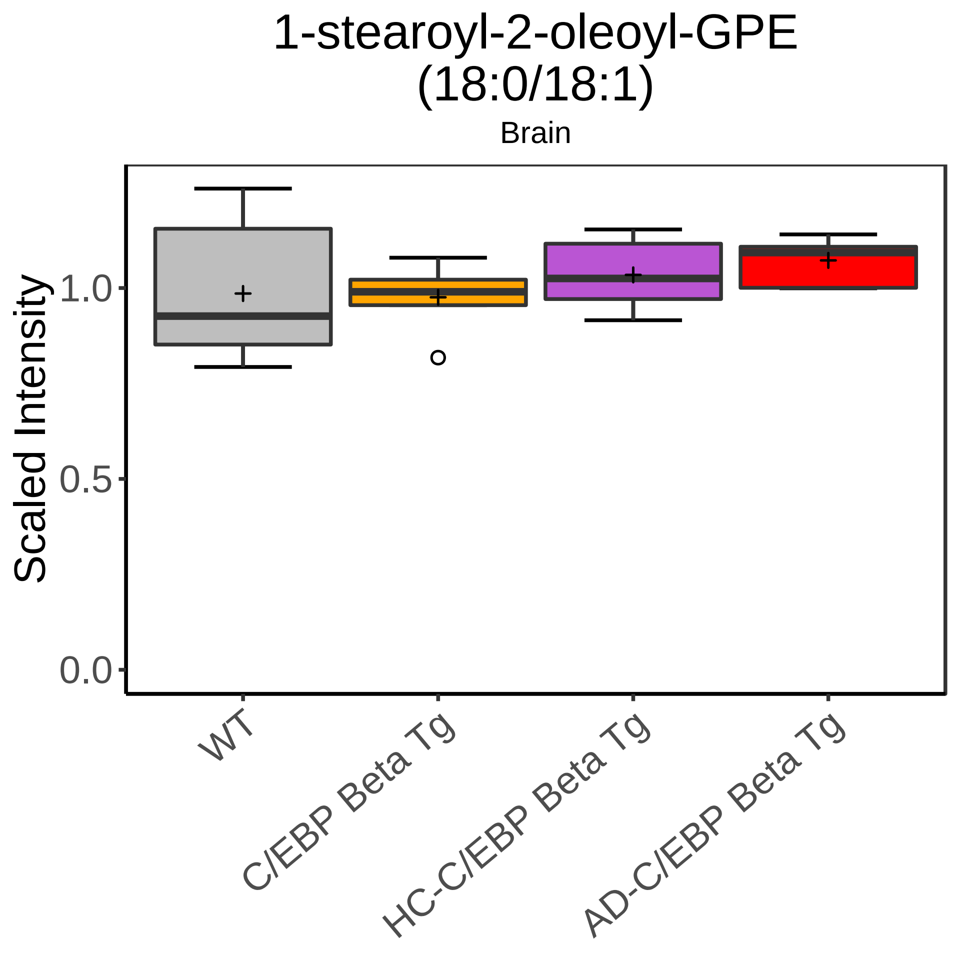
1-stearoyl-2-oleoyl-GPE
(18:0/18:1)
Brain
Scaled Intensity
1.0
0.5
0.0
WT
C/EBP Beta Tg
HC-C/EBP Beta Tg
AD-C/EBP Beta Tg

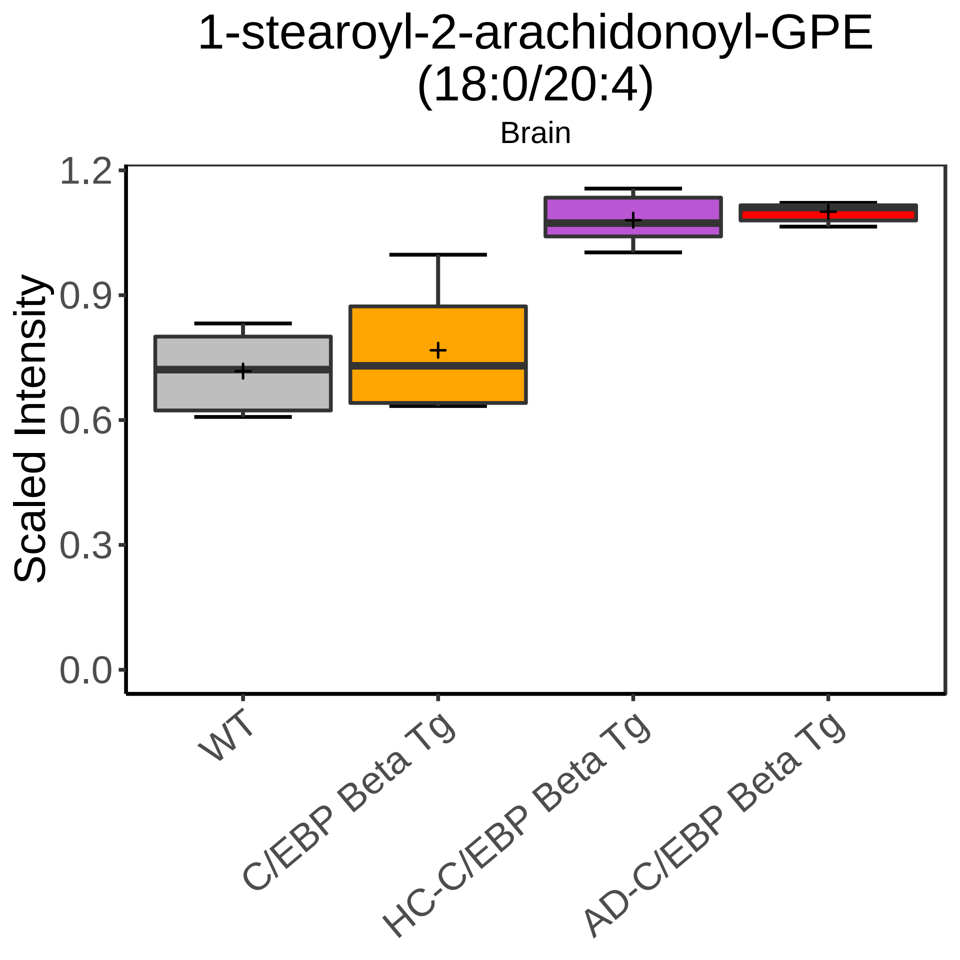

1-stearoyl-2-arachidonoyl-GPE
(18:0/20:4)
Brain
Scaled Intensity
1.2
0.9
0.6
0.3
0.0
WT
C/EBP Beta Tg
HC-C/EBP Beta Tg
AD-C/EBP Beta Tg

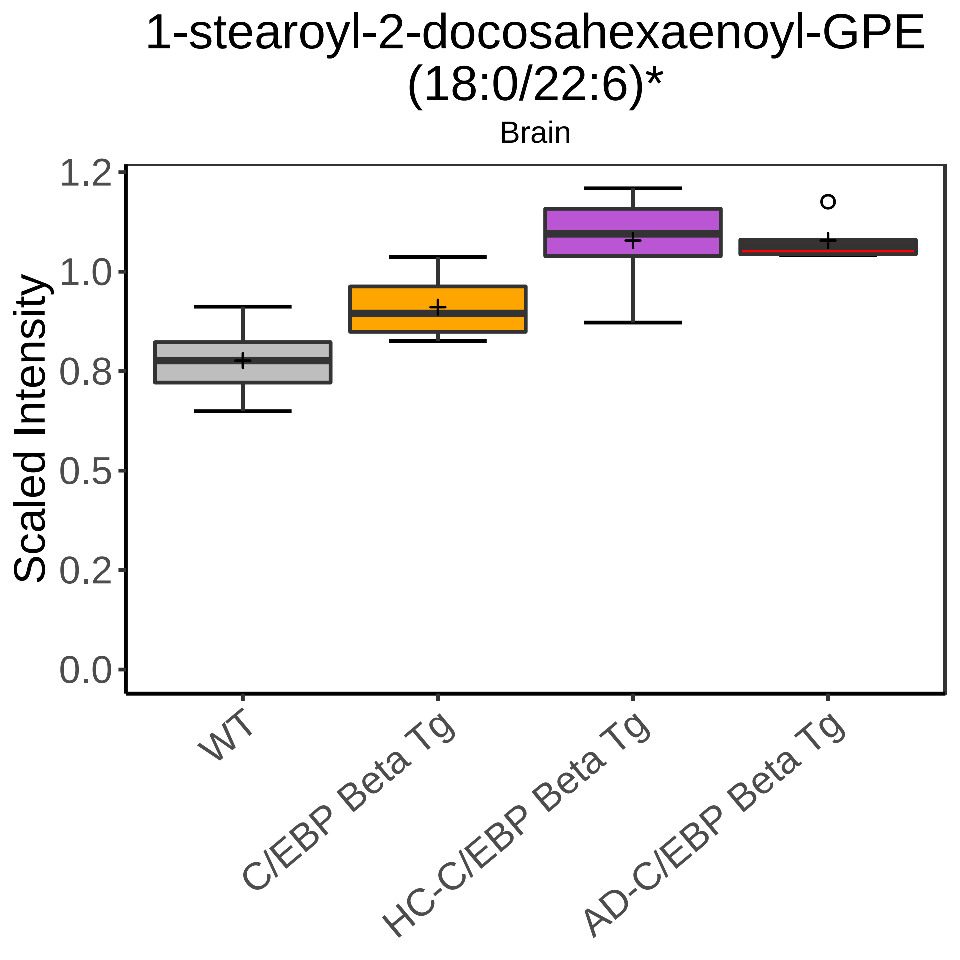
1-stearoyl-2-docosahexaenoyl-GPE
(18:0/22:6)*
Brain
Scaled Intensity
1.2
1.0
0.8
0.5
0.2
0.0
WT
C/EBP Beta Tg
HC-C/EBP Beta Tg
AD-C/EBP Beta Tg

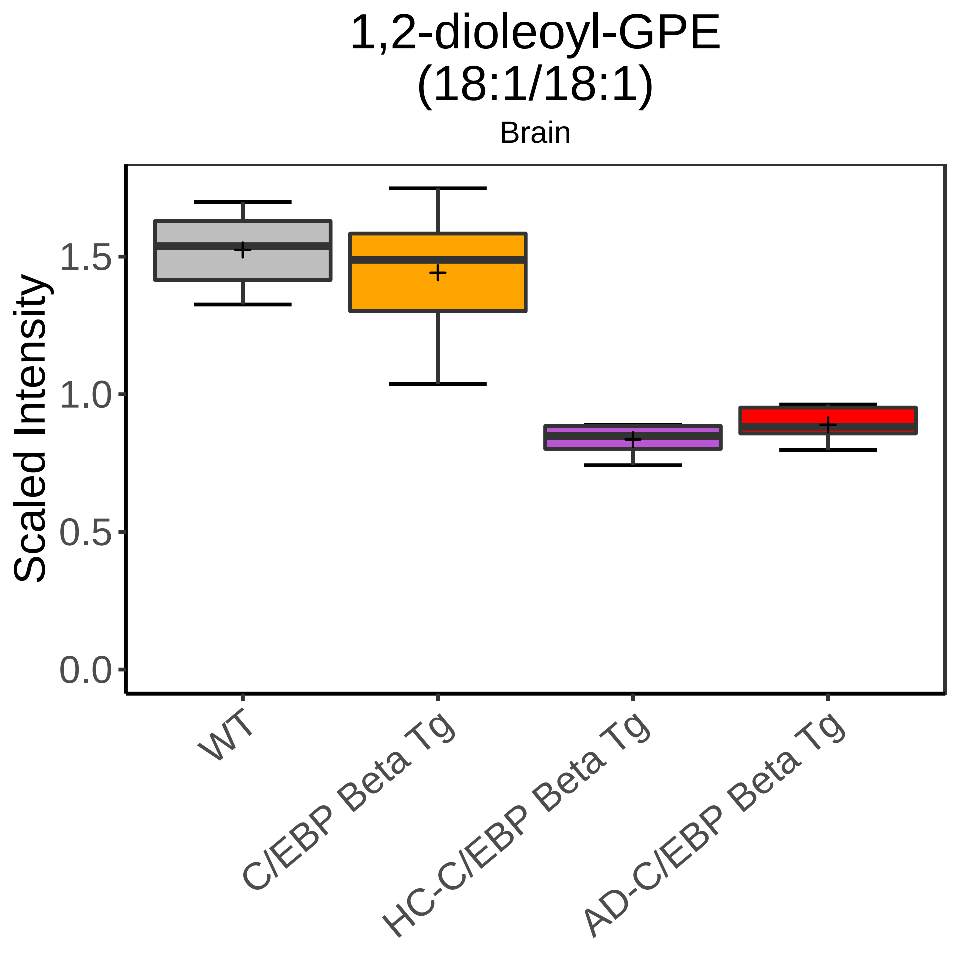
1,2-dioleoyl-GPE
(18:1/18:1)
Brain
Scaled Intensity
0.0
0.5
1.0
1.5
WT
C/EBP Beta Tg
HC-C/EBP Beta Tg
AD-C/EBP Beta Tg

1-oleoyl-2-linoleoyl-GPE (18:1/18:2)*

Brain

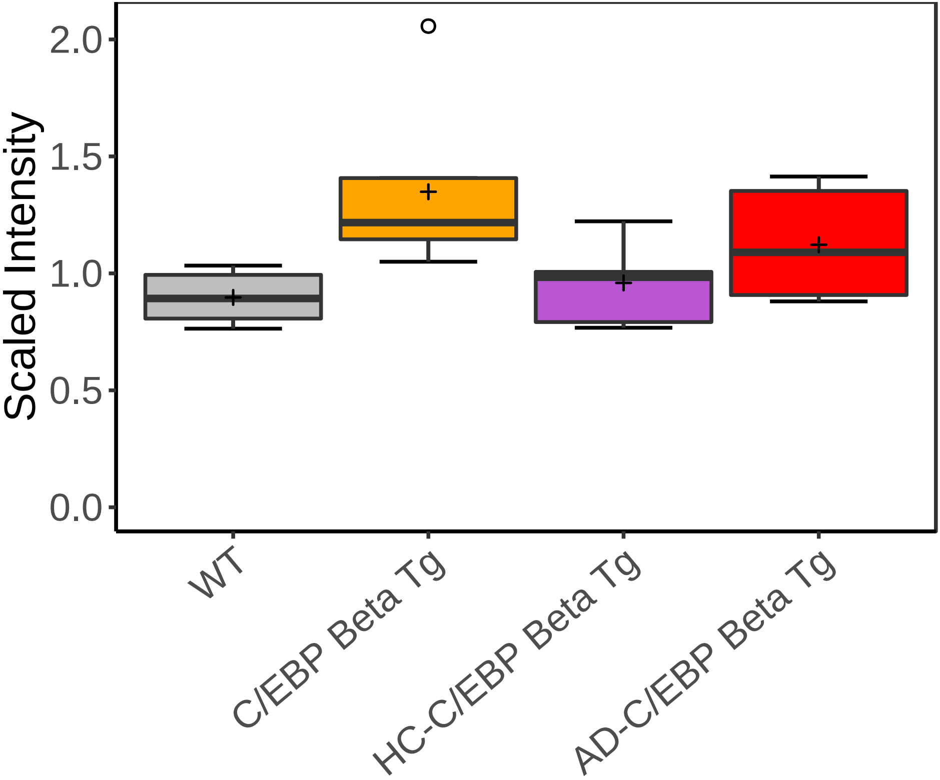

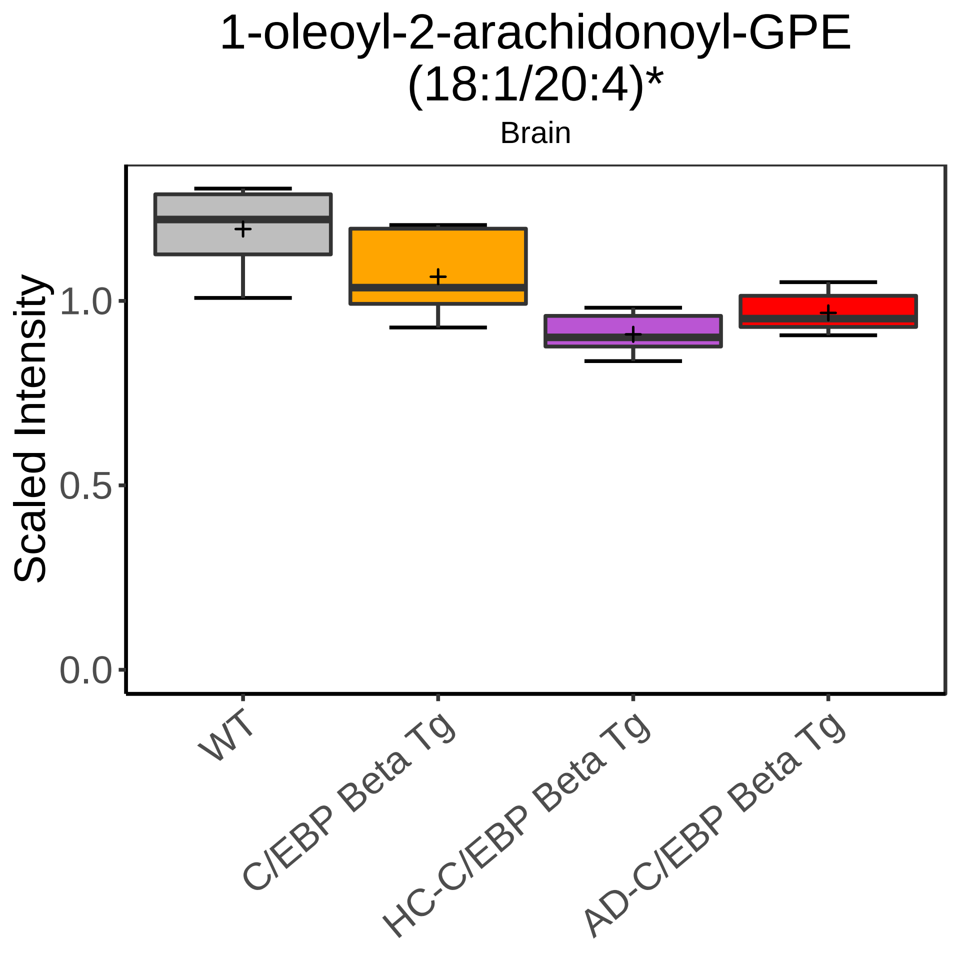
1-oleoyl-2-arachidonoyl-GPE (18:1/20:4)*
Brain
Scaled Intensity
0.0
0.5
1.0
WT
C/EBP Beta Tg
HC-C/EBP Beta Tg
AD-C/EBP Beta Tg

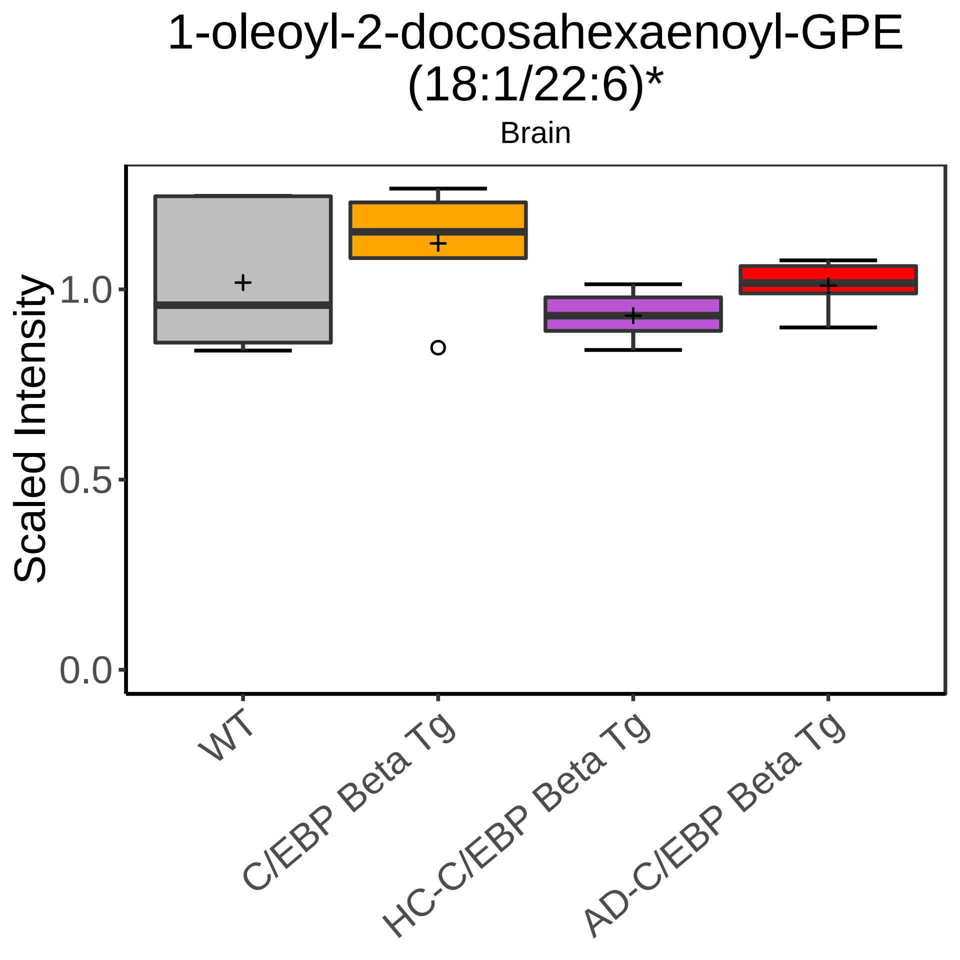
1-oleoyl-2-docosahexaenoyl-GPE
(18:1/22:6)*
Brain
Scaled Intensity
0.0
0.5
1.0
WT
C/EBP Beta Tg
HC-C/EBP Beta Tg
AD-C/EBP Beta Tg

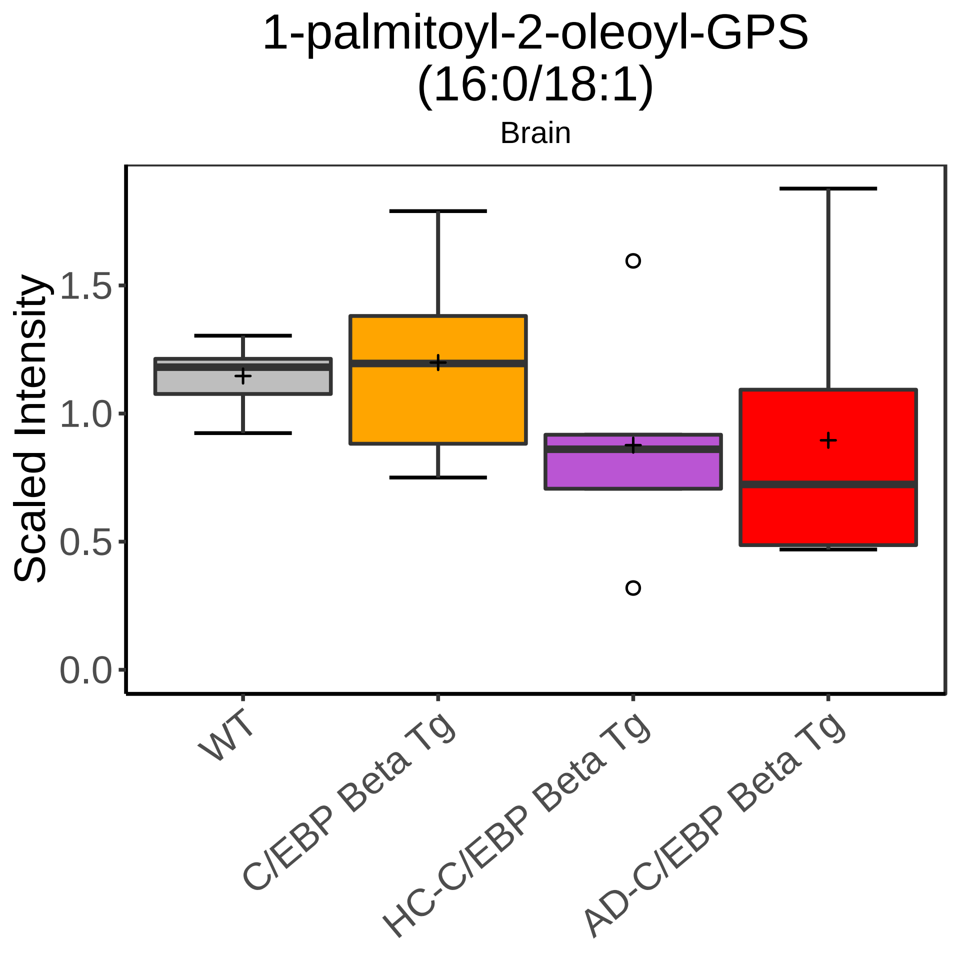

1-palmitoyl-2-oleoyl-GPS
(16:0/18:1)
Brain
Scaled Intensity
0.0
0.5
1.0
1.5
WT
C/EBP Beta Tg
HC-C/EBP Beta Tg
AD-C/EBP Beta Tg

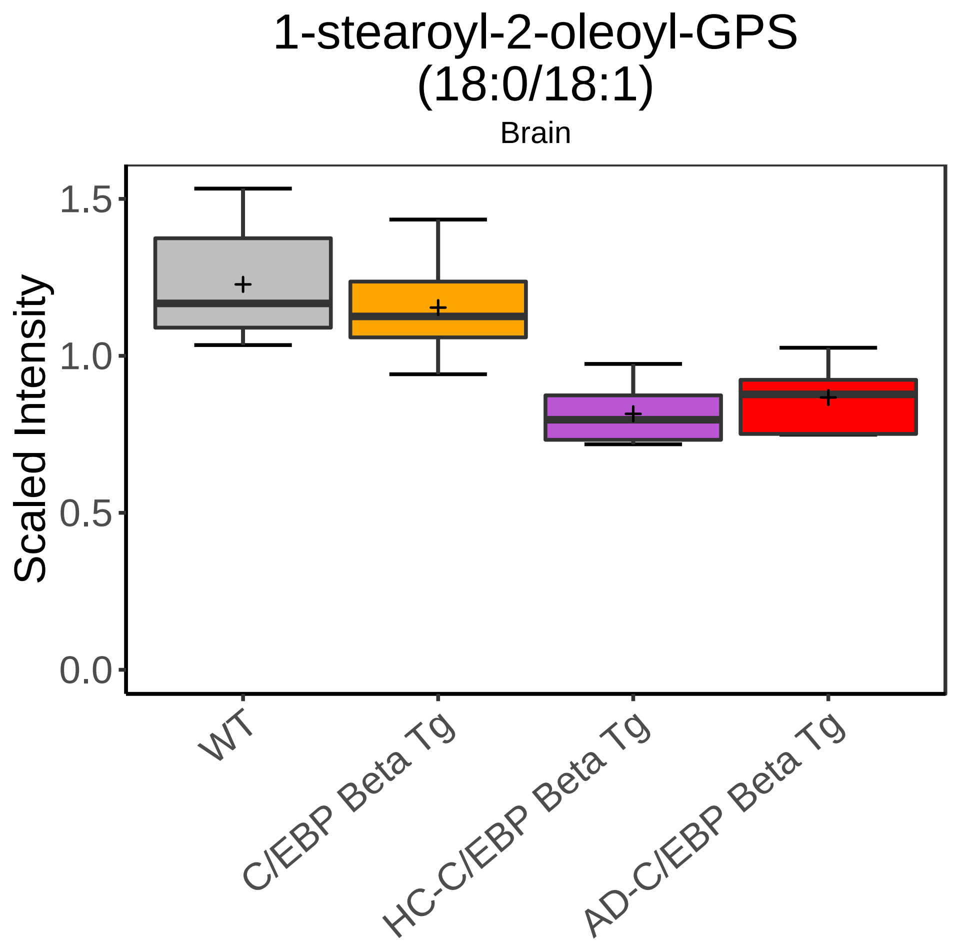

1-stearoyl-2-oleoyl-GPS
(18:0/18:1)
Brain
Scaled Intensity
1.5
1.0
0.5
0.0
WT
C/EBP Beta Tg
HC-C/EBP Beta Tg
AD-C/EBP Beta Tg

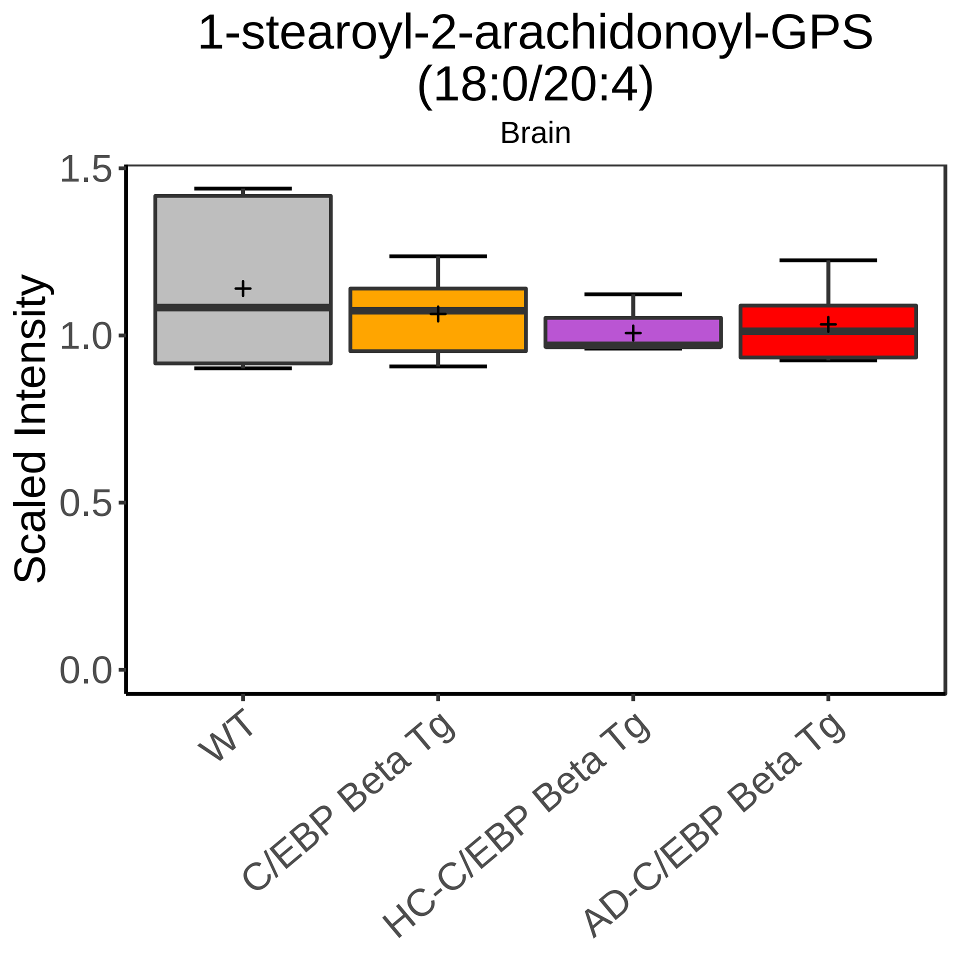
1-stearoyl-2-arachidonoyl-GPS
(18:0/20:4)
Brain
Scaled Intensity
0.0
0.5
1.0
1.5
WT
C/EBP Beta Tg
HC-C/EBP Beta Tg
AD-C/EBP Beta Tg

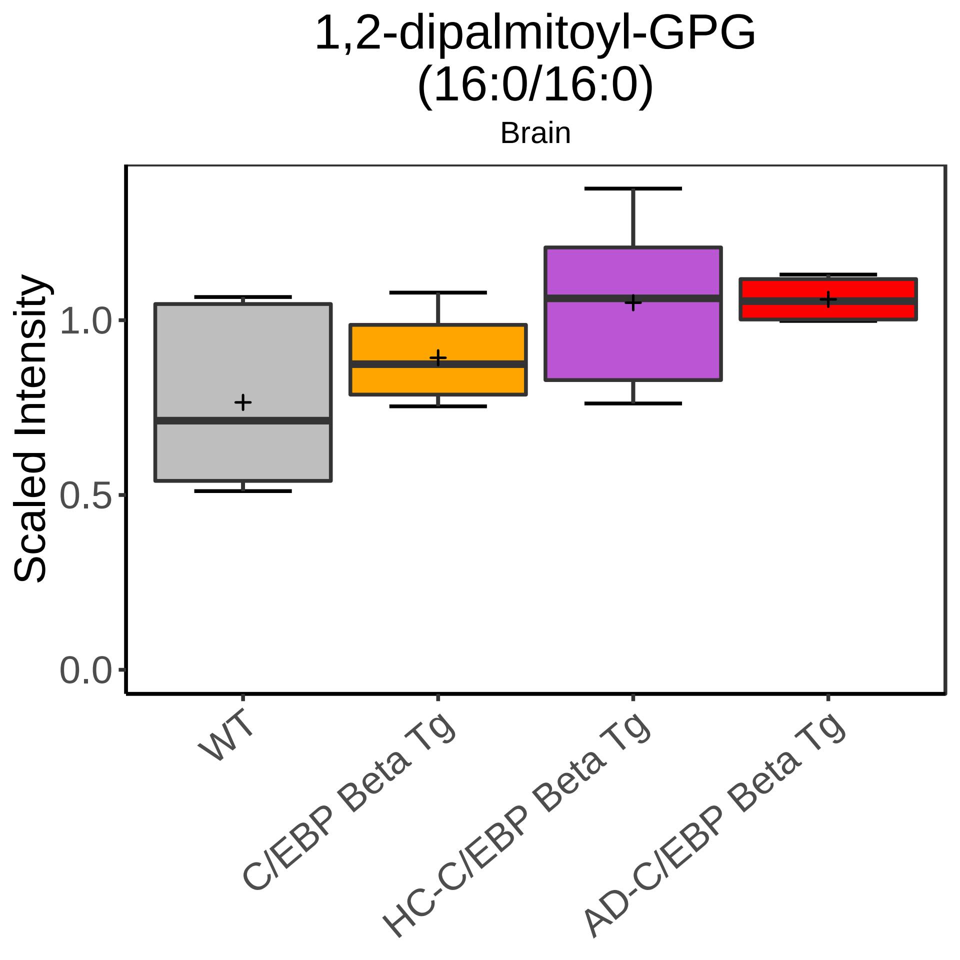
1,2-dipalmitoyl-GPG
(16:0/16:0)
Brain
Scaled Intensity
0.0
0.5
1.0
WT
C/EBP Beta Tg
HC-C/EBP Beta Tg
AD-C/EBP Beta Tg

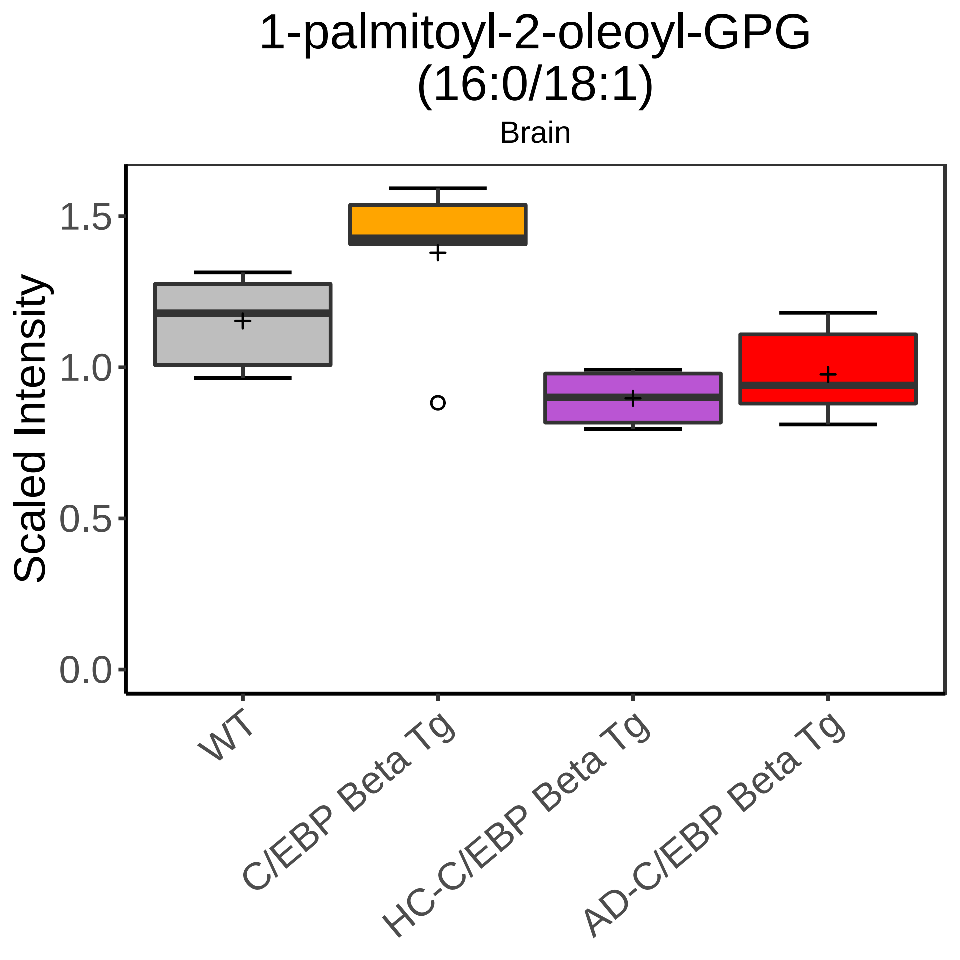

1-palmitoyl-2-oleoyl-GPG
(16:0/18:1)
Brain
Scaled Intensity
1.5
1.0
0.5
0.0
WT
C/EBP Beta Tg
HC-C/EBP Beta Tg
AD-C/EBP Beta Tg

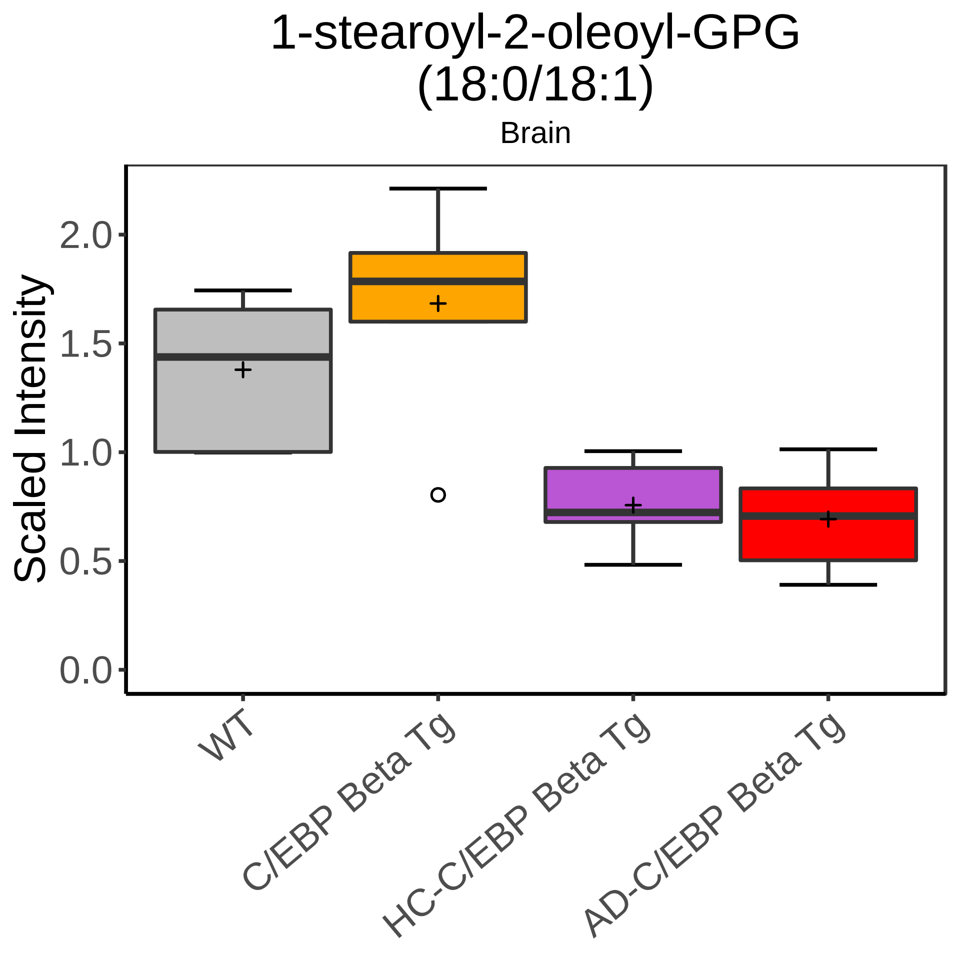

1-stearoyl-2-oleoyl-GPG
(18:0/18:1)
Brain
Scaled Intensity
2.0
1.5
1.0
0.5
0.0
WT
C/EBP Beta Tg
HC-C/EBP Beta Tg
AD-C/EBP Beta Tg

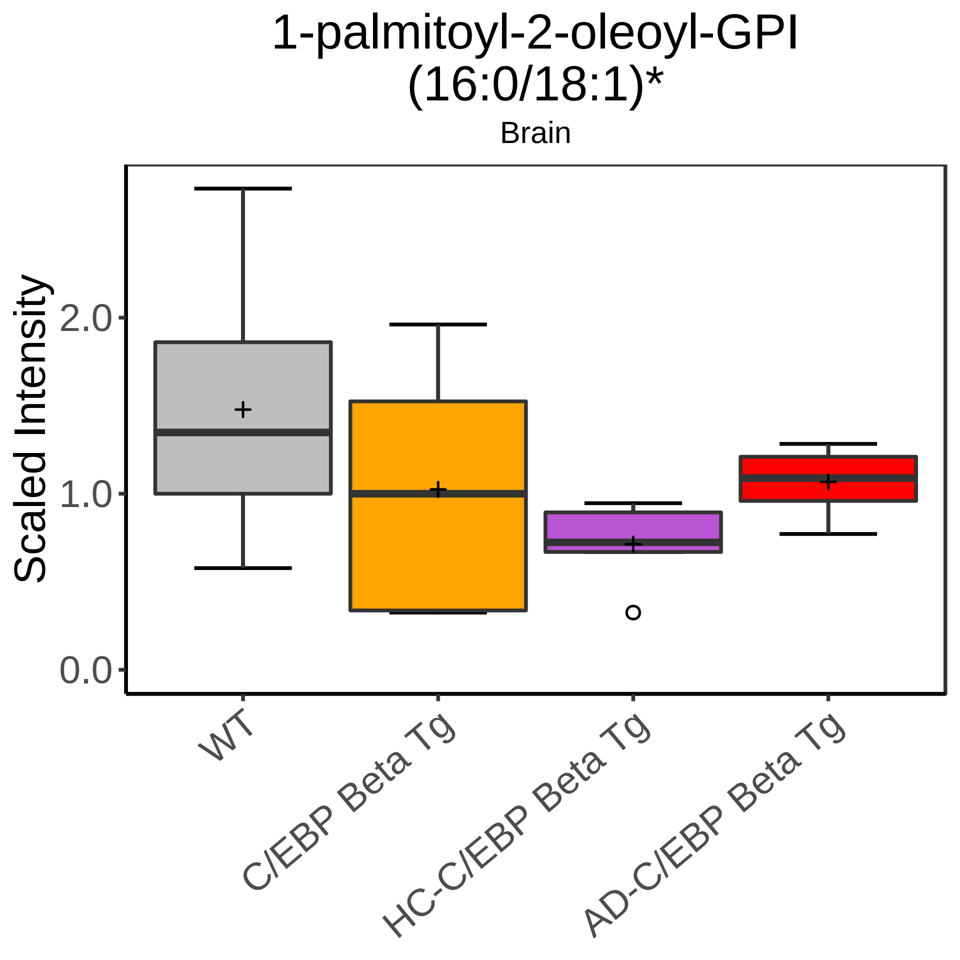

1-palmitoyl-2-oleoyl-GPI
(16:0/18:1)*
Brain
Scaled Intensity
0.0
1.0
2.0
WT
C/EBP Beta Tg
HC-C/EBP Beta Tg
AD-C/EBP Beta Tg

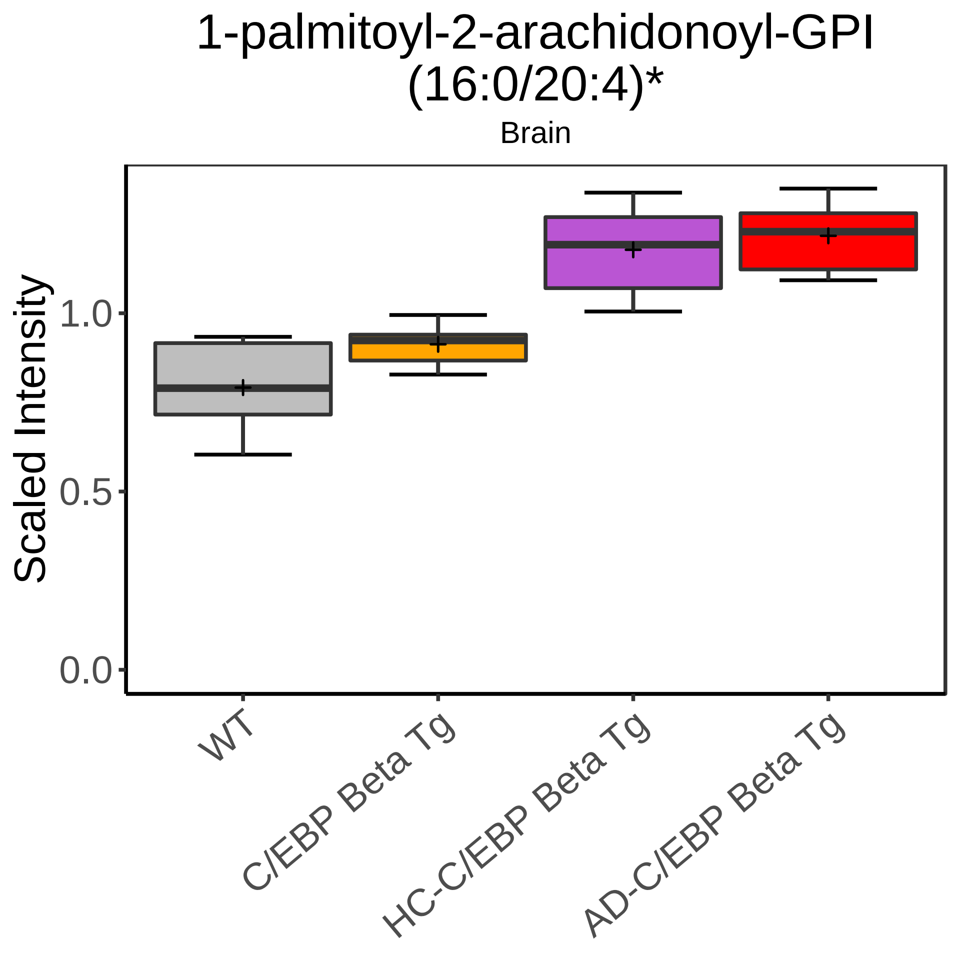
1-palmitoyl-2-arachidonoyl-GPI
(16:0/20:4)*
Brain
Scaled Intensity
0.0
0.5
1.0
WT
C/EBP Beta Tg
HC-C/EBP Beta Tg
AD-C/EBP Beta Tg

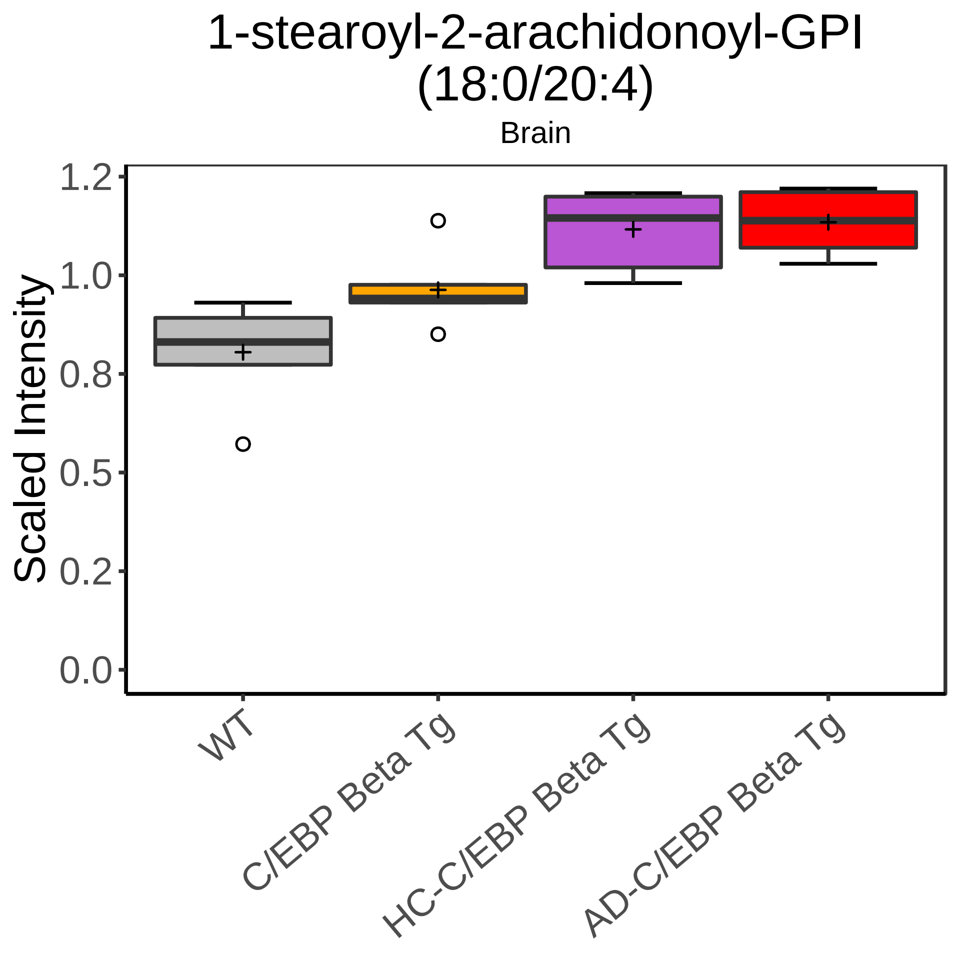
1-stearoyl-2-arachidonoyl-GPI
(18:0/20:4)
Brain
Scaled Intensity
1.2
1.0
0.8
0.5
0.2
0.0
WT
C/EBP Beta Tg
HC-C/EBP Beta Tg
AD-C/EBP Beta Tg

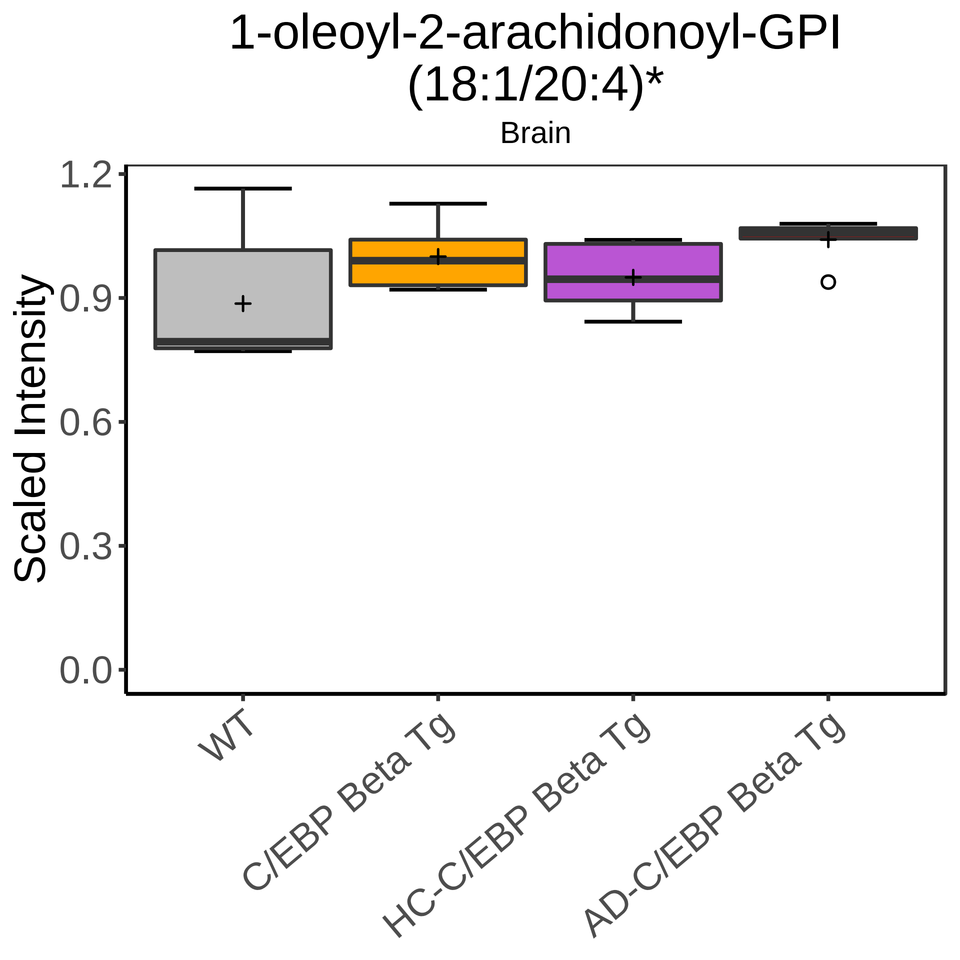
1-oleoyl-2-arachidonoyl-GPI
(18:1/20:4)*
Brain
Scaled Intensity
1.2
0.9
0.6
0.3
0.0
WT
C/EBP Beta Tg
HC-C/EBP Beta Tg
AD-C/EBP Beta Tg

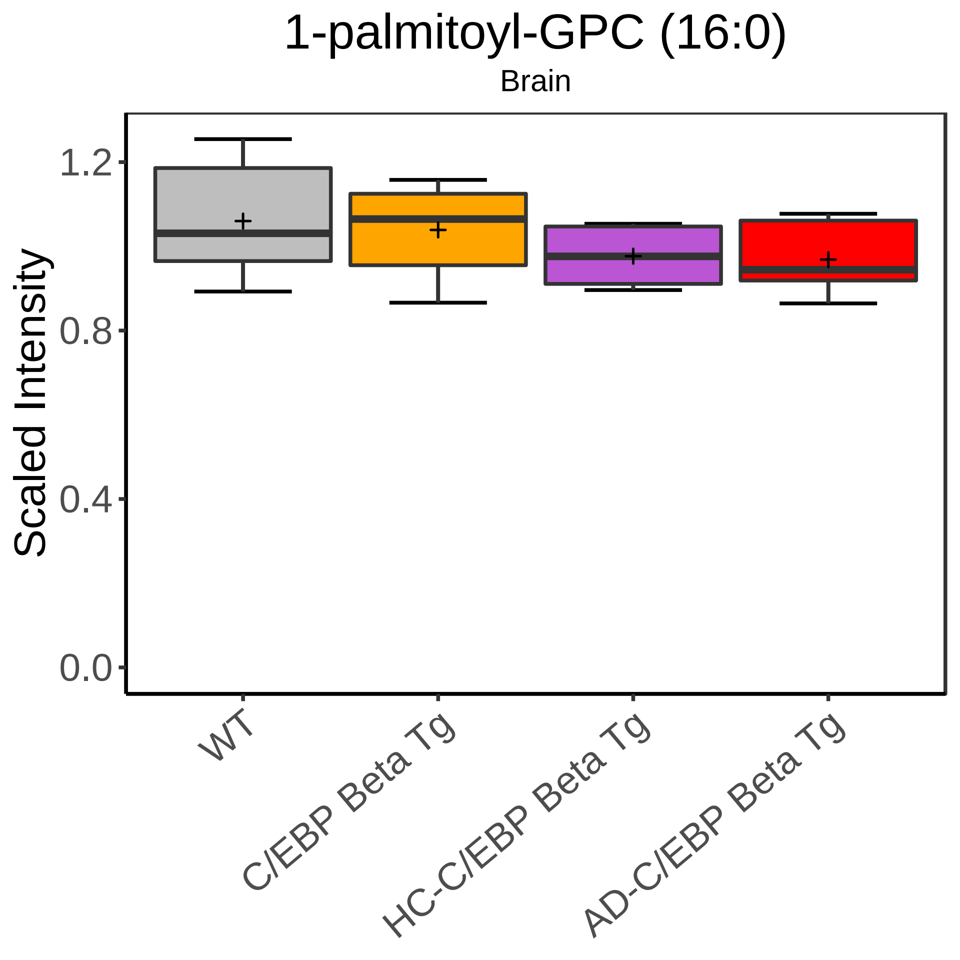

1-palmitoyl-GPC (16:0)
Brain
Scaled Intensity
1.2
0.8
0.4
0.0
WT
C/EBP Beta Tg
HC-C/EBP Beta Tg
AD-C/EBP Beta Tg

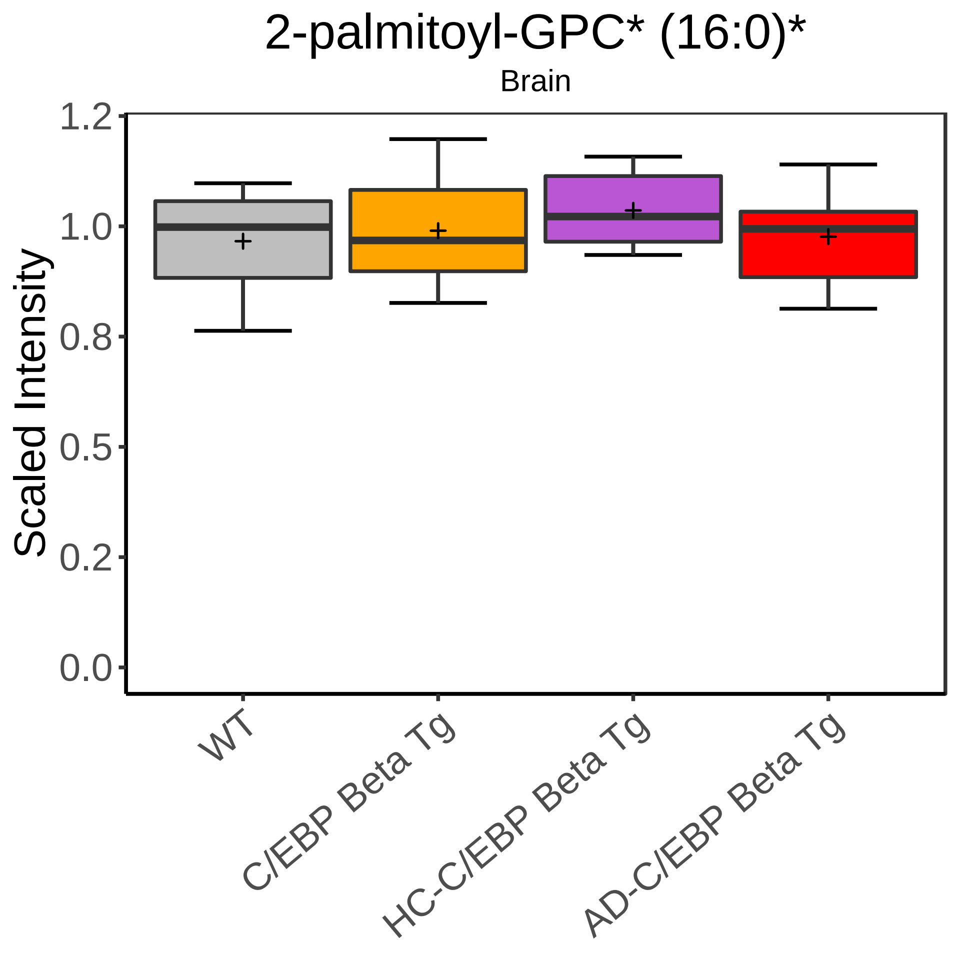

2-palmitoyl-GPC* (16:0)*
Brain
Scaled Intensity
1.2
1.0
0.8
0.5
0.2
0.0
WT
C/EBP Beta Tg
HC-C/EBP Beta Tg
AD-C/EBP Beta Tg

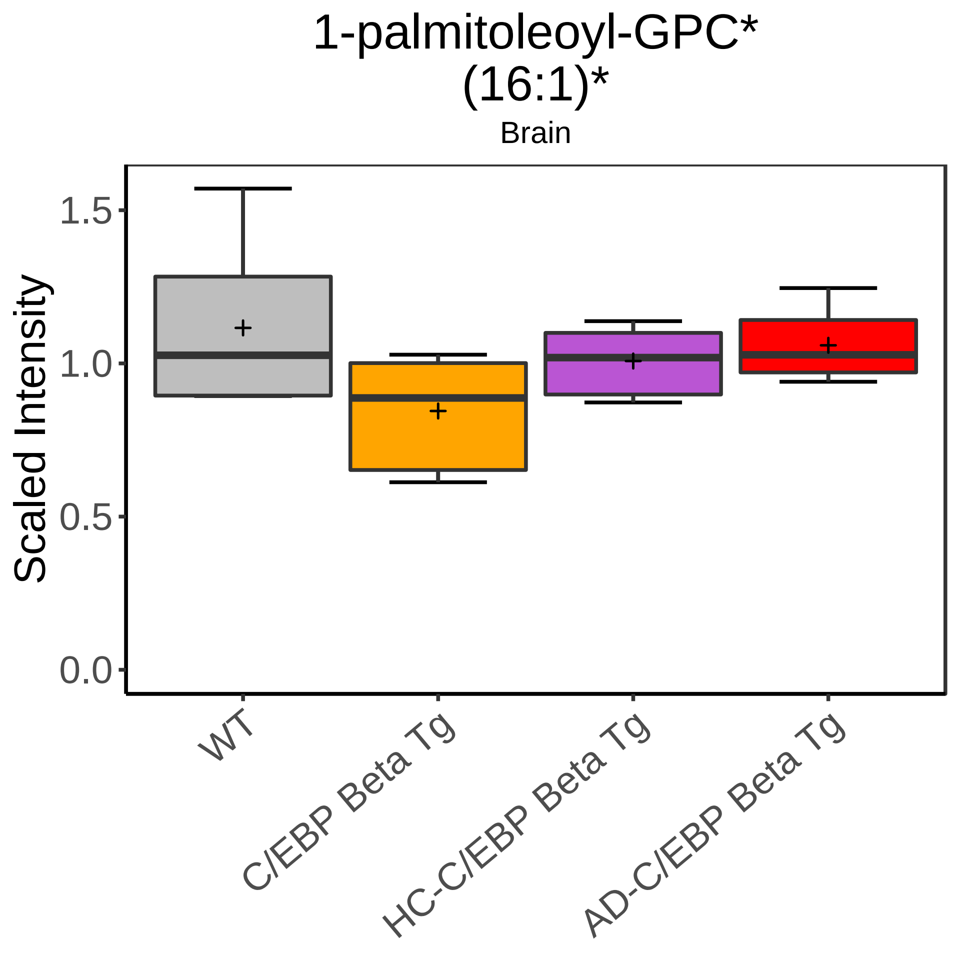

1-palmitoleoyl-GPC*
(16:1)*
Brain
Scaled Intensity
0.0
0.5
1.0
1.5
WT
C/EBP Beta Tg
HC-C/EBP Beta Tg
AD-C/EBP Beta Tg

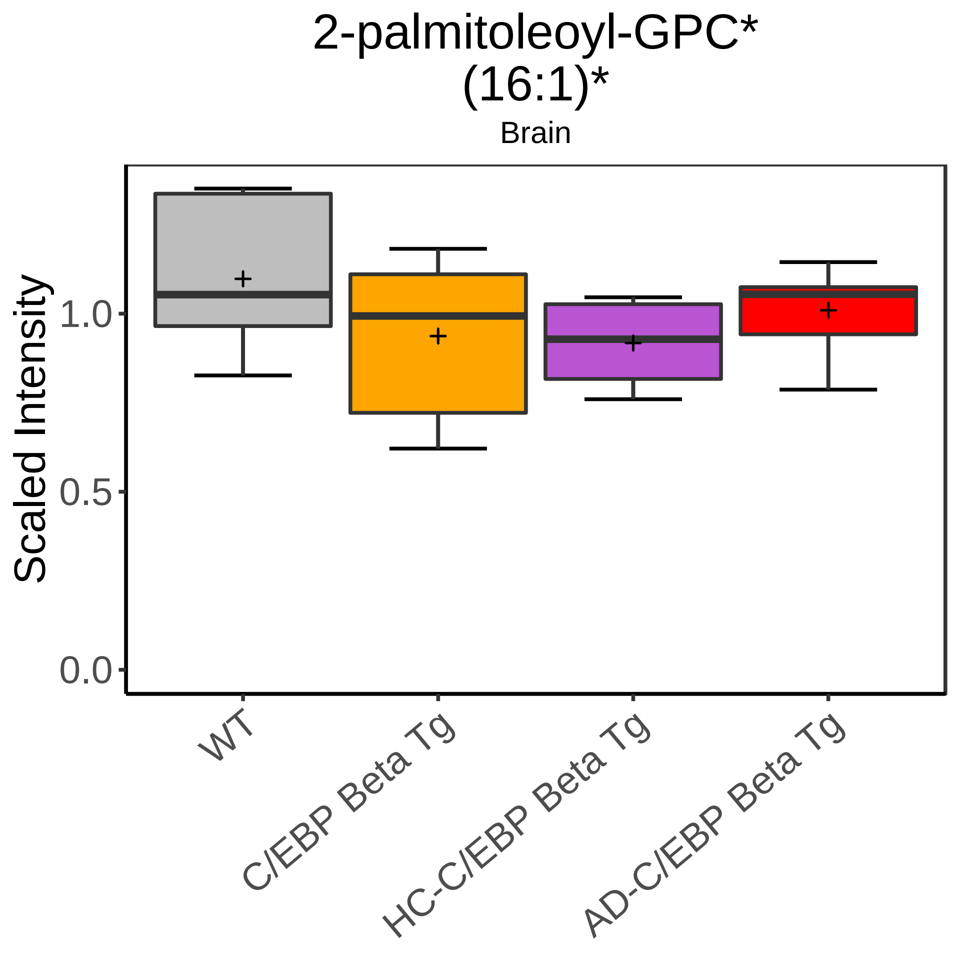
2-palmitoleoyl-GPC*
(16:1)*
Brain
Scaled Intensity
0.0
0.5
1.0
WT
C/EBP Beta Tg
HC-C/EBP Beta Tg
AD-C/EBP Beta Tg

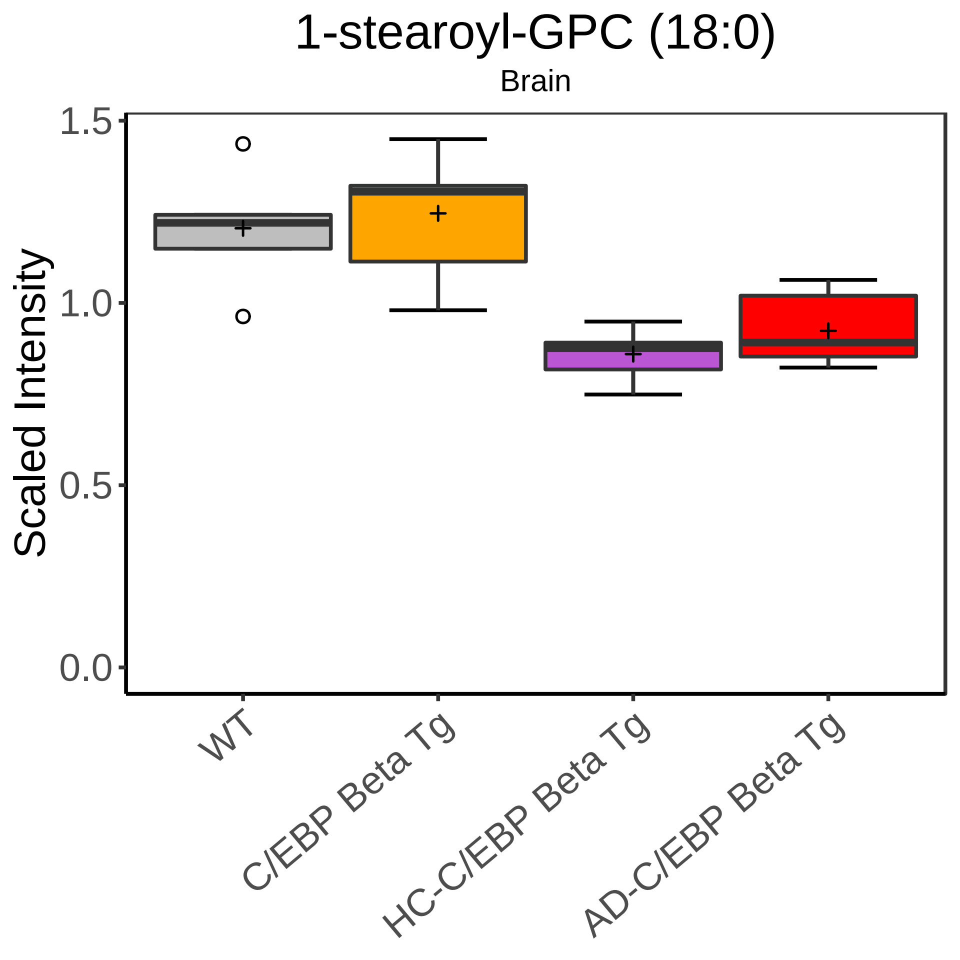
1-stearoyl-GPC (18:0)
Brain
Scaled Intensity
1.5
1.0
0.5
0.0
WT
C/EBP Beta Tg
HC-C/EBP Beta Tg
AD-C/EBP Beta Tg

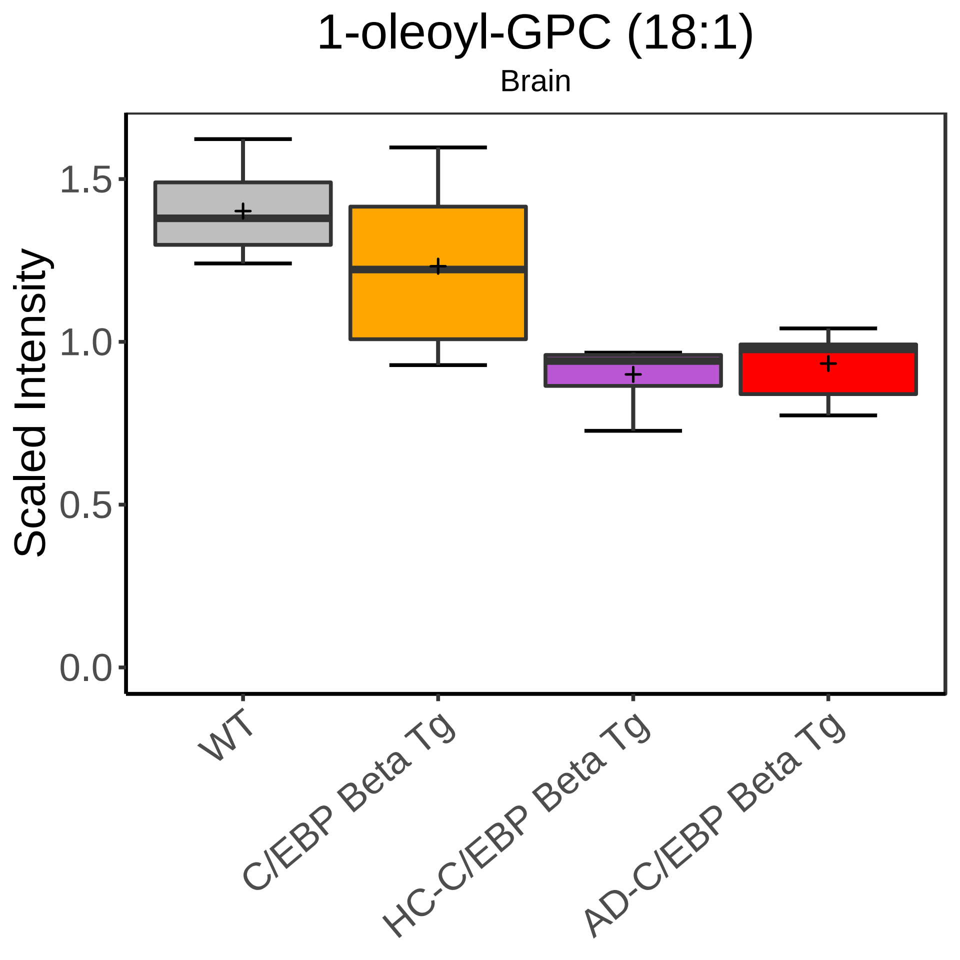

1-oleoyl-GPC (18:1)
Brain
Scaled Intensity
1.5
1.0
0.5
0.0
WT
C/EBP Beta Tg
HC-C/EBP Beta Tg
AD-C/EBP Beta Tg

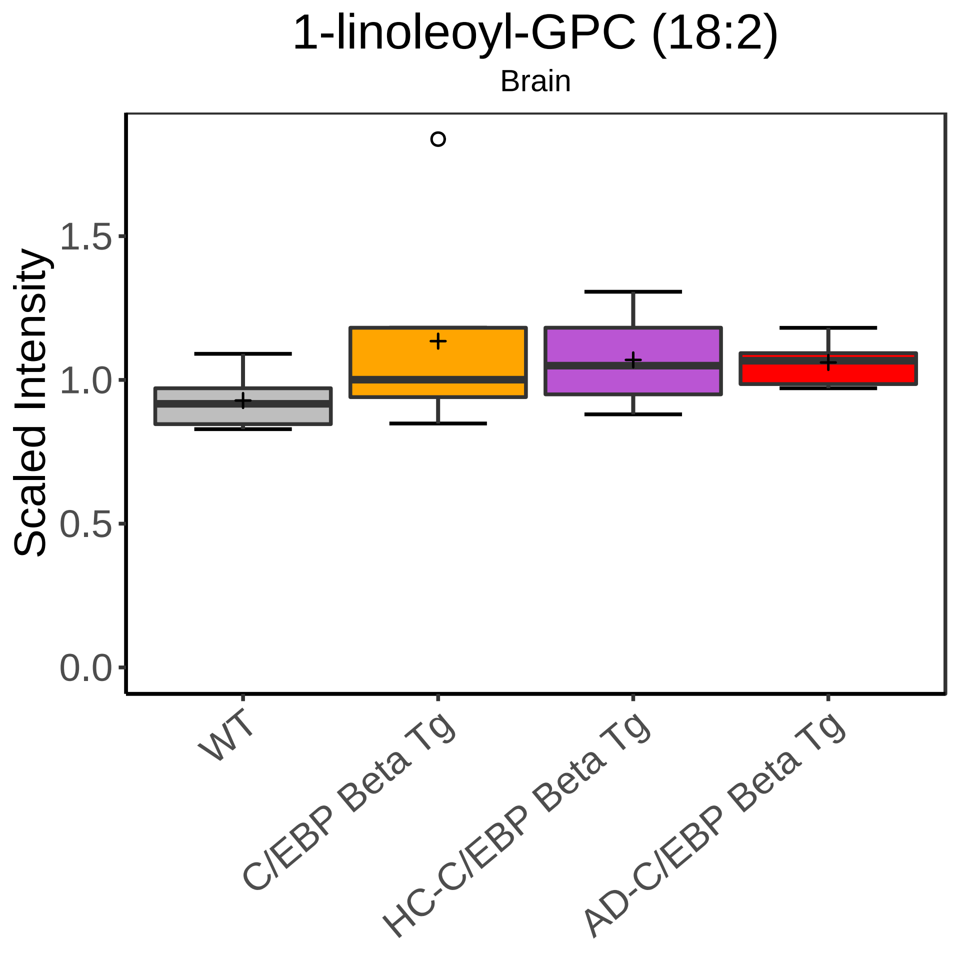

1-linoleoyl-GPC (18:2)
Brain
Scaled Intensity
1.5
1.0
0.5
0.0
WT
C/EBP Beta Tg
HC-C/EBP Beta Tg
AD-C/EBP Beta Tg

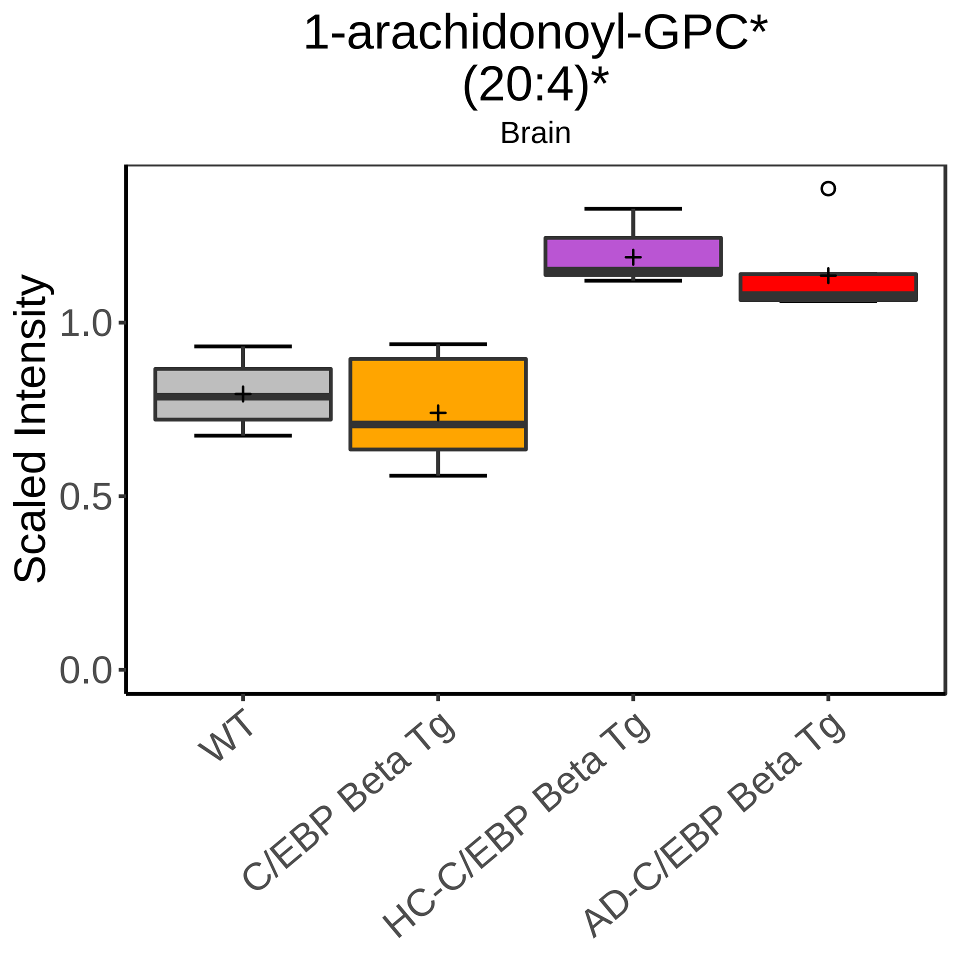
1-arachidonoyl-GPC*
(20:4)*
Brain
Scaled Intensity
1.0
0.5
0.0
WT
C/EBP Beta Tg
HC-C/EBP Beta Tg
AD-C/EBP Beta Tg

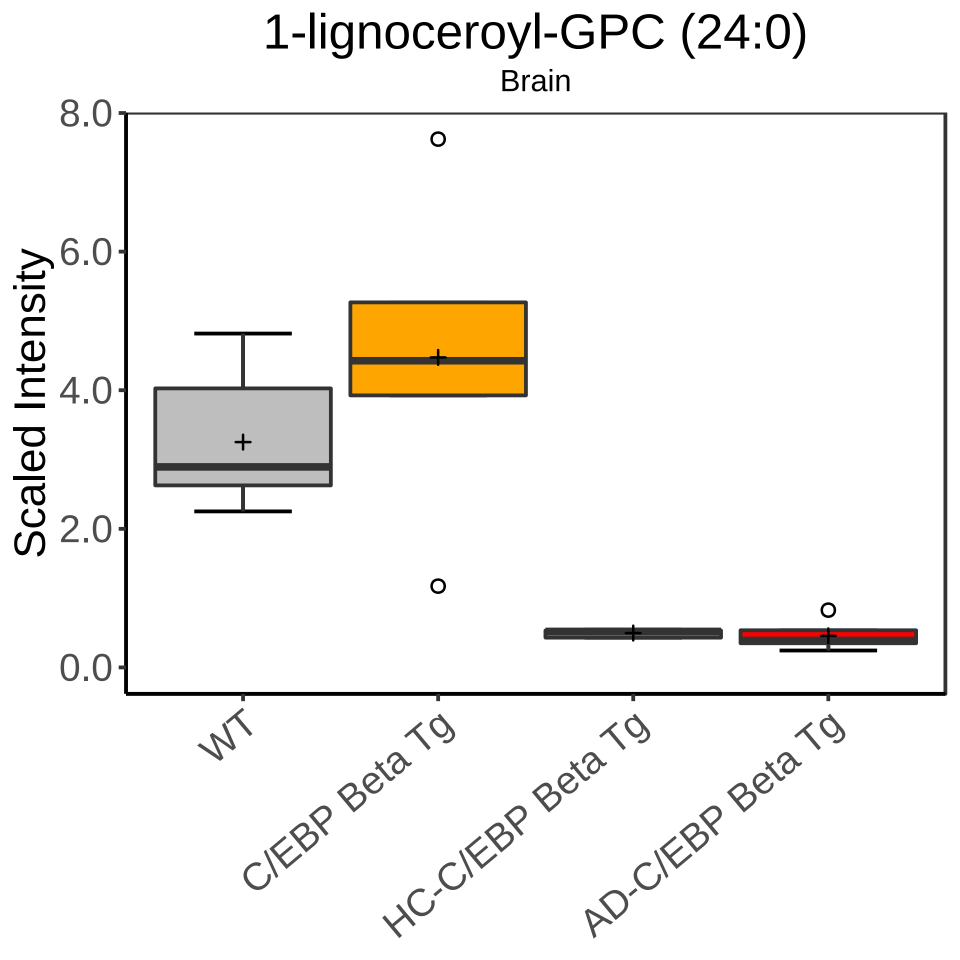

1-lignoceroyl-GPC (24:0)
Brain
Scaled Intensity
8.0
6.0
4.0
2.0
0.0
WT
C/EBP Beta Tg
HC-C/EBP Beta Tg
AD-C/EBP Beta Tg

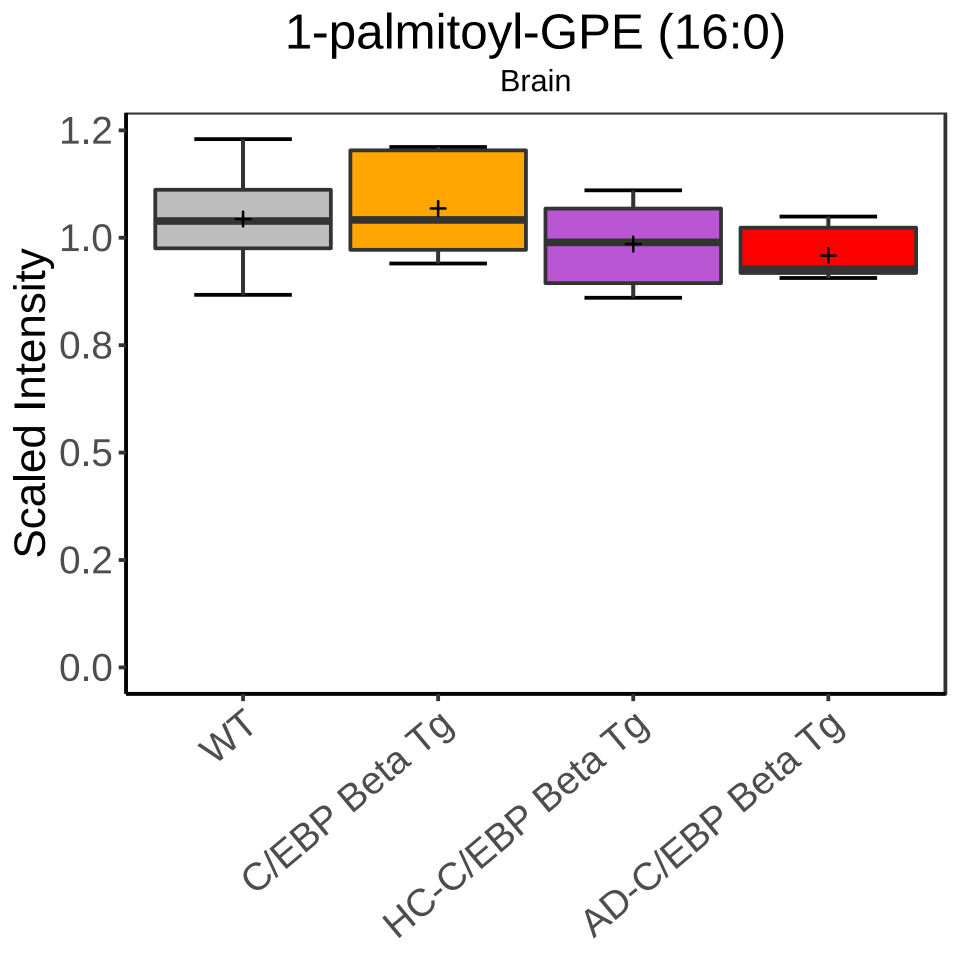
1-palmitoyl-GPE (16:0)
Brain
Scaled Intensity
1.2
1.0
0.8
0.5
0.2
0.0
WT
C/EBP Beta Tg
HC-C/EBP Beta Tg
AD-C/EBP Beta Tg

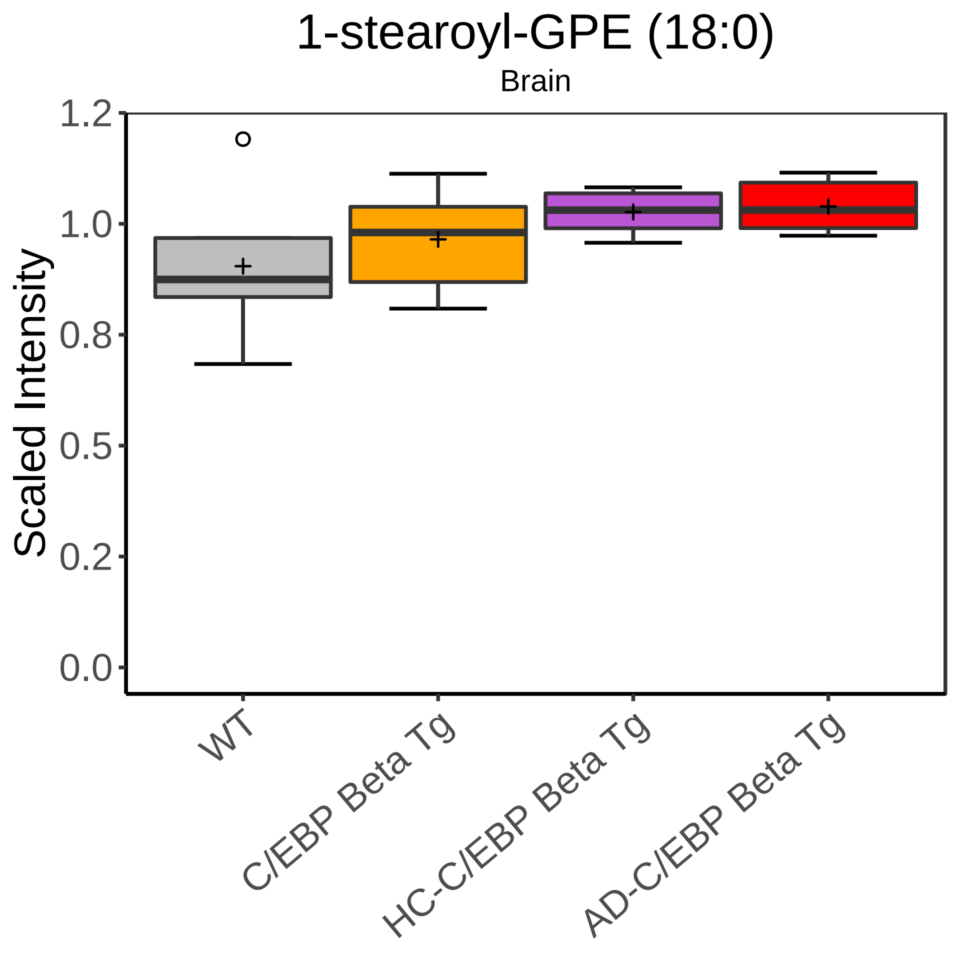
1-stearoyl-GPE (18:0)
Brain
Scaled Intensity
1.2
1.0
0.8
0.5
0.2
0.0
WT
C/EBP Beta Tg
HC-C/EBP Beta Tg
AD-C/EBP Beta Tg

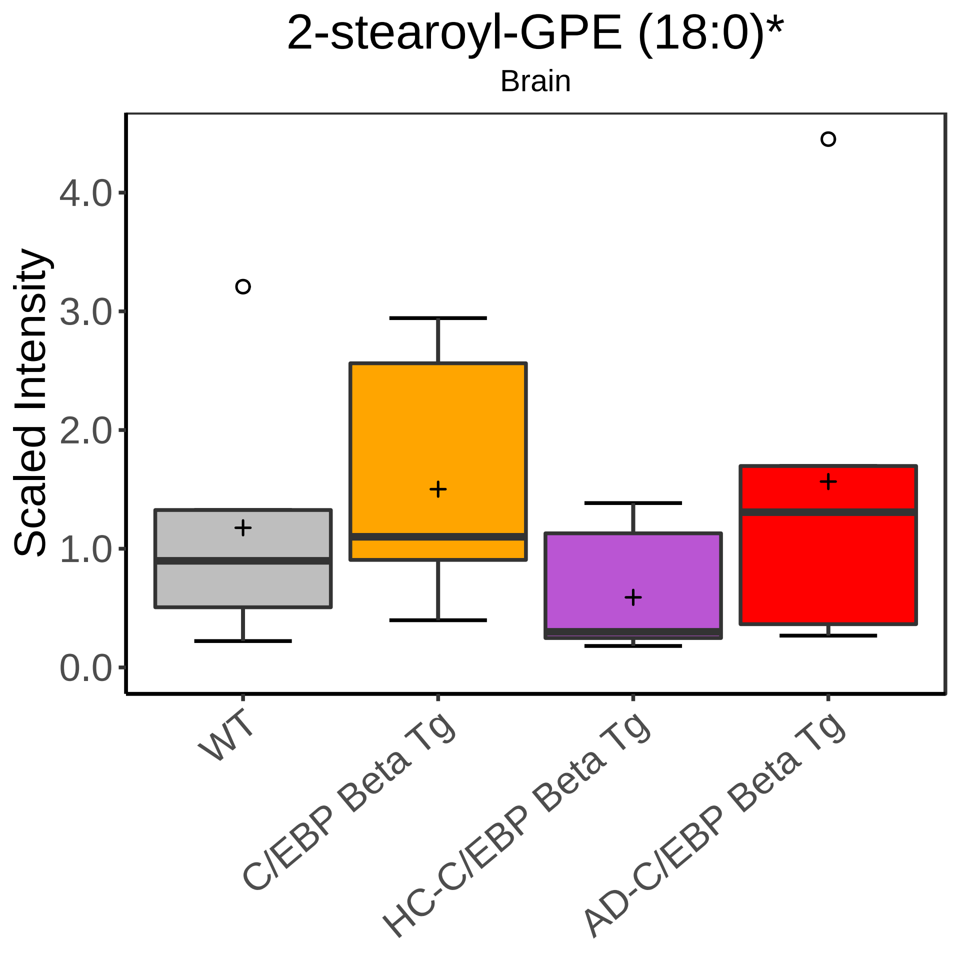
2-stearoyl-GPE (18:0)*
Brain
Scaled Intensity
4.0
3.0
2.0
1.0
0.0
WT
C/EBP Beta Tg
HC-C/EBP Beta Tg
AD-C/EBP Beta Tg

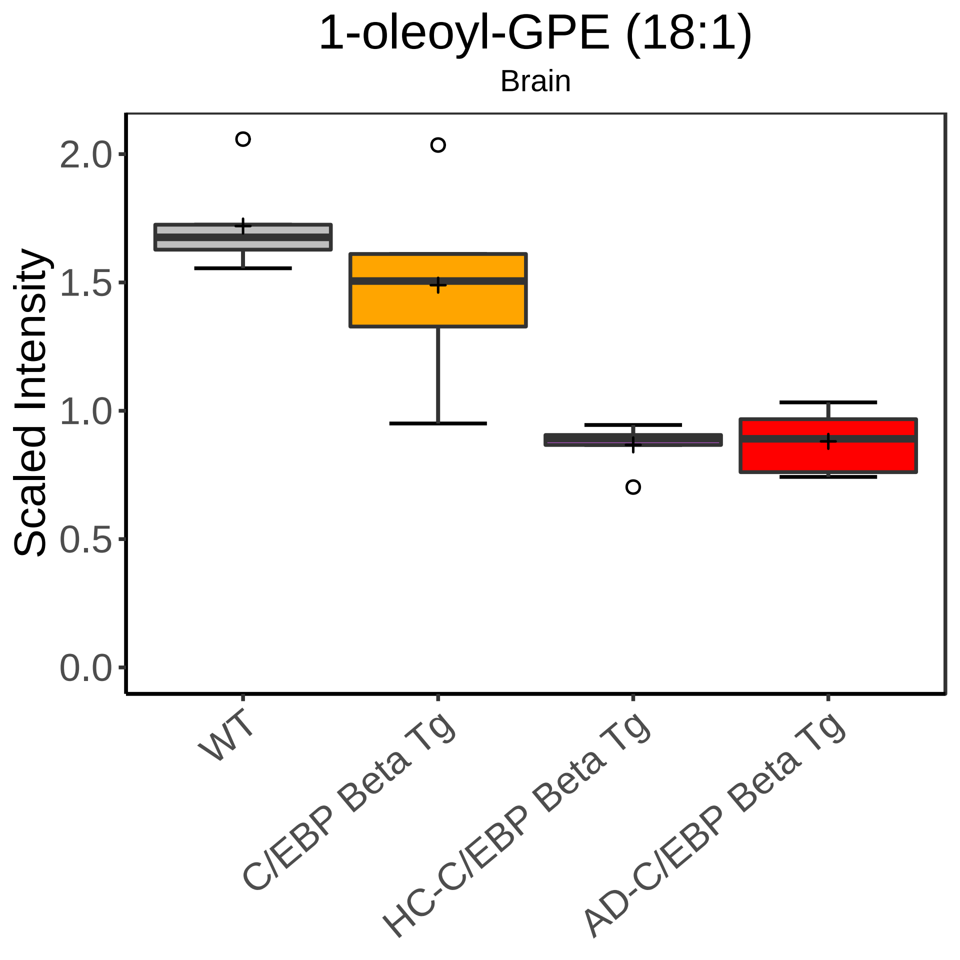
1-oleoyl-GPE (18:1)
Brain
Scaled Intensity
2.0
1.5
1.0
0.5
0.0
WT
C/EBP Beta Tg
HC-C/EBP Beta Tg
AD-C/EBP Beta Tg

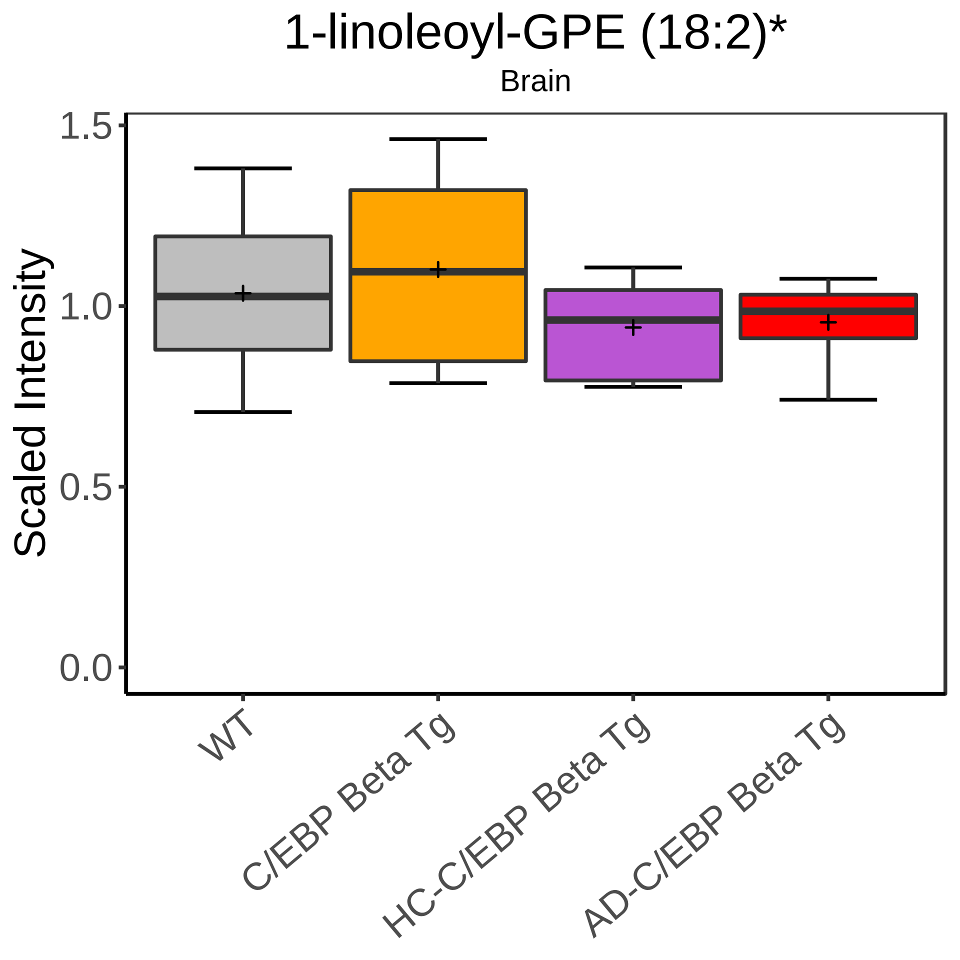
1-linoleoyl-GPE (18:2)*
Brain
Scaled Intensity
1.5
1.0
0.5
0.0
WT
C/EBP Beta Tg
HC-C/EBP Beta Tg
AD-C/EBP Beta Tg

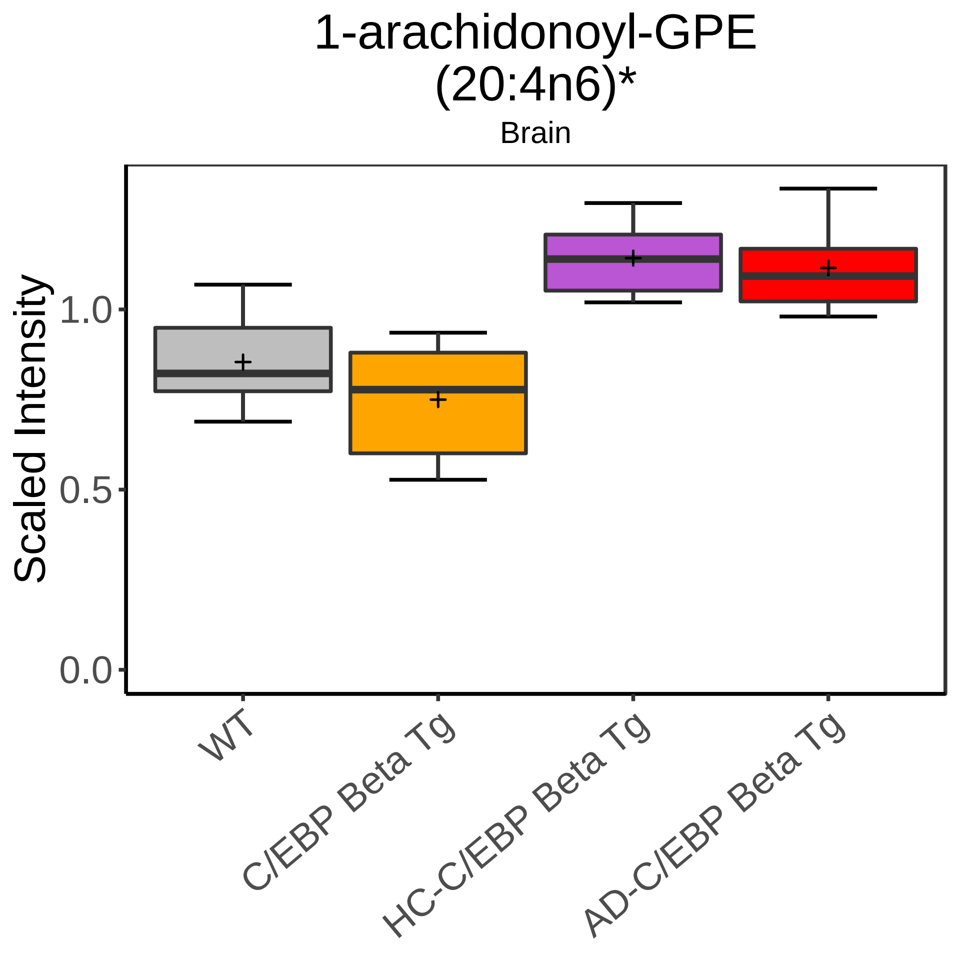
1-arachidonoyl-GPE
(20:4n6)*
Brain
Scaled Intensity
1.0
0.5
0.0
WT
C/EBP Beta Tg
HC-C/EBP Beta Tg
AD-C/EBP Beta Tg

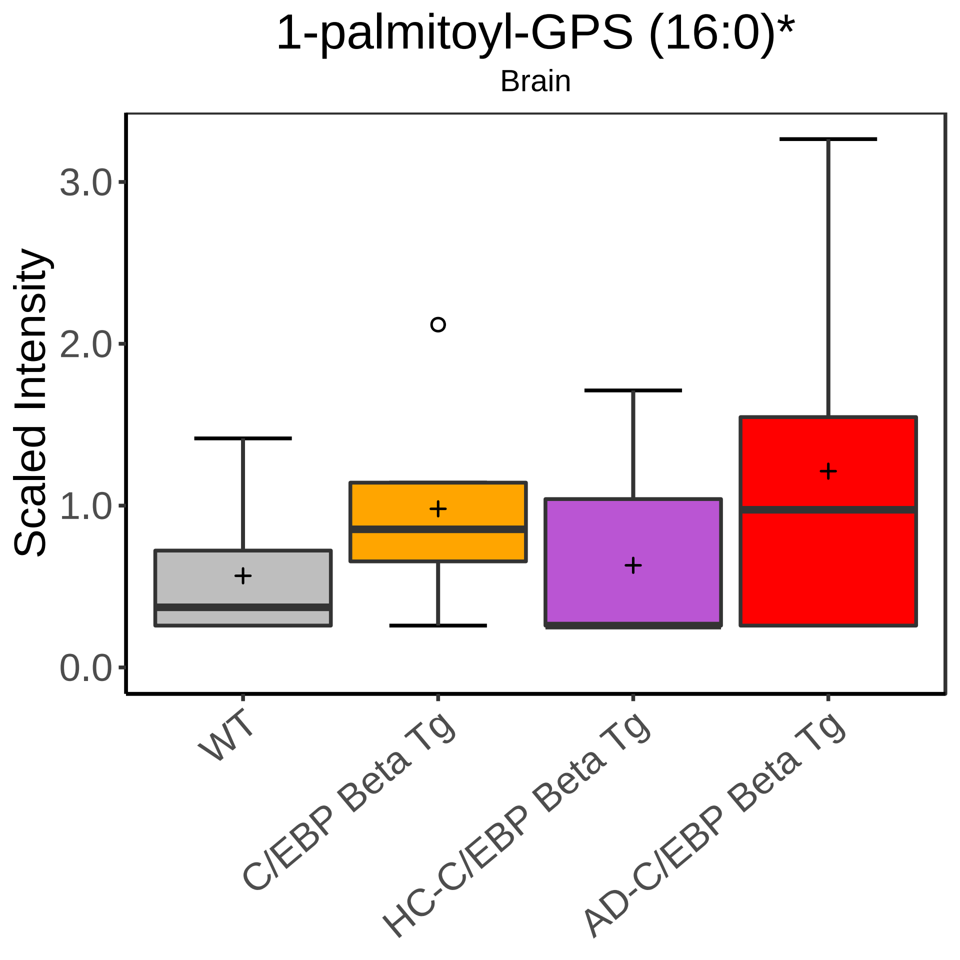
1-palmitoyl-GPS (16:0)*
Brain
Scaled Intensity
3.0
2.0
1.0
0.0
WT
C/EBP Beta Tg
HC-C/EBP Beta Tg
AD-C/EBP Beta Tg

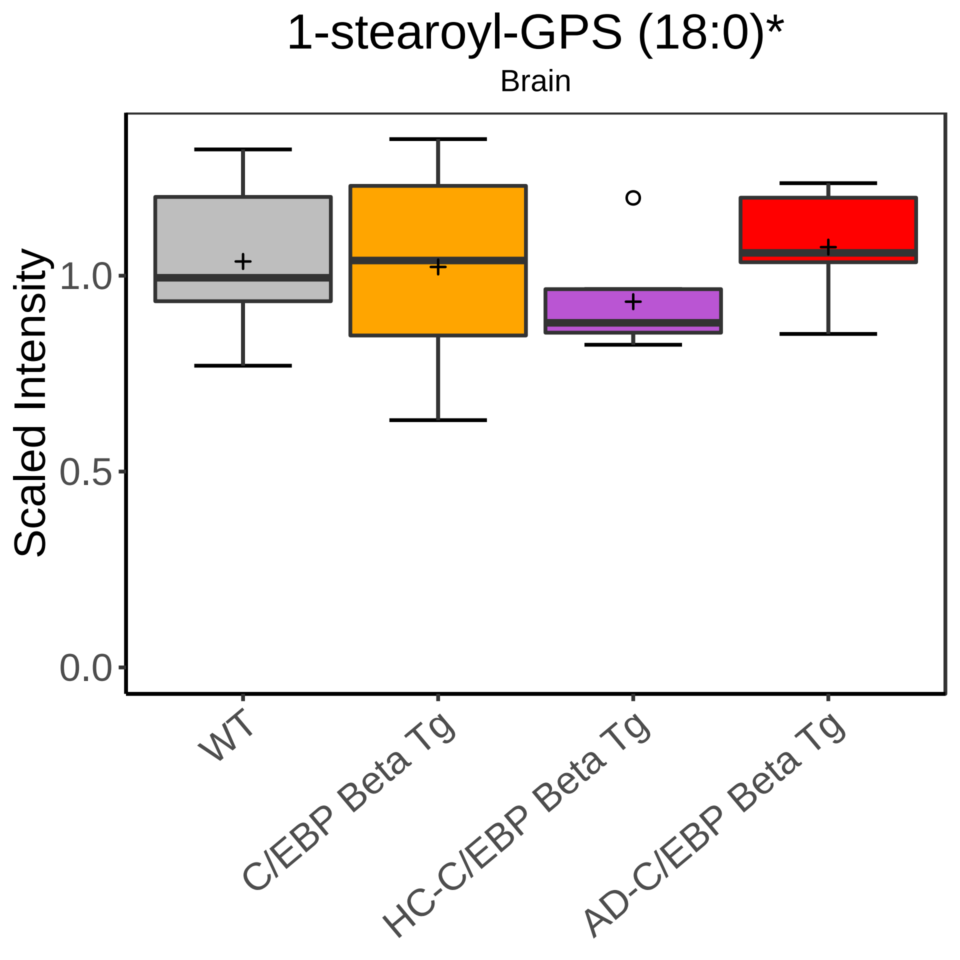
1-stearoyl-GPS (18:0)*
Brain
Scaled Intensity
1.0
0.5
0.0
WT
C/EBP Beta Tg
HC-C/EBP Beta Tg
AD-C/EBP Beta Tg

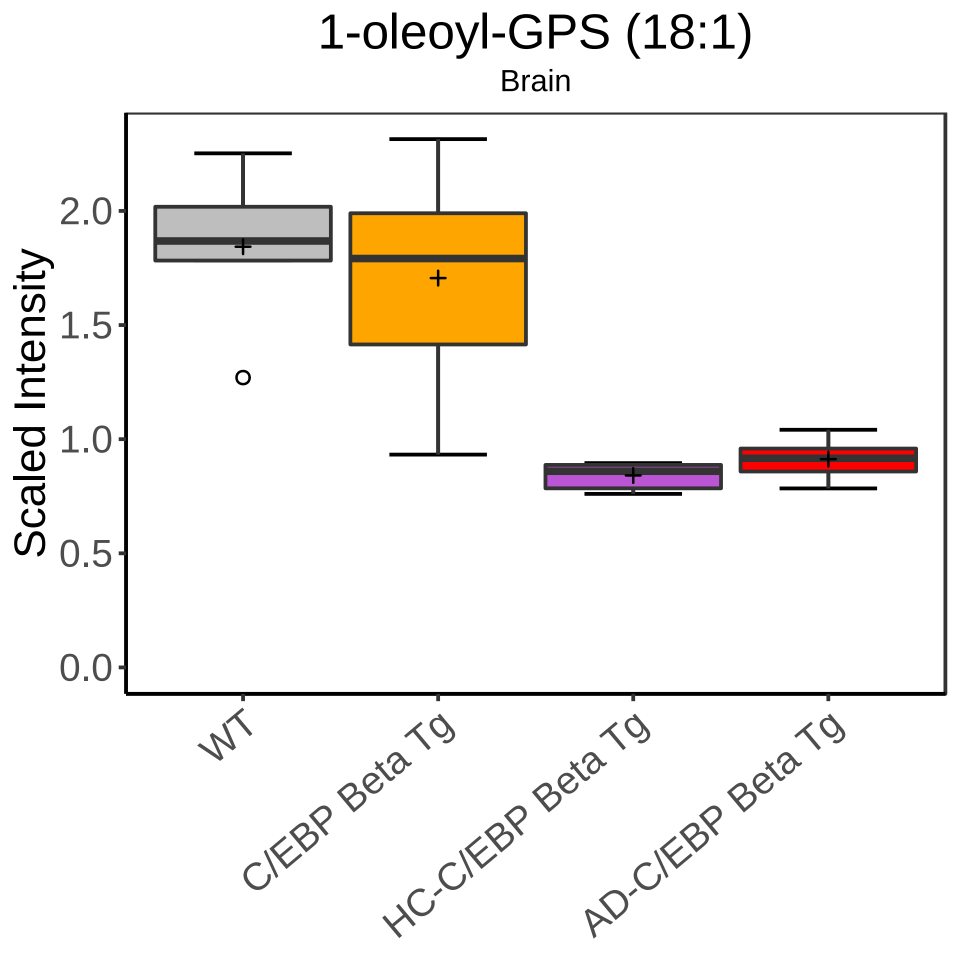
1-oleoyl-GPS (18:1)
Brain
Scaled Intensity
0.0
0.5
1.0
1.5
2.0
WT
C/EBP Beta Tg
HC-C/EBP Beta Tg
AD-C/EBP Beta Tg

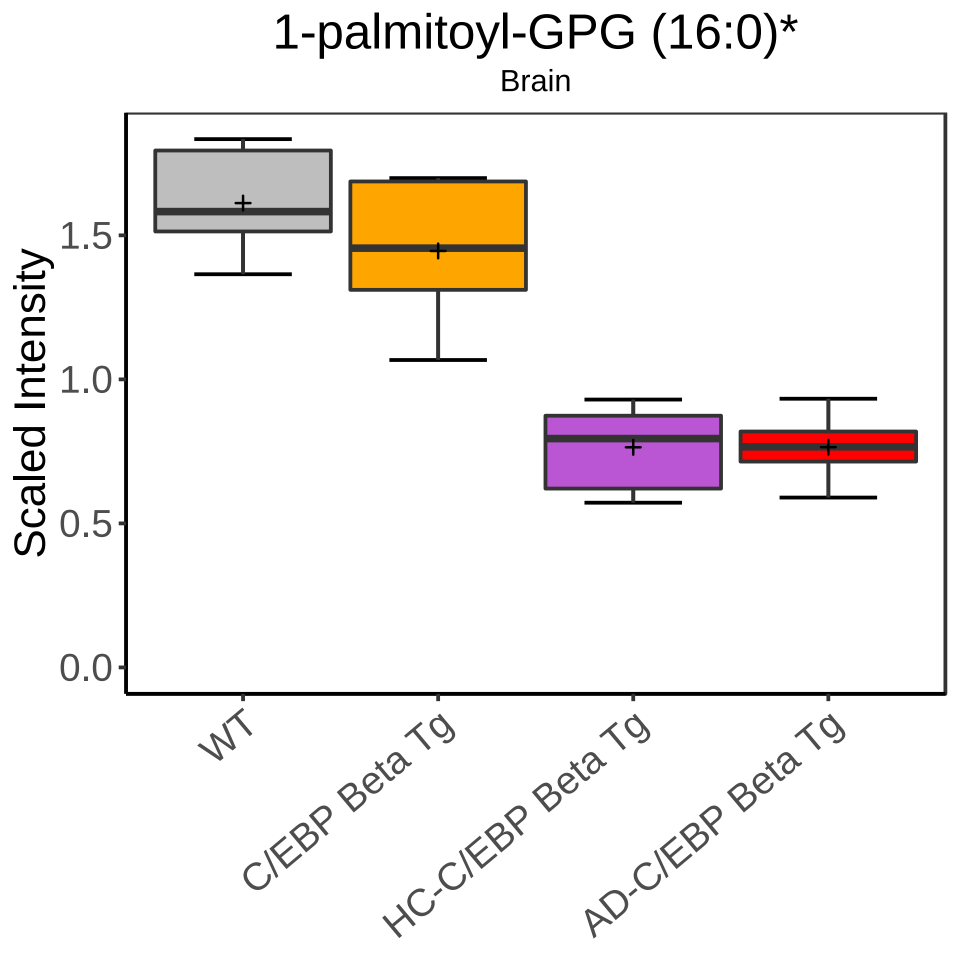

1-palmitoyl-GPG (16:0)*
Brain
Scaled Intensity
0.0
0.5
1.0
1.5
WT
C/EBP Beta Tg
HC-C/EBP Beta Tg
AD-C/EBP Beta Tg

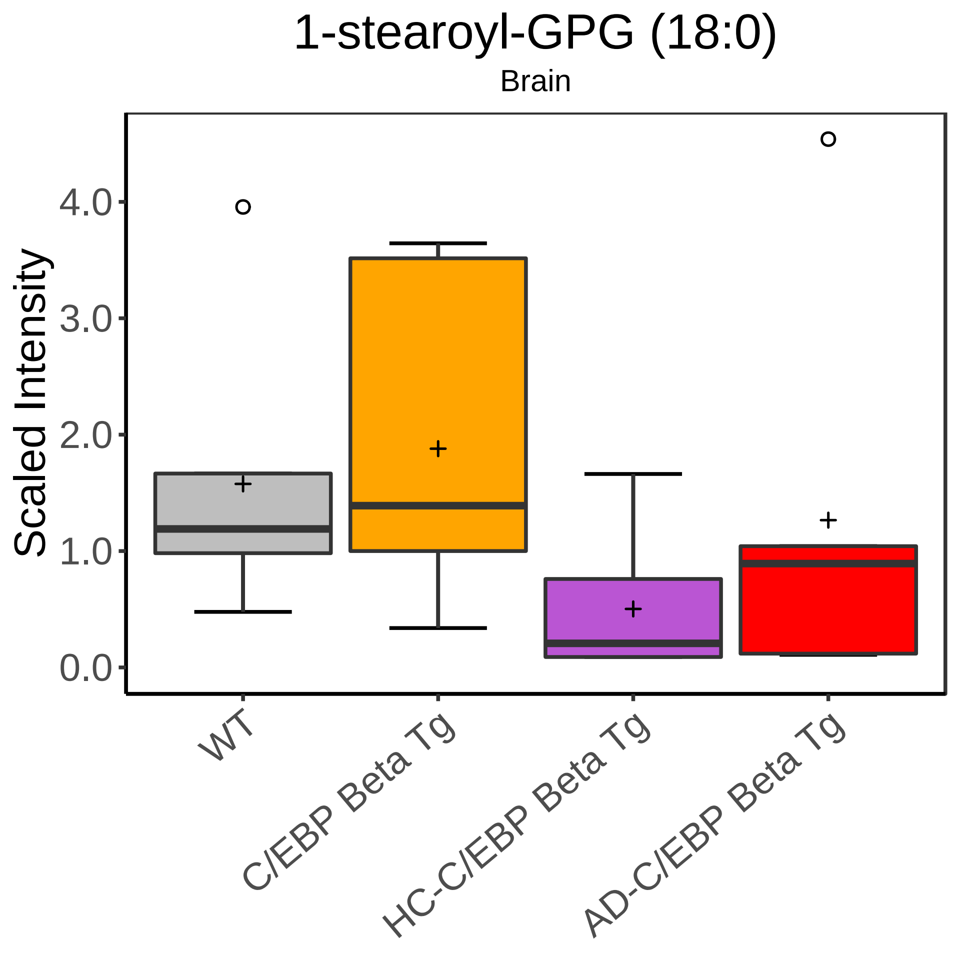

1-stearoyl-GPG (18:0)
Brain
Scaled Intensity
0.0
1.0
2.0
3.0
4.0
WT
C/EBP Beta Tg
HC-C/EBP Beta Tg
AD-C/EBP Beta Tg

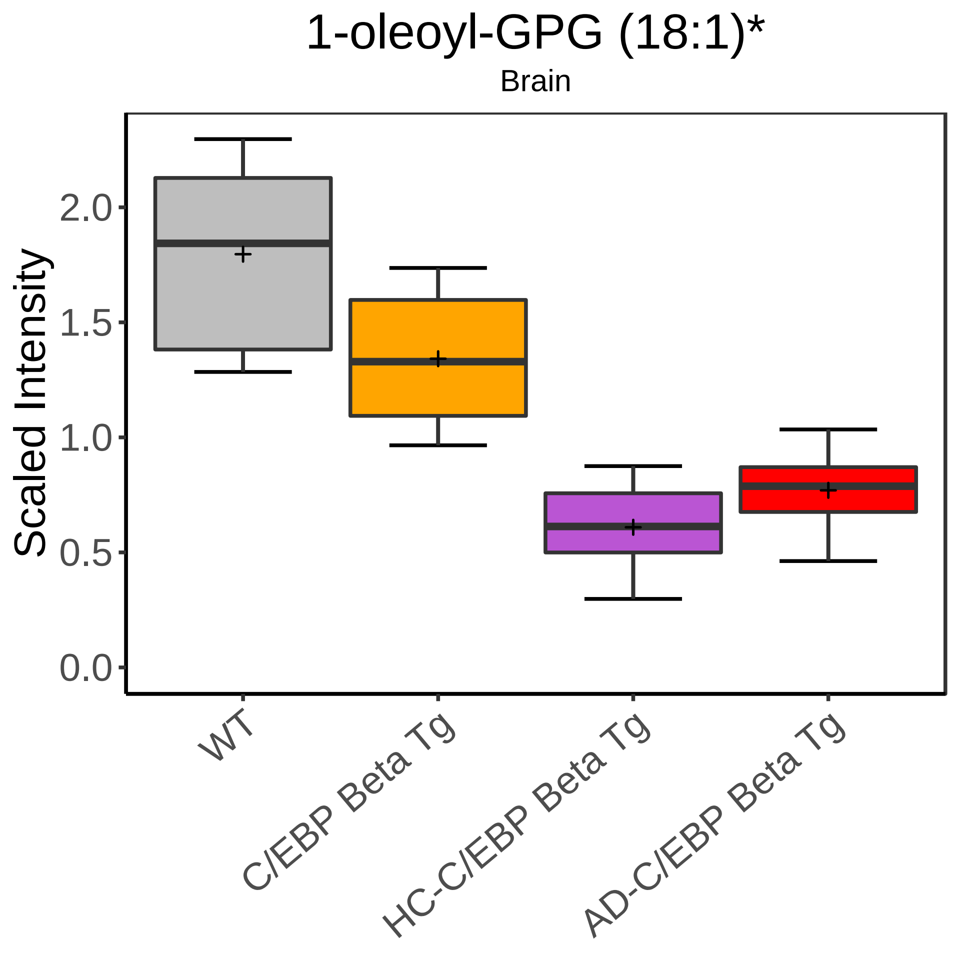

1-oleoyl-GPG (18:1)*
Brain
Scaled Intensity
2.0
1.5
1.0
0.5
0.0
WT
C/EBP Beta Tg
HC-C/EBP Beta Tg
AD-C/EBP Beta Tg

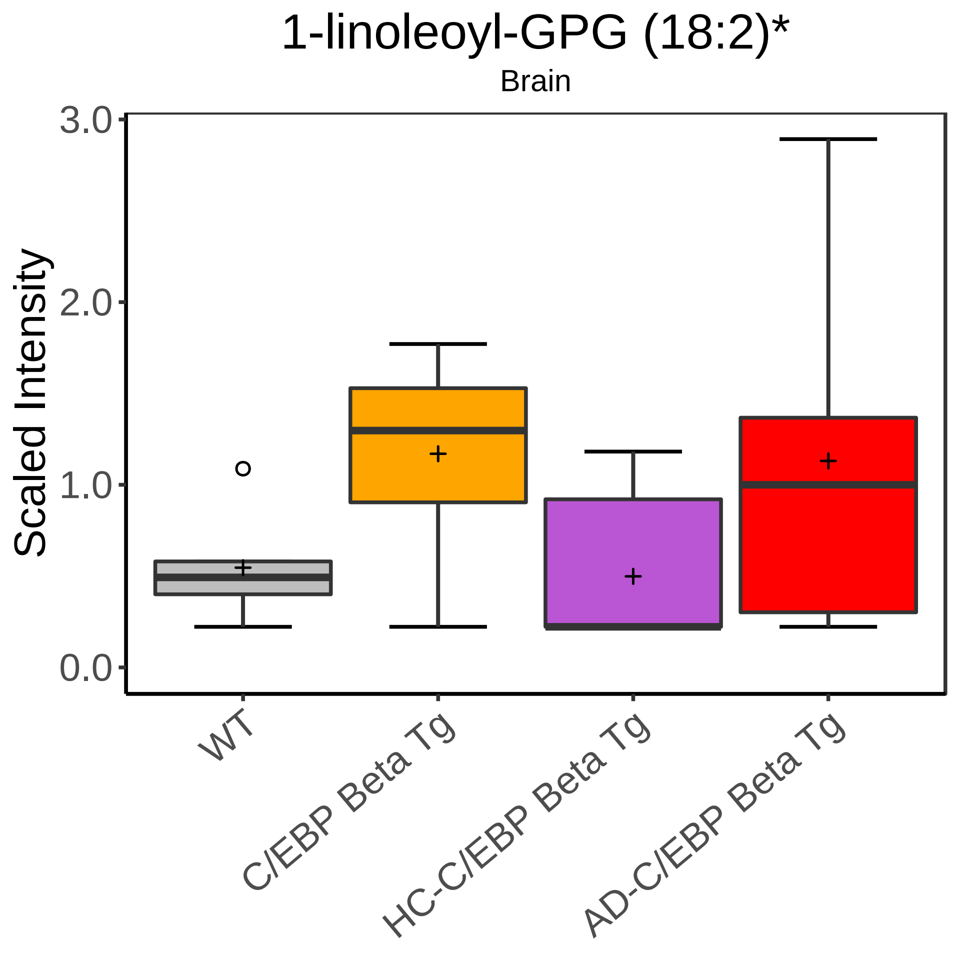

1-linoleoyl-GPG (18:2)*
Brain
Scaled Intensity
3.0
2.0
1.0
0.0
WT
C/EBP Beta Tg
HC-C/EBP Beta Tg
AD-C/EBP Beta Tg

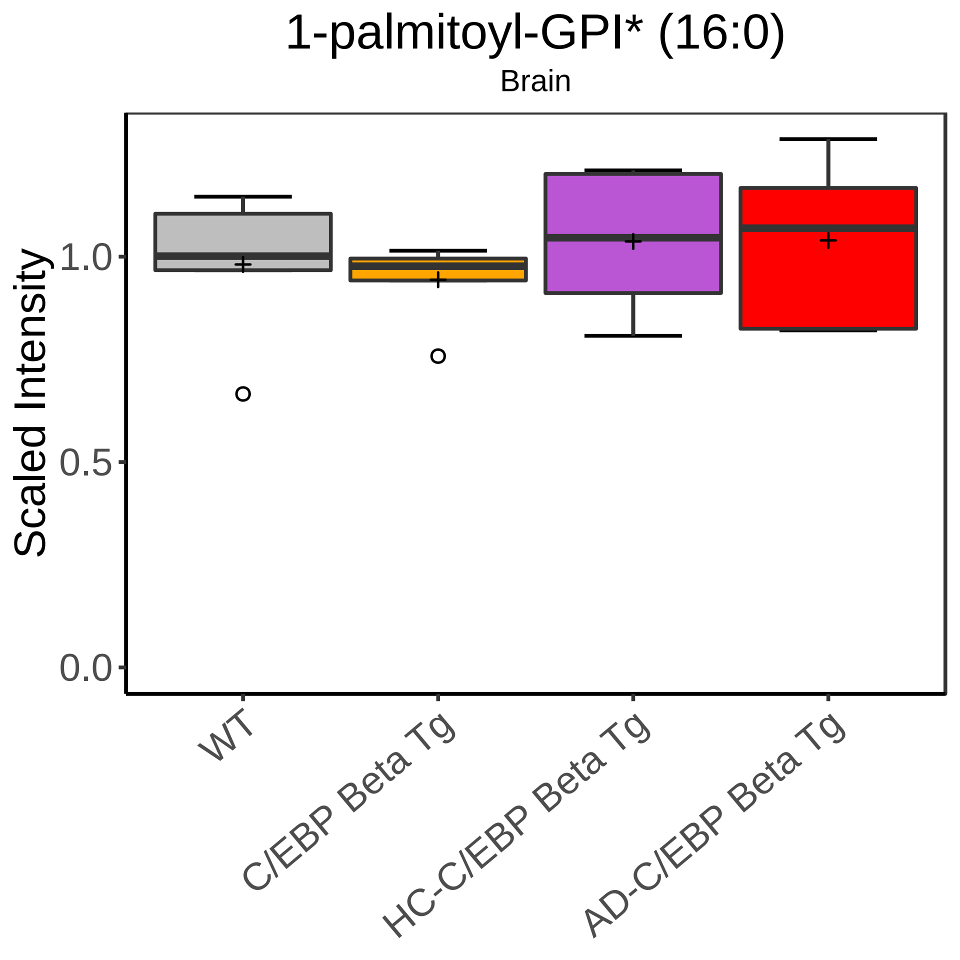
1-palmitoyl-GPI* (16:0)
Brain
Scaled Intensity
1.0
0.5
0.0
WT
C/EBP Beta Tg
HC-C/EBP Beta Tg
AD-C/EBP Beta Tg

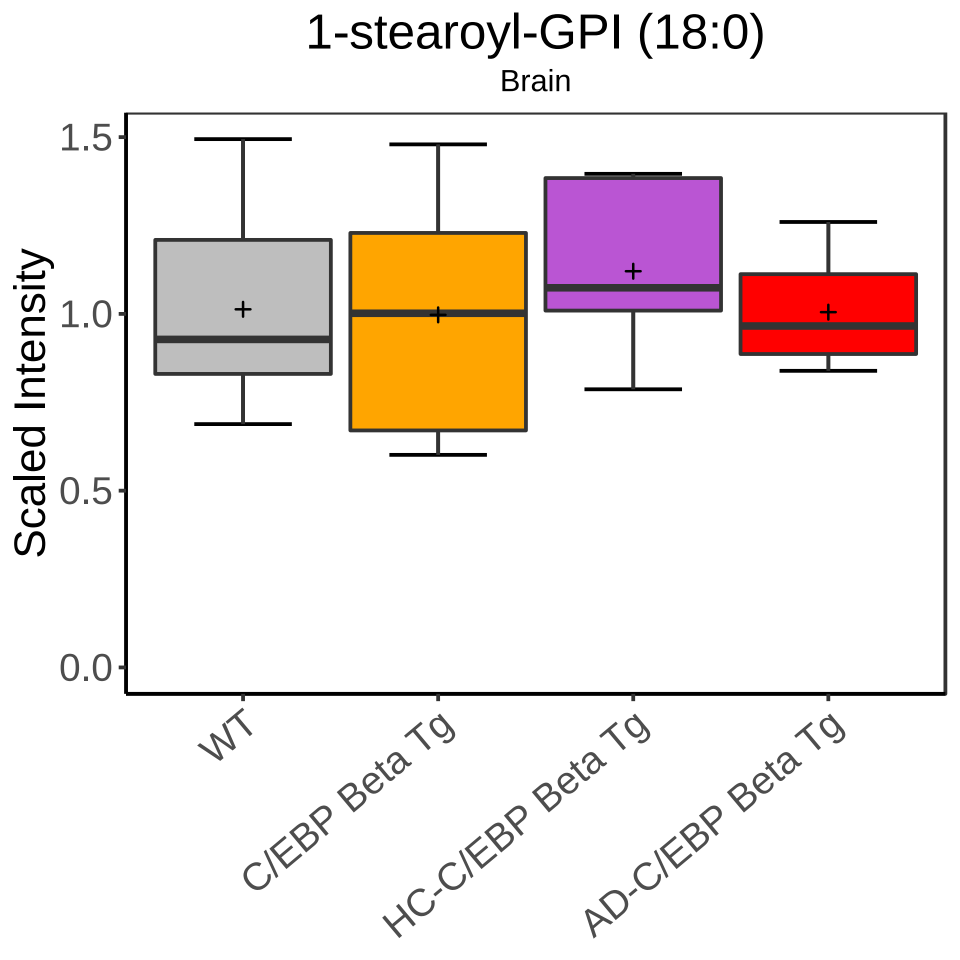
1-stearoyl-GPI (18:0)
Brain
Scaled Intensity
1.5
1.0
0.5
0.0
WT
C/EBP Beta Tg
HC-C/EBP Beta Tg
AD-C/EBP Beta Tg

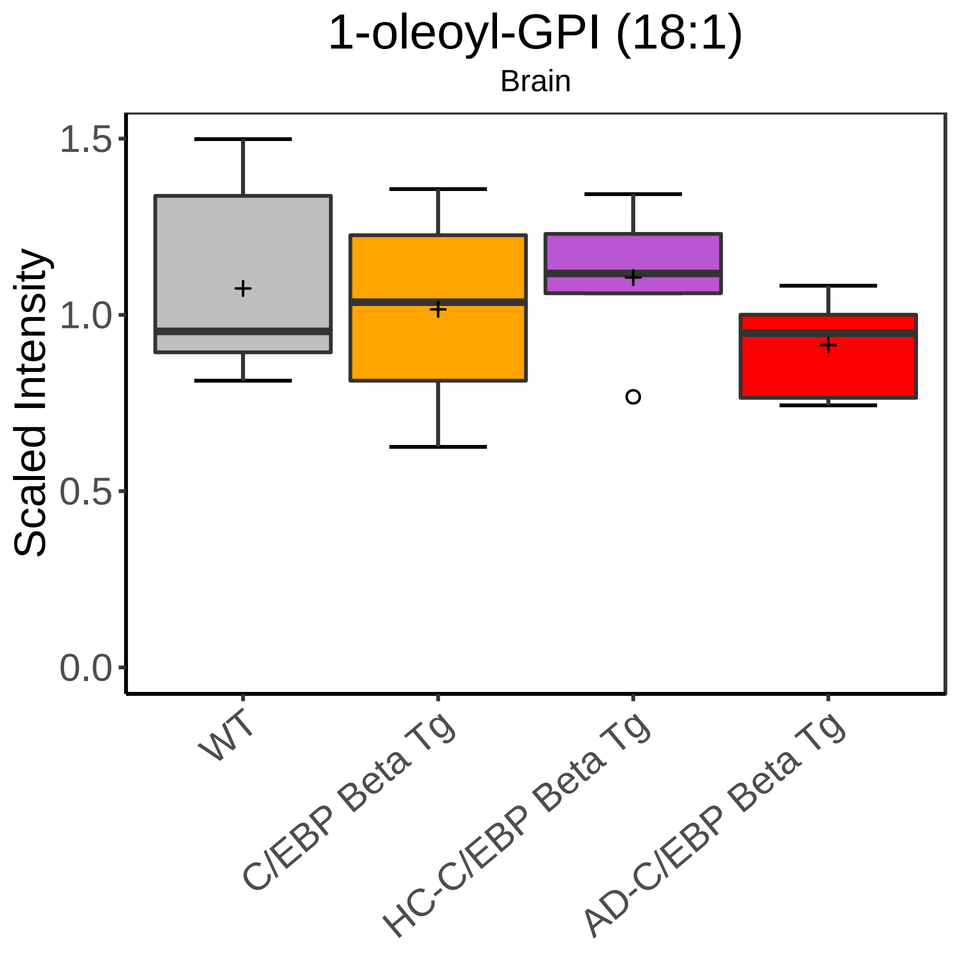

1-oleoyl-GPI (18:1)
Brain
Scaled Intensity
1.5
1.0
0.5
0.0
WT
C/EBP Beta Tg
HC-C/EBP Beta Tg
AD-C/EBP Beta Tg

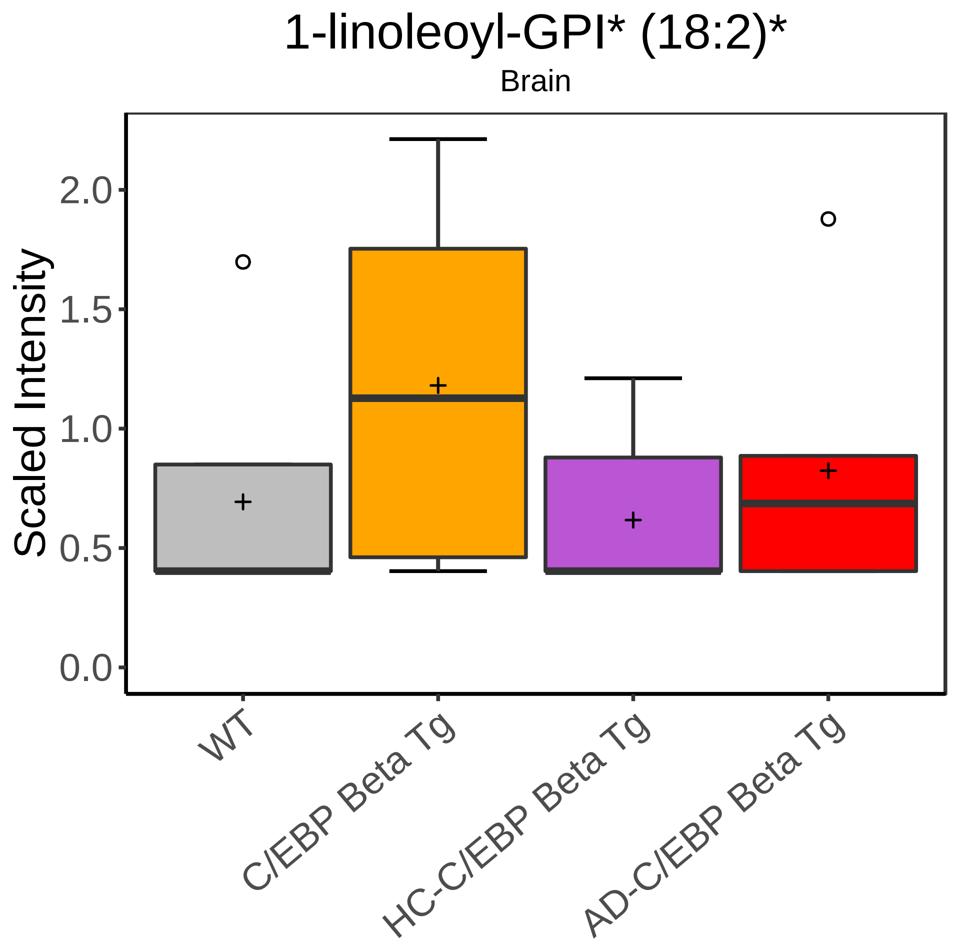

1-linoleoyl-GPI* (18:2)*
Brain
Scaled Intensity
0.0
0.5
1.0
1.5
2.0
WT
C/EBP Beta Tg
HC-C/EBP Beta Tg
AD-C/EBP Beta Tg

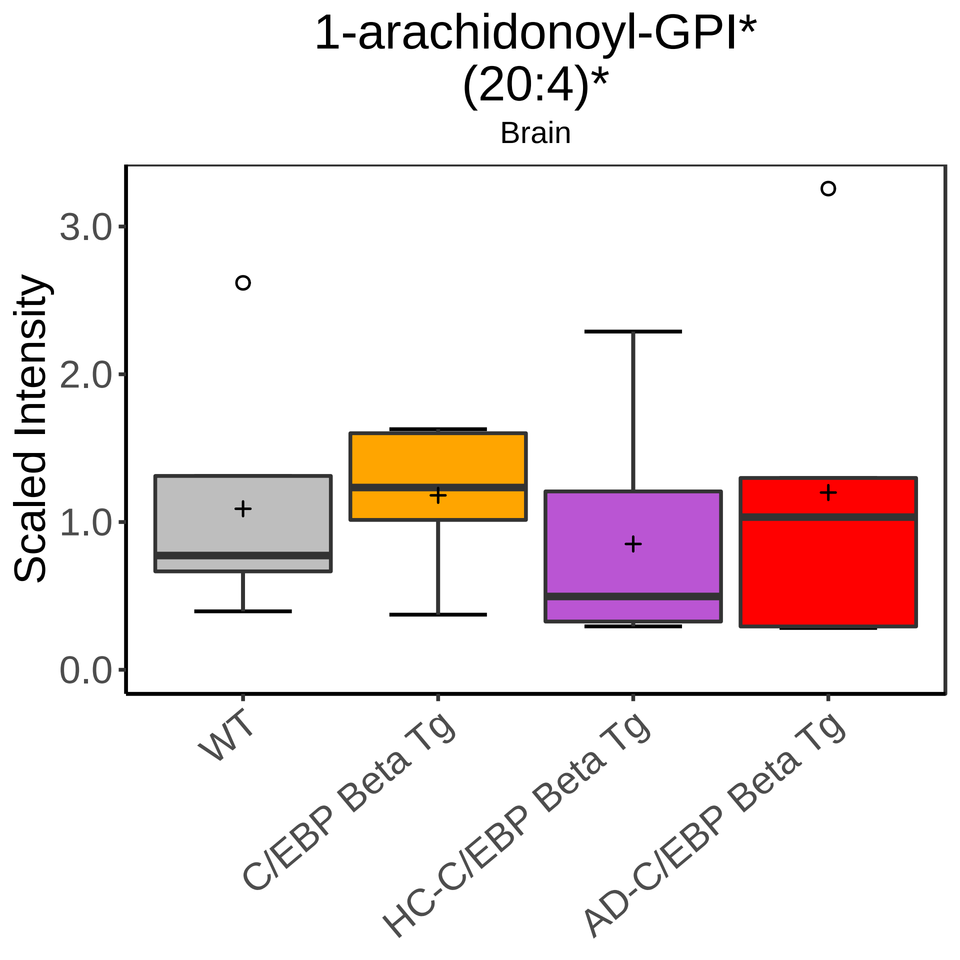
1-arachidonoyl-GPI*
(20:4)*
Brain
Scaled Intensity
3.0
2.0
1.0
0.0
WT
C/EBP Beta Tg
HC-C/EBP Beta Tg
AD-C/EBP Beta Tg

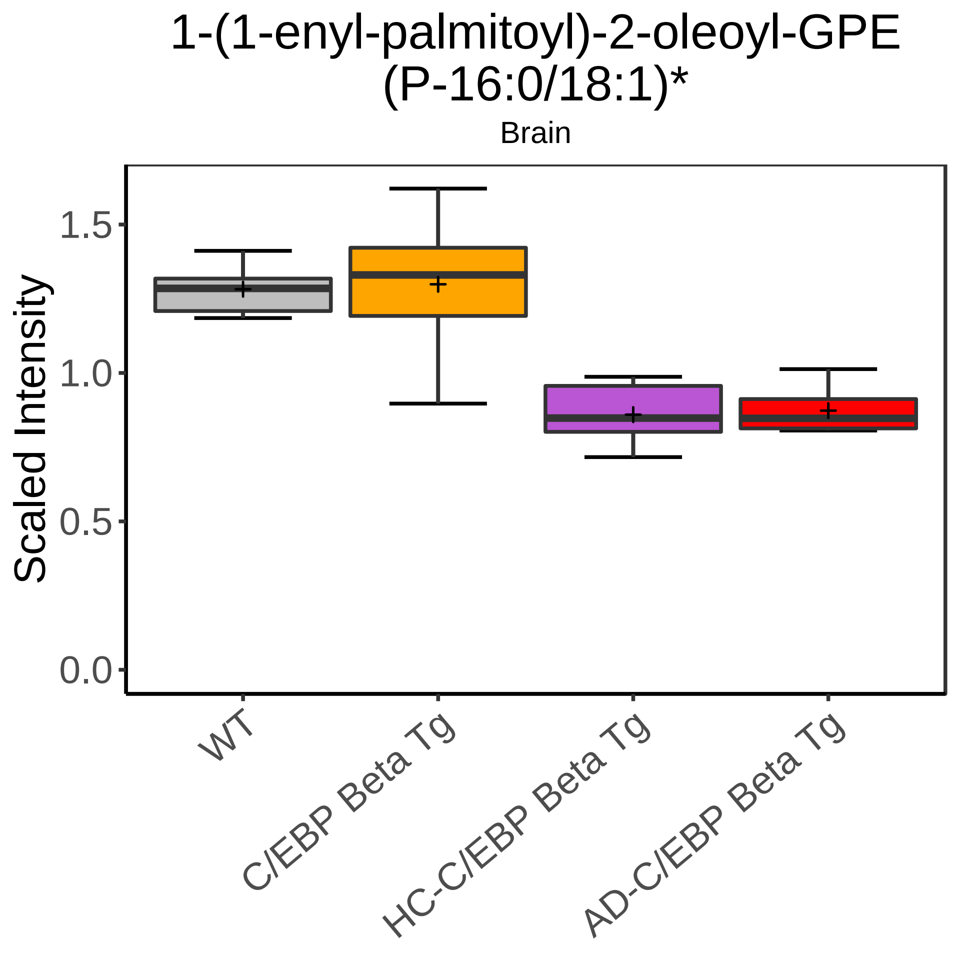
1-(1-enyl-palmitoyl)-2-oleoyl-GPE
(P-16:0/18:1)*
Brain
Scaled Intensity
1.5
1.0
0.5
0.0
WT
C/EBP Beta Tg
HC-C/EBP Beta Tg
AD-C/EBP Beta Tg

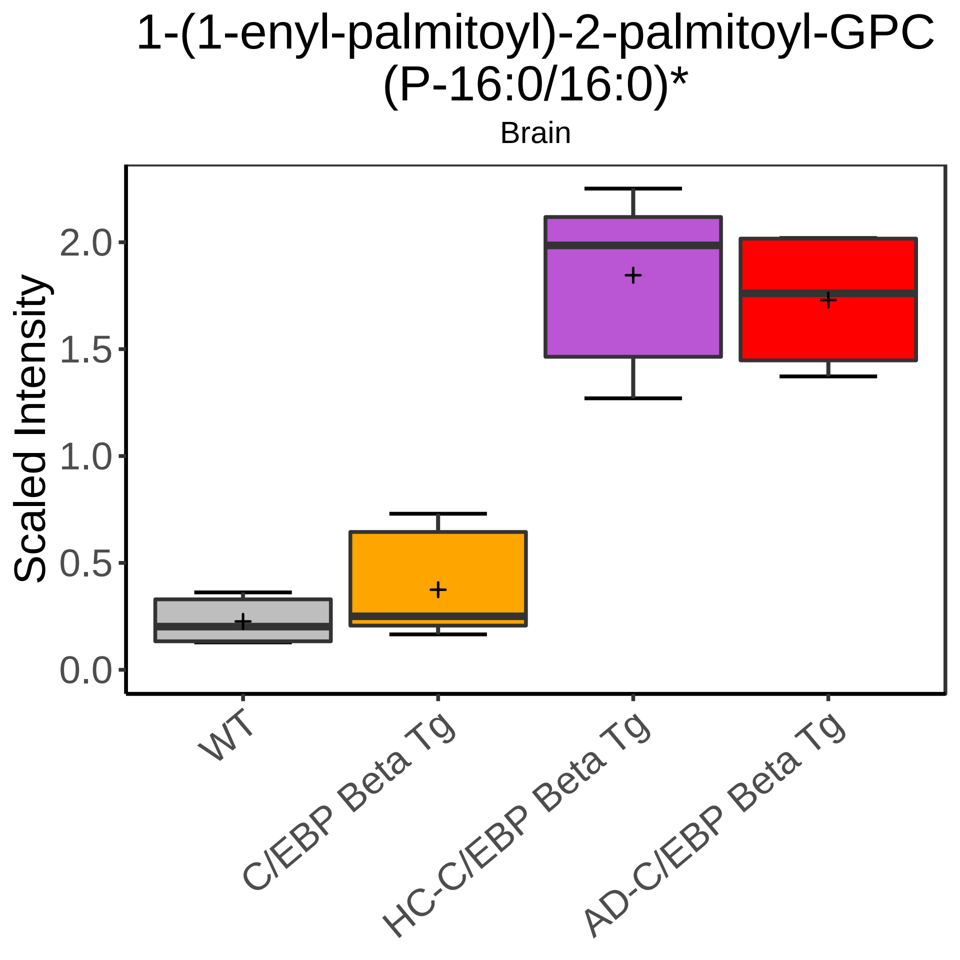
1-(1-enyl-palmitoyl)-2-palmitoyl-GPC
(P-16:0/16:0)*
Brain
Scaled Intensity
2.0
1.5
1.0
0.5
0.0
WT
C/EBP Beta Tg
HC-C/EBP Beta Tg
AD-C/EBP Beta Tg

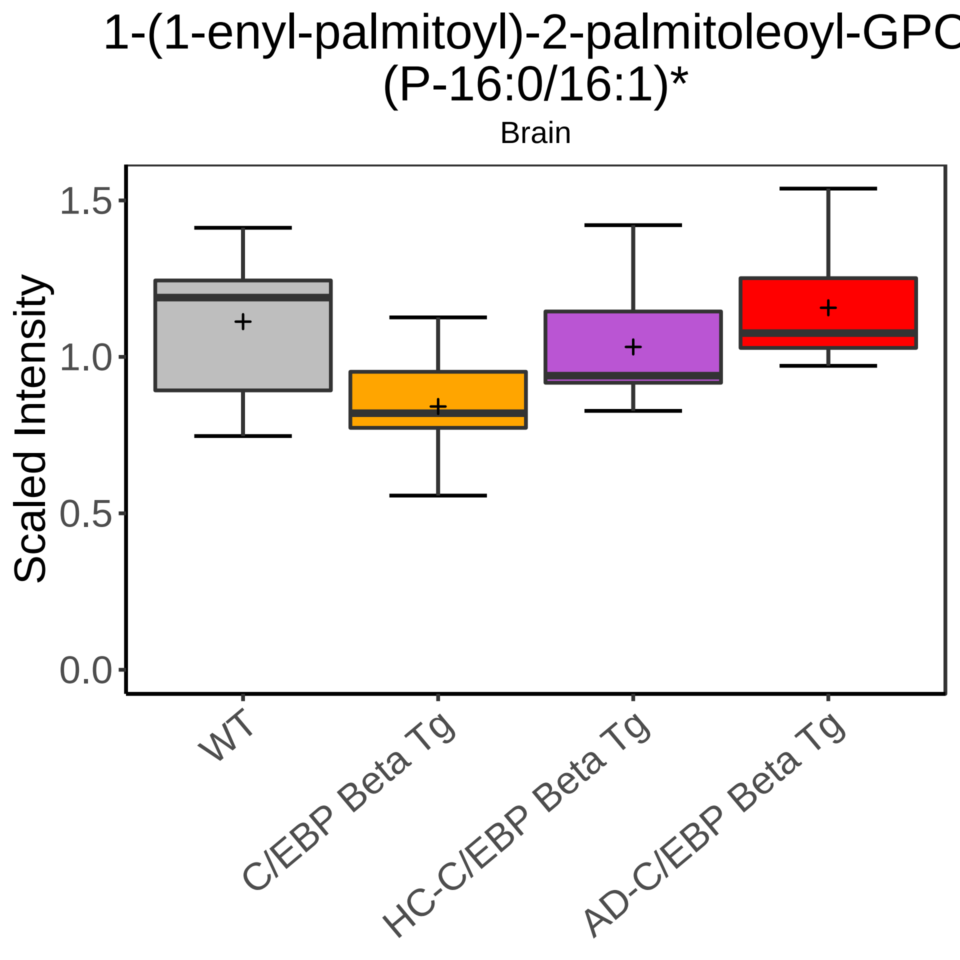

1-(1-enyl-palmitoyl)-2-palmitoleoyl-GPC
(P-16:0/16:1)*
Brain
Scaled Intensity
1.5
1.0
0.5
0.0
WT
C/EBP Beta Tg
HC-C/EBP Beta Tg
AD-C/EBP Beta Tg

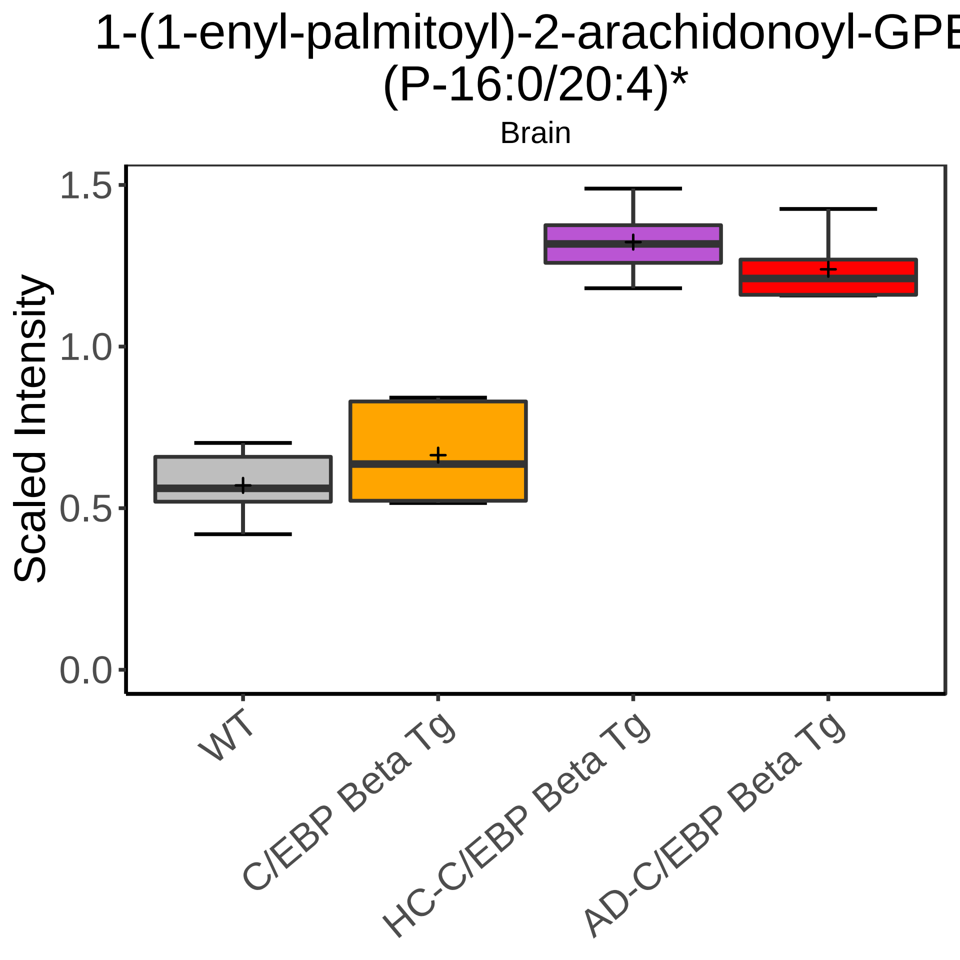

1-(1-enyl-palmitoyl)-2-arachidonoyl-GPE
(P-16:0/20:4)*
Brain
Scaled Intensity
1.5
1.0
0.5
0.0
WT
C/EBP Beta Tg
HC-C/EBP Beta Tg
AD-C/EBP Beta Tg

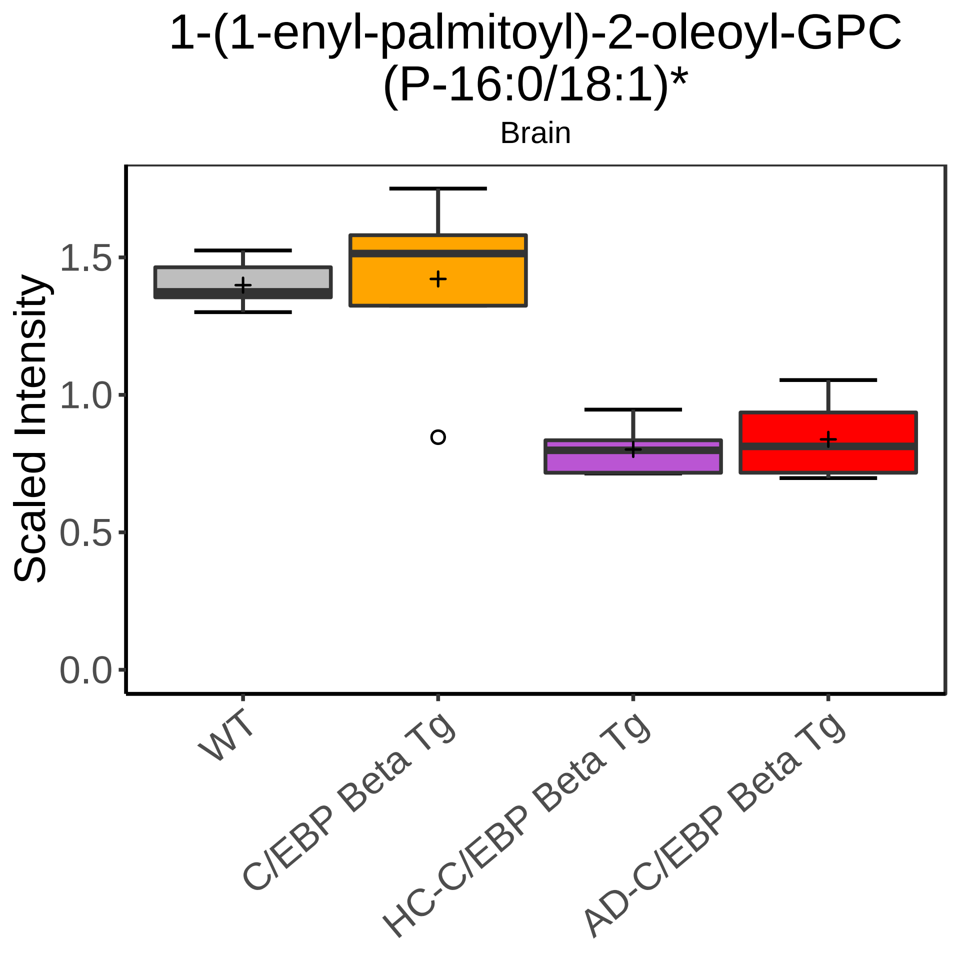
1-(1-enyl-palmitoyl)-2-oleoyl-GPC
(P-16:0/18:1)*
Brain
Scaled Intensity
0.0
0.5
1.0
1.5
WT
C/EBP Beta Tg
HC-C/EBP Beta Tg
AD-C/EBP Beta Tg

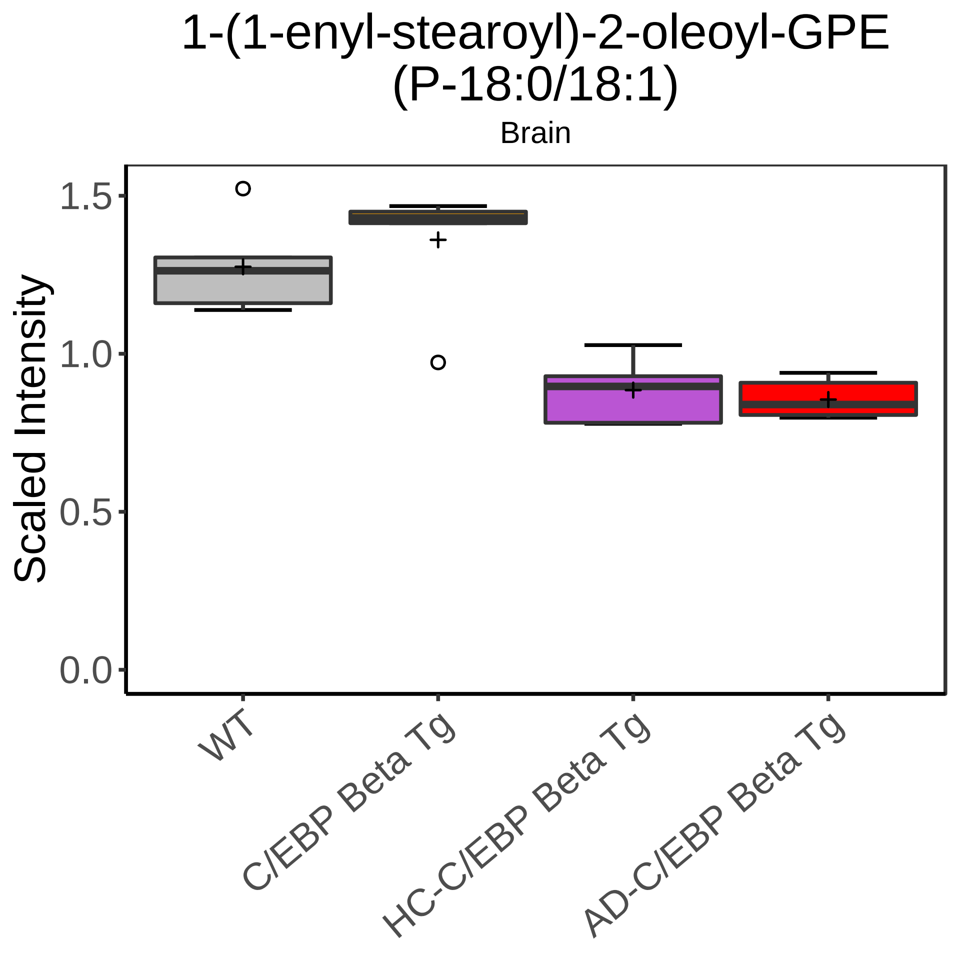
1-(1-enyl-stearoyl)-2-oleoyl-GPE
(P-18:0/18:1)
Brain
Scaled Intensity
1.5
1.0
0.5
0.0
WT
C/EBP Beta Tg
HC-C/EBP Beta Tg
AD-C/EBP Beta Tg

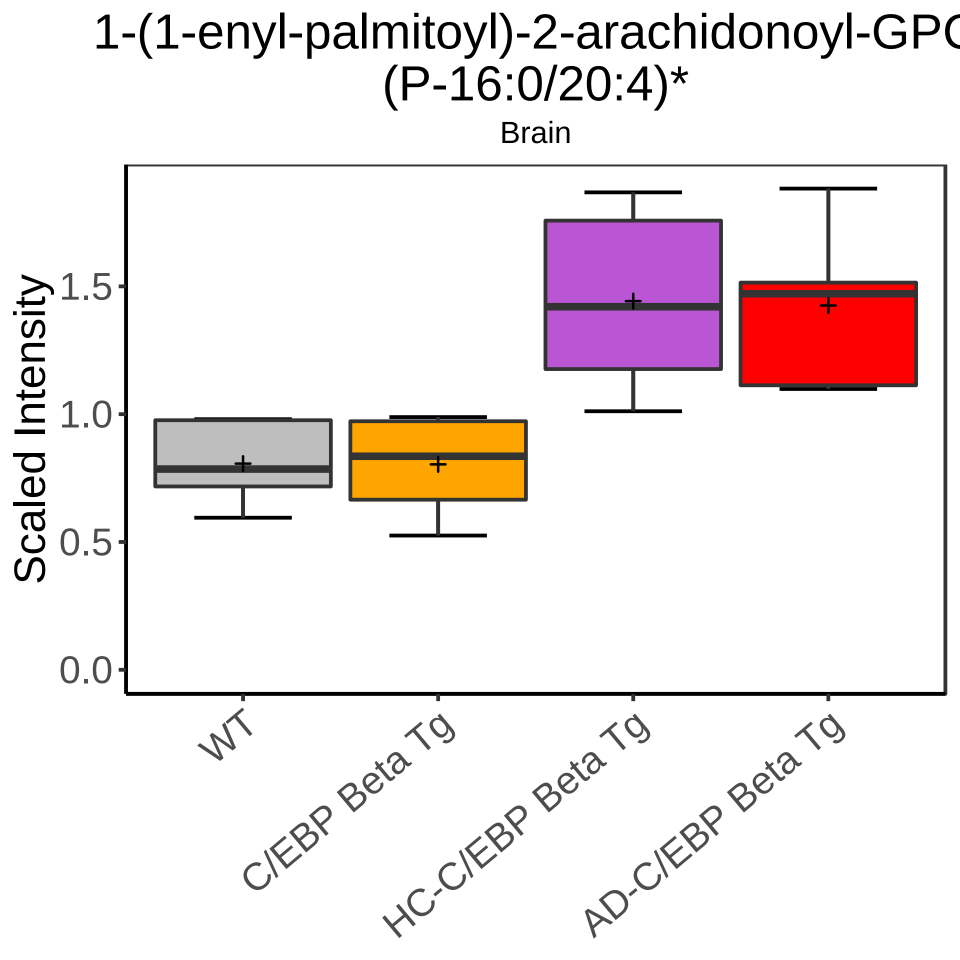
1-(1-enyl-palmitoyl)-2-arachidonoyl-GPC
(P-16:0/20:4)*
Brain
Scaled Intensity
0.0
0.5
1.0
1.5
WT
C/EBP Beta Tg
HC-C/EBP Beta Tg
AD-C/EBP Beta Tg

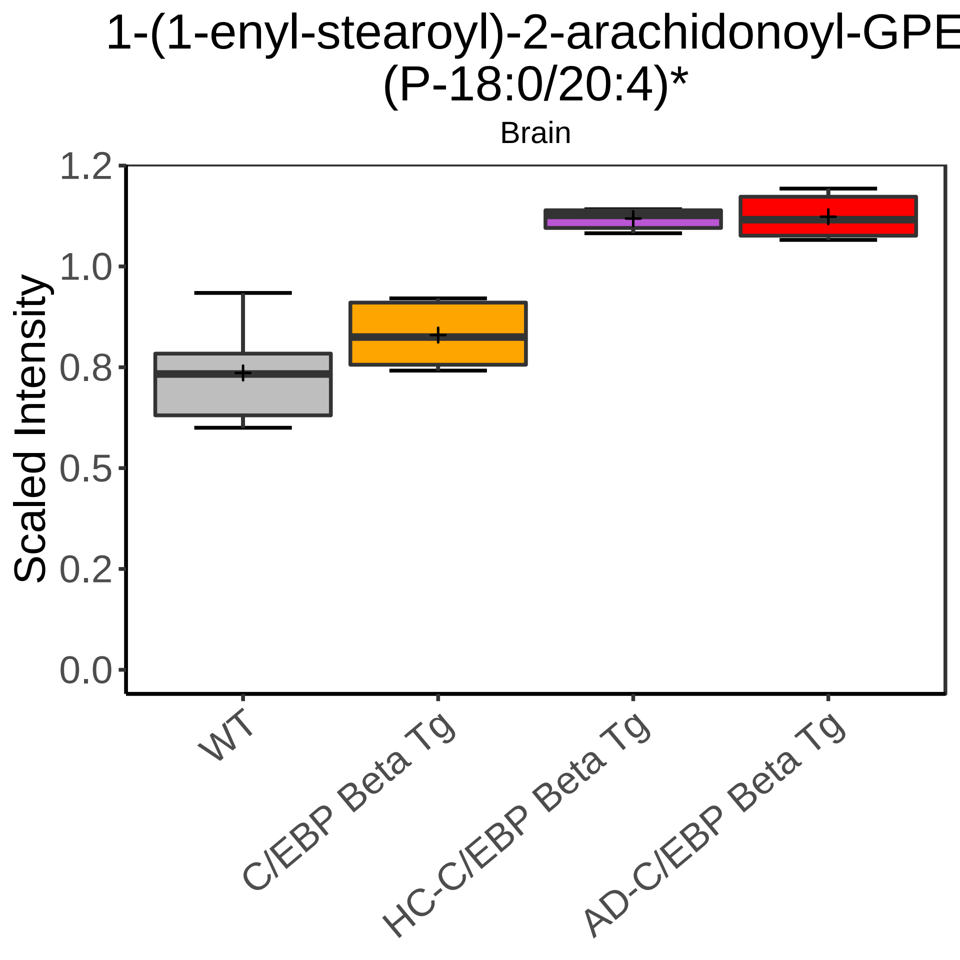
1-(1-enyl-stearoyl)-2-arachidonoyl-GPE
(P-18:0/20:4)*
Brain
Scaled Intensity
1.2
1.0
0.8
0.5
0.2
0.0
WT
C/EBP Beta Tg
HC-C/EBP Beta Tg
AD-C/EBP Beta Tg

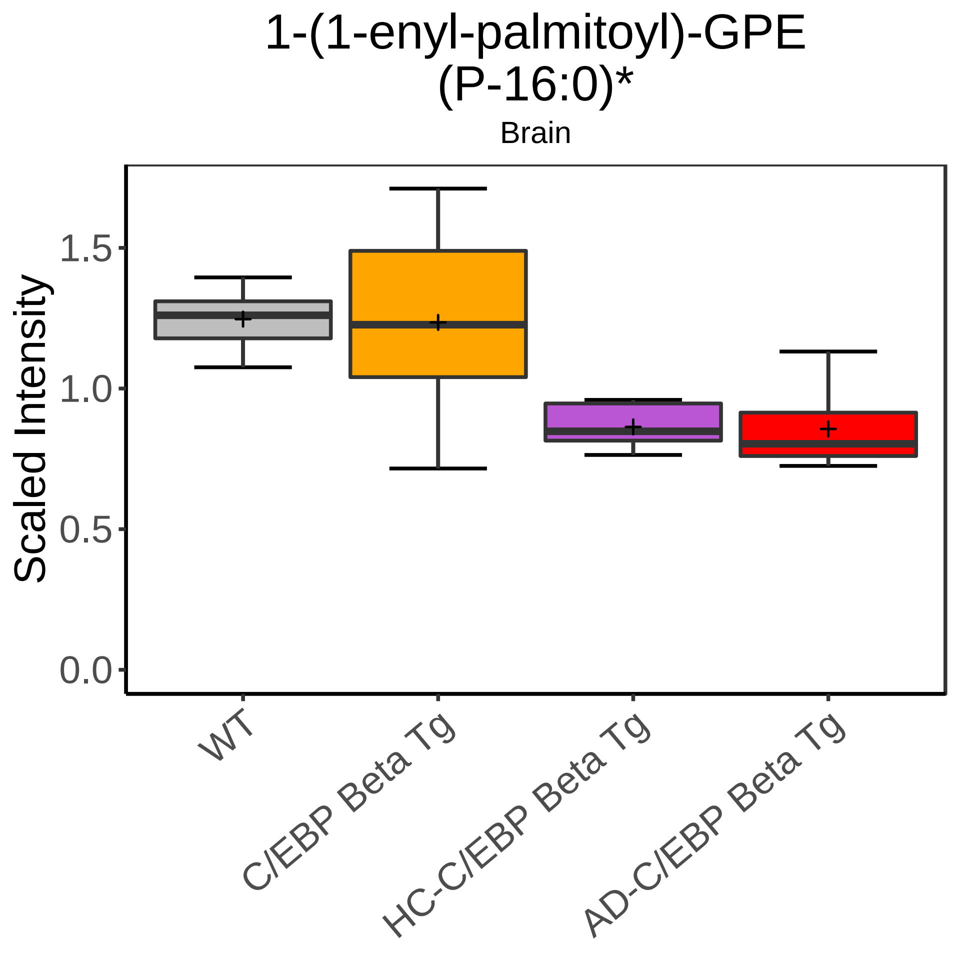
1-(1-enyl-palmitoyl)-GPE
(P-16:0)*
Brain
Scaled Intensity
0.0
0.5
1.0
1.5
WT
C/EBP Beta Tg
HC-C/EBP Beta Tg
AD-C/EBP Beta Tg

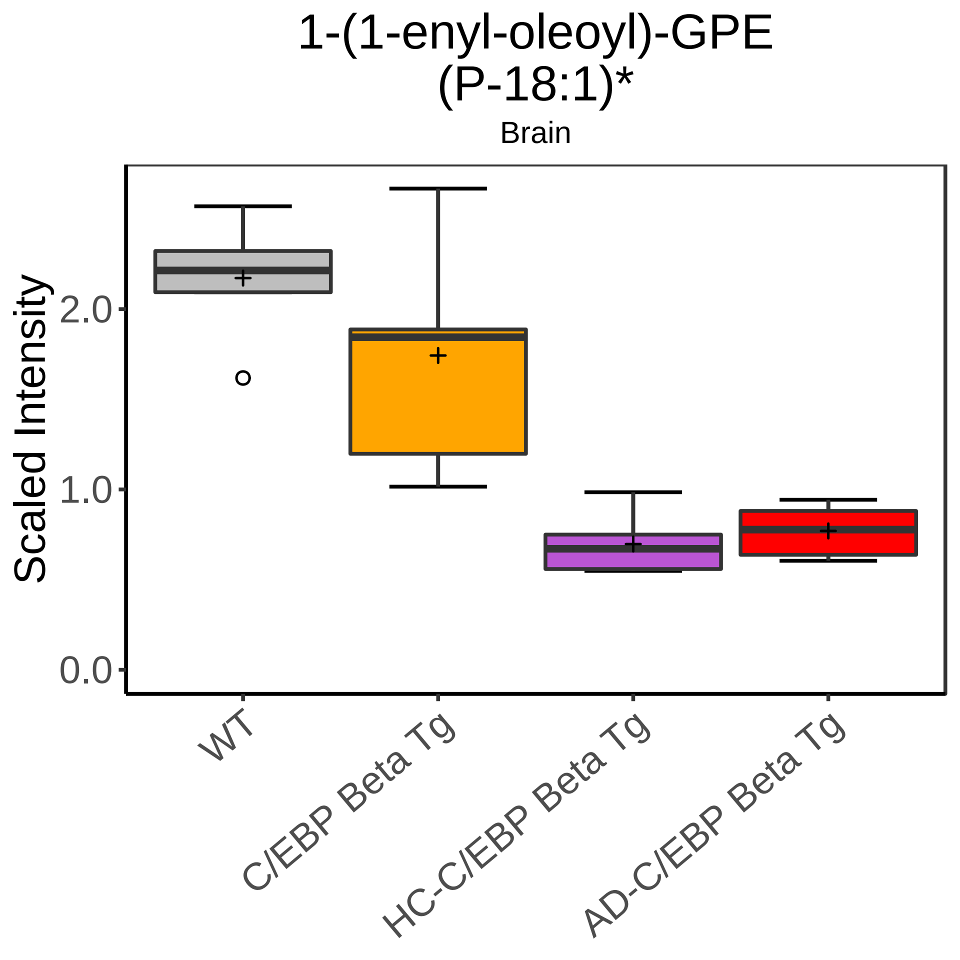
1-(1-enyl-oleoyl)-GPE
(P-18:1)*
Brain
Scaled Intensity
2.0
1.0
0.0
WT
C/EBP Beta Tg
HC-C/EBP Beta Tg
AD-C/EBP Beta Tg

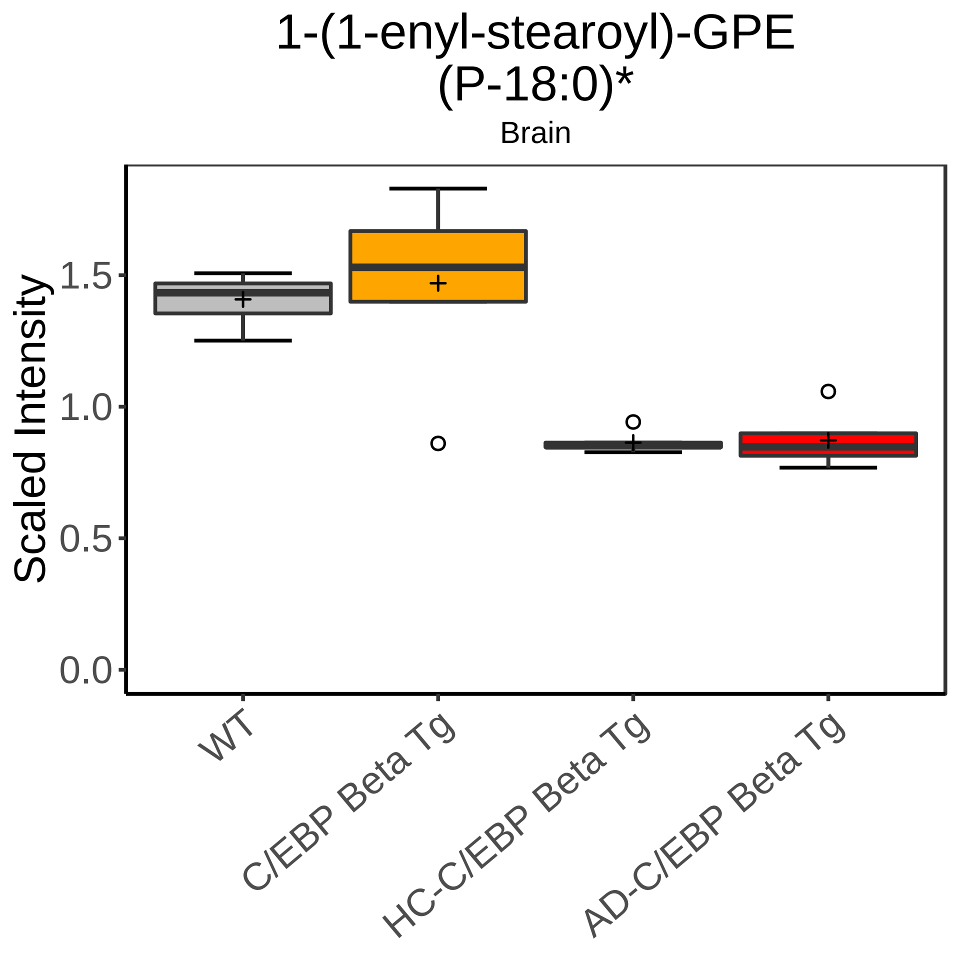

1-(1-enyl-stearoyl)-GPE
(P-18:0)*
Brain
Scaled Intensity
0.0
0.5
1.0
1.5
WT
C/EBP Beta Tg
HC-C/EBP Beta Tg
AD-C/EBP Beta Tg

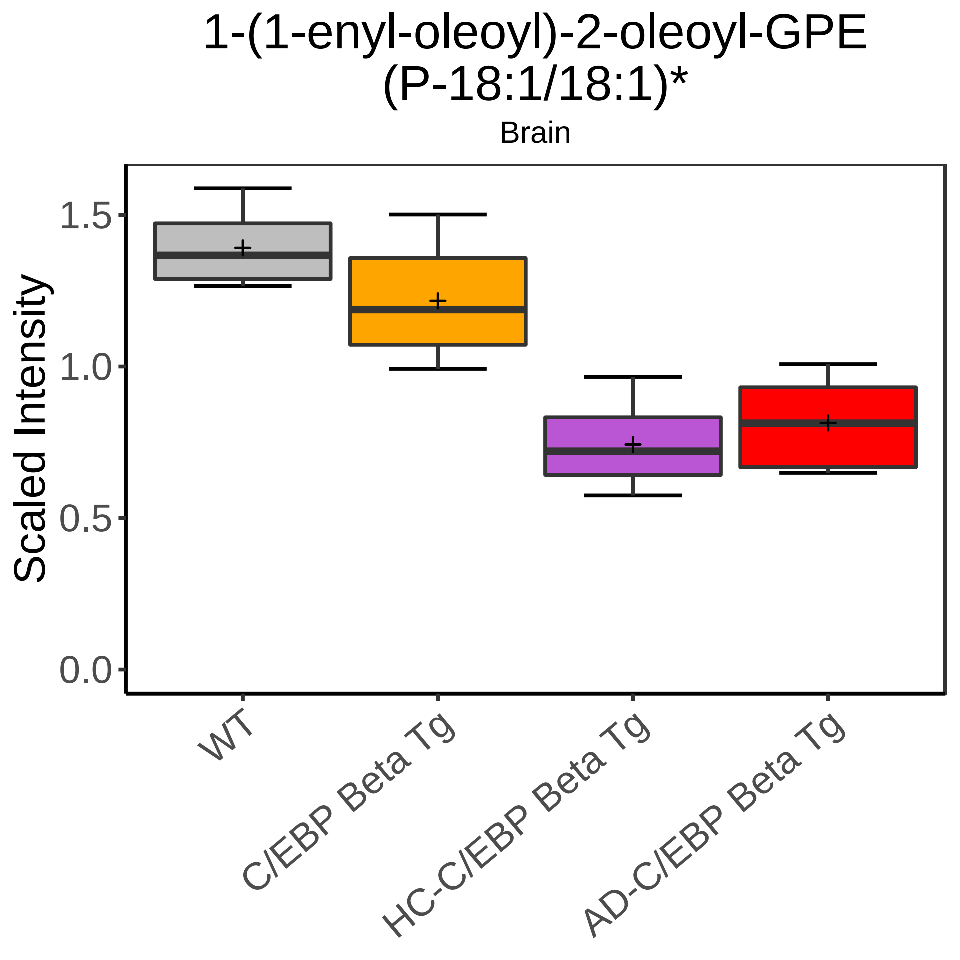
1-(1-enyl-oleoyl)-2-oleoyl-GPE
(P-18:1/18:1)*
Brain
Scaled Intensity
1.5
1.0
0.5
0.0
WT
C/EBP Beta Tg
HC-C/EBP Beta Tg
AD-C/EBP Beta Tg

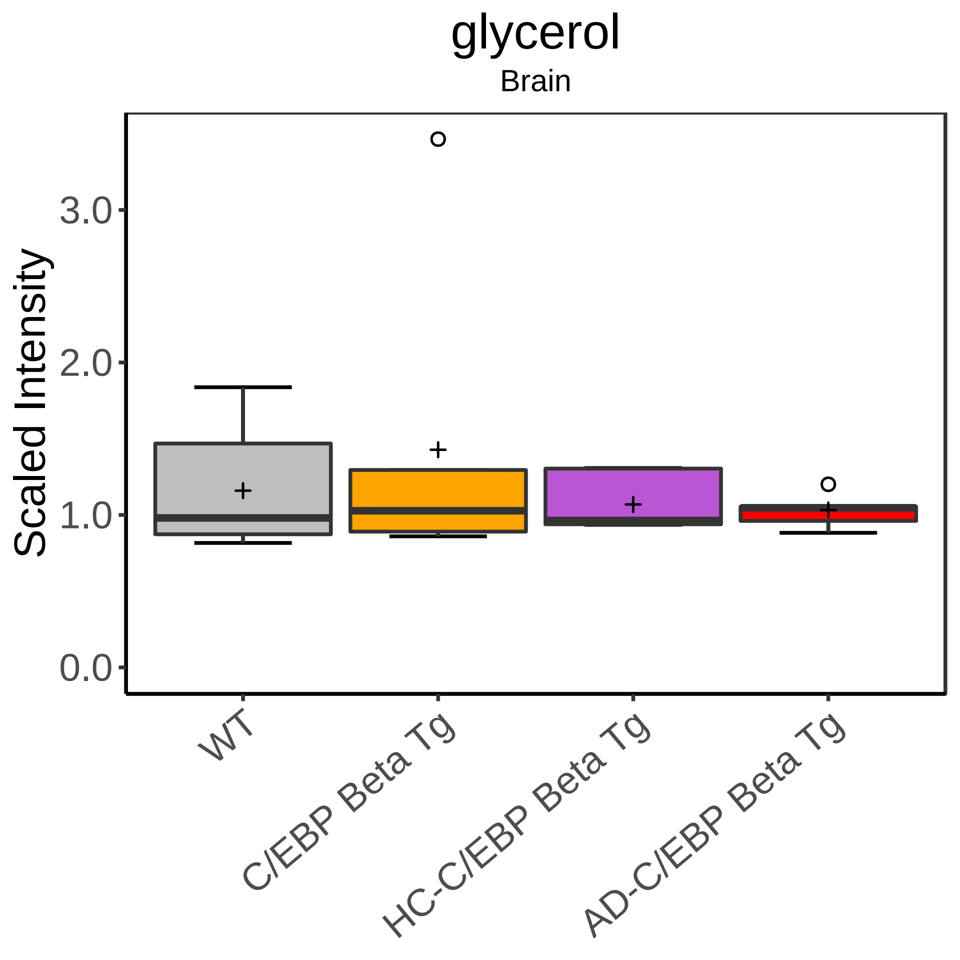

glycerol
Brain
Scaled Intensity
3.0
2.0
1.0
0.0
WT
C/EBP Beta Tg
HC-C/EBP Beta Tg
AD-C/EBP Beta Tg

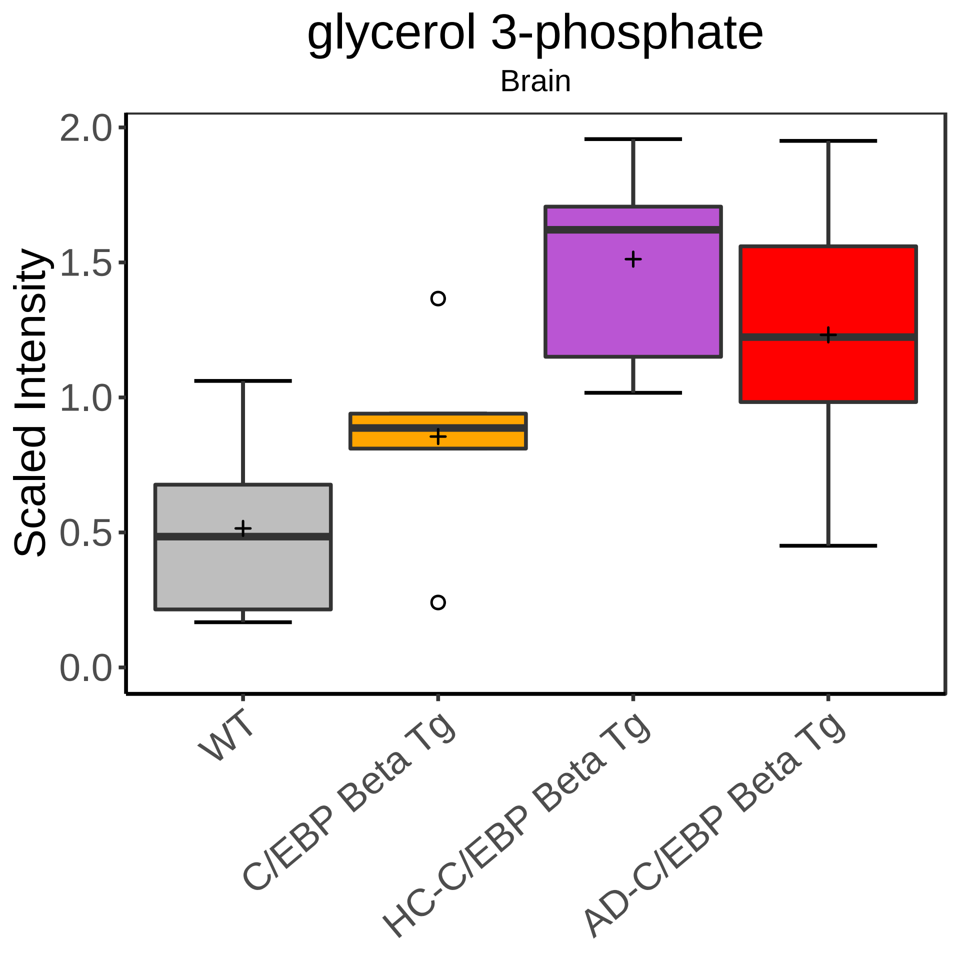
glycerol 3-phosphate
Brain
Scaled Intensity
2.0
1.5
1.0
0.5
0.0
WT
C/EBP Beta Tg
HC-C/EBP Beta Tg
AD-C/EBP Beta Tg

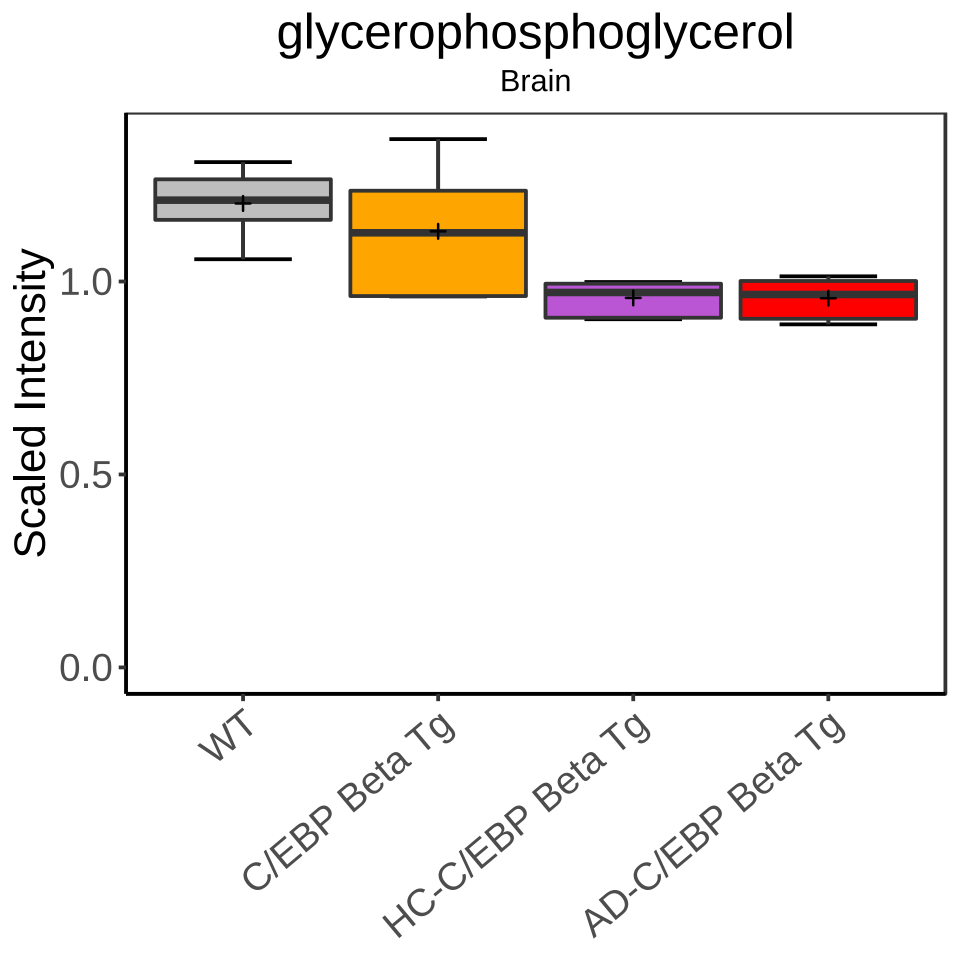
glycerophosphoglycerol
Brain
Scaled Intensity
0.0
0.5
1.0
WT
C/EBP Beta Tg
HC-C/EBP Beta Tg
AD-C/EBP Beta Tg

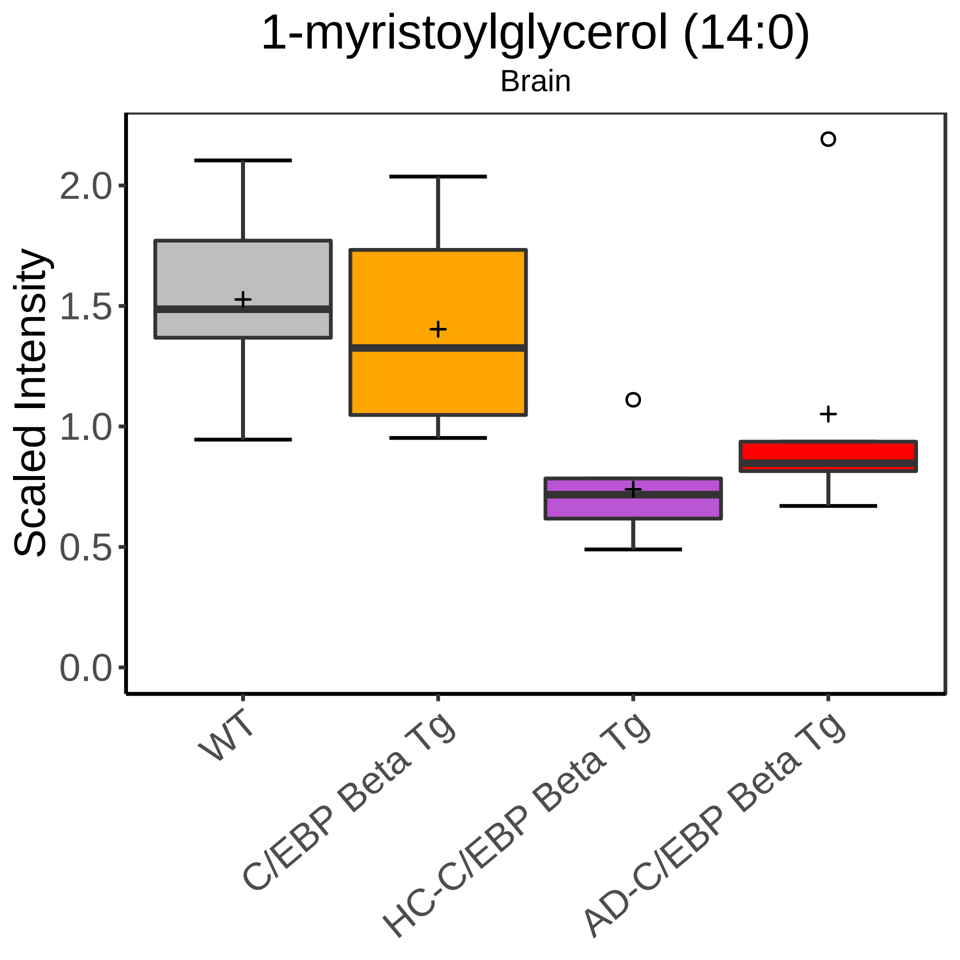
1-myristoylglycerol (14:0)
Brain
Scaled Intensity
2.0
1.5
1.0
0.5
0.0
WT
C/EBP Beta Tg
HC-C/EBP Beta Tg
AD-C/EBP Beta Tg

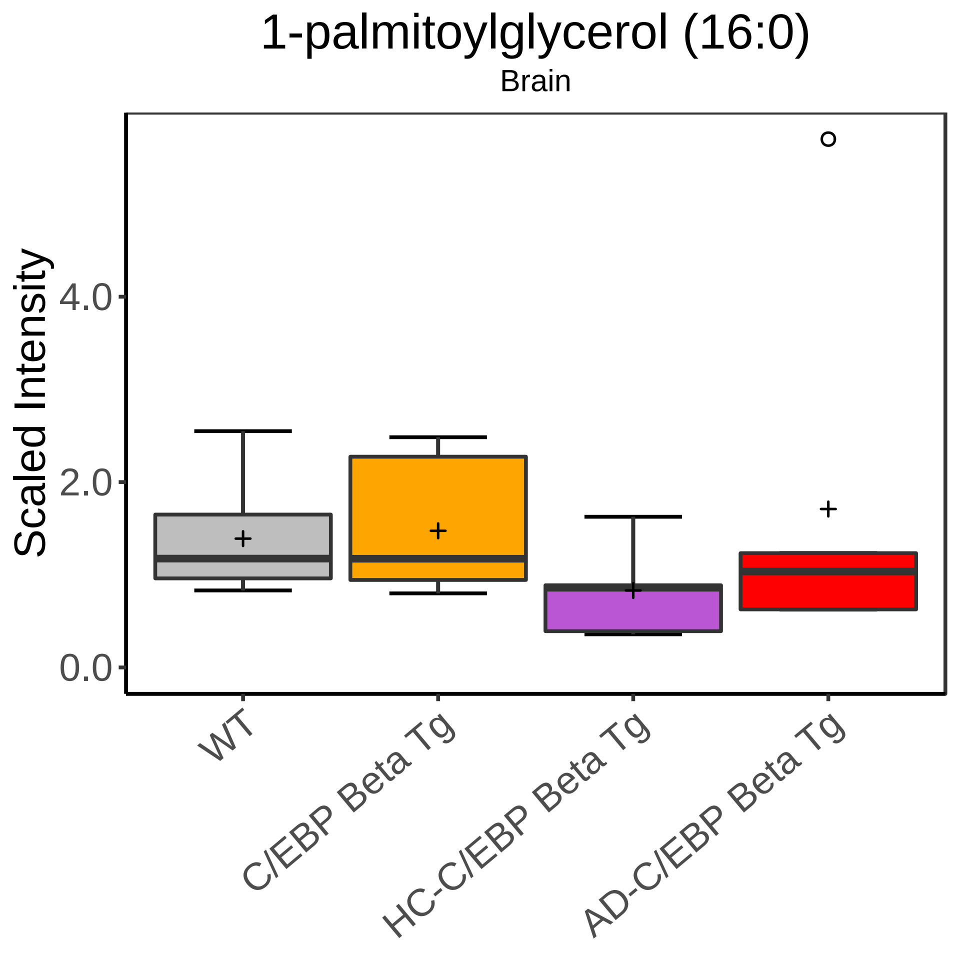

1-palmitoylglycerol (16:0)
Brain
Scaled Intensity
4.0
2.0
0.0
WT
C/EBP Beta Tg
HC-C/EBP Beta Tg
AD-C/EBP Beta Tg

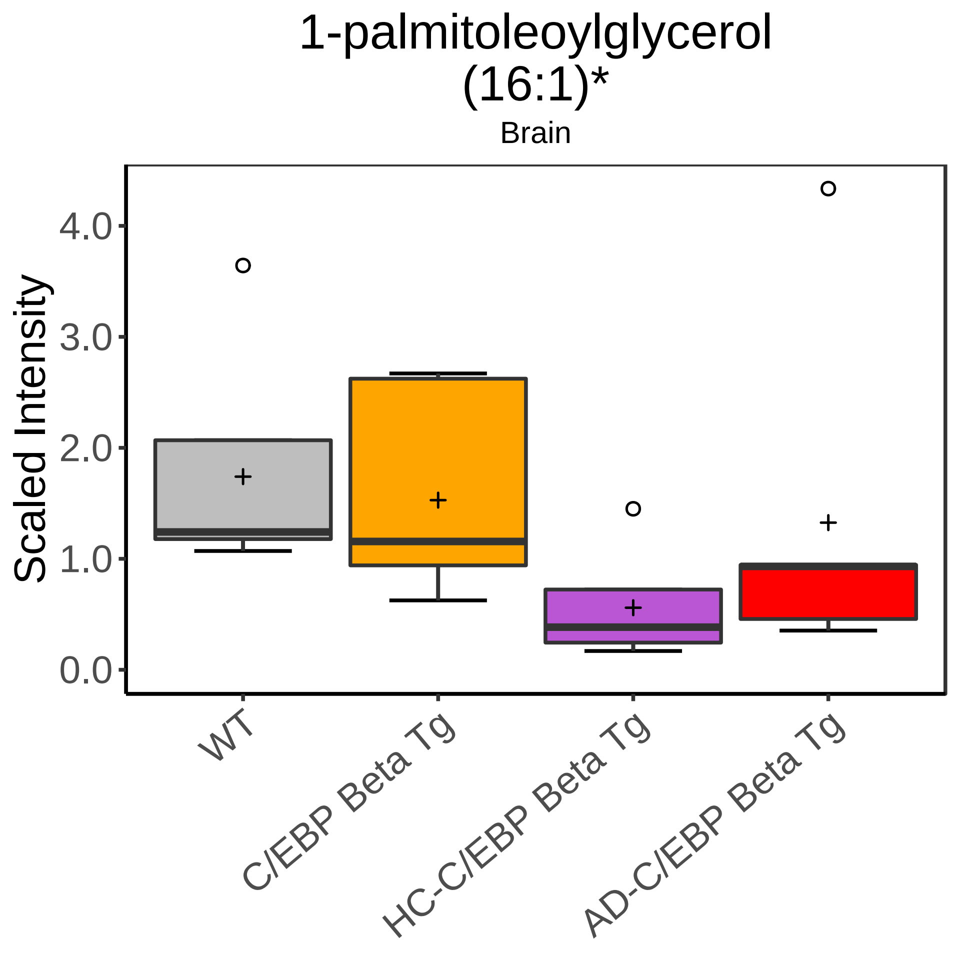
1-palmitoleoylglycerol
(16:1)*
Brain
Scaled Intensity
0.0
1.0
2.0
3.0
4.0
WT
C/EBP Beta Tg
HC-C/EBP Beta Tg
AD-C/EBP Beta Tg

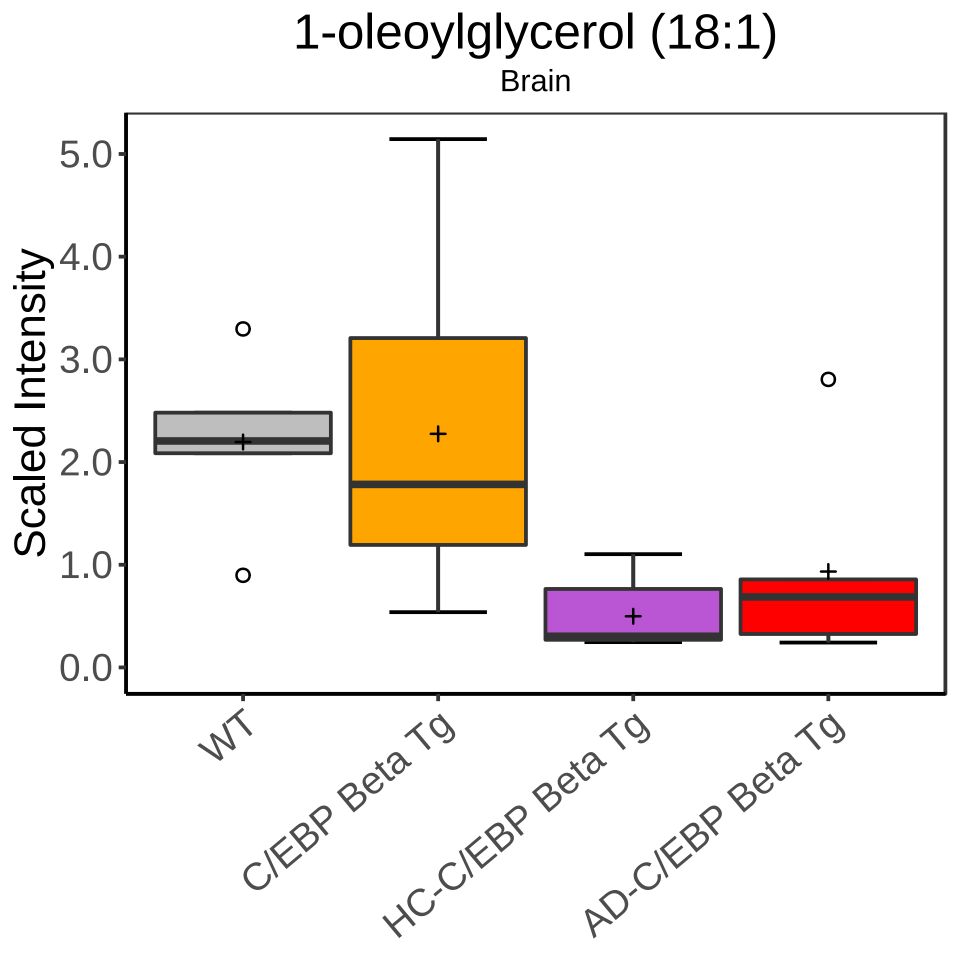

1-oleoylglycerol (18:1)
Brain
Scaled Intensity
5.0
4.0
3.0
2.0
1.0
0.0
WT
C/EBP Beta Tg
HC-C/EBP Beta Tg
AD-C/EBP Beta Tg

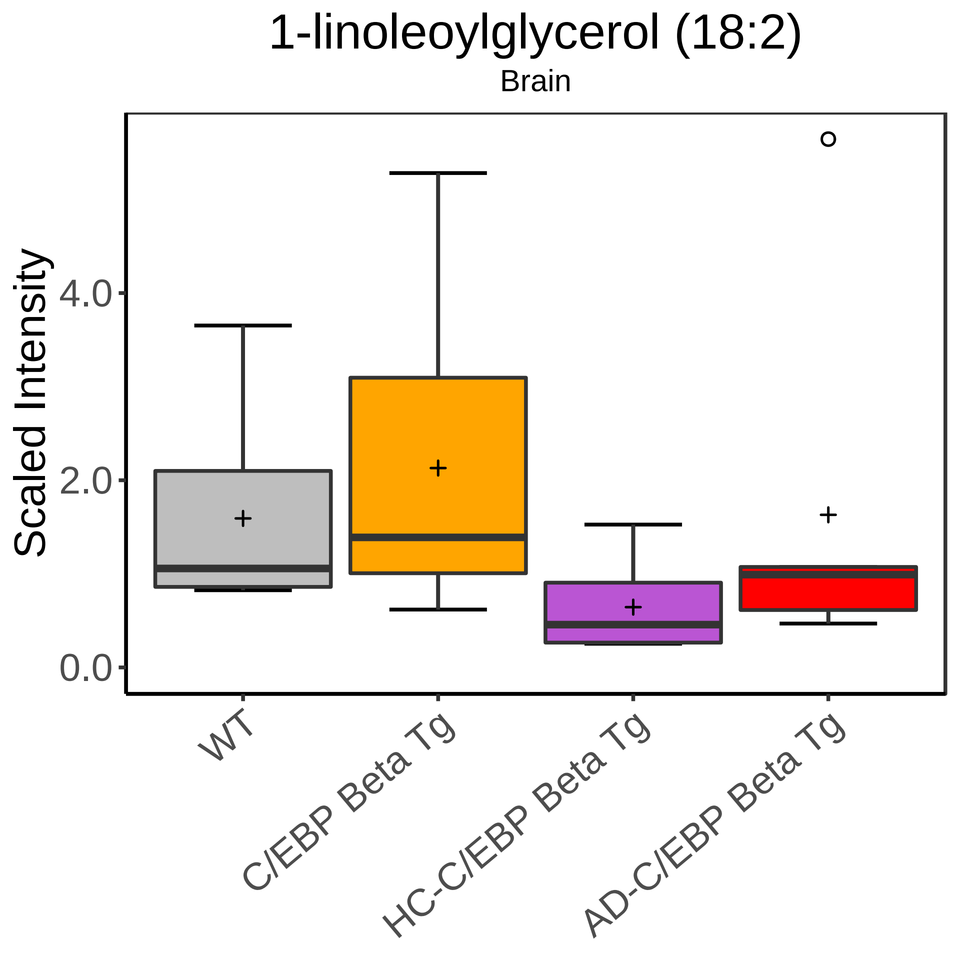

1-linoleoylglycerol (18:2)
Brain
Scaled Intensity
4.0
2.0
0.0
WT
C/EBP Beta Tg
HC-C/EBP Beta Tg
AD-C/EBP Beta Tg

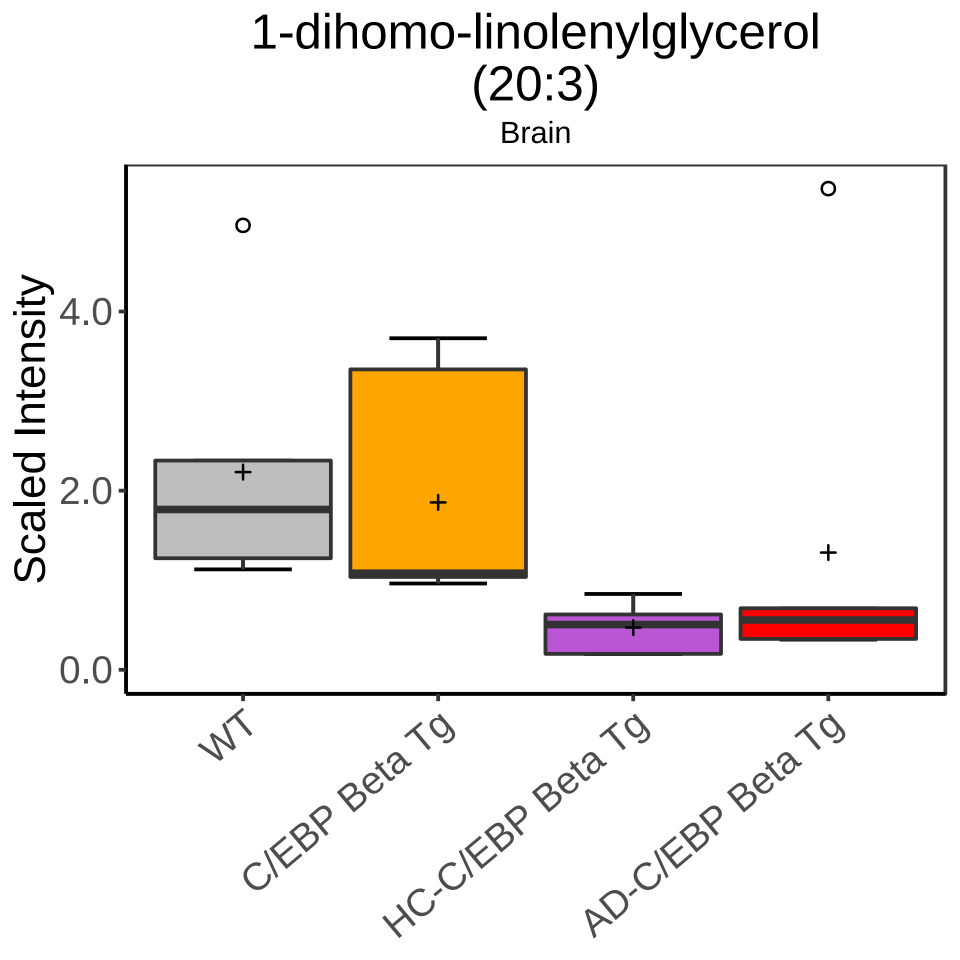
1-dihomo-linolenylglycerol
(20:3)
Brain
Scaled Intensity
0.0
2.0
4.0
WT
C/EBP Beta Tg
HC-C/EBP Beta Tg
AD-C/EBP Beta Tg

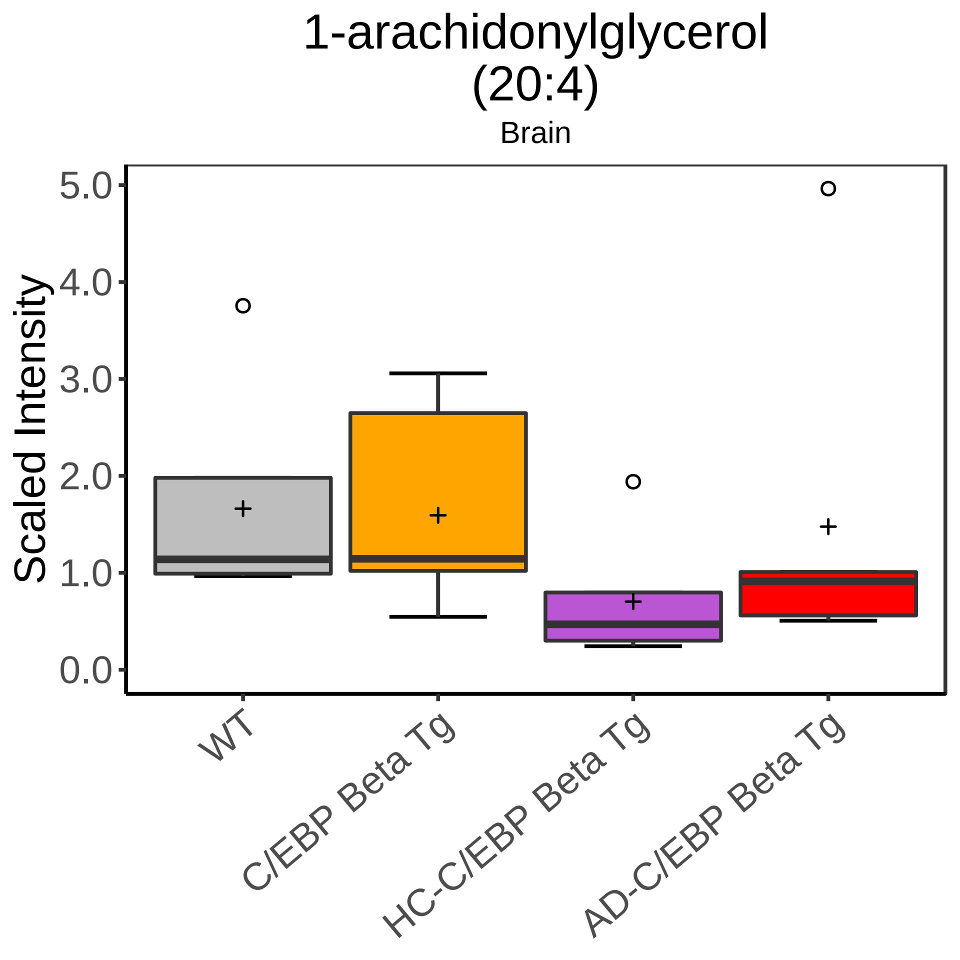

1-arachidonylglycerol
(20:4)
Brain
Scaled Intensity
5.0
4.0
3.0
2.0
1.0
0.0
WT
C/EBP Beta Tg
HC-C/EBP Beta Tg
AD-C/EBP Beta Tg

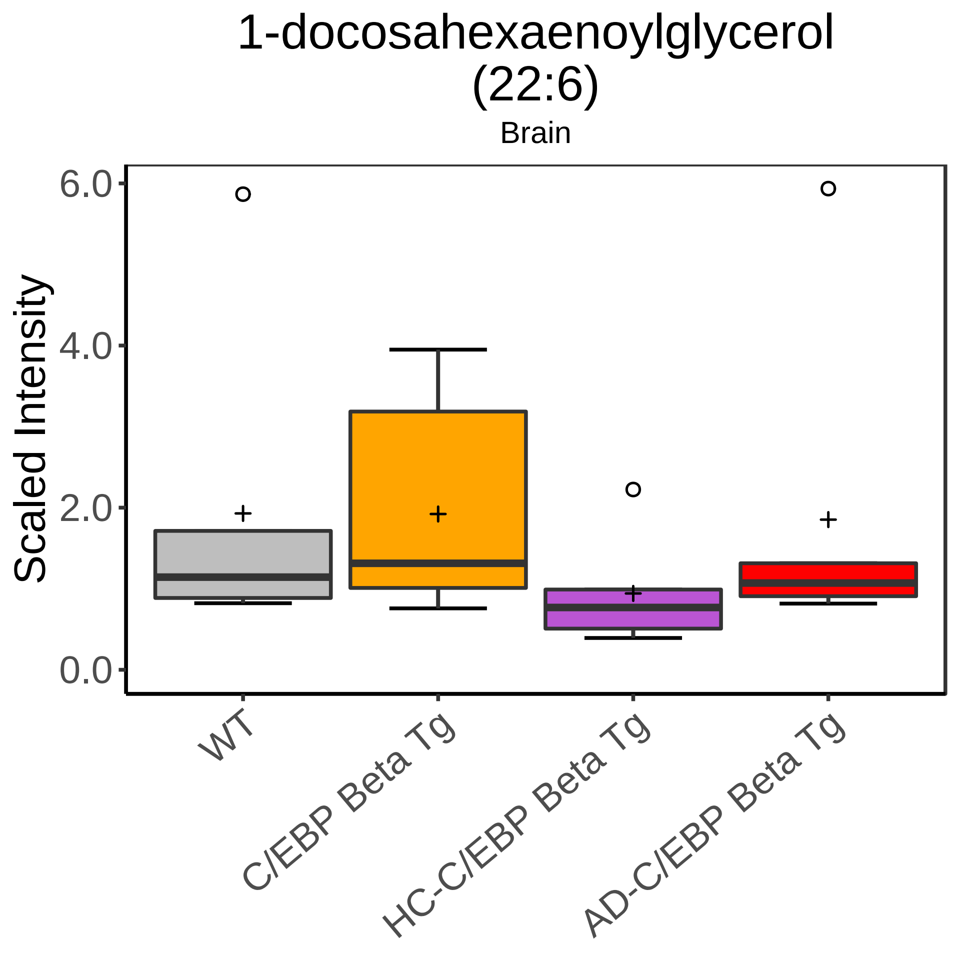
1-docosahexaenoylglycerol
(22:6)
Brain
Scaled Intensity
6.0
4.0
2.0
0.0
WT
C/EBP Beta Tg
HC-C/EBP Beta Tg
AD-C/EBP Beta Tg

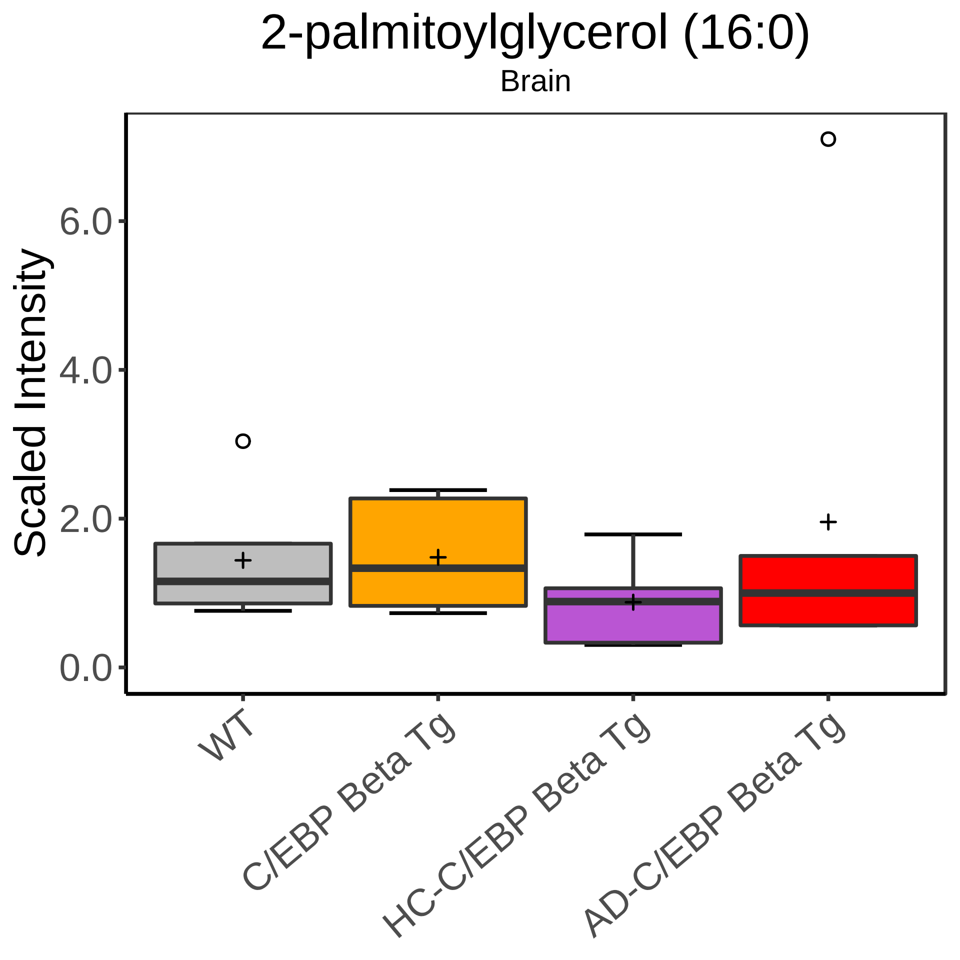

2-palmitoylglycerol (16:0)
Brain
Scaled Intensity
6.0
4.0
2.0
0.0
WT
C/EBP Beta Tg
HC-C/EBP Beta Tg
AD-C/EBP Beta Tg

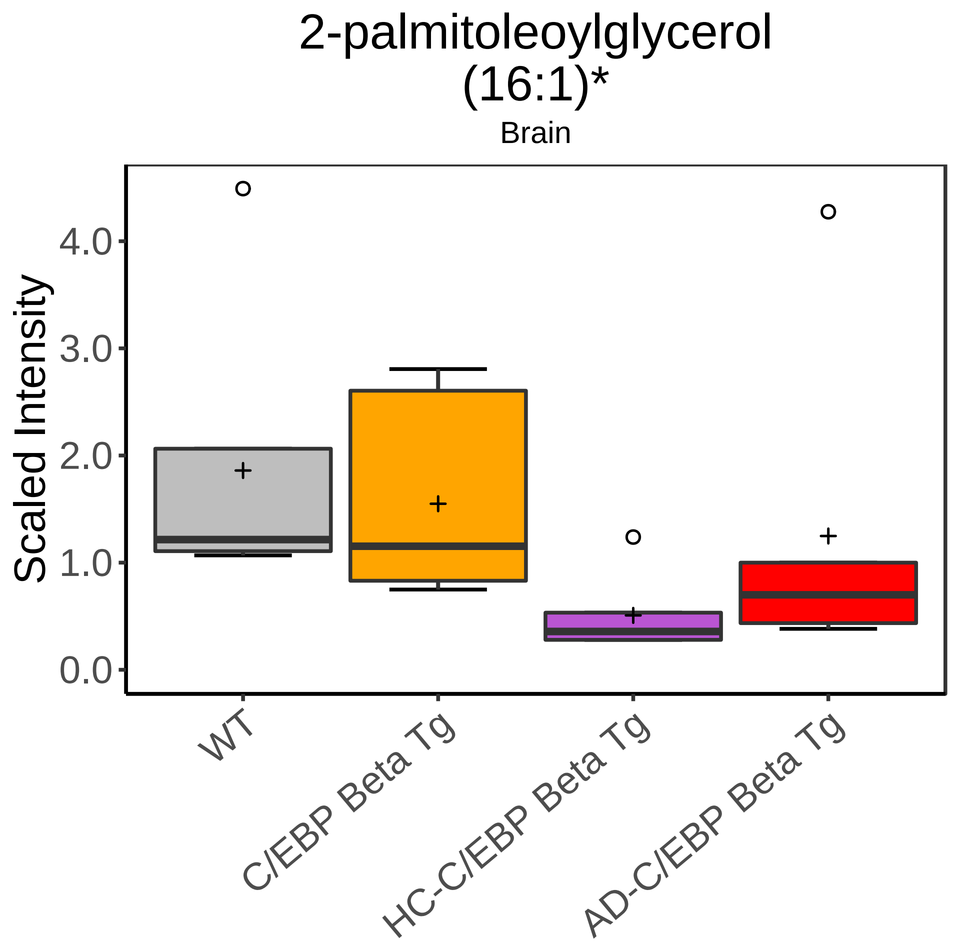

2-palmitoleoylglycerol (16:1)*
Brain
Scaled Intensity
4.0
3.0
2.0
1.0
0.0
WT
C/EBP Beta Tg
HC-C/EBP Beta Tg
AD-C/EBP Beta Tg

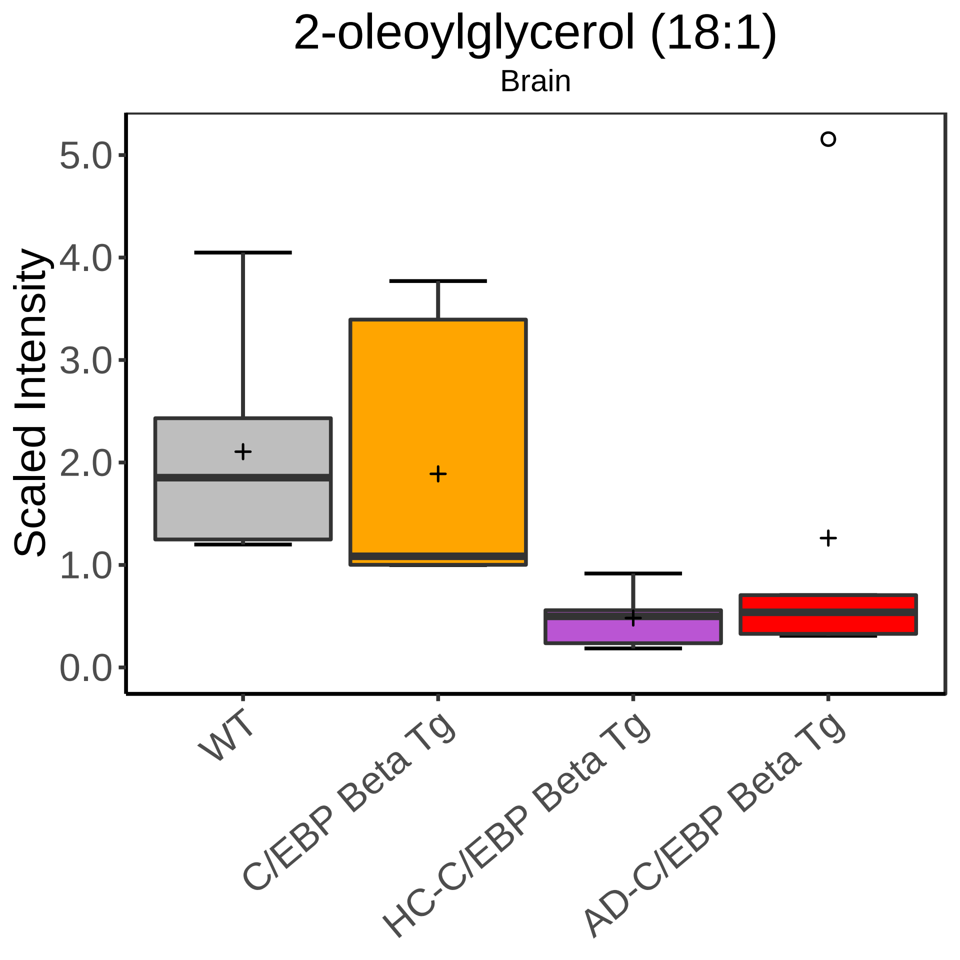
2-oleoylglycerol (18:1)
Brain
Scaled Intensity
5.0
4.0
3.0
2.0
1.0
0.0
WT
C/EBP Beta Tg
HC-C/EBP Beta Tg
AD-C/EBP Beta Tg

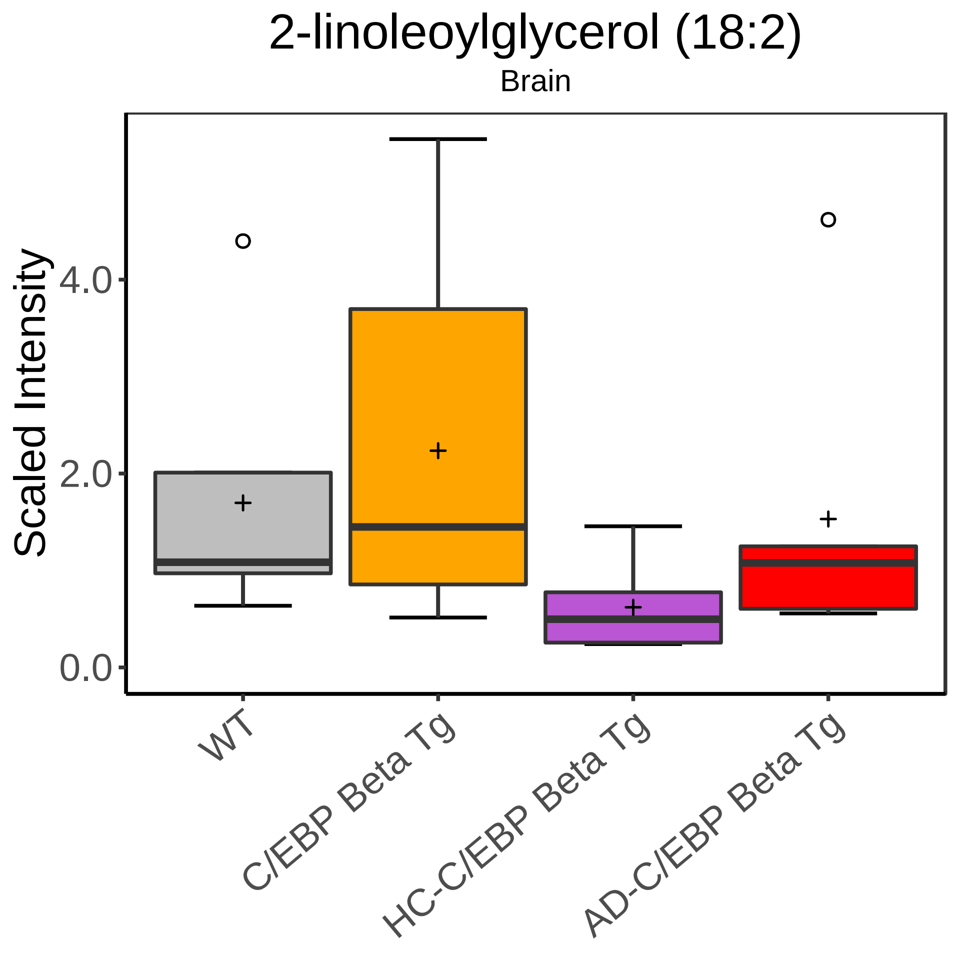
2-linoleoylglycerol (18:2)
Brain
Scaled Intensity
0.0
2.0
4.0
WT
C/EBP Beta Tg
HC-C/EBP Beta Tg
AD-C/EBP Beta Tg

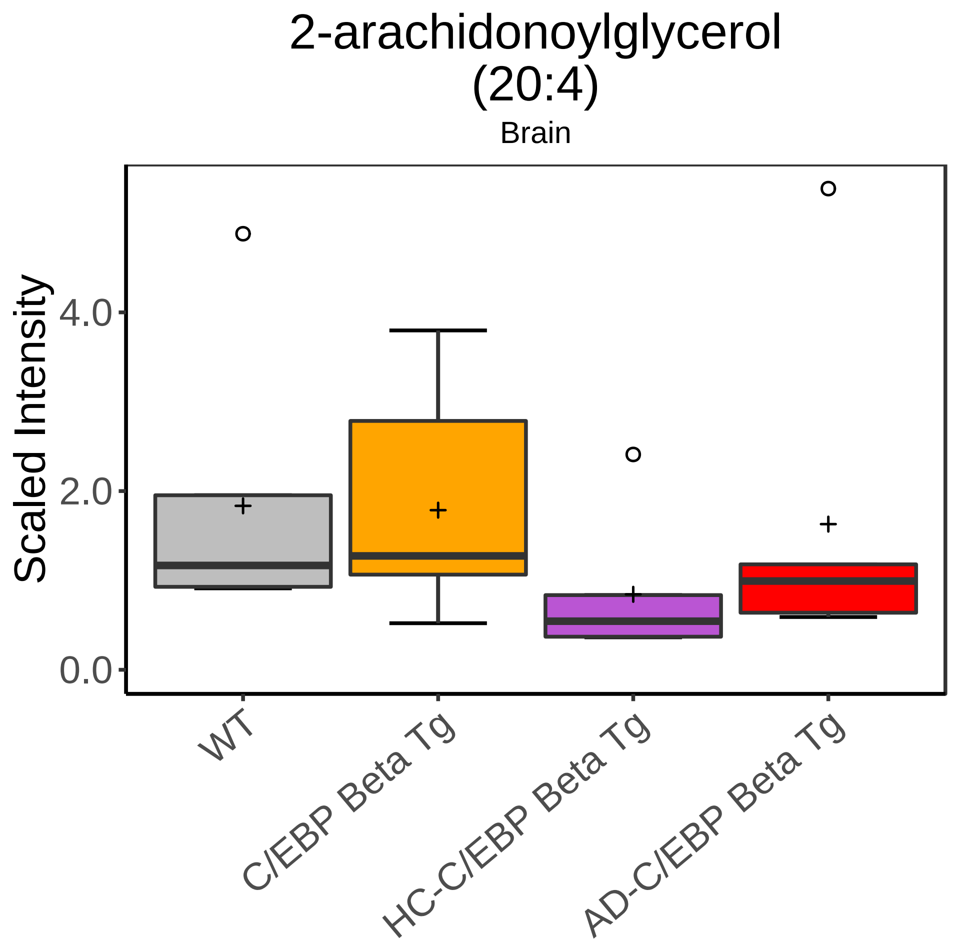

2-arachidonoylglycerol
(20:4)
Brain
Scaled Intensity
4.0
2.0
0.0
WT
C/EBP Beta Tg
HC-C/EBP Beta Tg
AD-C/EBP Beta Tg

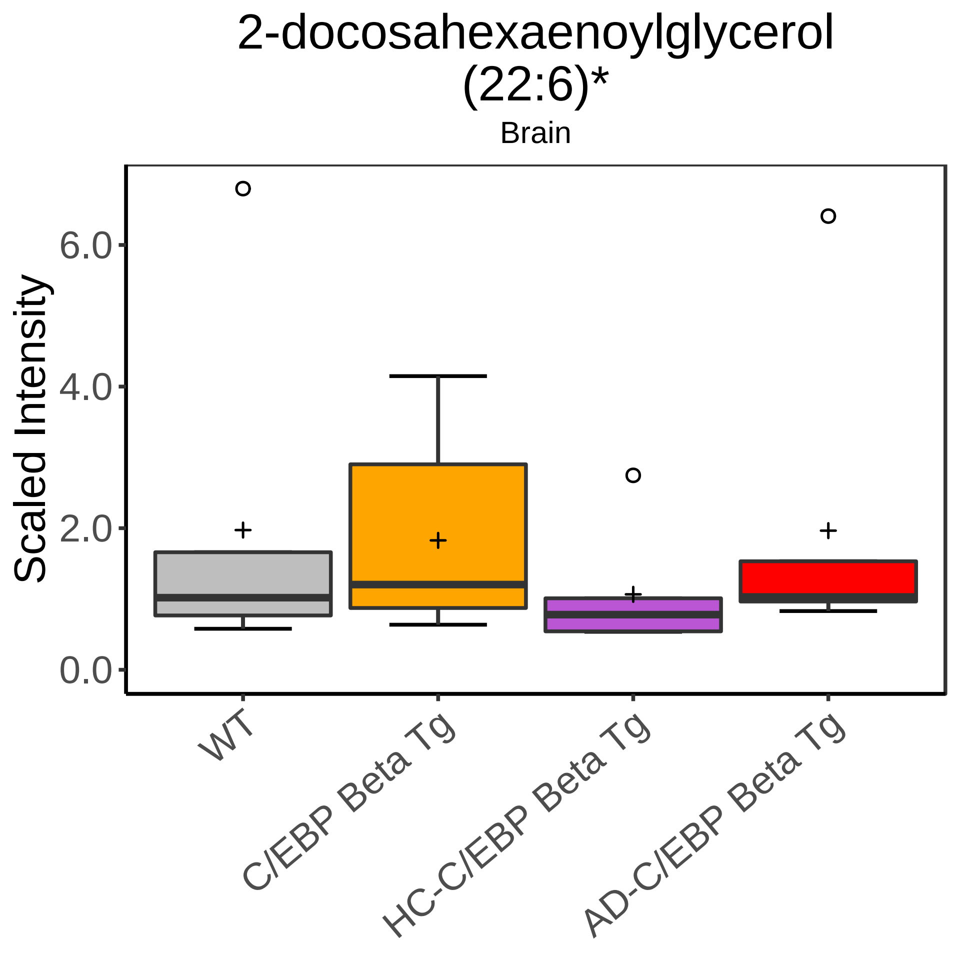

2-docosahexaenoylglycerol
(22:6)*
Brain
Scaled Intensity
6.0
4.0
2.0
0.0
WT
C/EBP Beta Tg
HC-C/EBP Beta Tg
AD-C/EBP Beta Tg

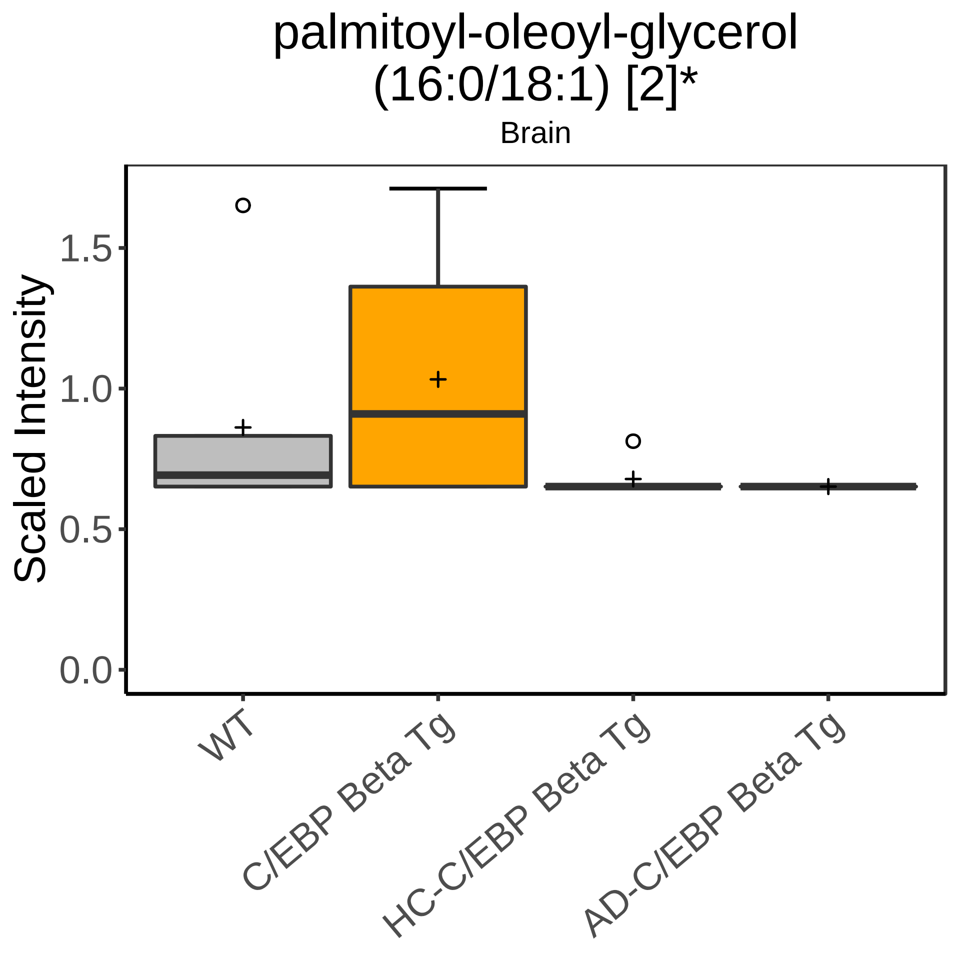
palmitoyl-oleoyl-glycerol
(16:0/18:1) [2]*
Brain
Scaled Intensity
1.5
1.0
0.5
0.0
WT
C/EBP Beta Tg
HC-C/EBP Beta Tg
AD-C/EBP Beta Tg

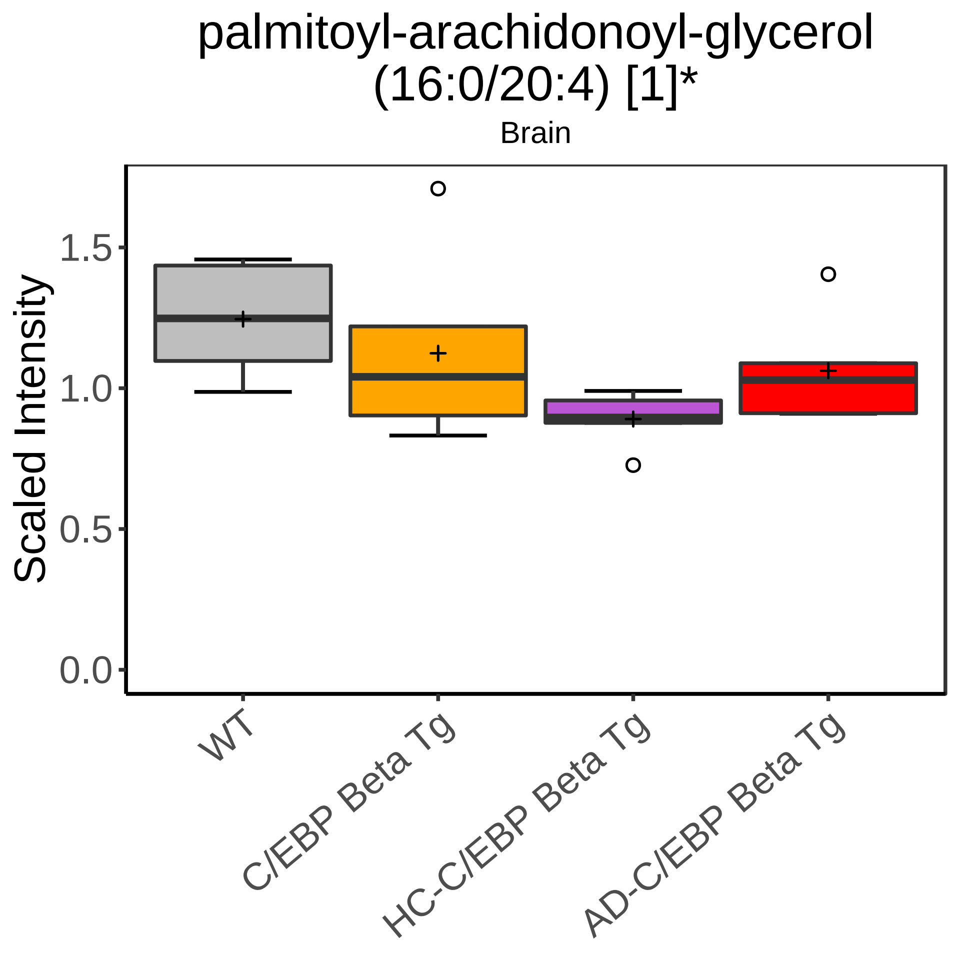

palmitoyl-arachidonoyl-glycerol
(16:0/20:4) [1]*
Brain
Scaled Intensity
0.0
0.5
1.0
1.5
WT
C/EBP Beta Tg
HC-C/EBP Beta Tg
AD-C/EBP Beta Tg

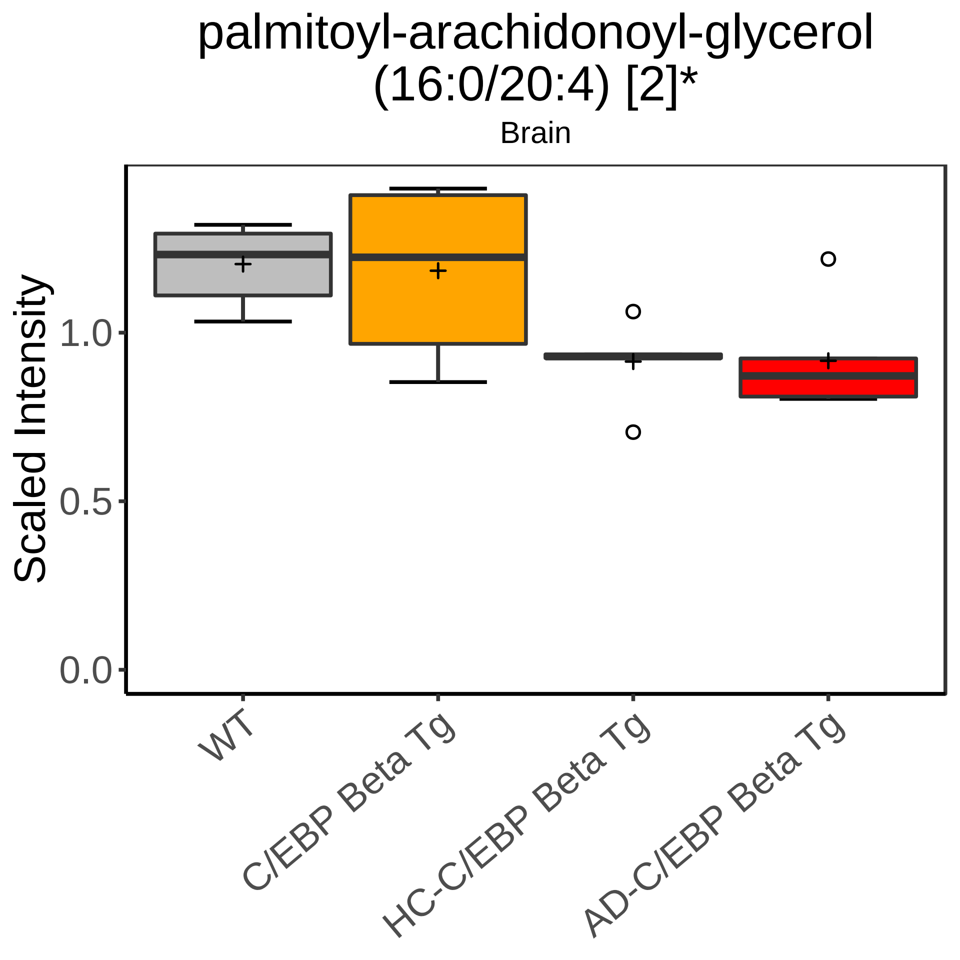
palmitoyl-arachidonoyl-glycerol
(16:0/20:4) [2]*
Brain
Scaled Intensity
0.0
0.5
1.0
WT
C/EBP Beta Tg
HC-C/EBP Beta Tg
AD-C/EBP Beta Tg

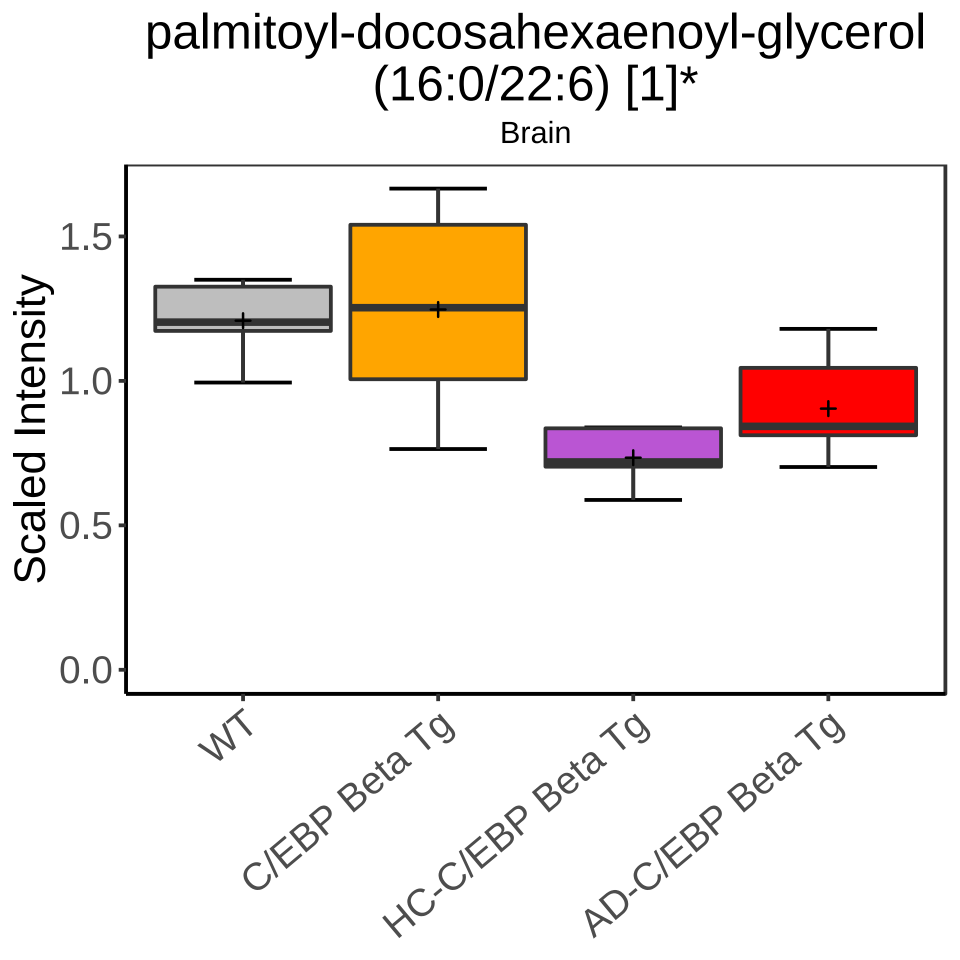

palmitoyl-docosahexaenoyl-glycerol
(16:0/22:6) [1]*
Brain
Scaled Intensity
0.0
0.5
1.0
1.5
WT
C/EBP Beta Tg
HC-C/EBP Beta Tg
AD-C/EBP Beta Tg

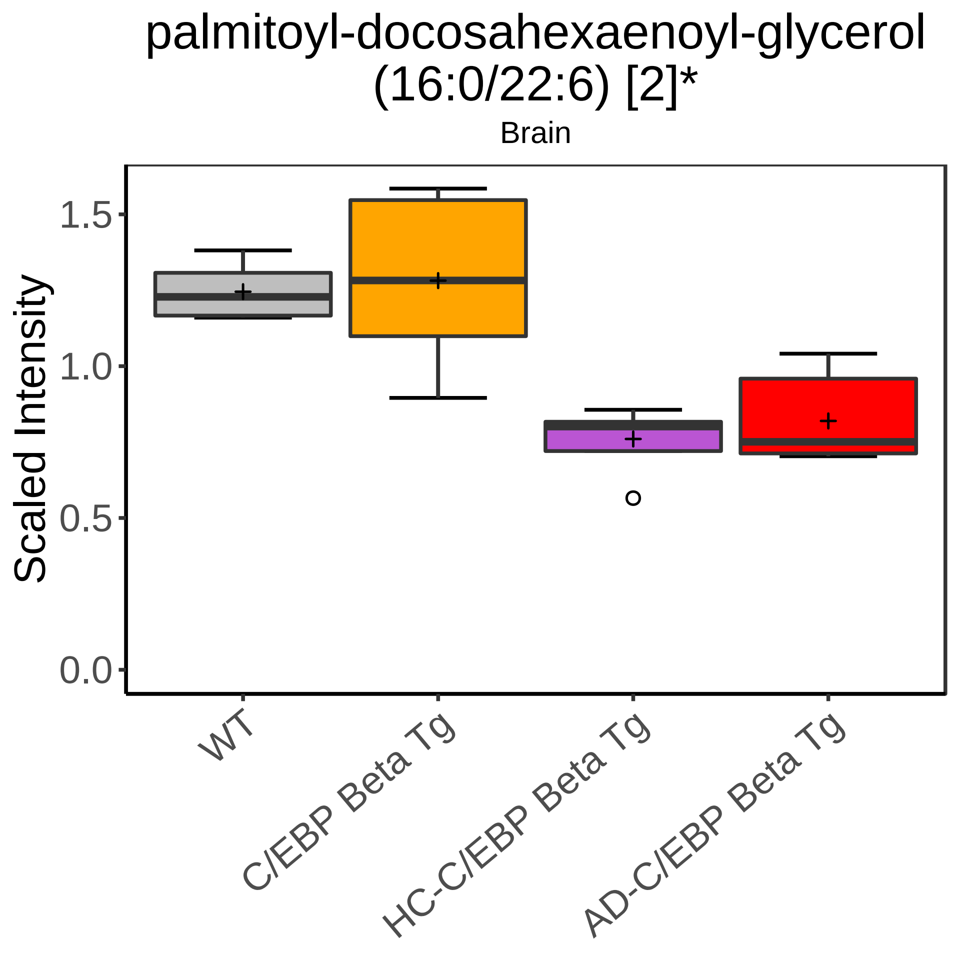
palmitoyl-docosahexaenoyl-glycerol
(16:0/22:6) [2]*
Brain
Scaled Intensity
0.0
0.5
1.0
1.5
WT
C/EBP Beta Tg
HC-C/EBP Beta Tg
AD-C/EBP Beta Tg

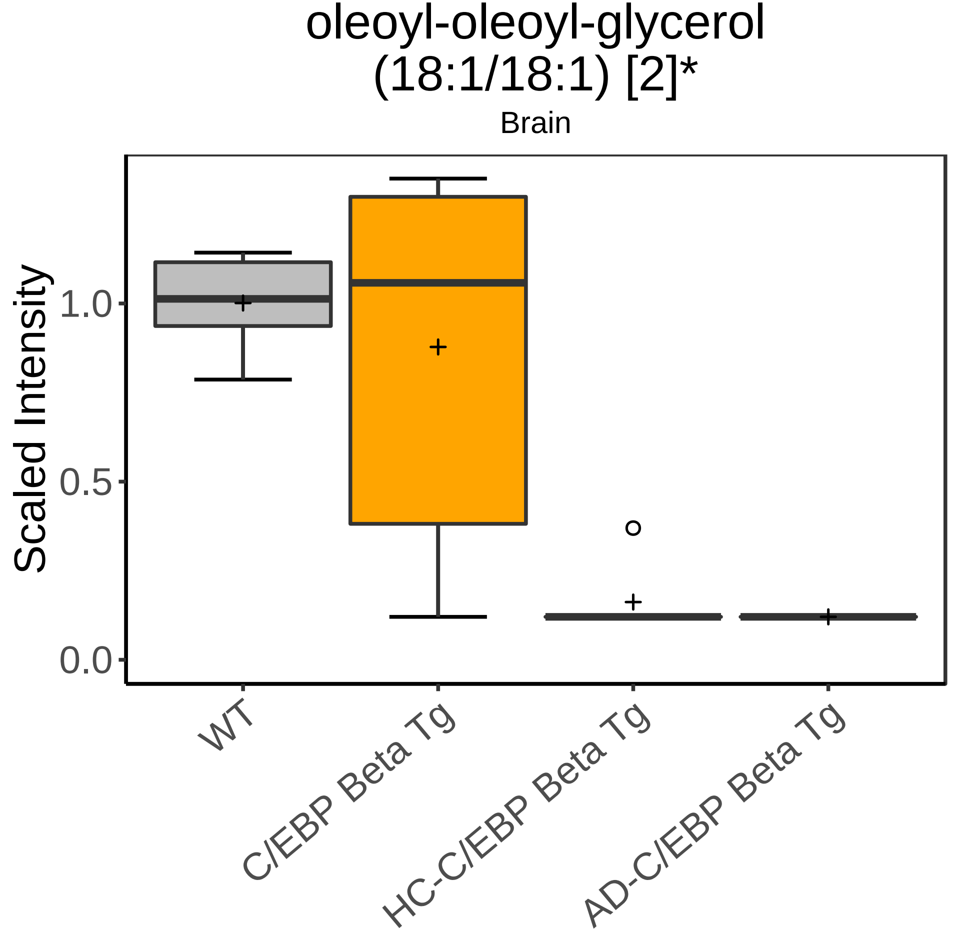
oleoyl-oleoyl-glycerol
(18:1/18:1) [2]*
Brain
Scaled Intensity
0.0
0.5
1.0
WT
C/EBP Beta Tg
HC-C/EBP Beta Tg
AD-C/EBP Beta Tg

oleoyl-linoleoyl-glycerol (18:1/18:2) [2]

Brain

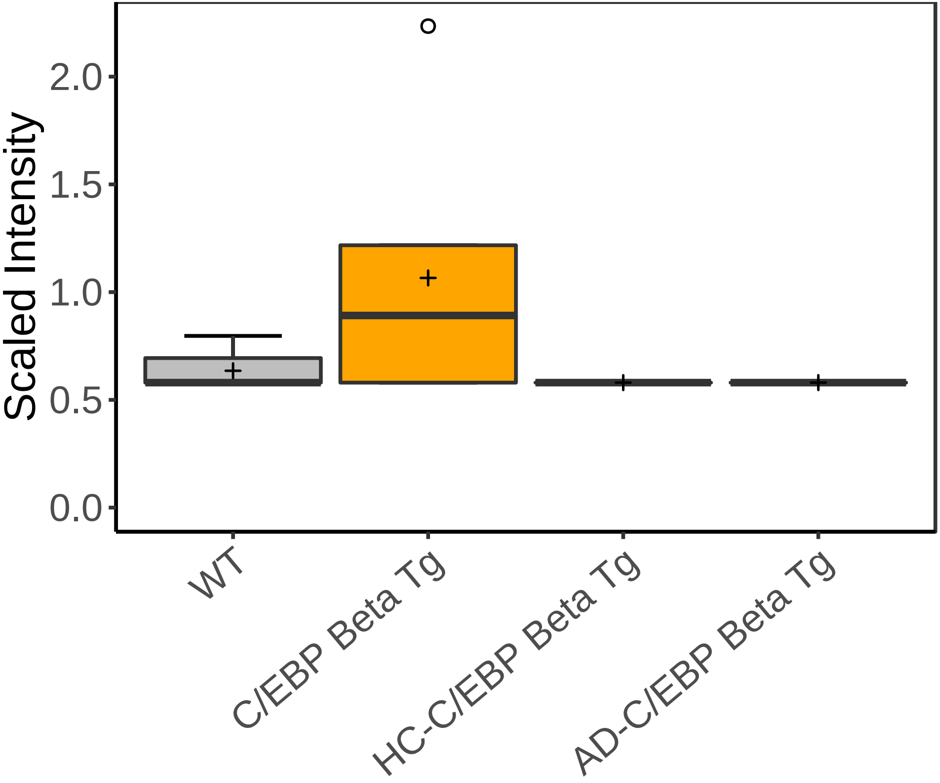

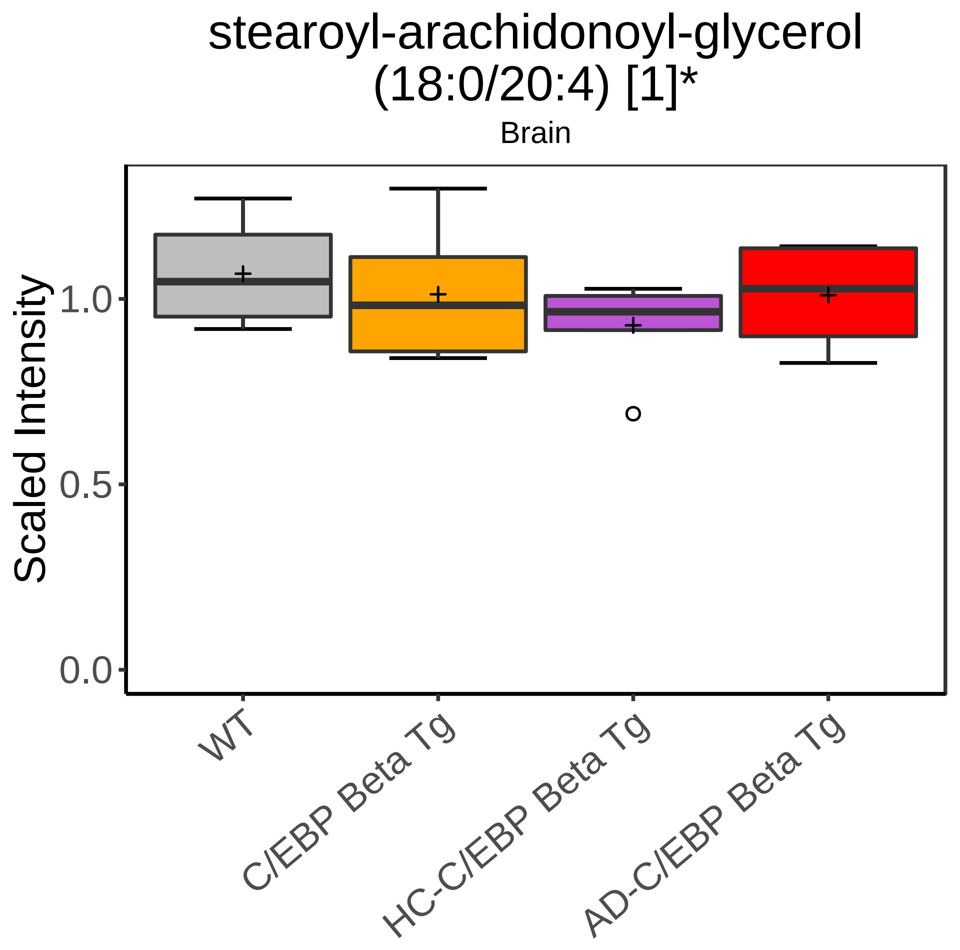
stearoyl-arachidonoyl-glycerol
(18:0/20:4) [1]*
Brain
Scaled Intensity
1.0
0.5
0.0
WT
C/EBP Beta Tg
HC-C/EBP Beta Tg
AD-C/EBP Beta Tg

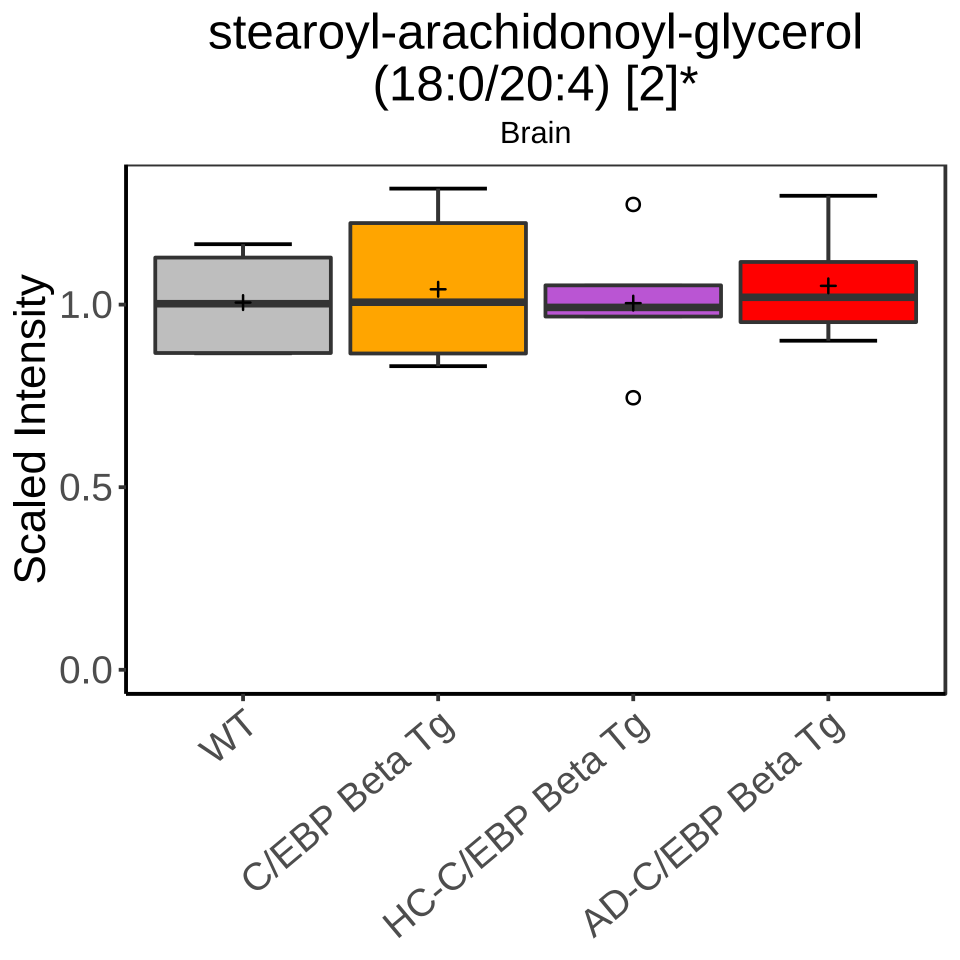
stearoyl-arachidonoyl-glycerol (18:0/20:4) [2]*
Brain
Scaled Intensity
0.0
0.5
1.0
WT
C/EBP Beta Tg
HC-C/EBP Beta Tg
AD-C/EBP Beta Tg

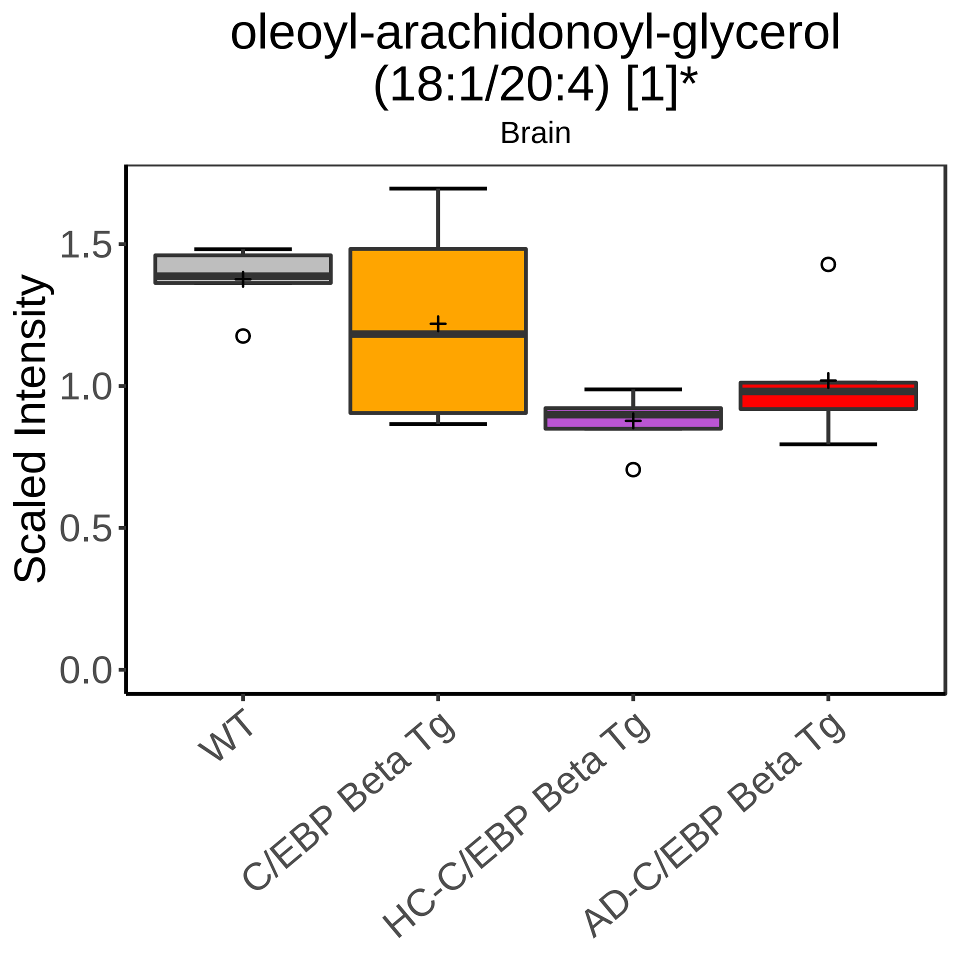

oleoyl-arachidonoyl-glycerol
(18:1/20:4) [1]*
Brain
Scaled Intensity
1.5
1.0
0.5
0.0
WT
C/EBP Beta Tg
HC-C/EBP Beta Tg
AD-C/EBP Beta Tg

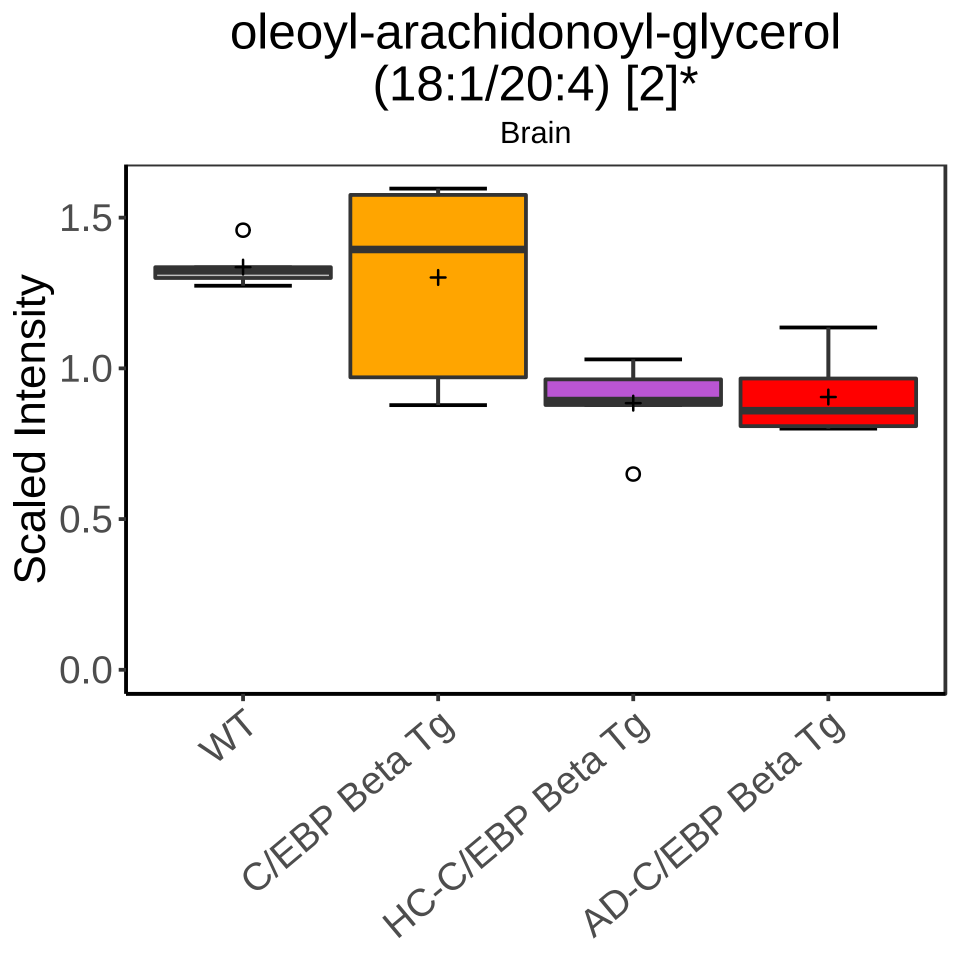

oleoyl-arachidonoyl-glycerol
(18:1/20:4) [2]*
Brain
Scaled Intensity
1.5
1.0
0.5
0.0
WT
C/EBP Beta Tg
HC-C/EBP Beta Tg
AD-C/EBP Beta Tg

linoleoyl-arachidonoyl-glycerol (18:2/20:4) [2]*

Brain

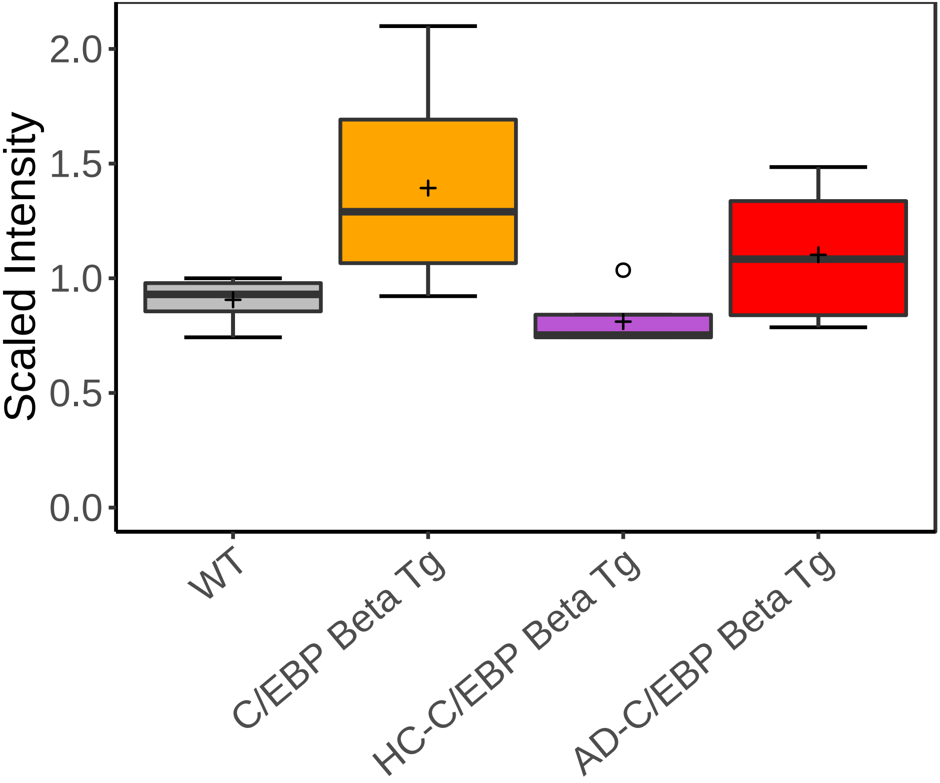

stearoyl-docosahexaenoyl-glycerol (18:0/22:6) [1]*

Brain

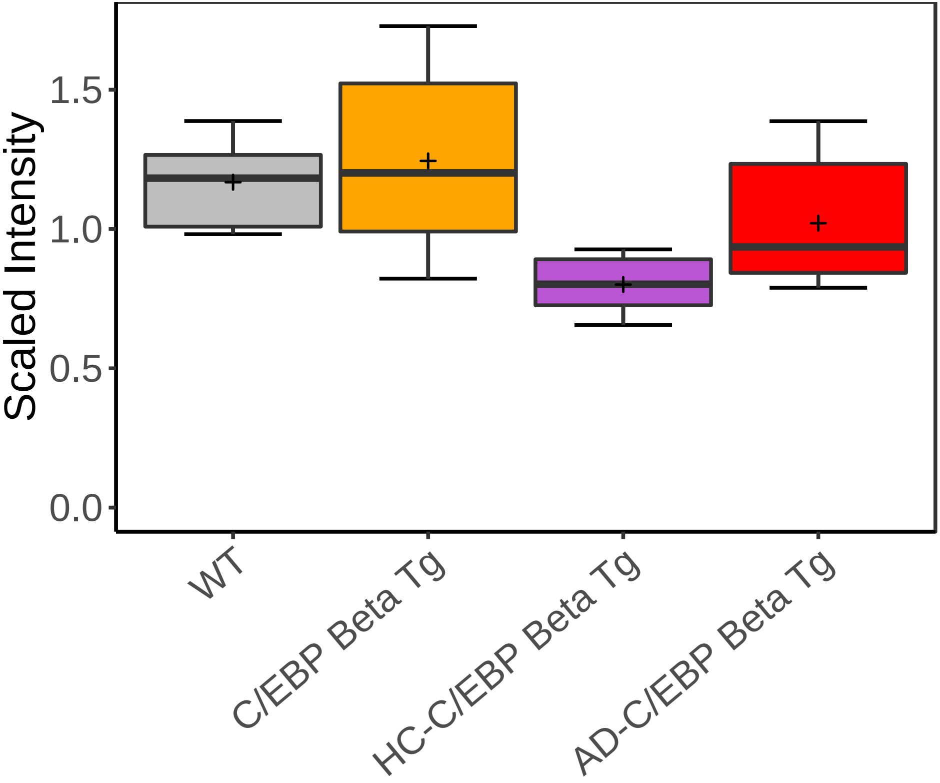

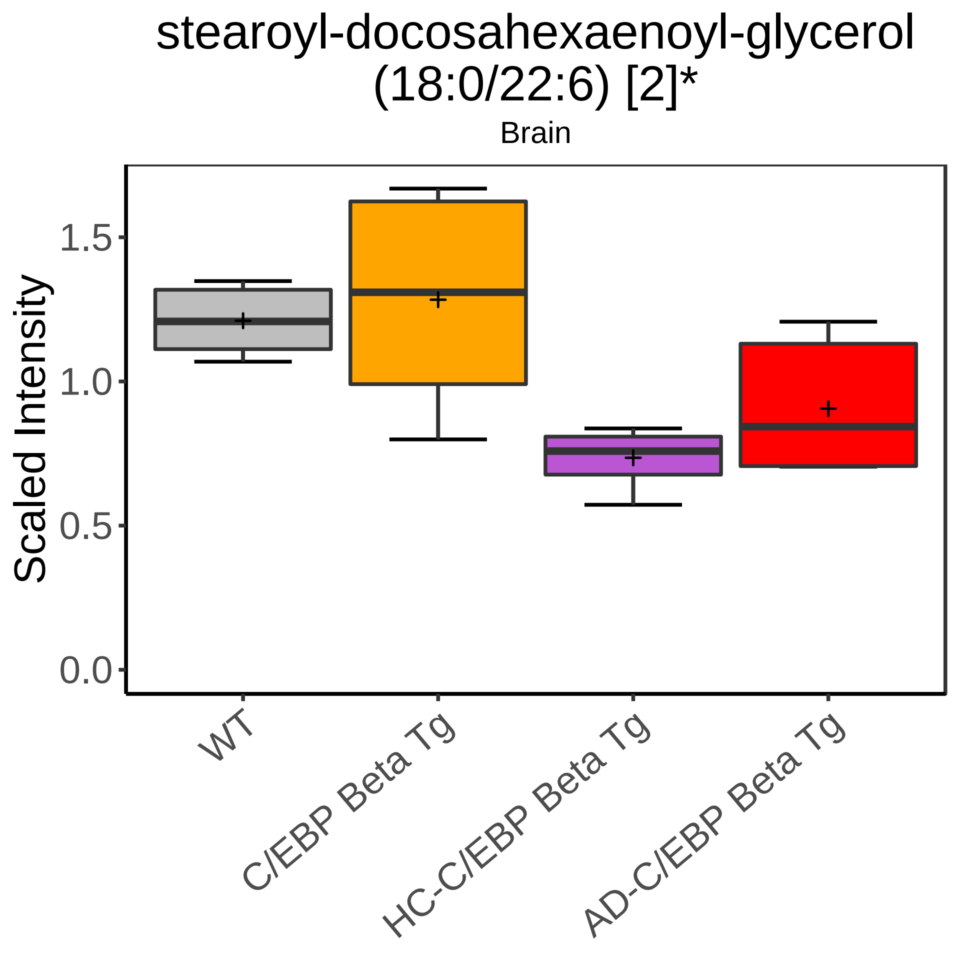
stearoyl-docosahexaenoyl-glycerol
(18:0/22:6) [2]*
Brain
Scaled Intensity
1.5
1.0
0.5
0.0
WT
C/EBP Beta Tg
HC-C/EBP Beta Tg
AD-C/EBP Beta Tg

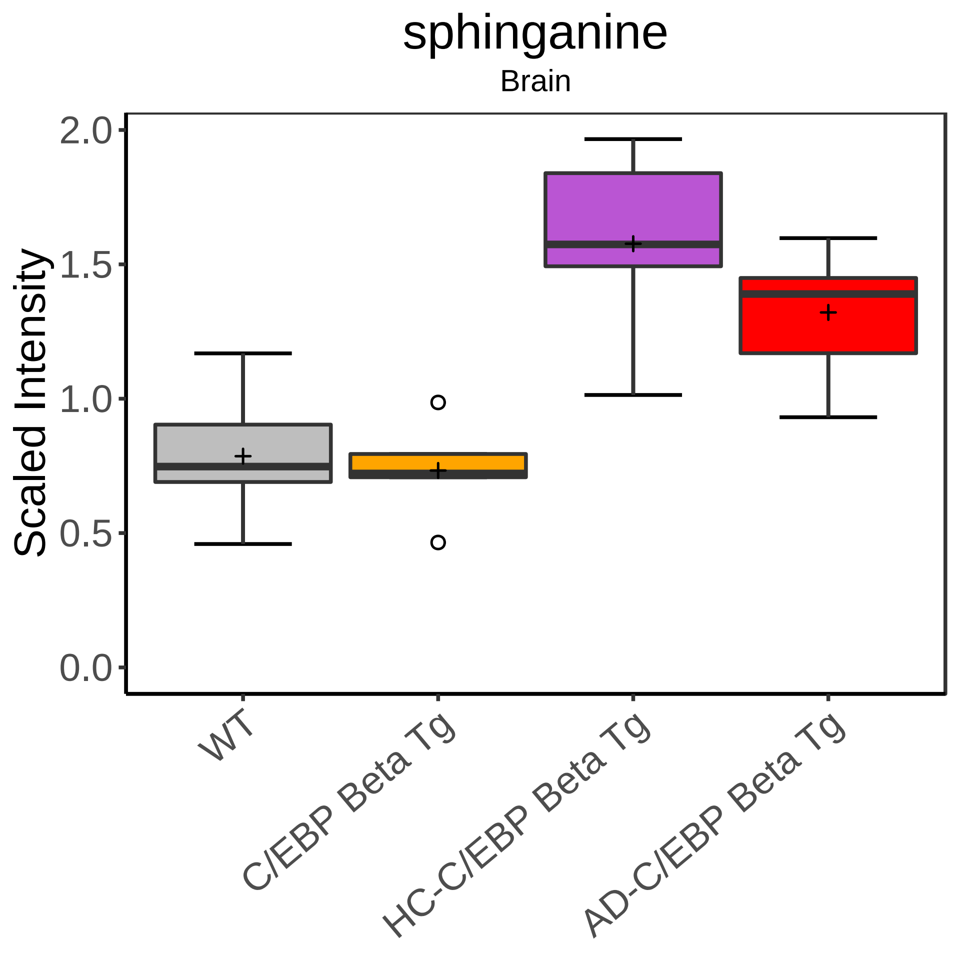
sphinganine
Brain
Scaled Intensity
2.0
1.5
1.0
0.5
0.0
WT
C/EBP Beta Tg
HC-C/EBP Beta Tg
AD-C/EBP Beta Tg

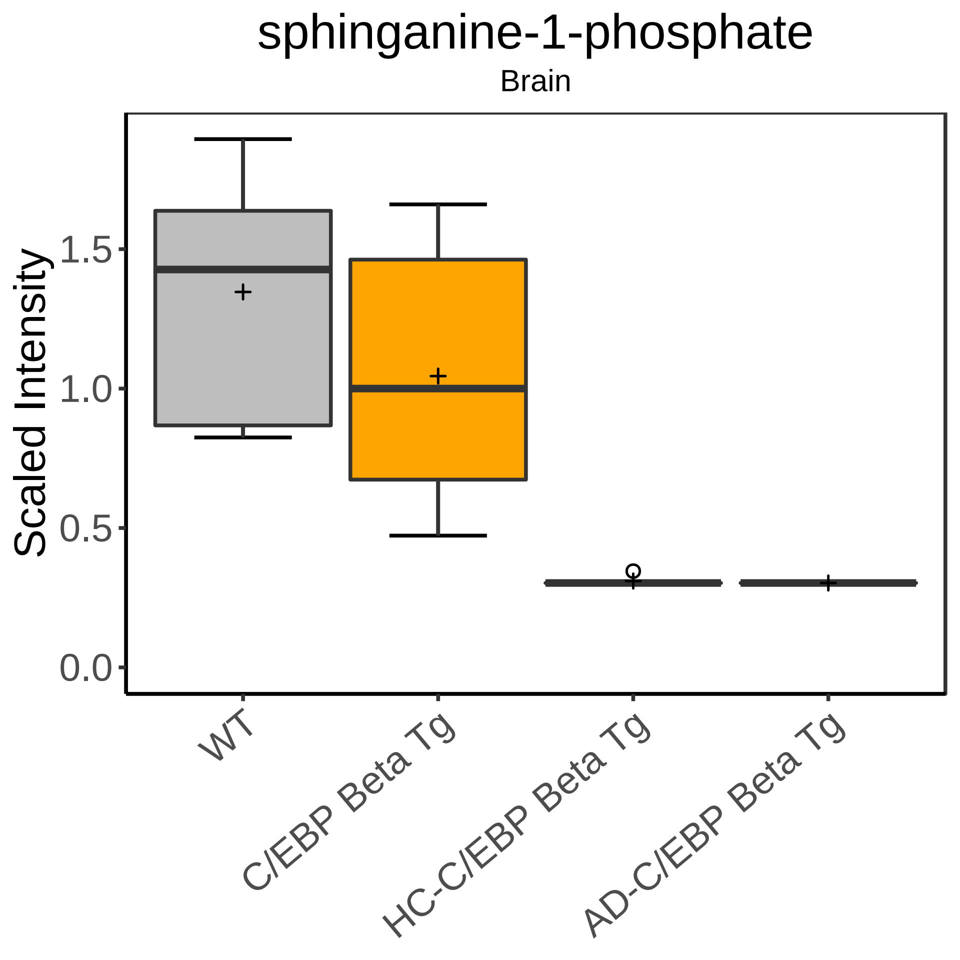
sphinganine-1-phosphate
Brain
Scaled Intensity
0.0
0.5
1.0
1.5
WT
C/EBP Beta Tg
HC-C/EBP Beta Tg
AD-C/EBP Beta Tg

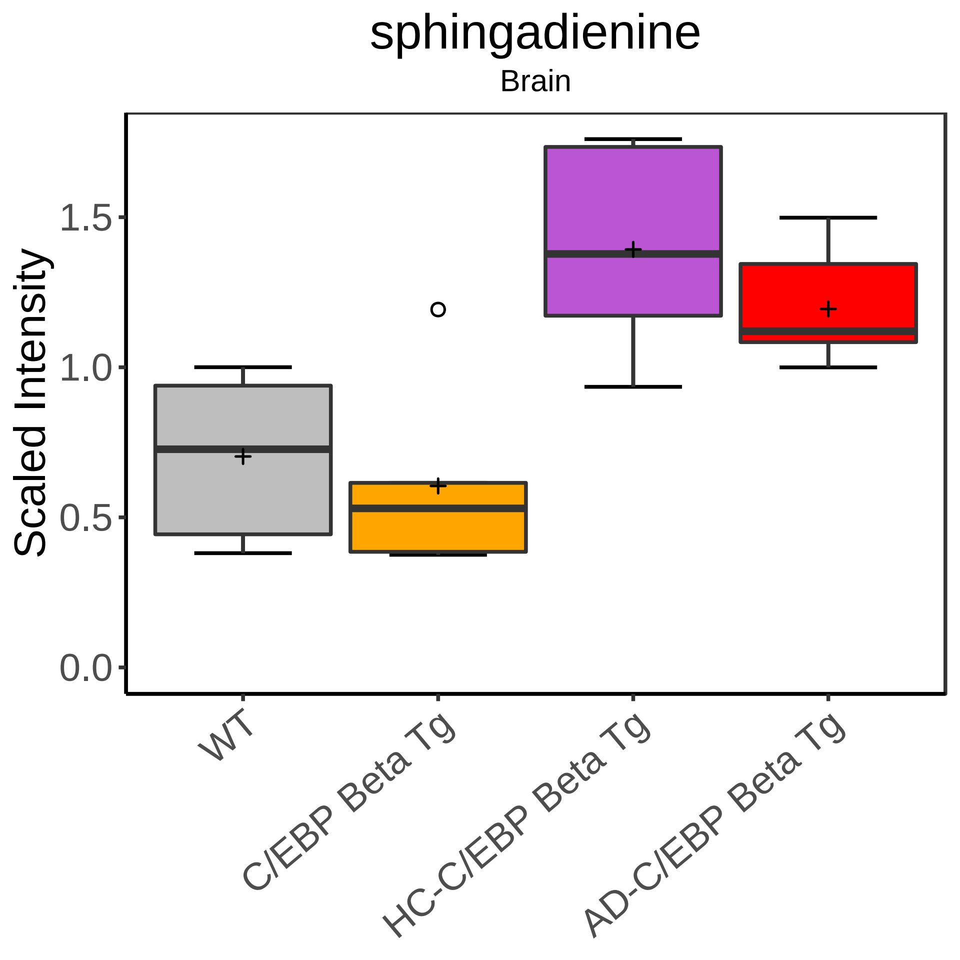

sphingadienine
Brain
Scaled Intensity
1.5
1.0
0.5
0.0
WT
C/EBP Beta Tg
HC-C/EBP Beta Tg
AD-C/EBP Beta Tg

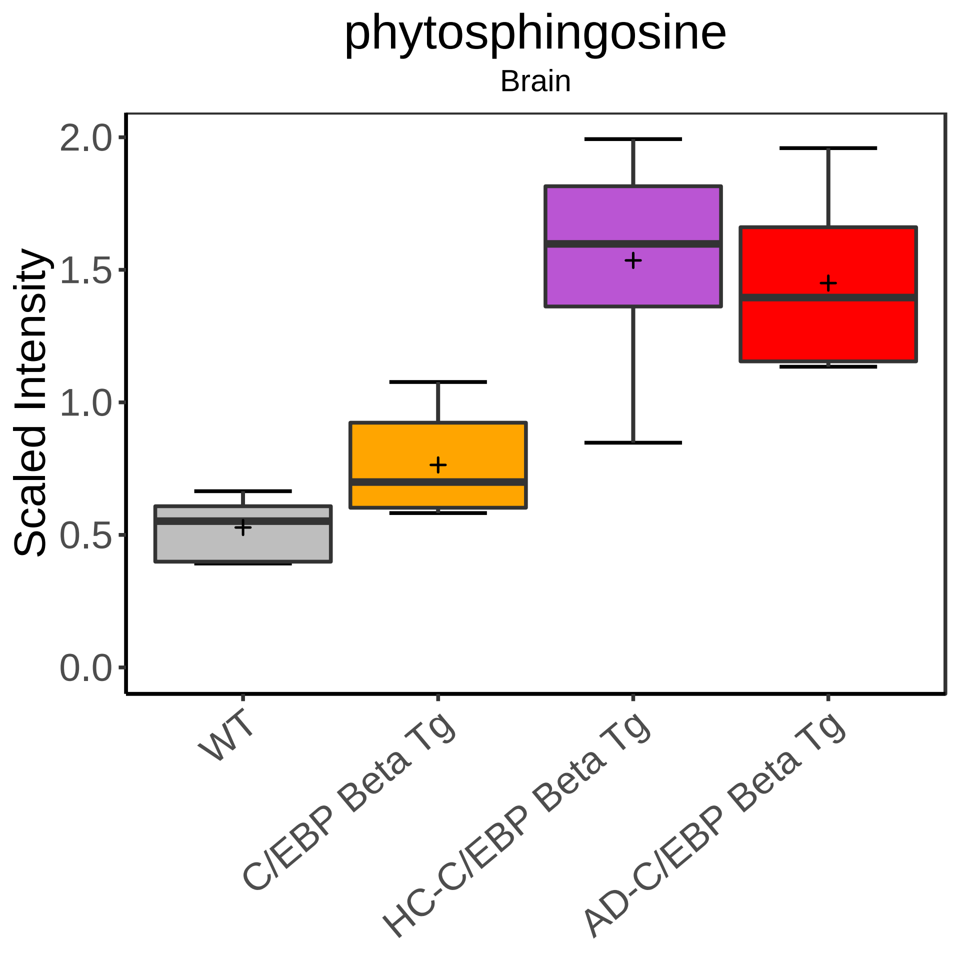
phytosphingosine
Brain
Scaled Intensity
2.0
1.5
1.0
0.5
0.0
WT
C/EBP Beta Tg
HC-C/EBP Beta Tg
AD-C/EBP Beta Tg

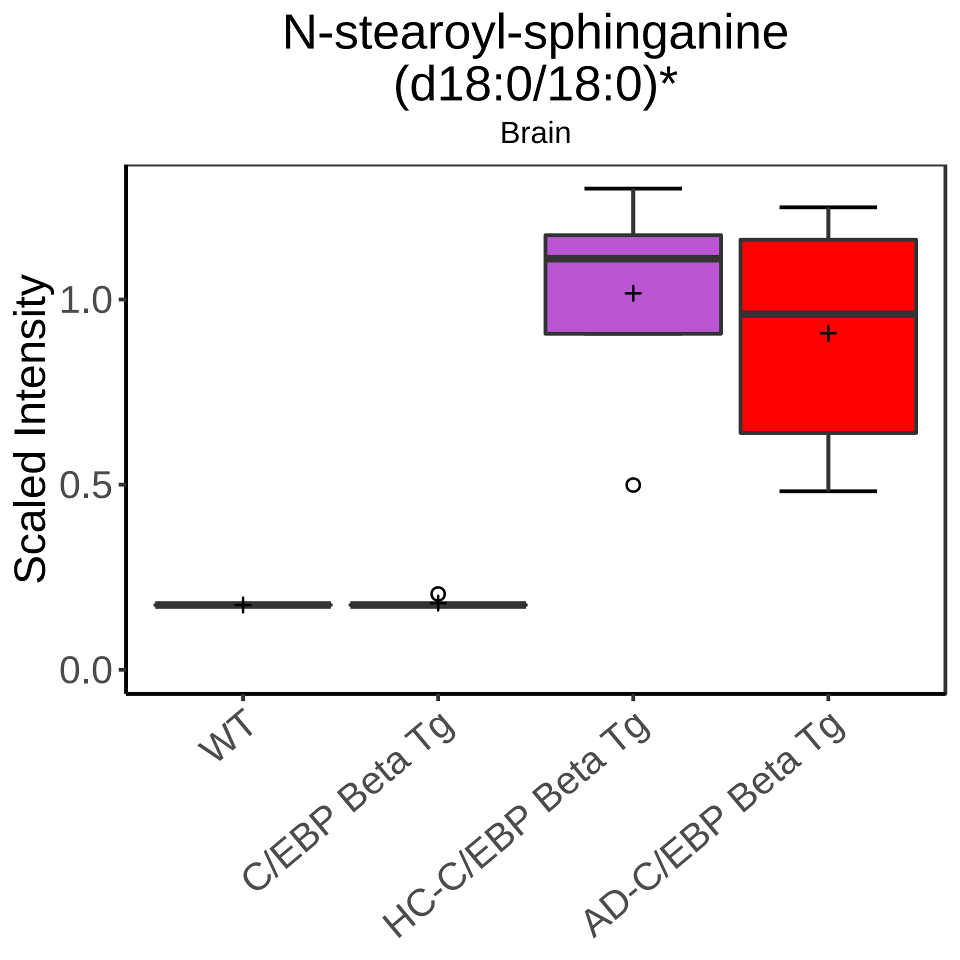
N-stearoyl-sphinganine
(d18:0/18:0)*
Brain
Scaled Intensity
1.0
0.5
0.0
WT
C/EBP Beta Tg
HC-C/EBP Beta Tg
AD-C/EBP Beta Tg

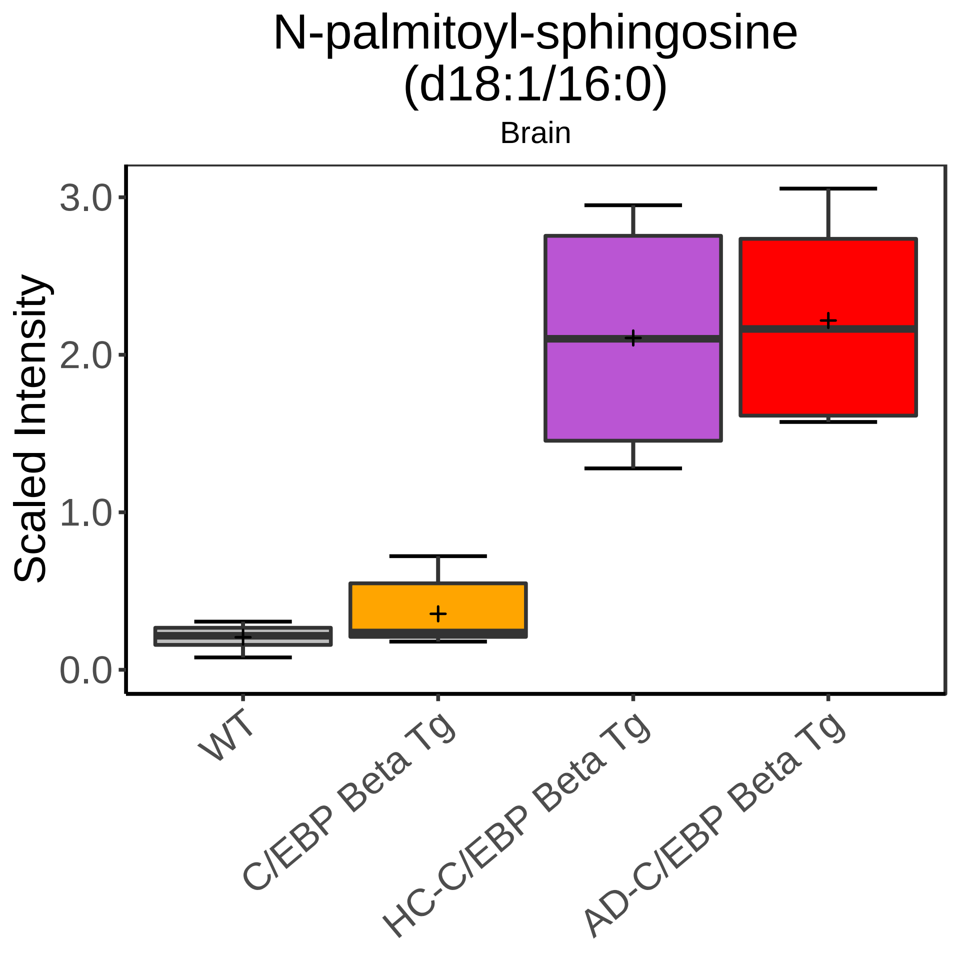

N-palmitoyl-sphingosine
(d18:1/16:0)
Brain
Scaled Intensity
3.0
2.0
1.0
0.0
WT
C/EBP Beta Tg
HC-C/EBP Beta Tg
AD-C/EBP Beta Tg

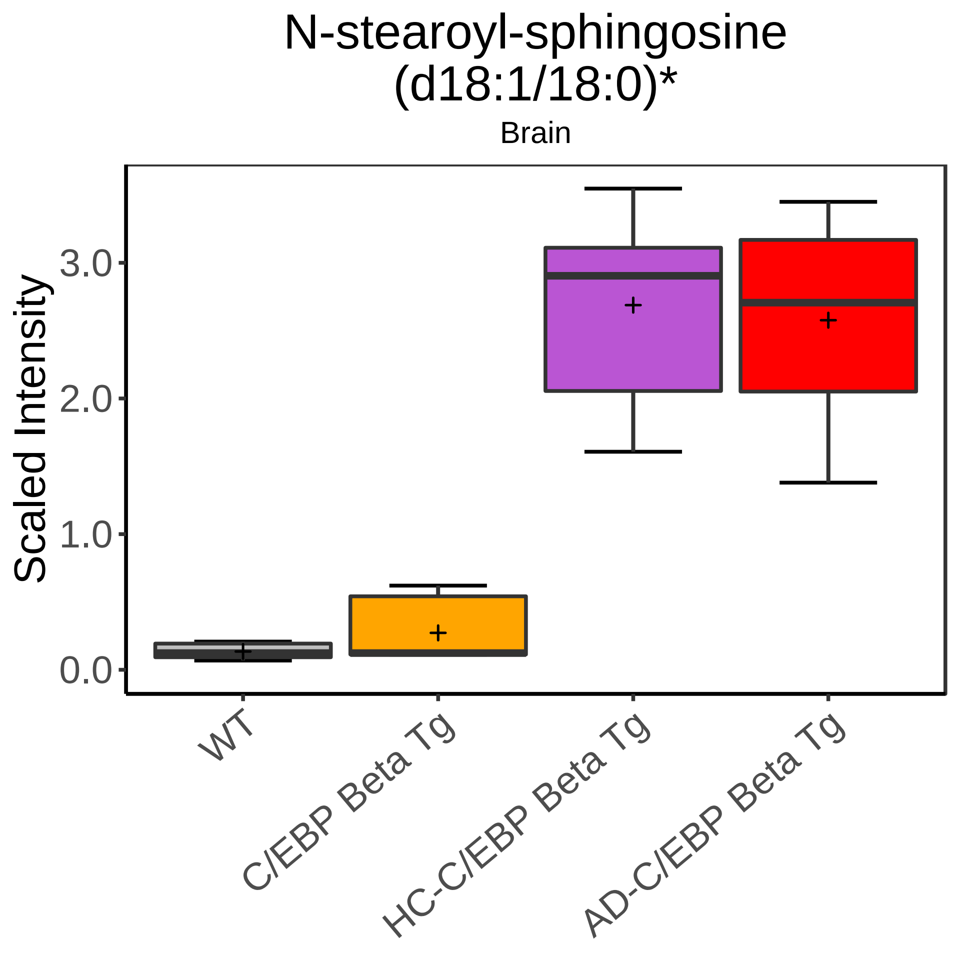
N-stearoyl-sphingosine
(d18:1/18:0)*
Brain
Scaled Intensity
3.0
2.0
1.0
0.0
WT
C/EBP Beta Tg
HC-C/EBP Beta Tg
AD-C/EBP Beta Tg

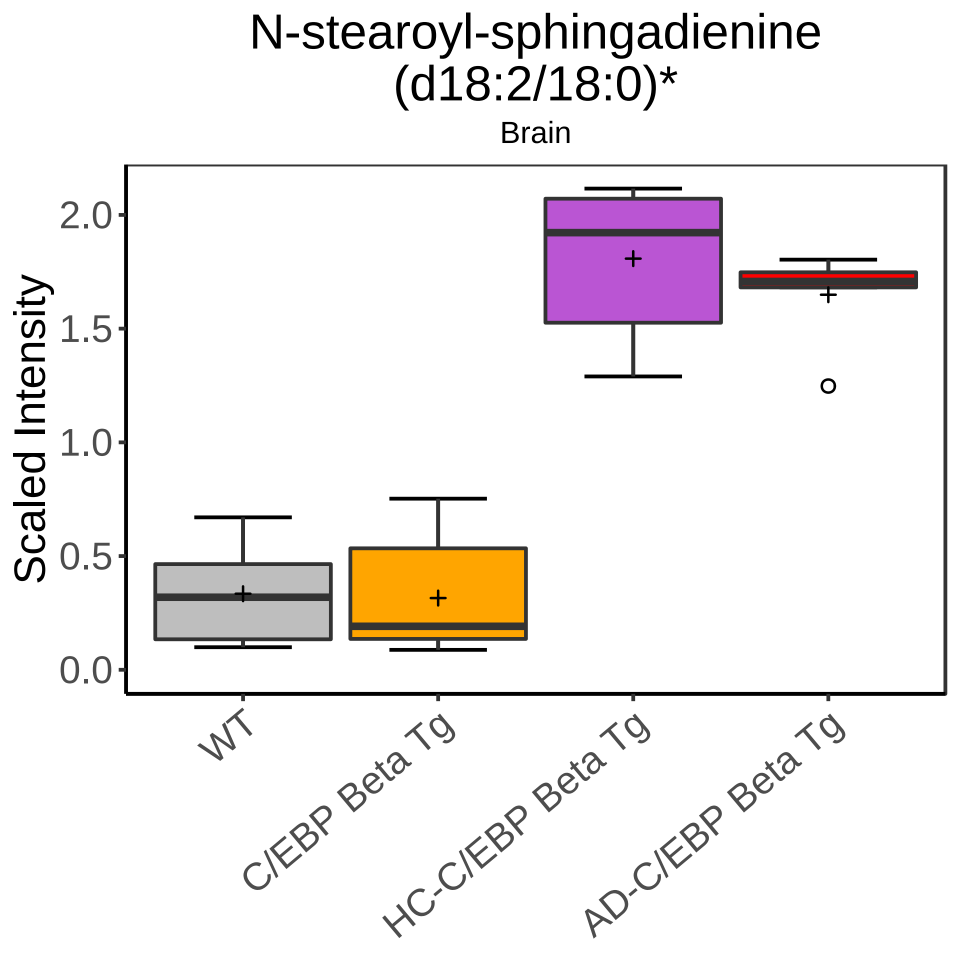
N-stearoyl-sphingadienine (d18:2/18:0)*
Brain
Scaled Intensity
2.0
1.5
1.0
0.5
0.0
WT
C/EBP Beta Tg
HC-C/EBP Beta Tg
AD-C/EBP Beta Tg

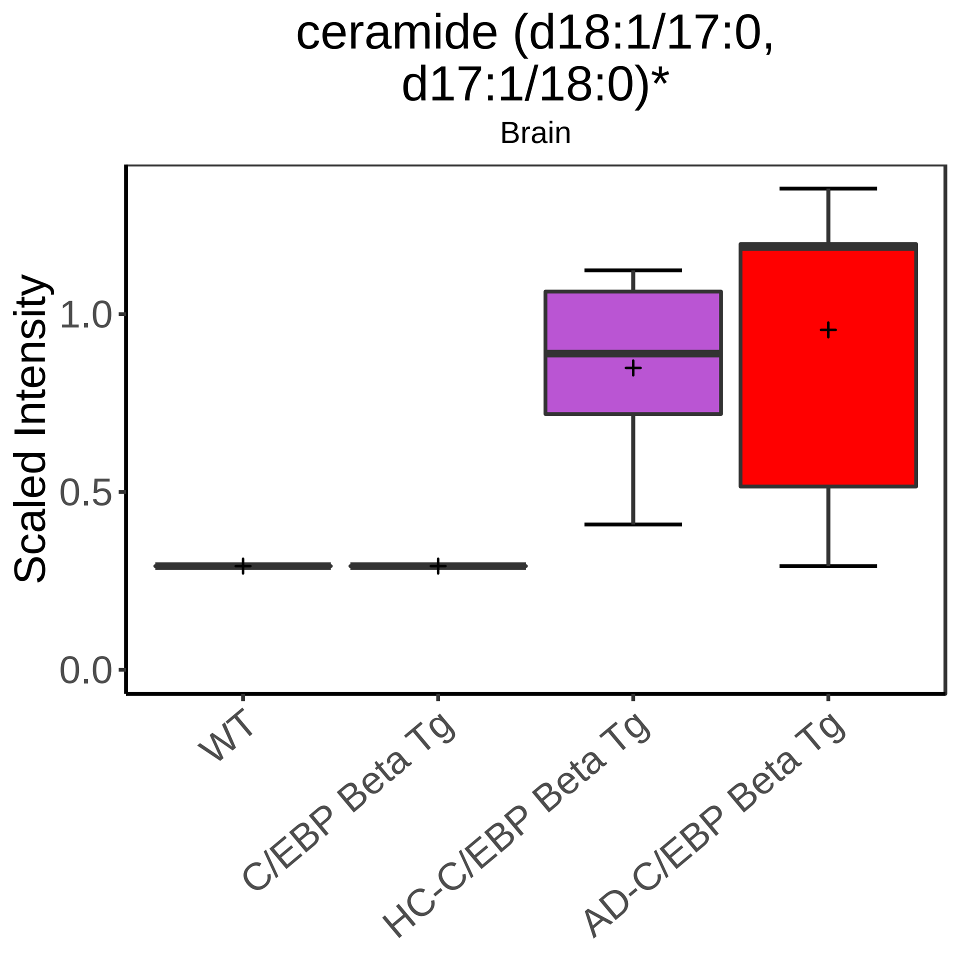

ceramide (d18:1/17:0, d17:1/18:0)*
Brain
Scaled Intensity
1.0
0.5
0.0
WT
C/EBP Beta Tg
HC-C/EBP Beta Tg
AD-C/EBP Beta Tg

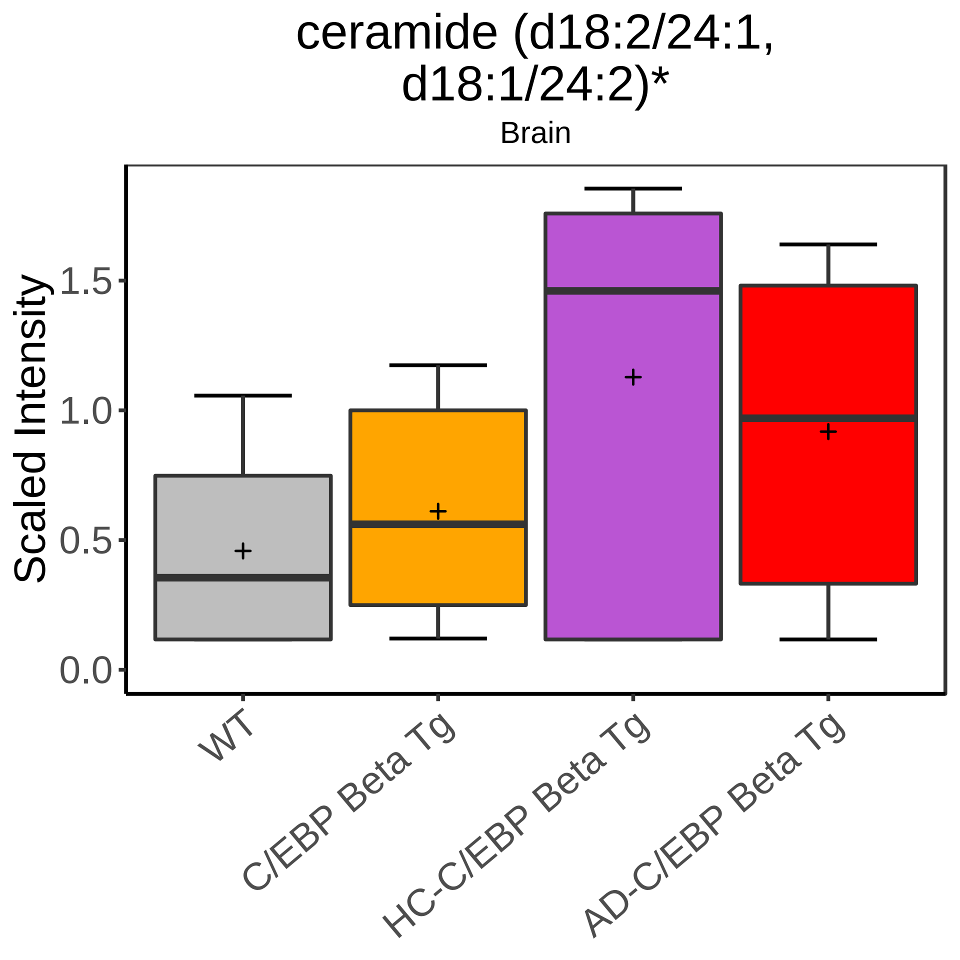

ceramide (d18:2/24:1, d18:1/24:2)*
Brain
Scaled Intensity
0.0
0.5
1.0
1.5
WT
C/EBP Beta Tg
HC-C/EBP Beta Tg
AD-C/EBP Beta Tg

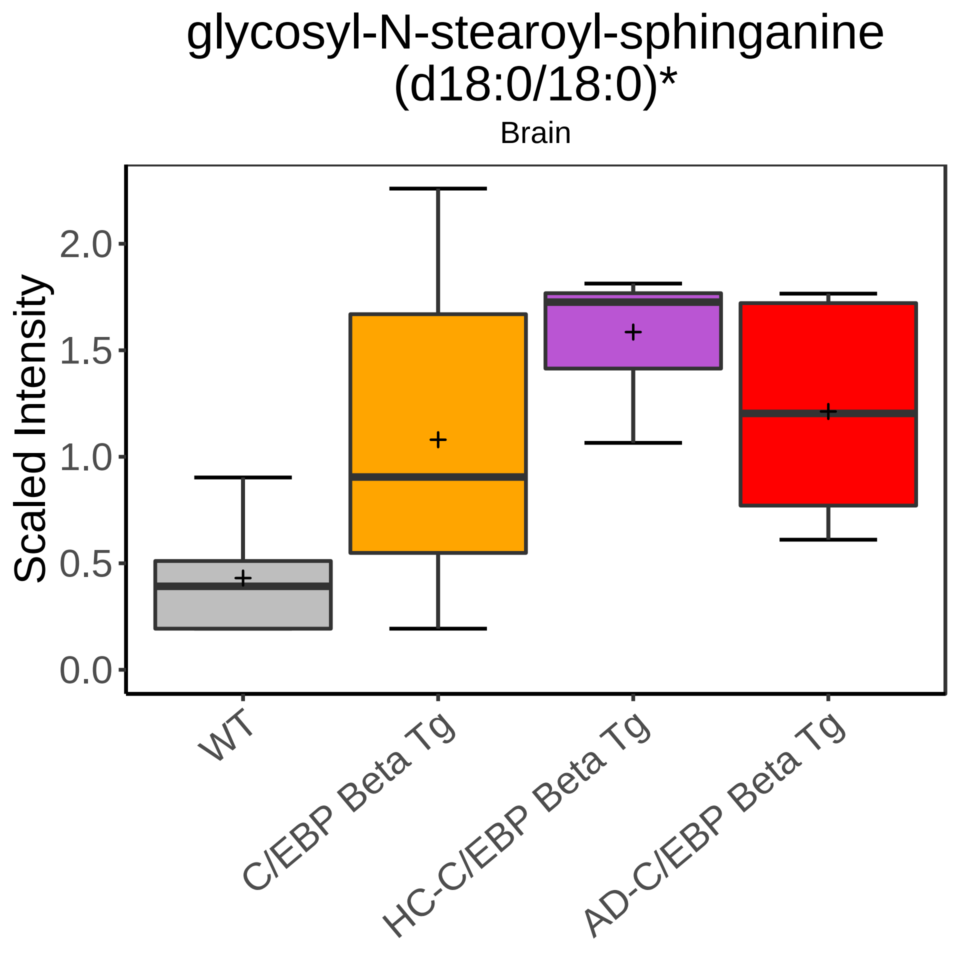
glycosyl-N-stearoyl-sphinganine
(d18:0/18:0)*
Brain
Scaled Intensity
2.0
1.5
1.0
0.5
0.0
WT
C/EBP Beta Tg
HC-C/EBP Beta Tg
AD-C/EBP Beta Tg

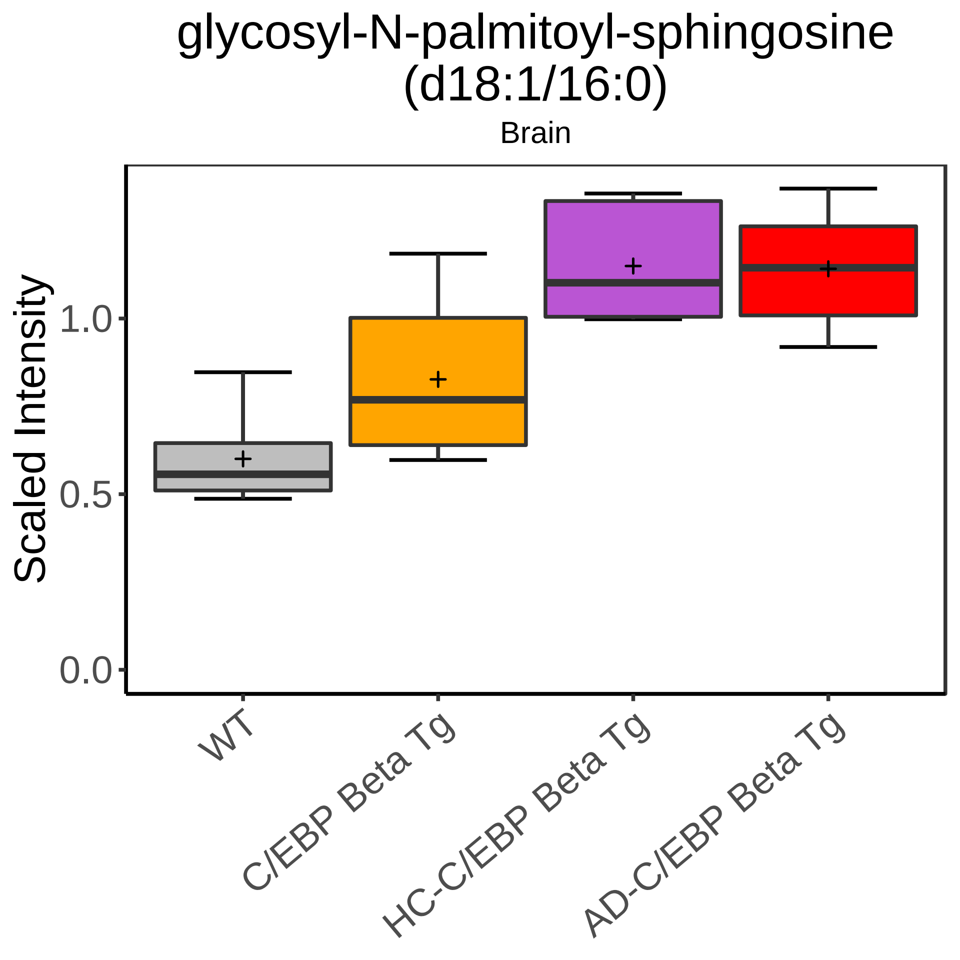
glycosyl-N-palmitoyl-sphingosine
(d18:1/16:0)
Brain
Scaled Intensity
1.0
0.5
0.0
WT
C/EBP Beta Tg
HC-C/EBP Beta Tg
AD-C/EBP Beta Tg

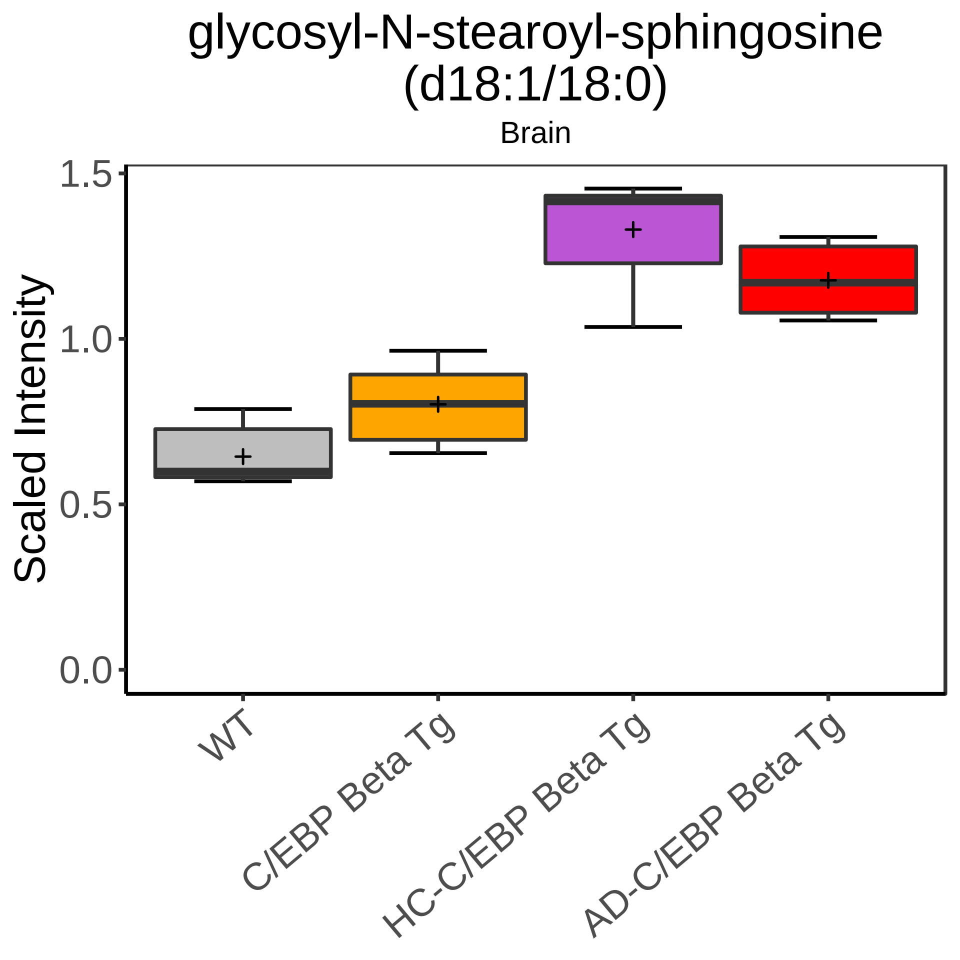

glycosyl-N-stearoyl-sphingosine
(d18:1/18:0)
Brain
Scaled Intensity
1.5
1.0
0.5
0.0
WT
C/EBP Beta Tg
HC-C/EBP Beta Tg
AD-C/EBP Beta Tg

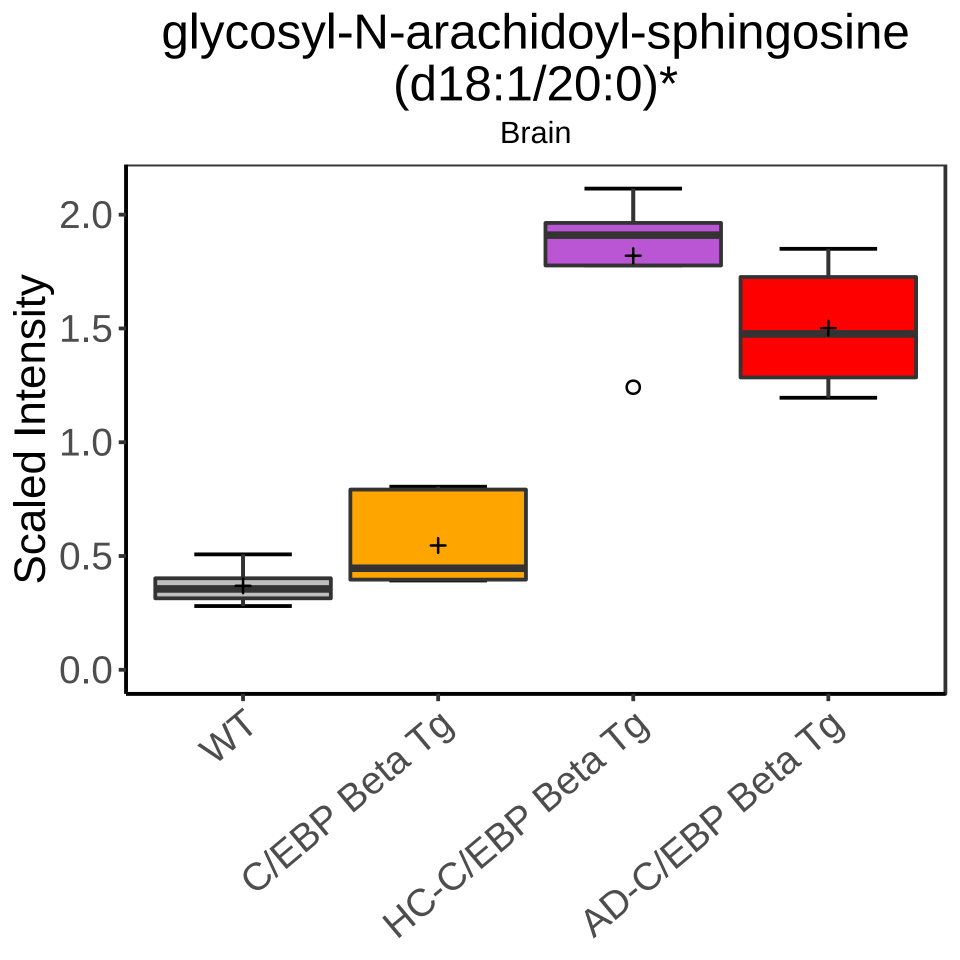

glycosyl-N-arachidoyl-sphingosine
(d18:1/20:0)*
Brain
Scaled Intensity
2.0
1.5
1.0
0.5
0.0
WT
C/EBP Beta Tg
HC-C/EBP Beta Tg
AD-C/EBP Beta Tg

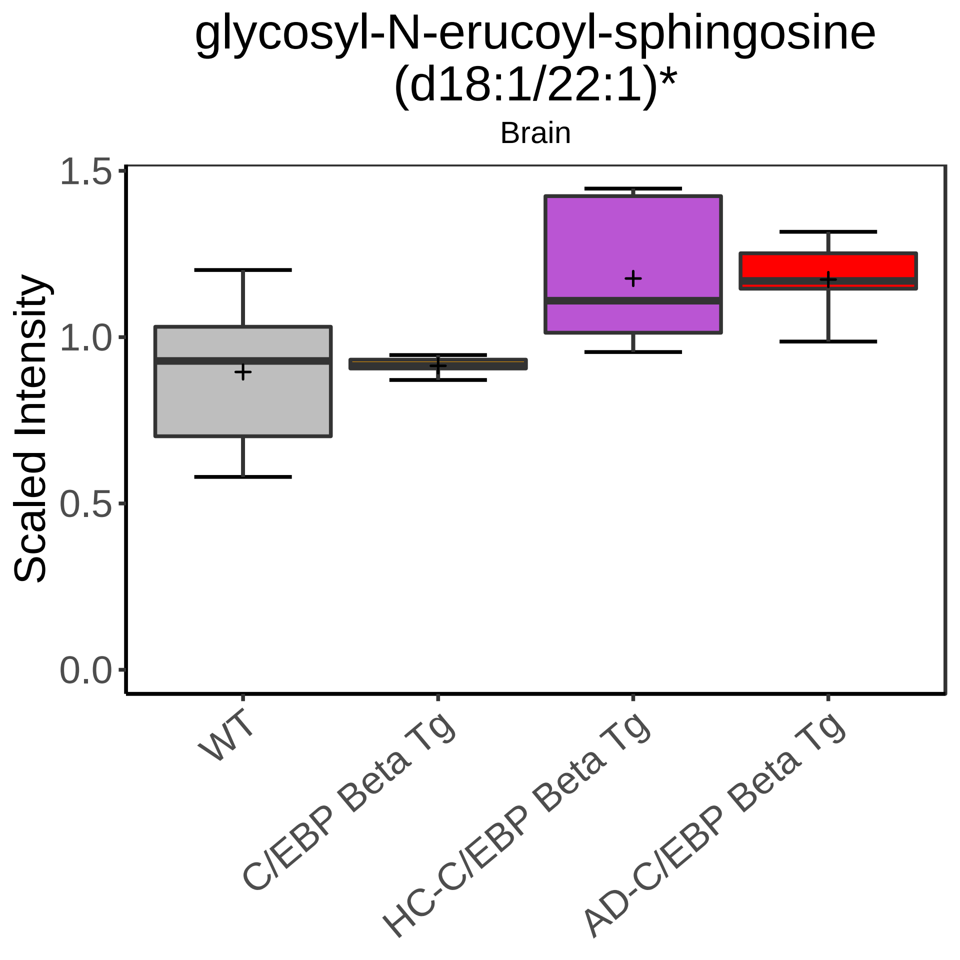

glycosyl-N-erucoyl-sphingosine
(d18:1/22:1)*
Brain
Scaled Intensity
1.5
1.0
0.5
0.0
WT
C/EBP Beta Tg
HC-C/EBP Beta Tg
AD-C/EBP Beta Tg

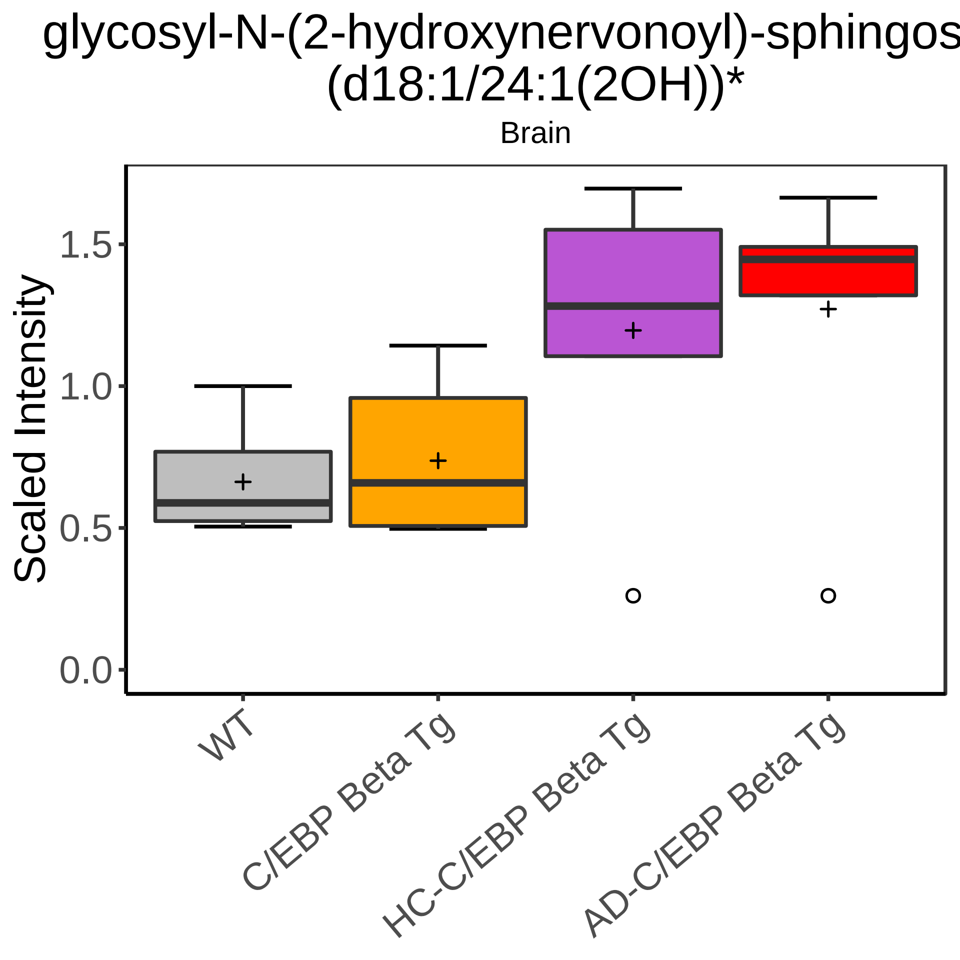
glycosyl-N-(2-hydroxynervonoyl)-sphingos
(d18:1/24:1(2OH))*
Brain
Scaled Intensity
1.5
1.0
0.5
0.0
WT
C/EBP Beta Tg
HC-C/EBP Beta Tg
AD-C/EBP Beta Tg

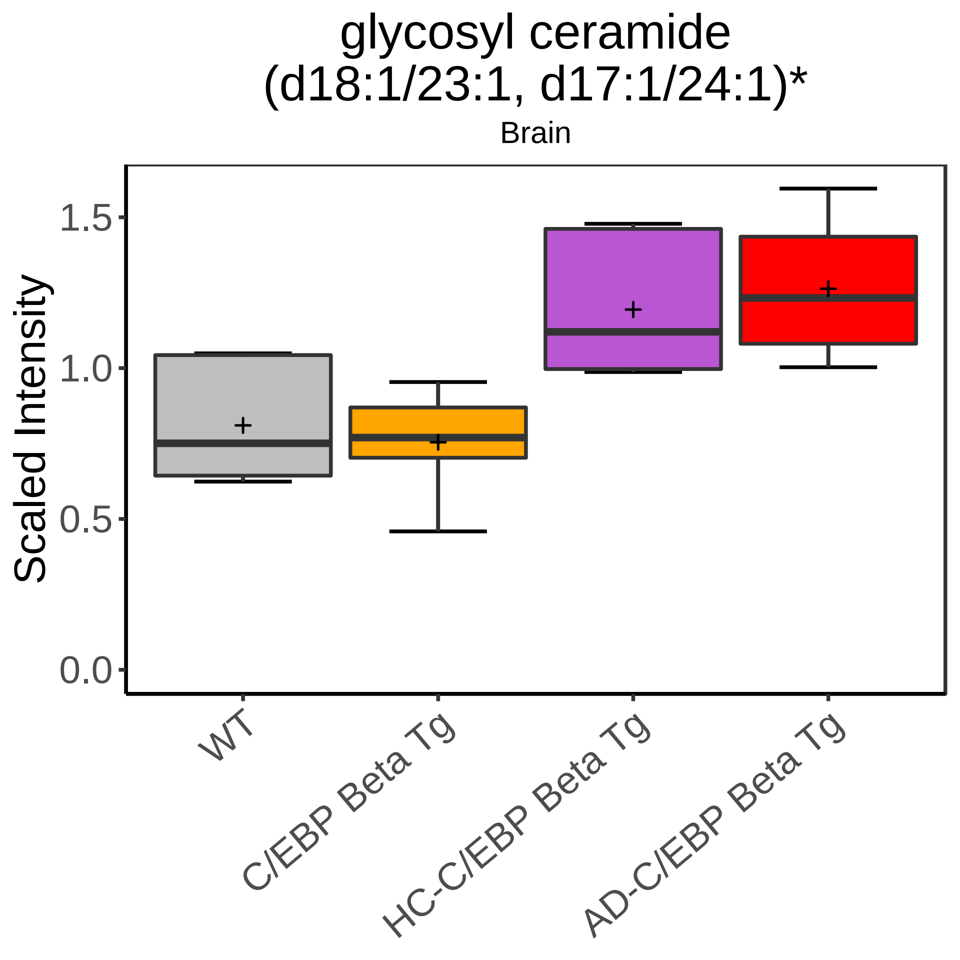
glycosyl ceramide
(d18:1/23:1, d17:1/24:1)*
Brain
Scaled Intensity
1.5
1.0
0.5
0.0
WT
C/EBP Beta Tg
HC-C/EBP Beta Tg
AD-C/EBP Beta Tg

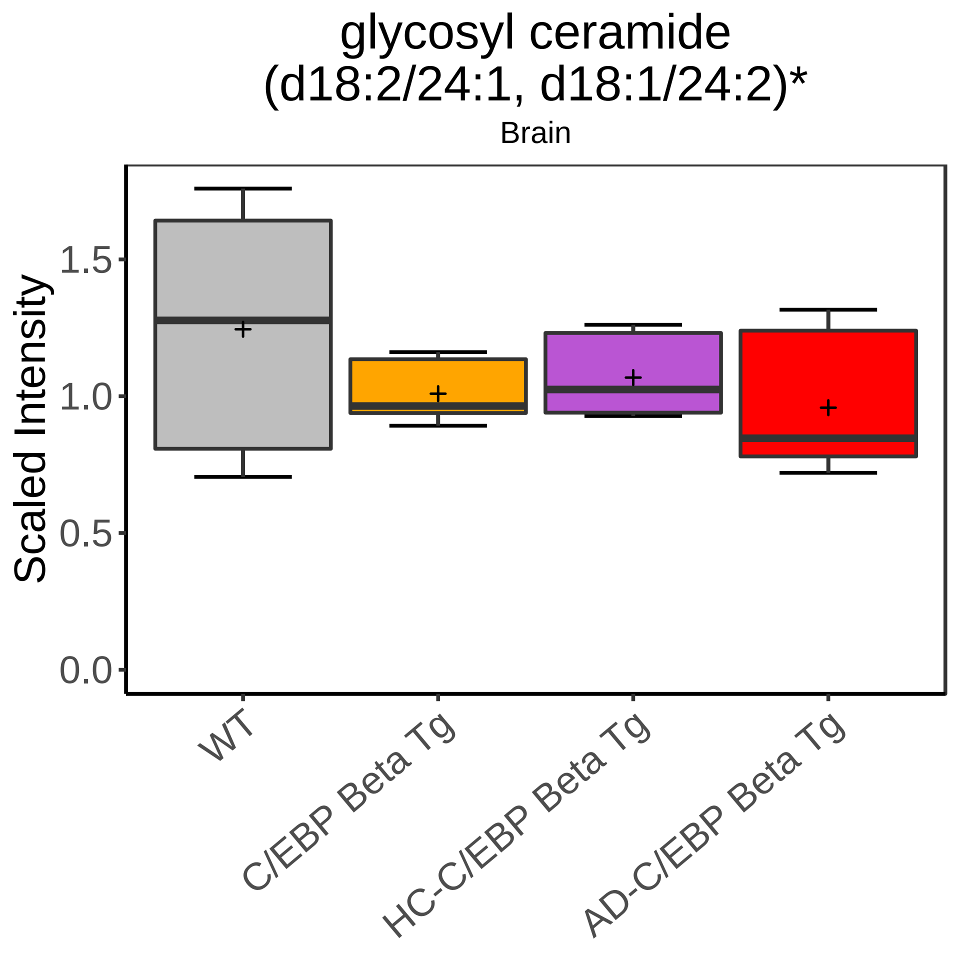
glycosyl ceramide
(d18:2/24:1, d18:1/24:2)*
Brain
Scaled Intensity
1.5
1.0
0.5
0.0
WT
C/EBP Beta Tg
HC-C/EBP Beta Tg
AD-C/EBP Beta Tg

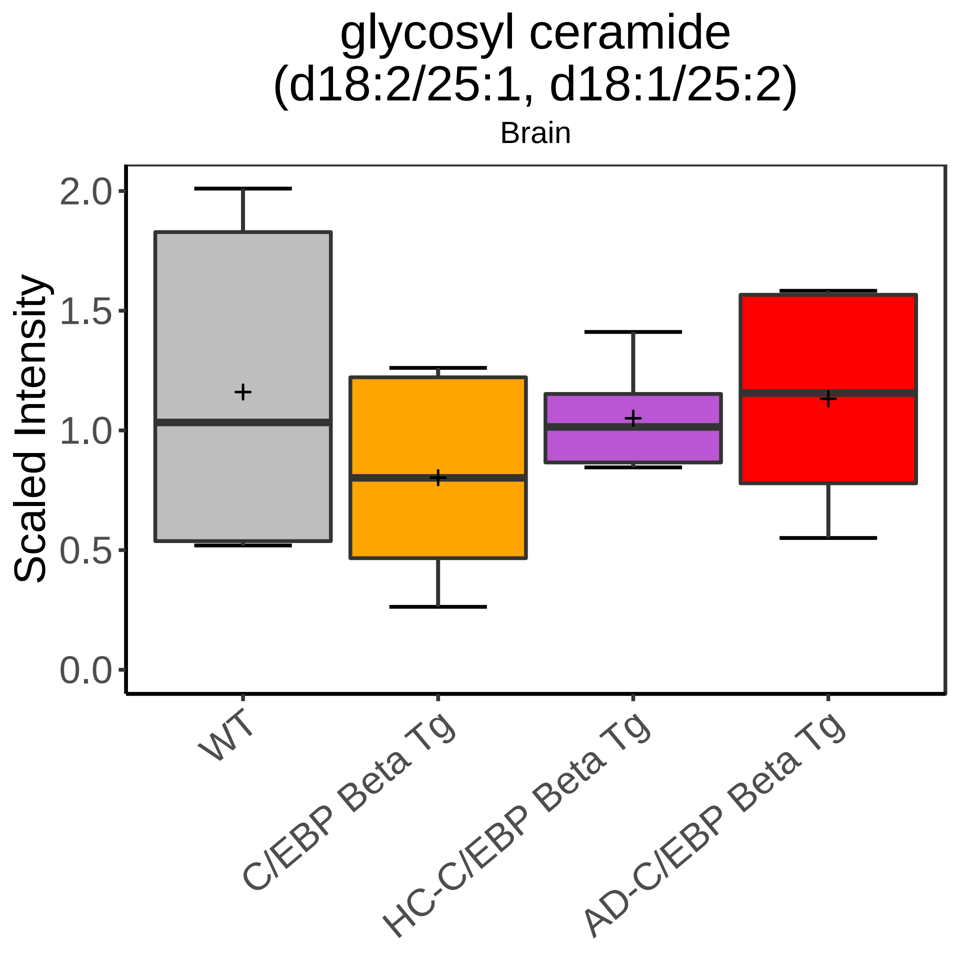
glycosyl ceramide
(d18:2/25:1, d18:1/25:2)
Brain
Scaled Intensity
2.0
1.5
1.0
0.5
0.0
WT
C/EBP Beta Tg
HC-C/EBP Beta Tg
AD-C/EBP Beta Tg

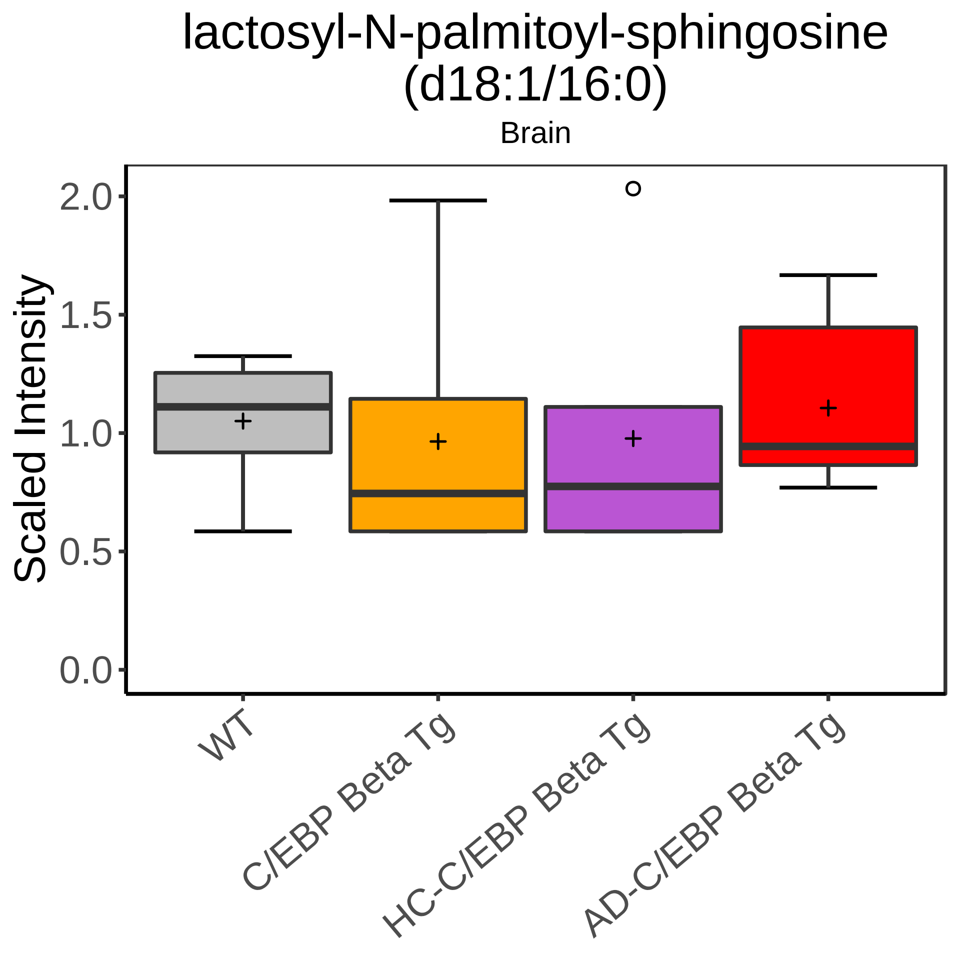

lactosyl-N-palmitoyl-sphingosine
(d18:1/16:0)
Brain
Scaled Intensity
2.0
1.5
1.0
0.5
0.0
WT
C/EBP Beta Tg
HC-C/EBP Beta Tg
AD-C/EBP Beta Tg

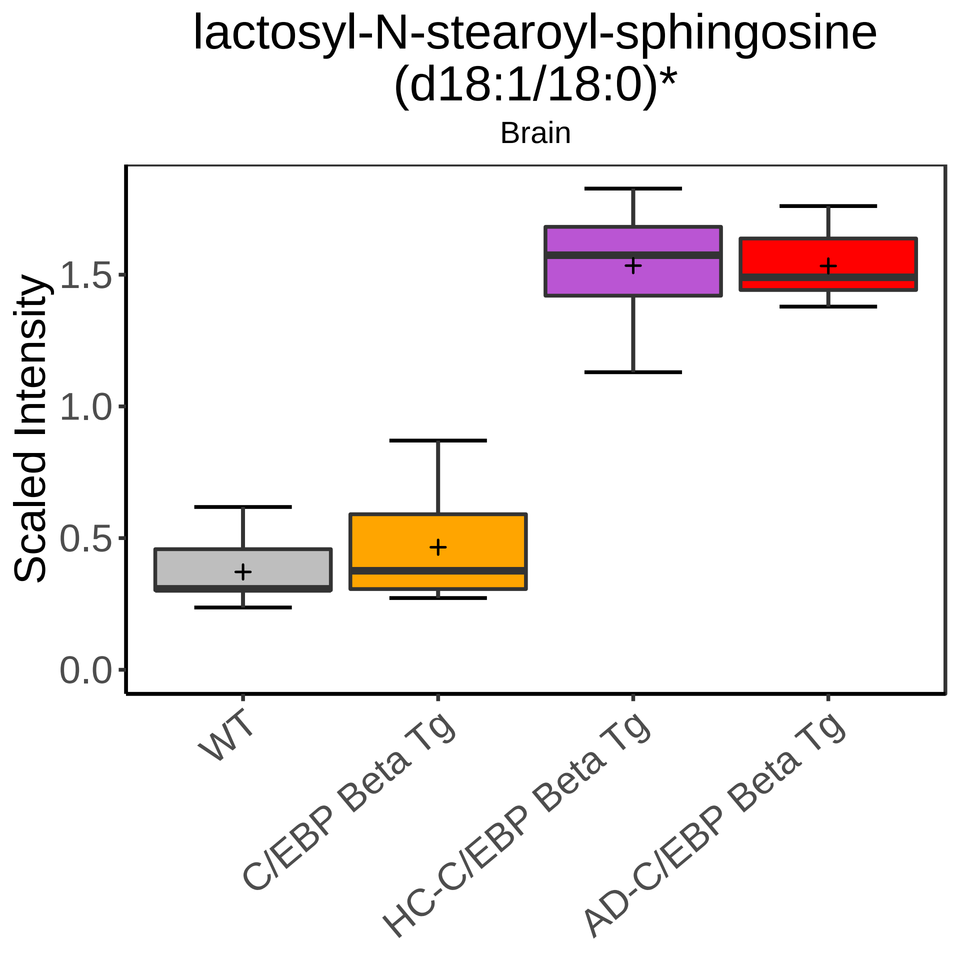
lactosyl-N-stearoyl-sphingosine
(d18:1/18:0)*
Brain
Scaled Intensity
0.0
0.5
1.0
1.5
WT
C/EBP Beta Tg
HC-C/EBP Beta Tg
AD-C/EBP Beta Tg

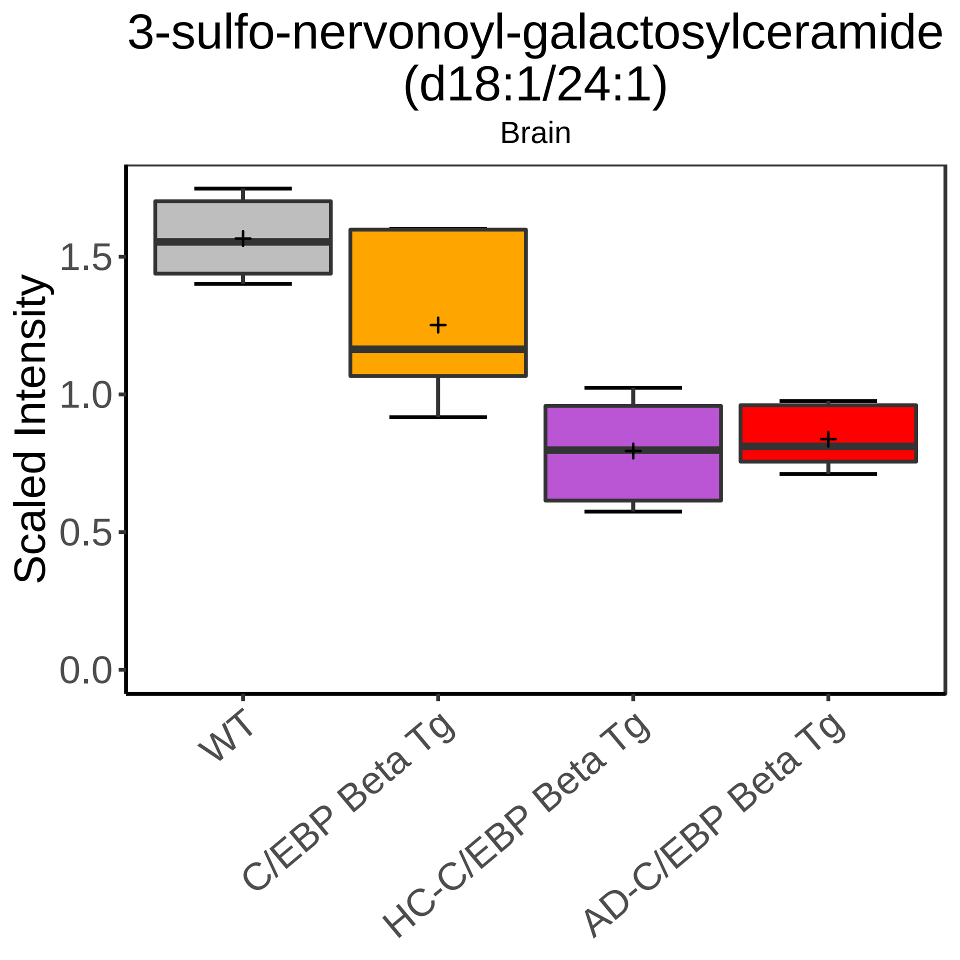

3-sulfo-nervonoyl-galactosylceramide (d18:1/24:1)
Brain
Scaled Intensity
0.0
0.5
1.0
1.5
WT
C/EBP Beta Tg
HC-C/EBP Beta Tg
AD-C/EBP Beta Tg

palmitoyl dihydrosphingomyelin (d18:0/16:0)*

Brain

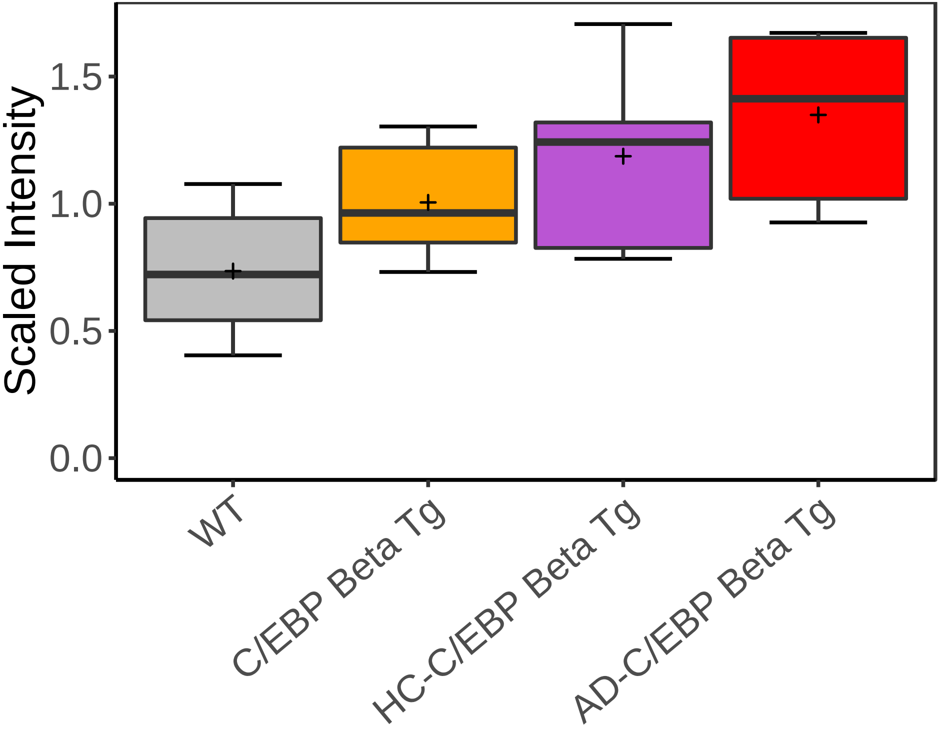

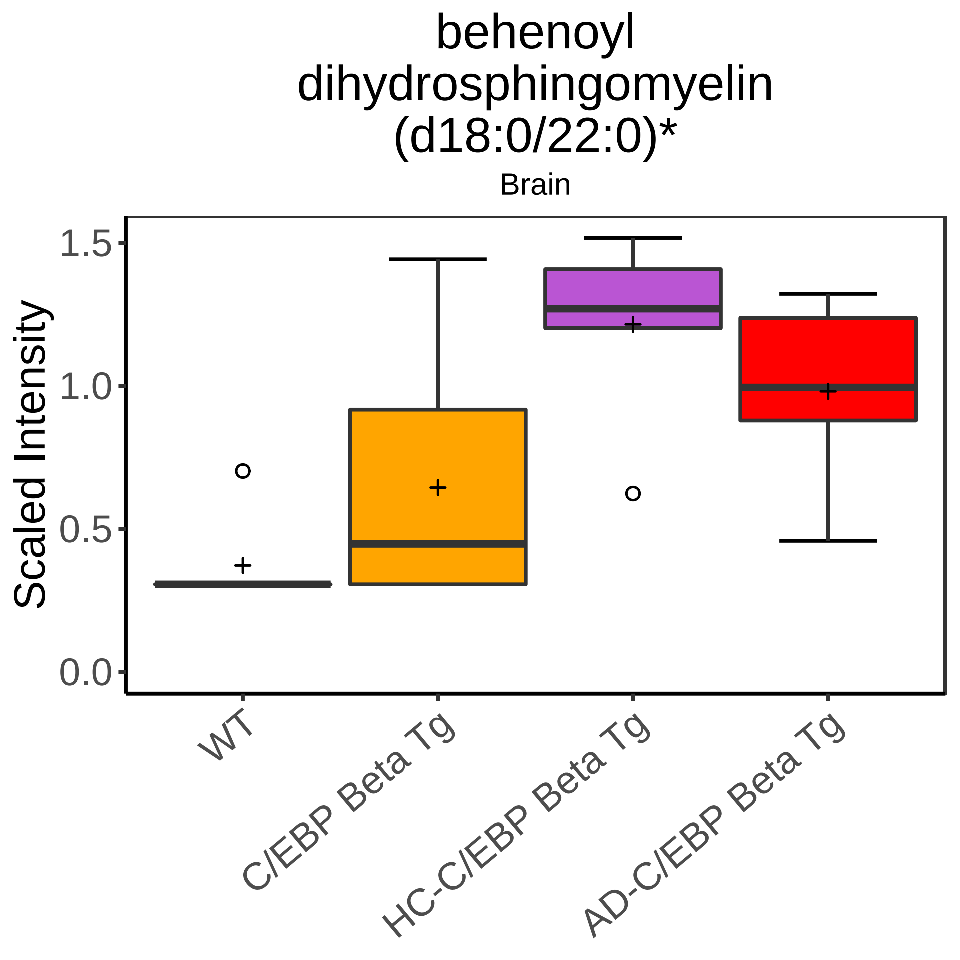

behenoyl
dihydrosphingomyelin
(d18:0/22:0)*
Brain
Scaled Intensity
1.5
1.0
0.5
0.0
WT
C/EBP Beta Tg
HC-C/EBP Beta Tg
AD-C/EBP Beta Tg

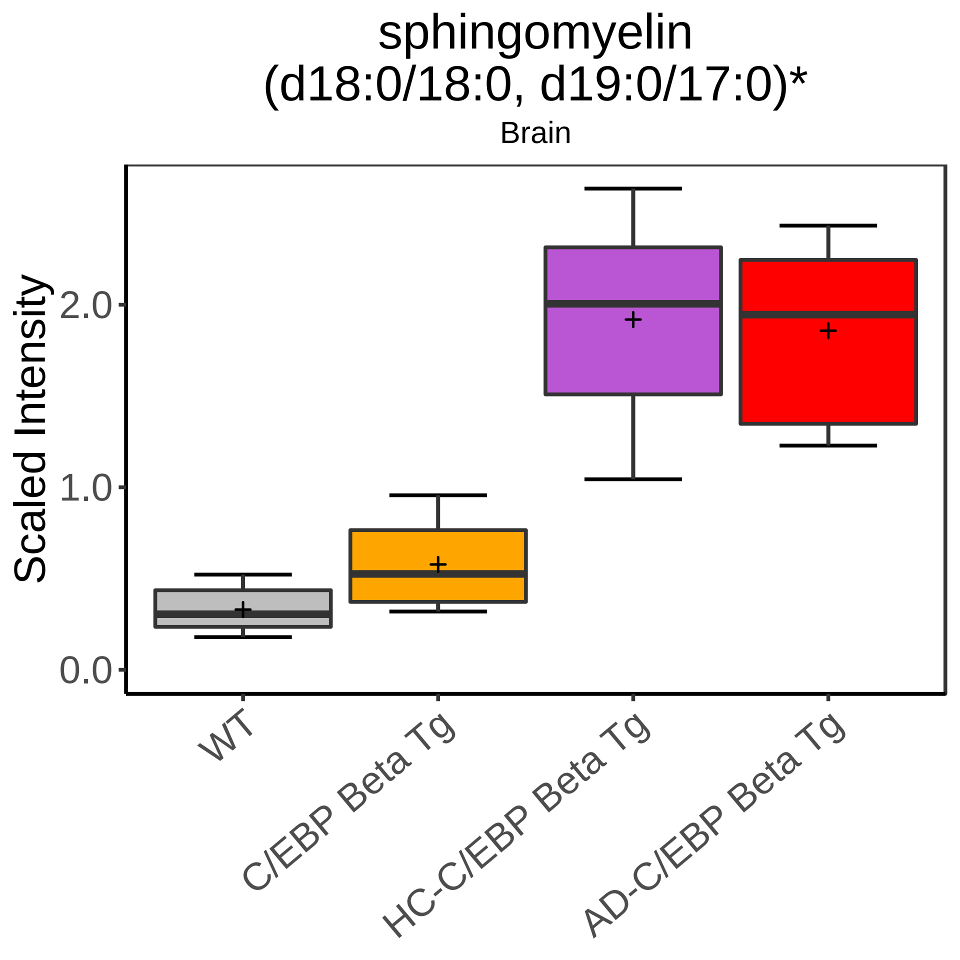
sphingomyelin
(d18:0/18:0, d19:0/17:0)*
Brain
Scaled Intensity
2.0
1.0
0.0
WT
C/EBP Beta Tg
HC-C/EBP Beta Tg
AD-C/EBP Beta Tg

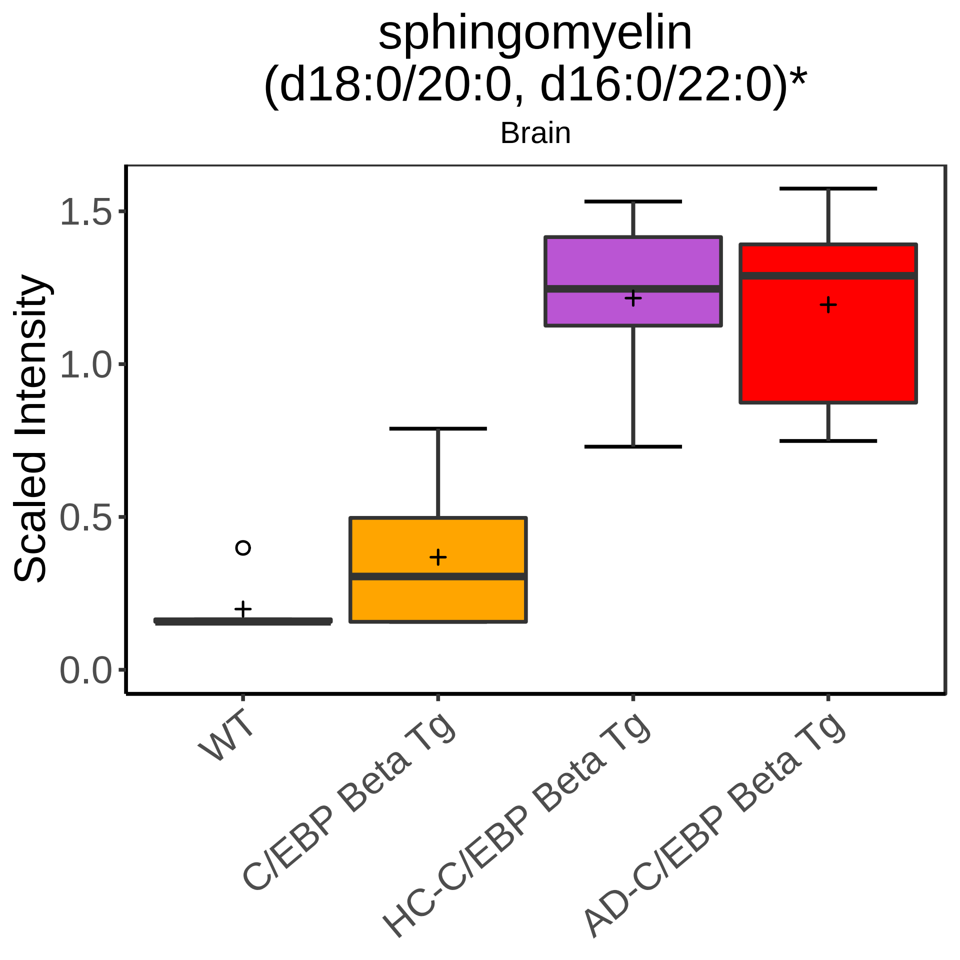
sphingomyelin
(d18:0/20:0, d16:0/22:0)*
Brain
Scaled Intensity
0.0
0.5
1.0
1.5
WT
C/EBP Beta Tg
HC-C/EBP Beta Tg
AD-C/EBP Beta Tg

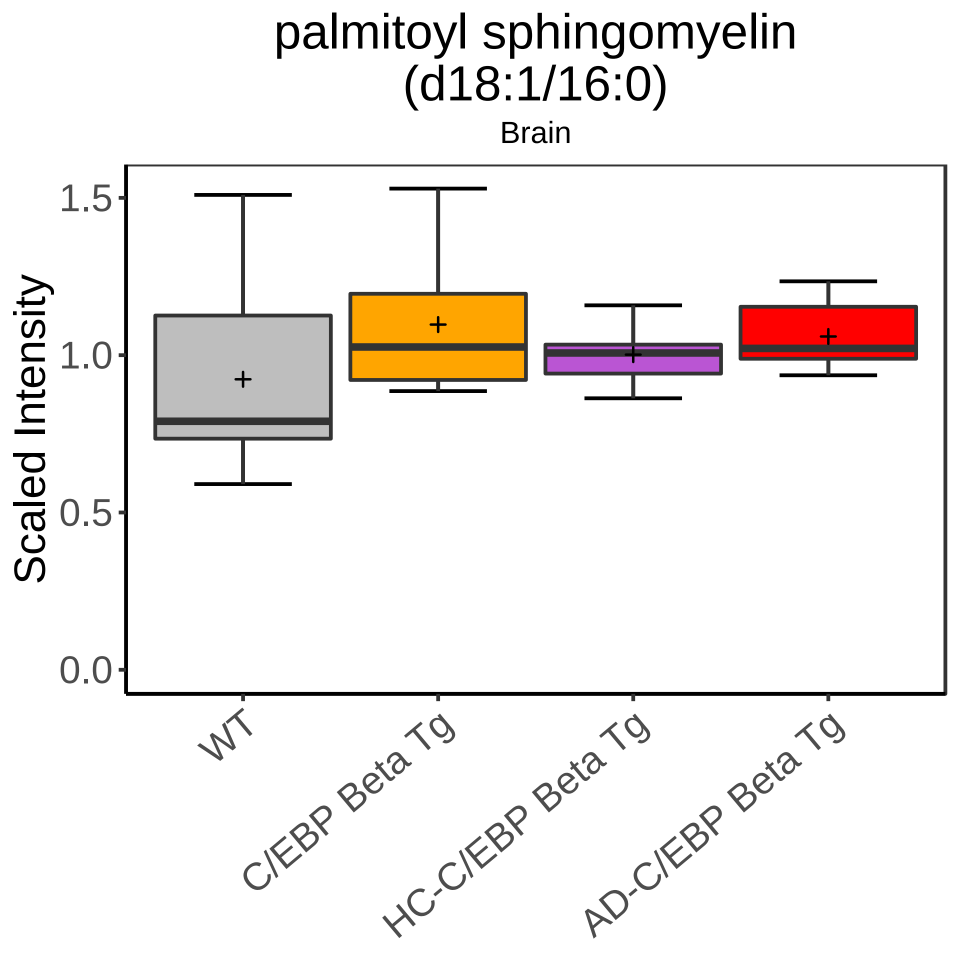
palmitoyl sphingomyelin
(d18:1/16:0)
Brain
Scaled Intensity
1.5
1.0
0.5
0.0
WT
C/EBP Beta Tg
HC-C/EBP Beta Tg
AD-C/EBP Beta Tg

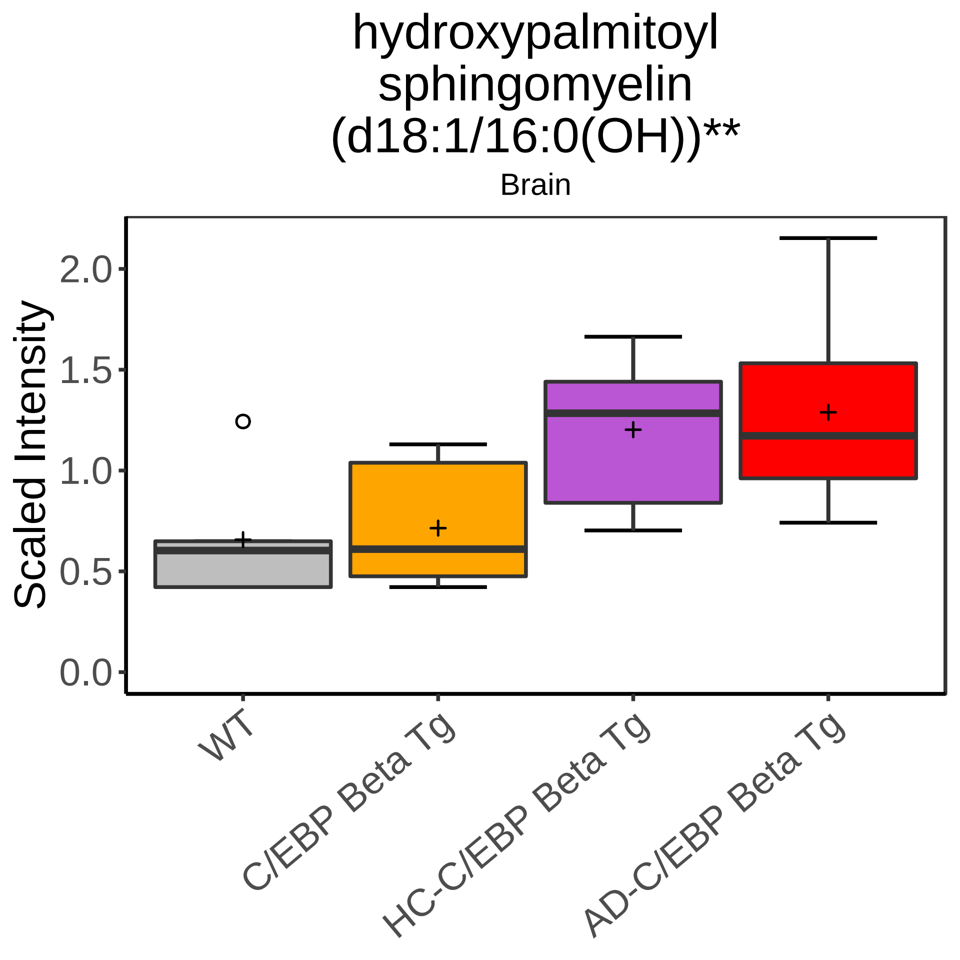

hydroxypalmitoyl sphingomyelin (d18:1/16:0(OH))**
Brain
Scaled Intensity
2.0
1.5
1.0
0.5
0.0
WT
C/EBP Beta Tg
HC-C/EBP Beta Tg
AD-C/EBP Beta Tg

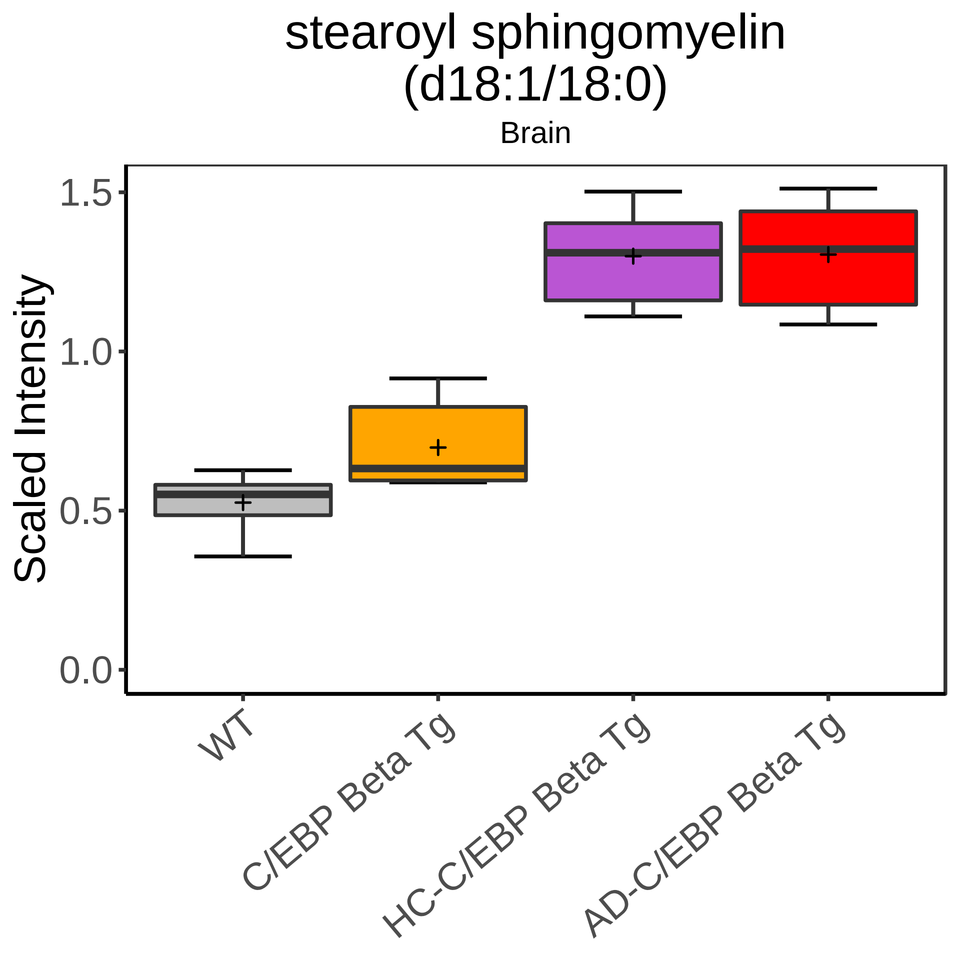
stearoyl sphingomyelin
(d18:1/18:0)
Brain
Scaled Intensity
1.5
1.0
0.5
0.0
WT
C/EBP Beta Tg
HC-C/EBP Beta Tg
AD-C/EBP Beta Tg

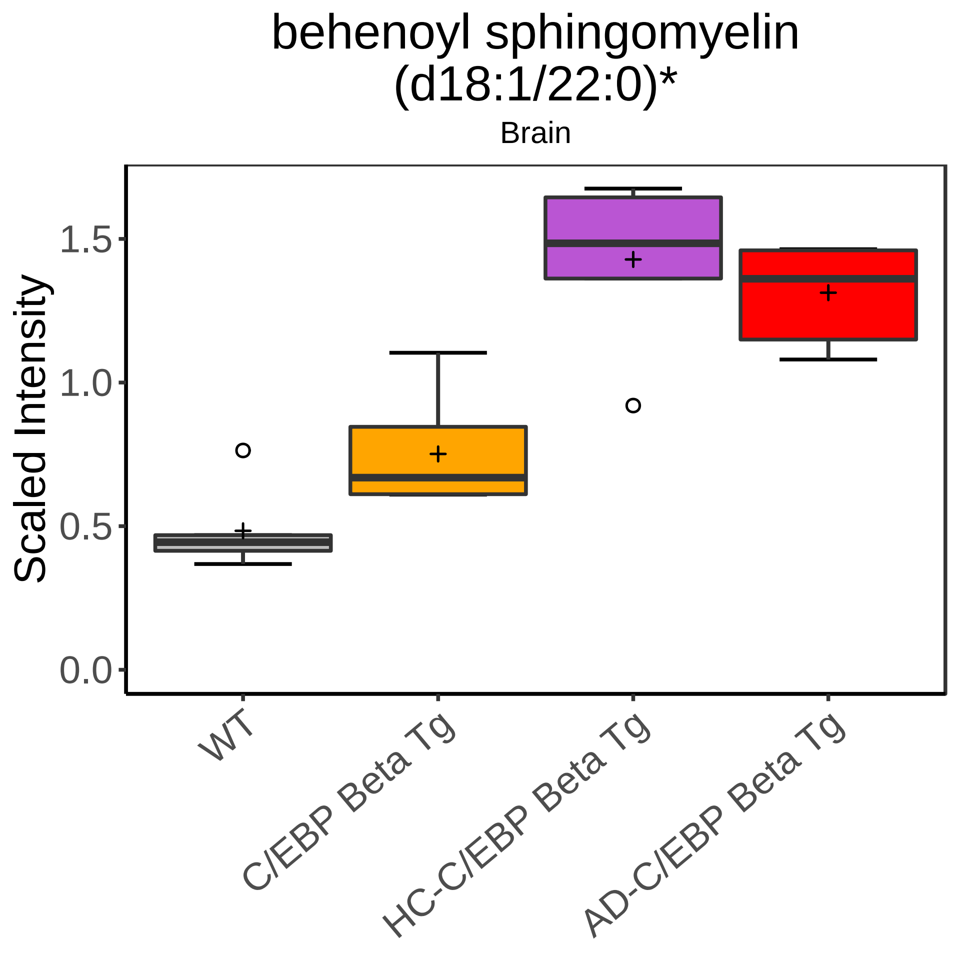

behenoyl sphingomyelin
(d18:1/22:0)*
Brain
Scaled Intensity
1.5
1.0
0.5
0.0
WT
C/EBP Beta Tg
HC-C/EBP Beta Tg
AD-C/EBP Beta Tg

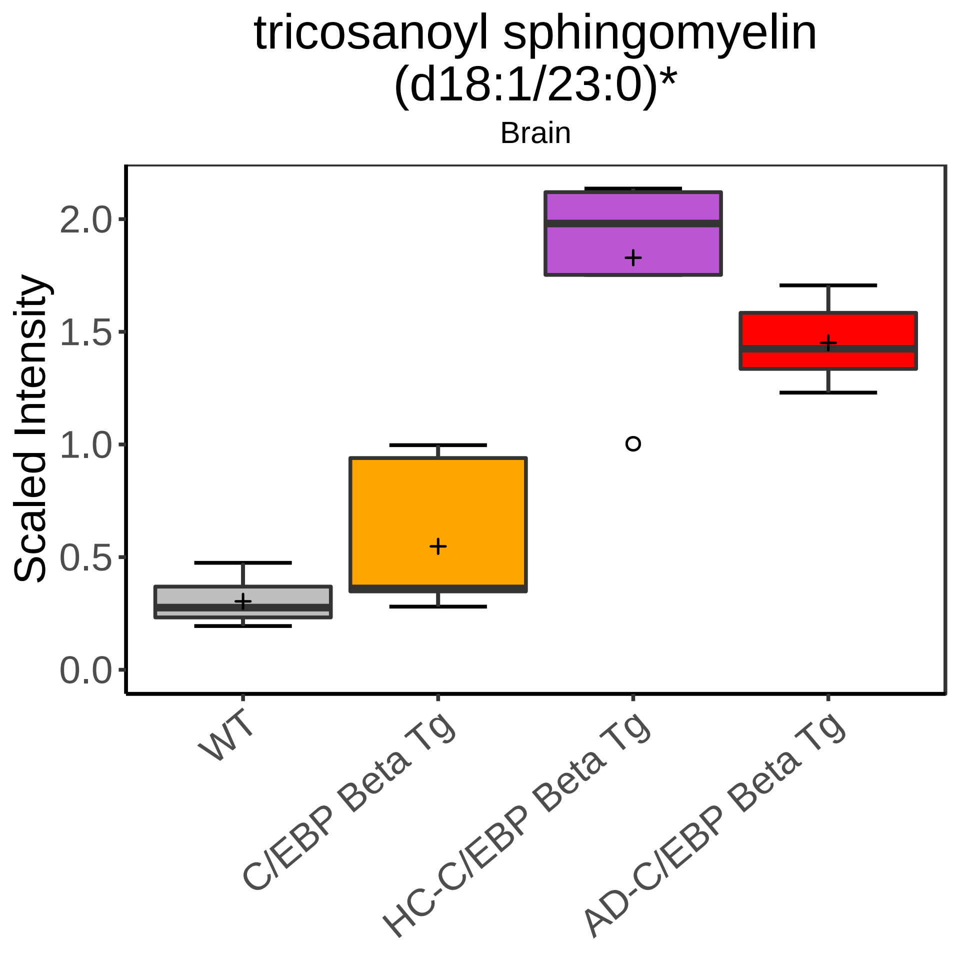

tricosanoyl sphingomyelin
(d18:1/23:0)*
Brain
Scaled Intensity
2.0
1.5
1.0
0.5
0.0
WT
C/EBP Beta Tg
HC-C/EBP Beta Tg
AD-C/EBP Beta Tg

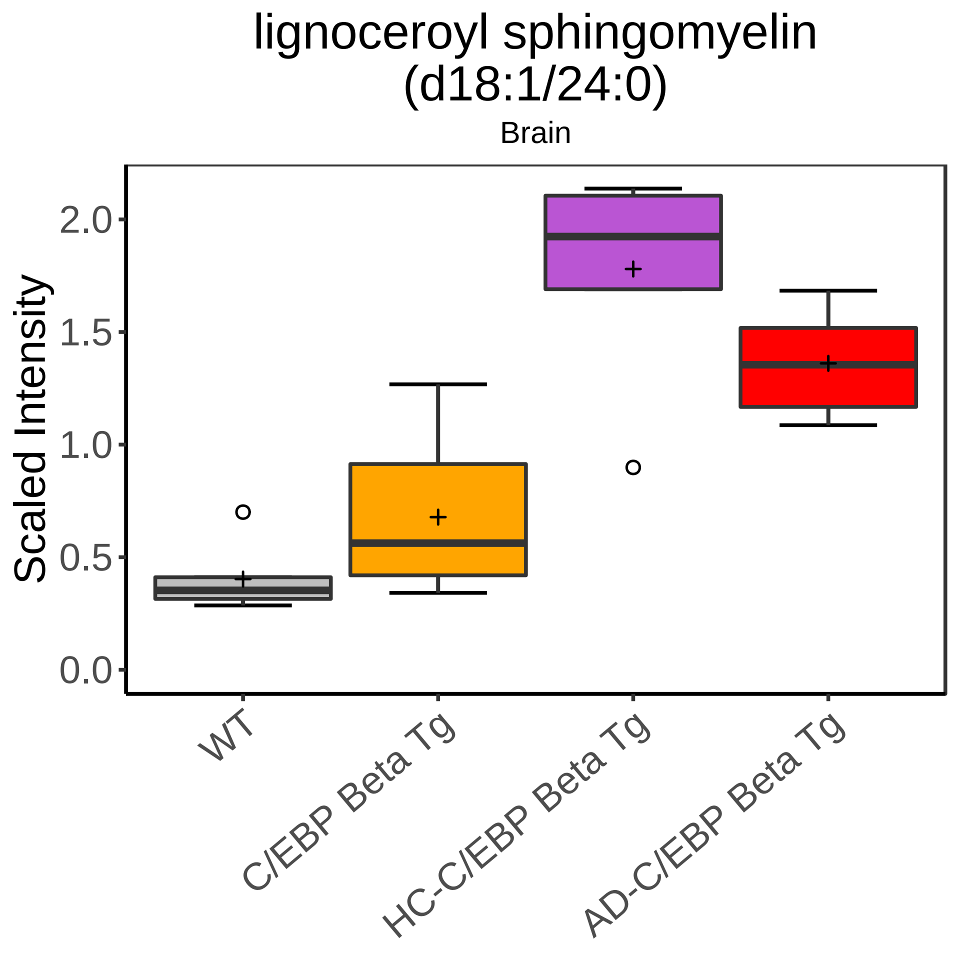

lignoceroyl sphingomyelin
(d18:1/24:0)
Brain
Scaled Intensity
2.0
1.5
1.0
0.5
0.0
WT
C/EBP Beta Tg
HC-C/EBP Beta Tg
AD-C/EBP Beta Tg

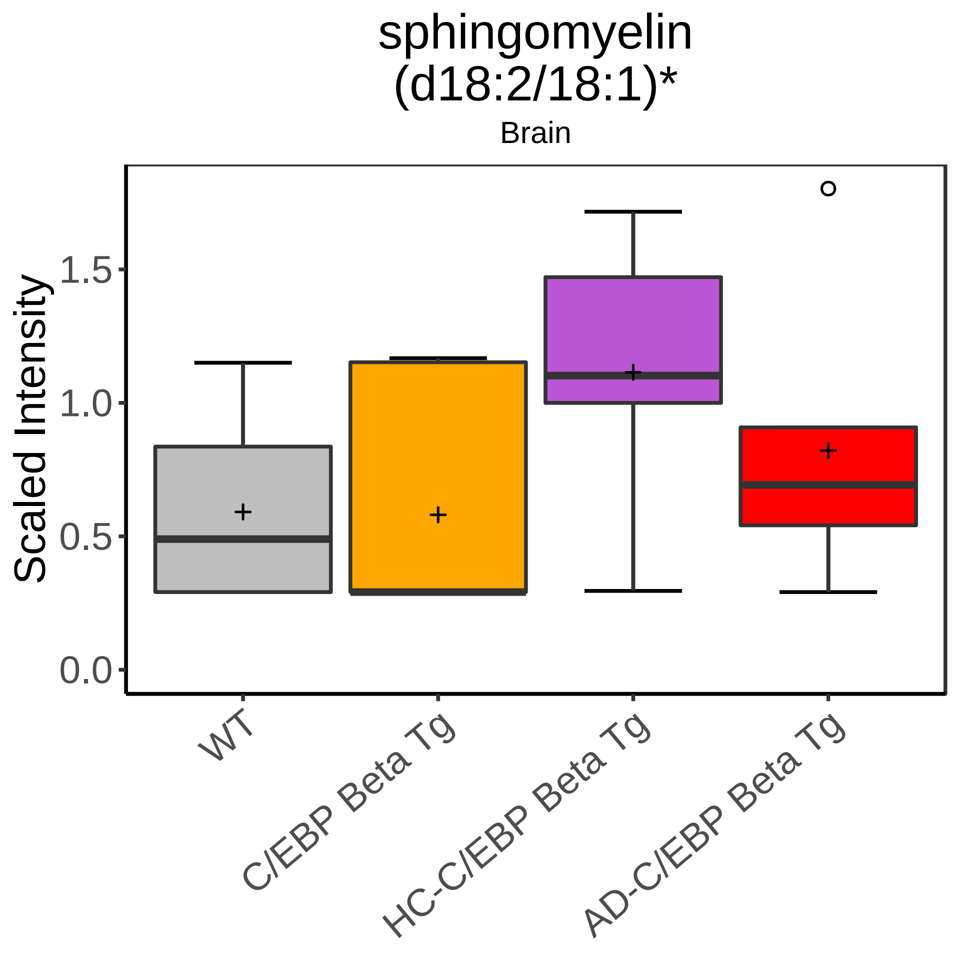
sphingomyelin
(d18:2/18:1)*
Brain
Scaled Intensity
0.0
0.5
1.0
1.5
WT
C/EBP Beta Tg
HC-C/EBP Beta Tg
AD-C/EBP Beta Tg

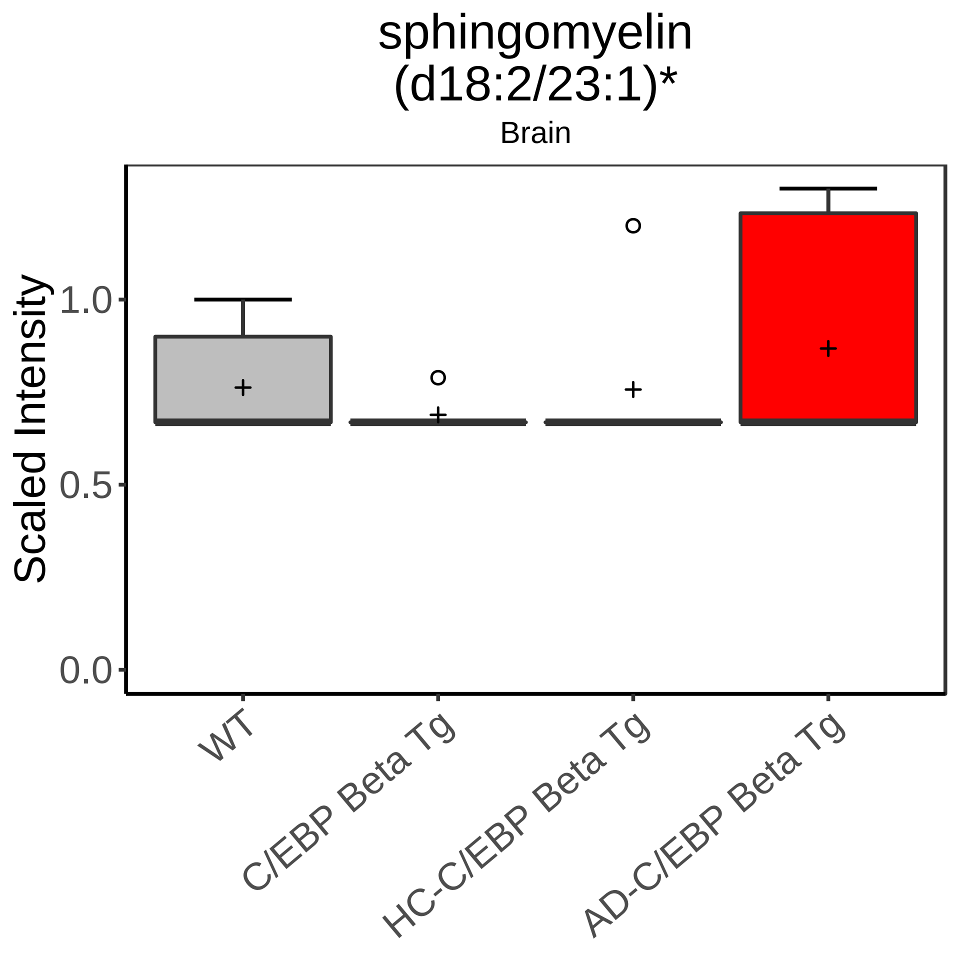
sphingomyelin
(d18:2/23:1)*
Brain
Scaled Intensity
0.0
0.5
1.0
WT
C/EBP Beta Tg
HC-C/EBP Beta Tg
AD-C/EBP Beta Tg

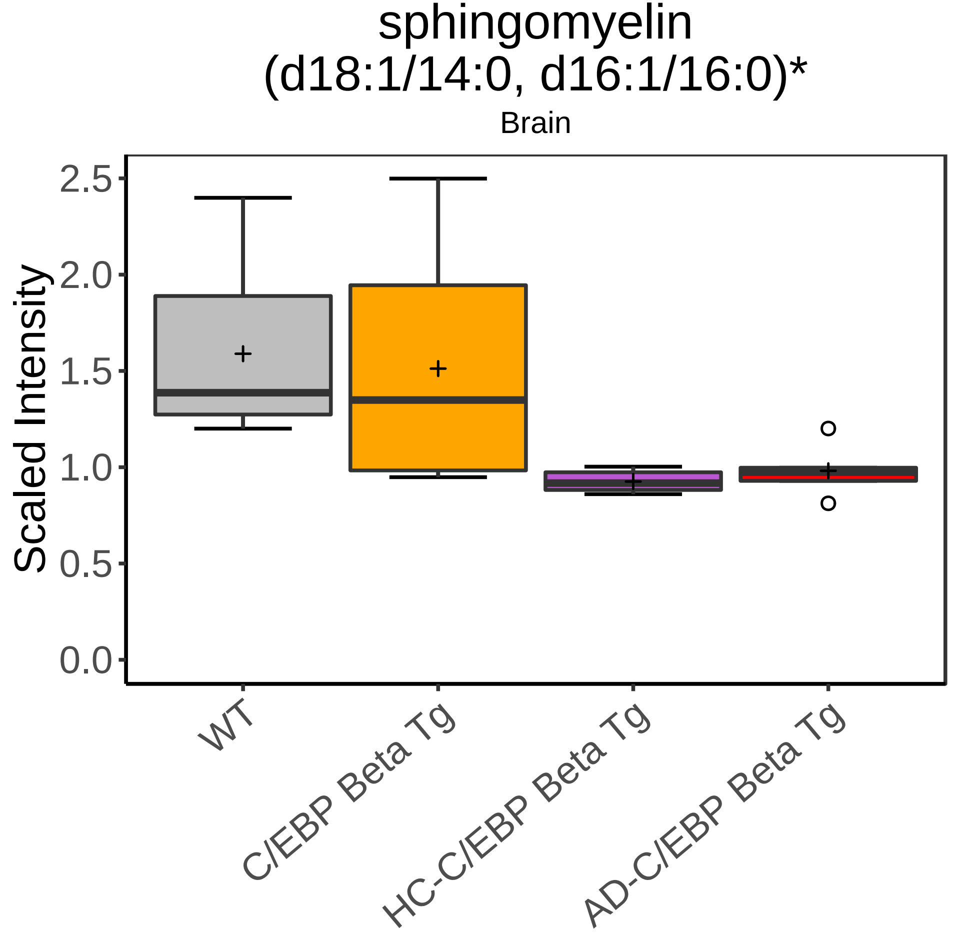
sphingomyelin
(d18:1/14:0, d16:1/16:0)*
Brain
Scaled Intensity
0.0
0.5
1.0
1.5
2.0
2.5
WT
C/EBP Beta Tg
HC-C/EBP Beta Tg
AD-C/EBP Beta Tg

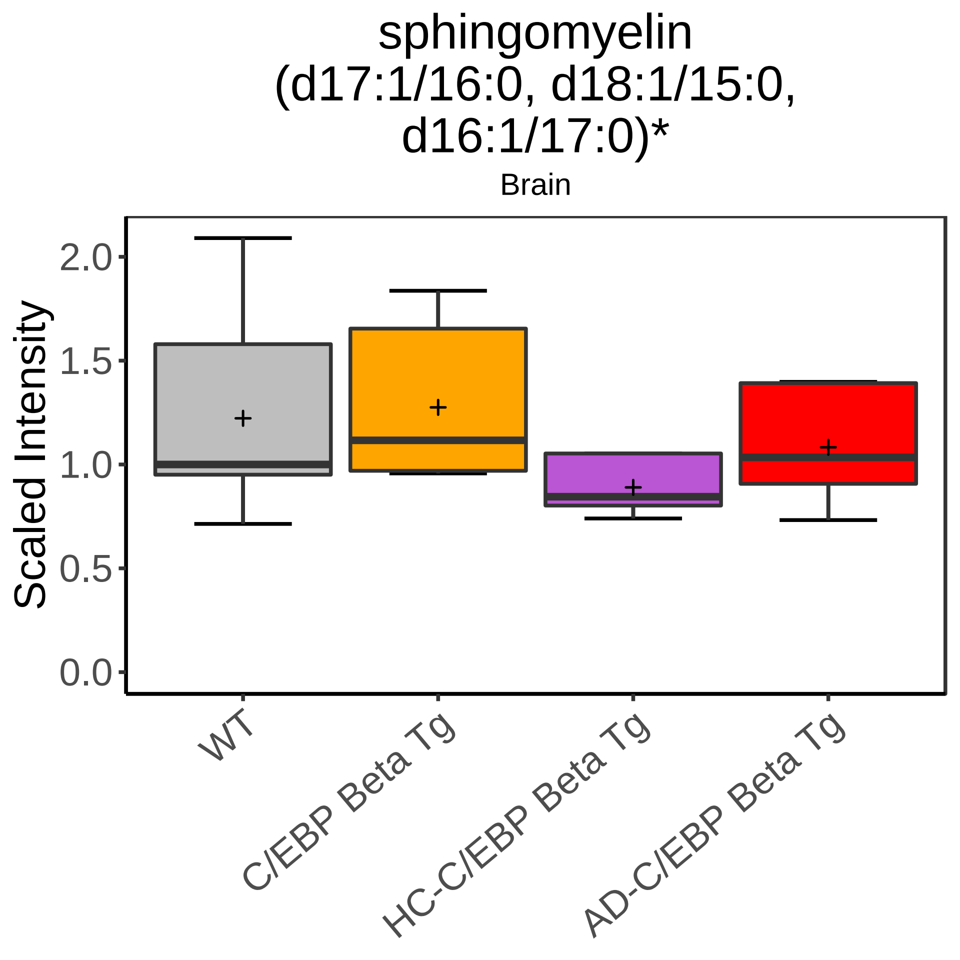
sphingomyelin
(d17:1/16:0, d18:1/15:0,
d16:1/17:0)*
Brain
Scaled Intensity
2.0
1.5
1.0
0.5
0.0
WT
C/EBP Beta Tg
HC-C/EBP Beta Tg
AD-C/EBP Beta Tg

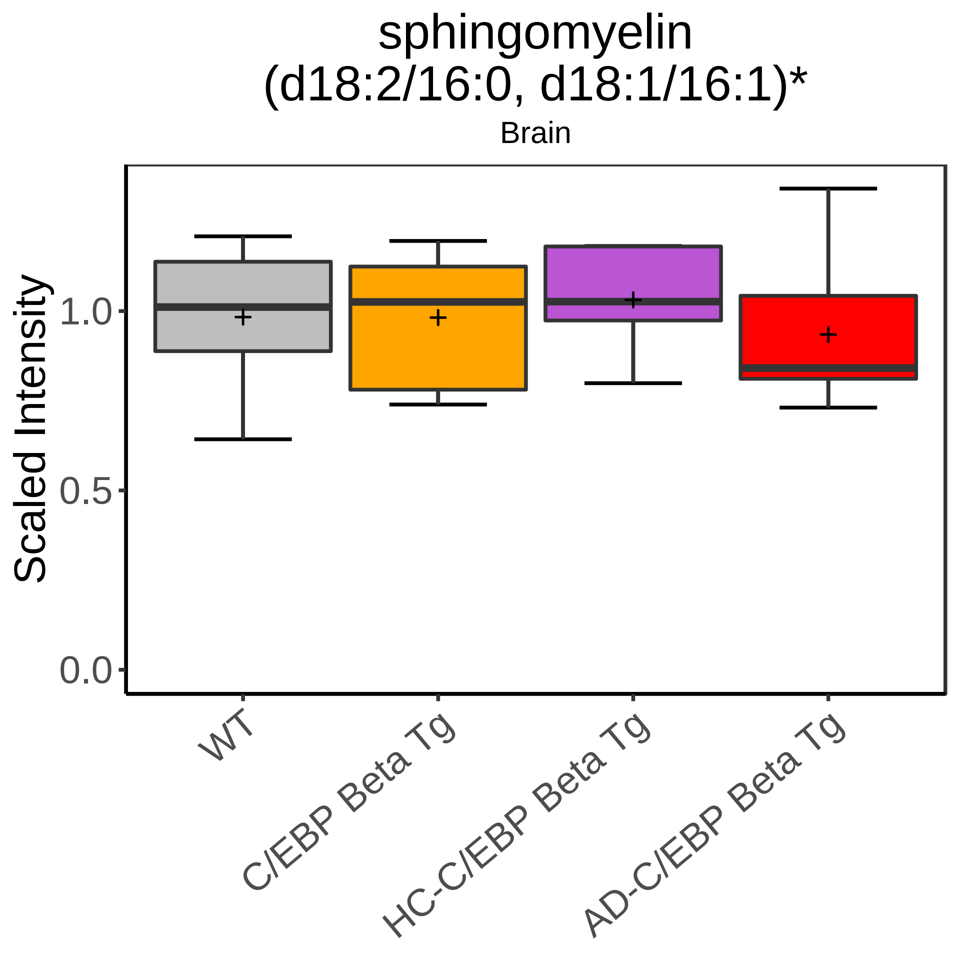
sphingomyelin
(d18:2/16:0, d18:1/16:1)*
Brain
Scaled Intensity
0.0
0.5
1.0
WT
C/EBP Beta Tg
HC-C/EBP Beta Tg
AD-C/EBP Beta Tg

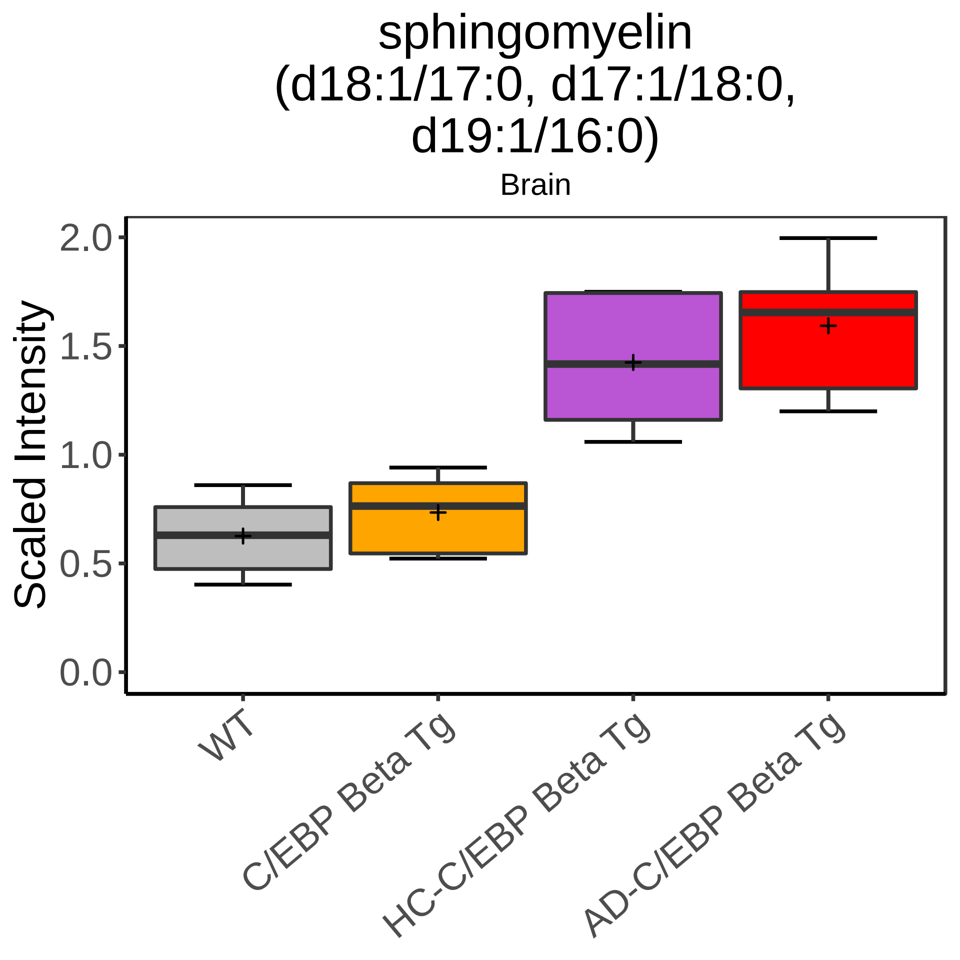
sphingomyelin
(d18:1/17:0, d17:1/18:0,
d19:1/16:0)
Brain
Scaled Intensity
2.0
1.5
1.0
0.5
0.0
WT
C/EBP Beta Tg
HC-C/EBP Beta Tg
AD-C/EBP Beta Tg

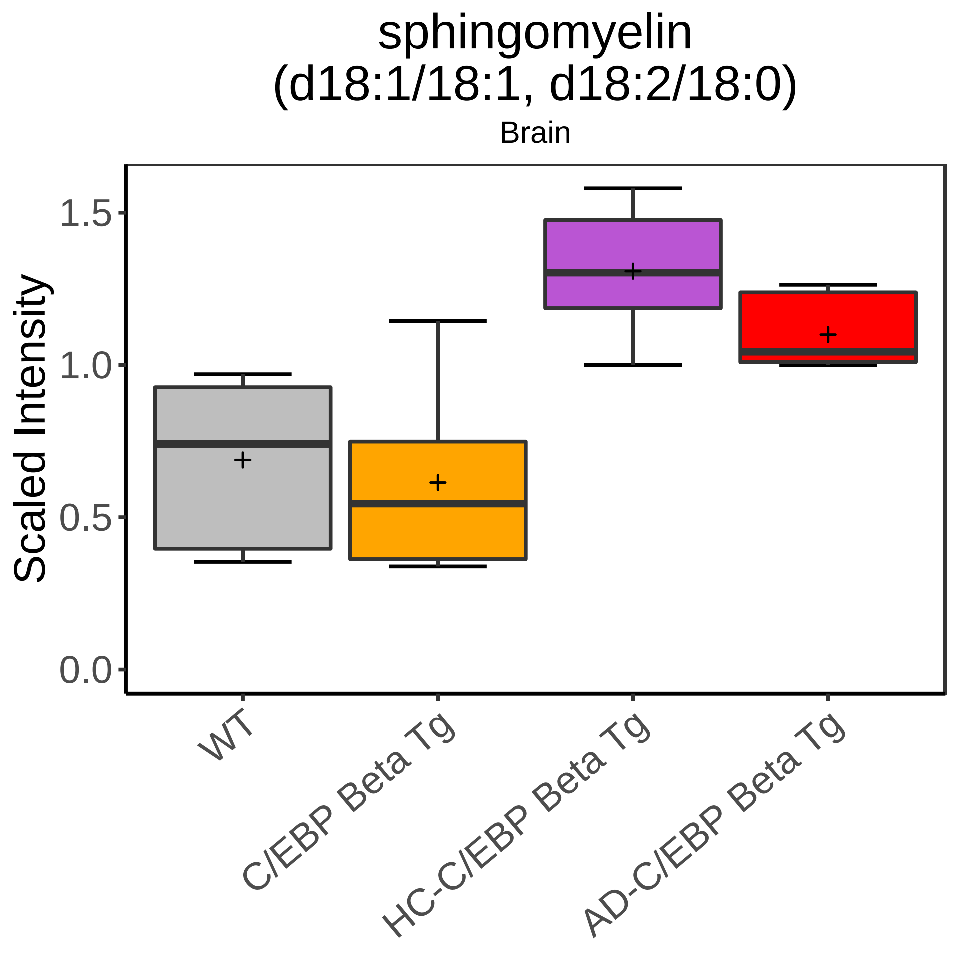
sphingomyelin
(d18:1/18:1, d18:2/18:0)
Brain
Scaled Intensity
1.5
1.0
0.5
0.0
WT
C/EBP Beta Tg
HC-C/EBP Beta Tg
AD-C/EBP Beta Tg

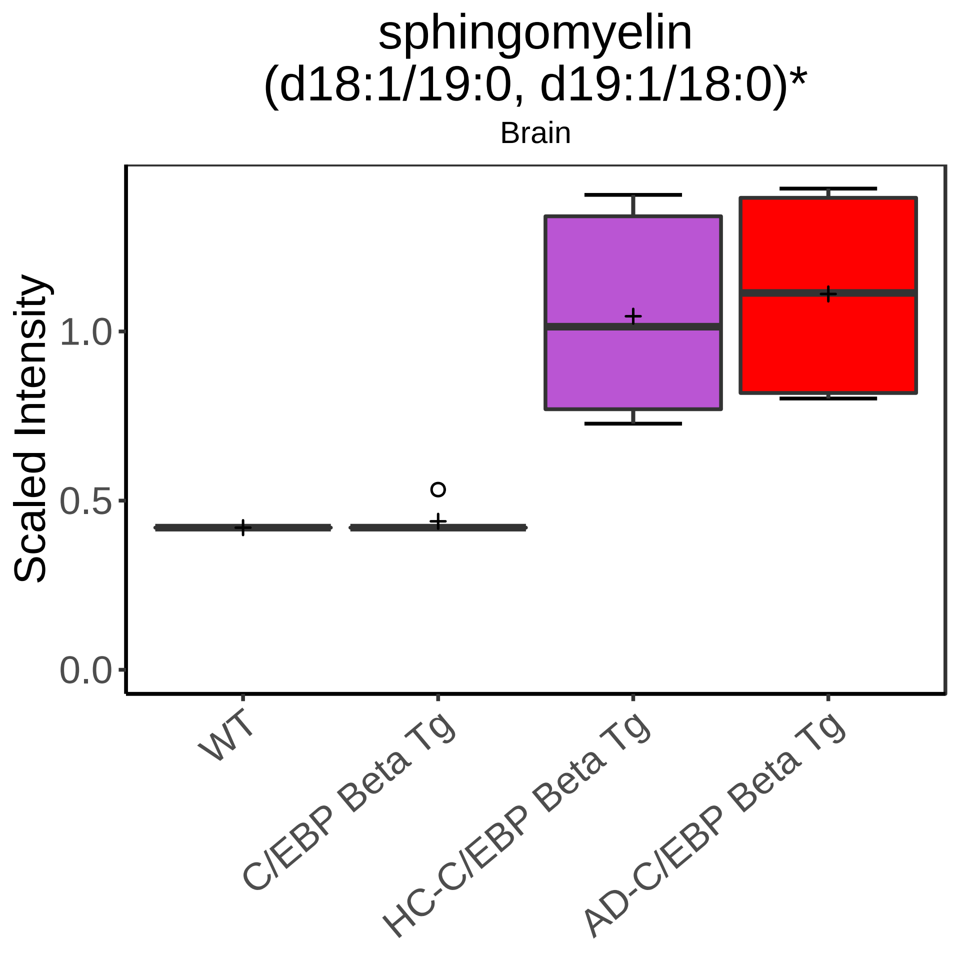
sphingomyelin
(d18:1/19:0, d19:1/18:0)*
Brain
Scaled Intensity
0.0
0.5
1.0
WT
C/EBP Beta Tg
HC-C/EBP Beta Tg
AD-C/EBP Beta Tg

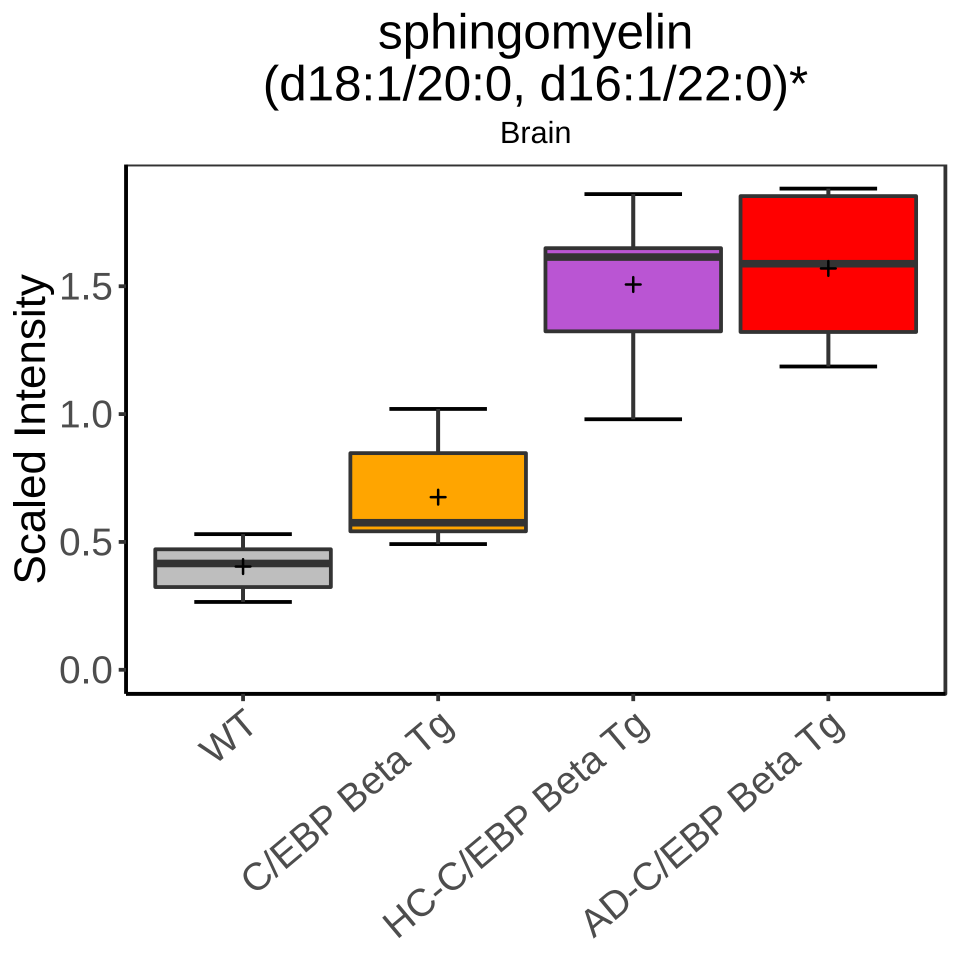

sphingomyelin
(d18:1/20:0, d16:1/22:0)*
Brain
Scaled Intensity
0.0
0.5
1.0
1.5
WT
C/EBP Beta Tg
HC-C/EBP Beta Tg
AD-C/EBP Beta Tg

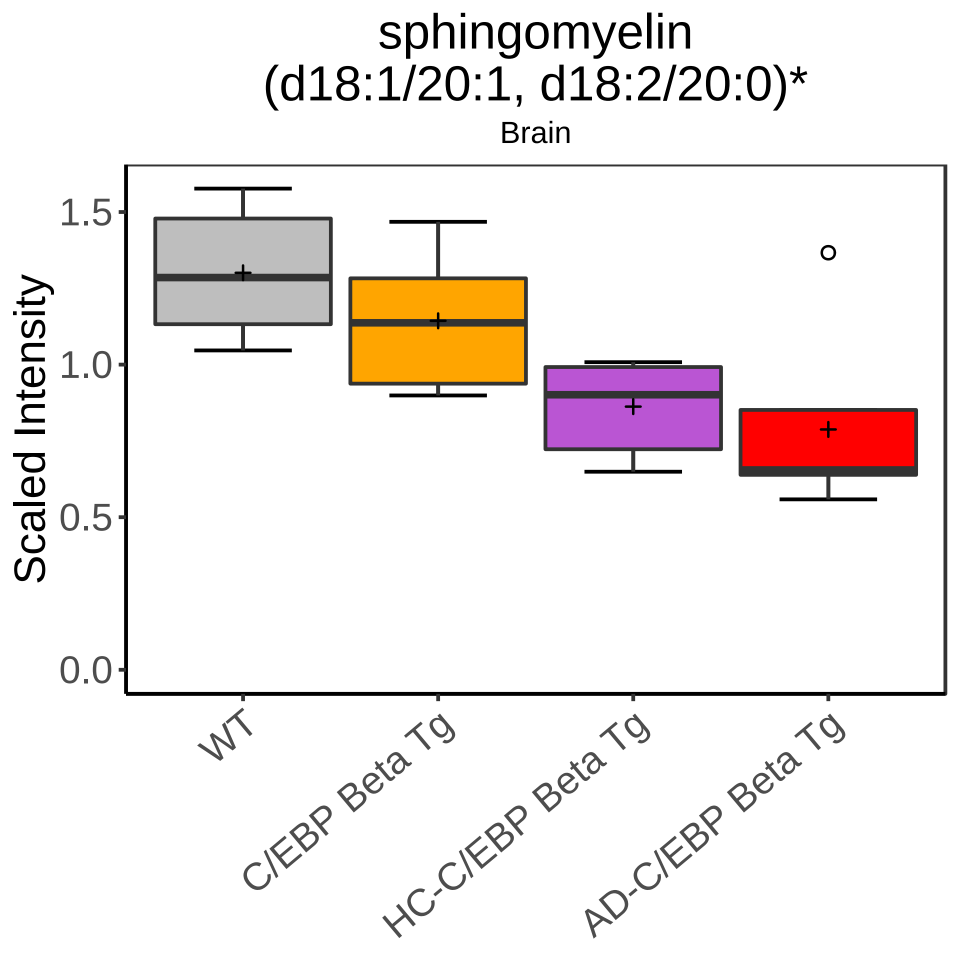
sphingomyelin
(d18:1/20:1, d18:2/20:0)*
Brain
Scaled Intensity
0.0
0.5
1.0
1.5
WT
C/EBP Beta Tg
HC-C/EBP Beta Tg
AD-C/EBP Beta Tg

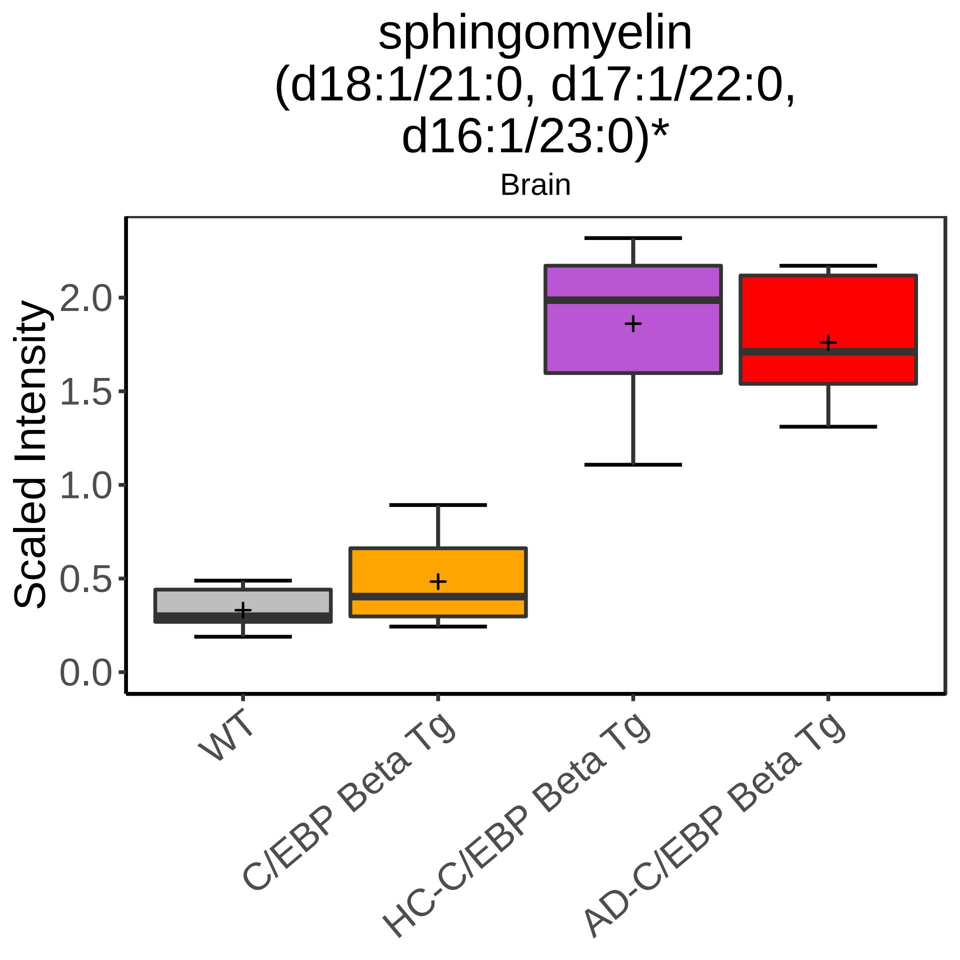
sphingomyelin
(d18:1/21:0, d17:1/22:0,
d16:1/23:0)*
Brain
Scaled Intensity
0.0
0.5
1.0
1.5
2.0
WT
C/EBP Beta Tg
HC-C/EBP Beta Tg
AD-C/EBP Beta Tg

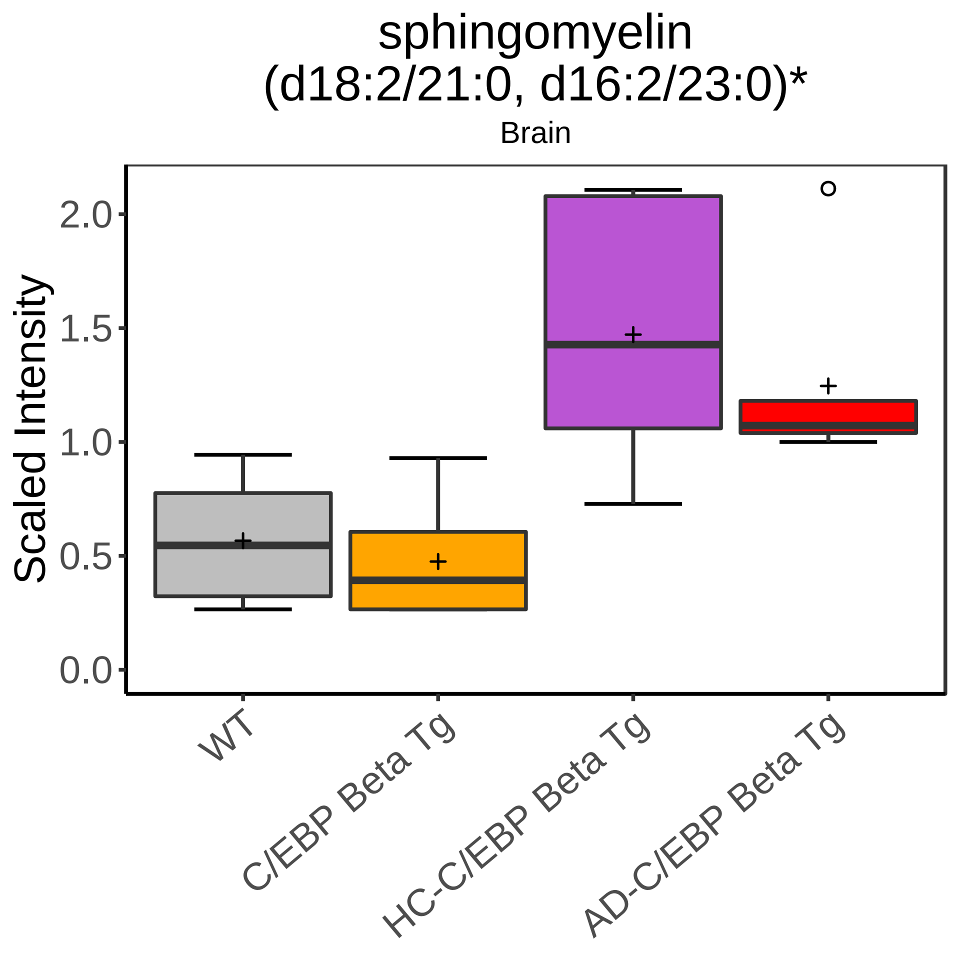
sphingomyelin
(d18:2/21:0, d16:2/23:0)*
Brain
Scaled Intensity
0.0
0.5
1.0
1.5
2.0
WT
C/EBP Beta Tg
HC-C/EBP Beta Tg
AD-C/EBP Beta Tg

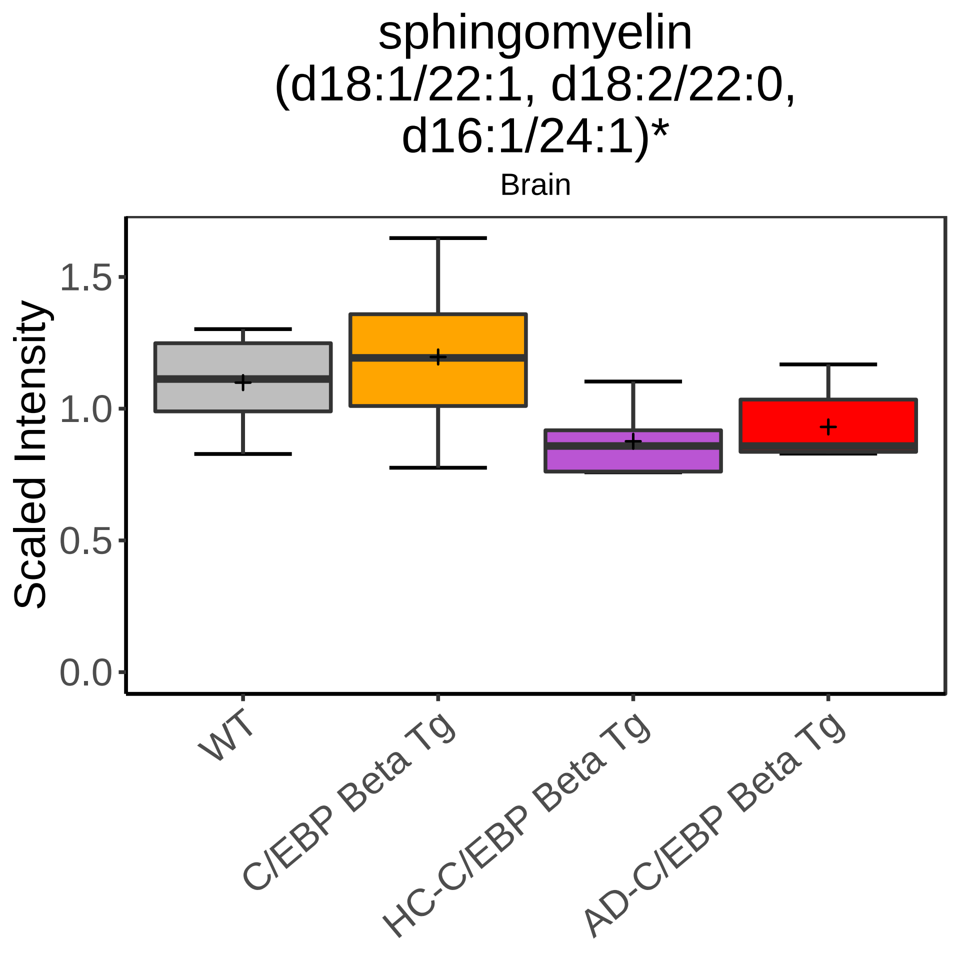
sphingomyelin
(d18:1/22:1, d18:2/22:0,
d16:1/24:1)*
Brain
Scaled Intensity
1.5
1.0
0.5
0.0
WT
C/EBP Beta Tg
HC-C/EBP Beta Tg
AD-C/EBP Beta Tg

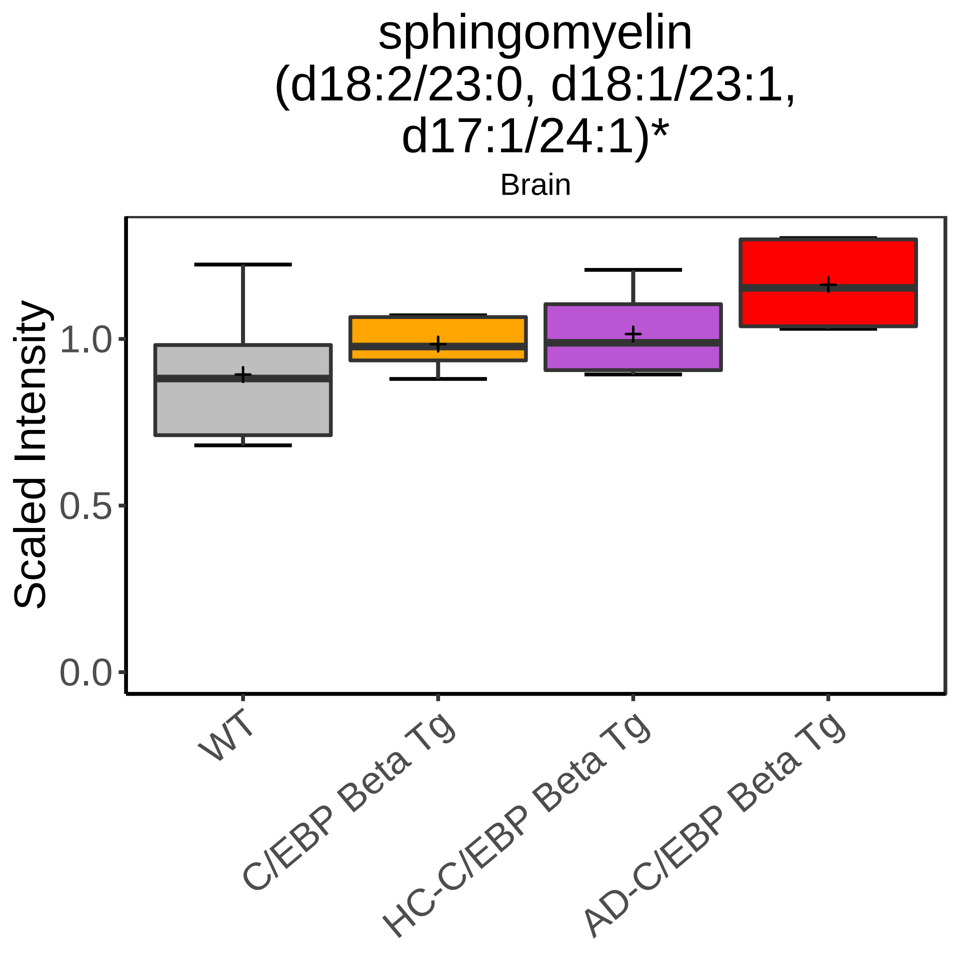
sphingomyelin
(d18:2/23:0, d18:1/23:1,
d17:1/24:1)*
Brain
Scaled Intensity
0.0
0.5
1.0
WT
C/EBP Beta Tg
HC-C/EBP Beta Tg
AD-C/EBP Beta Tg

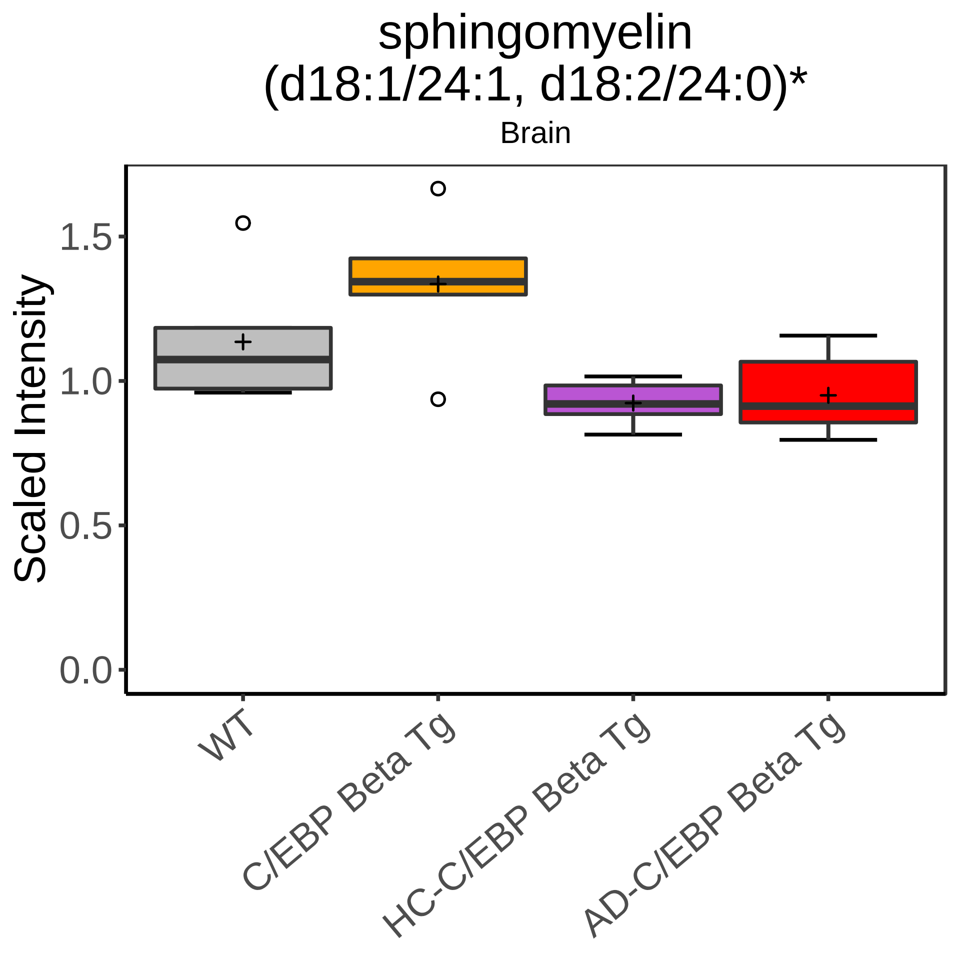

sphingomyelin
(d18:1/24:1, d18:2/24:0)*
Brain
Scaled Intensity
0.0
0.5
1.0
1.5
WT
C/EBP Beta Tg
HC-C/EBP Beta Tg
AD-C/EBP Beta Tg

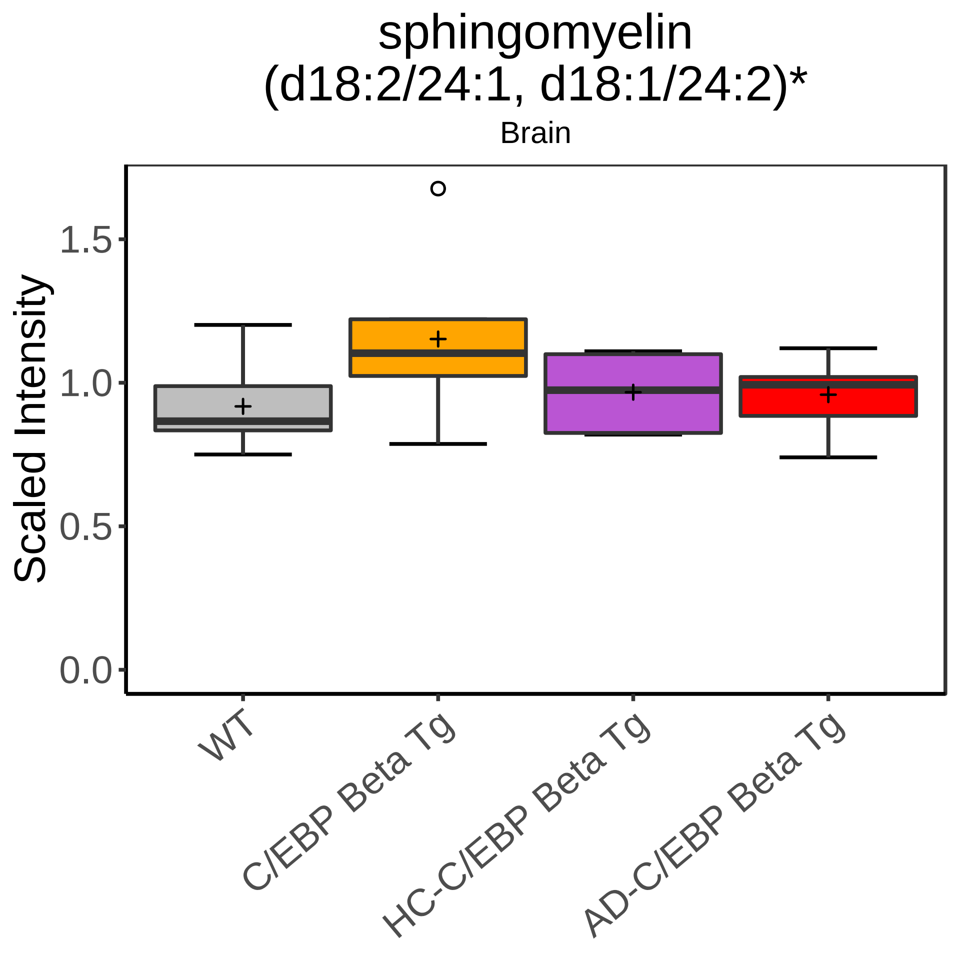
sphingomyelin
(d18:2/24:1, d18:1/24:2)*
Brain
Scaled Intensity
1.5
1.0
0.5
0.0
WT
C/EBP Beta Tg
HC-C/EBP Beta Tg
AD-C/EBP Beta Tg

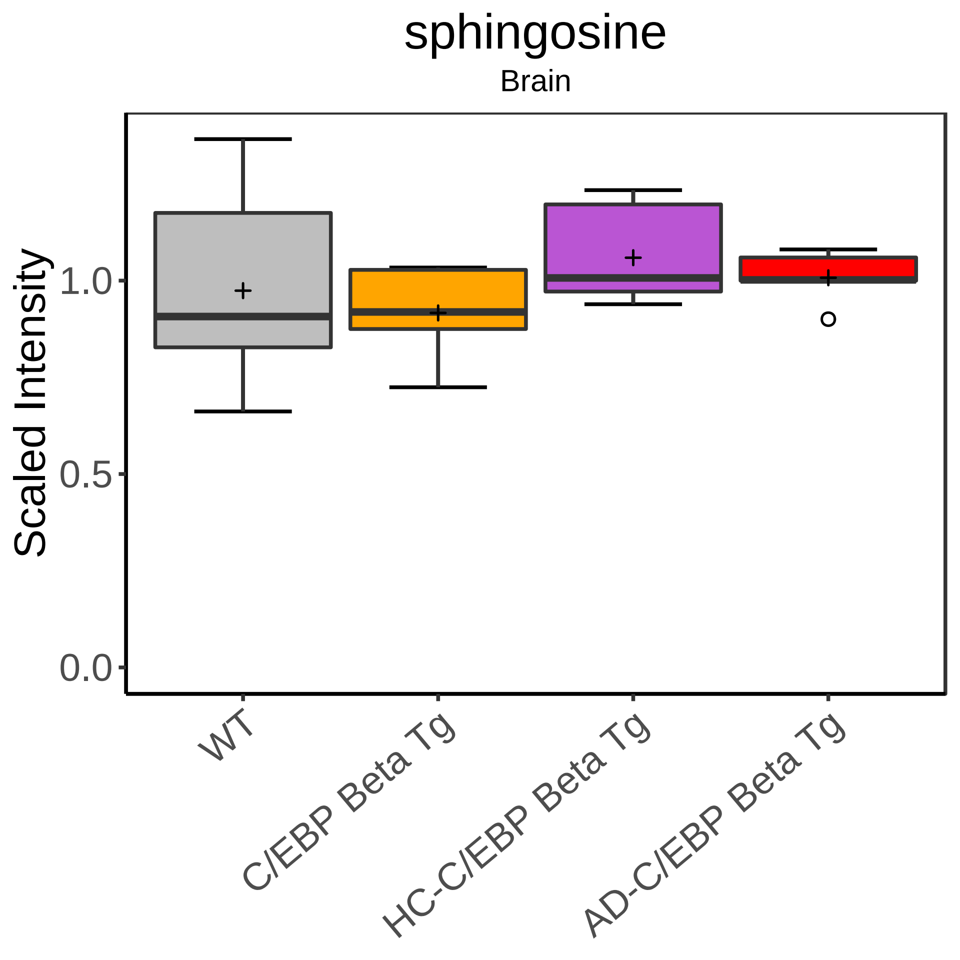
sphingosine
Brain
Scaled Intensity
1.0
0.5
0.0
WT
C/EBP Beta Tg
HC-C/EBP Beta Tg
AD-C/EBP Beta Tg

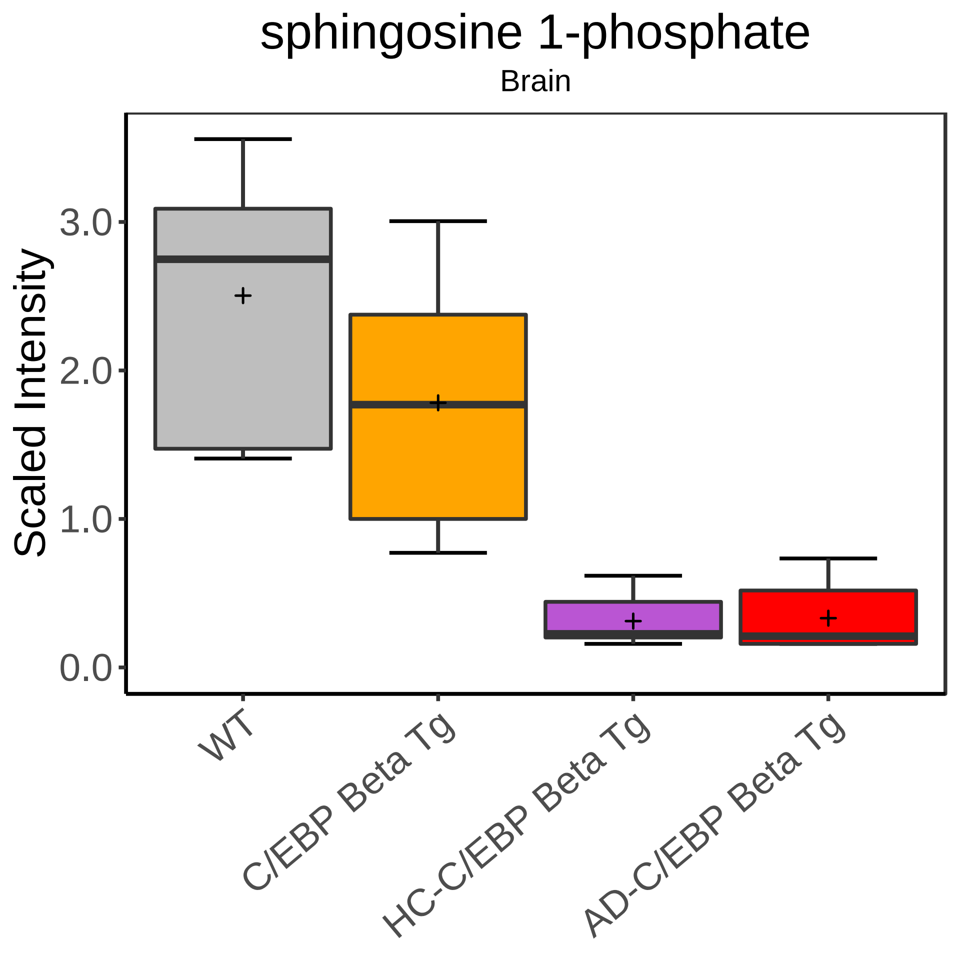
sphingosine 1-phosphate
Brain
Scaled Intensity
3.0
2.0
1.0
0.0
WT
C/EBP Beta Tg
HC-C/EBP Beta Tg
AD-C/EBP Beta Tg

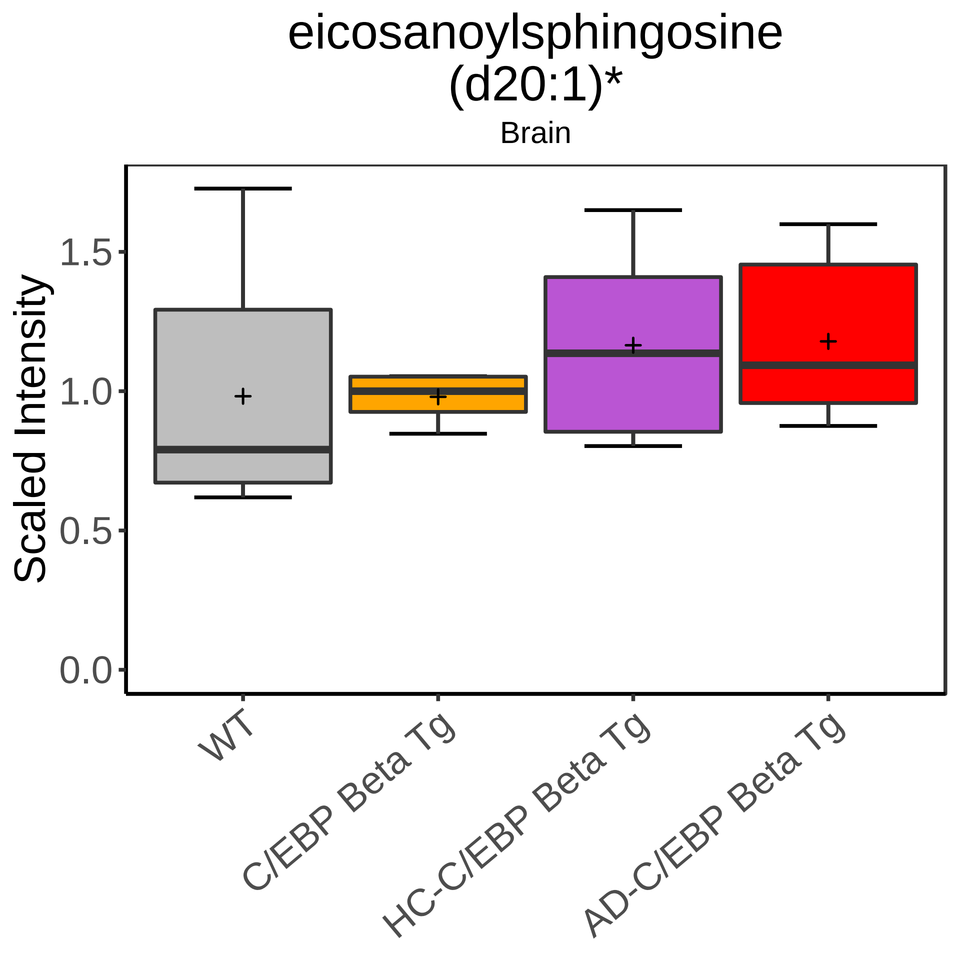
eicosanoylsphingosine
(d20:1)*
Brain
Scaled Intensity
1.5
1.0
0.5
0.0
WT
C/EBP Beta Tg
HC-C/EBP Beta Tg
AD-C/EBP Beta Tg

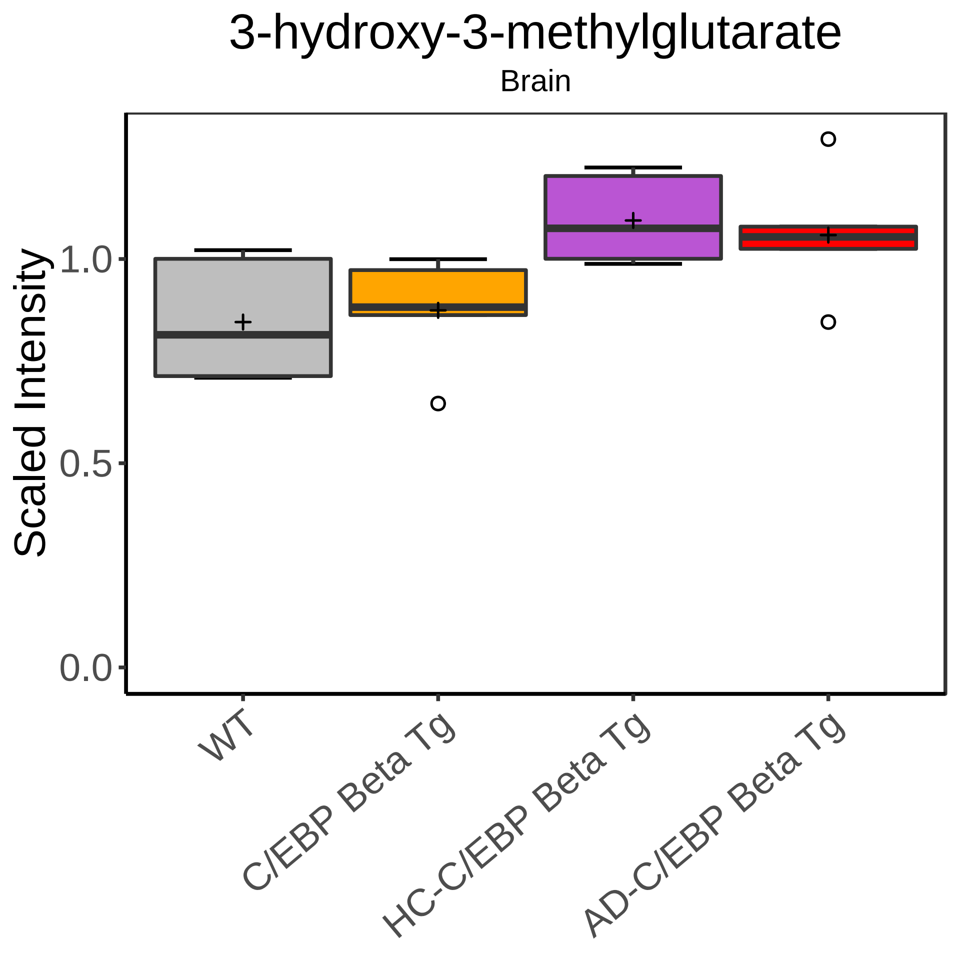
3-hydroxy-3-methylglutarate
Brain
Scaled Intensity
0.0
0.5
1.0
WT
C/EBP Beta Tg
HC-C/EBP Beta Tg
AD-C/EBP Beta Tg

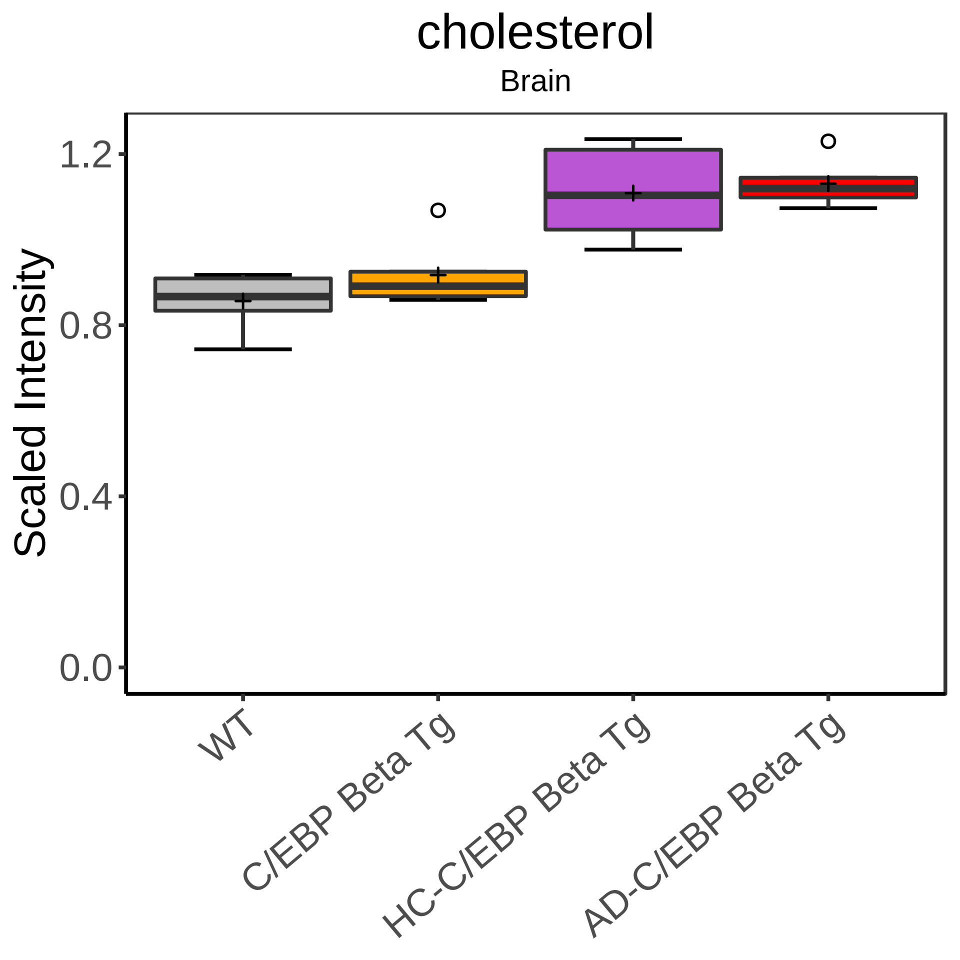
cholesterol
Brain
Scaled Intensity
1.2
0.8
0.4
0.0
WT
C/EBP Beta Tg
HC-C/EBP Beta Tg
AD-C/EBP Beta Tg

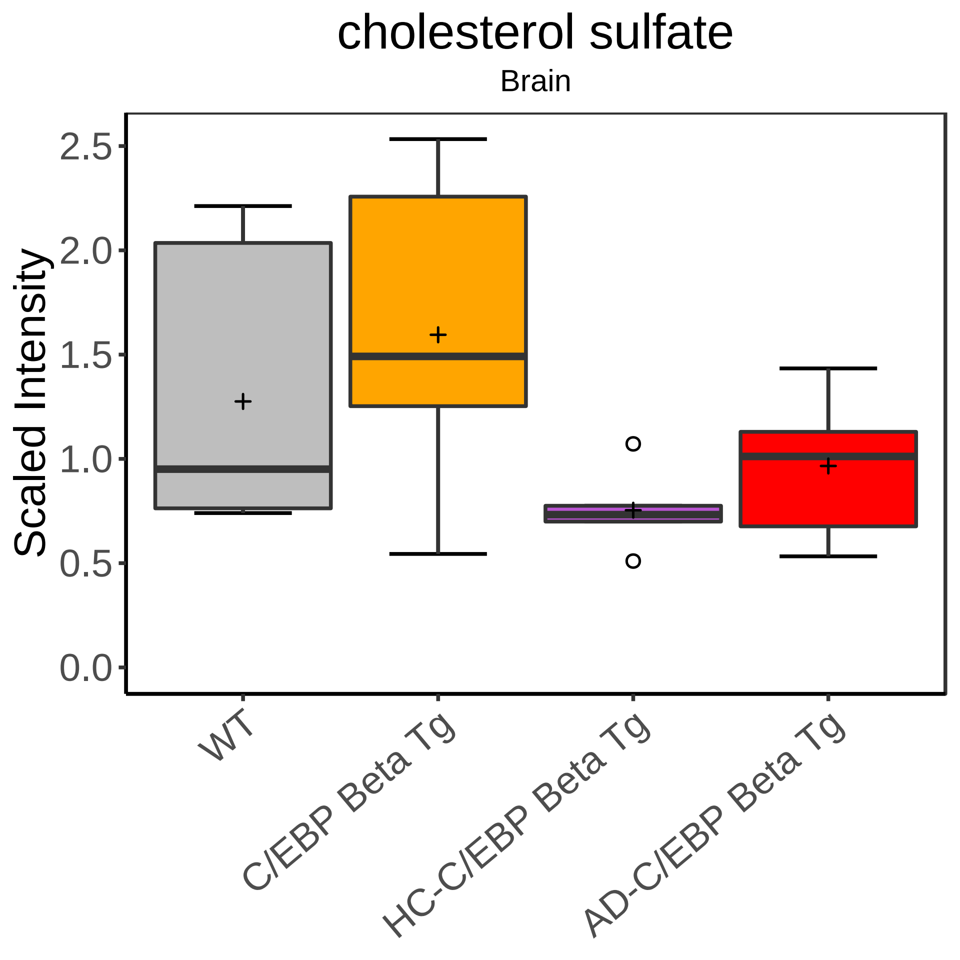

cholesterol sulfate
Brain
Scaled Intensity
2.5
2.0
1.5
1.0
0.5
0.0
WT
C/EBP Beta Tg
HC-C/EBP Beta Tg
AD-C/EBP Beta Tg

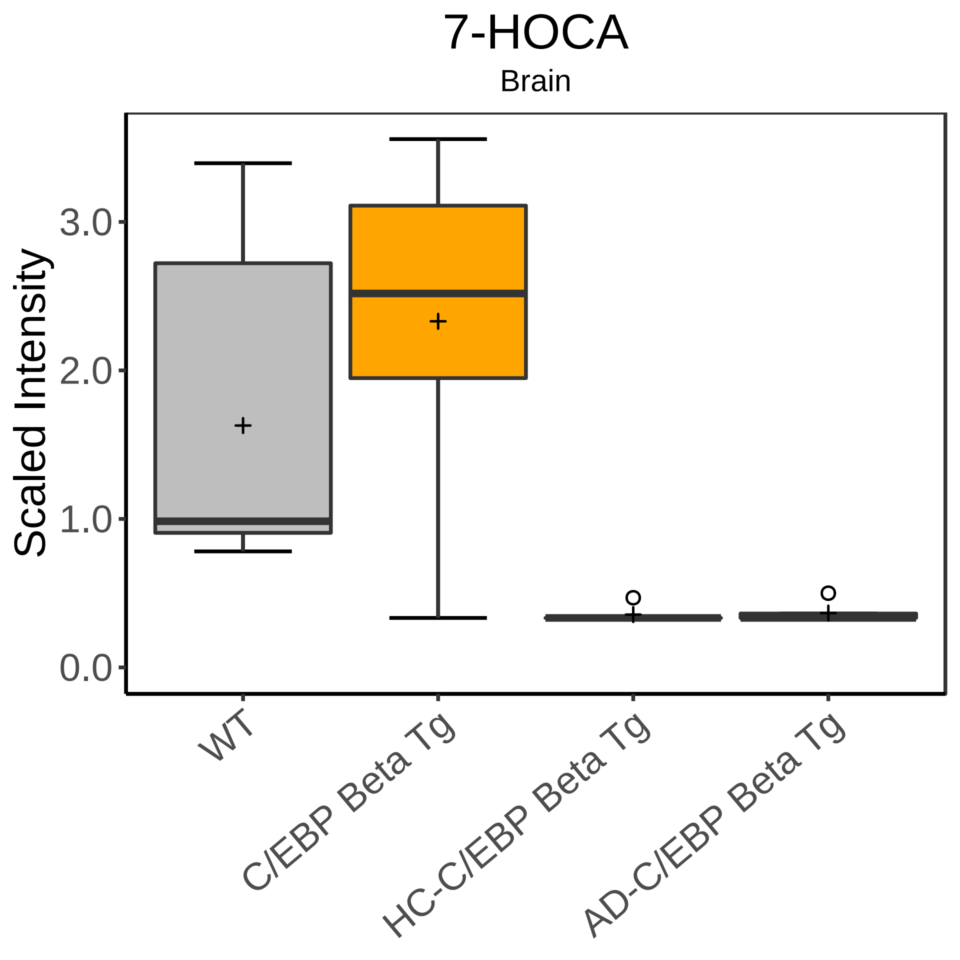

7-HOCA
Brain
Scaled Intensity
0.0
1.0
2.0
3.0
WT
C/EBP Beta Tg
HC-C/EBP Beta Tg
AD-C/EBP Beta Tg

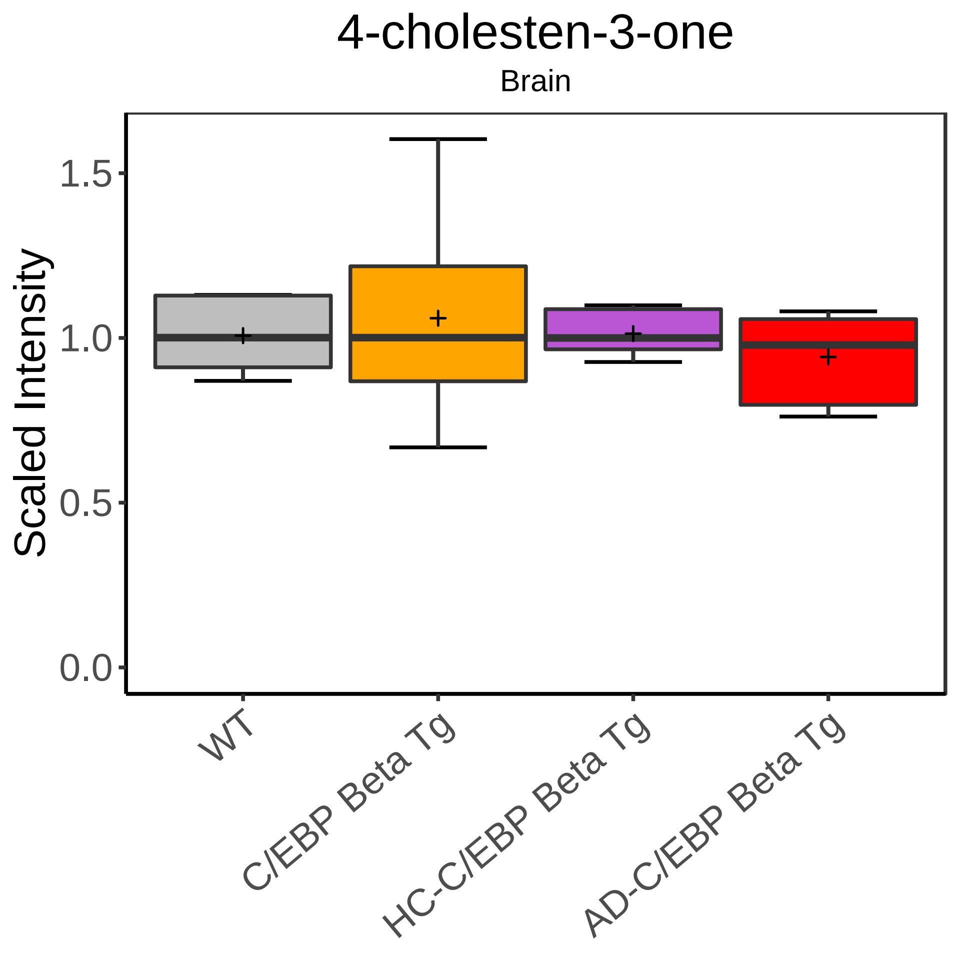
4-cholesten-3-one
Brain
Scaled Intensity
1.5
1.0
0.5
0.0
WT
C/EBP Beta Tg
HC-C/EBP Beta Tg
AD-C/EBP Beta Tg

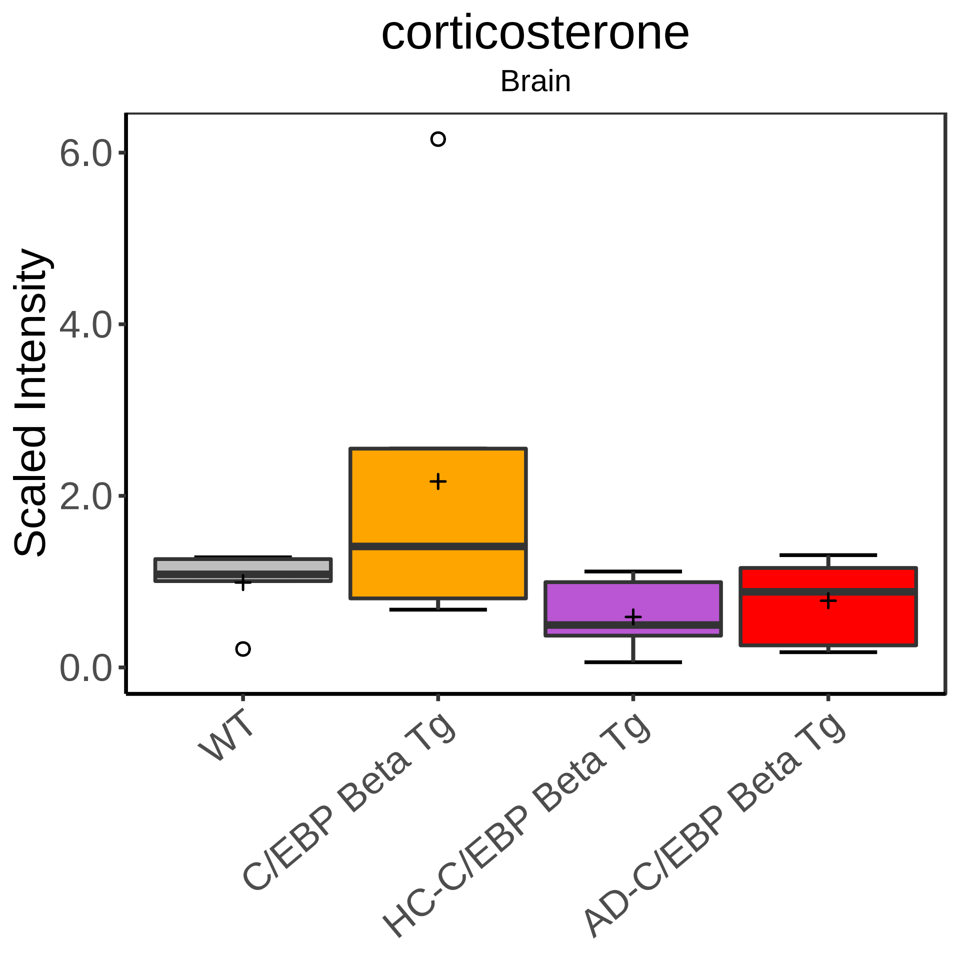
corticosterone
Brain
Scaled Intensity
6.0
4.0
2.0
0.0
WT
C/EBP Beta Tg
HC-C/EBP Beta Tg
AD-C/EBP Beta Tg

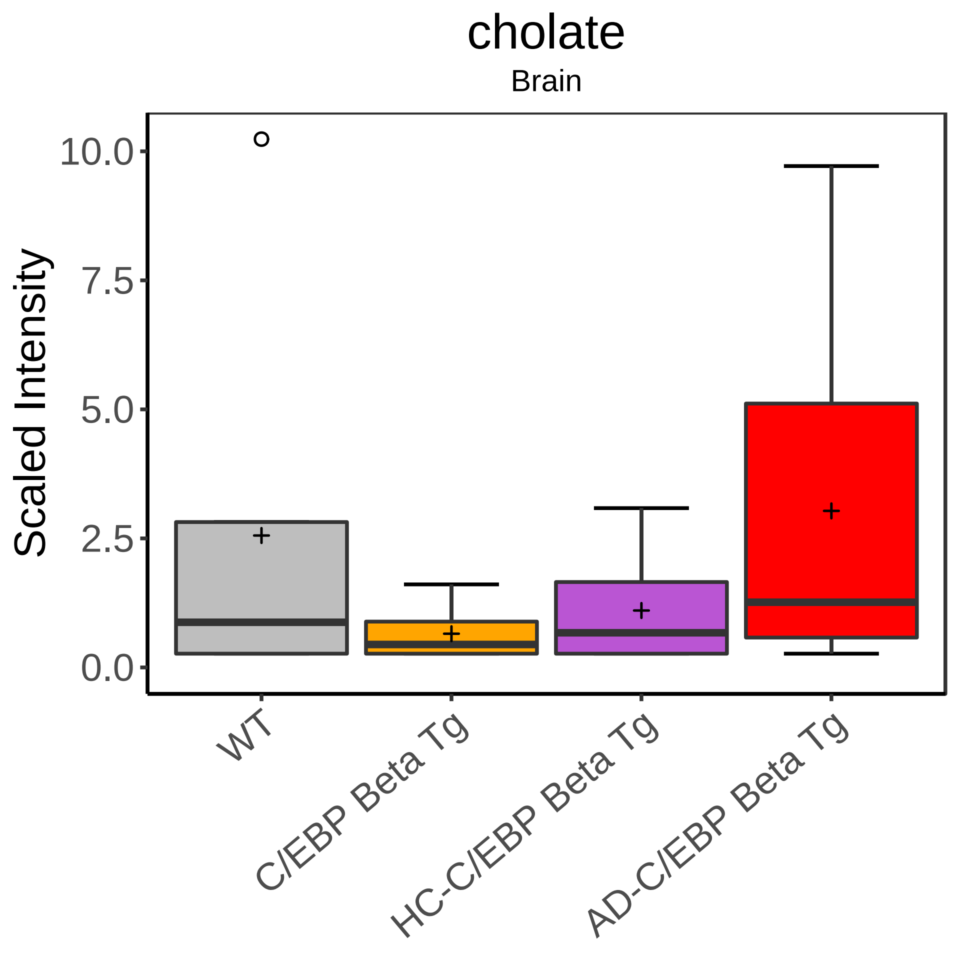
cholate
Brain
Scaled Intensity
10.0
7.5
5.0
2.5
0.0
WT
C/EBP Beta Tg
HC-C/EBP Beta Tg
AD-C/EBP Beta Tg

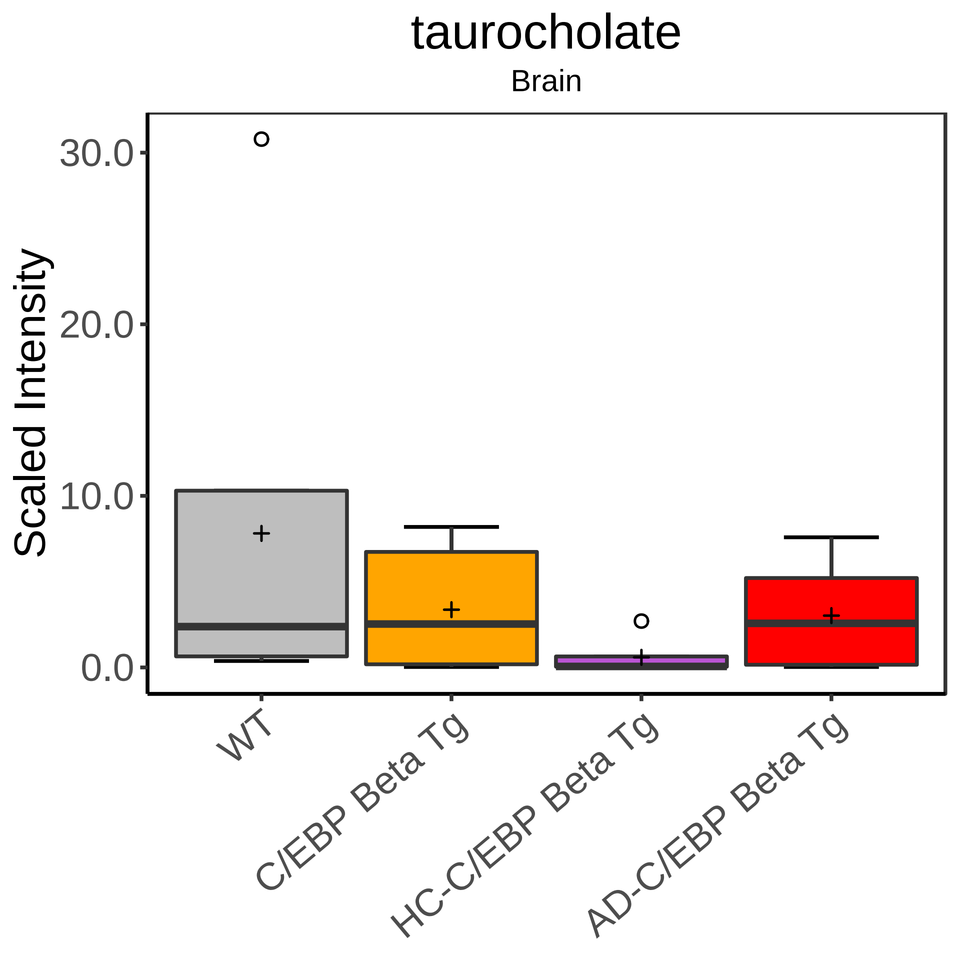
taurocholate
Brain
Scaled Intensity
30.0
20.0
10.0
0.0
WT
C/EBP Beta Tg
HC-C/EBP Beta Tg
AD-C/EBP Beta Tg

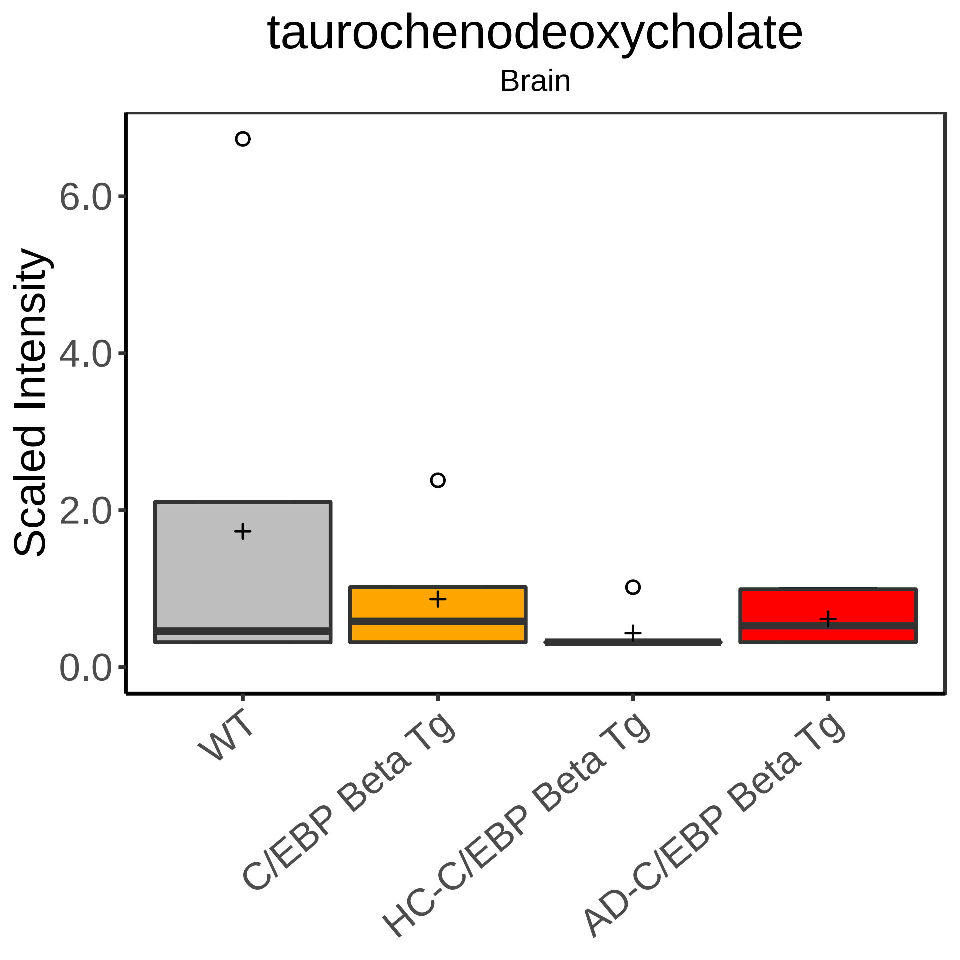

taurochenodeoxycholate
Brain
Scaled Intensity
6.0
4.0
2.0
0.0
WT
C/EBP Beta Tg
HC-C/EBP Beta Tg
AD-C/EBP Beta Tg

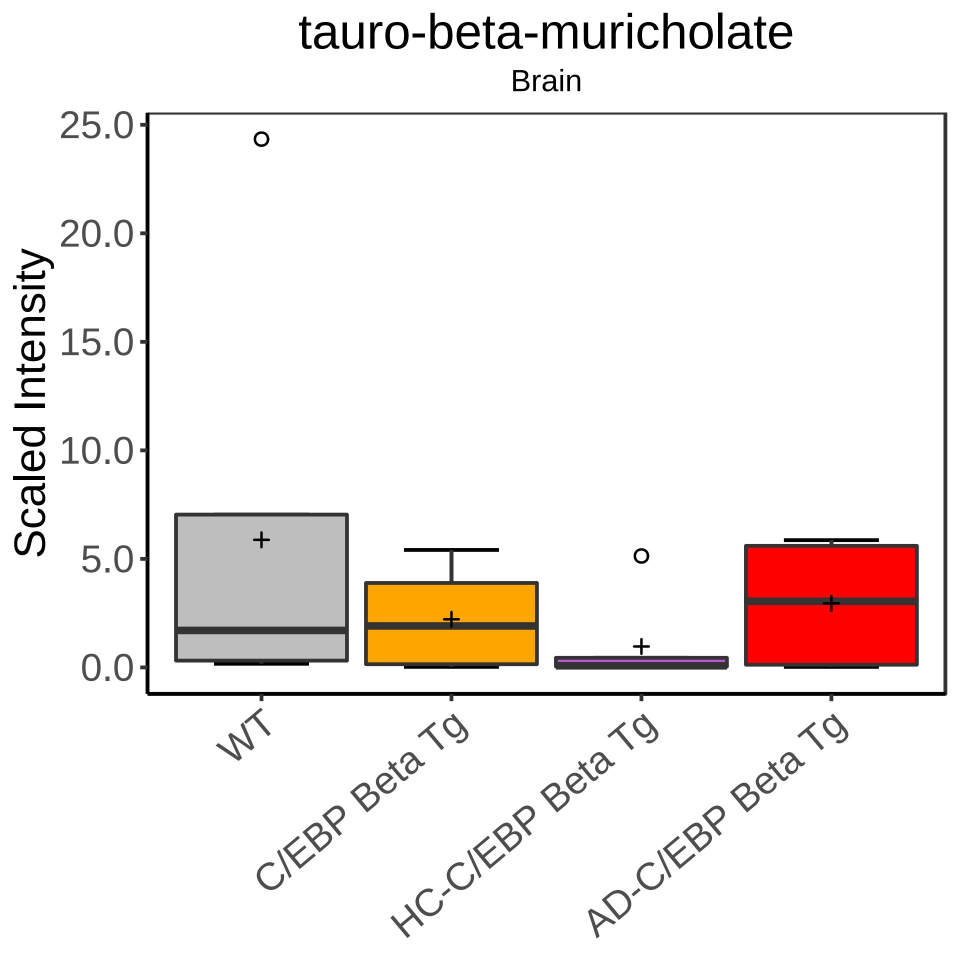

tauro-beta-muricholate
Brain
Scaled Intensity
25.0
20.0
15.0
10.0
5.0
0.0
WT
C/EBP Beta Tg
HC-C/EBP Beta Tg
AD-C/EBP Beta Tg

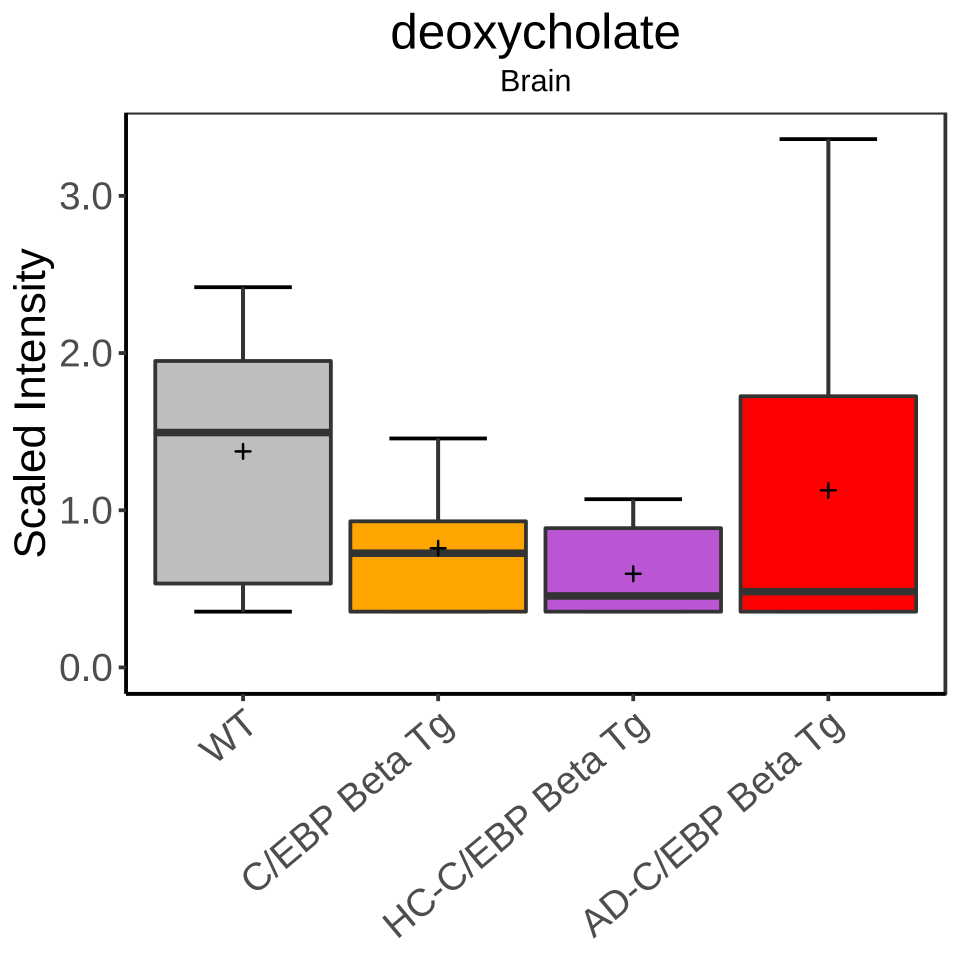
deoxycholate
Brain
Scaled Intensity
3.0
2.0
1.0
0.0
WT
C/EBP Beta Tg
HC-C/EBP Beta Tg
AD-C/EBP Beta Tg

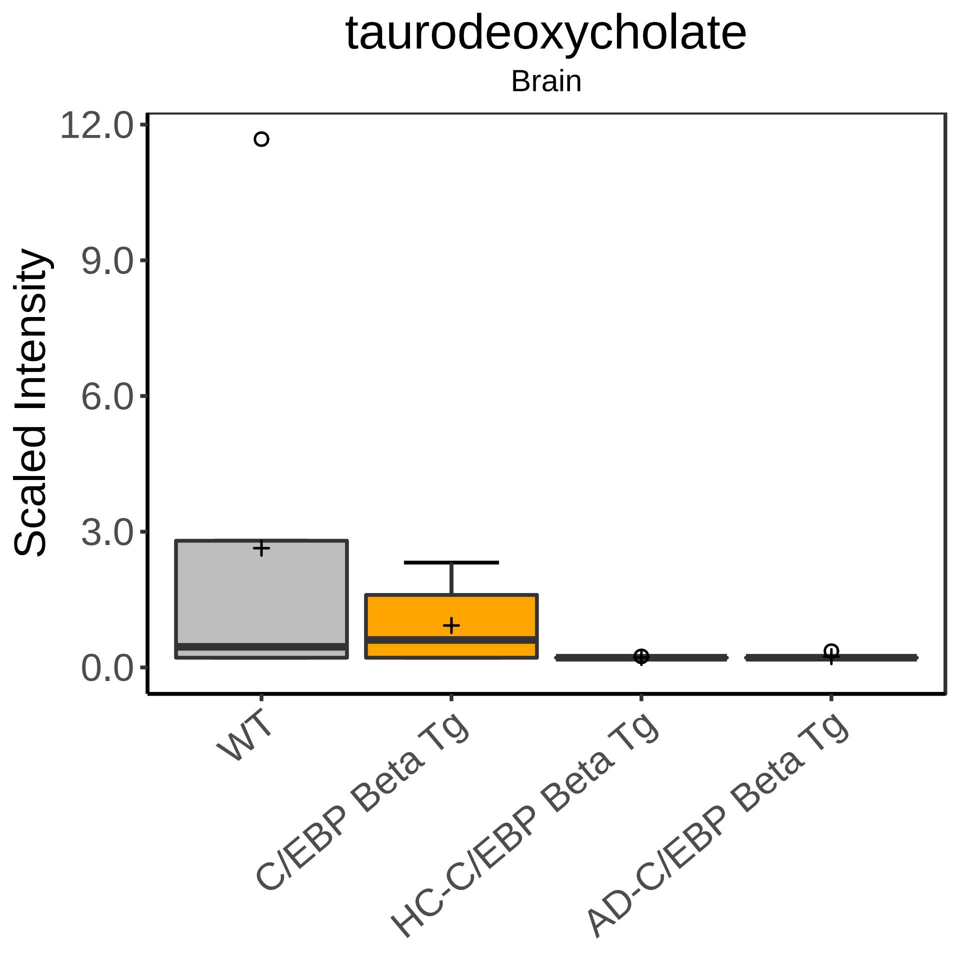

taurodeoxycholate
Brain
Scaled Intensity
12.0
9.0
6.0
3.0
0.0
WT
C/EBP Beta Tg
HC-C/EBP Beta Tg
AD-C/EBP Beta Tg

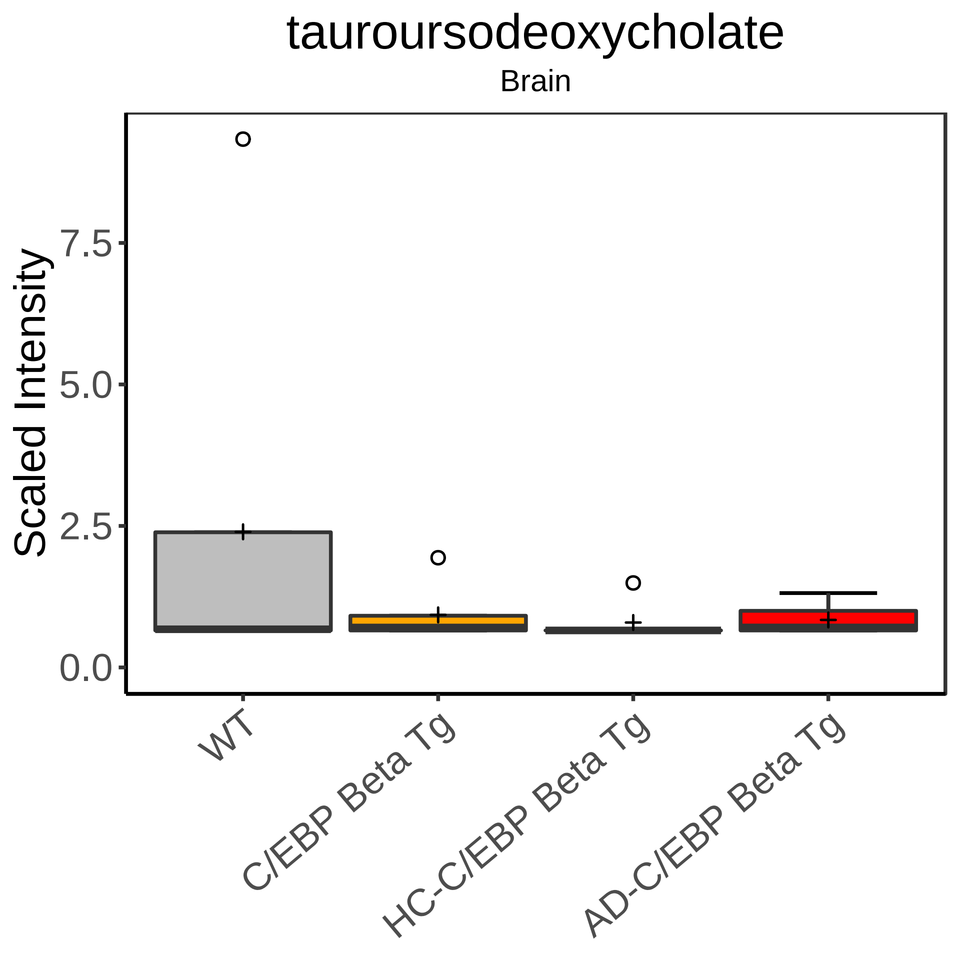

tauroursodeoxycholate
Brain
Scaled Intensity
7.5
5.0
2.5
0.0
WT
C/EBP Beta Tg
HC-C/EBP Beta Tg
AD-C/EBP Beta Tg

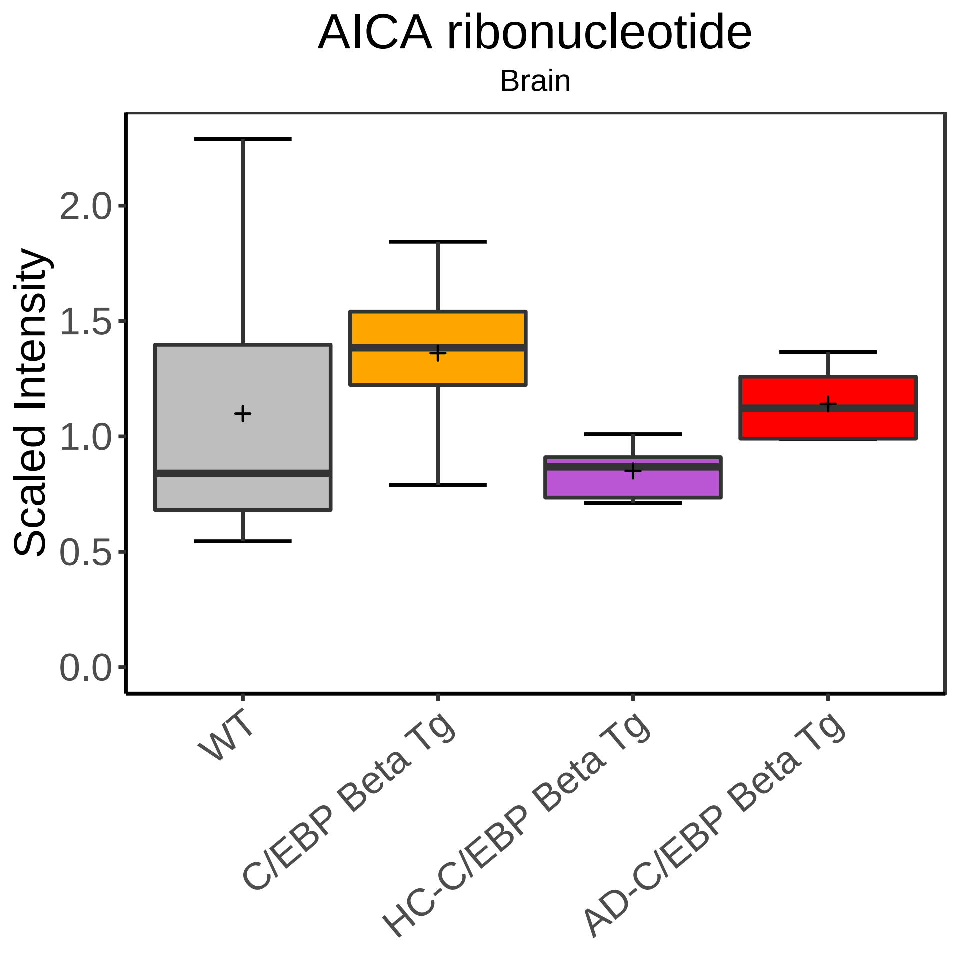
AICA ribonucleotide
Brain
Scaled Intensity
2.0
1.5
1.0
0.5
0.0
WT
C/EBP Beta Tg
HC-C/EBP Beta Tg
AD-C/EBP Beta Tg

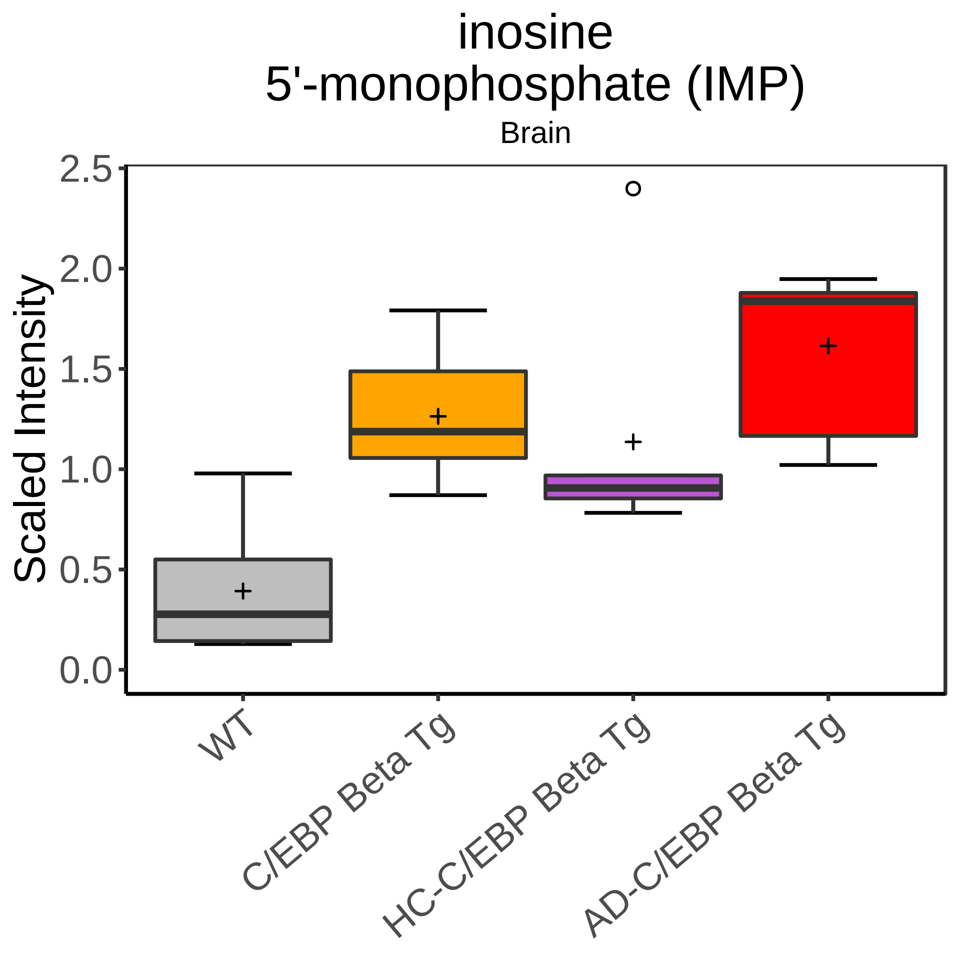

inosine
5'-monophosphate (IMP)
Brain
Scaled Intensity
2.5
2.0
1.5
1.0
0.5
0.0
WT
C/EBP Beta Tg
HC-C/EBP Beta Tg
AD-C/EBP Beta Tg

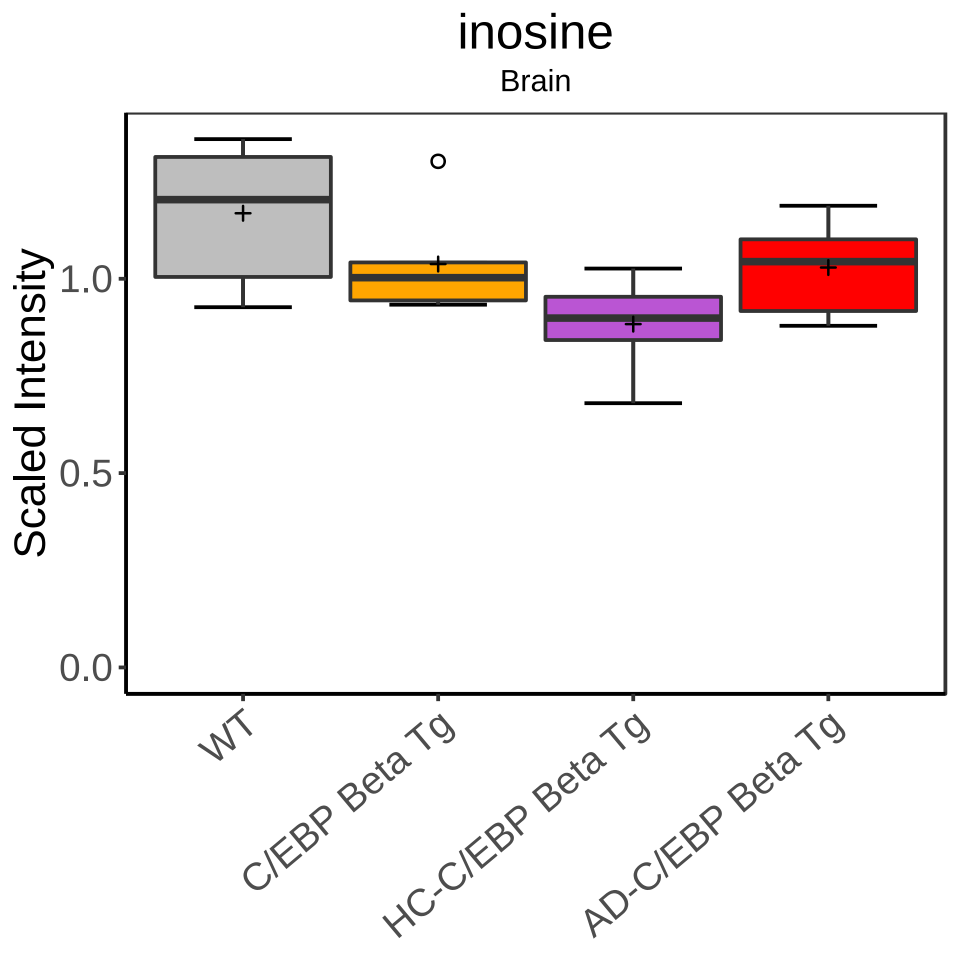

inosine
Brain
Scaled Intensity
1.0
0.5
0.0
WT
C/EBP Beta Tg
HC-C/EBP Beta Tg
AD-C/EBP Beta Tg

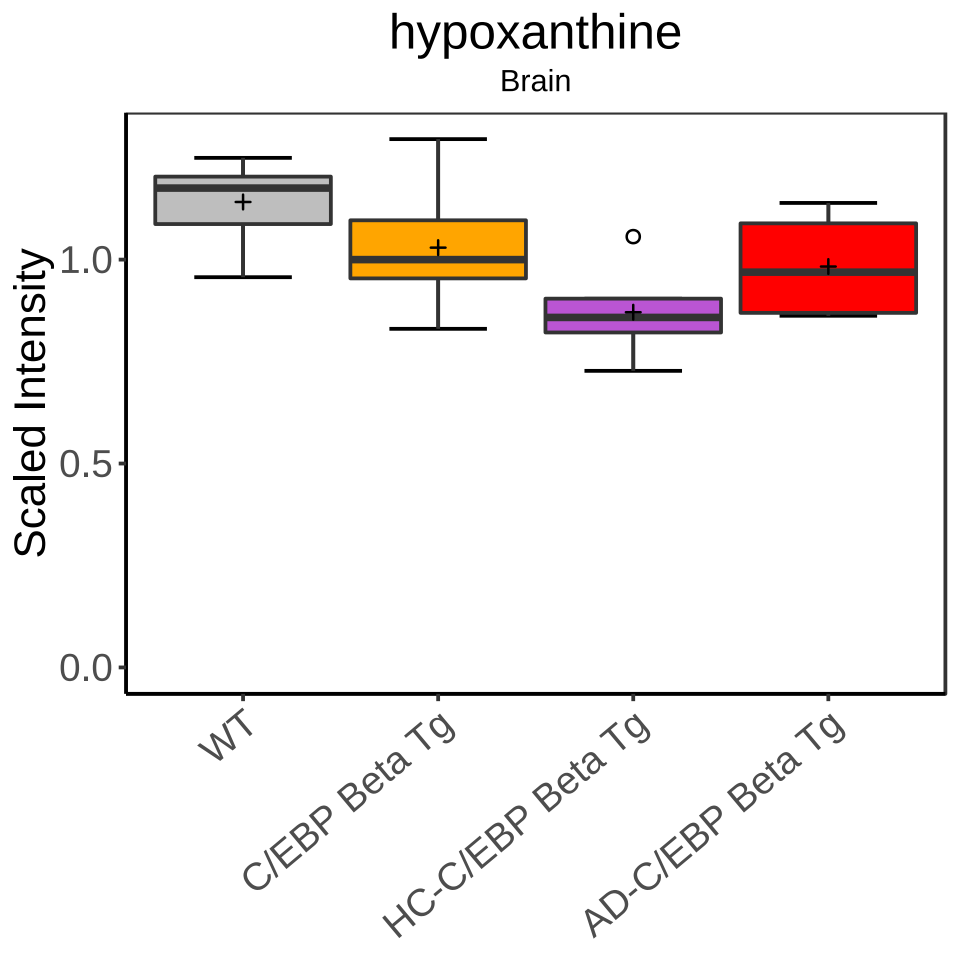
hypoxanthine
Brain
Scaled Intensity
1.0
0.5
0.0
WT
C/EBP Beta Tg
HC-C/EBP Beta Tg
AD-C/EBP Beta Tg

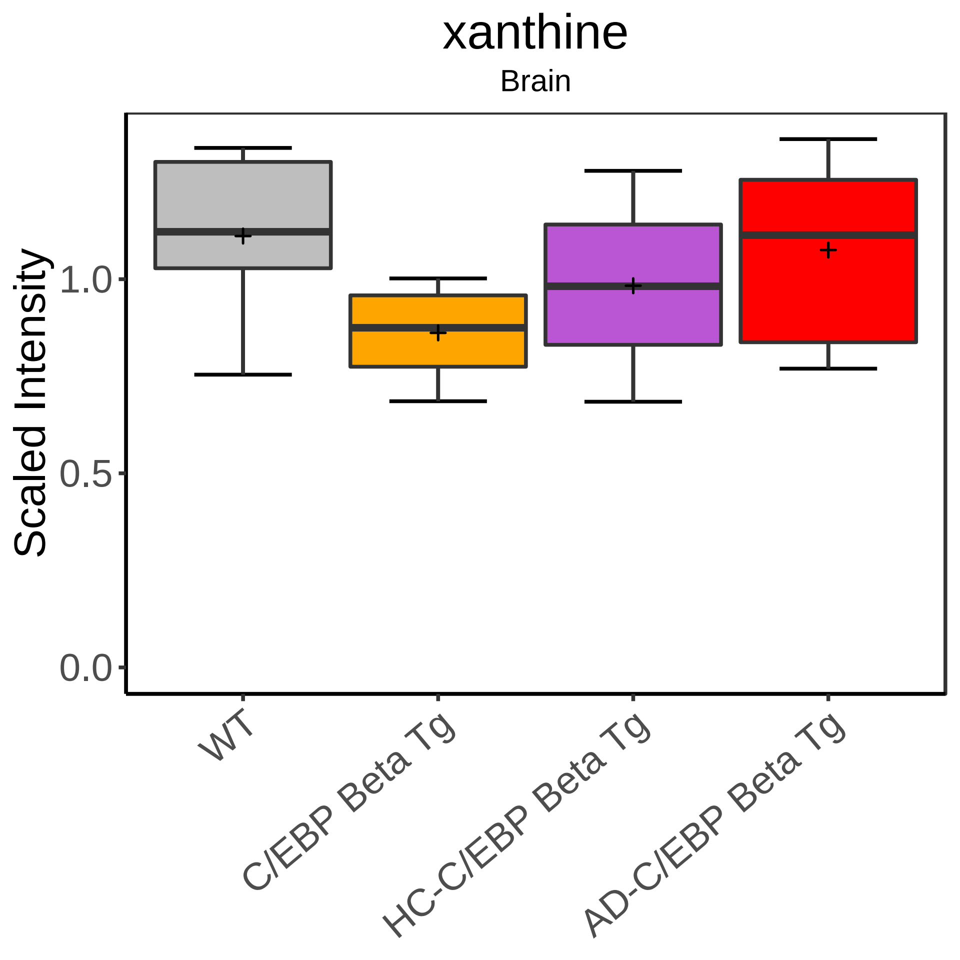
xanthine
Brain
Scaled Intensity
0.0
0.5
1.0
WT
C/EBP Beta Tg
HC-C/EBP Beta Tg
AD-C/EBP Beta Tg

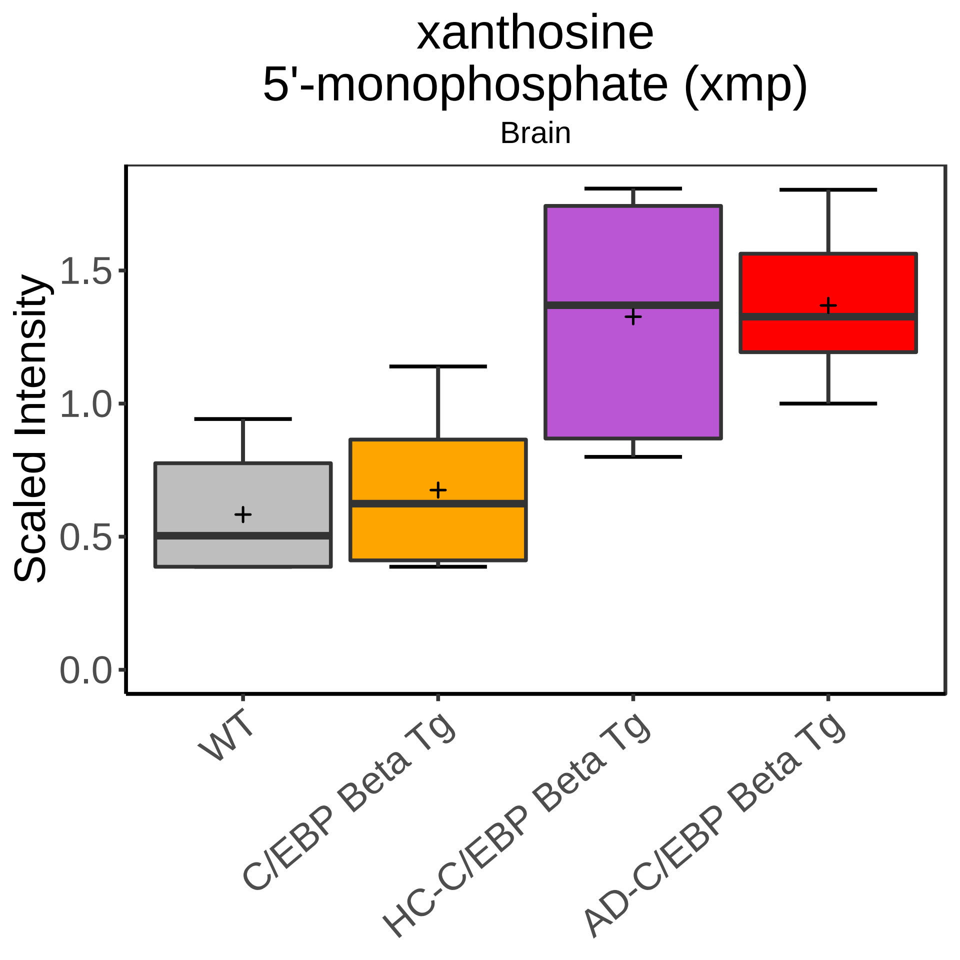
xanthosine
5'-monophosphate (xmp)
Brain
Scaled Intensity
0.0
0.5
1.0
1.5
WT
C/EBP Beta Tg
HC-C/EBP Beta Tg
AD-C/EBP Beta Tg

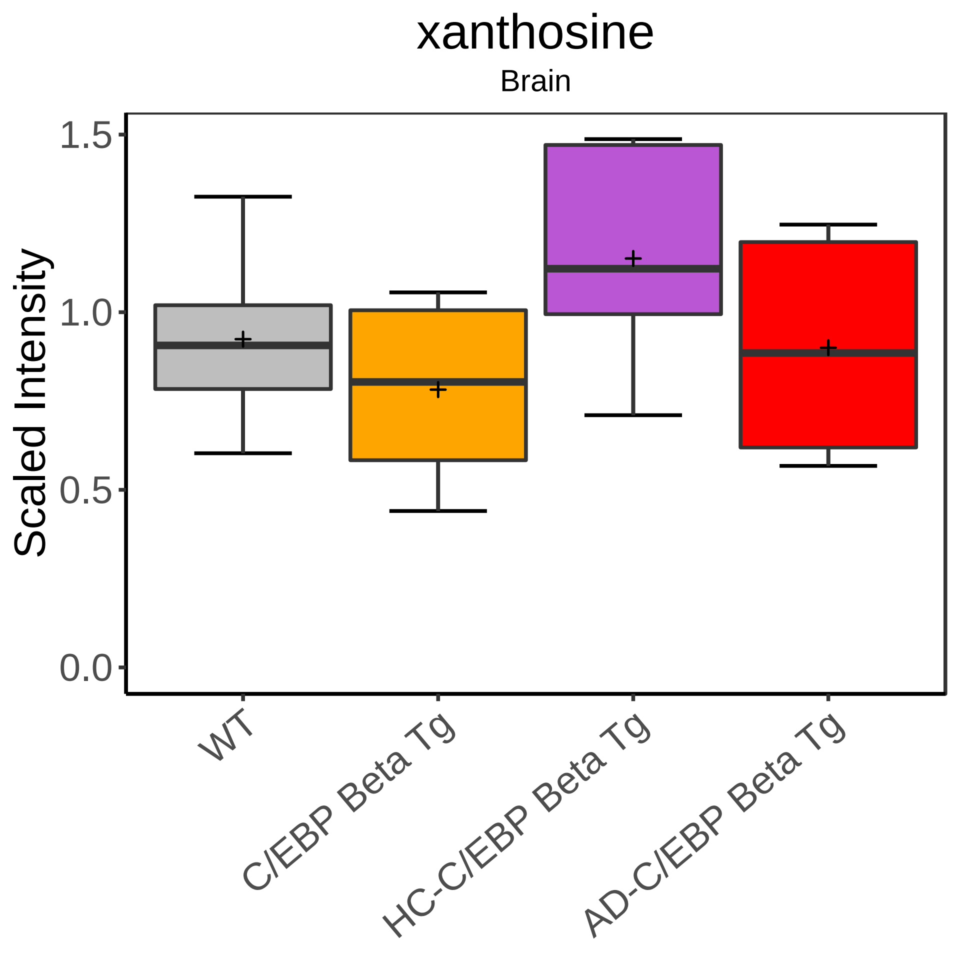

xanthosine
Brain
Scaled Intensity
1.5
1.0
0.5
0.0
WT
C/EBP Beta Tg
HC-C/EBP Beta Tg
AD-C/EBP Beta Tg

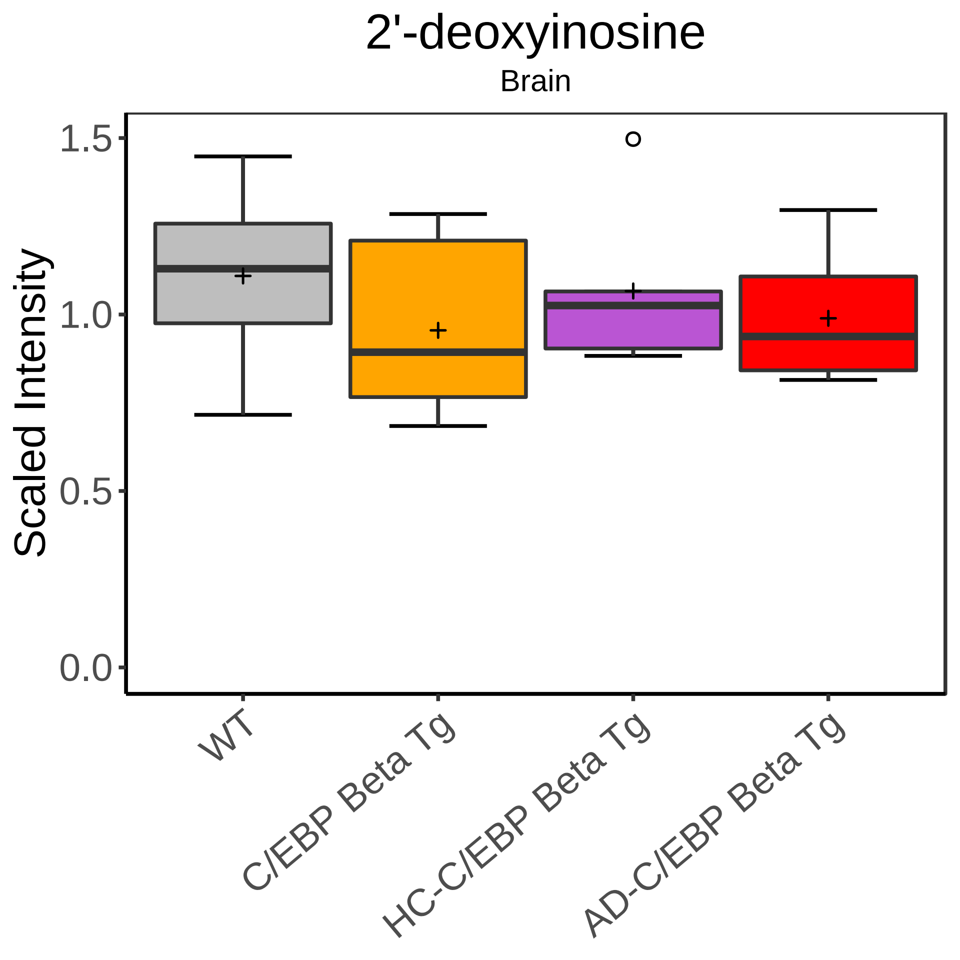

2'-deoxyinosine
Brain
Scaled Intensity
0.0
0.5
1.0
1.5
WT
C/EBP Beta Tg
HC-C/EBP Beta Tg
AD-C/EBP Beta Tg

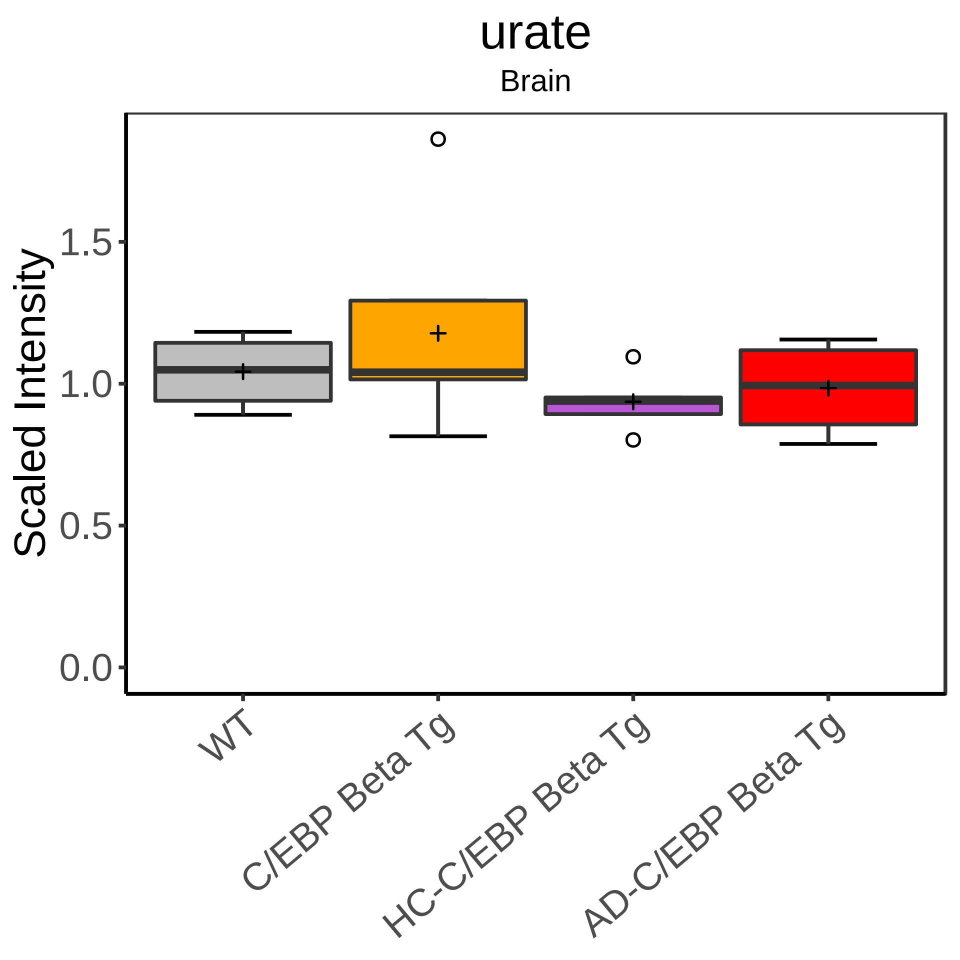

urate
Brain
Scaled Intensity
0.0
0.5
1.0
1.5
WT
C/EBP Beta Tg
HC-C/EBP Beta Tg
AD-C/EBP Beta Tg

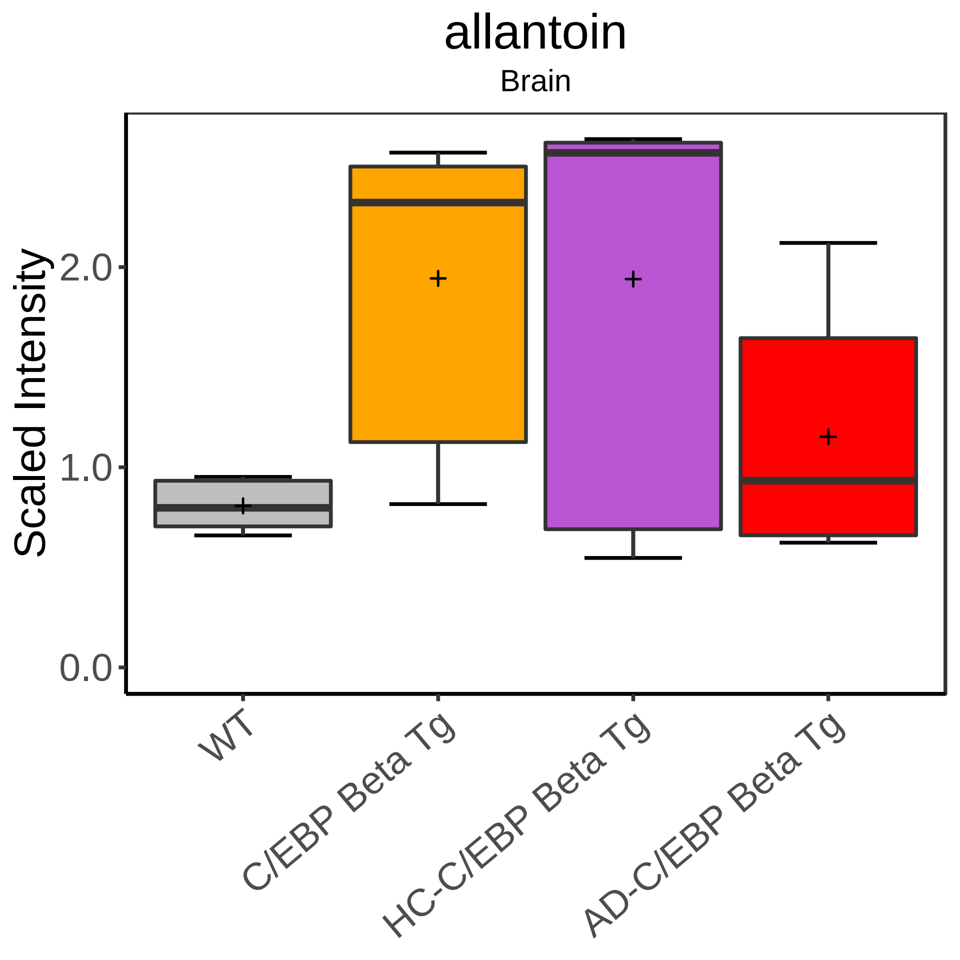

allantoin
Brain
Scaled Intensity
2.0
1.0
0.0
WT
C/EBP Beta Tg
HC-C/EBP Beta Tg
AD-C/EBP Beta Tg

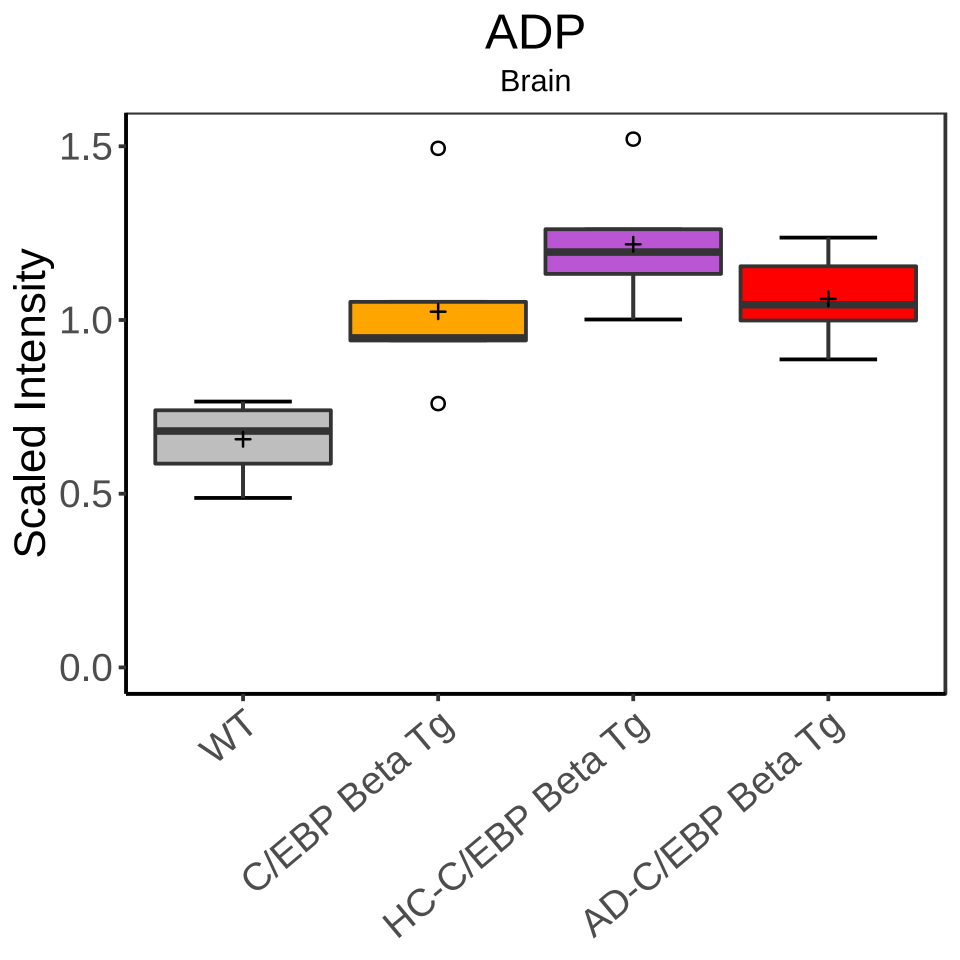

ADP
Brain
Scaled Intensity
1.5
1.0
0.5
0.0
WT
C/EBP Beta Tg
HC-C/EBP Beta Tg
AD-C/EBP Beta Tg

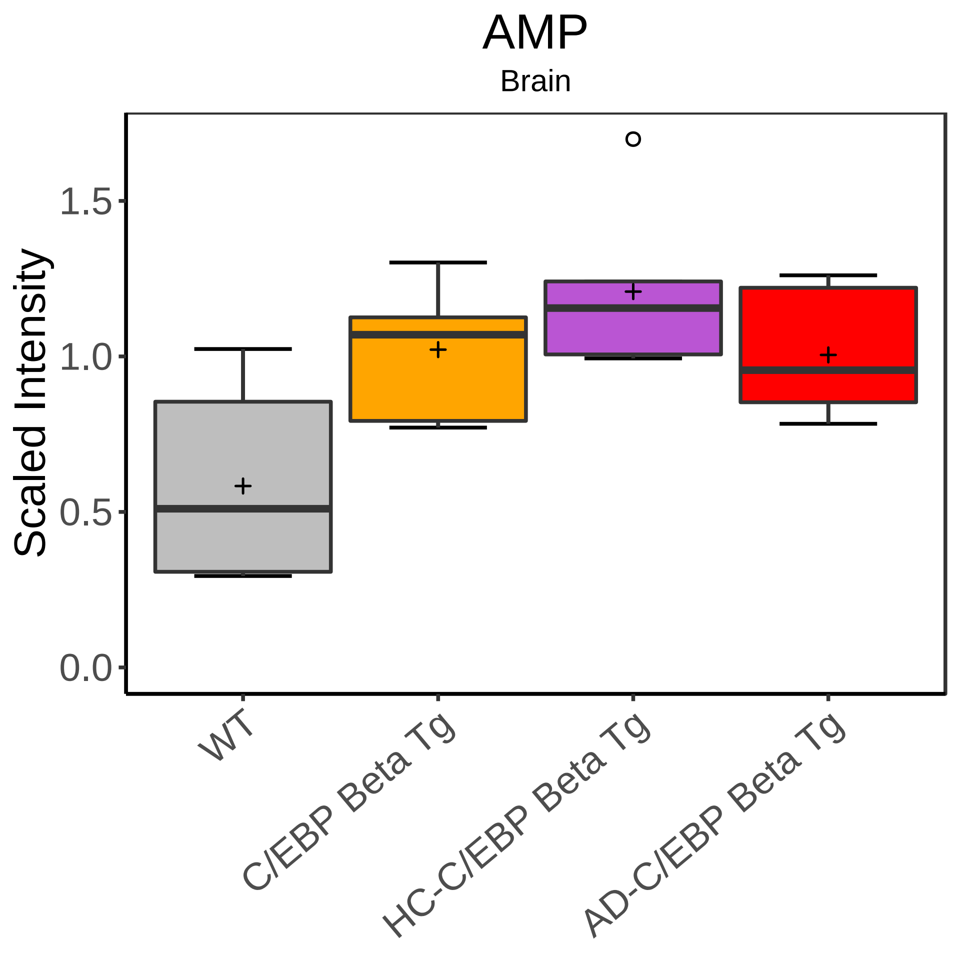
AMP
Brain
Scaled Intensity
0.0
0.5
1.0
1.5
WT
C/EBP Beta Tg
HC-C/EBP Beta Tg
AD-C/EBP Beta Tg

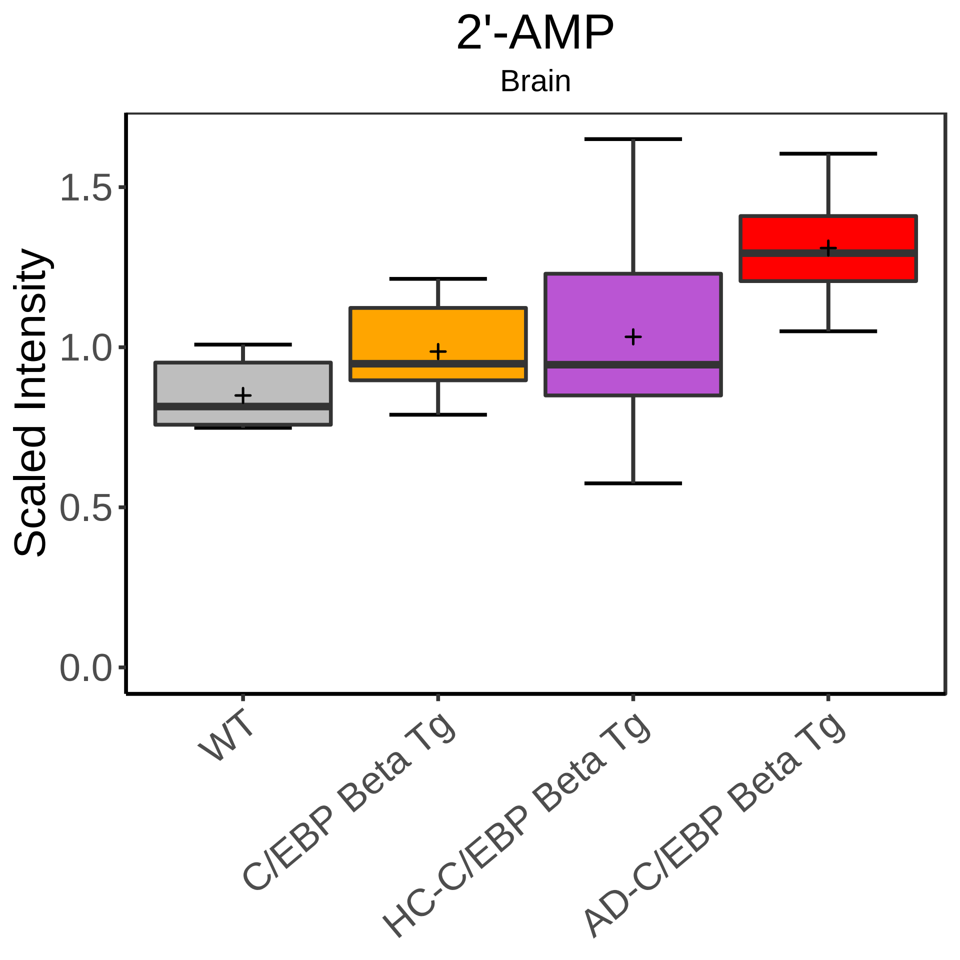
2'-AMP
Brain
Scaled Intensity
0.0
0.5
1.0
1.5
WT
C/EBP Beta Tg
HC-C/EBP Beta Tg
AD-C/EBP Beta Tg

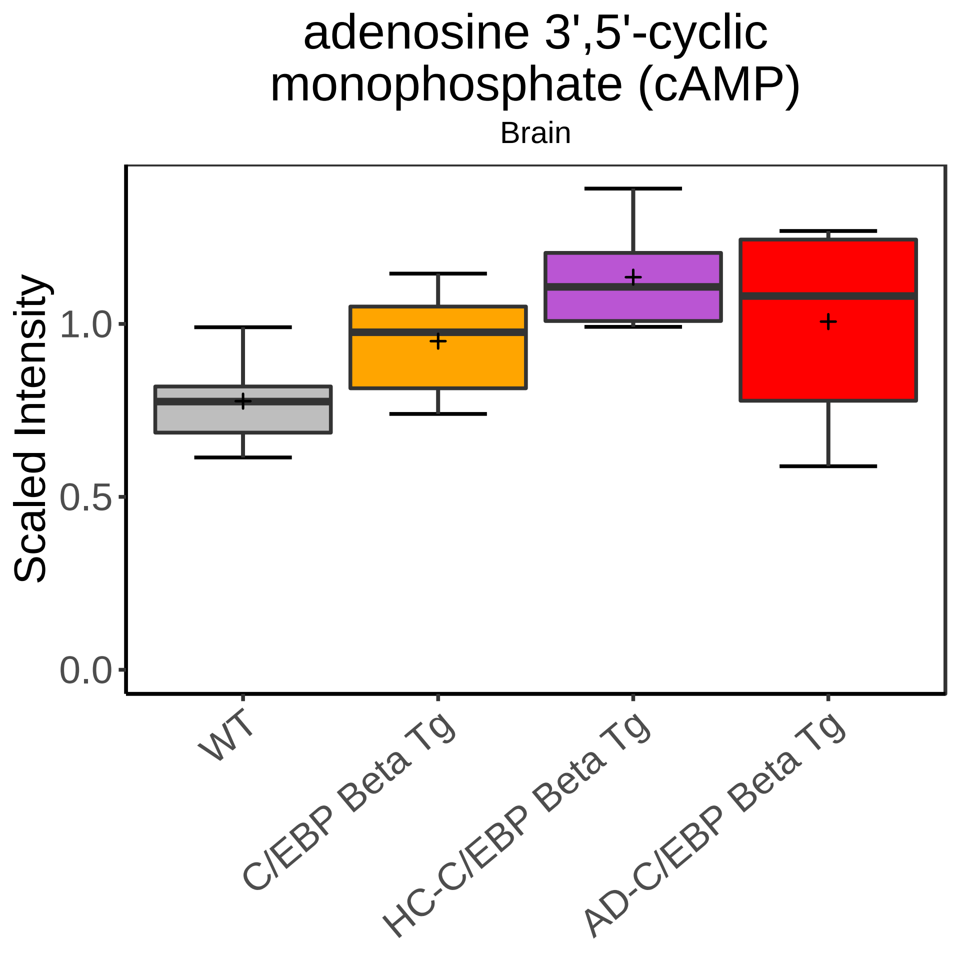
adenosine 3',5'-cyclic monophosphate (cAMP)
Brain
Scaled Intensity
1.0
0.5
0.0
WT
C/EBP Beta Tg
HC-C/EBP Beta Tg
AD-C/EBP Beta Tg

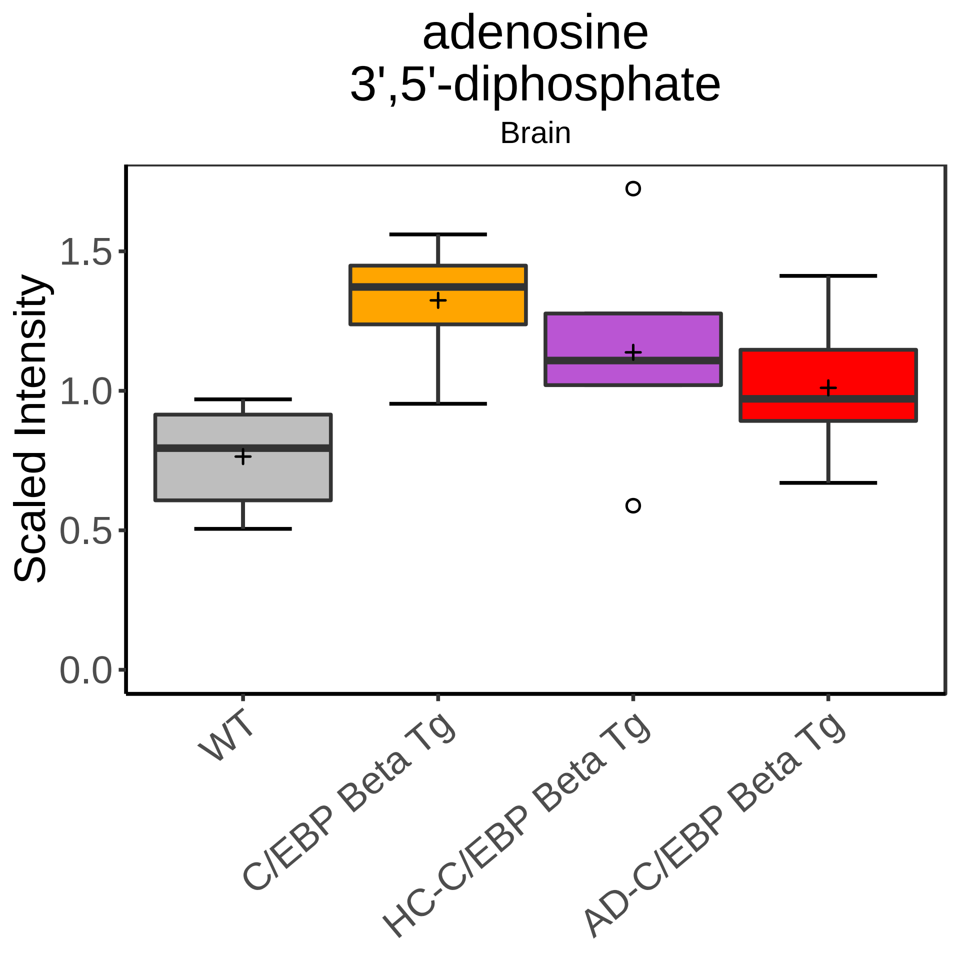

adenosine
3',5'-diphosphate
Brain
Scaled Intensity
1.5
1.0
0.5
0.0
WT
C/EBP Beta Tg
HC-C/EBP Beta Tg
AD-C/EBP Beta Tg

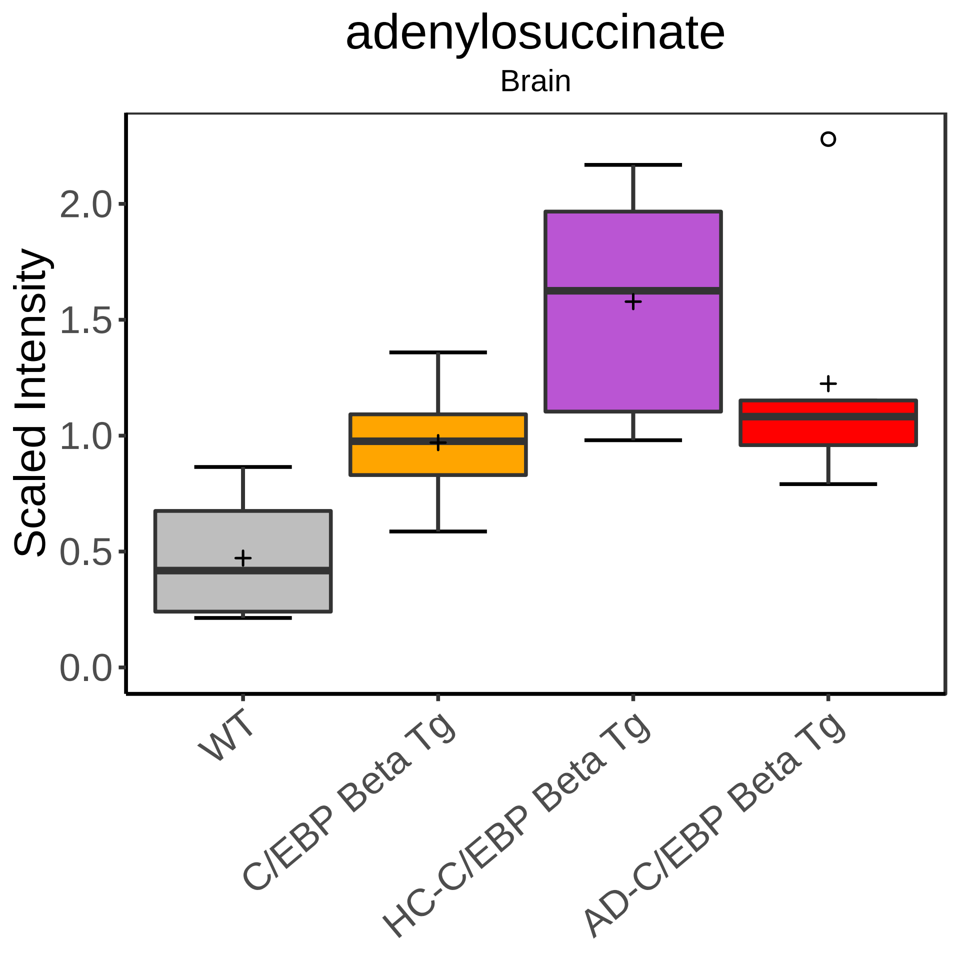

adenylosuccinate
Brain
Scaled Intensity
2.0
1.5
1.0
0.5
0.0
WT
C/EBP Beta Tg
HC-C/EBP Beta Tg
AD-C/EBP Beta Tg

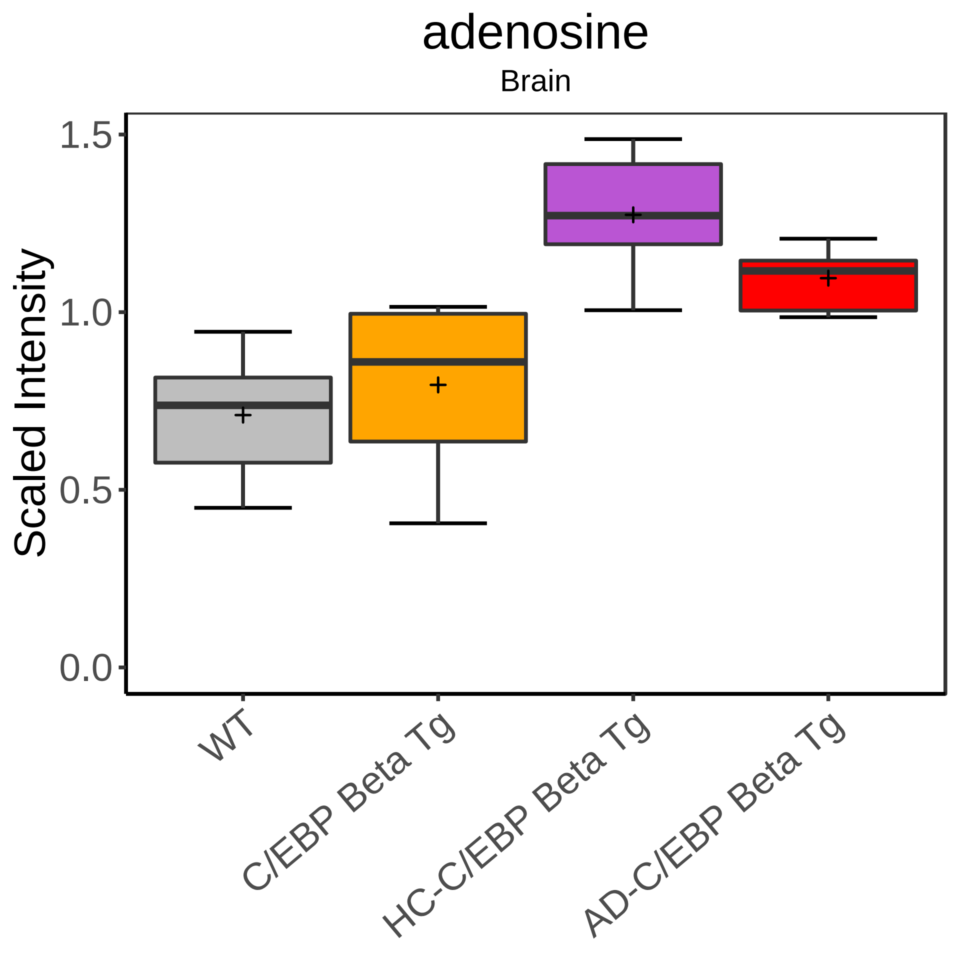
adenosine
Brain
Scaled Intensity
1.5
1.0
0.5
0.0
WT
C/EBP Beta Tg
HC-C/EBP Beta Tg
AD-C/EBP Beta Tg

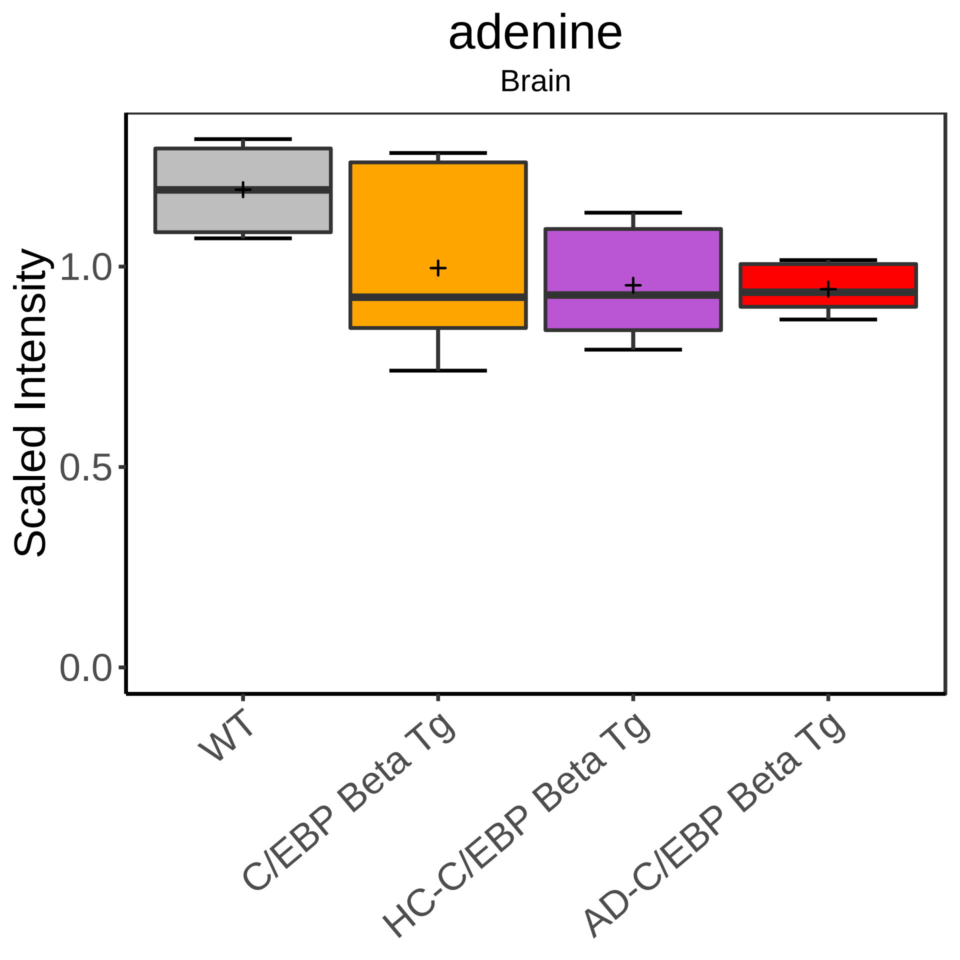
adenine
Brain
Scaled Intensity
1.0
0.5
0.0
WT
C/EBP Beta Tg
HC-C/EBP Beta Tg
AD-C/EBP Beta Tg

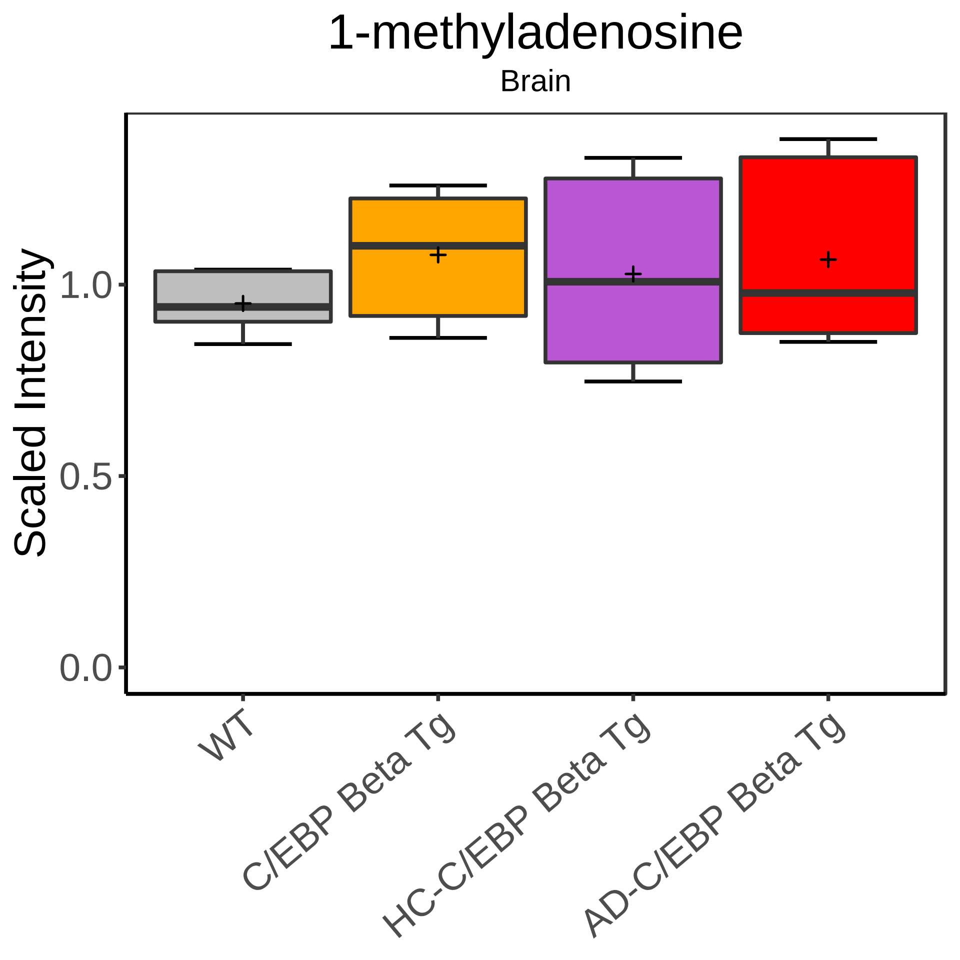
1-methyladenosine
Brain
Scaled Intensity
1.0
0.5
0.0
WT
C/EBP Beta Tg
HC-C/EBP Beta Tg
AD-C/EBP Beta Tg

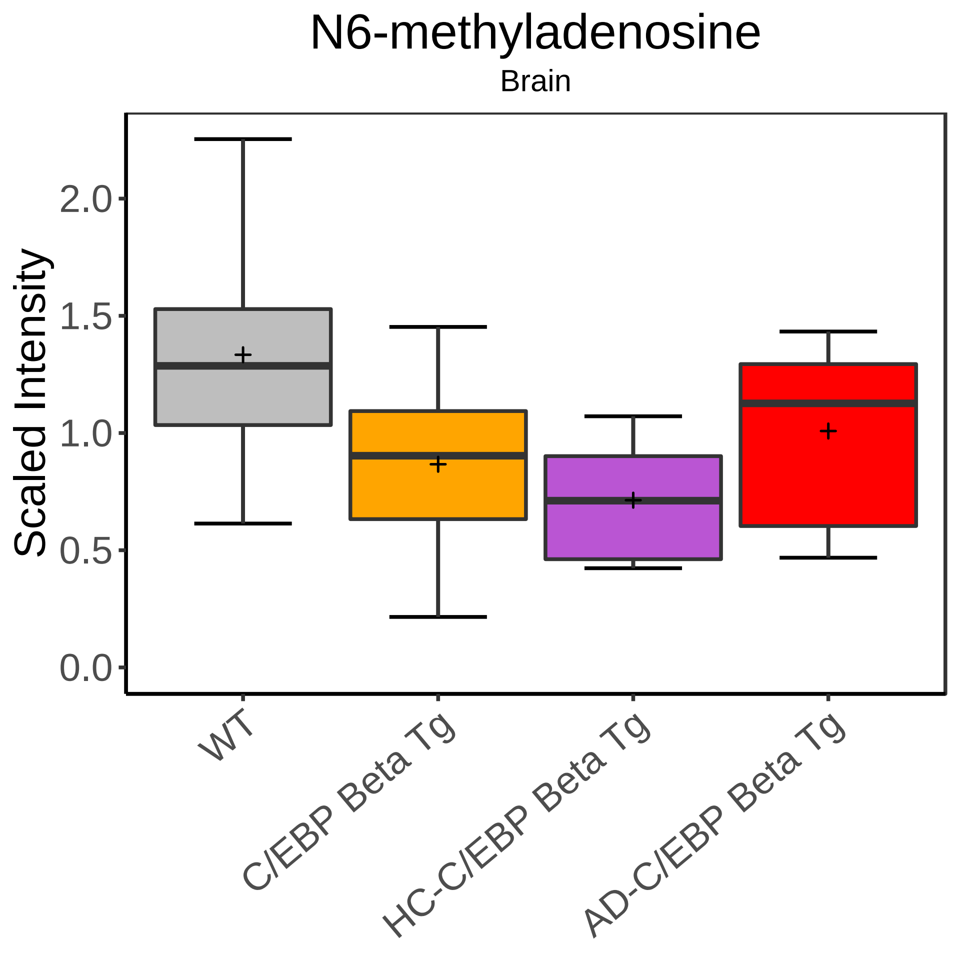
N6-methyladenosine
Brain
Scaled Intensity
0.0
0.5
1.0
1.5
2.0
WT
C/EBP Beta Tg
HC-C/EBP Beta Tg
AD-C/EBP Beta Tg

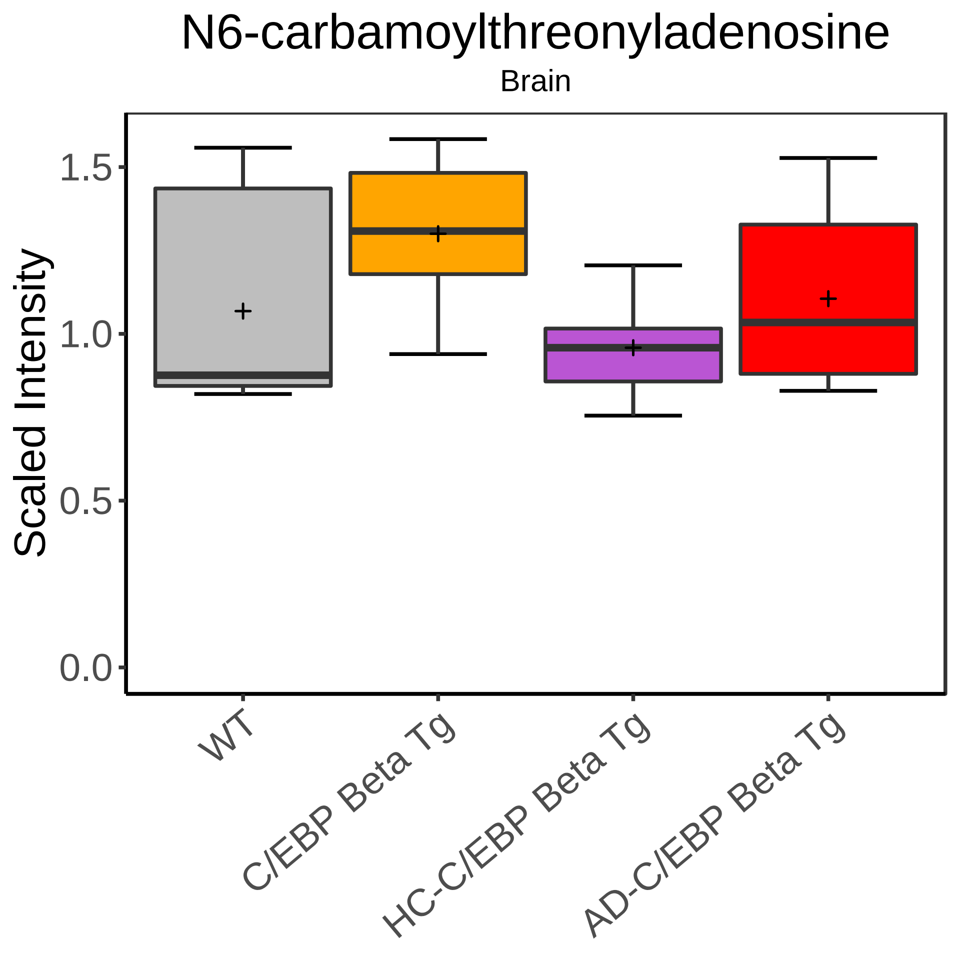
N6-carbamoylthreonyladenosine
Brain
Scaled Intensity
1.5
1.0
0.5
0.0
WT
C/EBP Beta Tg
HC-C/EBP Beta Tg
AD-C/EBP Beta Tg

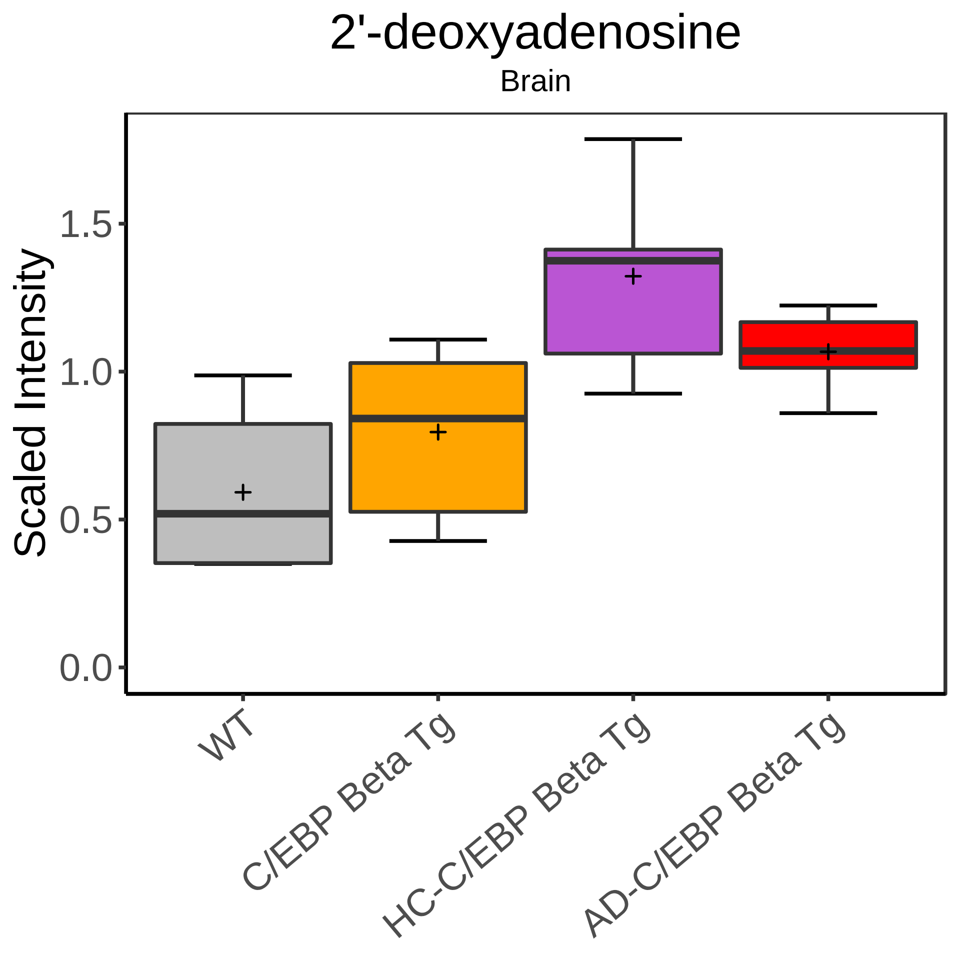
2'-deoxyadenosine
Brain
Scaled Intensity
1.5
1.0
0.5
0.0
WT
C/EBP Beta Tg
HC-C/EBP Beta Tg
AD-C/EBP Beta Tg

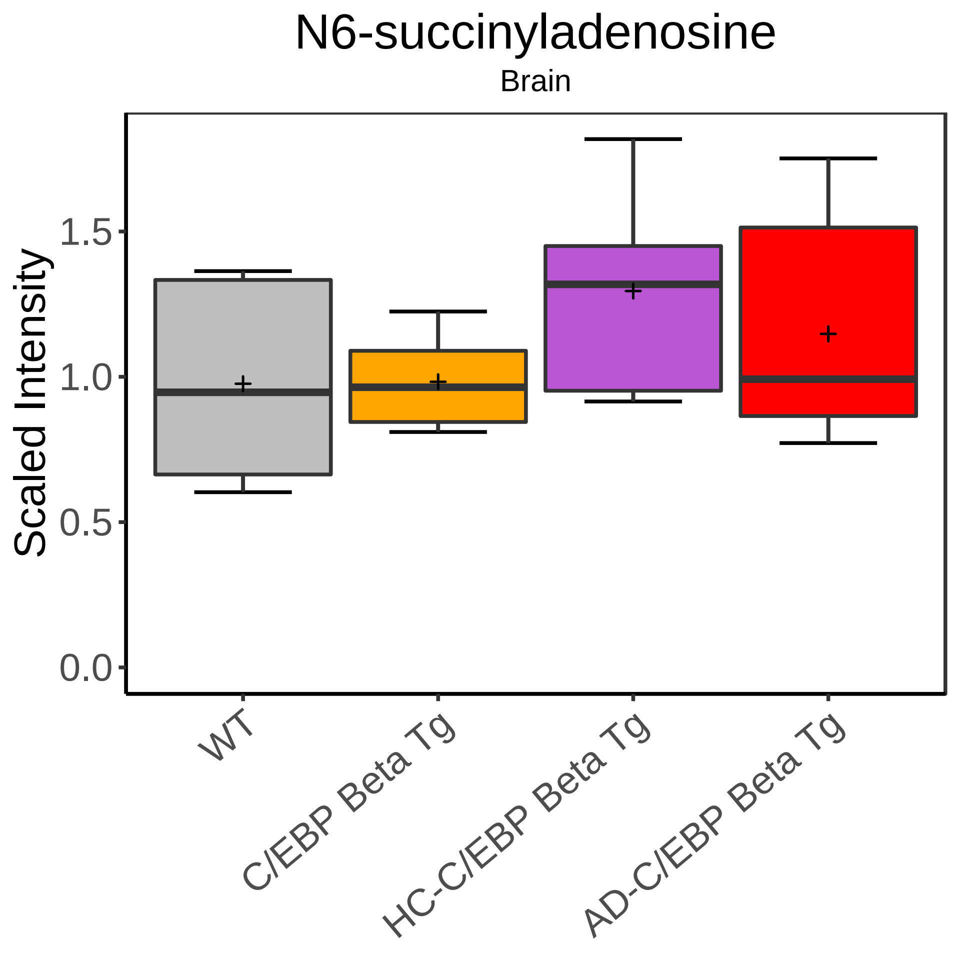

N6-succinyladenosine
Brain
Scaled Intensity
0.0
0.5
1.0
1.5
WT
C/EBP Beta Tg
HC-C/EBP Beta Tg
AD-C/EBP Beta Tg

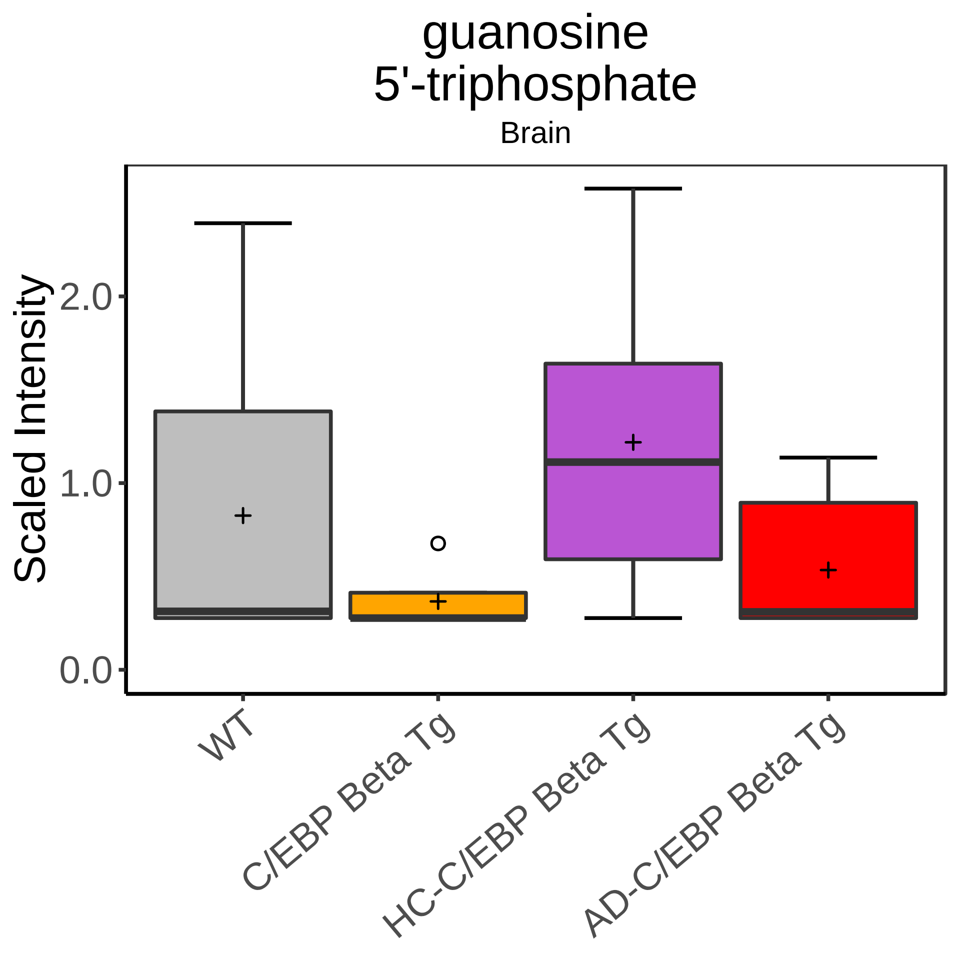
guanosine
5'-triphosphate
Brain
Scaled Intensity
0.0
1.0
2.0
WT
C/EBP Beta Tg
HC-C/EBP Beta Tg
AD-C/EBP Beta Tg

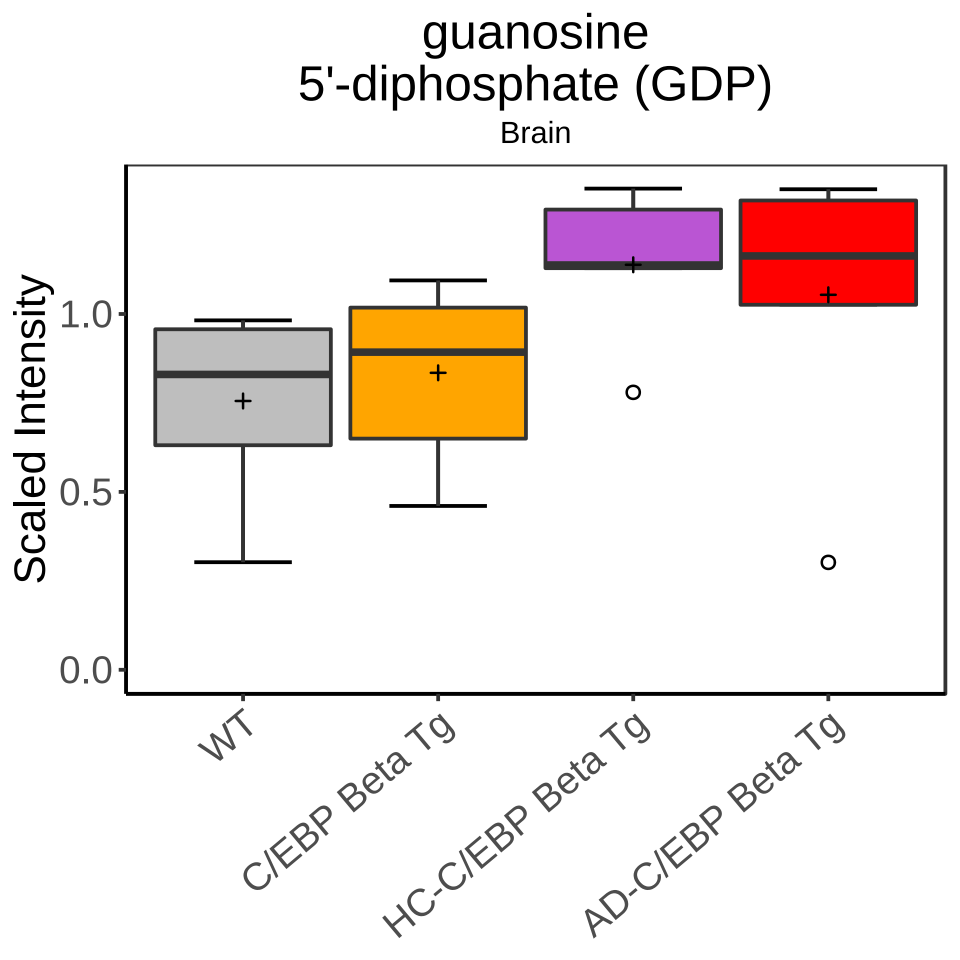
guanosine
5'-diphosphate (GDP)
Brain
Scaled Intensity
0.0
0.5
1.0
WT
C/EBP Beta Tg
HC-C/EBP Beta Tg
AD-C/EBP Beta Tg

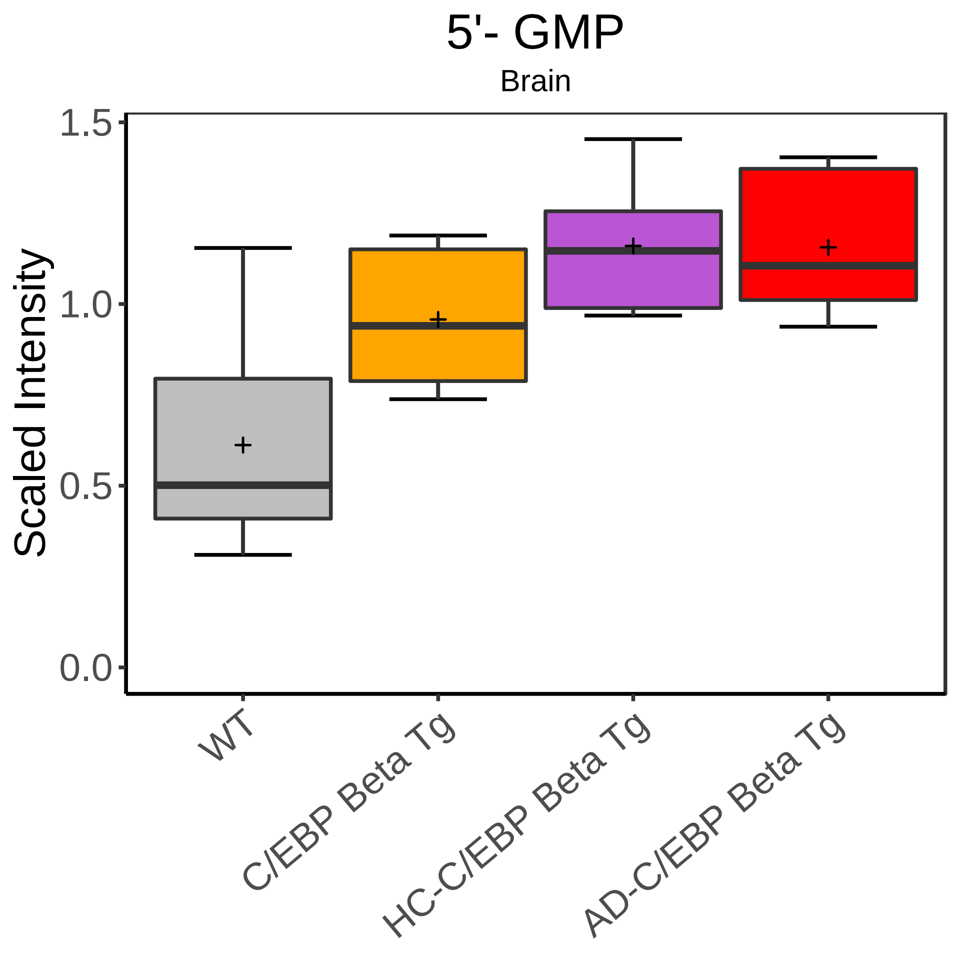
5'- GMP
Brain
Scaled Intensity
1.5
1.0
0.5
0.0
WT
C/EBP Beta Tg
HC-C/EBP Beta Tg
AD-C/EBP Beta Tg

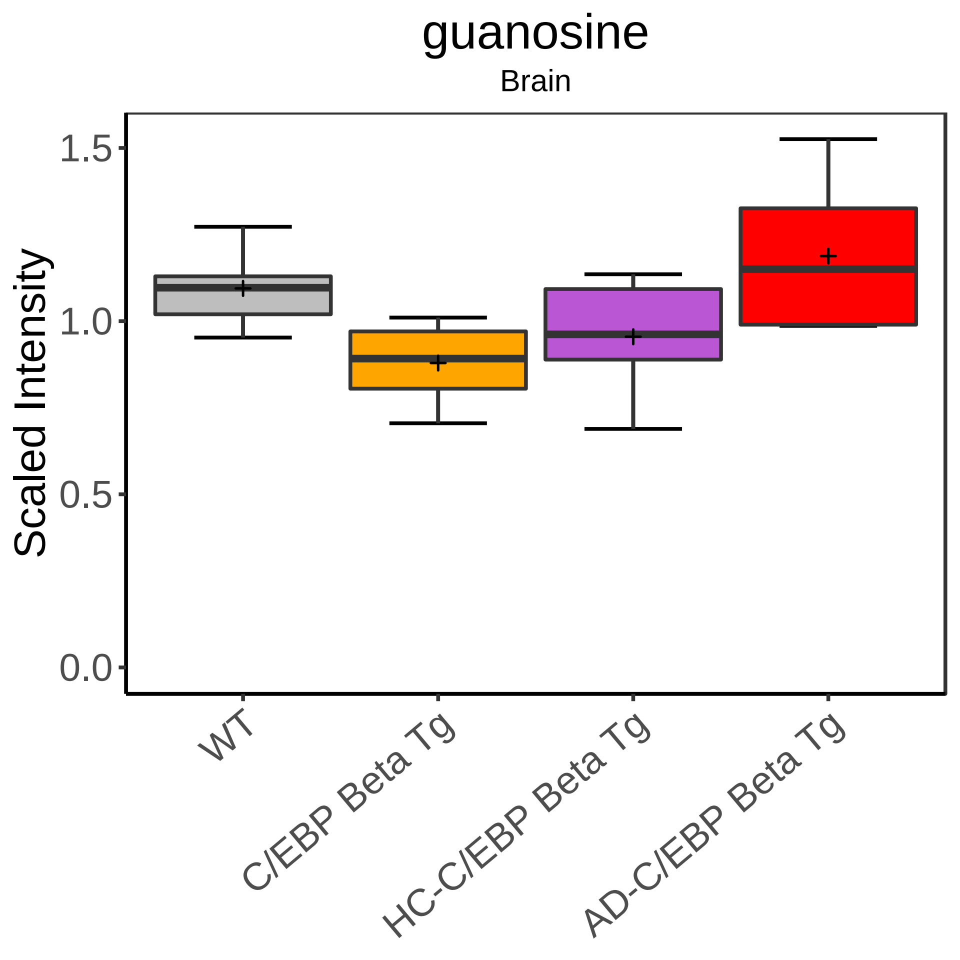
guanosine
Brain
Scaled Intensity
1.5
1.0
0.5
0.0
WT
C/EBP Beta Tg
HC-C/EBP Beta Tg
AD-C/EBP Beta Tg

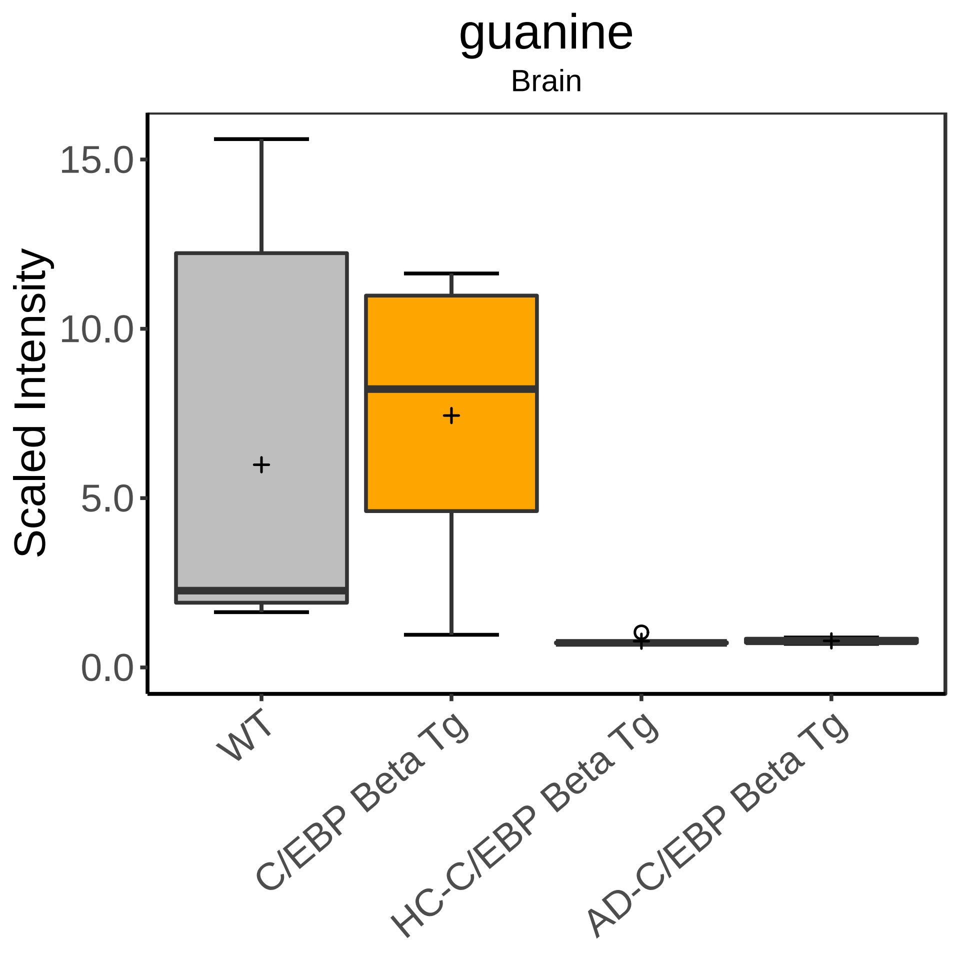

guanine
Brain
Scaled Intensity
15.0
10.0
5.0
0.0
WT
C/EBP Beta Tg
HC-C/EBP Beta Tg
AD-C/EBP Beta Tg

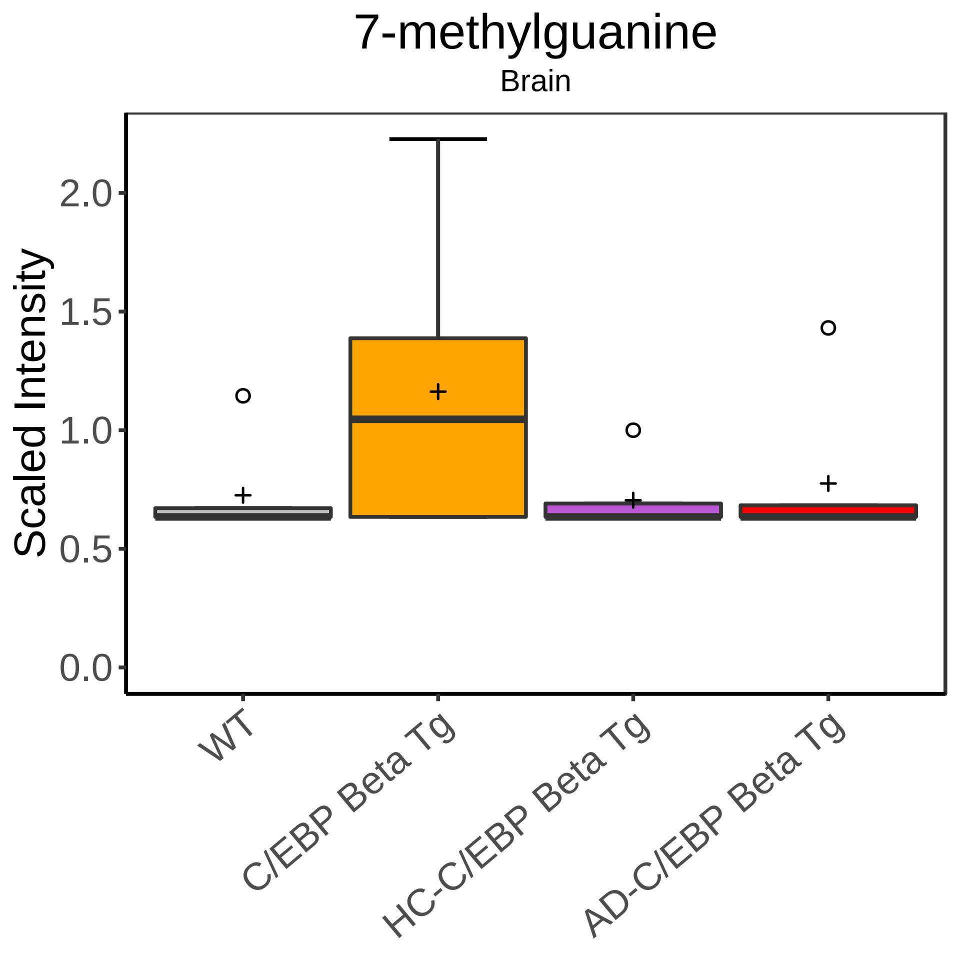

7-methylguanine
Brain
Scaled Intensity
2.0
1.5
1.0
0.5
0.0
WT
C/EBP Beta Tg
HC-C/EBP Beta Tg
AD-C/EBP Beta Tg

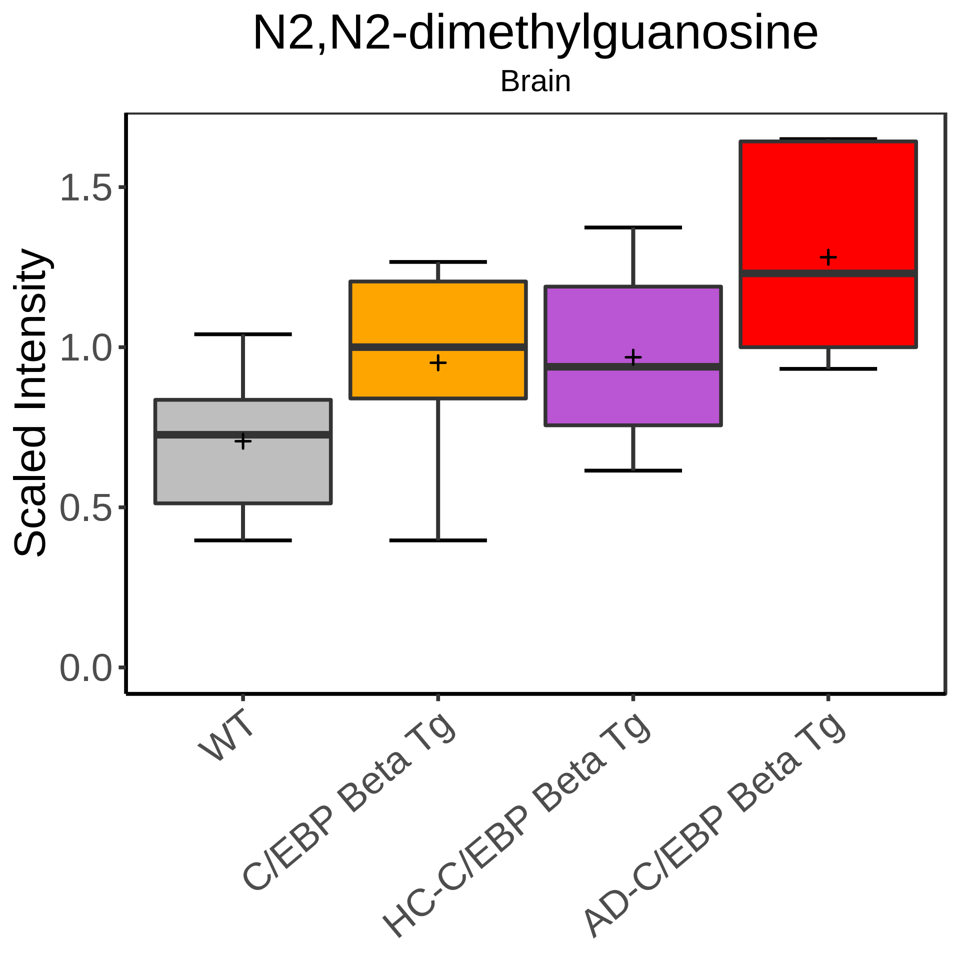

N2,N2-dimethylguanosine
Brain
Scaled Intensity
0.0
0.5
1.0
1.5
WT
C/EBP Beta Tg
HC-C/EBP Beta Tg
AD-C/EBP Beta Tg

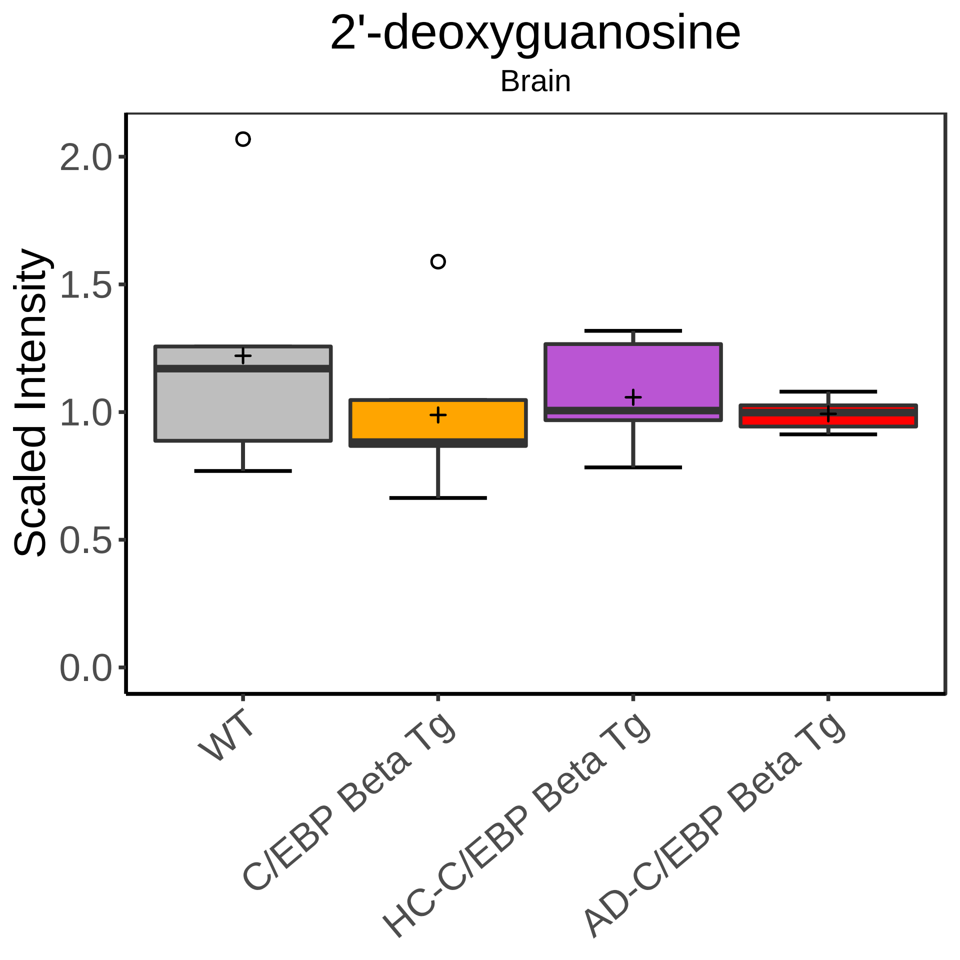

2'-deoxyguanosine
Brain
Scaled Intensity
2.0
1.5
1.0
0.5
0.0
WT
C/EBP Beta Tg
HC-C/EBP Beta Tg
AD-C/EBP Beta Tg

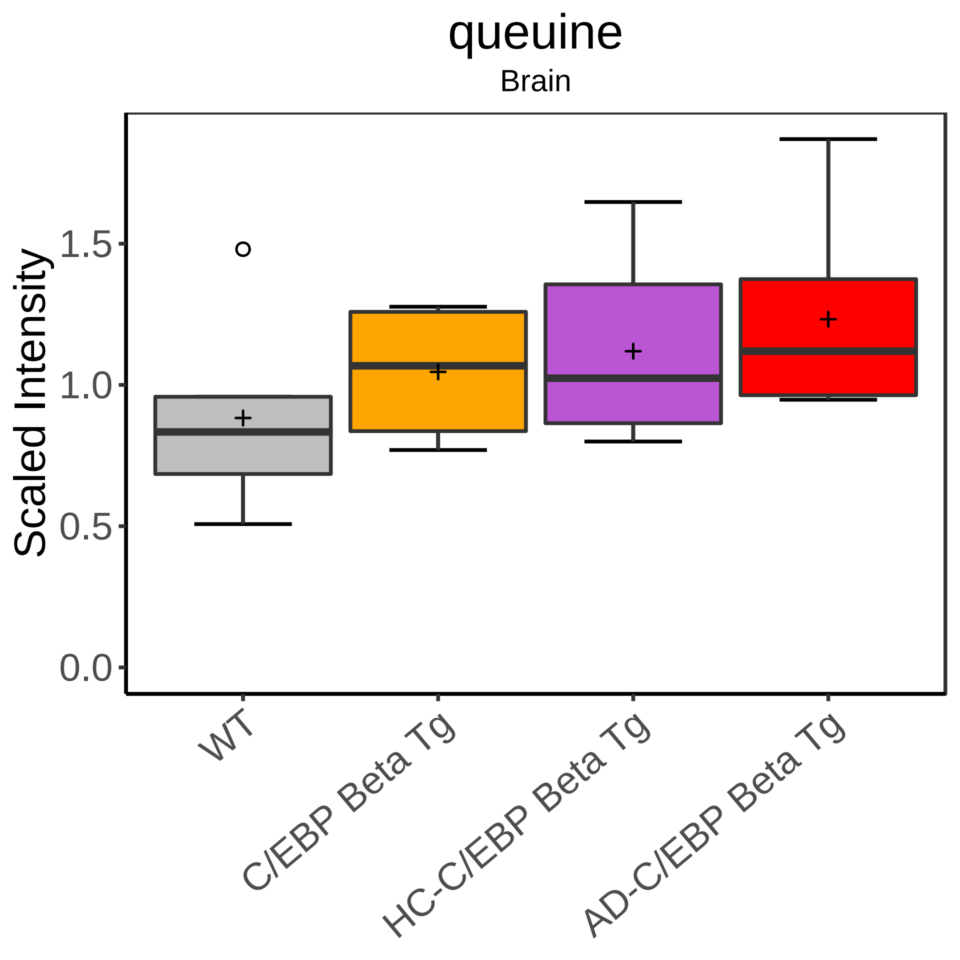
queuine
Brain
Scaled Intensity
0.0
0.5
1.0
1.5
WT
C/EBP Beta Tg
HC-C/EBP Beta Tg
AD-C/EBP Beta Tg

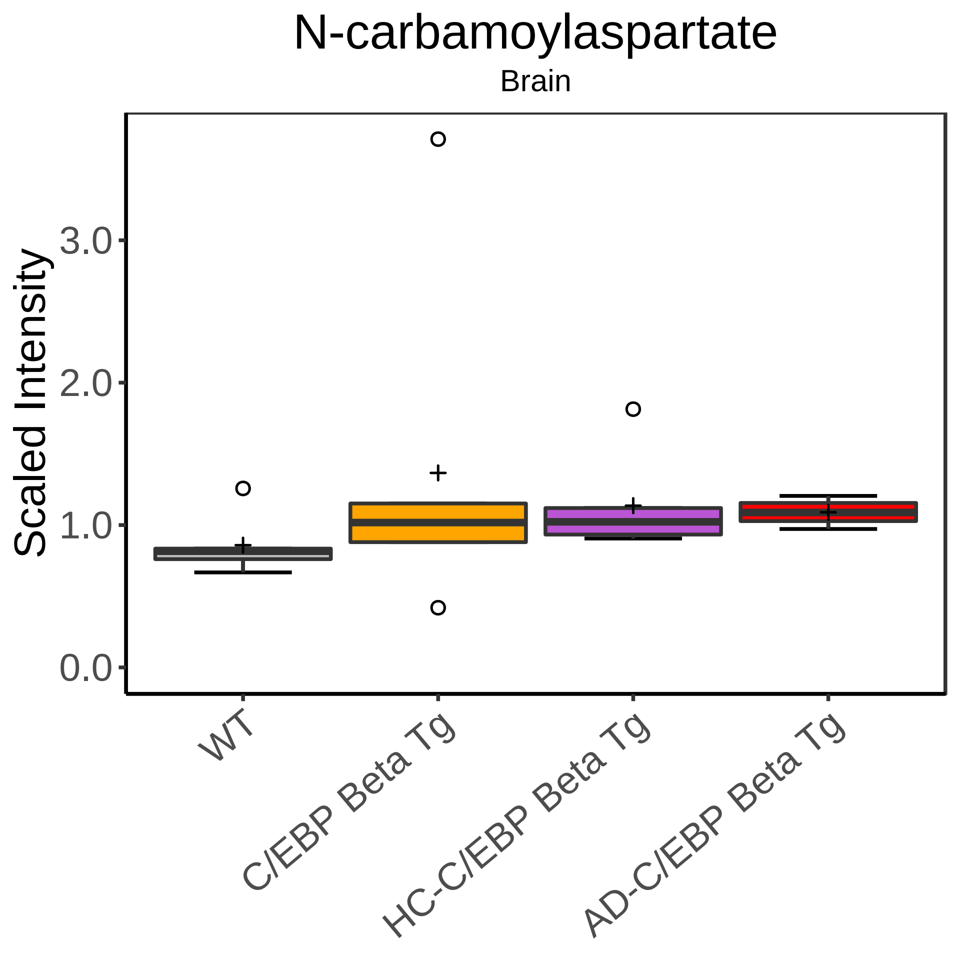
N-carbamoylaspartate
Brain
Scaled Intensity
3.0
2.0
1.0
0.0
WT
C/EBP Beta Tg
HC-C/EBP Beta Tg
AD-C/EBP Beta Tg

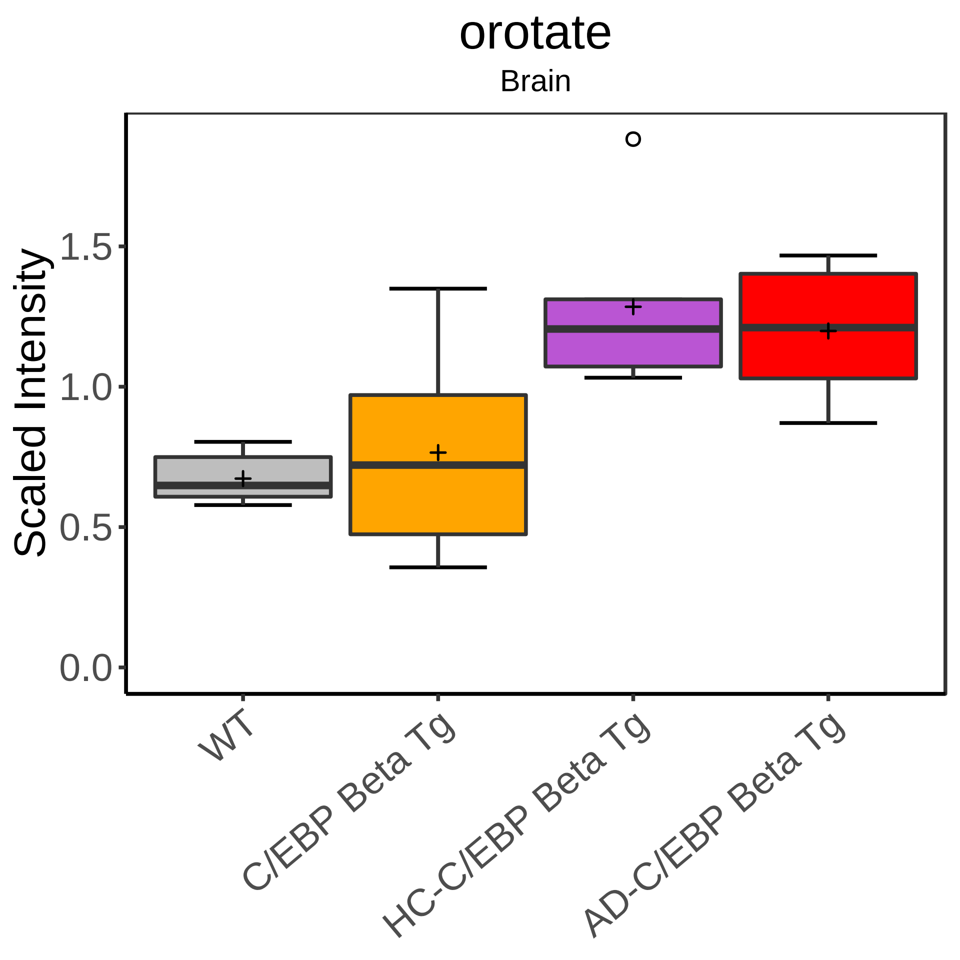

orotate
Brain
Scaled Intensity
0.0
0.5
1.0
1.5
WT
C/EBP Beta Tg
HC-C/EBP Beta Tg
AD-C/EBP Beta Tg

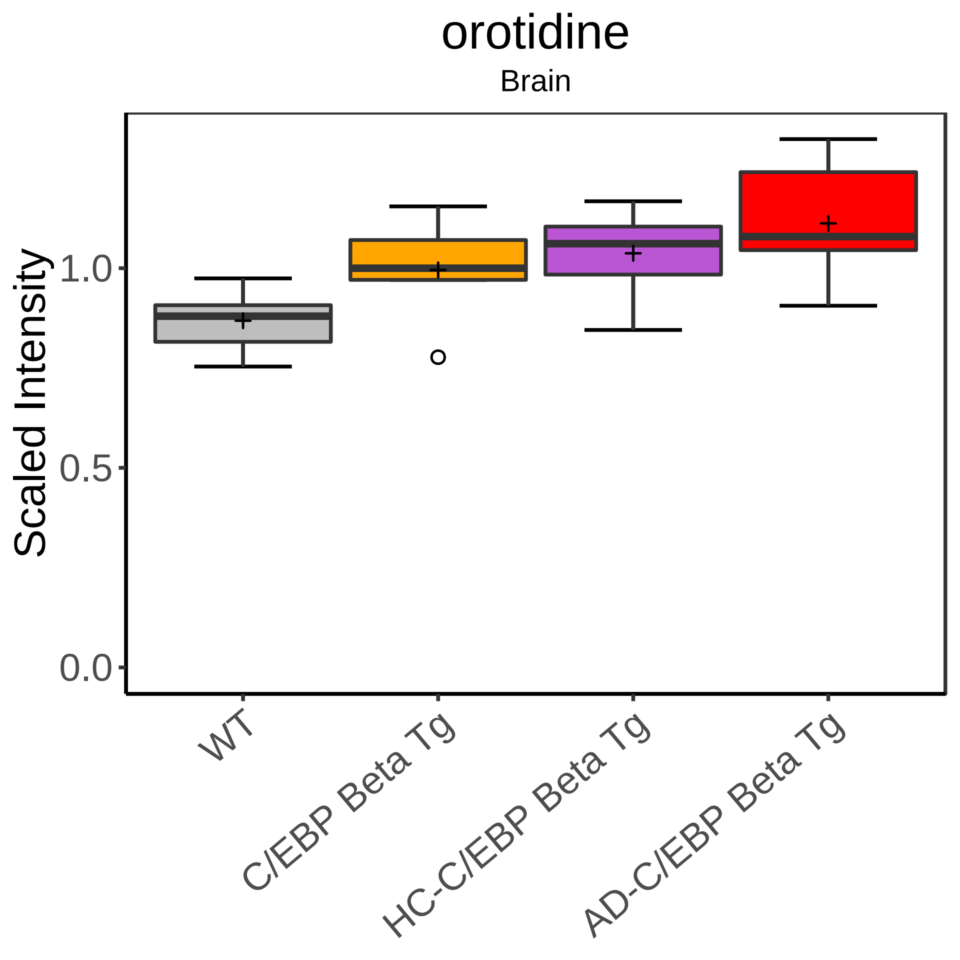
orotidine
Brain
Scaled Intensity
1.0
0.5
0.0
WT
C/EBP Beta Tg
HC-C/EBP Beta Tg
AD-C/EBP Beta Tg

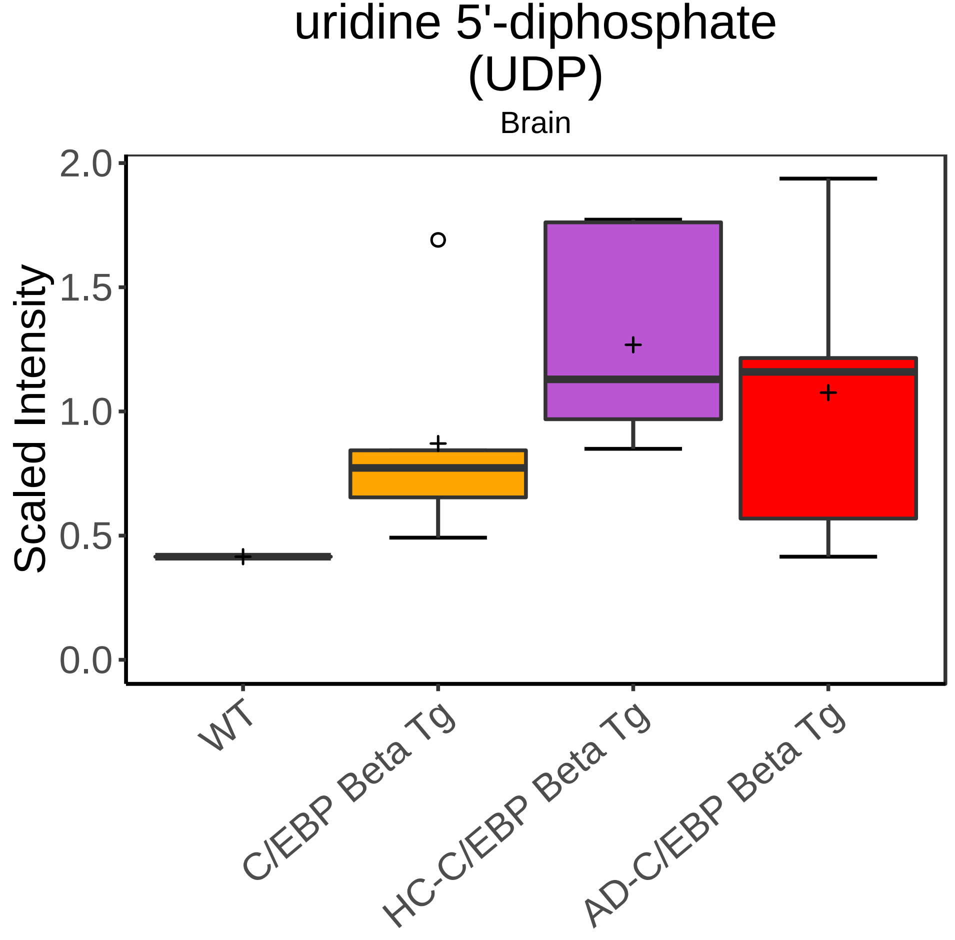
uridine 5'-diphosphate
(UDP)
Brain
Scaled Intensity
2.0
1.5
1.0
0.5
0.0
WT
C/EBP Beta Tg
HC-C/EBP Beta Tg
AD-C/EBP Beta Tg

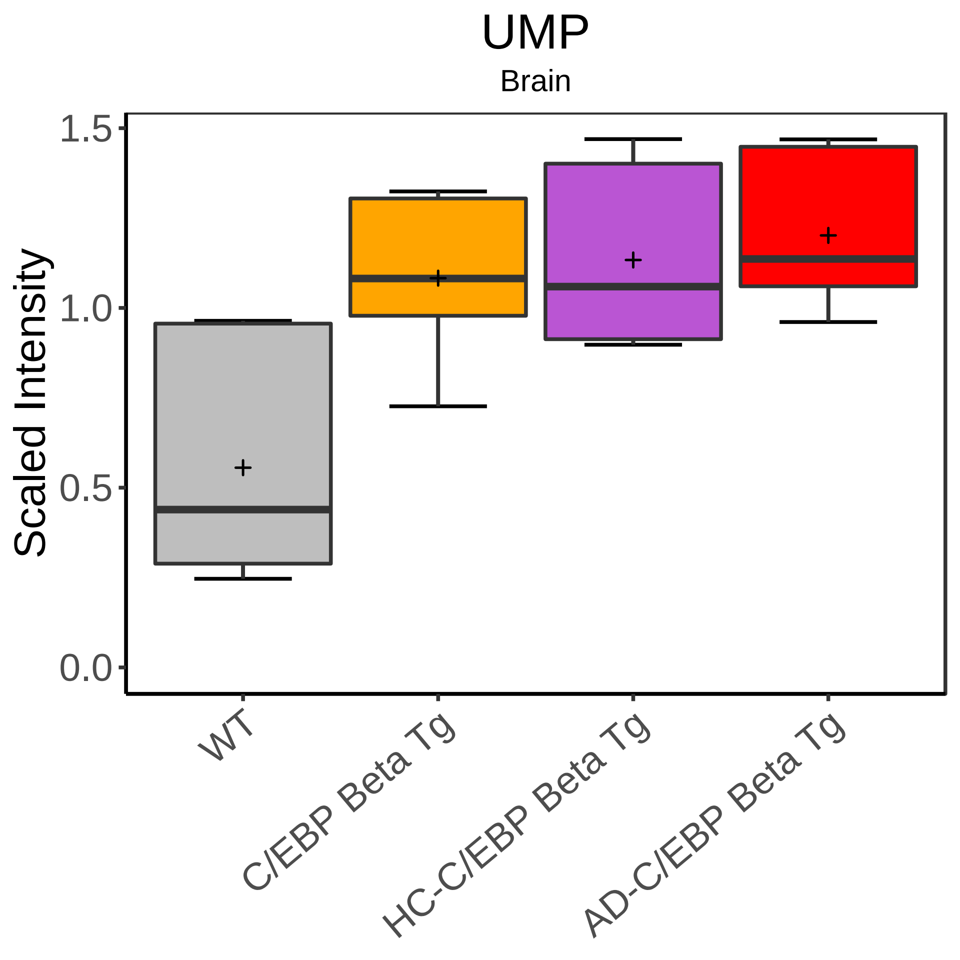
UMP
Brain
Scaled Intensity
0.0
0.5
1.0
1.5
WT
C/EBP Beta Tg
HC-C/EBP Beta Tg
AD-C/EBP Beta Tg

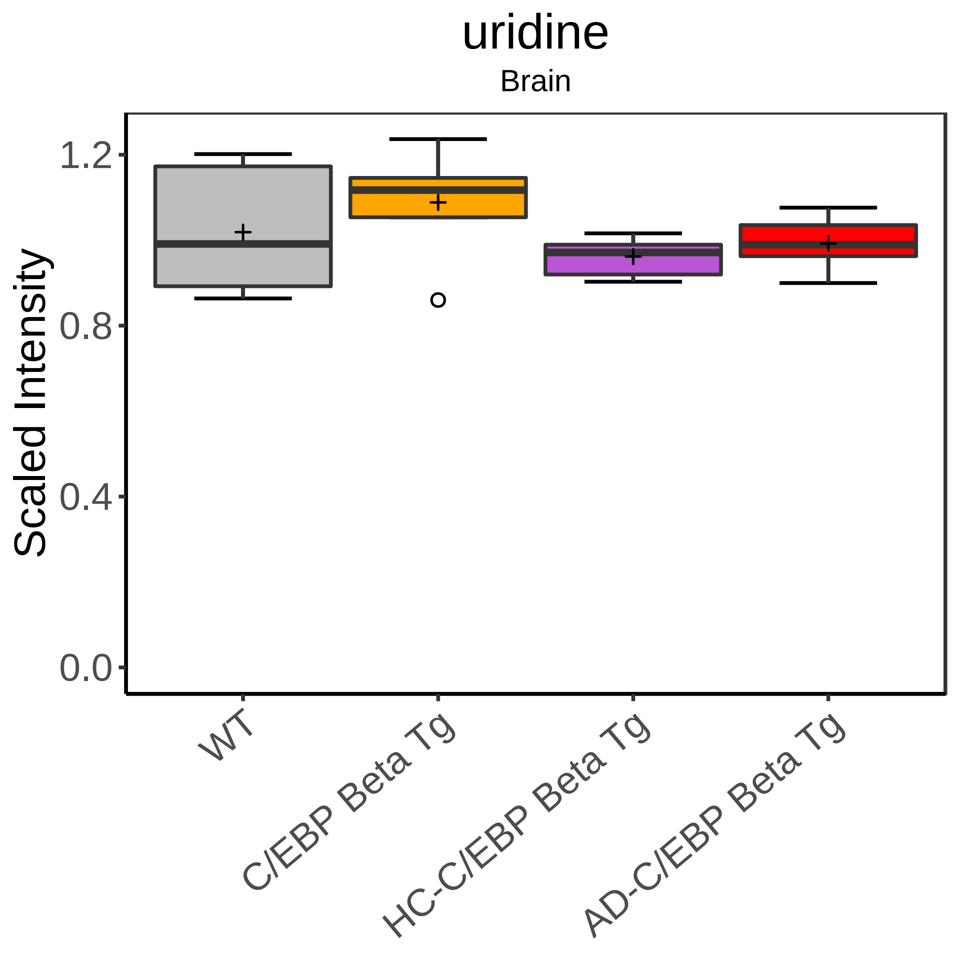

uridine
Brain
Scaled Intensity
1.2
0.8
0.4
0.0
WT
C/EBP Beta Tg
HC-C/EBP Beta Tg
AD-C/EBP Beta Tg

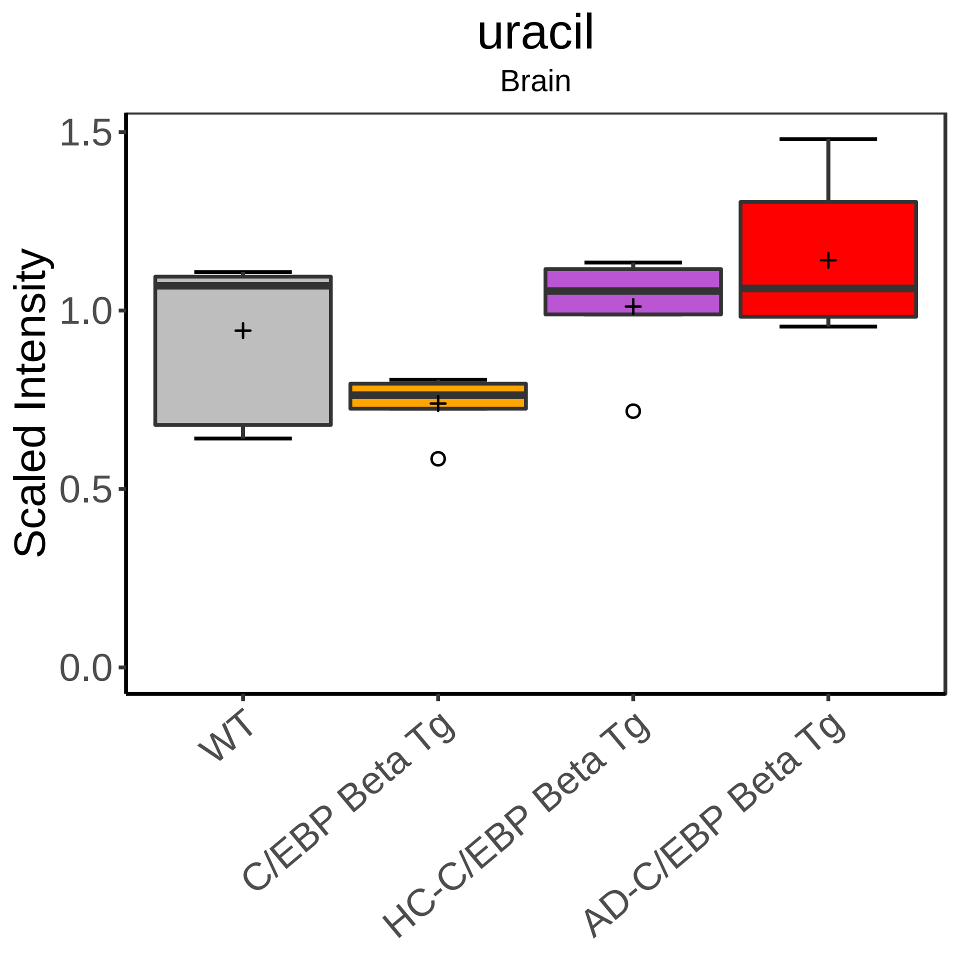
uracil
Brain
Scaled Intensity
1.5
1.0
0.5
0.0
WT
C/EBP Beta Tg
HC-C/EBP Beta Tg
AD-C/EBP Beta Tg

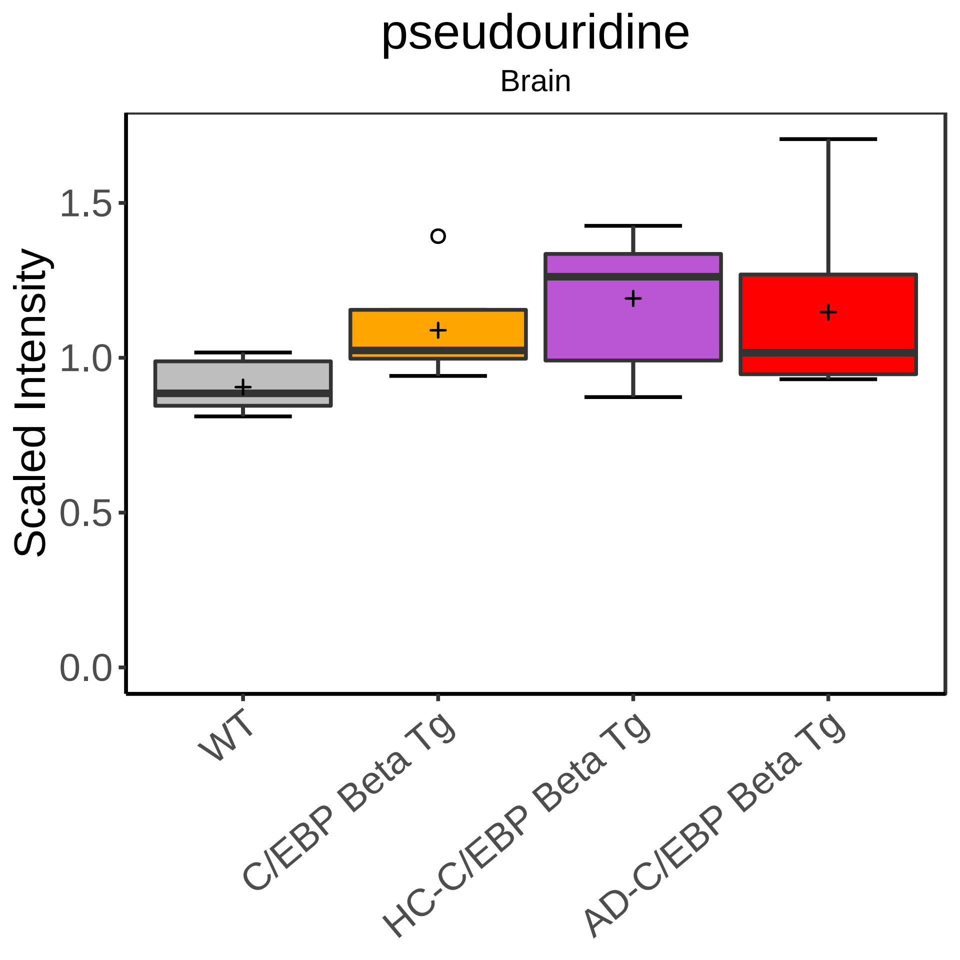

pseudouridine
Brain
Scaled Intensity
1.5
1.0
0.5
0.0
WT
C/EBP Beta Tg
HC-C/EBP Beta Tg
AD-C/EBP Beta Tg

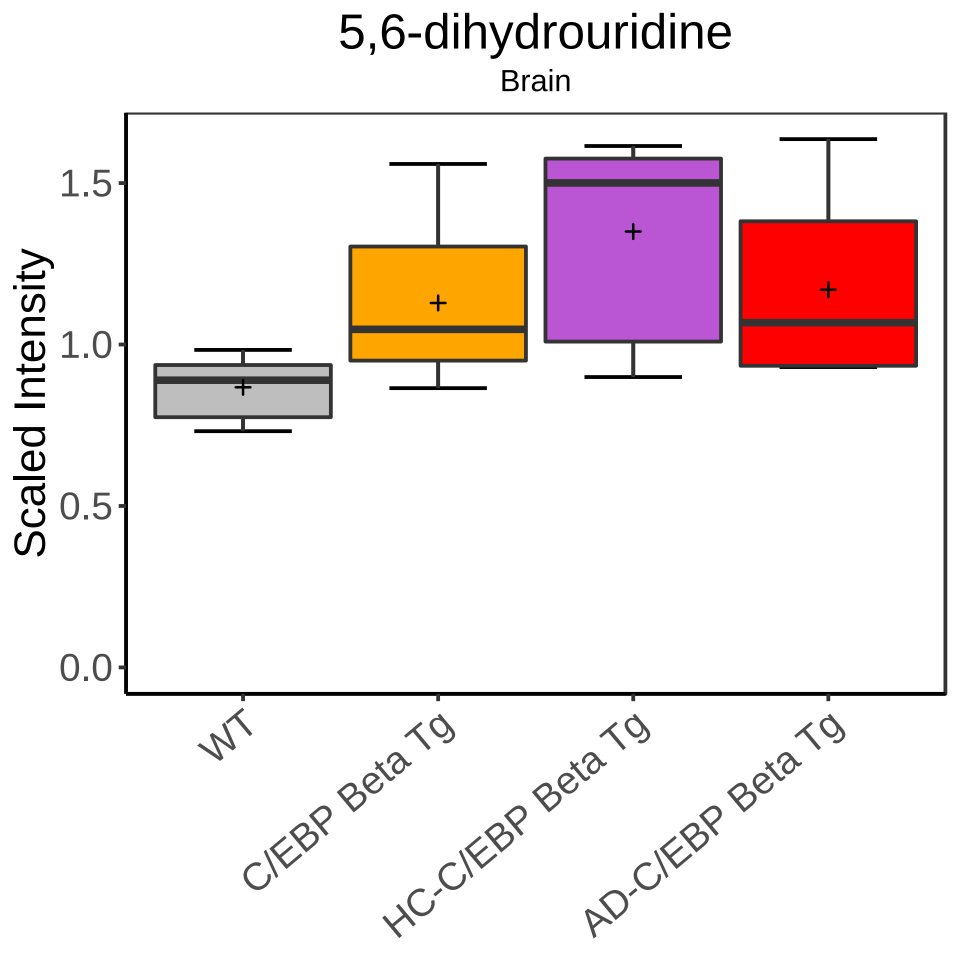

5,6-dihydrouridine
Brain
Scaled Intensity
0.0
0.5
1.0
1.5
WT
C/EBP Beta Tg
HC-C/EBP Beta Tg
AD-C/EBP Beta Tg

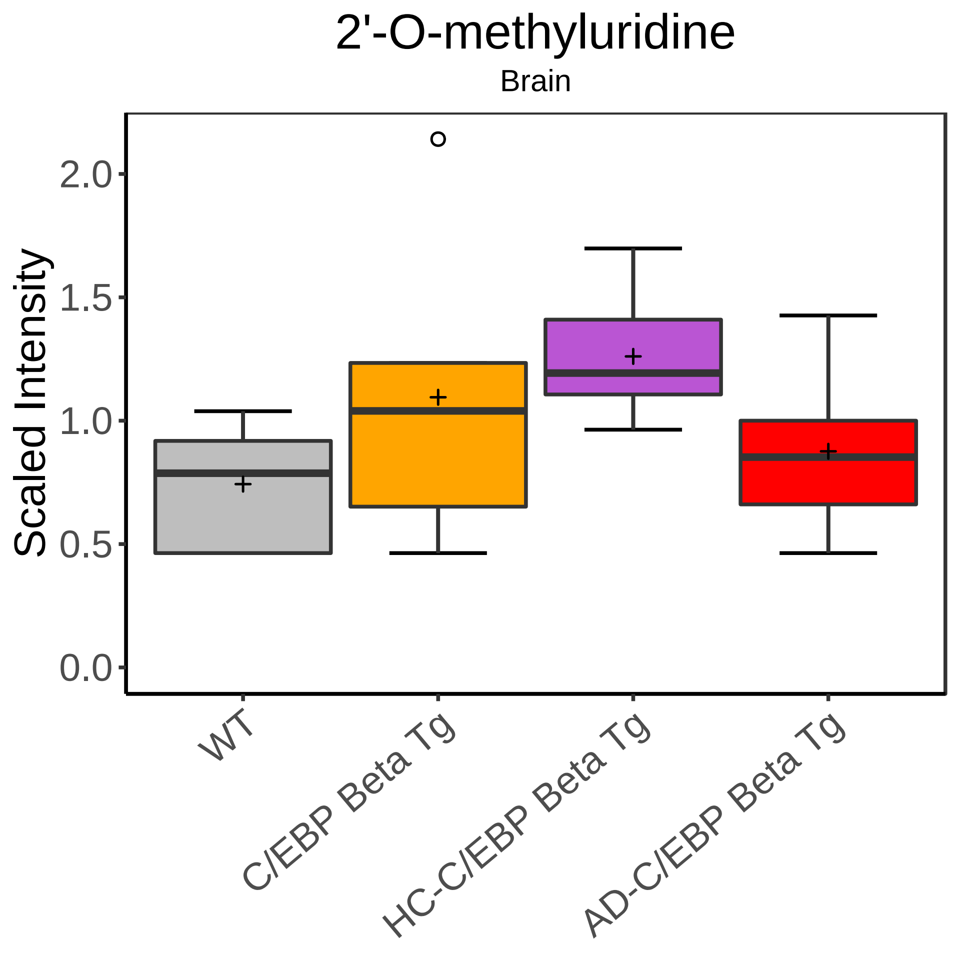
2'-O-methyluridine
Brain
Scaled Intensity
2.0
1.5
1.0
0.5
0.0
WT
C/EBP Beta Tg
HC-C/EBP Beta Tg
AD-C/EBP Beta Tg

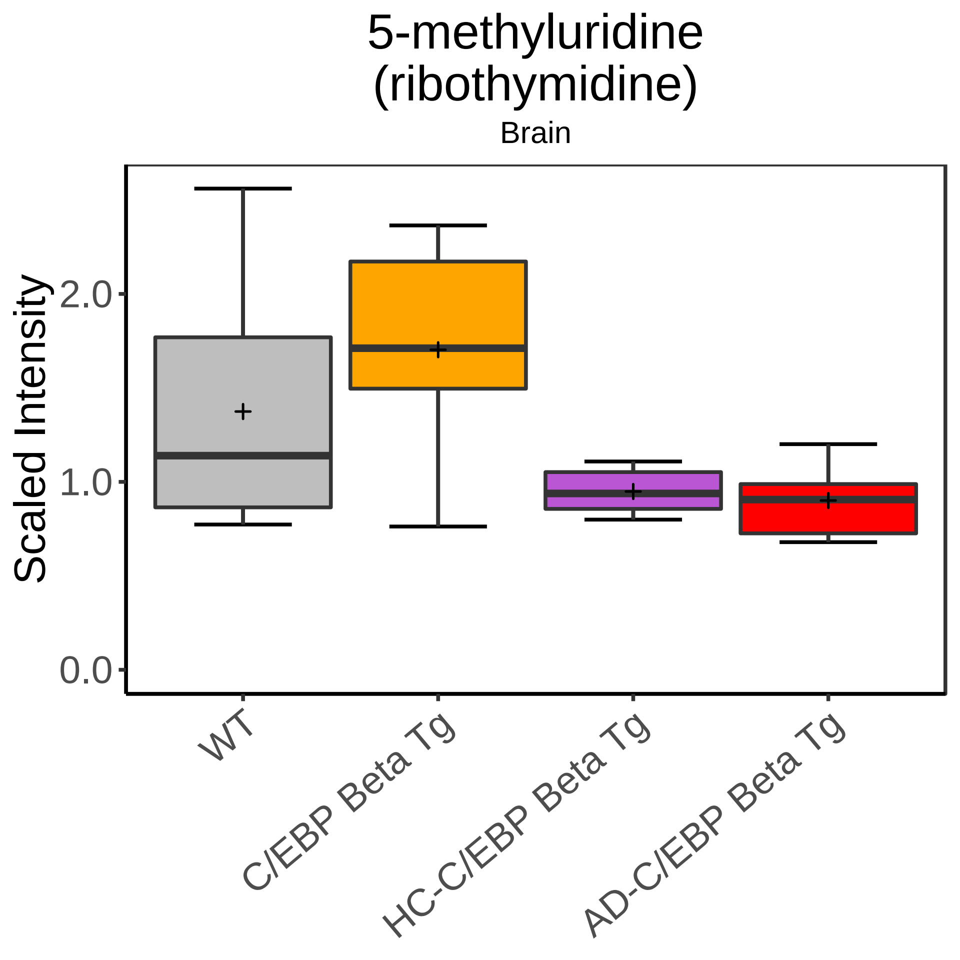

5-methyluridine
(ribothymidine)
Brain
Scaled Intensity
2.0
1.0
0.0
WT
C/EBP Beta Tg
HC-C/EBP Beta Tg
AD-C/EBP Beta Tg

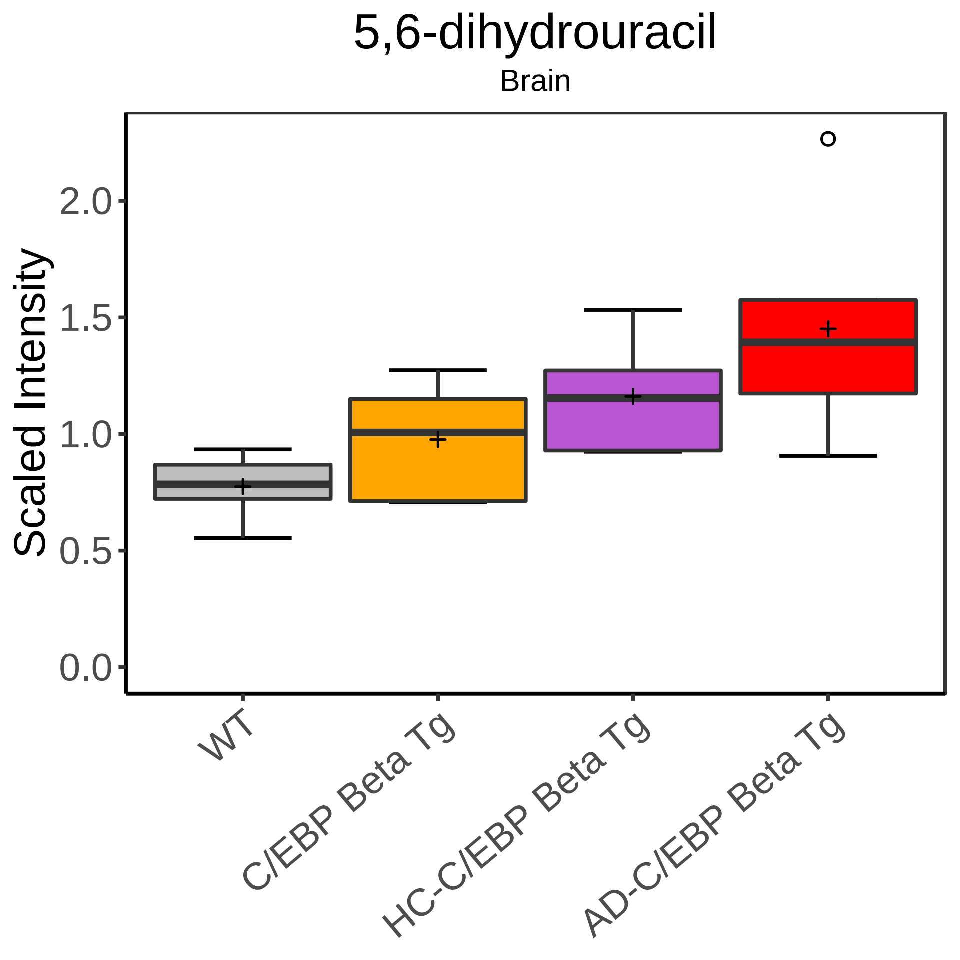
5,6-dihydrouracil
Brain
Scaled Intensity
2.0
1.5
1.0
0.5
0.0
WT
C/EBP Beta Tg
HC-C/EBP Beta Tg
AD-C/EBP Beta Tg

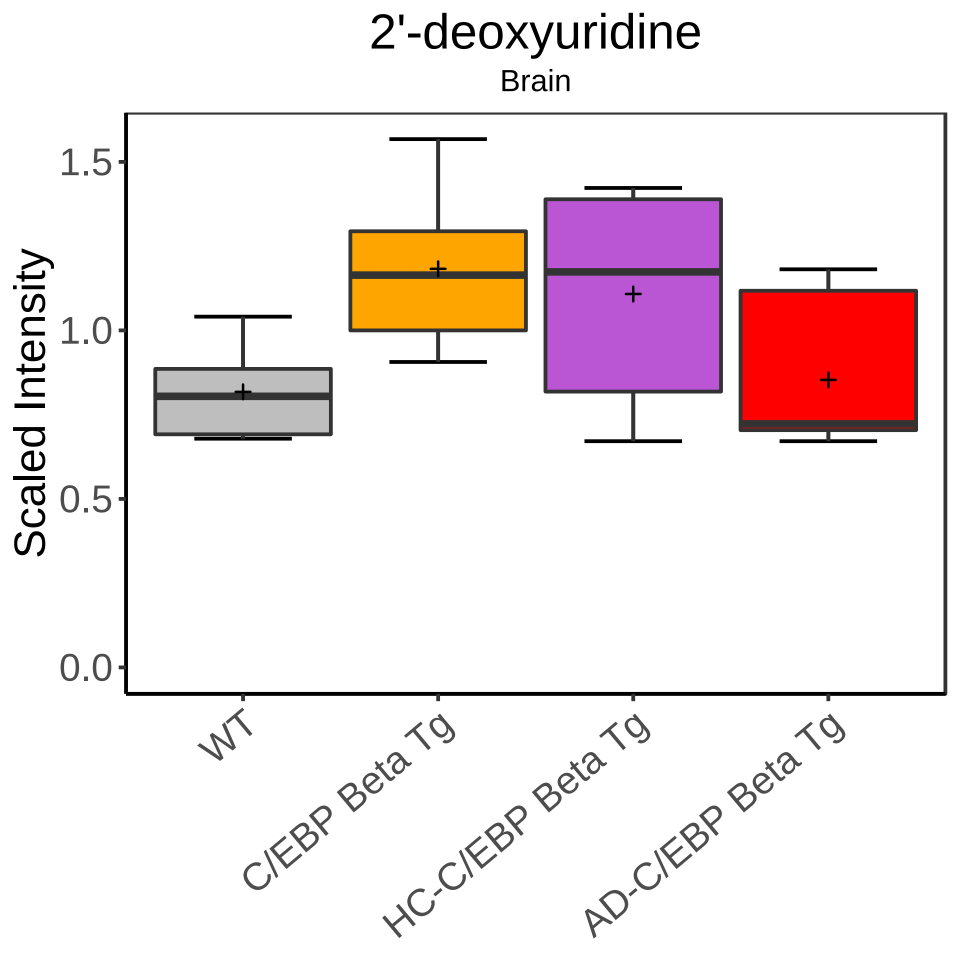
2'-deoxyuridine
Brain
Scaled Intensity
1.5
1.0
0.5
0.0
WT
C/EBP Beta Tg
HC-C/EBP Beta Tg
AD-C/EBP Beta Tg

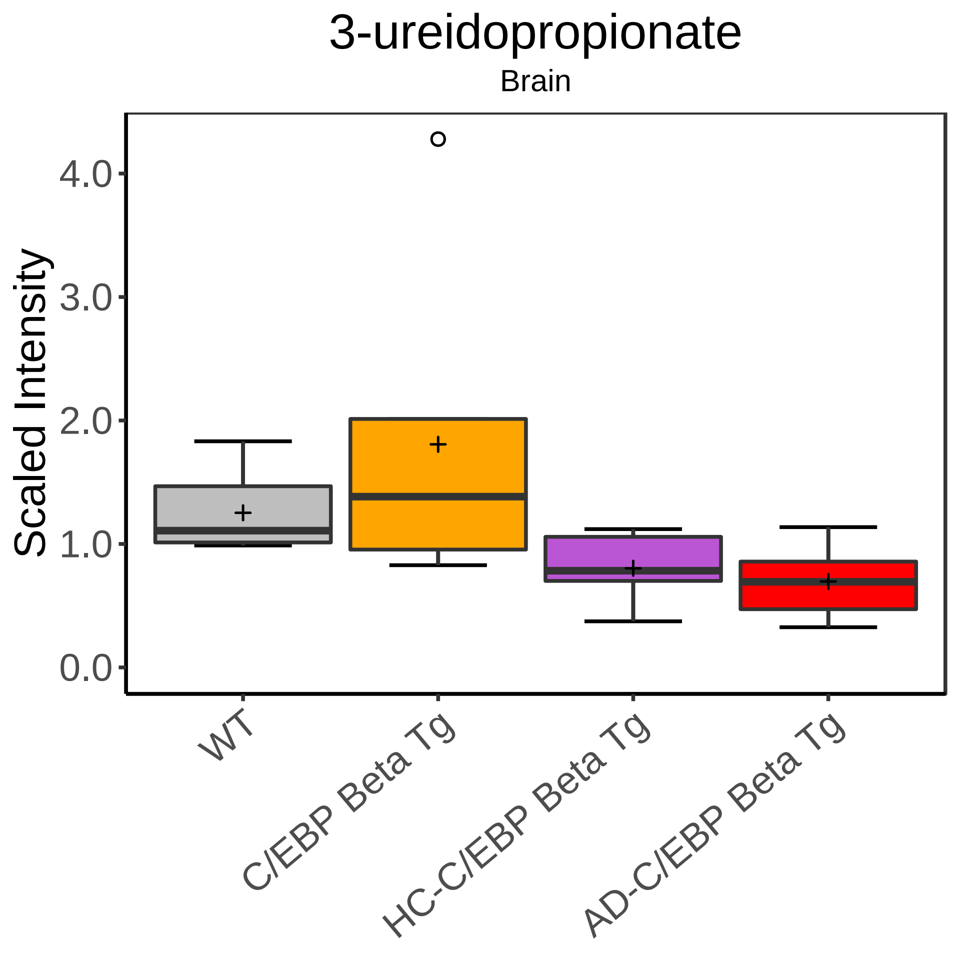
3-ureidopropionate
Brain
Scaled Intensity
4.0
3.0
2.0
1.0
0.0
WT
C/EBP Beta Tg
HC-C/EBP Beta Tg
AD-C/EBP Beta Tg

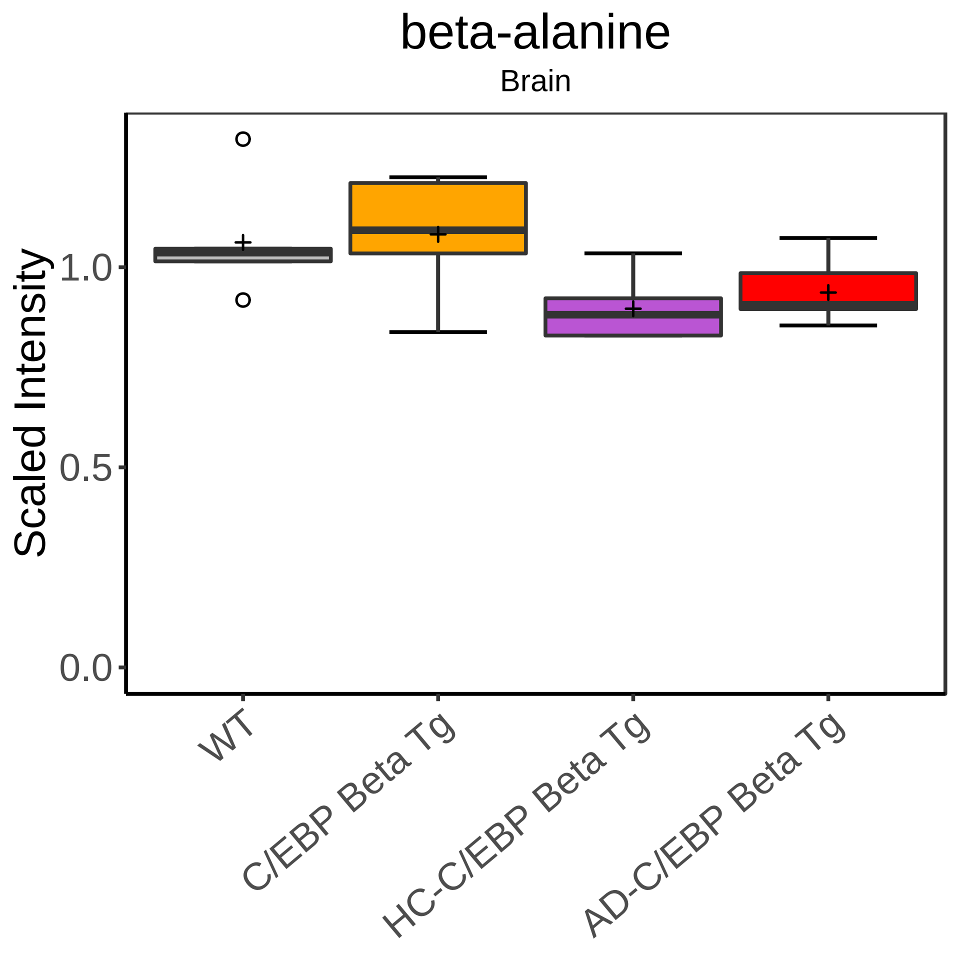
beta-alanine
Brain
Scaled Intensity
1.0
0.5
0.0
WT
C/EBP Beta Tg
HC-C/EBP Beta Tg
AD-C/EBP Beta Tg

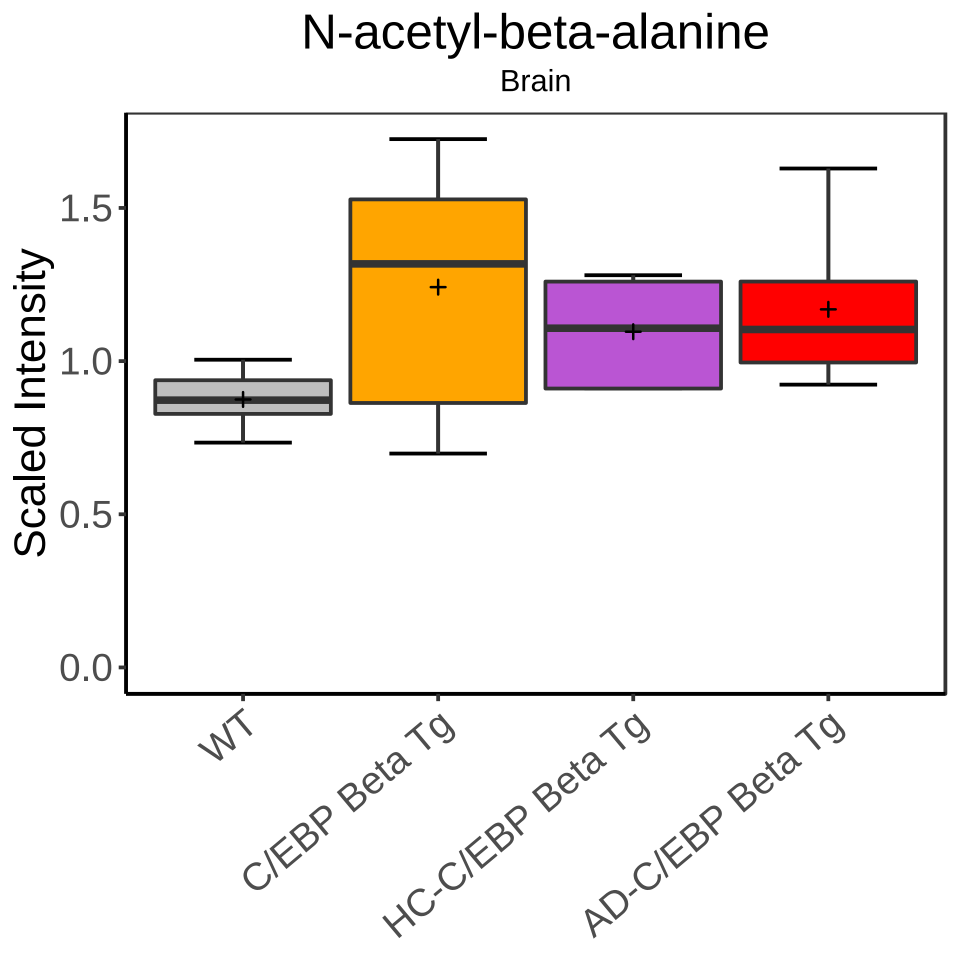
N-acetyl-beta-alanine
Brain
Scaled Intensity
1.5
1.0
0.5
0.0
WT
C/EBP Beta Tg
HC-C/EBP Beta Tg
AD-C/EBP Beta Tg

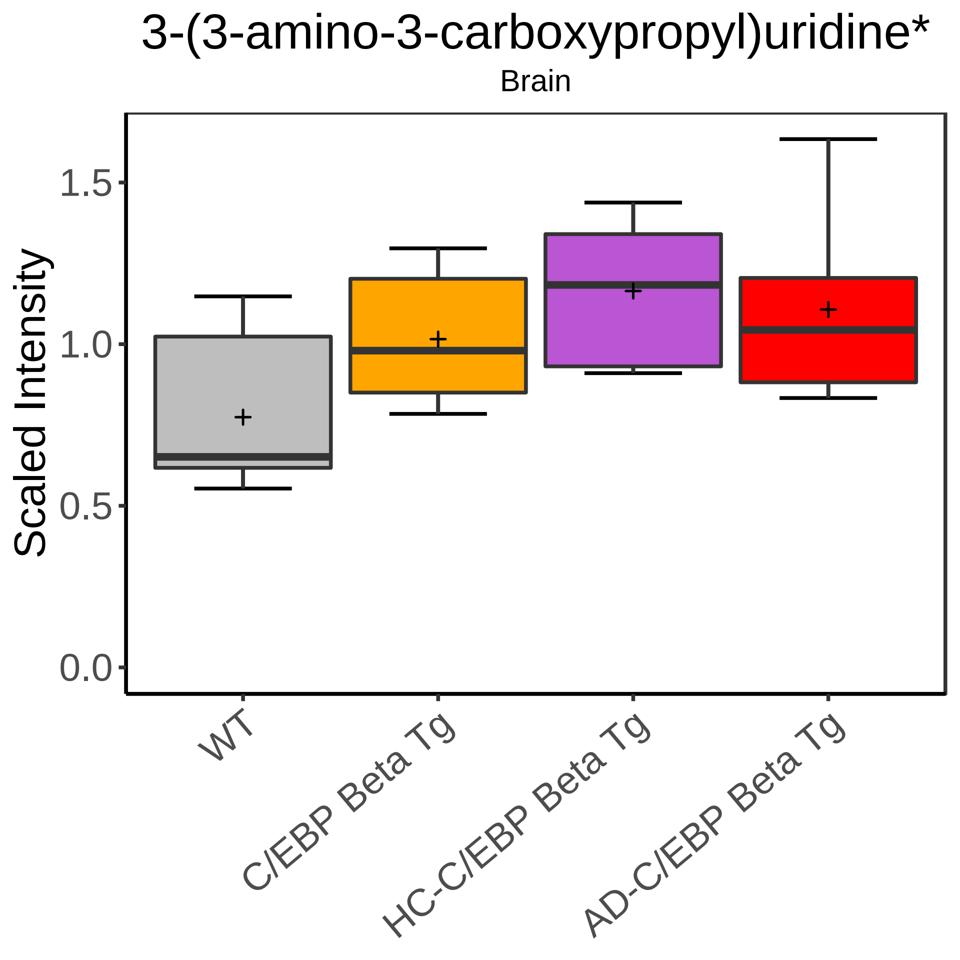

3-(3-amino-3-carboxypropyl)uridine*
Brain
Scaled Intensity
0.0
0.5
1.0
1.5
WT
C/EBP Beta Tg
HC-C/EBP Beta Tg
AD-C/EBP Beta Tg

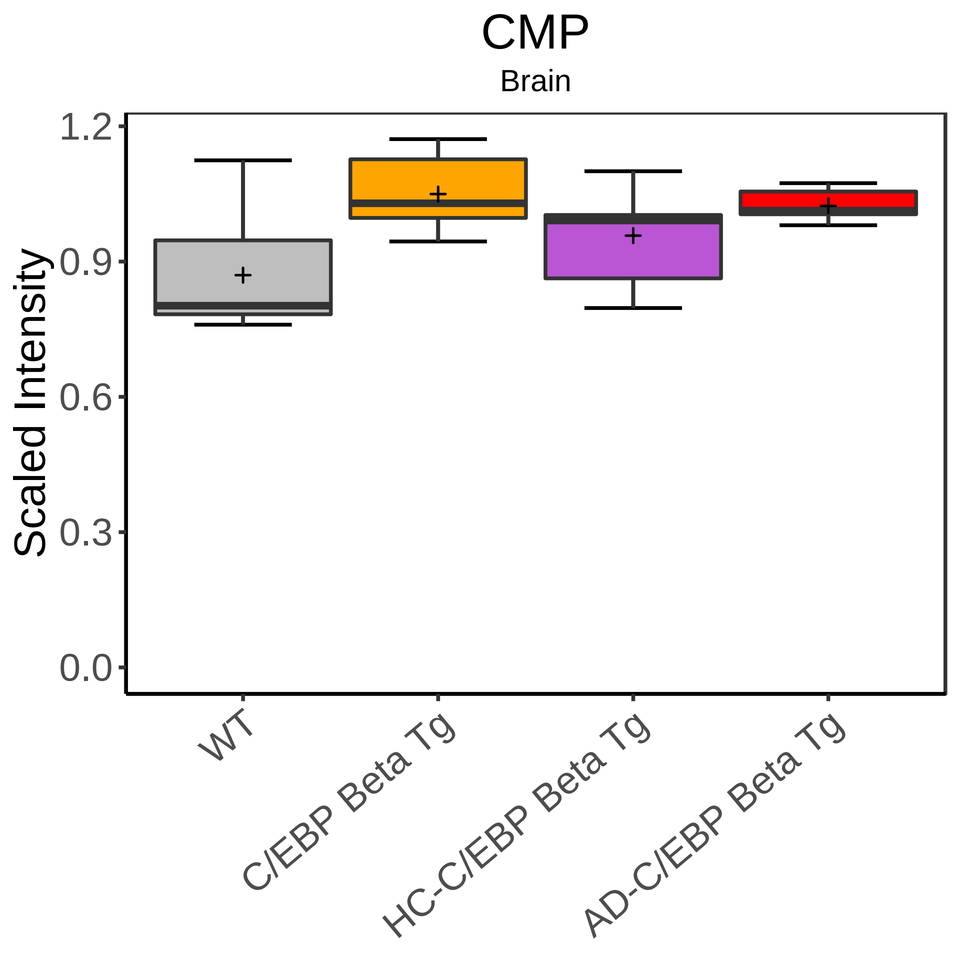
CMP
Brain
Scaled Intensity
1.2
0.9
0.6
0.3
0.0
WT
C/EBP Beta Tg
HC-C/EBP Beta Tg
AD-C/EBP Beta Tg

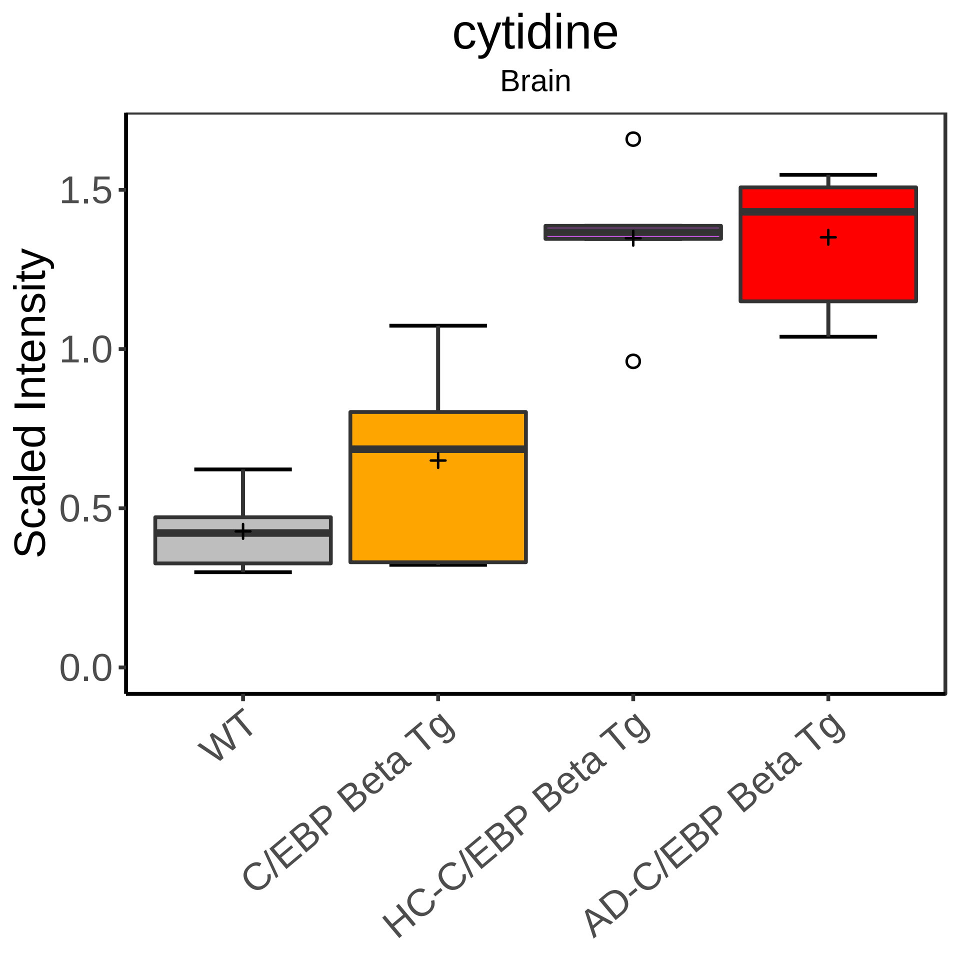
cytidine
Brain
Scaled Intensity
0.0
0.5
1.0
1.5
WT
C/EBP Beta Tg
HC-C/EBP Beta Tg
AD-C/EBP Beta Tg

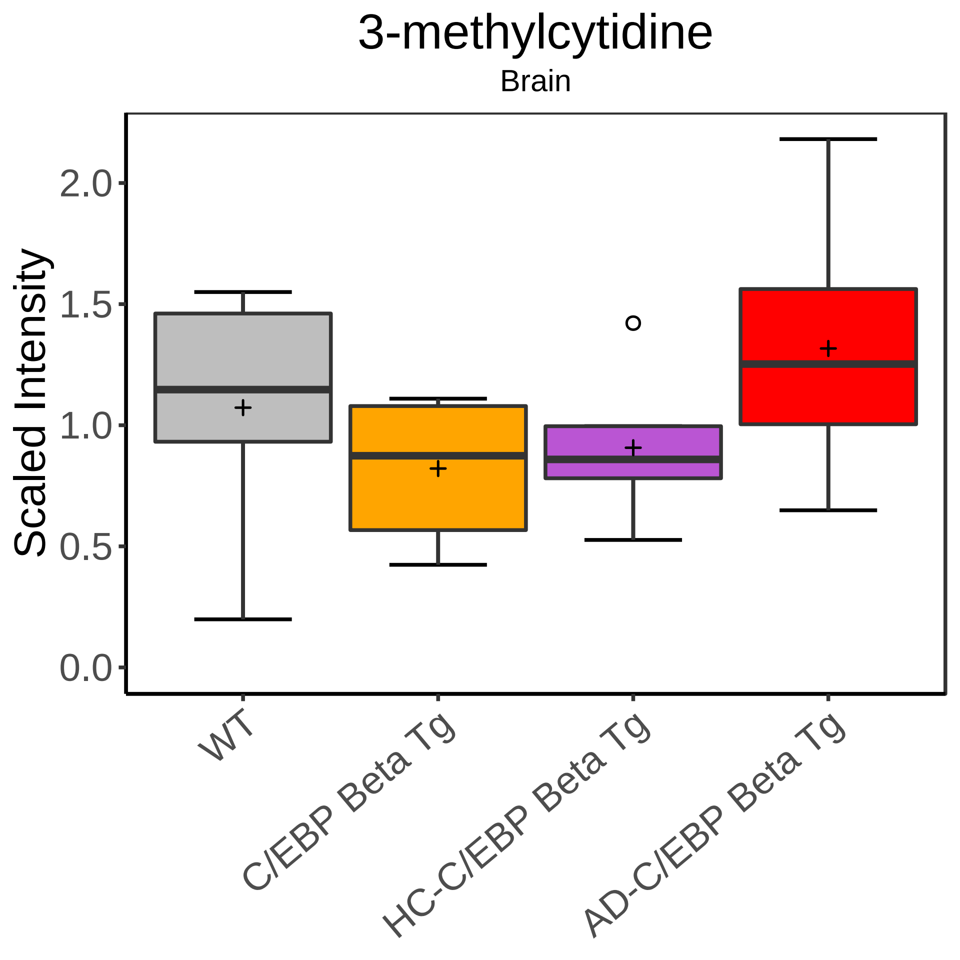

3-methylcytidine
Brain
Scaled Intensity
2.0
1.5
1.0
0.5
0.0
WT
C/EBP Beta Tg
HC-C/EBP Beta Tg
AD-C/EBP Beta Tg

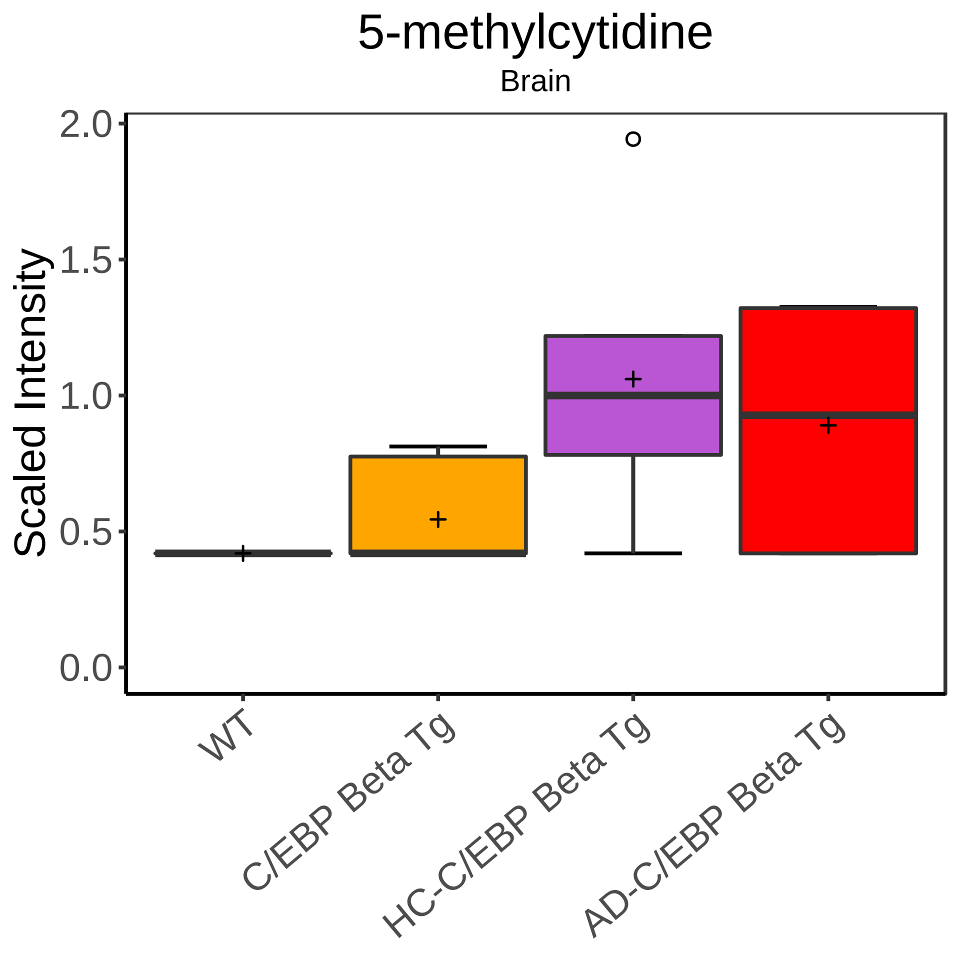
5-methylcytidine
Brain
Scaled Intensity
2.0
1.5
1.0
0.5
0.0
WT
C/EBP Beta Tg
HC-C/EBP Beta Tg
AD-C/EBP Beta Tg

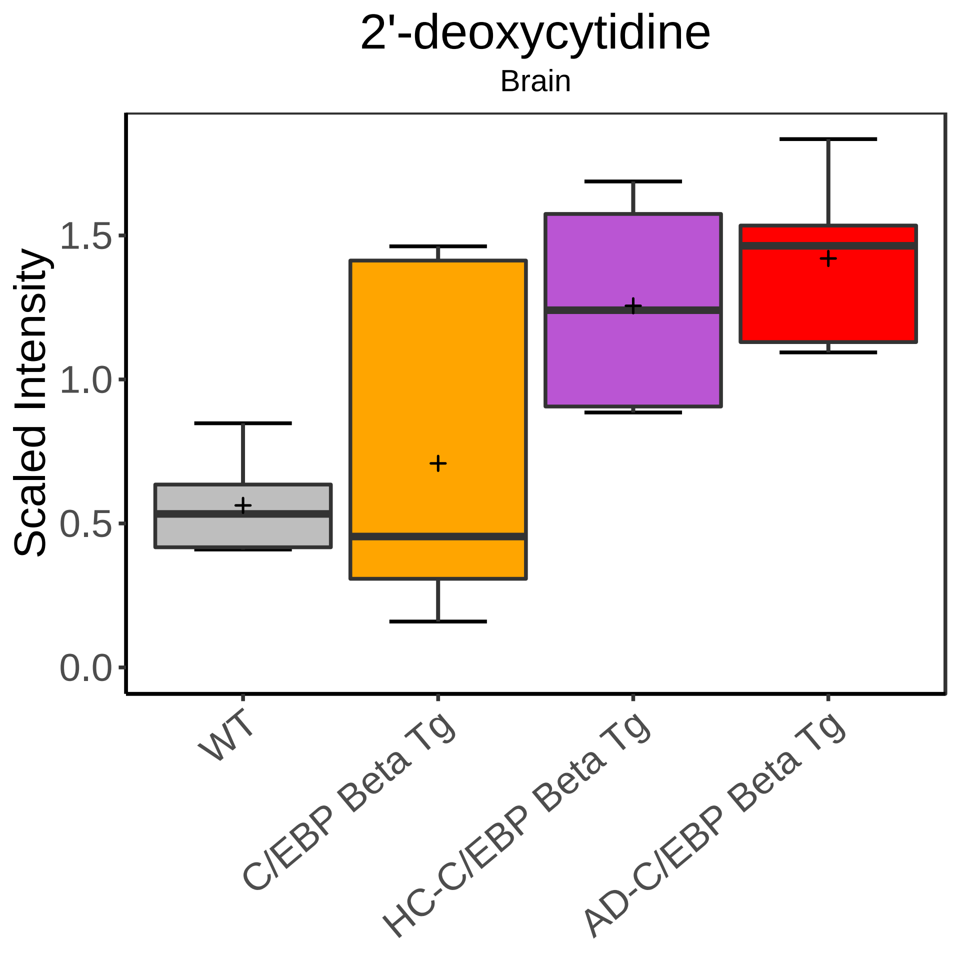
2'-deoxycytidine
Brain
Scaled Intensity
0.0
0.5
1.0
1.5
WT
C/EBP Beta Tg
HC-C/EBP Beta Tg
AD-C/EBP Beta Tg

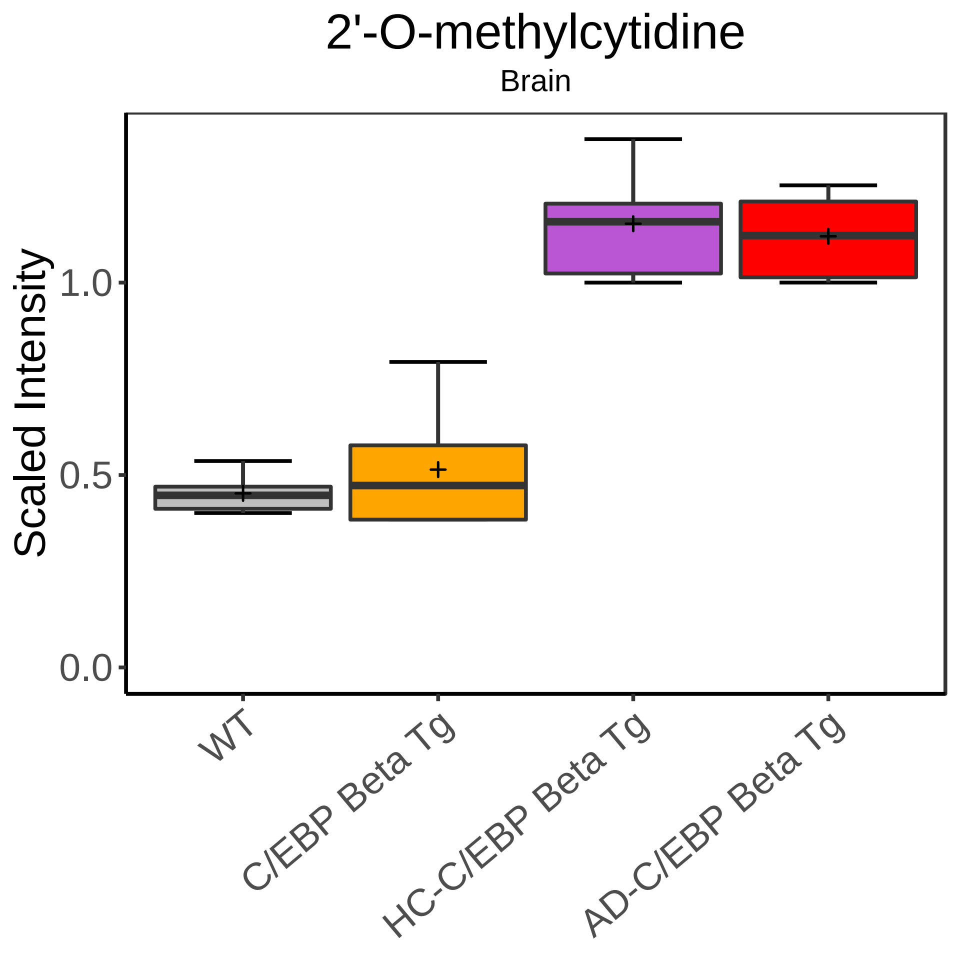
2'-O-methylcytidine
Brain
Scaled Intensity
1.0
0.5
0.0
WT
C/EBP Beta Tg
HC-C/EBP Beta Tg
AD-C/EBP Beta Tg

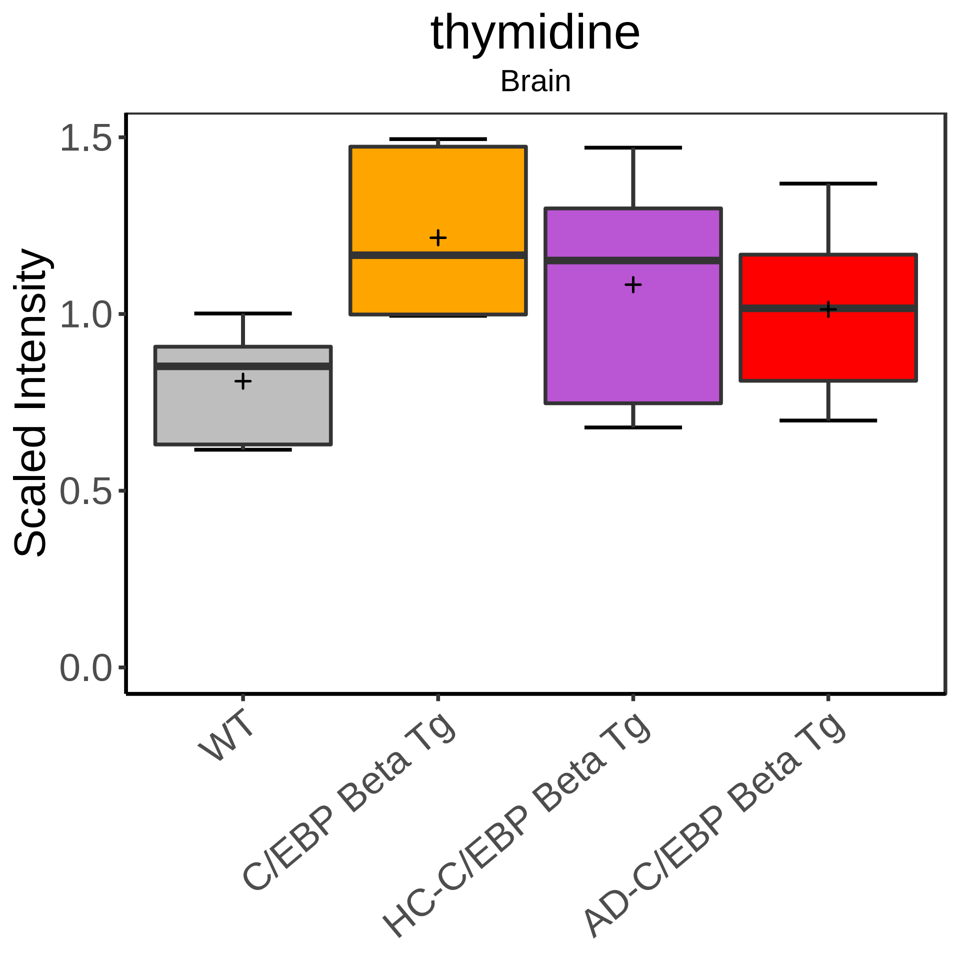

thymidine
Brain
Scaled Intensity
0.0
0.5
1.0
1.5
WT
C/EBP Beta Tg
HC-C/EBP Beta Tg
AD-C/EBP Beta Tg

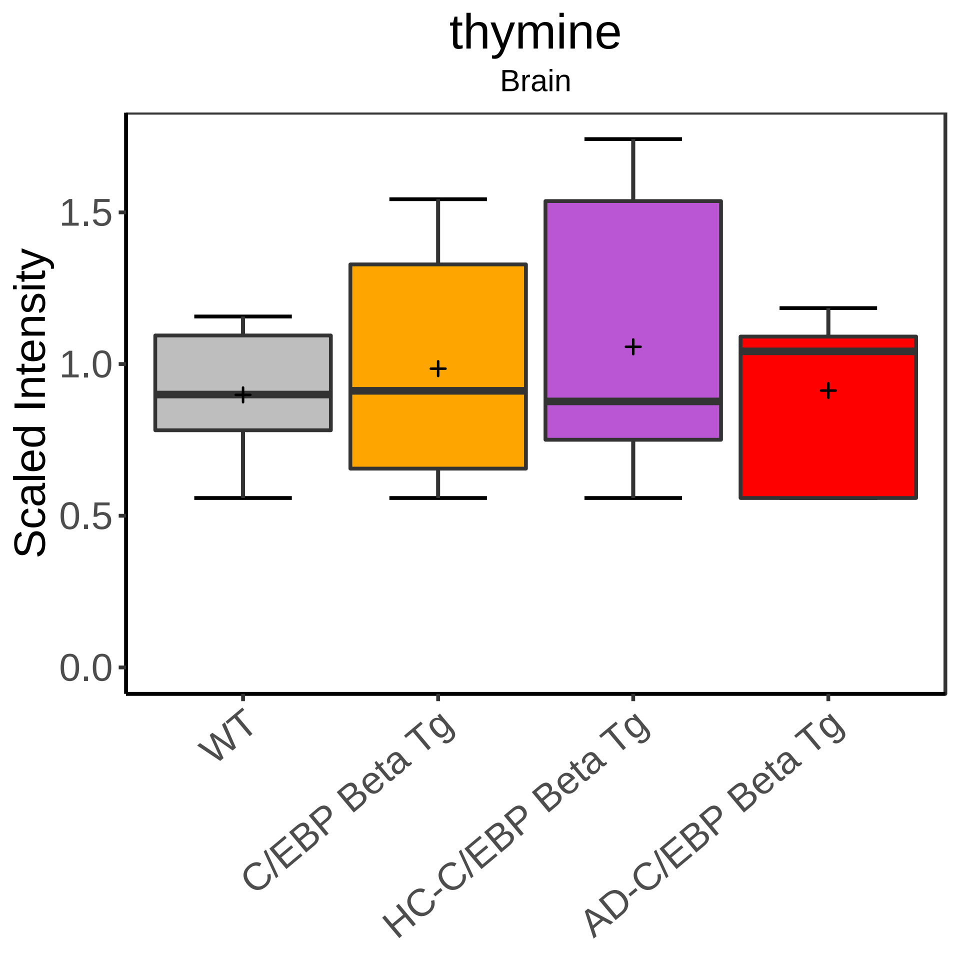

thymine
Brain
Scaled Intensity
0.0
0.5
1.0
1.5
WT
C/EBP Beta Tg
HC-C/EBP Beta Tg
AD-C/EBP Beta Tg

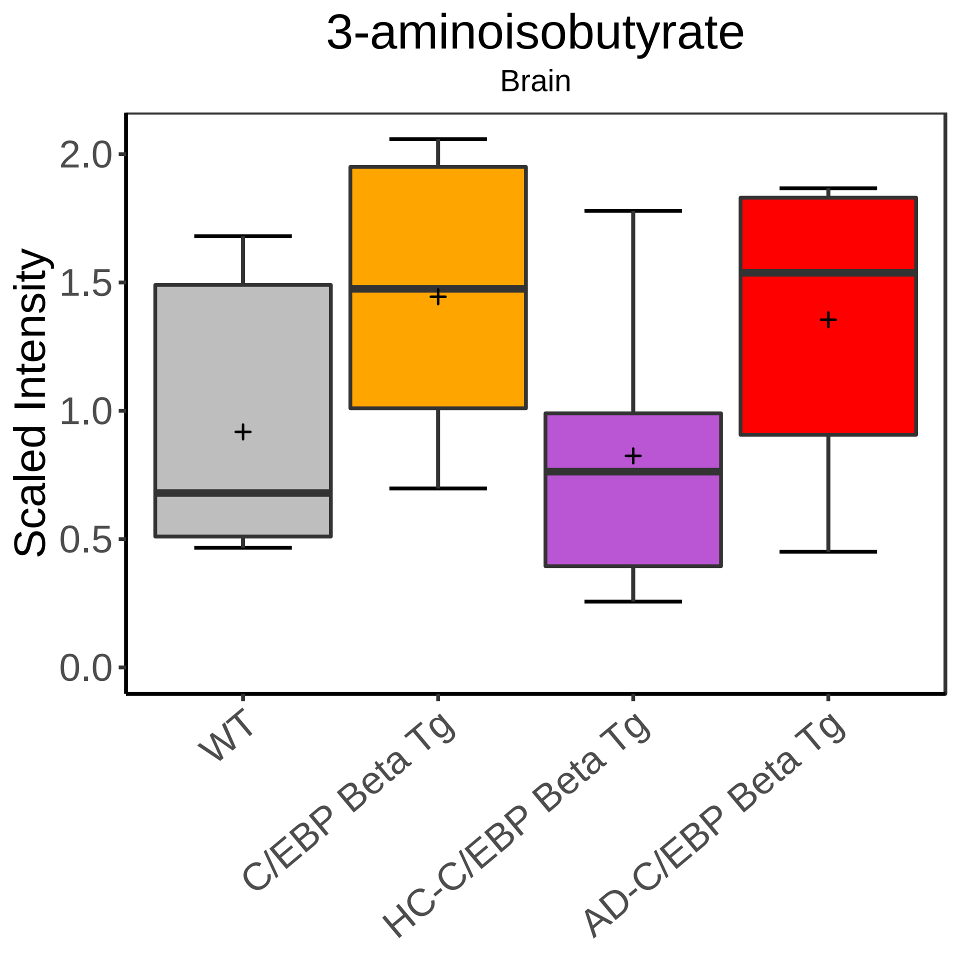
3-aminoisobutyrate
Brain
Scaled Intensity
2.0
1.5
1.0
0.5
0.0
WT
C/EBP Beta Tg
HC-C/EBP Beta Tg
AD-C/EBP Beta Tg

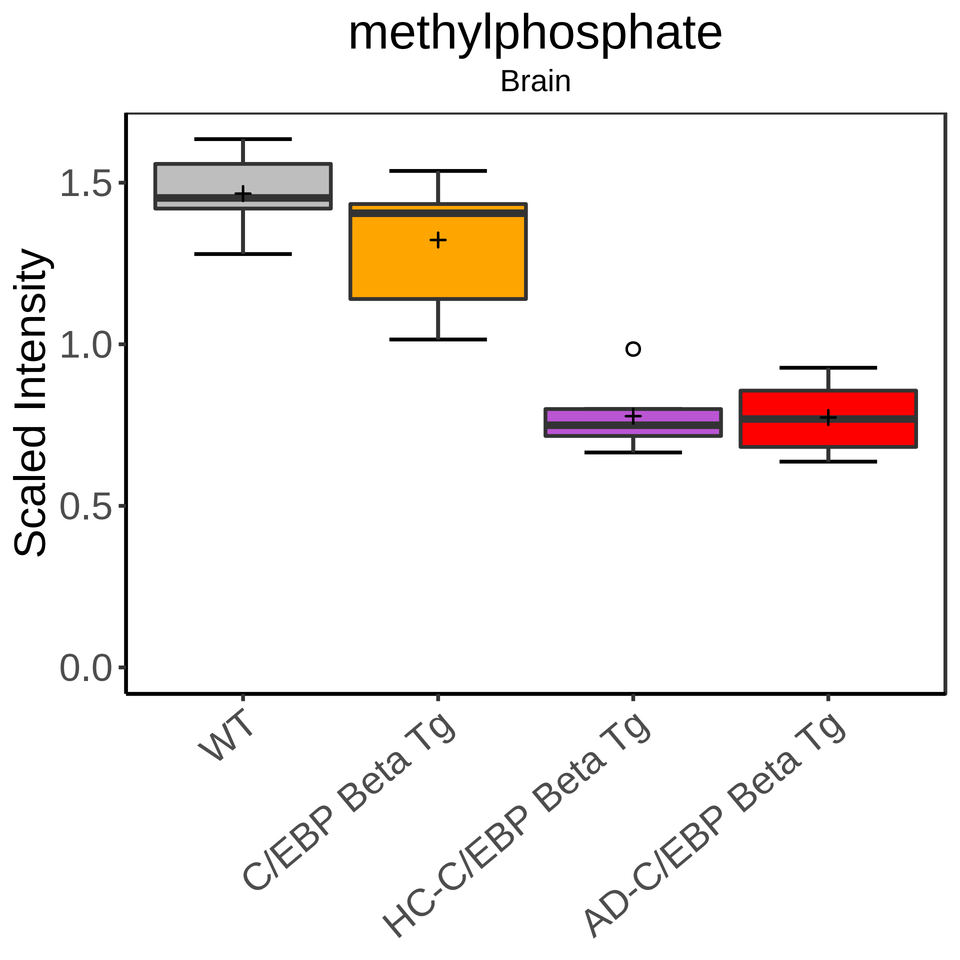

methylphosphate
Brain
Scaled Intensity
0.0
0.5
1.0
1.5
WT
C/EBP Beta Tg
HC-C/EBP Beta Tg
AD-C/EBP Beta Tg

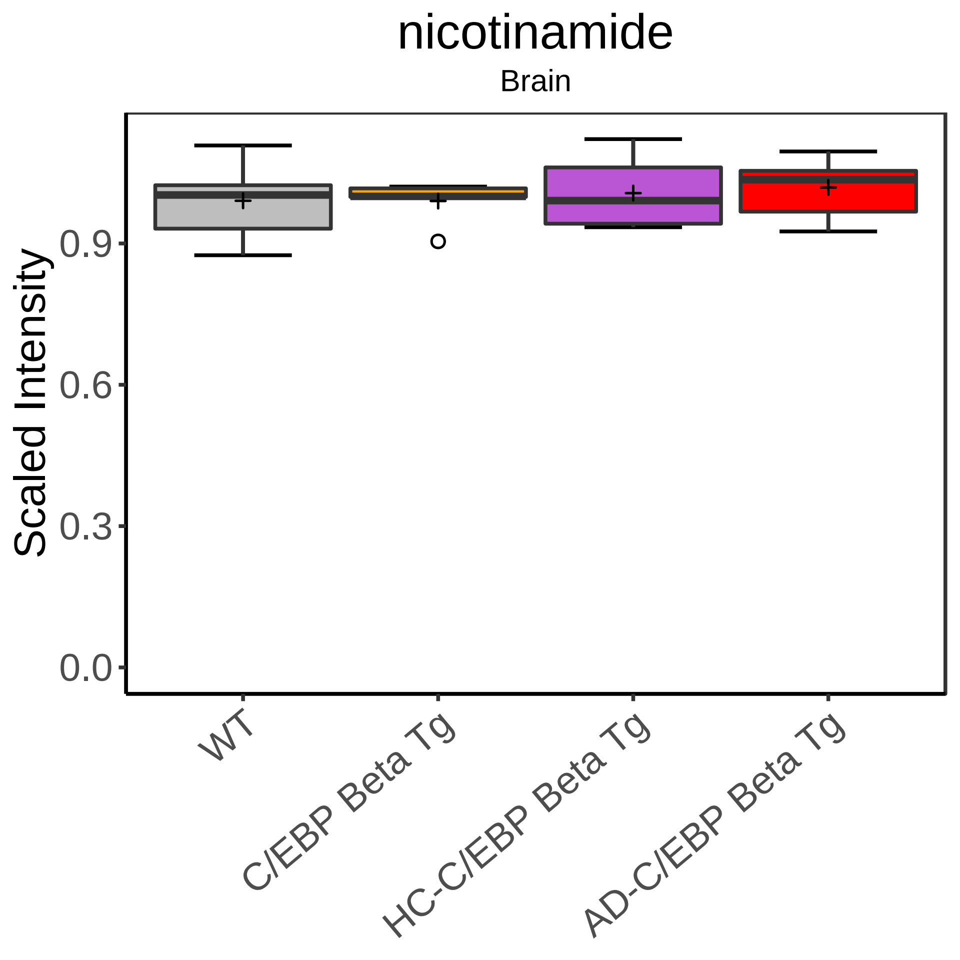
nicotinamide
Brain
Scaled Intensity
0.0
0.3
0.6
0.9
WT
C/EBP Beta Tg
HC-C/EBP Beta Tg
AD-C/EBP Beta Tg

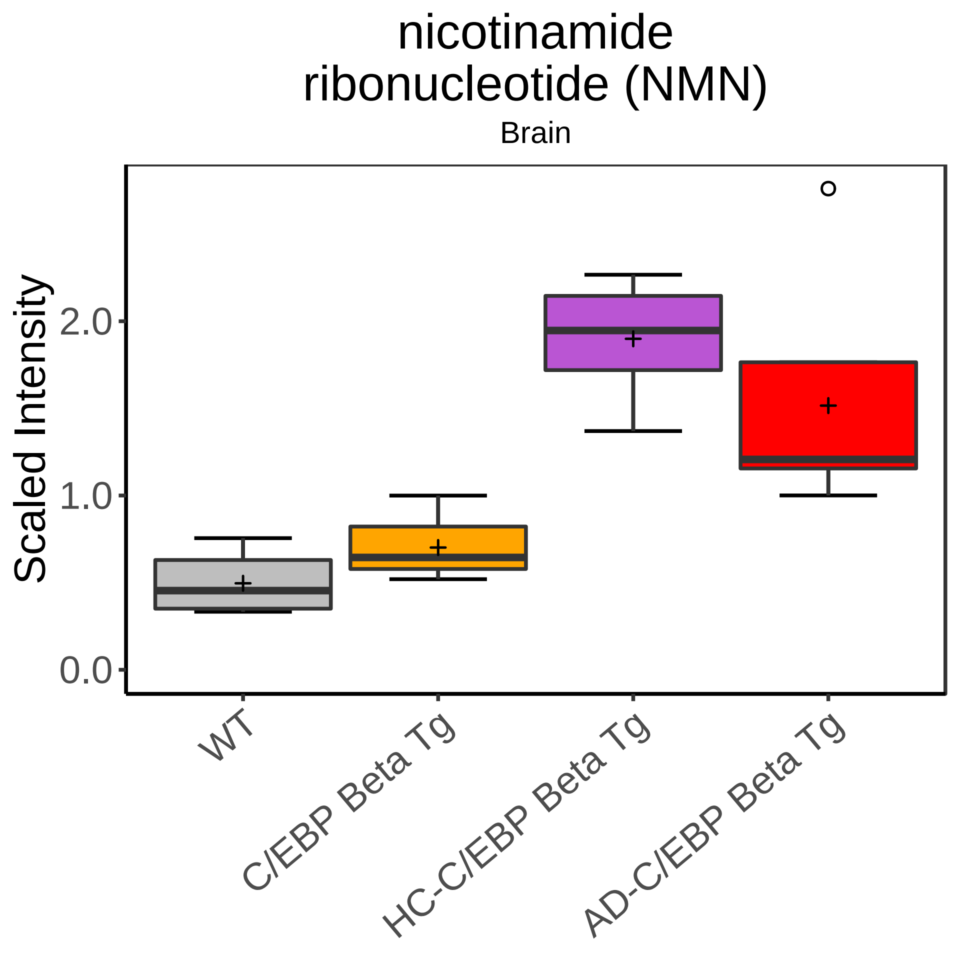
nicotinamide
ribonucleotide (NMN)
Brain
Scaled Intensity
2.0
1.0
0.0
WT
C/EBP Beta Tg
HC-C/EBP Beta Tg
AD-C/EBP Beta Tg

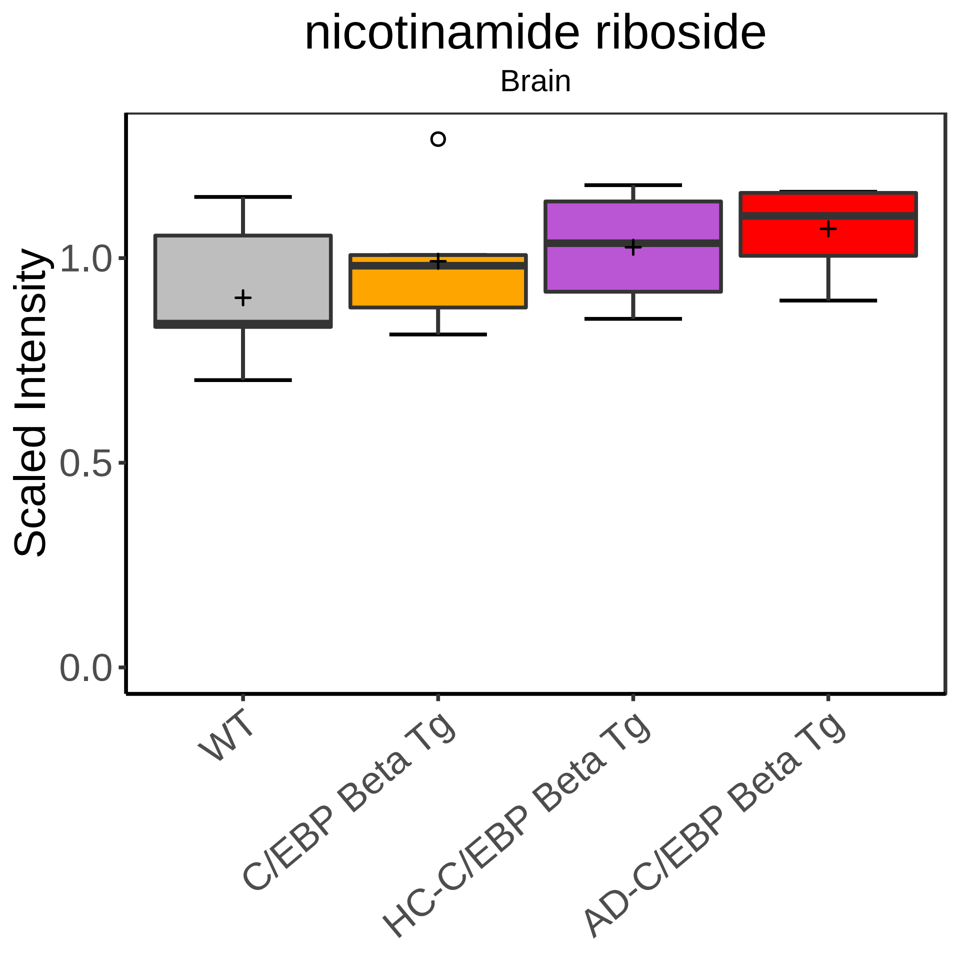
nicotinamide riboside
Brain
Scaled Intensity
1.0
0.5
0.0
WT
C/EBP Beta Tg
HC-C/EBP Beta Tg
AD-C/EBP Beta Tg

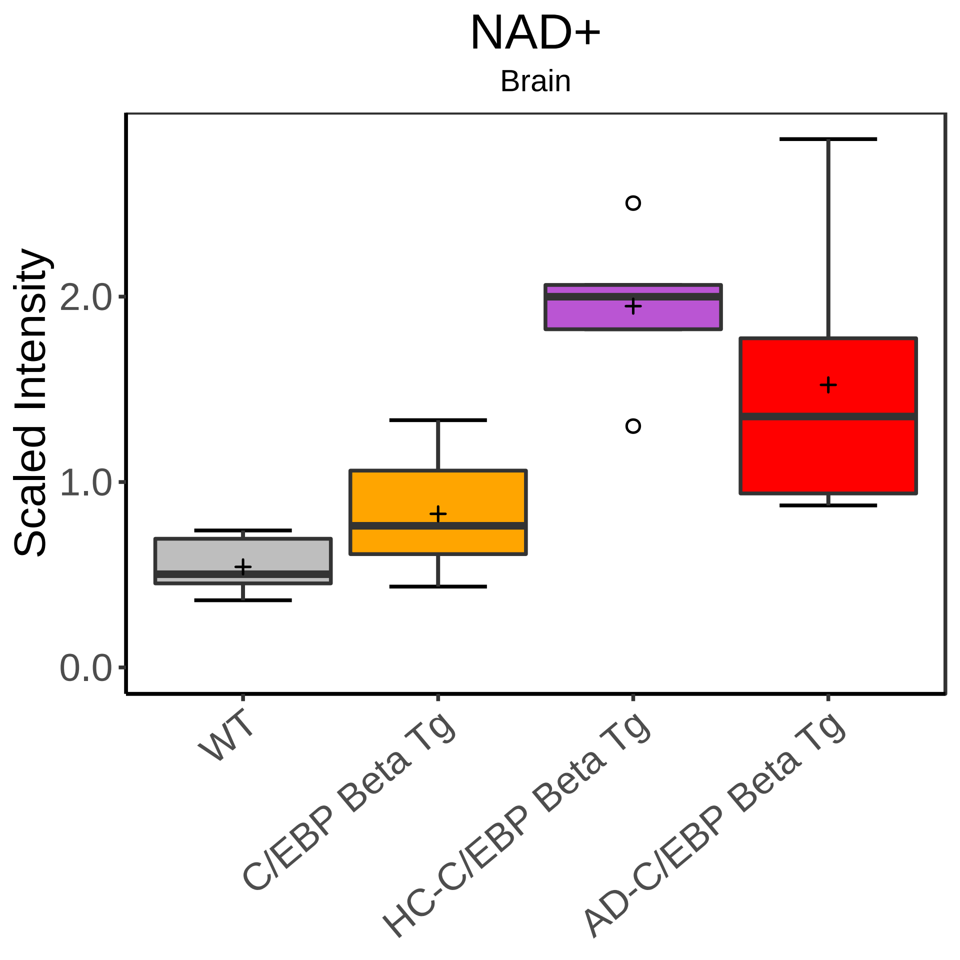
NAD+
Brain
Scaled Intensity
2.0
1.0
0.0
WT
C/EBP Beta Tg
HC-C/EBP Beta Tg
AD-C/EBP Beta Tg

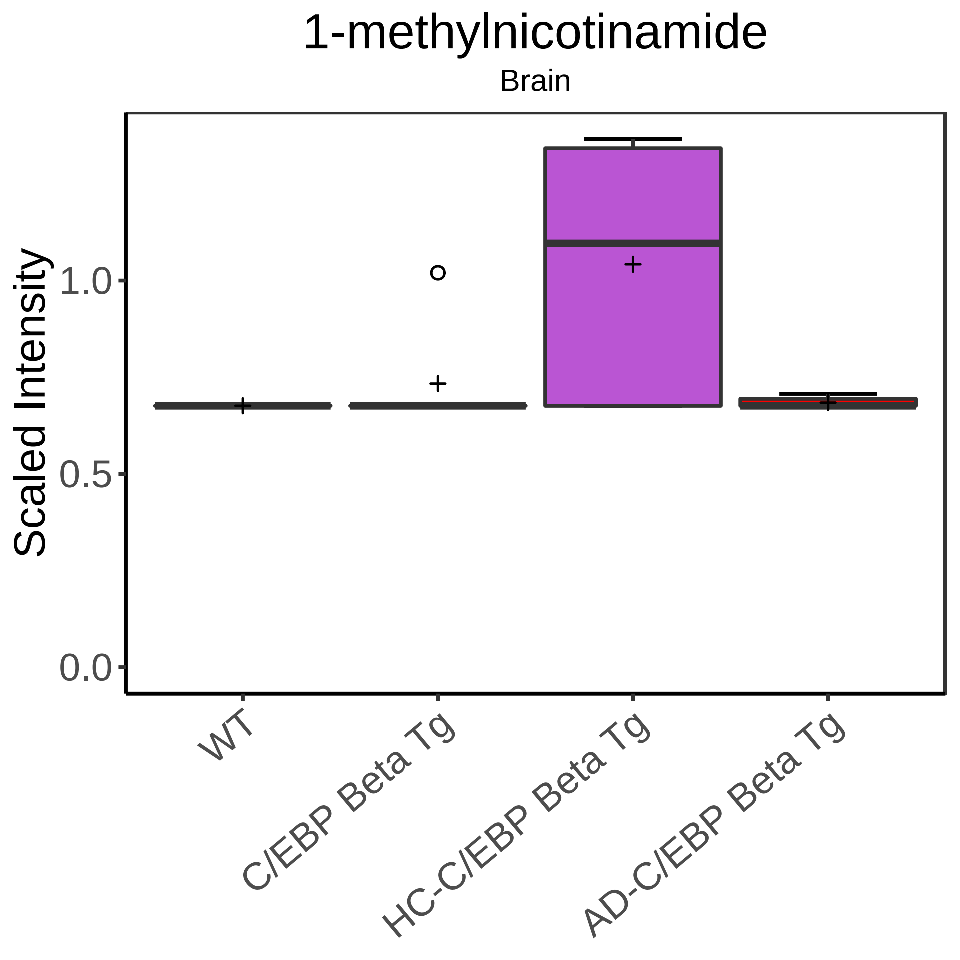

1-methylnicotinamide
Brain
Scaled Intensity
1.0
0.5
0.0
WT
C/EBP Beta Tg
HC-C/EBP Beta Tg
AD-C/EBP Beta Tg

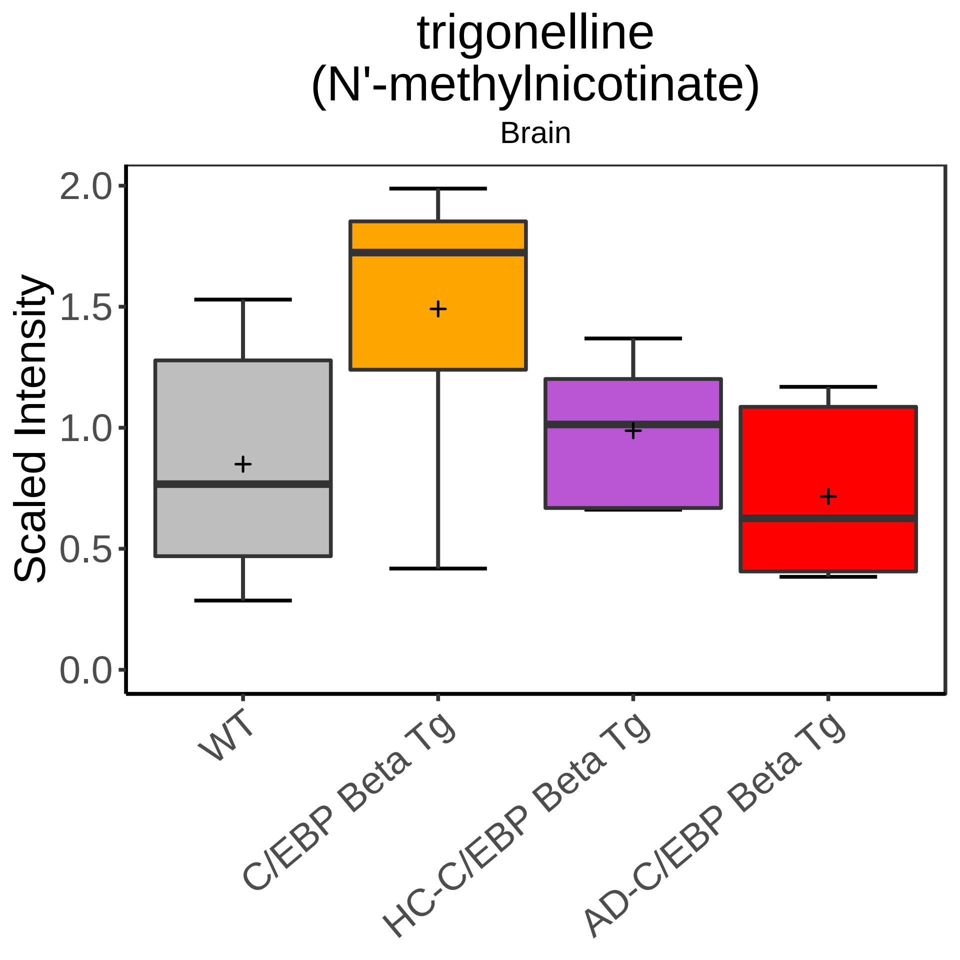
trigonelline
(N'-methylnicotinate)
Brain
Scaled Intensity
2.0
1.5
1.0
0.5
0.0
WT
C/EBP Beta Tg
HC-C/EBP Beta Tg
AD-C/EBP Beta Tg

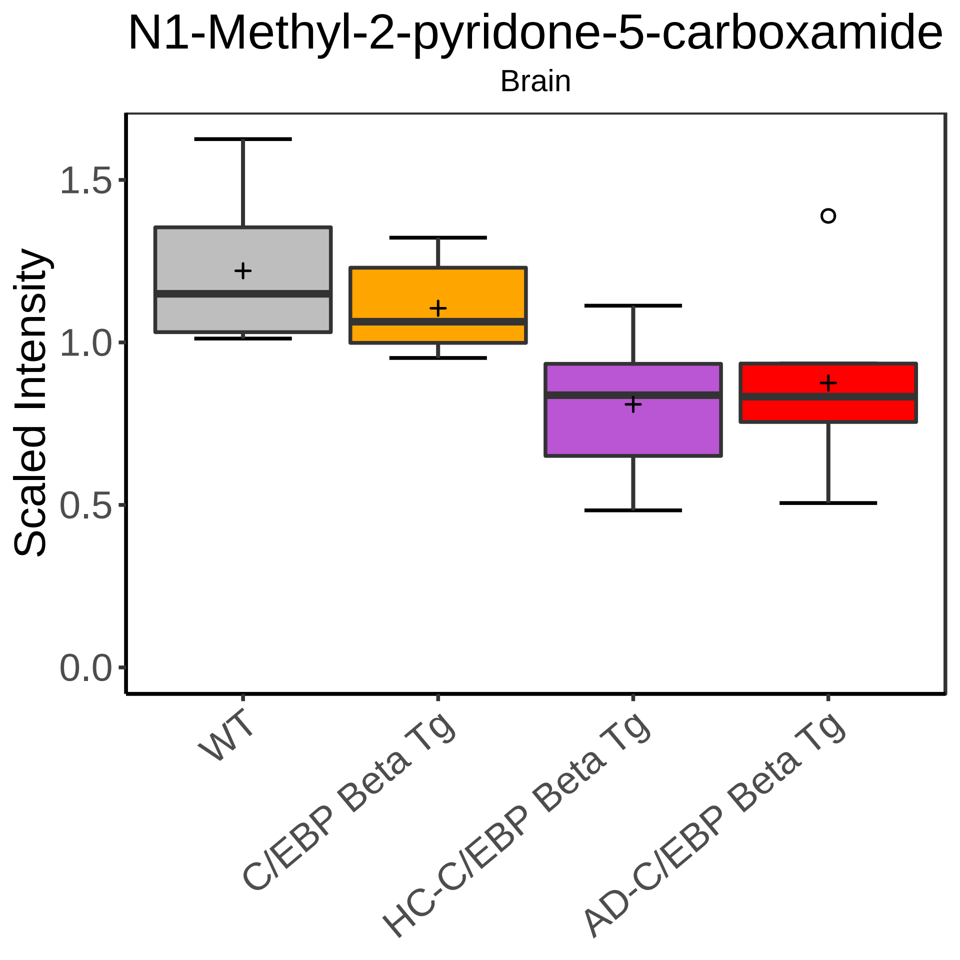
N1-Methyl-2-pyridone-5-carboxamide
Brain
Scaled Intensity
1.5
1.0
0.5
0.0
WT
C/EBP Beta Tg
HC-C/EBP Beta Tg
AD-C/EBP Beta Tg

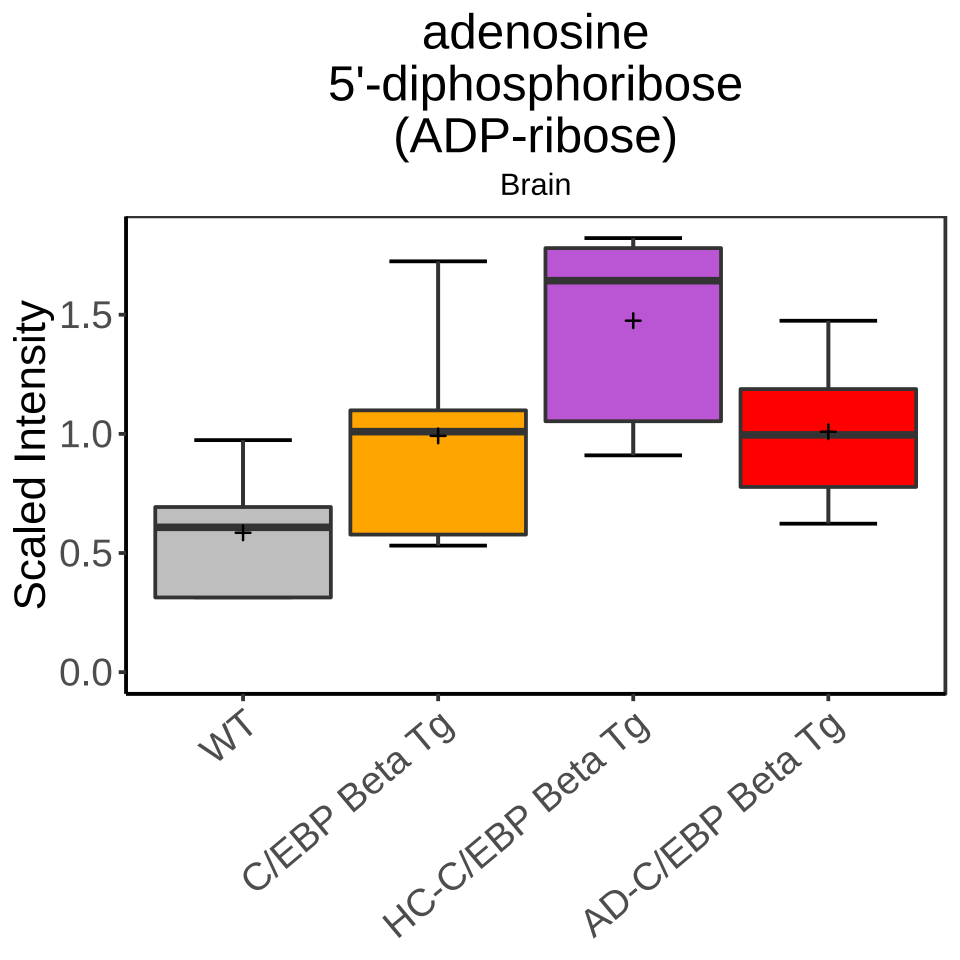

adenosine
5'-diphosphoribose
(ADP-ribose)
Brain
Scaled Intensity
0.0
0.5
1.0
1.5
WT
C/EBP Beta Tg
HC-C/EBP Beta Tg
AD-C/EBP Beta Tg

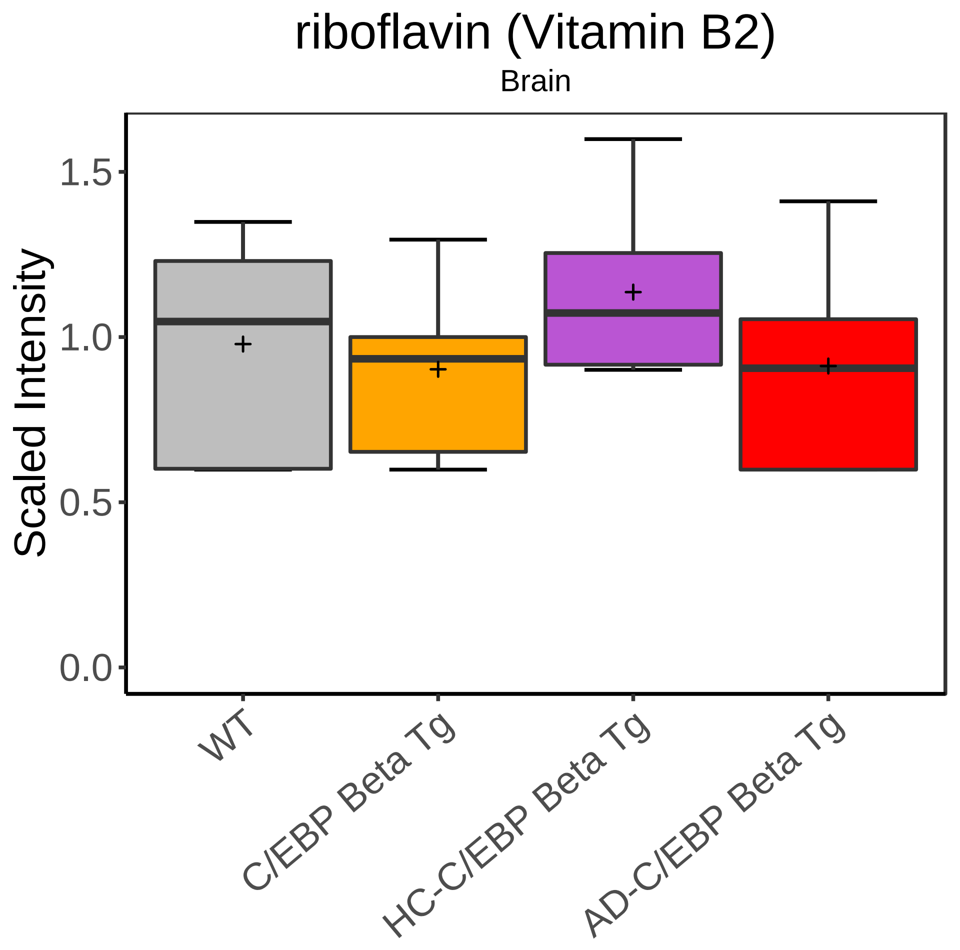

riboflavin (Vitamin B2)
Brain
Scaled Intensity
1.5
1.0
0.5
0.0
WT
C/EBP Beta Tg
HC-C/EBP Beta Tg
AD-C/EBP Beta Tg

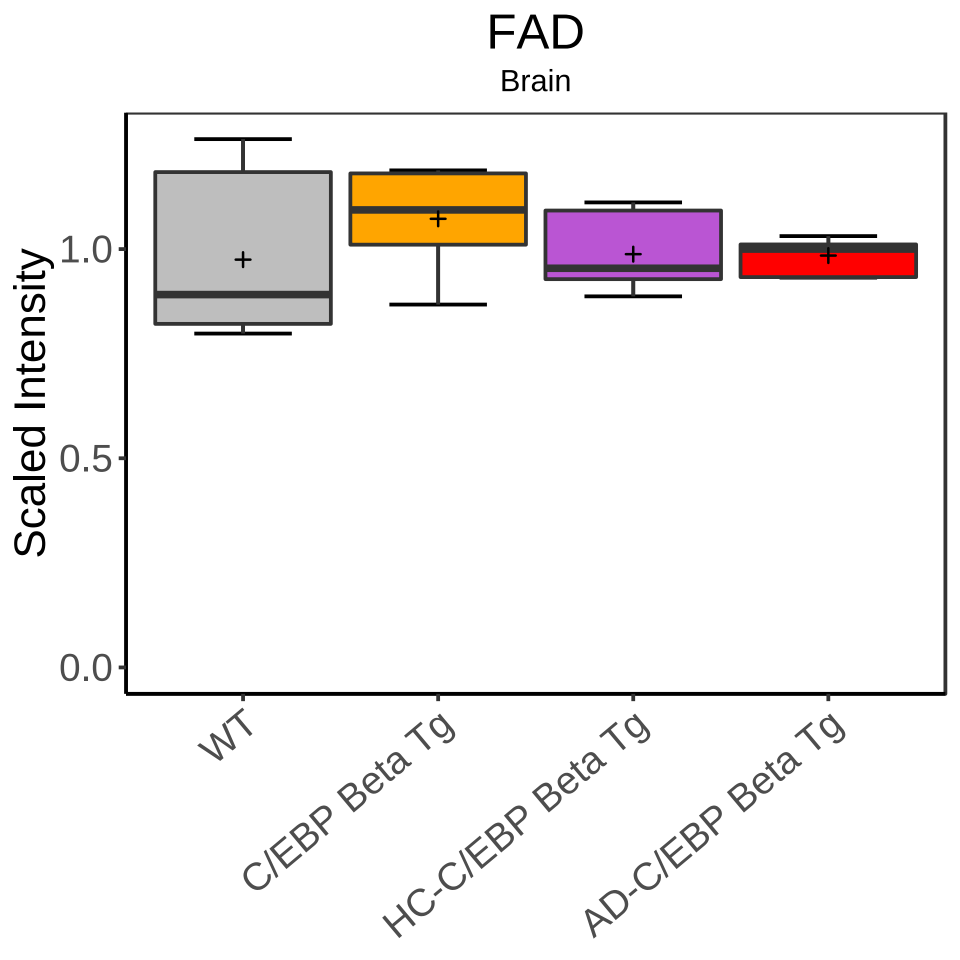
FAD
Brain
Scaled Intensity
0.0
0.5
1.0
WT
C/EBP Beta Tg
HC-C/EBP Beta Tg
AD-C/EBP Beta Tg

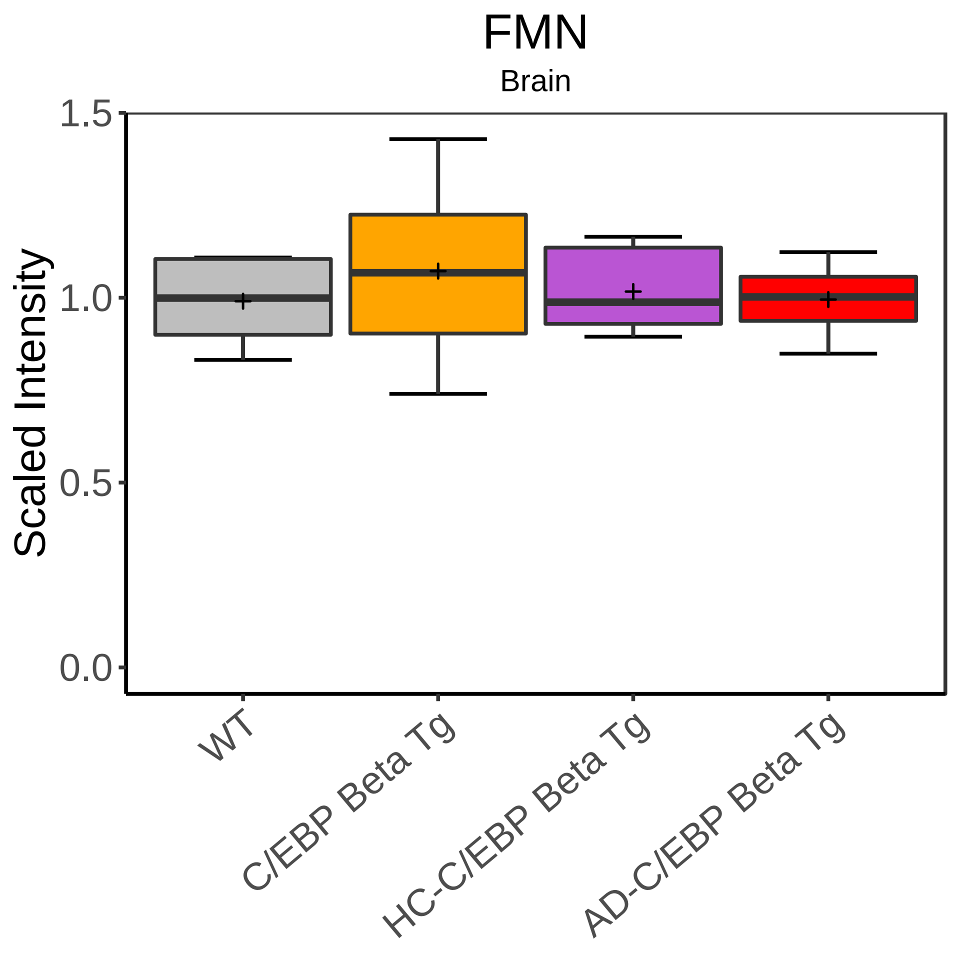

FMN
Brain
Scaled Intensity
1.5
1.0
0.5
0.0
WT
C/EBP Beta Tg
HC-C/EBP Beta Tg
AD-C/EBP Beta Tg

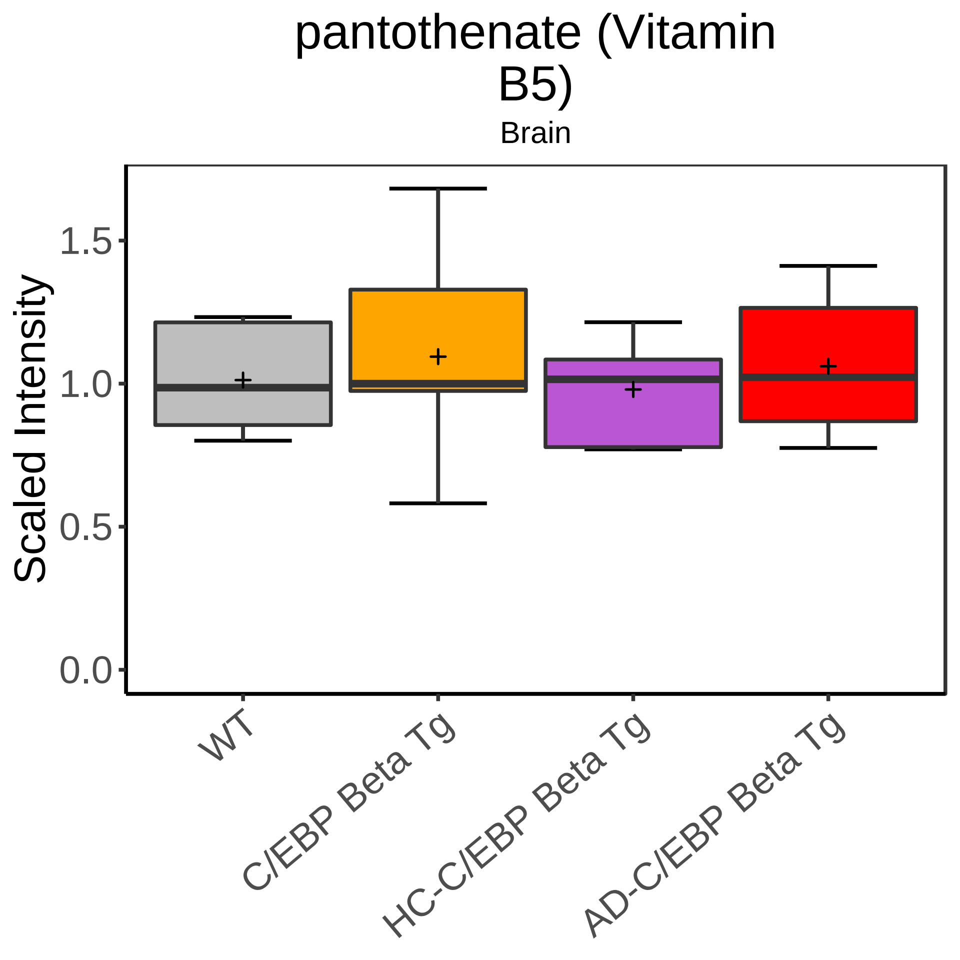
pantothenate (Vitamin B5)
Brain
Scaled Intensity
0.0
0.5
1.0
1.5
WT
C/EBP Beta Tg
HC-C/EBP Beta Tg
AD-C/EBP Beta Tg

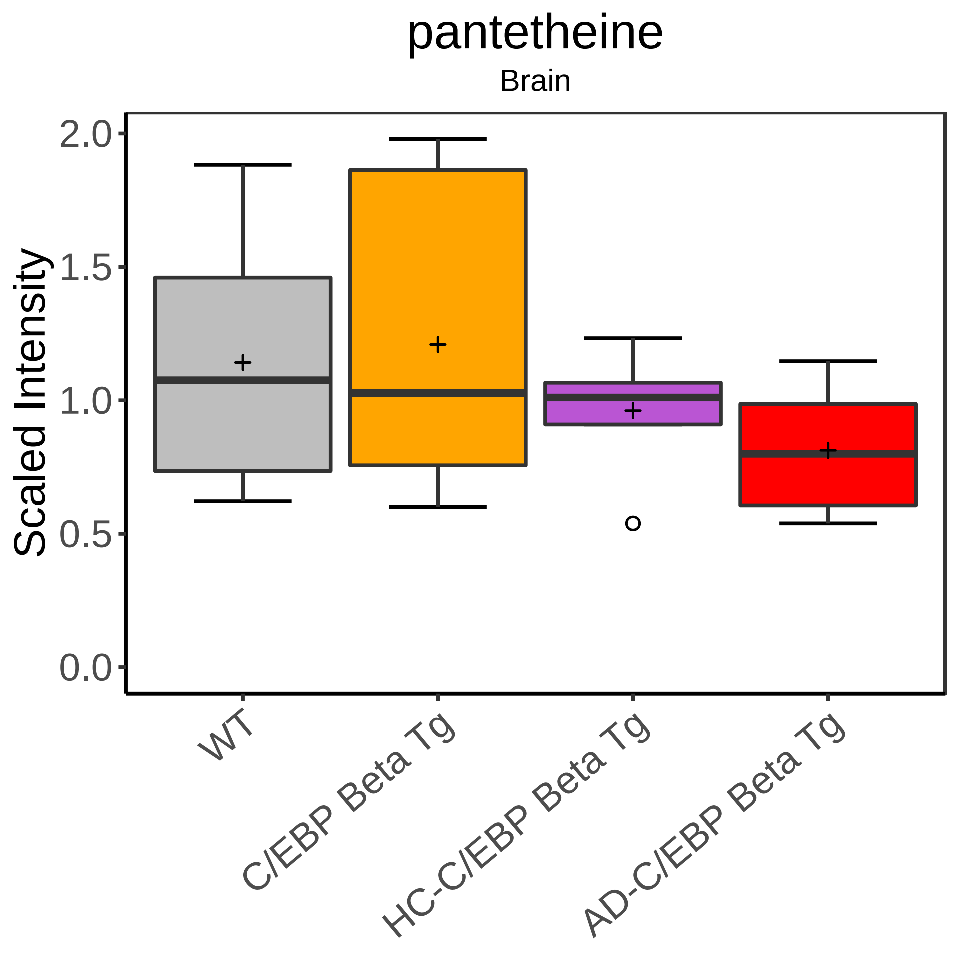

pantetheine
Brain
Scaled Intensity
2.0
1.5
1.0
0.5
0.0
WT
C/EBP Beta Tg
HC-C/EBP Beta Tg
AD-C/EBP Beta Tg

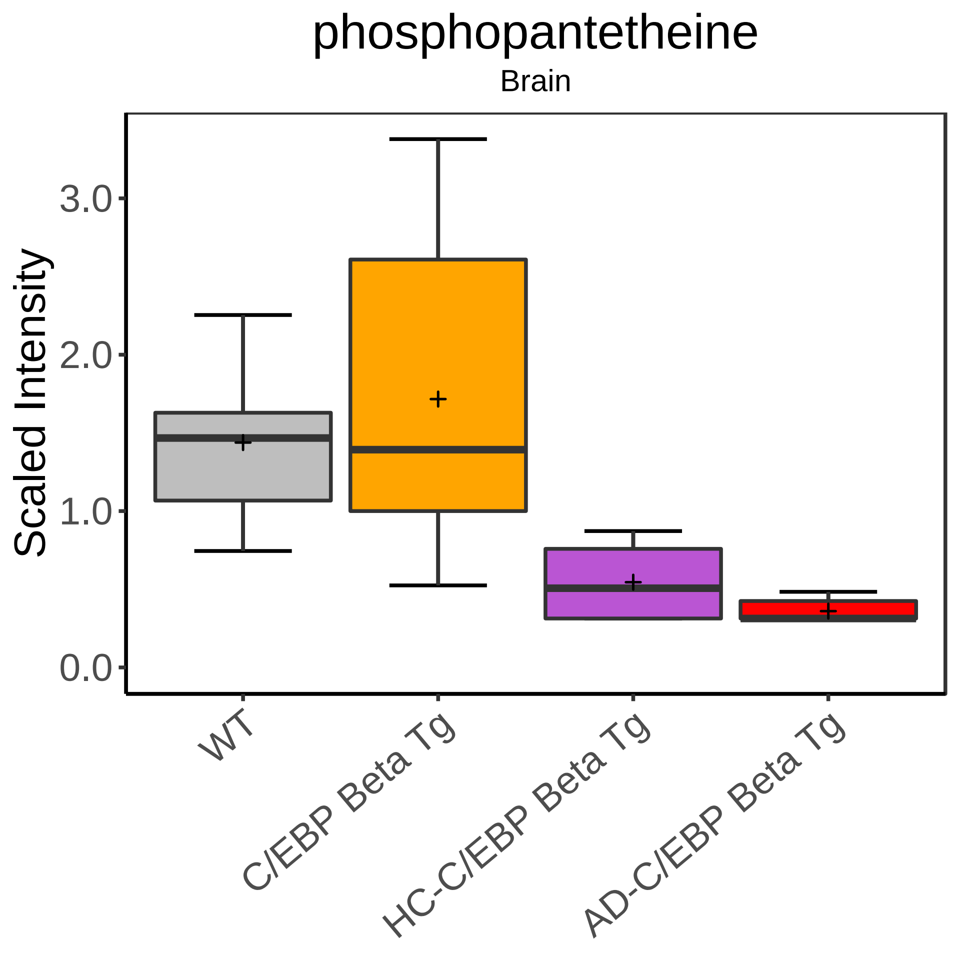
phosphopantetheine
Brain
Scaled Intensity
3.0
2.0
1.0
0.0
WT
C/EBP Beta Tg
HC-C/EBP Beta Tg
AD-C/EBP Beta Tg

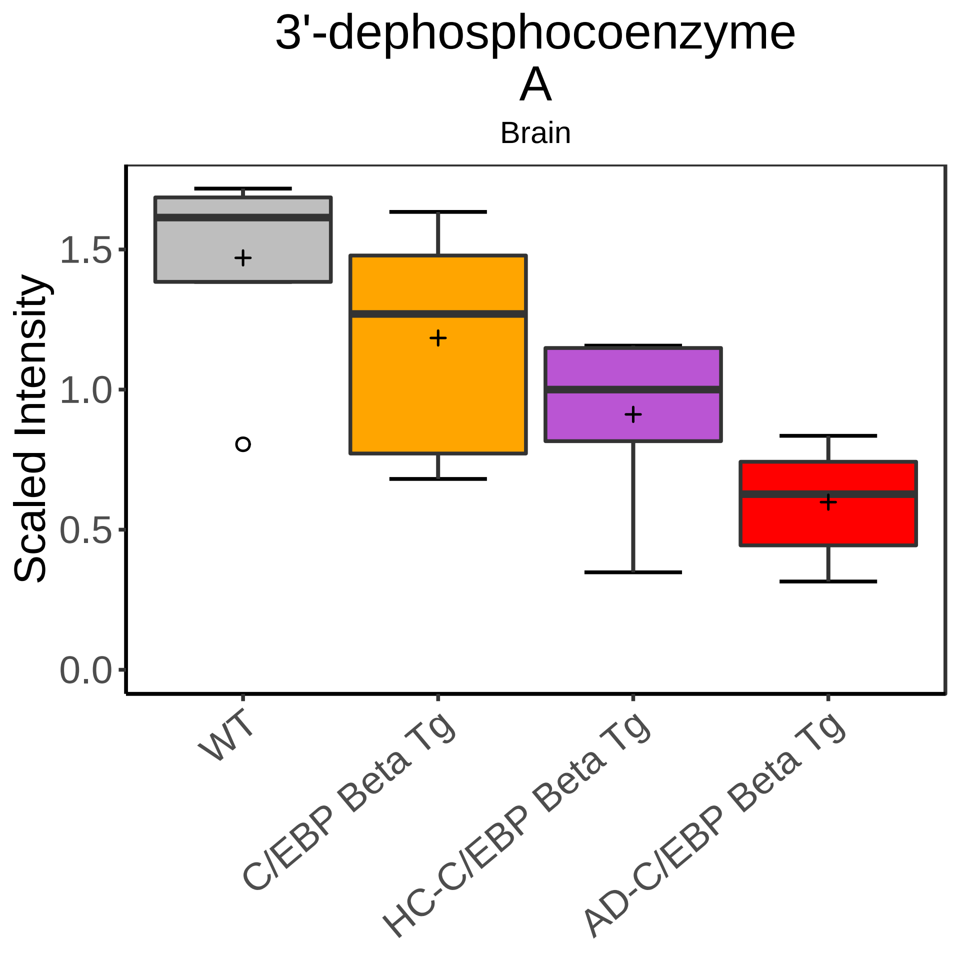
3'-dephosphocoenzyme A
Brain
Scaled Intensity
0.0
0.5
1.0
1.5
WT
C/EBP Beta Tg
HC-C/EBP Beta Tg
AD-C/EBP Beta Tg

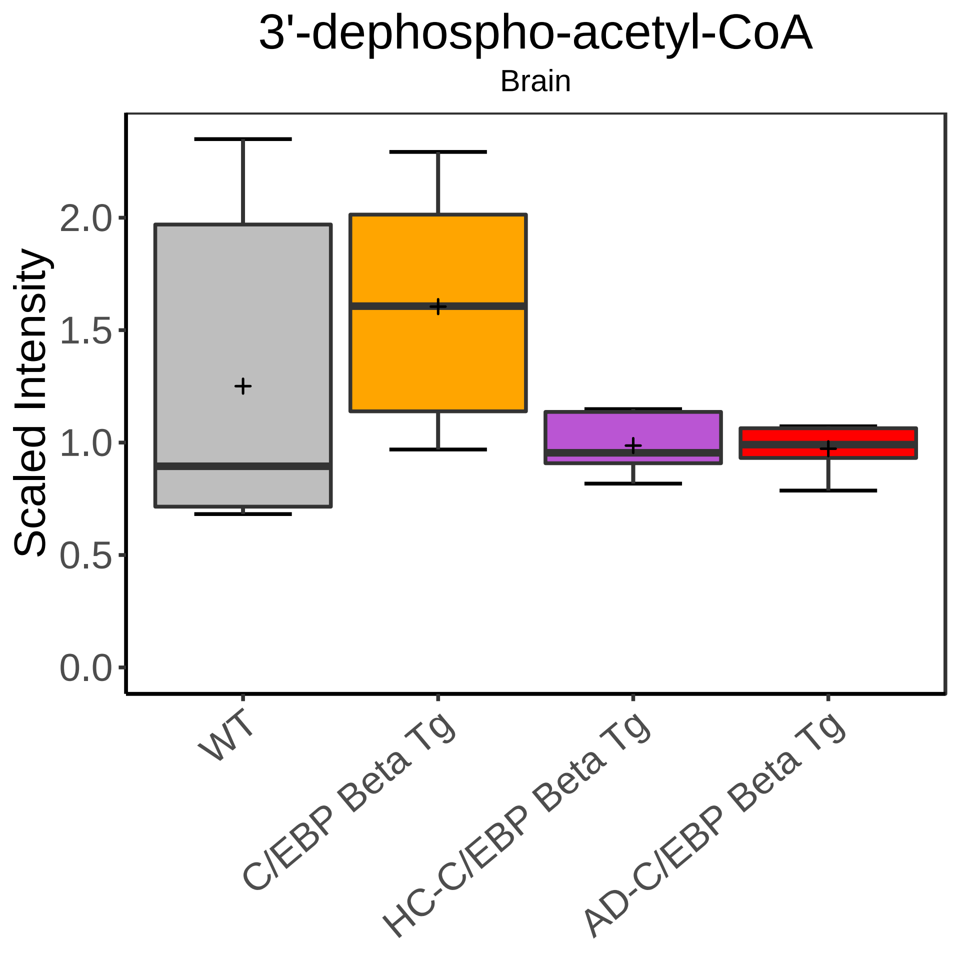

3'-dephospho-acetyl-CoA
Brain
Scaled Intensity
0.0
0.5
1.0
1.5
2.0
WT
C/EBP Beta Tg
HC-C/EBP Beta Tg
AD-C/EBP Beta Tg

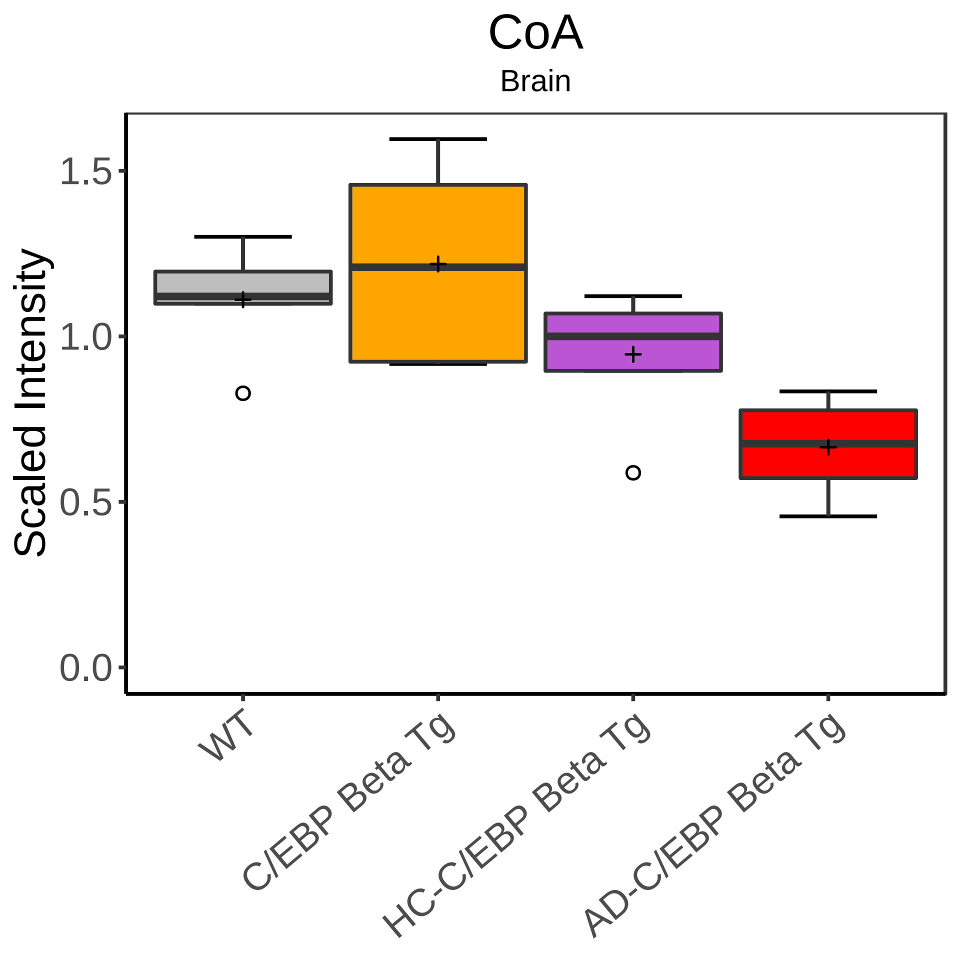
CoA
Brain
Scaled Intensity
1.5
1.0
0.5
0.0
WT
C/EBP Beta Tg
HC-C/EBP Beta Tg
AD-C/EBP Beta Tg

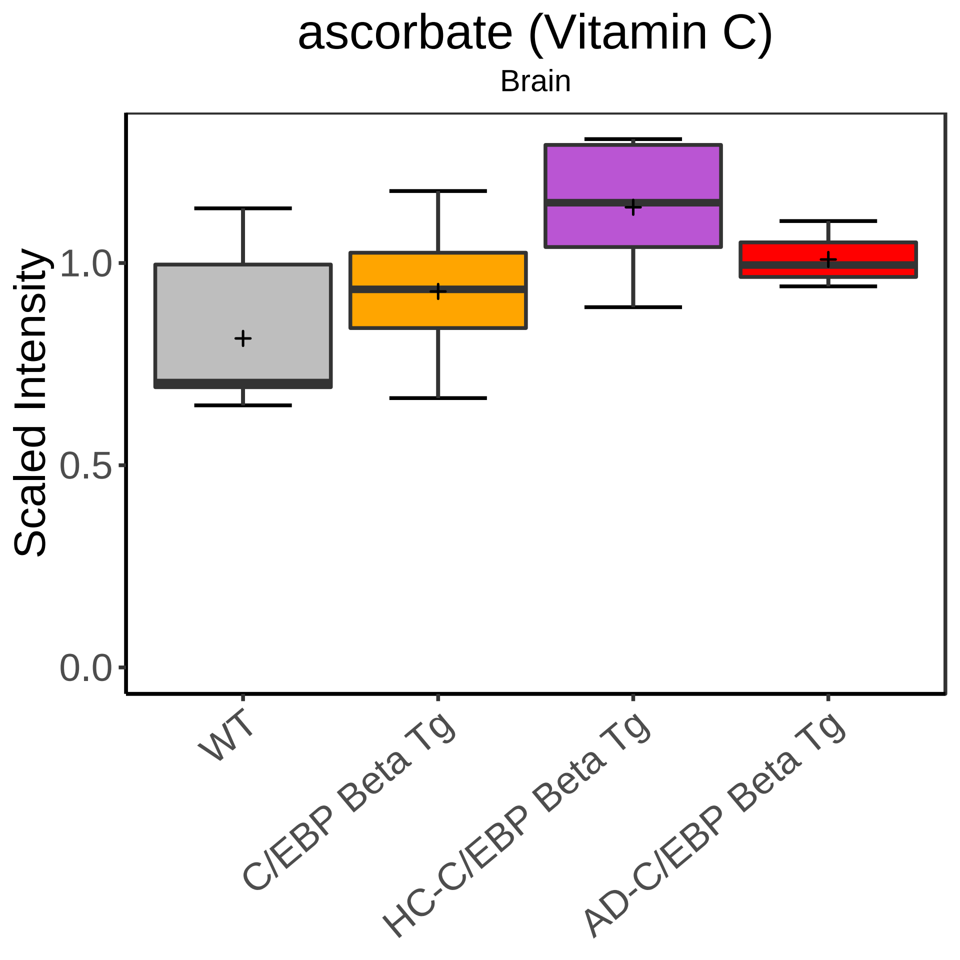
ascorbate (Vitamin C)
Brain
Scaled Intensity
0.0
0.5
1.0
WT
C/EBP Beta Tg
HC-C/EBP Beta Tg
AD-C/EBP Beta Tg

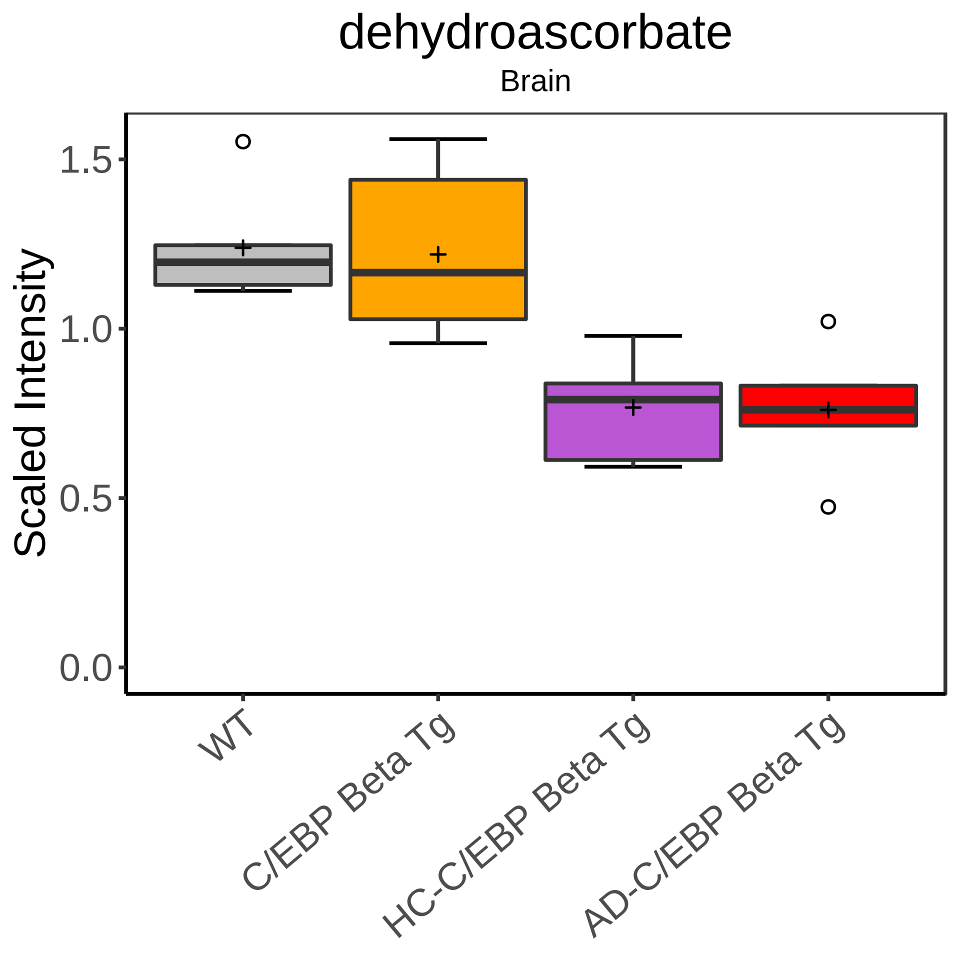
dehydroascorbate
Brain
Scaled Intensity
1.5
1.0
0.5
0.0
WT
C/EBP Beta Tg
HC-C/EBP Beta Tg
AD-C/EBP Beta Tg

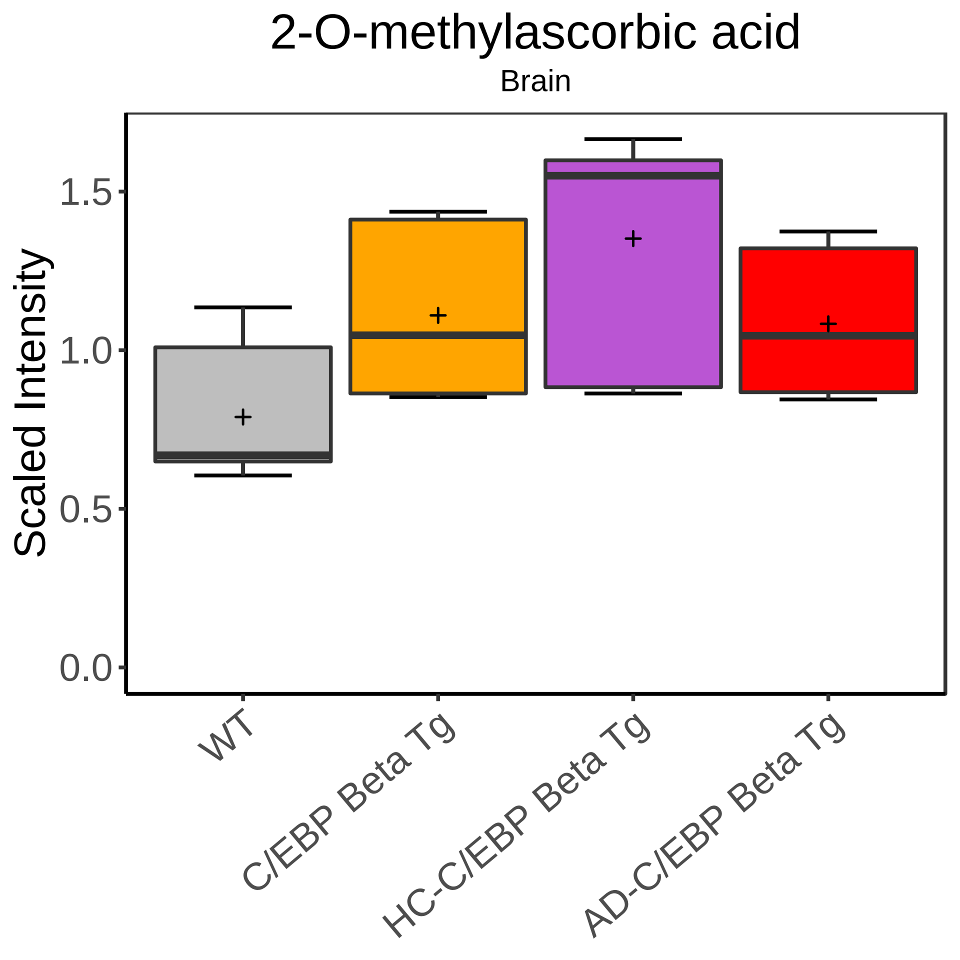

2-O-methylascorbic acid
Brain
Scaled Intensity
0.0
0.5
1.0
1.5
WT
C/EBP Beta Tg
HC-C/EBP Beta Tg
AD-C/EBP Beta Tg

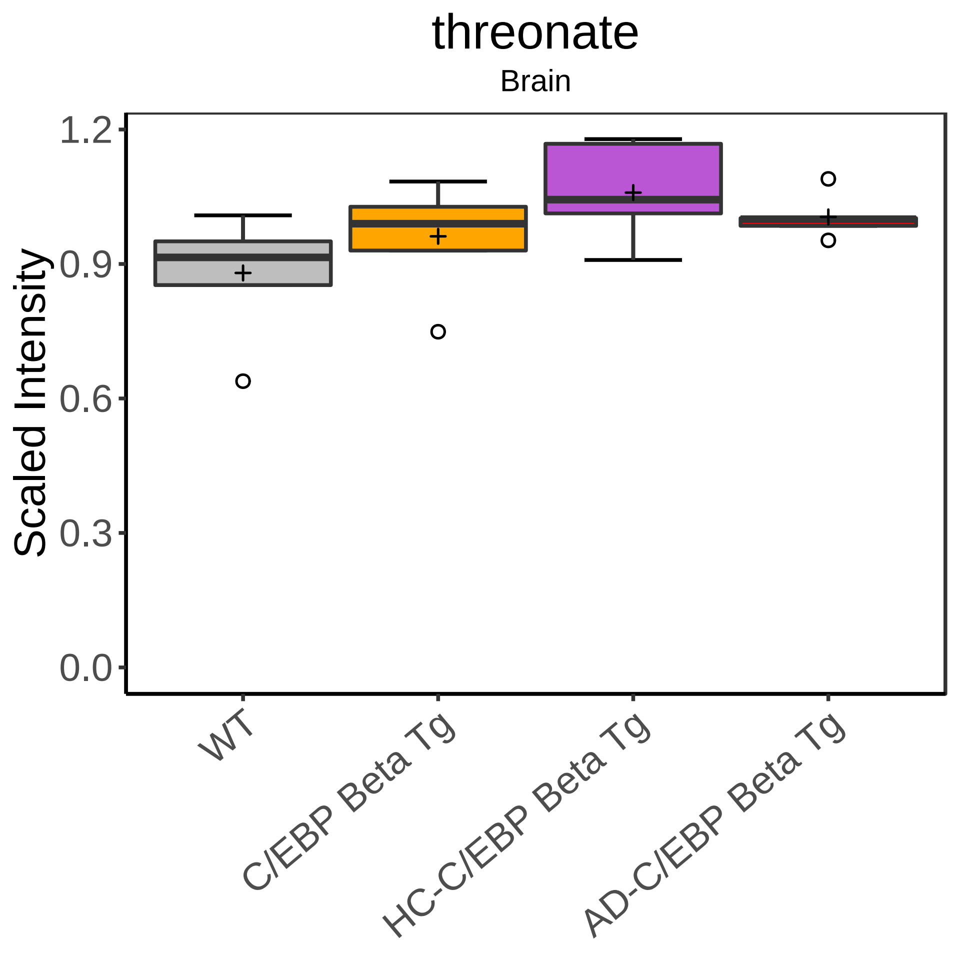

threonate
Brain
Scaled Intensity
1.2
0.9
0.6
0.3
0.0
WT
C/EBP Beta Tg
HC-C/EBP Beta Tg
AD-C/EBP Beta Tg

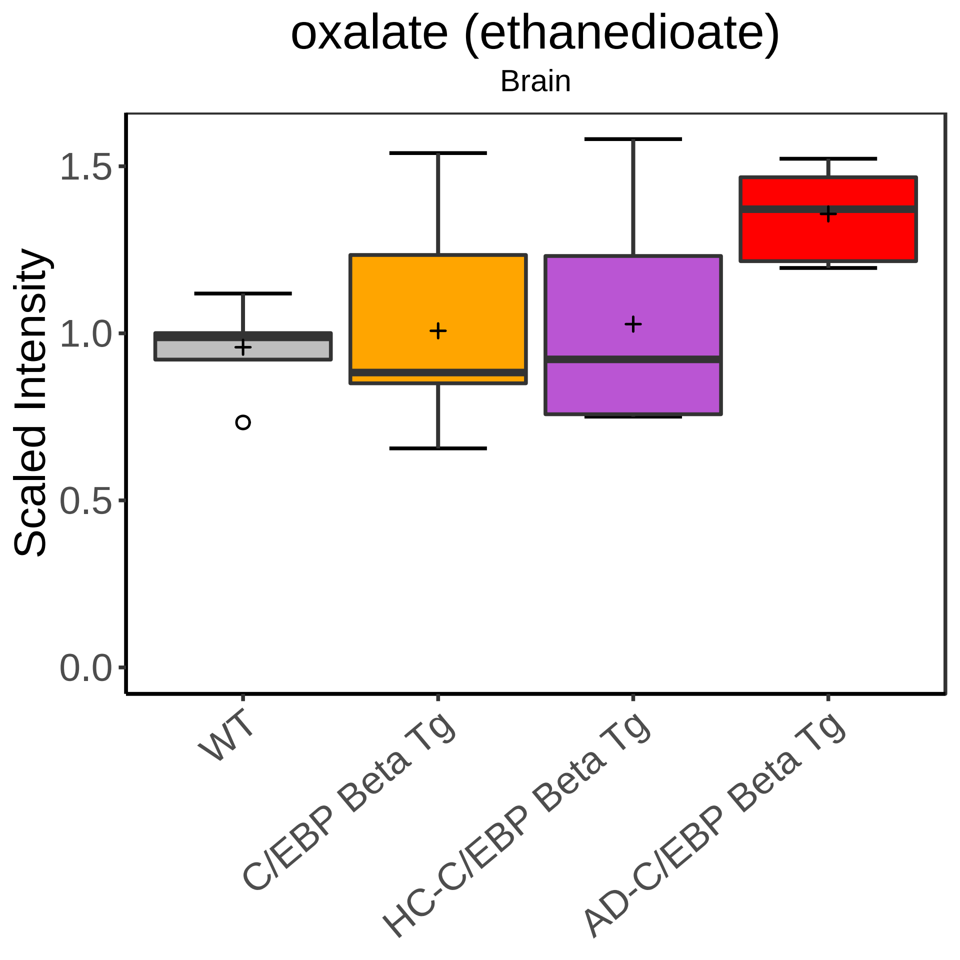

oxalate (ethanedioate)
Brain
Scaled Intensity
0.0
0.5
1.0
1.5
WT
C/EBP Beta Tg
HC-C/EBP Beta Tg
AD-C/EBP Beta Tg

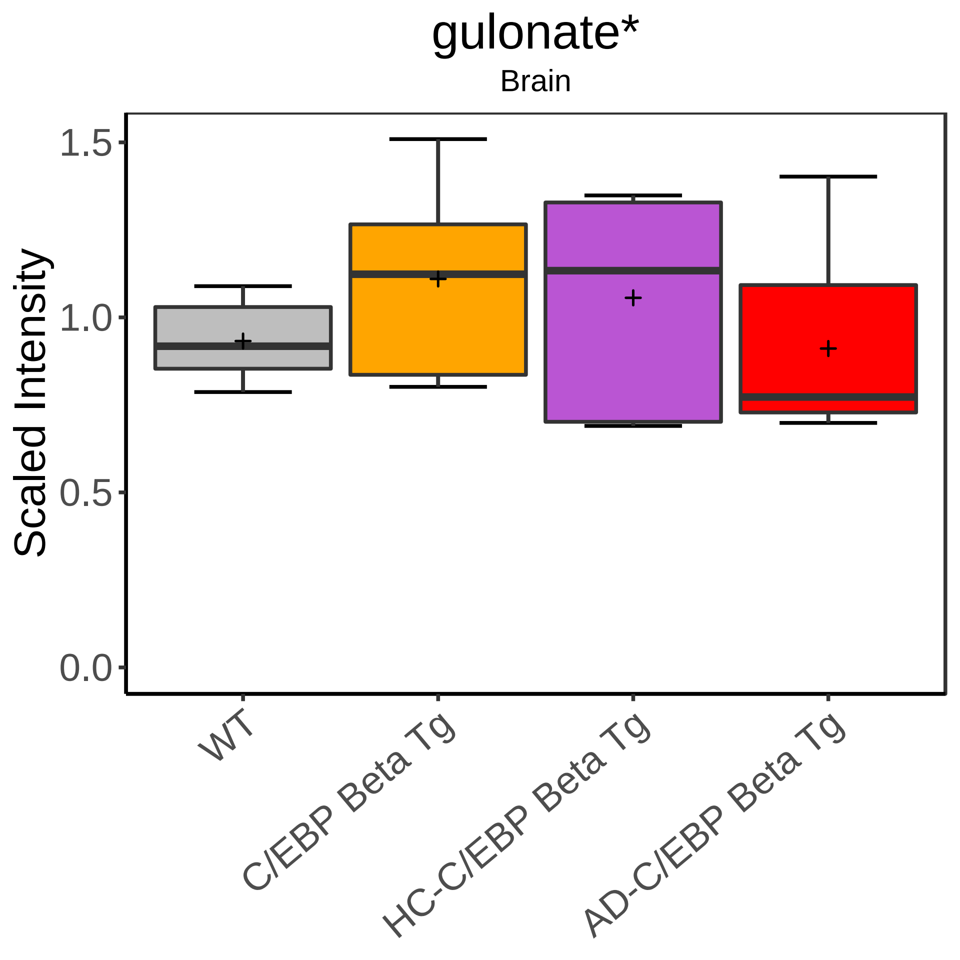
gulonate*
Brain
Scaled Intensity
1.5
1.0
0.5
0.0
WT
C/EBP Beta Tg
HC-C/EBP Beta Tg
AD-C/EBP Beta Tg

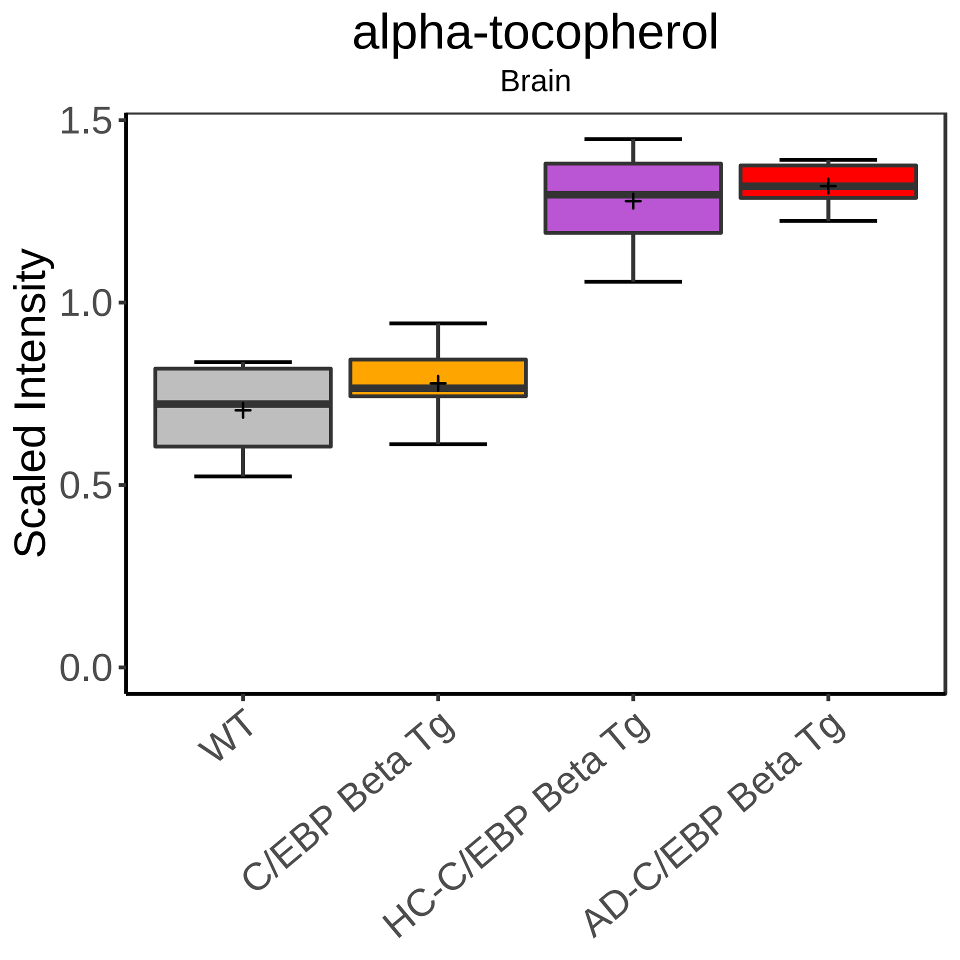
alpha-tocopherol
Brain
Scaled Intensity
1.5
1.0
0.5
0.0
WT
C/EBP Beta Tg
HC-C/EBP Beta Tg
AD-C/EBP Beta Tg

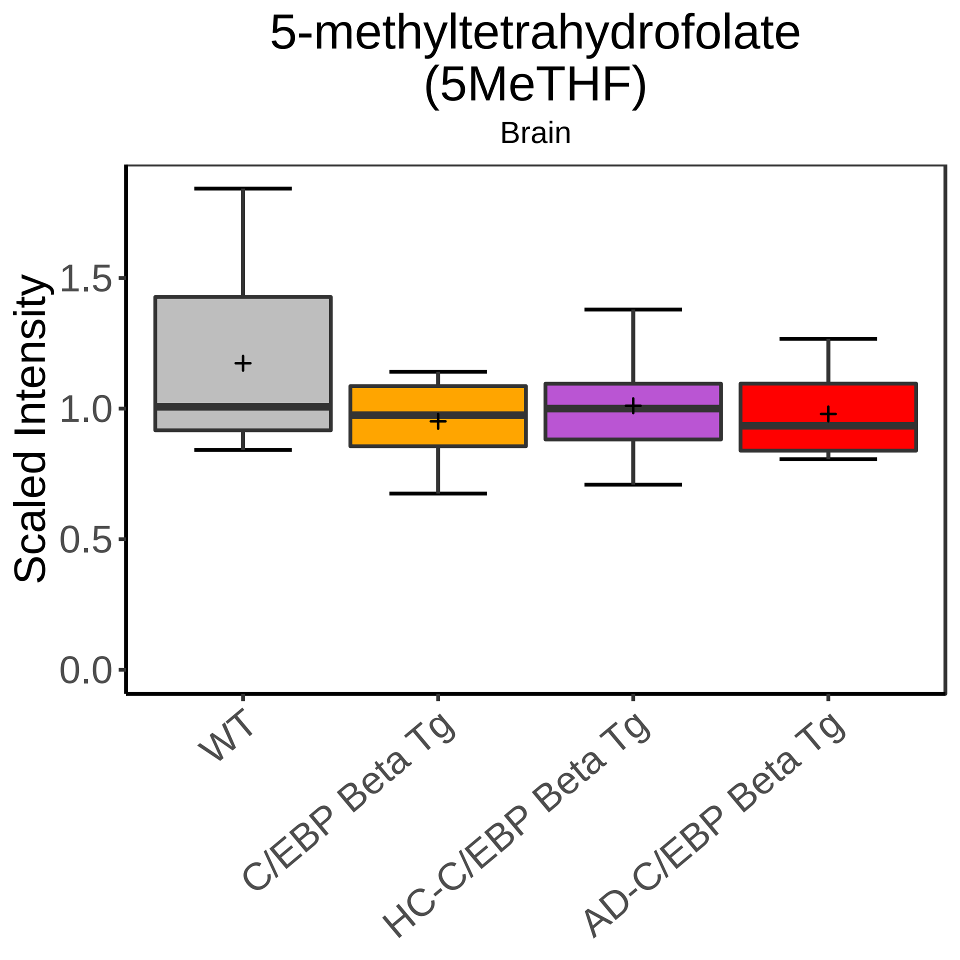
5-methyltetrahydrofolate
(5MeTHF)
Brain
Scaled Intensity
1.5
1.0
0.5
0.0
WT
C/EBP Beta Tg
HC-C/EBP Beta Tg
AD-C/EBP Beta Tg

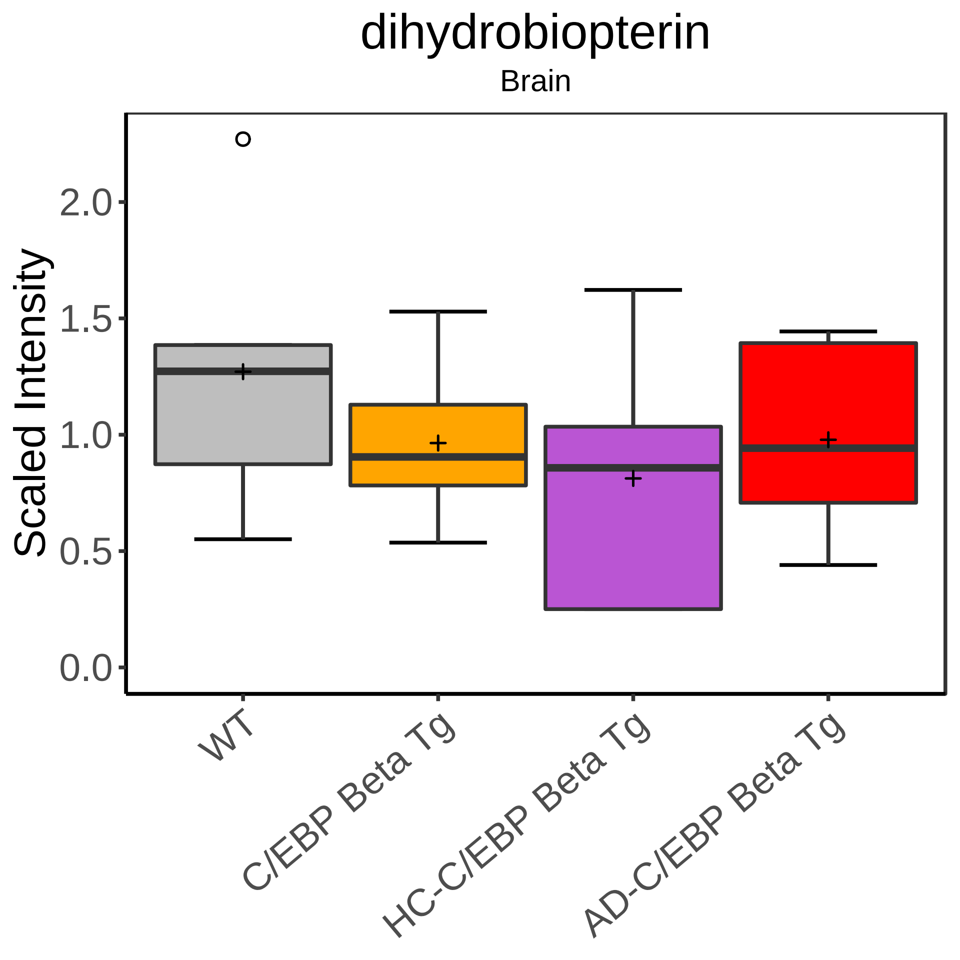
dihydrobiopterin
Brain
Scaled Intensity
0.0
0.5
1.0
1.5
2.0
WT
C/EBP Beta Tg
HC-C/EBP Beta Tg
AD-C/EBP Beta Tg

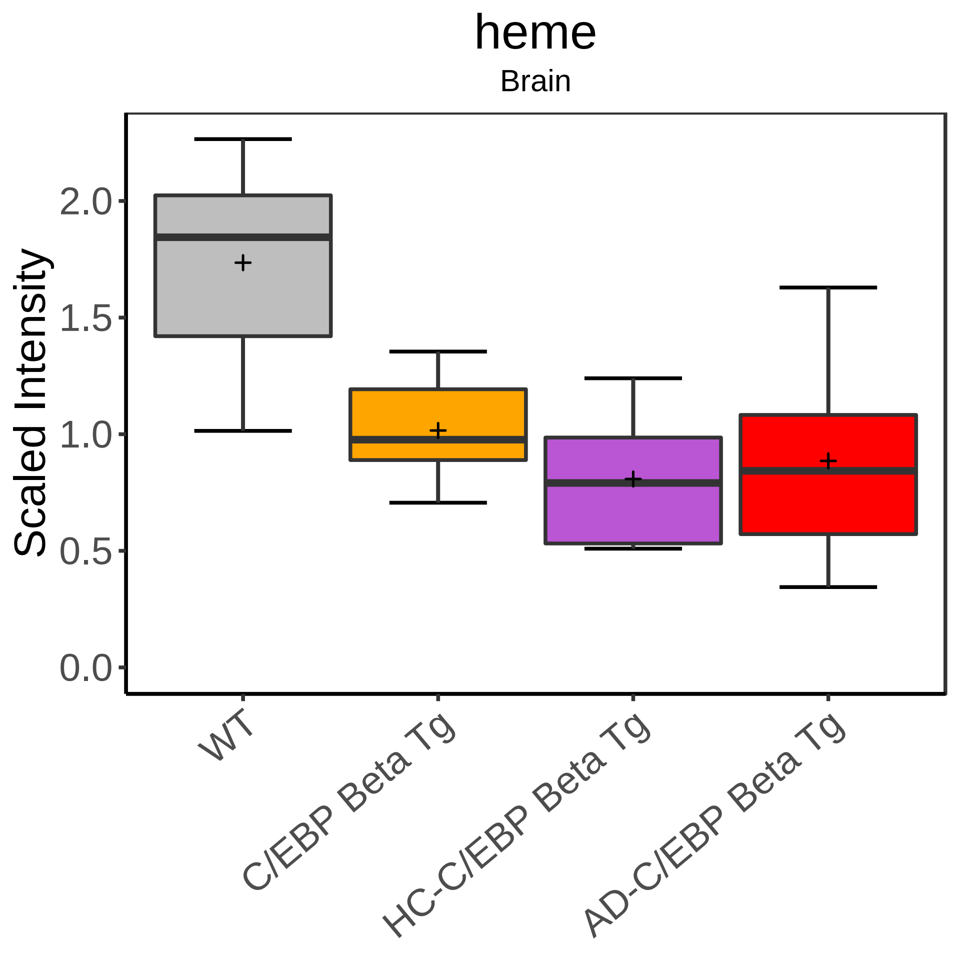
heme
Brain
Scaled Intensity
2.0
1.5
1.0
0.5
0.0
WT
C/EBP Beta Tg
HC-C/EBP Beta Tg
AD-C/EBP Beta Tg

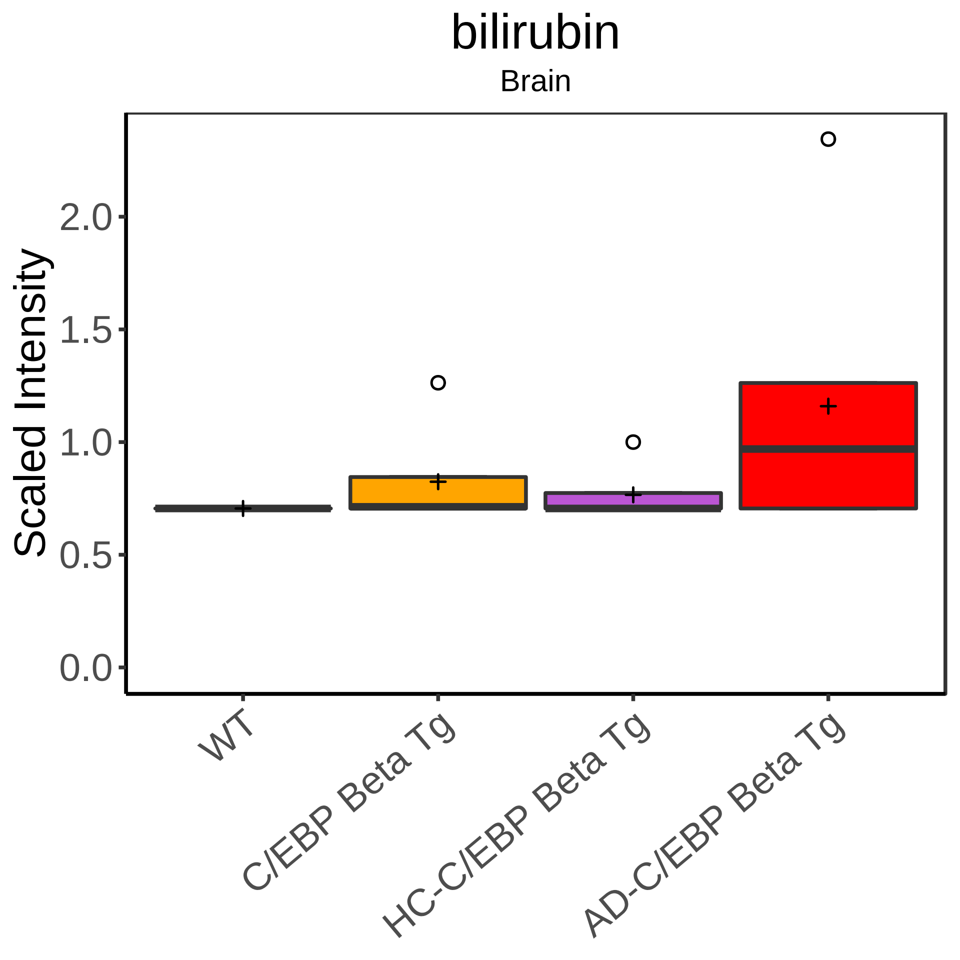

bilirubin
Brain
Scaled Intensity
0.0
0.5
1.0
1.5
2.0
WT
C/EBP Beta Tg
HC-C/EBP Beta Tg
AD-C/EBP Beta Tg

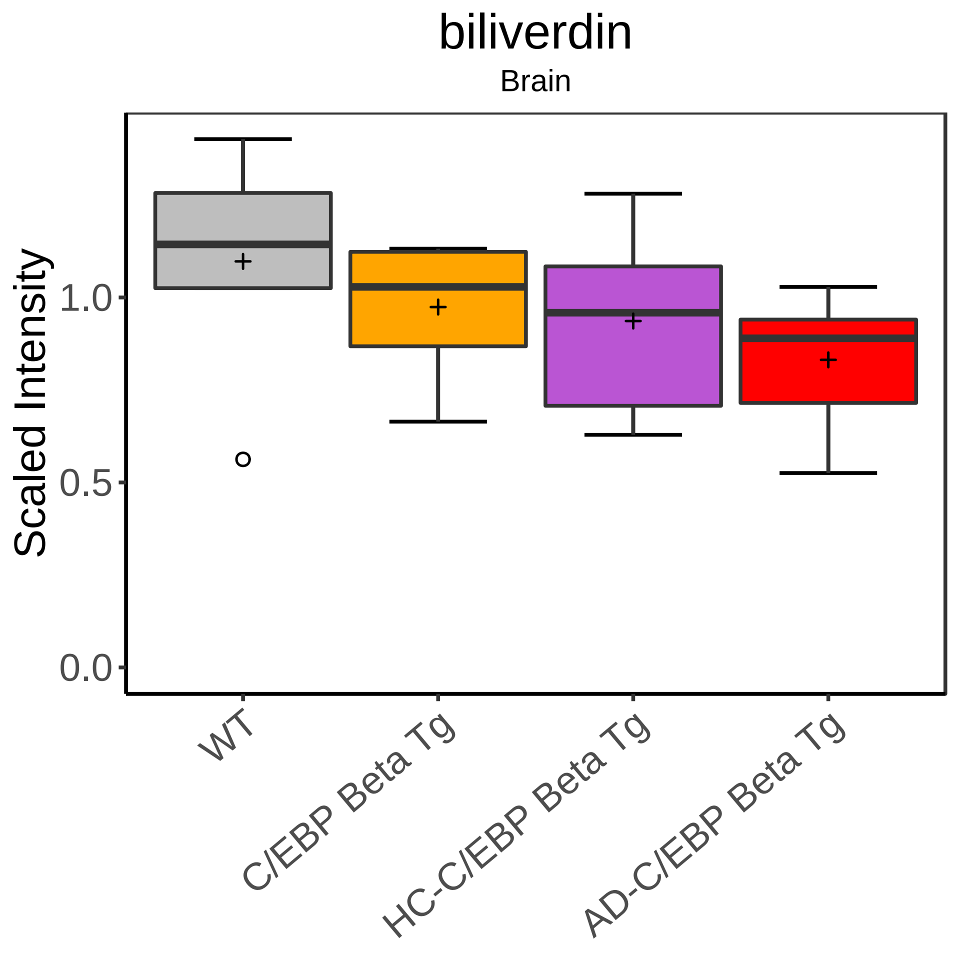
biliverdin
Brain
Scaled Intensity
1.0
0.5
0.0
WT
C/EBP Beta Tg
HC-C/EBP Beta Tg
AD-C/EBP Beta Tg

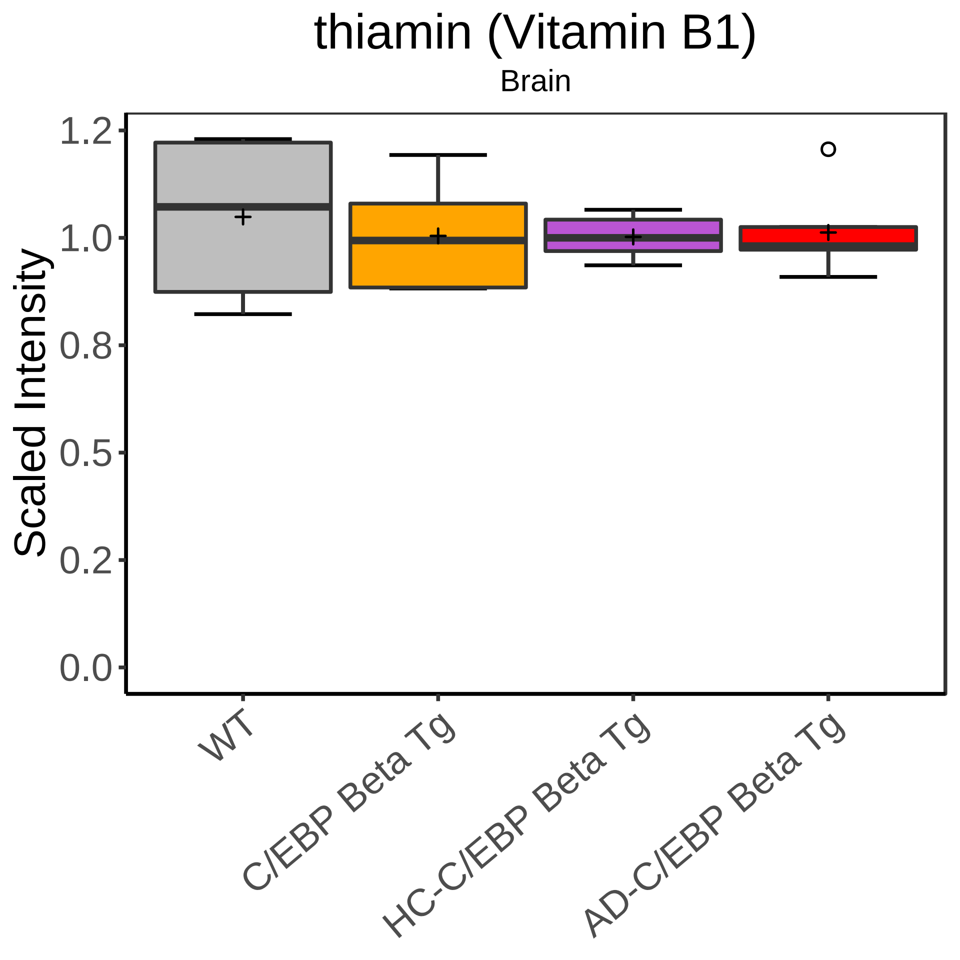

thiamin (Vitamin B1)
Brain
Scaled Intensity
1.2
1.0
0.8
0.5
0.2
0.0
WT
C/EBP Beta Tg
HC-C/EBP Beta Tg
AD-C/EBP Beta Tg

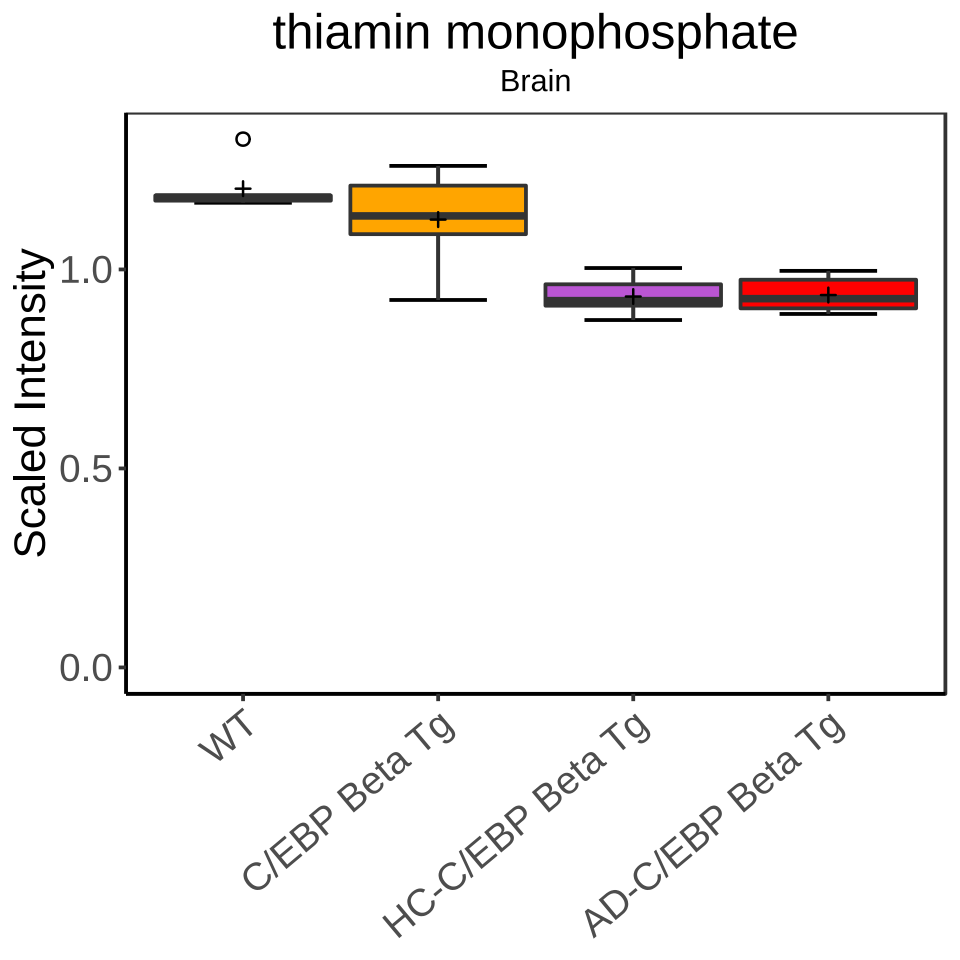

thiamin monophosphate
Brain
Scaled Intensity
1.0
0.5
0.0
WT
C/EBP Beta Tg
HC-C/EBP Beta Tg
AD-C/EBP Beta Tg

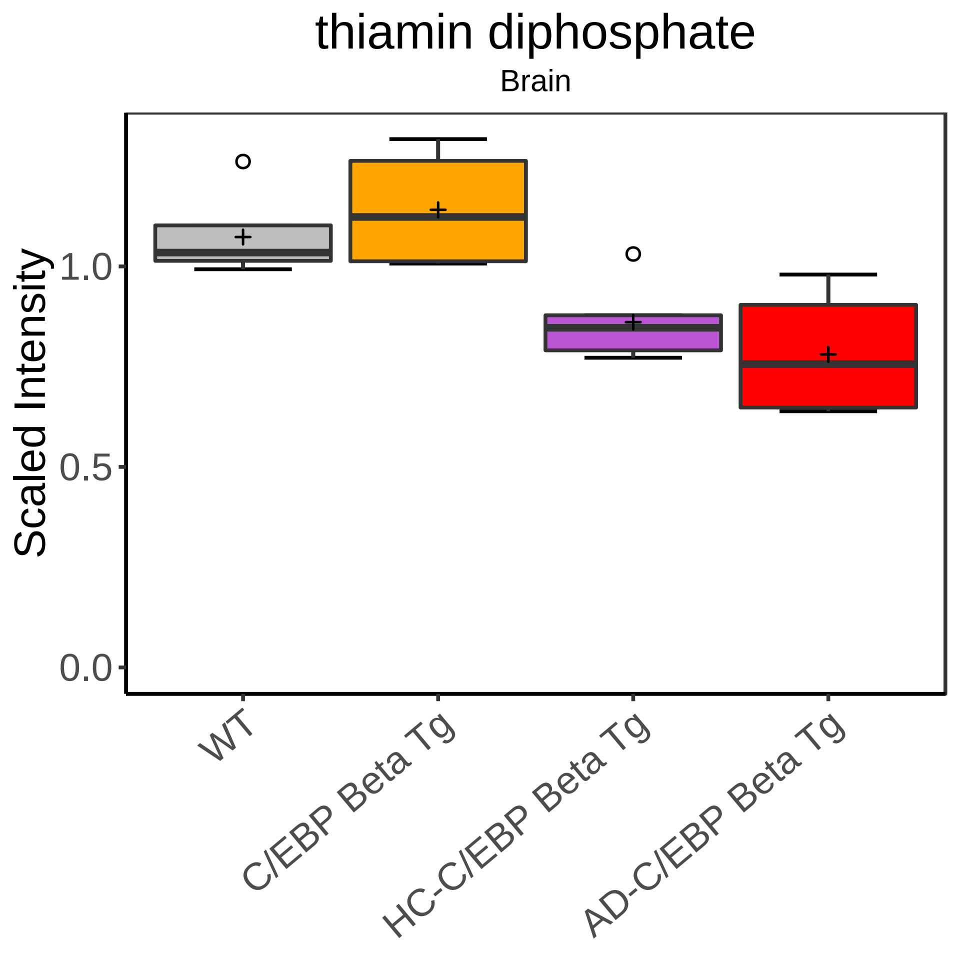
thiamin diphosphate
Brain
Scaled Intensity
1.0
0.5
0.0
WT
C/EBP Beta Tg
HC-C/EBP Beta Tg
AD-C/EBP Beta Tg

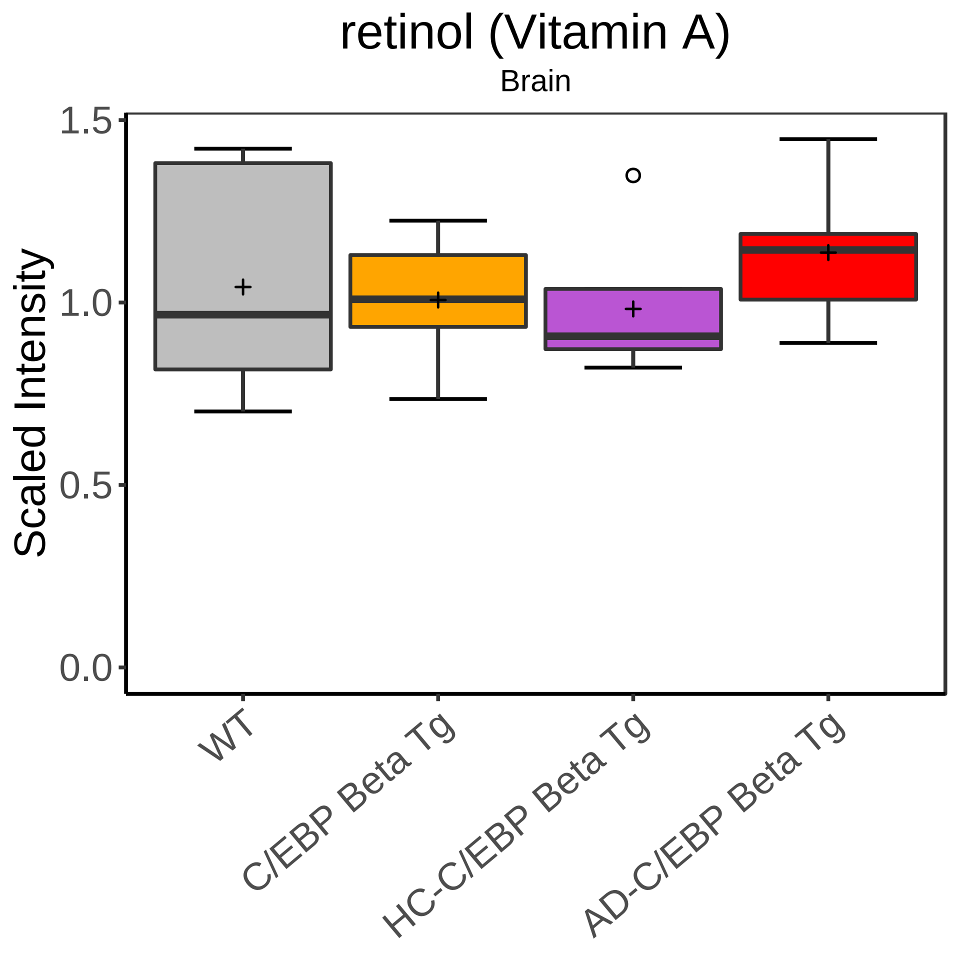

retinol (Vitamin A)
Brain
Scaled Intensity
1.5
1.0
0.5
0.0
WT
C/EBP Beta Tg
HC-C/EBP Beta Tg
AD-C/EBP Beta Tg

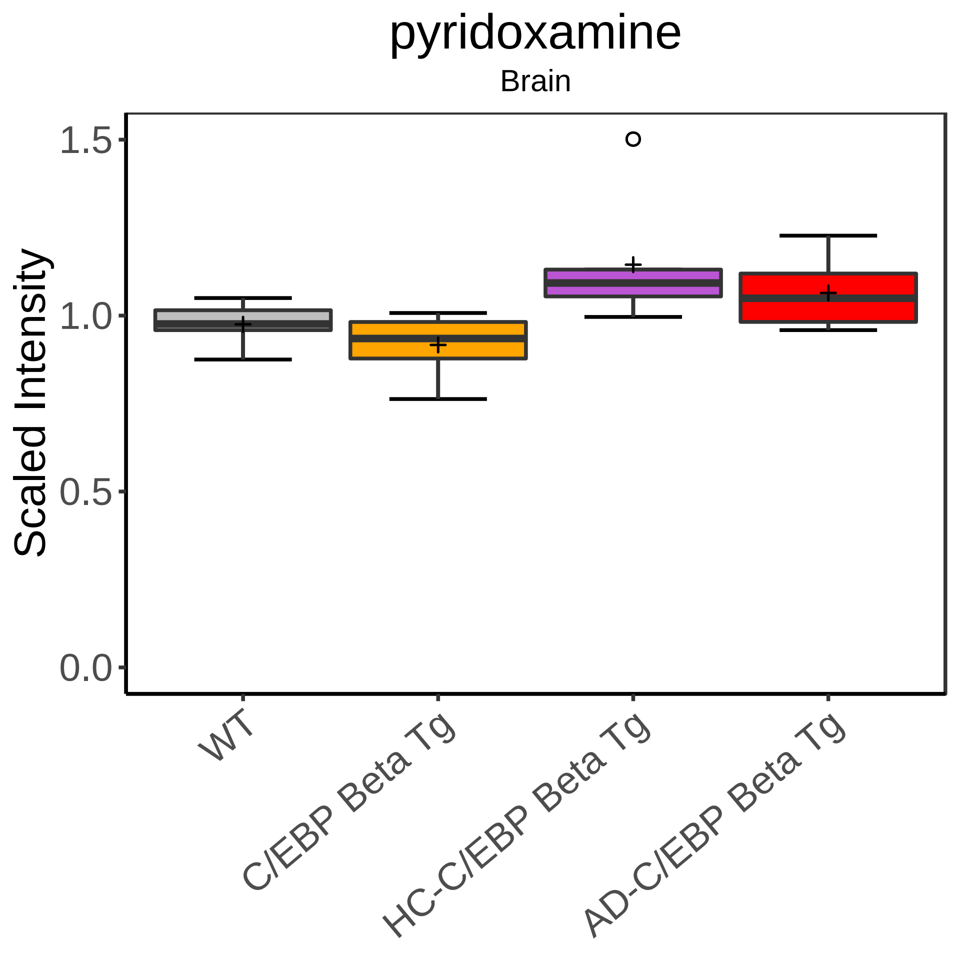
pyridoxamine
Brain
Scaled Intensity
1.5
1.0
0.5
0.0
WT
C/EBP Beta Tg
HC-C/EBP Beta Tg
AD-C/EBP Beta Tg

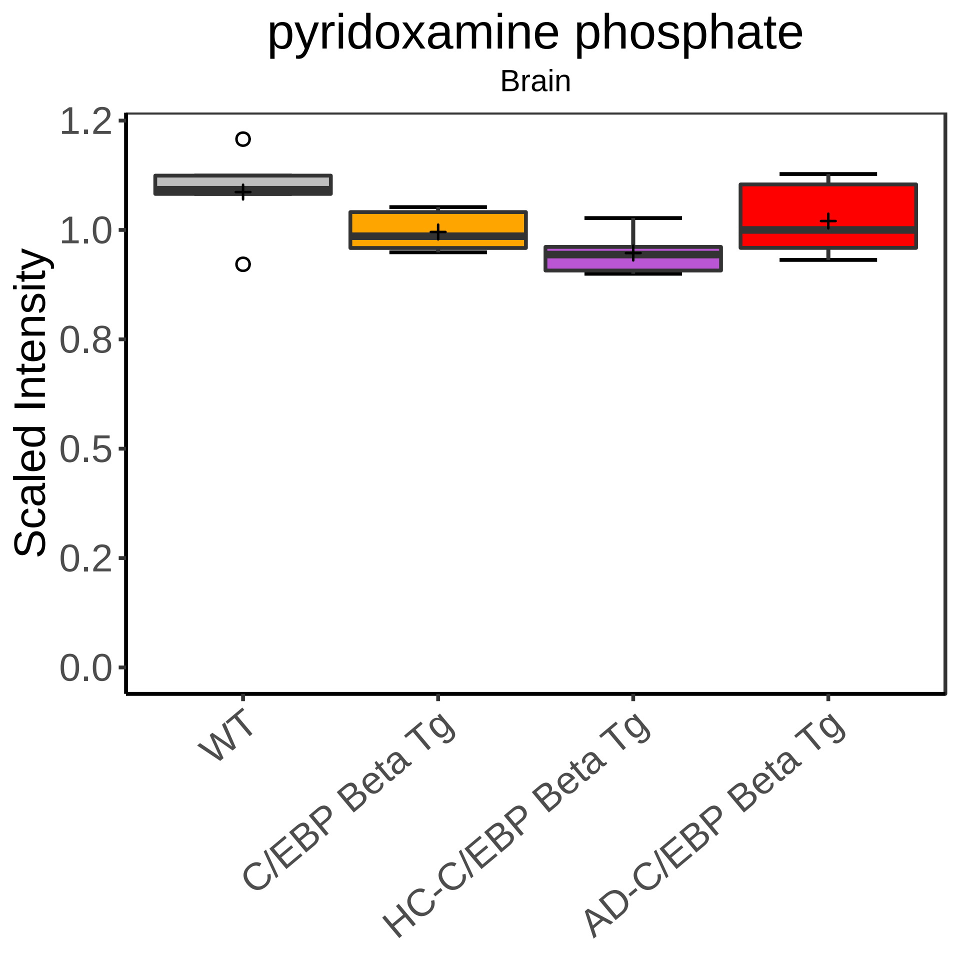

pyridoxamine phosphate
Brain
Scaled Intensity
1.2
1.0
0.8
0.5
0.2
0.0
WT
C/EBP Beta Tg
HC-C/EBP Beta Tg
AD-C/EBP Beta Tg

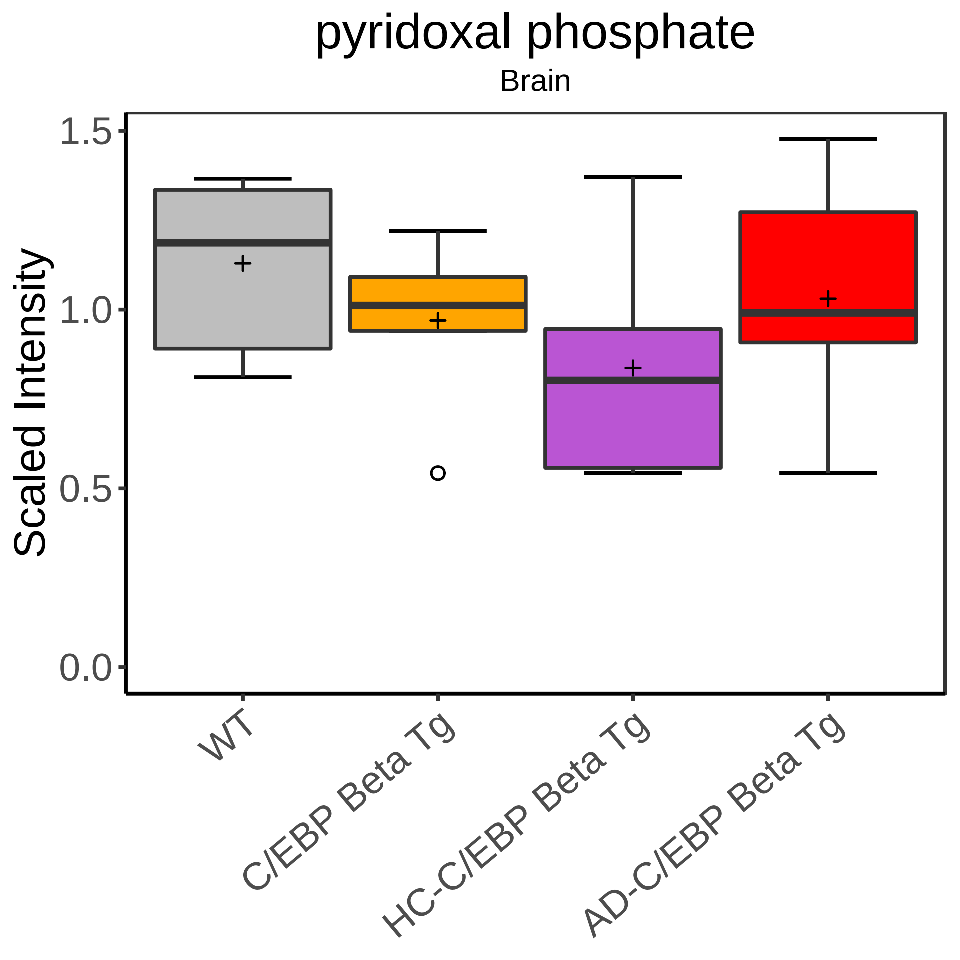
pyridoxal phosphate
Brain
Scaled Intensity
1.5
1.0
0.5
0.0
WT
C/EBP Beta Tg
HC-C/EBP Beta Tg
AD-C/EBP Beta Tg

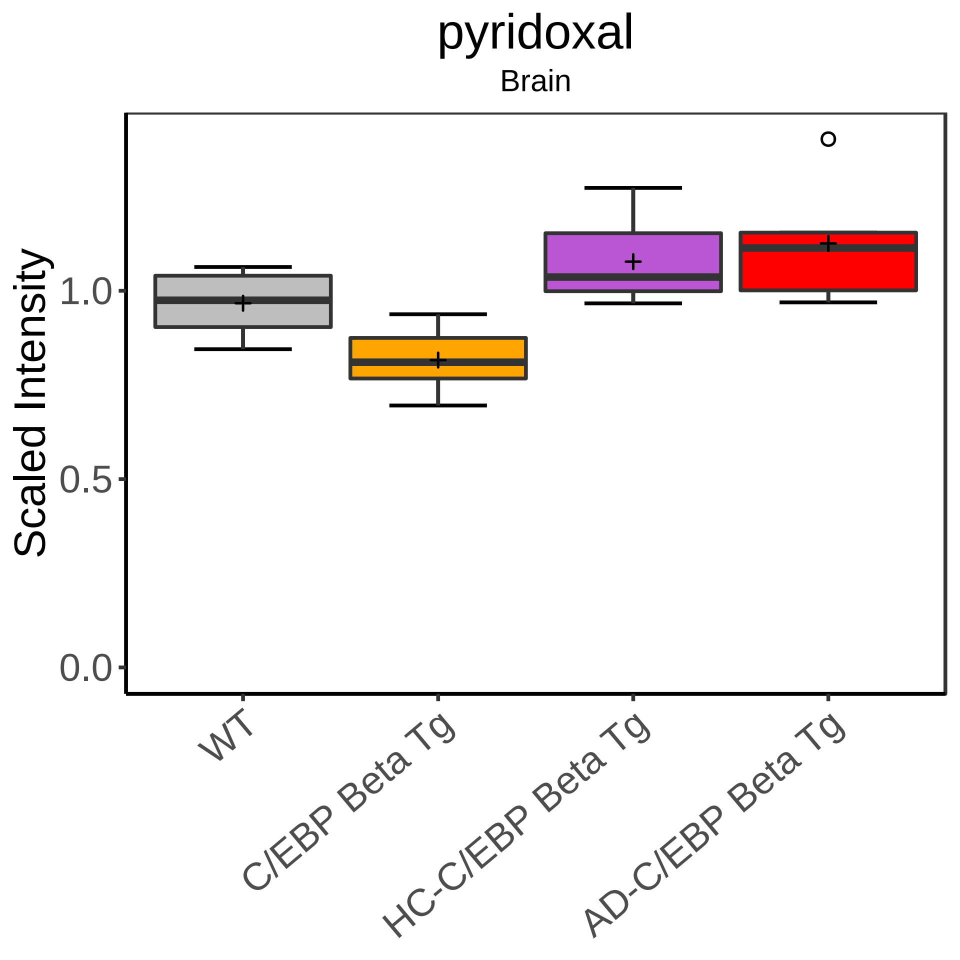

pyridoxal
Brain
Scaled Intensity
1.0
0.5
0.0
WT
C/EBP Beta Tg
HC-C/EBP Beta Tg
AD-C/EBP Beta Tg

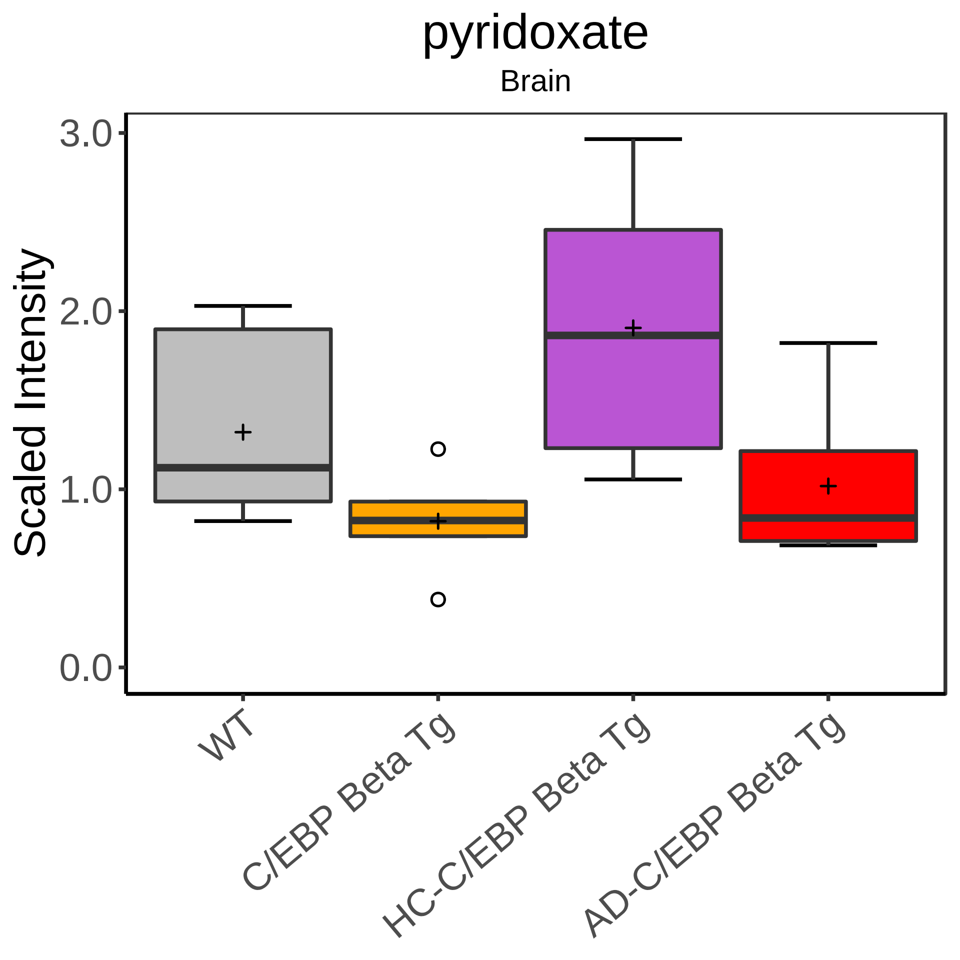
pyridoxate
Brain
Scaled Intensity
3.0
2.0
1.0
0.0
WT
C/EBP Beta Tg
HC-C/EBP Beta Tg
AD-C/EBP Beta Tg

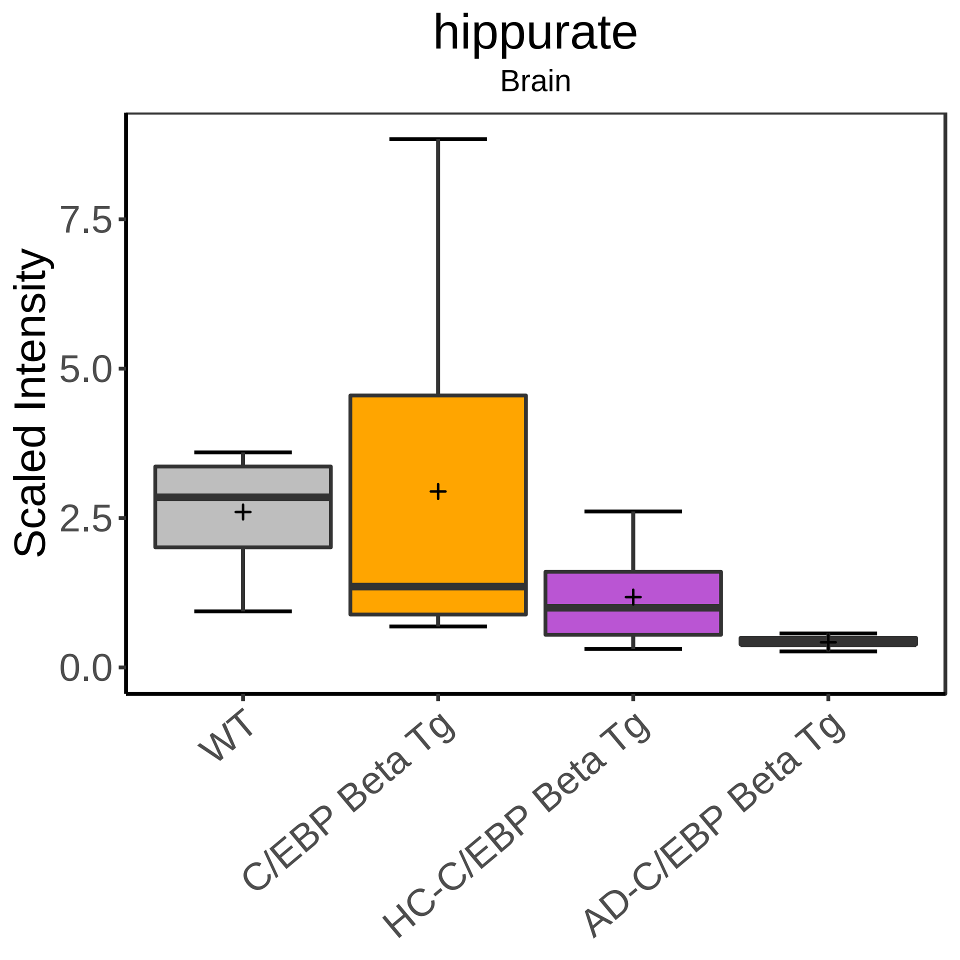

hippurate
Brain
Scaled Intensity
7.5
5.0
2.5
0.0
WT
C/EBP Beta Tg
HC-C/EBP Beta Tg
AD-C/EBP Beta Tg

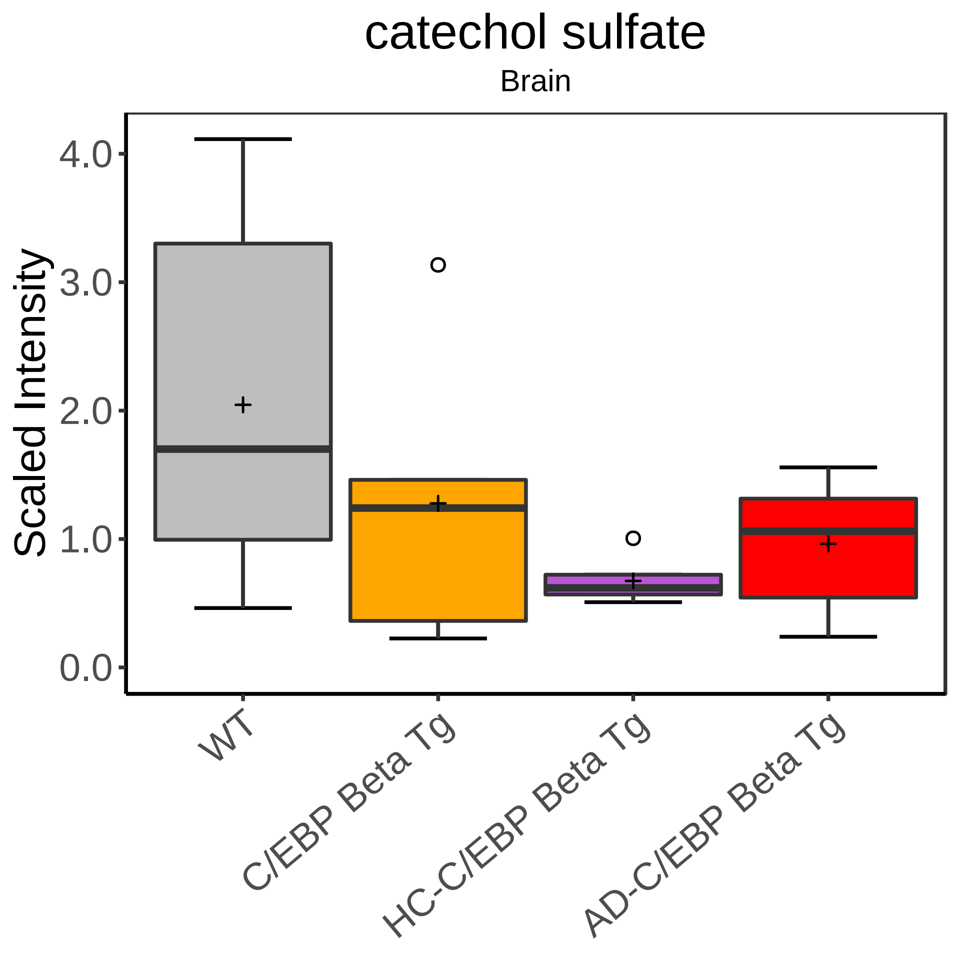
catechol sulfate
Brain
Scaled Intensity
4.0
3.0
2.0
1.0
0.0
WT
C/EBP Beta Tg
HC-C/EBP Beta Tg
AD-C/EBP Beta Tg

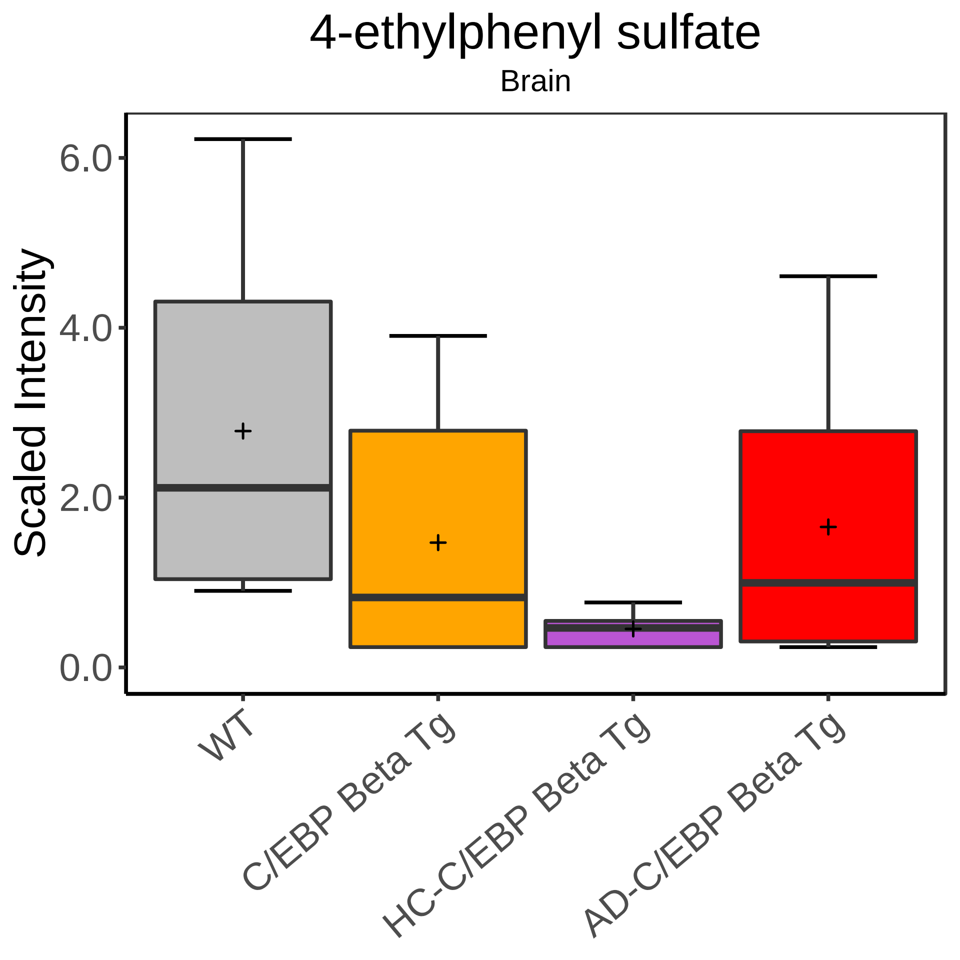

4-ethylphenyl sulfate
Brain
Scaled Intensity
6.0
4.0
2.0
0.0
WT
C/EBP Beta Tg
HC-C/EBP Beta Tg
AD-C/EBP Beta Tg

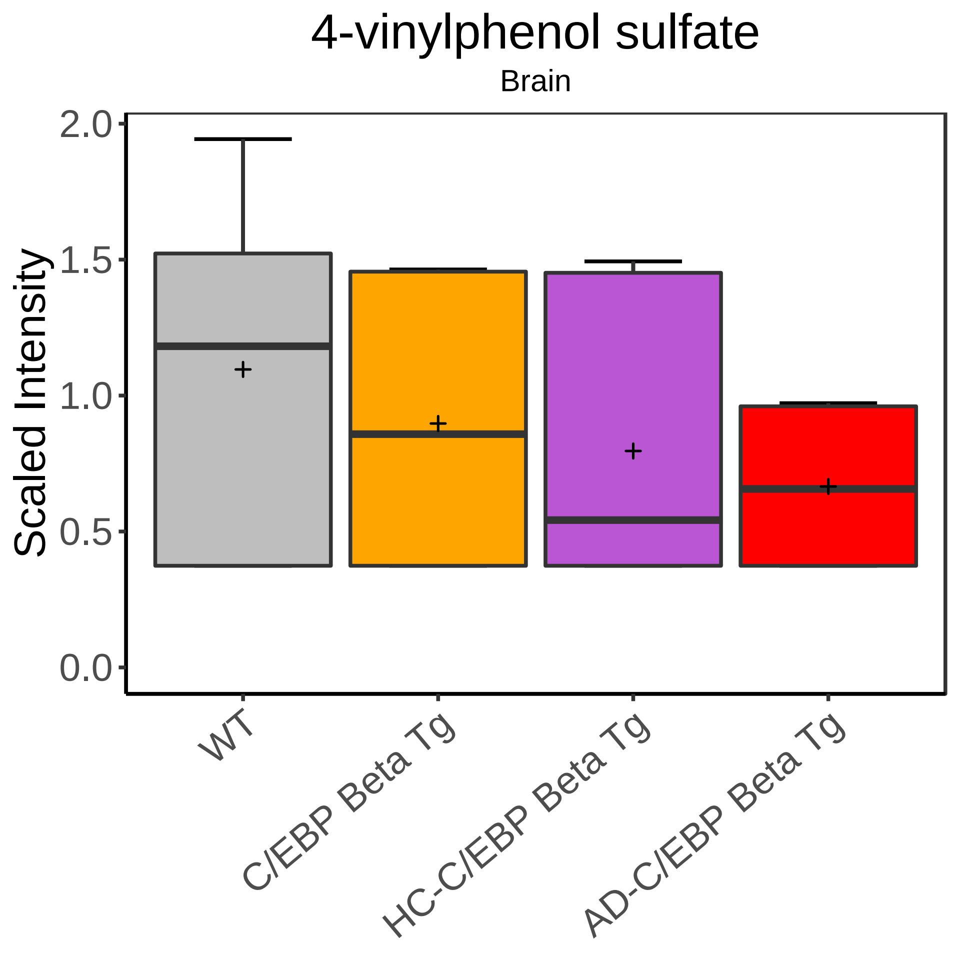

4-vinylphenol sulfate
Brain
Scaled Intensity
2.0
1.5
1.0
0.5
0.0
WT
C/EBP Beta Tg
HC-C/EBP Beta Tg
AD-C/EBP Beta Tg

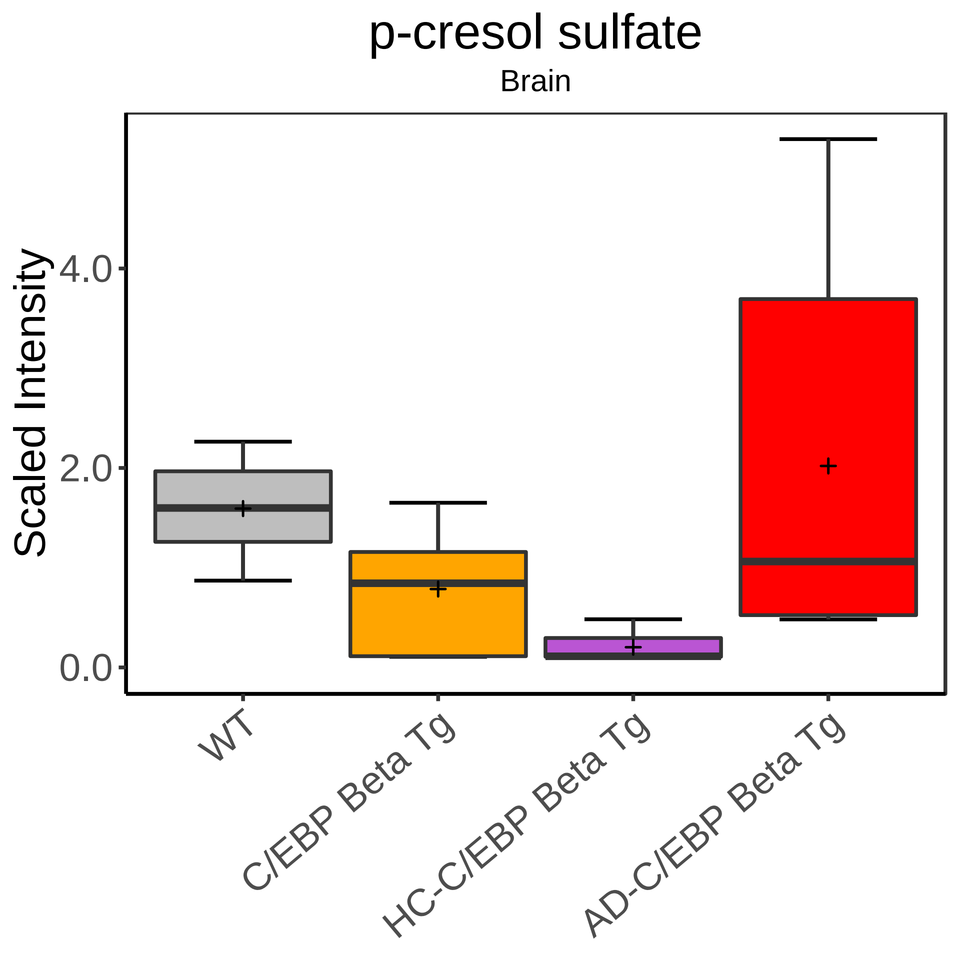

p-cresol sulfate
Brain
Scaled Intensity
4.0
2.0
0.0
WT
C/EBP Beta Tg
HC-C/EBP Beta Tg
AD-C/EBP Beta Tg

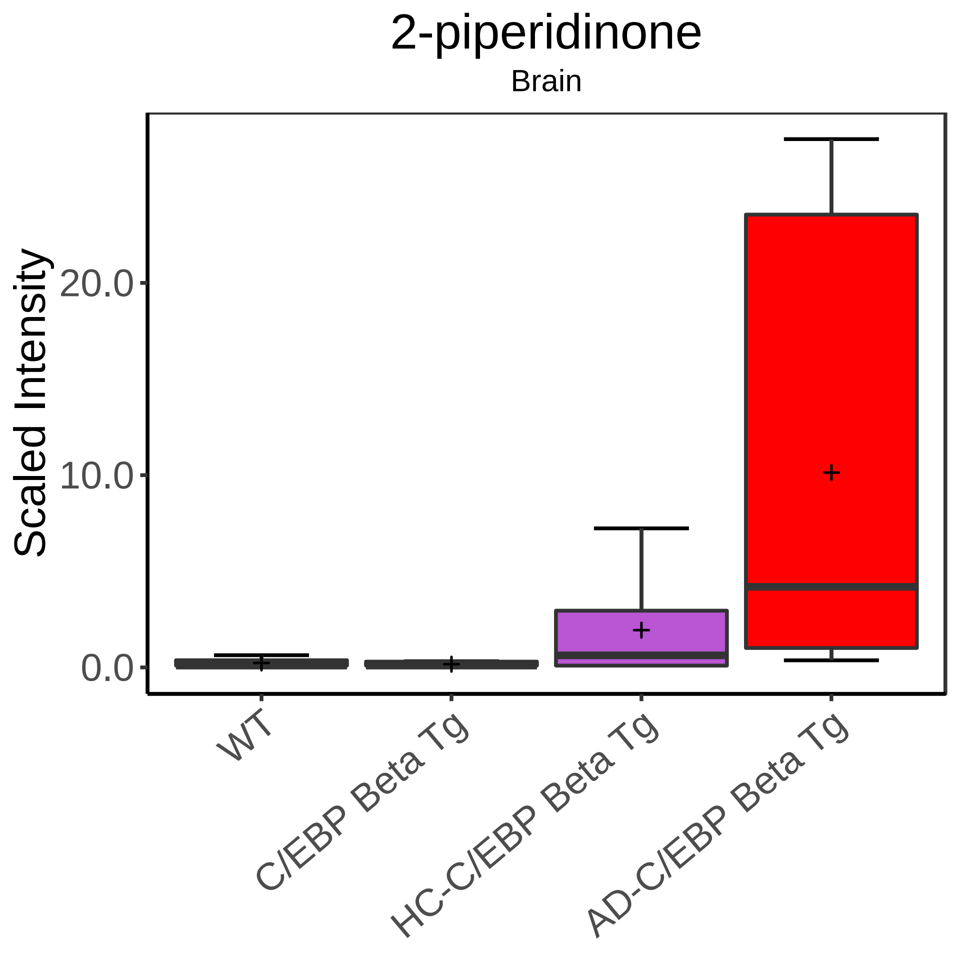
2-piperidinone
Brain
Scaled Intensity
20.0
10.0
0.0
WT
C/EBP Beta Tg
HC-C/EBP Beta Tg
AD-C/EBP Beta Tg

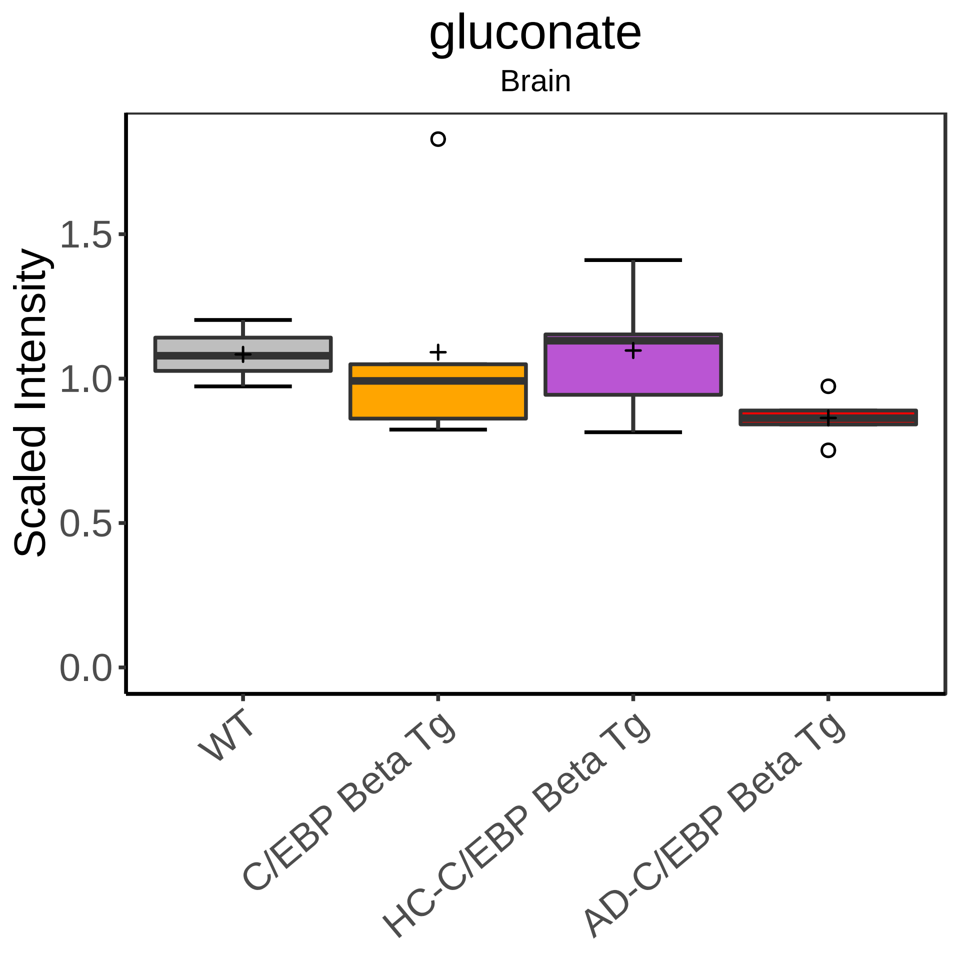
gluconate
Brain
Scaled Intensity
0.0
0.5
1.0
1.5
WT
C/EBP Beta Tg
HC-C/EBP Beta Tg
AD-C/EBP Beta Tg

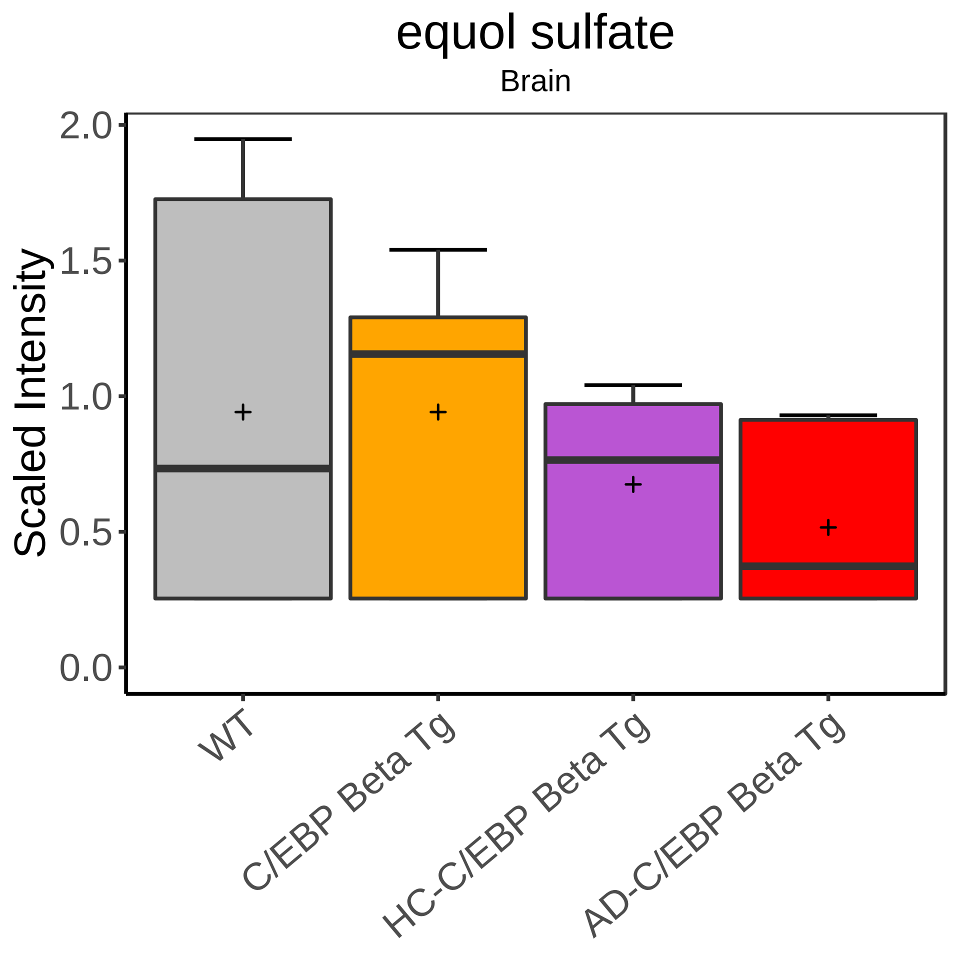

equol sulfate
Brain
Scaled Intensity
2.0
1.5
1.0
0.5
0.0
WT
C/EBP Beta Tg
HC-C/EBP Beta Tg
AD-C/EBP Beta Tg

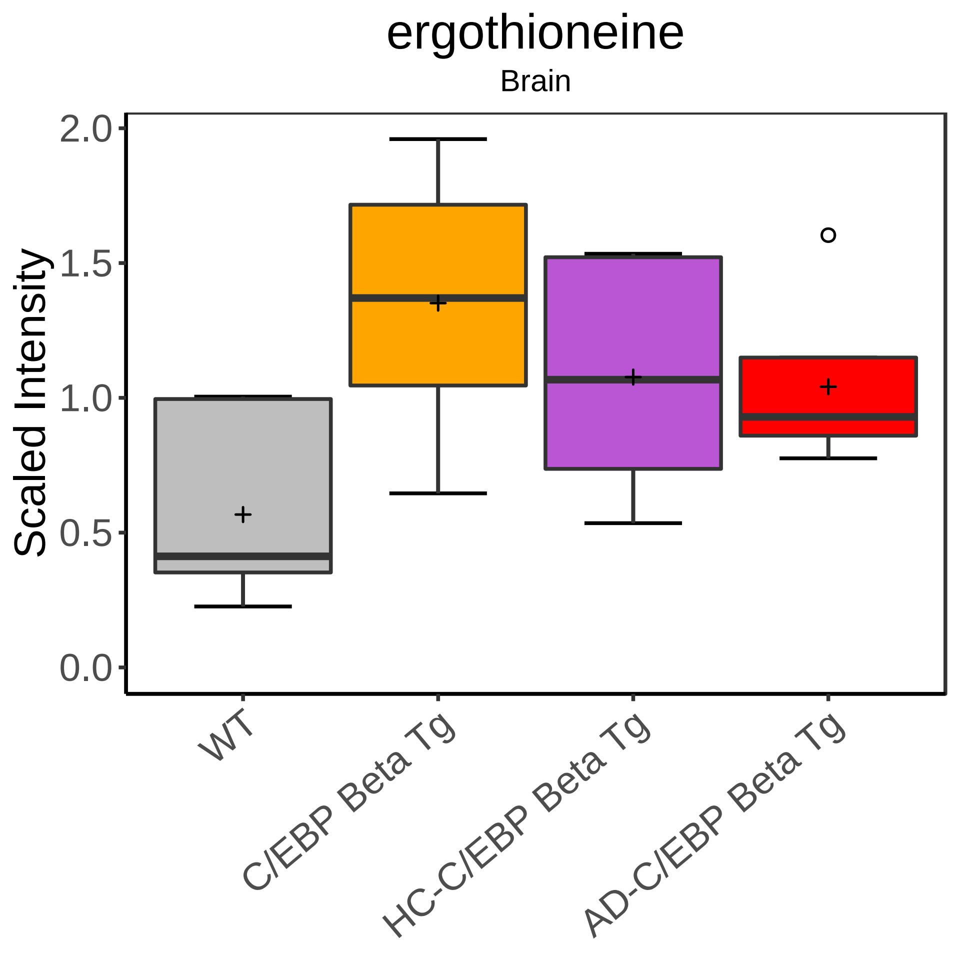
ergothioneine
Brain
Scaled Intensity
2.0
1.5
1.0
0.5
0.0
WT
C/EBP Beta Tg
HC-C/EBP Beta Tg
AD-C/EBP Beta Tg

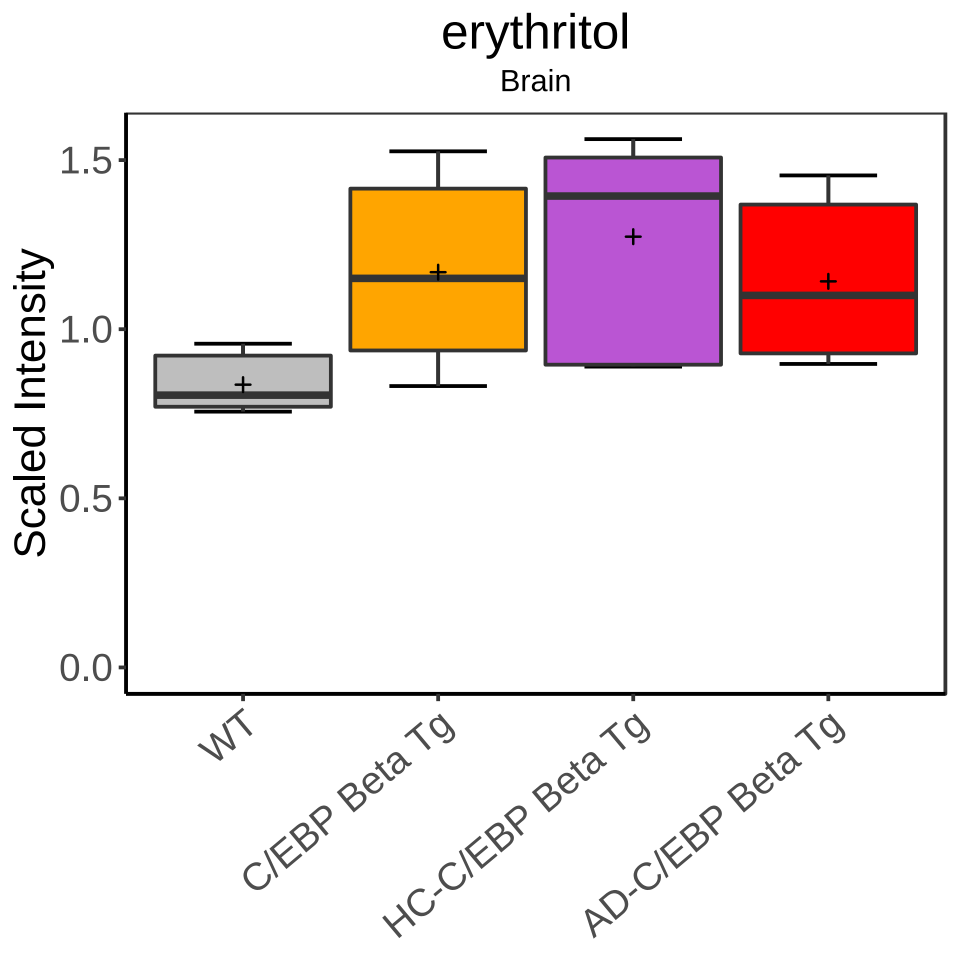

erythritol
Brain
Scaled Intensity
1.5
1.0
0.5
0.0
WT
C/EBP Beta Tg
HC-C/EBP Beta Tg
AD-C/EBP Beta Tg

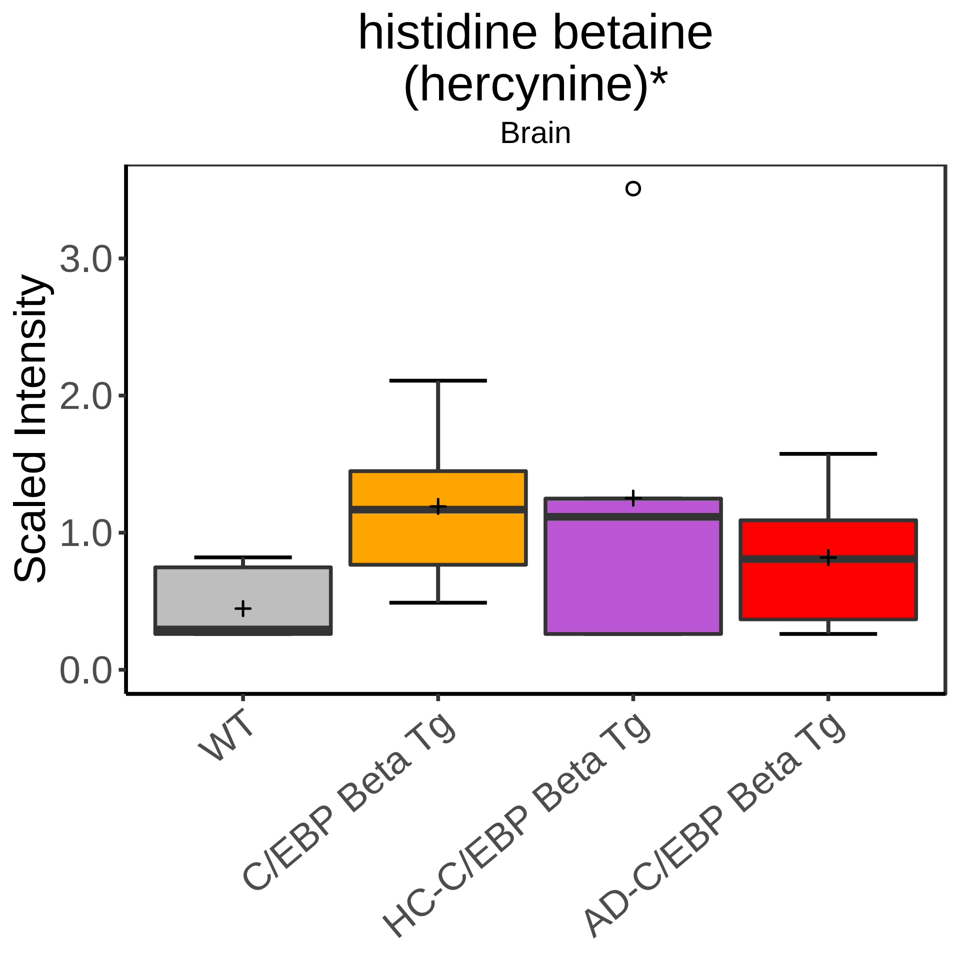

histidine betaine
(hercynine)*
Brain
Scaled Intensity
3.0
2.0
1.0
0.0
WT
C/EBP Beta Tg
HC-C/EBP Beta Tg
AD-C/EBP Beta Tg

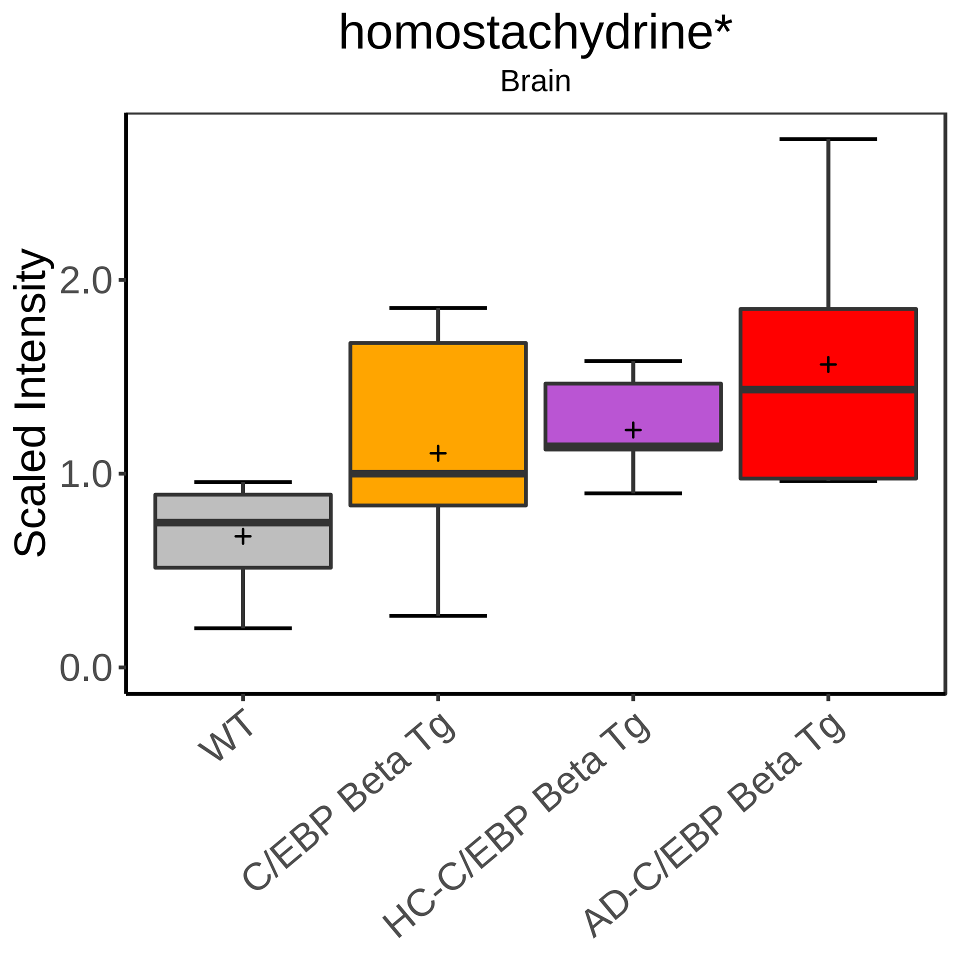
homostachydrine*
Brain
Scaled Intensity
2.0
1.0
0.0
WT
C/EBP Beta Tg
HC-C/EBP Beta Tg
AD-C/EBP Beta Tg

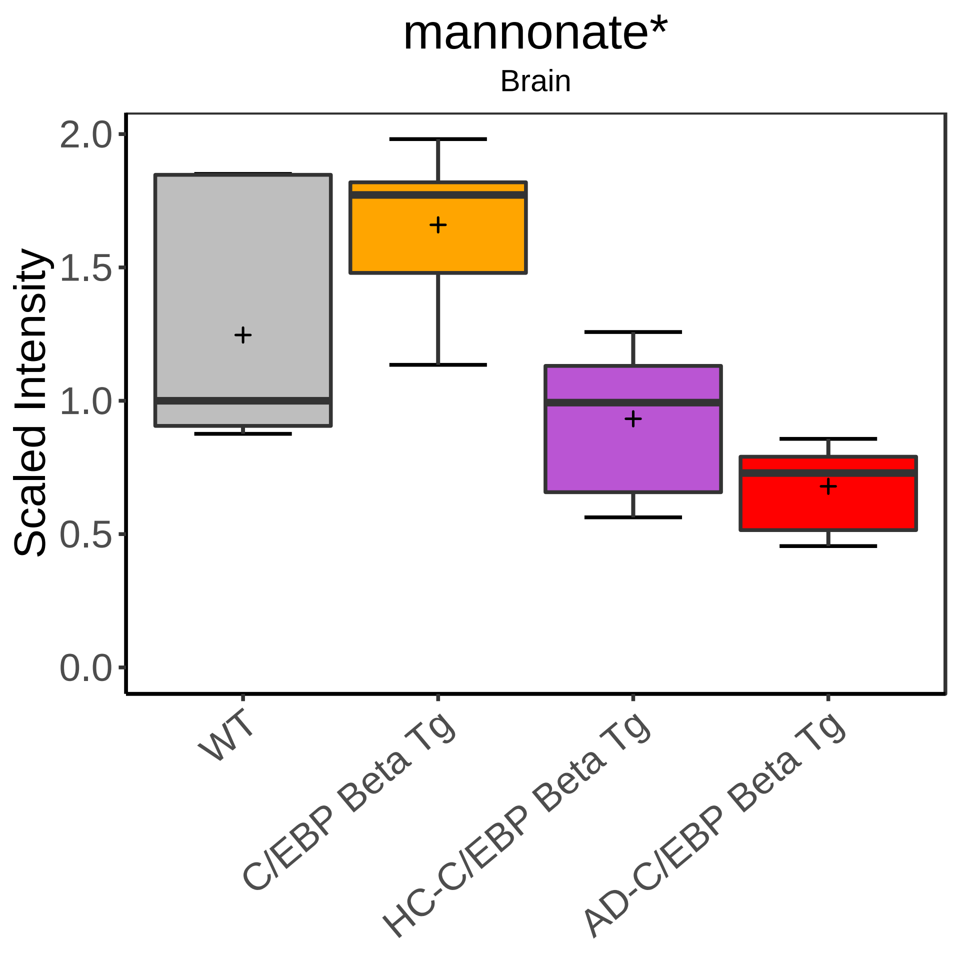

mannonate*
Brain
Scaled Intensity
2.0
1.5
1.0
0.5
0.0
WT
C/EBP Beta Tg
HC-C/EBP Beta Tg
AD-C/EBP Beta Tg

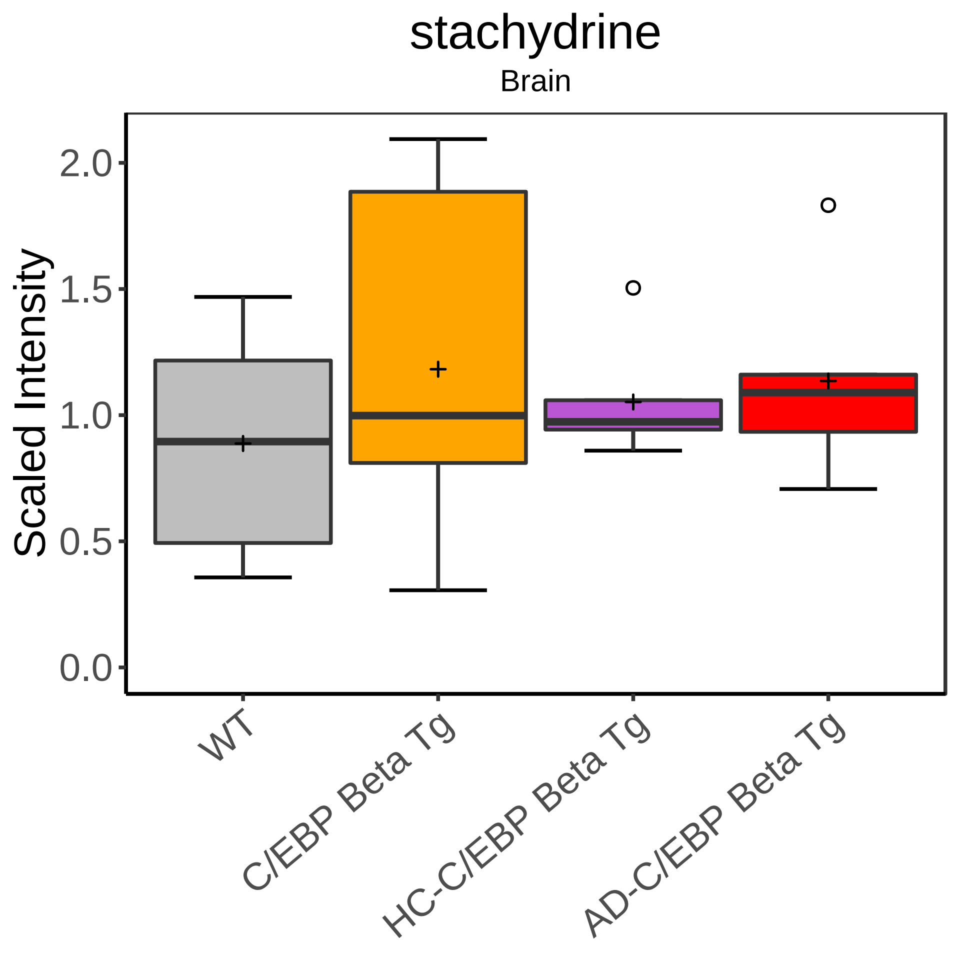
stachydrine
Brain
Scaled Intensity
2.0
1.5
1.0
0.5
0.0
WT
C/EBP Beta Tg
HC-C/EBP Beta Tg
AD-C/EBP Beta Tg

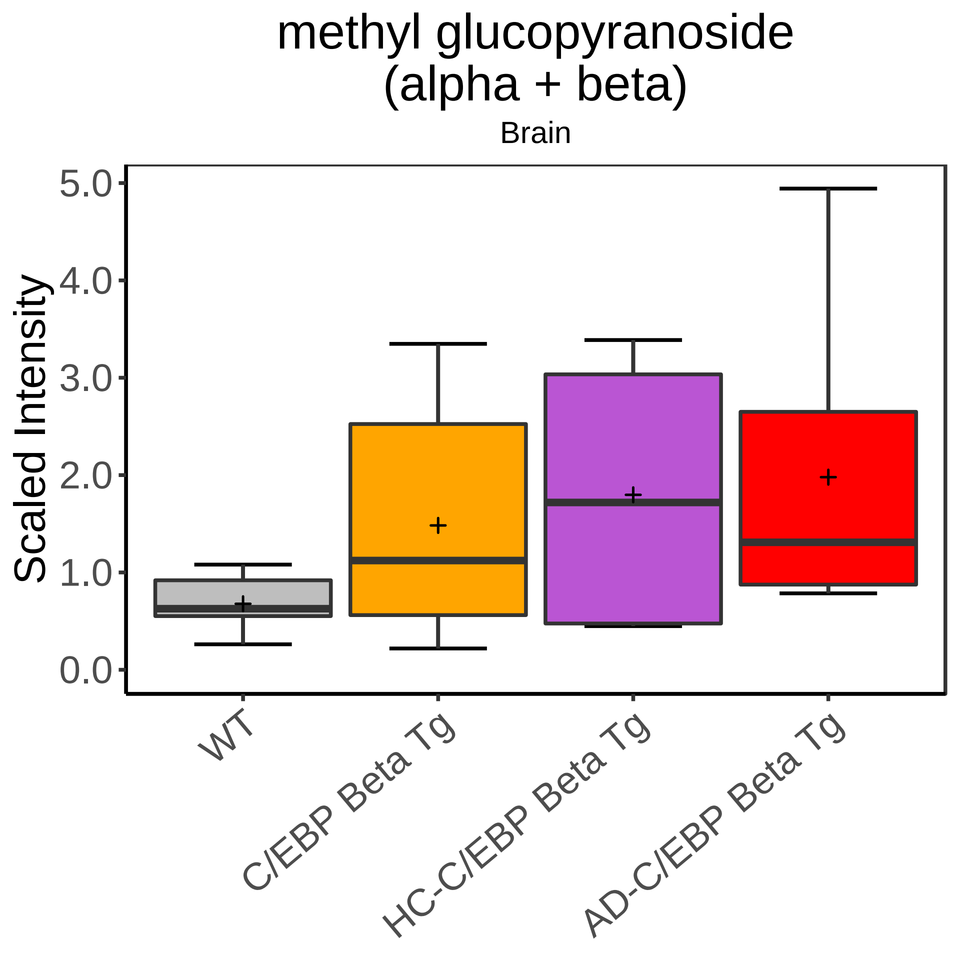
methyl glucopyranoside
(alpha + beta)
Brain
Scaled Intensity
5.0
4.0
3.0
2.0
1.0
0.0
WT
C/EBP Beta Tg
HC-C/EBP Beta Tg
AD-C/EBP Beta Tg

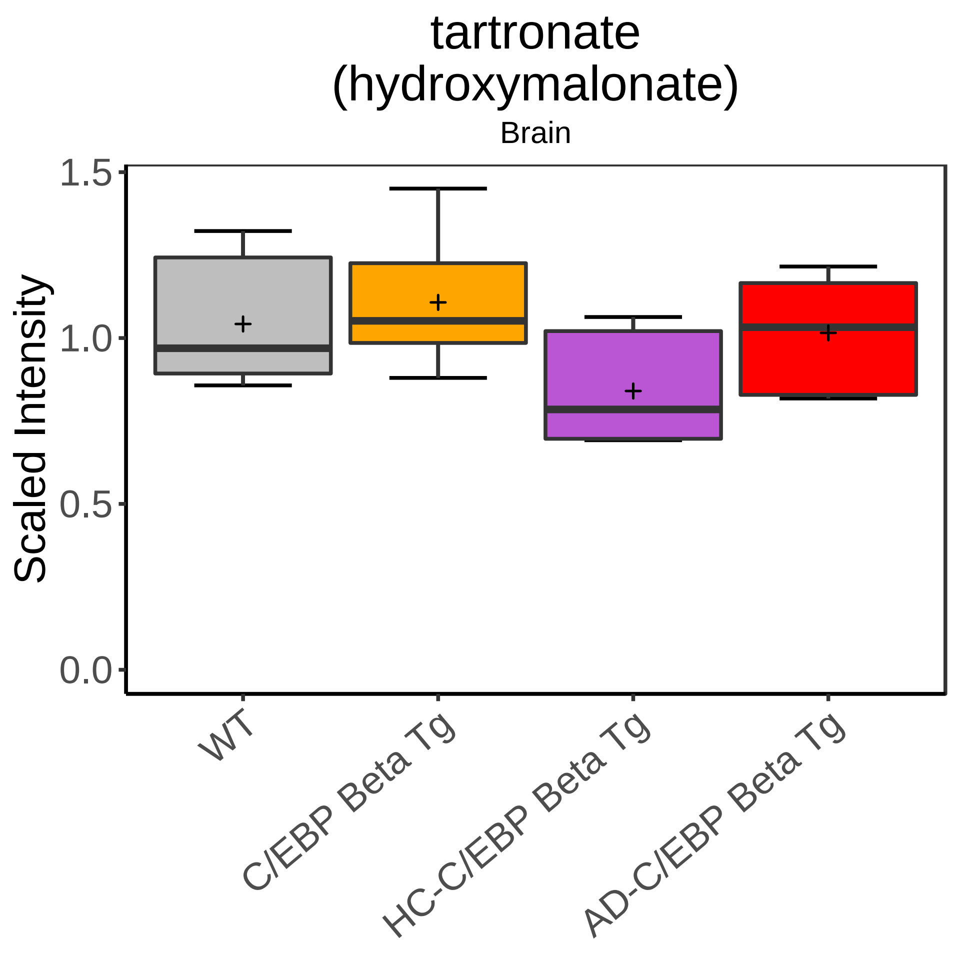
tartronate
(hydroxymalonate)
Brain
Scaled Intensity
1.5
1.0
0.5
0.0
WT
C/EBP Beta Tg
HC-C/EBP Beta Tg
AD-C/EBP Beta Tg

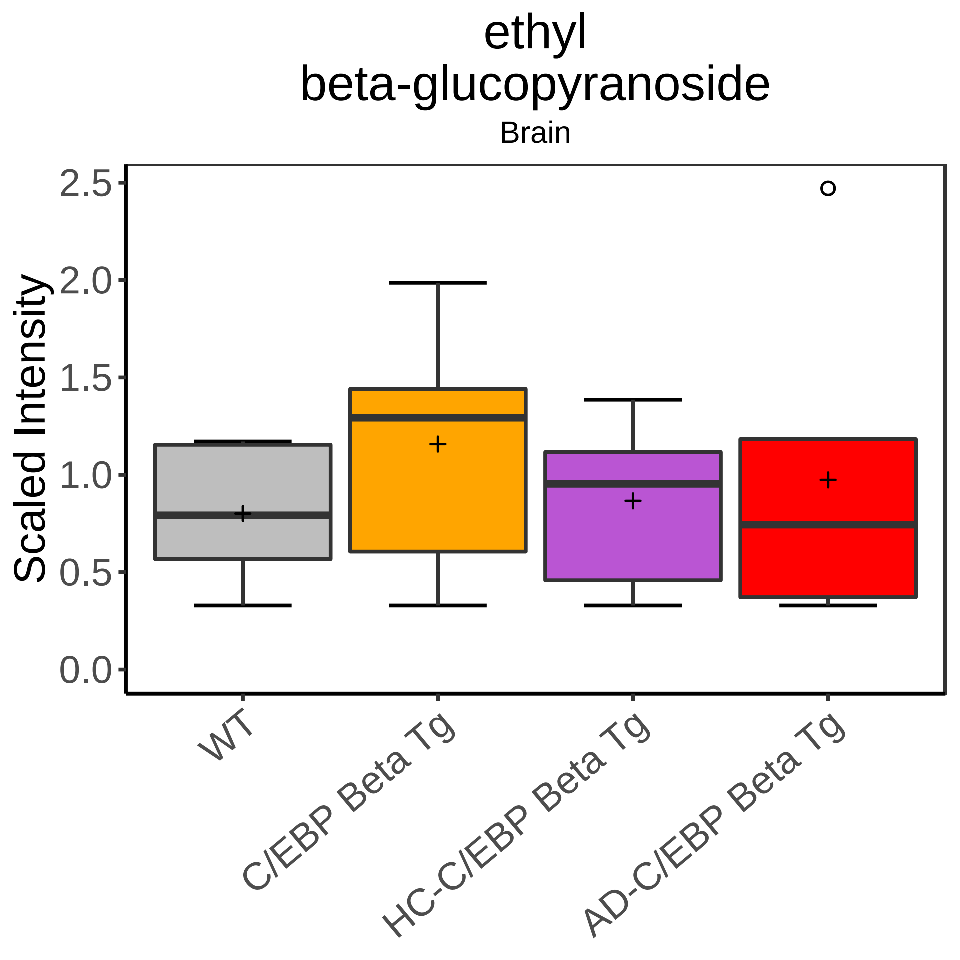
ethyl
beta-glucopyranoside
Brain
Scaled Intensity
2.5
2.0
1.5
1.0
0.5
0.0
WT
C/EBP Beta Tg
HC-C/EBP Beta Tg
AD-C/EBP Beta Tg

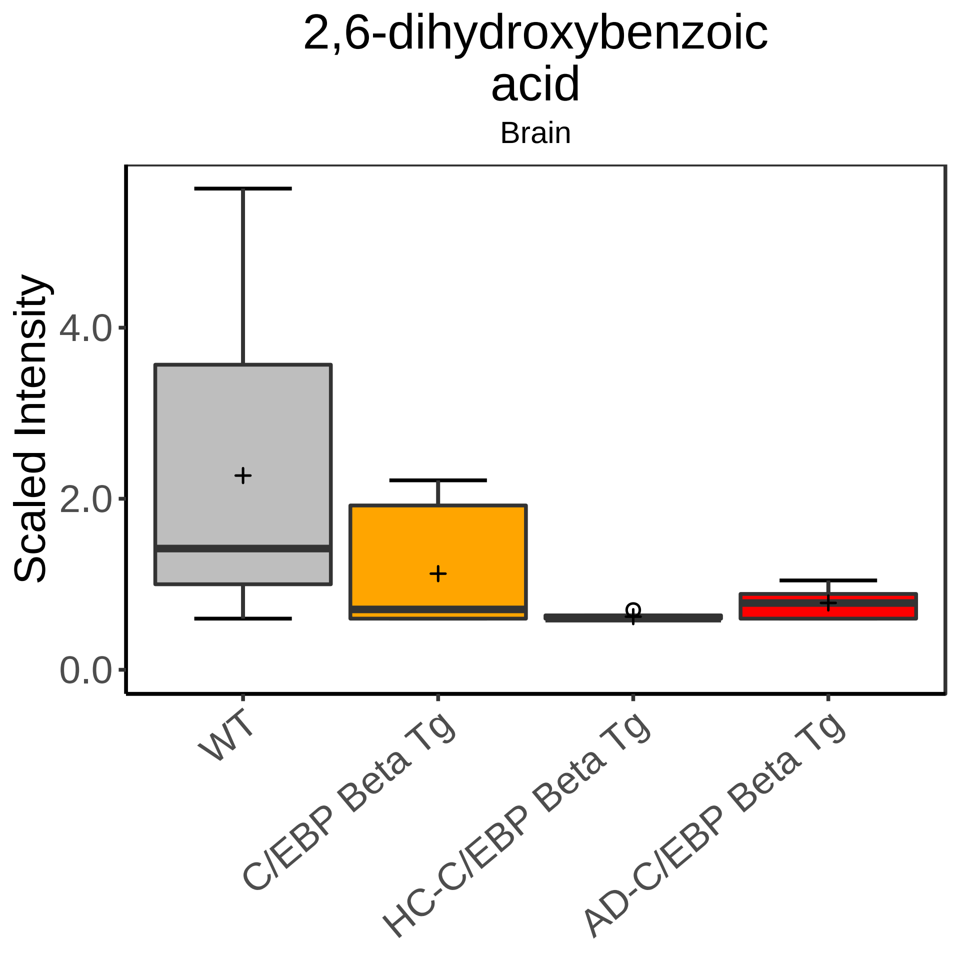
2,6-dihydroxybenzoic acid
Brain
Scaled Intensity
0.0
2.0
4.0
WT
C/EBP Beta Tg
HC-C/EBP Beta Tg
AD-C/EBP Beta Tg

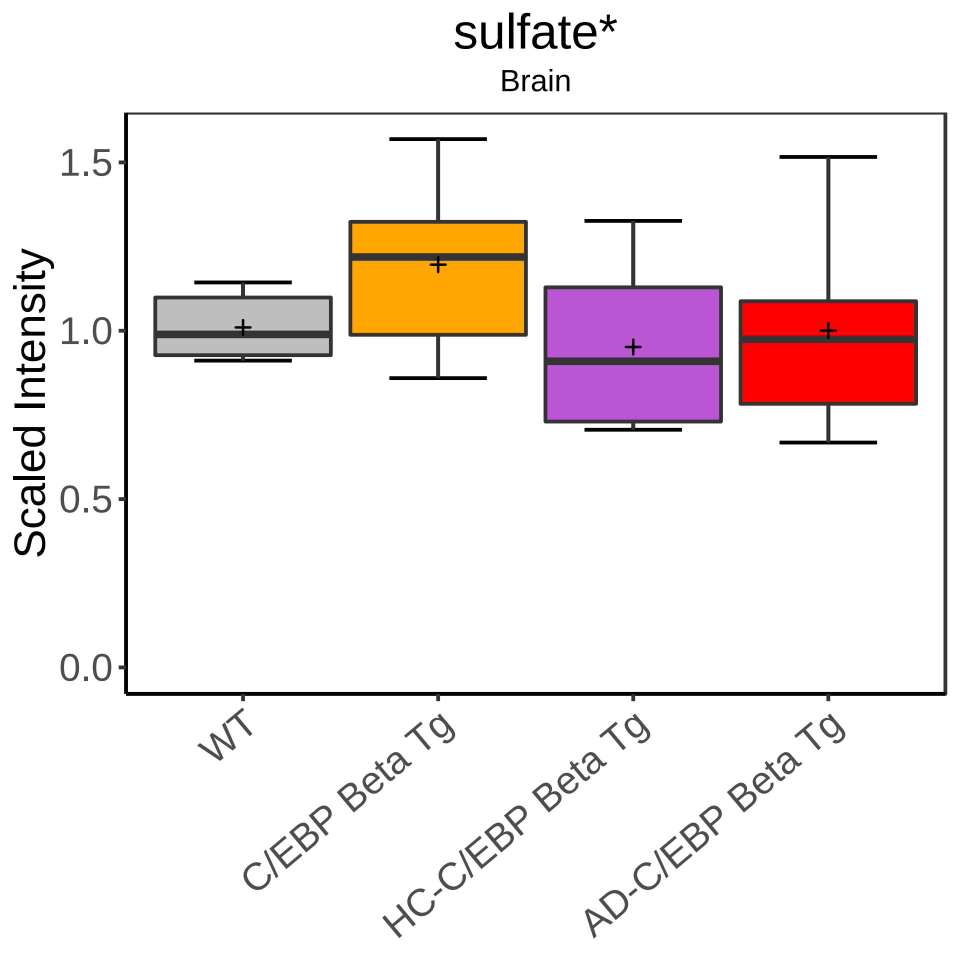

sulfate*
Brain
Scaled Intensity
0.0
0.5
1.0
1.5
WT
C/EBP Beta Tg
HC-C/EBP Beta Tg
AD-C/EBP Beta Tg

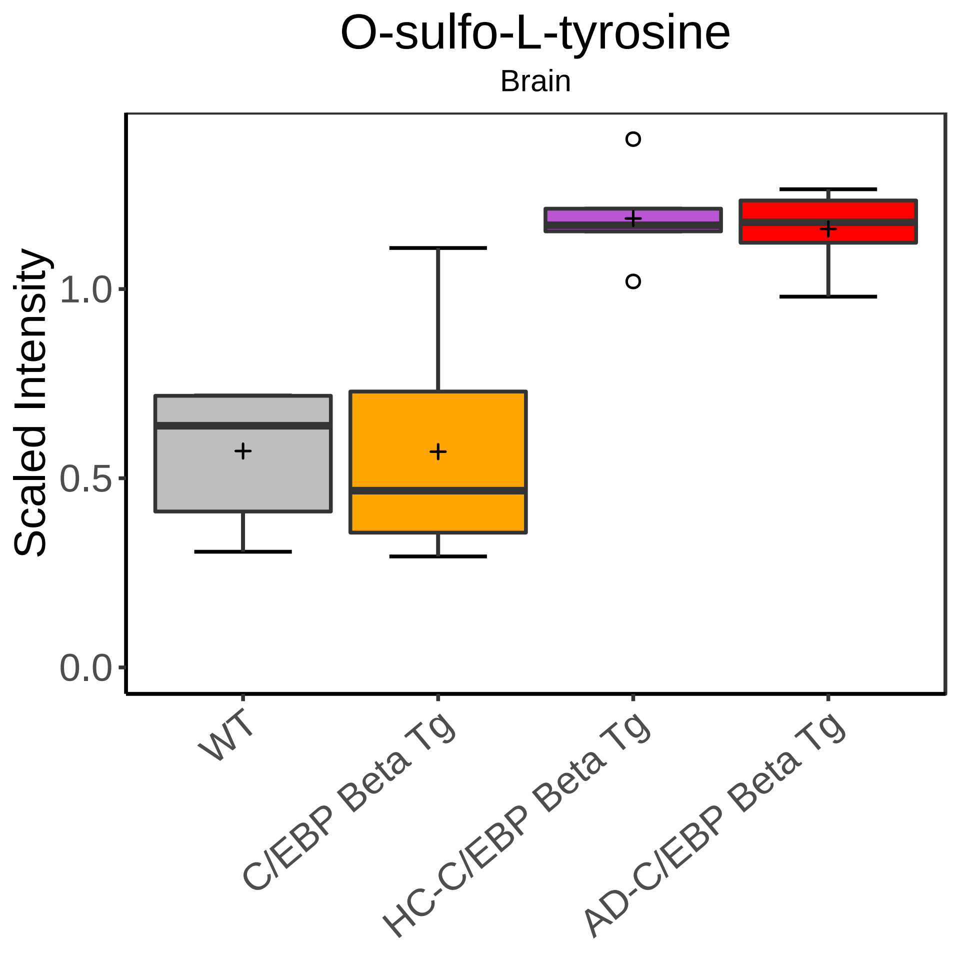
O-sulfo-L-tyrosine
Brain
Scaled Intensity
1.0
0.5
0.0
WT
C/EBP Beta Tg
HC-C/EBP Beta Tg
AD-C/EBP Beta Tg

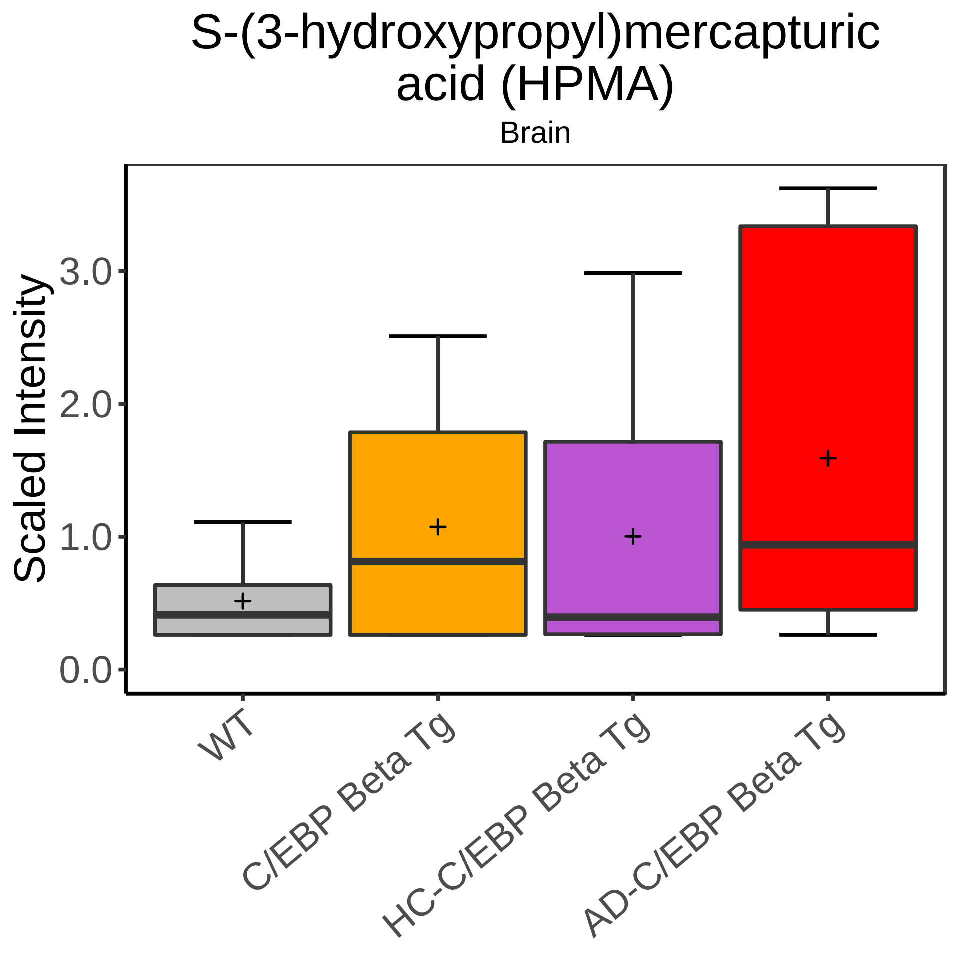
S-(3-hydroxypropyl)mercapturic acid (HPMA)
Brain
Scaled Intensity
3.0
2.0
1.0
0.0
WT
C/EBP Beta Tg
HC-C/EBP Beta Tg
AD-C/EBP Beta Tg

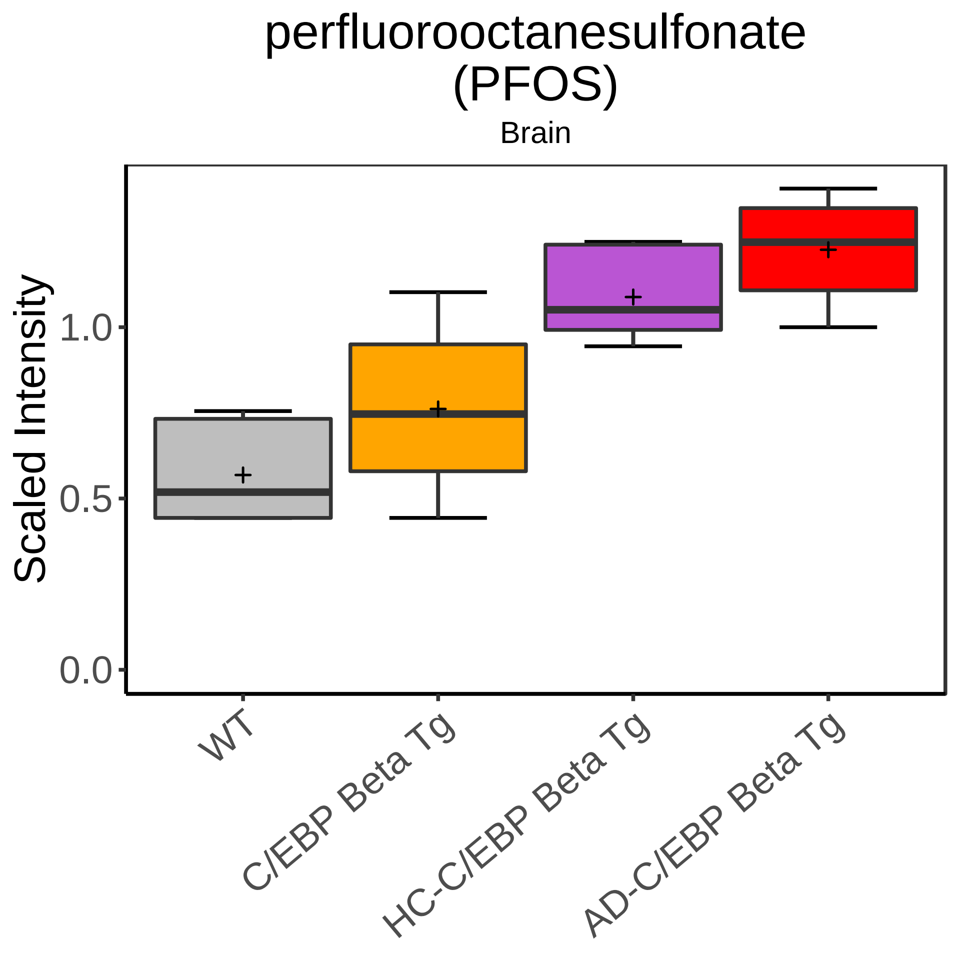
perfluorooctanesulfonate
(PFOS)
Brain
Scaled Intensity
1.0
0.5
0.0
WT
C/EBP Beta Tg
HC-C/EBP Beta Tg
AD-C/EBP Beta Tg

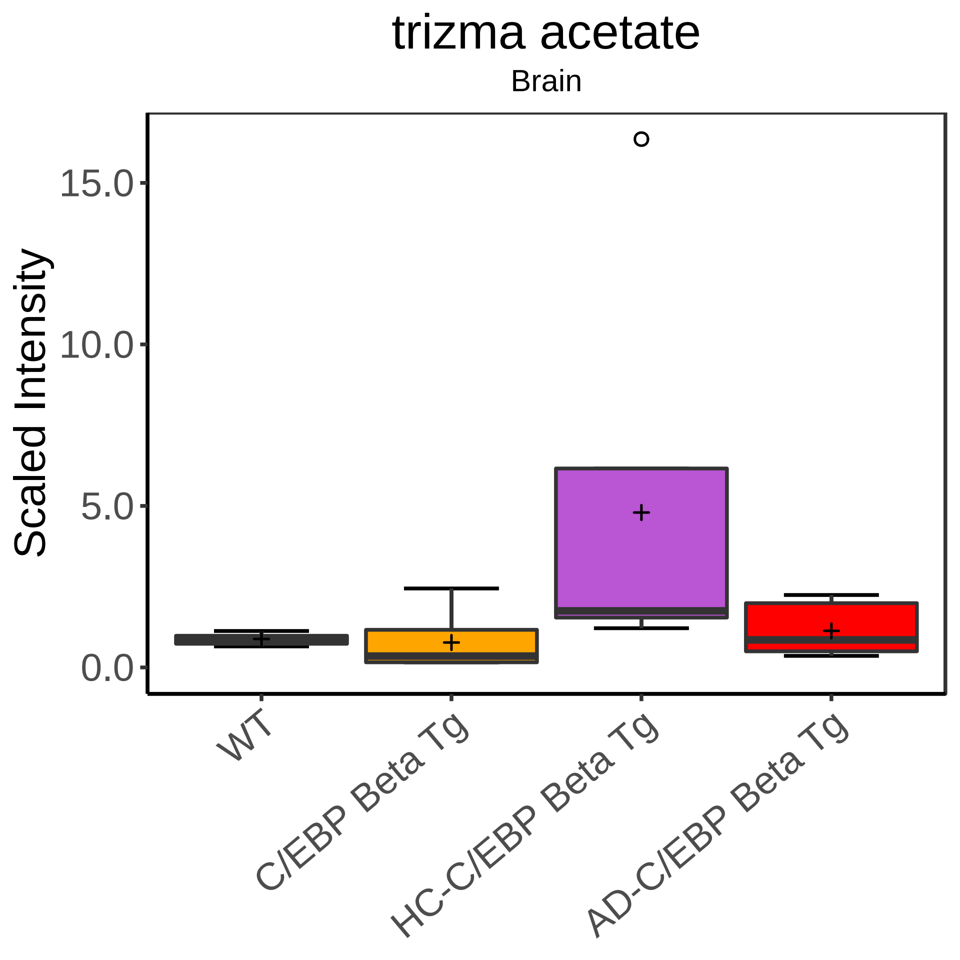
trizma acetate
Brain
Scaled Intensity
15.0
10.0
5.0
0.0
WT
C/EBP Beta Tg
HC-C/EBP Beta Tg
AD-C/EBP Beta Tg

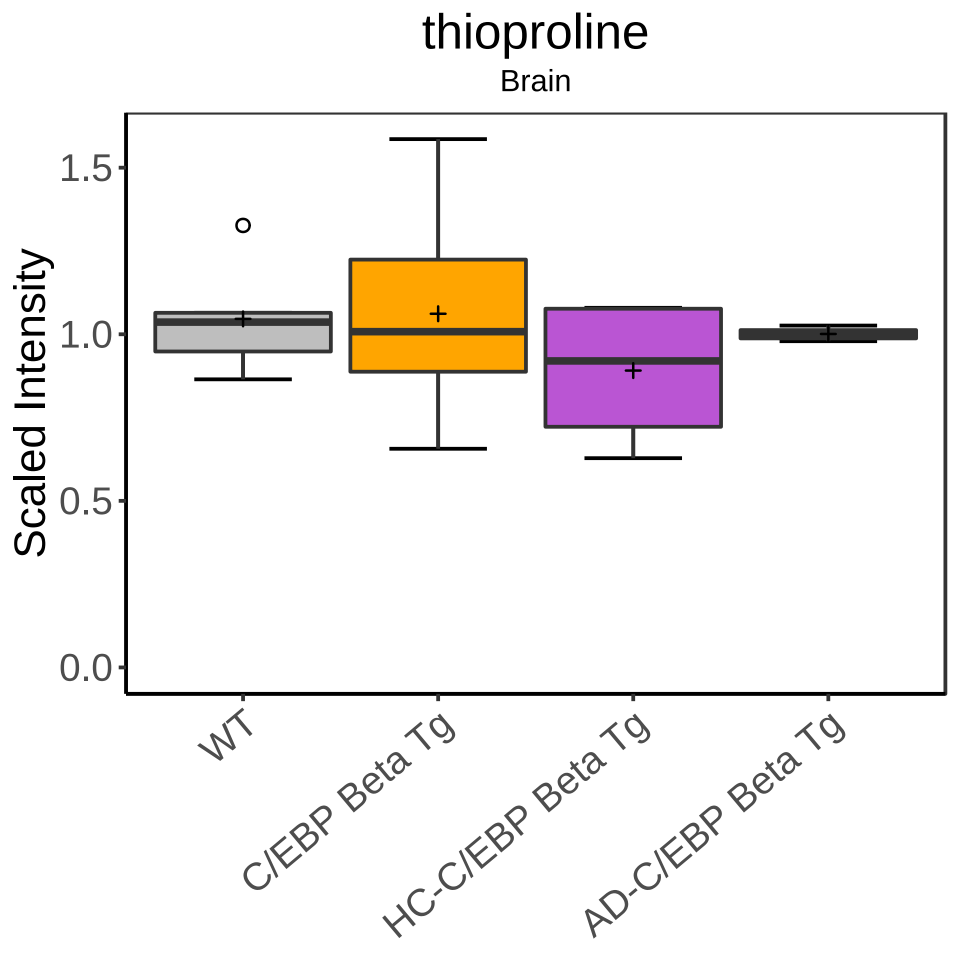

thioproline
Brain
Scaled Intensity
1.5
1.0
0.5
0.0
WT
C/EBP Beta Tg
HC-C/EBP Beta Tg
AD-C/EBP Beta Tg

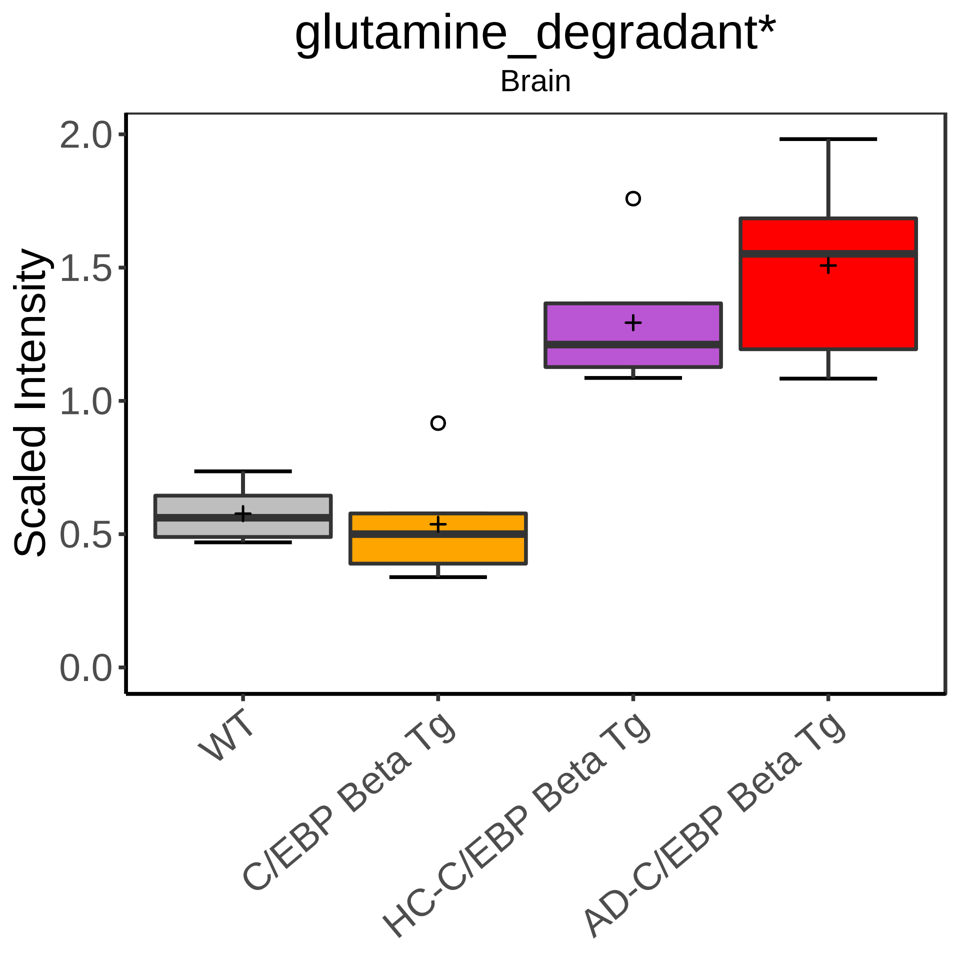

glutamine_degradant*
Brain
Scaled Intensity
2.0
1.5
1.0
0.5
0.0
WT
C/EBP Beta Tg
HC-C/EBP Beta Tg
AD-C/EBP Beta Tg

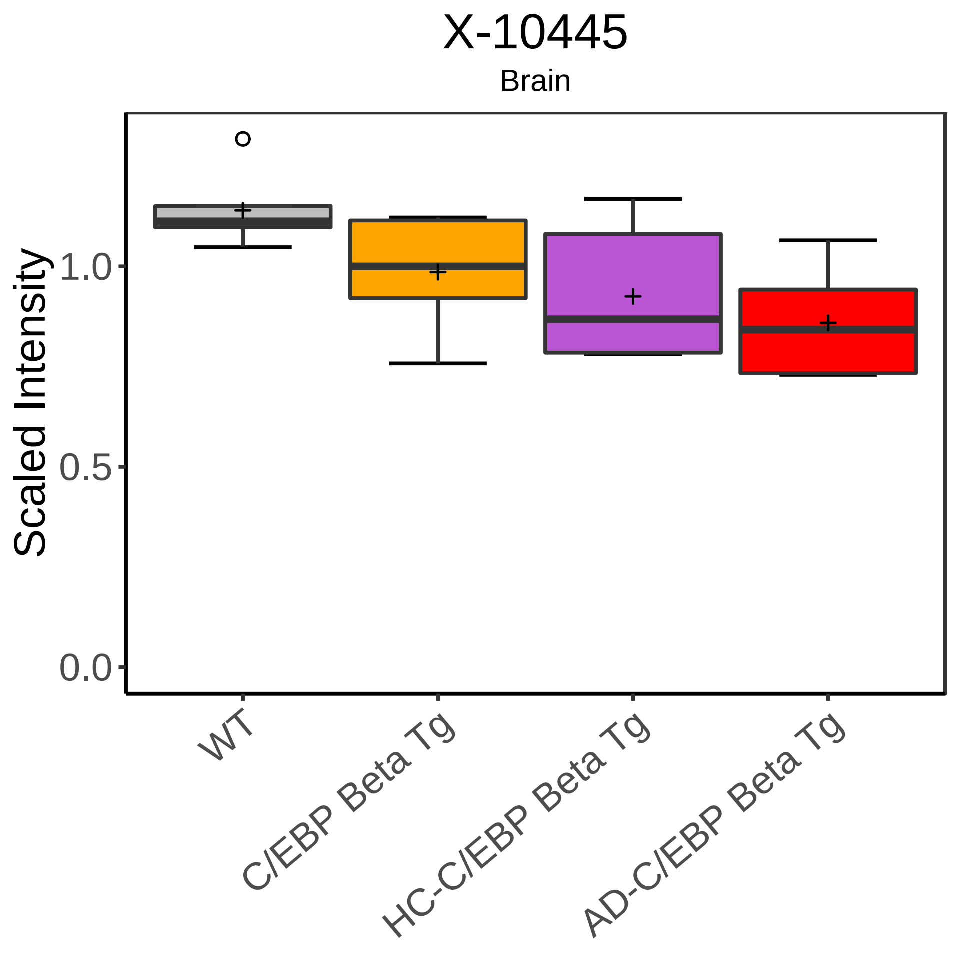

X-10445
Brain
Scaled Intensity
1.0
0.5
0.0
WT
C/EBP Beta Tg
HC-C/EBP Beta Tg
AD-C/EBP Beta Tg

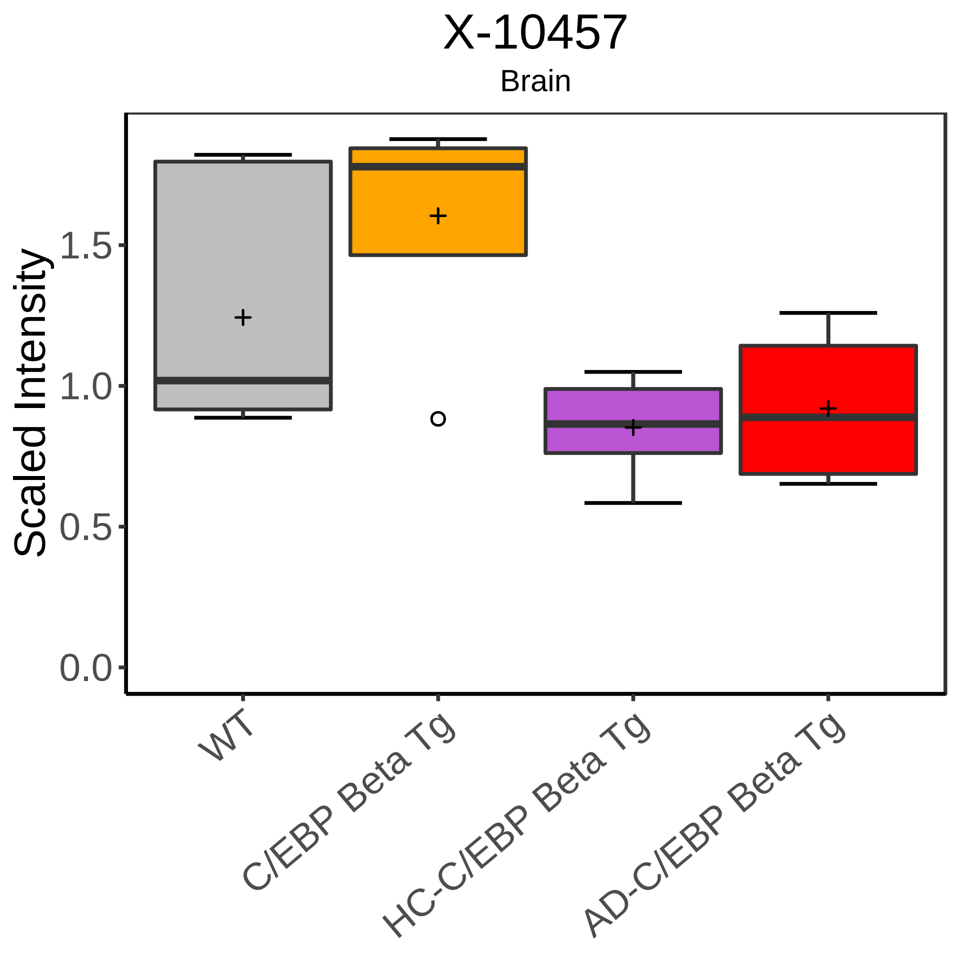
X-10457
Brain
Scaled Intensity
1.5
1.0
0.5
0.0
WT
C/EBP Beta Tg
HC-C/EBP Beta Tg
AD-C/EBP Beta Tg

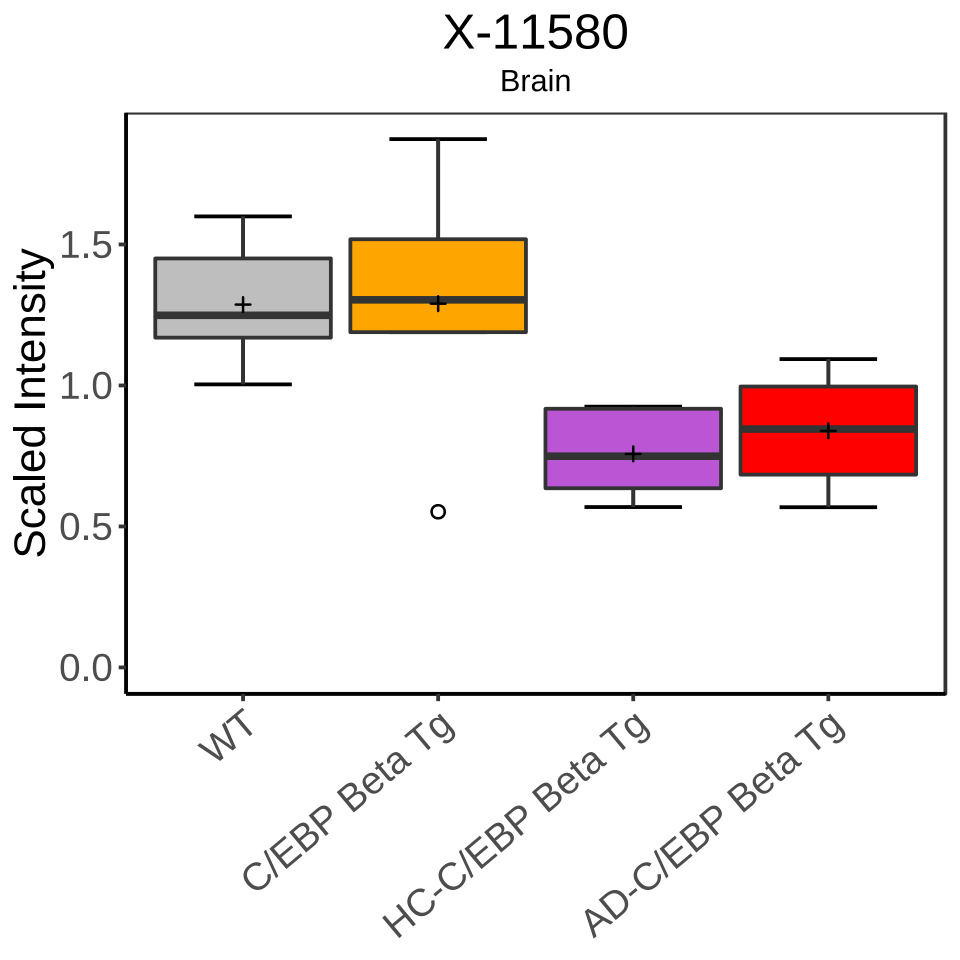
X-11580
Brain
Scaled Intensity
1.5
1.0
0.5
0.0
WT
C/EBP Beta Tg
HC-C/EBP Beta Tg
AD-C/EBP Beta Tg

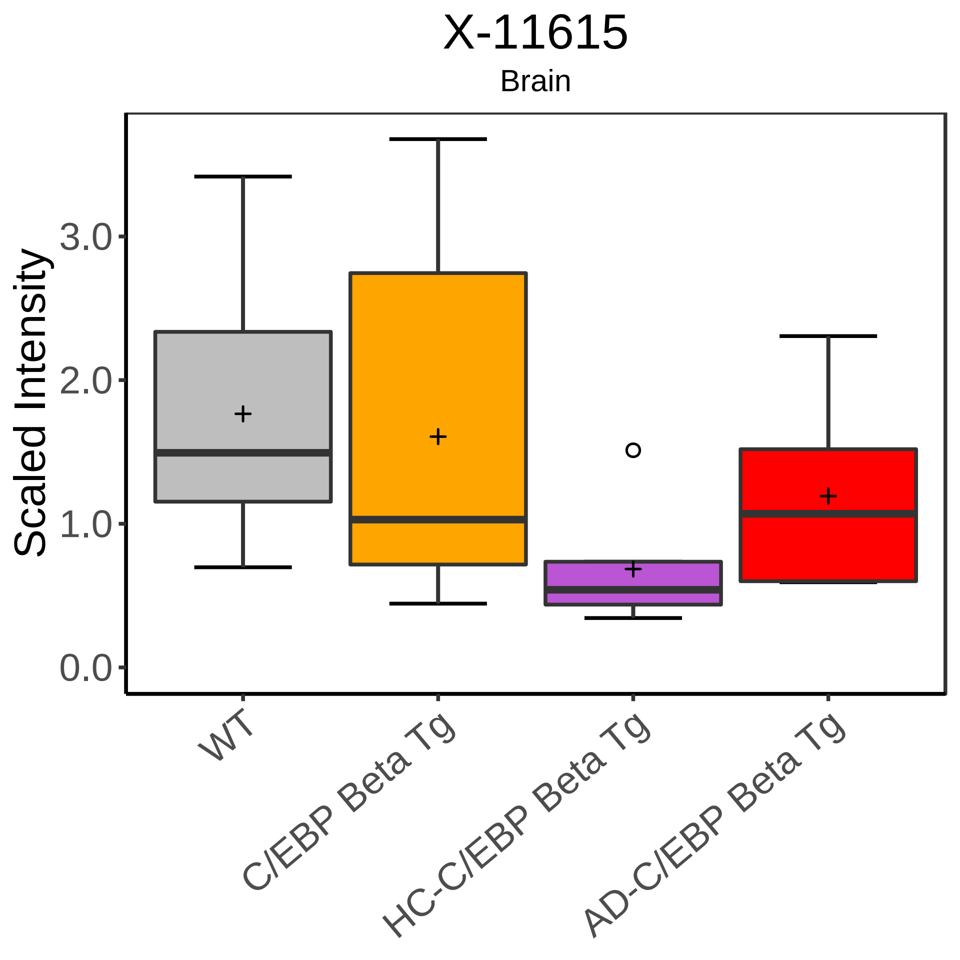
X-11615
Brain
Scaled Intensity
0.0
1.0
2.0
3.0
WT
C/EBP Beta Tg
HC-C/EBP Beta Tg
AD-C/EBP Beta Tg

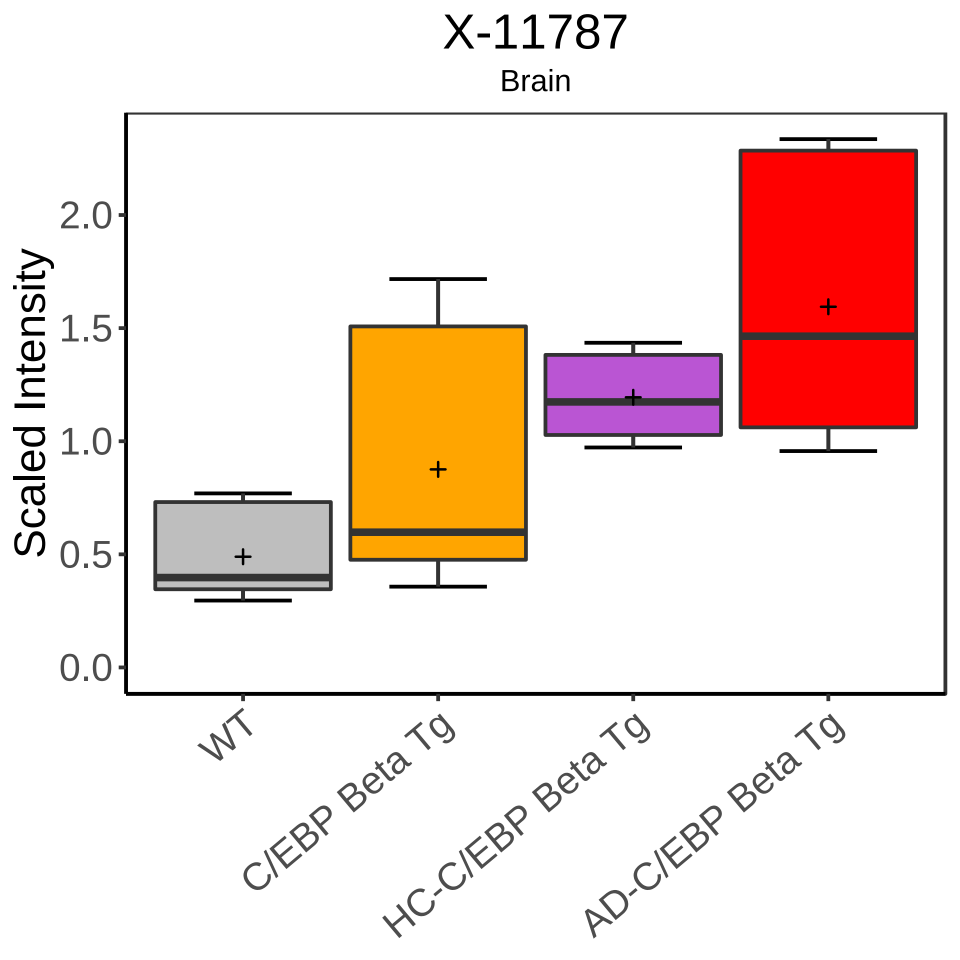

X-11787
Brain
Scaled Intensity
2.0
1.5
1.0
0.5
0.0
WT
C/EBP Beta Tg
HC-C/EBP Beta Tg
AD-C/EBP Beta Tg

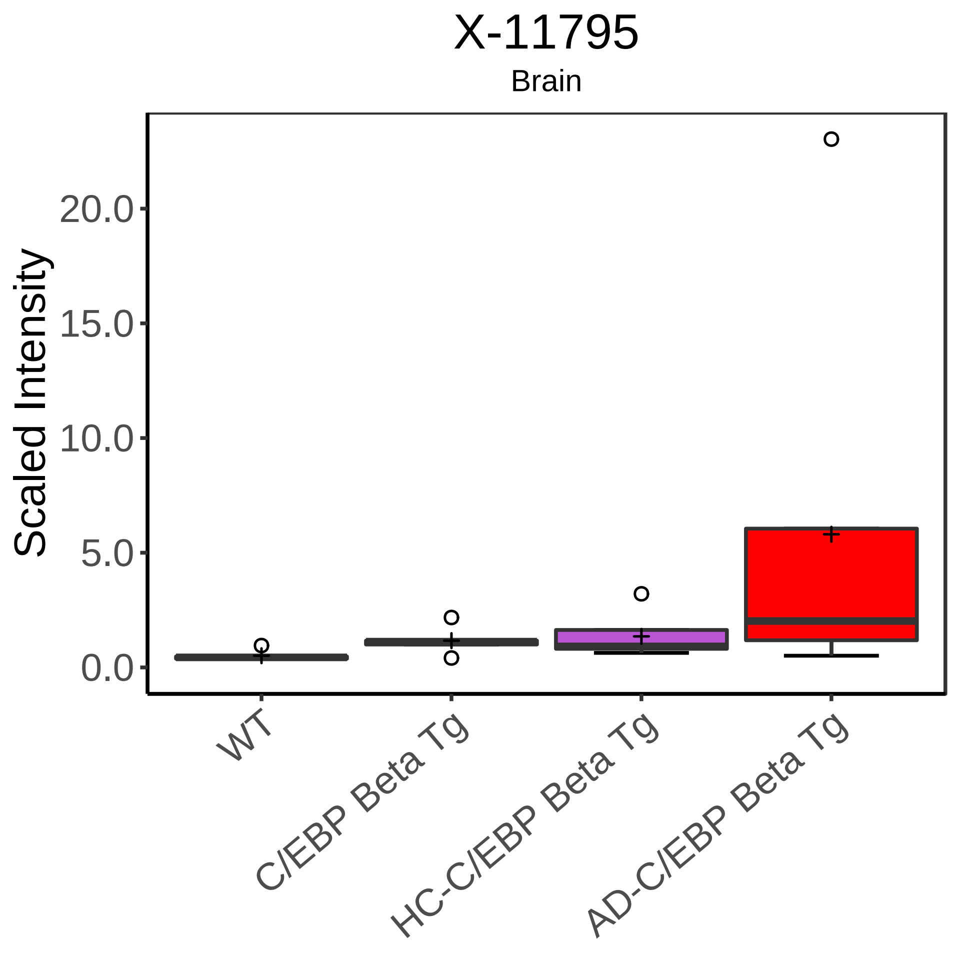
X-11795
Brain
Scaled Intensity
20.0
15.0
10.0
5.0
0.0
WT
C/EBP Beta Tg
HC-C/EBP Beta Tg
AD-C/EBP Beta Tg

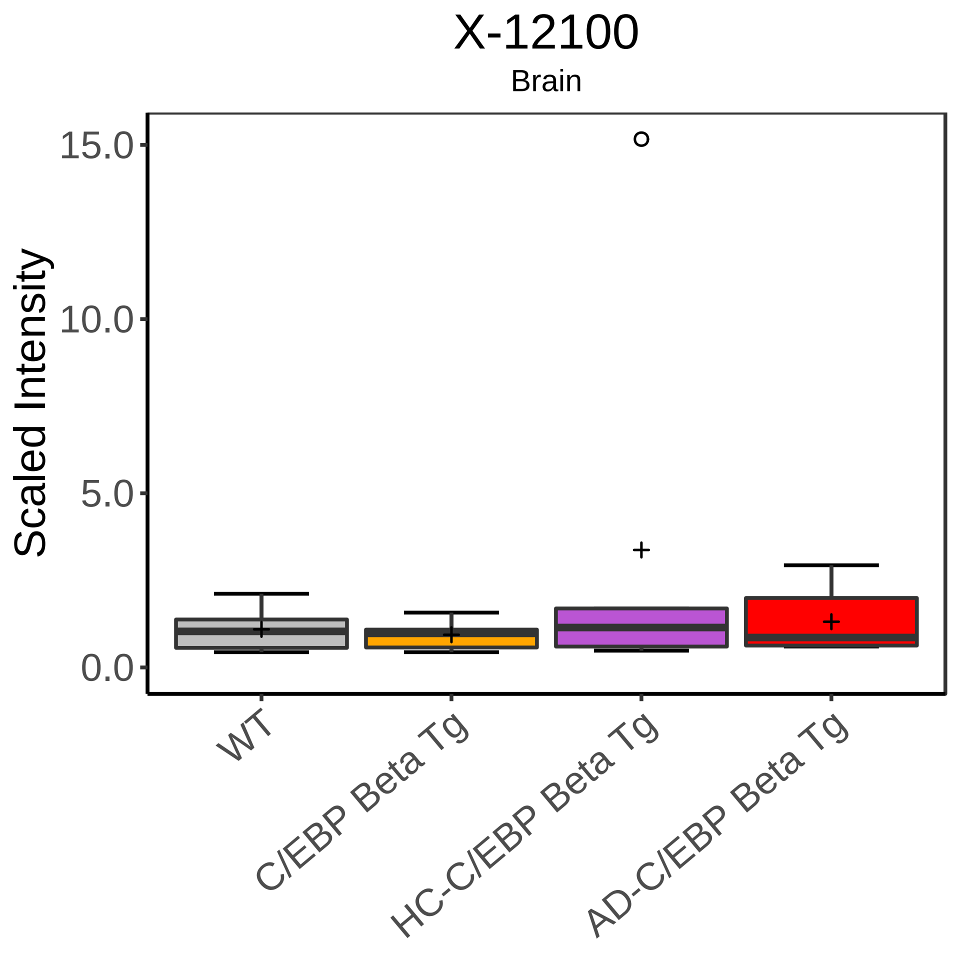
X-12100
Brain
Scaled Intensity
15.0
10.0
5.0
0.0
WT
C/EBP Beta Tg
HC-C/EBP Beta Tg
AD-C/EBP Beta Tg

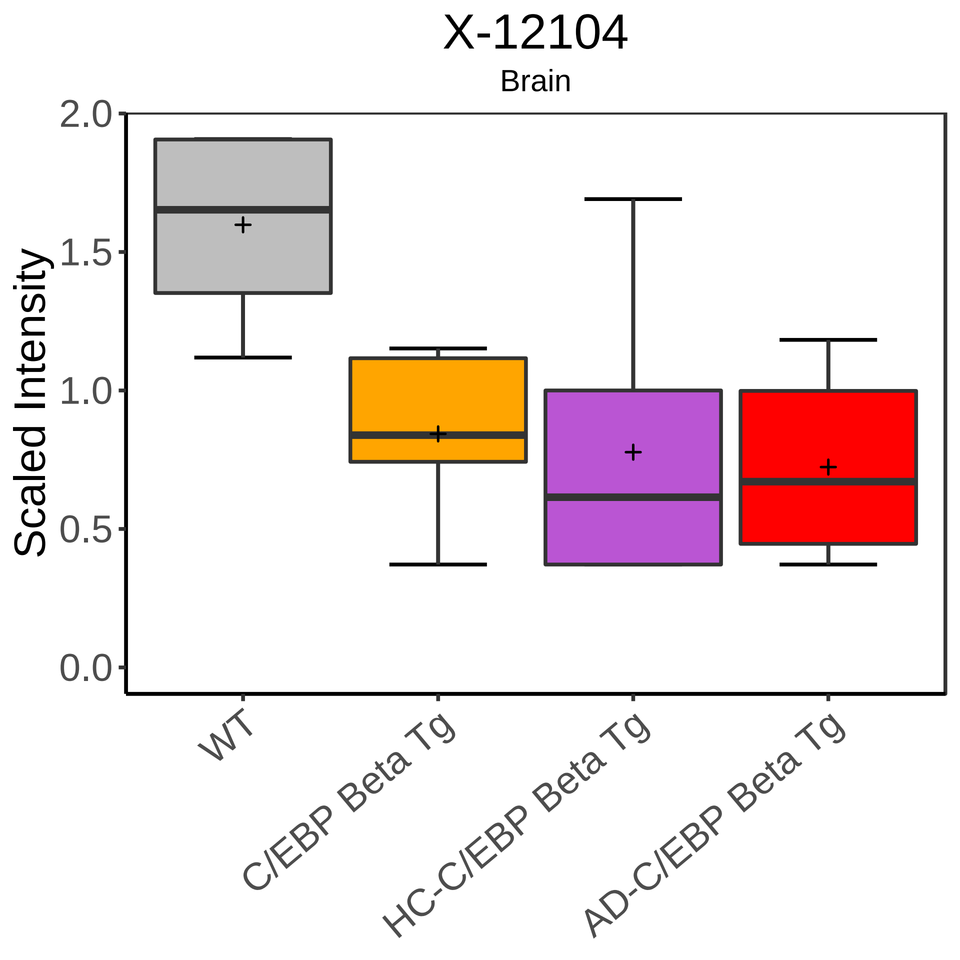
X-12104
Brain
Scaled Intensity
2.0
1.5
1.0
0.5
0.0
WT
C/EBP Beta Tg
HC-C/EBP Beta Tg
AD-C/EBP Beta Tg

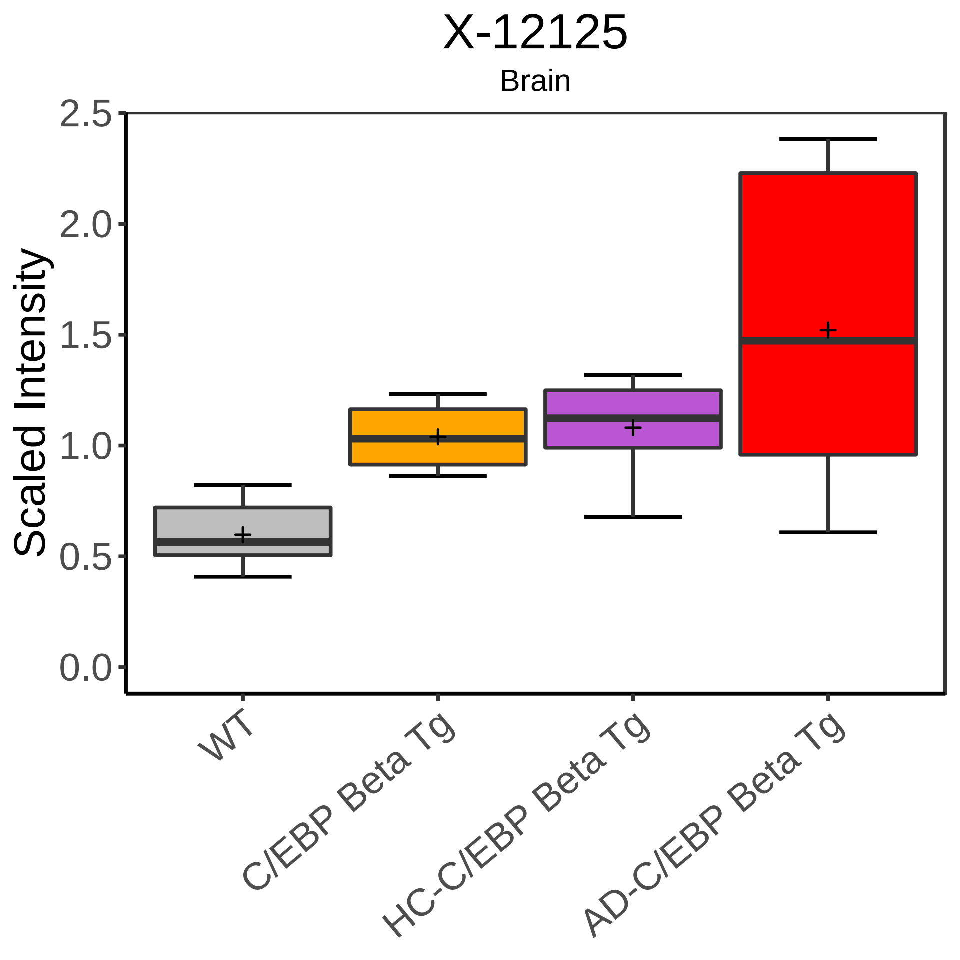

X-12125
Brain
Scaled Intensity
2.5
2.0
1.5
1.0
0.5
0.0
WT
C/EBP Beta Tg
HC-C/EBP Beta Tg
AD-C/EBP Beta Tg

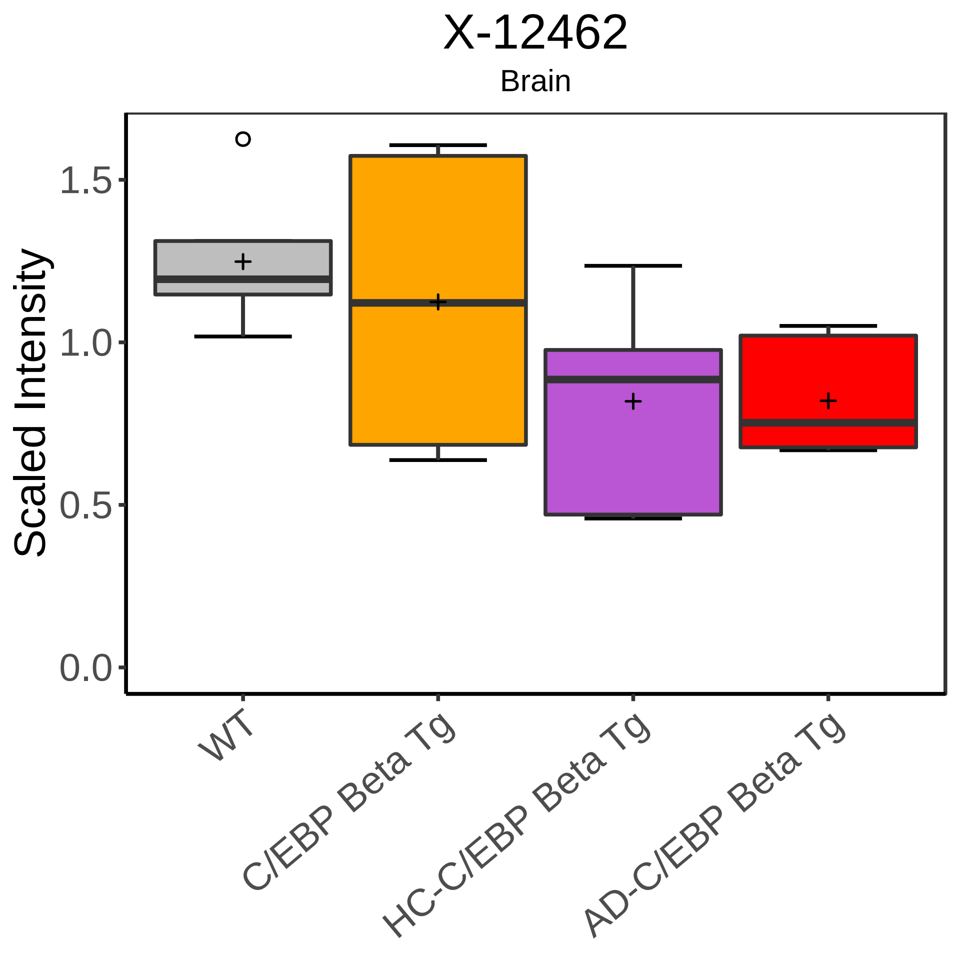
X-12462
Brain
Scaled Intensity
1.5
1.0
0.5
0.0
WT
C/EBP Beta Tg
HC-C/EBP Beta Tg
AD-C/EBP Beta Tg

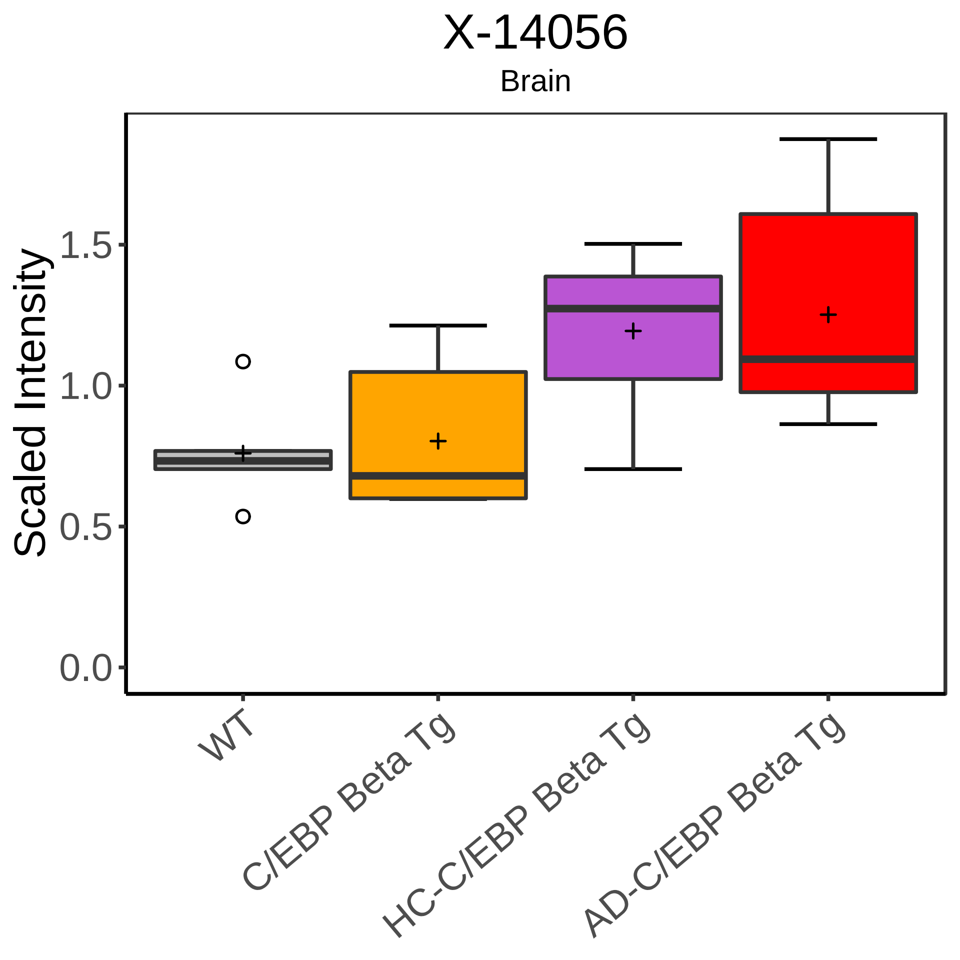

X-14056
Brain
Scaled Intensity
0.0
0.5
1.0
1.5
WT
C/EBP Beta Tg
HC-C/EBP Beta Tg
AD-C/EBP Beta Tg

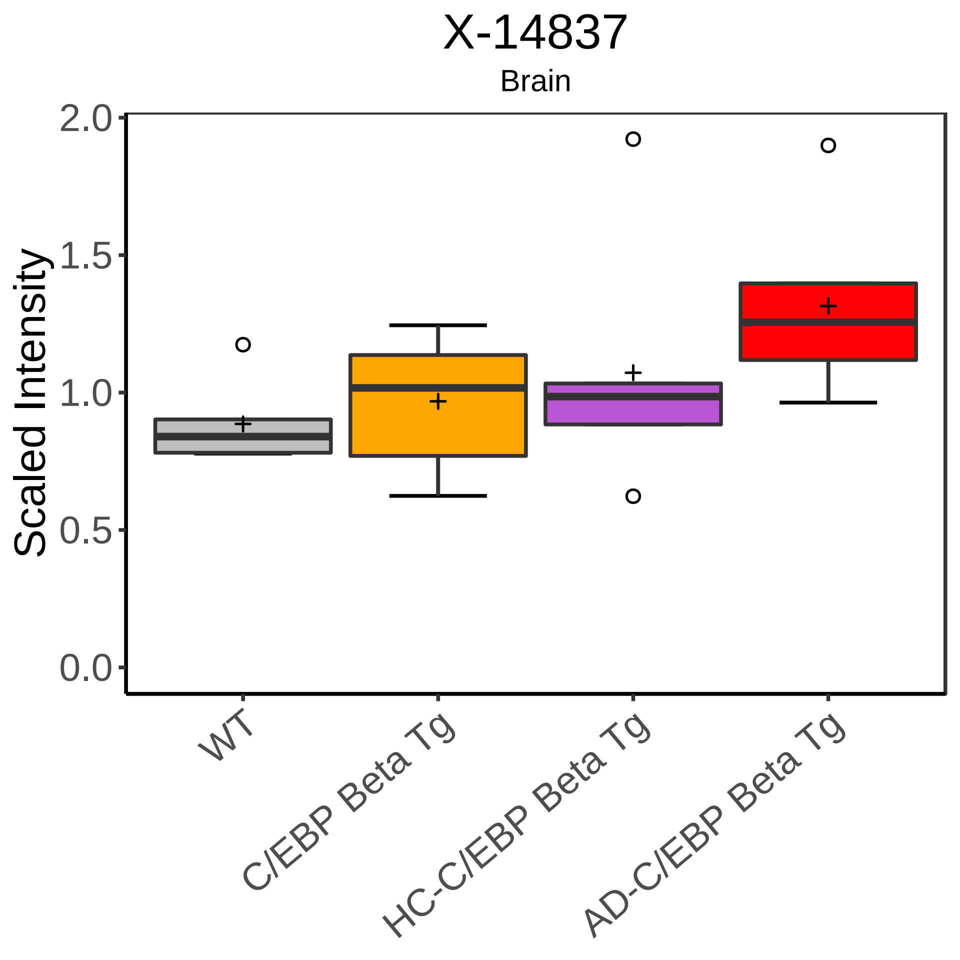

X-14837
Brain
Scaled Intensity
2.0
1.5
1.0
0.5
0.0
WT
C/EBP Beta Tg
HC-C/EBP Beta Tg
AD-C/EBP Beta Tg

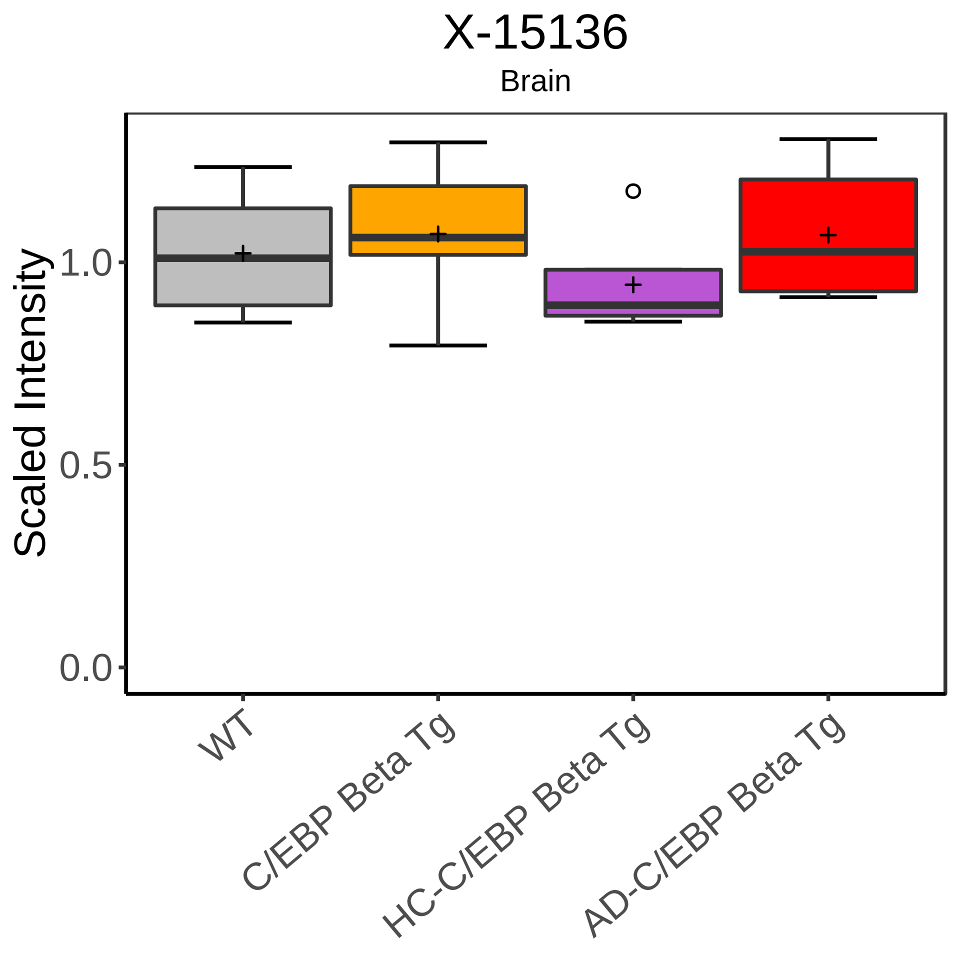
X-15136
Brain
Scaled Intensity
1.0
0.5
0.0
WT
C/EBP Beta Tg
HC-C/EBP Beta Tg
AD-C/EBP Beta Tg

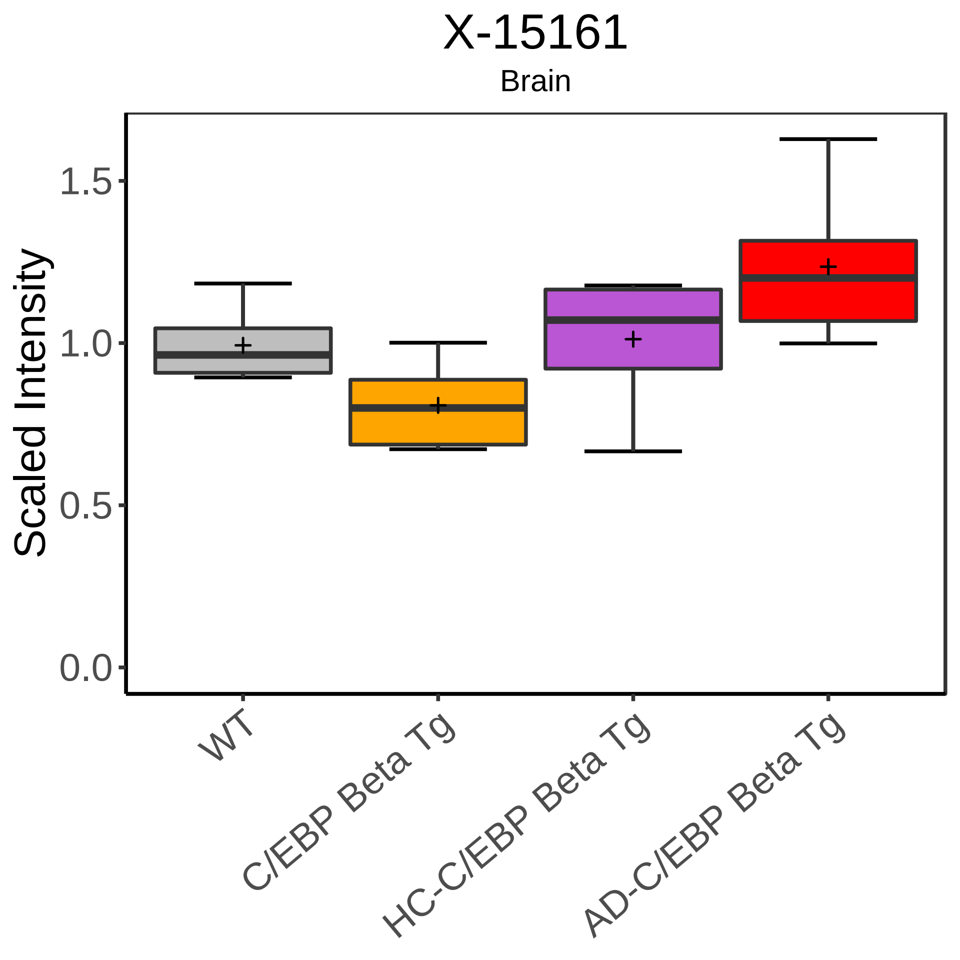
X-15161
Brain
Scaled Intensity
1.5
1.0
0.5
0.0
WT
C/EBP Beta Tg
HC-C/EBP Beta Tg
AD-C/EBP Beta Tg

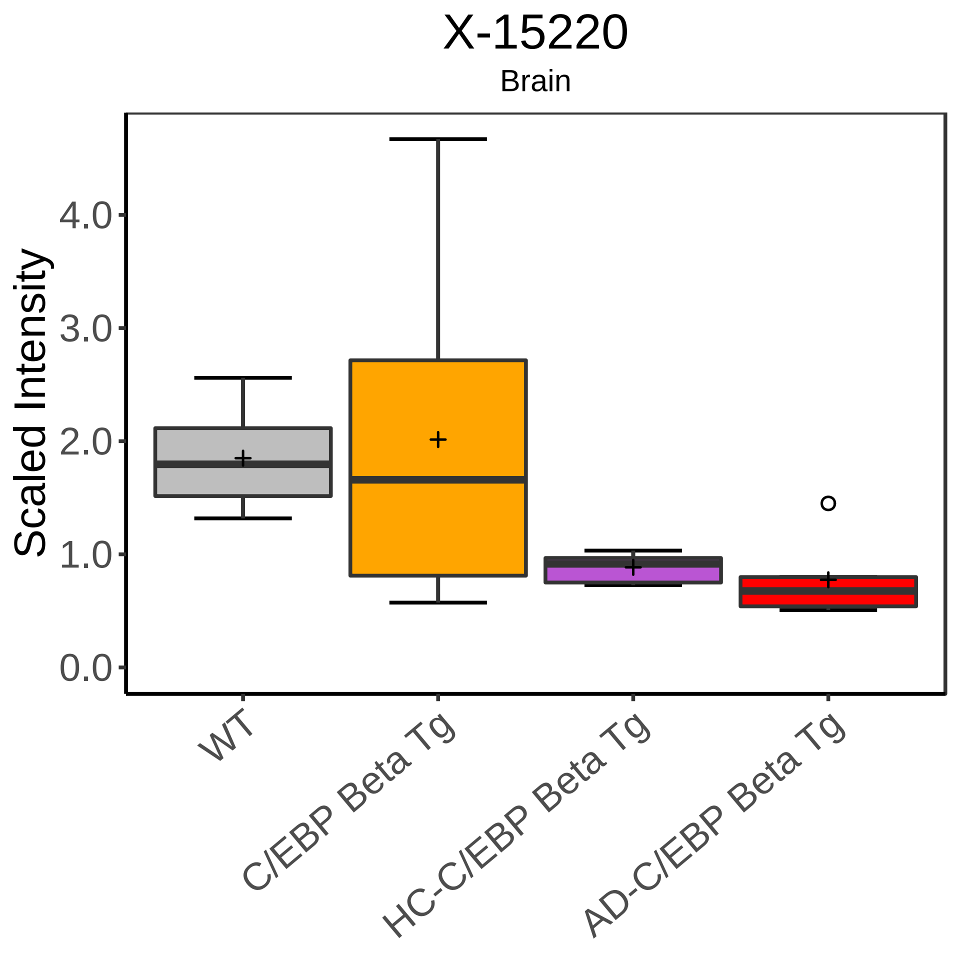

X-15220
Brain
Scaled Intensity
4.0
3.0
2.0
1.0
0.0
WT
C/EBP Beta Tg
HC-C/EBP Beta Tg
AD-C/EBP Beta Tg

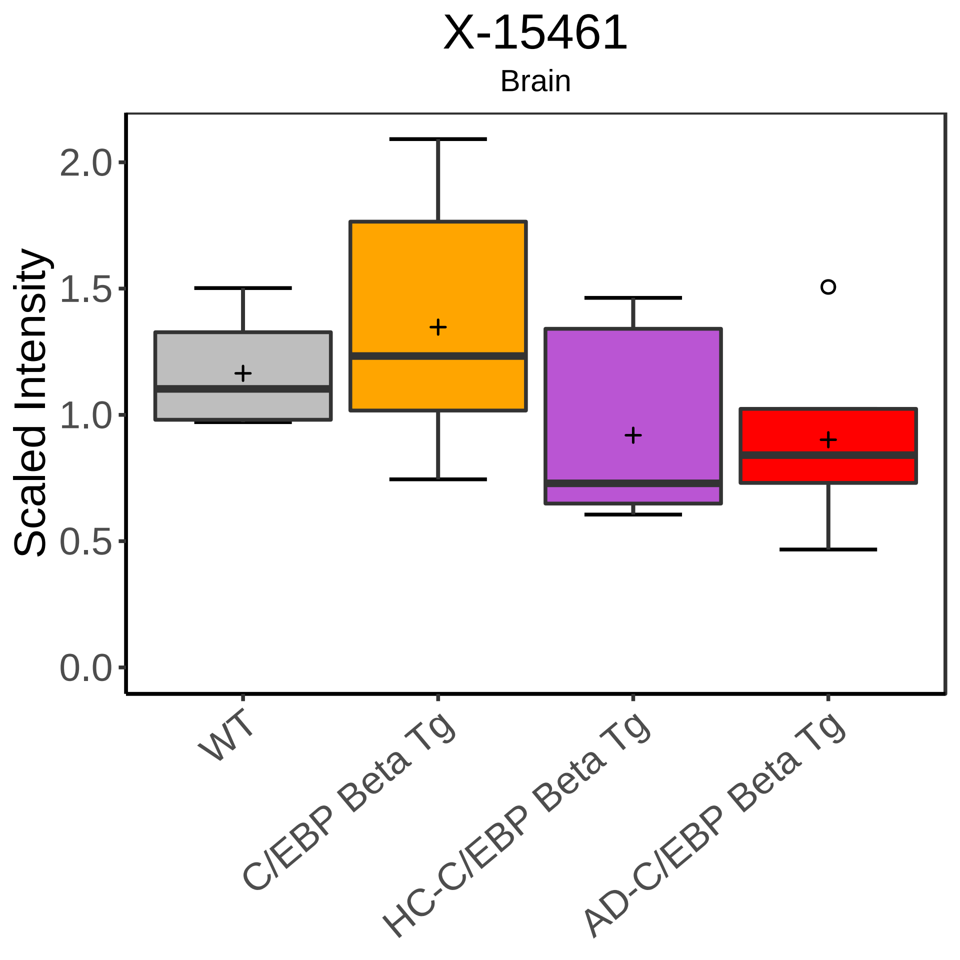
X-15461
Brain
Scaled Intensity
2.0
1.5
1.0
0.5
0.0
WT
C/EBP Beta Tg
HC-C/EBP Beta Tg
AD-C/EBP Beta Tg

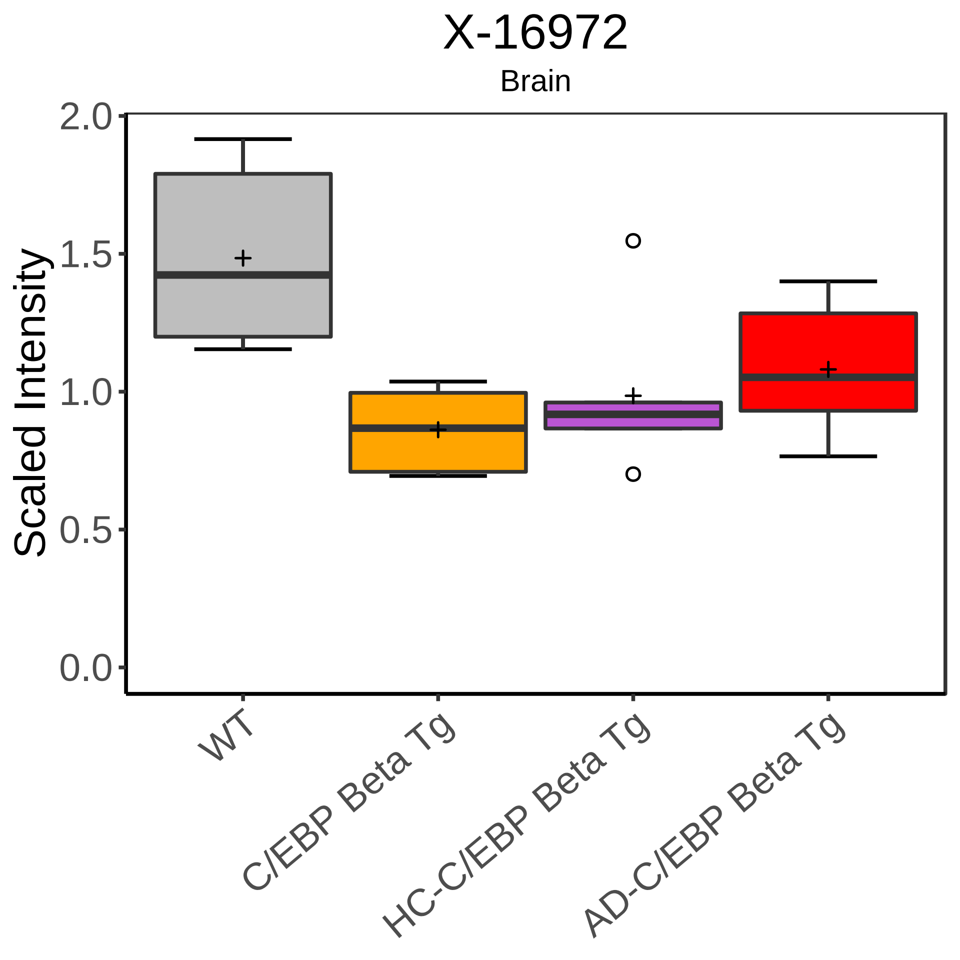
X-16972
Brain
Scaled Intensity
2.0
1.5
1.0
0.5
0.0
WT
C/EBP Beta Tg
HC-C/EBP Beta Tg
AD-C/EBP Beta Tg

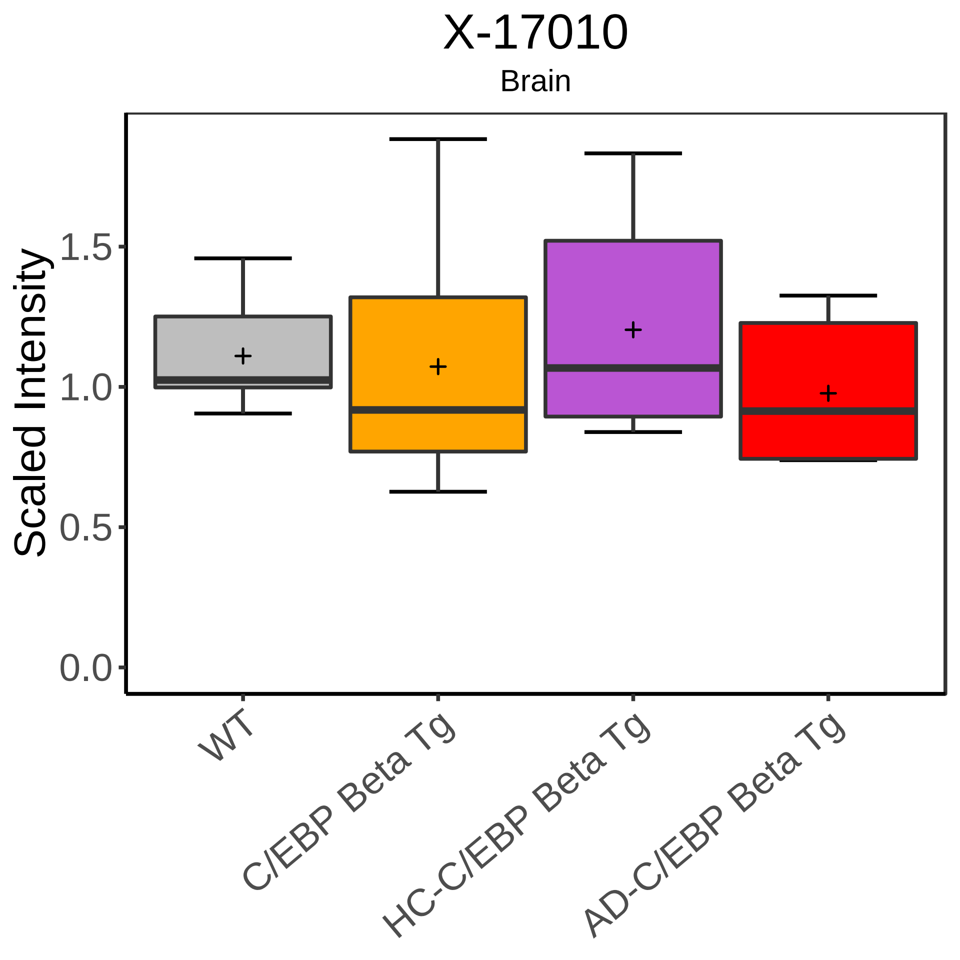
X-17010
Brain
Scaled Intensity
1.5
1.0
0.5
0.0
WT
C/EBP Beta Tg
HC-C/EBP Beta Tg
AD-C/EBP Beta Tg

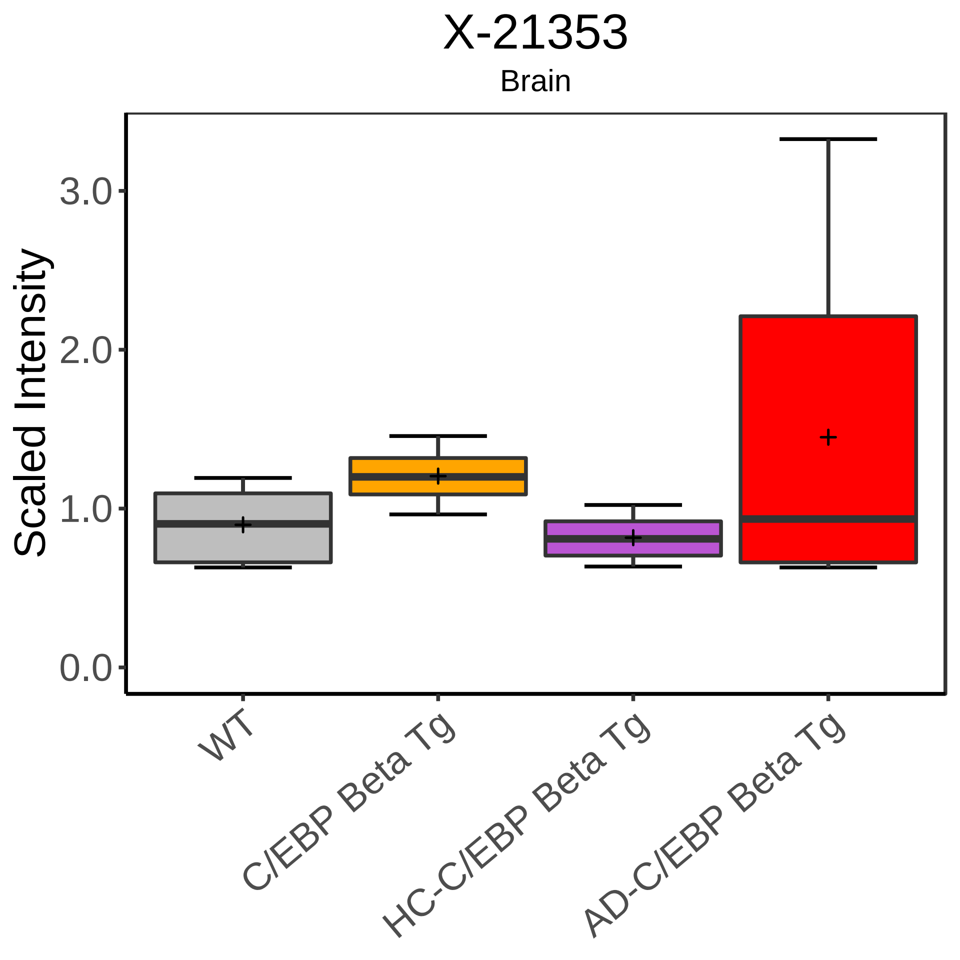

X-21353
Brain
Scaled Intensity
3.0
2.0
1.0
0.0
WT
C/EBP Beta Tg
HC-C/EBP Beta Tg
AD-C/EBP Beta Tg

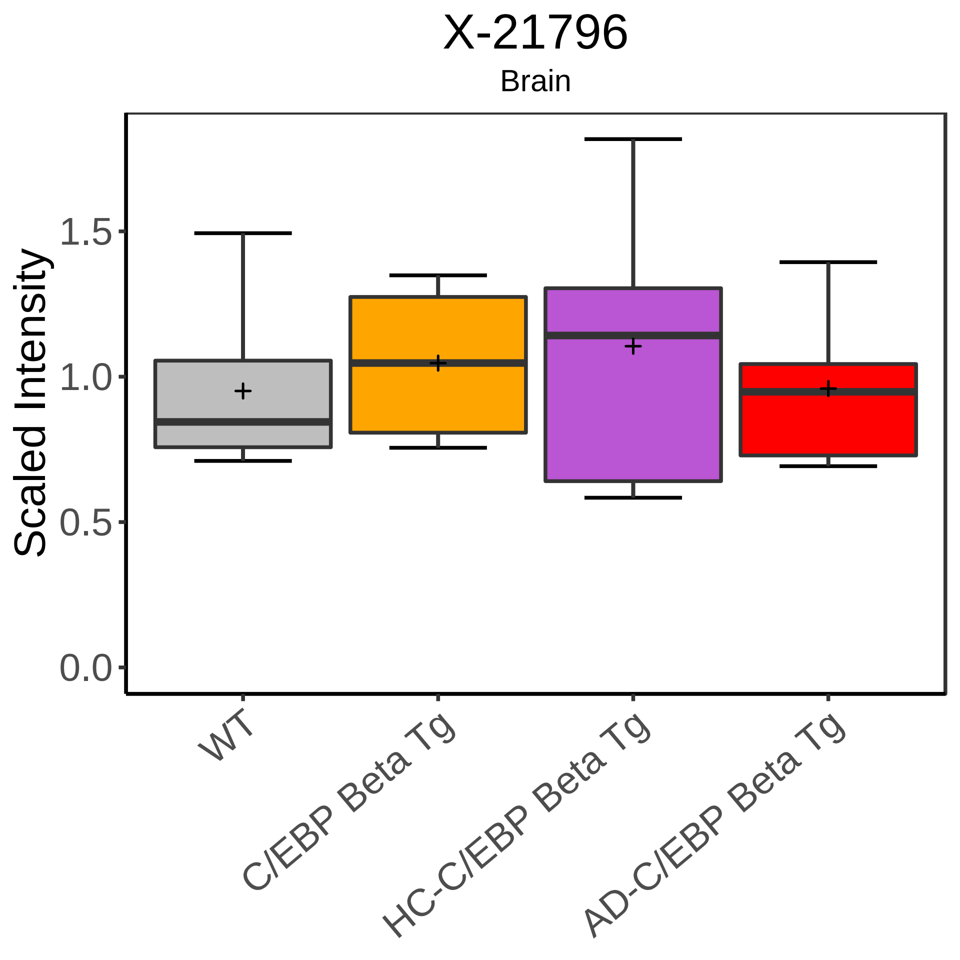
X-21796
Brain
Scaled Intensity
0.0
0.5
1.0
1.5
WT
C/EBP Beta Tg
HC-C/EBP Beta Tg
AD-C/EBP Beta Tg

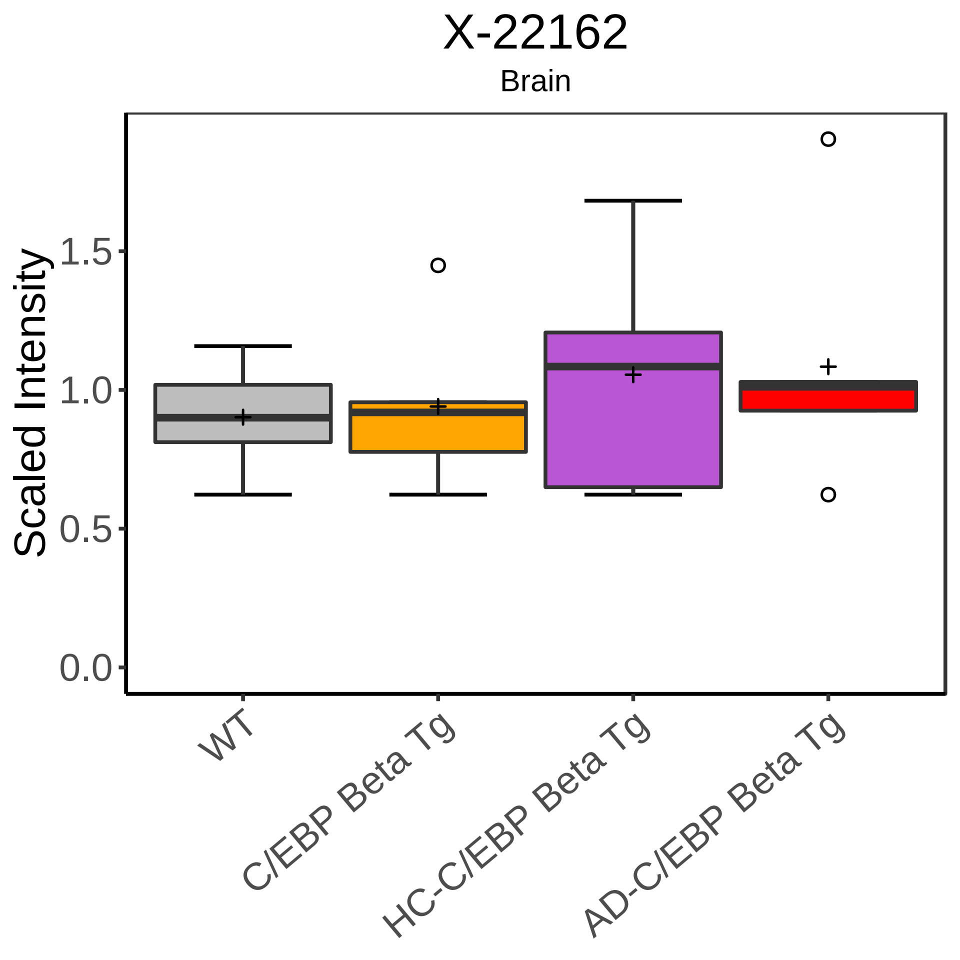
X-22162
Brain
Scaled Intensity
0.0
0.5
1.0
1.5
WT
C/EBP Beta Tg
HC-C/EBP Beta Tg
AD-C/EBP Beta Tg

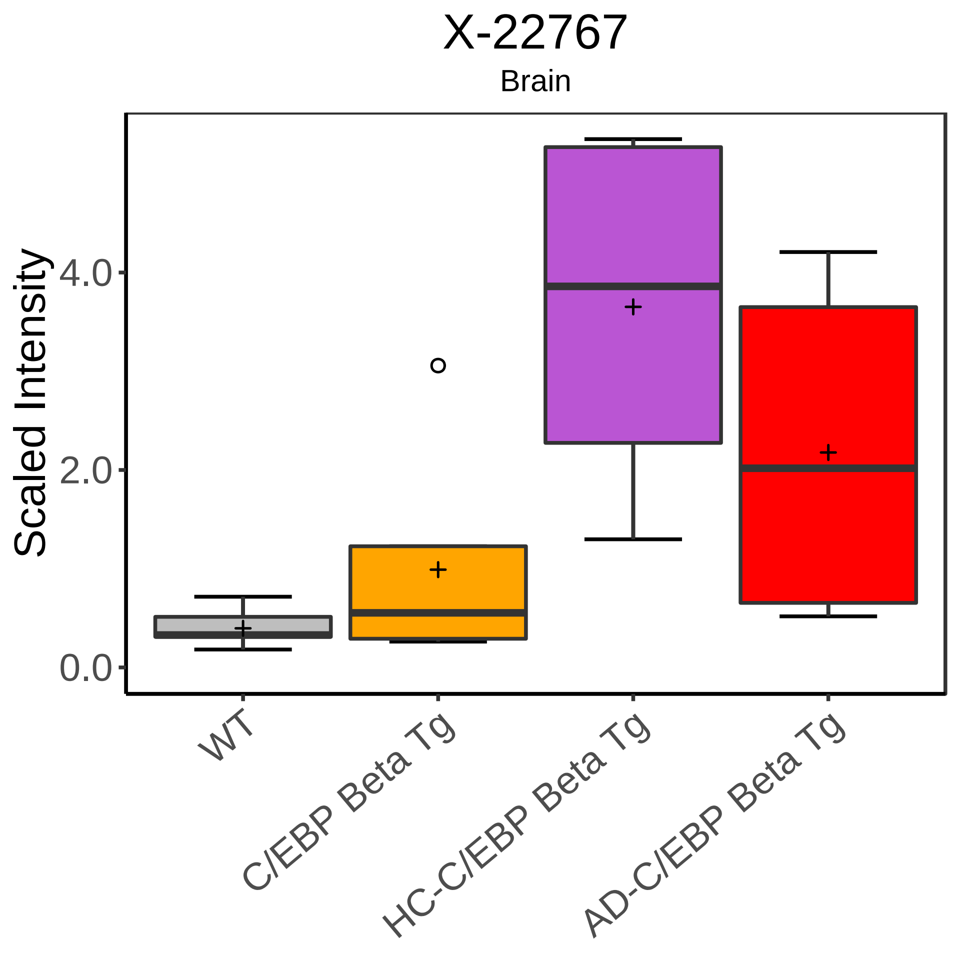

X-22767
Brain
Scaled Intensity
0.0
2.0
4.0
WT
C/EBP Beta Tg
HC-C/EBP Beta Tg
AD-C/EBP Beta Tg

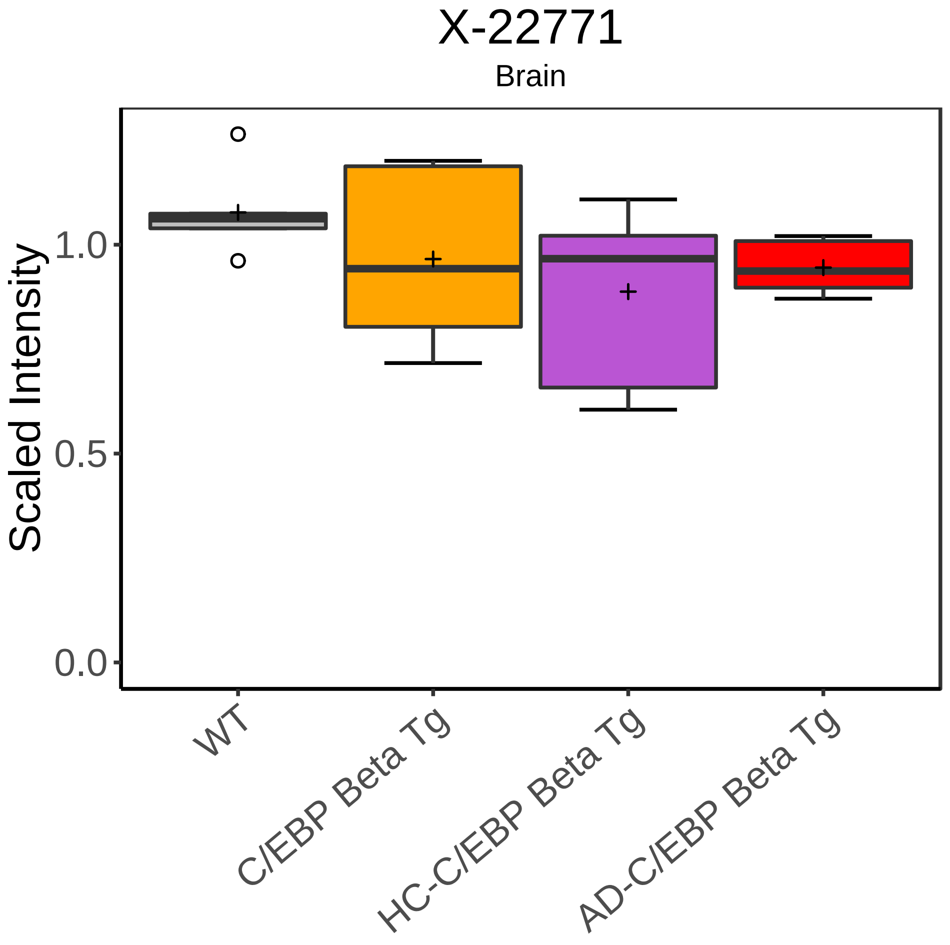

X-22771
Brain
Scaled Intensity
1.0
0.5
0.0
WT
C/EBP Beta Tg
HC-C/EBP Beta Tg
AD-C/EBP Beta Tg

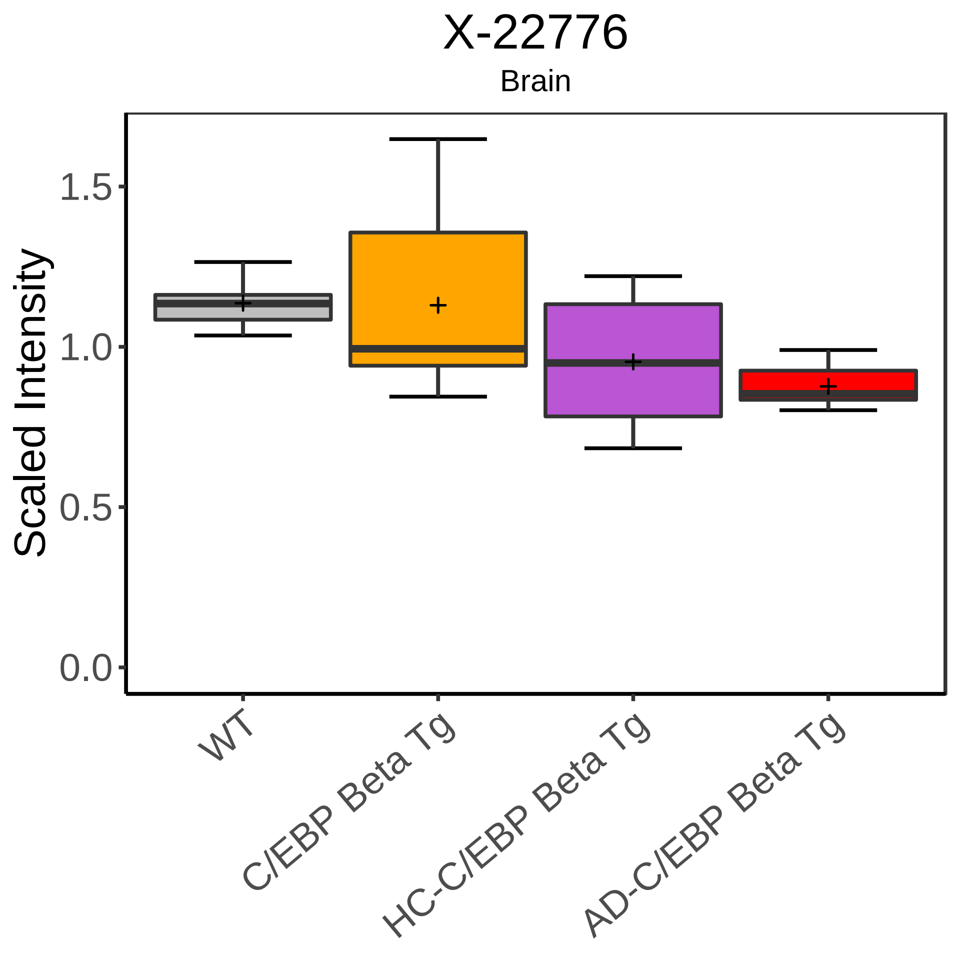

X-22776
Brain
Scaled Intensity
1.5
1.0
0.5
0.0
WT
C/EBP Beta Tg
HC-C/EBP Beta Tg
AD-C/EBP Beta Tg

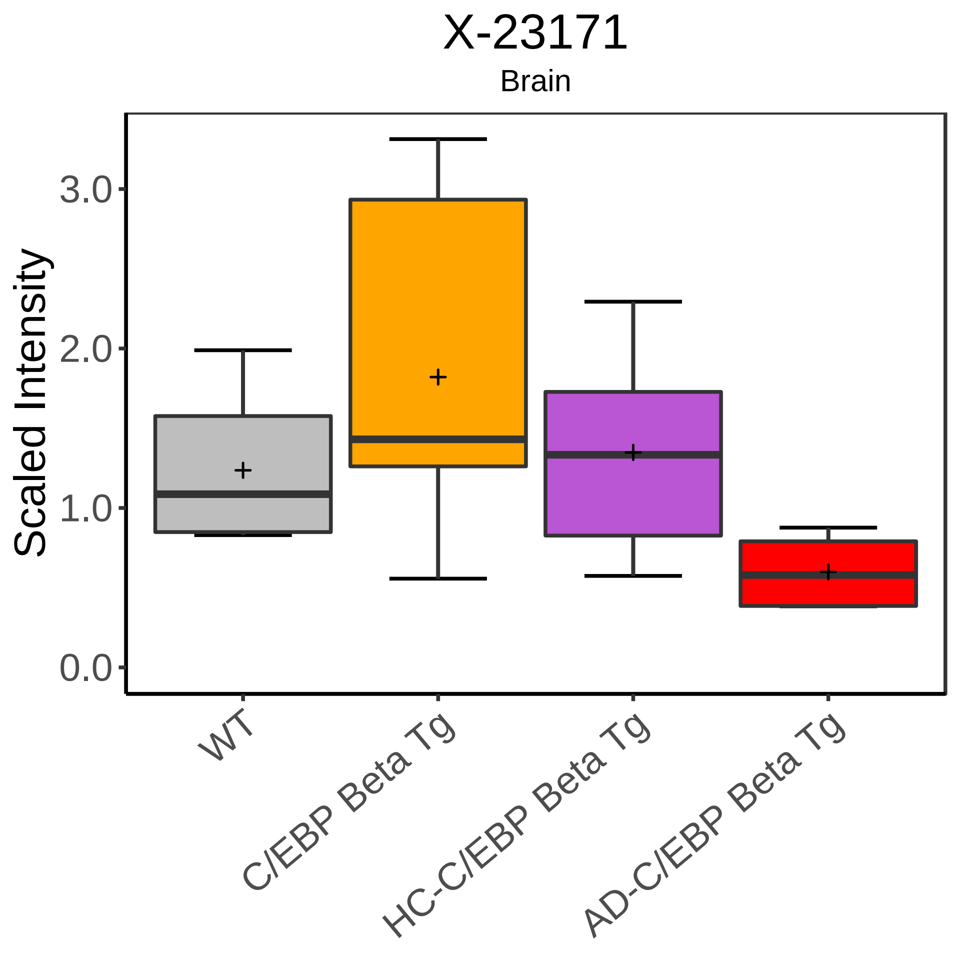

X-23171
Brain
Scaled Intensity
3.0
2.0
1.0
0.0
WT
C/EBP Beta Tg
HC-C/EBP Beta Tg
AD-C/EBP Beta Tg

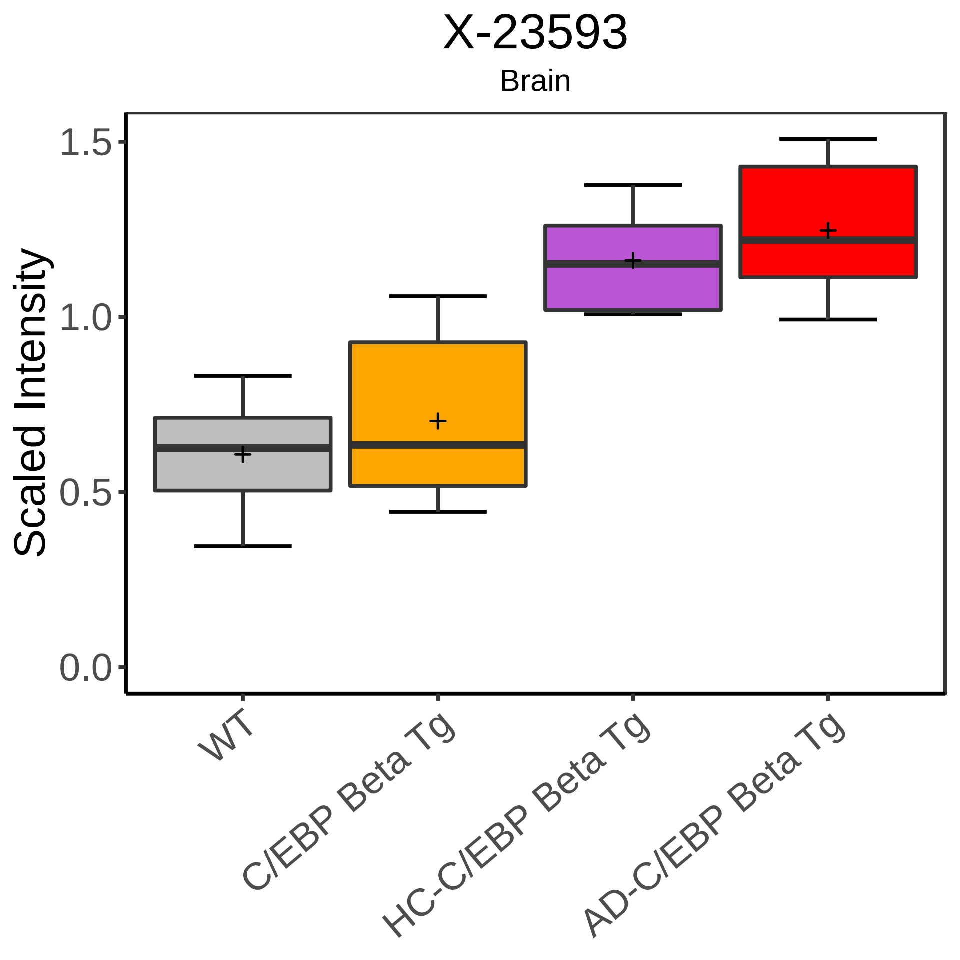

X-23593
Brain
Scaled Intensity
1.5
1.0
0.5
0.0
WT
C/EBP Beta Tg
HC-C/EBP Beta Tg
AD-C/EBP Beta Tg

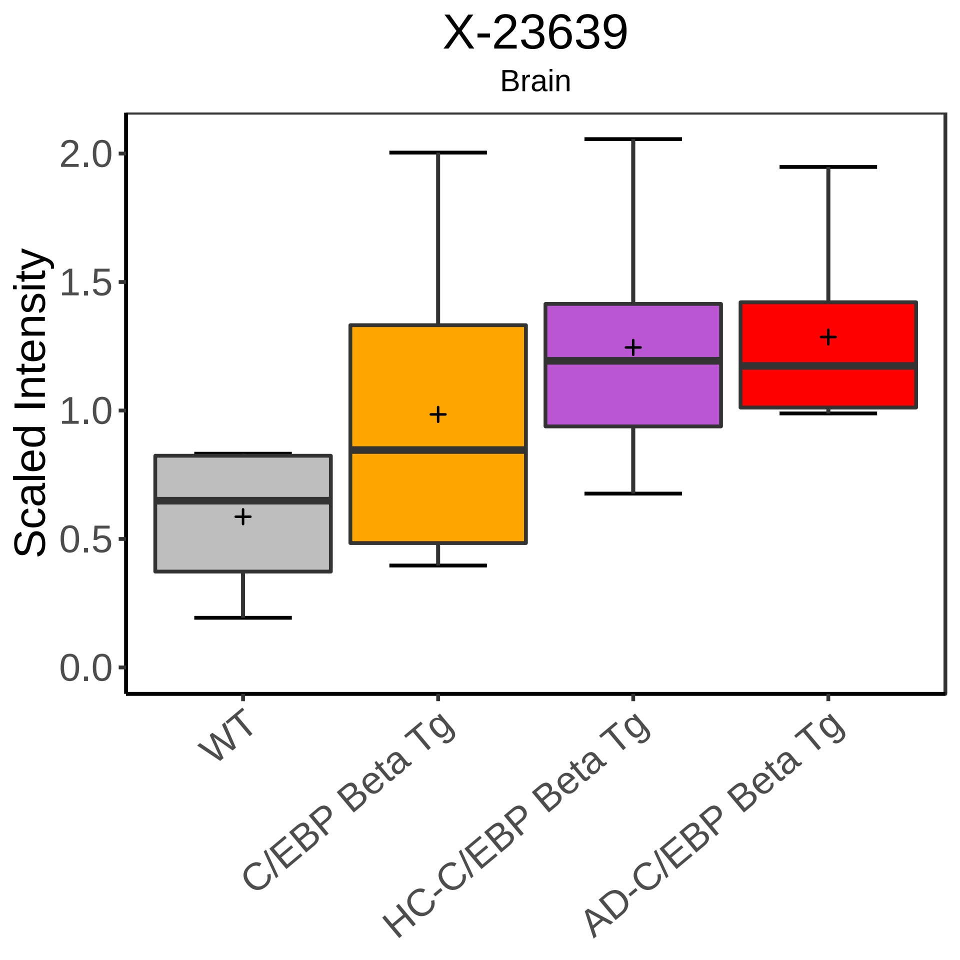
X-23639
Brain
Scaled Intensity
2.0
1.5
1.0
0.5
0.0
WT
C/EBP Beta Tg
HC-C/EBP Beta Tg
AD-C/EBP Beta Tg

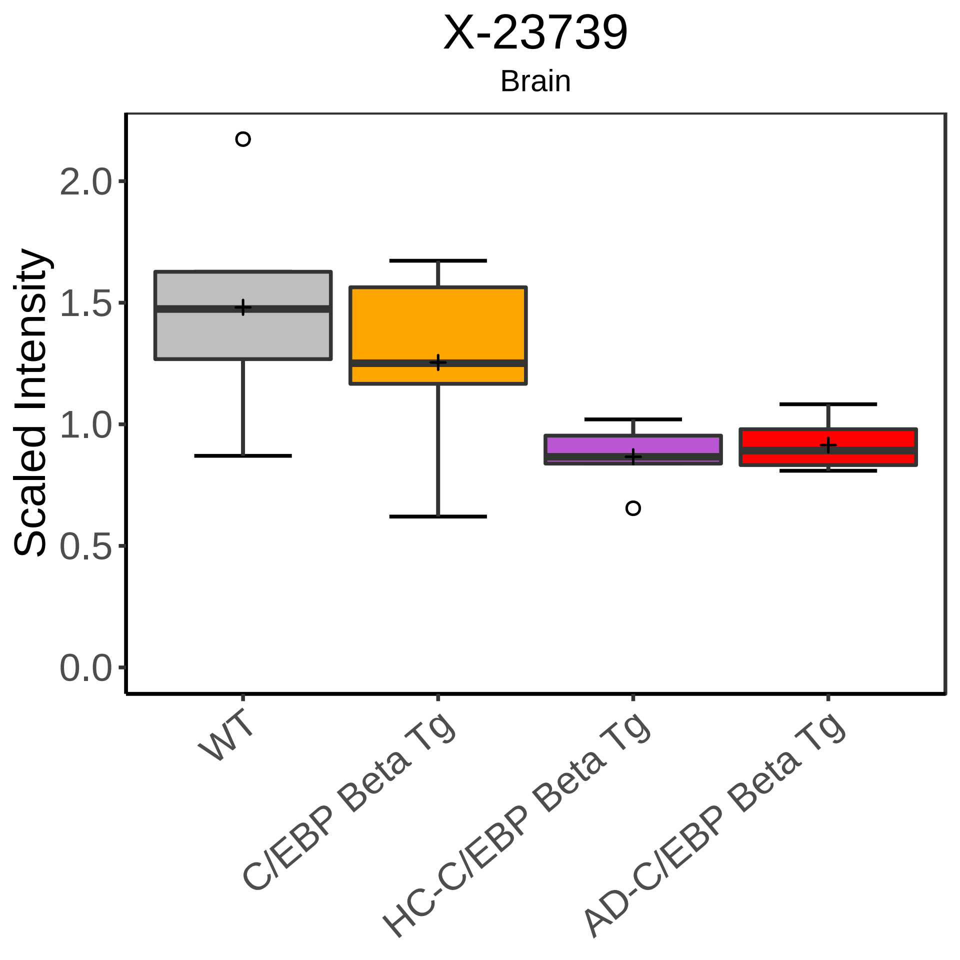

X-23739
Brain
Scaled Intensity
2.0
1.5
1.0
0.5
0.0
WT
C/EBP Beta Tg
HC-C/EBP Beta Tg
AD-C/EBP Beta Tg

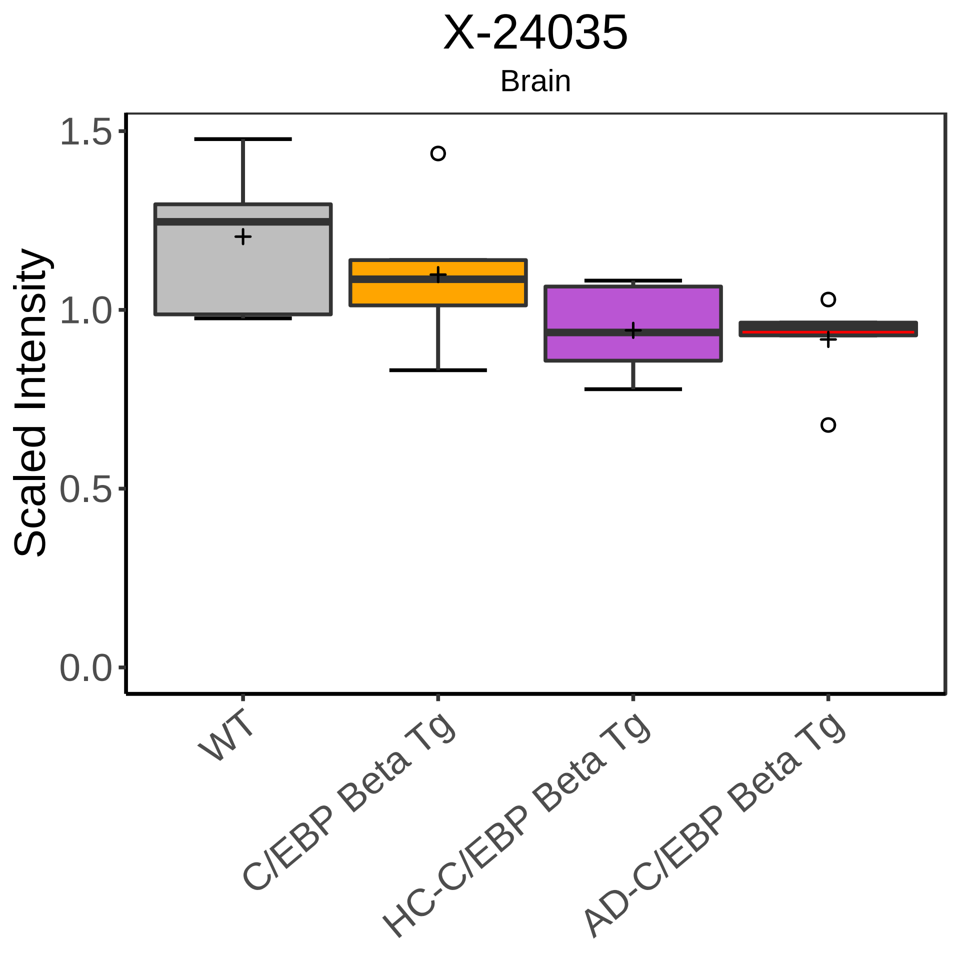
X-24035
Brain
Scaled Intensity
1.5
1.0
0.5
0.0
WT
C/EBP Beta Tg
HC-C/EBP Beta Tg
AD-C/EBP Beta Tg

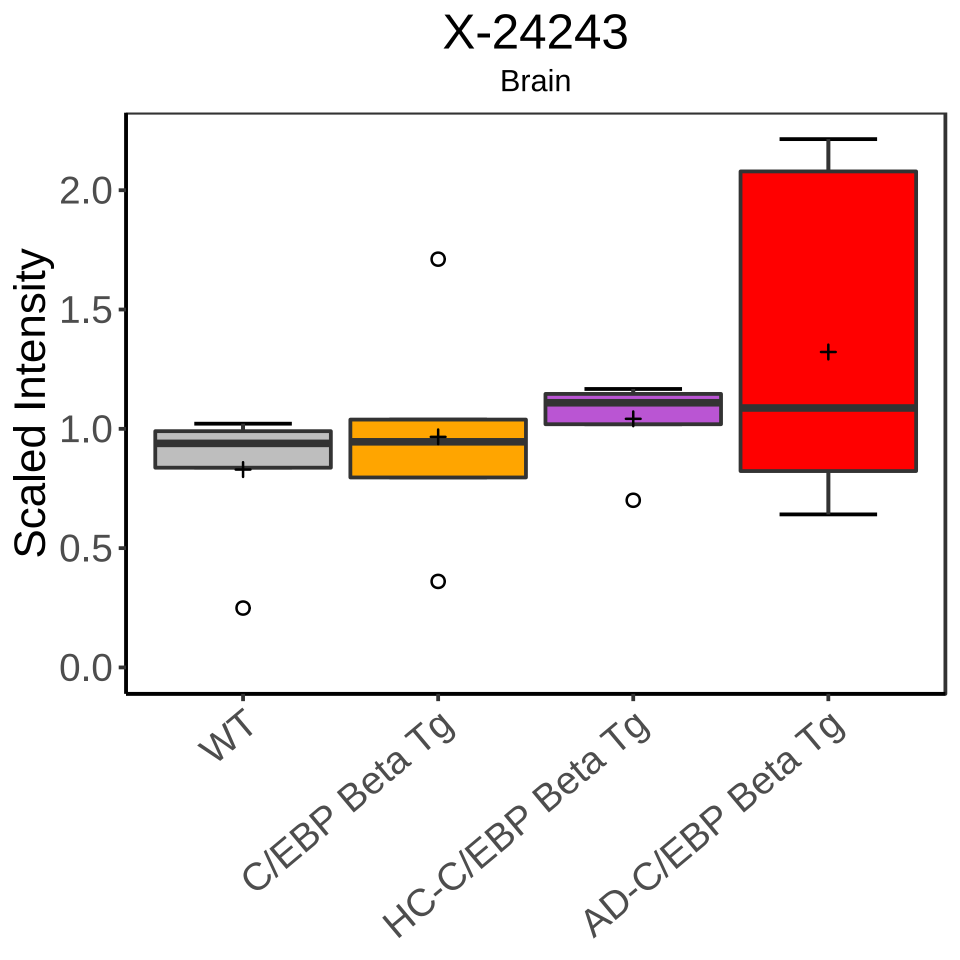
X-24243
Brain
Scaled Intensity
2.0
1.5
1.0
0.5
0.0
WT
C/EBP Beta Tg
HC-C/EBP Beta Tg
AD-C/EBP Beta Tg

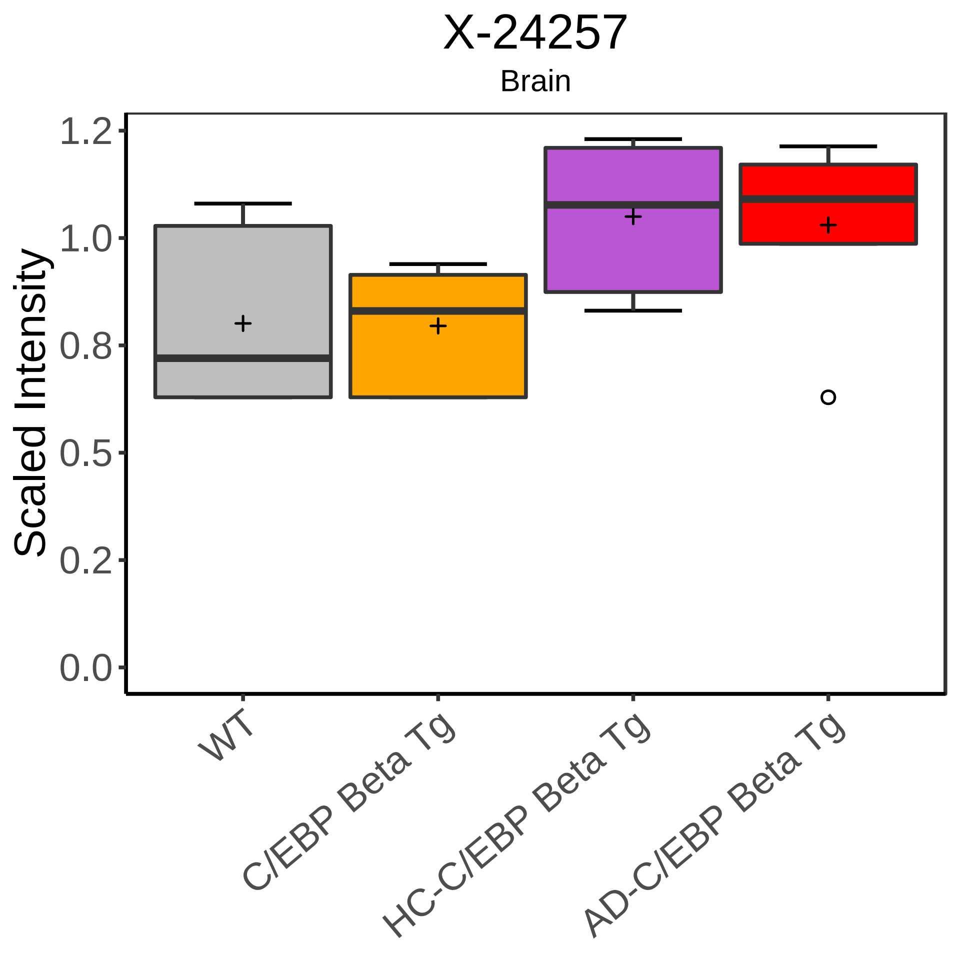

X-24257
Brain
Scaled Intensity
1.2
1.0
0.8
0.5
0.2
0.0
WT
C/EBP Beta Tg
HC-C/EBP Beta Tg
AD-C/EBP Beta Tg

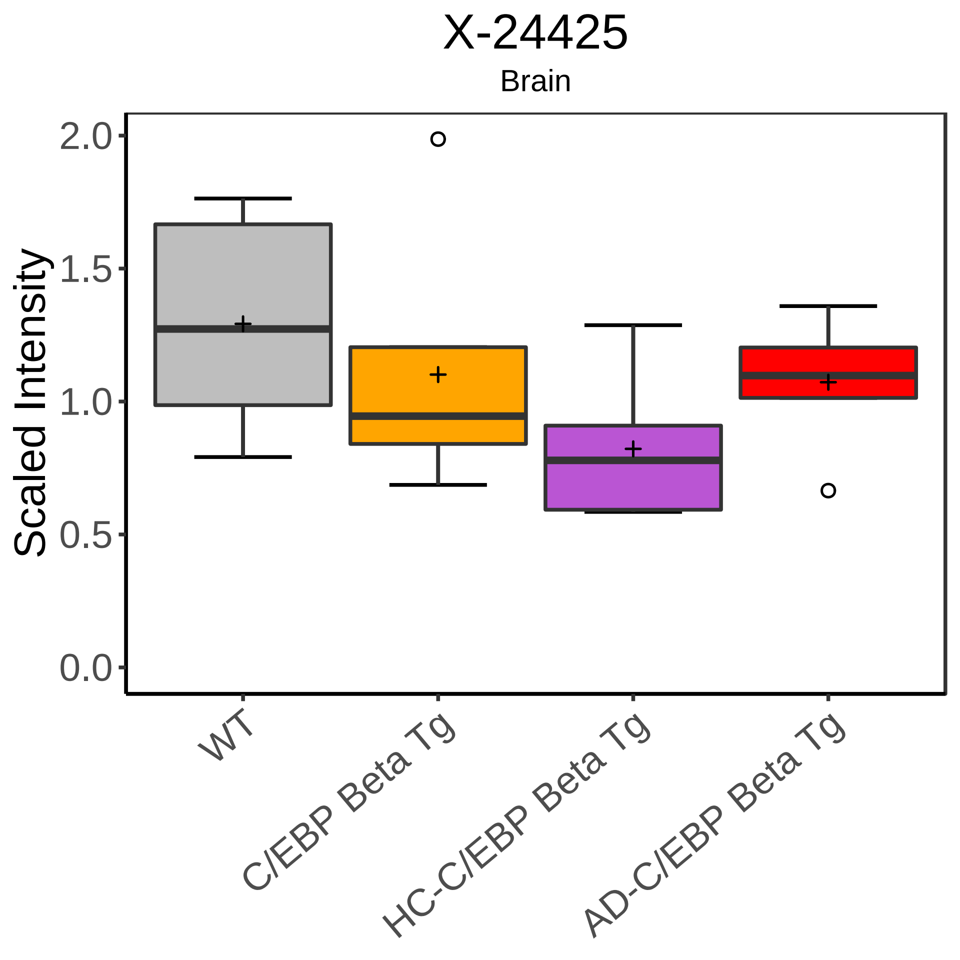

X-24425
Brain
Scaled Intensity
2.0
1.5
1.0
0.5
0.0
WT
C/EBP Beta Tg
HC-C/EBP Beta Tg
AD-C/EBP Beta Tg

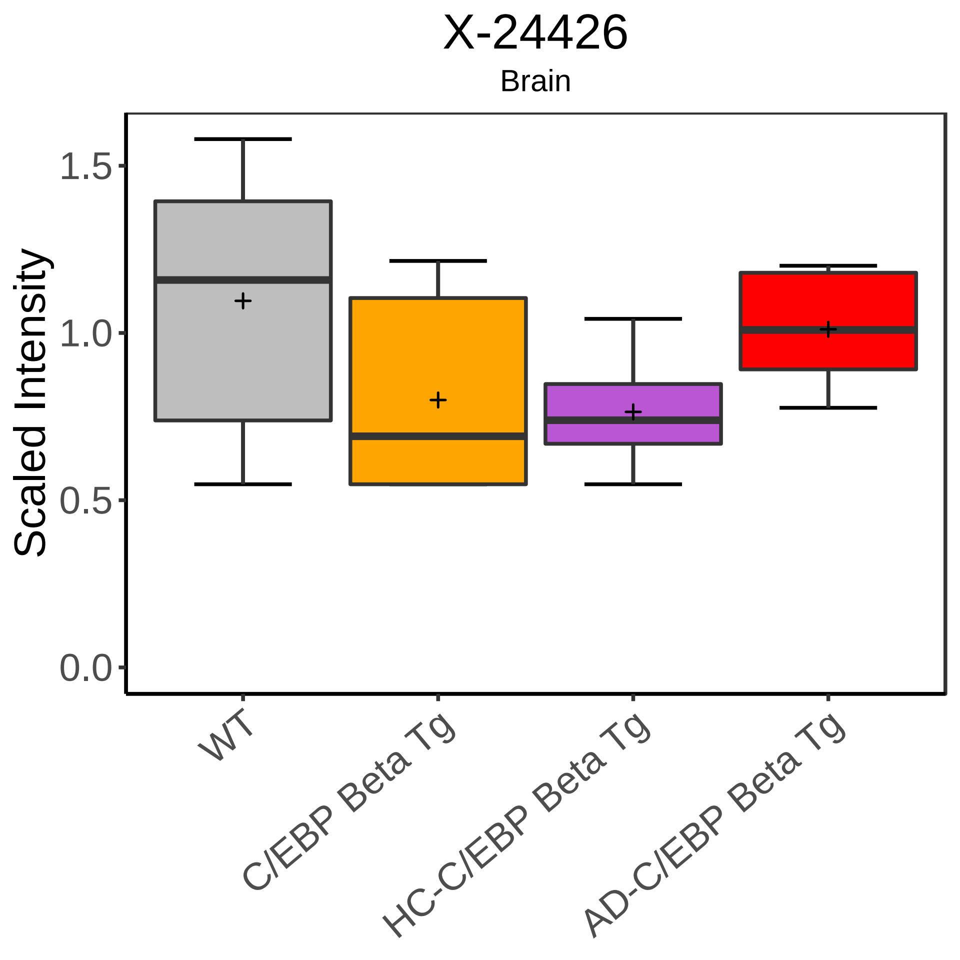

X-24426
Brain
Scaled Intensity
1.5
1.0
0.5
0.0
WT
C/EBP Beta Tg
HC-C/EBP Beta Tg
AD-C/EBP Beta Tg

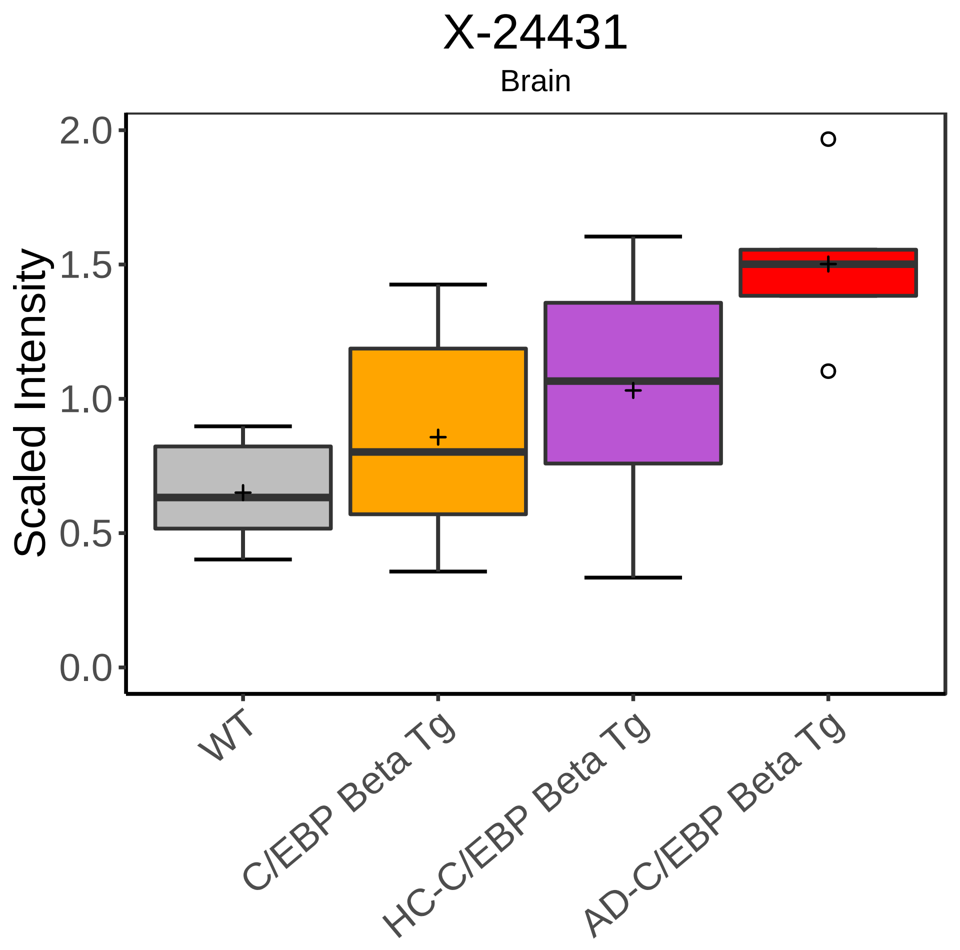
X-24431
Brain
Scaled Intensity
2.0
1.5
1.0
0.5
0.0
WT
C/EBP Beta Tg
HC-C/EBP Beta Tg
AD-C/EBP Beta Tg

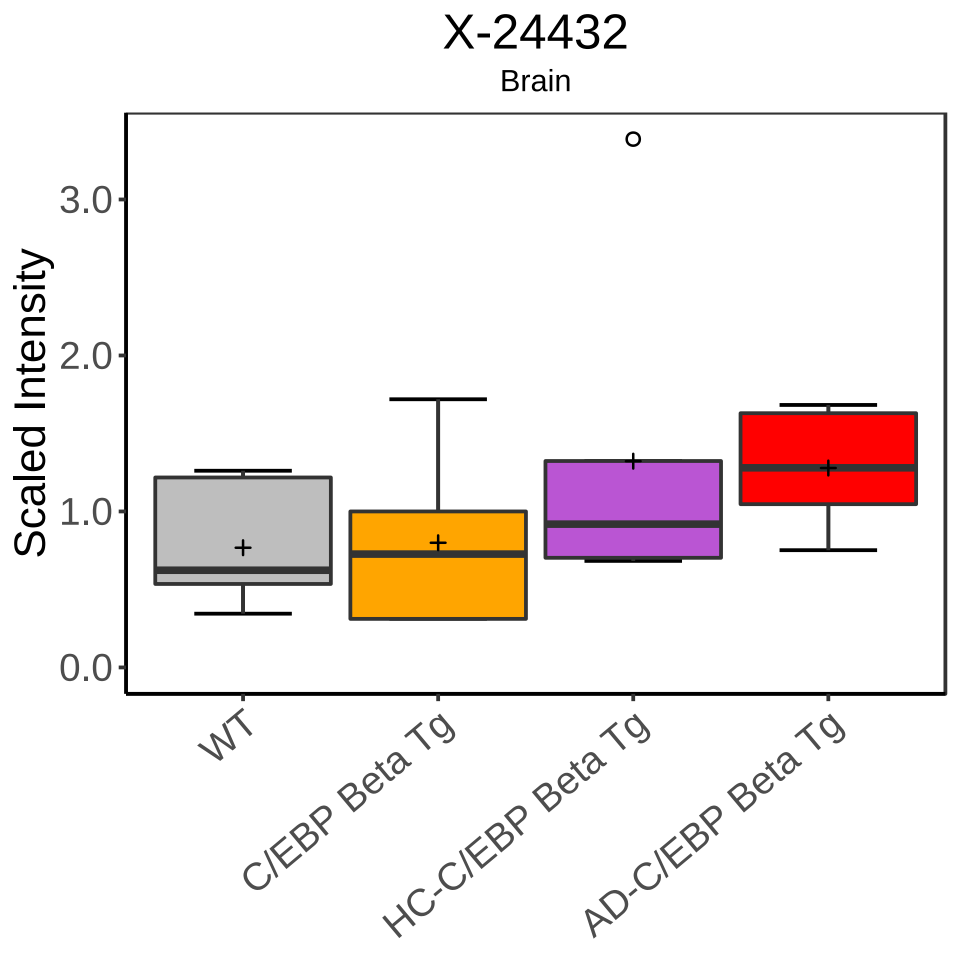
X-24432
Brain
Scaled Intensity
3.0
2.0
1.0
0.0
WT
C/EBP Beta Tg
HC-C/EBP Beta Tg
AD-C/EBP Beta Tg

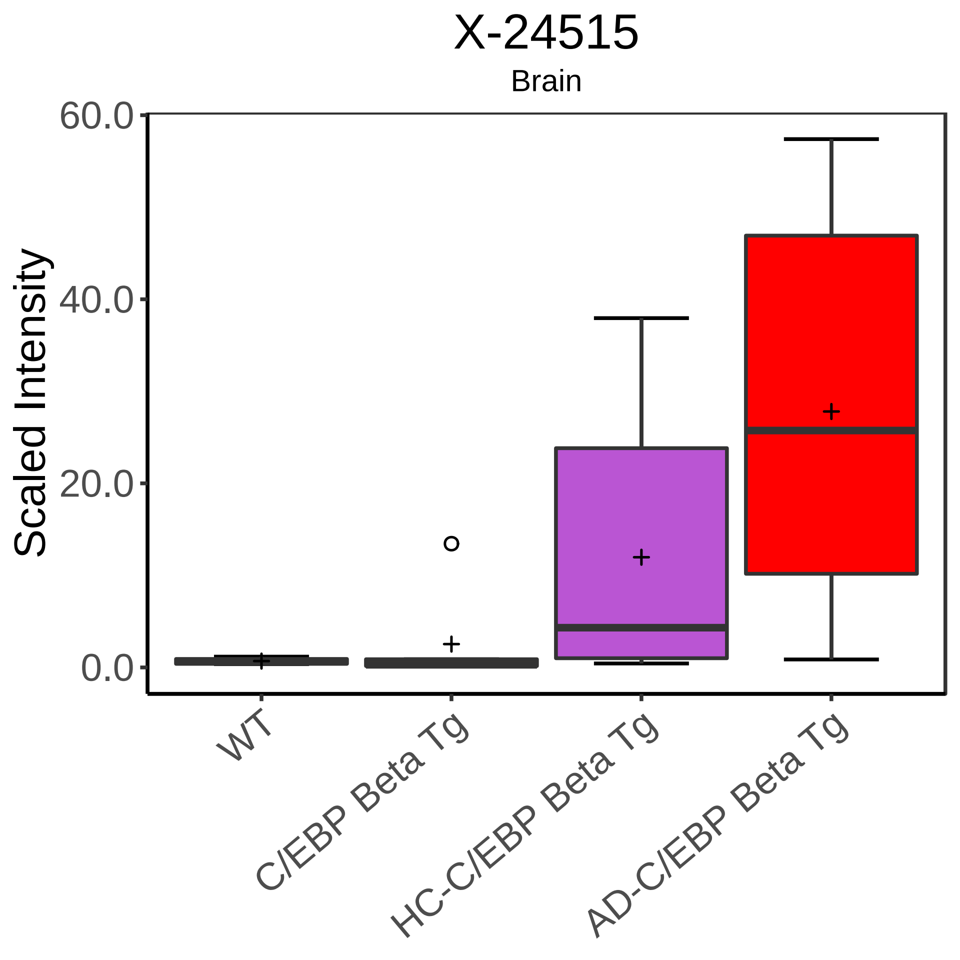
X-24515
Brain
Scaled Intensity
60.0
40.0
20.0
0.0
WT
C/EBP Beta Tg
HC-C/EBP Beta Tg
AD-C/EBP Beta Tg

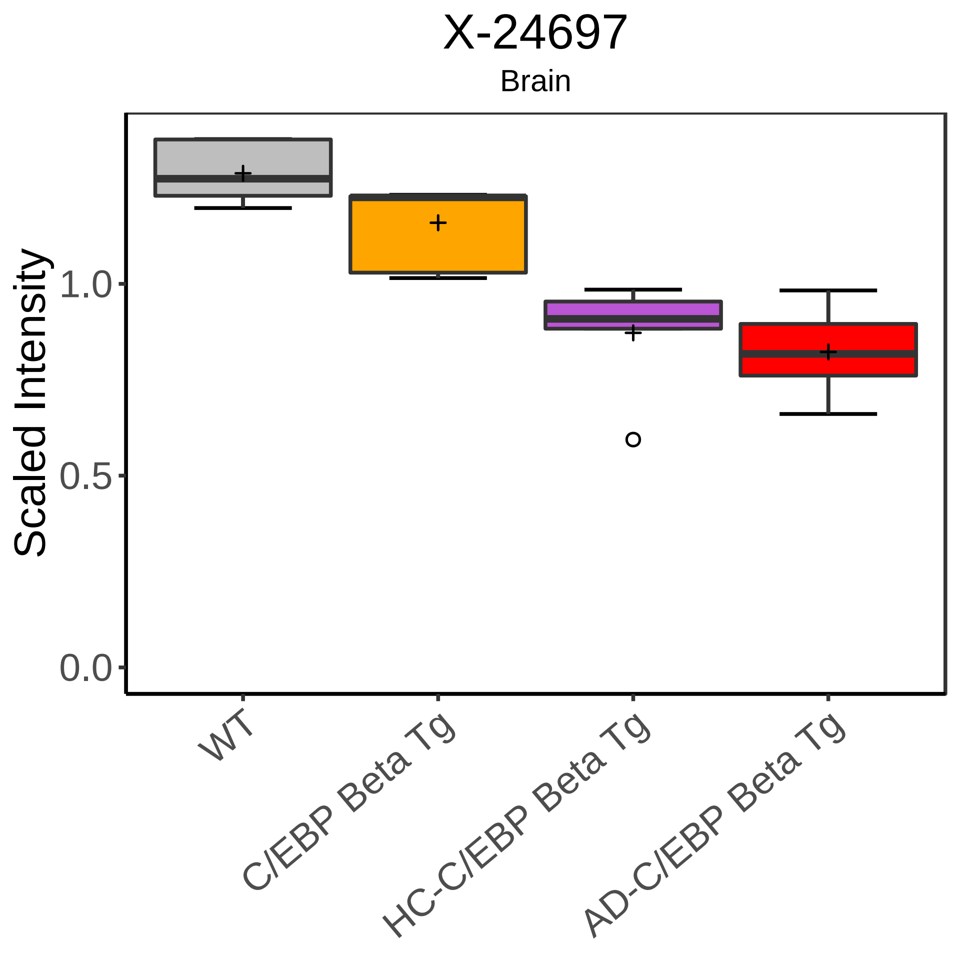
X-24697
Brain
Scaled Intensity
1.0
0.5
0.0
WT
C/EBP Beta Tg
HC-C/EBP Beta Tg
AD-C/EBP Beta Tg

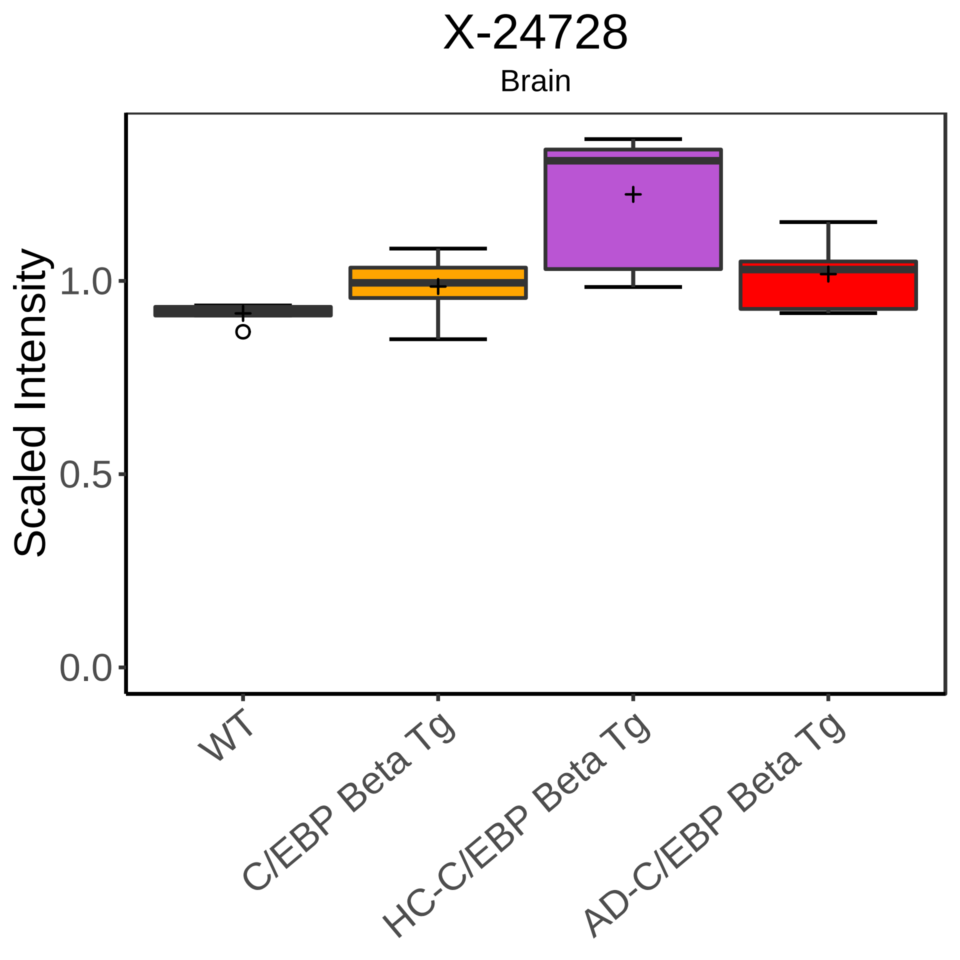
X-24728
Brain
Scaled Intensity
1.0
0.5
0.0
WT
C/EBP Beta Tg
HC-C/EBP Beta Tg
AD-C/EBP Beta Tg

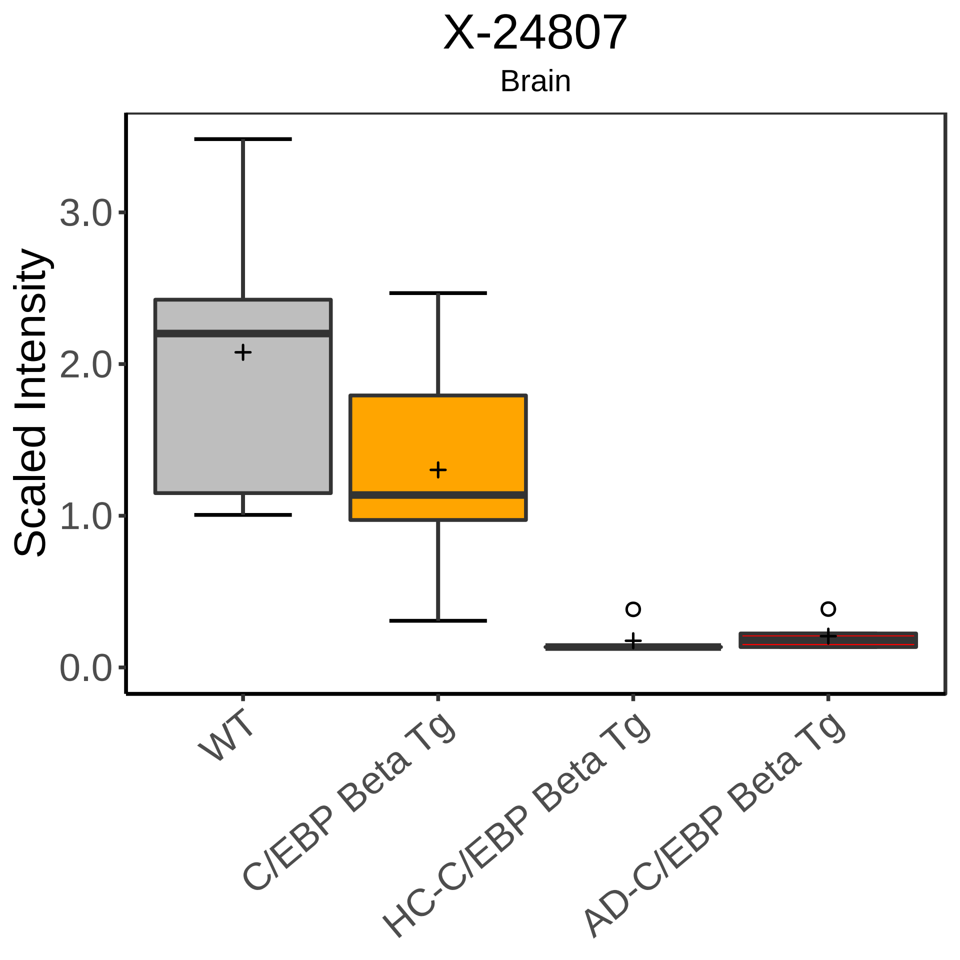
X-24807
Brain
Scaled Intensity
0.0
1.0
2.0
3.0
WT
C/EBP Beta Tg
HC-C/EBP Beta Tg
AD-C/EBP Beta Tg

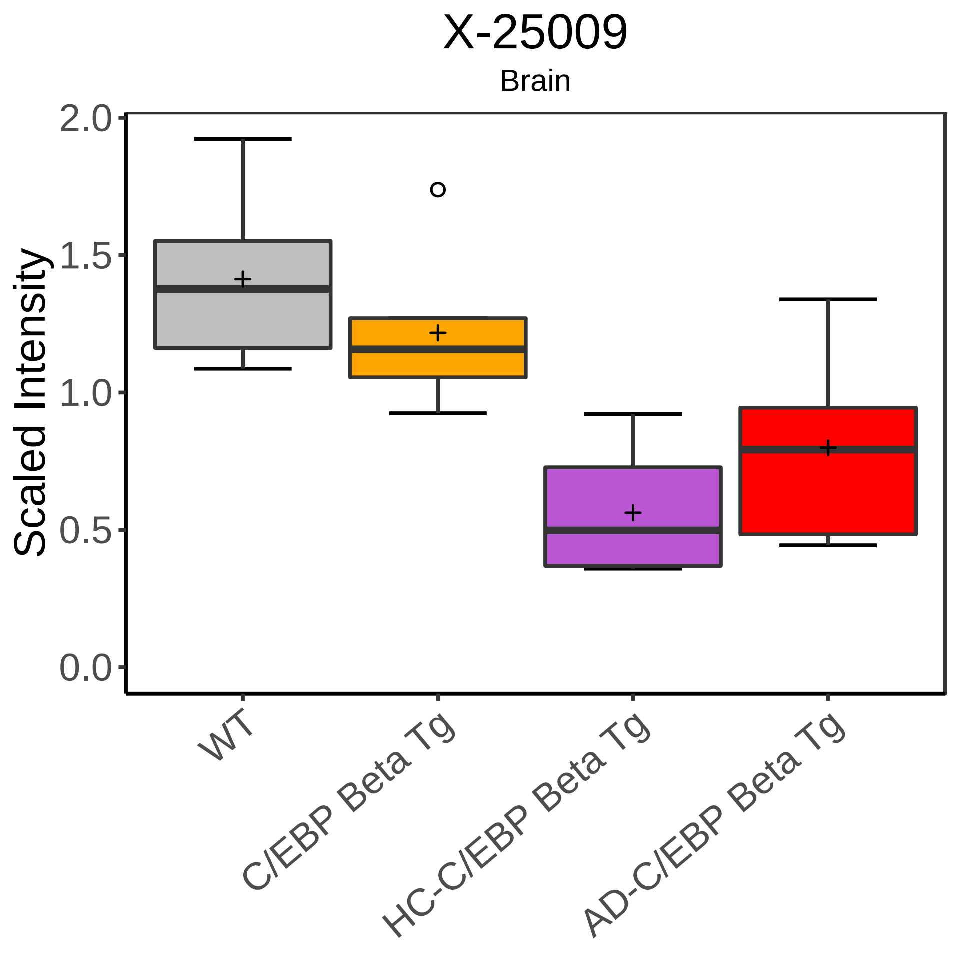
X-25009
Brain
Scaled Intensity
2.0
1.5
1.0
0.5
0.0
WT
C/EBP Beta Tg
HC-C/EBP Beta Tg
AD-C/EBP Beta Tg

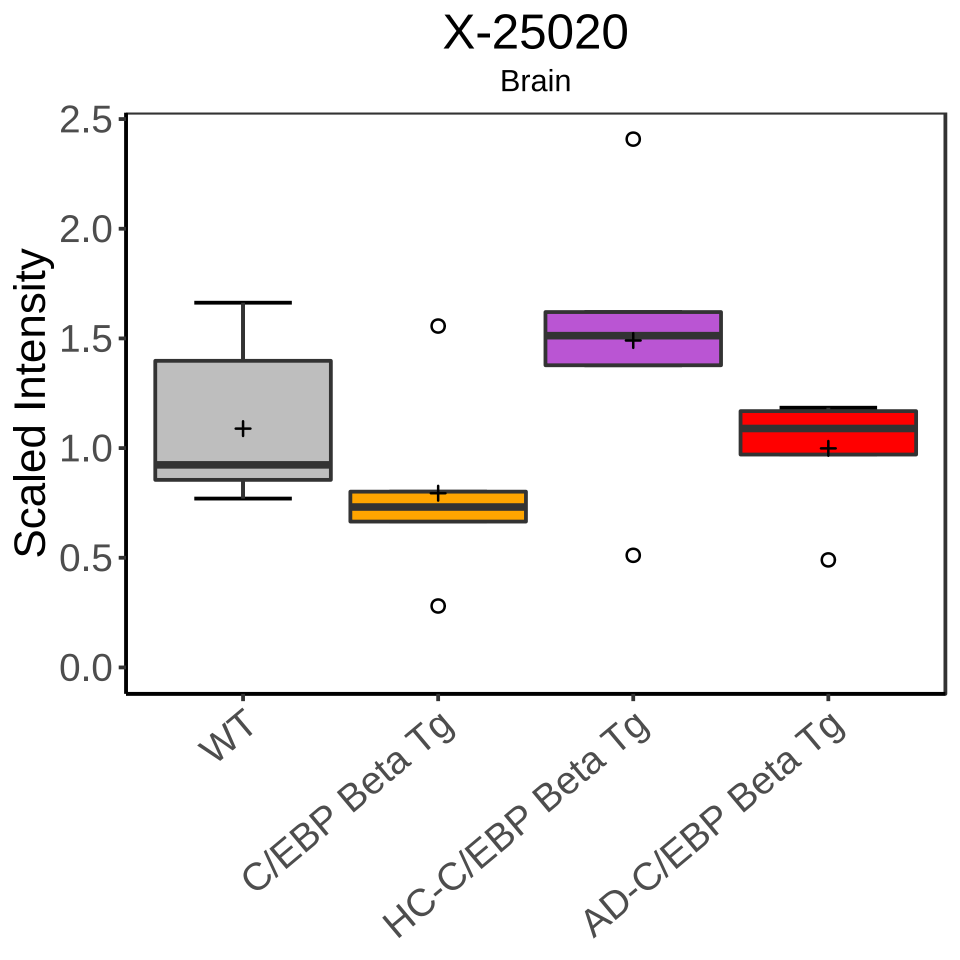

X-25020
Brain
Scaled Intensity
2.5
2.0
1.5
1.0
0.5
0.0
WT
C/EBP Beta Tg
HC-C/EBP Beta Tg
AD-C/EBP Beta Tg

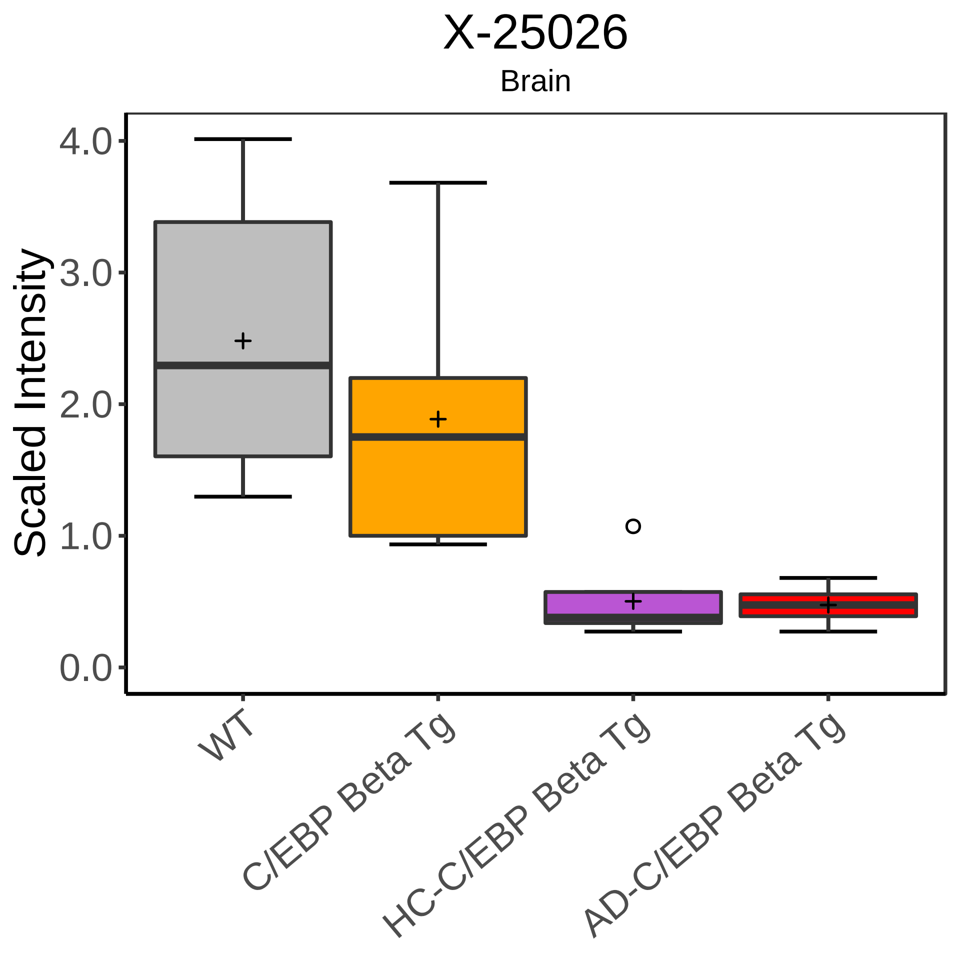
X-25026
Brain
Scaled Intensity
4.0
3.0
2.0
1.0
0.0
WT
C/EBP Beta Tg
HC-C/EBP Beta Tg
AD-C/EBP Beta Tg

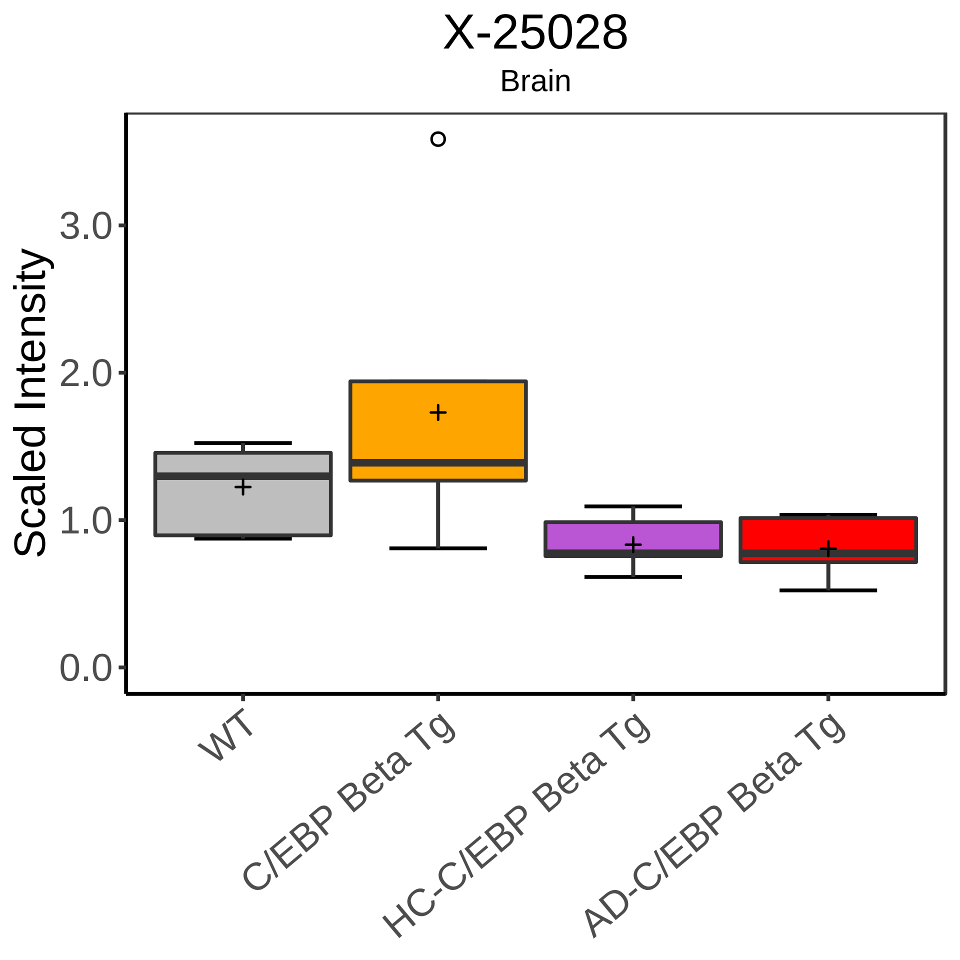
X-25028
Brain
Scaled Intensity
0.0
1.0
2.0
3.0
WT
C/EBP Beta Tg
HC-C/EBP Beta Tg
AD-C/EBP Beta Tg

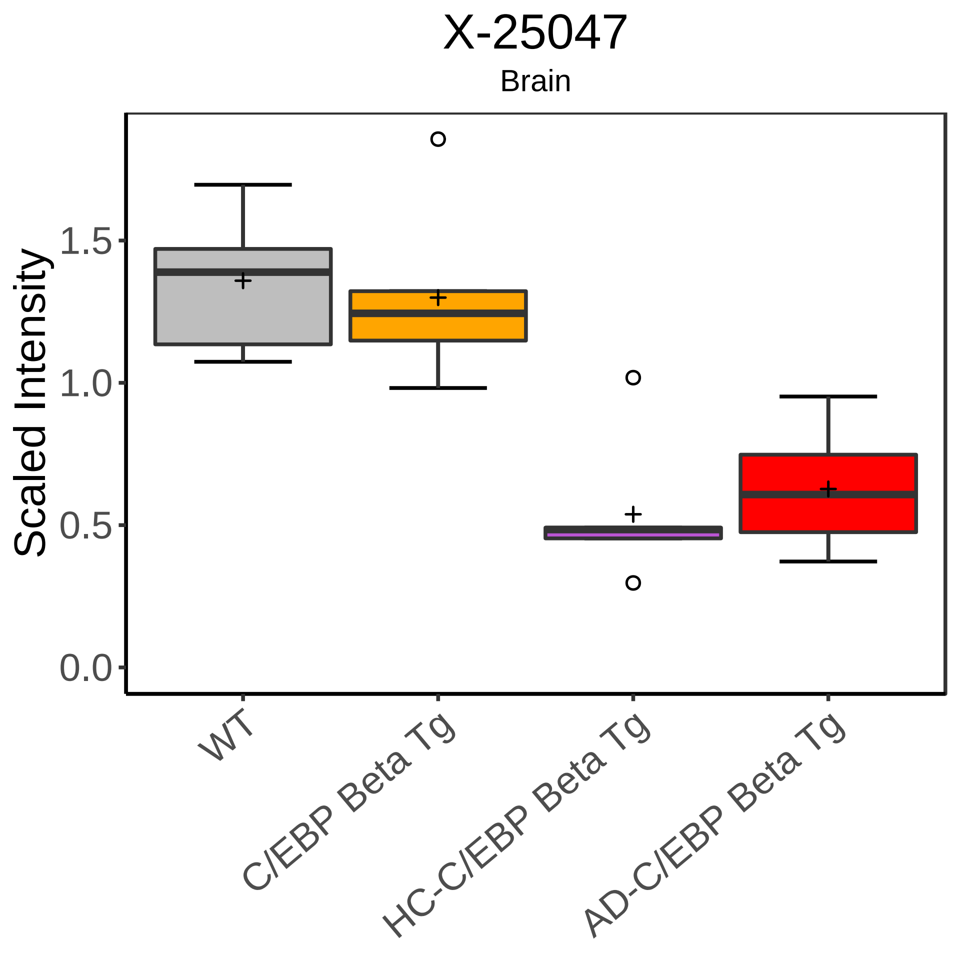
X-25047
Brain
Scaled Intensity
0.0
0.5
1.0
1.5
WT
C/EBP Beta Tg
HC-C/EBP Beta Tg
AD-C/EBP Beta Tg

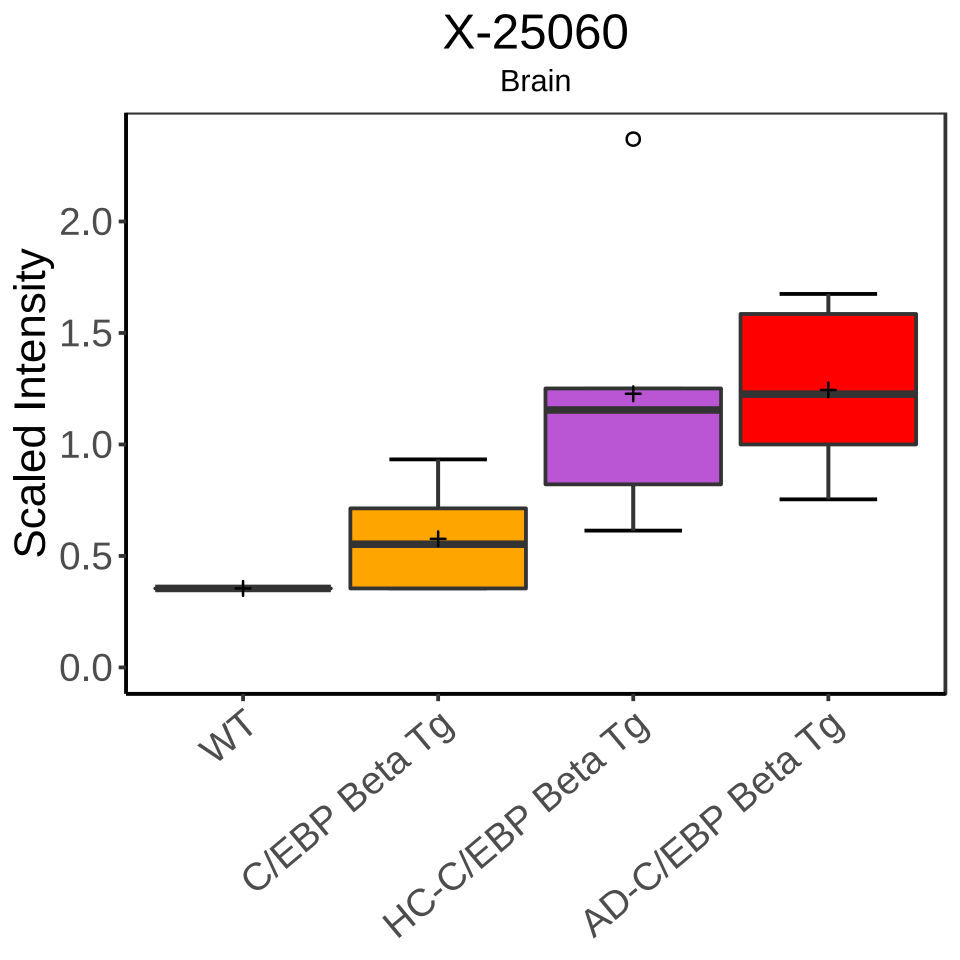

X-25060
Brain
Scaled Intensity
2.0
1.5
1.0
0.5
0.0
WT
C/EBP Beta Tg
HC-C/EBP Beta Tg
AD-C/EBP Beta Tg

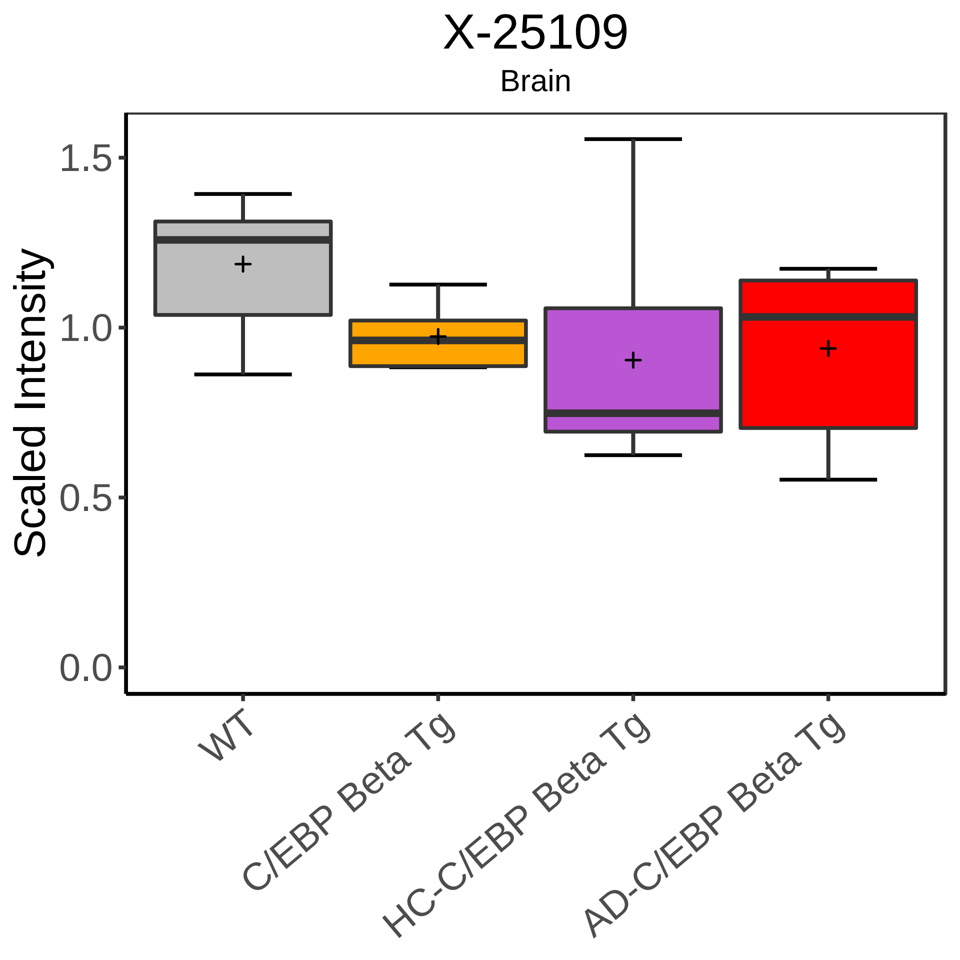

X-25109
Brain
Scaled Intensity
1.5
1.0
0.5
0.0
WT
C/EBP Beta Tg
HC-C/EBP Beta Tg
AD-C/EBP Beta Tg

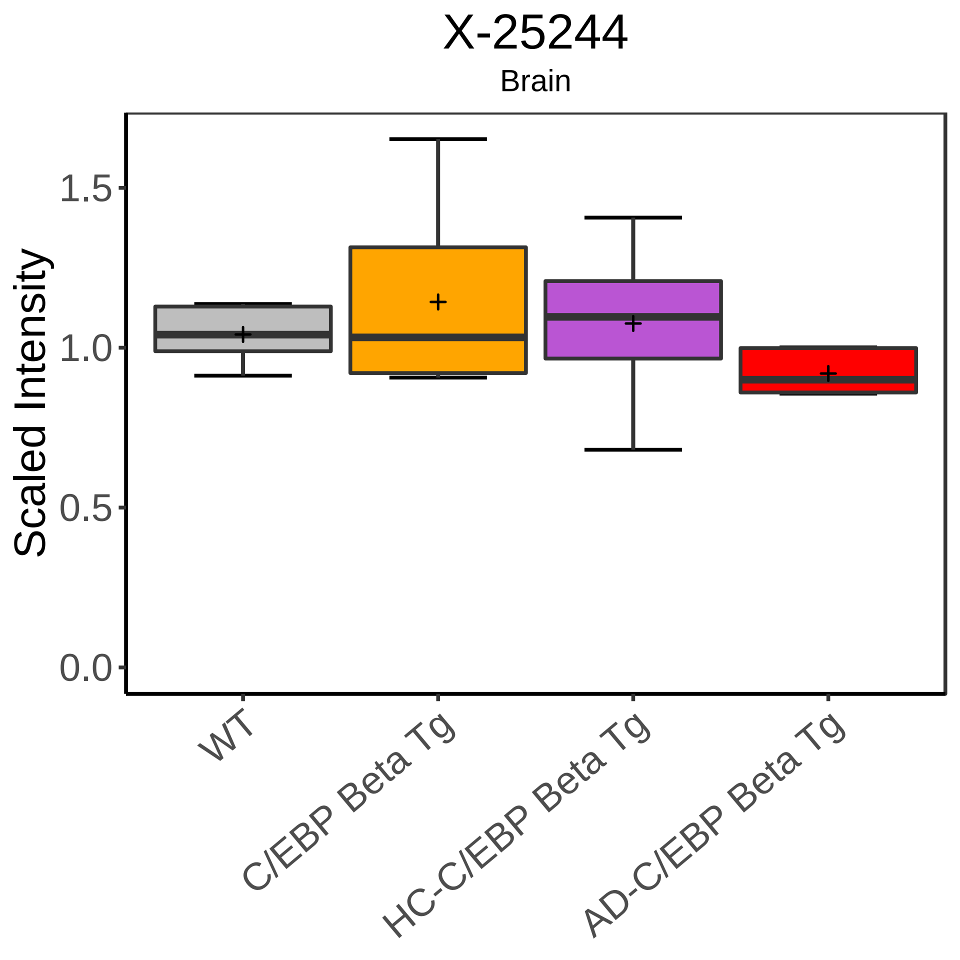

X-25244
Brain
Scaled Intensity
1.5
1.0
0.5
0.0
WT
C/EBP Beta Tg
HC-C/EBP Beta Tg
AD-C/EBP Beta Tg

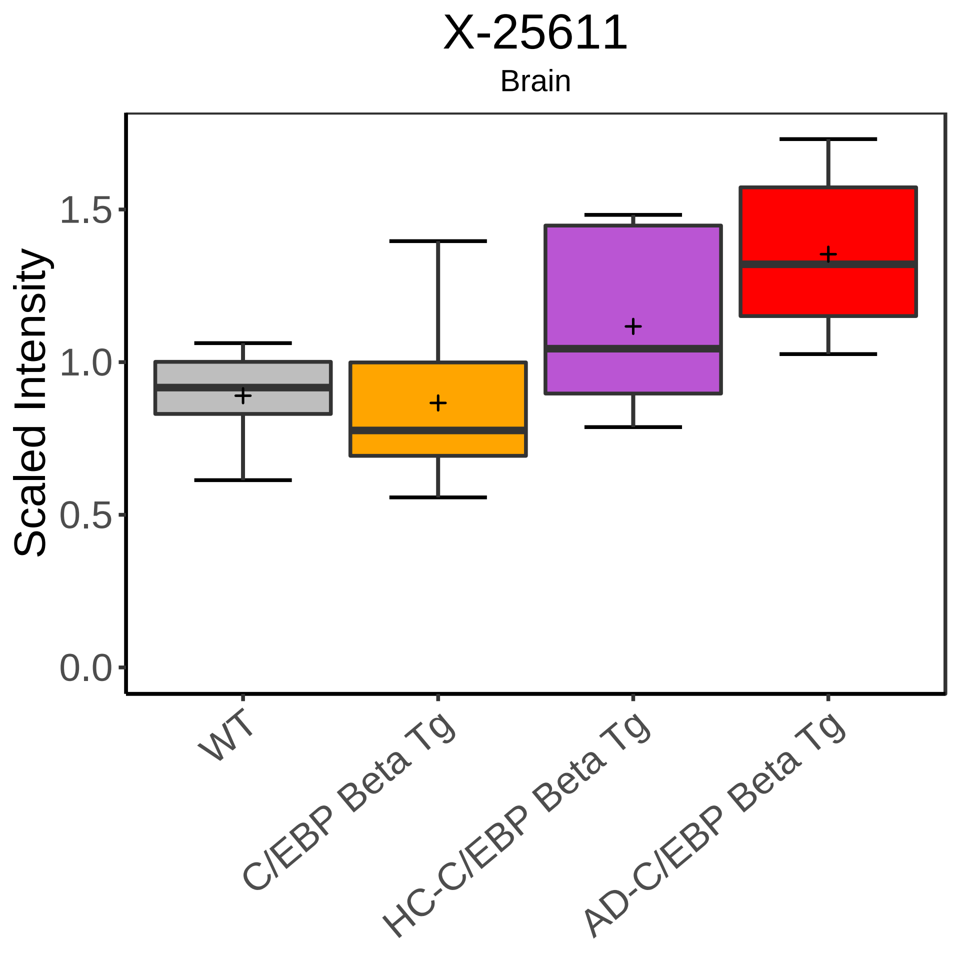

X-25611
Brain
Scaled Intensity
1.5
1.0
0.5
0.0
WT
C/EBP Beta Tg
HC-C/EBP Beta Tg
AD-C/EBP Beta Tg

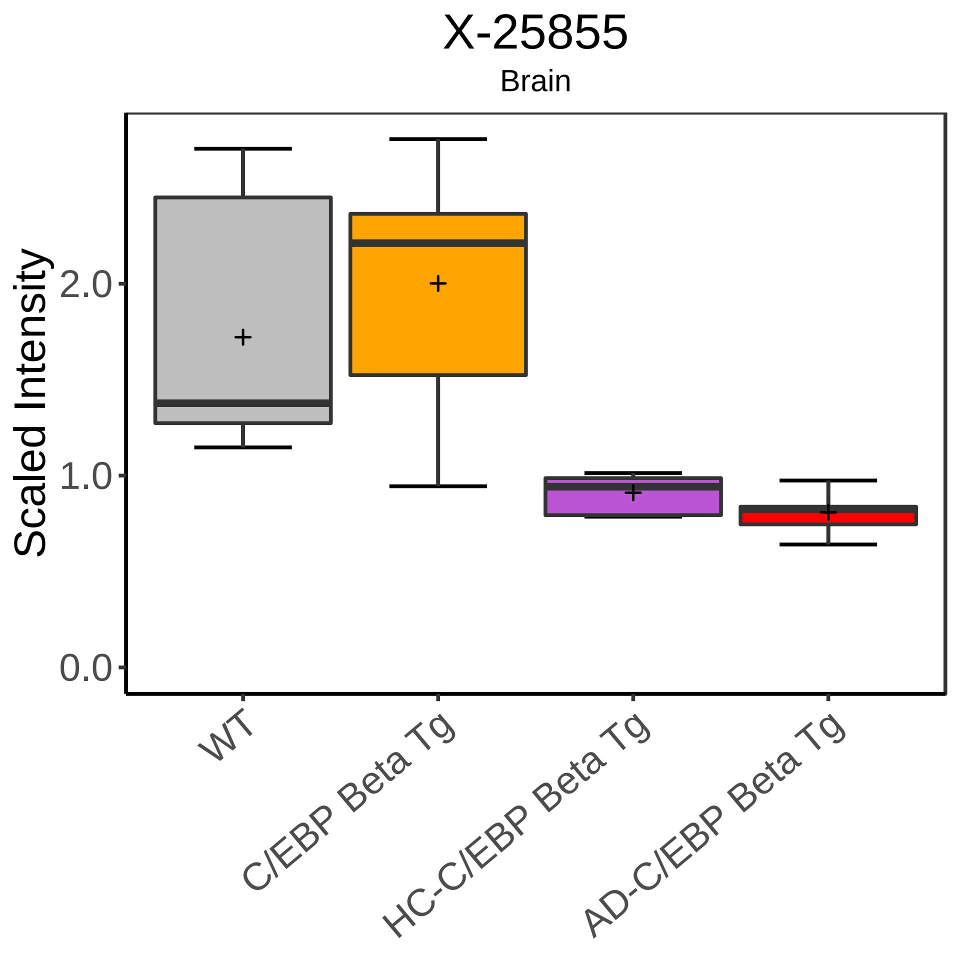
X-25855
Brain
Scaled Intensity
2.0
1.0
0.0
WT
C/EBP Beta Tg
HC-C/EBP Beta Tg
AD-C/EBP Beta Tg

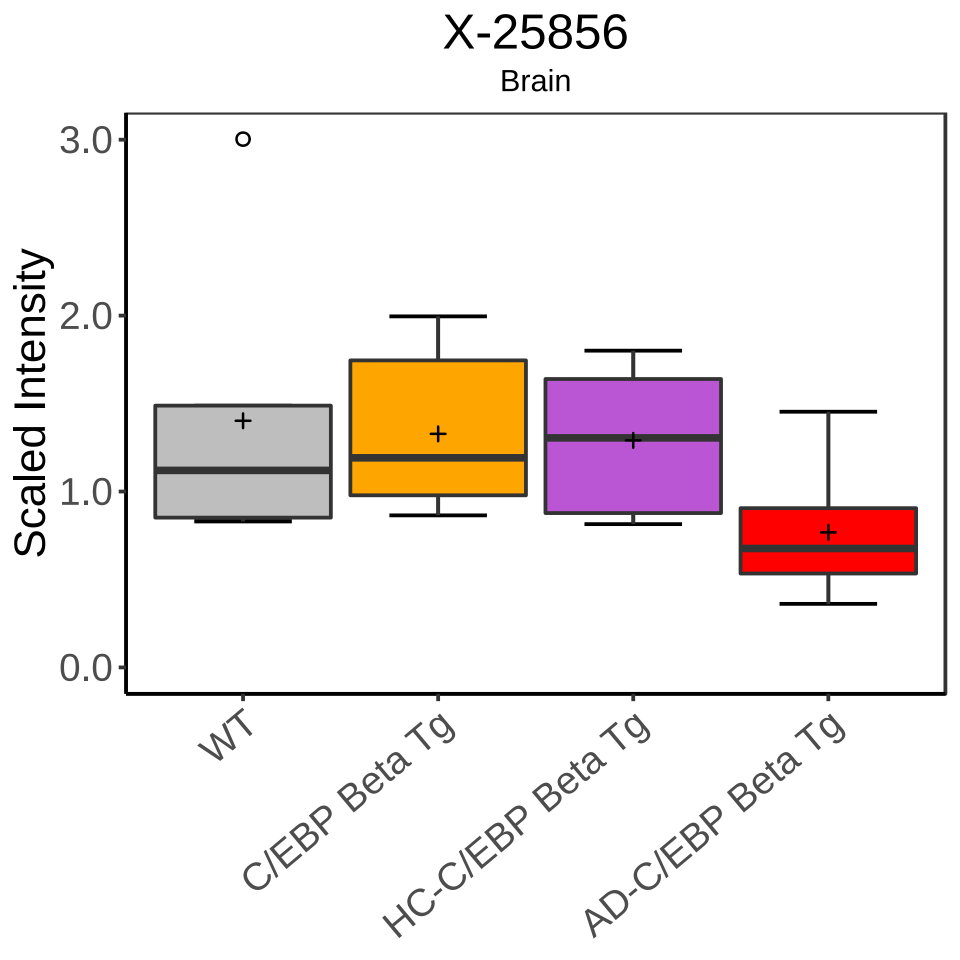
X-25856
Brain
Scaled Intensity
3.0
2.0
1.0
0.0
WT
C/EBP Beta Tg
HC-C/EBP Beta Tg
AD-C/EBP Beta Tg

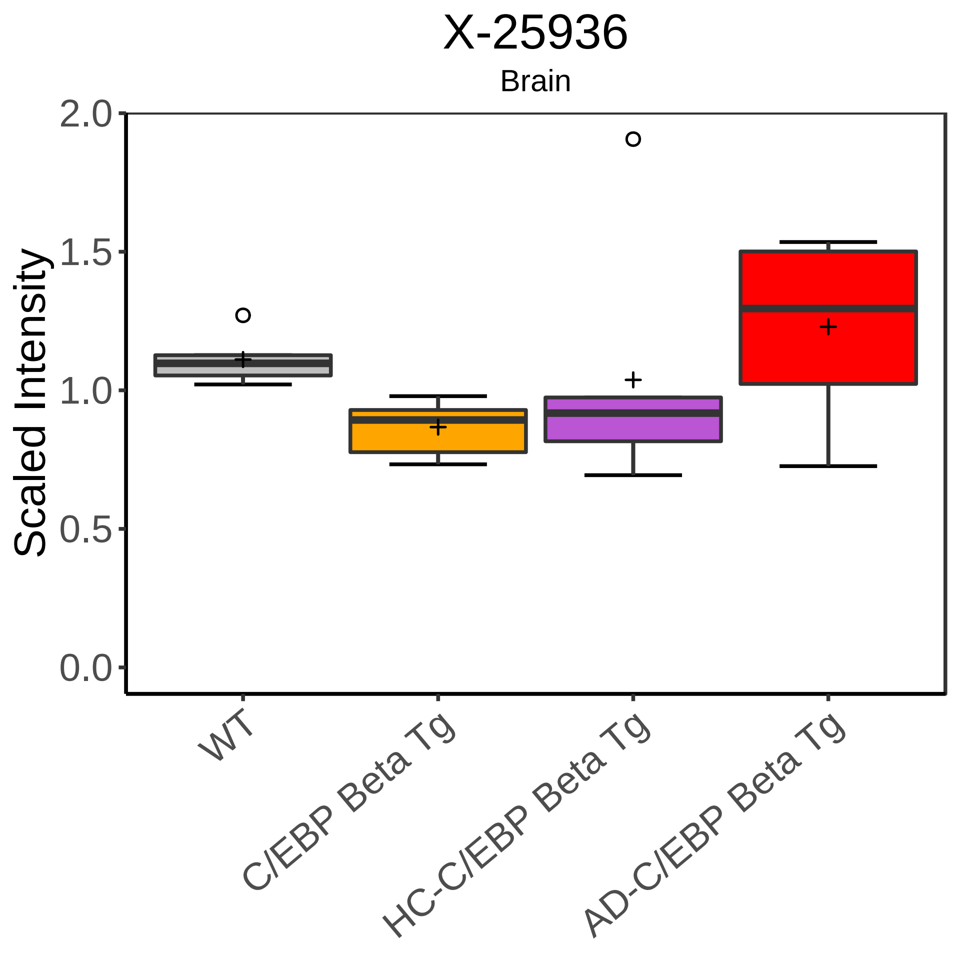
X-25936
Brain
Scaled Intensity
2.0
1.5
1.0
0.5
0.0
WT
C/EBP Beta Tg
HC-C/EBP Beta Tg
AD-C/EBP Beta Tg

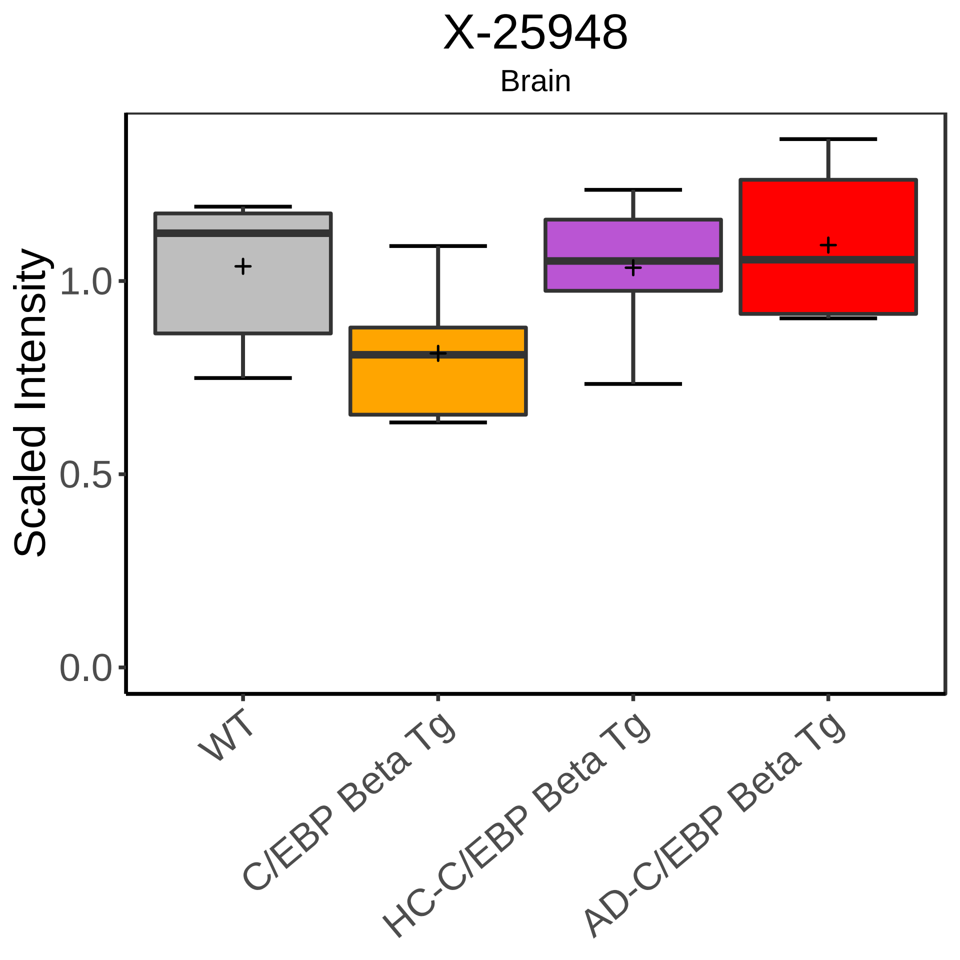
X-25948
Brain
Scaled Intensity
1.0
0.5
0.0
WT
C/EBP Beta Tg
HC-C/EBP Beta Tg
AD-C/EBP Beta Tg

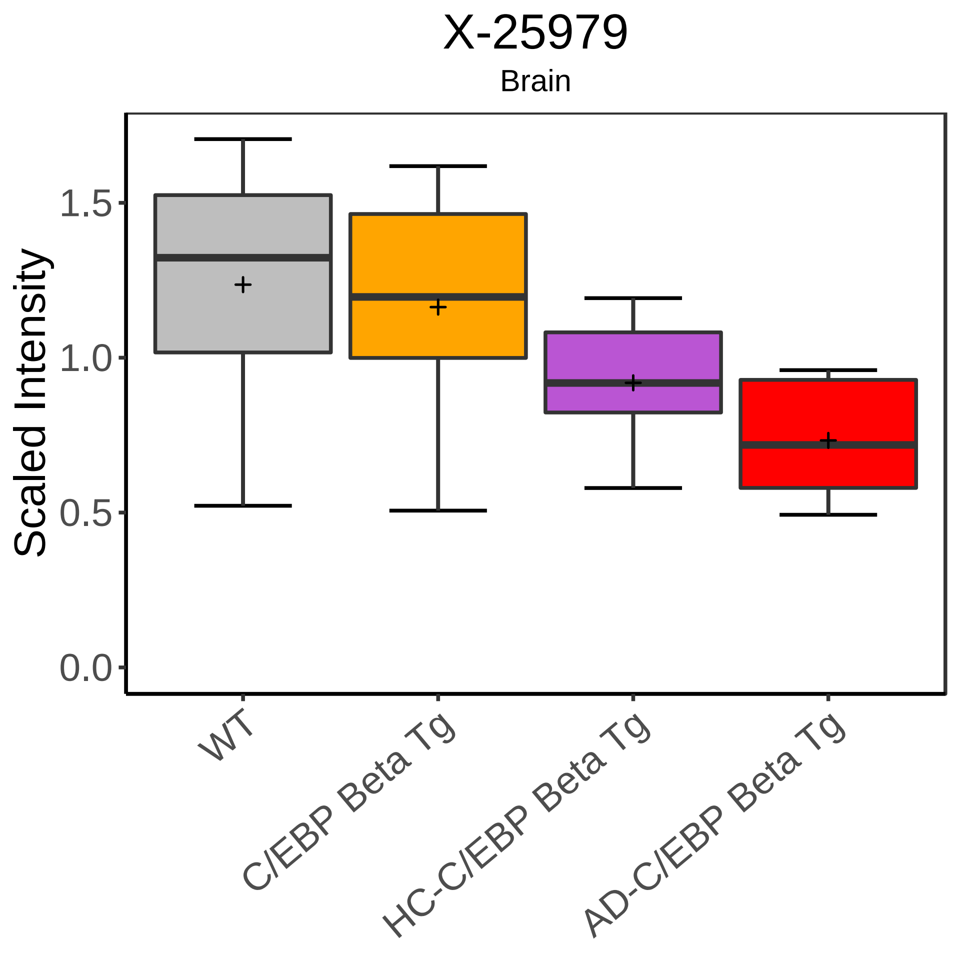

X-25979
Brain
Scaled Intensity
1.5
1.0
0.5
0.0
WT
C/EBP Beta Tg
HC-C/EBP Beta Tg
AD-C/EBP Beta Tg

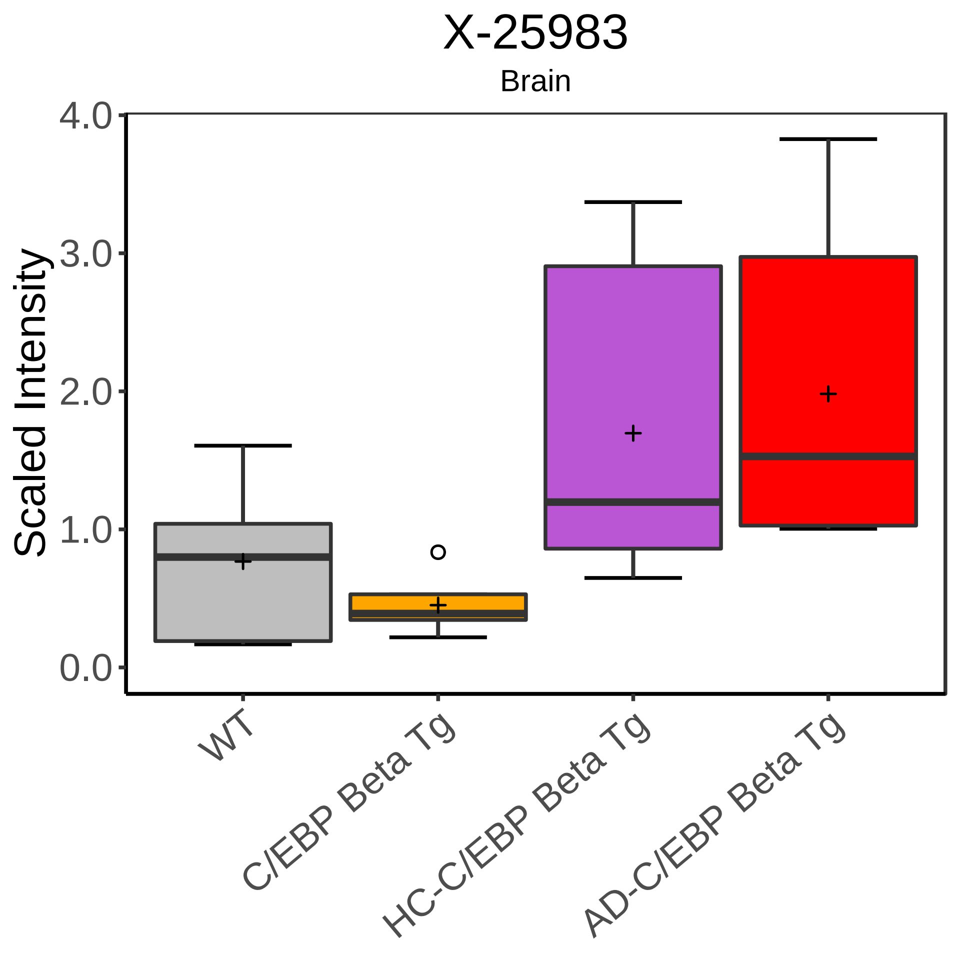
X-25983
Brain
Scaled Intensity
4.0
3.0
2.0
1.0
0.0
WT
C/EBP Beta Tg
HC-C/EBP Beta Tg
AD-C/EBP Beta Tg
