# Supplementary material for: Bacteroides Fragilis in the gut microbiomes of Alzheimer’s disease activates microglia and triggers pathogenesis in neuronal C/EBPβ transgenic mice
Source: Nat Commun. 2023 Sep 6;14:5471. doi: 10.1038/s41467-023-41283-w (PMC10482867; doi:10.1038/s41467-023-41283-w)
Supplement: Supplementary file 4 — Supplementary Data 1 [file 41467_2023_41283_MOESM4_ESM.zip › EMOR-0201-20VW+/EMOR-0201-20VW+ BOX PLOTS (FECES).PDF]

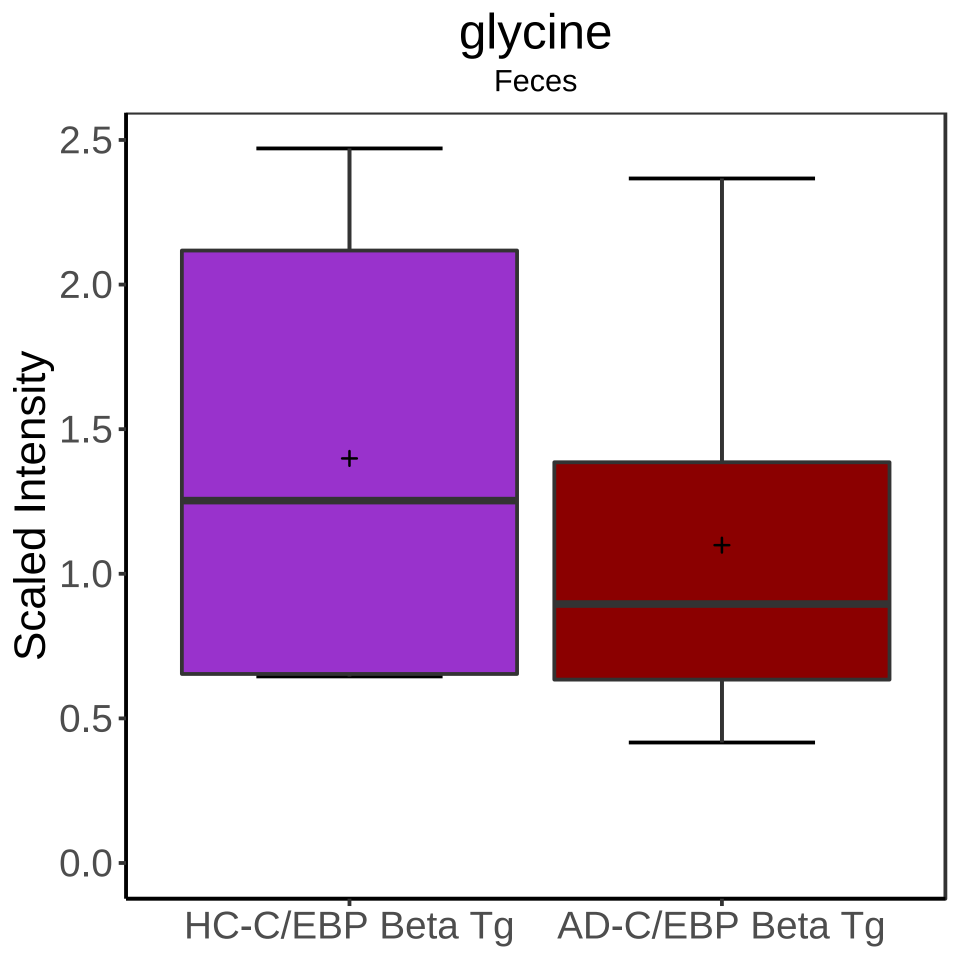
glycine
Feces
Scaled Intensity
2.5
2.0
1.5
1.0
0.5
0.0
HC-C/EBP Beta Tg
AD-C/EBP Beta Tg

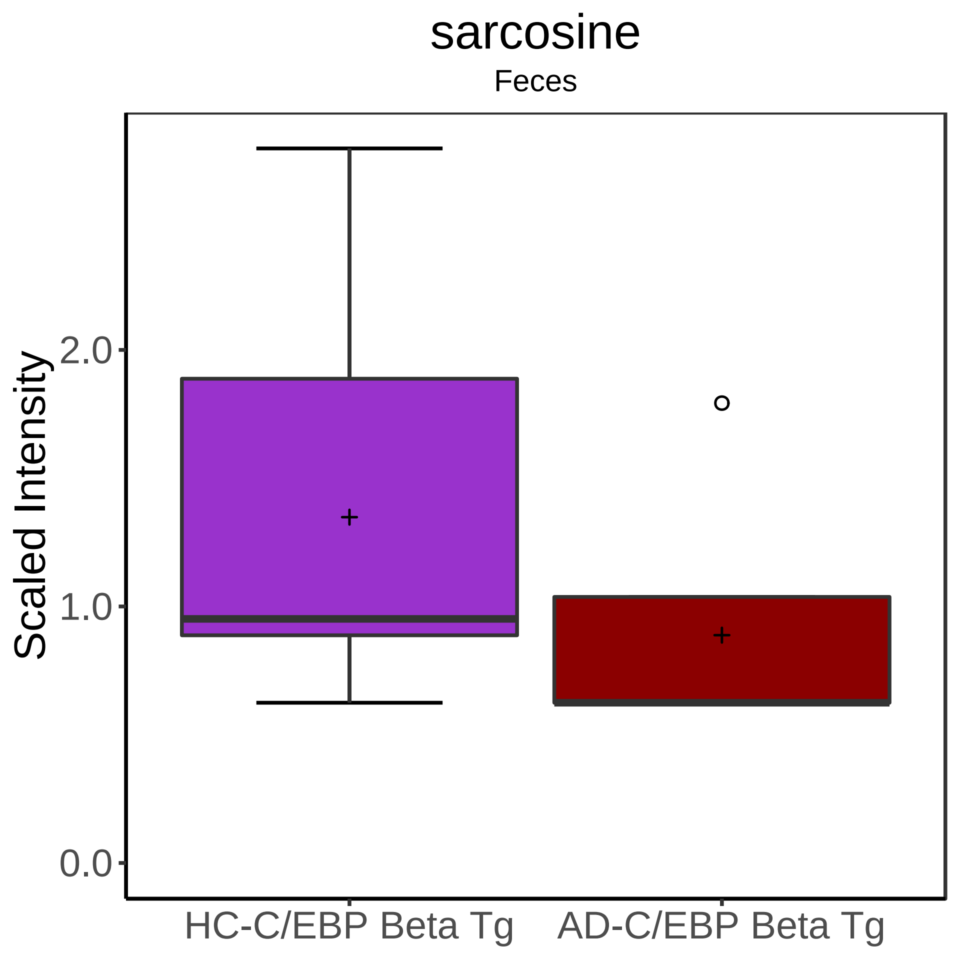
sarcosine
Feces
Scaled Intensity
2.0
1.0
0.0
HC-C/EBP Beta Tg
AD-C/EBP Beta Tg

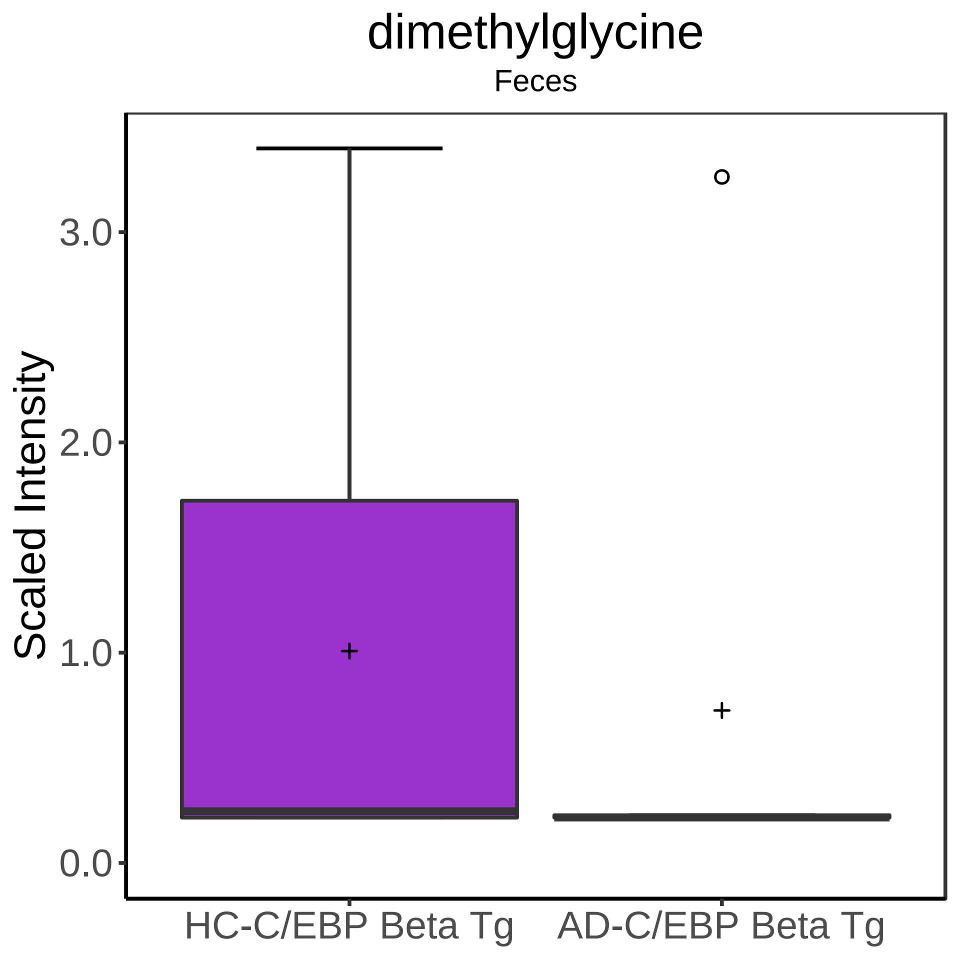
dimethylglycine
Feces
Scaled Intensity
3.0
2.0
1.0
0.0
HC-C/EBP Beta Tg
AD-C/EBP Beta Tg

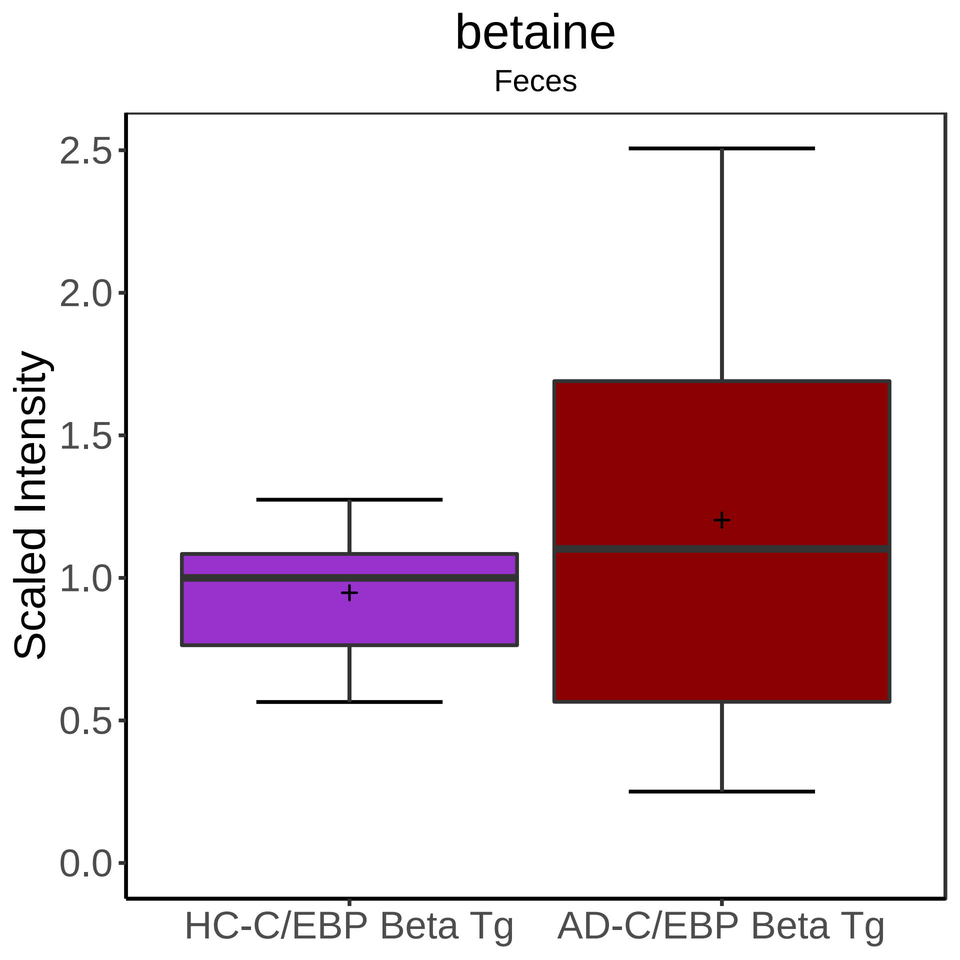
betaine
Feces
Scaled Intensity
2.5
2.0
1.5
1.0
0.5
0.0
HC-C/EBP Beta Tg
AD-C/EBP Beta Tg

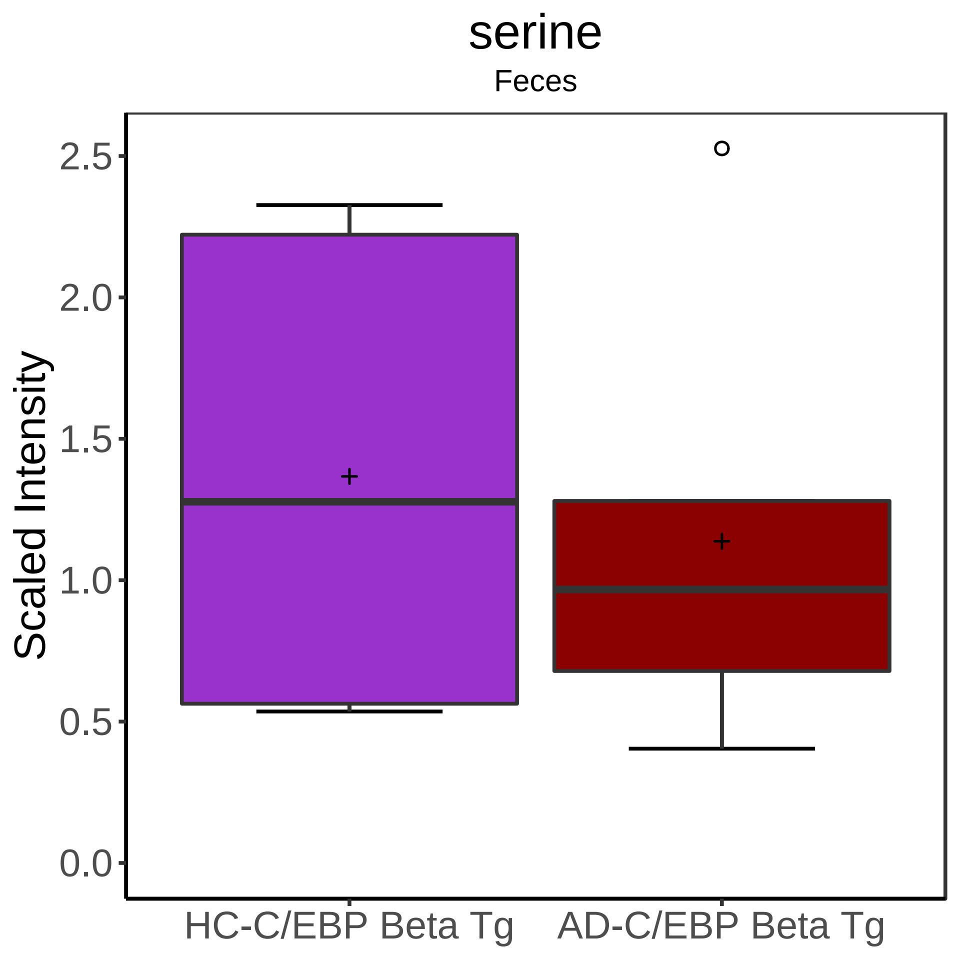

serine
Feces
Scaled Intensity
2.5
2.0
1.5
1.0
0.5
0.0
HC-C/EBP Beta Tg
AD-C/EBP Beta Tg

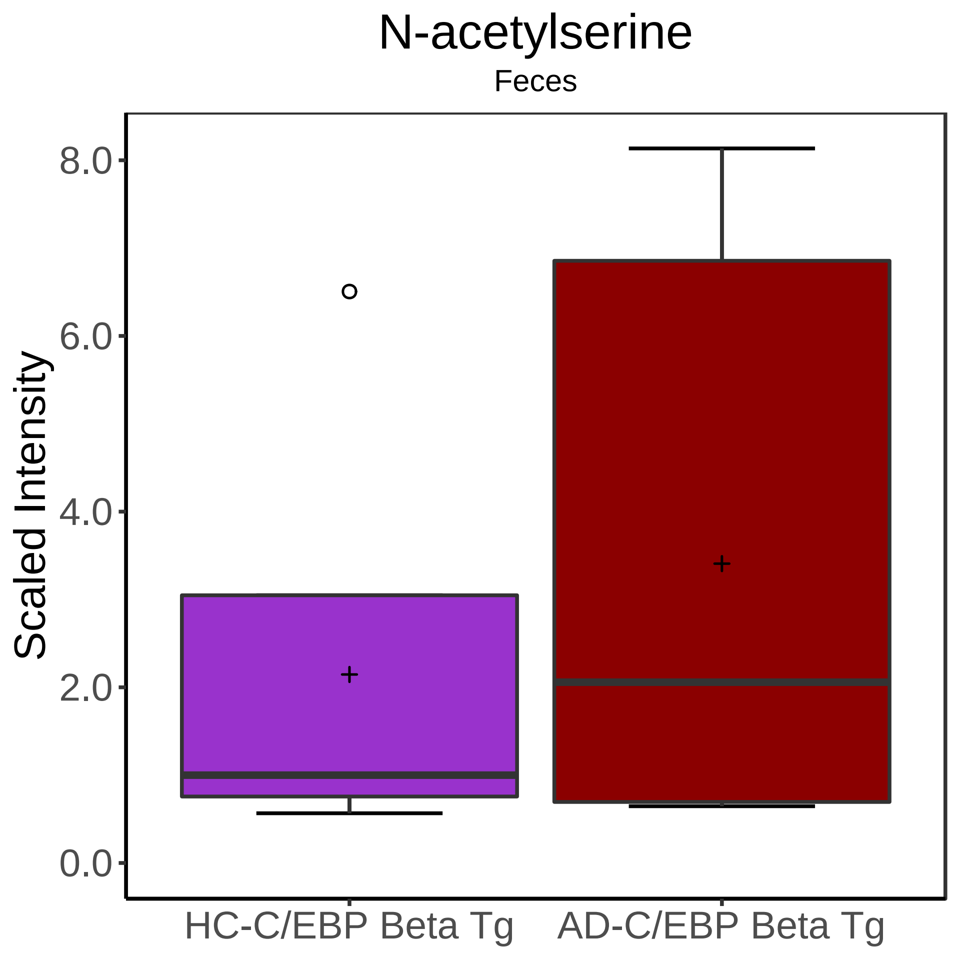

N-acetylserine
Feces
Scaled Intensity
8.0
6.0
4.0
2.0
0.0
HC-C/EBP Beta Tg
AD-C/EBP Beta Tg

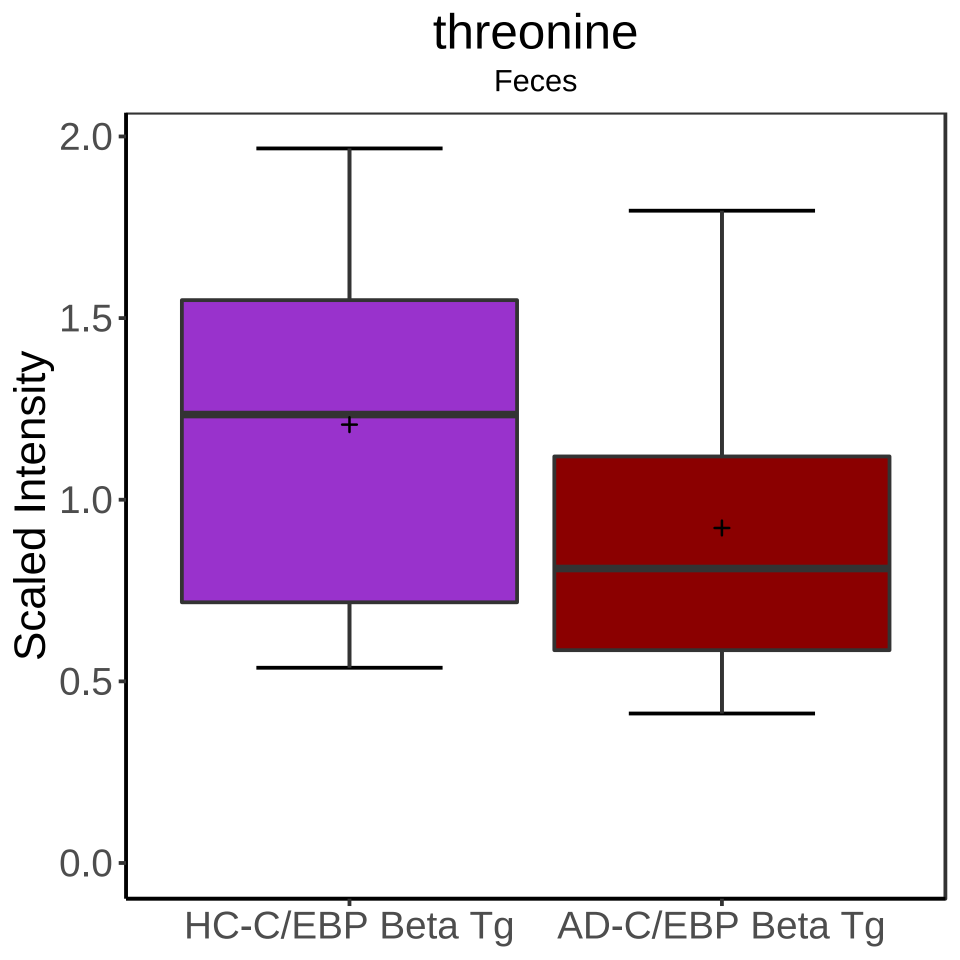
threonine
Feces
Scaled Intensity
2.0
1.5
1.0
0.5
0.0
HC-C/EBP Beta Tg
AD-C/EBP Beta Tg

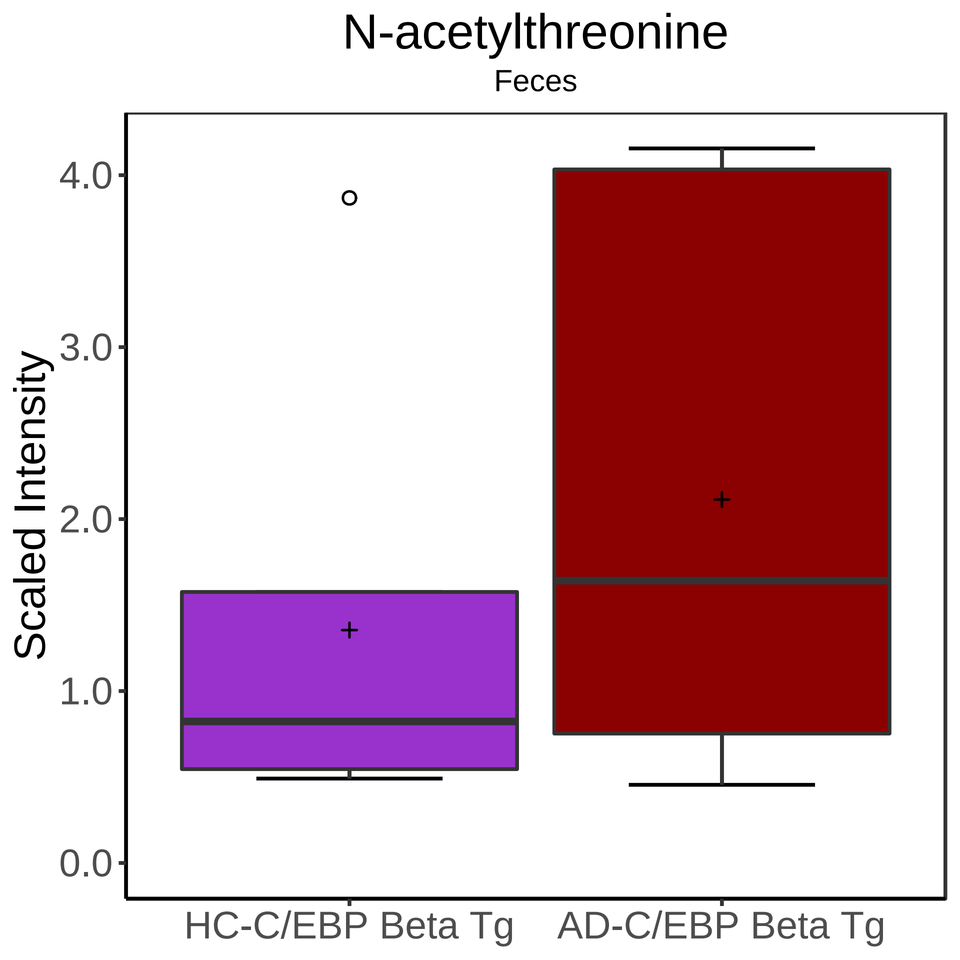
N-acetylthreonine
Feces
Scaled Intensity
4.0
3.0
2.0
1.0
0.0
HC-C/EBP Beta Tg
AD-C/EBP Beta Tg

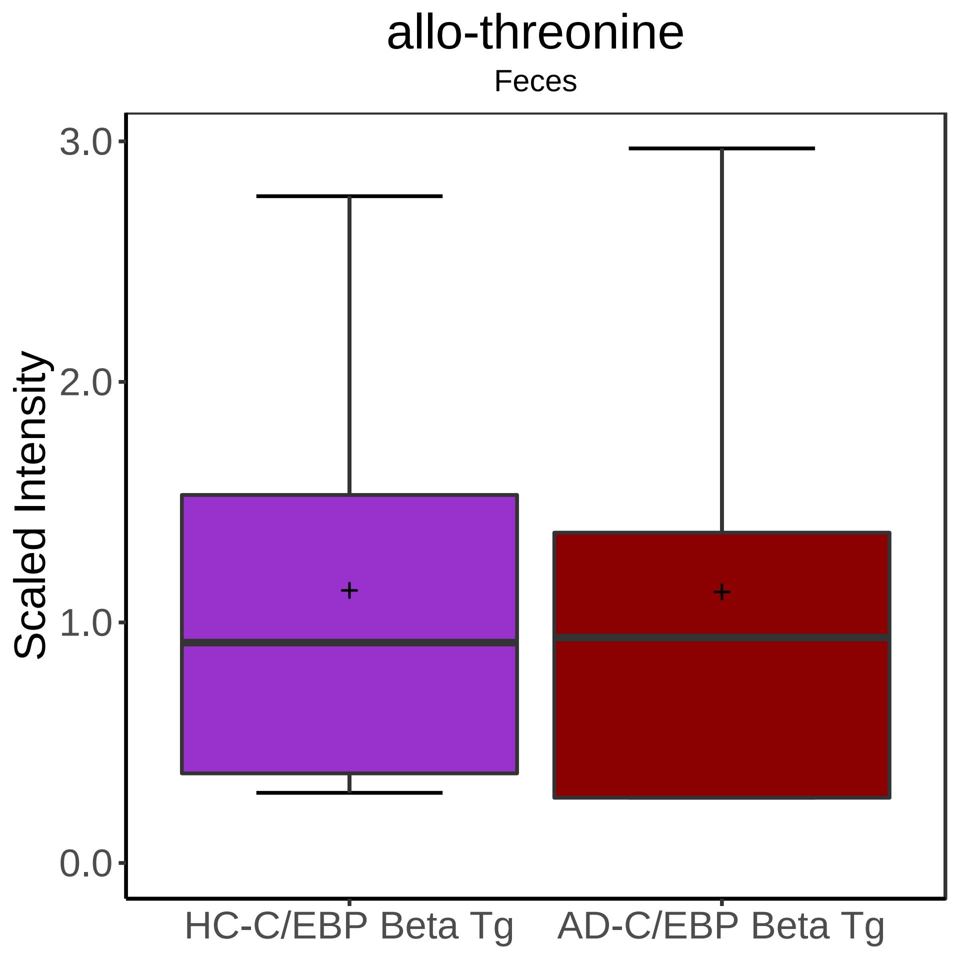
allo-threonine
Feces
Scaled Intensity
3.0
2.0
1.0
0.0
HC-C/EBP Beta Tg
AD-C/EBP Beta Tg

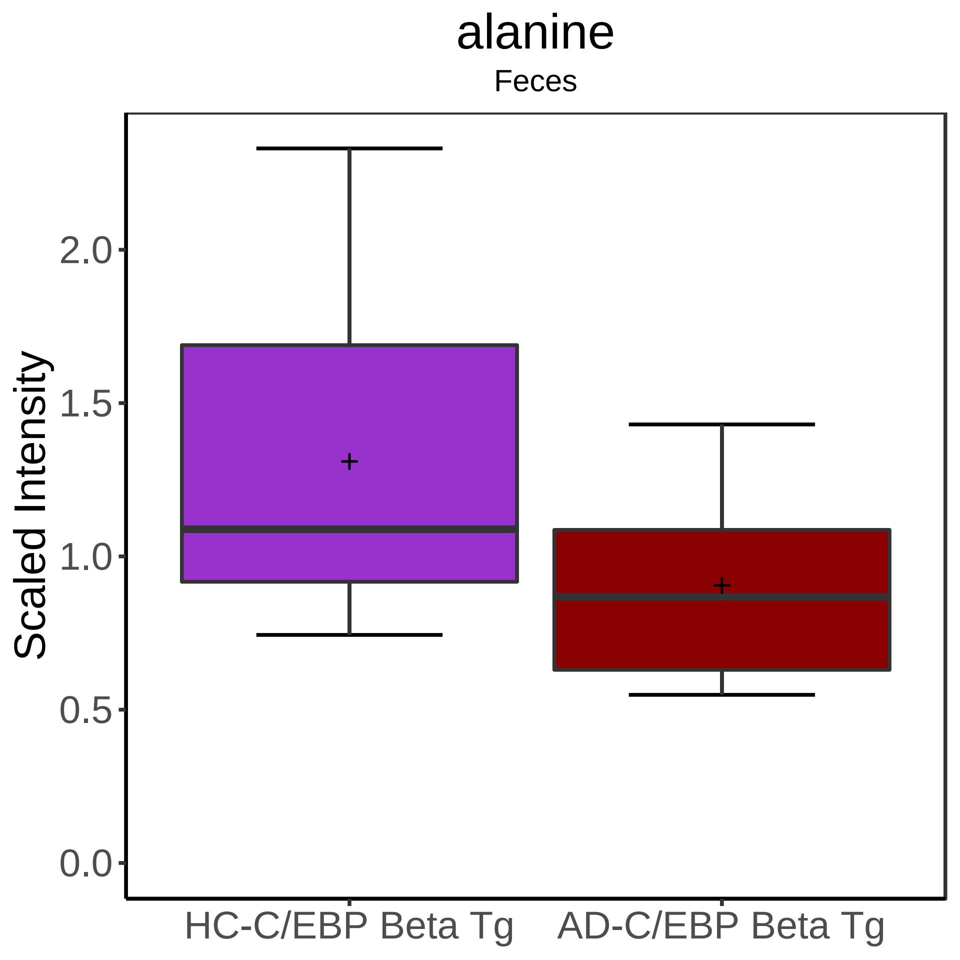

alanine
Feces
Scaled Intensity
2.0
1.5
1.0
0.5
0.0
HC-C/EBP Beta Tg
AD-C/EBP Beta Tg

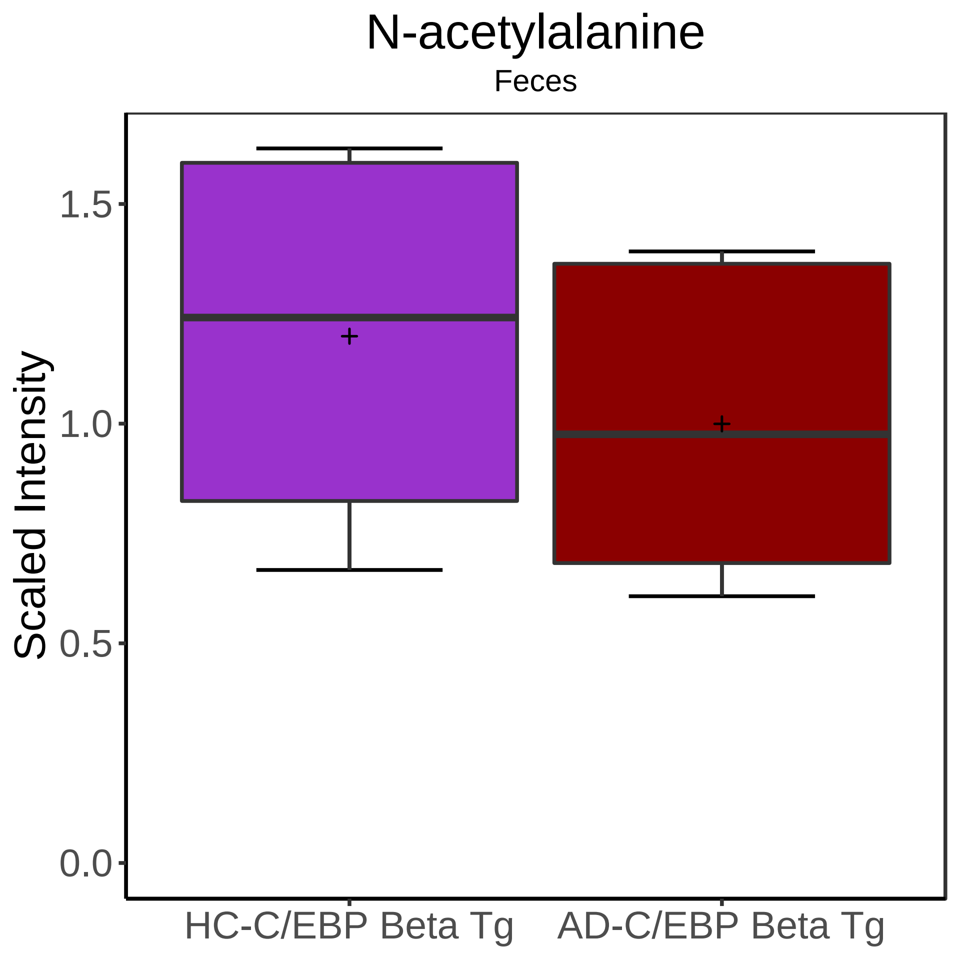
N-acetylalanine
Feces
Scaled Intensity
1.5
1.0
0.5
0.0
HC-C/EBP Beta Tg
AD-C/EBP Beta Tg

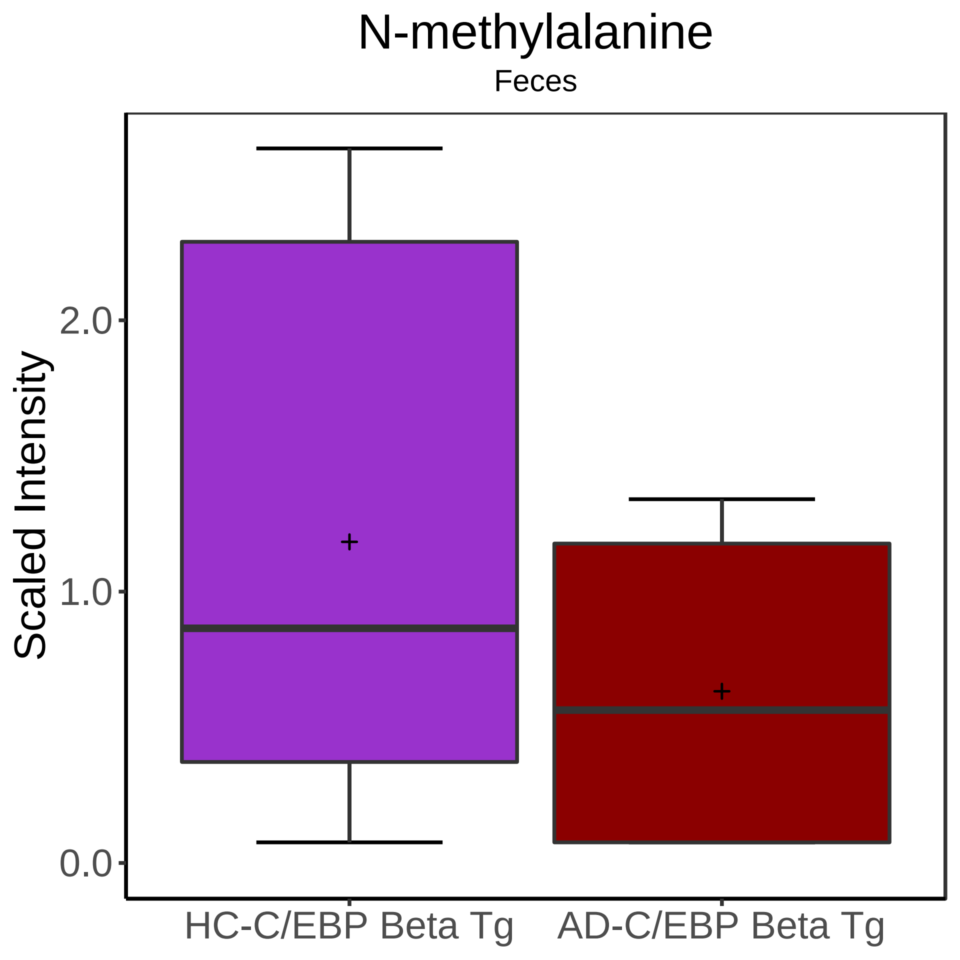

N-methylalanine
Feces
Scaled Intensity
2.0
1.0
0.0
HC-C/EBP Beta Tg
AD-C/EBP Beta Tg

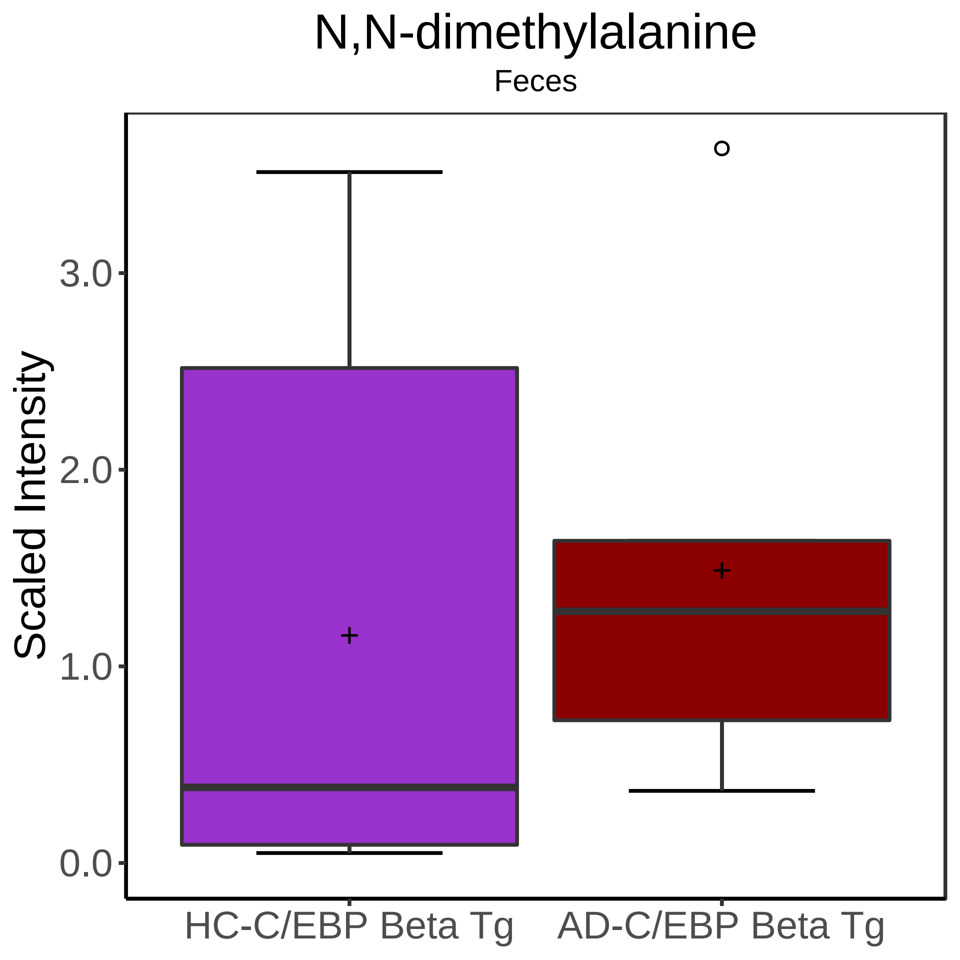

N,N-dimethylalalanine
Feces
Scaled Intensity
3.0
2.0
1.0
0.0
HC-C/EBP Beta Tg
AD-C/EBP Beta Tg

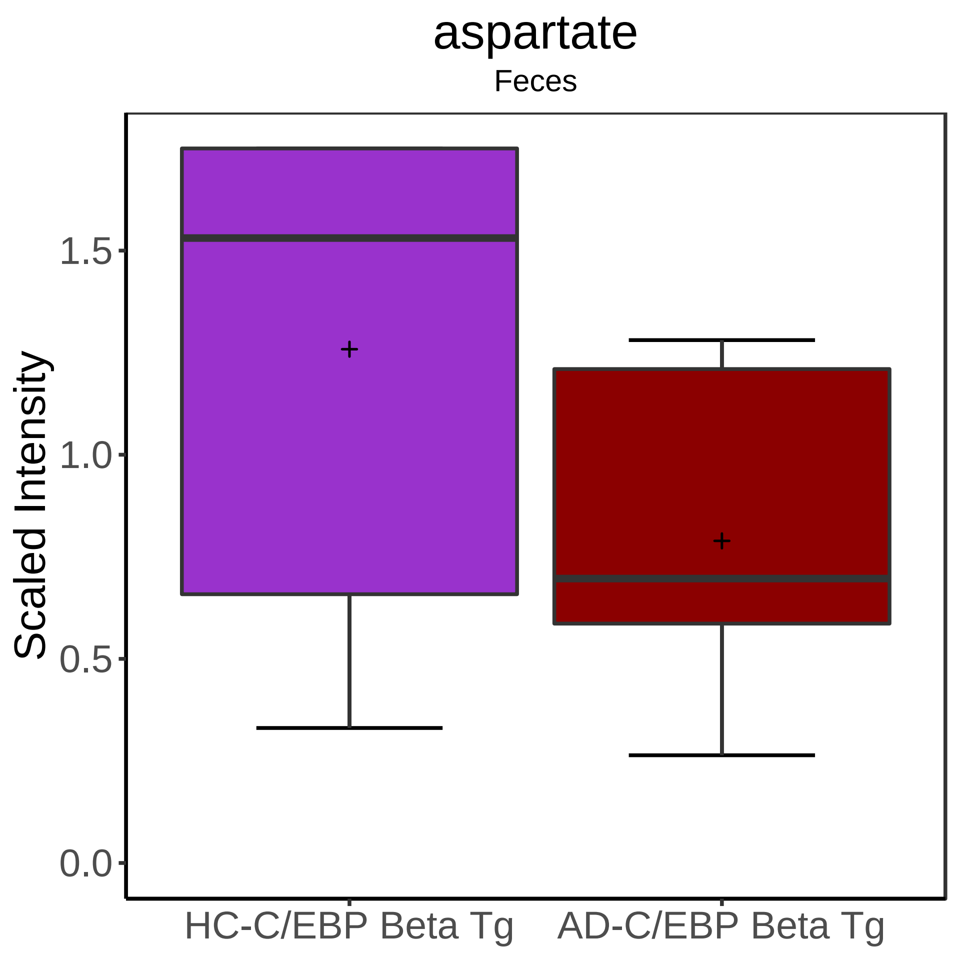
aspartate
Feces
Scaled Intensity
1.5
1.0
0.5
0.0
HC-C/EBP Beta Tg
AD-C/EBP Beta Tg

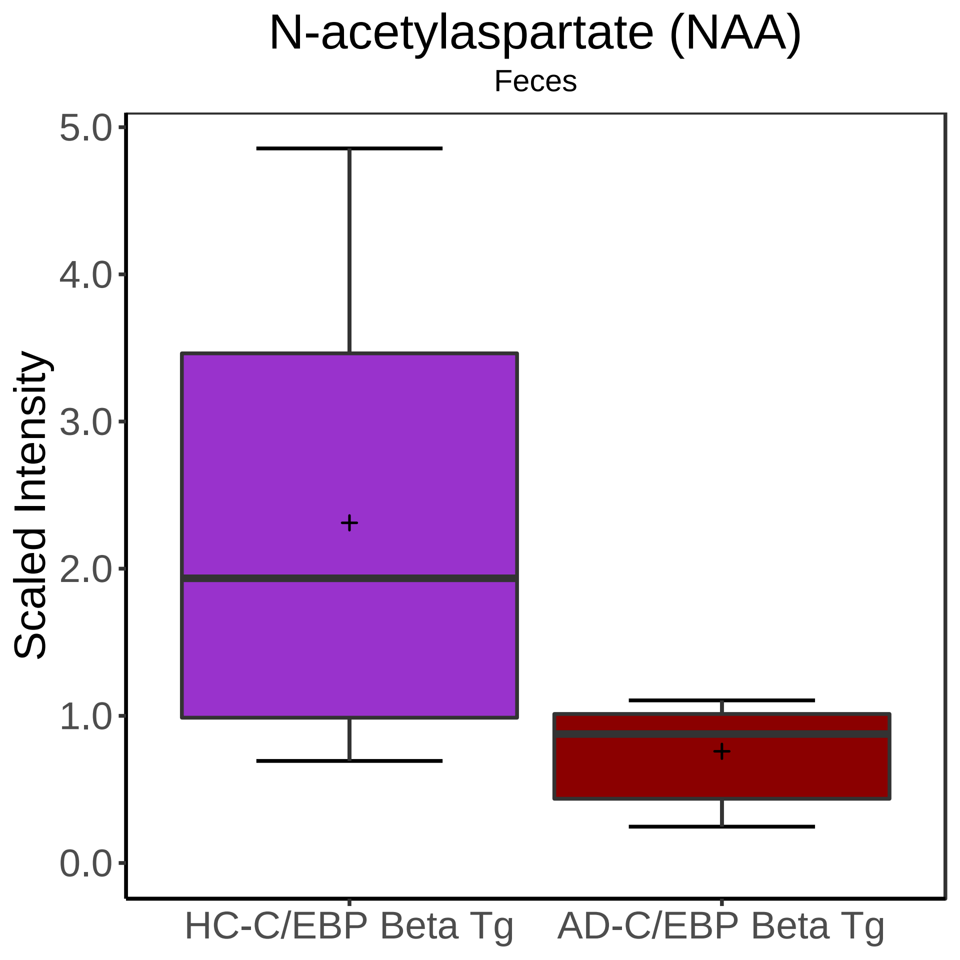
N-acetylaspartate (NAA)
Feces
Scaled Intensity
5.0
4.0
3.0
2.0
1.0
0.0
HC-C/EBP Beta Tg
AD-C/EBP Beta Tg

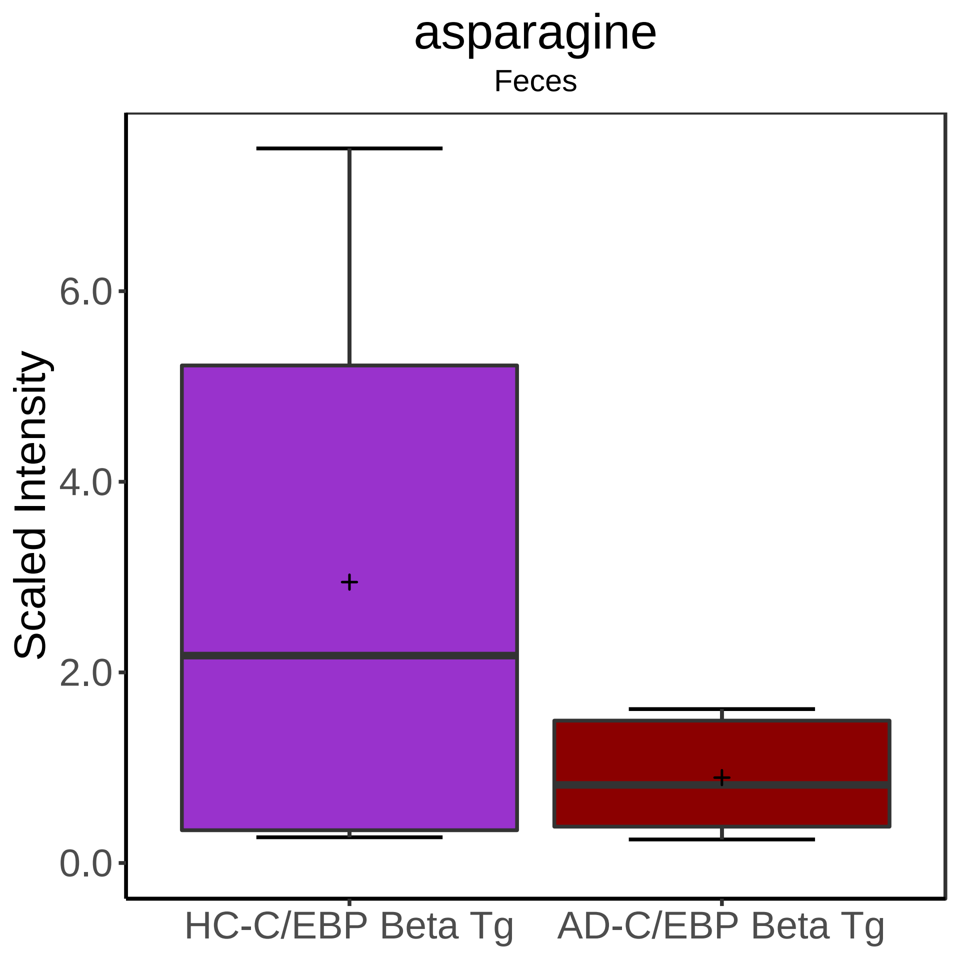

asparagine
Feces
Scaled Intensity
6.0
4.0
2.0
0.0
HC-C/EBP Beta Tg
AD-C/EBP Beta Tg

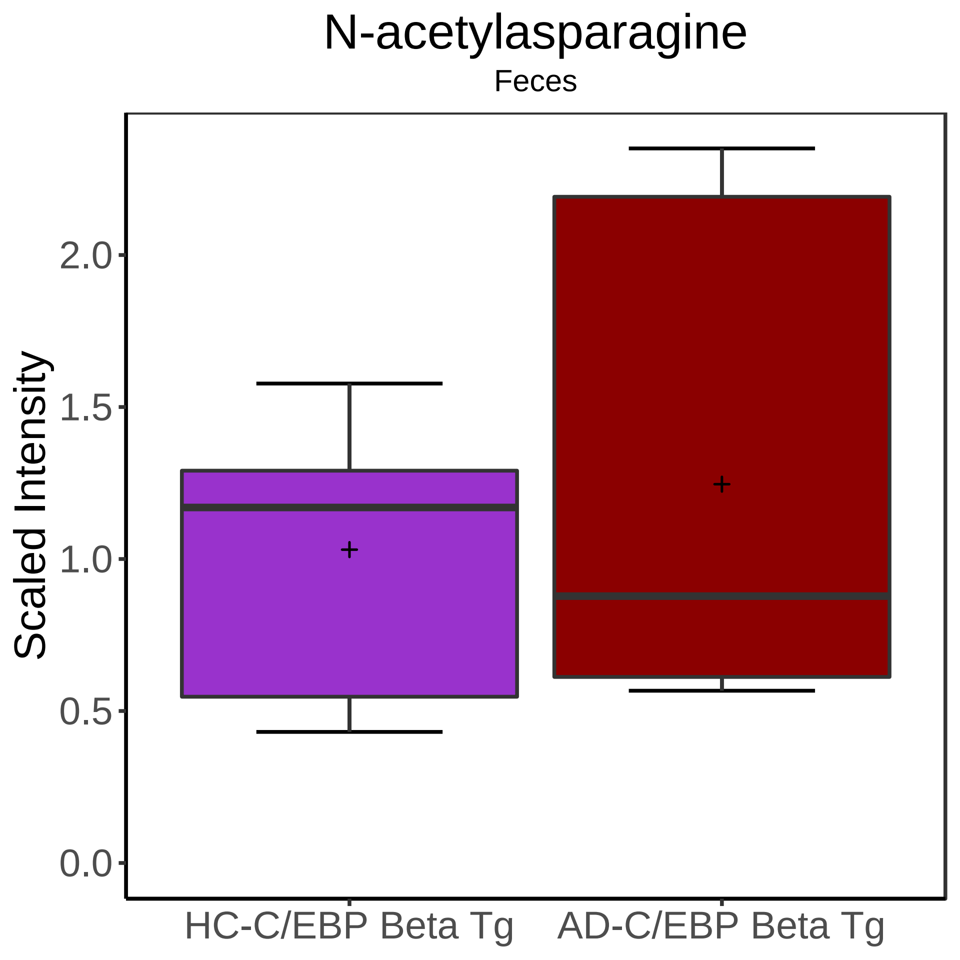

N-acetylasparagine
Feces
Scaled Intensity
2.0
1.5
1.0
0.5
0.0
HC-C/EBP Beta Tg
AD-C/EBP Beta Tg

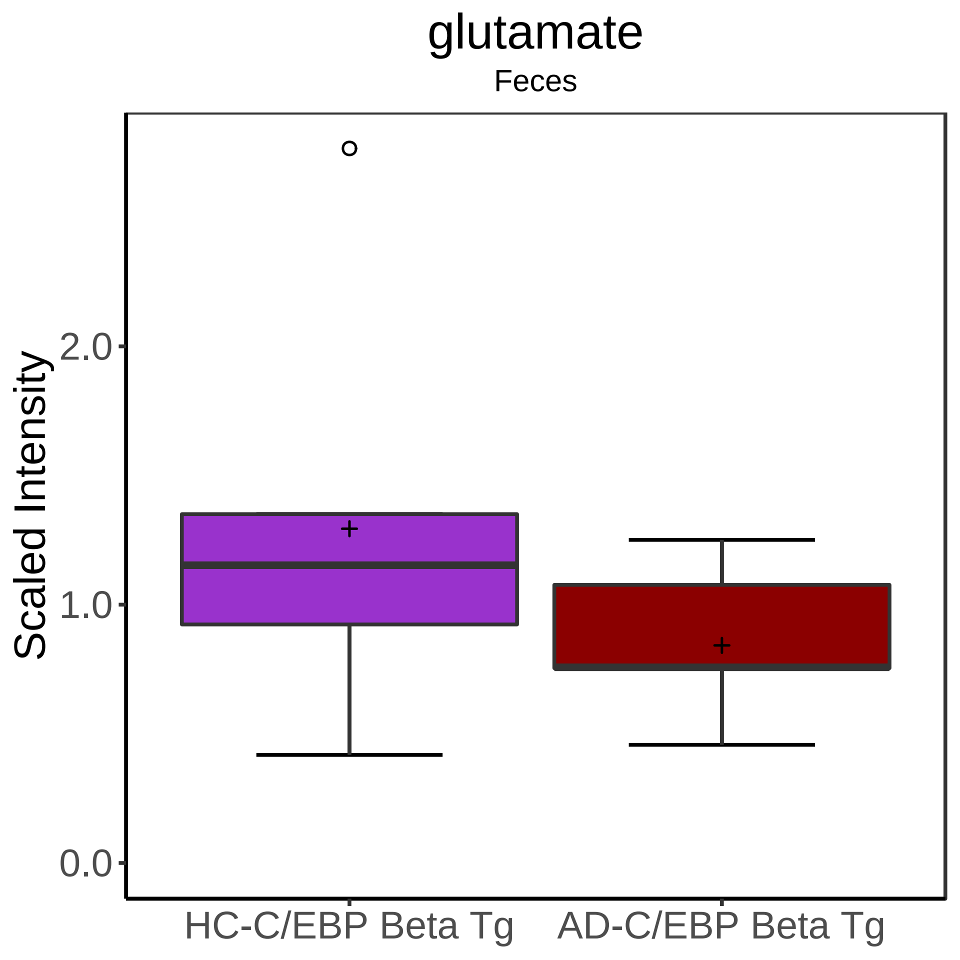
glutamate
Feces
Scaled Intensity
2.0
1.0
0.0
HC-C/EBP Beta Tg
AD-C/EBP Beta Tg

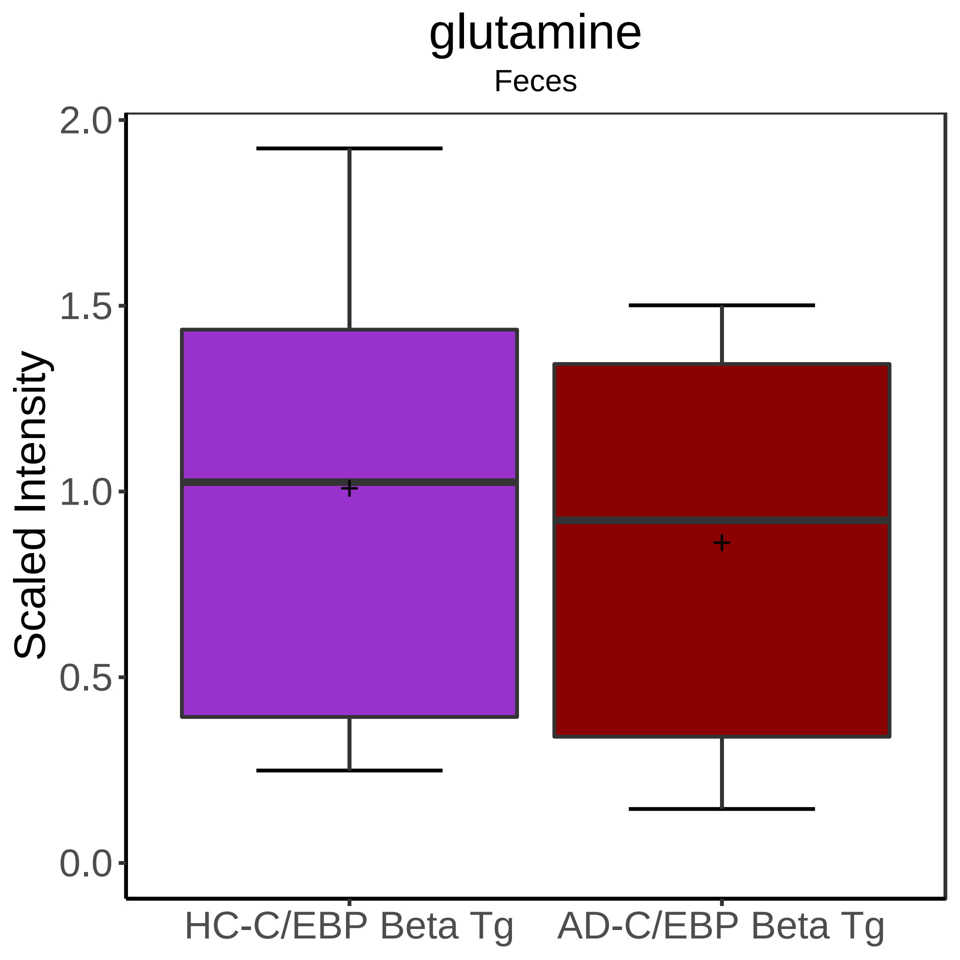

glutamine
Feces
Scaled Intensity
2.0
1.5
1.0
0.5
0.0
HC-C/EBP Beta Tg
AD-C/EBP Beta Tg

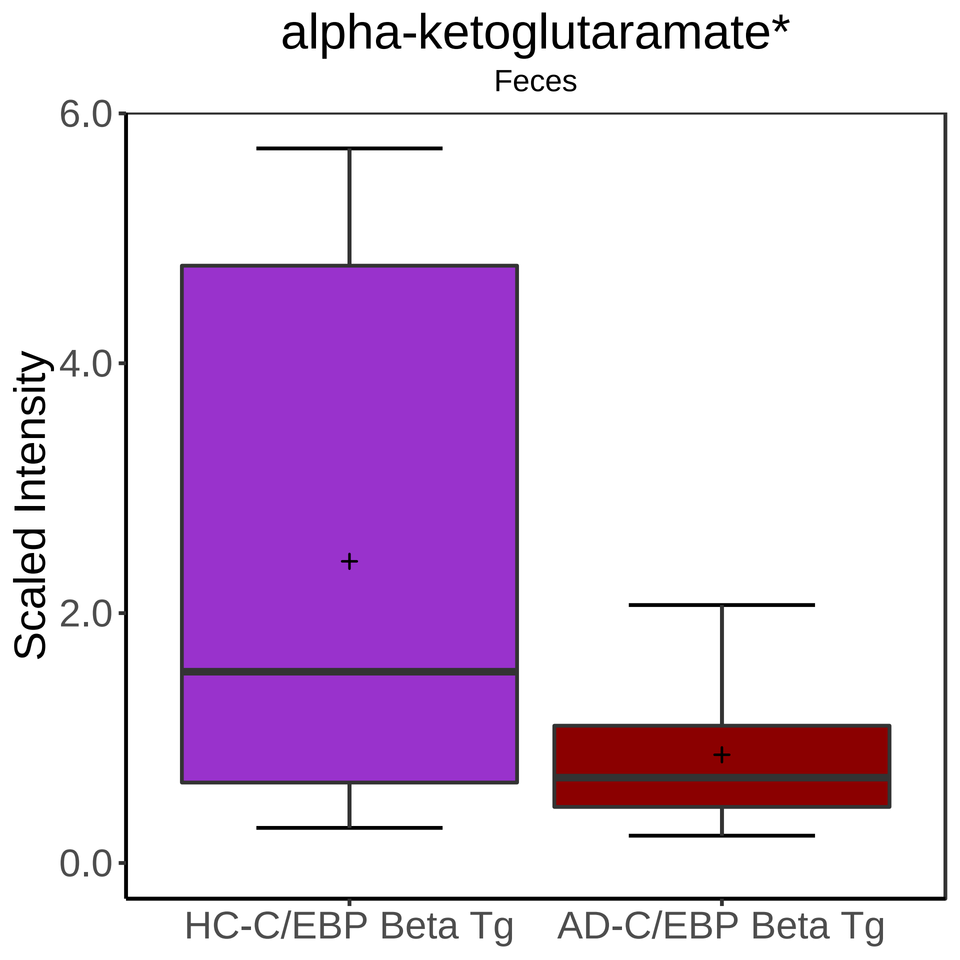
alpha-ketoglutaramate*
Feces
Scaled Intensity
6.0
4.0
2.0
0.0
HC-C/EBP Beta Tg
AD-C/EBP Beta Tg

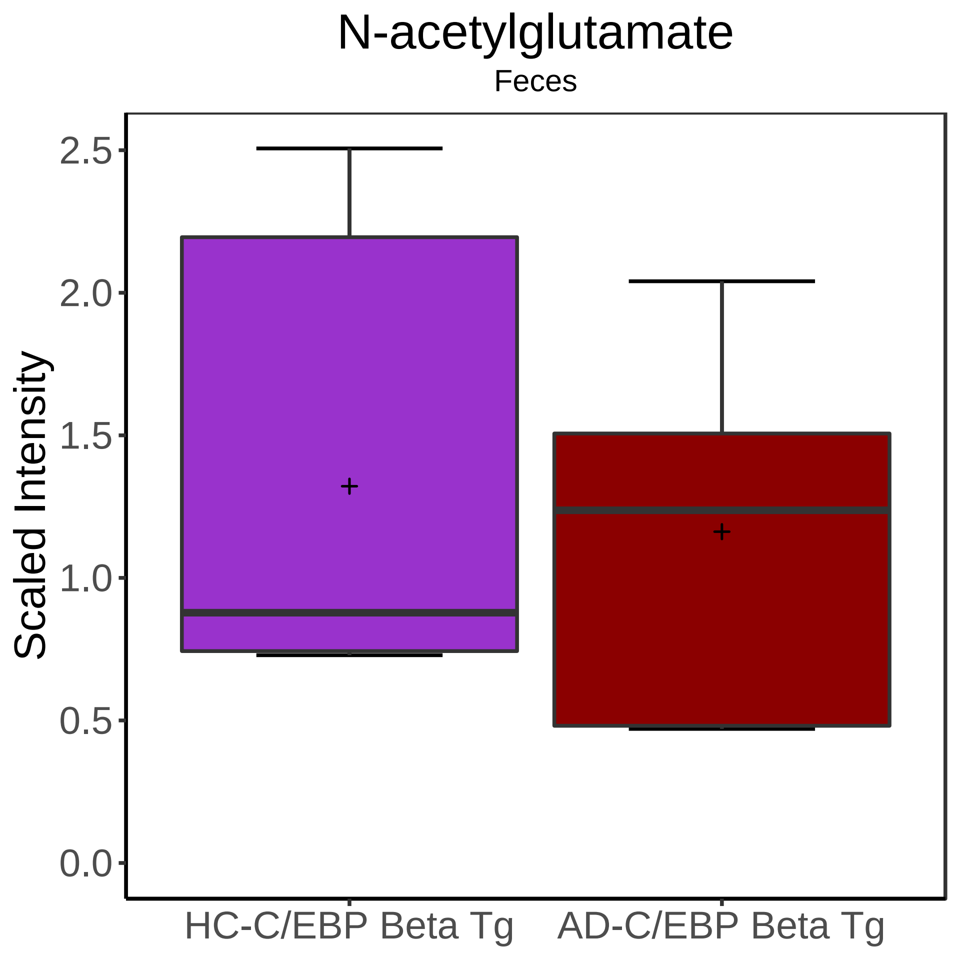
N-acetylglutamate
Feces
Scaled Intensity
2.5
2.0
1.5
1.0
0.5
0.0
HC-C/EBP Beta Tg
AD-C/EBP Beta Tg

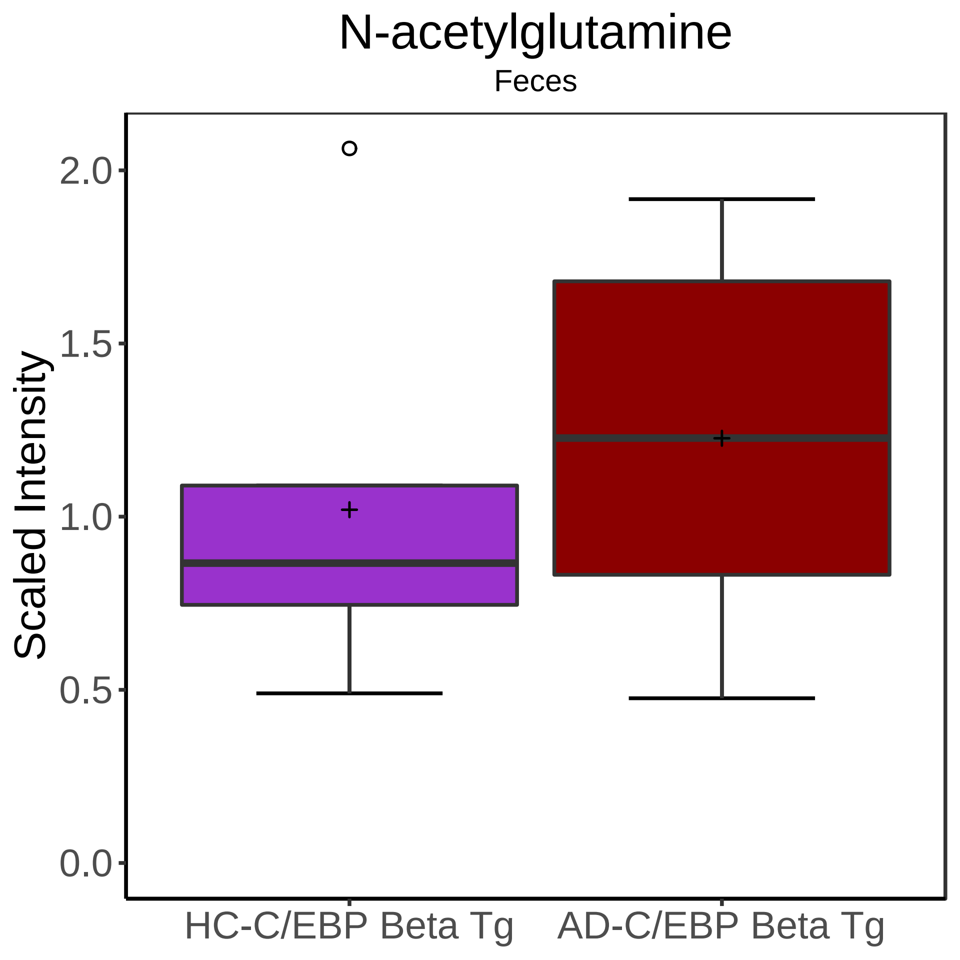
N-acetylglutamine
Feces
Scaled Intensity
2.0
1.5
1.0
0.5
0.0
HC-C/EBP Beta Tg
AD-C/EBP Beta Tg

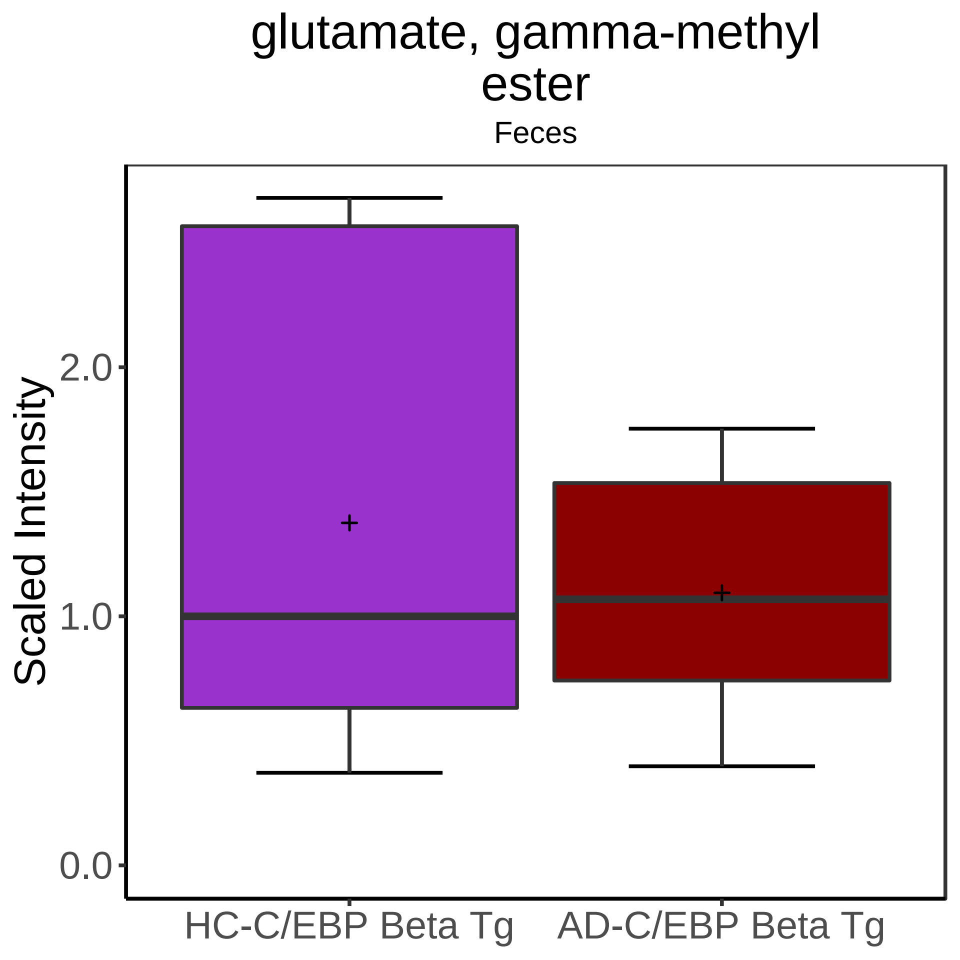
glutamate, gamma-methyl ester
Feces
Scaled Intensity
2.0
1.0
0.0
HC-C/EBP Beta Tg
AD-C/EBP Beta Tg

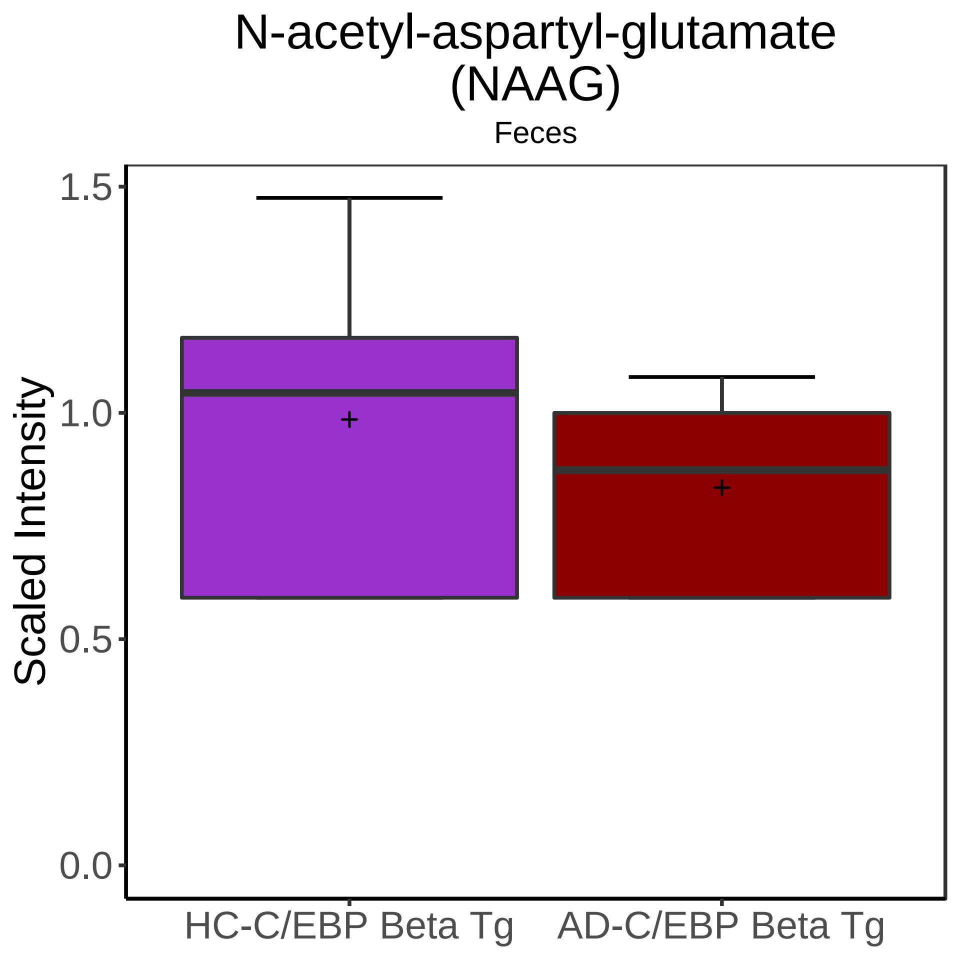
N-acetyl-aspartyl-glutamate
(NAAG)
Feces
Scaled Intensity
1.5
1.0
0.5
0.0
HC-C/EBP Beta Tg
AD-C/EBP Beta Tg

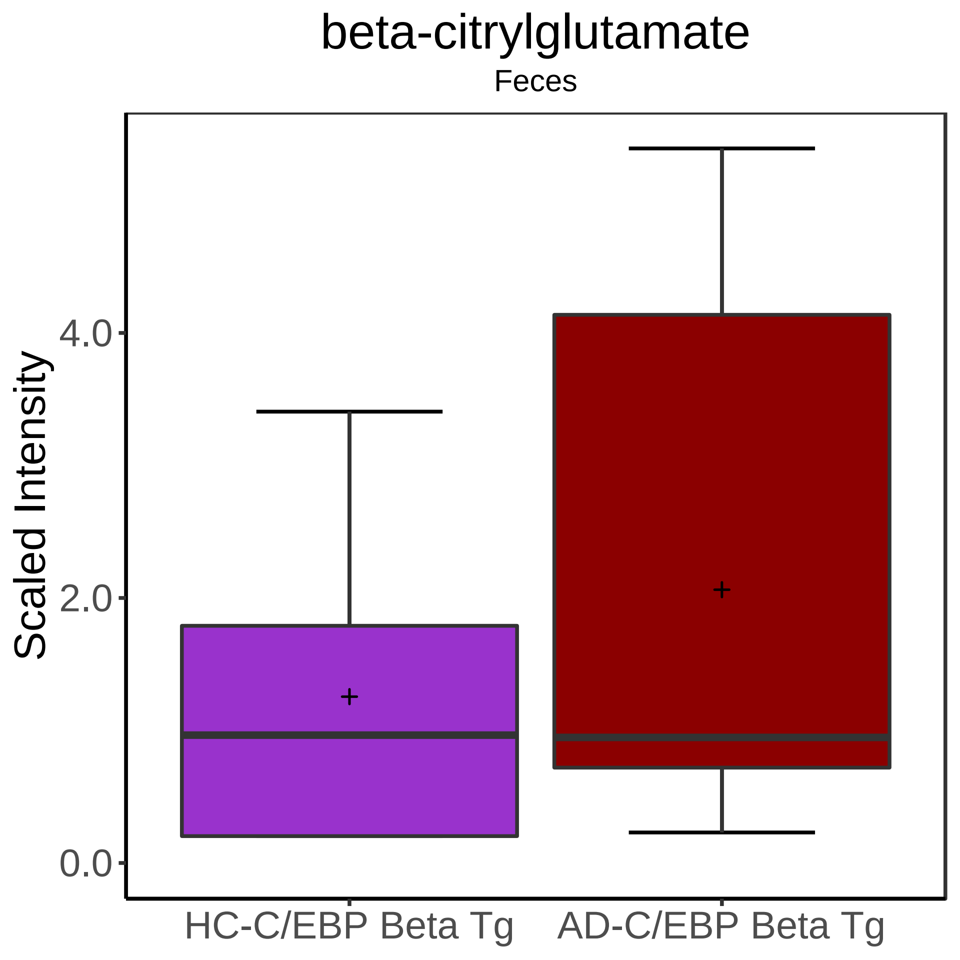
beta-citrylglutamate
Feces
Scaled Intensity
0.0
2.0
4.0
HC-C/EBP Beta Tg
AD-C/EBP Beta Tg

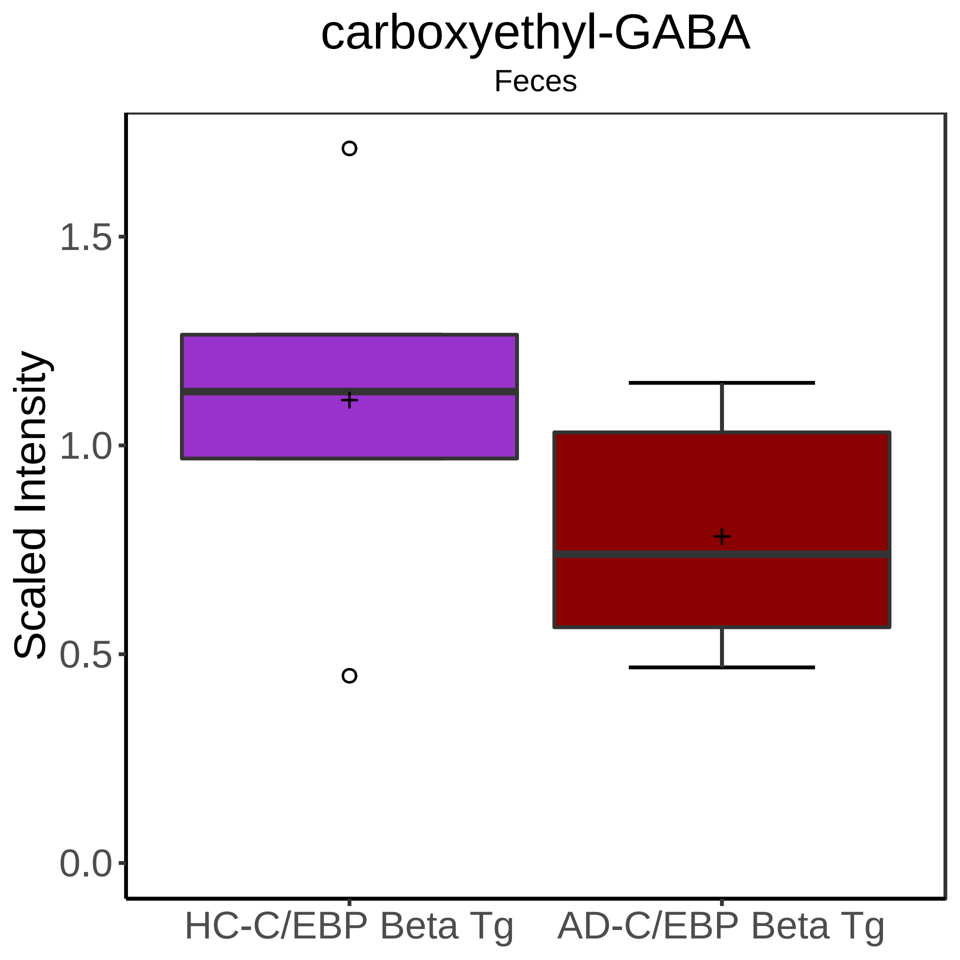
carboxyethyl-GABA
Feces
Scaled Intensity
1.5
1.0
0.5
0.0
HC-C/EBP Beta Tg
AD-C/EBP Beta Tg

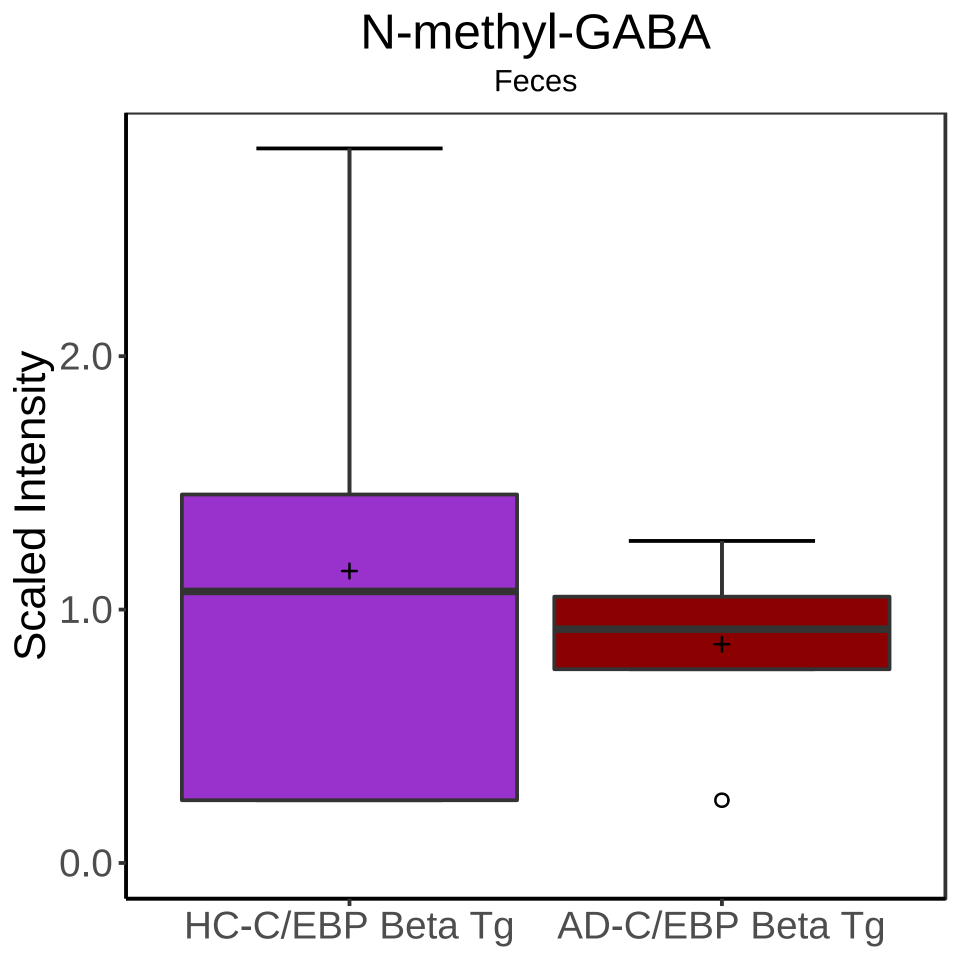
N-methyl-GABA
Feces
Scaled Intensity
2.0
1.0
0.0
HC-C/EBP Beta Tg
AD-C/EBP Beta Tg

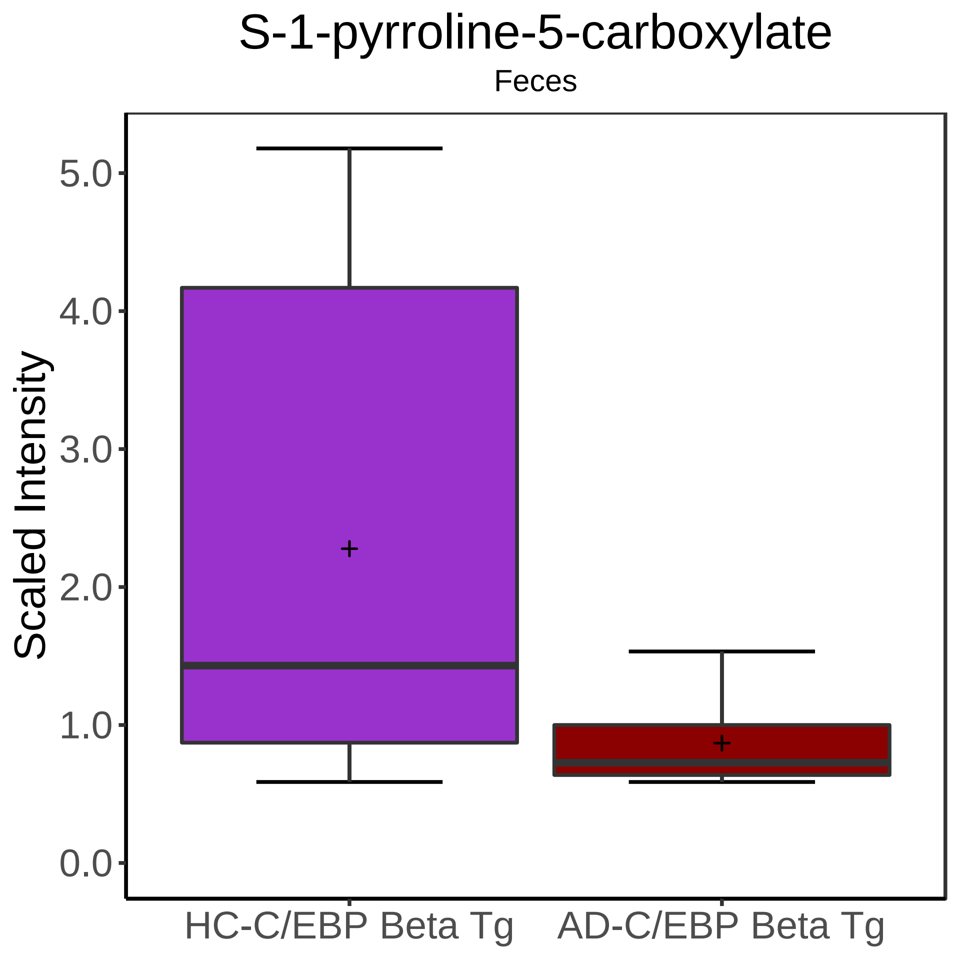
S-1-pyrroline-5-carboxylate
Feces
Scaled Intensity
5.0
4.0
3.0
2.0
1.0
0.0
HC-C/EBP Beta Tg
AD-C/EBP Beta Tg

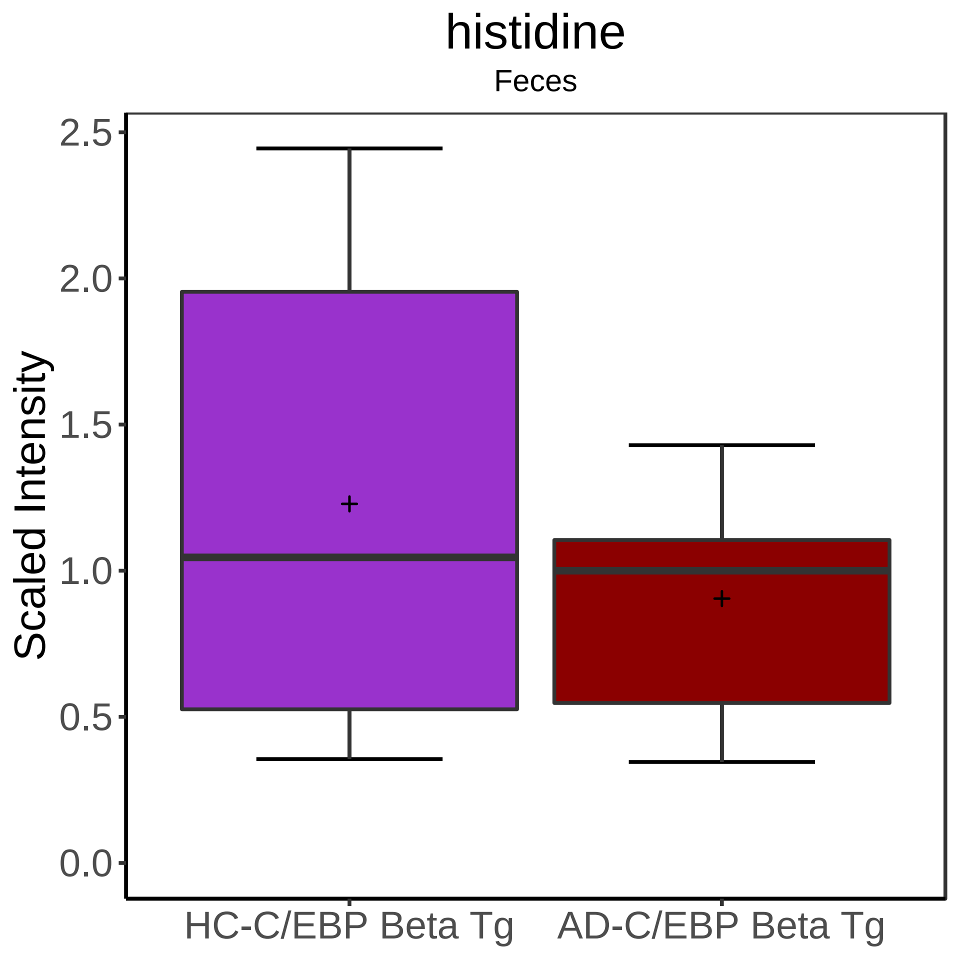
histidine
Feces
Scaled Intensity
2.5
2.0
1.5
1.0
0.5
0.0
HC-C/EBP Beta Tg
AD-C/EBP Beta Tg

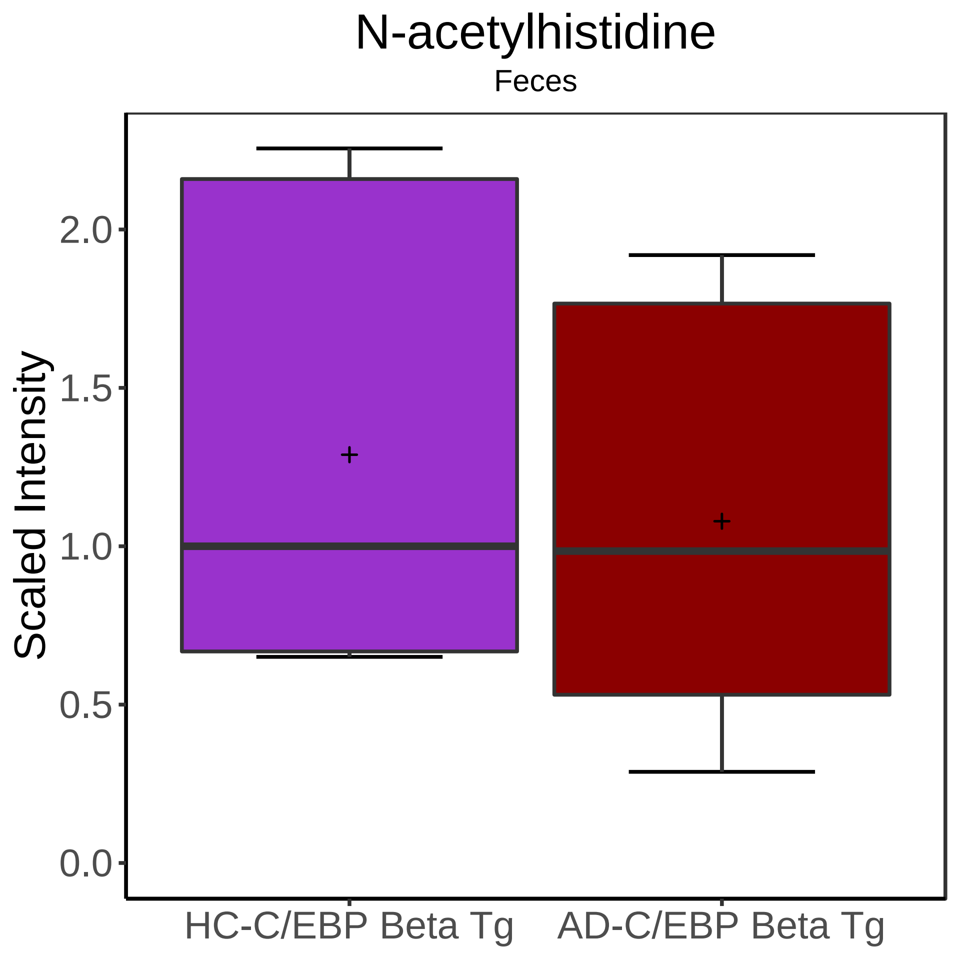
N-acetylhistidine
Feces
Scaled Intensity
2.0
1.5
1.0
0.5
0.0
HC-C/EBP Beta Tg
AD-C/EBP Beta Tg

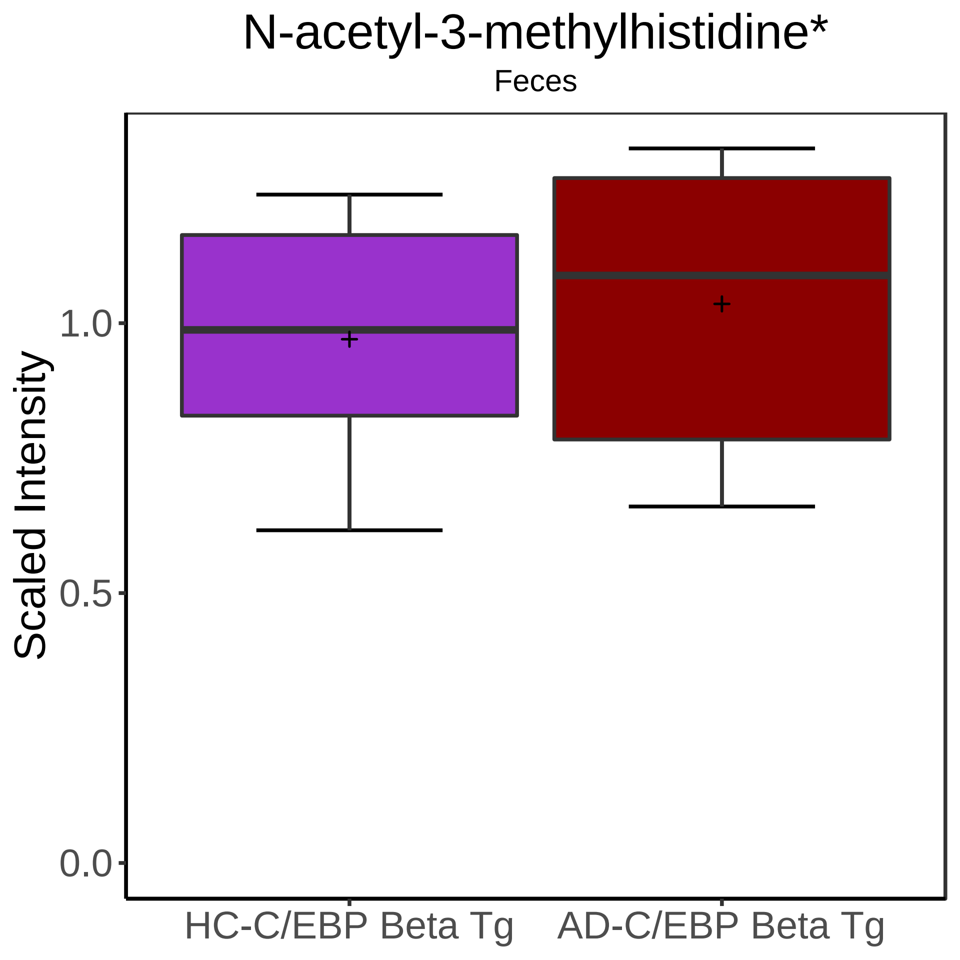
N-acetyl-3-methylhistidine*
Feces
Scaled Intensity
1.0
0.5
0.0
HC-C/EBP Beta Tg
AD-C/EBP Beta Tg

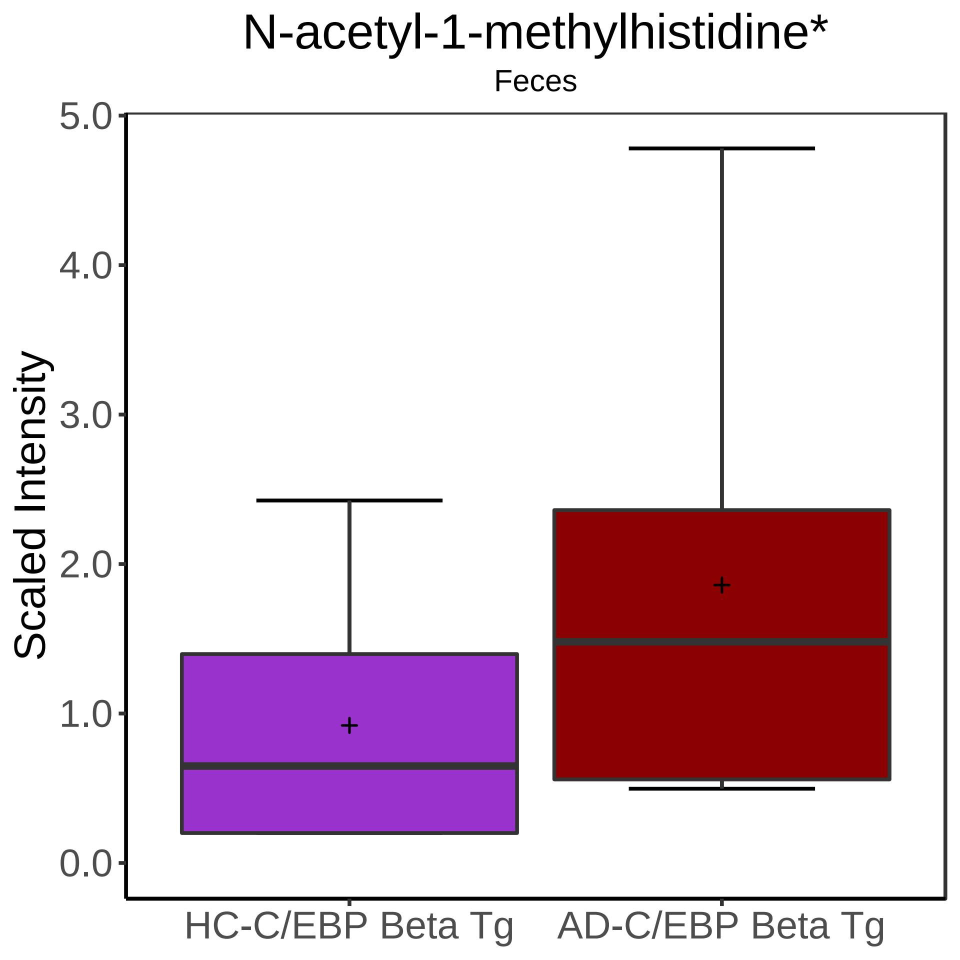
N-acetyl-1-methylhistidine*
Feces
Scaled Intensity
5.0
4.0
3.0
2.0
1.0
0.0
HC-C/EBP Beta Tg
AD-C/EBP Beta Tg

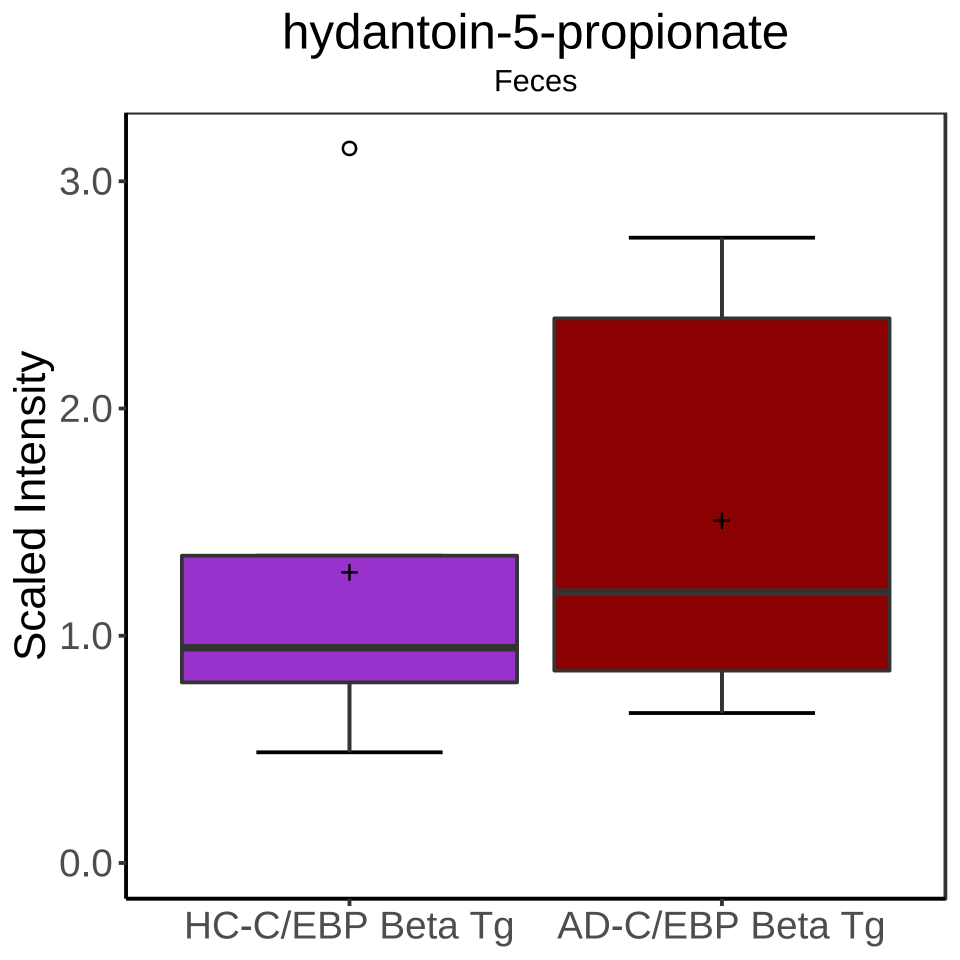

hydantoin-5-propionate
Feces
Scaled Intensity
3.0
2.0
1.0
0.0
HC-C/EBP Beta Tg
AD-C/EBP Beta Tg

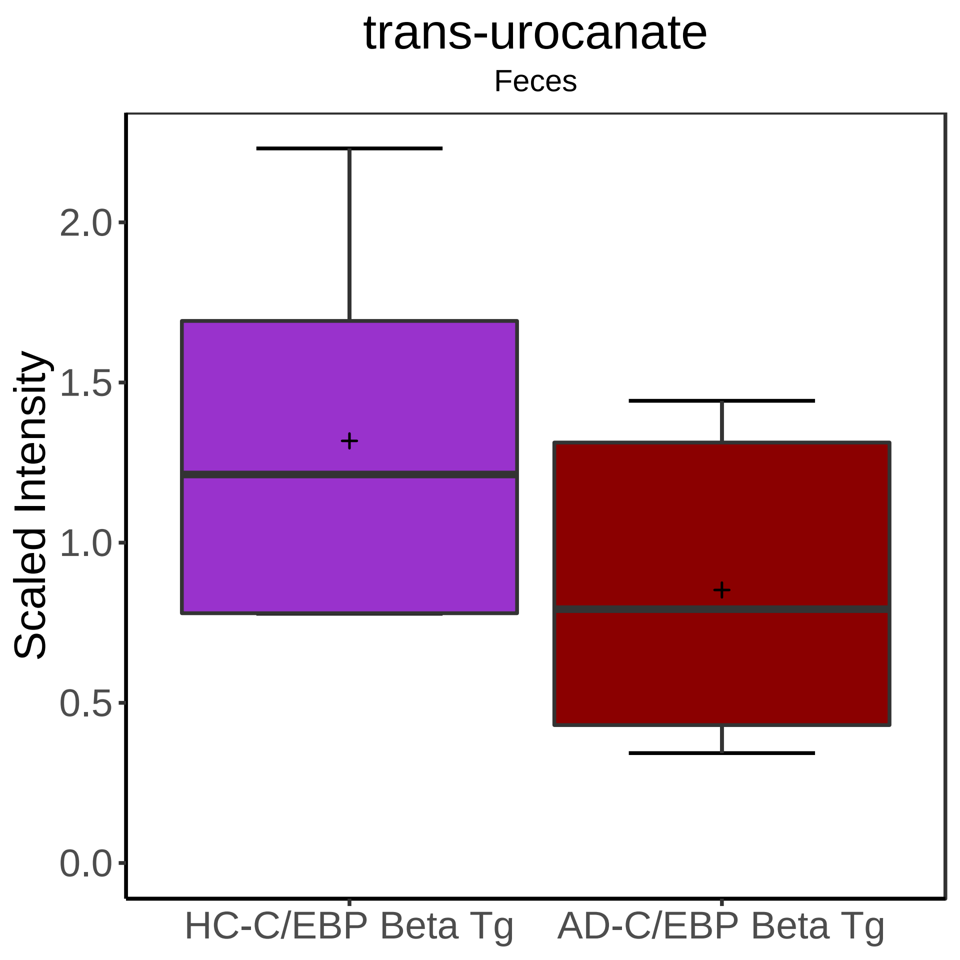
trans-urocanate
Feces
Scaled Intensity
2.0
1.5
1.0
0.5
0.0
HC-C/EBP Beta Tg
AD-C/EBP Beta Tg

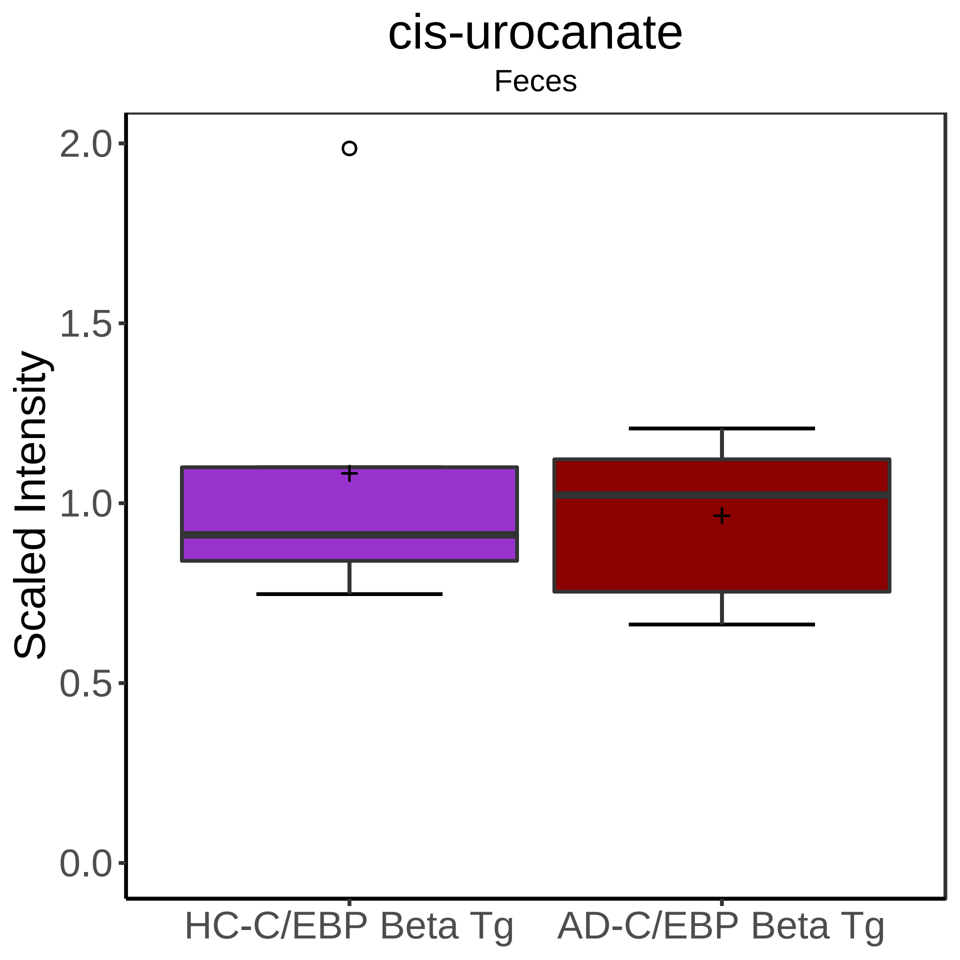
cis-urocanate
Feces
Scaled Intensity
2.0
1.5
1.0
0.5
0.0
HC-C/EBP Beta Tg
AD-C/EBP Beta Tg

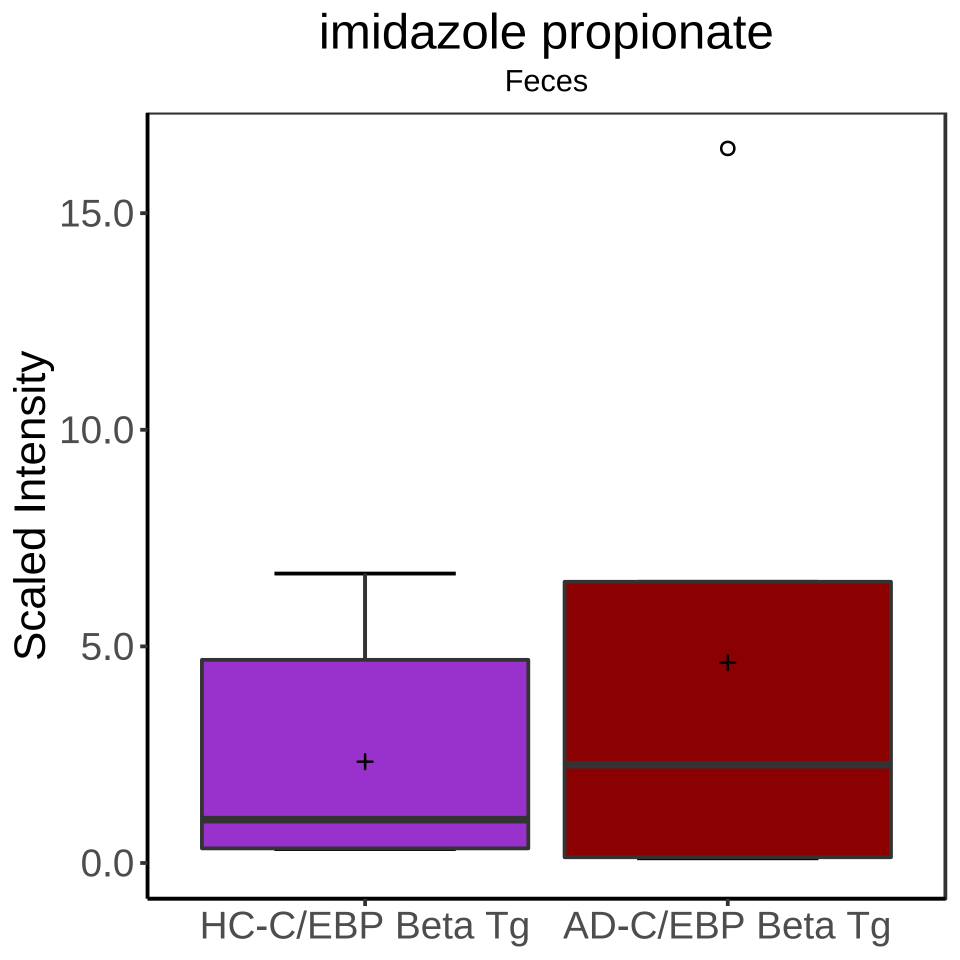

imidazole propionate
Feces
Scaled Intensity
15.0
10.0
5.0
0.0
HC-C/EBP Beta Tg
AD-C/EBP Beta Tg

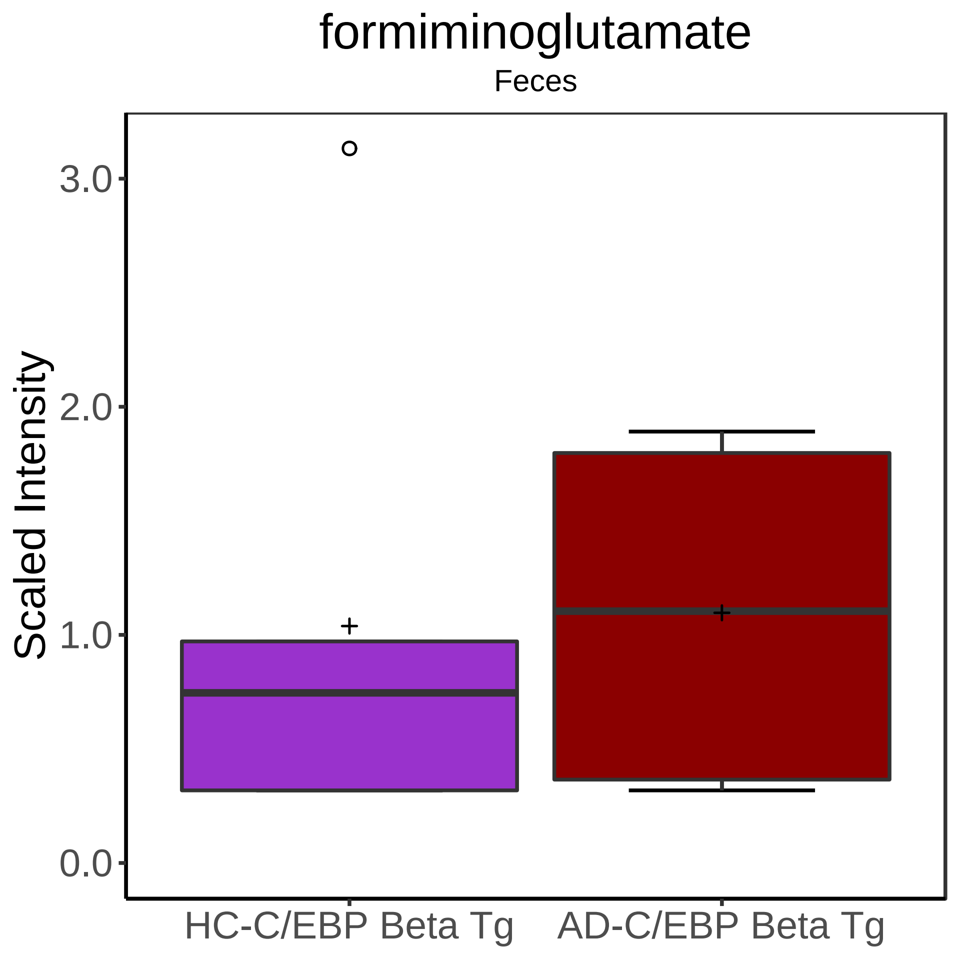
formiminoglutamate
Feces
Scaled Intensity
3.0
2.0
1.0
0.0
HC-C/EBP Beta Tg
AD-C/EBP Beta Tg

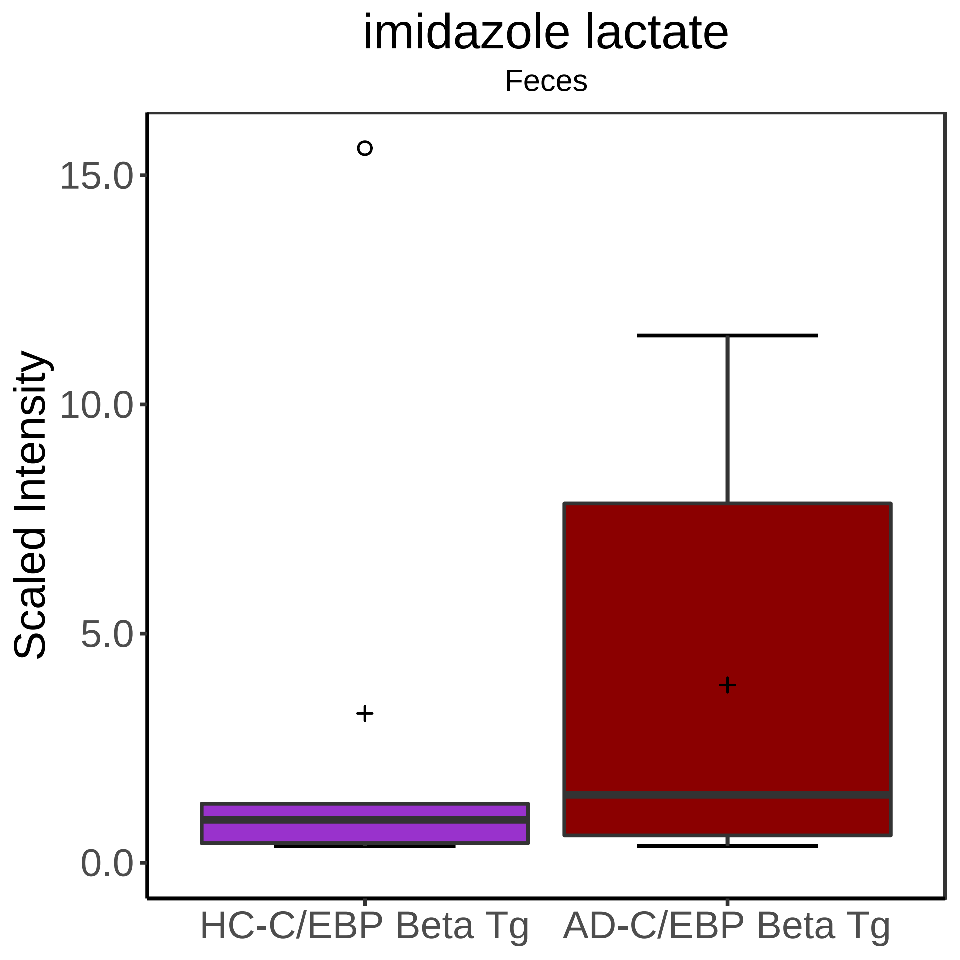

imidazole lactate
Feces
Scaled Intensity
15.0
10.0
5.0
0.0
HC-C/EBP Beta Tg
AD-C/EBP Beta Tg

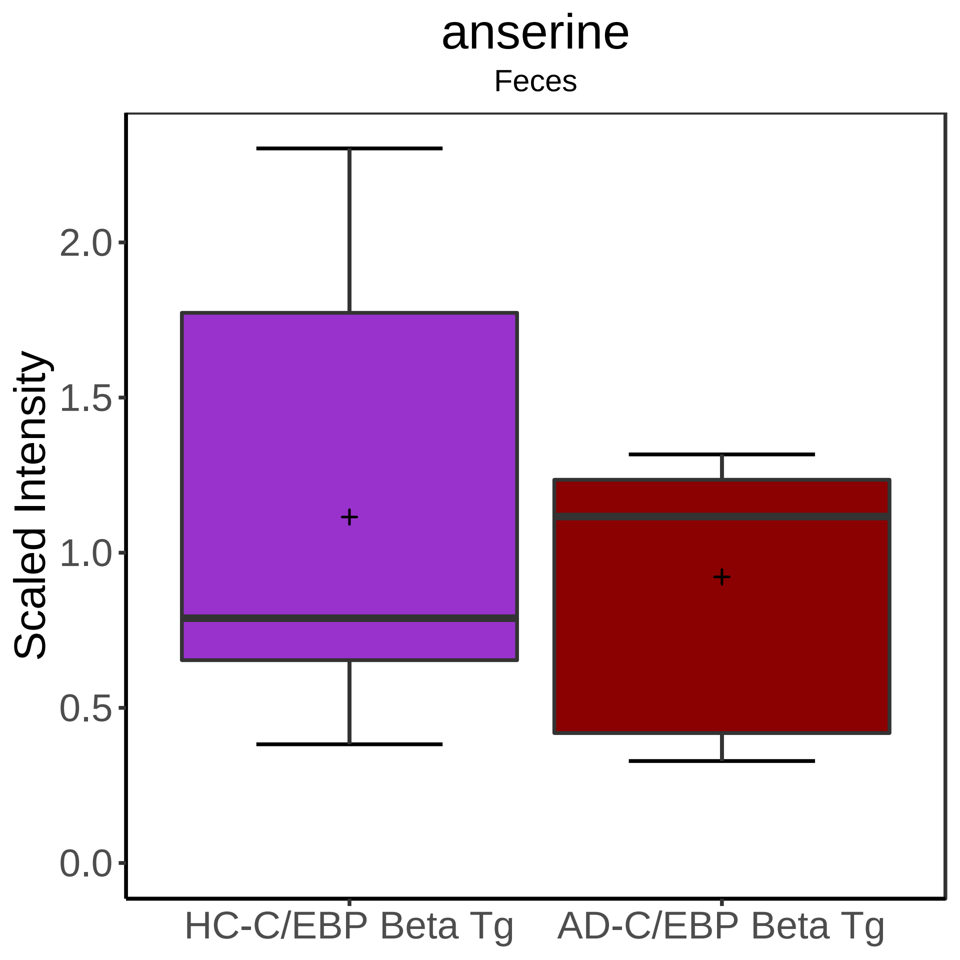
anserine
Feces
Scaled Intensity
2.0
1.5
1.0
0.5
0.0
HC-C/EBP Beta Tg
AD-C/EBP Beta Tg

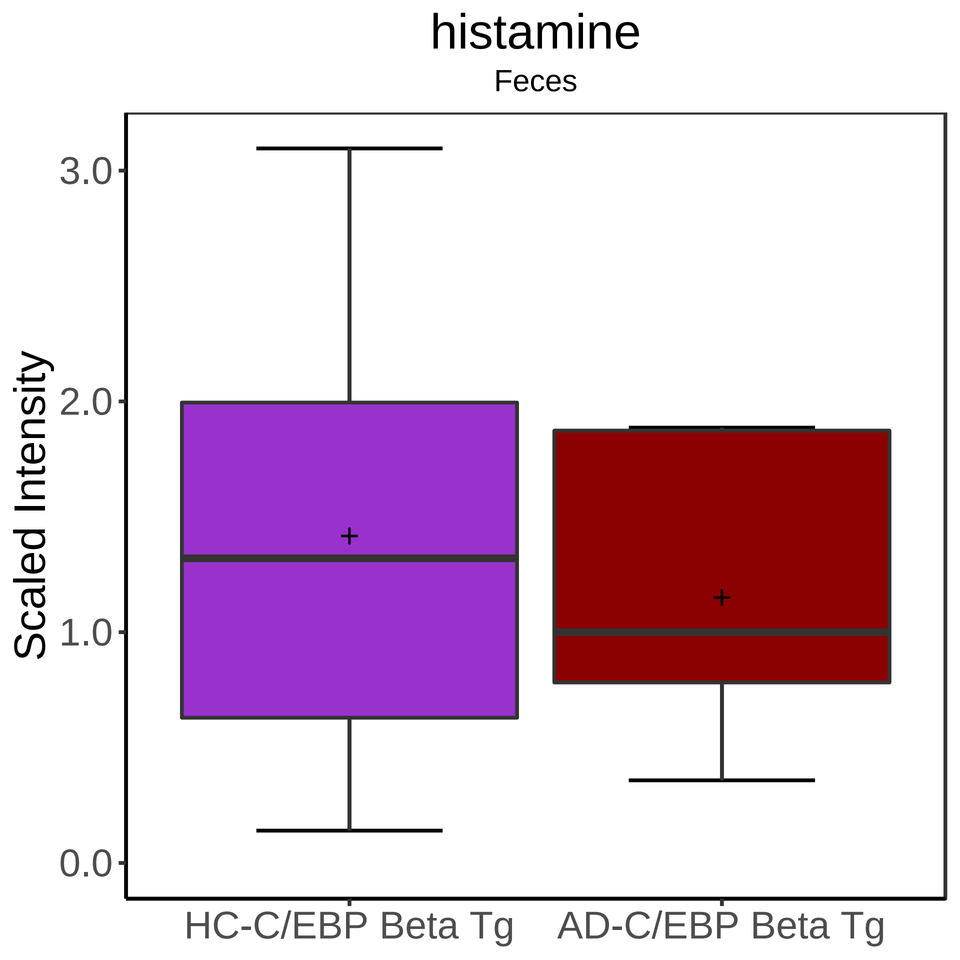

histamine
Feces
Scaled Intensity
3.0
2.0
1.0
0.0
HC-C/EBP Beta Tg
AD-C/EBP Beta Tg

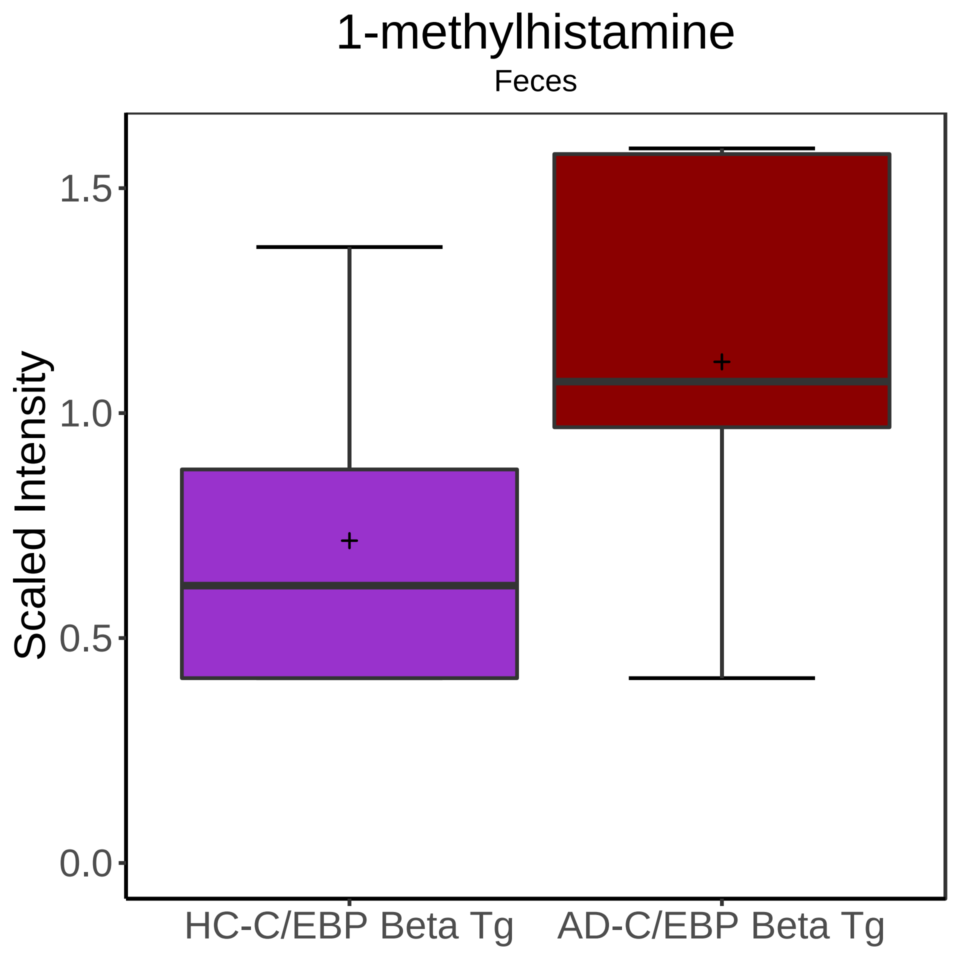
1-methylhistamine
Feces
Scaled Intensity
1.5
1.0
0.5
0.0
HC-C/EBP Beta Tg
AD-C/EBP Beta Tg

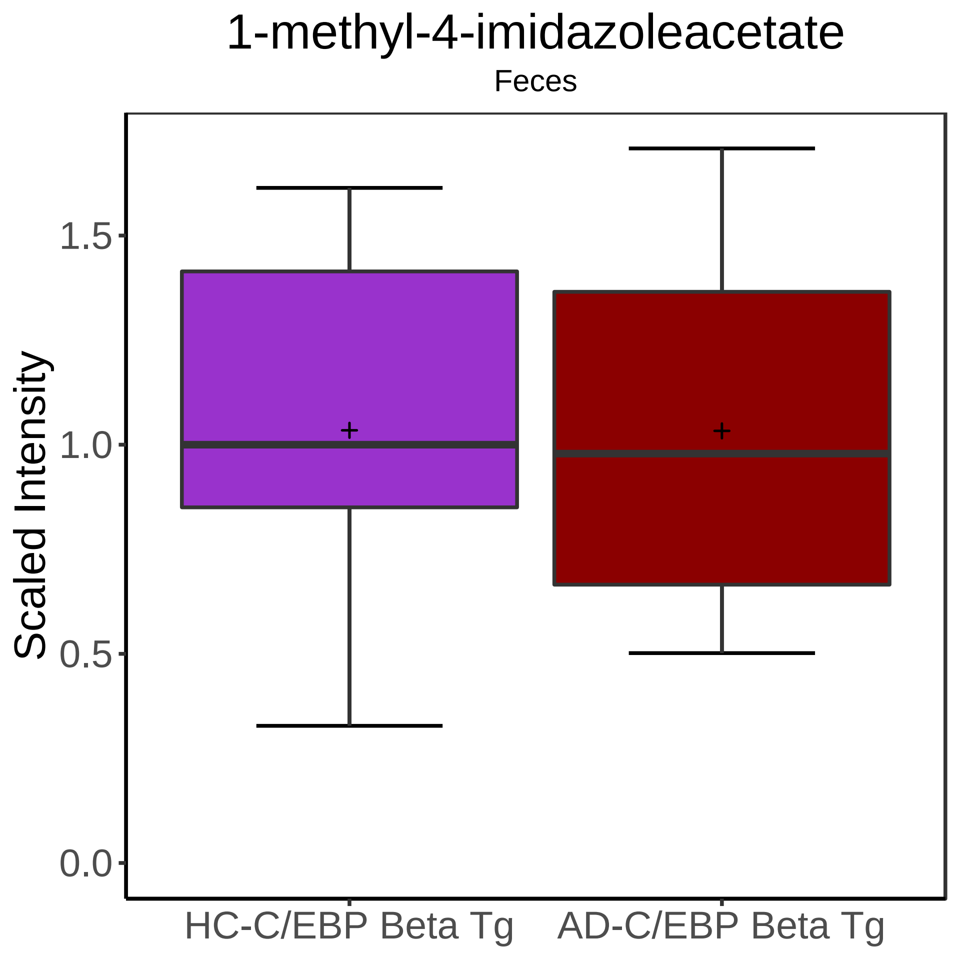

1-methyl-4-imidazoleacetate
Feces
Scaled Intensity
1.5
1.0
0.5
0.0
HC-C/EBP Beta Tg
AD-C/EBP Beta Tg

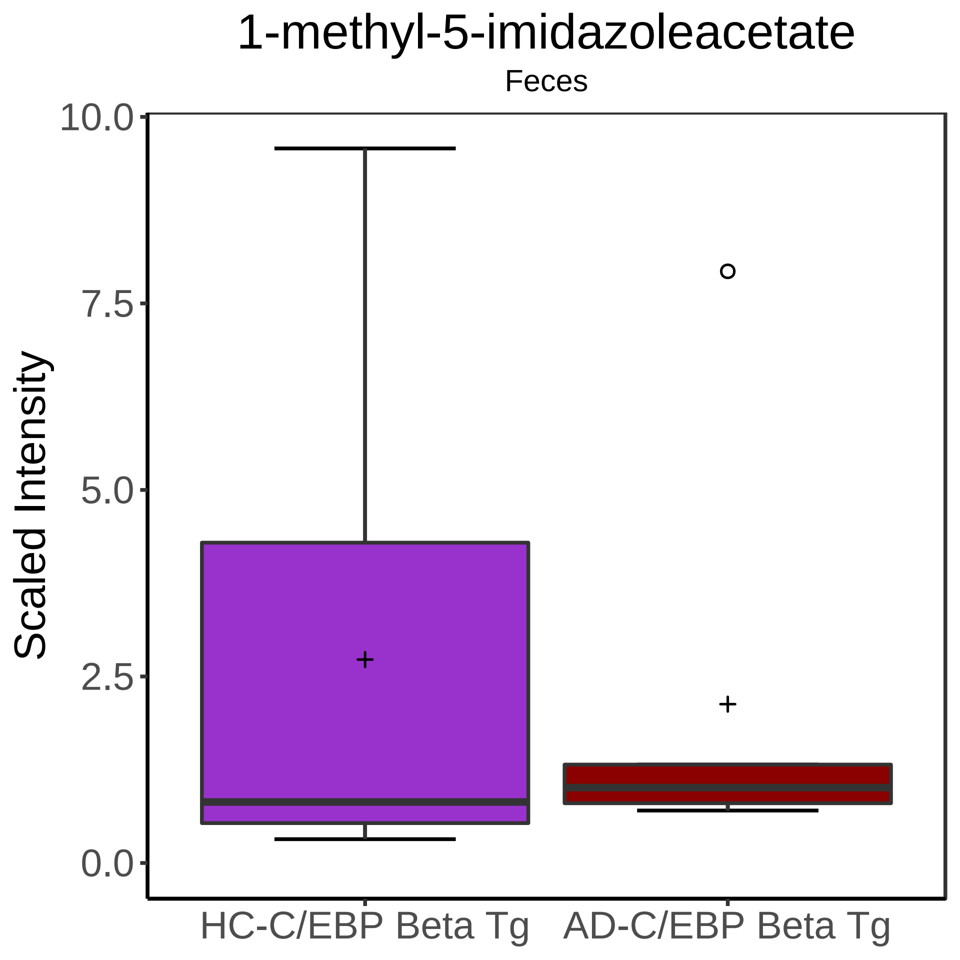
1-methyl-5-imidazoleacetate
Feces
Scaled Intensity
10.0
7.5
5.0
2.5
0.0
HC-C/EBP Beta Tg
AD-C/EBP Beta Tg

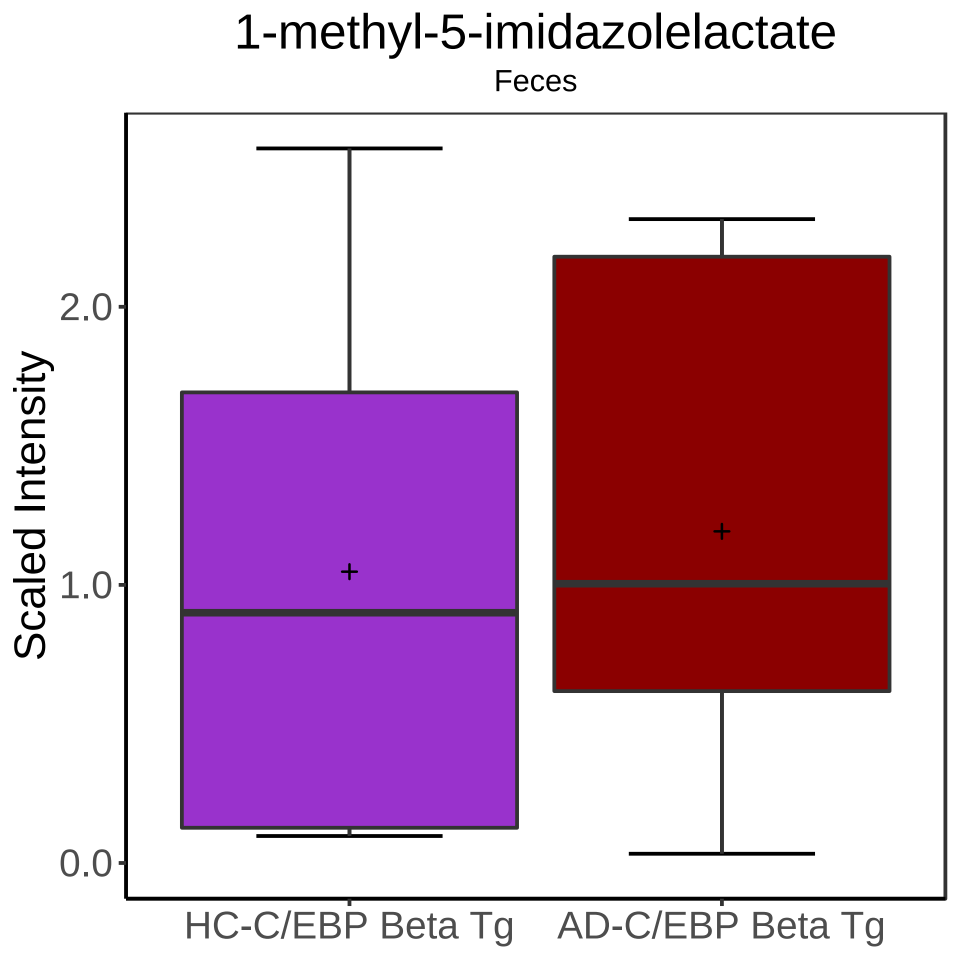

1-methyl-5-imidazolelactate
Feces
Scaled Intensity
2.0
1.0
0.0
HC-C/EBP Beta Tg
AD-C/EBP Beta Tg

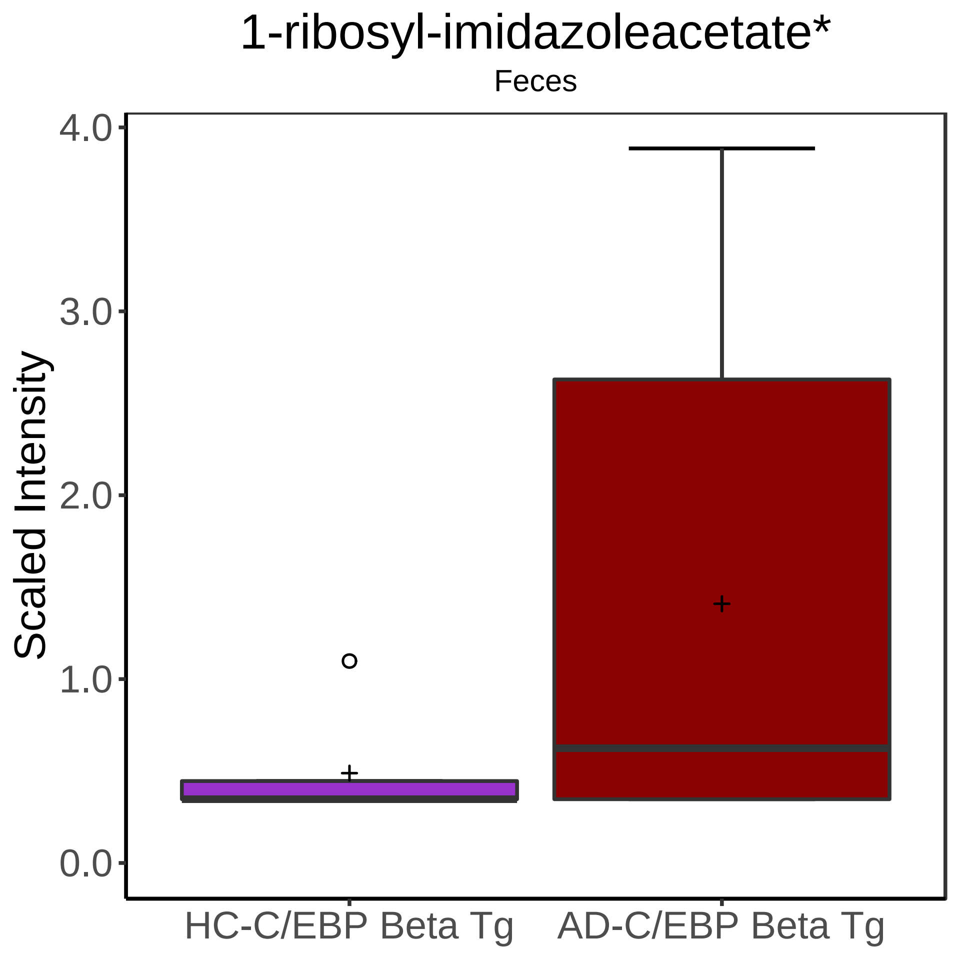

1-ribosyl-imidazoleacetate*
Feces
Scaled Intensity
4.0
3.0
2.0
1.0
0.0
HC-C/EBP Beta Tg
AD-C/EBP Beta Tg

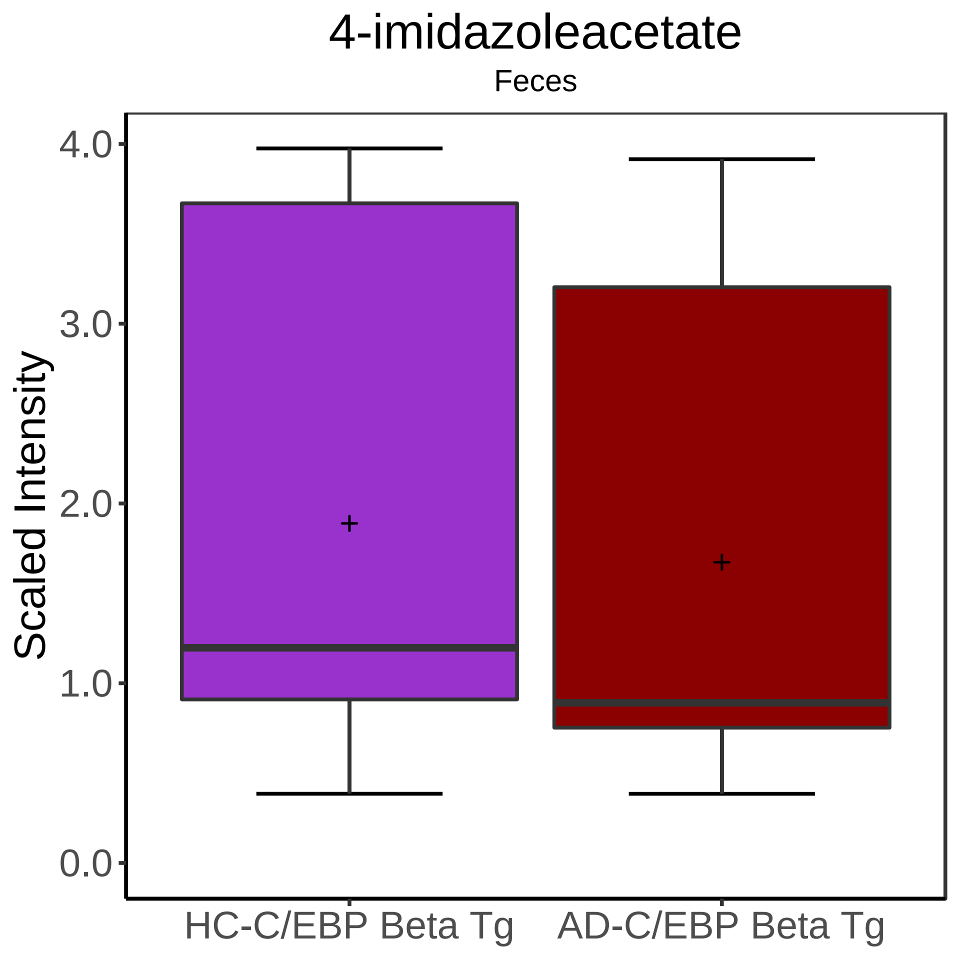

4-imidazoleacetate
Feces
Scaled Intensity
4.0
3.0
2.0
1.0
0.0
HC-C/EBP Beta Tg
AD-C/EBP Beta Tg

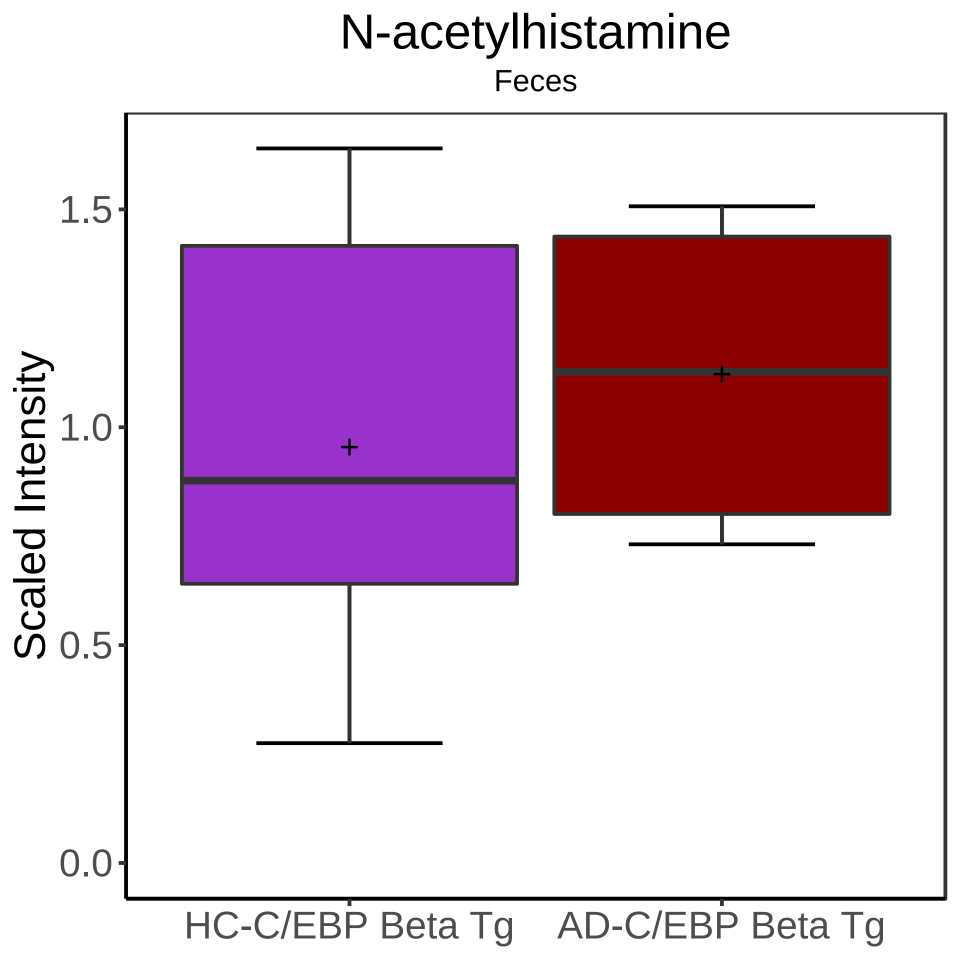
N-acetylhistamine
Feces
Scaled Intensity
1.5
1.0
0.5
0.0
HC-C/EBP Beta Tg
AD-C/EBP Beta Tg

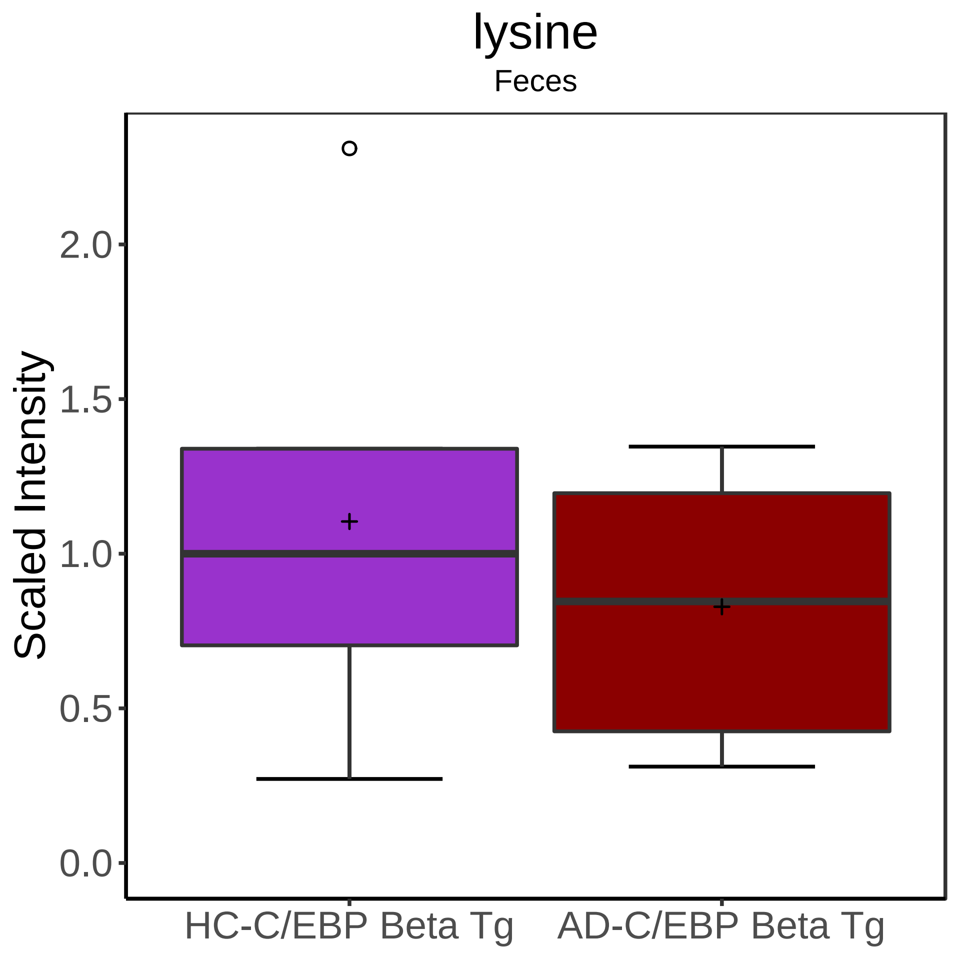

lysine
Feces
Scaled Intensity
2.0
1.5
1.0
0.5
0.0
HC-C/EBP Beta Tg
AD-C/EBP Beta Tg

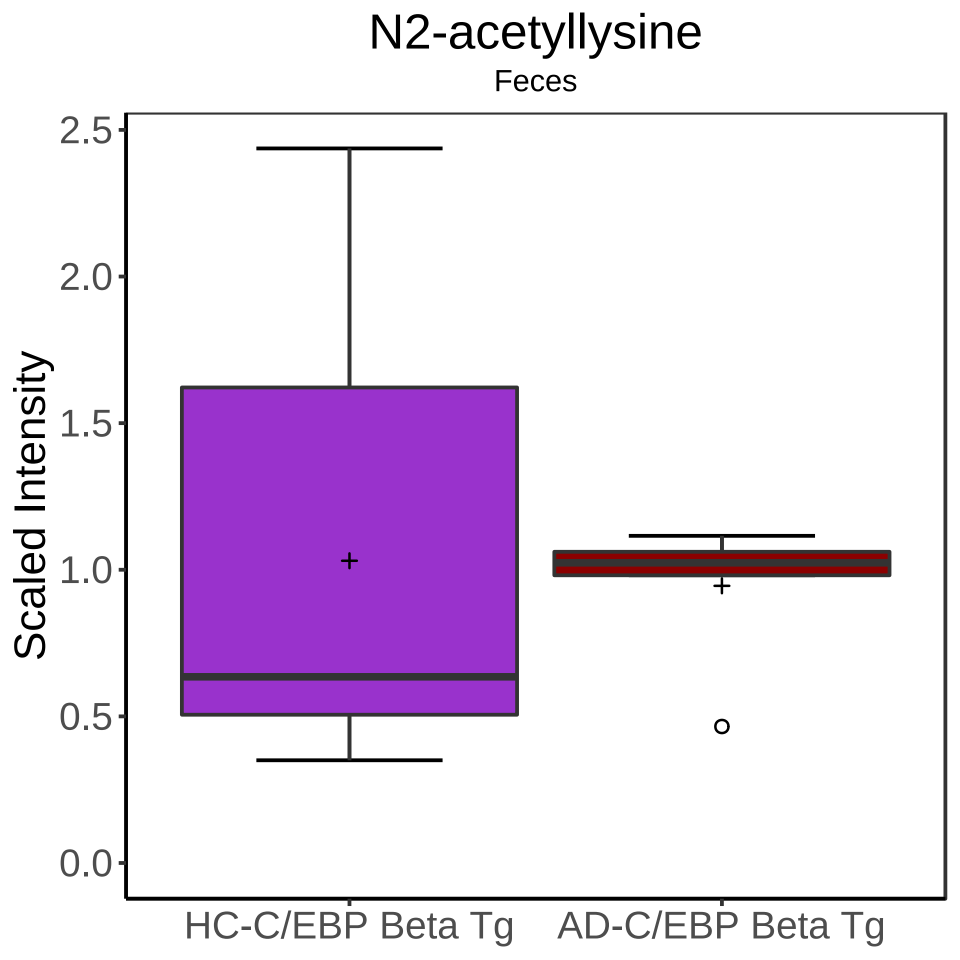
N2-acetyllysine
Feces
Scaled Intensity
2.5
2.0
1.5
1.0
0.5
0.0
HC-C/EBP Beta Tg
AD-C/EBP Beta Tg

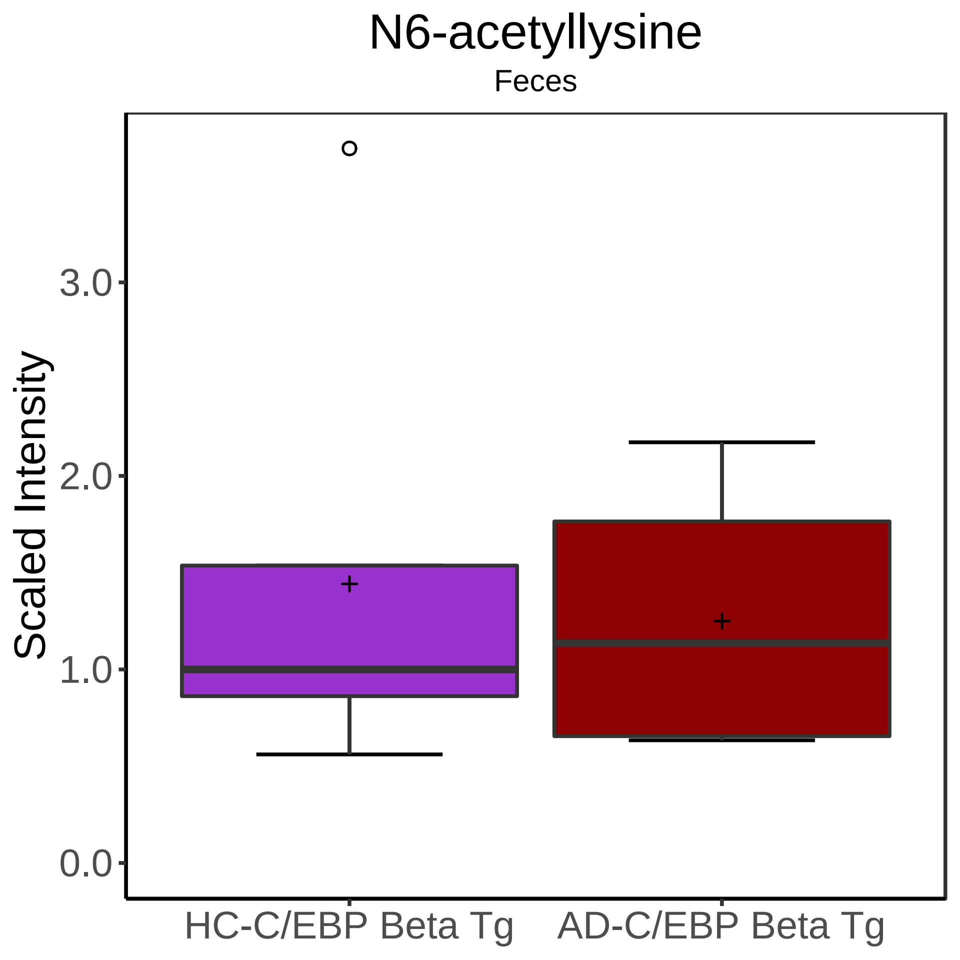

N6-acetyllysine
Feces
Scaled Intensity
3.0
2.0
1.0
0.0
HC-C/EBP Beta Tg
AD-C/EBP Beta Tg

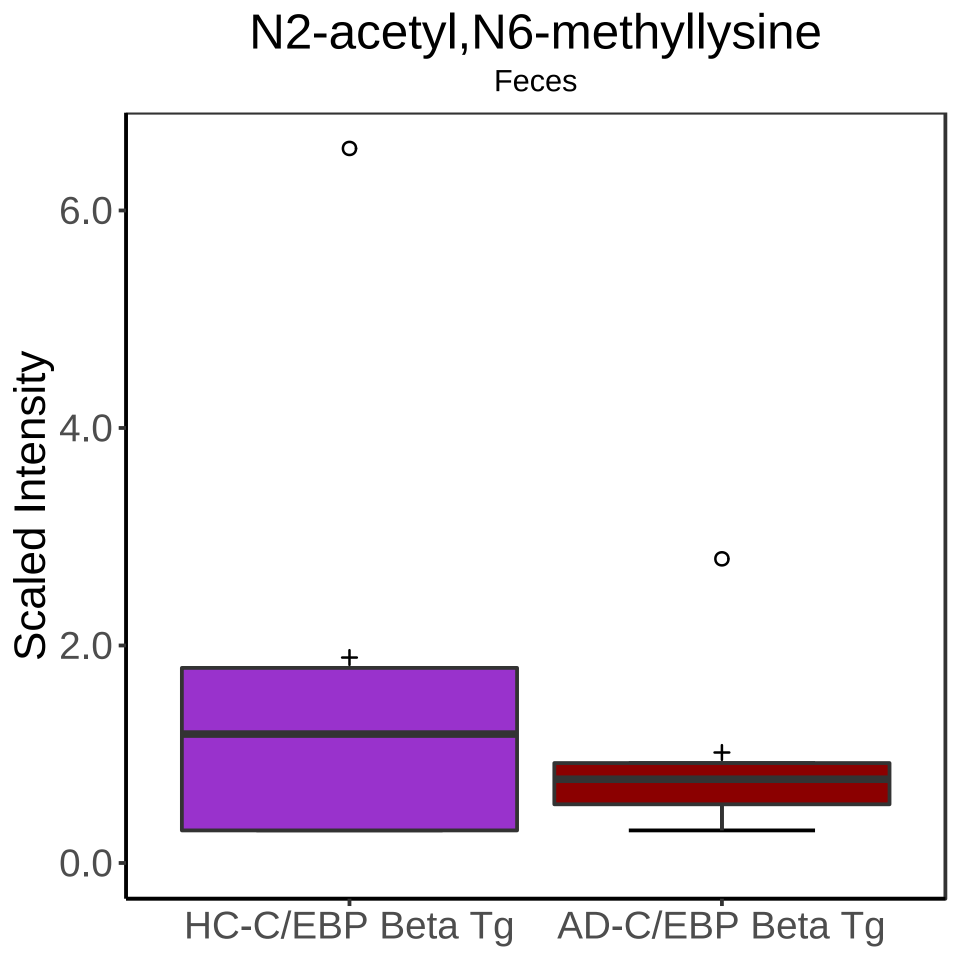
N2-acetyl,N6-methyllysine
Feces
Scaled Intensity
6.0
4.0
2.0
0.0
HC-C/EBP Beta Tg
AD-C/EBP Beta Tg

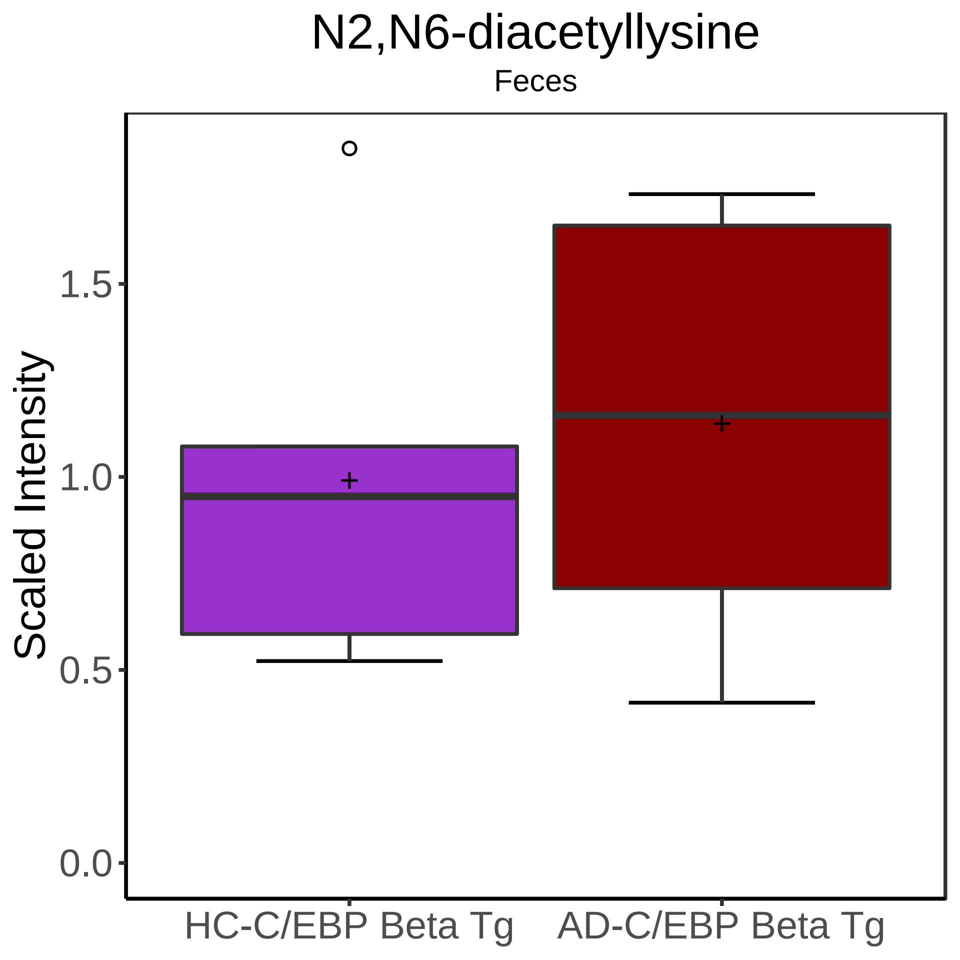
N2,N6-diacetyllysine
Feces
Scaled Intensity
0.0
0.5
1.0
1.5
HC-C/EBP Beta Tg
AD-C/EBP Beta Tg

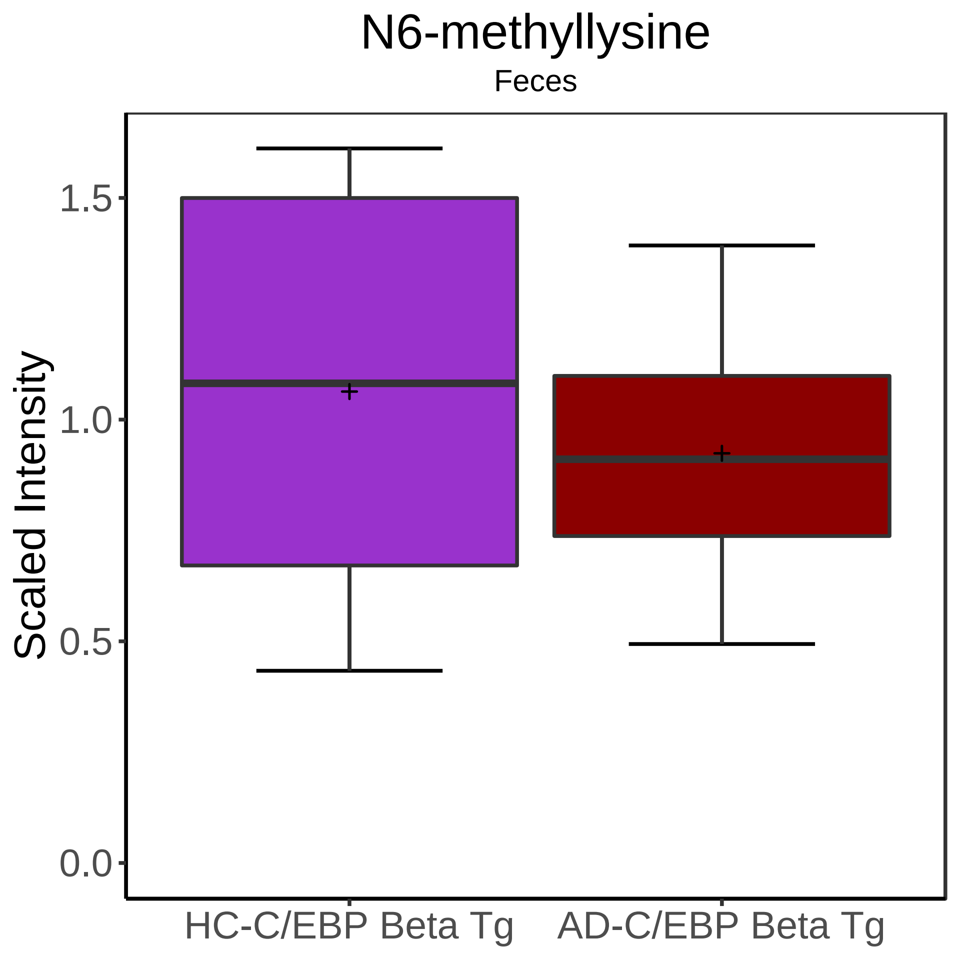
N6-methyllysine
Feces
Scaled Intensity
1.5
1.0
0.5
0.0
HC-C/EBP Beta Tg
AD-C/EBP Beta Tg

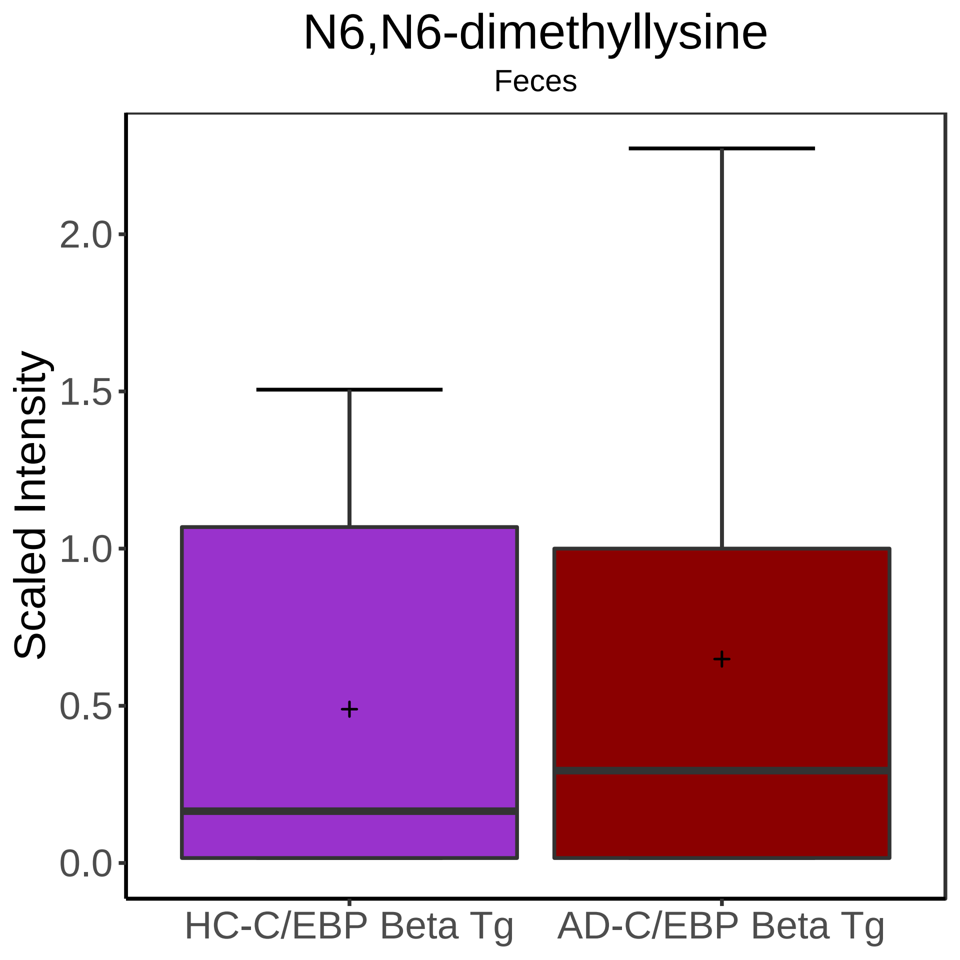
N6,N6-dimethyllysine
Feces
Scaled Intensity
2.0
1.5
1.0
0.5
0.0
HC-C/EBP Beta Tg
AD-C/EBP Beta Tg

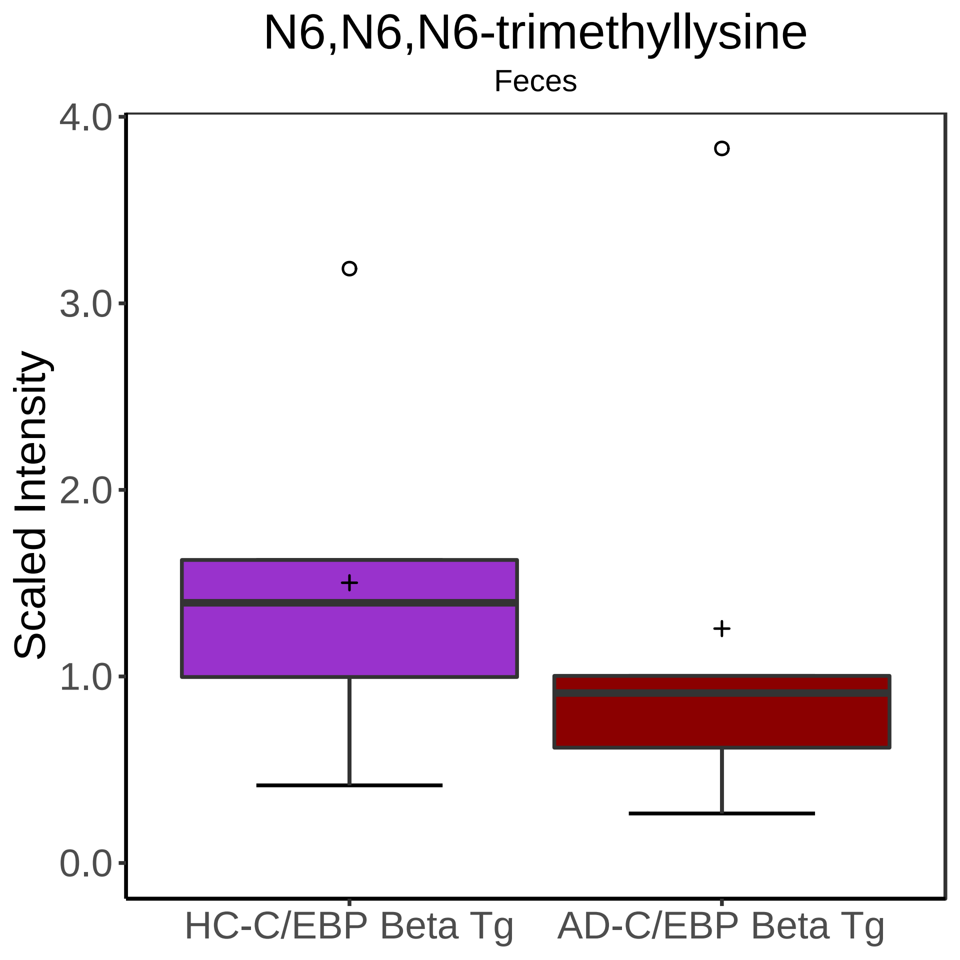
N6,N6,N6-trimethyllysine
Feces
Scaled Intensity
4.0
3.0
2.0
1.0
0.0
HC-C/EBP Beta Tg
AD-C/EBP Beta Tg

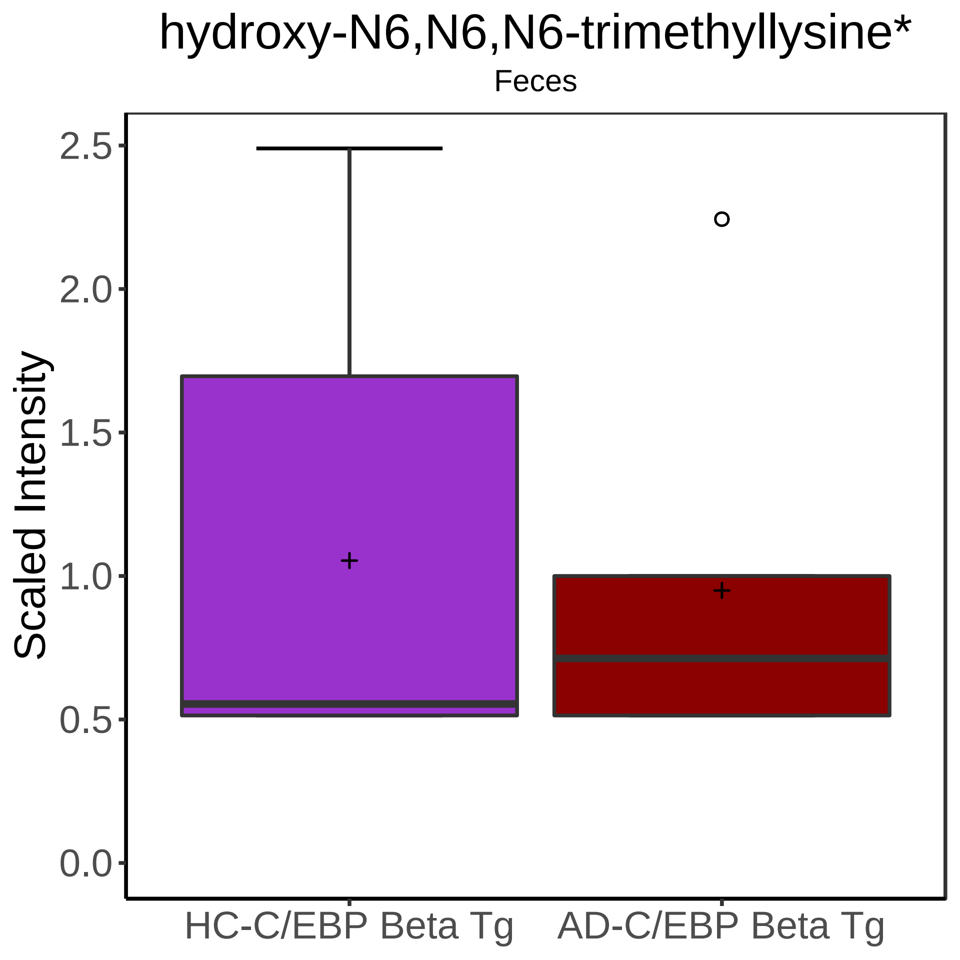
hydroxy-N6,N6,N6-trimethyllysine*
Feces
Scaled Intensity
2.5
2.0
1.5
1.0
0.5
0.0
HC-C/EBP Beta Tg
AD-C/EBP Beta Tg

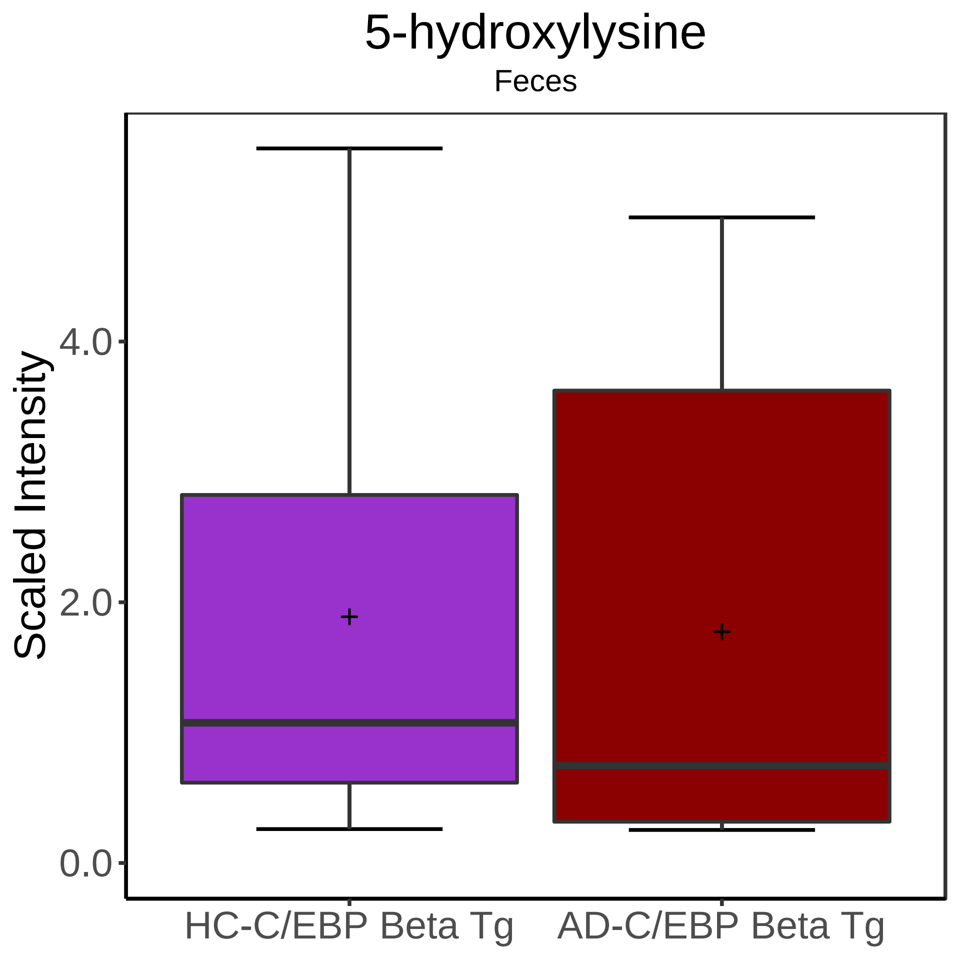
5-hydroxylysine
Feces
Scaled Intensity
4.0
2.0
0.0
HC-C/EBP Beta Tg
AD-C/EBP Beta Tg

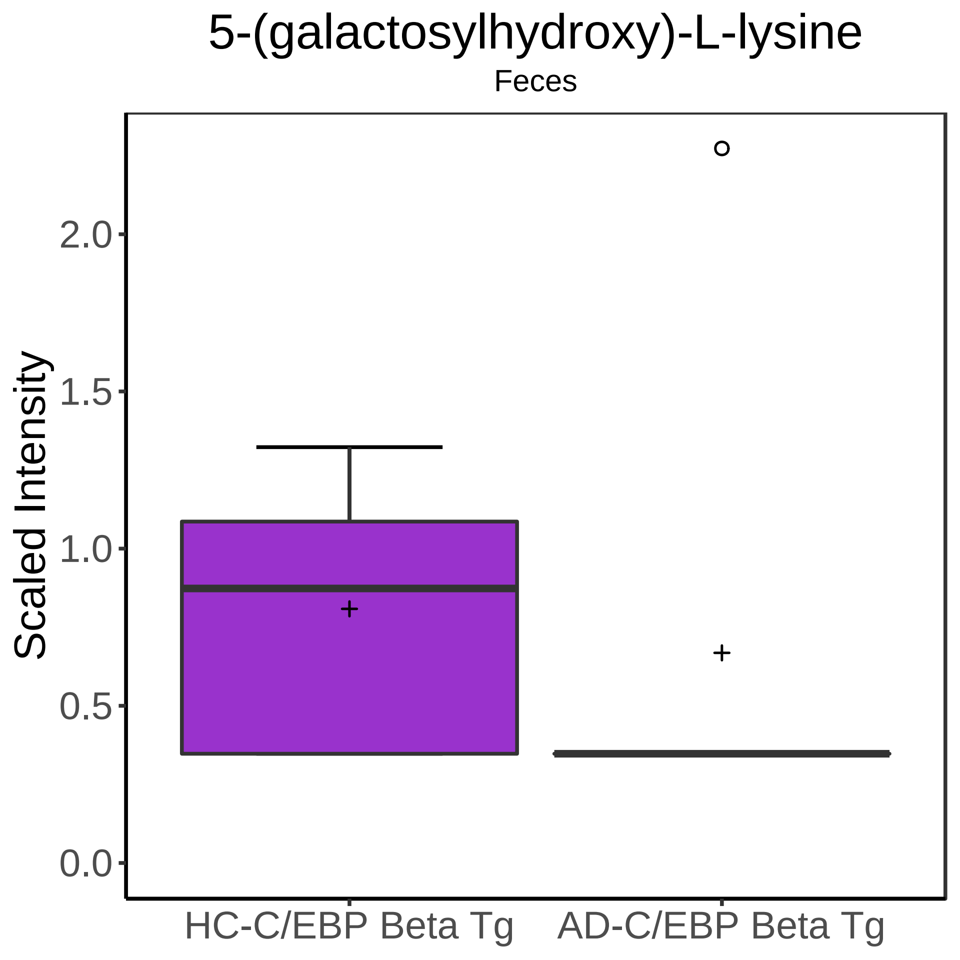
5-(galactosylhydroxy)-L-lysine
Feces
Scaled Intensity
2.0
1.5
1.0
0.5
0.0
HC-C/EBP Beta Tg
AD-C/EBP Beta Tg

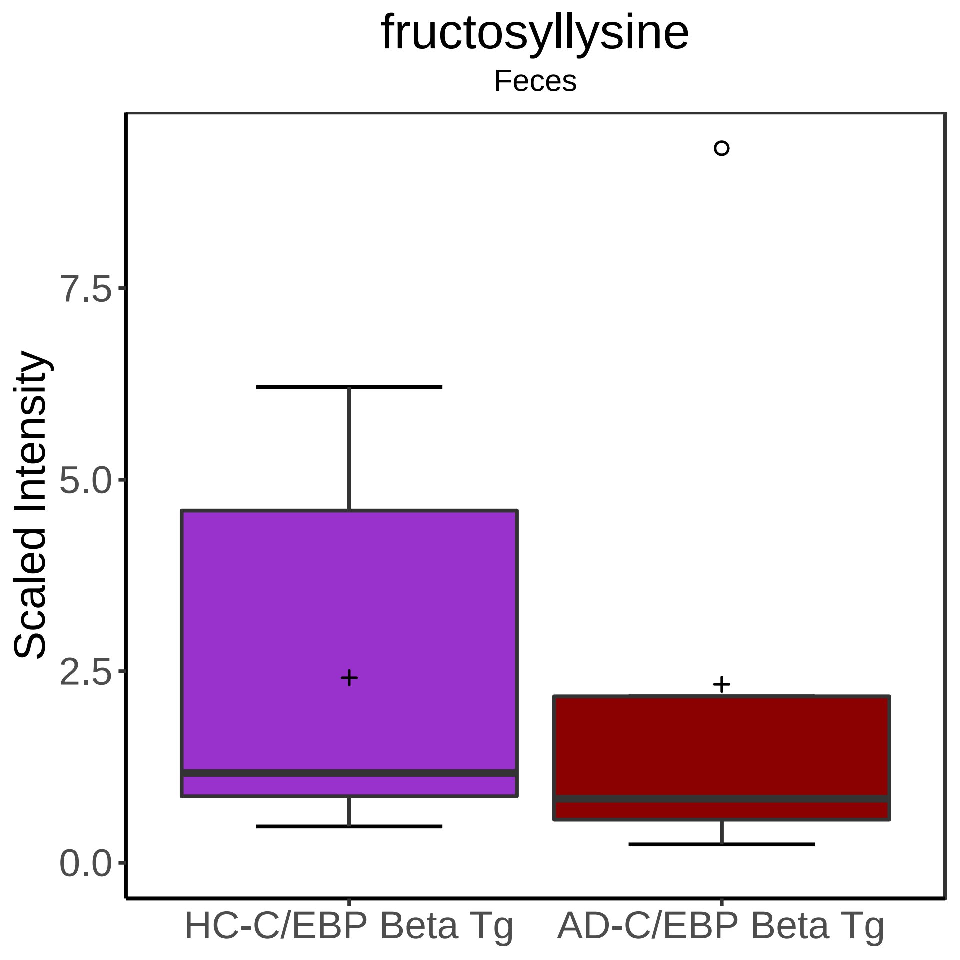
fructosyllysine
Feces
Scaled Intensity
7.5
5.0
2.5
0.0
HC-C/EBP Beta Tg
AD-C/EBP Beta Tg

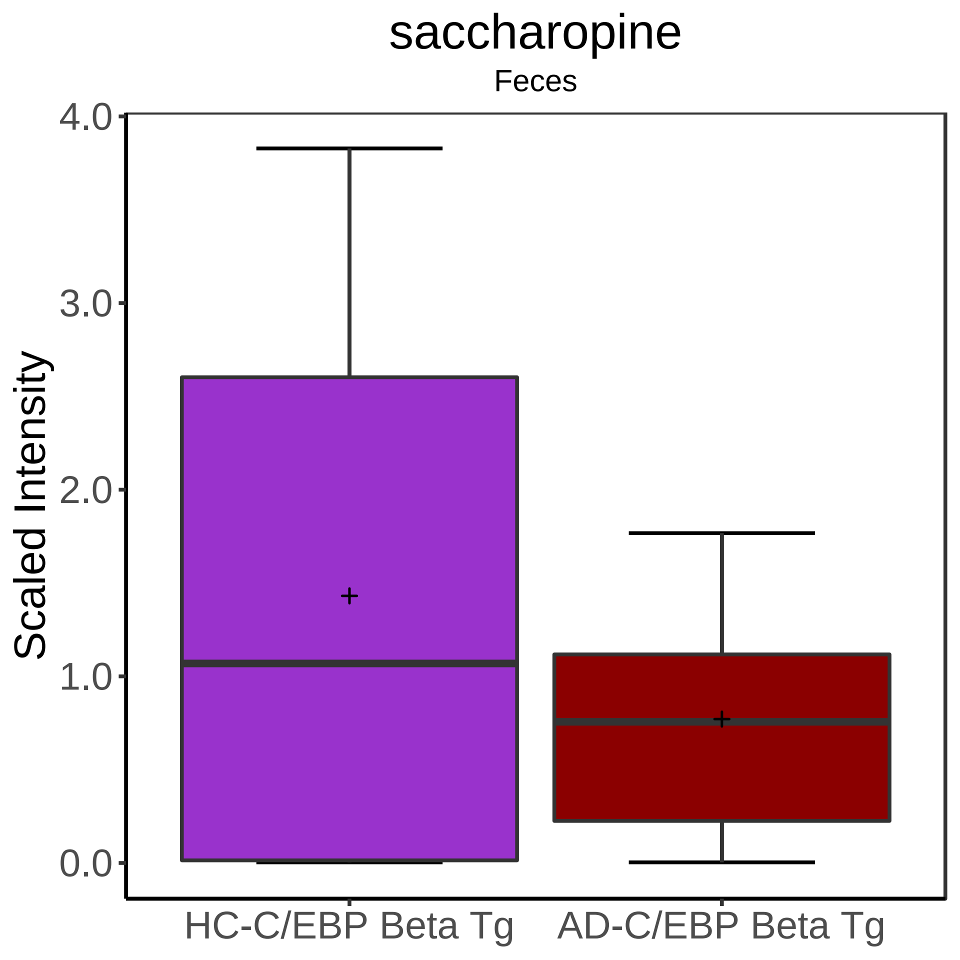
saccharopine
Feces
Scaled Intensity
4.0
3.0
2.0
1.0
0.0
HC-C/EBP Beta Tg
AD-C/EBP Beta Tg

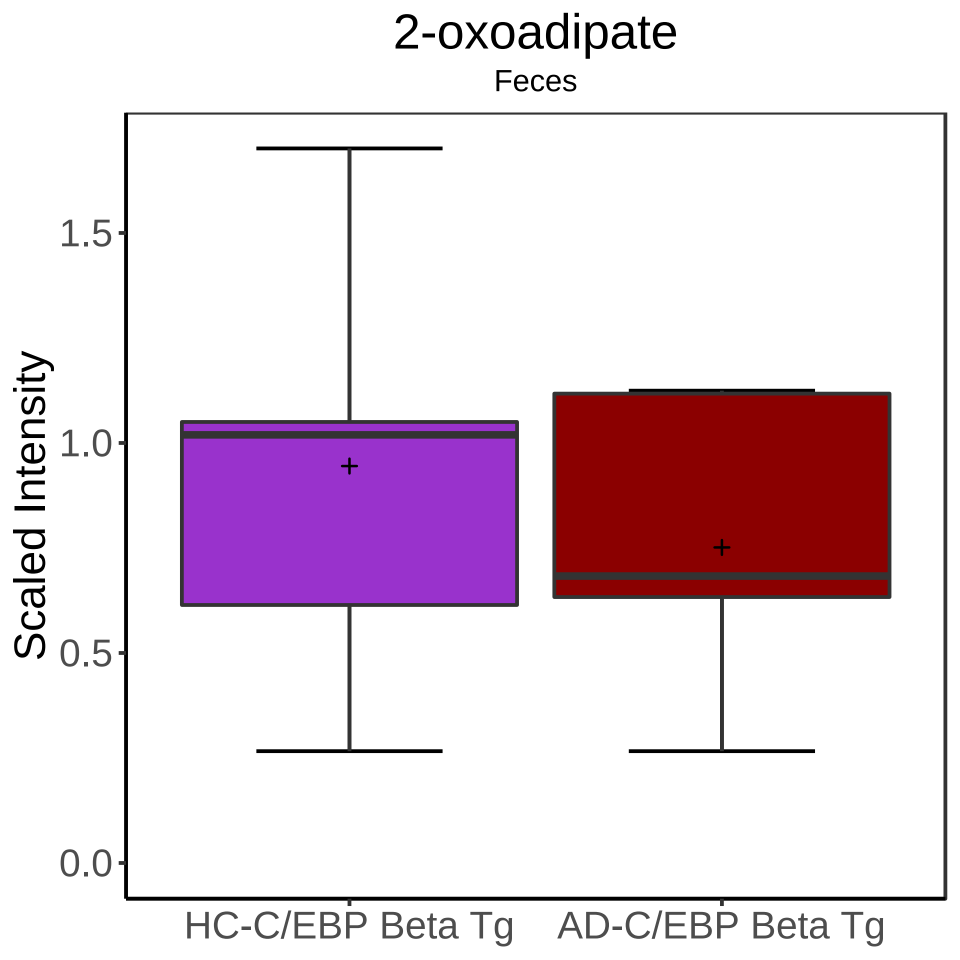
2-oxoadipate
Feces
Scaled Intensity
1.5
1.0
0.5
0.0
HC-C/EBP Beta Tg
AD-C/EBP Beta Tg

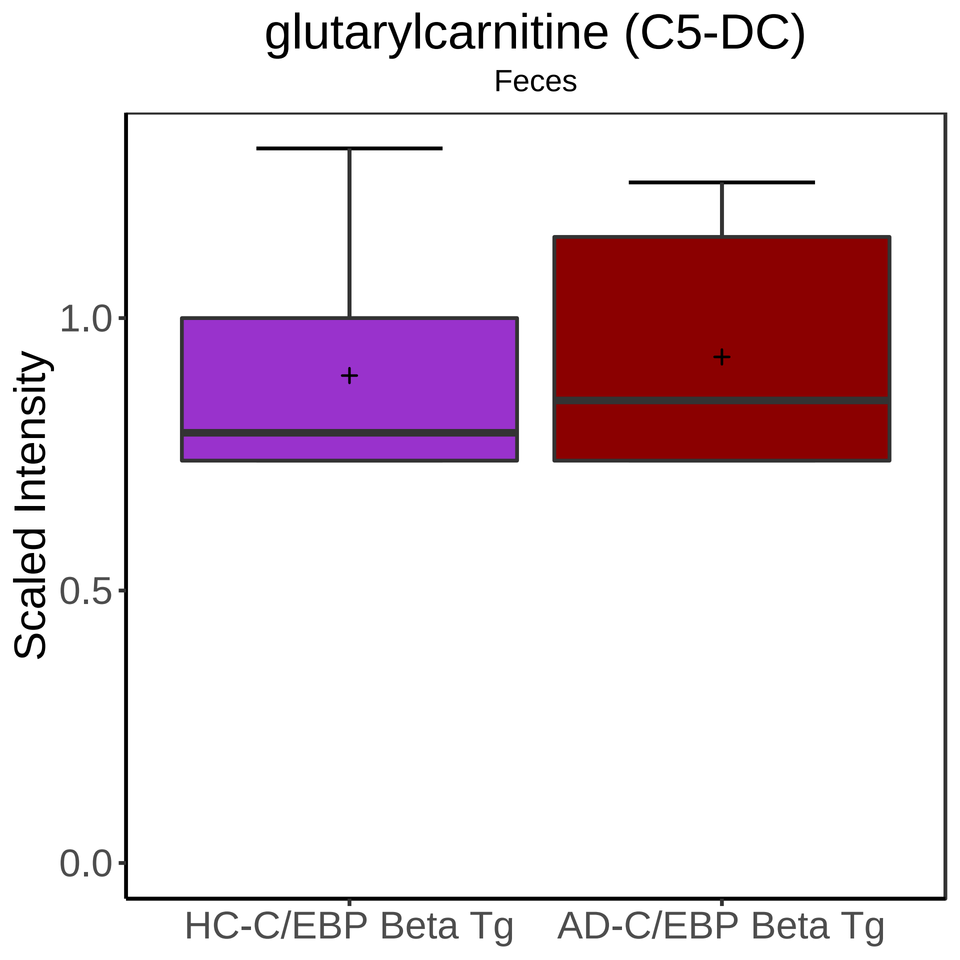
glutarylcarnitine (C5-DC)
Feces
Scaled Intensity
1.0
0.5
0.0
HC-C/EBP Beta Tg
AD-C/EBP Beta Tg

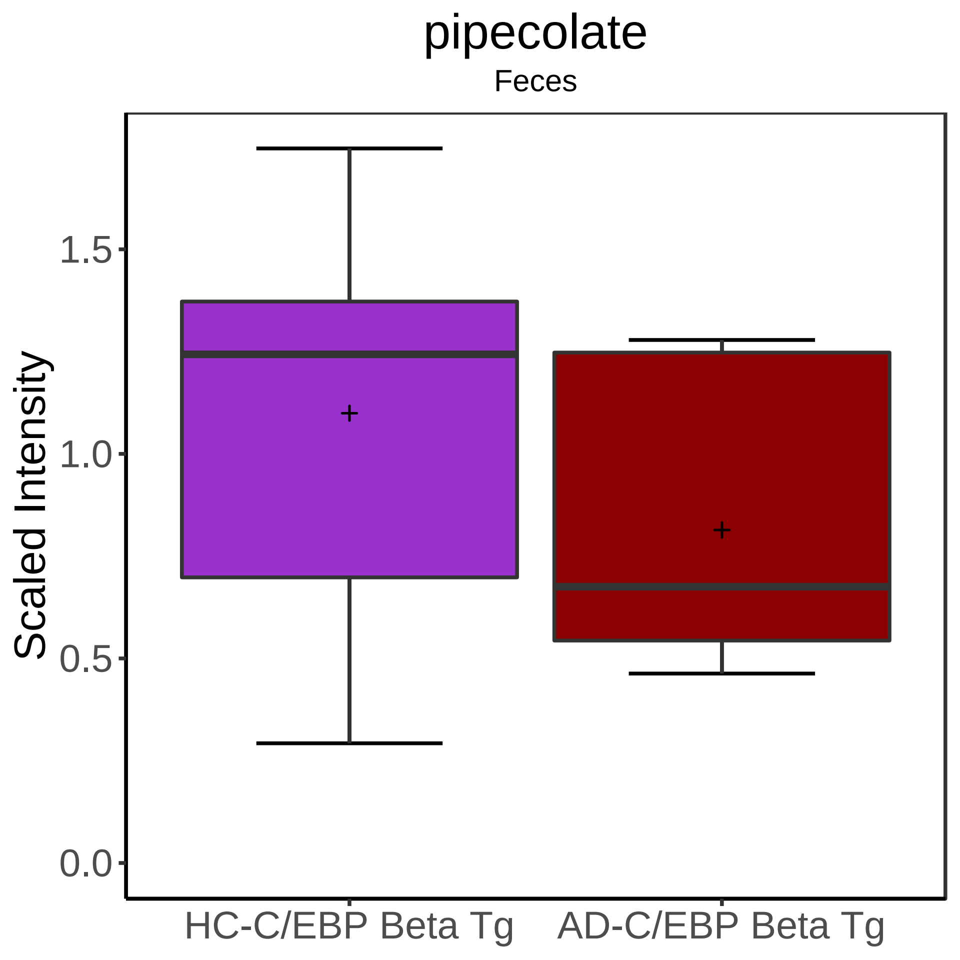

pipecolate
Feces
Scaled Intensity
1.5
1.0
0.5
0.0
HC-C/EBP Beta Tg
AD-C/EBP Beta Tg

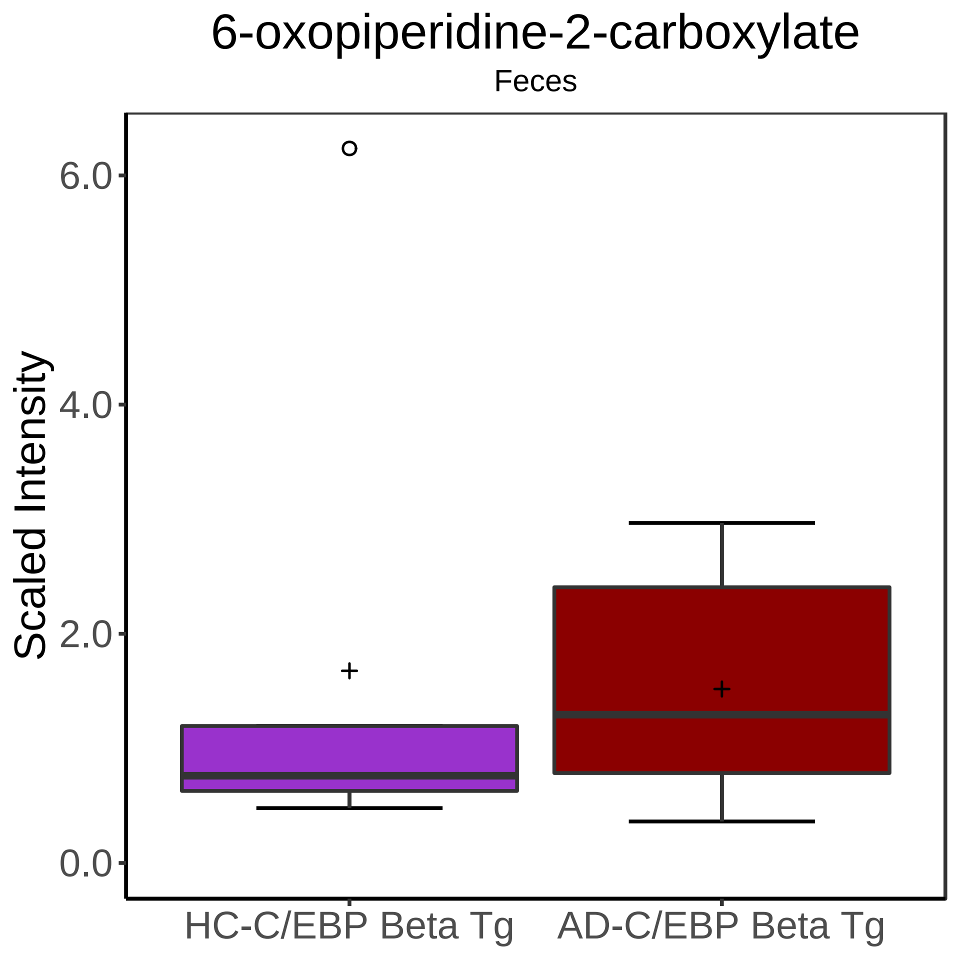
6-oxopiperidine-2-carboxylate
Feces
Scaled Intensity
6.0
4.0
2.0
0.0
HC-C/EBP Beta Tg
AD-C/EBP Beta Tg

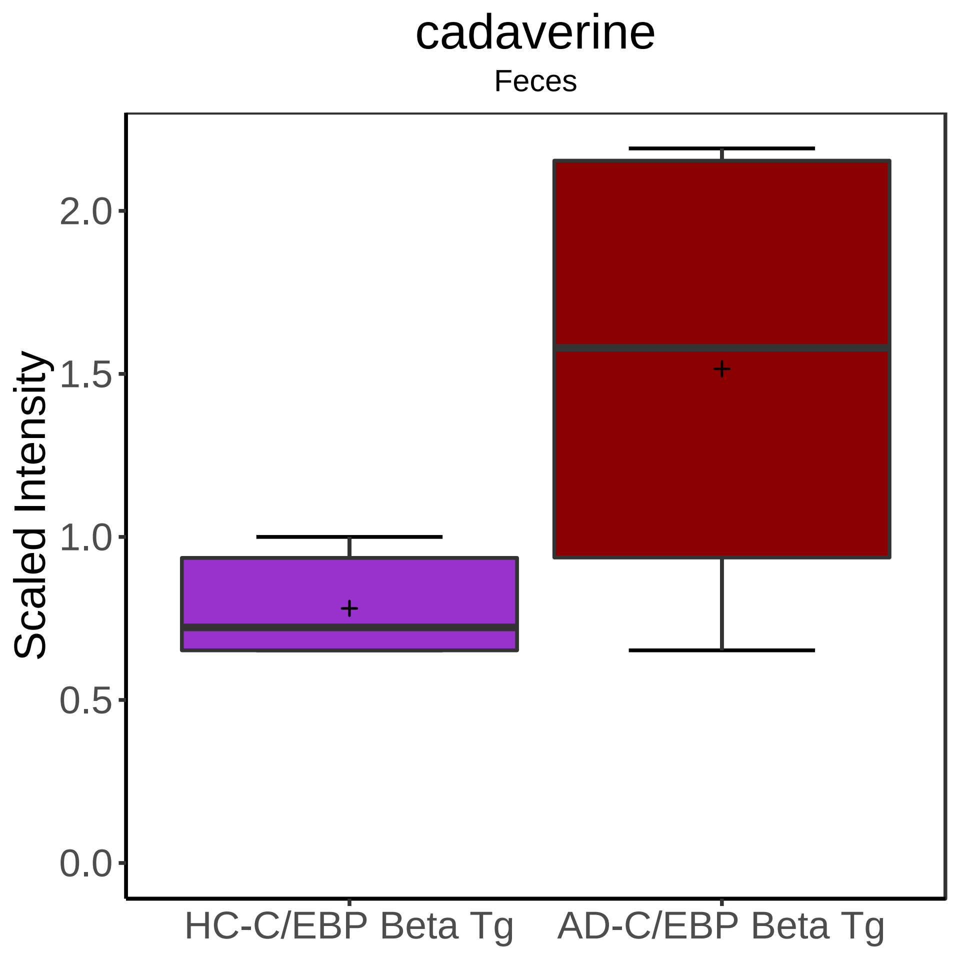

cadaverine
Feces
Scaled Intensity
2.0
1.5
1.0
0.5
0.0
HC-C/EBP Beta Tg
AD-C/EBP Beta Tg

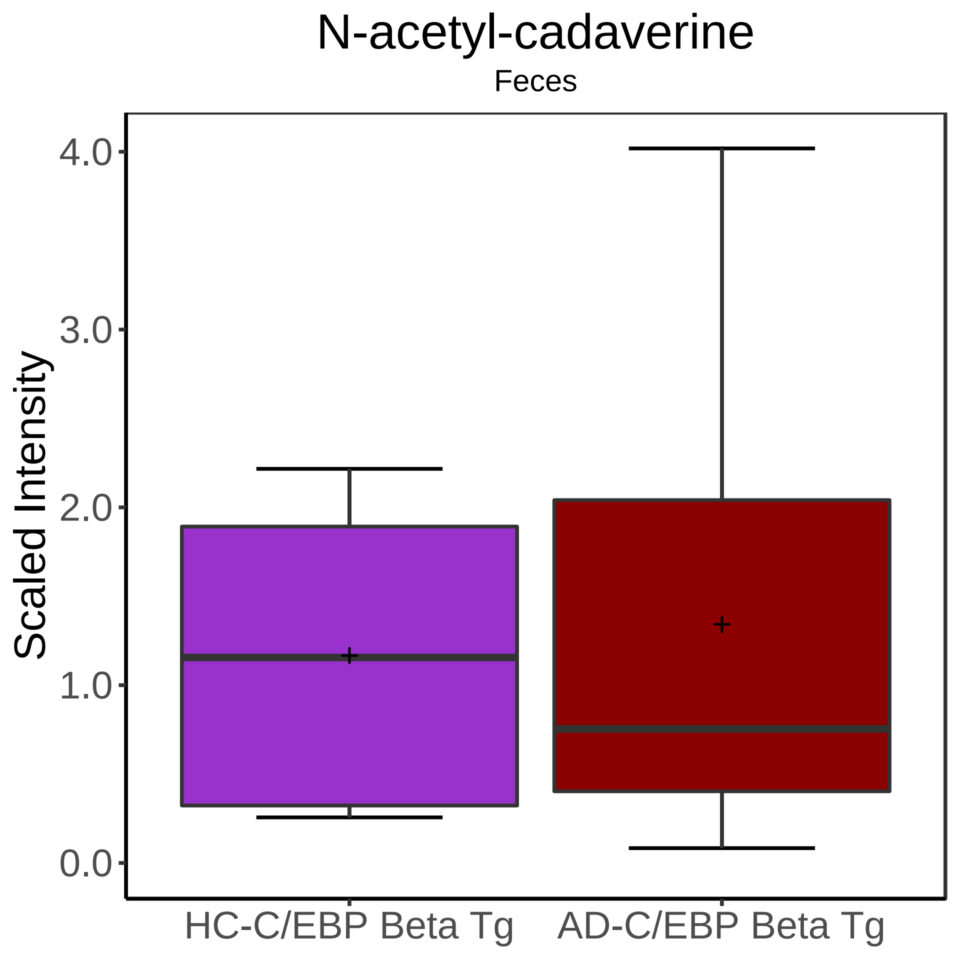
N-acetyl-cadaverine
Feces
Scaled Intensity
4.0
3.0
2.0
1.0
0.0
HC-C/EBP Beta Tg
AD-C/EBP Beta Tg

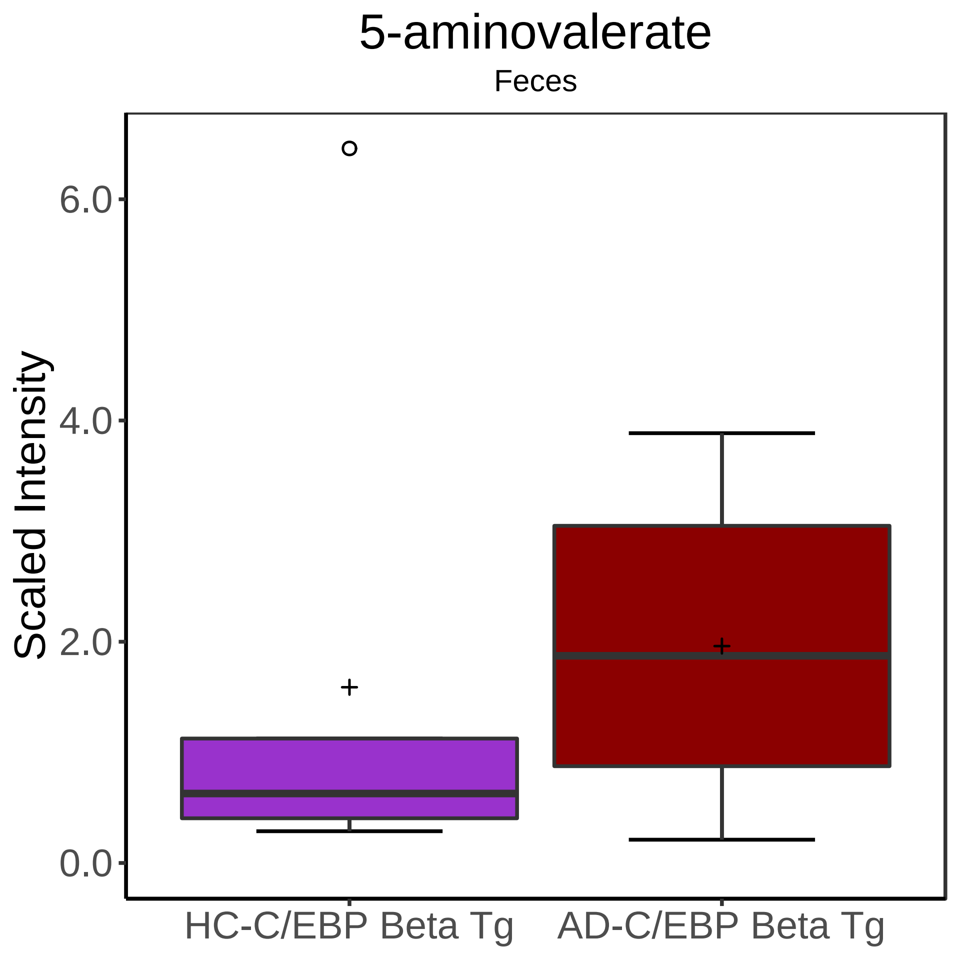

5-aminovalerate
Feces
Scaled Intensity
6.0
4.0
2.0
0.0
HC-C/EBP Beta Tg
AD-C/EBP Beta Tg

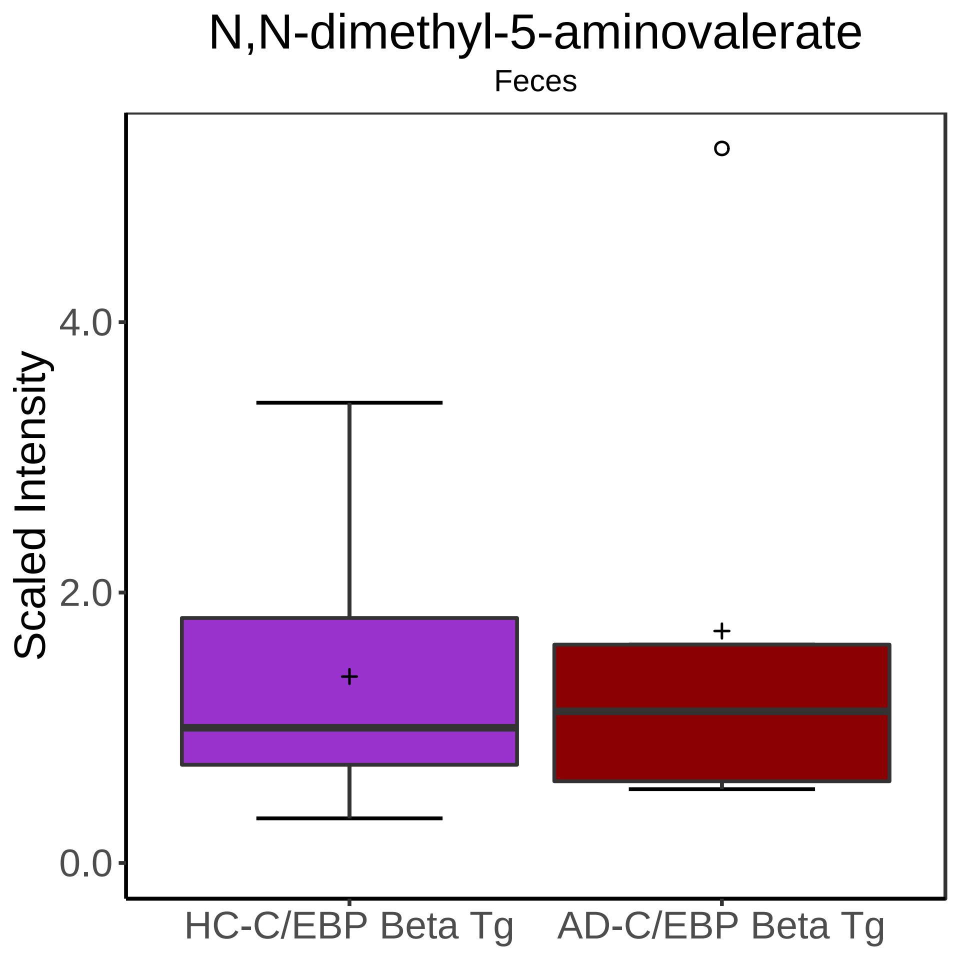
N,N-dimethyl-5-aminovalerate
Feces
Scaled Intensity
4.0
2.0
0.0
HC-C/EBP Beta Tg
AD-C/EBP Beta Tg

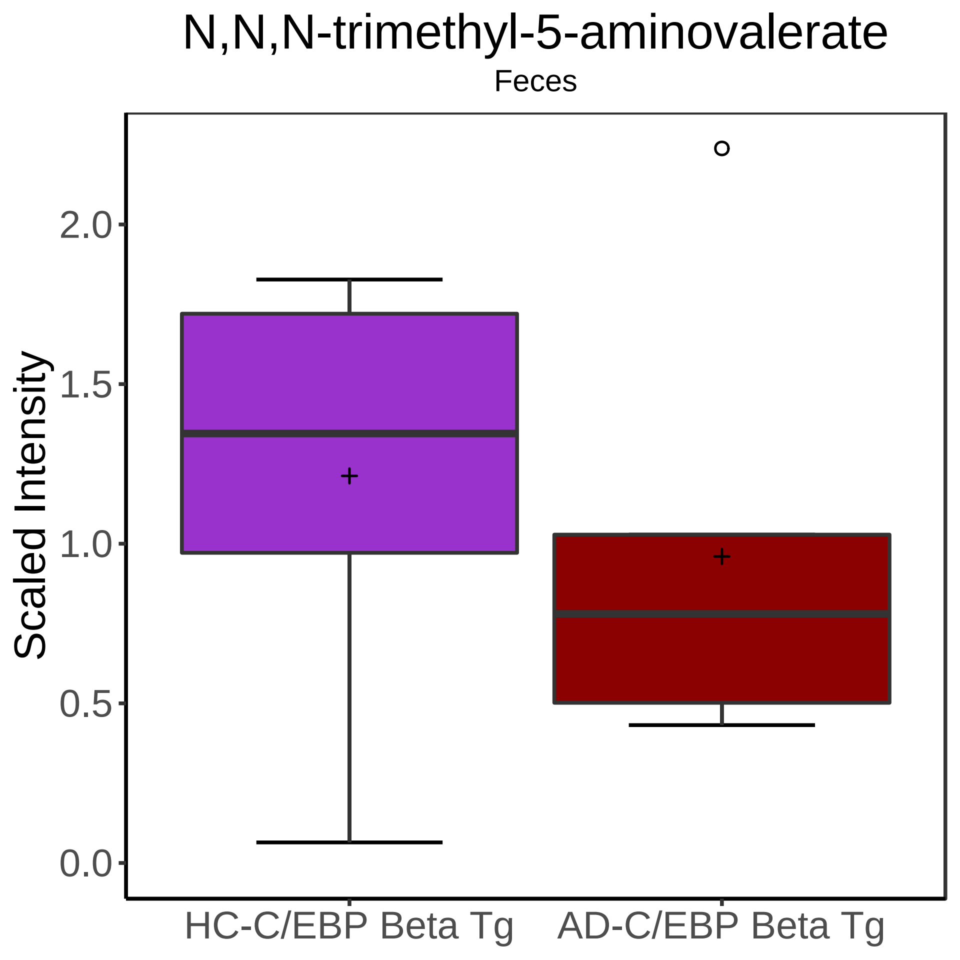

N,N,N-trimethyl-5-aminovalerate
Feces
Scaled Intensity
2.0
1.5
1.0
0.5
0.0
HC-C/EBP Beta Tg
AD-C/EBP Beta Tg

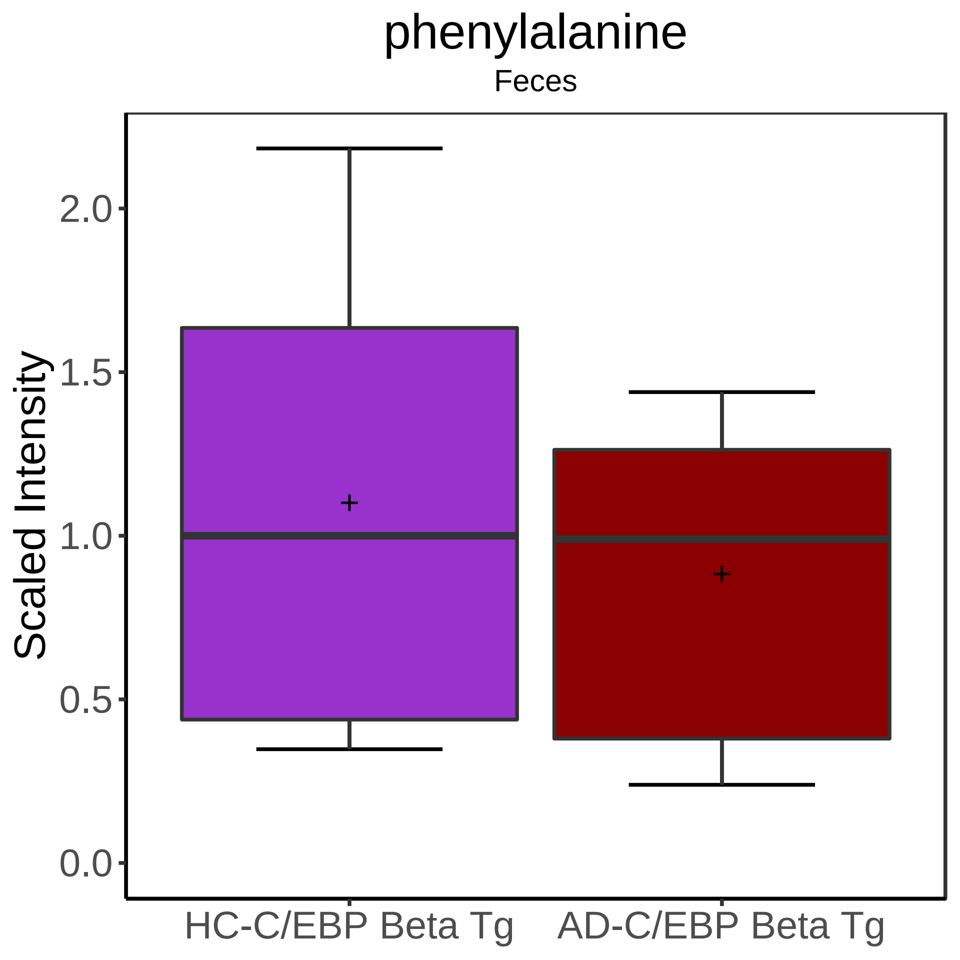
phenylalanine
Feces
Scaled Intensity
2.0
1.5
1.0
0.5
0.0
HC-C/EBP Beta Tg
AD-C/EBP Beta Tg

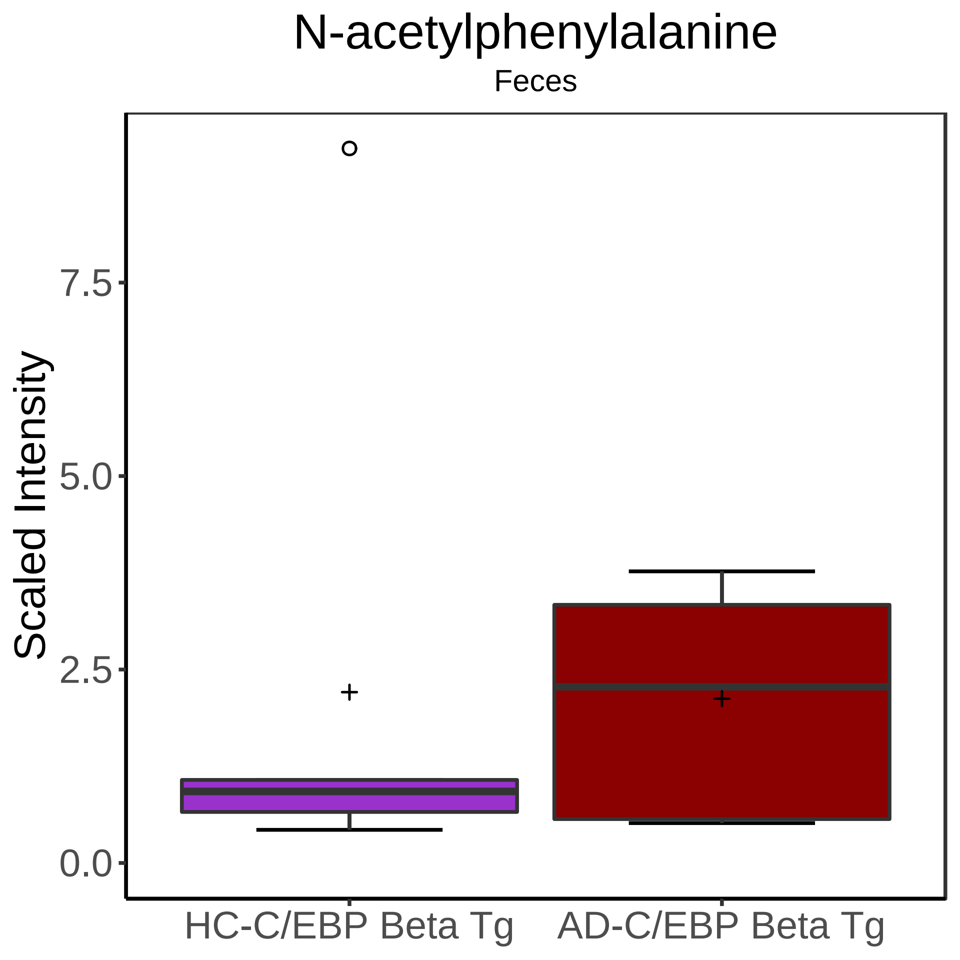

N-acetylphenylalanine
Feces
Scaled Intensity
7.5
5.0
2.5
0.0
HC-C/EBP Beta Tg
AD-C/EBP Beta Tg

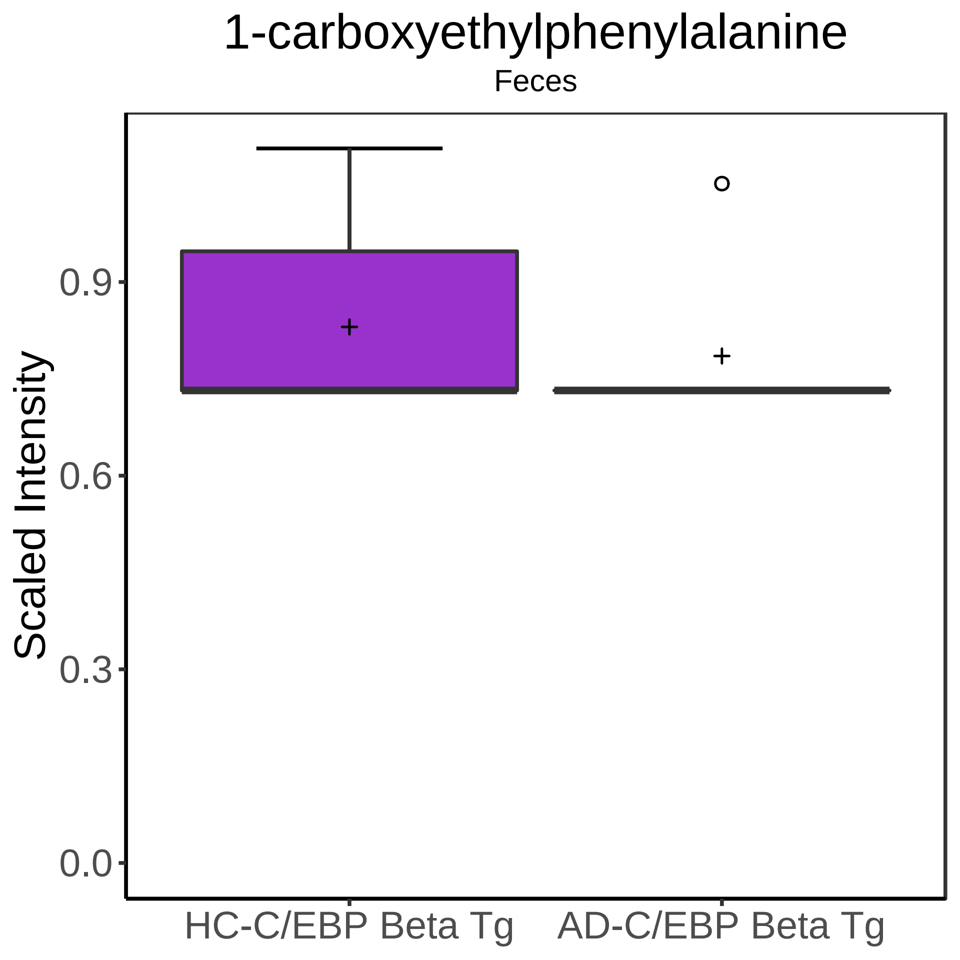
1-carboxyethylphenylalanine
Feces
Scaled Intensity
0.9
0.6
0.3
0.0
HC-C/EBP Beta Tg
AD-C/EBP Beta Tg

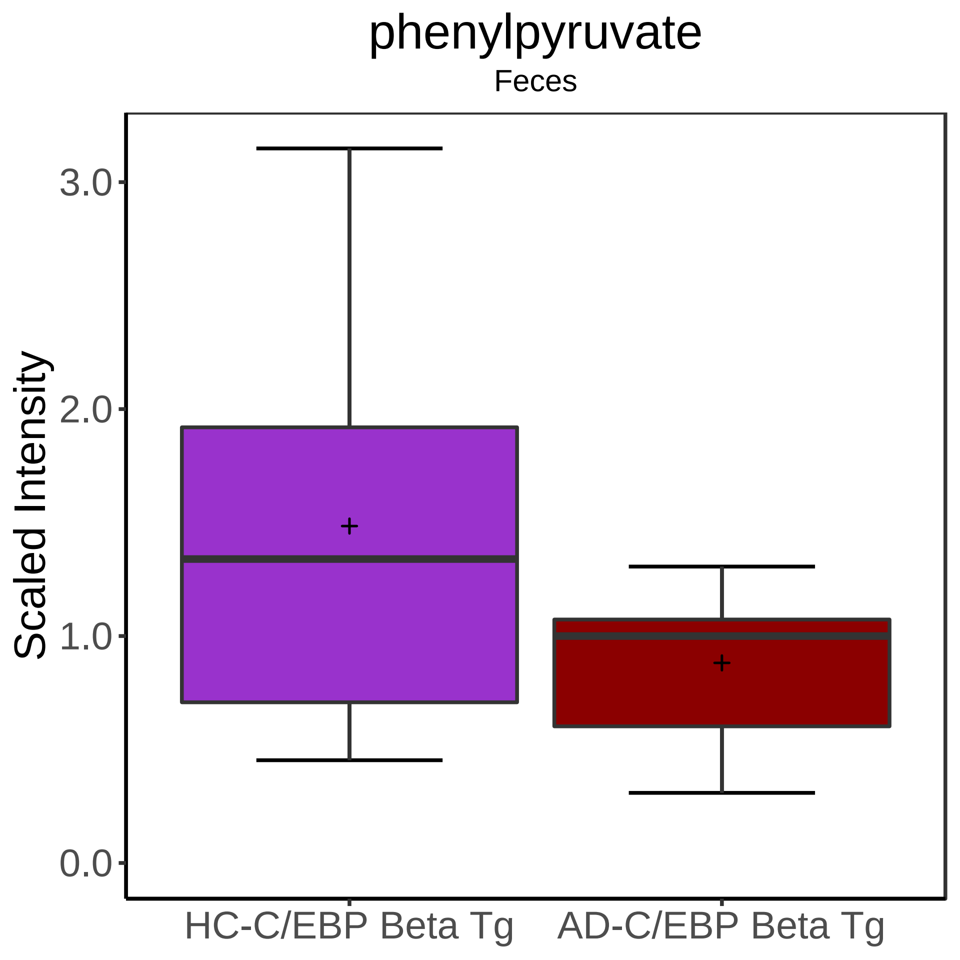
phenylpyruvate
Feces
Scaled Intensity
3.0
2.0
1.0
0.0
HC-C/EBP Beta Tg
AD-C/EBP Beta Tg

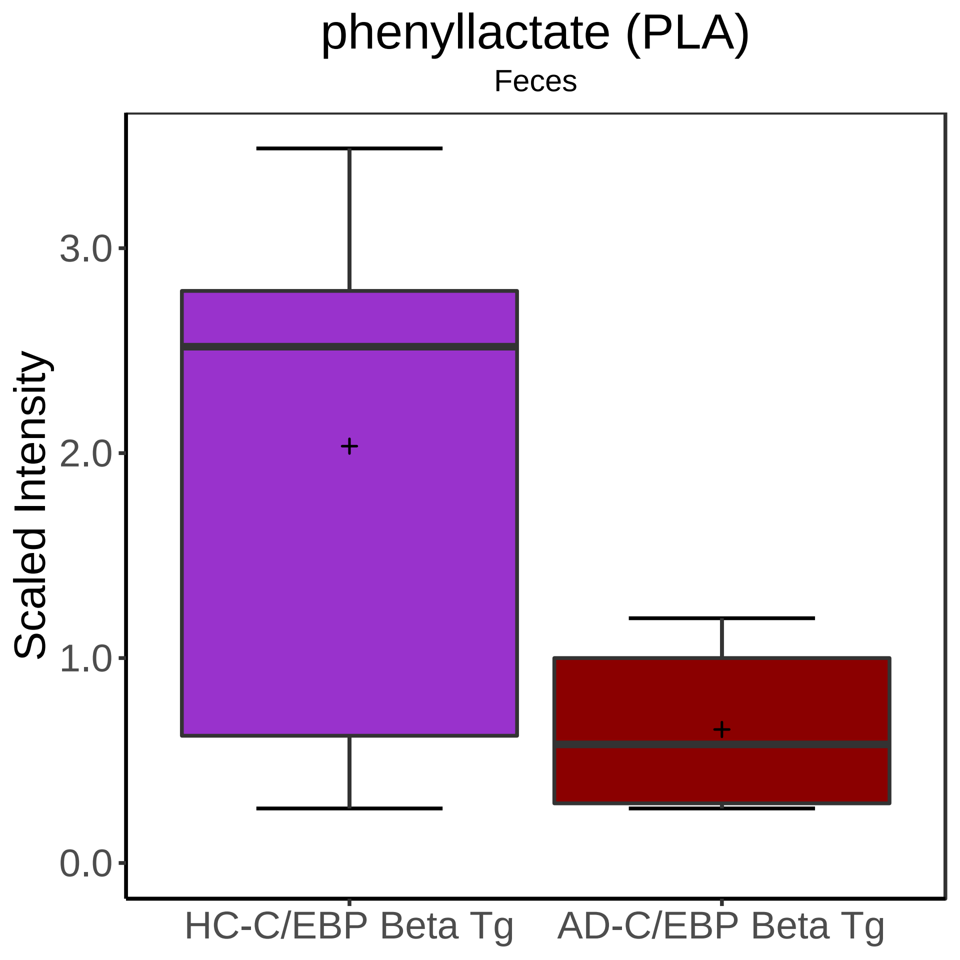
phenyllactate (PLA)
Feces
Scaled Intensity
3.0
2.0
1.0
0.0
HC-C/EBP Beta Tg
AD-C/EBP Beta Tg

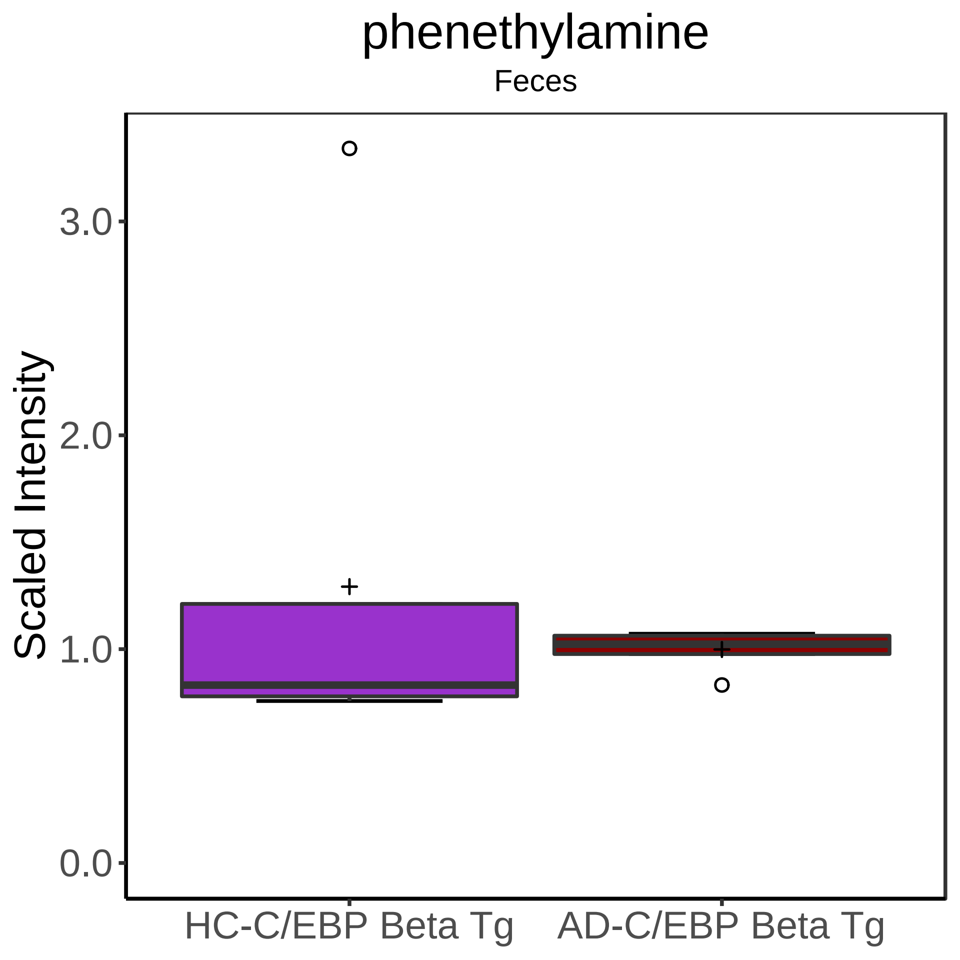
phenethylamine
Feces
Scaled Intensity
3.0
2.0
1.0
0.0
HC-C/EBP Beta Tg
AD-C/EBP Beta Tg

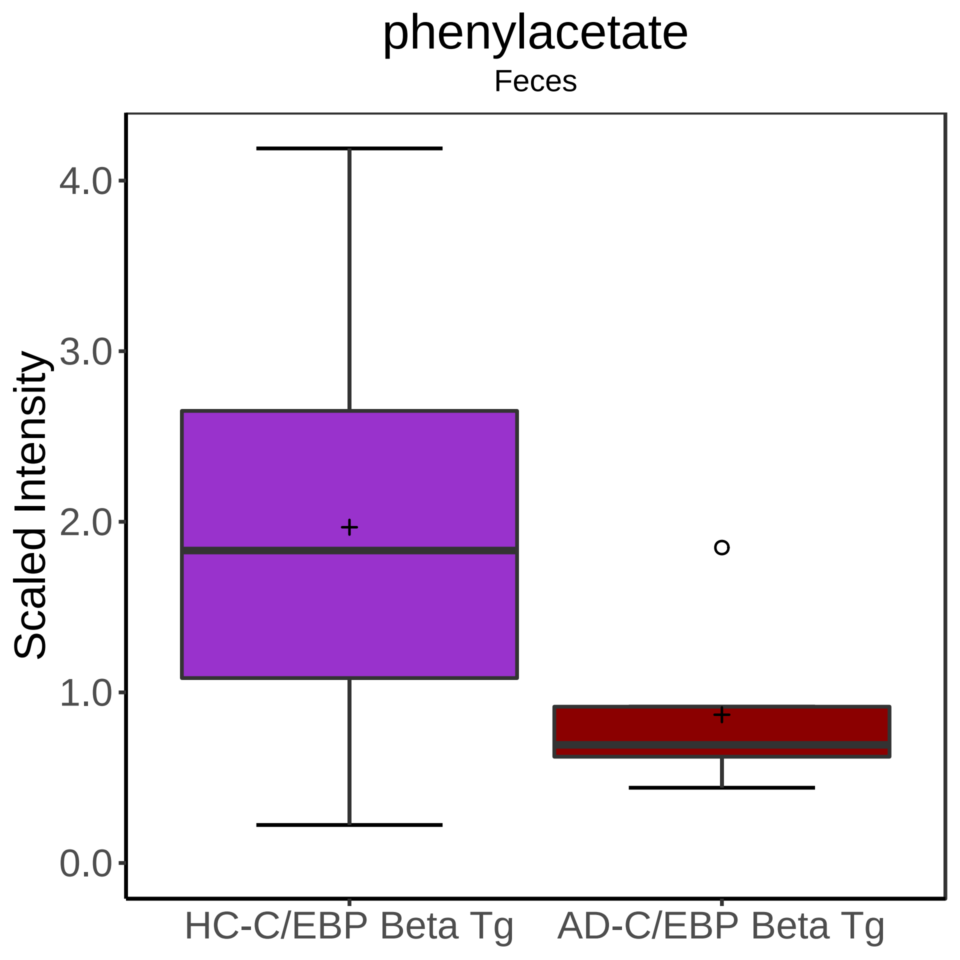
phenylacetate
Feces
Scaled Intensity
4.0
3.0
2.0
1.0
0.0
HC-C/EBP Beta Tg
AD-C/EBP Beta Tg

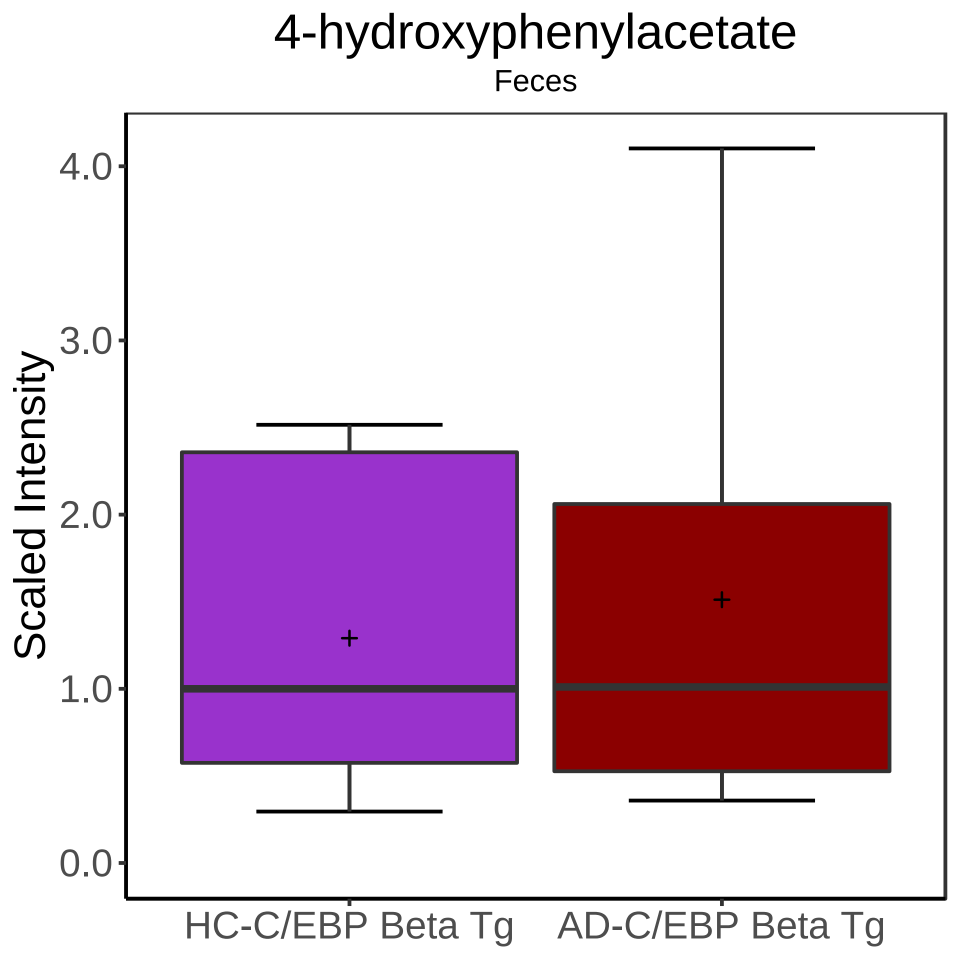

4-hydroxyphenylacetate
Feces
Scaled Intensity
4.0
3.0
2.0
1.0
0.0
HC-C/EBP Beta Tg
AD-C/EBP Beta Tg

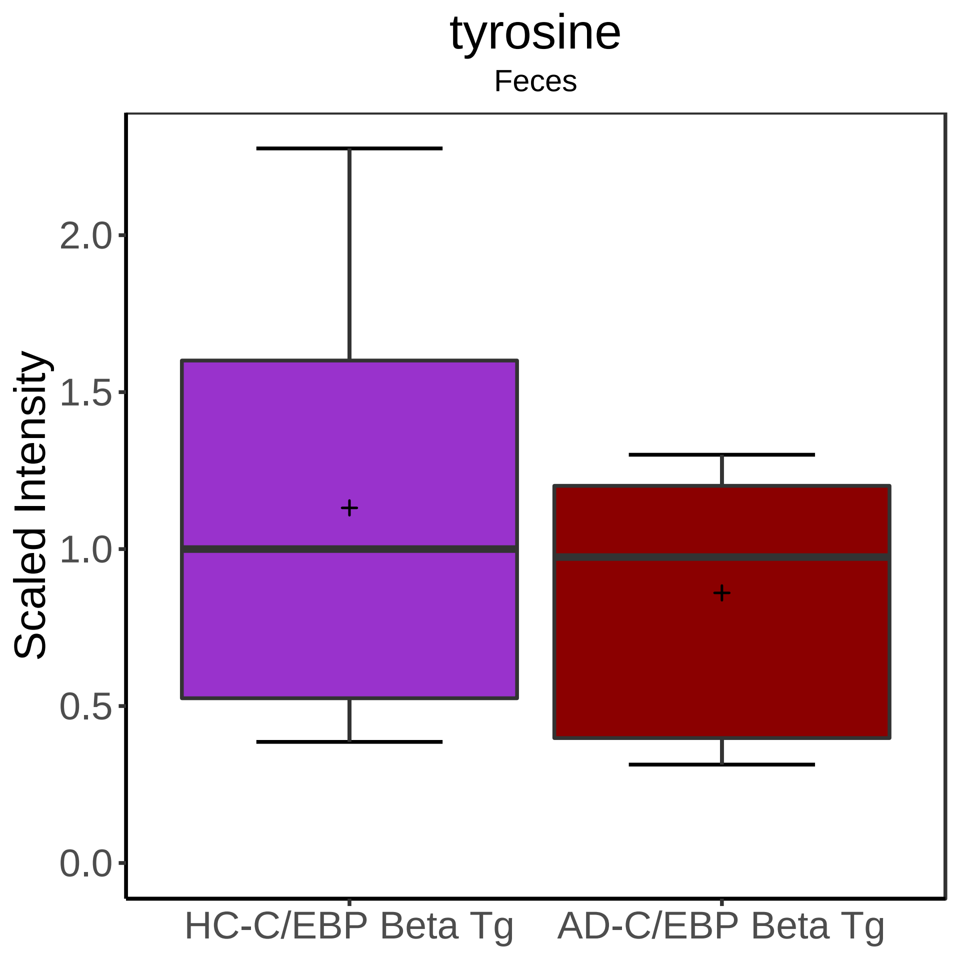
tyrosine
Feces
Scaled Intensity
2.0
1.5
1.0
0.5
0.0
HC-C/EBP Beta Tg
AD-C/EBP Beta Tg

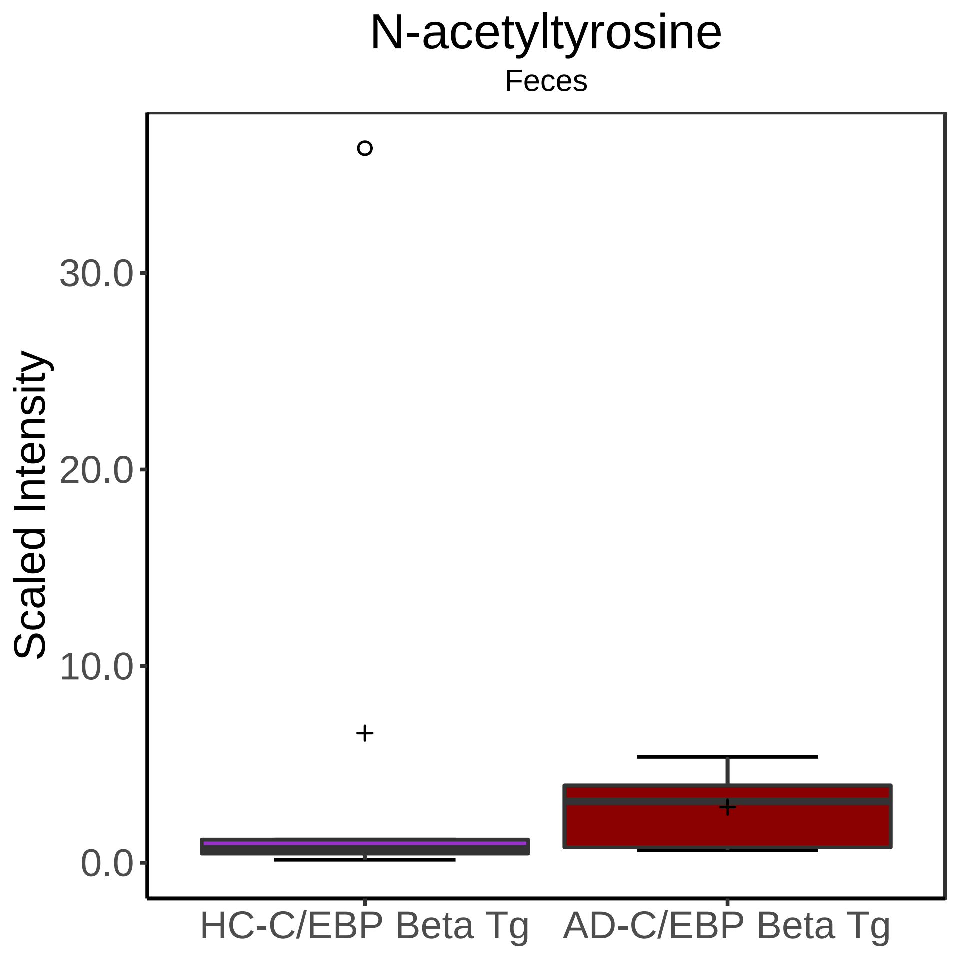

N-acetyltyrosine
Feces
Scaled Intensity
30.0
20.0
10.0
0.0
HC-C/EBP Beta Tg
AD-C/EBP Beta Tg

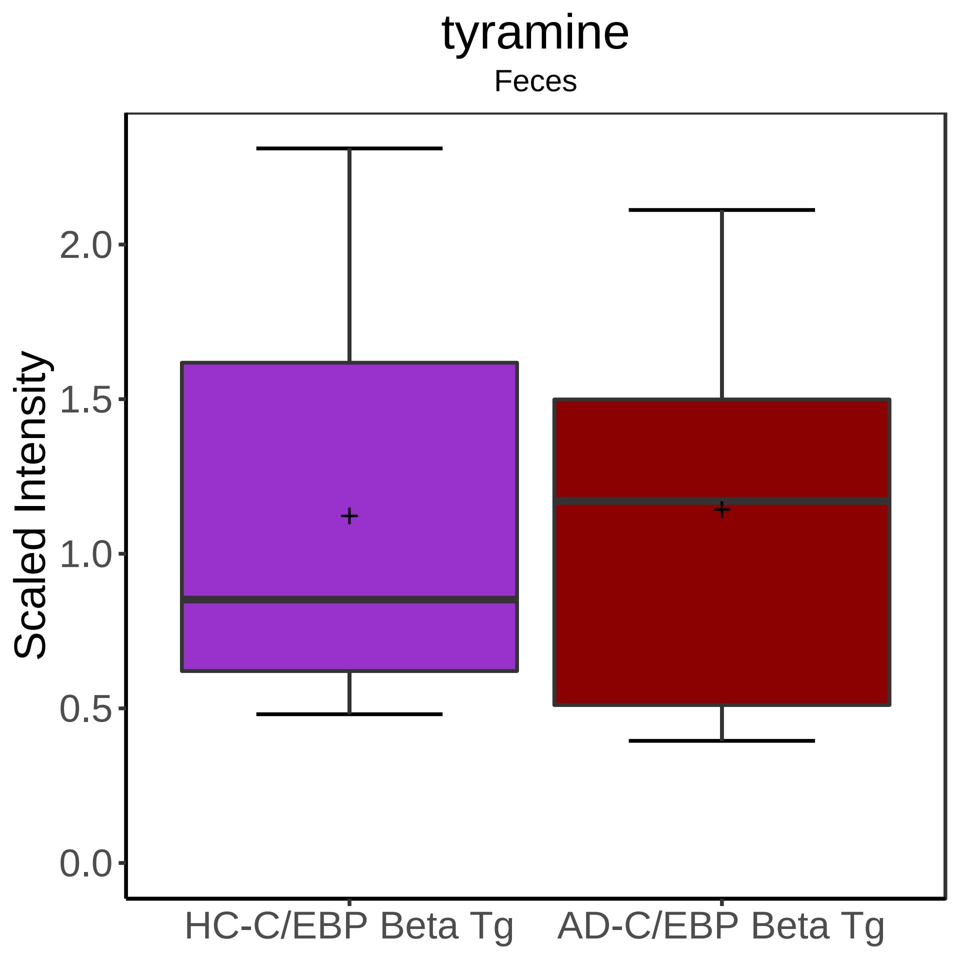
tyramine
Feces
Scaled Intensity
2.0
1.5
1.0
0.5
0.0
HC-C/EBP Beta Tg
AD-C/EBP Beta Tg

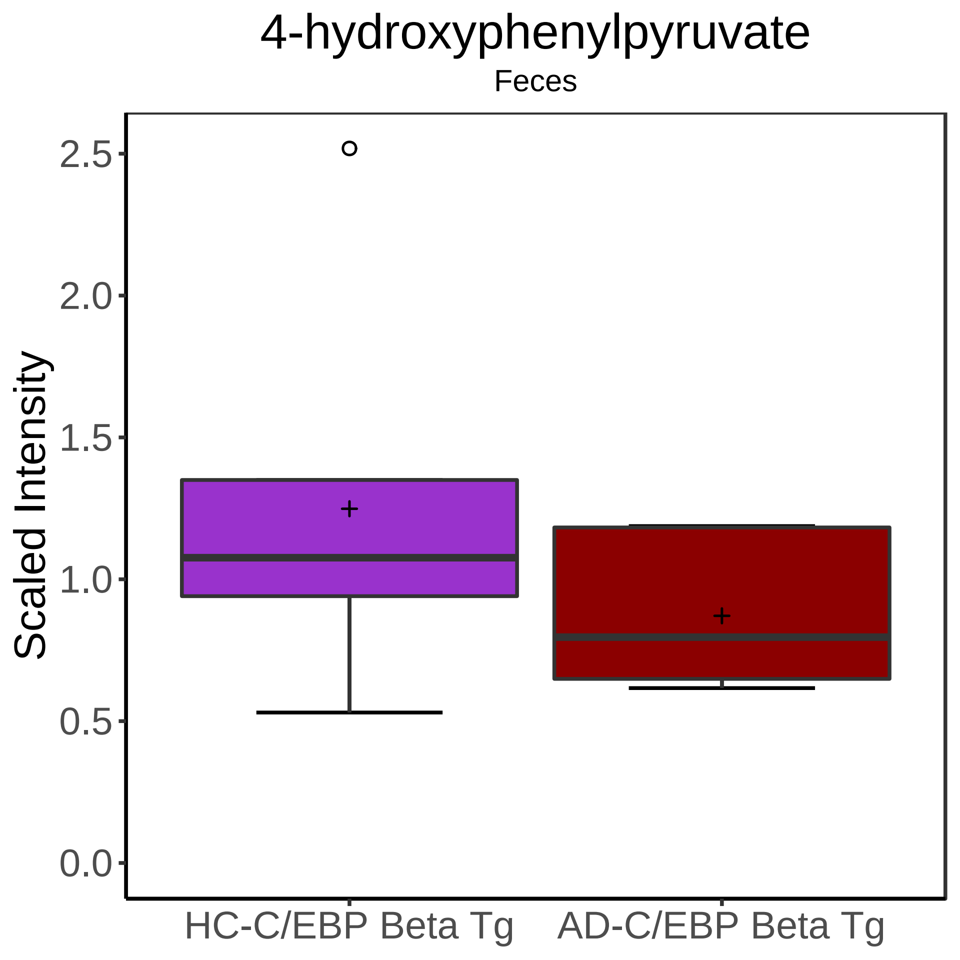

4-hydroxyphenylpyruvate
Feces
Scaled Intensity
2.5
2.0
1.5
1.0
0.5
0.0
HC-C/EBP Beta Tg
AD-C/EBP Beta Tg

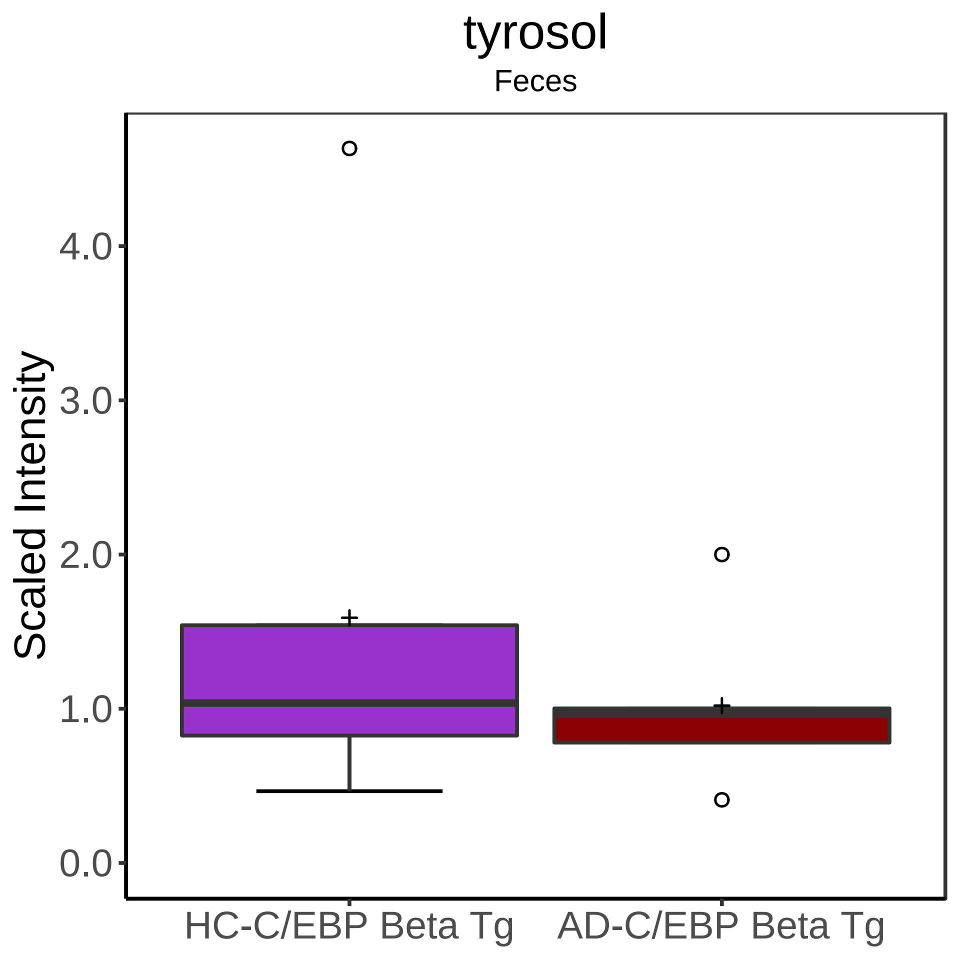

tyrosol
Feces
Scaled Intensity
4.0
3.0
2.0
1.0
0.0
HC-C/EBP Beta Tg
AD-C/EBP Beta Tg

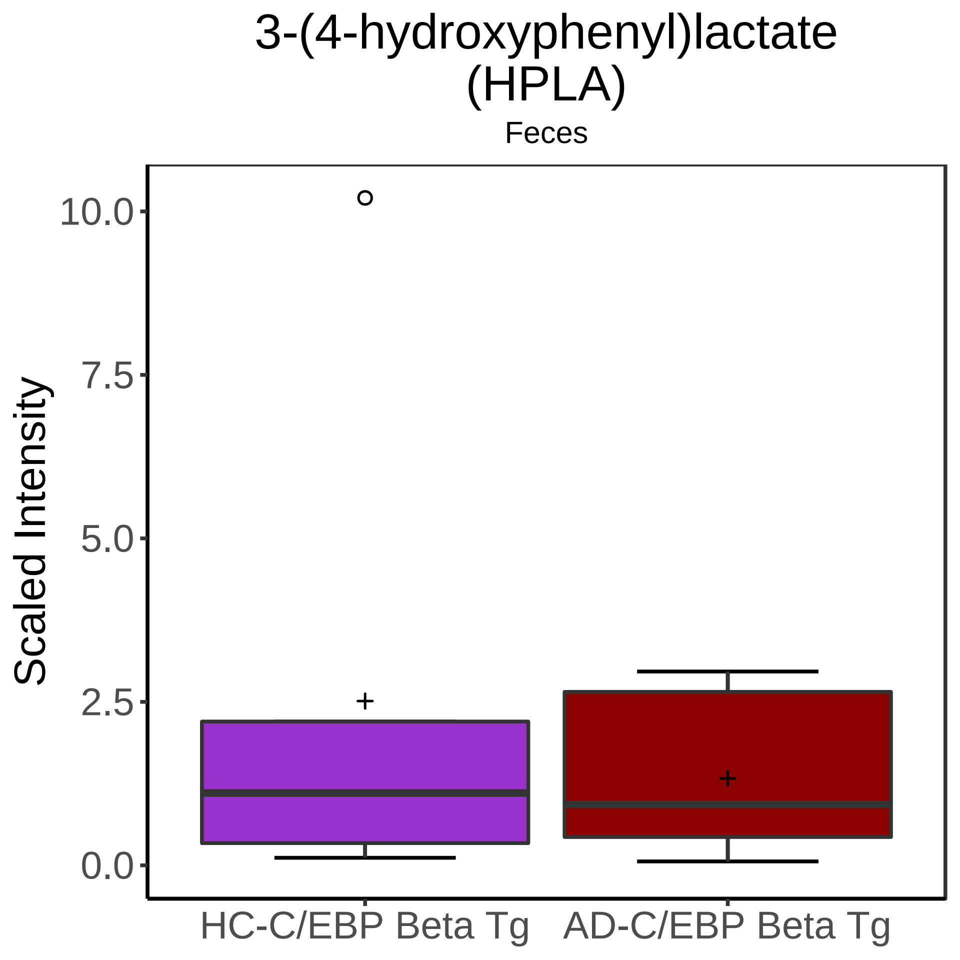

3-(4-hydroxyphenyl)lactate
(HPLA)
Feces
Scaled Intensity
10.0
7.5
5.0
2.5
0.0
HC-C/EBP Beta Tg
AD-C/EBP Beta Tg

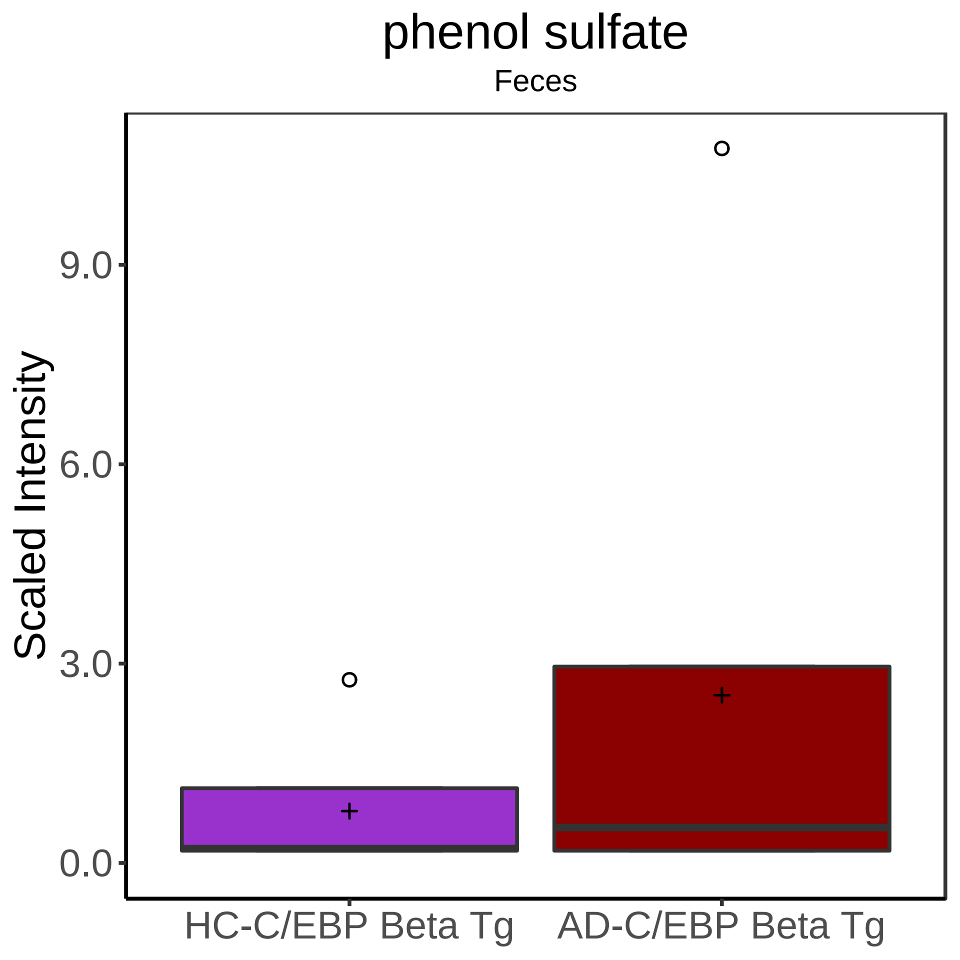

phenol sulfate
Feces
Scaled Intensity
9.0
6.0
3.0
0.0
HC-C/EBP Beta Tg
AD-C/EBP Beta Tg

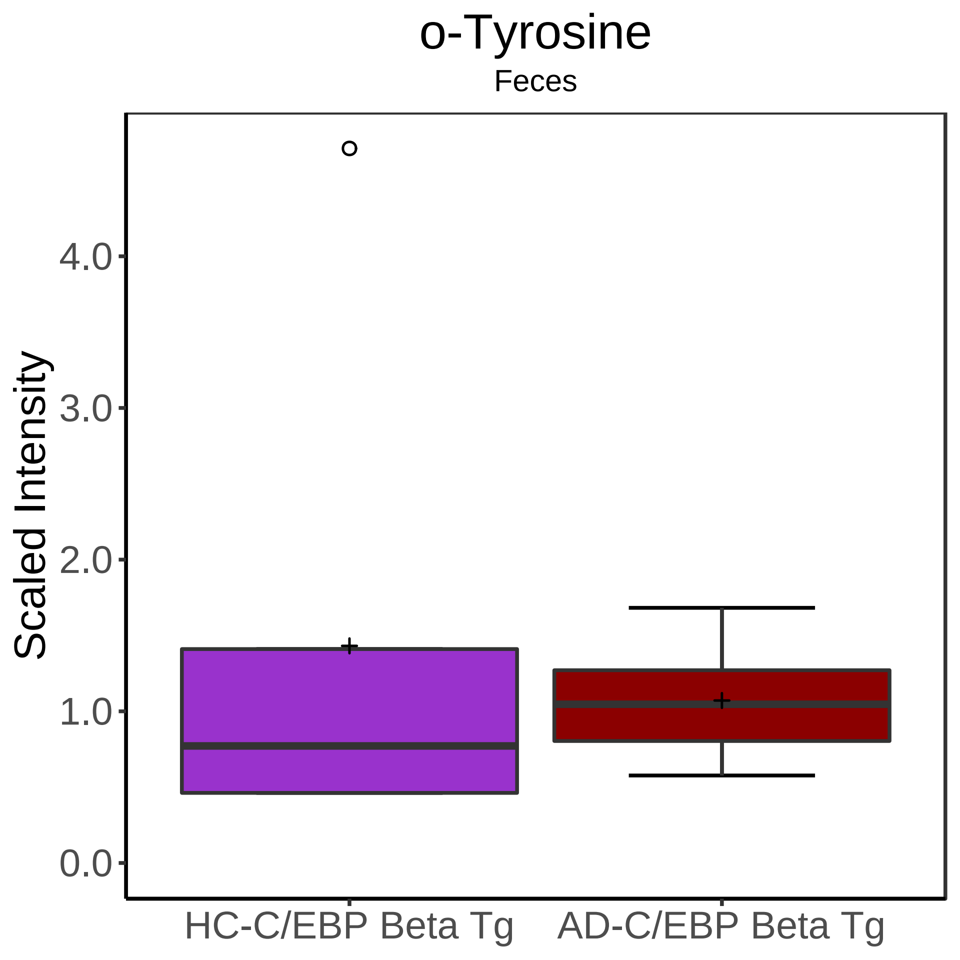
o-Tyrosine
Feces
Scaled Intensity
4.0
3.0
2.0
1.0
0.0
HC-C/EBP Beta Tg
AD-C/EBP Beta Tg

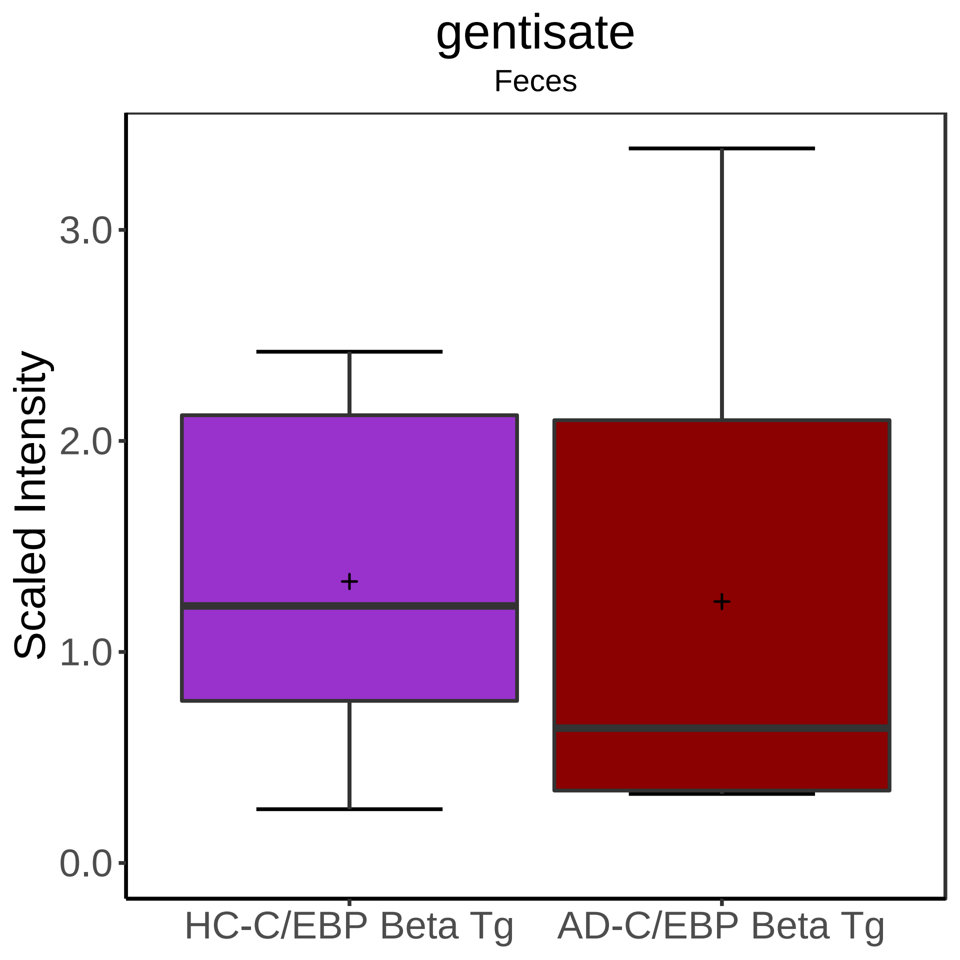

gentisate
Feces
Scaled Intensity
3.0
2.0
1.0
0.0
HC-C/EBP Beta Tg
AD-C/EBP Beta Tg

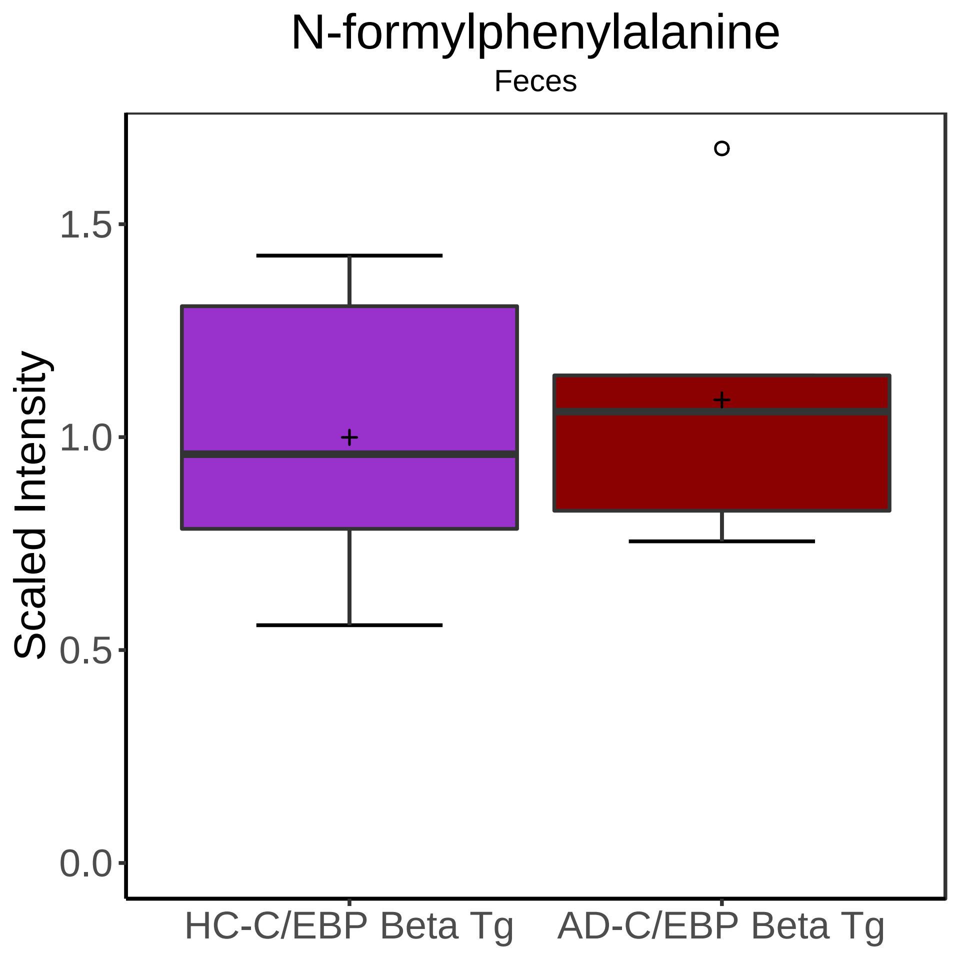

N-formylphenylalanine
Feces
Scaled Intensity
1.5
1.0
0.5
0.0
HC-C/EBP Beta Tg
AD-C/EBP Beta Tg

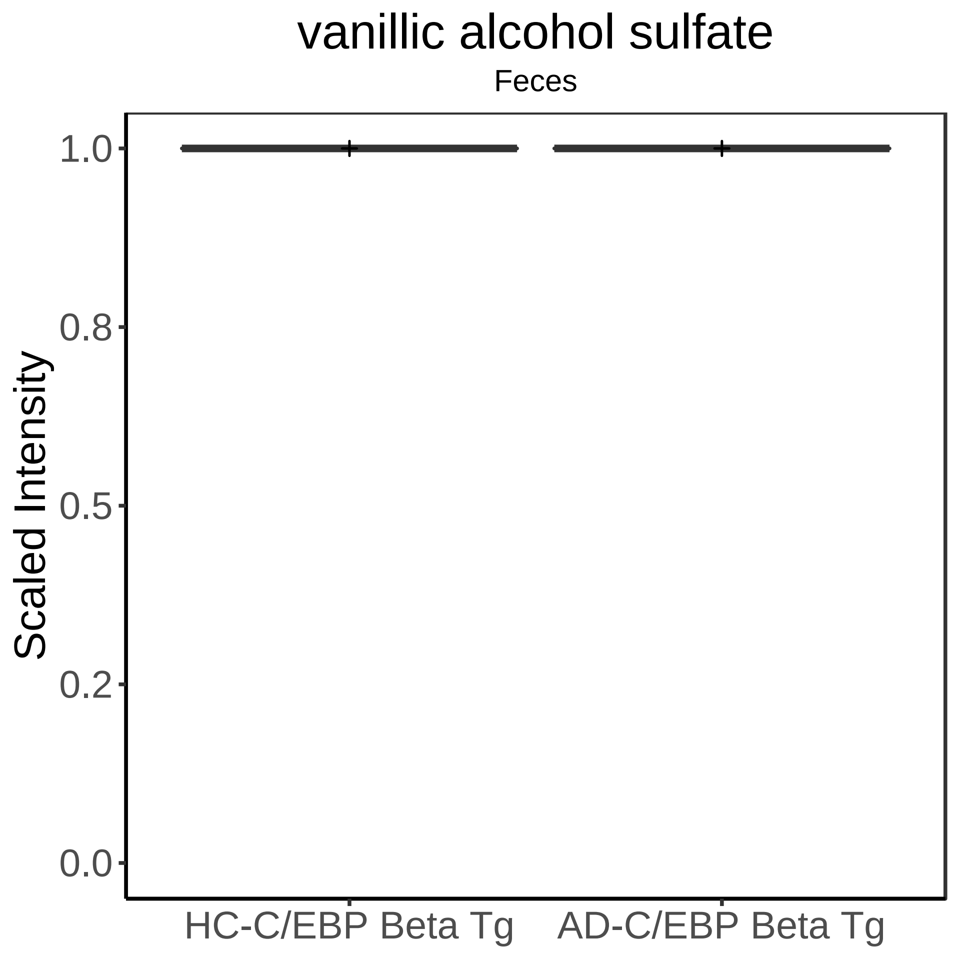
vanillic alcohol sulfate
Feces
Scaled Intensity
1.0
0.8
0.5
0.2
0.0
HC-C/EBP Beta Tg
AD-C/EBP Beta Tg

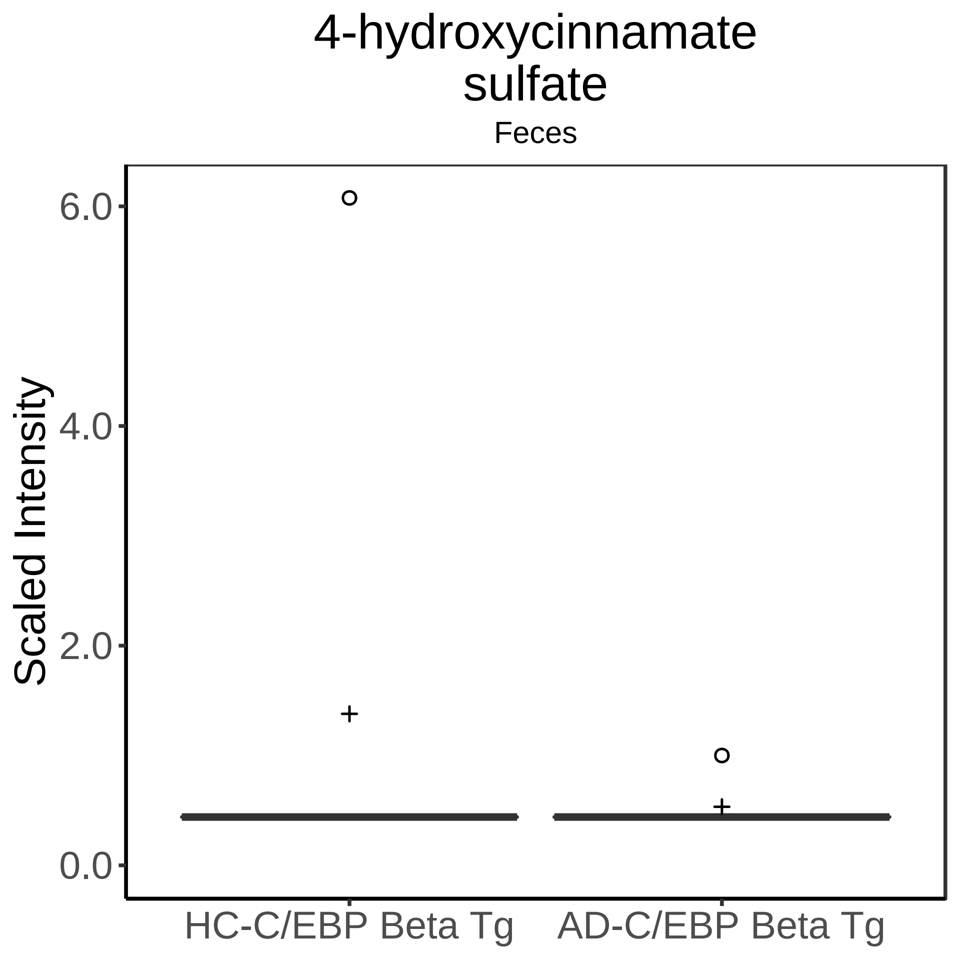
4-hydroxycinnamate sulfate
Feces
Scaled Intensity
6.0
4.0
2.0
0.0
HC-C/EBP Beta Tg
AD-C/EBP Beta Tg

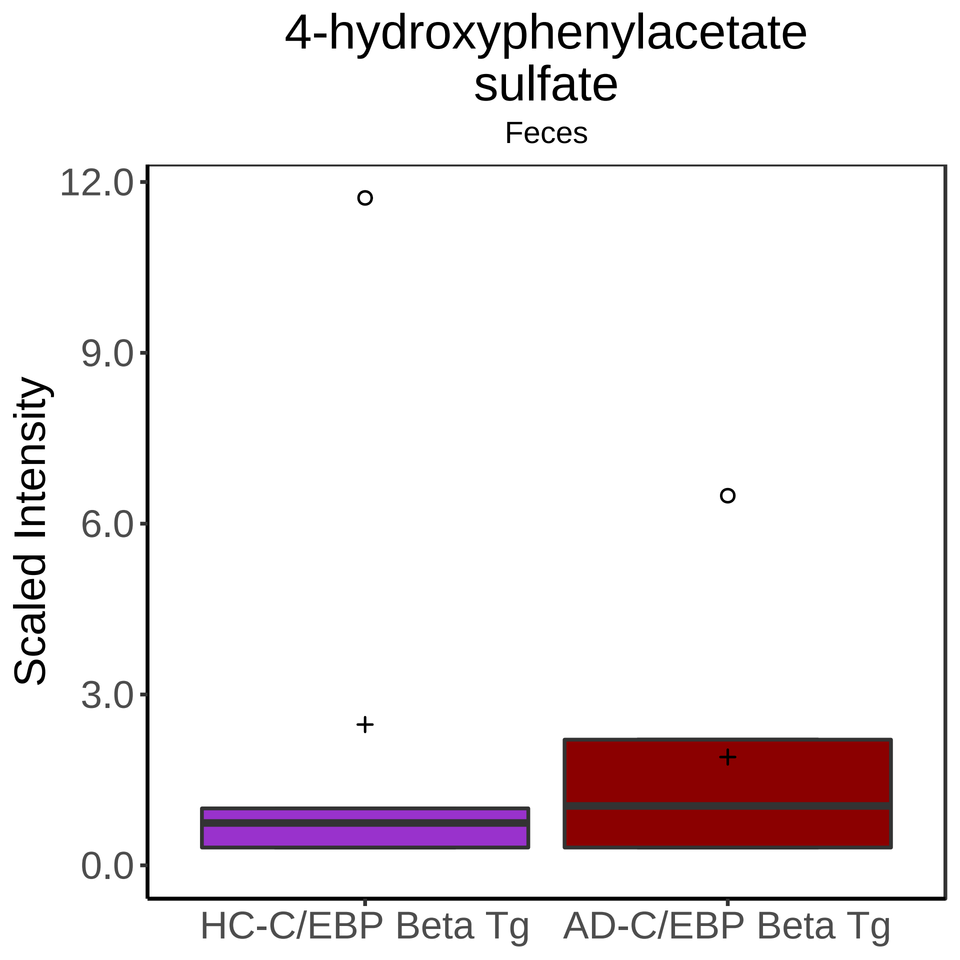
4-hydroxyphenylacetate sulfate
Feces
Scaled Intensity
12.0
9.0
6.0
3.0
0.0
HC-C/EBP Beta Tg
AD-C/EBP Beta Tg

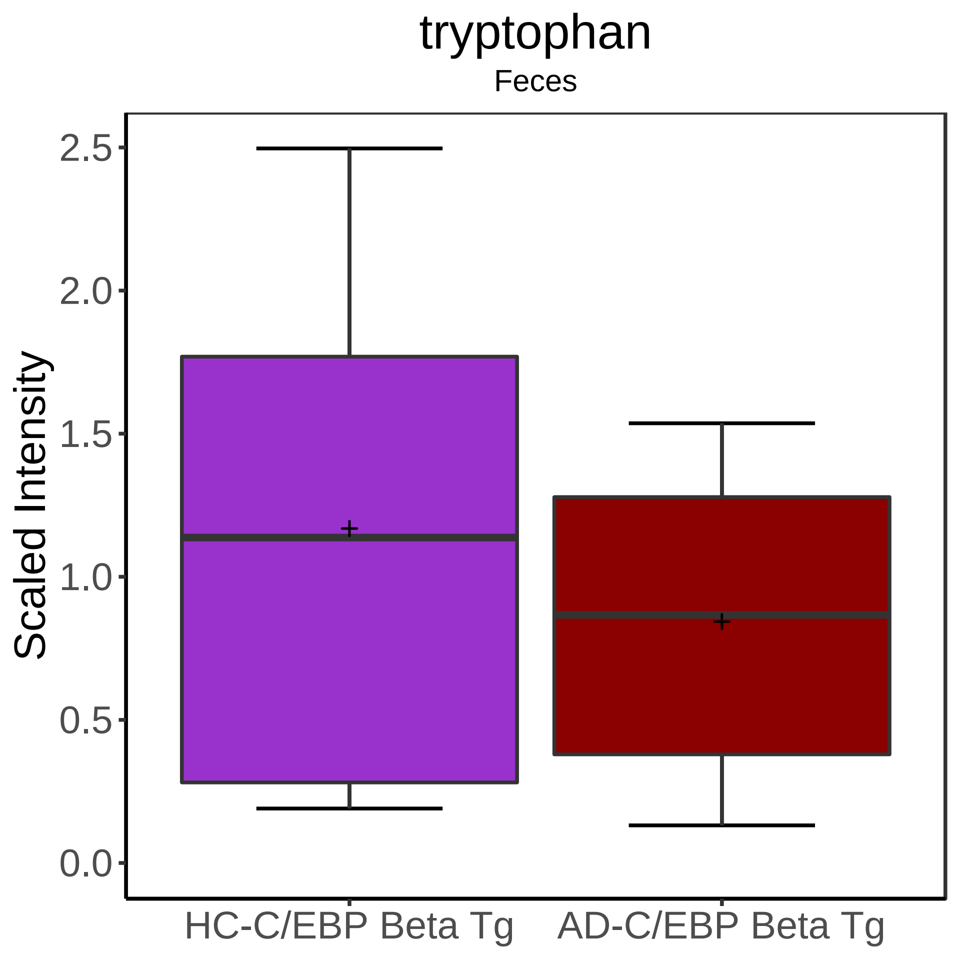
tryptophan
Feces
Scaled Intensity
2.5
2.0
1.5
1.0
0.5
0.0
HC-C/EBP Beta Tg
AD-C/EBP Beta Tg

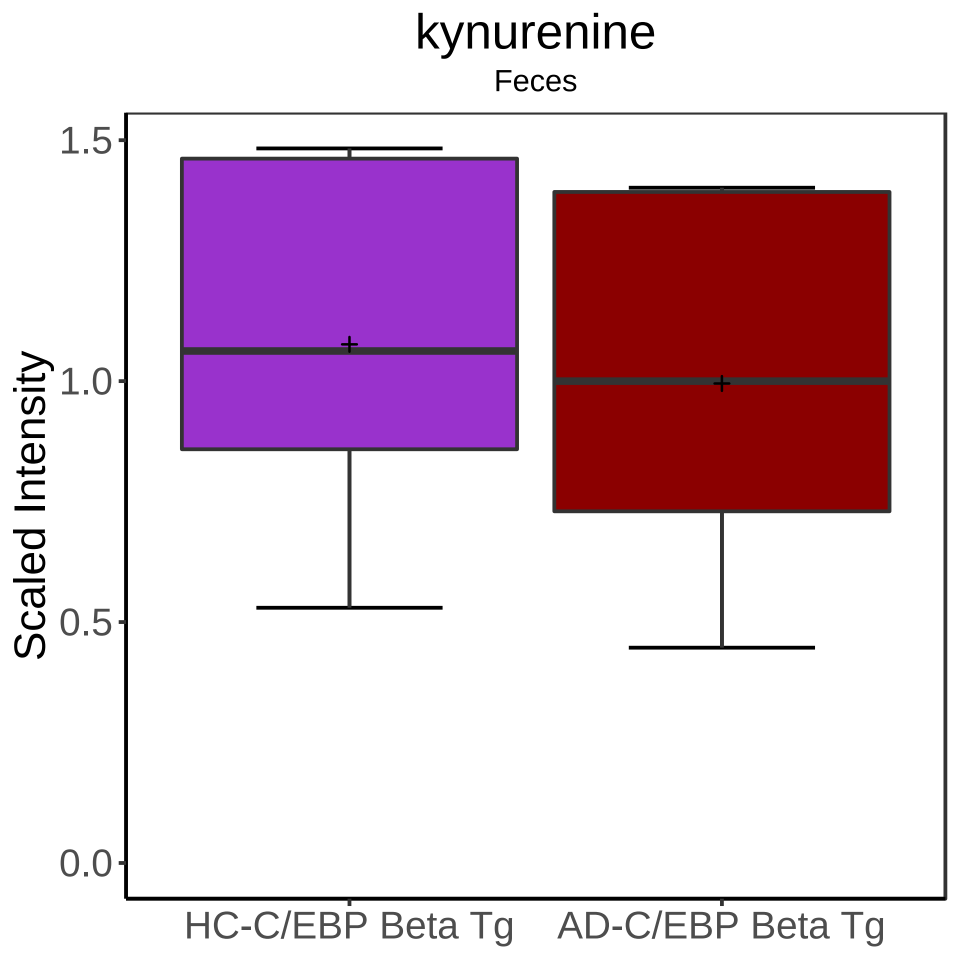
kynurenine
Feces
Scaled Intensity
1.5
1.0
0.5
0.0
HC-C/EBP Beta Tg
AD-C/EBP Beta Tg

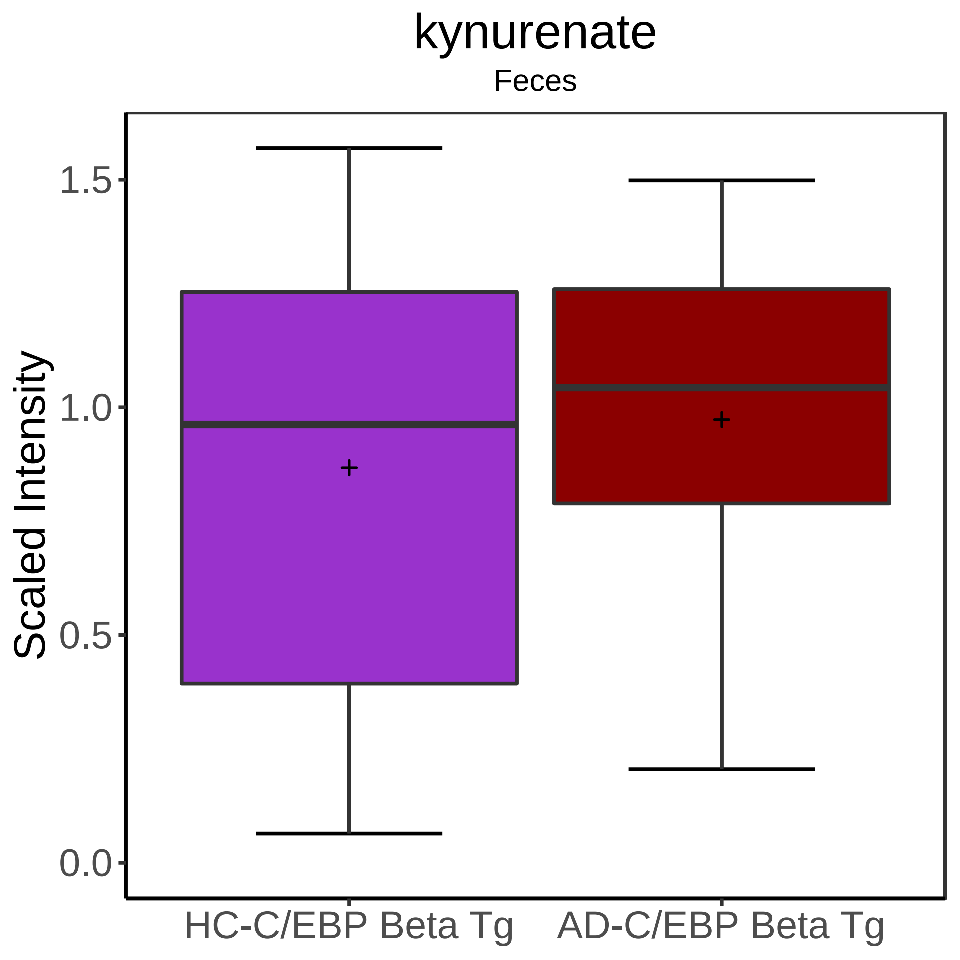

kynurenate
Feces
Scaled Intensity
1.5
1.0
0.5
0.0
HC-C/EBP Beta Tg
AD-C/EBP Beta Tg

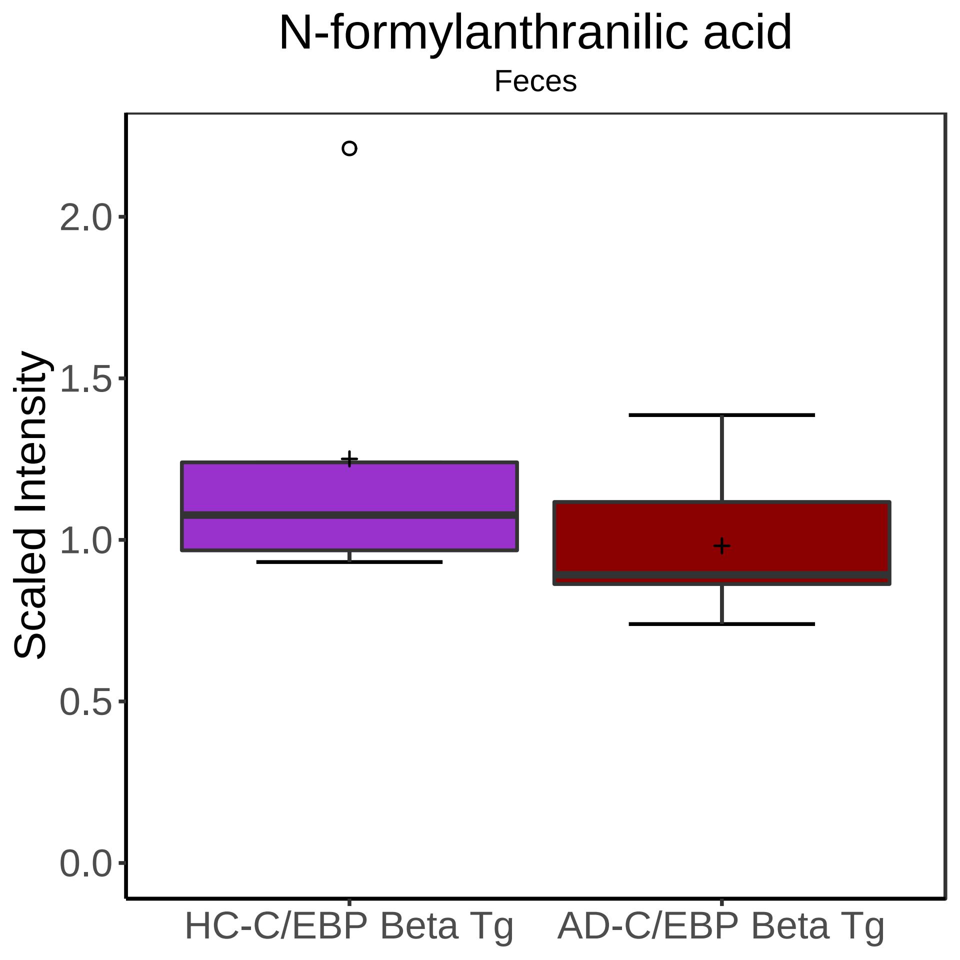
N-formylanthranilic acid
Feces
Scaled Intensity
2.0
1.5
1.0
0.5
0.0
HC-C/EBP Beta Tg
AD-C/EBP Beta Tg

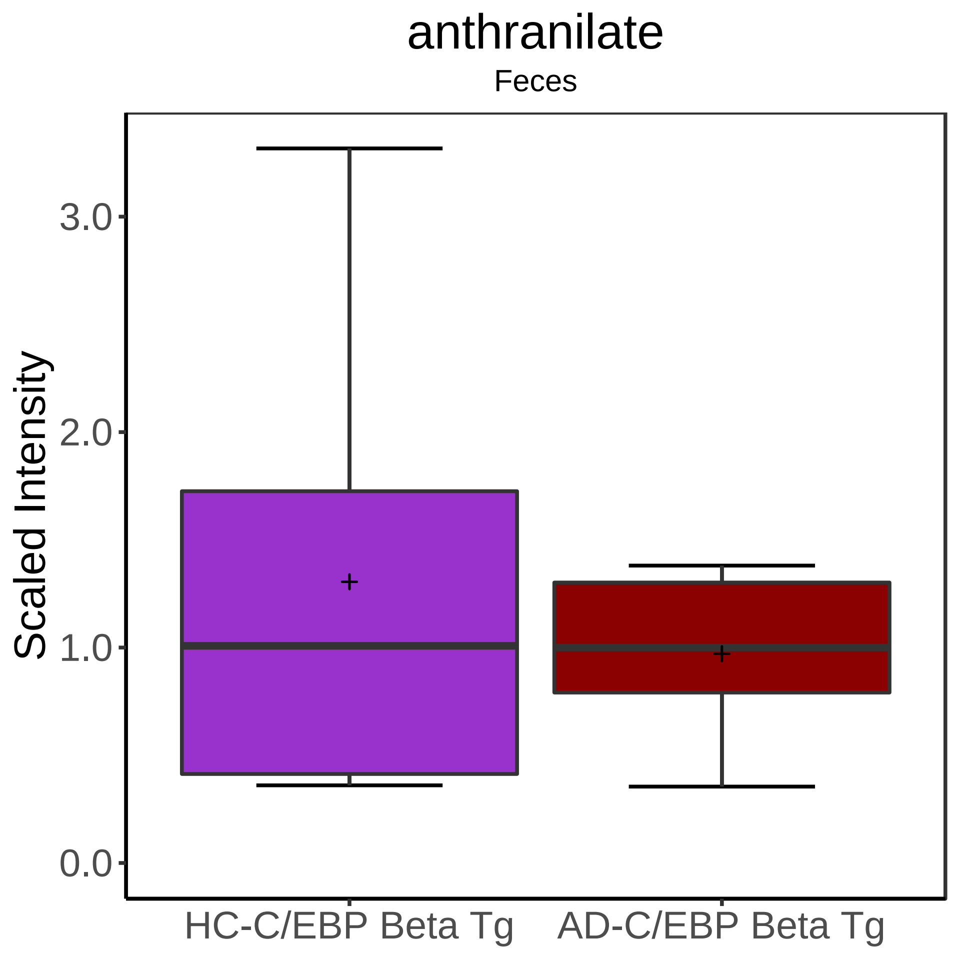
anthranilate
Feces
Scaled Intensity
3.0
2.0
1.0
0.0
HC-C/EBP Beta Tg
AD-C/EBP Beta Tg

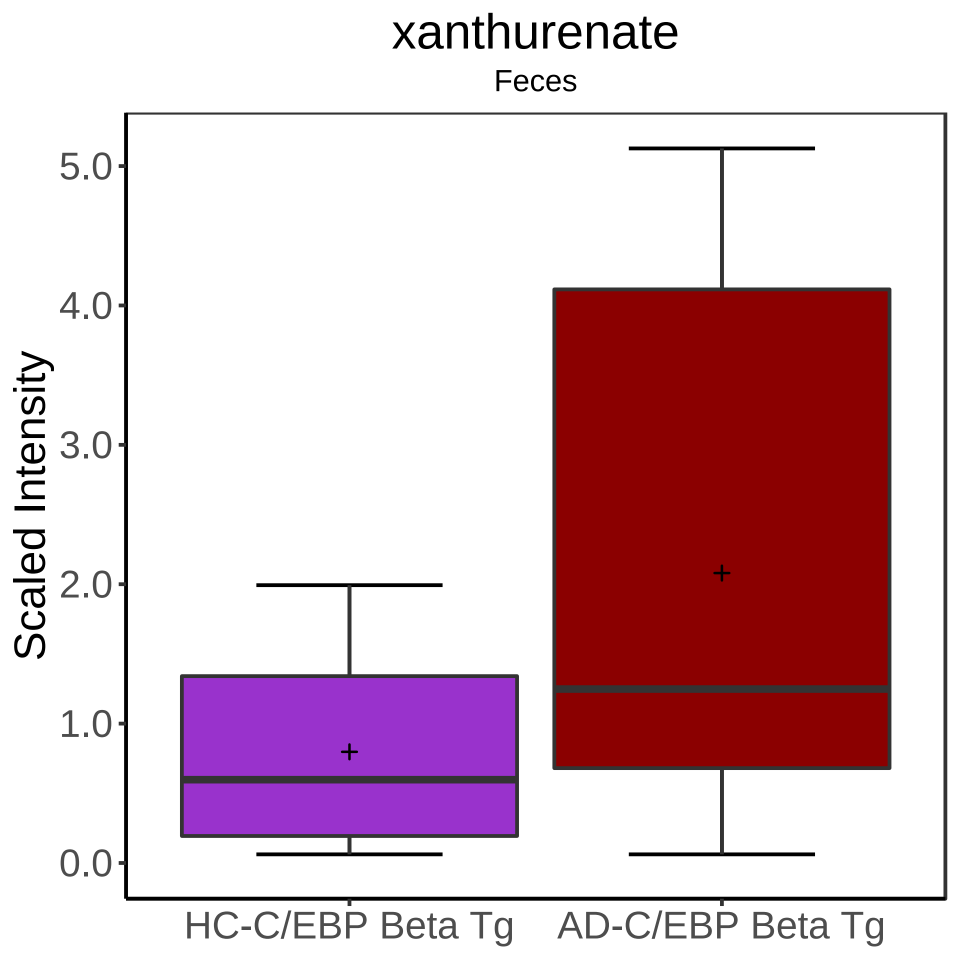
xanthurenate
Feces
Scaled Intensity
5.0
4.0
3.0
2.0
1.0
0.0
HC-C/EBP Beta Tg
AD-C/EBP Beta Tg

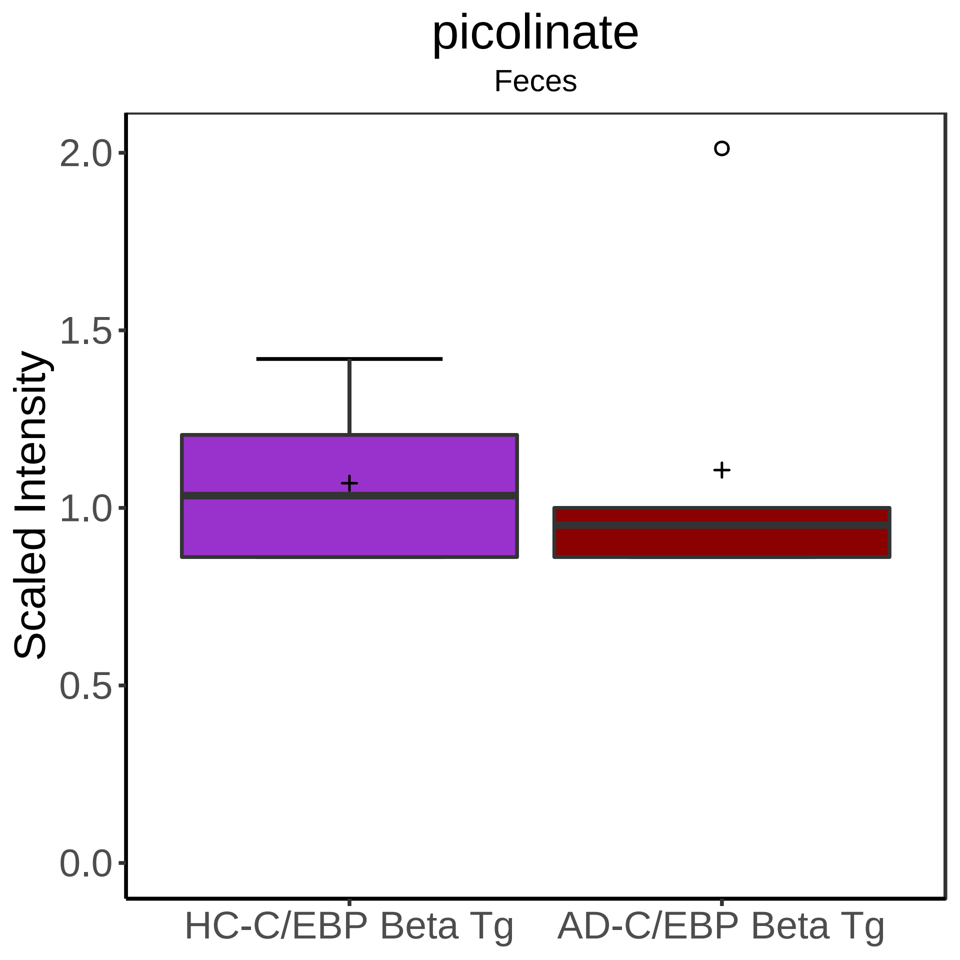

picolinate
Feces
Scaled Intensity
2.0
1.5
1.0
0.5
0.0
HC-C/EBP Beta Tg
AD-C/EBP Beta Tg

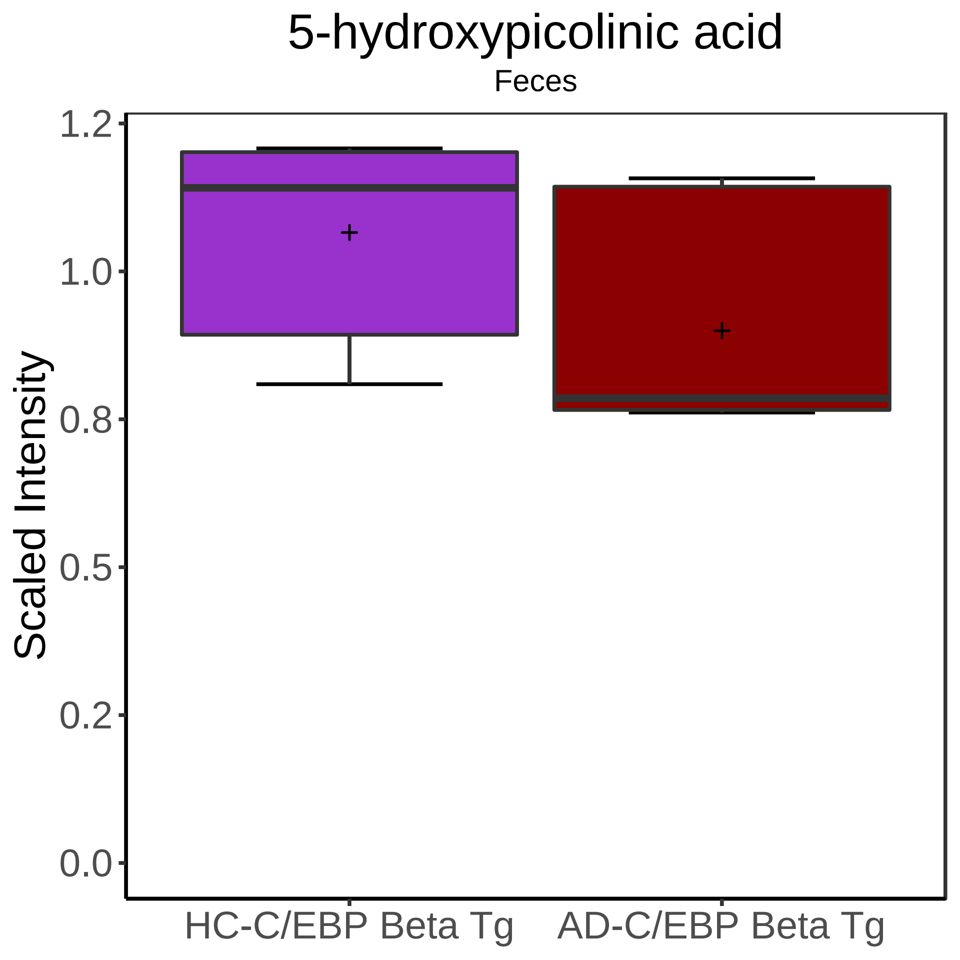
5-hydroxypicolinic acid
Feces
Scaled Intensity
1.2
1.0
0.8
0.5
0.2
0.0
HC-C/EBP Beta Tg
AD-C/EBP Beta Tg

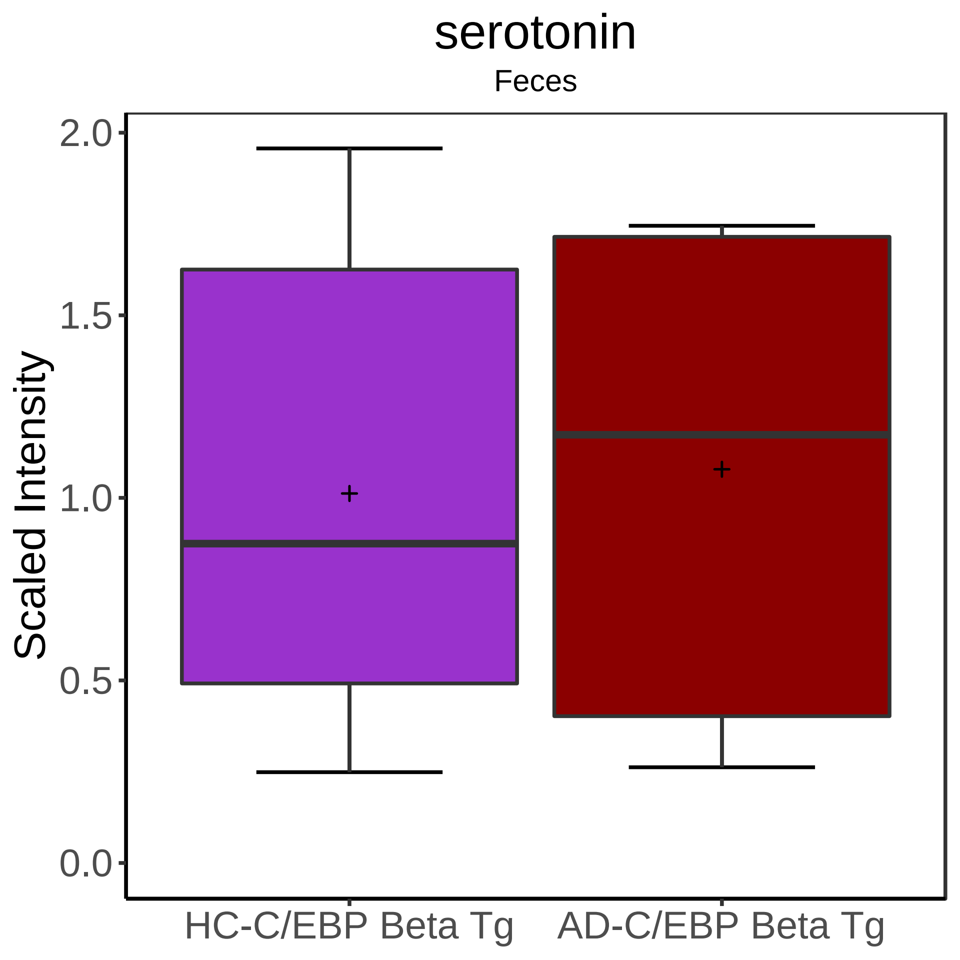
serotonin
Feces
Scaled Intensity
2.0
1.5
1.0
0.5
0.0
HC-C/EBP Beta Tg
AD-C/EBP Beta Tg

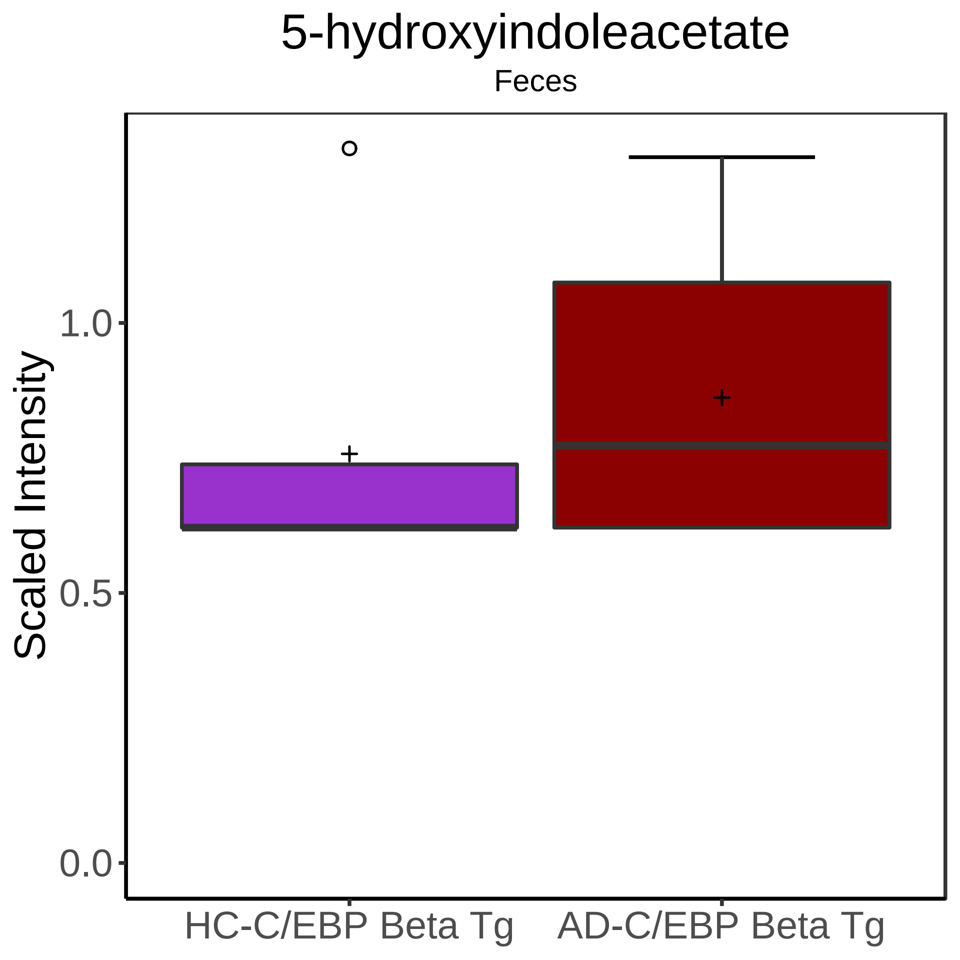

5-hydroxyindoleacetate
Feces
Scaled Intensity
1.0
0.5
0.0
HC-C/EBP Beta Tg
AD-C/EBP Beta Tg

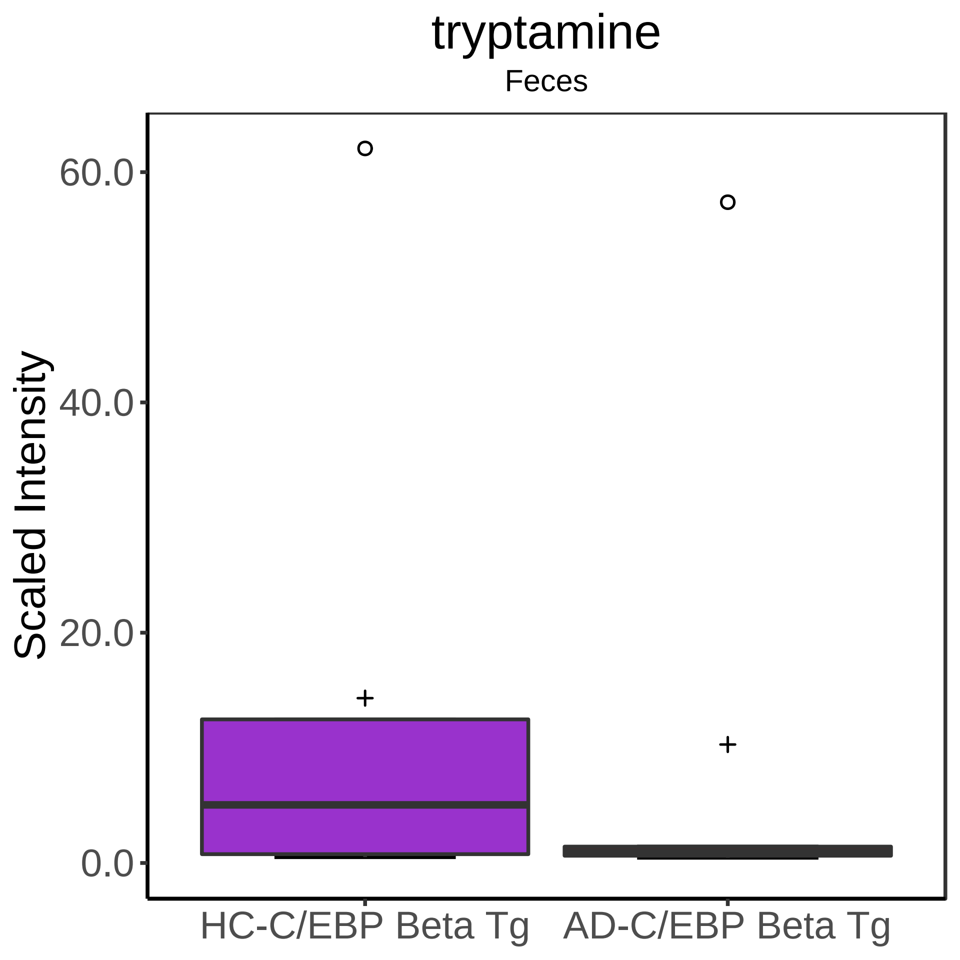
tryptamine
Feces
Scaled Intensity
60.0
40.0
20.0
0.0
HC-C/EBP Beta Tg
AD-C/EBP Beta Tg

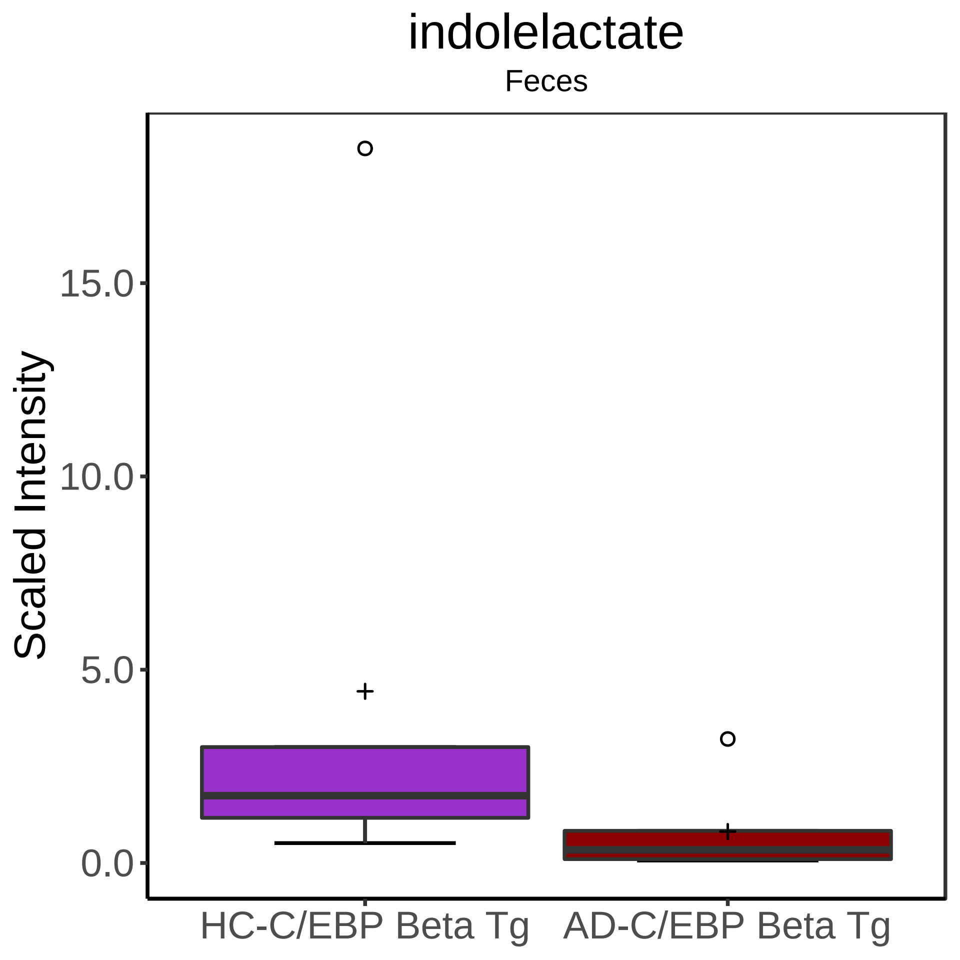
indolelactate
Feces
Scaled Intensity
15.0
10.0
5.0
0.0
HC-C/EBP Beta Tg
AD-C/EBP Beta Tg

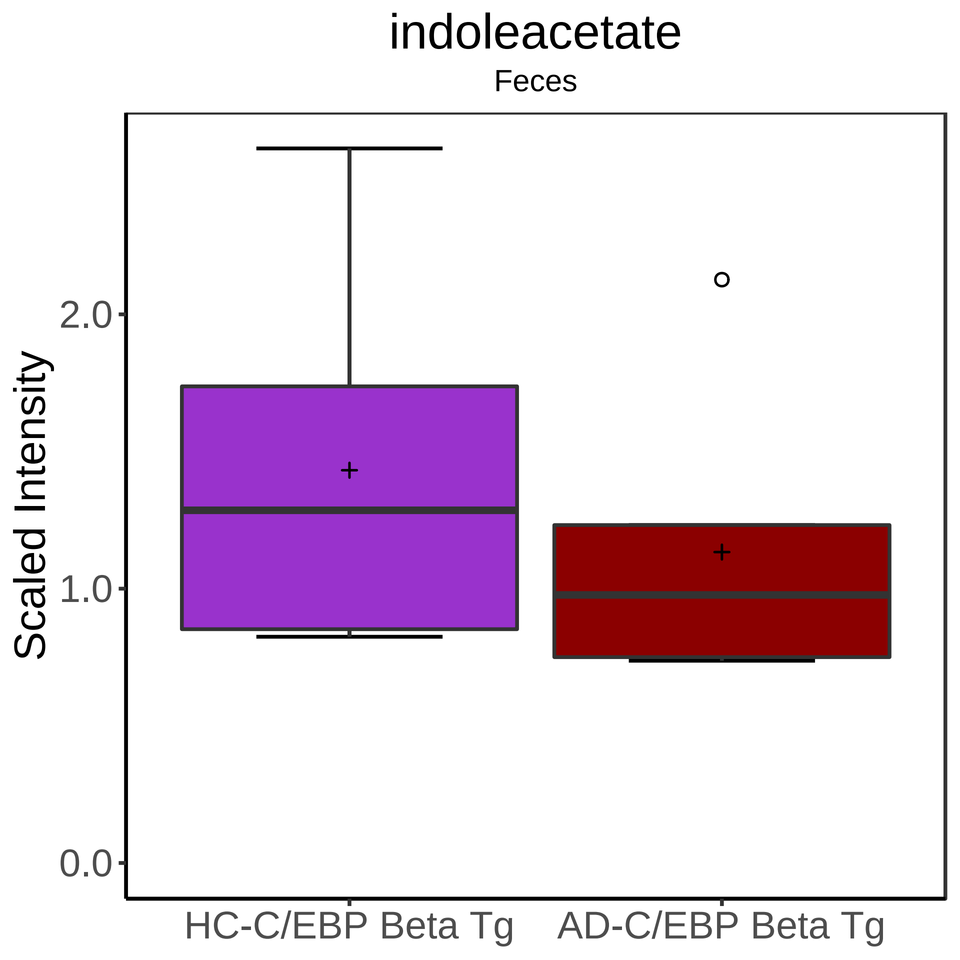
indoleacetate
Feces
Scaled Intensity
2.0
1.0
0.0
HC-C/EBP Beta Tg
AD-C/EBP Beta Tg

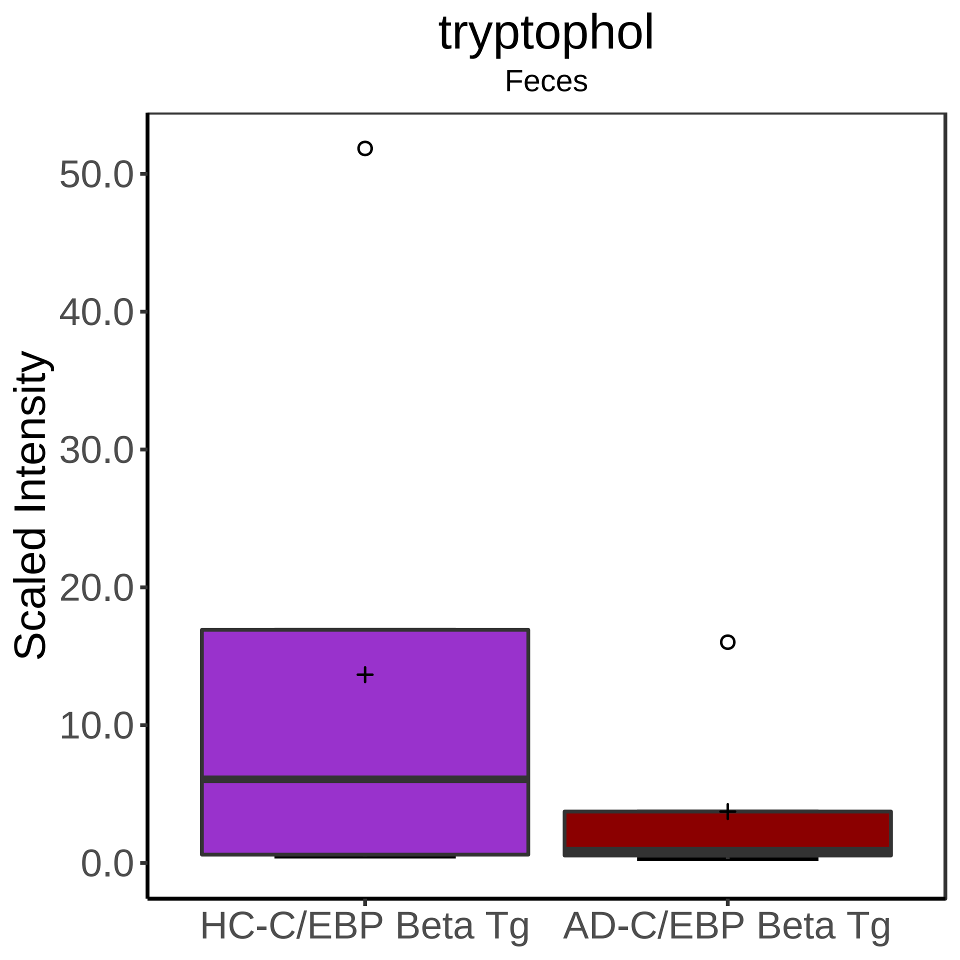
tryptophol
Feces
Scaled Intensity
50.0
40.0
30.0
20.0
10.0
0.0
HC-C/EBP Beta Tg
AD-C/EBP Beta Tg

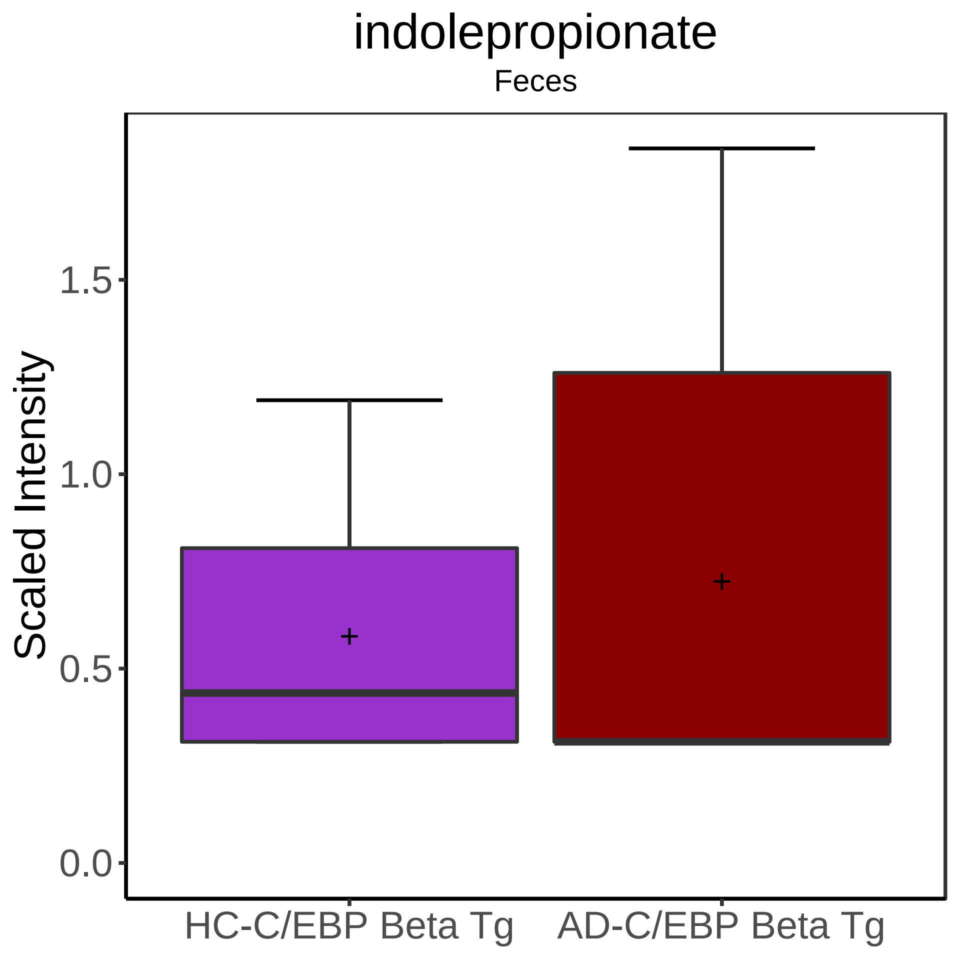
indolepropionate
Feces
Scaled Intensity
0.0
0.5
1.0
1.5
HC-C/EBP Beta Tg
AD-C/EBP Beta Tg

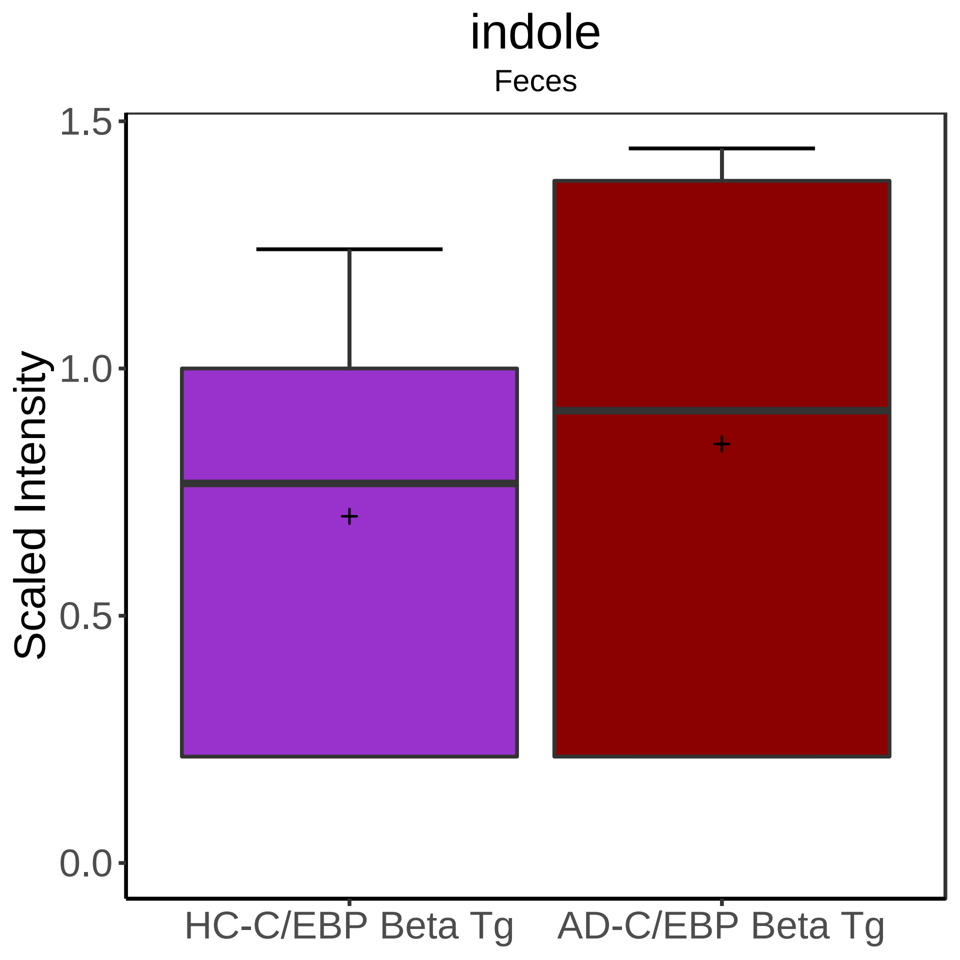

indole
Feces
Scaled Intensity
1.5
1.0
0.5
0.0
HC-C/EBP Beta Tg
AD-C/EBP Beta Tg

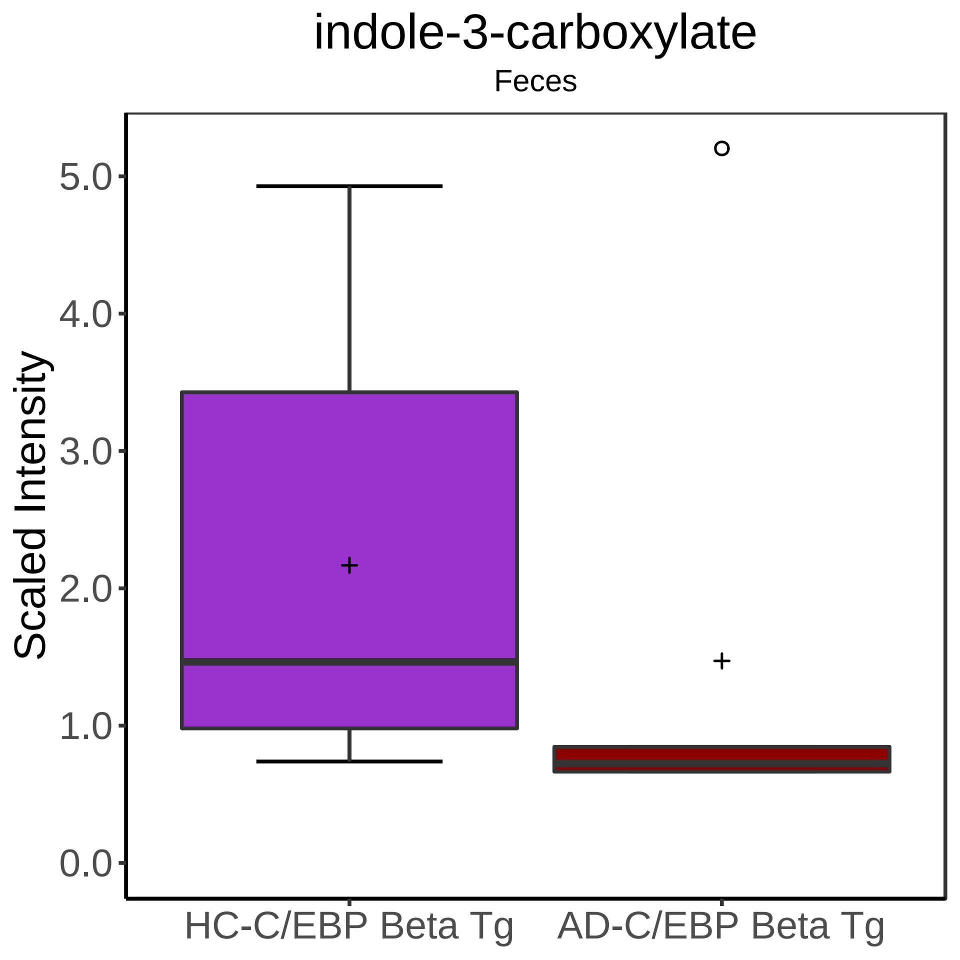
indole-3-carboxylate
Feces
Scaled Intensity
5.0
4.0
3.0
2.0
1.0
0.0
HC-C/EBP Beta Tg
AD-C/EBP Beta Tg

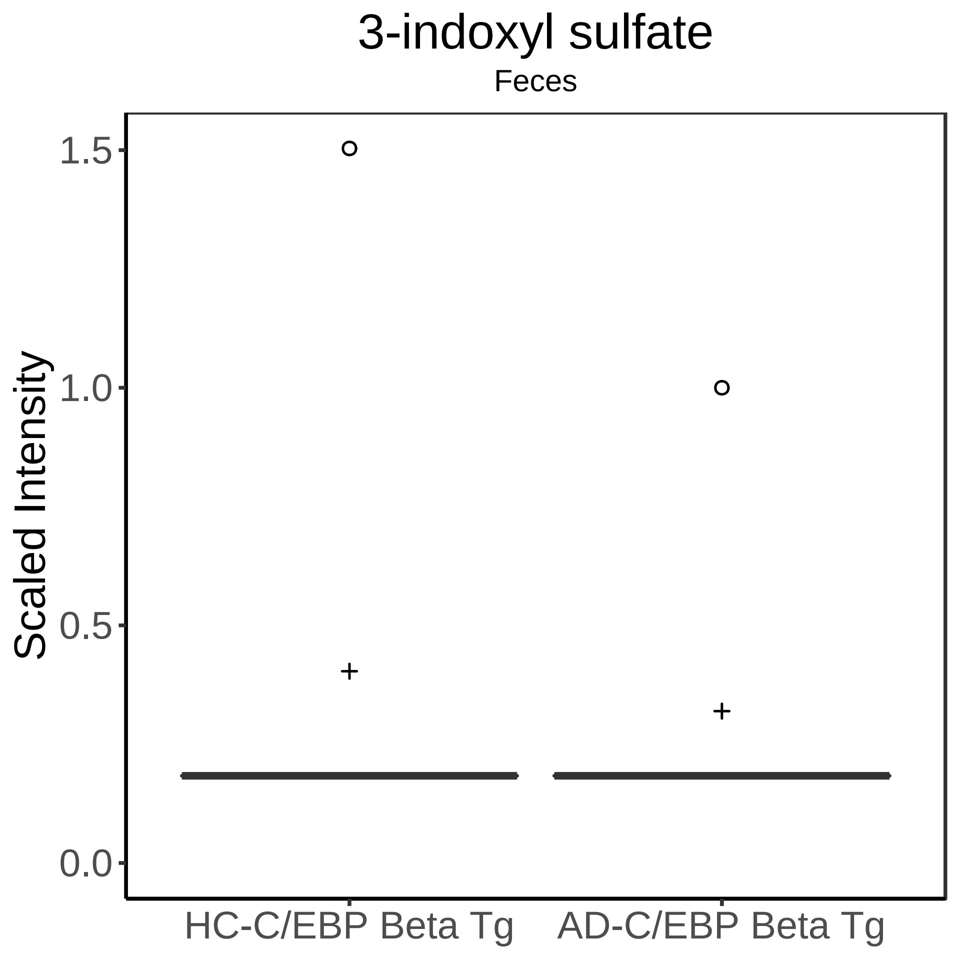
3-indoxyl sulfate
Feces
Scaled Intensity
1.5
1.0
0.5
0.0
HC-C/EBP Beta Tg
AD-C/EBP Beta Tg

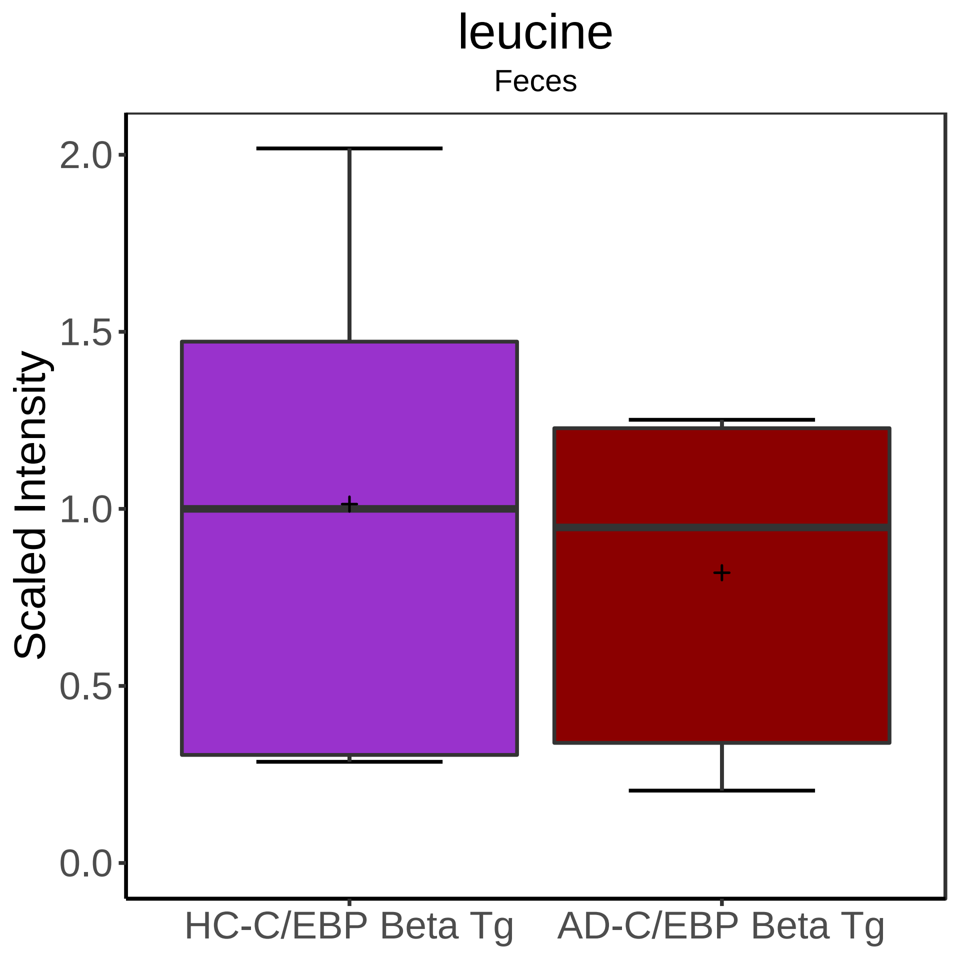

leucine
Feces
Scaled Intensity
2.0
1.5
1.0
0.5
0.0
HC-C/EBP Beta Tg
AD-C/EBP Beta Tg

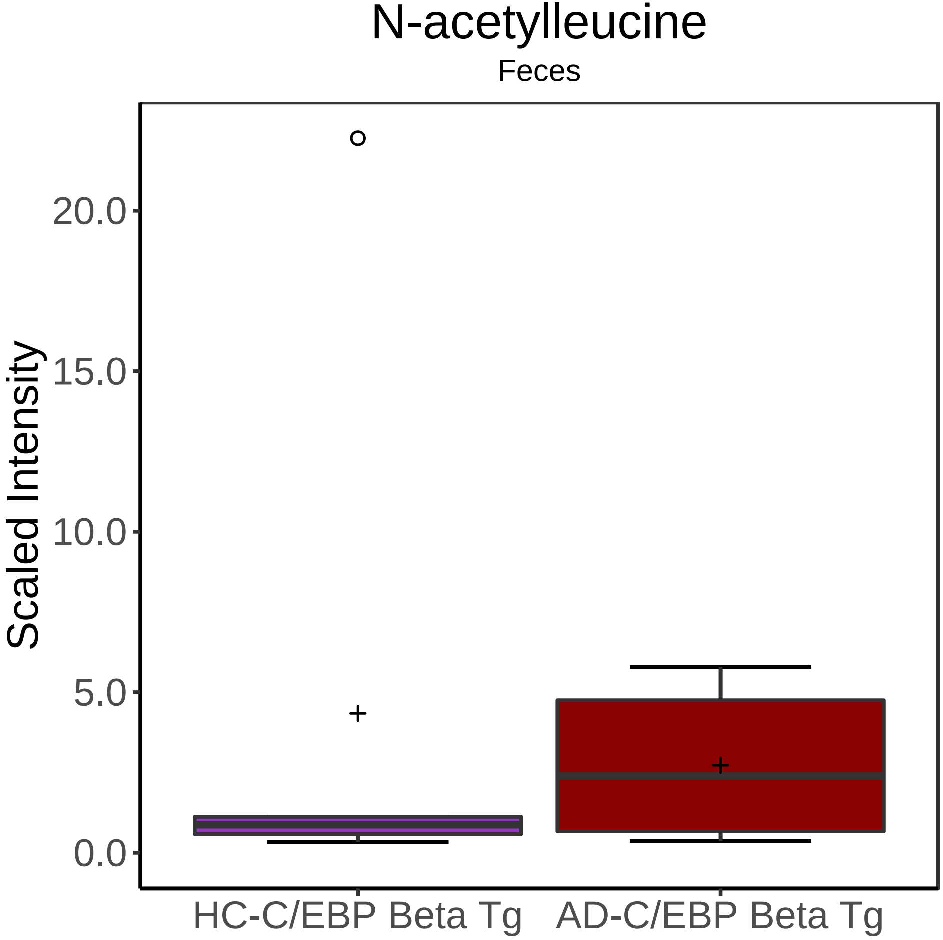

N-acetylleucine
Feces
Scaled Intensity
20.0
15.0
10.0
5.0
0.0
HC-C/EBP Beta Tg
AD-C/EBP Beta Tg

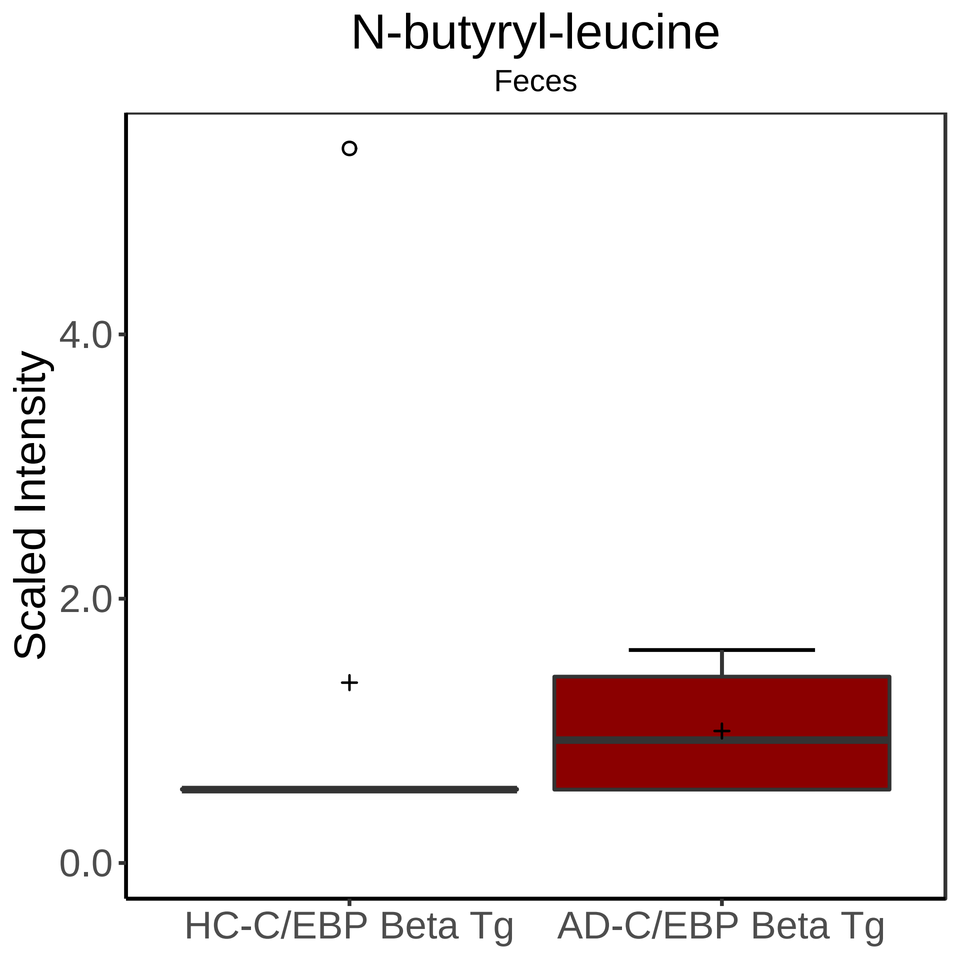
N-butyryl-leucine
Feces
Scaled Intensity
4.0
2.0
0.0
HC-C/EBP Beta Tg
AD-C/EBP Beta Tg

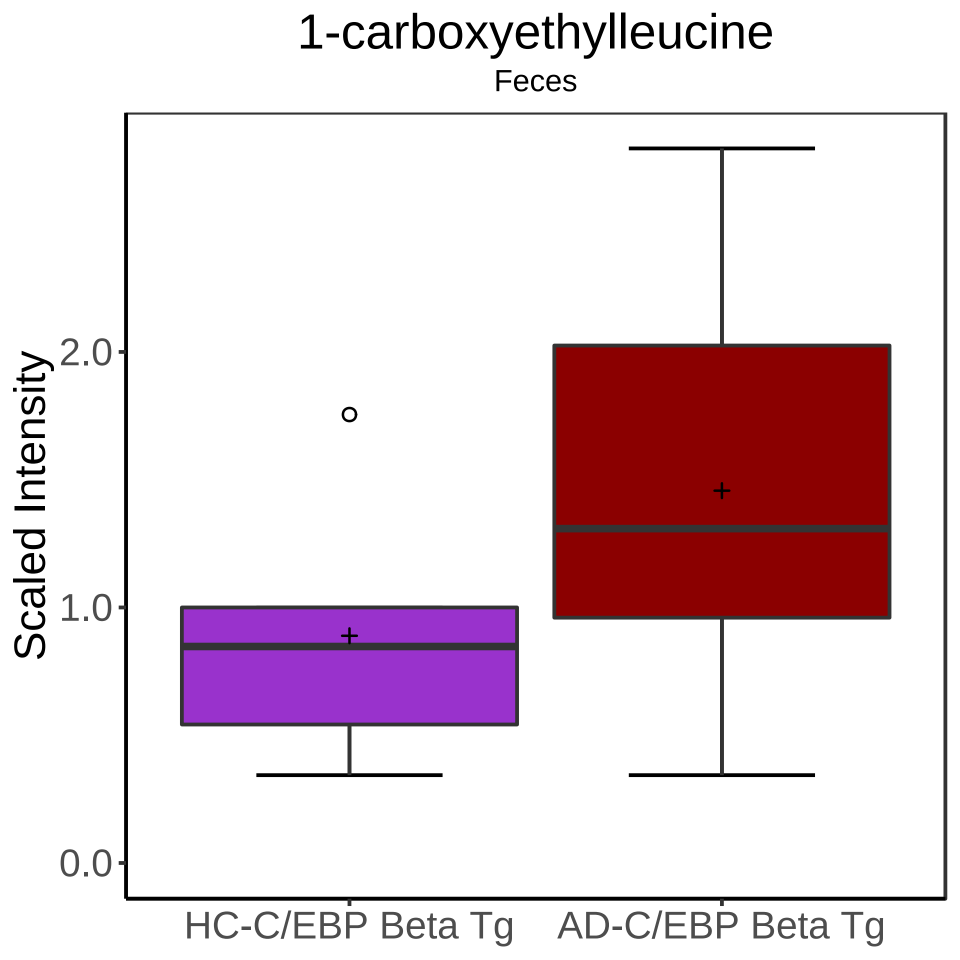
1-carboxyethylleucine
Feces
Scaled Intensity
2.0
1.0
0.0
HC-C/EBP Beta Tg
AD-C/EBP Beta Tg

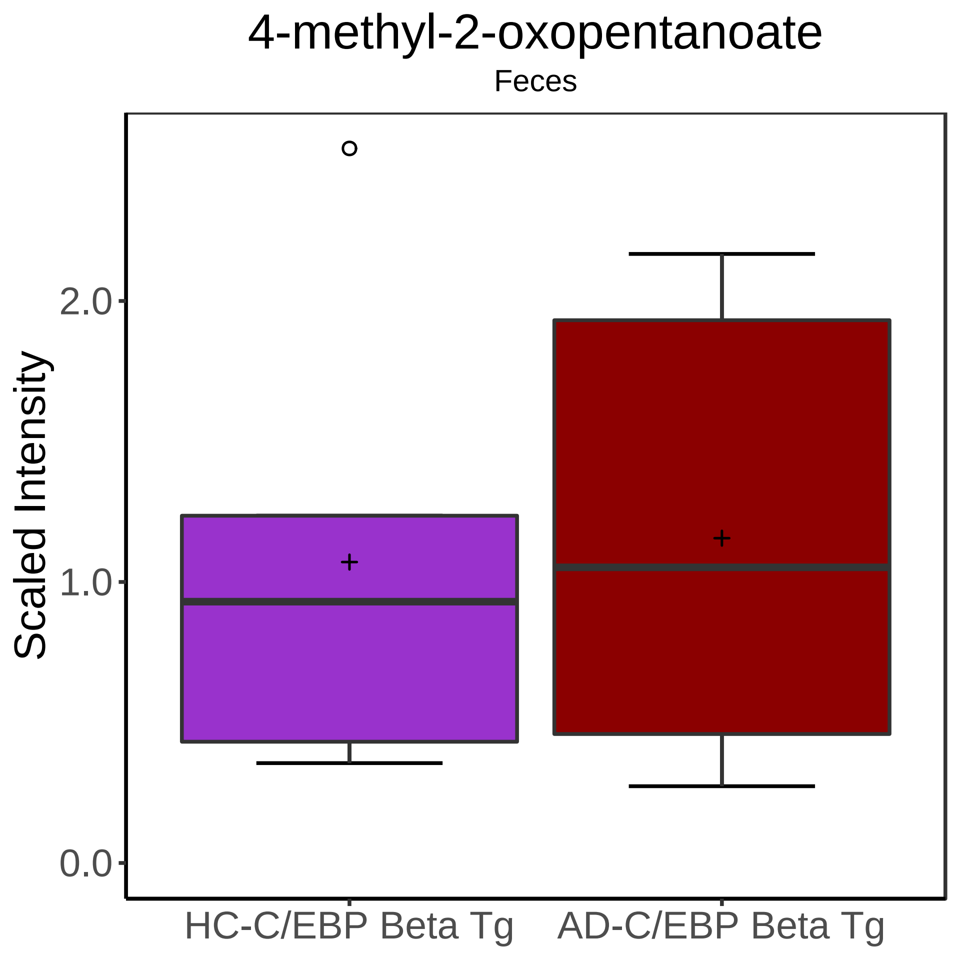

4-methyl-2-oxopentanoate
Feces
Scaled Intensity
2.0
1.0
0.0
HC-C/EBP Beta Tg
AD-C/EBP Beta Tg

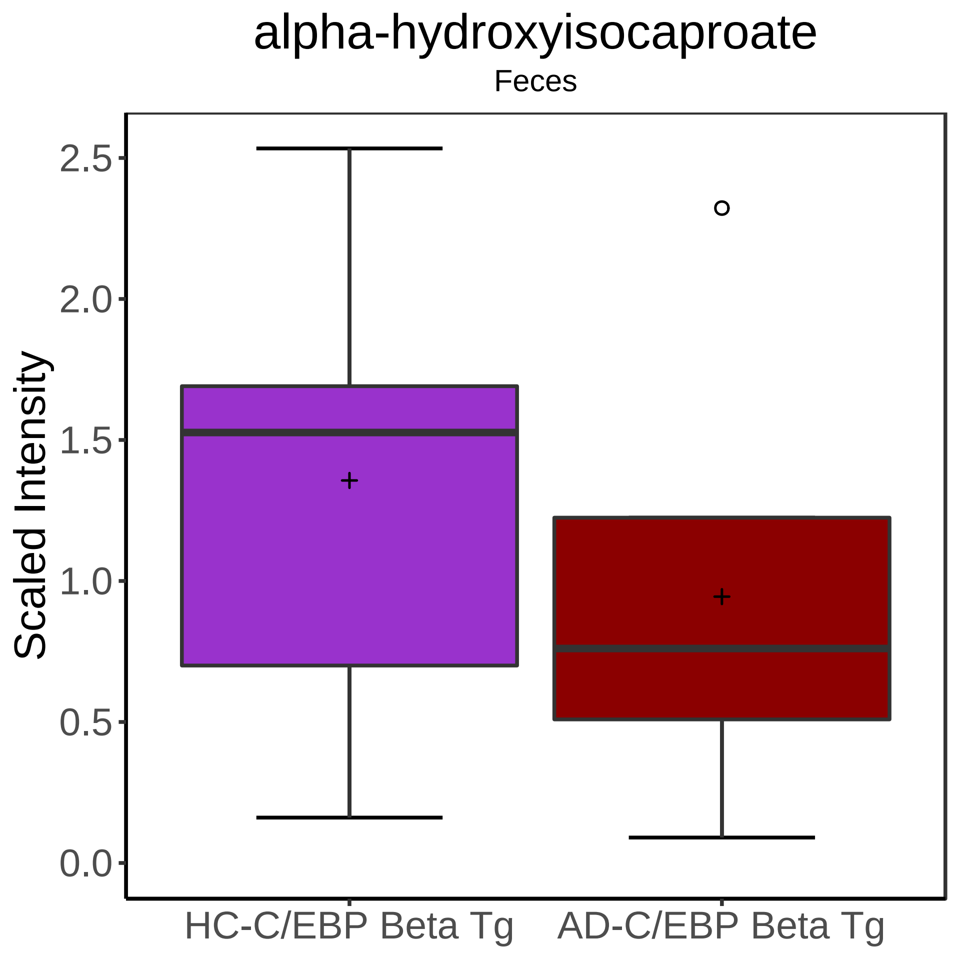
alpha-hydroxyisocaproate
Feces
Scaled Intensity
2.5
2.0
1.5
1.0
0.5
0.0
HC-C/EBP Beta Tg
AD-C/EBP Beta Tg

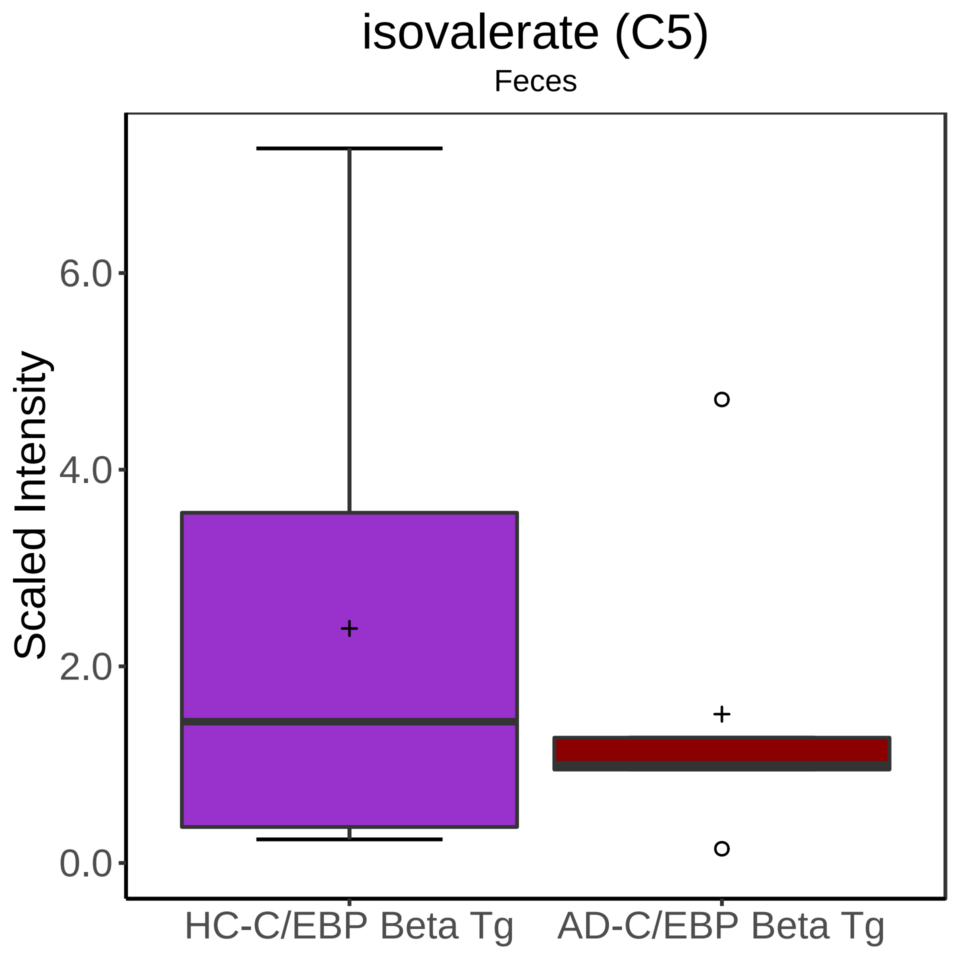
isovalerate (C5)
Feces
Scaled Intensity
6.0
4.0
2.0
0.0
HC-C/EBP Beta Tg
AD-C/EBP Beta Tg

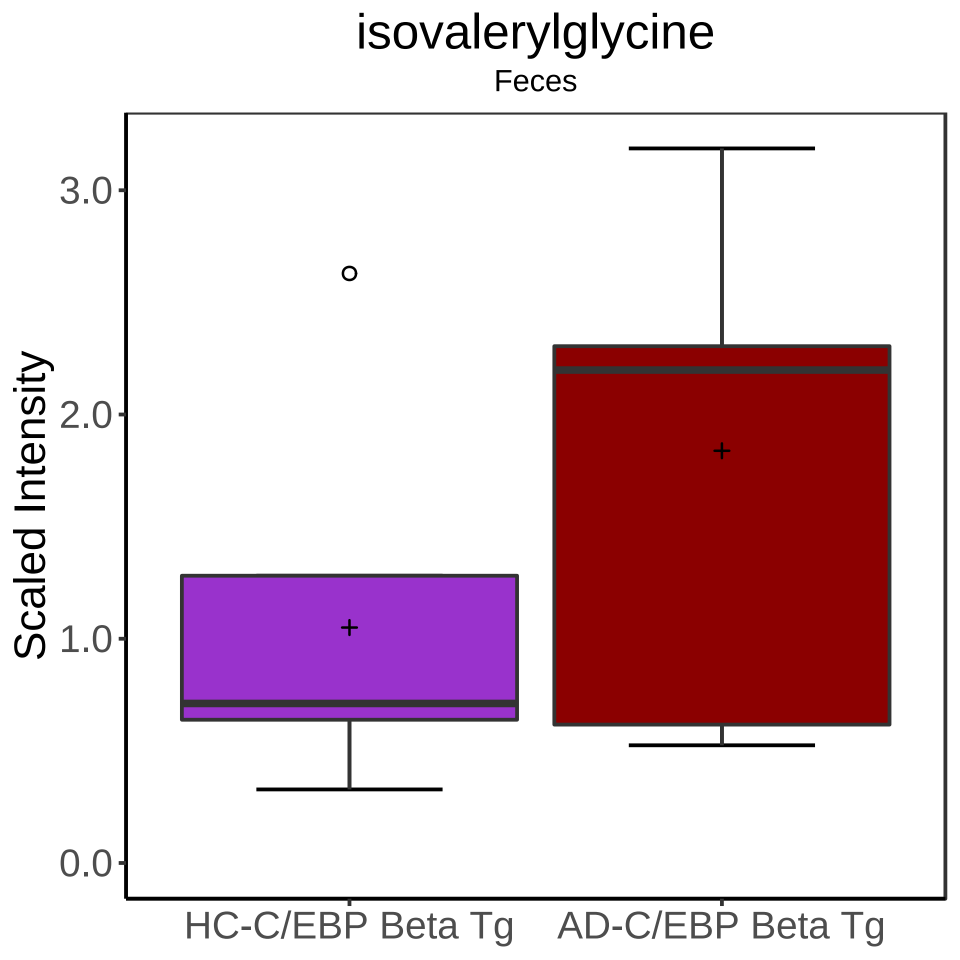
isovalerylglycine
Feces
Scaled Intensity
3.0
2.0
1.0
0.0
HC-C/EBP Beta Tg
AD-C/EBP Beta Tg

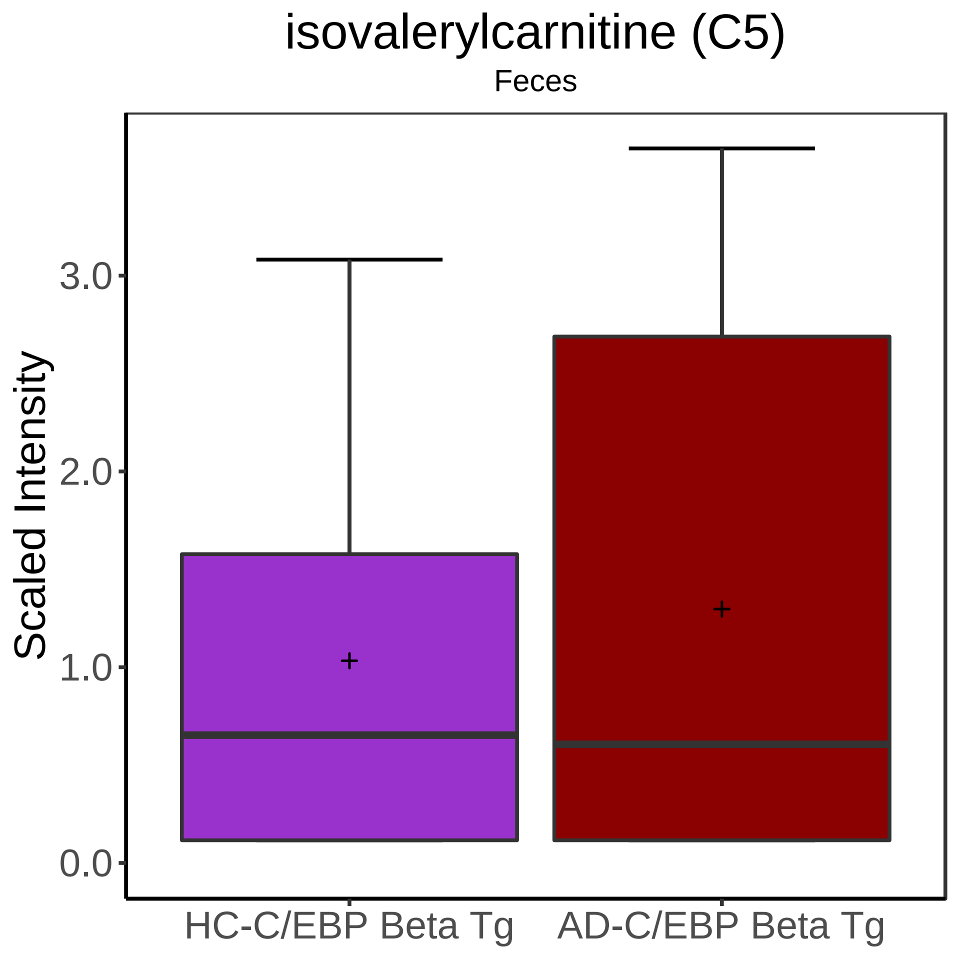
isovalerylcarnitine (C5)
Feces
Scaled Intensity
3.0
2.0
1.0
0.0
HC-C/EBP Beta Tg
AD-C/EBP Beta Tg

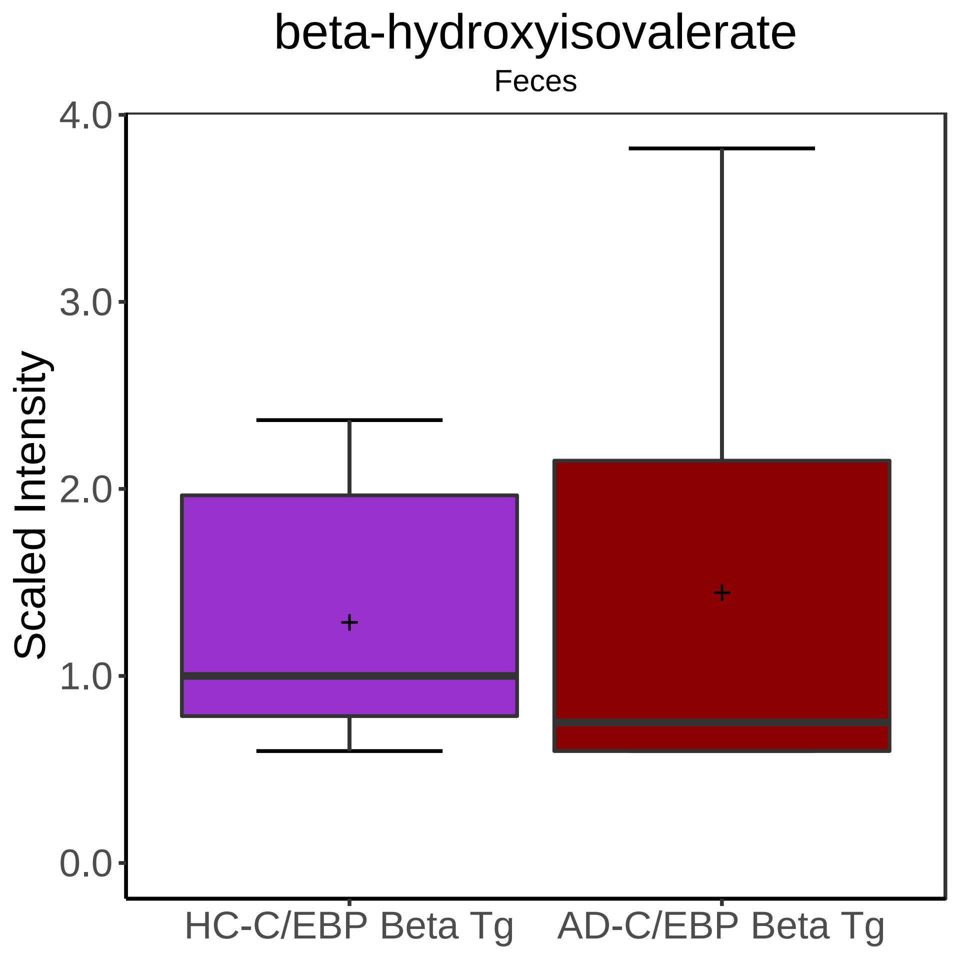
beta-hydroxyisovalerate
Feces
Scaled Intensity
4.0
3.0
2.0
1.0
0.0
HC-C/EBP Beta Tg
AD-C/EBP Beta Tg

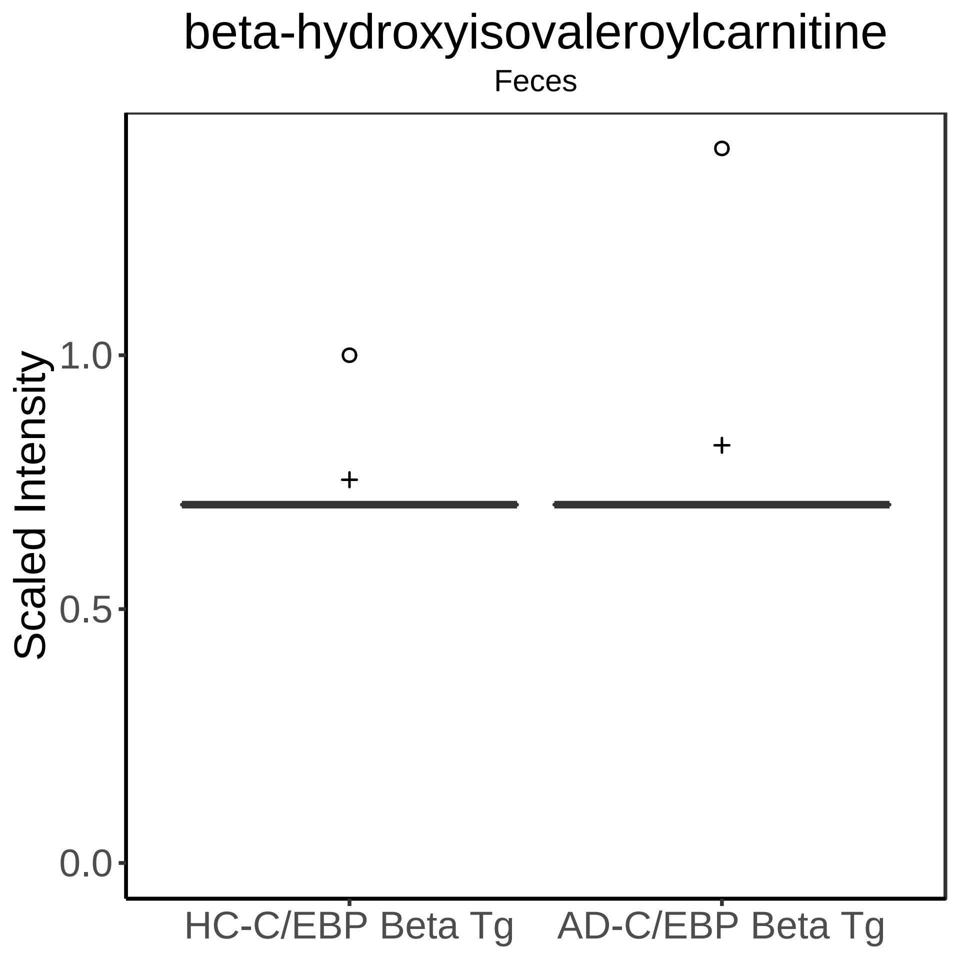

beta-hydroxyisovaleroylcarnitine
Feces
Scaled Intensity
1.0
0.5
0.0
HC-C/EBP Beta Tg
AD-C/EBP Beta Tg

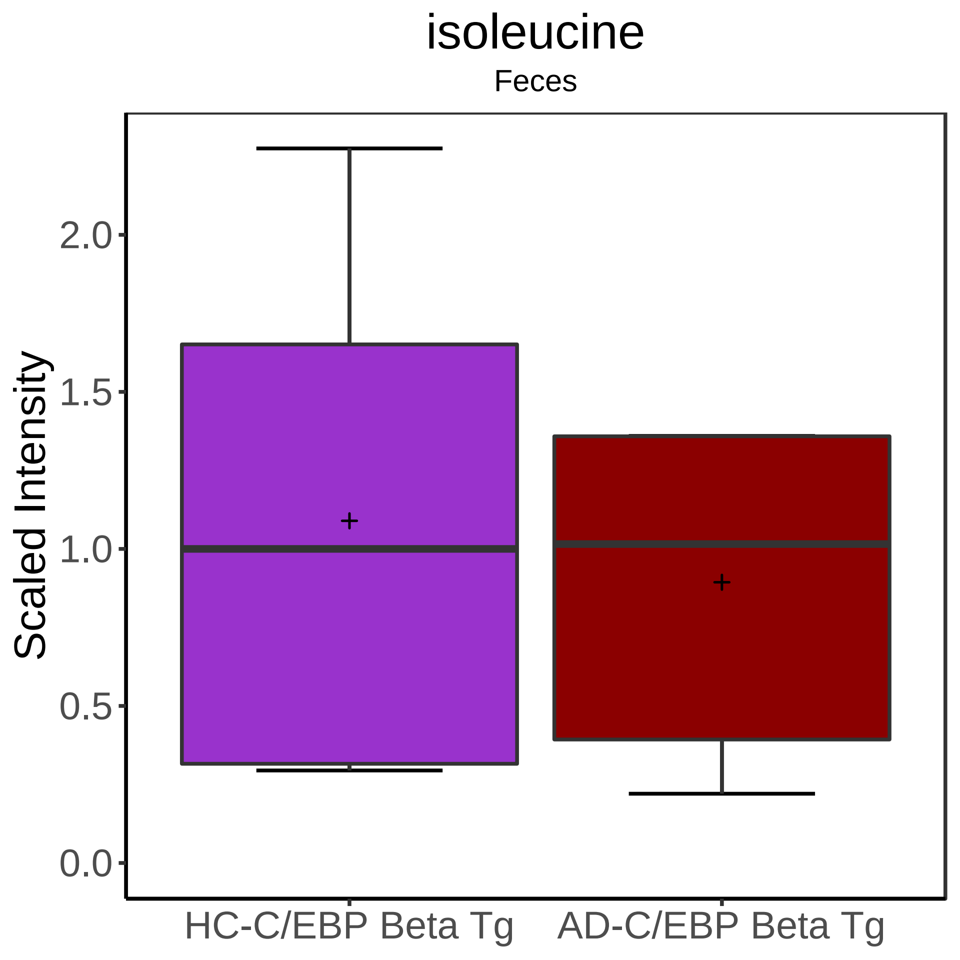

isoleucine
Feces
Scaled Intensity
2.0
1.5
1.0
0.5
0.0
HC-C/EBP Beta Tg
AD-C/EBP Beta Tg

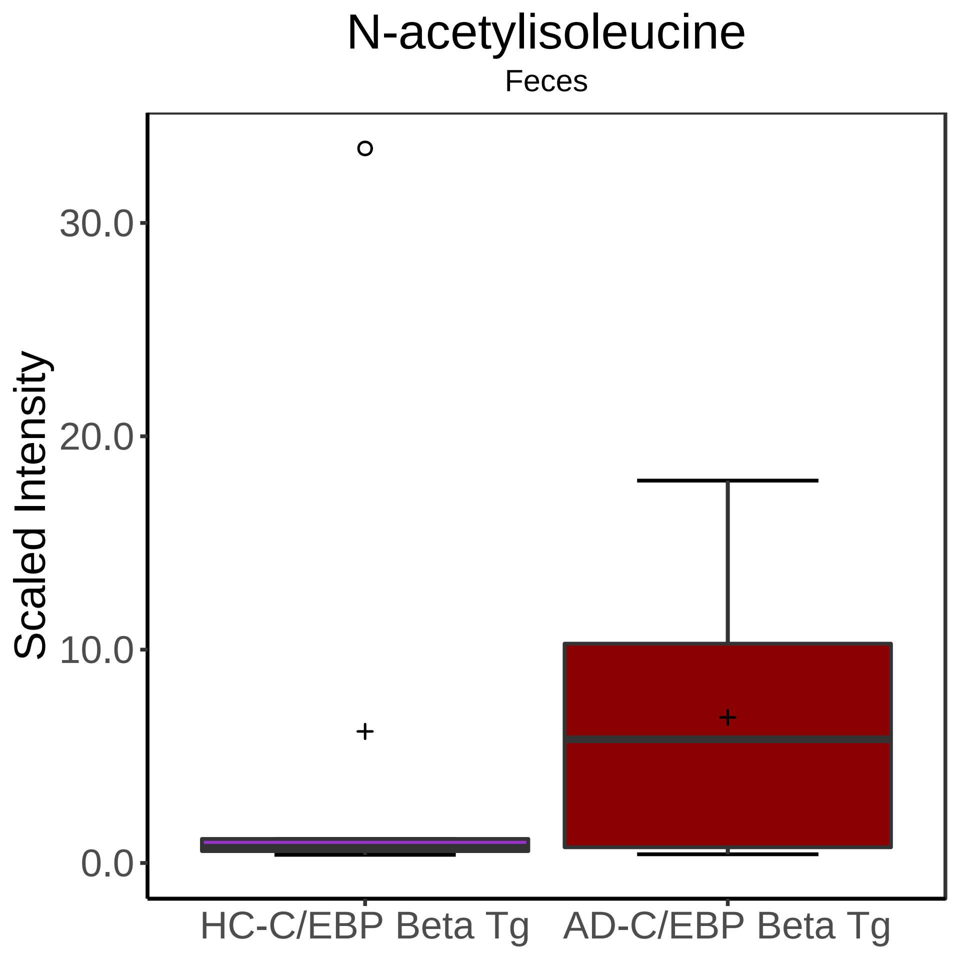

N-acetylisoleucine
Feces
Scaled Intensity
30.0
20.0
10.0
0.0
HC-C/EBP Beta Tg
AD-C/EBP Beta Tg

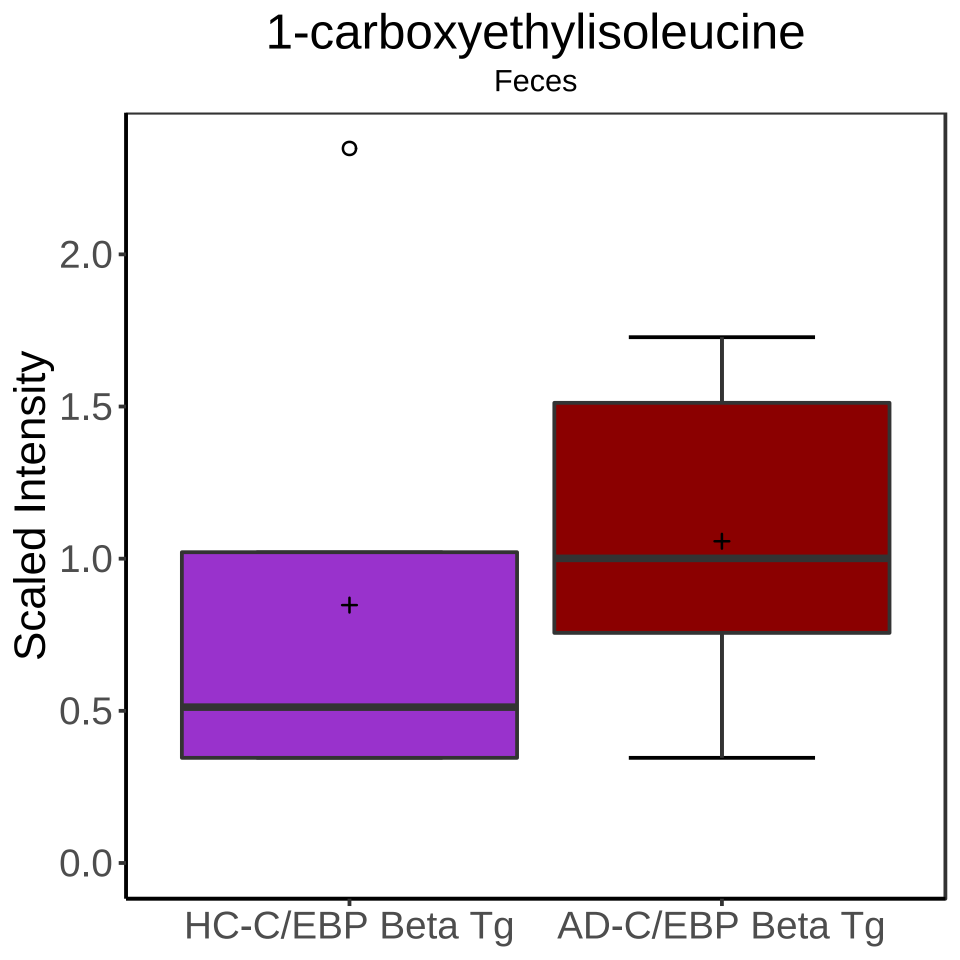
1-carboxyethylisoleucine
Feces
Scaled Intensity
2.0
1.5
1.0
0.5
0.0
HC-C/EBP Beta Tg
AD-C/EBP Beta Tg

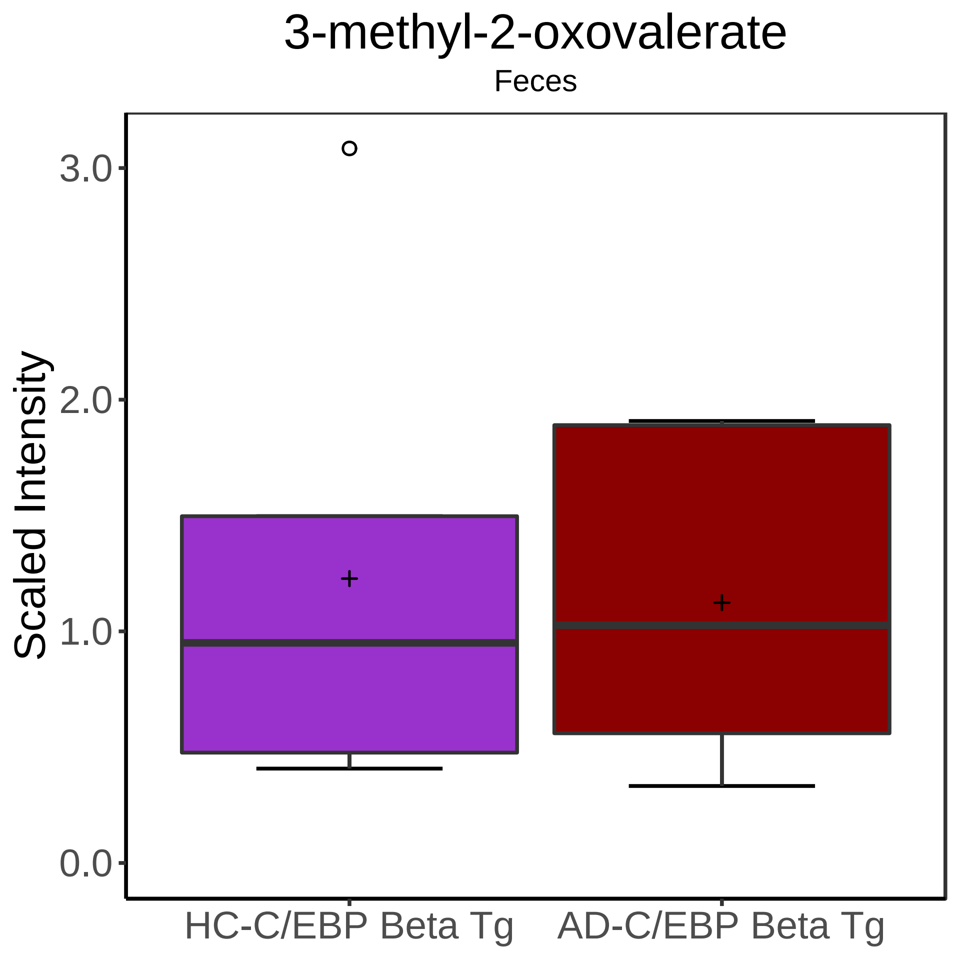

3-methyl-2-oxovalerate
Feces
Scaled Intensity
3.0
2.0
1.0
0.0
HC-C/EBP Beta Tg
AD-C/EBP Beta Tg

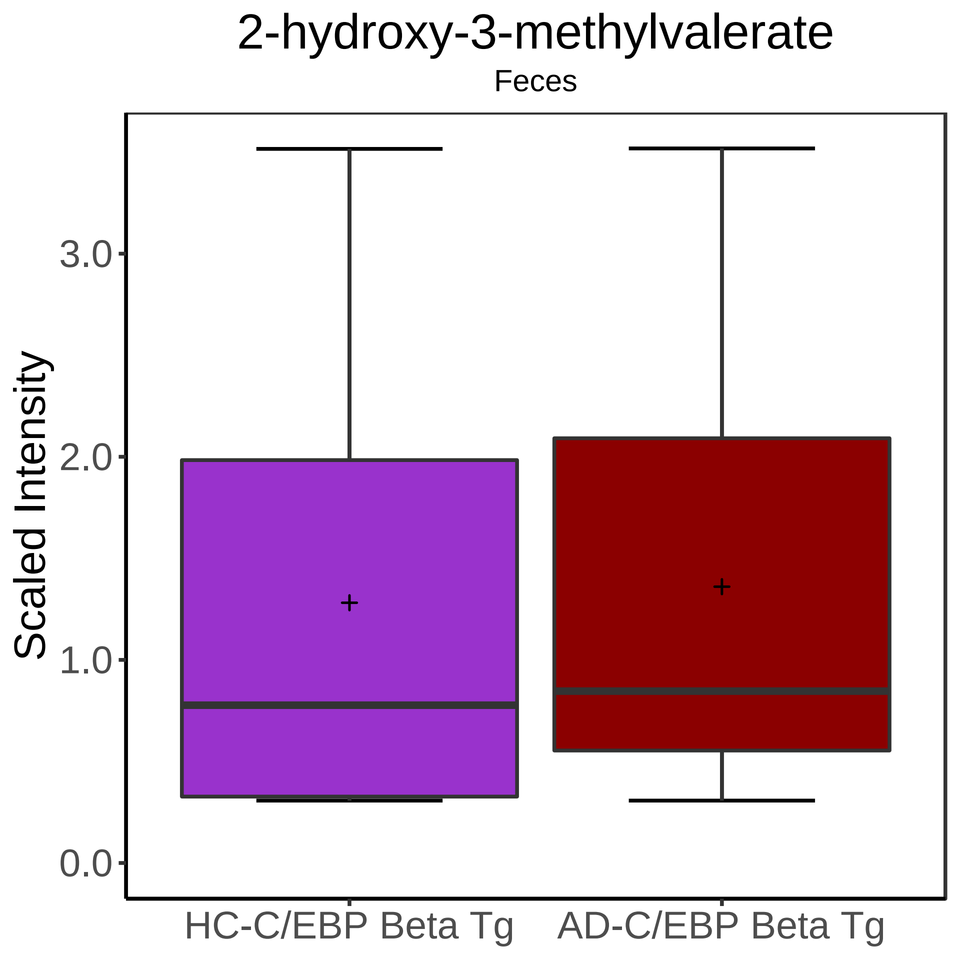
2-hydroxy-3-methylvalerate
Feces
Scaled Intensity
3.0
2.0
1.0
0.0
HC-C/EBP Beta Tg
AD-C/EBP Beta Tg

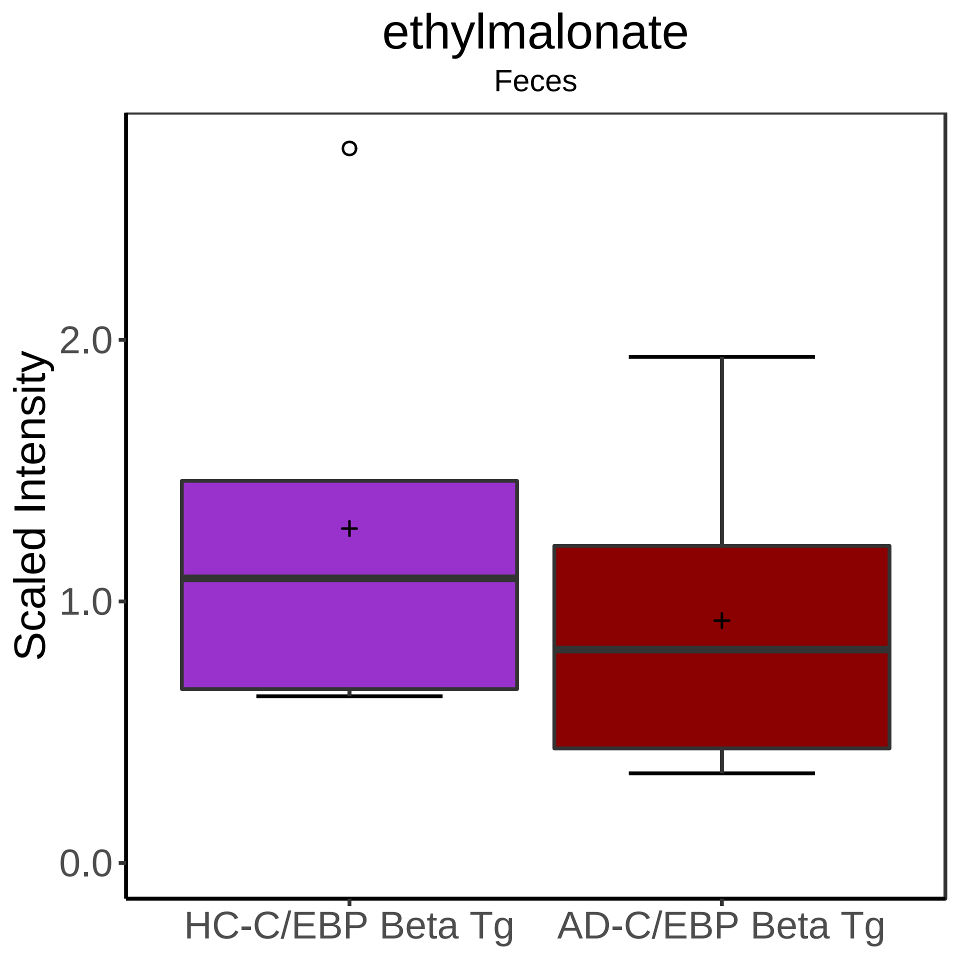

ethylmalonate
Feces
Scaled Intensity
2.0
1.0
0.0
HC-C/EBP Beta Tg
AD-C/EBP Beta Tg

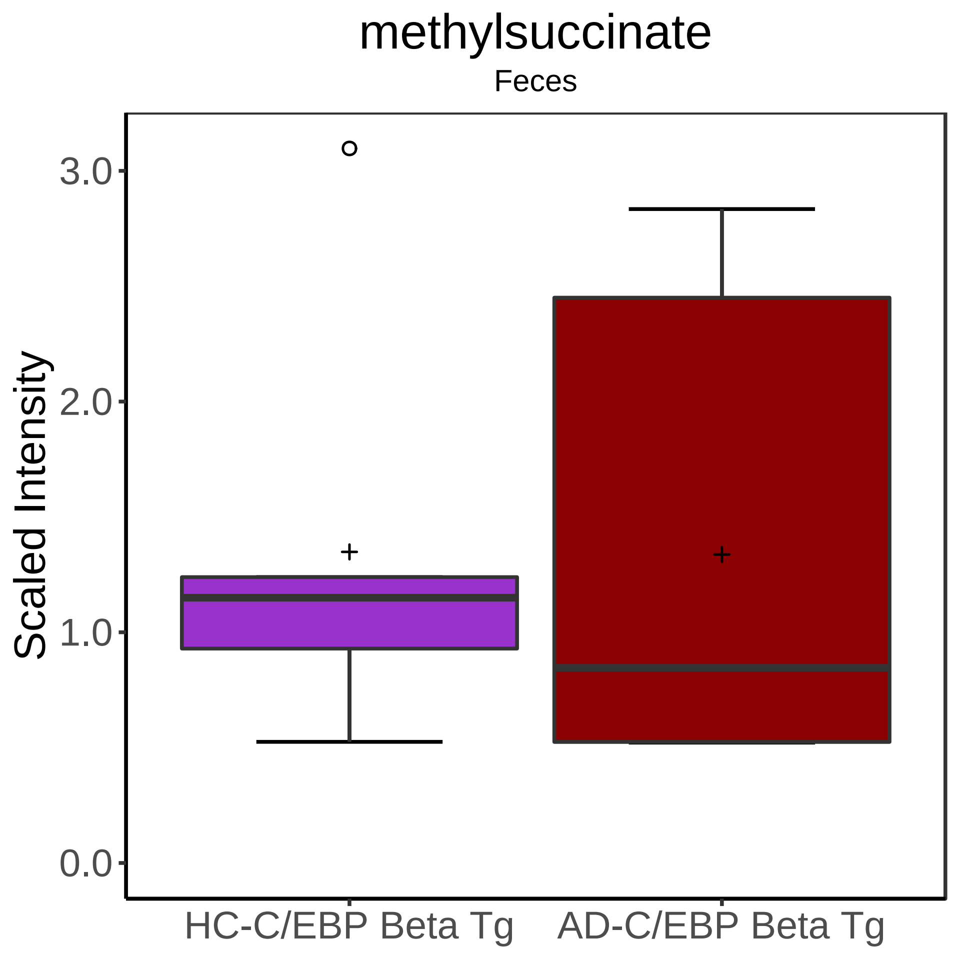
methylsuccinate
Feces
Scaled Intensity
3.0
2.0
1.0
0.0
HC-C/EBP Beta Tg
AD-C/EBP Beta Tg

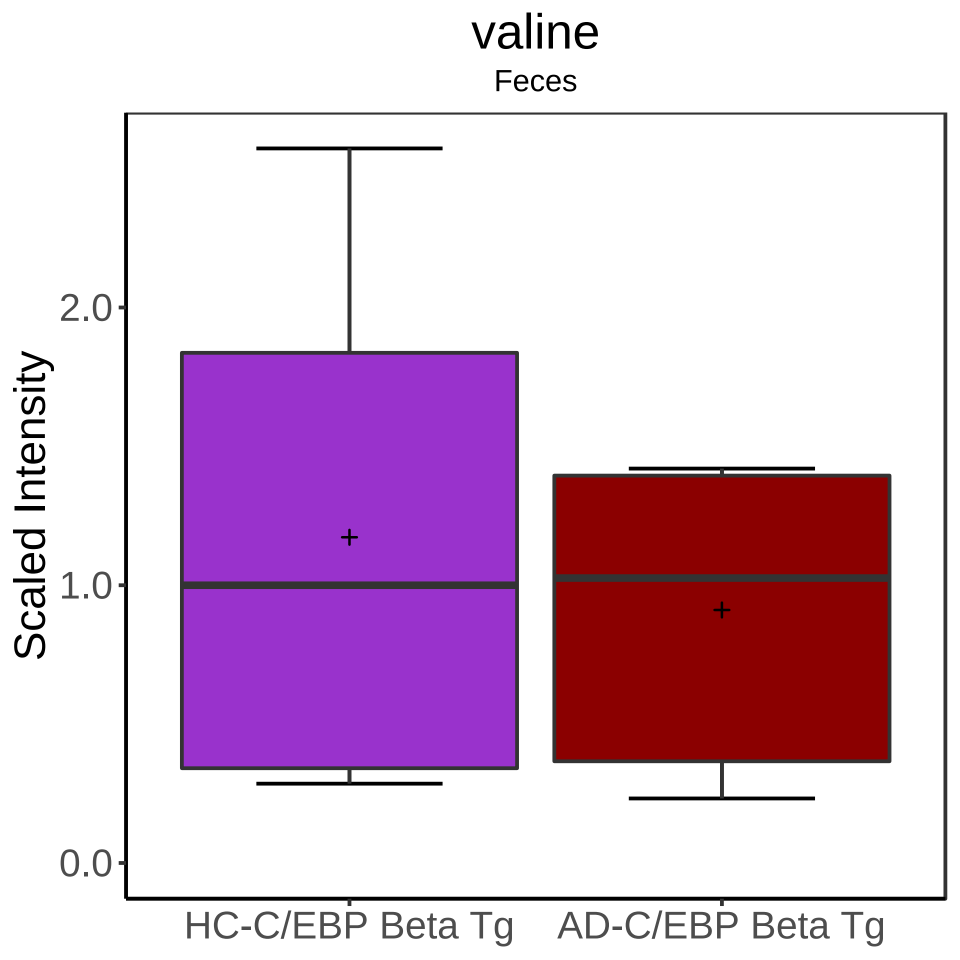
valine
Feces
Scaled Intensity
2.0
1.0
0.0
HC-C/EBP Beta Tg
AD-C/EBP Beta Tg

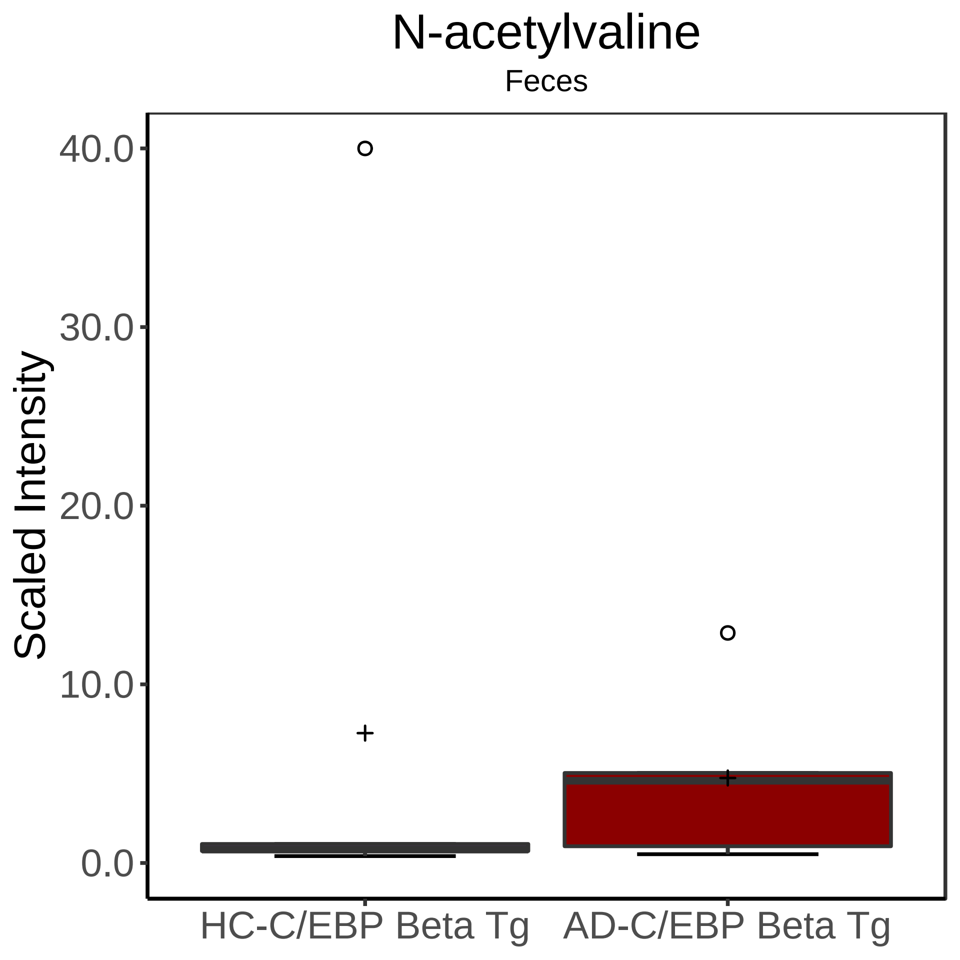

N-acetylvaline
Feces
Scaled Intensity
40.0
30.0
20.0
10.0
0.0
HC-C/EBP Beta Tg
AD-C/EBP Beta Tg

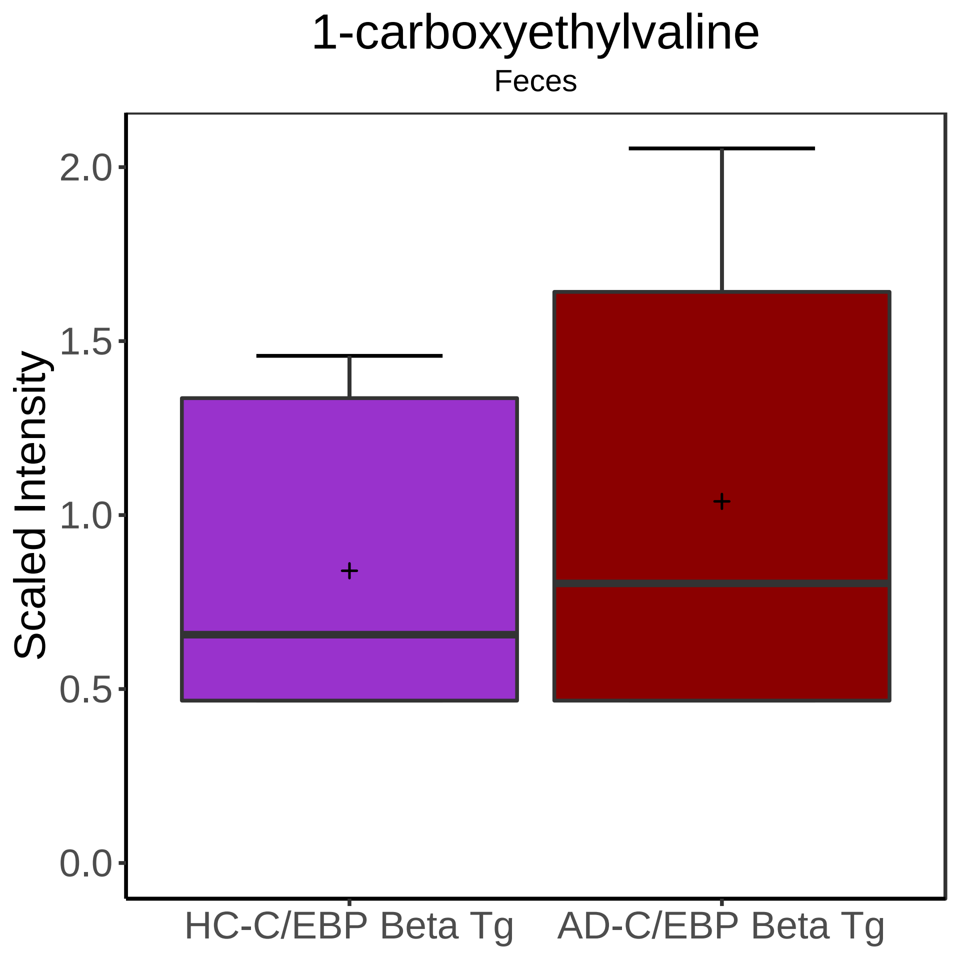
1-carboxyethylvaline
Feces
Scaled Intensity
2.0
1.5
1.0
0.5
0.0
HC-C/EBP Beta Tg
AD-C/EBP Beta Tg

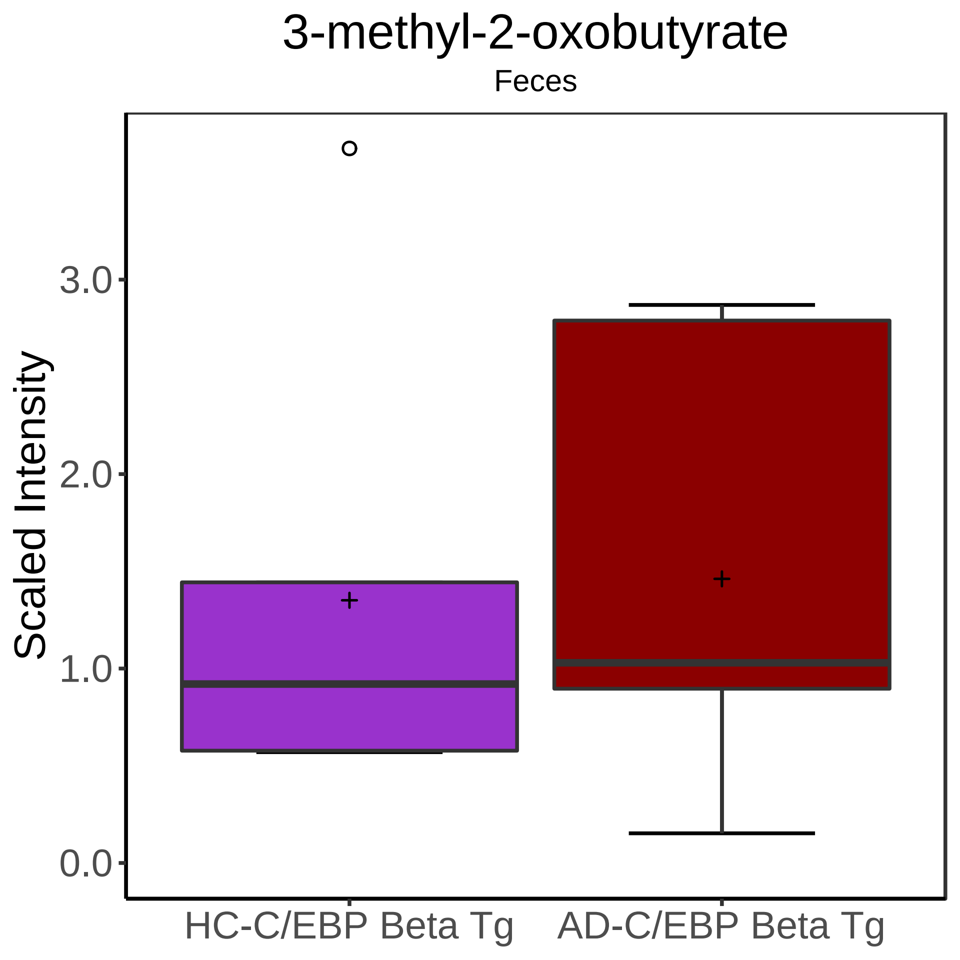

3-methyl-2-oxobutyrate
Feces
Scaled Intensity
3.0
2.0
1.0
0.0
HC-C/EBP Beta Tg
AD-C/EBP Beta Tg

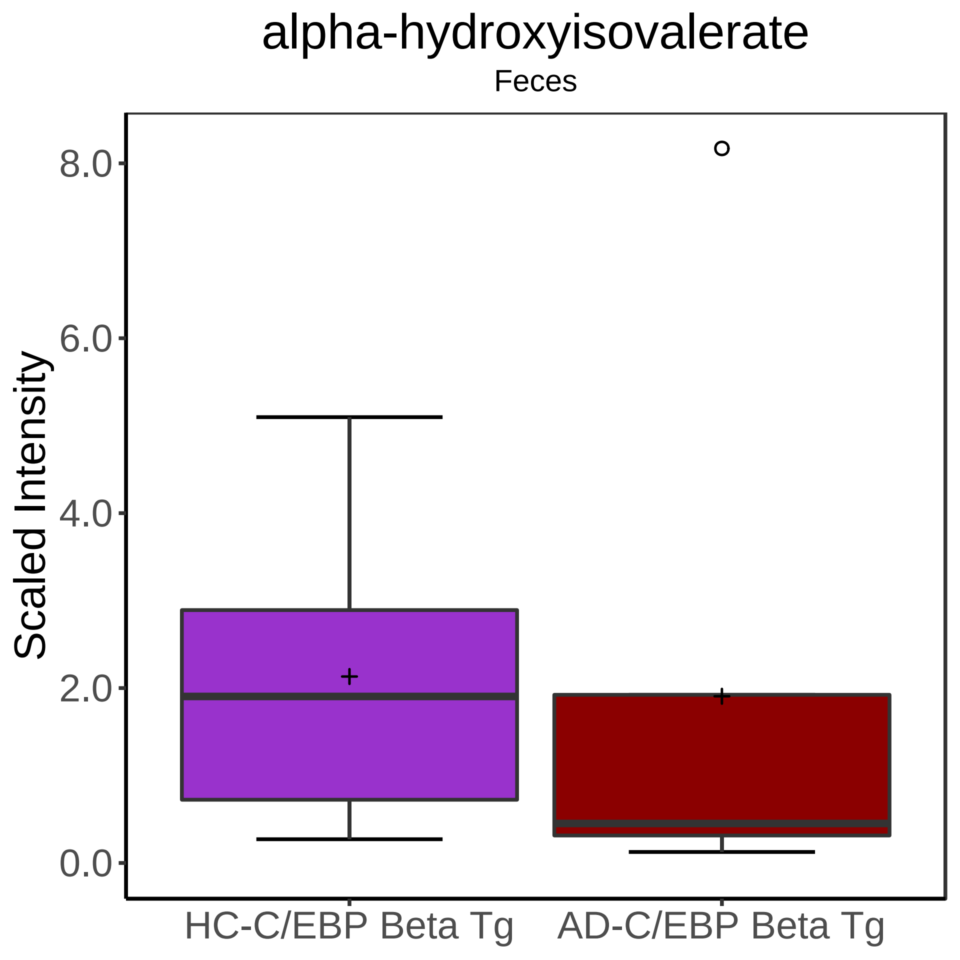
alpha-hydroxyisovalerate
Feces
Scaled Intensity
8.0
6.0
4.0
2.0
0.0
HC-C/EBP Beta Tg
AD-C/EBP Beta Tg

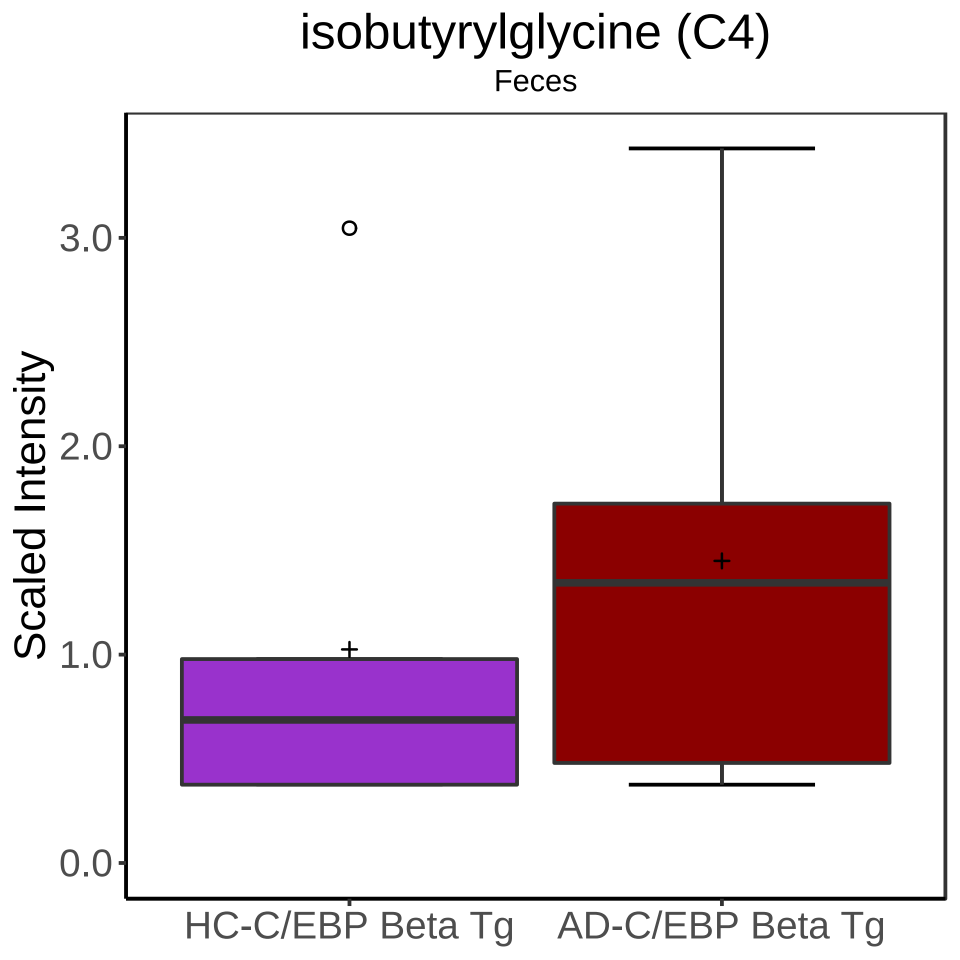

isobutyrylglycine (C4)
Feces
Scaled Intensity
3.0
2.0
1.0
0.0
HC-C/EBP Beta Tg
AD-C/EBP Beta Tg

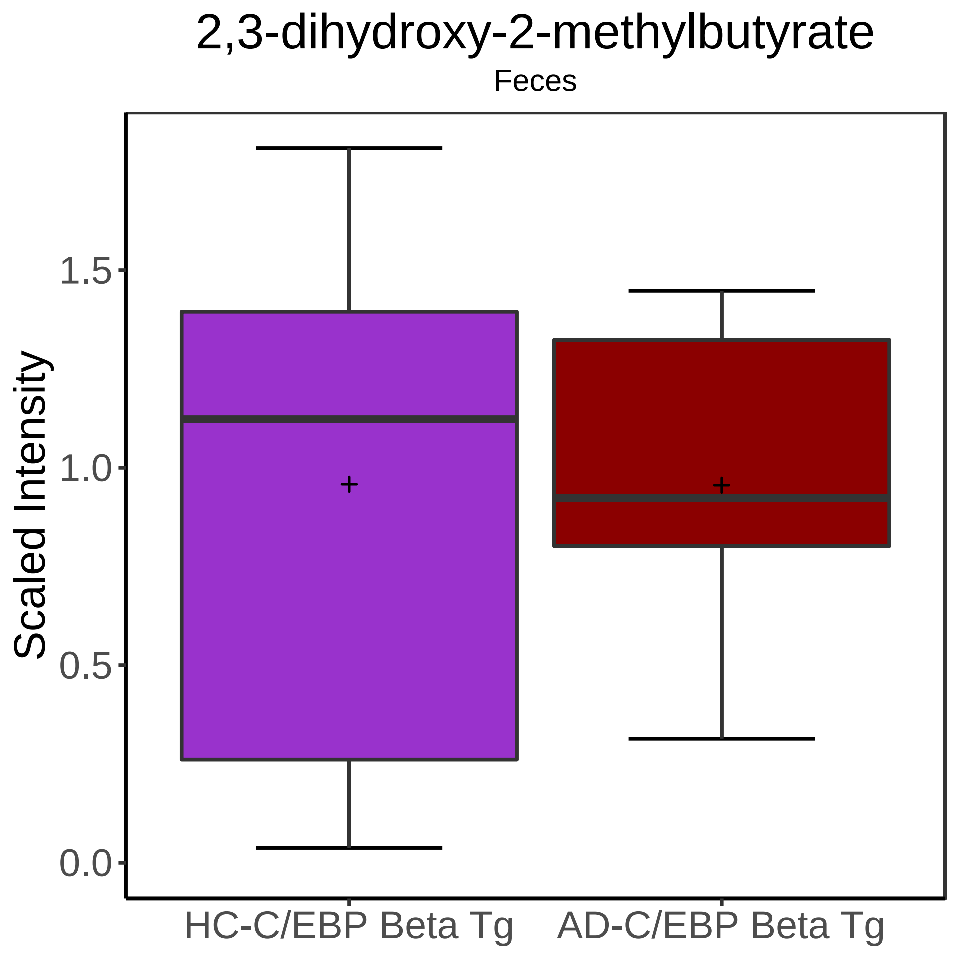

2,3-dihydroxy-2-methylbutyrate
Feces
Scaled Intensity
1.5
1.0
0.5
0.0
HC-C/EBP Beta Tg
AD-C/EBP Beta Tg

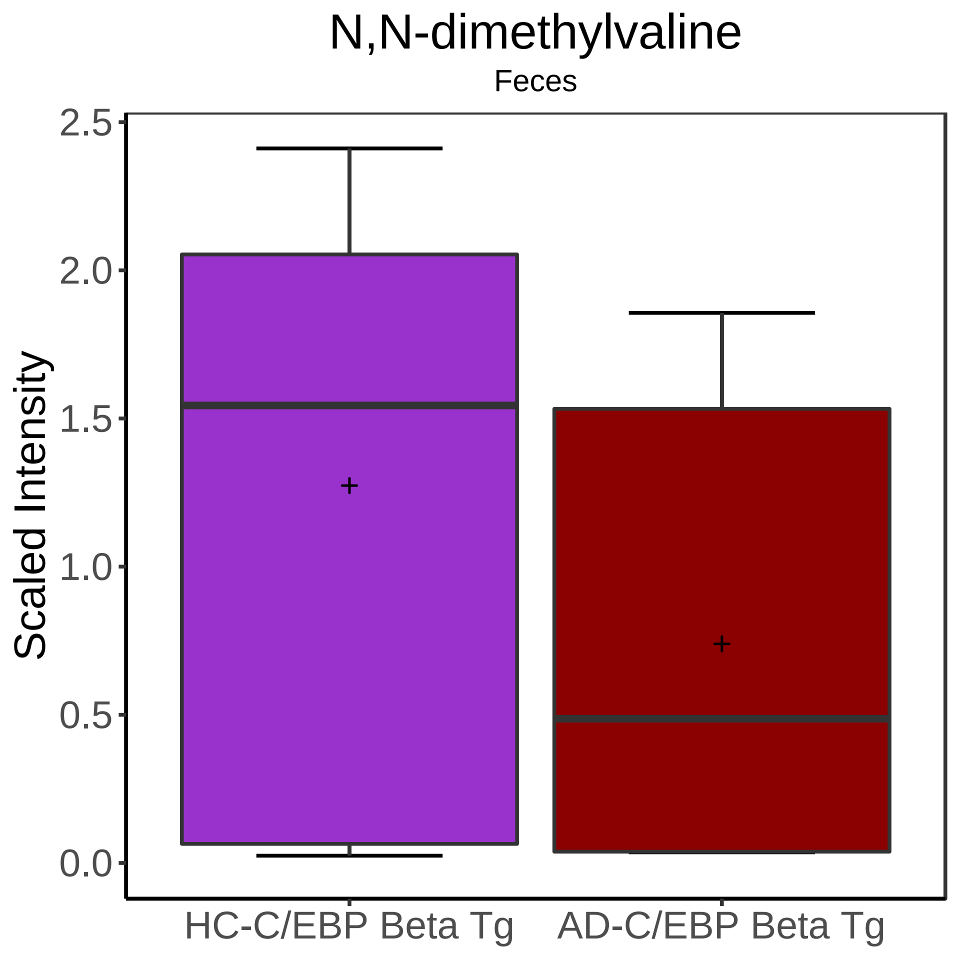
N,N-dimethylvaline
Feces
Scaled Intensity
2.5
2.0
1.5
1.0
0.5
0.0
HC-C/EBP Beta Tg
AD-C/EBP Beta Tg

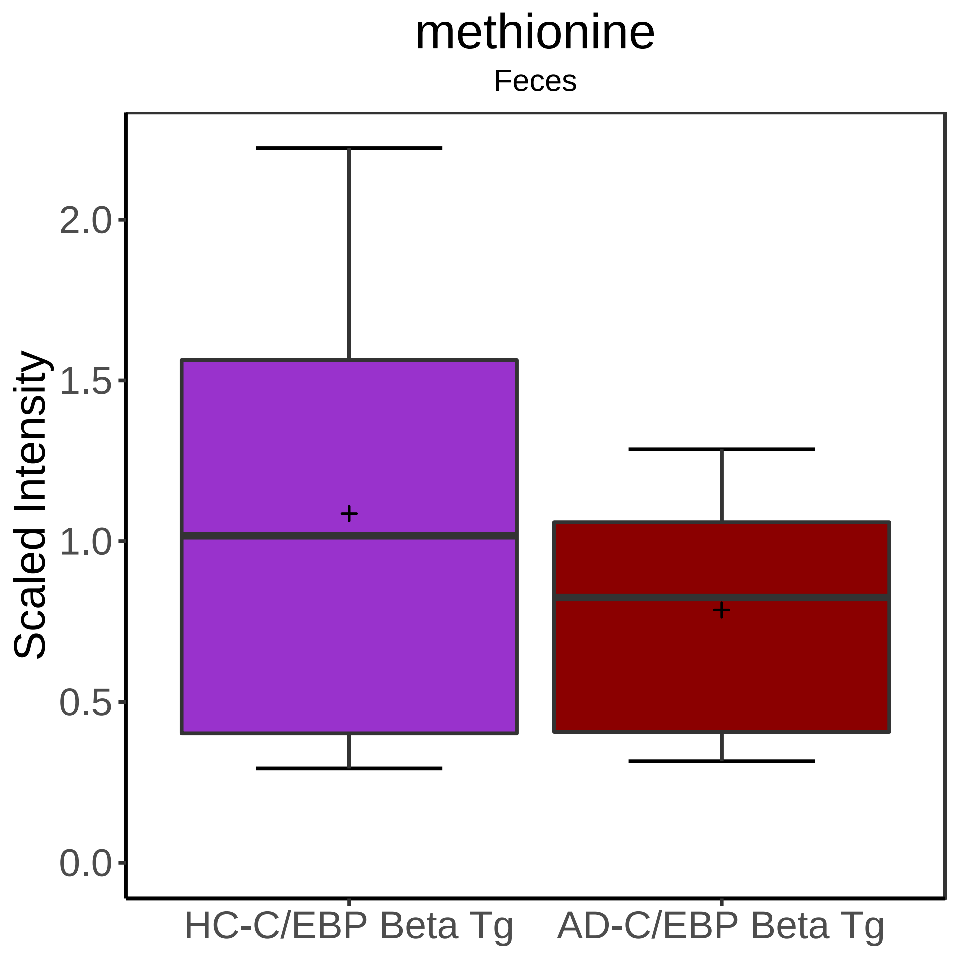

methionine
Feces
Scaled Intensity
2.0
1.5
1.0
0.5
0.0
HC-C/EBP Beta Tg
AD-C/EBP Beta Tg

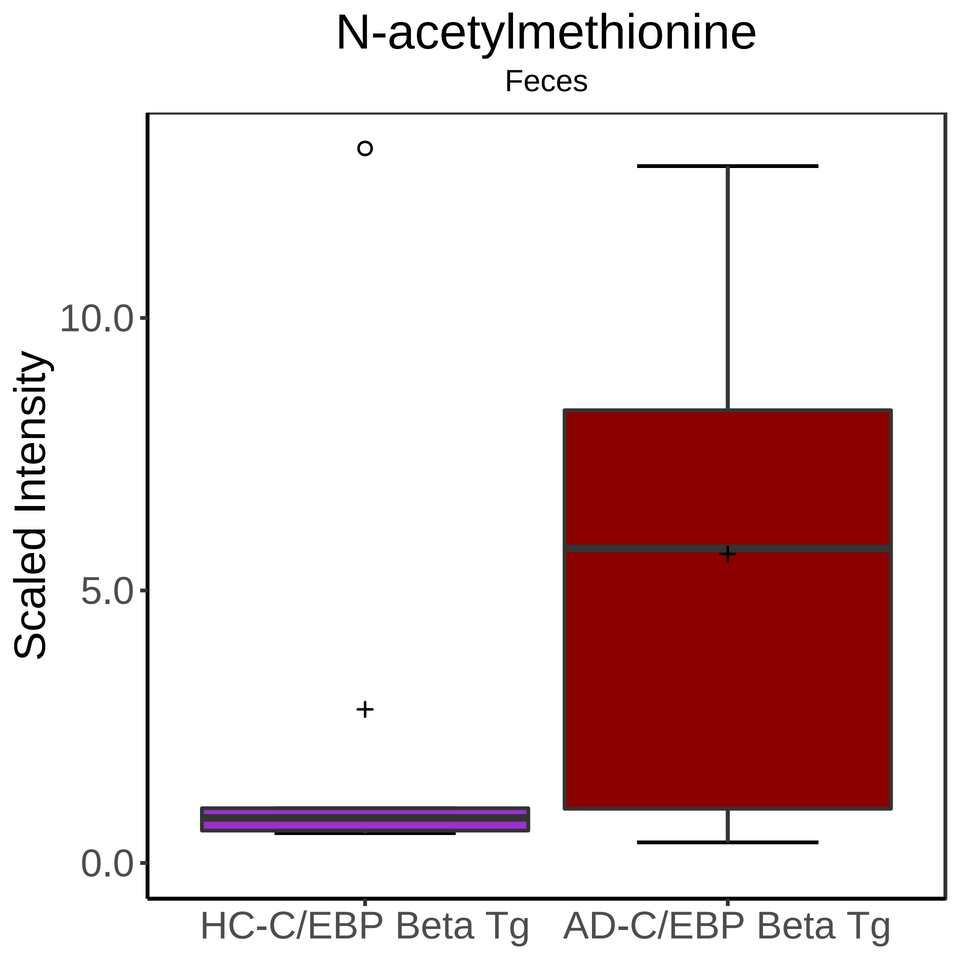
N-acetylmethionine
Feces
Scaled Intensity
10.0
5.0
0.0
HC-C/EBP Beta Tg
AD-C/EBP Beta Tg

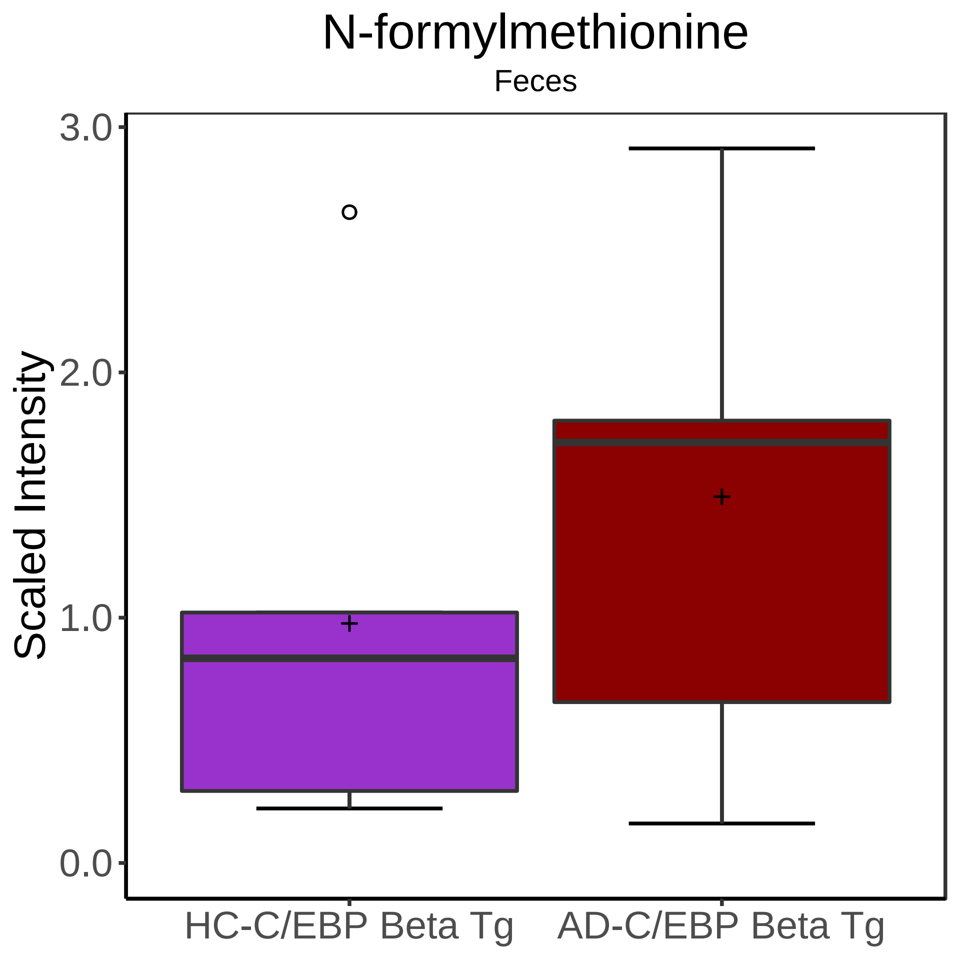
N-formylmethionine
Feces
Scaled Intensity
3.0
2.0
1.0
0.0
HC-C/EBP Beta Tg
AD-C/EBP Beta Tg

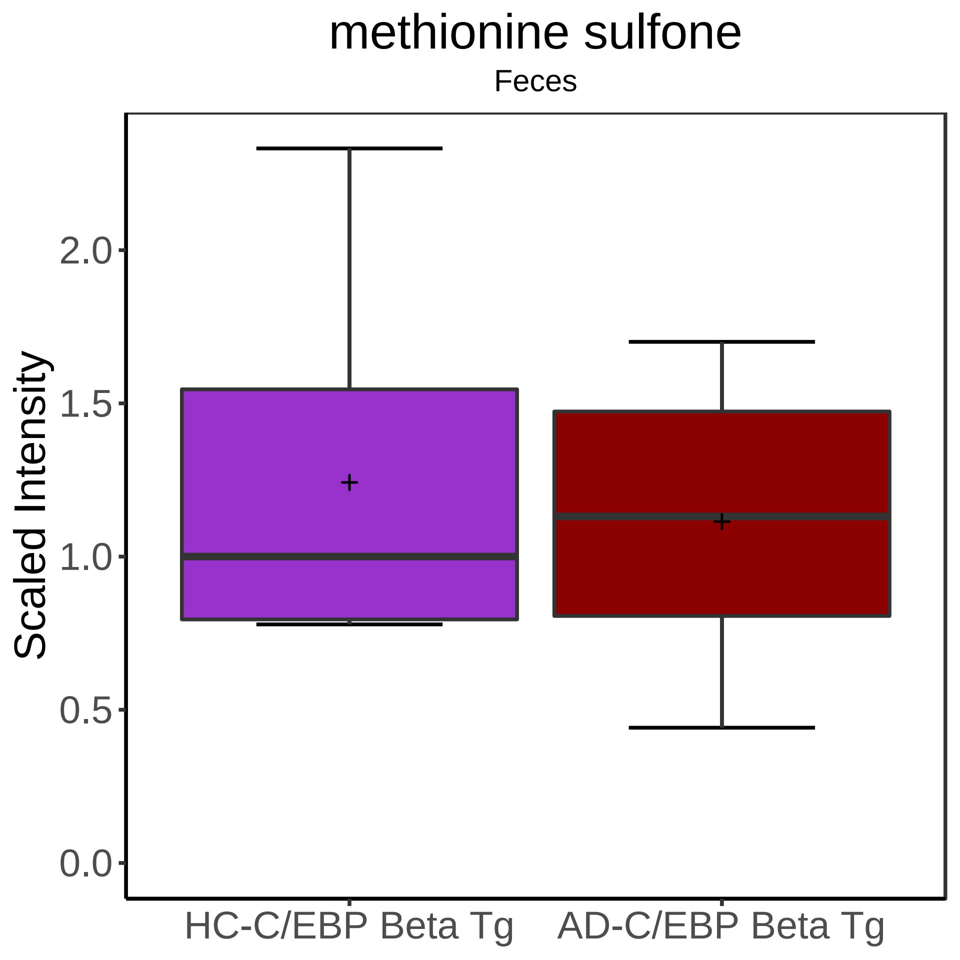
methionine sulfone
Feces
Scaled Intensity
2.0
1.5
1.0
0.5
0.0
HC-C/EBP Beta Tg
AD-C/EBP Beta Tg

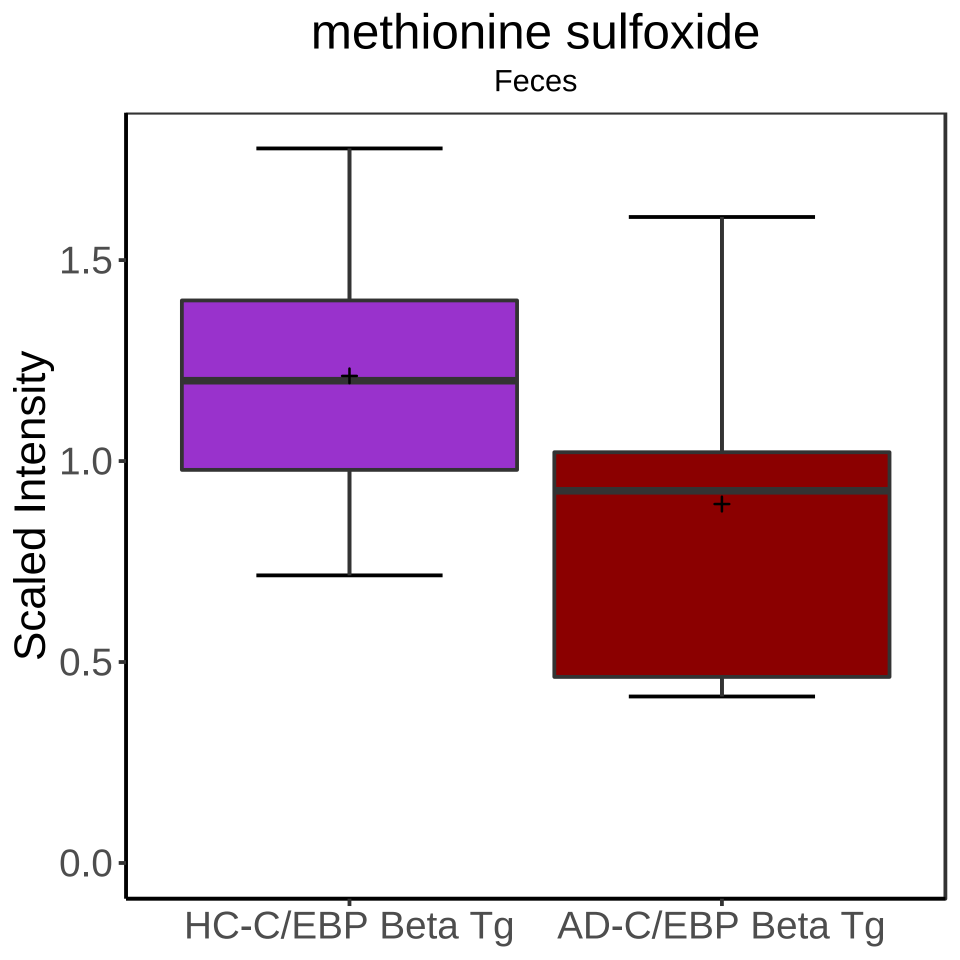

methionine sulfoxide
Feces
Scaled Intensity
1.5
1.0
0.5
0.0
HC-C/EBP Beta Tg
AD-C/EBP Beta Tg

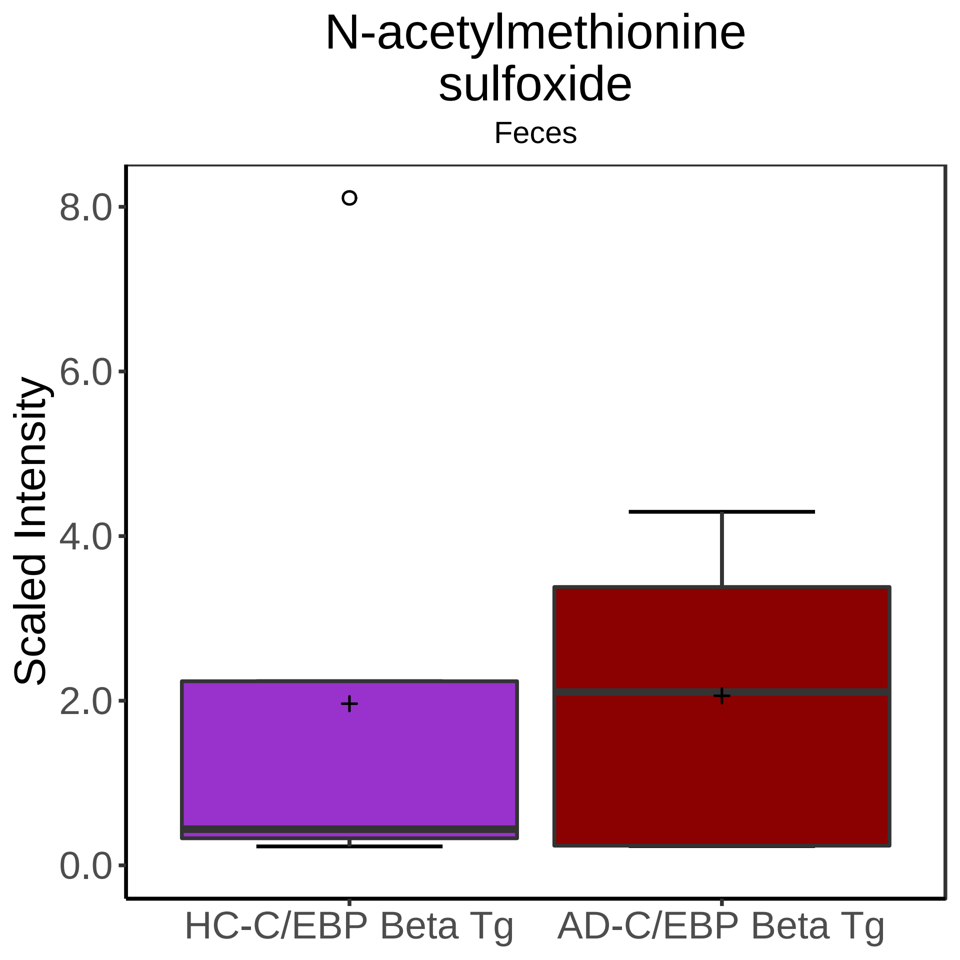
N-acetylmethionine
sulfoxide
Feces
Scaled Intensity
8.0
6.0
4.0
2.0
0.0
HC-C/EBP Beta Tg
AD-C/EBP Beta Tg

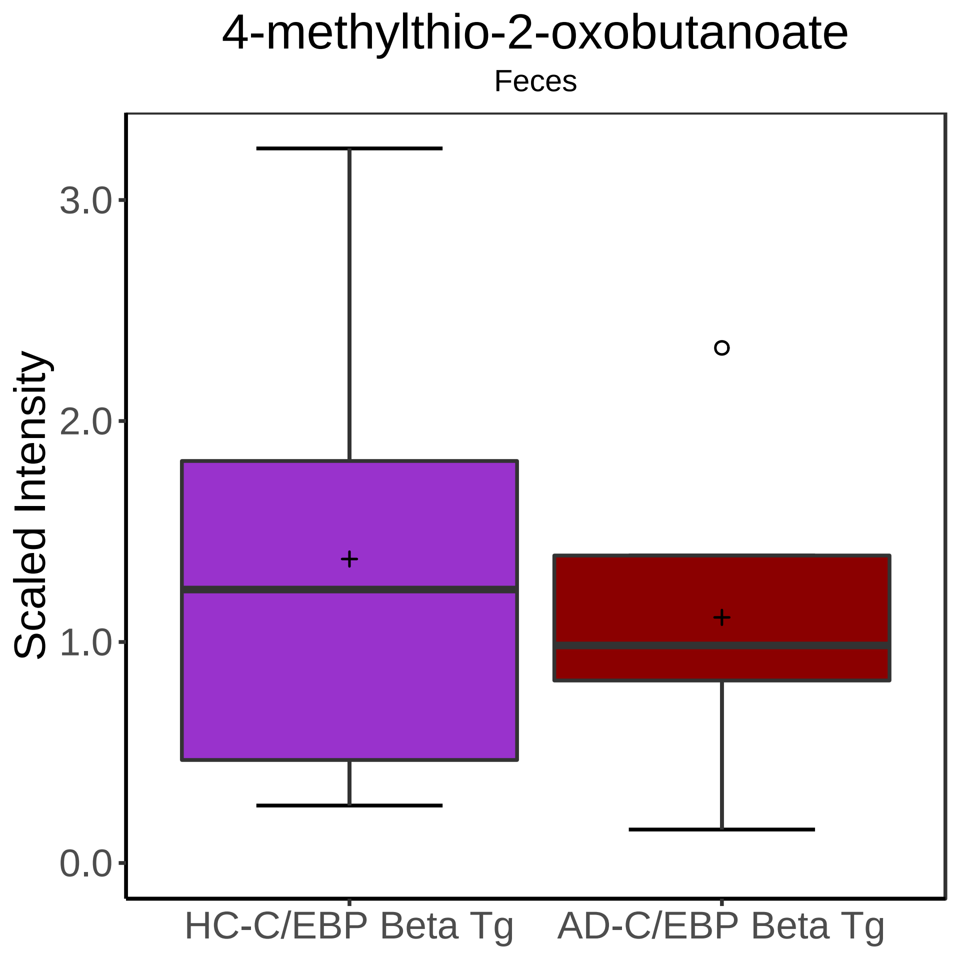
4-methylthio-2-oxobutanoate
Feces
Scaled Intensity
3.0
2.0
1.0
0.0
HC-C/EBP Beta Tg
AD-C/EBP Beta Tg

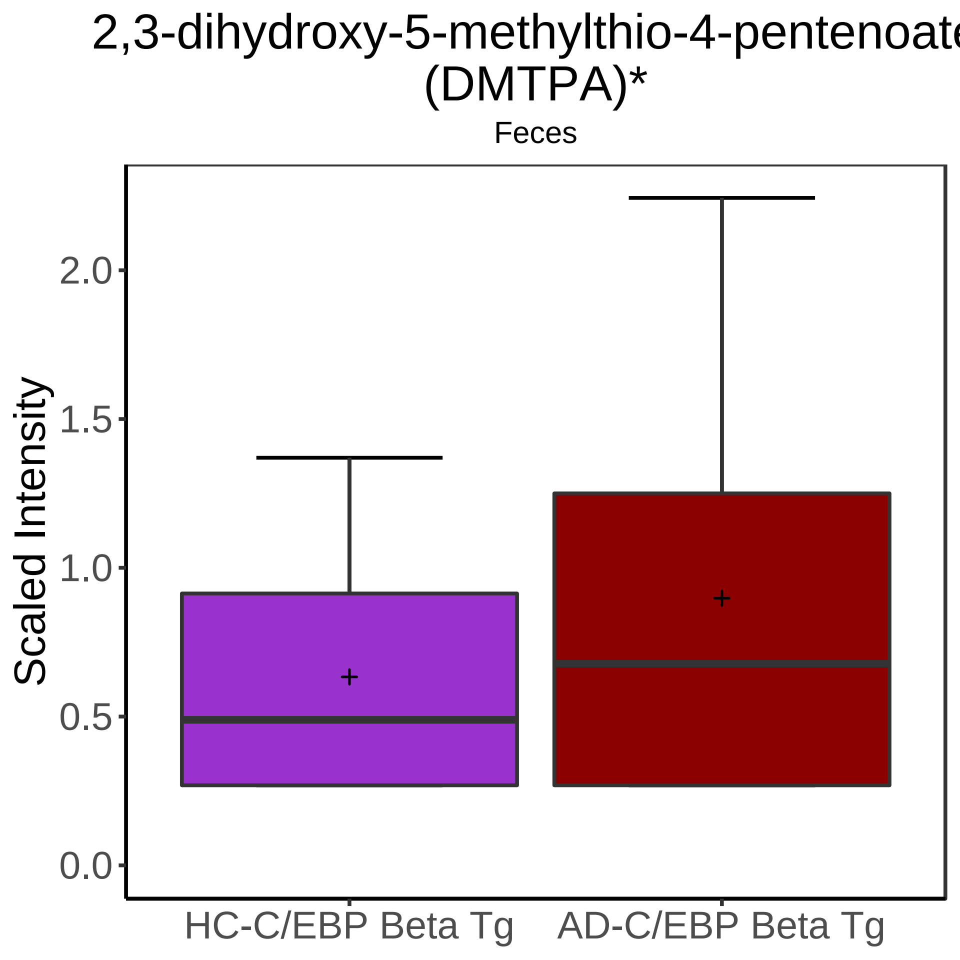
2,3-dihydroxy-5-methylthio-4-pentenoate
(DMTPA)*
Feces
Scaled Intensity
0.0
0.5
1.0
1.5
2.0
HC-C/EBP Beta Tg
AD-C/EBP Beta Tg

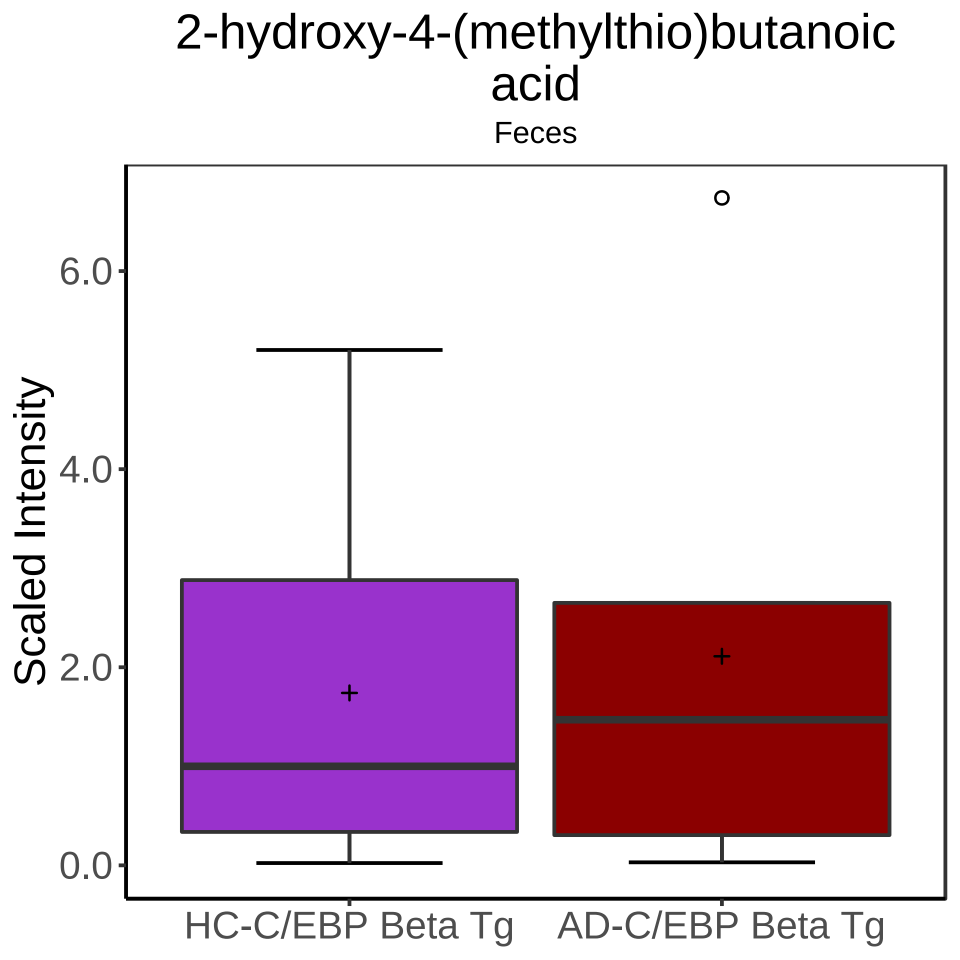
2-hydroxy-4-(methylthio)butanoic acid
Feces
Scaled Intensity
6.0
4.0
2.0
0.0
HC-C/EBP Beta Tg
AD-C/EBP Beta Tg

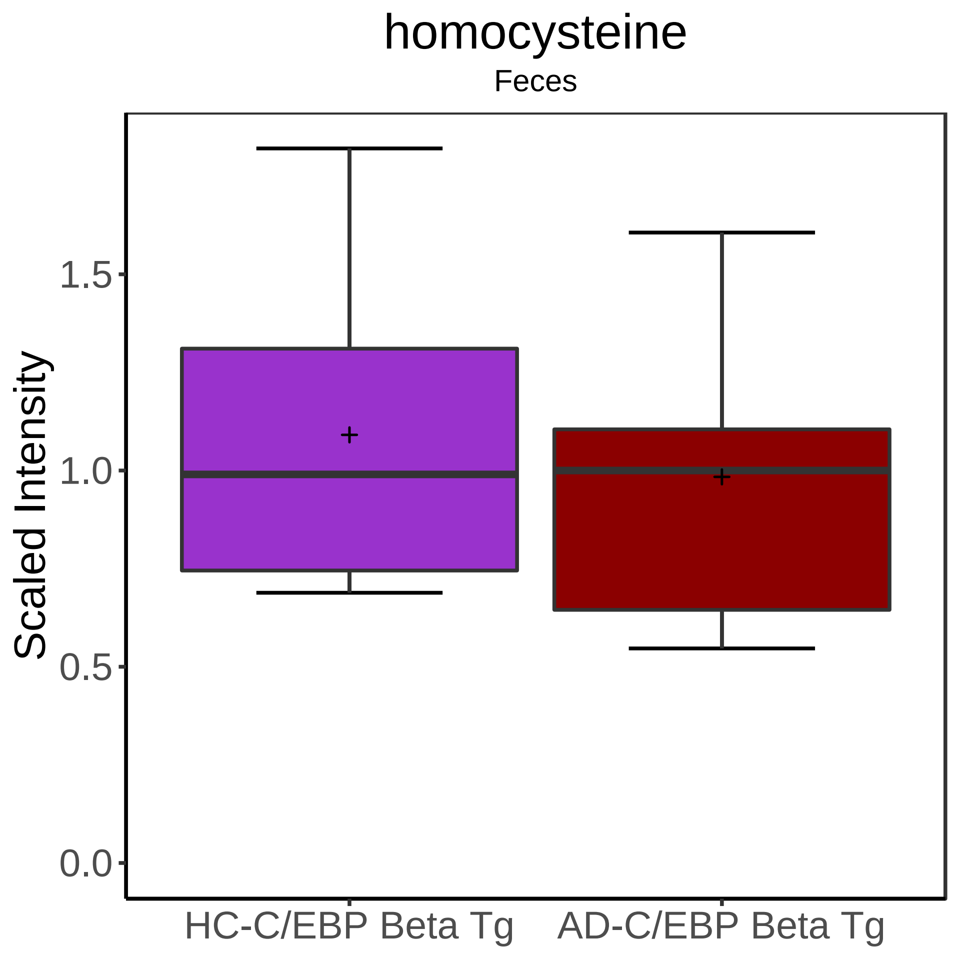
homocysteine
Feces
Scaled Intensity
1.5
1.0
0.5
0.0
HC-C/EBP Beta Tg
AD-C/EBP Beta Tg

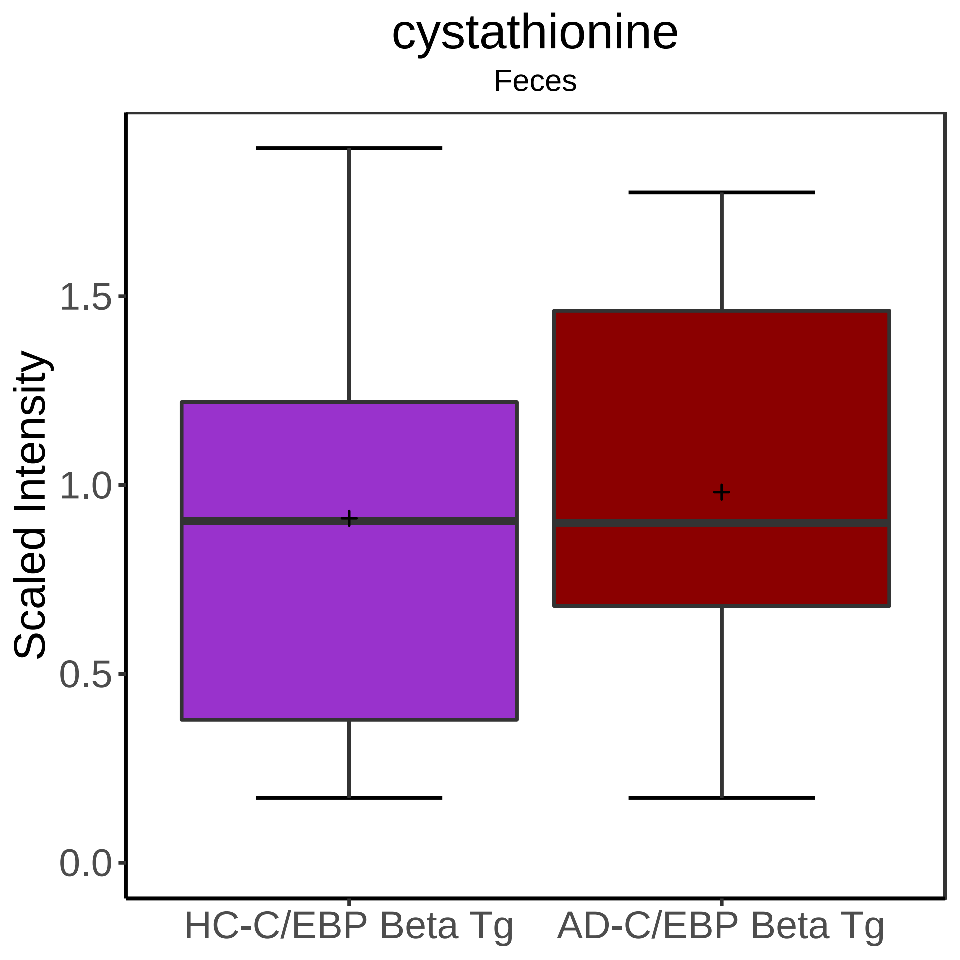
cystathionine
Feces
Scaled Intensity
1.5
1.0
0.5
0.0
HC-C/EBP Beta Tg
AD-C/EBP Beta Tg

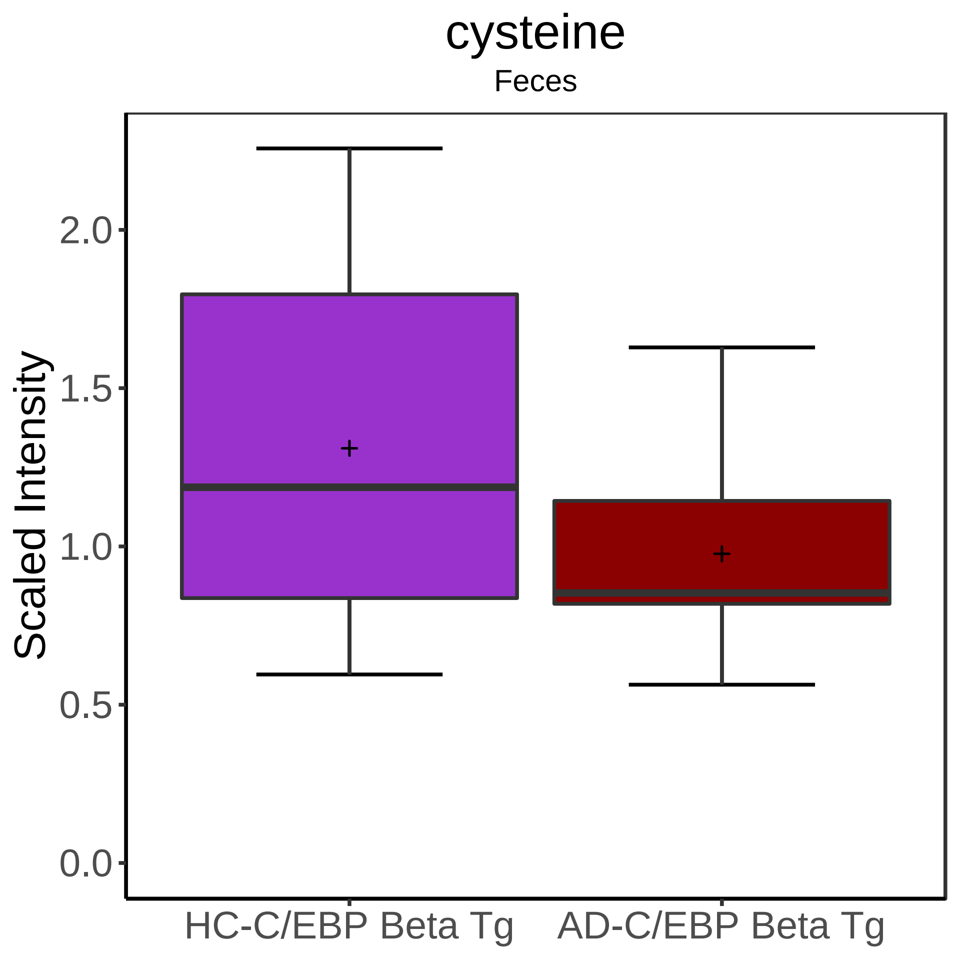

cysteine
Feces
Scaled Intensity
2.0
1.5
1.0
0.5
0.0
HC-C/EBP Beta Tg
AD-C/EBP Beta Tg

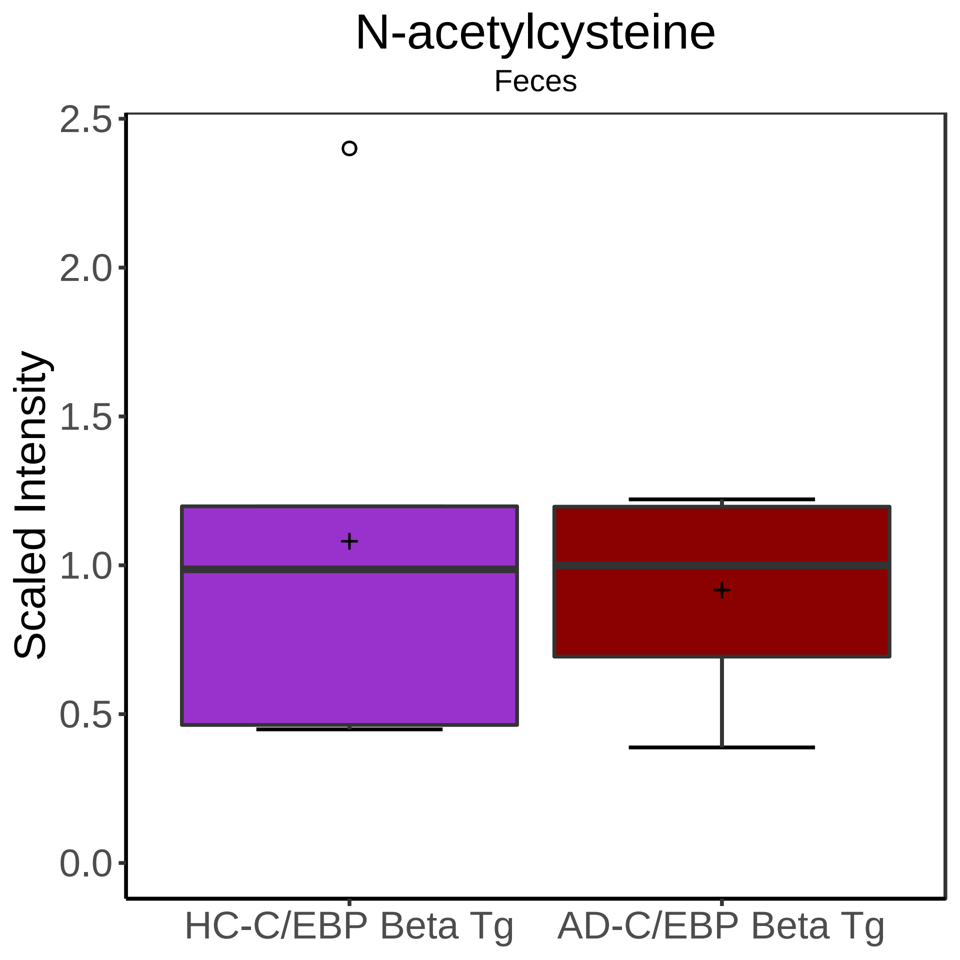

N-acetylcysteine
Feces
Scaled Intensity
2.5
2.0
1.5
1.0
0.5
0.0
HC-C/EBP Beta Tg
AD-C/EBP Beta Tg

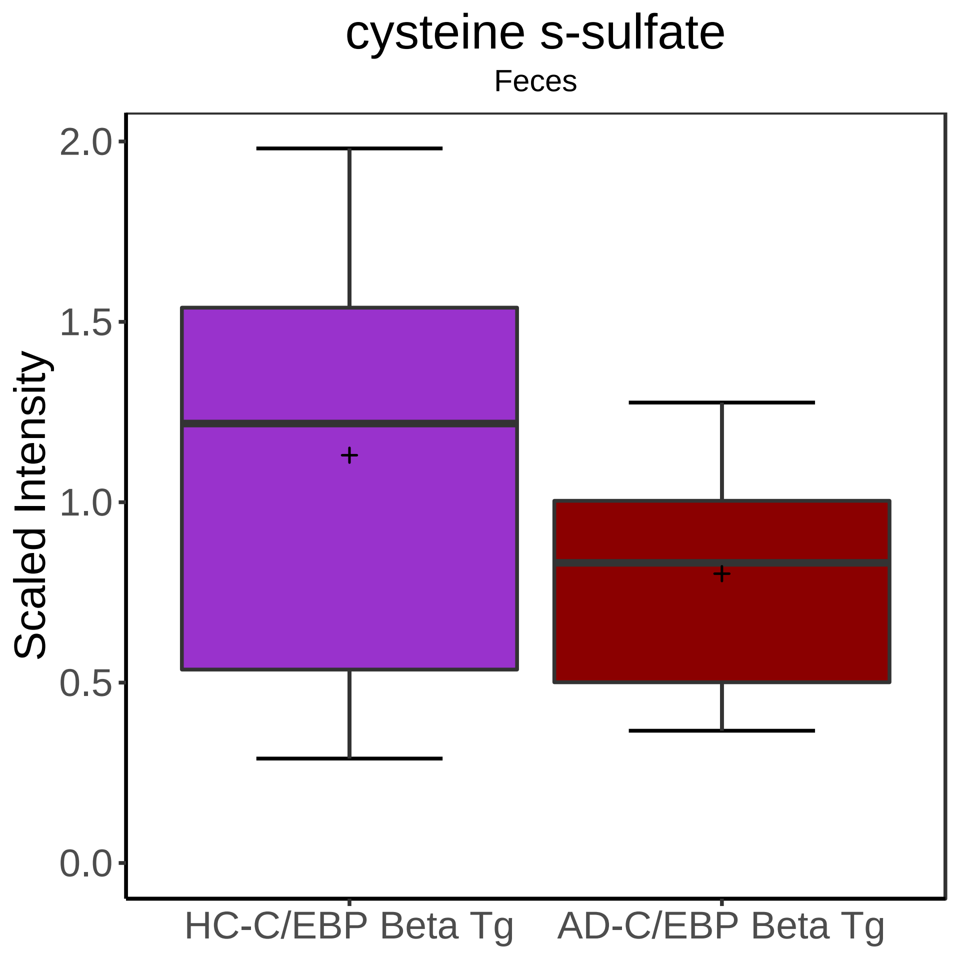
cysteine s-sulfate
Feces
Scaled Intensity
2.0
1.5
1.0
0.5
0.0
HC-C/EBP Beta Tg
AD-C/EBP Beta Tg

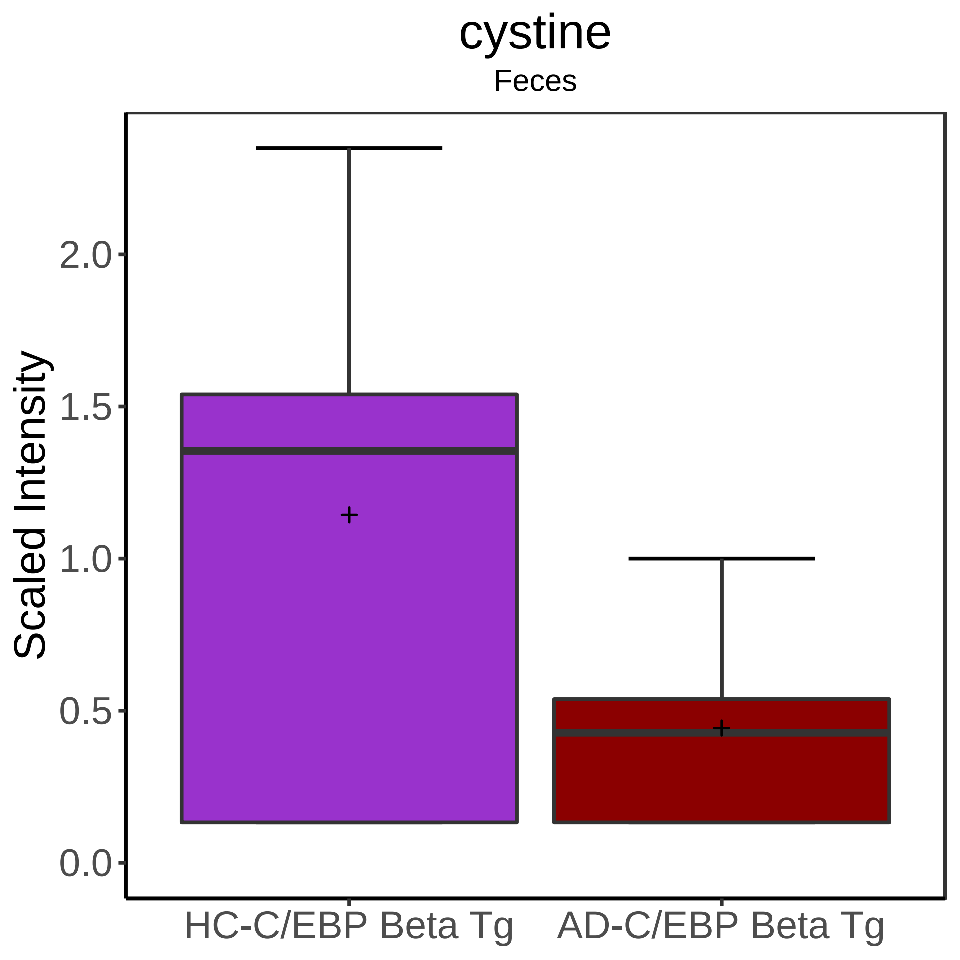
cystine
Feces
Scaled Intensity
2.0
1.5
1.0
0.5
0.0
HC-C/EBP Beta Tg
AD-C/EBP Beta Tg

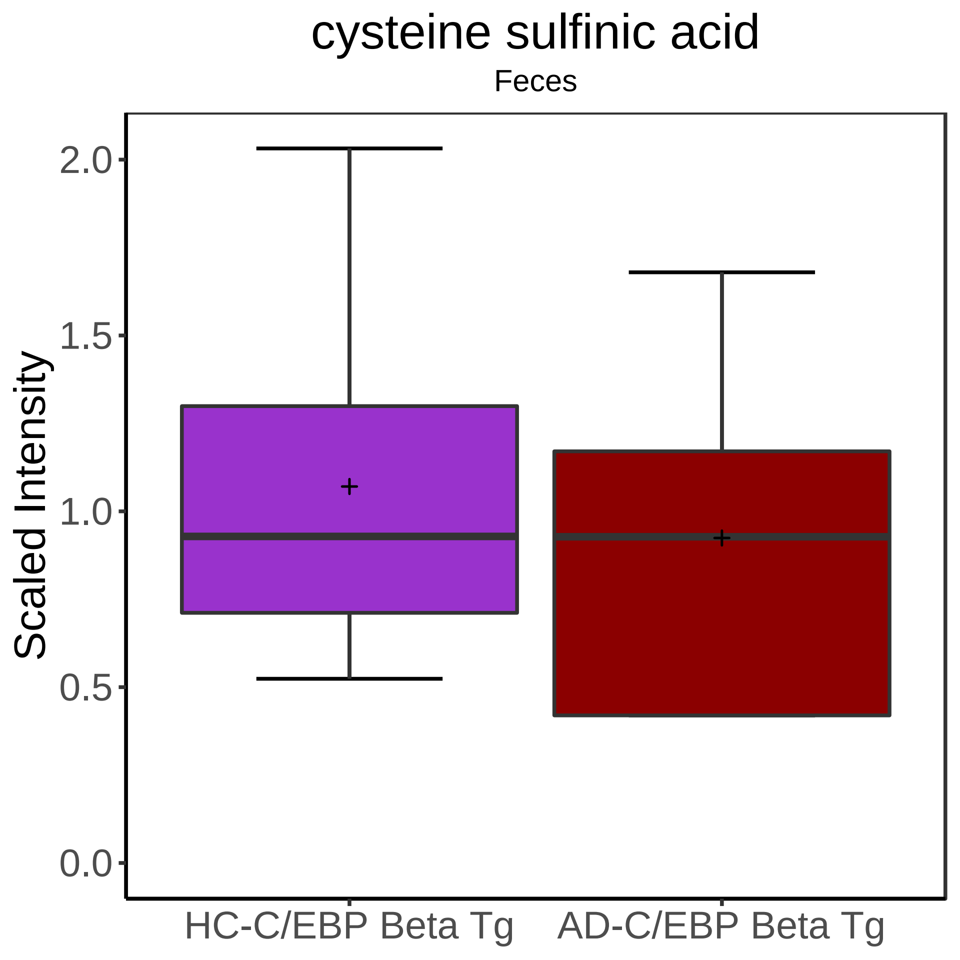
cysteine sulfinic acid
Feces
Scaled Intensity
2.0
1.5
1.0
0.5
0.0
HC-C/EBP Beta Tg
AD-C/EBP Beta Tg

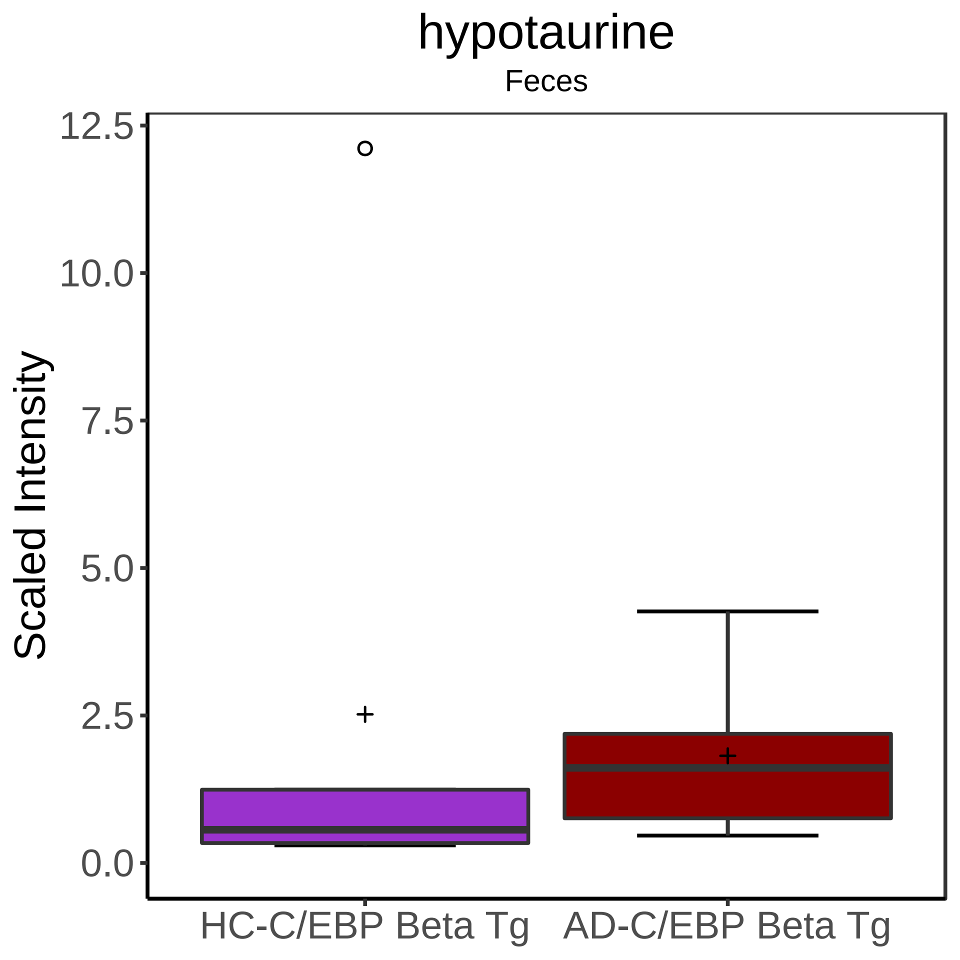
hypotaurine
Feces
Scaled Intensity
12.5
10.0
7.5
5.0
2.5
0.0
HC-C/EBP Beta Tg
AD-C/EBP Beta Tg

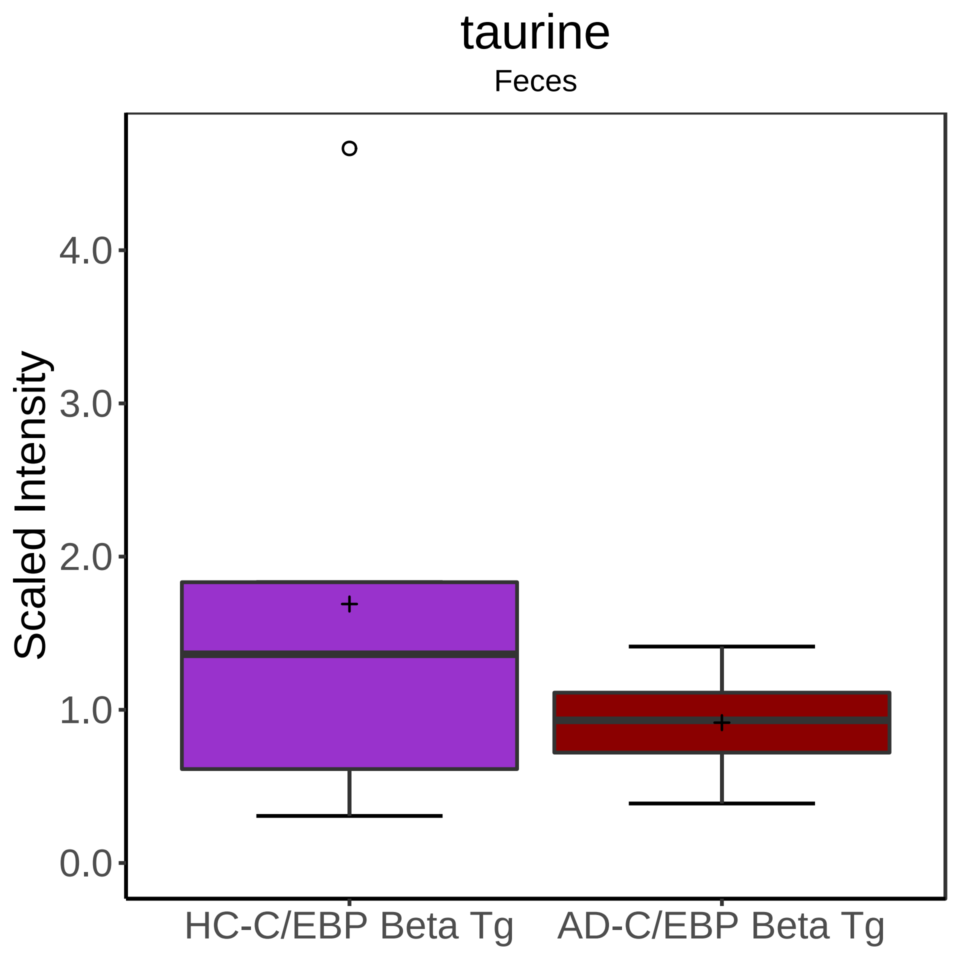
taurine
Feces
Scaled Intensity
4.0
3.0
2.0
1.0
0.0
HC-C/EBP Beta Tg
AD-C/EBP Beta Tg

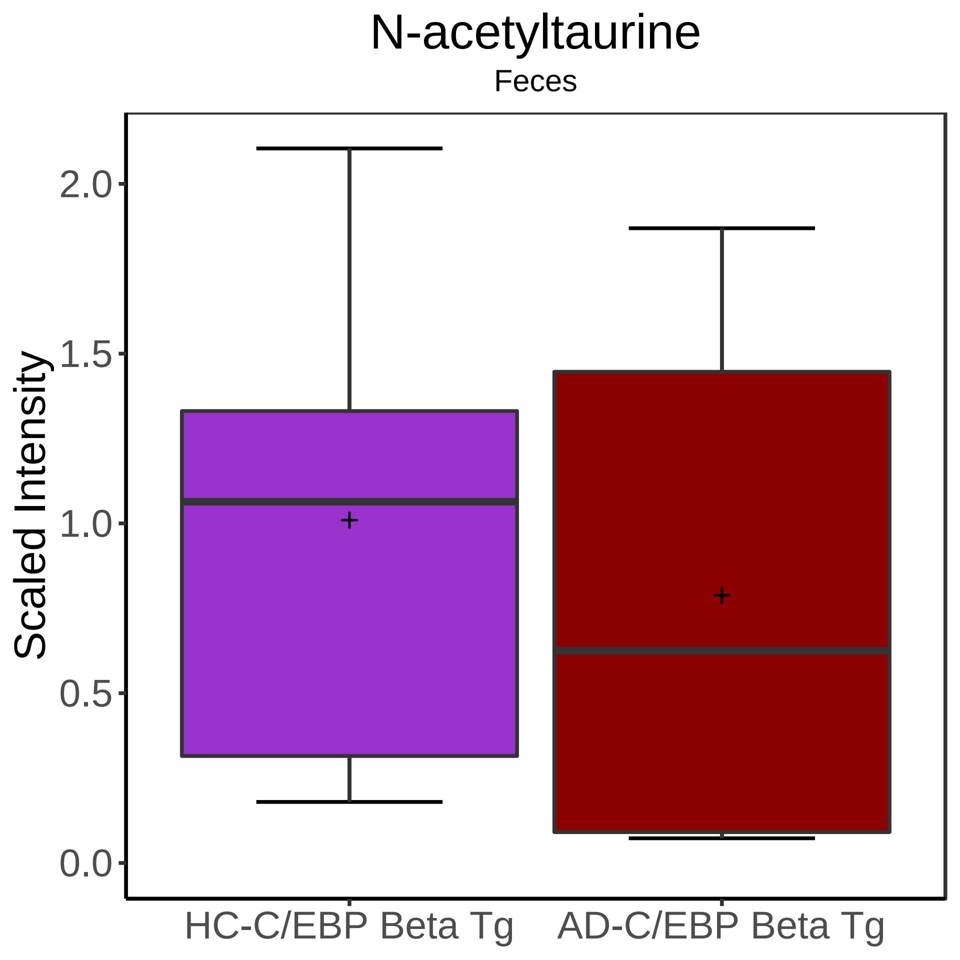

N-acetyltaurine
Feces
Scaled Intensity
2.0
1.5
1.0
0.5
0.0
HC-C/EBP Beta Tg
AD-C/EBP Beta Tg

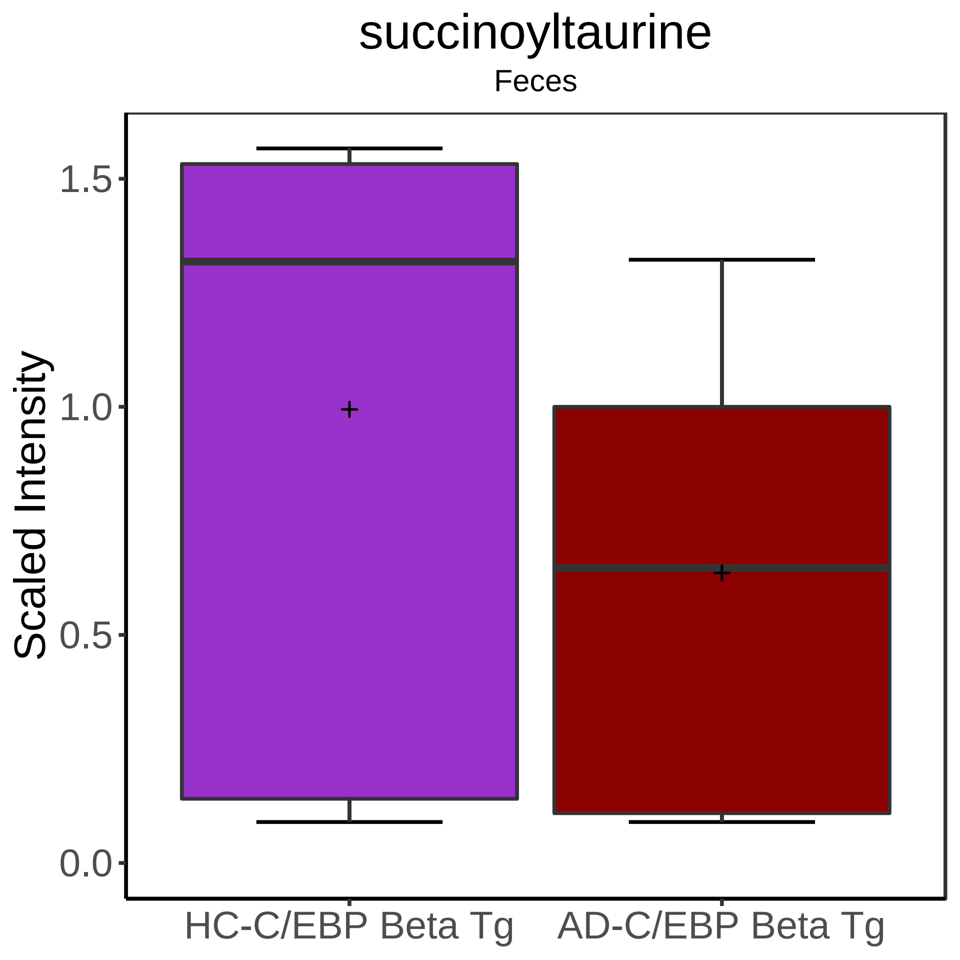
succinoyltaurine
Feces
Scaled Intensity
1.5
1.0
0.5
0.0
HC-C/EBP Beta Tg
AD-C/EBP Beta Tg

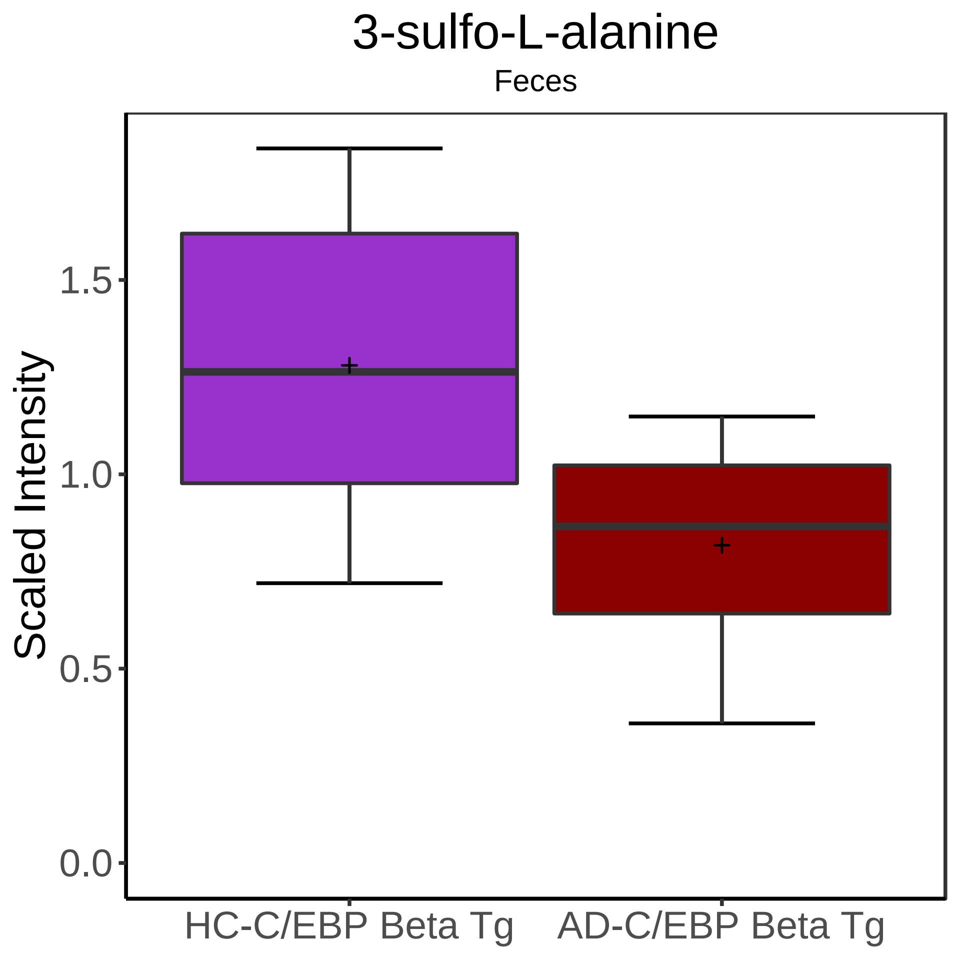
3-sulfo-L-alanine
Feces
Scaled Intensity
1.5
1.0
0.5
0.0
HC-C/EBP Beta Tg
AD-C/EBP Beta Tg

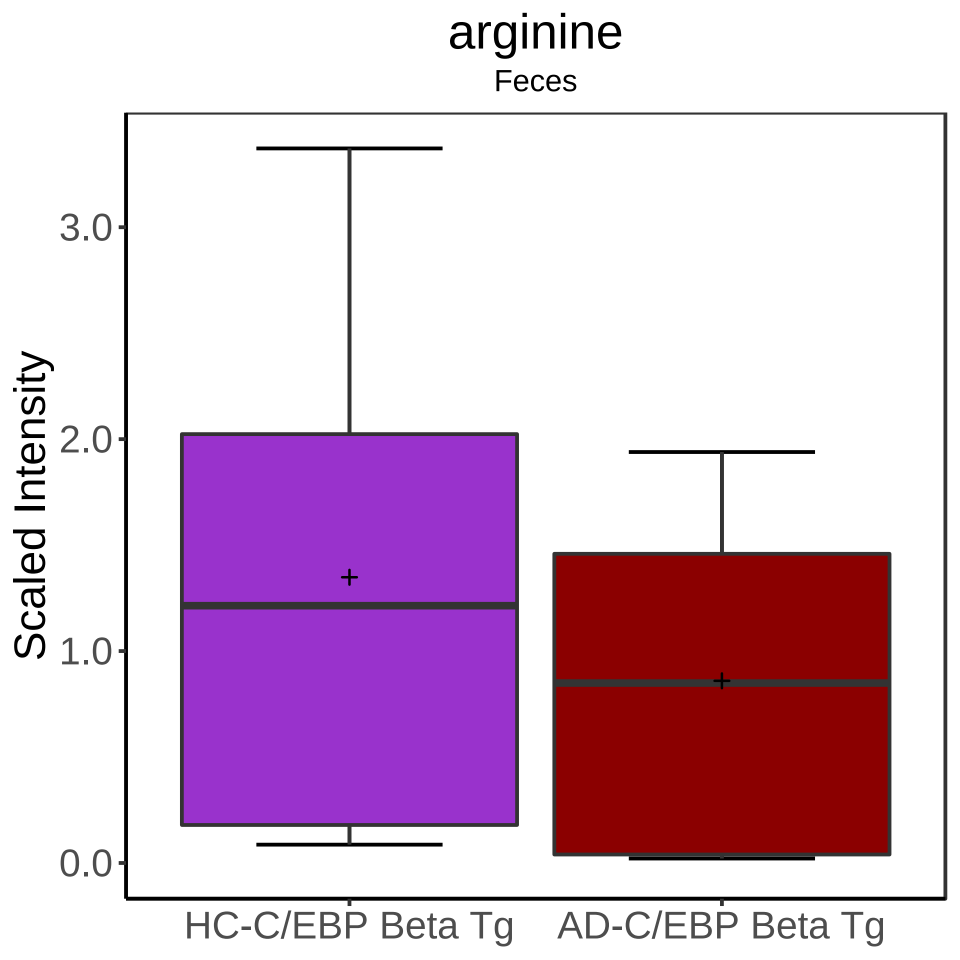
arginine
Feces
Scaled Intensity
3.0
2.0
1.0
0.0
HC-C/EBP Beta Tg
AD-C/EBP Beta Tg

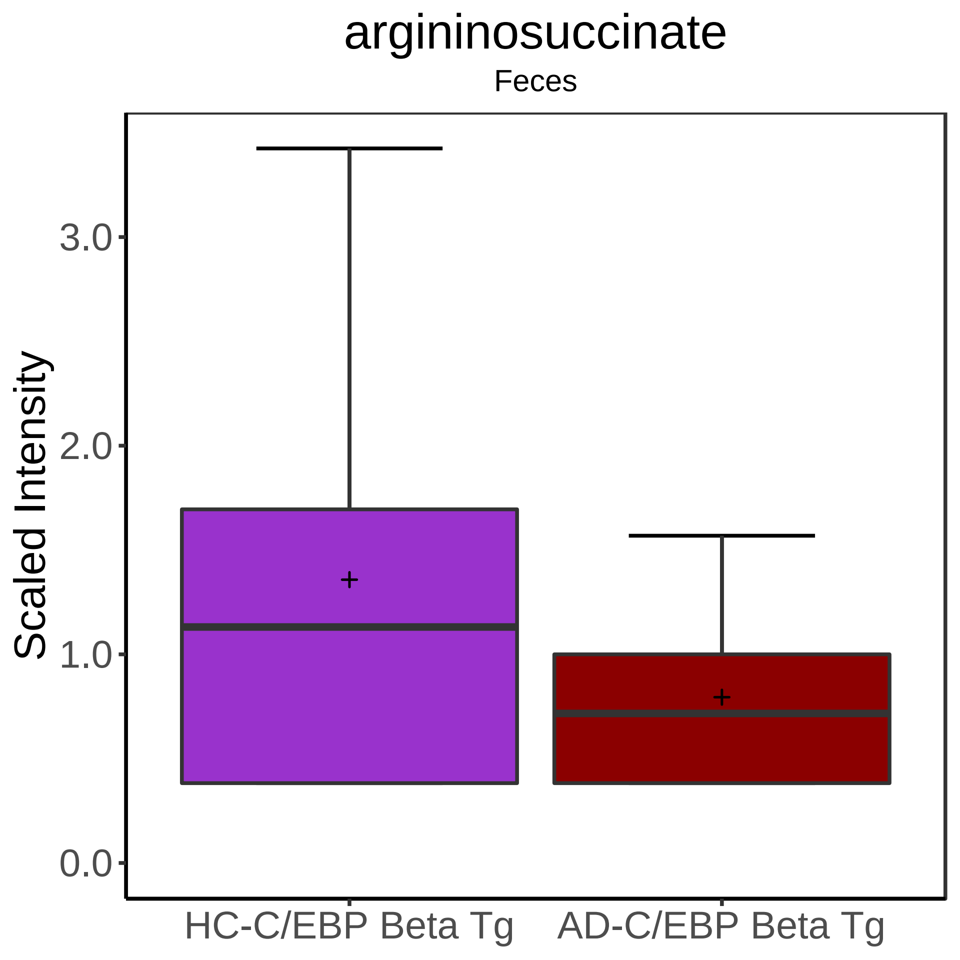
argininosuccinate
Feces
Scaled Intensity
3.0
2.0
1.0
0.0
HC-C/EBP Beta Tg
AD-C/EBP Beta Tg

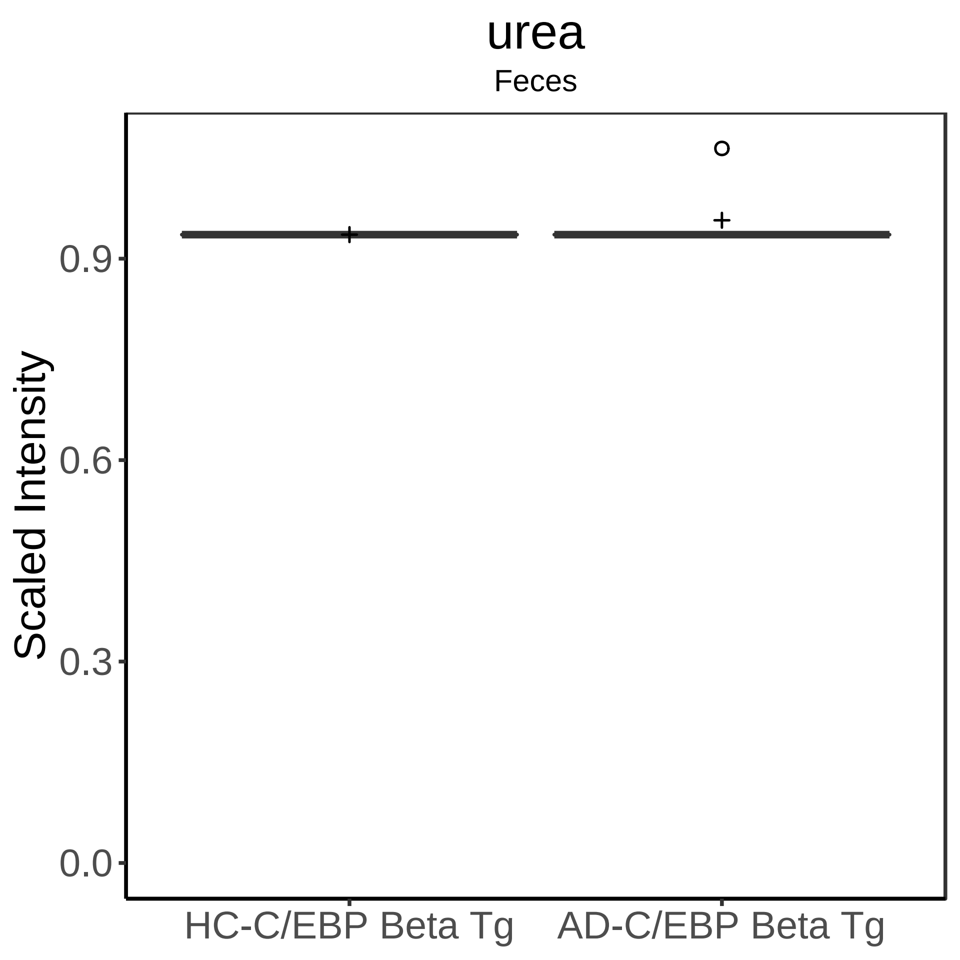

urea
Feces
Scaled Intensity
0.9
0.6
0.3
0.0
HC-C/EBP Beta Tg
AD-C/EBP Beta Tg

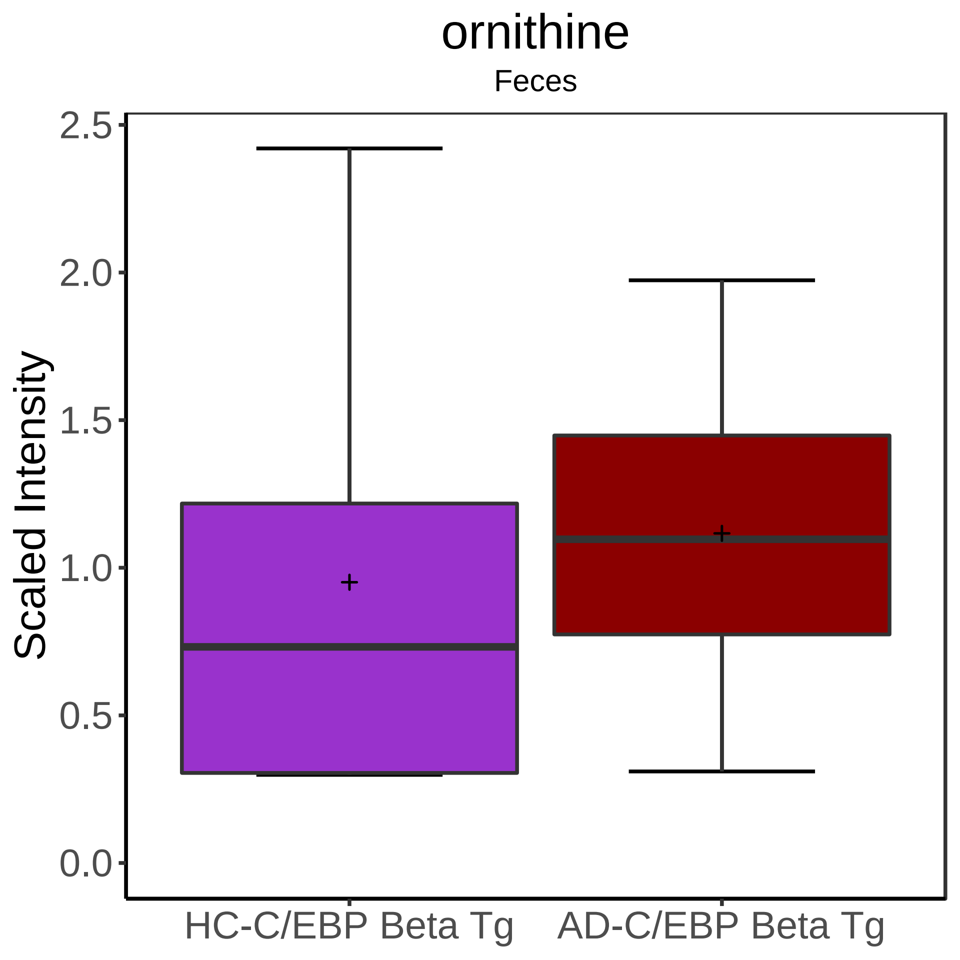
ornithine
Feces
Scaled Intensity
2.5
2.0
1.5
1.0
0.5
0.0
HC-C/EBP Beta Tg
AD-C/EBP Beta Tg

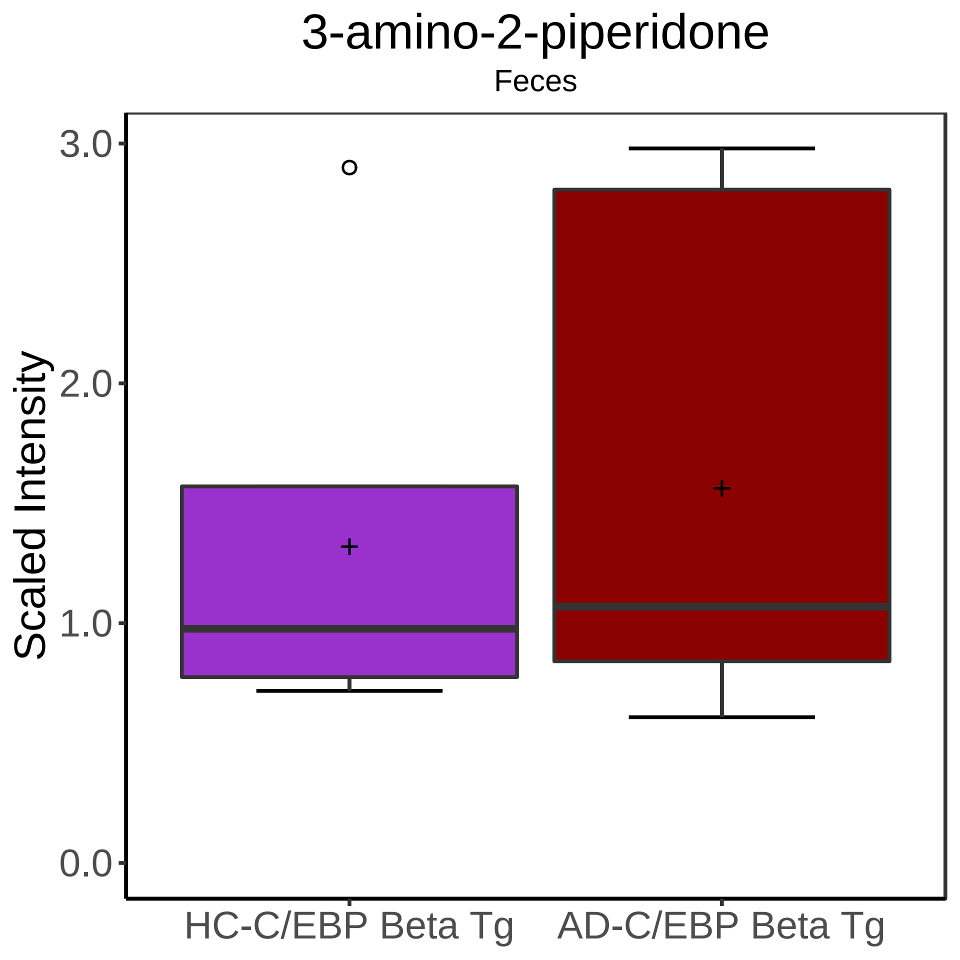

3-amino-2-piperidone
Feces
Scaled Intensity
3.0
2.0
1.0
0.0
HC-C/EBP Beta Tg
AD-C/EBP Beta Tg

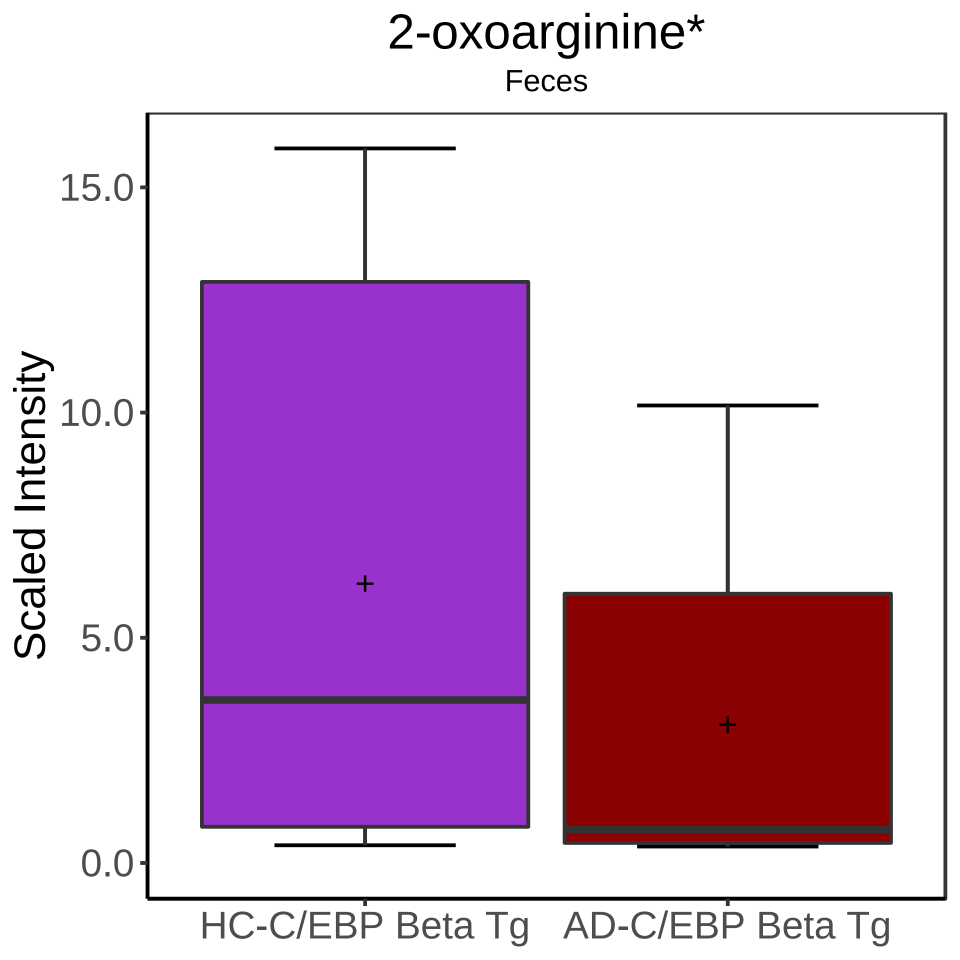
2-oxoarginine*
Feces
Scaled Intensity
15.0
10.0
5.0
0.0
HC-C/EBP Beta Tg
AD-C/EBP Beta Tg

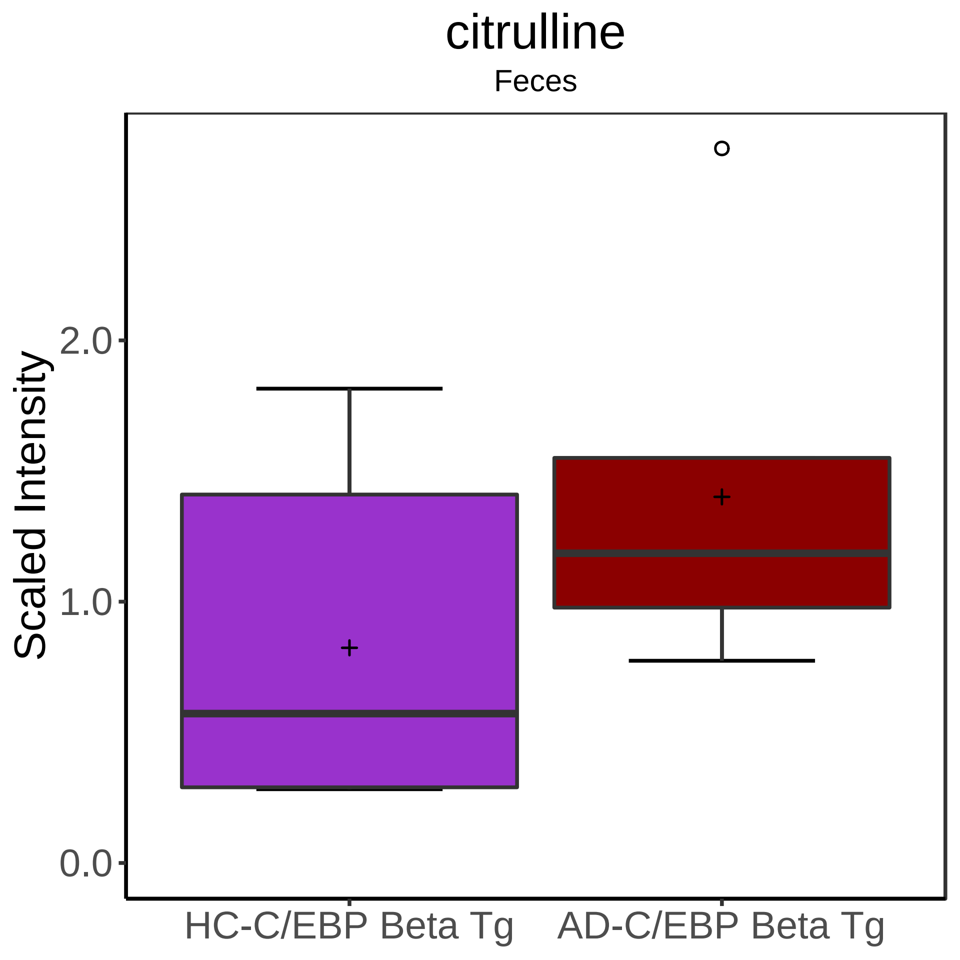
citrulline
Feces
Scaled Intensity
2.0
1.0
0.0
HC-C/EBP Beta Tg
AD-C/EBP Beta Tg

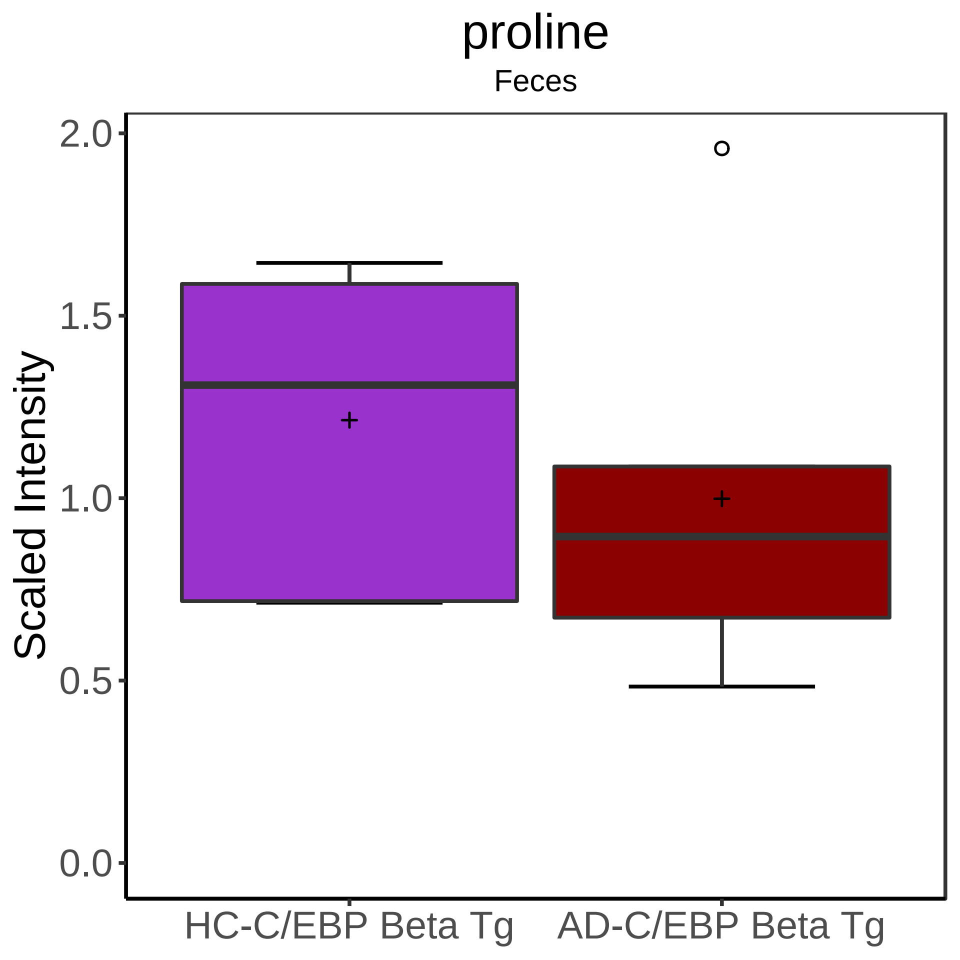

proline
Feces
Scaled Intensity
2.0
1.5
1.0
0.5
0.0
HC-C/EBP Beta Tg
AD-C/EBP Beta Tg

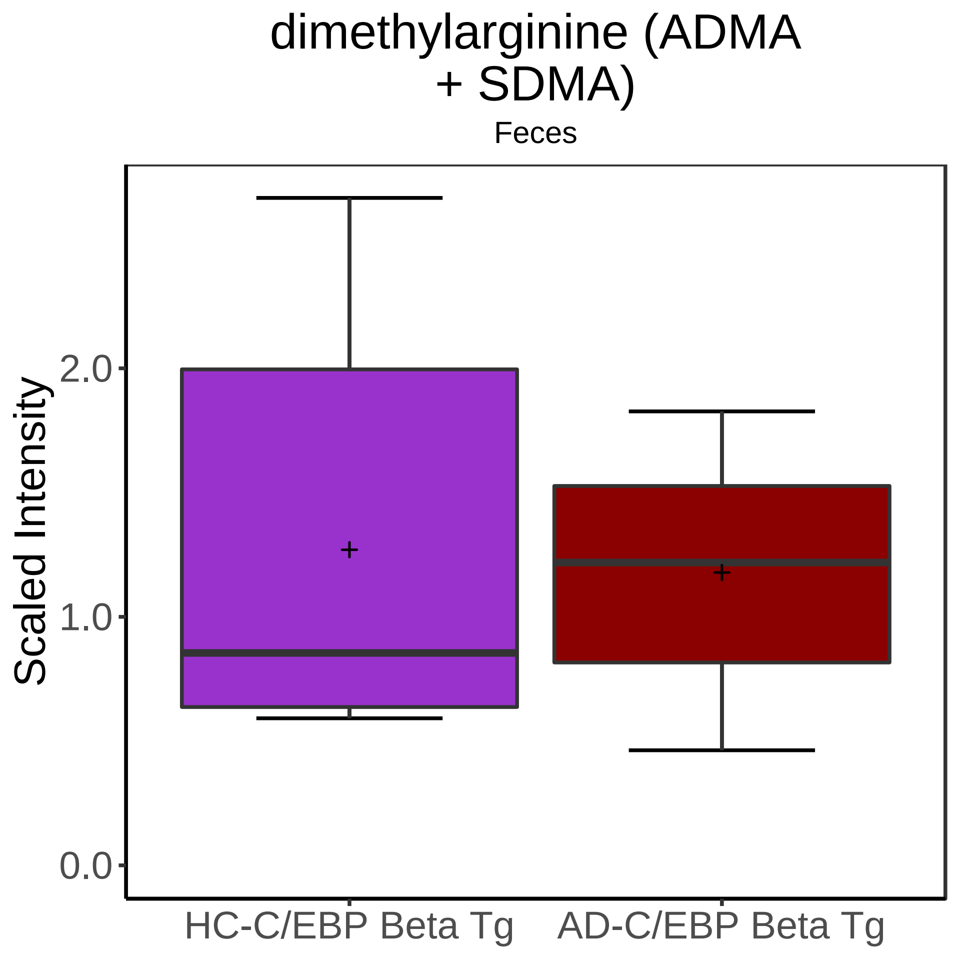

dimethylarginine (ADMA + SDMA)
Feces
Scaled Intensity
2.0
1.0
0.0
HC-C/EBP Beta Tg
AD-C/EBP Beta Tg

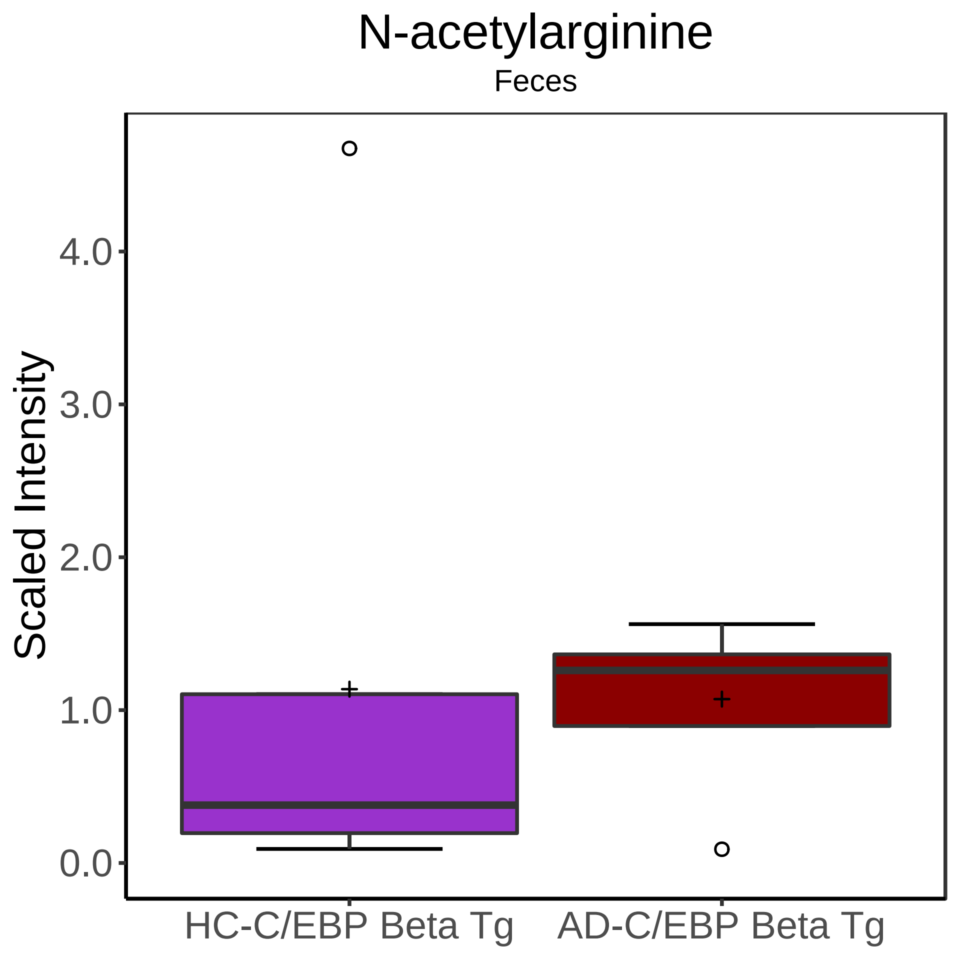

N-acetylarginine
Feces
Scaled Intensity
4.0
3.0
2.0
1.0
0.0
HC-C/EBP Beta Tg
AD-C/EBP Beta Tg

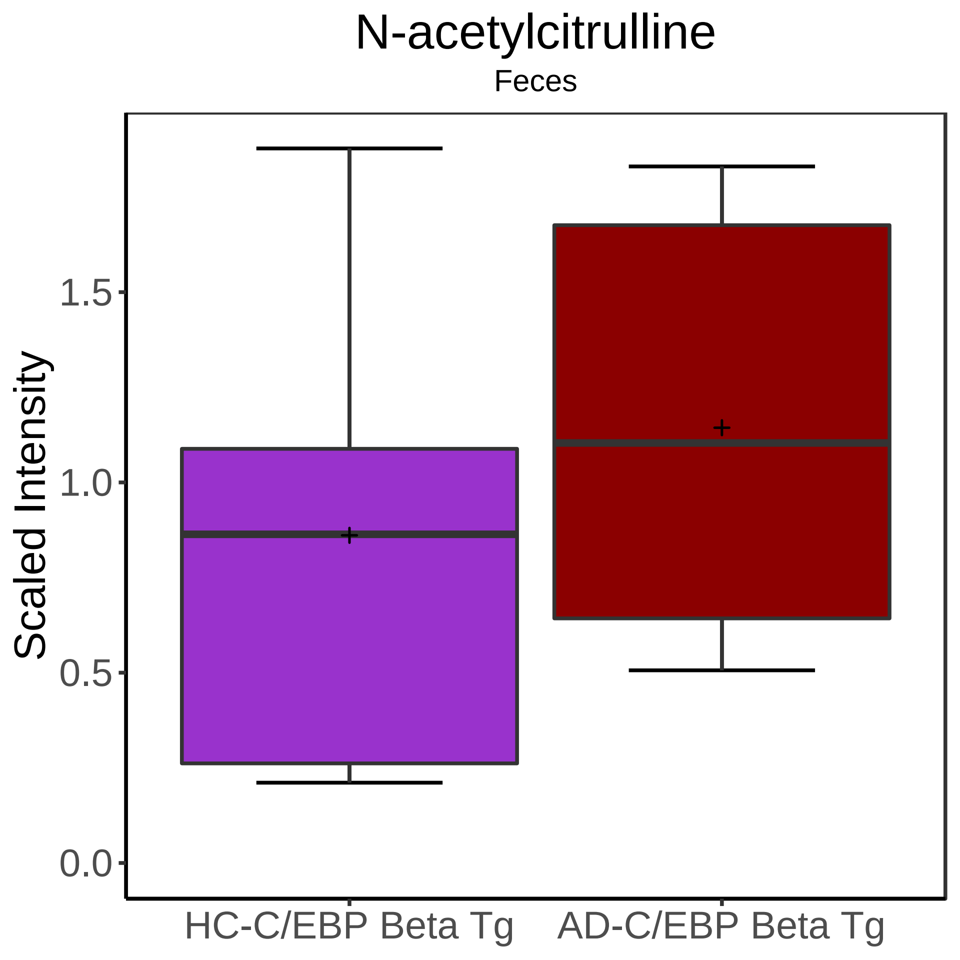
N-acetylcitrulline
Feces
Scaled Intensity
0.0
0.5
1.0
1.5
HC-C/EBP Beta Tg
AD-C/EBP Beta Tg

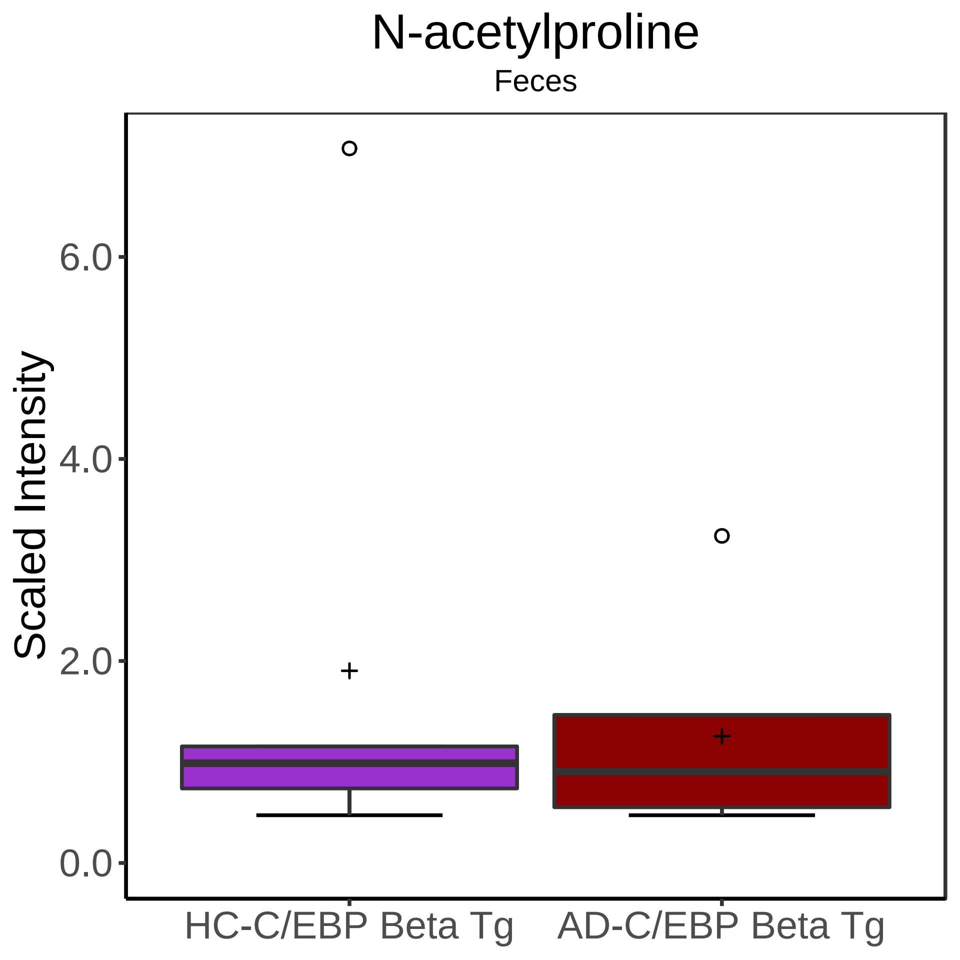
N-acetylproline
Feces
Scaled Intensity
6.0
4.0
2.0
0.0
HC-C/EBP Beta Tg
AD-C/EBP Beta Tg

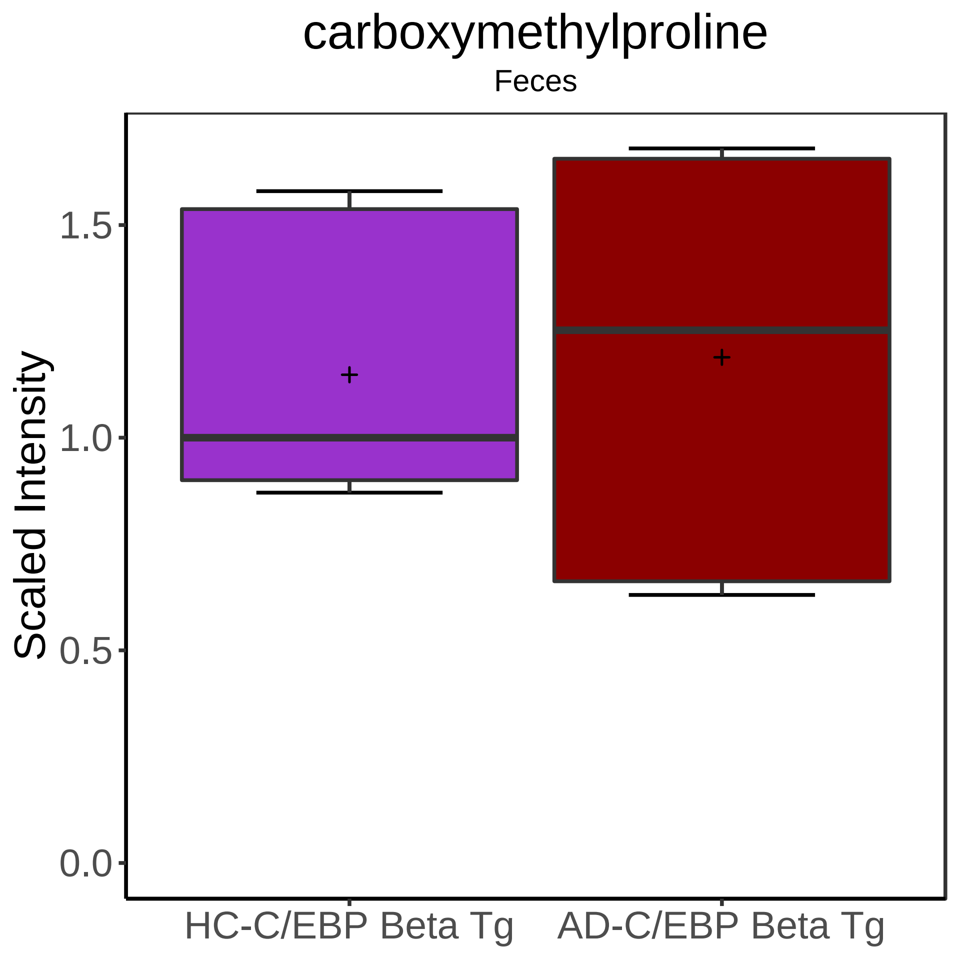
carboxymethylproline
Feces
Scaled Intensity
1.5
1.0
0.5
0.0
HC-C/EBP Beta Tg
AD-C/EBP Beta Tg

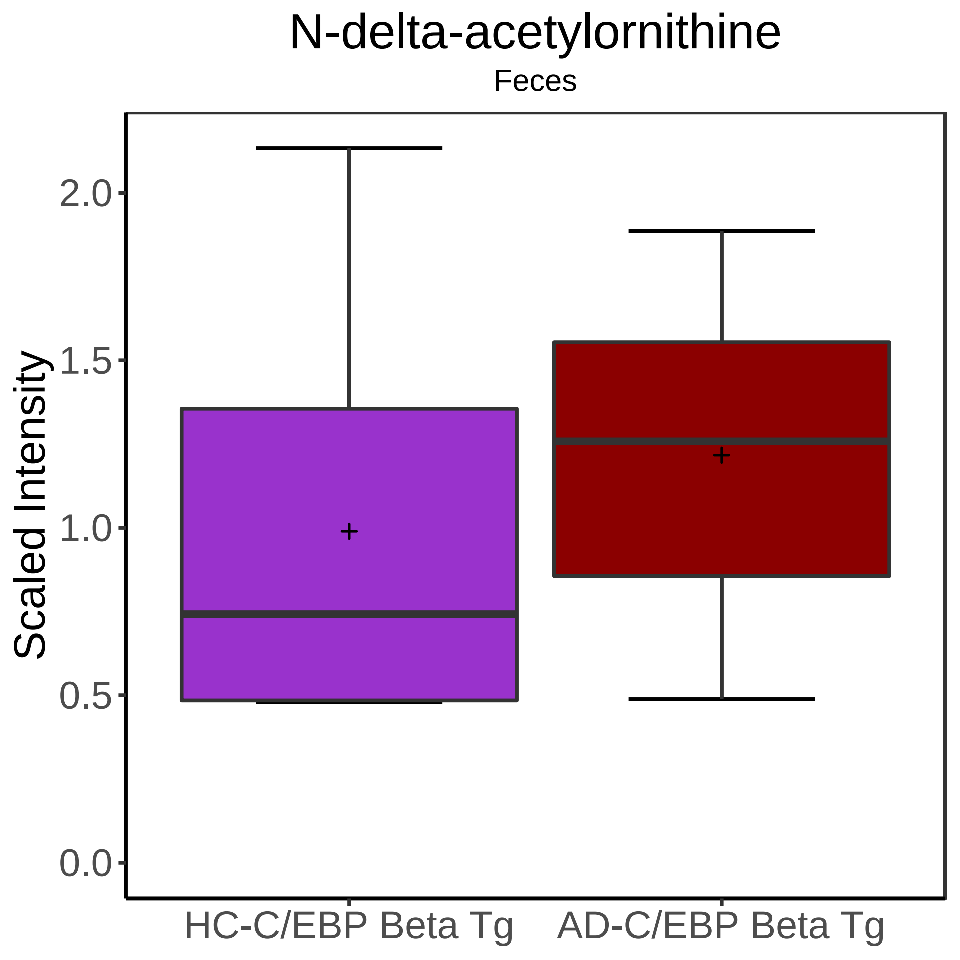

N-delta-acetylornithine
Feces
Scaled Intensity
2.0
1.5
1.0
0.5
0.0
HC-C/EBP Beta Tg
AD-C/EBP Beta Tg

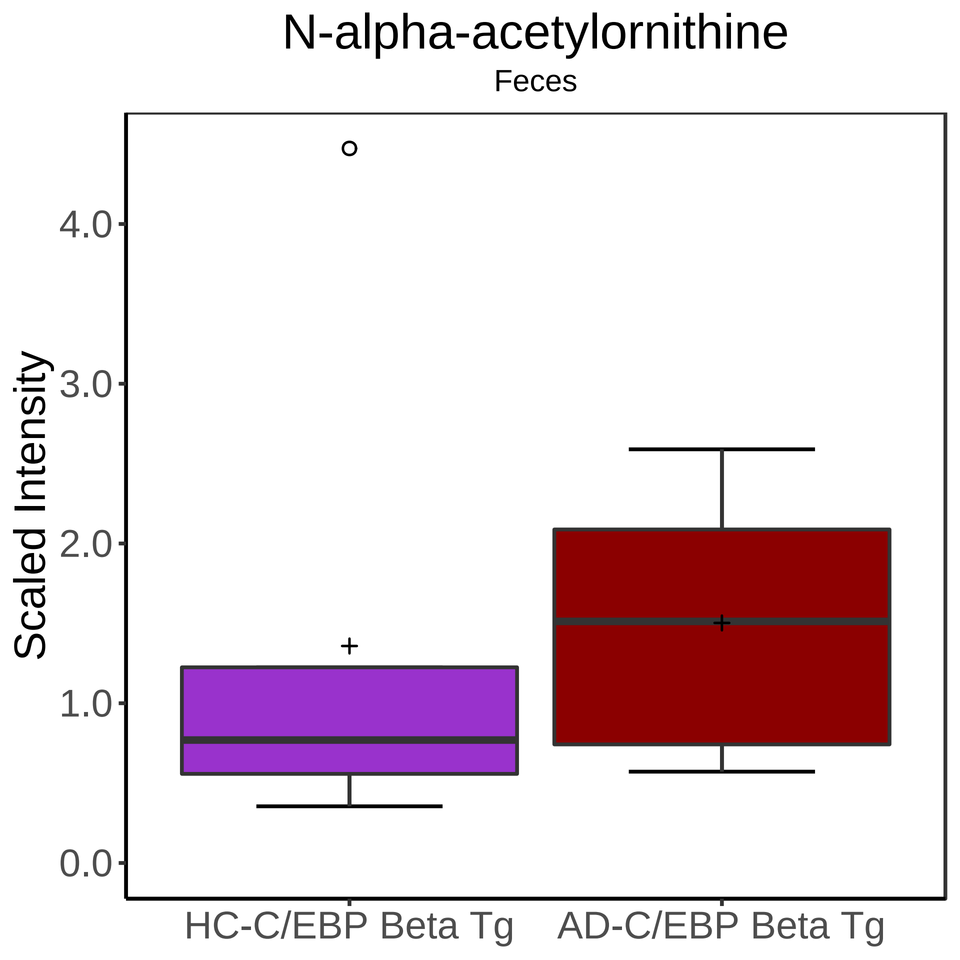
N-alpha-acetylornithine
Feces
Scaled Intensity
4.0
3.0
2.0
1.0
0.0
HC-C/EBP Beta Tg
AD-C/EBP Beta Tg

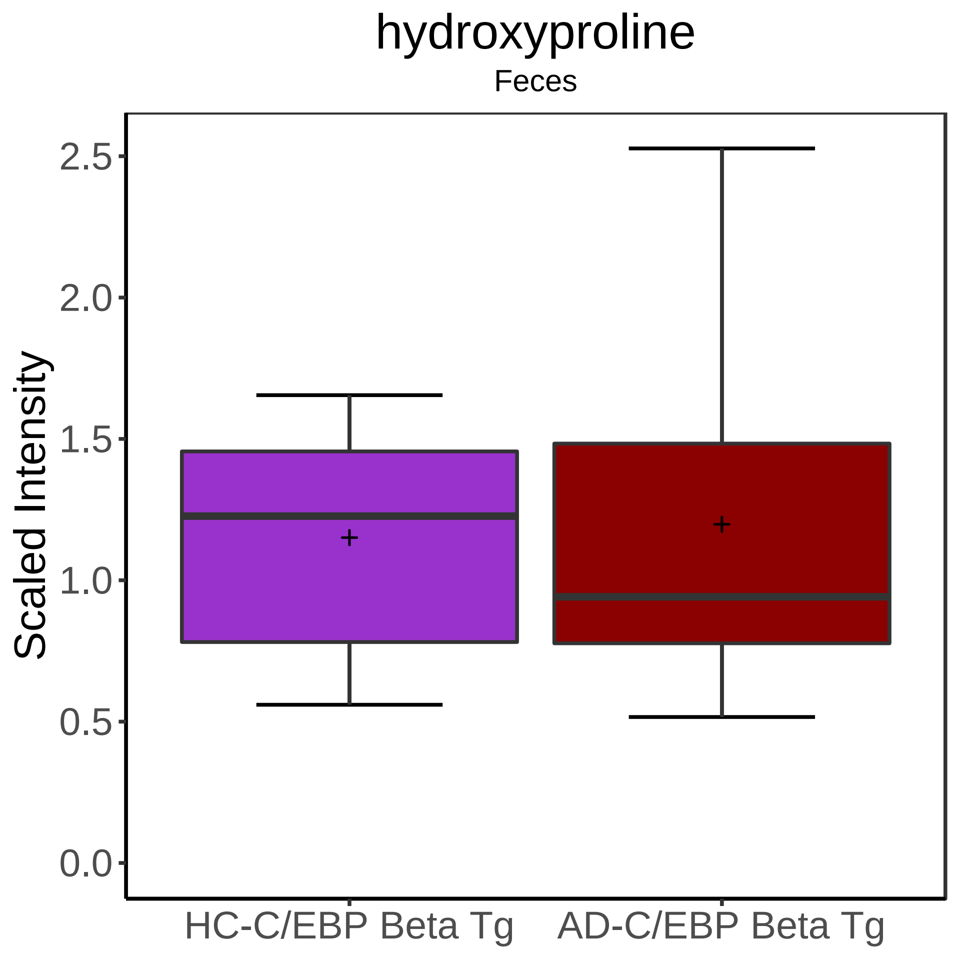
hydroxyproline
Feces
Scaled Intensity
2.5
2.0
1.5
1.0
0.5
0.0
HC-C/EBP Beta Tg
AD-C/EBP Beta Tg

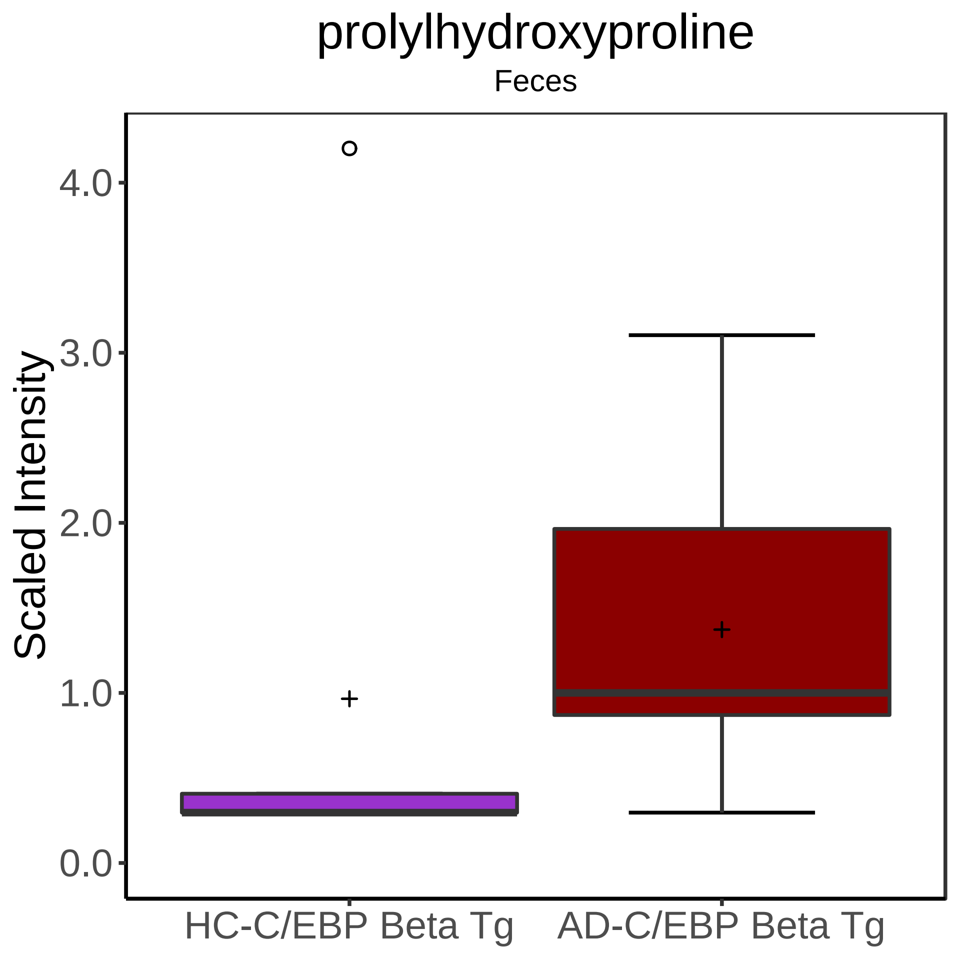

prolylhydroxyproline
Feces
Scaled Intensity
4.0
3.0
2.0
1.0
0.0
HC-C/EBP Beta Tg
AD-C/EBP Beta Tg

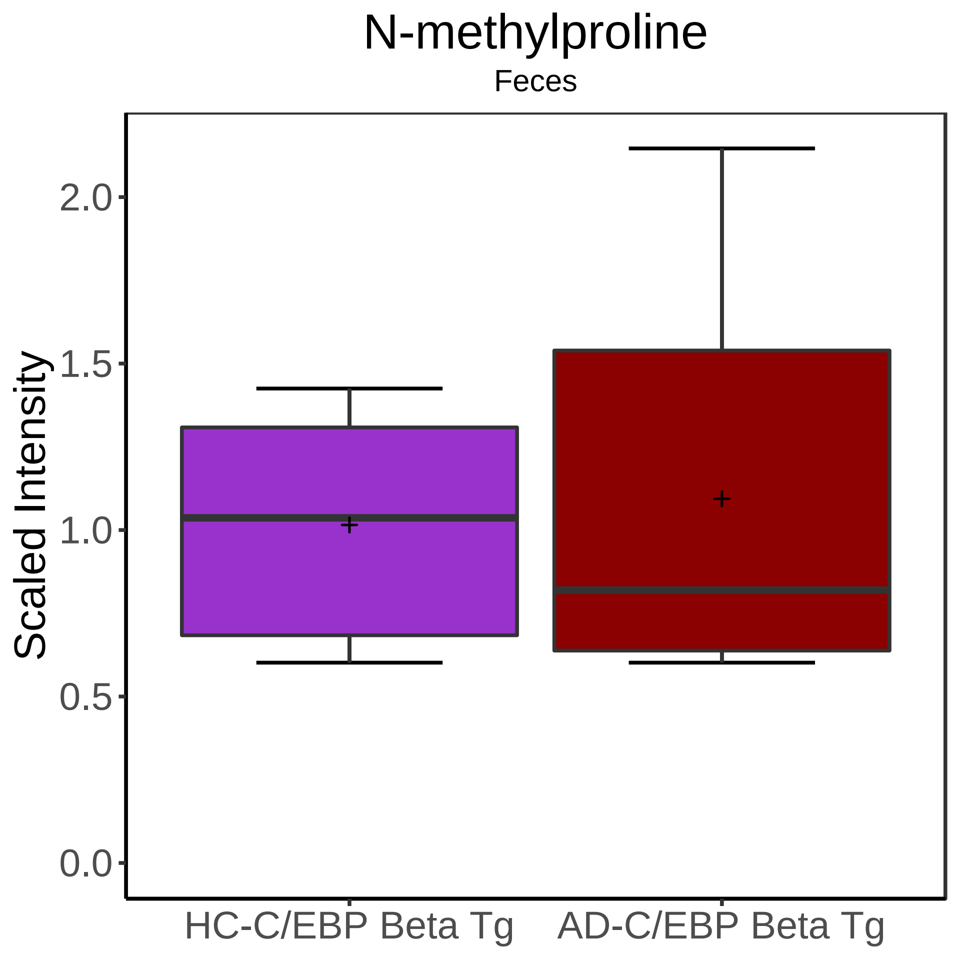

N-methylproline
Feces
Scaled Intensity
2.0
1.5
1.0
0.5
0.0
HC-C/EBP Beta Tg
AD-C/EBP Beta Tg

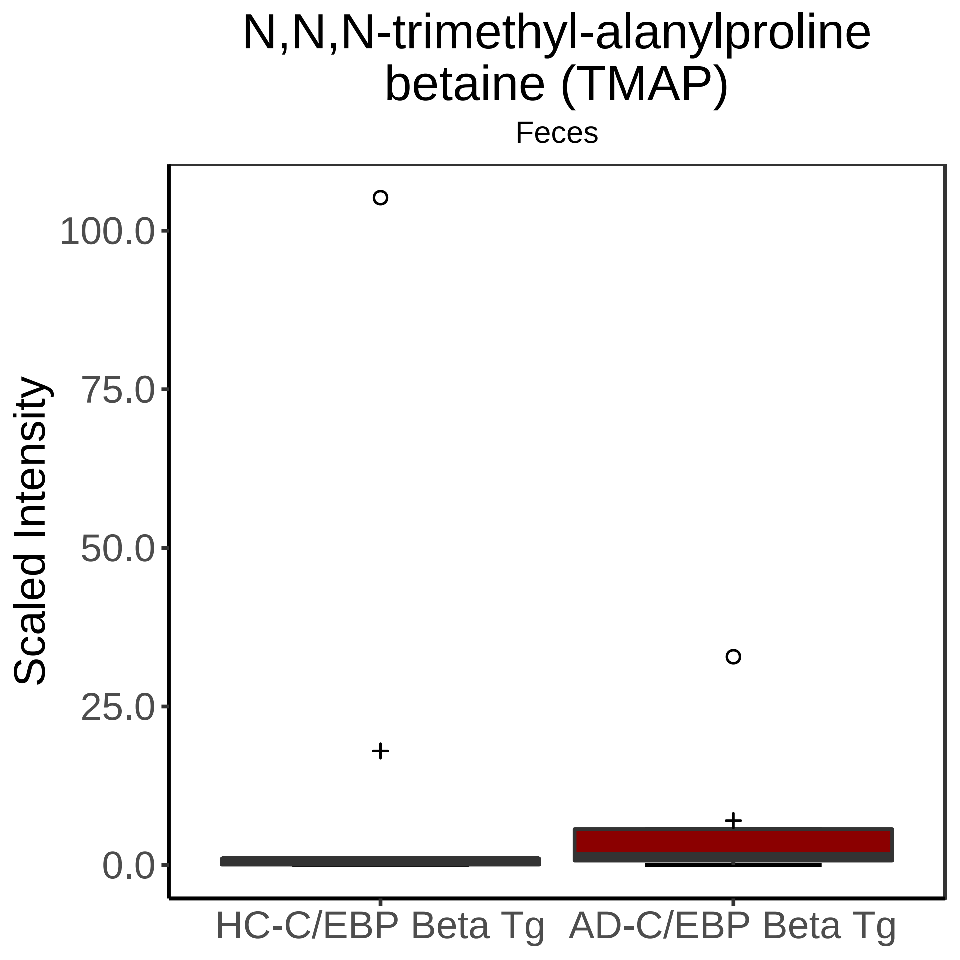

N,N,N-trimethyl-alanylproline betaine (TMAP)
Feces
Scaled Intensity
100.0
75.0
50.0
25.0
0.0
HC-C/EBP Beta Tg
AD-C/EBP Beta Tg

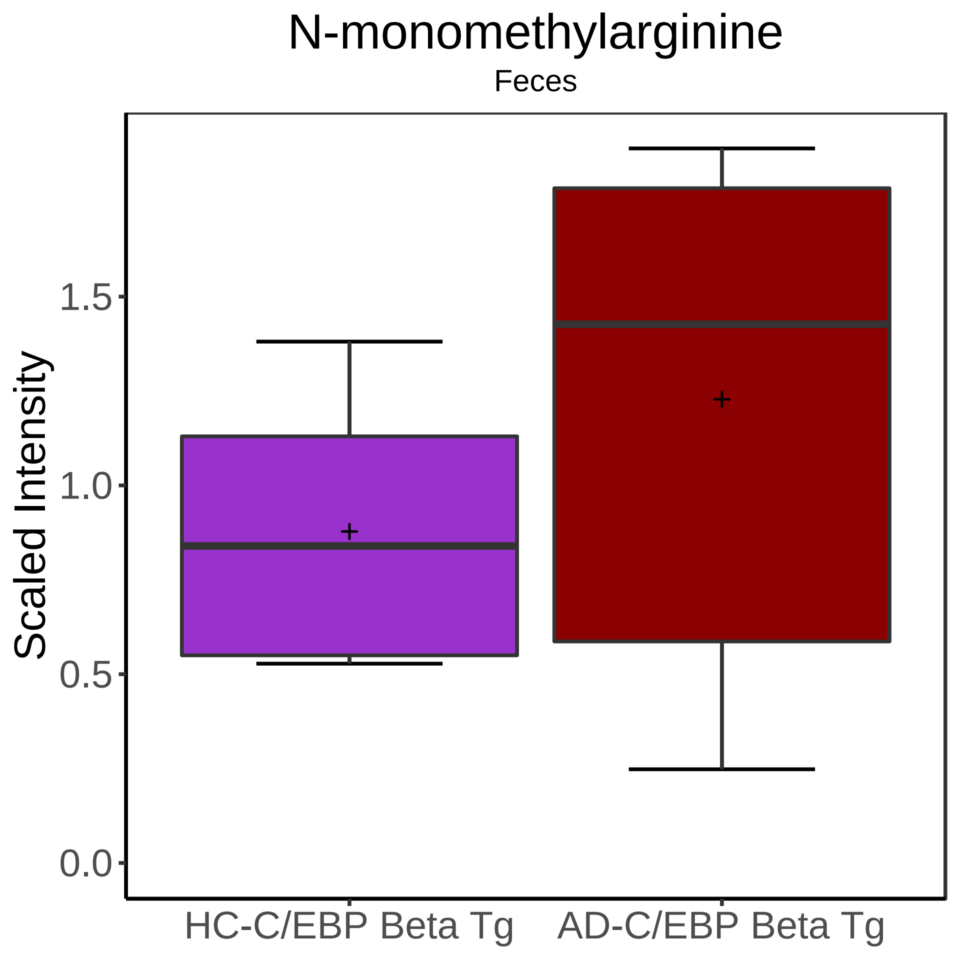
N-monomethylarginine
Feces
Scaled Intensity
0.0
0.5
1.0
1.5
HC-C/EBP Beta Tg
AD-C/EBP Beta Tg

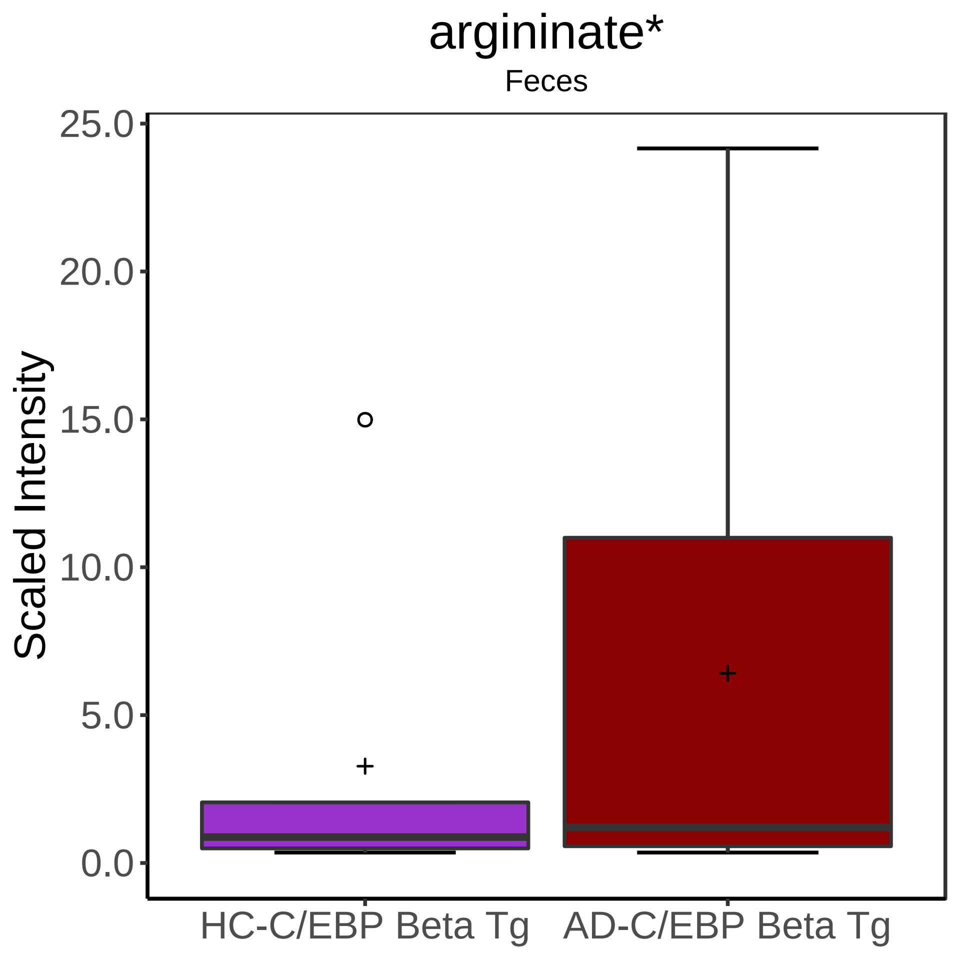

arginate*
Feces
Scaled Intensity
25.0
20.0
15.0
10.0
5.0
0.0
HC-C/EBP Beta Tg
AD-C/EBP Beta Tg

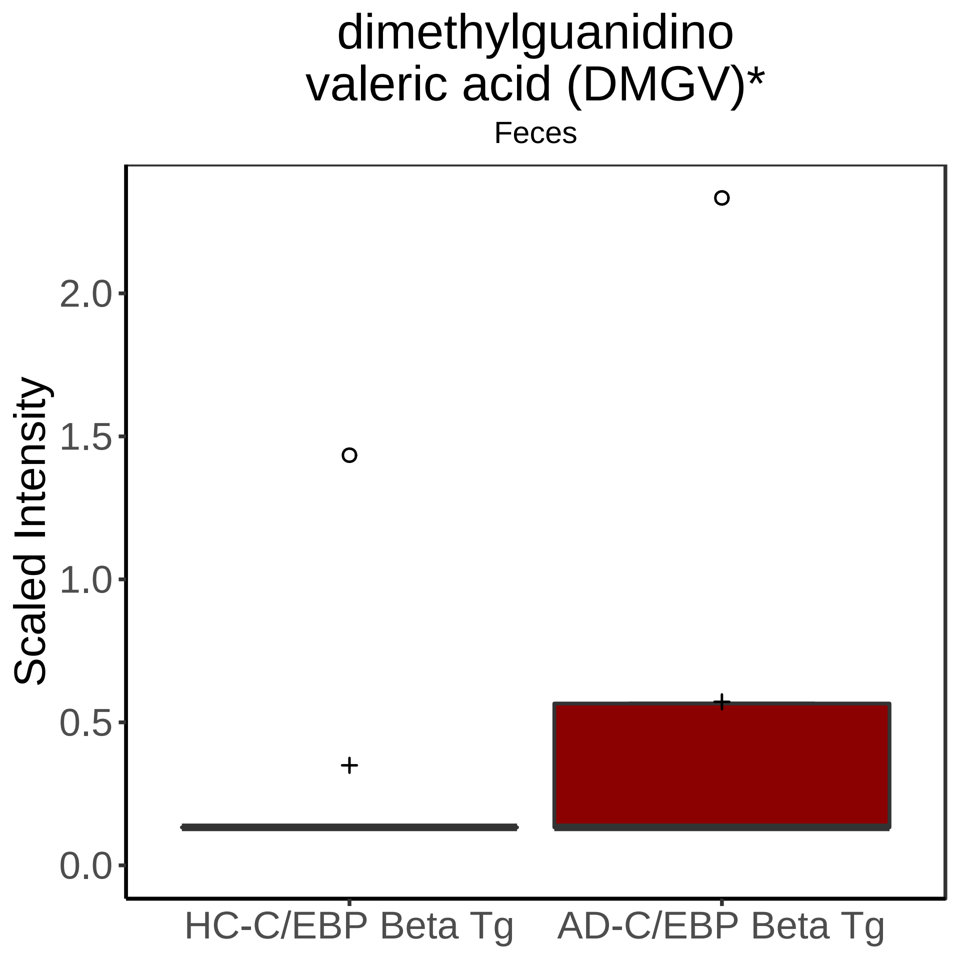
dimethylguanidino
valeric acid (DMGV)*
Feces
Scaled Intensity
0.0
0.5
1.0
1.5
2.0
HC-C/EBP Beta Tg
AD-C/EBP Beta Tg

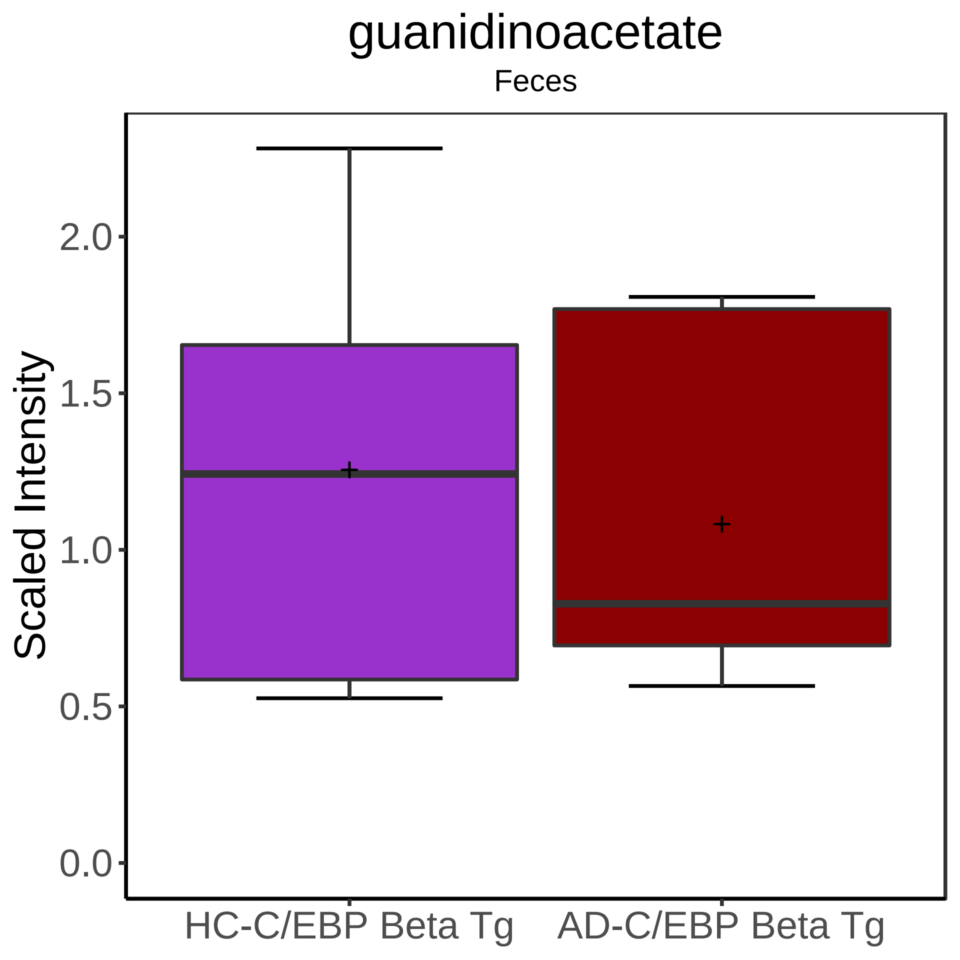

guanidinoacetate
Feces
Scaled Intensity
2.0
1.5
1.0
0.5
0.0
HC-C/EBP Beta Tg
AD-C/EBP Beta Tg

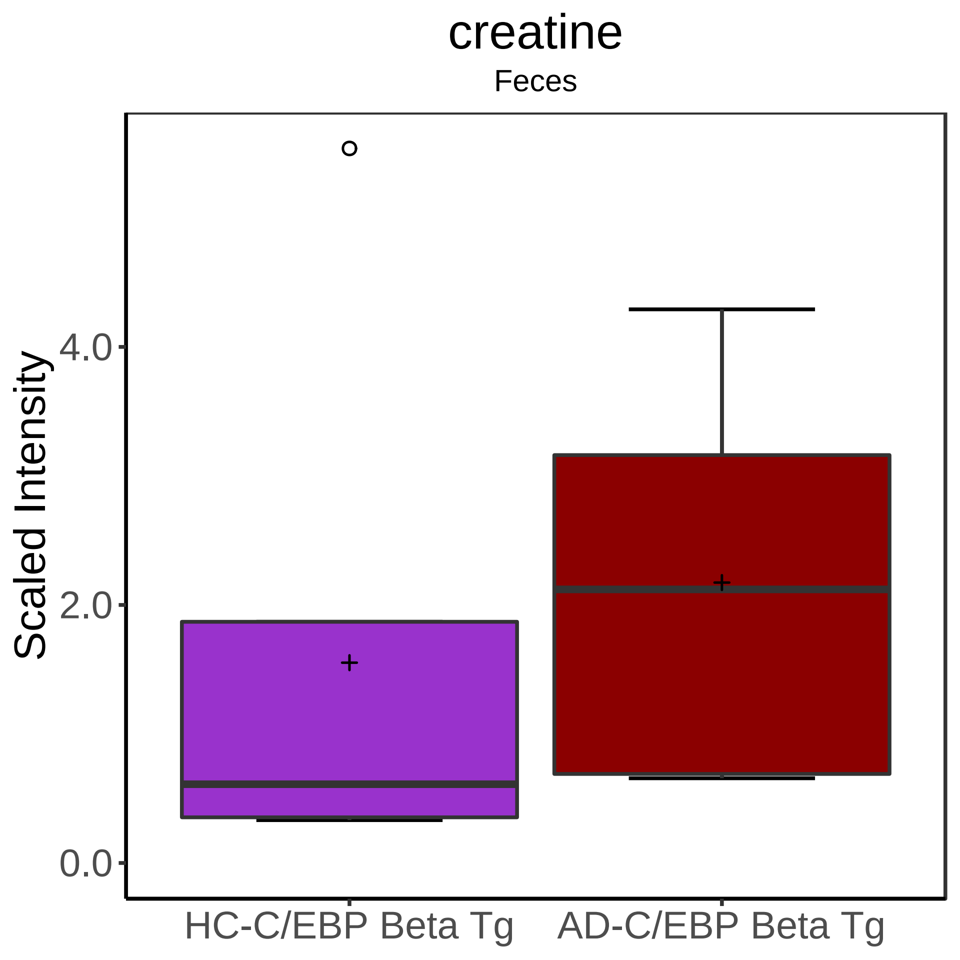

creatine
Feces
Scaled Intensity
4.0
2.0
0.0
HC-C/EBP Beta Tg
AD-C/EBP Beta Tg

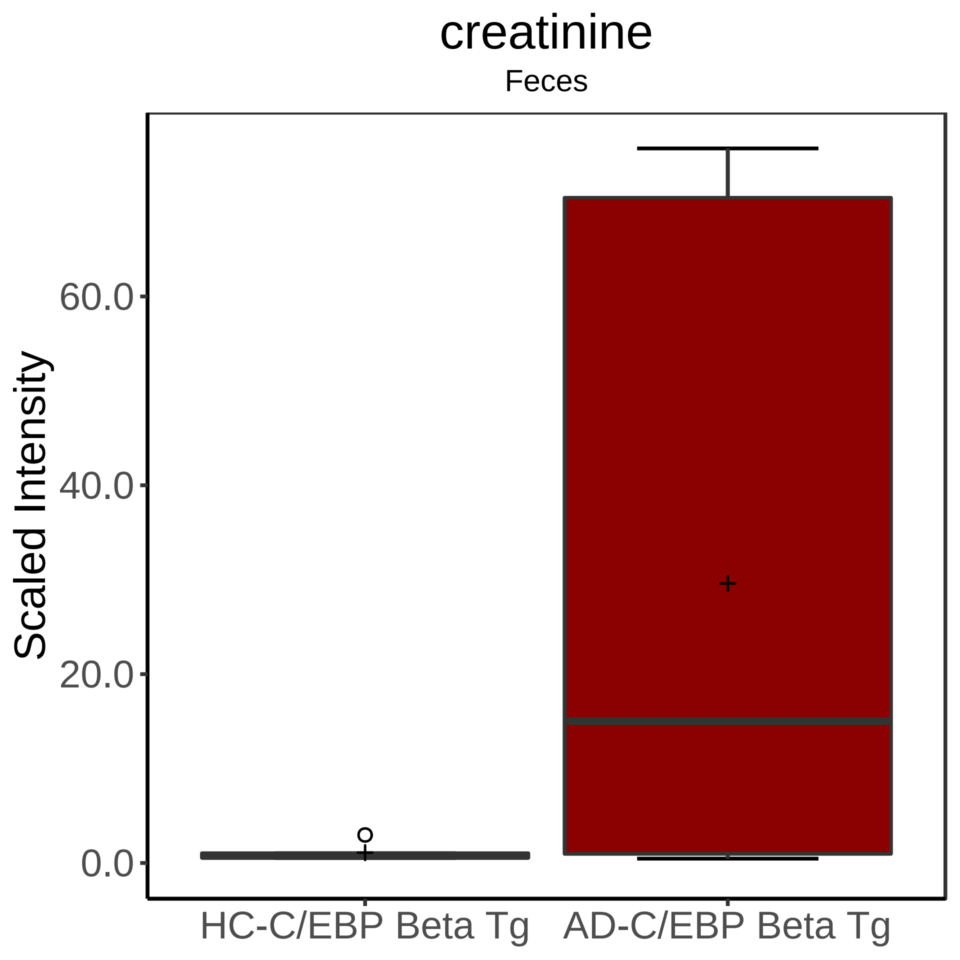

creatinine
Feces
Scaled Intensity
60.0
40.0
20.0
0.0
HC-C/EBP Beta Tg
AD-C/EBP Beta Tg

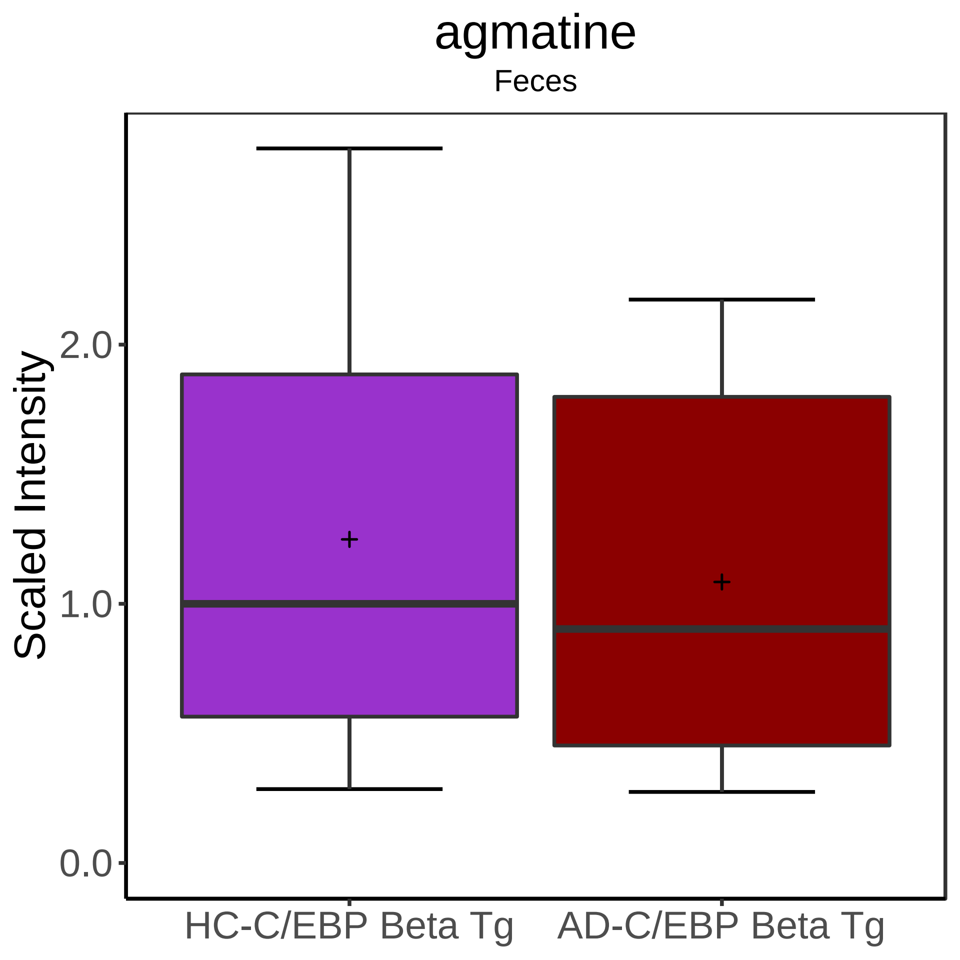
agmatine
Feces
Scaled Intensity
2.0
1.0
0.0
HC-C/EBP Beta Tg
AD-C/EBP Beta Tg

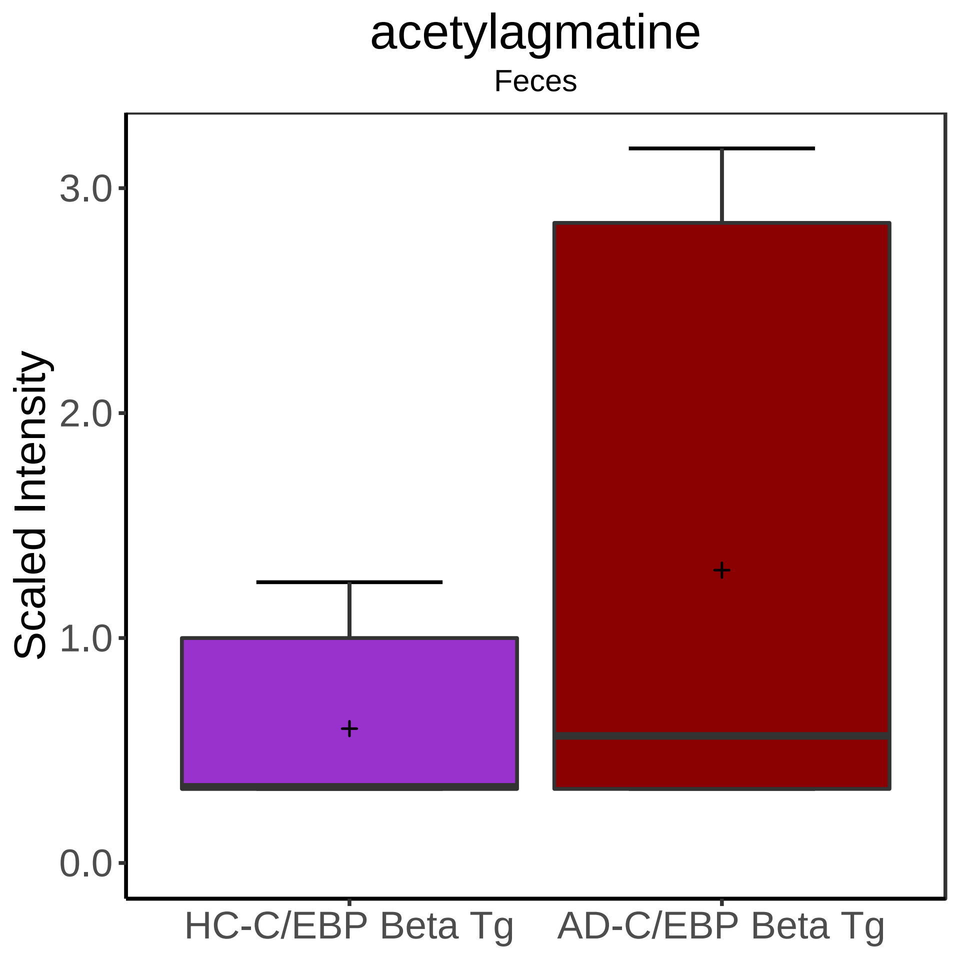
acetylagmatine
Feces
Scaled Intensity
3.0
2.0
1.0
0.0
HC-C/EBP Beta Tg
AD-C/EBP Beta Tg

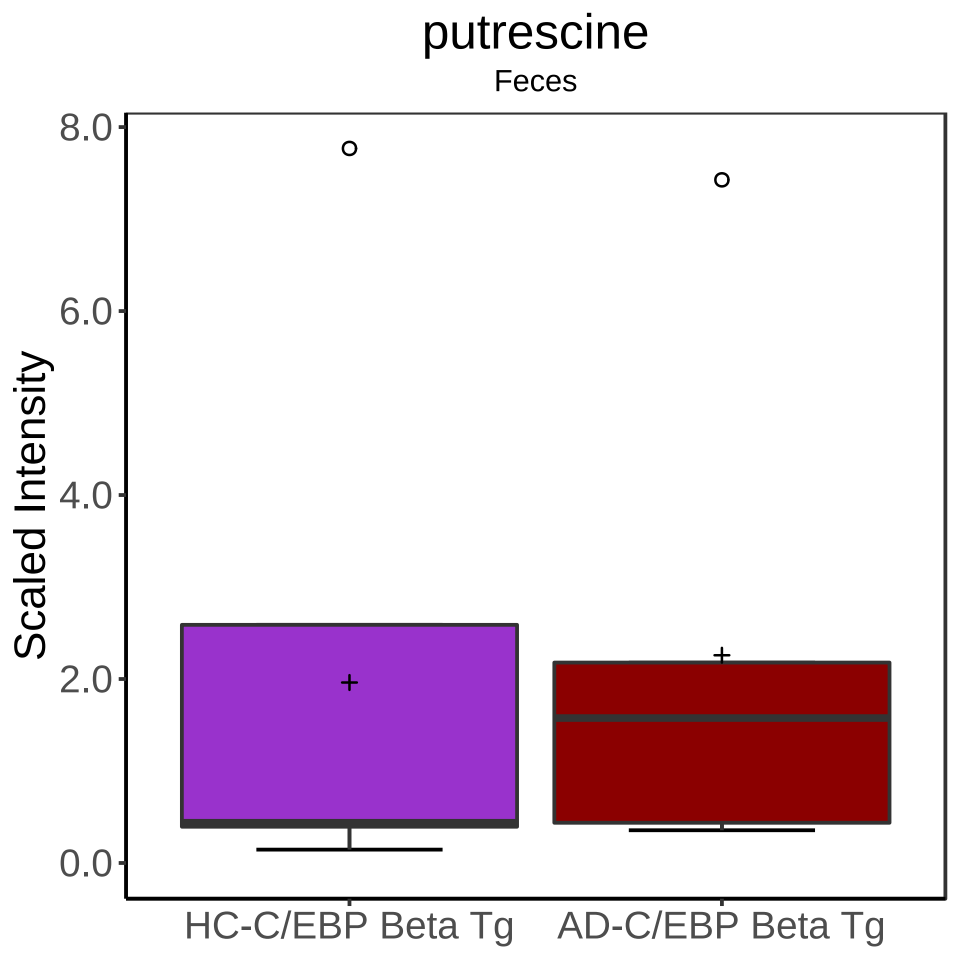
putrescine
Feces
Scaled Intensity
8.0
6.0
4.0
2.0
0.0
HC-C/EBP Beta Tg
AD-C/EBP Beta Tg

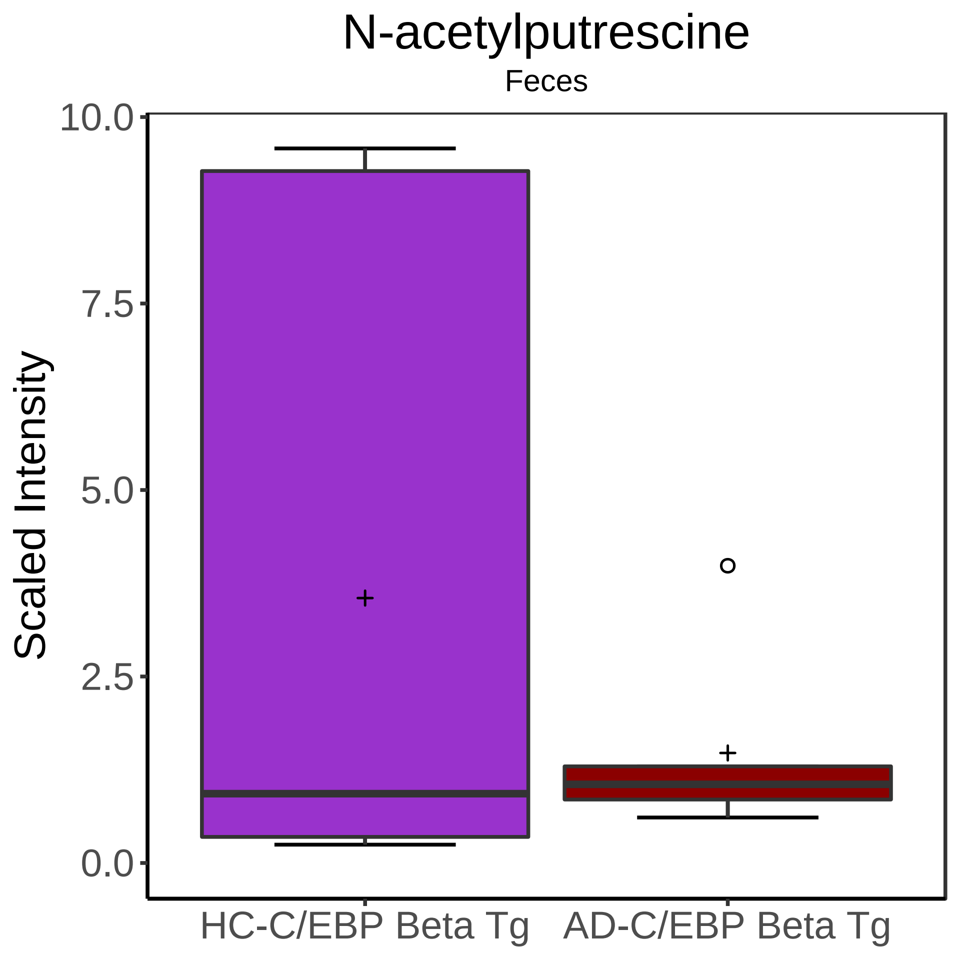
N-acetylputrescine
Feces
Scaled Intensity
10.0
7.5
5.0
2.5
0.0
HC-C/EBP Beta Tg
AD-C/EBP Beta Tg

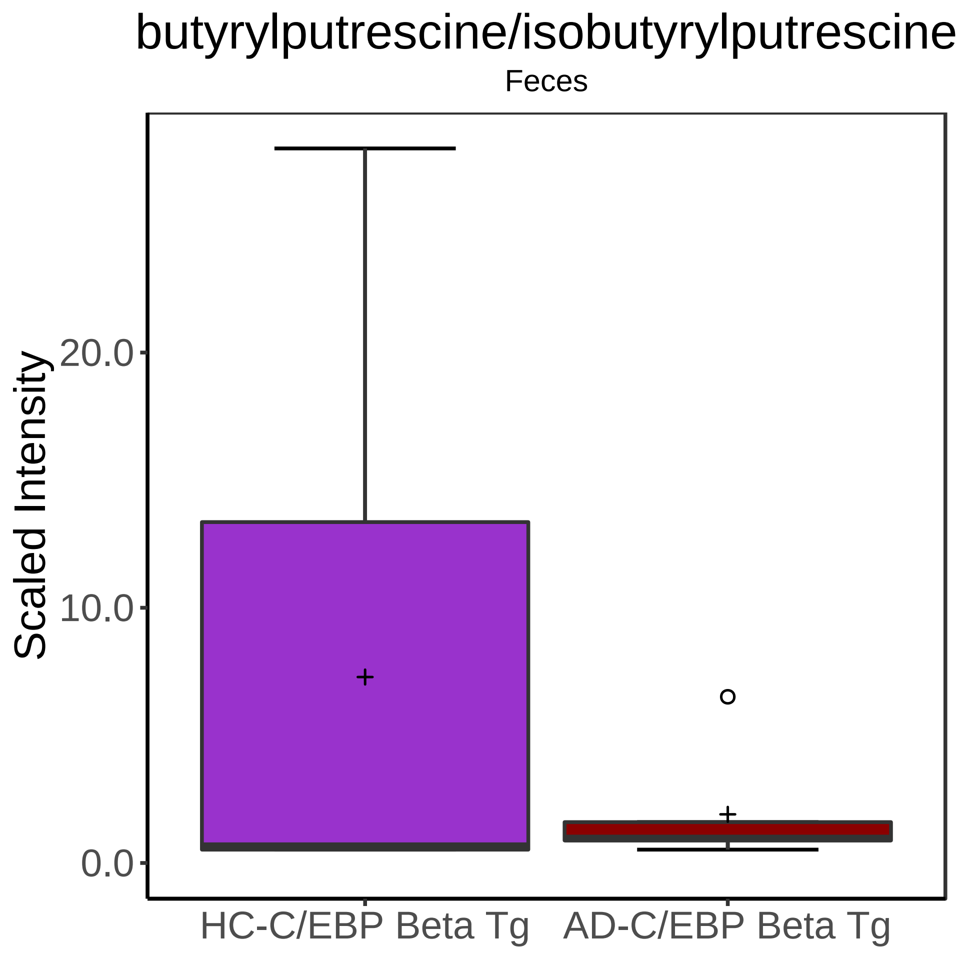

butyrylputrescine/isobutyrylputrescine
Feces
Scaled Intensity
20.0
10.0
0.0
HC-C/EBP Beta Tg
AD-C/EBP Beta Tg

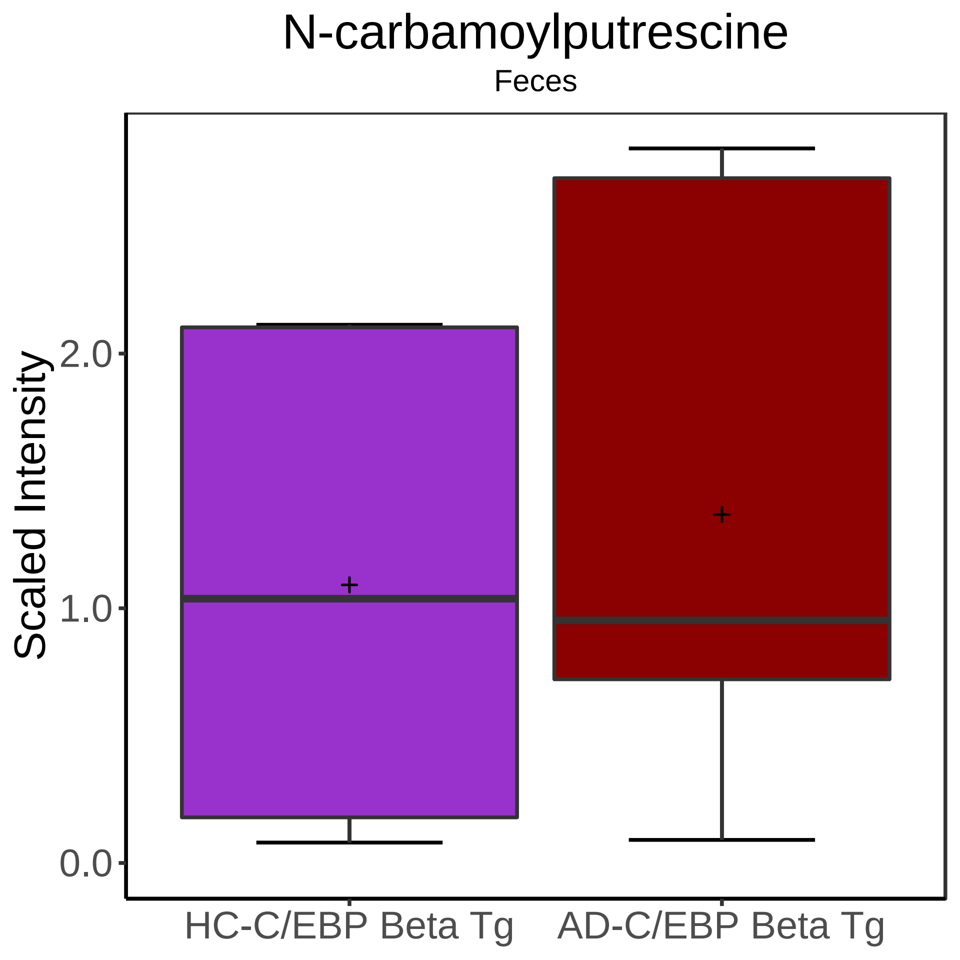
N-carbamoylputrescine
Feces
Scaled Intensity
2.0
1.0
0.0
HC-C/EBP Beta Tg
AD-C/EBP Beta Tg

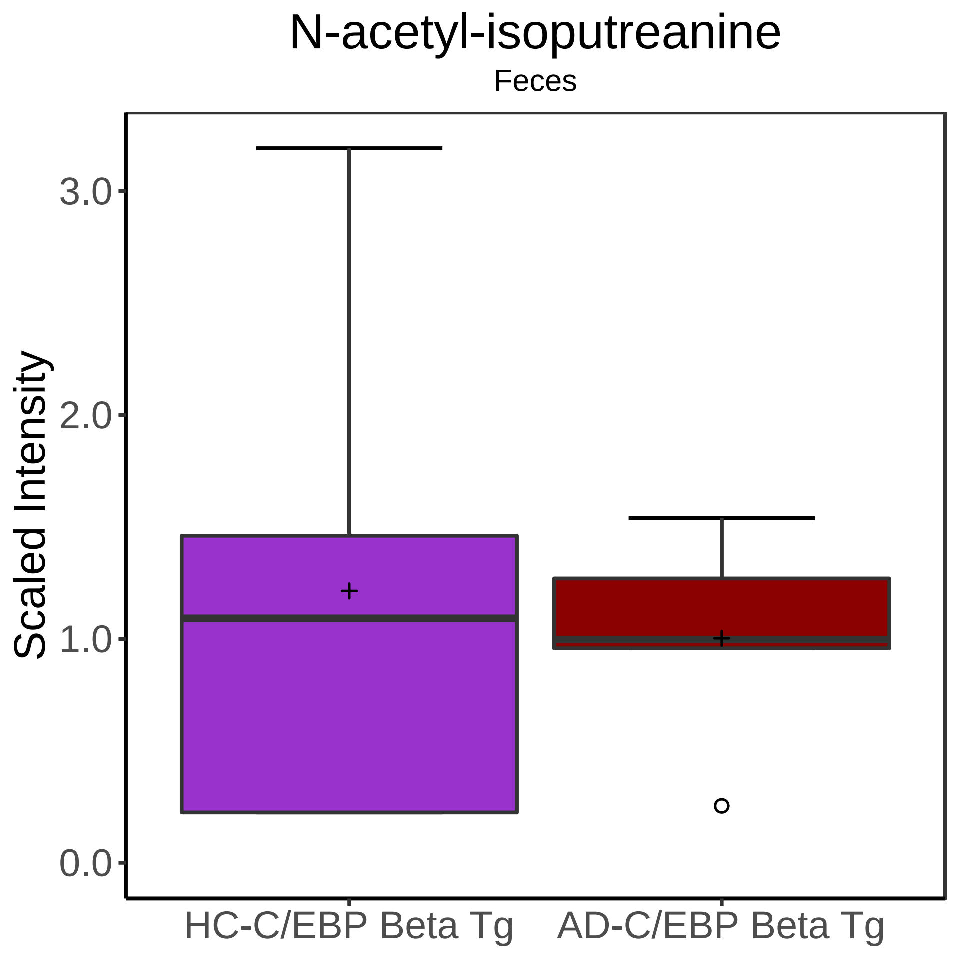

N-acetyl-isoputreanine
Feces
Scaled Intensity
3.0
2.0
1.0
0.0
HC-C/EBP Beta Tg
AD-C/EBP Beta Tg

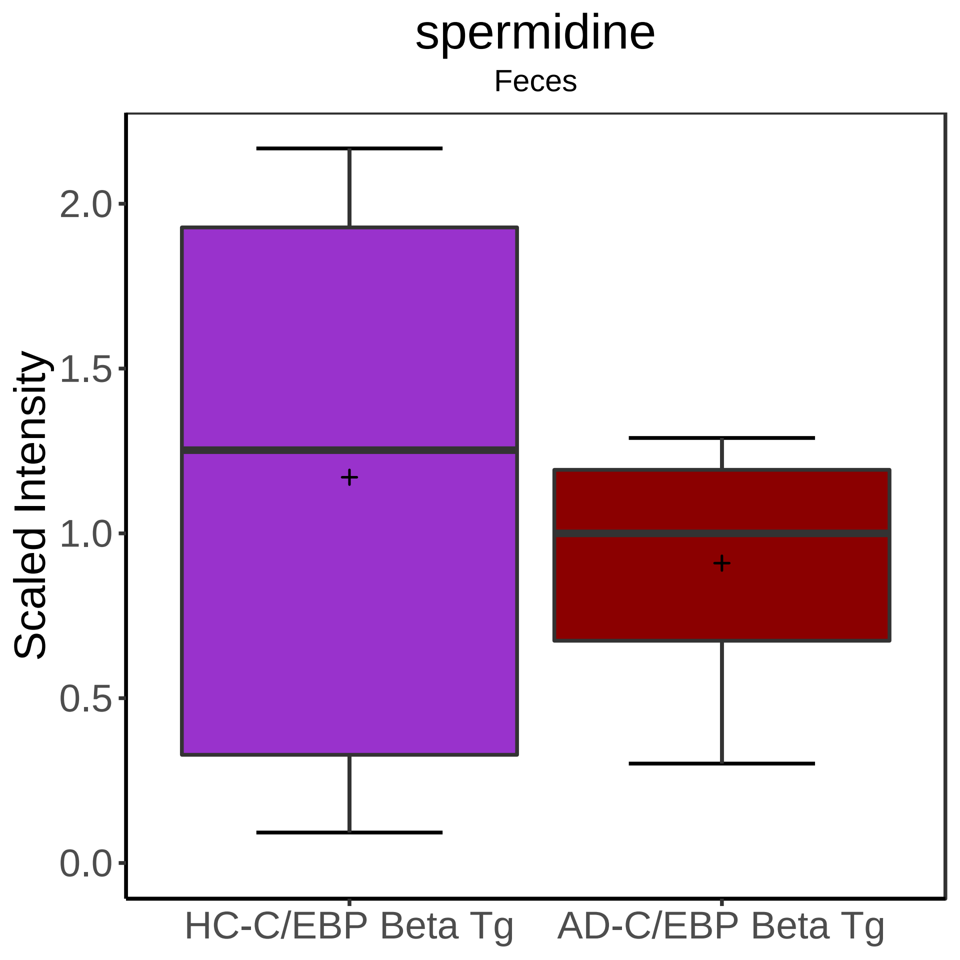

spermidine
Feces
Scaled Intensity
2.0
1.5
1.0
0.5
0.0
HC-C/EBP Beta Tg
AD-C/EBP Beta Tg

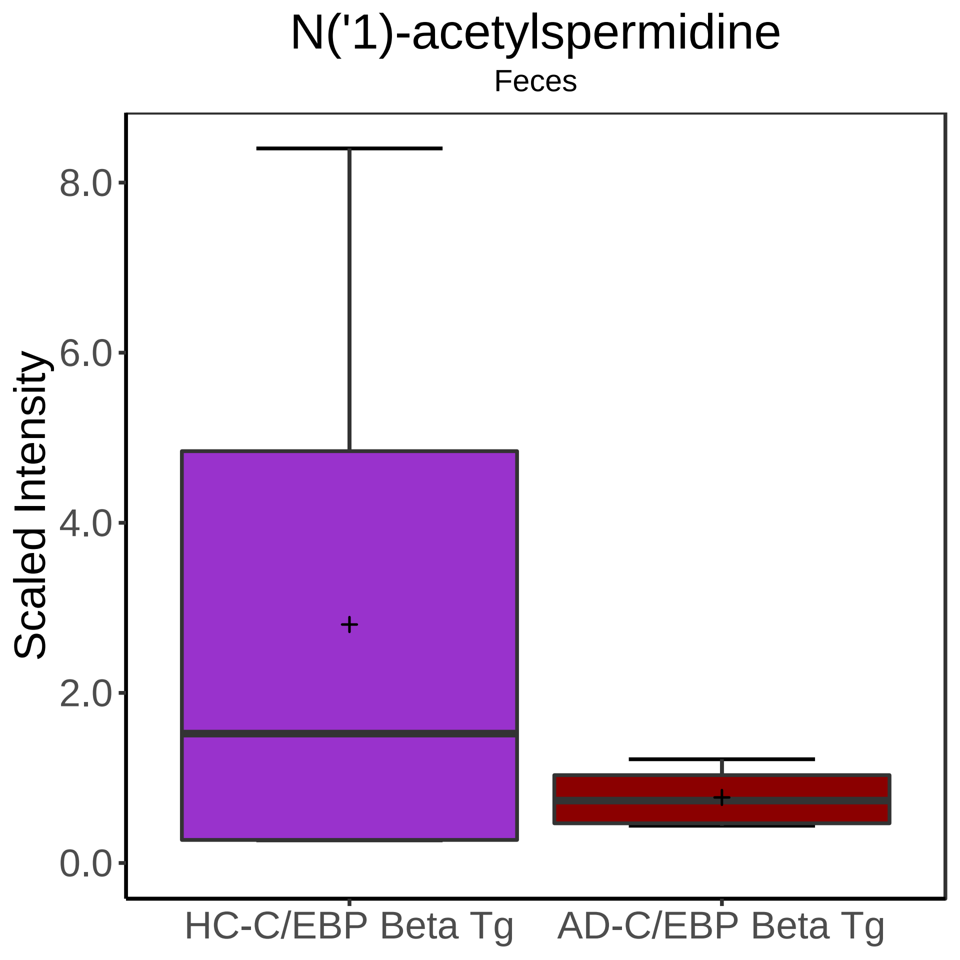
N('1)-acetylspermidine
Feces
Scaled Intensity
8.0
6.0
4.0
2.0
0.0
HC-C/EBP Beta Tg
AD-C/EBP Beta Tg

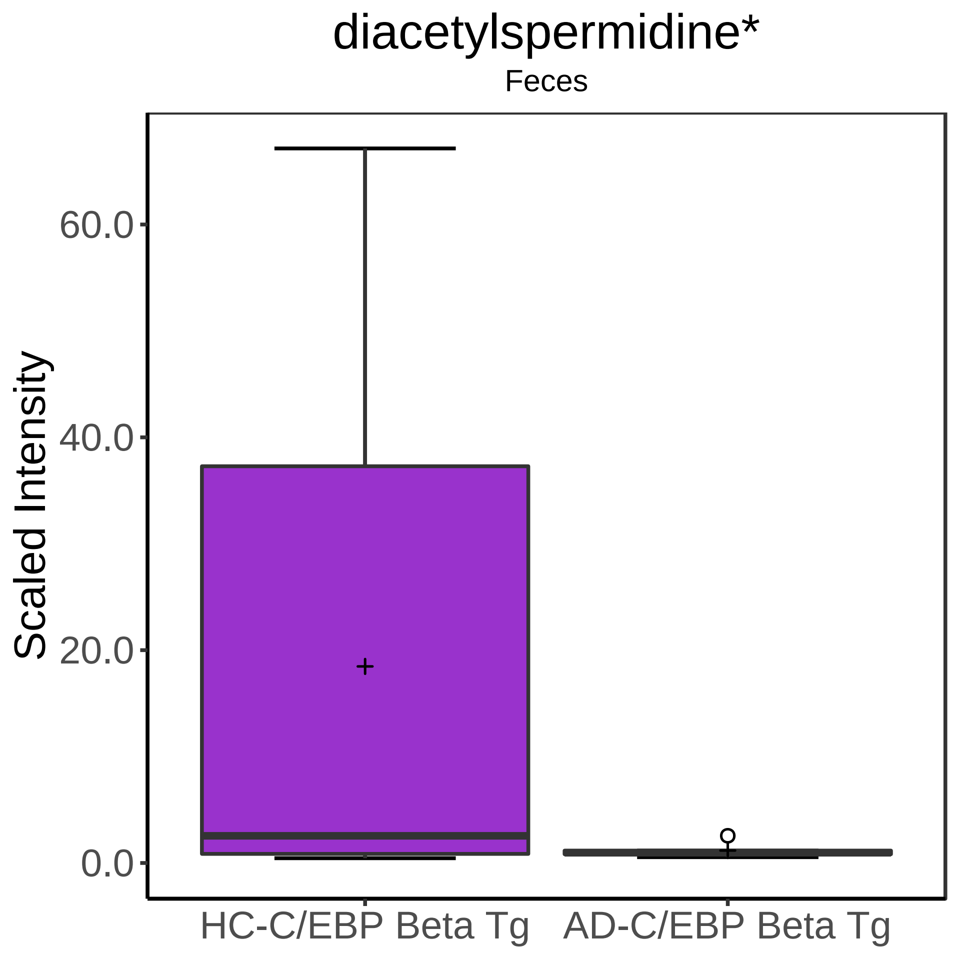

diacetylspermidine*
Feces
Scaled Intensity
60.0
40.0
20.0
0.0
HC-C/EBP Beta Tg
AD-C/EBP Beta Tg

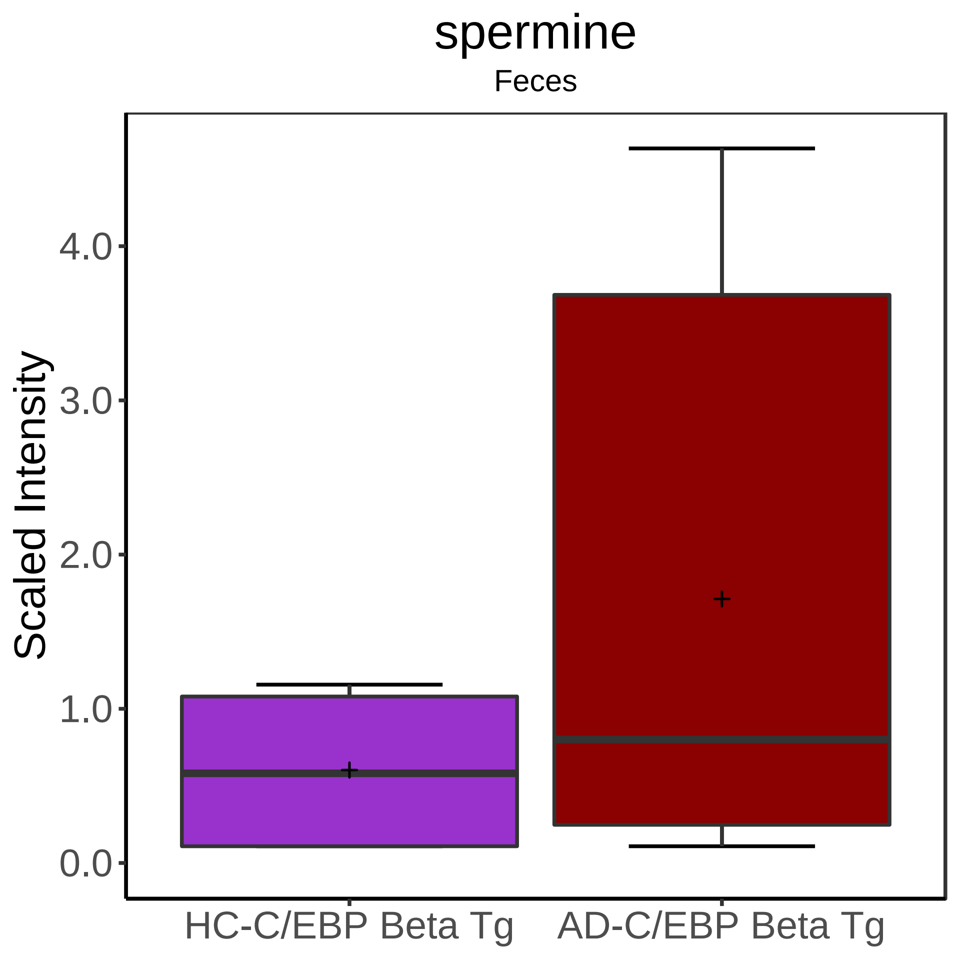
spermine
Feces
Scaled Intensity
4.0
3.0
2.0
1.0
0.0
HC-C/EBP Beta Tg
AD-C/EBP Beta Tg

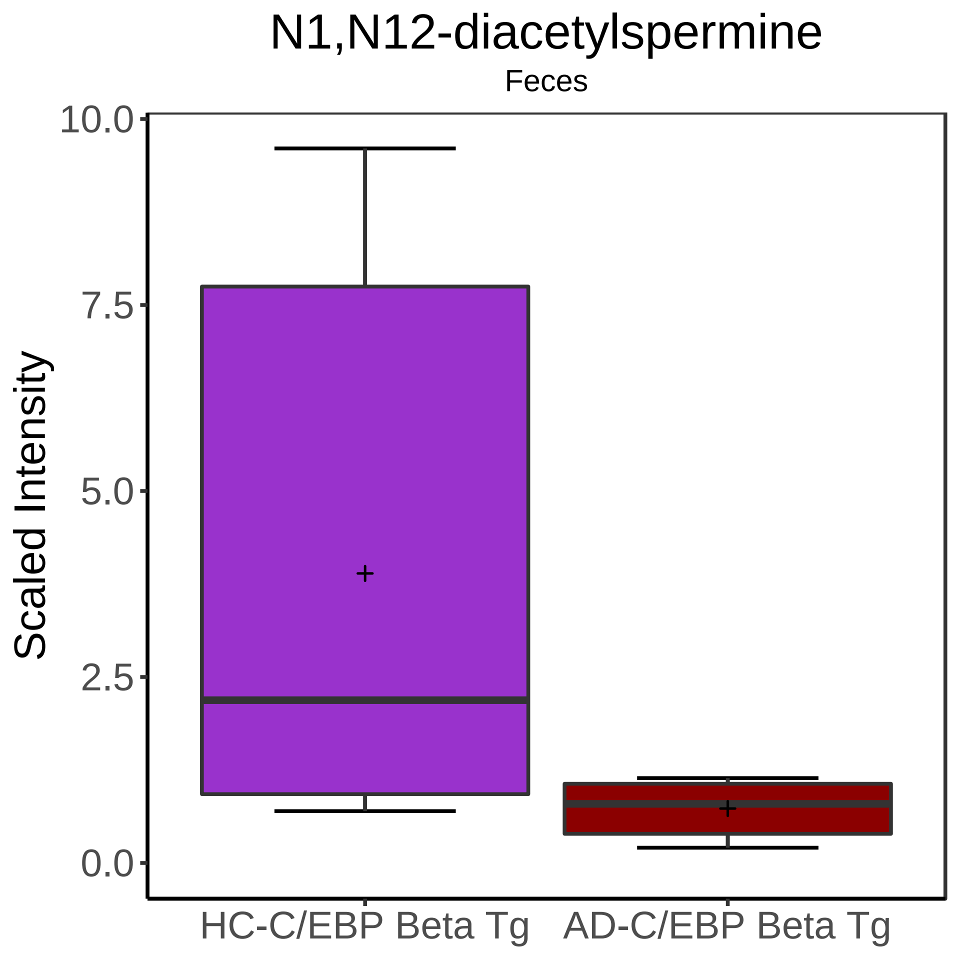
N1,N12-diacetylspermine
Feces
Scaled Intensity
10.0
7.5
5.0
2.5
0.0
HC-C/EBP Beta Tg
AD-C/EBP Beta Tg

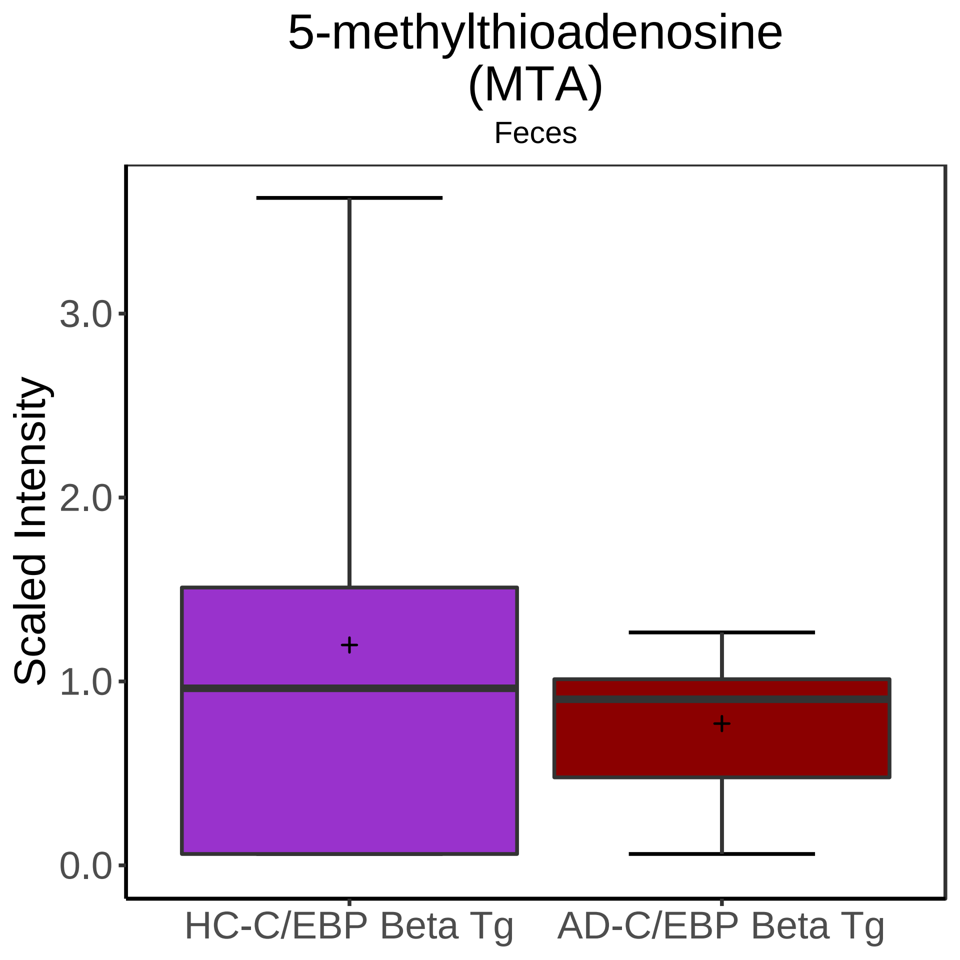

5-methylthioadenosine
(MTA)
Feces
Scaled Intensity
3.0
2.0
1.0
0.0
HC-C/EBP Beta Tg
AD-C/EBP Beta Tg

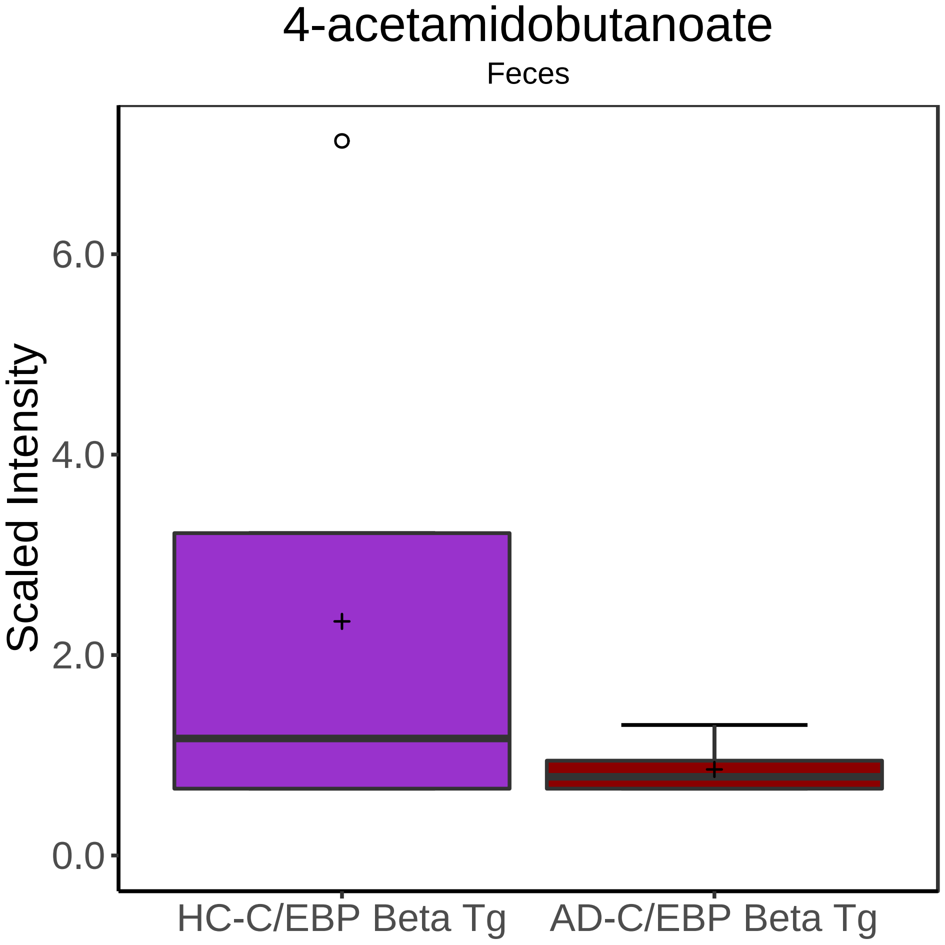

4-acetamidobutanoate
Feces
Scaled Intensity
6.0
4.0
2.0
0.0
HC-C/EBP Beta Tg
AD-C/EBP Beta Tg

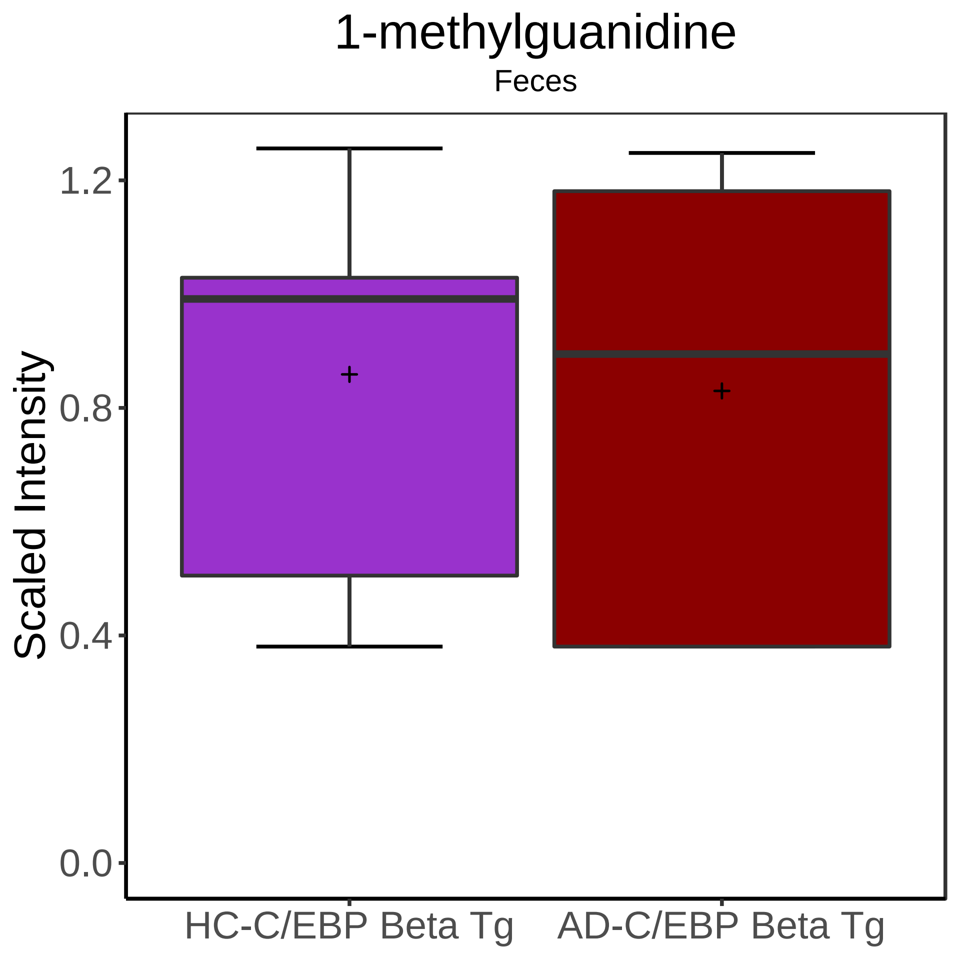
1-methylguanidine
Feces
Scaled Intensity
1.2
0.8
0.4
0.0
HC-C/EBP Beta Tg
AD-C/EBP Beta Tg

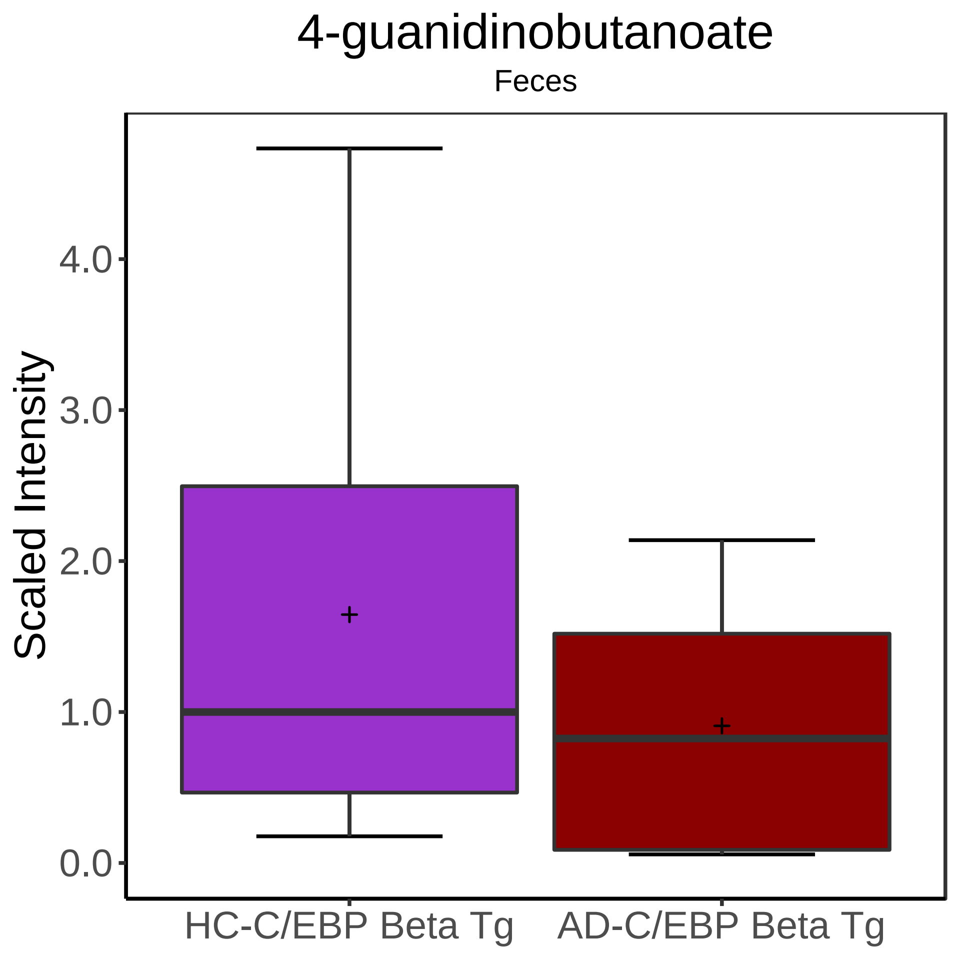
4-guanidinobutanoate
Feces
Scaled Intensity
0.0
1.0
2.0
3.0
4.0
HC-C/EBP Beta Tg
AD-C/EBP Beta Tg

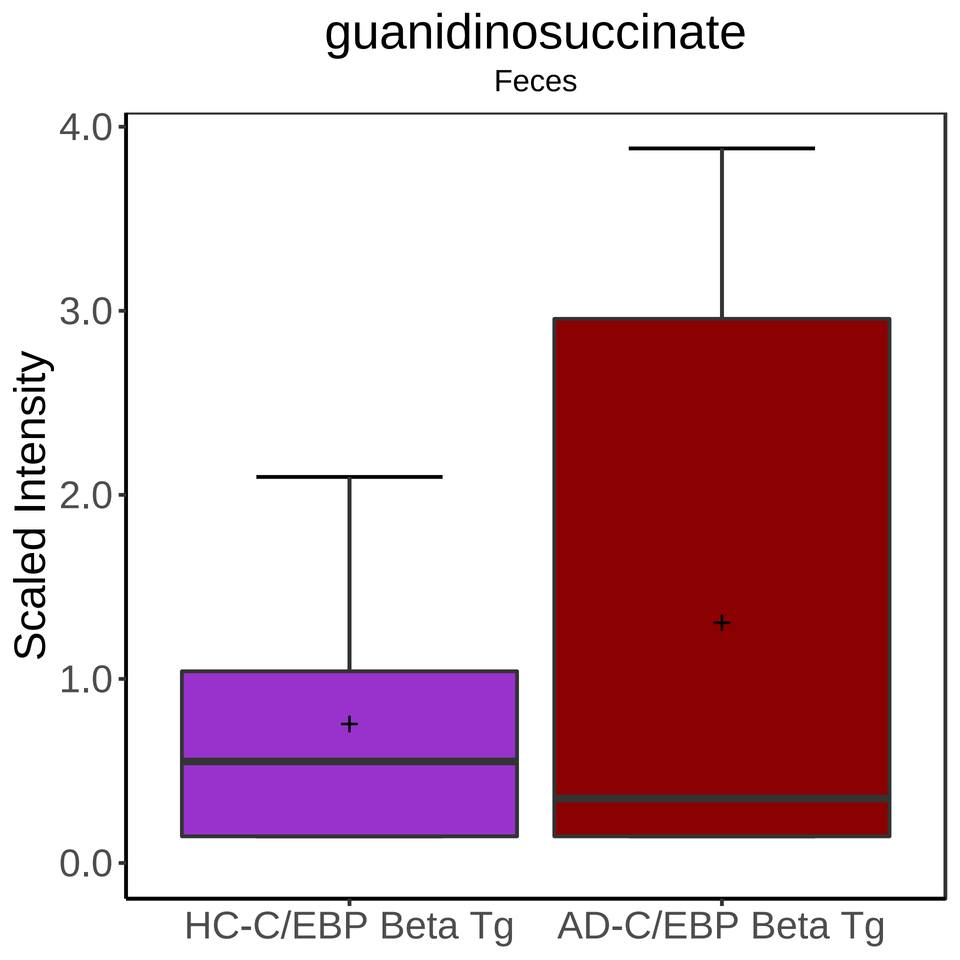
guanidinosuccinate
Feces
Scaled Intensity
4.0
3.0
2.0
1.0
0.0
HC-C/EBP Beta Tg
AD-C/EBP Beta Tg

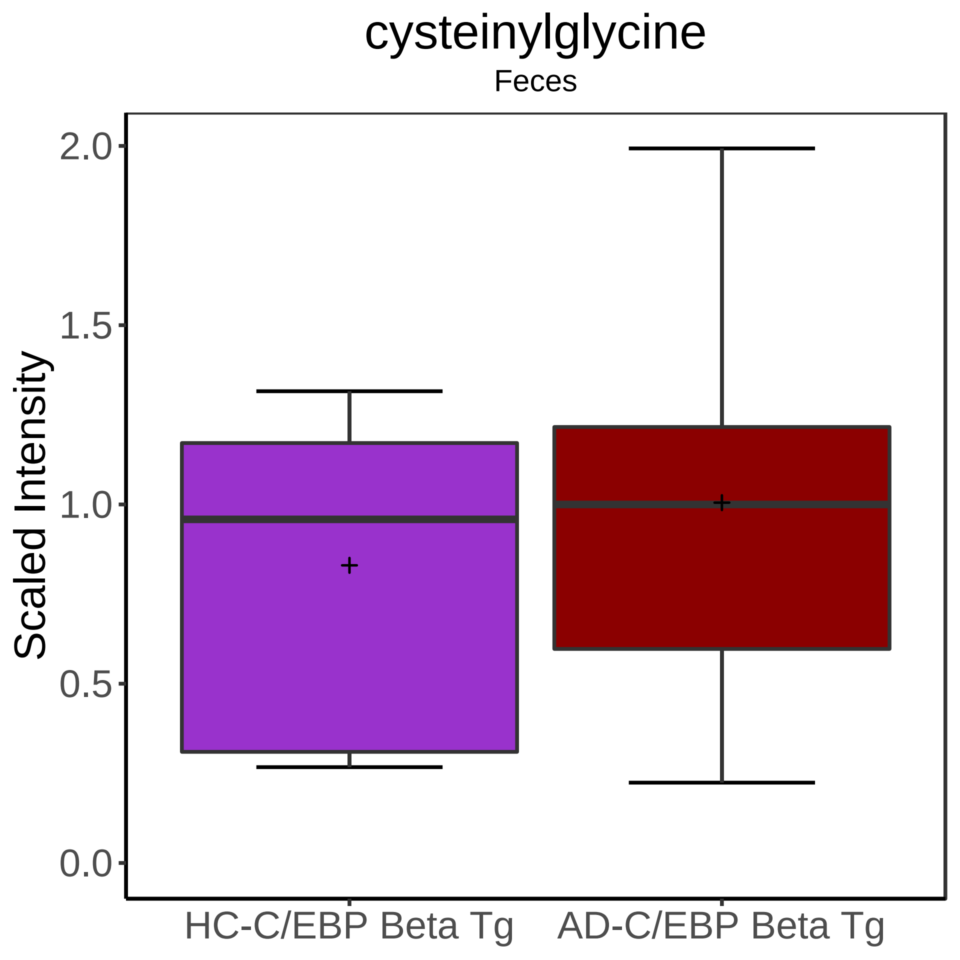
cysteinylglycine
Feces
Scaled Intensity
2.0
1.5
1.0
0.5
0.0
HC-C/EBP Beta Tg
AD-C/EBP Beta Tg

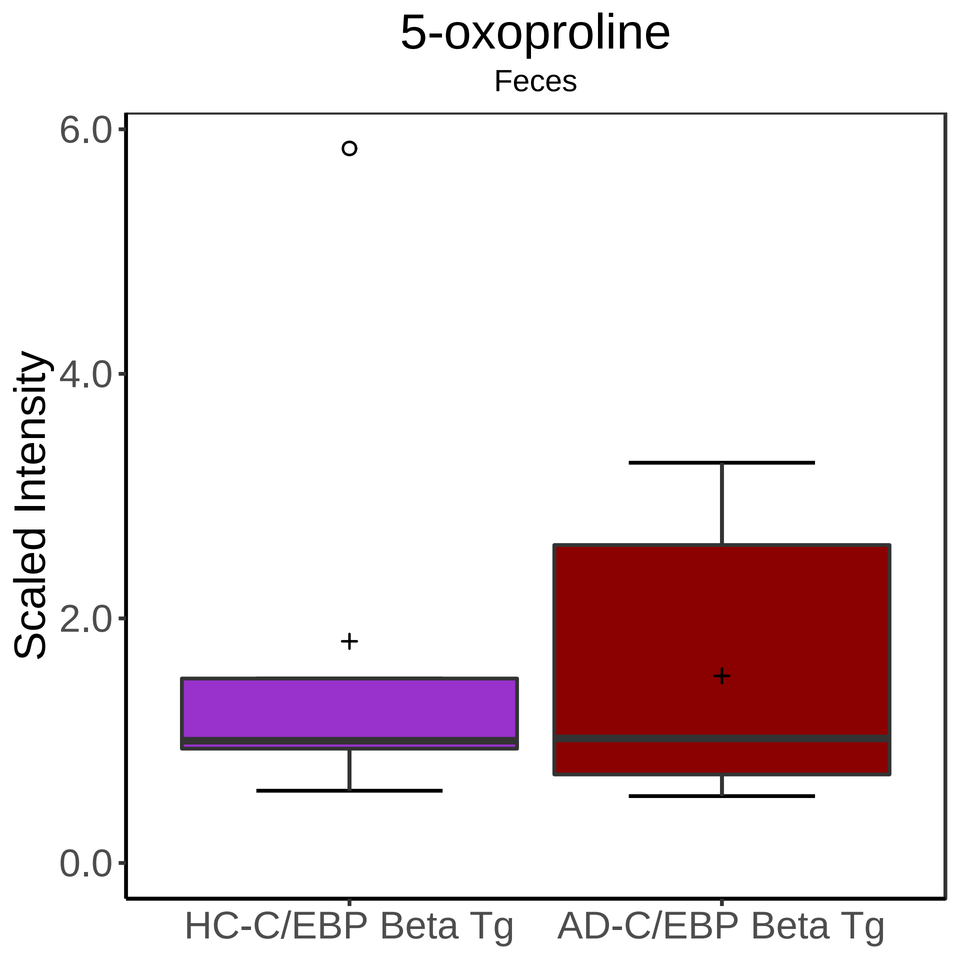
5-oxoproline
Feces
Scaled Intensity
6.0
4.0
2.0
0.0
HC-C/EBP Beta Tg
AD-C/EBP Beta Tg

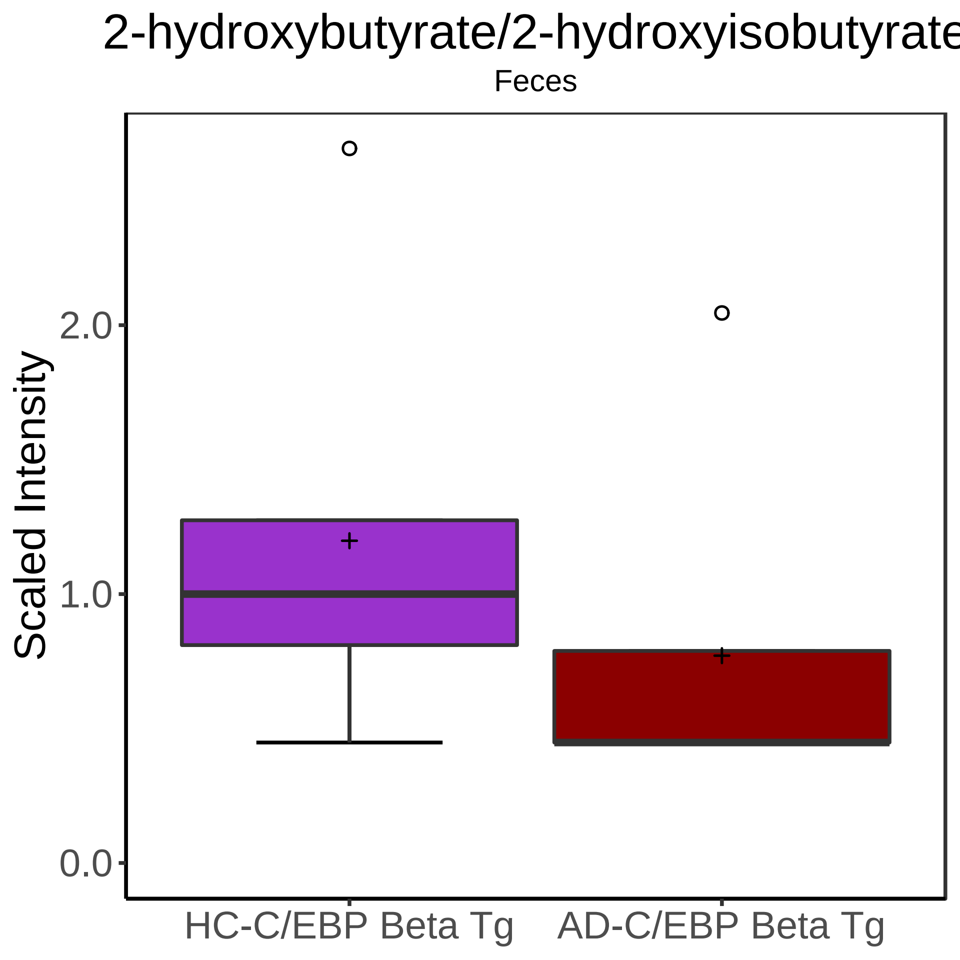
2-hydroxybutyrate/2-hydroxyisobutyrate
Feces
Scaled Intensity
2.0
1.0
0.0
HC-C/EBP Beta Tg
AD-C/EBP Beta Tg

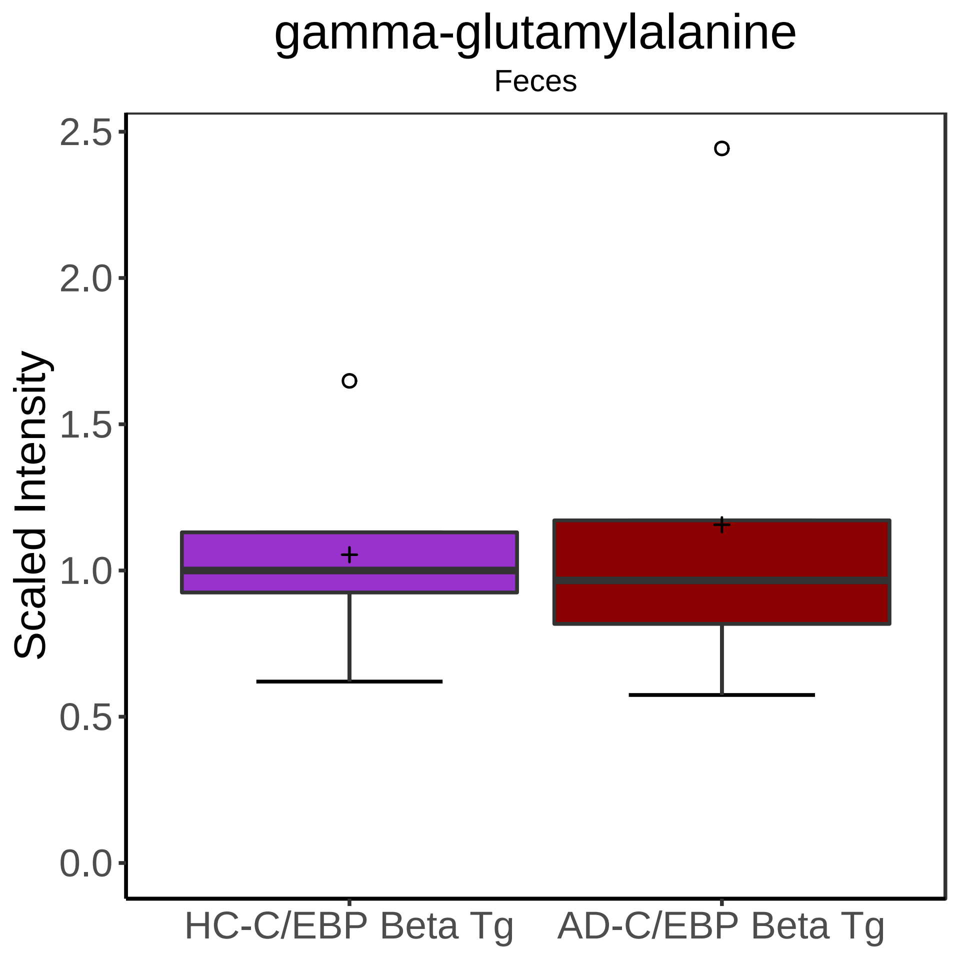

gamma-glutamylalanine
Feces
Scaled Intensity
0.0
0.5
1.0
1.5
2.0
2.5
HC-C/EBP Beta Tg
AD-C/EBP Beta Tg

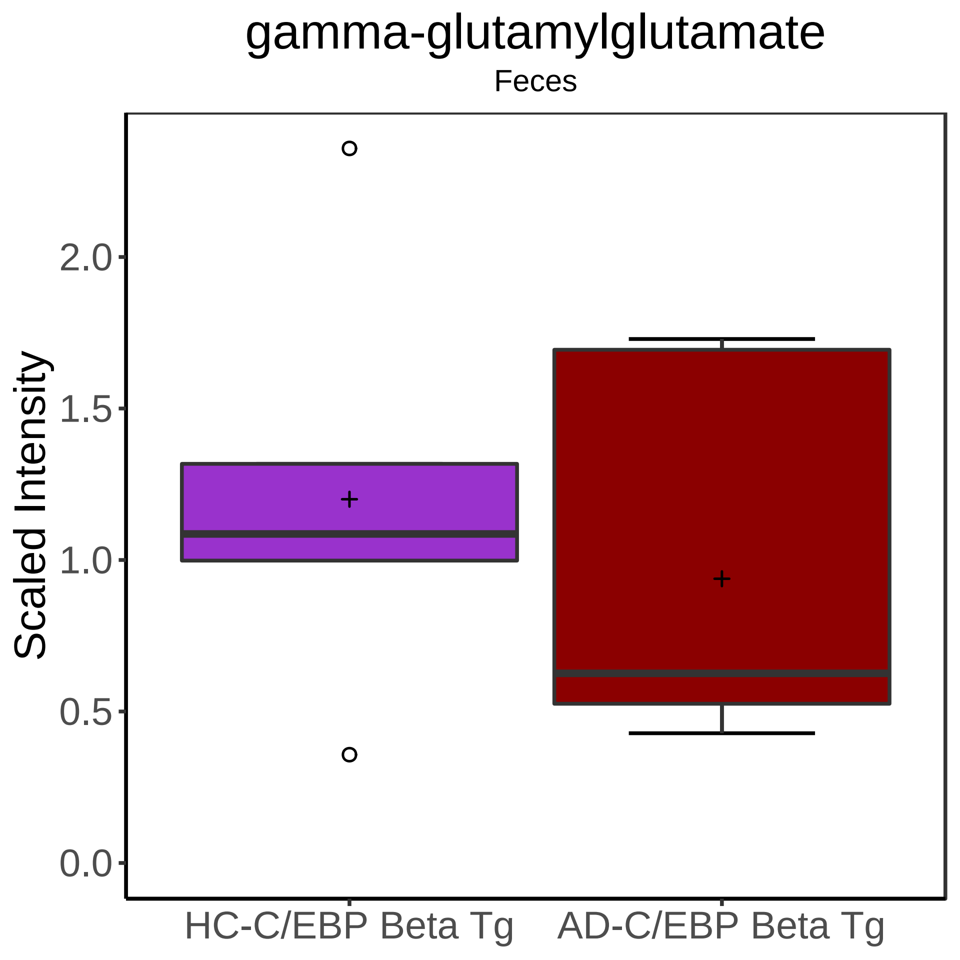

gamma-glutamylglutamate
Feces
Scaled Intensity
2.0
1.5
1.0
0.5
0.0
HC-C/EBP Beta Tg
AD-C/EBP Beta Tg

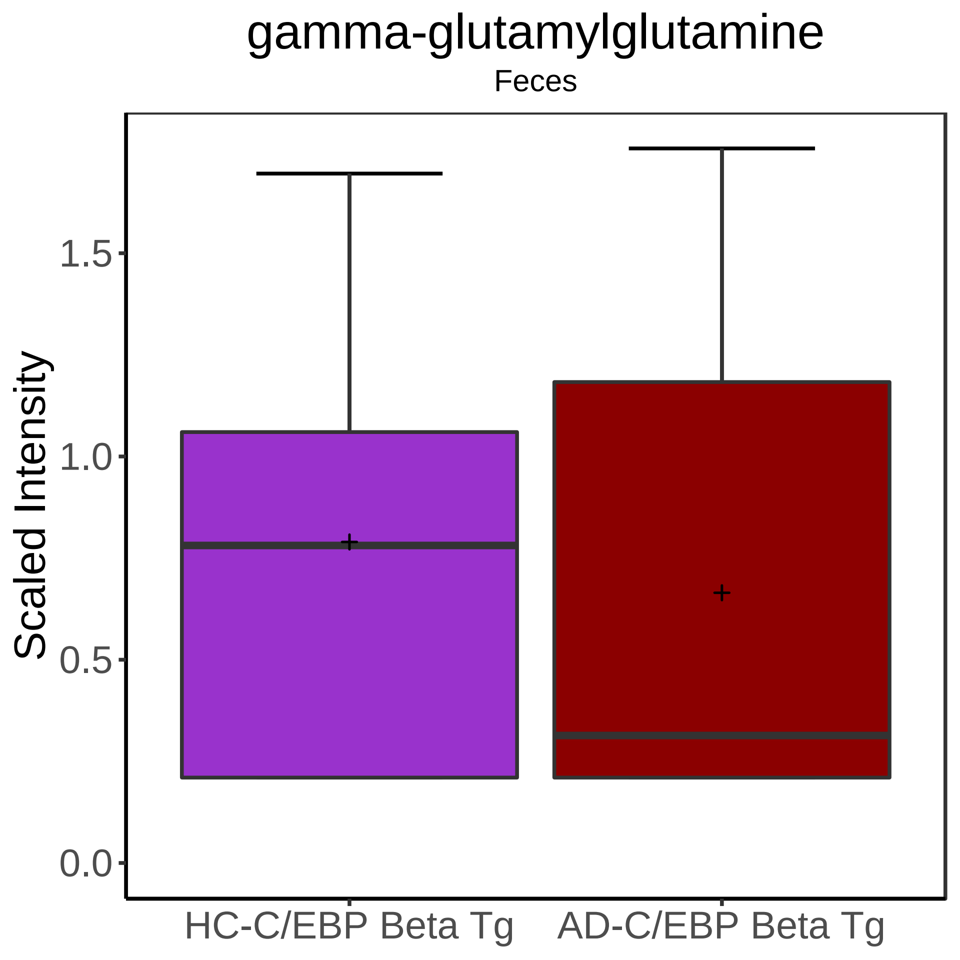
gamma-glutamylglutamine
Feces
Scaled Intensity
0.0
0.5
1.0
1.5
HC-C/EBP Beta Tg
AD-C/EBP Beta Tg

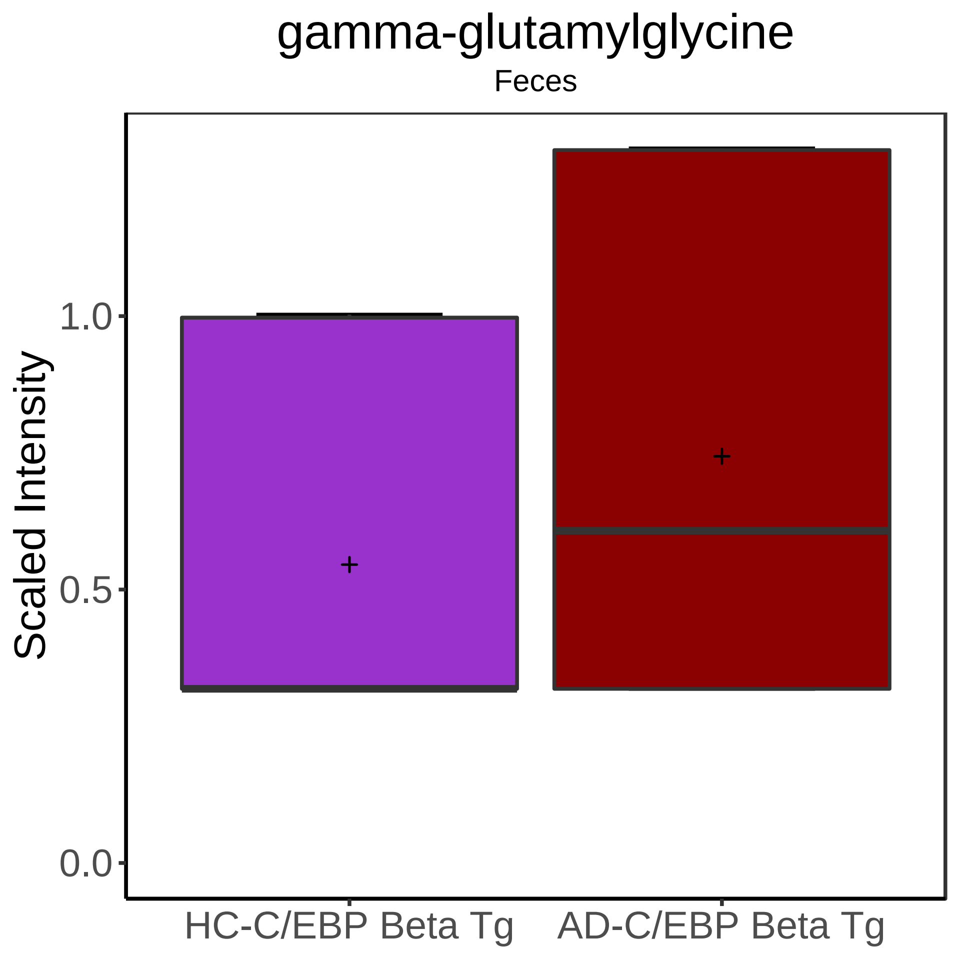
gamma-glutamylglycine
Feces
Scaled Intensity
1.0
0.5
0.0
HC-C/EBP Beta Tg
AD-C/EBP Beta Tg

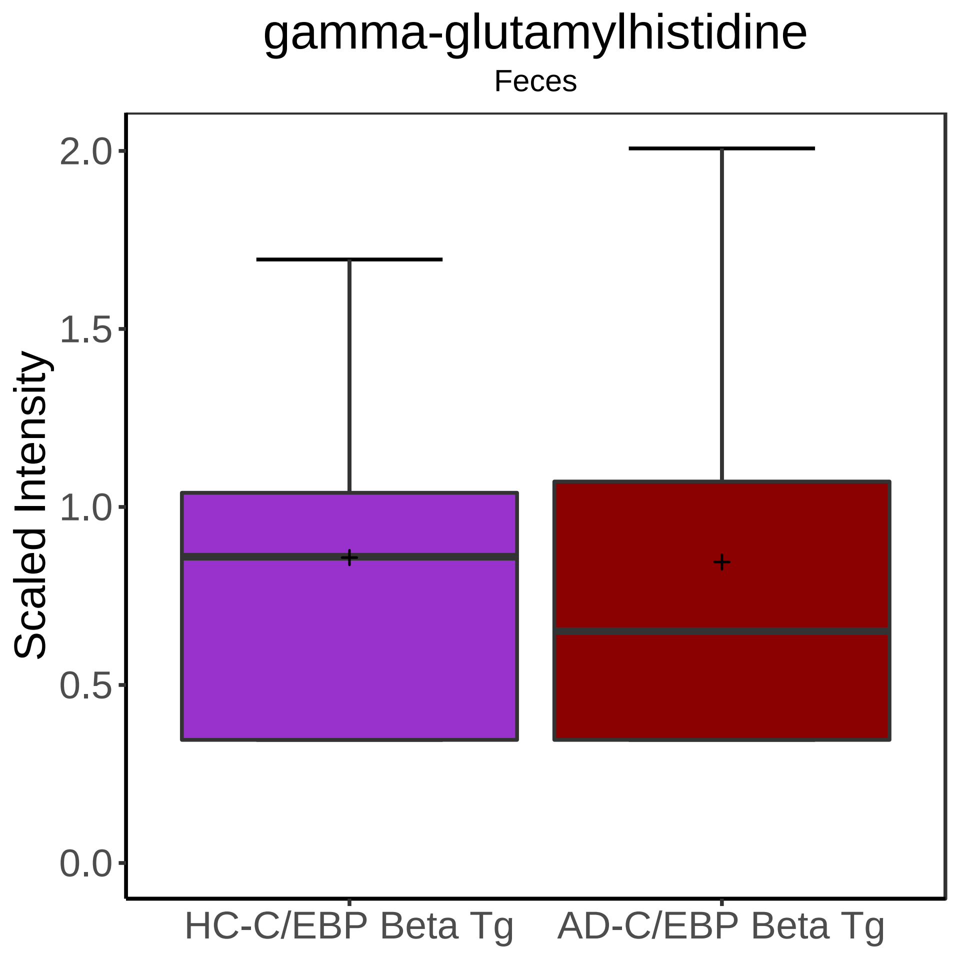
gamma-glutamylhistidine
Feces
Scaled Intensity
2.0
1.5
1.0
0.5
0.0
HC-C/EBP Beta Tg
AD-C/EBP Beta Tg

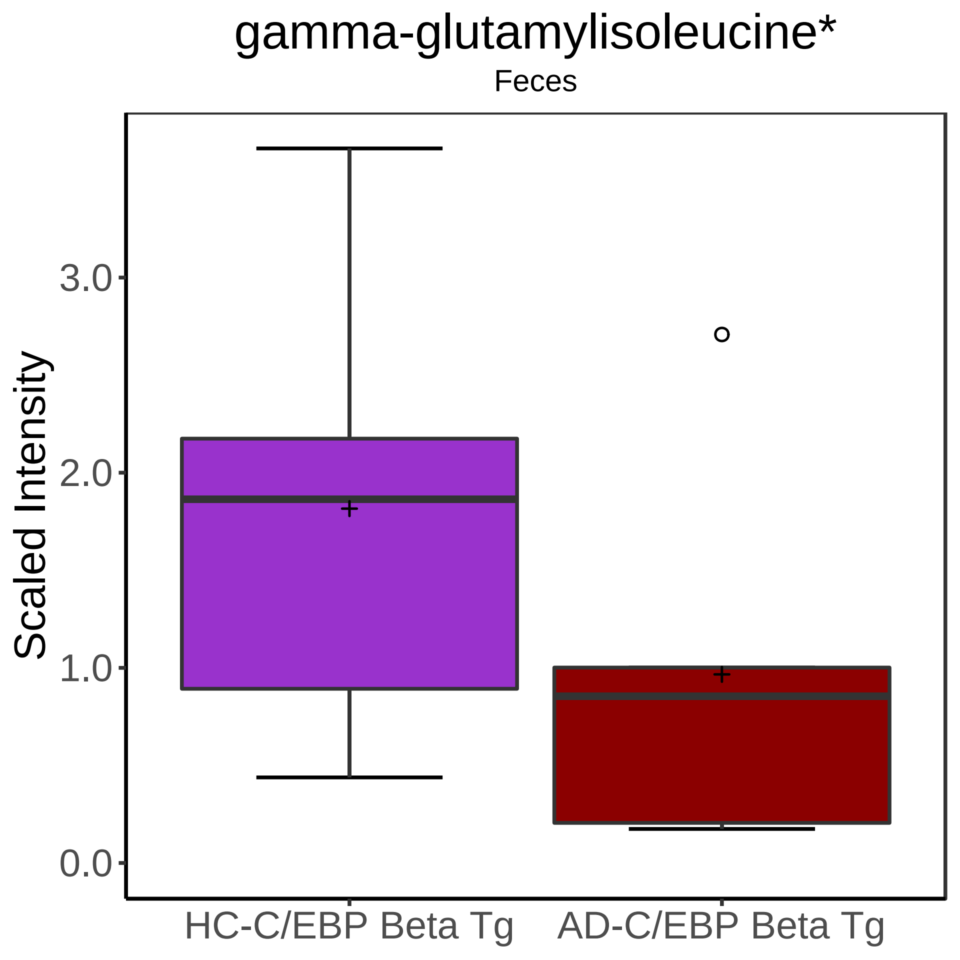
gamma-glutamylisoleucine*
Feces
Scaled Intensity
3.0
2.0
1.0
0.0
HC-C/EBP Beta Tg
AD-C/EBP Beta Tg

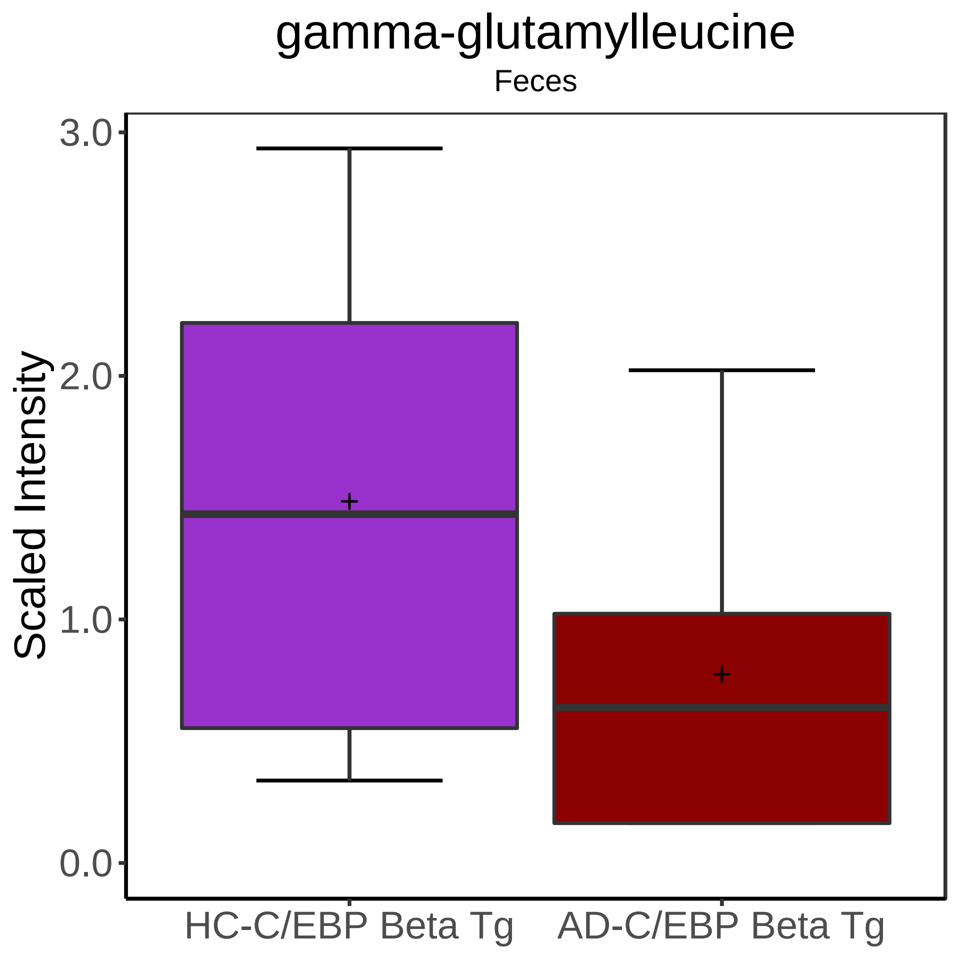

gamma-glutamylleucine
Feces
Scaled Intensity
3.0
2.0
1.0
0.0
HC-C/EBP Beta Tg
AD-C/EBP Beta Tg

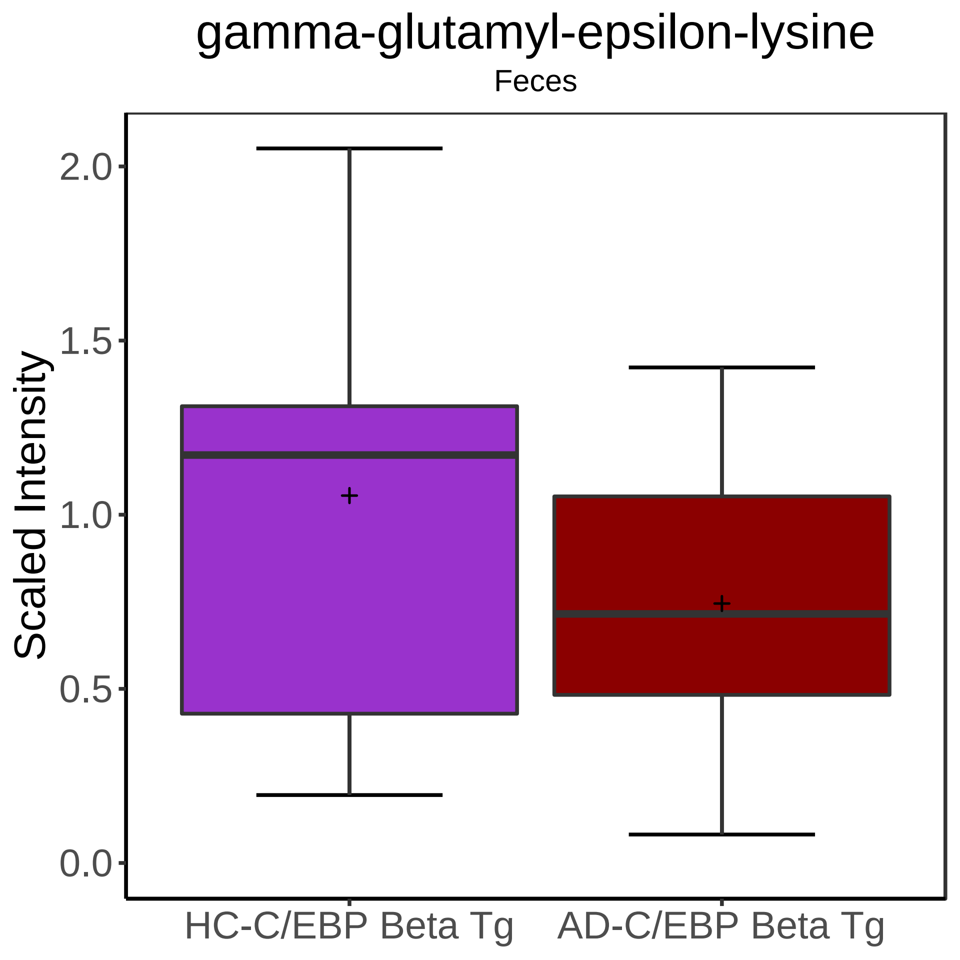

gamma-glutamyl-epsilon-lysine
Feces
Scaled Intensity
2.0
1.5
1.0
0.5
0.0
HC-C/EBP Beta Tg
AD-C/EBP Beta Tg

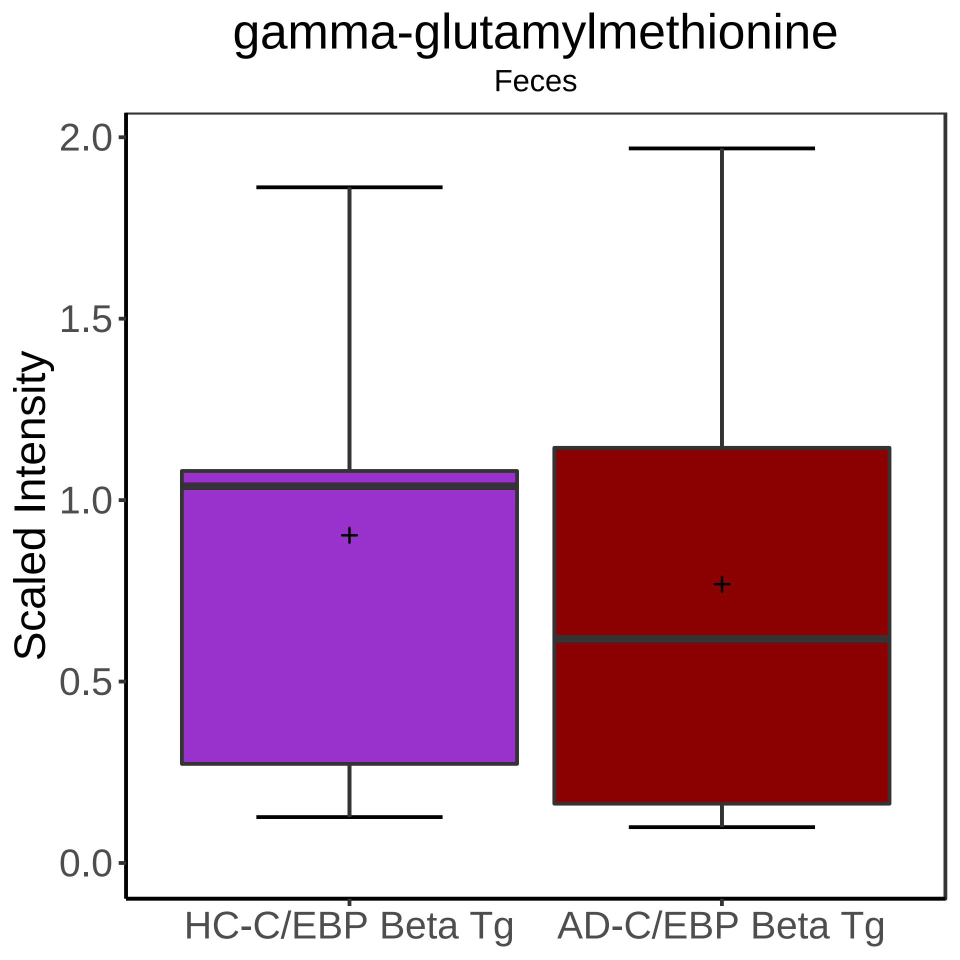
gamma-glutamylmethionine
Feces
Scaled Intensity
2.0
1.5
1.0
0.5
0.0
HC-C/EBP Beta Tg
AD-C/EBP Beta Tg

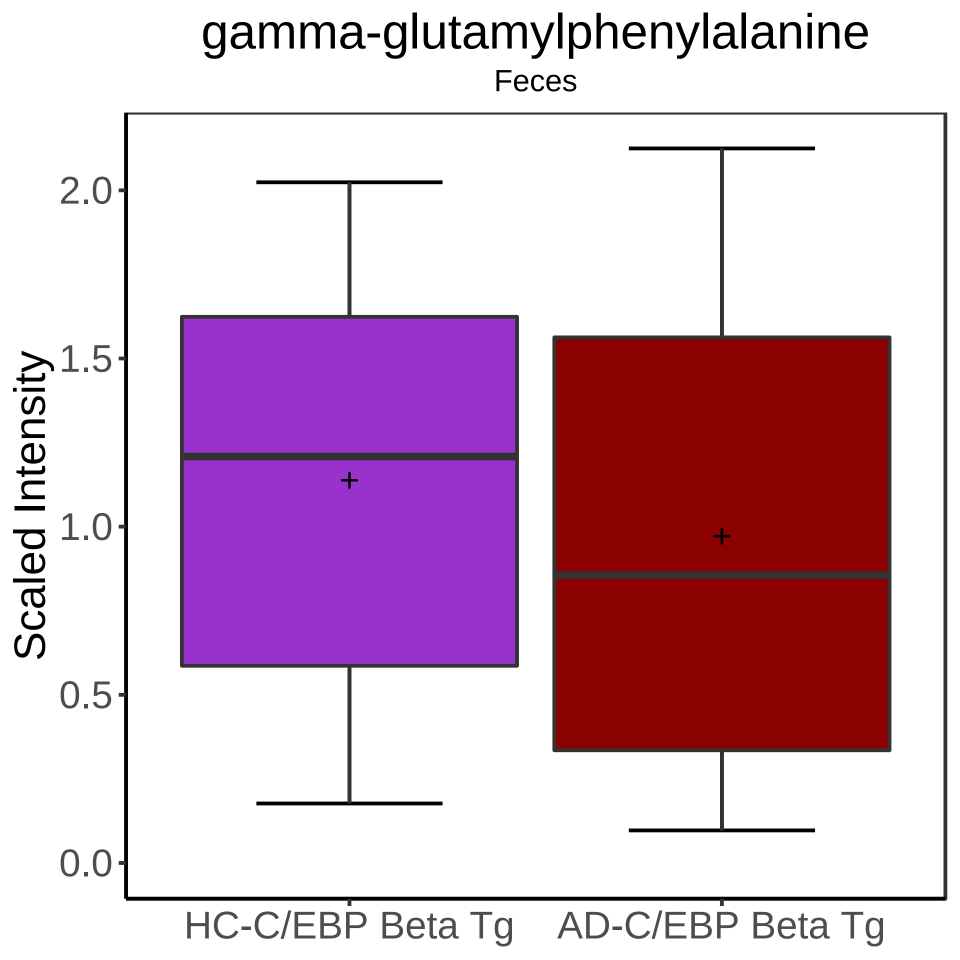
gamma-glutamylphenylalanine
Feces
Scaled Intensity
2.0
1.5
1.0
0.5
0.0
HC-C/EBP Beta Tg
AD-C/EBP Beta Tg

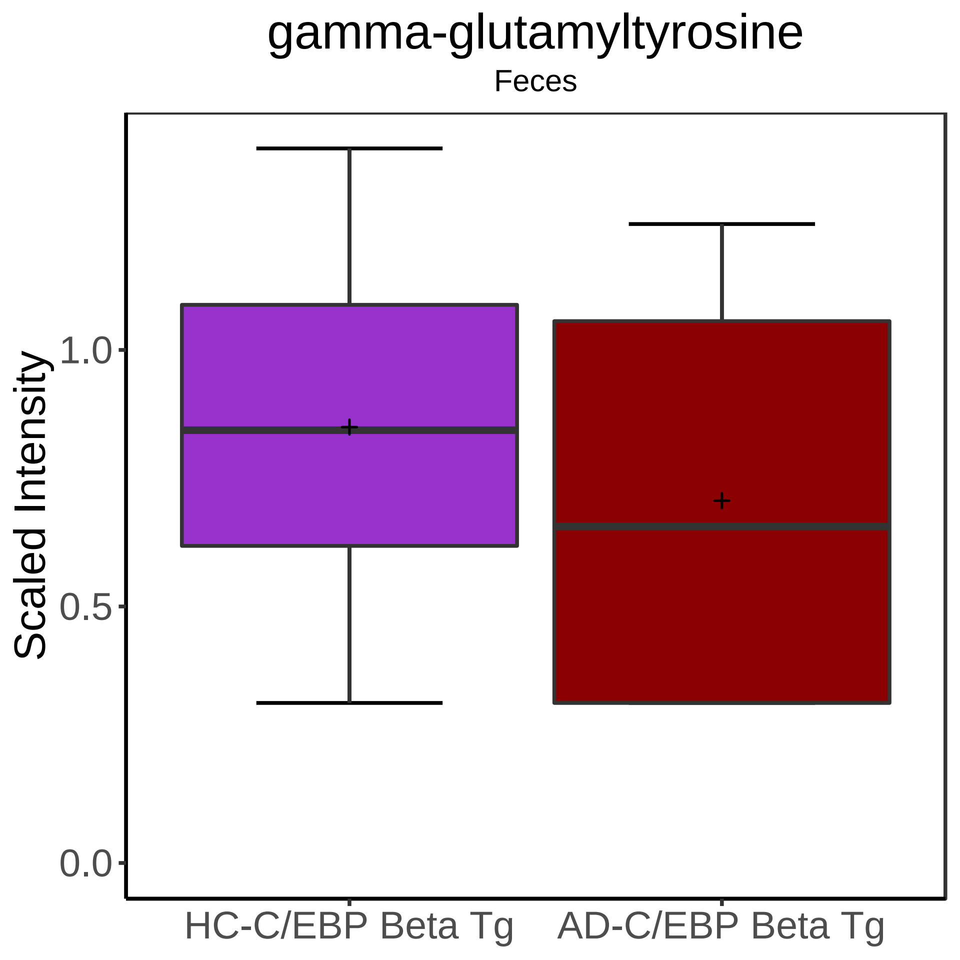
gamma-glutamyltyrosine
Feces
Scaled Intensity
1.0
0.5
0.0
HC-C/EBP Beta Tg
AD-C/EBP Beta Tg

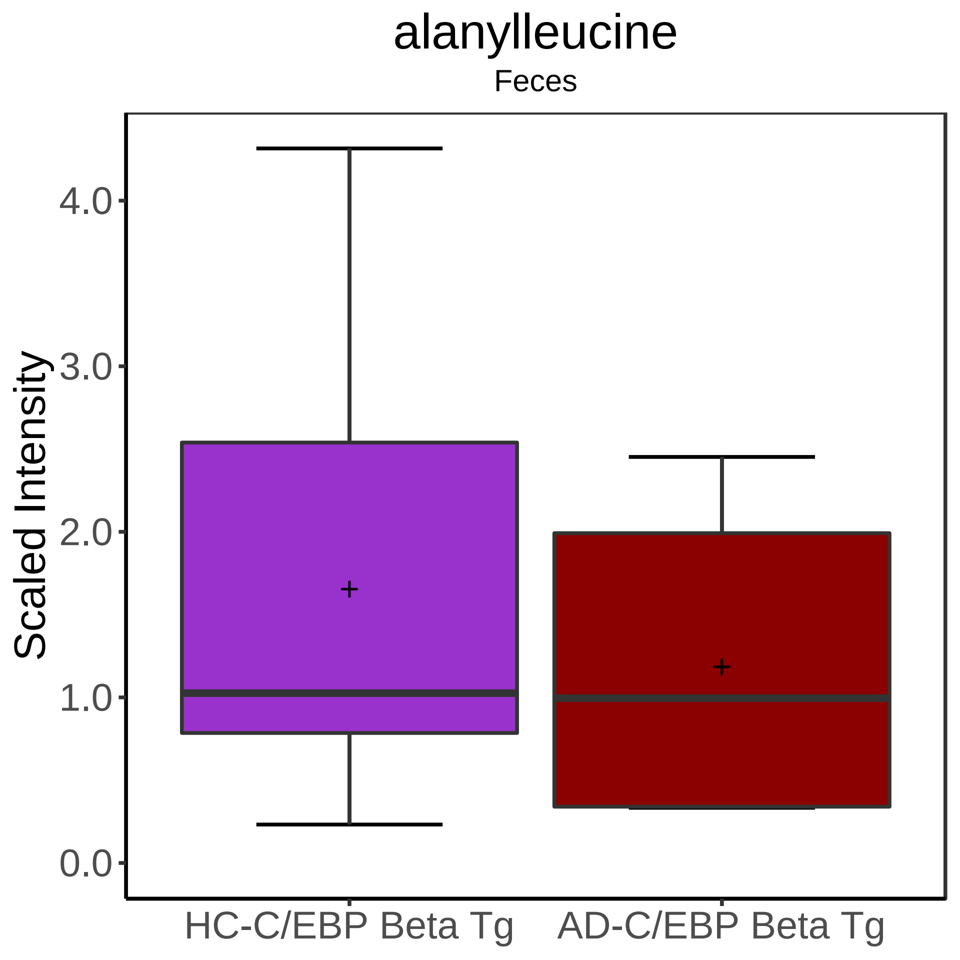
alanylleucine
Feces
Scaled Intensity
4.0
3.0
2.0
1.0
0.0
HC-C/EBP Beta Tg
AD-C/EBP Beta Tg

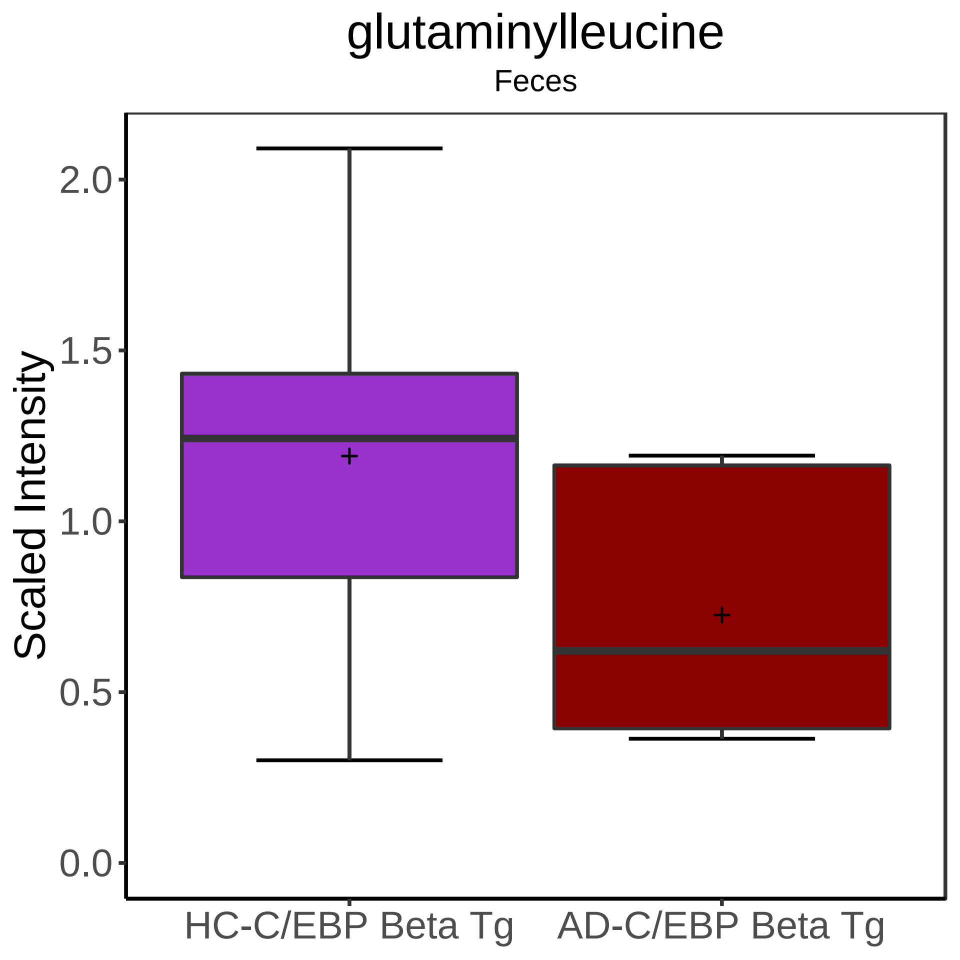

glutaminylleucine
Feces
Scaled Intensity
2.0
1.5
1.0
0.5
0.0
HC-C/EBP Beta Tg
AD-C/EBP Beta Tg

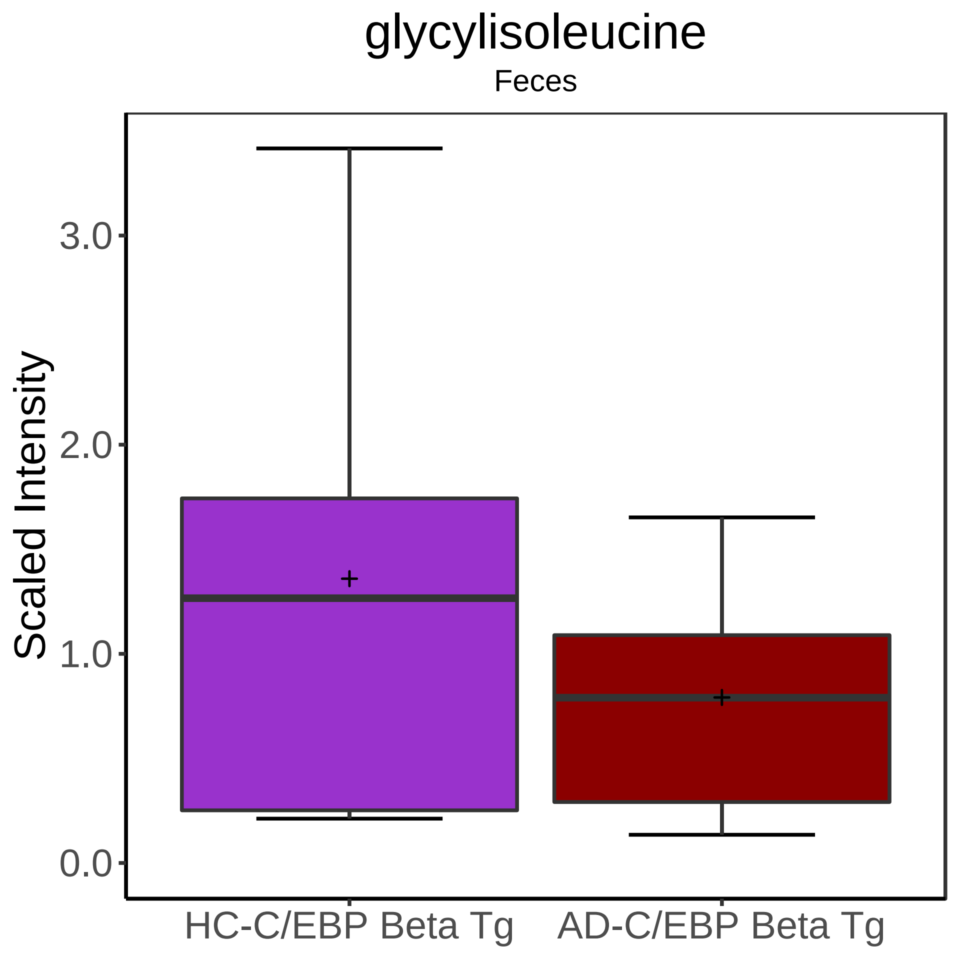
glycylisoleucine
Feces
Scaled Intensity
3.0
2.0
1.0
0.0
HC-C/EBP Beta Tg
AD-C/EBP Beta Tg

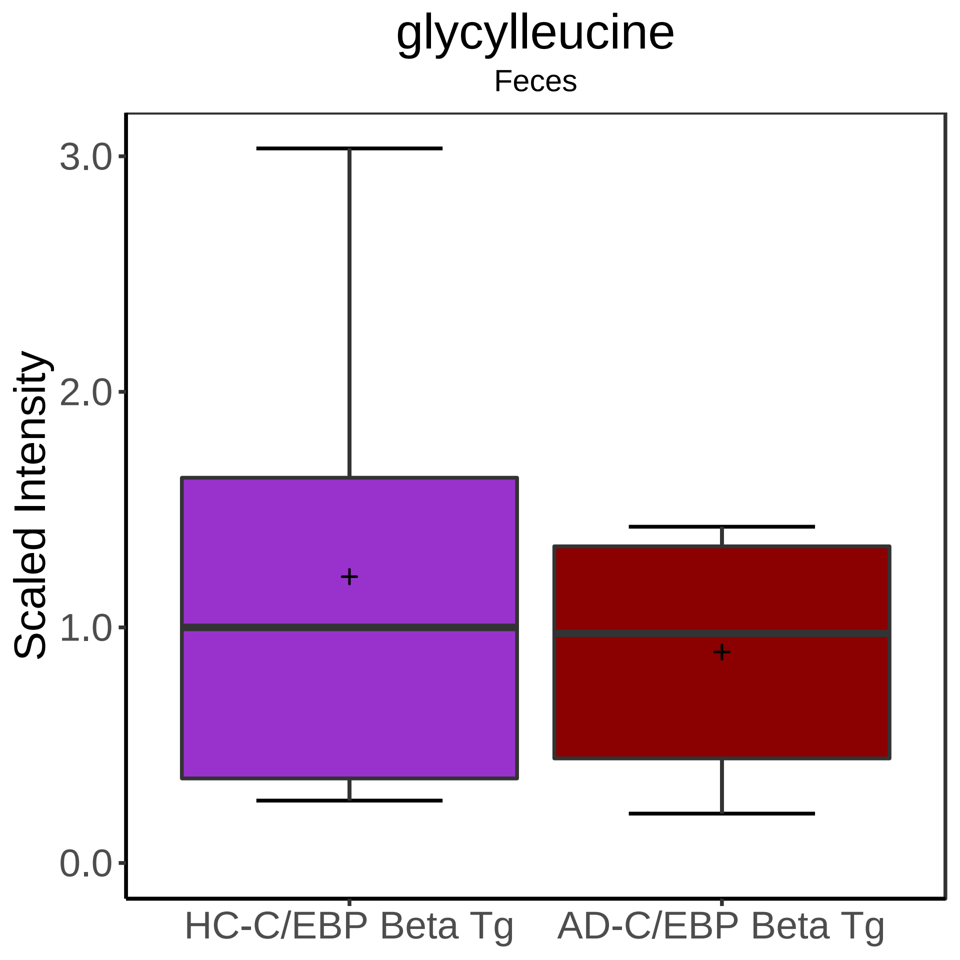

glycylleucine
Feces
Scaled Intensity
3.0
2.0
1.0
0.0
HC-C/EBP Beta Tg
AD-C/EBP Beta Tg

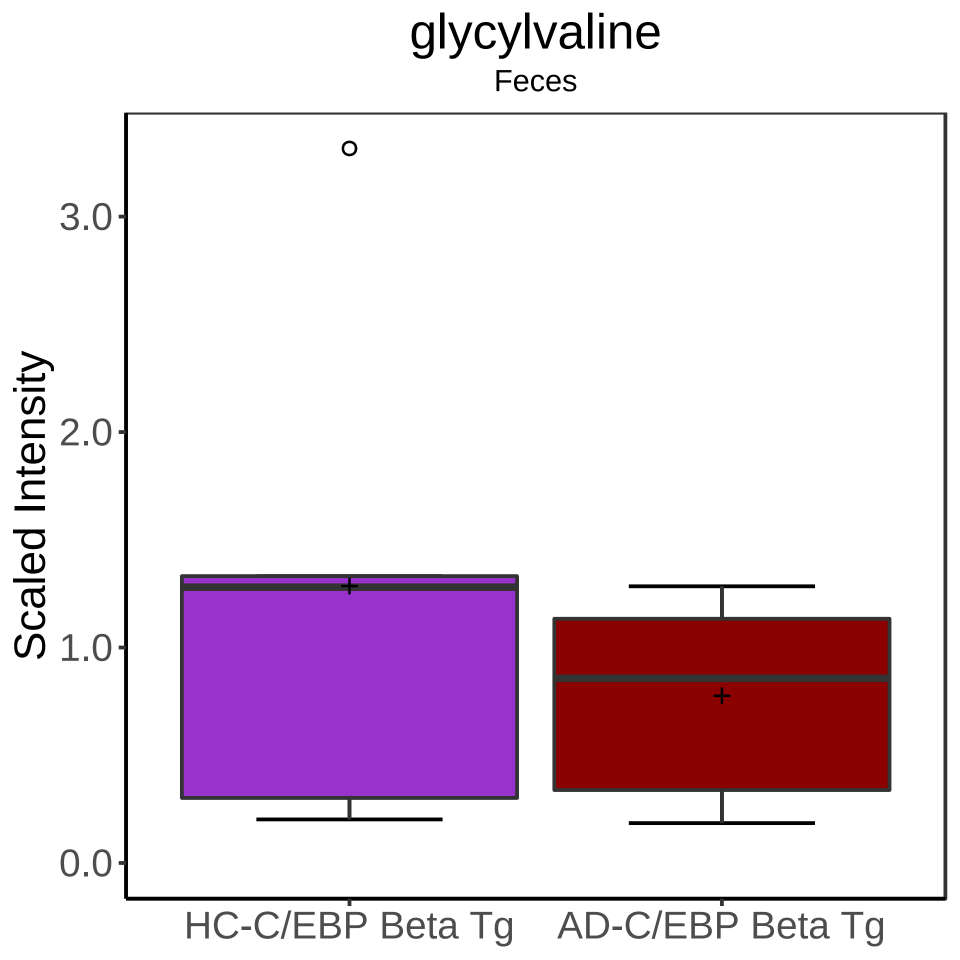
glycylvaline
Feces
Scaled Intensity
3.0
2.0
1.0
0.0
HC-C/EBP Beta Tg
AD-C/EBP Beta Tg

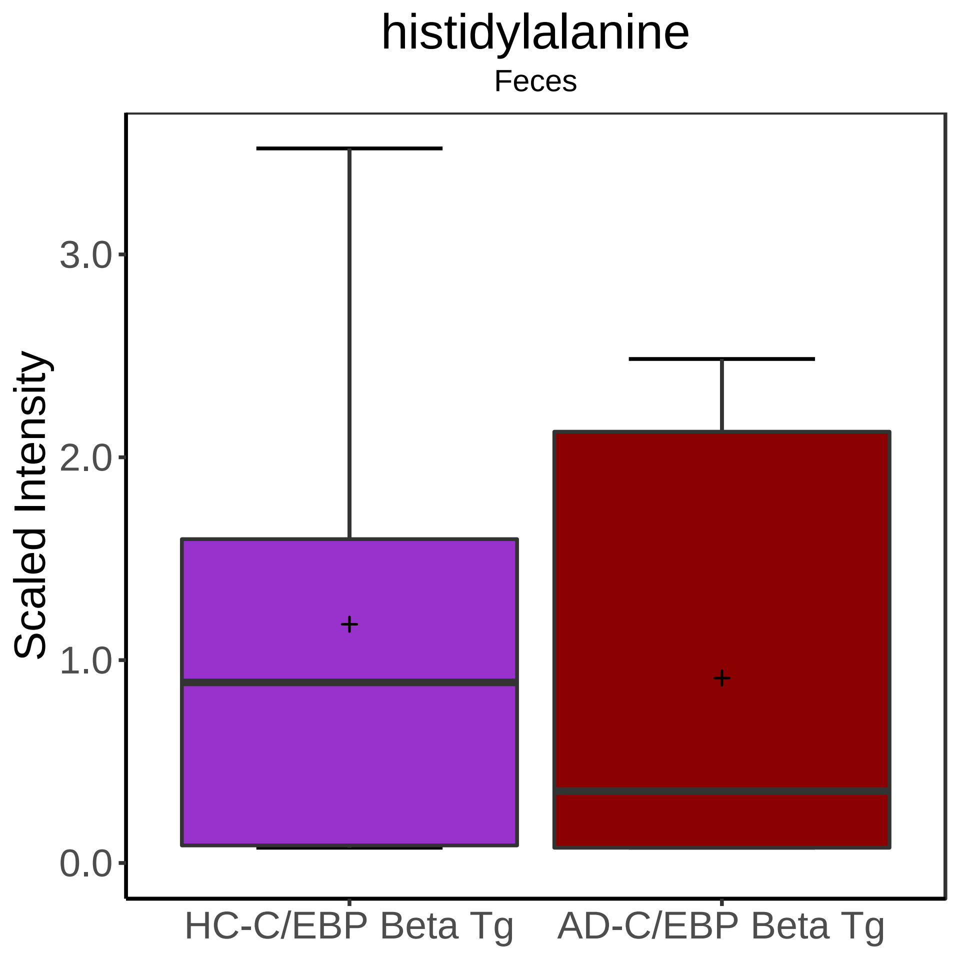

histidylalanine
Feces
Scaled Intensity
3.0
2.0
1.0
0.0
HC-C/EBP Beta Tg
AD-C/EBP Beta Tg

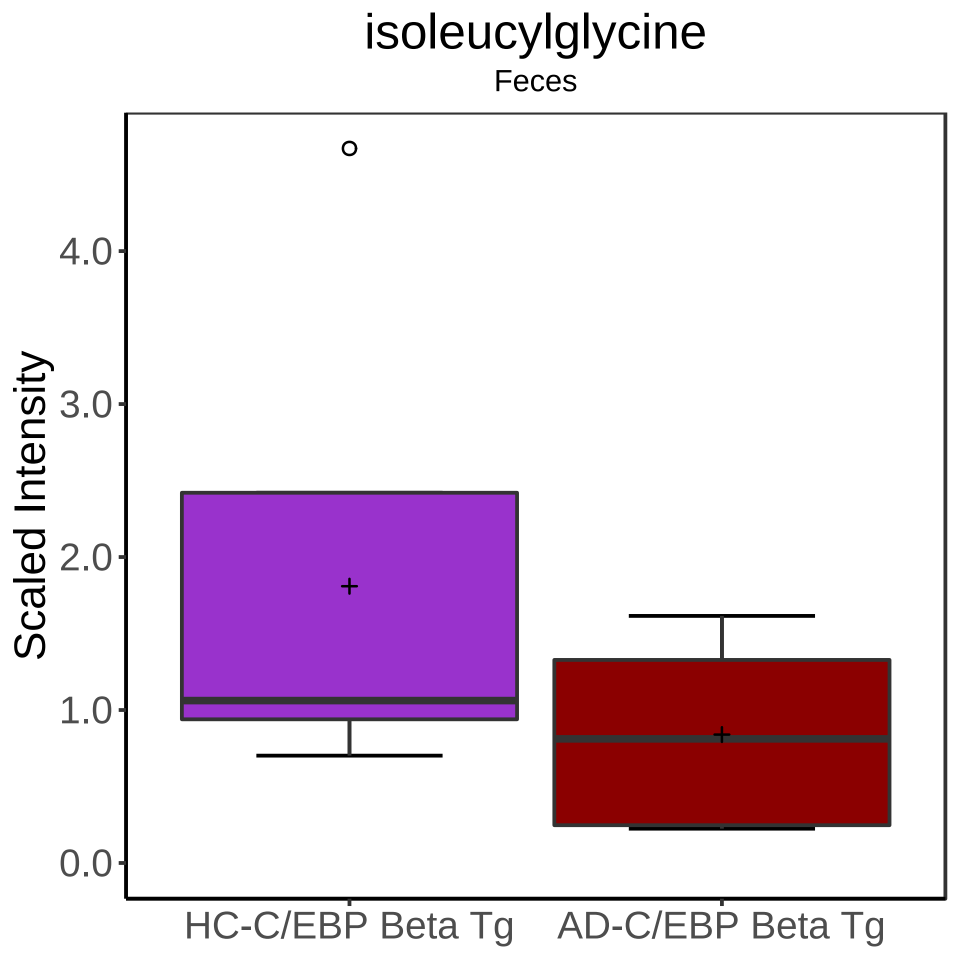

isoleucylglycine
Feces
Scaled Intensity
4.0
3.0
2.0
1.0
0.0
HC-C/EBP Beta Tg
AD-C/EBP Beta Tg

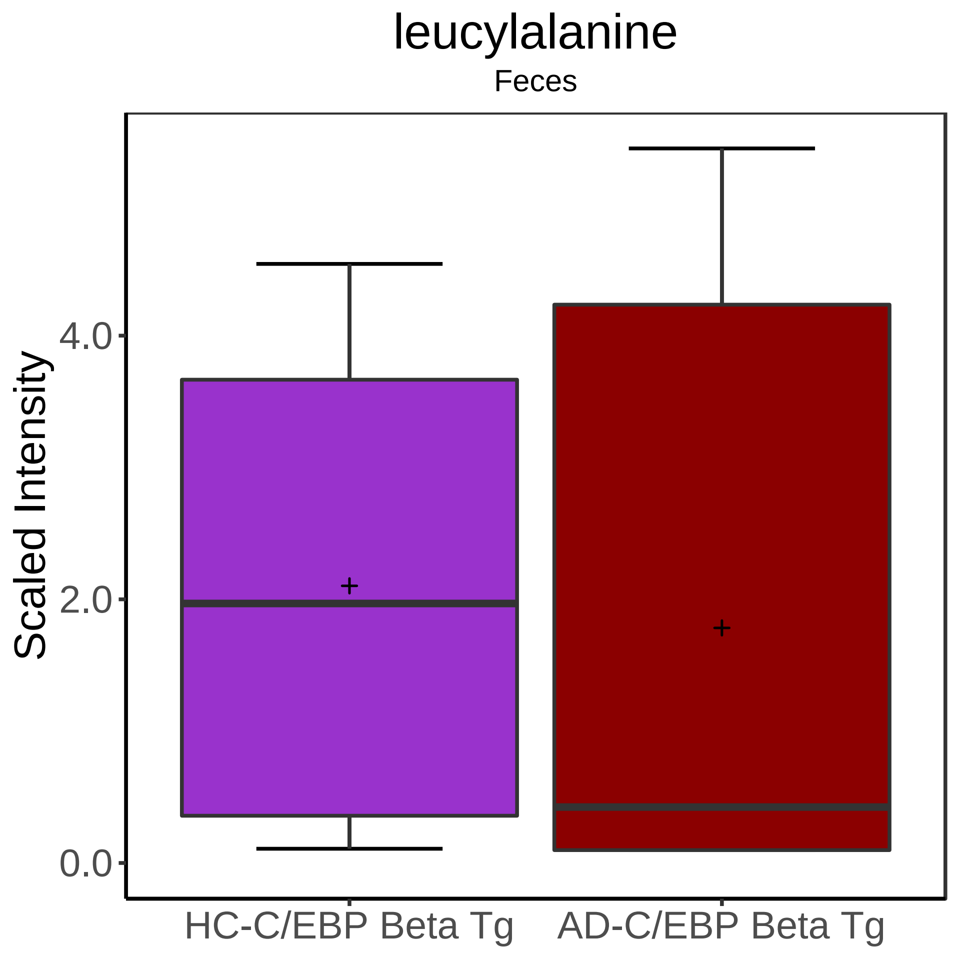

leucylalanine
Feces
Scaled Intensity
4.0
2.0
0.0
HC-C/EBP Beta Tg
AD-C/EBP Beta Tg

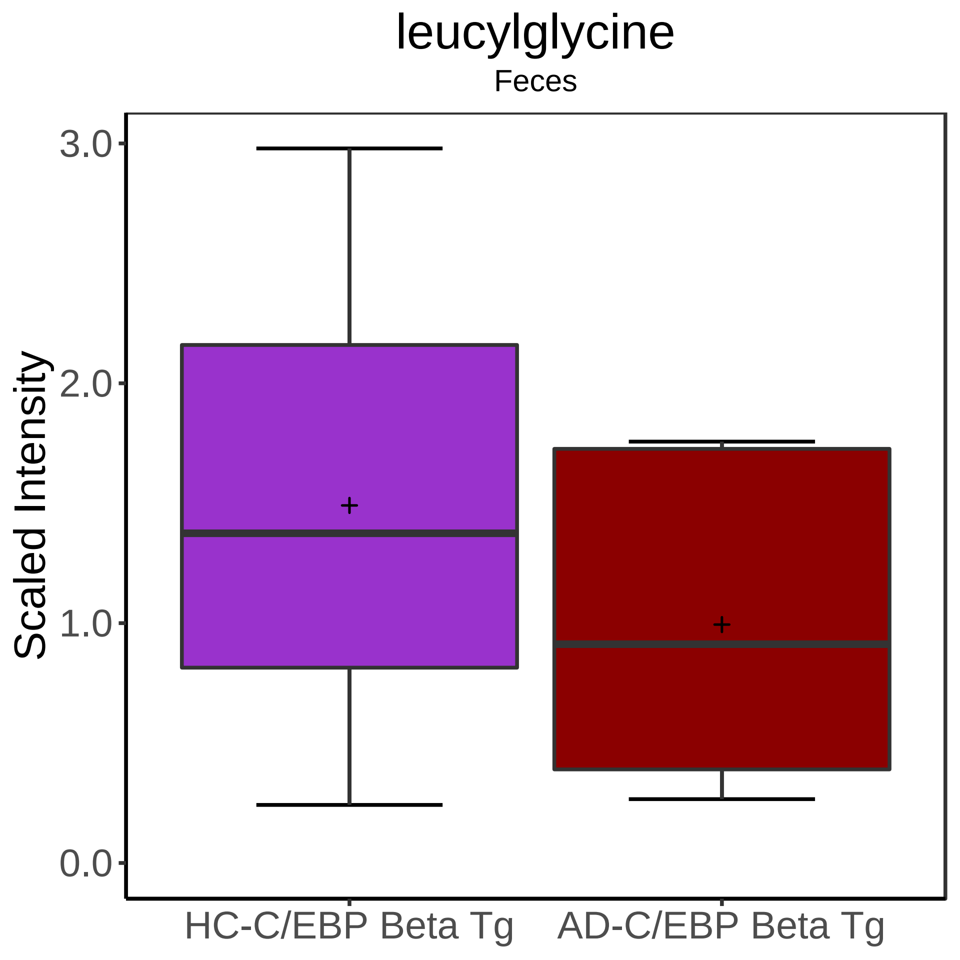
leucylglycine
Feces
Scaled Intensity
3.0
2.0
1.0
0.0
HC-C/EBP Beta Tg
AD-C/EBP Beta Tg

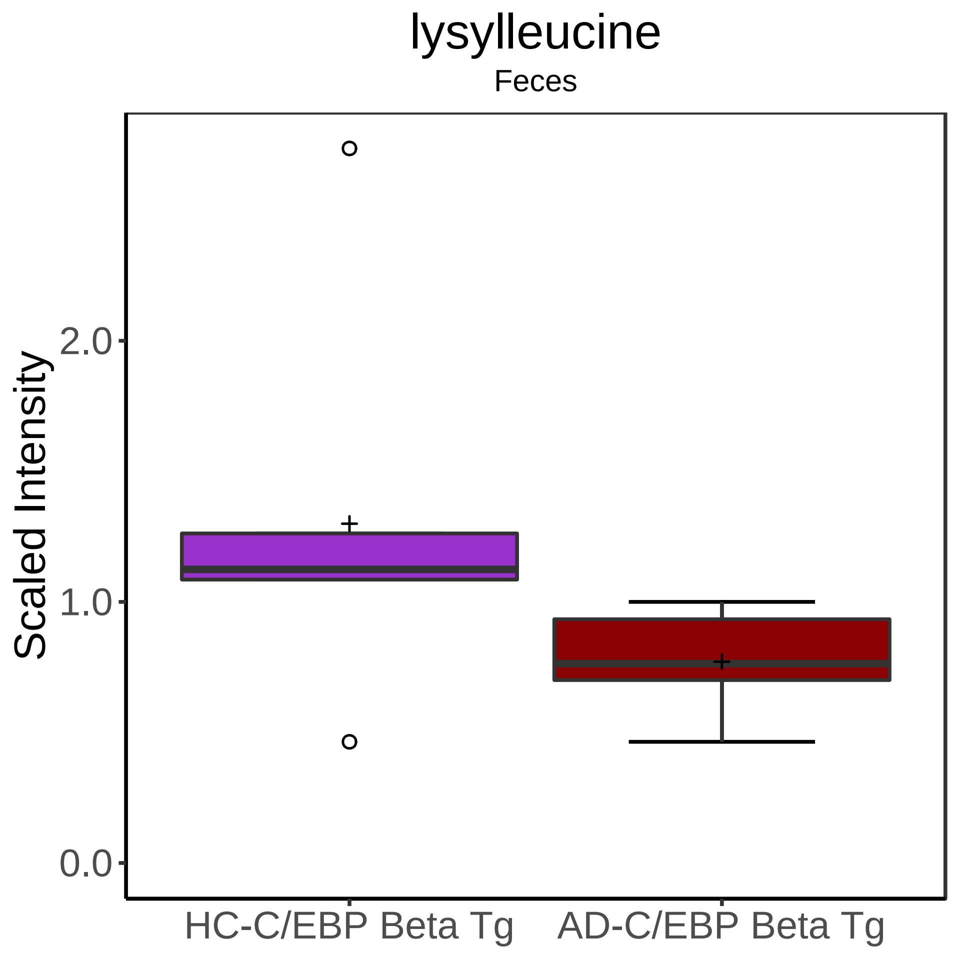

lysylleucine
Feces
Scaled Intensity
2.0
1.0
0.0
HC-C/EBP Beta Tg
AD-C/EBP Beta Tg

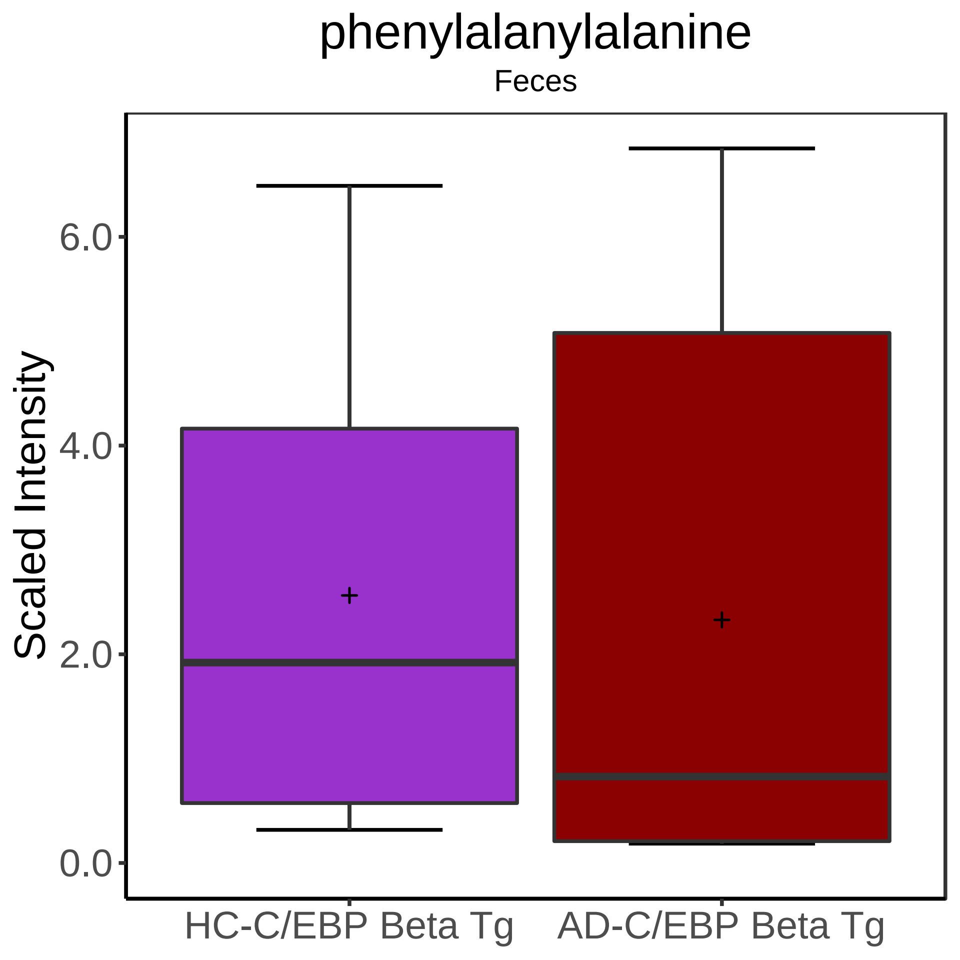
phenylalanylalanine
Feces
Scaled Intensity
6.0
4.0
2.0
0.0
HC-C/EBP Beta Tg
AD-C/EBP Beta Tg

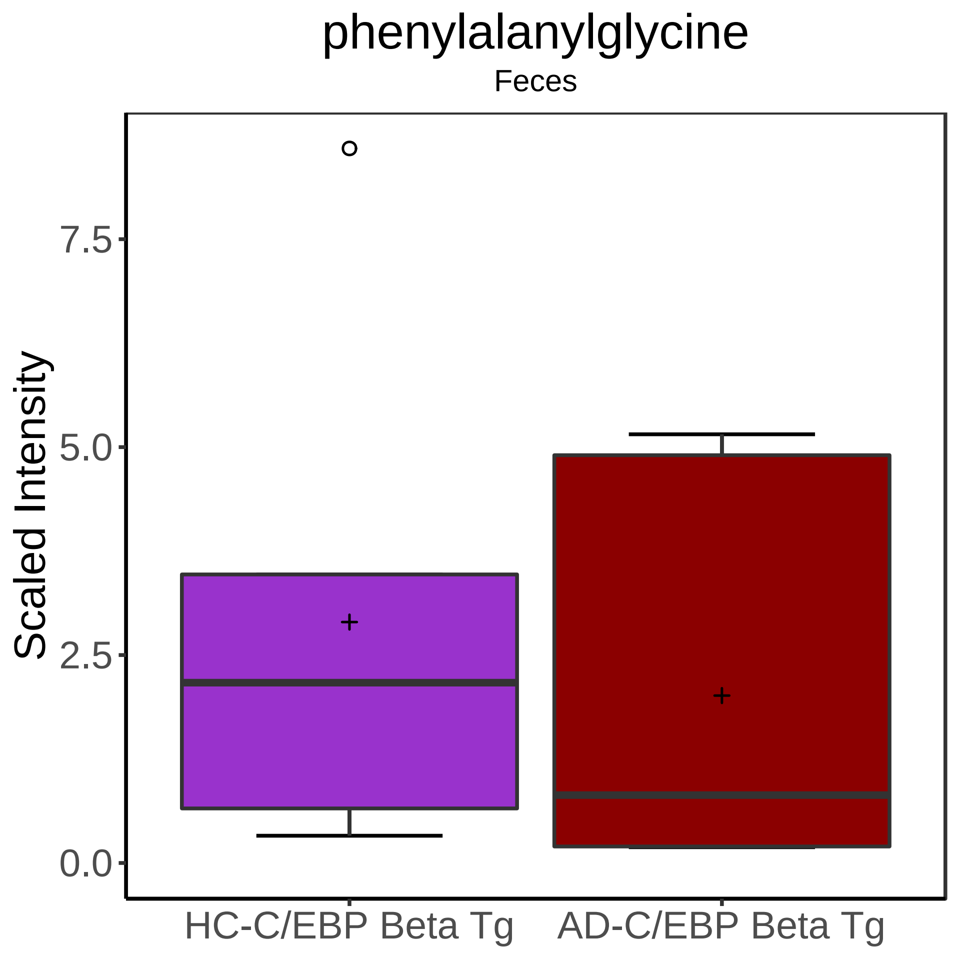
phenylalanylglycine
Feces
Scaled Intensity
7.5
5.0
2.5
0.0
HC-C/EBP Beta Tg
AD-C/EBP Beta Tg

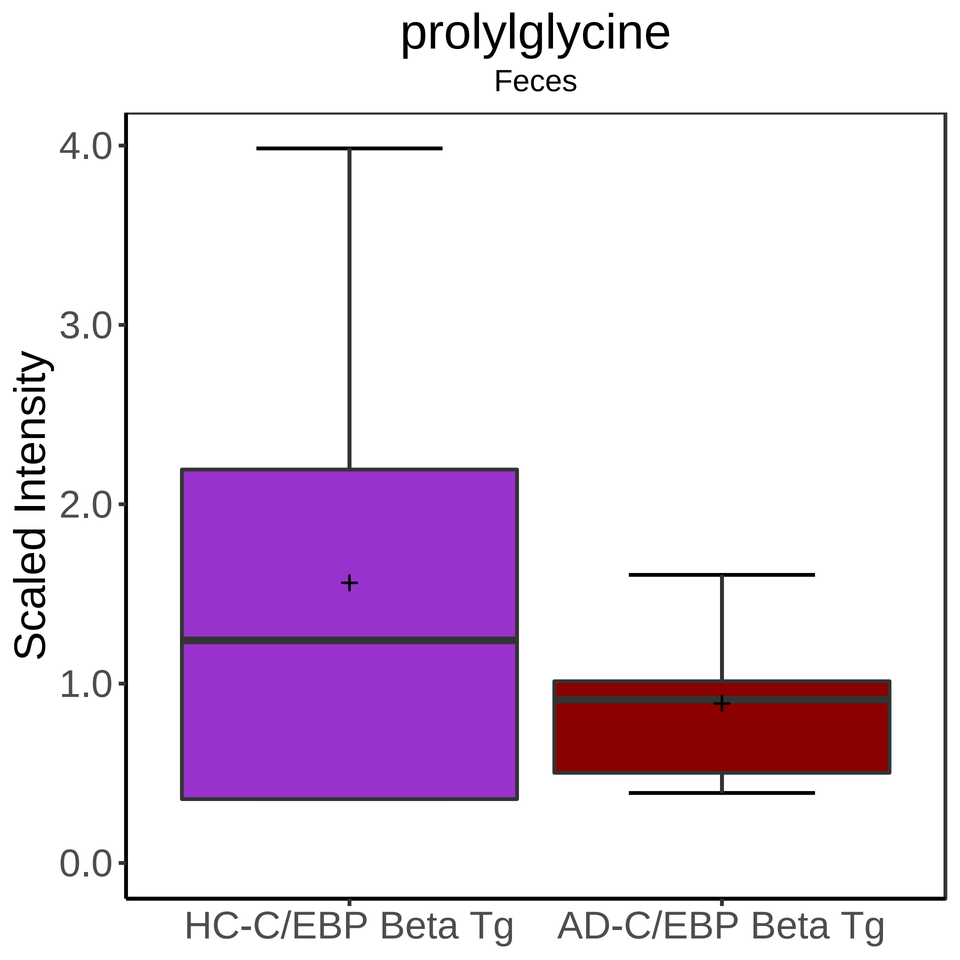
prolylglycine
Feces
Scaled Intensity
4.0
3.0
2.0
1.0
0.0
HC-C/EBP Beta Tg
AD-C/EBP Beta Tg

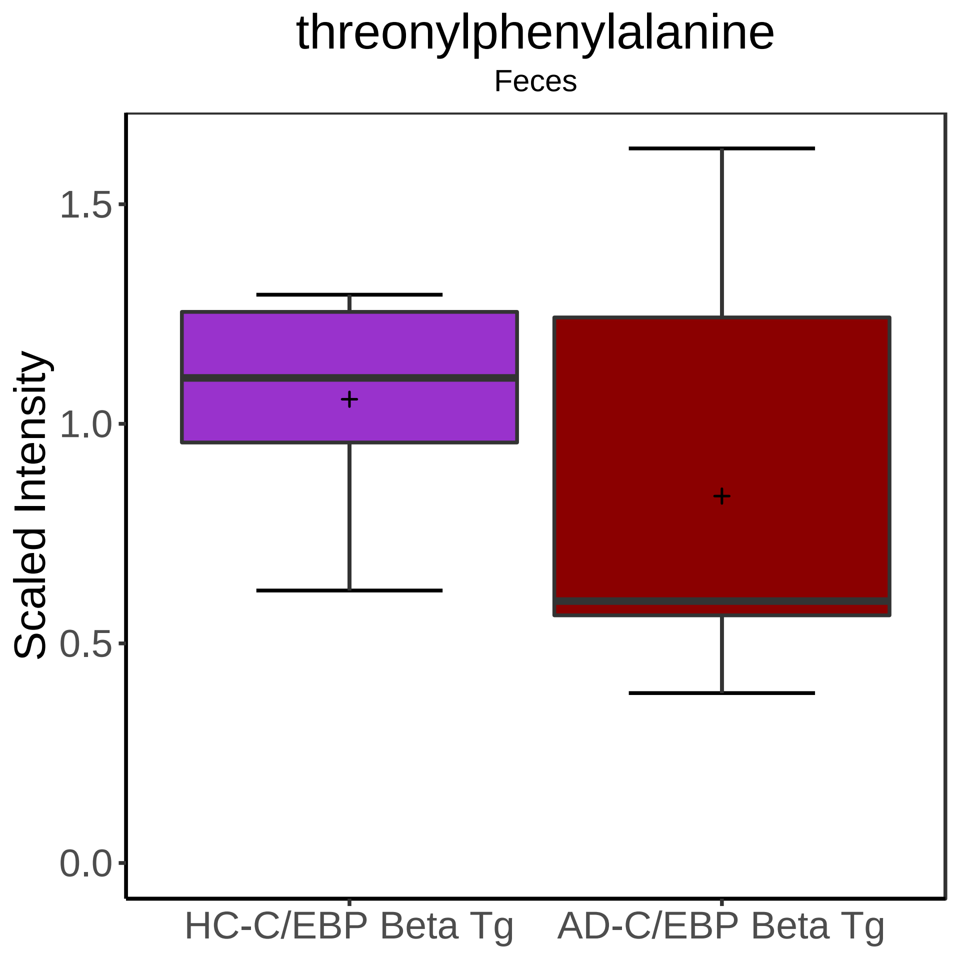

threonylphenylalanine
Feces
Scaled Intensity
1.5
1.0
0.5
0.0
HC-C/EBP Beta Tg
AD-C/EBP Beta Tg

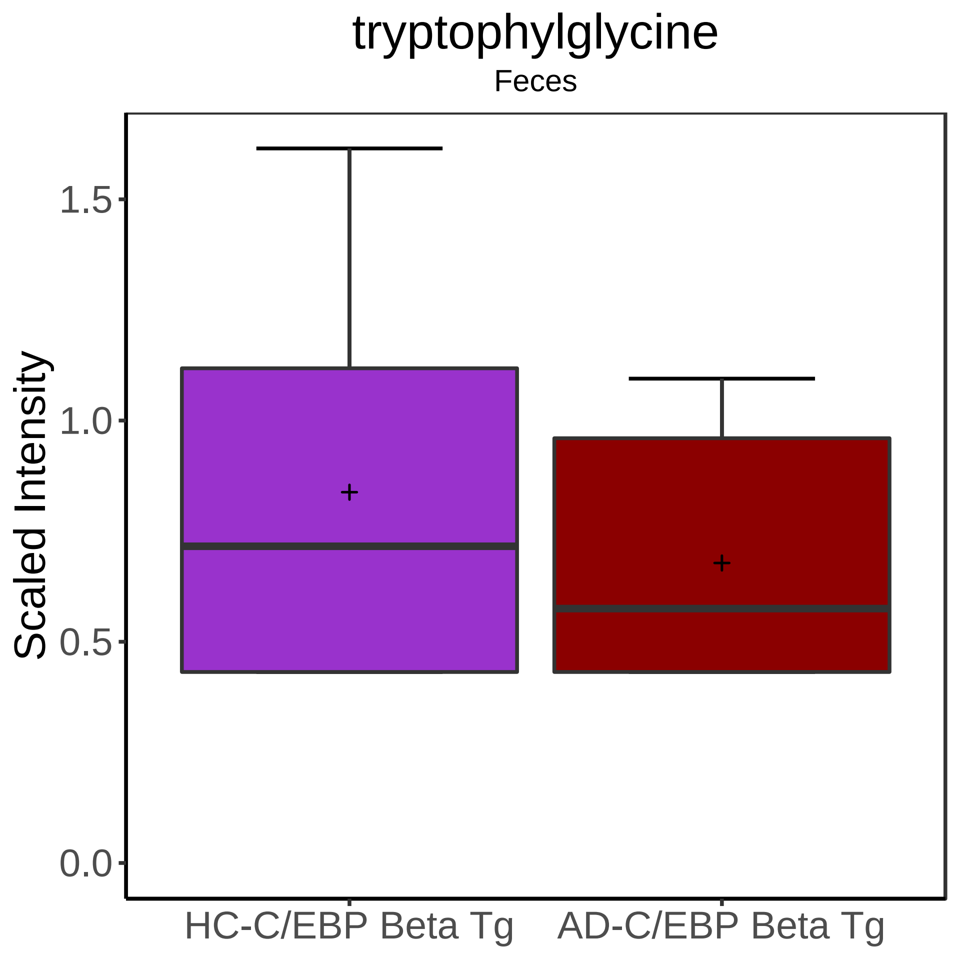

tryptophylglycine
Feces
Scaled Intensity
1.5
1.0
0.5
0.0
HC-C/EBP Beta Tg
AD-C/EBP Beta Tg

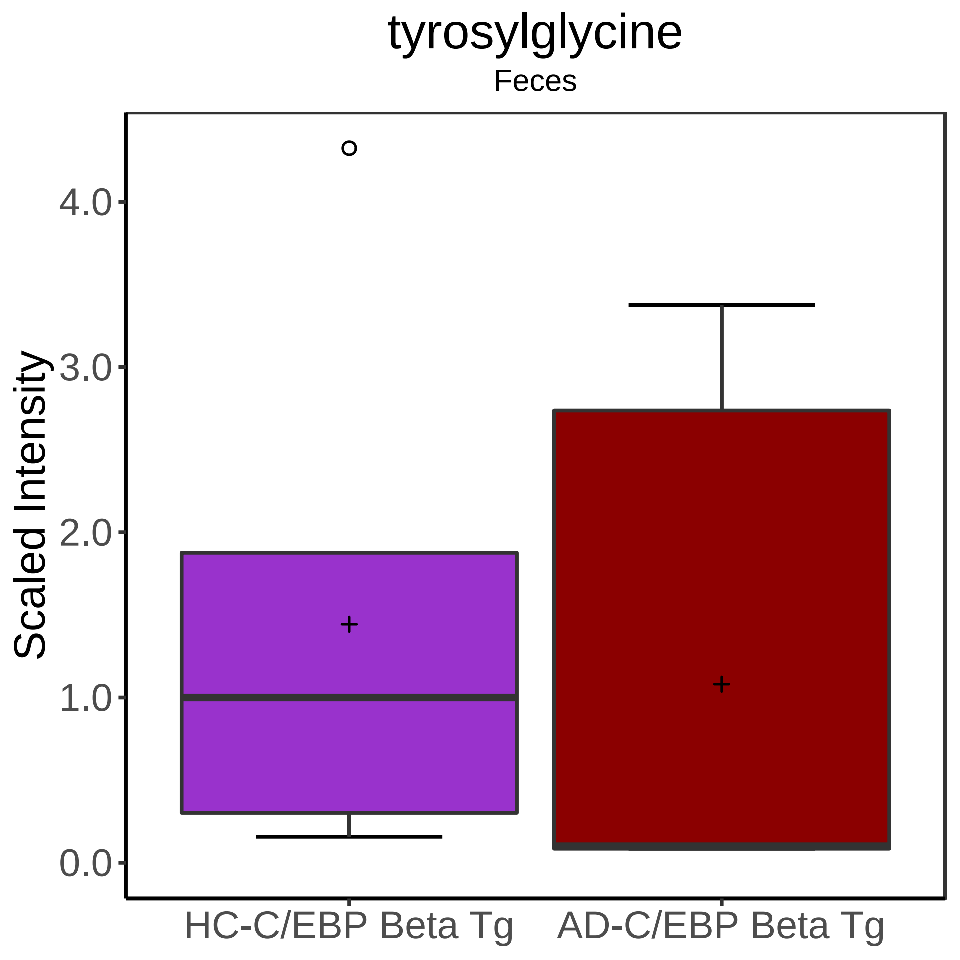
tyrosylglycine
Feces
Scaled Intensity
4.0
3.0
2.0
1.0
0.0
HC-C/EBP Beta Tg
AD-C/EBP Beta Tg

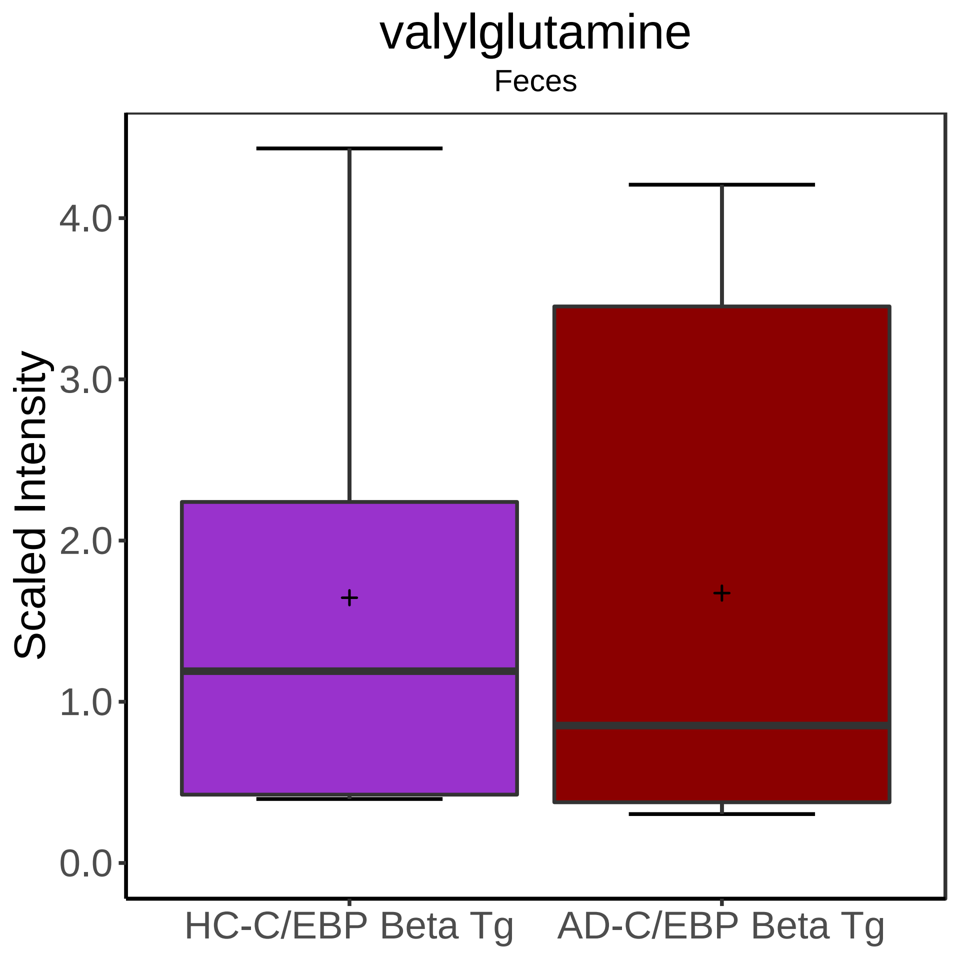

valylglutamine
Feces
Scaled Intensity
4.0
3.0
2.0
1.0
0.0
HC-C/EBP Beta Tg
AD-C/EBP Beta Tg

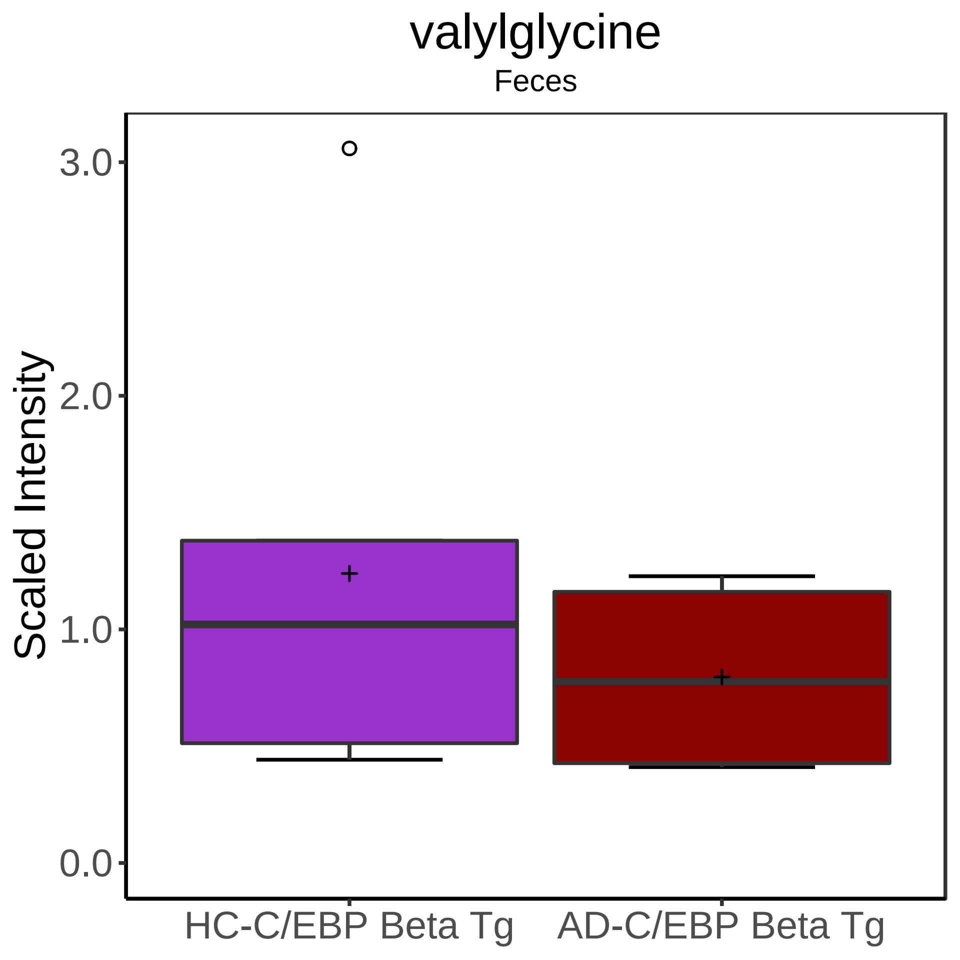
valylglycine
Feces
Scaled Intensity
3.0
2.0
1.0
0.0
HC-C/EBP Beta Tg
AD-C/EBP Beta Tg

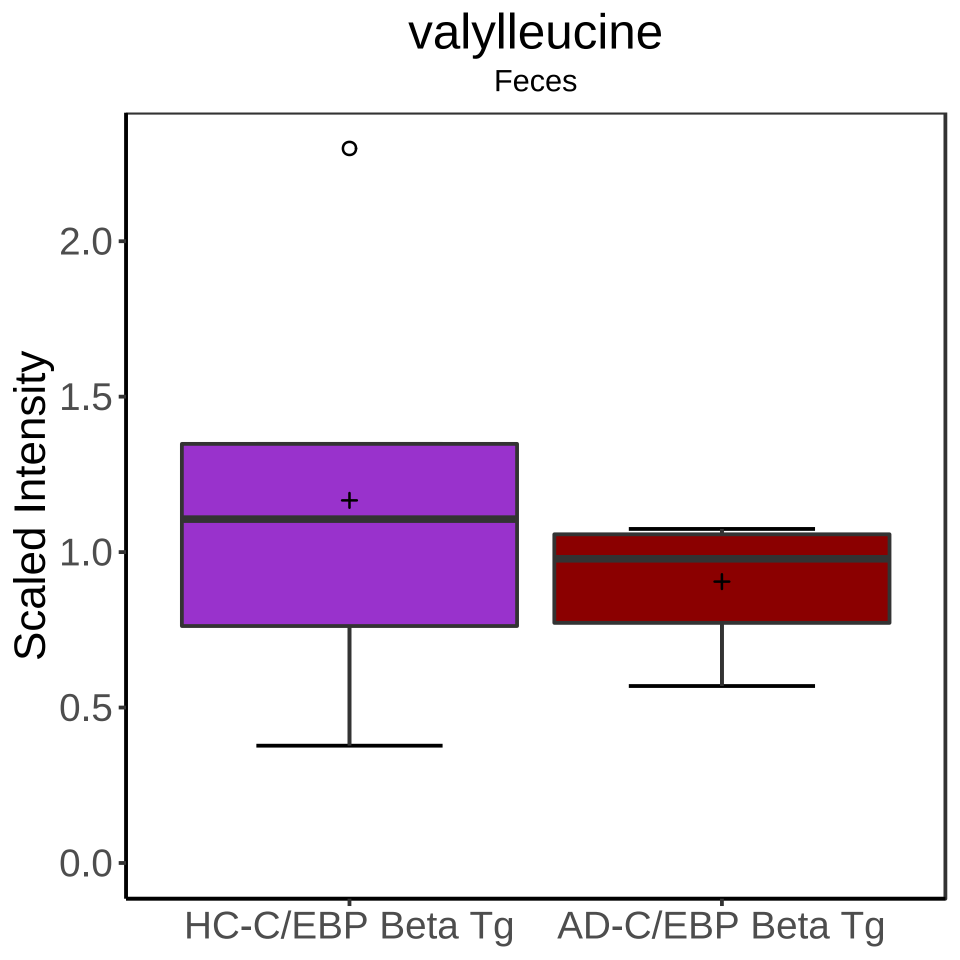
valylleucine
Feces
Scaled Intensity
2.0
1.5
1.0
0.5
0.0
HC-C/EBP Beta Tg
AD-C/EBP Beta Tg

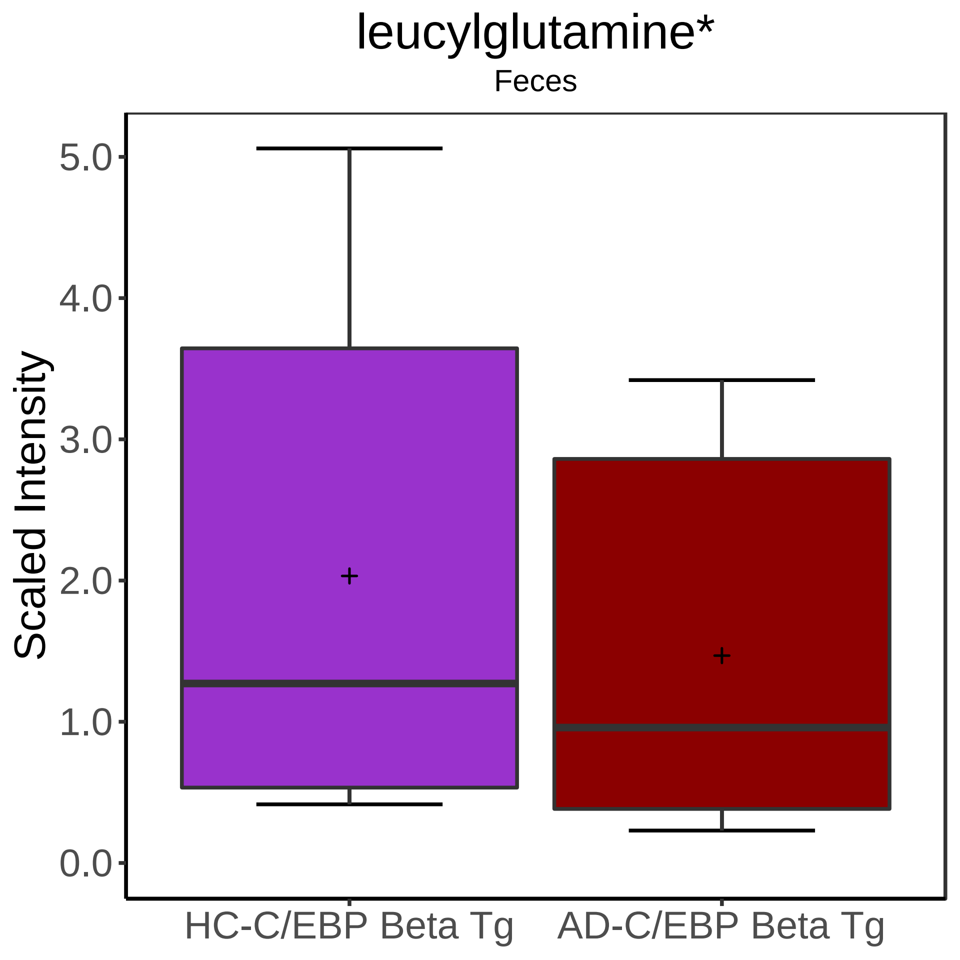

leucylglutamine*
Feces
Scaled Intensity
5.0
4.0
3.0
2.0
1.0
0.0
HC-C/EBP Beta Tg
AD-C/EBP Beta Tg

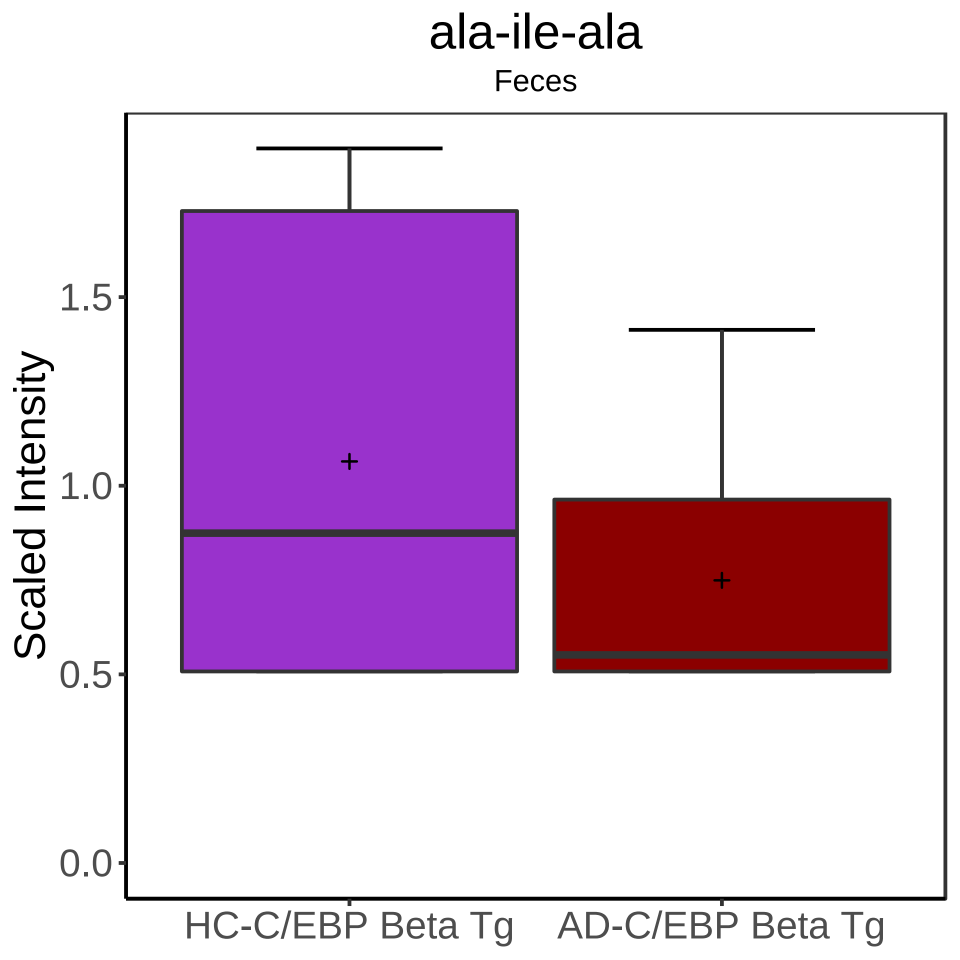
ala-ile-ala
Feces
Scaled Intensity
1.5
1.0
0.5
0.0
HC-C/EBP Beta Tg
AD-C/EBP Beta Tg

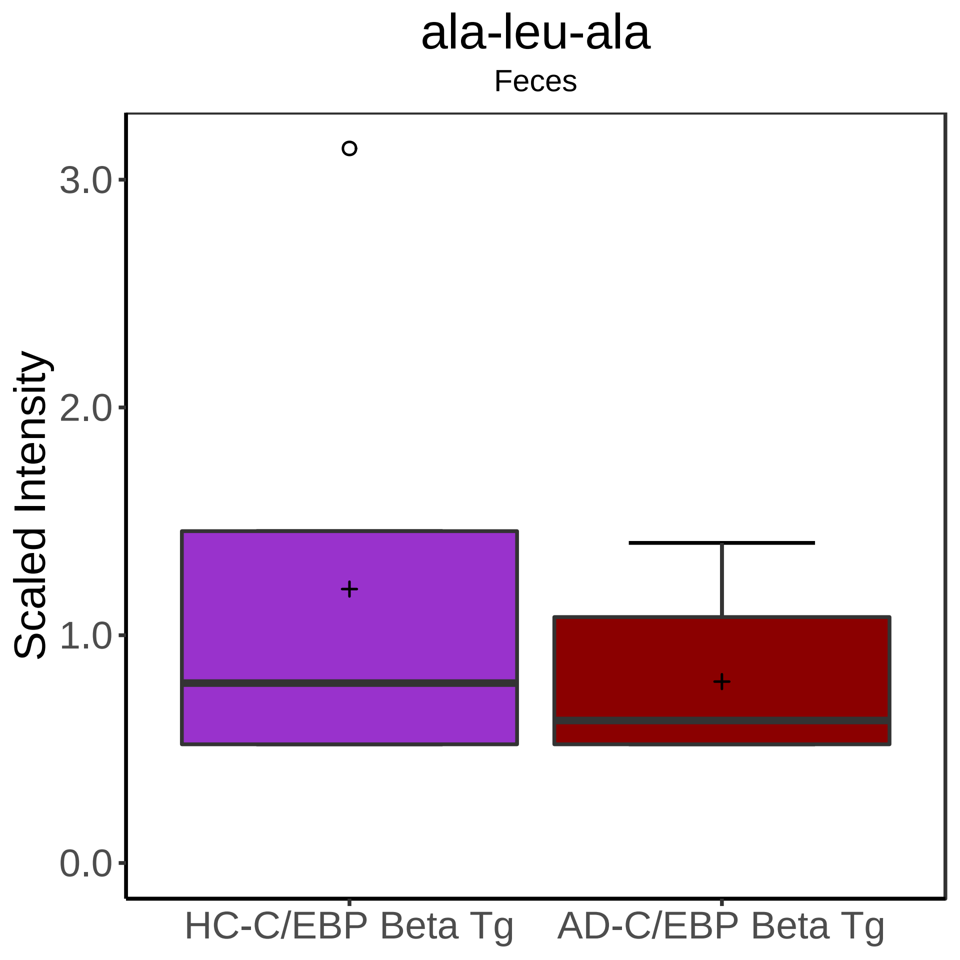

ala-leu-ala
Feces
Scaled Intensity
3.0
2.0
1.0
0.0
HC-C/EBP Beta Tg
AD-C/EBP Beta Tg

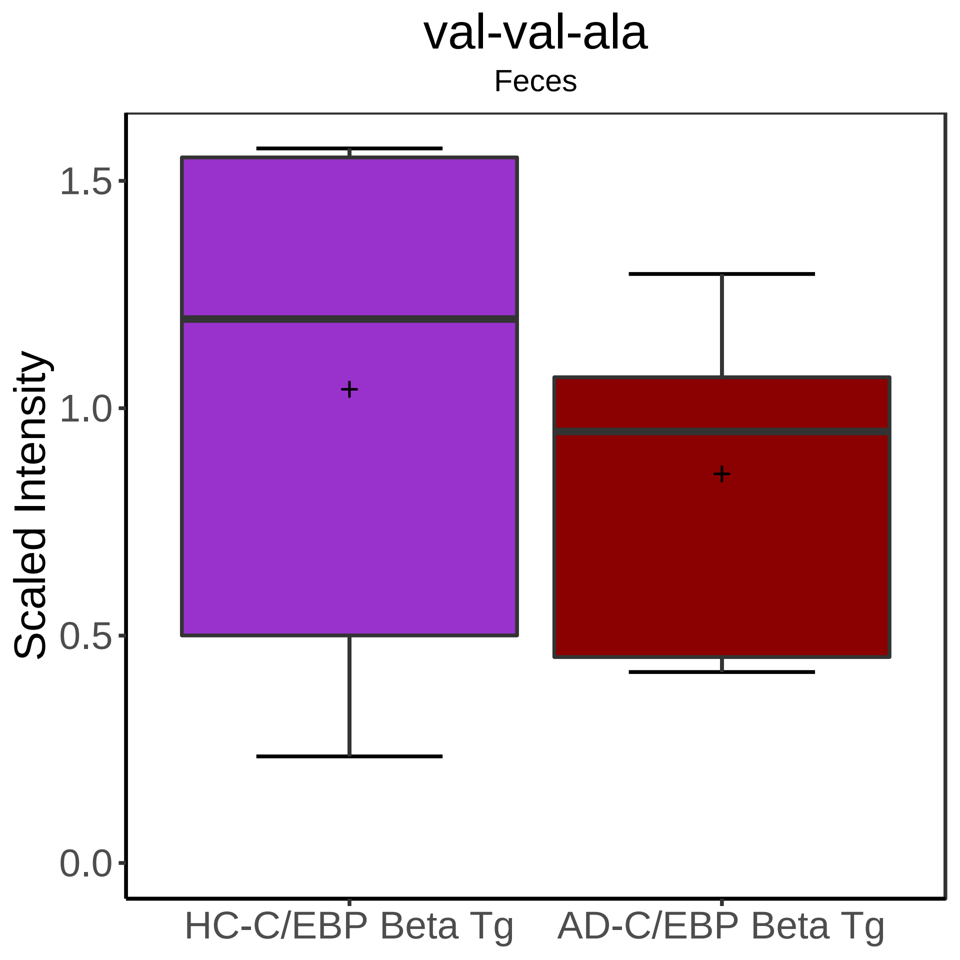

val-val-ala
Feces
Scaled Intensity
1.5
1.0
0.5
0.0
HC-C/EBP Beta Tg
AD-C/EBP Beta Tg

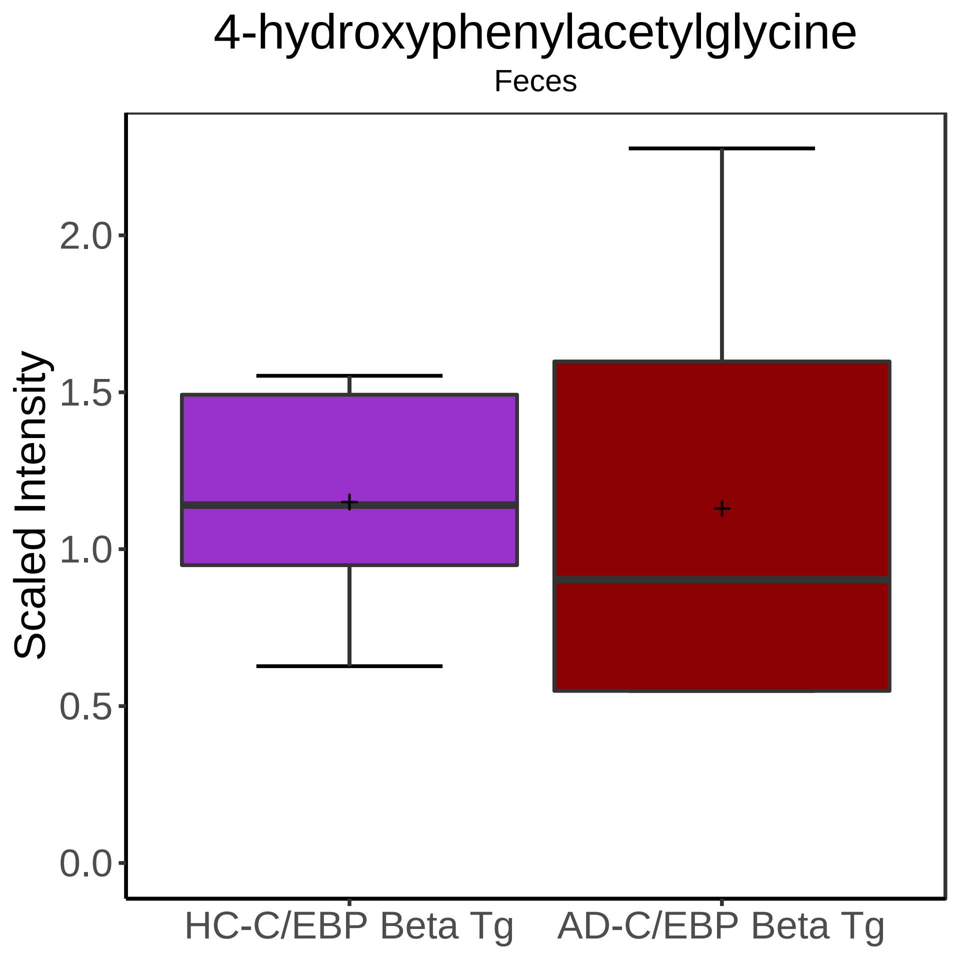
4-hydroxyphenylacetylglycine
Feces
Scaled Intensity
2.0
1.5
1.0
0.5
0.0
HC-C/EBP Beta Tg
AD-C/EBP Beta Tg

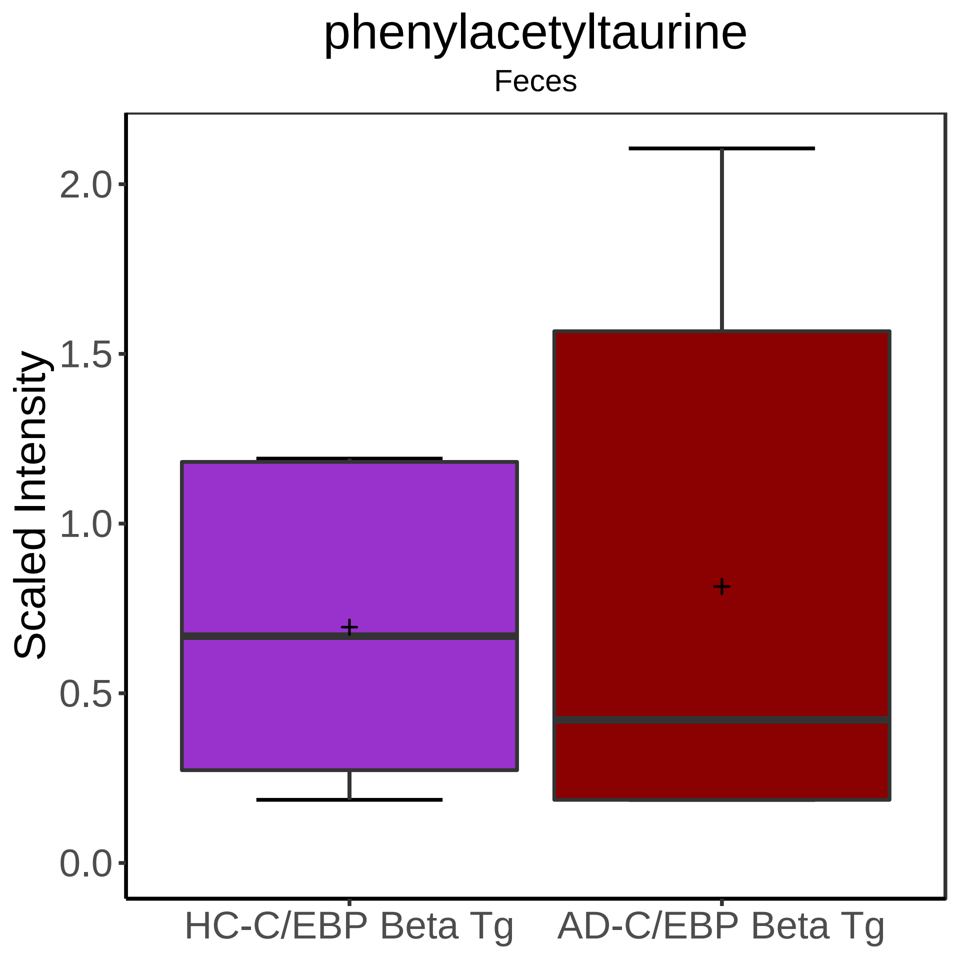
phenylacetyltaurine
Feces
Scaled Intensity
2.0
1.5
1.0
0.5
0.0
HC-C/EBP Beta Tg
AD-C/EBP Beta Tg

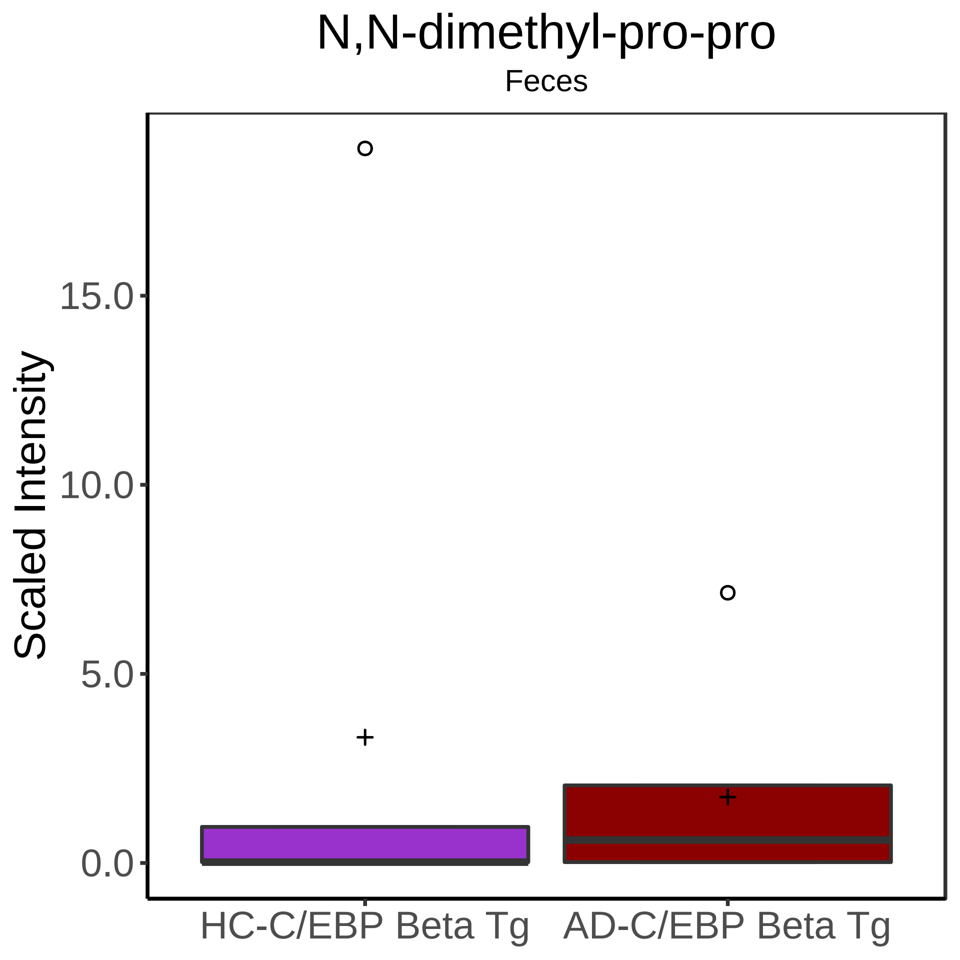

N,N-dimethyl-pro-pro
Feces
Scaled Intensity
15.0
10.0
5.0
0.0
HC-C/EBP Beta Tg
AD-C/EBP Beta Tg

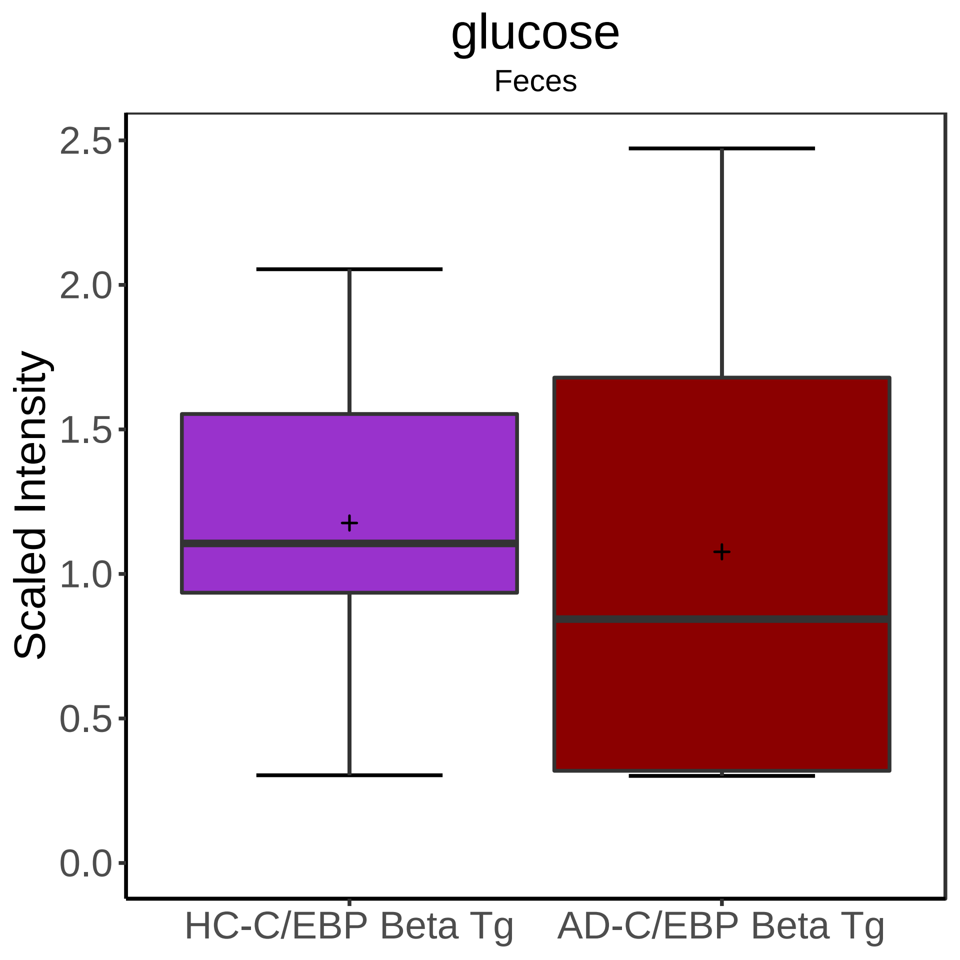

glucose
Feces
Scaled Intensity
0.0
0.5
1.0
1.5
2.0
2.5
HC-C/EBP Beta Tg
AD-C/EBP Beta Tg

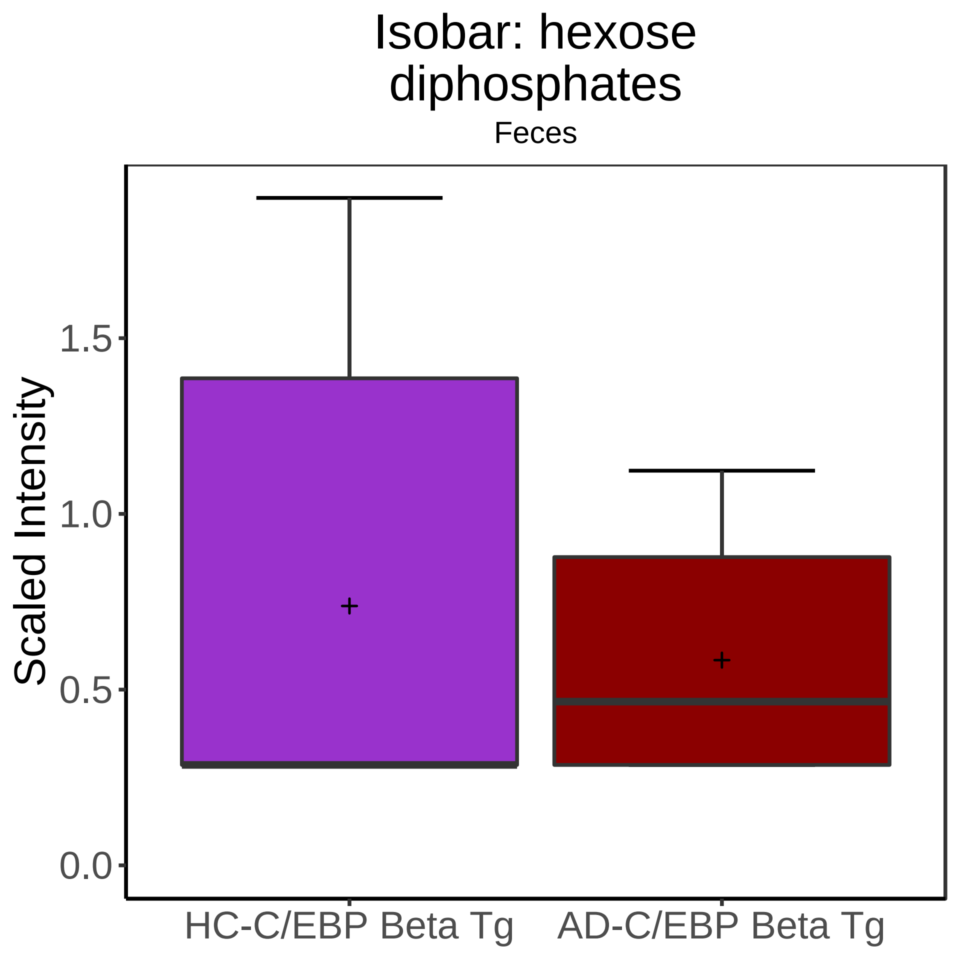
Isobar: hexose diphosphates
Feces
Scaled Intensity
1.5
1.0
0.5
0.0
HC-C/EBP Beta Tg
AD-C/EBP Beta Tg

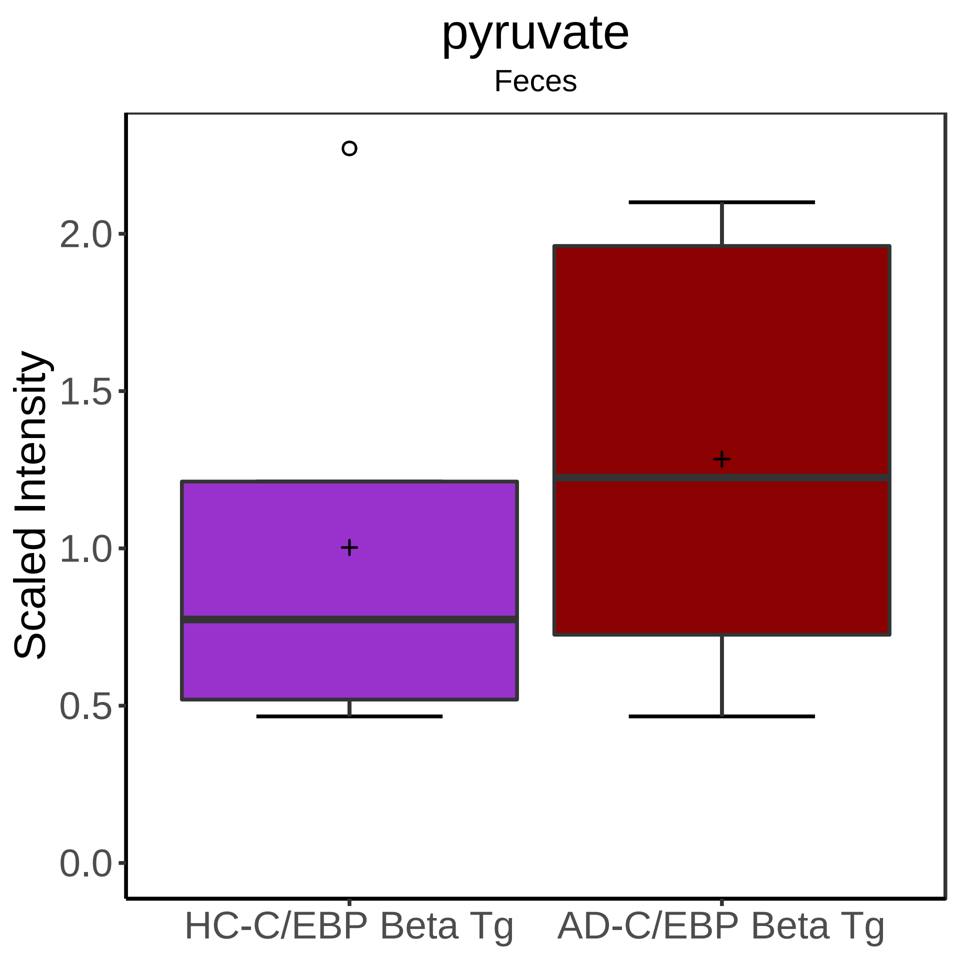
pyruvate
Feces
Scaled Intensity
2.0
1.5
1.0
0.5
0.0
HC-C/EBP Beta Tg
AD-C/EBP Beta Tg

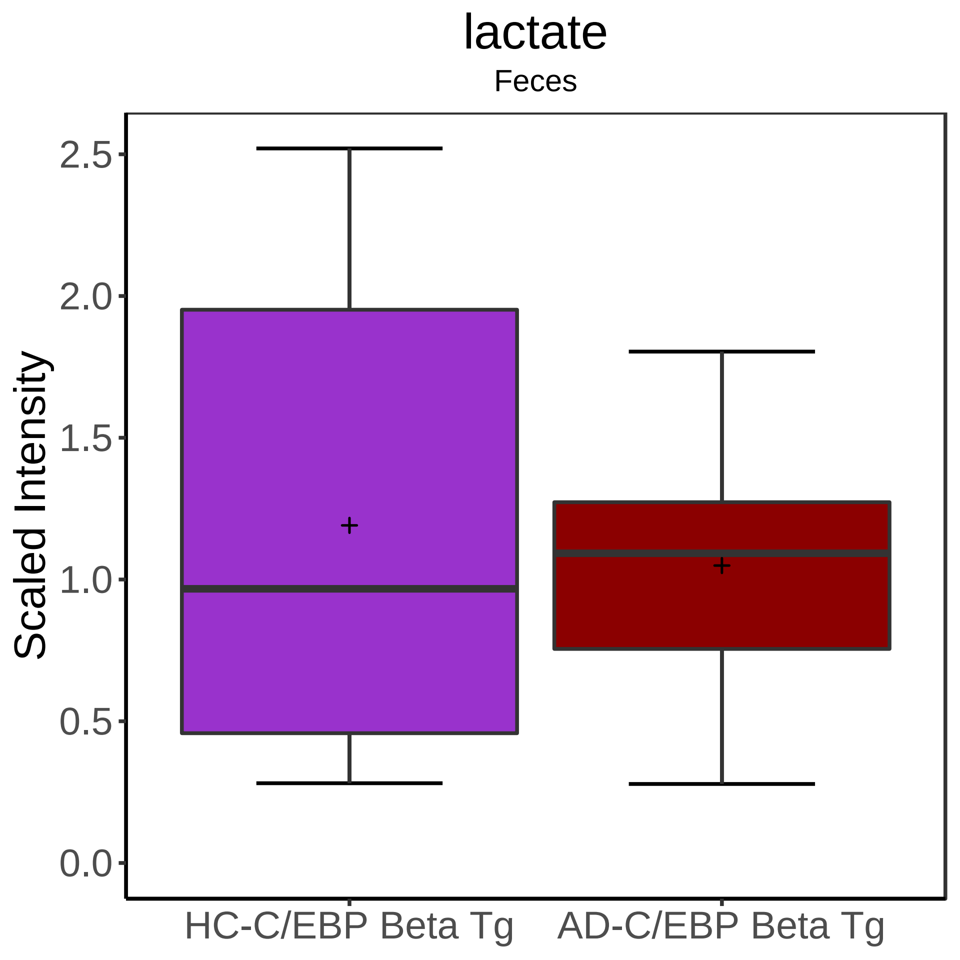
lactate
Feces
Scaled Intensity
2.5
2.0
1.5
1.0
0.5
0.0
HC-C/EBP Beta Tg
AD-C/EBP Beta Tg

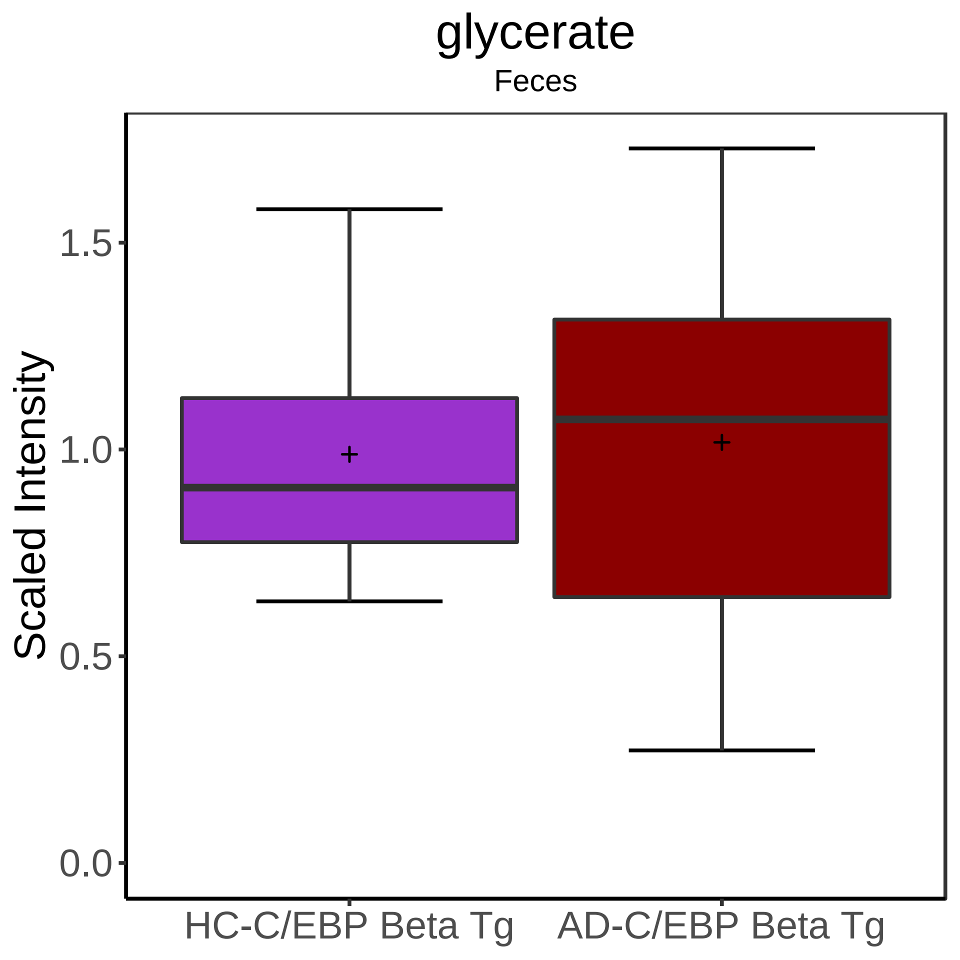
glycerate
Feces
Scaled Intensity
1.5
1.0
0.5
0.0
HC-C/EBP Beta Tg
AD-C/EBP Beta Tg

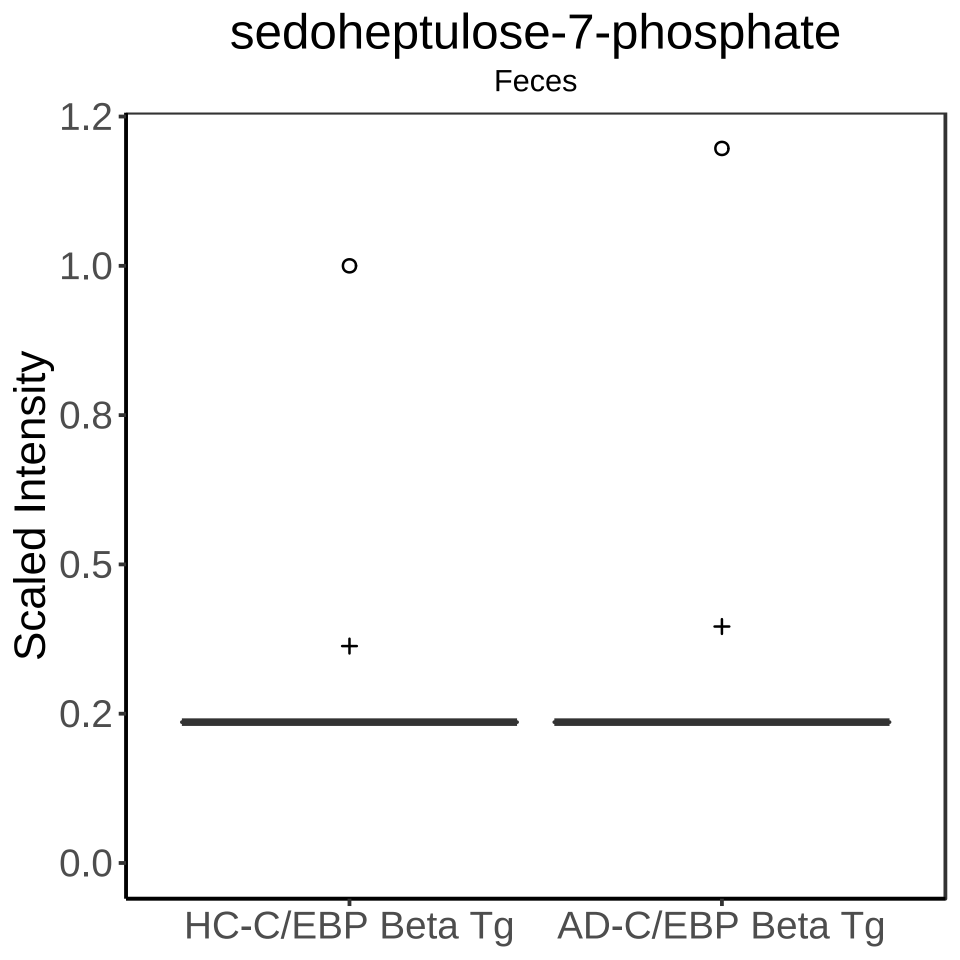

sedoheptulose-7-phosphate
Feces
Scaled Intensity
1.2
1.0
0.8
0.5
0.2
0.0
HC-C/EBP Beta Tg
AD-C/EBP Beta Tg

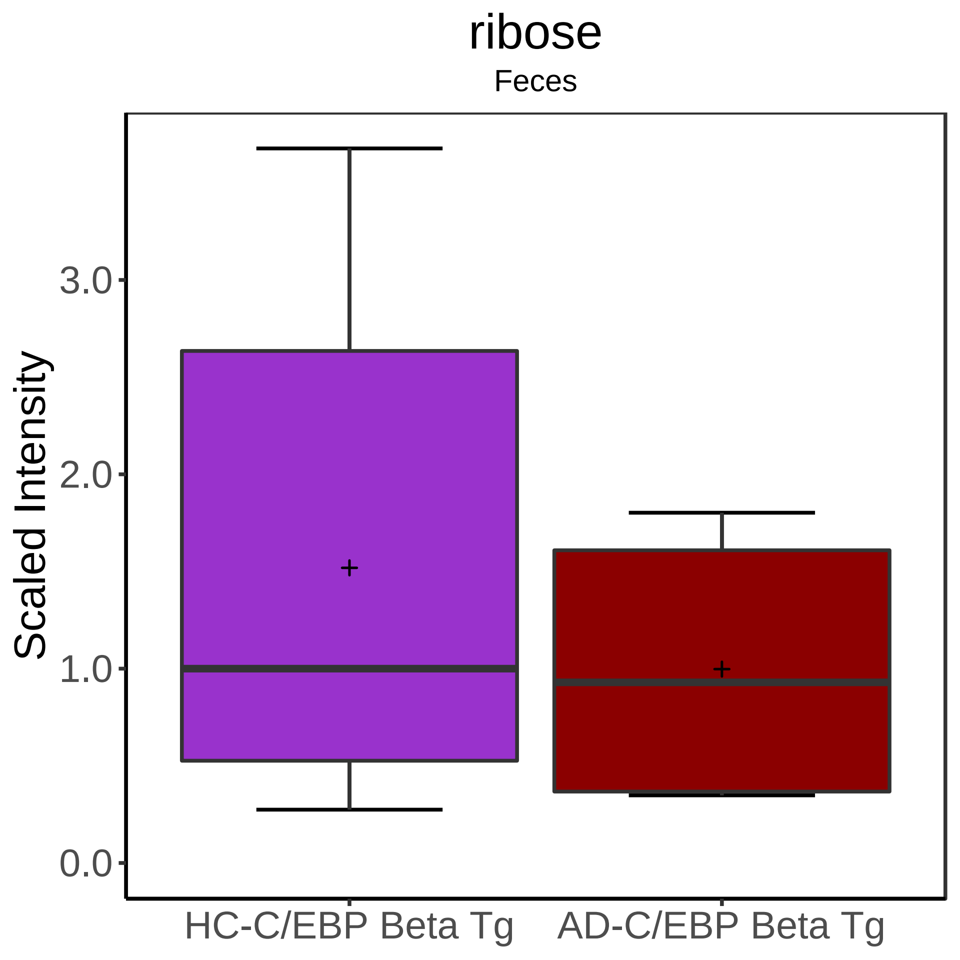
ribose
Feces
Scaled Intensity
3.0
2.0
1.0
0.0
HC-C/EBP Beta Tg
AD-C/EBP Beta Tg

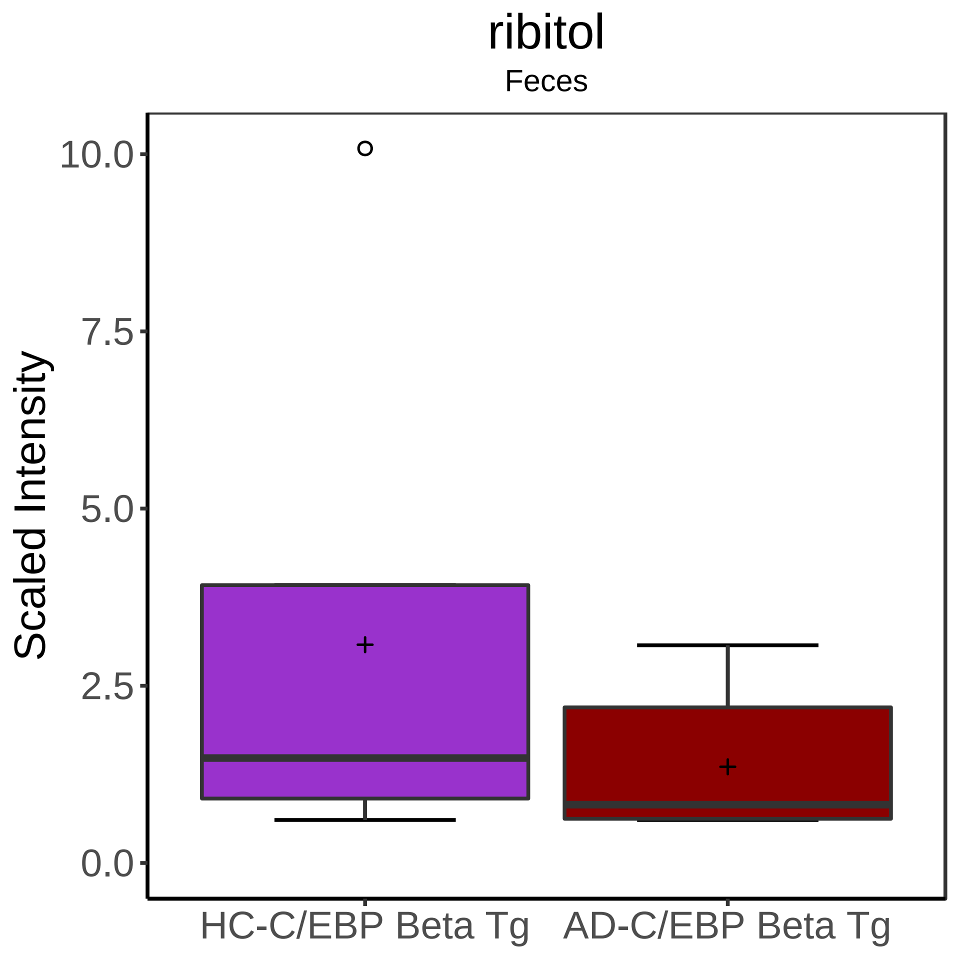
ribitol
Feces
Scaled Intensity
10.0
7.5
5.0
2.5
0.0
HC-C/EBP Beta Tg
AD-C/EBP Beta Tg

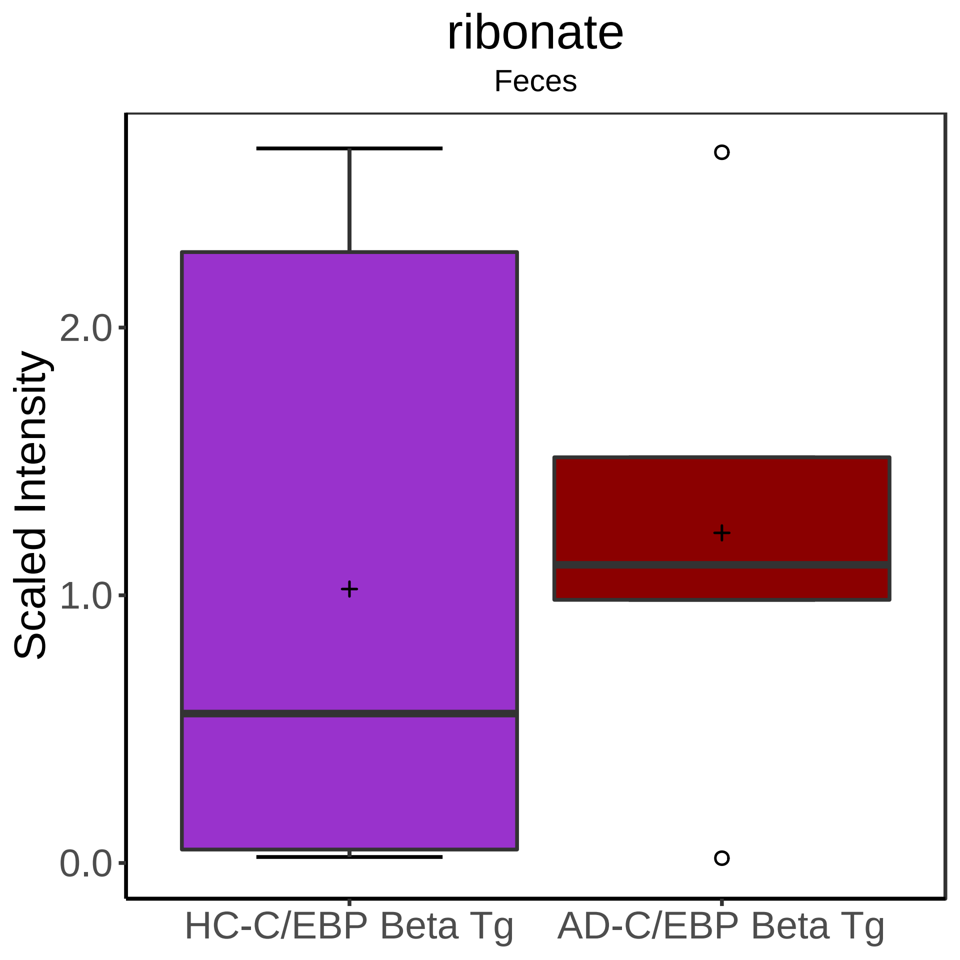

ribonate
Feces
Scaled Intensity
2.0
1.0
0.0
HC-C/EBP Beta Tg
AD-C/EBP Beta Tg

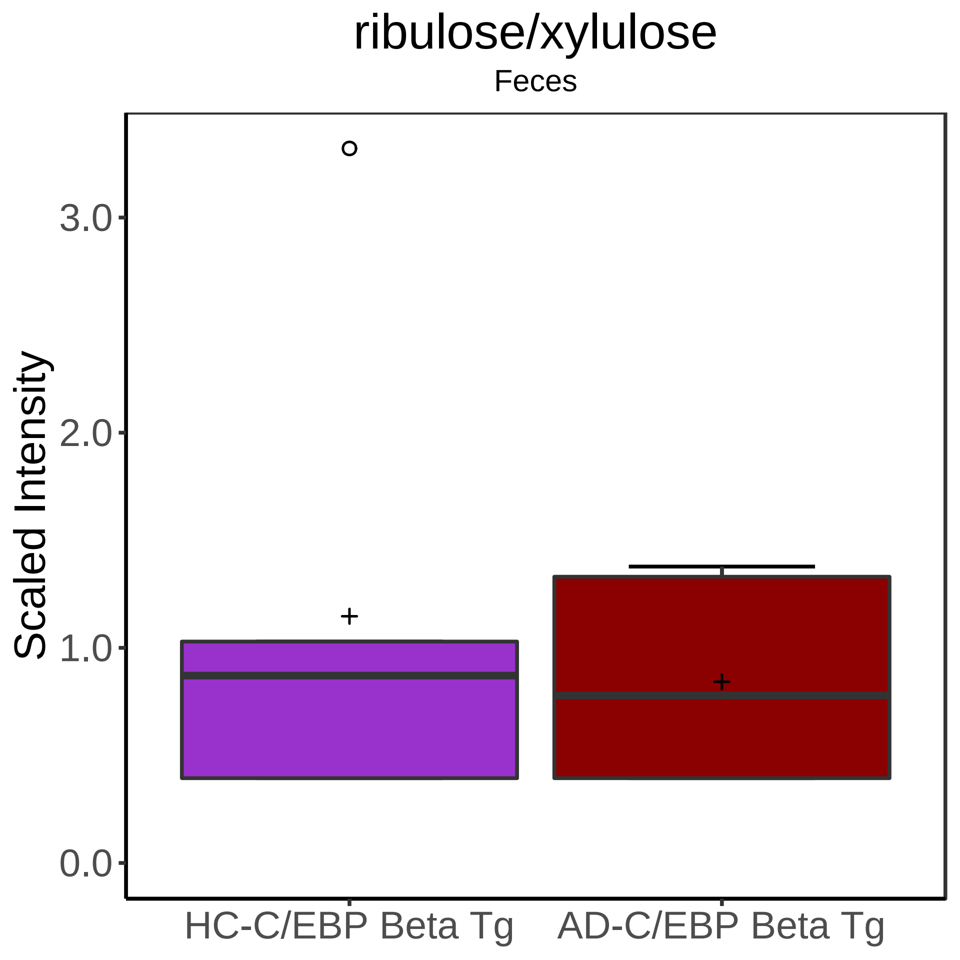
ribulose/xylulose
Feces
Scaled Intensity
3.0
2.0
1.0
0.0
HC-C/EBP Beta Tg
AD-C/EBP Beta Tg

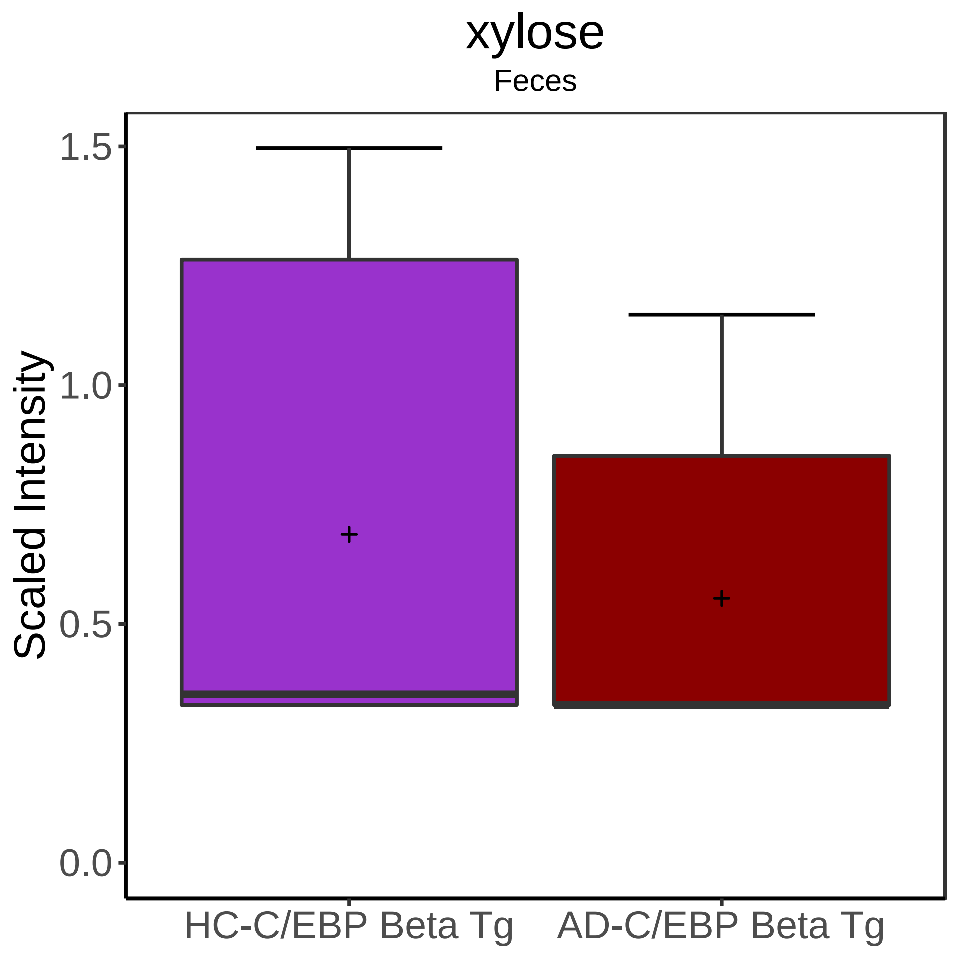

xylose
Feces
Scaled Intensity
1.5
1.0
0.5
0.0
HC-C/EBP Beta Tg
AD-C/EBP Beta Tg

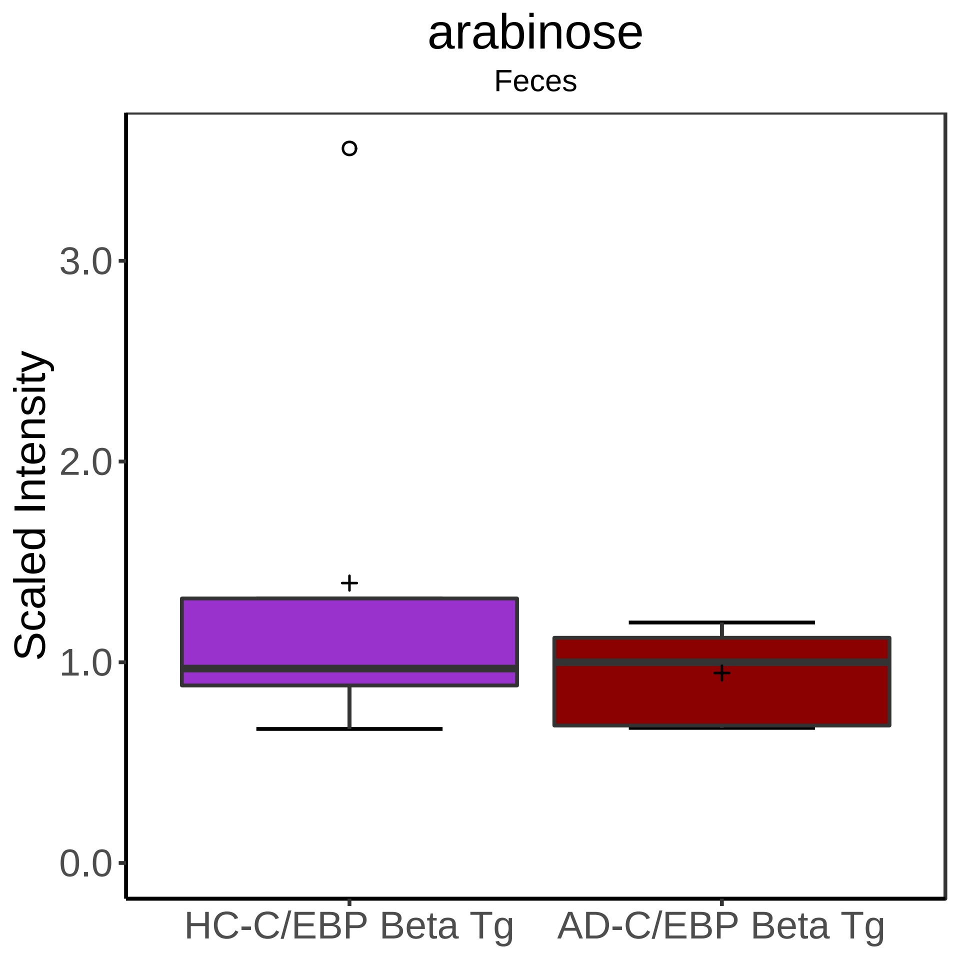
arabinose
Feces
Scaled Intensity
3.0
2.0
1.0
0.0
HC-C/EBP Beta Tg
AD-C/EBP Beta Tg

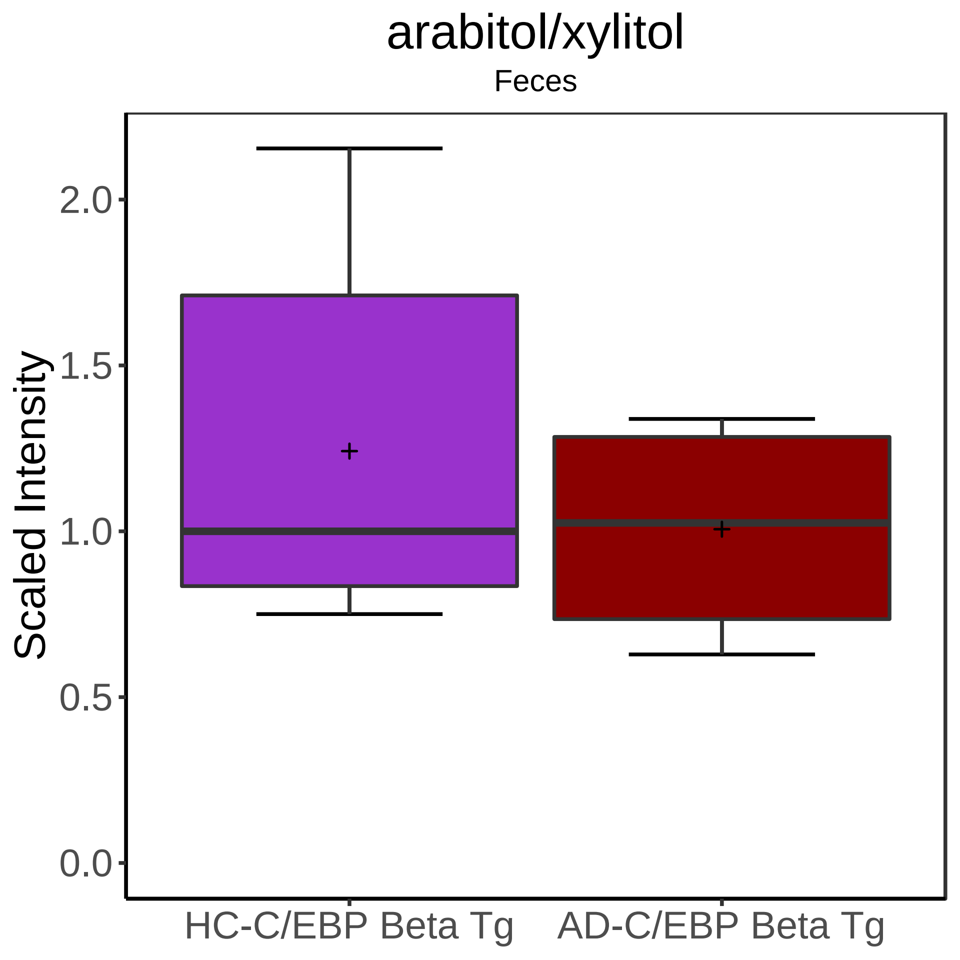
arabitol/xylitol
Feces
Scaled Intensity
2.0
1.5
1.0
0.5
0.0
HC-C/EBP Beta Tg
AD-C/EBP Beta Tg

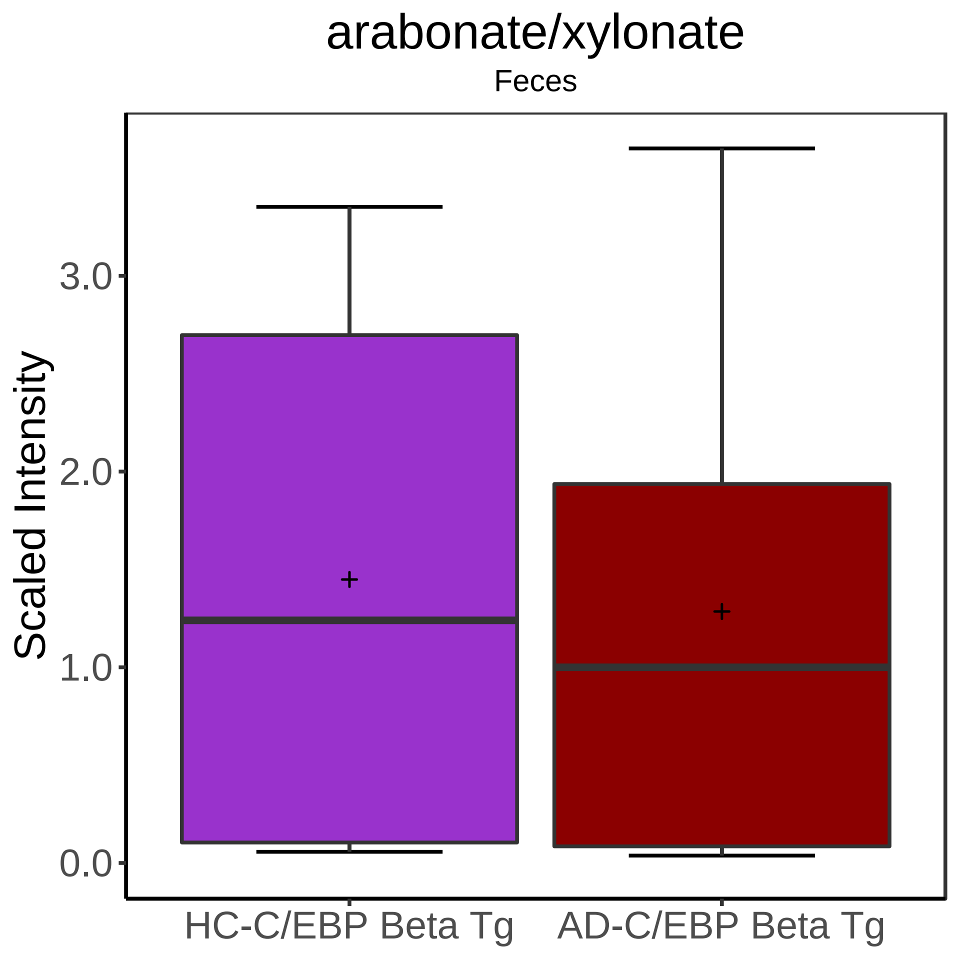
arabonate/xylonate
Feces
Scaled Intensity
3.0
2.0
1.0
0.0
HC-C/EBP Beta Tg
AD-C/EBP Beta Tg

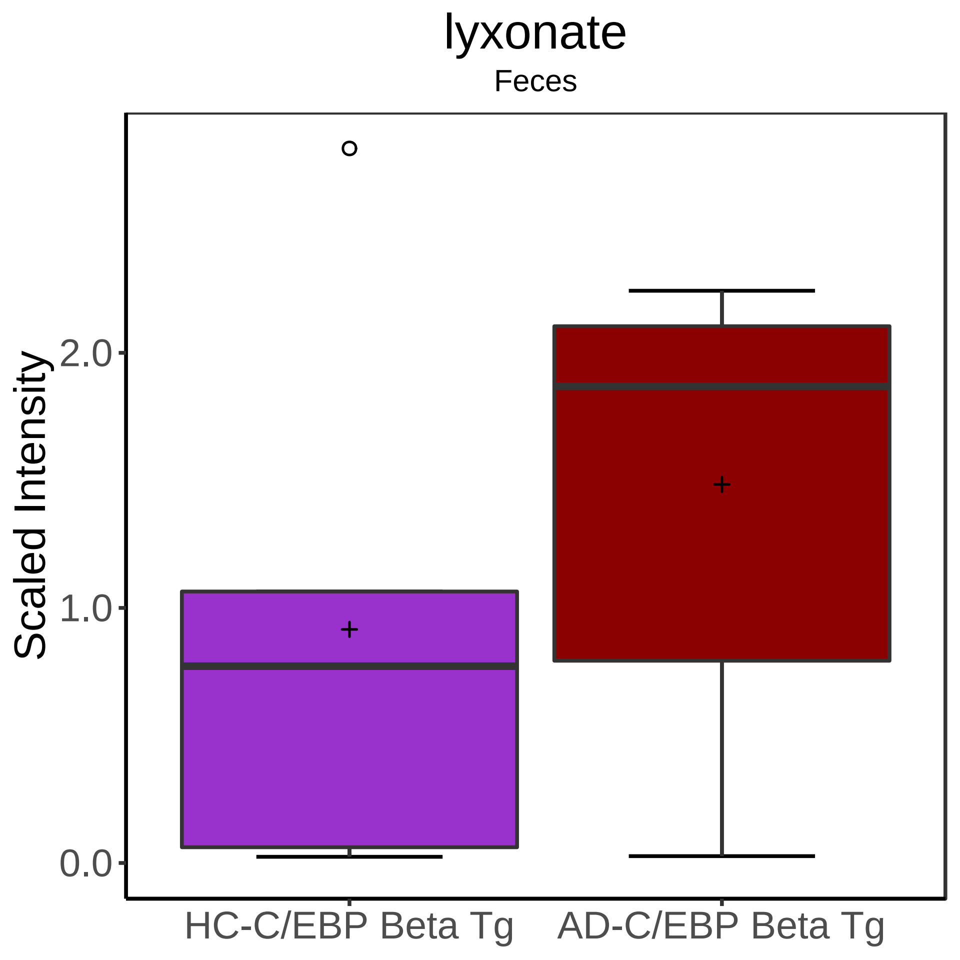

lyxonate
Feces
Scaled Intensity
2.0
1.0
0.0
HC-C/EBP Beta Tg
AD-C/EBP Beta Tg

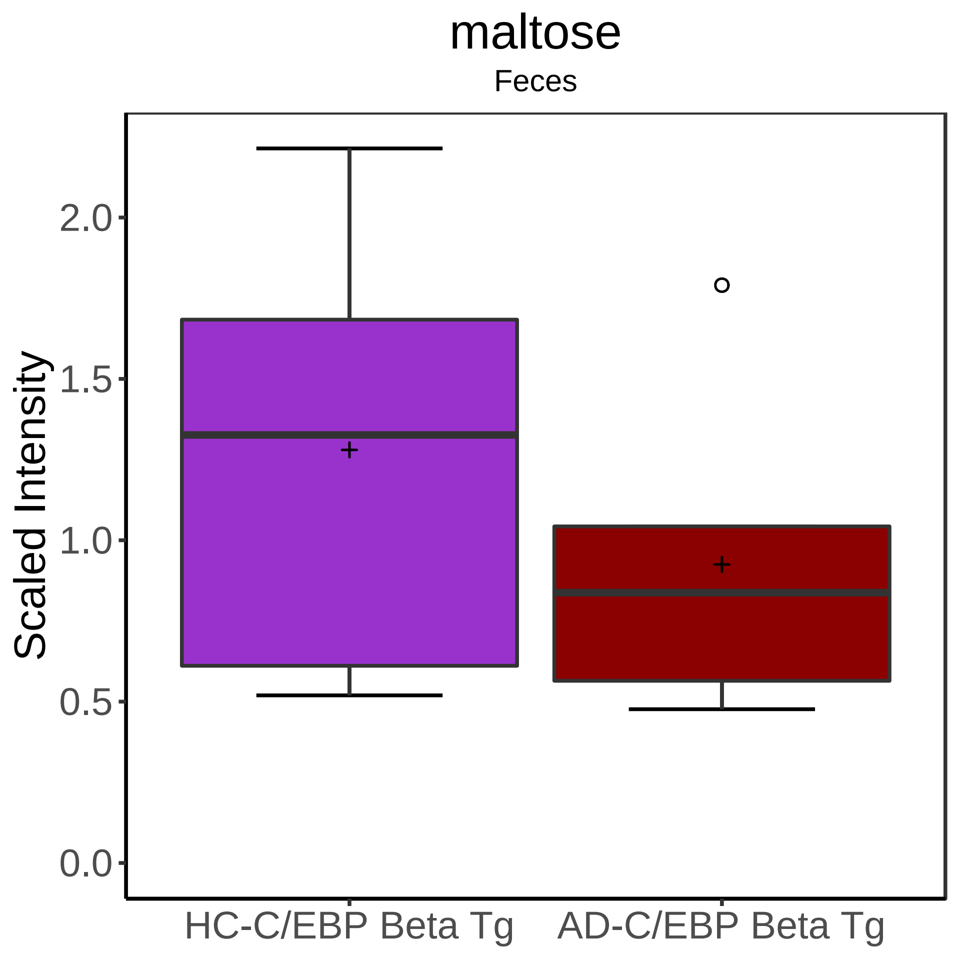

maltose
Feces
Scaled Intensity
2.0
1.5
1.0
0.5
0.0
HC-C/EBP Beta Tg
AD-C/EBP Beta Tg

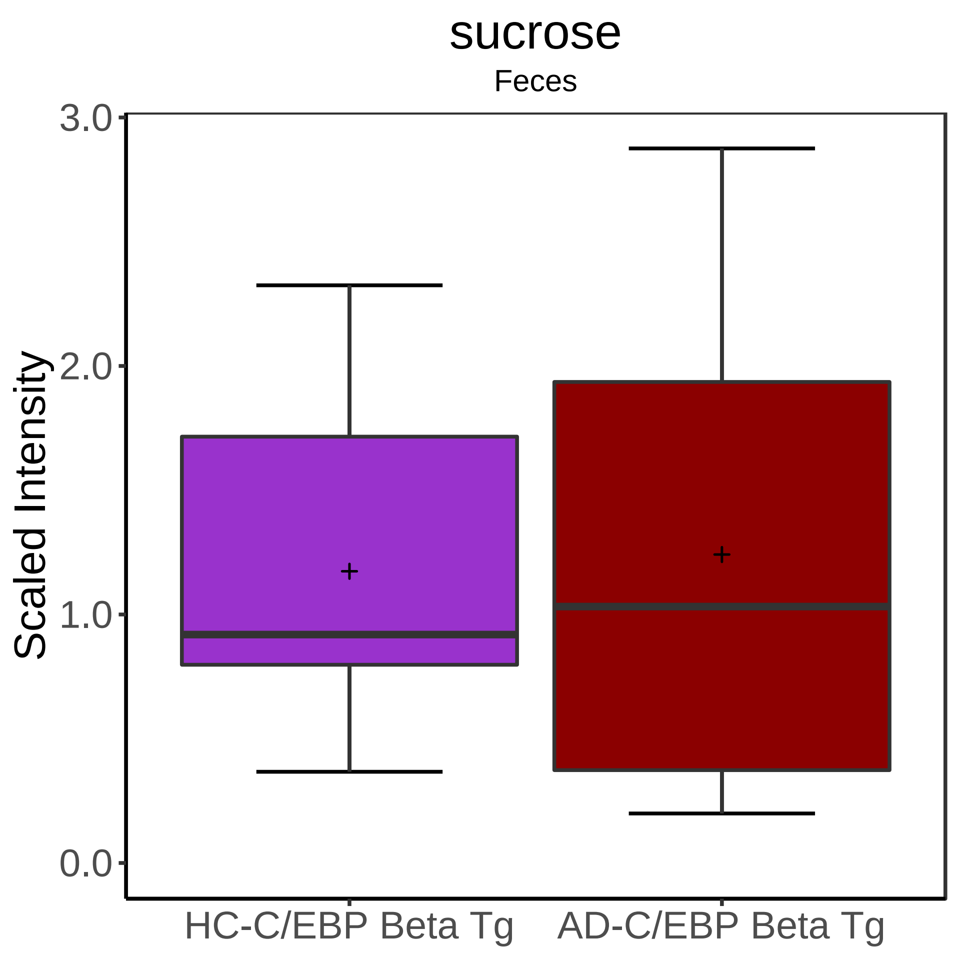
sucrose
Feces
Scaled Intensity
3.0
2.0
1.0
0.0
HC-C/EBP Beta Tg
AD-C/EBP Beta Tg

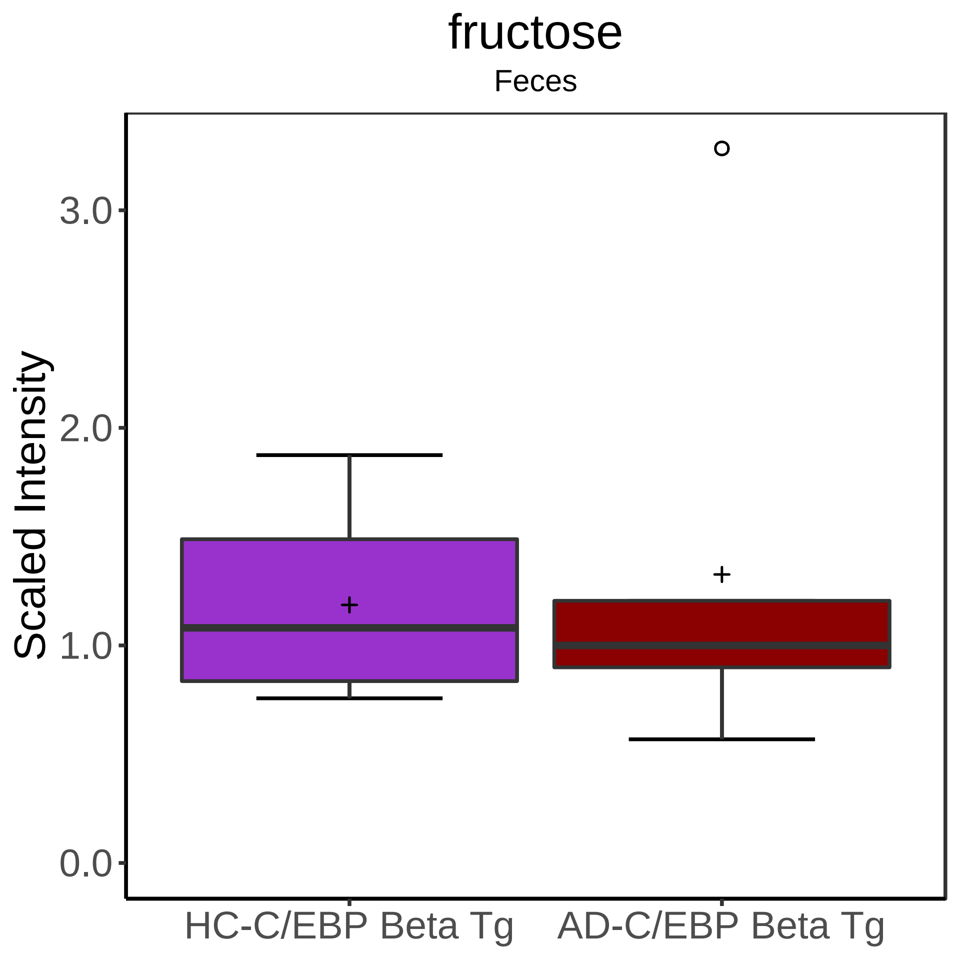
fructose
Feces
Scaled Intensity
3.0
2.0
1.0
0.0
HC-C/EBP Beta Tg
AD-C/EBP Beta Tg

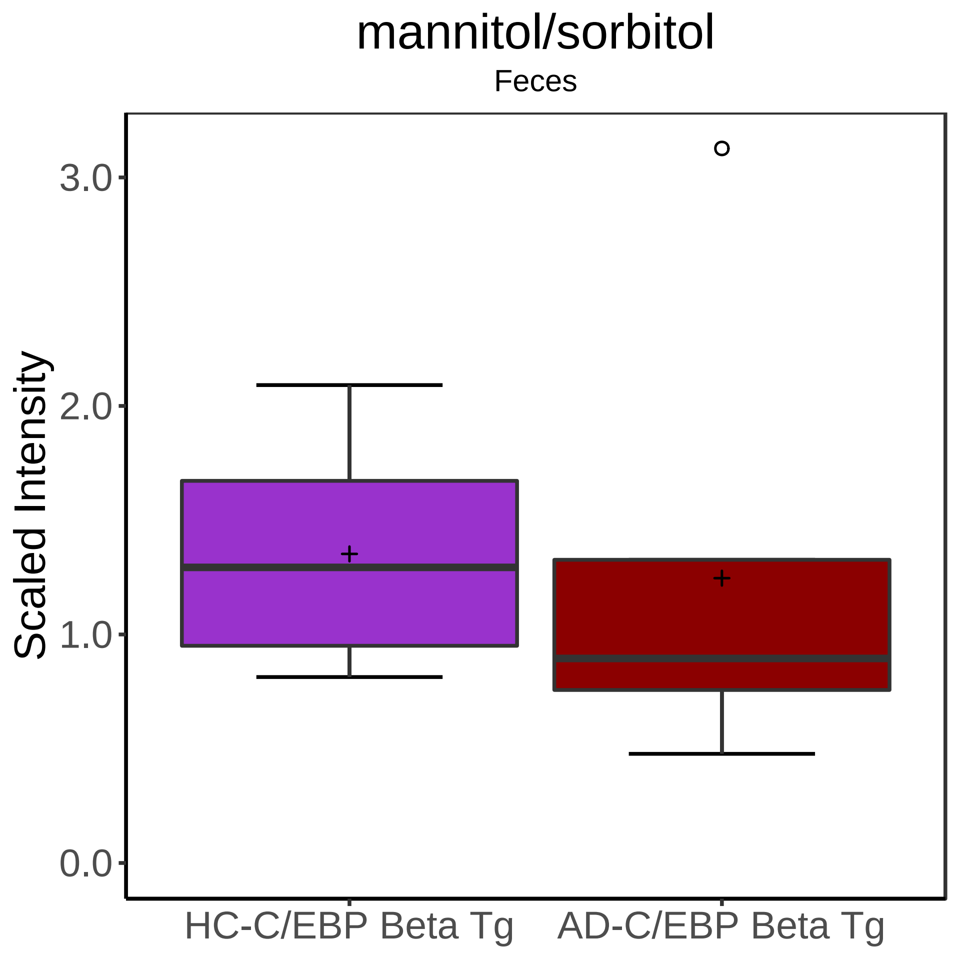
mannitol/sorbitol
Feces
Scaled Intensity
3.0
2.0
1.0
0.0
HC-C/EBP Beta Tg
AD-C/EBP Beta Tg

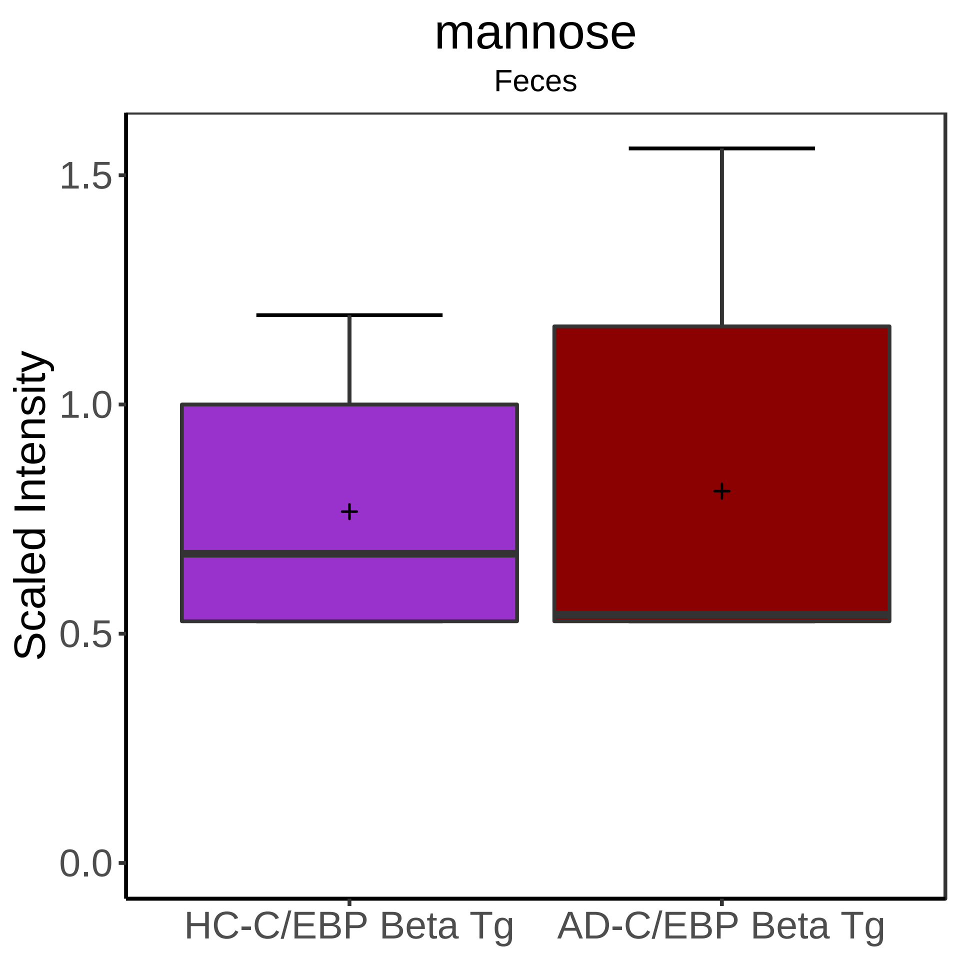
mannose
Feces
Scaled Intensity
1.5
1.0
0.5
0.0
HC-C/EBP Beta Tg
AD-C/EBP Beta Tg

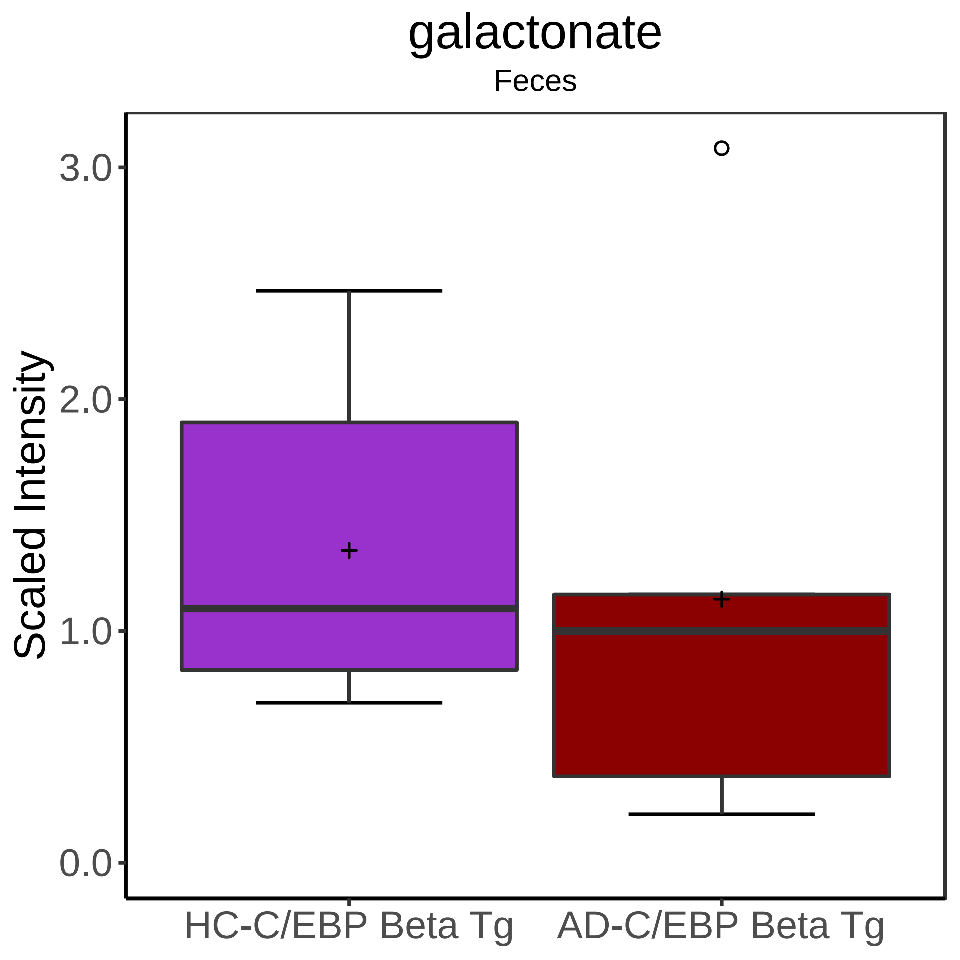
galactonate
Feces
Scaled Intensity
3.0
2.0
1.0
0.0
HC-C/EBP Beta Tg
AD-C/EBP Beta Tg

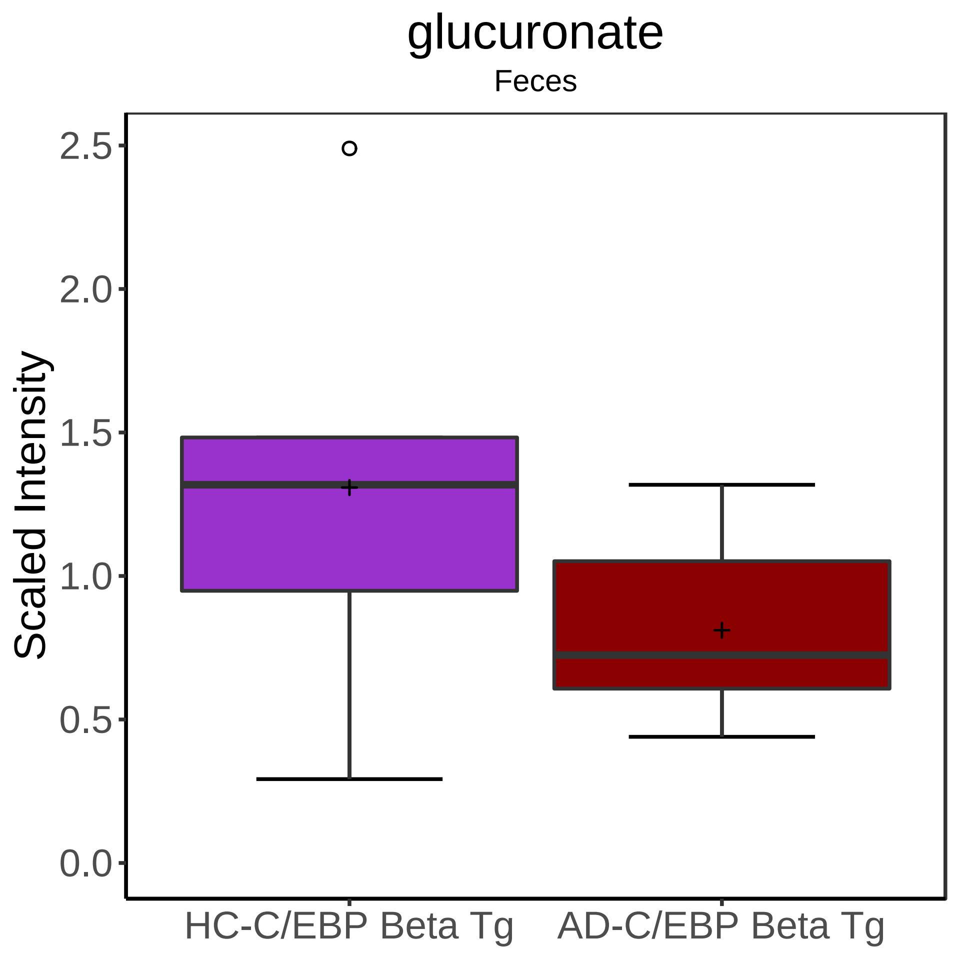
glucuronate
Feces
Scaled Intensity
2.5
2.0
1.5
1.0
0.5
0.0
HC-C/EBP Beta Tg
AD-C/EBP Beta Tg

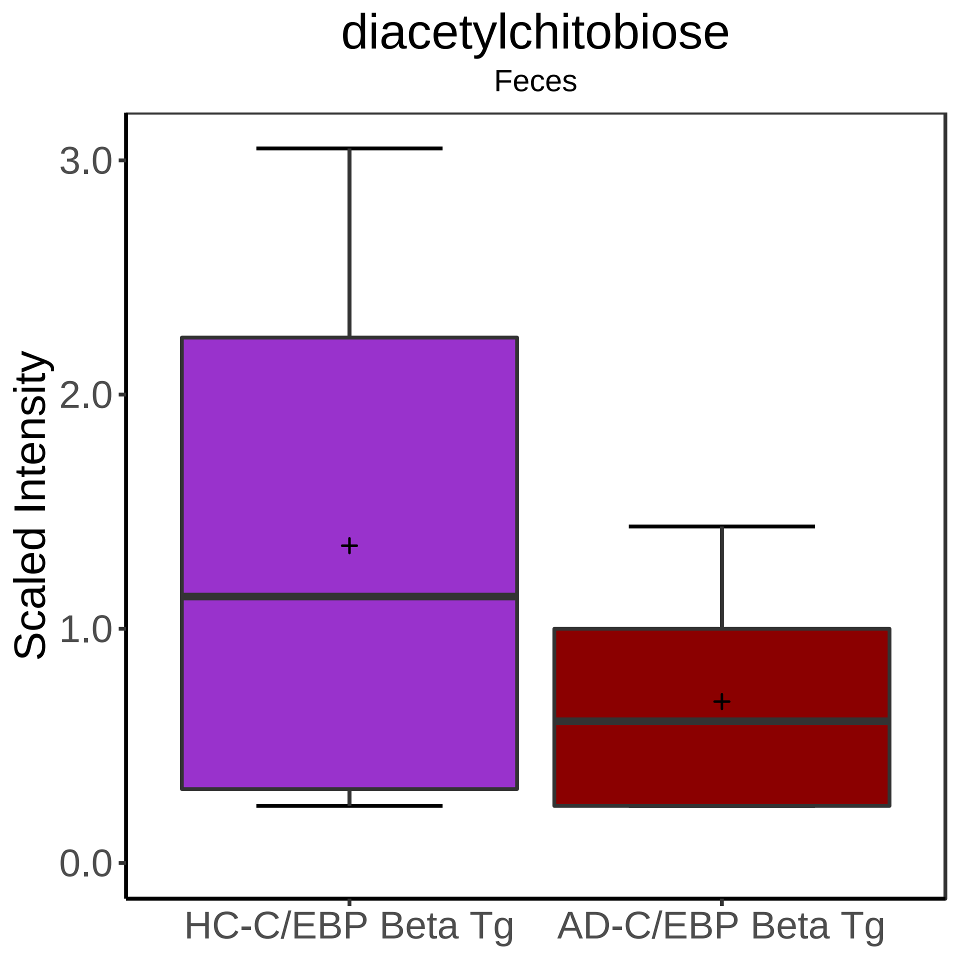
diacetylchitobiose
Feces
Scaled Intensity
3.0
2.0
1.0
0.0
HC-C/EBP Beta Tg
AD-C/EBP Beta Tg

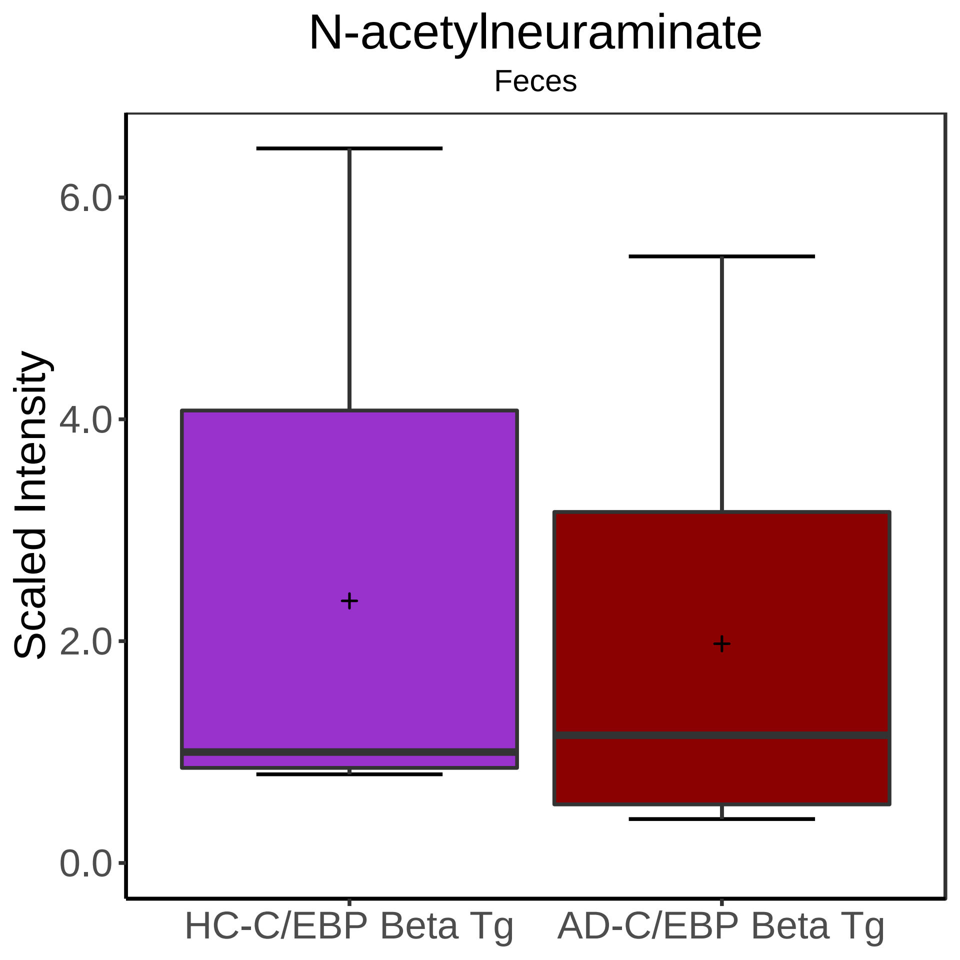

N-acetylneuraminate
Feces
Scaled Intensity
6.0
4.0
2.0
0.0
HC-C/EBP Beta Tg
AD-C/EBP Beta Tg

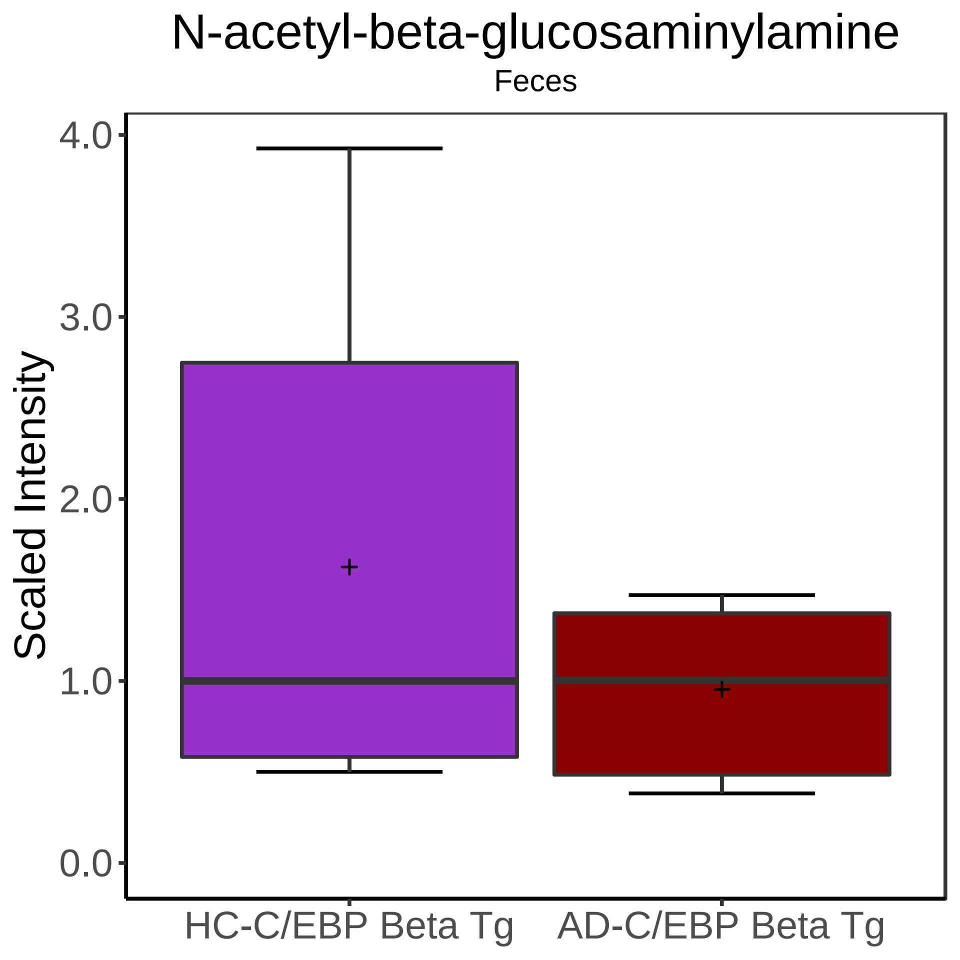

N-acetyl-beta-glucosaminylamine
Feces
Scaled Intensity
4.0
3.0
2.0
1.0
0.0
HC-C/EBP Beta Tg
AD-C/EBP Beta Tg

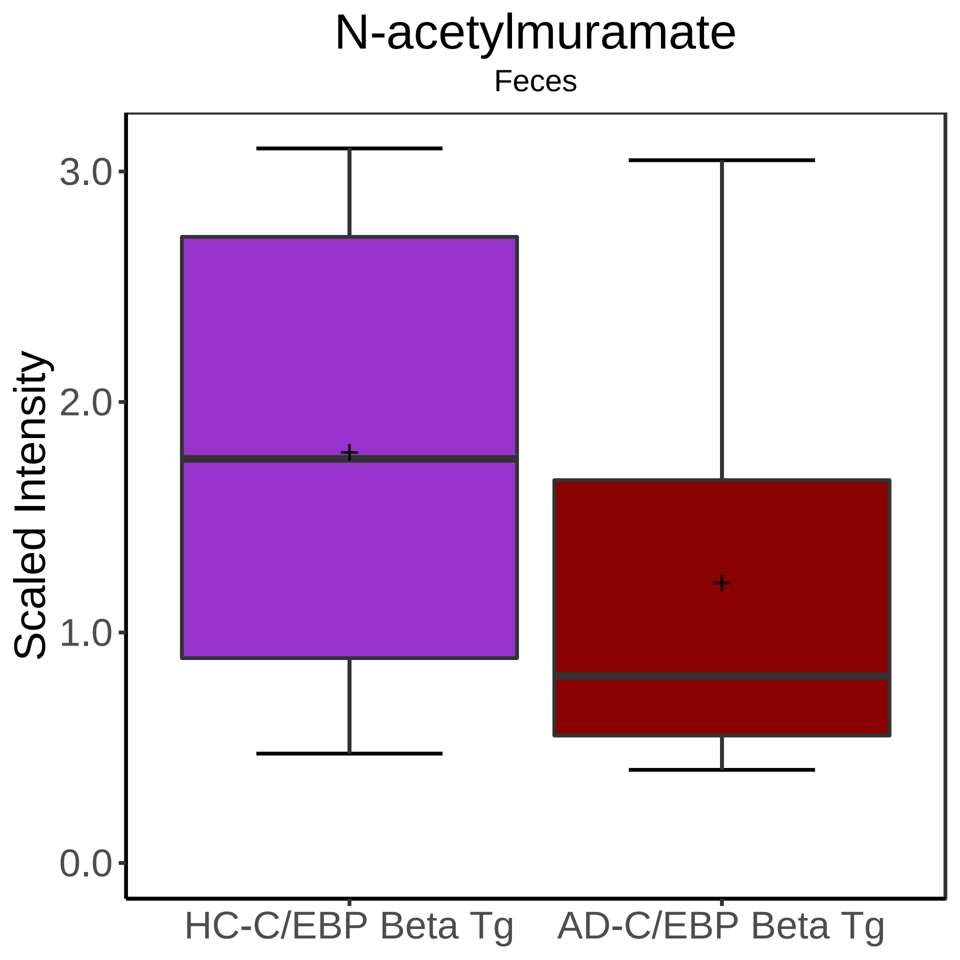

N-acetylmuramate
Feces
Scaled Intensity
3.0
2.0
1.0
0.0
HC-C/EBP Beta Tg
AD-C/EBP Beta Tg

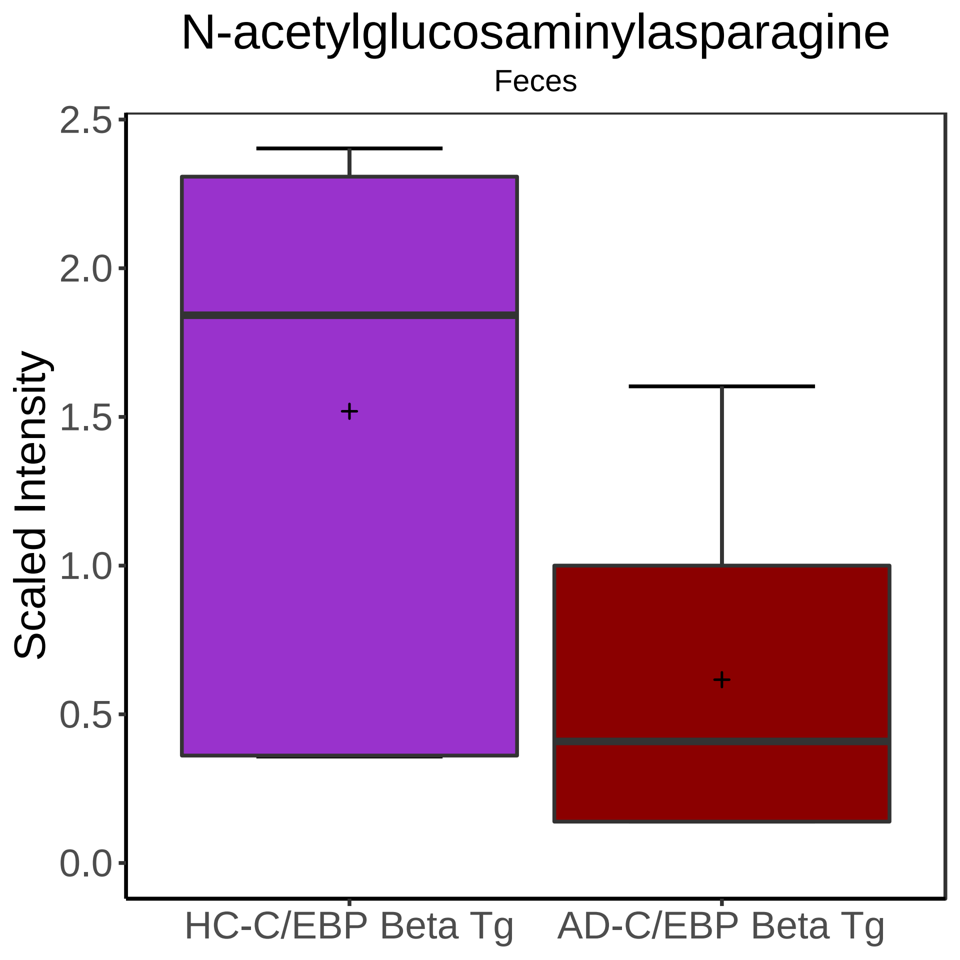
N-acetylglucosaminylasparagine
Feces
Scaled Intensity
2.5
2.0
1.5
1.0
0.5
0.0
HC-C/EBP Beta Tg
AD-C/EBP Beta Tg

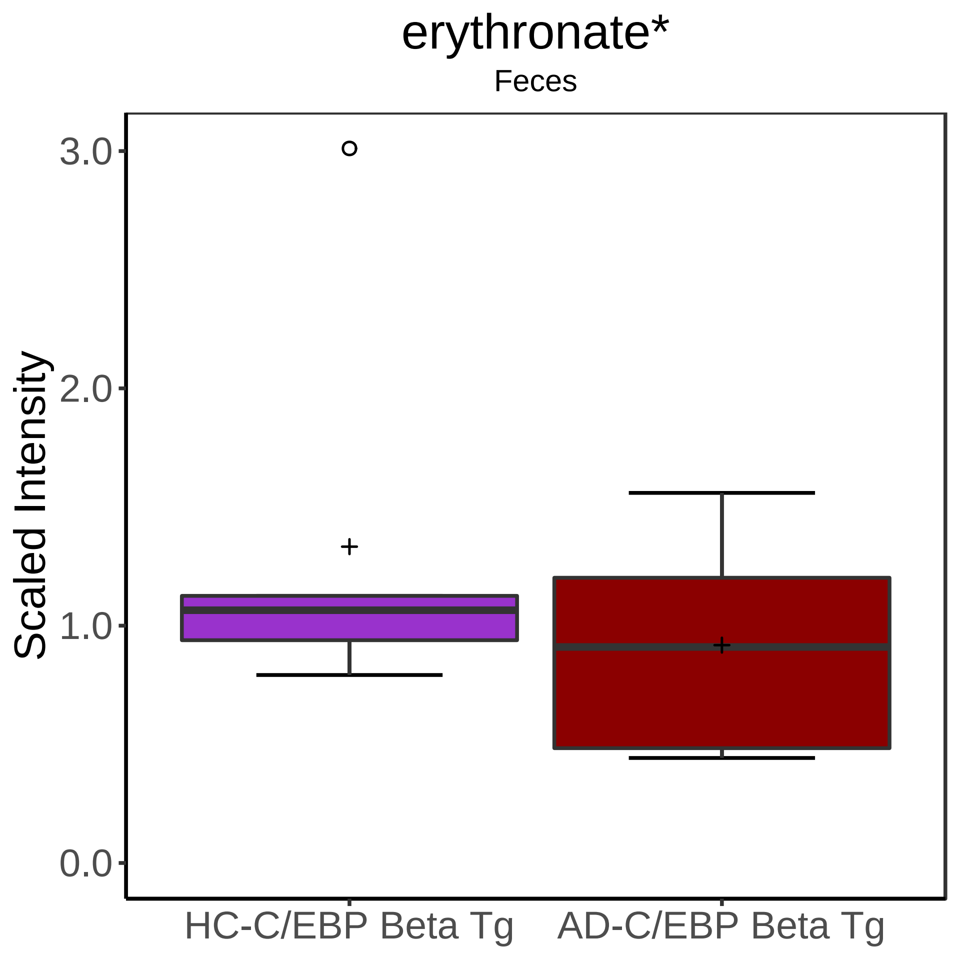

erythronate*
Feces
Scaled Intensity
3.0
2.0
1.0
0.0
HC-C/EBP Beta Tg
AD-C/EBP Beta Tg

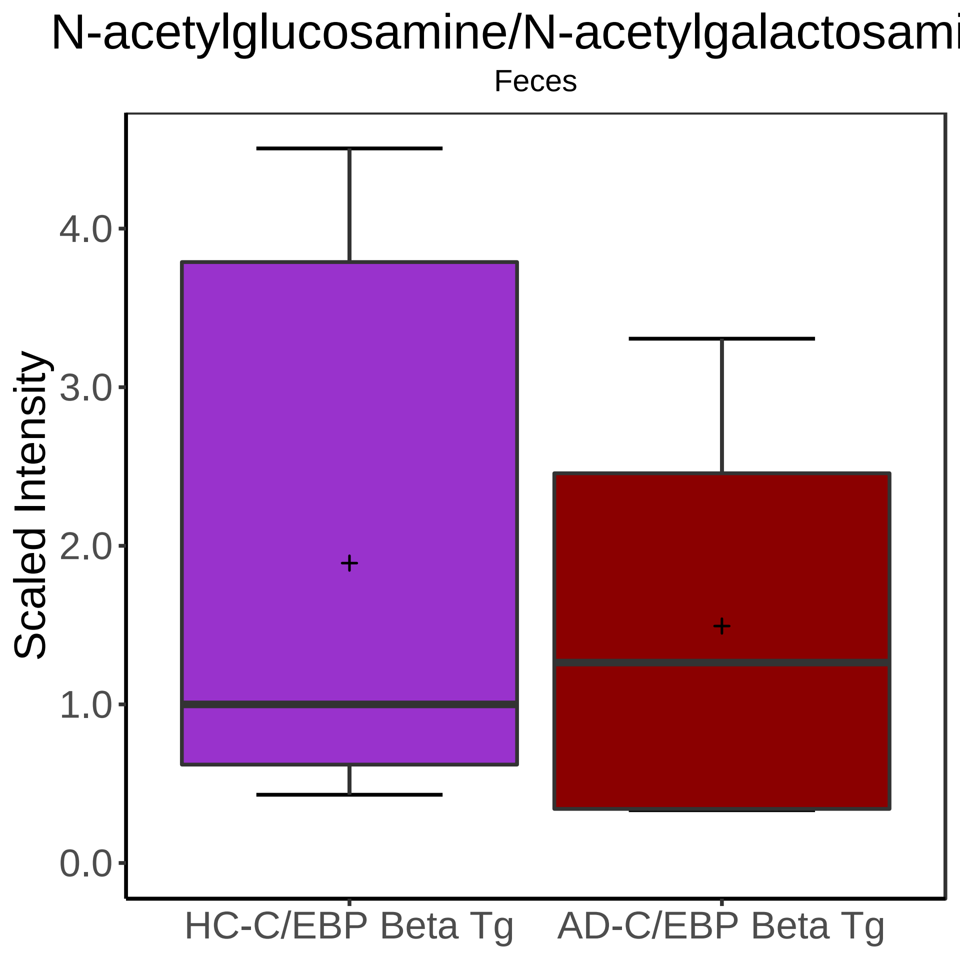
N-acetylglucosamine/N-acetylgalactosami
Feces
Scaled Intensity
0.0
1.0
2.0
3.0
4.0
HC-C/EBP Beta Tg
AD-C/EBP Beta Tg

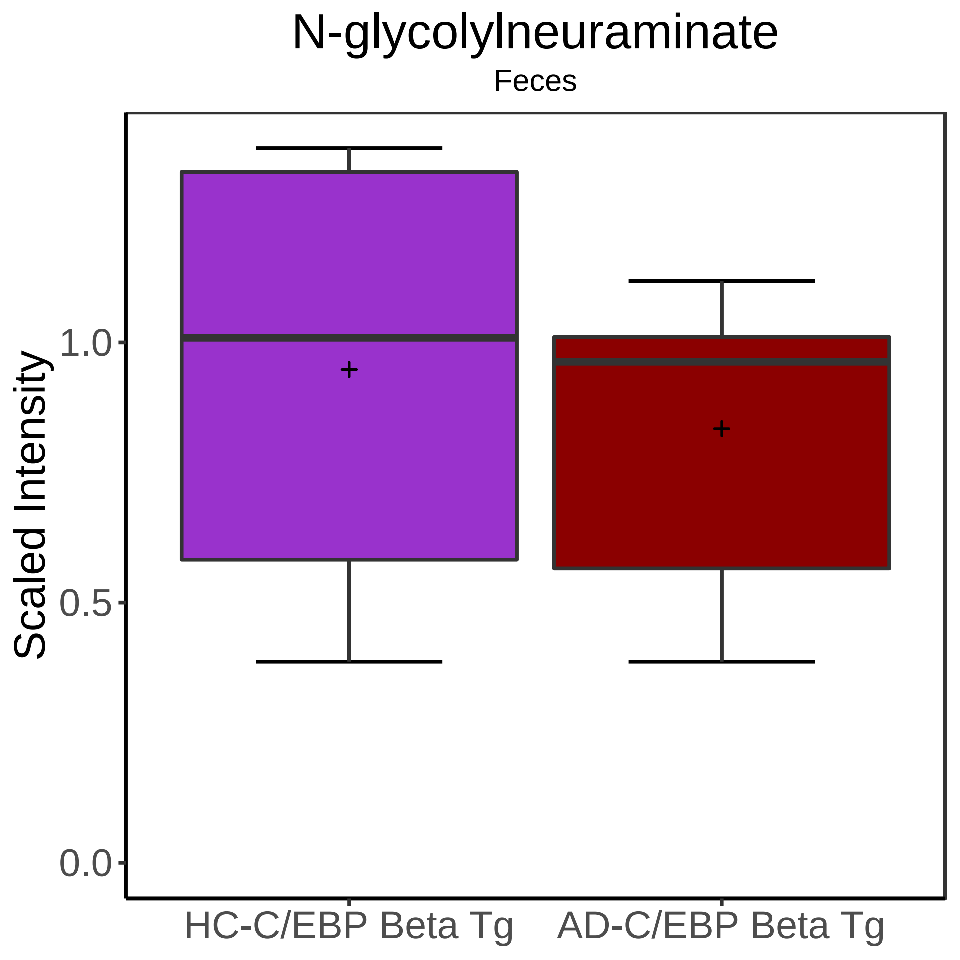
N-glycolylneuraminate
Feces
Scaled Intensity
1.0
0.5
0.0
HC-C/EBP Beta Tg
AD-C/EBP Beta Tg

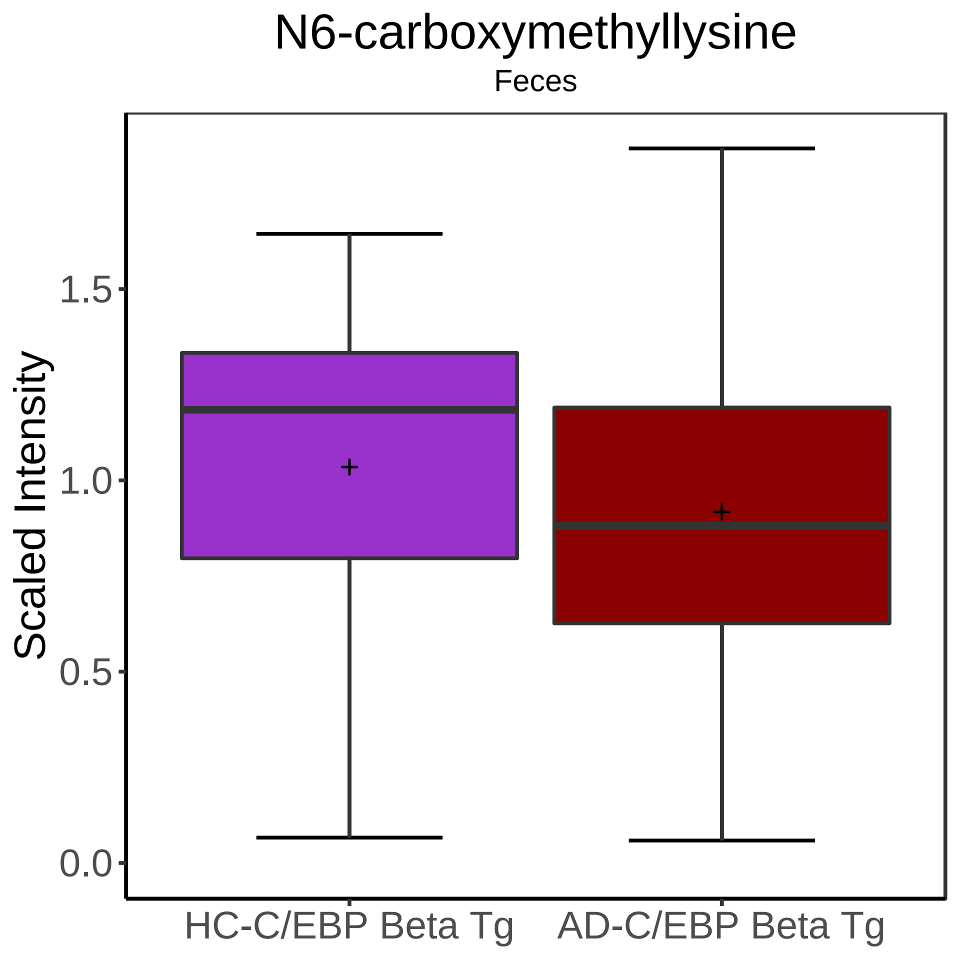

N6-carboxymethyllysine
Feces
Scaled Intensity
1.5
1.0
0.5
0.0
HC-C/EBP Beta Tg
AD-C/EBP Beta Tg

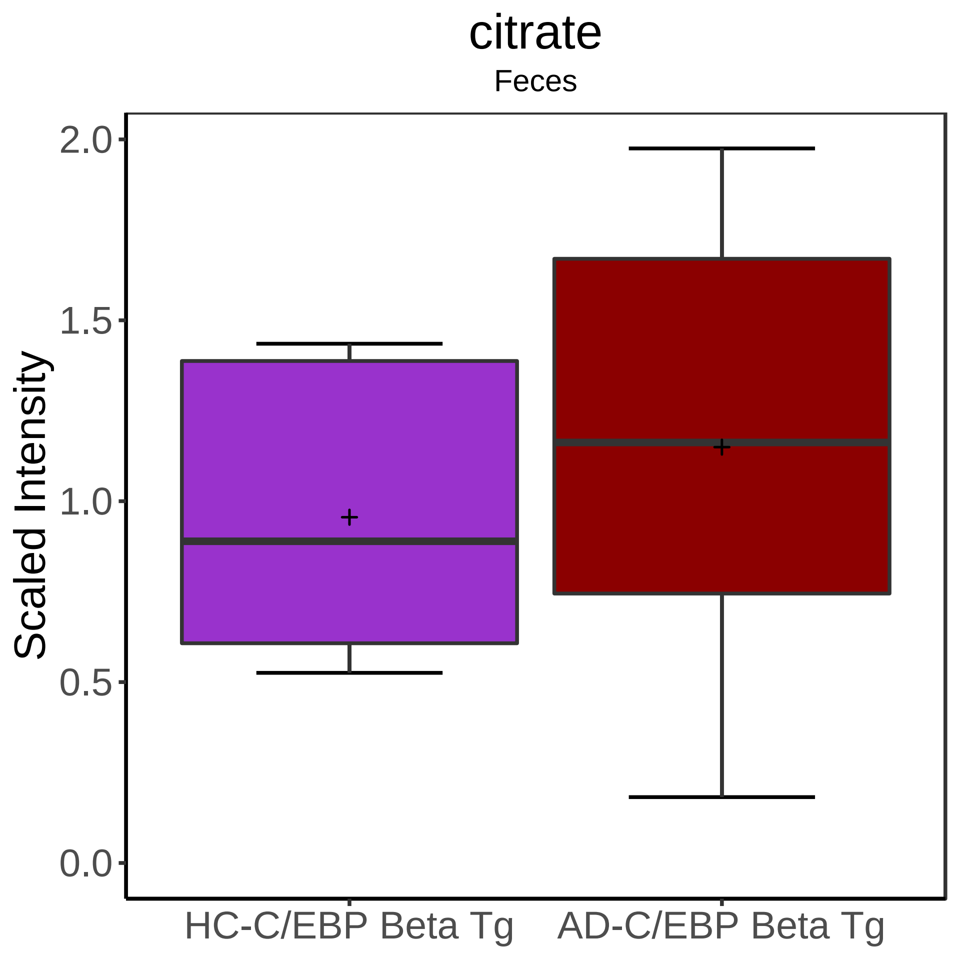

citrate
Feces
Scaled Intensity
2.0
1.5
1.0
0.5
0.0
HC-C/EBP Beta Tg
AD-C/EBP Beta Tg

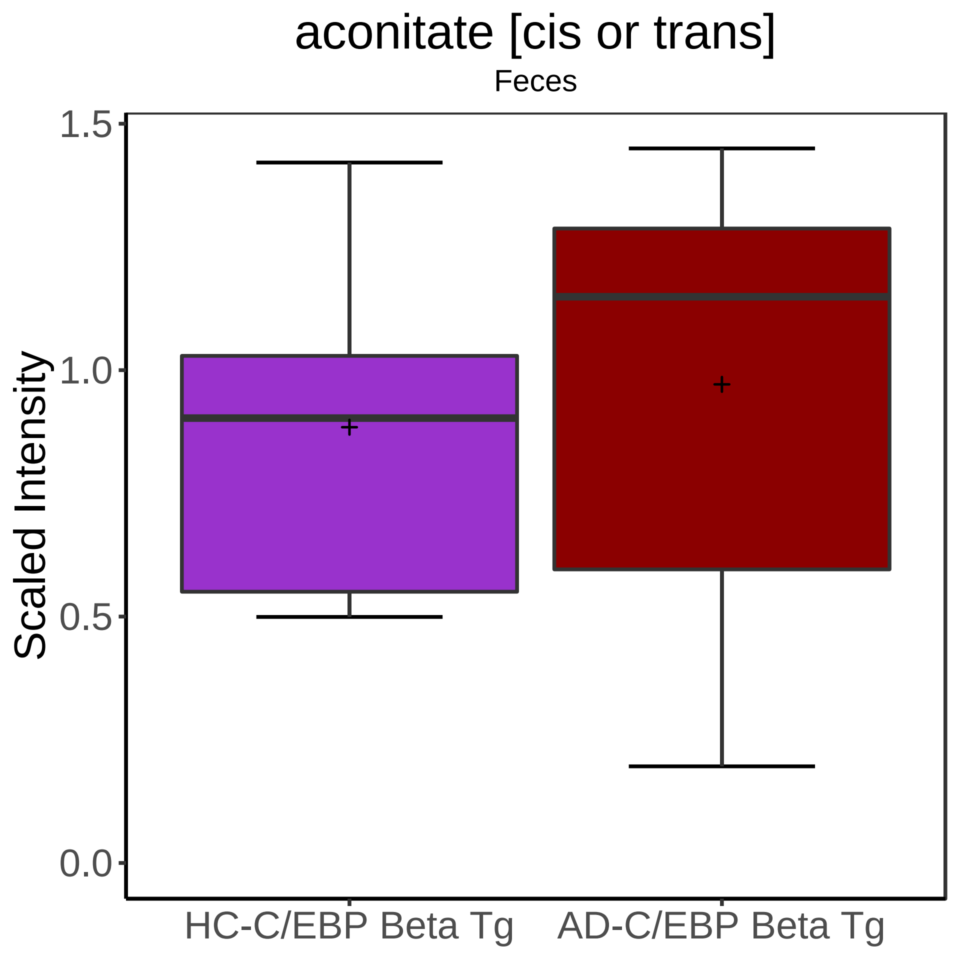
aconitate [cis or trans]
Feces
Scaled Intensity
1.5
1.0
0.5
0.0
HC-C/EBP Beta Tg
AD-C/EBP Beta Tg

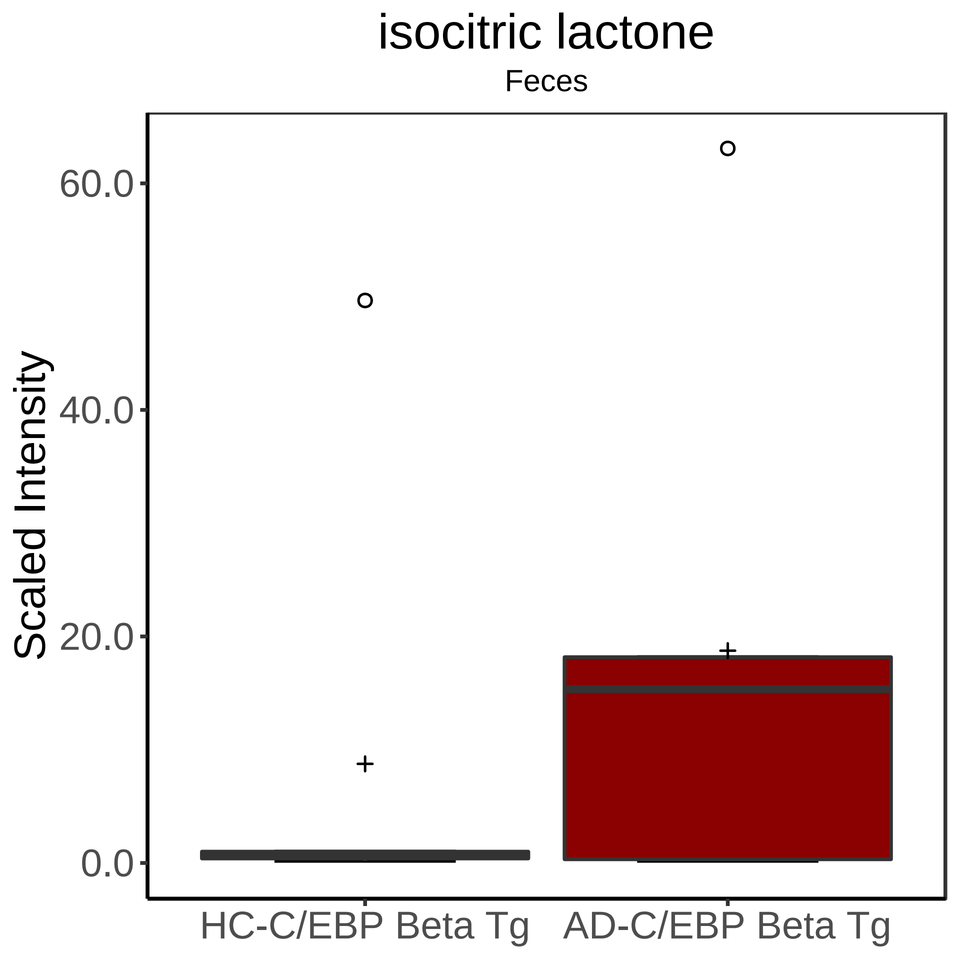

isocitric lactone
Feces
Scaled Intensity
60.0
40.0
20.0
0.0
HC-C/EBP Beta Tg
AD-C/EBP Beta Tg

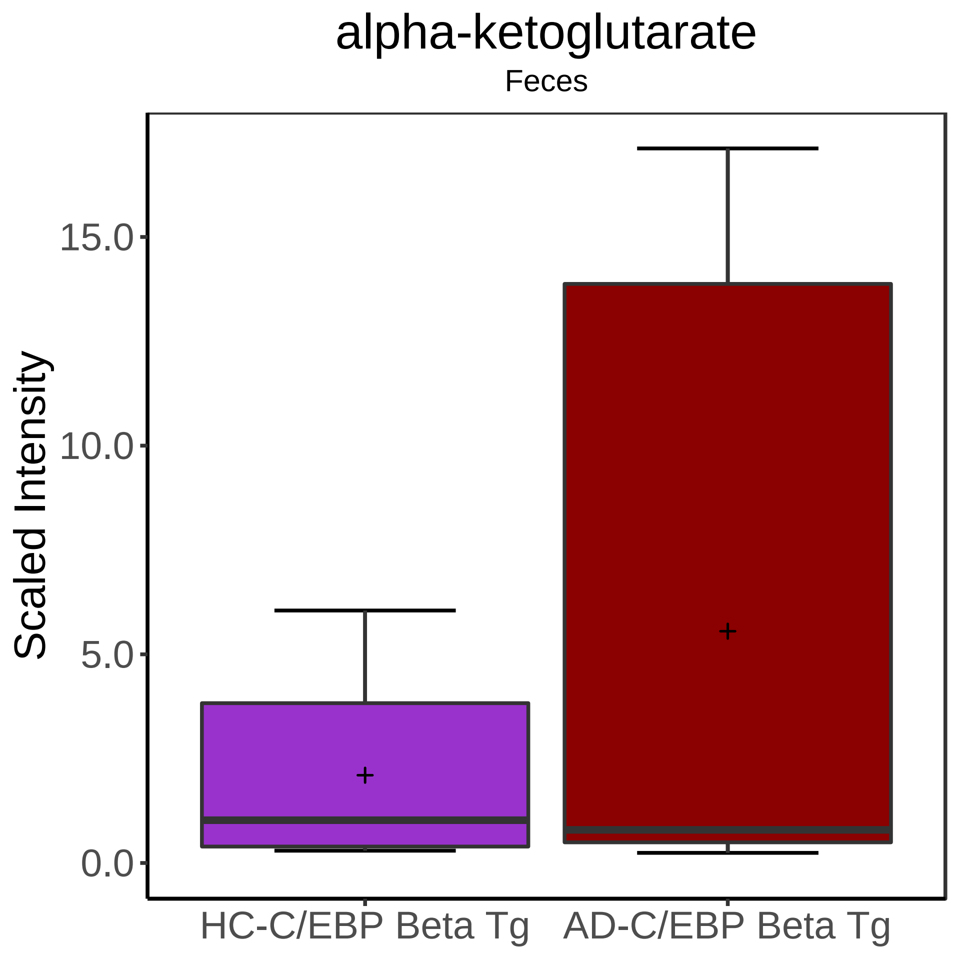
alpha-ketoglutarate
Feces
Scaled Intensity
15.0
10.0
5.0
0.0
HC-C/EBP Beta Tg
AD-C/EBP Beta Tg

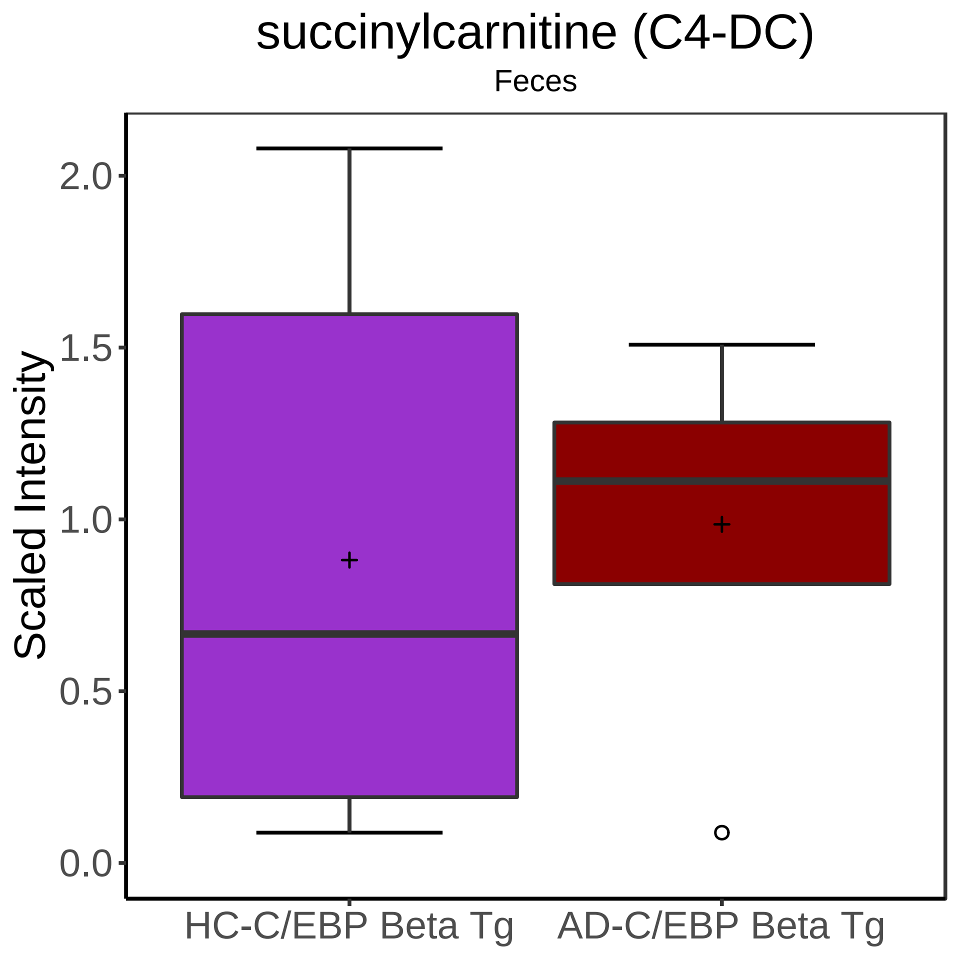

succinylcarnitine (C4-DC)
Feces
Scaled Intensity
2.0
1.5
1.0
0.5
0.0
HC-C/EBP Beta Tg
AD-C/EBP Beta Tg

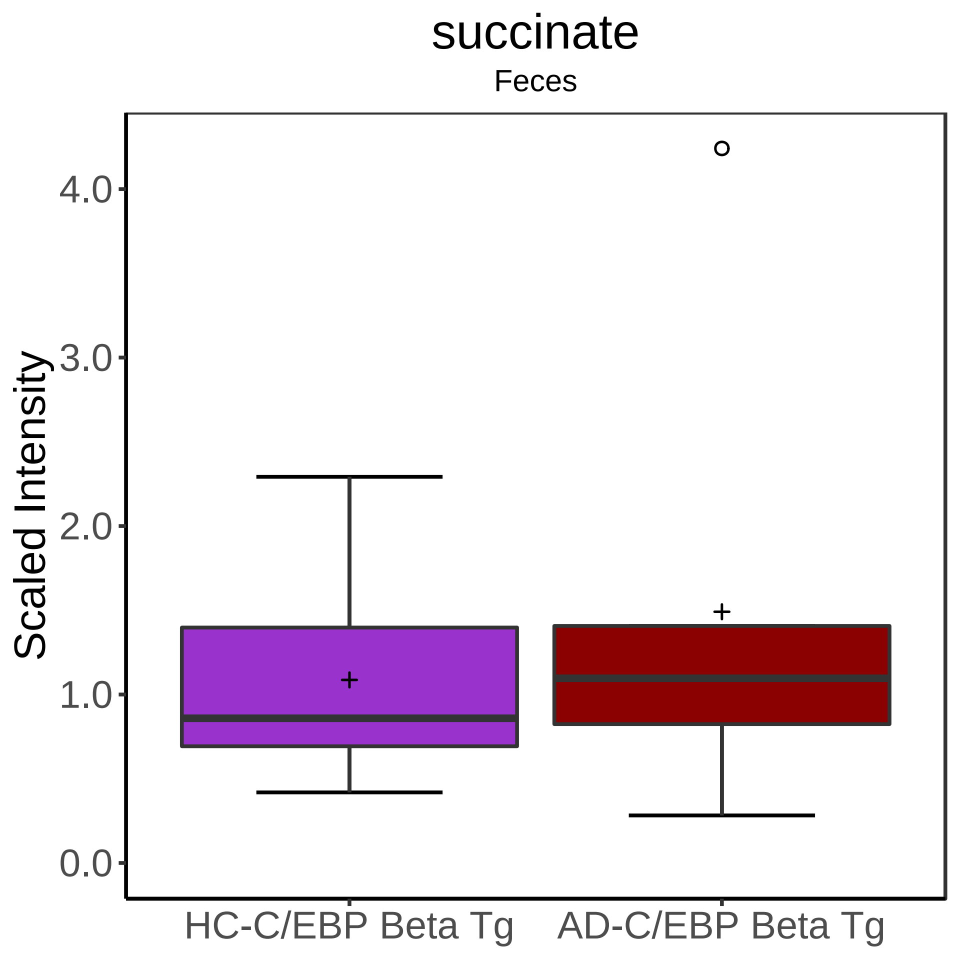
succinate
Feces
Scaled Intensity
4.0
3.0
2.0
1.0
0.0
HC-C/EBP Beta Tg
AD-C/EBP Beta Tg

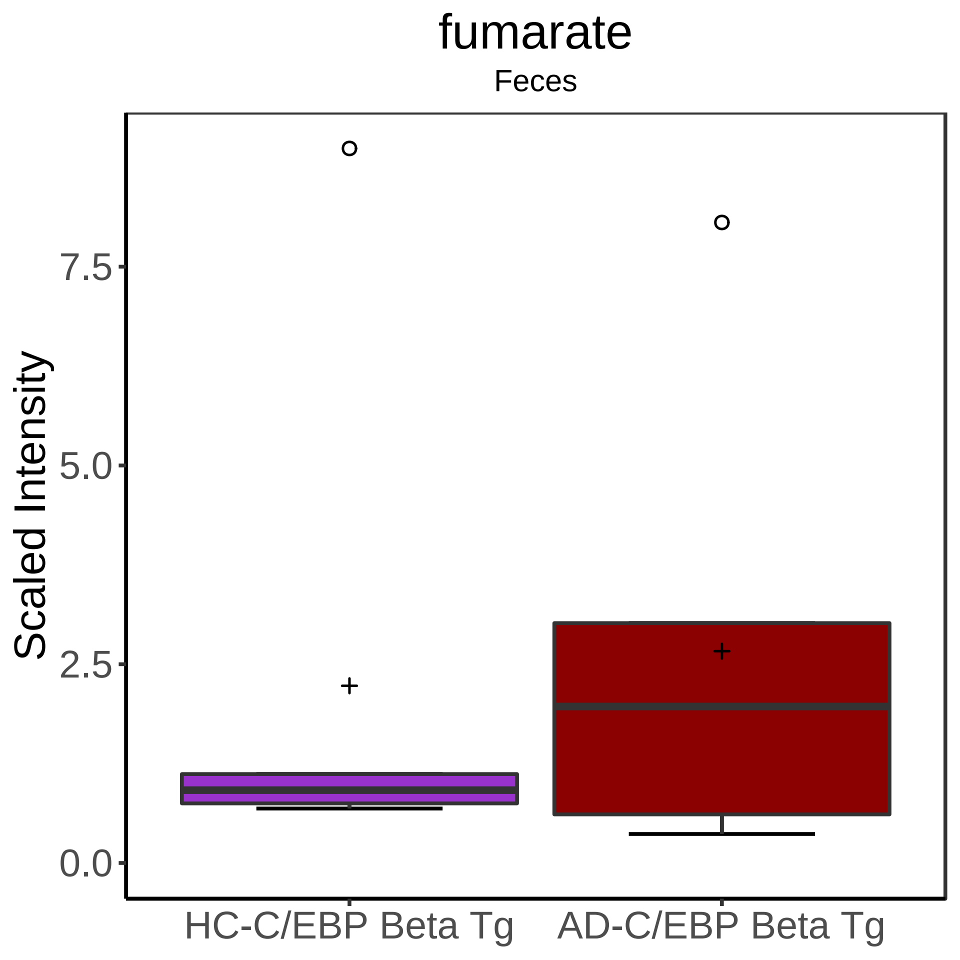
fumarate
Feces
Scaled Intensity
7.5
5.0
2.5
0.0
HC-C/EBP Beta Tg
AD-C/EBP Beta Tg

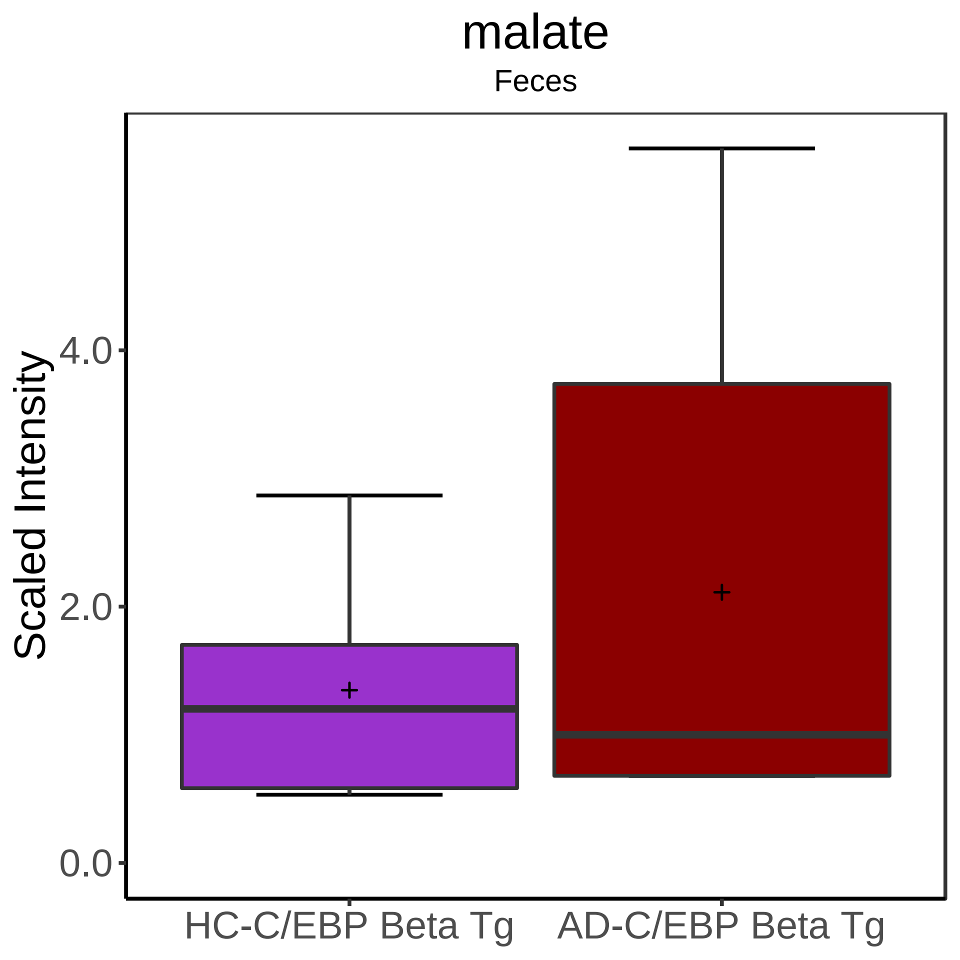
malate
Feces
Scaled Intensity
4.0
2.0
0.0
HC-C/EBP Beta Tg
AD-C/EBP Beta Tg

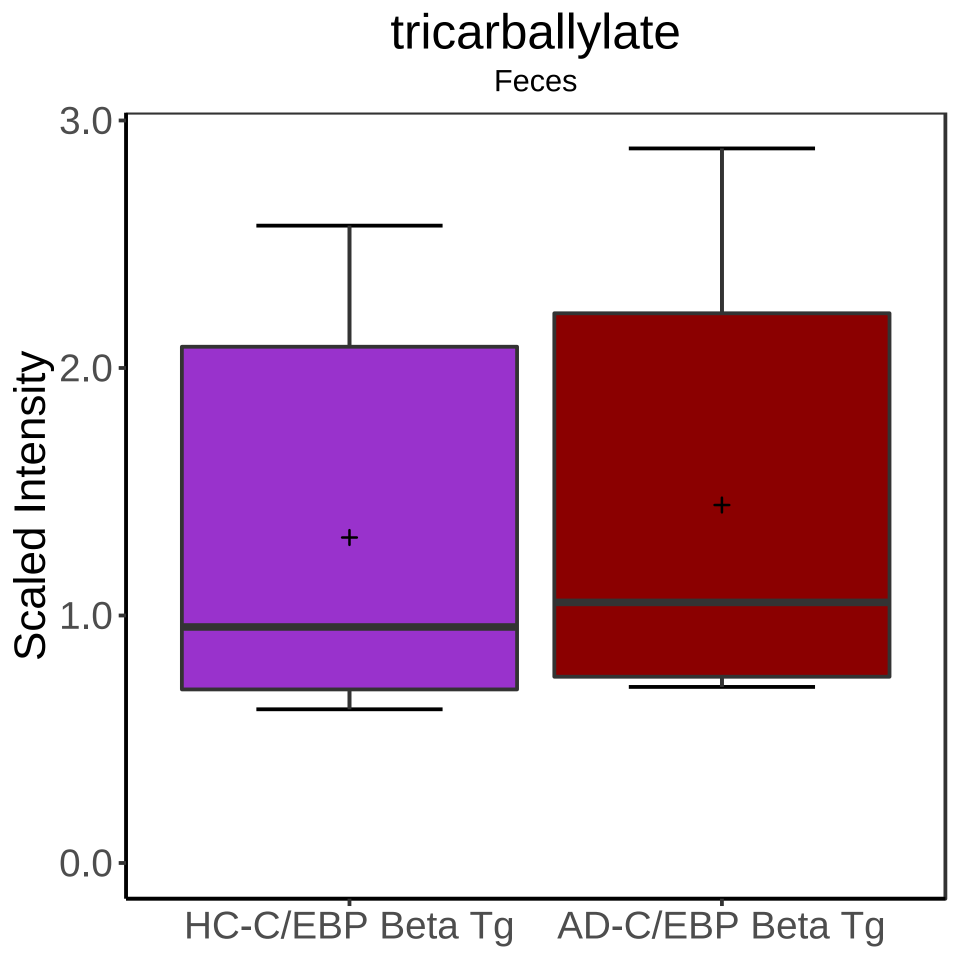
tricarballylate
Feces
Scaled Intensity
3.0
2.0
1.0
0.0
HC-C/EBP Beta Tg
AD-C/EBP Beta Tg

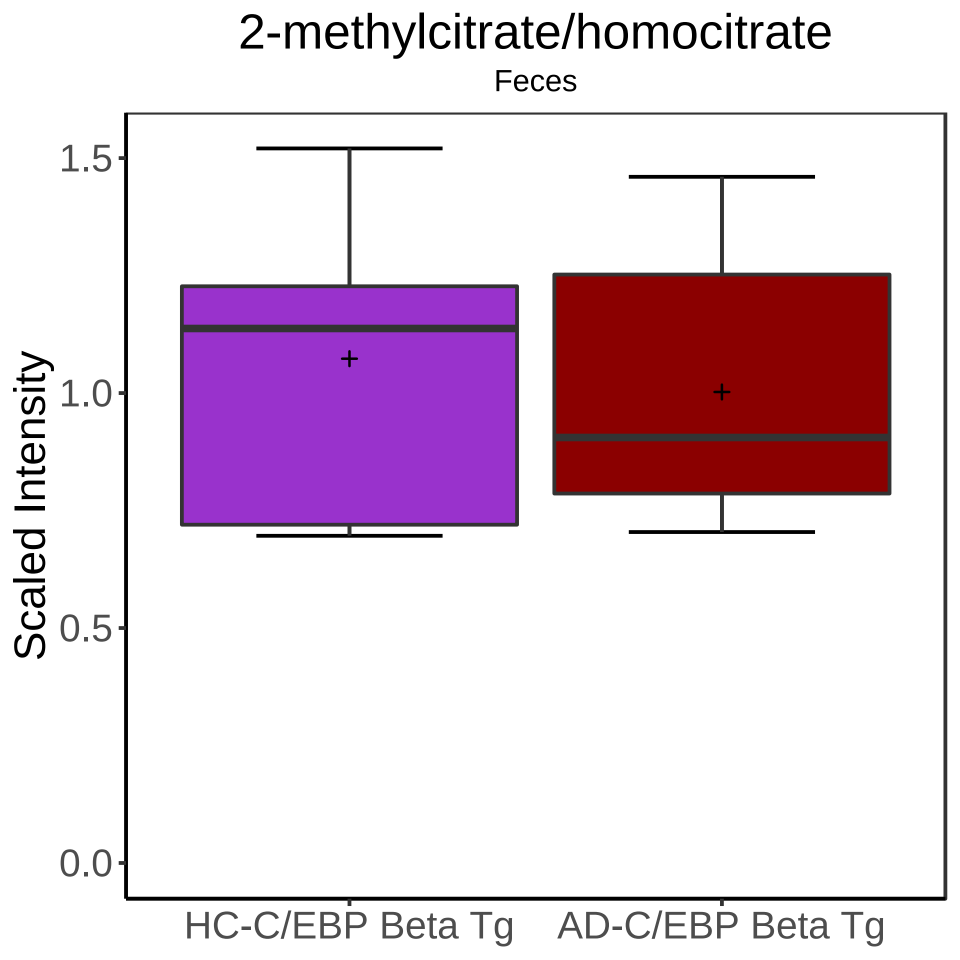
2-methylcitrate/homocitrate
Feces
Scaled Intensity
1.5
1.0
0.5
0.0
HC-C/EBP Beta Tg
AD-C/EBP Beta Tg

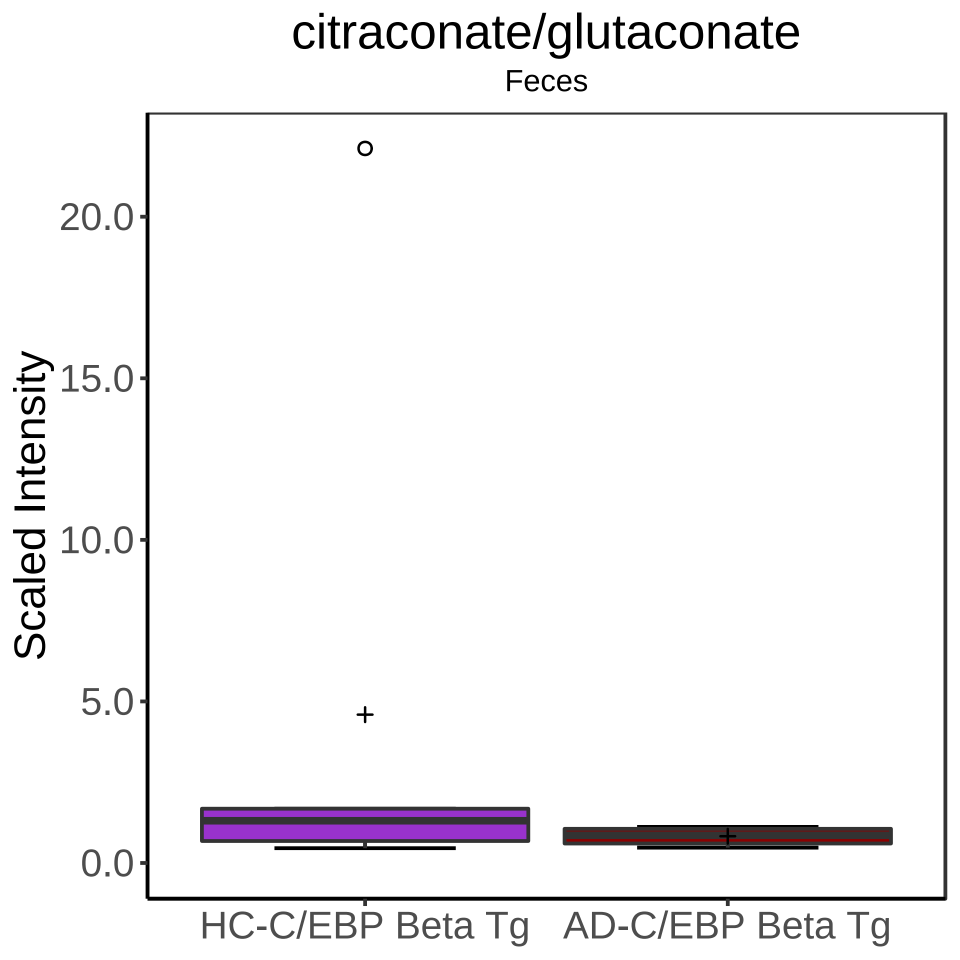
citraconate/glutaconate
Feces
Scaled Intensity
20.0
15.0
10.0
5.0
0.0
HC-C/EBP Beta Tg
AD-C/EBP Beta Tg

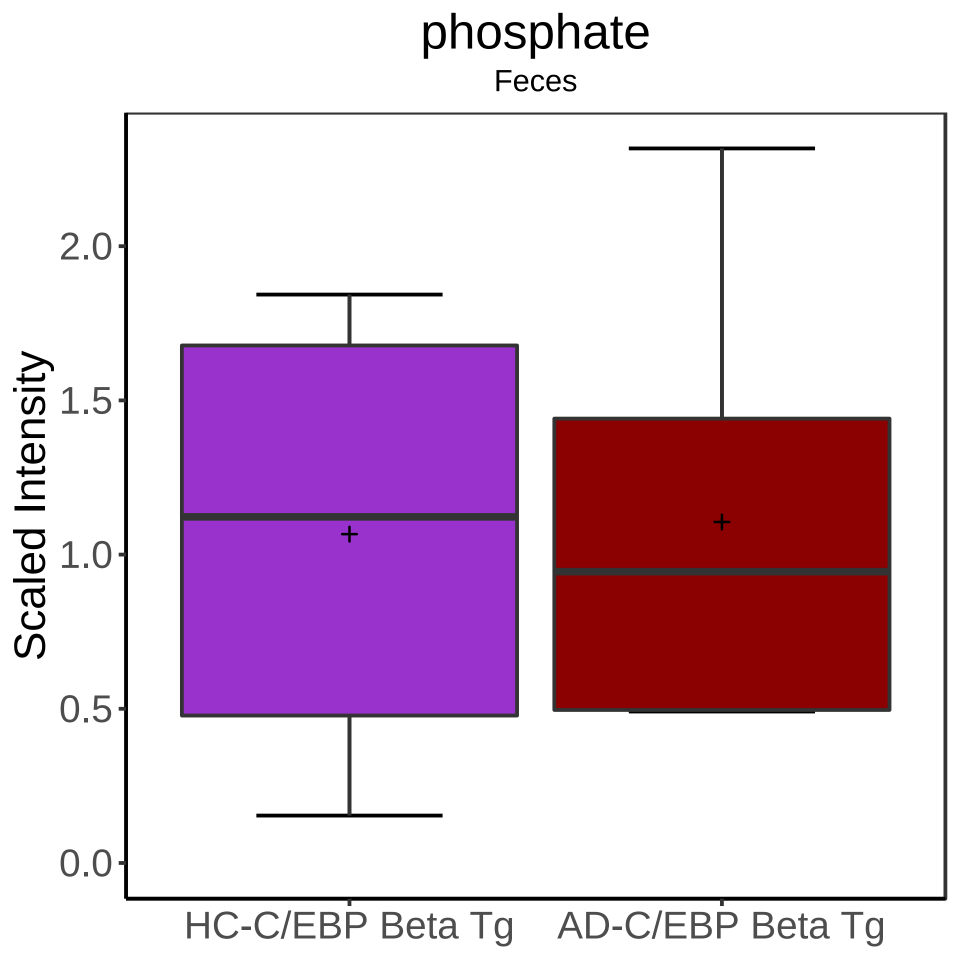
phosphate
Feces
Scaled Intensity
0.0
0.5
1.0
1.5
2.0
HC-C/EBP Beta Tg
AD-C/EBP Beta Tg

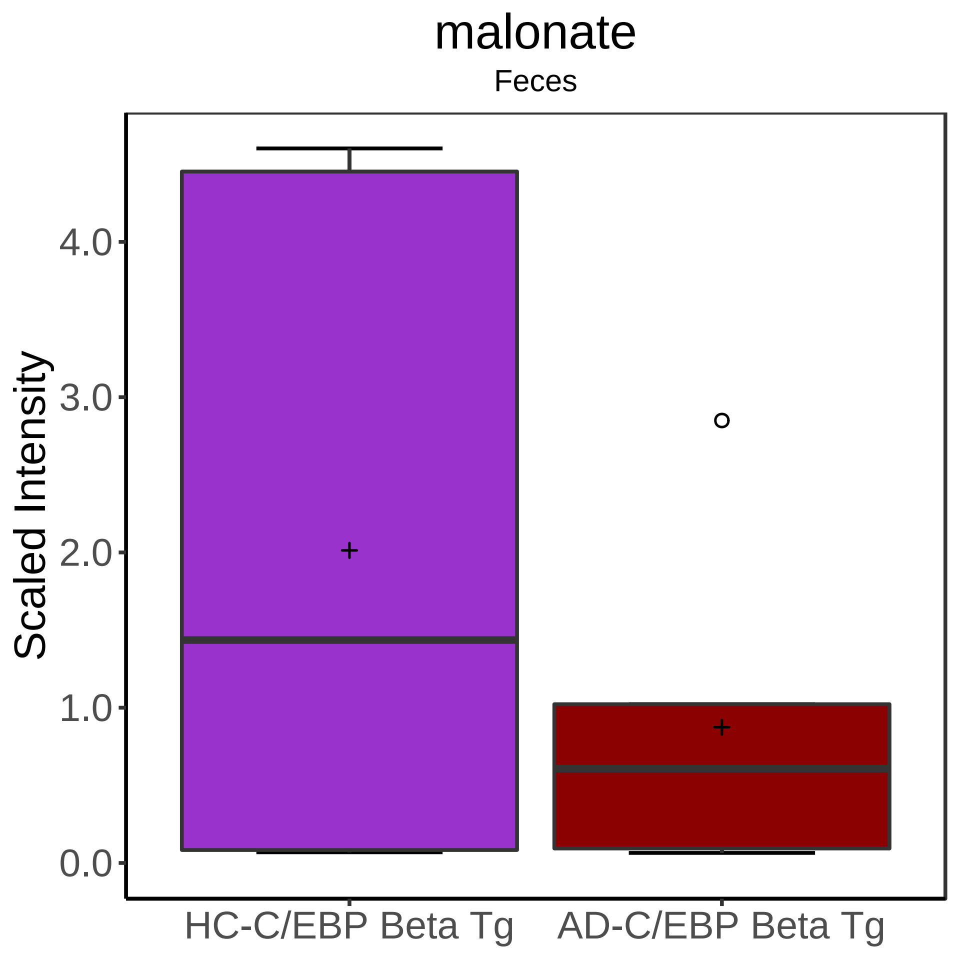
malonate
Feces
Scaled Intensity
0.0
1.0
2.0
3.0
4.0
HC-C/EBP Beta Tg
AD-C/EBP Beta Tg

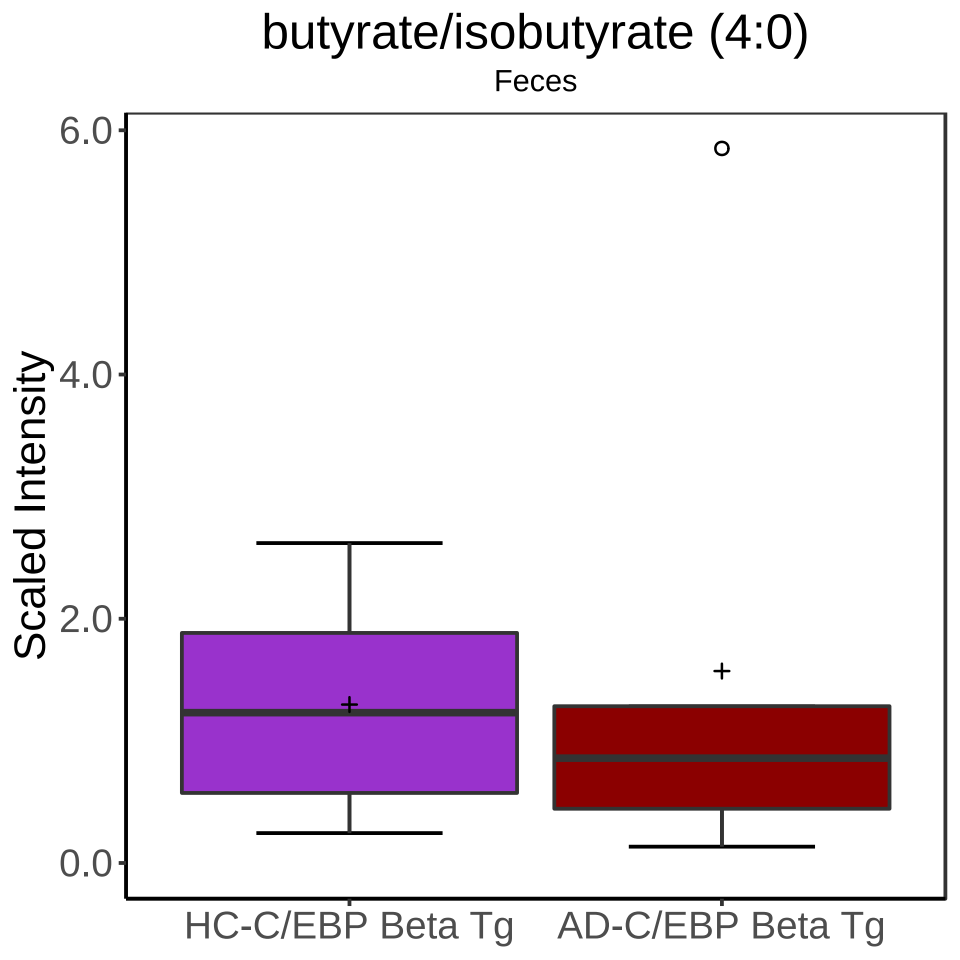
butyrate/isobutyrate (4:0)
Feces
Scaled Intensity
6.0
4.0
2.0
0.0
HC-C/EBP Beta Tg
AD-C/EBP Beta Tg

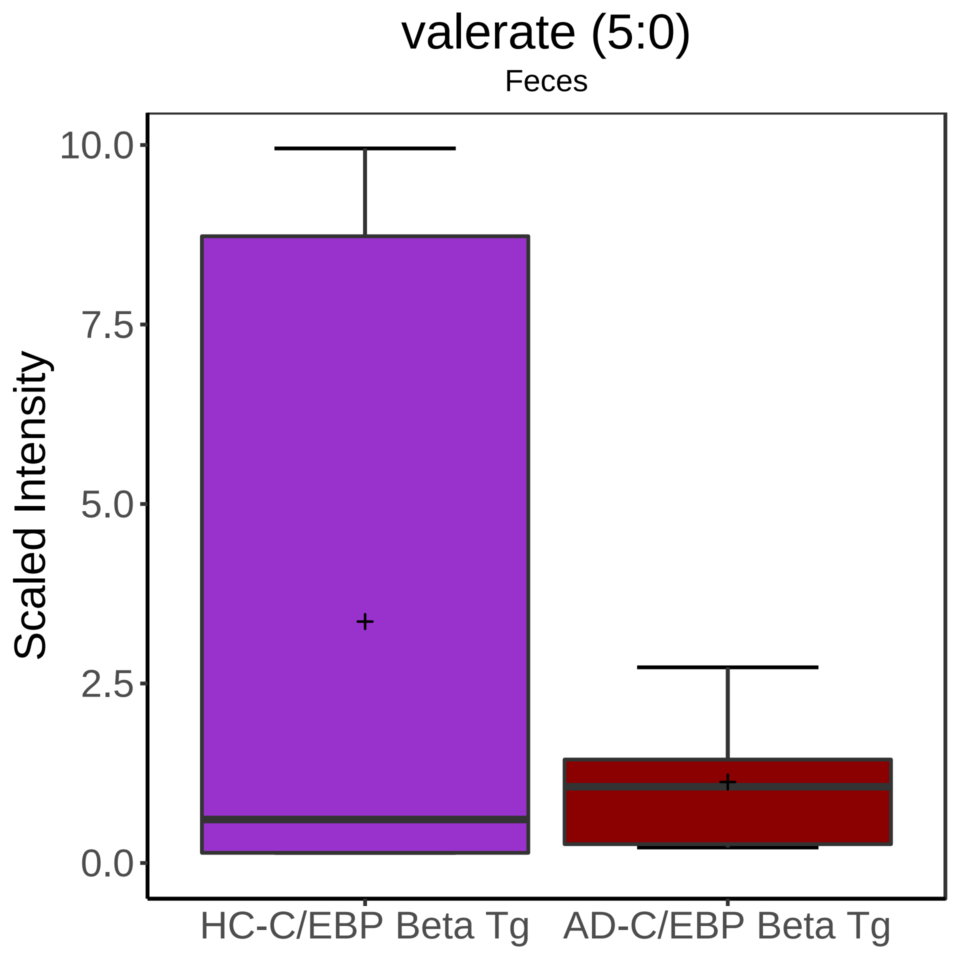
valerate (5:0)
Feces
Scaled Intensity
10.0
7.5
5.0
2.5
0.0
HC-C/EBP Beta Tg
AD-C/EBP Beta Tg

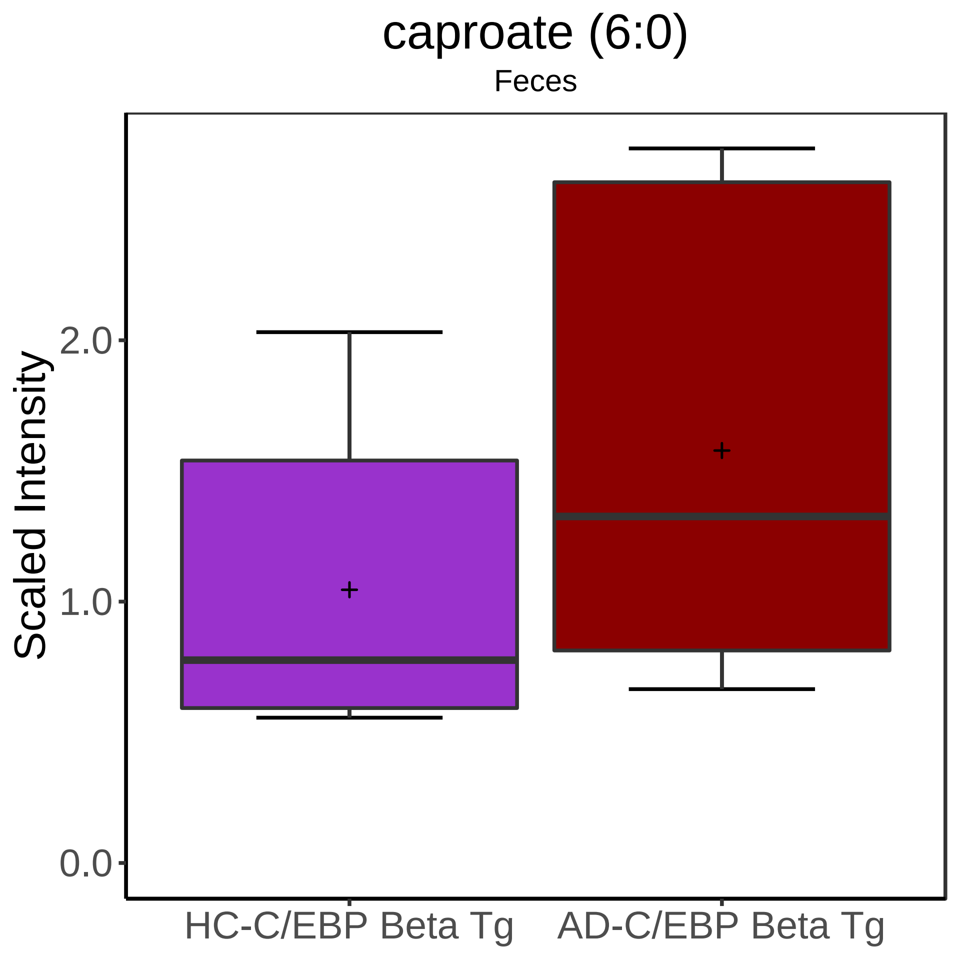
caproate (6:0)
Feces
Scaled Intensity
2.0
1.0
0.0
HC-C/EBP Beta Tg
AD-C/EBP Beta Tg

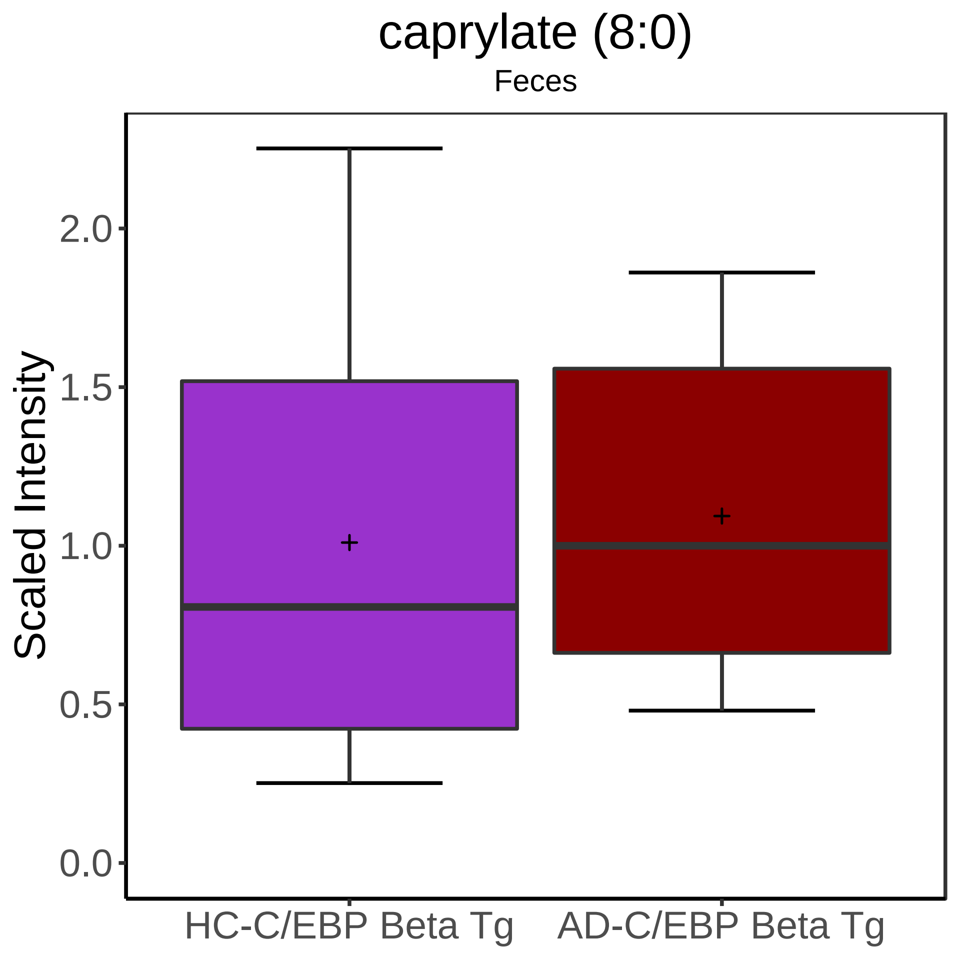
caprylate (8:0)
Feces
Scaled Intensity
2.0
1.5
1.0
0.5
0.0
HC-C/EBP Beta Tg
AD-C/EBP Beta Tg

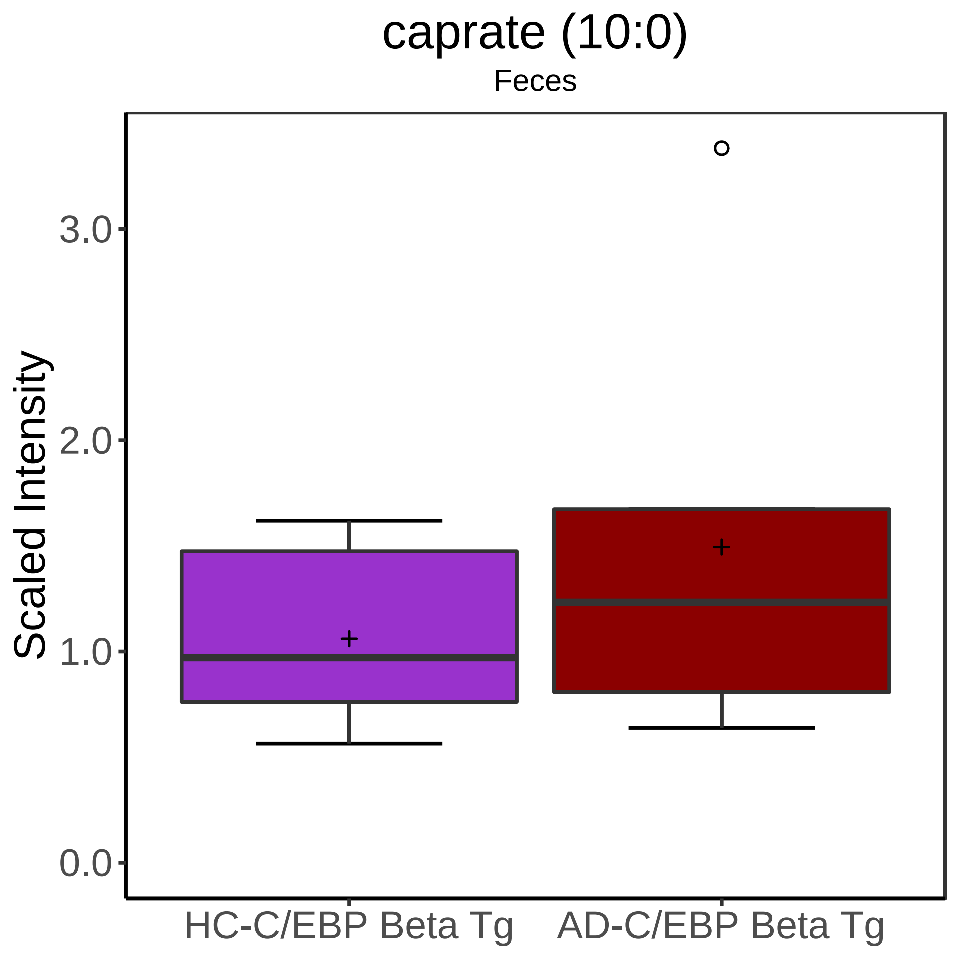
caprate (10:0)
Feces
Scaled Intensity
3.0
2.0
1.0
0.0
HC-C/EBP Beta Tg
AD-C/EBP Beta Tg

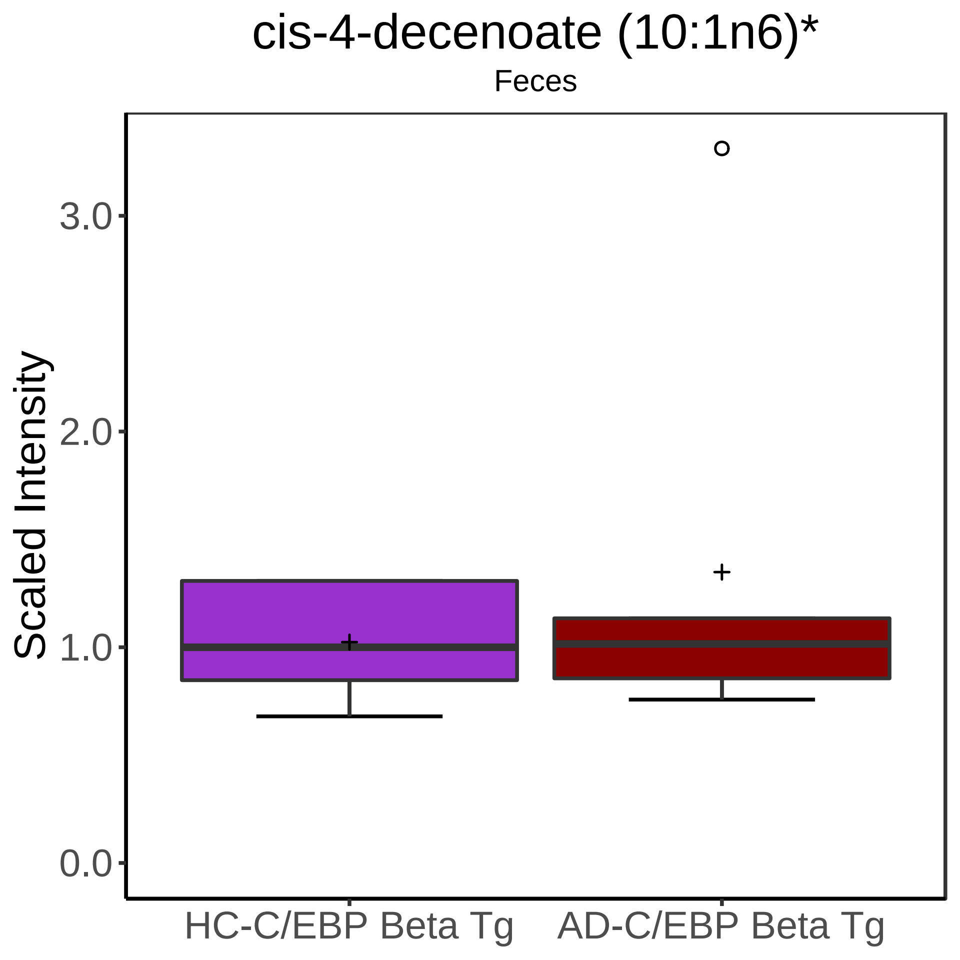

cis-4-decenoate (10:1n6)*
Feces
Scaled Intensity
3.0
2.0
1.0
0.0
HC-C/EBP Beta Tg
AD-C/EBP Beta Tg

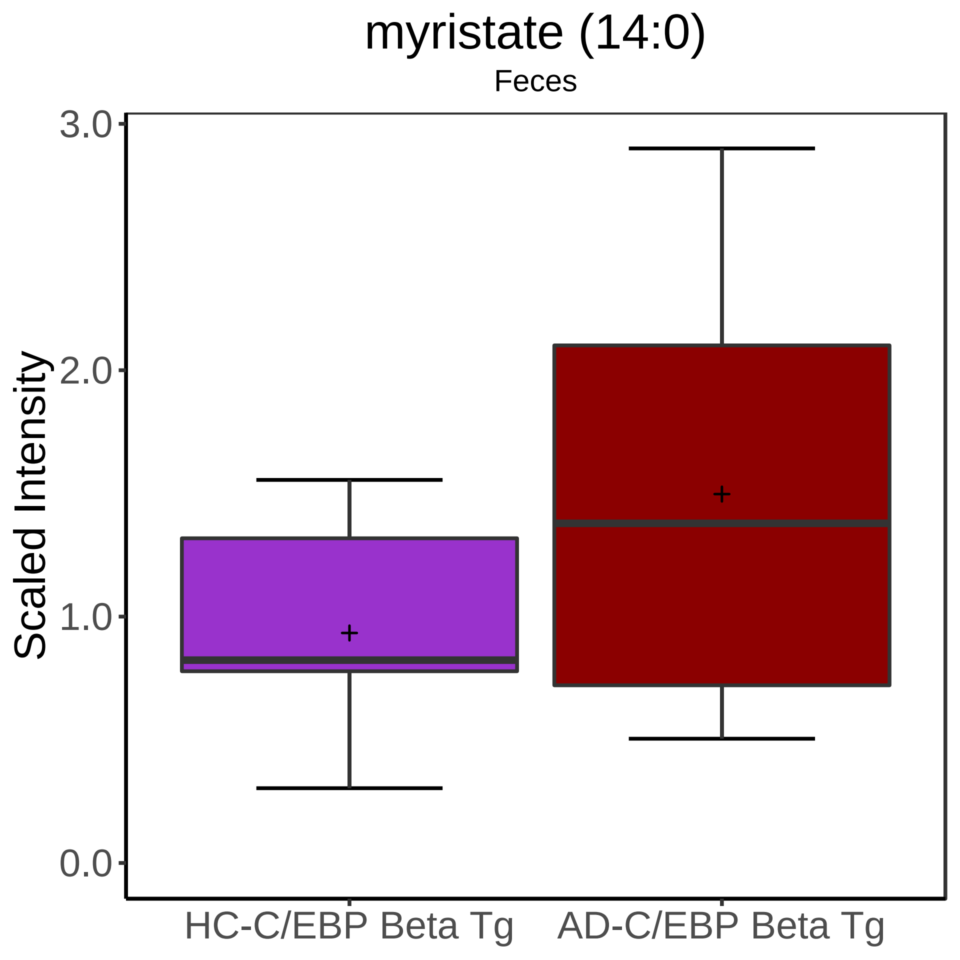
myristate (14:0)
Feces
Scaled Intensity
3.0
2.0
1.0
0.0
HC-C/EBP Beta Tg
AD-C/EBP Beta Tg

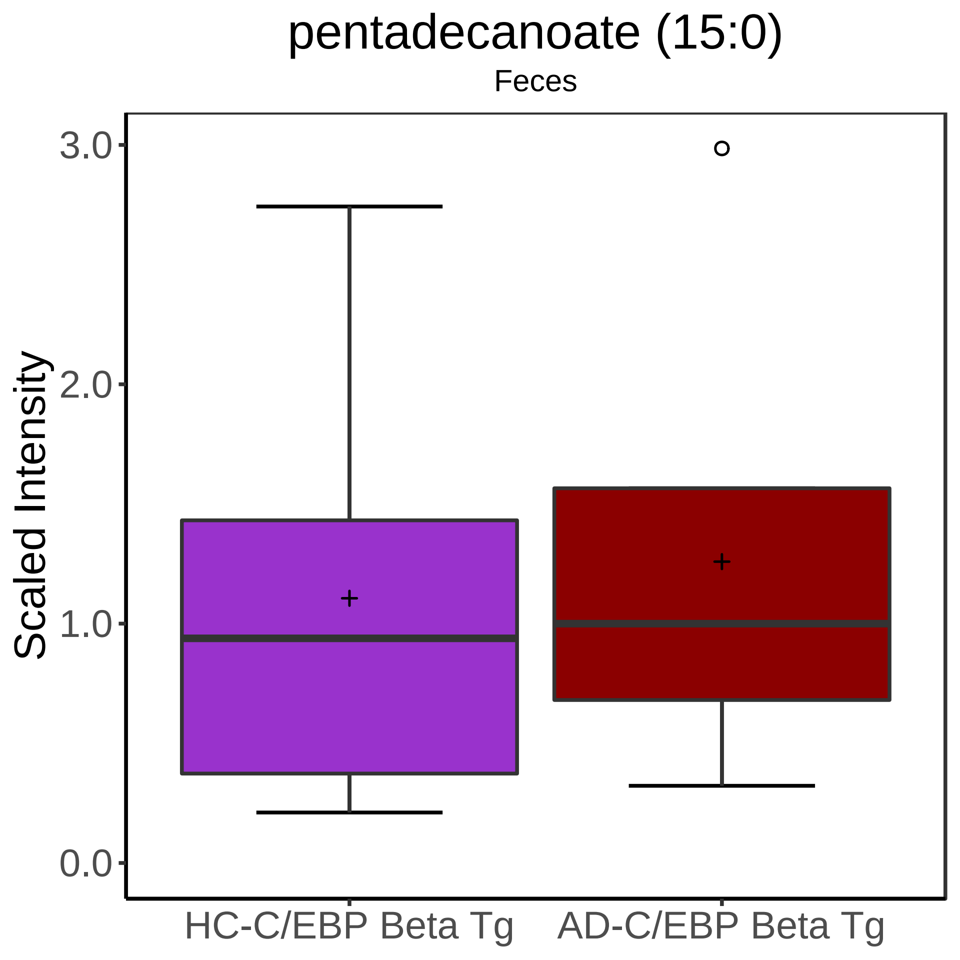
pentadecanoate (15:0)
Feces
Scaled Intensity
3.0
2.0
1.0
0.0
HC-C/EBP Beta Tg
AD-C/EBP Beta Tg

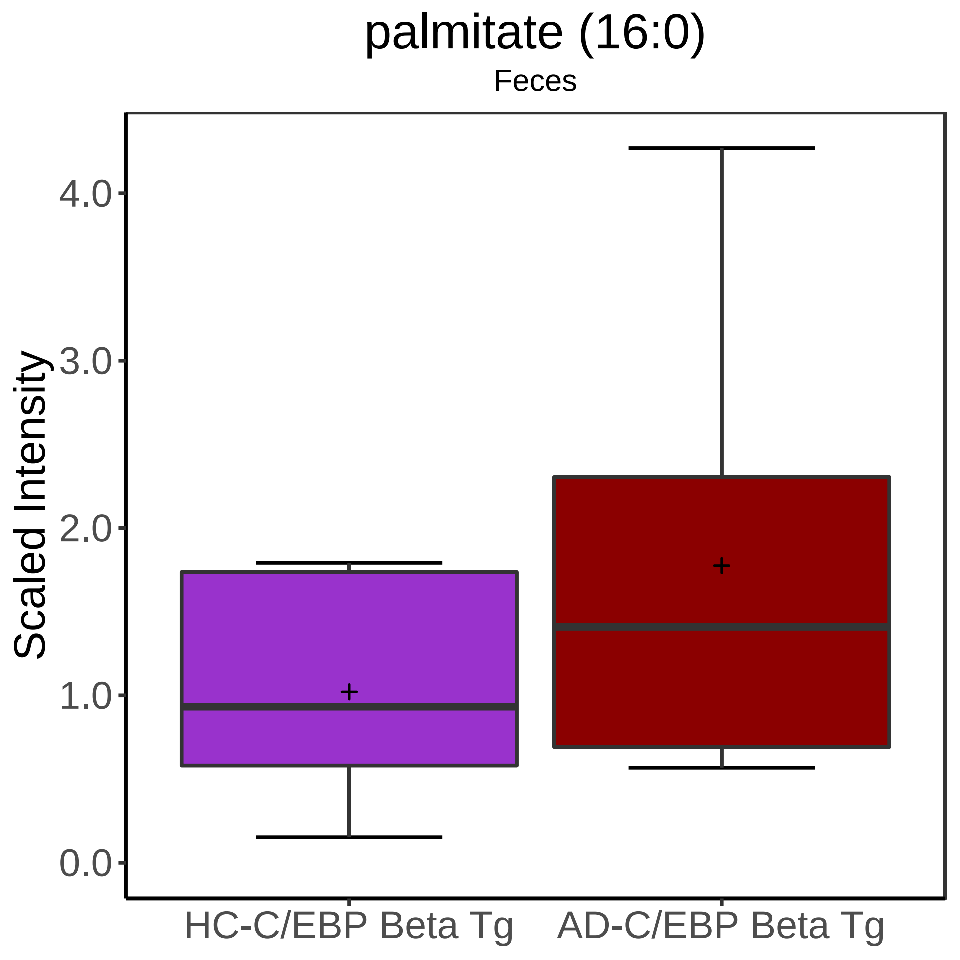

palmitate (16:0)
Feces
Scaled Intensity
0.0
1.0
2.0
3.0
4.0
HC-C/EBP Beta Tg
AD-C/EBP Beta Tg

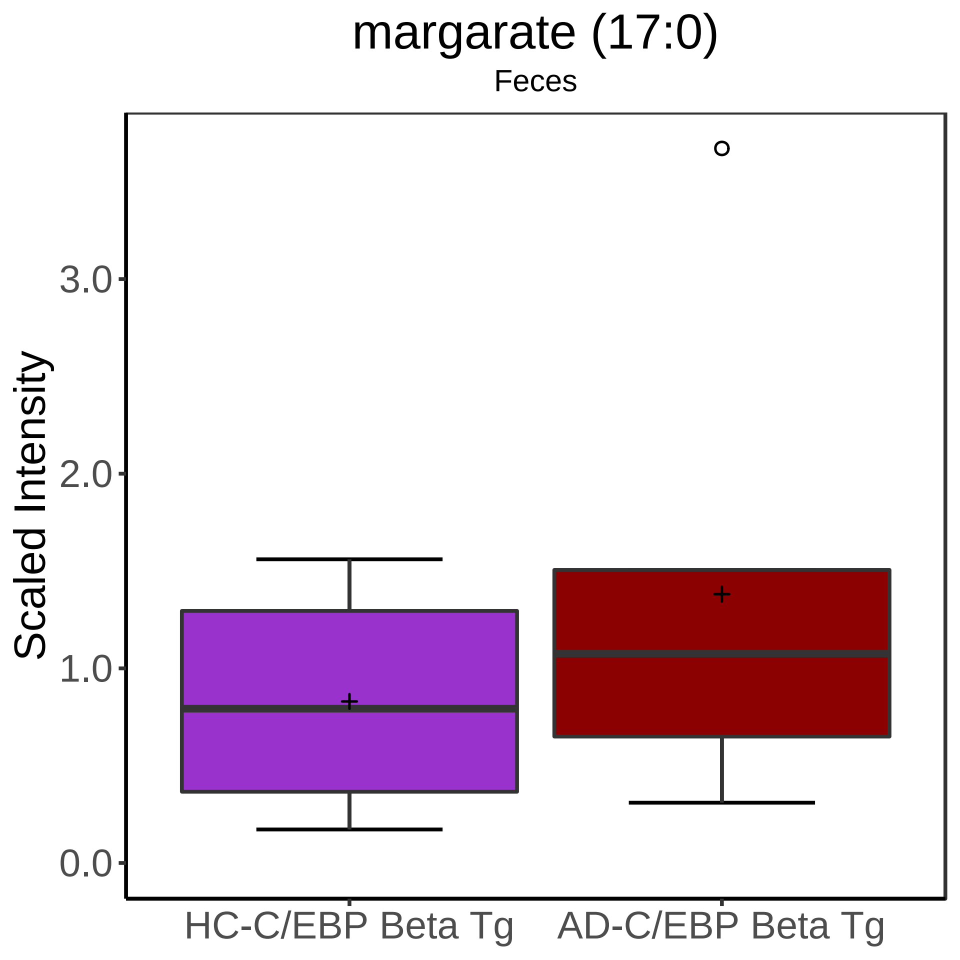

margarate (17:0)
Feces
Scaled Intensity
3.0
2.0
1.0
0.0
HC-C/EBP Beta Tg
AD-C/EBP Beta Tg

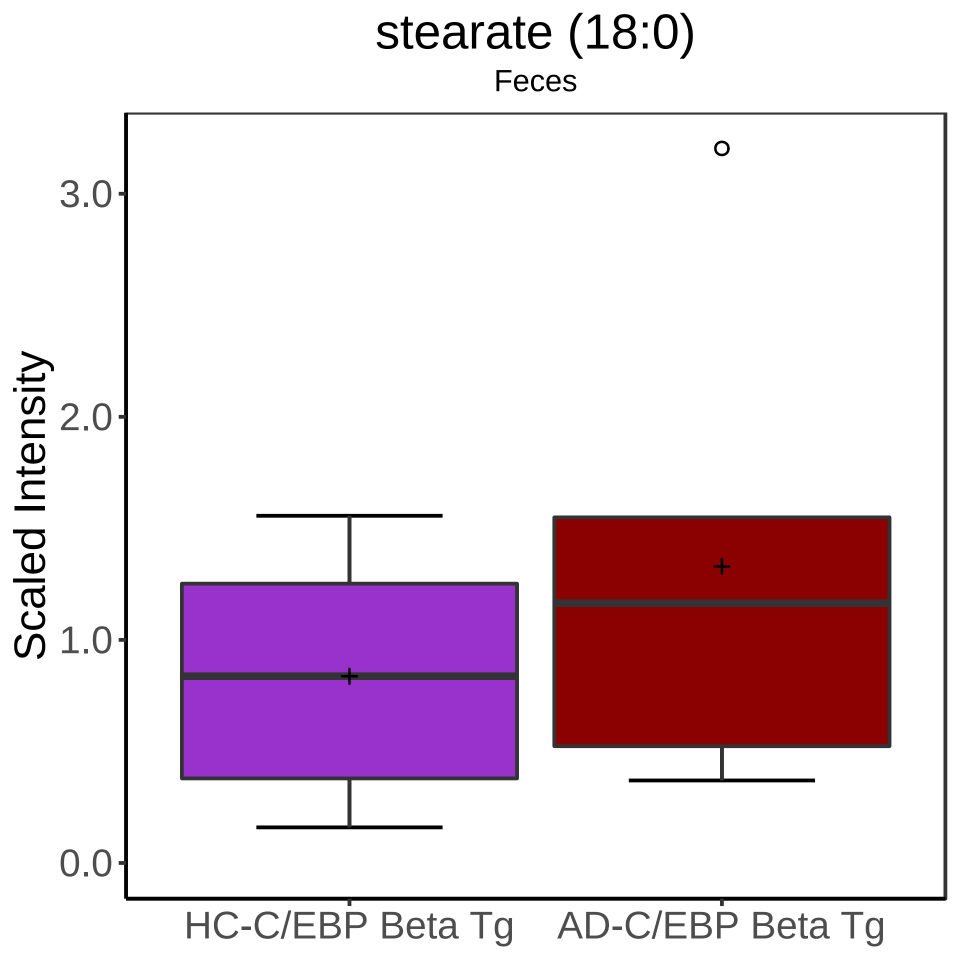
stearate (18:0)
Feces
Scaled Intensity
3.0
2.0
1.0
0.0
HC-C/EBP Beta Tg
AD-C/EBP Beta Tg

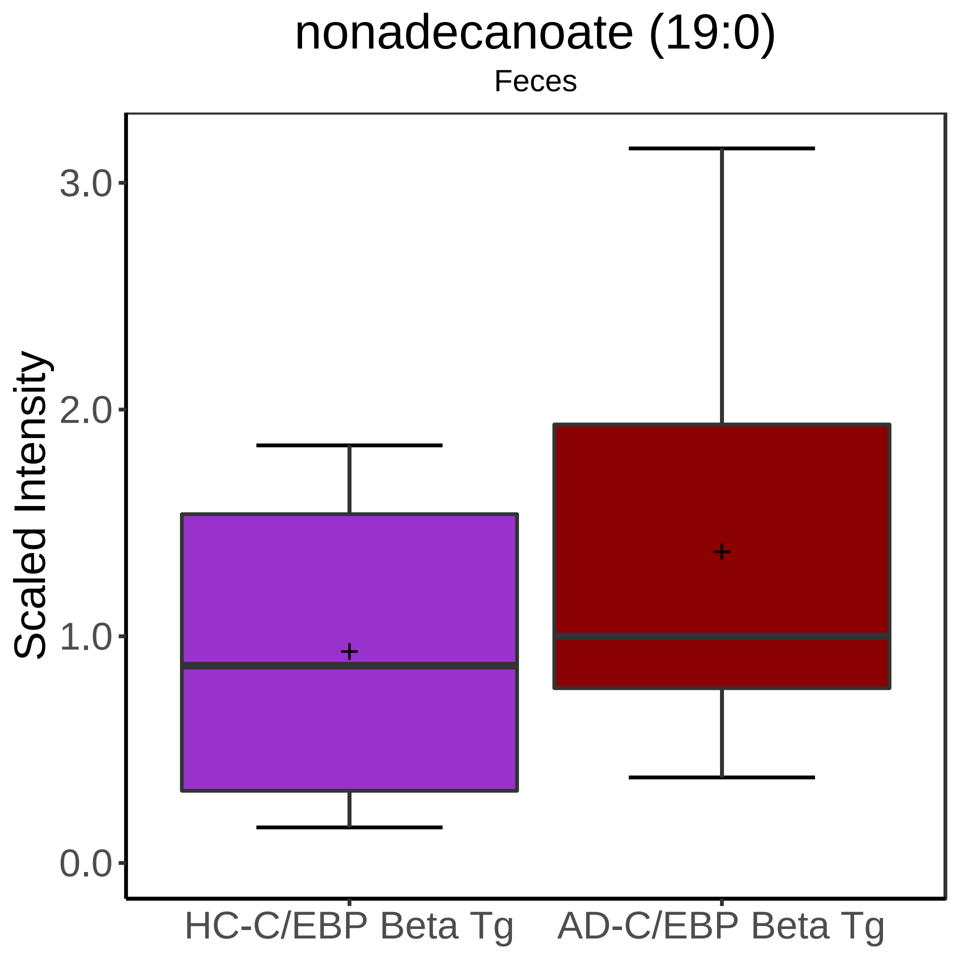
nonadecanoate (19:0)
Feces
Scaled Intensity
3.0
2.0
1.0
0.0
HC-C/EBP Beta Tg
AD-C/EBP Beta Tg

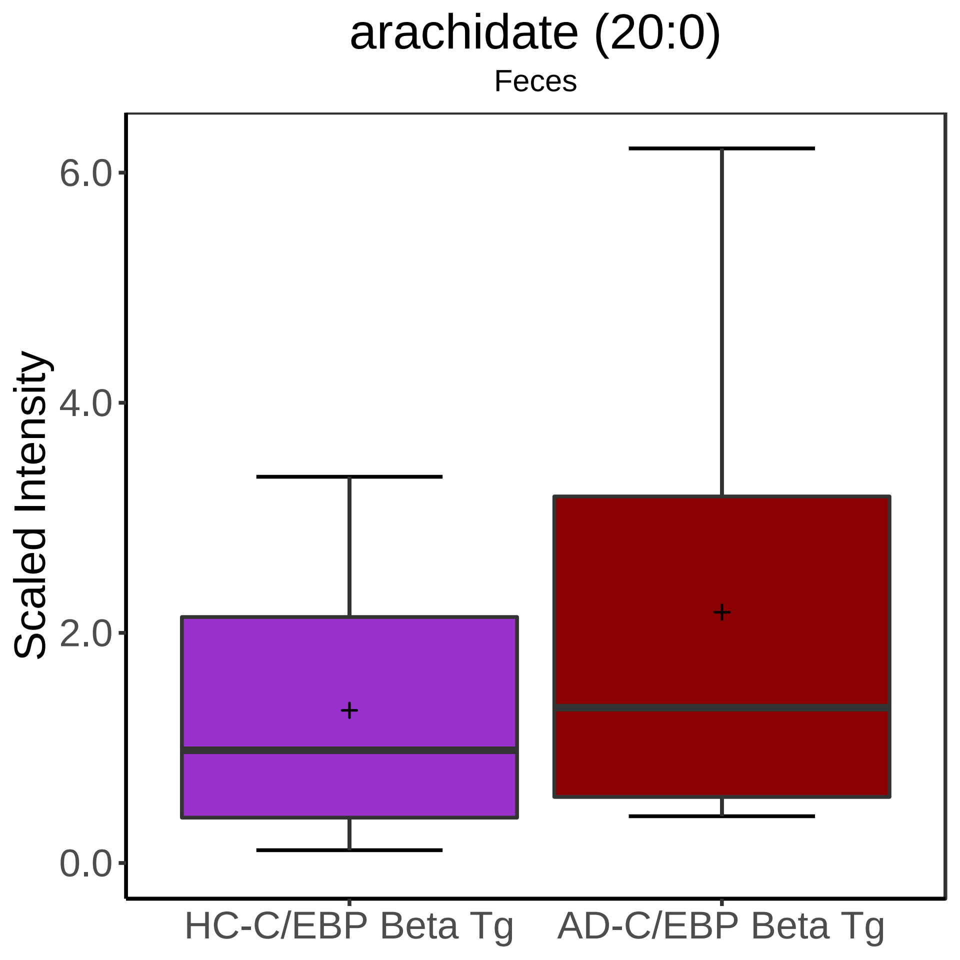

arachidate (20:0)
Feces
Scaled Intensity
6.0
4.0
2.0
0.0
HC-C/EBP Beta Tg
AD-C/EBP Beta Tg

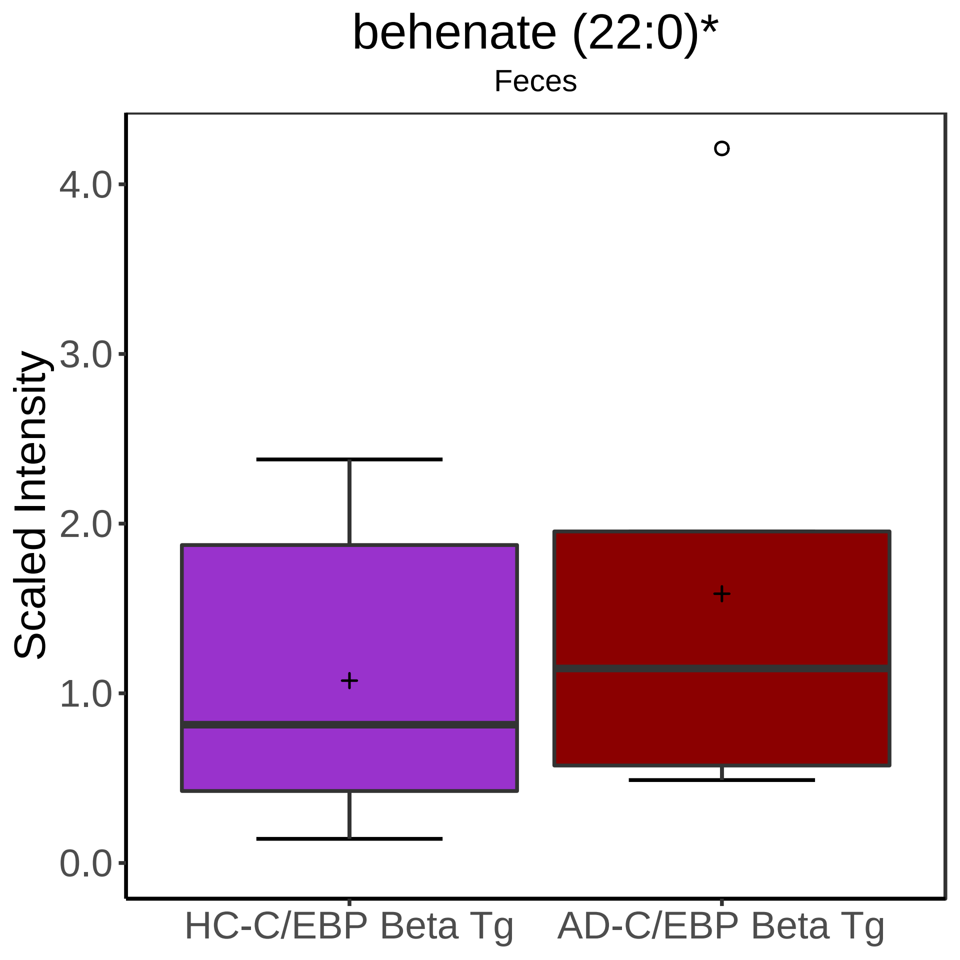
behenate (22:0)*
Feces
Scaled Intensity
4.0
3.0
2.0
1.0
0.0
HC-C/EBP Beta Tg
AD-C/EBP Beta Tg

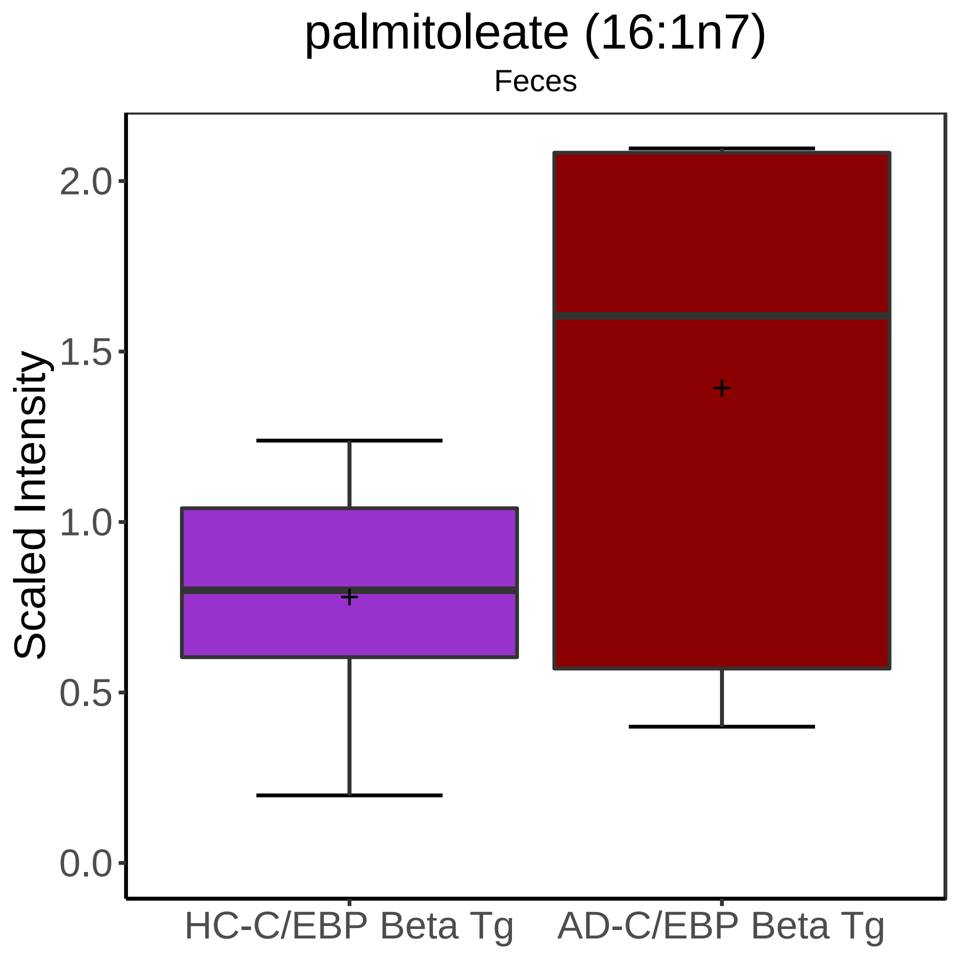

palmitoleate (16:1n7)
Feces
Scaled Intensity
2.0
1.5
1.0
0.5
0.0
HC-C/EBP Beta Tg
AD-C/EBP Beta Tg

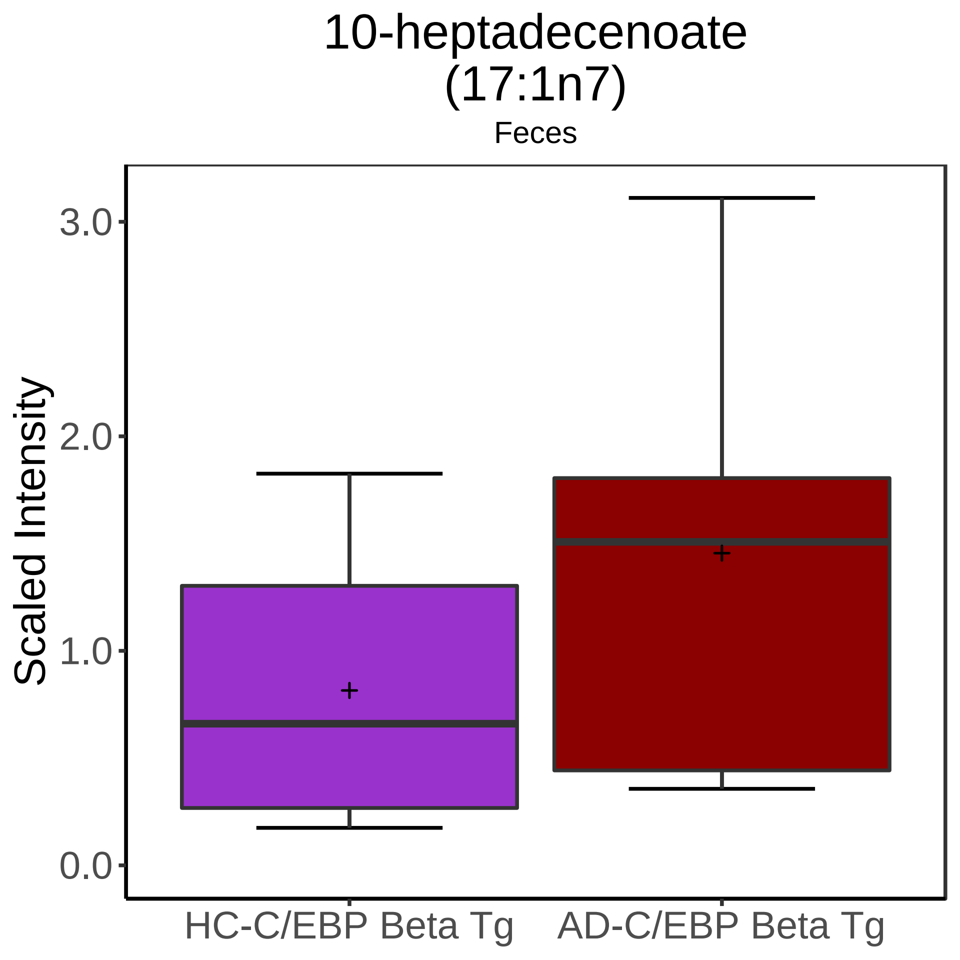
10-heptadecenoate
(17:1n7)
Feces
Scaled Intensity
3.0
2.0
1.0
0.0
HC-C/EBP Beta Tg
AD-C/EBP Beta Tg

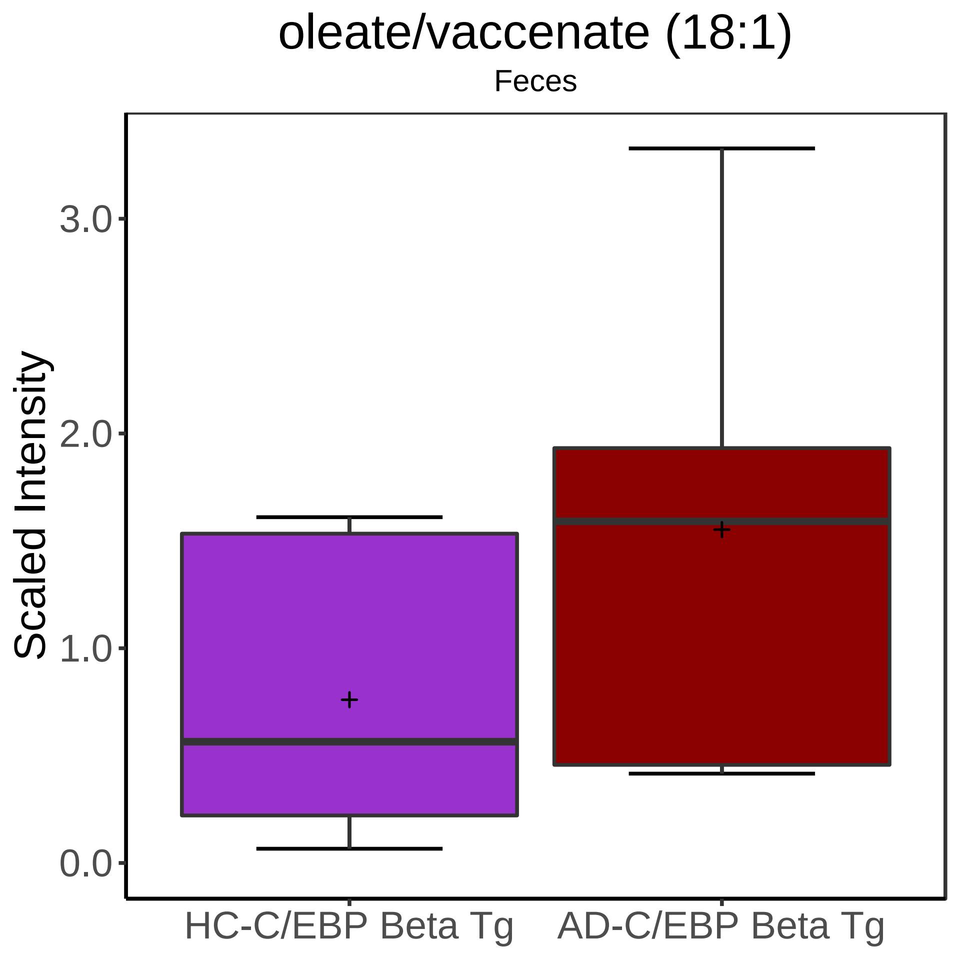

oleate/vaccenate (18:1)
Feces
Scaled Intensity
3.0
2.0
1.0
0.0
HC-C/EBP Beta Tg
AD-C/EBP Beta Tg

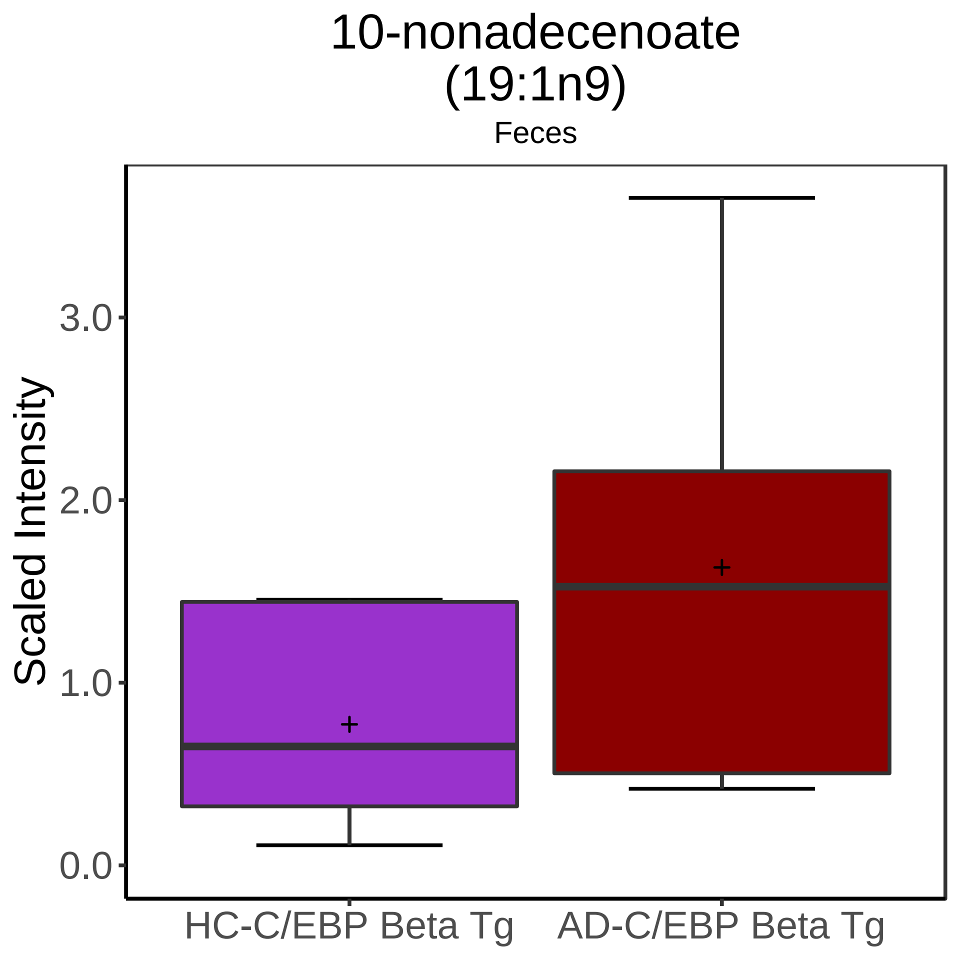

10-nonadecenoate
(19:1n9)
Feces
Scaled Intensity
3.0
2.0
1.0
0.0
HC-C/EBP Beta Tg
AD-C/EBP Beta Tg

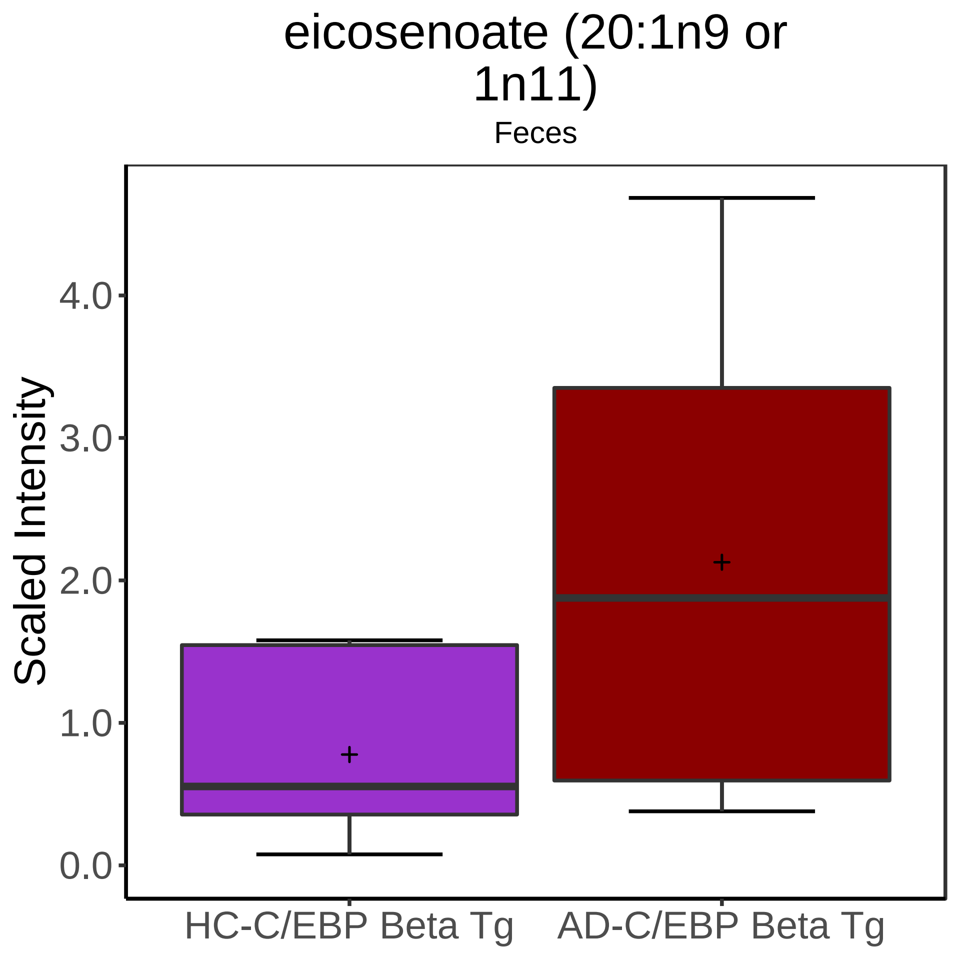
eicosenoate (20:1n9 or 1n11)
Feces
Scaled Intensity
4.0
3.0
2.0
1.0
0.0
HC-C/EBP Beta Tg
AD-C/EBP Beta Tg

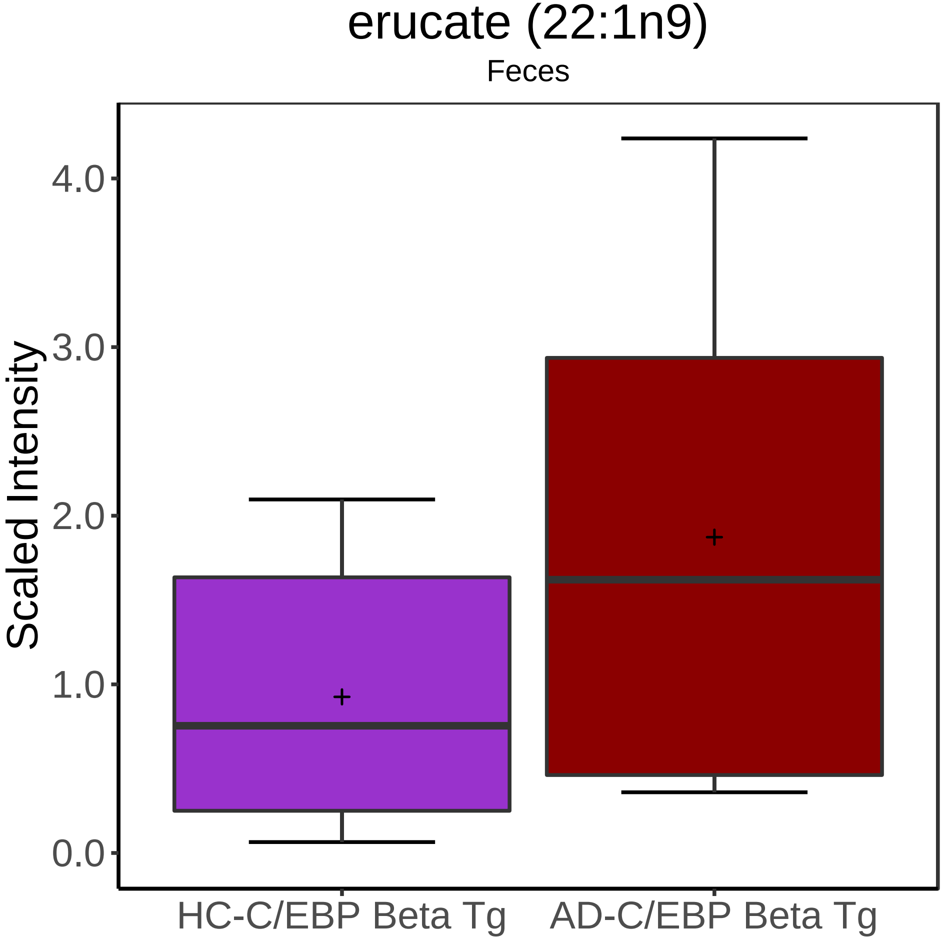
erucate (22:1n9)
Feces
Scaled Intensity
4.0
3.0
2.0
1.0
0.0
HC-C/EBP Beta Tg
AD-C/EBP Beta Tg

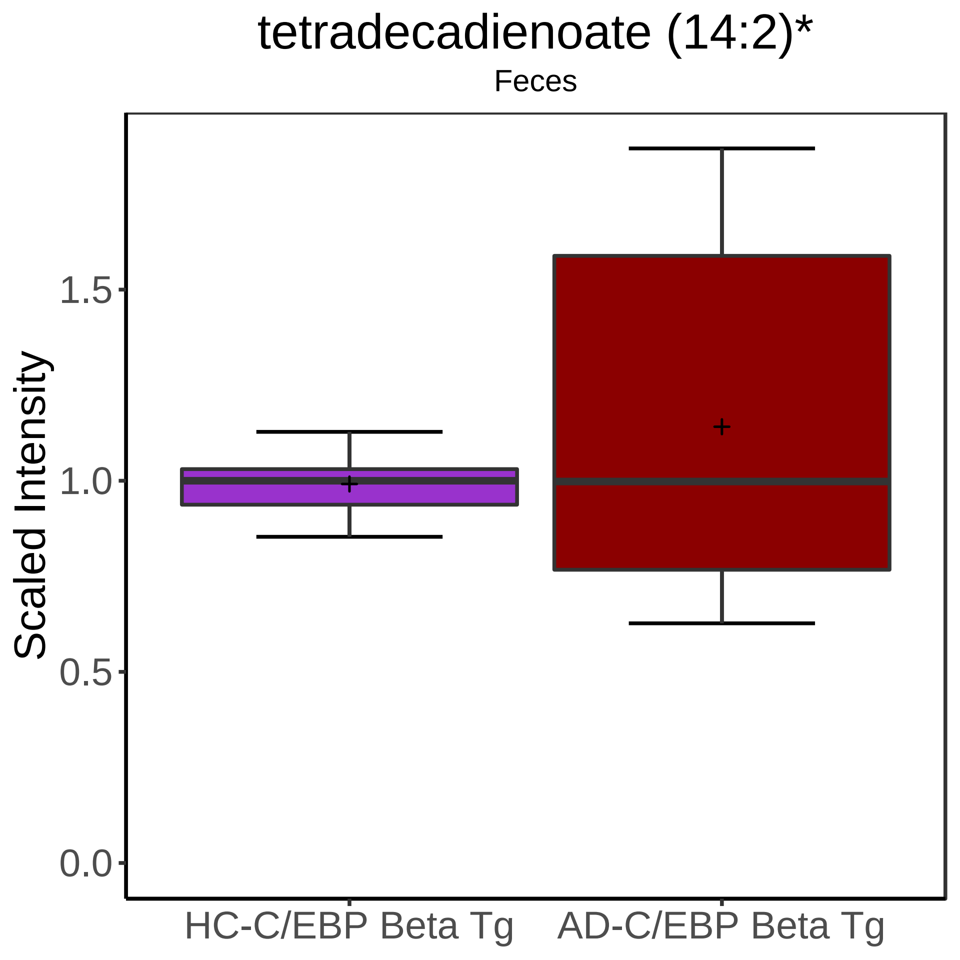

tetradecadienoate (14:2)*
Feces
Scaled Intensity
0.0
0.5
1.0
1.5
HC-C/EBP Beta Tg
AD-C/EBP Beta Tg

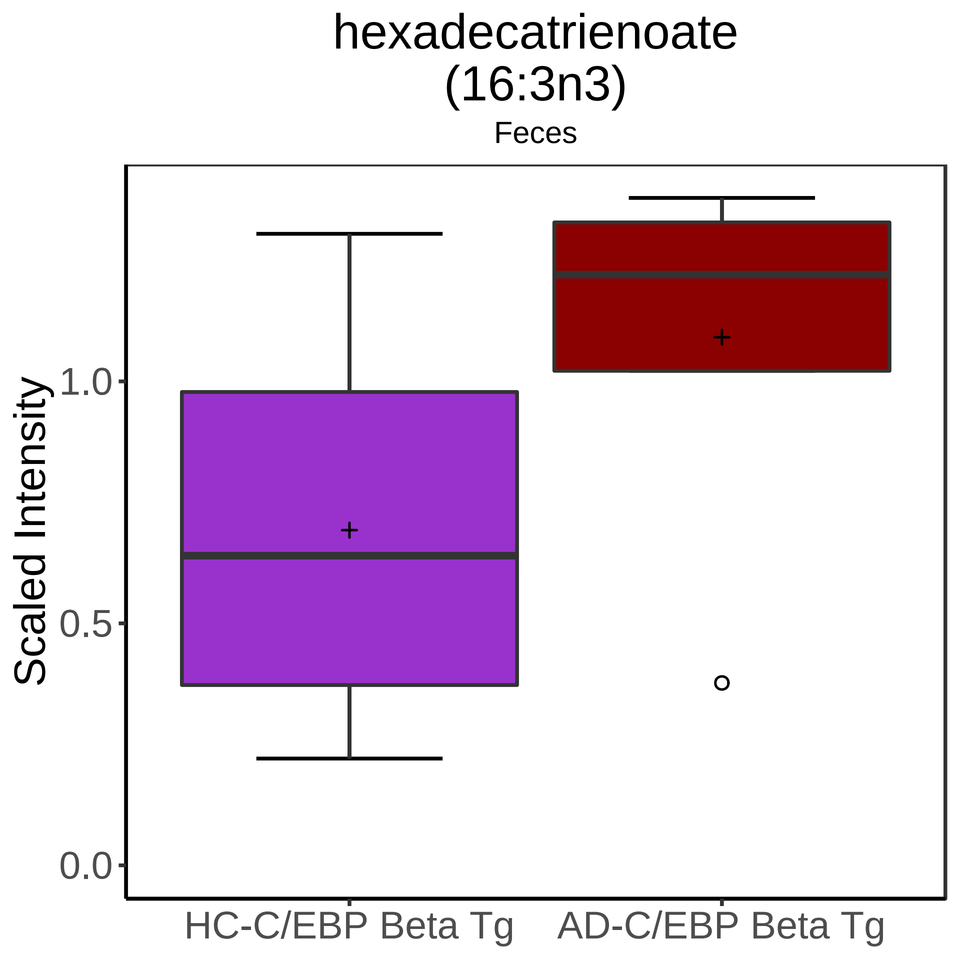

hexadecatrienoate
(16:3n3)
Feces
Scaled Intensity
1.0
0.5
0.0
HC-C/EBP Beta Tg
AD-C/EBP Beta Tg

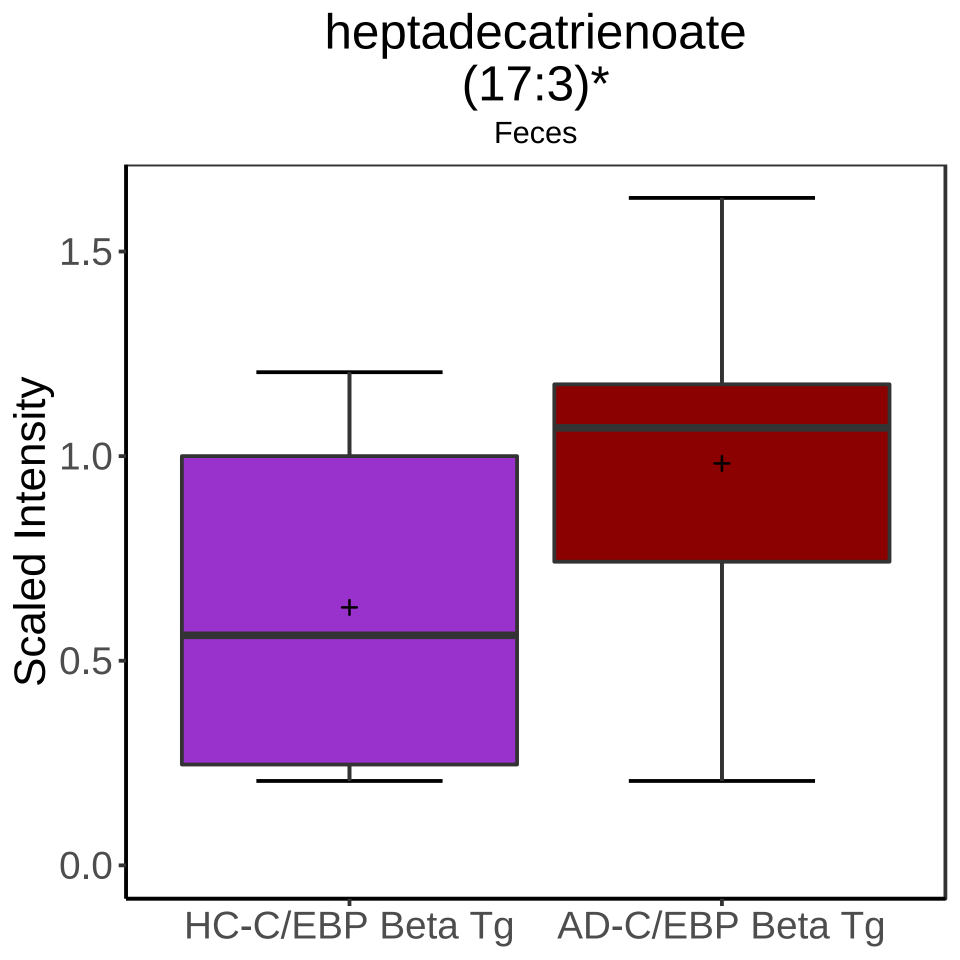
heptadecatrienoate
(17:3)*
Feces
Scaled Intensity
1.5
1.0
0.5
0.0
HC-C/EBP Beta Tg
AD-C/EBP Beta Tg

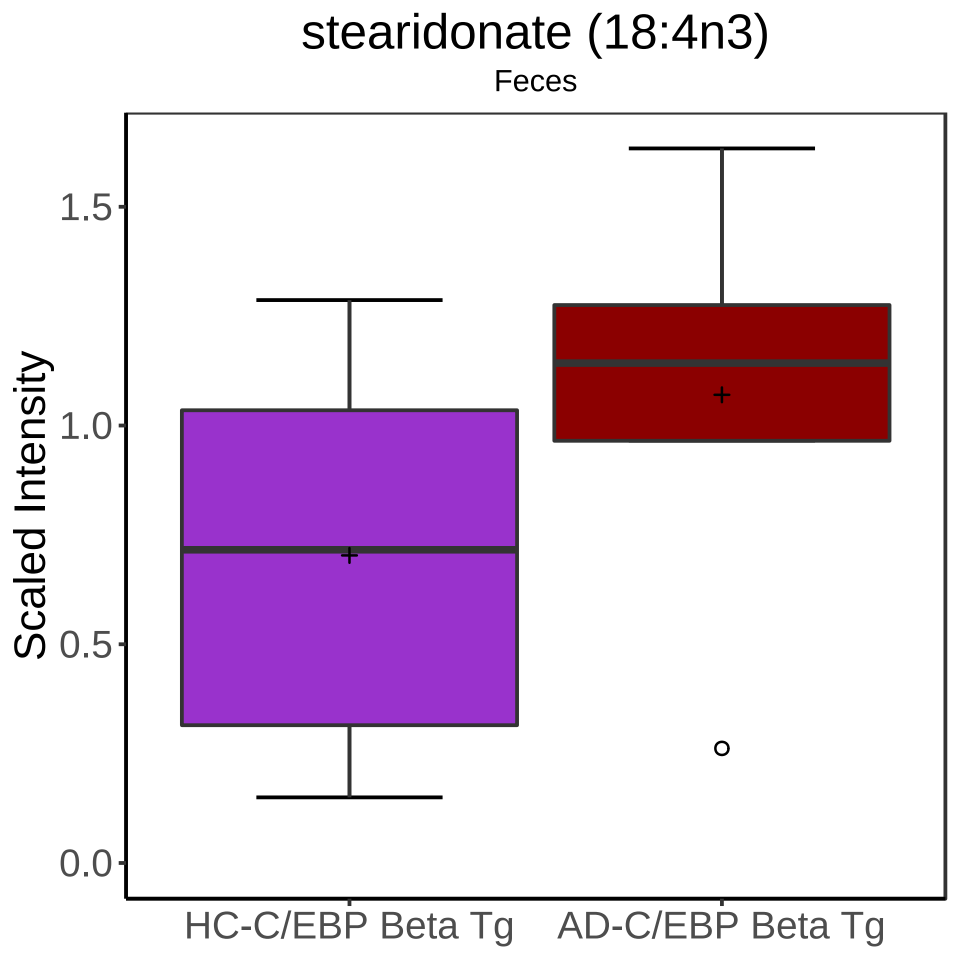
stearidonate (18:4n3)
Feces
Scaled Intensity
1.5
1.0
0.5
0.0
HC-C/EBP Beta Tg
AD-C/EBP Beta Tg

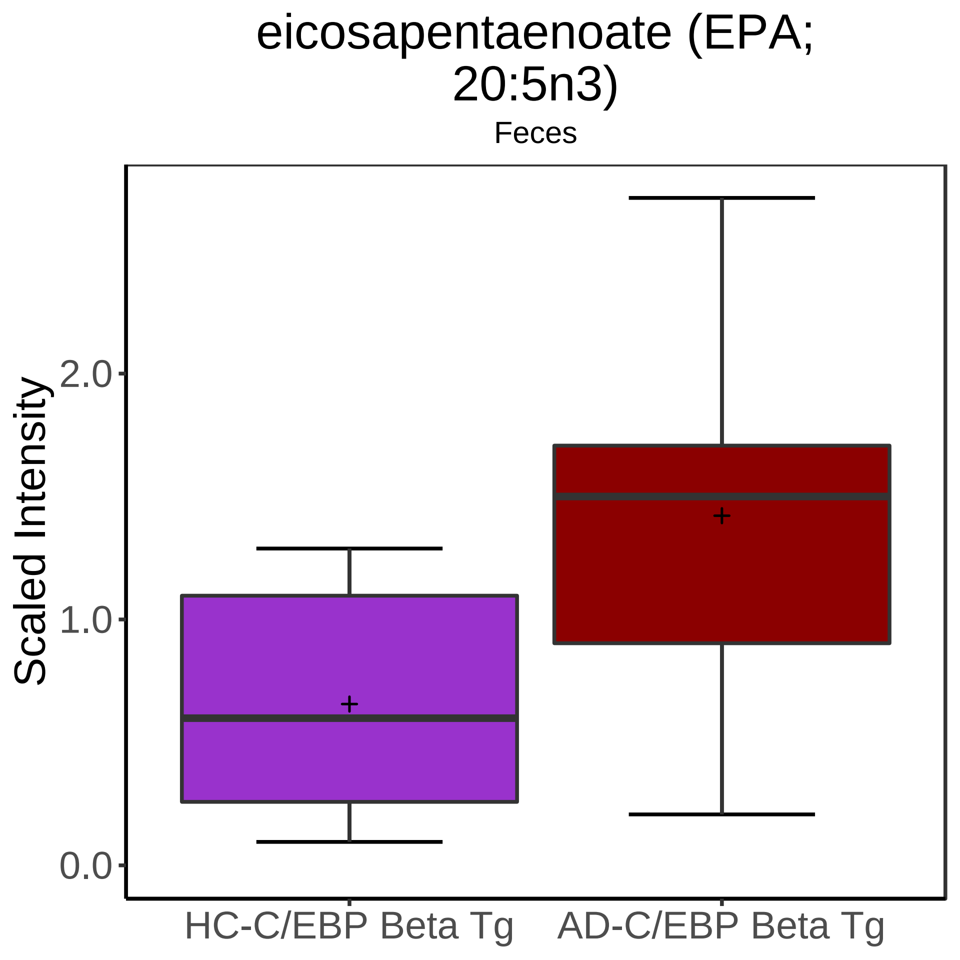

eicosapentaenoate (EPA; 20:5n3)
Feces
Scaled Intensity
2.0
1.0
0.0
HC-C/EBP Beta Tg
AD-C/EBP Beta Tg

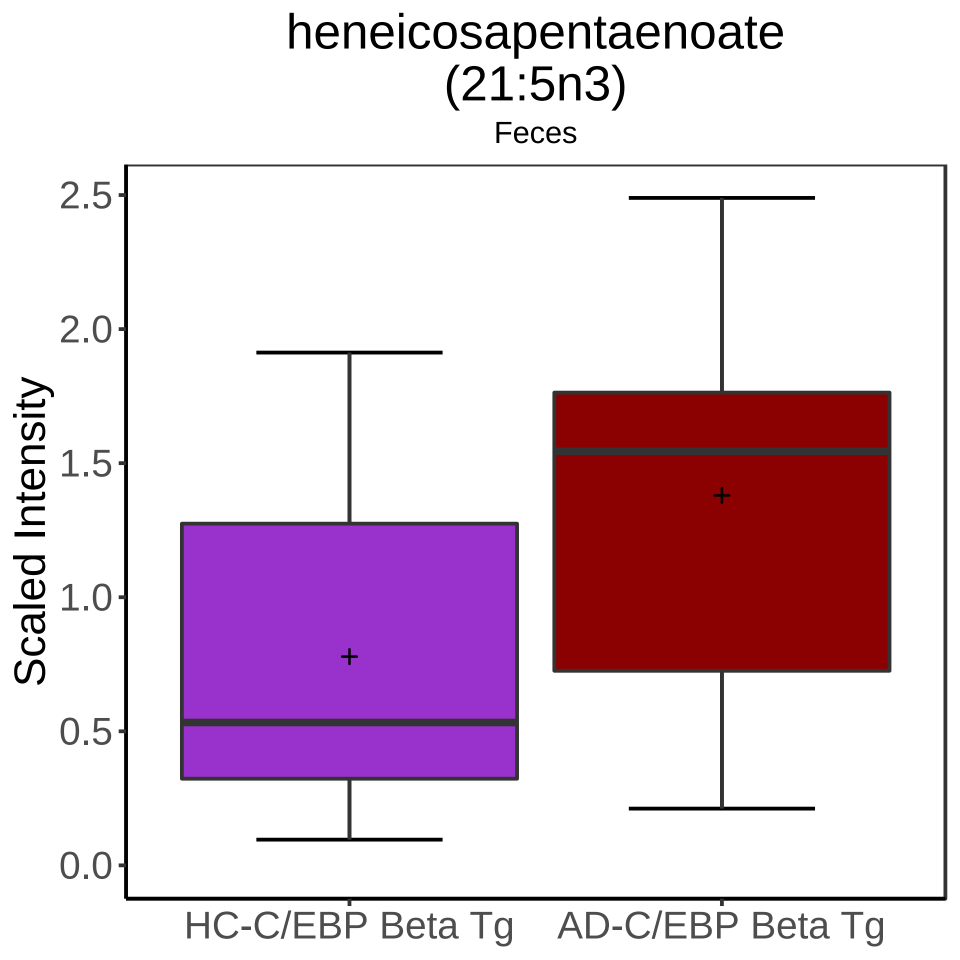
heneicosapentaenoate
(21:5n3)
Feces
Scaled Intensity
2.5
2.0
1.5
1.0
0.5
0.0
HC-C/EBP Beta Tg
AD-C/EBP Beta Tg

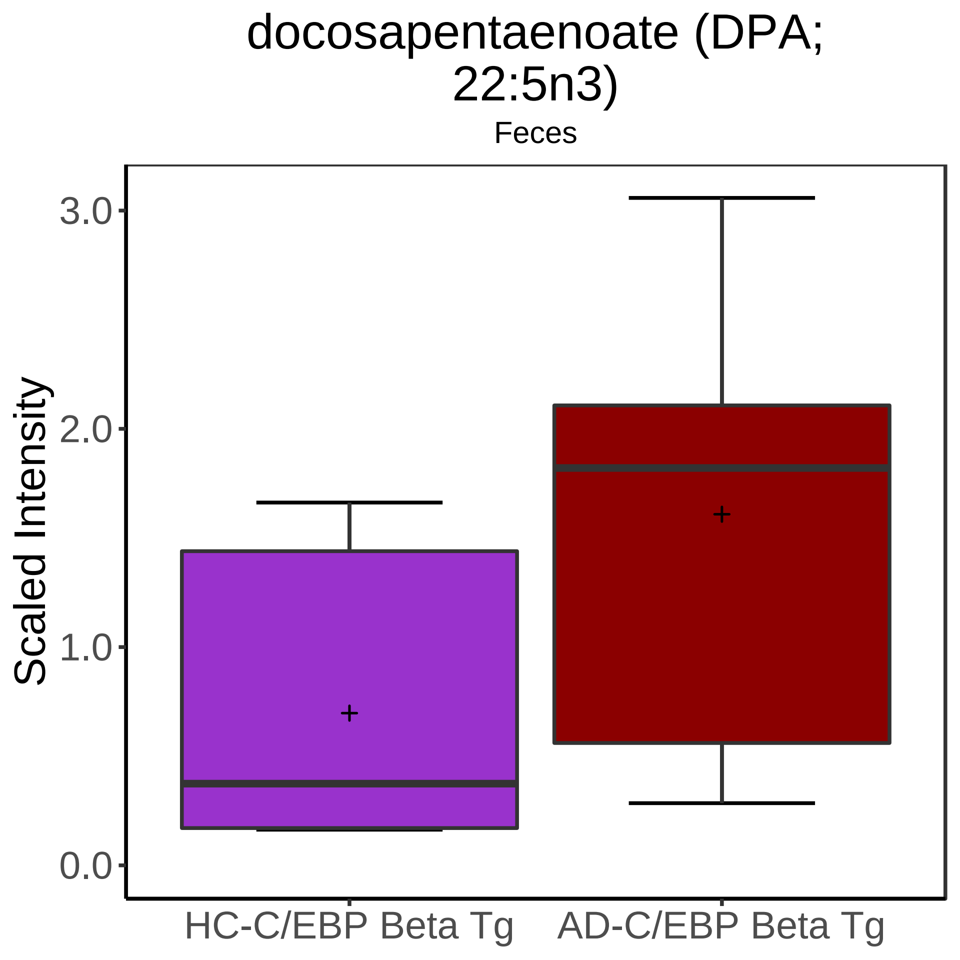

docosapentaenoate (DPA; 22:5n3)
Feces
Scaled Intensity
3.0
2.0
1.0
0.0
HC-C/EBP Beta Tg
AD-C/EBP Beta Tg

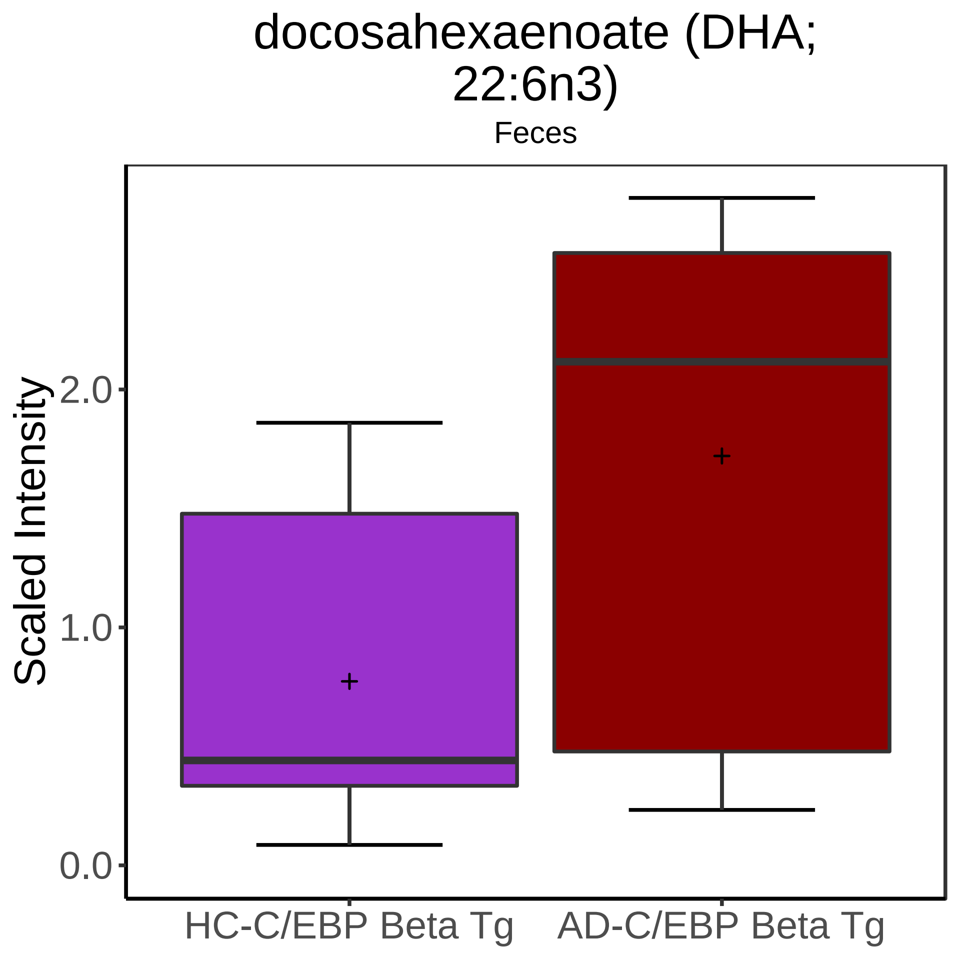
docosahexaenoate (DHA; 22:6n3)
Feces
Scaled Intensity
2.0
1.0
0.0
HC-C/EBP Beta Tg
AD-C/EBP Beta Tg

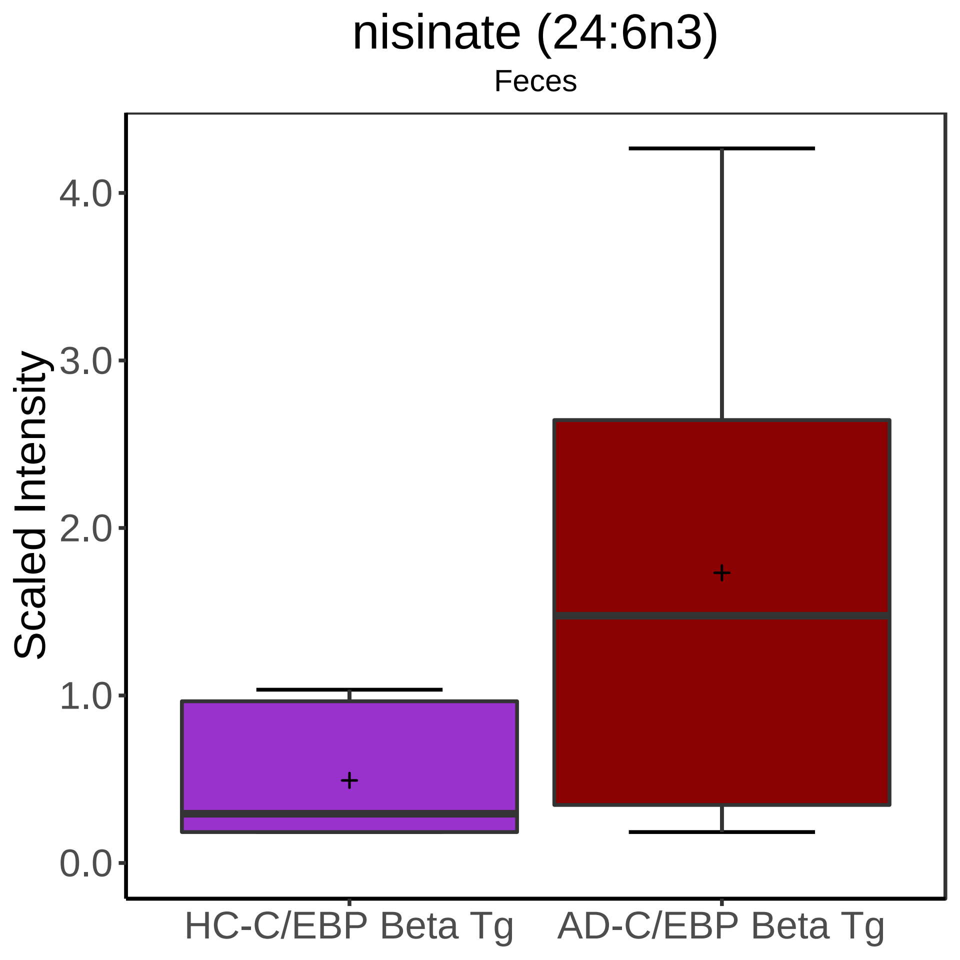
nisinate (24:6n3)
Feces
Scaled Intensity
4.0
3.0
2.0
1.0
0.0
HC-C/EBP Beta Tg
AD-C/EBP Beta Tg

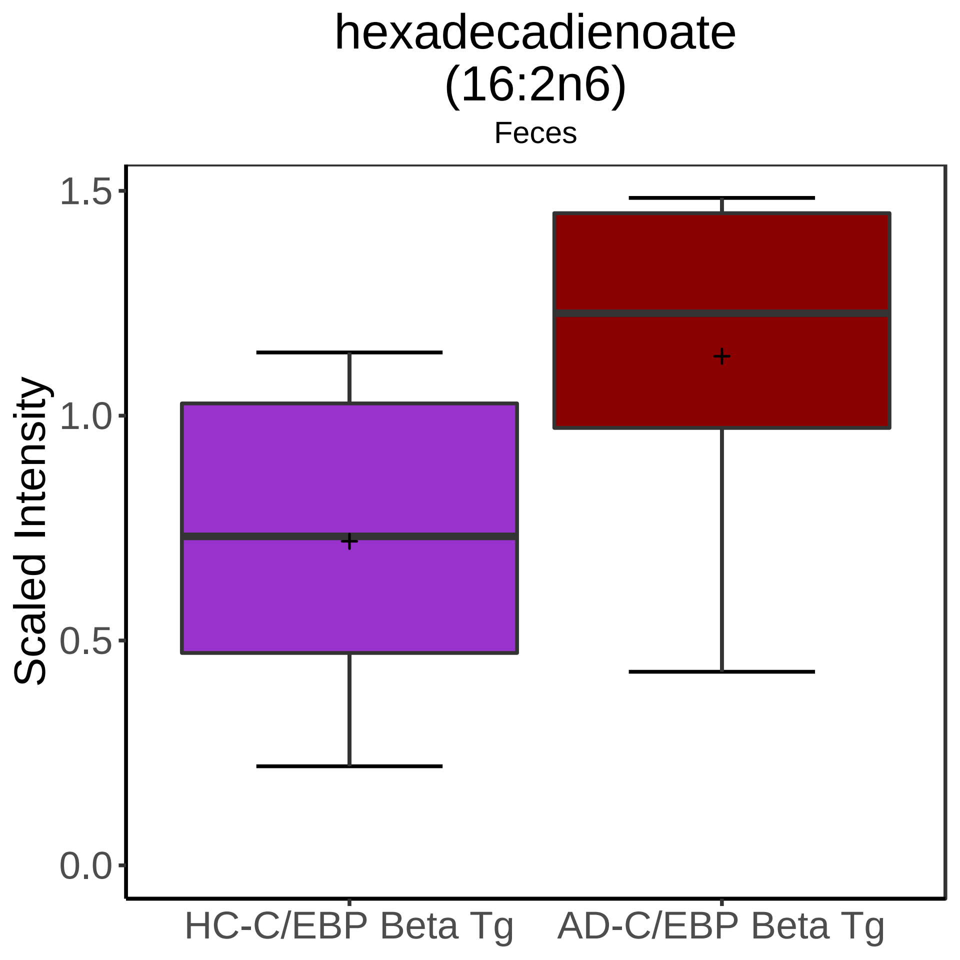

hexadecadienoate
(16:2n6)
Feces
Scaled Intensity
1.5
1.0
0.5
0.0
HC-C/EBP Beta Tg
AD-C/EBP Beta Tg

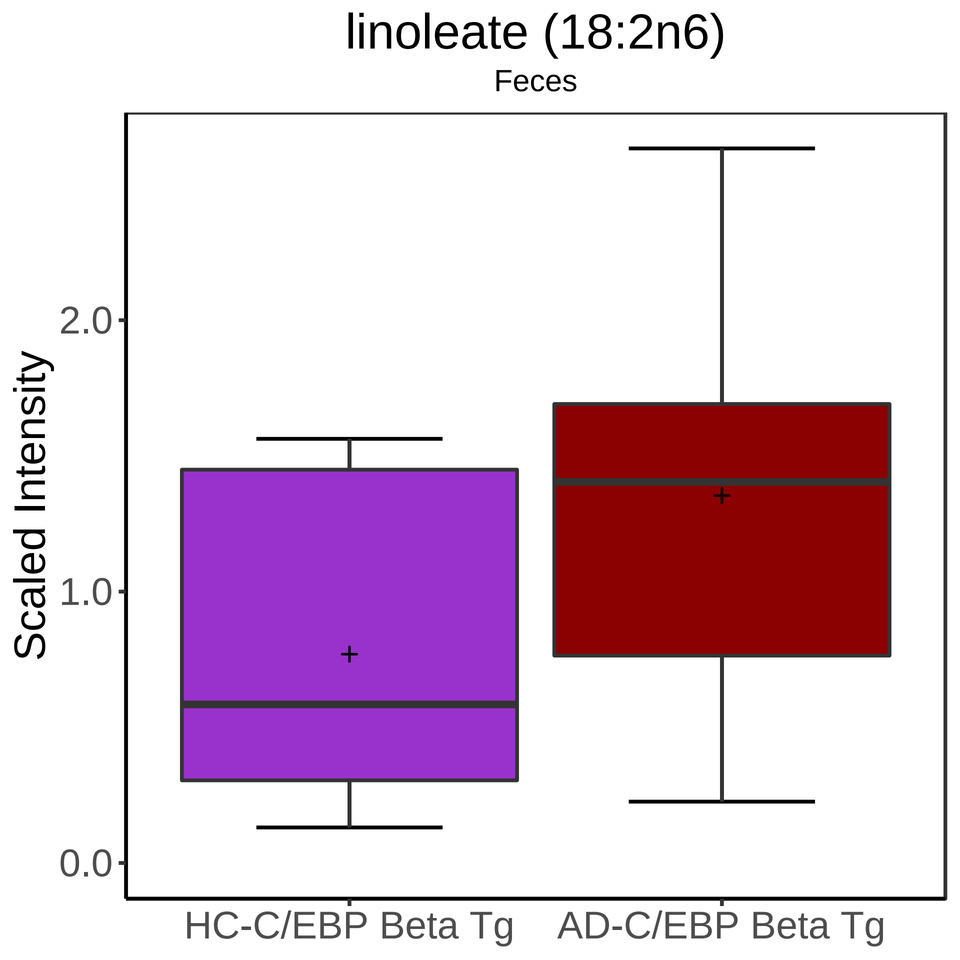
linoleate (18:2n6)
Feces
Scaled Intensity
2.0
1.0
0.0
HC-C/EBP Beta Tg
AD-C/EBP Beta Tg

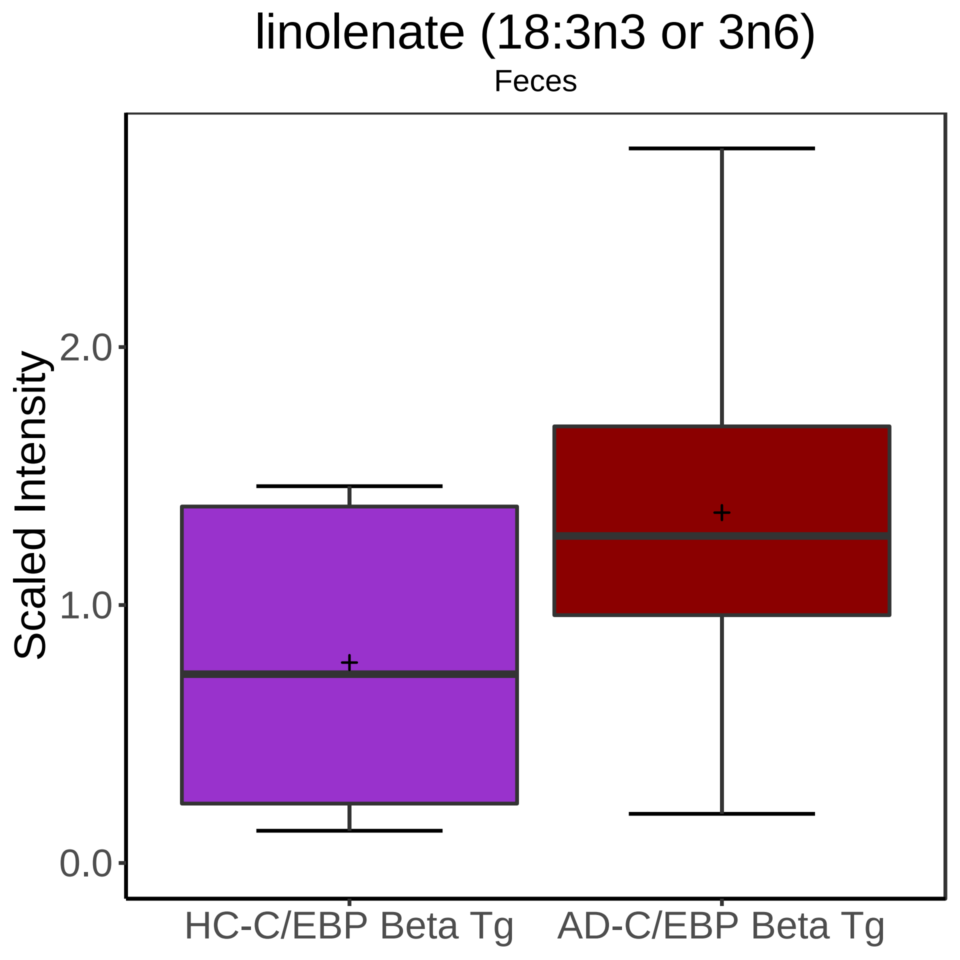
linolenate (18:3n3 or 3n6)
Feces
Scaled Intensity
2.0
1.0
0.0
HC-C/EBP Beta Tg
AD-C/EBP Beta Tg

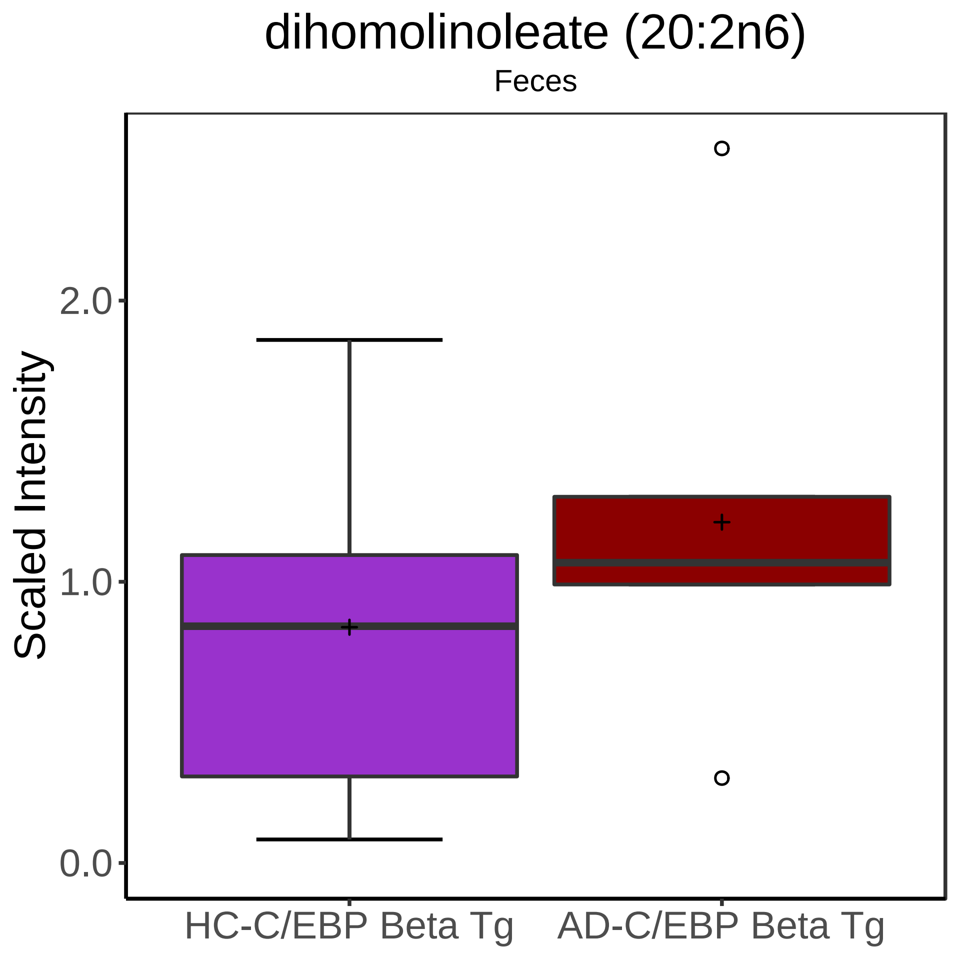
dihomolinoleate (20:2n6)
Feces
Scaled Intensity
2.0
1.0
0.0
HC-C/EBP Beta Tg
AD-C/EBP Beta Tg

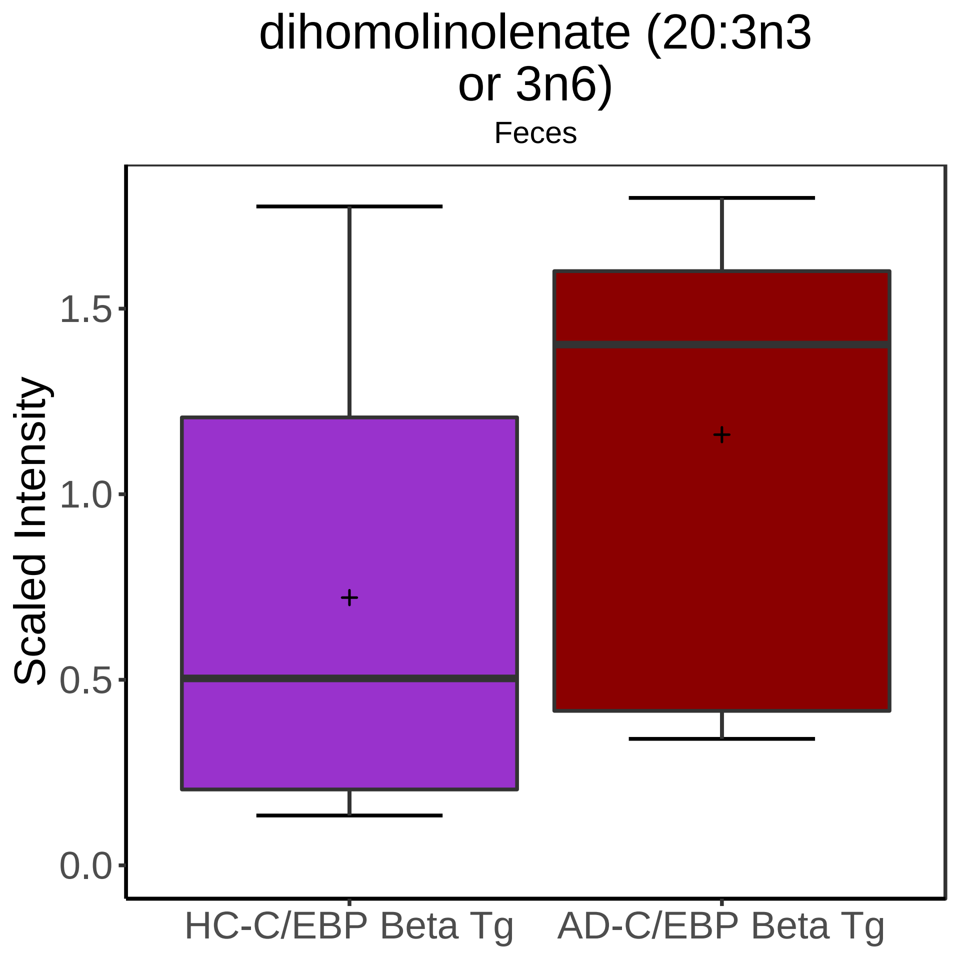
dihomolinolenate (20:3n3
or 3n6)
Feces
Scaled Intensity
0.0
0.5
1.0
1.5
HC-C/EBP Beta Tg
AD-C/EBP Beta Tg

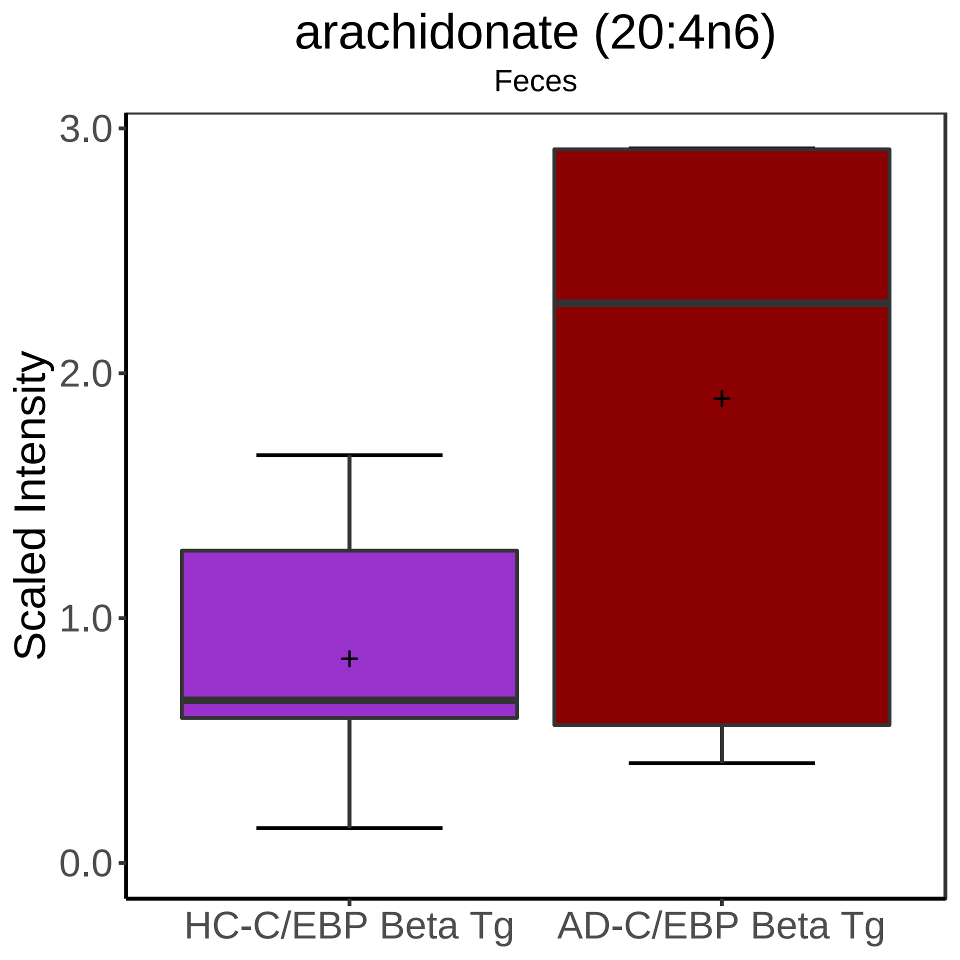

arachidonate (20:4n6)
Feces
Scaled Intensity
3.0
2.0
1.0
0.0
HC-C/EBP Beta Tg
AD-C/EBP Beta Tg

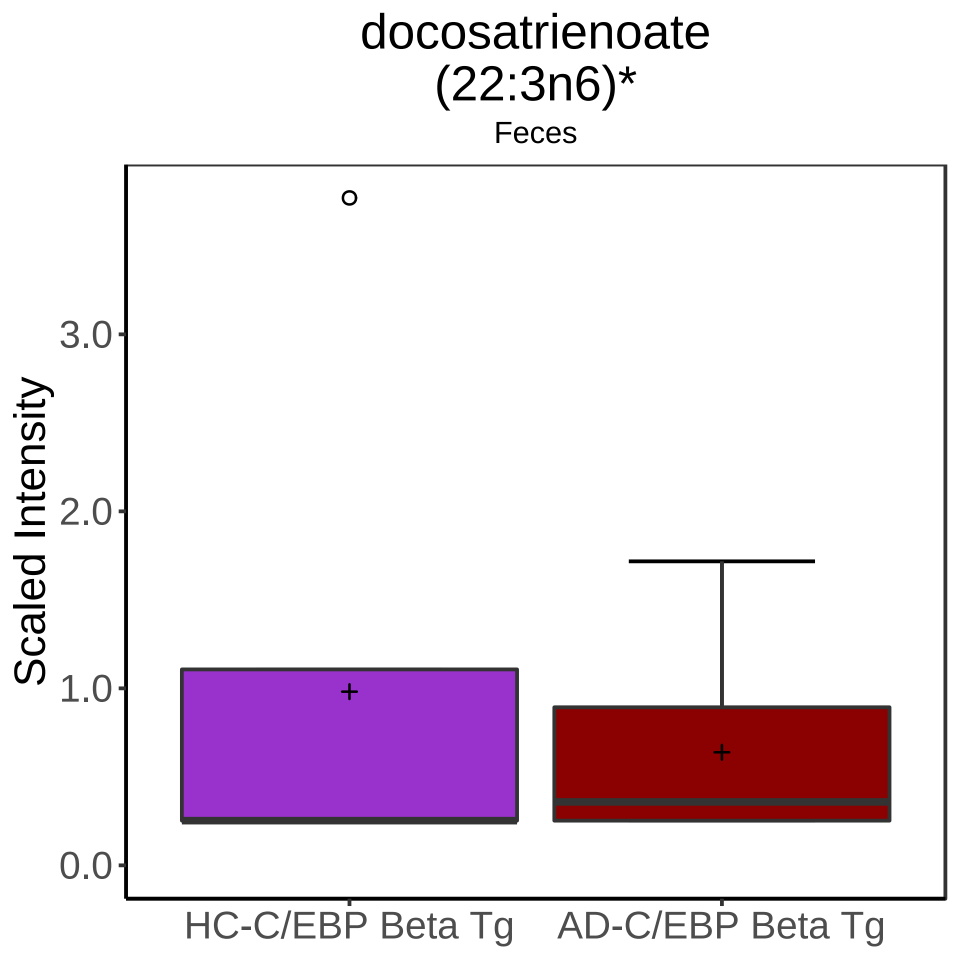
docosatrienoate
(22:3n6)*
Feces
Scaled Intensity
3.0
2.0
1.0
0.0
HC-C/EBP Beta Tg
AD-C/EBP Beta Tg

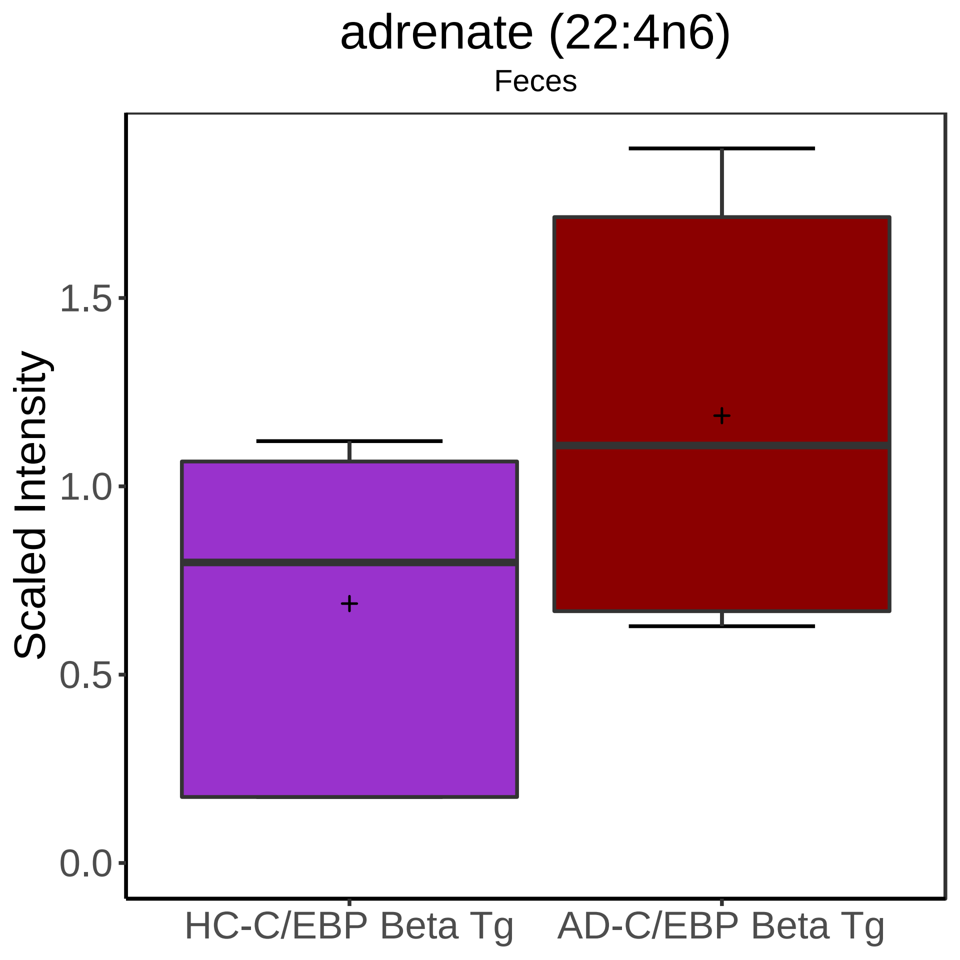
adrenate (22:4n6)
Feces
Scaled Intensity
1.5
1.0
0.5
0.0
HC-C/EBP Beta Tg
AD-C/EBP Beta Tg

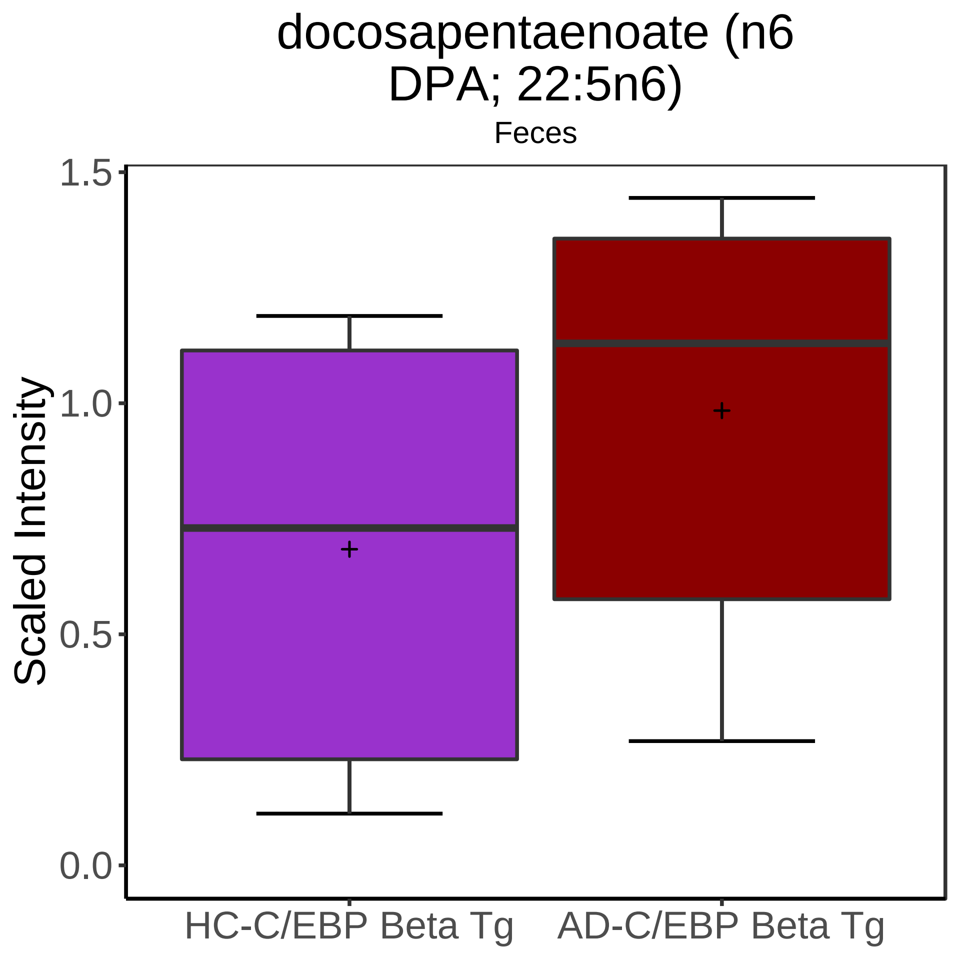

docosapentaenoate (n6
DPA; 22:5n6)
Feces
Scaled Intensity
1.5
1.0
0.5
0.0
HC-C/EBP Beta Tg
AD-C/EBP Beta Tg

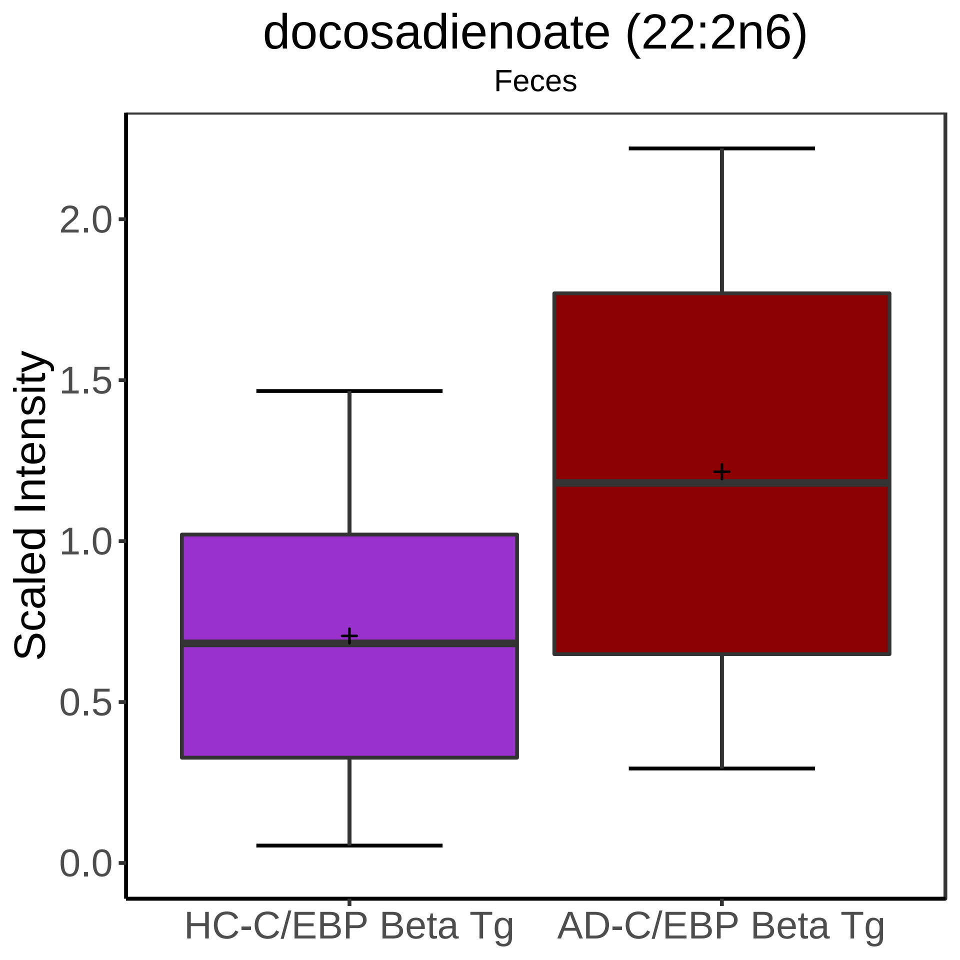
docosadienoate (22:2n6)
Feces
Scaled Intensity
0.0
0.5
1.0
1.5
2.0
HC-C/EBP Beta Tg
AD-C/EBP Beta Tg

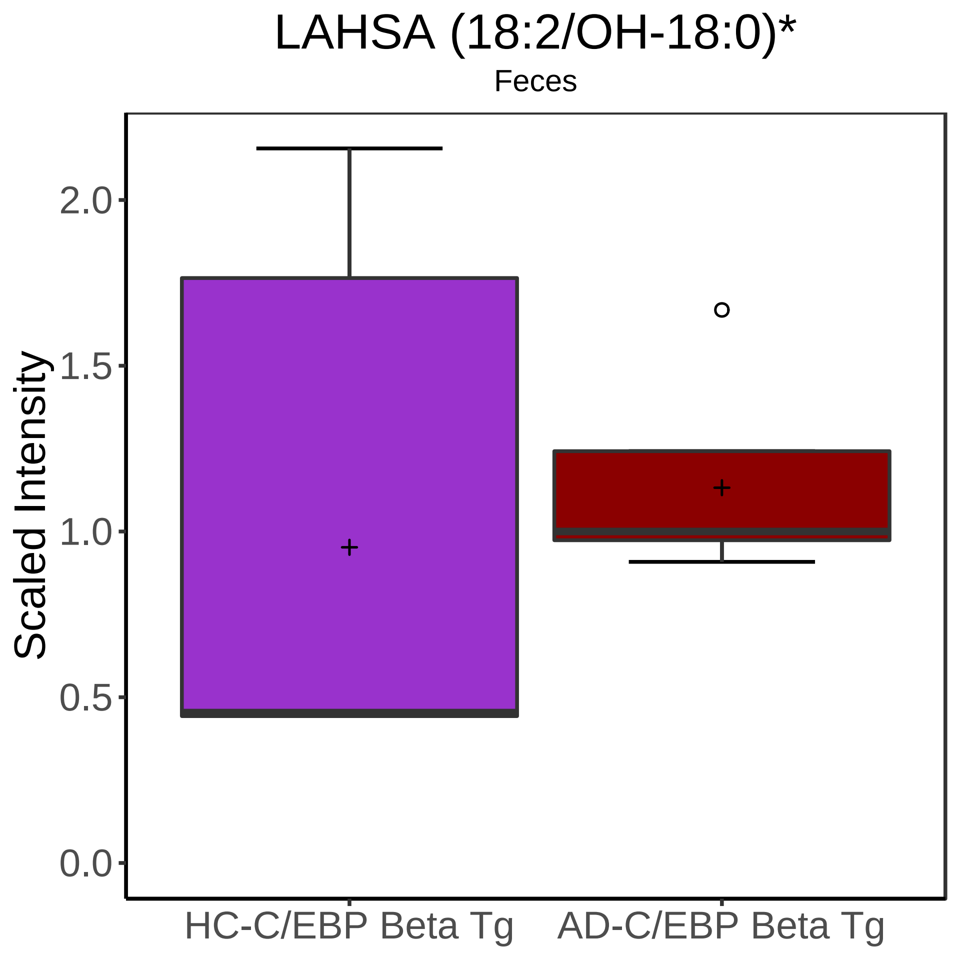
LAHSA (18:2/OH-18:0)*
Feces
Scaled Intensity
2.0
1.5
1.0
0.5
0.0
HC-C/EBP Beta Tg
AD-C/EBP Beta Tg

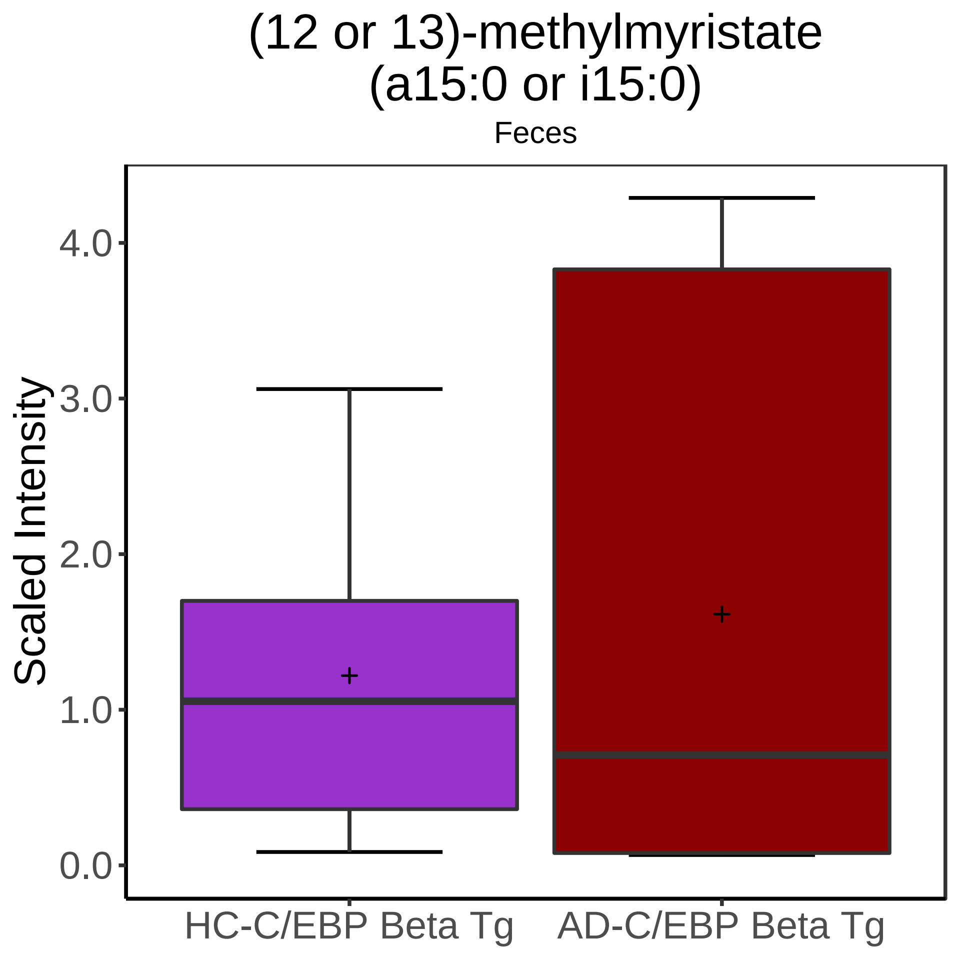

(12 or 13)-methylmyristate
(a15:0 or i15:0)
Feces
Scaled Intensity
4.0
3.0
2.0
1.0
0.0
HC-C/EBP Beta Tg
AD-C/EBP Beta Tg

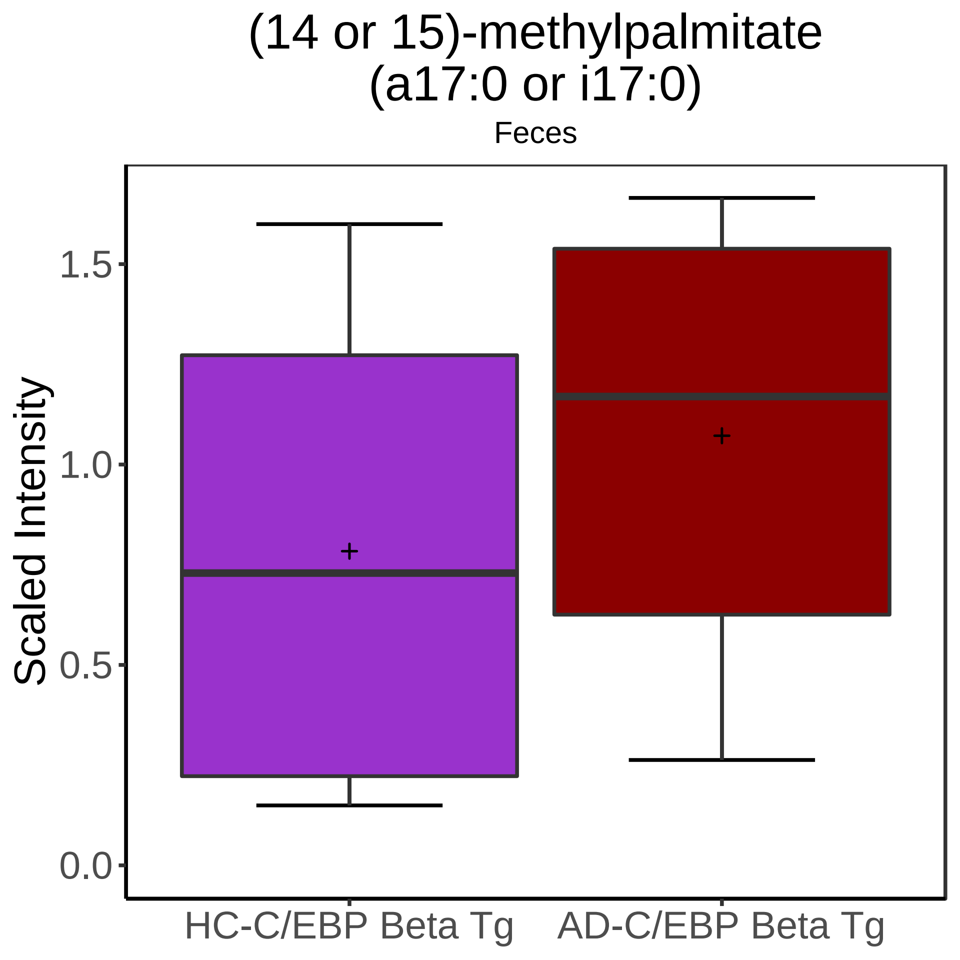
(14 or 15)-methylpalmitate
(a17:0 or i17:0)
Feces
Scaled Intensity
1.5
1.0
0.5
0.0
HC-C/EBP Beta Tg
AD-C/EBP Beta Tg

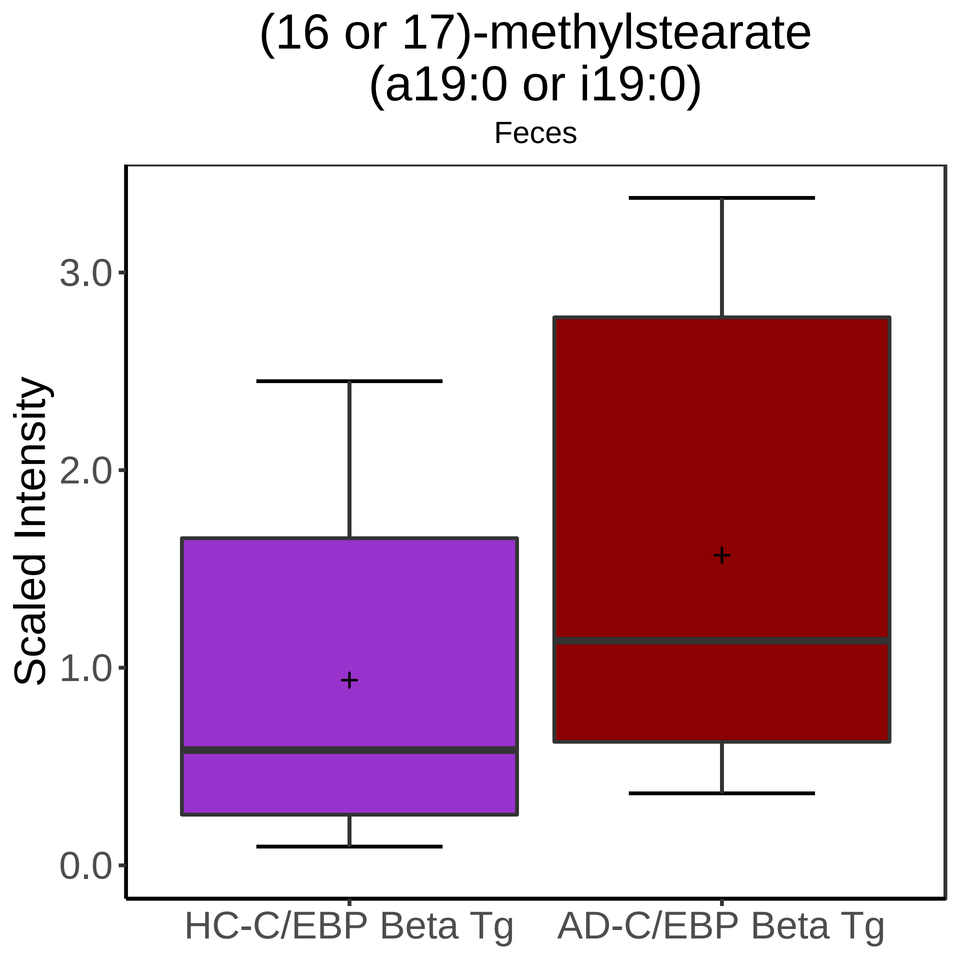

(16 or 17)-methylstearate
(a19:0 or i19:0)
Feces
Scaled Intensity
3.0
2.0
1.0
0.0
HC-C/EBP Beta Tg
AD-C/EBP Beta Tg

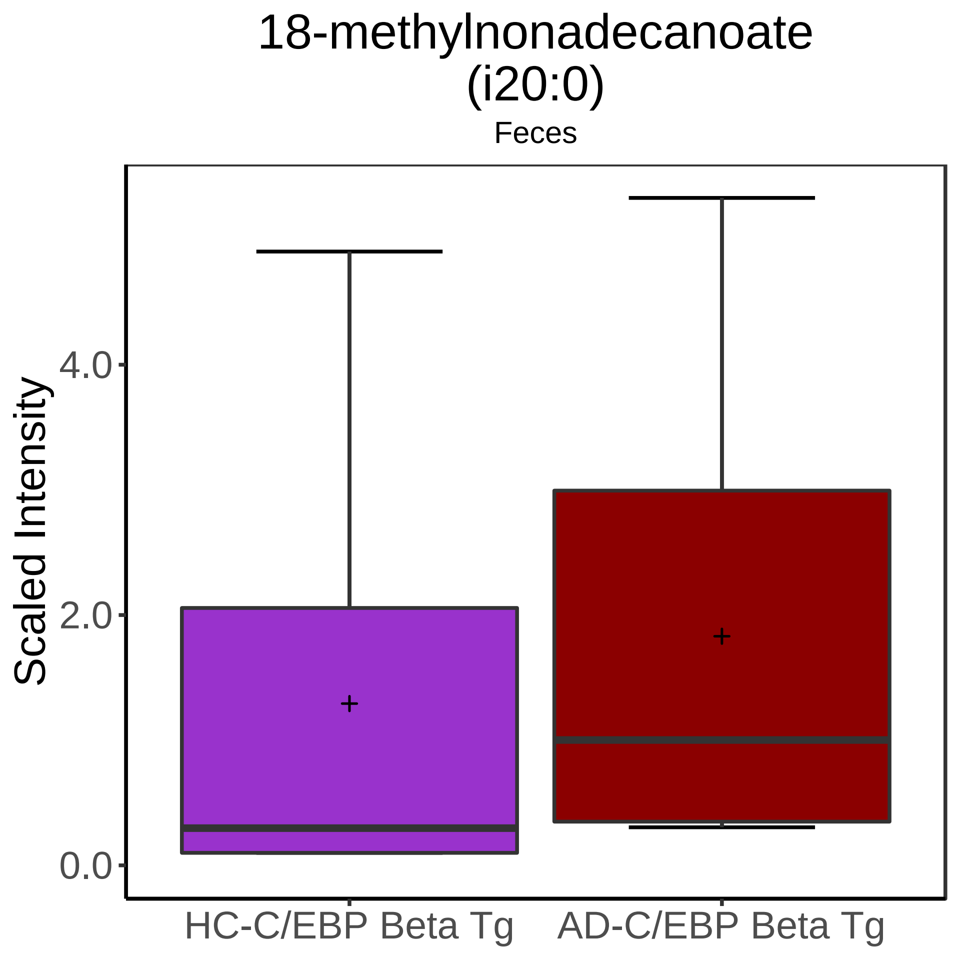

18-methylnonadecanoate
(i20:0)
Feces
Scaled Intensity
4.0
2.0
0.0
HC-C/EBP Beta Tg
AD-C/EBP Beta Tg

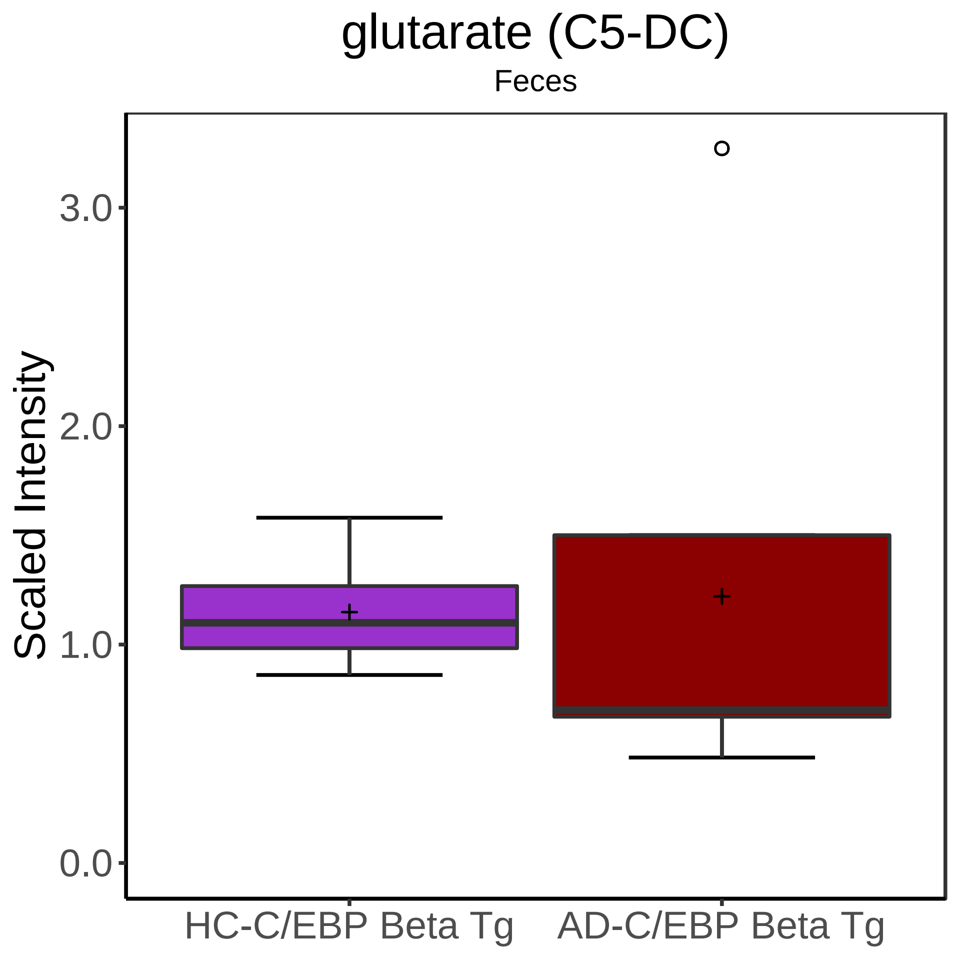

glutarate (C5-DC)
Feces
Scaled Intensity
3.0
2.0
1.0
0.0
HC-C/EBP Beta Tg
AD-C/EBP Beta Tg

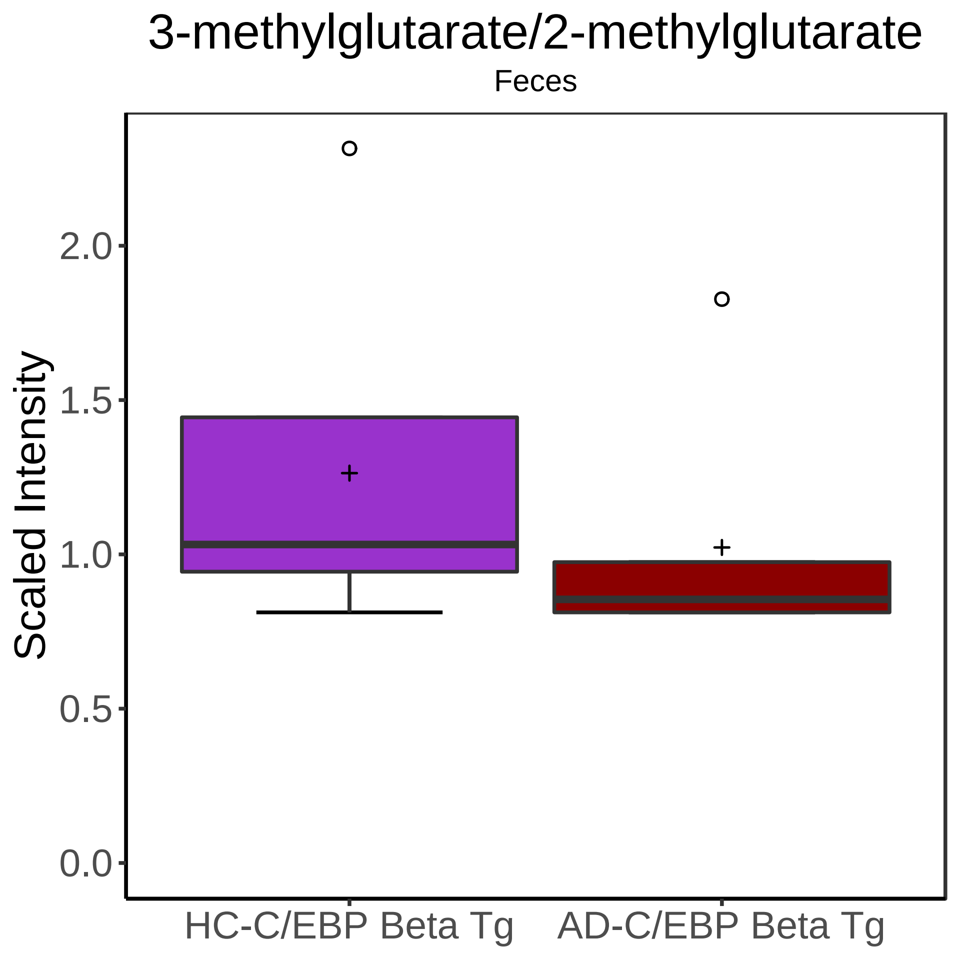

3-methylglutarate/2-methylglutarate
Feces
Scaled Intensity
2.0
1.5
1.0
0.5
0.0
HC-C/EBP Beta Tg
AD-C/EBP Beta Tg

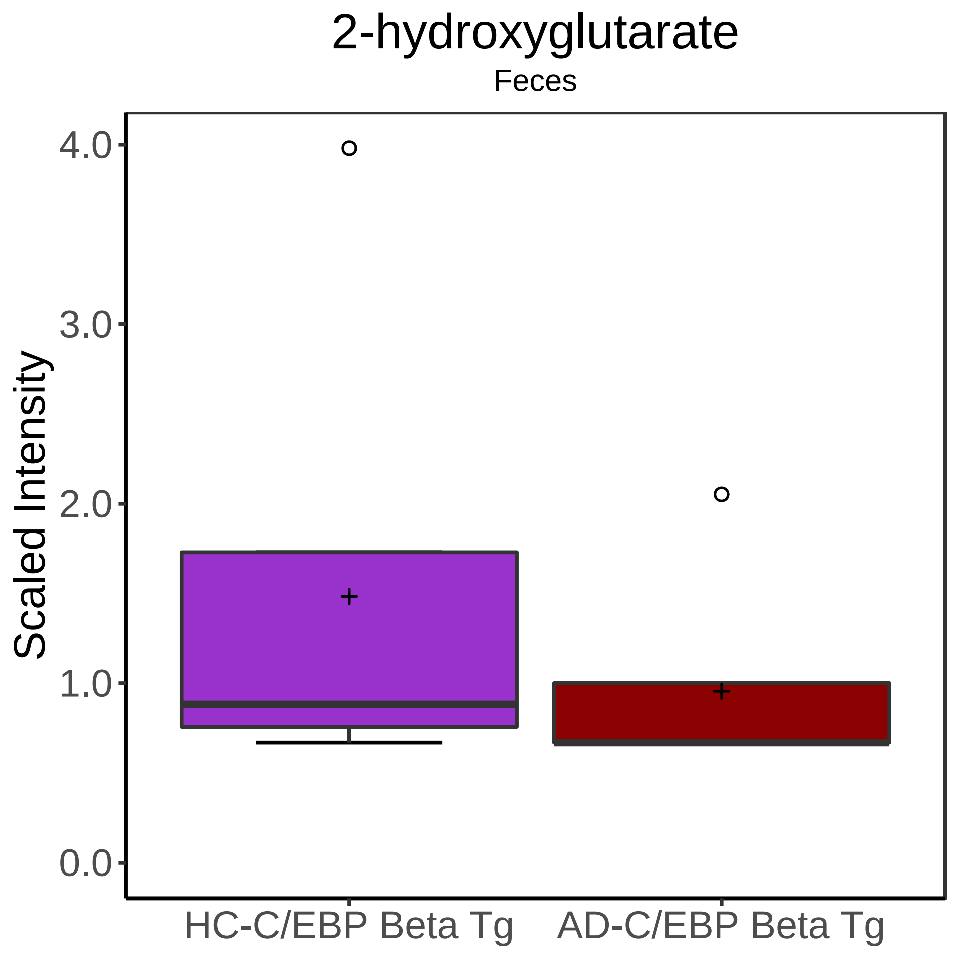
2-hydroxyglutarate
Feces
Scaled Intensity
4.0
3.0
2.0
1.0
0.0
HC-C/EBP Beta Tg
AD-C/EBP Beta Tg

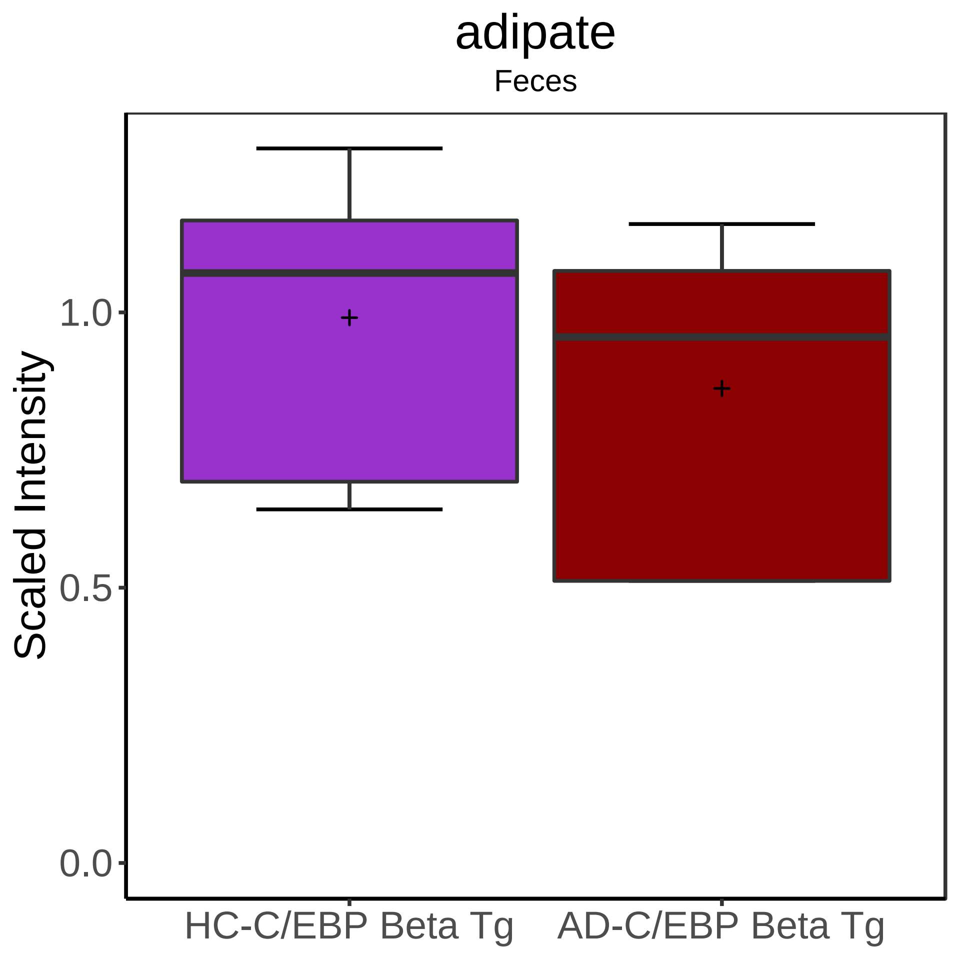
adipate
Feces
Scaled Intensity
1.0
0.5
0.0
HC-C/EBP Beta Tg
AD-C/EBP Beta Tg

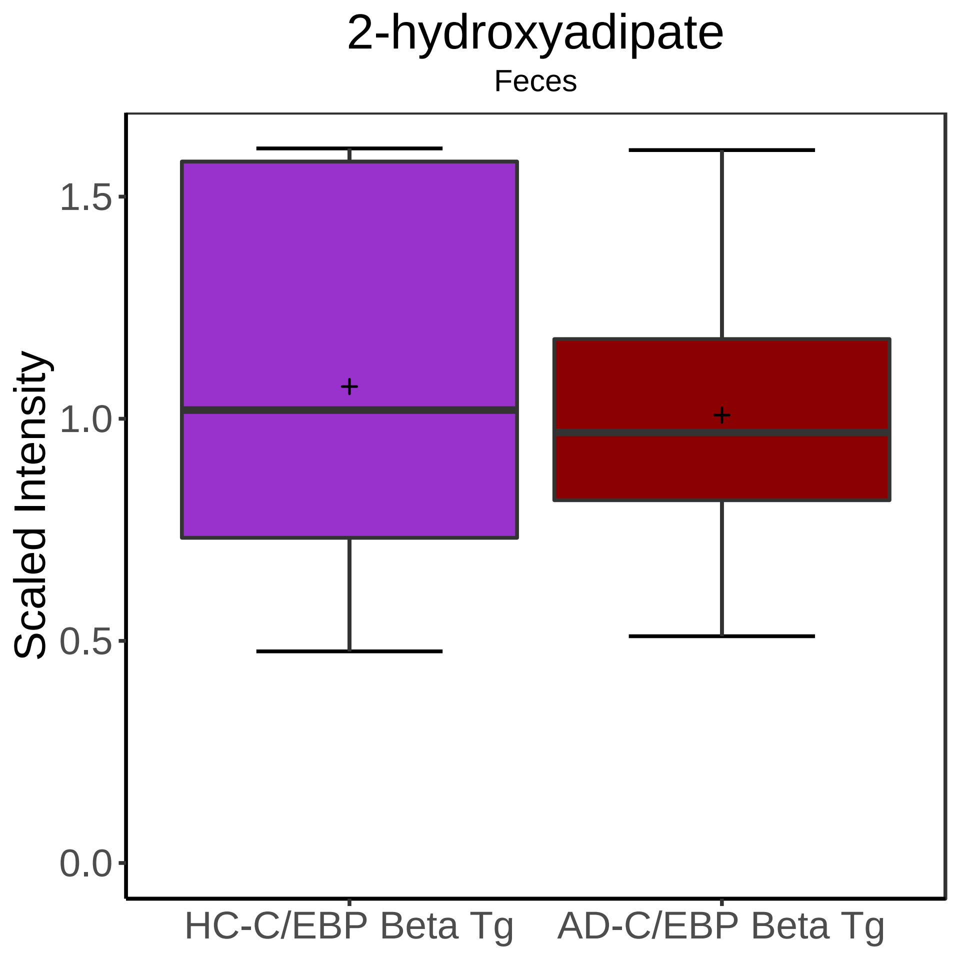
2-hydroxyadipate
Feces
Scaled Intensity
1.5
1.0
0.5
0.0
HC-C/EBP Beta Tg
AD-C/EBP Beta Tg

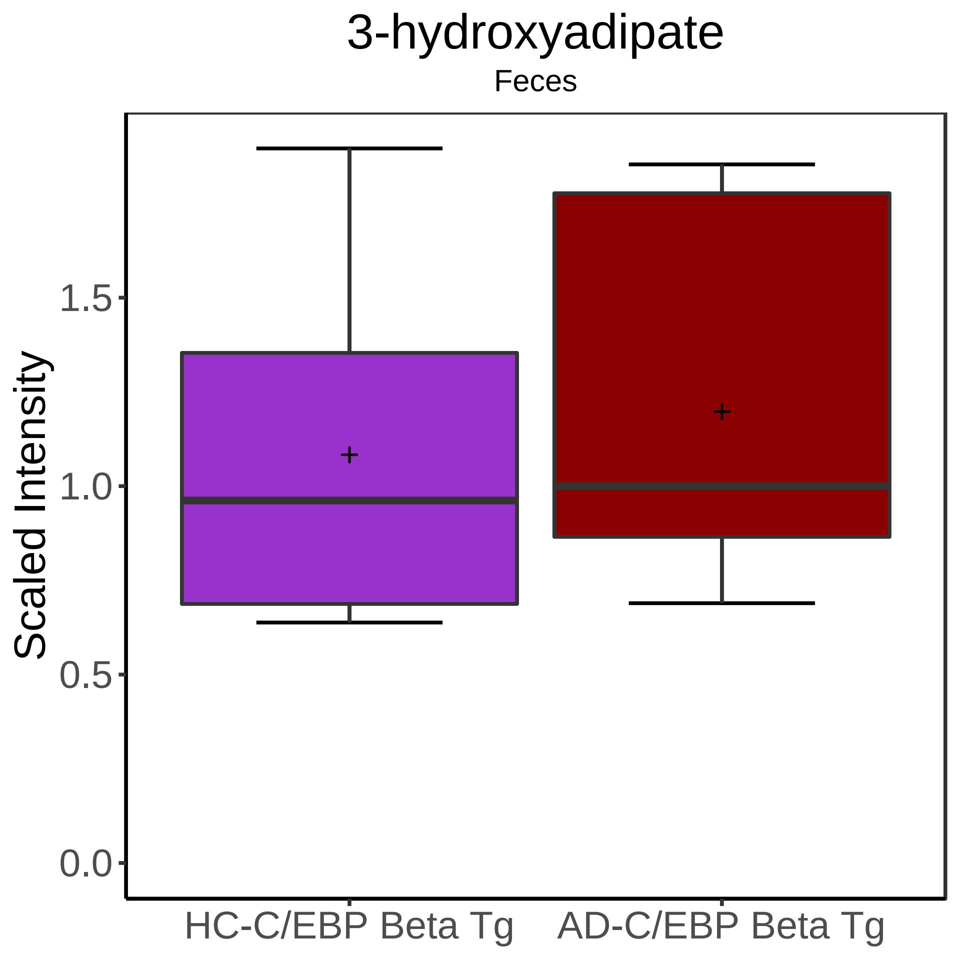
3-hydroxyadipate
Feces
Scaled Intensity
1.5
1.0
0.5
0.0
HC-C/EBP Beta Tg
AD-C/EBP Beta Tg

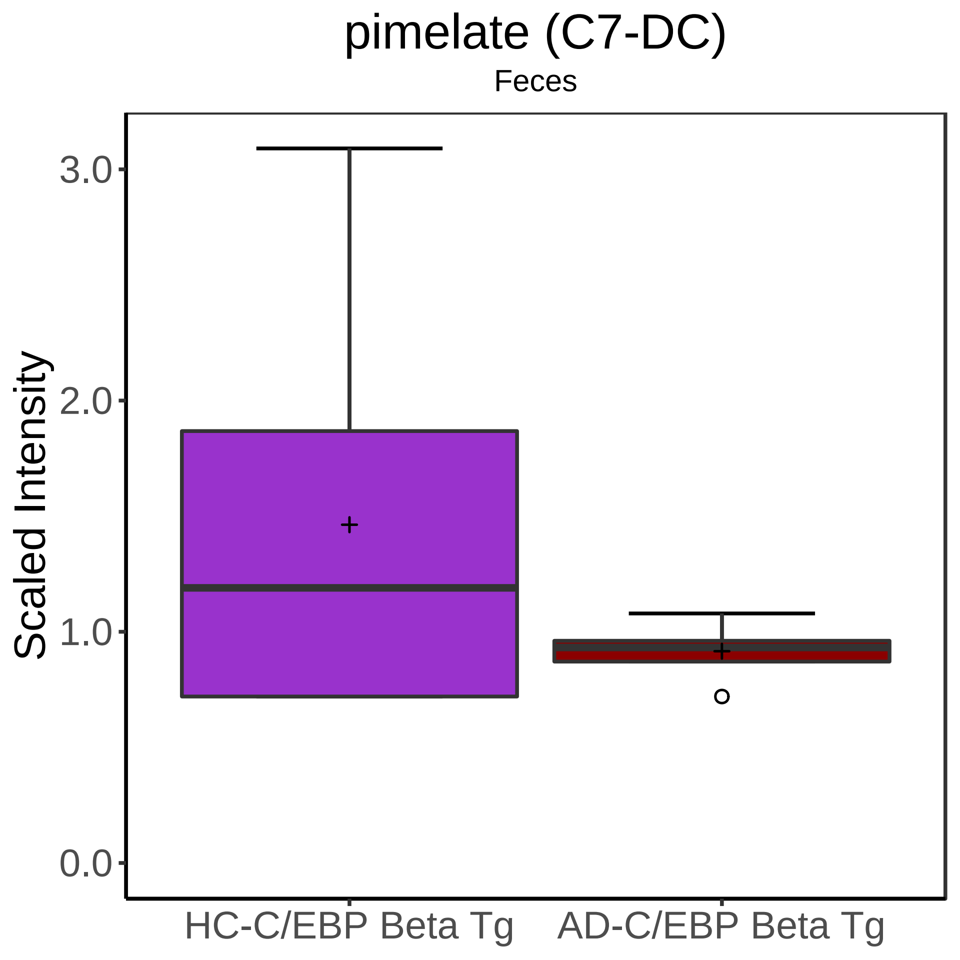
pimelate (C7-DC)
Feces
Scaled Intensity
3.0
2.0
1.0
0.0
HC-C/EBP Beta Tg
AD-C/EBP Beta Tg

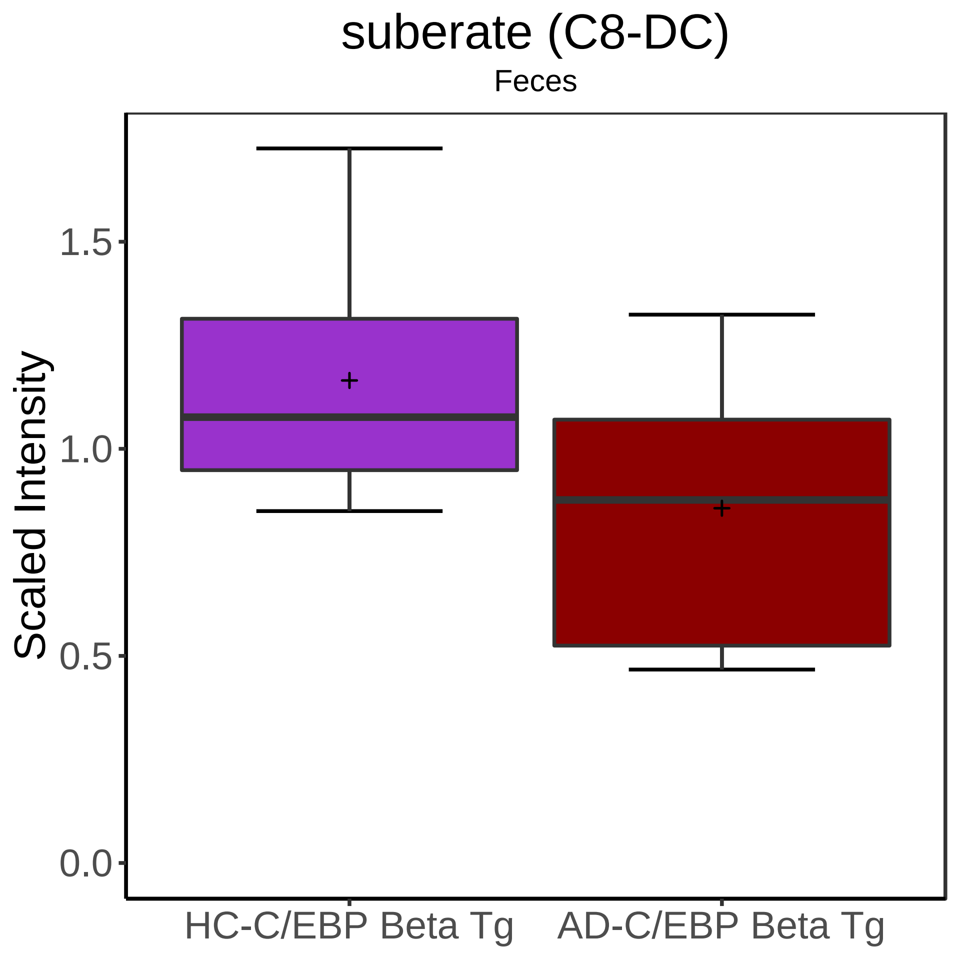
suberate (C8-DC)
Feces
Scaled Intensity
1.5
1.0
0.5
0.0
HC-C/EBP Beta Tg
AD-C/EBP Beta Tg

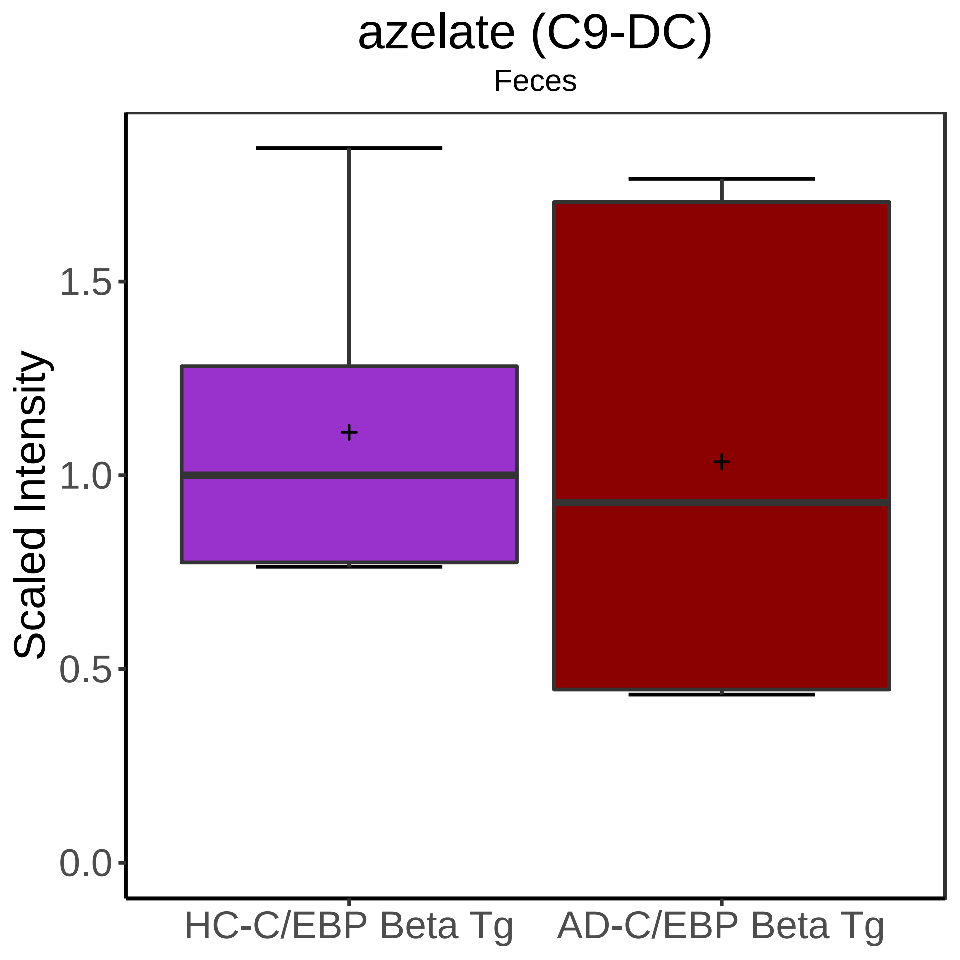
azelate (C9-DC)
Feces
Scaled Intensity
1.5
1.0
0.5
0.0
HC-C/EBP Beta Tg
AD-C/EBP Beta Tg

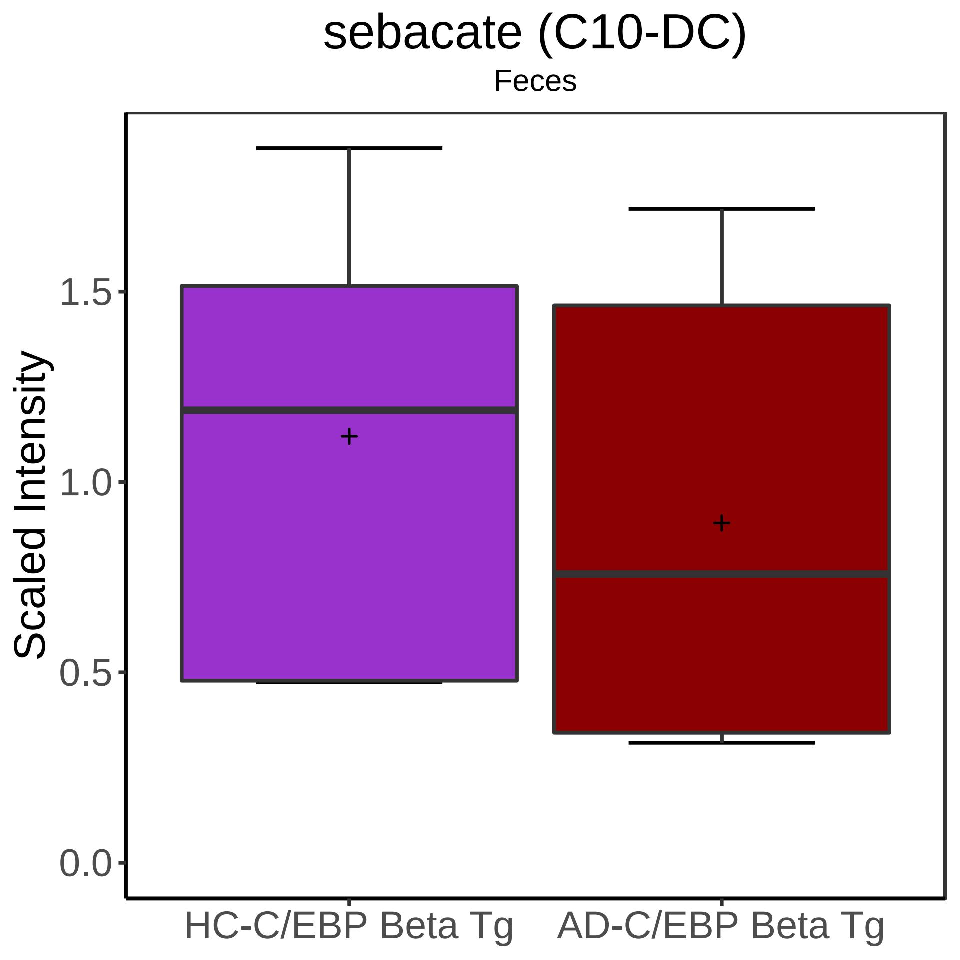
sebacate (C10-DC)
Feces
Scaled Intensity
0.0
0.5
1.0
1.5
HC-C/EBP Beta Tg
AD-C/EBP Beta Tg

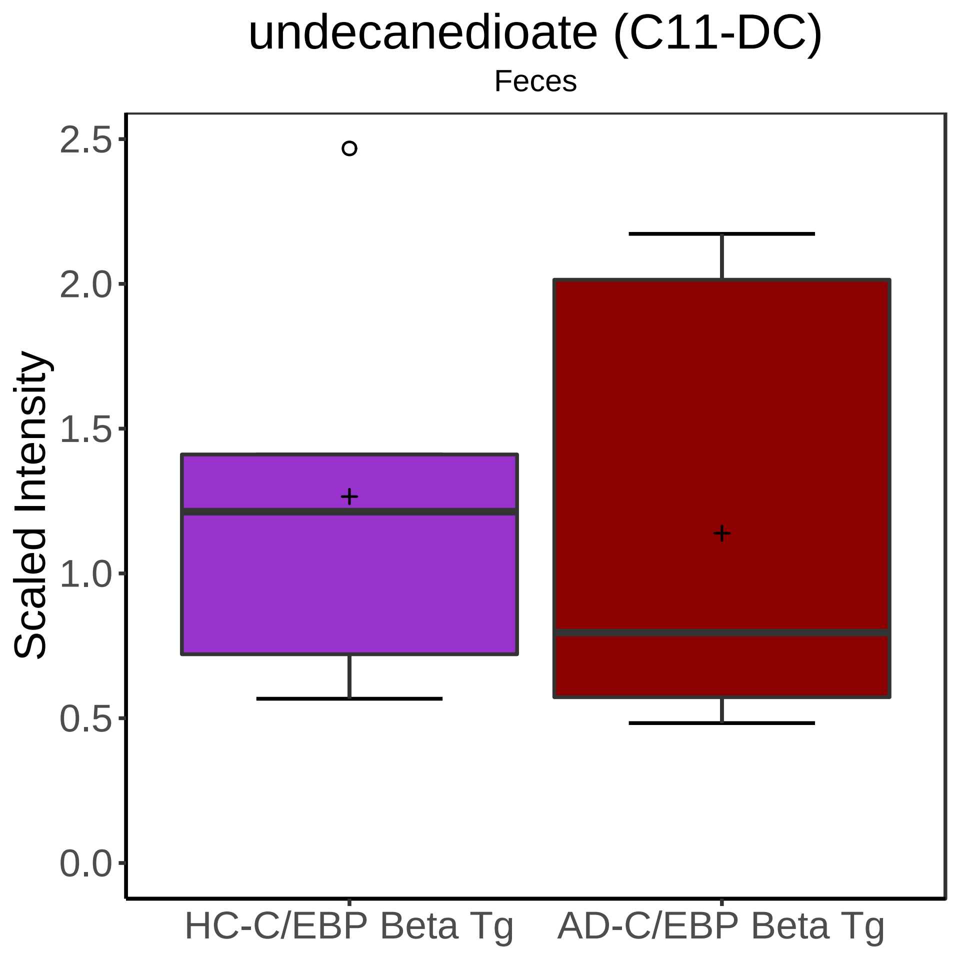
undecanedioate (C11-DC)
Feces
Scaled Intensity
2.5
2.0
1.5
1.0
0.5
0.0
HC-C/EBP Beta Tg
AD-C/EBP Beta Tg

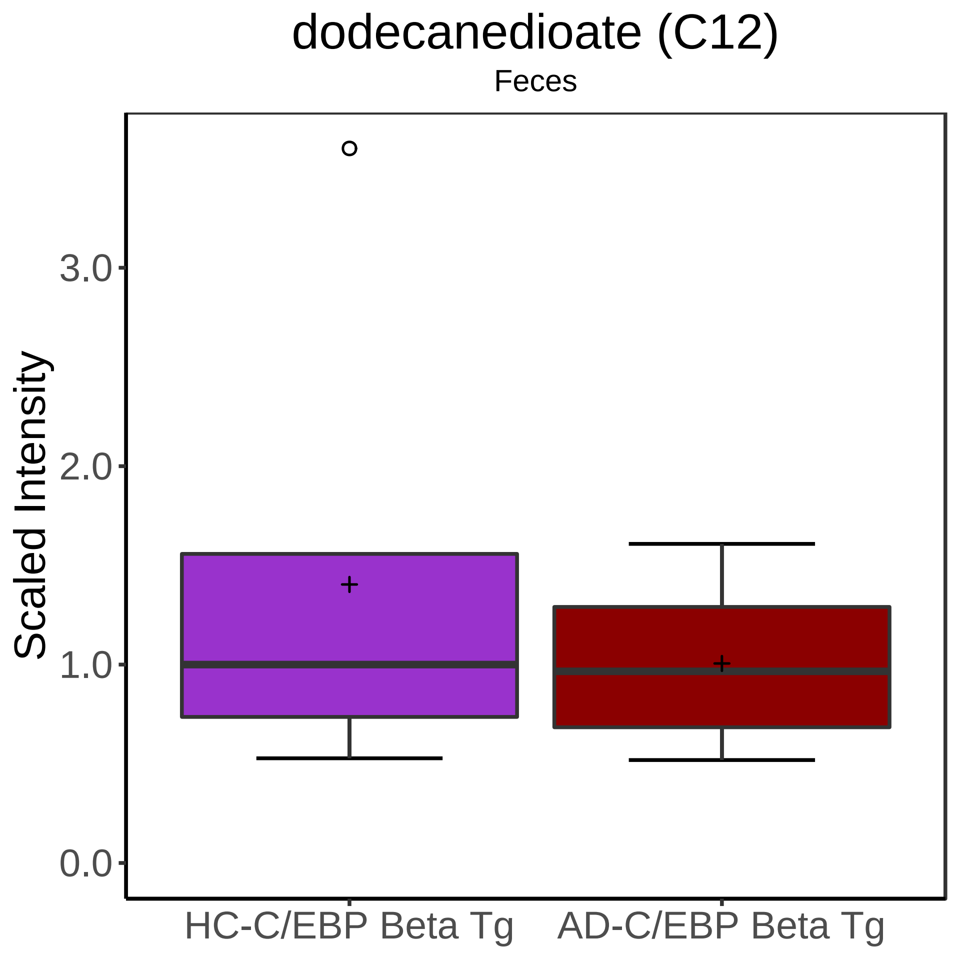

dodecanedioate (C12)
Feces
Scaled Intensity
3.0
2.0
1.0
0.0
HC-C/EBP Beta Tg
AD-C/EBP Beta Tg

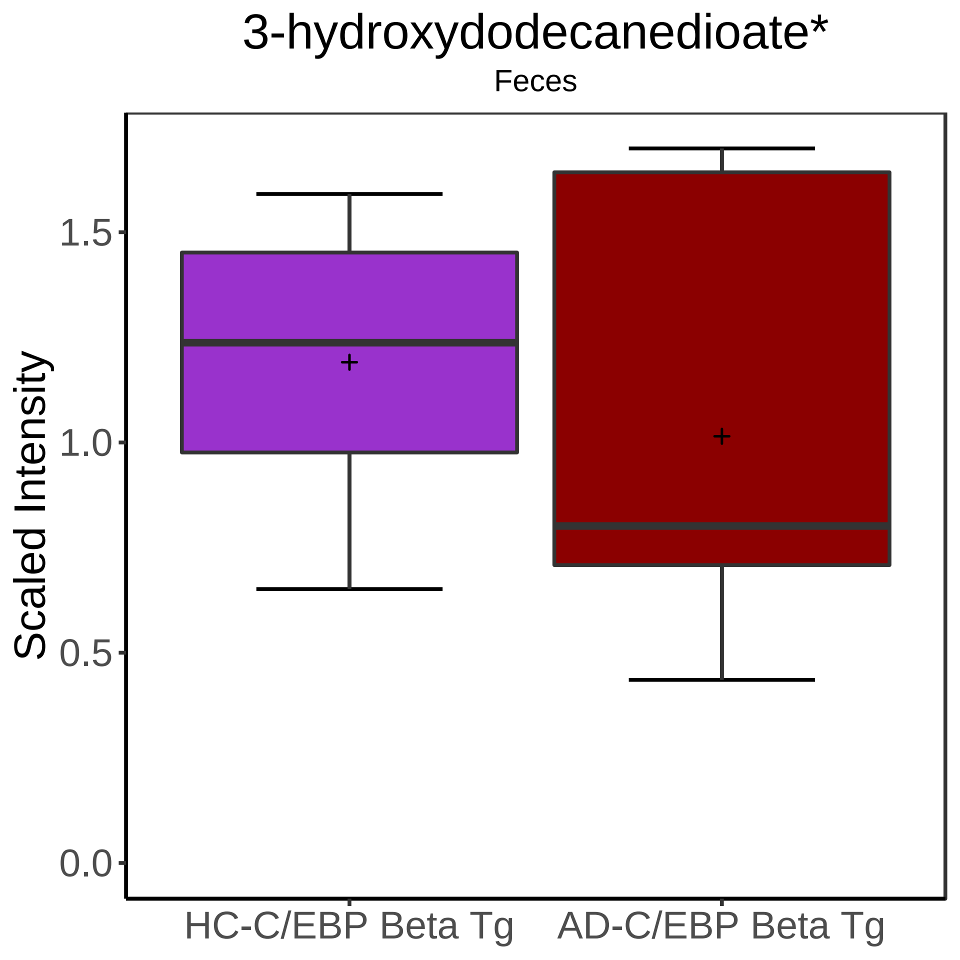
3-hydroxydodecanedioate*
Feces
Scaled Intensity
0.0
0.5
1.0
1.5
HC-C/EBP Beta Tg
AD-C/EBP Beta Tg

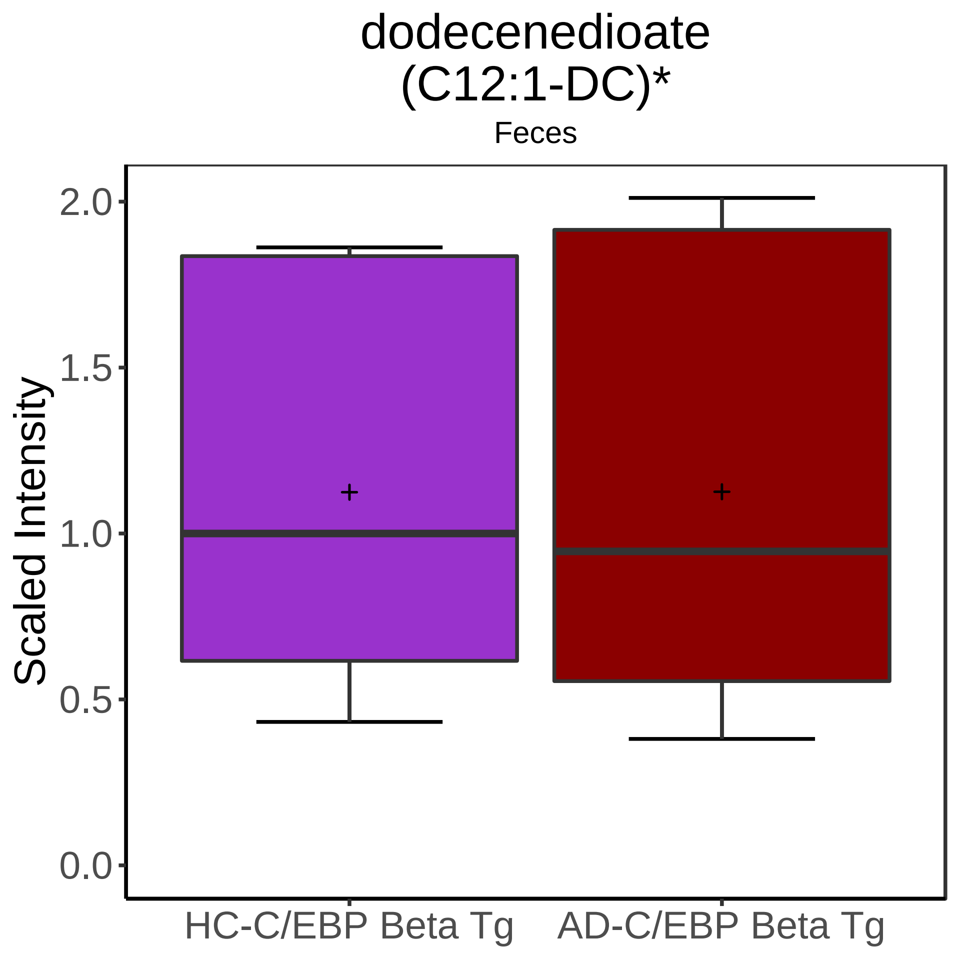

dodecenedioate
(C12:1-DC)*
Feces
Scaled Intensity
2.0
1.5
1.0
0.5
0.0
HC-C/EBP Beta Tg
AD-C/EBP Beta Tg

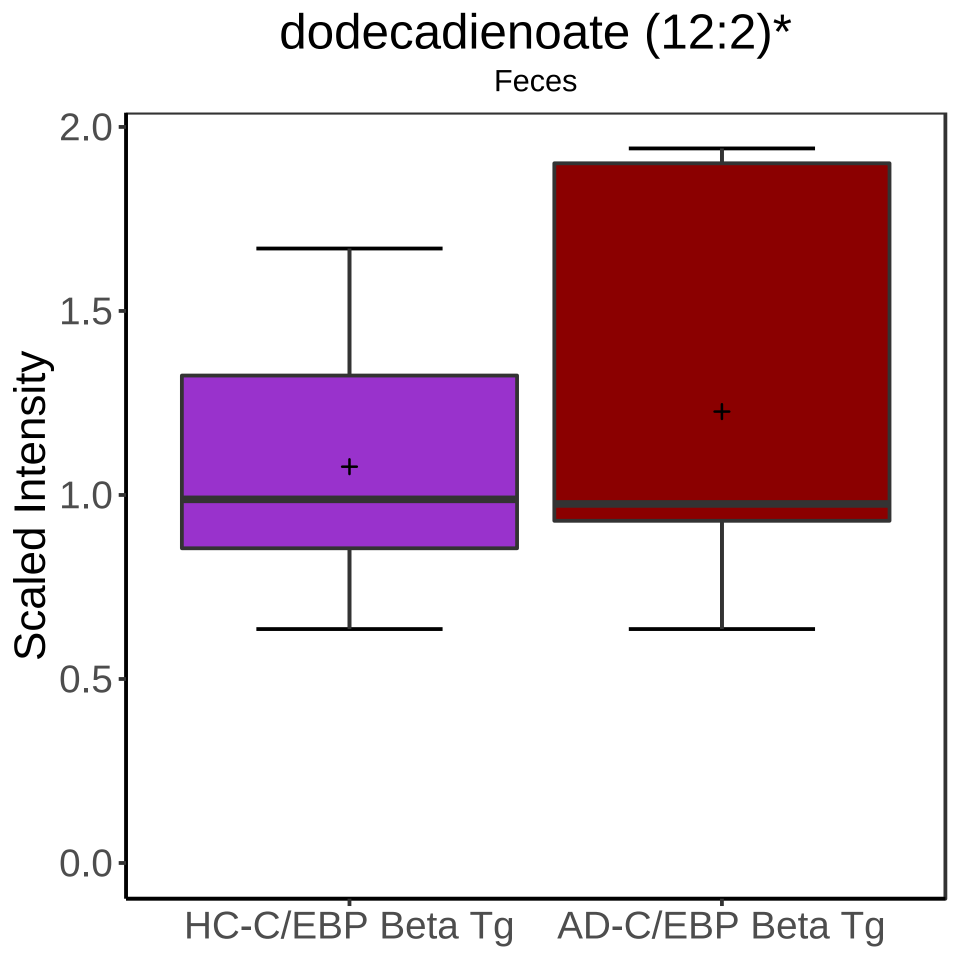

dodecadienoate (12:2)*
Feces
Scaled Intensity
2.0
1.5
1.0
0.5
0.0
HC-C/EBP Beta Tg
AD-C/EBP Beta Tg

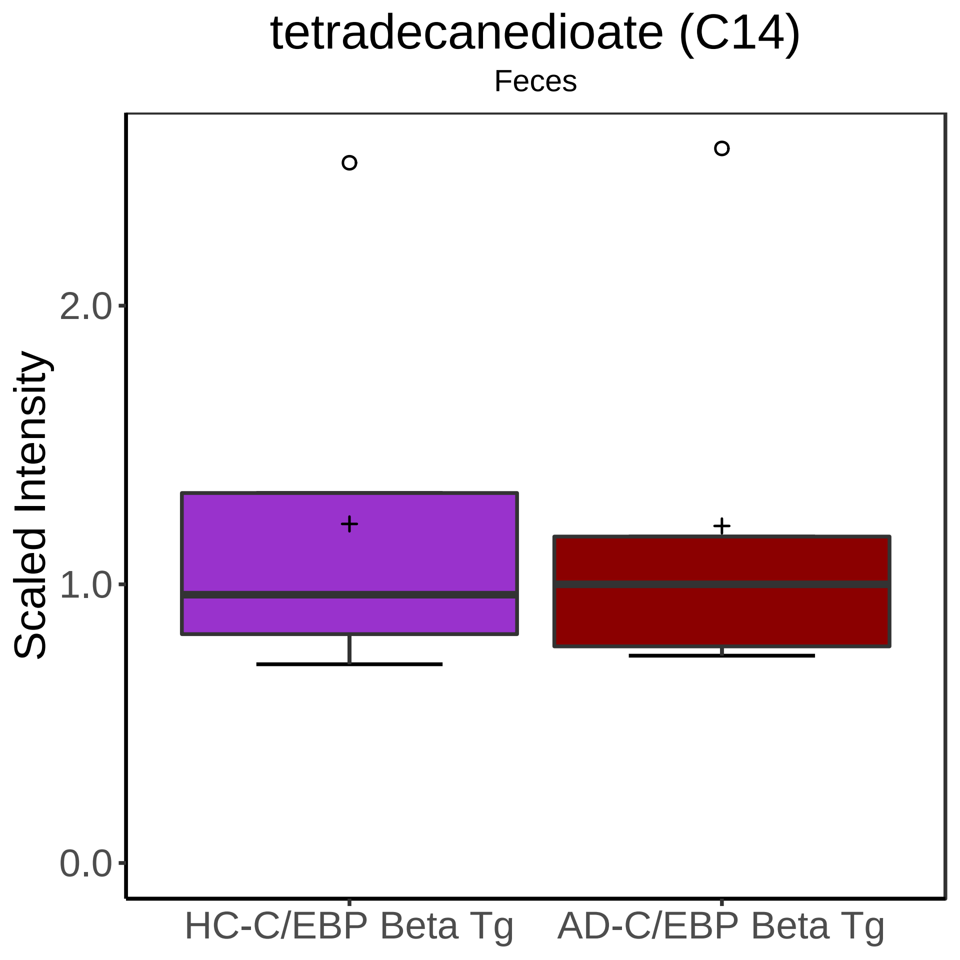

tetradecanedioate (C14)
Feces
Scaled Intensity
2.0
1.0
0.0
HC-C/EBP Beta Tg
AD-C/EBP Beta Tg

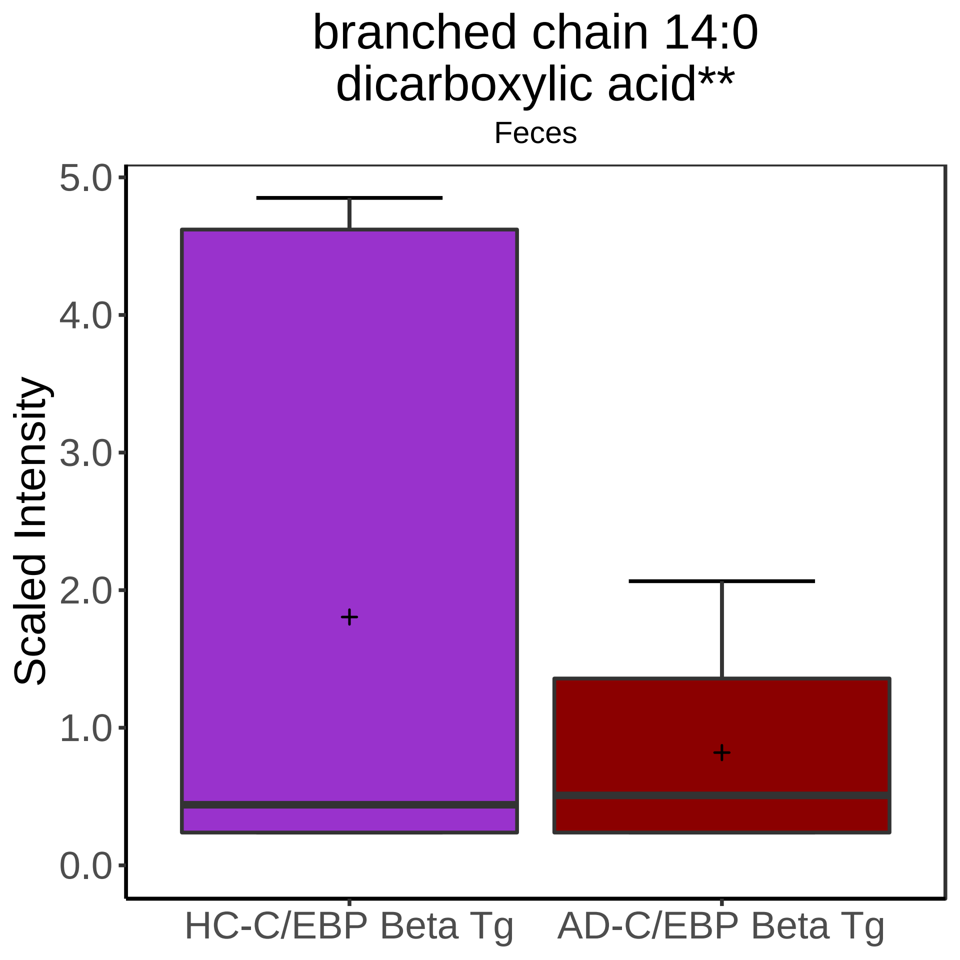
branched chain 14:0
dicarboxylic acid**
Feces
Scaled Intensity
5.0
4.0
3.0
2.0
1.0
0.0
HC-C/EBP Beta Tg
AD-C/EBP Beta Tg

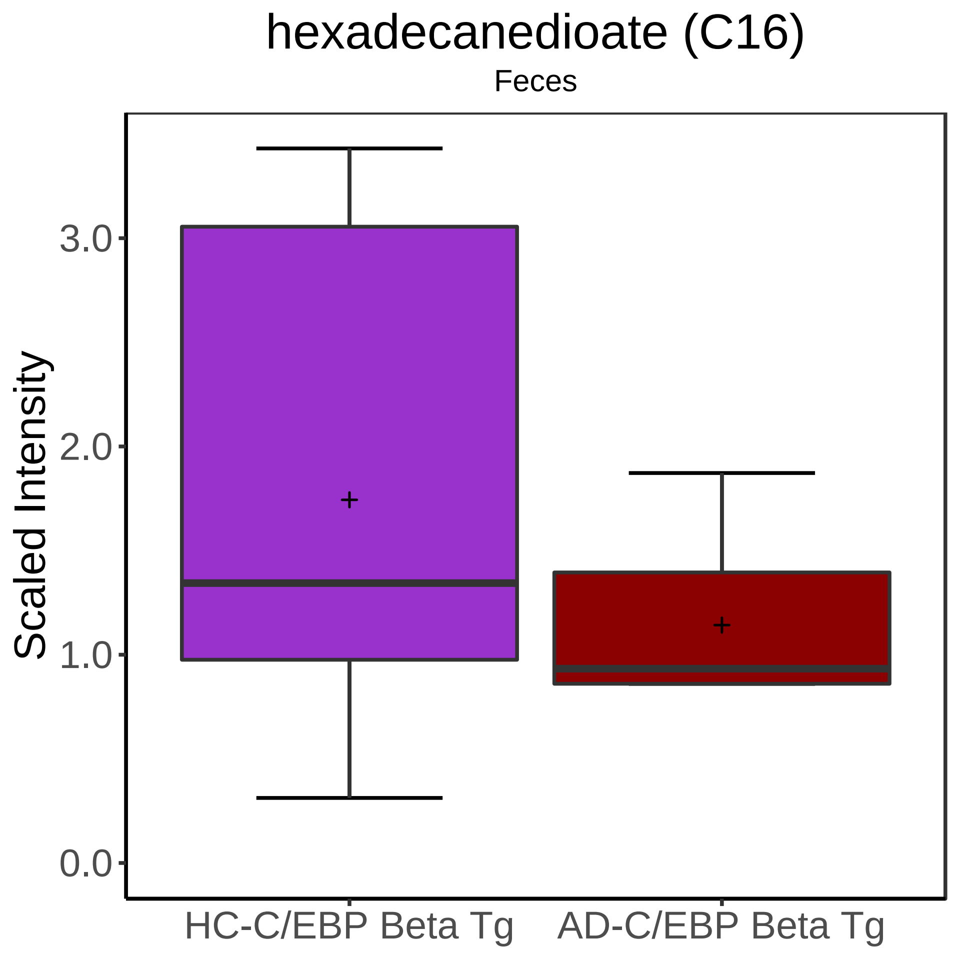
hexadecanedioate (C16)
Feces
Scaled Intensity
3.0
2.0
1.0
0.0
HC-C/EBP Beta Tg
AD-C/EBP Beta Tg

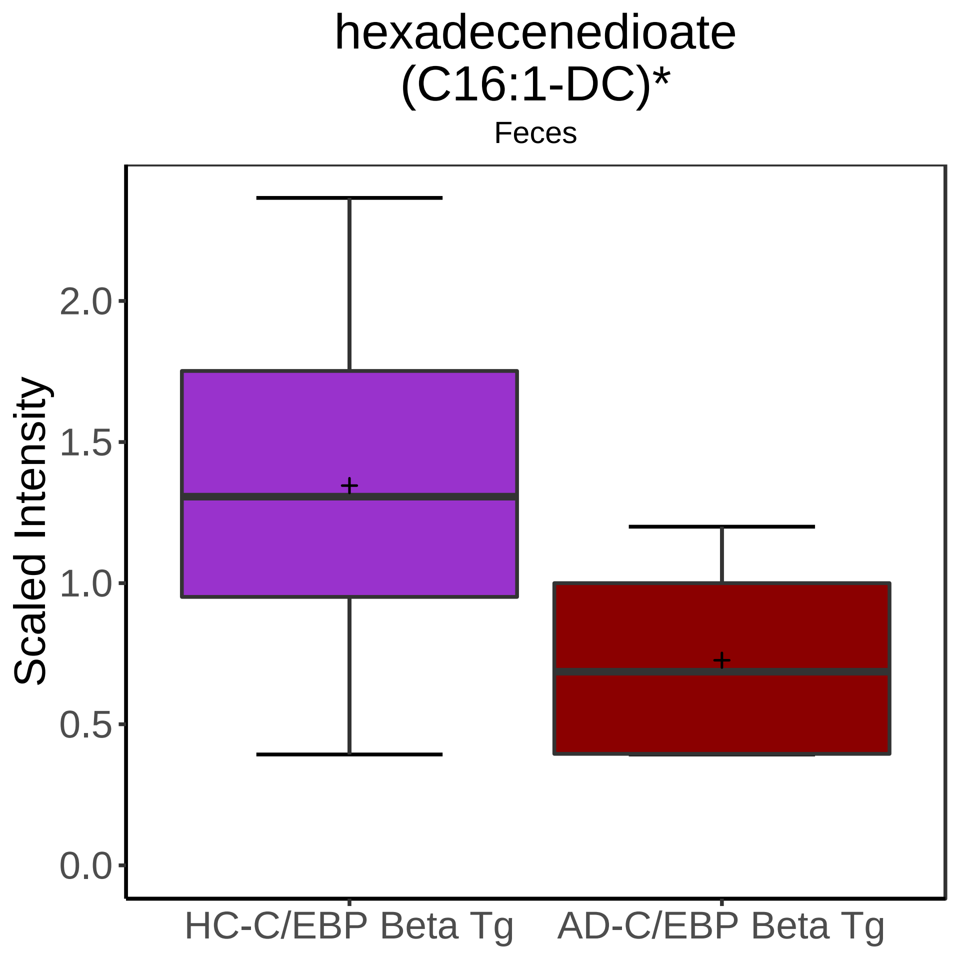
hexadecenedioate
(C16:1-DC)*
Feces
Scaled Intensity
2.0
1.5
1.0
0.5
0.0
HC-C/EBP Beta Tg
AD-C/EBP Beta Tg

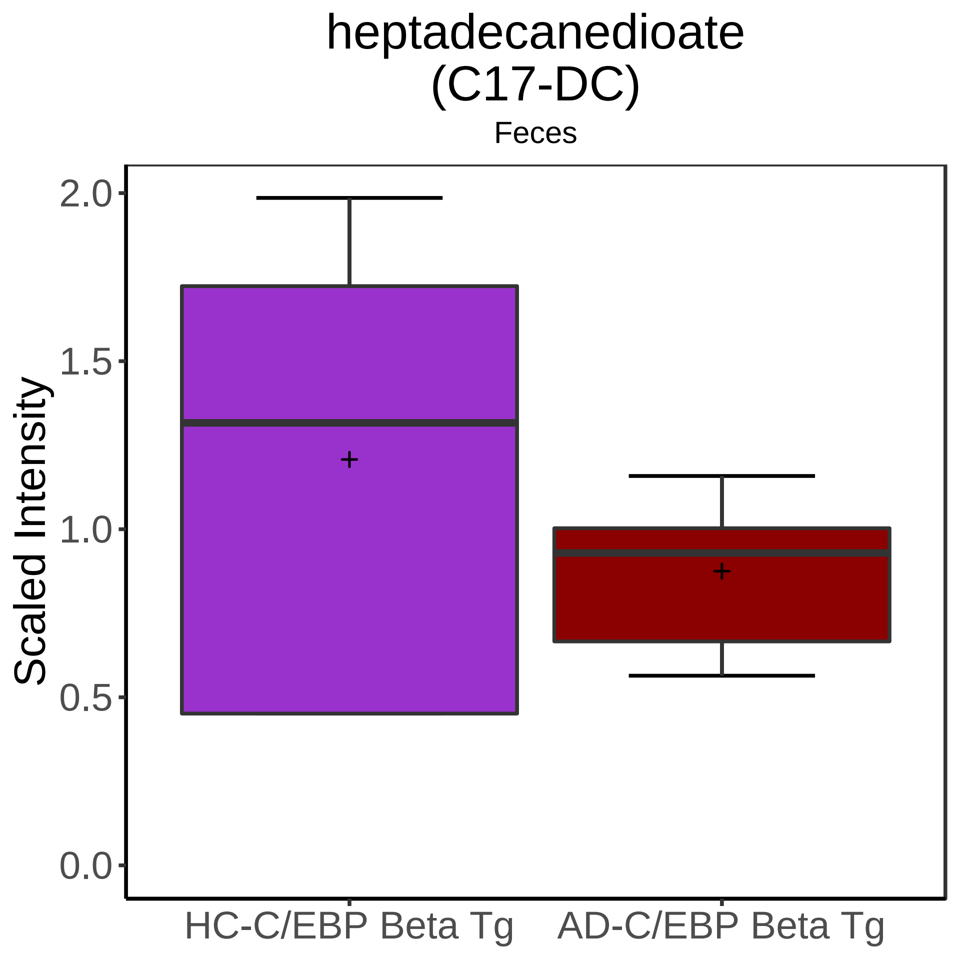
heptadecanedioate
(C17-DC)
Feces
Scaled Intensity
2.0
1.5
1.0
0.5
0.0
HC-C/EBP Beta Tg
AD-C/EBP Beta Tg

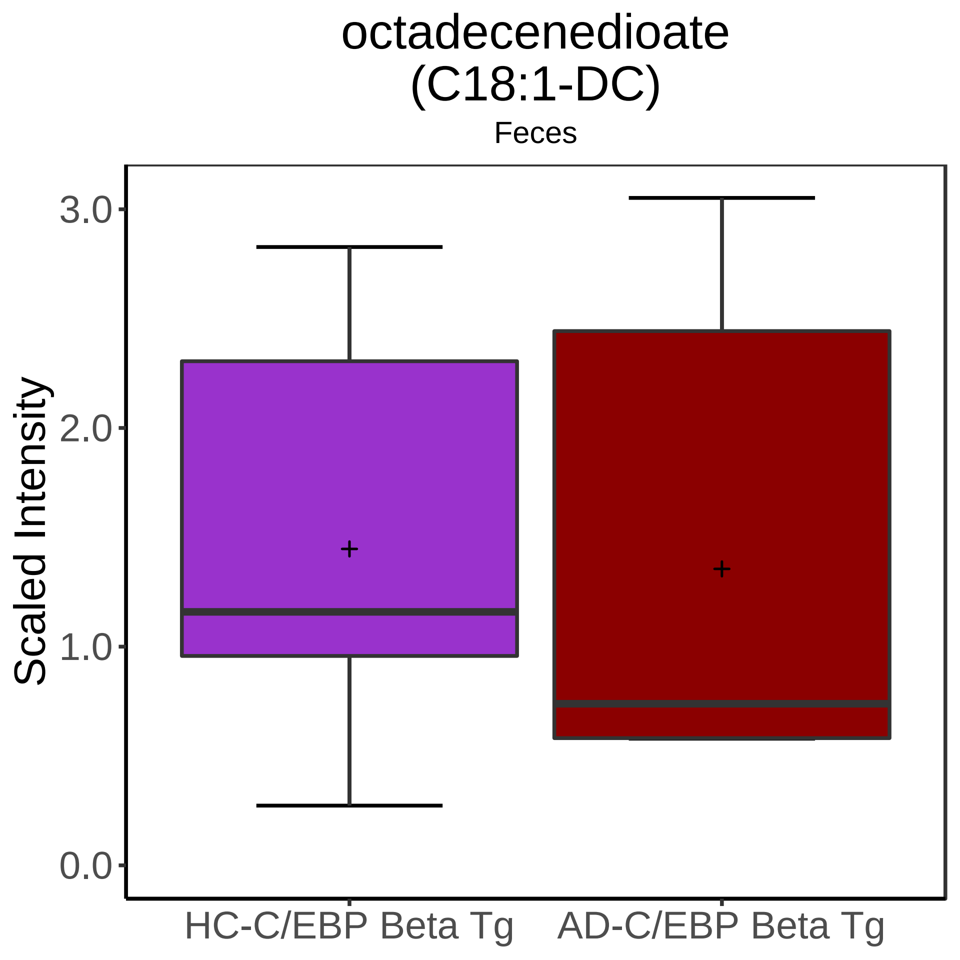

octadecenedioate
(C18:1-DC)
Feces
Scaled Intensity
3.0
2.0
1.0
0.0
HC-C/EBP Beta Tg
AD-C/EBP Beta Tg

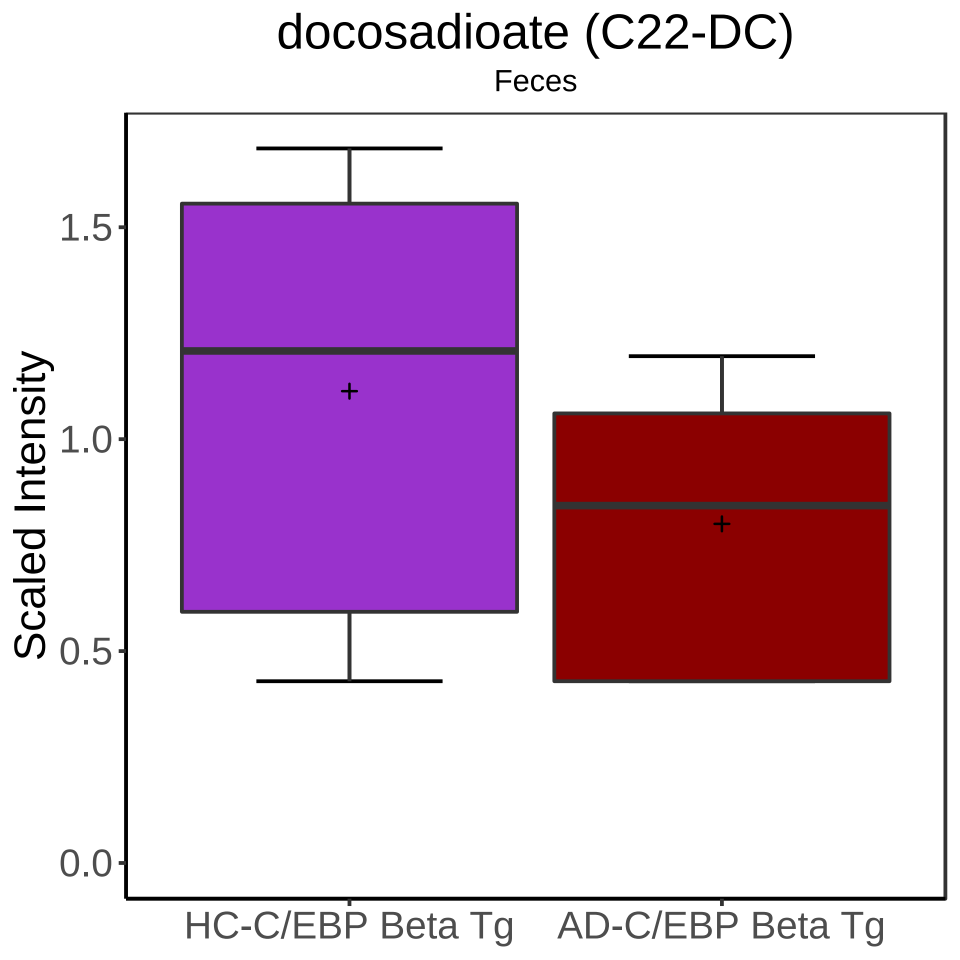
docosadioate (C22-DC)
Feces
Scaled Intensity
1.5
1.0
0.5
0.0
HC-C/EBP Beta Tg
AD-C/EBP Beta Tg

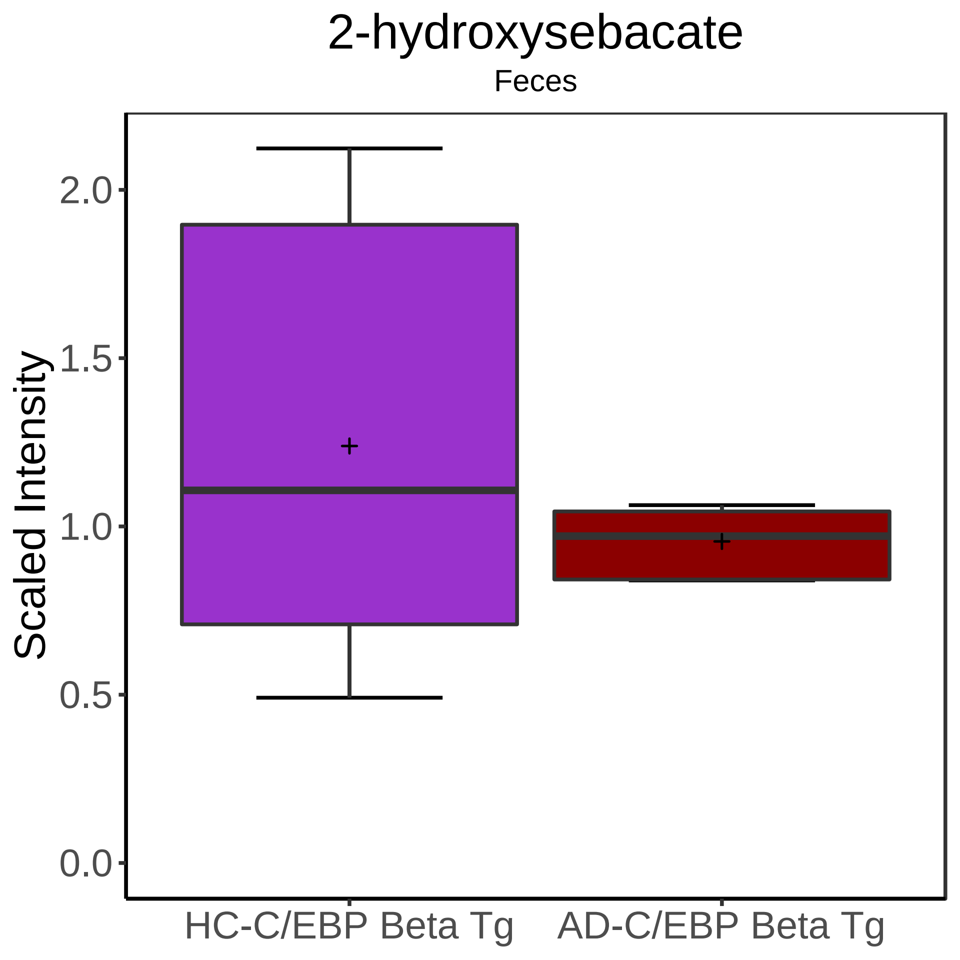

2-hydroxysebacate
Feces
Scaled Intensity
2.0
1.5
1.0
0.5
0.0
HC-C/EBP Beta Tg
AD-C/EBP Beta Tg

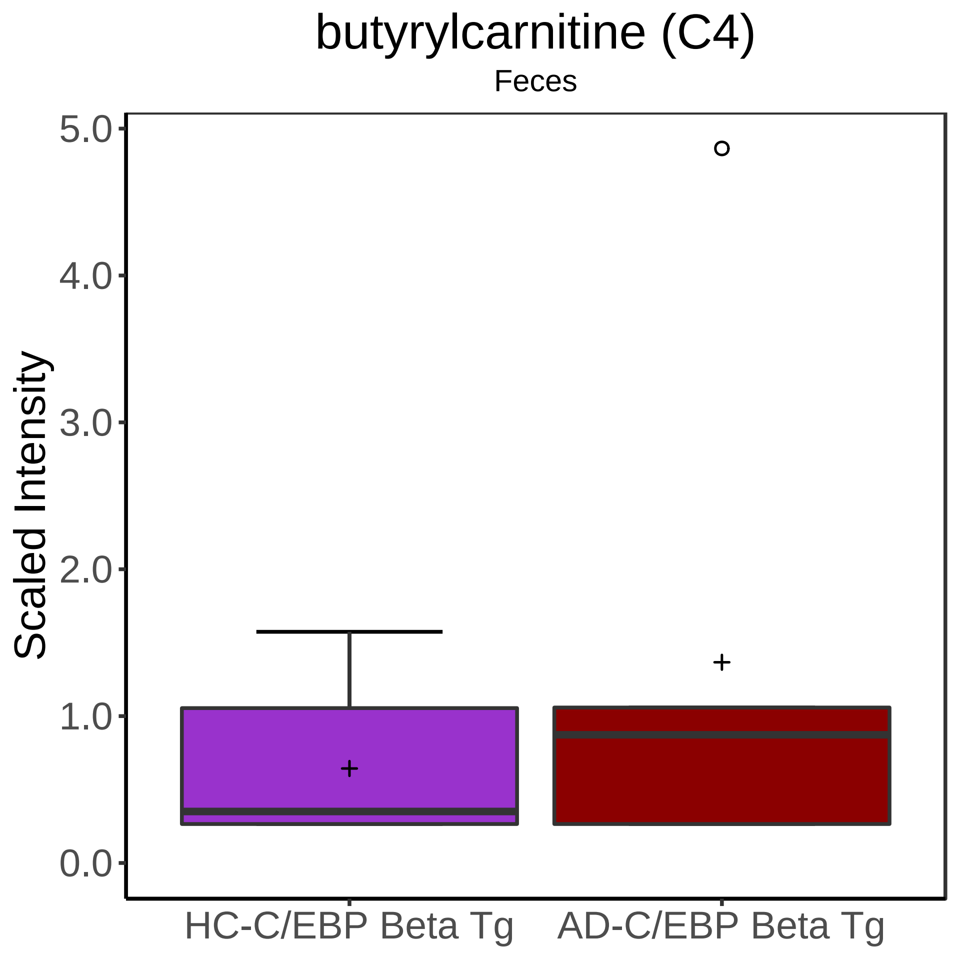

butyrylcarnitine (C4)
Feces
Scaled Intensity
5.0
4.0
3.0
2.0
1.0
0.0
HC-C/EBP Beta Tg
AD-C/EBP Beta Tg

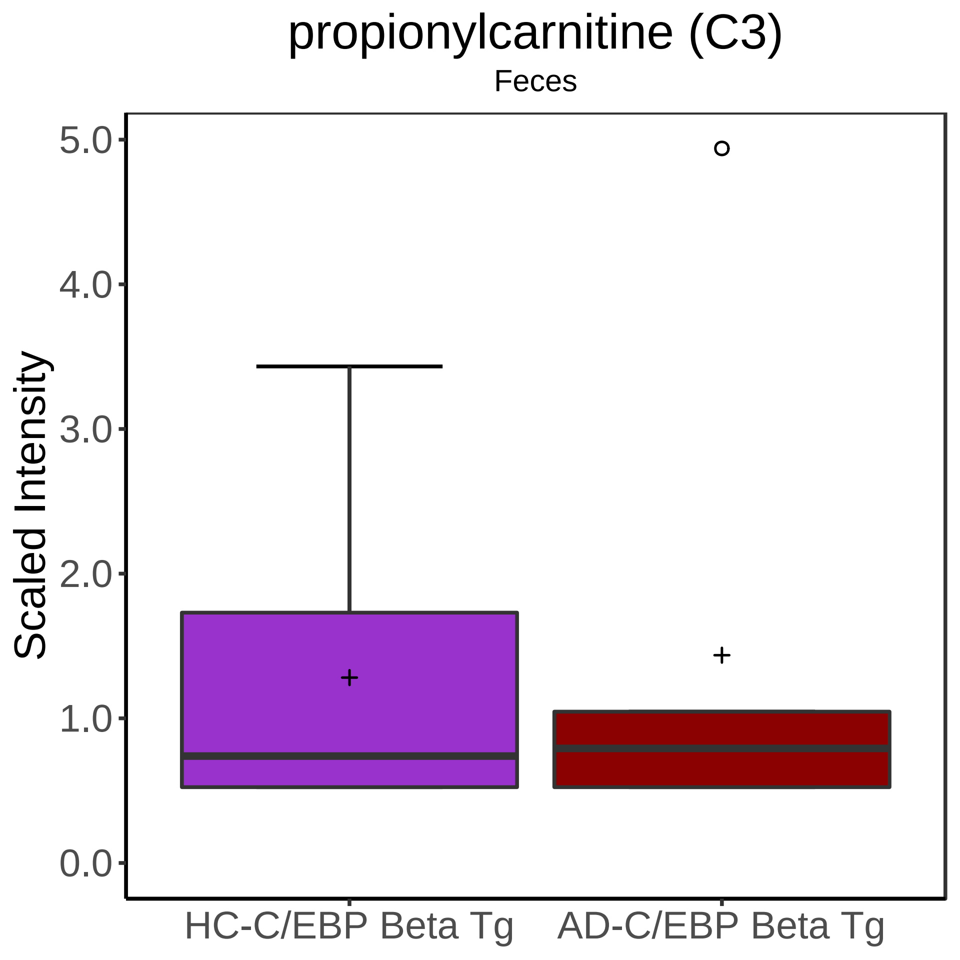
propionylcarnitine (C3)
Feces
Scaled Intensity
5.0
4.0
3.0
2.0
1.0
0.0
HC-C/EBP Beta Tg
AD-C/EBP Beta Tg

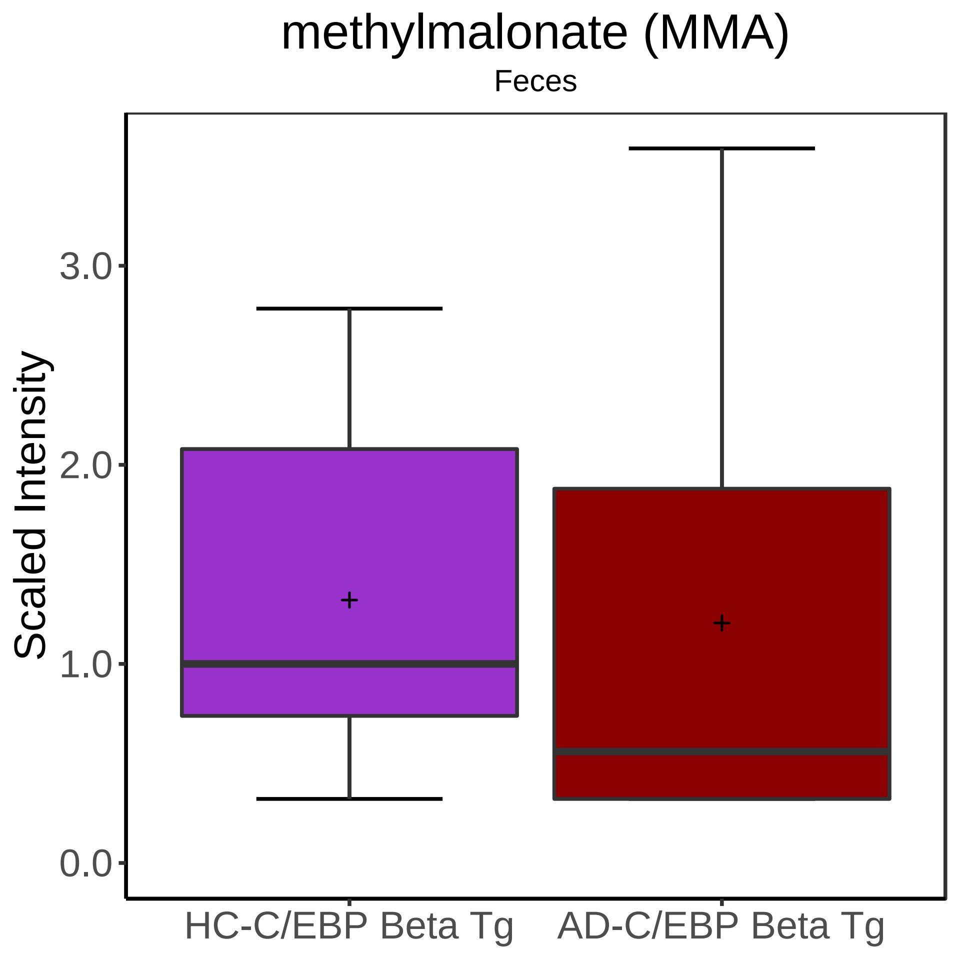
methylmalonate (MMA)
Feces
Scaled Intensity
3.0
2.0
1.0
0.0
HC-C/EBP Beta Tg
AD-C/EBP Beta Tg

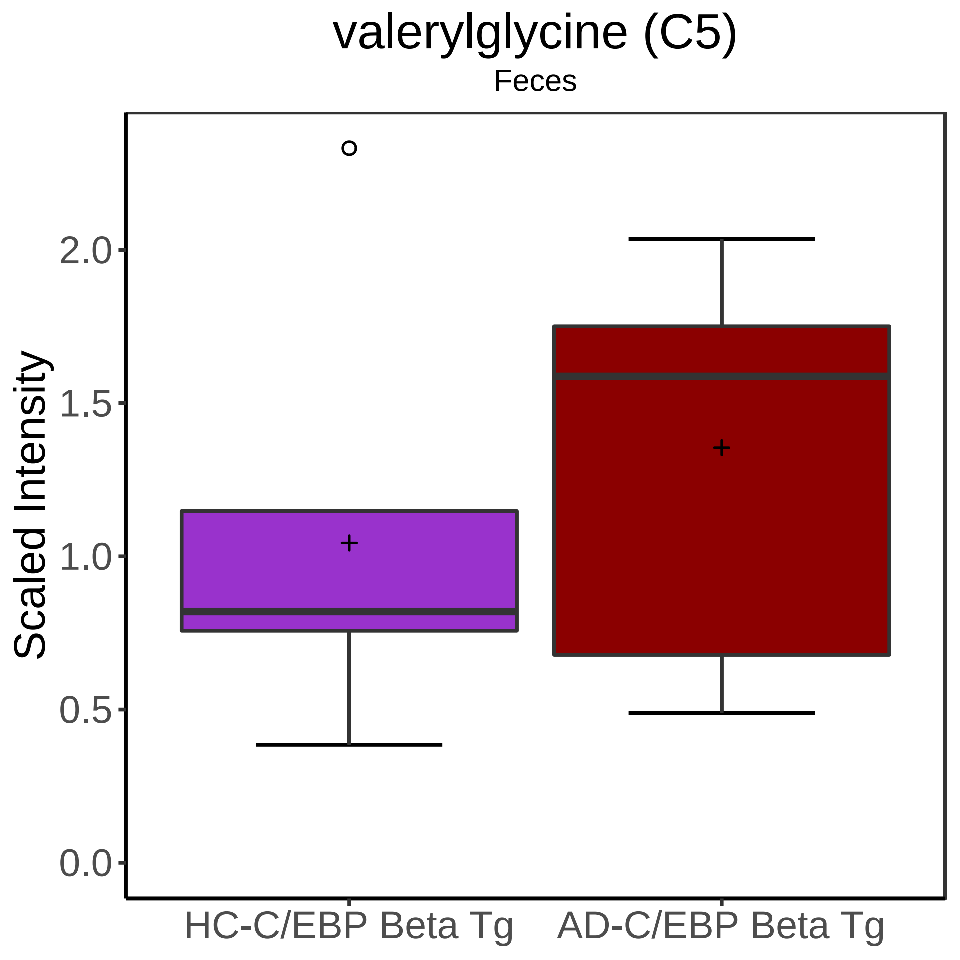

valerylglycine (C5)
Feces
Scaled Intensity
2.0
1.5
1.0
0.5
0.0
HC-C/EBP Beta Tg
AD-C/EBP Beta Tg

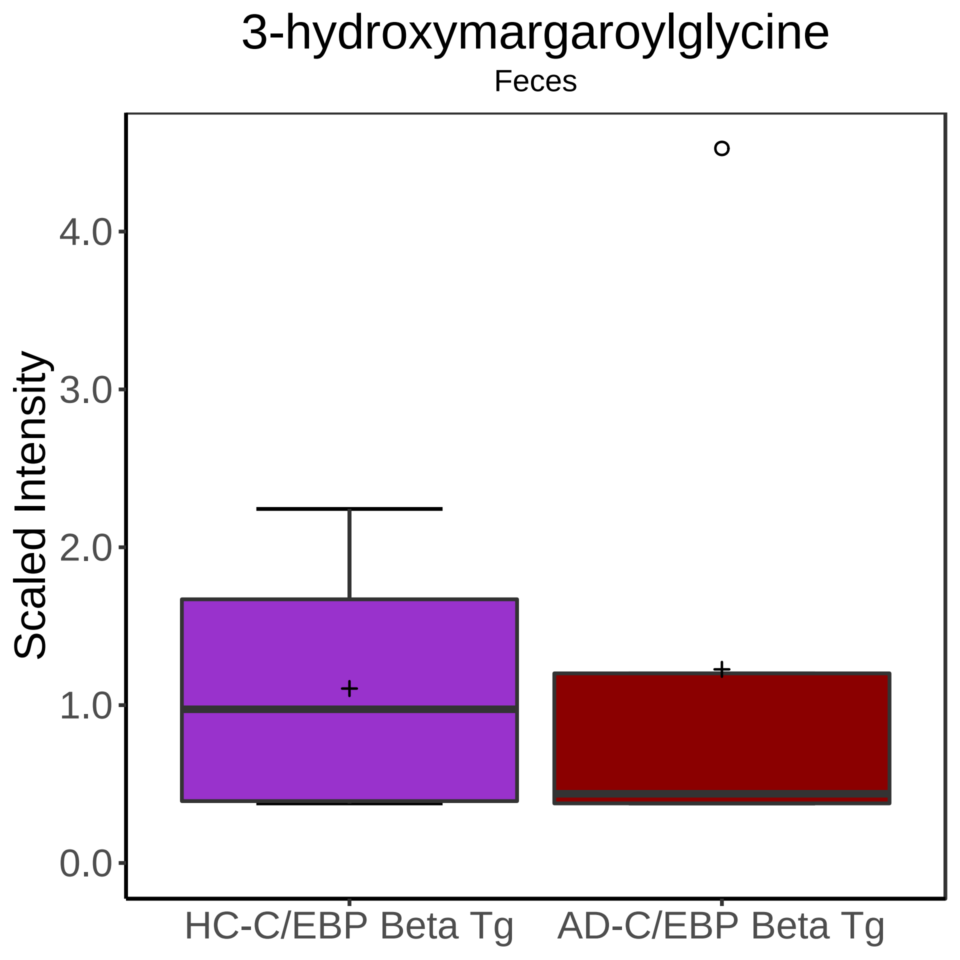
3-hydroxymargaroylglycine
Feces
Scaled Intensity
4.0
3.0
2.0
1.0
0.0
HC-C/EBP Beta Tg
AD-C/EBP Beta Tg

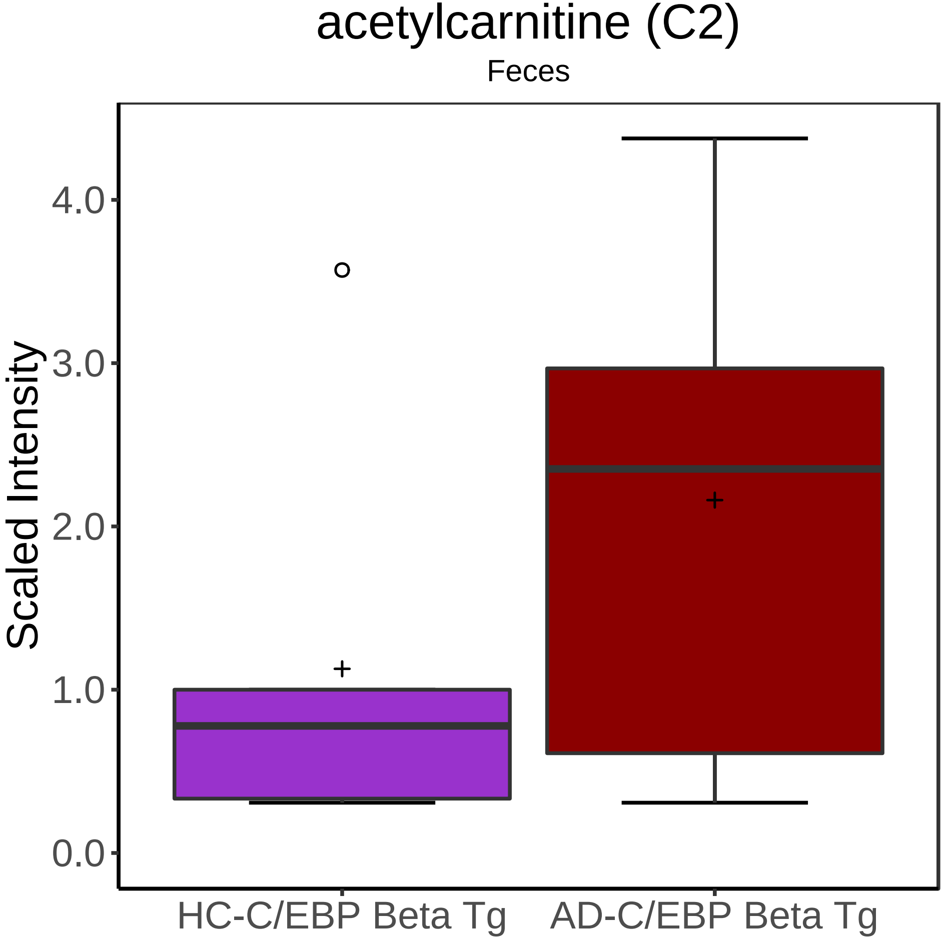
acetylcarnitine (C2)
Feces
Scaled Intensity
4.0
3.0
2.0
1.0
0.0
HC-C/EBP Beta Tg
AD-C/EBP Beta Tg

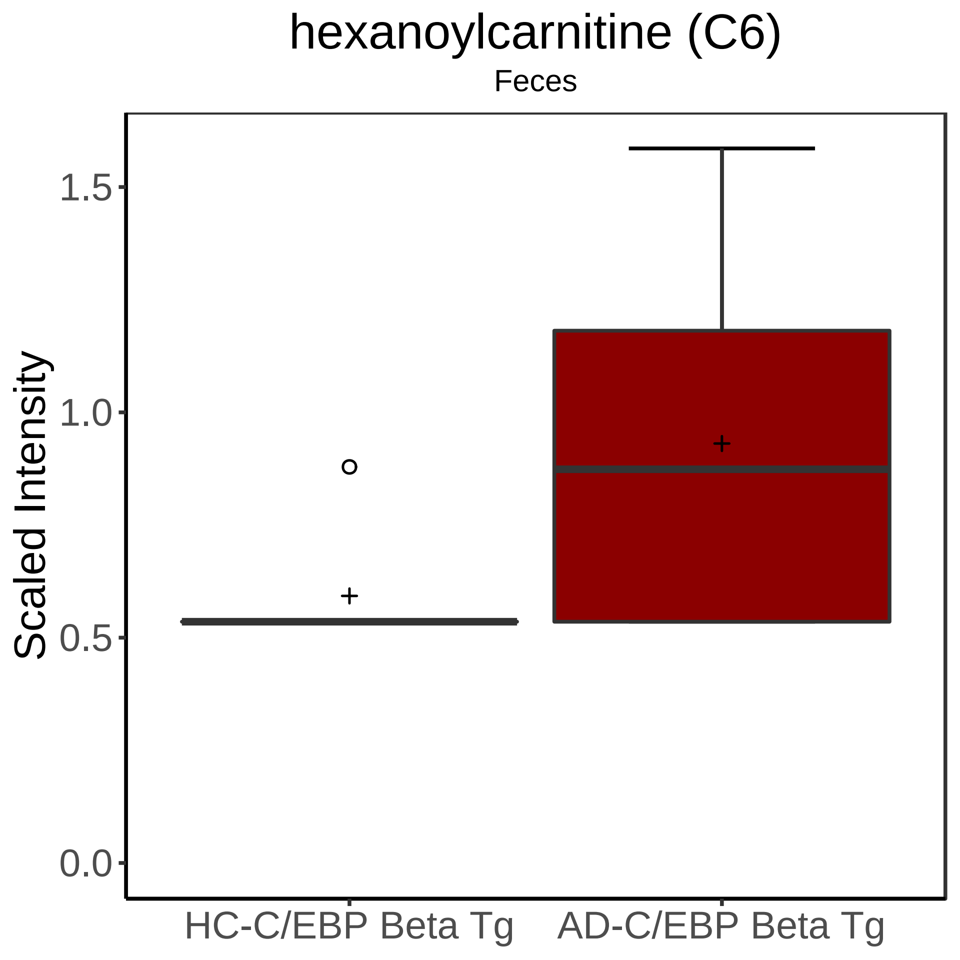

hexanoylcarnitine (C6)
Feces
Scaled Intensity
1.5
1.0
0.5
0.0
HC-C/EBP Beta Tg
AD-C/EBP Beta Tg

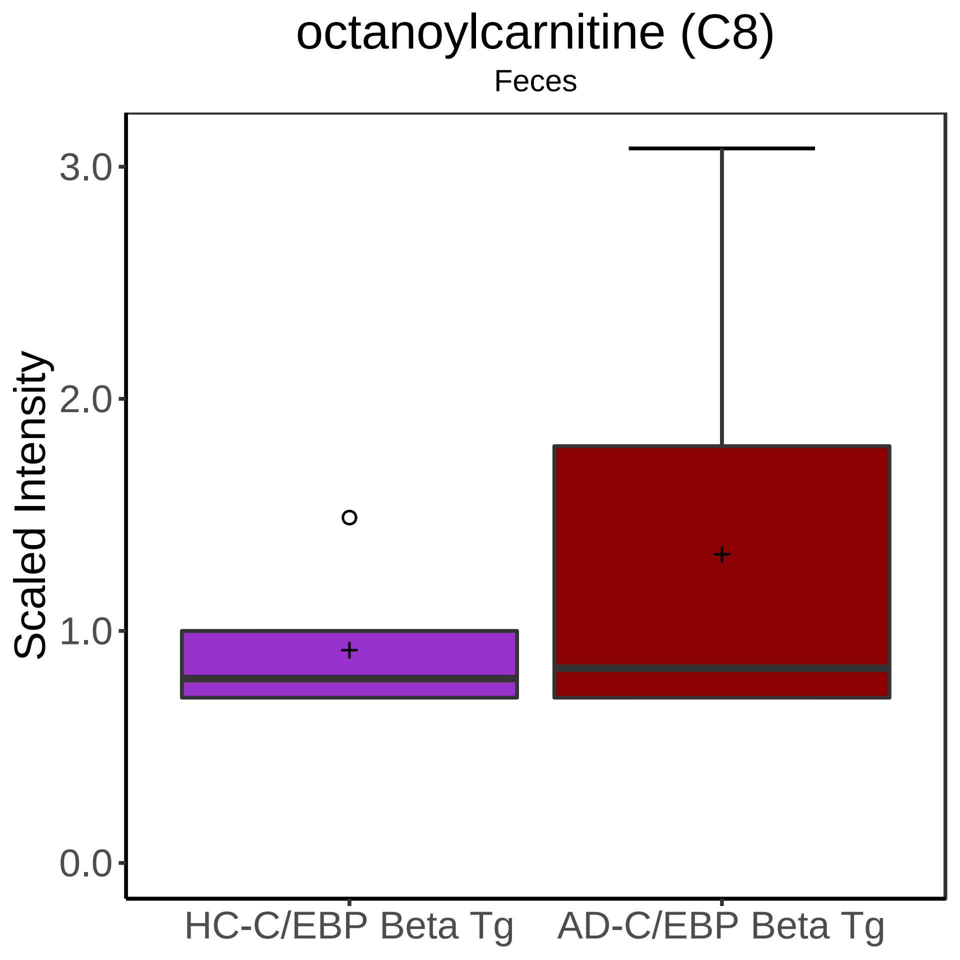
octanoylcarnitine (C8)
Feces
Scaled Intensity
3.0
2.0
1.0
0.0
HC-C/EBP Beta Tg
AD-C/EBP Beta Tg

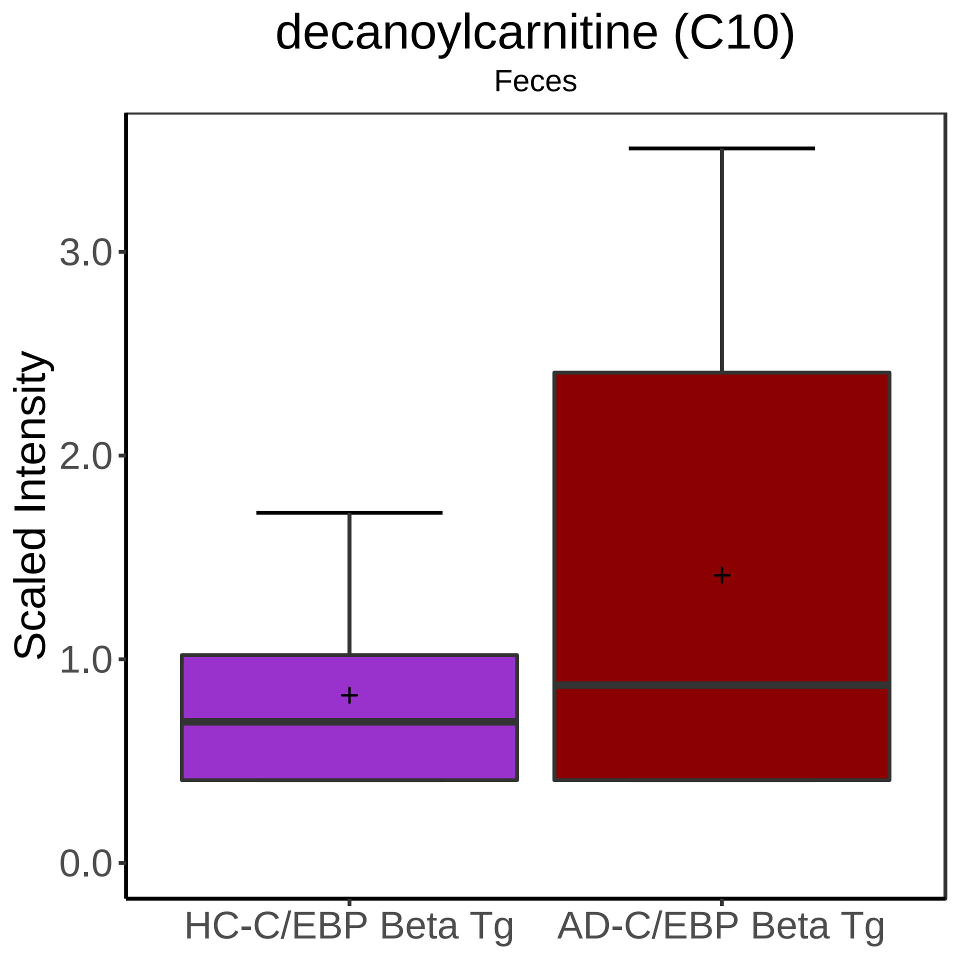
decanoylcarnitine (C10)
Feces
Scaled Intensity
3.0
2.0
1.0
0.0
HC-C/EBP Beta Tg
AD-C/EBP Beta Tg

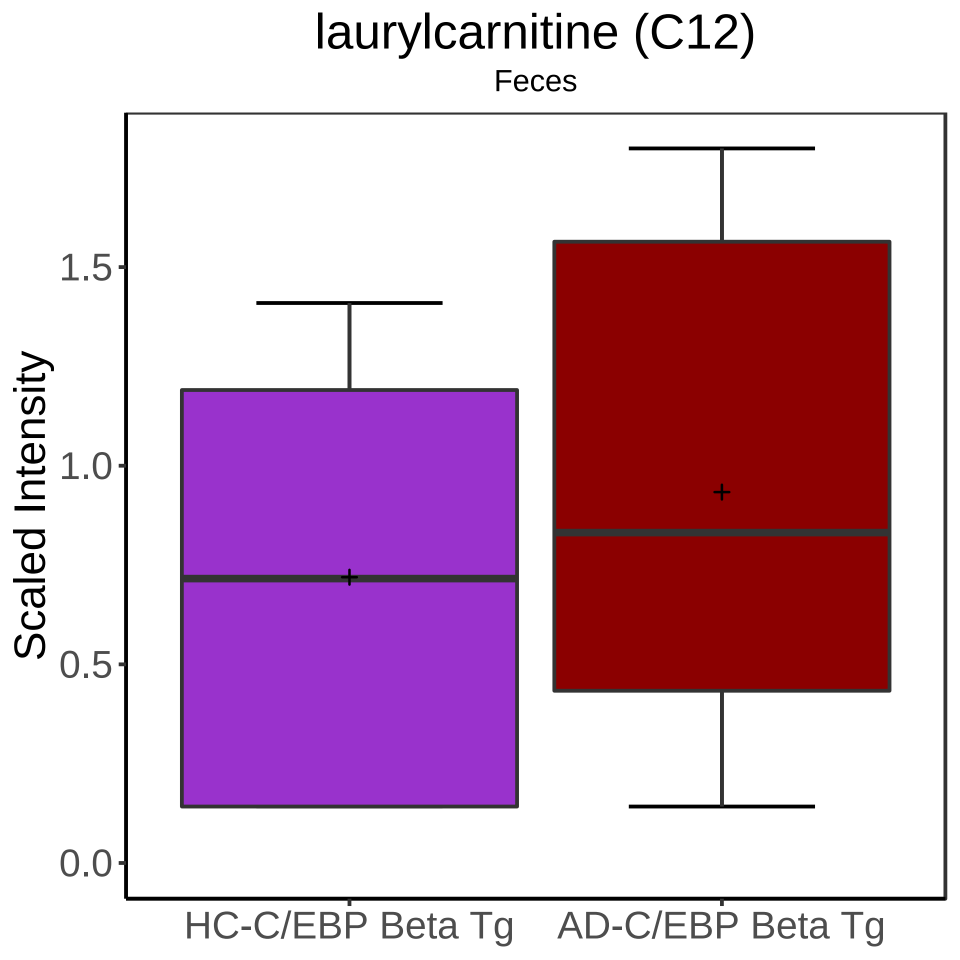
laurylcarnitine (C12)
Feces
Scaled Intensity
1.5
1.0
0.5
0.0
HC-C/EBP Beta Tg
AD-C/EBP Beta Tg

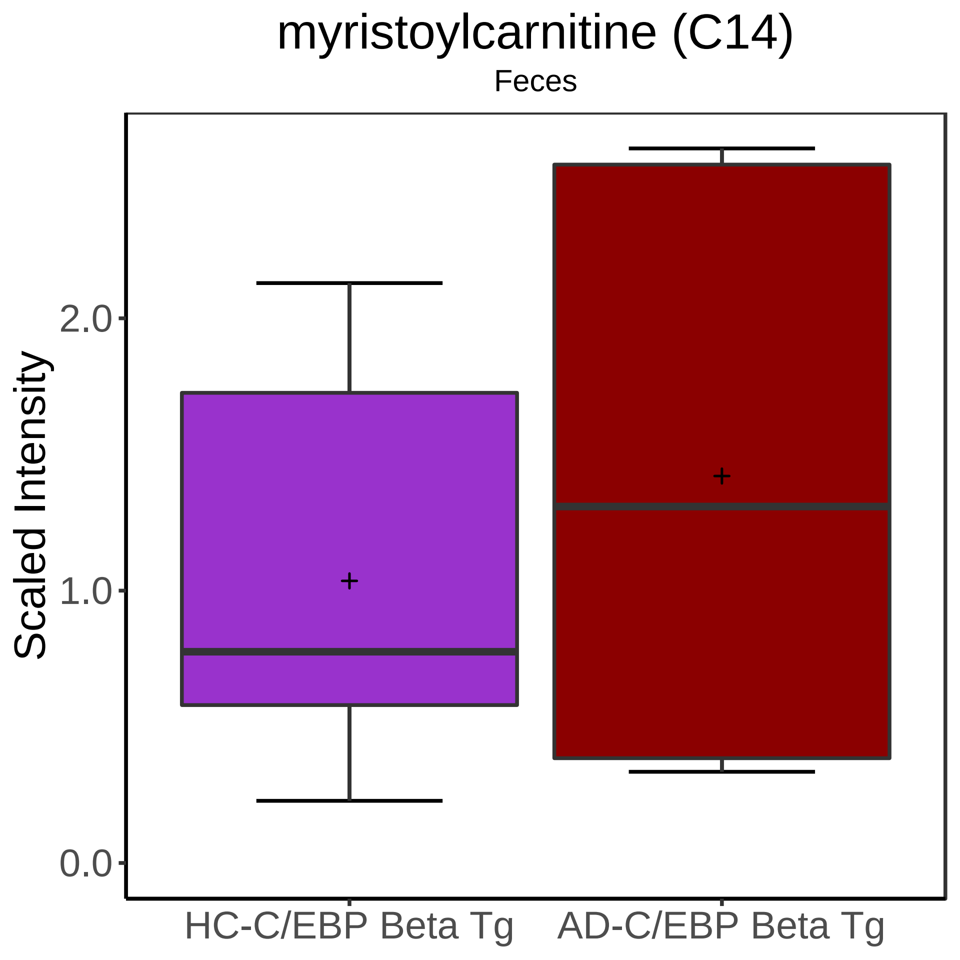
myristoylcarnitine (C14)
Feces
Scaled Intensity
2.0
1.0
0.0
HC-C/EBP Beta Tg
AD-C/EBP Beta Tg

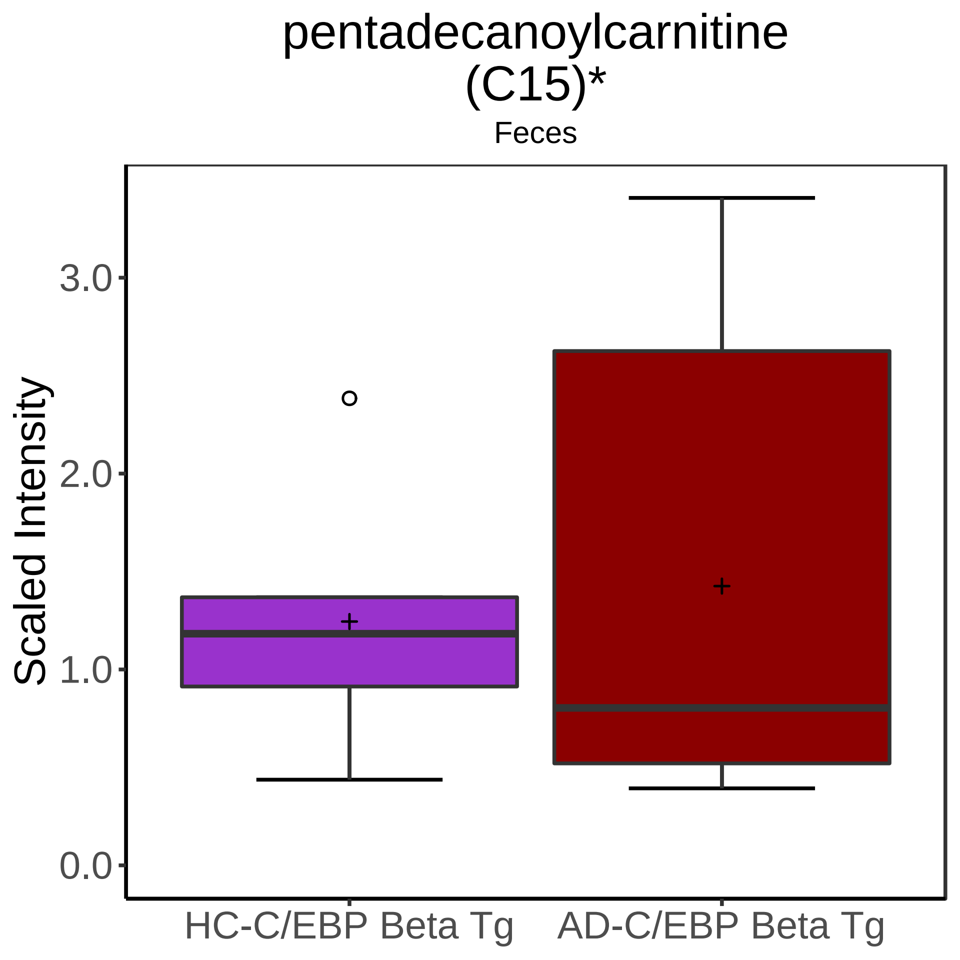

pentadecanoylcarnitine
(C15)*
Feces
Scaled Intensity
3.0
2.0
1.0
0.0
HC-C/EBP Beta Tg
AD-C/EBP Beta Tg

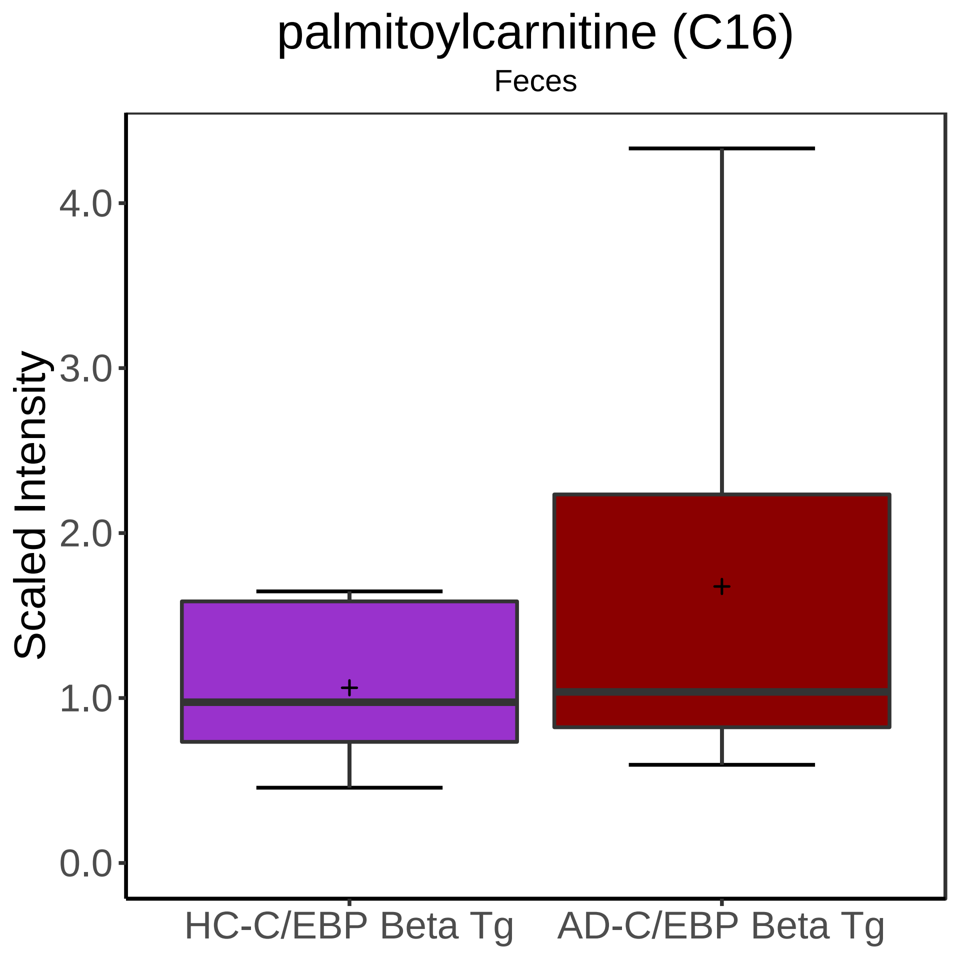
palmitoylcarnitine (C16)
Feces
Scaled Intensity
0.0
1.0
2.0
3.0
4.0
HC-C/EBP Beta Tg
AD-C/EBP Beta Tg

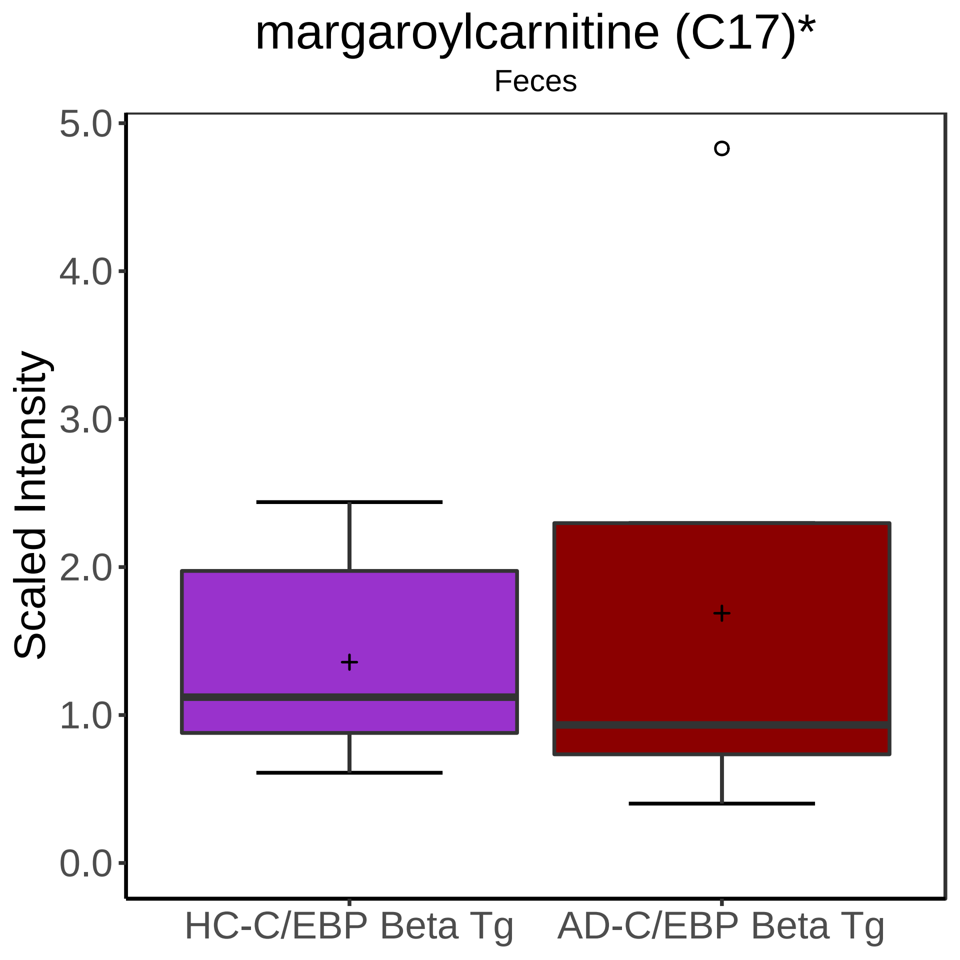
margaroylcarnitine (C17)*
Feces
Scaled Intensity
5.0
4.0
3.0
2.0
1.0
0.0
HC-C/EBP Beta Tg
AD-C/EBP Beta Tg

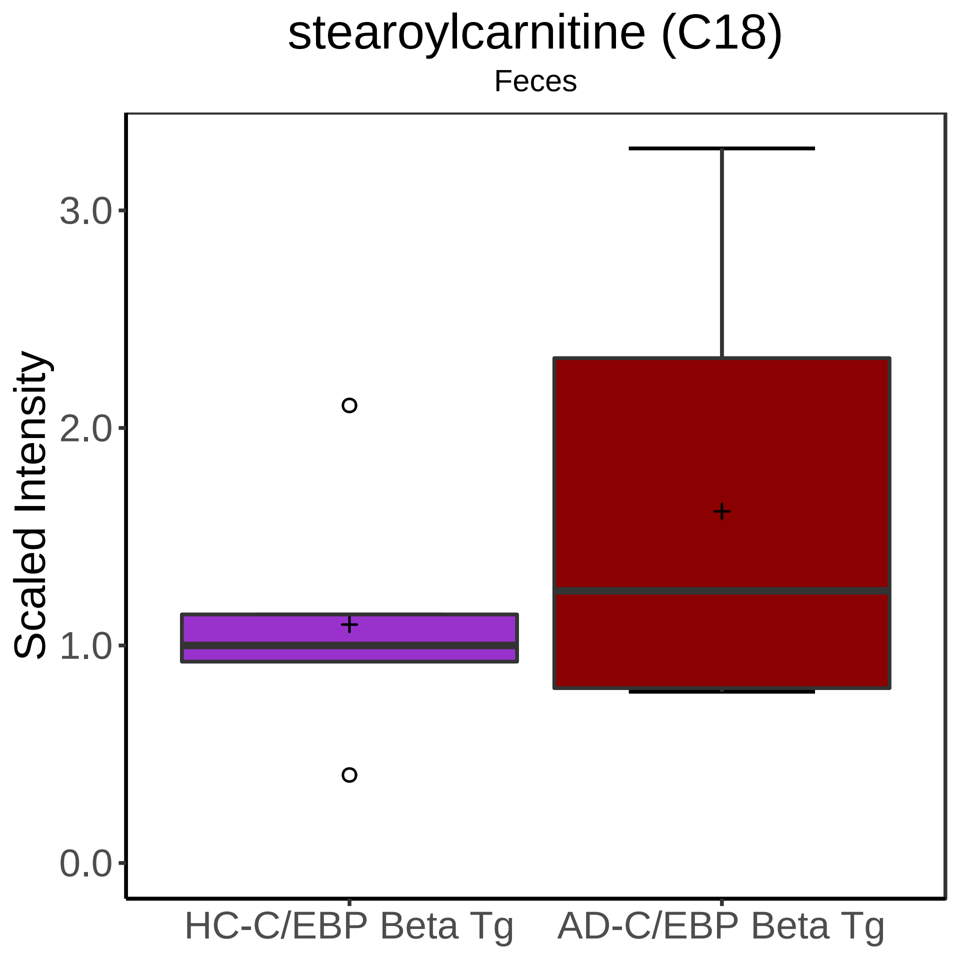

stearoylcarnitine (C18)
Feces
Scaled Intensity
3.0
2.0
1.0
0.0
HC-C/EBP Beta Tg
AD-C/EBP Beta Tg

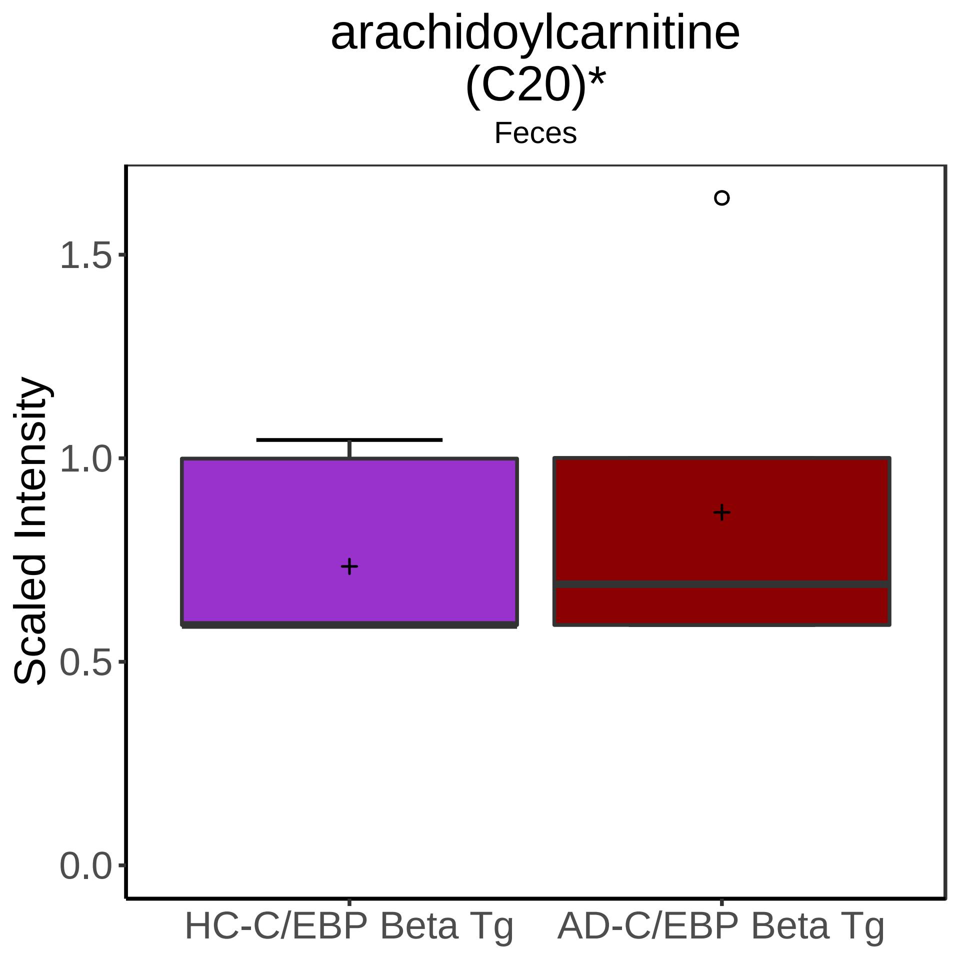

arachidoylcarnitine
(C20)*
Feces
Scaled Intensity
1.5
1.0
0.5
0.0
HC-C/EBP Beta Tg
AD-C/EBP Beta Tg

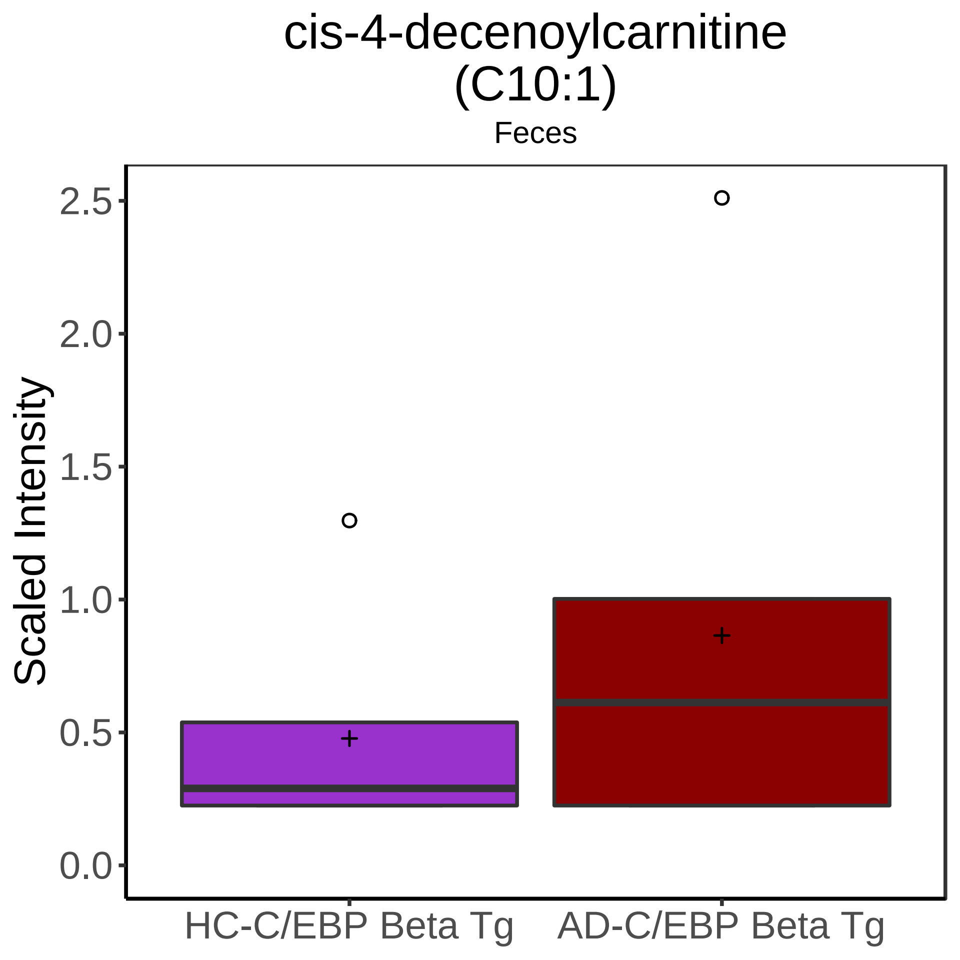
cis-4-decenoylcarnitine
(C10:1)
Feces
Scaled Intensity
0.0
0.5
1.0
1.5
2.0
2.5
HC-C/EBP Beta Tg
AD-C/EBP Beta Tg

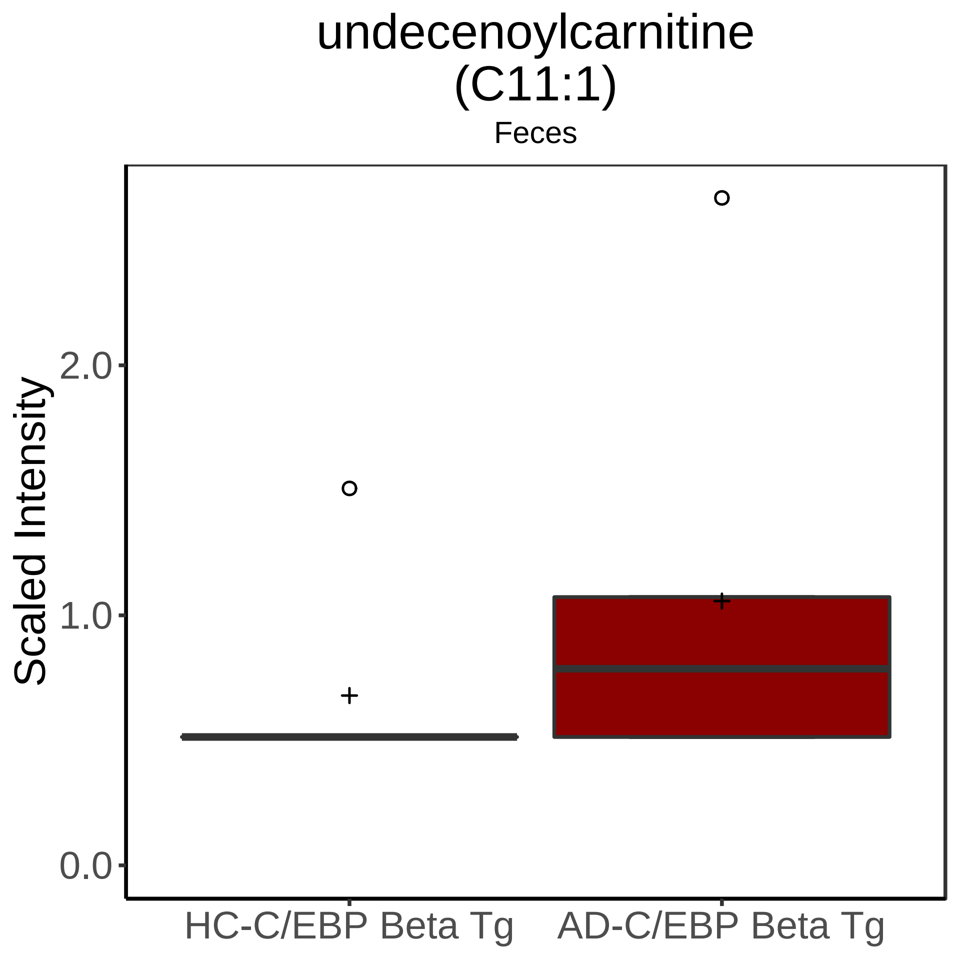

undecenoylcarnitine
(C11:1)
Feces
Scaled Intensity
2.0
1.0
0.0
HC-C/EBP Beta Tg
AD-C/EBP Beta Tg

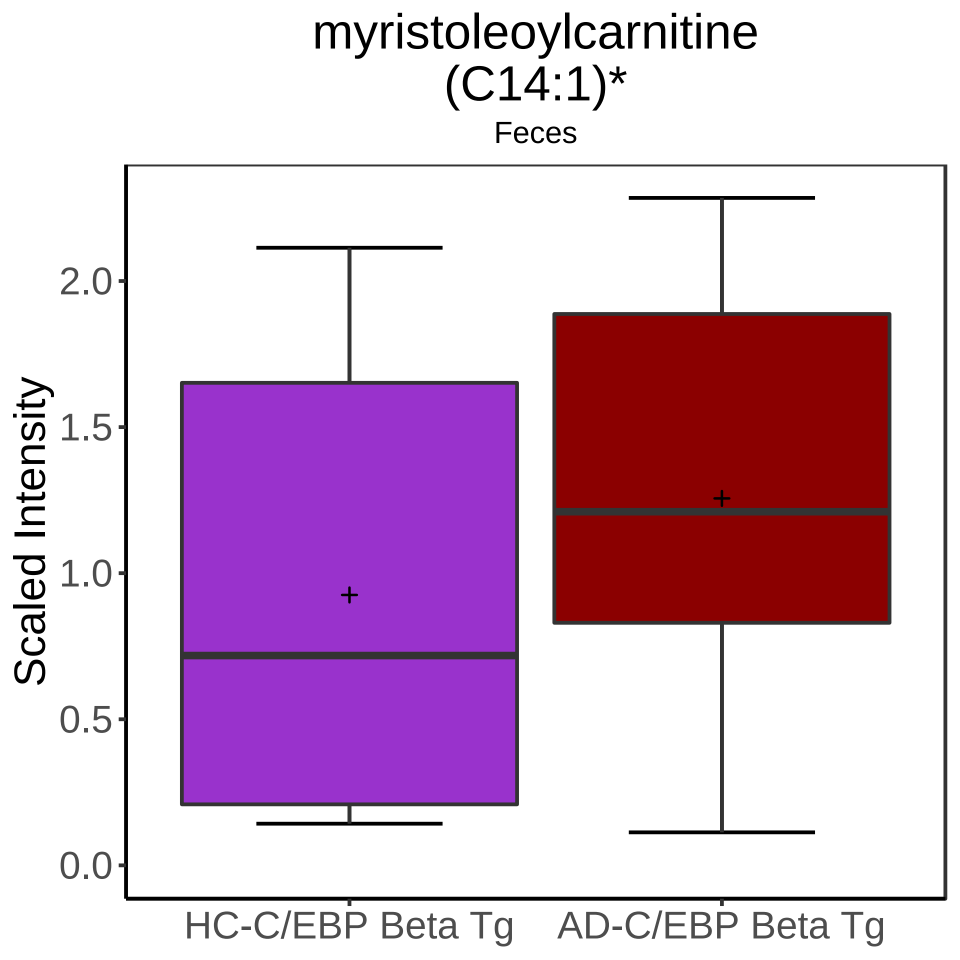

myristoleoylcarnitine
(C14:1)*
Feces
Scaled Intensity
2.0
1.5
1.0
0.5
0.0
HC-C/EBP Beta Tg
AD-C/EBP Beta Tg

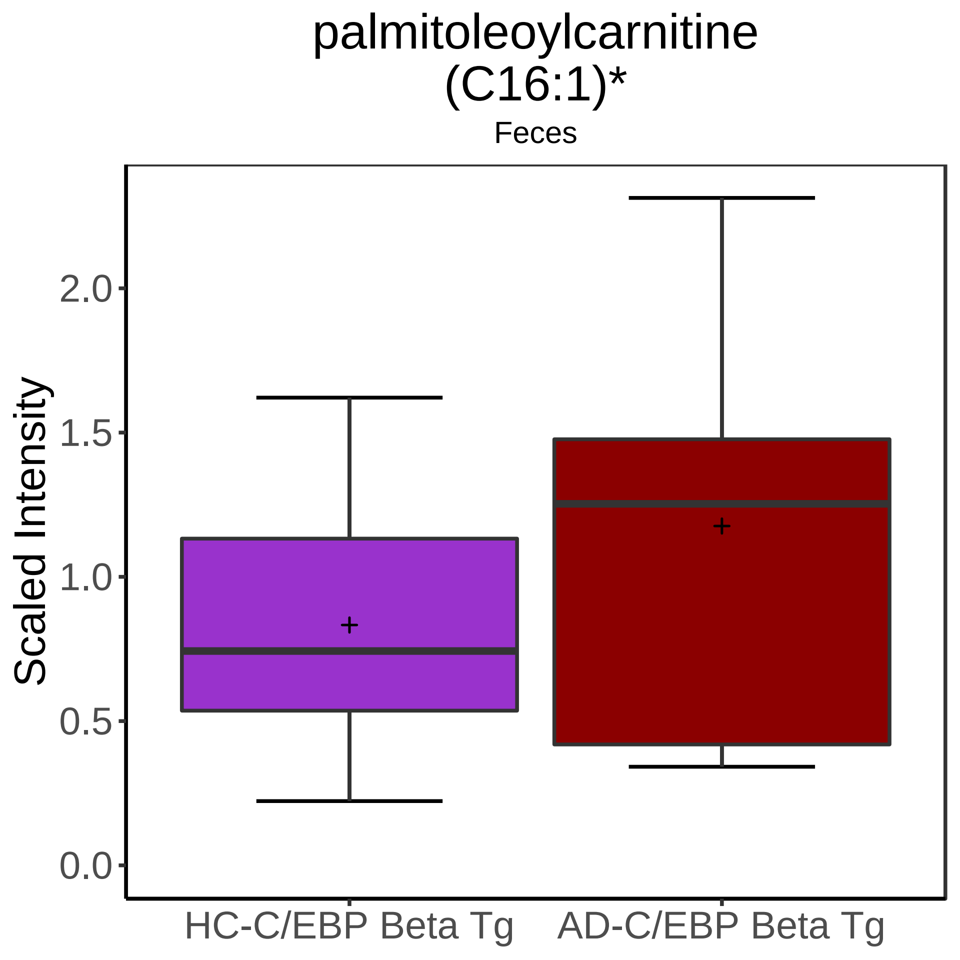
palmitoleoylcarnitine
(C16:1)*
Feces
Scaled Intensity
2.0
1.5
1.0
0.5
0.0
HC-C/EBP Beta Tg
AD-C/EBP Beta Tg

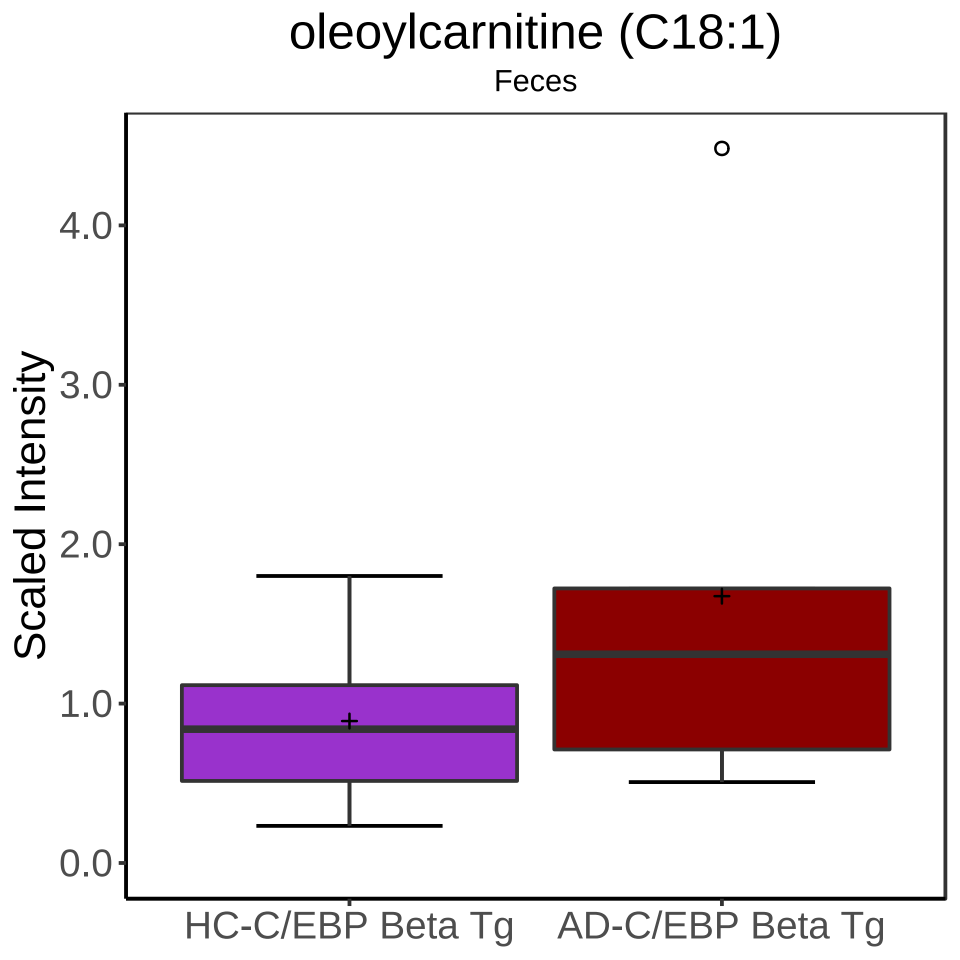
oleoylcarnitine (C18:1)
Feces
Scaled Intensity
4.0
3.0
2.0
1.0
0.0
HC-C/EBP Beta Tg
AD-C/EBP Beta Tg

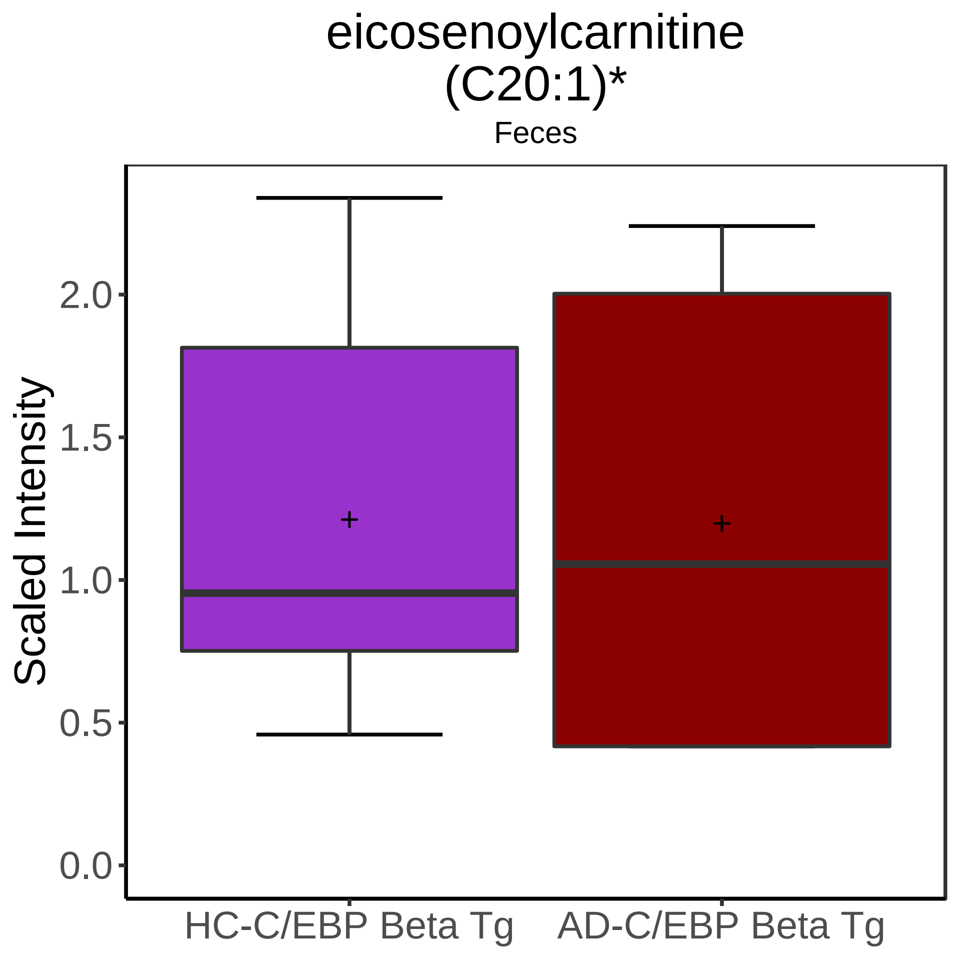

eicosenoylcarnitine
(C20:1)*
Feces
Scaled Intensity
0.0
0.5
1.0
1.5
2.0
HC-C/EBP Beta Tg
AD-C/EBP Beta Tg

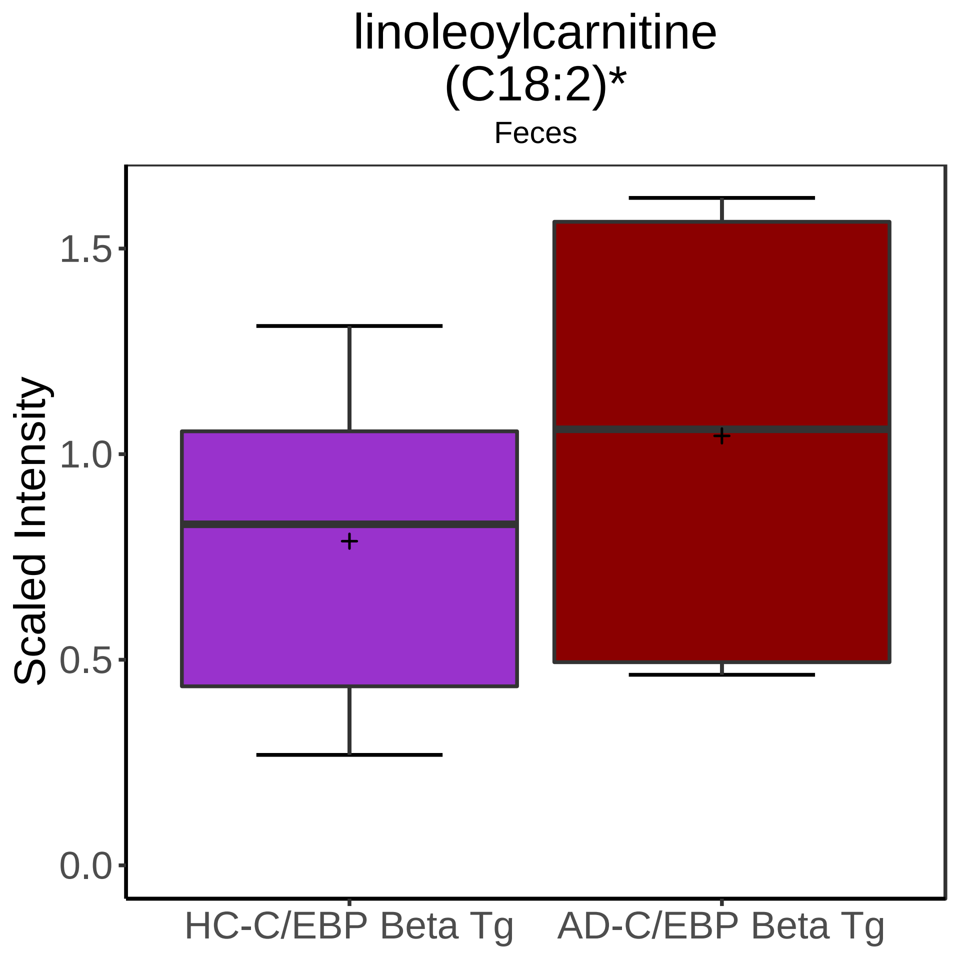
linoleoylcarnitine
(C18:2)*
Feces
Scaled Intensity
0.0
0.5
1.0
1.5
HC-C/EBP Beta Tg
AD-C/EBP Beta Tg

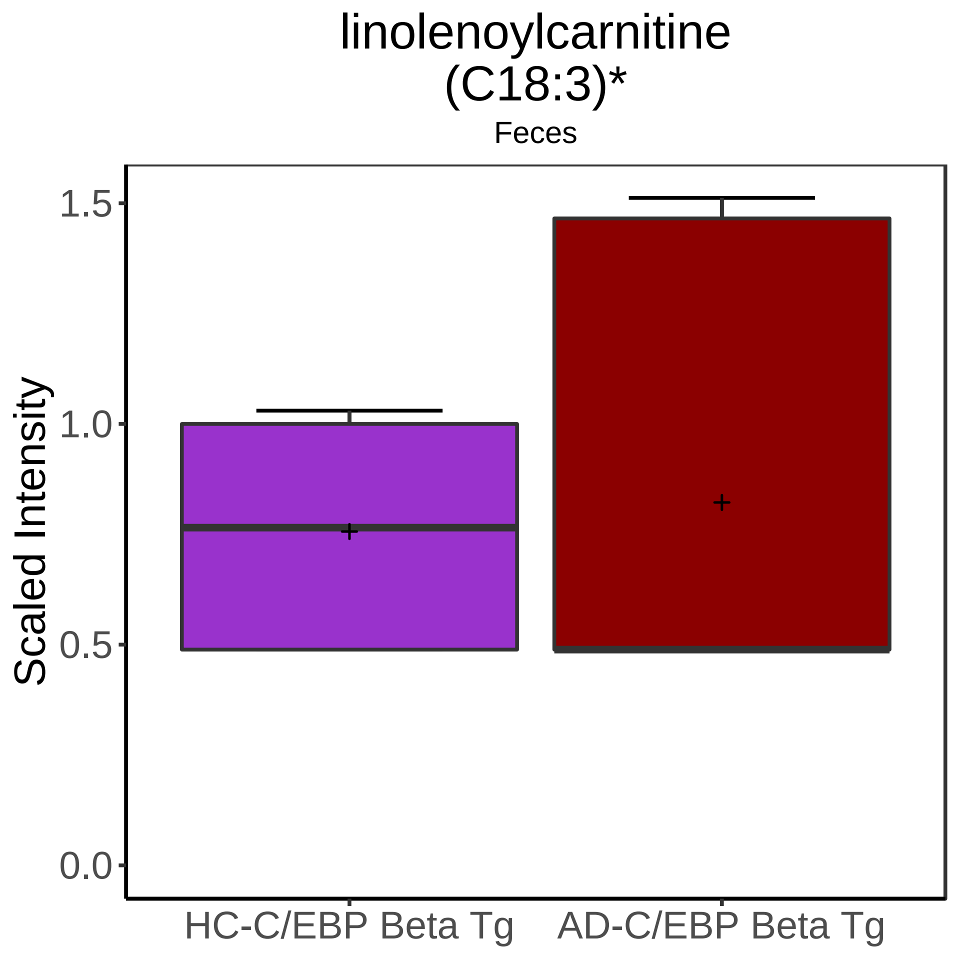
linolenoylcarnitine
(C18:3)*
Feces
Scaled Intensity
1.5
1.0
0.5
0.0
HC-C/EBP Beta Tg
AD-C/EBP Beta Tg

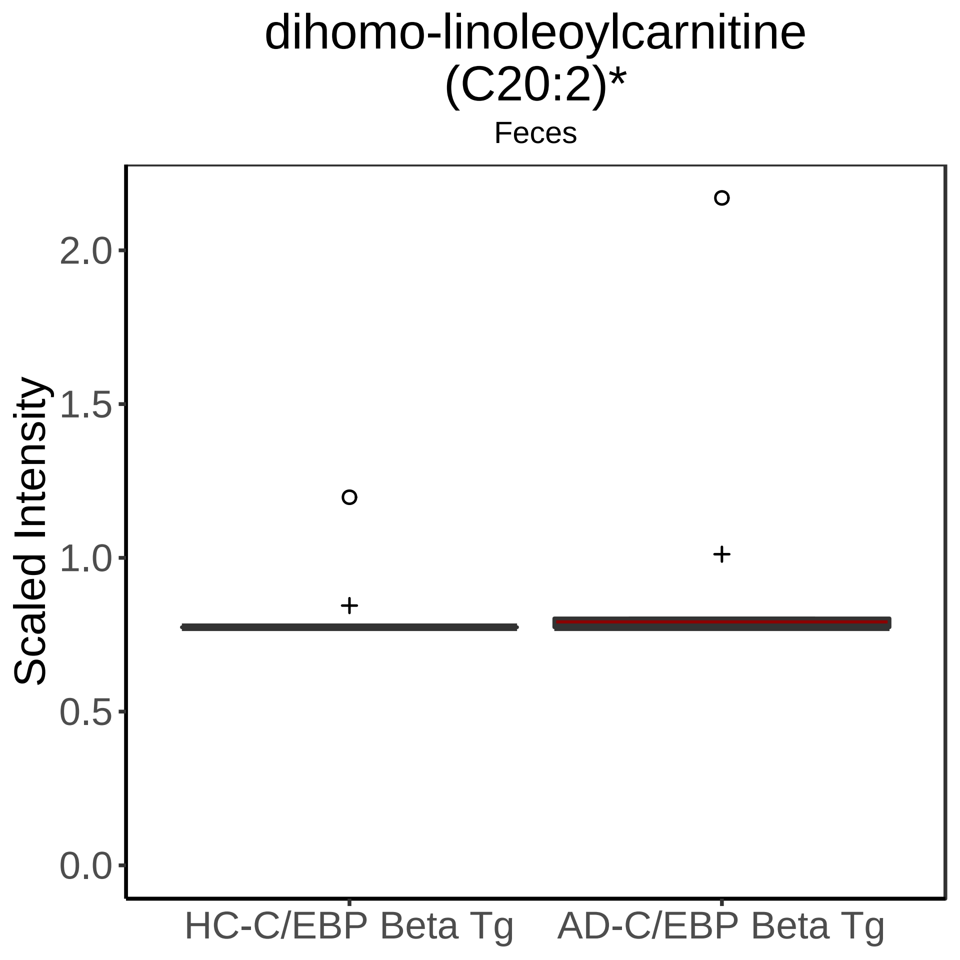
dihomo-linoleoylcarnitine
(C20:2)*
Feces
Scaled Intensity
2.0
1.5
1.0
0.5
0.0
HC-C/EBP Beta Tg
AD-C/EBP Beta Tg

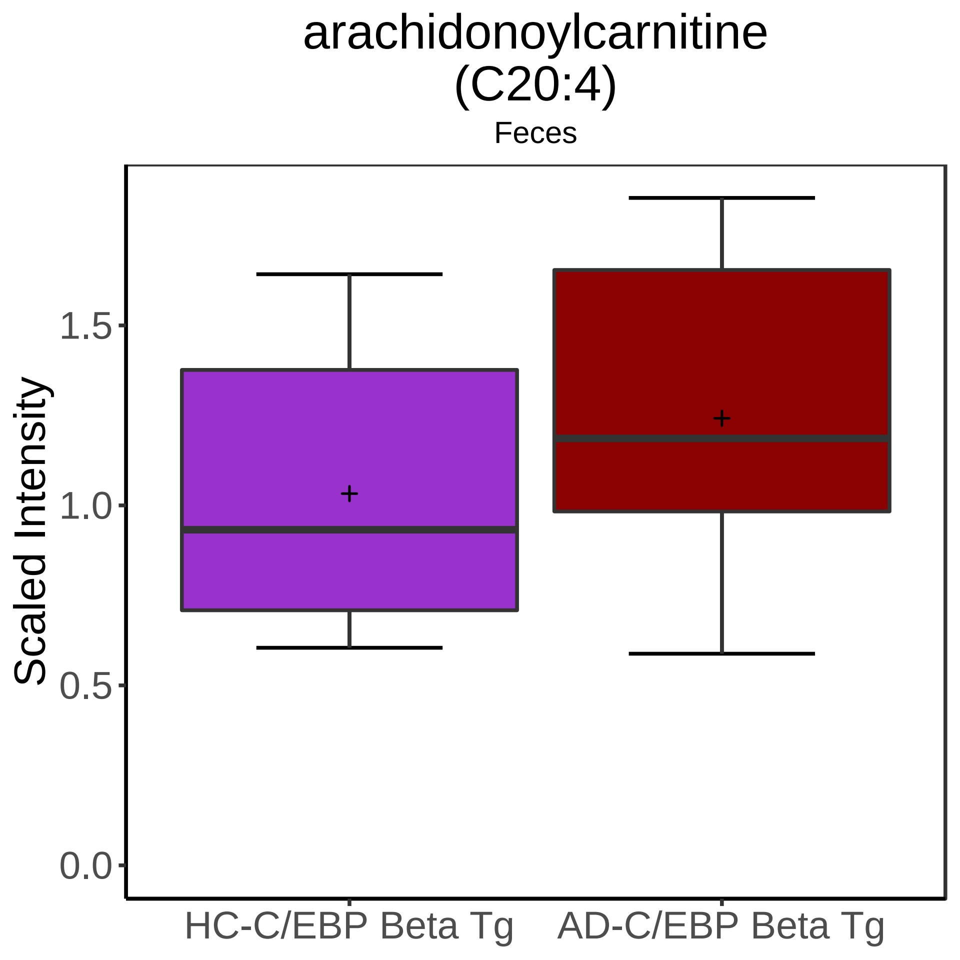
arachidonoylcarnitine
(C20:4)
Feces
Scaled Intensity
0.0
0.5
1.0
1.5
HC-C/EBP Beta Tg
AD-C/EBP Beta Tg

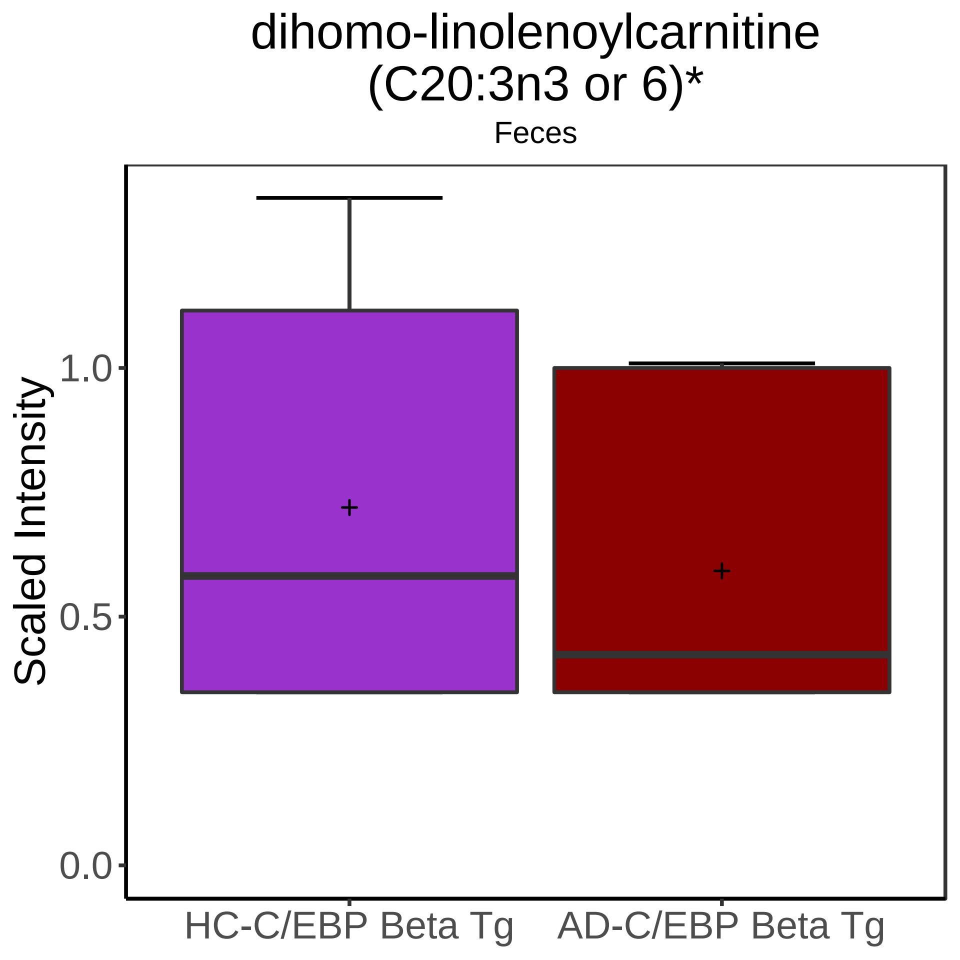
dihomo-linolenoylcarnitine
(C20:3n3 or 6)*
Feces
Scaled Intensity
1.0
0.5
0.0
HC-C/EBP Beta Tg
AD-C/EBP Beta Tg

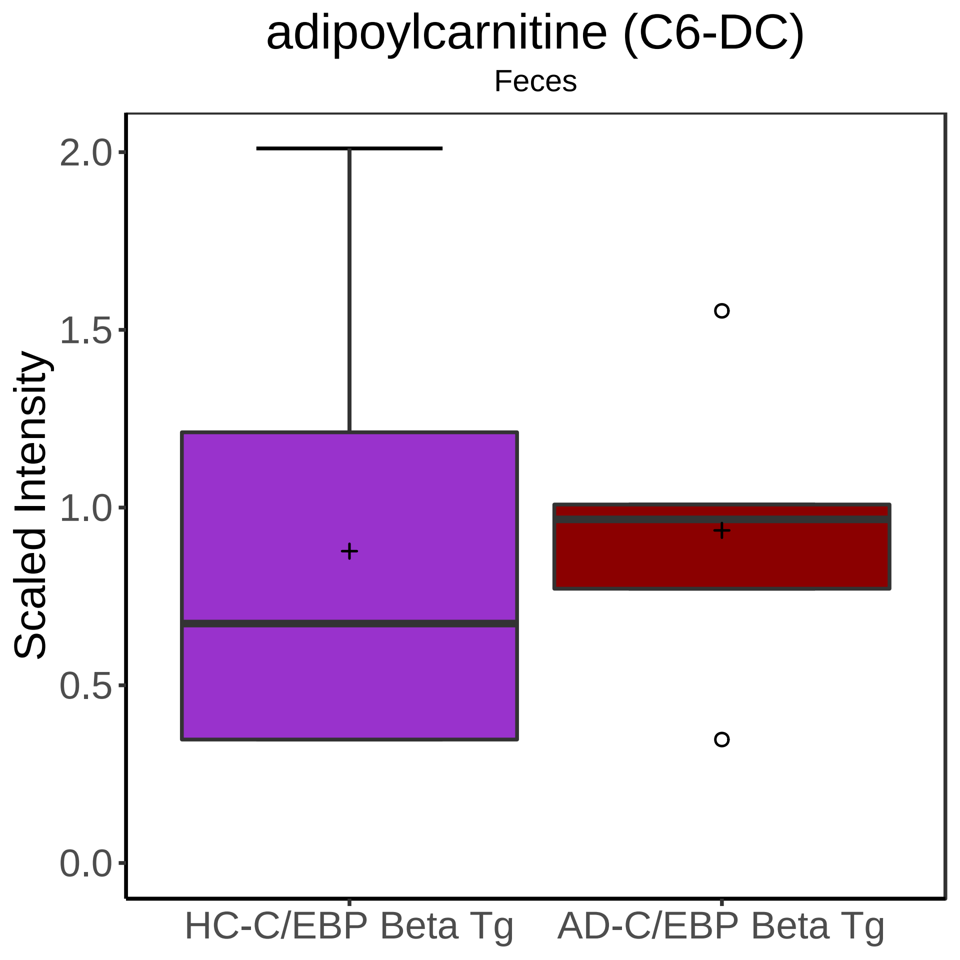

adipoylcarnitine (C6-DC)
Feces
Scaled Intensity
2.0
1.5
1.0
0.5
0.0
HC-C/EBP Beta Tg
AD-C/EBP Beta Tg

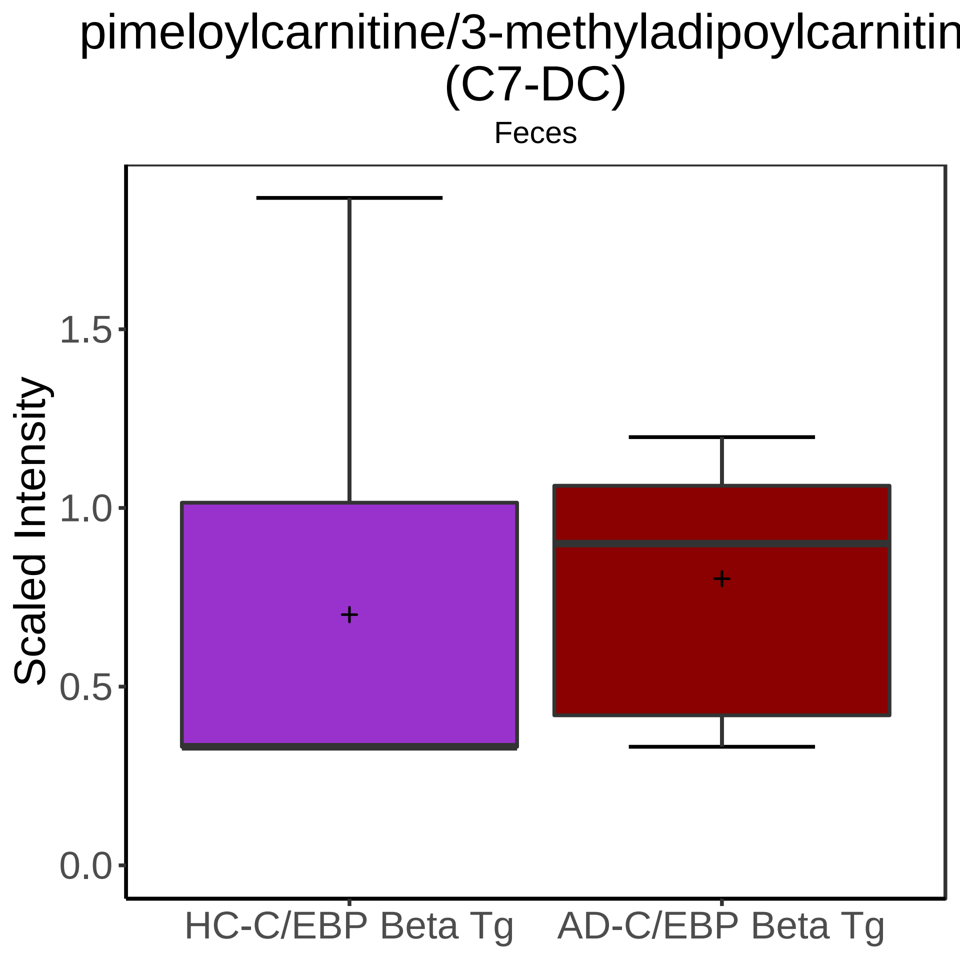

pimeloylcarnitine/3-methyladipoylcarnitin
(C7-DC)
Feces
Scaled Intensity
1.5
1.0
0.5
0.0
HC-C/EBP Beta Tg
AD-C/EBP Beta Tg

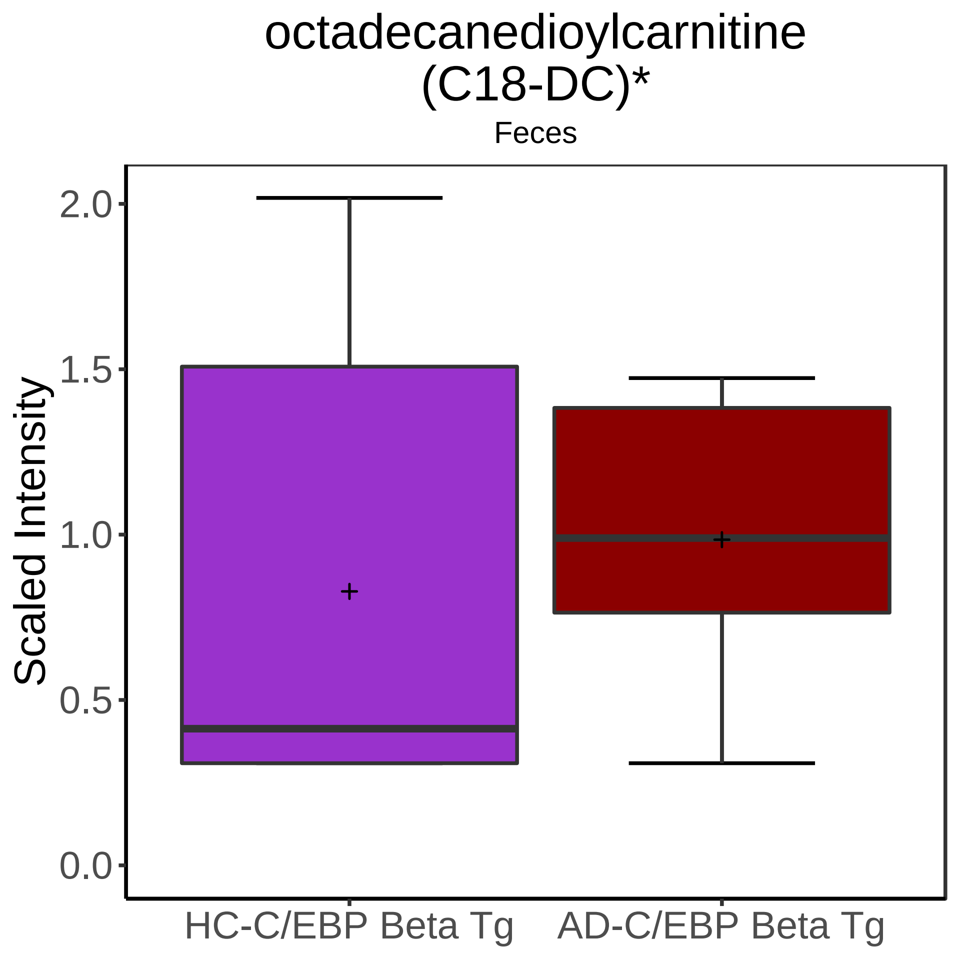

octadecanedioylcarnitine
(C18-DC)*
Feces
Scaled Intensity
2.0
1.5
1.0
0.5
0.0
HC-C/EBP Beta Tg
AD-C/EBP Beta Tg

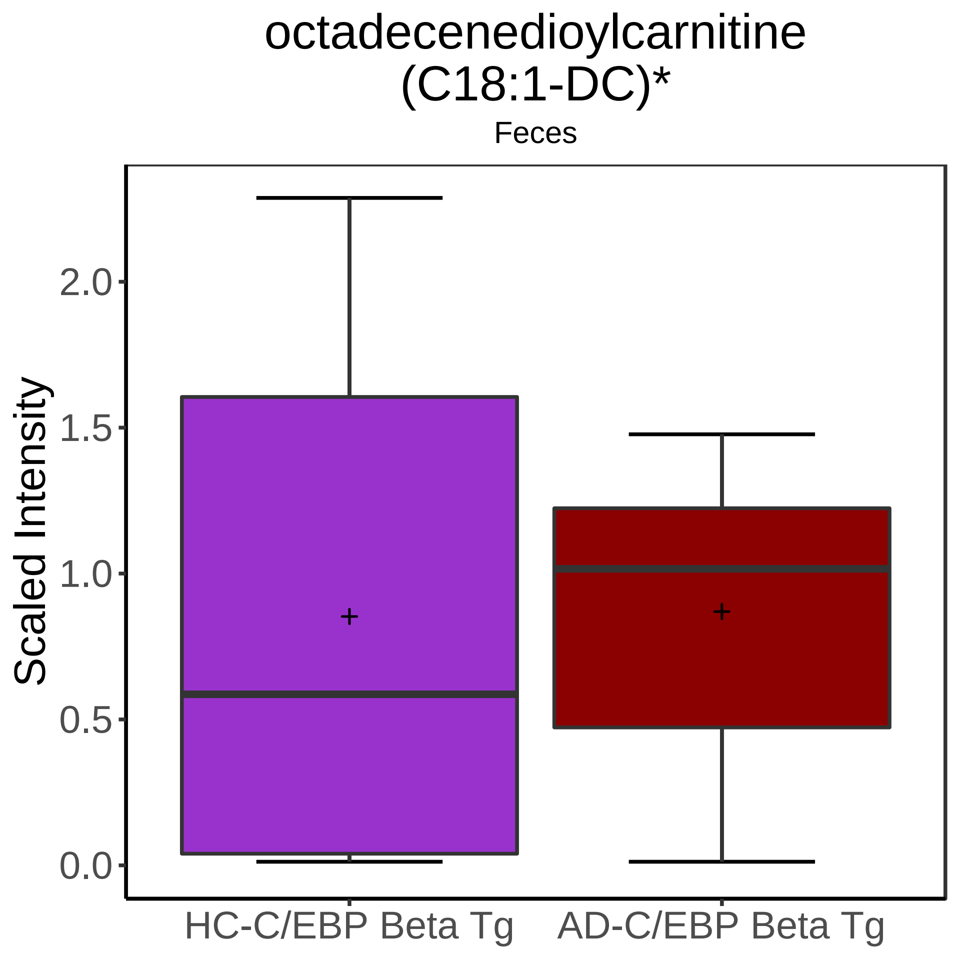

octadecenedioylcarnitine
(C18:1-DC)*
Feces
Scaled Intensity
2.0
1.5
1.0
0.5
0.0
HC-C/EBP Beta Tg
AD-C/EBP Beta Tg

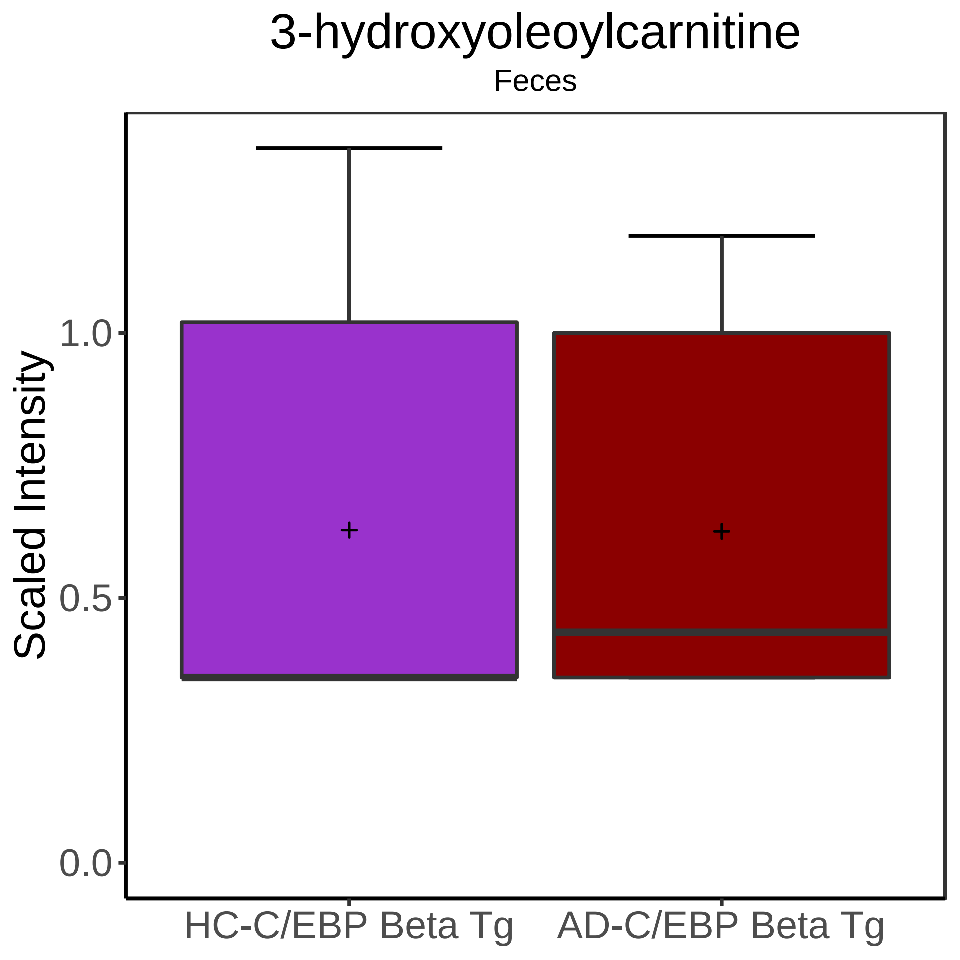
3-hydroxyoleoylcarnitine
Feces
Scaled Intensity
1.0
0.5
0.0
HC-C/EBP Beta Tg
AD-C/EBP Beta Tg

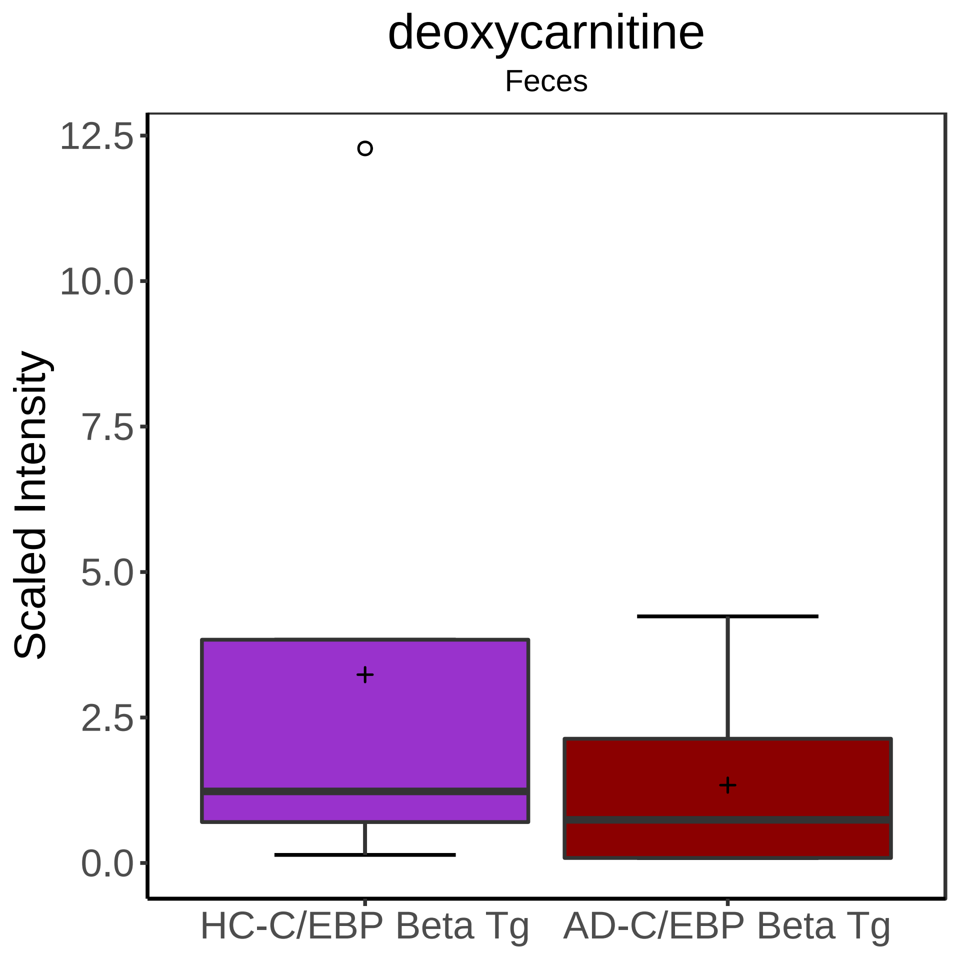

deoxycarnitine
Feces
Scaled Intensity
12.5
10.0
7.5
5.0
2.5
0.0
HC-C/EBP Beta Tg
AD-C/EBP Beta Tg

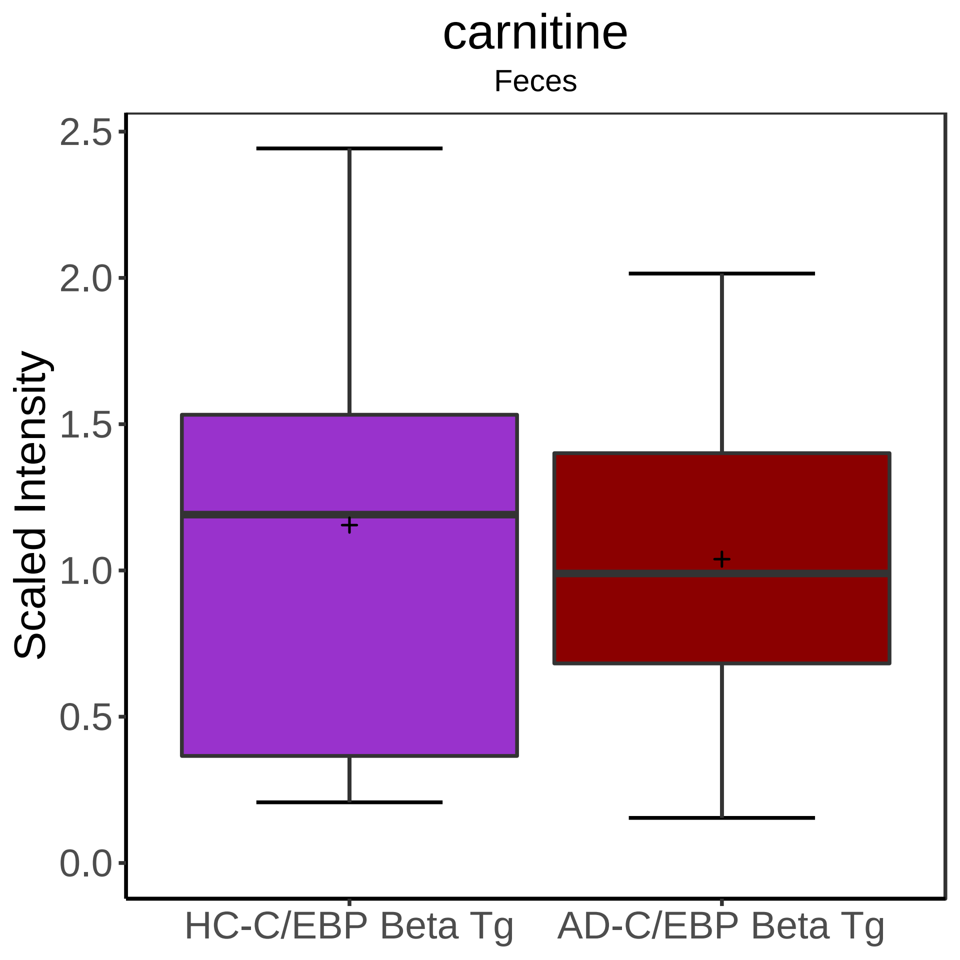
carnitine
Feces
Scaled Intensity
2.5
2.0
1.5
1.0
0.5
0.0
HC-C/EBP Beta Tg
AD-C/EBP Beta Tg

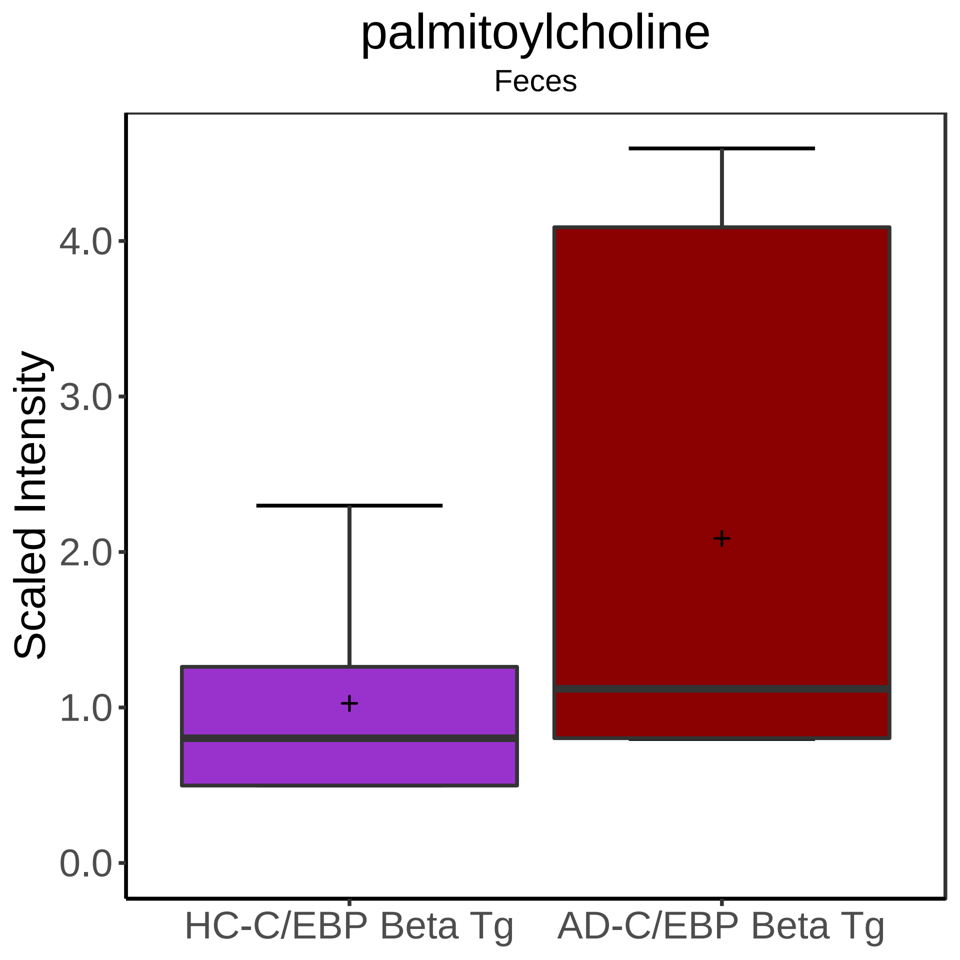
palmitoylcholine
Feces
Scaled Intensity
4.0
3.0
2.0
1.0
0.0
HC-C/EBP Beta Tg
AD-C/EBP Beta Tg

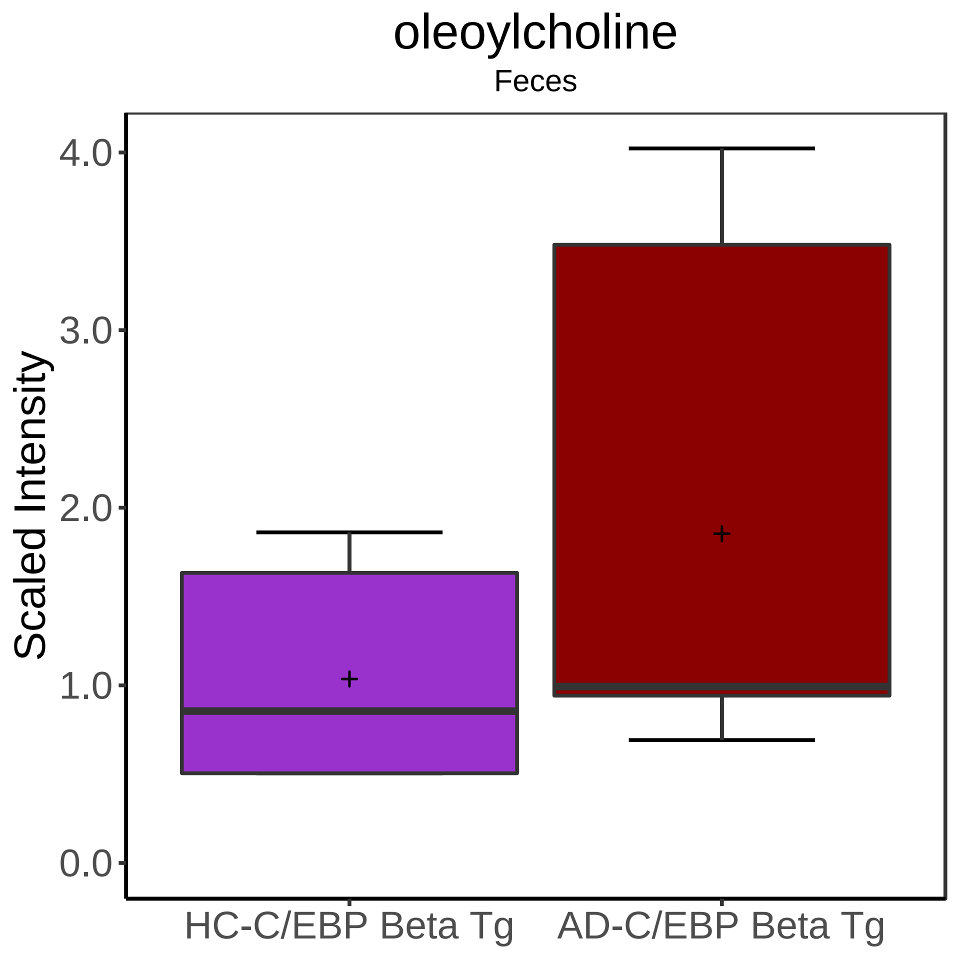

oleoylcholine
Feces
Scaled Intensity
4.0
3.0
2.0
1.0
0.0
HC-C/EBP Beta Tg
AD-C/EBP Beta Tg

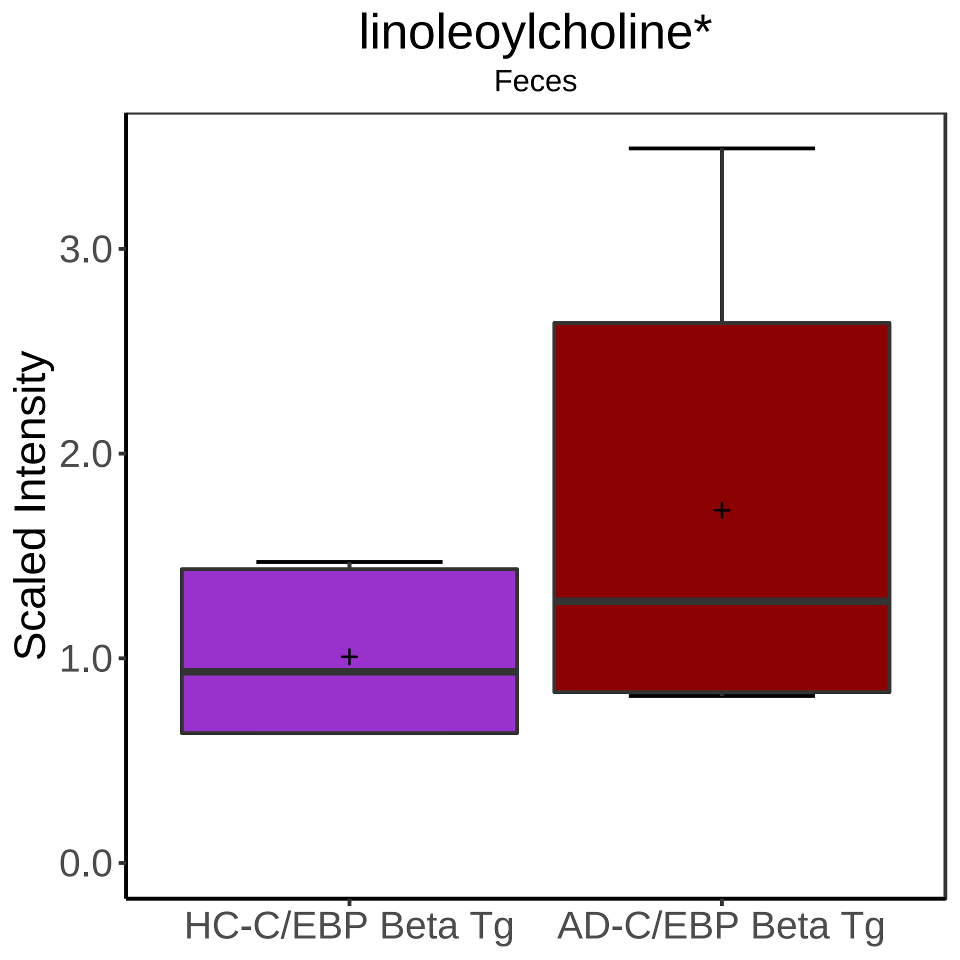

linoleoylcholine*
Feces
Scaled Intensity
3.0
2.0
1.0
0.0
HC-C/EBP Beta Tg
AD-C/EBP Beta Tg

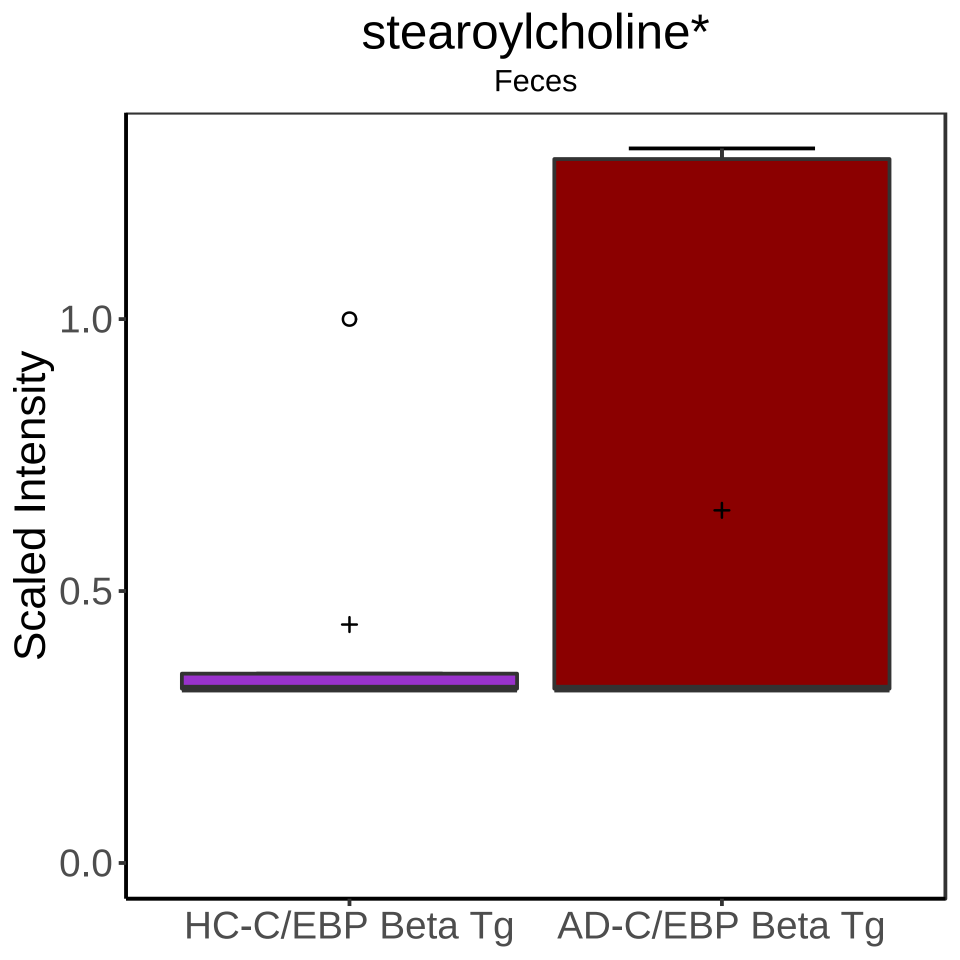
stearoylcholine*
Feces
Scaled Intensity
1.0
0.5
0.0
HC-C/EBP Beta Tg
AD-C/EBP Beta Tg

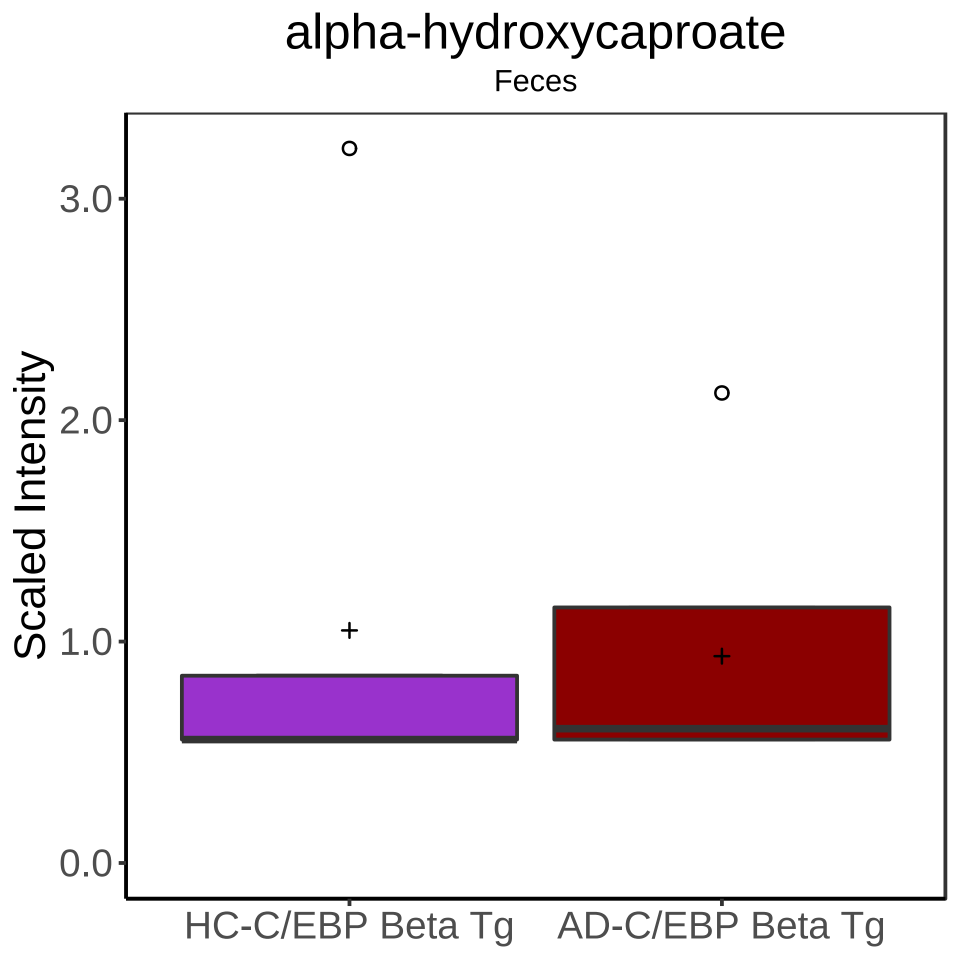

alpha-hydroxycaproate
Feces
Scaled Intensity
3.0
2.0
1.0
0.0
HC-C/EBP Beta Tg
AD-C/EBP Beta Tg

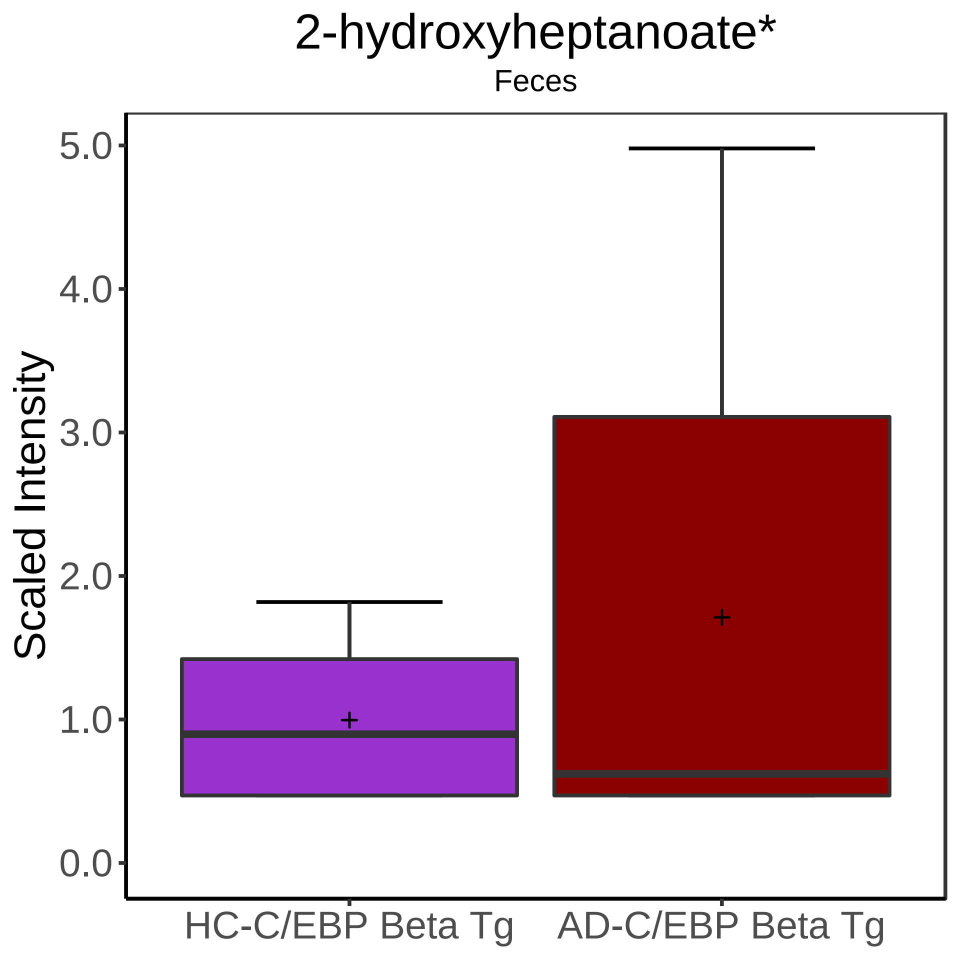
2-hydroxyheptanoate*
Feces
Scaled Intensity
5.0
4.0
3.0
2.0
1.0
0.0
HC-C/EBP Beta Tg
AD-C/EBP Beta Tg

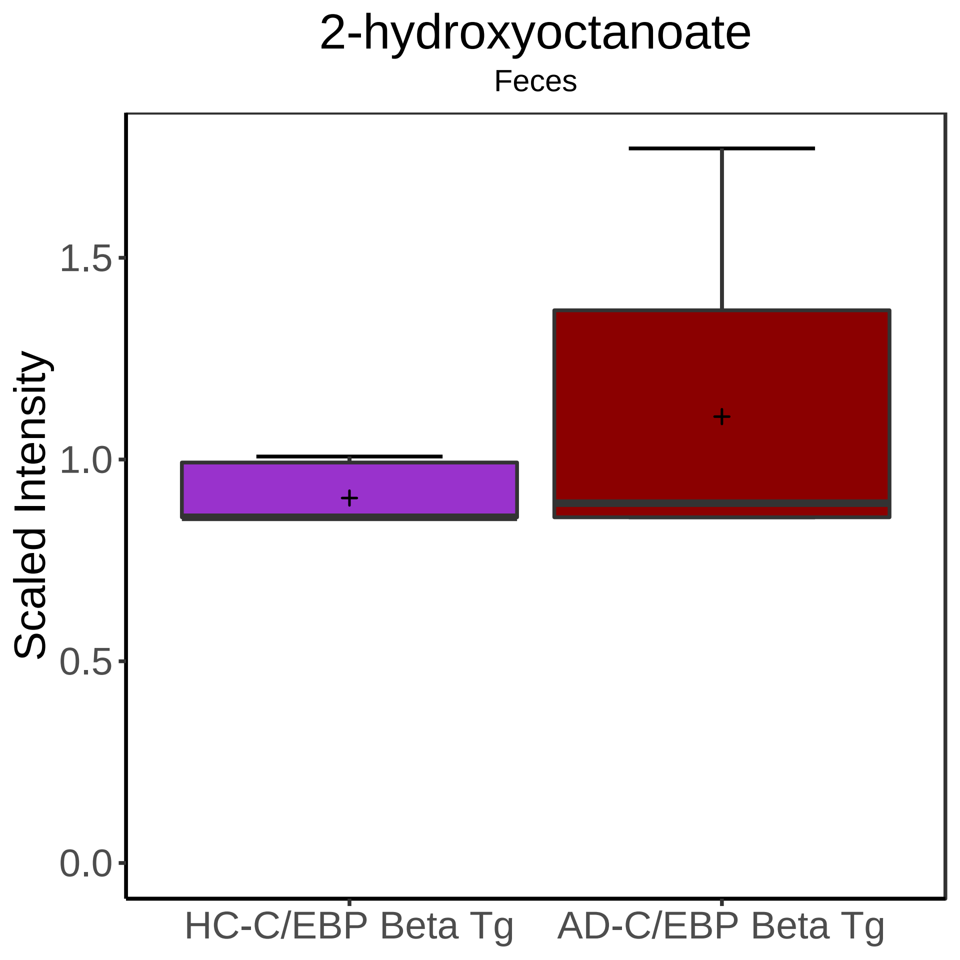
2-hydroxyoctanoate
Feces
Scaled Intensity
0.0
0.5
1.0
1.5
HC-C/EBP Beta Tg
AD-C/EBP Beta Tg

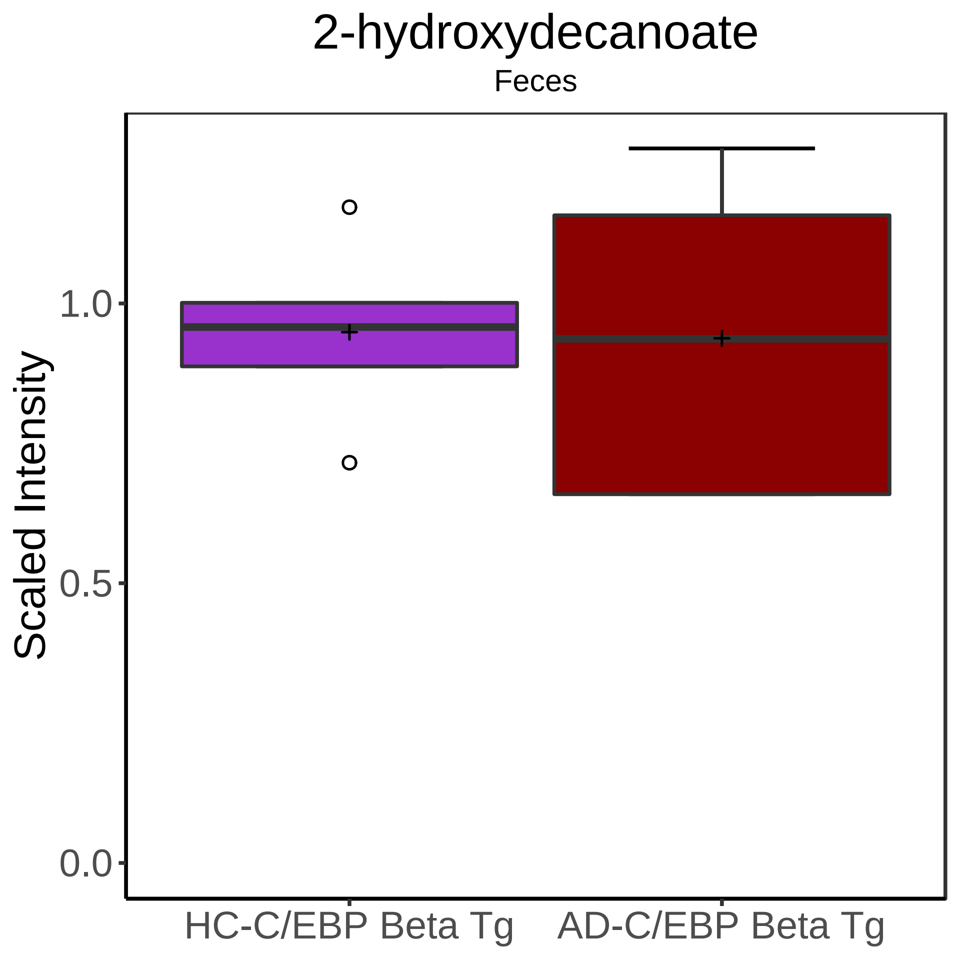

2-hydroxydecanoate
Feces
Scaled Intensity
1.0
0.5
0.0
HC-C/EBP Beta Tg
AD-C/EBP Beta Tg

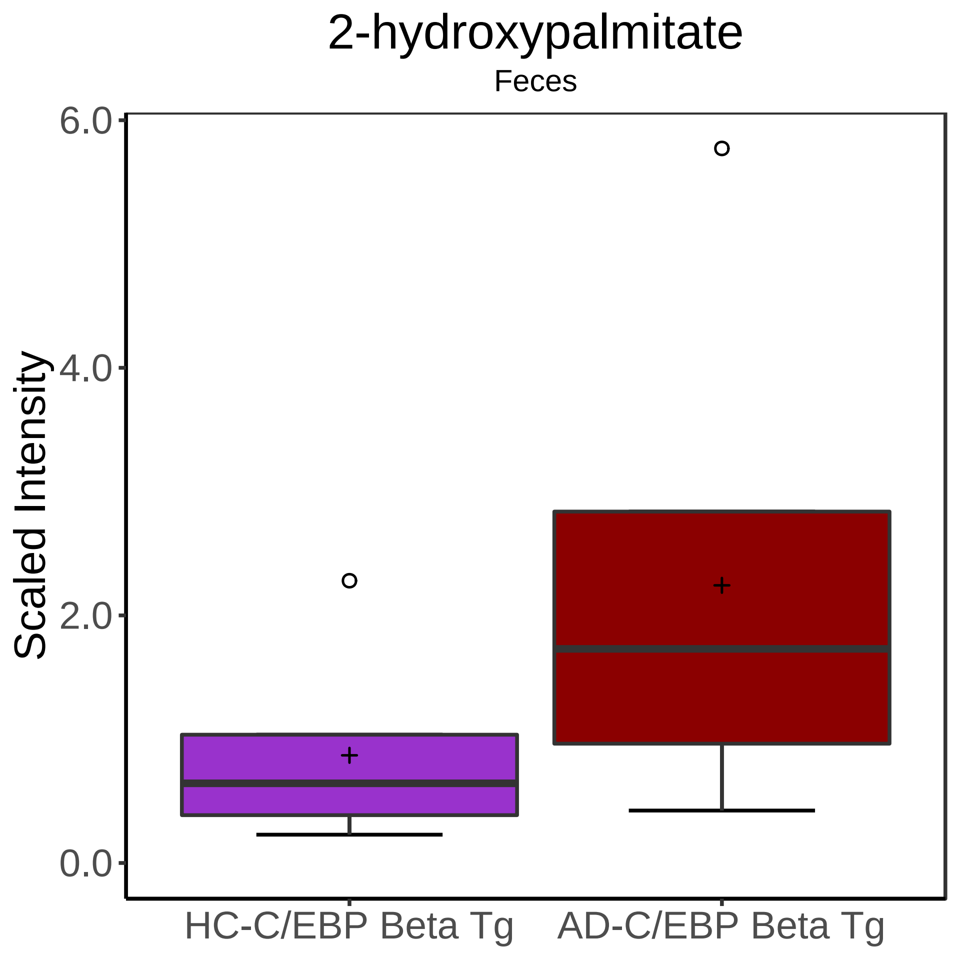
2-hydroxypalmitate
Feces
Scaled Intensity
6.0
4.0
2.0
0.0
HC-C/EBP Beta Tg
AD-C/EBP Beta Tg

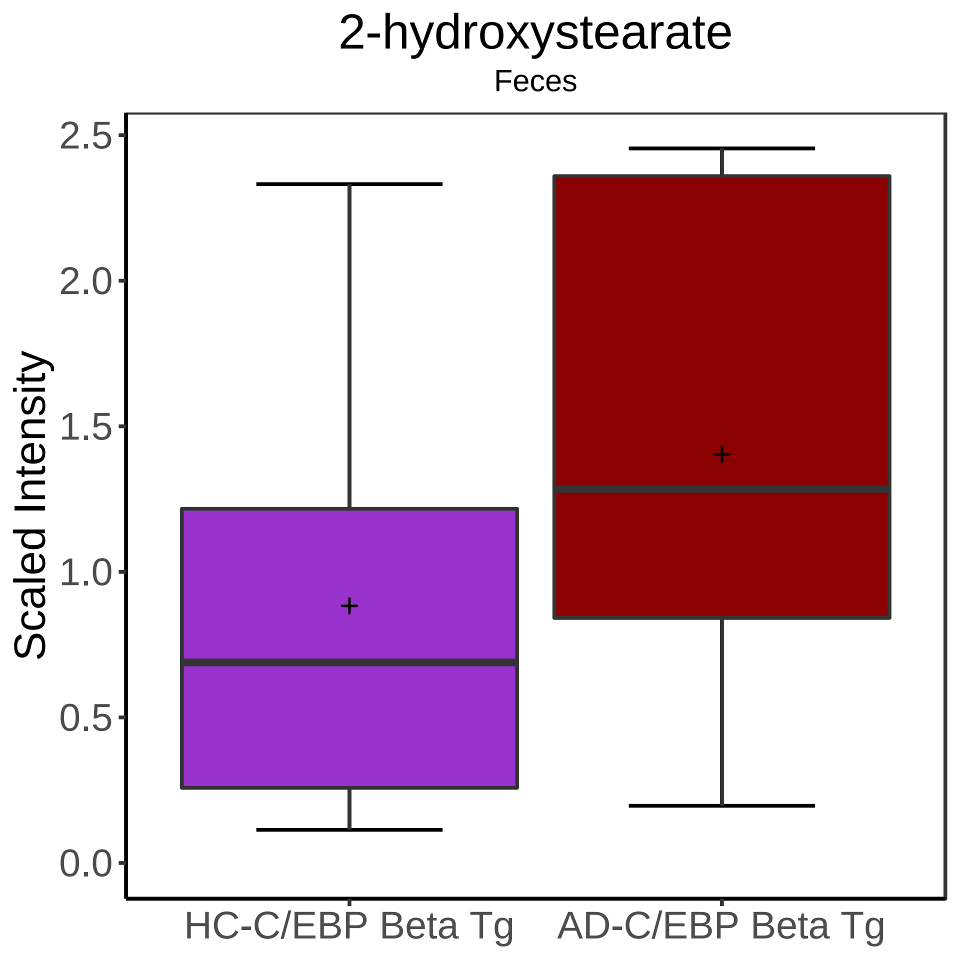
2-hydroxystearate
Feces
Scaled Intensity
0.0
0.5
1.0
1.5
2.0
2.5
HC-C/EBP Beta Tg
AD-C/EBP Beta Tg

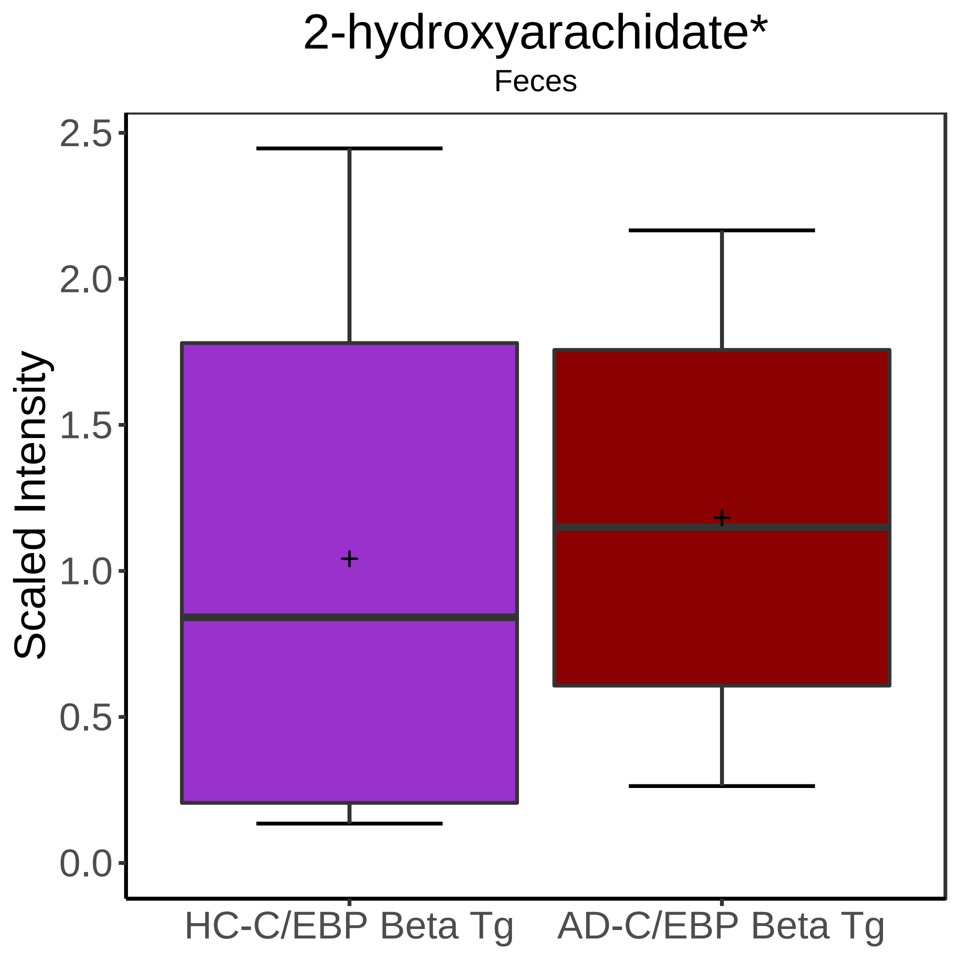
2-hydroxyarachidate*
Feces
Scaled Intensity
2.5
2.0
1.5
1.0
0.5
0.0
HC-C/EBP Beta Tg
AD-C/EBP Beta Tg

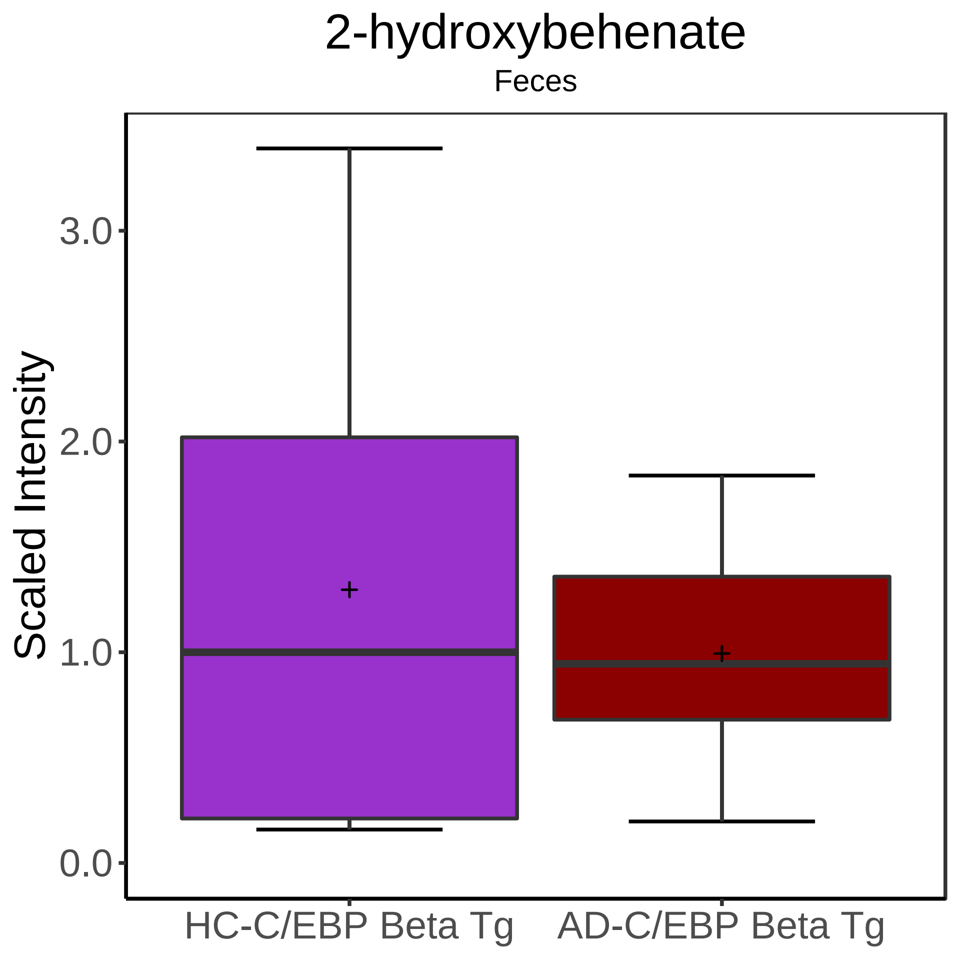

2-hydroxybehenate
Feces
Scaled Intensity
3.0
2.0
1.0
0.0
HC-C/EBP Beta Tg
AD-C/EBP Beta Tg

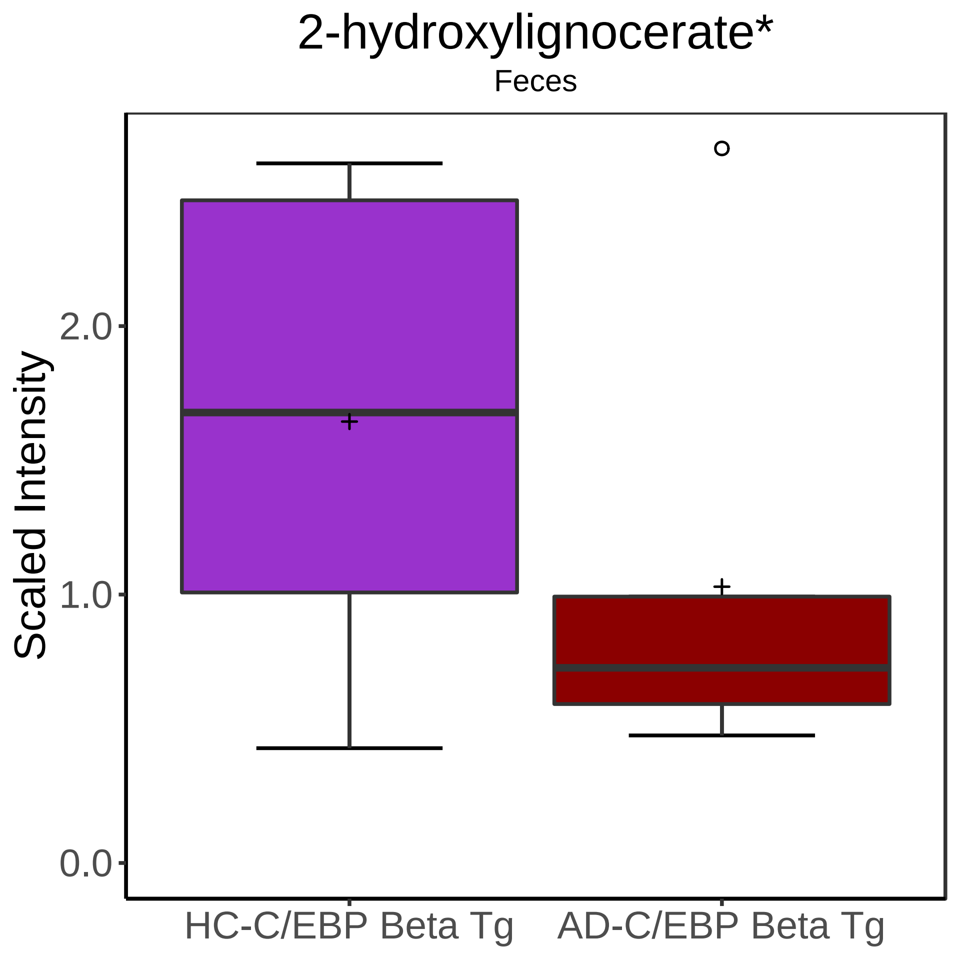

2-hydroxylignocerate*
Feces
Scaled Intensity
2.0
1.0
0.0
HC-C/EBP Beta Tg
AD-C/EBP Beta Tg

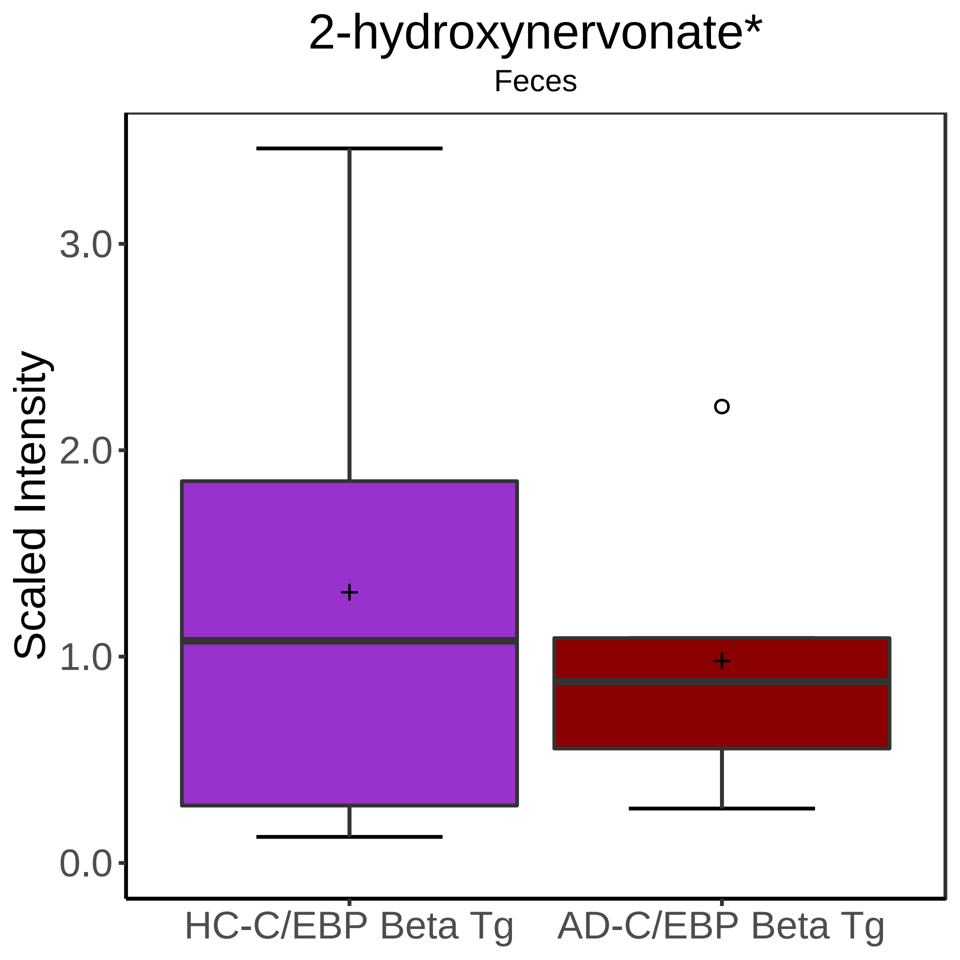
2-hydroxynervonate*
Feces
Scaled Intensity
3.0
2.0
1.0
0.0
HC-C/EBP Beta Tg
AD-C/EBP Beta Tg

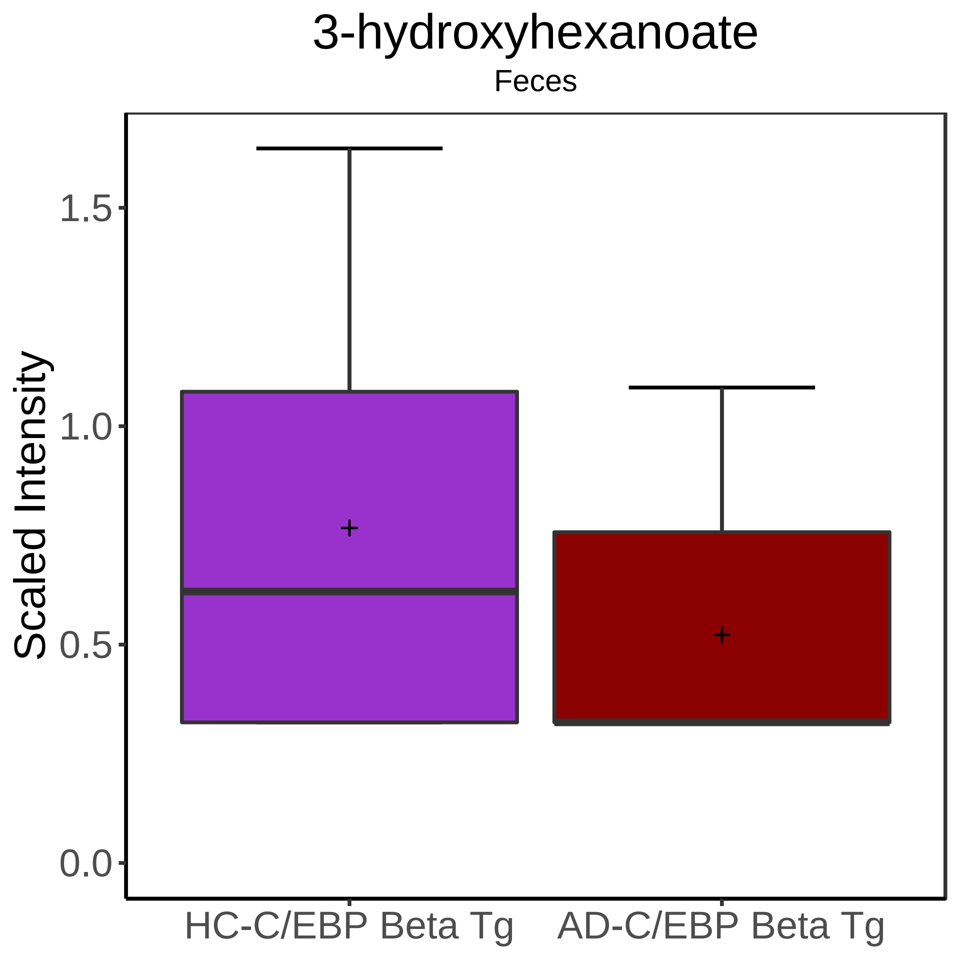
3-hydroxyhexanoate
Feces
Scaled Intensity
1.5
1.0
0.5
0.0
HC-C/EBP Beta Tg
AD-C/EBP Beta Tg

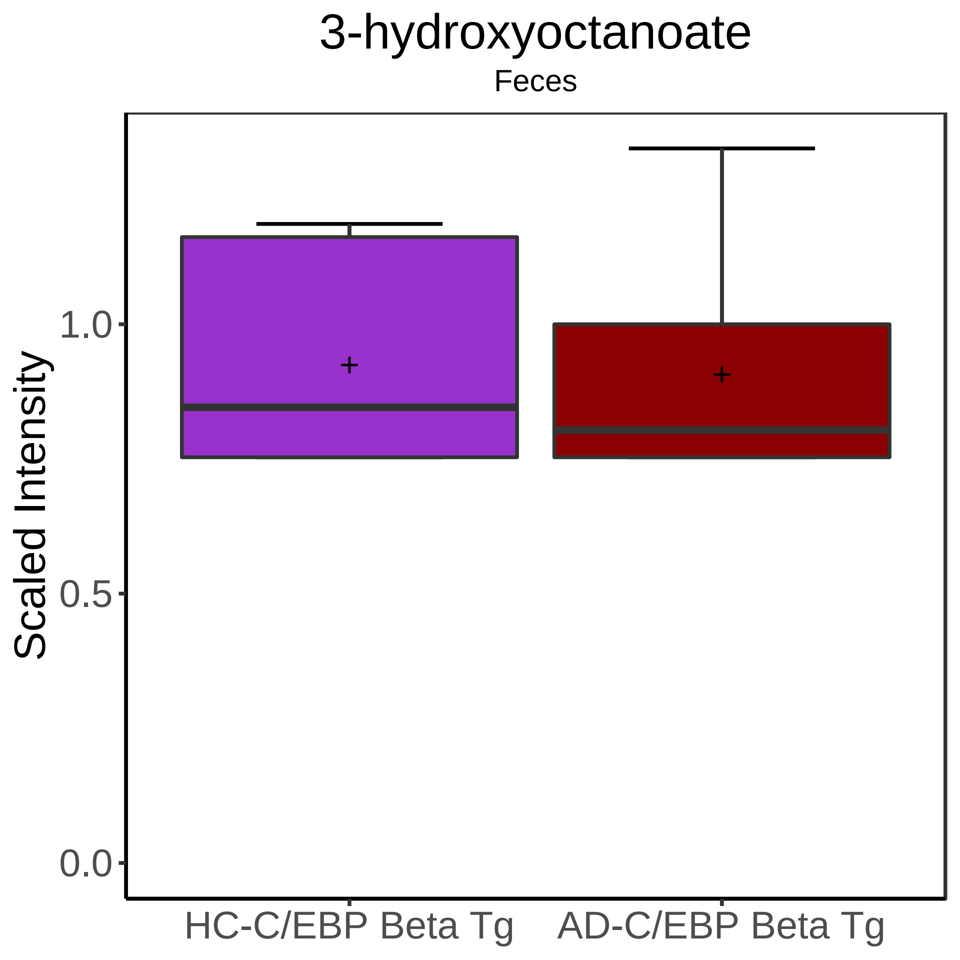
3-hydroxyoctanoate
Feces
Scaled Intensity
1.0
0.5
0.0
HC-C/EBP Beta Tg
AD-C/EBP Beta Tg

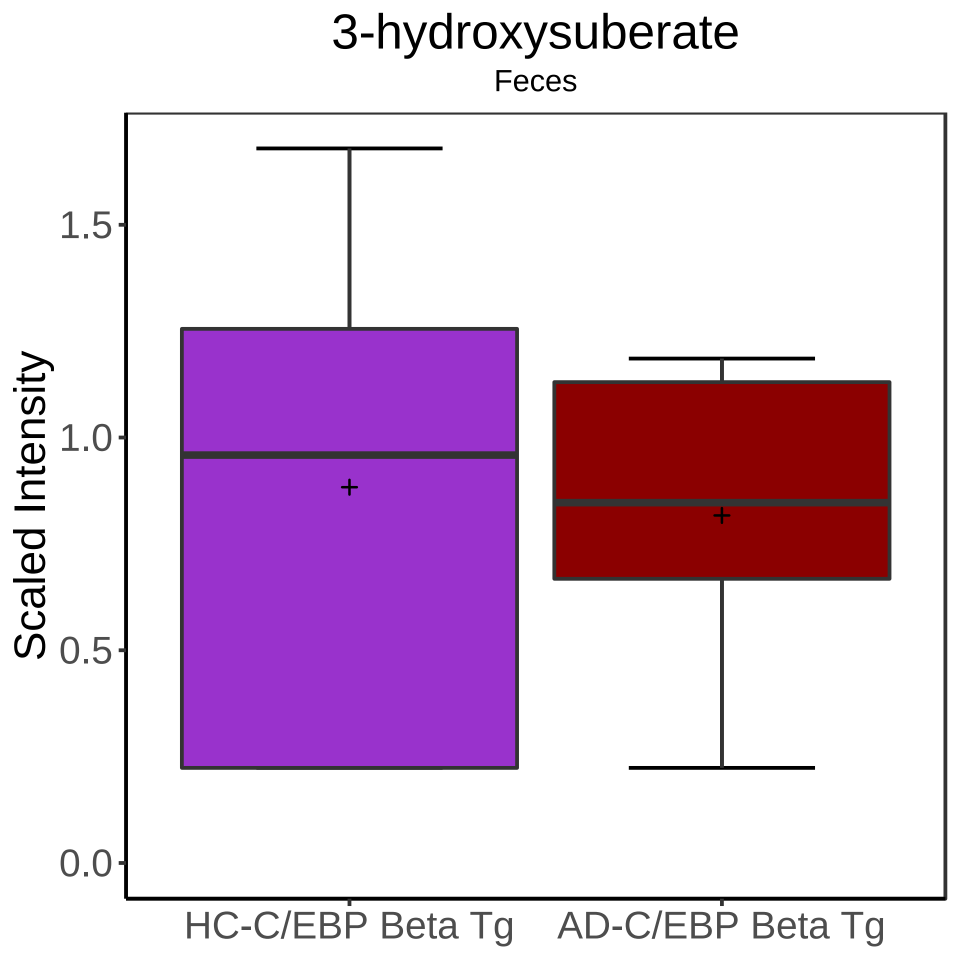
3-hydroxysuberate
Feces
Scaled Intensity
1.5
1.0
0.5
0.0
HC-C/EBP Beta Tg
AD-C/EBP Beta Tg

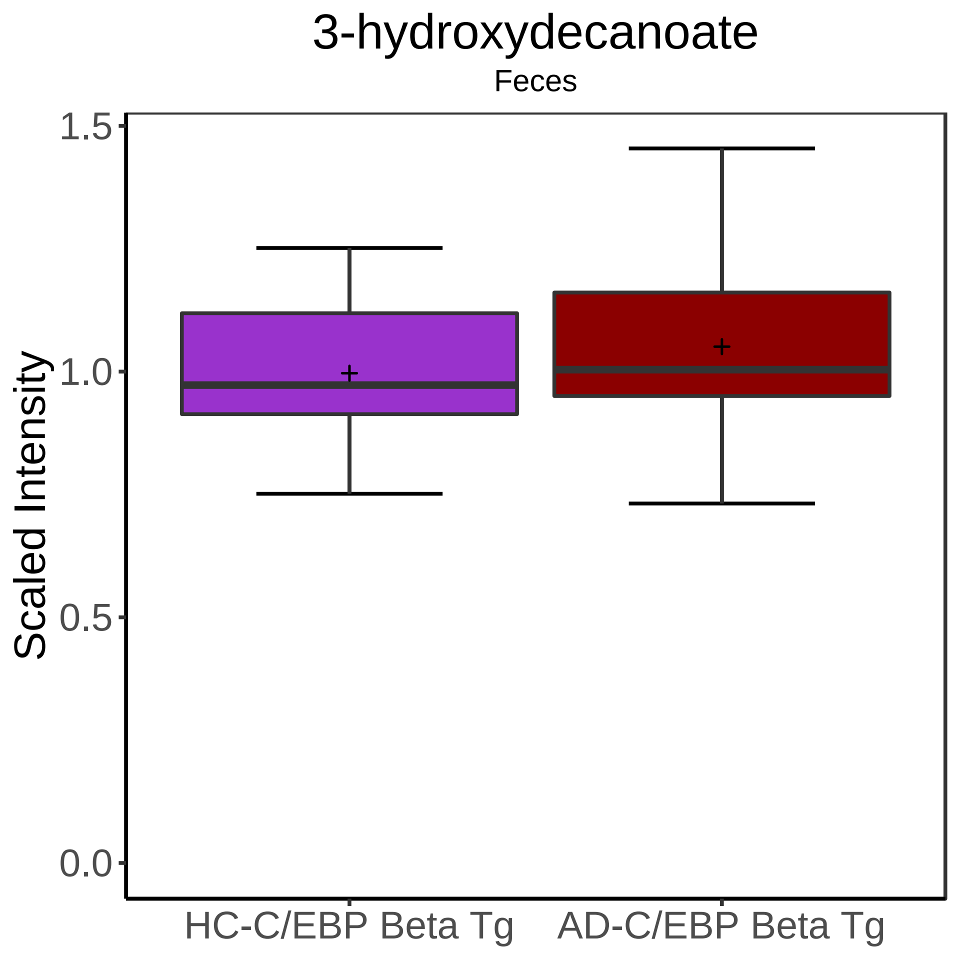
3-hydroxydecanoate
Feces
Scaled Intensity
1.5
1.0
0.5
0.0
HC-C/EBP Beta Tg
AD-C/EBP Beta Tg

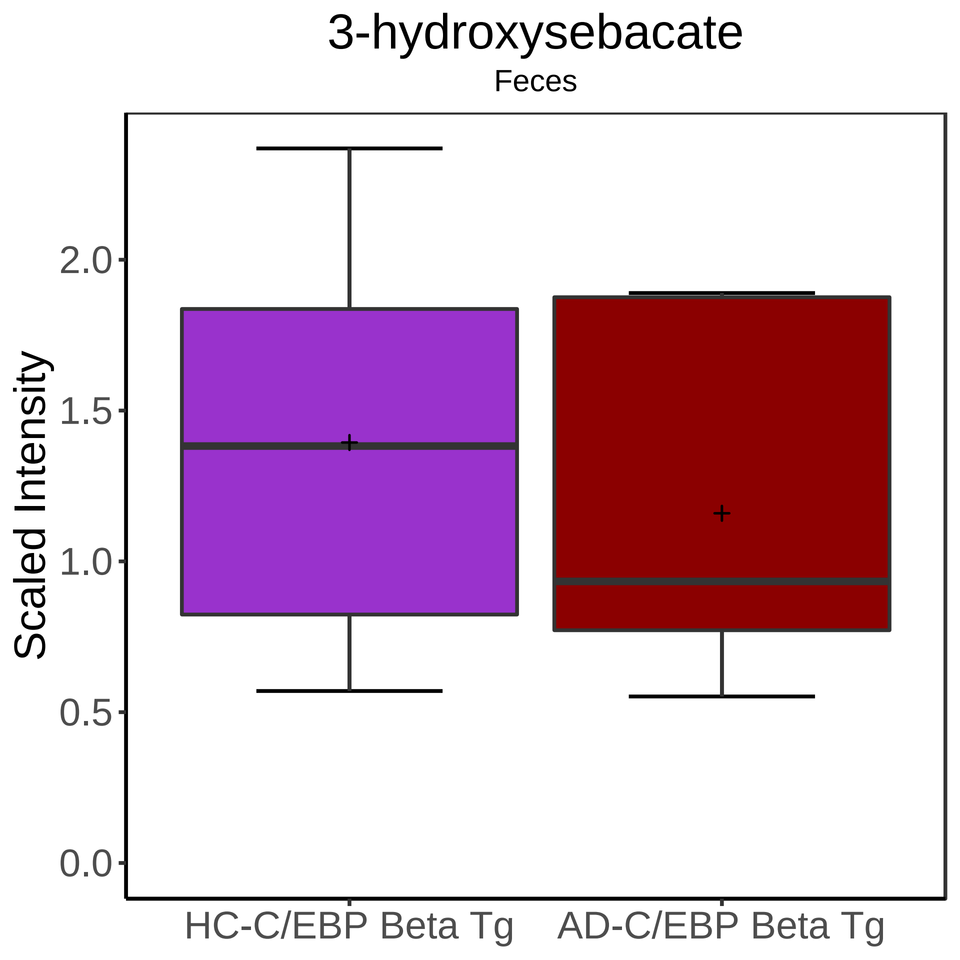
3-hydroxysebacate
Feces
Scaled Intensity
2.0
1.5
1.0
0.5
0.0
HC-C/EBP Beta Tg
AD-C/EBP Beta Tg

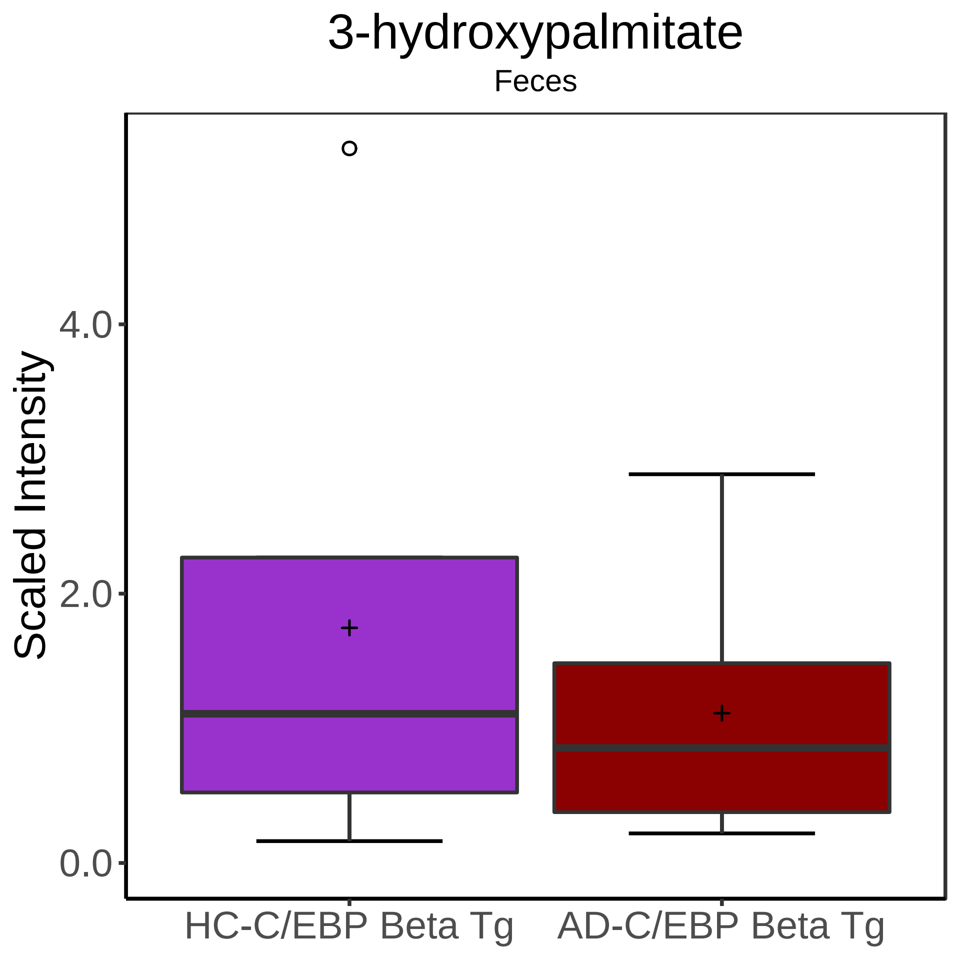

3-hydroxypalmitate
Feces
Scaled Intensity
4.0
2.0
0.0
HC-C/EBP Beta Tg
AD-C/EBP Beta Tg

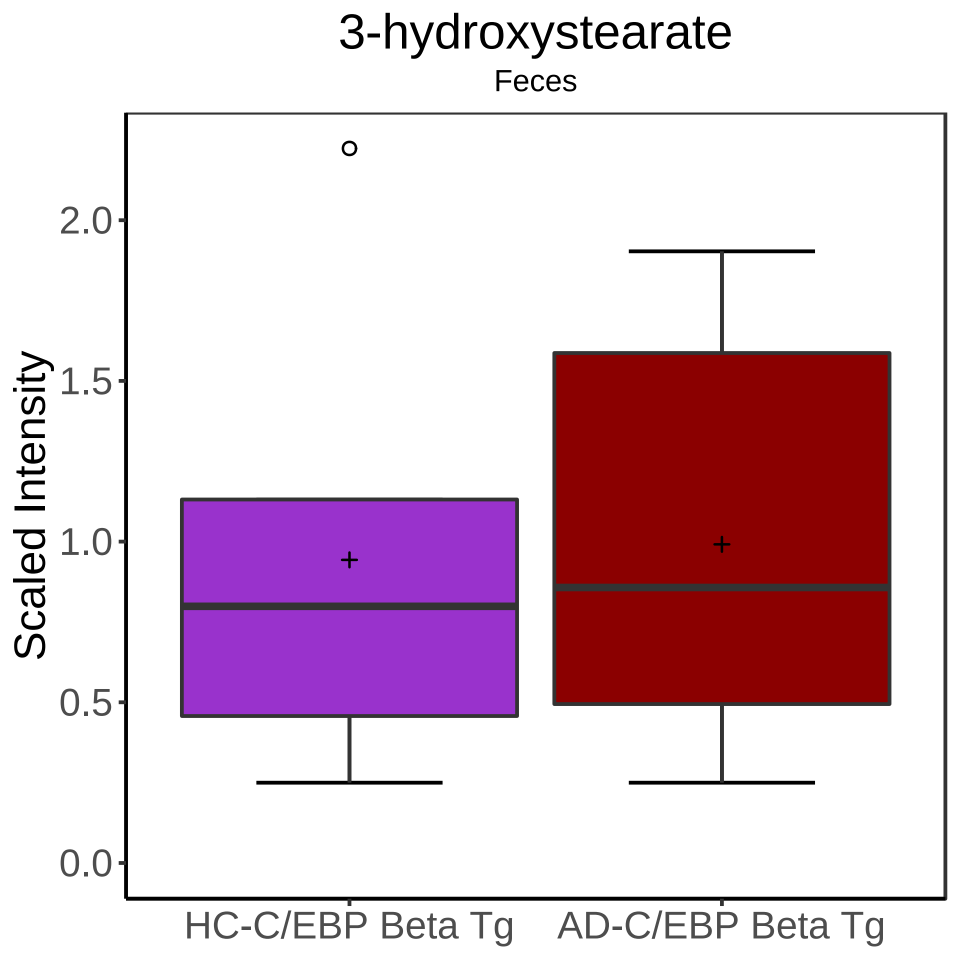

3-hydroxystearate
Feces
Scaled Intensity
0.0
0.5
1.0
1.5
2.0
HC-C/EBP Beta Tg
AD-C/EBP Beta Tg

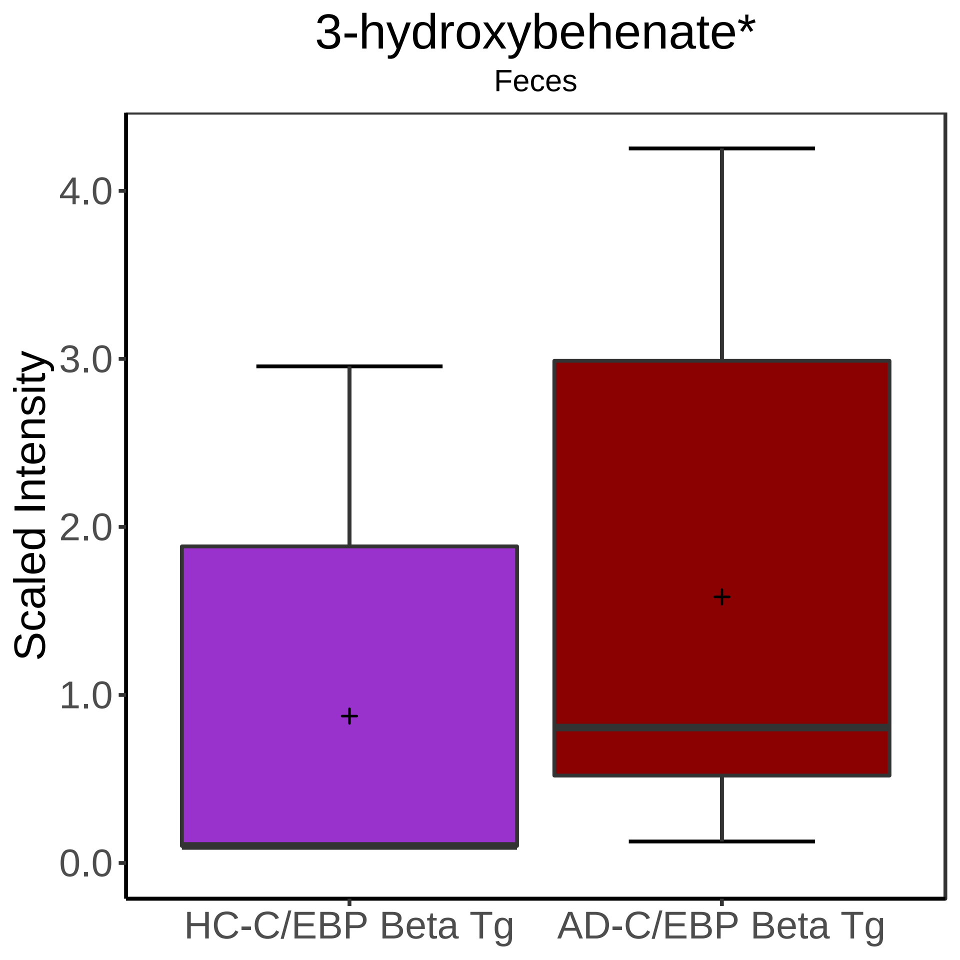
3-hydroxybehenate*
Feces
Scaled Intensity
4.0
3.0
2.0
1.0
0.0
HC-C/EBP Beta Tg
AD-C/EBP Beta Tg

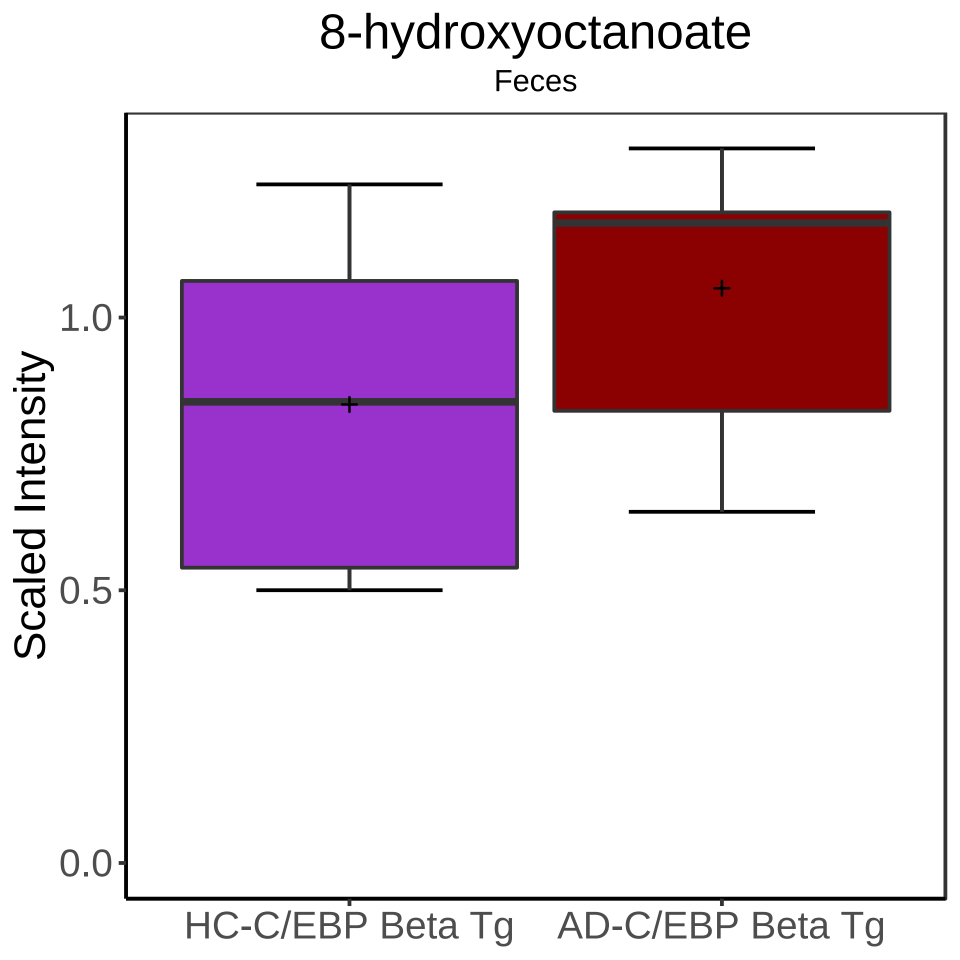
8-hydroxyoctanoate
Feces
Scaled Intensity
1.0
0.5
0.0
HC-C/EBP Beta Tg
AD-C/EBP Beta Tg

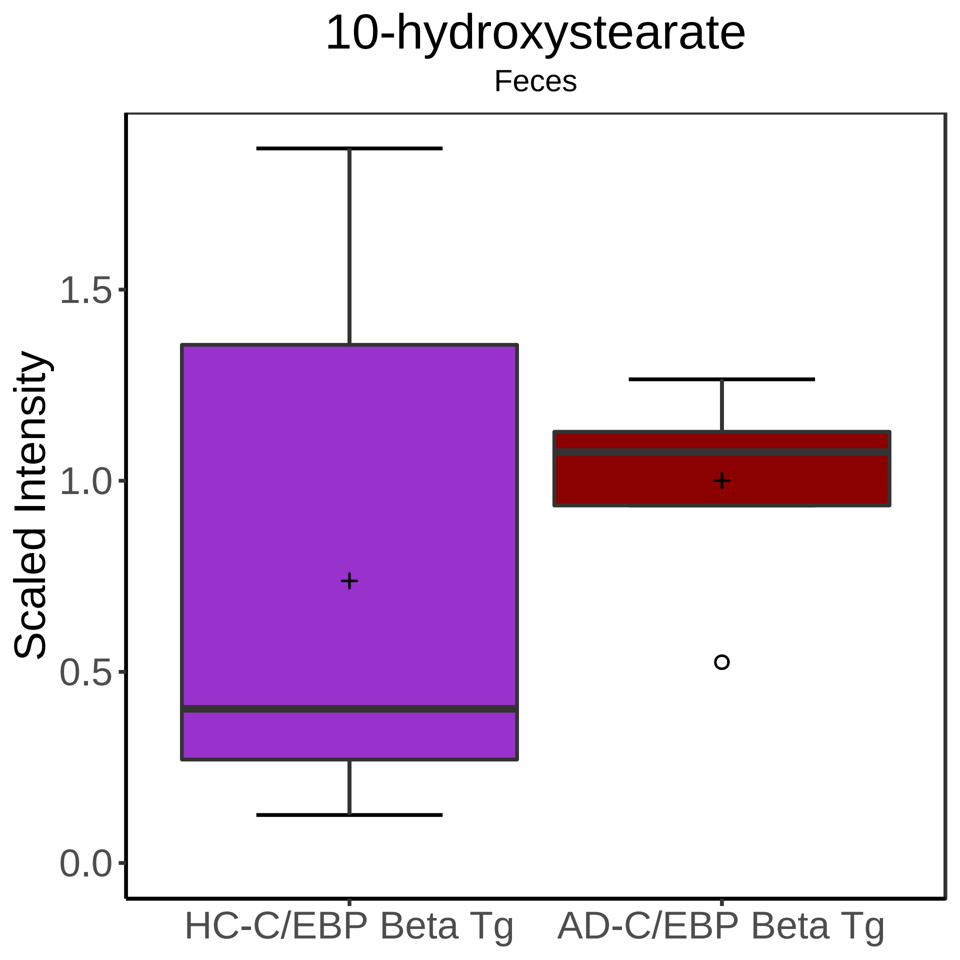
10-hydroxystearate
Feces
Scaled Intensity
0.0
0.5
1.0
1.5
HC-C/EBP Beta Tg
AD-C/EBP Beta Tg

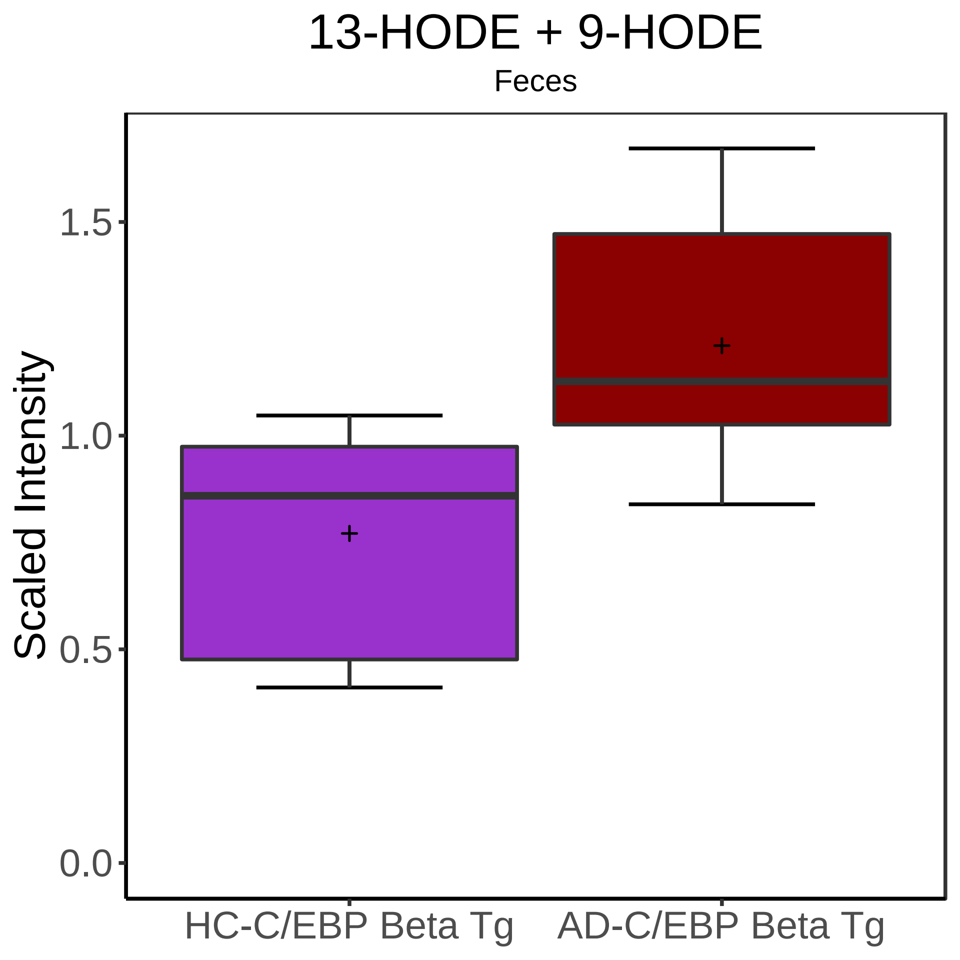
13-HODE + 9-HODE
Feces
Scaled Intensity
1.5
1.0
0.5
0.0
HC-C/EBP Beta Tg
AD-C/EBP Beta Tg

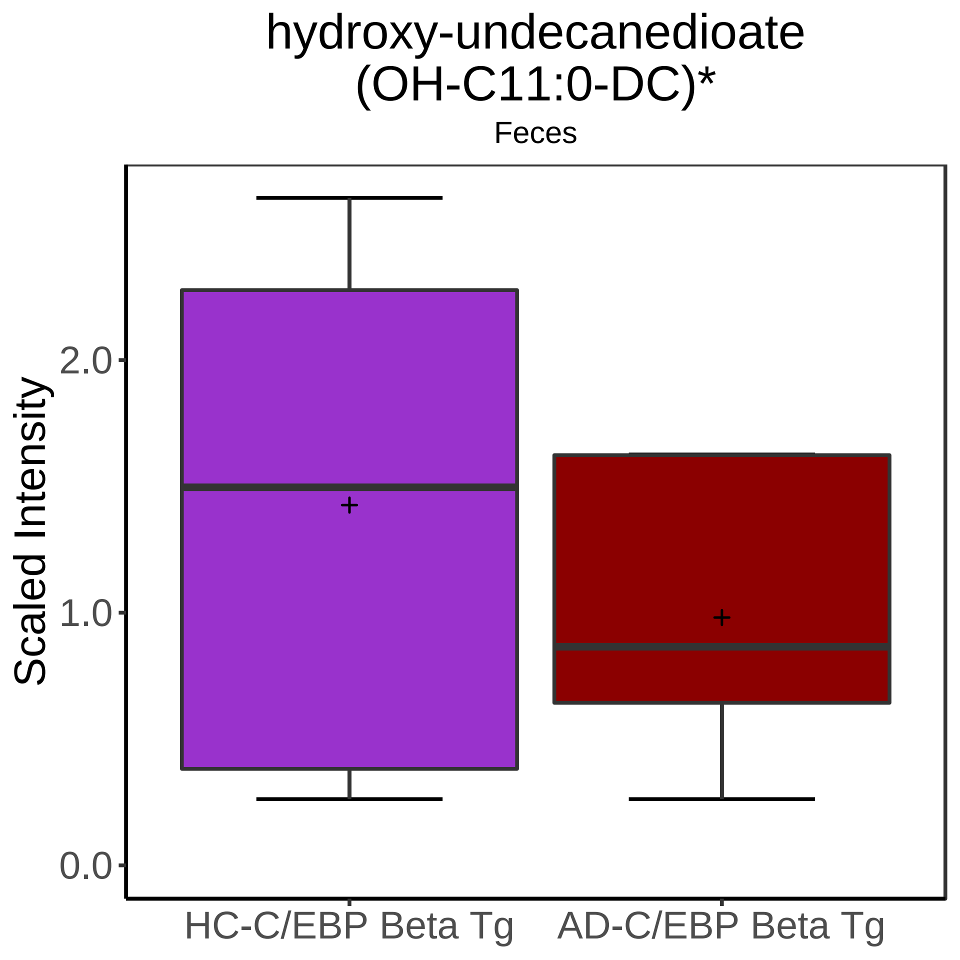
hydroxy-undecanedioate
(OH-C11:0-DC)*
Feces
Scaled Intensity
2.0
1.0
0.0
HC-C/EBP Beta Tg
AD-C/EBP Beta Tg

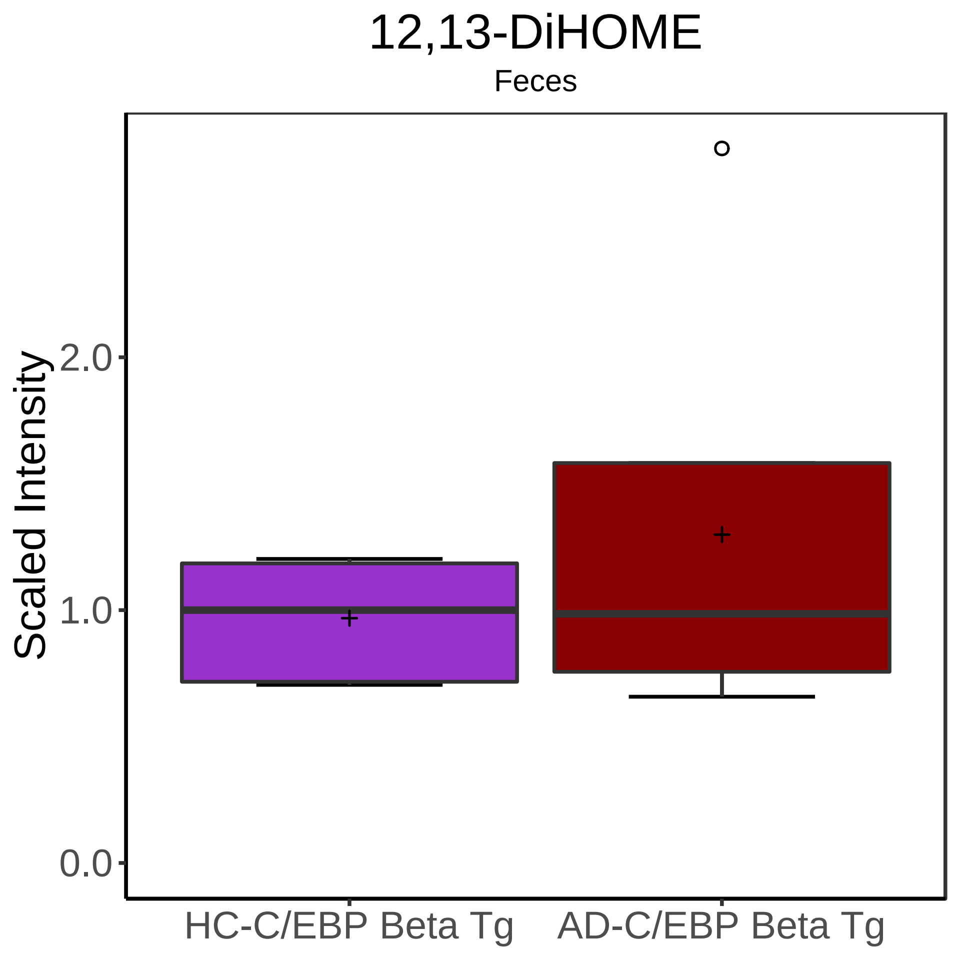

12,13-DiHOME
Feces
Scaled Intensity
2.0
1.0
0.0
HC-C/EBP Beta Tg
AD-C/EBP Beta Tg

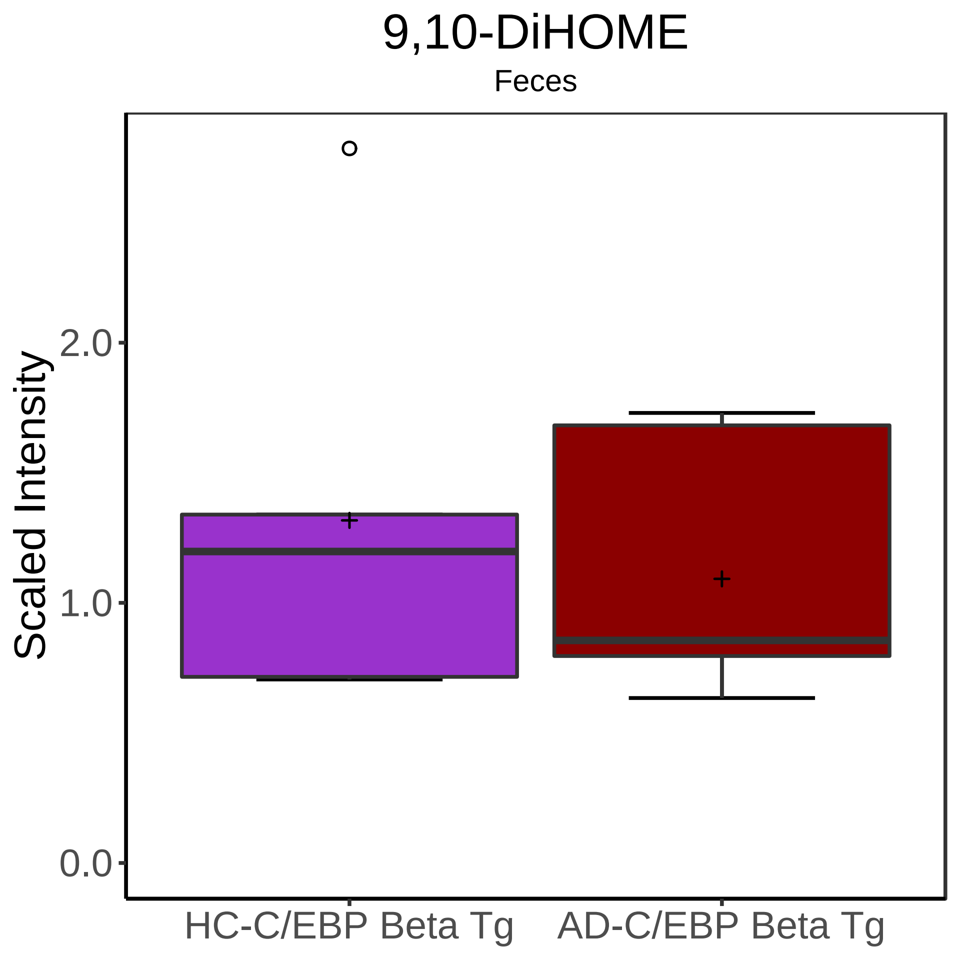
9,10-DiHOME
Feces
Scaled Intensity
2.0
1.0
0.0
HC-C/EBP Beta Tg
AD-C/EBP Beta Tg

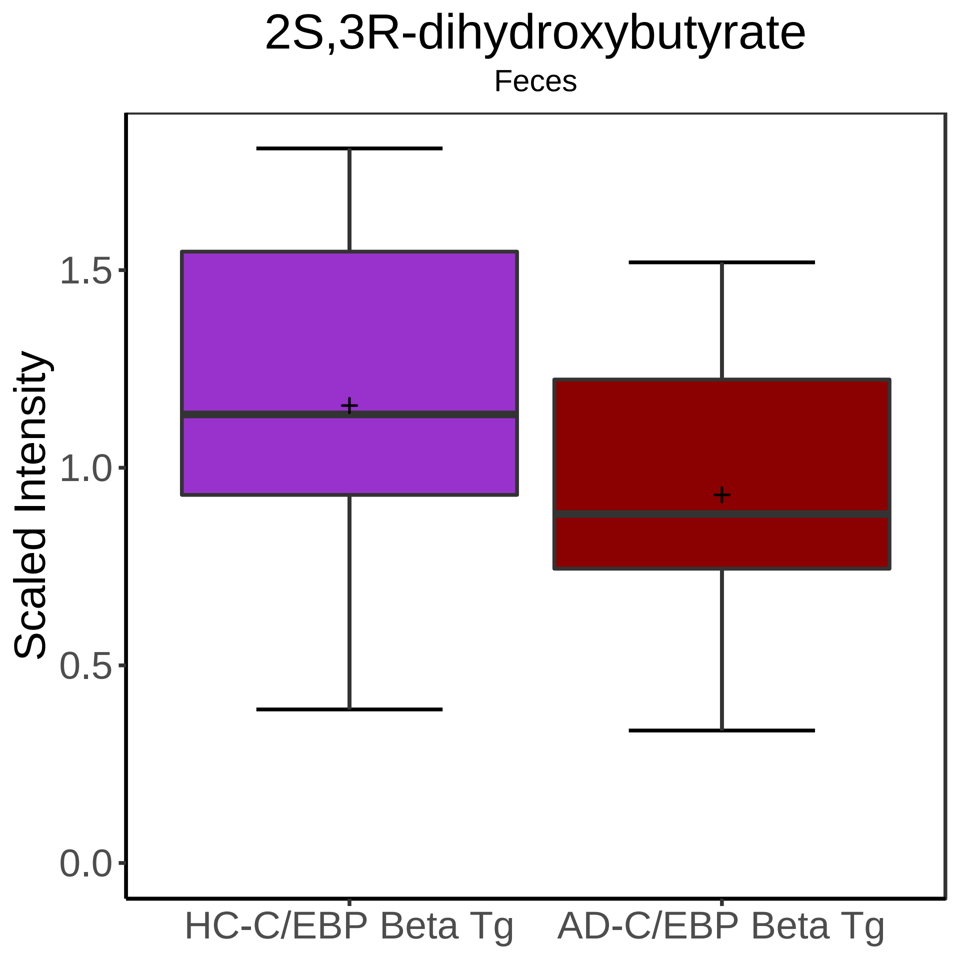
2S,3R-dihydroxybutyrate
Feces
Scaled Intensity
1.5
1.0
0.5
0.0
HC-C/EBP Beta Tg
AD-C/EBP Beta Tg

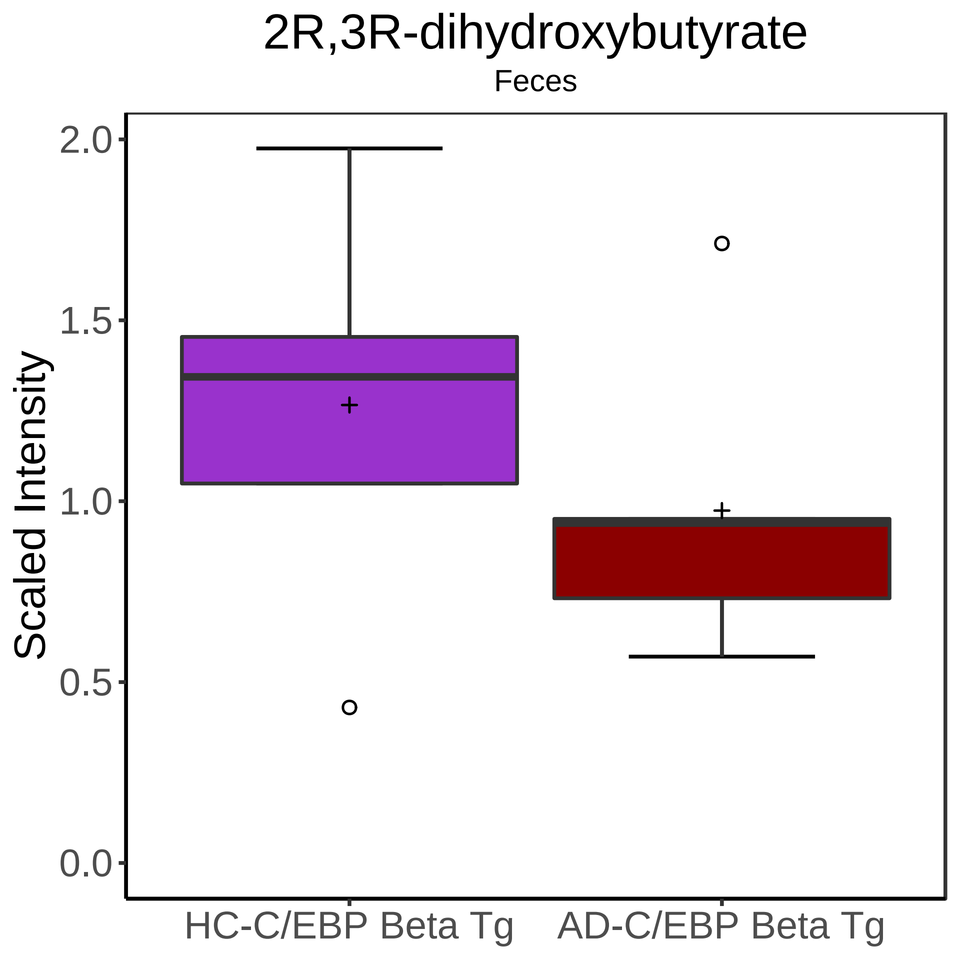
2R,3R-dihydroxybutyrate
Feces
Scaled Intensity
2.0
1.5
1.0
0.5
0.0
HC-C/EBP Beta Tg
AD-C/EBP Beta Tg

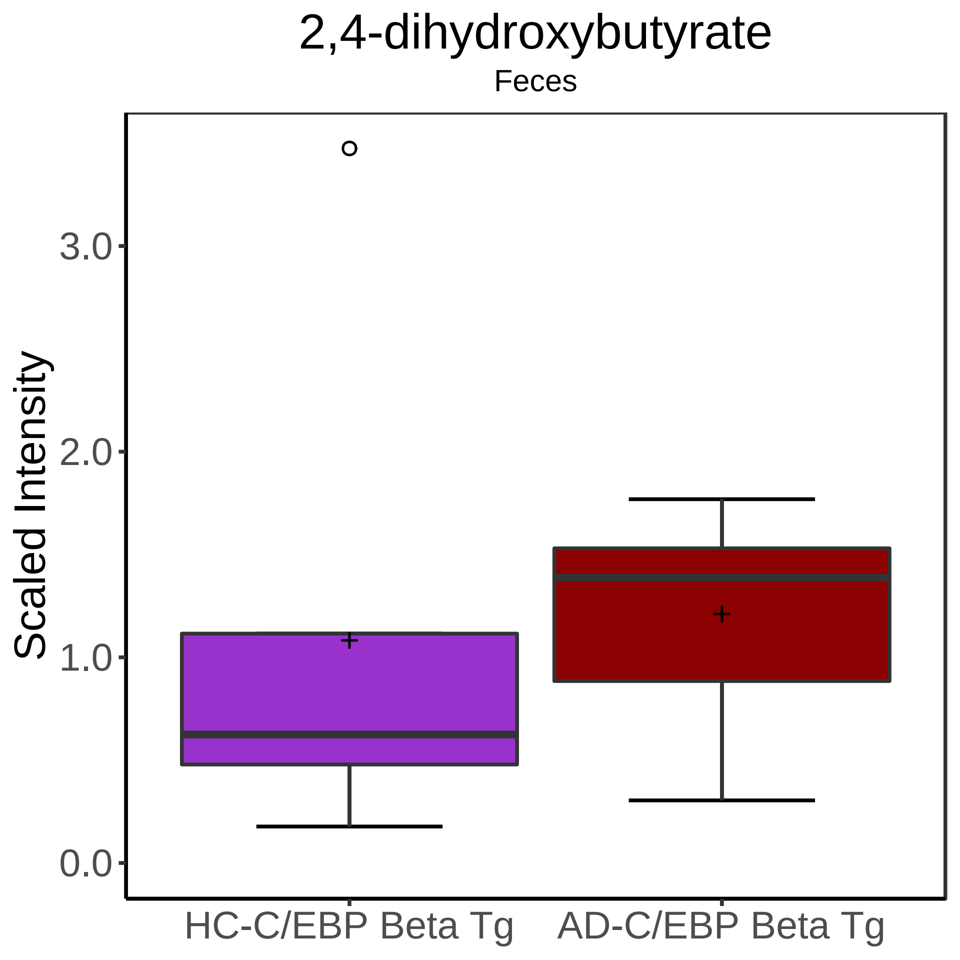
2,4-dihydroxybutyrate
Feces
Scaled Intensity
3.0
2.0
1.0
0.0
HC-C/EBP Beta Tg
AD-C/EBP Beta Tg

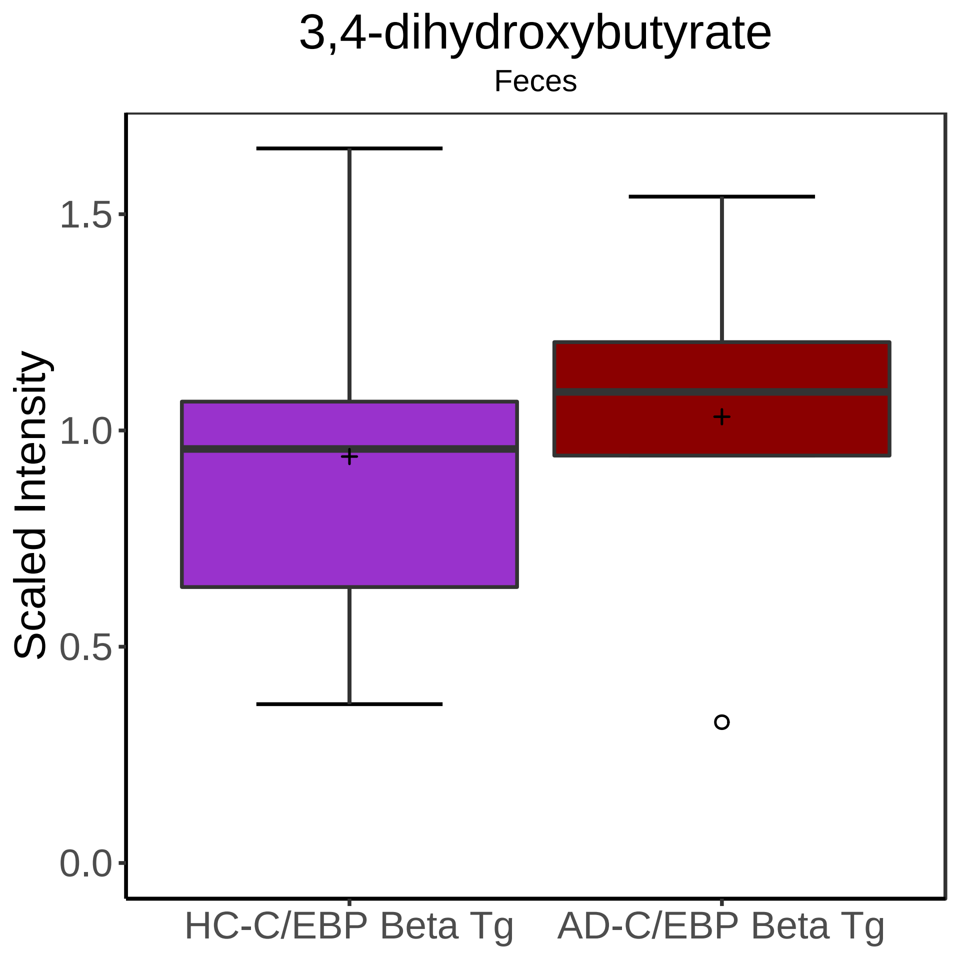
3,4-dihydroxybutyrate
Feces
Scaled Intensity
1.5
1.0
0.5
0.0
HC-C/EBP Beta Tg
AD-C/EBP Beta Tg

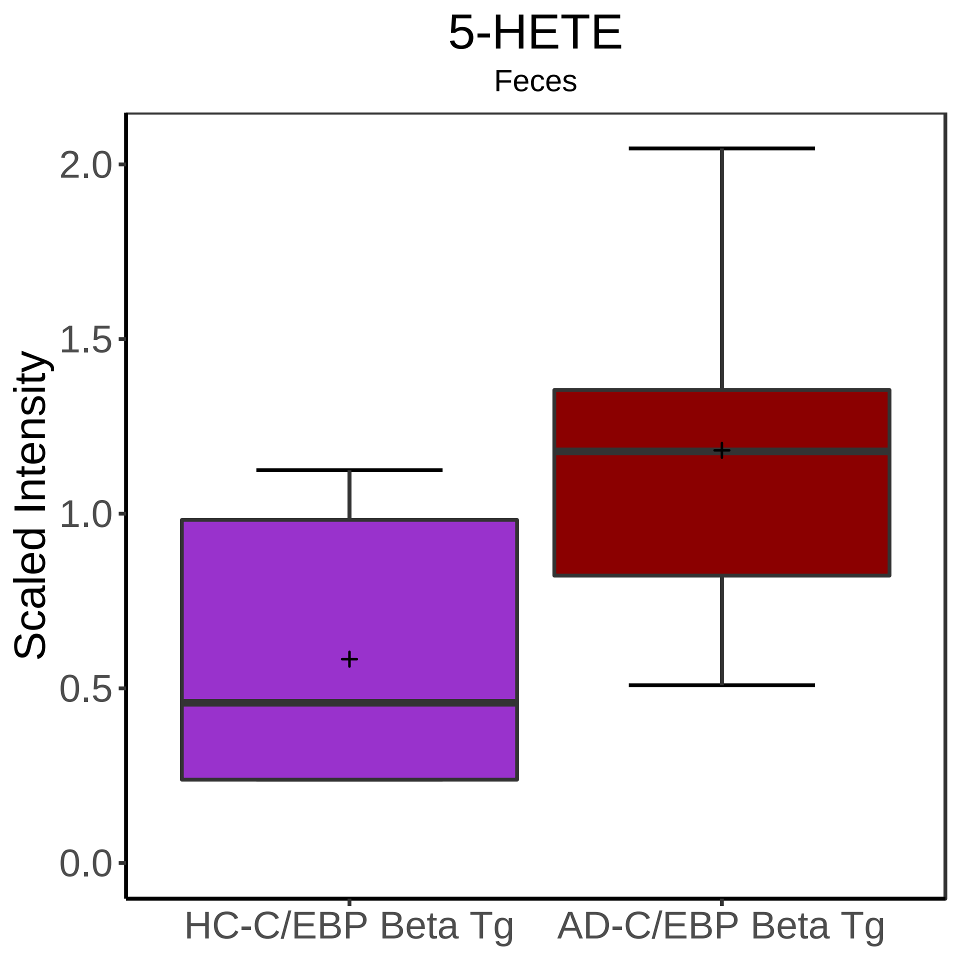

5-HETE
Feces
Scaled Intensity
2.0
1.5
1.0
0.5
0.0
HC-C/EBP Beta Tg
AD-C/EBP Beta Tg

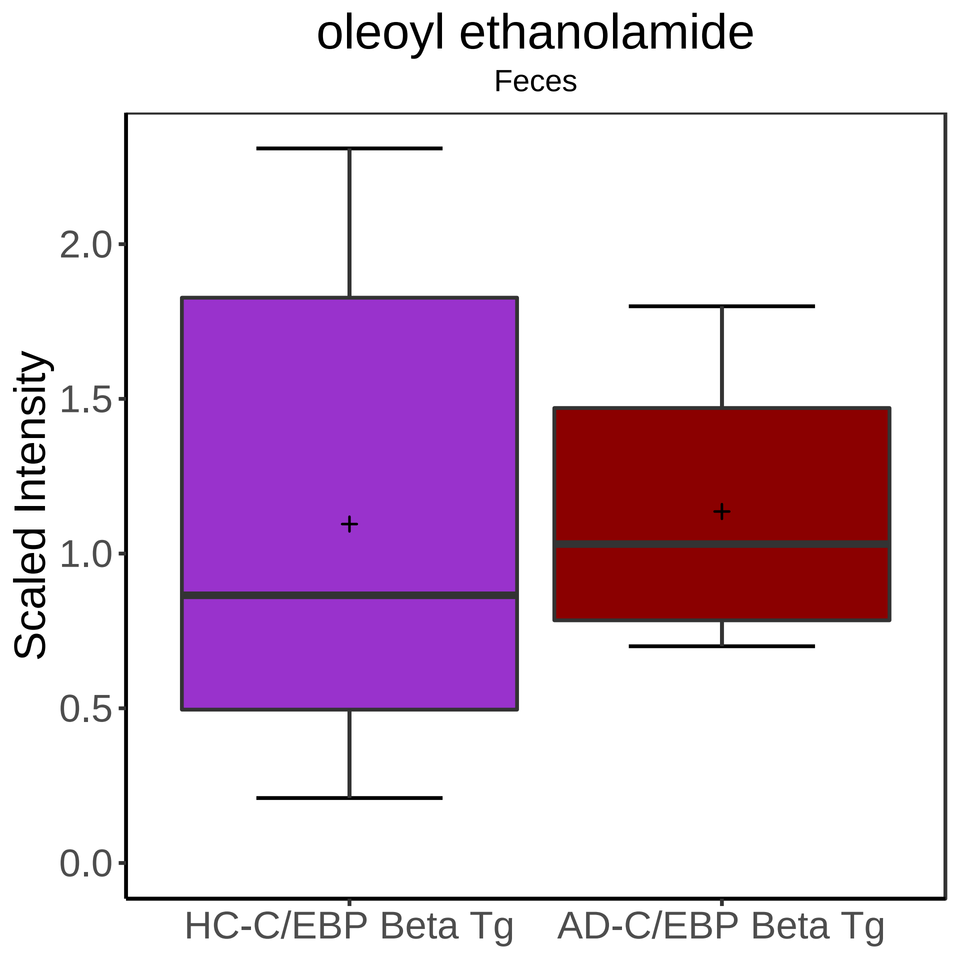
oleoyl ethanolamide
Feces
Scaled Intensity
2.0
1.5
1.0
0.5
0.0
HC-C/EBP Beta Tg
AD-C/EBP Beta Tg

## palmitoyl ethanolamide

Feces

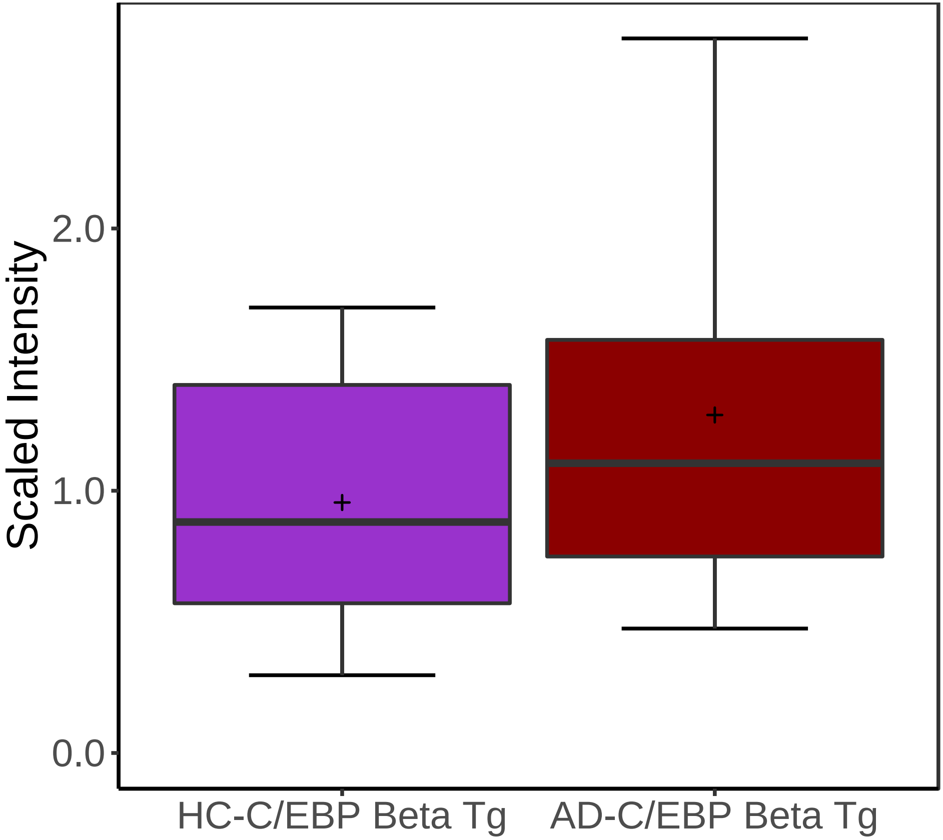

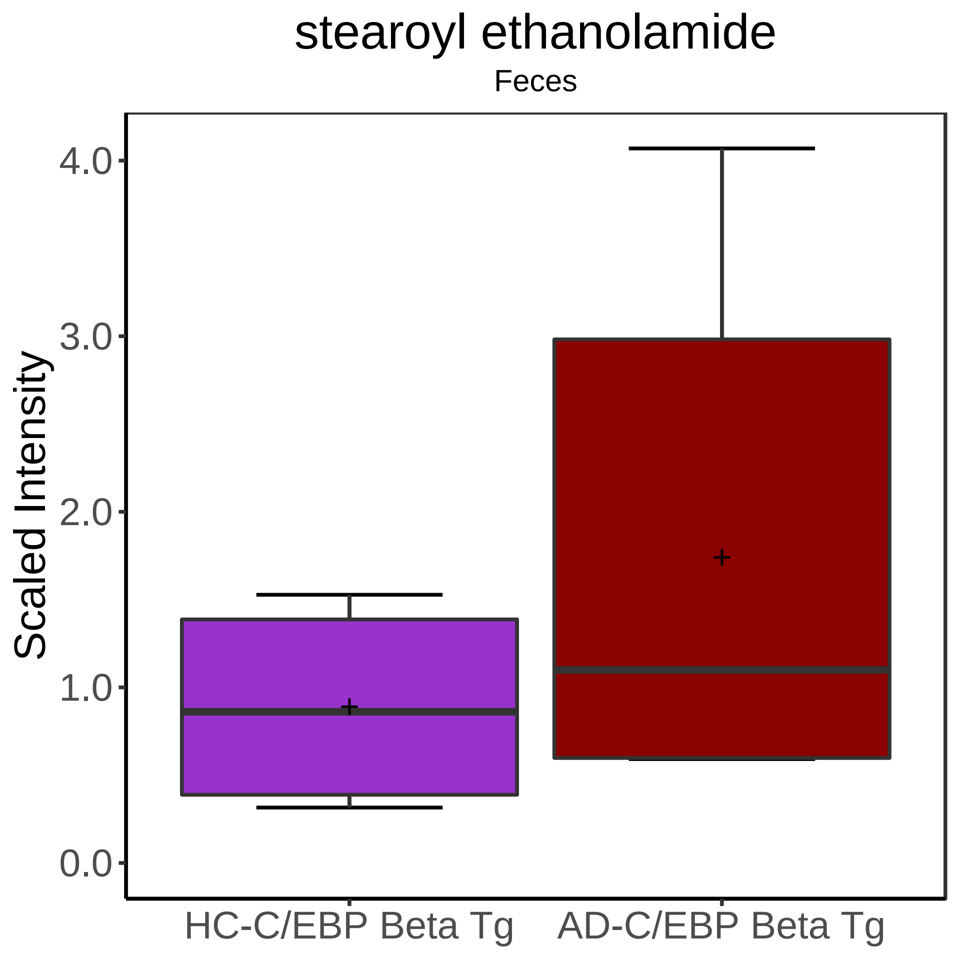

stearoyl ethanolamide
Feces
Scaled Intensity
4.0
3.0
2.0
1.0
0.0
HC-C/EBP Beta Tg
AD-C/EBP Beta Tg

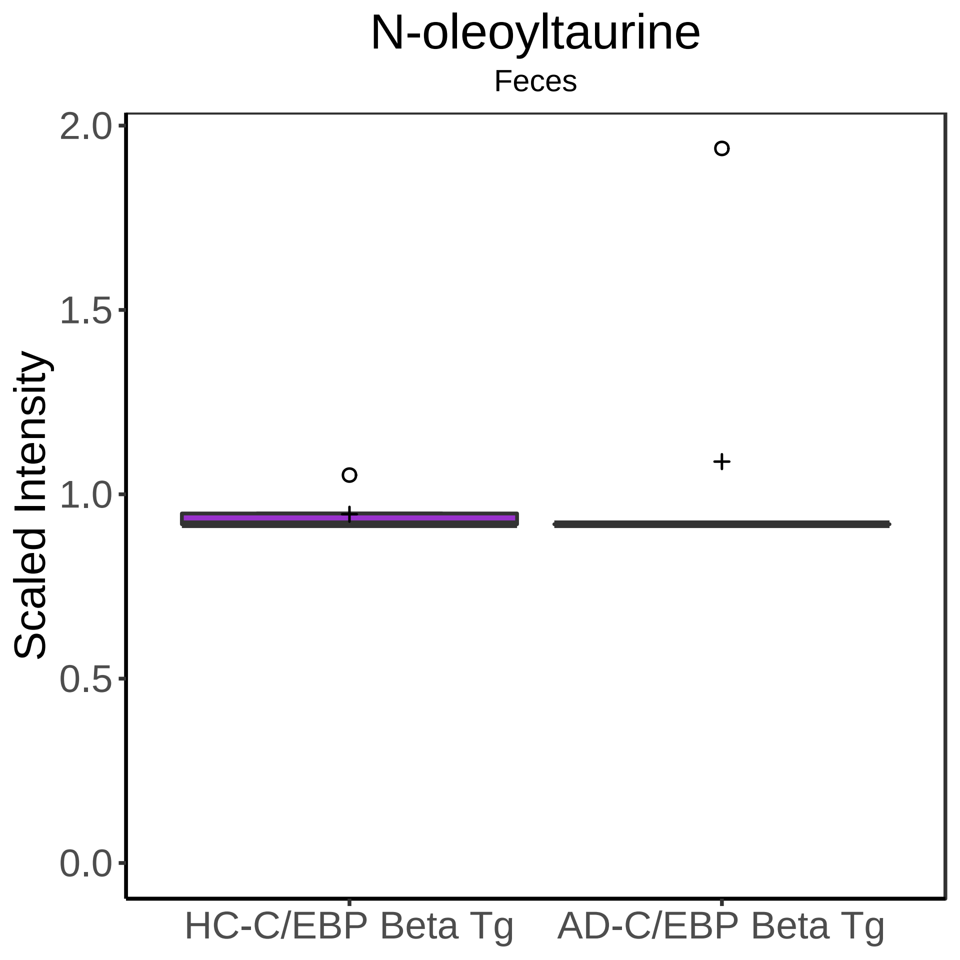
N-oleoyltaurine
Feces
Scaled Intensity
2.0
1.5
1.0
0.5
0.0
HC-C/EBP Beta Tg
AD-C/EBP Beta Tg

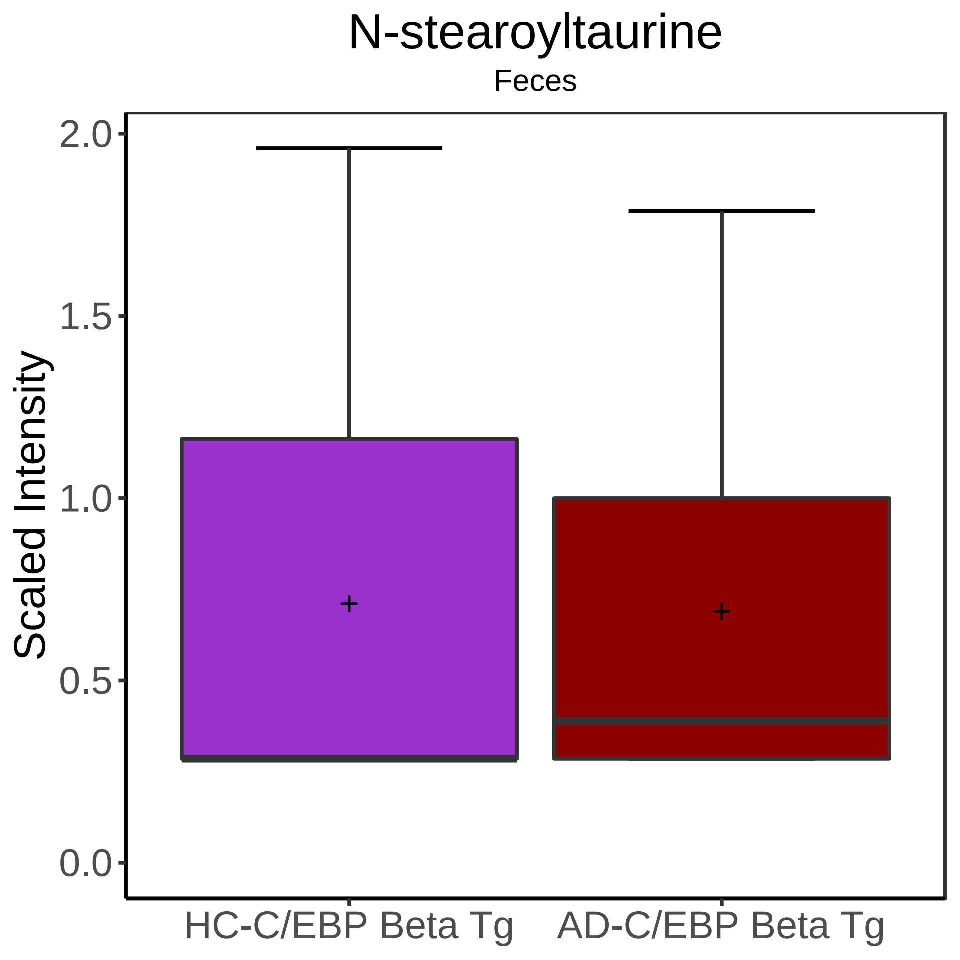
N-stearoyltaurine
Feces
Scaled Intensity
2.0
1.5
1.0
0.5
0.0
HC-C/EBP Beta Tg
AD-C/EBP Beta Tg

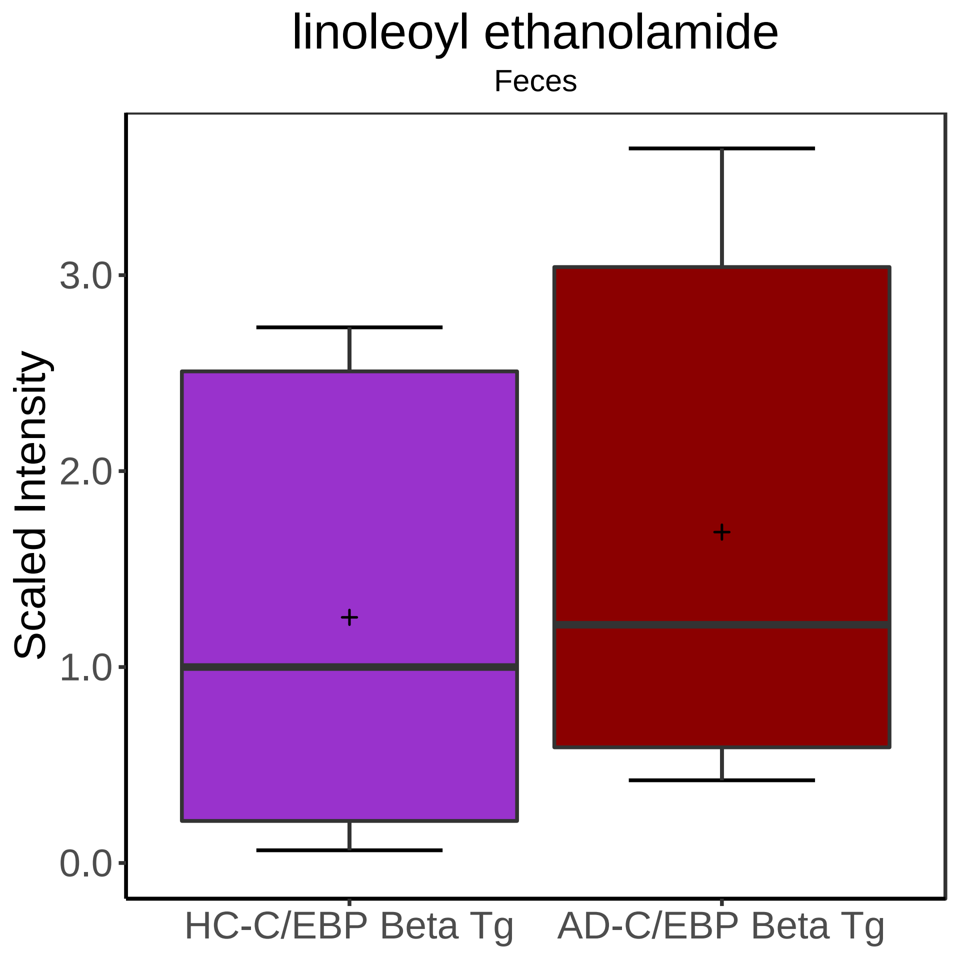
linoleoyl ethanolamide
Feces
Scaled Intensity
3.0
2.0
1.0
0.0
HC-C/EBP Beta Tg
AD-C/EBP Beta Tg

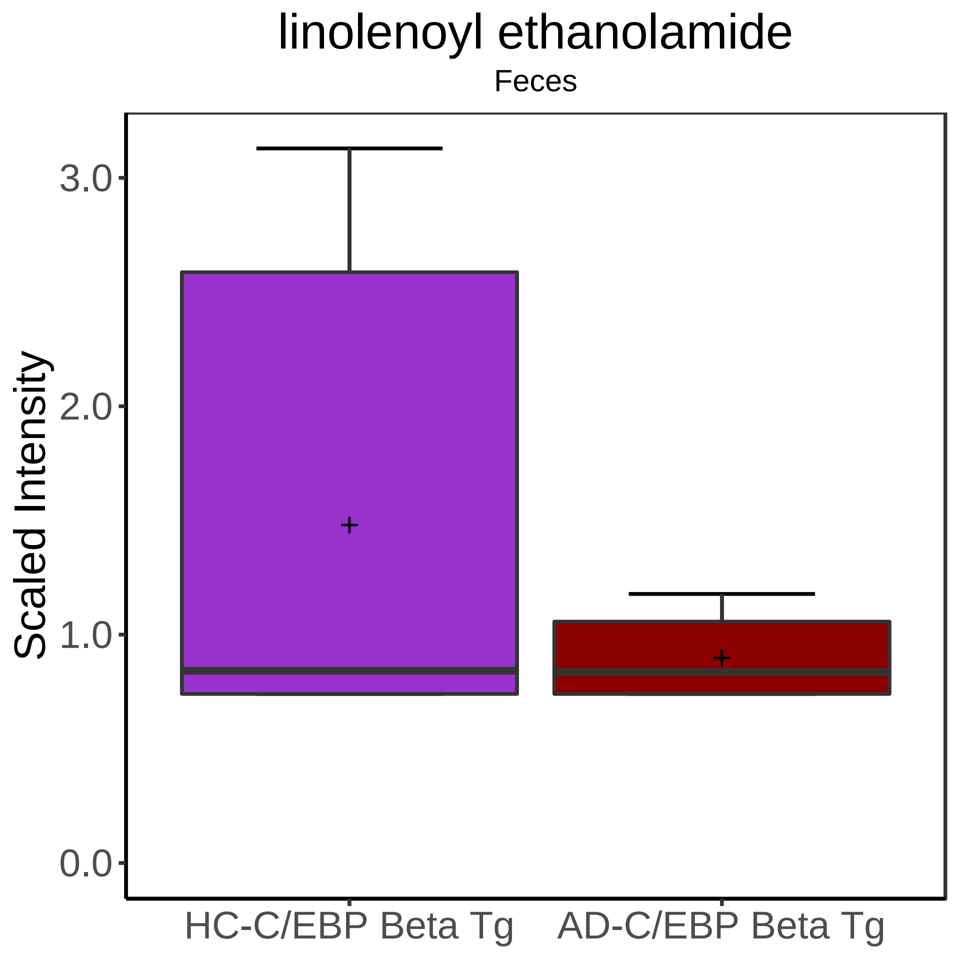
linolenoyl ethanolamide
Feces
Scaled Intensity
3.0
2.0
1.0
0.0
HC-C/EBP Beta Tg
AD-C/EBP Beta Tg

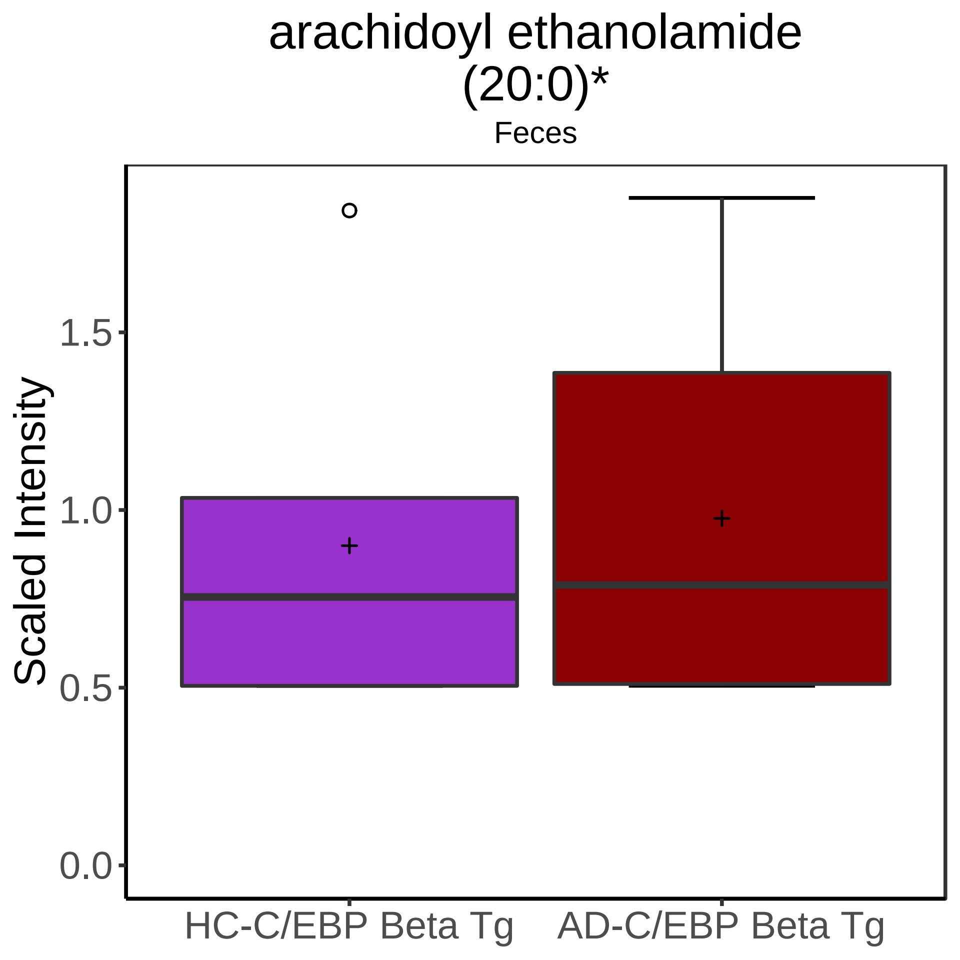

arachidoyl ethanolamide
(20:0)*
Feces
Scaled Intensity
0.0
0.5
1.0
1.5
HC-C/EBP Beta Tg
AD-C/EBP Beta Tg

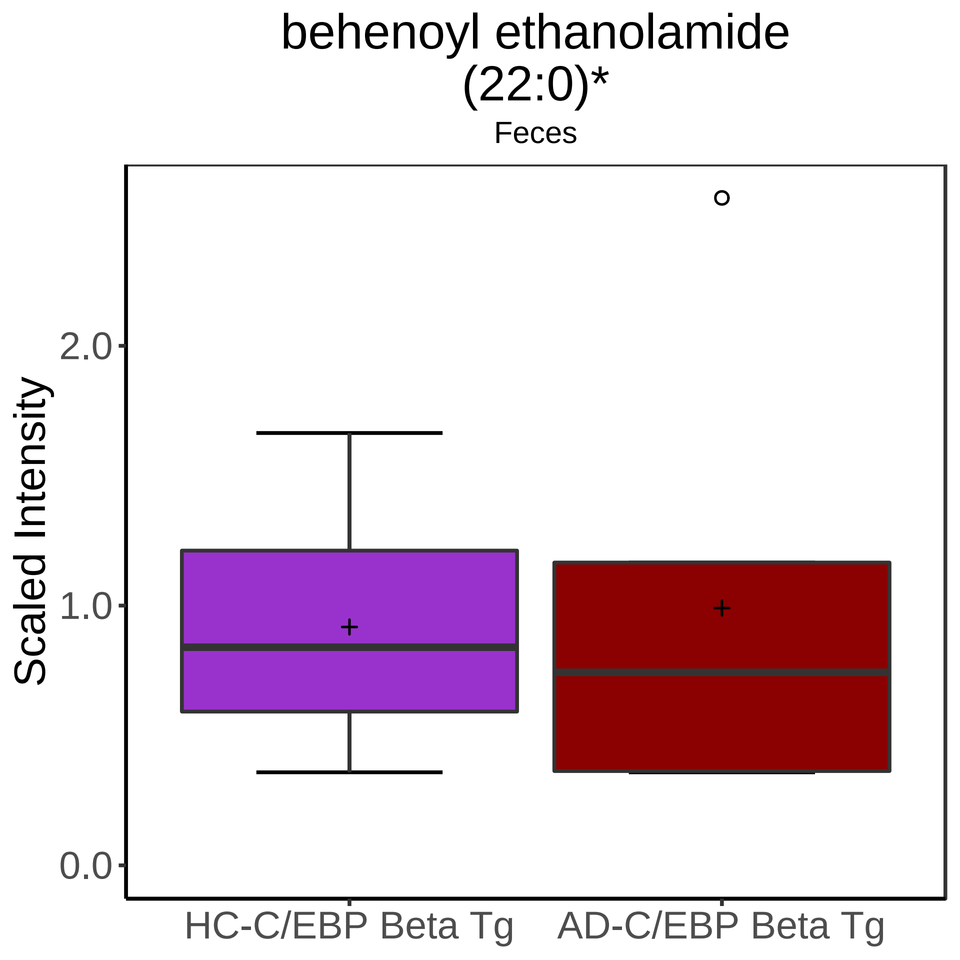
behenoyl ethanolamide
(22:0)*
Feces
Scaled Intensity
2.0
1.0
0.0
HC-C/EBP Beta Tg
AD-C/EBP Beta Tg

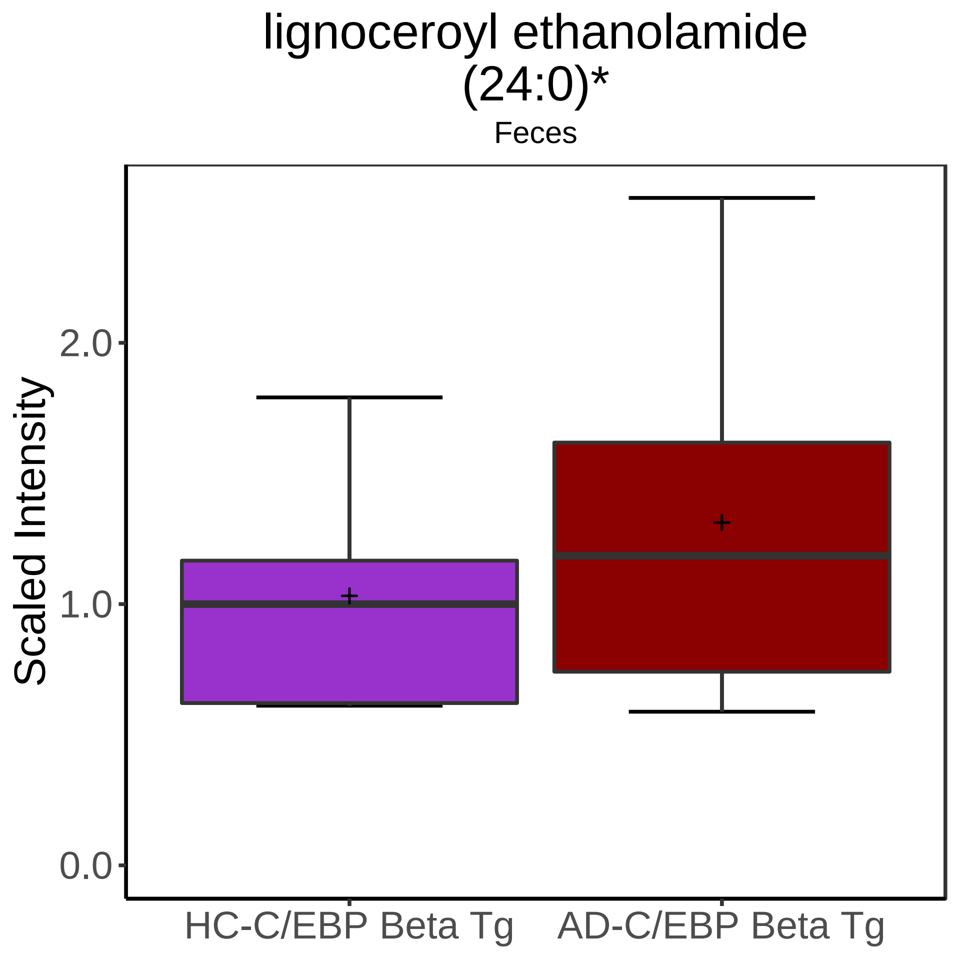
lignoceroyl ethanolamide
(24:0)*
Feces
Scaled Intensity
2.0
1.0
0.0
HC-C/EBP Beta Tg
AD-C/EBP Beta Tg

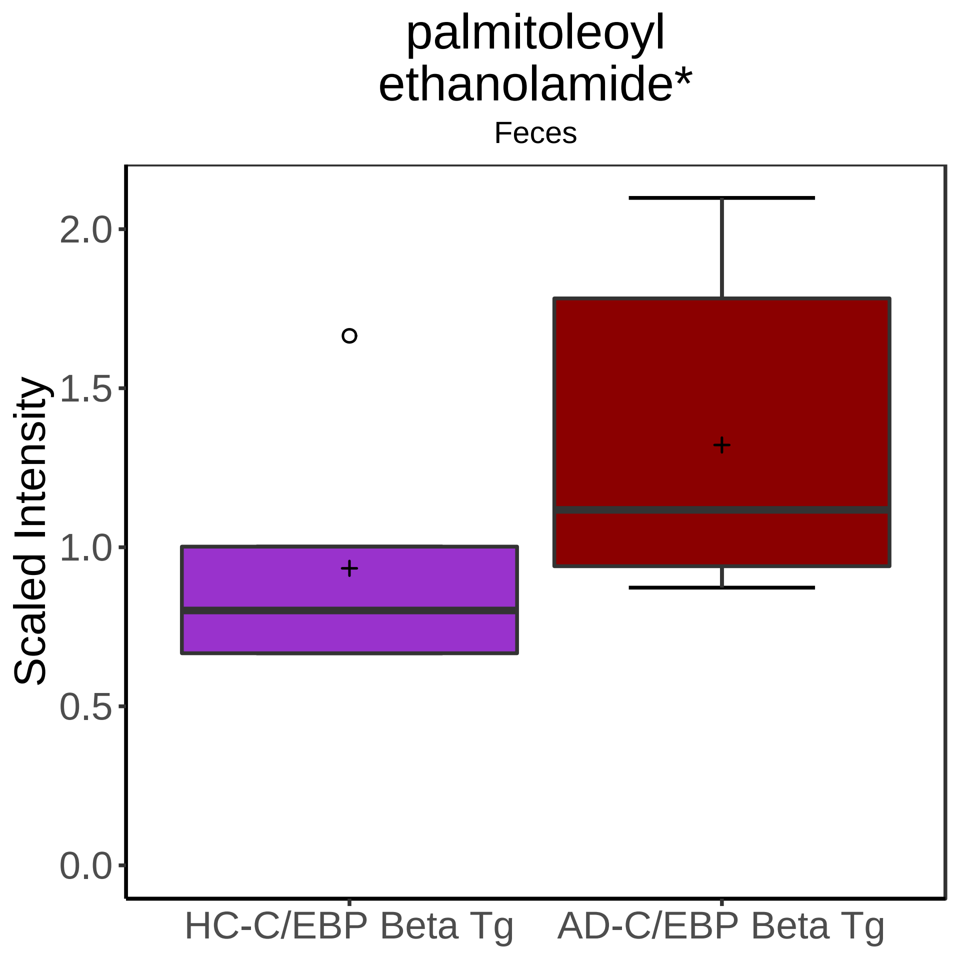

palmitoleoyl
ethanolamide*
Feces
Scaled Intensity
2.0
1.5
1.0
0.5
0.0
HC-C/EBP Beta Tg
AD-C/EBP Beta Tg

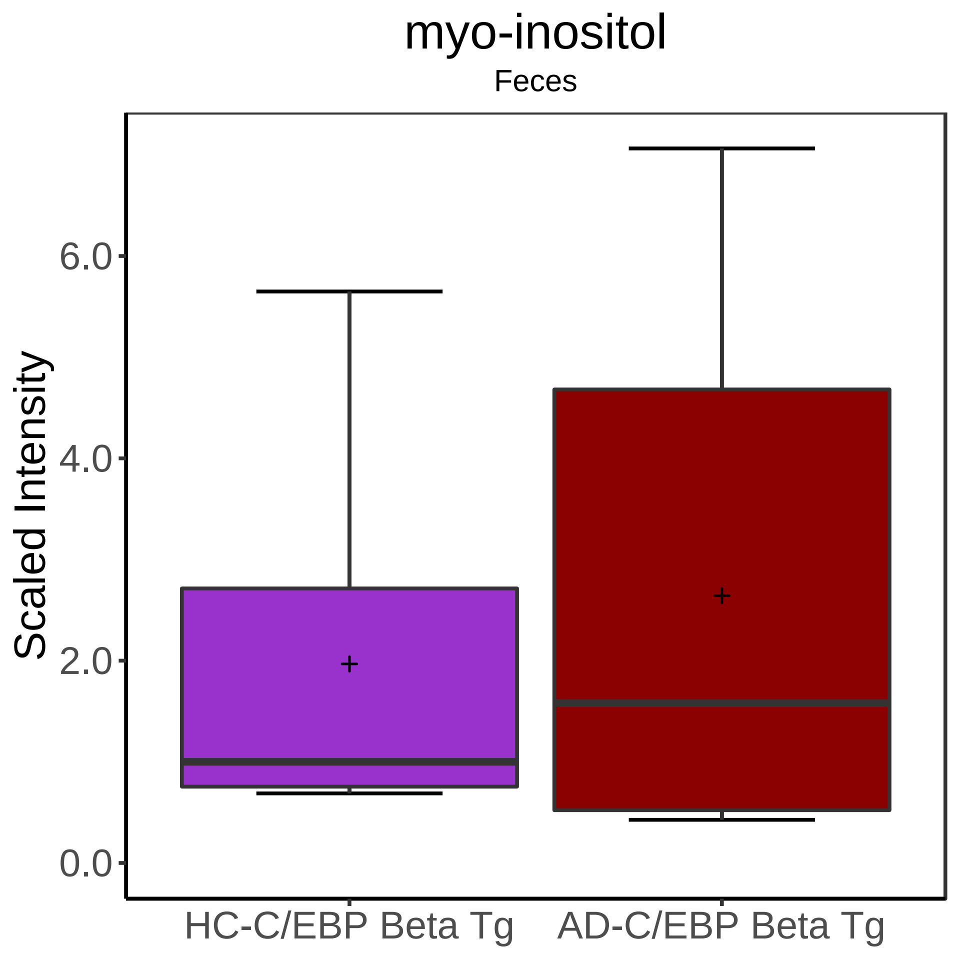
myo-inositol
Feces
Scaled Intensity
6.0
4.0
2.0
0.0
HC-C/EBP Beta Tg
AD-C/EBP Beta Tg

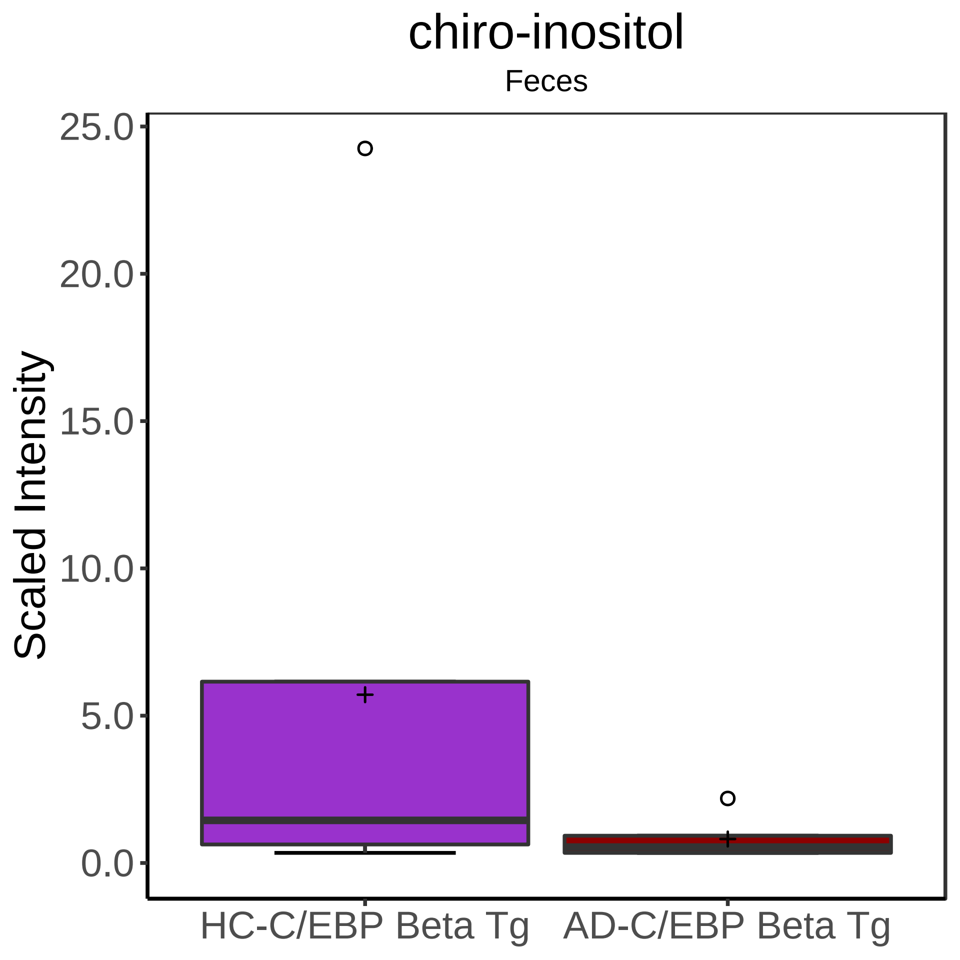
chiro-inositol
Feces
Scaled Intensity
25.0
20.0
15.0
10.0
5.0
0.0
HC-C/EBP Beta Tg
AD-C/EBP Beta Tg

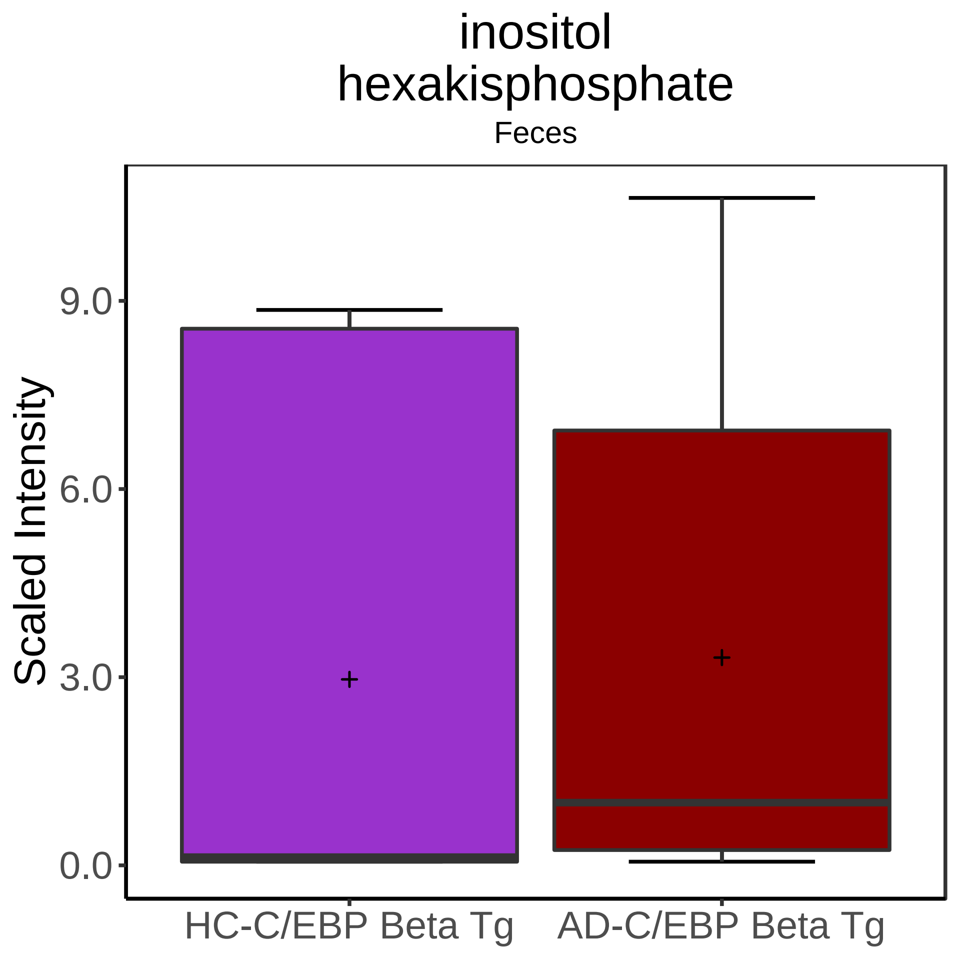
inositol
hexakisphosphate
Feces
Scaled Intensity
9.0
6.0
3.0
0.0
HC-C/EBP Beta Tg
AD-C/EBP Beta Tg

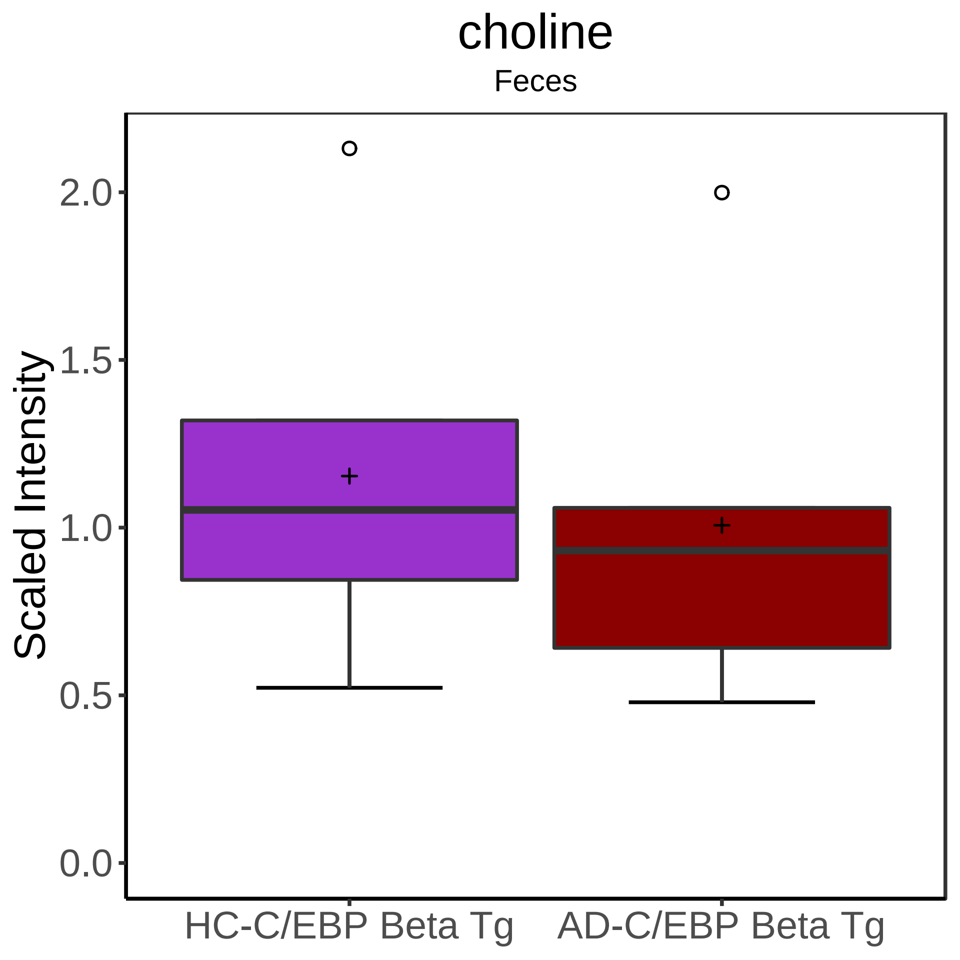

choline
Feces
Scaled Intensity
2.0
1.5
1.0
0.5
0.0
HC-C/EBP Beta Tg
AD-C/EBP Beta Tg

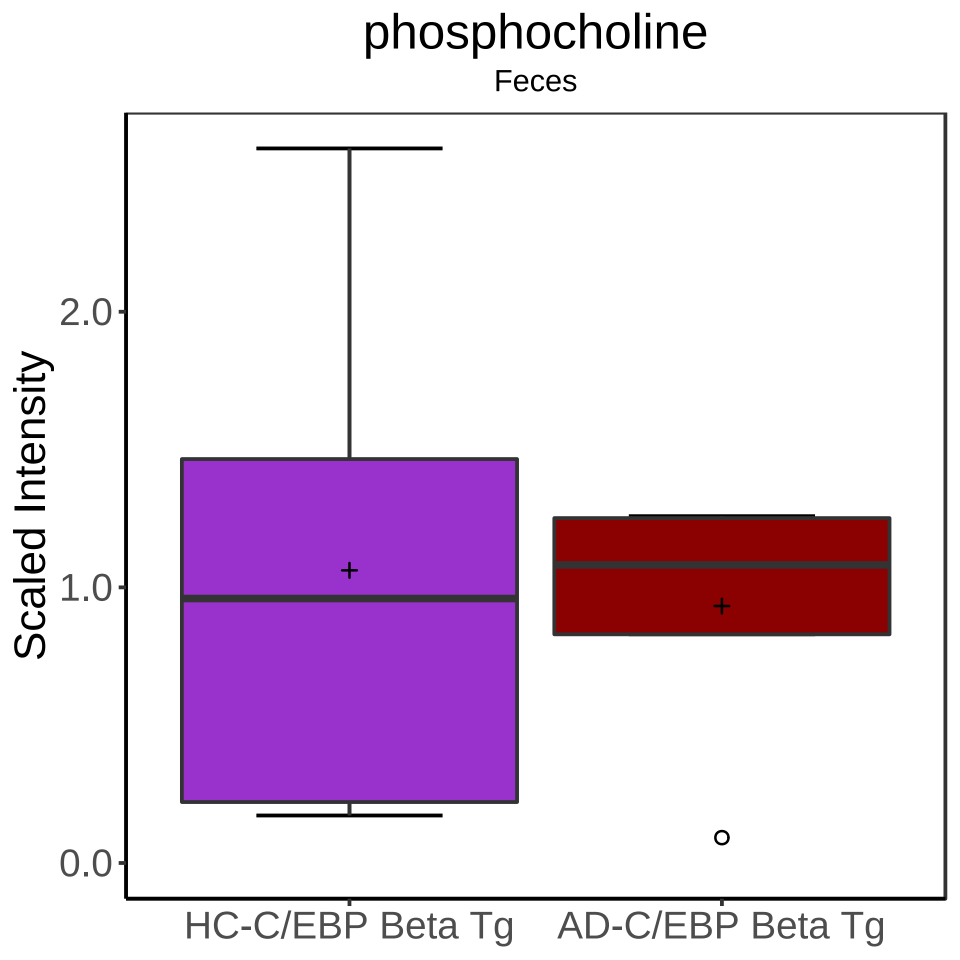
phosphocholine
Feces
Scaled Intensity
2.0
1.0
0.0
HC-C/EBP Beta Tg
AD-C/EBP Beta Tg

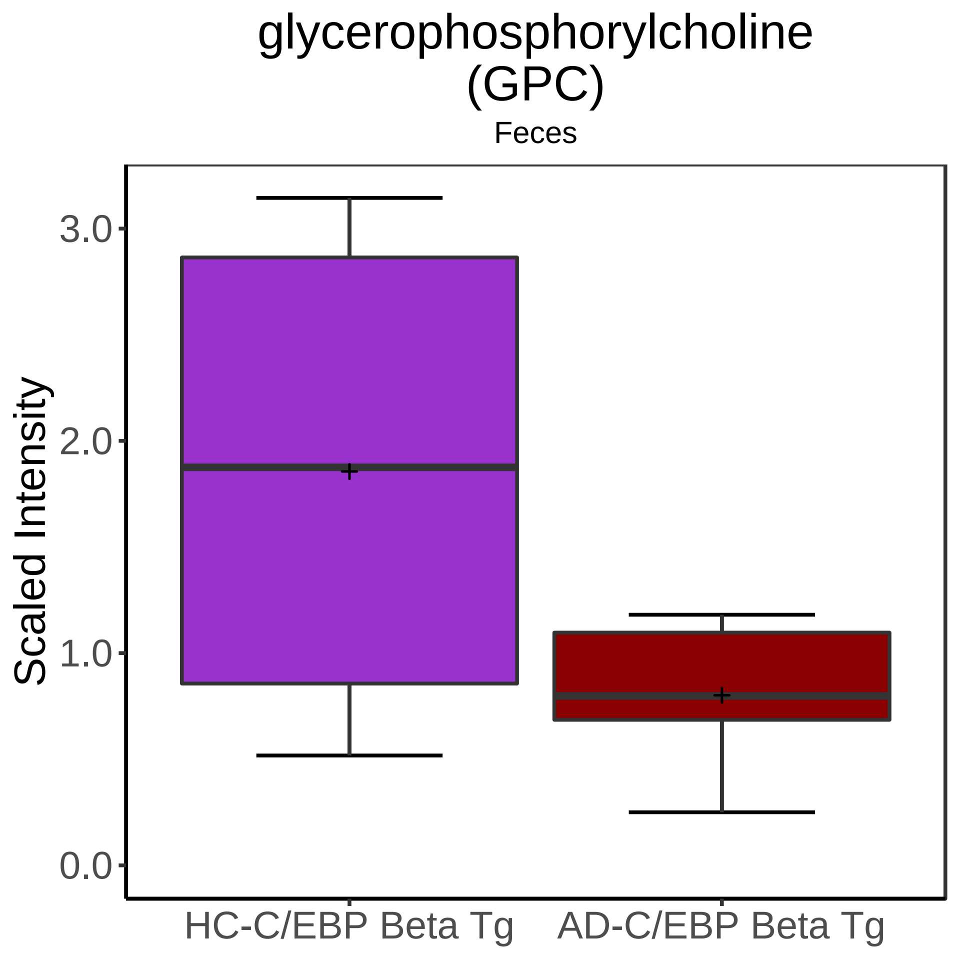
glycerophosphorylcholine
(GPC)
Feces
Scaled Intensity
3.0
2.0
1.0
0.0
HC-C/EBP Beta Tg
AD-C/EBP Beta Tg

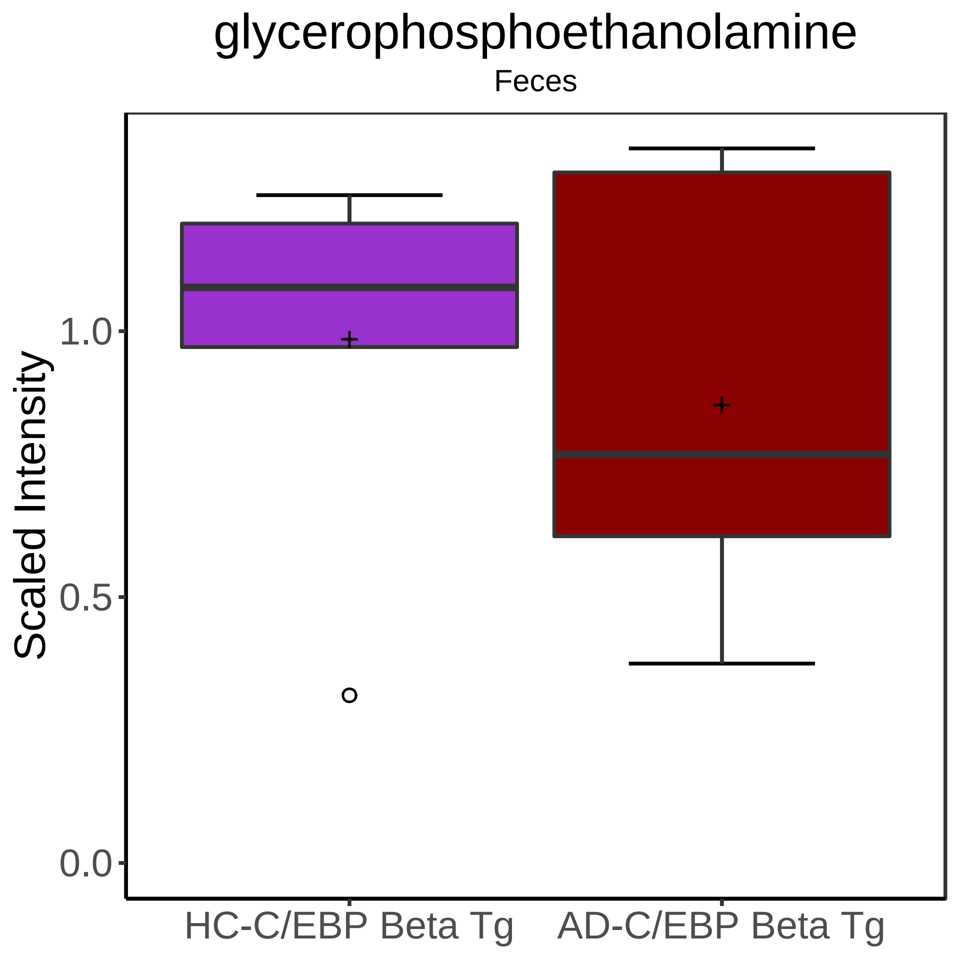
glycerophosphoethanolamine
Feces
Scaled Intensity
1.0
0.5
0.0
HC-C/EBP Beta Tg
AD-C/EBP Beta Tg

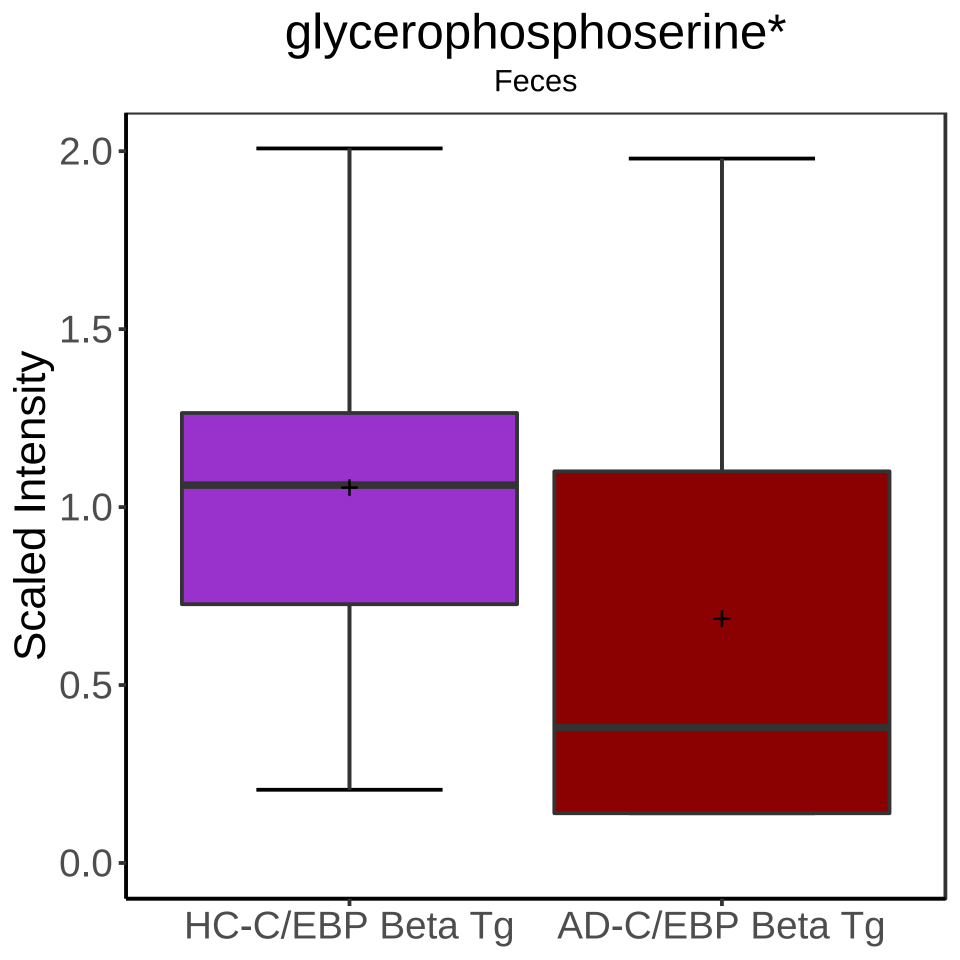
glycerophosphoserine*
Feces
Scaled Intensity
2.0
1.5
1.0
0.5
0.0
HC-C/EBP Beta Tg
AD-C/EBP Beta Tg

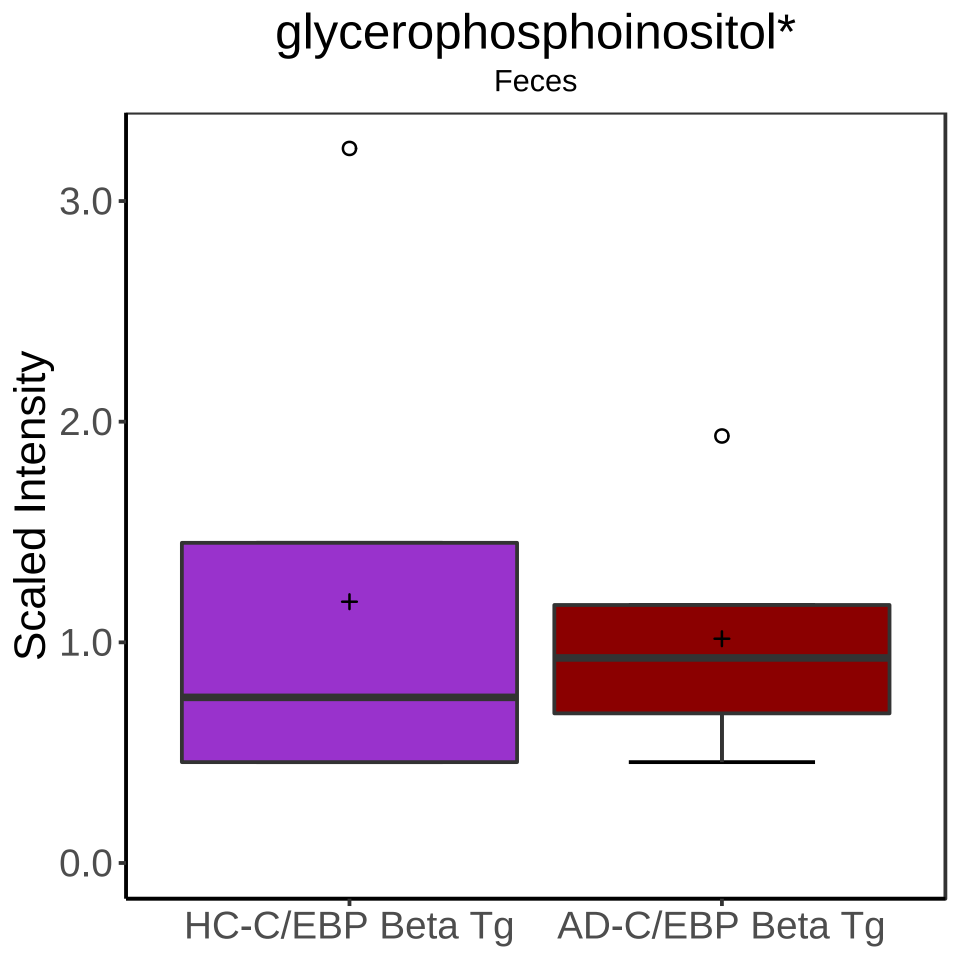

glycerophosphoinositol*
Feces
Scaled Intensity
3.0
2.0
1.0
0.0
HC-C/EBP Beta Tg
AD-C/EBP Beta Tg

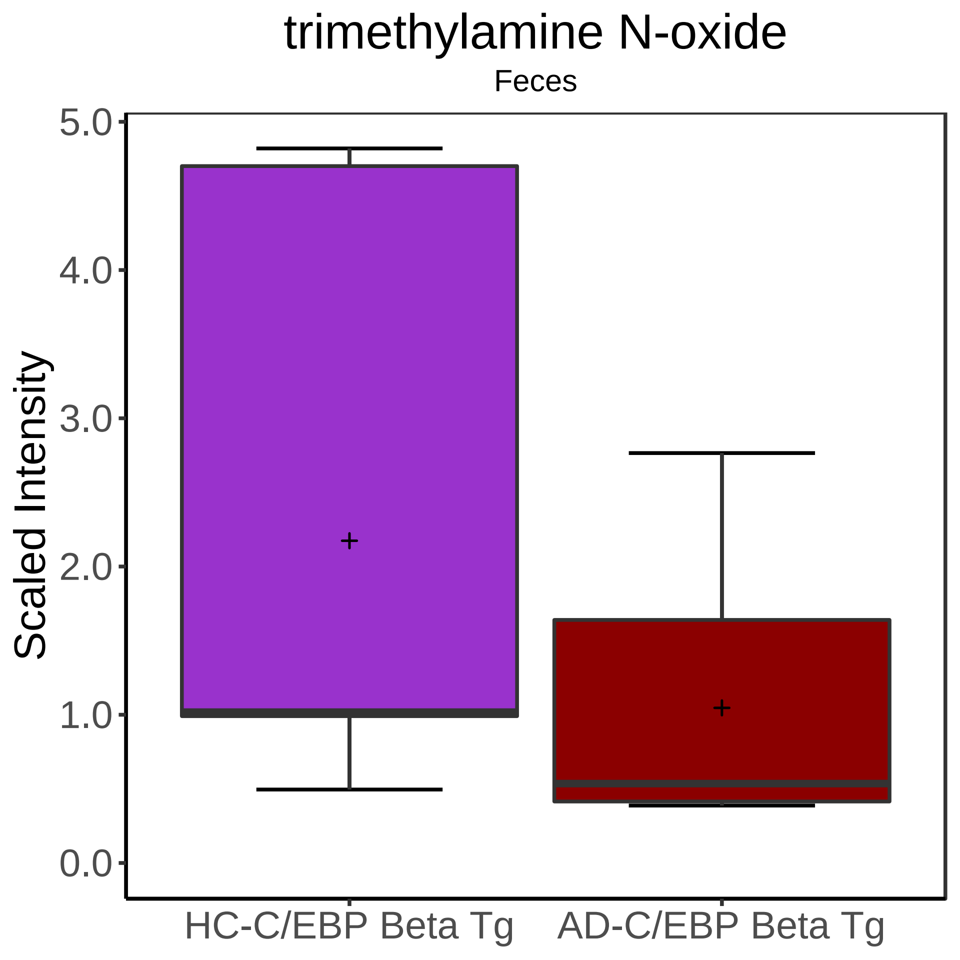

trimethylamine N-oxide
Feces
Scaled Intensity
5.0
4.0
3.0
2.0
1.0
0.0
HC-C/EBP Beta Tg
AD-C/EBP Beta Tg

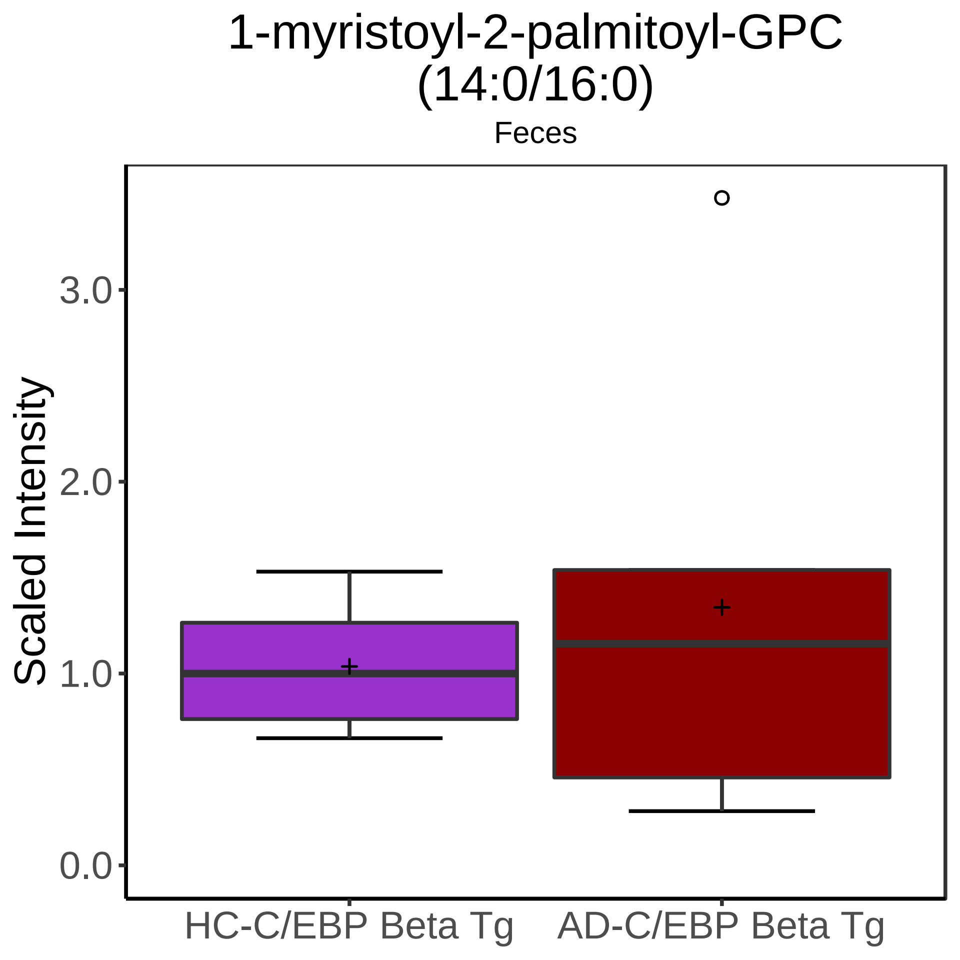
1-myristoyl-2-palmitoyl-GPC
(14:0/16:0)
Feces
Scaled Intensity
3.0
2.0
1.0
0.0
HC-C/EBP Beta Tg
AD-C/EBP Beta Tg

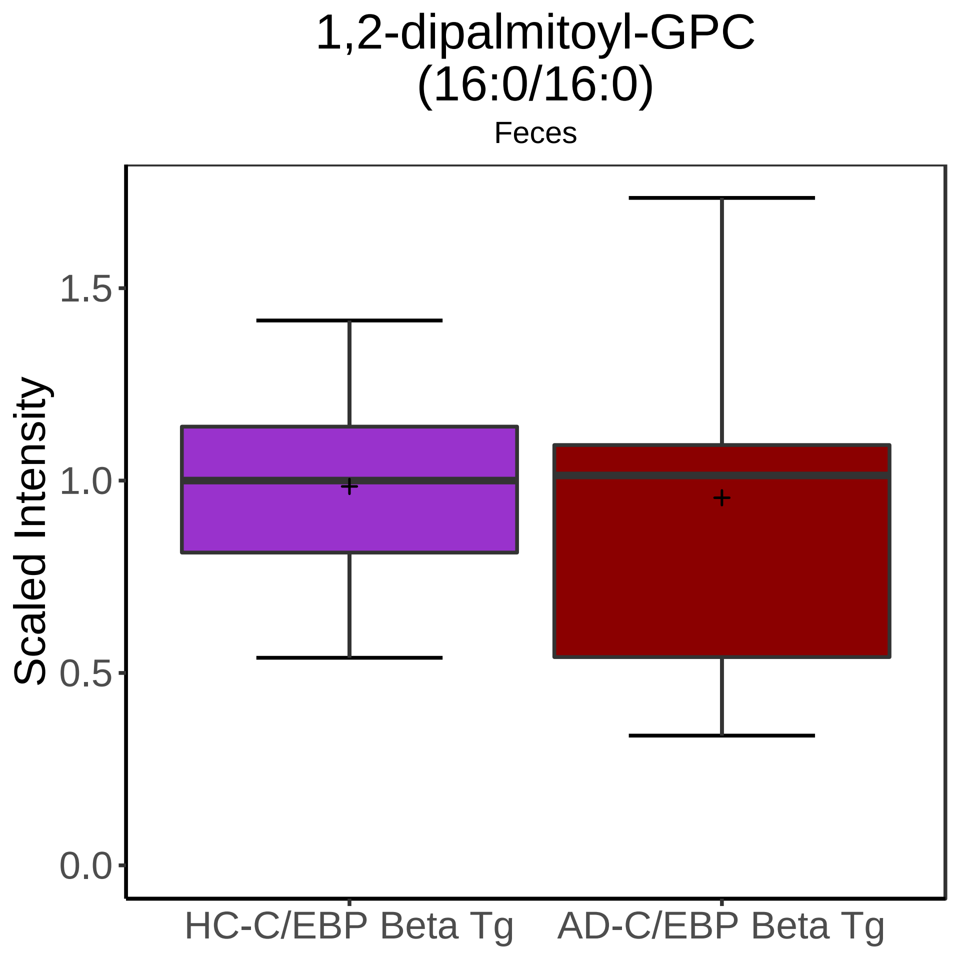
1,2-dipalmitoyl-GPC
(16:0/16:0)
Feces
Scaled Intensity
1.5
1.0
0.5
0.0
HC-C/EBP Beta Tg
AD-C/EBP Beta Tg

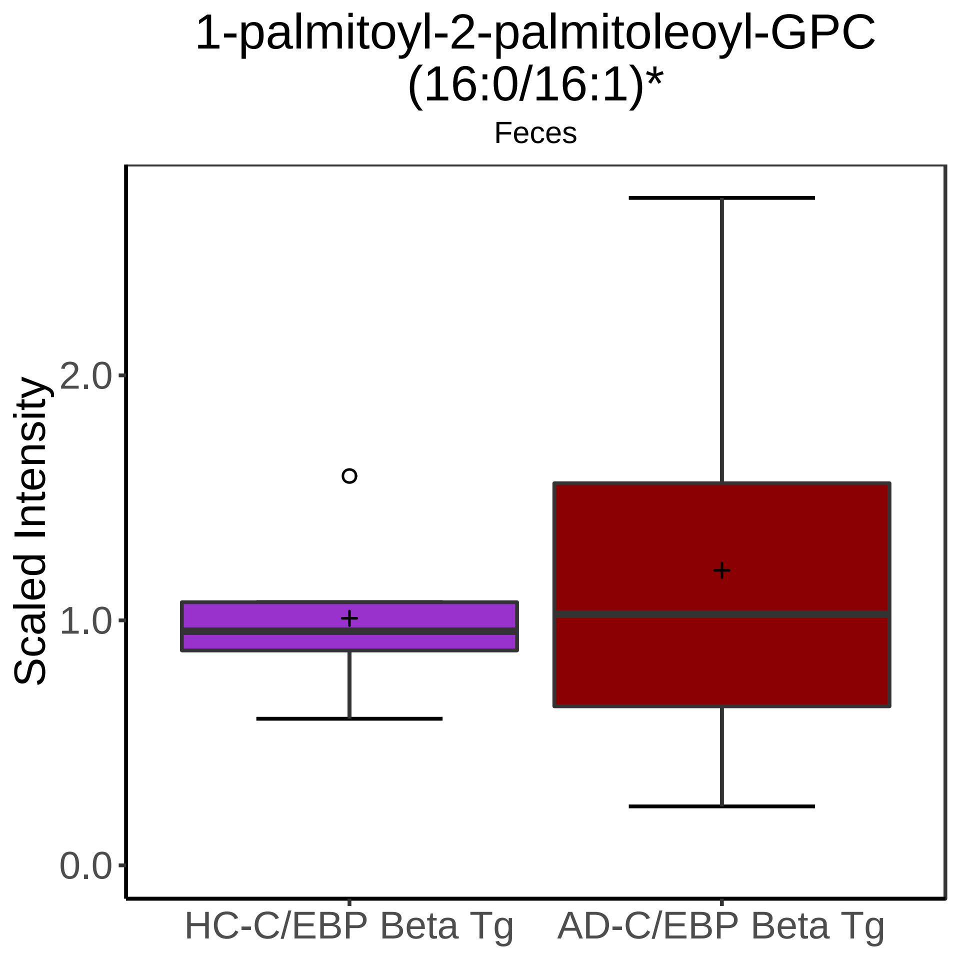

1-palmitoyl-2-palmitoleoyl-GPC
(16:0/16:1)*
Feces
Scaled Intensity
2.0
1.0
0.0
HC-C/EBP Beta Tg
AD-C/EBP Beta Tg

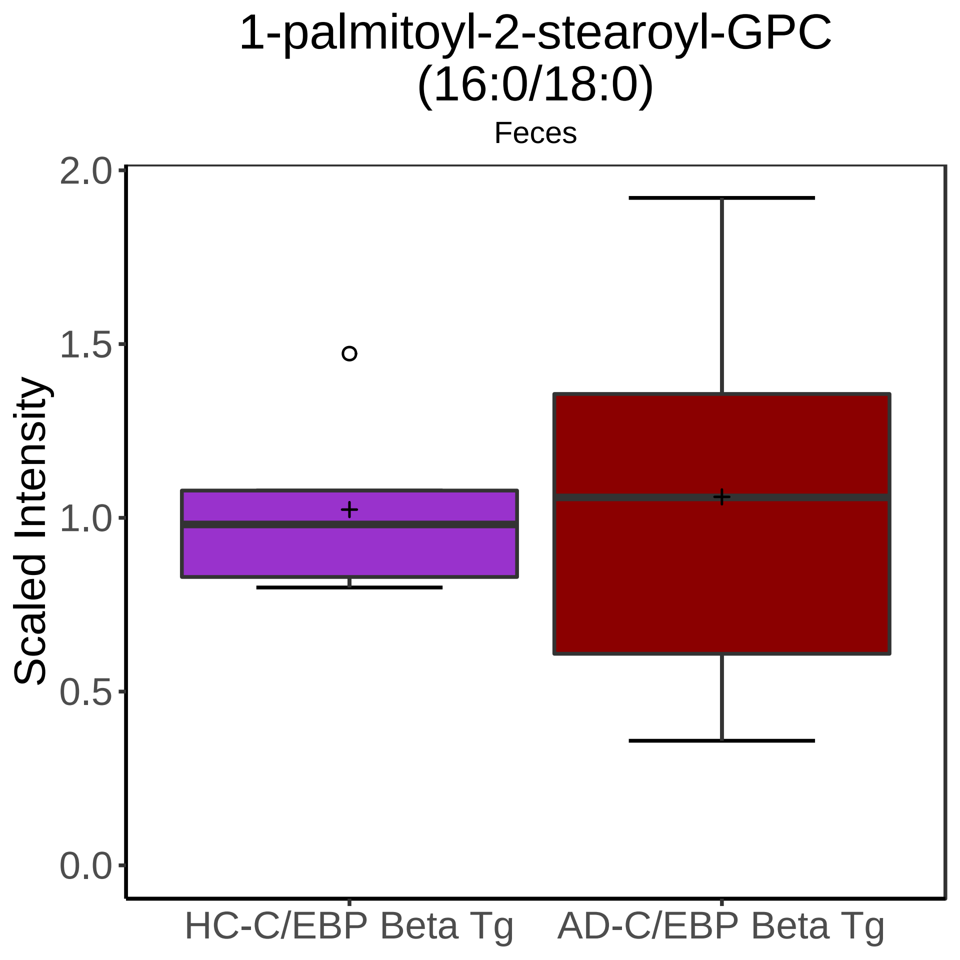

1-palmitoyl-2-stearoyl-GPC
(16:0/18:0)
Feces
Scaled Intensity
2.0
1.5
1.0
0.5
0.0
HC-C/EBP Beta Tg
AD-C/EBP Beta Tg

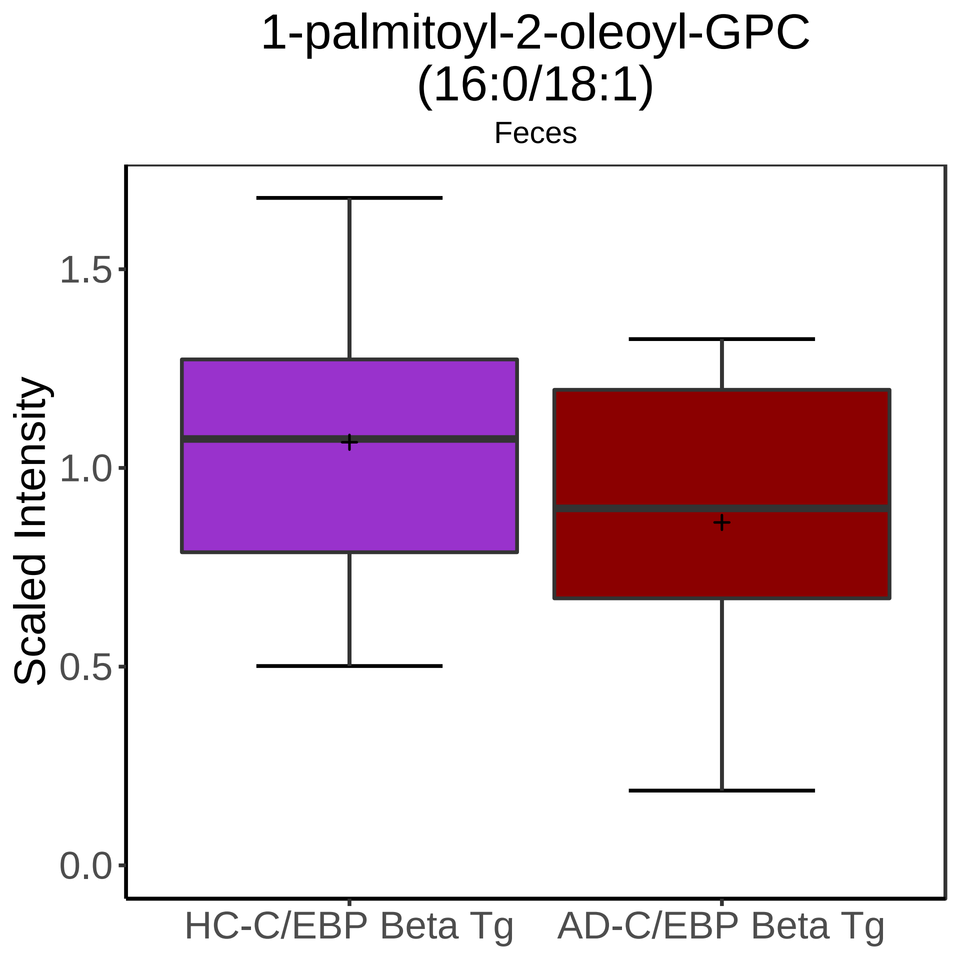

1-palmitoyl-2-oleoyl-GPC
(16:0/18:1)
Feces
Scaled Intensity
1.5
1.0
0.5
0.0
HC-C/EBP Beta Tg
AD-C/EBP Beta Tg

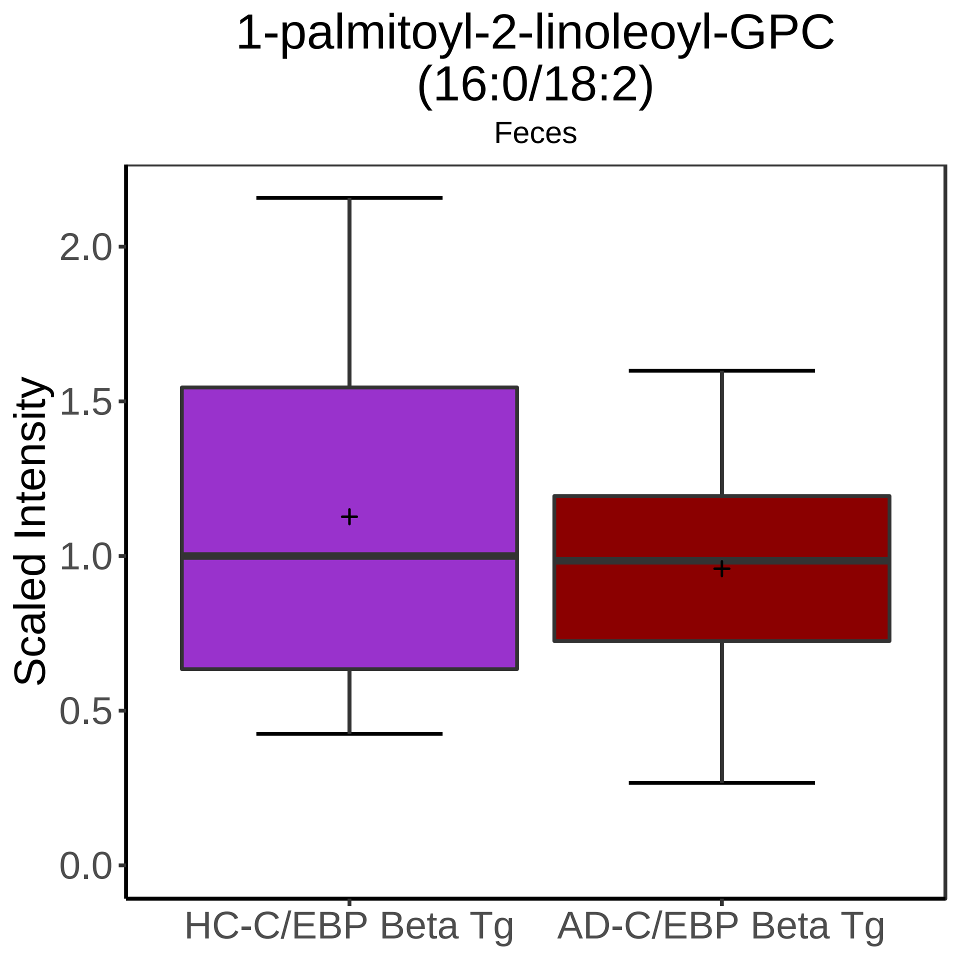
1-palmitoyl-2-linoleoyl-GPC
(16:0/18:2)
Feces
Scaled Intensity
2.0
1.5
1.0
0.5
0.0
HC-C/EBP Beta Tg
AD-C/EBP Beta Tg

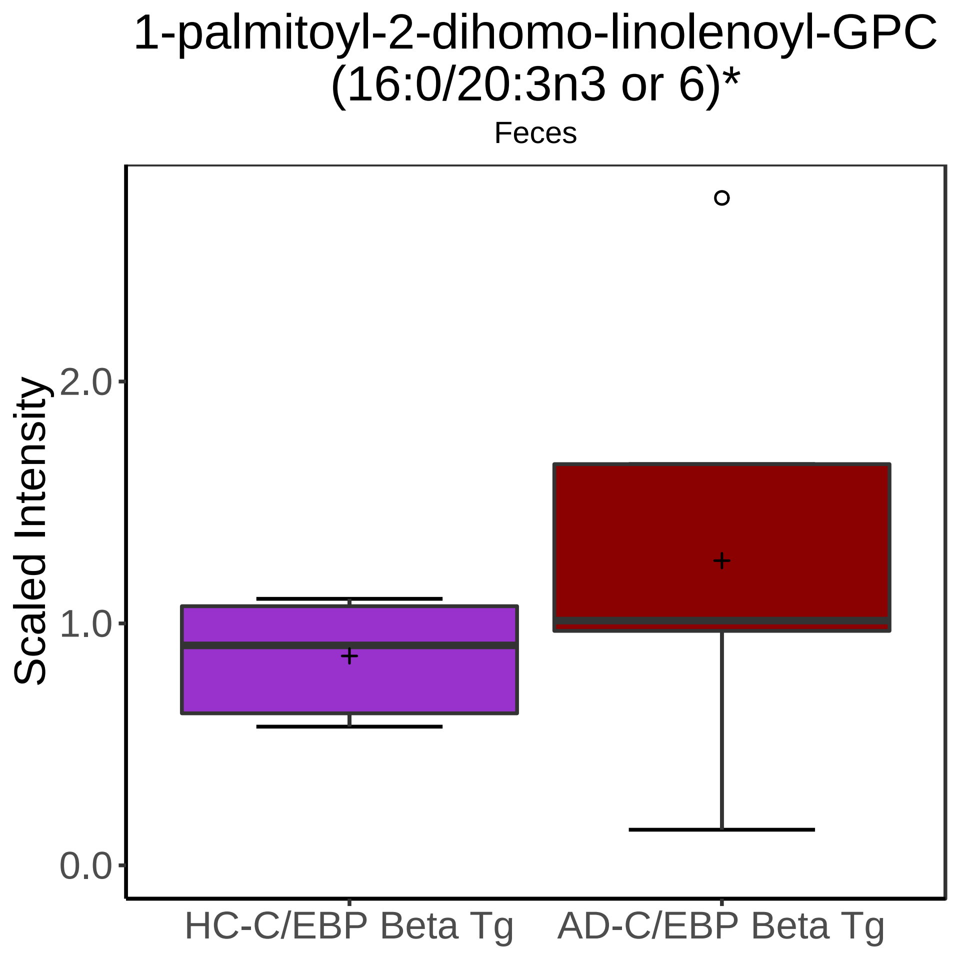
1-palmitoyl-2-dihomo-linolenoyl-GPC
(16:0/20:3n3 or 6)*
Feces
Scaled Intensity
2.0
1.0
0.0
HC-C/EBP Beta Tg
AD-C/EBP Beta Tg

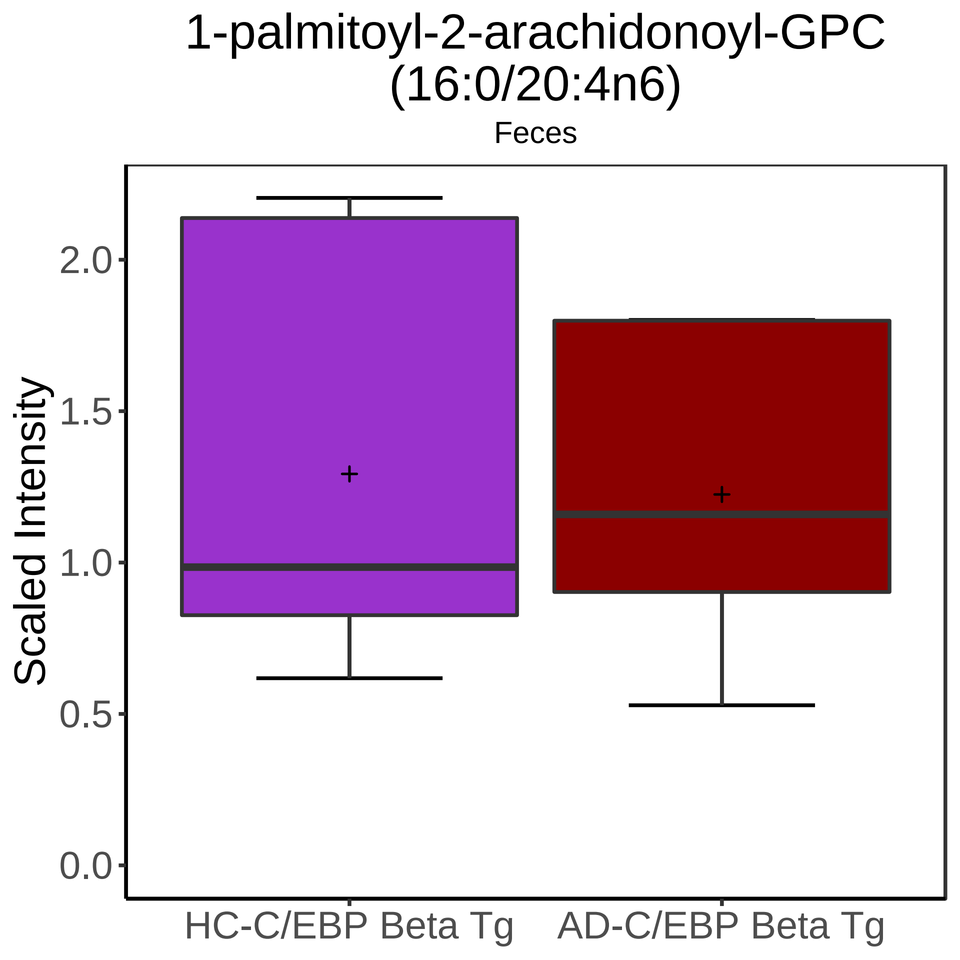
1-palmitoyl-2-arachidonoyl-GPC
(16:0/20:4n6)
Feces
Scaled Intensity
2.0
1.5
1.0
0.5
0.0
HC-C/EBP Beta Tg
AD-C/EBP Beta Tg

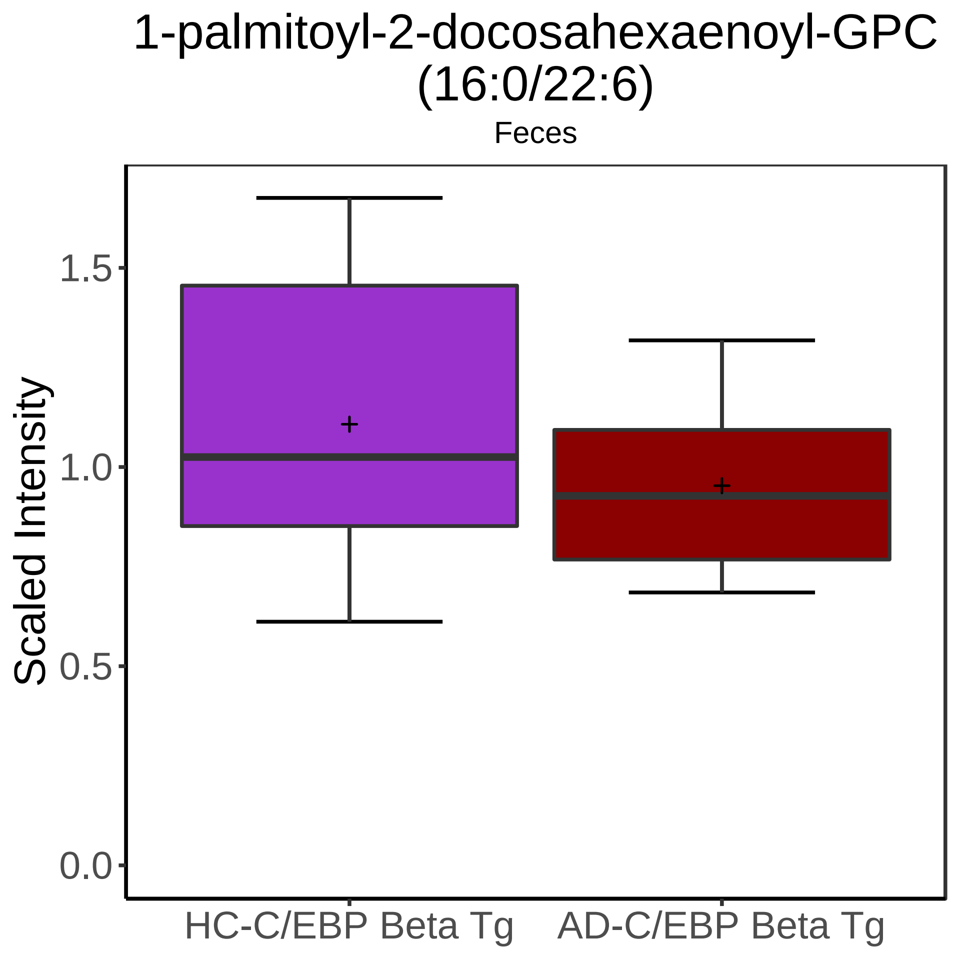
1-palmitoyl-2-docosahexaenoyl-GPC
(16:0/22:6)
Feces
Scaled Intensity
0.0
0.5
1.0
1.5
HC-C/EBP Beta Tg
AD-C/EBP Beta Tg

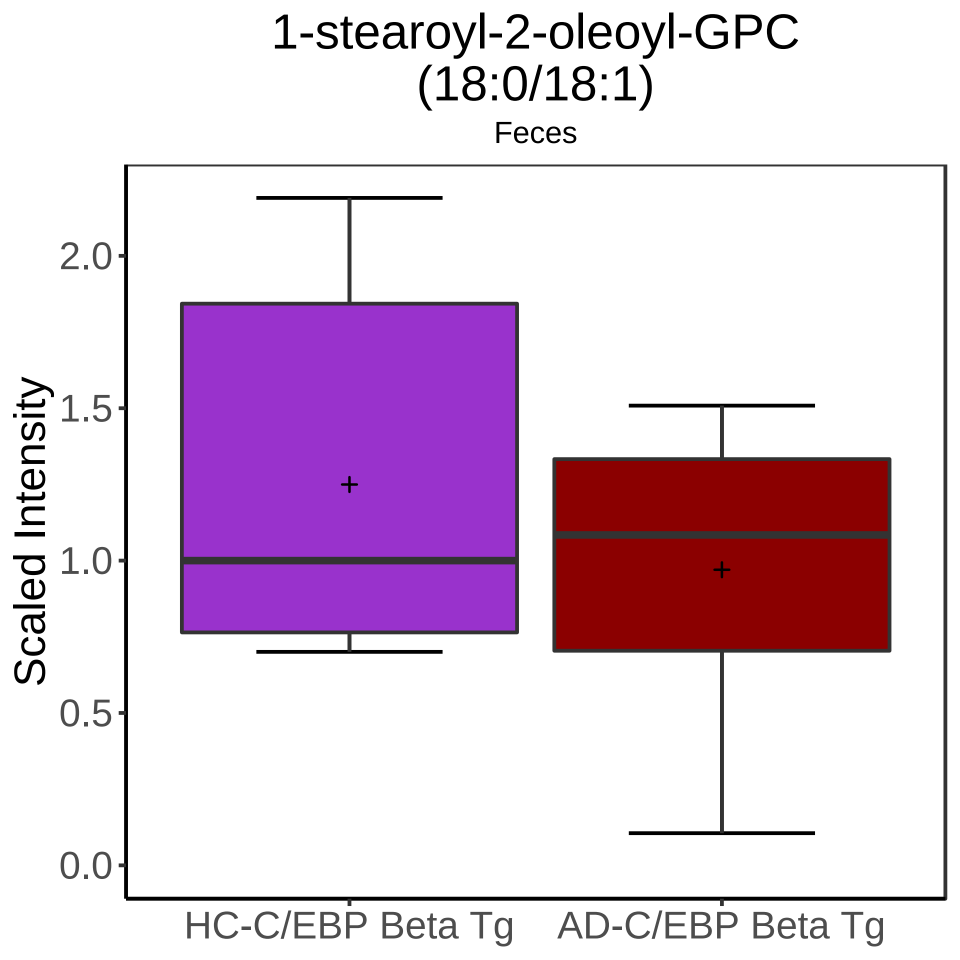

1-stearoyl-2-oleoyl-GPC
(18:0/18:1)
Feces
Scaled Intensity
2.0
1.5
1.0
0.5
0.0
HC-C/EBP Beta Tg
AD-C/EBP Beta Tg

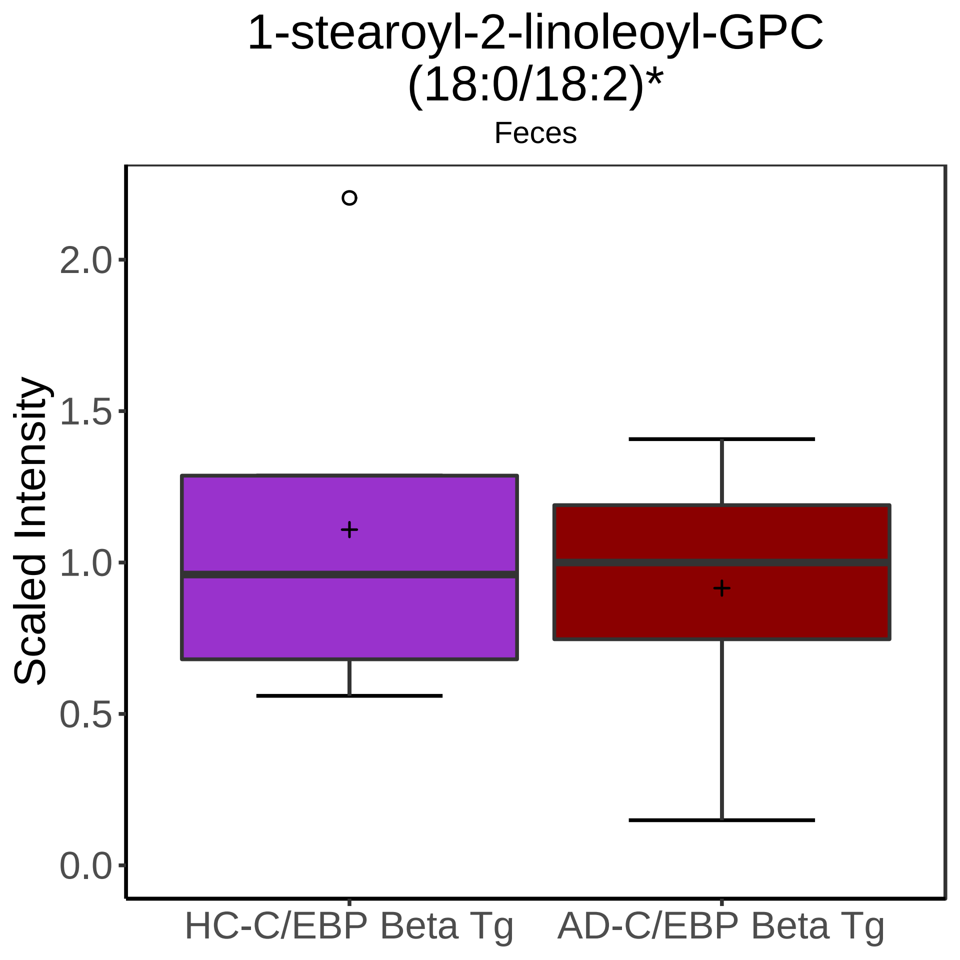

1-stearoyl-2-linoleoyl-GPC
(18:0/18:2)*
Feces
Scaled Intensity
2.0
1.5
1.0
0.5
0.0
HC-C/EBP Beta Tg
AD-C/EBP Beta Tg

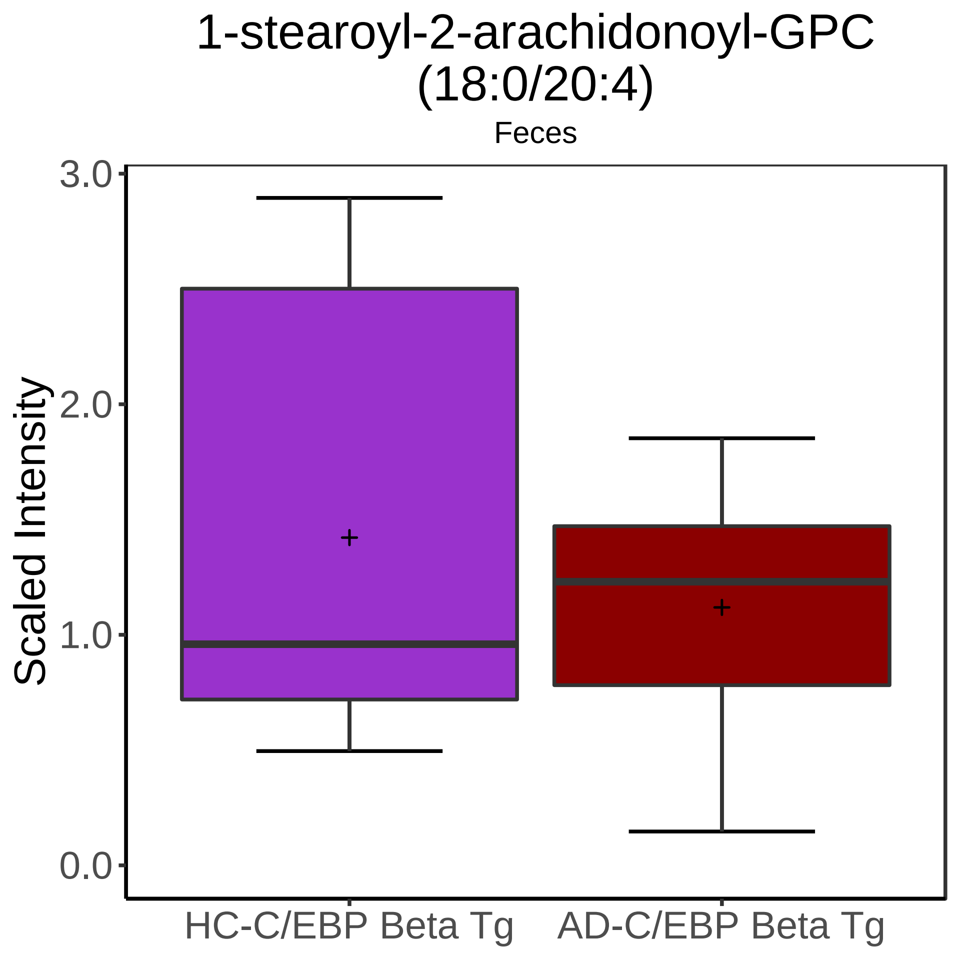
1-stearoyl-2-arachidonoyl-GPC
(18:0/20:4)
Feces
Scaled Intensity
3.0
2.0
1.0
0.0
HC-C/EBP Beta Tg
AD-C/EBP Beta Tg

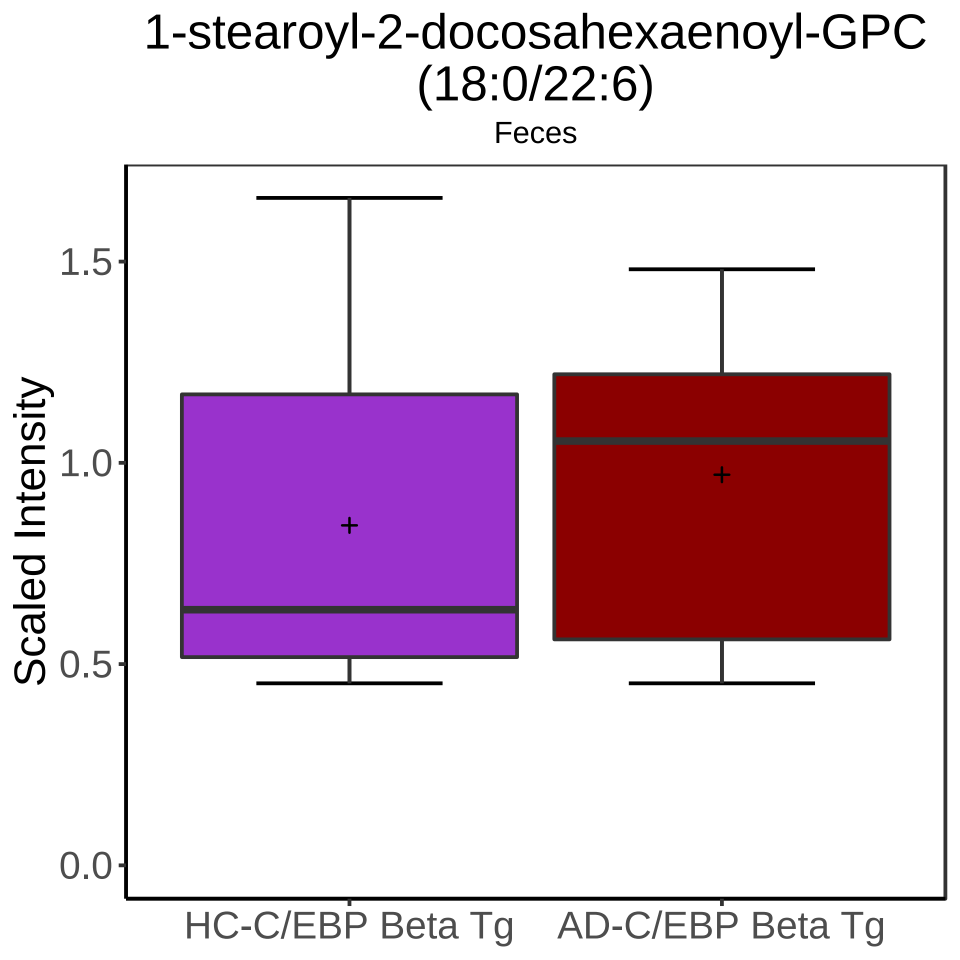

1-stearoyl-2-docosahexaenoyl-GPC
(18:0/22:6)
Feces
Scaled Intensity
1.5
1.0
0.5
0.0
HC-C/EBP Beta Tg
AD-C/EBP Beta Tg

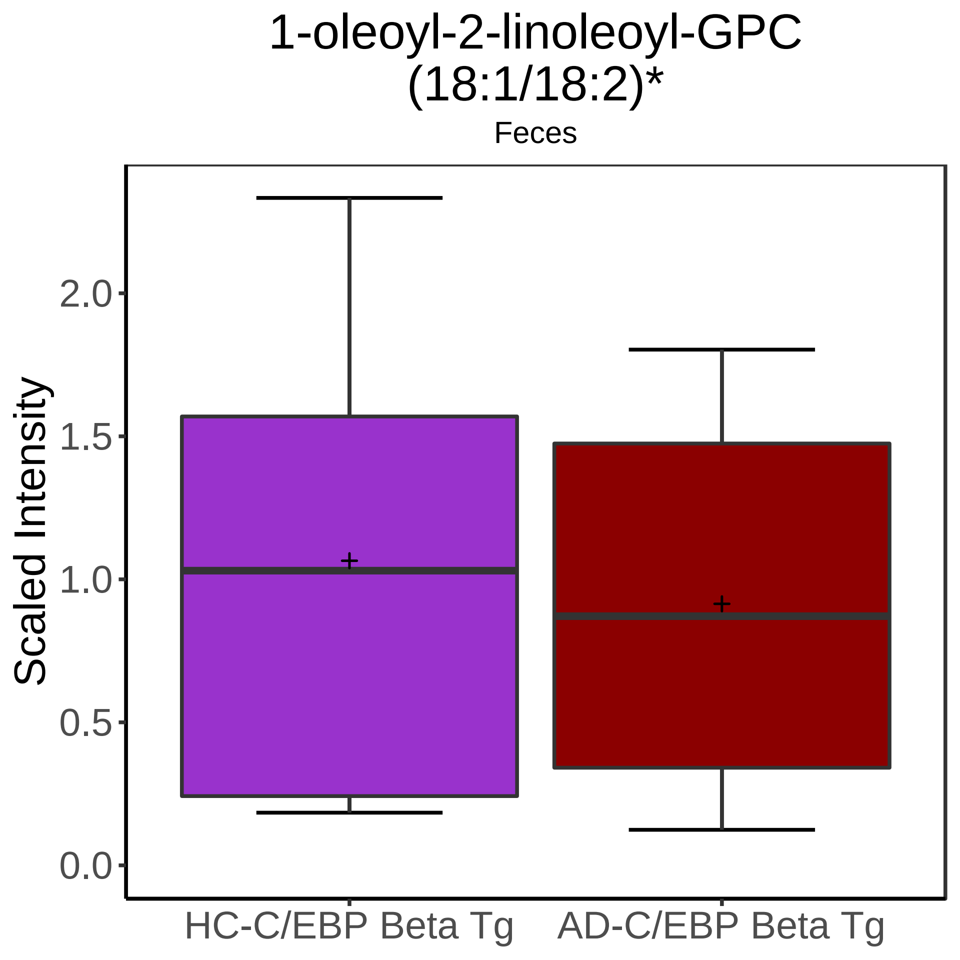

1-oleoyl-2-linoleoyl-GPC
(18:1/18:2)*
Feces
Scaled Intensity
0.0
0.5
1.0
1.5
2.0
HC-C/EBP Beta Tg
AD-C/EBP Beta Tg

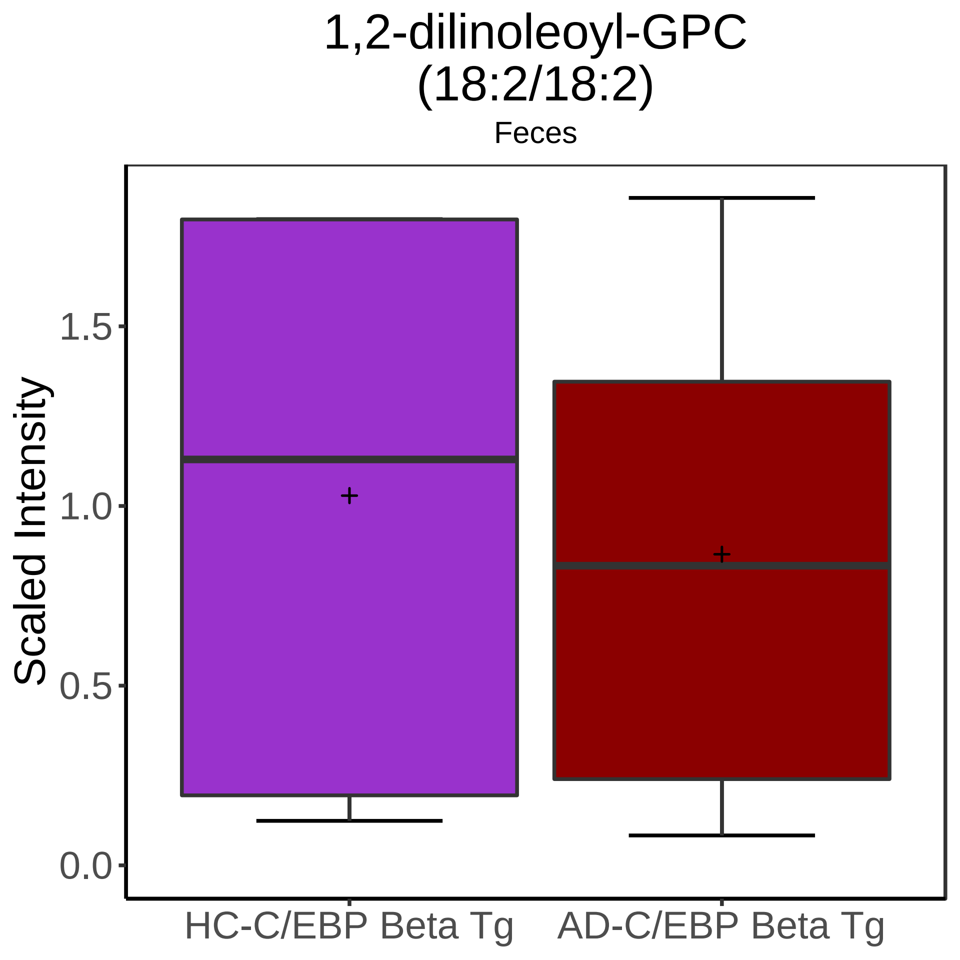

1,2-dilinoleoyl-GPC
(18:2/18:2)
Feces
Scaled Intensity
1.5
1.0
0.5
0.0
HC-C/EBP Beta Tg
AD-C/EBP Beta Tg

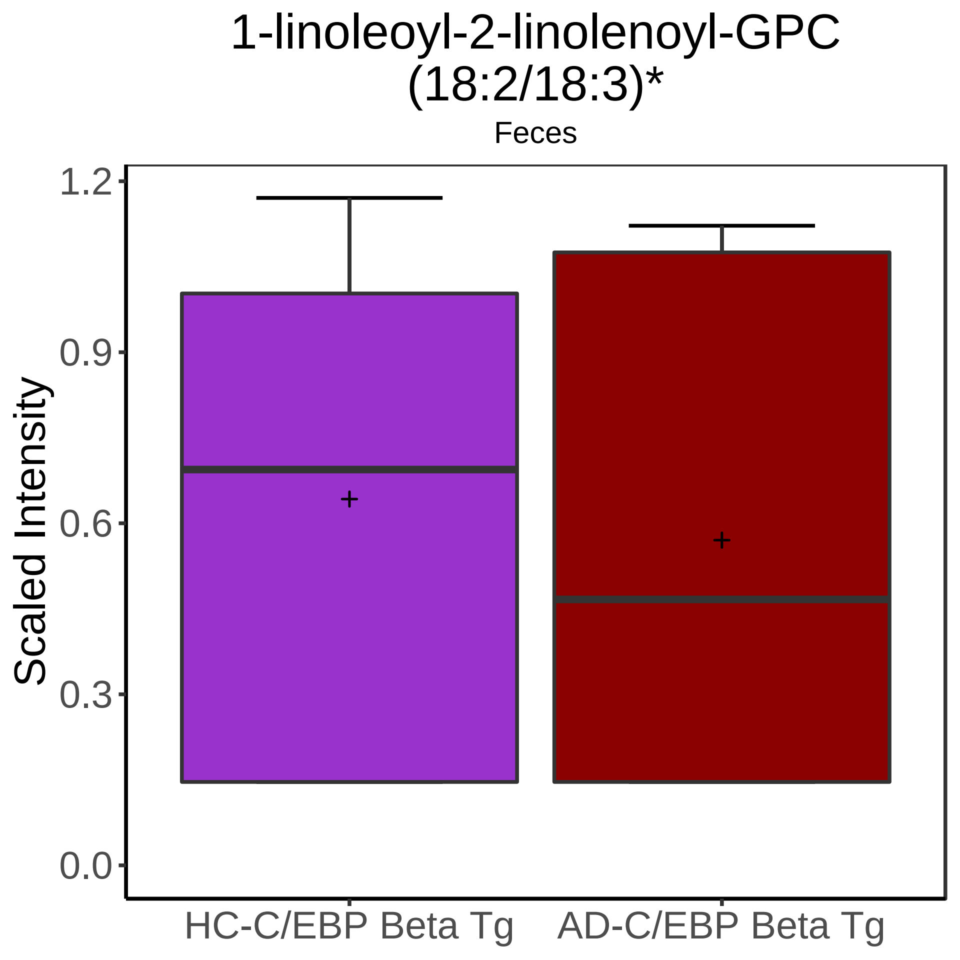
1-linoleoyl-2-linolenoyl-GPC
(18:2/18:3)*
Feces
Scaled Intensity
1.2
0.9
0.6
0.3
0.0
HC-C/EBP Beta Tg
AD-C/EBP Beta Tg

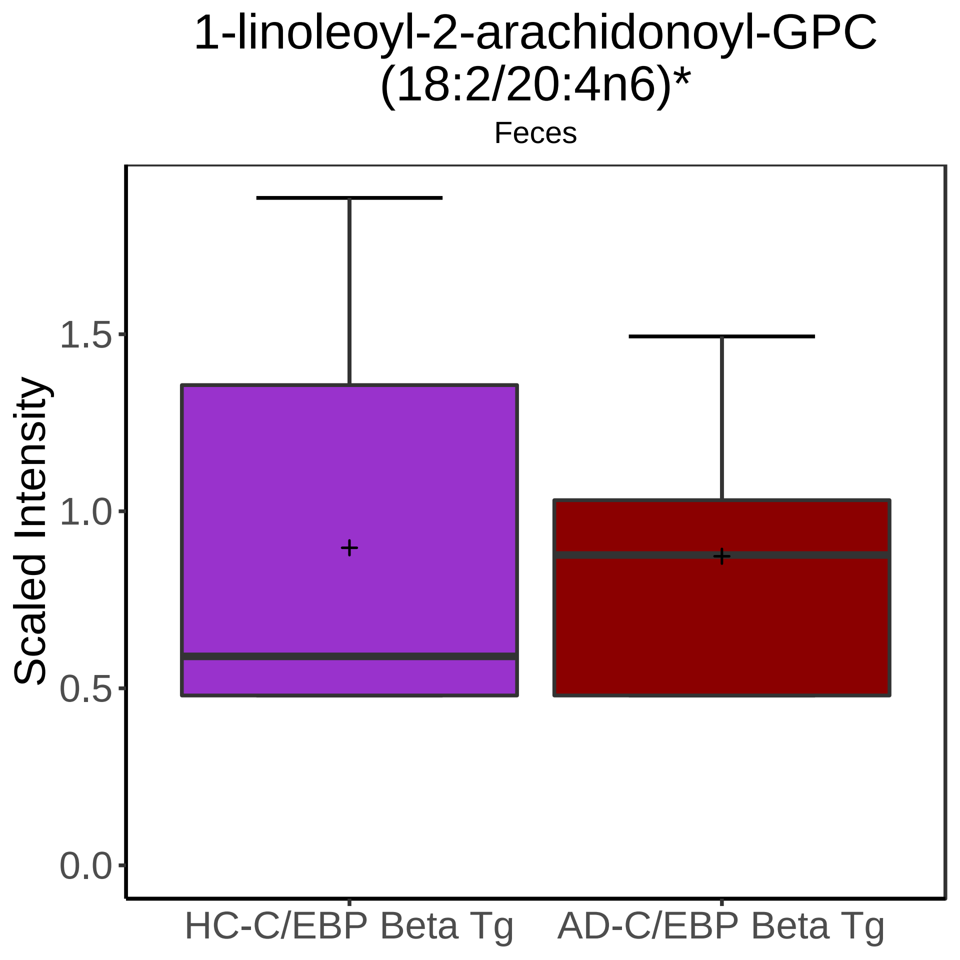
1-linoleoyl-2-arachidonoyl-GPC
(18:2/20:4n6)*
Feces
Scaled Intensity
0.0
0.5
1.0
1.5
HC-C/EBP Beta Tg
AD-C/EBP Beta Tg

# 1,2-dipalmitoyl-GPE (16:0/16:0)*

Feces

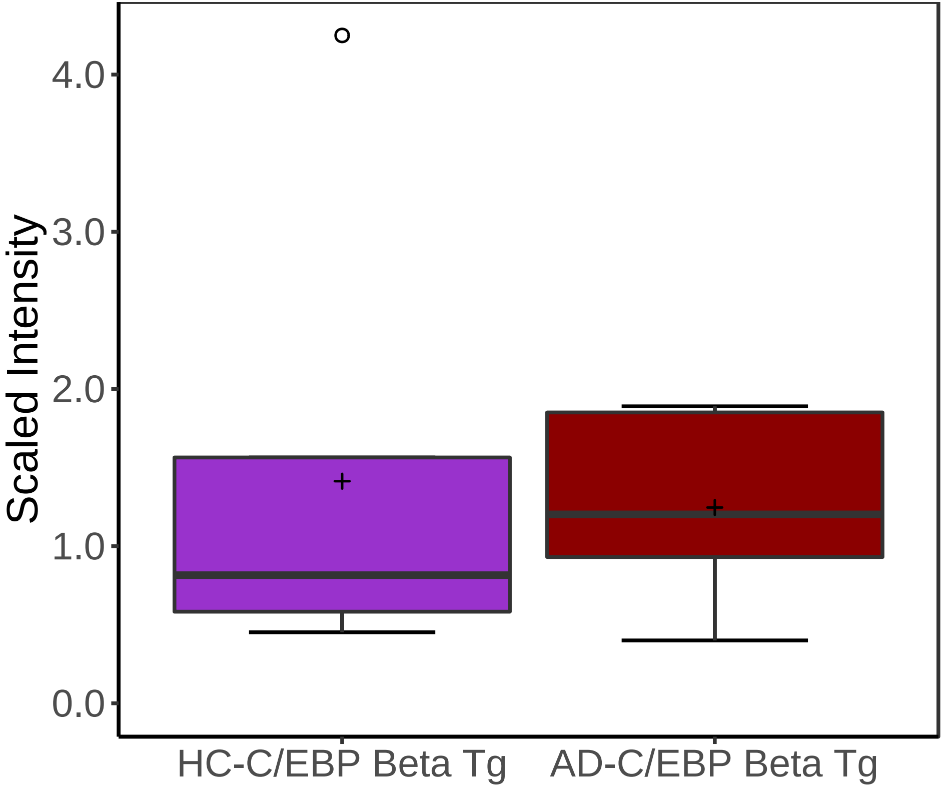

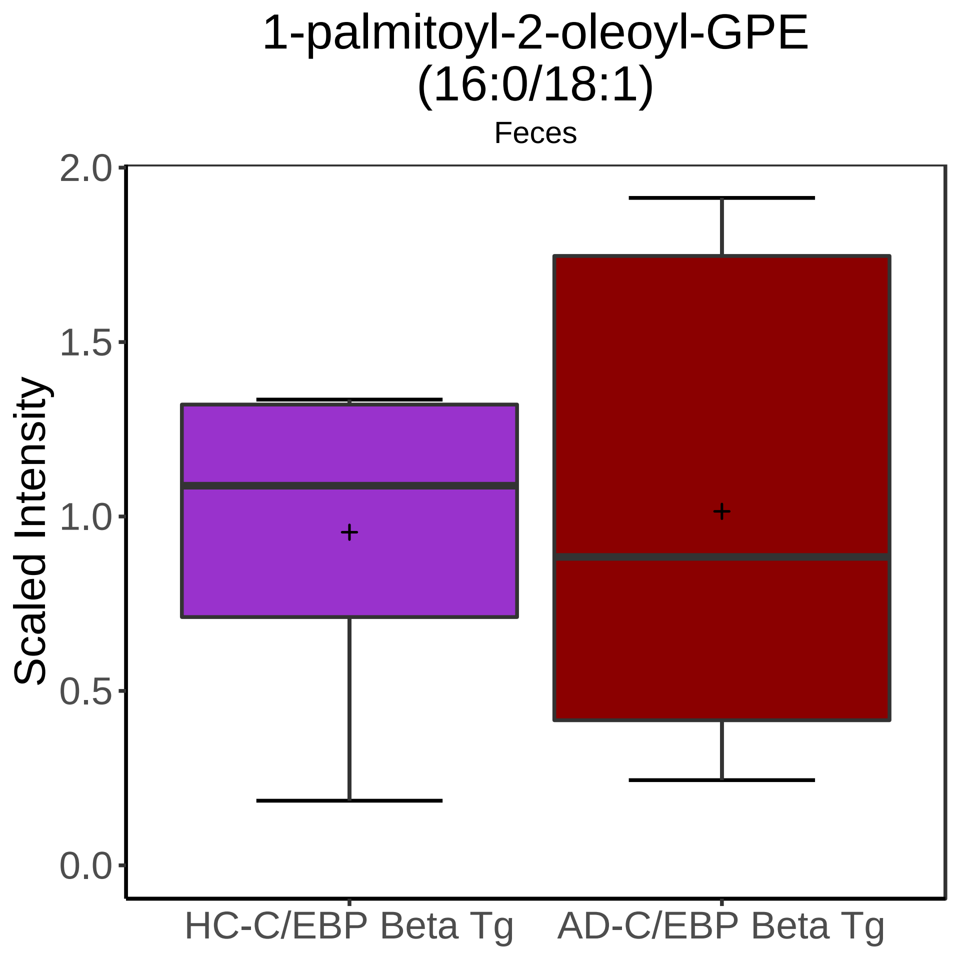

1-palmitoyl-2-oleoyl-GPE
(16:0/18:1)
Feces
Scaled Intensity
2.0
1.5
1.0
0.5
0.0
HC-C/EBP Beta Tg
AD-C/EBP Beta Tg

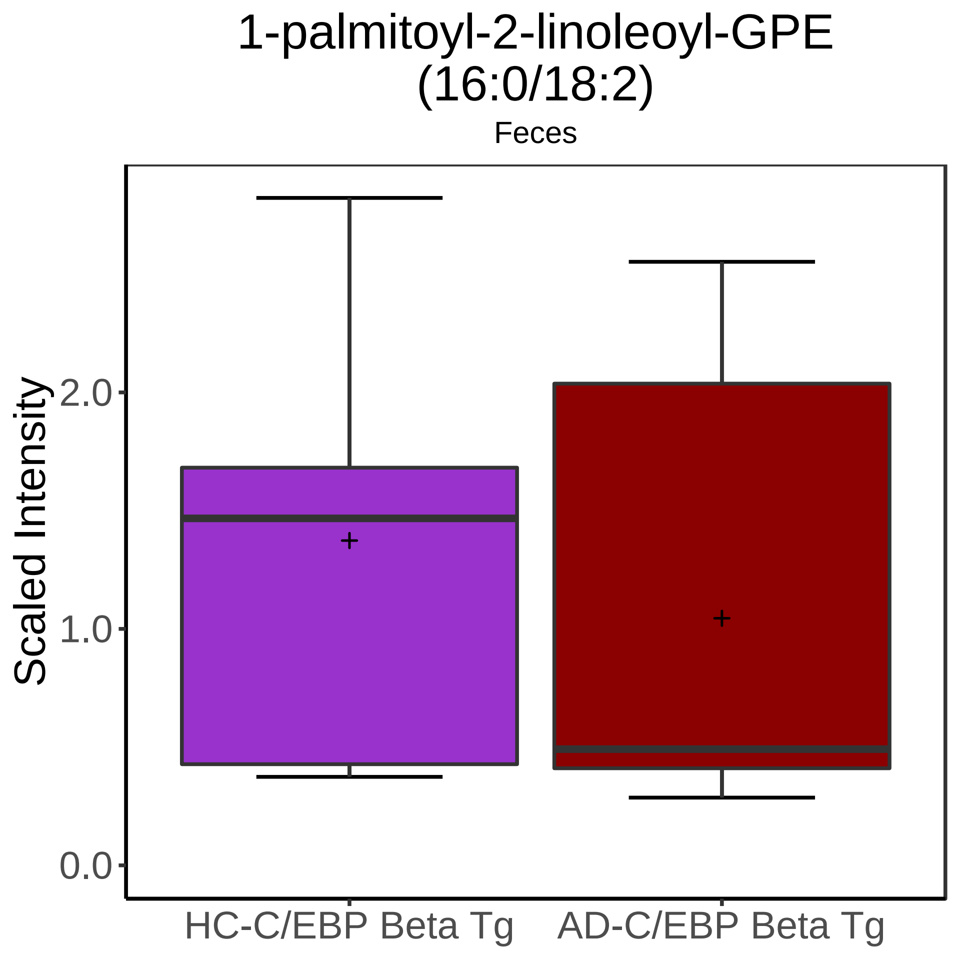
1-palmitoyl-2-linoleoyl-GPE
(16:0/18:2)
Feces
Scaled Intensity
2.0
1.0
0.0
HC-C/EBP Beta Tg
AD-C/EBP Beta Tg

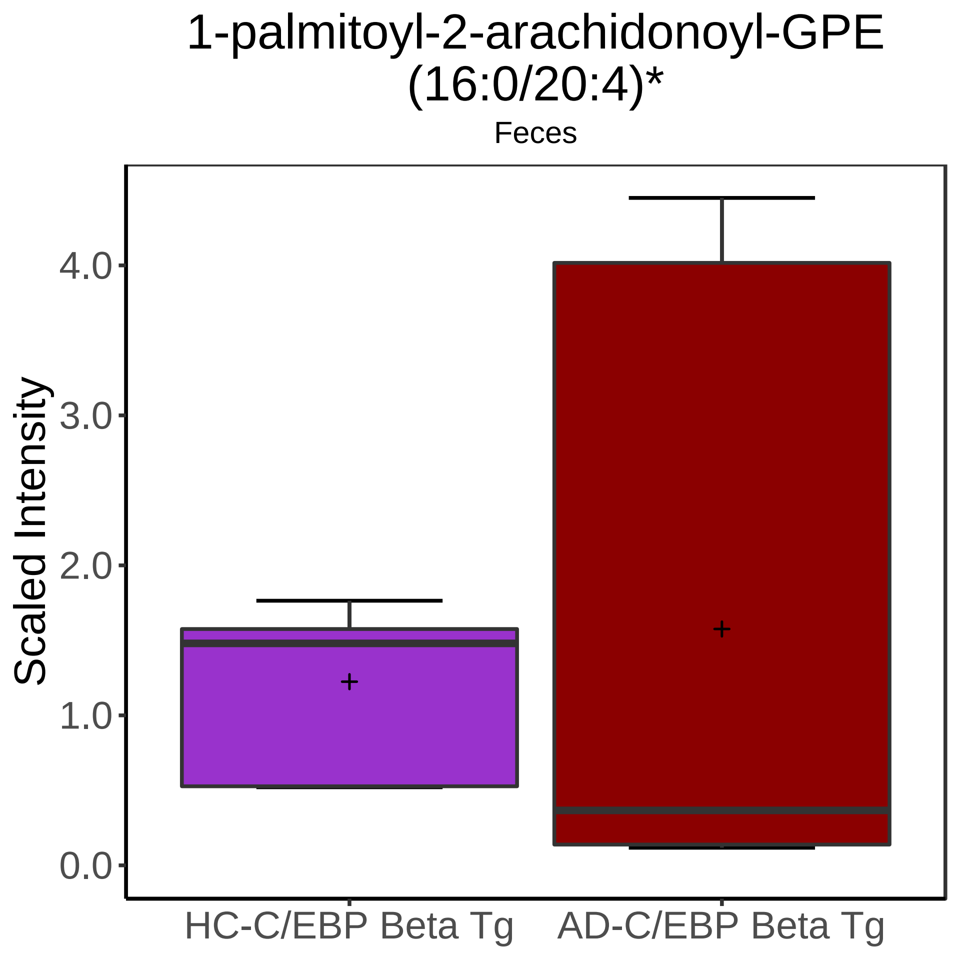

1-palmitoyl-2-arachidonoyl-GPE
(16:0/20:4)*
Feces
Scaled Intensity
4.0
3.0
2.0
1.0
0.0
HC-C/EBP Beta Tg
AD-C/EBP Beta Tg

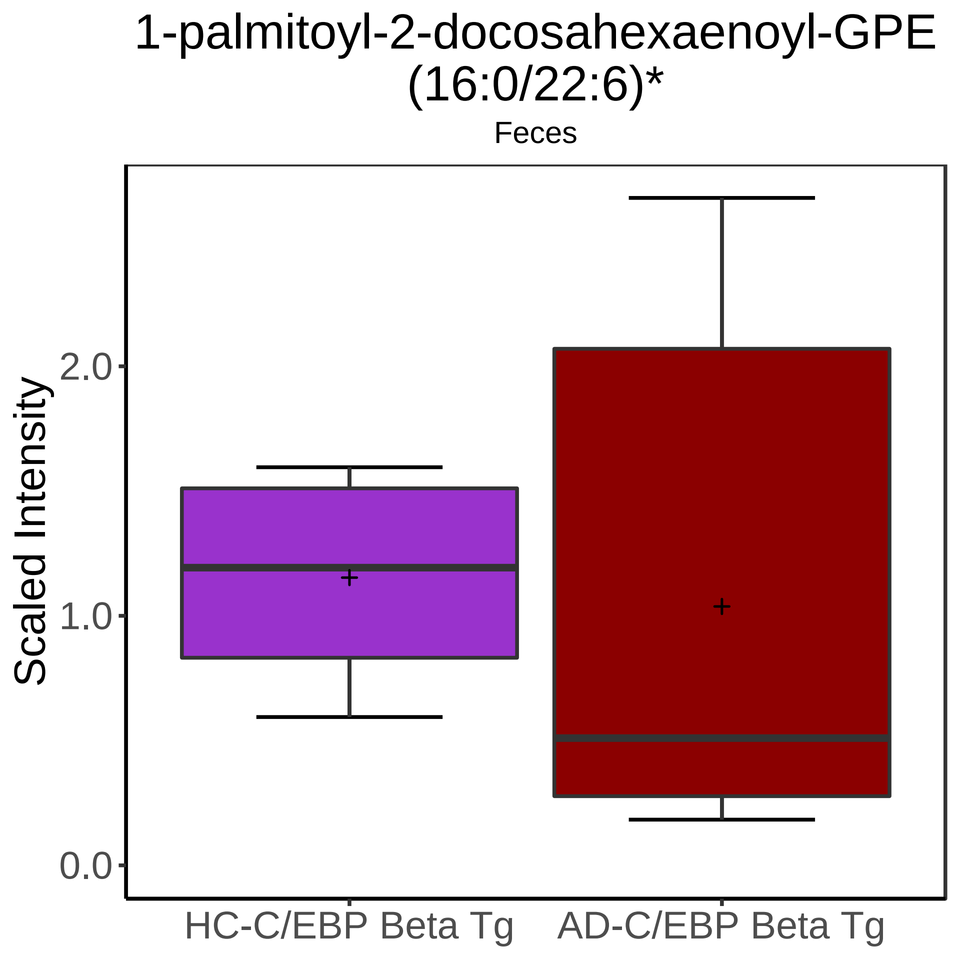
1-palmitoyl-2-docosahexaenoyl-GPE
(16:0/22:6)*
Feces
Scaled Intensity
2.0
1.0
0.0
HC-C/EBP Beta Tg
AD-C/EBP Beta Tg

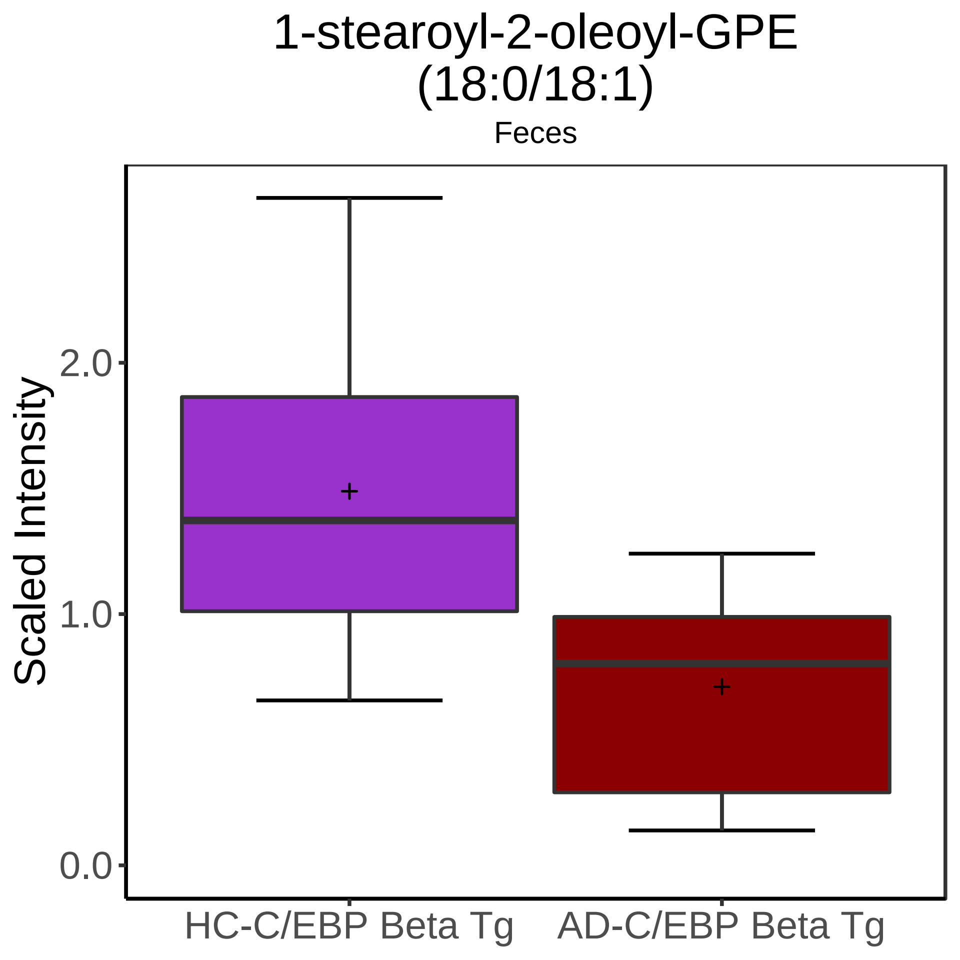
1-stearoyl-2-oleoyl-GPE
(18:0/18:1)
Feces
Scaled Intensity
2.0
1.0
0.0
HC-C/EBP Beta Tg
AD-C/EBP Beta Tg

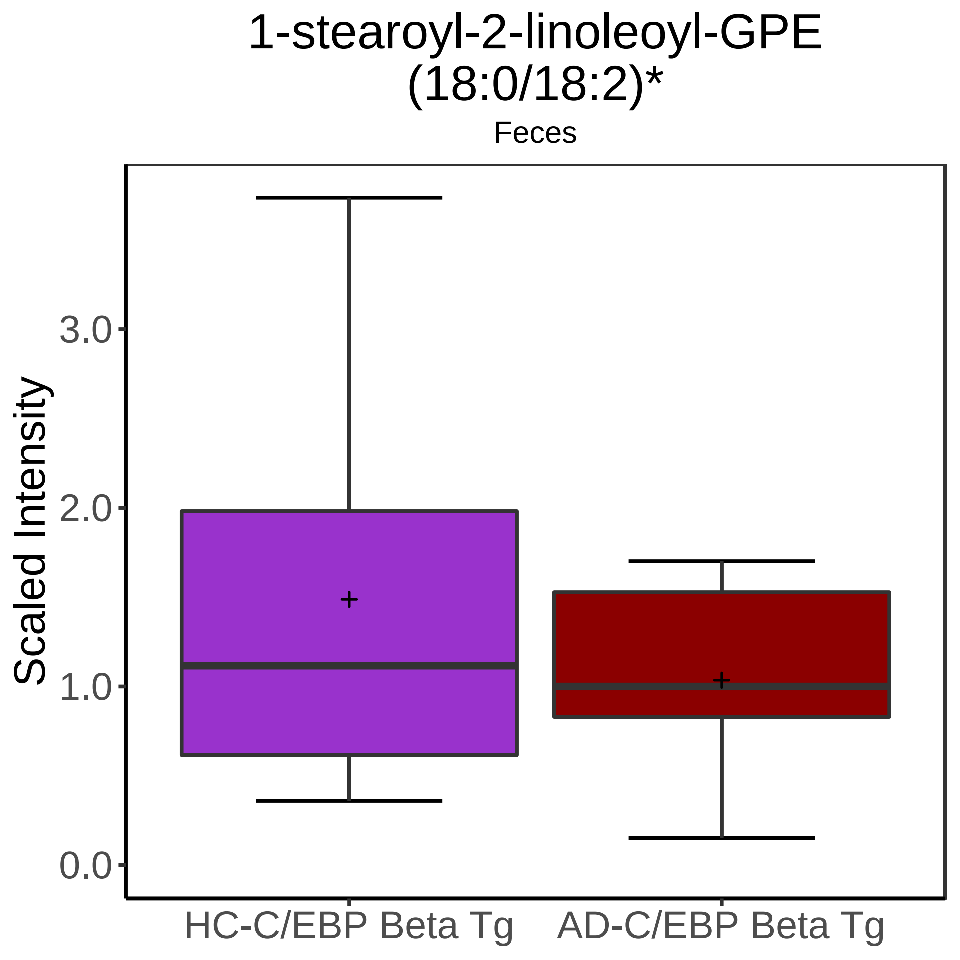
1-stearoyl-2-linoleoyl-GPE
(18:0/18:2)*
Feces
Scaled Intensity
3.0
2.0
1.0
0.0
HC-C/EBP Beta Tg
AD-C/EBP Beta Tg

# 1-stearoyl-2-arachidonoyl-GPE (18:0/20:4)

Feces

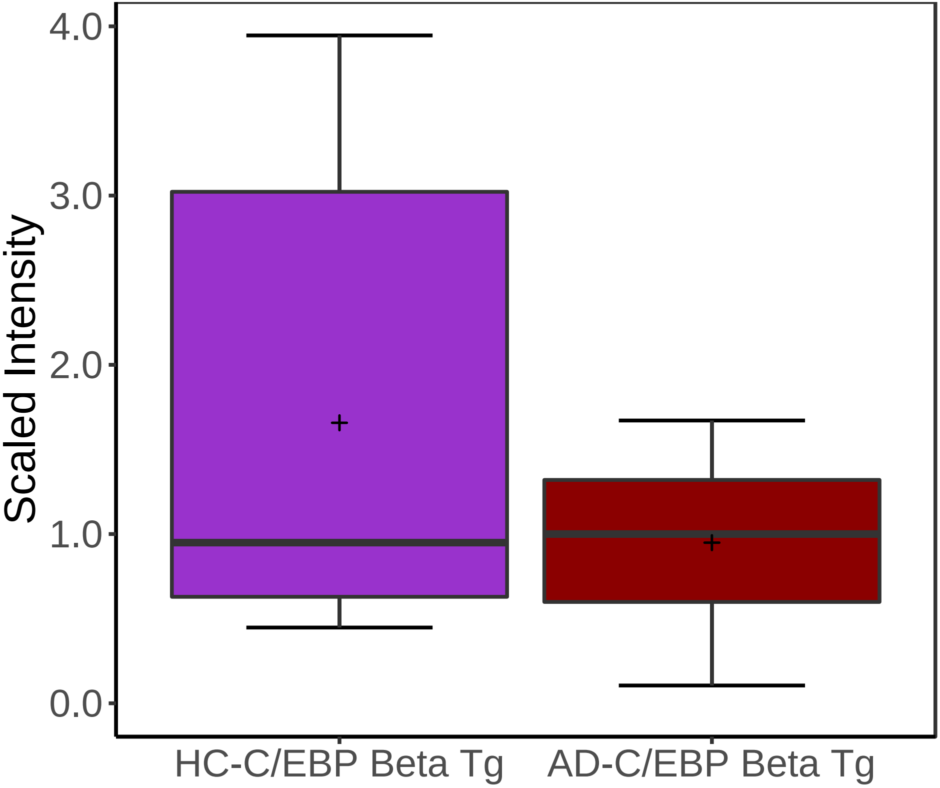

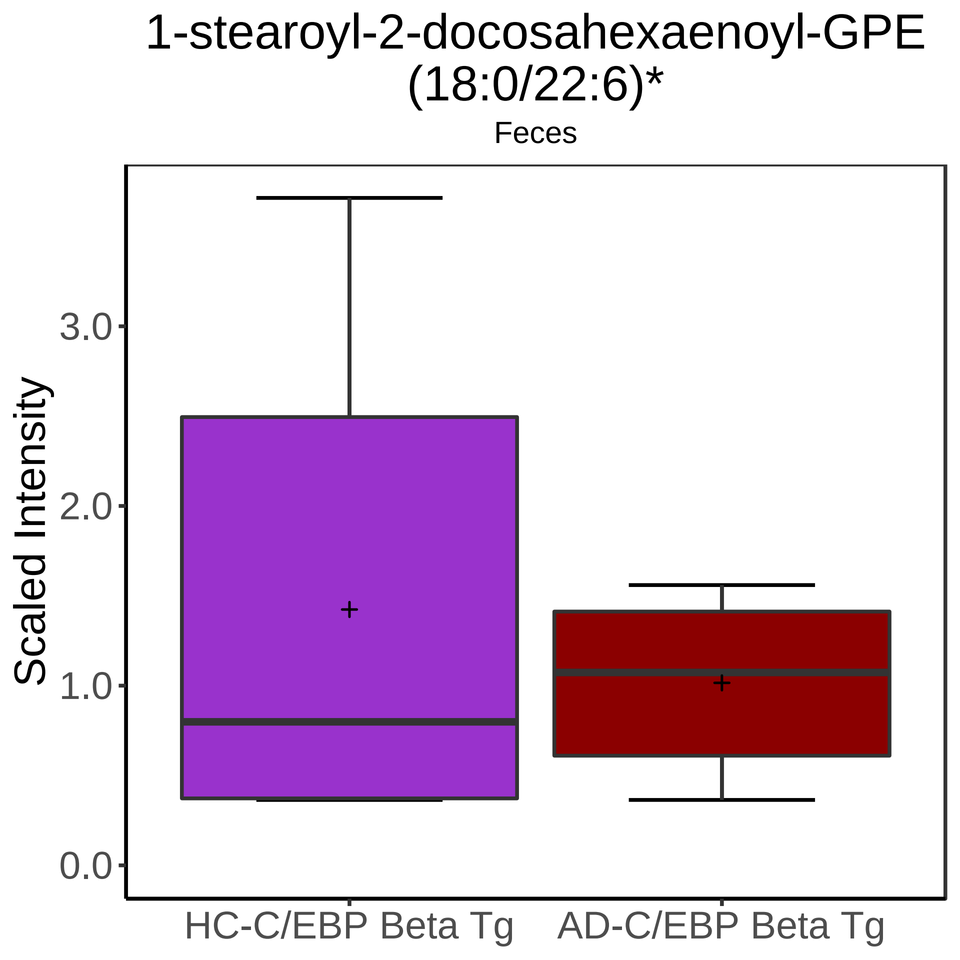
1-stearoyl-2-docosahexaenoyl-GPE (18:0/22:6)*
Feces
Scaled Intensity
3.0
2.0
1.0
0.0
HC-C/EBP Beta Tg
AD-C/EBP Beta Tg

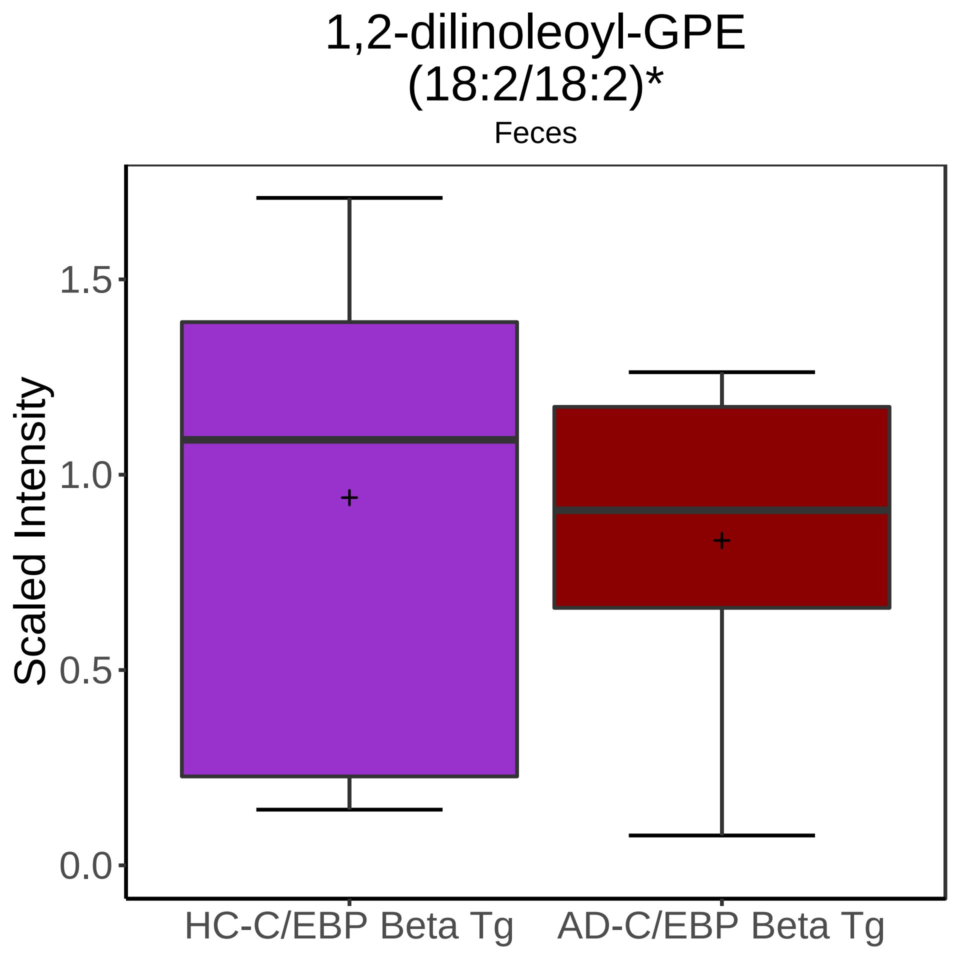

1,2-dilinoleoyl-GPE
(18:2/18:2)*
Feces
Scaled Intensity
1.5
1.0
0.5
0.0
HC-C/EBP Beta Tg
AD-C/EBP Beta Tg

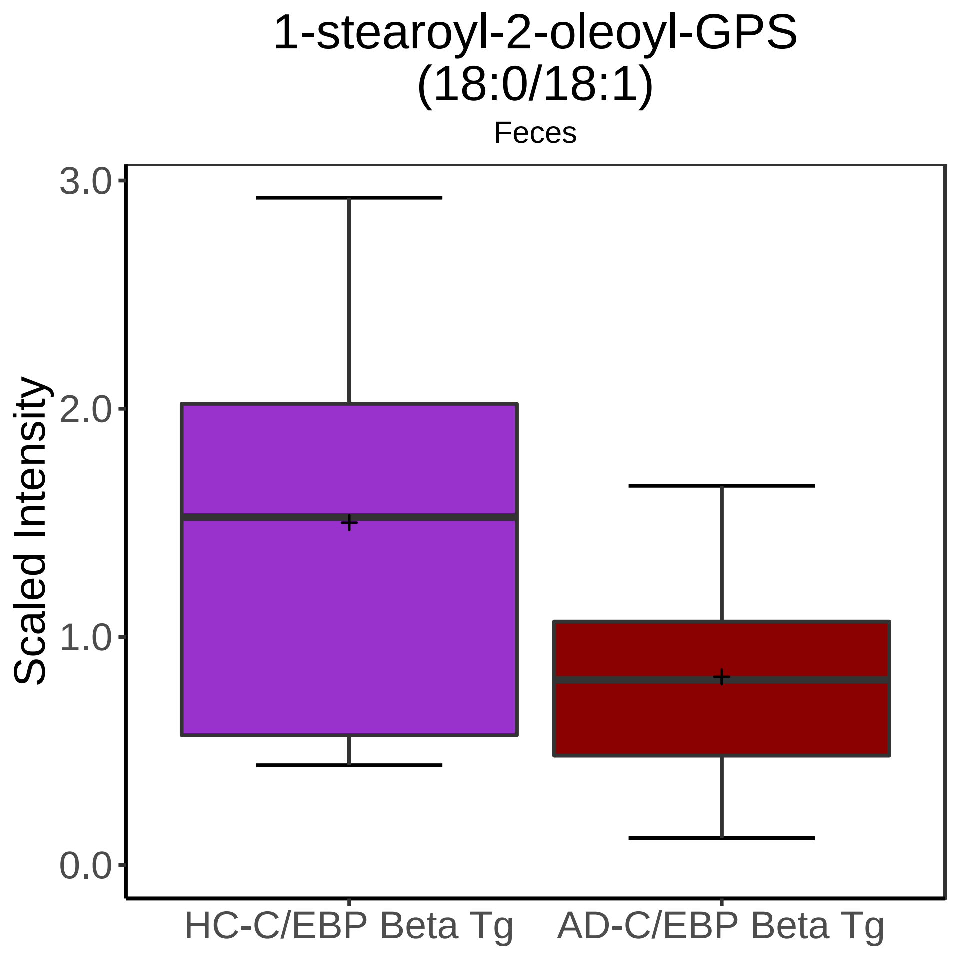
1-stearoyl-2-oleoyl-GPS
(18:0/18:1)
Feces
Scaled Intensity
3.0
2.0
1.0
0.0
HC-C/EBP Beta Tg
AD-C/EBP Beta Tg

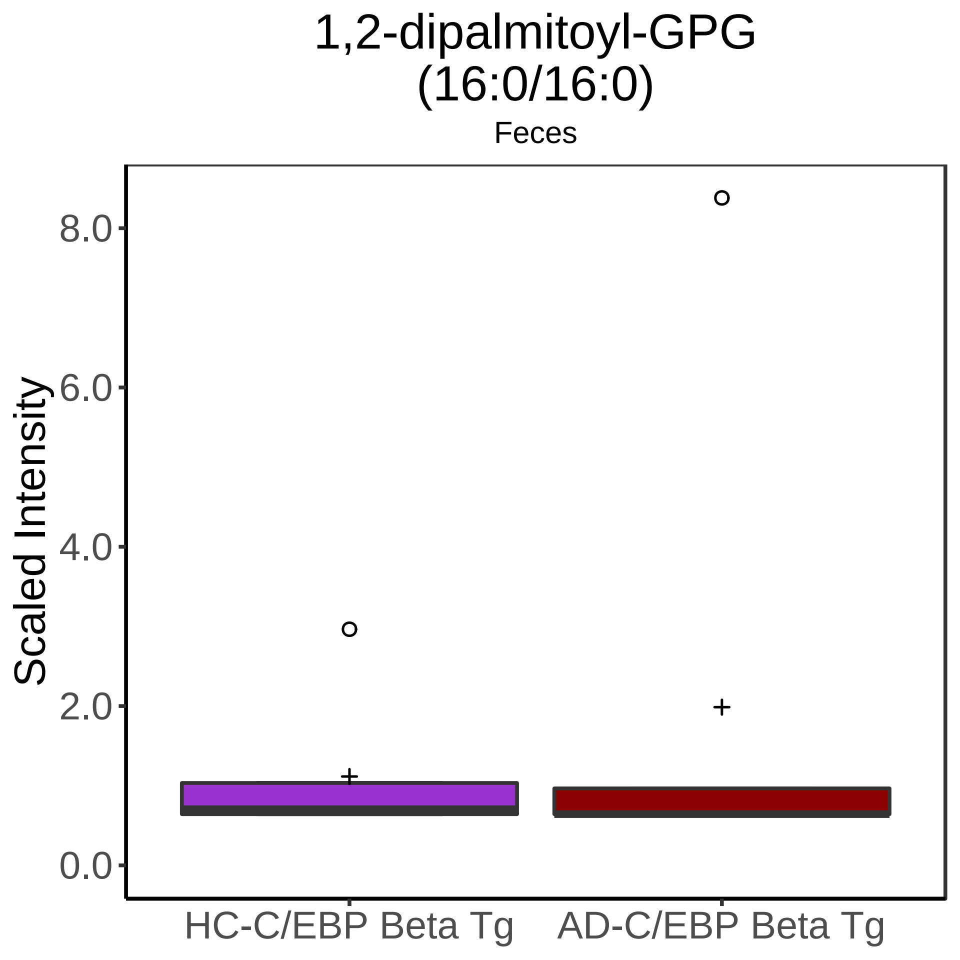

1,2-dipalmitoyl-GPG
(16:0/16:0)
Feces
Scaled Intensity
8.0
6.0
4.0
2.0
0.0
HC-C/EBP Beta Tg
AD-C/EBP Beta Tg

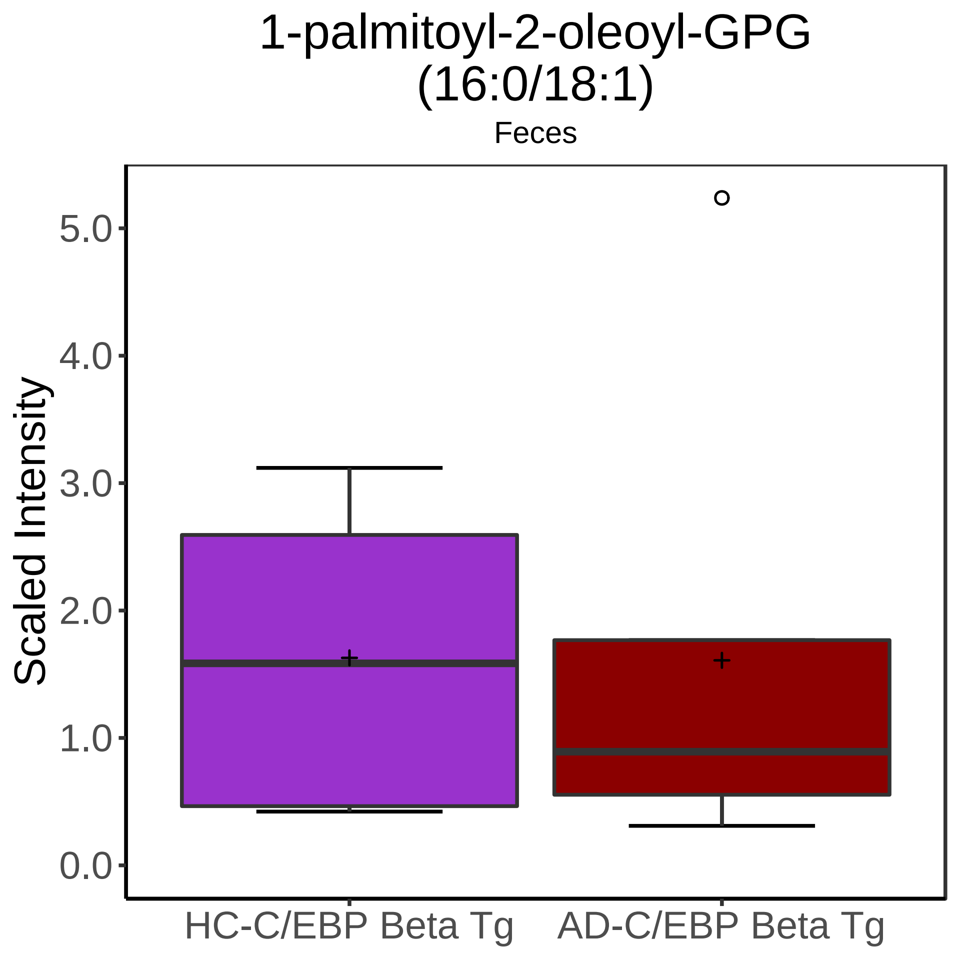
1-palmitoyl-2-oleoyl-GPG
(16:0/18:1)
Feces
Scaled Intensity
5.0
4.0
3.0
2.0
1.0
0.0
HC-C/EBP Beta Tg
AD-C/EBP Beta Tg

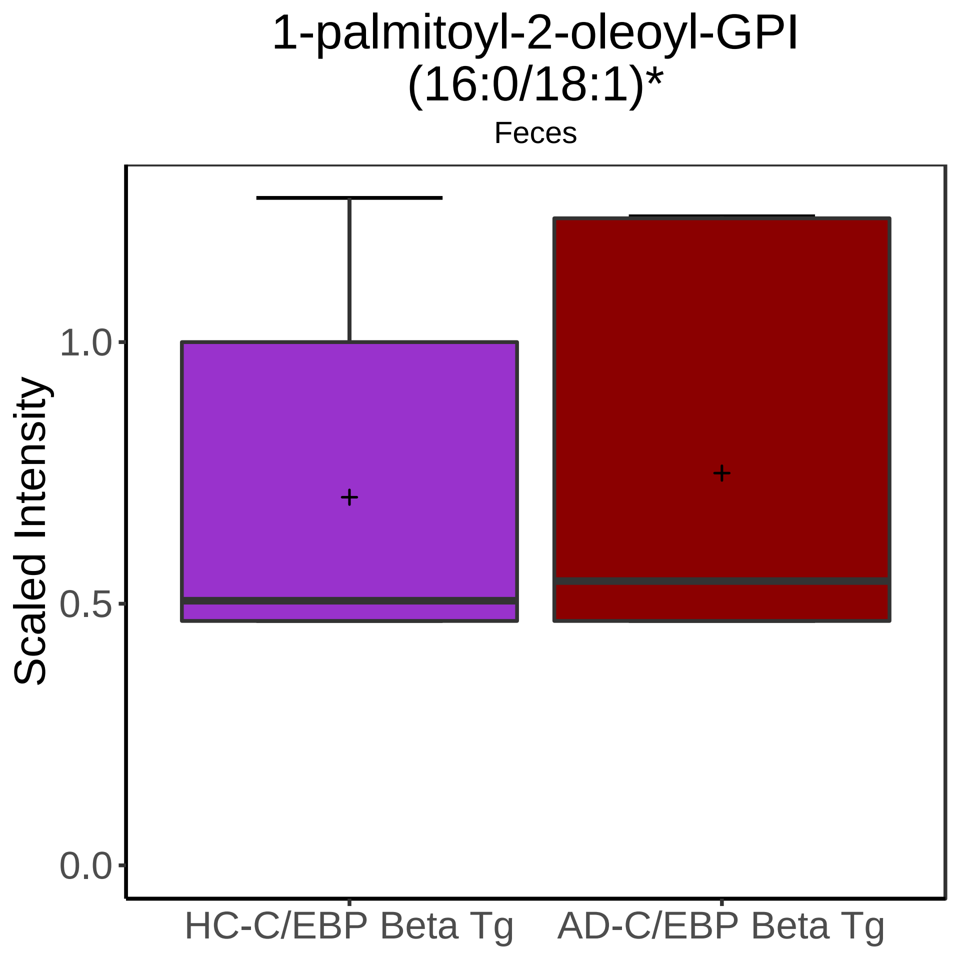

1-palmitoyl-2-oleoyl-GPI
(16:0/18:1)*
Feces
Scaled Intensity
1.0
0.5
0.0
HC-C/EBP Beta Tg
AD-C/EBP Beta Tg

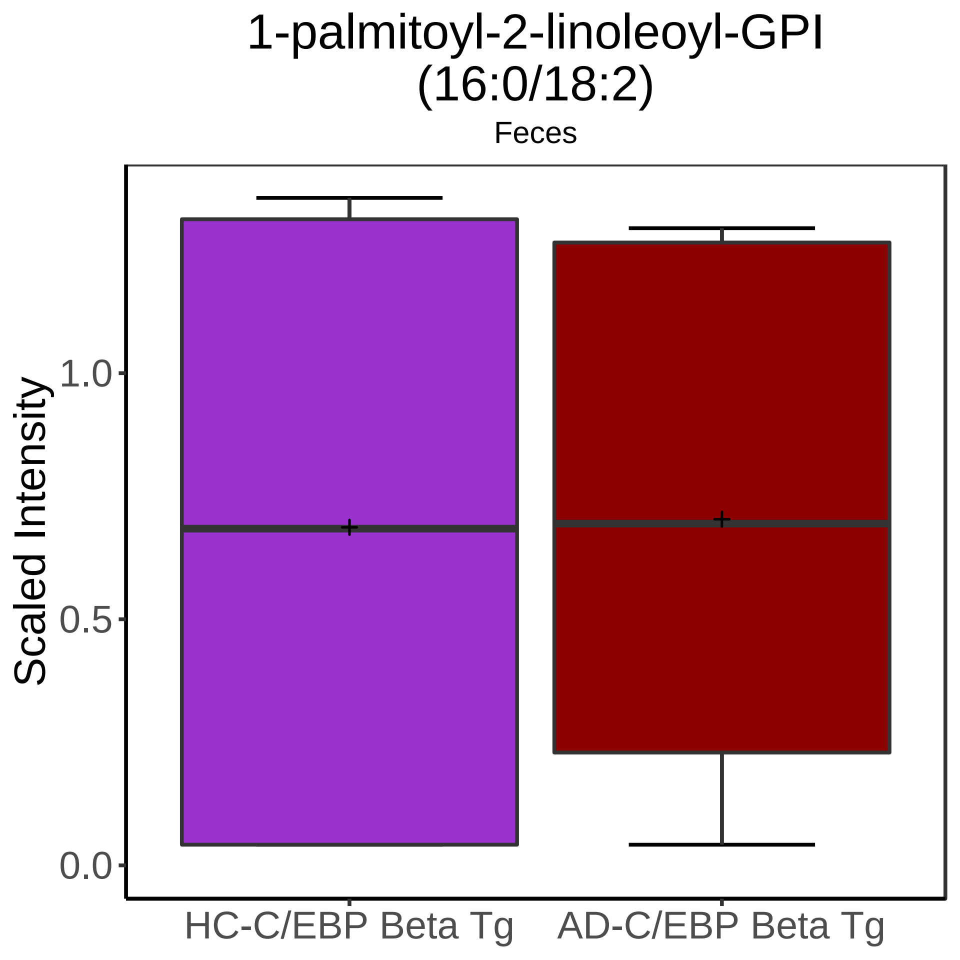

1-palmitoyl-2-linoleoyl-GPI
(16:0/18:2)
Feces
Scaled Intensity
1.0
0.5
0.0
HC-C/EBP Beta Tg
AD-C/EBP Beta Tg

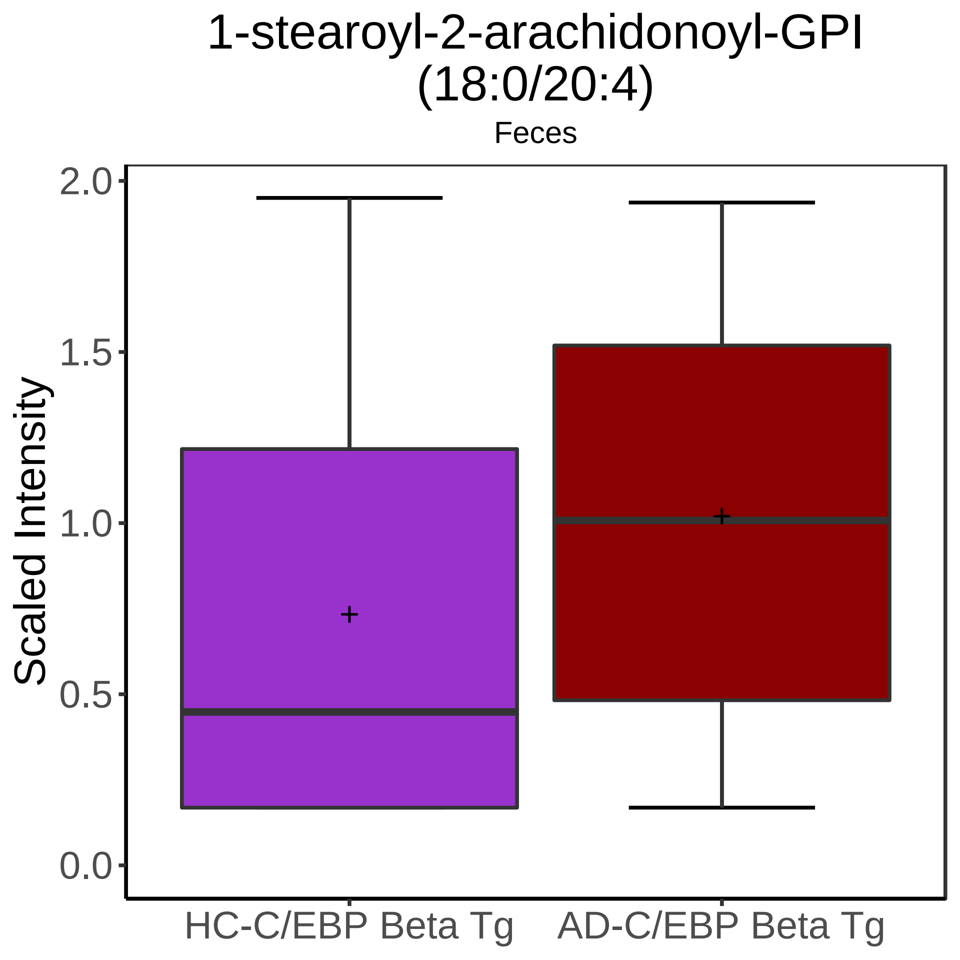
1-stearoyl-2-arachidonoyl-GPI
(18:0/20:4)
Feces
Scaled Intensity
2.0
1.5
1.0
0.5
0.0
HC-C/EBP Beta Tg
AD-C/EBP Beta Tg

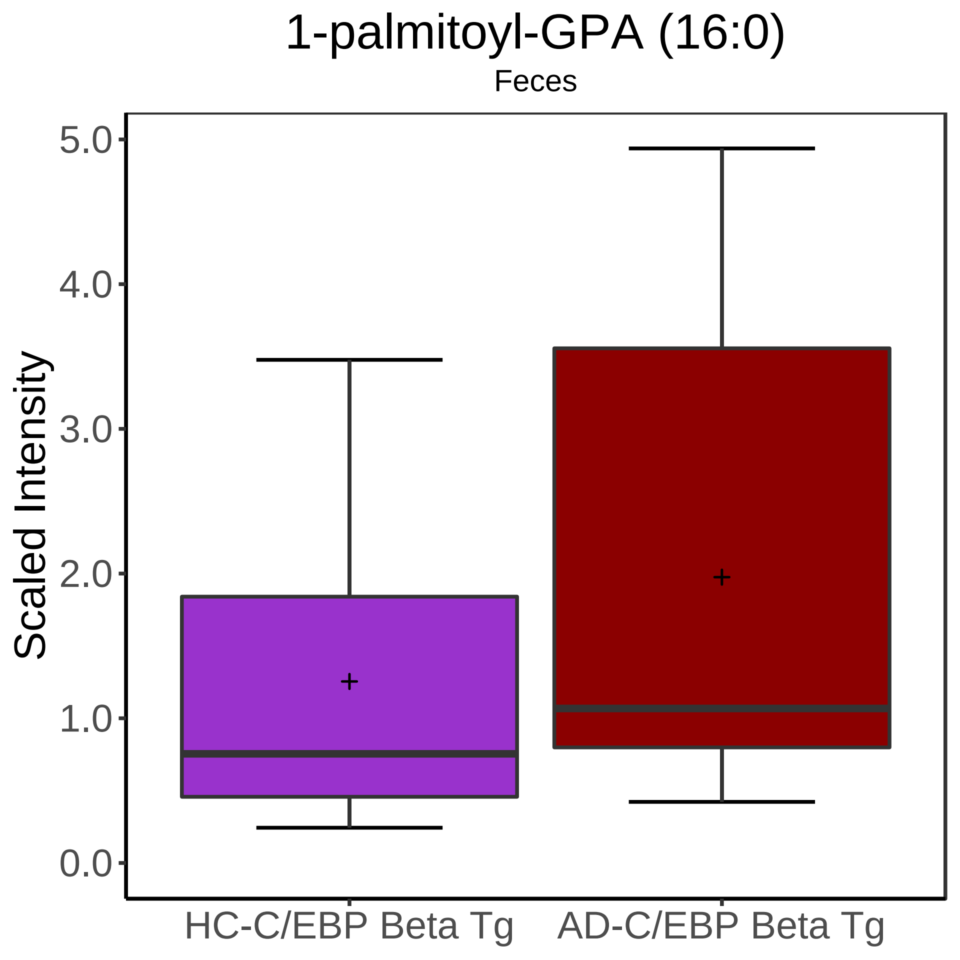
1-palmitoyl-GPA (16:0)
Feces
Scaled Intensity
5.0
4.0
3.0
2.0
1.0
0.0
HC-C/EBP Beta Tg
AD-C/EBP Beta Tg

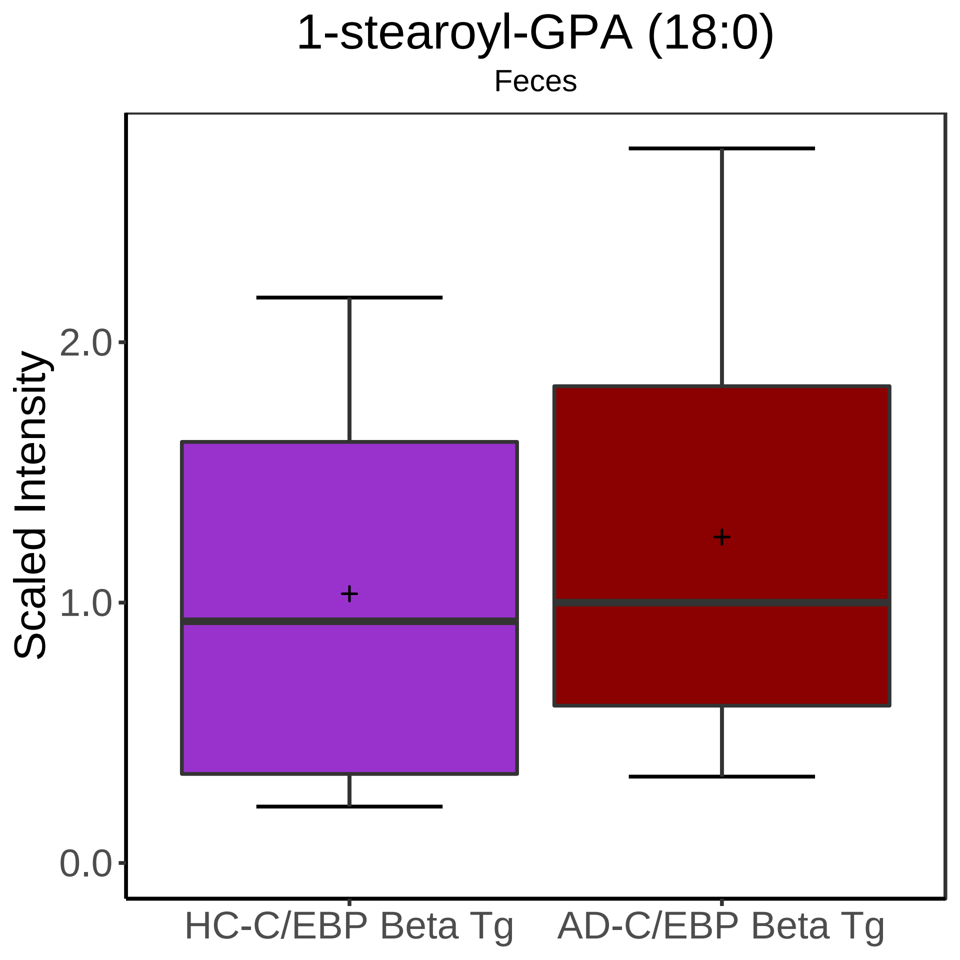

1-stearoyl-GPA (18:0)
Feces
Scaled Intensity
2.0
1.0
0.0
HC-C/EBP Beta Tg
AD-C/EBP Beta Tg

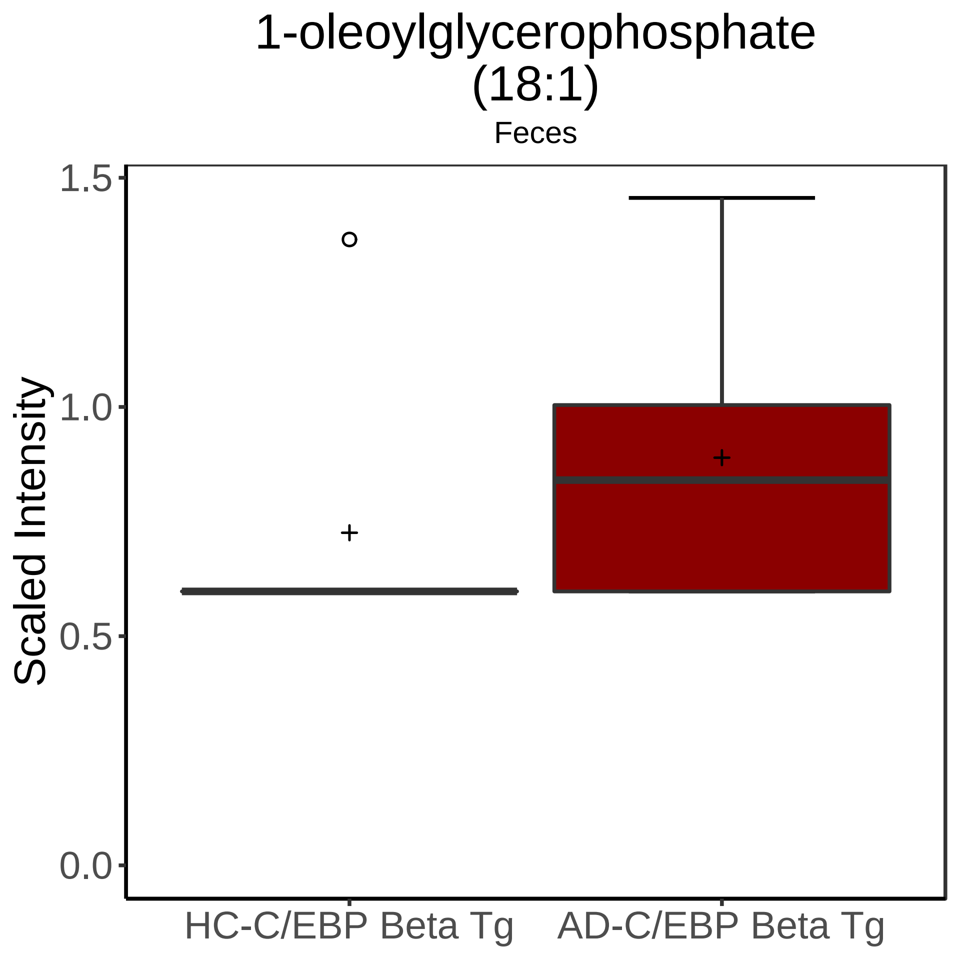

1-oleoylglycerophosphate
(18:1)
Feces
Scaled Intensity
1.5
1.0
0.5
0.0
HC-C/EBP Beta Tg
AD-C/EBP Beta Tg

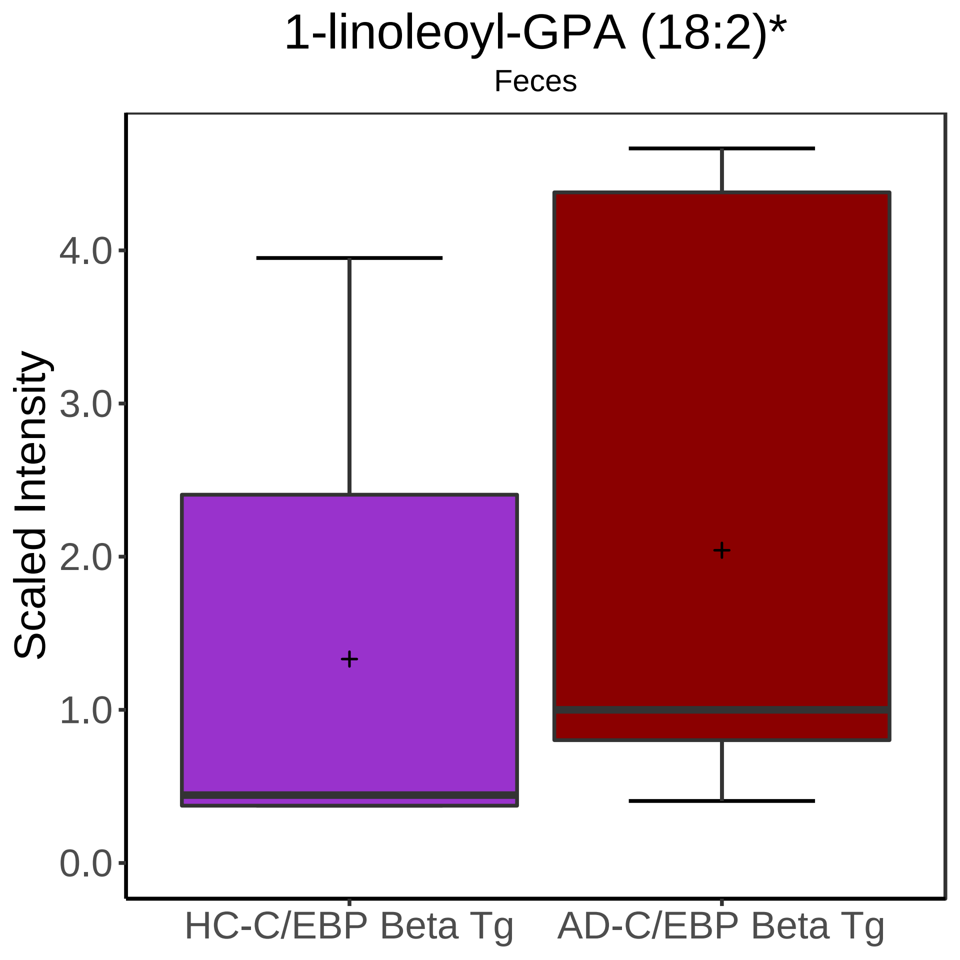
1-linoleoyl-GPA (18:2)*
Feces
Scaled Intensity
4.0
3.0
2.0
1.0
0.0
HC-C/EBP Beta Tg
AD-C/EBP Beta Tg

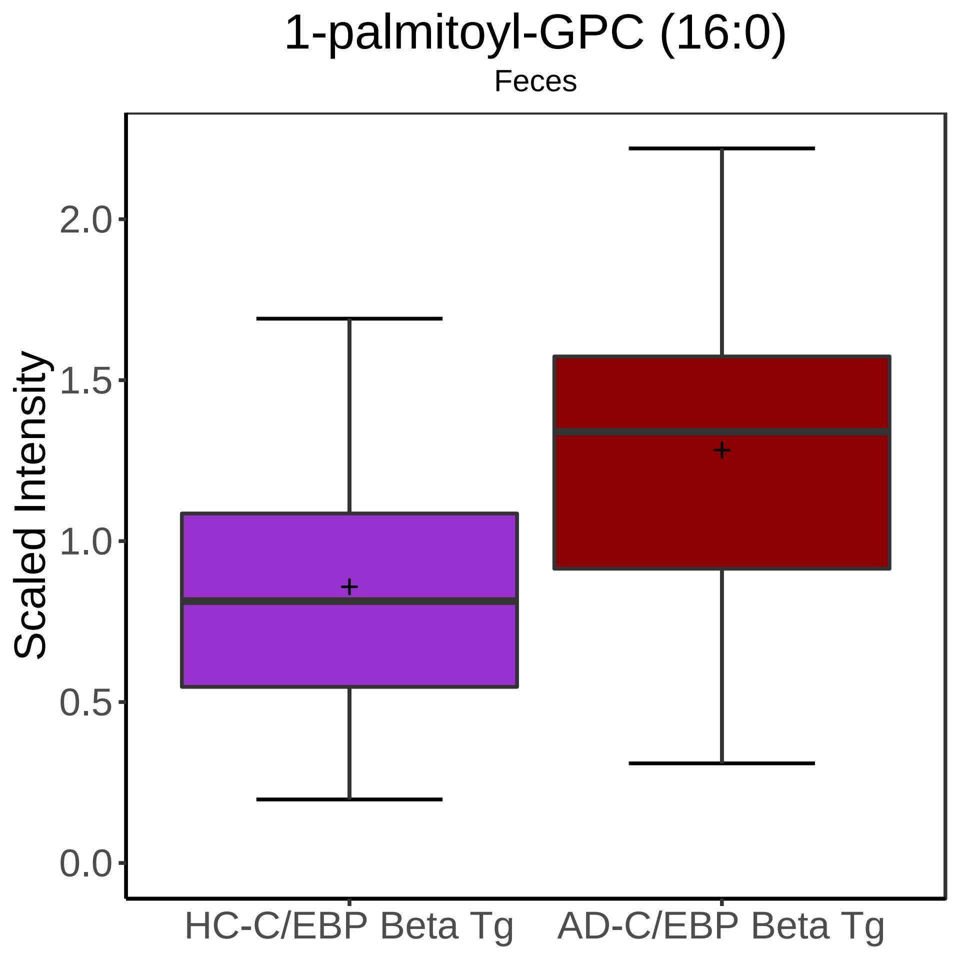
1-palmitoyl-GPC (16:0)
Feces
Scaled Intensity
0.0
0.5
1.0
1.5
2.0
HC-C/EBP Beta Tg
AD-C/EBP Beta Tg

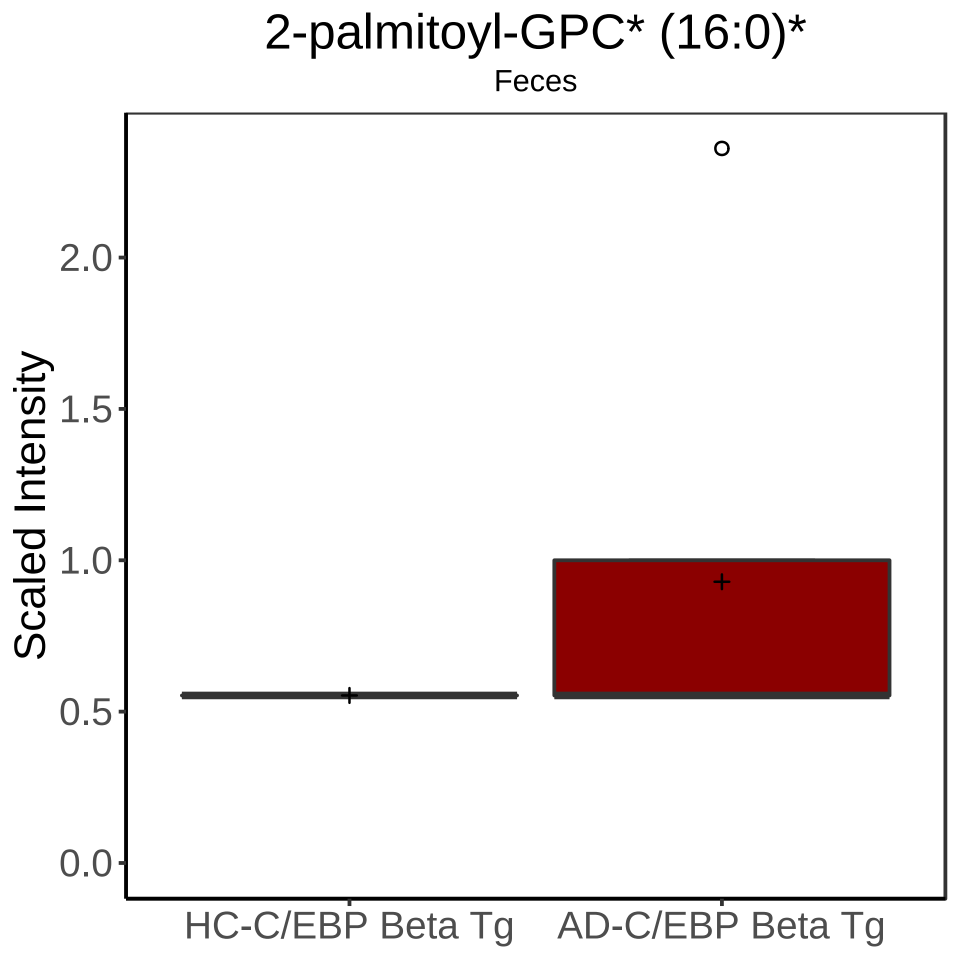

2-palmitoyl-GPC* (16:0)*
Feces
Scaled Intensity
0.0
0.5
1.0
1.5
2.0
HC-C/EBP Beta Tg
AD-C/EBP Beta Tg

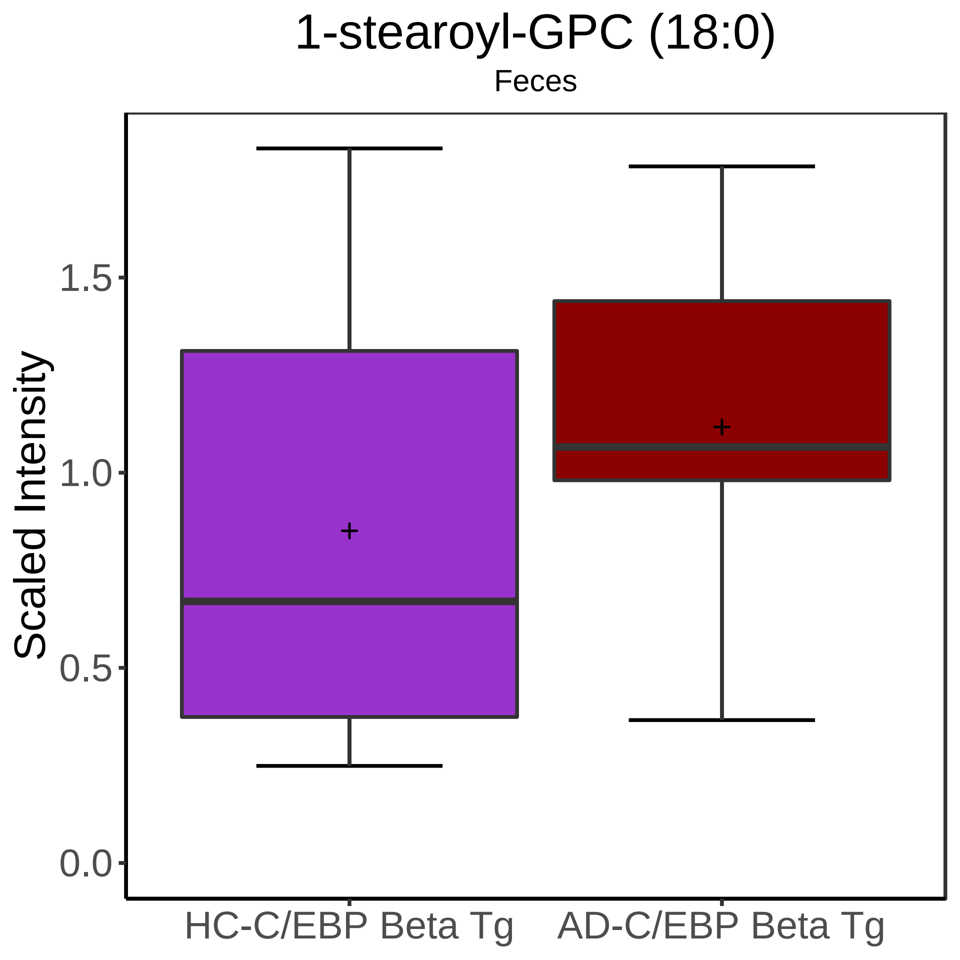
1-stearoyl-GPC (18:0)
Feces
Scaled Intensity
0.0
0.5
1.0
1.5
HC-C/EBP Beta Tg
AD-C/EBP Beta Tg

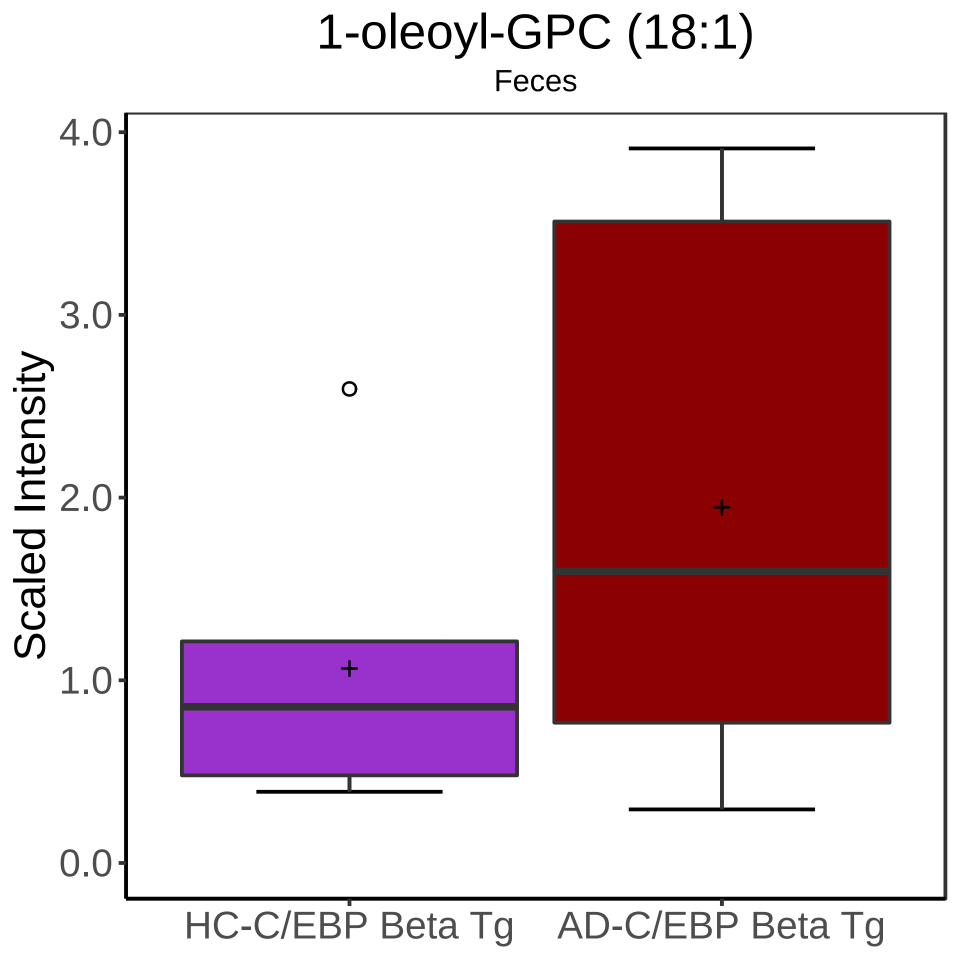
1-oleoyl-GPC (18:1)
Feces
Scaled Intensity
4.0
3.0
2.0
1.0
0.0
HC-C/EBP Beta Tg
AD-C/EBP Beta Tg

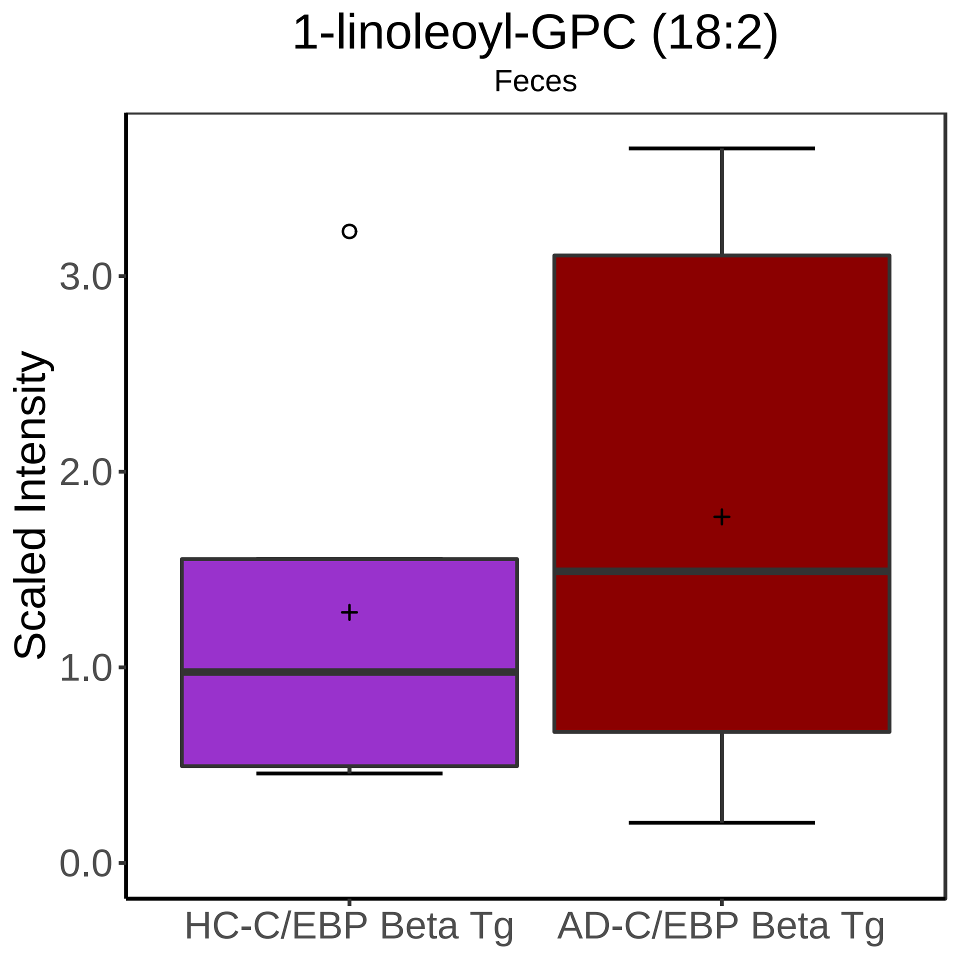

1-linoleoyl-GPC (18:2)
Feces
Scaled Intensity
3.0
2.0
1.0
0.0
HC-C/EBP Beta Tg
AD-C/EBP Beta Tg

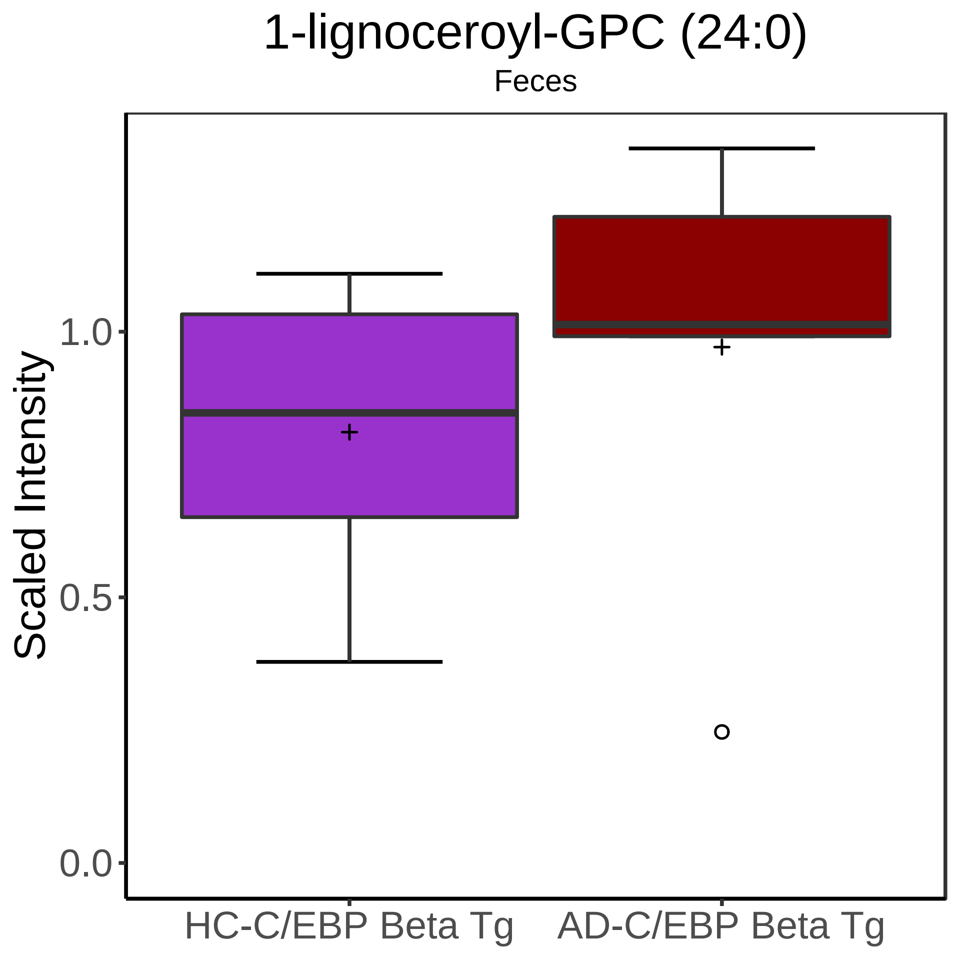
1-lignoceroyl-GPC (24:0)
Feces
Scaled Intensity
1.0
0.5
0.0
HC-C/EBP Beta Tg
AD-C/EBP Beta Tg

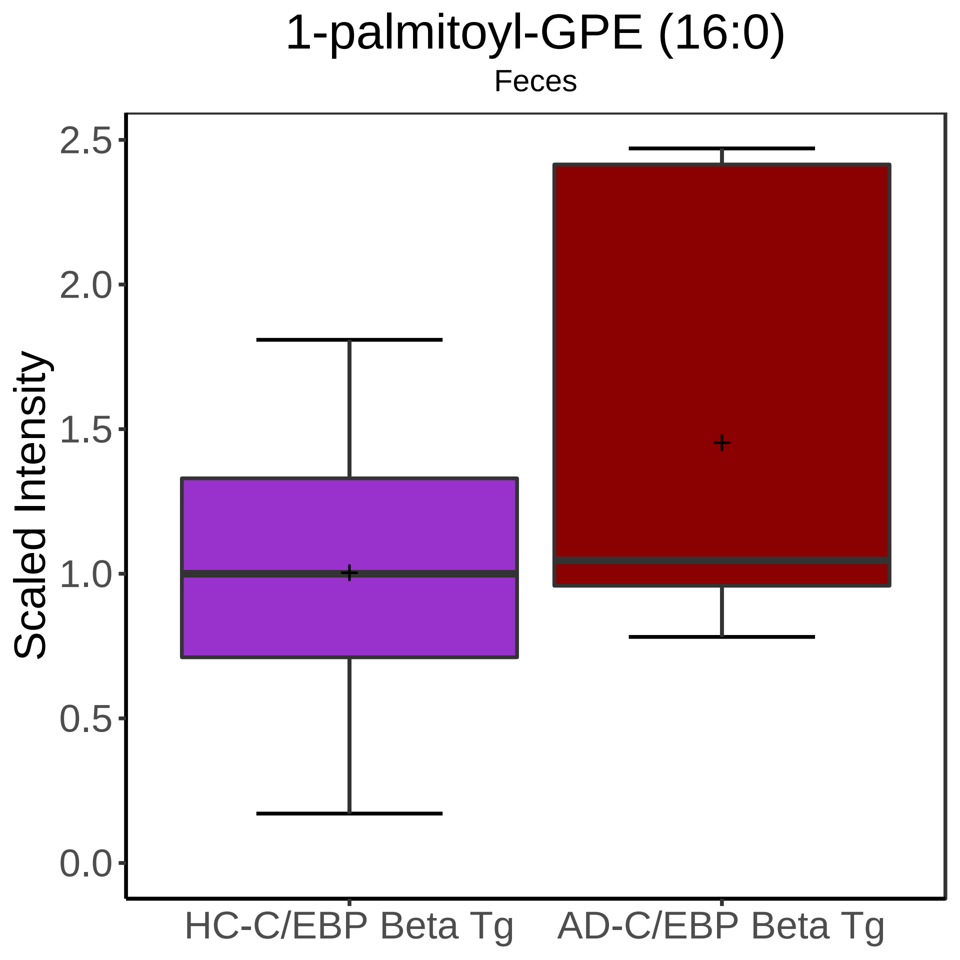
1-palmitoyl-GPE (16:0)
Feces
Scaled Intensity
2.5
2.0
1.5
1.0
0.5
0.0
HC-C/EBP Beta Tg
AD-C/EBP Beta Tg

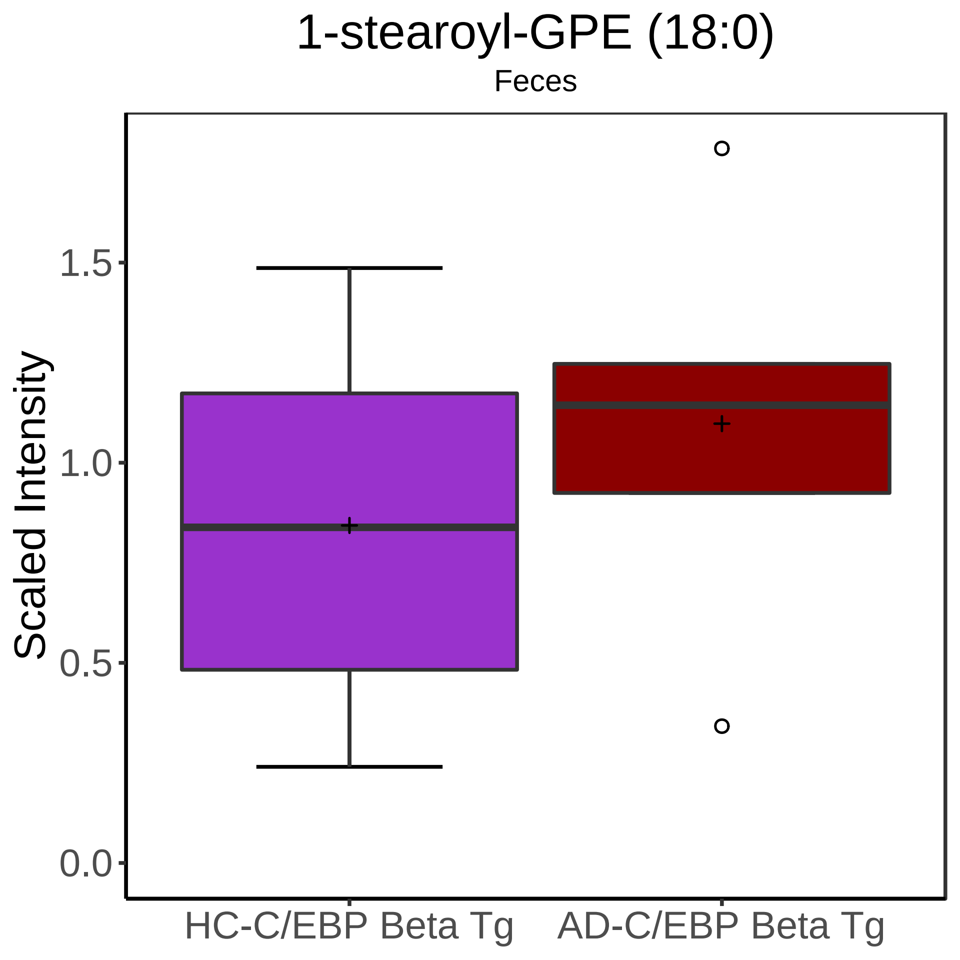
1-stearoyl-GPE (18:0)
Feces
Scaled Intensity
0.0
0.5
1.0
1.5
HC-C/EBP Beta Tg
AD-C/EBP Beta Tg

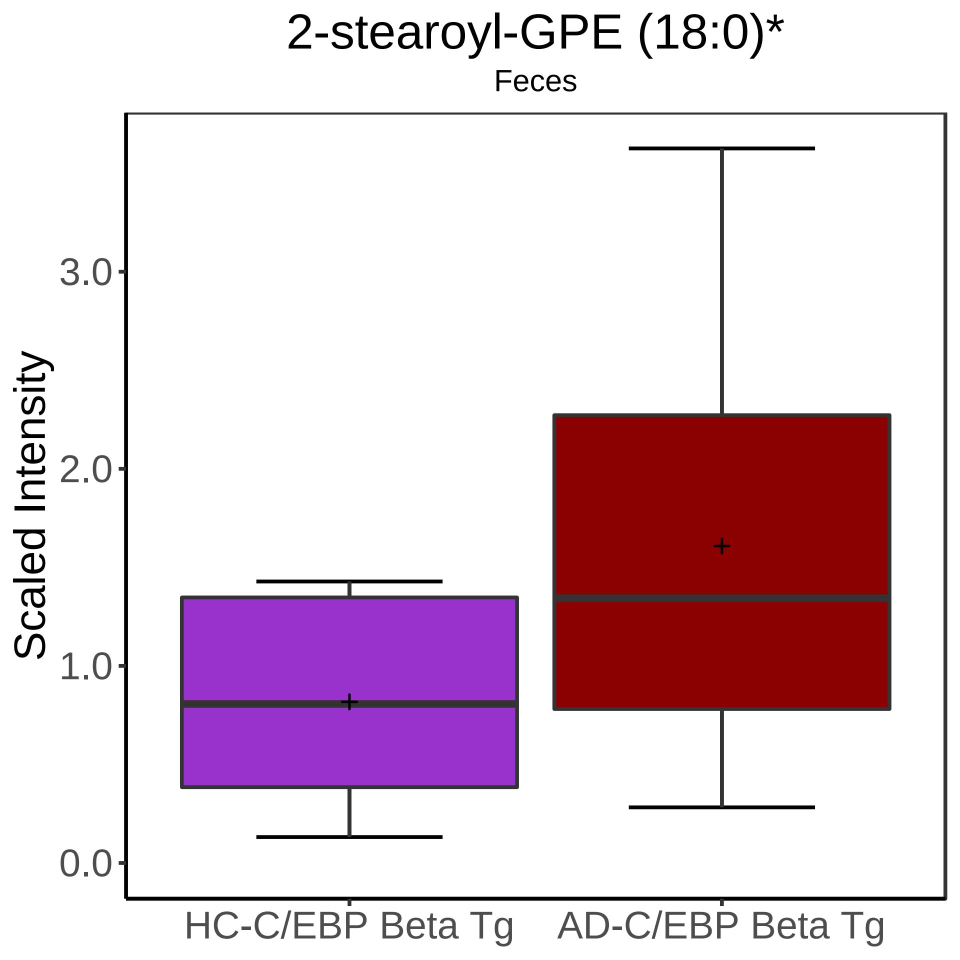

2-stearoyl-GPE (18:0)*
Feces
Scaled Intensity
3.0
2.0
1.0
0.0
HC-C/EBP Beta Tg
AD-C/EBP Beta Tg

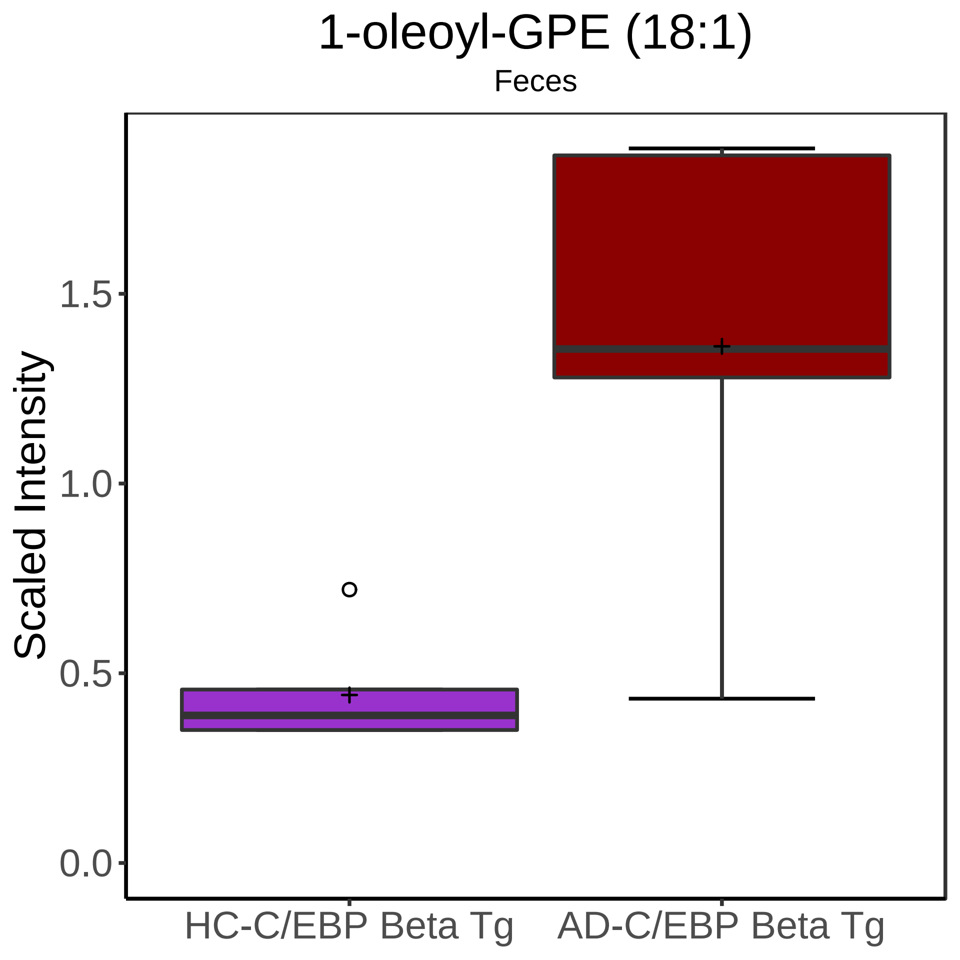
1-oleoyl-GPE (18:1)
Feces
Scaled Intensity
0.0
0.5
1.0
1.5
HC-C/EBP Beta Tg
AD-C/EBP Beta Tg

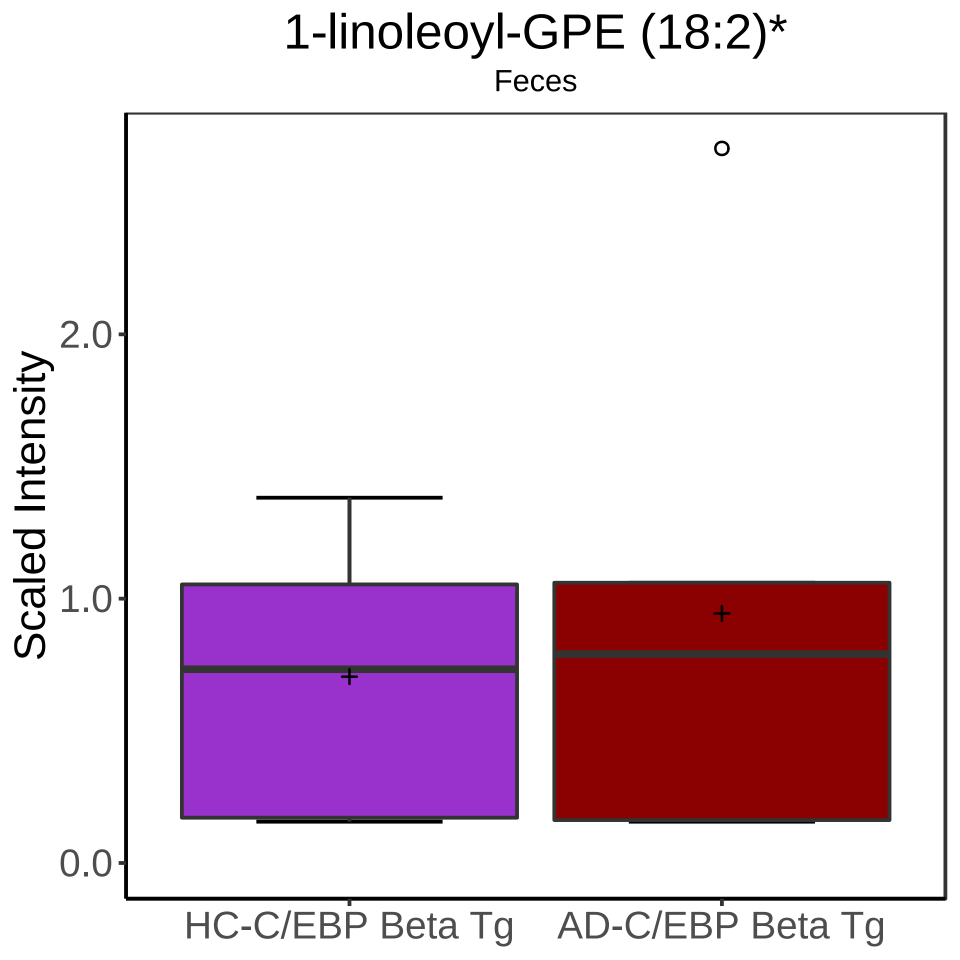
1-linoleoyl-GPE (18:2)*
Feces
Scaled Intensity
2.0
1.0
0.0
HC-C/EBP Beta Tg
AD-C/EBP Beta Tg

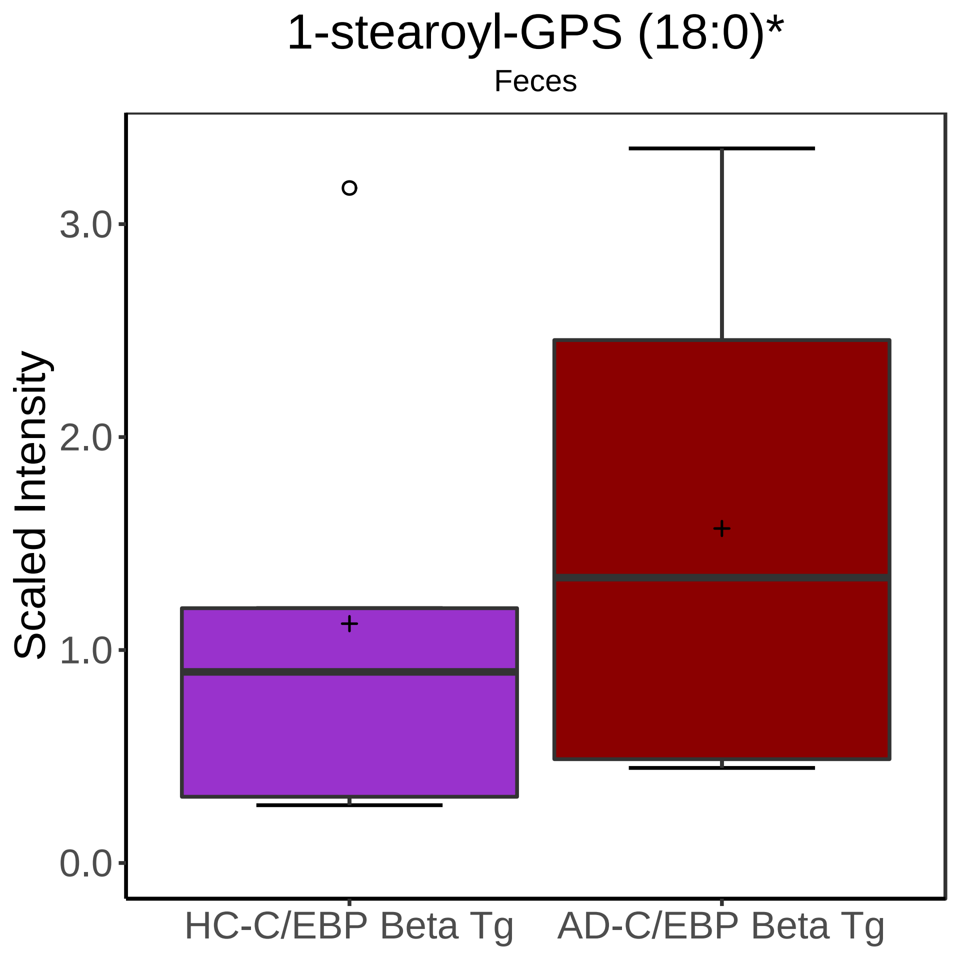

1-stearoyl-GPS (18:0)*
Feces
Scaled Intensity
3.0
2.0
1.0
0.0
HC-C/EBP Beta Tg
AD-C/EBP Beta Tg

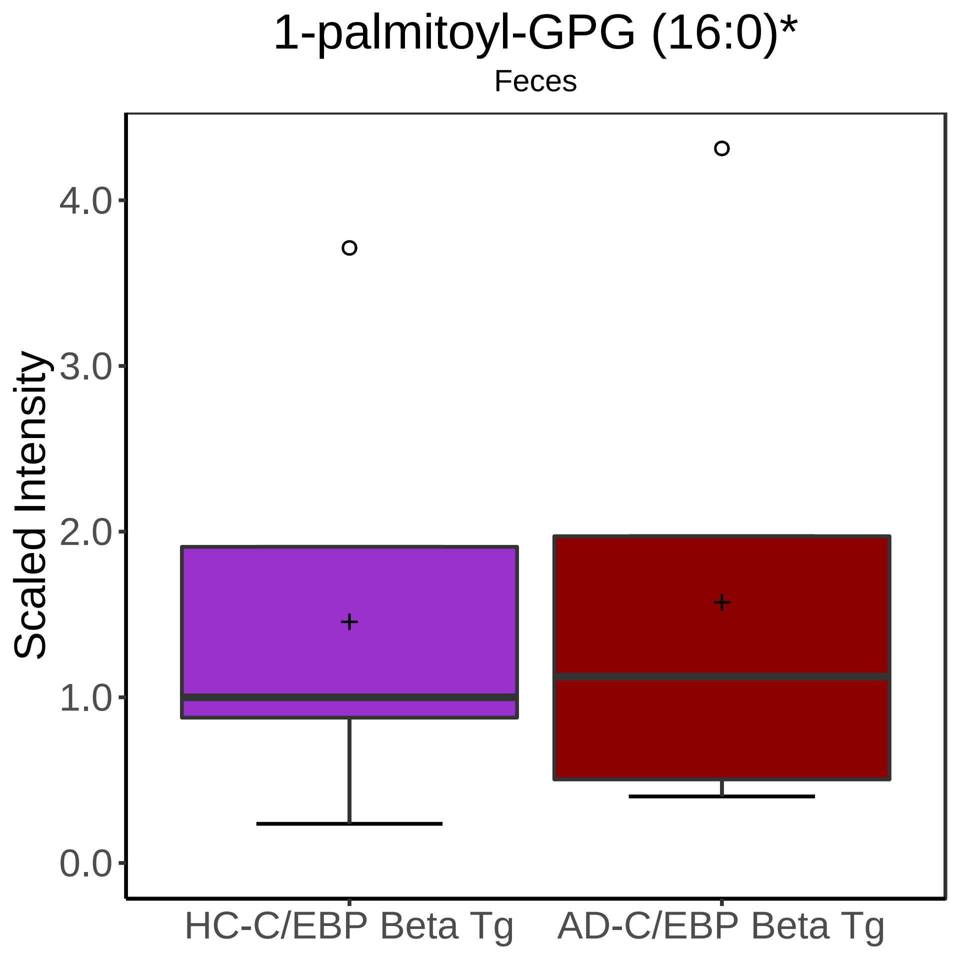
1-palmitoyl-GPG (16:0)*
Feces
Scaled Intensity
4.0
3.0
2.0
1.0
0.0
HC-C/EBP Beta Tg
AD-C/EBP Beta Tg

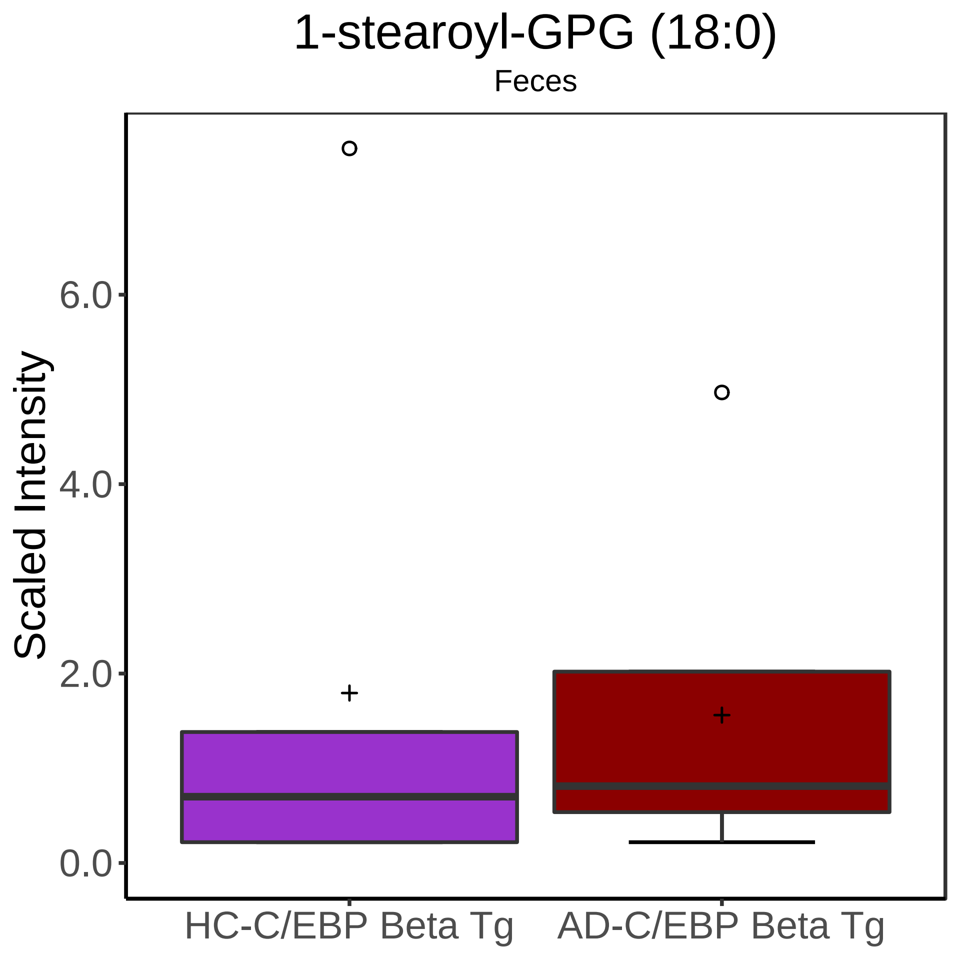

1-stearoyl-GPG (18:0)
Feces
Scaled Intensity
6.0
4.0
2.0
0.0
HC-C/EBP Beta Tg
AD-C/EBP Beta Tg

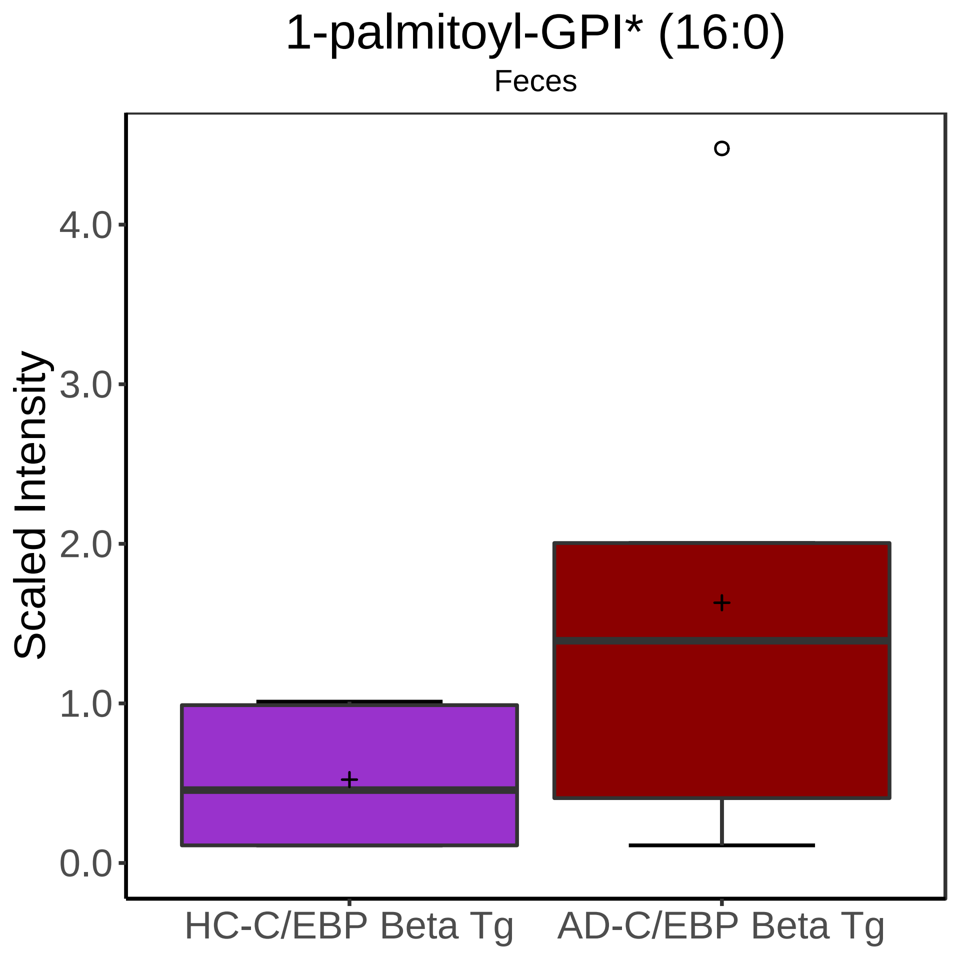

1-palmitoyl-GPI* (16:0)
Feces
Scaled Intensity
4.0
3.0
2.0
1.0
0.0
HC-C/EBP Beta Tg
AD-C/EBP Beta Tg

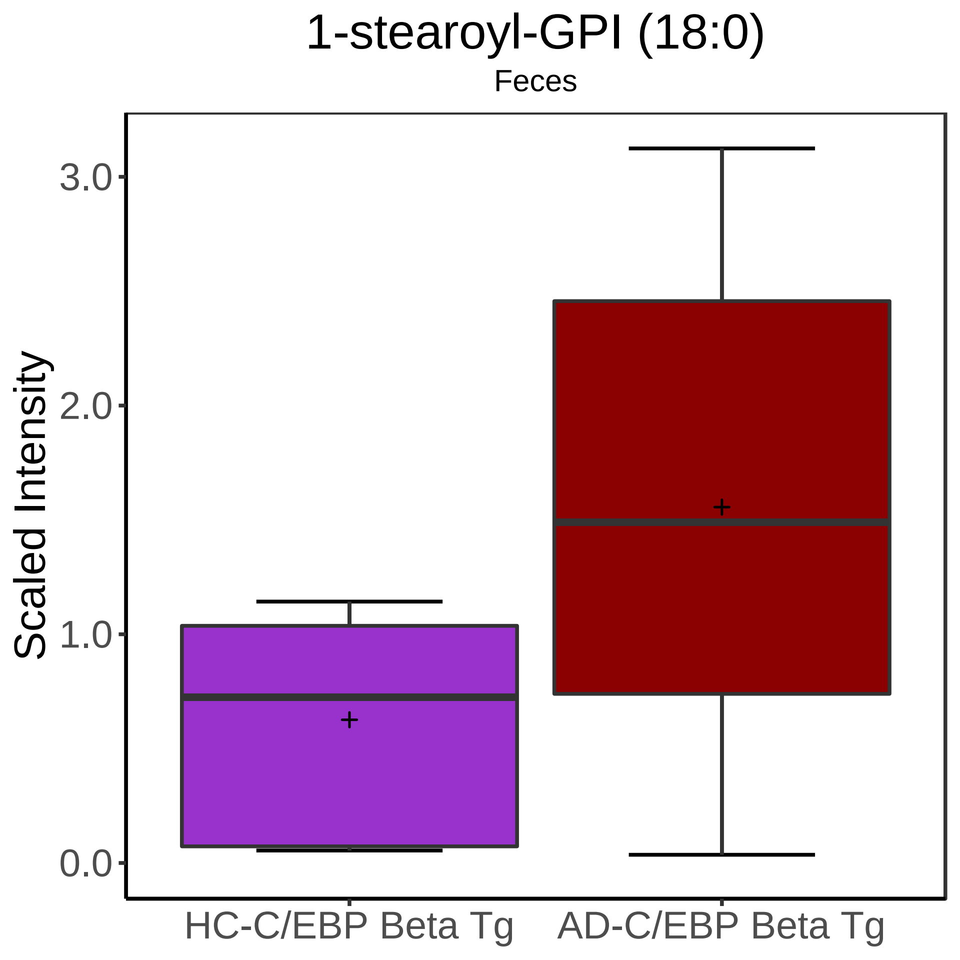
1-stearoyl-GPI (18:0)
Feces
Scaled Intensity
3.0
2.0
1.0
0.0
HC-C/EBP Beta Tg
AD-C/EBP Beta Tg

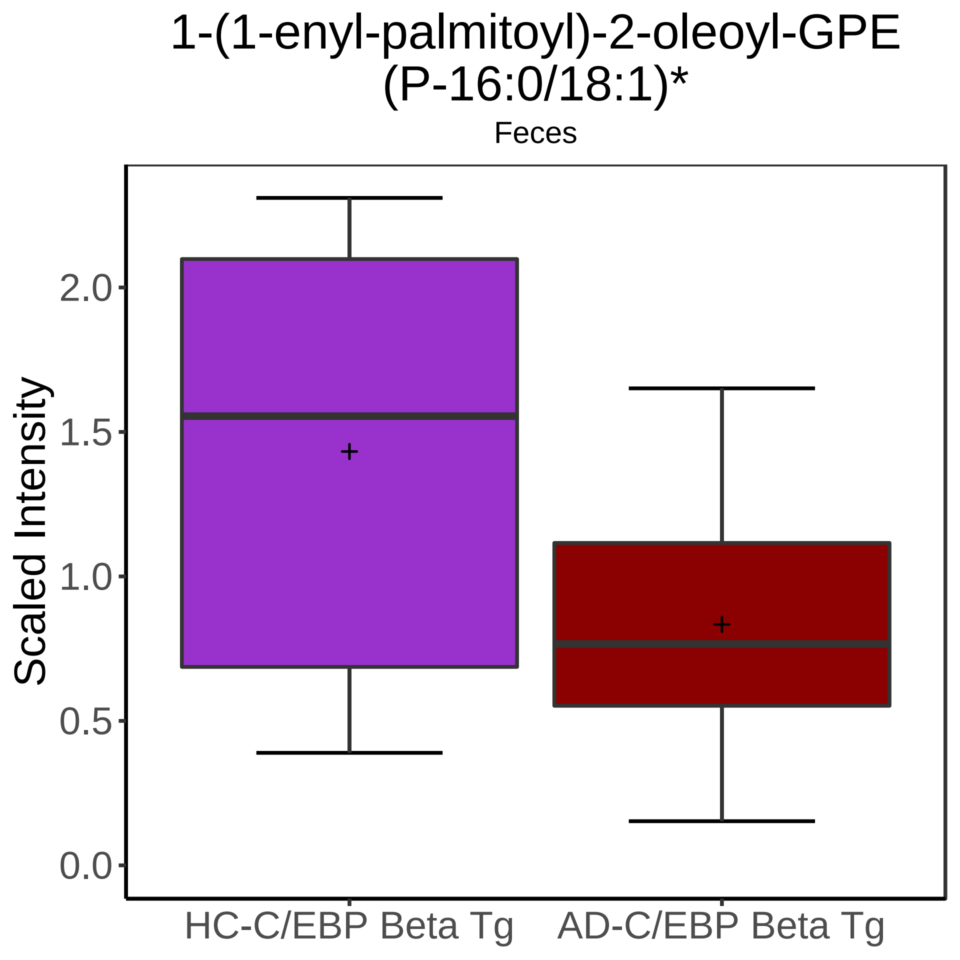
1-(1-enyl-palmitoyl)-2-oleoyl-GPE
(P-16:0/18:1)*
Feces
Scaled Intensity
2.0
1.5
1.0
0.5
0.0
HC-C/EBP Beta Tg
AD-C/EBP Beta Tg

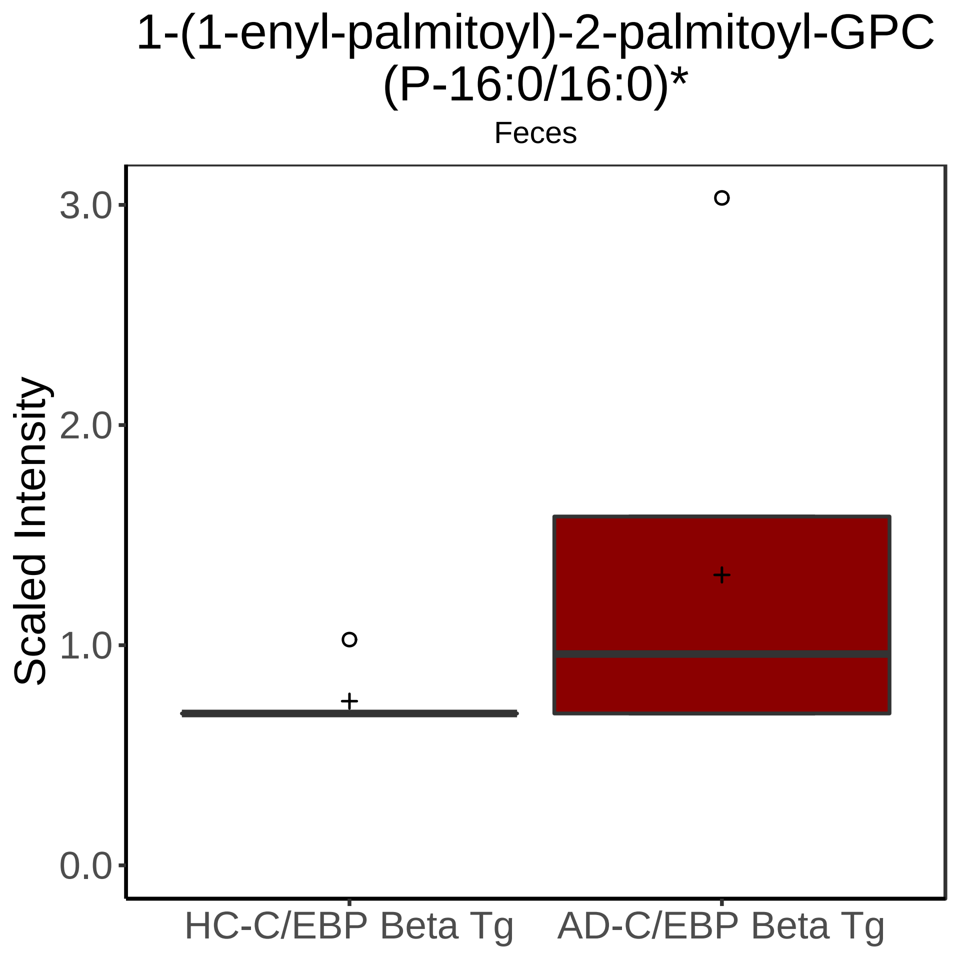
1-(1-enyl-palmitoyl)-2-palmitoyl-GPC
(P-16:0/16:0)*
Feces
Scaled Intensity
3.0
2.0
1.0
0.0
HC-C/EBP Beta Tg
AD-C/EBP Beta Tg

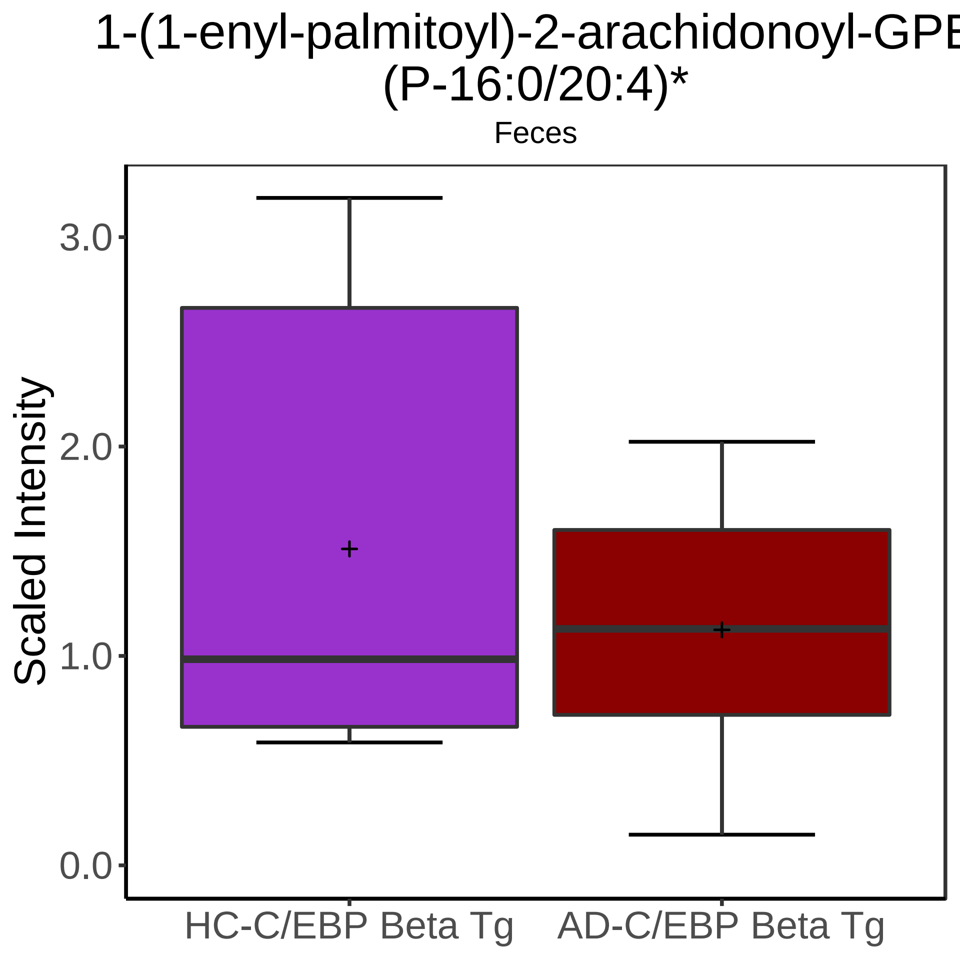

1-(1-enyl-palmitoyl)-2-arachidonoyl-GPE
(P-16:0/20:4)*
Feces
Scaled Intensity
3.0
2.0
1.0
0.0
HC-C/EBP Beta Tg
AD-C/EBP Beta Tg

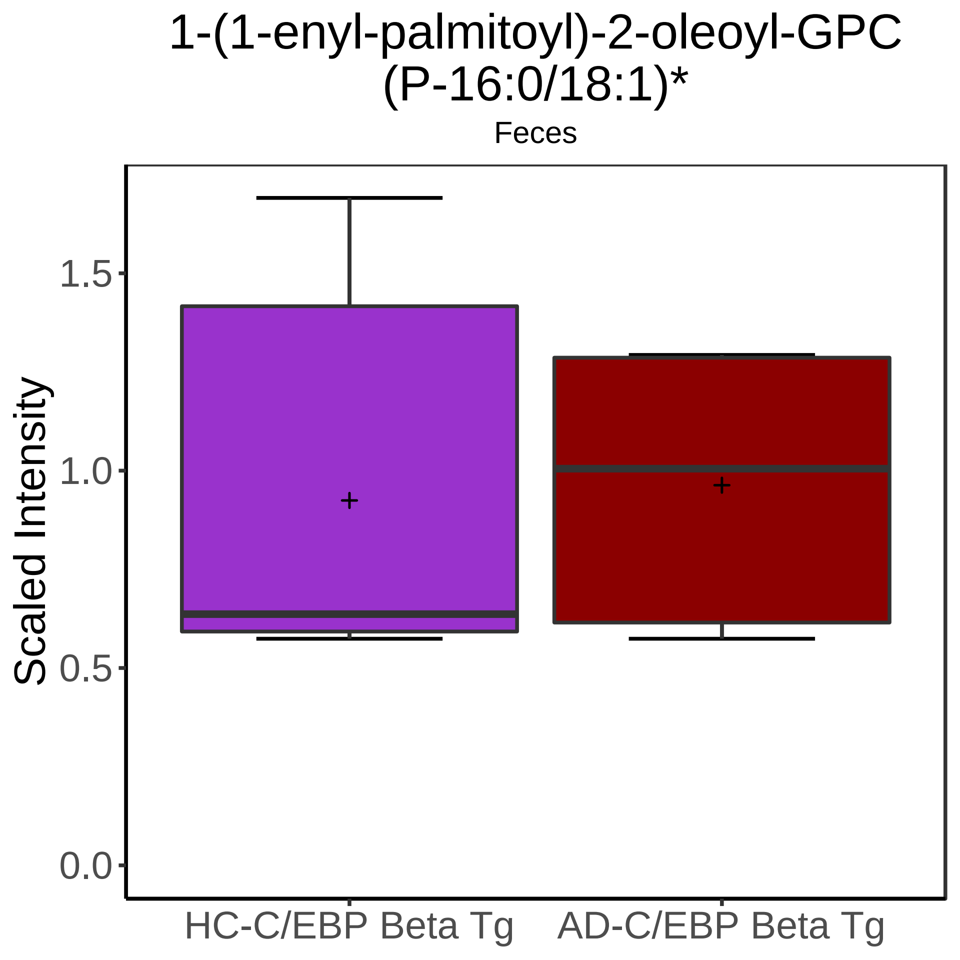
1-(1-enyl-palmitoyl)-2-oleoyl-GPC
(P-16:0/18:1)*
Feces
Scaled Intensity
0.0
0.5
1.0
1.5
HC-C/EBP Beta Tg
AD-C/EBP Beta Tg

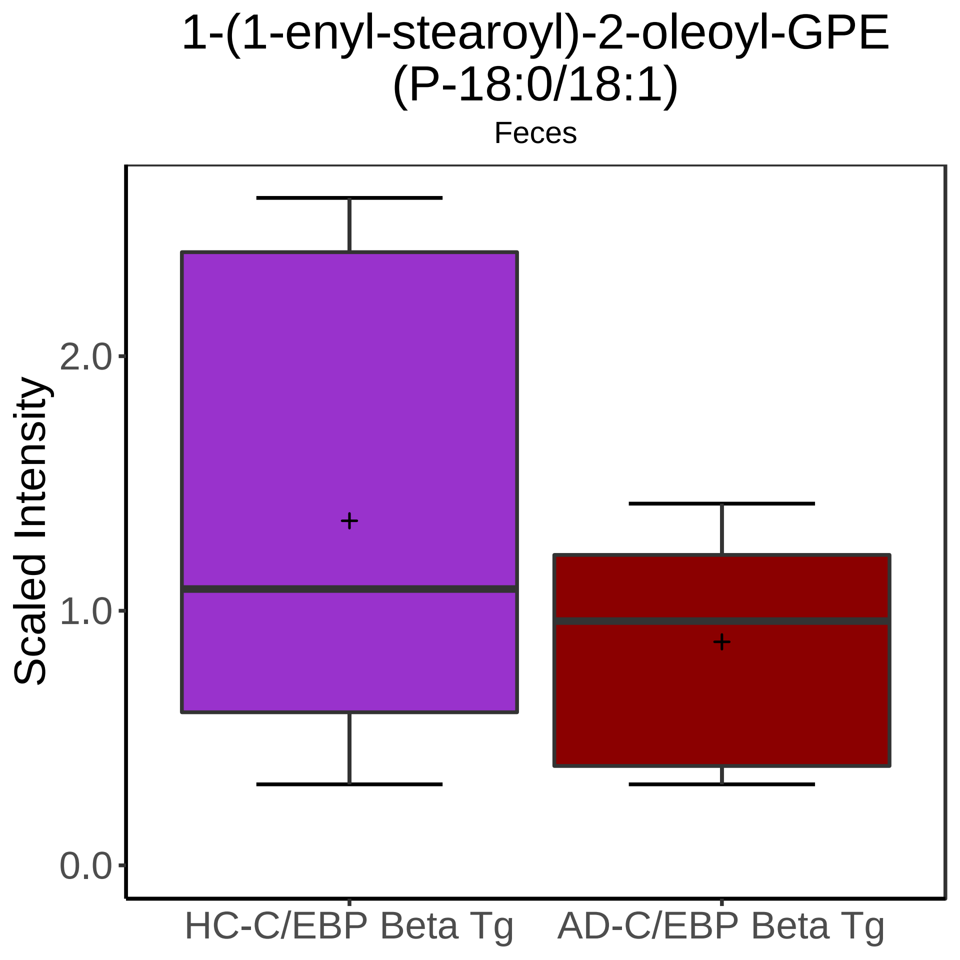

1-(1-enyl-stearoyl)-2-oleoyl-GPE
(P-18:0/18:1)
Feces
Scaled Intensity
2.0
1.0
0.0
HC-C/EBP Beta Tg
AD-C/EBP Beta Tg

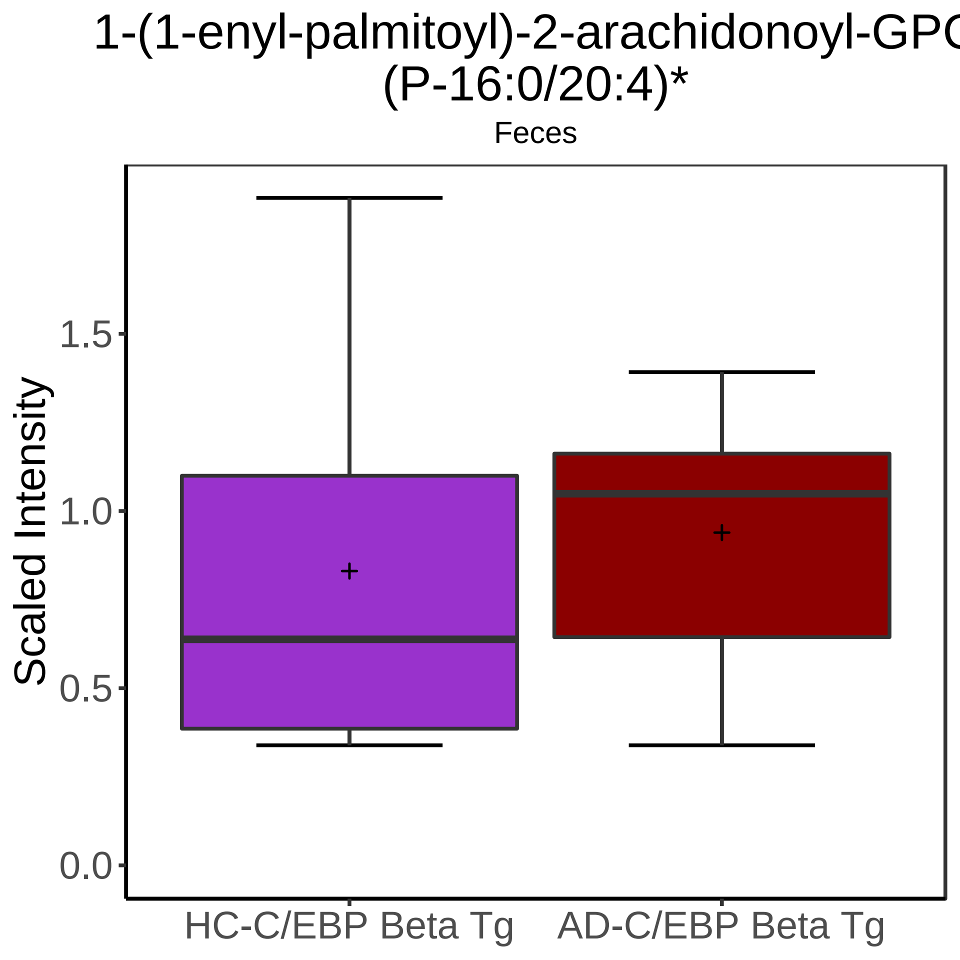
1-(1-enyl-palmitoyl)-2-arachidonoyl-GPC
(P-16:0/20:4)*
Feces
Scaled Intensity
0.0
0.5
1.0
1.5
HC-C/EBP Beta Tg
AD-C/EBP Beta Tg

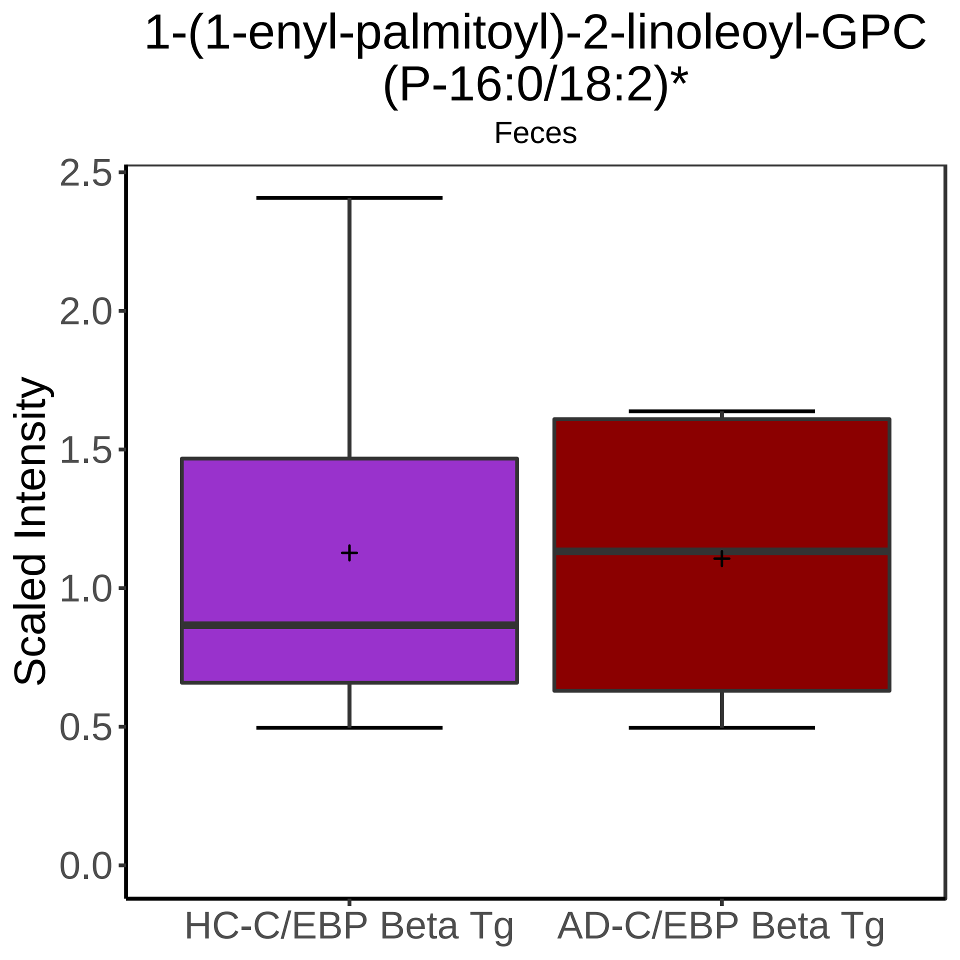
1-(1-enyl-palmitoyl)-2-linoleoyl-GPC (P-16:0/18:2)*
Feces
Scaled Intensity
2.5
2.0
1.5
1.0
0.5
0.0
HC-C/EBP Beta Tg
AD-C/EBP Beta Tg

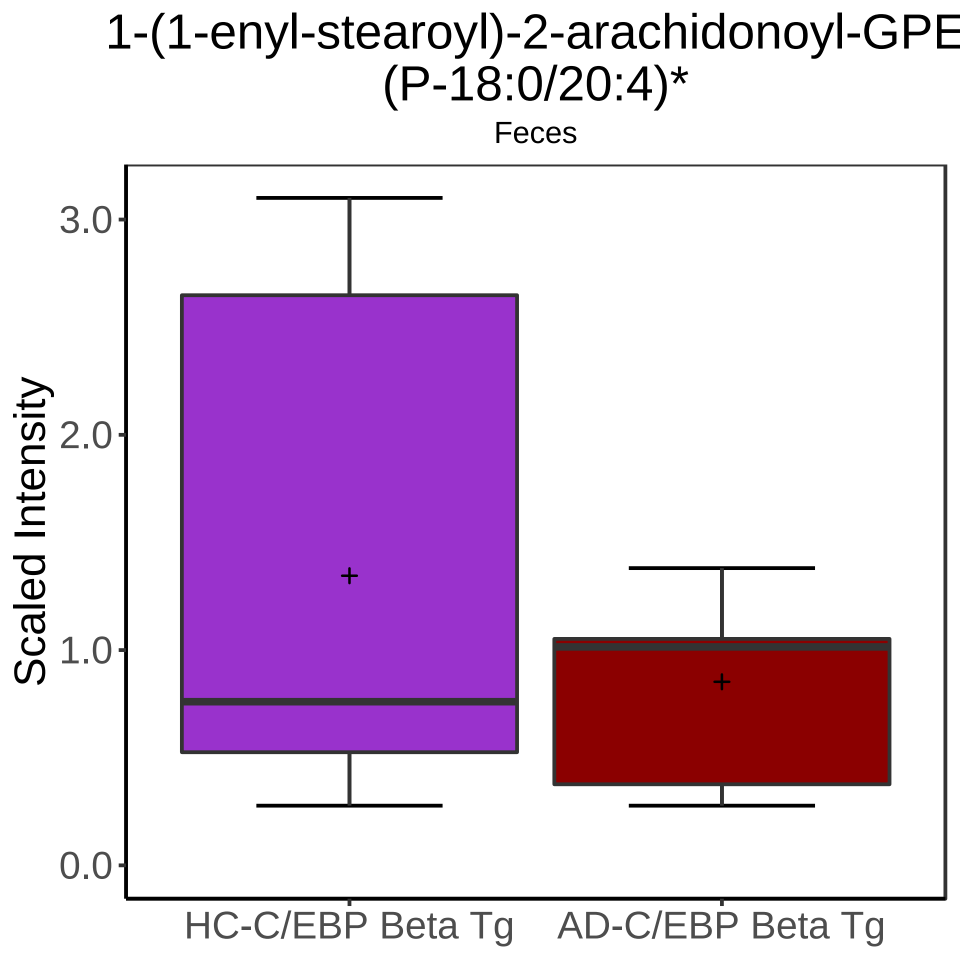

1-(1-enyl-stearoyl)-2-arachidonoyl-GPE
(P-18:0/20:4)*
Feces
Scaled Intensity
3.0
2.0
1.0
0.0
HC-C/EBP Beta Tg
AD-C/EBP Beta Tg

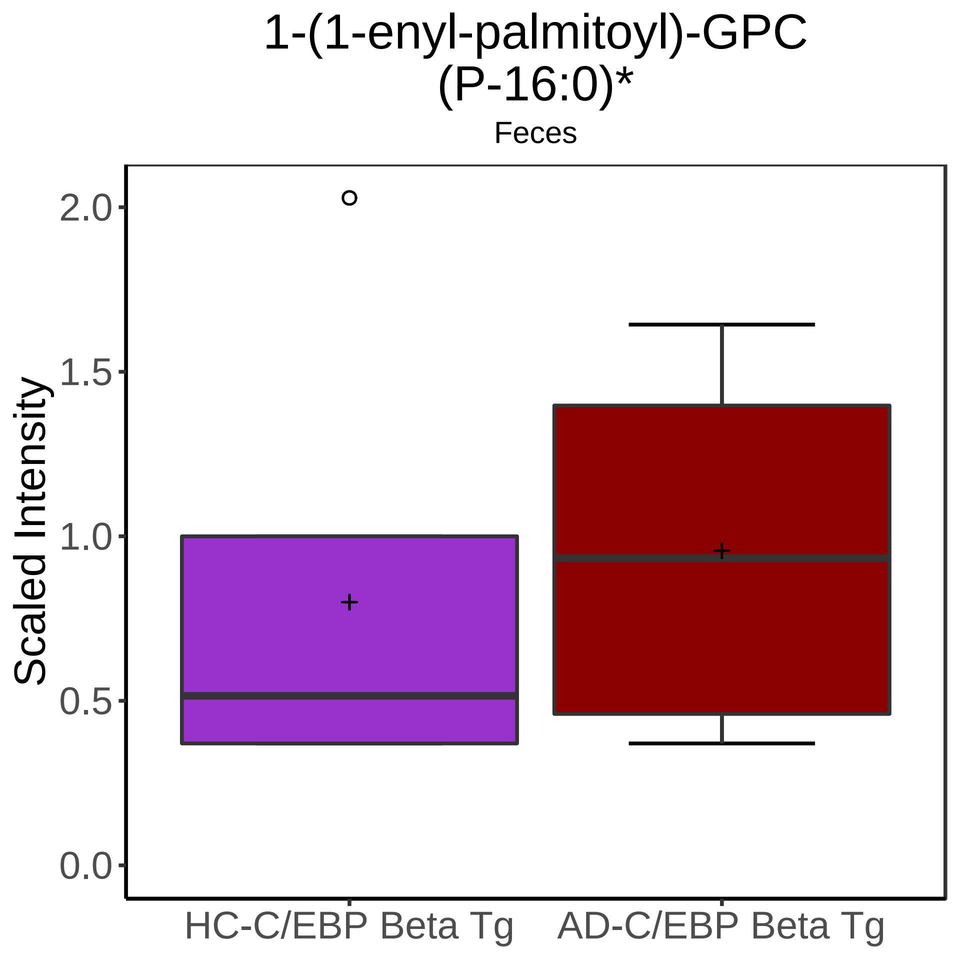

1-(1-enyl-palmitoyl)-GPC
(P-16:0)*
Feces
Scaled Intensity
2.0
1.5
1.0
0.5
0.0
HC-C/EBP Beta Tg
AD-C/EBP Beta Tg

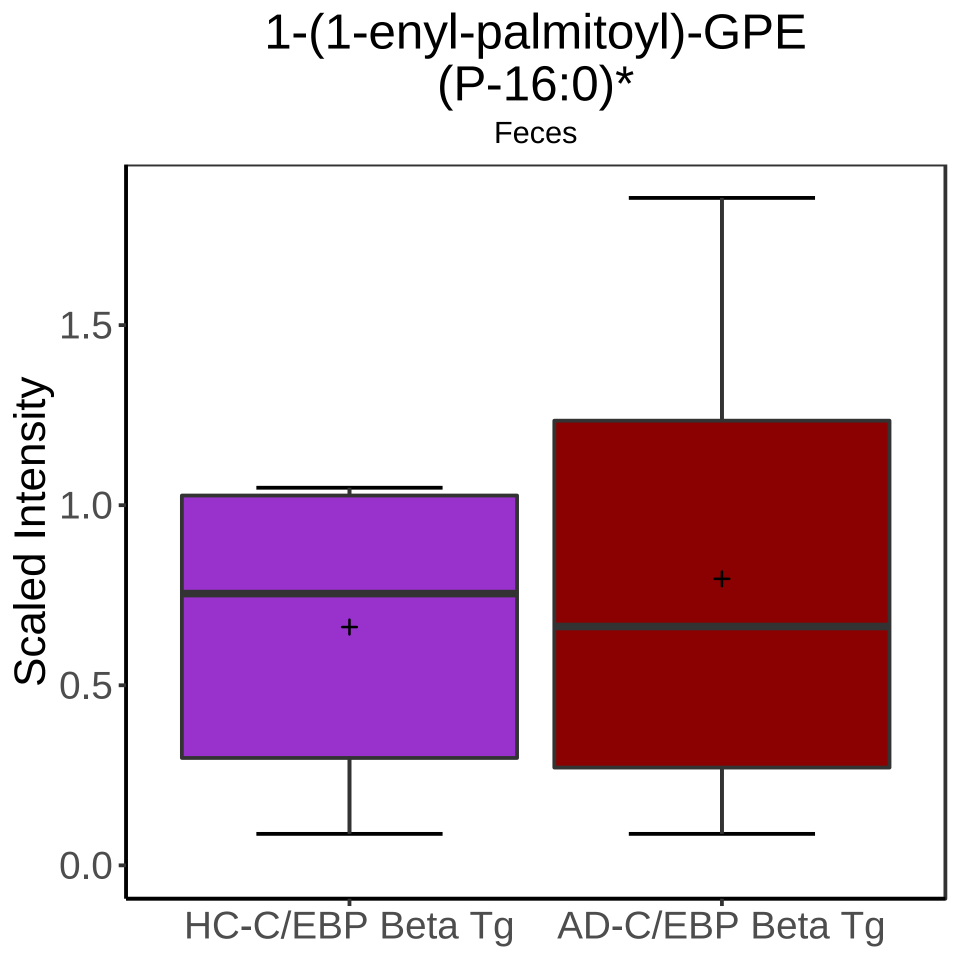
1-(1-enyl-palmitoyl)-GPE
(P-16:0)*
Feces
Scaled Intensity
0.0
0.5
1.0
1.5
HC-C/EBP Beta Tg
AD-C/EBP Beta Tg

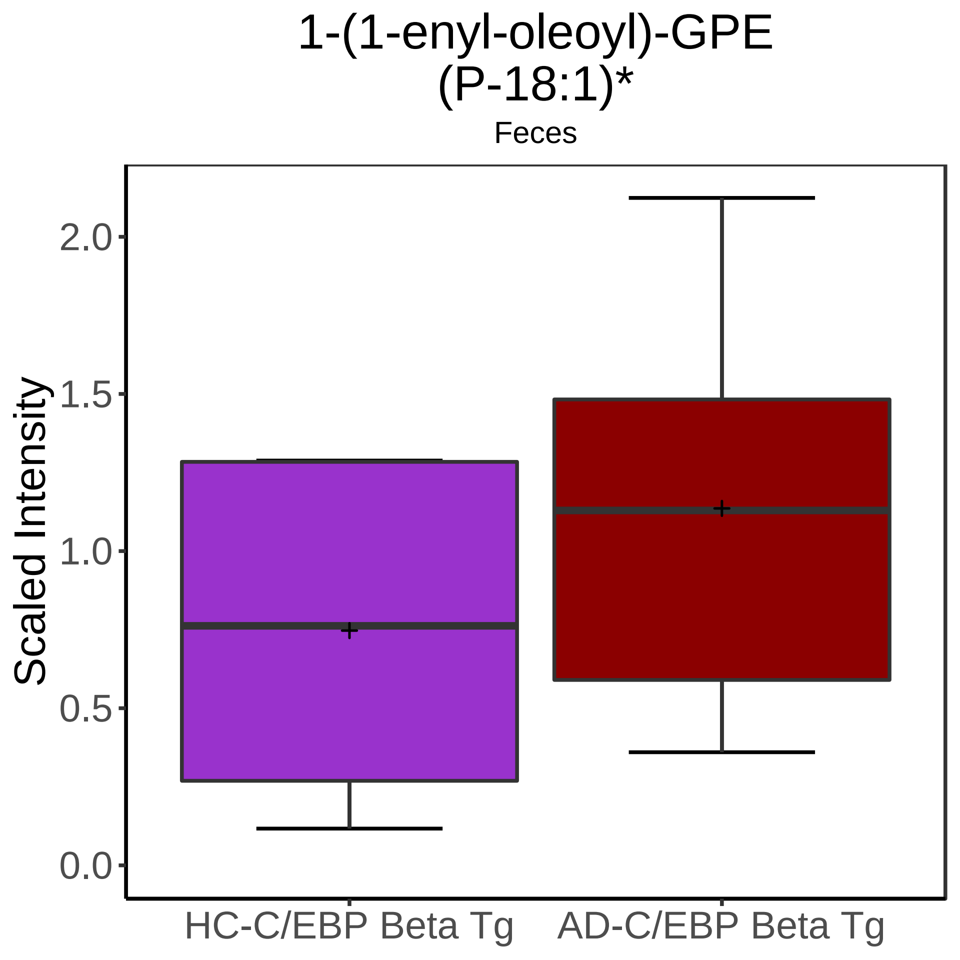

1-(1-enyl-oleoyl)-GPE
(P-18:1)*
Feces
Scaled Intensity
2.0
1.5
1.0
0.5
0.0
HC-C/EBP Beta Tg
AD-C/EBP Beta Tg

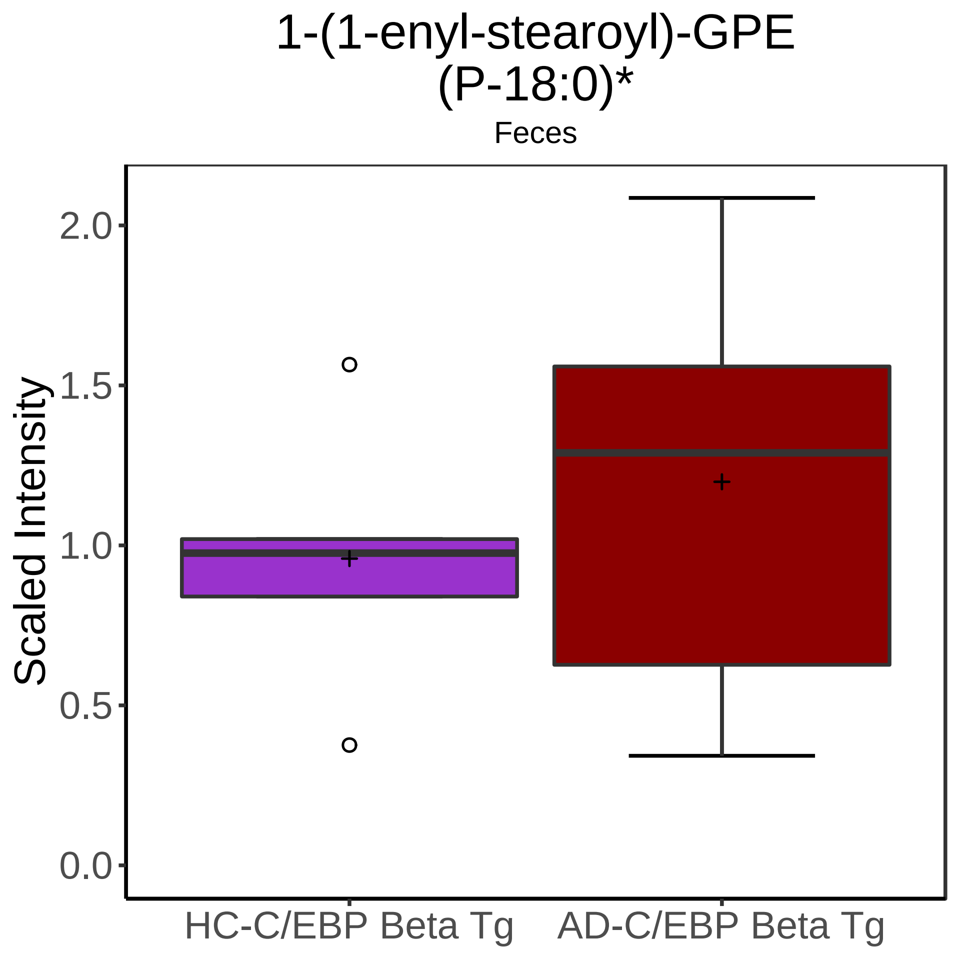
1-(1-enyl-stearoyl)-GPE
(P-18:0)*
Feces
Scaled Intensity
2.0
1.5
1.0
0.5
0.0
HC-C/EBP Beta Tg
AD-C/EBP Beta Tg

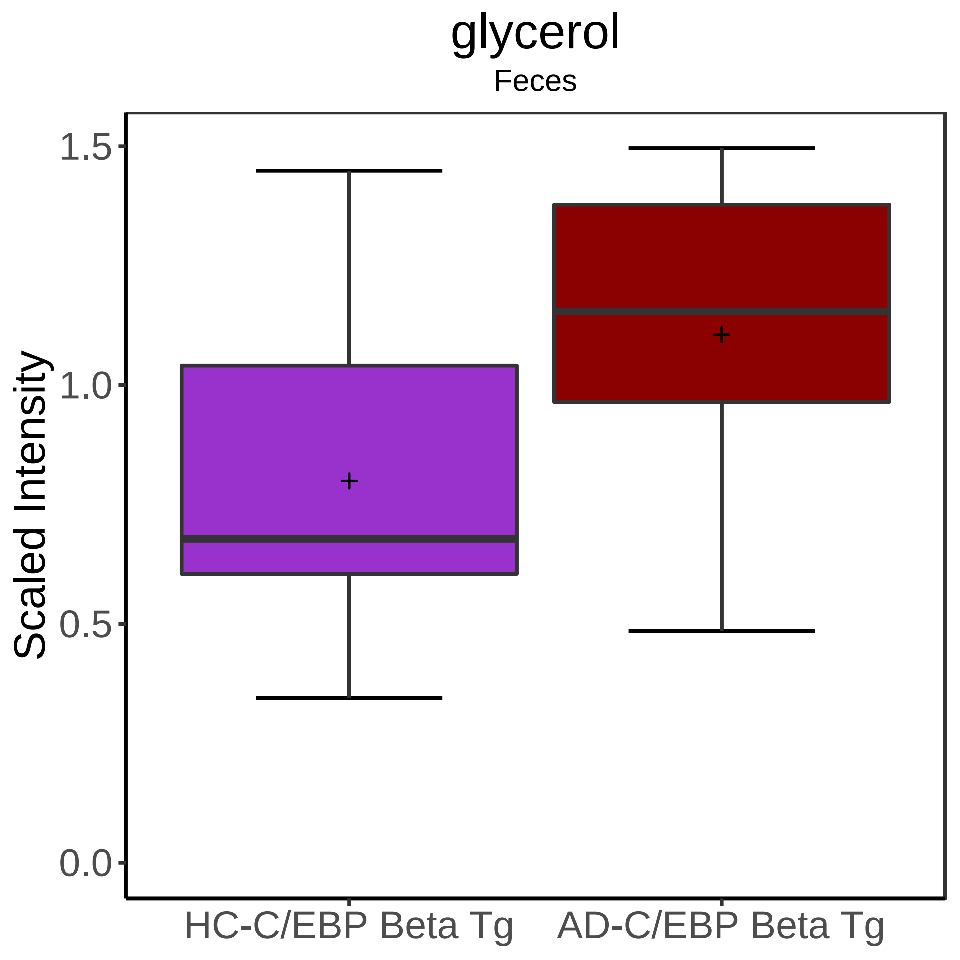
glycerol
Feces
Scaled Intensity
1.5
1.0
0.5
0.0
HC-C/EBP Beta Tg
AD-C/EBP Beta Tg

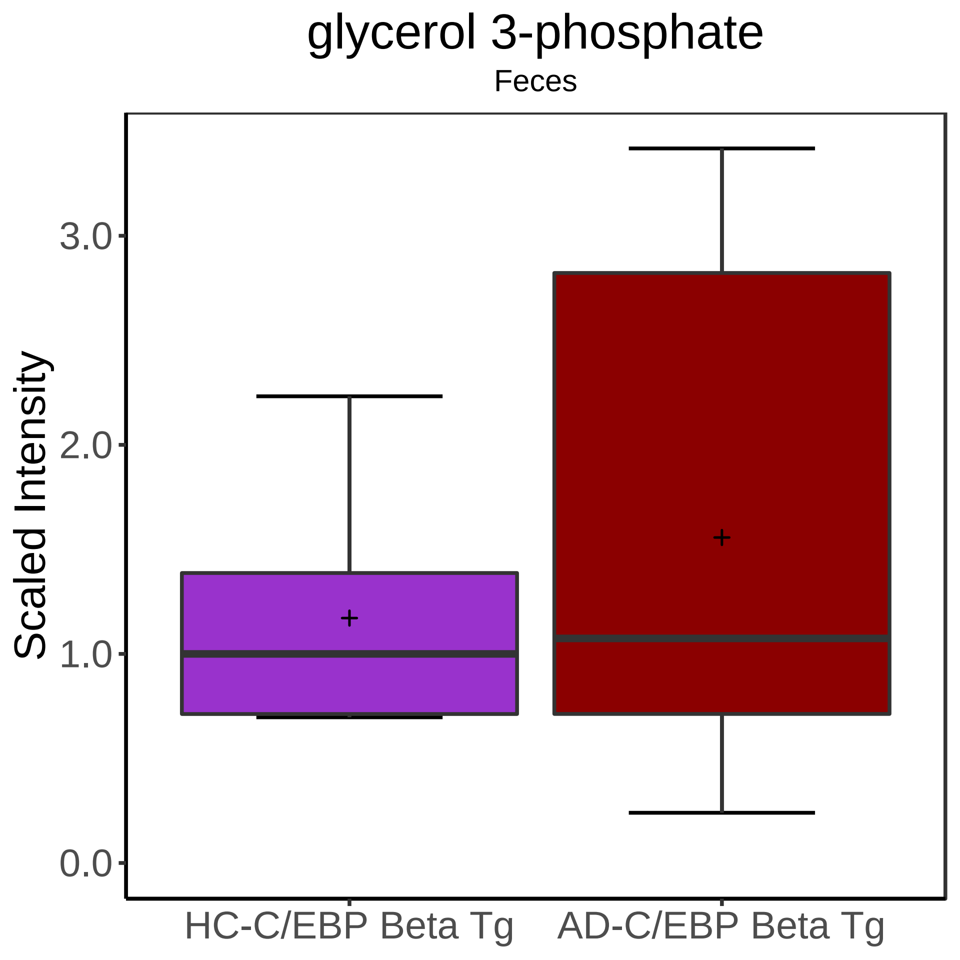

glycerol 3-phosphate
Feces
Scaled Intensity
3.0
2.0
1.0
0.0
HC-C/EBP Beta Tg
AD-C/EBP Beta Tg

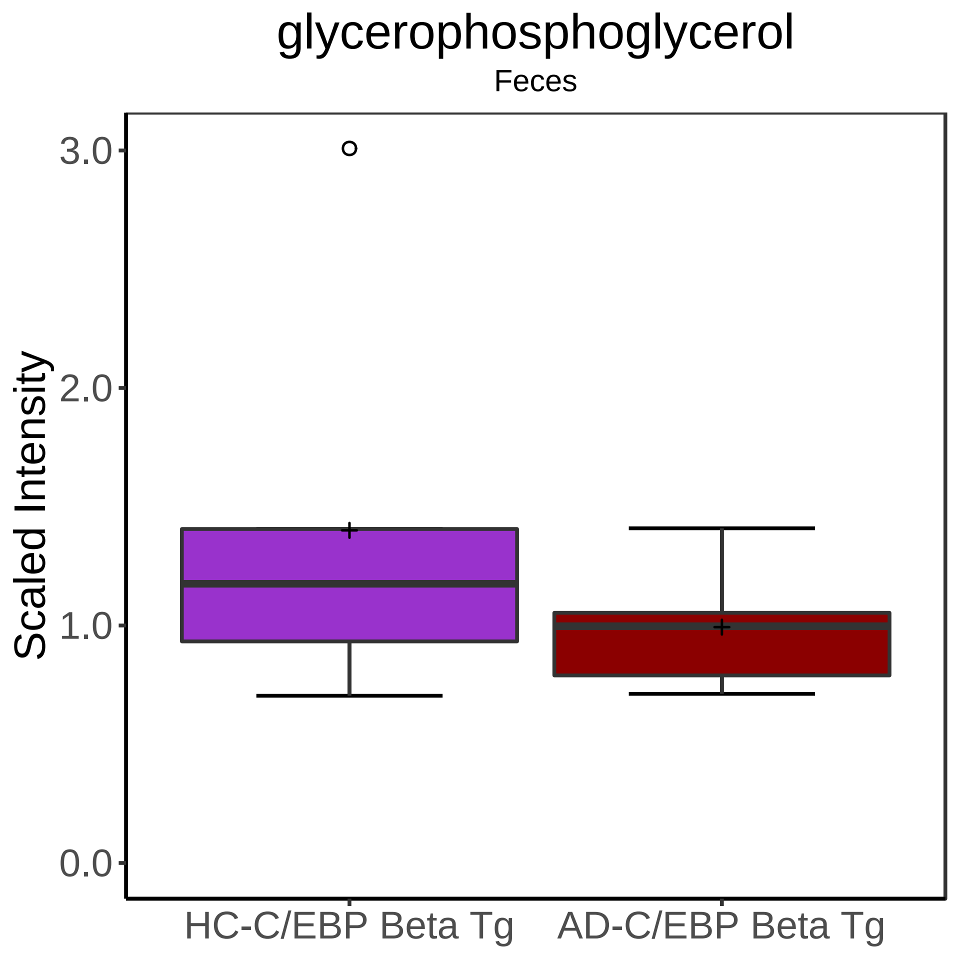
glycerophosphoglycerol
Feces
Scaled Intensity
3.0
2.0
1.0
0.0
HC-C/EBP Beta Tg
AD-C/EBP Beta Tg

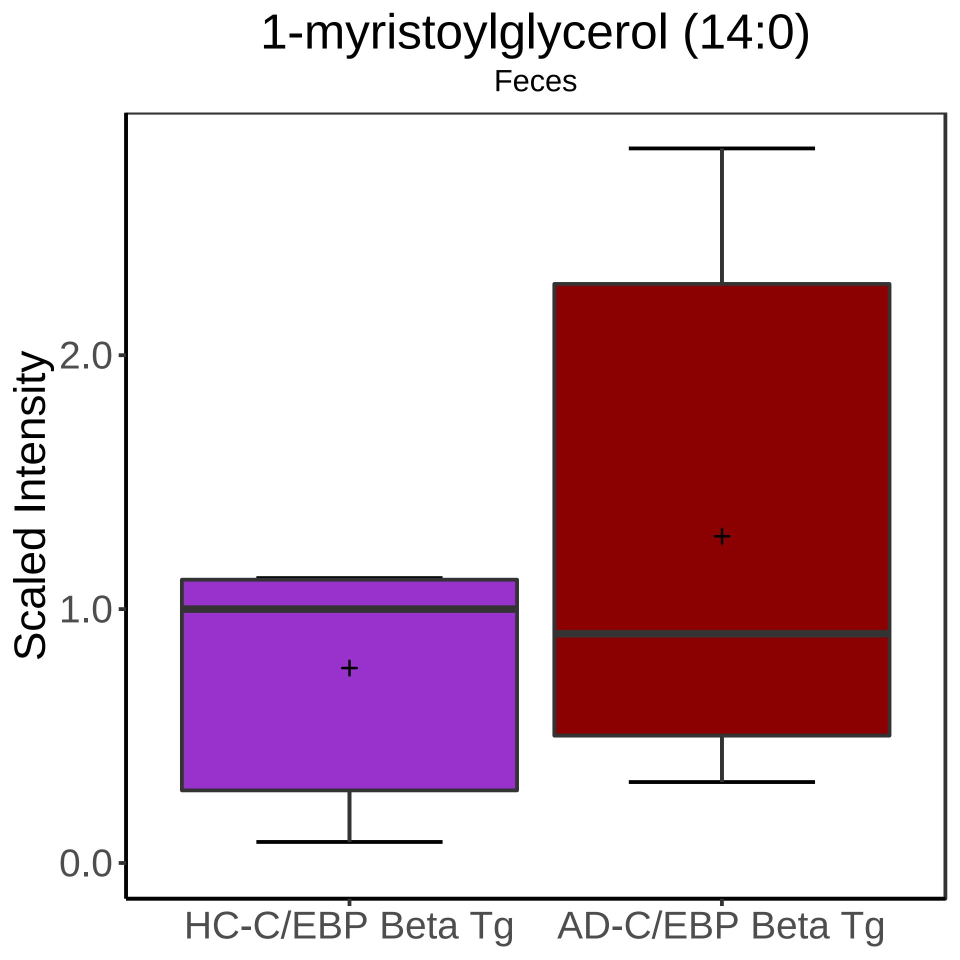

1-myristoylglycerol (14:0)
Feces
Scaled Intensity
2.0
1.0
0.0
HC-C/EBP Beta Tg
AD-C/EBP Beta Tg

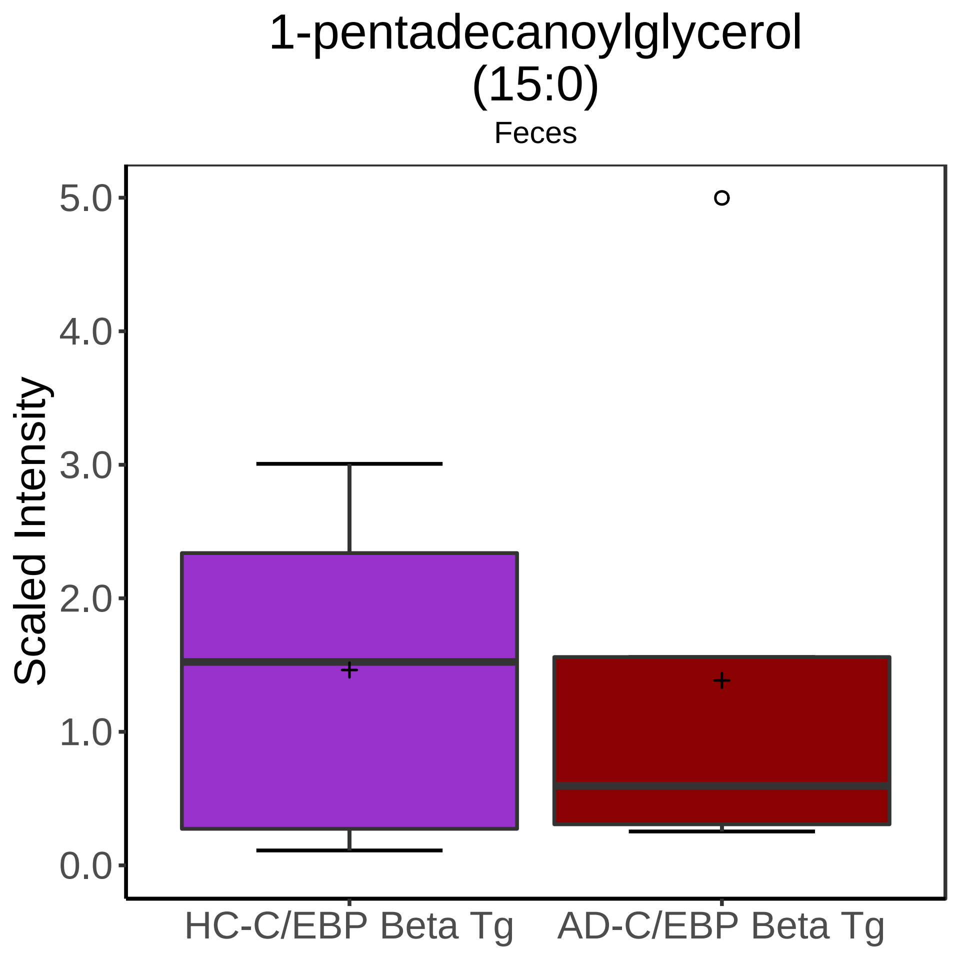
1-pentadecanoylglycerol
(15:0)
Feces
Scaled Intensity
5.0
4.0
3.0
2.0
1.0
0.0
HC-C/EBP Beta Tg
AD-C/EBP Beta Tg

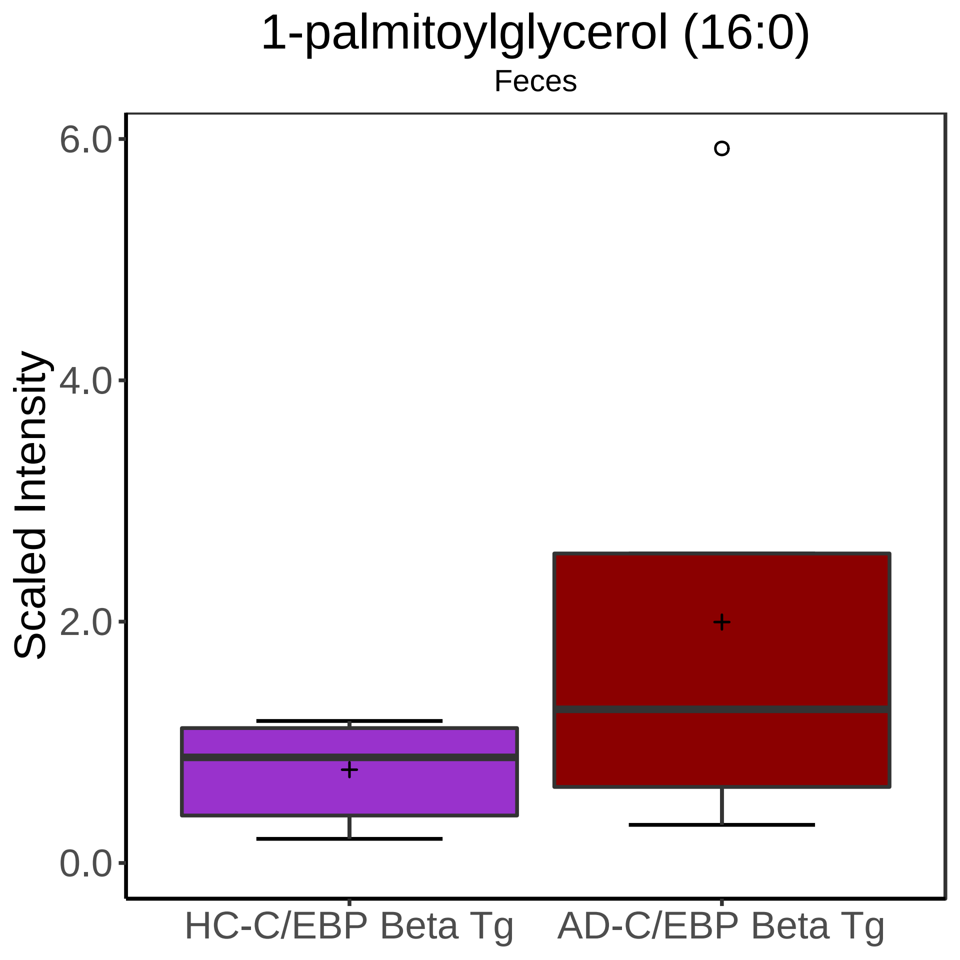
1-palmitoylglycerol (16:0)
Feces
Scaled Intensity
6.0
4.0
2.0
0.0
HC-C/EBP Beta Tg
AD-C/EBP Beta Tg

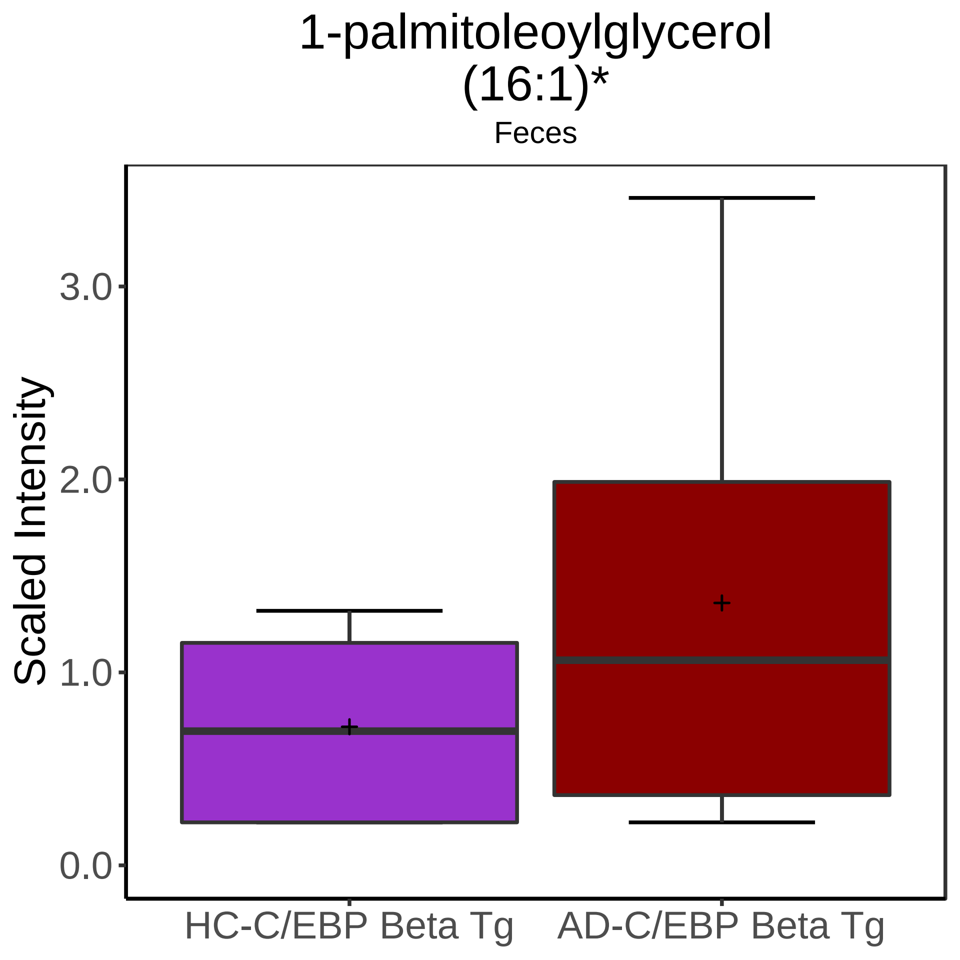
1-palmitoleoylglycerol
(16:1)*
Feces
Scaled Intensity
3.0
2.0
1.0
0.0
HC-C/EBP Beta Tg
AD-C/EBP Beta Tg

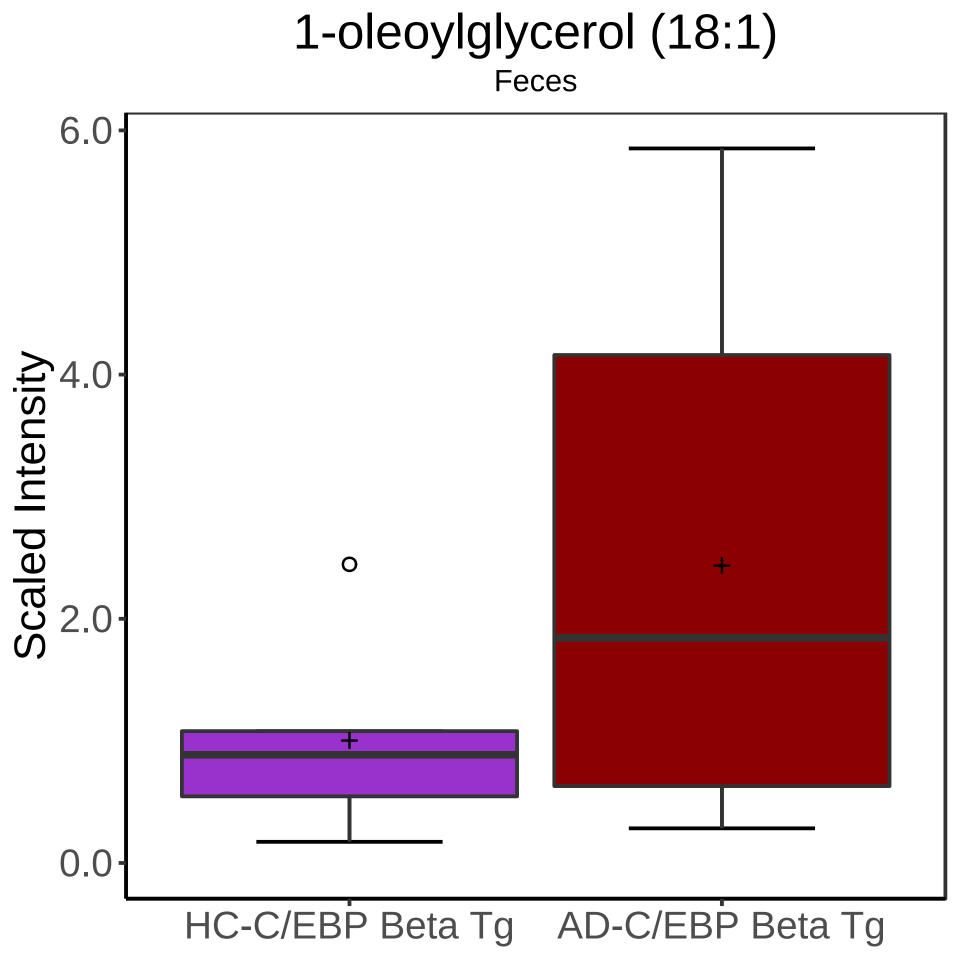

1-oleoylglycerol (18:1)
Feces
Scaled Intensity
6.0
4.0
2.0
0.0
HC-C/EBP Beta Tg
AD-C/EBP Beta Tg

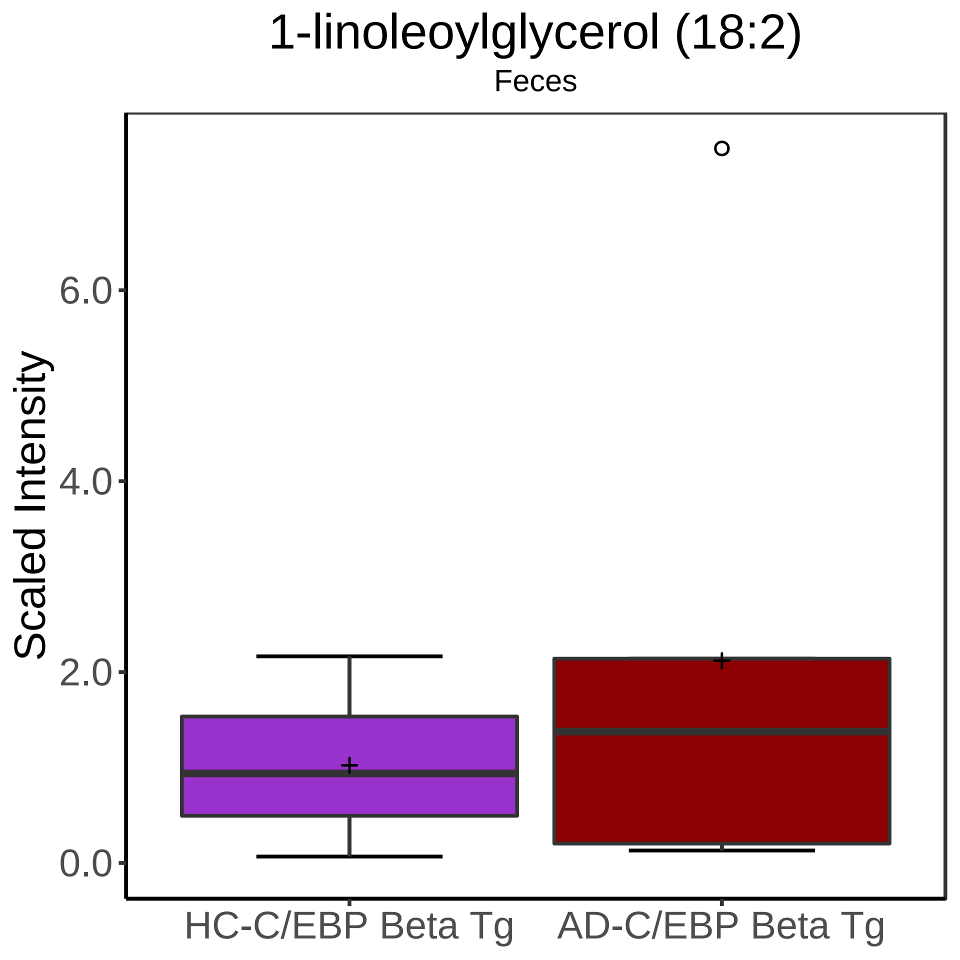

1-linoleoylglycerol (18:2)
Feces
Scaled Intensity
6.0
4.0
2.0
0.0
HC-C/EBP Beta Tg
AD-C/EBP Beta Tg

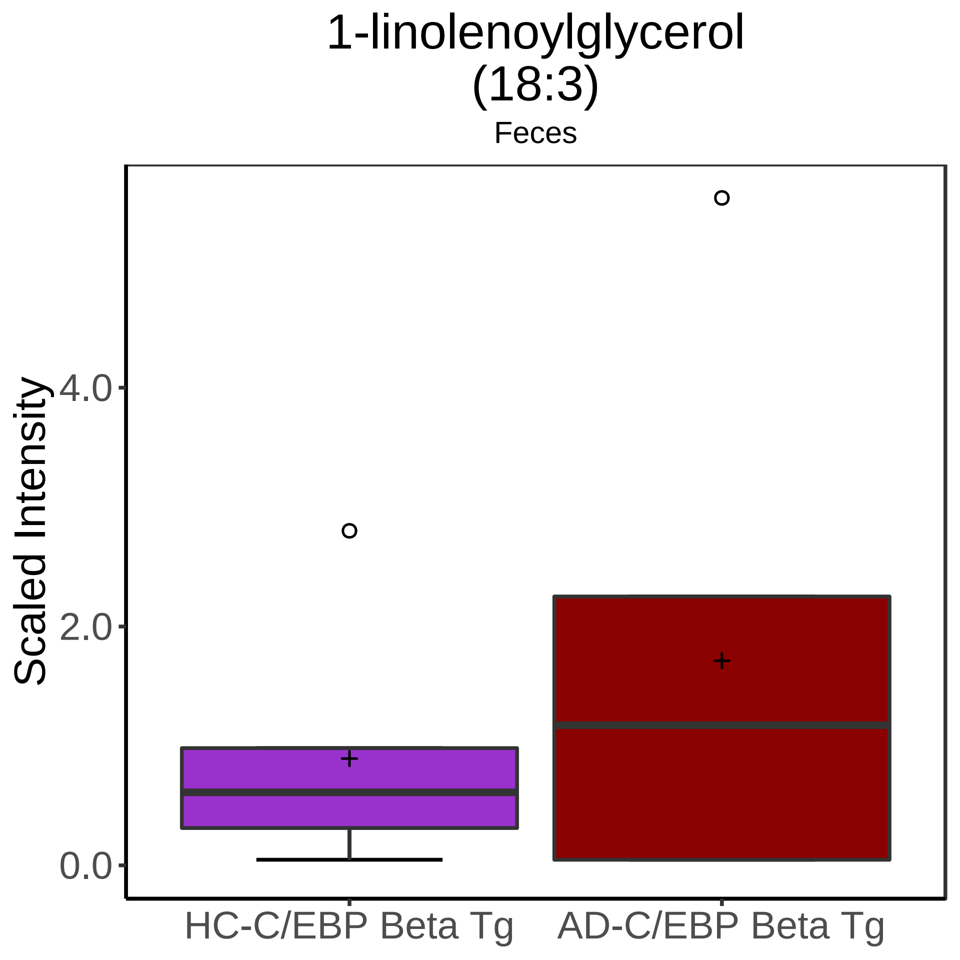
1-linolenoylglycerol
(18:3)
Feces
Scaled Intensity
4.0
2.0
0.0
HC-C/EBP Beta Tg
AD-C/EBP Beta Tg

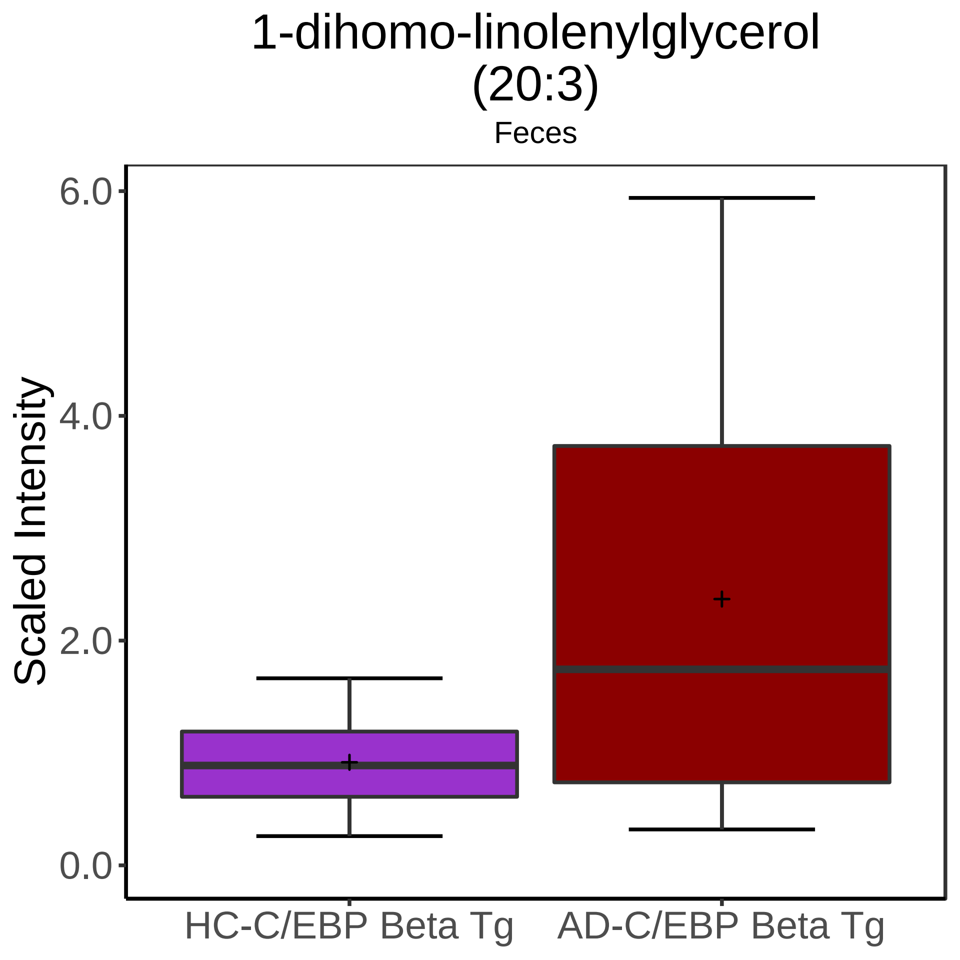
1-dihomo-linolenylglycerol
(20:3)
Feces
Scaled Intensity
6.0
4.0
2.0
0.0
HC-C/EBP Beta Tg
AD-C/EBP Beta Tg

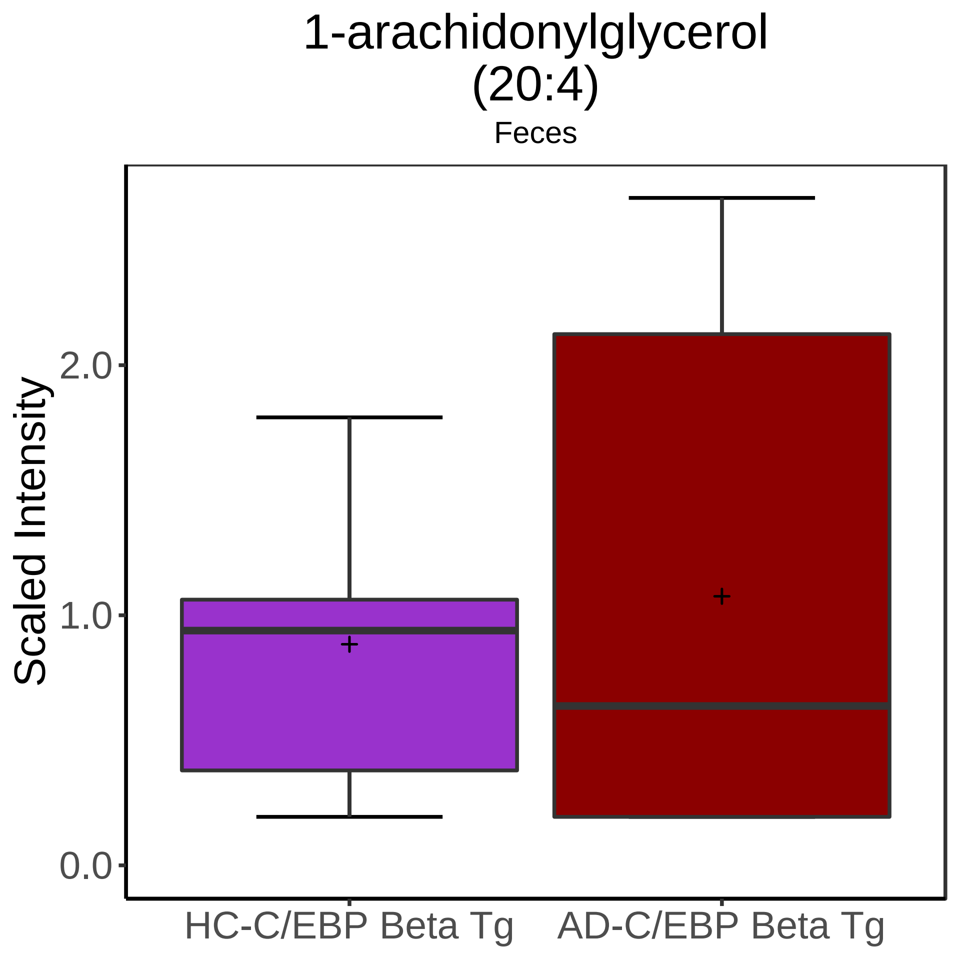
1-arachidonylglycerol
(20:4)
Feces
Scaled Intensity
2.0
1.0
0.0
HC-C/EBP Beta Tg
AD-C/EBP Beta Tg

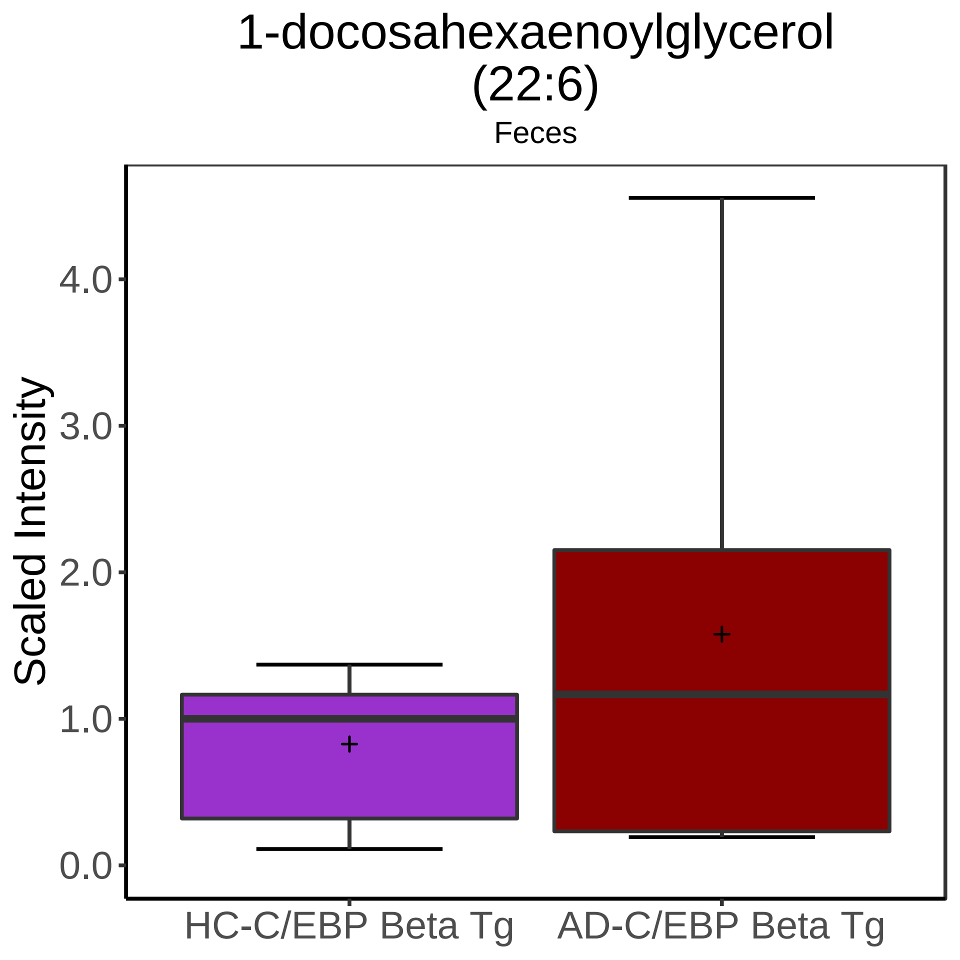

1-docosahexaenoylglycerol (22:6)
Feces
Scaled Intensity
4.0
3.0
2.0
1.0
0.0
HC-C/EBP Beta Tg
AD-C/EBP Beta Tg

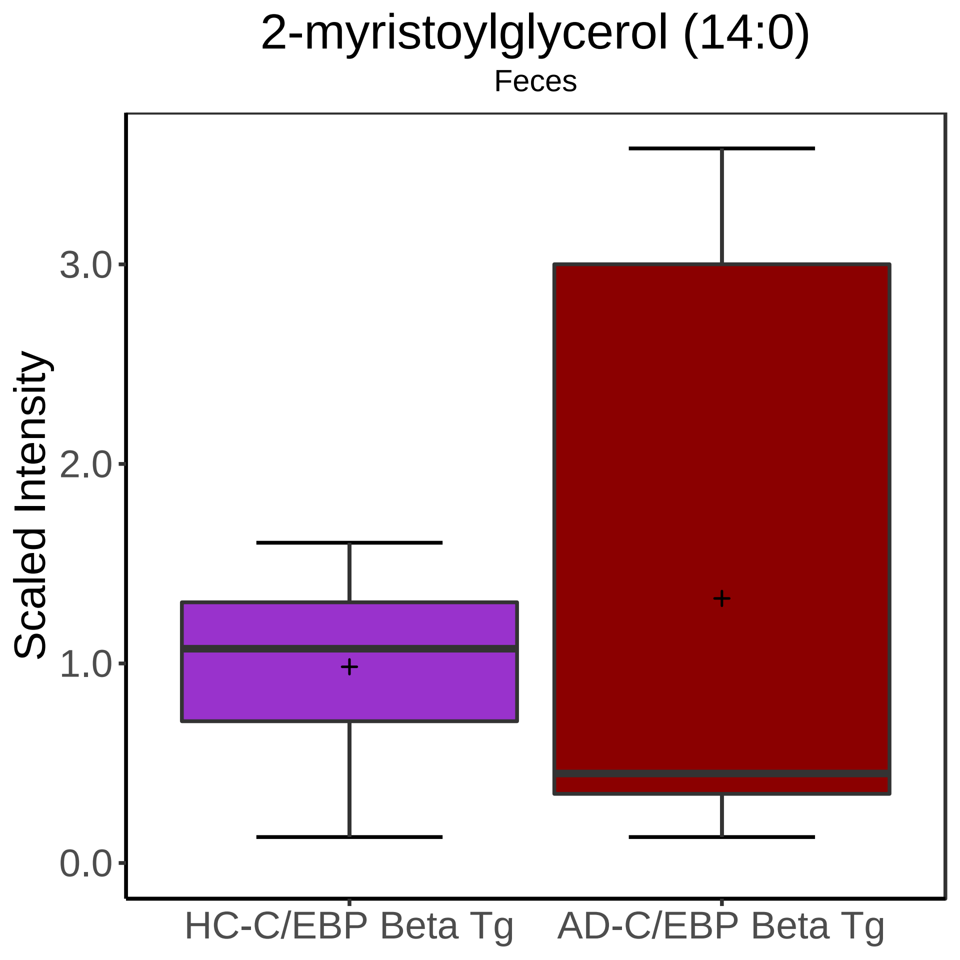
2-myristoylglycerol (14:0)
Feces
Scaled Intensity
3.0
2.0
1.0
0.0
HC-C/EBP Beta Tg
AD-C/EBP Beta Tg

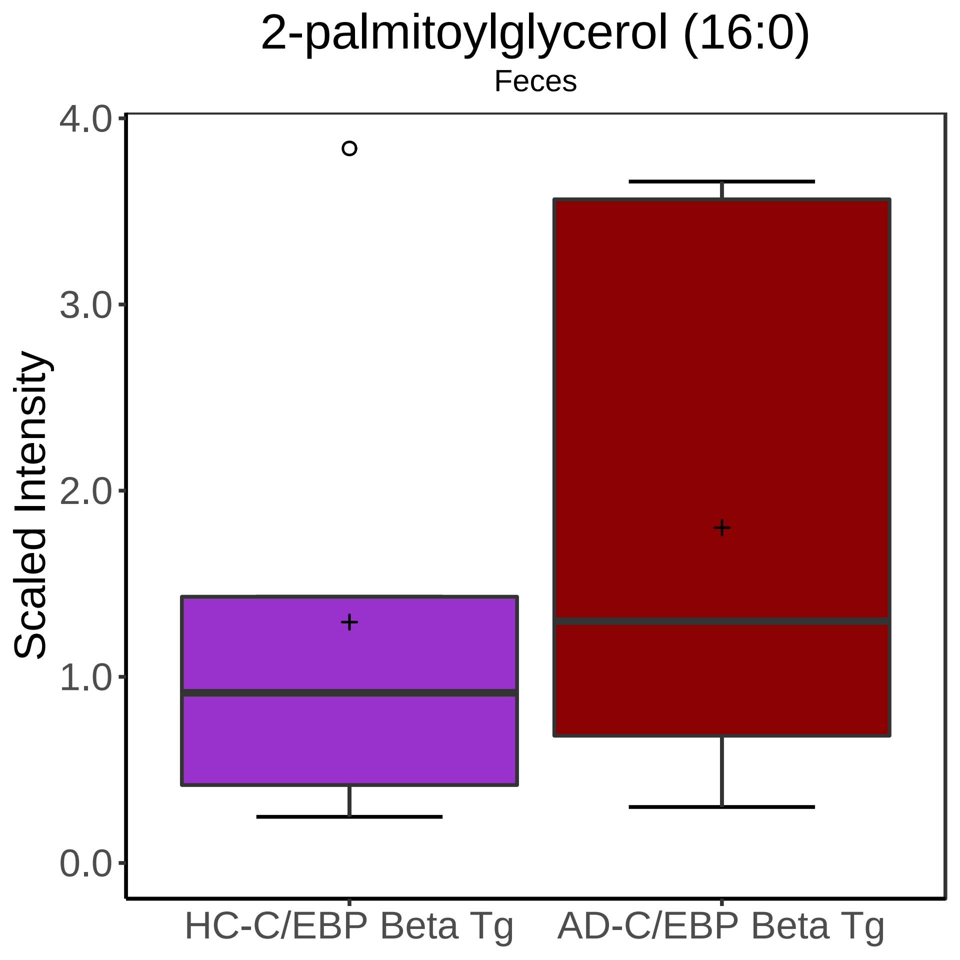

2-palmitoylglycerol (16:0)
Feces
Scaled Intensity
4.0
3.0
2.0
1.0
0.0
HC-C/EBP Beta Tg
AD-C/EBP Beta Tg

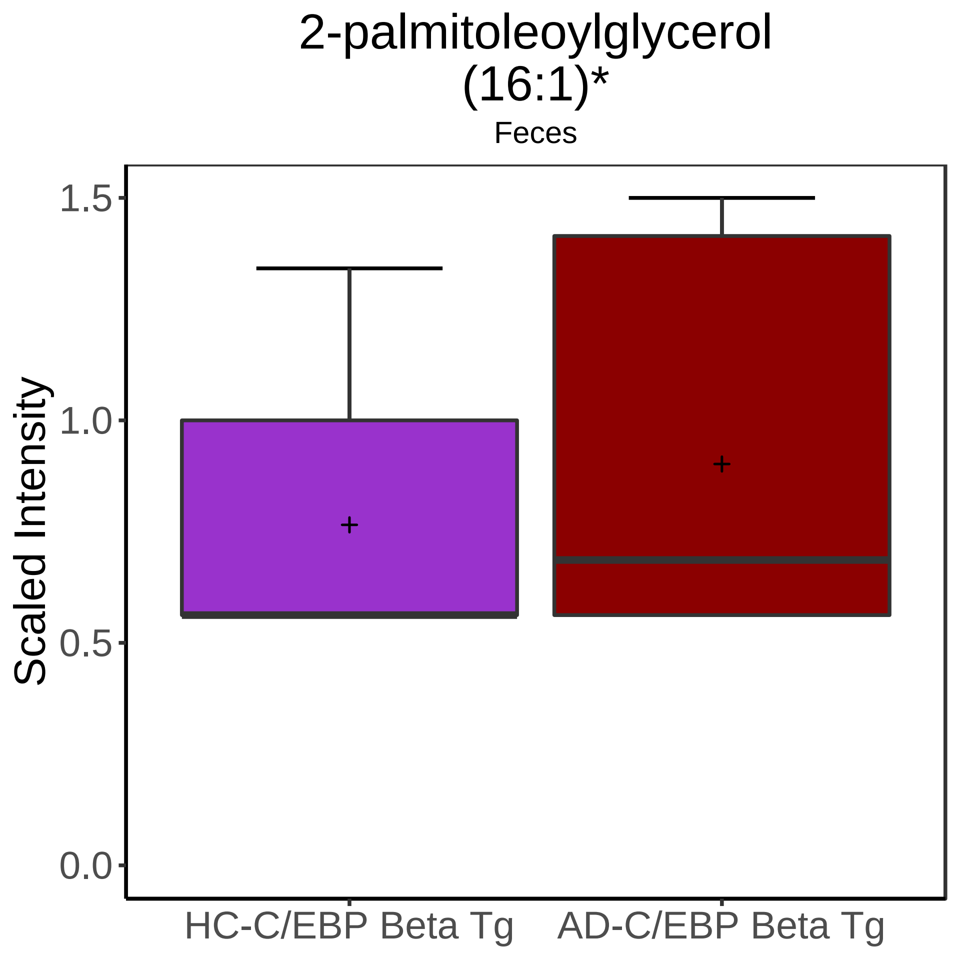
2-palmitoleoylglycerol
(16:1)*
Feces
Scaled Intensity
1.5
1.0
0.5
0.0
HC-C/EBP Beta Tg
AD-C/EBP Beta Tg

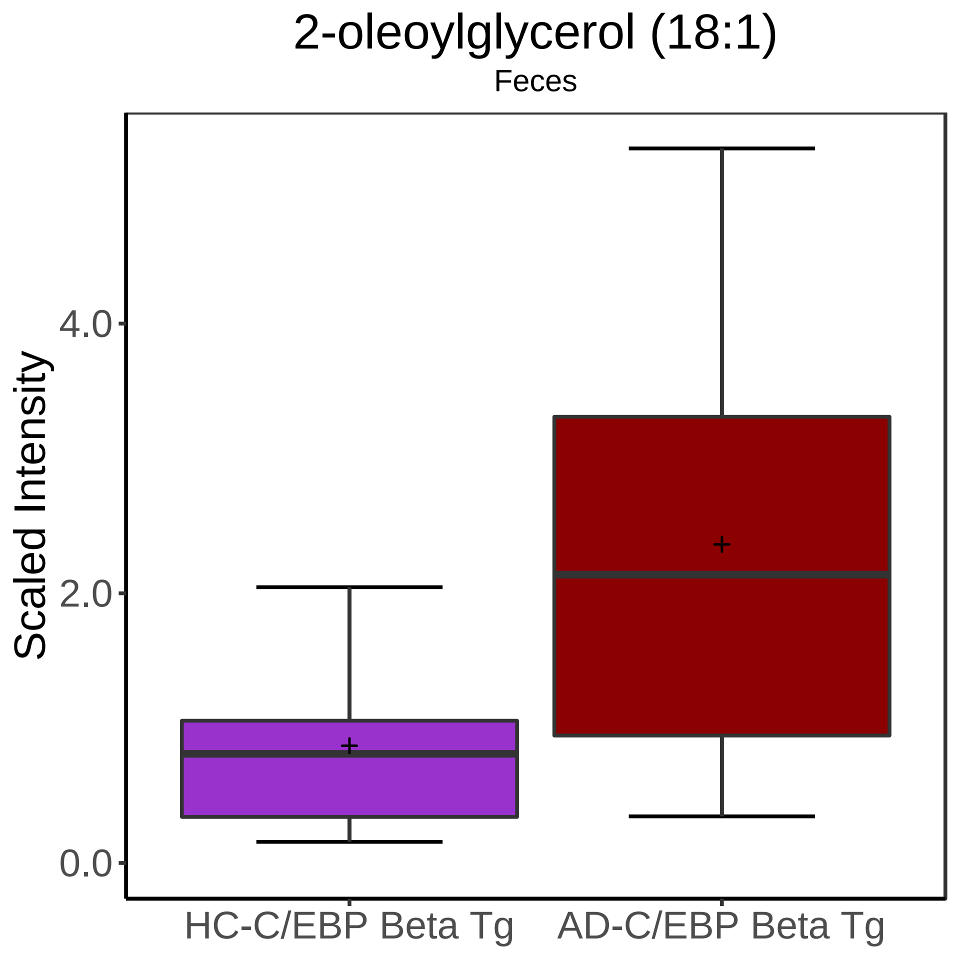

2-oleoylglycerol (18:1)
Feces
Scaled Intensity
4.0
2.0
0.0
HC-C/EBP Beta Tg
AD-C/EBP Beta Tg

# 2-linoleoylglycerol (18:2)

Feces

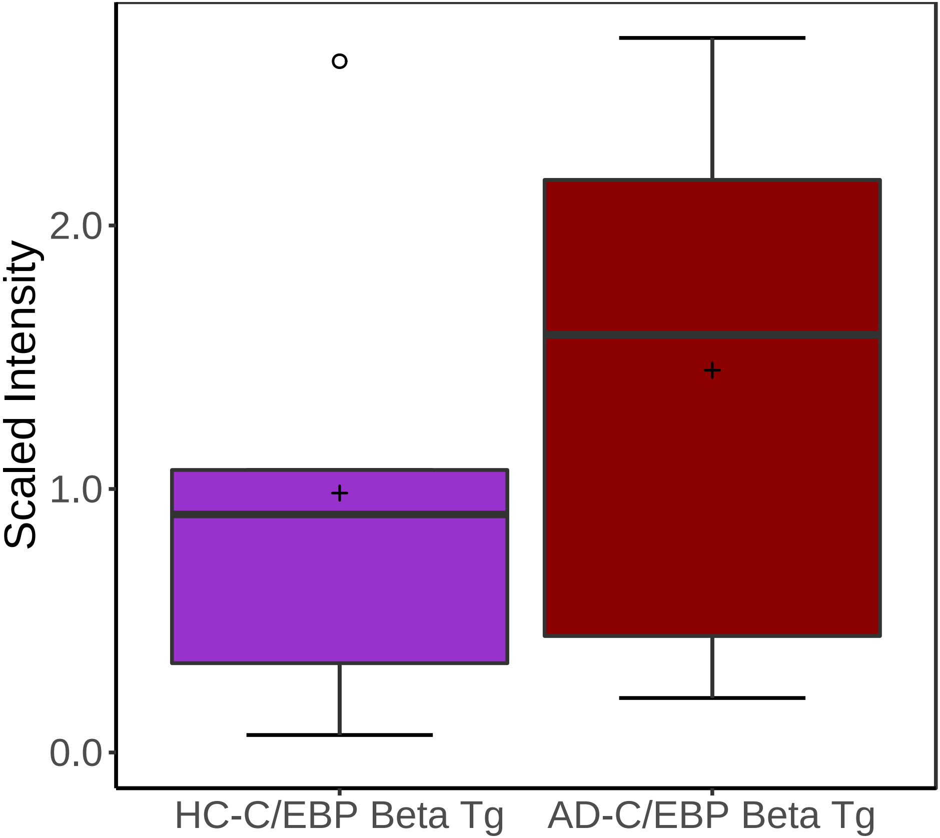

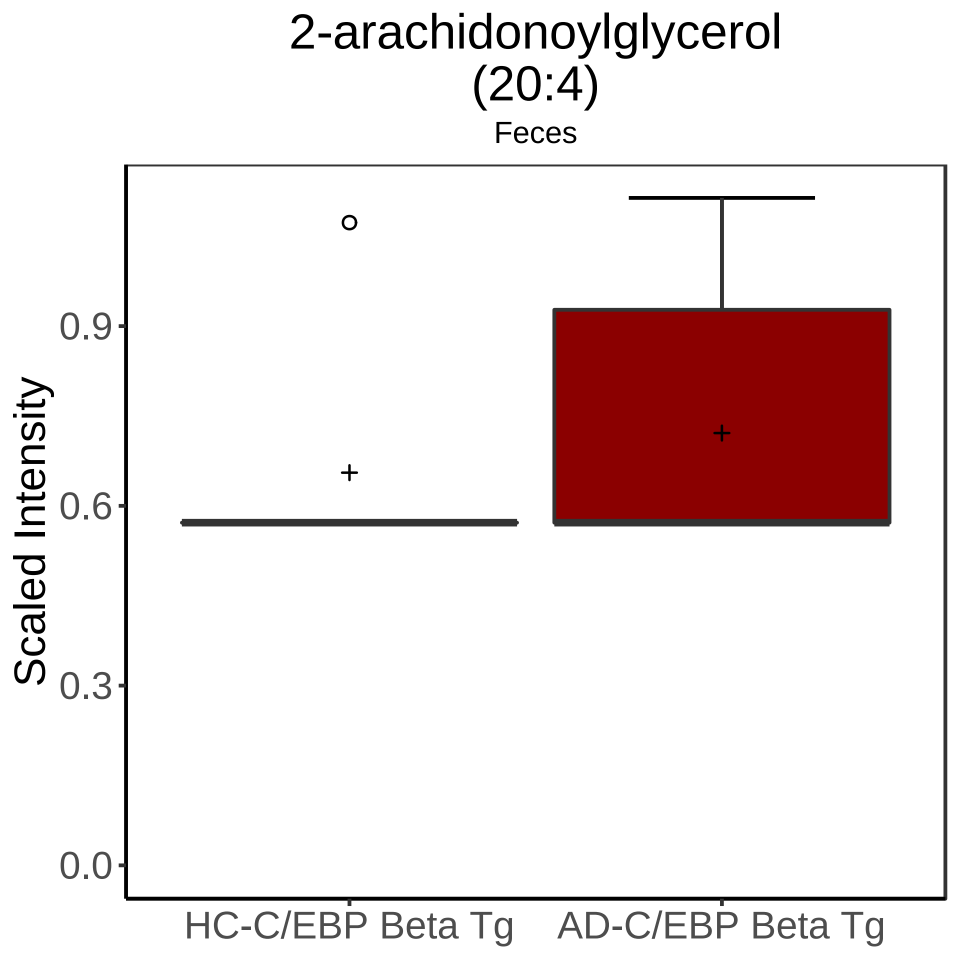

2-arachidonoylglycerol
(20:4)
Feces
Scaled Intensity
0.0
0.3
0.6
0.9
HC-C/EBP Beta Tg
AD-C/EBP Beta Tg

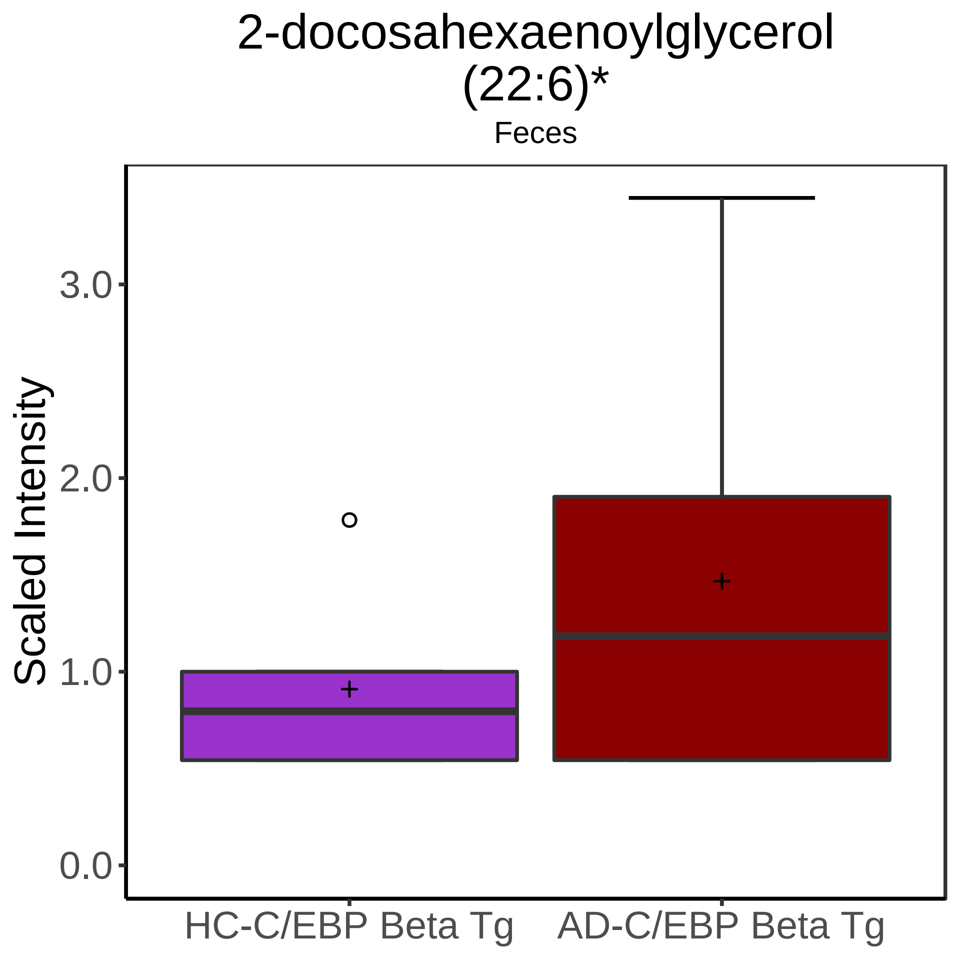
2-docosahexaenoylglycerol (22:6)*
Feces
Scaled Intensity
0.0
1.0
2.0
3.0
HC-C/EBP Beta Tg
AD-C/EBP Beta Tg

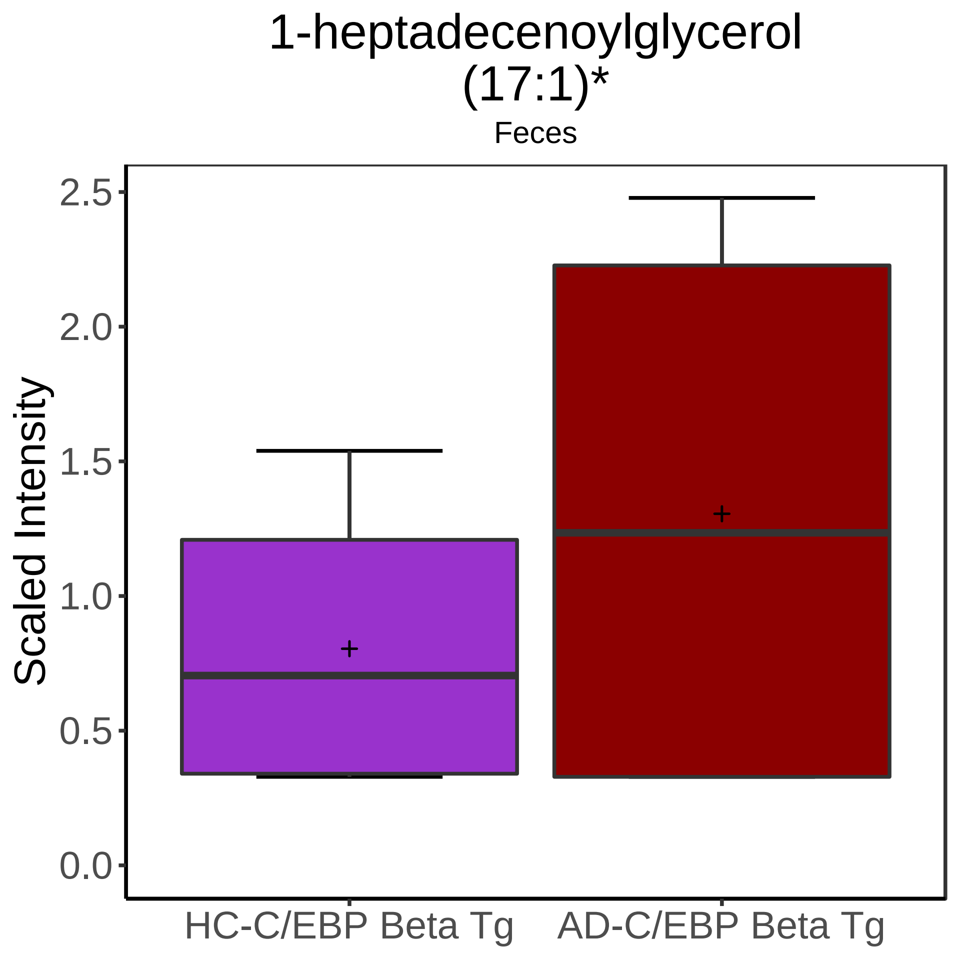
1-heptadecenoylglycerol (17:1)*
Feces
Scaled Intensity
2.5
2.0
1.5
1.0
0.5
0.0
HC-C/EBP Beta Tg
AD-C/EBP Beta Tg

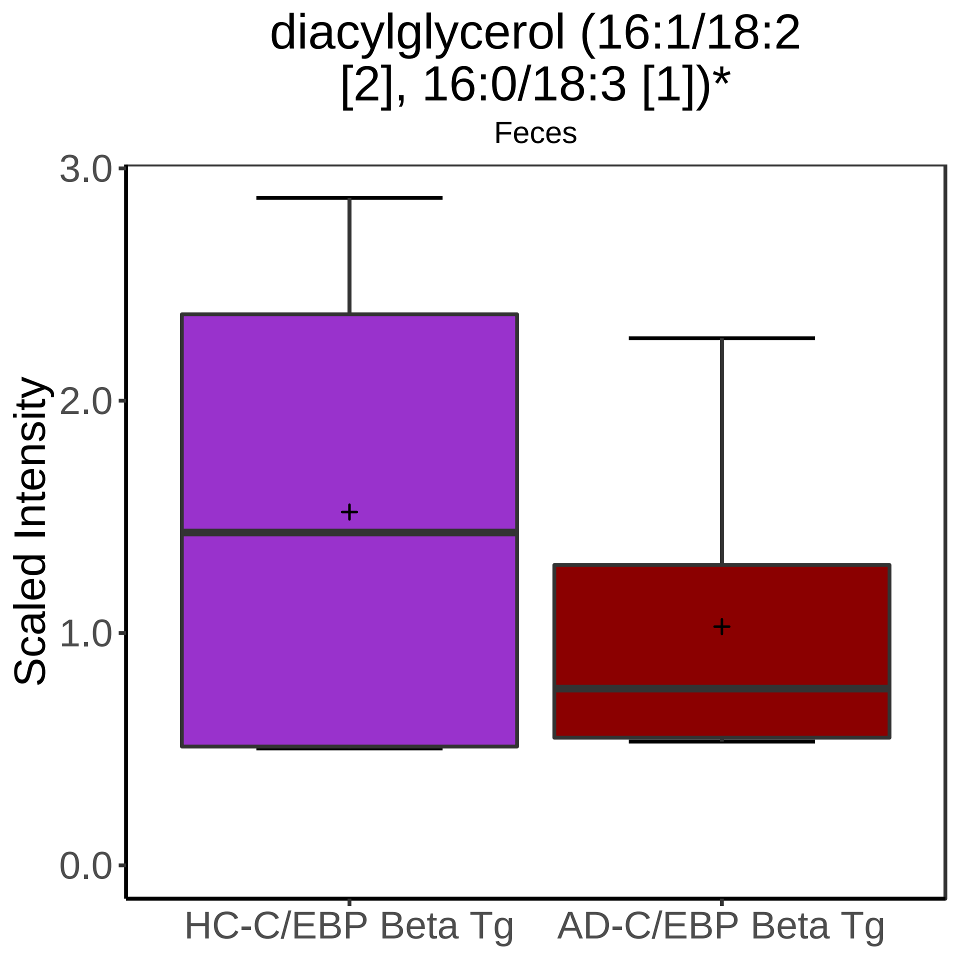

diacylglycerol (16:1/18:2
[2], 16:0/18:3 [1])*
Feces
Scaled Intensity
3.0
2.0
1.0
0.0
HC-C/EBP Beta Tg
AD-C/EBP Beta Tg

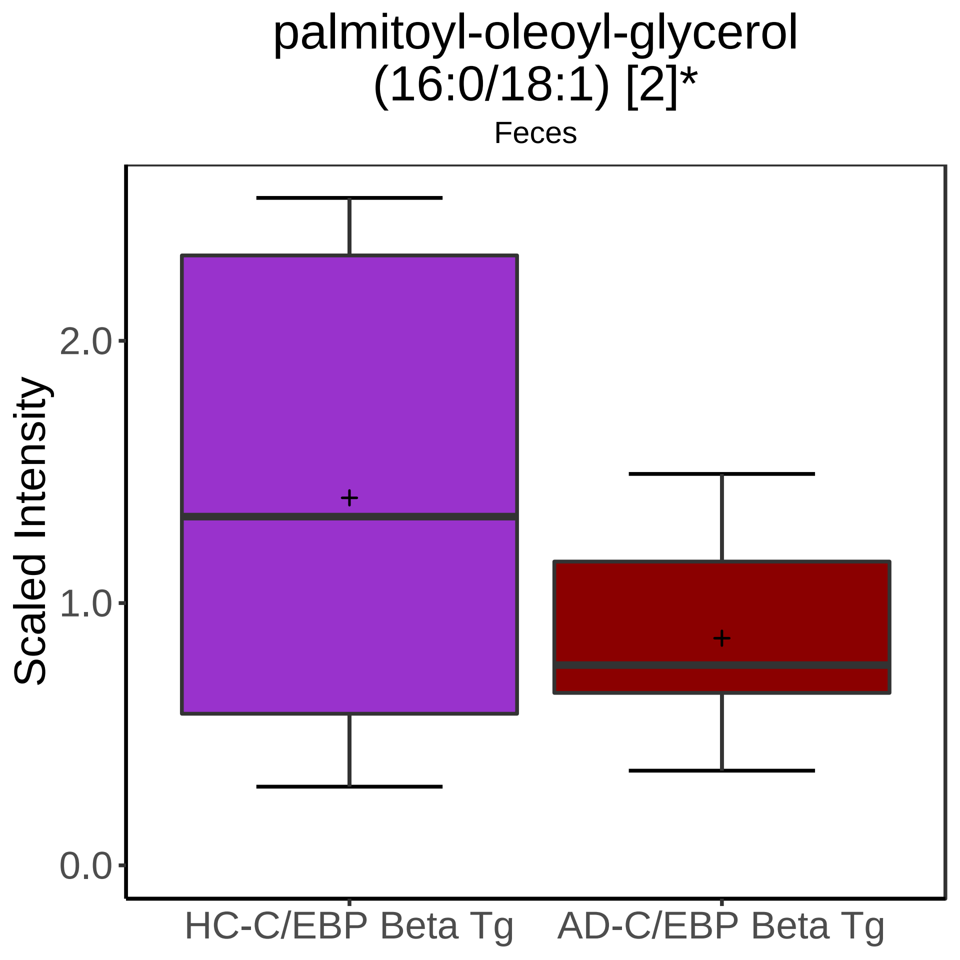
palmitoyl-oleoyl-glycerol
(16:0/18:1) [2]*
Feces
Scaled Intensity
2.0
1.0
0.0
HC-C/EBP Beta Tg
AD-C/EBP Beta Tg

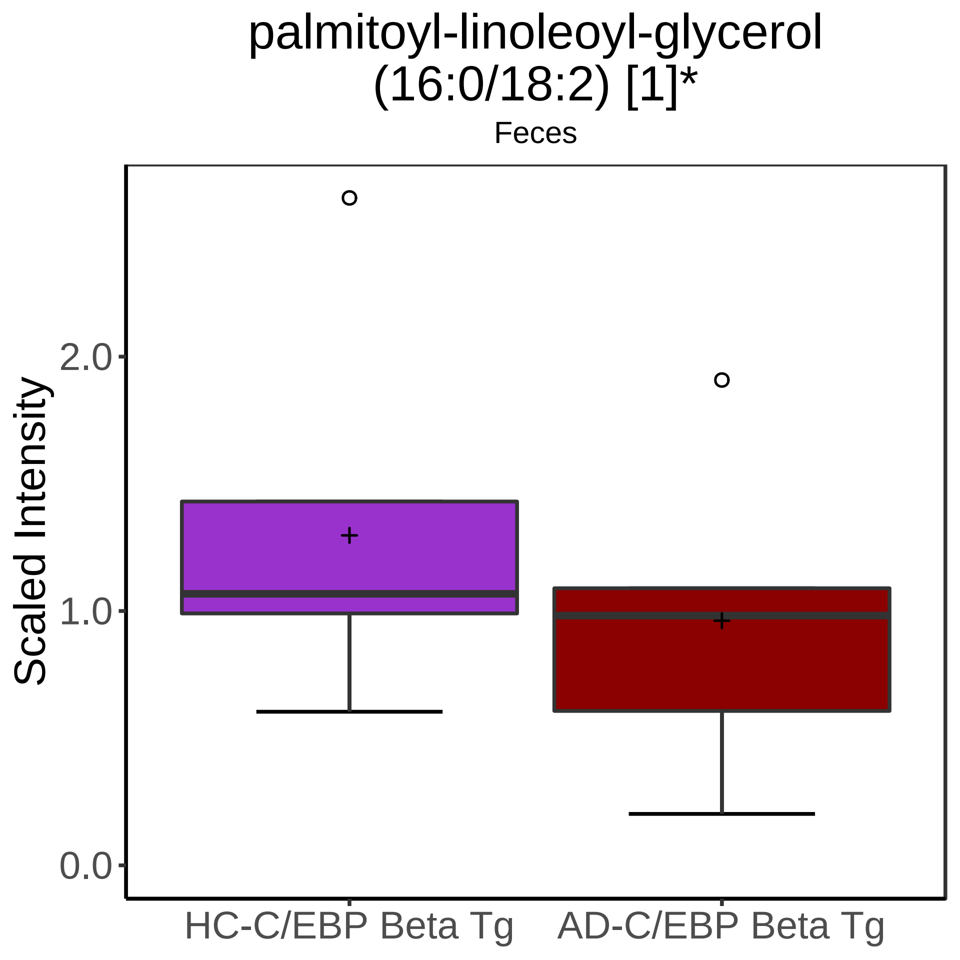
palmitoyl-linoleoyl-glycerol
(16:0/18:2) [1]*
Feces
Scaled Intensity
2.0
1.0
0.0
HC-C/EBP Beta Tg
AD-C/EBP Beta Tg

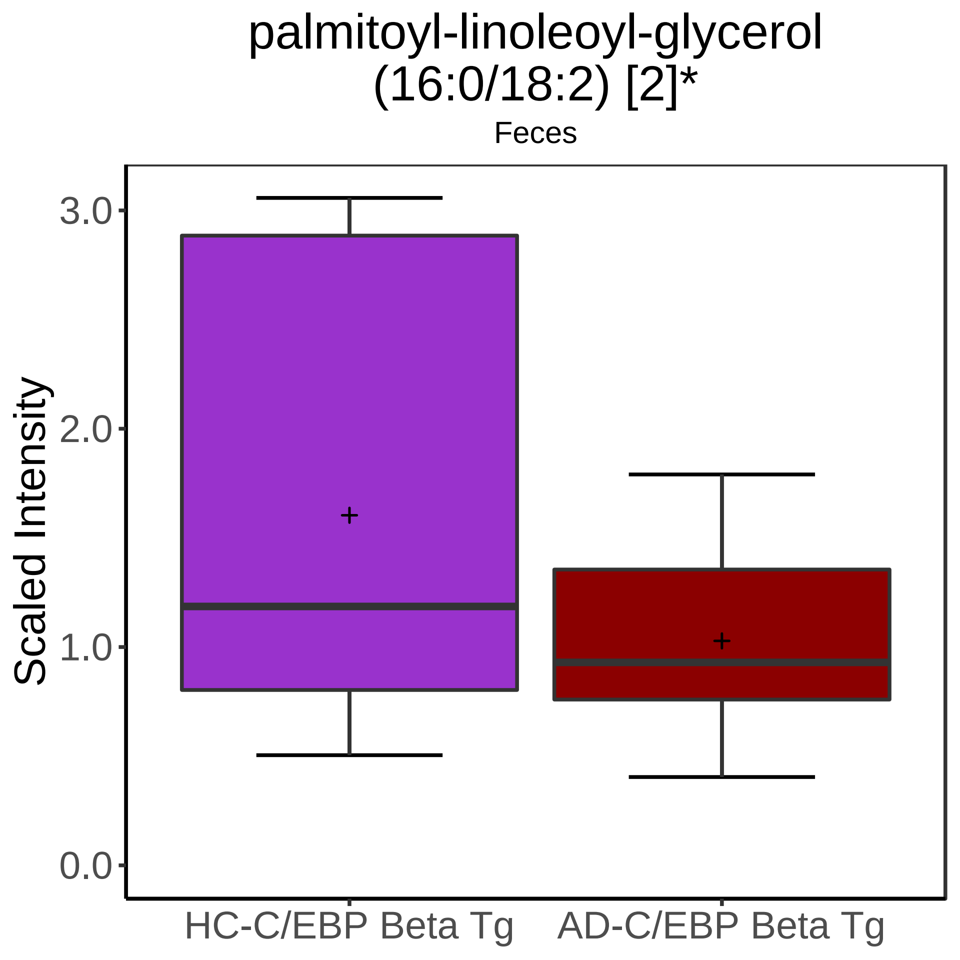
palmitoyl-linoleoyl-glycerol
(16:0/18:2) [2]*
Feces
Scaled Intensity
3.0
2.0
1.0
0.0
HC-C/EBP Beta Tg
AD-C/EBP Beta Tg

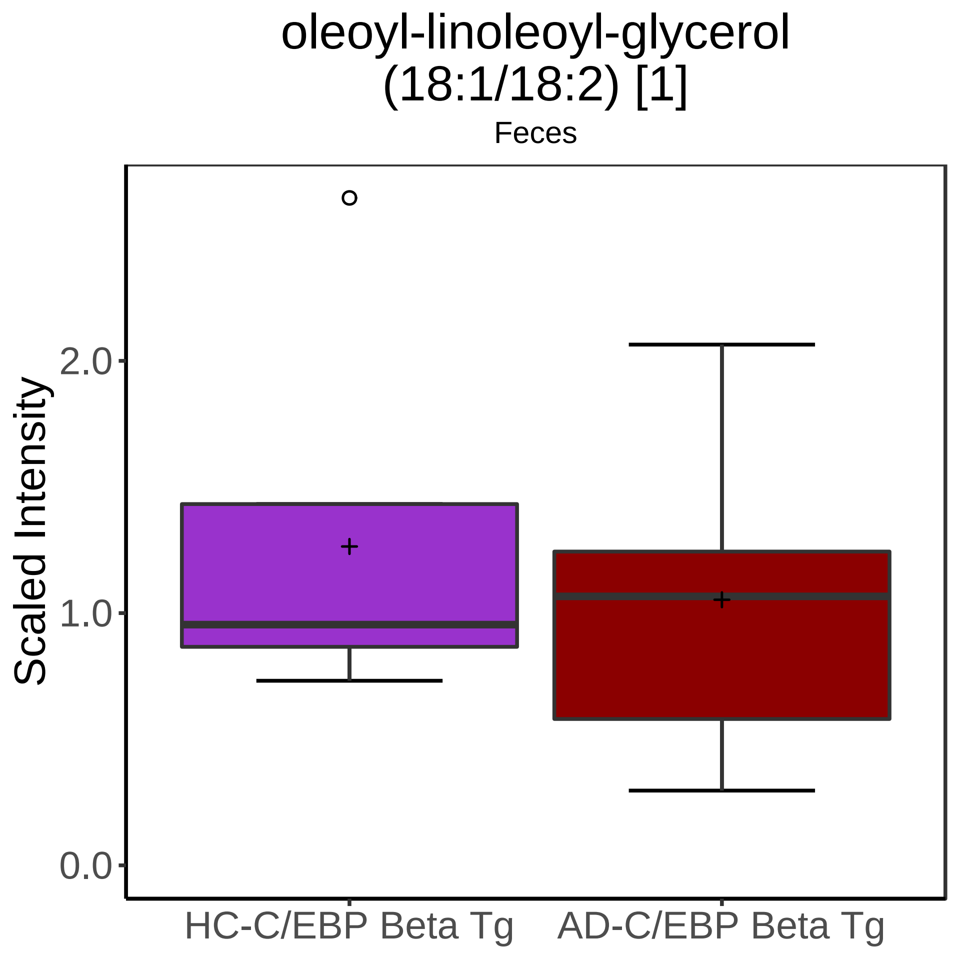

oleoyl-linoleoyl-glycerol
(18:1/18:2) [1]
Feces
Scaled Intensity
2.0
1.0
0.0
HC-C/EBP Beta Tg
AD-C/EBP Beta Tg

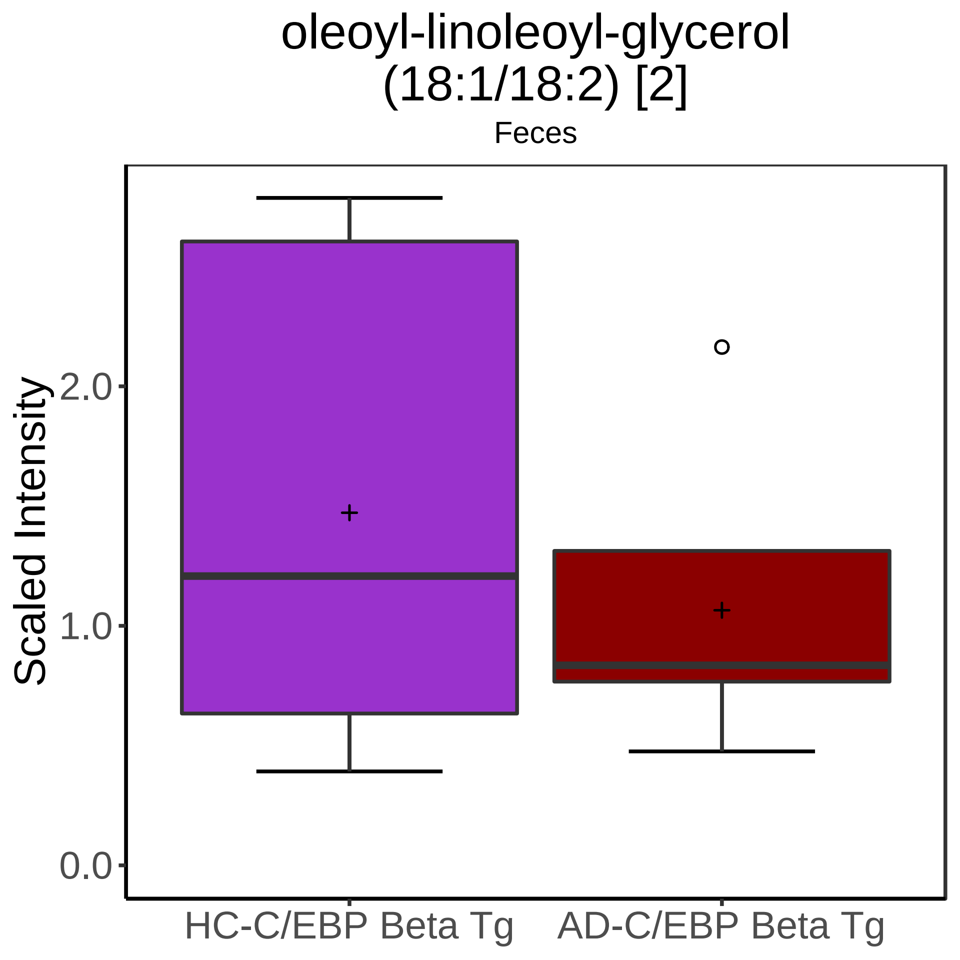

oleoyl-linoleoyl-glycerol
(18:1/18:2) [2]
Feces
Scaled Intensity
2.0
1.0
0.0
HC-C/EBP Beta Tg
AD-C/EBP Beta Tg

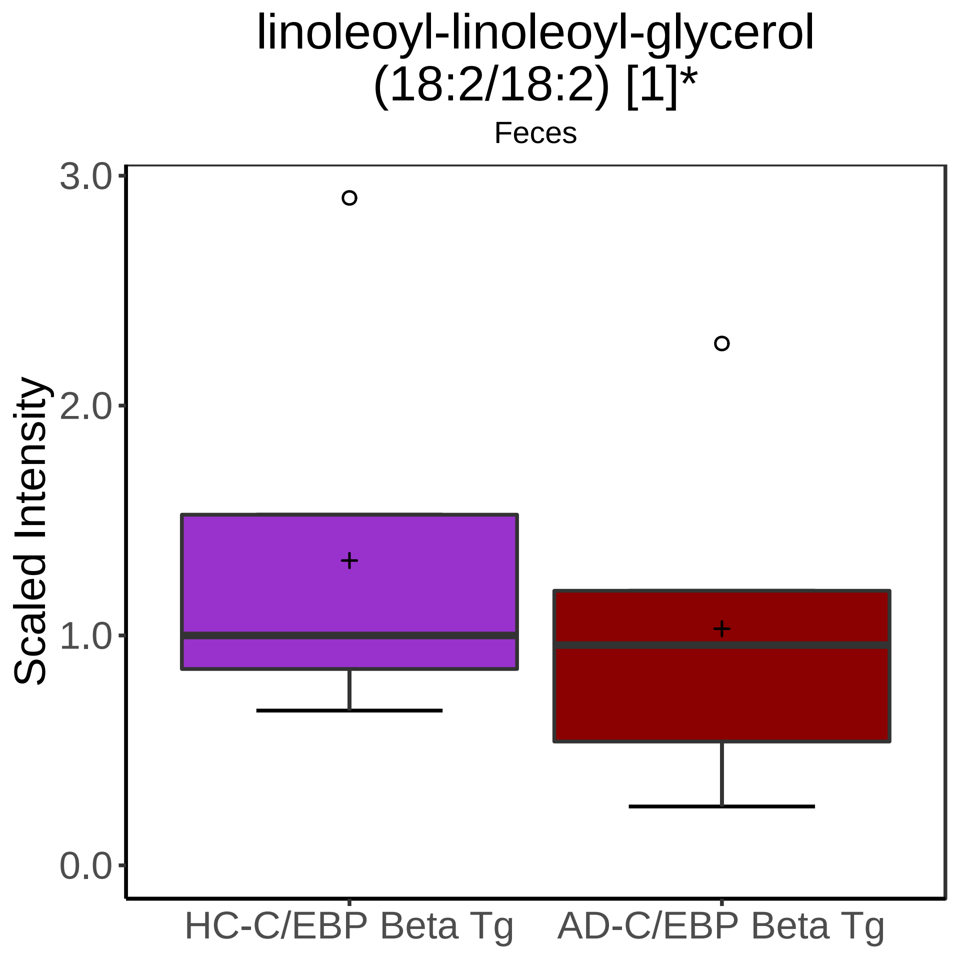
linoleoyl-linoleoyl-glycerol (18:2/18:2) [1]*
Feces
Scaled Intensity
3.0
2.0
1.0
0.0
HC-C/EBP Beta Tg
AD-C/EBP Beta Tg

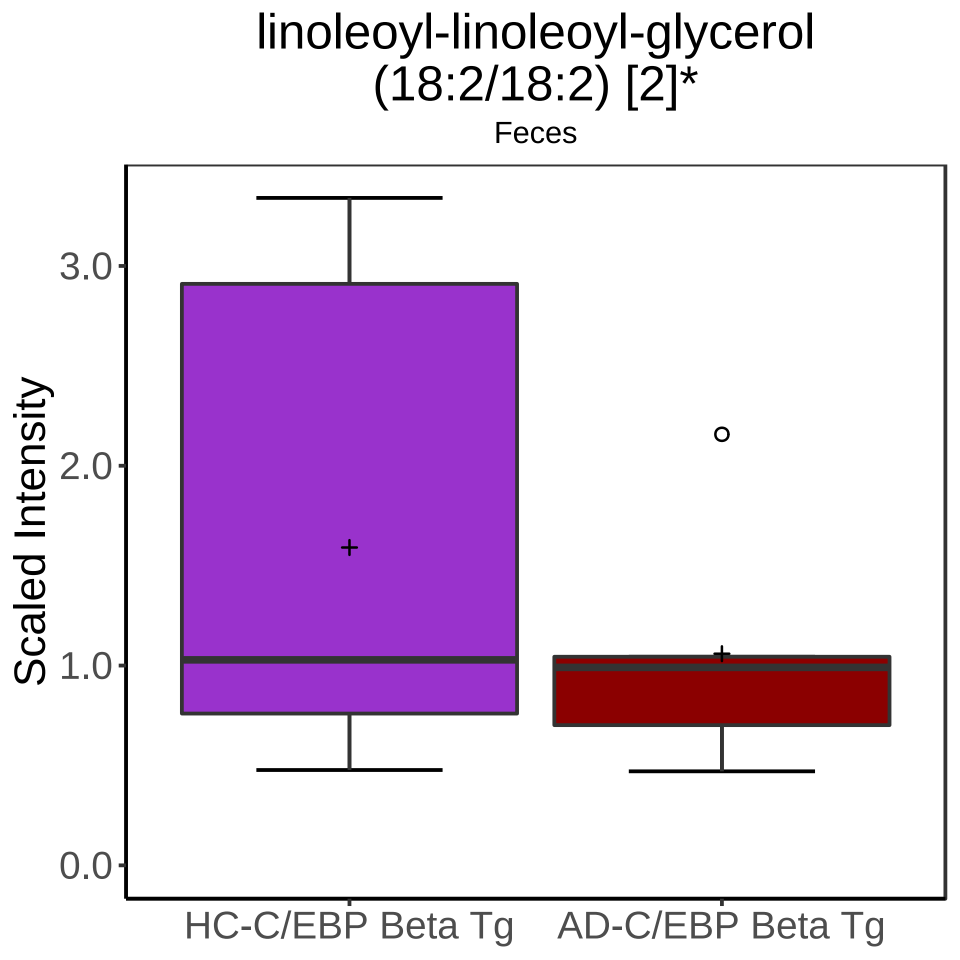
linoleoyl-linoleoyl-glycerol
(18:2/18:2) [2]*
Feces
Scaled Intensity
3.0
2.0
1.0
0.0
HC-C/EBP Beta Tg
AD-C/EBP Beta Tg

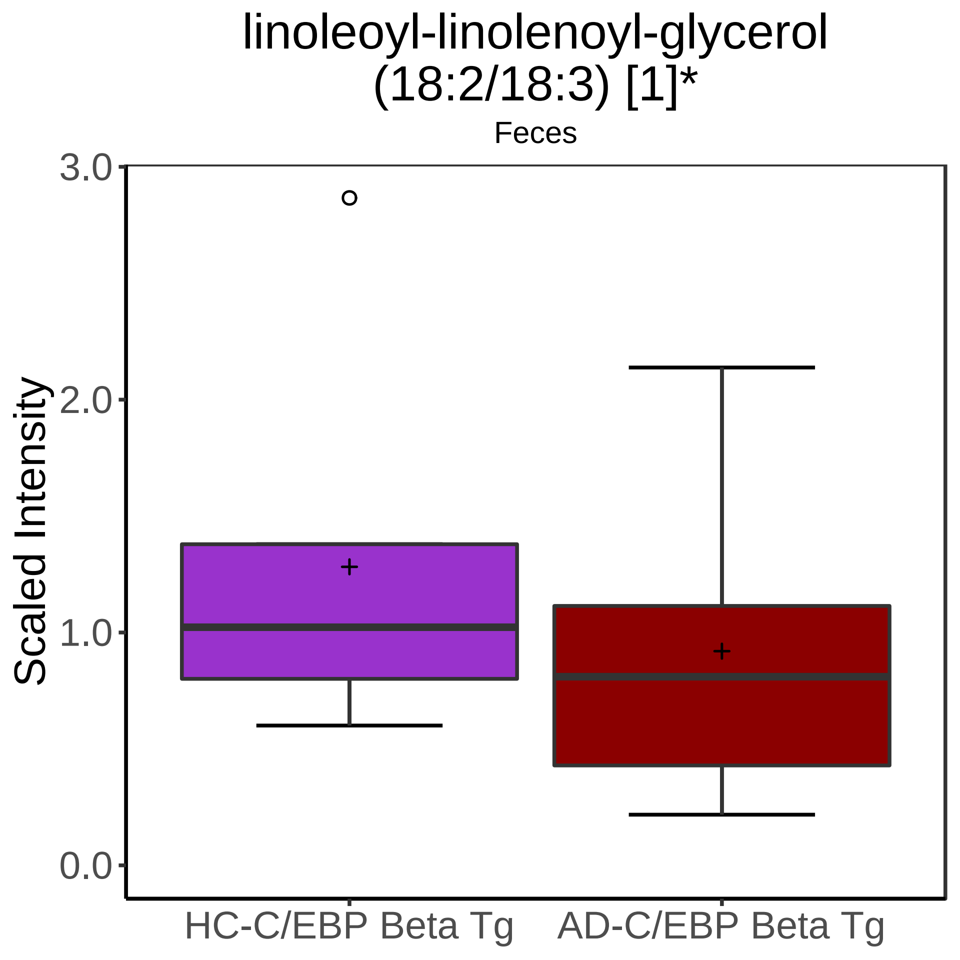
linoleoyl-linolenoyl-glycerol
(18:2/18:3) [1]*
Feces
Scaled Intensity
3.0
2.0
1.0
0.0
HC-C/EBP Beta Tg
AD-C/EBP Beta Tg

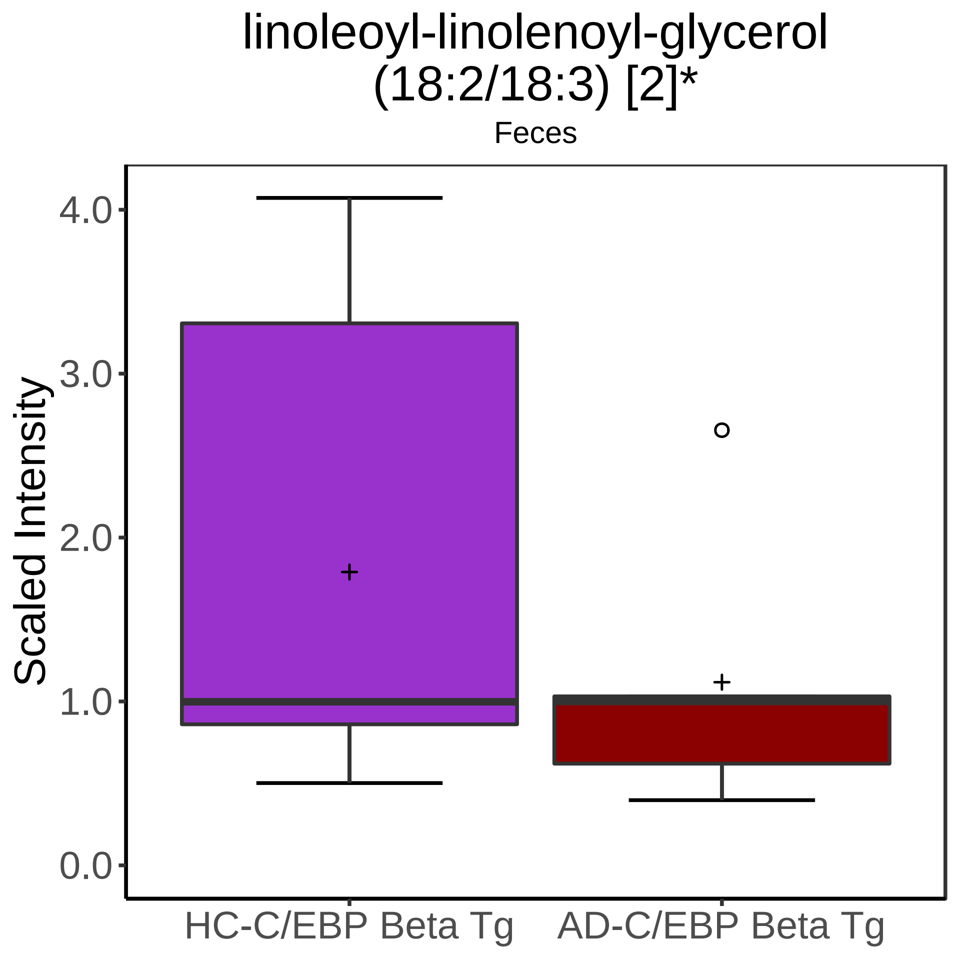

linoleoyl-linolenoyl-glycerol
(18:2/18:3) [2]*
Feces
Scaled Intensity
4.0
3.0
2.0
1.0
0.0
HC-C/EBP Beta Tg
AD-C/EBP Beta Tg

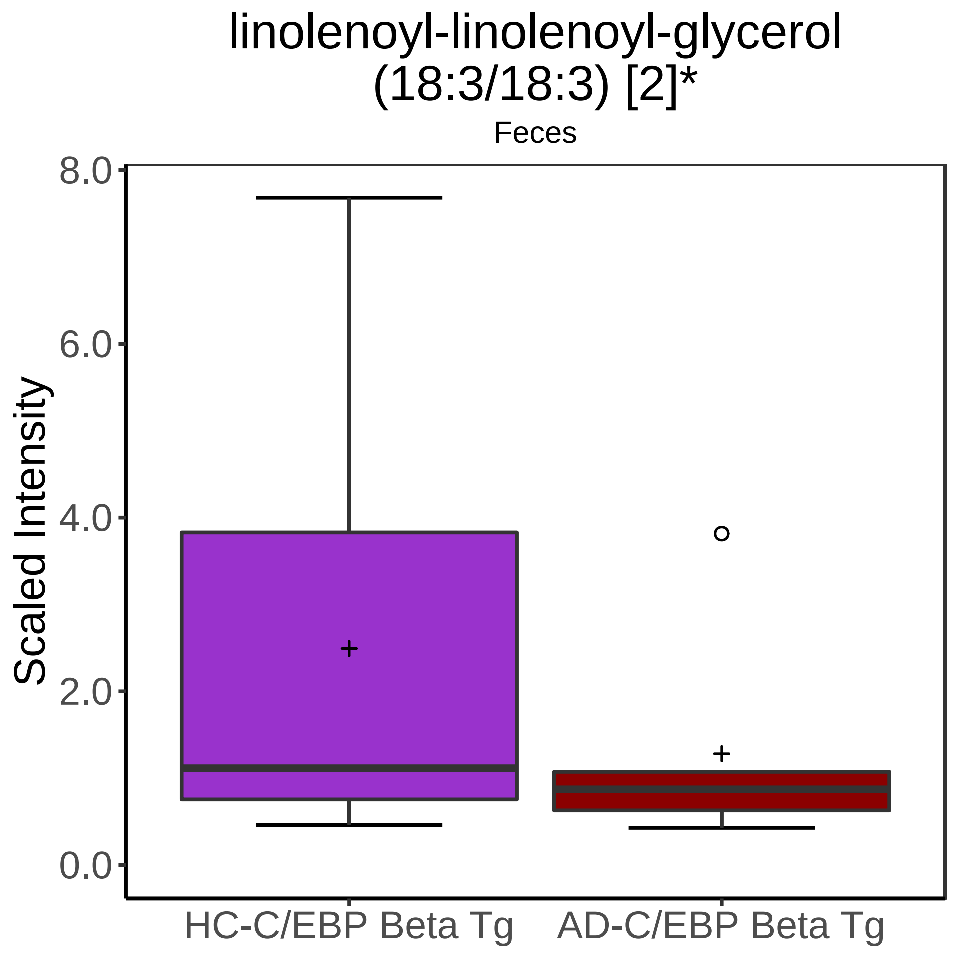
linolenoyl-linolenoyl-glycerol
(18:3/18:3) [2]*
Feces
Scaled Intensity
8.0
6.0
4.0
2.0
0.0
HC-C/EBP Beta Tg
AD-C/EBP Beta Tg

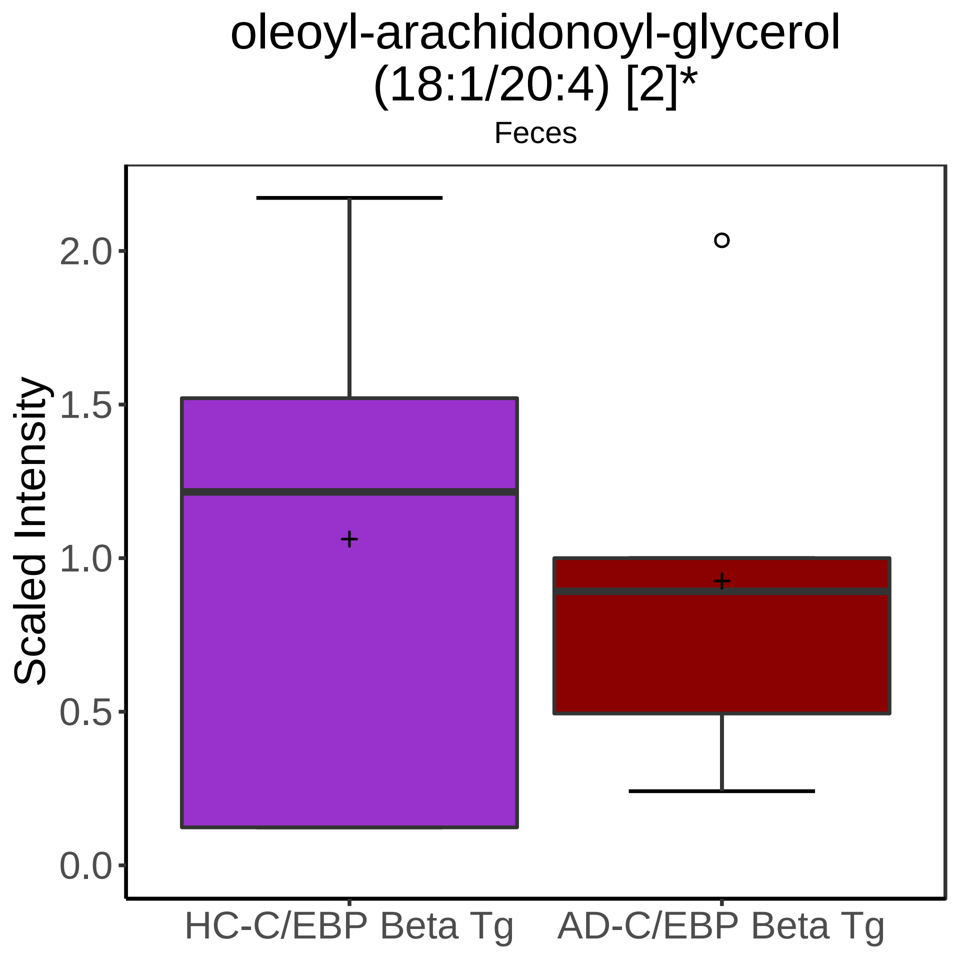
oleoyl-arachidonoyl-glycerol
(18:1/20:4) [2]*
Feces
Scaled Intensity
0.0
0.5
1.0
1.5
2.0
HC-C/EBP Beta Tg
AD-C/EBP Beta Tg

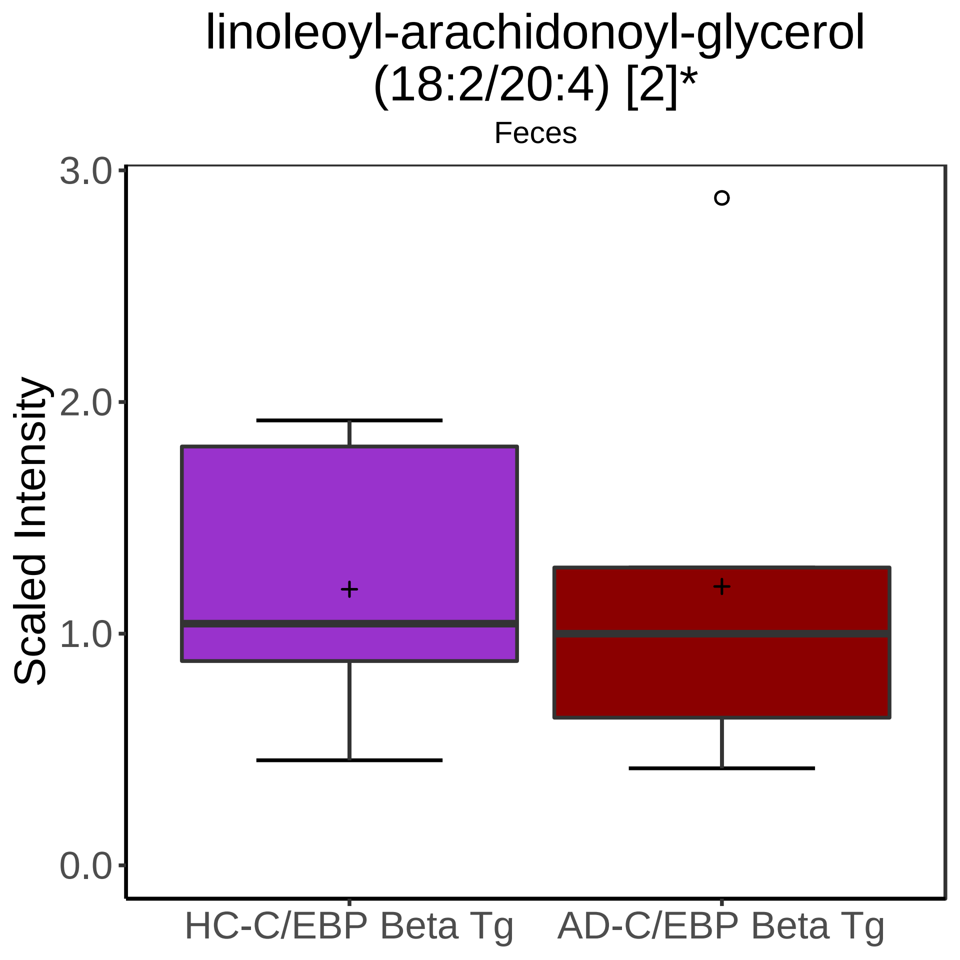

linoleoyl-arachidonoyl-glycerol
(18:2/20:4) [2]*
Feces
Scaled Intensity
3.0
2.0
1.0
0.0
HC-C/EBP Beta Tg
AD-C/EBP Beta Tg

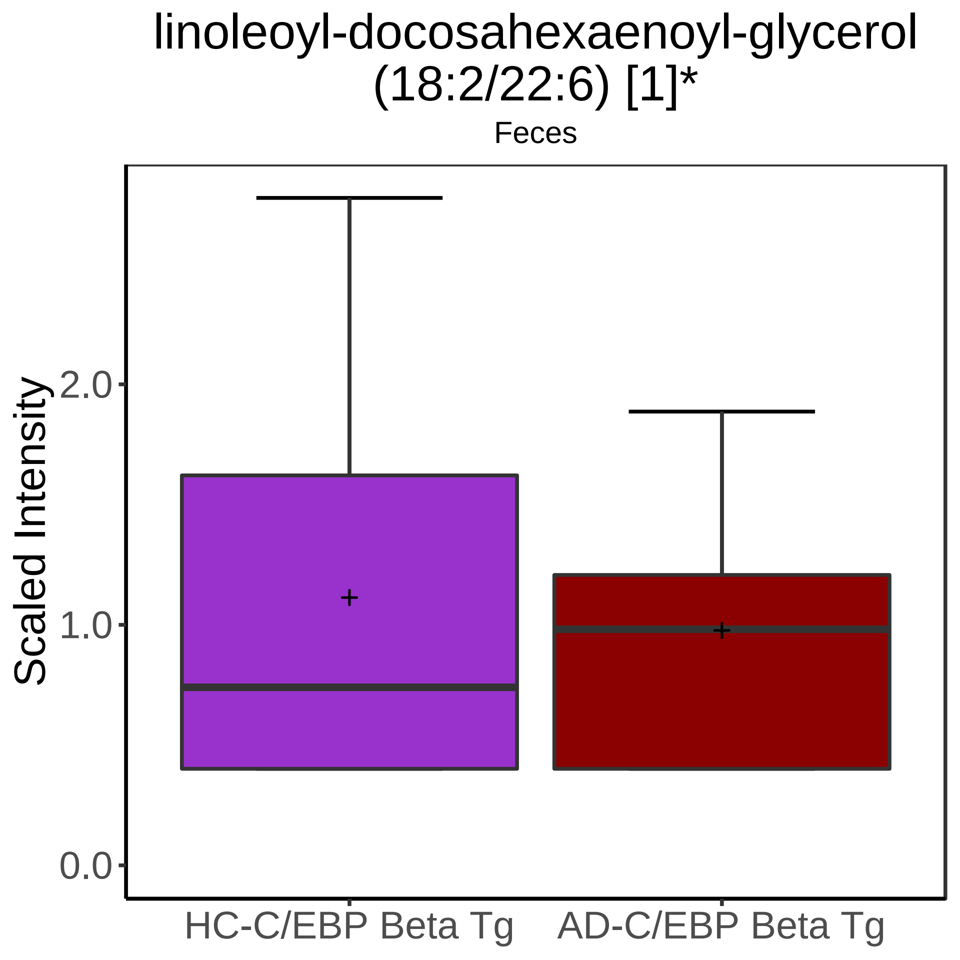
linoleoyl-docosahexaenoyl-glycerol
(18:2/22:6) [1]*
Feces
Scaled Intensity
2.0
1.0
0.0
HC-C/EBP Beta Tg
AD-C/EBP Beta Tg

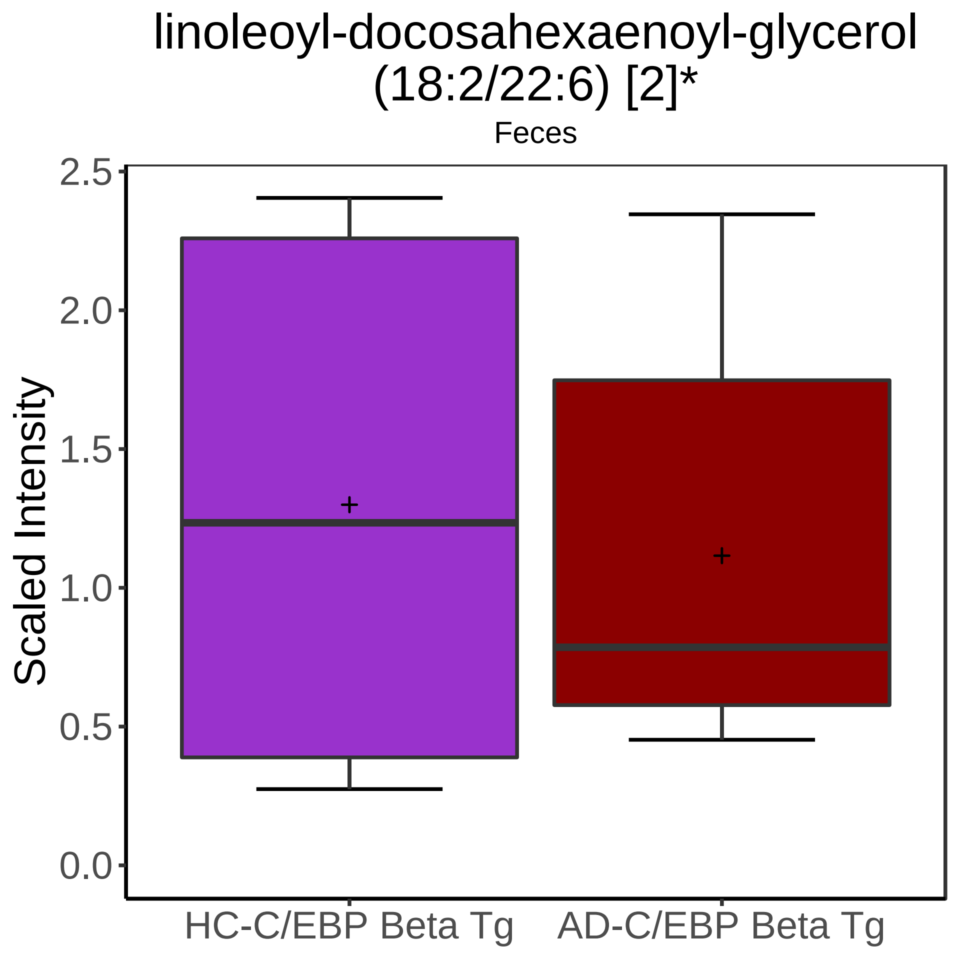

linoleoyl-docosahexaenoyl-glycerol
(18:2/22:6) [2]*
Feces
Scaled Intensity
2.5
2.0
1.5
1.0
0.5
0.0
HC-C/EBP Beta Tg
AD-C/EBP Beta Tg

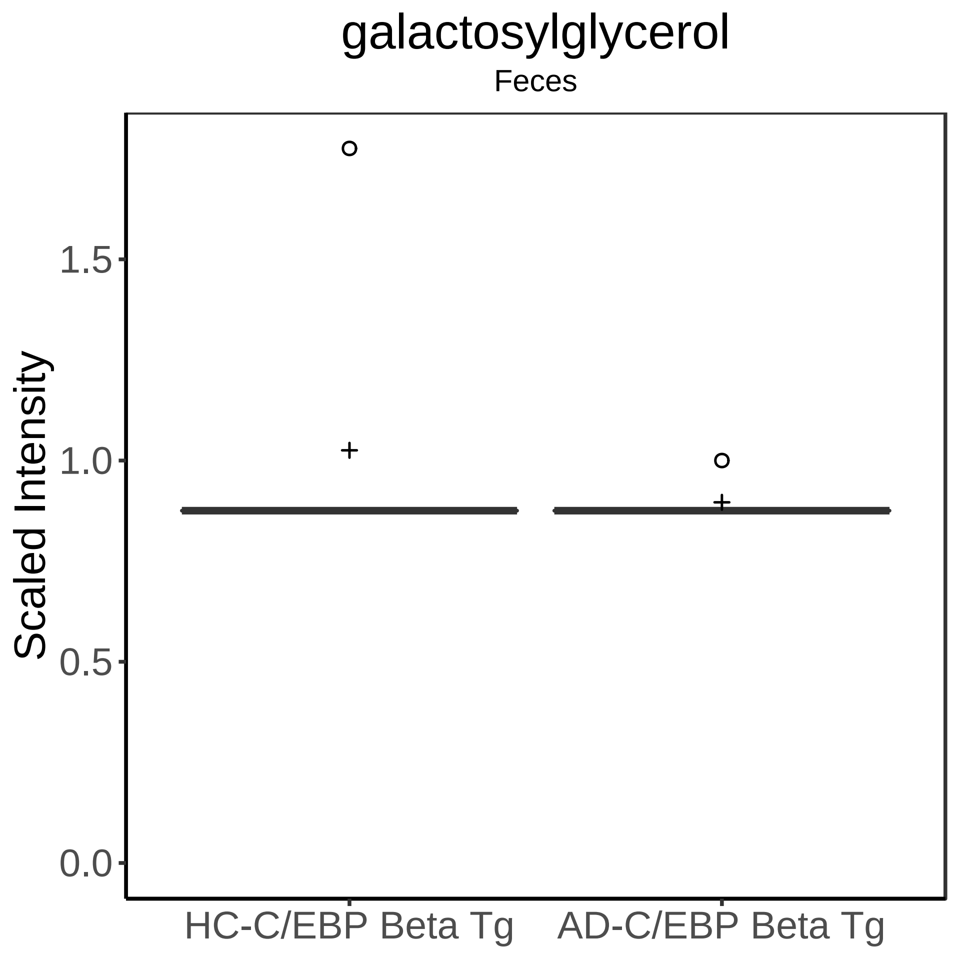

galactosylglycerol
Feces
Scaled Intensity
1.5
1.0
0.5
0.0
HC-C/EBP Beta Tg
AD-C/EBP Beta Tg

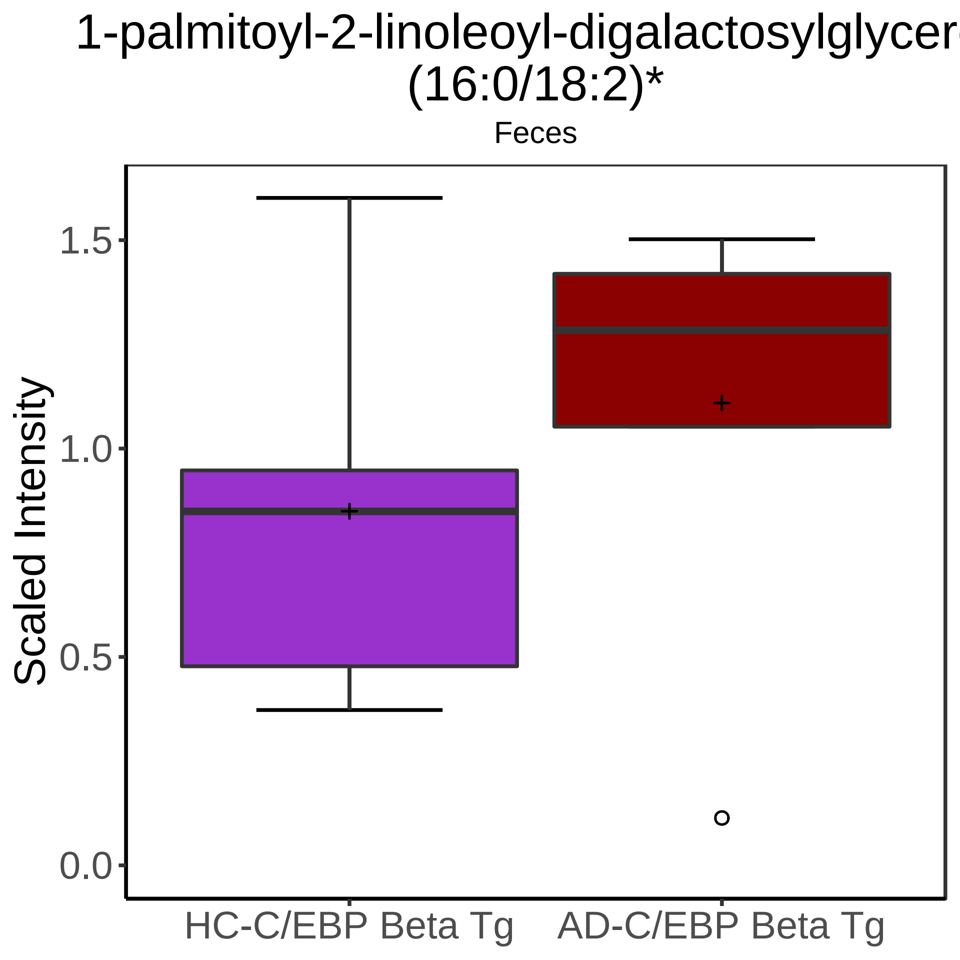

1-palmitoyl-2-linoleoyl-digalactosylglycer
(16:0/18:2)*
Feces
Scaled Intensity
1.5
1.0
0.5
0.0
HC-C/EBP Beta Tg
AD-C/EBP Beta Tg

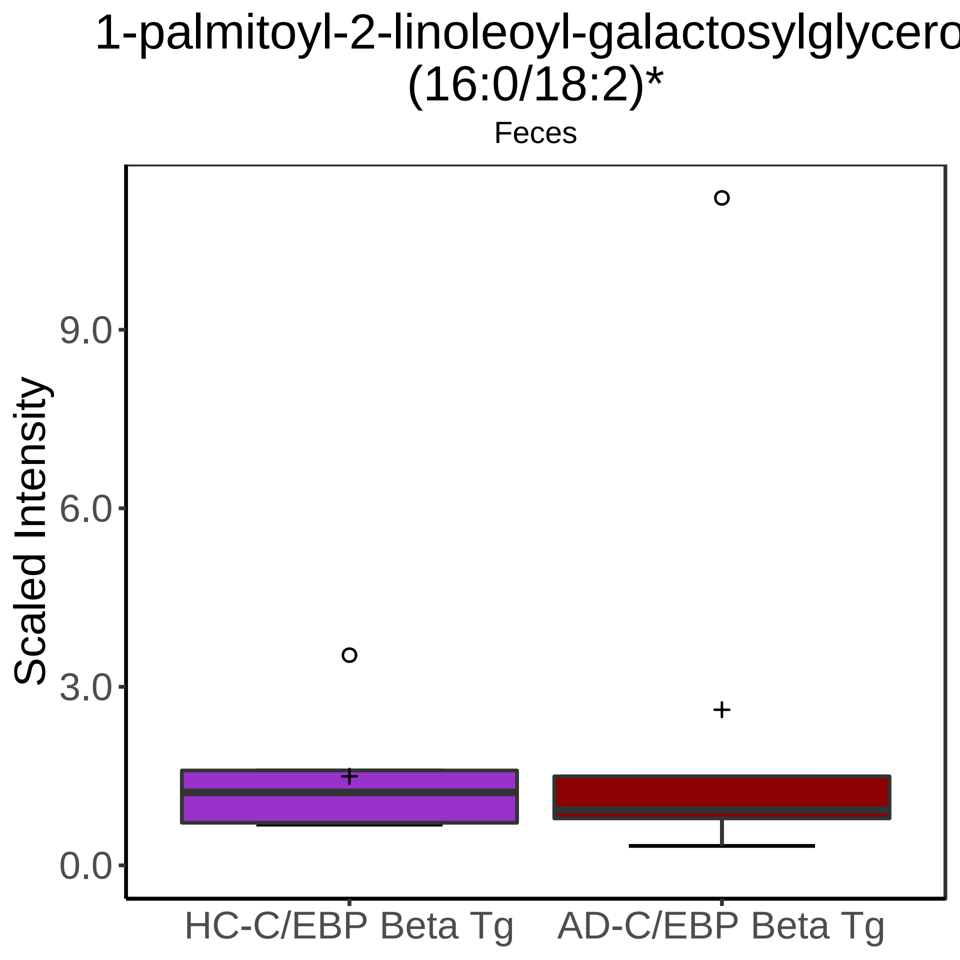
1-palmitoyl-2-linoleoyl-galactosylglycero
(16:0/18:2)*
Feces
Scaled Intensity
9.0
6.0
3.0
0.0
HC-C/EBP Beta Tg
AD-C/EBP Beta Tg

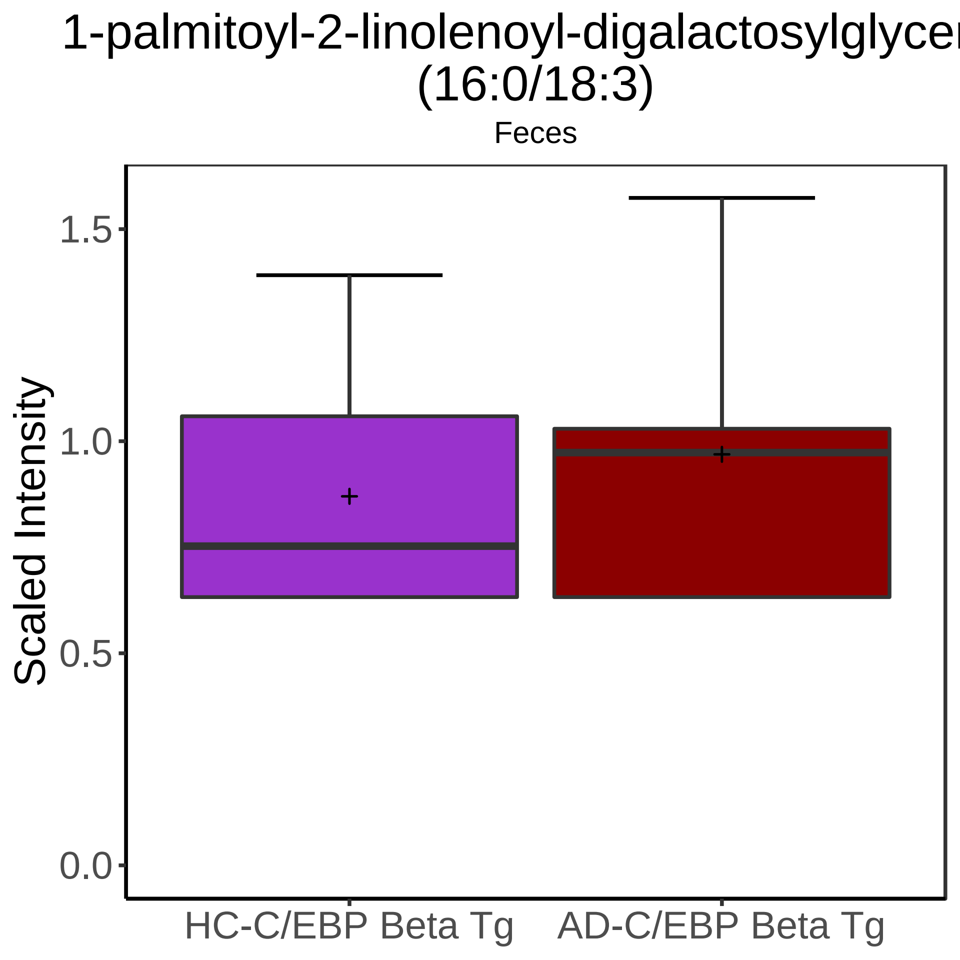

1-palmitoyl-2-linolenoyl-digalactosylglycer
(16:0/18:3)
Feces
Scaled Intensity
1.5
1.0
0.5
0.0
HC-C/EBP Beta Tg
AD-C/EBP Beta Tg

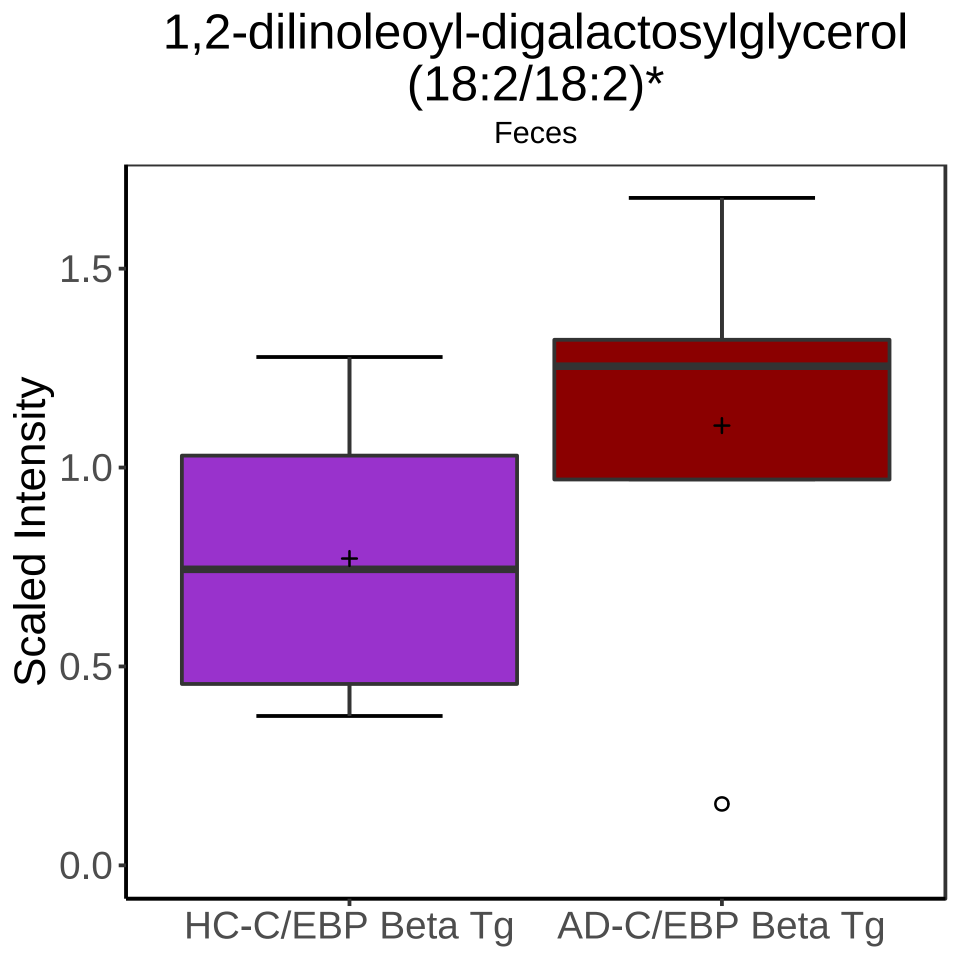
1,2-dilinoleoyl-digalactosylglycerol (18:2/18:2)*
Feces
Scaled Intensity
0.0
0.5
1.0
1.5
HC-C/EBP Beta Tg
AD-C/EBP Beta Tg

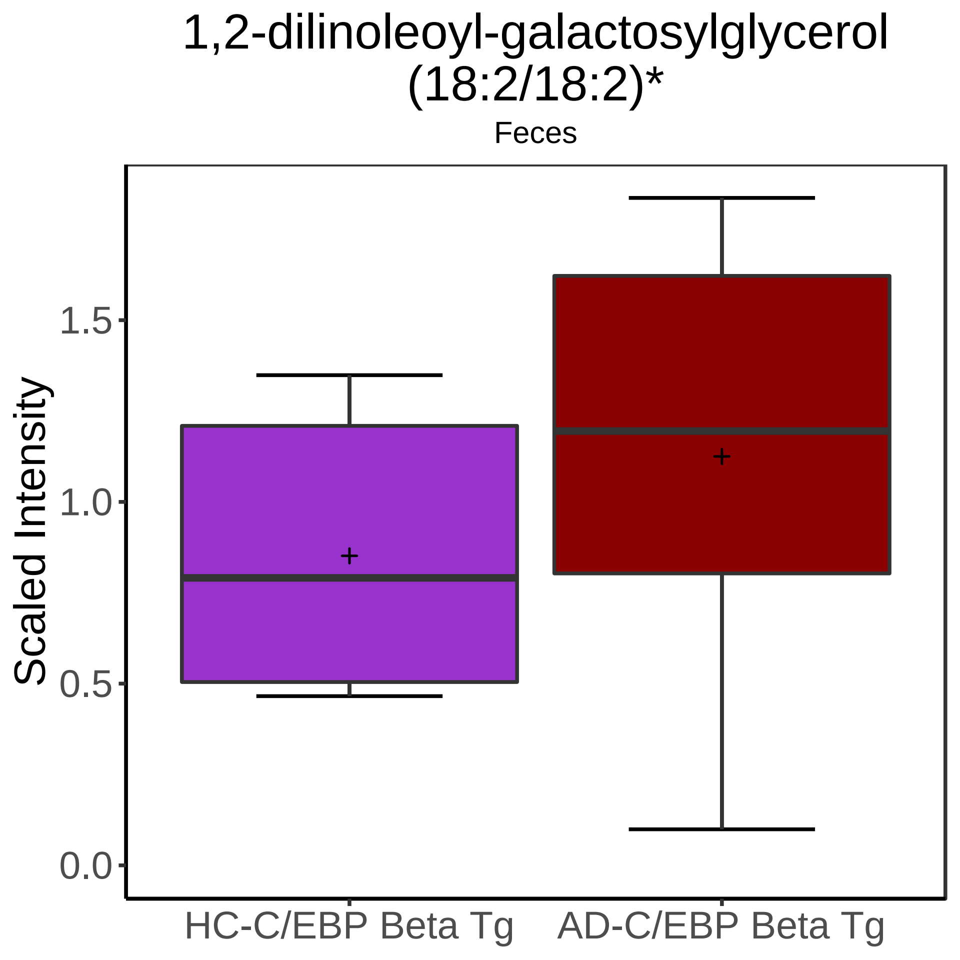

1,2-dilinoleoyl-galactosylglycerol
(18:2/18:2)*
Feces
Scaled Intensity
0.0
0.5
1.0
1.5
HC-C/EBP Beta Tg
AD-C/EBP Beta Tg

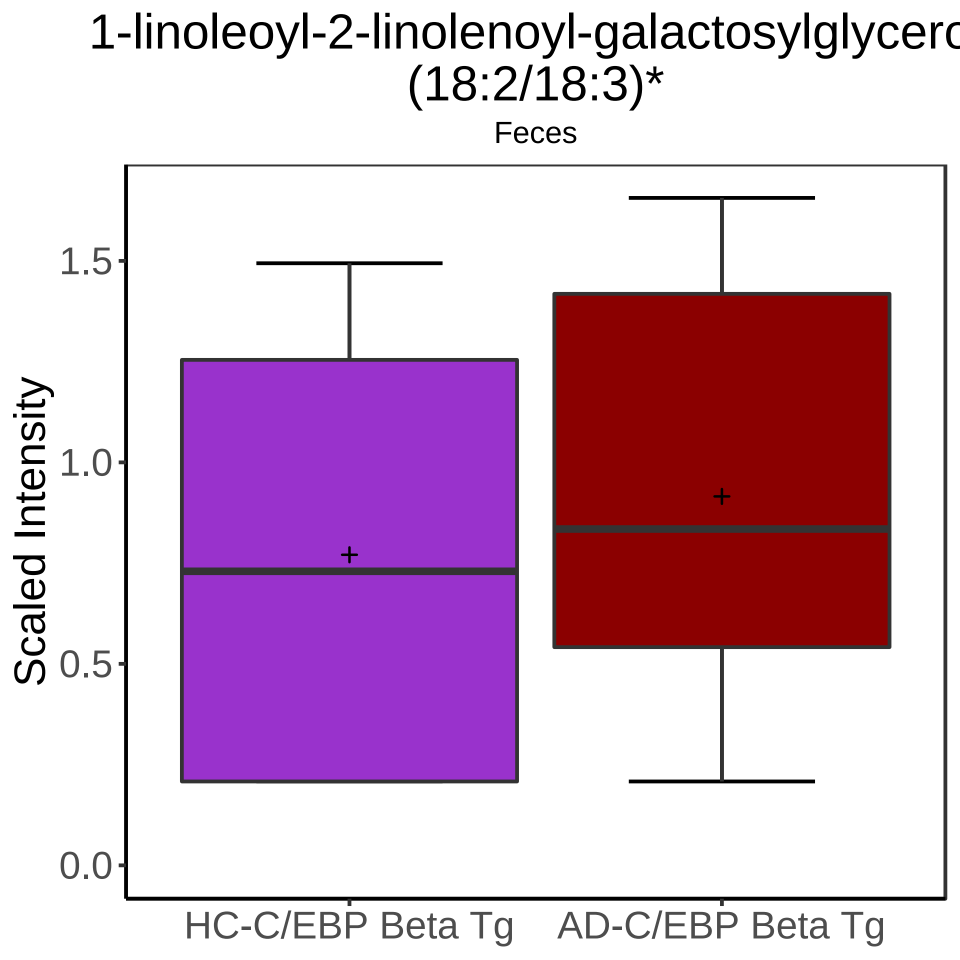
1-linoleoyl-2-linolenoyl-galactosylglycero
(18:2/18:3)*
Feces
Scaled Intensity
1.5
1.0
0.5
0.0
HC-C/EBP Beta Tg
AD-C/EBP Beta Tg

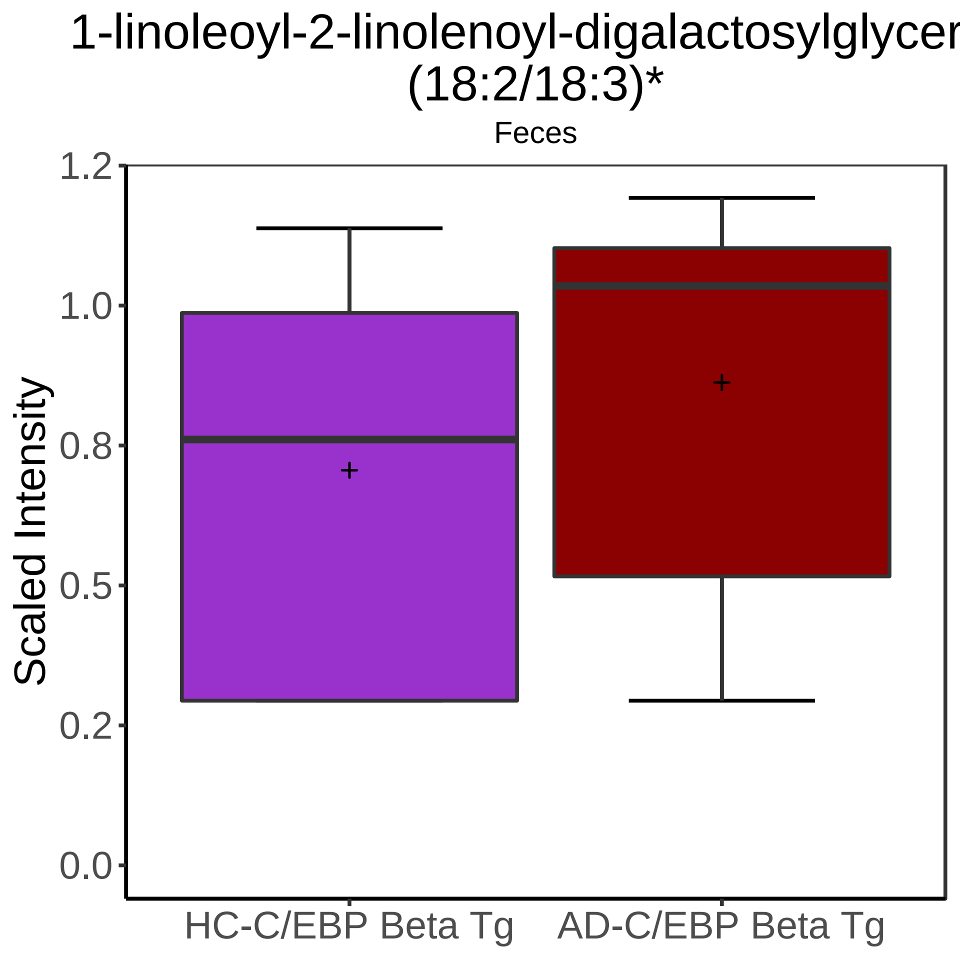
1-linoleoyl-2-linolenoyl-digalactosylglycer
(18:2/18:3)*
Feces
Scaled Intensity
1.2
1.0
0.8
0.5
0.2
0.0
HC-C/EBP Beta Tg
AD-C/EBP Beta Tg

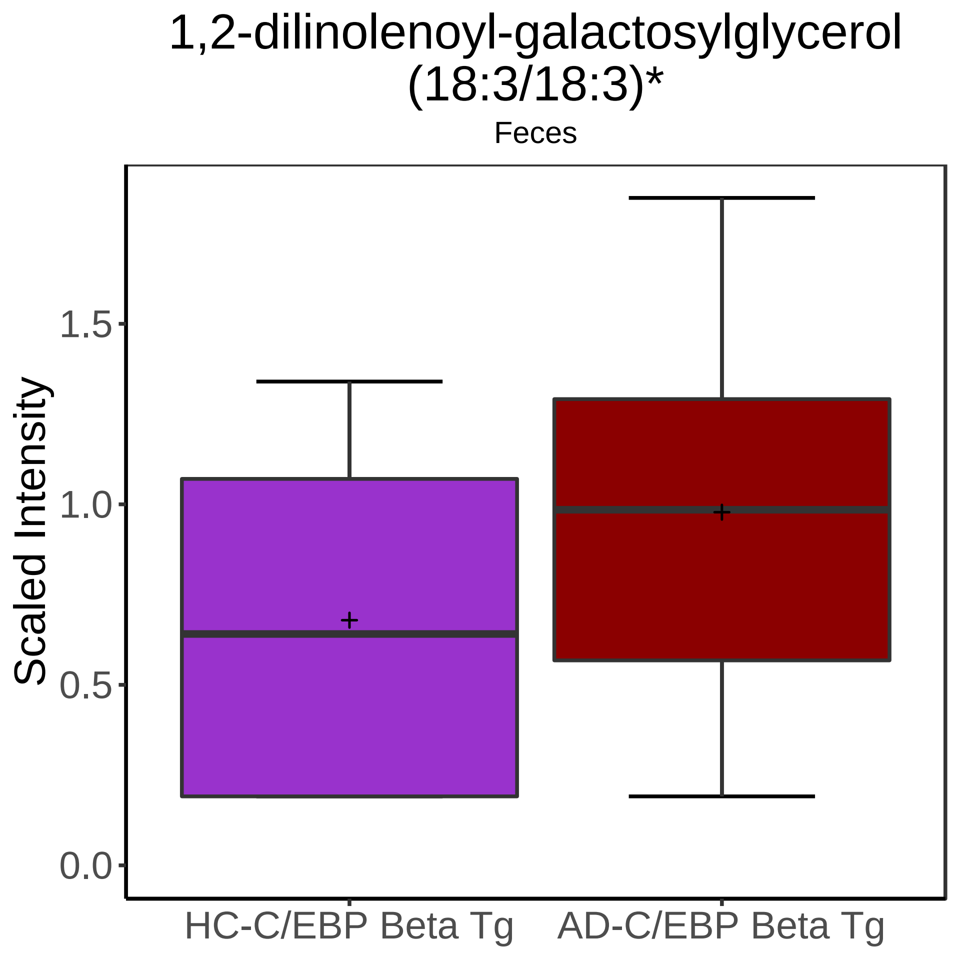

1,2-dilinolenoyl-galactosylglycerol
(18:3/18:3)*
Feces
Scaled Intensity
1.5
1.0
0.5
0.0
HC-C/EBP Beta Tg
AD-C/EBP Beta Tg

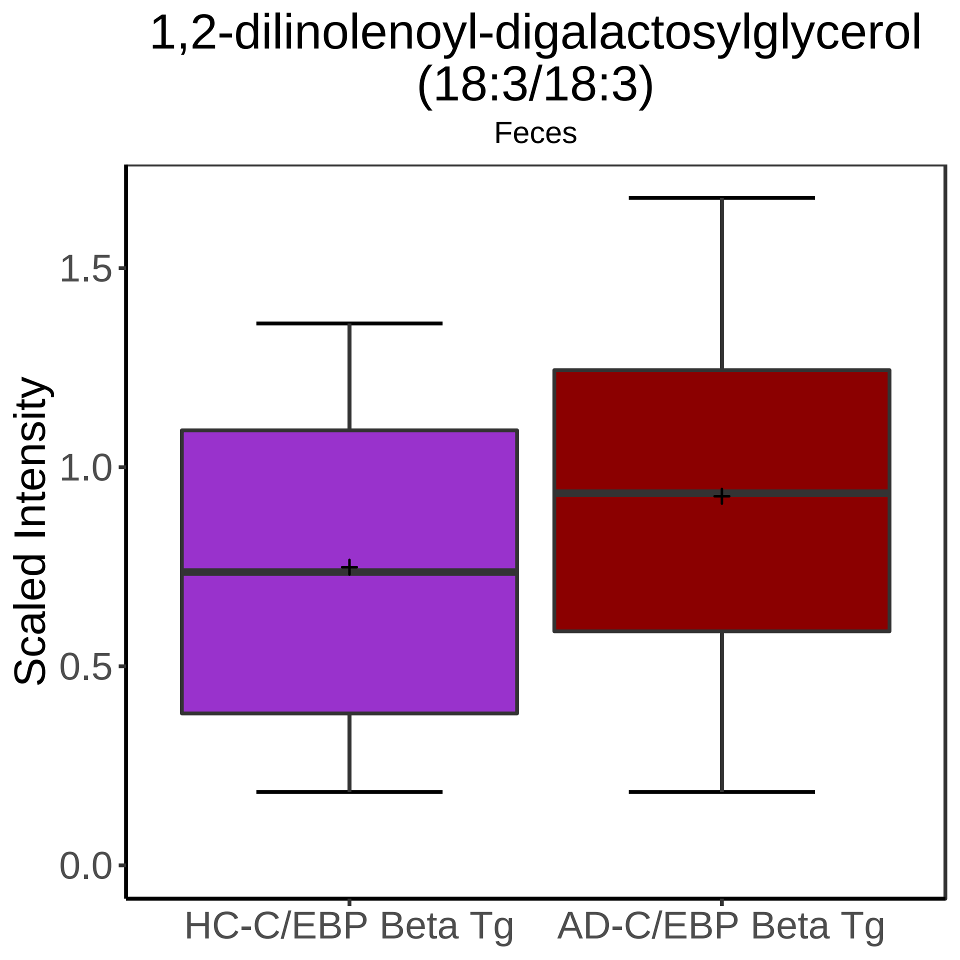

1,2-dilinolenoyl-digalactosylglycerol (18:3/18:3)
Feces
Scaled Intensity
0.0
0.5
1.0
1.5
HC-C/EBP Beta Tg
AD-C/EBP Beta Tg

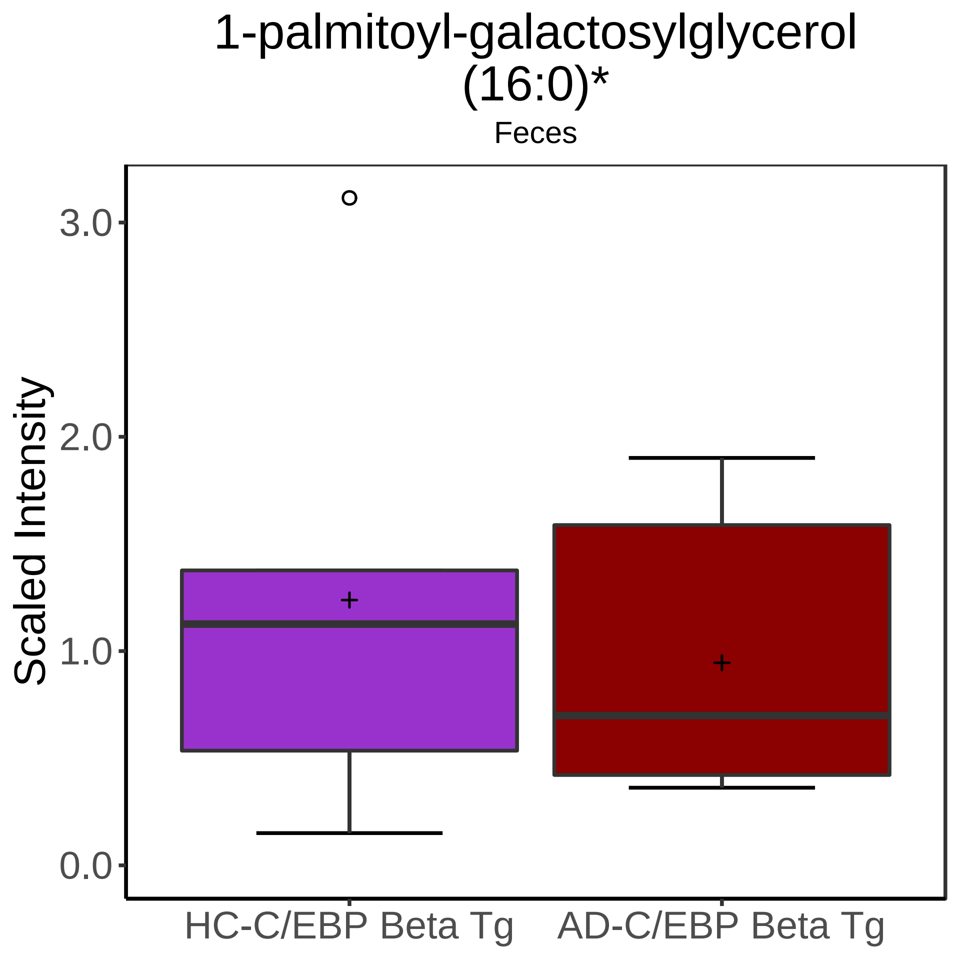

1-palmitoyl-galactosylglycerol
(16:0)*
Feces
Scaled Intensity
3.0
2.0
1.0
0.0
HC-C/EBP Beta Tg
AD-C/EBP Beta Tg

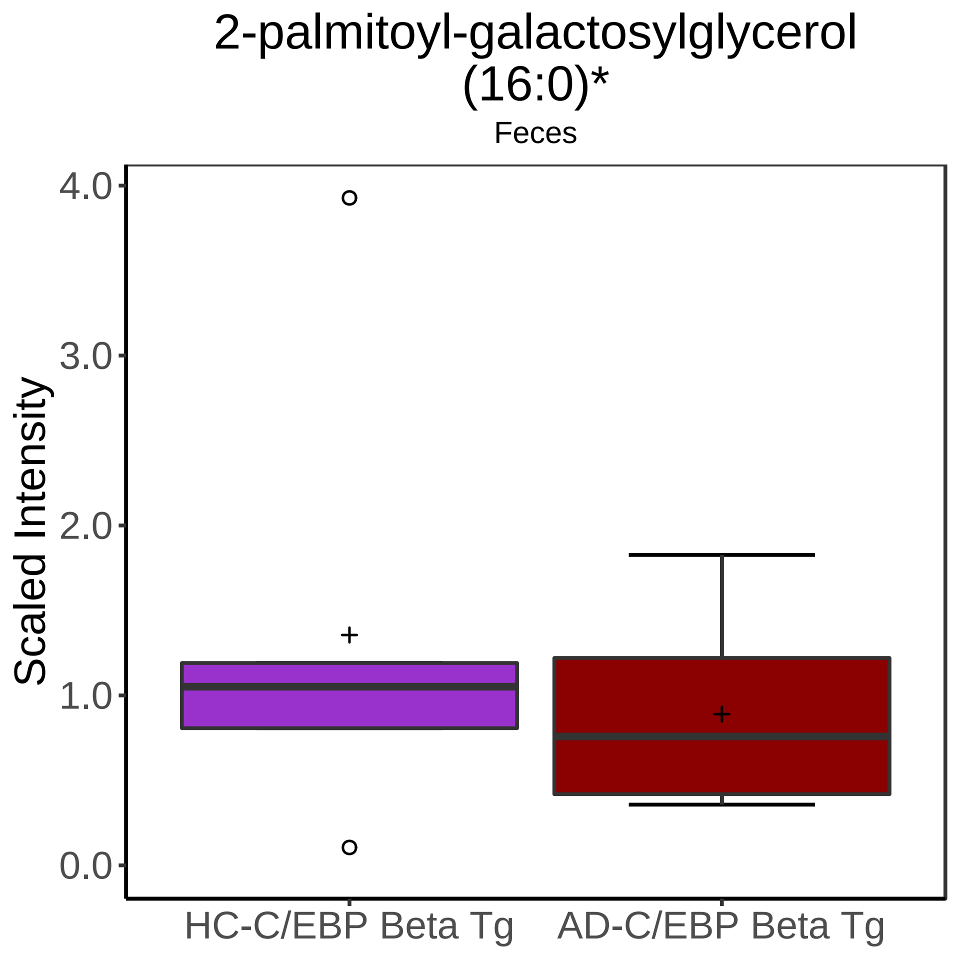
2-palmitoyl-galactosylglycerol
(16:0)*
Feces
Scaled Intensity
4.0
3.0
2.0
1.0
0.0
HC-C/EBP Beta Tg
AD-C/EBP Beta Tg

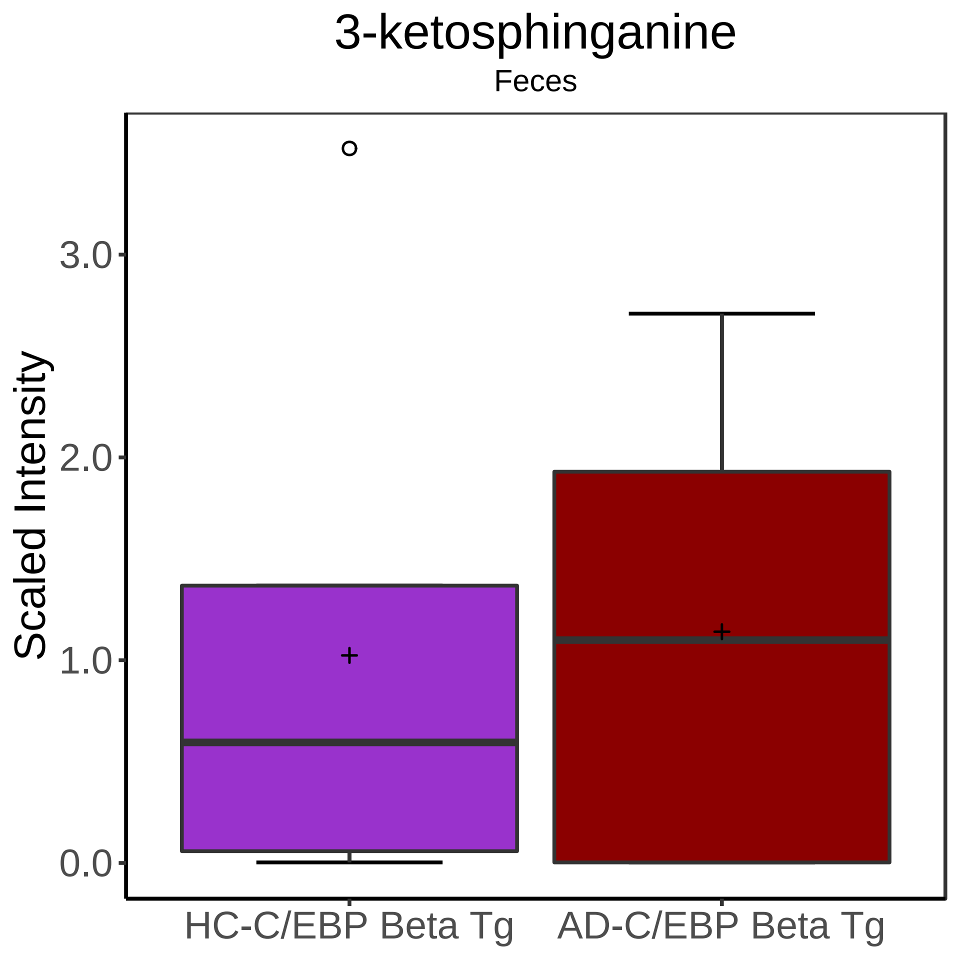

3-ketosphinganine
Feces
Scaled Intensity
3.0
2.0
1.0
0.0
HC-C/EBP Beta Tg
AD-C/EBP Beta Tg

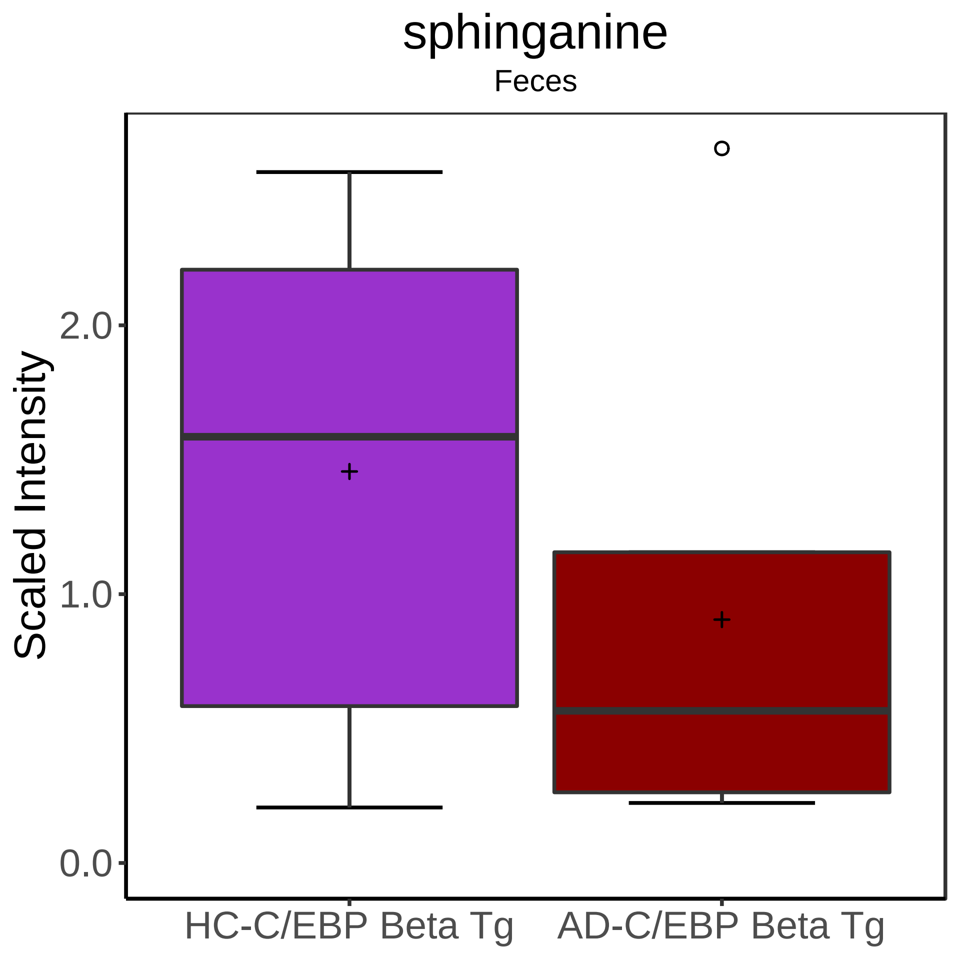
sphinganine
Feces
Scaled Intensity
2.0
1.0
0.0
HC-C/EBP Beta Tg
AD-C/EBP Beta Tg

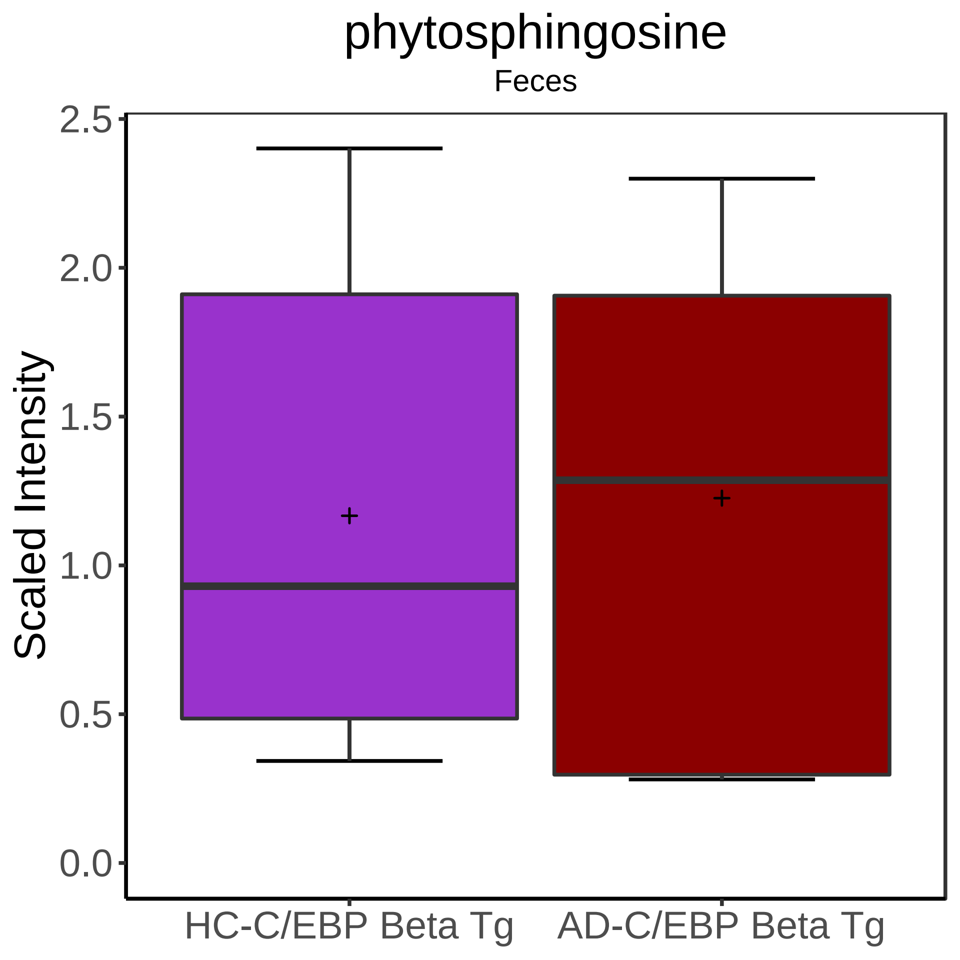
phytosphingosine
Feces
Scaled Intensity
2.5
2.0
1.5
1.0
0.5
0.0
HC-C/EBP Beta Tg
AD-C/EBP Beta Tg

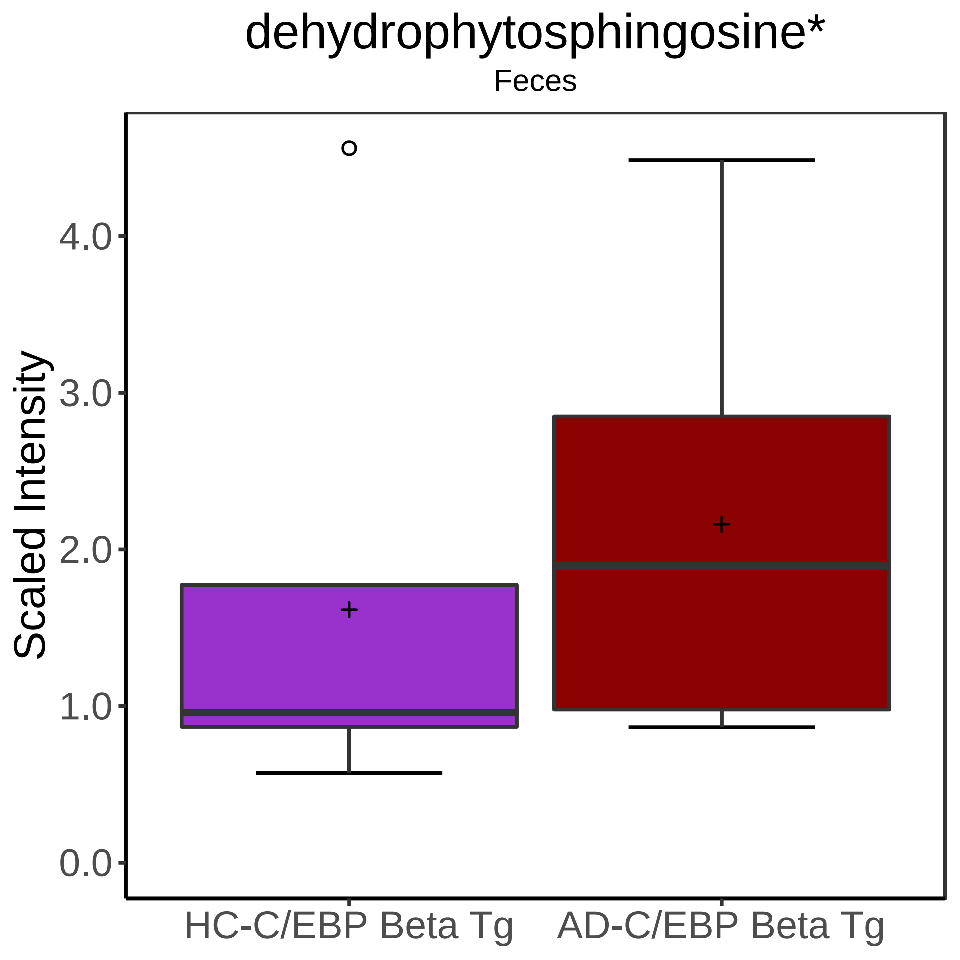
dehydrophytosphingosine*
Feces
Scaled Intensity
4.0
3.0
2.0
1.0
0.0
HC-C/EBP Beta Tg
AD-C/EBP Beta Tg

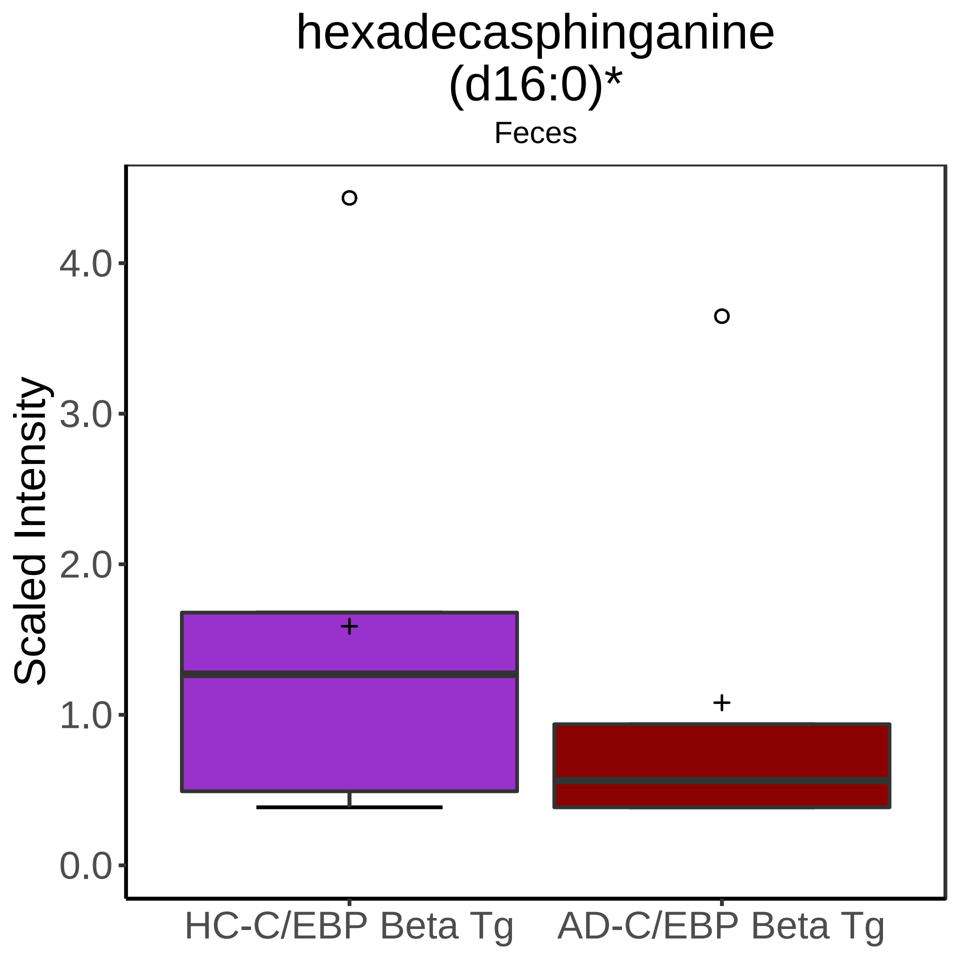

hexadecasphinganine
(d16:0)*
Feces
Scaled Intensity
4.0
3.0
2.0
1.0
0.0
HC-C/EBP Beta Tg
AD-C/EBP Beta Tg

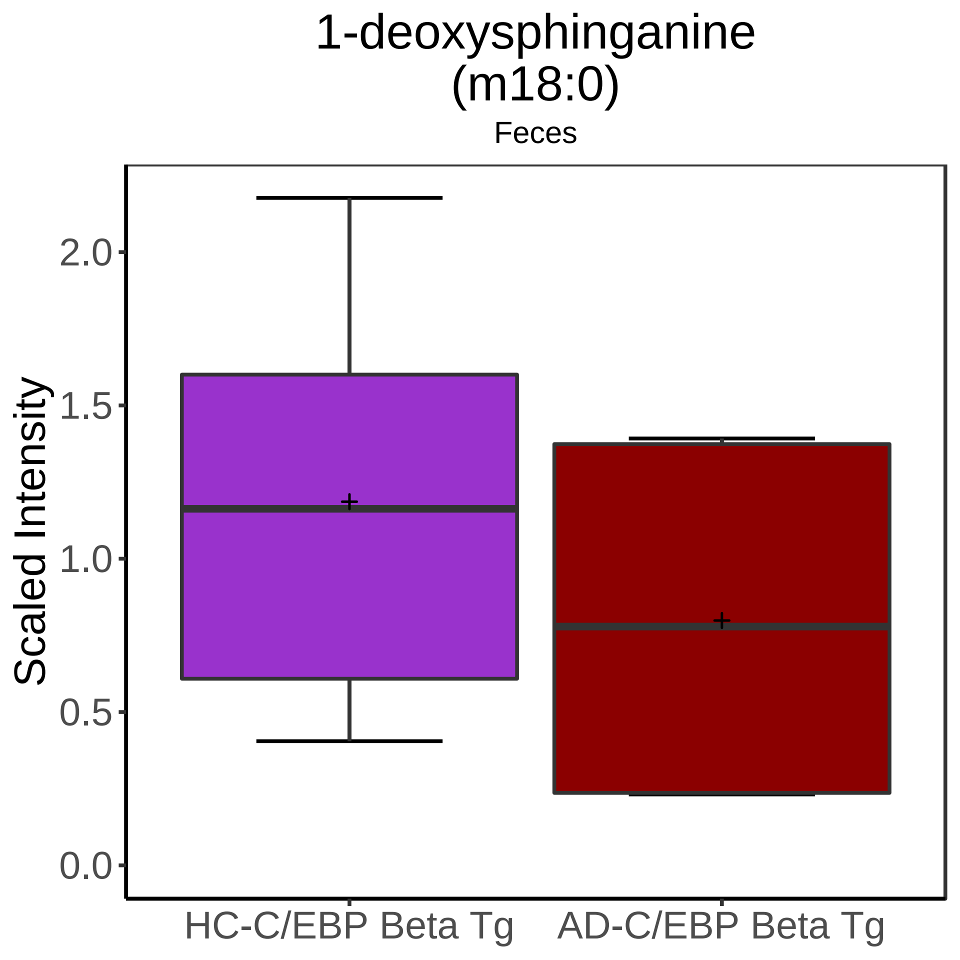
1-deoxysphinganine
(m18:0)
Feces
Scaled Intensity
2.0
1.5
1.0
0.5
0.0
HC-C/EBP Beta Tg
AD-C/EBP Beta Tg

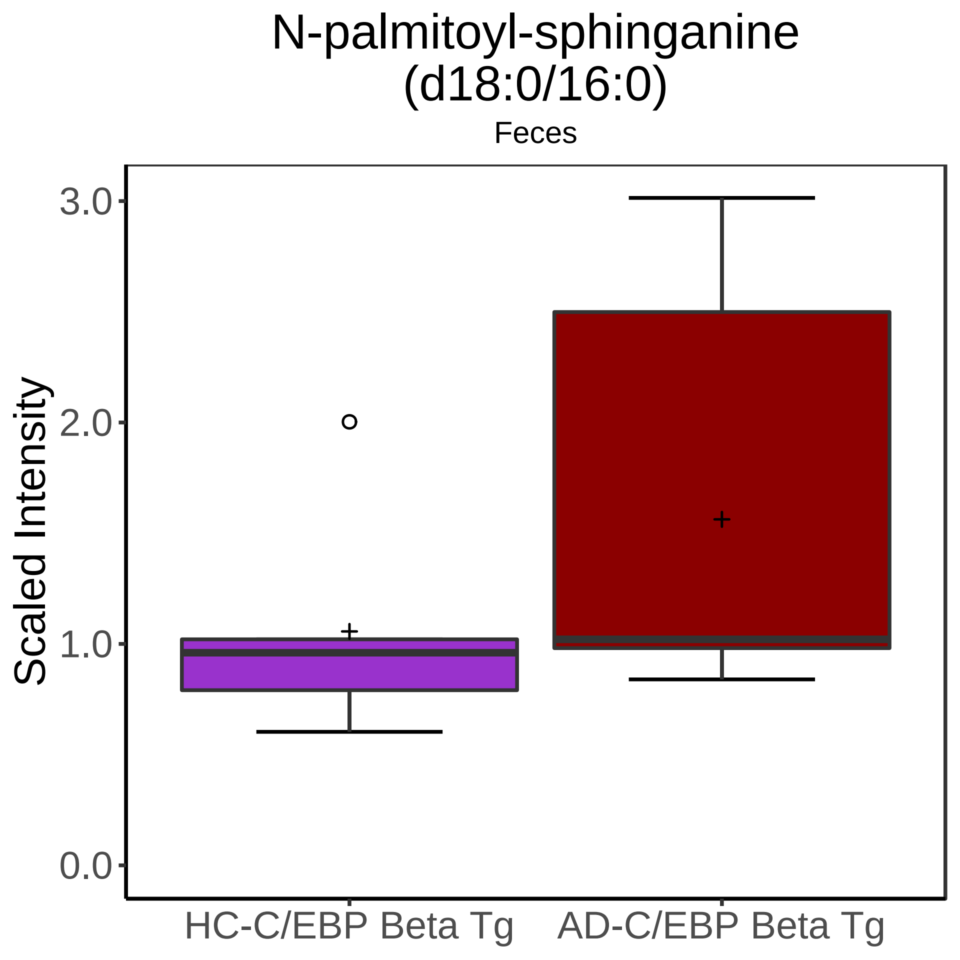
N-palmitoyl-sphinganine
(d18:0/16:0)
Feces
Scaled Intensity
3.0
2.0
1.0
0.0
HC-C/EBP Beta Tg
AD-C/EBP Beta Tg

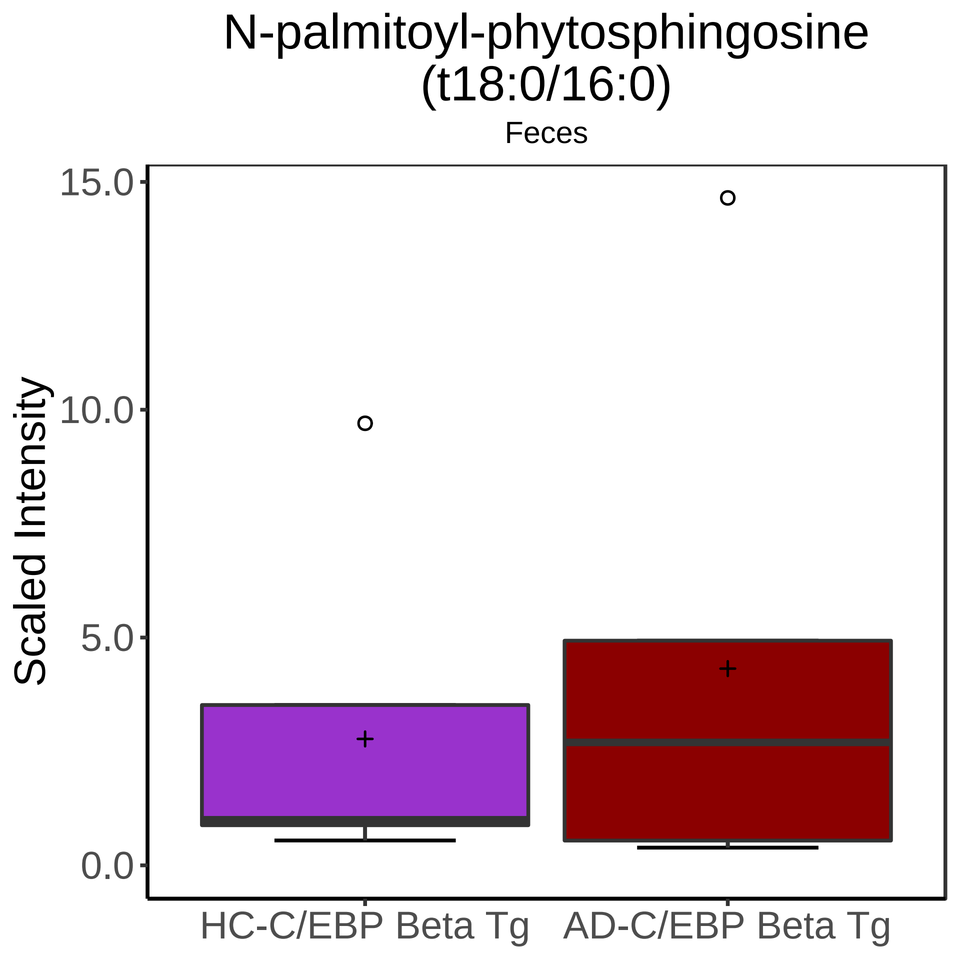

N-palmitoyl-phytosphingosine
(t18:0/16:0)
Feces
Scaled Intensity
15.0
10.0
5.0
0.0
HC-C/EBP Beta Tg
AD-C/EBP Beta Tg

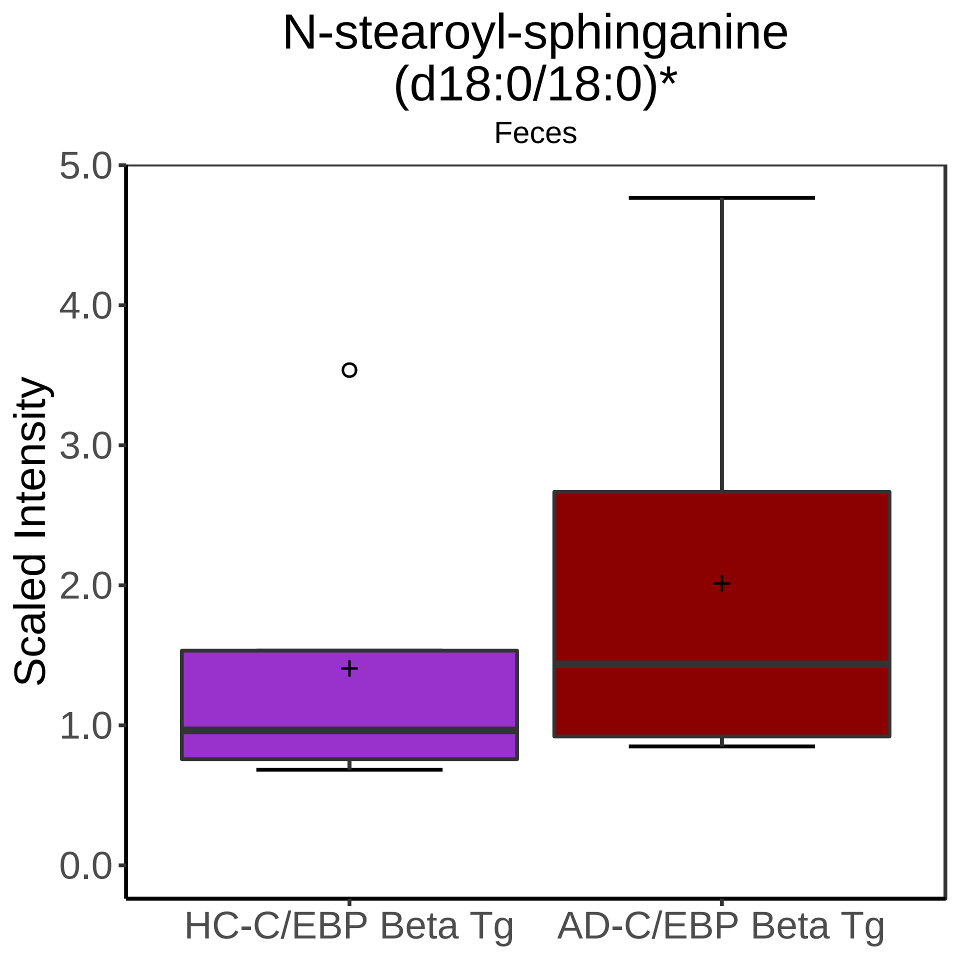
N-stearoyl-sphinganine
(d18:0/18:0)*
Feces
Scaled Intensity
5.0
4.0
3.0
2.0
1.0
0.0
HC-C/EBP Beta Tg
AD-C/EBP Beta Tg

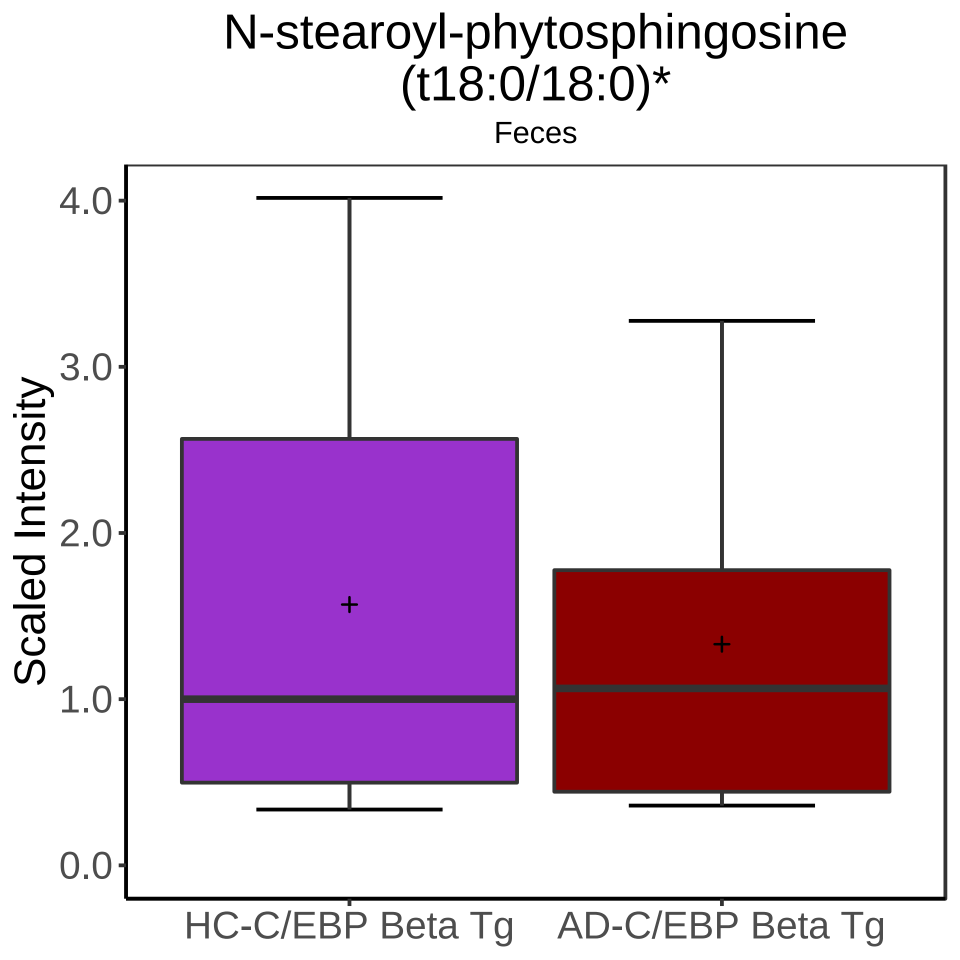

N-stearoyl-phytosphingosine
(t18:0/18:0)*
Feces
Scaled Intensity
4.0
3.0
2.0
1.0
0.0
HC-C/EBP Beta Tg
AD-C/EBP Beta Tg

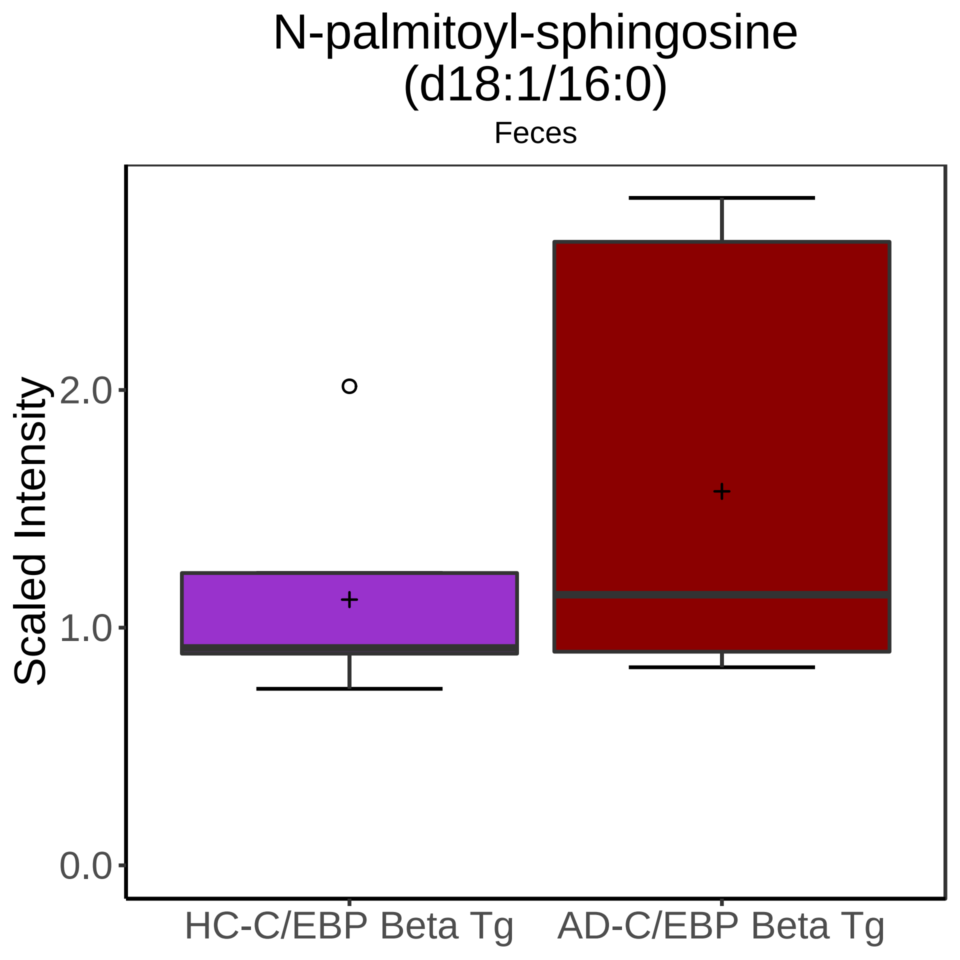

N-palmitoyl-sphingosine
(d18:1/16:0)
Feces
Scaled Intensity
2.0
1.0
0.0
HC-C/EBP Beta Tg
AD-C/EBP Beta Tg

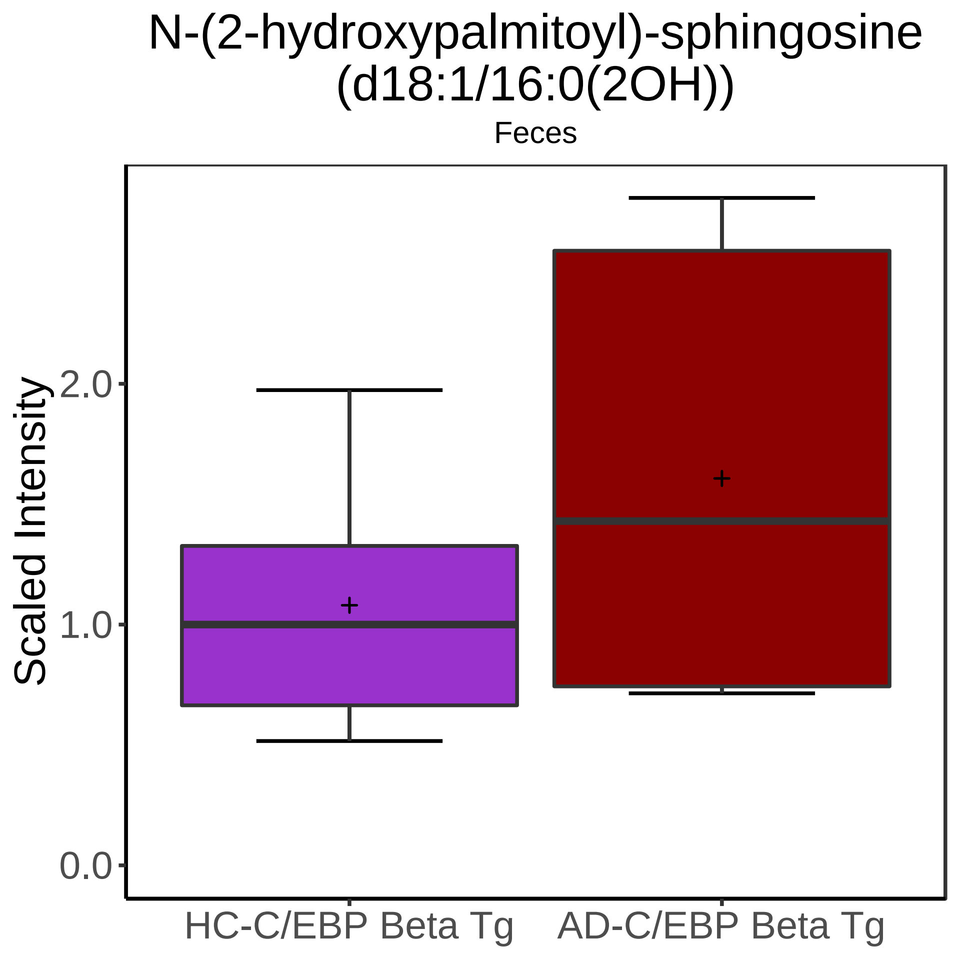

N-(2-hydroxypalmitoyl)-sphingosine
(d18:1/16:0(2OH))
Feces
Scaled Intensity
2.0
1.0
0.0
HC-C/EBP Beta Tg
AD-C/EBP Beta Tg

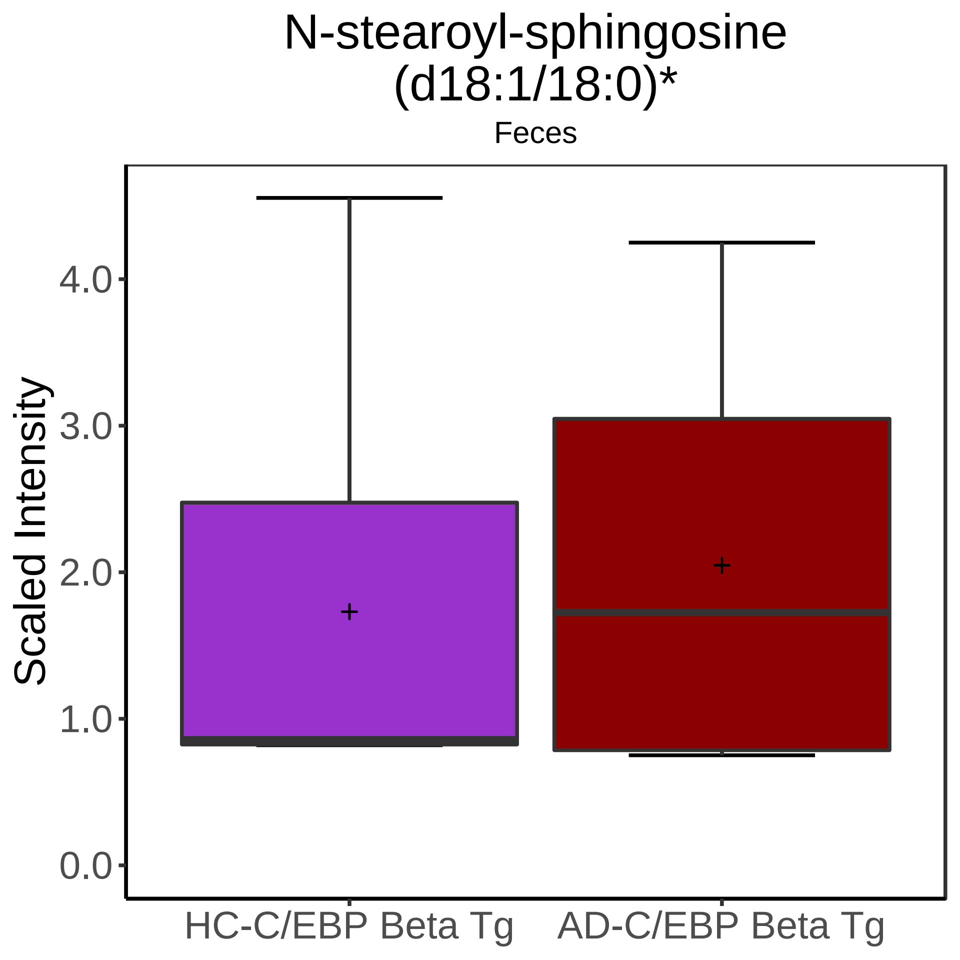

N-stearoyl-sphingosine (d18:1/18:0)*
Feces
Scaled Intensity
4.0
3.0
2.0
1.0
0.0
HC-C/EBP Beta Tg
AD-C/EBP Beta Tg

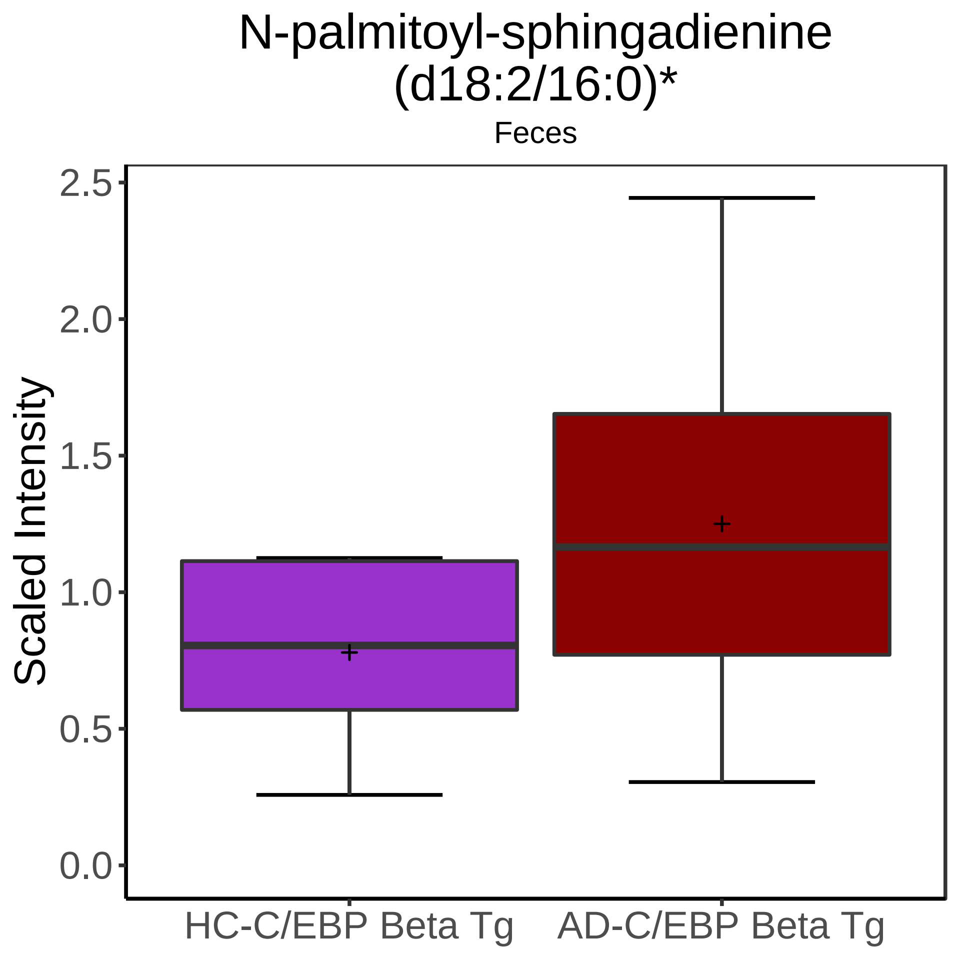
N-palmitoyl-sphingadienine
(d18:2/16:0)*
Feces
Scaled Intensity
2.5
2.0
1.5
1.0
0.5
0.0
HC-C/EBP Beta Tg
AD-C/EBP Beta Tg

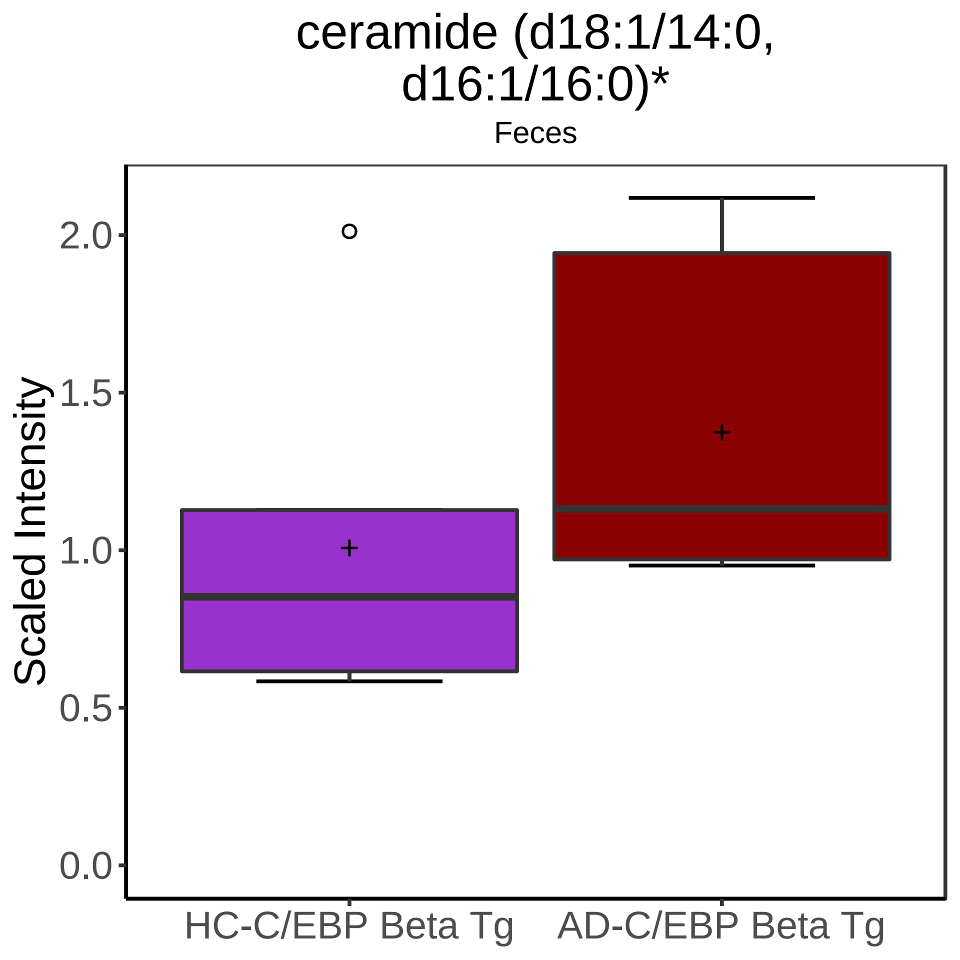
ceramide (d18:1/14:0,
d16:1/16:0)*
Feces
Scaled Intensity
2.0
1.5
1.0
0.5
0.0
HC-C/EBP Beta Tg
AD-C/EBP Beta Tg

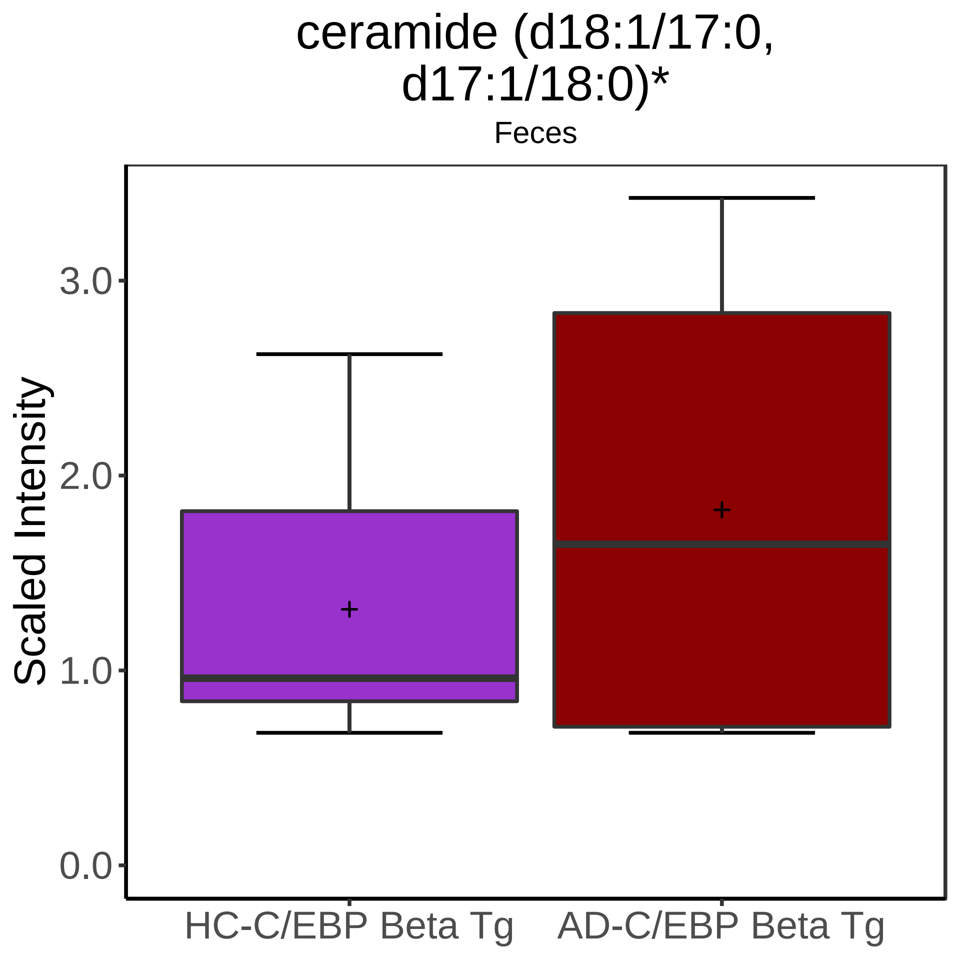
ceramide (d18:1/17:0,
d17:1/18:0)*
Feces
Scaled Intensity
3.0
2.0
1.0
0.0
HC-C/EBP Beta Tg
AD-C/EBP Beta Tg

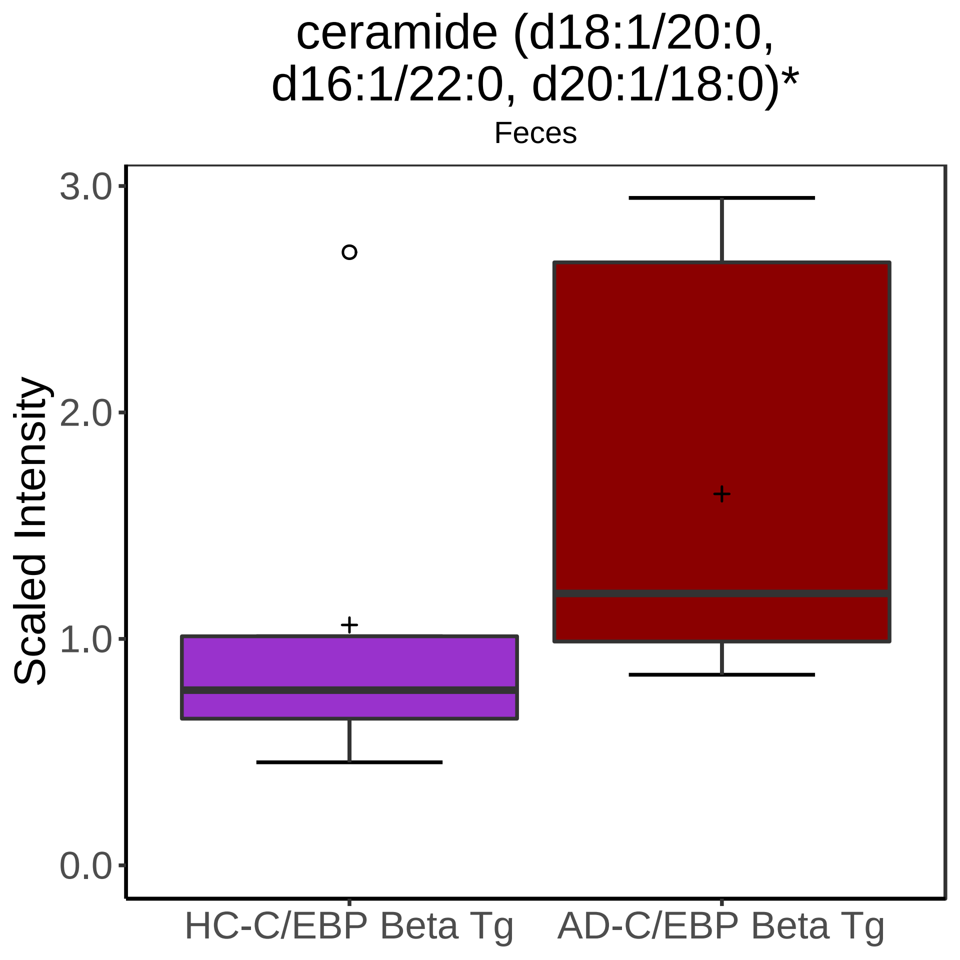

ceramide (d18:1/20:0,
d16:1/22:0, d20:1/18:0)*
Feces
Scaled Intensity
3.0
2.0
1.0
0.0
HC-C/EBP Beta Tg
AD-C/EBP Beta Tg

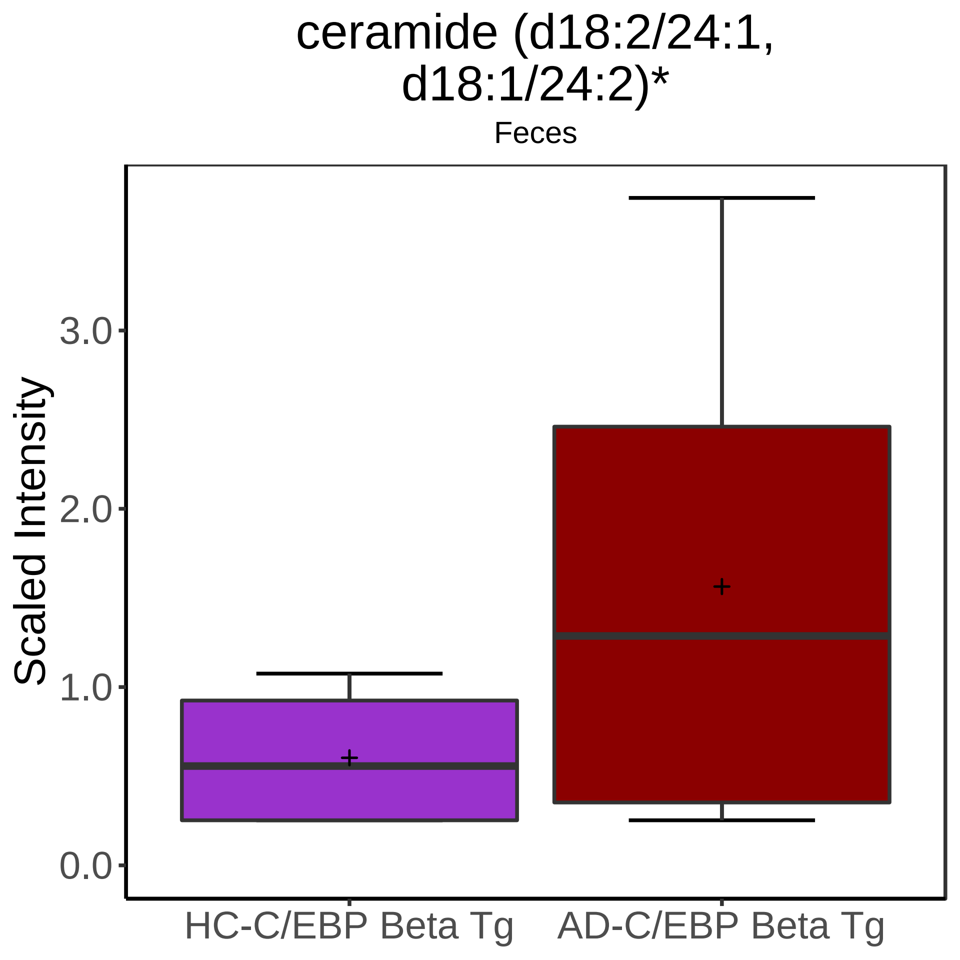
ceramide (d18:2/24:1, d18:1/24:2)*
Feces
Scaled Intensity
3.0
2.0
1.0
0.0
HC-C/EBP Beta Tg
AD-C/EBP Beta Tg

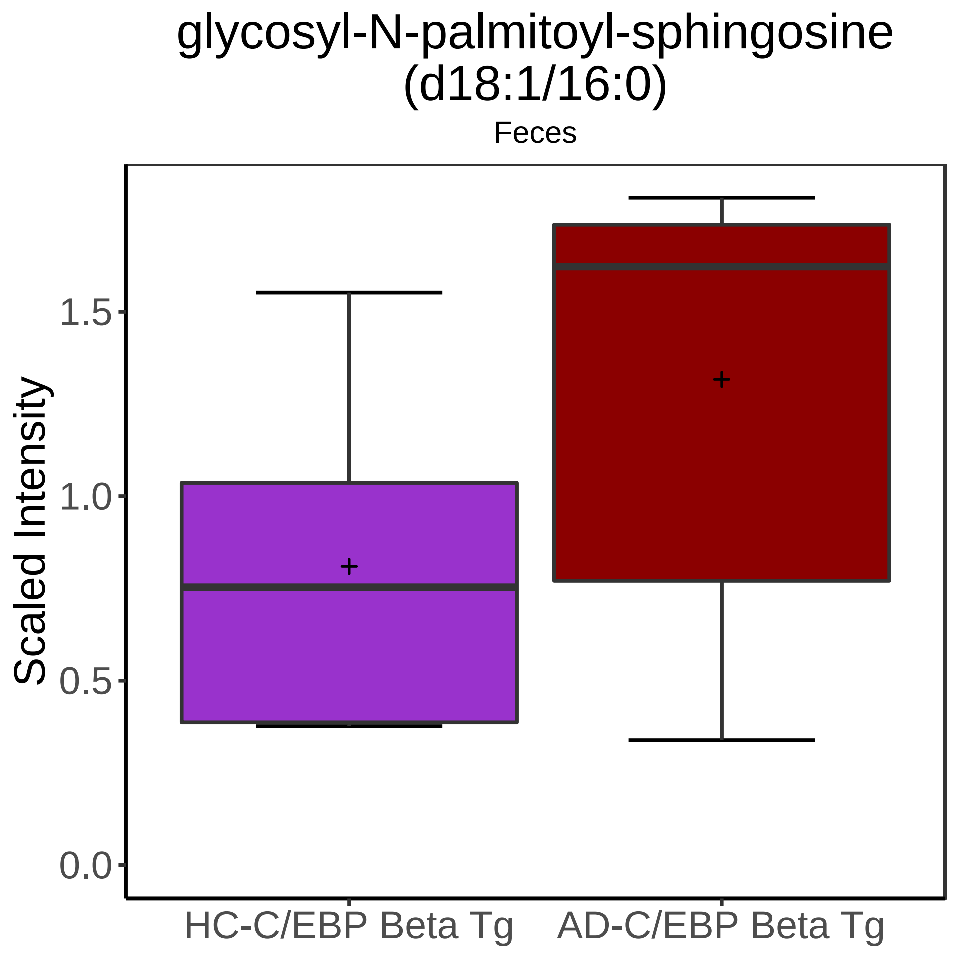

glycosyl-N-palmitoyl-sphingosine
(d18:1/16:0)
Feces
Scaled Intensity
1.5
1.0
0.5
0.0
HC-C/EBP Beta Tg
AD-C/EBP Beta Tg

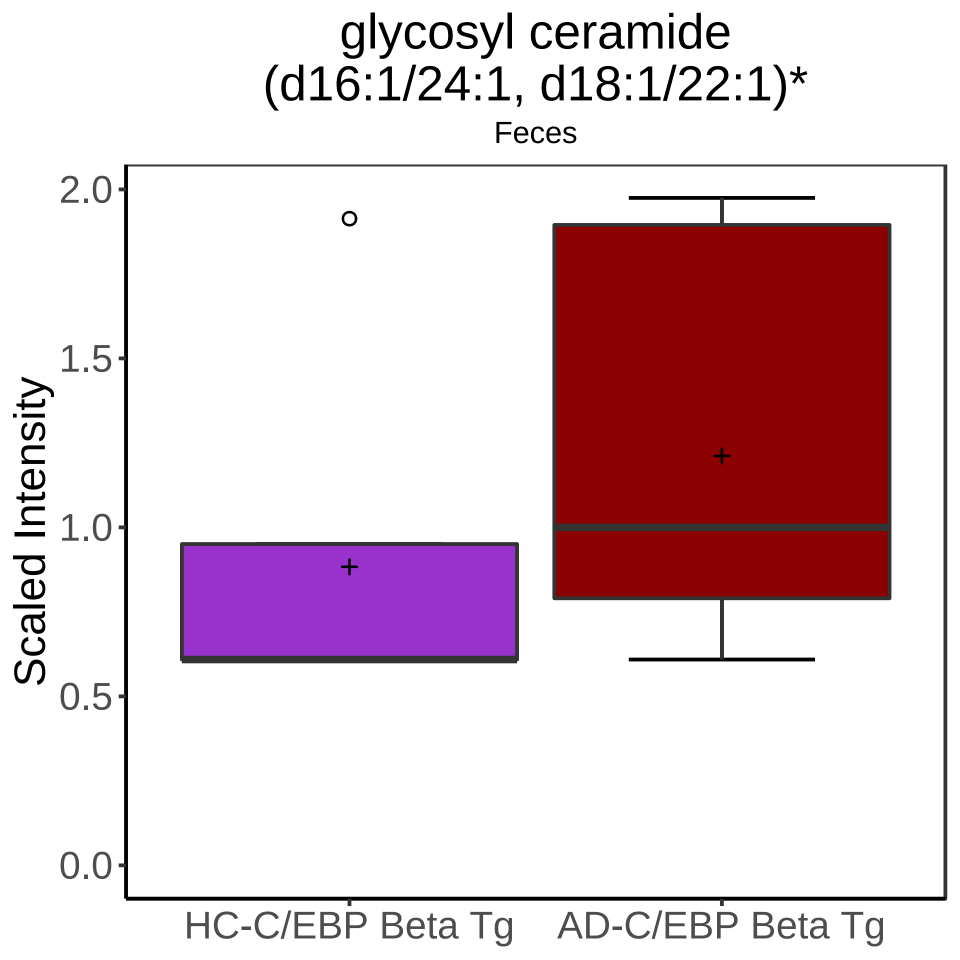

glycosyl ceramide
(d16:1/24:1, d18:1/22:1)*
Feces
Scaled Intensity
2.0
1.5
1.0
0.5
0.0
HC-C/EBP Beta Tg
AD-C/EBP Beta Tg

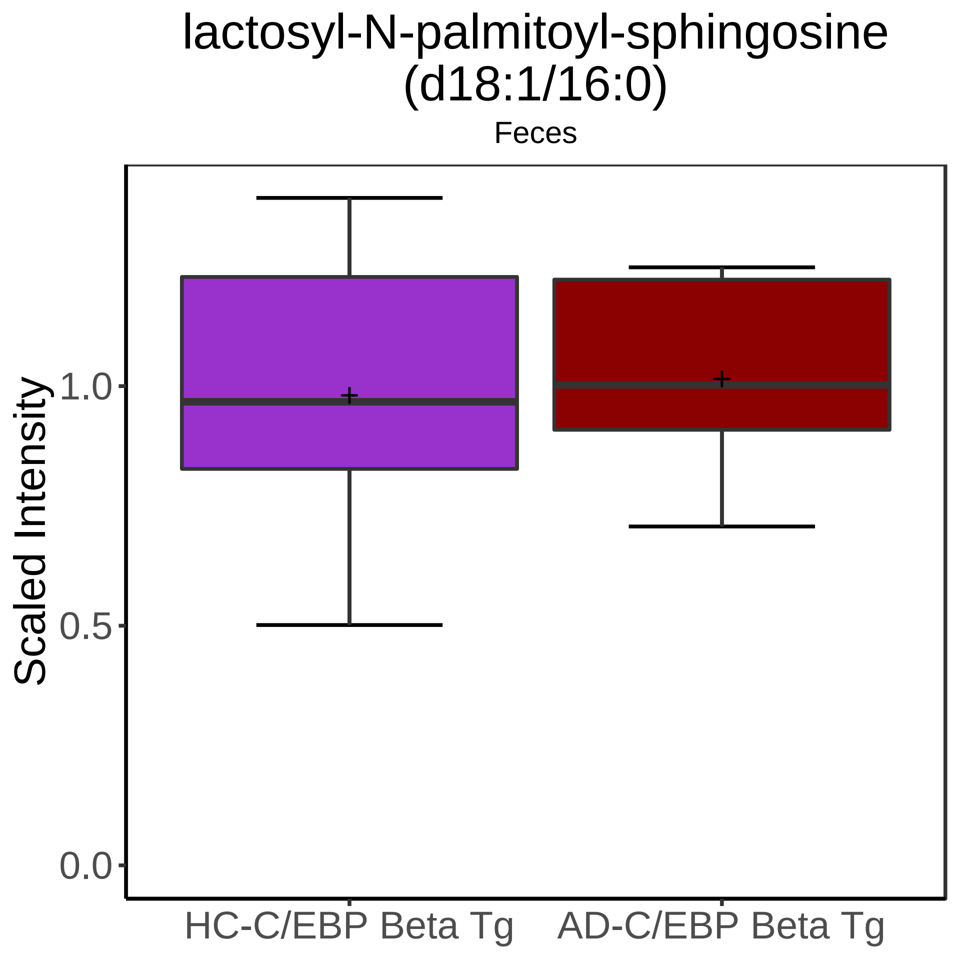
lactosyl-N-palmitoyl-sphingosine
(d18:1/16:0)
Feces
Scaled Intensity
1.0
0.5
0.0
HC-C/EBP Beta Tg
AD-C/EBP Beta Tg

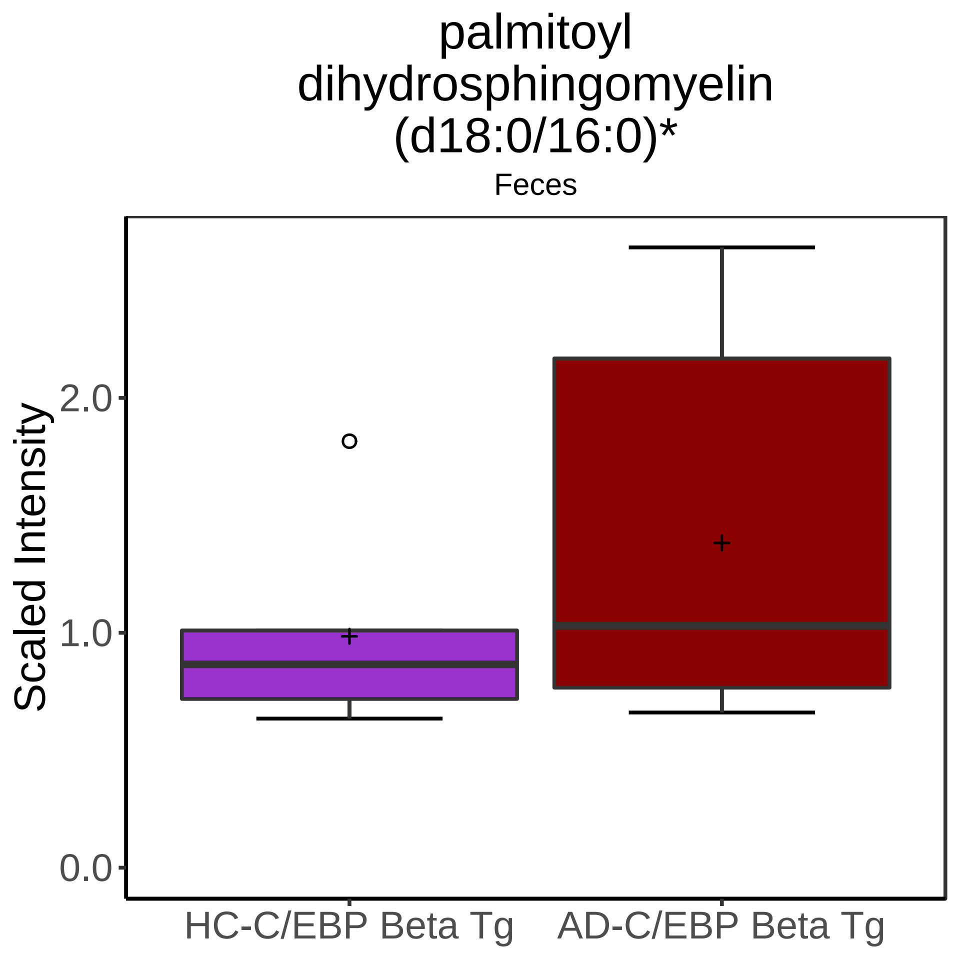

palmitoyl dihydrosphingomyelin (d18:0/16:0)*
Feces
Scaled Intensity
2.0
1.0
0.0
HC-C/EBP Beta Tg
AD-C/EBP Beta Tg

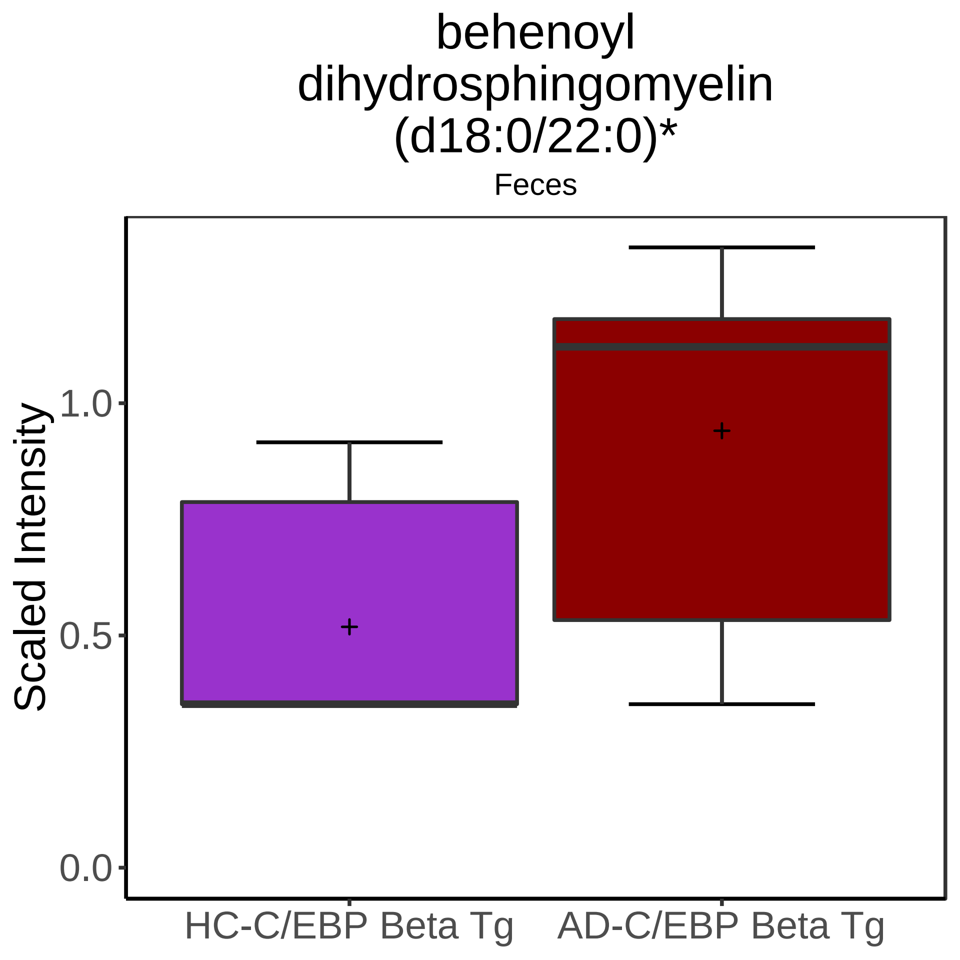
behenoyl
dihydrosphingomyelin
(d18:0/22:0)*
Feces
Scaled Intensity
1.0
0.5
0.0
HC-C/EBP Beta Tg
AD-C/EBP Beta Tg

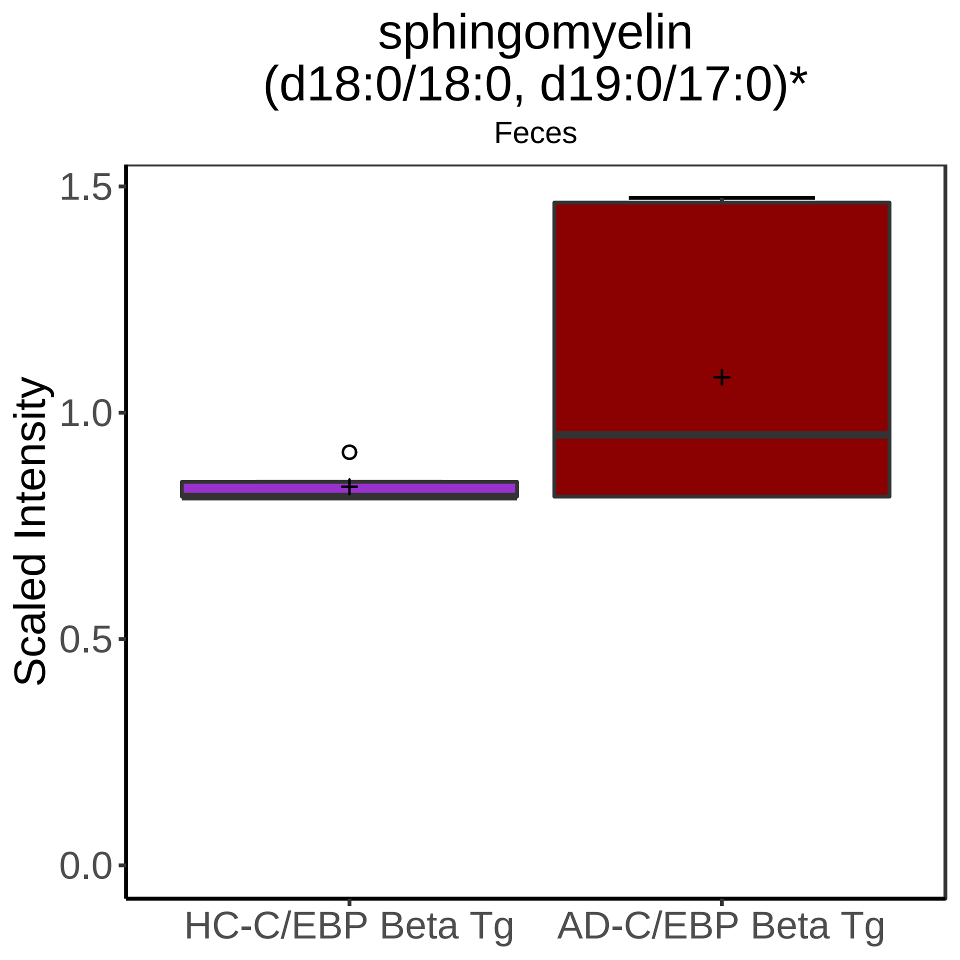
sphingomyelin
(d18:0/18:0, d19:0/17:0)*
Feces
Scaled Intensity
1.5
1.0
0.5
0.0
HC-C/EBP Beta Tg
AD-C/EBP Beta Tg

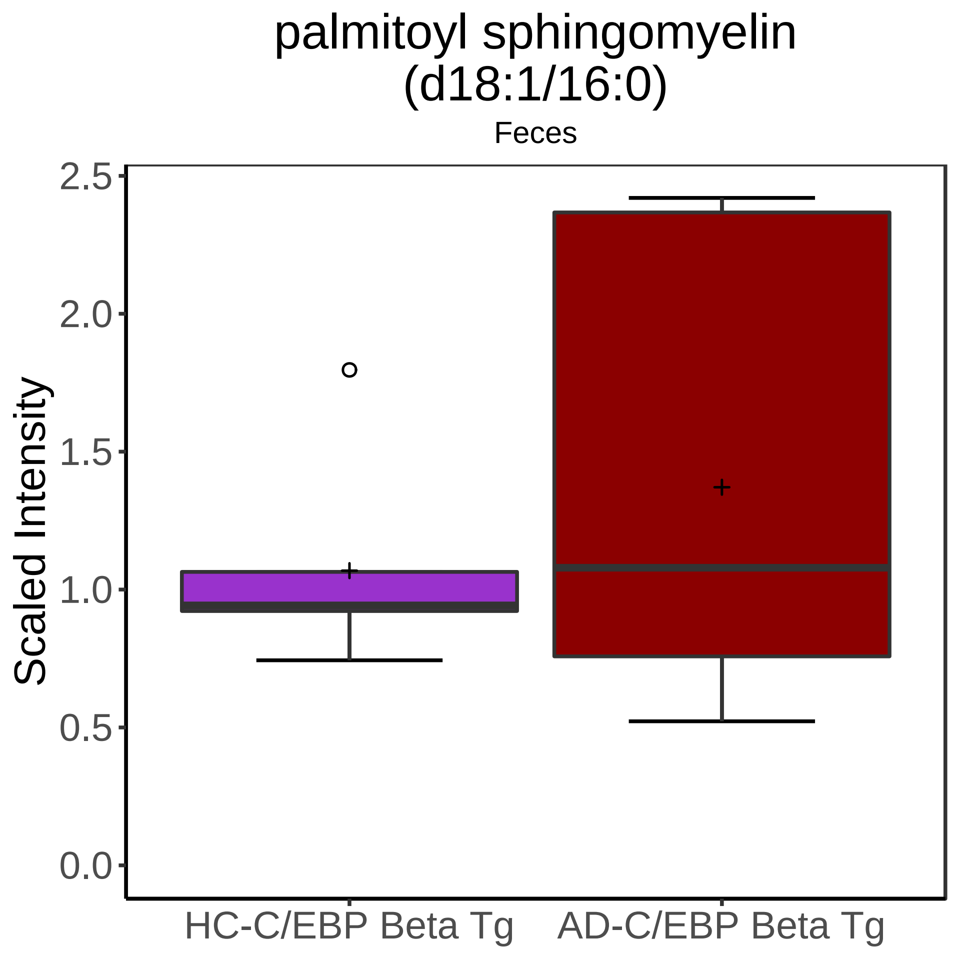
palmitoyl sphingomyelin
(d18:1/16:0)
Feces
Scaled Intensity
2.5
2.0
1.5
1.0
0.5
0.0
HC-C/EBP Beta Tg
AD-C/EBP Beta Tg

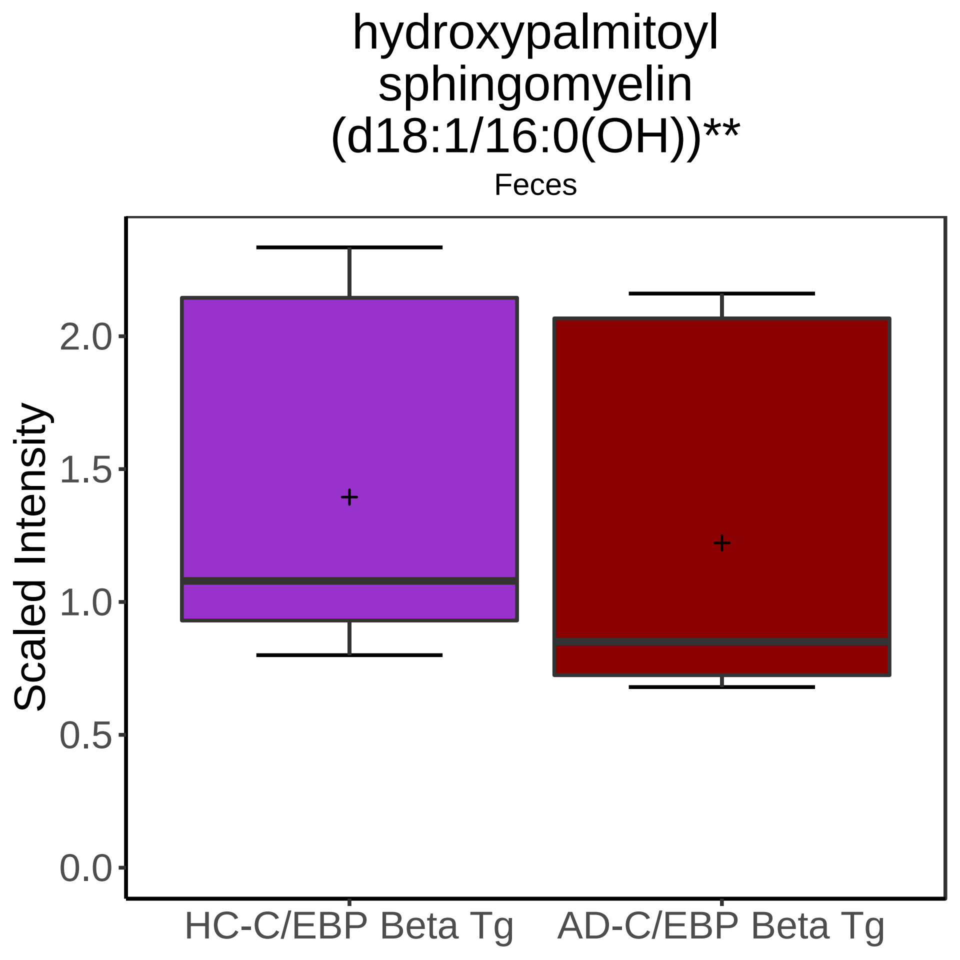
hydroxypalmitoyl
sphingomyelin
(d18:1/16:0(OH))**
Feces
Scaled Intensity
2.0
1.5
1.0
0.5
0.0
HC-C/EBP Beta Tg
AD-C/EBP Beta Tg

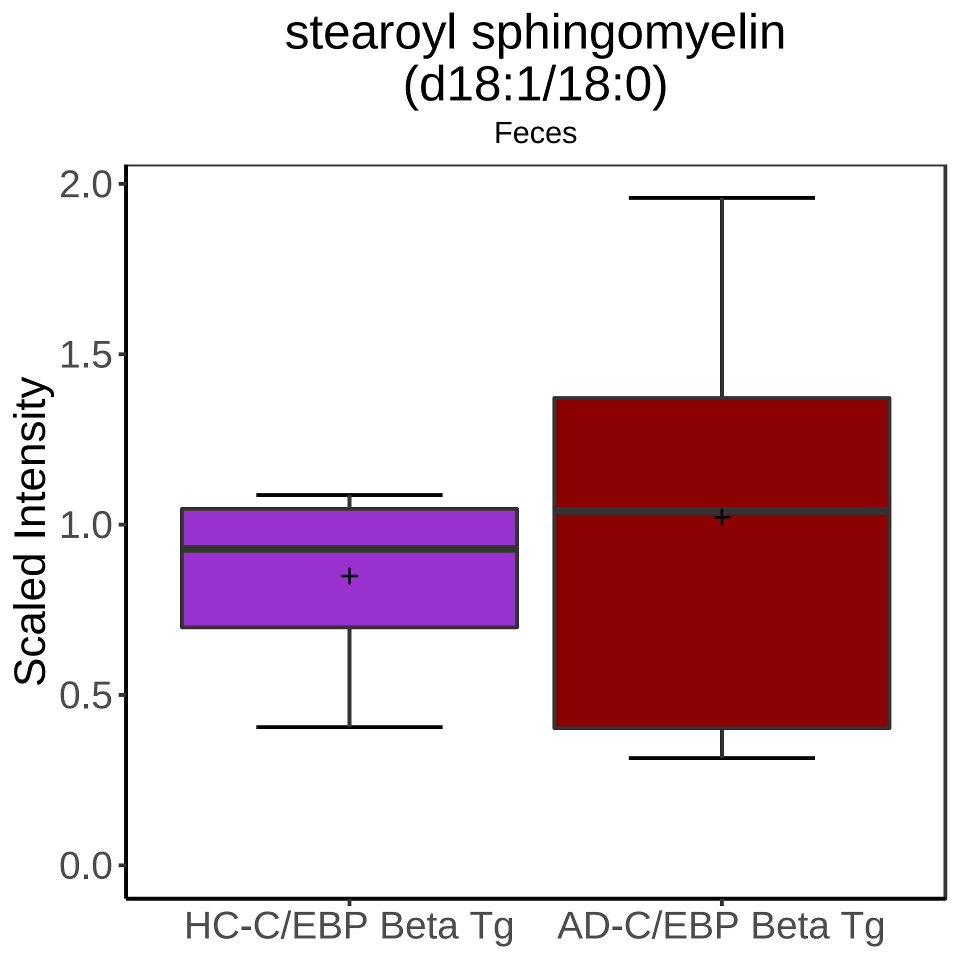

stearoyl sphingomyelin
(d18:1/18:0)
Feces
Scaled Intensity
2.0
1.5
1.0
0.5
0.0
HC-C/EBP Beta Tg
AD-C/EBP Beta Tg

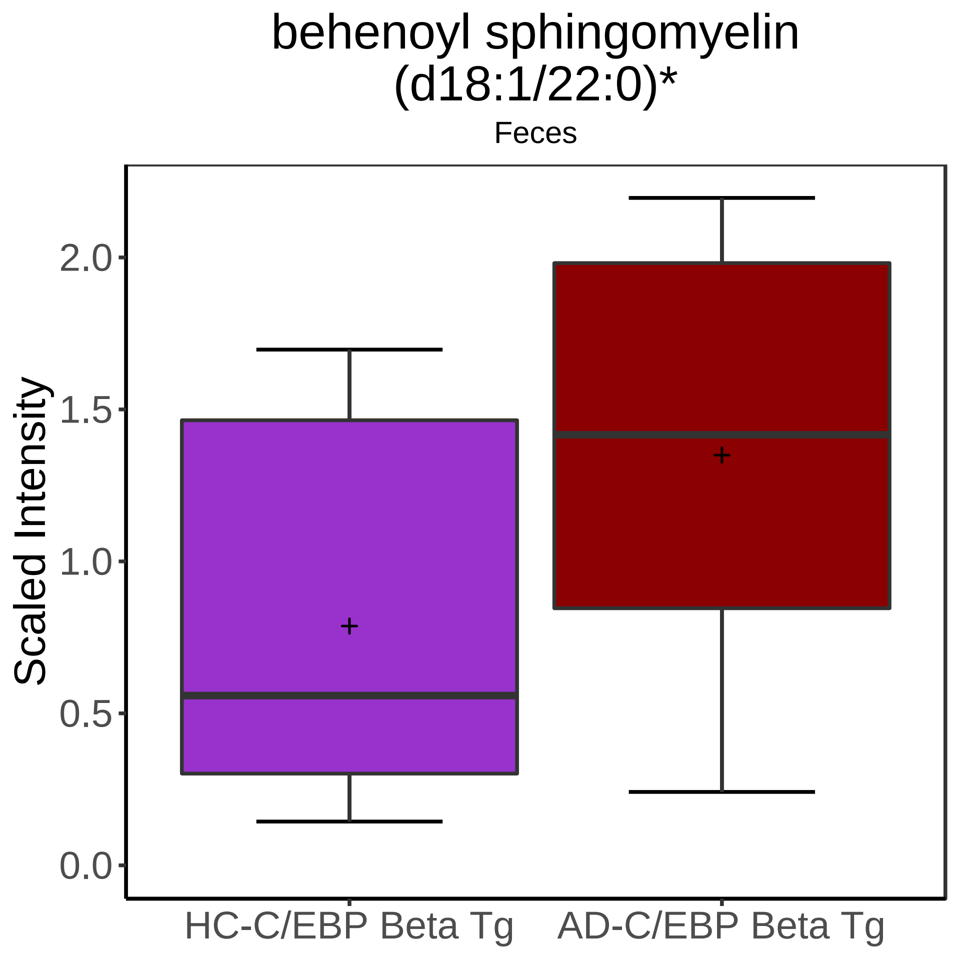

behenoyl sphingomyelin
(d18:1/22:0)*
Feces
Scaled Intensity
2.0
1.5
1.0
0.5
0.0
HC-C/EBP Beta Tg
AD-C/EBP Beta Tg

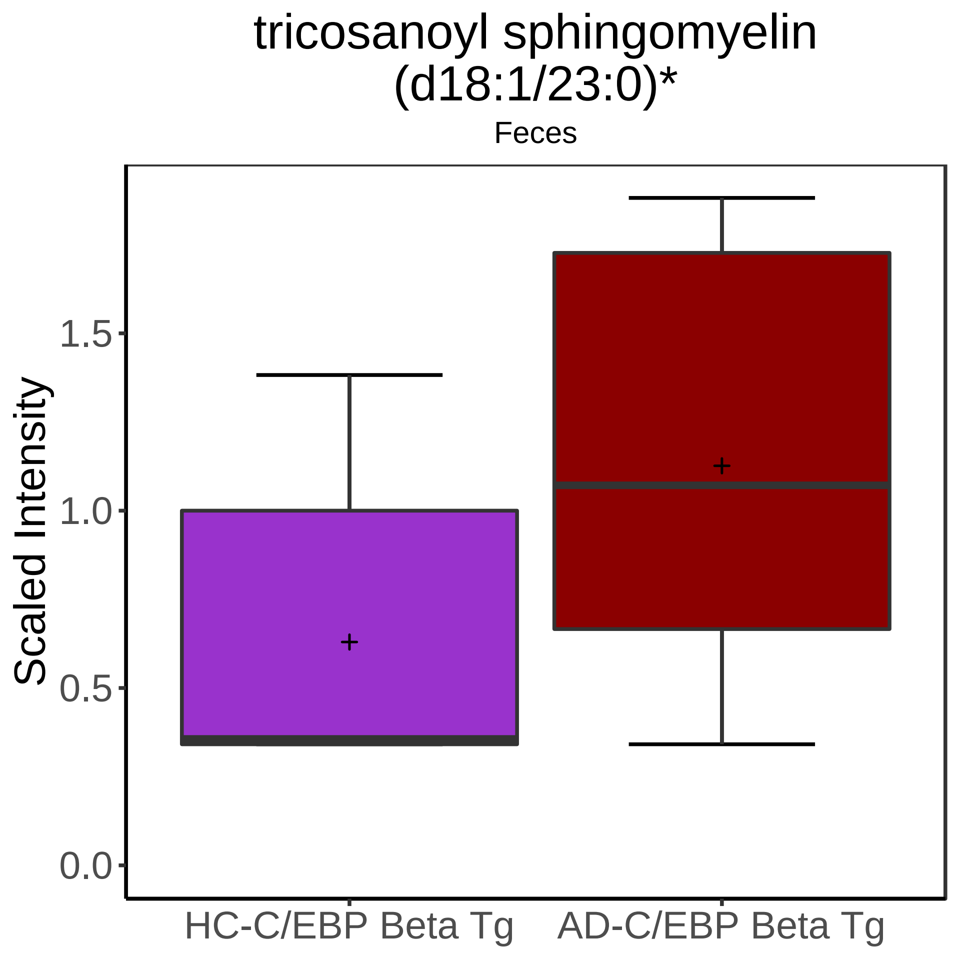

tricosanoyl sphingomyelin
(d18:1/23:0)*
Feces
Scaled Intensity
1.5
1.0
0.5
0.0
HC-C/EBP Beta Tg
AD-C/EBP Beta Tg

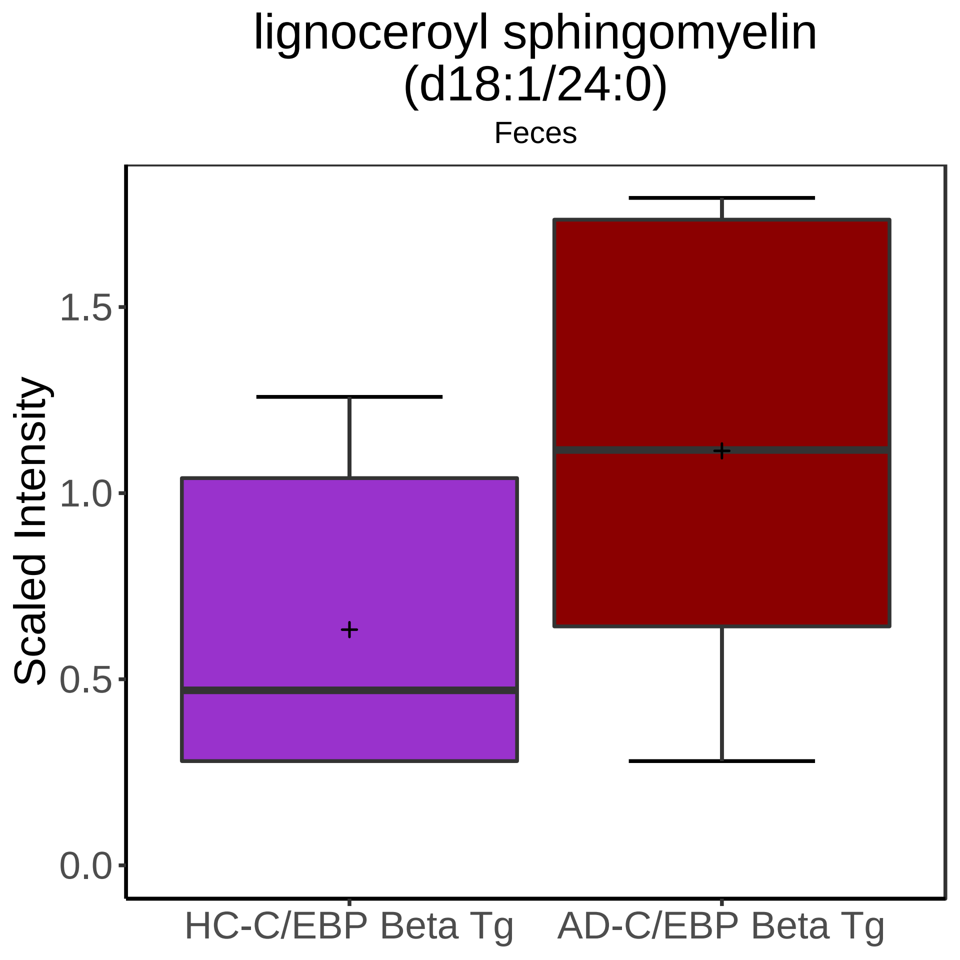

lignoceroyl sphingomyelin
(d18:1/24:0)
Feces
Scaled Intensity
1.5
1.0
0.5
0.0
HC-C/EBP Beta Tg
AD-C/EBP Beta Tg

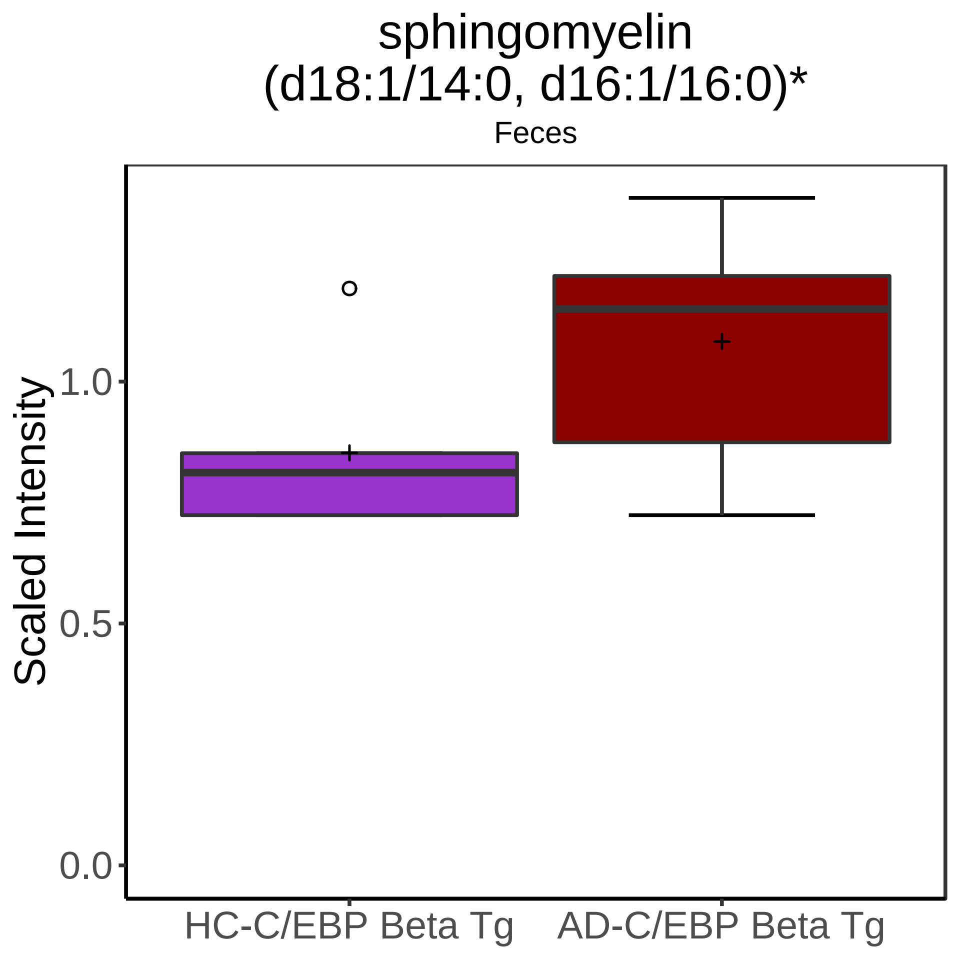

sphingomyelin
(d18:1/14:0, d16:1/16:0)*
Feces
Scaled Intensity
1.0
0.5
0.0
HC-C/EBP Beta Tg
AD-C/EBP Beta Tg

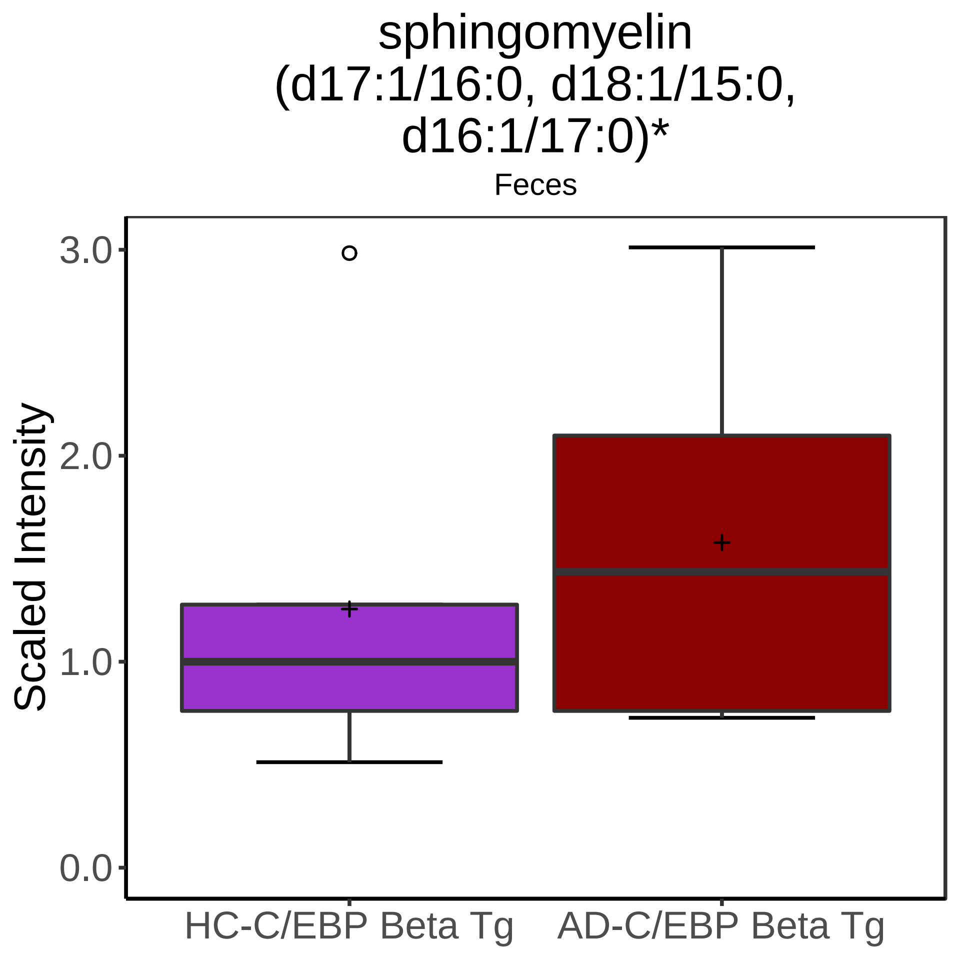
sphingomyelin
(d17:1/16:0, d18:1/15:0,
d16:1/17:0)*
Feces
Scaled Intensity
3.0
2.0
1.0
0.0
HC-C/EBP Beta Tg
AD-C/EBP Beta Tg

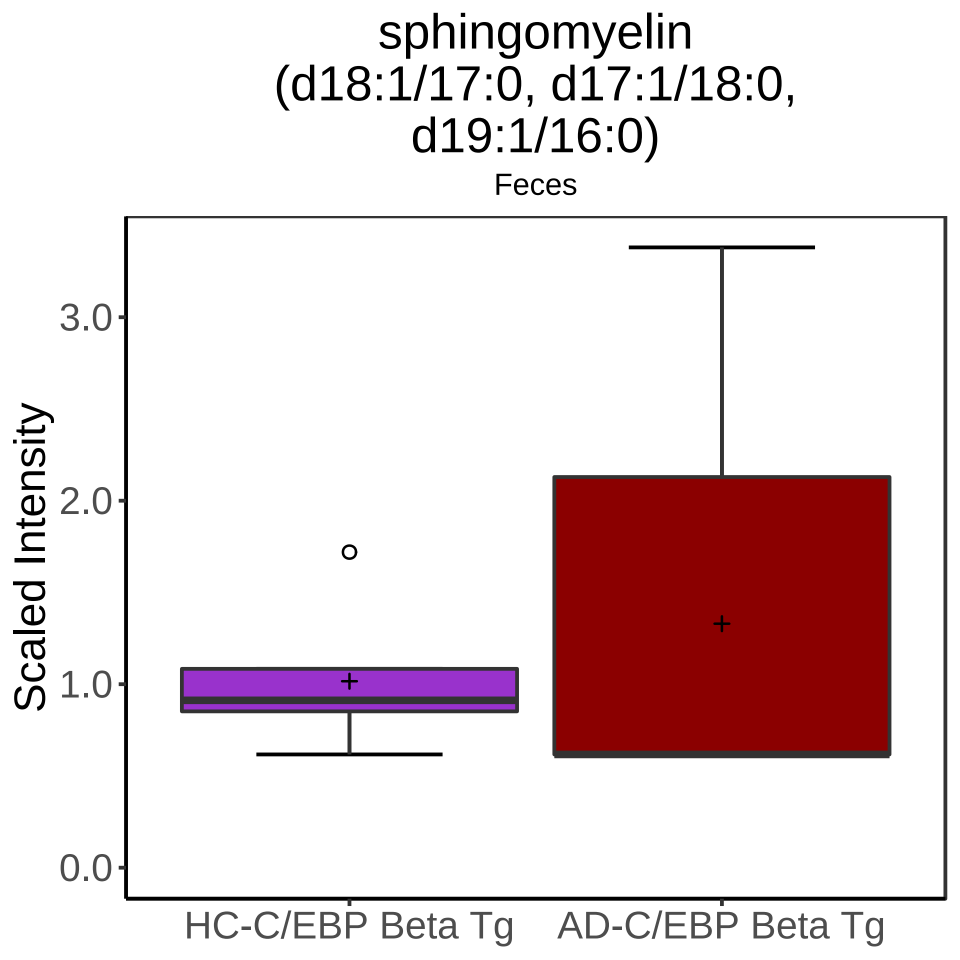
sphingomyelin
(d18:1/17:0, d17:1/18:0,
d19:1/16:0)
Feces
Scaled Intensity
3.0
2.0
1.0
0.0
HC-C/EBP Beta Tg
AD-C/EBP Beta Tg

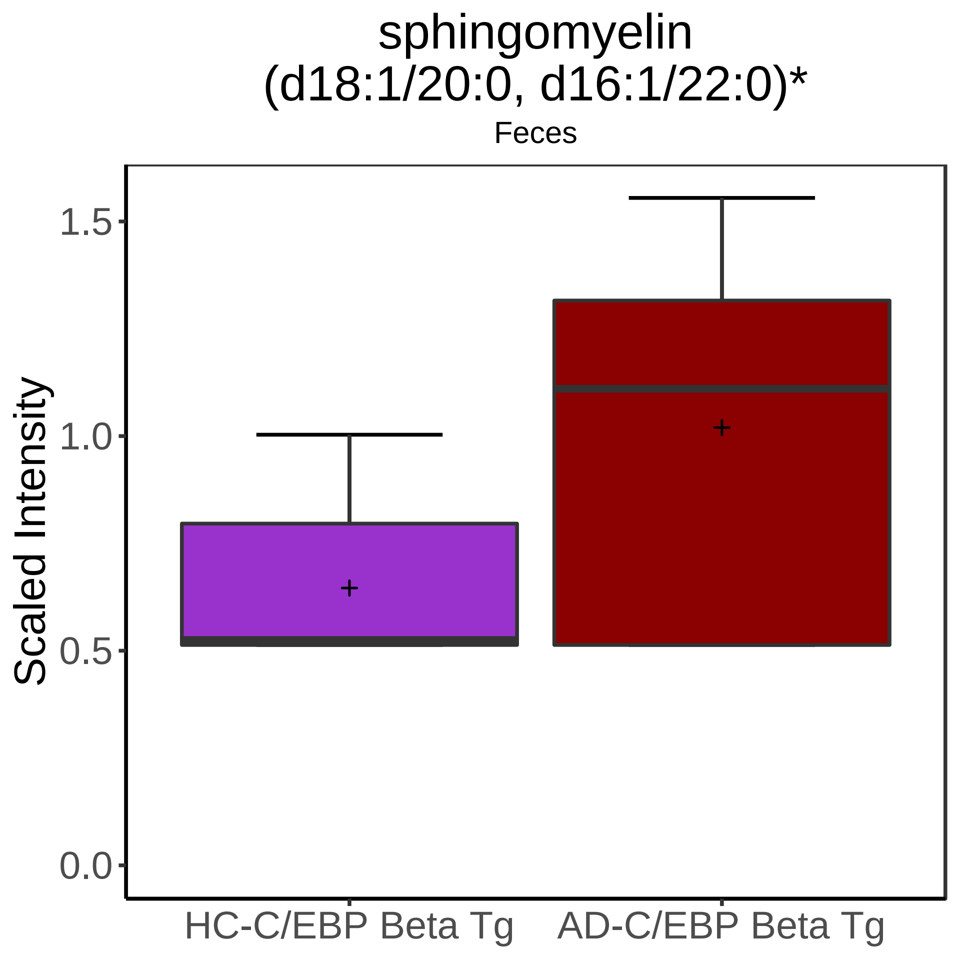

sphingomyelin
(d18:1/20:0, d16:1/22:0)*
Feces
Scaled Intensity
1.5
1.0
0.5
0.0
HC-C/EBP Beta Tg
AD-C/EBP Beta Tg

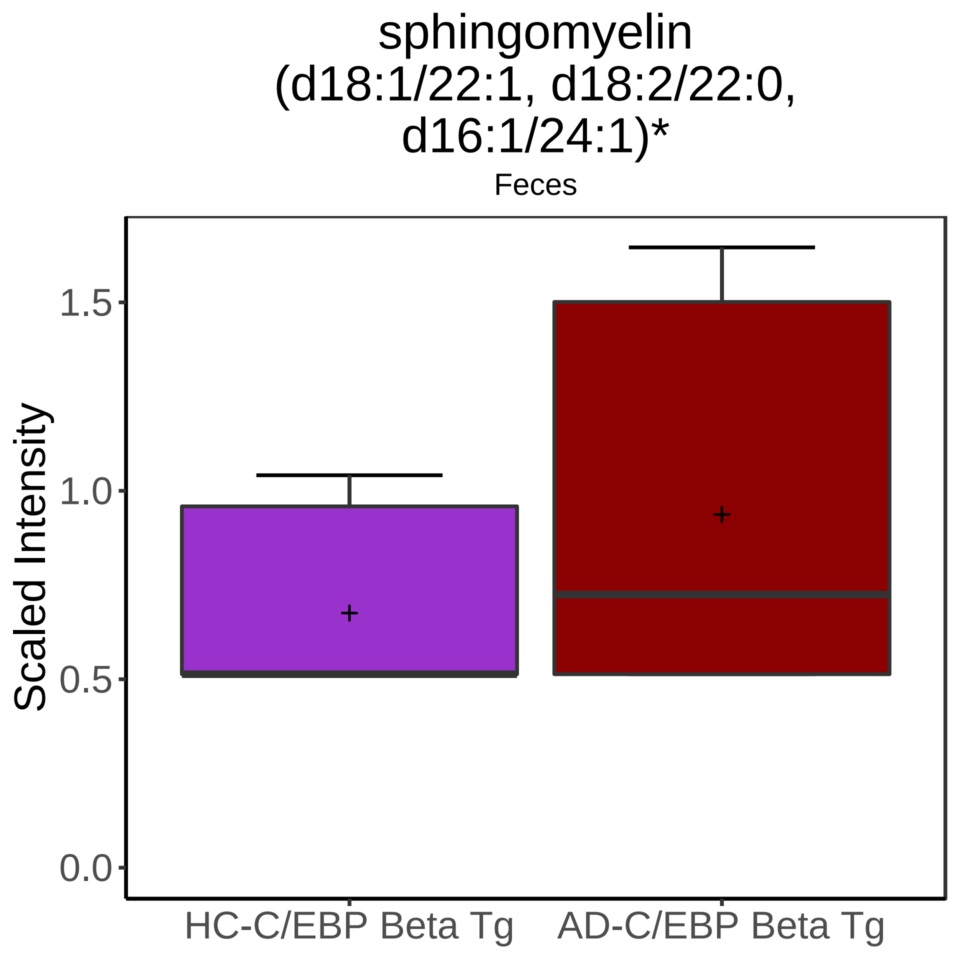
sphingomyelin
(d18:1/22:1, d18:2/22:0,
d16:1/24:1)*
Feces
Scaled Intensity
0.0
0.5
1.0
1.5
HC-C/EBP Beta Tg
AD-C/EBP Beta Tg

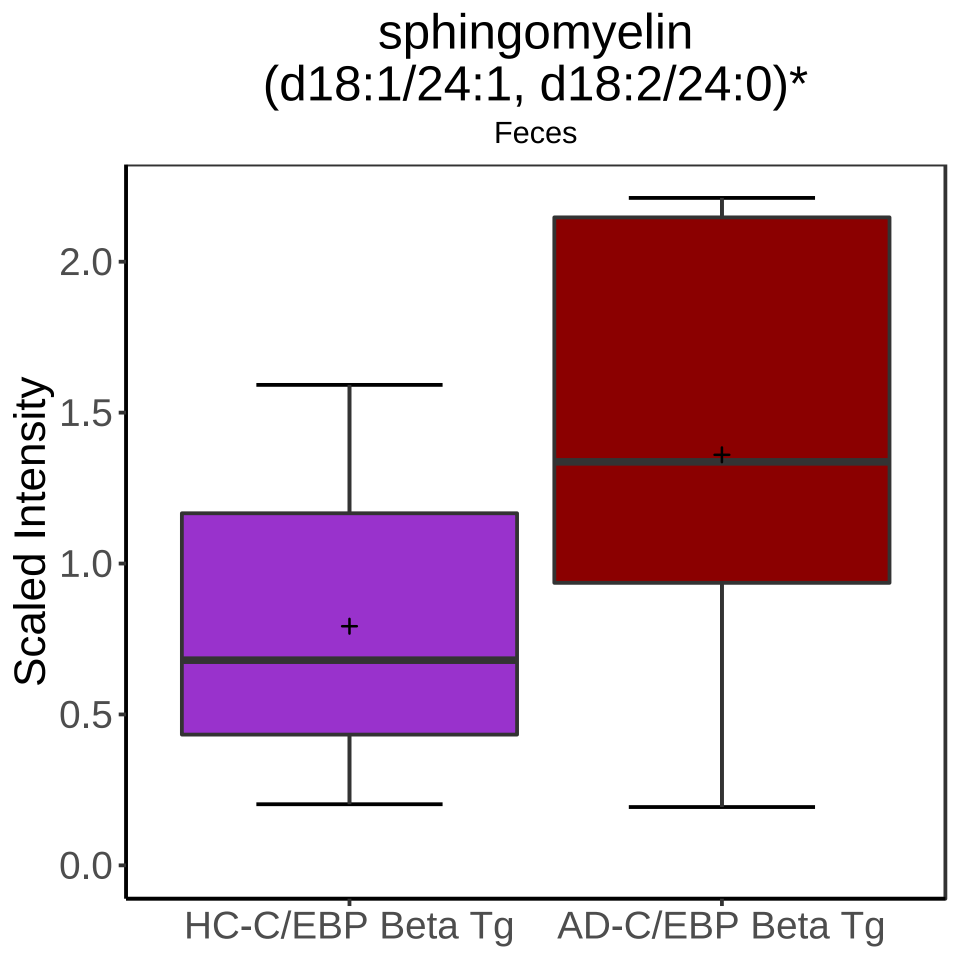
sphingomyelin
(d18:1/24:1, d18:2/24:0)*
Feces
Scaled Intensity
2.0
1.5
1.0
0.5
0.0
HC-C/EBP Beta Tg
AD-C/EBP Beta Tg

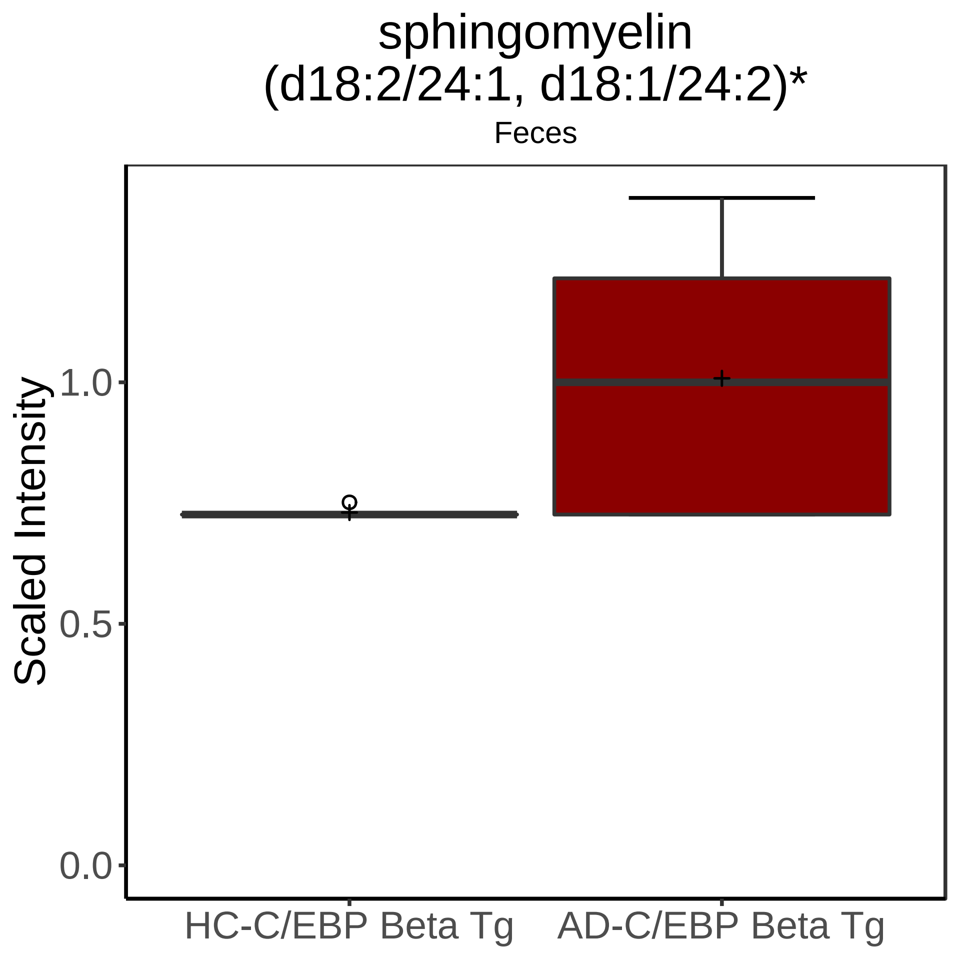
sphingomyelin
(d18:2/24:1, d18:1/24:2)*
Feces
Scaled Intensity
1.0
0.5
0.0
HC-C/EBP Beta Tg
AD-C/EBP Beta Tg

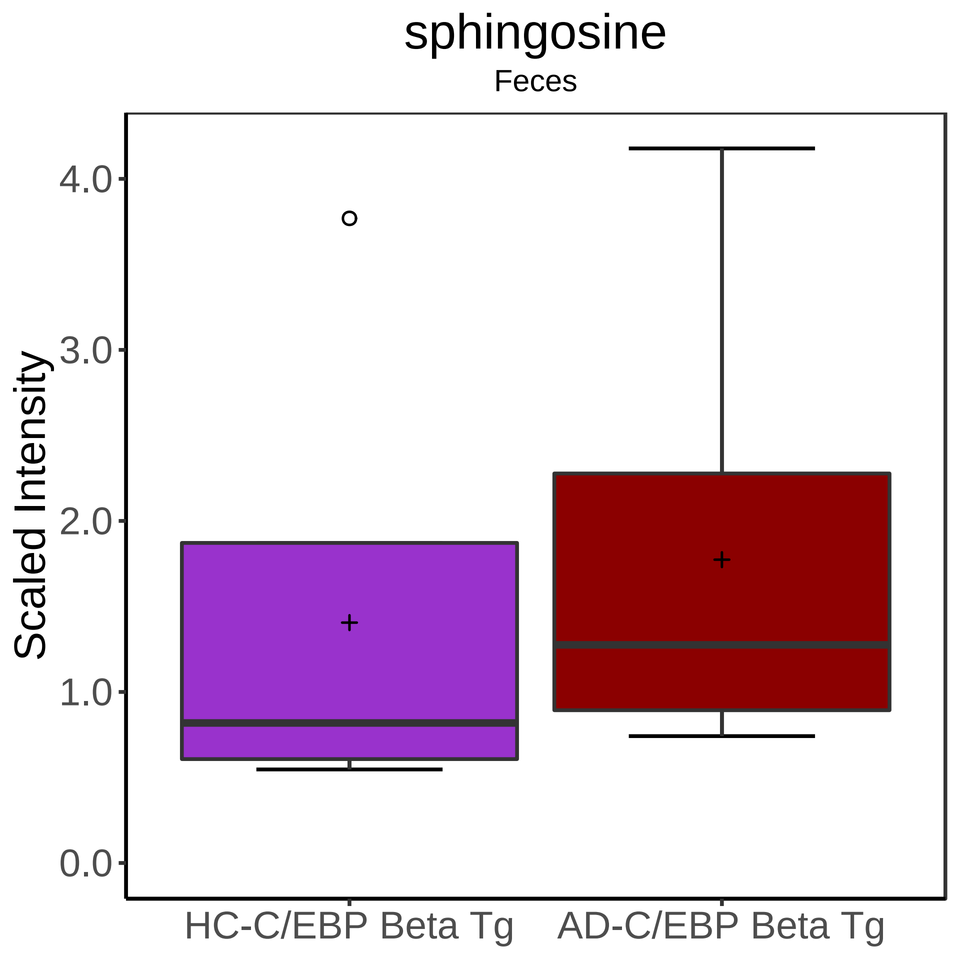
sphingosine
Feces
Scaled Intensity
4.0
3.0
2.0
1.0
0.0
HC-C/EBP Beta Tg
AD-C/EBP Beta Tg

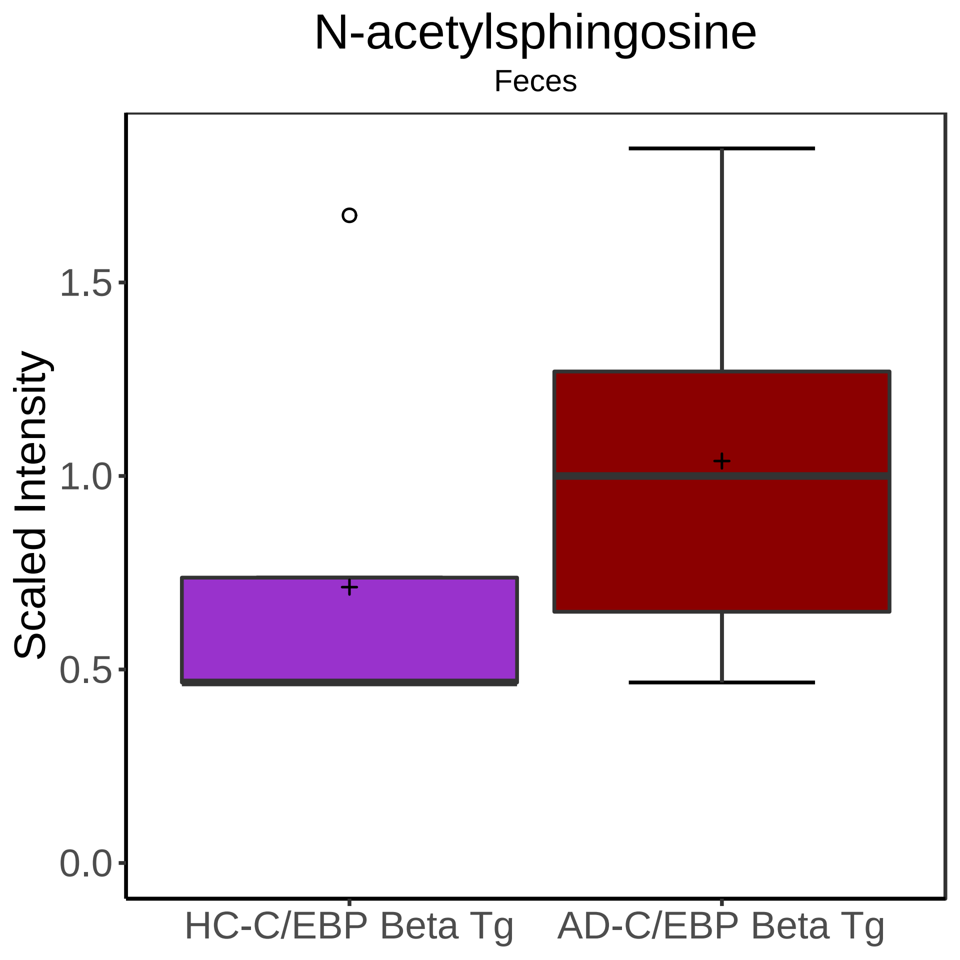

N-acetylsphingosine
Feces
Scaled Intensity
1.5
1.0
0.5
0.0
HC-C/EBP Beta Tg
AD-C/EBP Beta Tg

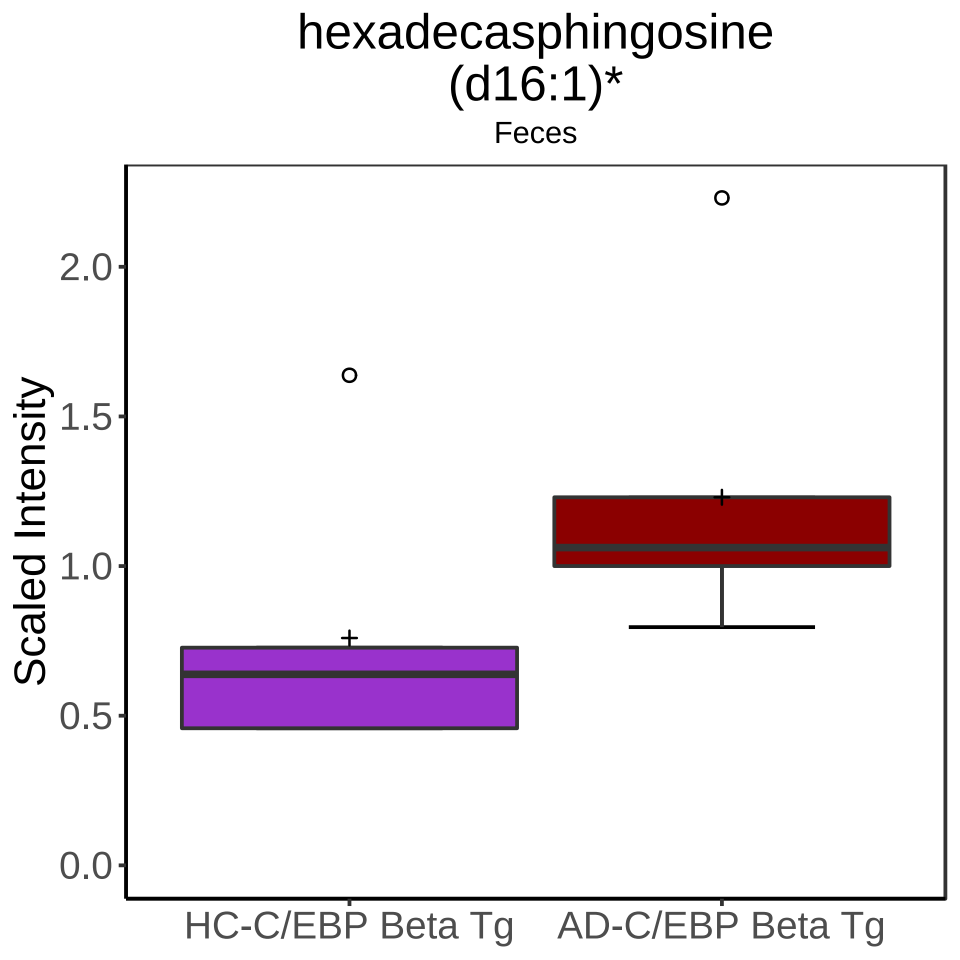

hexadecasphingosine
(d16:1)*
Feces
Scaled Intensity
2.0
1.5
1.0
0.5
0.0
HC-C/EBP Beta Tg
AD-C/EBP Beta Tg

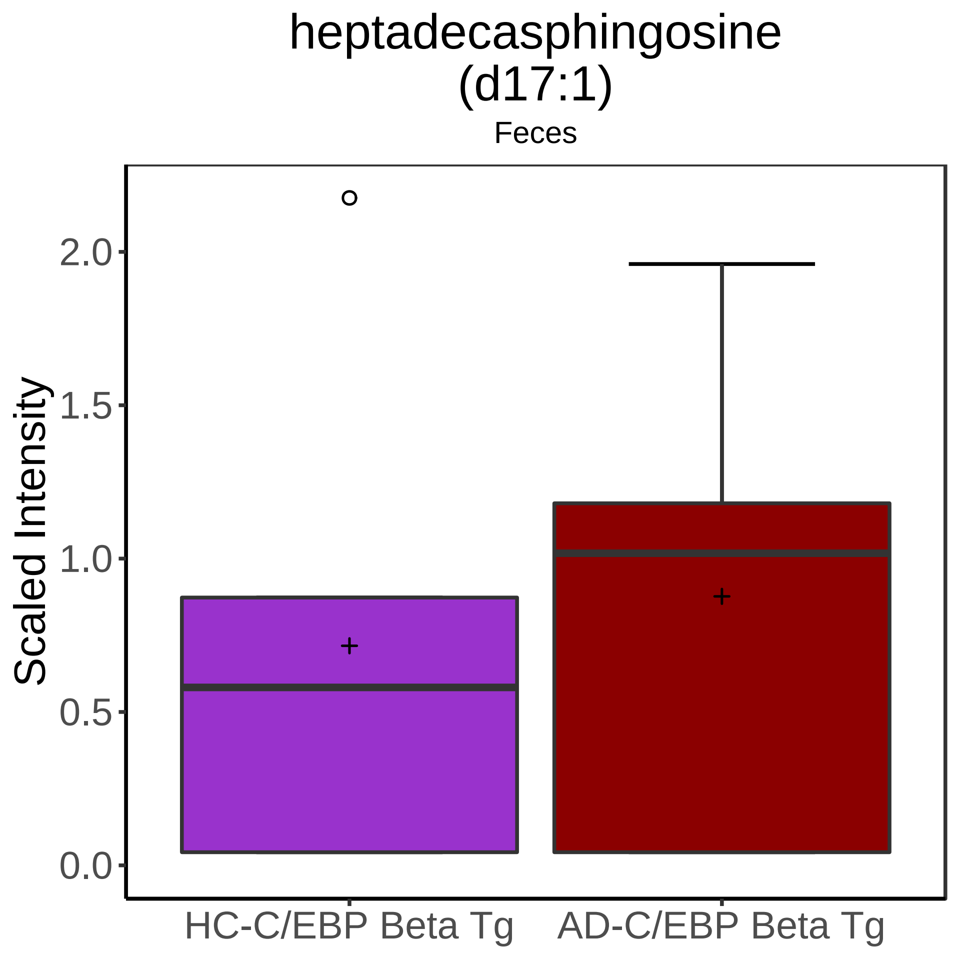

heptadecasphingosine
(d17:1)
Feces
Scaled Intensity
2.0
1.5
1.0
0.5
0.0
HC-C/EBP Beta Tg
AD-C/EBP Beta Tg

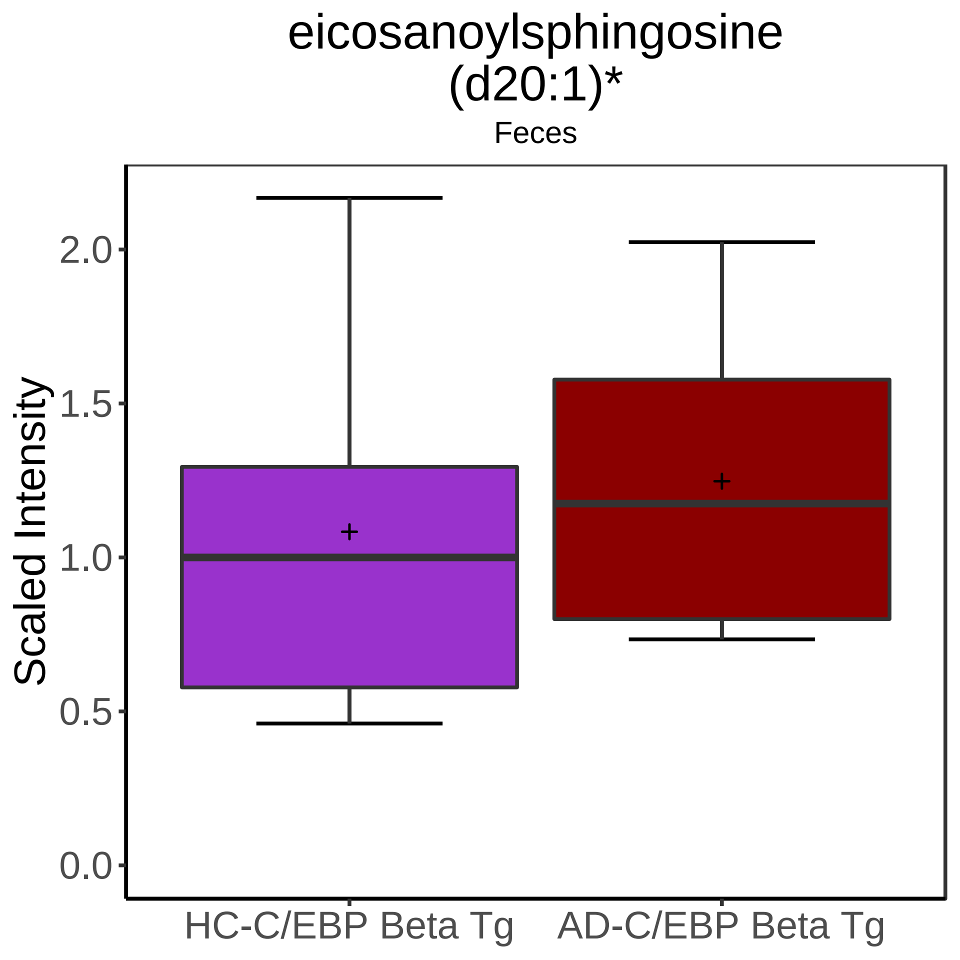
eicosanoylsphingosine
(d20:1)*
Feces
Scaled Intensity
2.0
1.5
1.0
0.5
0.0
HC-C/EBP Beta Tg
AD-C/EBP Beta Tg

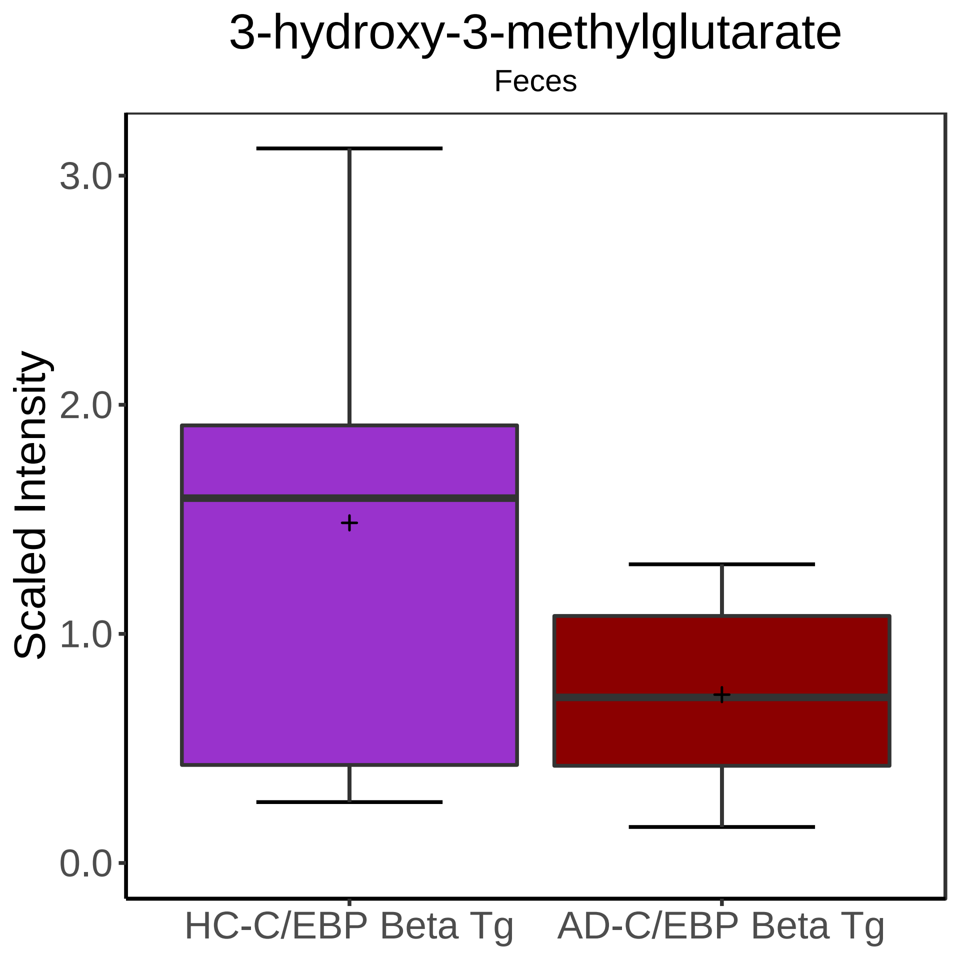
3-hydroxy-3-methylglutarate
Feces
Scaled Intensity
3.0
2.0
1.0
0.0
HC-C/EBP Beta Tg
AD-C/EBP Beta Tg

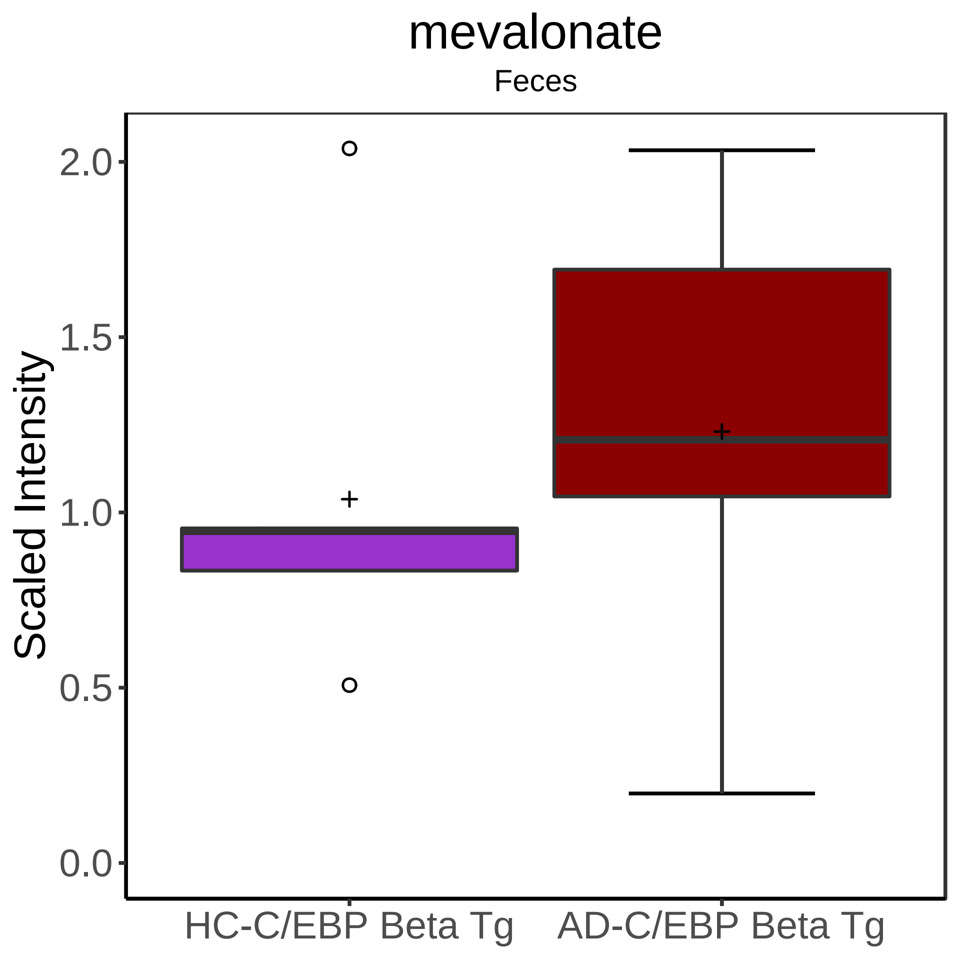

mevalonate
Feces
Scaled Intensity
2.0
1.5
1.0
0.5
0.0
HC-C/EBP Beta Tg
AD-C/EBP Beta Tg

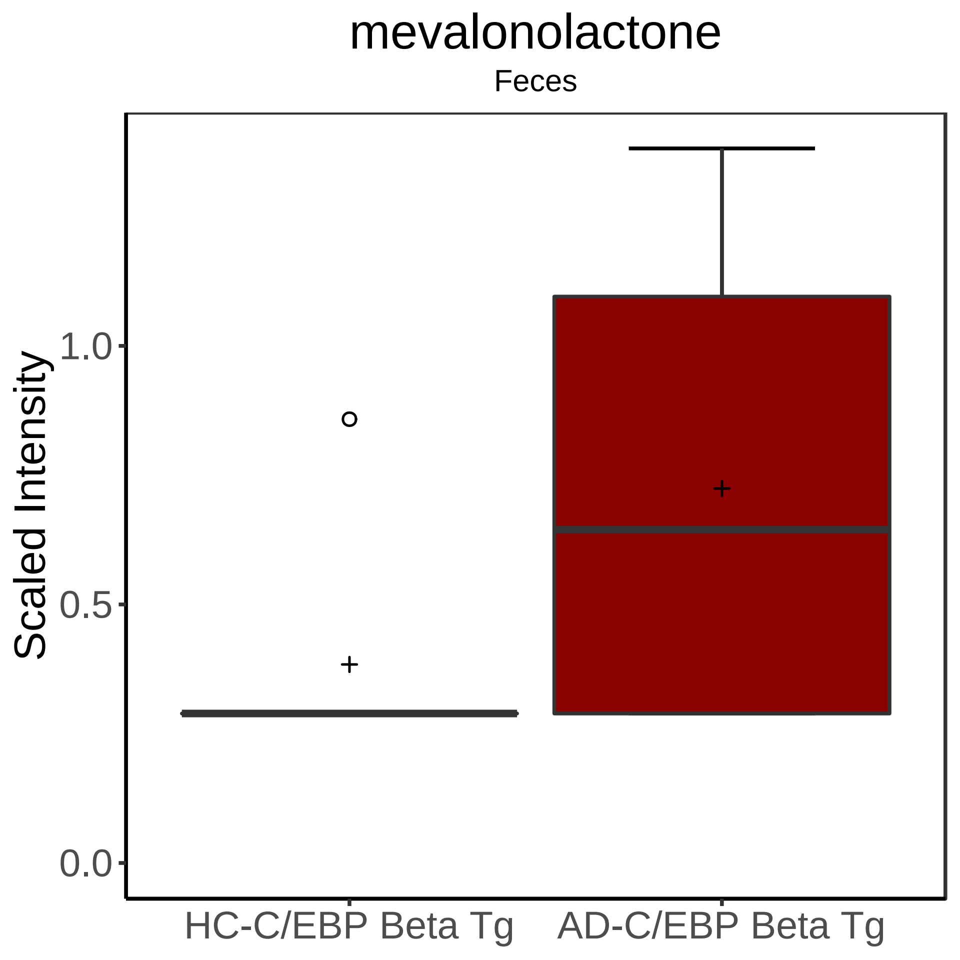
mevalonolactone
Feces
Scaled Intensity
1.0
0.5
0.0
HC-C/EBP Beta Tg
AD-C/EBP Beta Tg

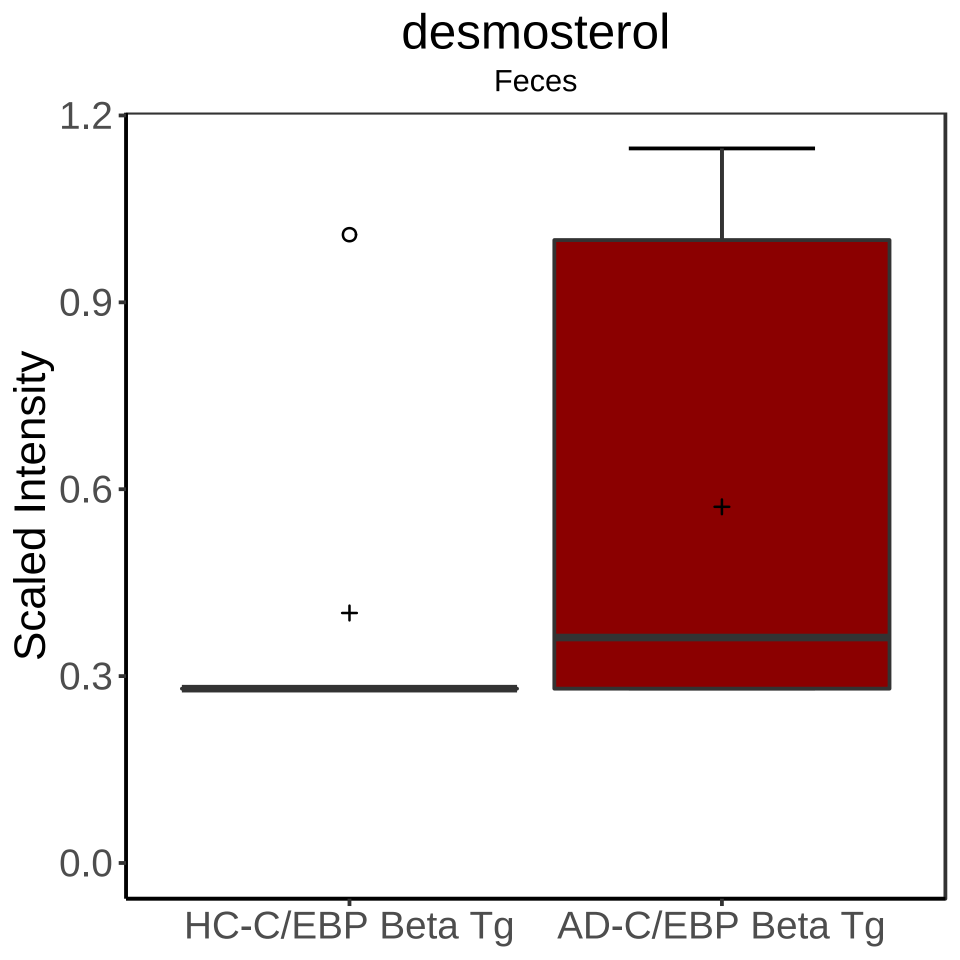
desmosterol
Feces
Scaled Intensity
1.2
0.9
0.6
0.3
0.0
HC-C/EBP Beta Tg
AD-C/EBP Beta Tg

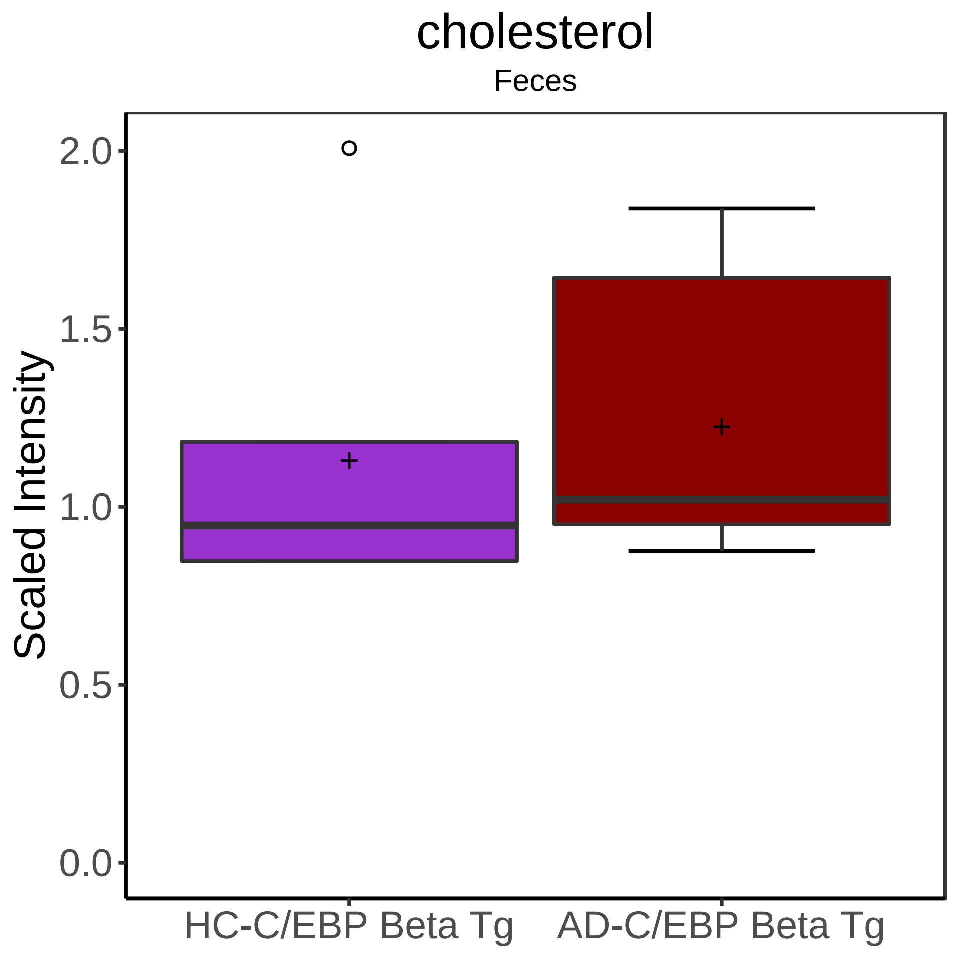
cholesterol
Feces
Scaled Intensity
2.0
1.5
1.0
0.5
0.0
HC-C/EBP Beta Tg
AD-C/EBP Beta Tg

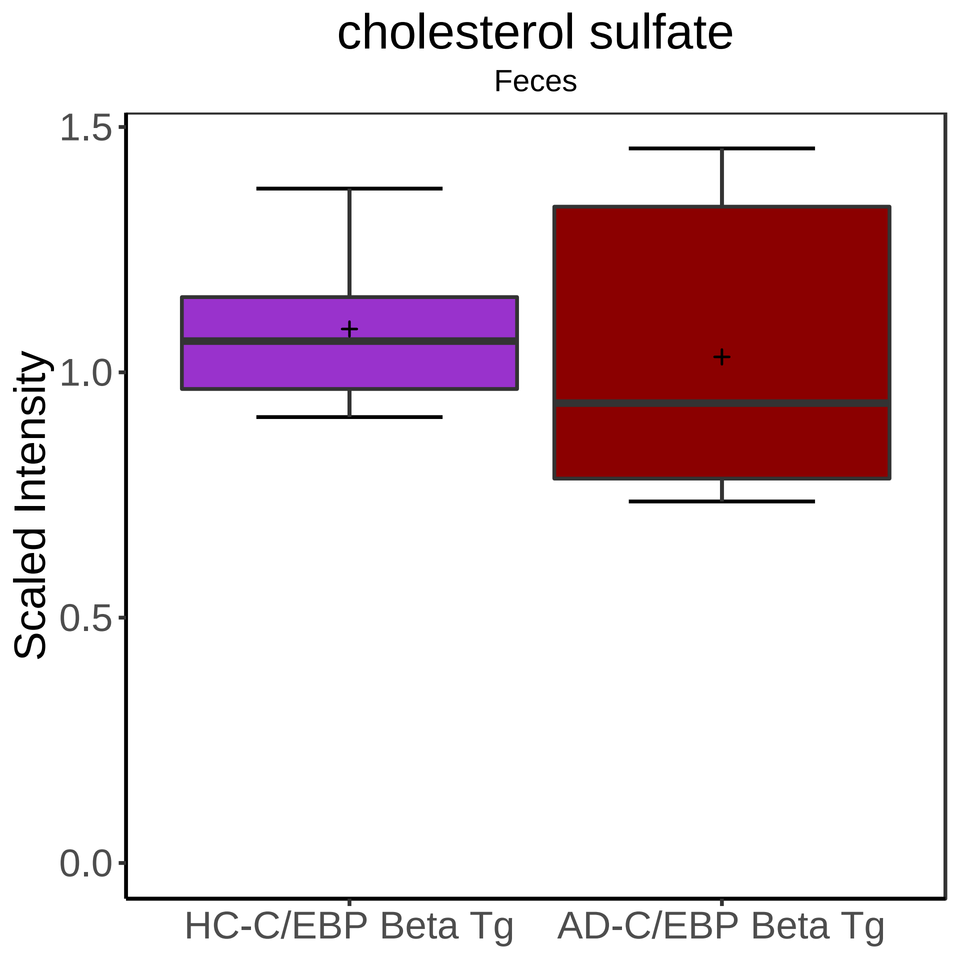

cholesterol sulfate
Feces
Scaled Intensity
1.5
1.0
0.5
0.0
HC-C/EBP Beta Tg
AD-C/EBP Beta Tg

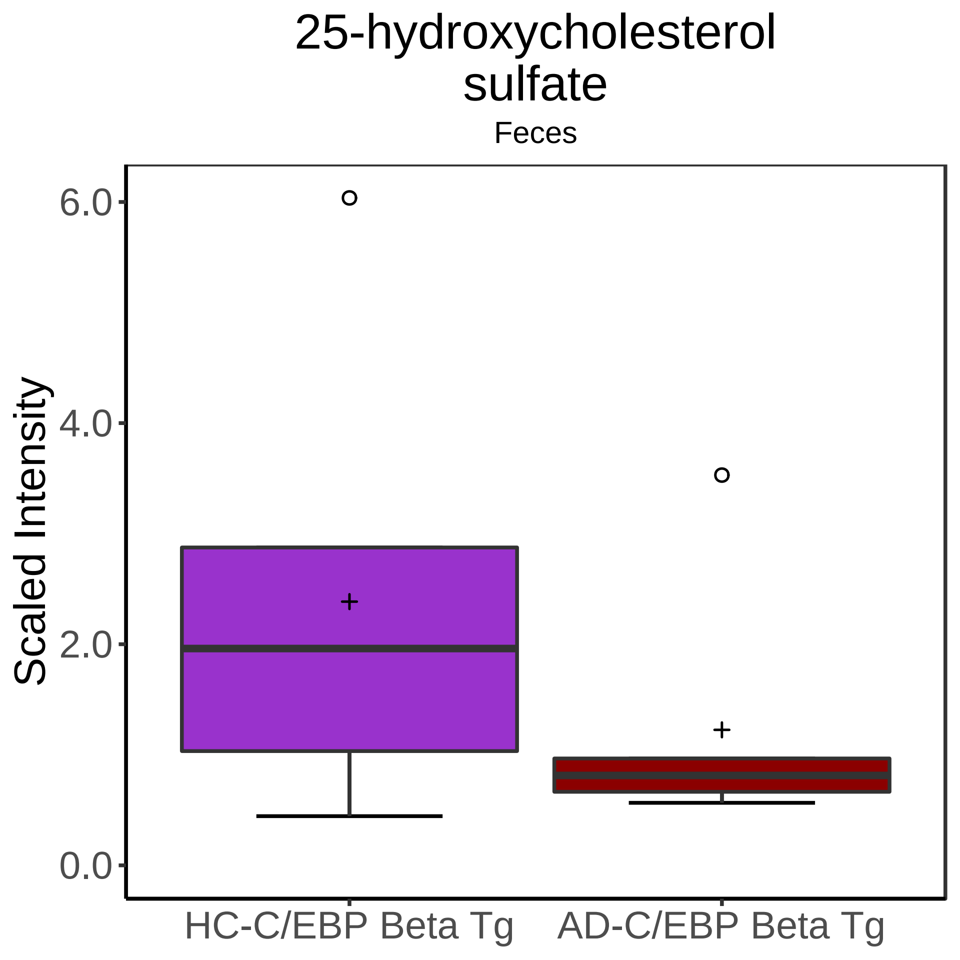
25-hydroxycholesterol sulfate
Feces
Scaled Intensity
6.0
4.0
2.0
0.0
HC-C/EBP Beta Tg
AD-C/EBP Beta Tg

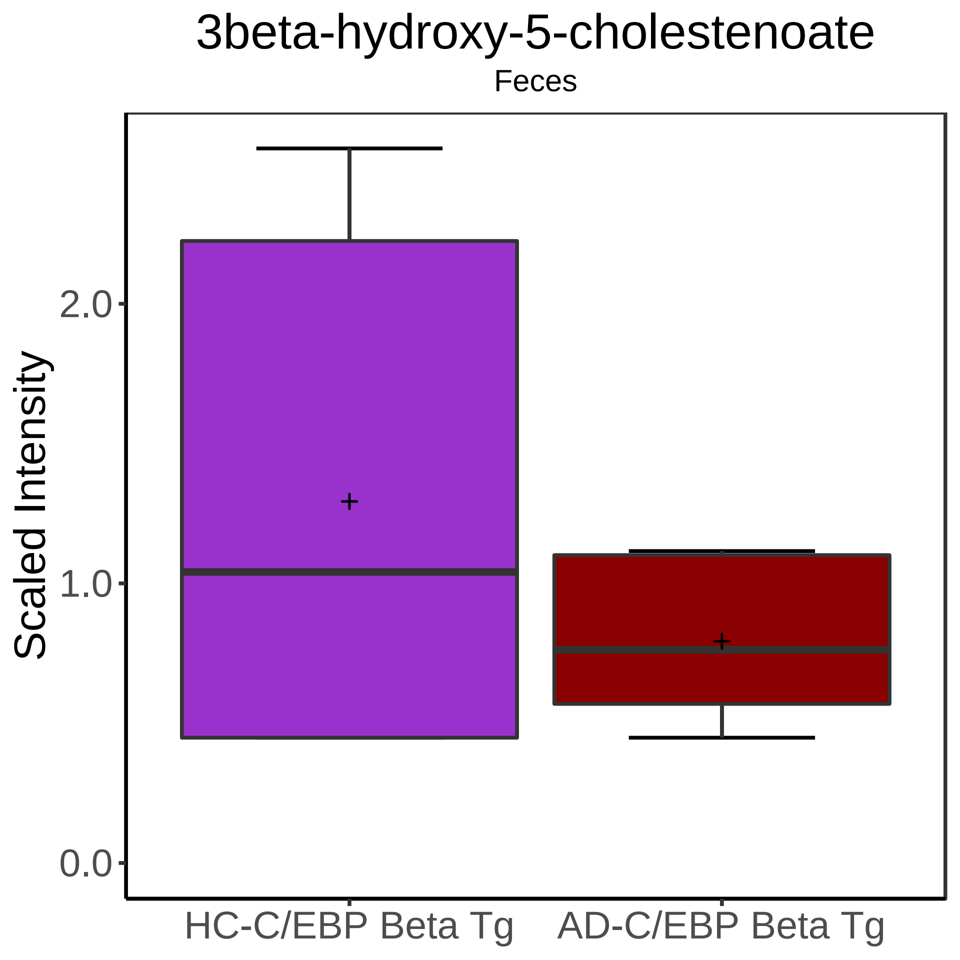

3beta-hydroxy-5-cholestenoate
Feces
Scaled Intensity
2.0
1.0
0.0
HC-C/EBP Beta Tg
AD-C/EBP Beta Tg

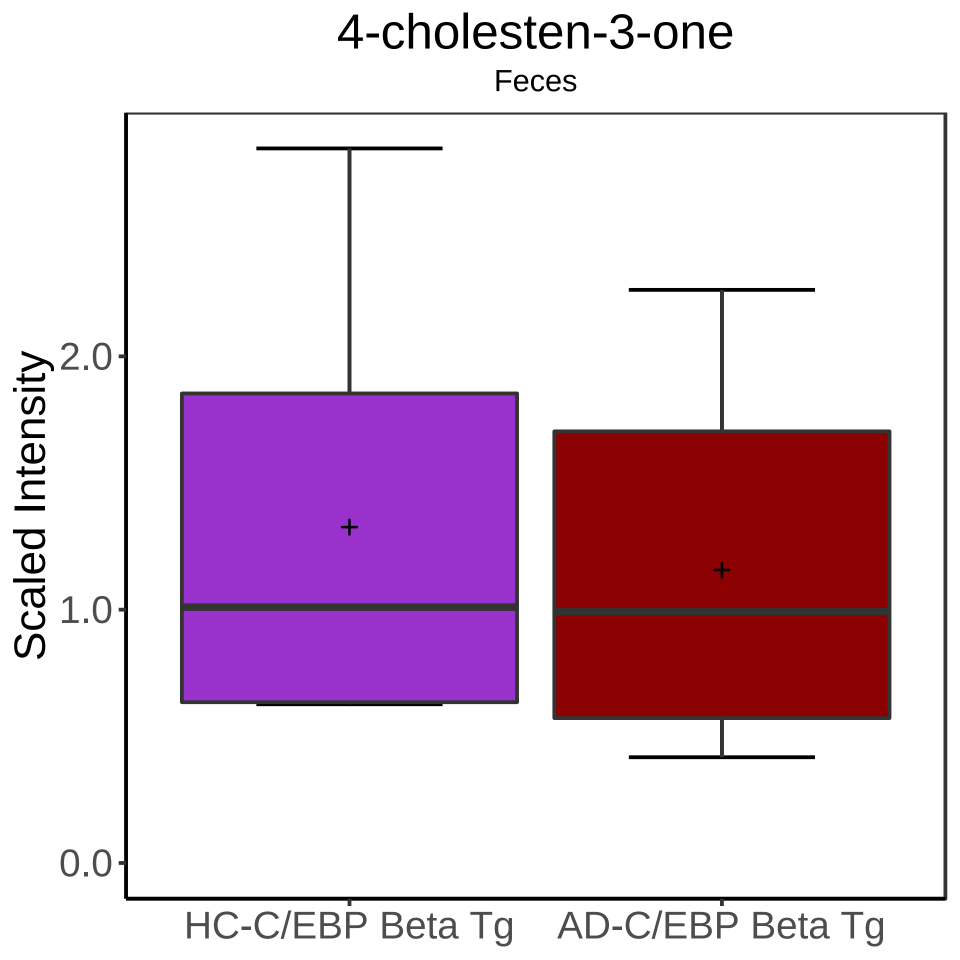
4-cholesten-3-one
Feces
Scaled Intensity
2.0
1.0
0.0
HC-C/EBP Beta Tg
AD-C/EBP Beta Tg

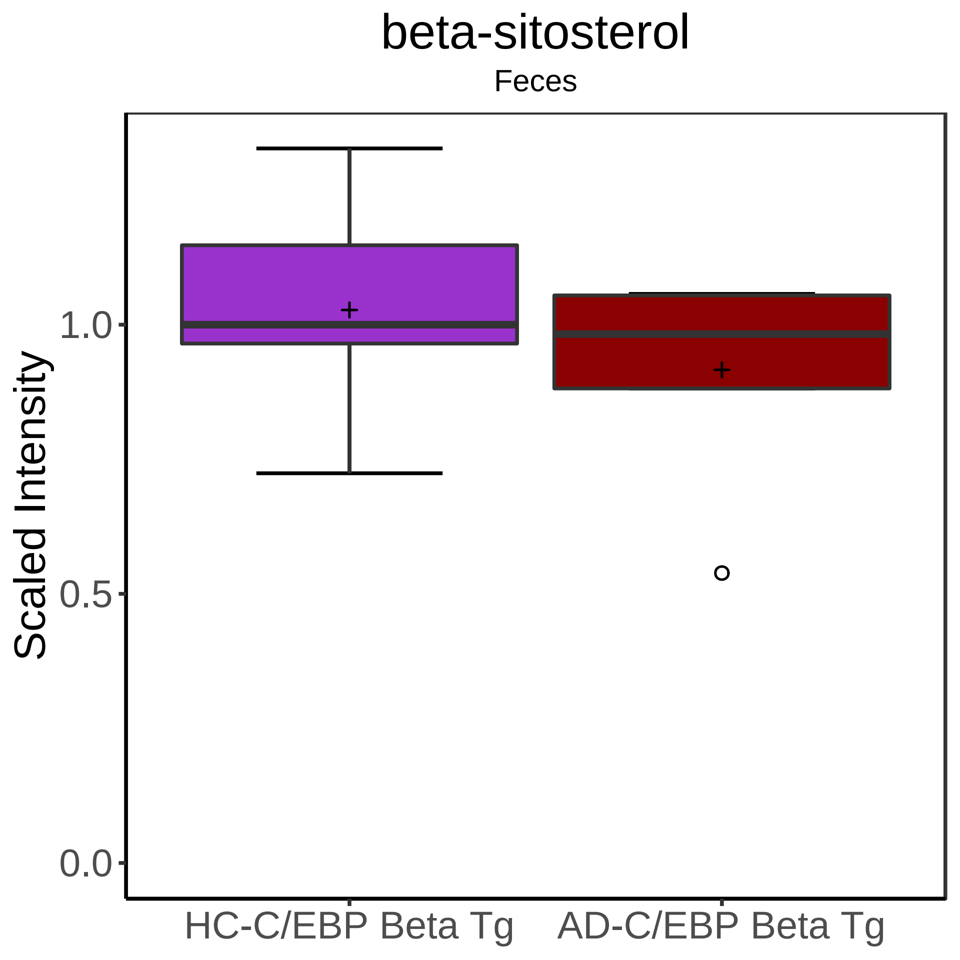
beta-sitosterol
Feces
Scaled Intensity
1.0
0.5
0.0
HC-C/EBP Beta Tg
AD-C/EBP Beta Tg

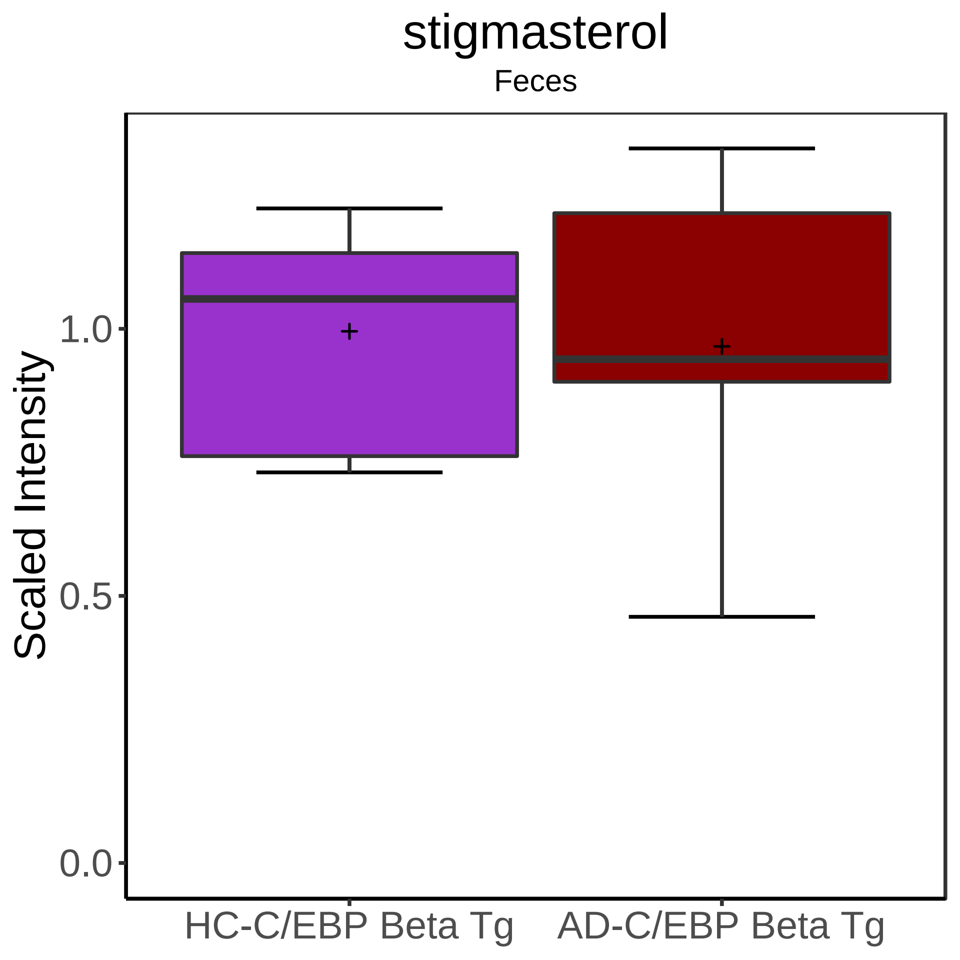
stigmasterol
Feces
Scaled Intensity
1.0
0.5
0.0
HC-C/EBP Beta Tg
AD-C/EBP Beta Tg

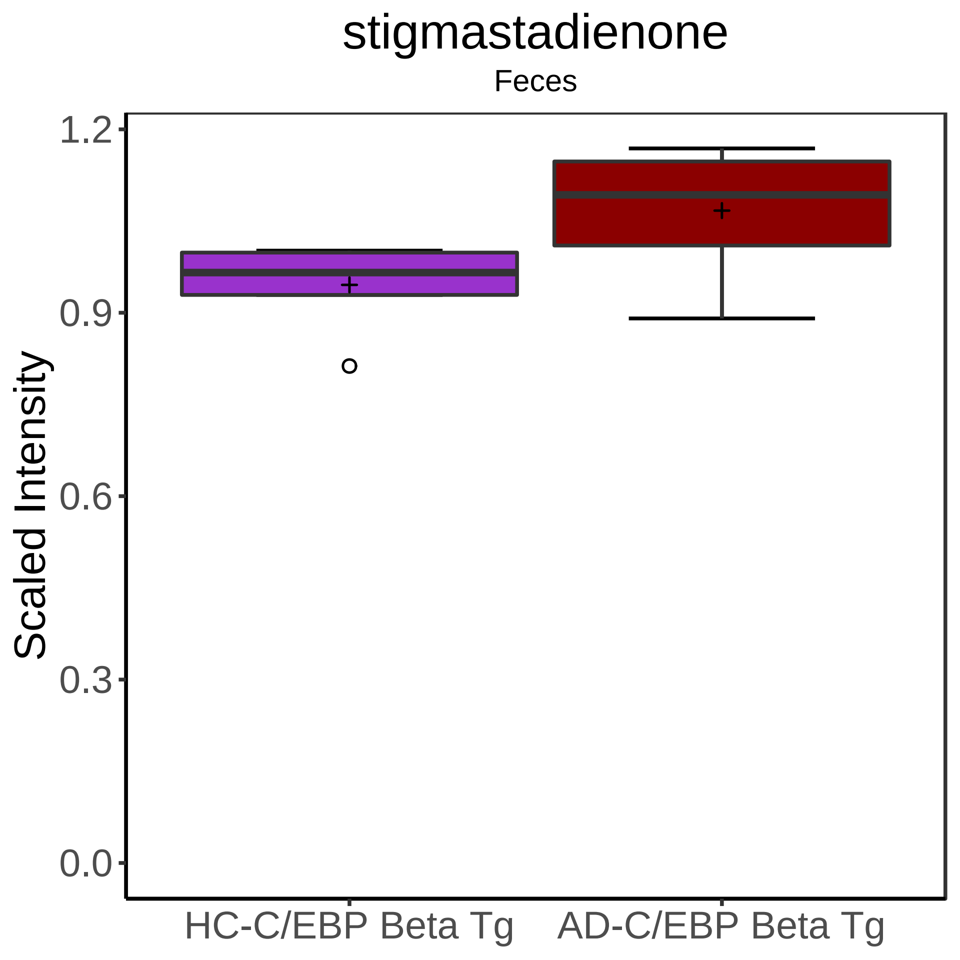

stigmastadienone
Feces
Scaled Intensity
1.2
0.9
0.6
0.3
0.0
HC-C/EBP Beta Tg
AD-C/EBP Beta Tg

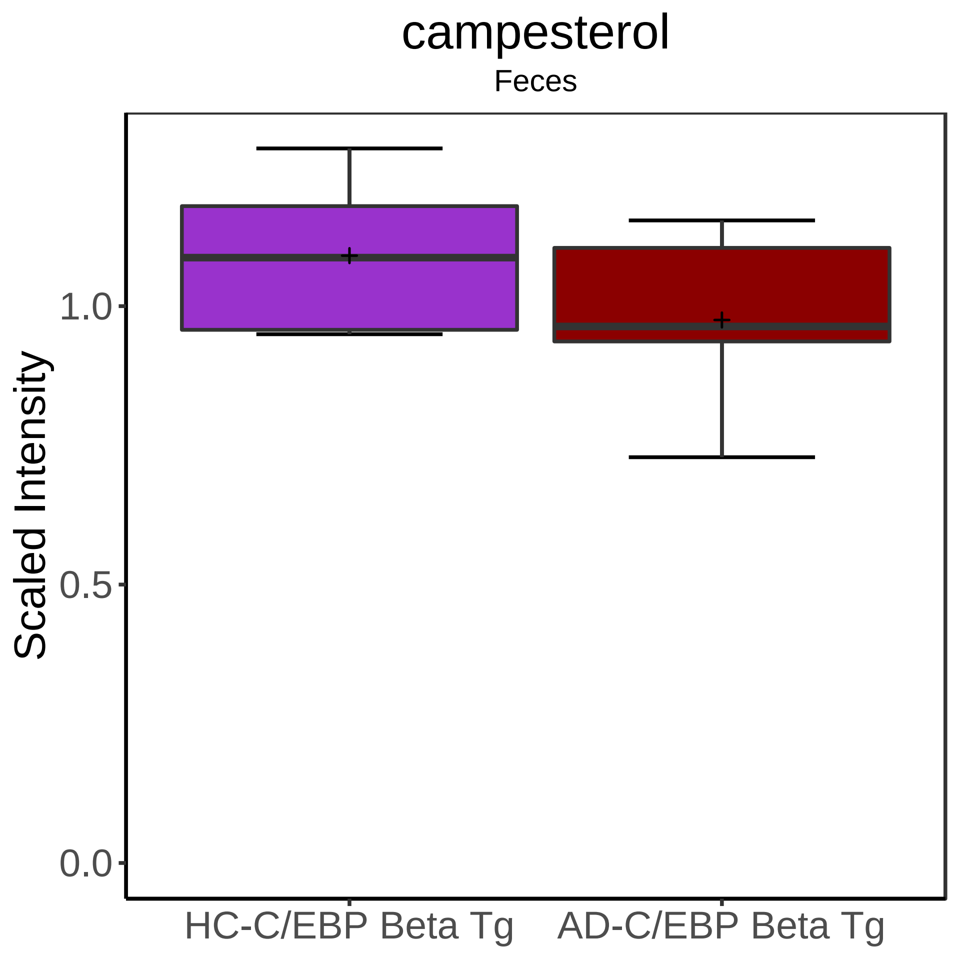
campesterol
Feces
Scaled Intensity
1.0
0.5
0.0
HC-C/EBP Beta Tg
AD-C/EBP Beta Tg

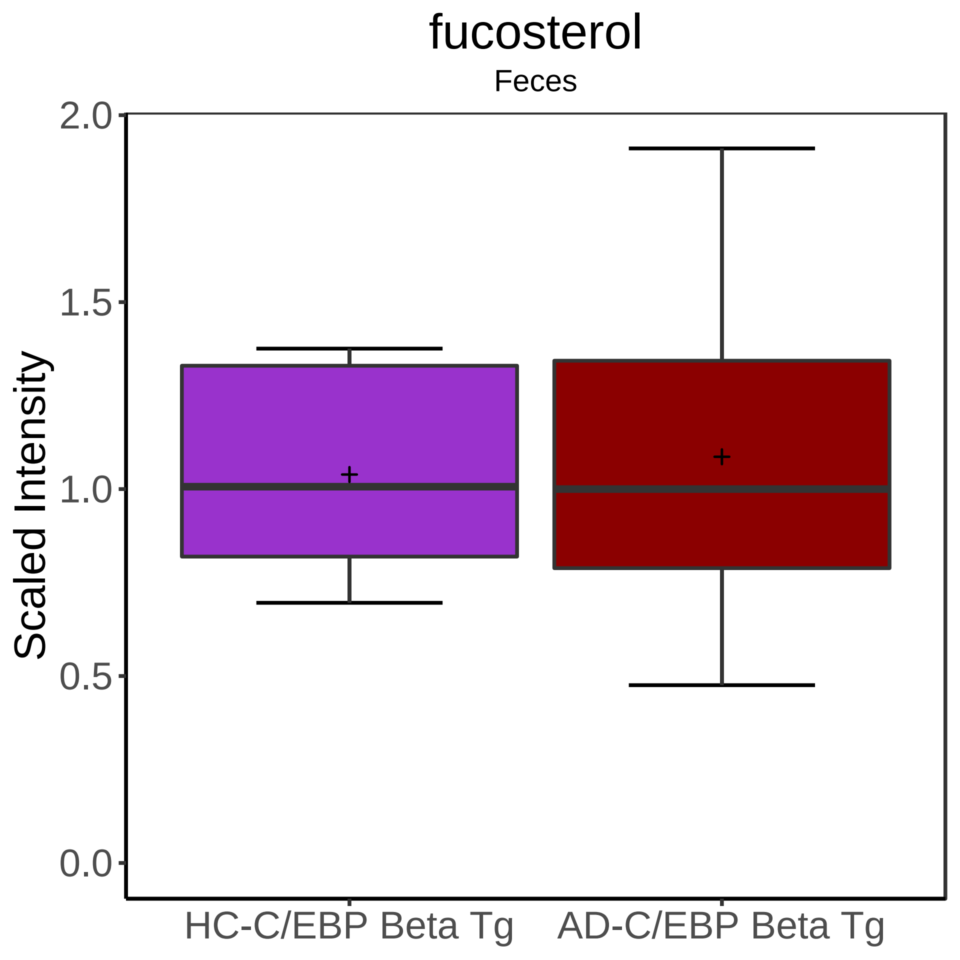
fucosterol
Feces
Scaled Intensity
2.0
1.5
1.0
0.5
0.0
HC-C/EBP Beta Tg
AD-C/EBP Beta Tg

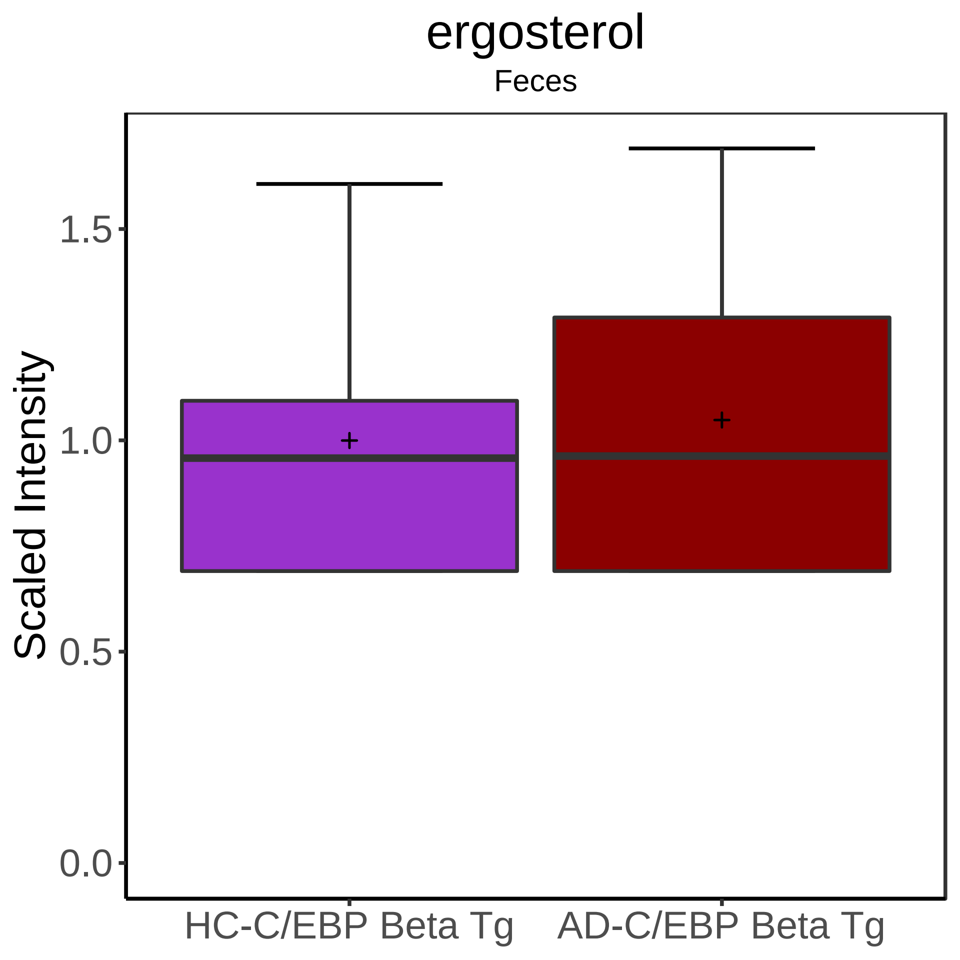

ergosterol
Feces
Scaled Intensity
1.5
1.0
0.5
0.0
HC-C/EBP Beta Tg
AD-C/EBP Beta Tg

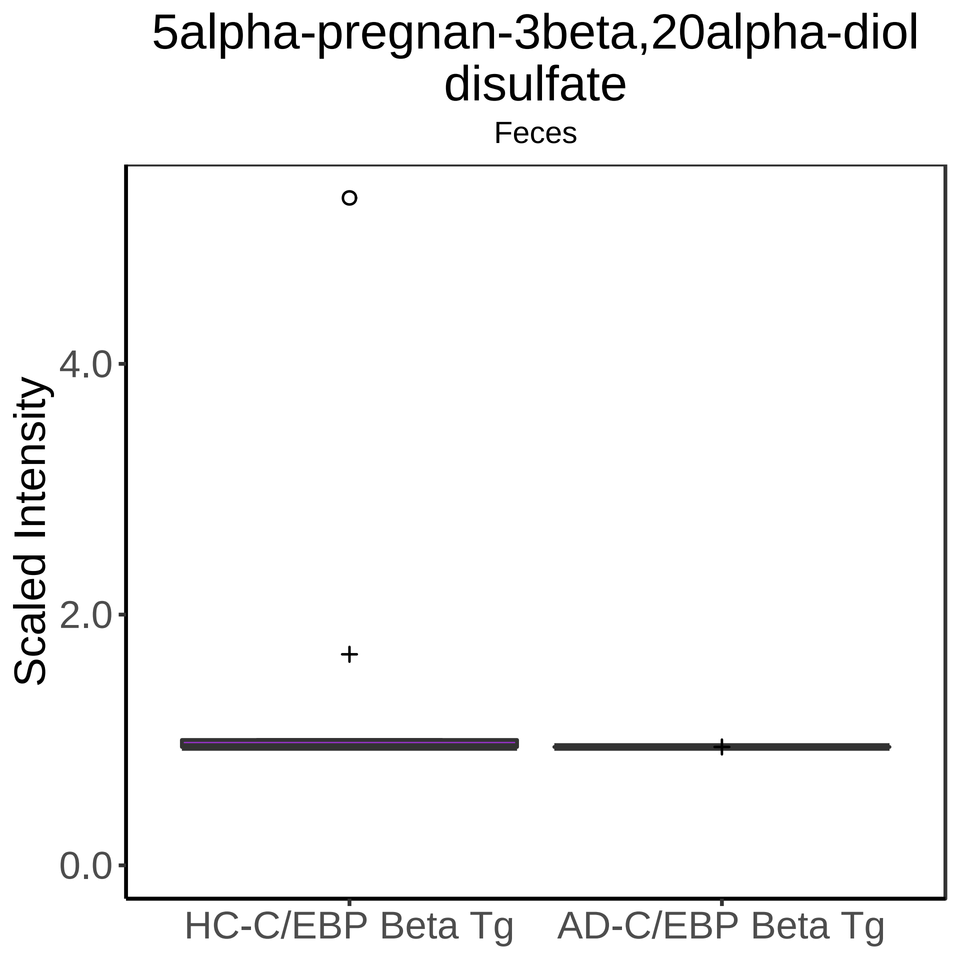

5alpha-pregnan-3beta,20alpha-diol disulfate
Feces
Scaled Intensity
4.0
2.0
0.0
HC-C/EBP Beta Tg
AD-C/EBP Beta Tg

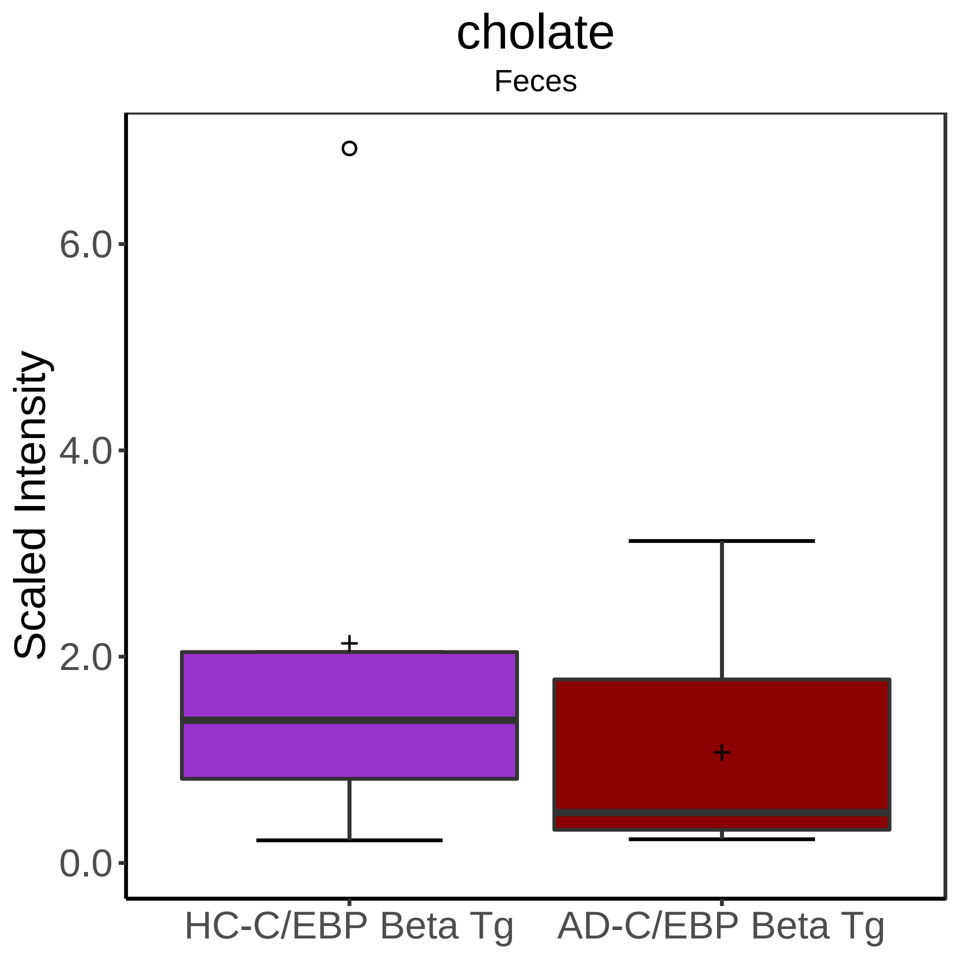
cholate
Feces
Scaled Intensity
6.0
4.0
2.0
0.0
HC-C/EBP Beta Tg
AD-C/EBP Beta Tg

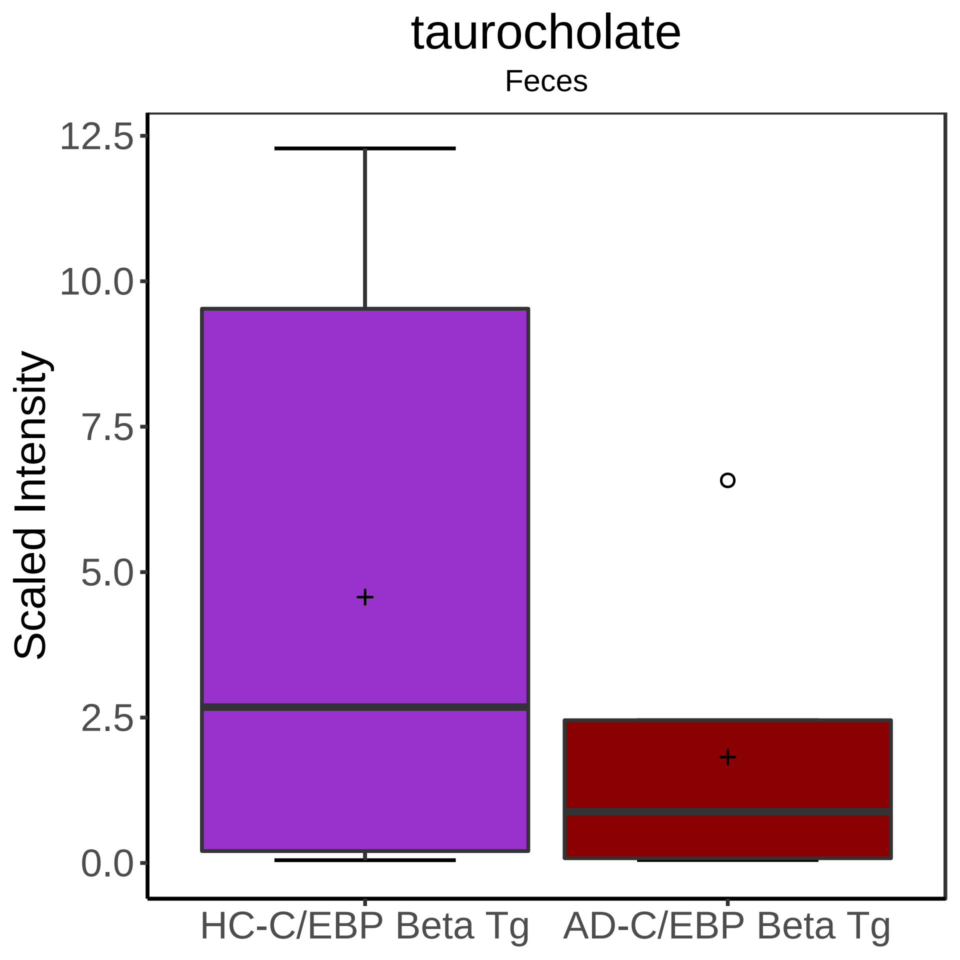
taurocholate
Feces
Scaled Intensity
12.5
10.0
7.5
5.0
2.5
0.0
HC-C/EBP Beta Tg
AD-C/EBP Beta Tg

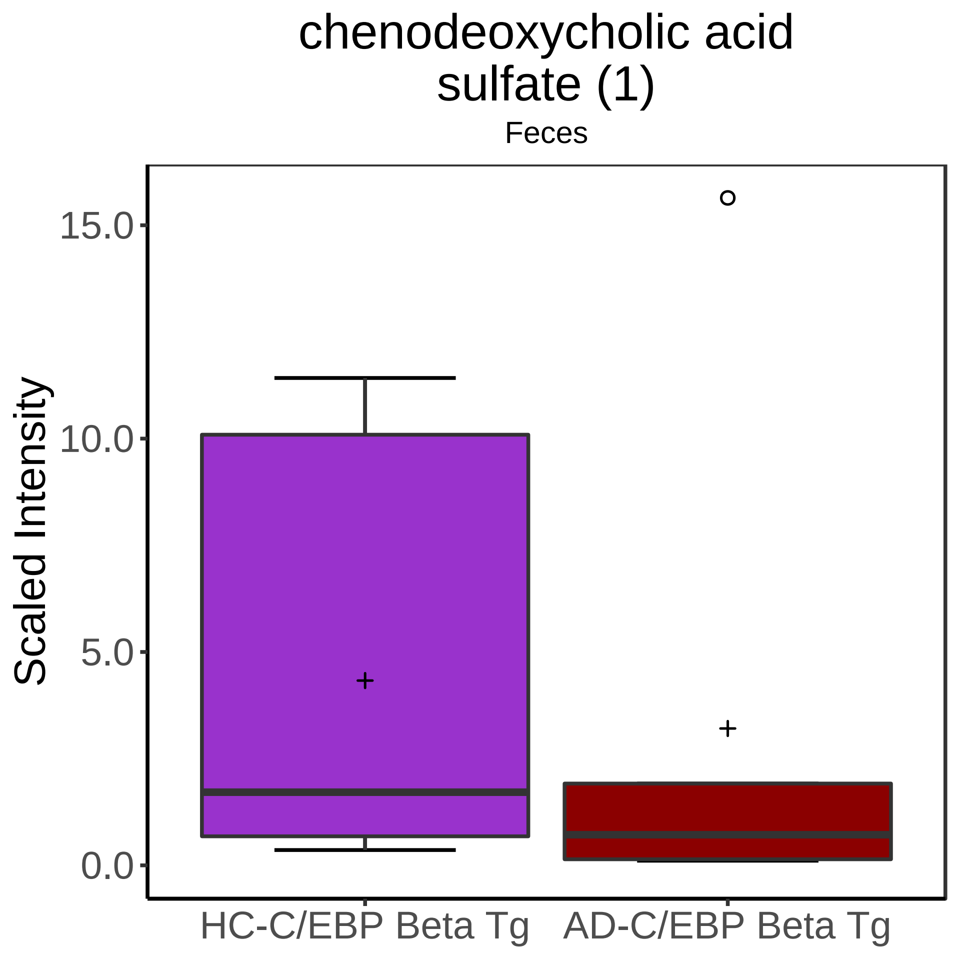
chenodeoxycholic acid
sulfate (1)
Feces
Scaled Intensity
15.0
10.0
5.0
0.0
HC-C/EBP Beta Tg
AD-C/EBP Beta Tg

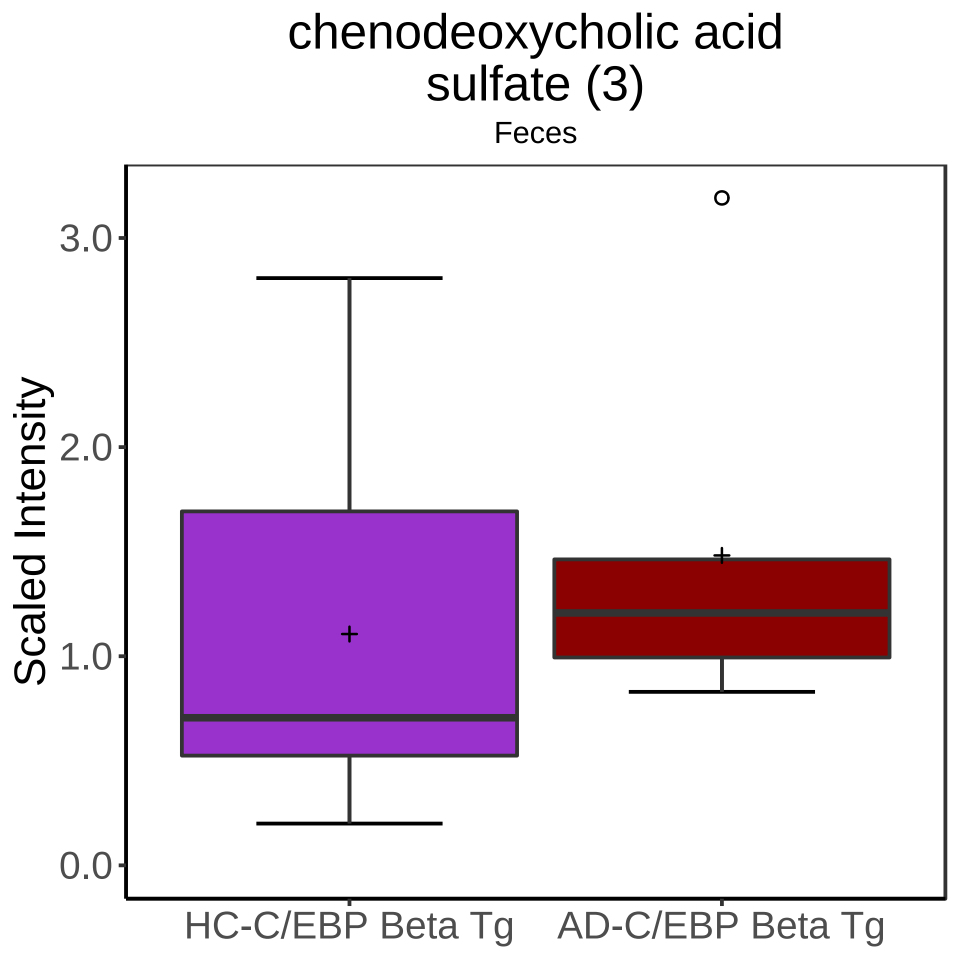
chenodeoxycholic acid
sulfate (3)
Feces
Scaled Intensity
3.0
2.0
1.0
0.0
HC-C/EBP Beta Tg
AD-C/EBP Beta Tg

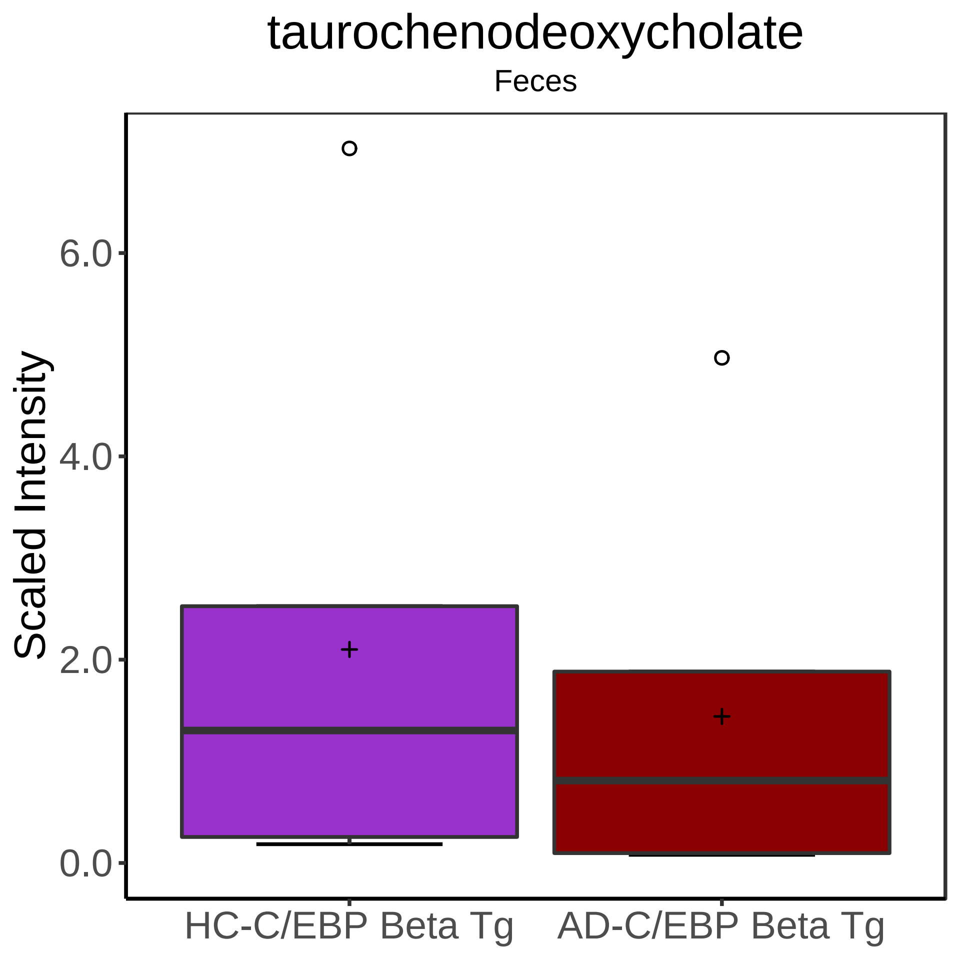
taurochenodeoxycholate
Feces
Scaled Intensity
6.0
4.0
2.0
0.0
HC-C/EBP Beta Tg
AD-C/EBP Beta Tg

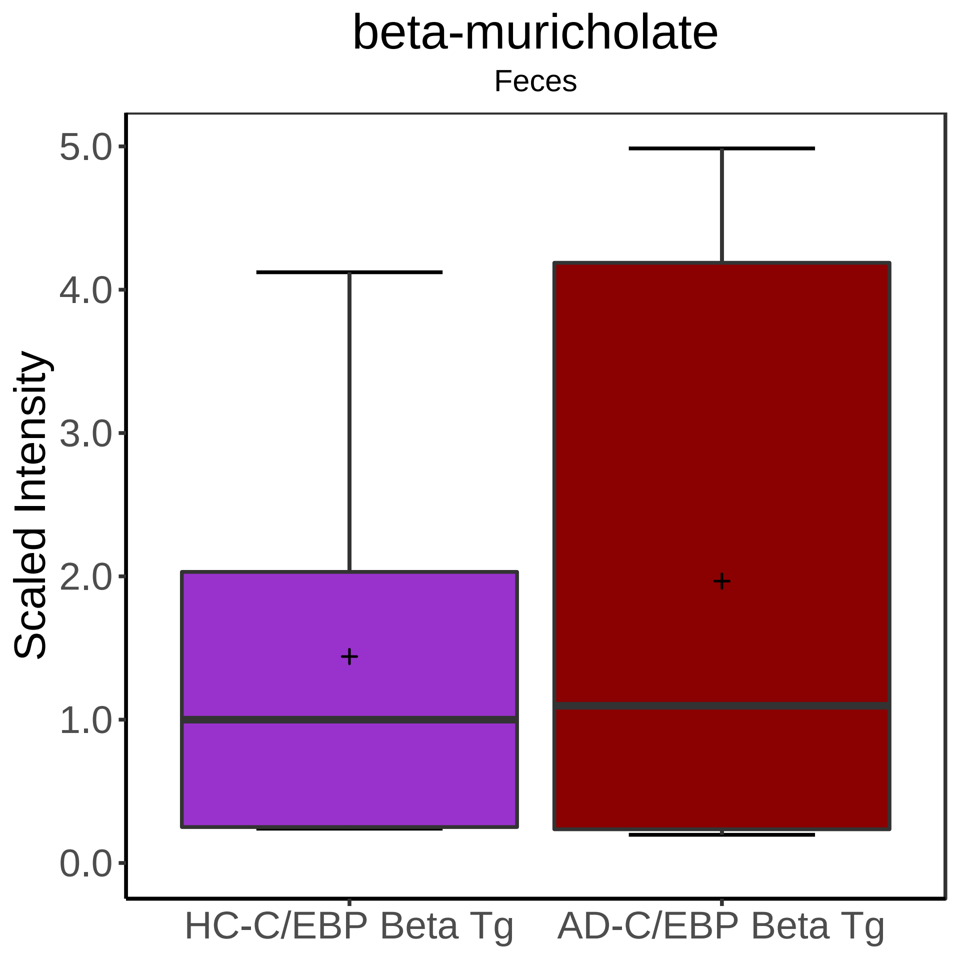

beta-muricholate
Feces
Scaled Intensity
5.0
4.0
3.0
2.0
1.0
0.0
HC-C/EBP Beta Tg
AD-C/EBP Beta Tg

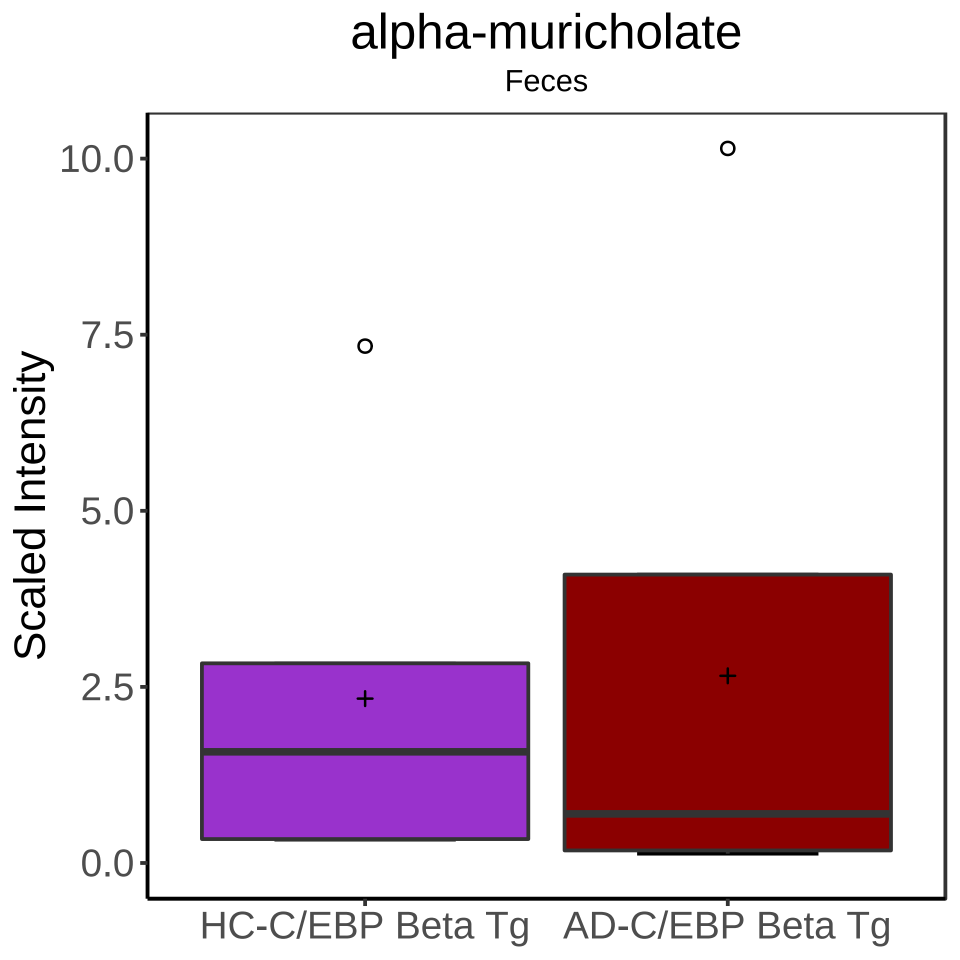
alpha-muricholate
Feces
Scaled Intensity
10.0
7.5
5.0
2.5
0.0
HC-C/EBP Beta Tg
AD-C/EBP Beta Tg

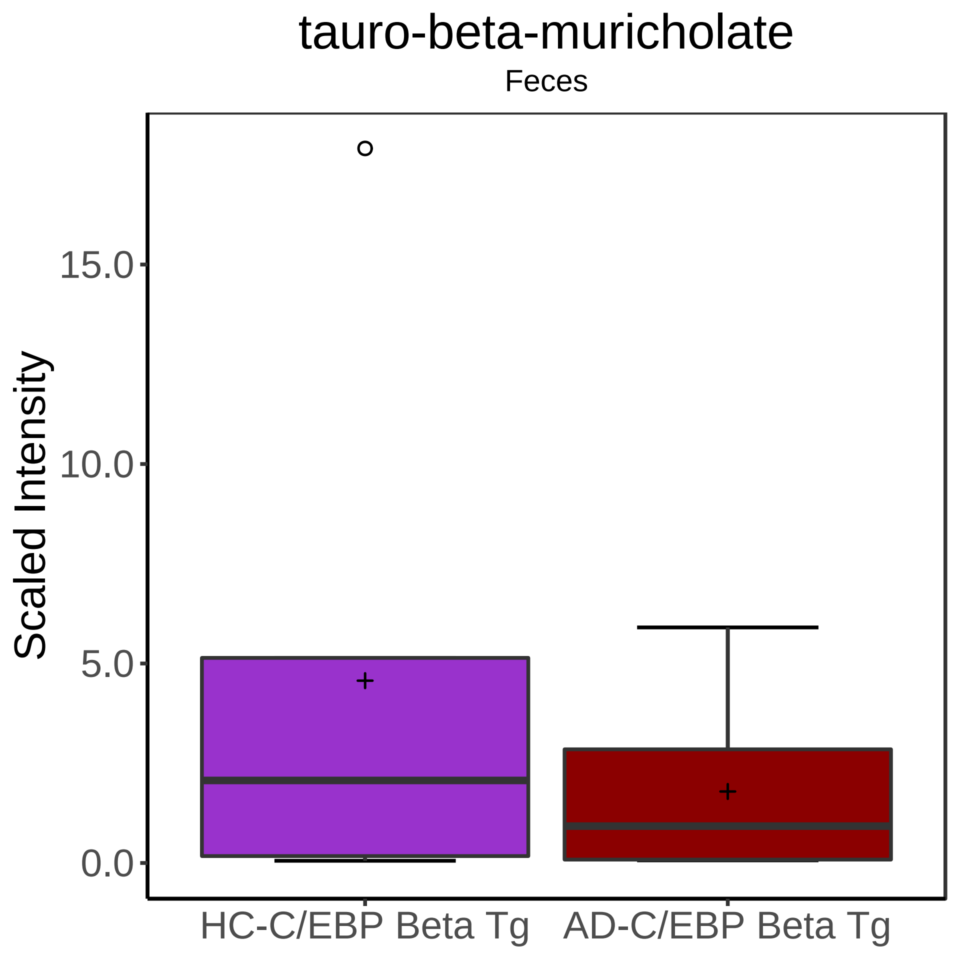

tauro-beta-muricholate
Feces
Scaled Intensity
15.0
10.0
5.0
0.0
HC-C/EBP Beta Tg
AD-C/EBP Beta Tg

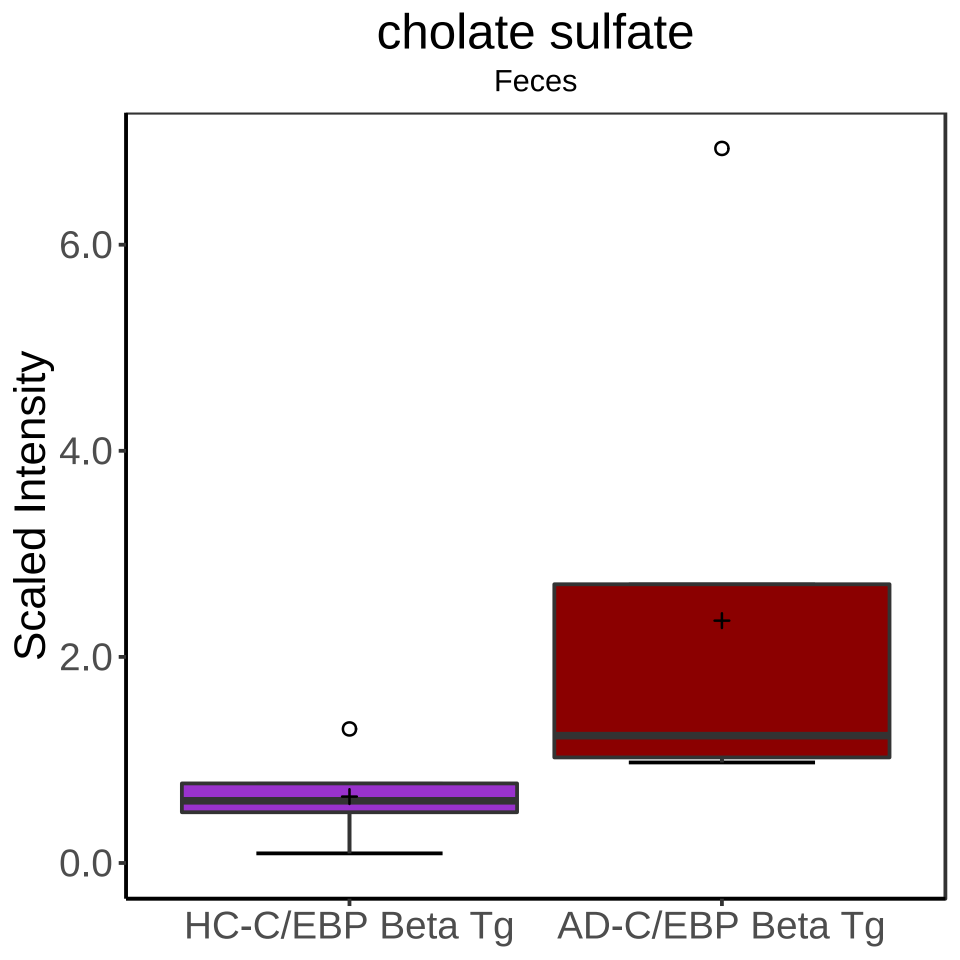

cholate sulfate
Feces
Scaled Intensity
6.0
4.0
2.0
0.0
HC-C/EBP Beta Tg
AD-C/EBP Beta Tg

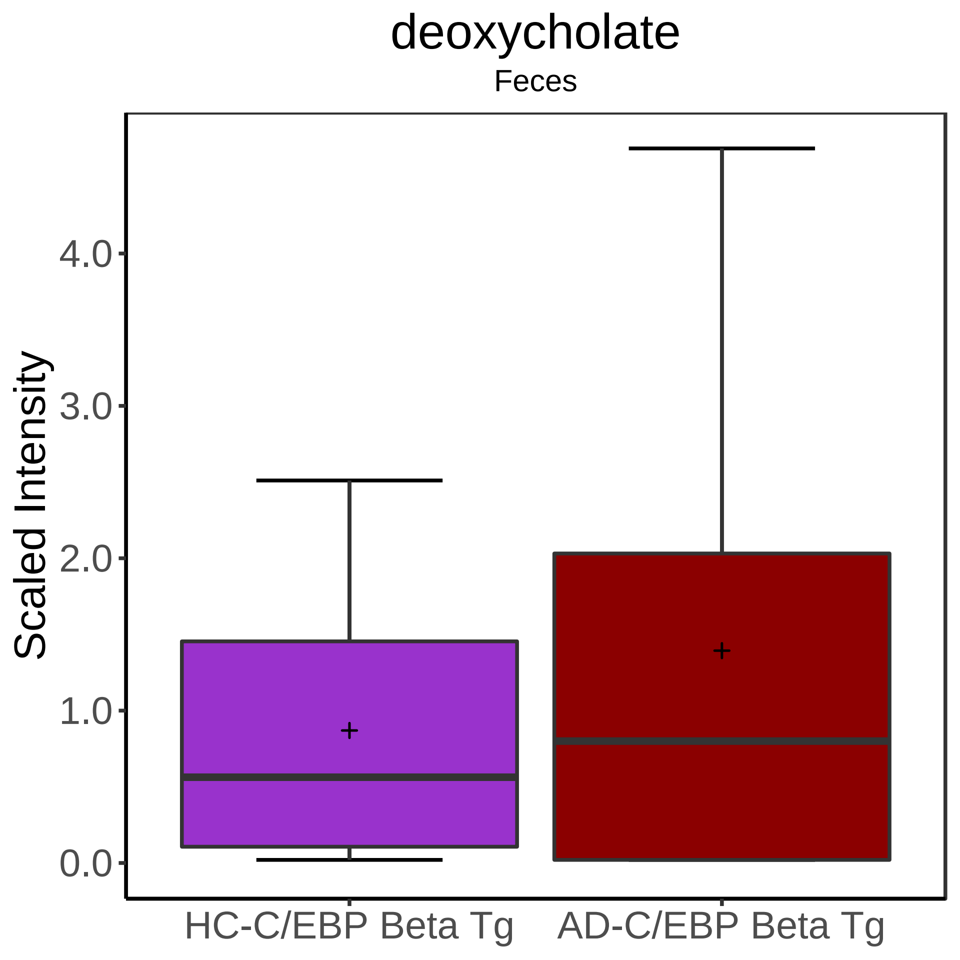
deoxycholate
Feces
Scaled Intensity
4.0
3.0
2.0
1.0
0.0
HC-C/EBP Beta Tg
AD-C/EBP Beta Tg

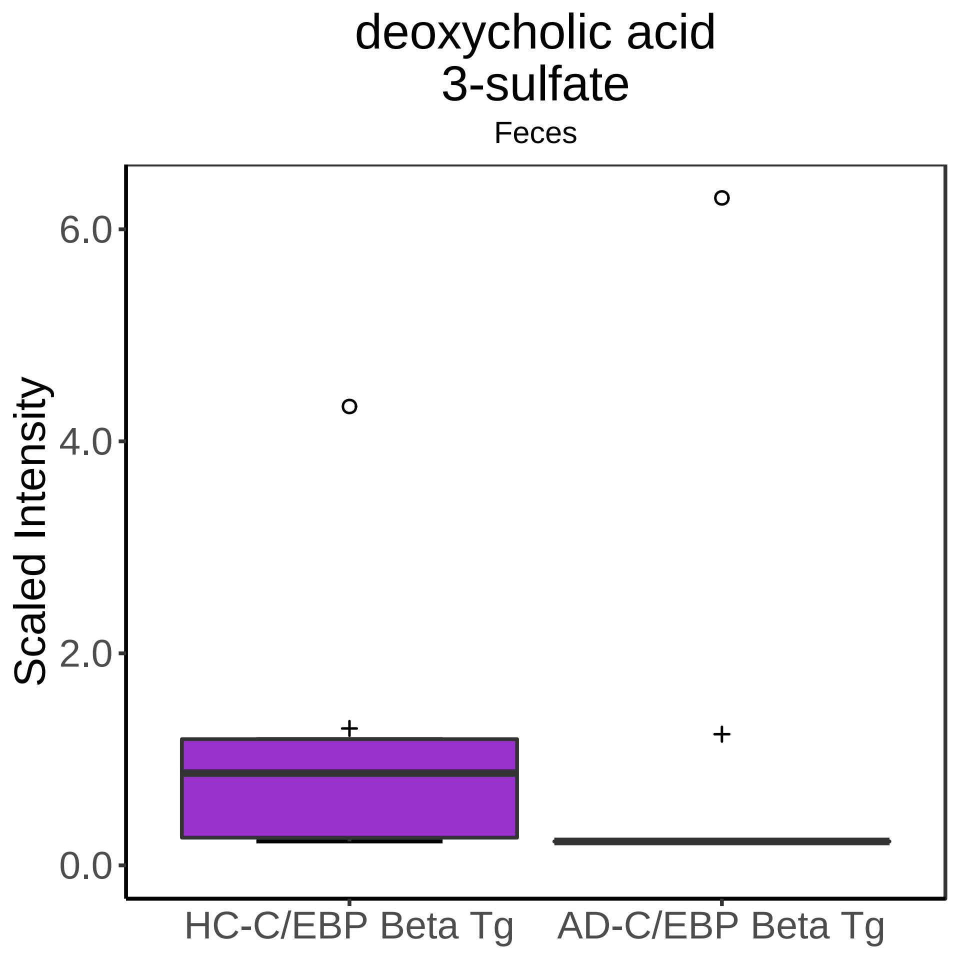

deoxycholic acid
3-sulfate
Feces
Scaled Intensity
6.0
4.0
2.0
0.0
HC-C/EBP Beta Tg
AD-C/EBP Beta Tg

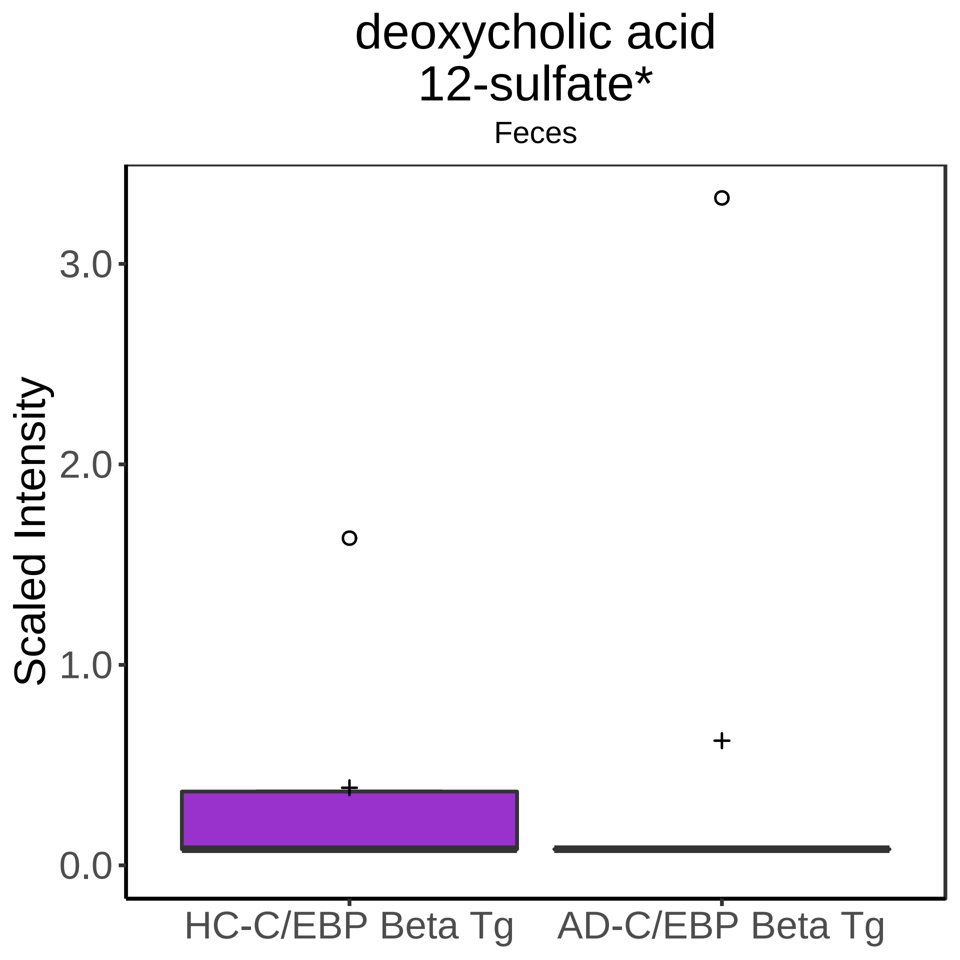
deoxycholic acid
12-sulfate*
Feces
Scaled Intensity
3.0
2.0
1.0
0.0
HC-C/EBP Beta Tg
AD-C/EBP Beta Tg

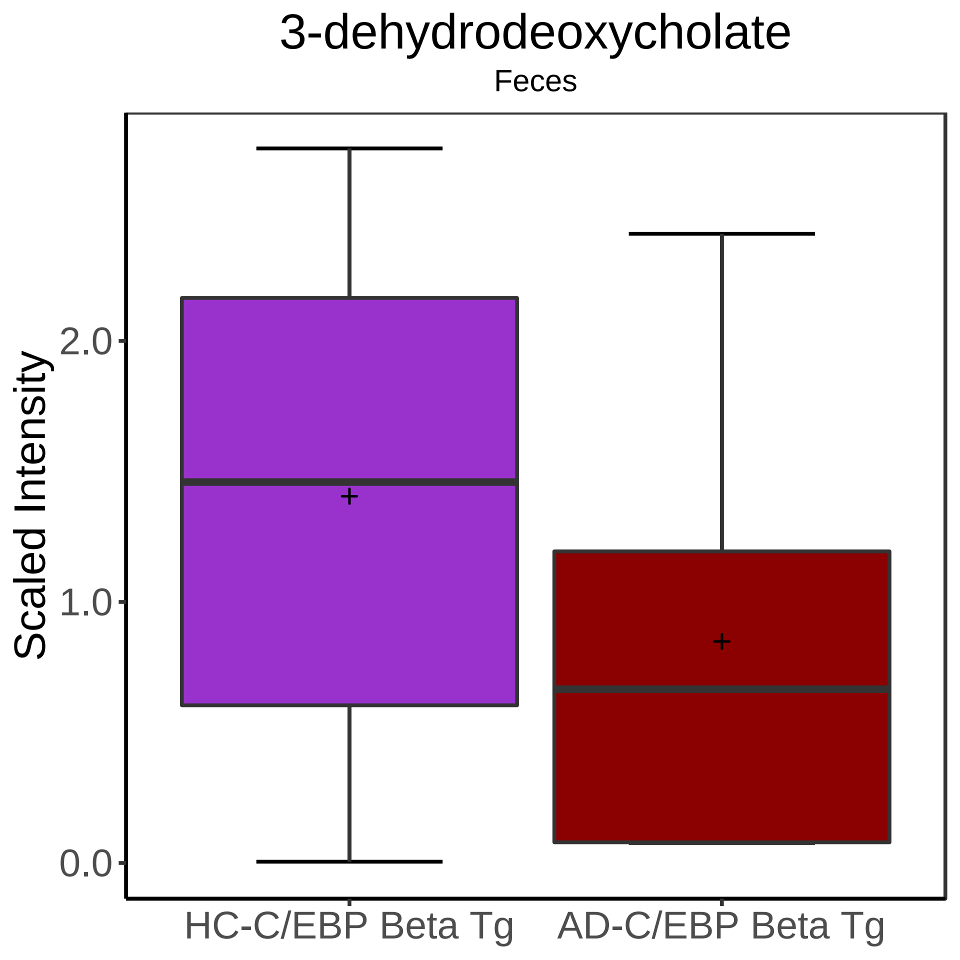
3-dehydrodeoxycholate
Feces
Scaled Intensity
2.0
1.0
0.0
HC-C/EBP Beta Tg
AD-C/EBP Beta Tg

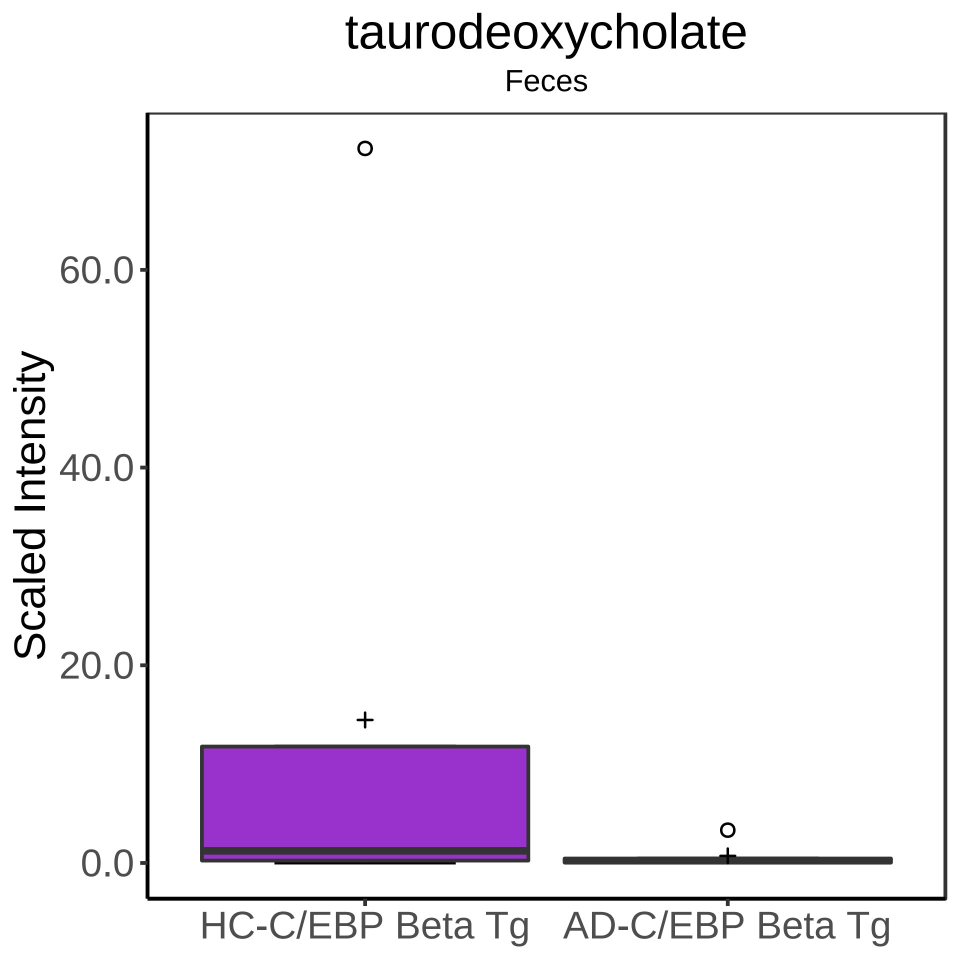

taurodeoxycholate
Feces
Scaled Intensity
60.0
40.0
20.0
0.0
HC-C/EBP Beta Tg
AD-C/EBP Beta Tg

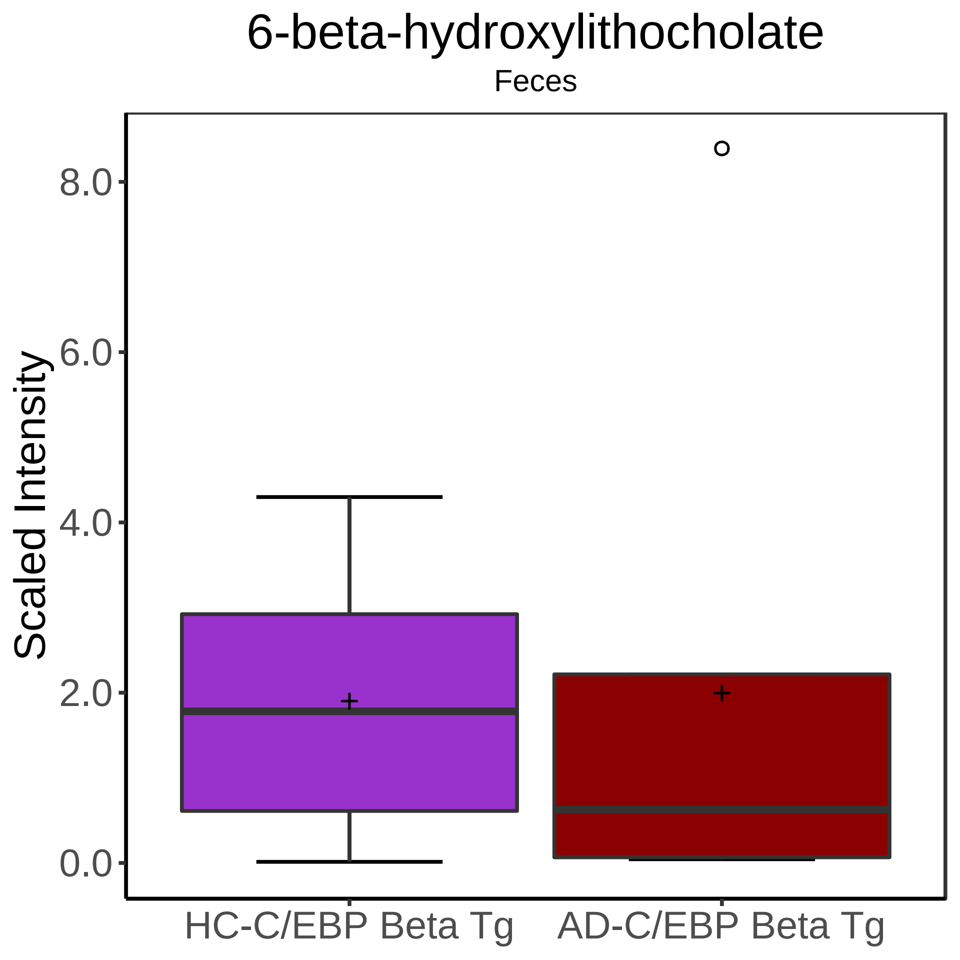
6-beta-hydroxylithocholate
Feces
Scaled Intensity
0.0
2.0
4.0
6.0
8.0
HC-C/EBP Beta Tg
AD-C/EBP Beta Tg

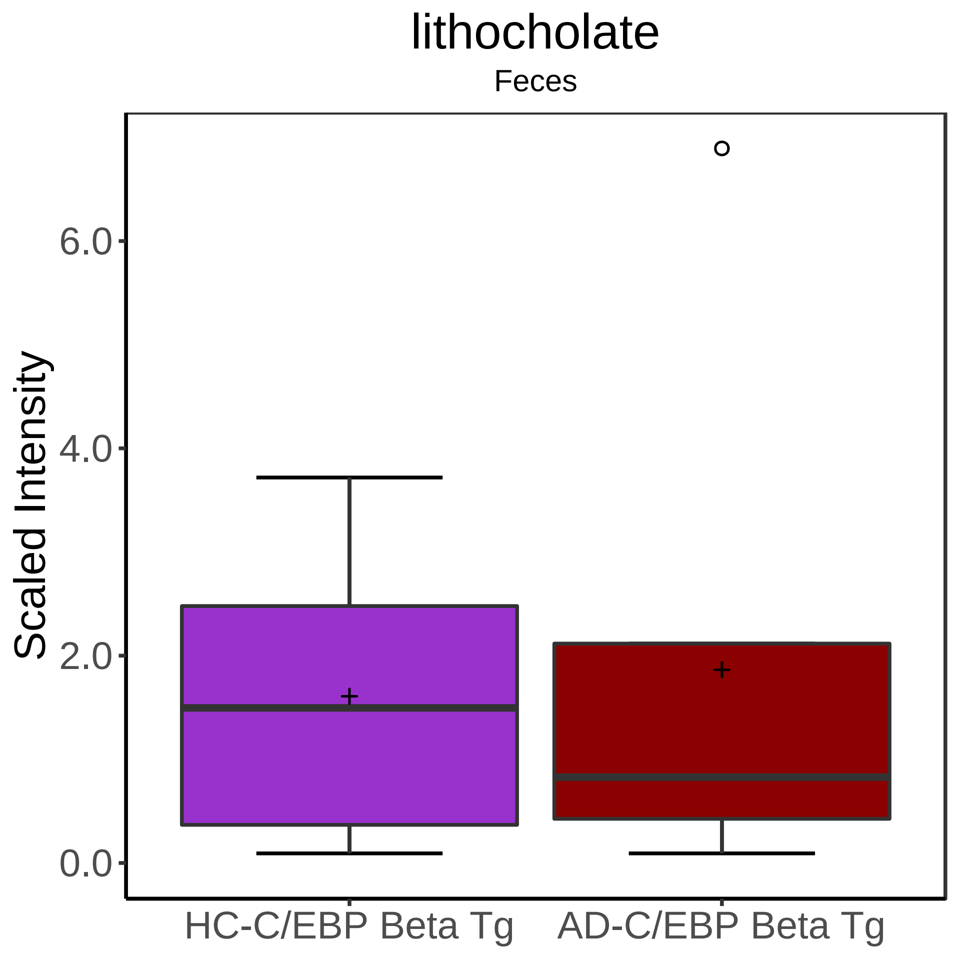
lithocholate
Feces
Scaled Intensity
6.0
4.0
2.0
0.0
HC-C/EBP Beta Tg
AD-C/EBP Beta Tg

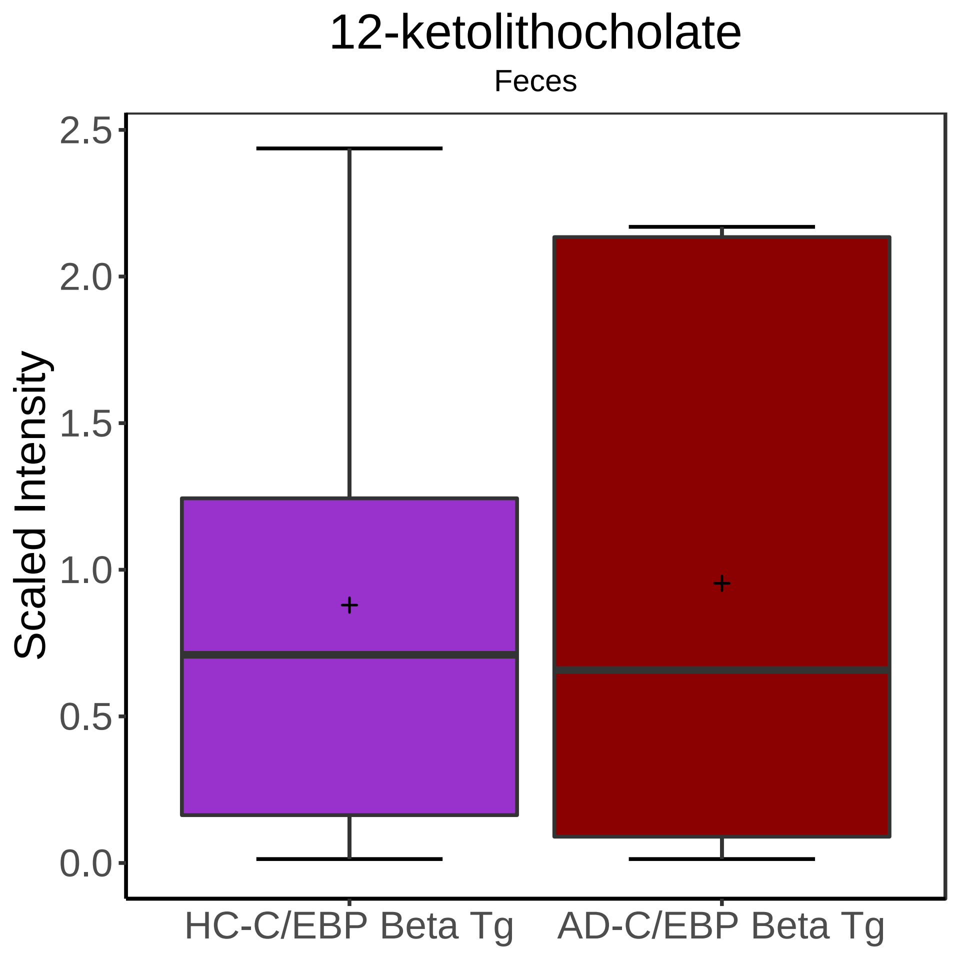
12-ketolithocholate
Feces
Scaled Intensity
2.5
2.0
1.5
1.0
0.5
0.0
HC-C/EBP Beta Tg
AD-C/EBP Beta Tg

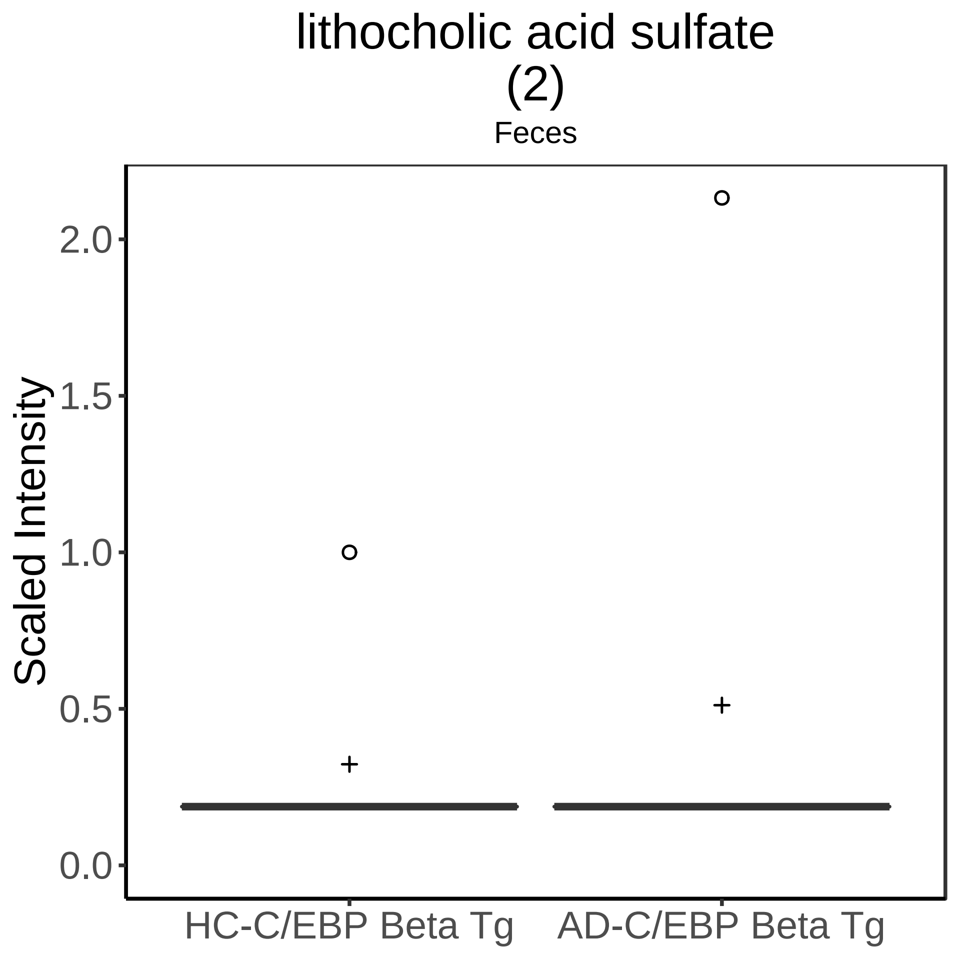
lithocholic acid sulfate
(2)
Feces
Scaled Intensity
2.0
1.5
1.0
0.5
0.0
HC-C/EBP Beta Tg
AD-C/EBP Beta Tg

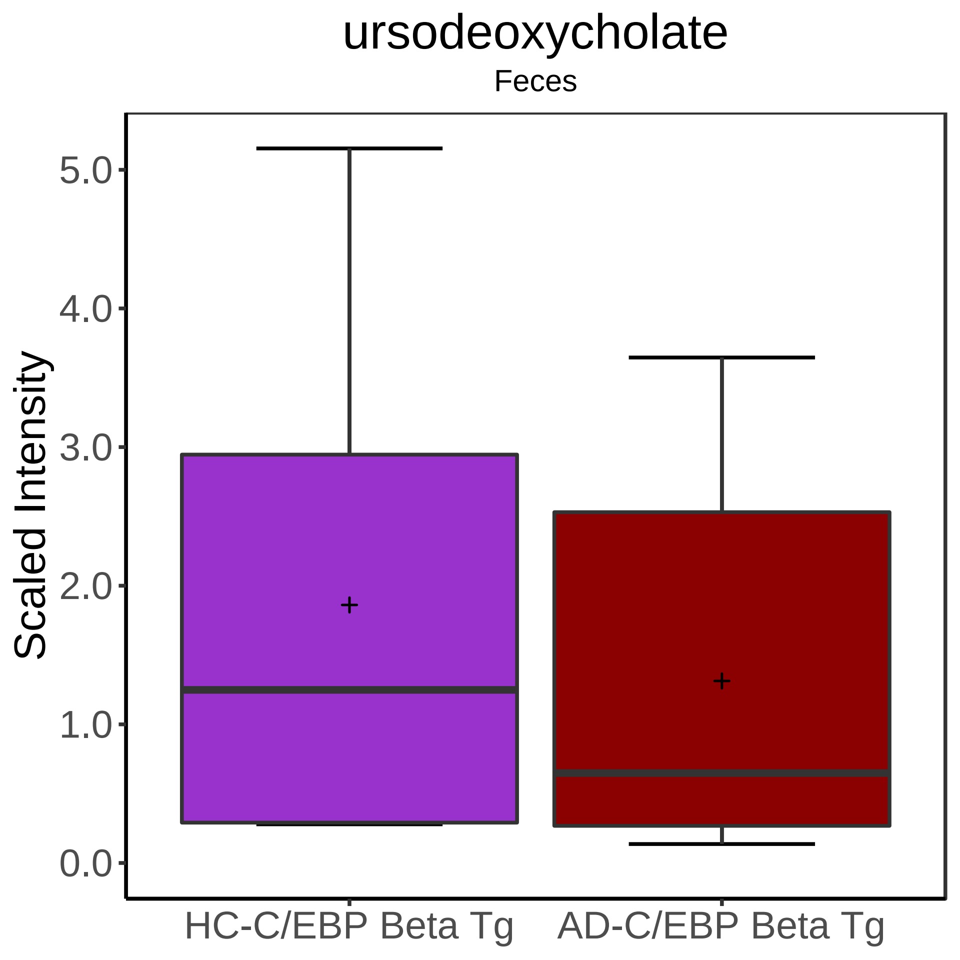

ursodeoxycholate
Feces
Scaled Intensity
5.0
4.0
3.0
2.0
1.0
0.0
HC-C/EBP Beta Tg
AD-C/EBP Beta Tg

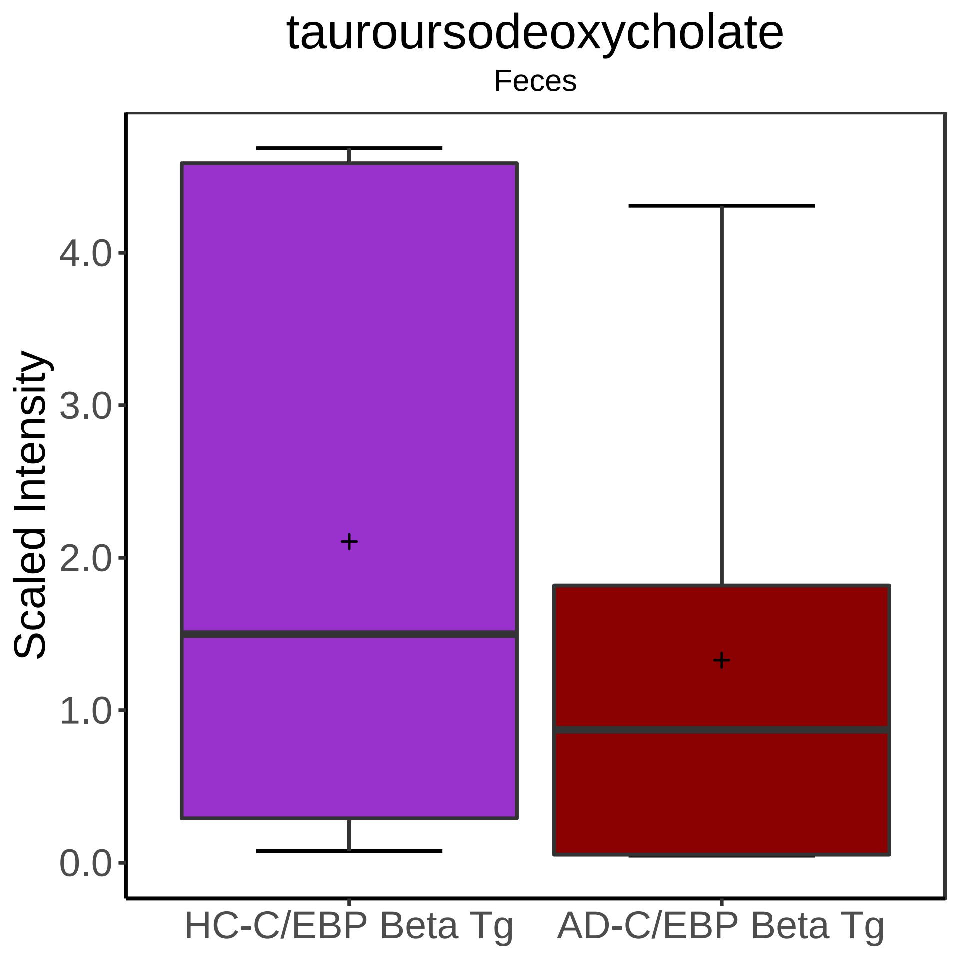

tauroursodeoxycholate
Feces
Scaled Intensity
0.0
1.0
2.0
3.0
4.0
HC-C/EBP Beta Tg
AD-C/EBP Beta Tg

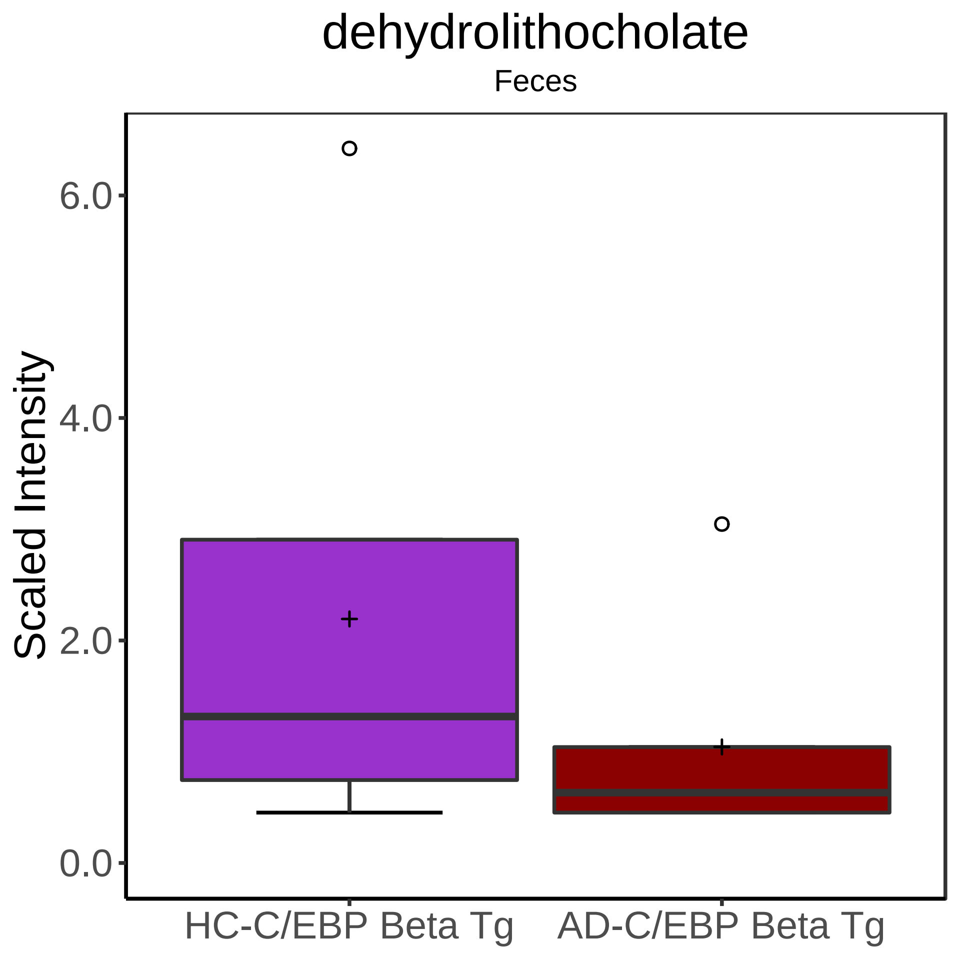
dehydrolithocholate
Feces
Scaled Intensity
6.0
4.0
2.0
0.0
HC-C/EBP Beta Tg
AD-C/EBP Beta Tg

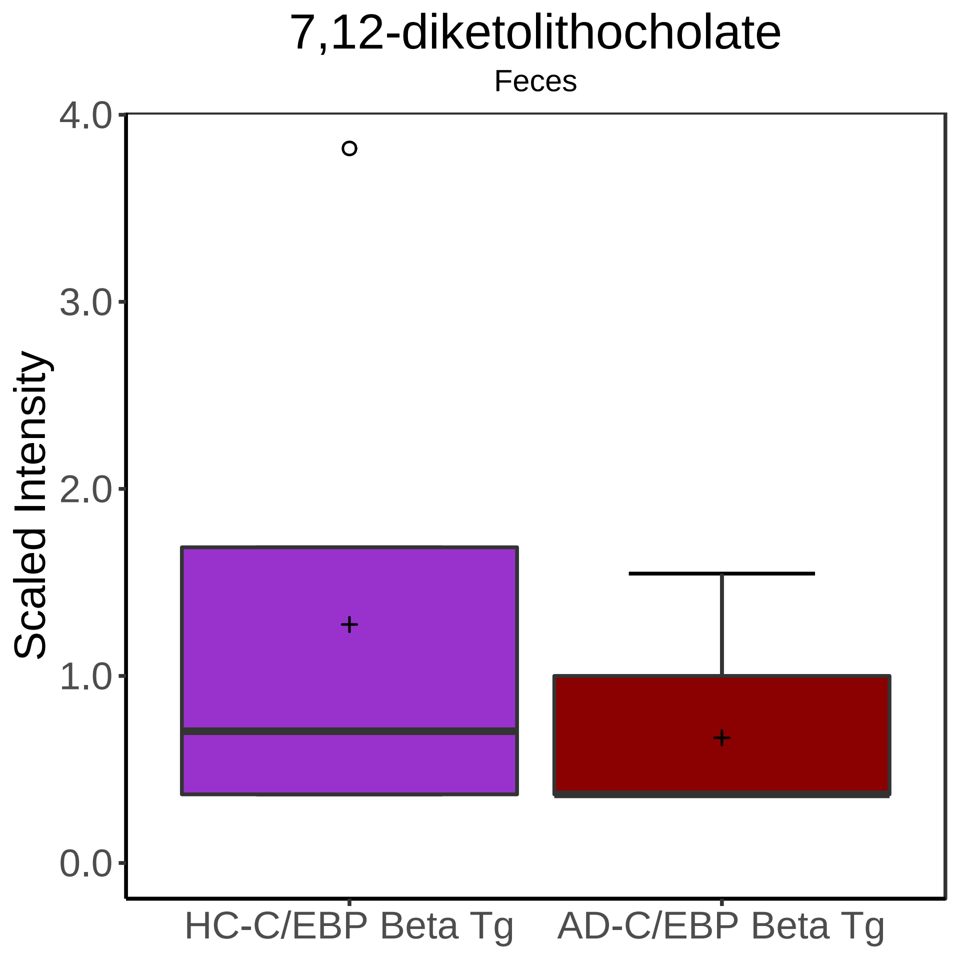
7,12-diketolithocholate
Feces
Scaled Intensity
4.0
3.0
2.0
1.0
0.0
HC-C/EBP Beta Tg
AD-C/EBP Beta Tg

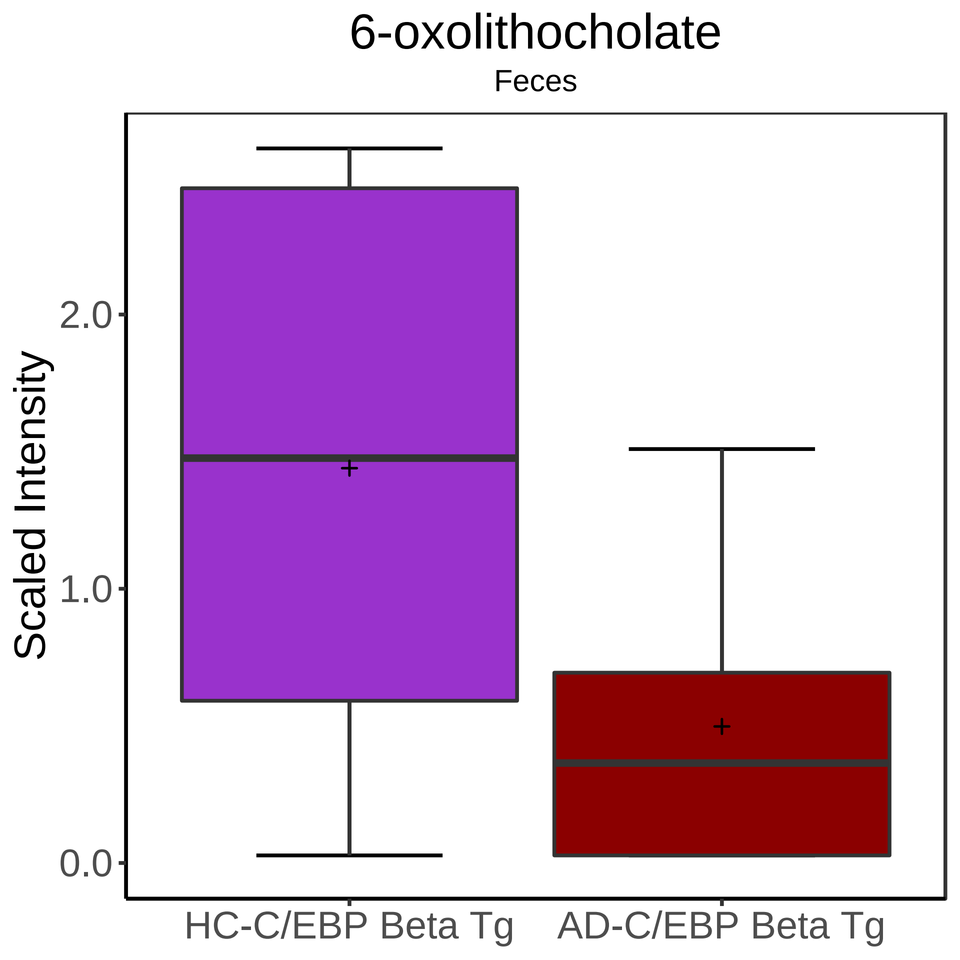

6-oxolithocholate
Feces
Scaled Intensity
2.0
1.0
0.0
HC-C/EBP Beta Tg
AD-C/EBP Beta Tg

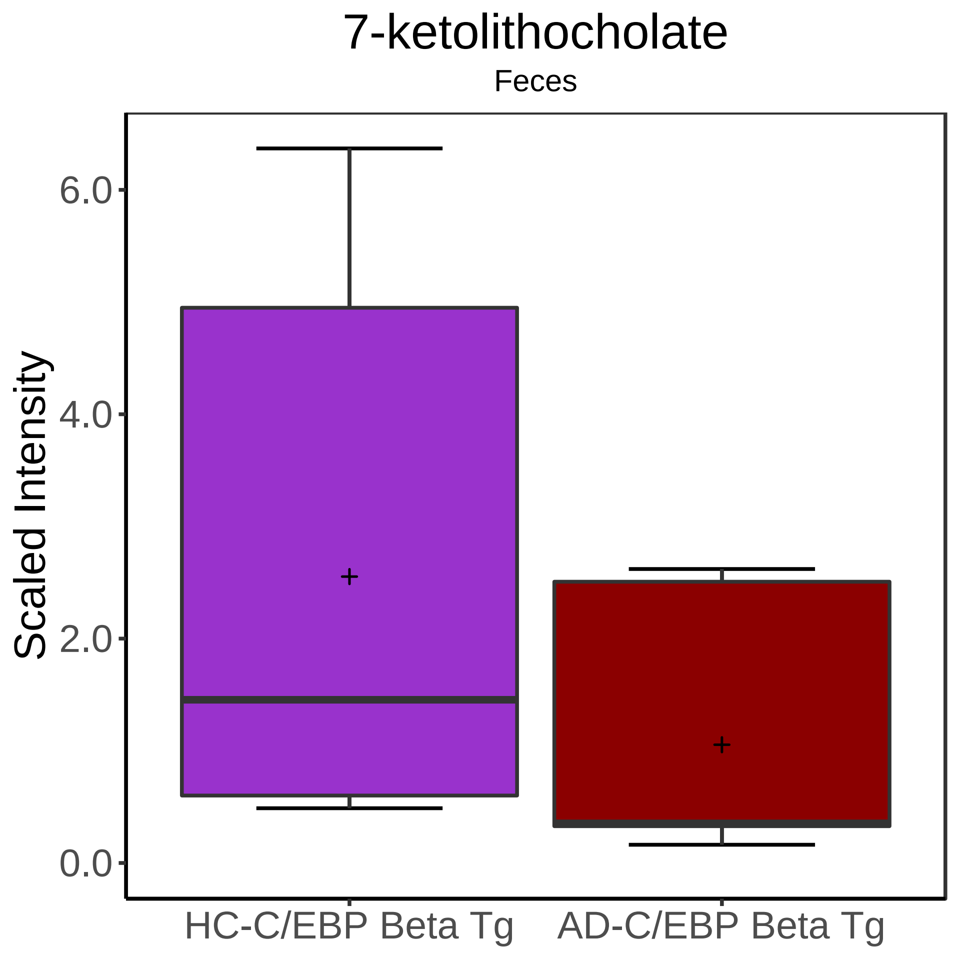
7-ketolithocholate
Feces
Scaled Intensity
6.0
4.0
2.0
0.0
HC-C/EBP Beta Tg
AD-C/EBP Beta Tg

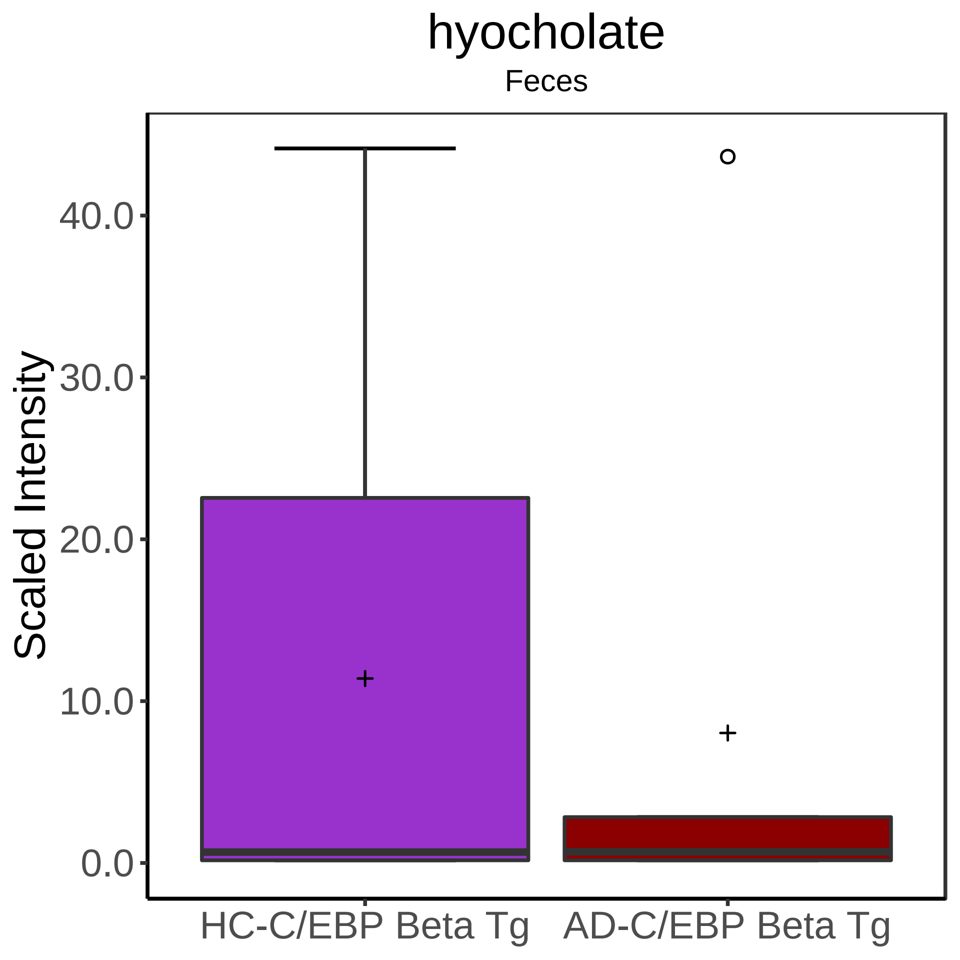
hyocholate
Feces
Scaled Intensity
40.0
30.0
20.0
10.0
0.0
HC-C/EBP Beta Tg
AD-C/EBP Beta Tg

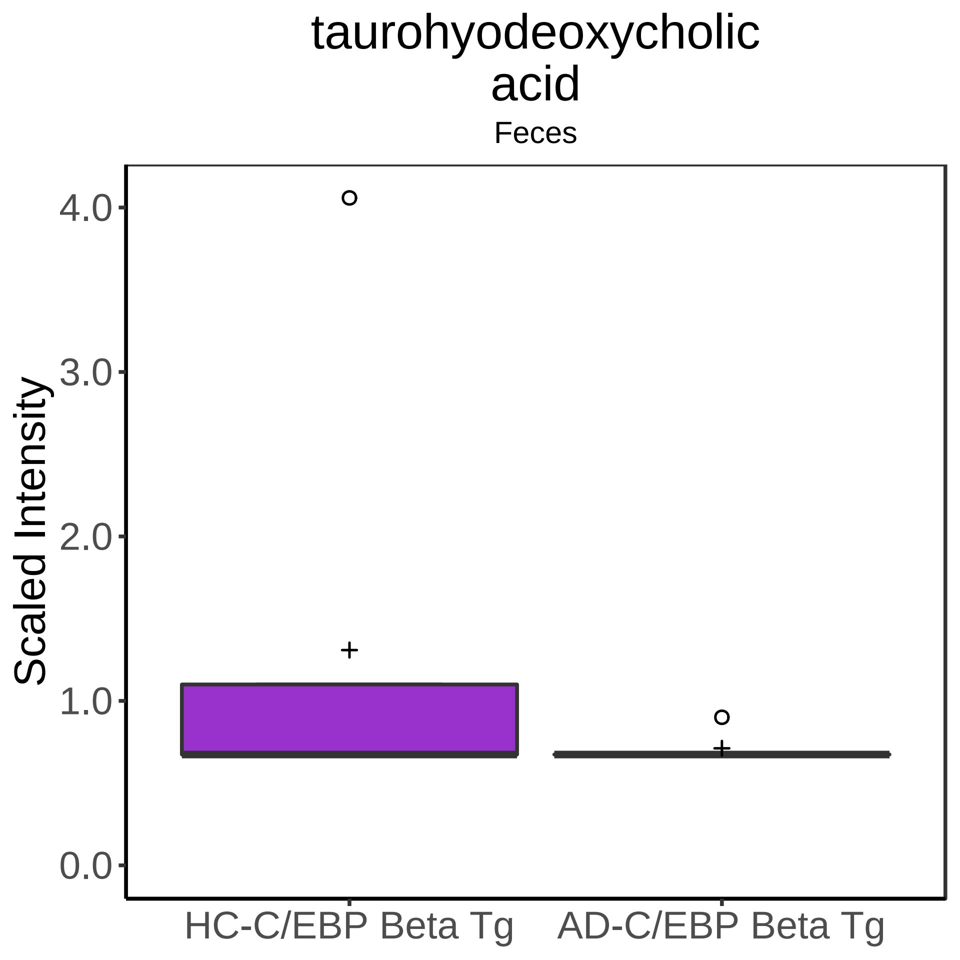
taurohyodeoxycholic
acid
Feces
Scaled Intensity
4.0
3.0
2.0
1.0
0.0
HC-C/EBP Beta Tg
AD-C/EBP Beta Tg

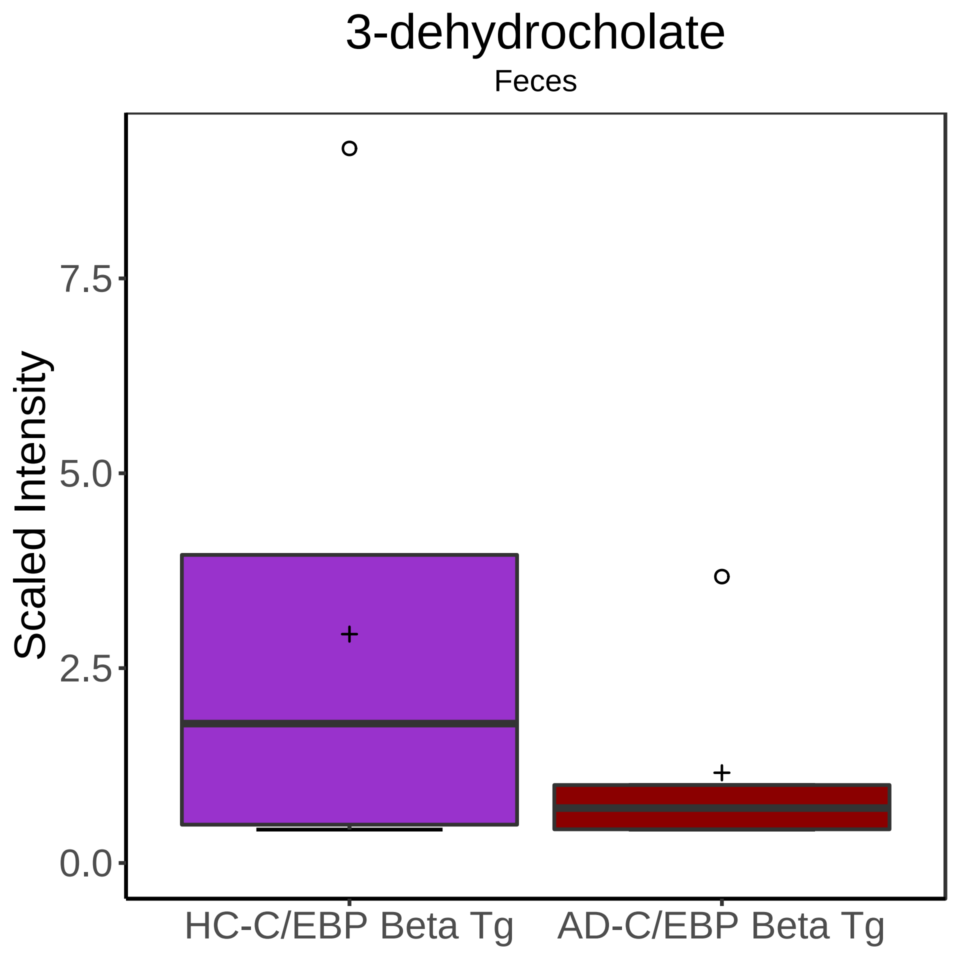
3-dehydrocholate
Feces
Scaled Intensity
7.5
5.0
2.5
0.0
HC-C/EBP Beta Tg
AD-C/EBP Beta Tg

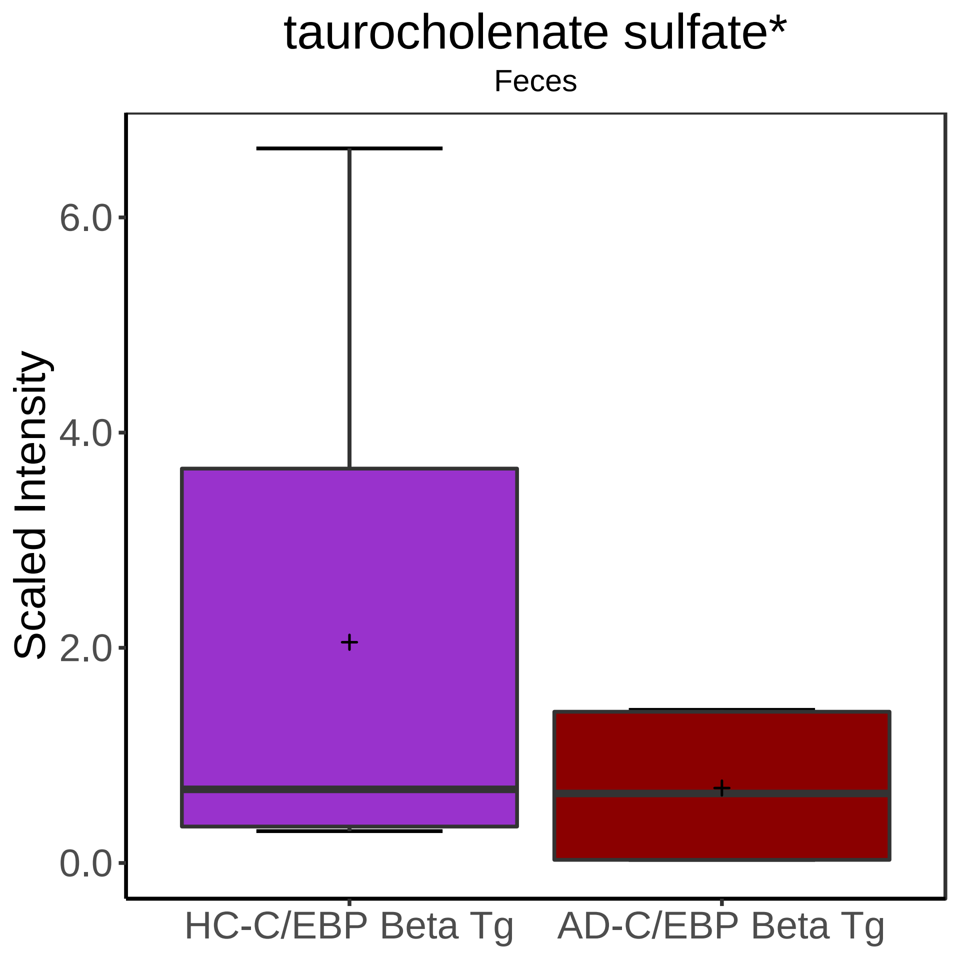
taurocholenate sulfate*
Feces
Scaled Intensity
6.0
4.0
2.0
0.0
HC-C/EBP Beta Tg
AD-C/EBP Beta Tg

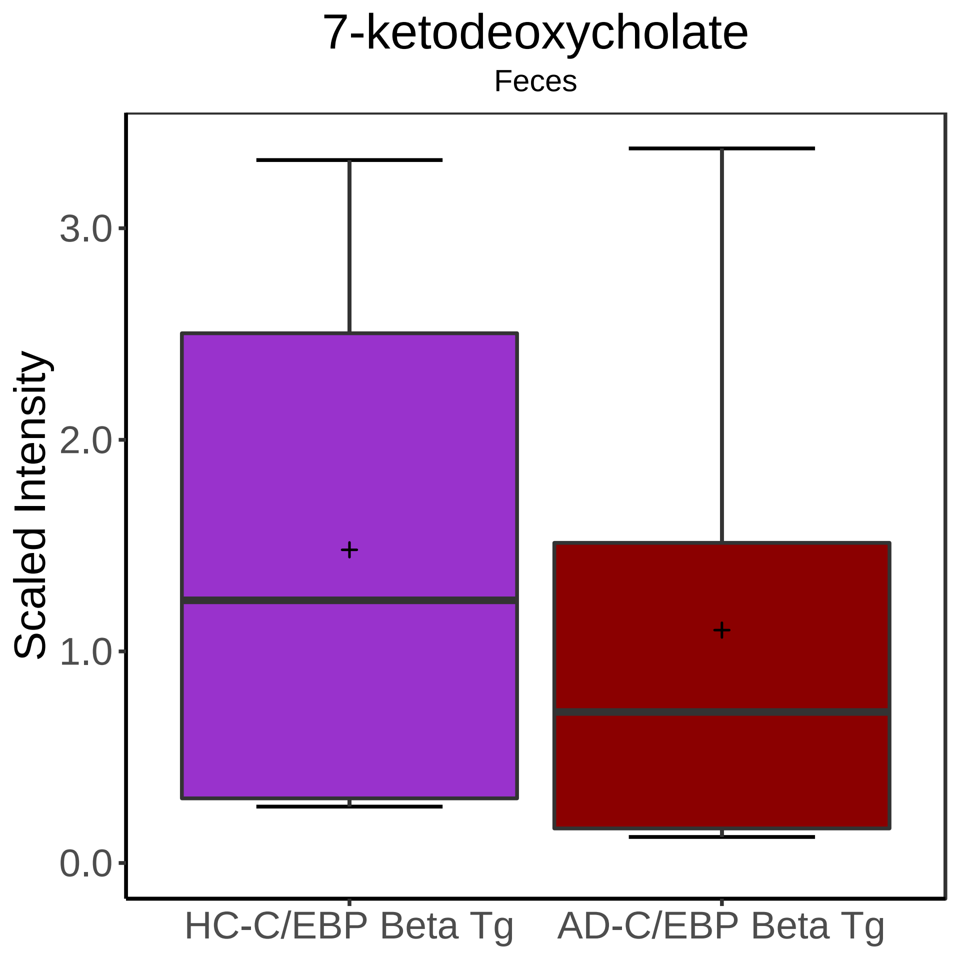

7-ketodeoxycholate
Feces
Scaled Intensity
3.0
2.0
1.0
0.0
HC-C/EBP Beta Tg
AD-C/EBP Beta Tg

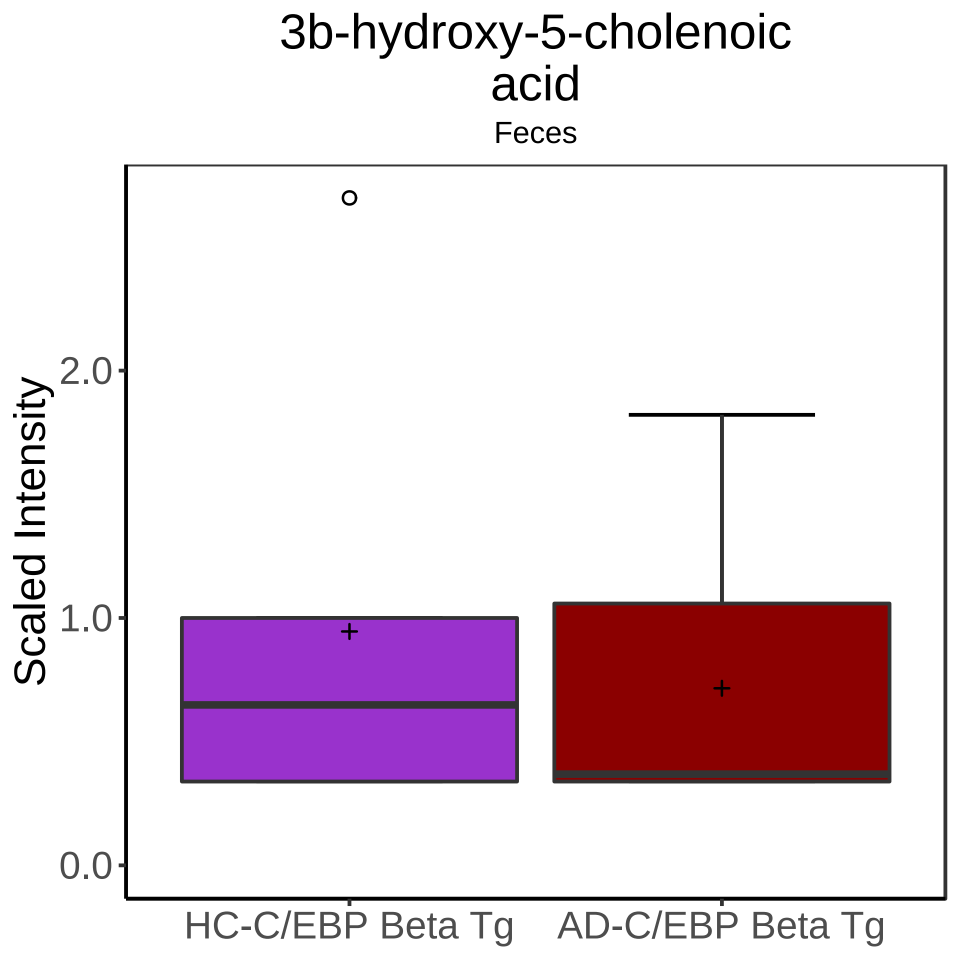
3b-hydroxy-5-cholenoic acid
Feces
Scaled Intensity
2.0
1.0
0.0
HC-C/EBP Beta Tg
AD-C/EBP Beta Tg

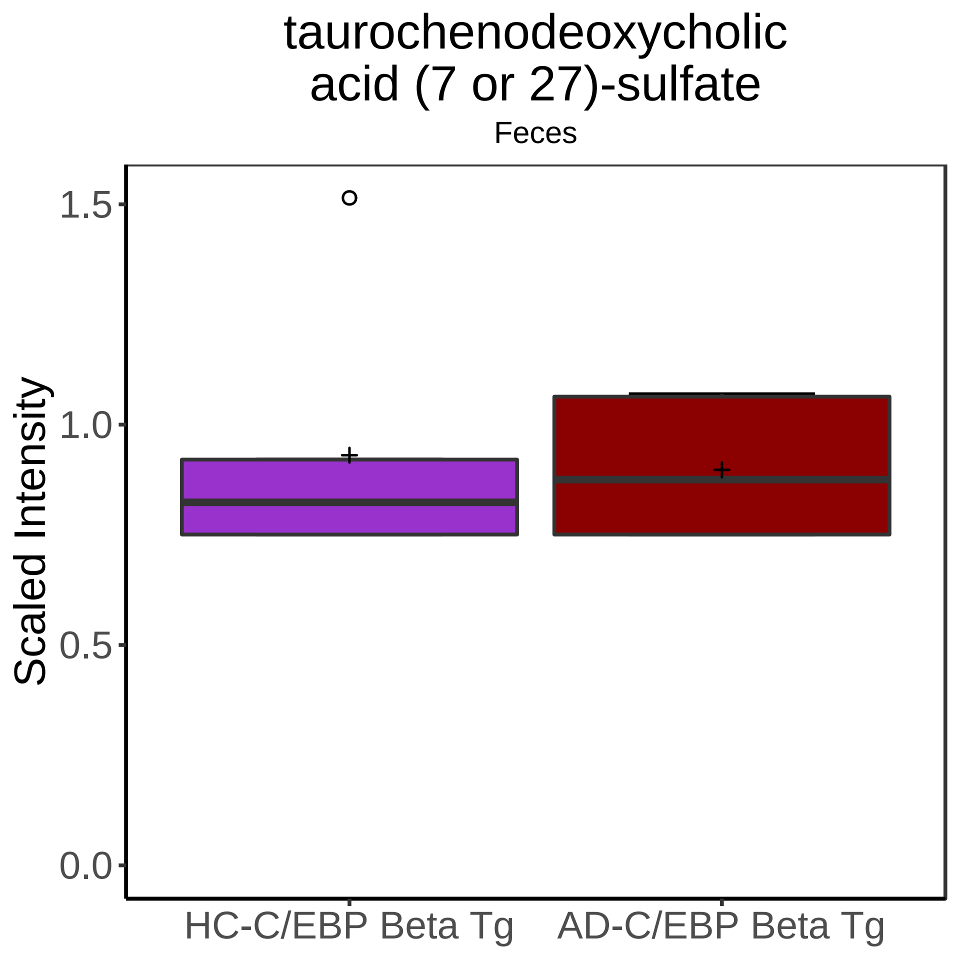
taurochenodeoxycholic
acid (7 or 27)-sulfate
Feces
Scaled Intensity
1.5
1.0
0.5
0.0
HC-C/EBP Beta Tg
AD-C/EBP Beta Tg

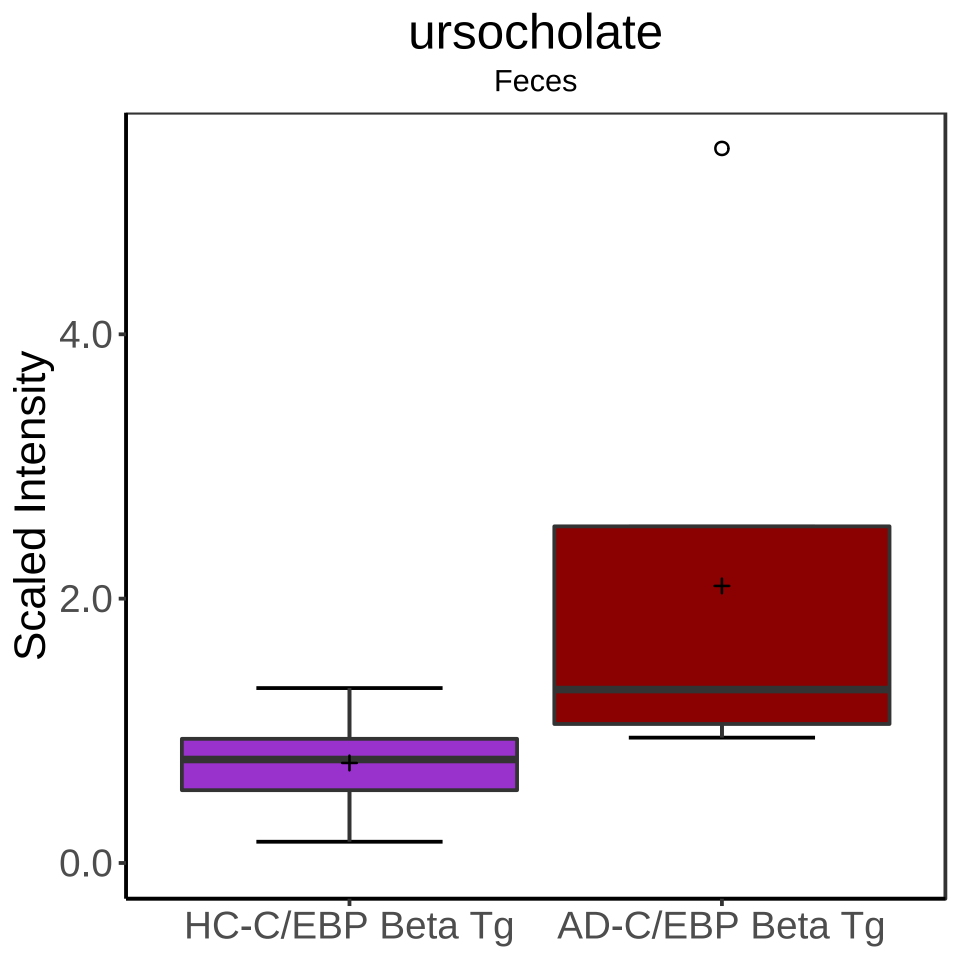
ursocholate
Feces
Scaled Intensity
4.0
2.0
0.0
HC-C/EBP Beta Tg
AD-C/EBP Beta Tg

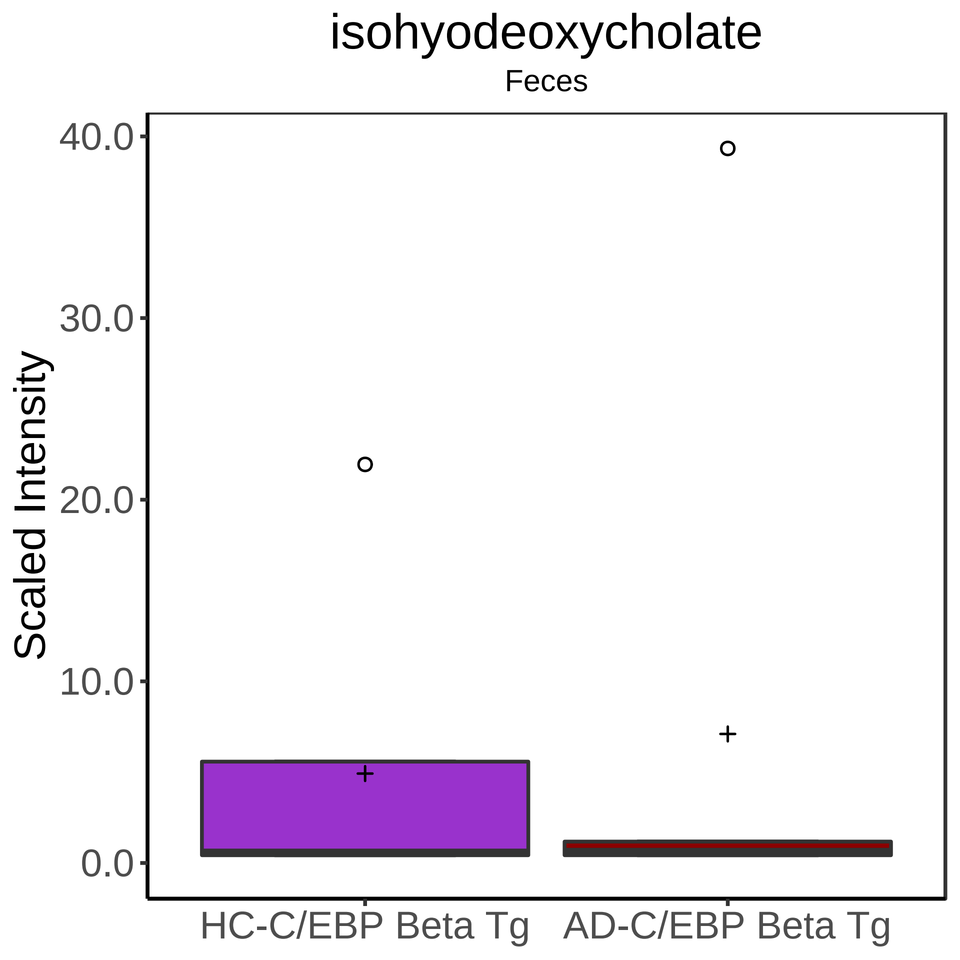

isohyodeoxycholate
Feces
Scaled Intensity
40.0
30.0
20.0
10.0
0.0
HC-C/EBP Beta Tg
AD-C/EBP Beta Tg

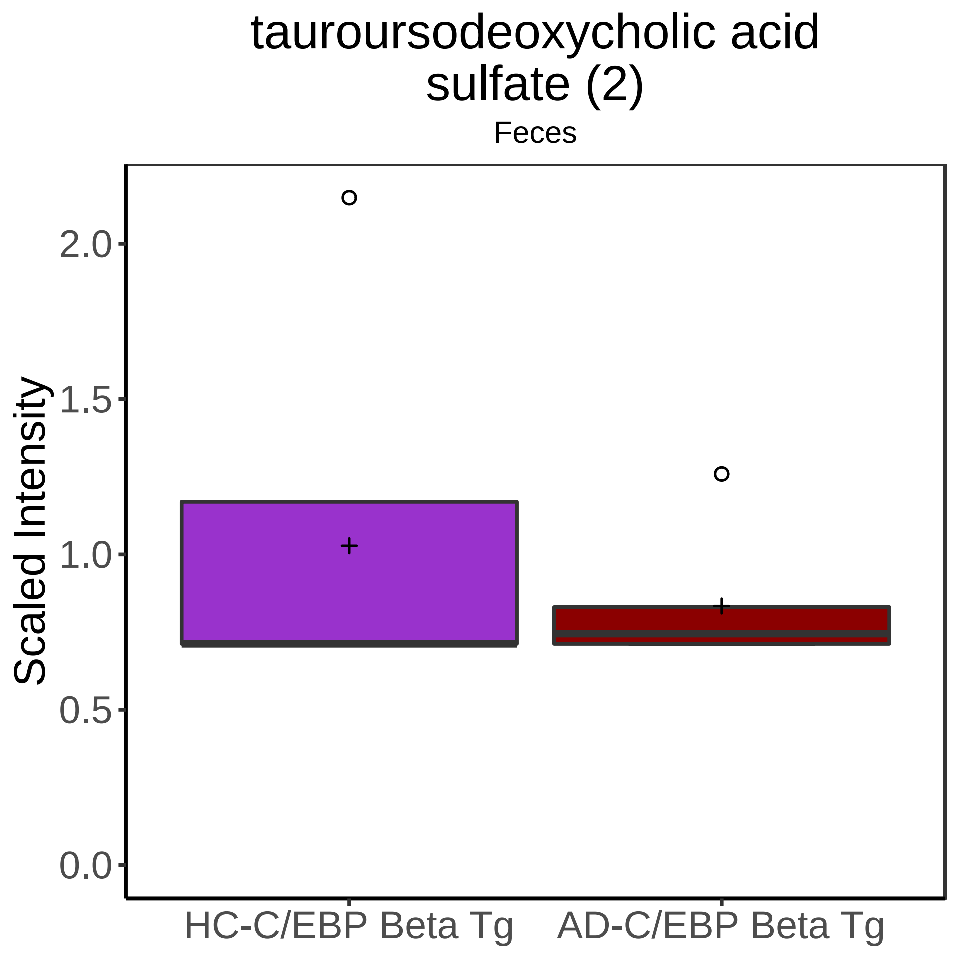

tauroursodeoxycholic acid sulfate (2)
Feces
Scaled Intensity
2.0
1.5
1.0
0.5
0.0
HC-C/EBP Beta Tg
AD-C/EBP Beta Tg

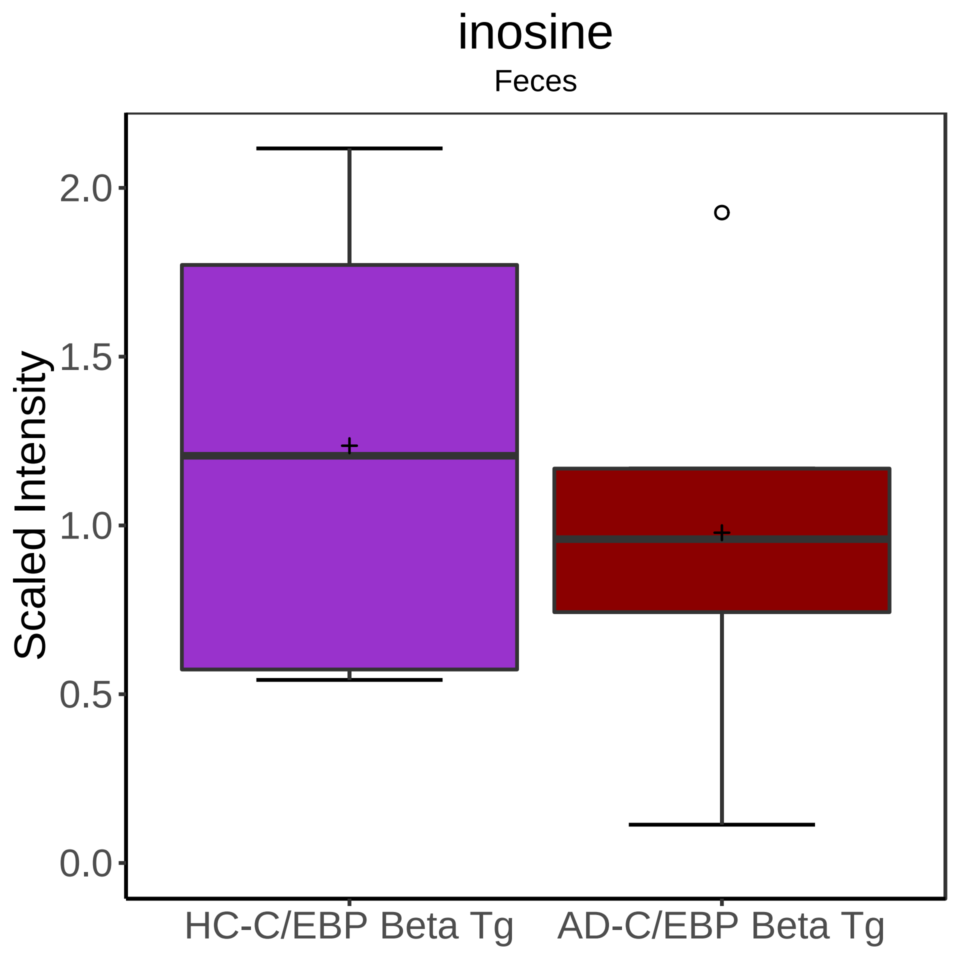
inosine
Feces
Scaled Intensity
2.0
1.5
1.0
0.5
0.0
HC-C/EBP Beta Tg
AD-C/EBP Beta Tg

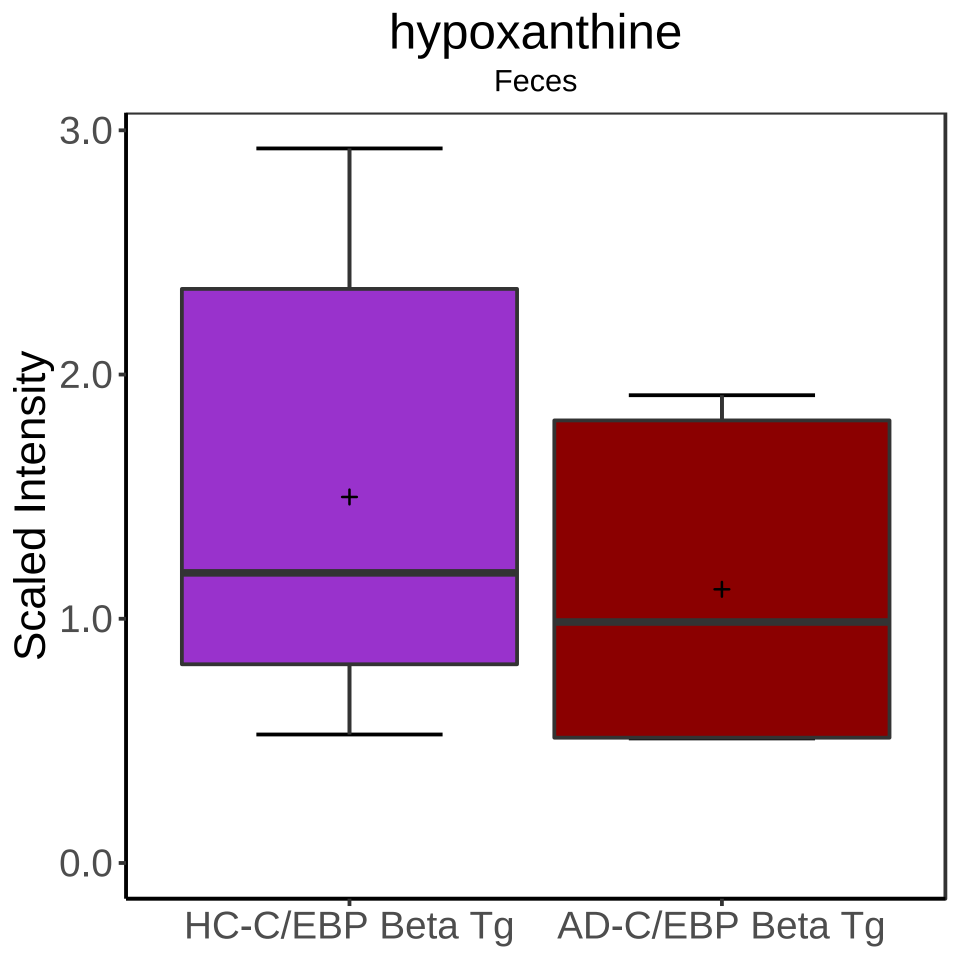
hypoxanthine
Feces
Scaled Intensity
3.0
2.0
1.0
0.0
HC-C/EBP Beta Tg
AD-C/EBP Beta Tg

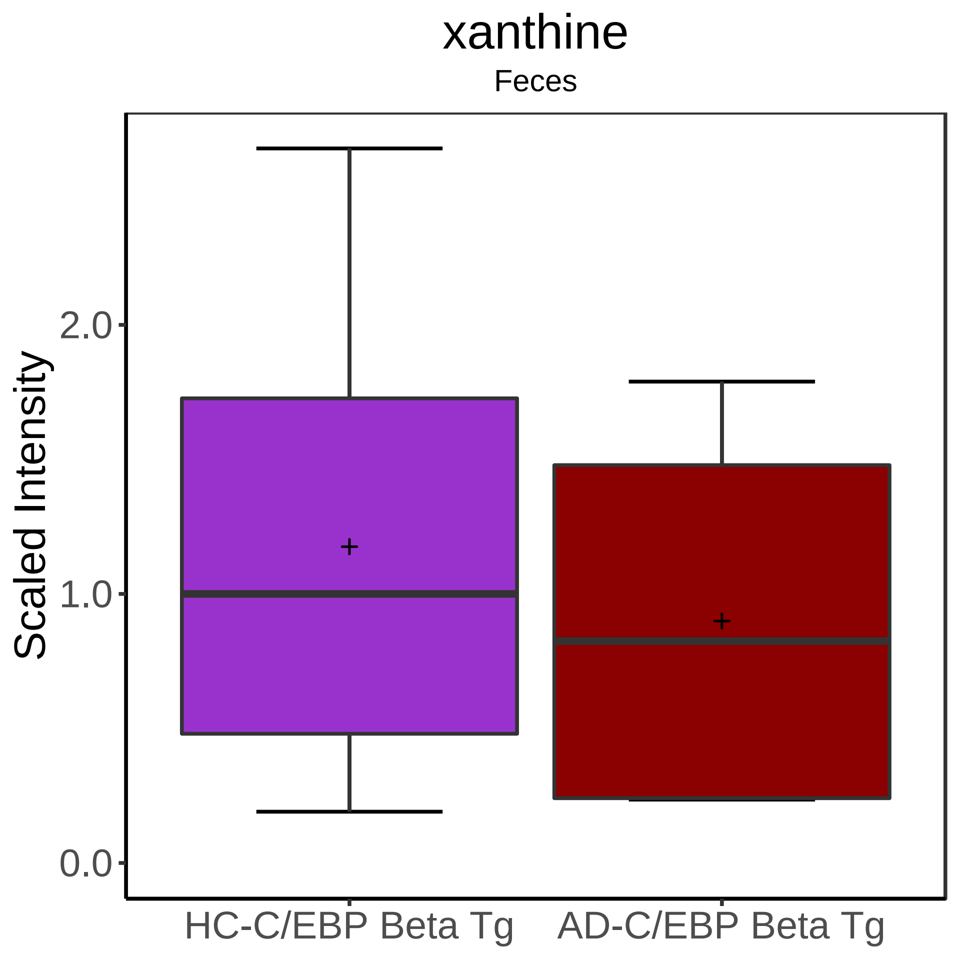

xanthine
Feces
Scaled Intensity
2.0
1.0
0.0
HC-C/EBP Beta Tg
AD-C/EBP Beta Tg

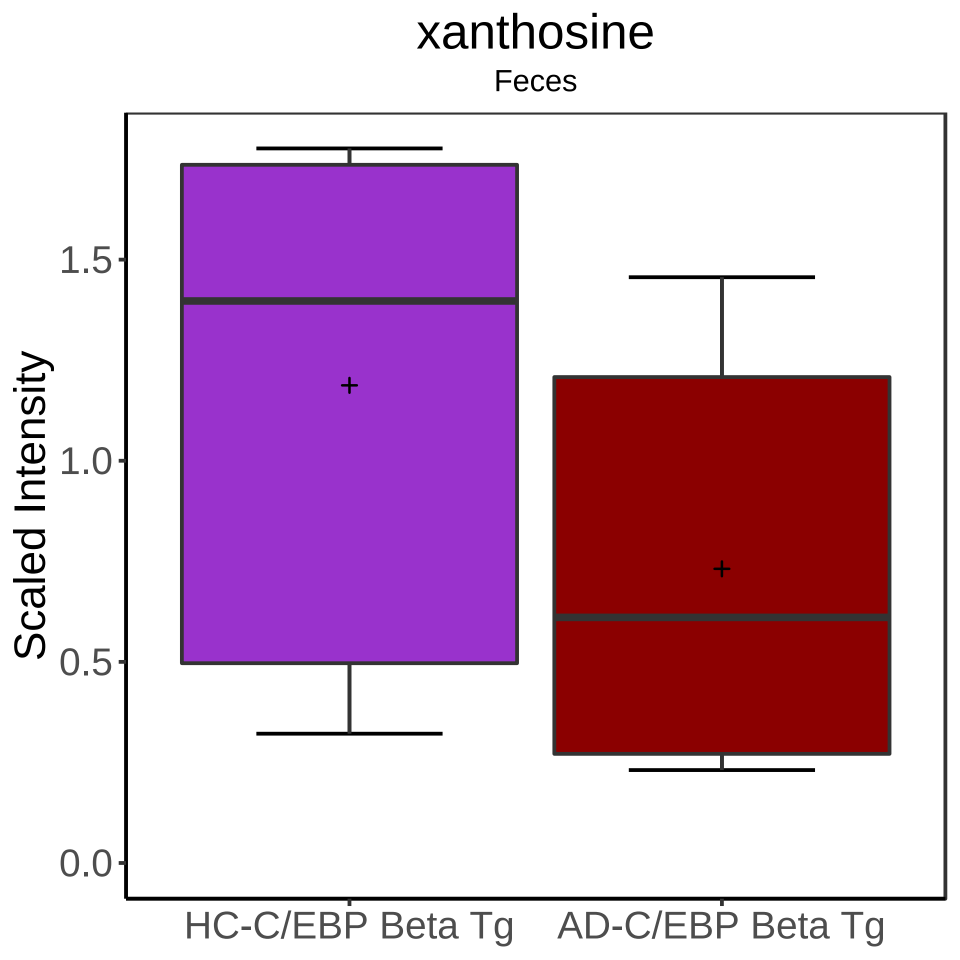
xanthosine
Feces
Scaled Intensity
1.5
1.0
0.5
0.0
HC-C/EBP Beta Tg
AD-C/EBP Beta Tg

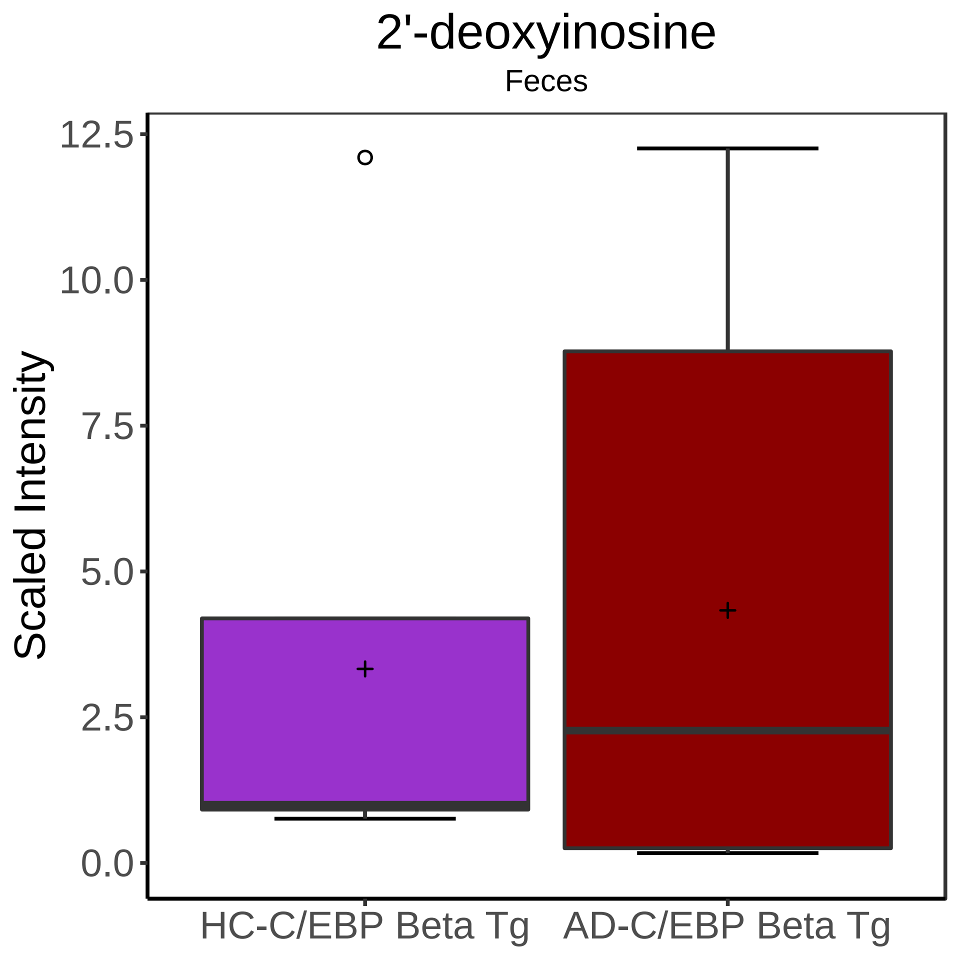

2'-deoxyinosine
Feces
Scaled Intensity
12.5
10.0
7.5
5.0
2.5
0.0
HC-C/EBP Beta Tg
AD-C/EBP Beta Tg

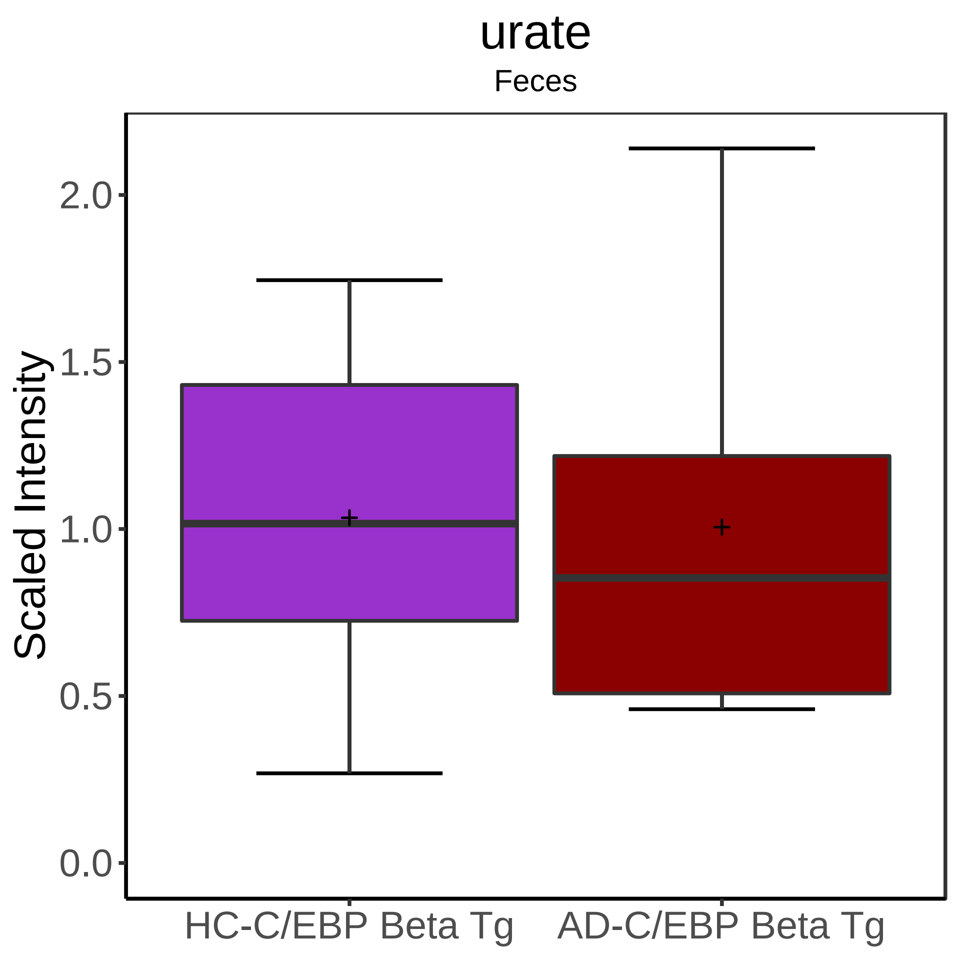
urate
Feces
Scaled Intensity
2.0
1.5
1.0
0.5
0.0
HC-C/EBP Beta Tg
AD-C/EBP Beta Tg

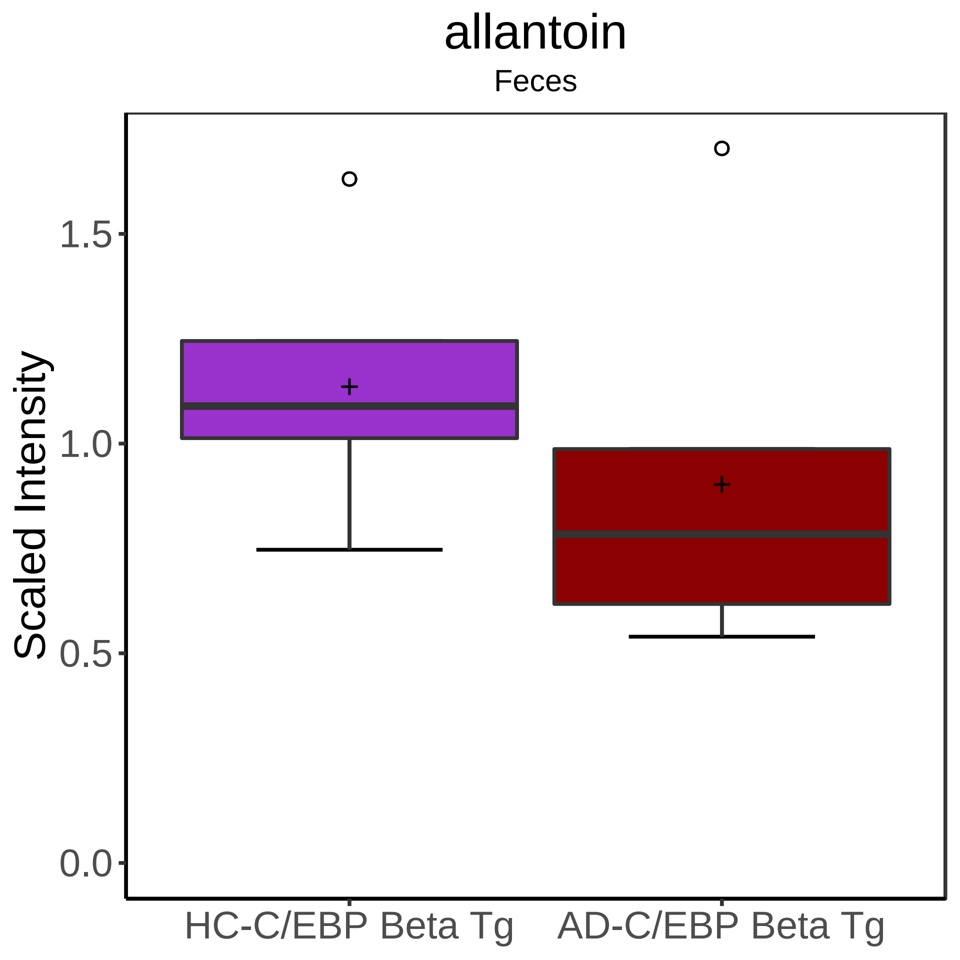
allantoin
Feces
Scaled Intensity
1.5
1.0
0.5
0.0
HC-C/EBP Beta Tg
AD-C/EBP Beta Tg

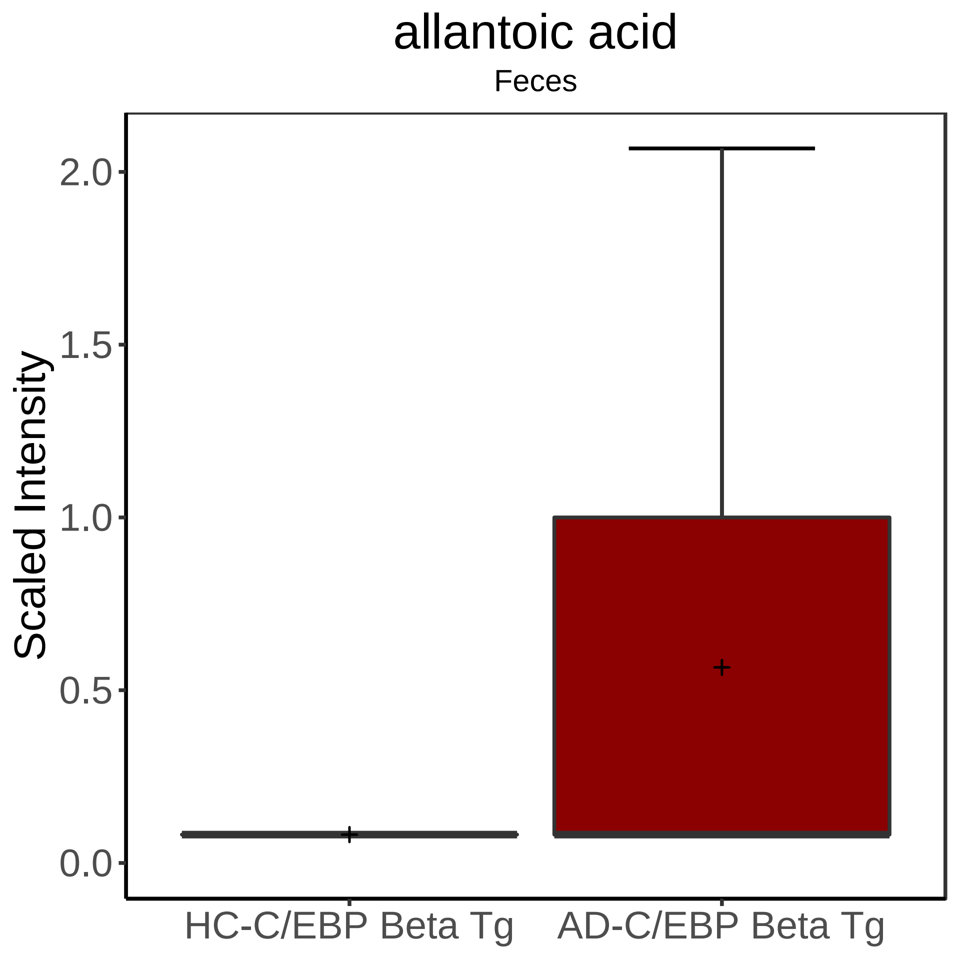
allantoic acid
Feces
Scaled Intensity
2.0
1.5
1.0
0.5
0.0
HC-C/EBP Beta Tg
AD-C/EBP Beta Tg

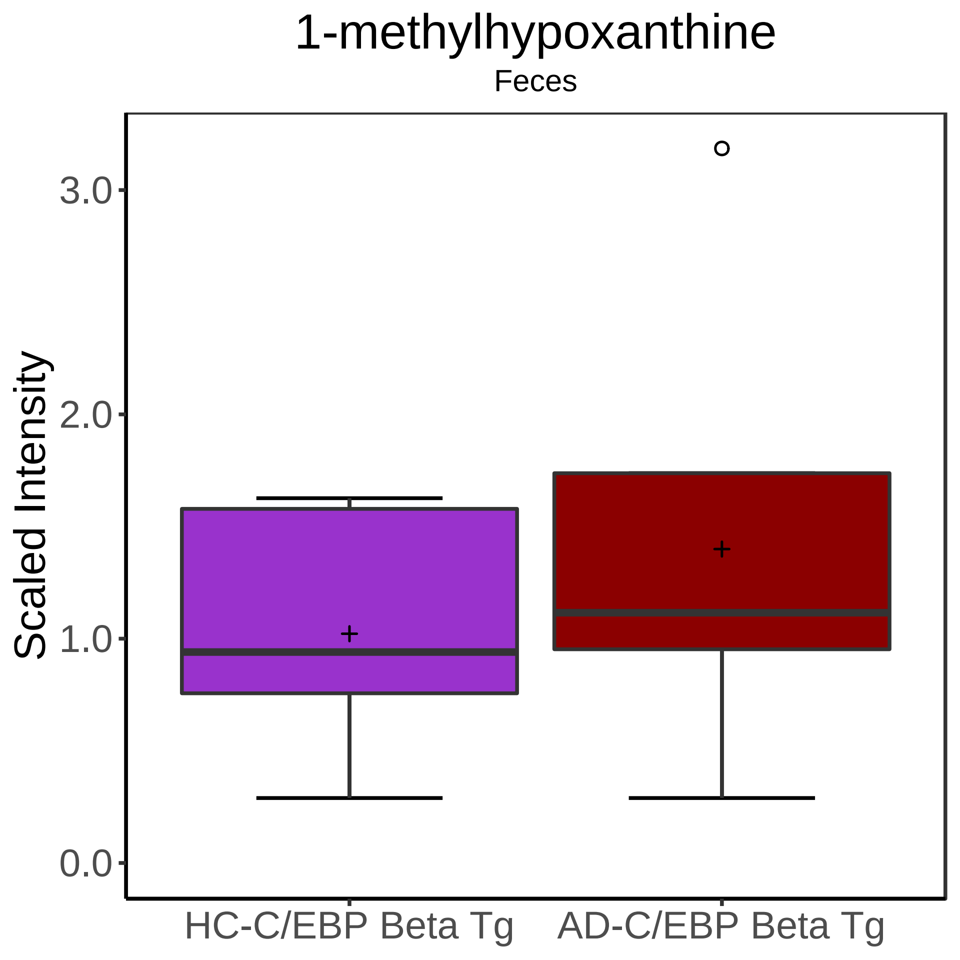

1-methylhypoxanthine
Feces
Scaled Intensity
3.0
2.0
1.0
0.0
HC-C/EBP Beta Tg
AD-C/EBP Beta Tg

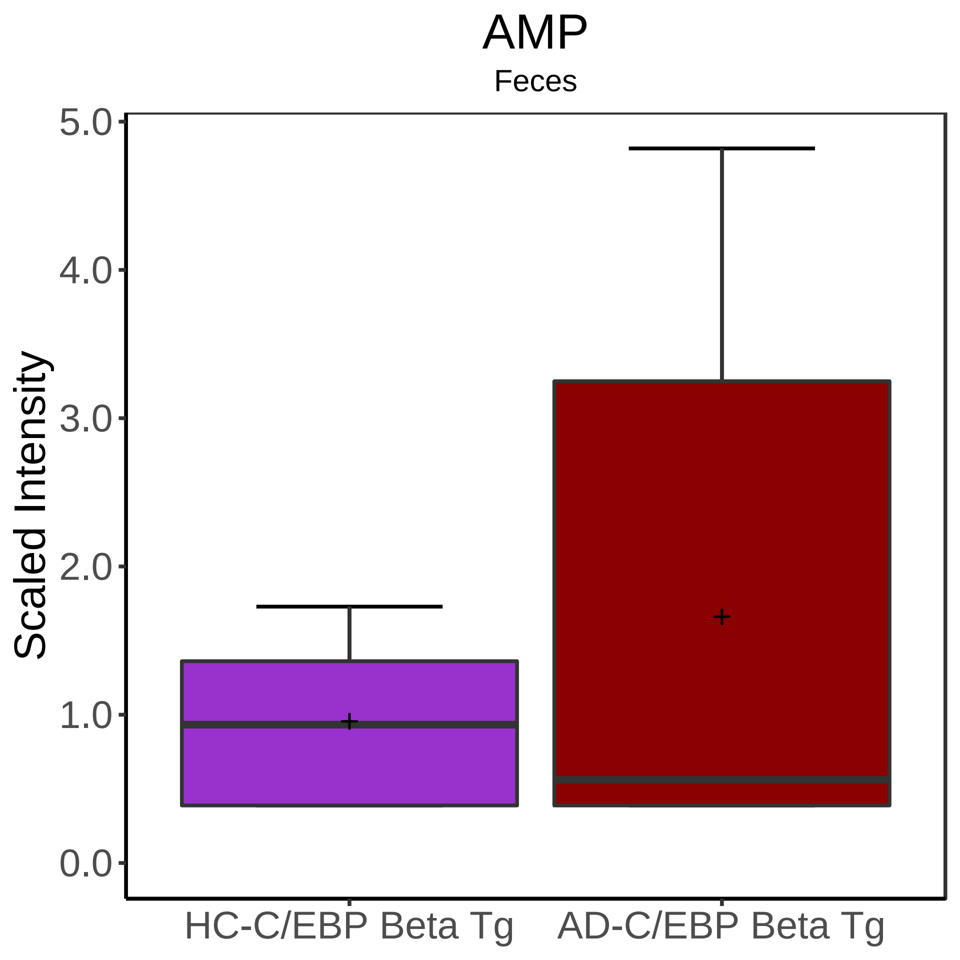

AMP
Feces
Scaled Intensity
5.0
4.0
3.0
2.0
1.0
0.0
HC-C/EBP Beta Tg
AD-C/EBP Beta Tg

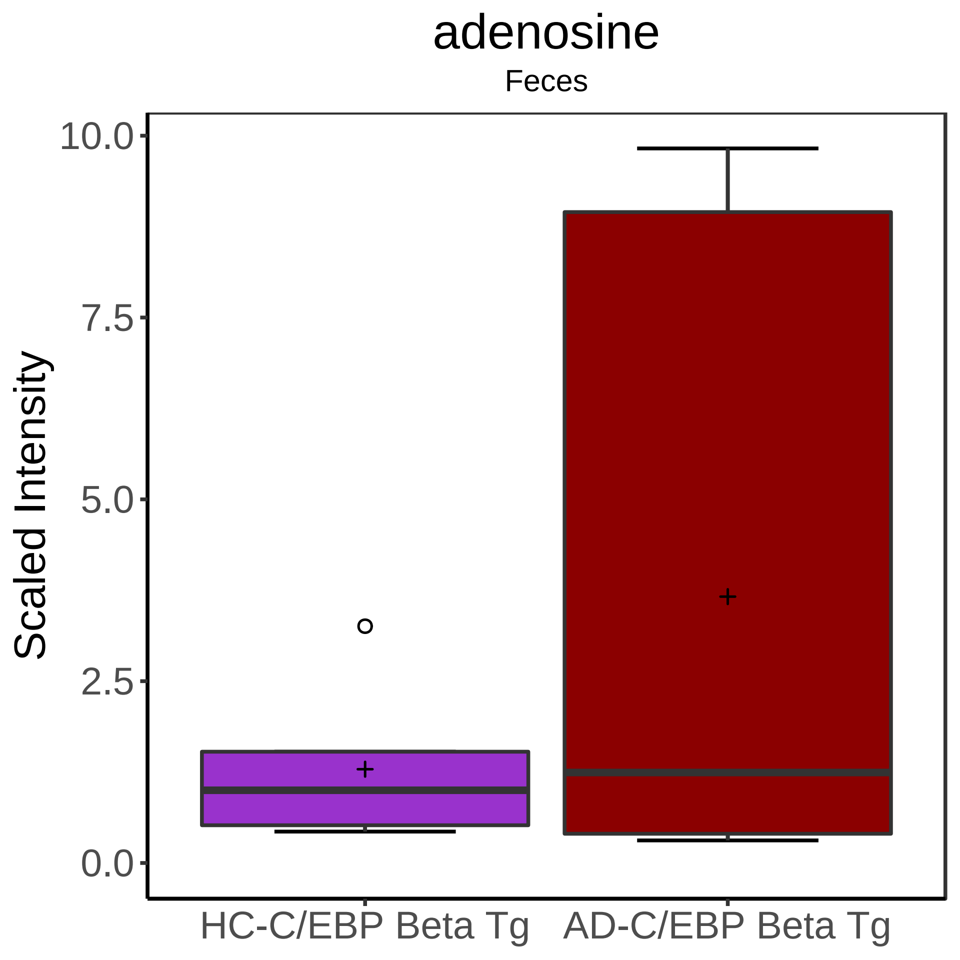

adenosine
Feces
Scaled Intensity
10.0
7.5
5.0
2.5
0.0
HC-C/EBP Beta Tg
AD-C/EBP Beta Tg

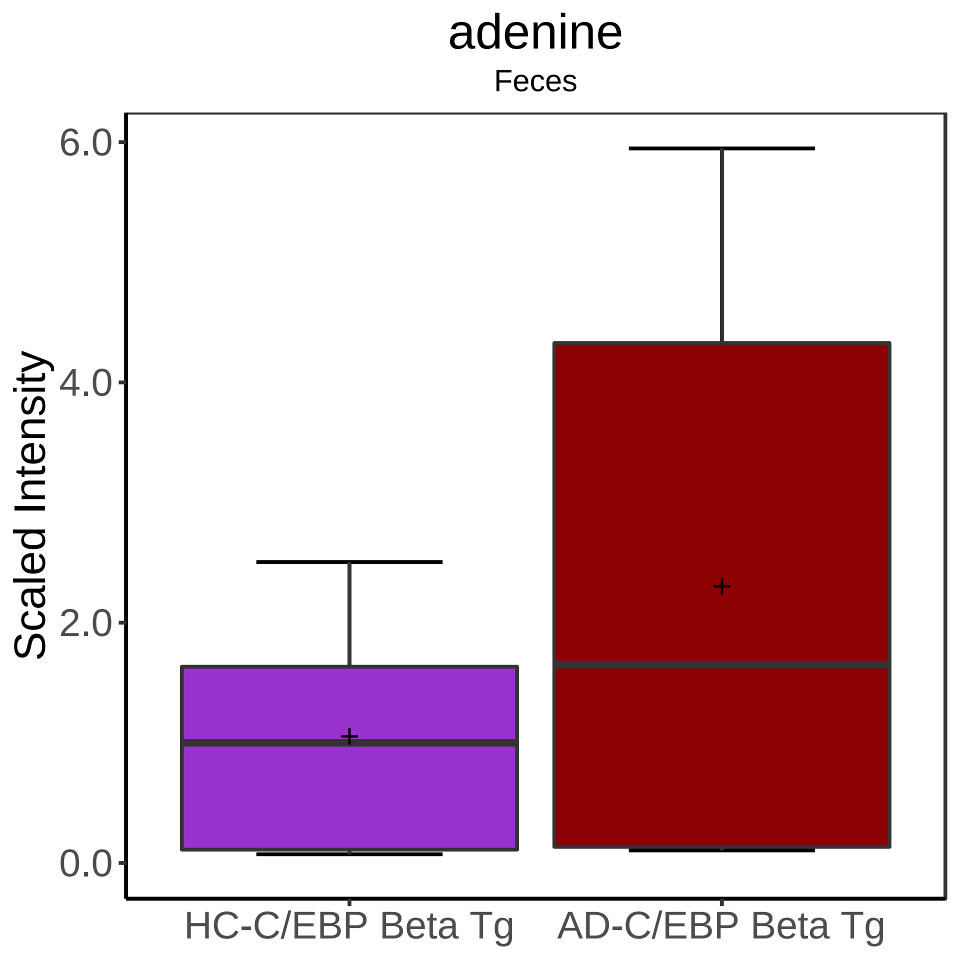
adenine
Feces
Scaled Intensity
6.0
4.0
2.0
0.0
HC-C/EBP Beta Tg
AD-C/EBP Beta Tg

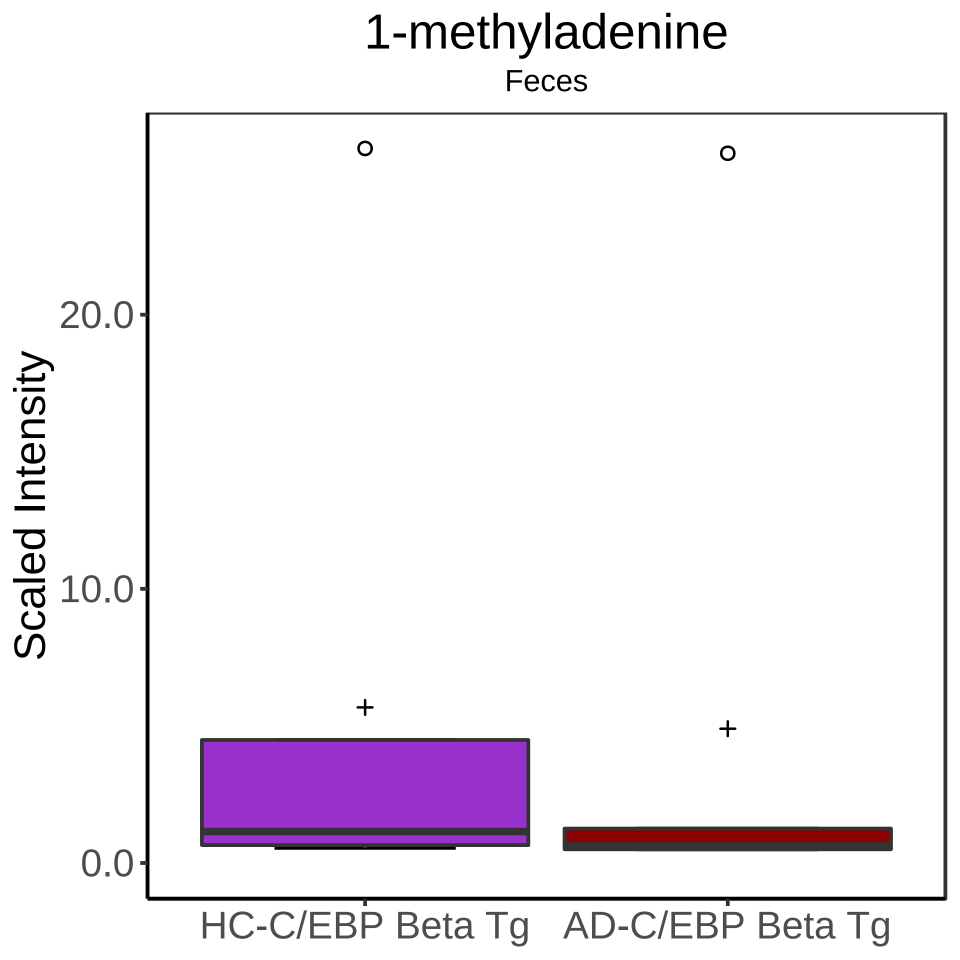
1-methyladenine
Feces
Scaled Intensity
20.0
10.0
0.0
HC-C/EBP Beta Tg
AD-C/EBP Beta Tg

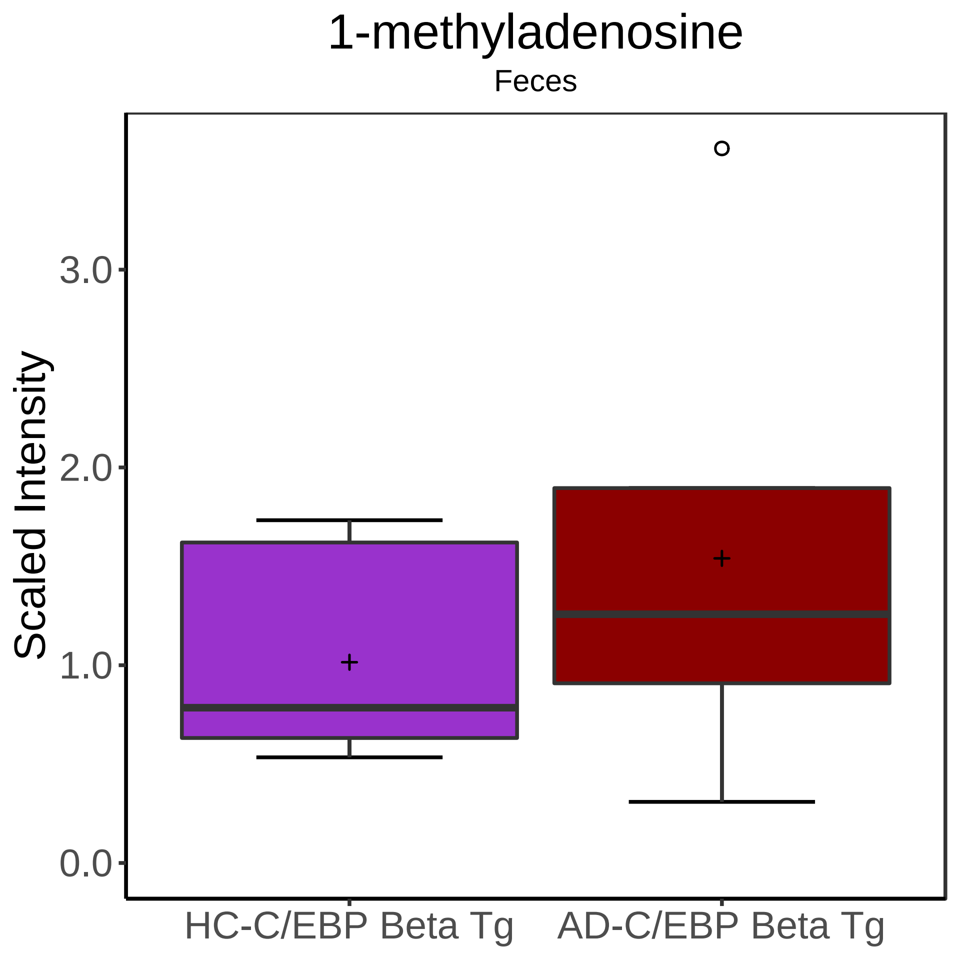
1-methyladenosine
Feces
Scaled Intensity
3.0
2.0
1.0
0.0
HC-C/EBP Beta Tg
AD-C/EBP Beta Tg

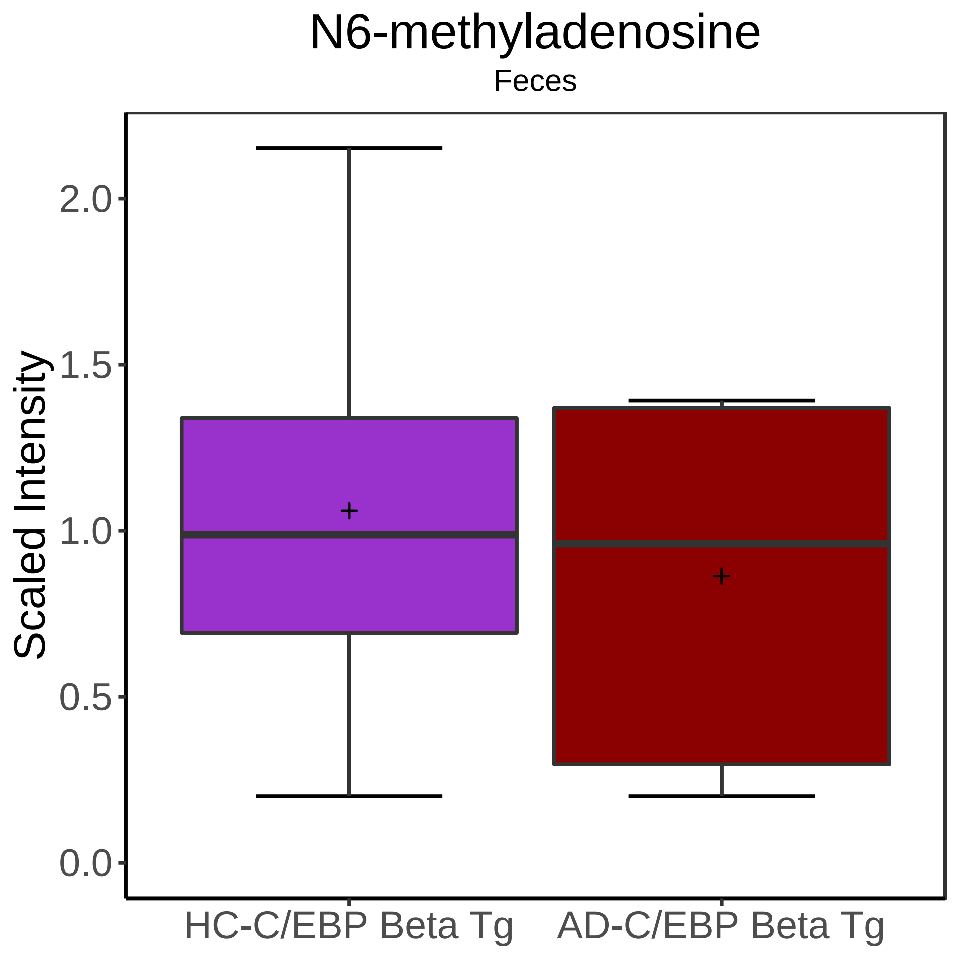
N6-methyladenosine
Feces
Scaled Intensity
2.0
1.5
1.0
0.5
0.0
HC-C/EBP Beta Tg
AD-C/EBP Beta Tg

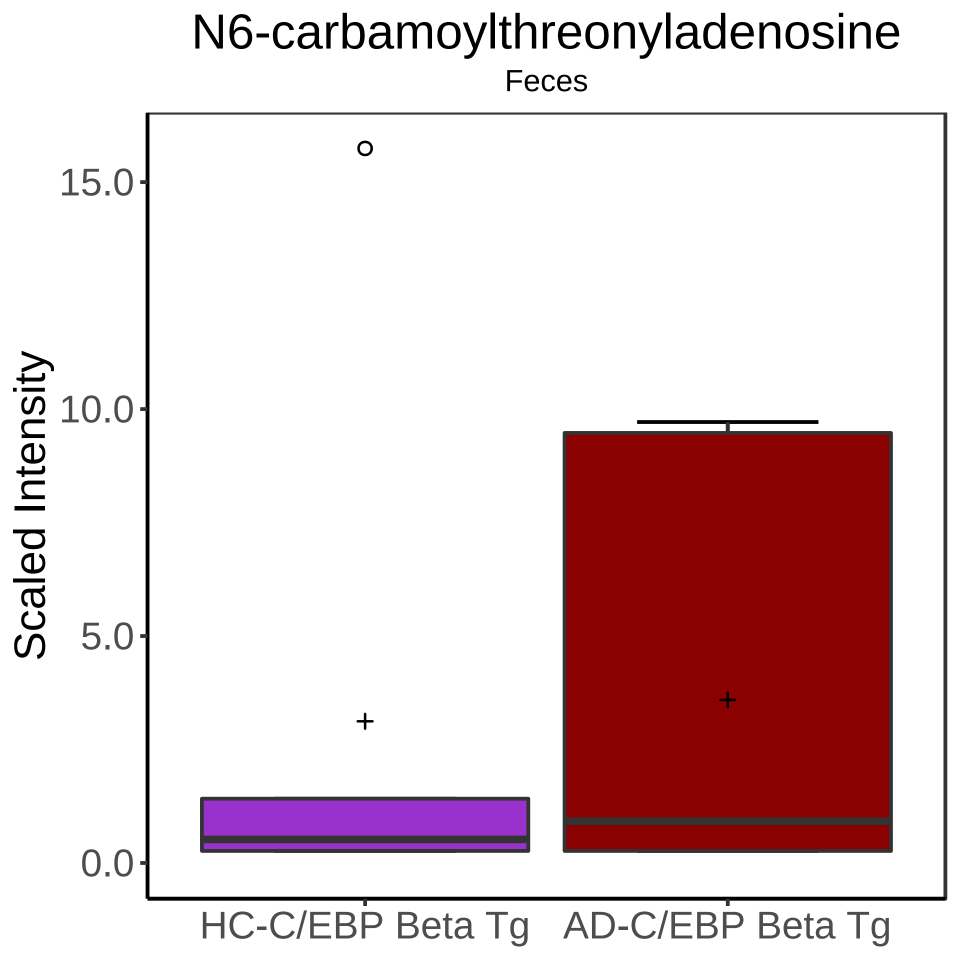
N6-carbamoylthreonyladenosine
Feces
Scaled Intensity
15.0
10.0
5.0
0.0
HC-C/EBP Beta Tg
AD-C/EBP Beta Tg

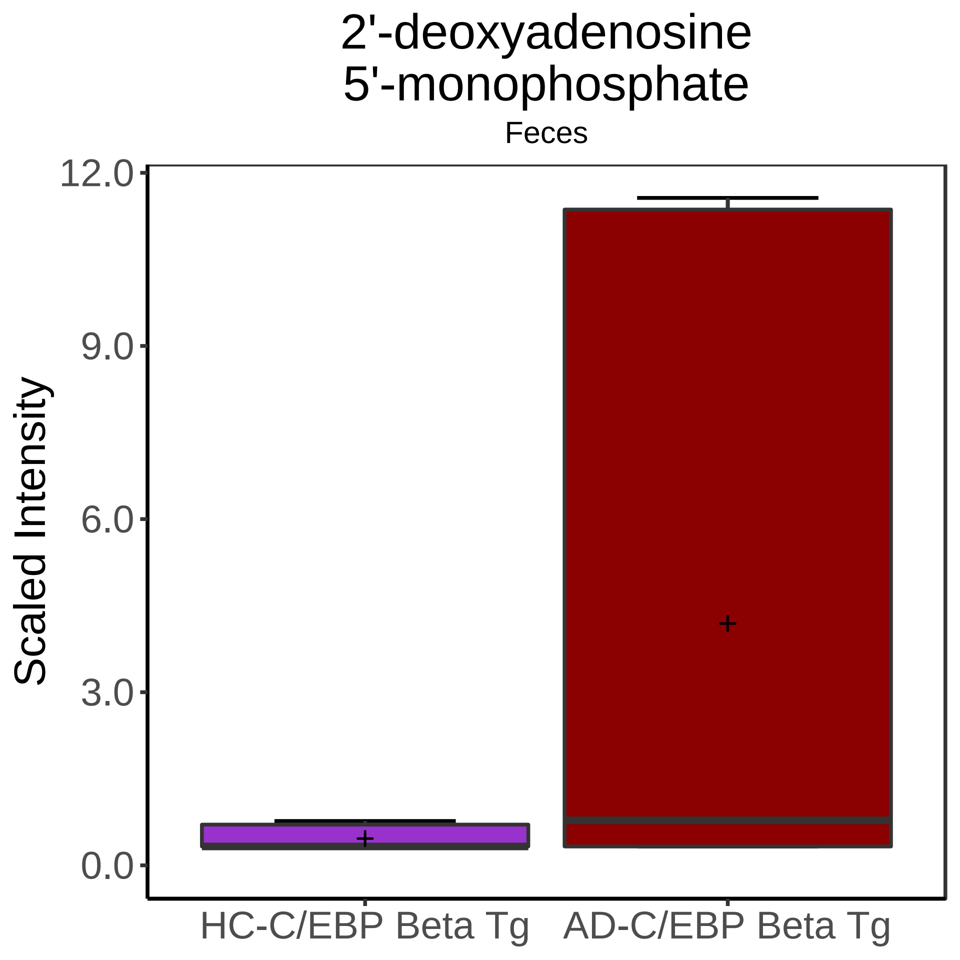
2'-deoxyadenosine
5'-monophosphate
Feces
Scaled Intensity
12.0
9.0
6.0
3.0
0.0
HC-C/EBP Beta Tg
AD-C/EBP Beta Tg

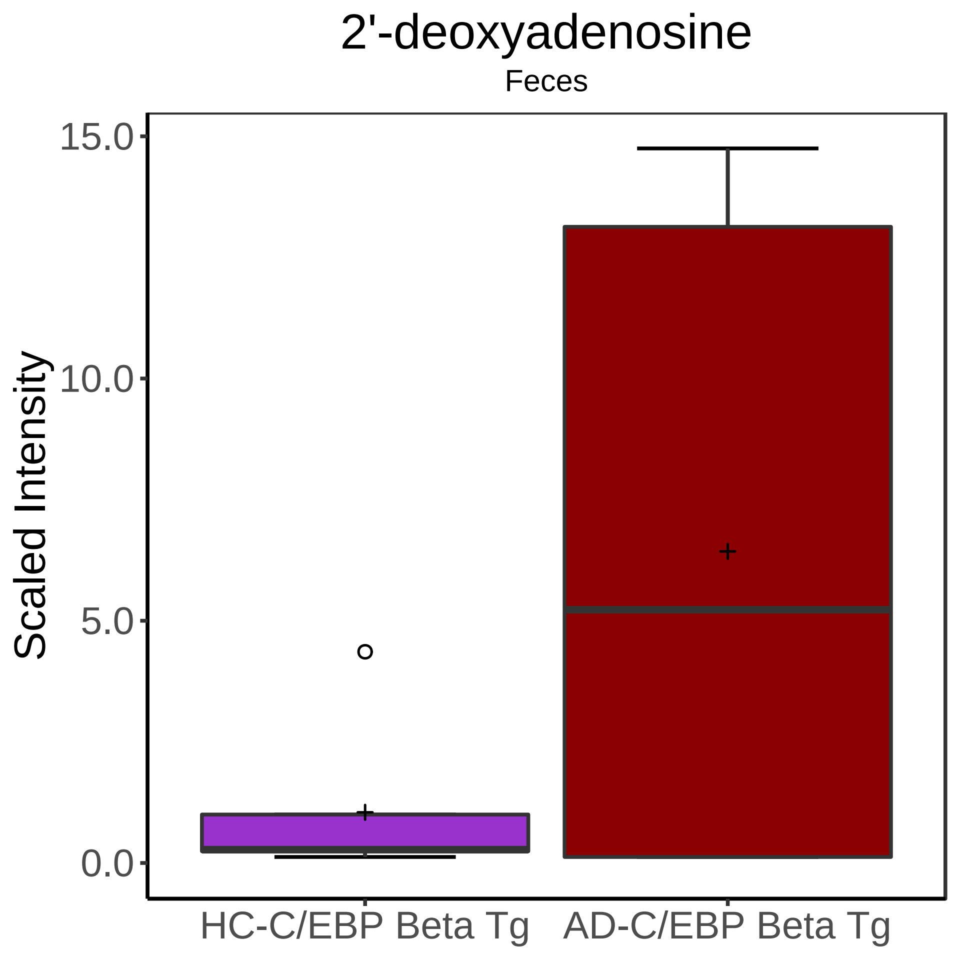
2'-deoxyadenosine
Feces
Scaled Intensity
15.0
10.0
5.0
0.0
HC-C/EBP Beta Tg
AD-C/EBP Beta Tg

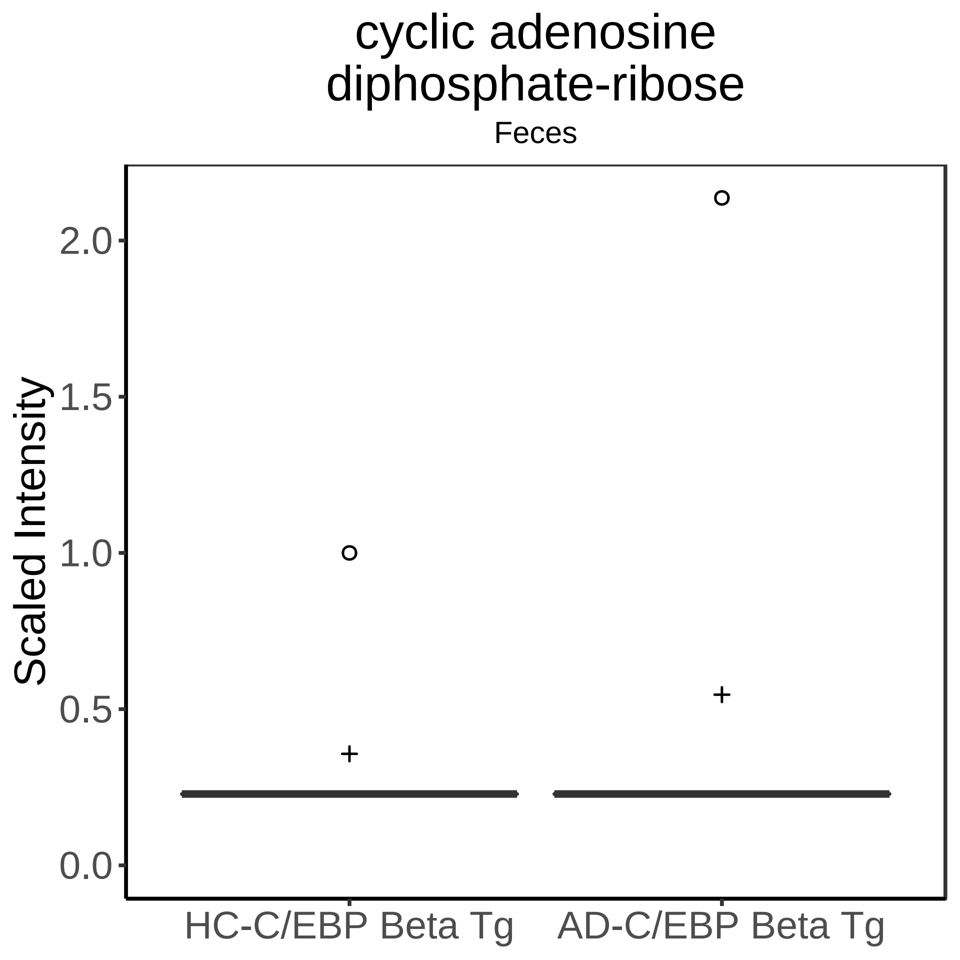
cyclic adenosine
diphosphate-ribose
Feces
Scaled Intensity
2.0
1.5
1.0
0.5
0.0
HC-C/EBP Beta Tg
AD-C/EBP Beta Tg

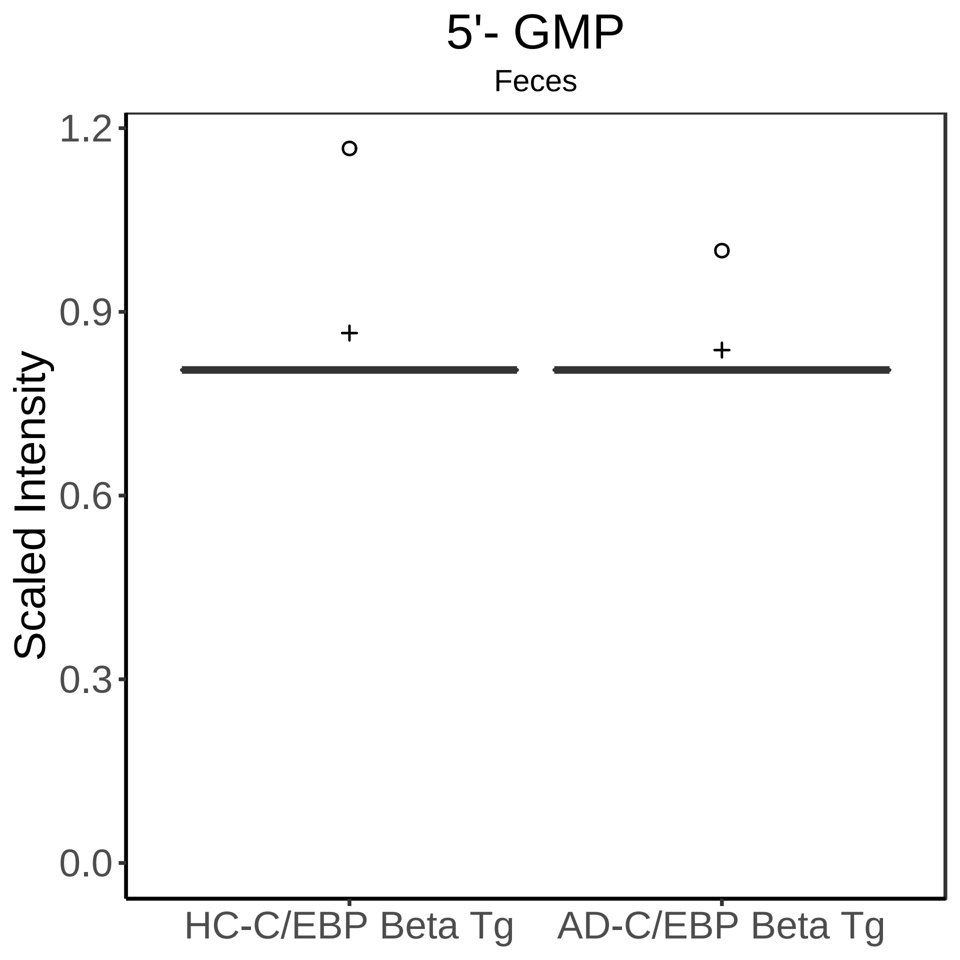
5'- GMP
Feces
Scaled Intensity
1.2
0.9
0.6
0.3
0.0
HC-C/EBP Beta Tg
AD-C/EBP Beta Tg

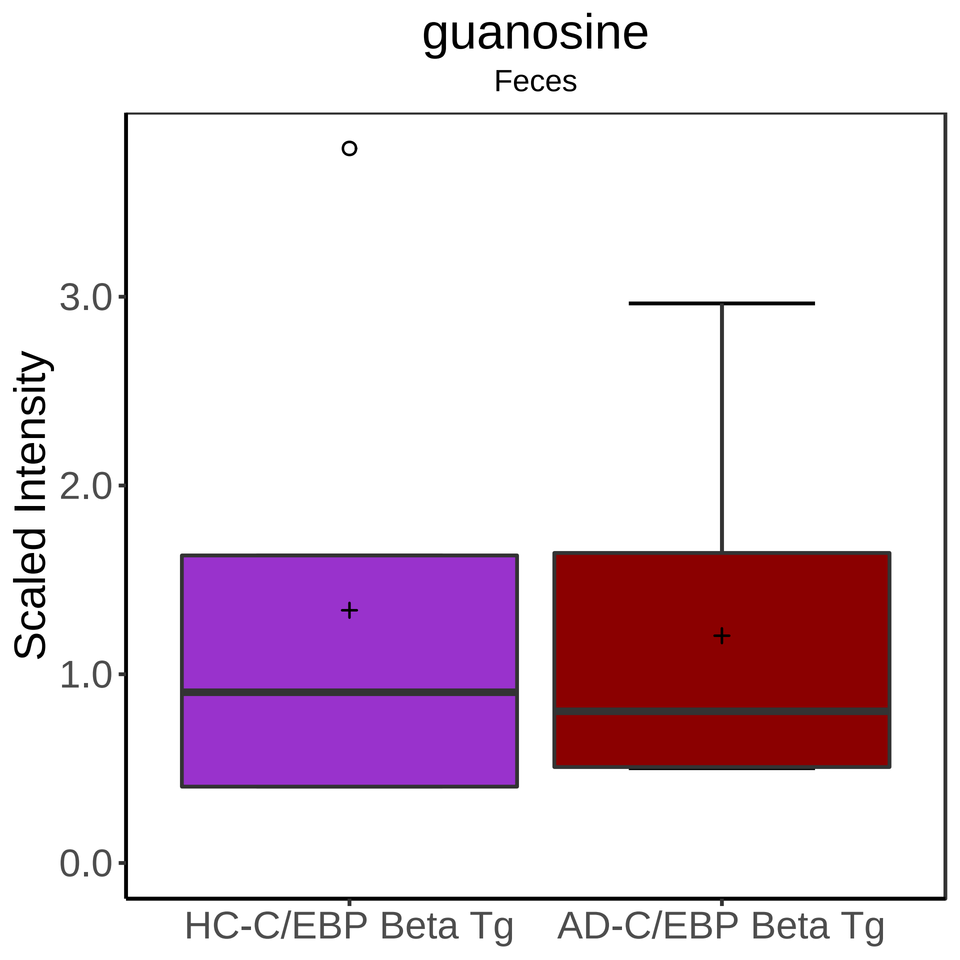

guanosine
Feces
Scaled Intensity
3.0
2.0
1.0
0.0
HC-C/EBP Beta Tg
AD-C/EBP Beta Tg

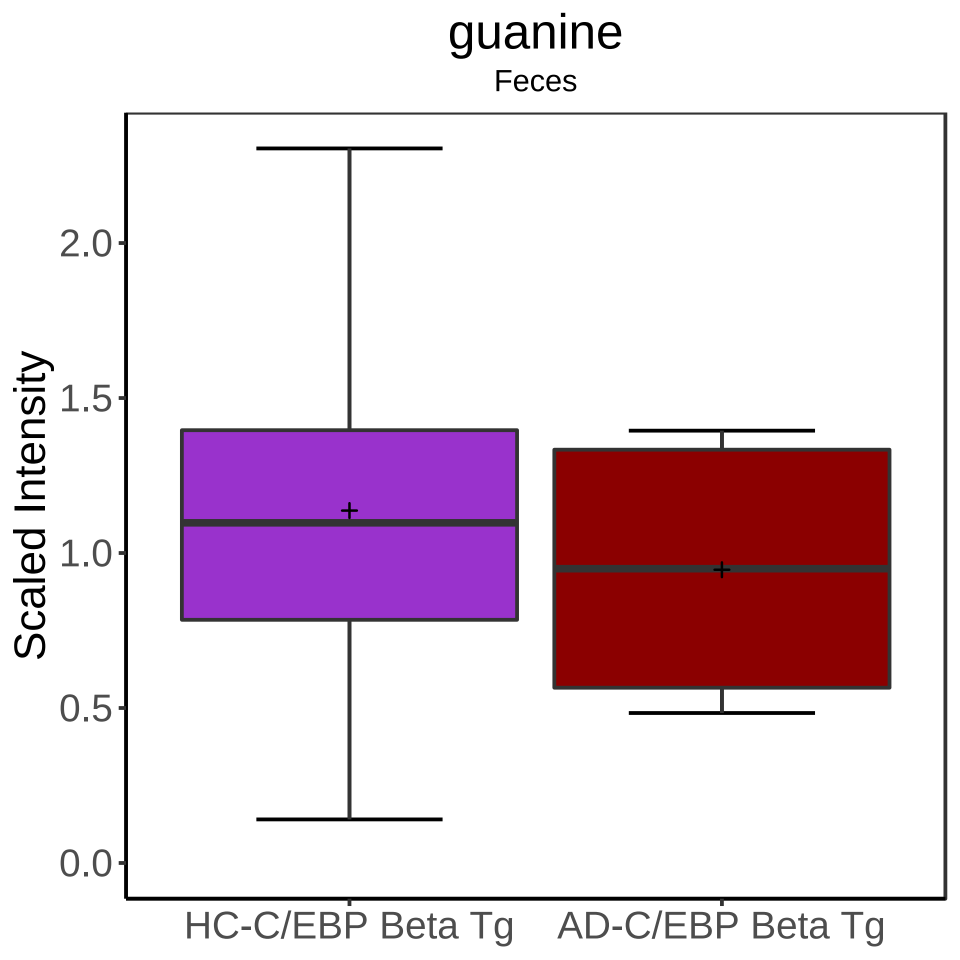
guanine
Feces
Scaled Intensity
2.0
1.5
1.0
0.5
0.0
HC-C/EBP Beta Tg
AD-C/EBP Beta Tg

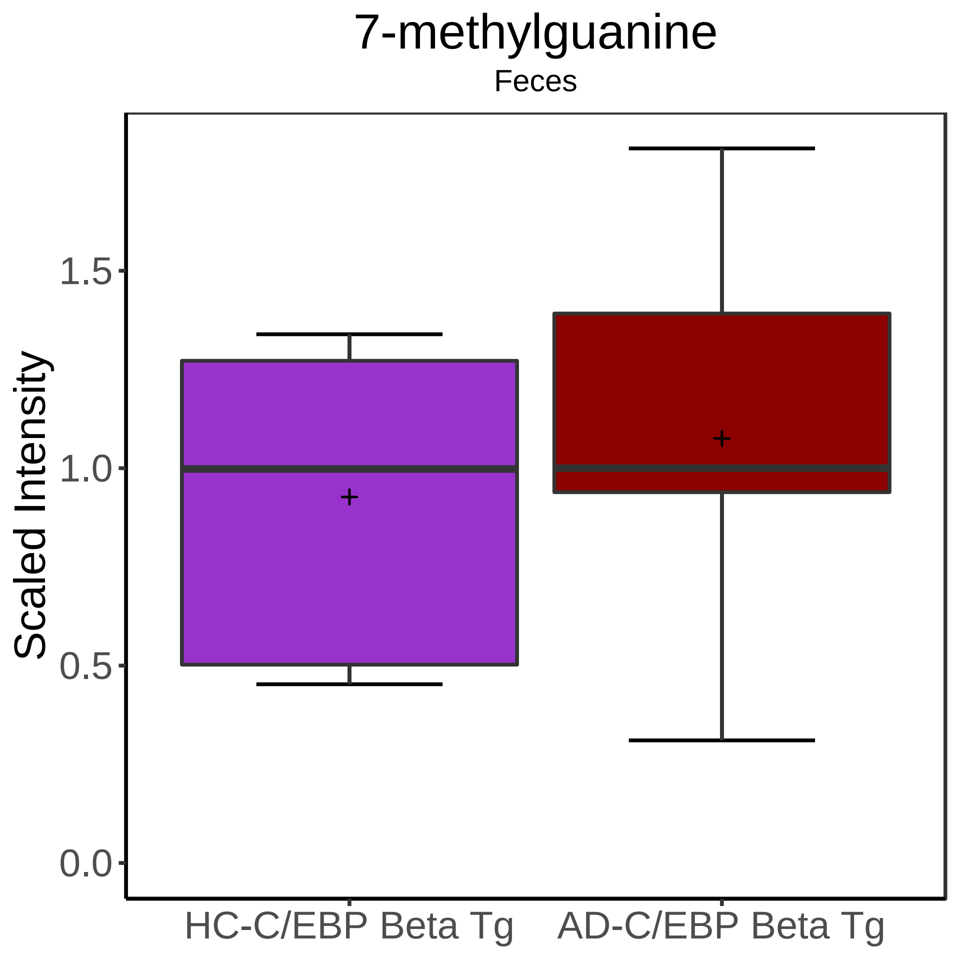
7-methylguanine
Feces
Scaled Intensity
1.5
1.0
0.5
0.0
HC-C/EBP Beta Tg
AD-C/EBP Beta Tg

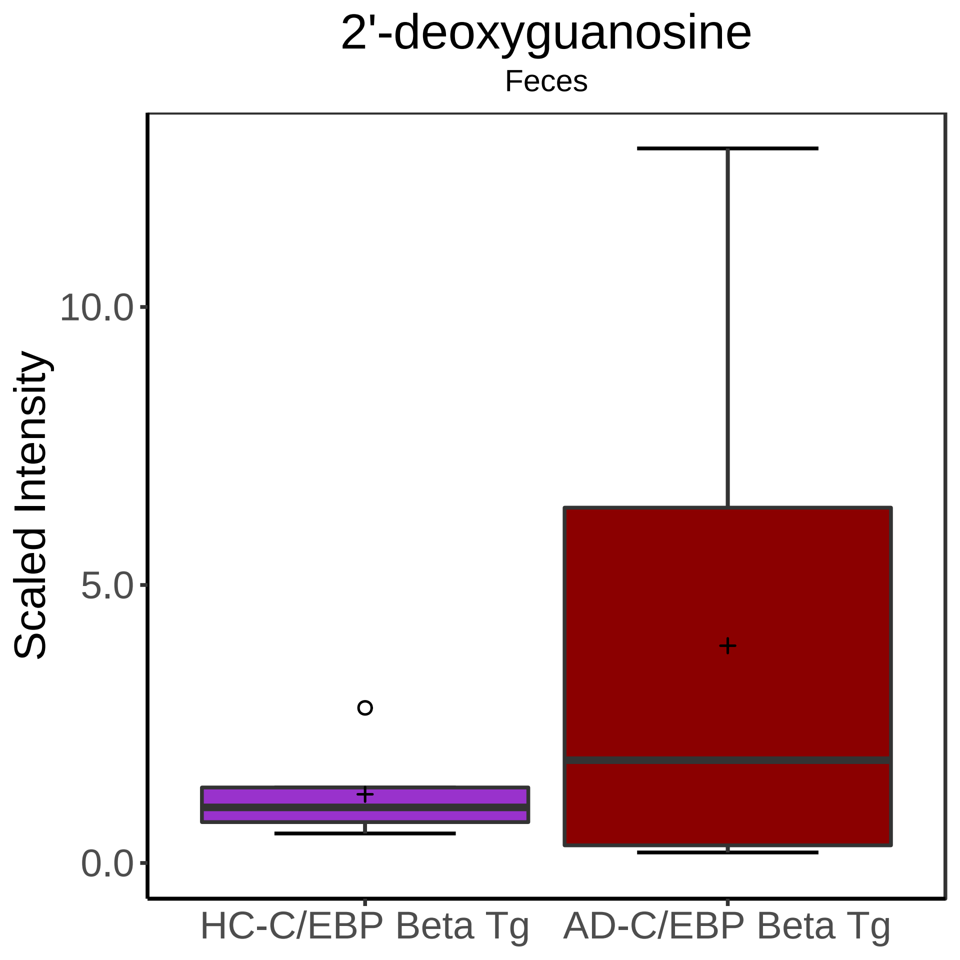

2'-deoxyguanosine
Feces
Scaled Intensity
10.0
5.0
0.0
HC-C/EBP Beta Tg
AD-C/EBP Beta Tg

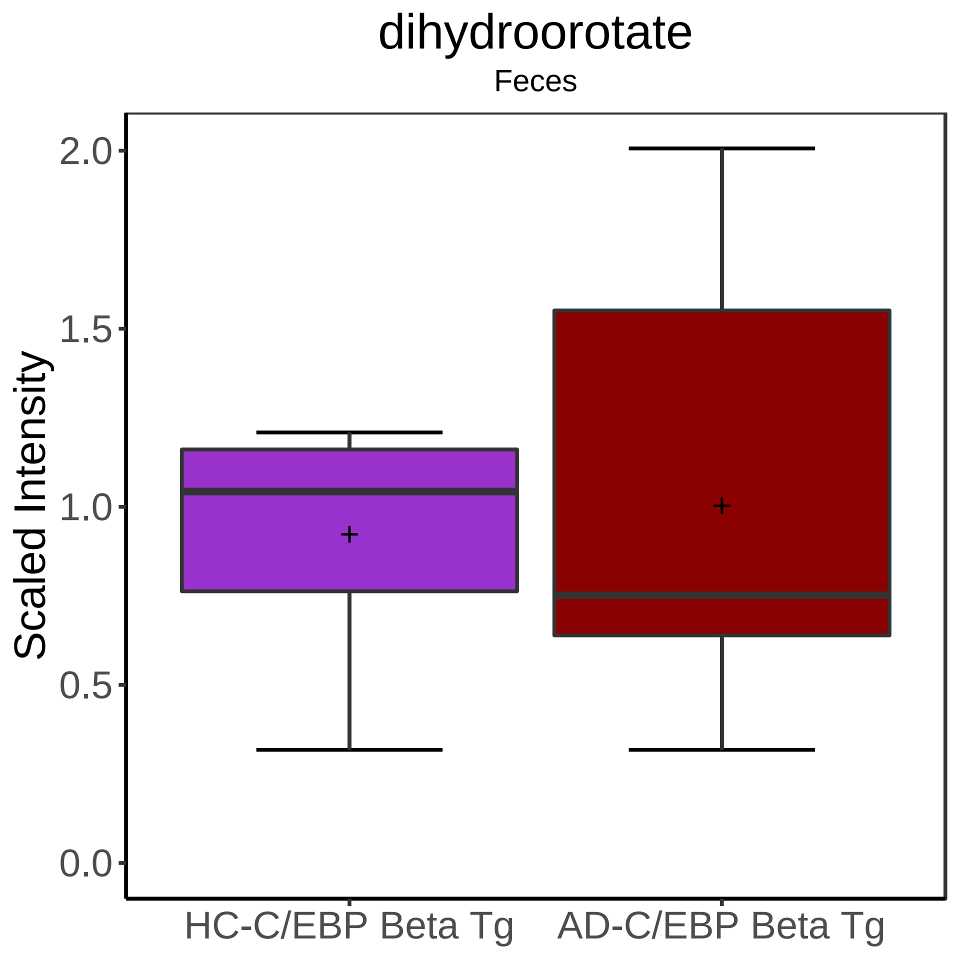
dihydroorotate
Feces
Scaled Intensity
2.0
1.5
1.0
0.5
0.0
HC-C/EBP Beta Tg
AD-C/EBP Beta Tg

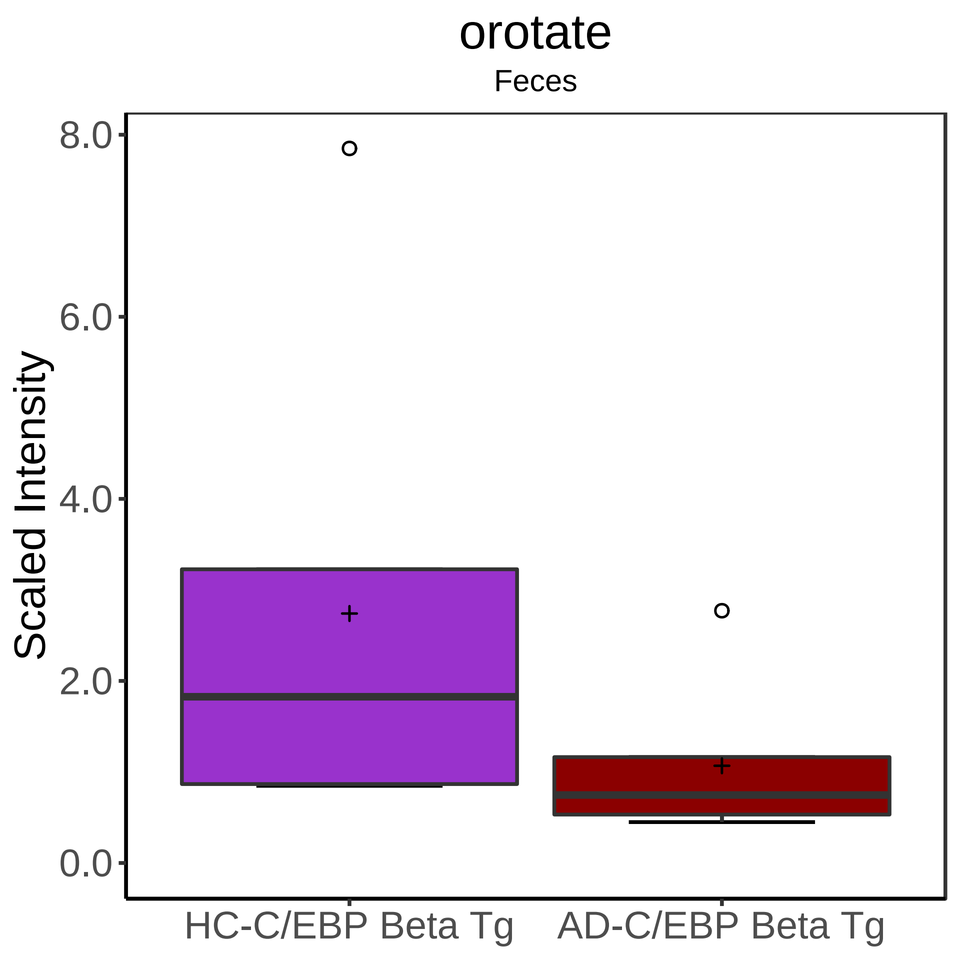

orotate
Feces
Scaled Intensity
8.0
6.0
4.0
2.0
0.0
HC-C/EBP Beta Tg
AD-C/EBP Beta Tg

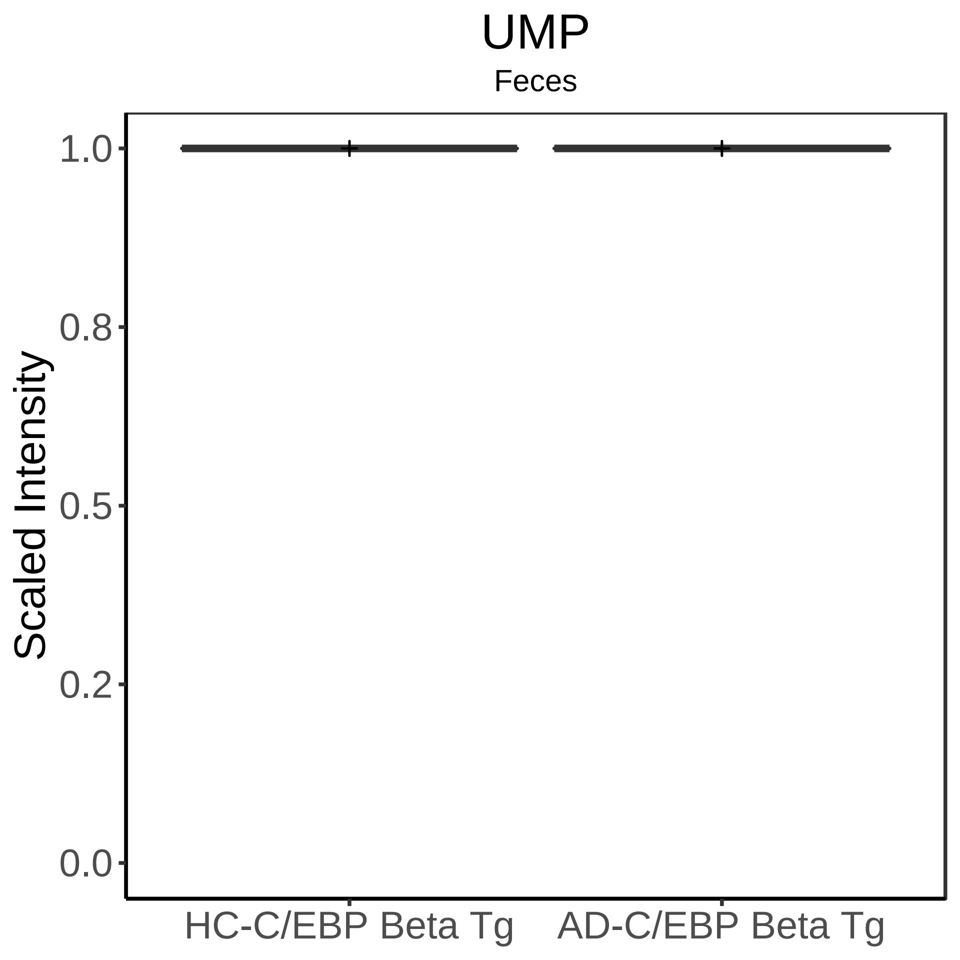
UMP
Feces
Scaled Intensity
1.0
0.8
0.5
0.2
0.0
HC-C/EBP Beta Tg
AD-C/EBP Beta Tg

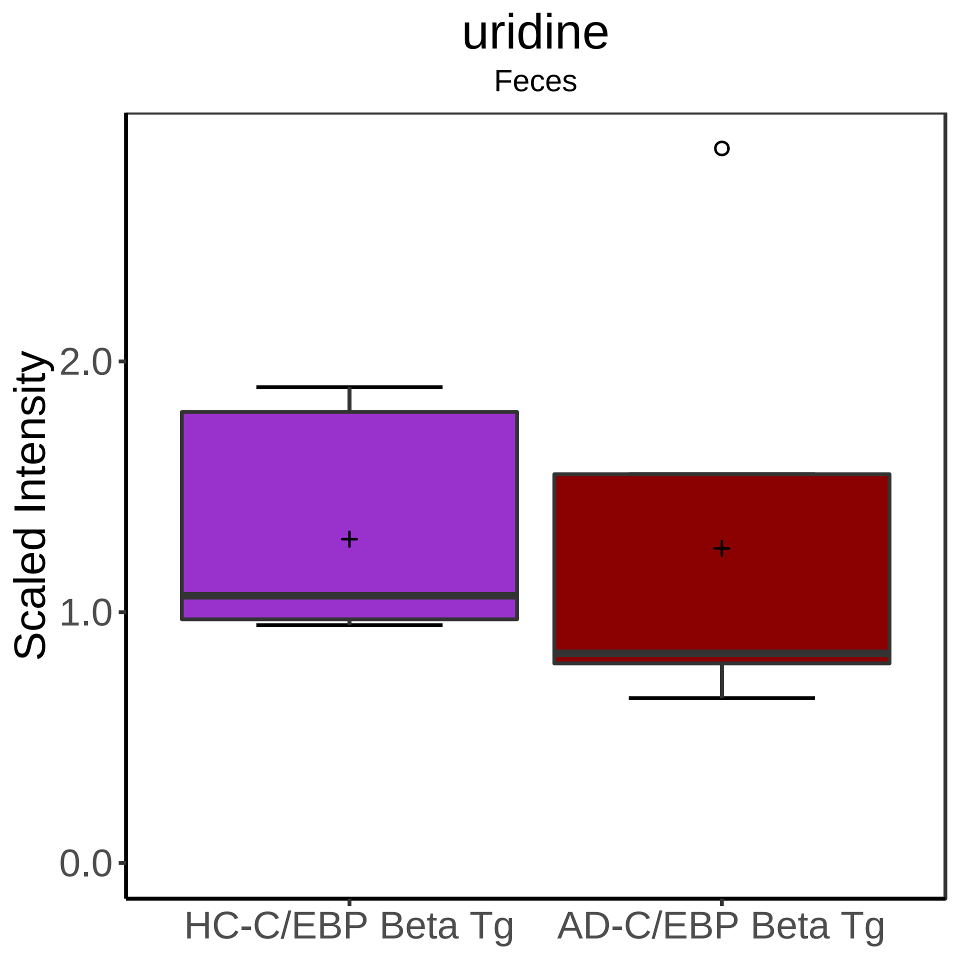

uridine
Feces
Scaled Intensity
2.0
1.0
0.0
HC-C/EBP Beta Tg
AD-C/EBP Beta Tg

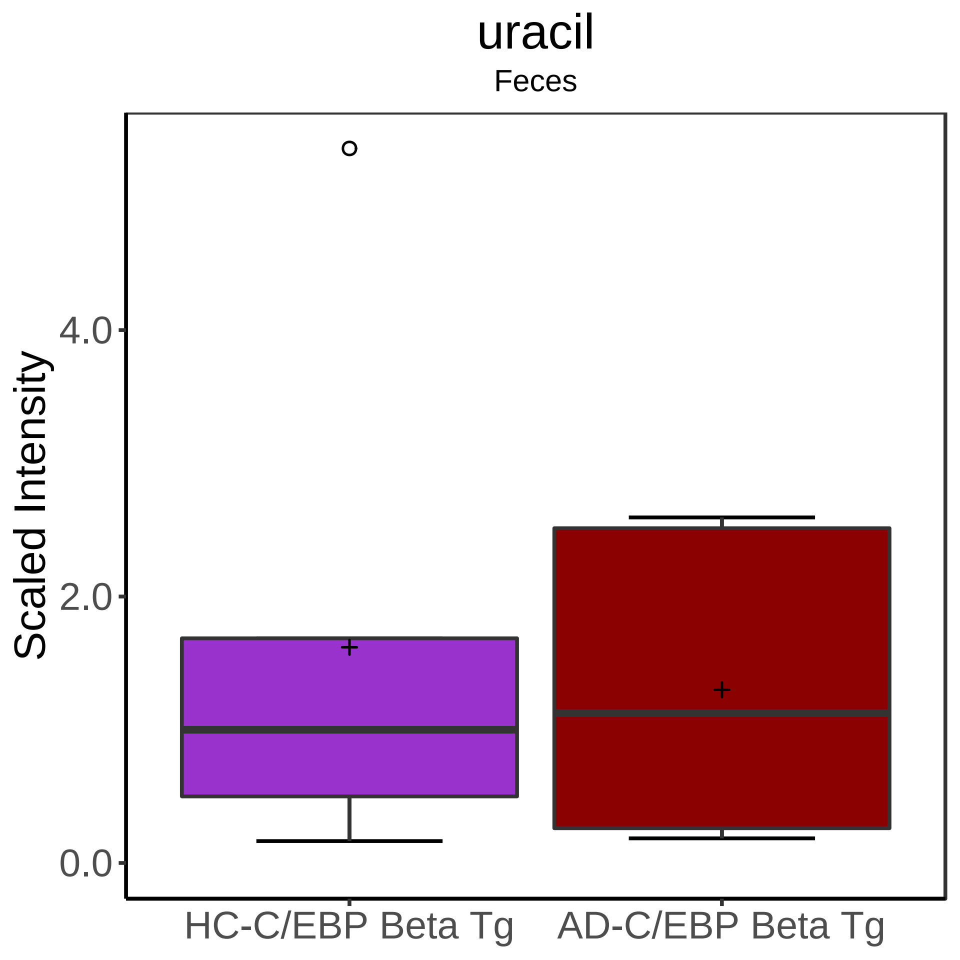

uracil
Feces
Scaled Intensity
4.0
2.0
0.0
HC-C/EBP Beta Tg
AD-C/EBP Beta Tg

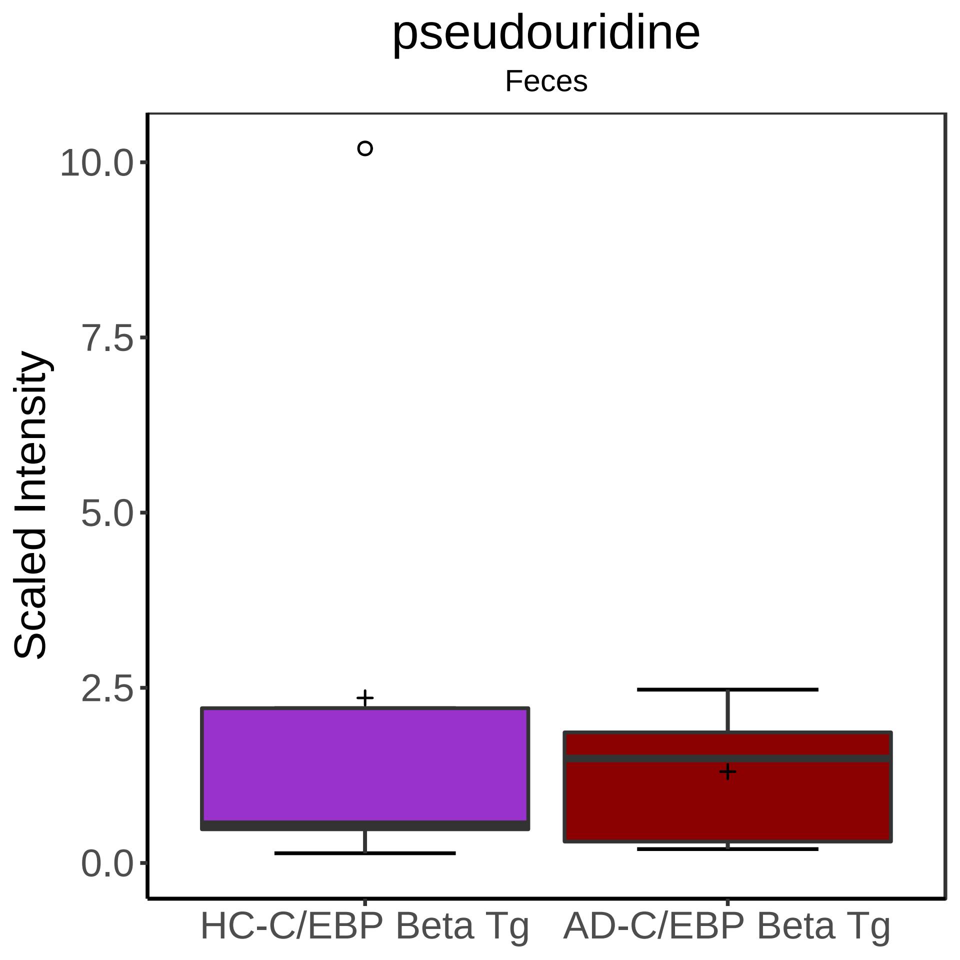

pseudouridine
Feces
Scaled Intensity
10.0
7.5
5.0
2.5
0.0
HC-C/EBP Beta Tg
AD-C/EBP Beta Tg

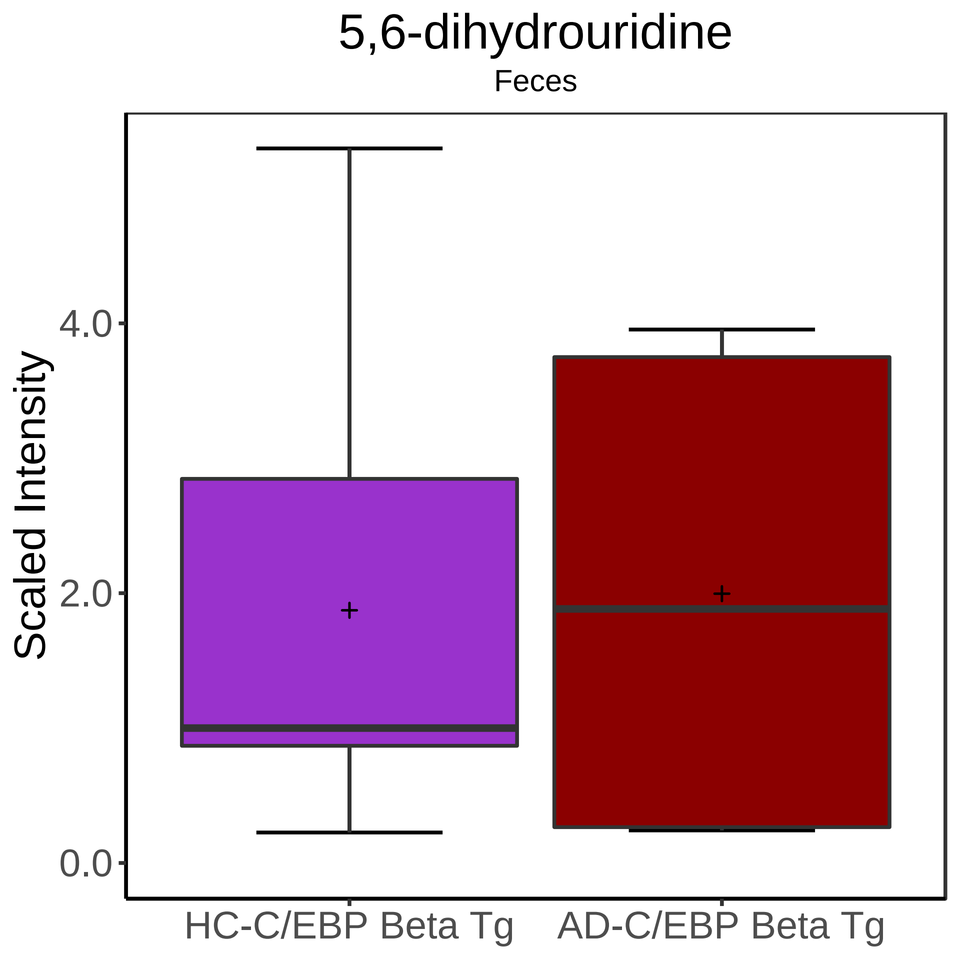

5,6-dihydrouridine
Feces
Scaled Intensity
4.0
2.0
0.0
HC-C/EBP Beta Tg
AD-C/EBP Beta Tg

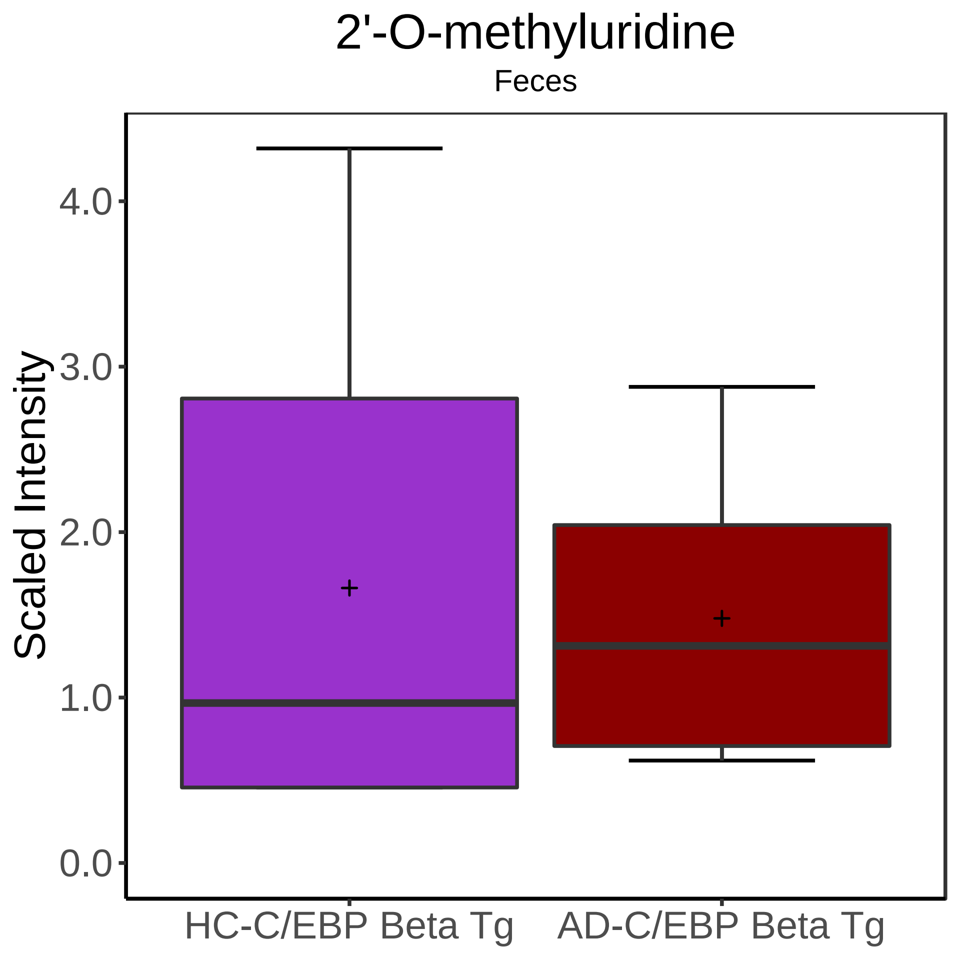

2'-O-methyluridine
Feces
Scaled Intensity
4.0
3.0
2.0
1.0
0.0
HC-C/EBP Beta Tg
AD-C/EBP Beta Tg

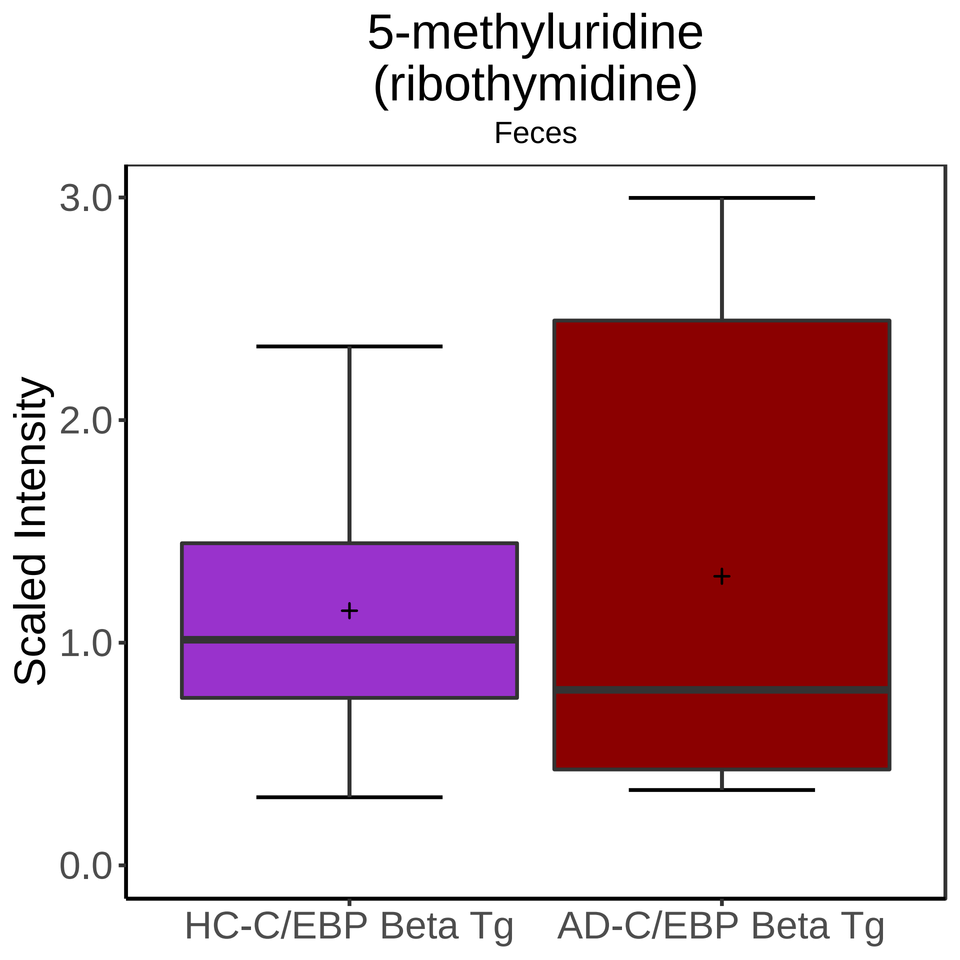
5-methyluridine
(ribothymidine)
Feces
Scaled Intensity
3.0
2.0
1.0
0.0
HC-C/EBP Beta Tg
AD-C/EBP Beta Tg

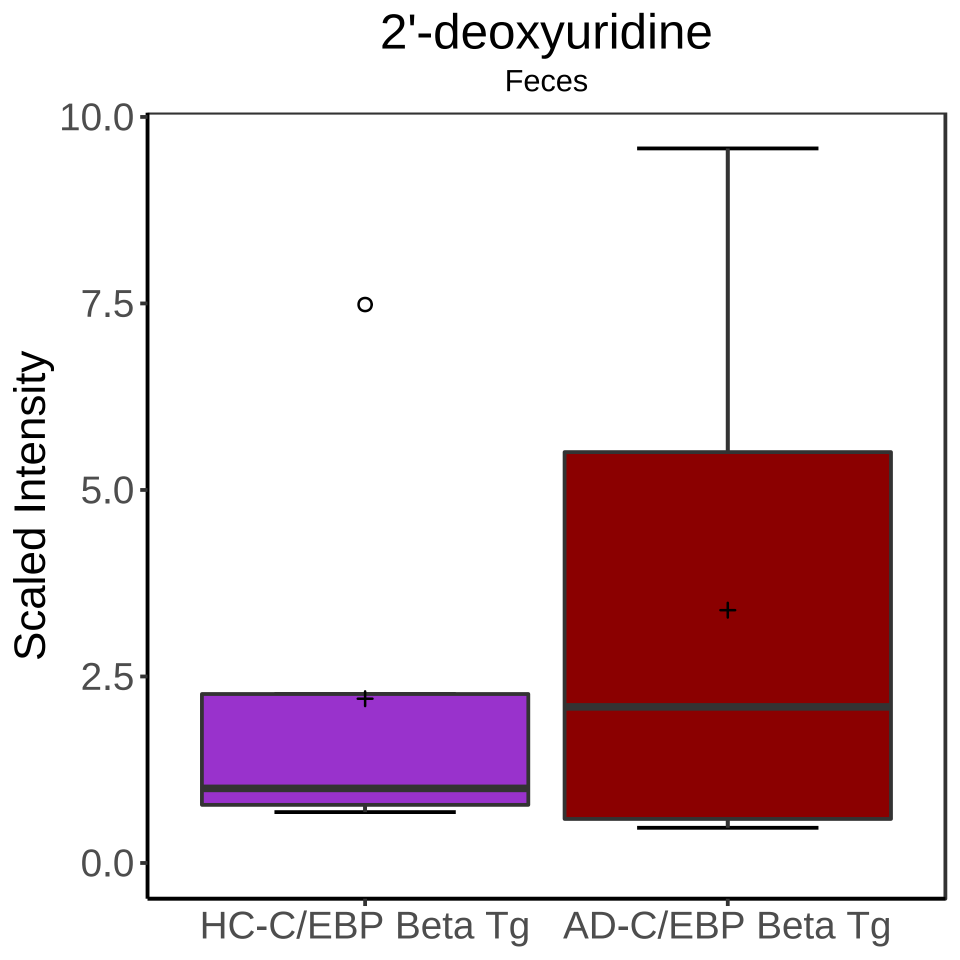
2'-deoxyuridine
Feces
Scaled Intensity
10.0
7.5
5.0
2.5
0.0
HC-C/EBP Beta Tg
AD-C/EBP Beta Tg

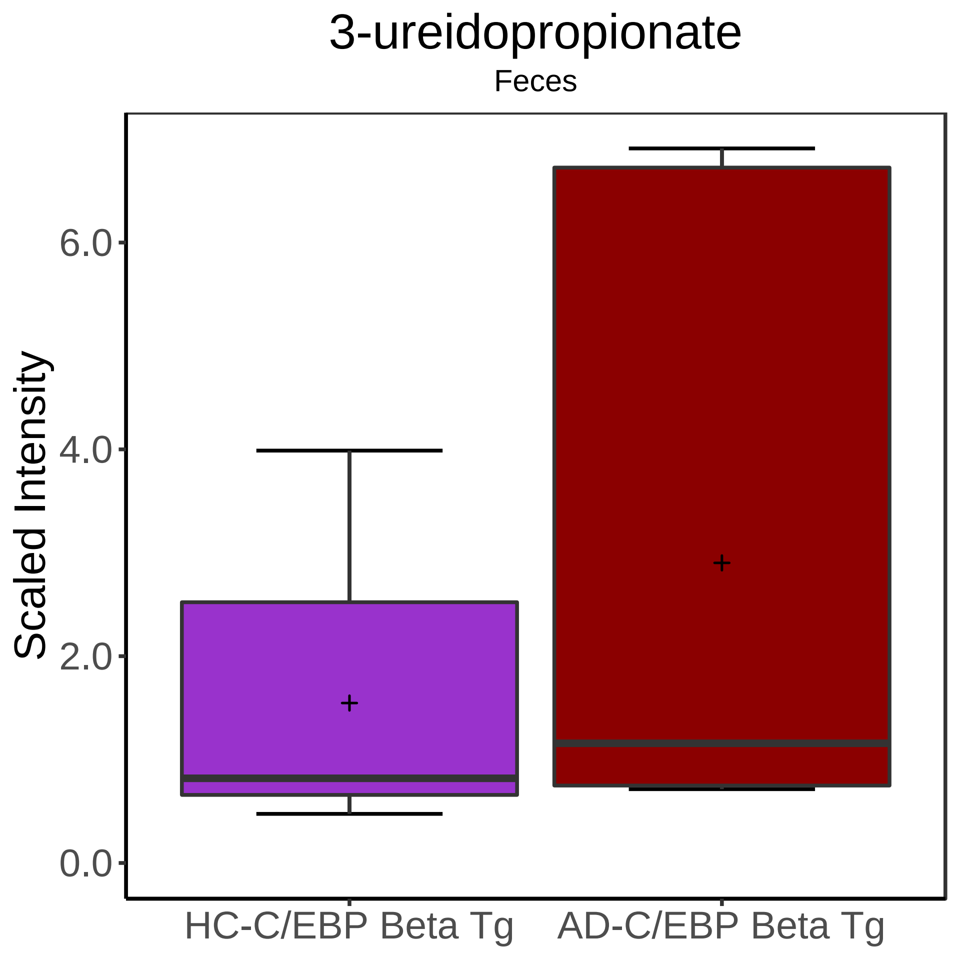

3-ureidopropionate
Feces
Scaled Intensity
6.0
4.0
2.0
0.0
HC-C/EBP Beta Tg
AD-C/EBP Beta Tg

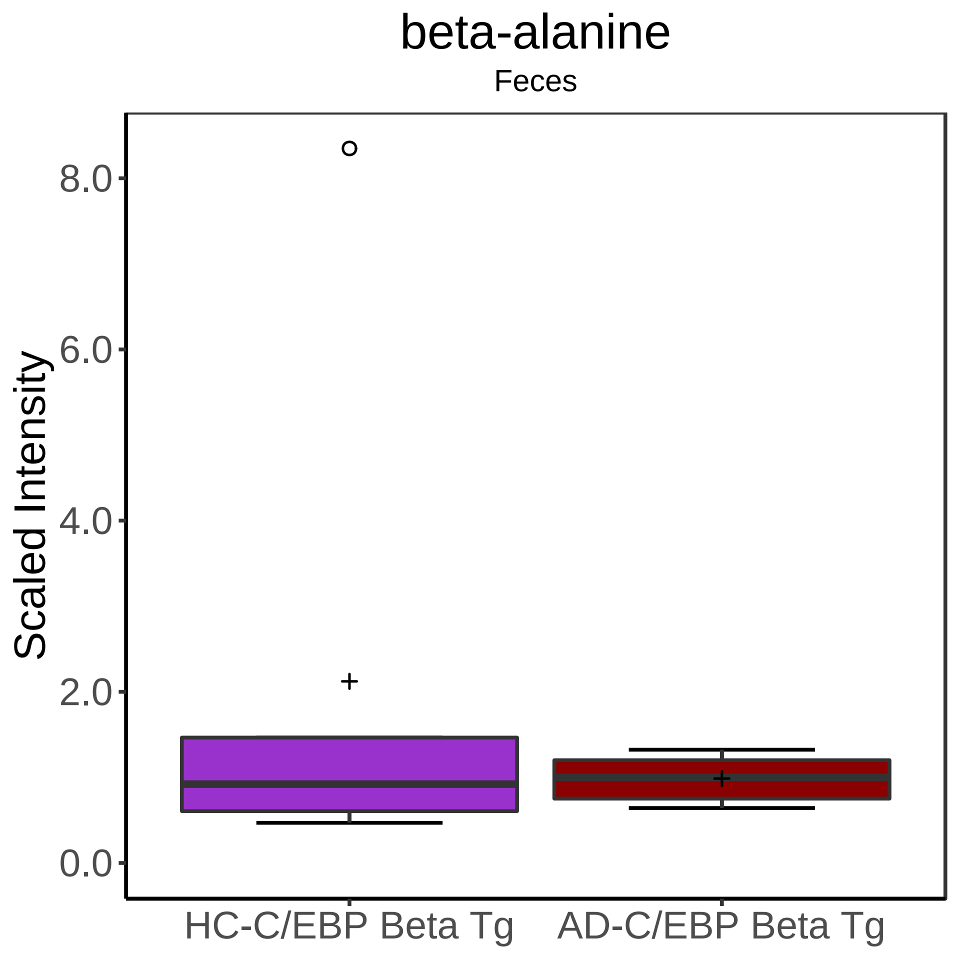
beta-alanine
Feces
Scaled Intensity
8.0
6.0
4.0
2.0
0.0
HC-C/EBP Beta Tg
AD-C/EBP Beta Tg

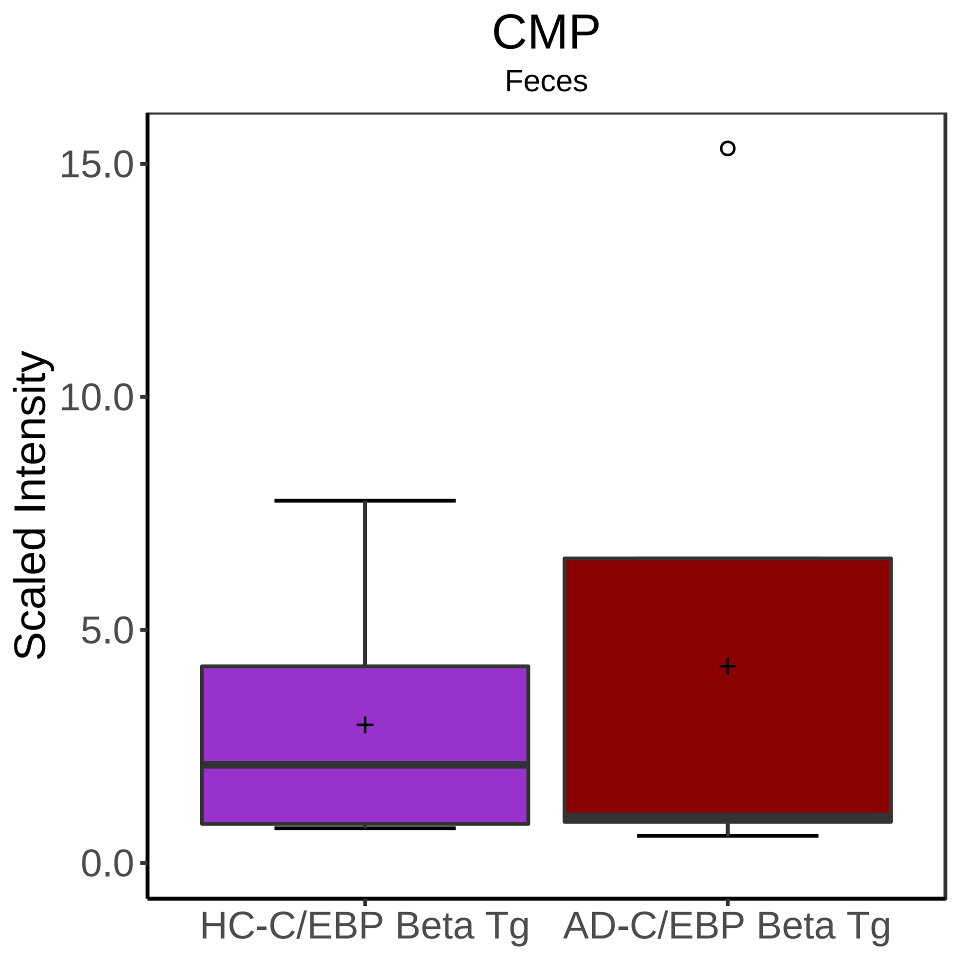

CMP
Feces
Scaled Intensity
15.0
10.0
5.0
0.0
HC-C/EBP Beta Tg
AD-C/EBP Beta Tg

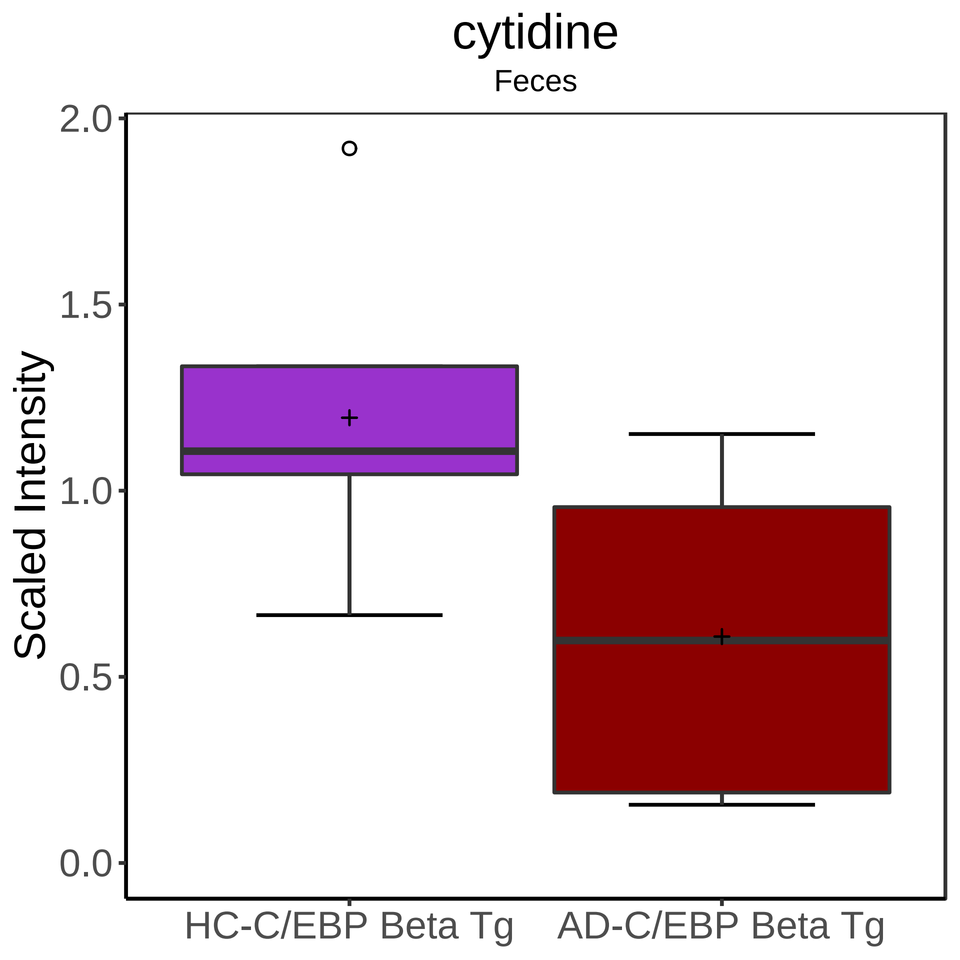

cytidine
Feces
Scaled Intensity
2.0
1.5
1.0
0.5
0.0
HC-C/EBP Beta Tg
AD-C/EBP Beta Tg

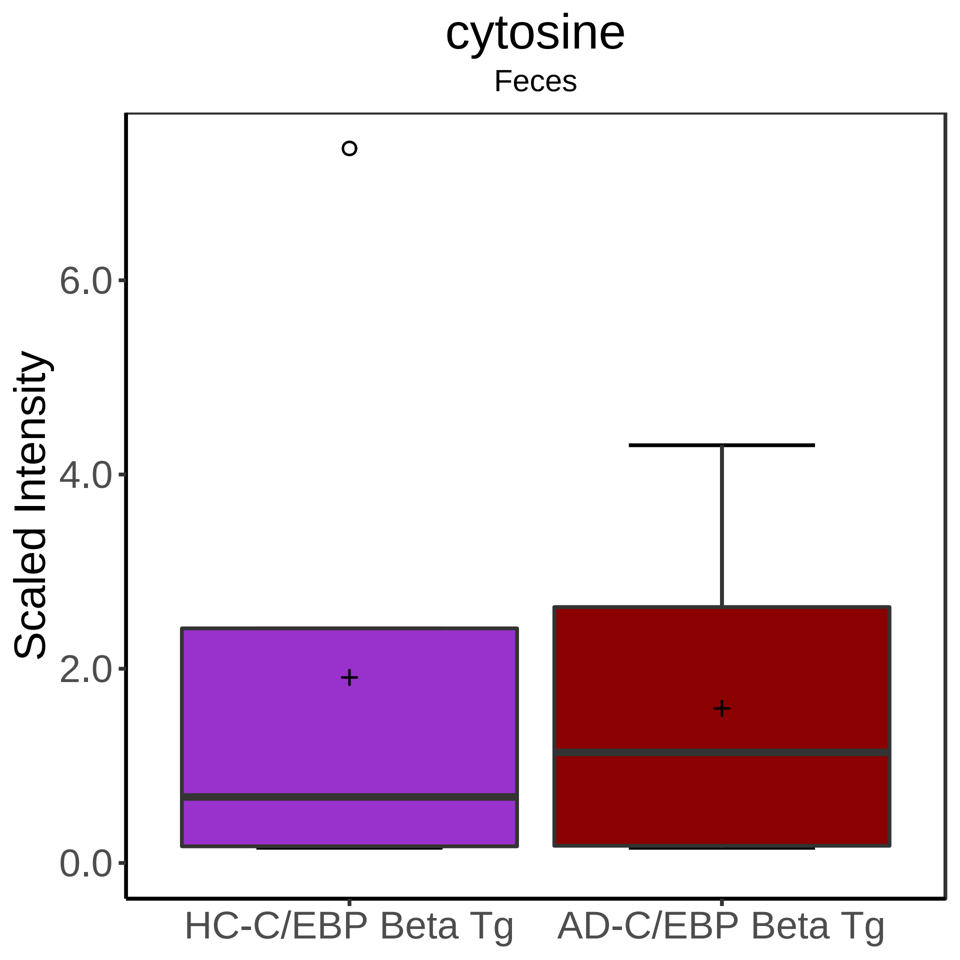
cytosine
Feces
Scaled Intensity
6.0
4.0
2.0
0.0
HC-C/EBP Beta Tg
AD-C/EBP Beta Tg

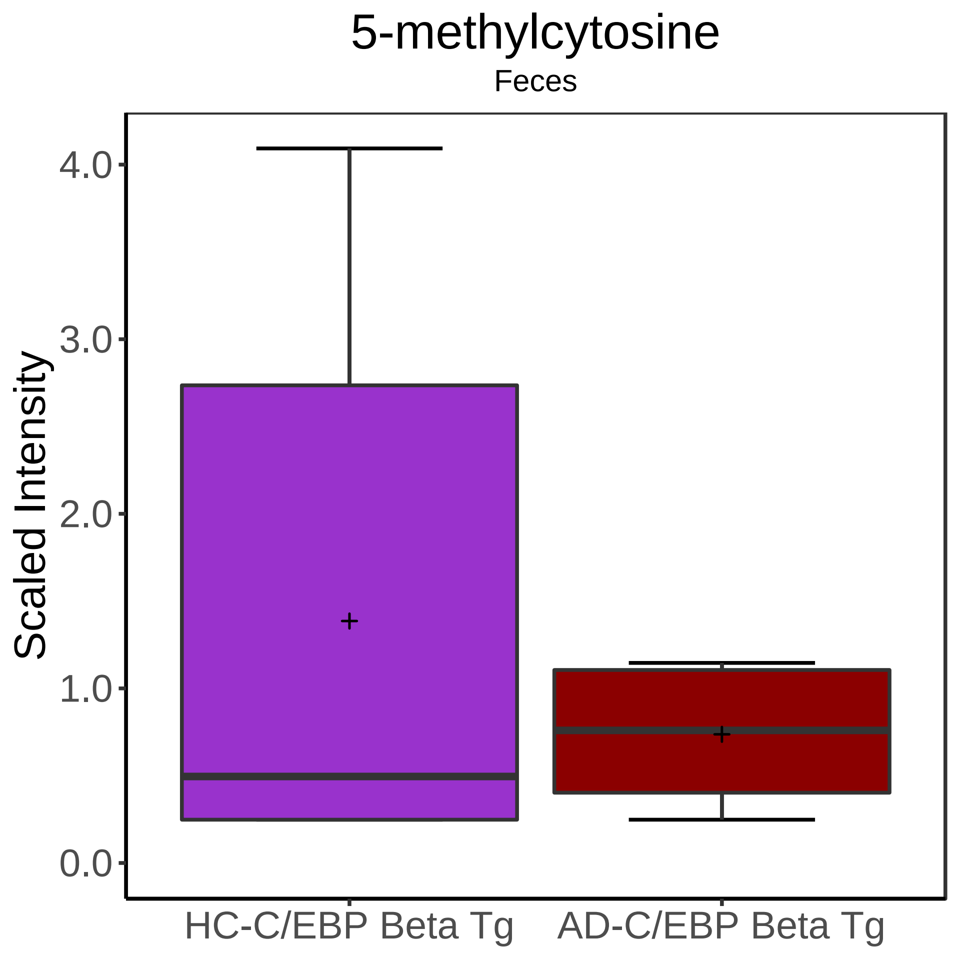
5-methylcytosine
Feces
Scaled Intensity
4.0
3.0
2.0
1.0
0.0
HC-C/EBP Beta Tg
AD-C/EBP Beta Tg

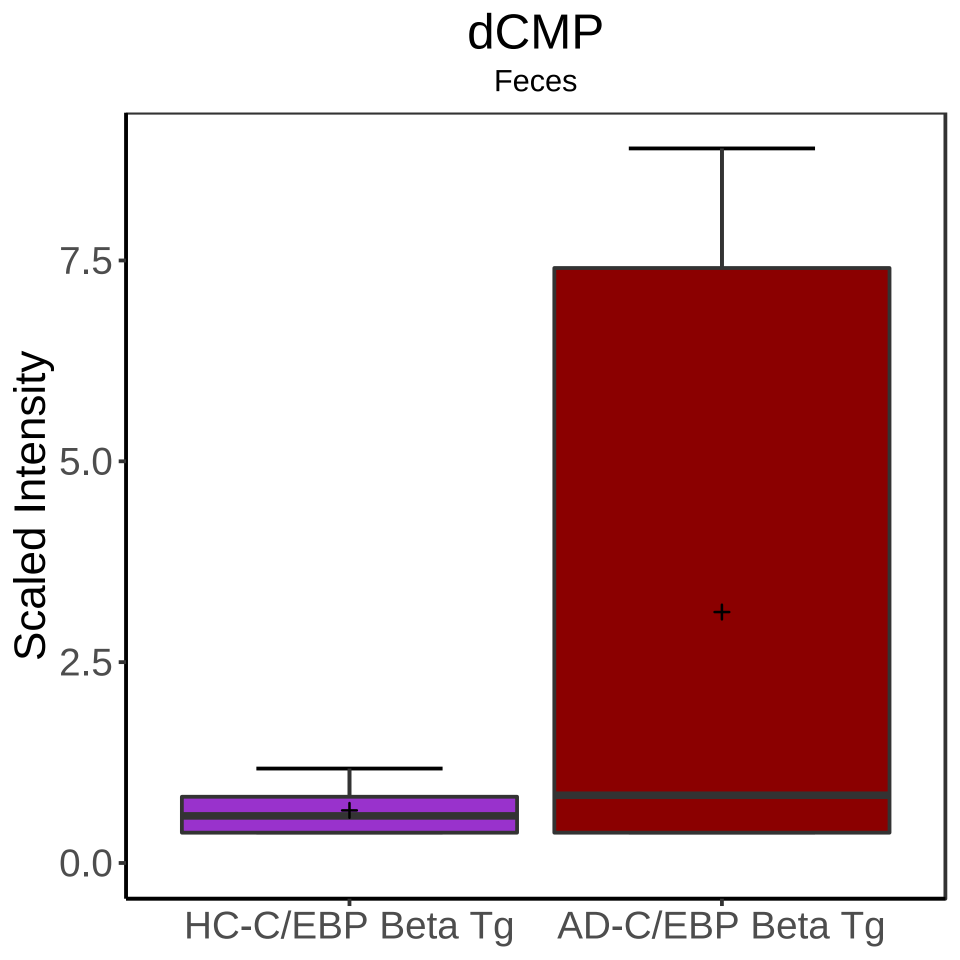

dCMP
Feces
Scaled Intensity
7.5
5.0
2.5
0.0
HC-C/EBP Beta Tg
AD-C/EBP Beta Tg

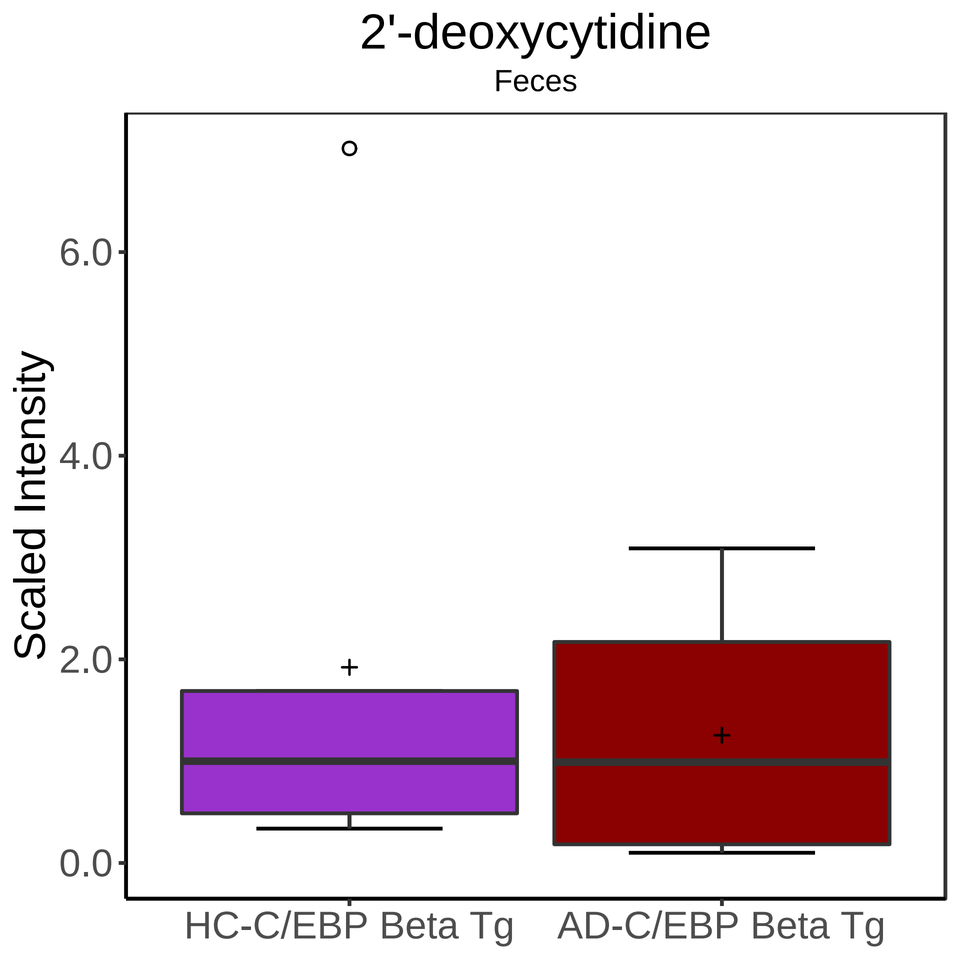

2'-deoxycytidine
Feces
Scaled Intensity
6.0
4.0
2.0
0.0
HC-C/EBP Beta Tg
AD-C/EBP Beta Tg

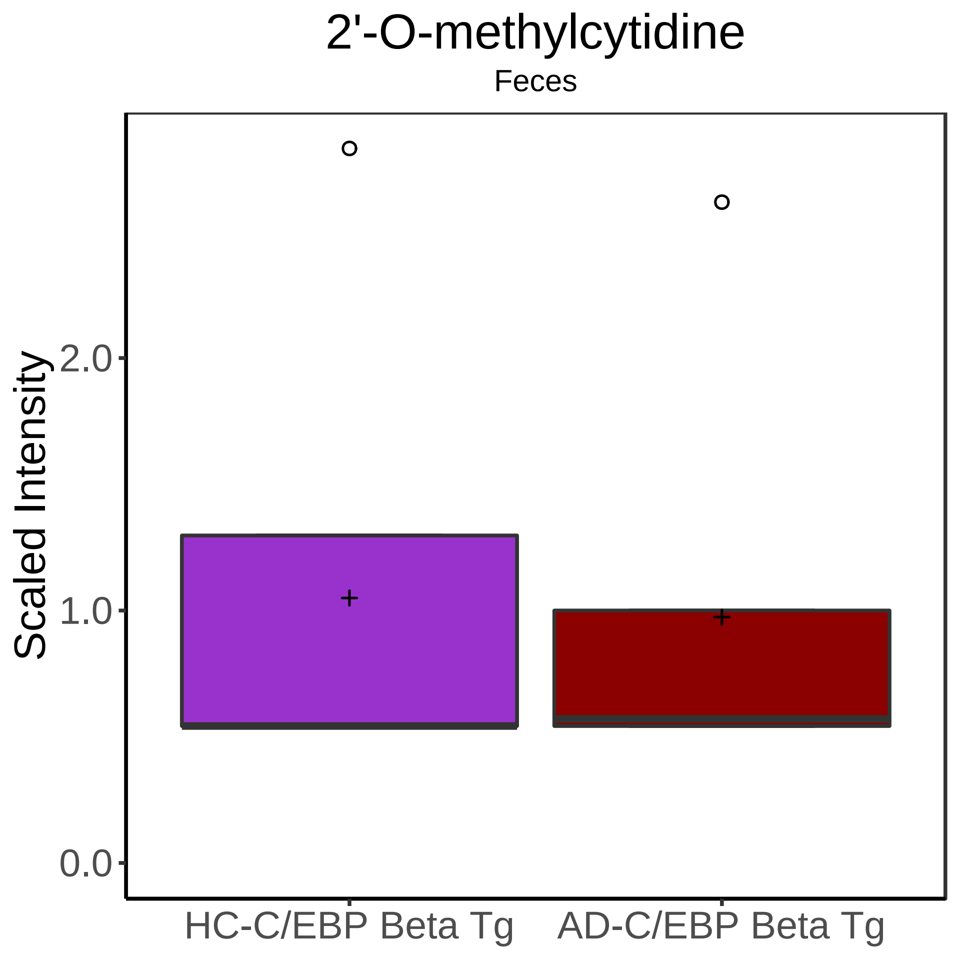
2'-O-methylcytidine
Feces
Scaled Intensity
2.0
1.0
0.0
HC-C/EBP Beta Tg
AD-C/EBP Beta Tg

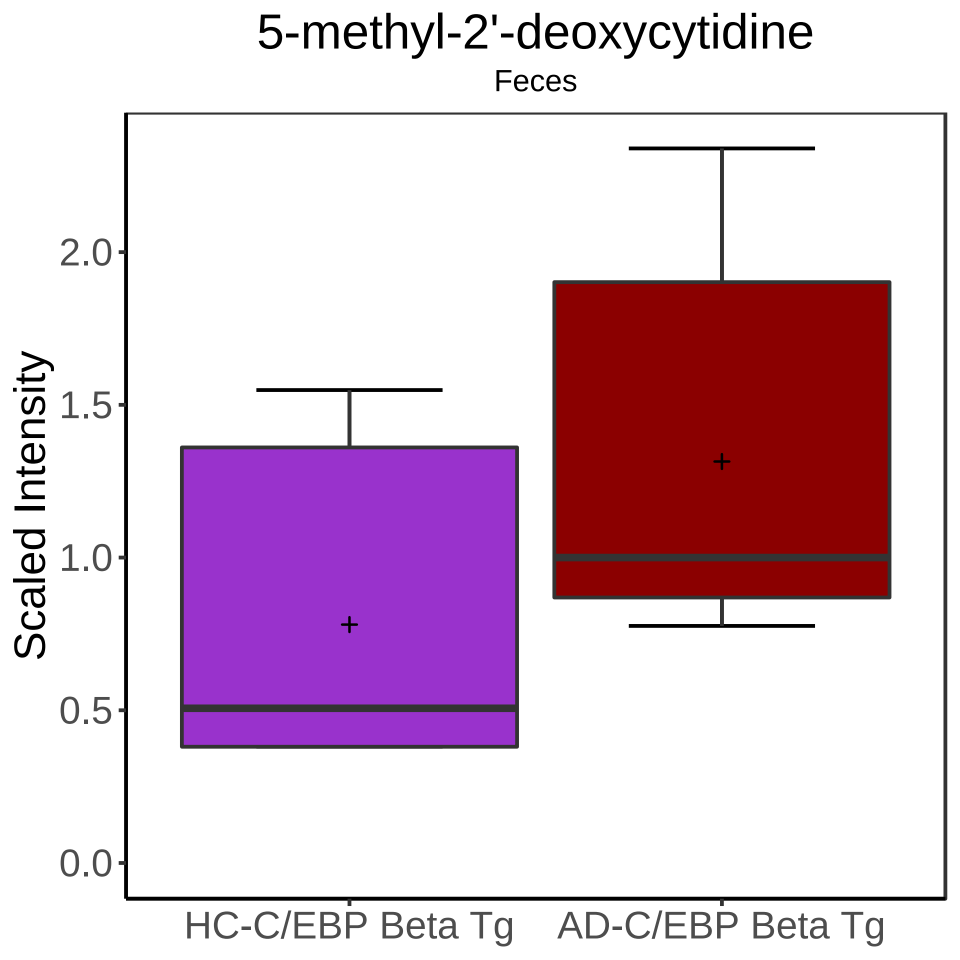
5-methyl-2'-deoxycytidine
Feces
Scaled Intensity
2.0
1.5
1.0
0.5
0.0
HC-C/EBP Beta Tg
AD-C/EBP Beta Tg

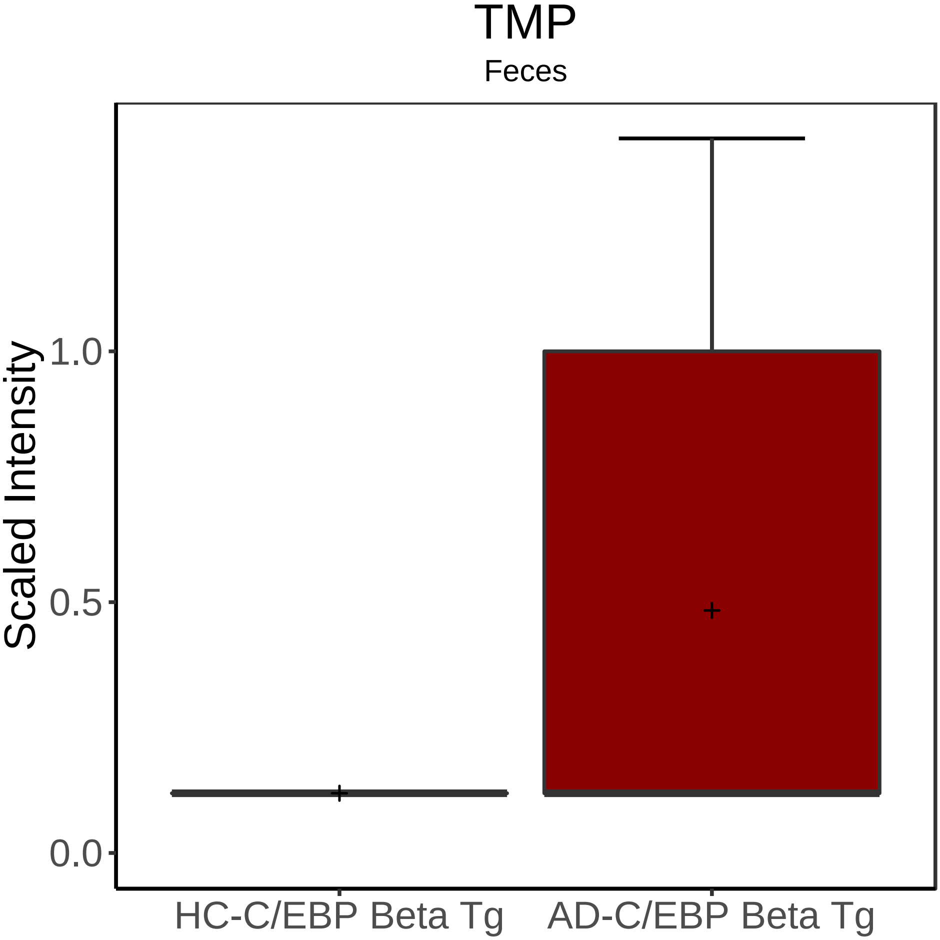

TMP
Feces
Scaled Intensity
1.0
0.5
0.0
HC-C/EBP Beta Tg
AD-C/EBP Beta Tg

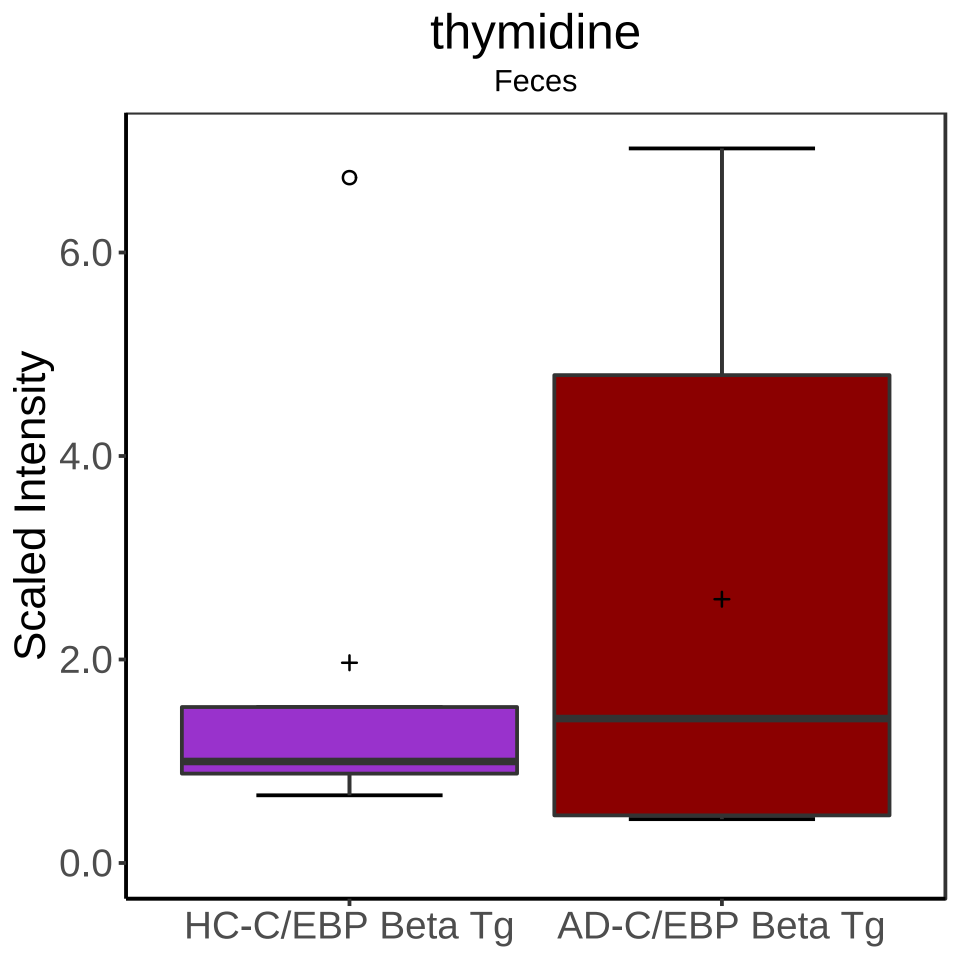
thymidine
Feces
Scaled Intensity
6.0
4.0
2.0
0.0
HC-C/EBP Beta Tg
AD-C/EBP Beta Tg

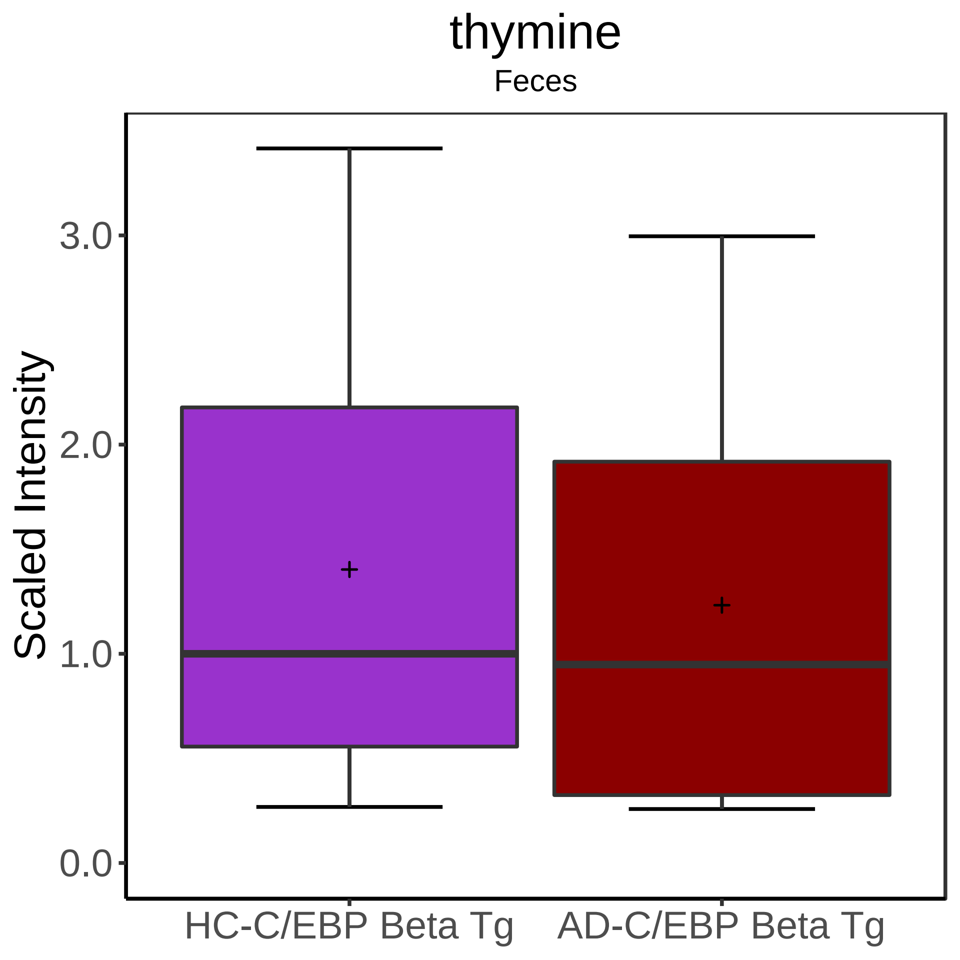

thymine
Feces
Scaled Intensity
3.0
2.0
1.0
0.0
HC-C/EBP Beta Tg
AD-C/EBP Beta Tg

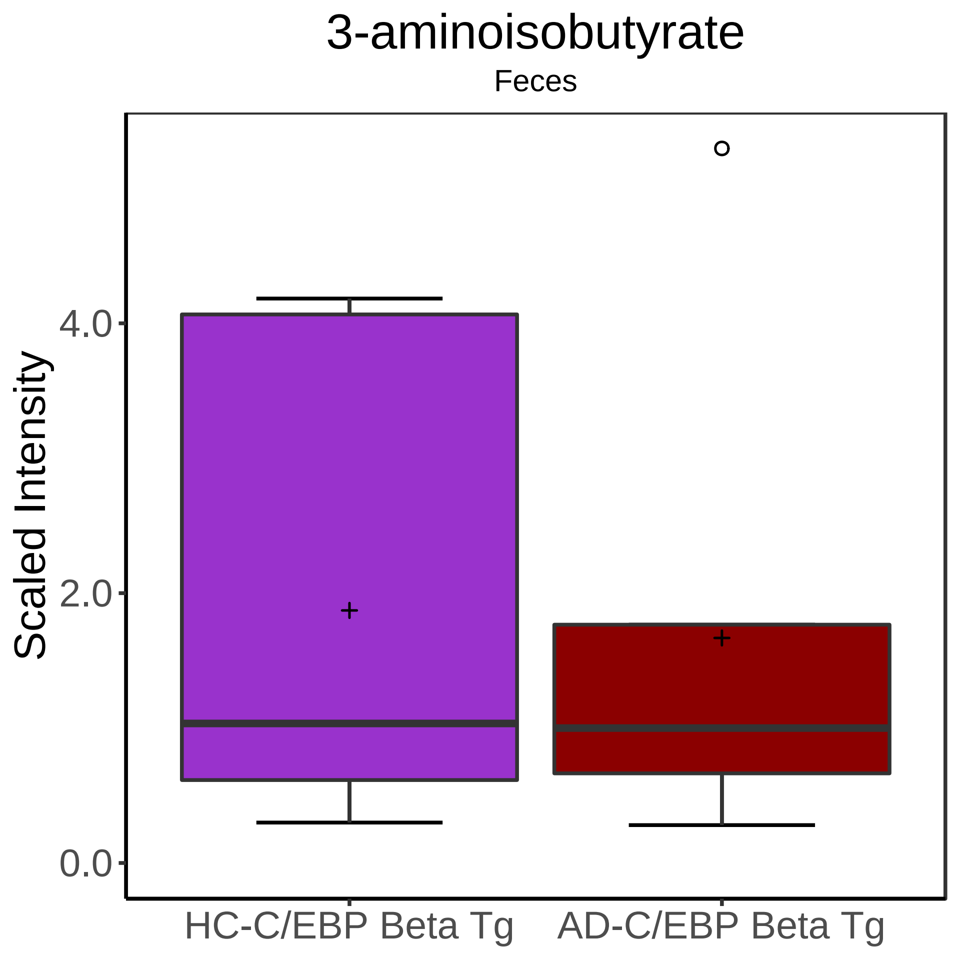

3-aminoisobutyrate
Feces
Scaled Intensity
4.0
2.0
0.0
HC-C/EBP Beta Tg
AD-C/EBP Beta Tg

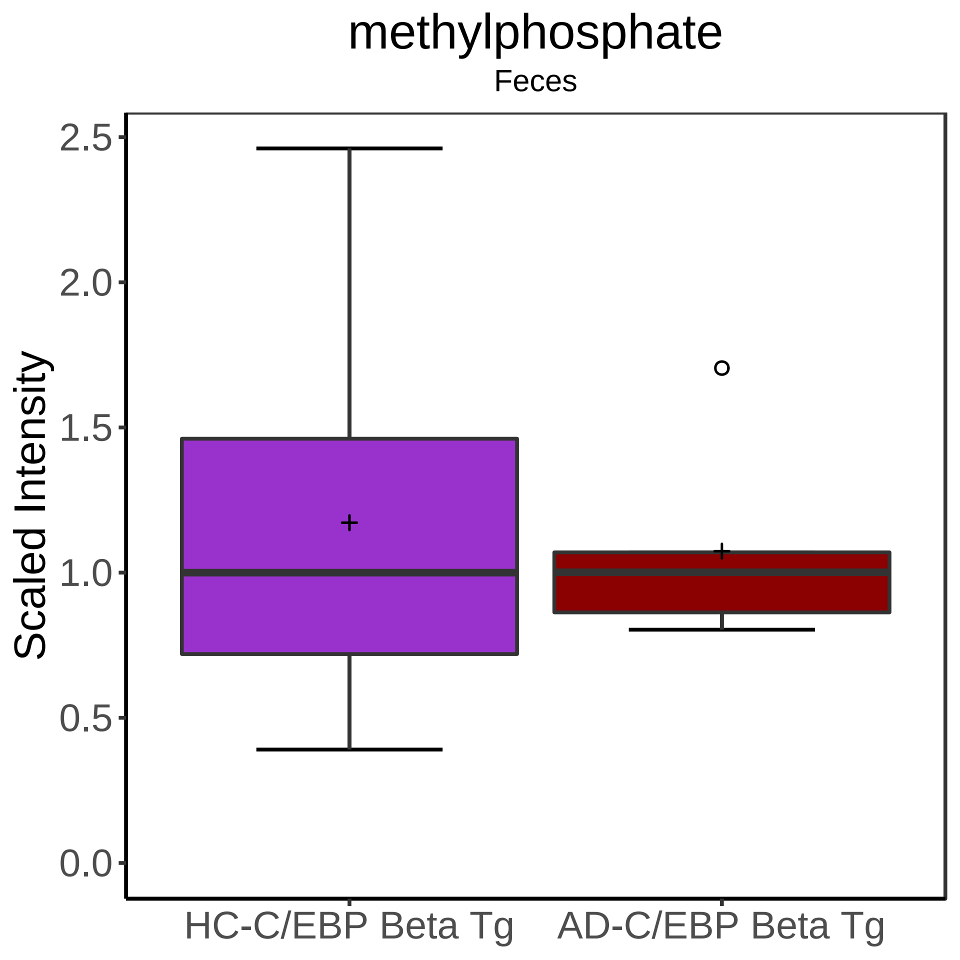
methylphosphate
Feces
Scaled Intensity
2.5
2.0
1.5
1.0
0.5
0.0
HC-C/EBP Beta Tg
AD-C/EBP Beta Tg

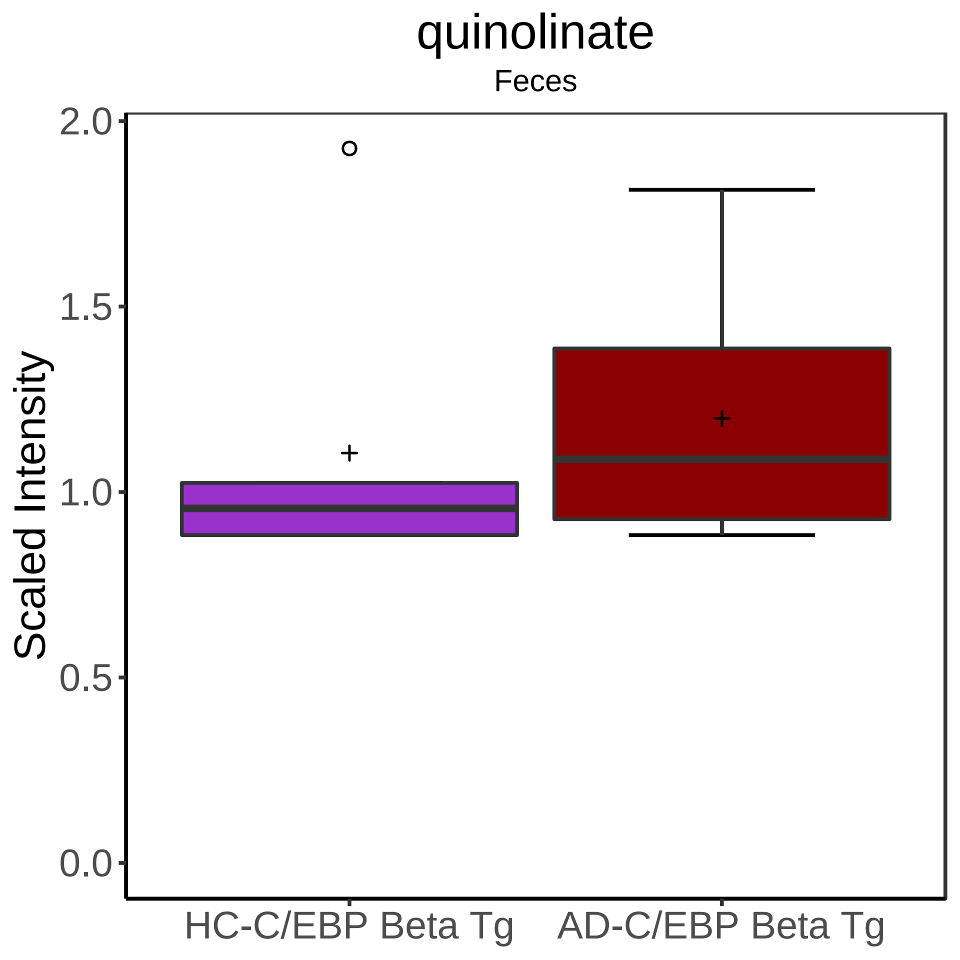
quinolinate
Feces
Scaled Intensity
2.0
1.5
1.0
0.5
0.0
HC-C/EBP Beta Tg
AD-C/EBP Beta Tg

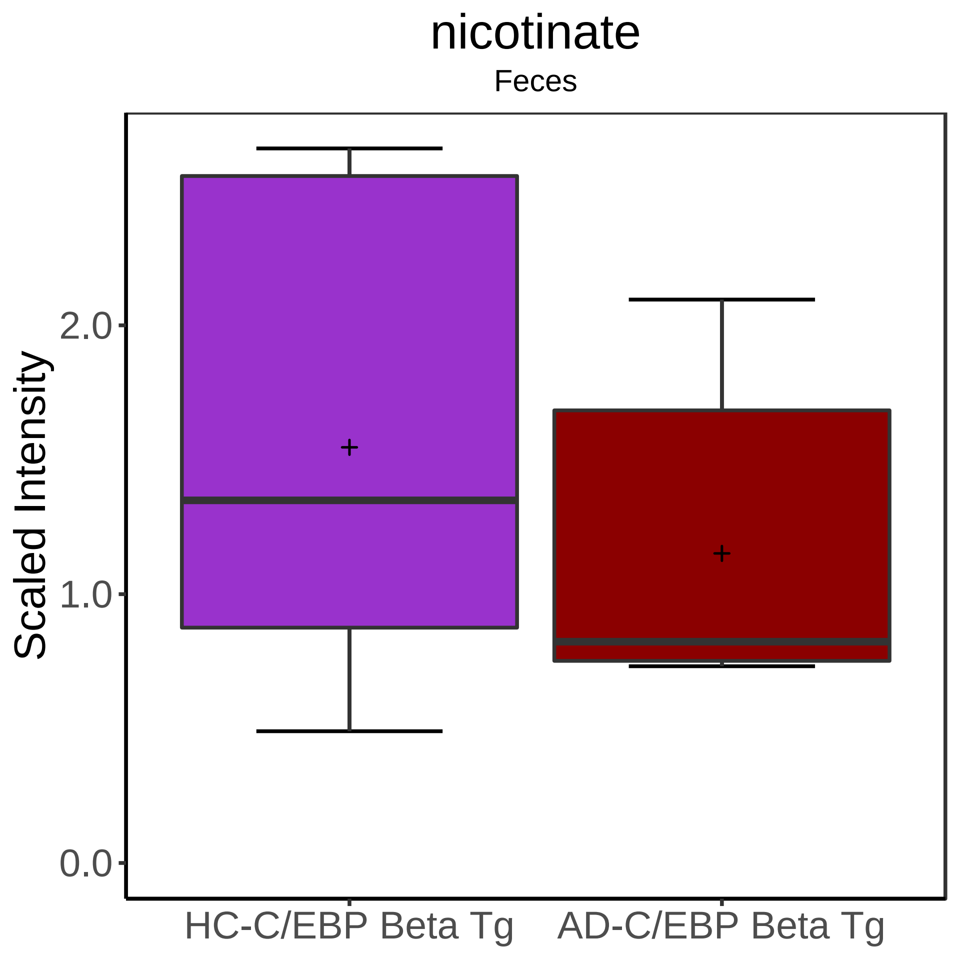

nicotinate
Feces
Scaled Intensity
2.0
1.0
0.0
HC-C/EBP Beta Tg
AD-C/EBP Beta Tg

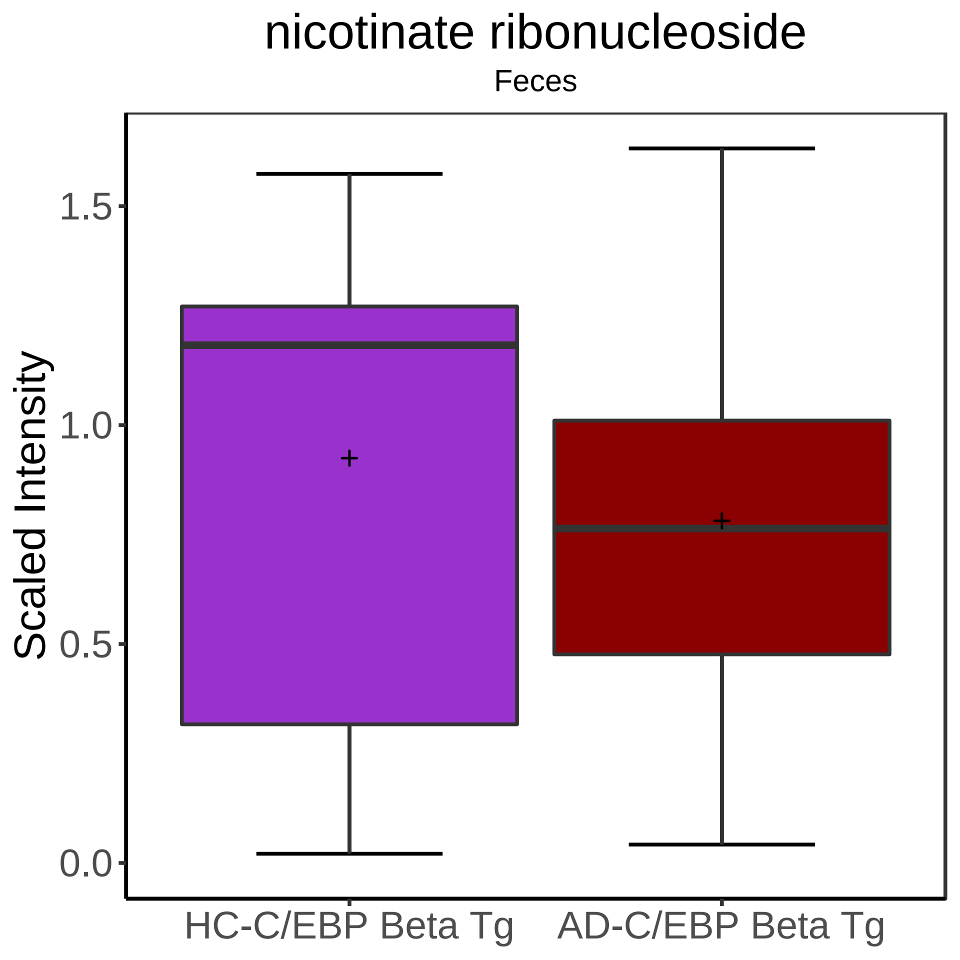
nicotinate ribonucleoside
Feces
Scaled Intensity
1.5
1.0
0.5
0.0
HC-C/EBP Beta Tg
AD-C/EBP Beta Tg

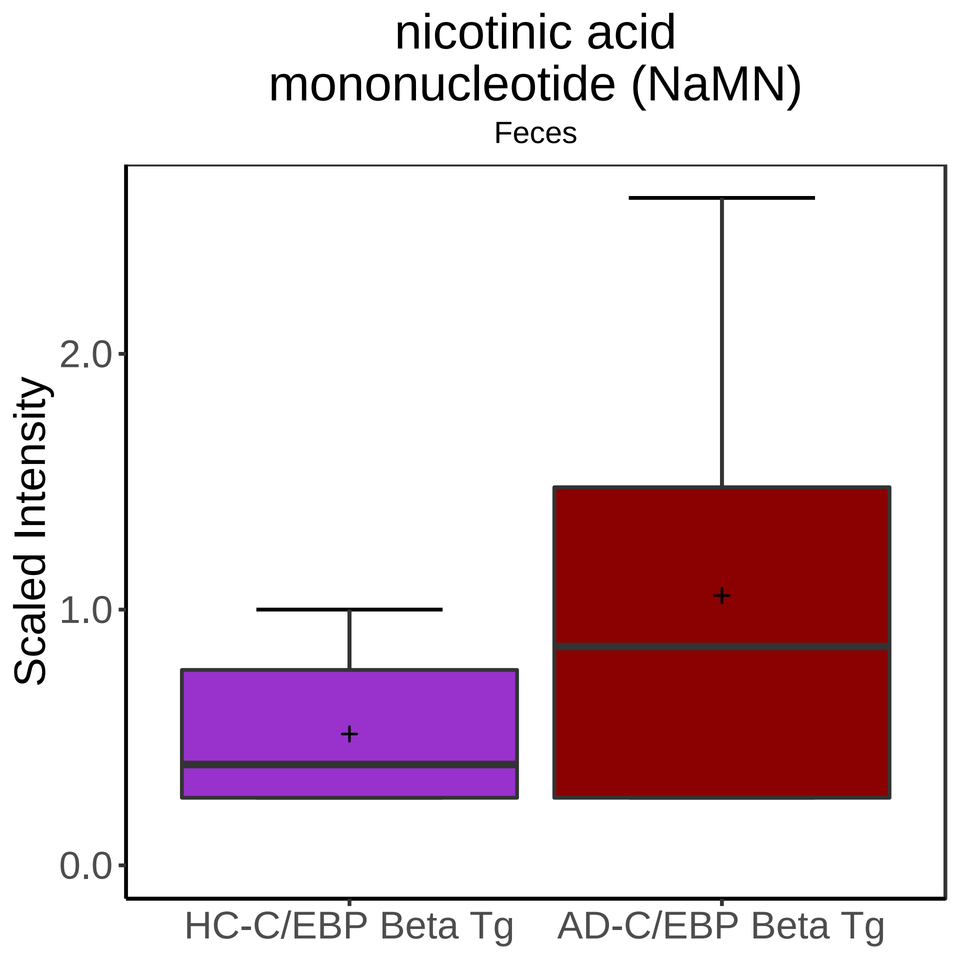
nicotinic acid mononucleotide (NaMN)
Feces
Scaled Intensity
2.0
1.0
0.0
HC-C/EBP Beta Tg
AD-C/EBP Beta Tg

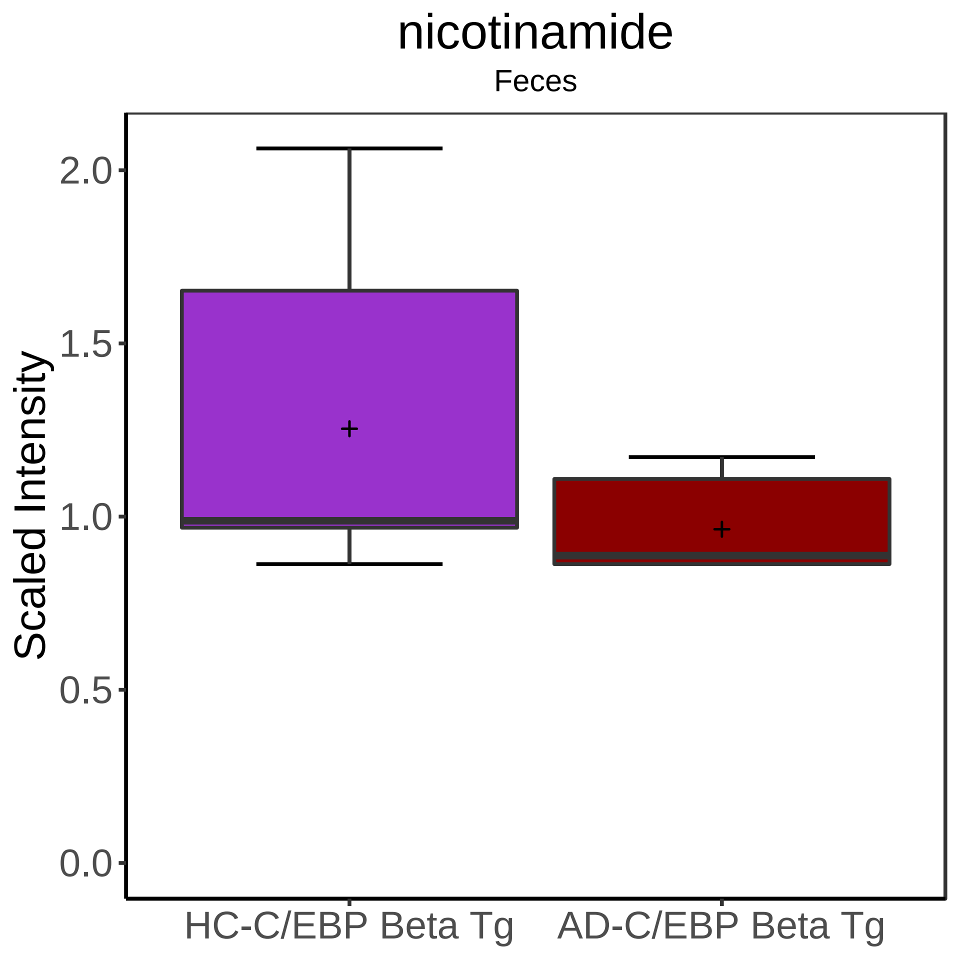
nicotinamide
Feces
Scaled Intensity
2.0
1.5
1.0
0.5
0.0
HC-C/EBP Beta Tg
AD-C/EBP Beta Tg

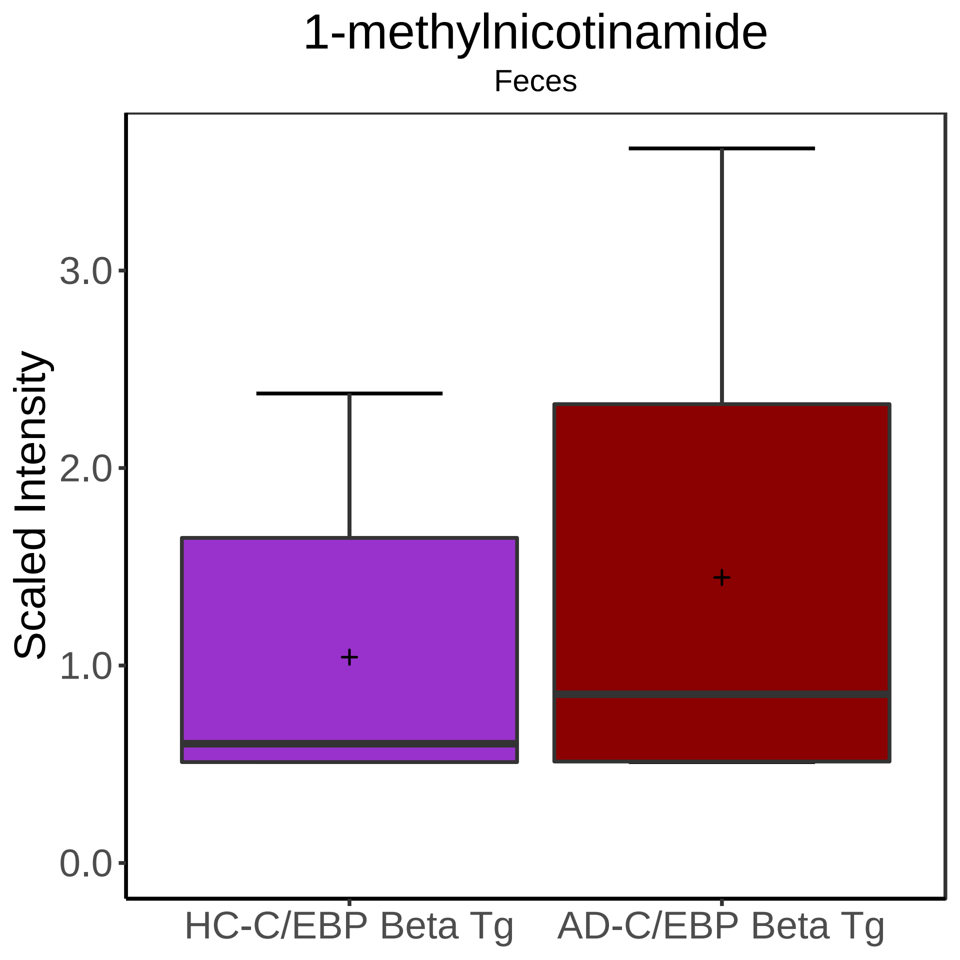
1-methylnicotinamide
Feces
Scaled Intensity
3.0
2.0
1.0
0.0
HC-C/EBP Beta Tg
AD-C/EBP Beta Tg

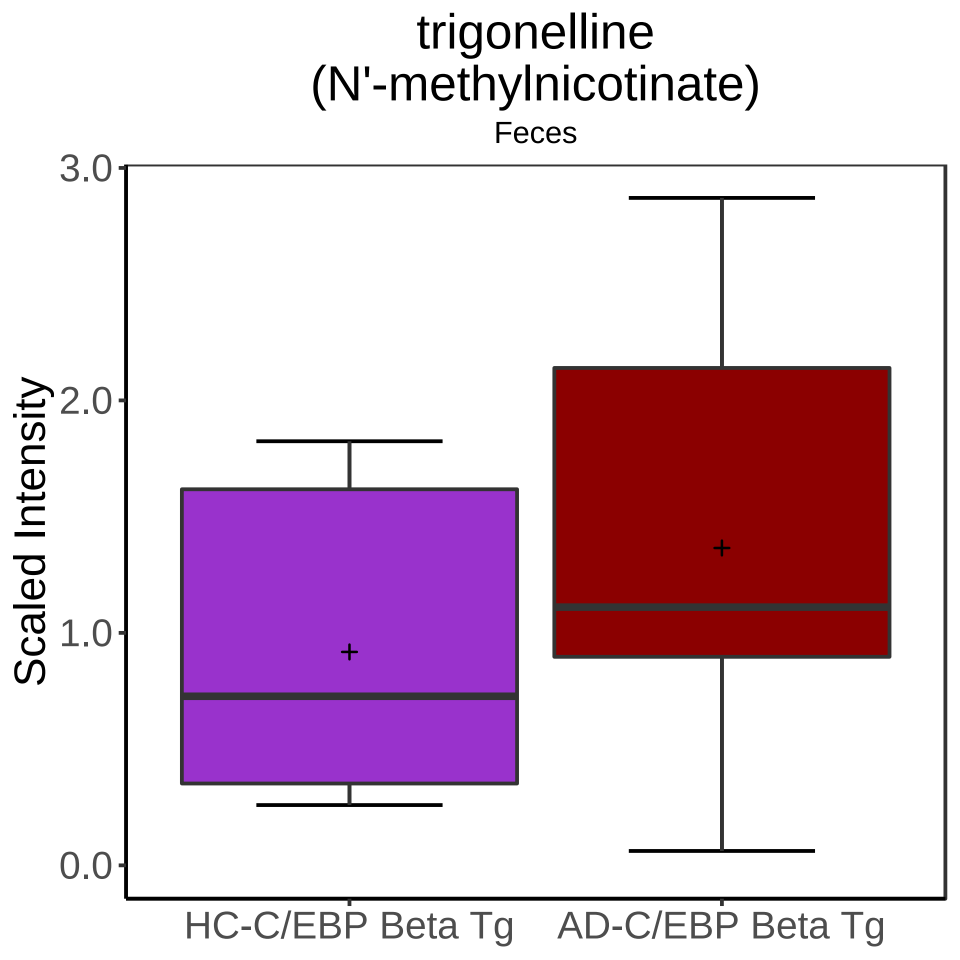
trigonelline
(N'-methylnicotinate)
Feces
Scaled Intensity
3.0
2.0
1.0
0.0
HC-C/EBP Beta Tg
AD-C/EBP Beta Tg

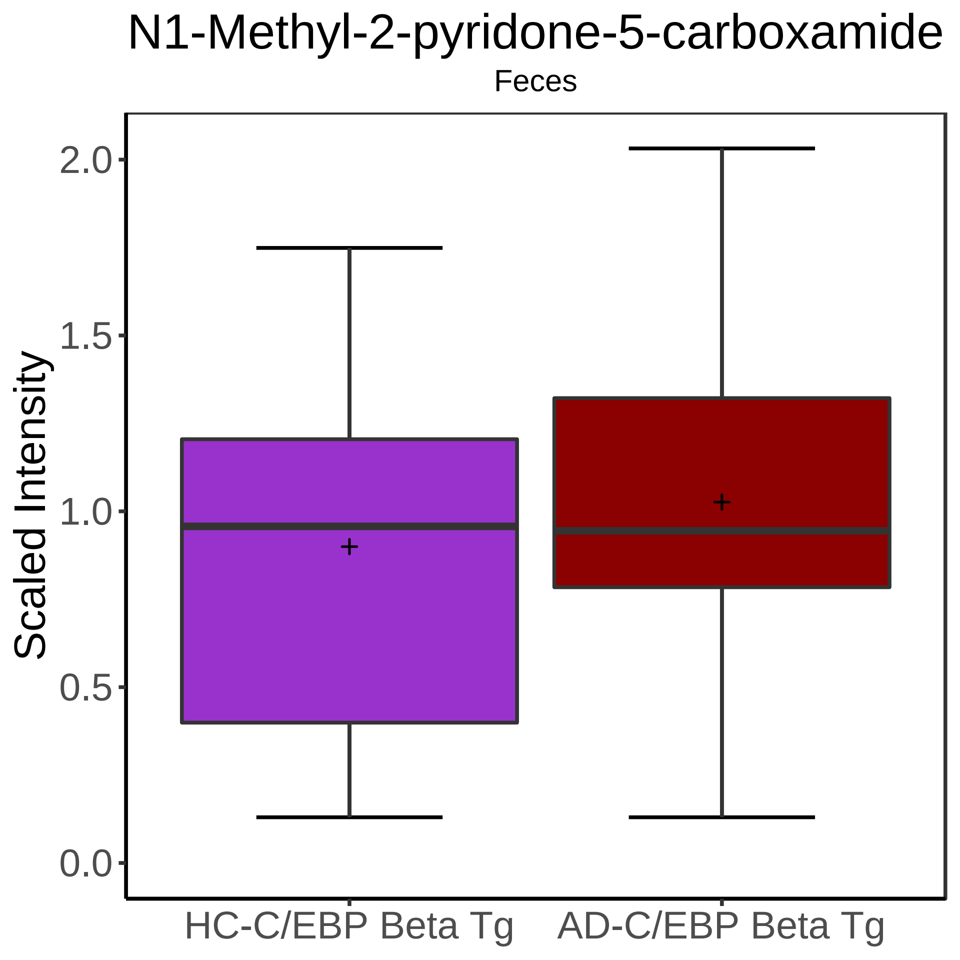
N1-Methyl-2-pyridone-5-carboxamide
Feces
Scaled Intensity
2.0
1.5
1.0
0.5
0.0
HC-C/EBP Beta Tg
AD-C/EBP Beta Tg

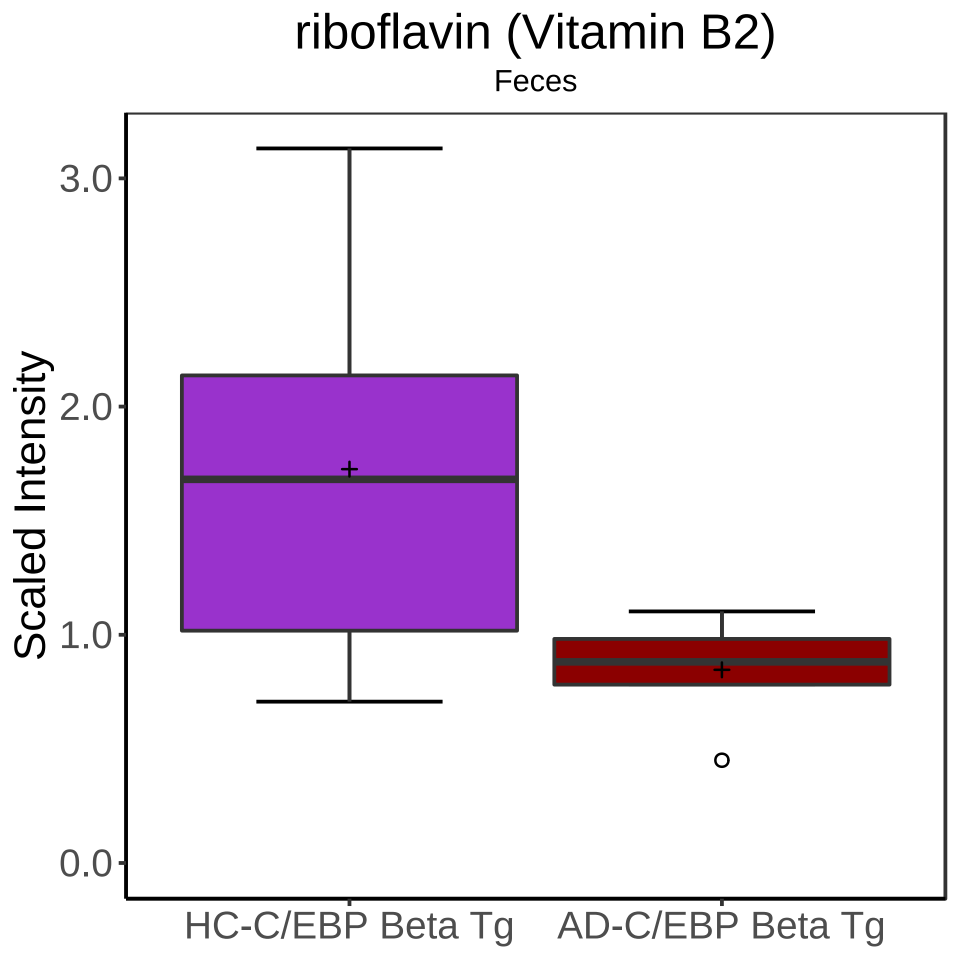
riboflavin (Vitamin B2)
Feces
Scaled Intensity
3.0
2.0
1.0
0.0
HC-C/EBP Beta Tg
AD-C/EBP Beta Tg

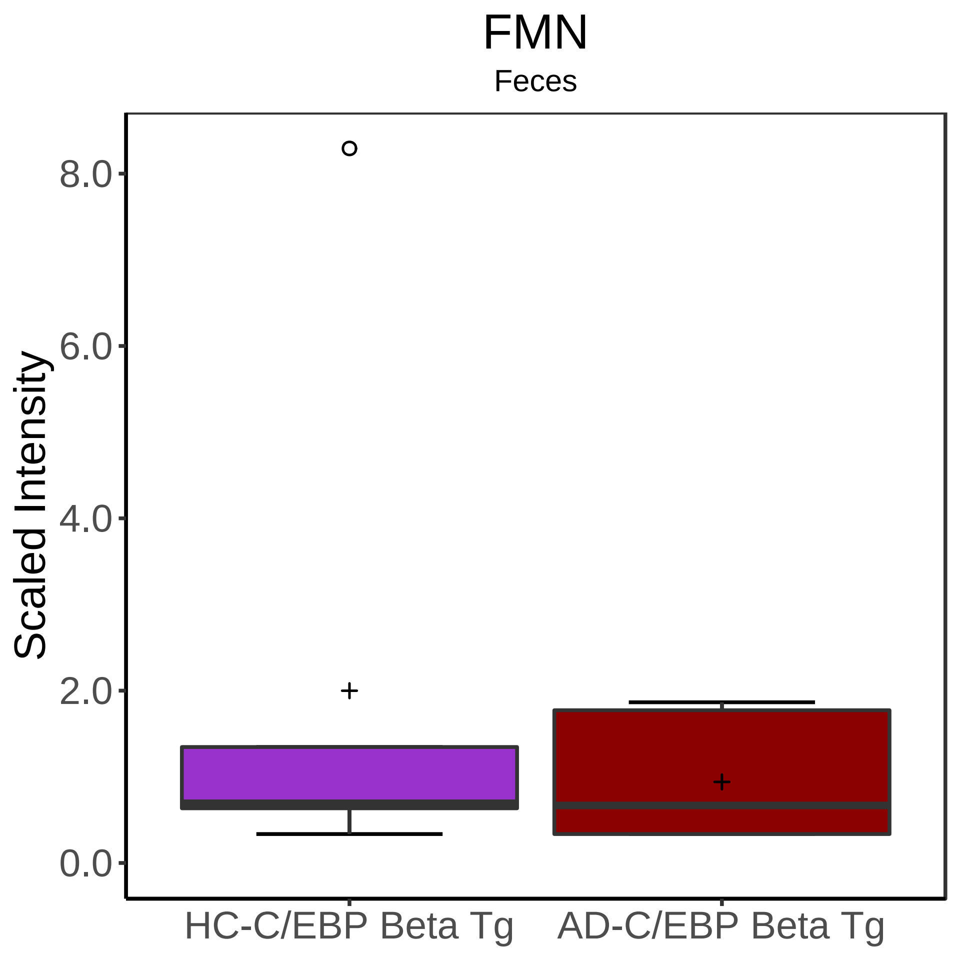

FMN
Feces
Scaled Intensity
8.0
6.0
4.0
2.0
0.0
HC-C/EBP Beta Tg
AD-C/EBP Beta Tg

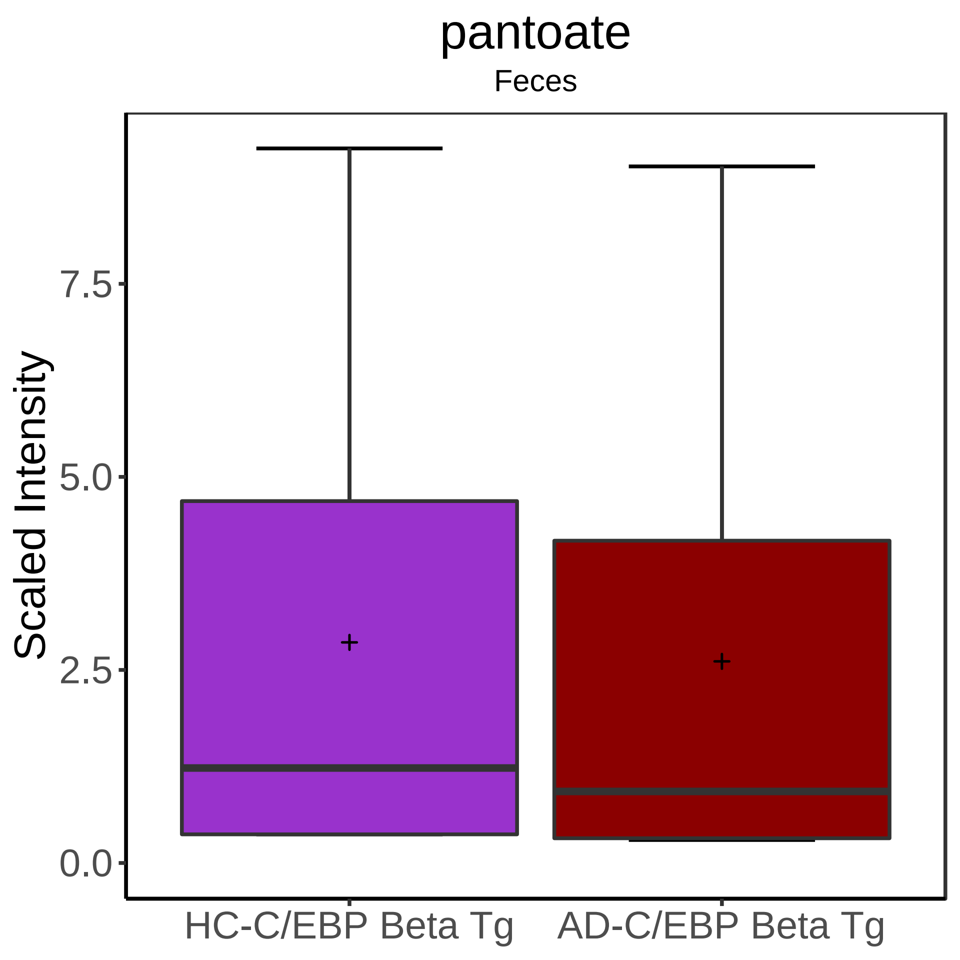

pantoate
Feces
Scaled Intensity
7.5
5.0
2.5
0.0
HC-C/EBP Beta Tg
AD-C/EBP Beta Tg

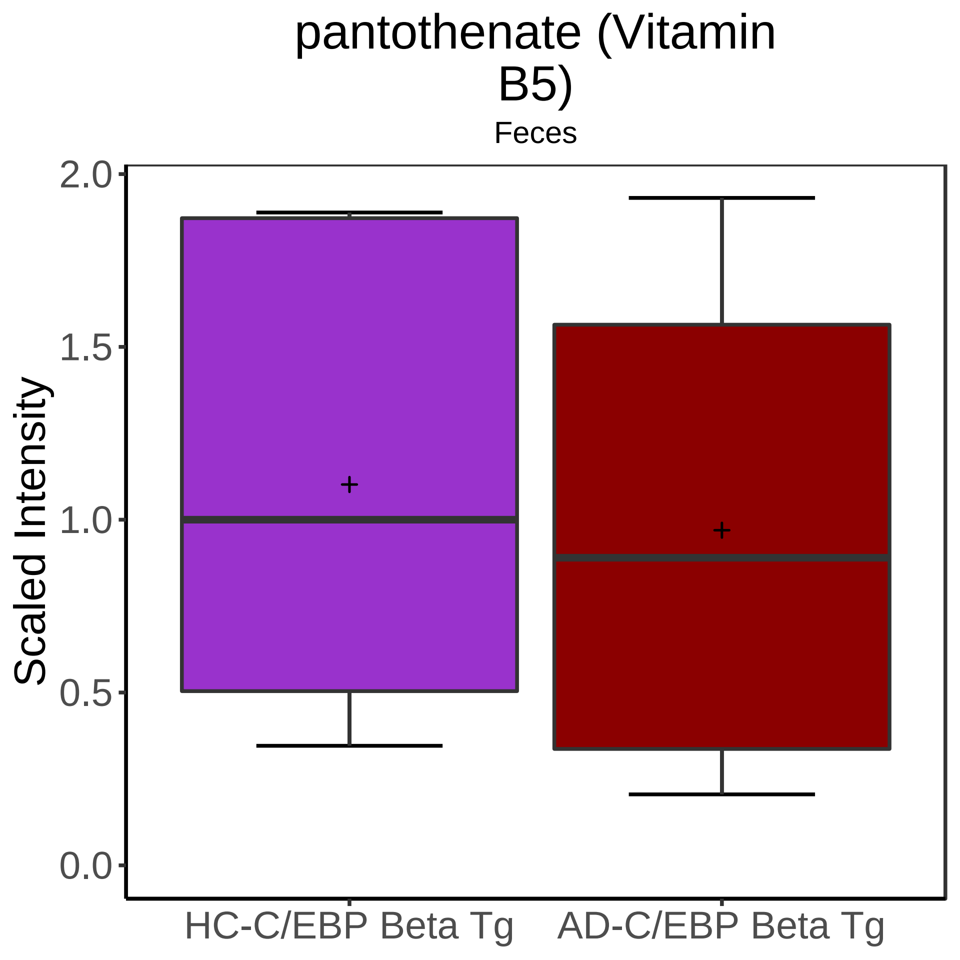

pantothenate (Vitamin B5)
Feces
Scaled Intensity
2.0
1.5
1.0
0.5
0.0
HC-C/EBP Beta Tg
AD-C/EBP Beta Tg

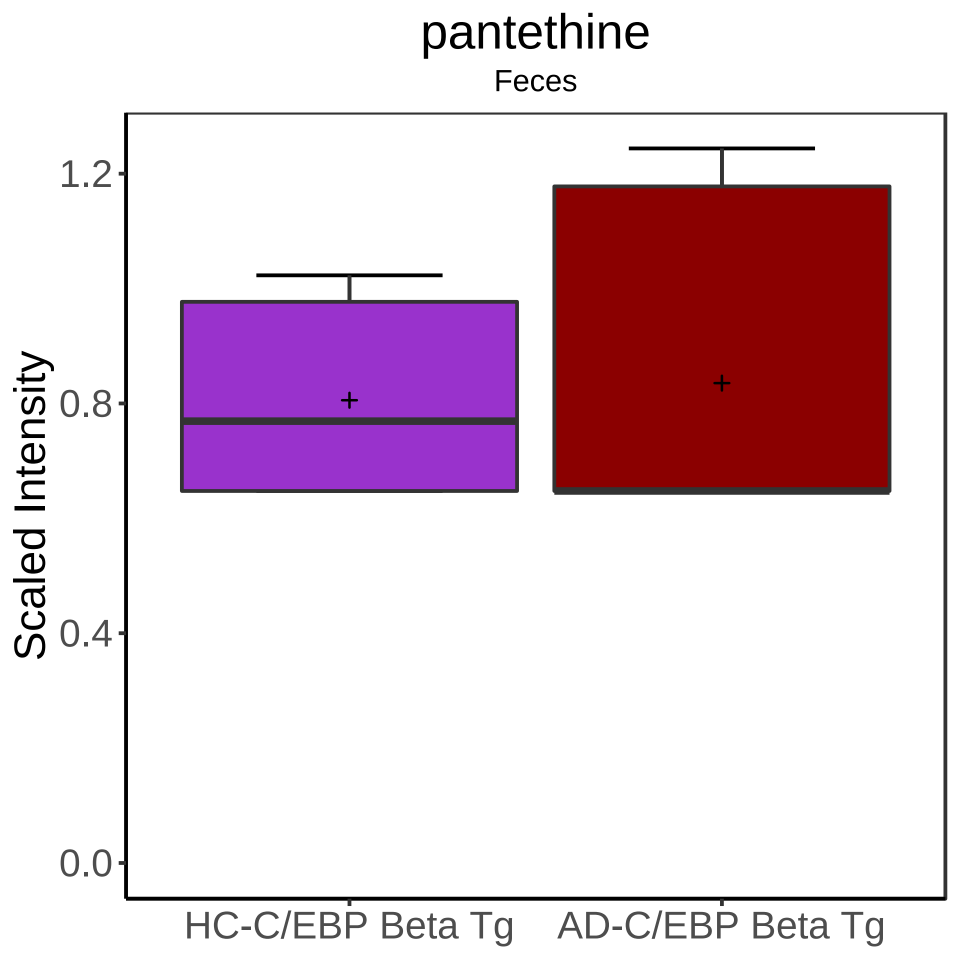

pantethine
Feces
Scaled Intensity
1.2
0.8
0.4
0.0
HC-C/EBP Beta Tg
AD-C/EBP Beta Tg

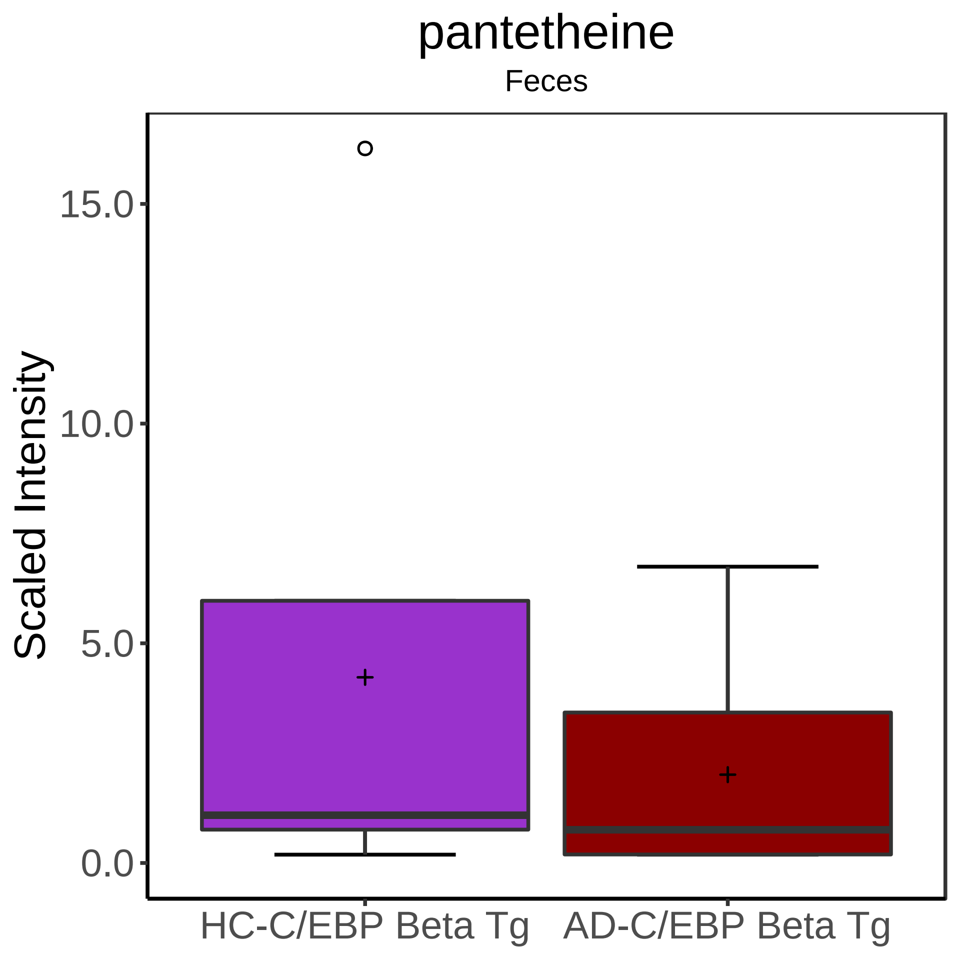
pantetheine
Feces
Scaled Intensity
15.0
10.0
5.0
0.0
HC-C/EBP Beta Tg
AD-C/EBP Beta Tg

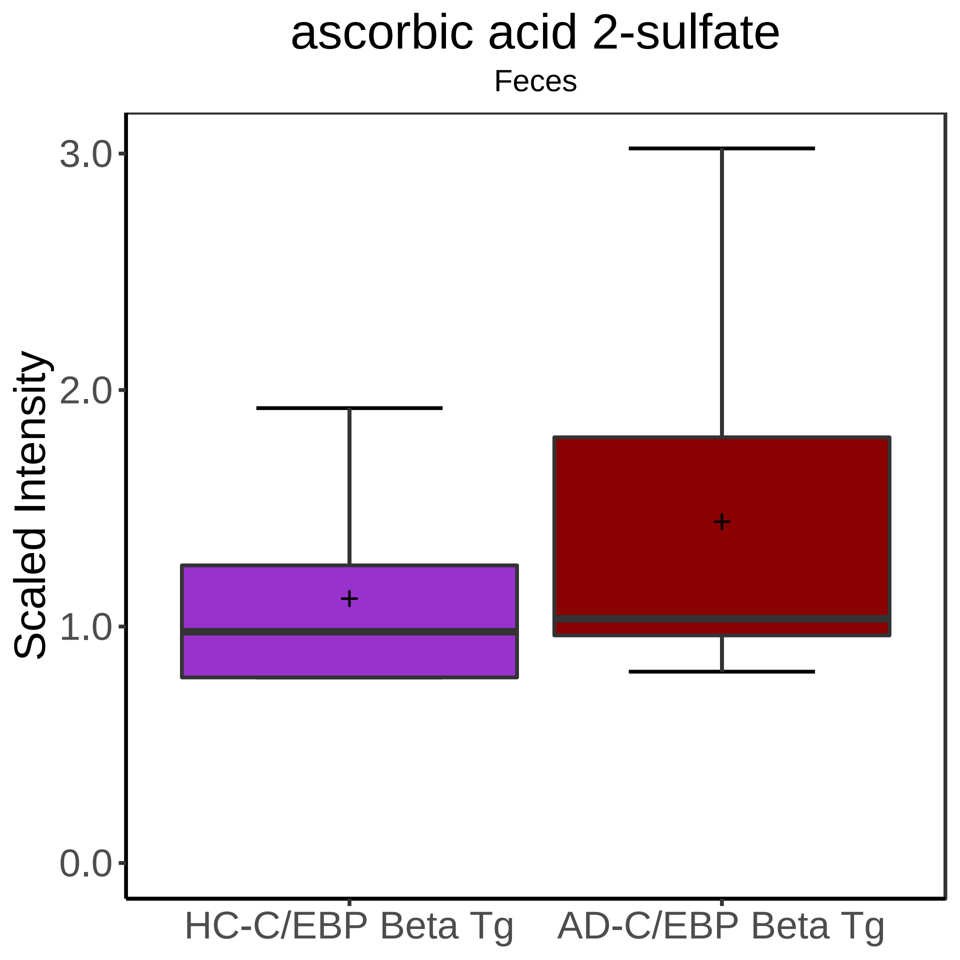
ascorbic acid 2-sulfate
Feces
Scaled Intensity
3.0
2.0
1.0
0.0
HC-C/EBP Beta Tg
AD-C/EBP Beta Tg

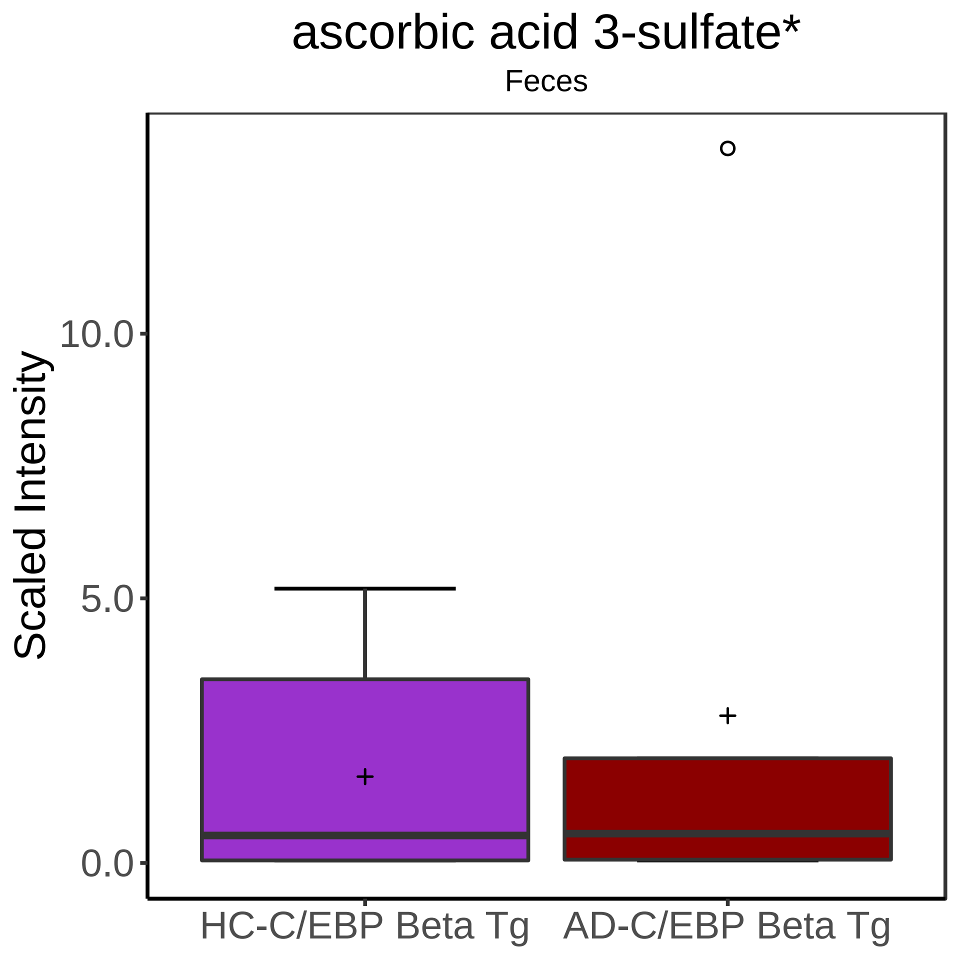
ascorbic acid 3-sulfate*
Feces
Scaled Intensity
10.0
5.0
0.0
HC-C/EBP Beta Tg
AD-C/EBP Beta Tg

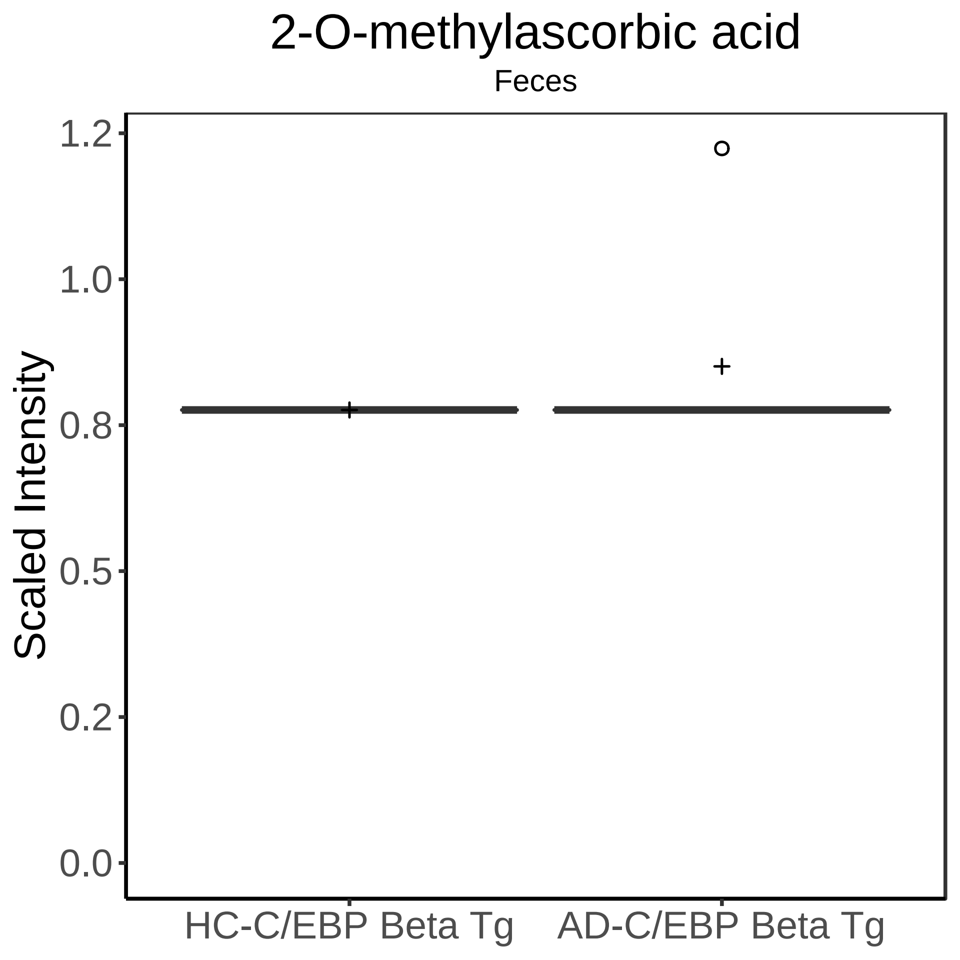
2-O-methylascorbic acid
Feces
Scaled Intensity
1.2
1.0
0.8
0.5
0.2
0.0
HC-C/EBP Beta Tg
AD-C/EBP Beta Tg

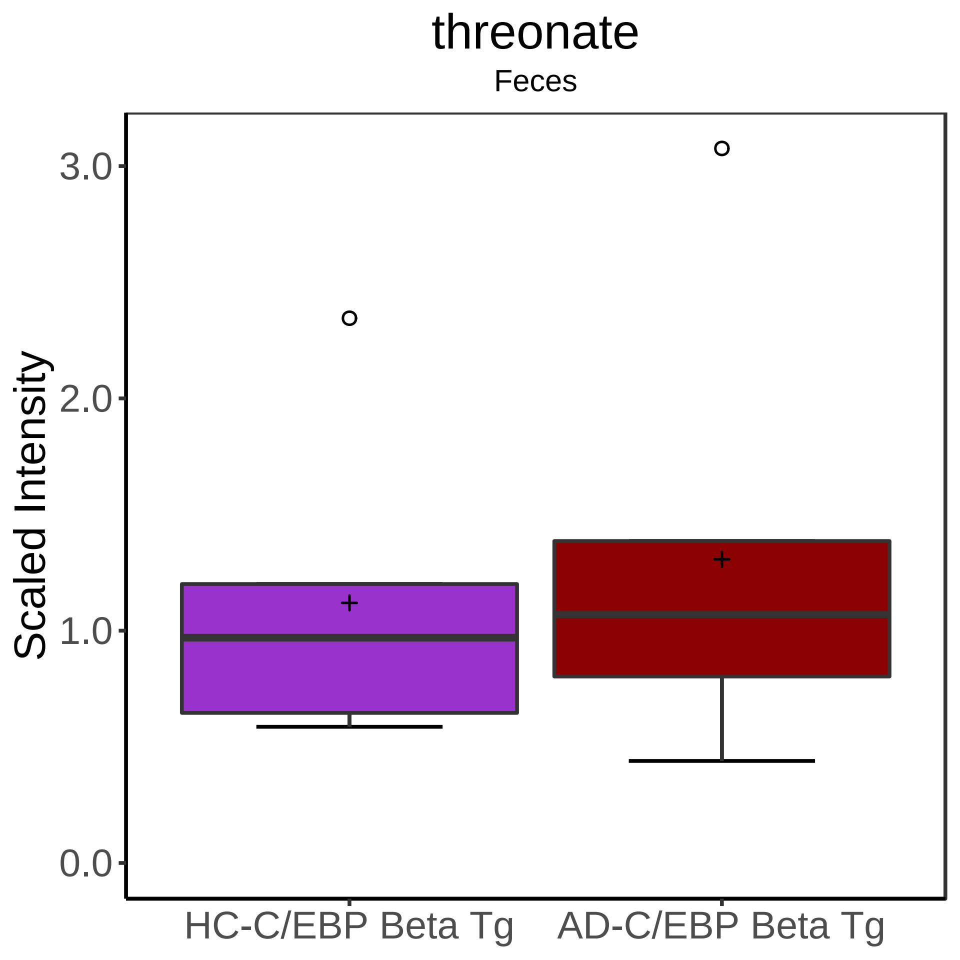
threonate
Feces
Scaled Intensity
3.0
2.0
1.0
0.0
HC-C/EBP Beta Tg
AD-C/EBP Beta Tg

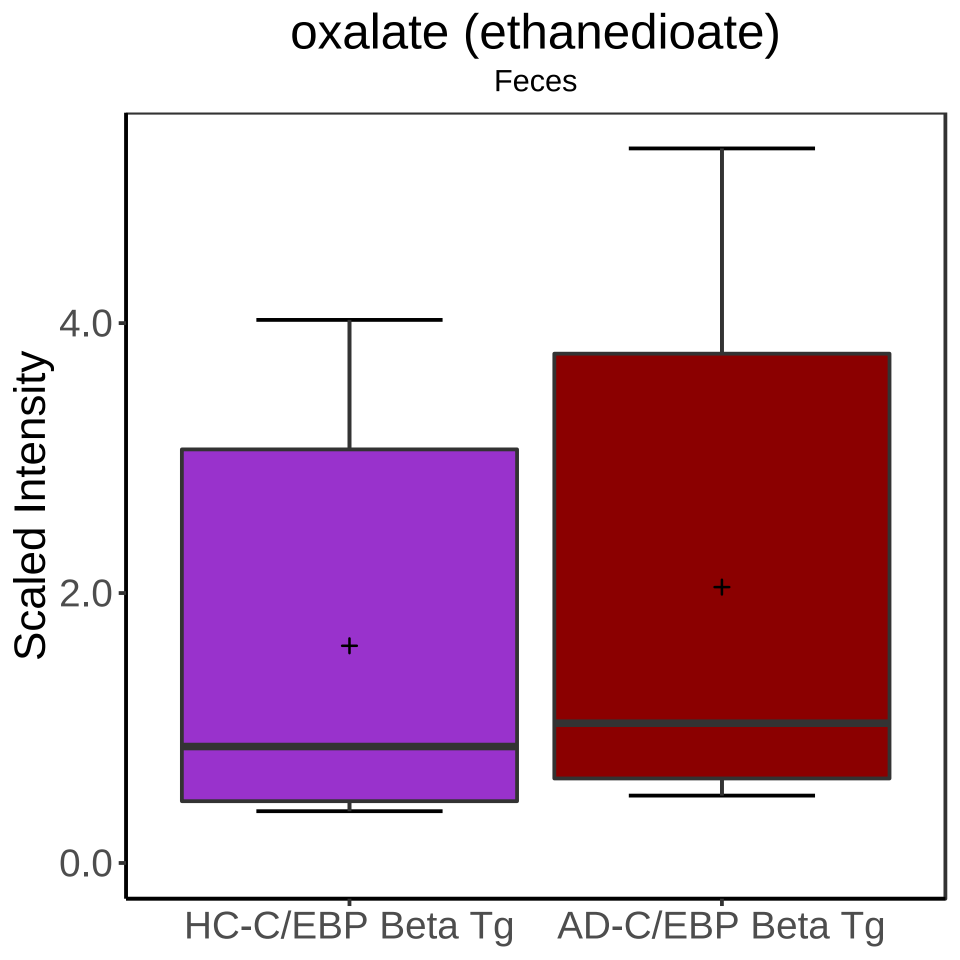

oxalate (ethanedioate)
Feces
Scaled Intensity
4.0
2.0
0.0
HC-C/EBP Beta Tg
AD-C/EBP Beta Tg

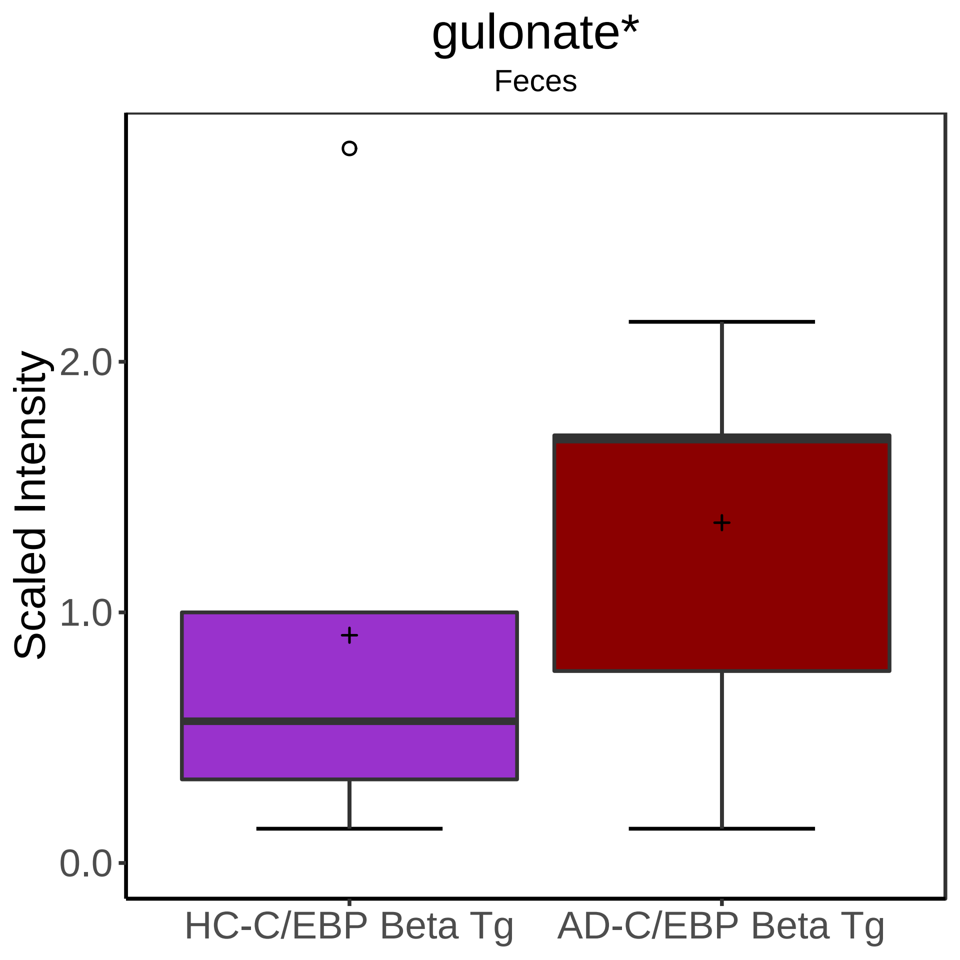
gulonate*
Feces
Scaled Intensity
2.0
1.0
0.0
HC-C/EBP Beta Tg
AD-C/EBP Beta Tg

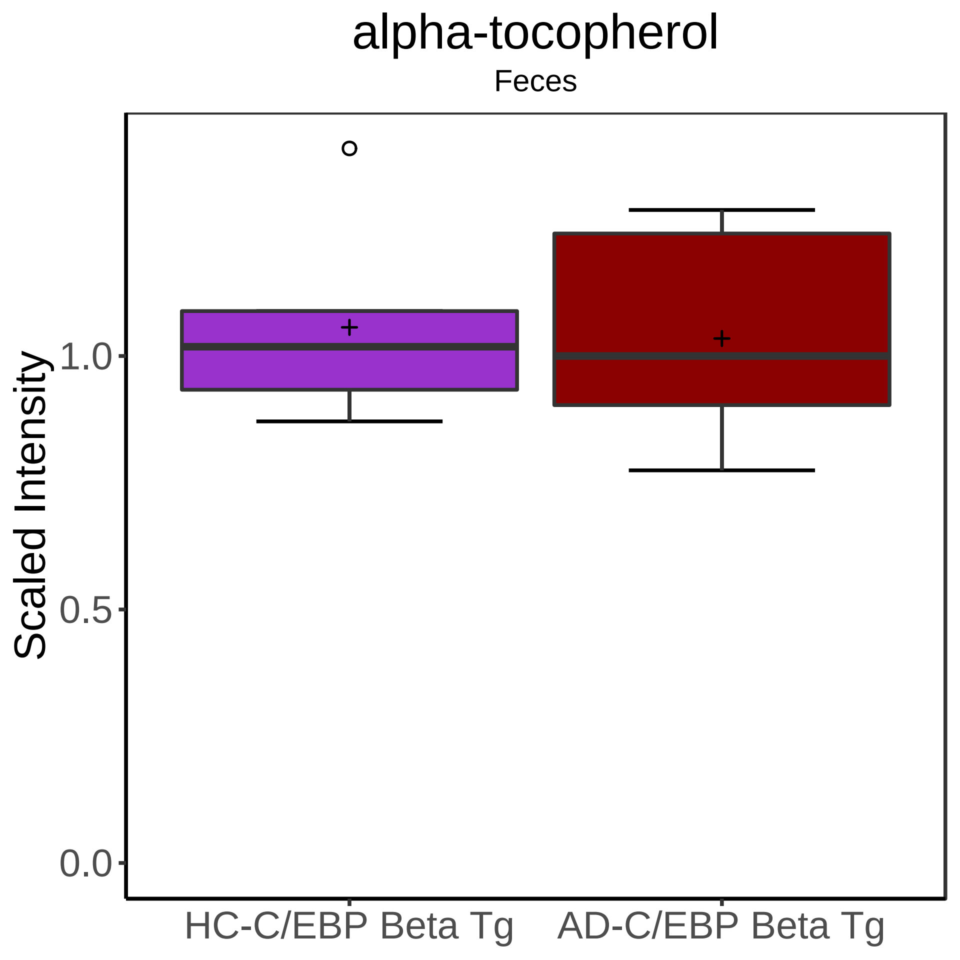
alpha-tocopherol
Feces
Scaled Intensity
1.0
0.5
0.0
HC-C/EBP Beta Tg
AD-C/EBP Beta Tg

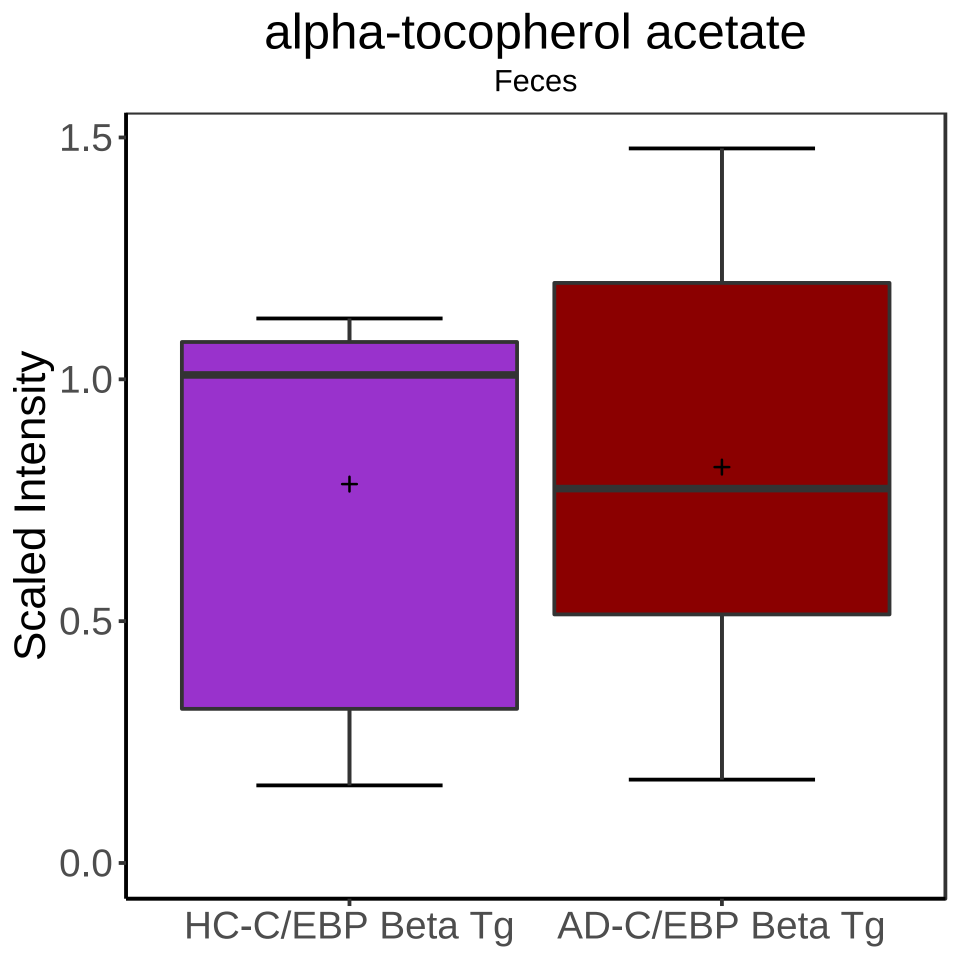
alpha-tocopherol acetate
Feces
Scaled Intensity
1.5
1.0
0.5
0.0
HC-C/EBP Beta Tg
AD-C/EBP Beta Tg

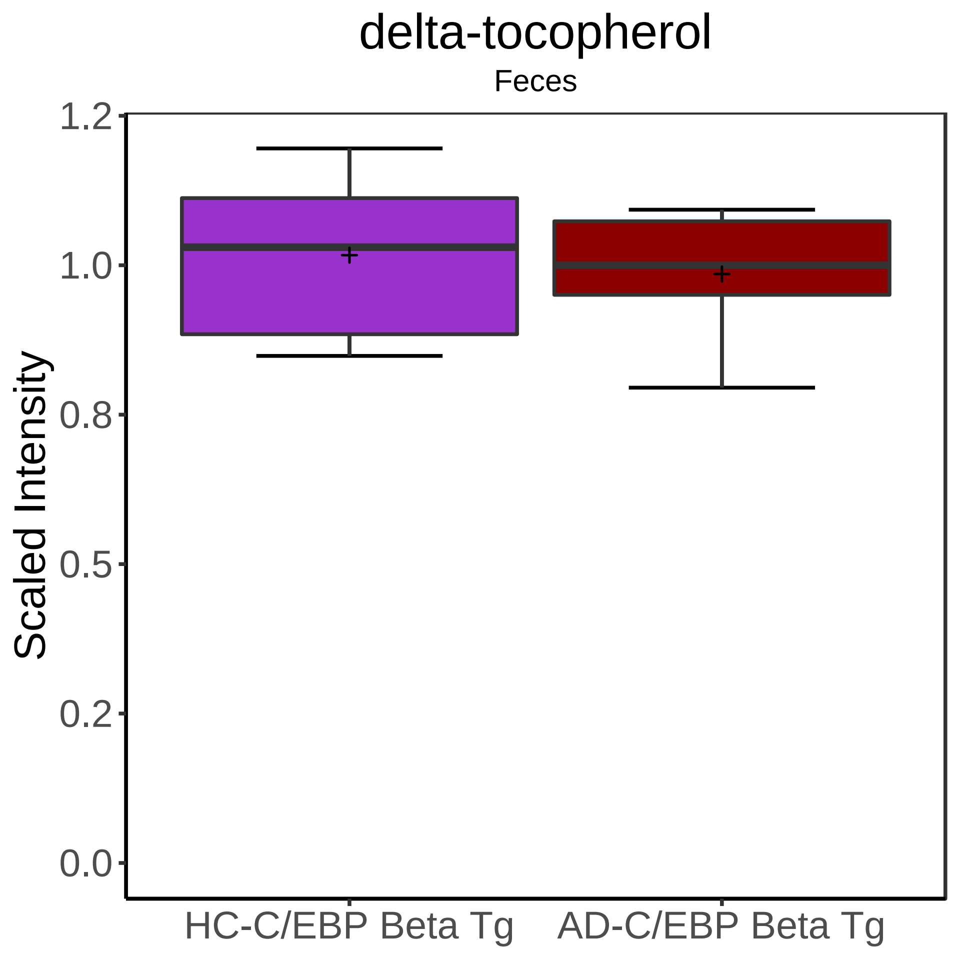
delta-tocopherol
Feces
Scaled Intensity
1.2
1.0
0.8
0.5
0.2
0.0
HC-C/EBP Beta Tg
AD-C/EBP Beta Tg

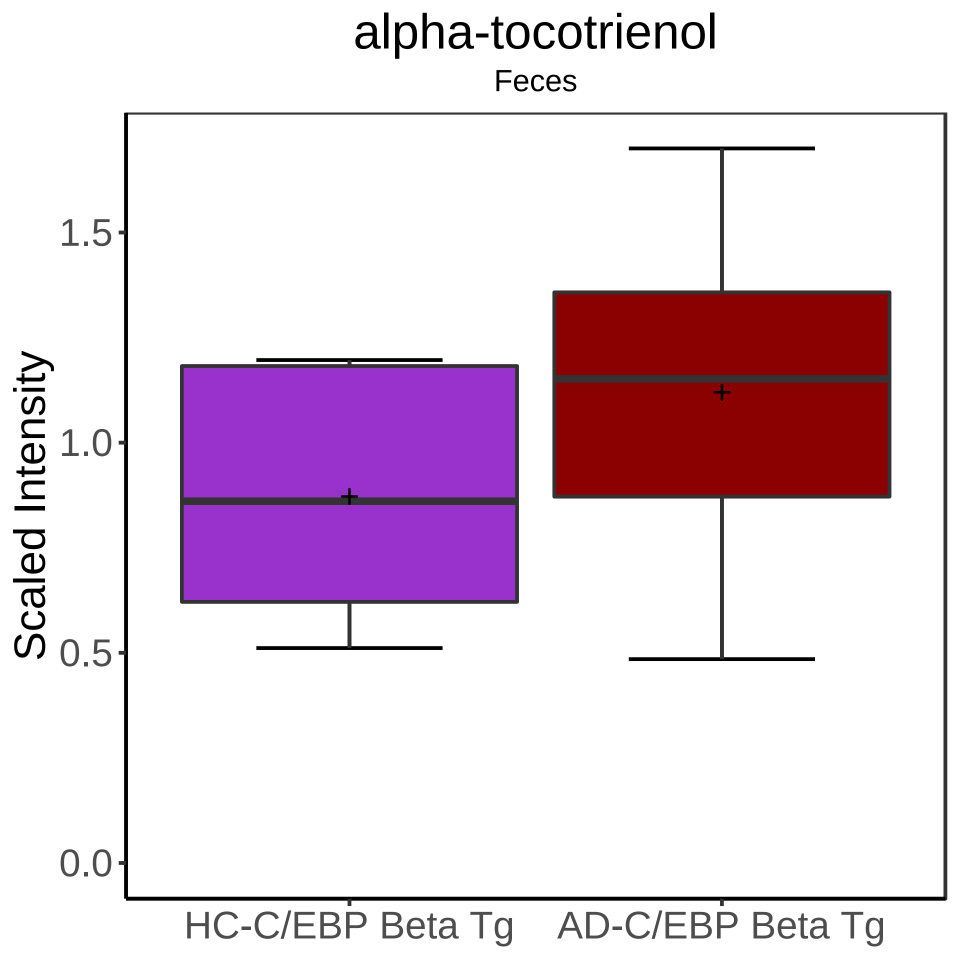
alpha-tocotrienol
Feces
Scaled Intensity
0.0
0.5
1.0
1.5
HC-C/EBP Beta Tg
AD-C/EBP Beta Tg

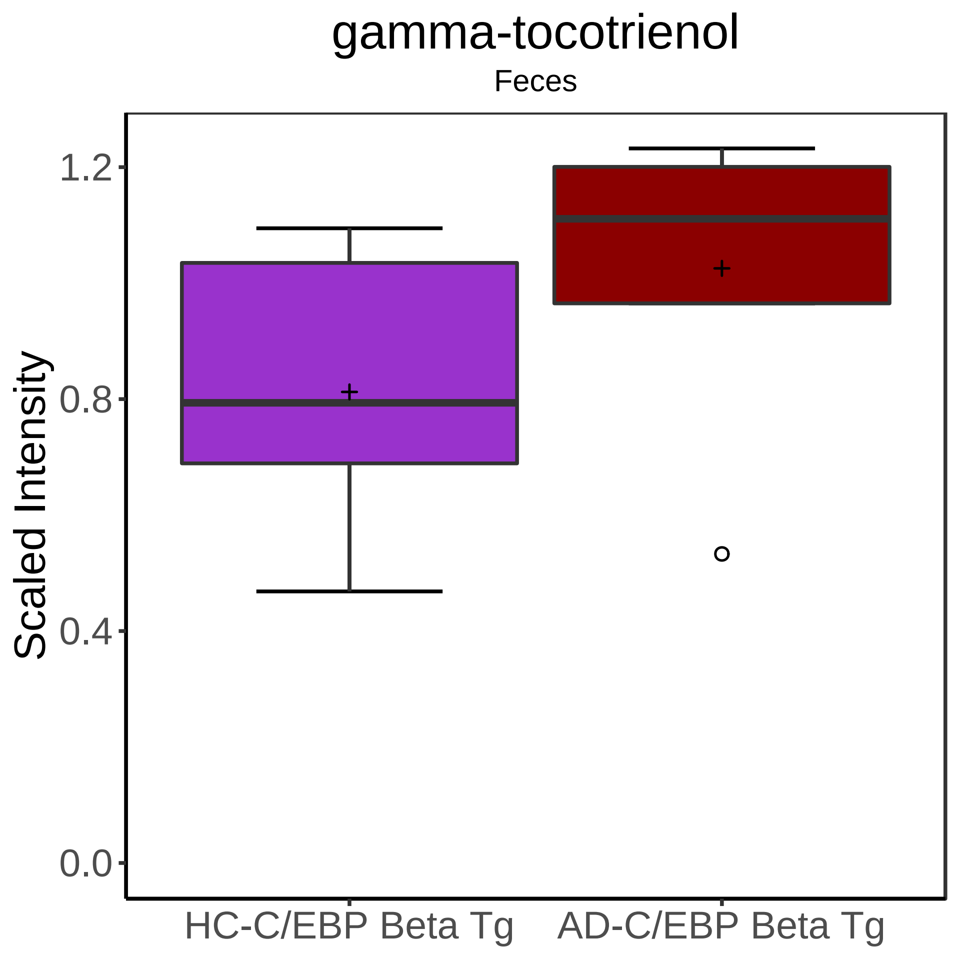

gamma-tocotrienol
Feces
Scaled Intensity
1.2
0.8
0.4
0.0
HC-C/EBP Beta Tg
AD-C/EBP Beta Tg

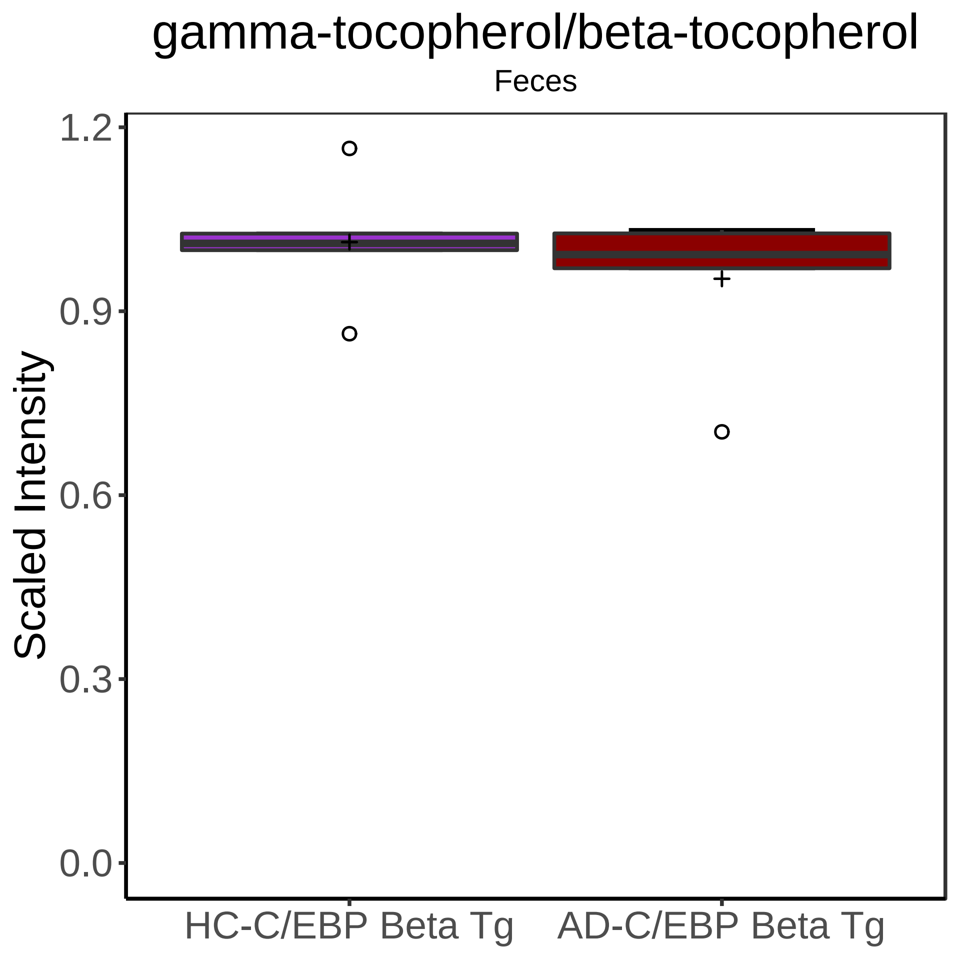
gamma-tocopherol/beta-tocopherol
Feces
Scaled Intensity
1.2
0.9
0.6
0.3
0.0
HC-C/EBP Beta Tg
AD-C/EBP Beta Tg

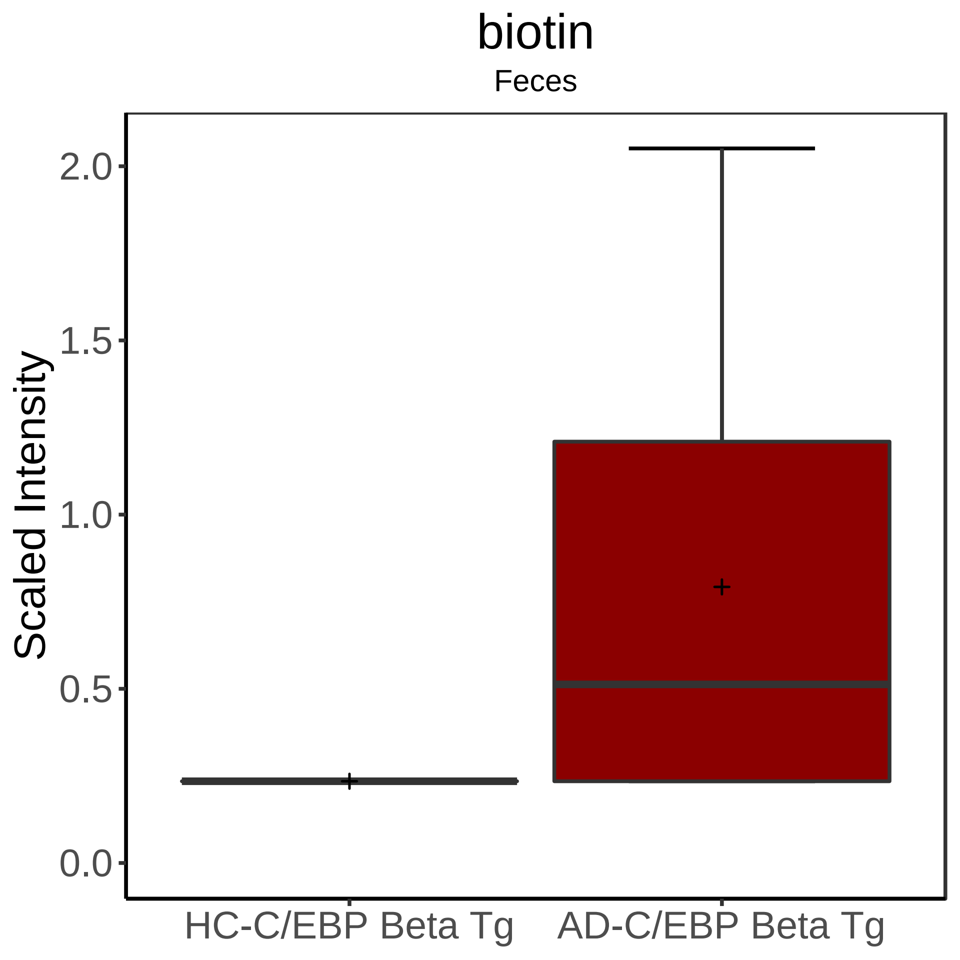
biotin
Feces
Scaled Intensity
2.0
1.5
1.0
0.5
0.0
HC-C/EBP Beta Tg
AD-C/EBP Beta Tg

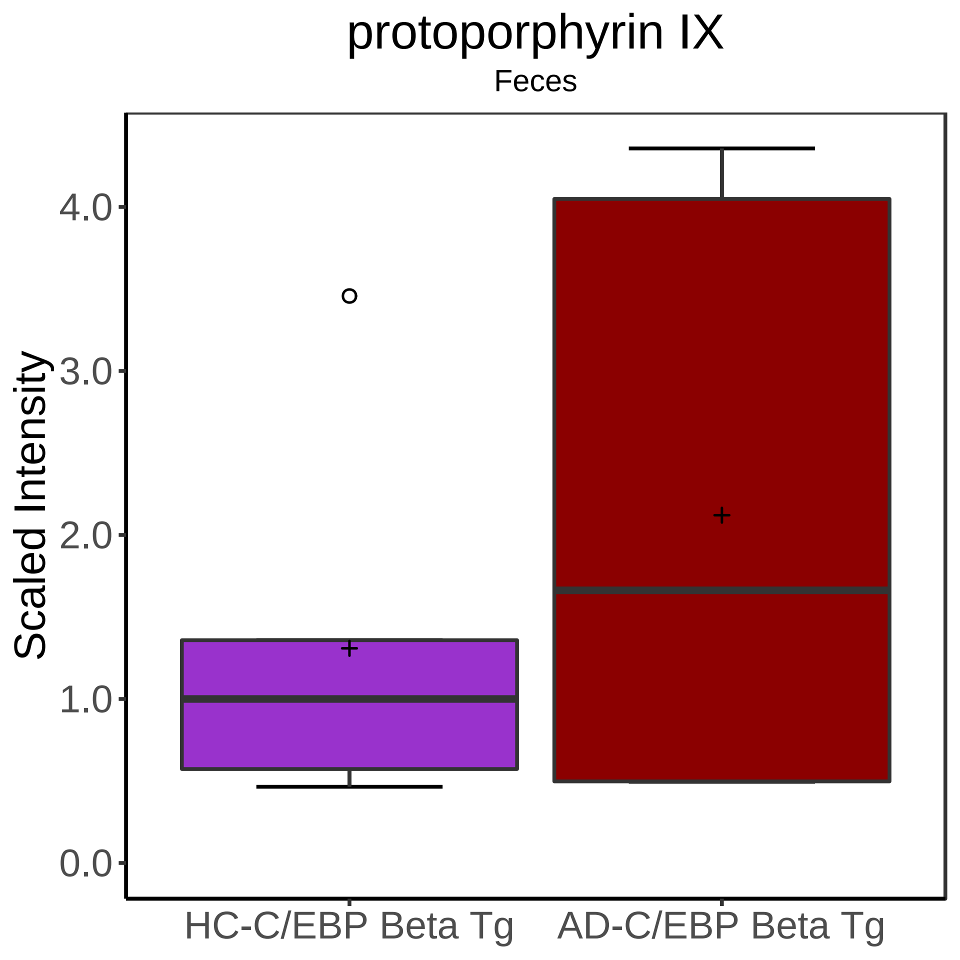
protoporphyrin IX
Feces
Scaled Intensity
4.0
3.0
2.0
1.0
0.0
HC-C/EBP Beta Tg
AD-C/EBP Beta Tg

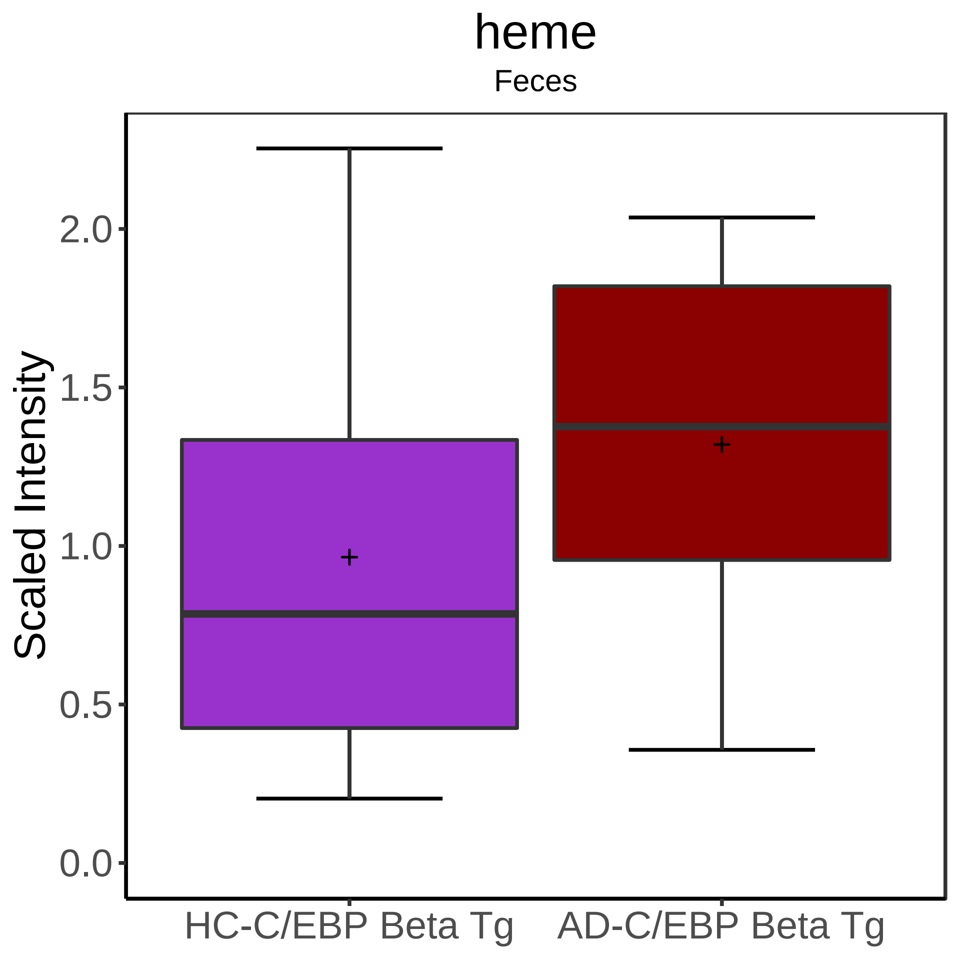
heme
Feces
Scaled Intensity
2.0
1.5
1.0
0.5
0.0
HC-C/EBP Beta Tg
AD-C/EBP Beta Tg

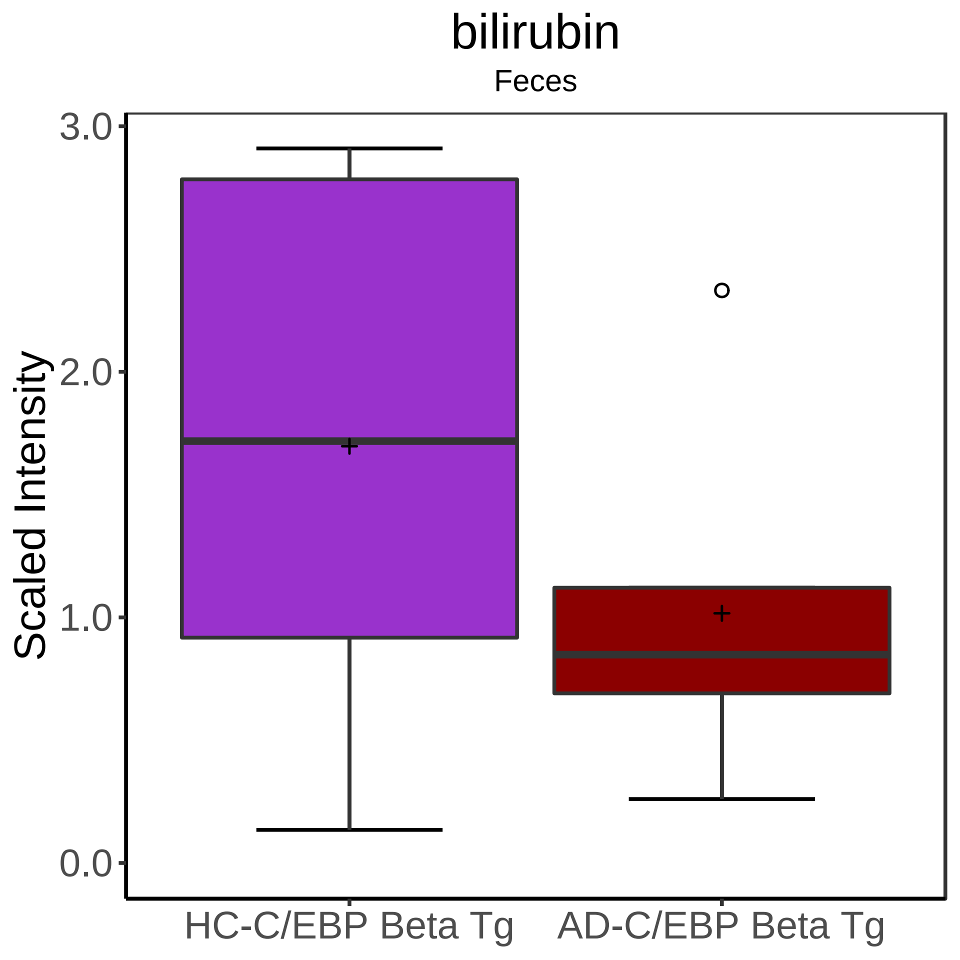
bilirubin
Feces
Scaled Intensity
3.0
2.0
1.0
0.0
HC-C/EBP Beta Tg
AD-C/EBP Beta Tg

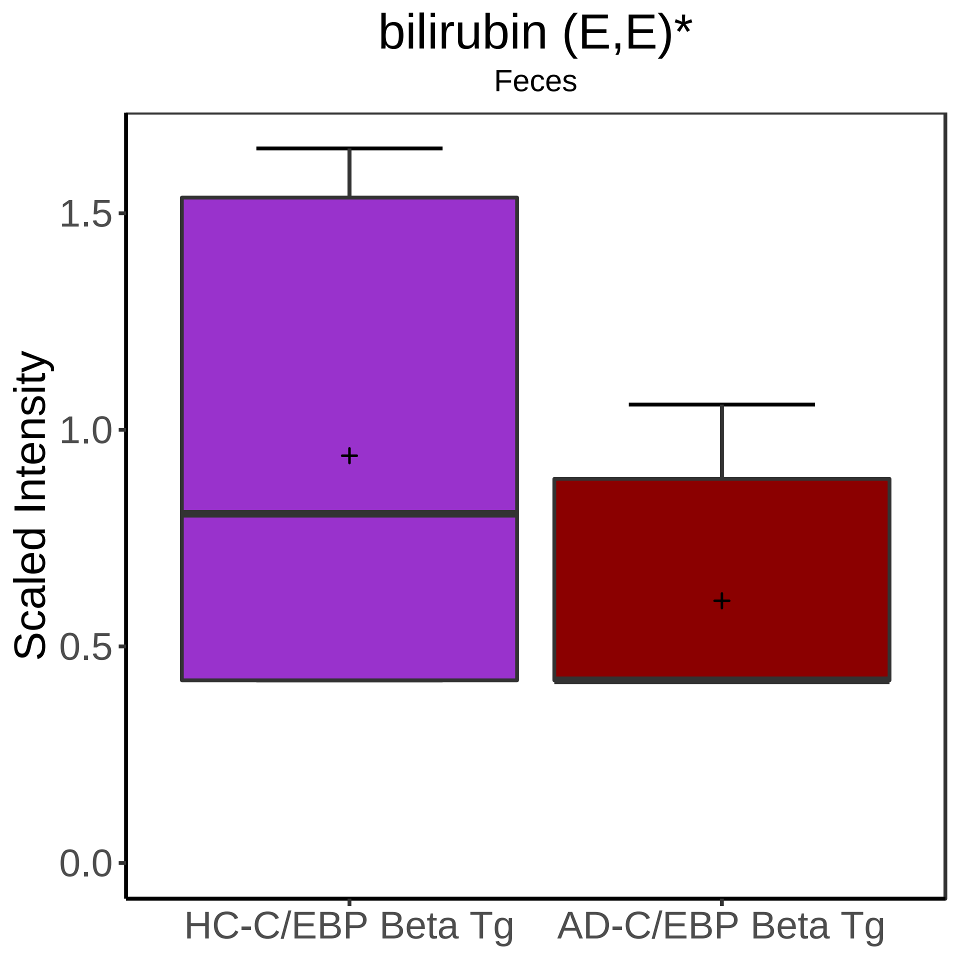

bilirubin (E,E)*
Feces
Scaled Intensity
1.5
1.0
0.5
0.0
HC-C/EBP Beta Tg
AD-C/EBP Beta Tg

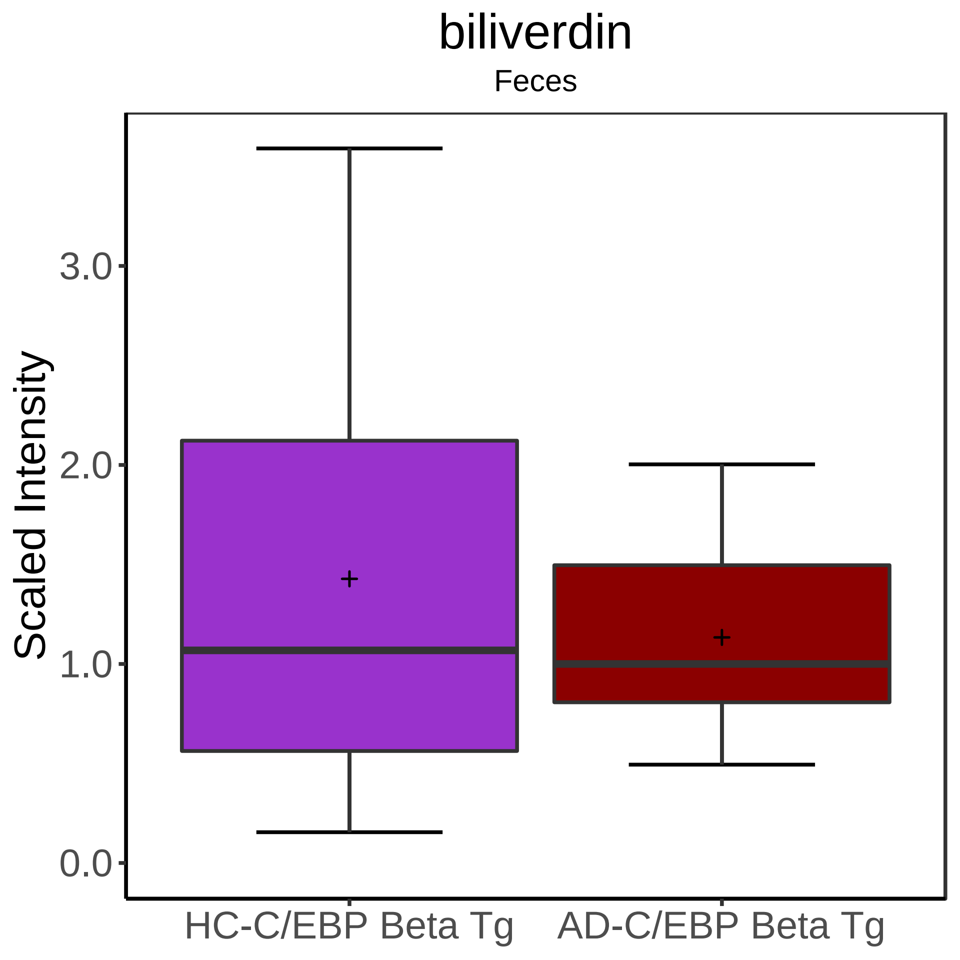
biliverdin
Feces
Scaled Intensity
3.0
2.0
1.0
0.0
HC-C/EBP Beta Tg
AD-C/EBP Beta Tg

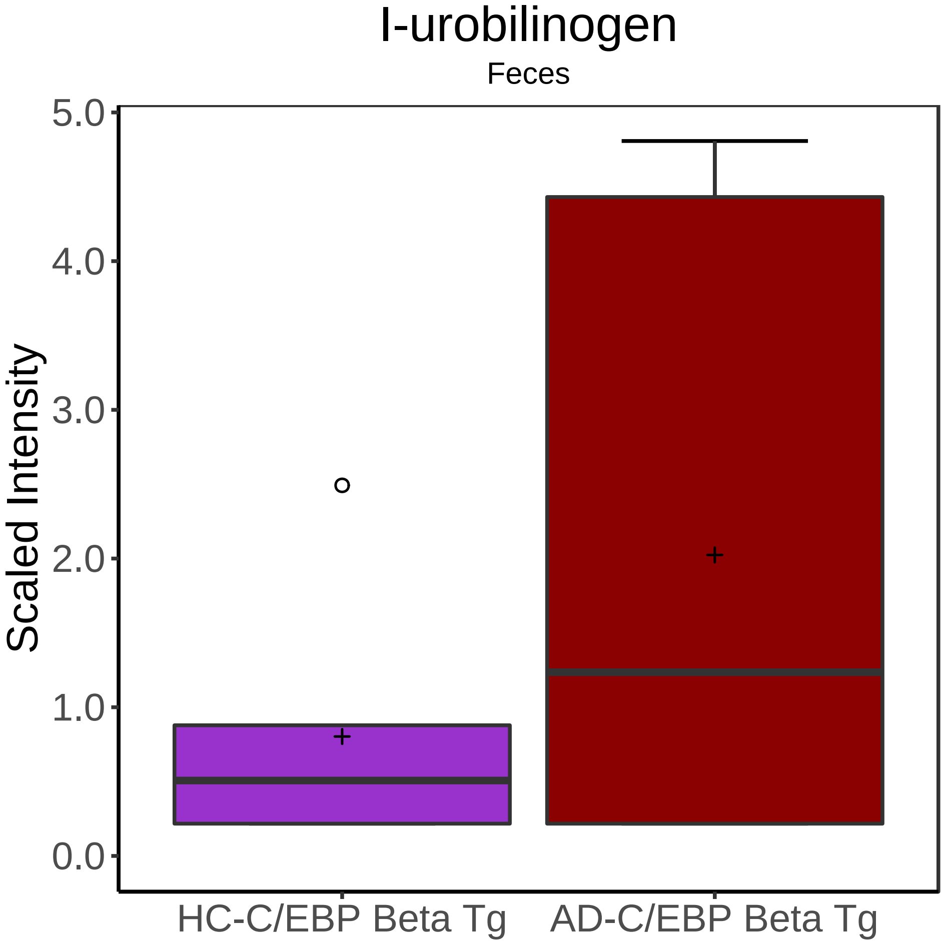
I-urobilinogen
Feces
Scaled Intensity
5.0
4.0
3.0
2.0
1.0
0.0
HC-C/EBP Beta Tg
AD-C/EBP Beta Tg

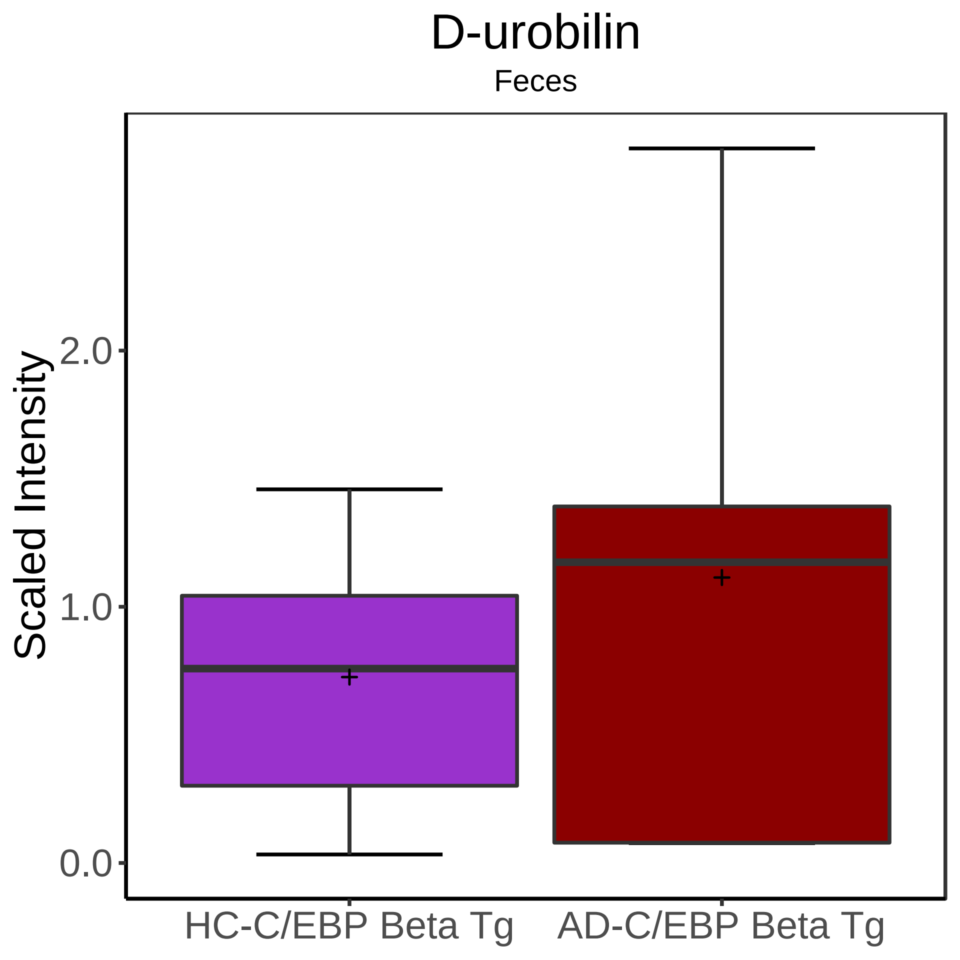
D-urobilin
Feces
Scaled Intensity
2.0
1.0
0.0
HC-C/EBP Beta Tg
AD-C/EBP Beta Tg

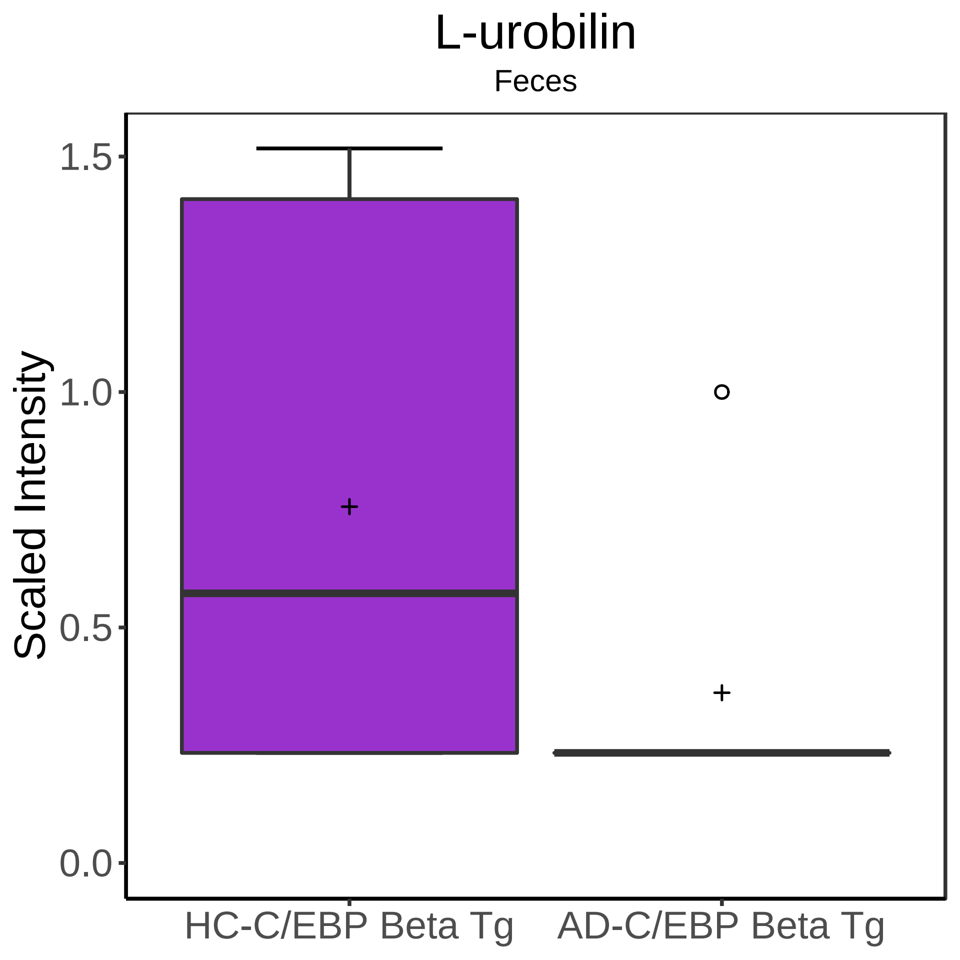

L-urobilin
Feces
Scaled Intensity
1.5
1.0
0.5
0.0
HC-C/EBP Beta Tg
AD-C/EBP Beta Tg

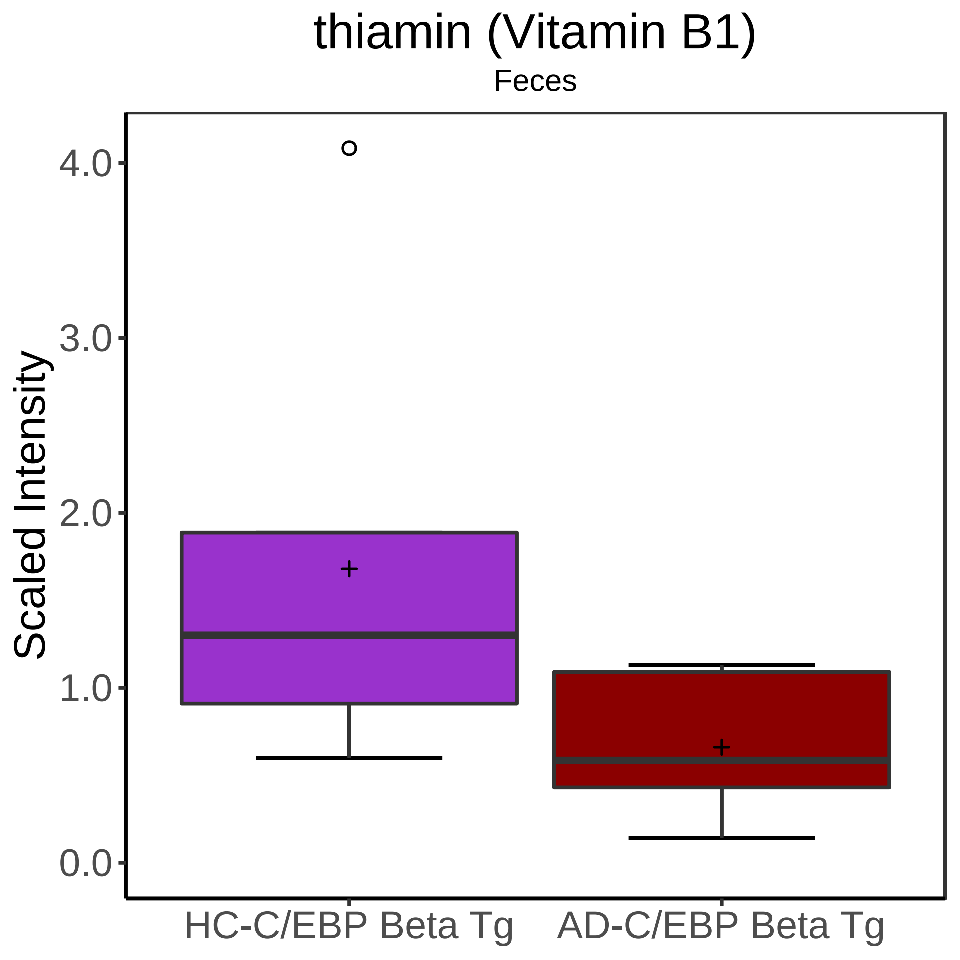

thiamin (Vitamin B1)
Feces
Scaled Intensity
4.0
3.0
2.0
1.0
0.0
HC-C/EBP Beta Tg
AD-C/EBP Beta Tg

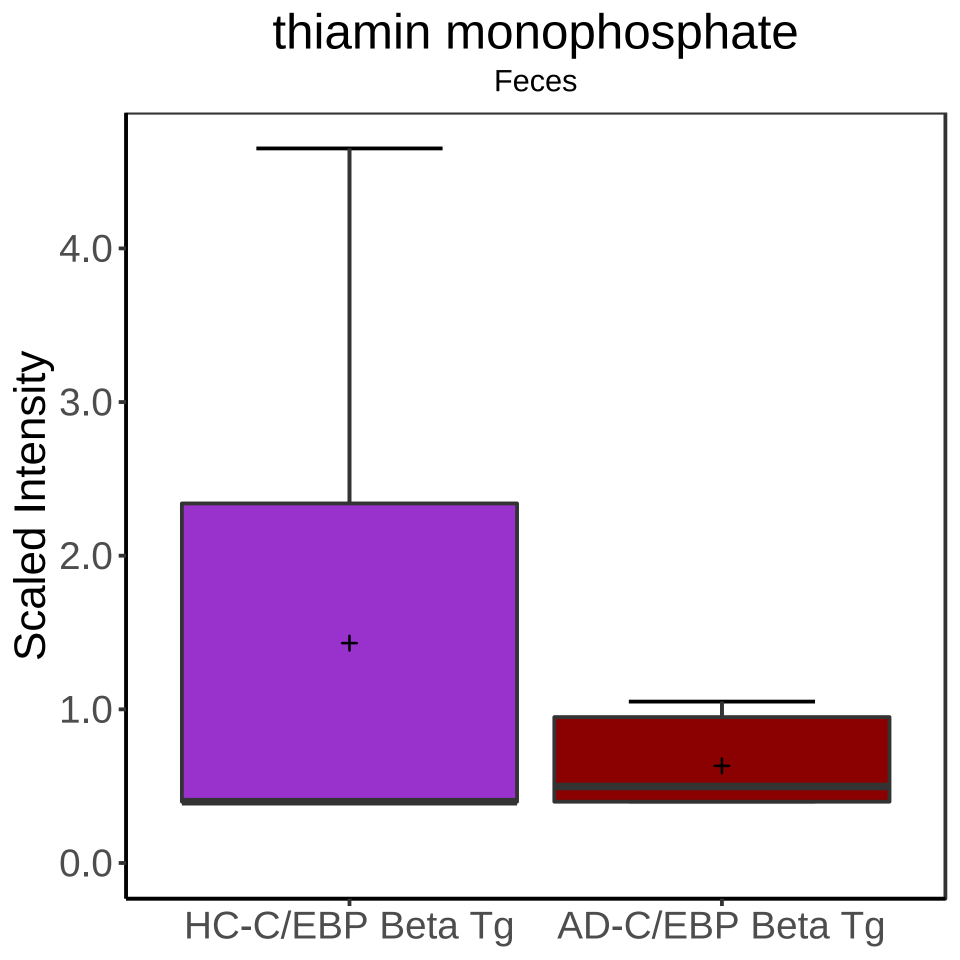
thiamin monophosphate
Feces
Scaled Intensity
4.0
3.0
2.0
1.0
0.0
HC-C/EBP Beta Tg
AD-C/EBP Beta Tg

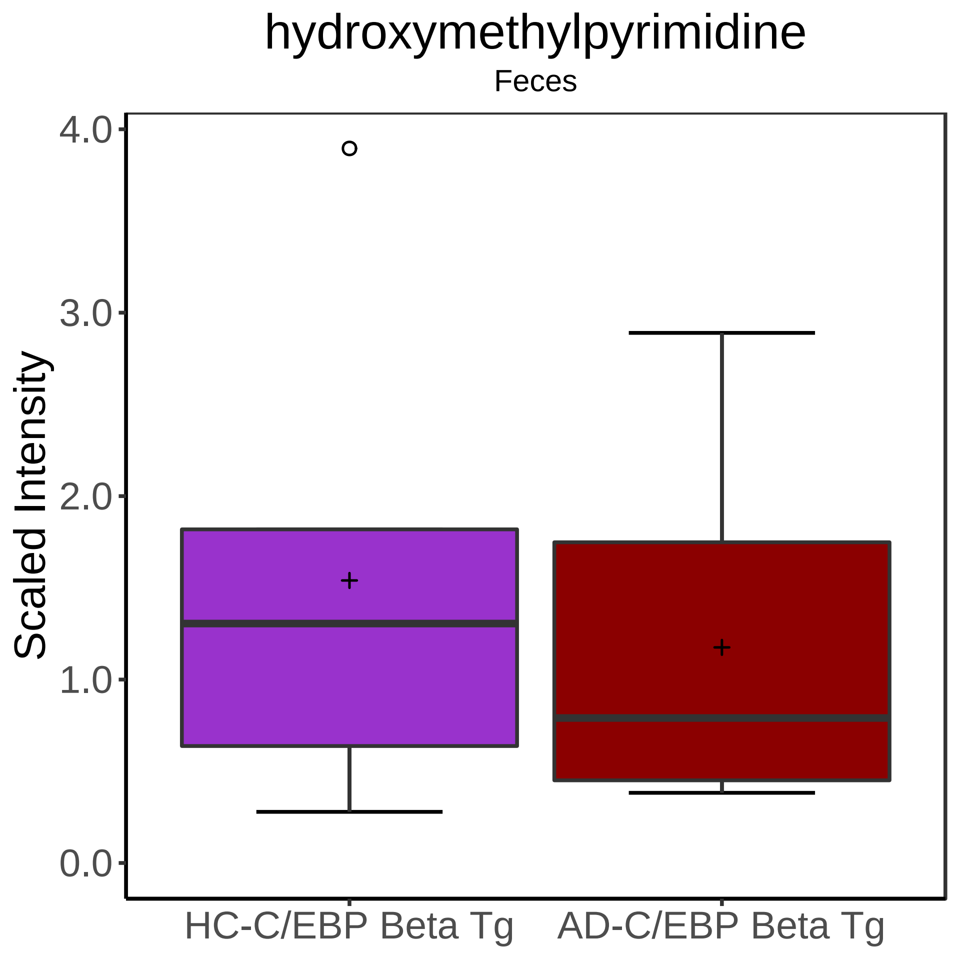
hydroxymethylpyrimidine
Feces
Scaled Intensity
4.0
3.0
2.0
1.0
0.0
HC-C/EBP Beta Tg
AD-C/EBP Beta Tg

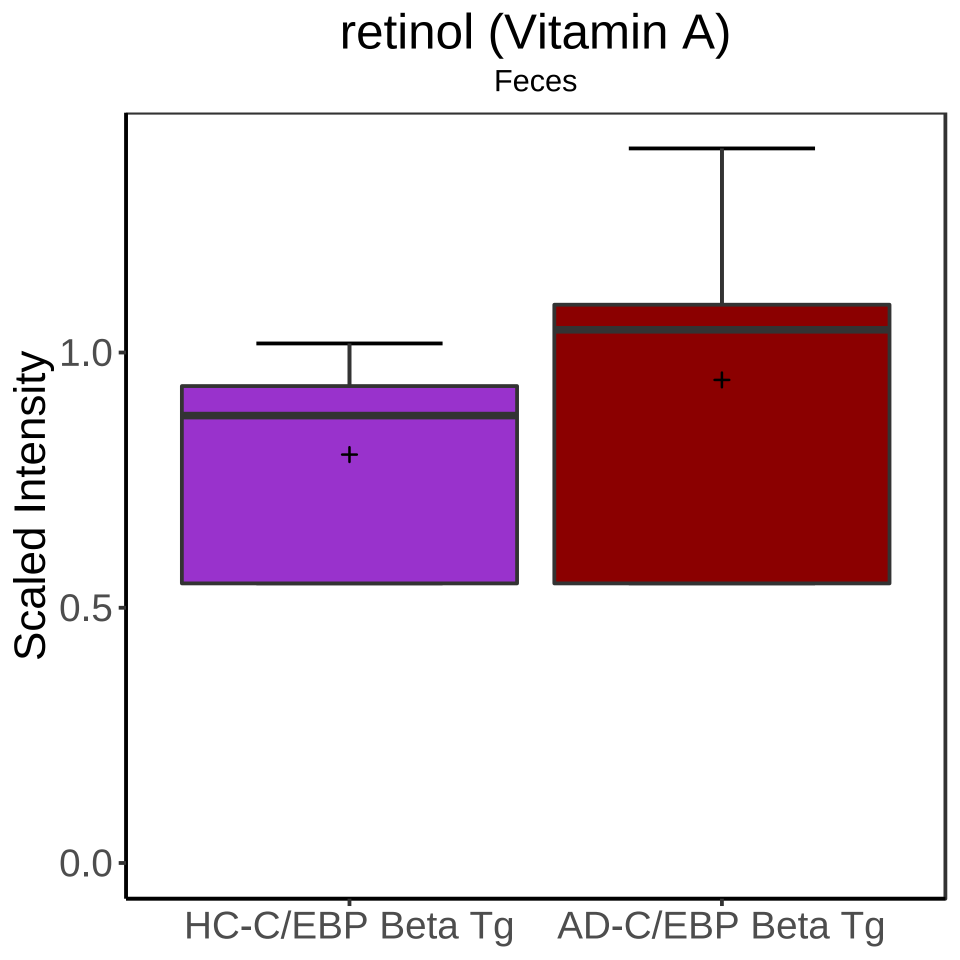
retinol (Vitamin A)
Feces
Scaled Intensity
1.0
0.5
0.0
HC-C/EBP Beta Tg
AD-C/EBP Beta Tg

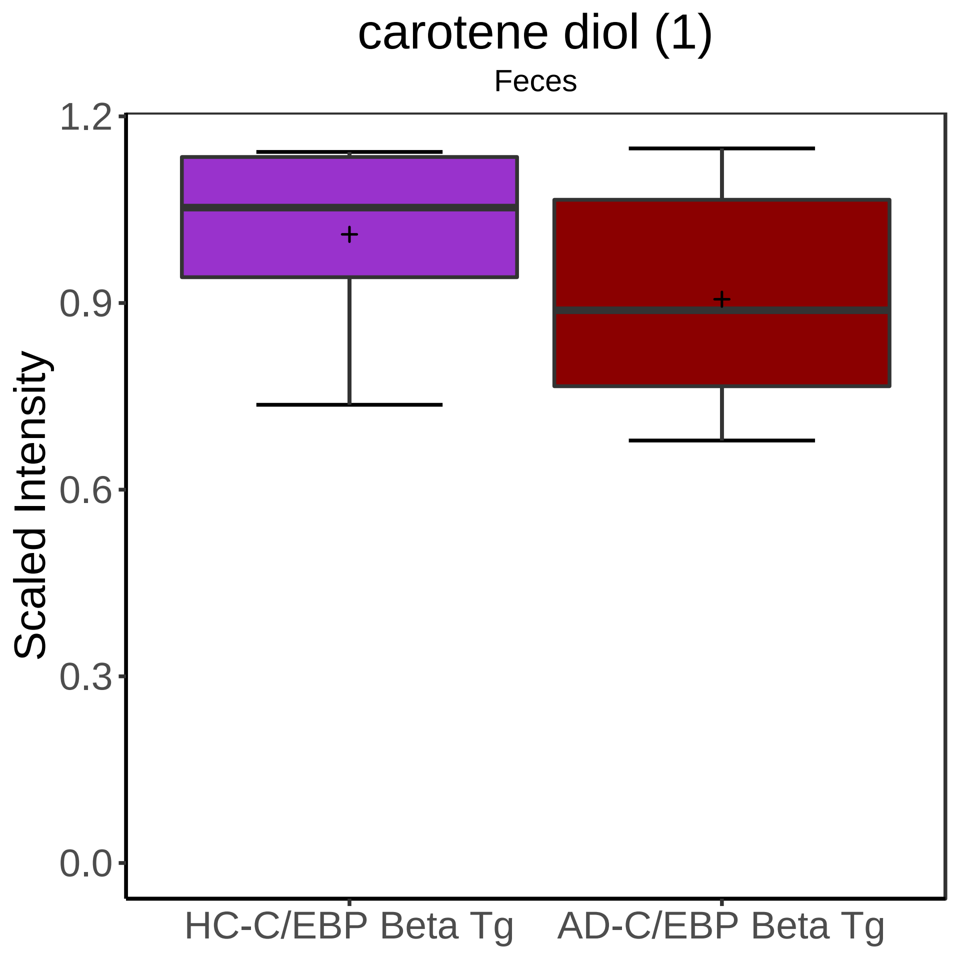
carotene diol (1)
Feces
Scaled Intensity
1.2
0.9
0.6
0.3
0.0
HC-C/EBP Beta Tg
AD-C/EBP Beta Tg

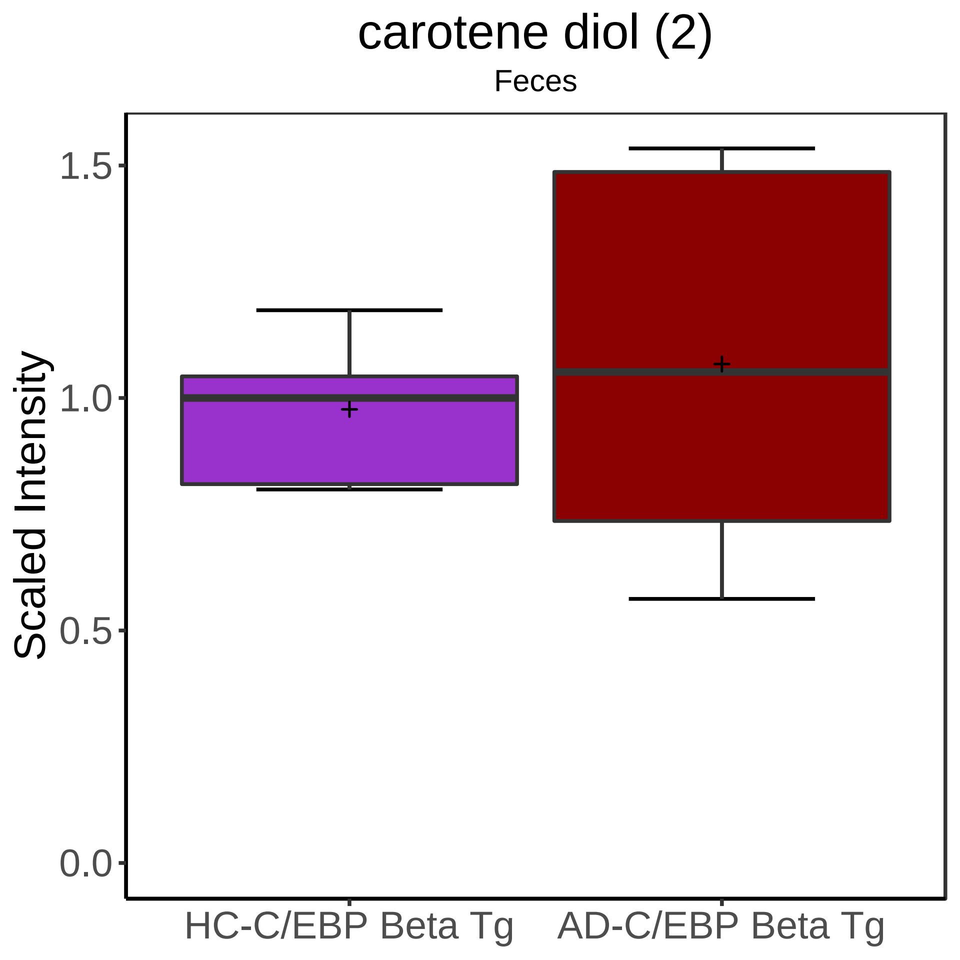
carotene diol (2)
Feces
Scaled Intensity
1.5
1.0
0.5
0.0
HC-C/EBP Beta Tg
AD-C/EBP Beta Tg

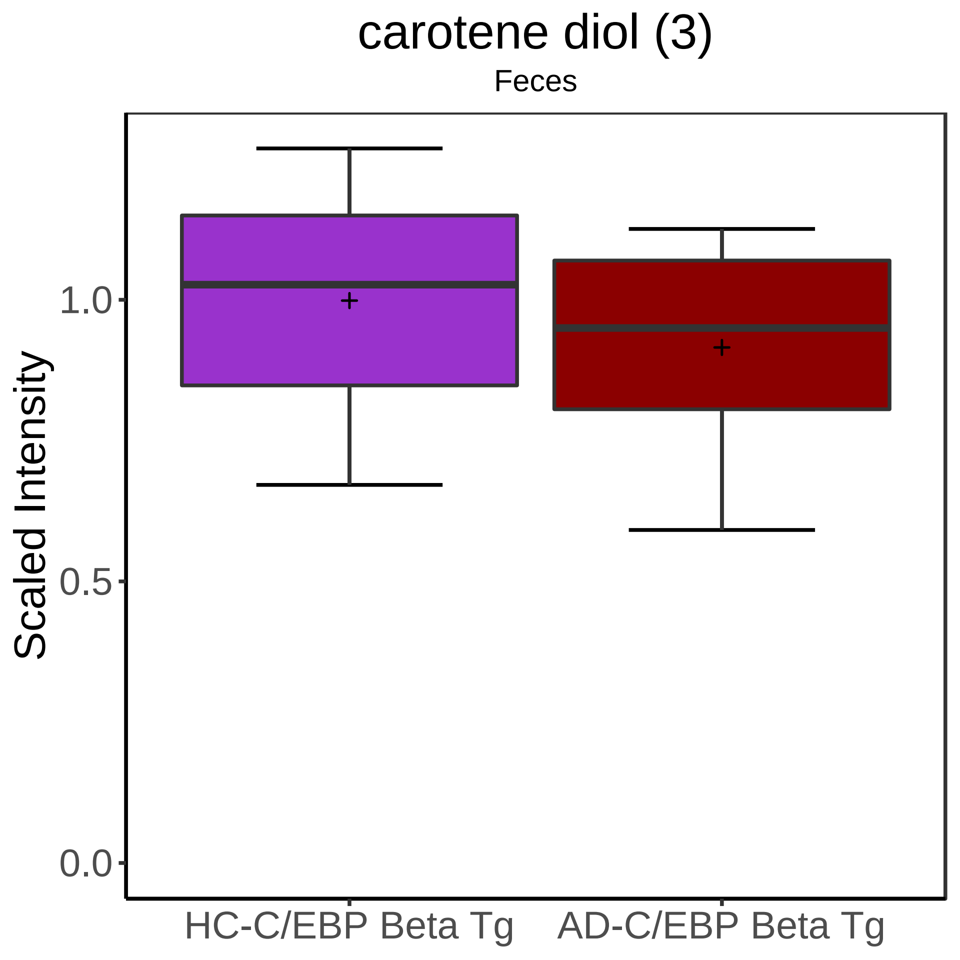
carotene diol (3)
Feces
Scaled Intensity
1.0
0.5
0.0
HC-C/EBP Beta Tg
AD-C/EBP Beta Tg

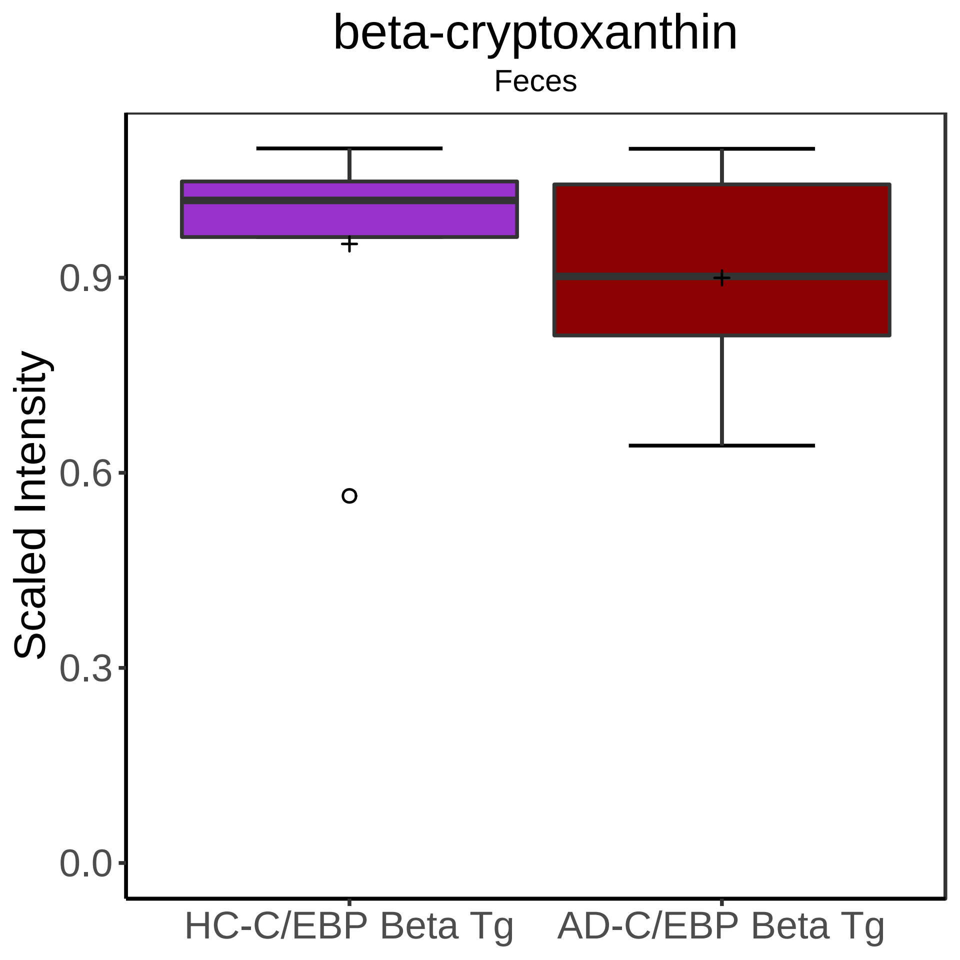

beta-cryptoxanthin
Feces
Scaled Intensity
0.9
0.6
0.3
0.0
HC-C/EBP Beta Tg
AD-C/EBP Beta Tg

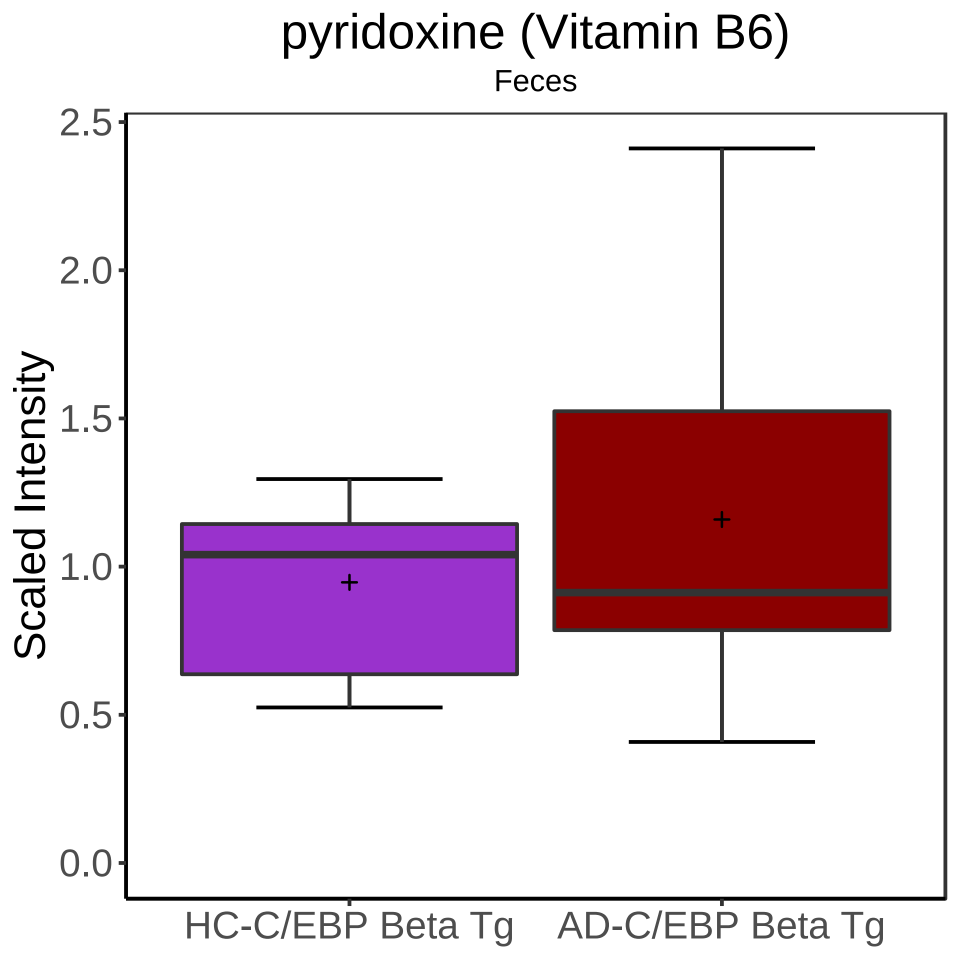

pyridoxine (Vitamin B6)
Feces
Scaled Intensity
2.5
2.0
1.5
1.0
0.5
0.0
HC-C/EBP Beta Tg
AD-C/EBP Beta Tg

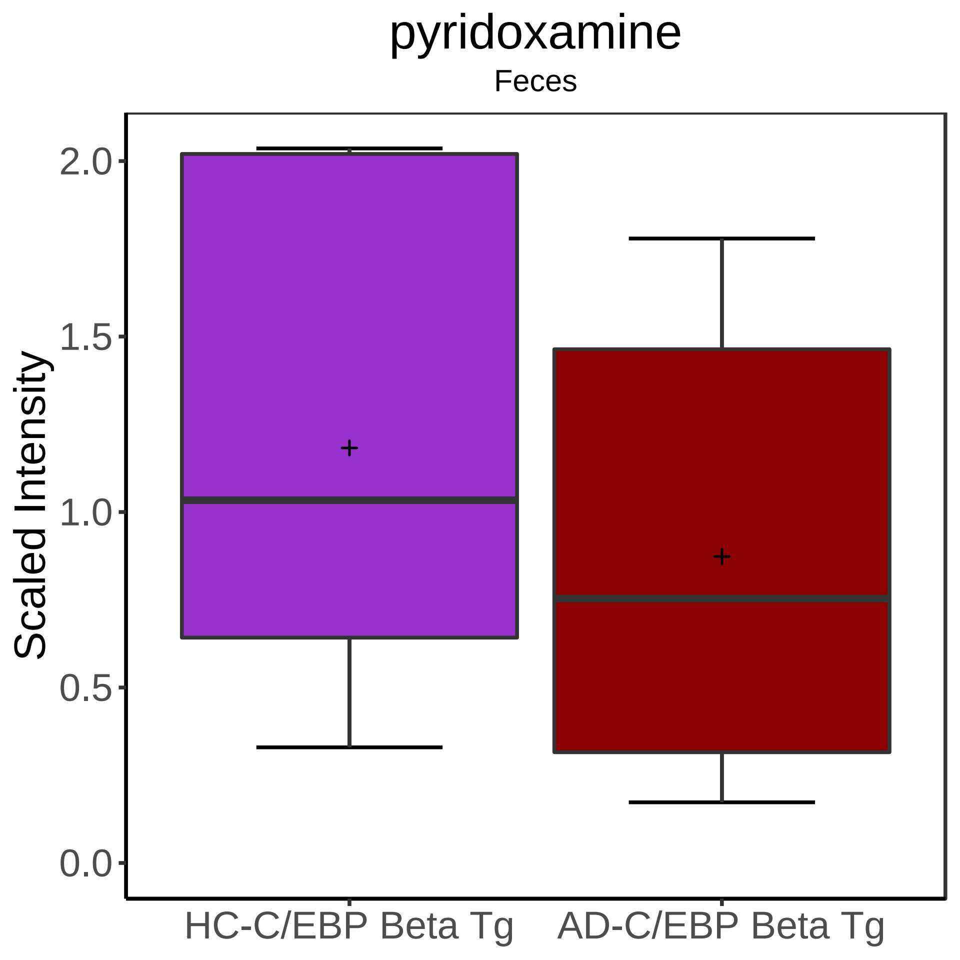
pyridoxamine
Feces
Scaled Intensity
2.0
1.5
1.0
0.5
0.0
HC-C/EBP Beta Tg
AD-C/EBP Beta Tg

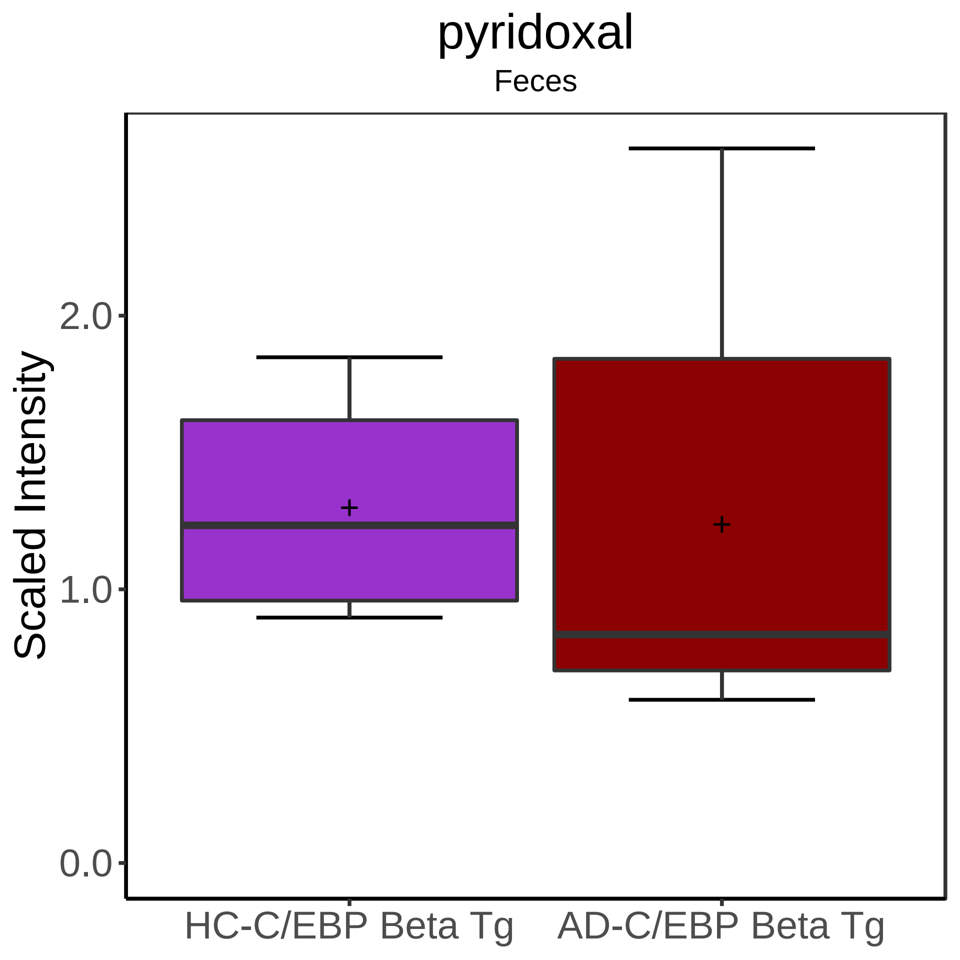
pyridoxal
Feces
Scaled Intensity
2.0
1.0
0.0
HC-C/EBP Beta Tg
AD-C/EBP Beta Tg

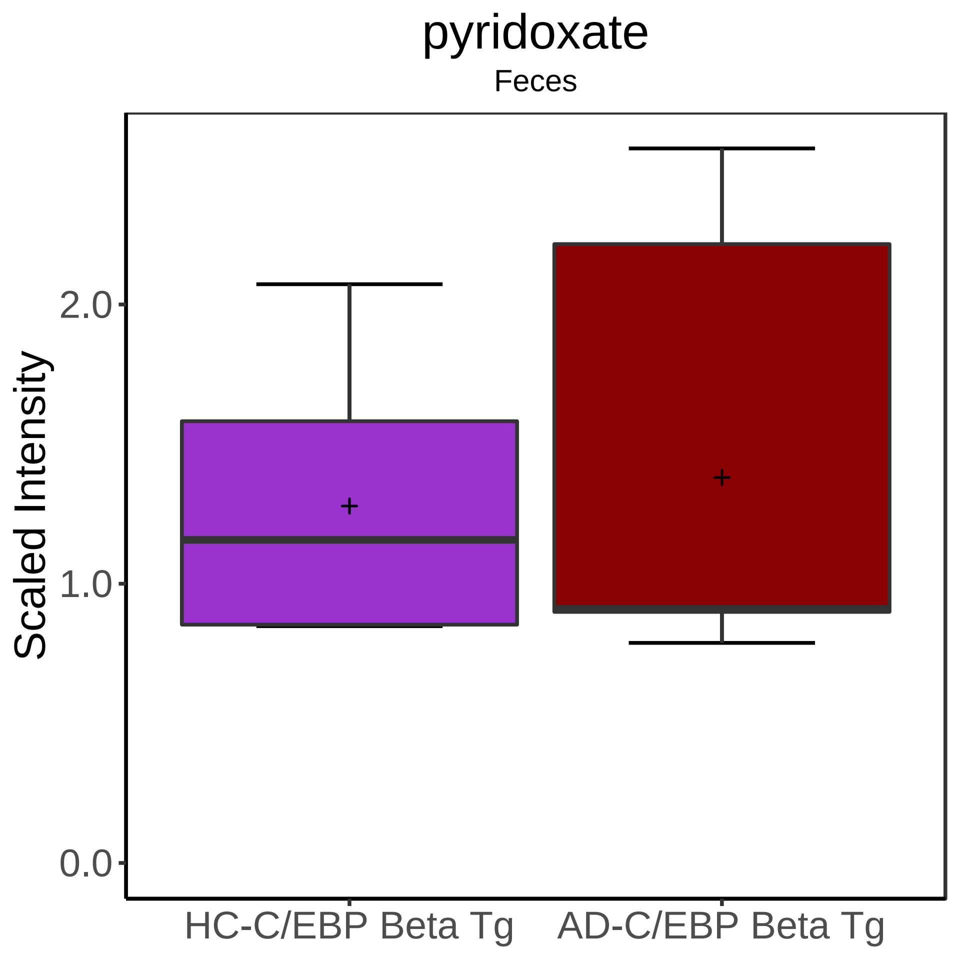
pyridoxate
Feces
Scaled Intensity
2.0
1.0
0.0
HC-C/EBP Beta Tg
AD-C/EBP Beta Tg

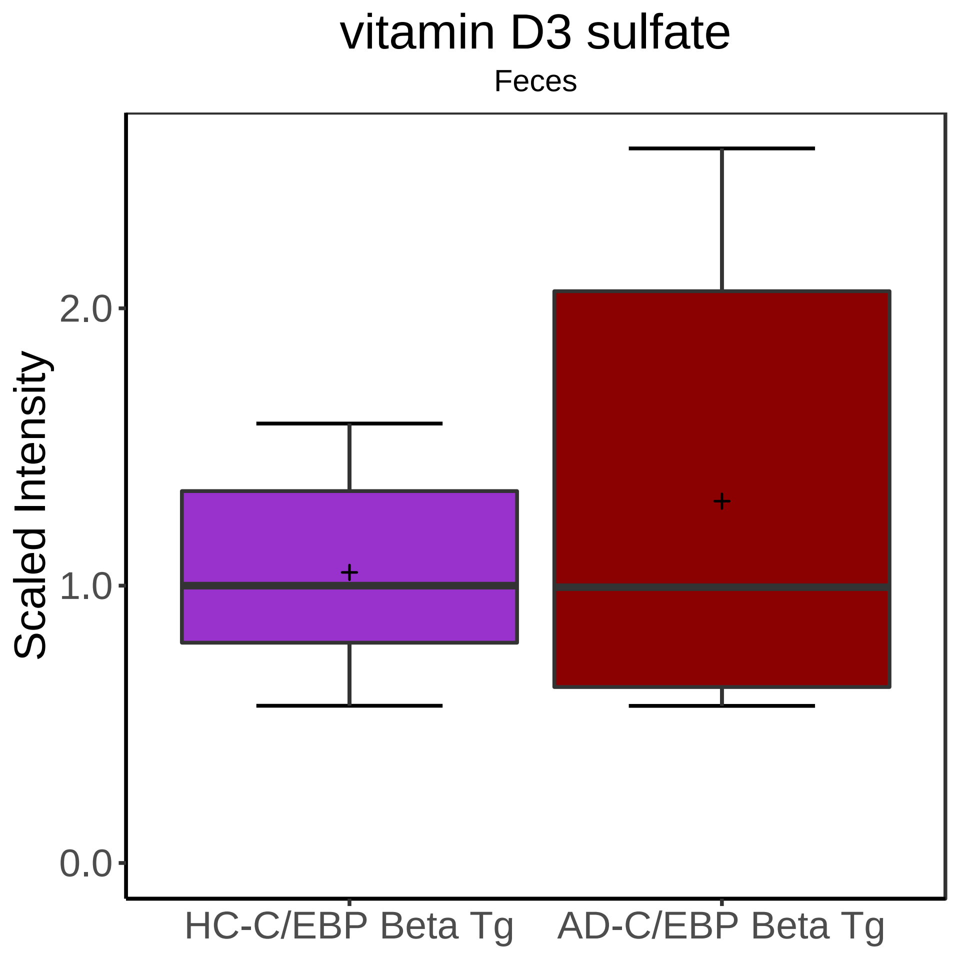
vitamin D3 sulfate
Feces
Scaled Intensity
2.0
1.0
0.0
HC-C/EBP Beta Tg
AD-C/EBP Beta Tg

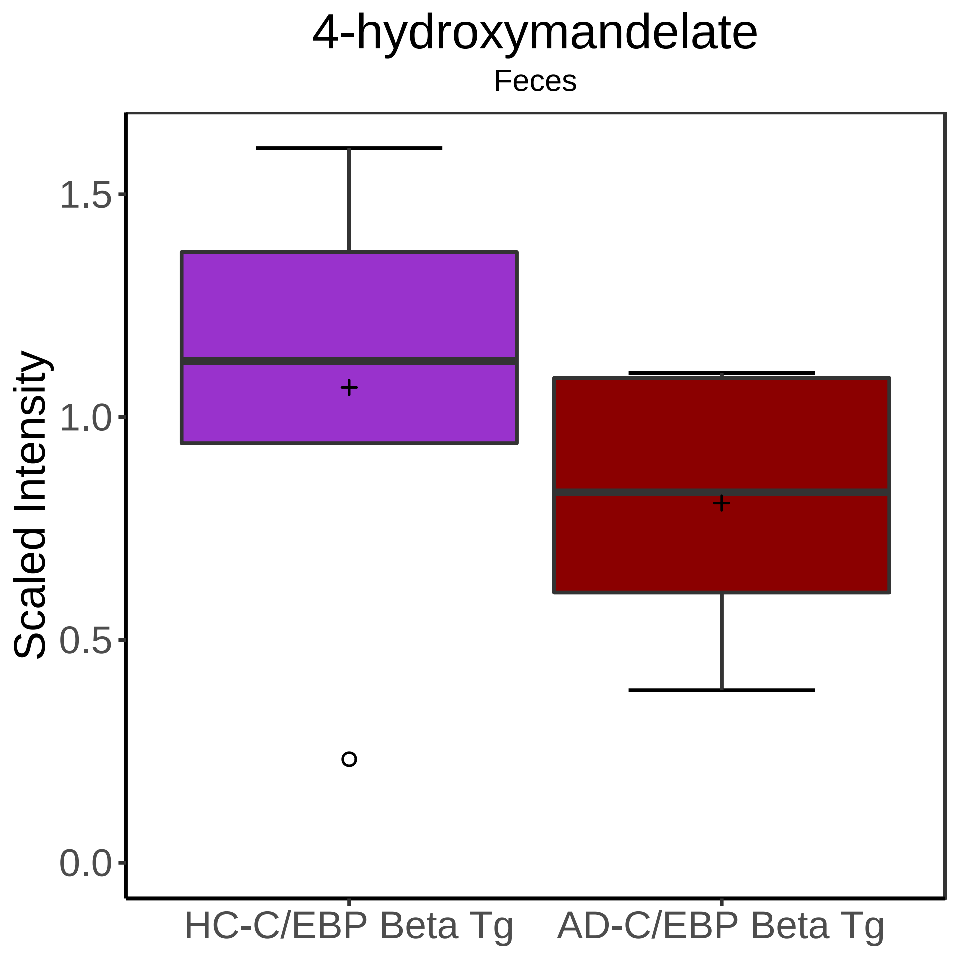

4-hydroxymandelate
Feces
Scaled Intensity
1.5
1.0
0.5
0.0
HC-C/EBP Beta Tg
AD-C/EBP Beta Tg

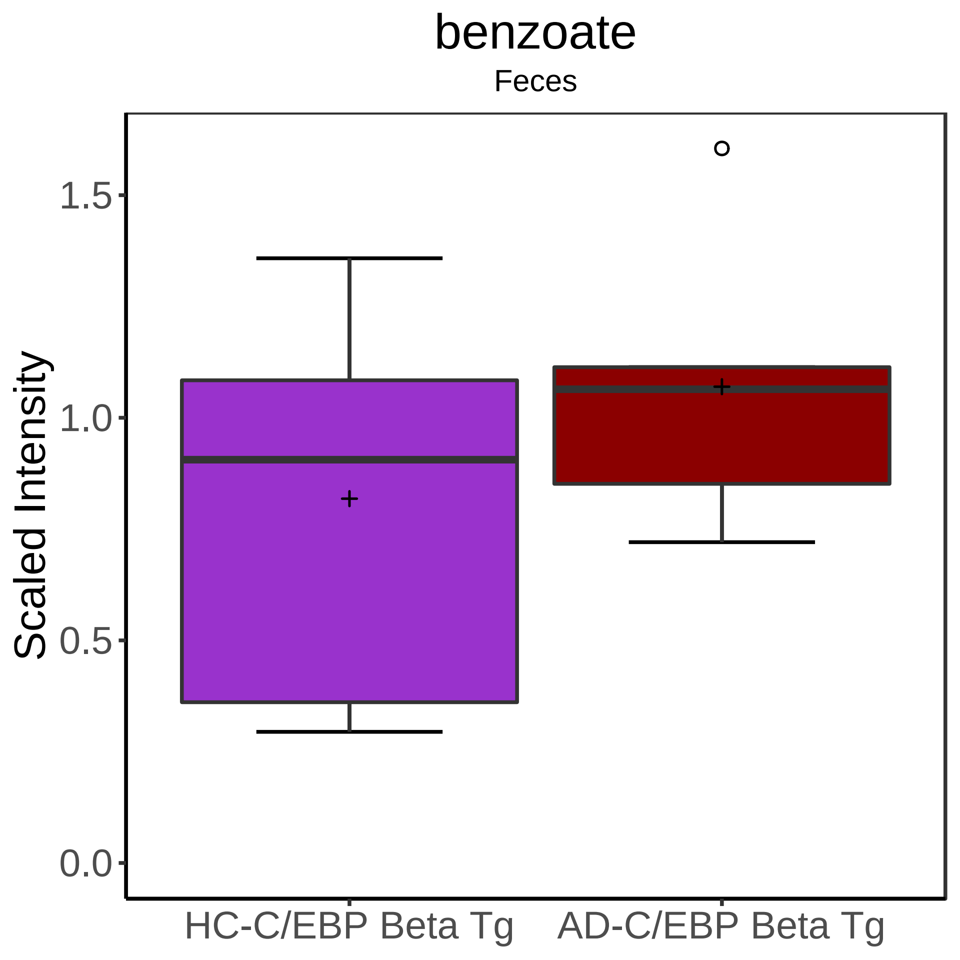
benzoate
Feces
Scaled Intensity
1.5
1.0
0.5
0.0
HC-C/EBP Beta Tg
AD-C/EBP Beta Tg

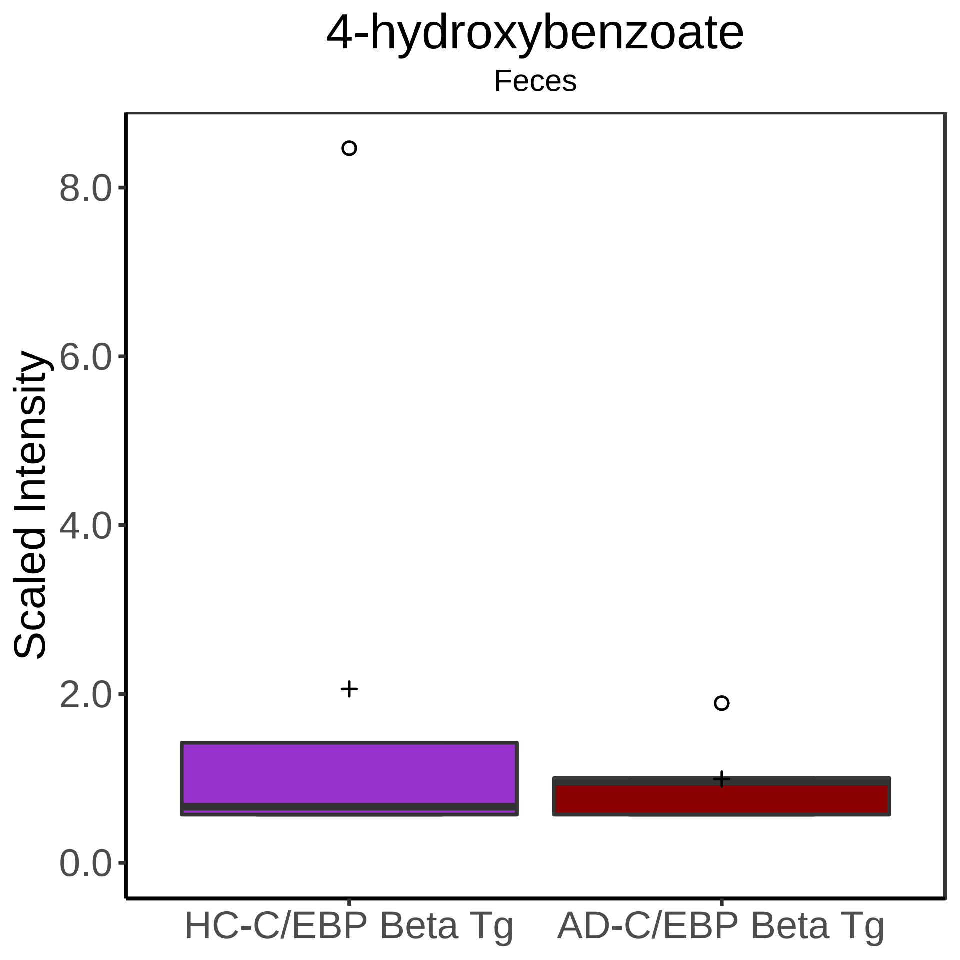

4-hydroxybenzoate
Feces
Scaled Intensity
8.0
6.0
4.0
2.0
0.0
HC-C/EBP Beta Tg
AD-C/EBP Beta Tg

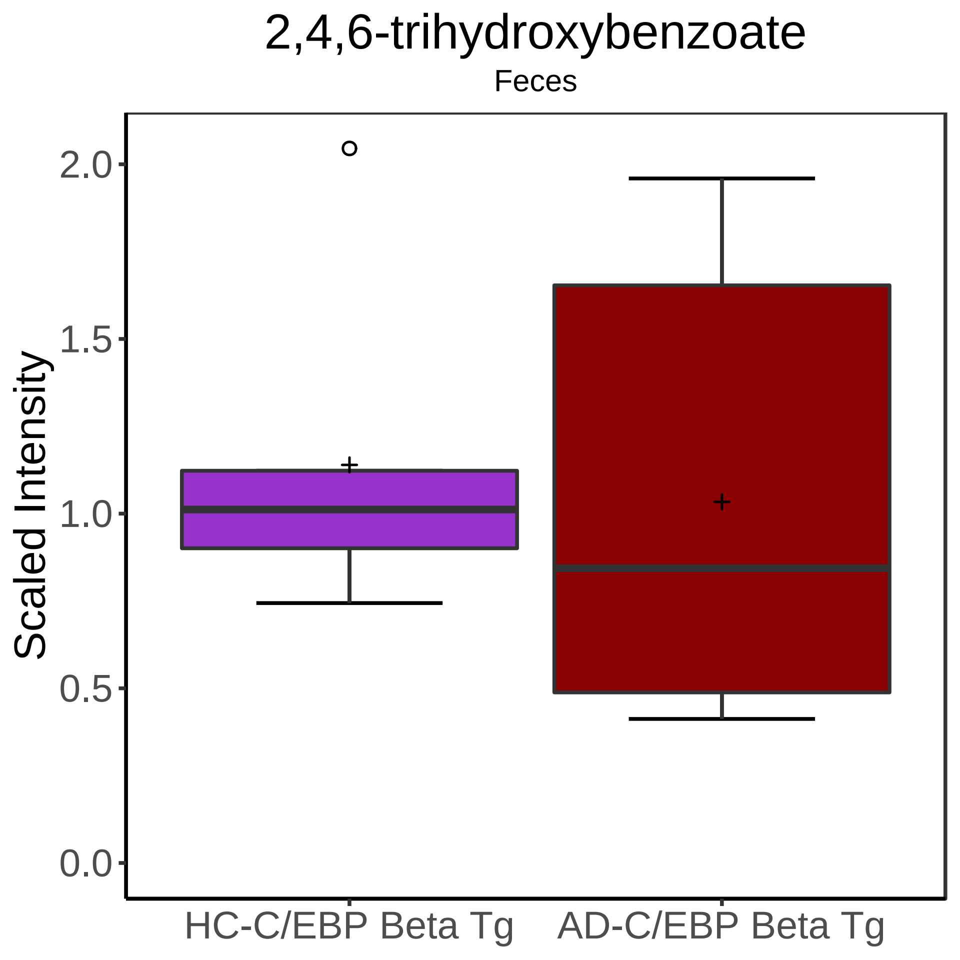
2,4,6-trihydroxybenzoate
Feces
Scaled Intensity
2.0
1.5
1.0
0.5
0.0
HC-C/EBP Beta Tg
AD-C/EBP Beta Tg

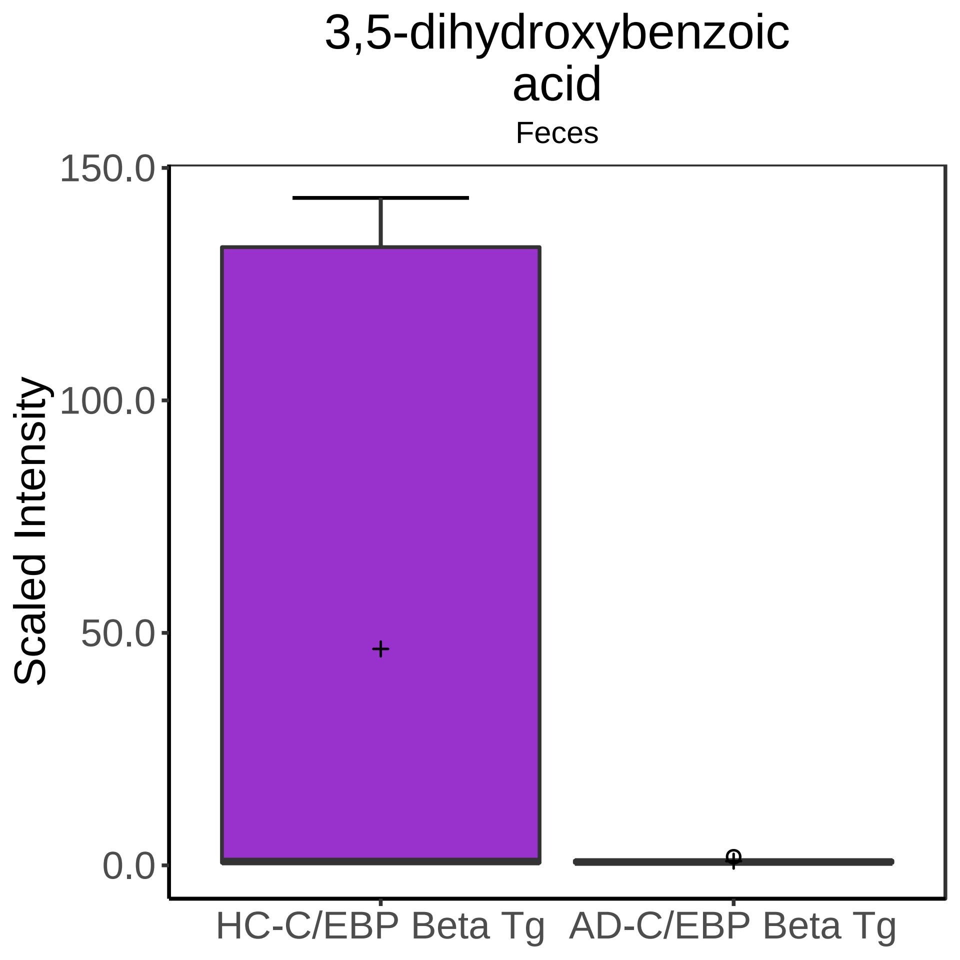
3,5-dihydroxybenzoic acid
Feces
Scaled Intensity
150.0
100.0
50.0
0.0
HC-C/EBP Beta Tg
AD-C/EBP Beta Tg

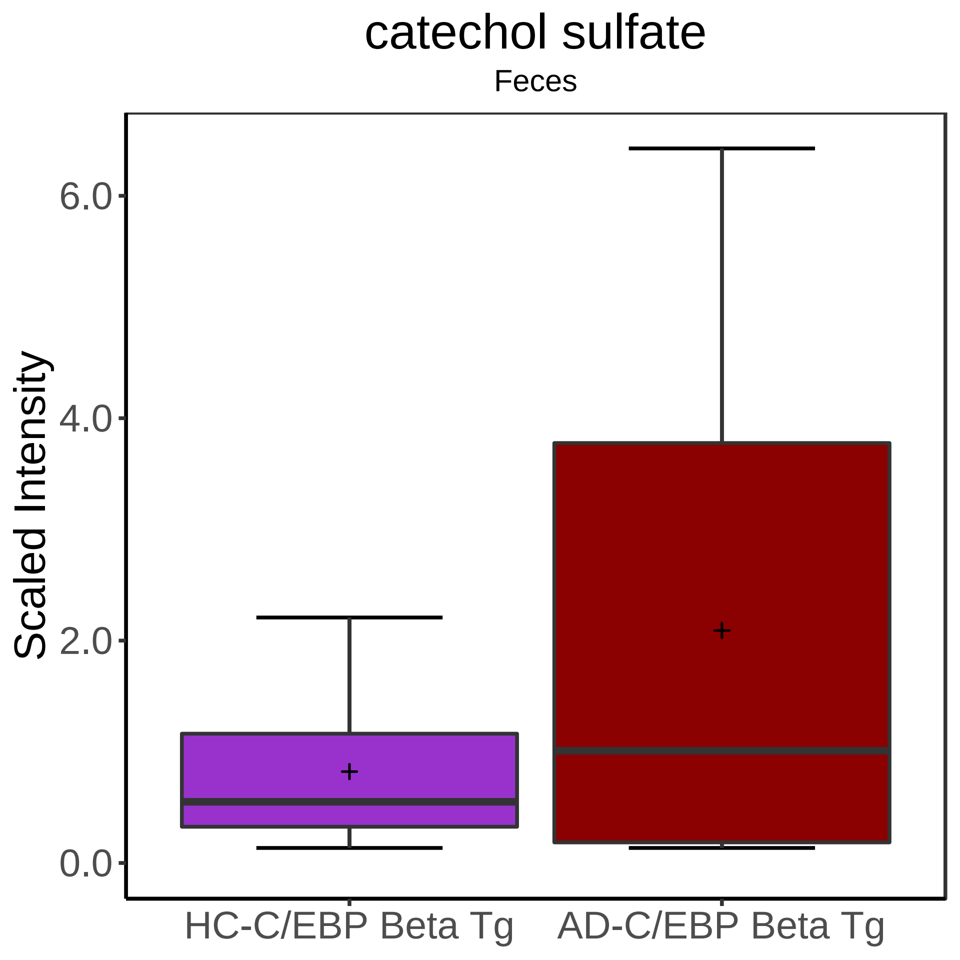

catechol sulfate
Feces
Scaled Intensity
6.0
4.0
2.0
0.0
HC-C/EBP Beta Tg
AD-C/EBP Beta Tg

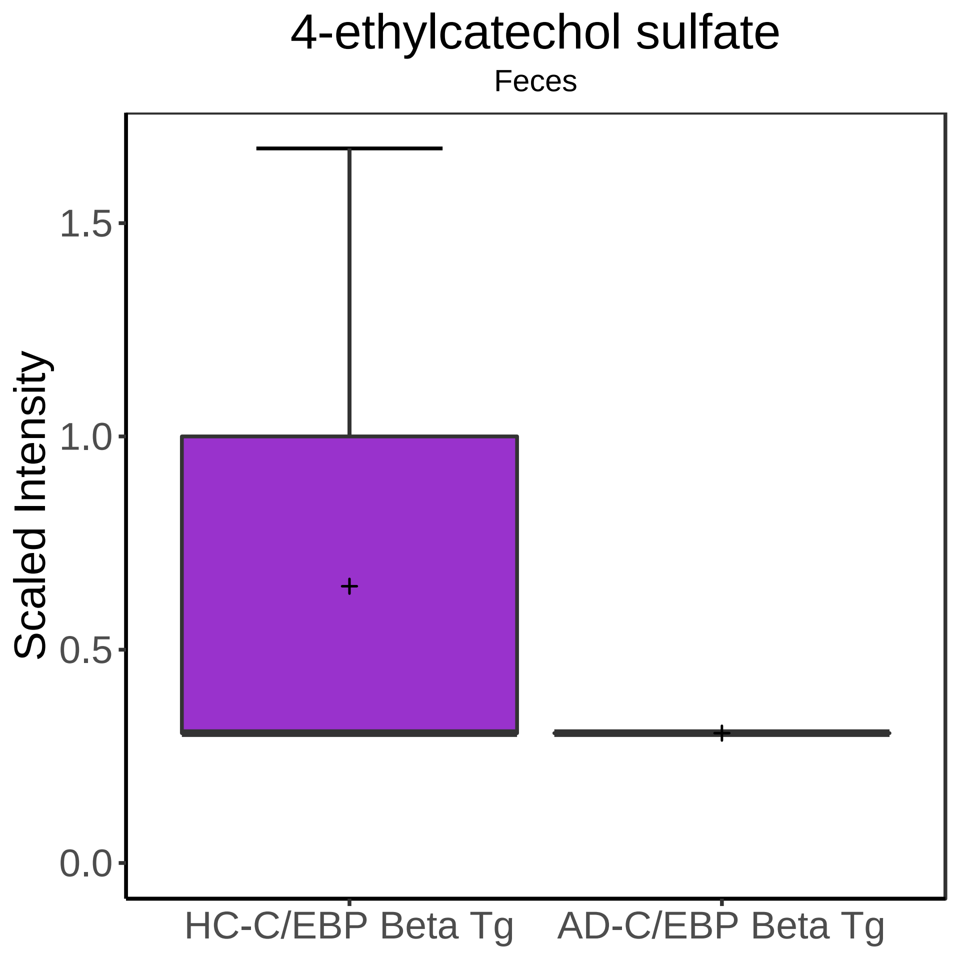
4-ethylcatechol sulfate
Feces
Scaled Intensity
1.5
1.0
0.5
0.0
HC-C/EBP Beta Tg
AD-C/EBP Beta Tg

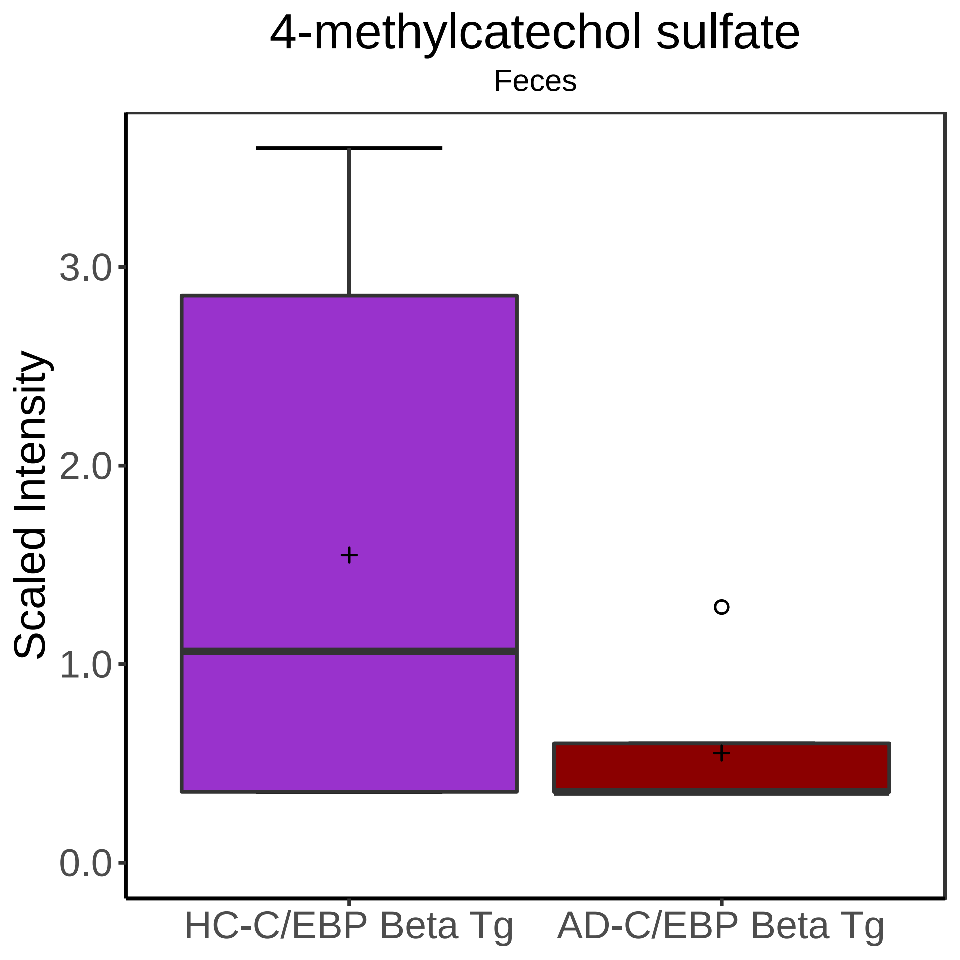

4-methylcatechol sulfate
Feces
Scaled Intensity
3.0
2.0
1.0
0.0
HC-C/EBP Beta Tg
AD-C/EBP Beta Tg

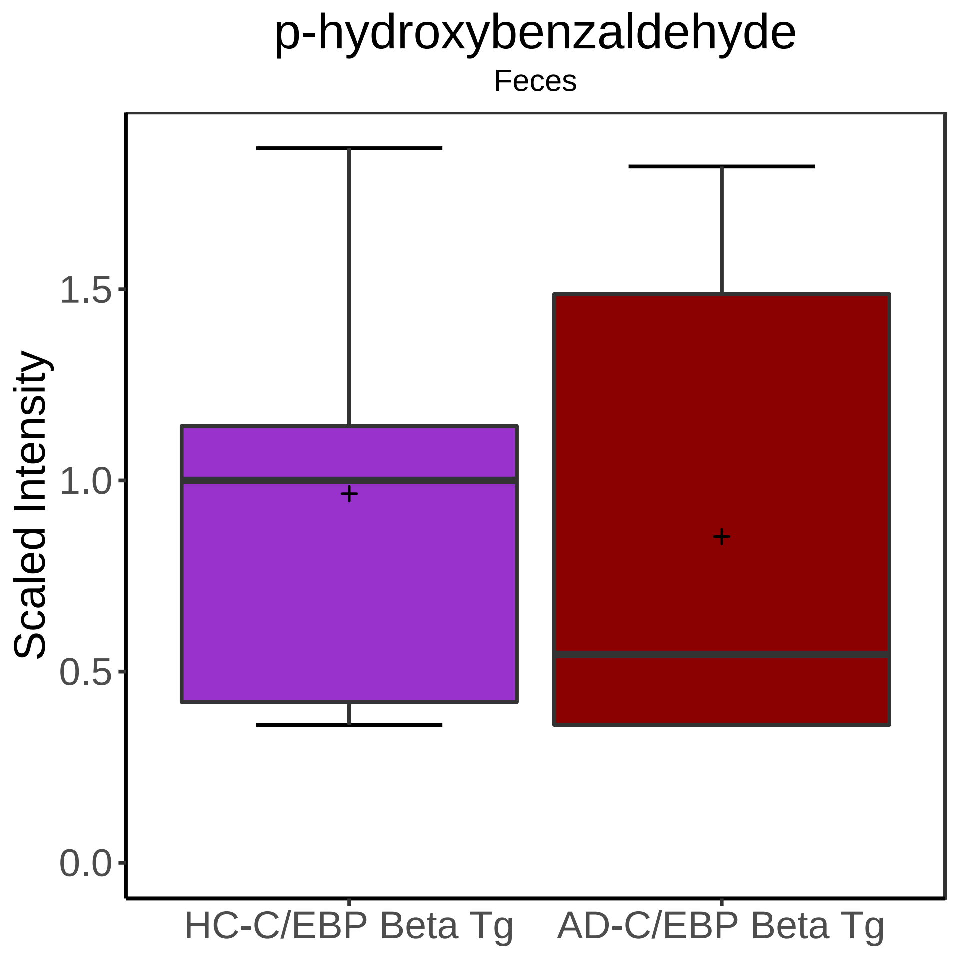
p-hydroxybenzaldehyde
Feces
Scaled Intensity
1.5
1.0
0.5
0.0
HC-C/EBP Beta Tg
AD-C/EBP Beta Tg

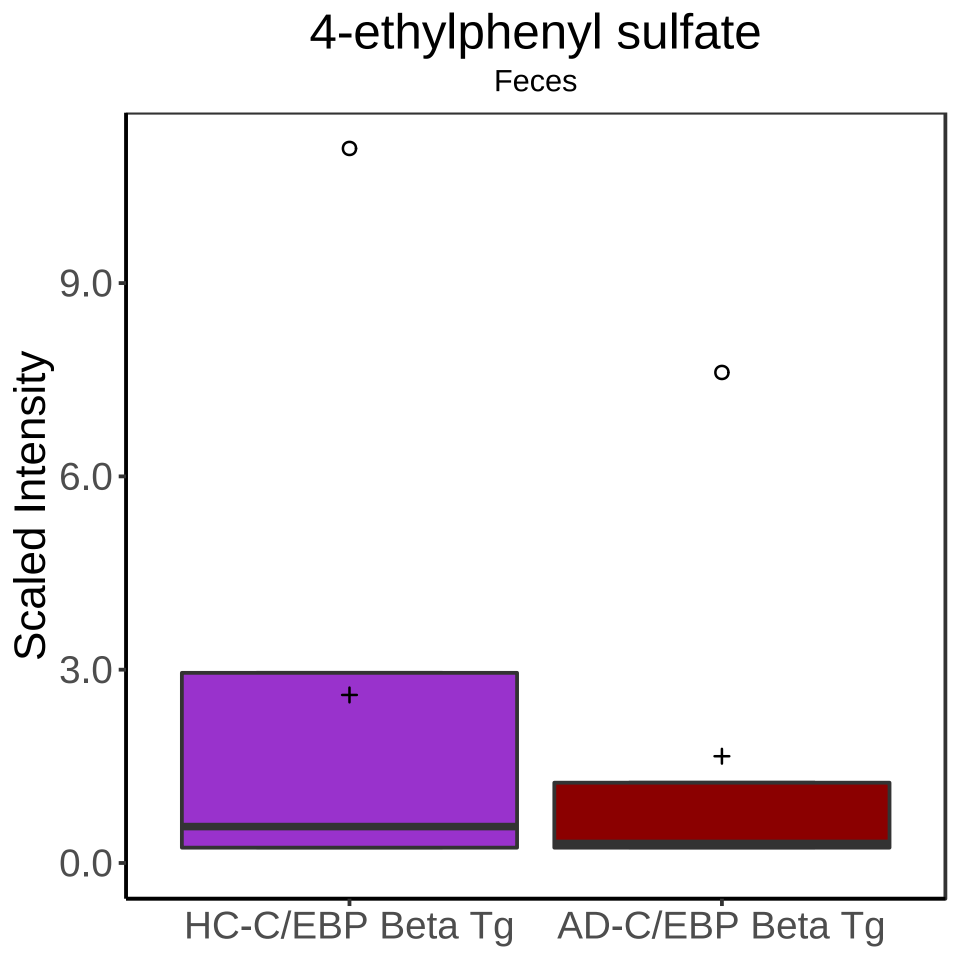

4-ethylphenyl sulfate
Feces
Scaled Intensity
9.0
6.0
3.0
0.0
HC-C/EBP Beta Tg
AD-C/EBP Beta Tg

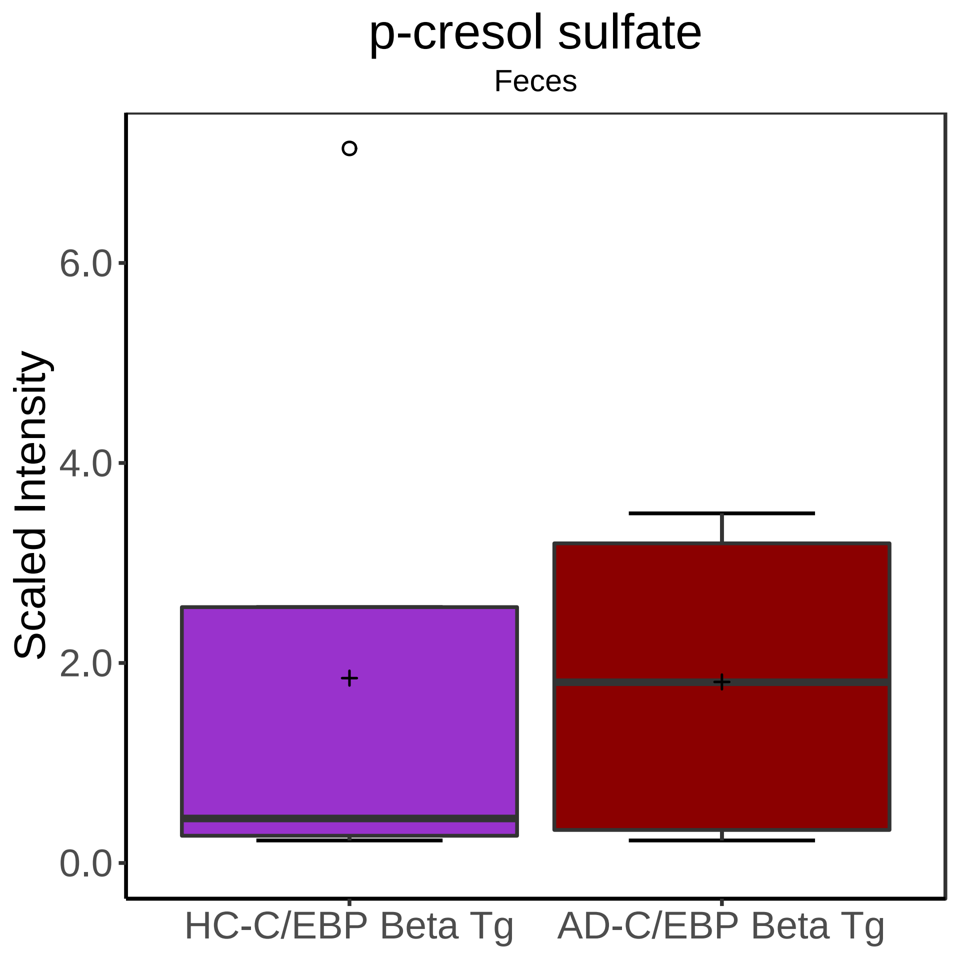
p-cresol sulfate
Feces
Scaled Intensity
6.0
4.0
2.0
0.0
HC-C/EBP Beta Tg
AD-C/EBP Beta Tg

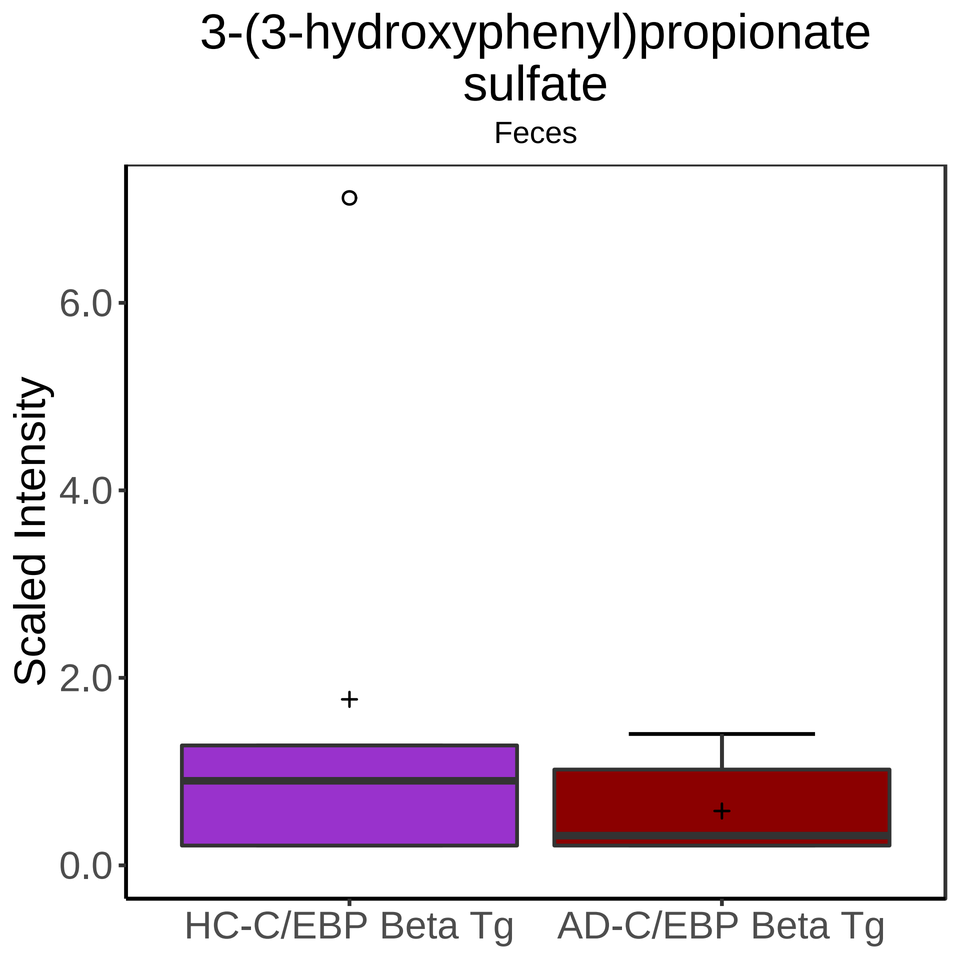
3-(3-hydroxyphenyl)propionate sulfate
Feces
Scaled Intensity
6.0
4.0
2.0
0.0
HC-C/EBP Beta Tg
AD-C/EBP Beta Tg

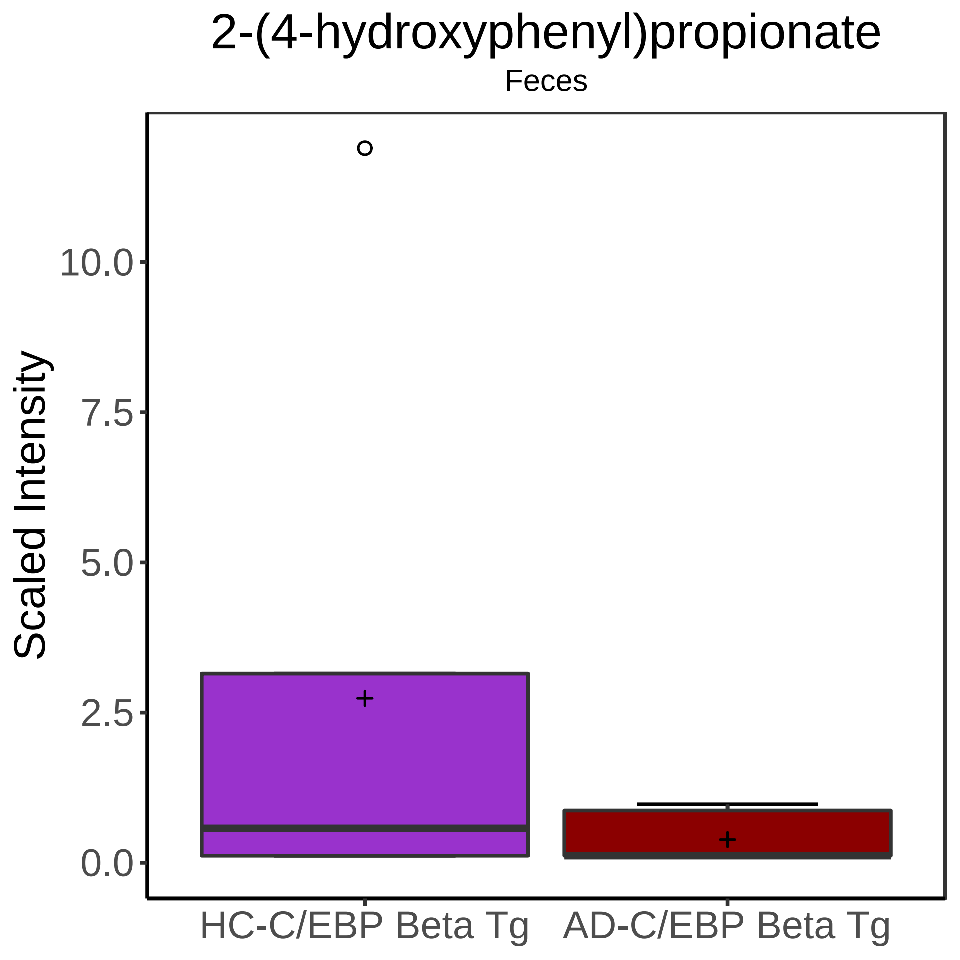
2-(4-hydroxyphenyl)propionate
Feces
Scaled Intensity
10.0
7.5
5.0
2.5
0.0
HC-C/EBP Beta Tg
AD-C/EBP Beta Tg

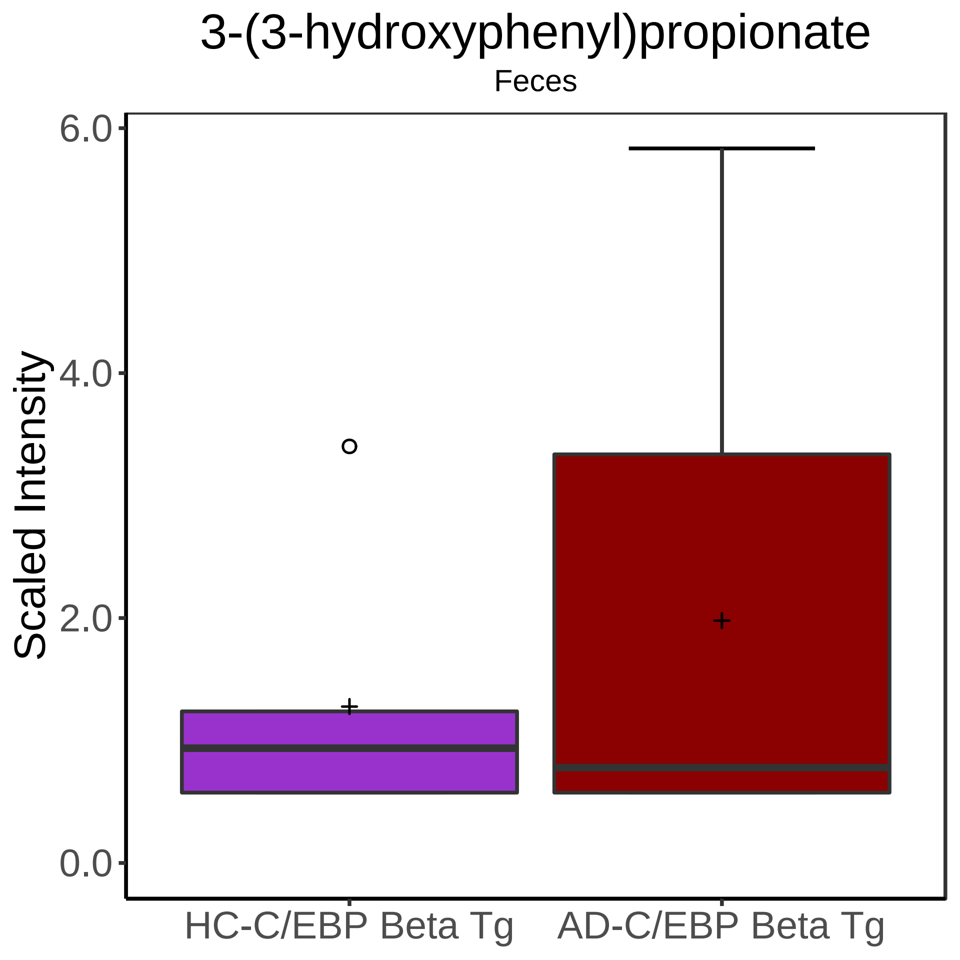

3-(3-hydroxyphenyl)propionate
Feces
Scaled Intensity
6.0
4.0
2.0
0.0
HC-C/EBP Beta Tg
AD-C/EBP Beta Tg

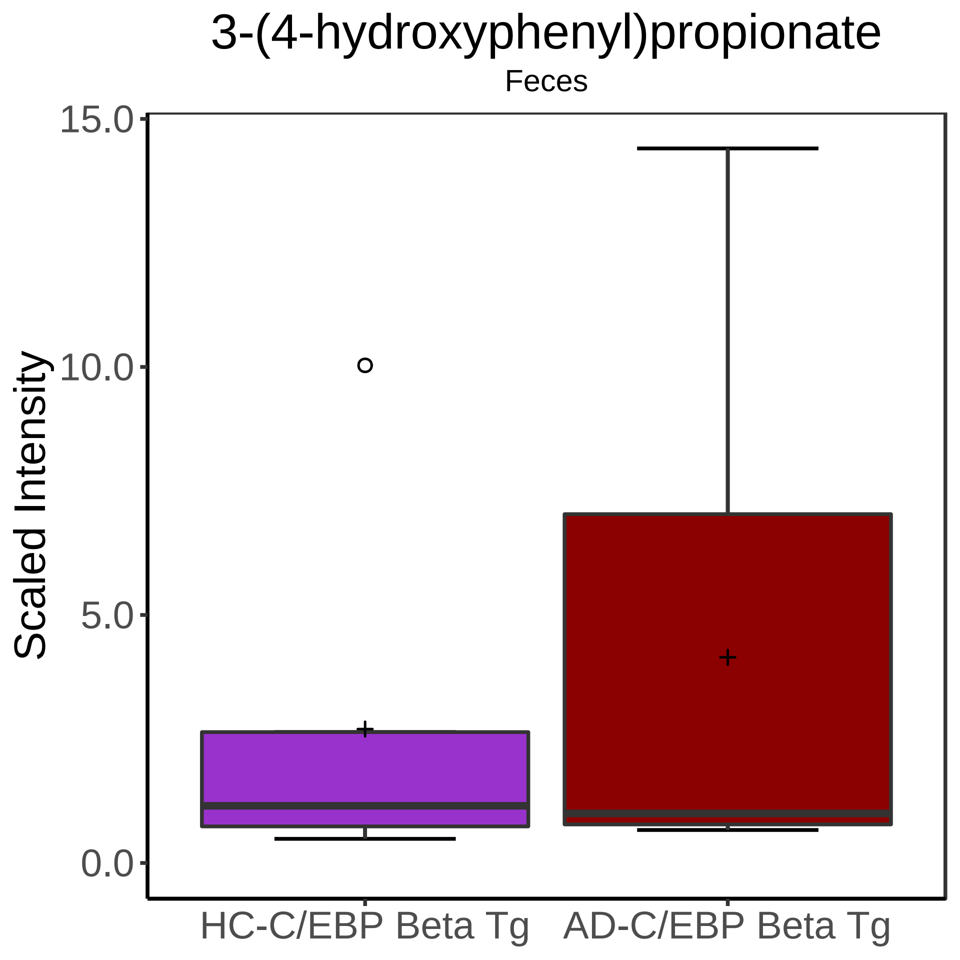
3-(4-hydroxyphenyl)propionate
Feces
Scaled Intensity
15.0
10.0
5.0
0.0
HC-C/EBP Beta Tg
AD-C/EBP Beta Tg

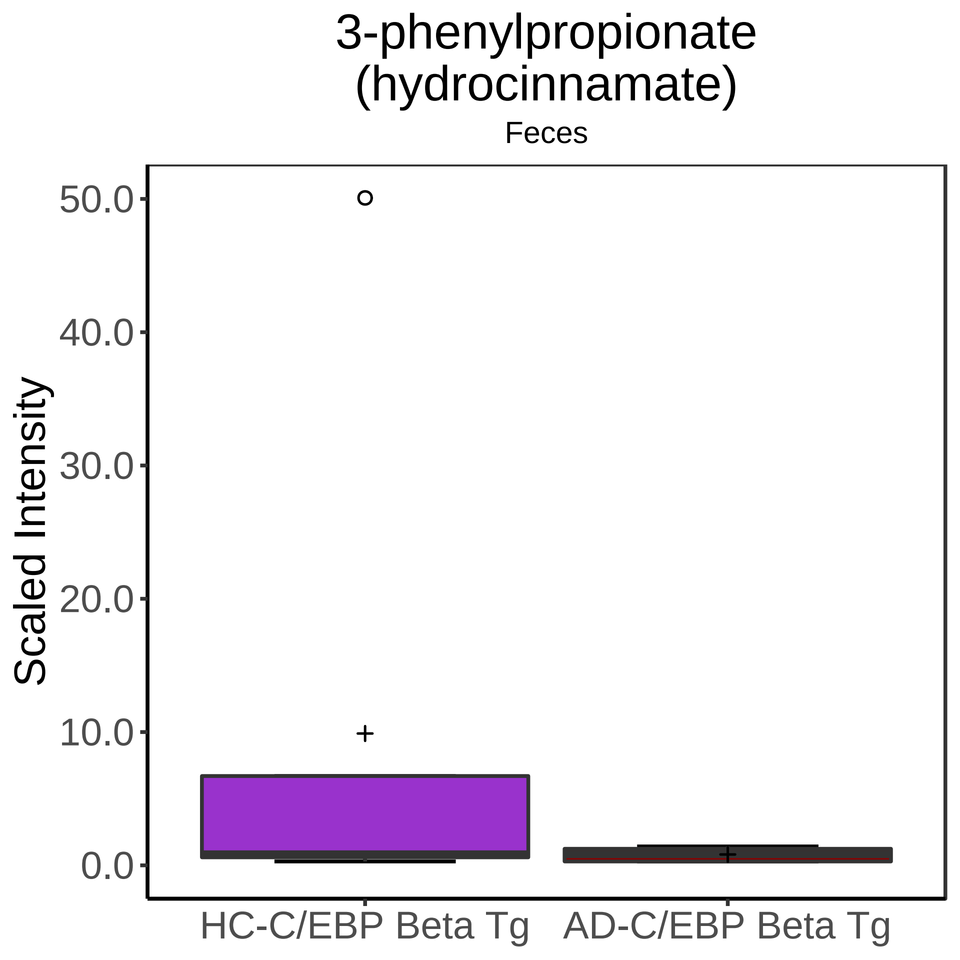
3-phenylpropionate
(hydrocinnamate)
Feces
Scaled Intensity
50.0
40.0
30.0
20.0
10.0
0.0
HC-C/EBP Beta Tg
AD-C/EBP Beta Tg

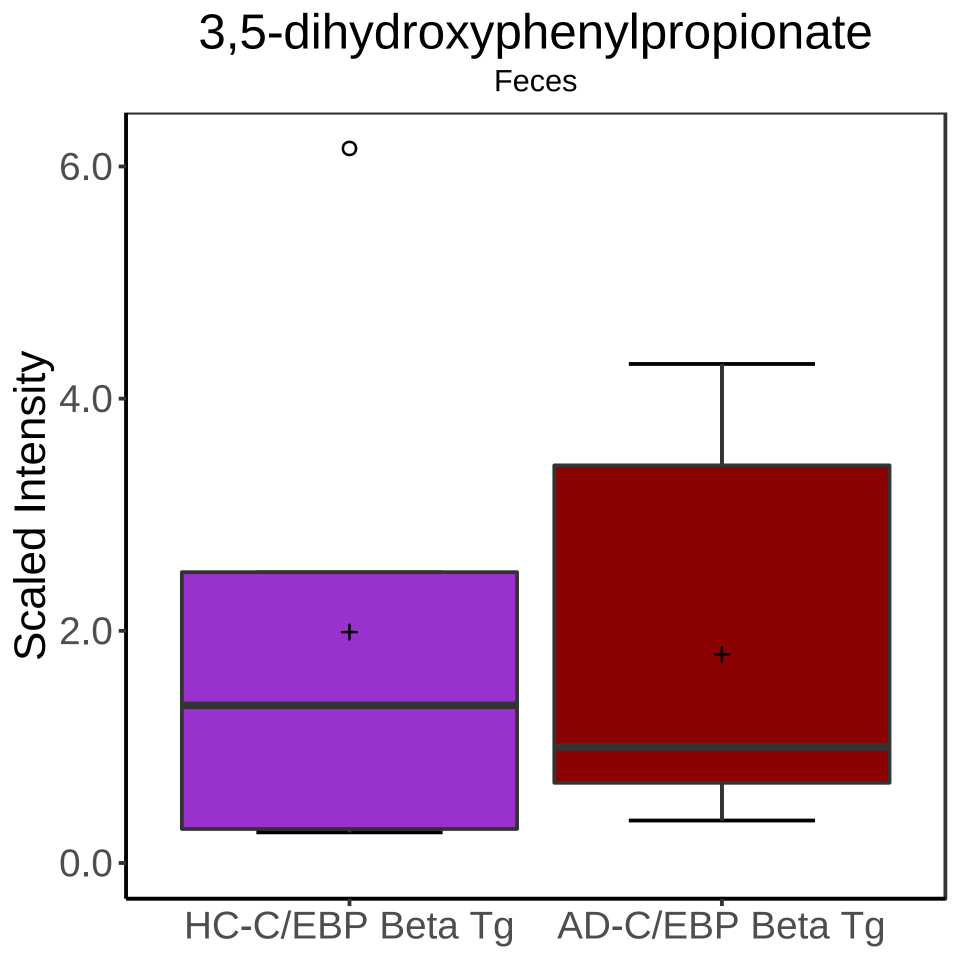
3,5-dihydroxyphenylpropionate
Feces
Scaled Intensity
6.0
4.0
2.0
0.0
HC-C/EBP Beta Tg
AD-C/EBP Beta Tg

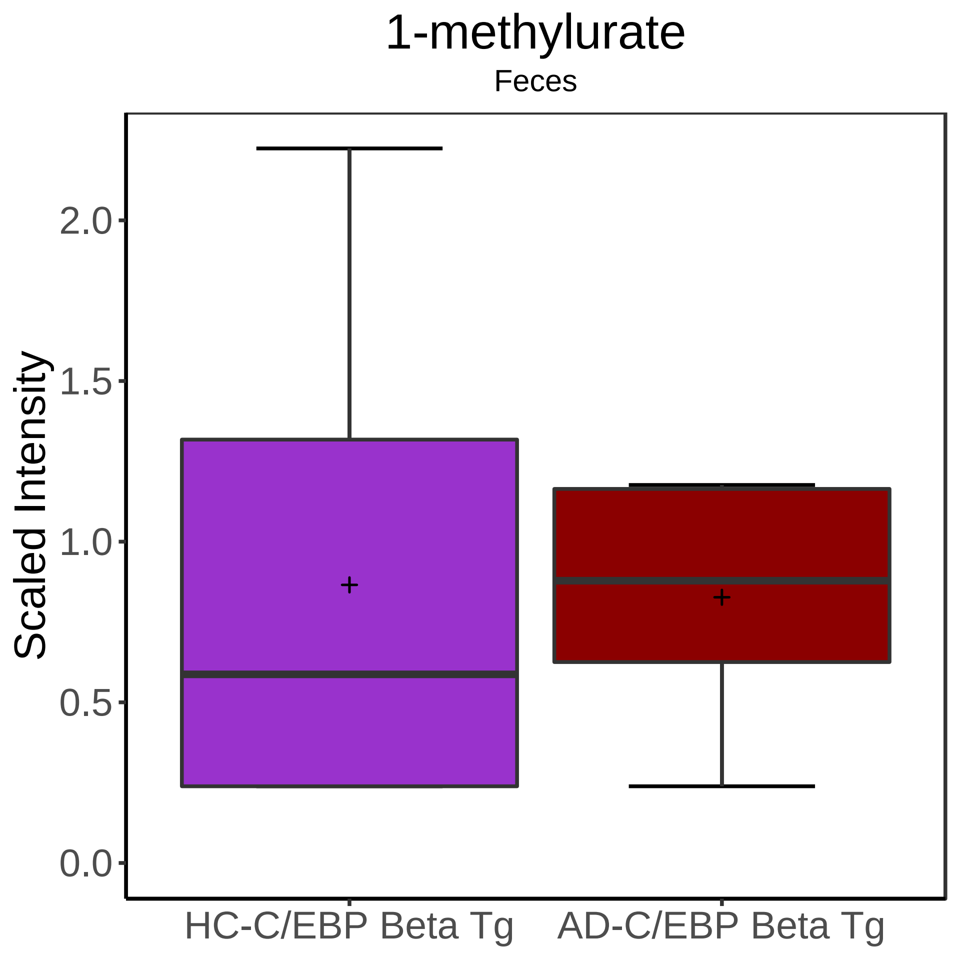
1-methylurate
Feces
Scaled Intensity
2.0
1.5
1.0
0.5
0.0
HC-C/EBP Beta Tg
AD-C/EBP Beta Tg

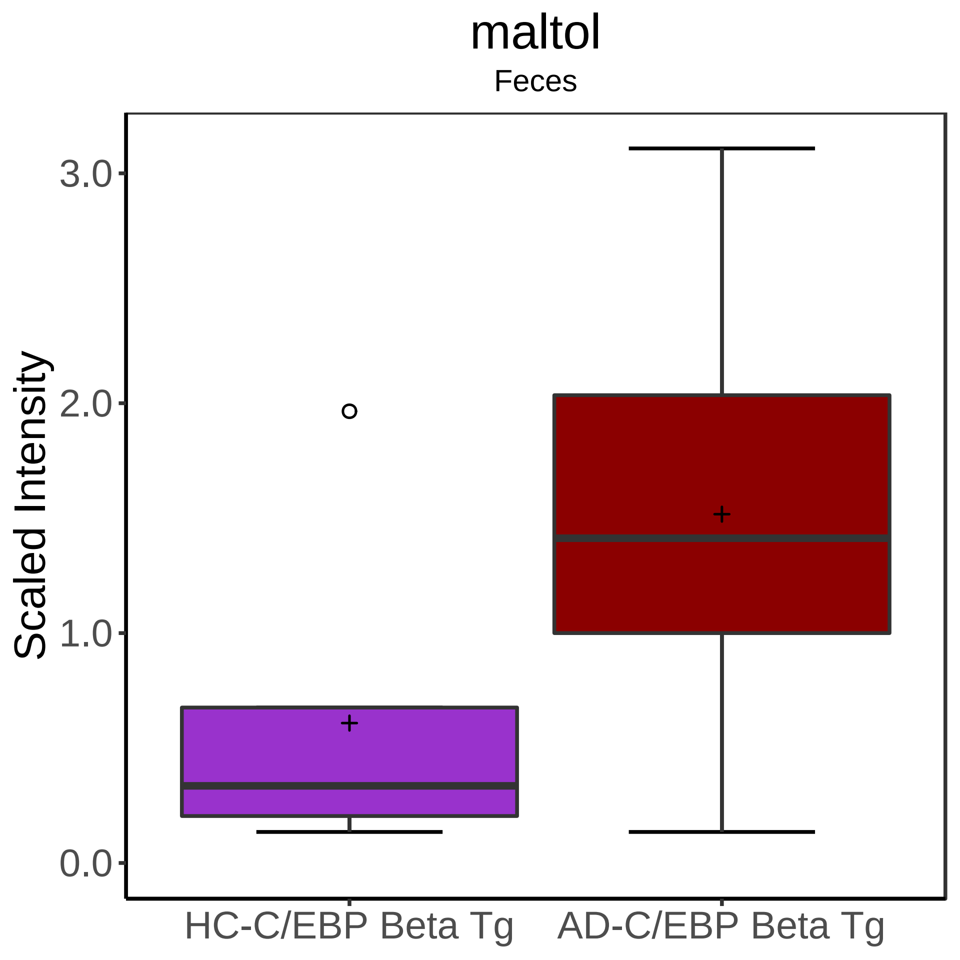

maltol
Feces
Scaled Intensity
3.0
2.0
1.0
0.0
HC-C/EBP Beta Tg
AD-C/EBP Beta Tg

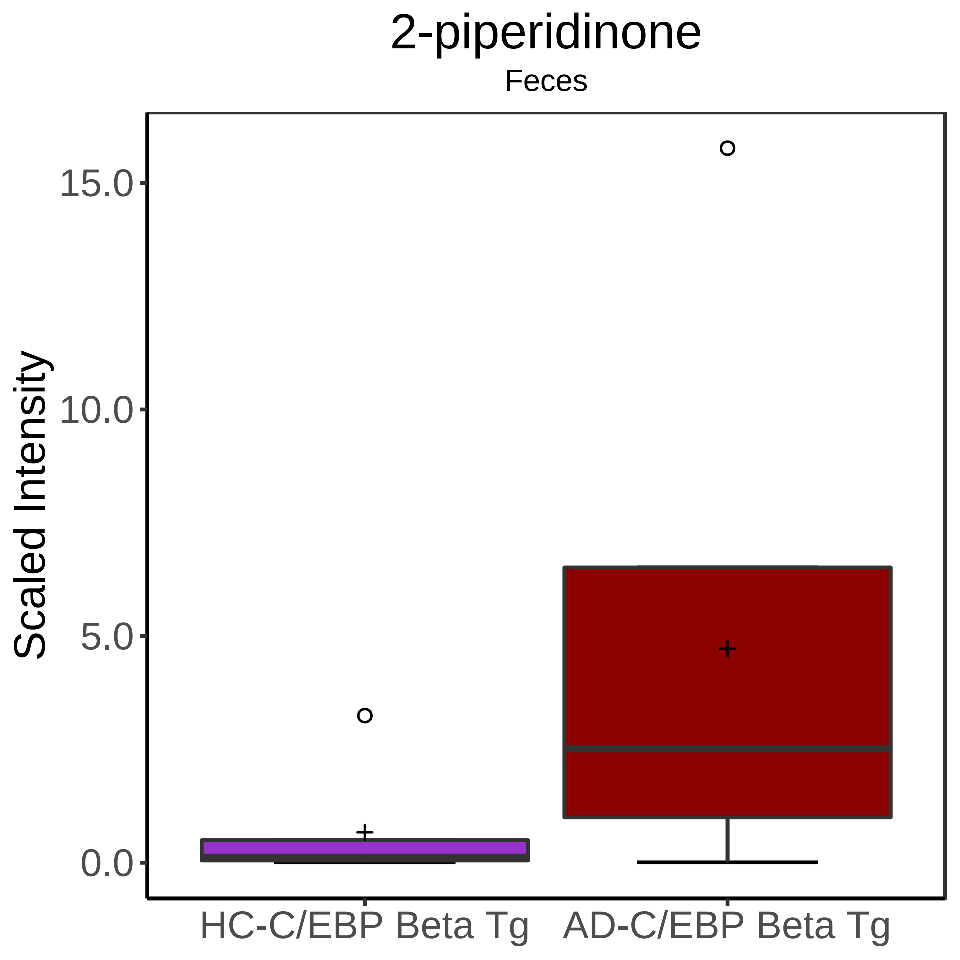
2-piperidinone
Feces
Scaled Intensity
15.0
10.0
5.0
0.0
HC-C/EBP Beta Tg
AD-C/EBP Beta Tg

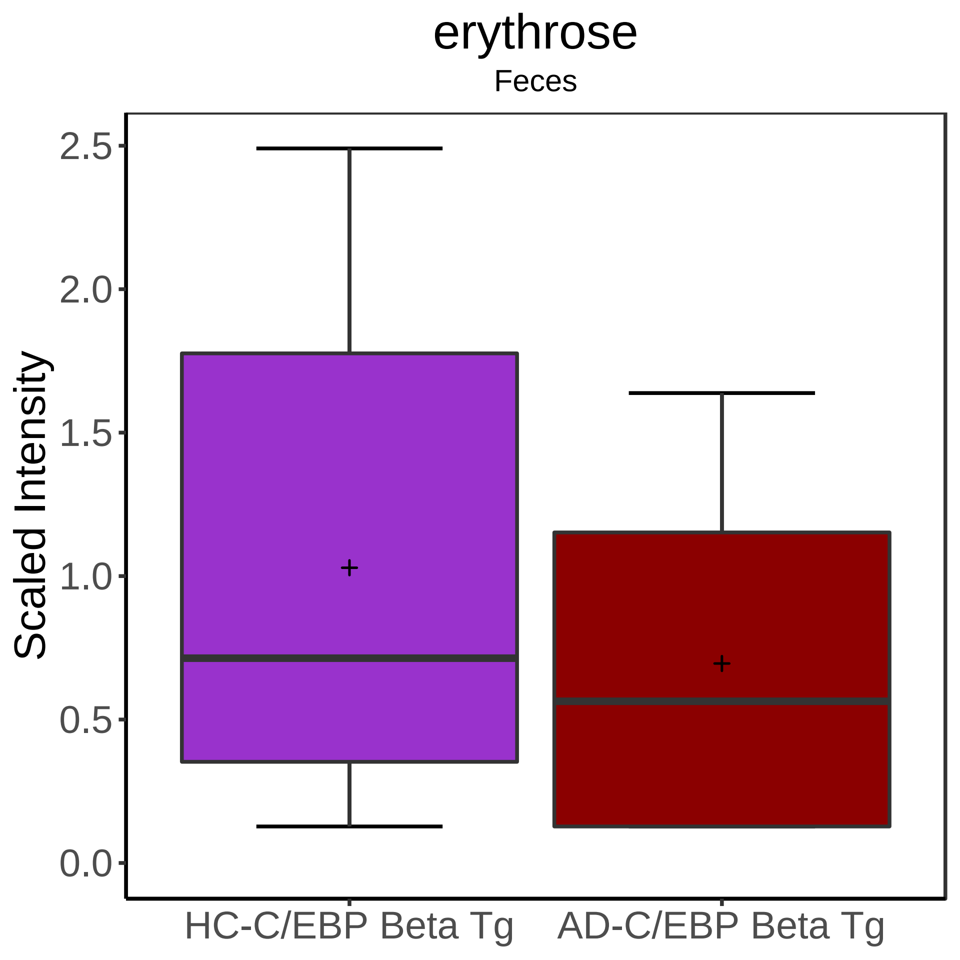
erythrose
Feces
Scaled Intensity
2.5
2.0
1.5
1.0
0.5
0.0
HC-C/EBP Beta Tg
AD-C/EBP Beta Tg

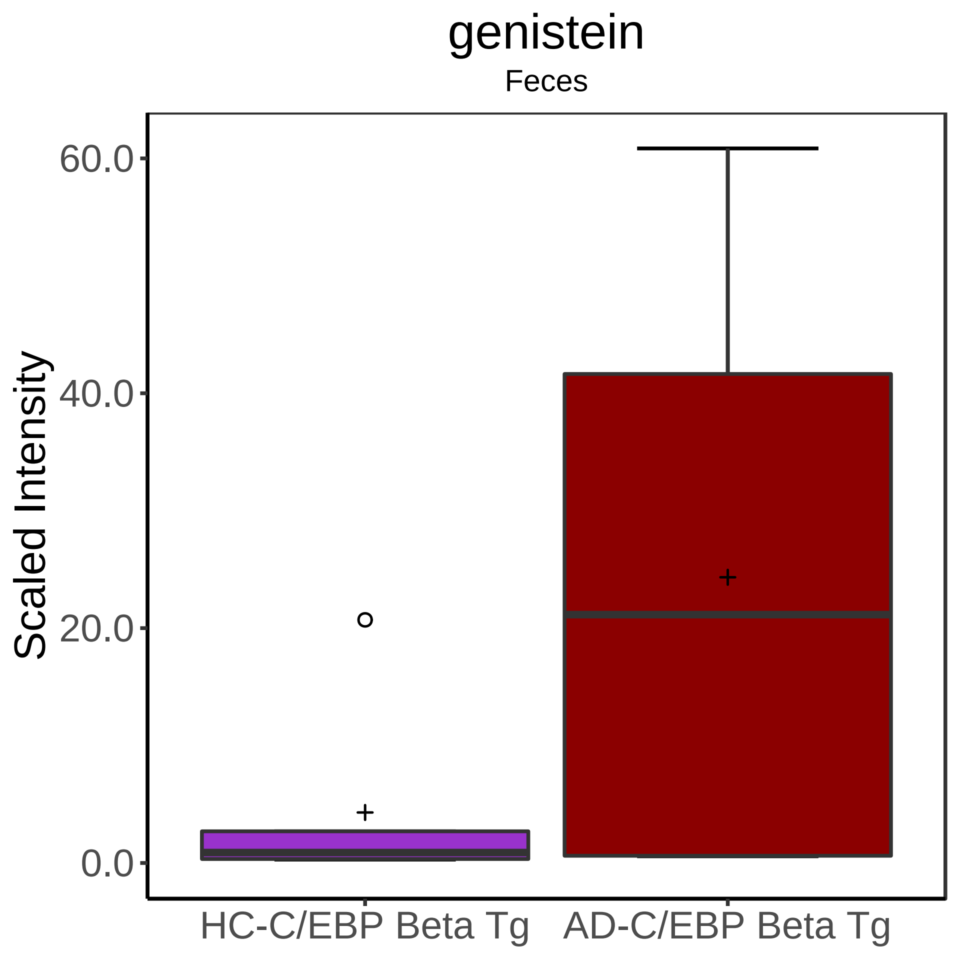
genistein
Feces
Scaled Intensity
60.0
40.0
20.0
0.0
HC-C/EBP Beta Tg
AD-C/EBP Beta Tg

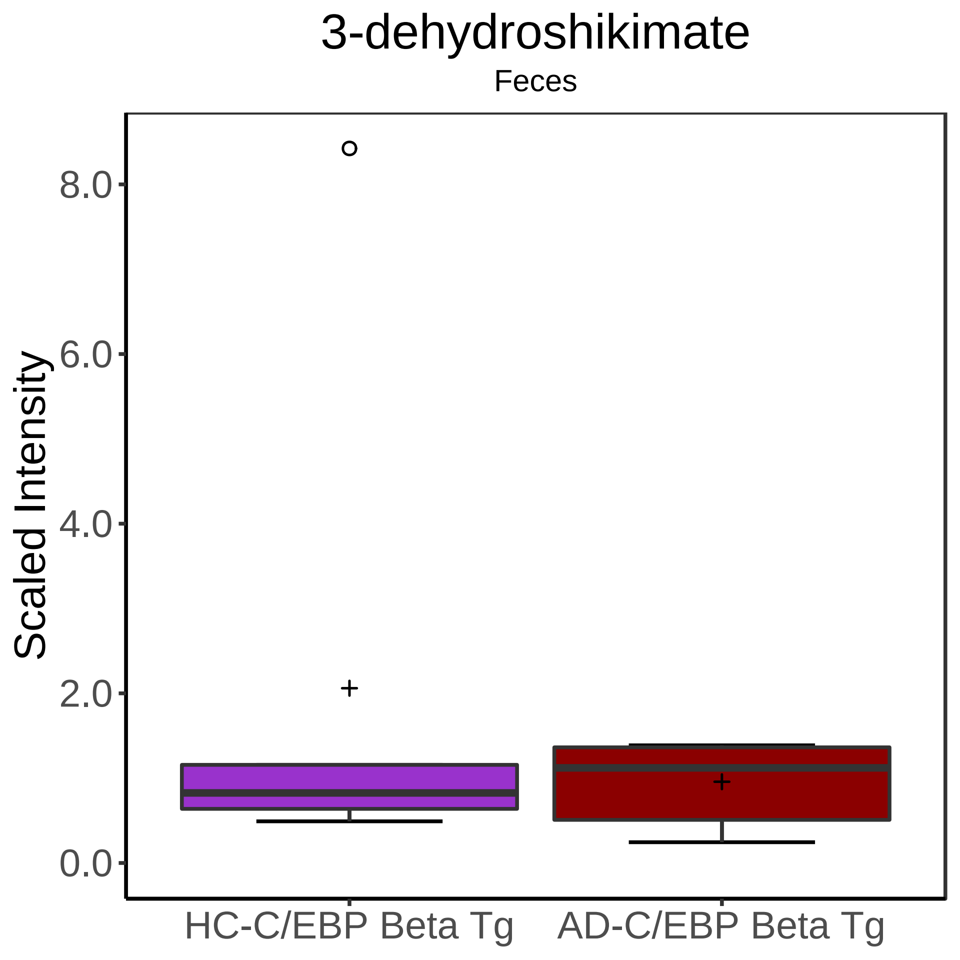
3-dehydroshikimate
Feces
Scaled Intensity
8.0
6.0
4.0
2.0
0.0
HC-C/EBP Beta Tg
AD-C/EBP Beta Tg

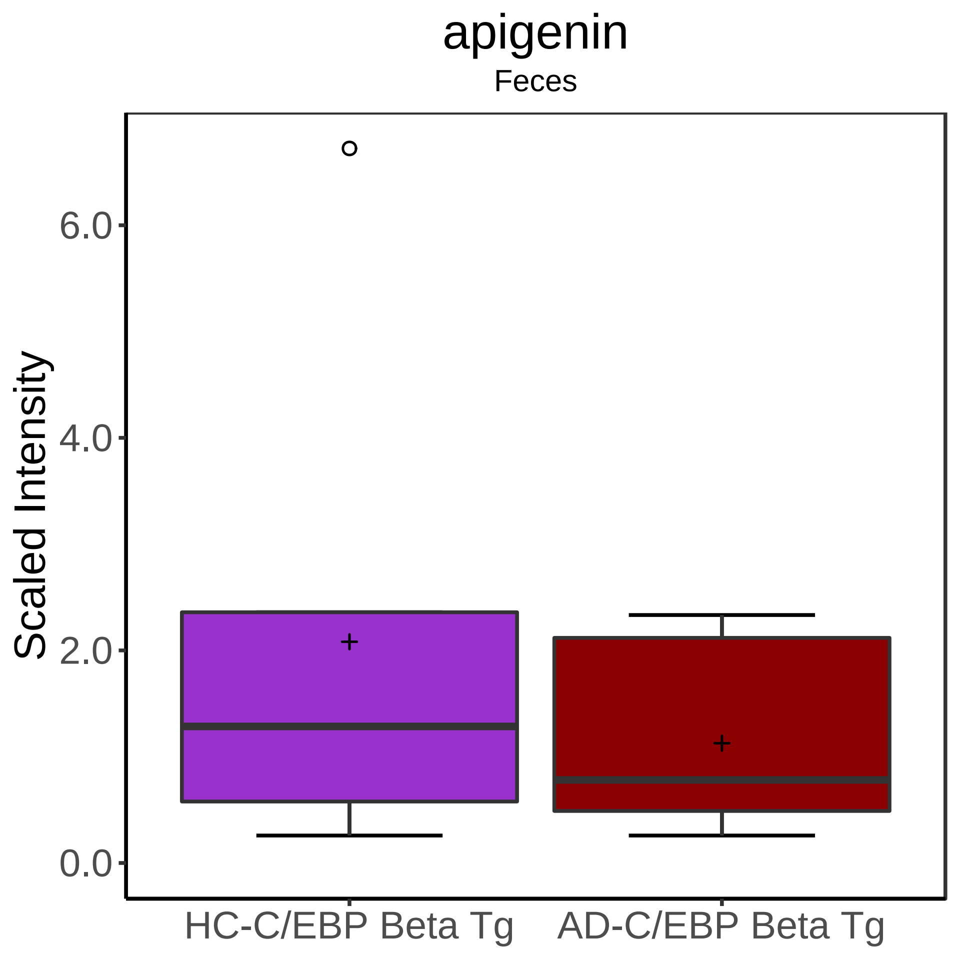

apigenin
Feces
Scaled Intensity
6.0
4.0
2.0
0.0
HC-C/EBP Beta Tg
AD-C/EBP Beta Tg

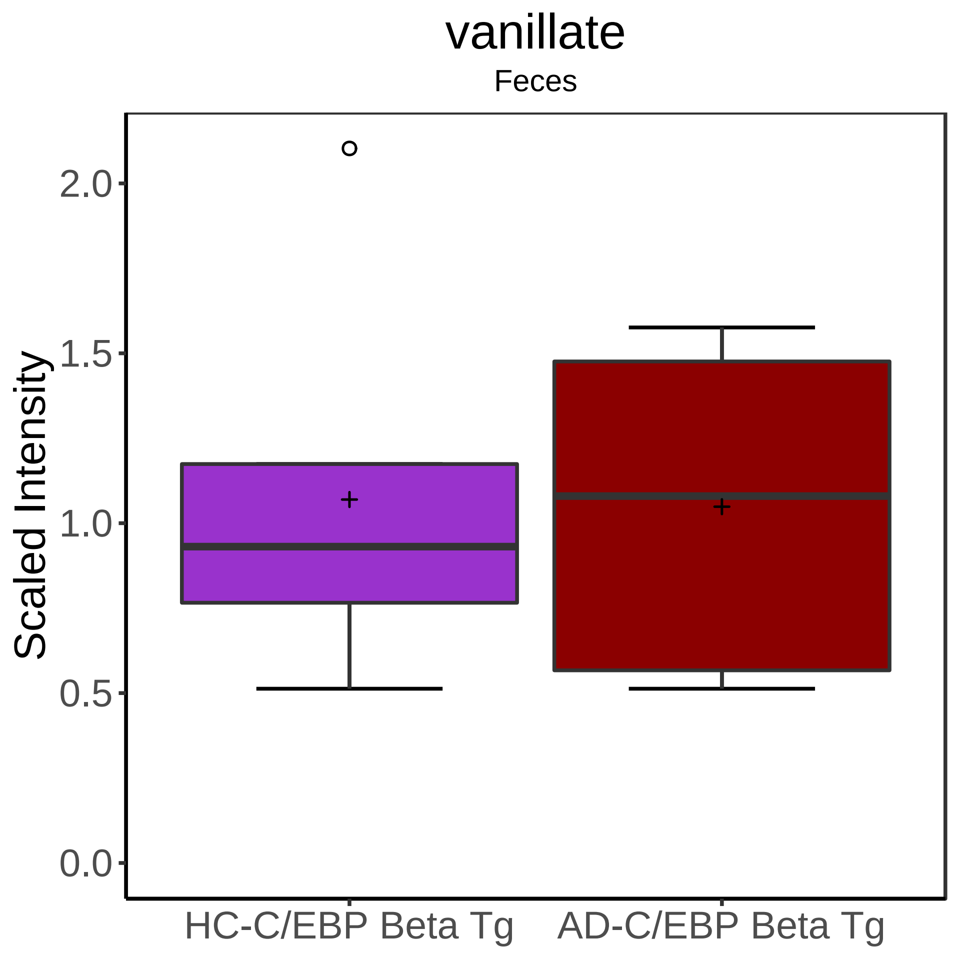
vanillate
Feces
Scaled Intensity
2.0
1.5
1.0
0.5
0.0
HC-C/EBP Beta Tg
AD-C/EBP Beta Tg

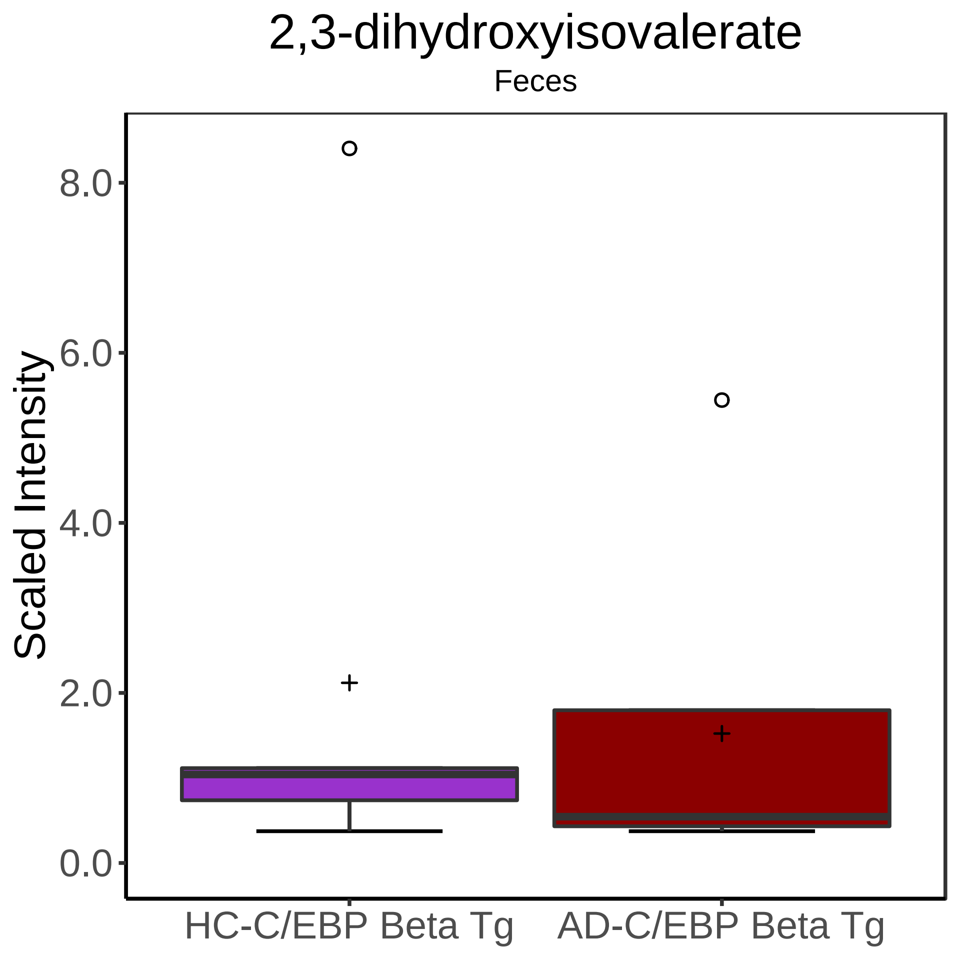

2,3-dihydroxyisovalerate
Feces
Scaled Intensity
8.0
6.0
4.0
2.0
0.0
HC-C/EBP Beta Tg
AD-C/EBP Beta Tg

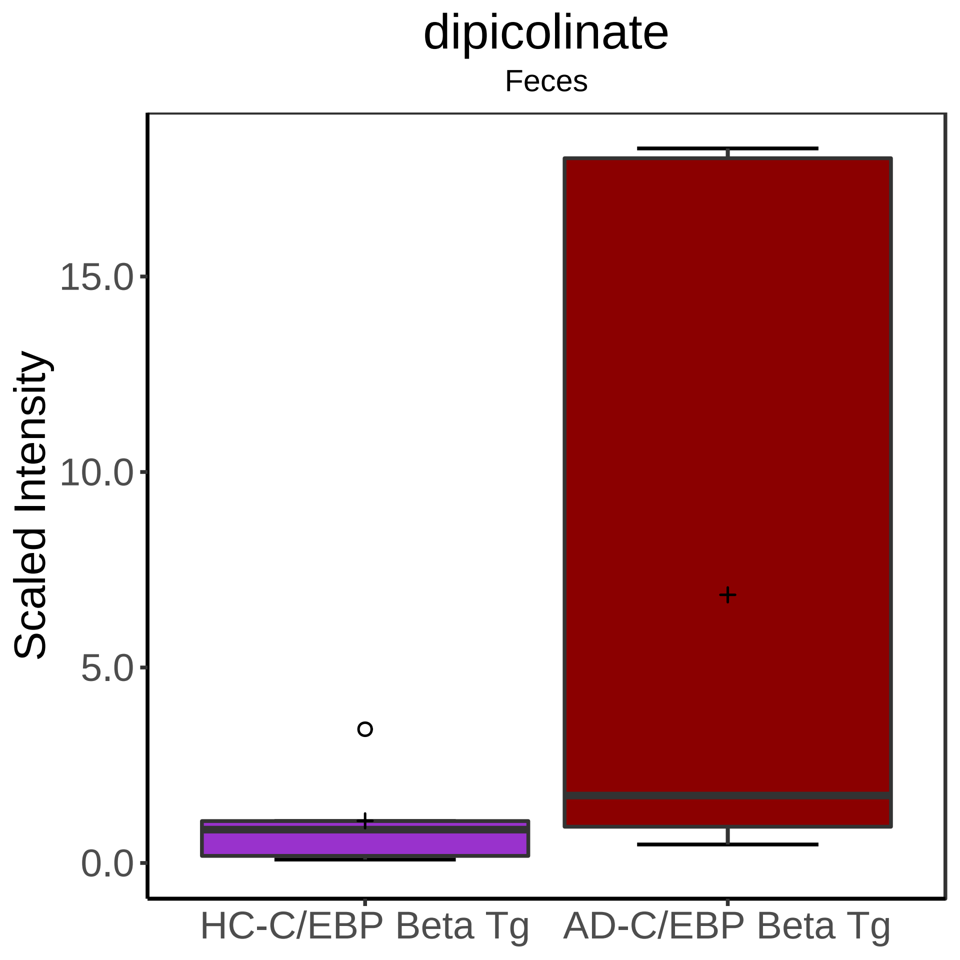

dipicolinate
Feces
Scaled Intensity
15.0
10.0
5.0
0.0
HC-C/EBP Beta Tg
AD-C/EBP Beta Tg

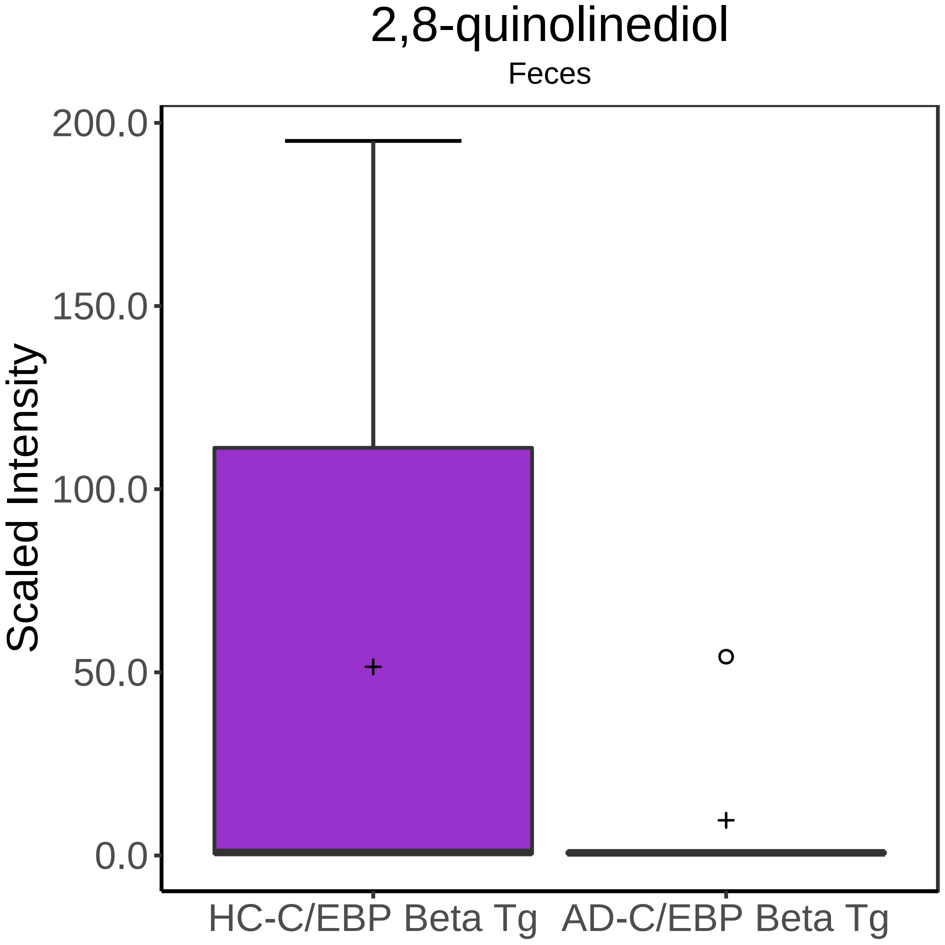

2,8-quinolinediol
Feces
Scaled Intensity
200.0
150.0
100.0
50.0
0.0
HC-C/EBP Beta Tg
AD-C/EBP Beta Tg

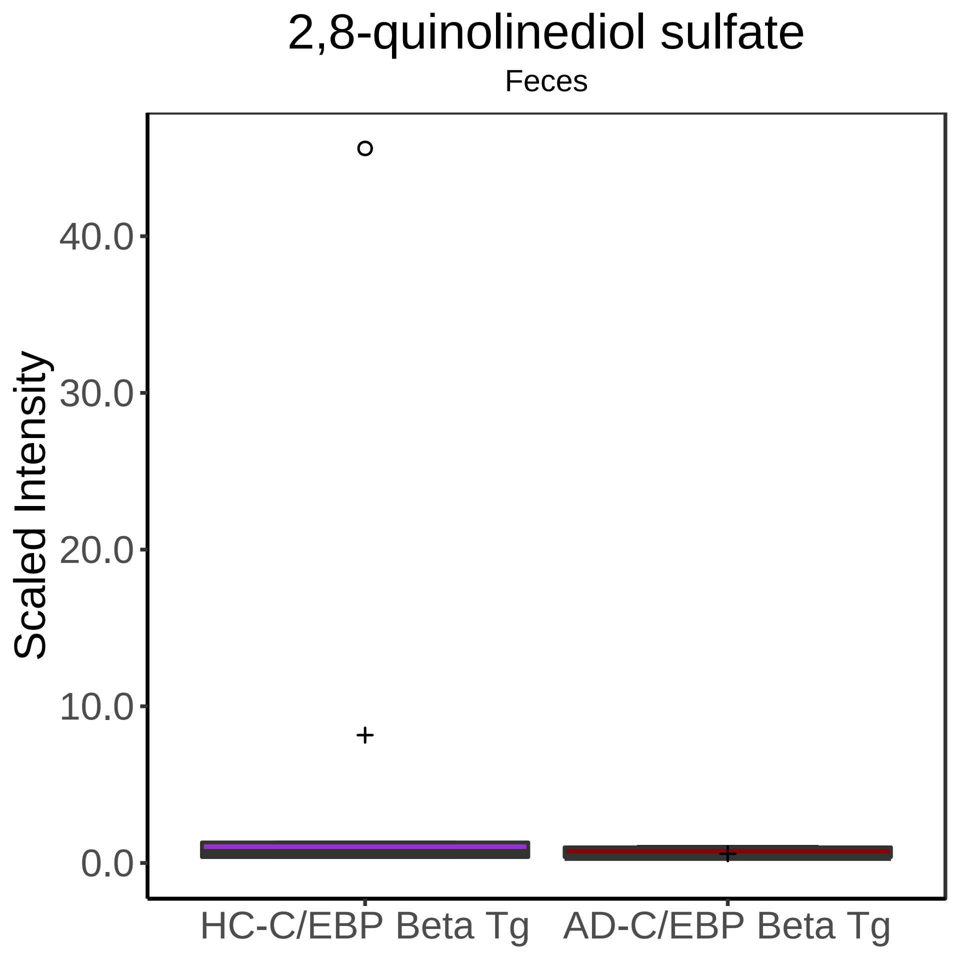
2,8-quinolinediol sulfate
Feces
Scaled Intensity
40.0
30.0
20.0
10.0
0.0
HC-C/EBP Beta Tg
AD-C/EBP Beta Tg

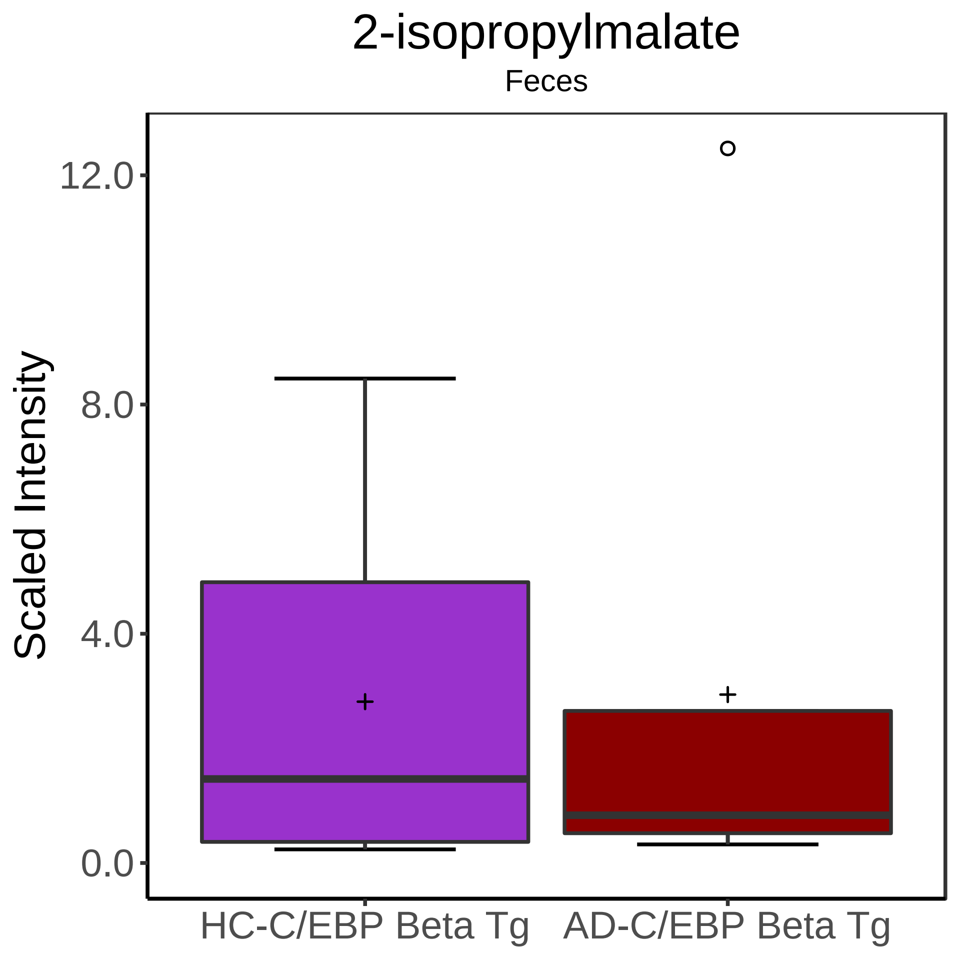

2-isopropylmalate
Feces
Scaled Intensity
12.0
8.0
4.0
0.0
HC-C/EBP Beta Tg
AD-C/EBP Beta Tg

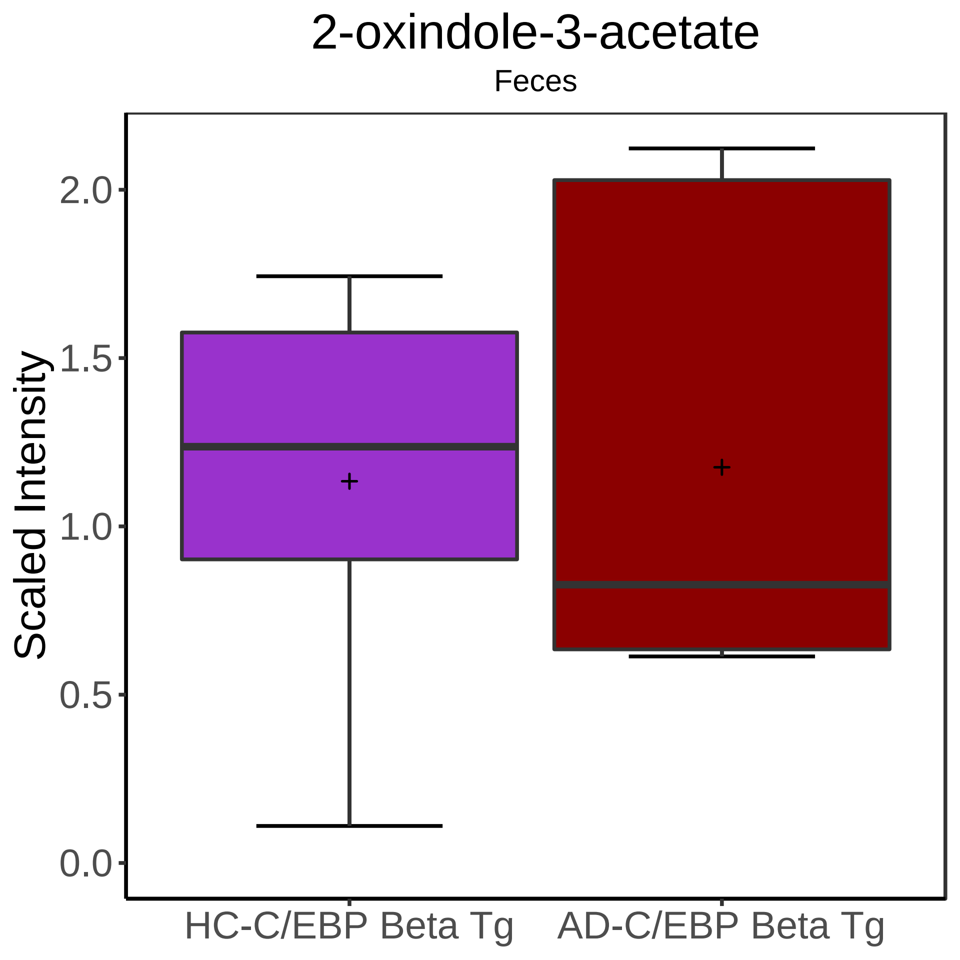
2-oxindole-3-acetate
Feces
Scaled Intensity
2.0
1.5
1.0
0.5
0.0
HC-C/EBP Beta Tg
AD-C/EBP Beta Tg

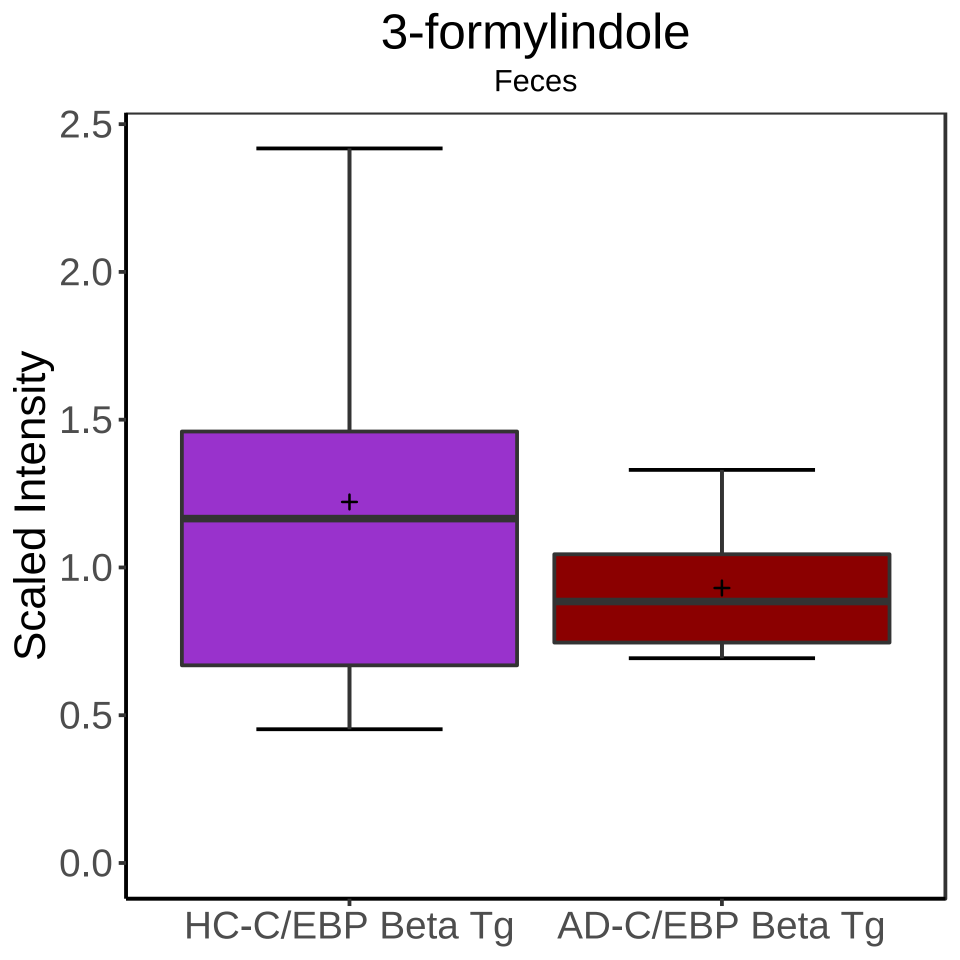

3-formylindole
Feces
Scaled Intensity
2.5
2.0
1.5
1.0
0.5
0.0
HC-C/EBP Beta Tg
AD-C/EBP Beta Tg

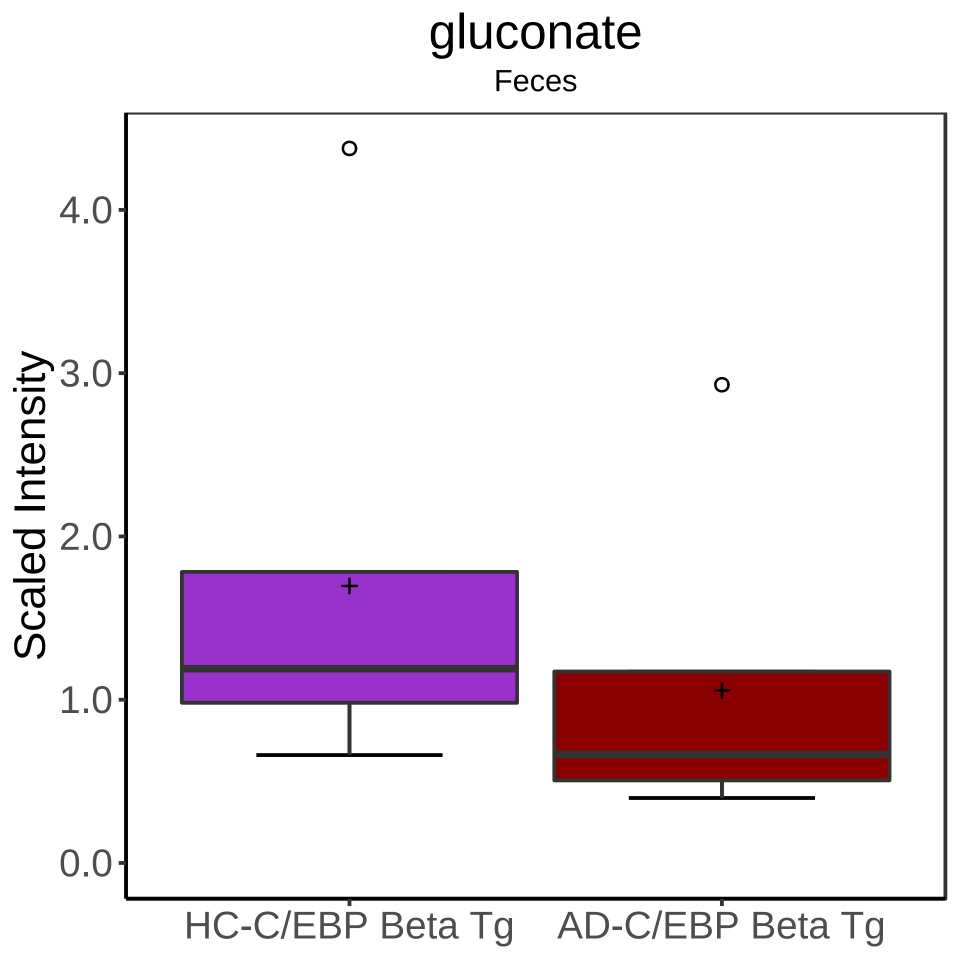

gluconate
Feces
Scaled Intensity
4.0
3.0
2.0
1.0
0.0
HC-C/EBP Beta Tg
AD-C/EBP Beta Tg

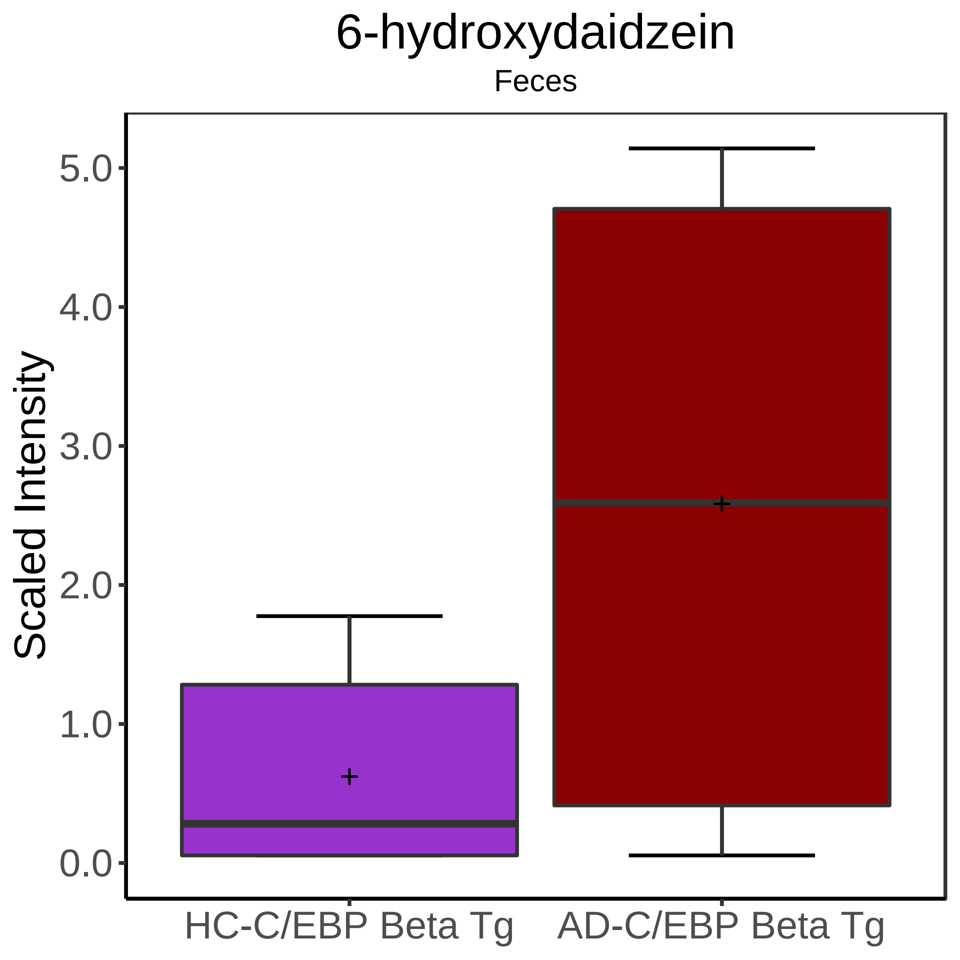
6-hydroxydaidzein
Feces
Scaled Intensity
5.0
4.0
3.0
2.0
1.0
0.0
HC-C/EBP Beta Tg
AD-C/EBP Beta Tg

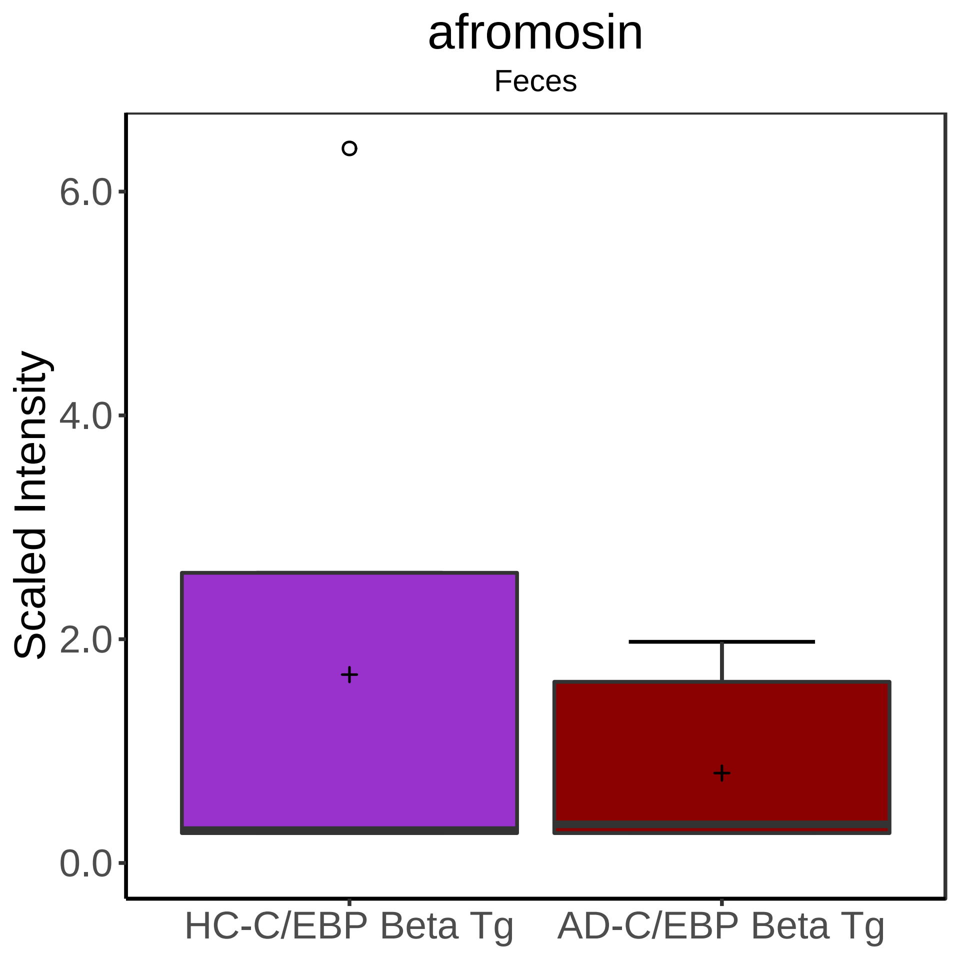
afromosin
Feces
Scaled Intensity
6.0
4.0
2.0
0.0
HC-C/EBP Beta Tg
AD-C/EBP Beta Tg

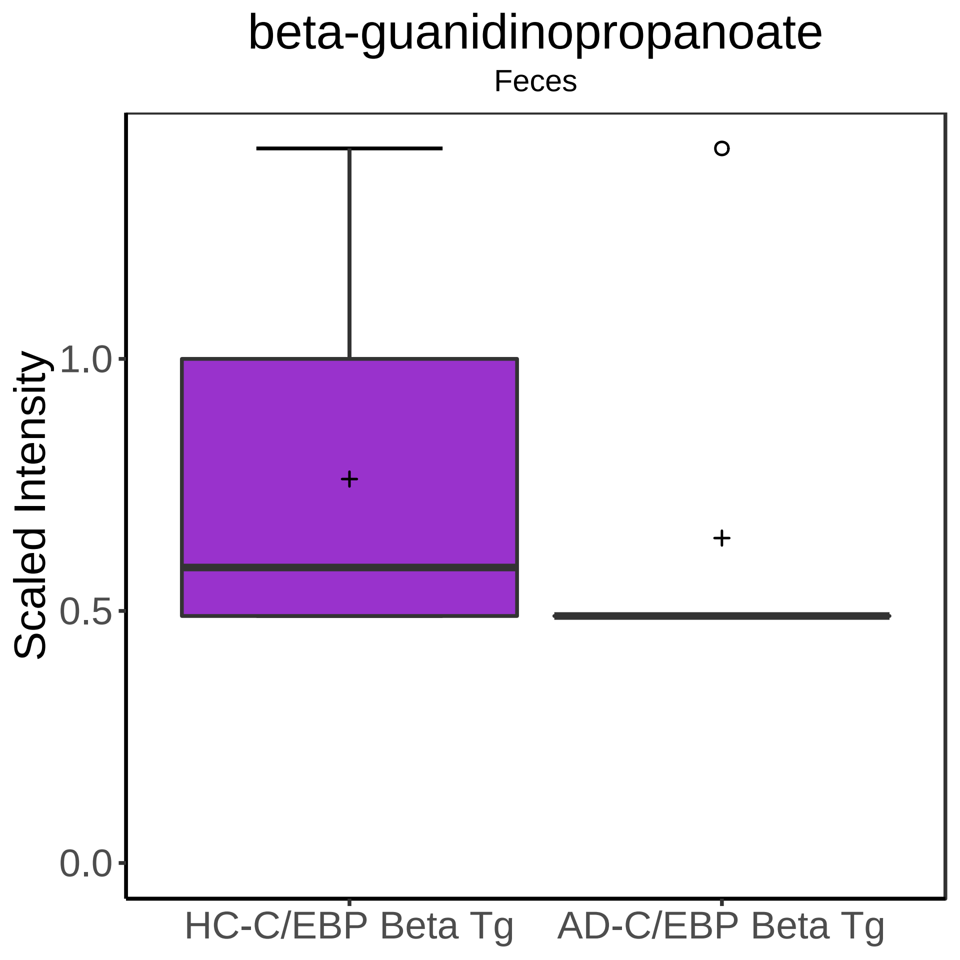

beta-guanidinopropanoate
Feces
Scaled Intensity
1.0
0.5
0.0
HC-C/EBP Beta Tg
AD-C/EBP Beta Tg

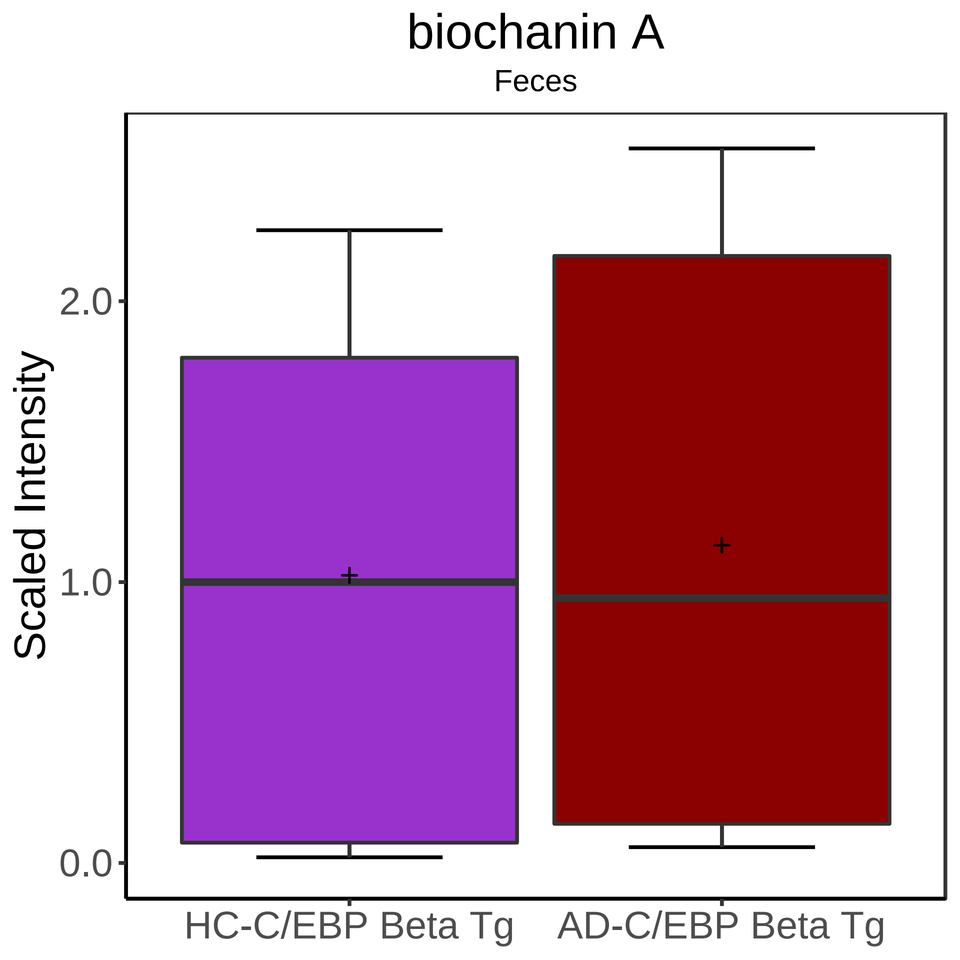
biochanin A
Feces
Scaled Intensity
2.0
1.0
0.0
HC-C/EBP Beta Tg
AD-C/EBP Beta Tg

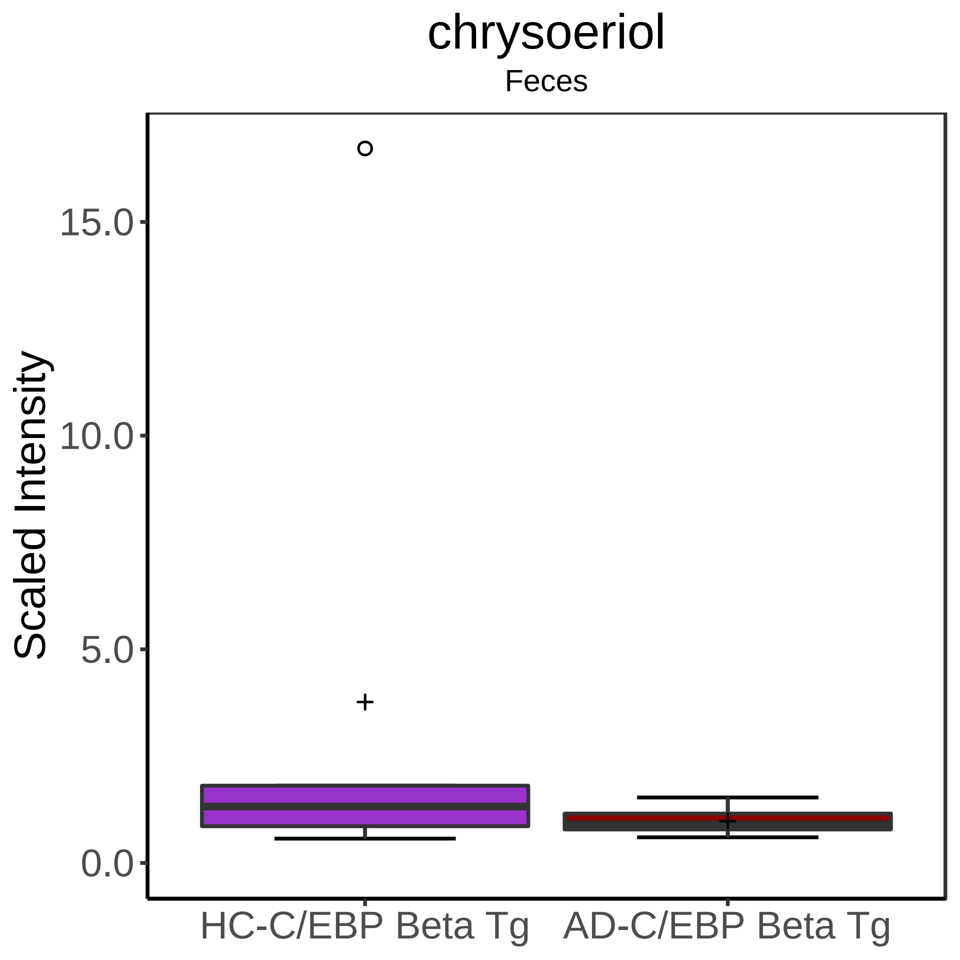

chrysoeriol
Feces
Scaled Intensity
15.0
10.0
5.0
0.0
HC-C/EBP Beta Tg
AD-C/EBP Beta Tg

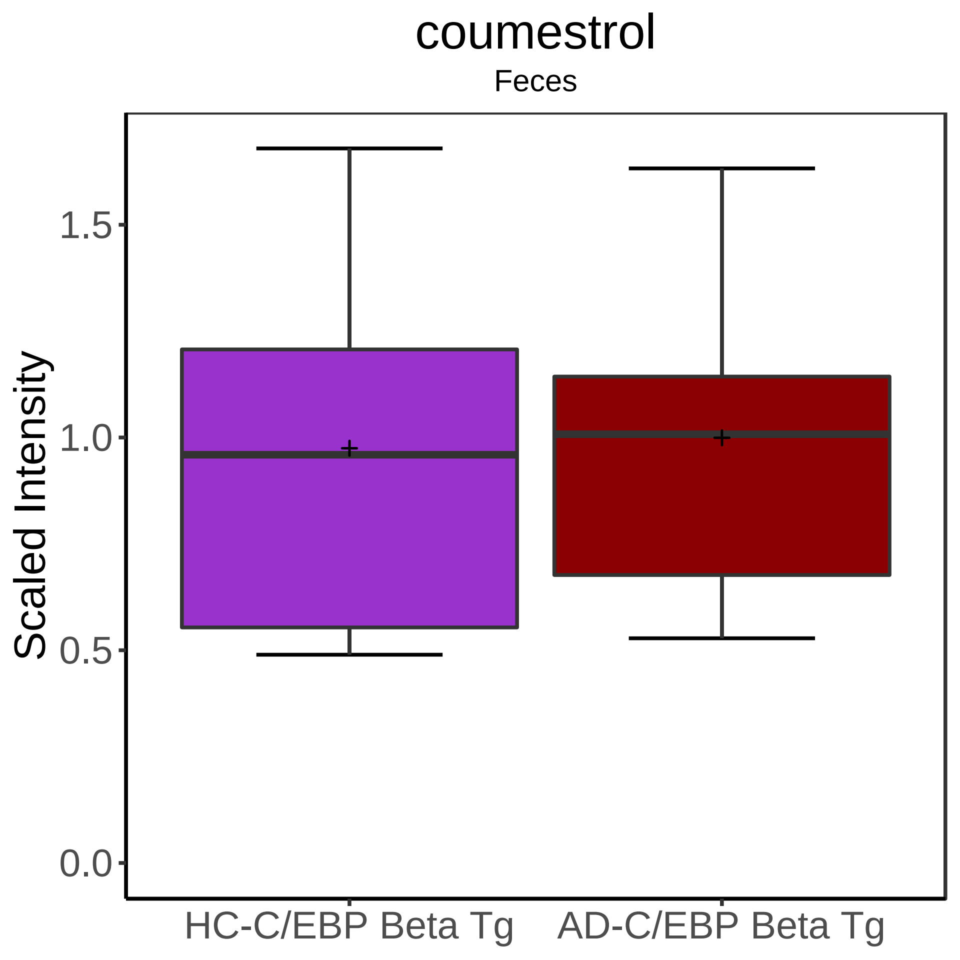
coumestrol
Feces
Scaled Intensity
1.5
1.0
0.5
0.0
HC-C/EBP Beta Tg
AD-C/EBP Beta Tg

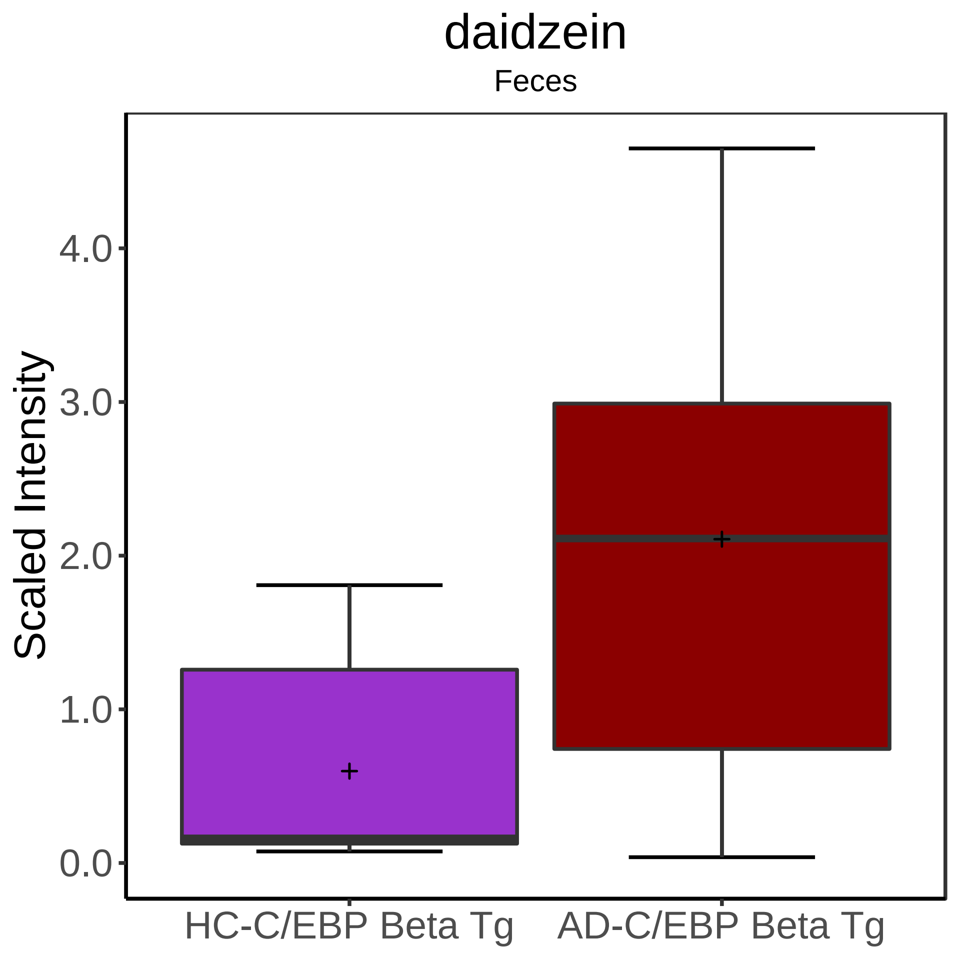
daidzein
Feces
Scaled Intensity
4.0
3.0
2.0
1.0
0.0
HC-C/EBP Beta Tg
AD-C/EBP Beta Tg

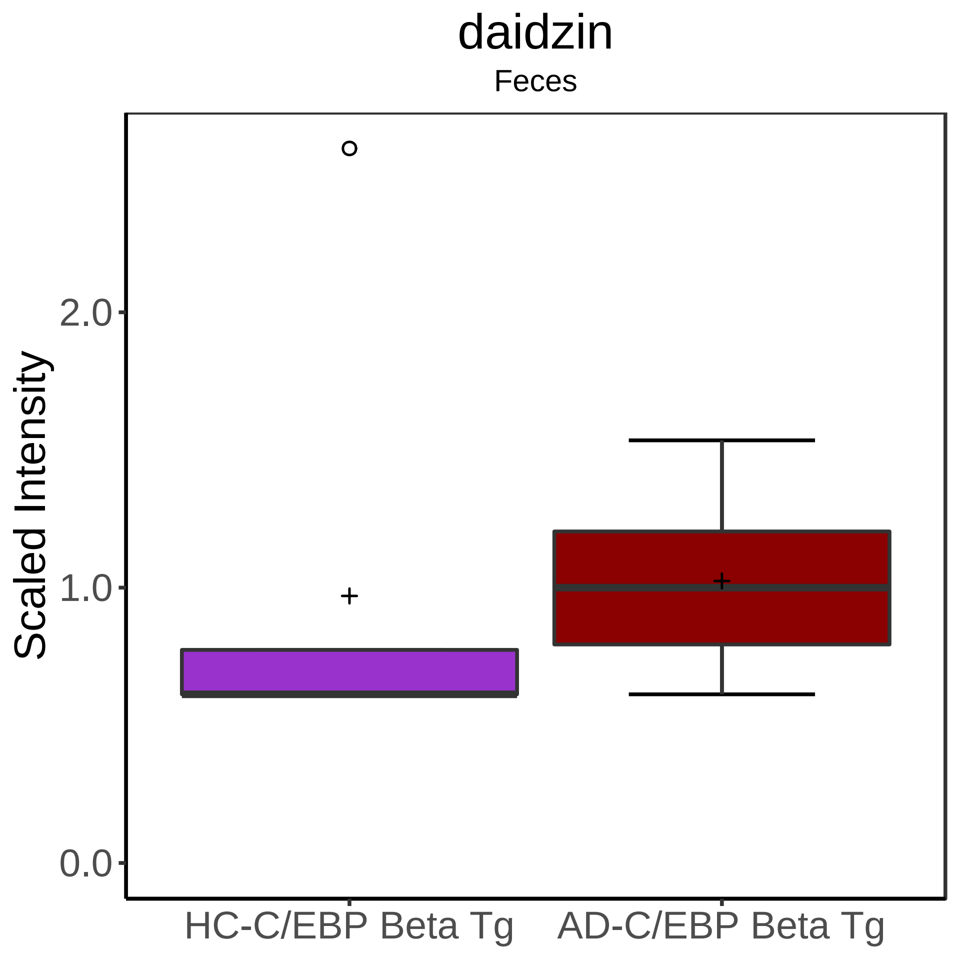

daidzin
Feces
Scaled Intensity
2.0
1.0
0.0
HC-C/EBP Beta Tg
AD-C/EBP Beta Tg

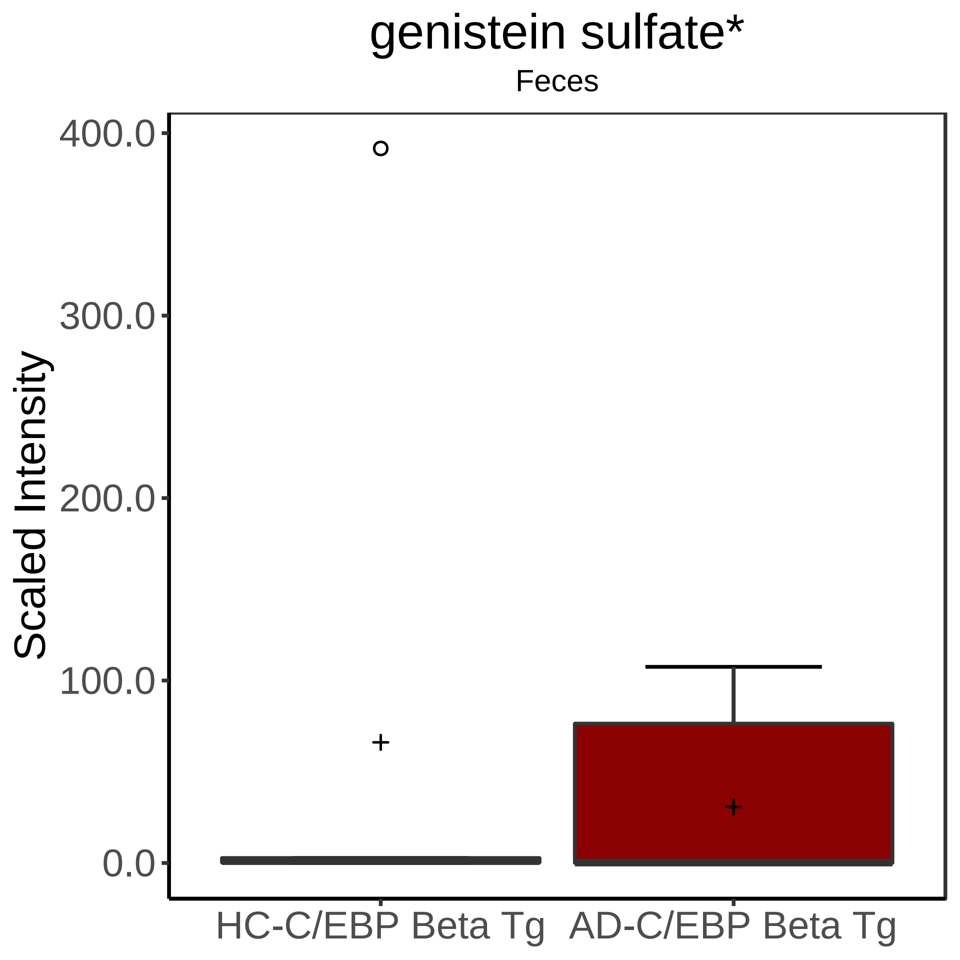
genistein sulfate*
Feces
Scaled Intensity
400.0
300.0
200.0
100.0
0.0
HC-C/EBP Beta Tg
AD-C/EBP Beta Tg

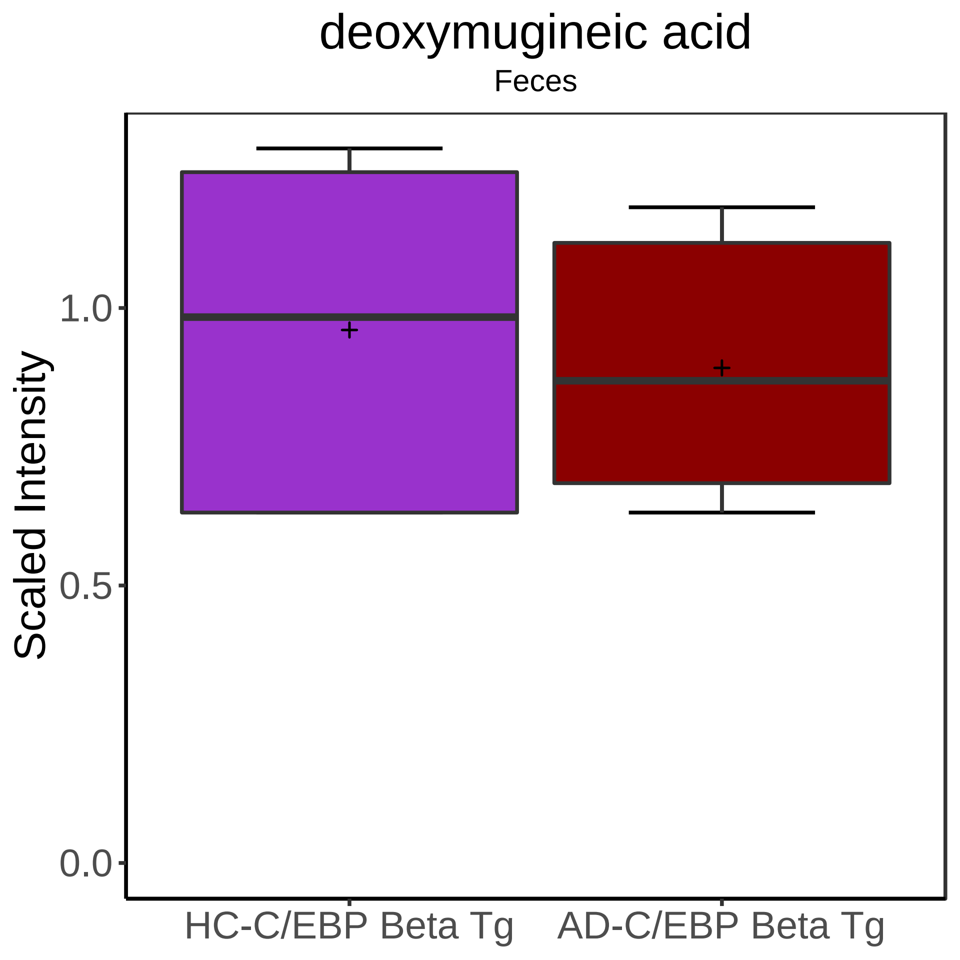

deoxymugineic acid
Feces
Scaled Intensity
1.0
0.5
0.0
HC-C/EBP Beta Tg
AD-C/EBP Beta Tg

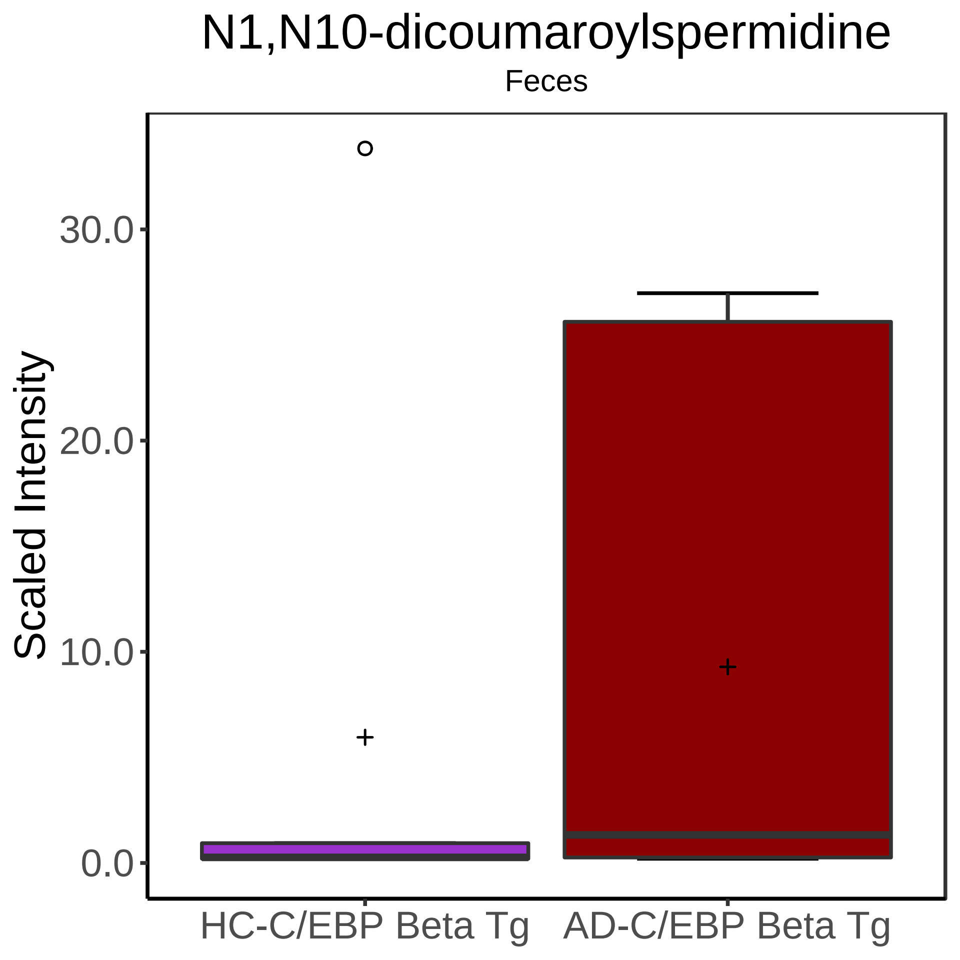
N1,N10-dicoumaroylspermidine
Feces
Scaled Intensity
30.0
20.0
10.0
0.0
HC-C/EBP Beta Tg
AD-C/EBP Beta Tg

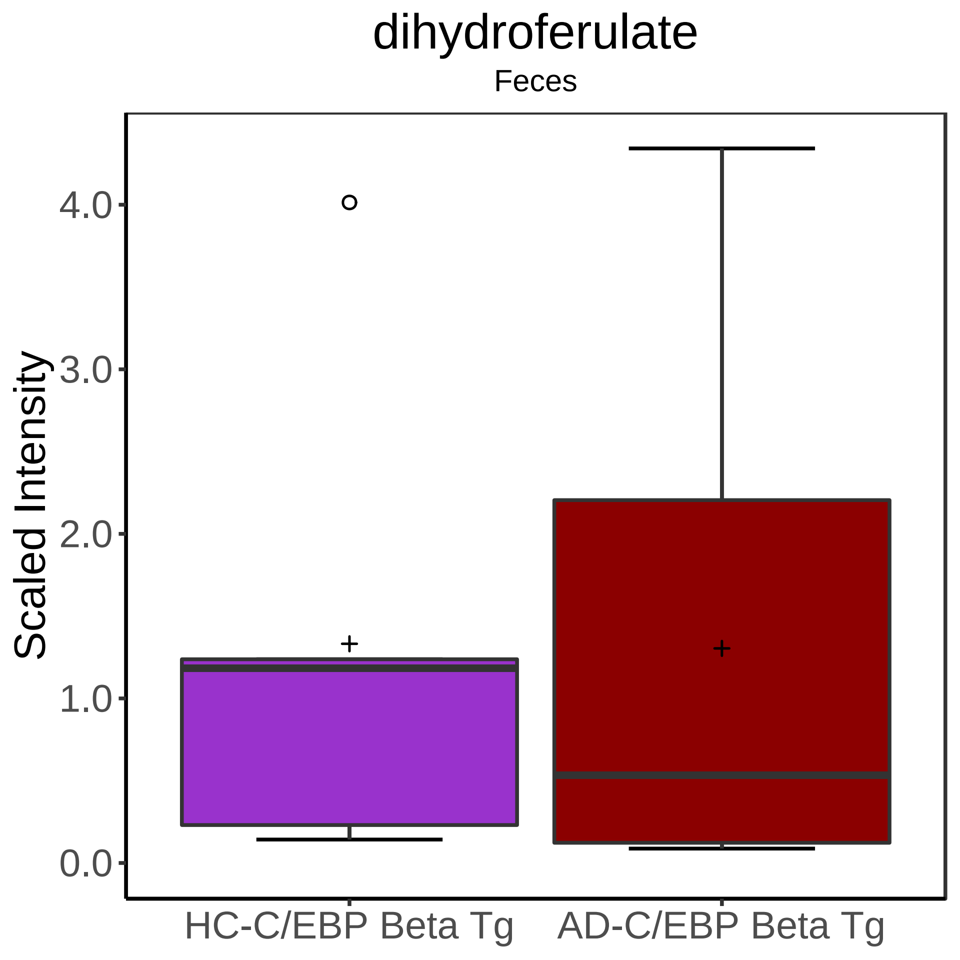

dihydroferulate
Feces
Scaled Intensity
4.0
3.0
2.0
1.0
0.0
HC-C/EBP Beta Tg
AD-C/EBP Beta Tg

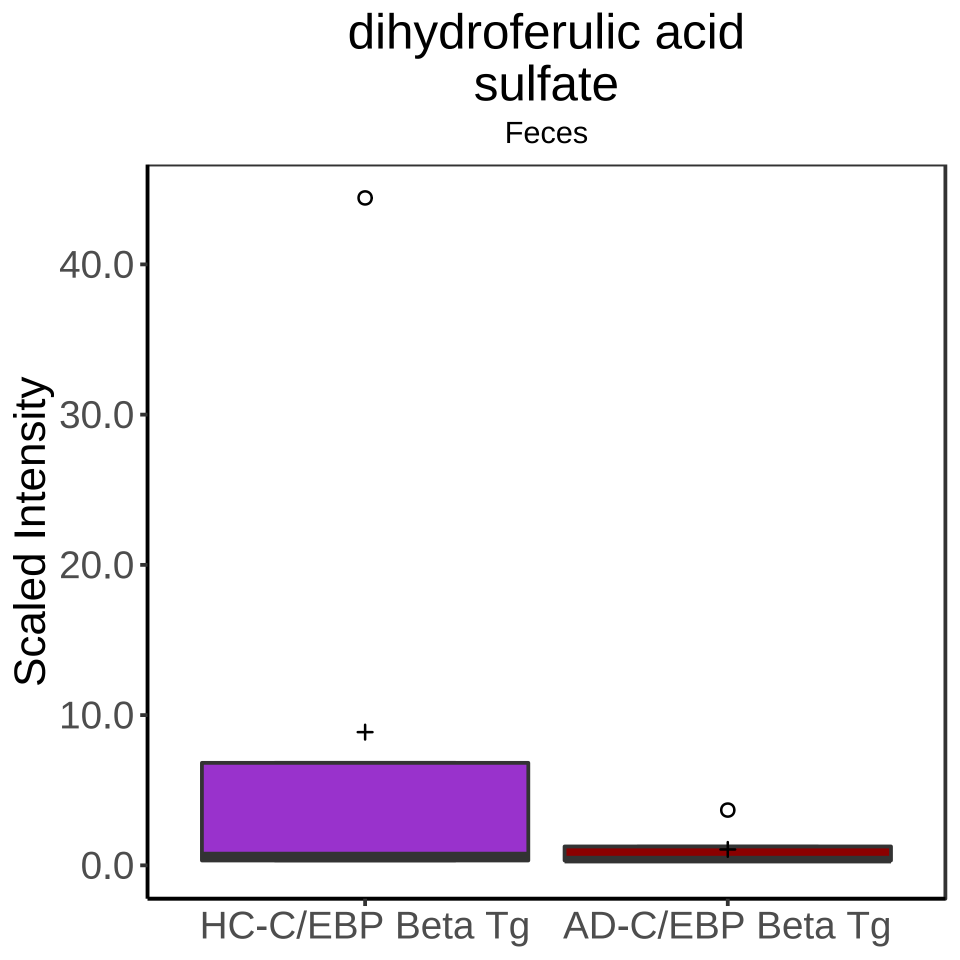
dihydroferulic acid
sulfate
Feces
Scaled Intensity
40.0
30.0
20.0
10.0
0.0
HC-C/EBP Beta Tg
AD-C/EBP Beta Tg

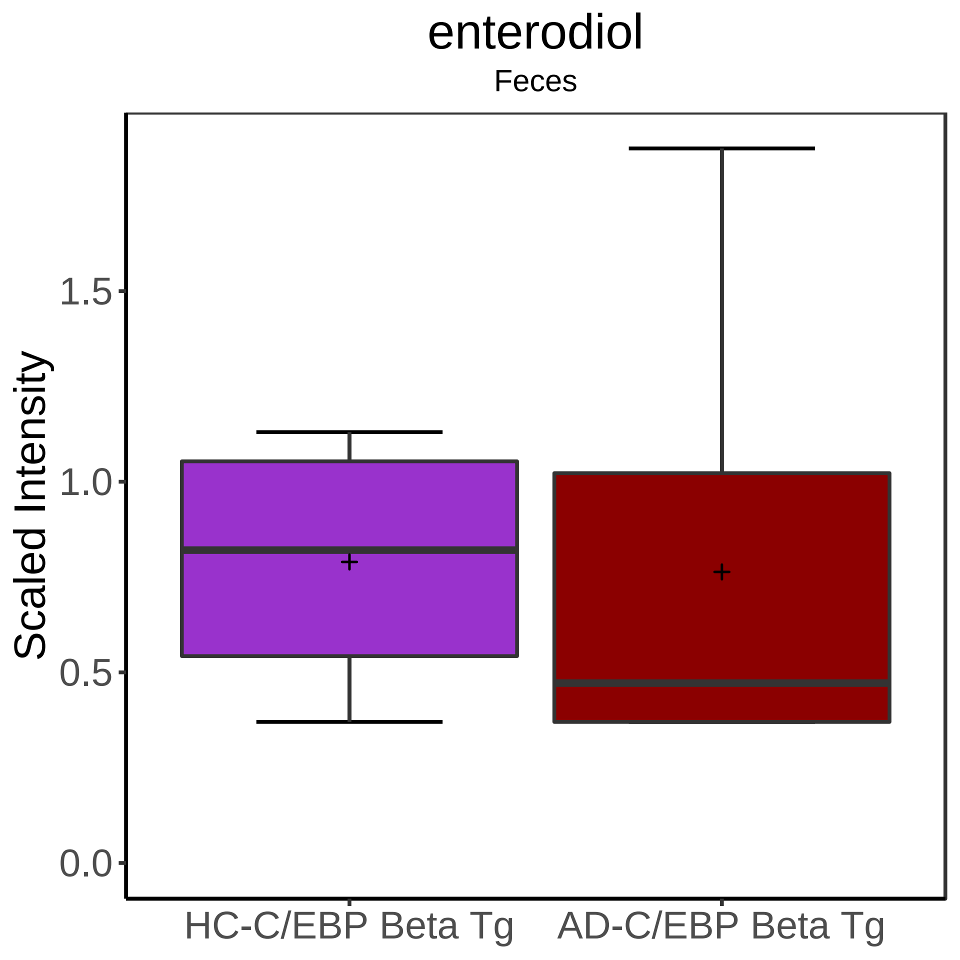

enterodiol
Feces
Scaled Intensity
1.5
1.0
0.5
0.0
HC-C/EBP Beta Tg
AD-C/EBP Beta Tg

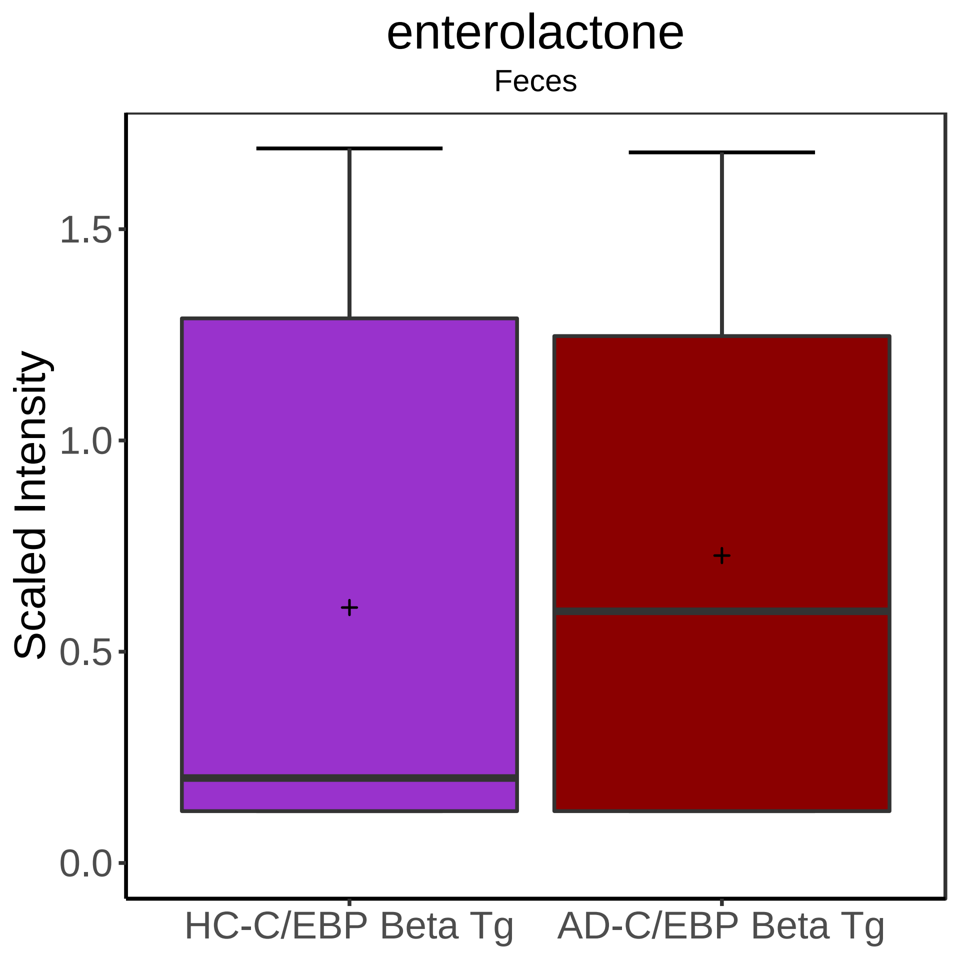

enterolactone
Feces
Scaled Intensity
1.5
1.0
0.5
0.0
HC-C/EBP Beta Tg
AD-C/EBP Beta Tg

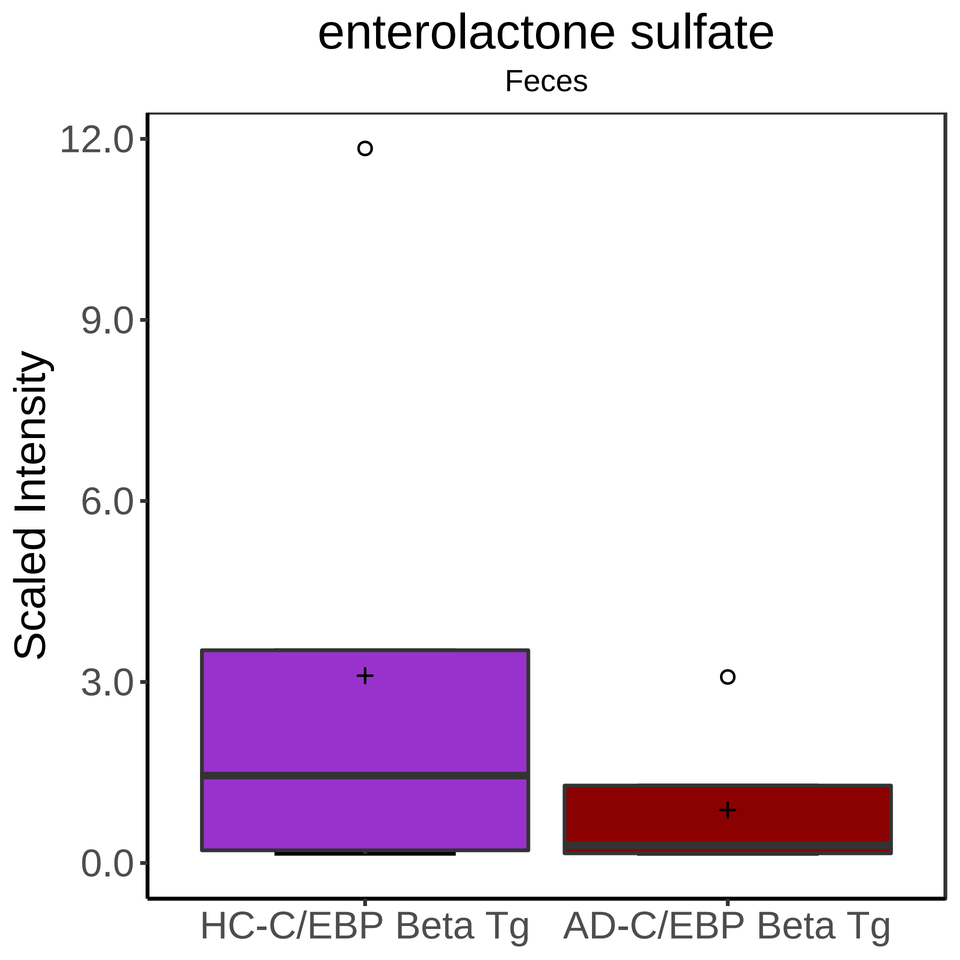

enterolactone sulfate
Feces
Scaled Intensity
12.0
9.0
6.0
3.0
0.0
HC-C/EBP Beta Tg
AD-C/EBP Beta Tg

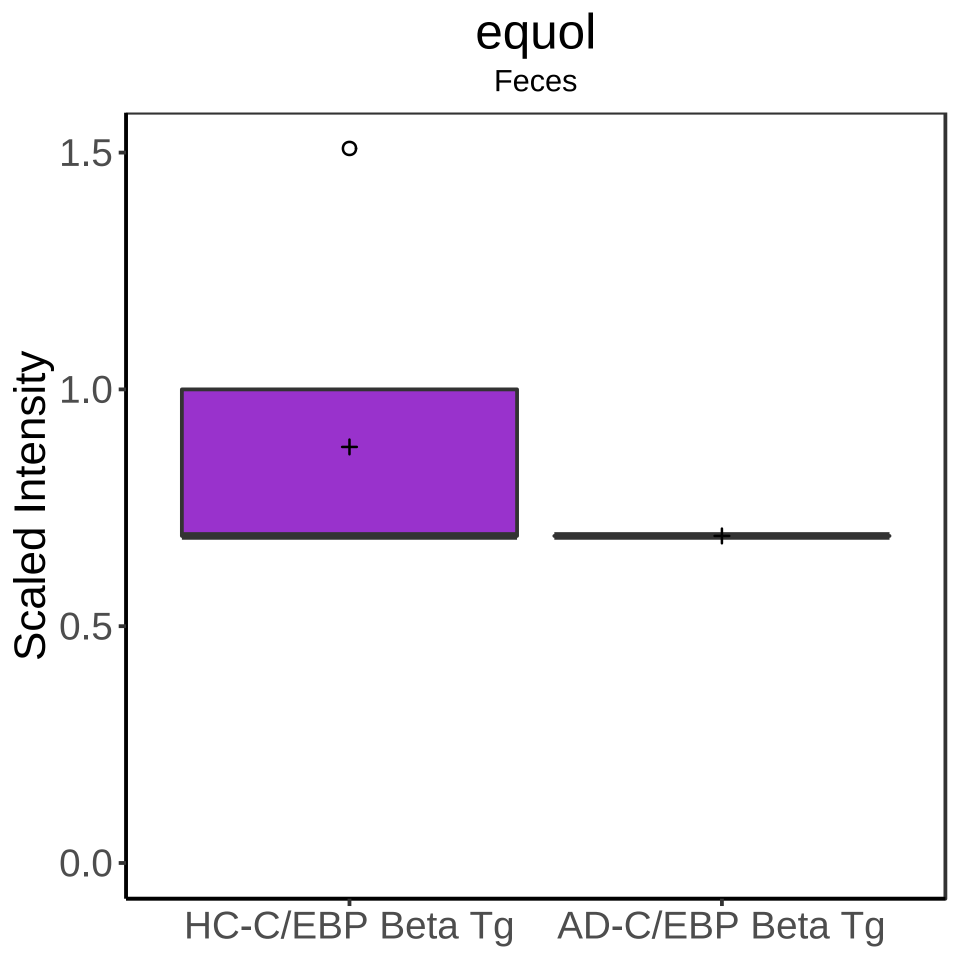

equol
Feces
Scaled Intensity
1.5
1.0
0.5
0.0
HC-C/EBP Beta Tg
AD-C/EBP Beta Tg

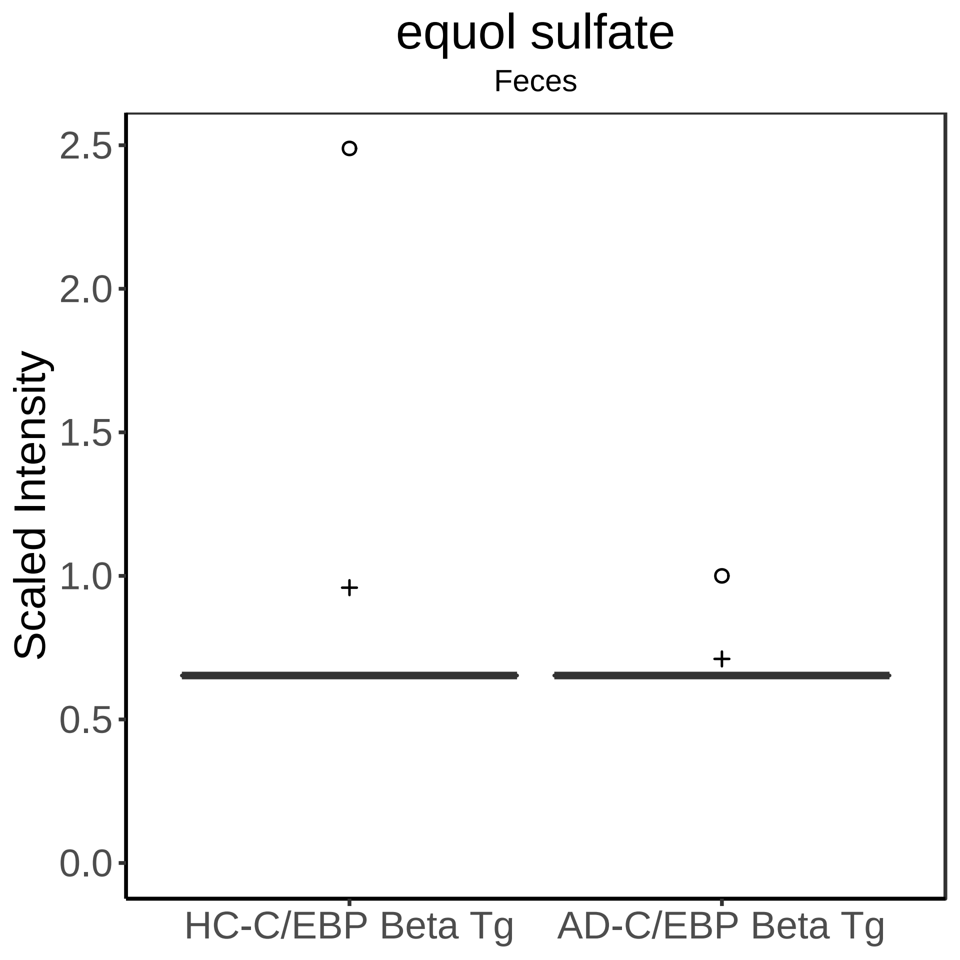

equol sulfate
Feces
Scaled Intensity
2.5
2.0
1.5
1.0
0.5
0.0
HC-C/EBP Beta Tg
AD-C/EBP Beta Tg

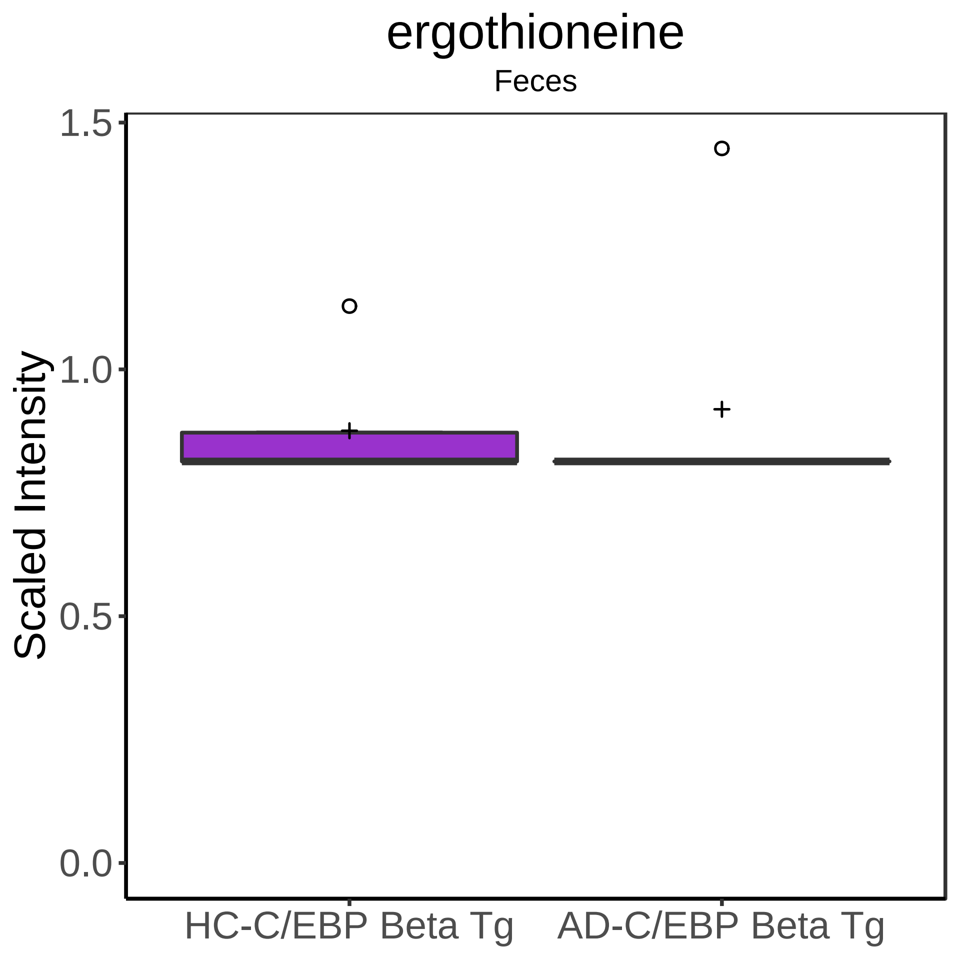
ergothioneine
Feces
Scaled Intensity
1.5
1.0
0.5
0.0
HC-C/EBP Beta Tg
AD-C/EBP Beta Tg

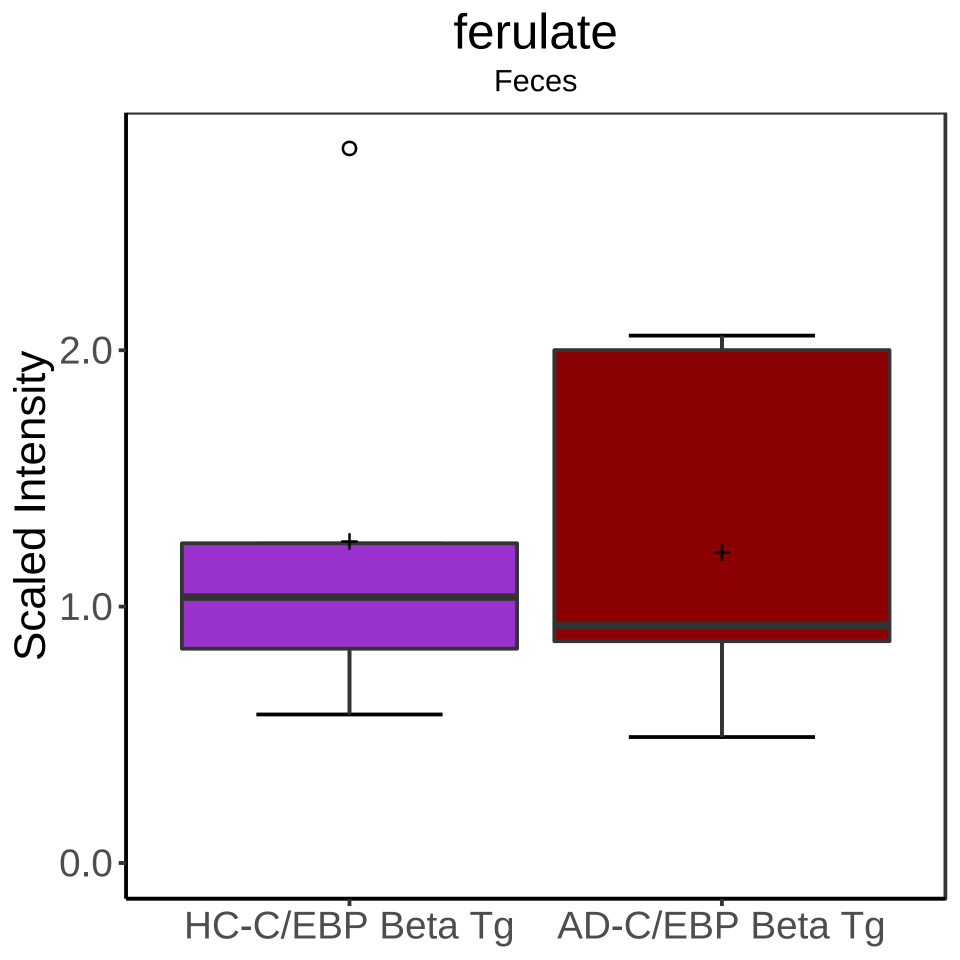
ferulate
Feces
Scaled Intensity
2.0
1.0
0.0
HC-C/EBP Beta Tg
AD-C/EBP Beta Tg

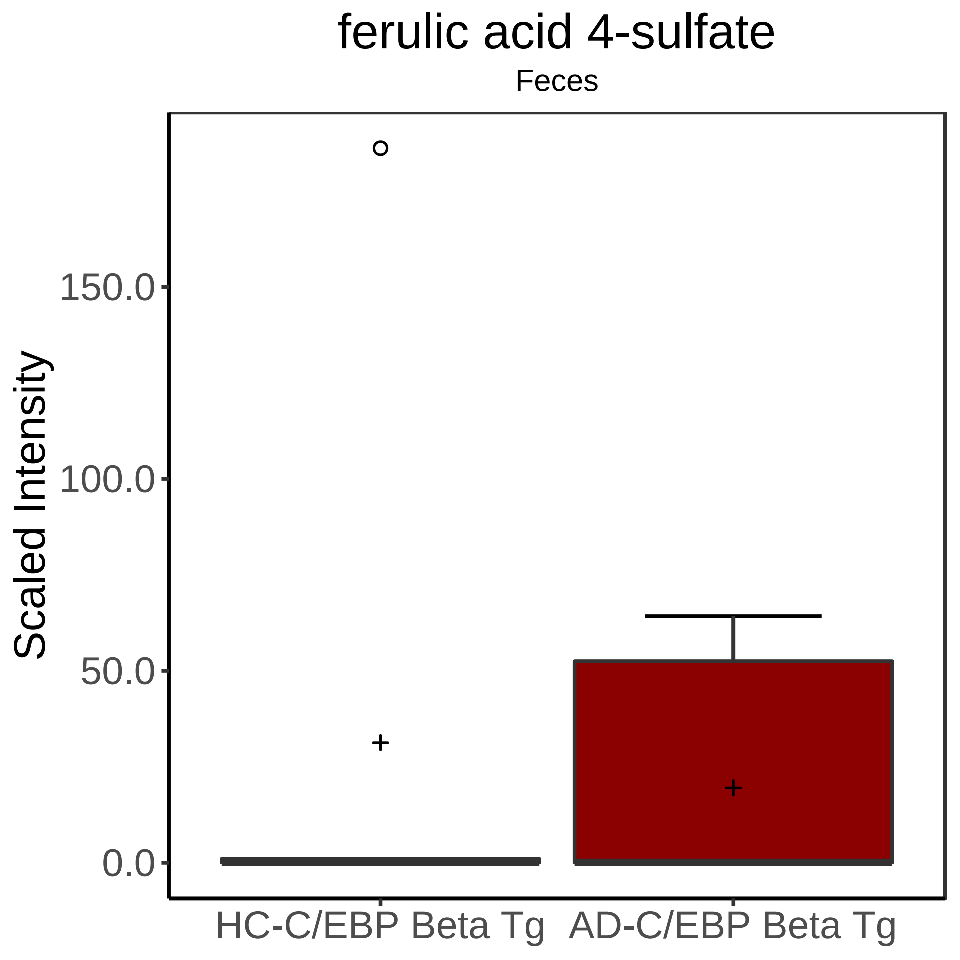
ferulic acid 4-sulfate
Feces
Scaled Intensity
150.0
100.0
50.0
0.0
HC-C/EBP Beta Tg
AD-C/EBP Beta Tg

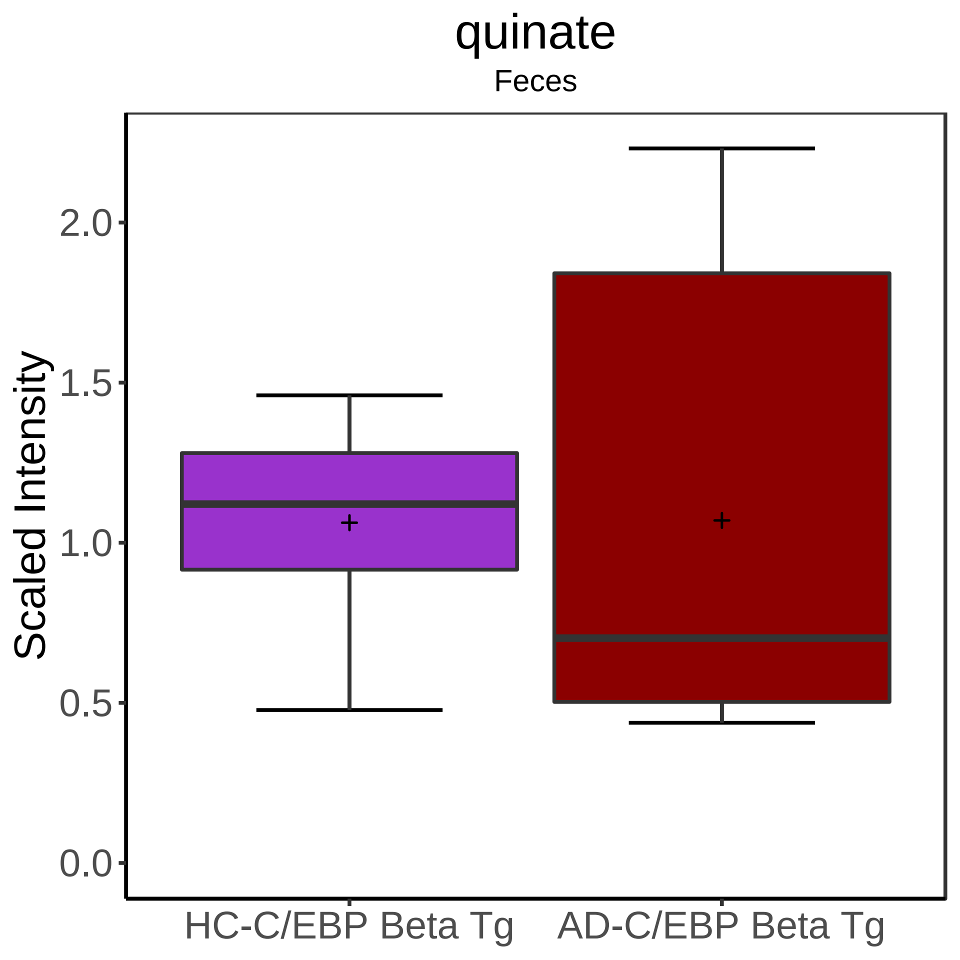

quinate
Feces
Scaled Intensity
2.0
1.5
1.0
0.5
0.0
HC-C/EBP Beta Tg
AD-C/EBP Beta Tg

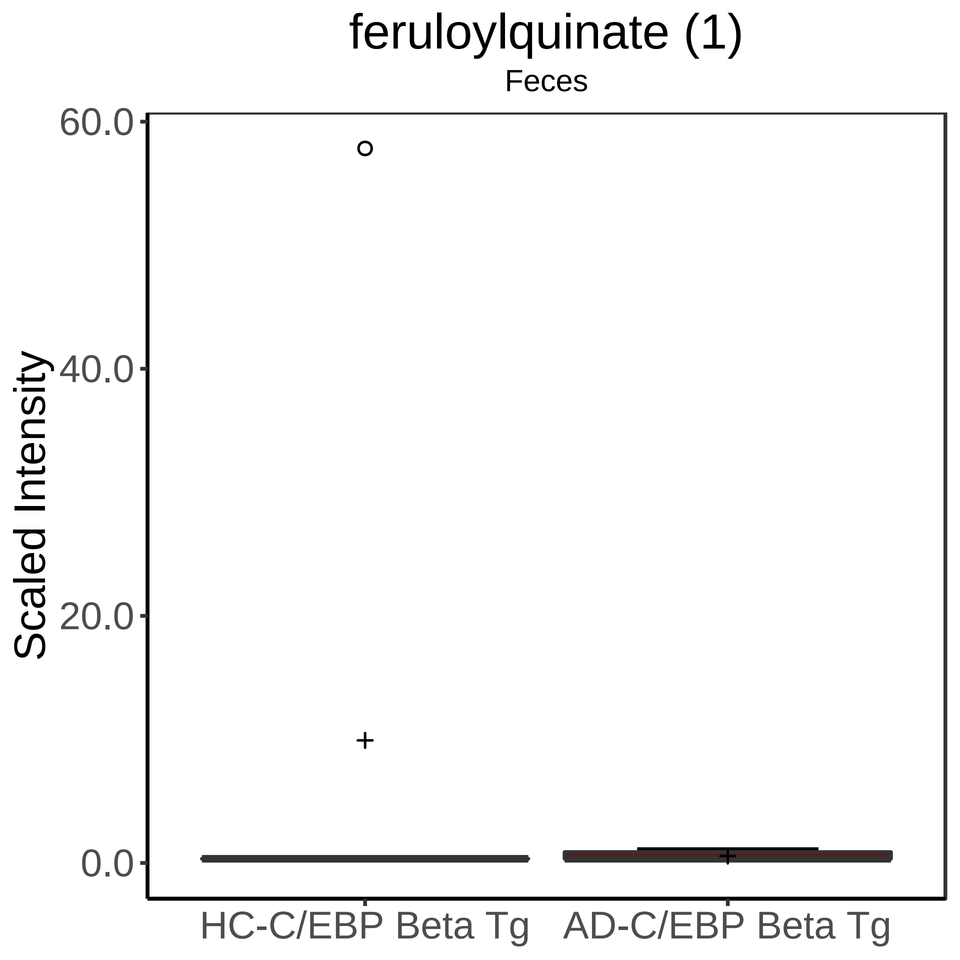

feruloylquinate (1)
Feces
Scaled Intensity
60.0
40.0
20.0
0.0
HC-C/EBP Beta Tg
AD-C/EBP Beta Tg

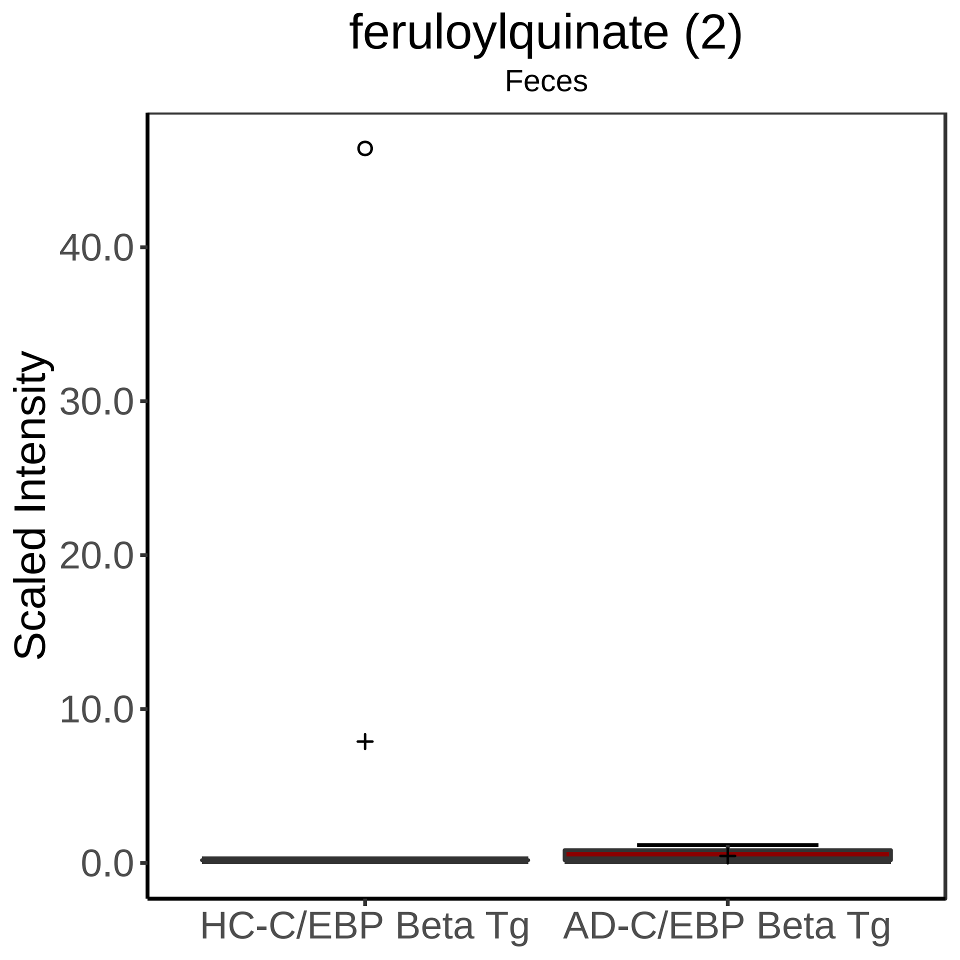

feruloylquinate (2)
Feces
Scaled Intensity
40.0
30.0
20.0
10.0
0.0
HC-C/EBP Beta Tg
AD-C/EBP Beta Tg

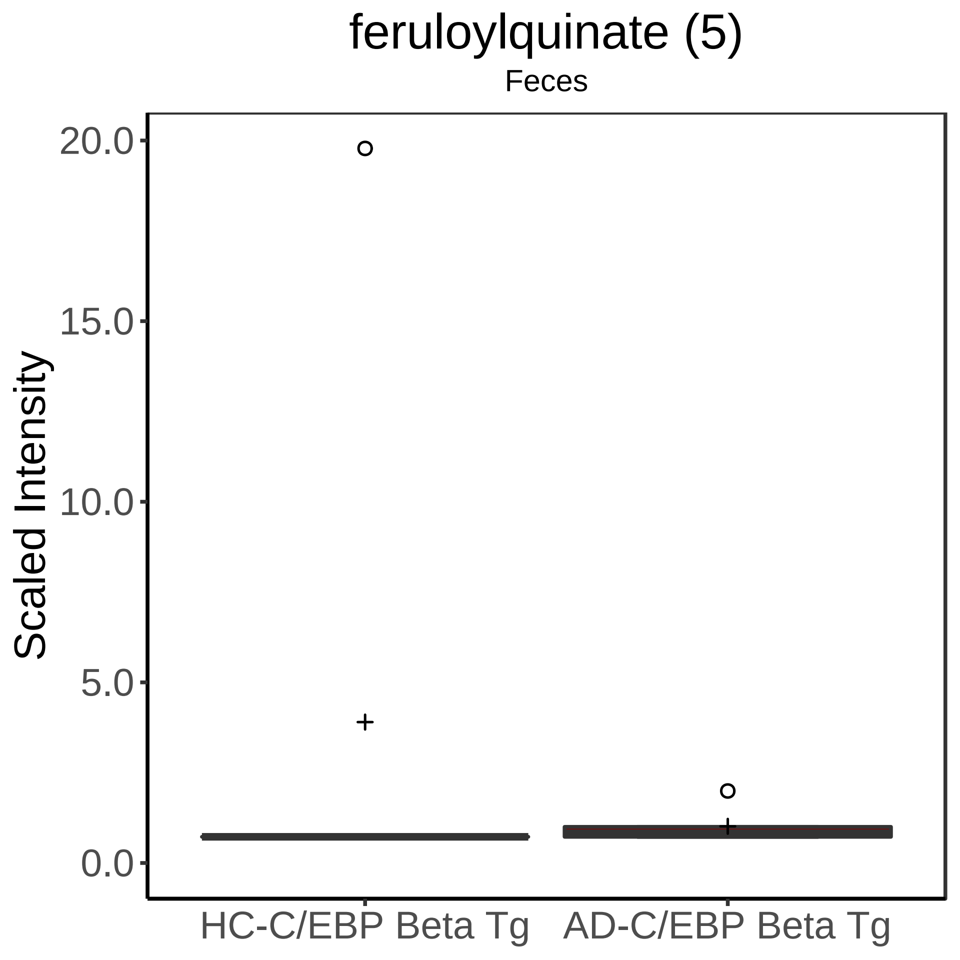
feruloylquinate (5)
Feces
Scaled Intensity
20.0
15.0
10.0
5.0
0.0
HC-C/EBP Beta Tg
AD-C/EBP Beta Tg

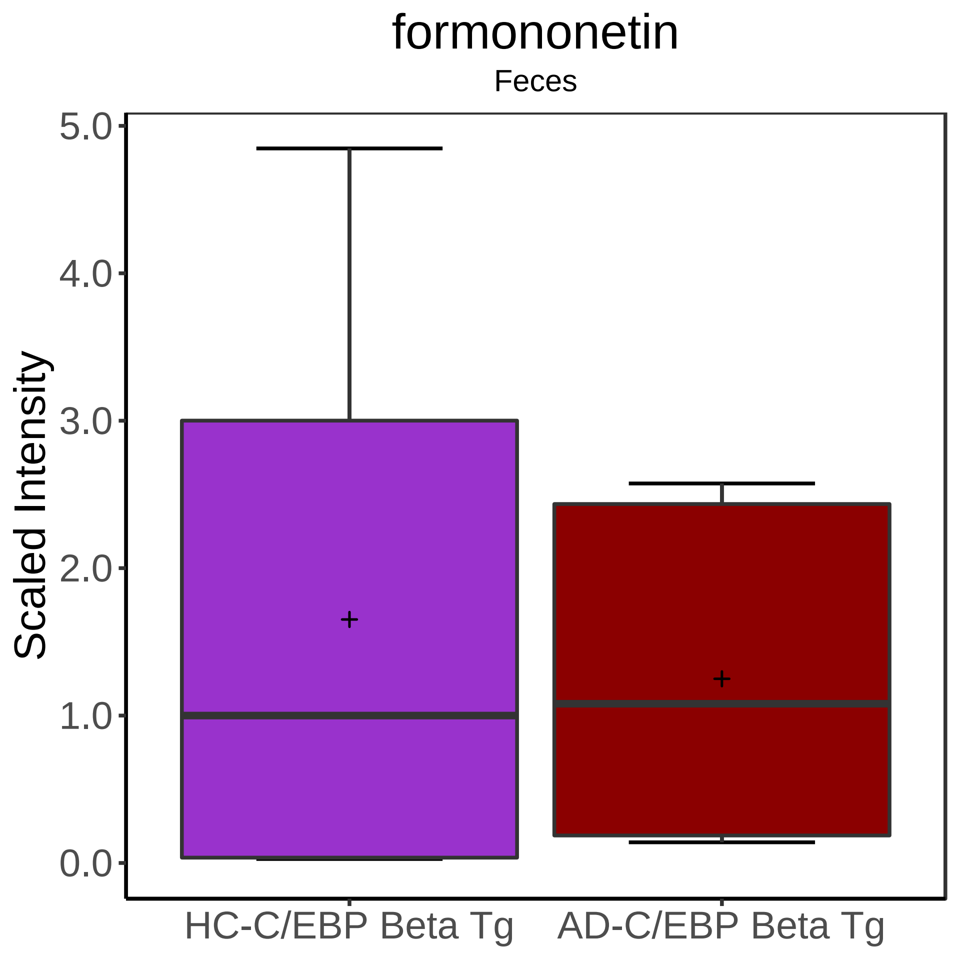
formononetin
Feces
Scaled Intensity
5.0
4.0
3.0
2.0
1.0
0.0
HC-C/EBP Beta Tg
AD-C/EBP Beta Tg

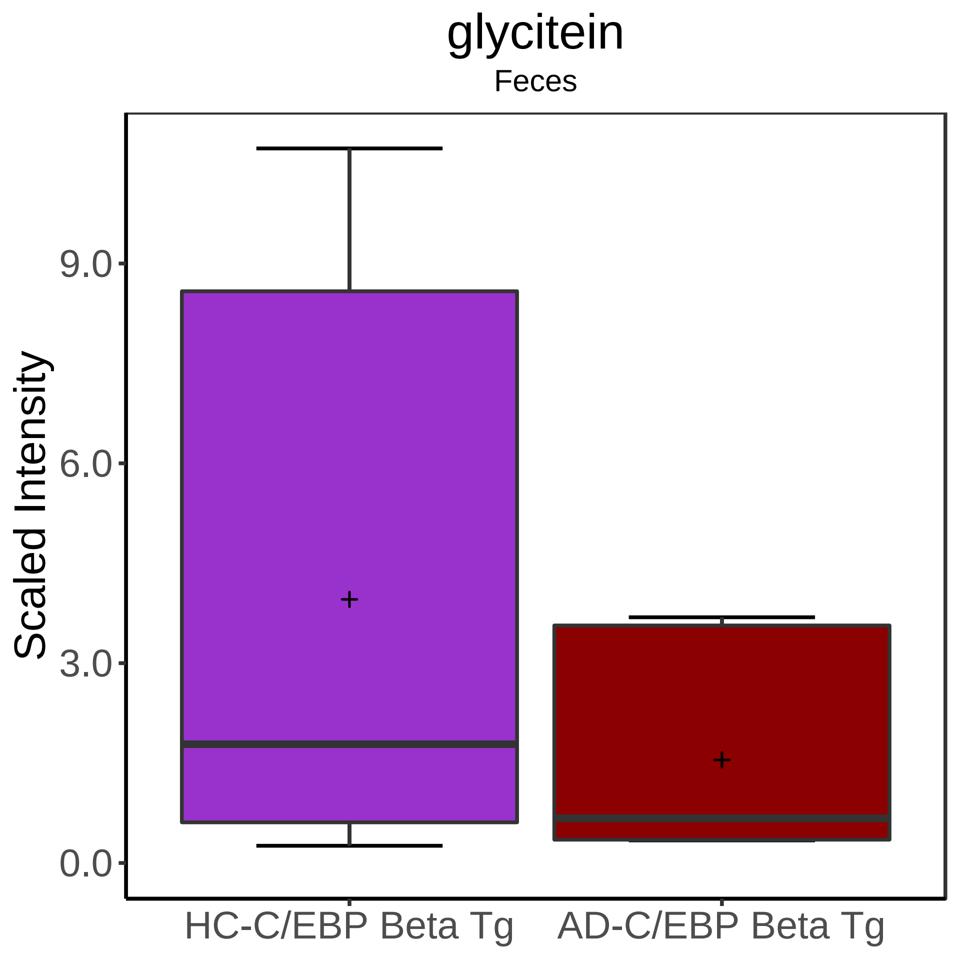
glycitein
Feces
Scaled Intensity
9.0
6.0
3.0
0.0
HC-C/EBP Beta Tg
AD-C/EBP Beta Tg

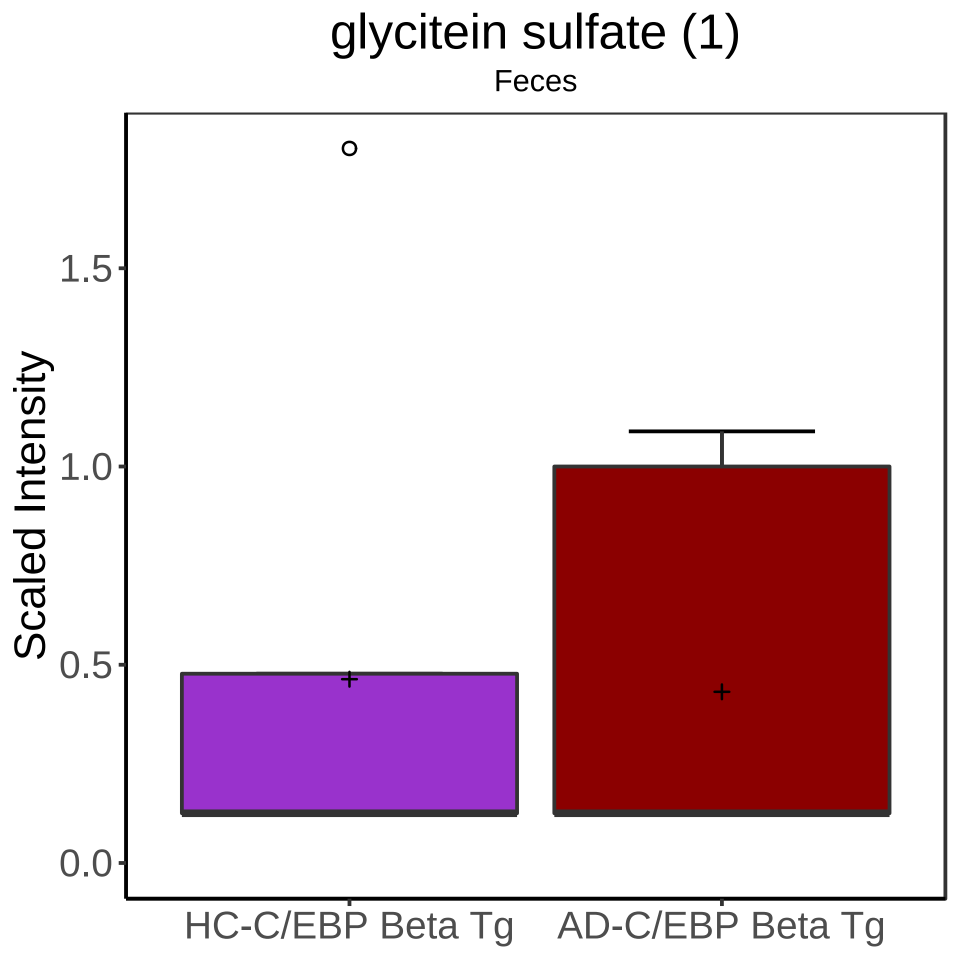
glycitein sulfate (1)
Feces
Scaled Intensity
1.5
1.0
0.5
0.0
HC-C/EBP Beta Tg
AD-C/EBP Beta Tg

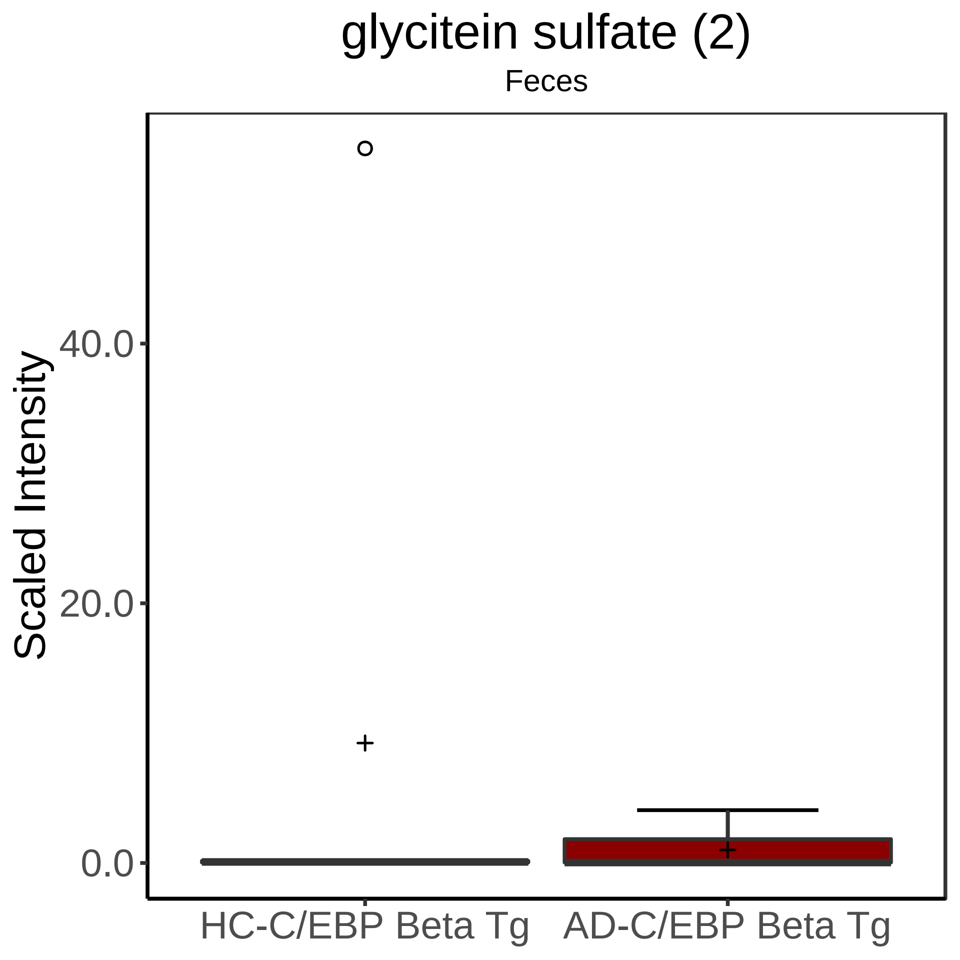

glycitein sulfate (2)
Feces
Scaled Intensity
40.0
20.0
0.0
HC-C/EBP Beta Tg
AD-C/EBP Beta Tg

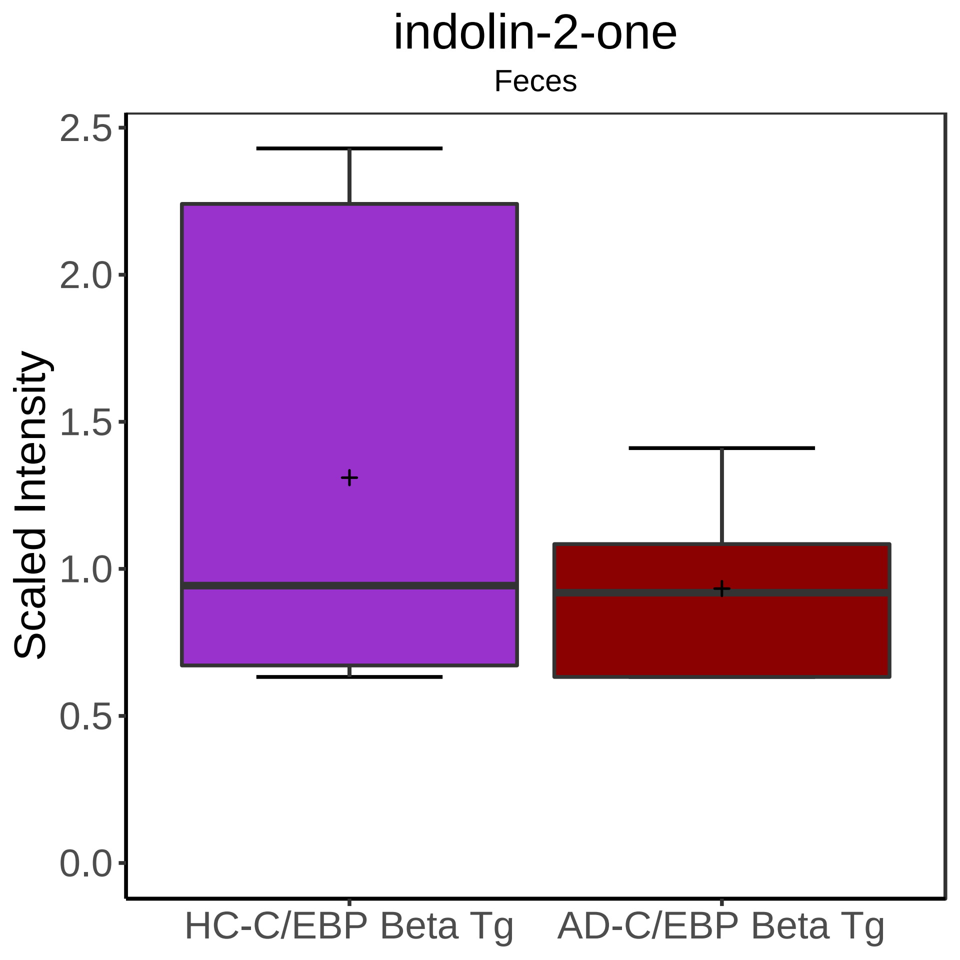

indolin-2-one
Feces
Scaled Intensity
2.5
2.0
1.5
1.0
0.5
0.0
HC-C/EBP Beta Tg
AD-C/EBP Beta Tg

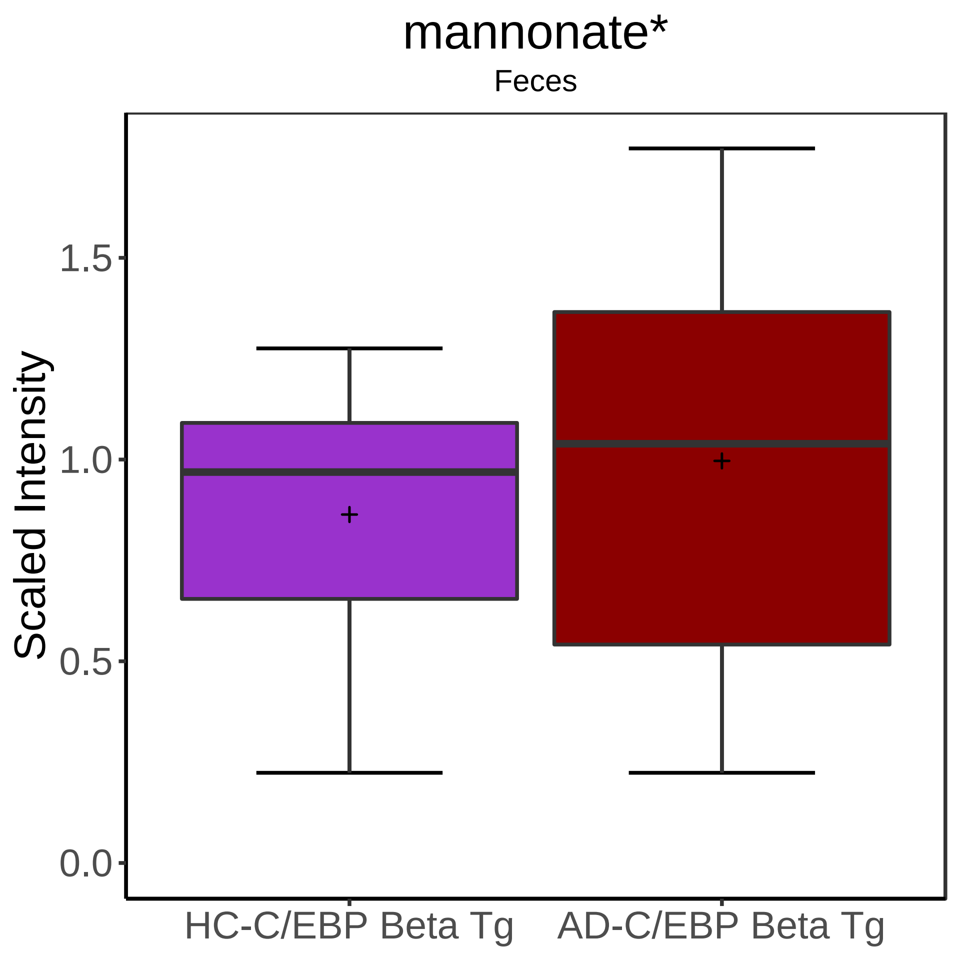
mannonate*
Feces
Scaled Intensity
1.5
1.0
0.5
0.0
HC-C/EBP Beta Tg
AD-C/EBP Beta Tg

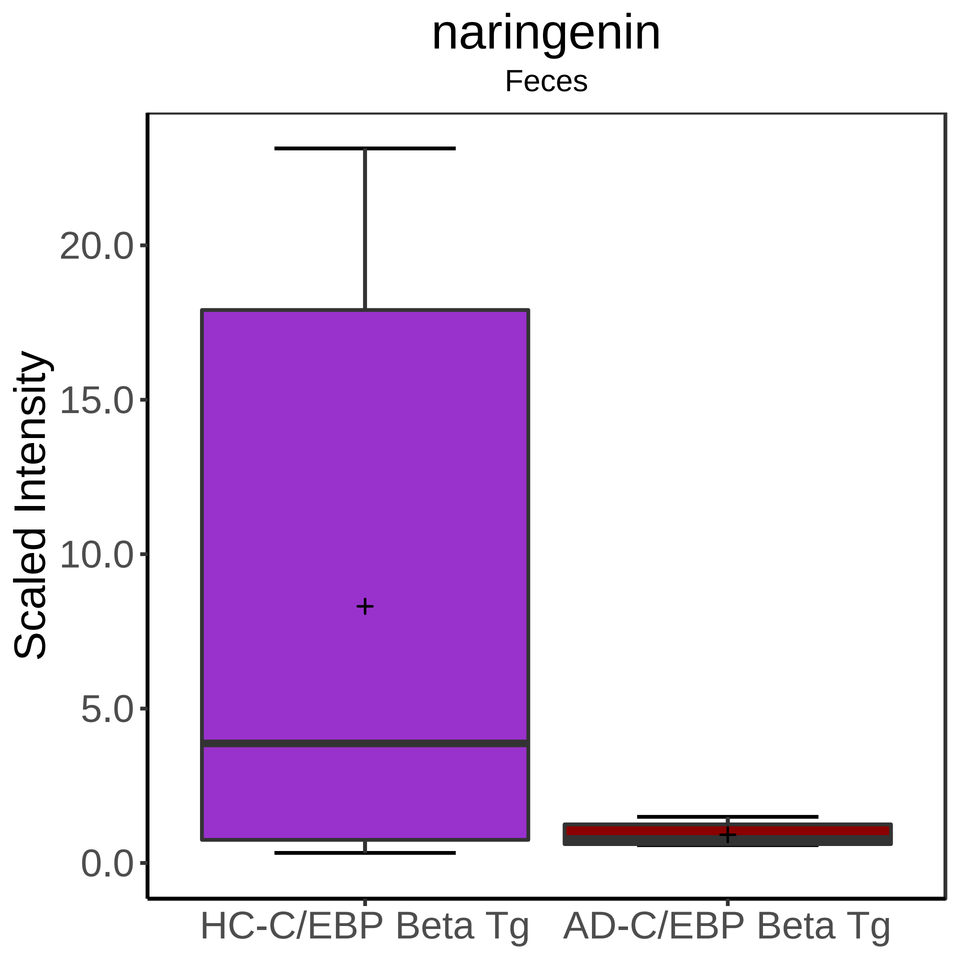

naringenin
Feces
Scaled Intensity
20.0
15.0
10.0
5.0
0.0
HC-C/EBP Beta Tg
AD-C/EBP Beta Tg

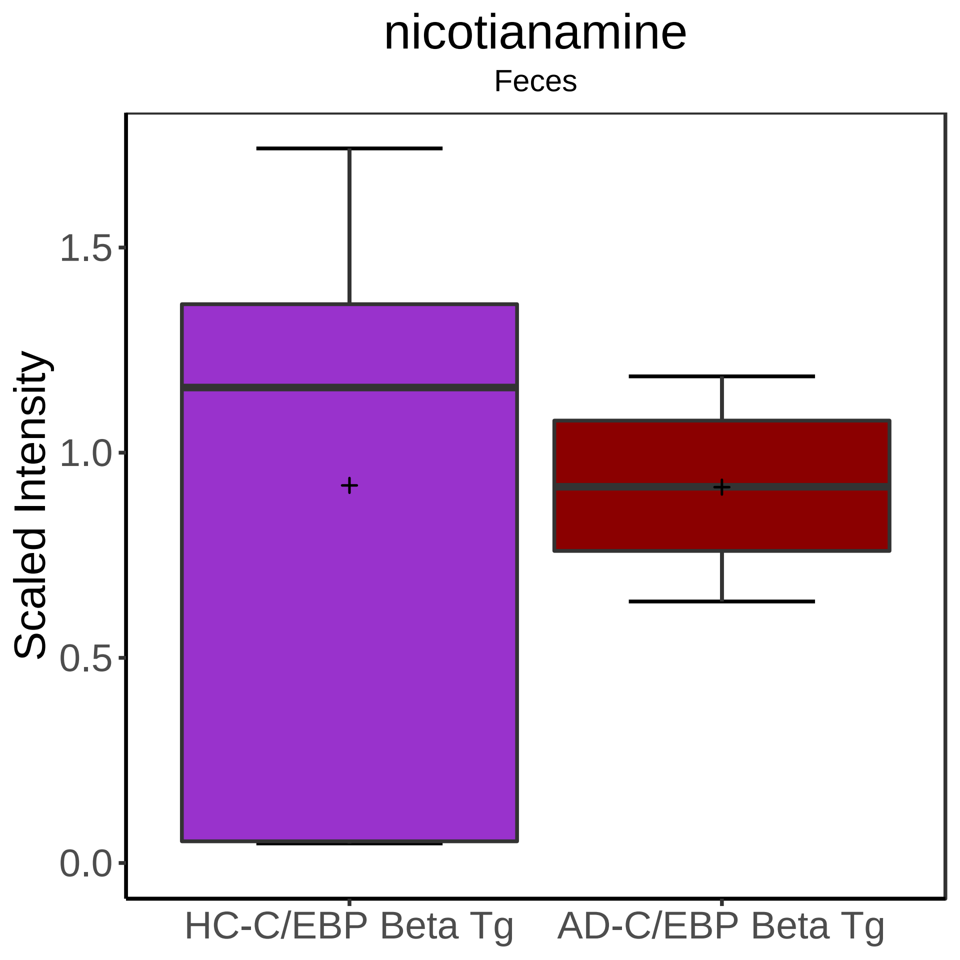

nicotianamine
Feces
Scaled Intensity
1.5
1.0
0.5
0.0
HC-C/EBP Beta Tg
AD-C/EBP Beta Tg

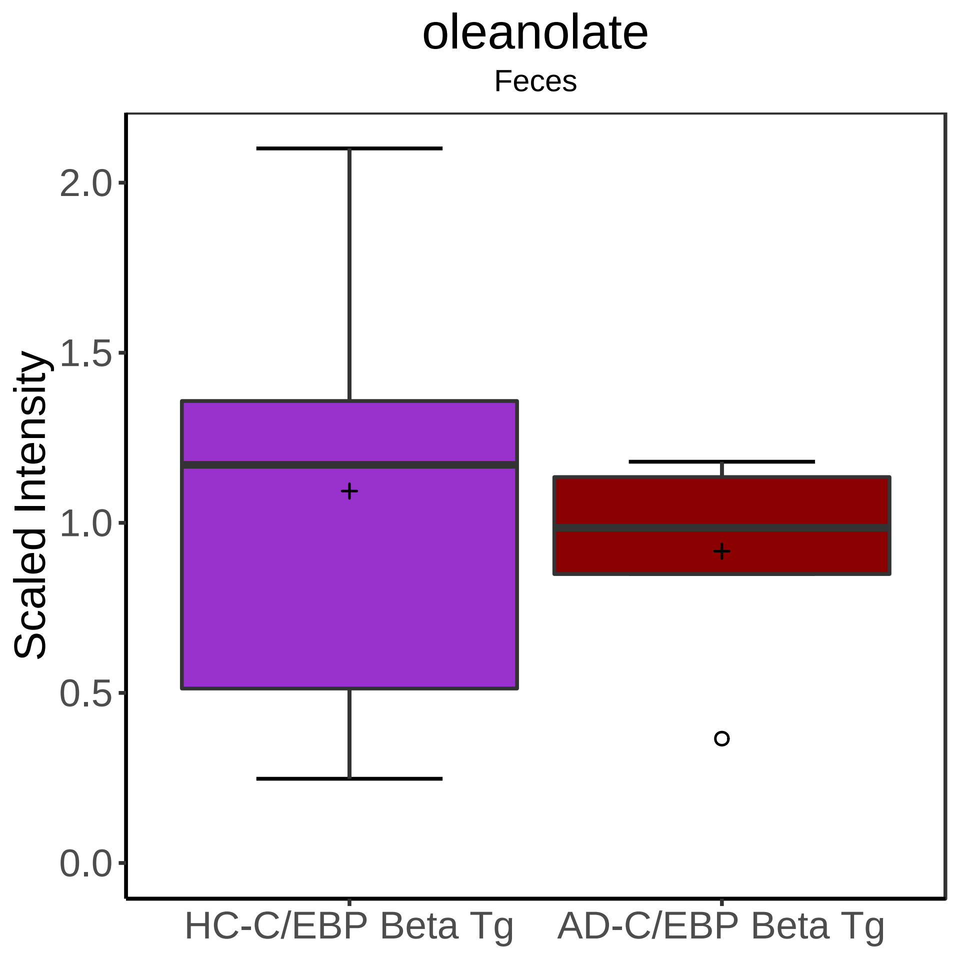
oleanolate
Feces
Scaled Intensity
2.0
1.5
1.0
0.5
0.0
HC-C/EBP Beta Tg
AD-C/EBP Beta Tg

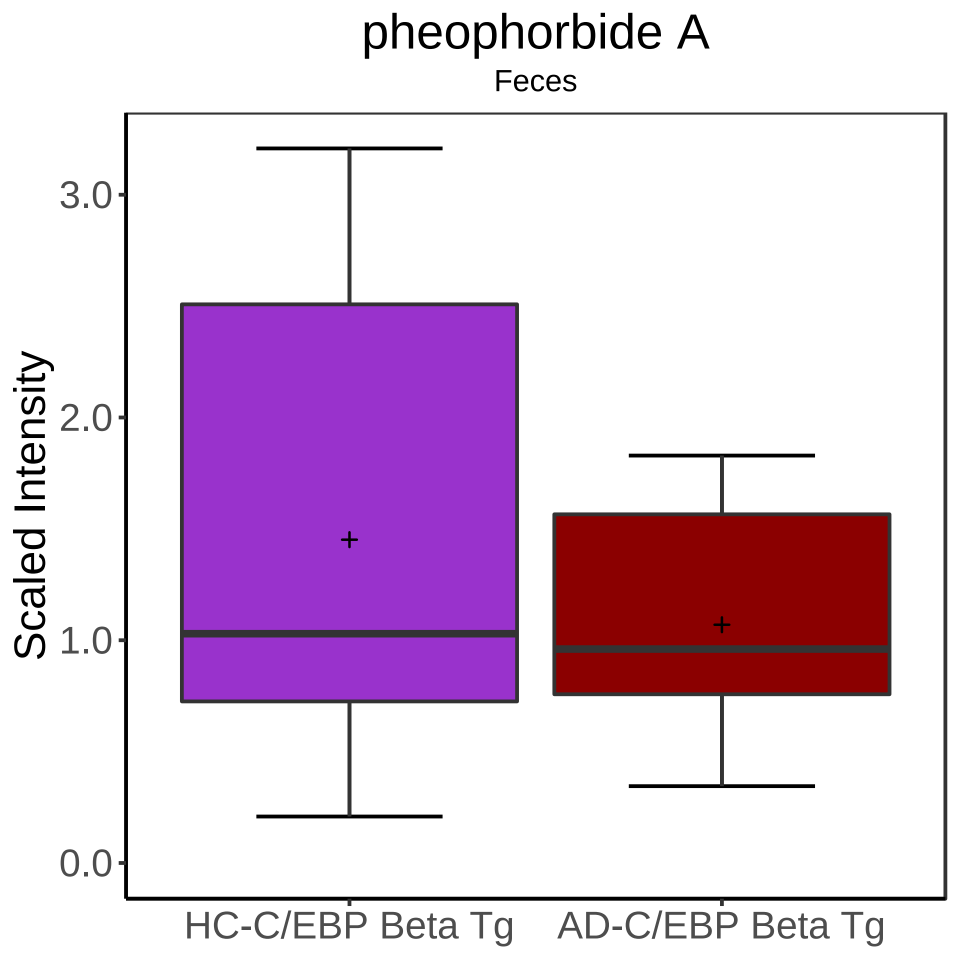
pheophorbide A
Feces
Scaled Intensity
3.0
2.0
1.0
0.0
HC-C/EBP Beta Tg
AD-C/EBP Beta Tg

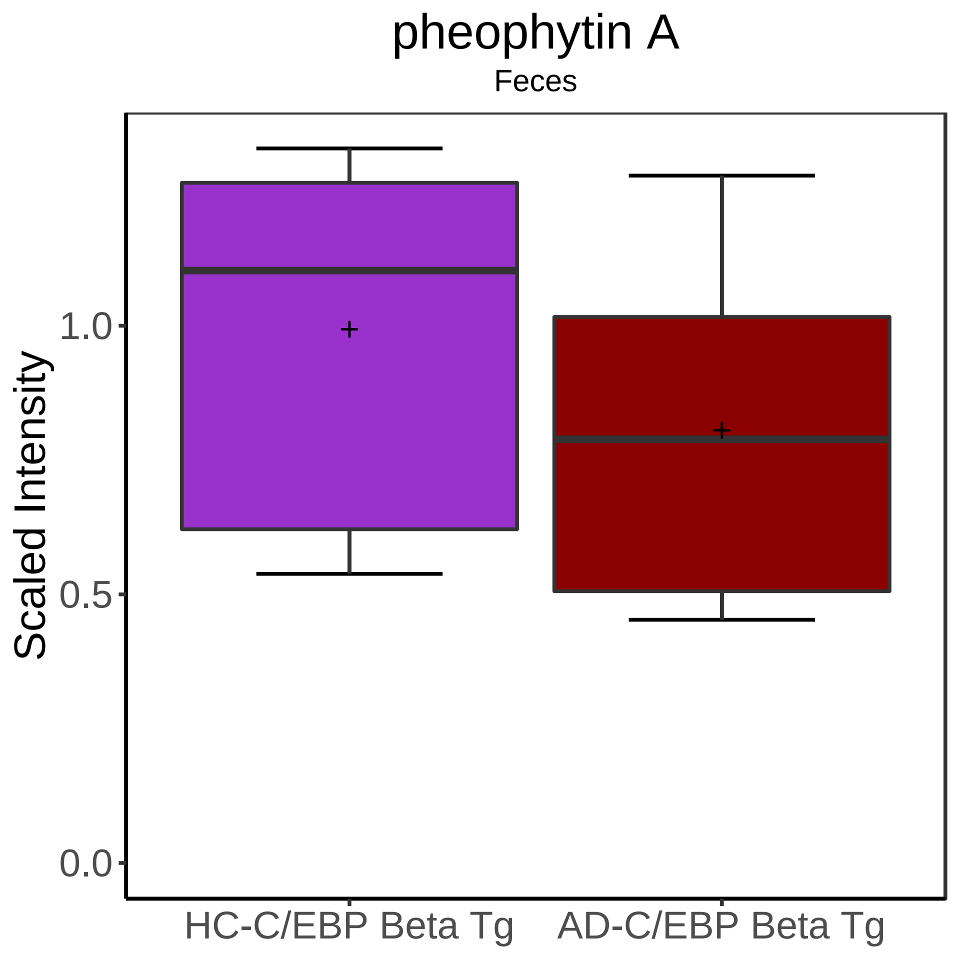
pheophytin A
Feces
Scaled Intensity
1.0
0.5
0.0
HC-C/EBP Beta Tg
AD-C/EBP Beta Tg

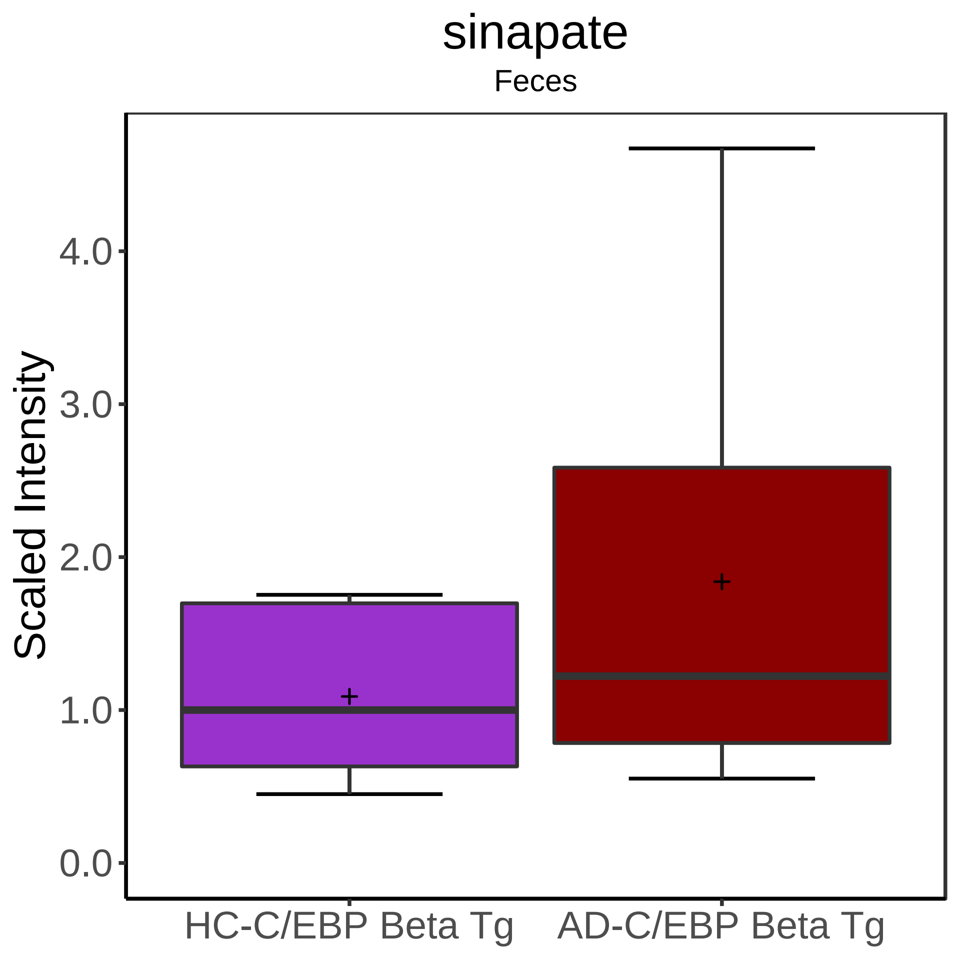

sinapate
Feces
Scaled Intensity
4.0
3.0
2.0
1.0
0.0
HC-C/EBP Beta Tg
AD-C/EBP Beta Tg

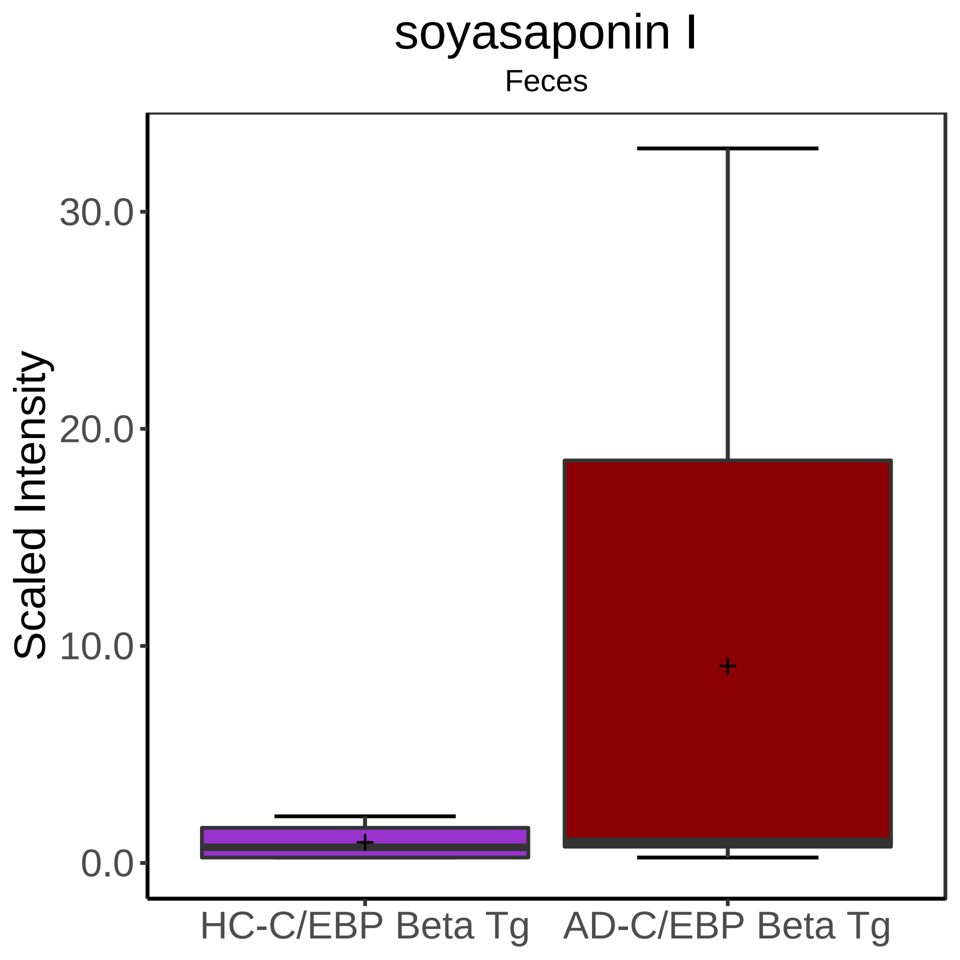
soyasaponin I
Feces
Scaled Intensity
30.0
20.0
10.0
0.0
HC-C/EBP Beta Tg
AD-C/EBP Beta Tg

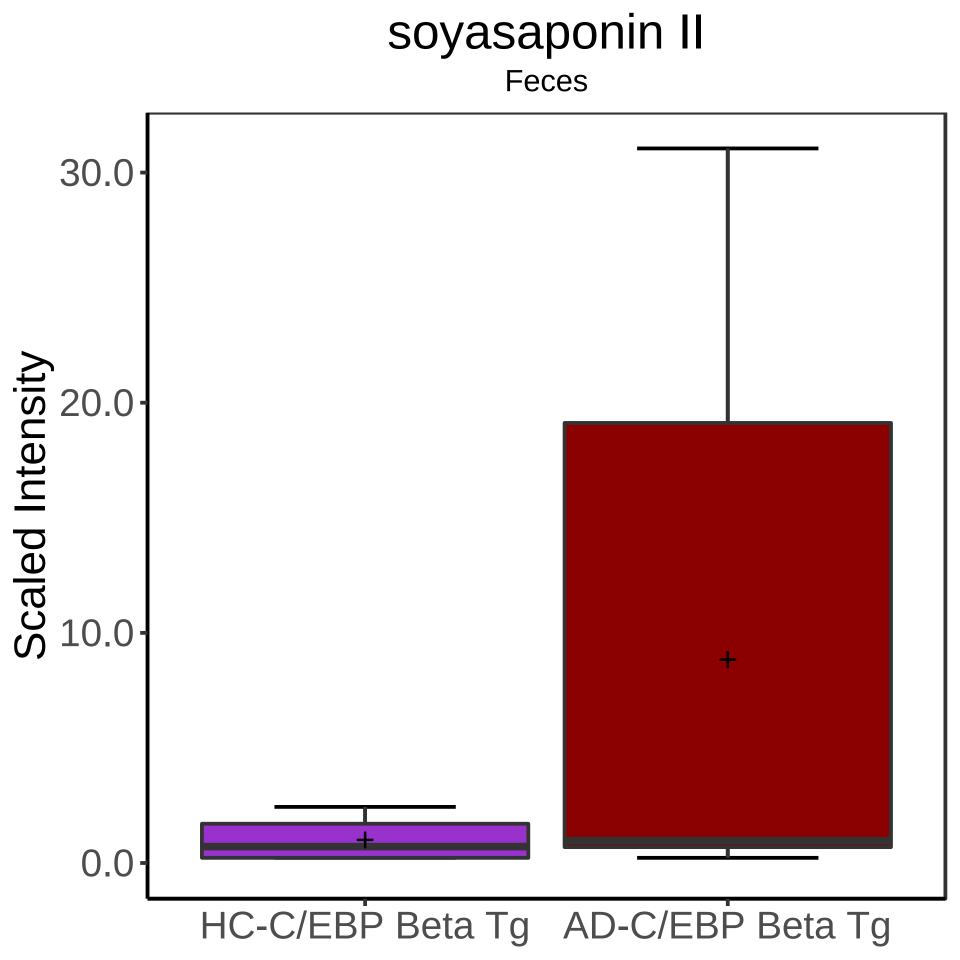

soyasaponin II
Feces
Scaled Intensity
30.0
20.0
10.0
0.0
HC-C/EBP Beta Tg
AD-C/EBP Beta Tg

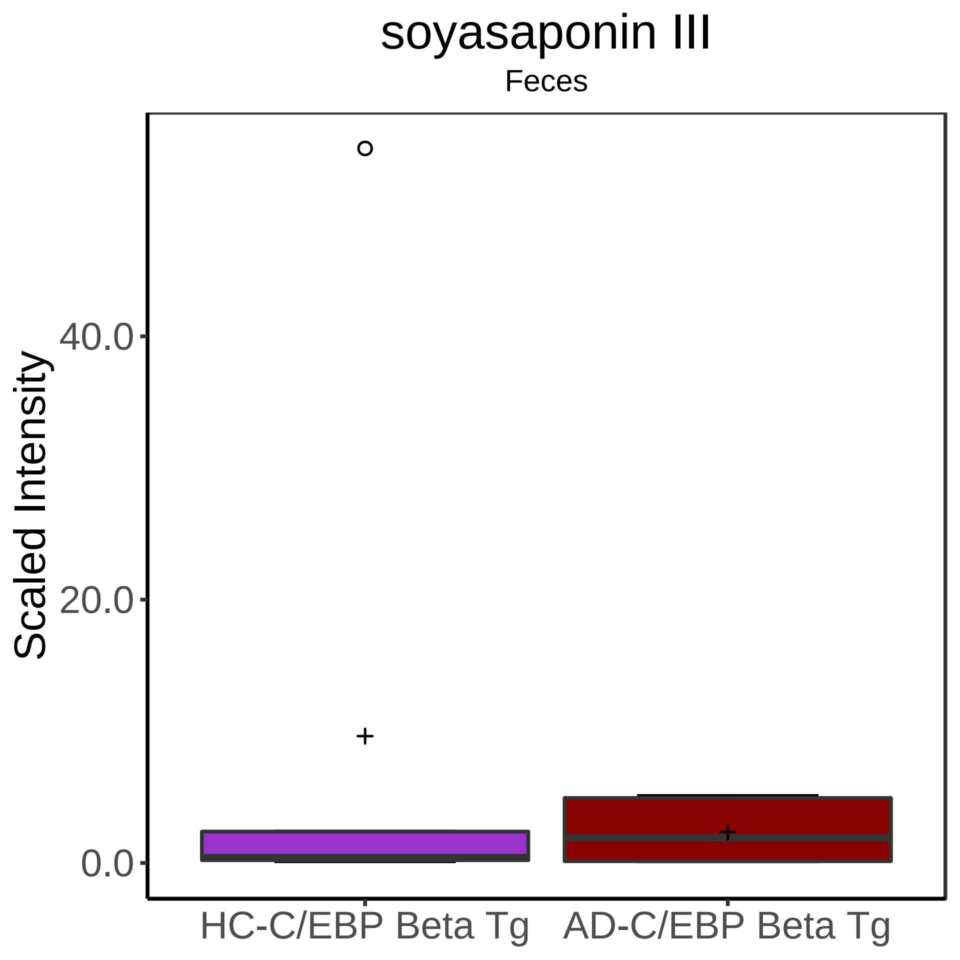

soyasaponin III
Feces
Scaled Intensity
40.0
20.0
0.0
HC-C/EBP Beta Tg
AD-C/EBP Beta Tg

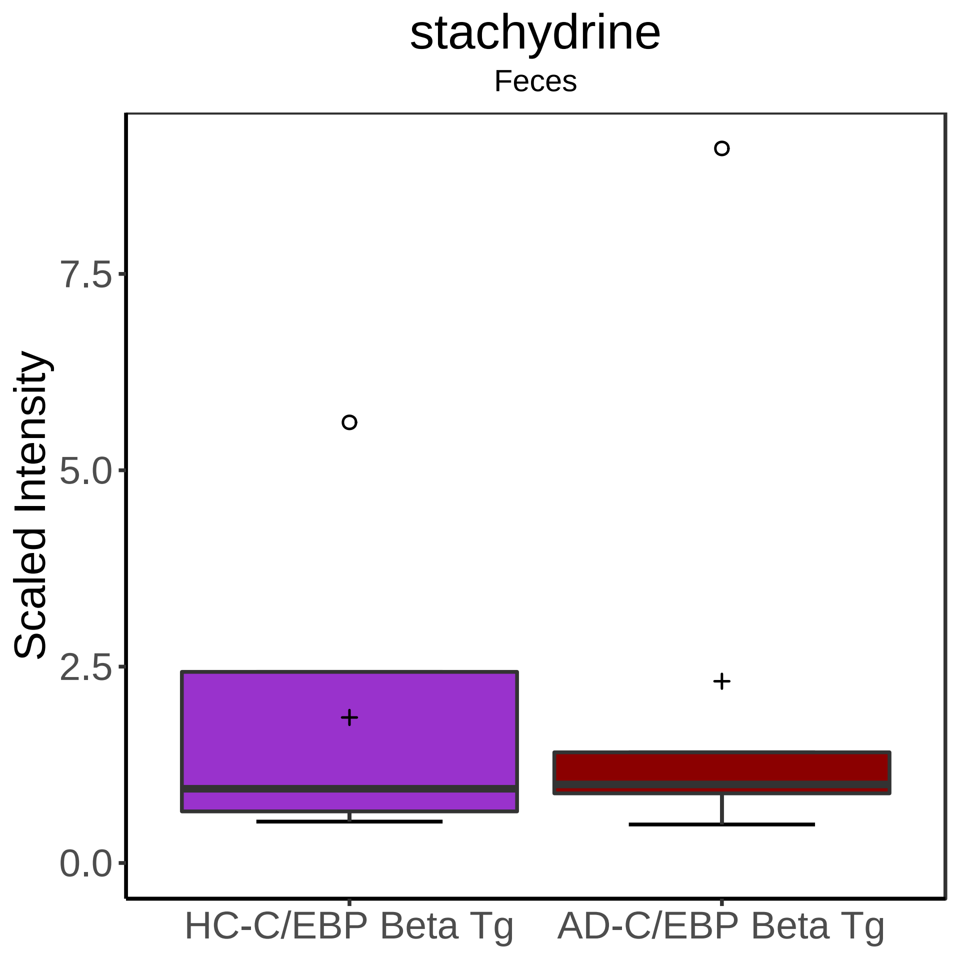
stachydrine
Feces
Scaled Intensity
7.5
5.0
2.5
0.0
HC-C/EBP Beta Tg
AD-C/EBP Beta Tg

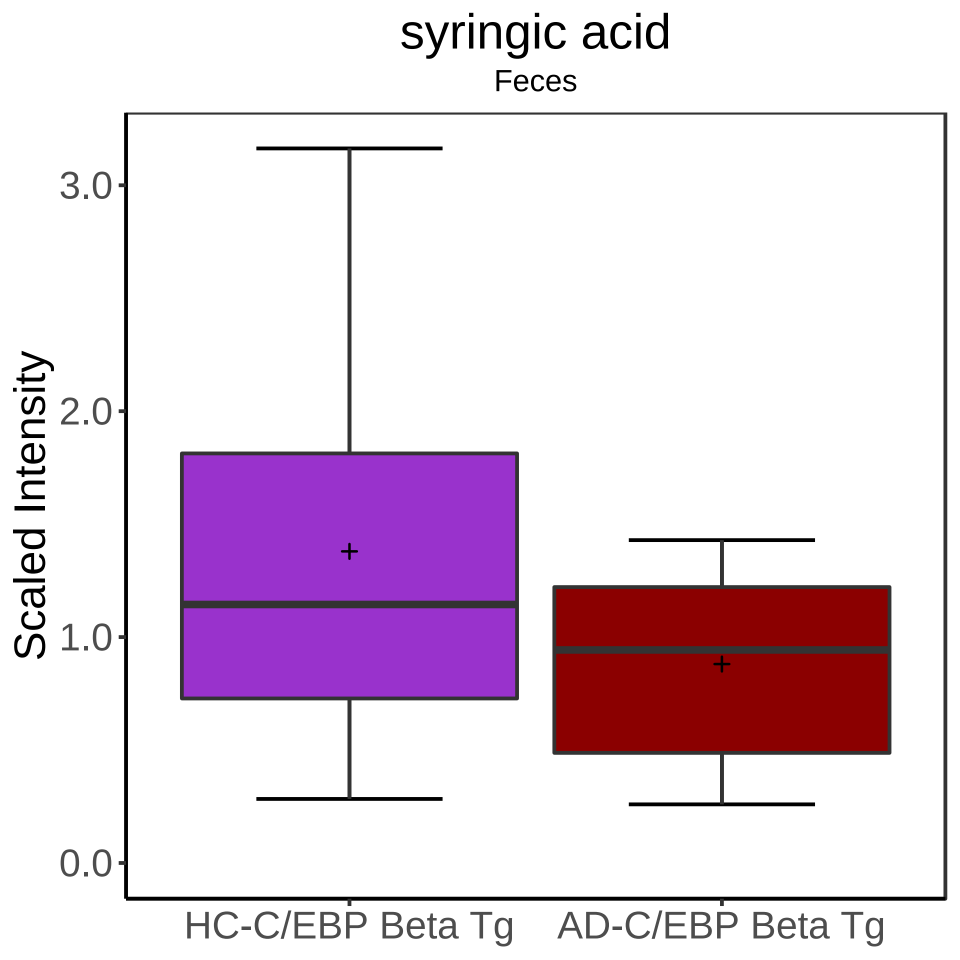

syringic acid
Feces
Scaled Intensity
3.0
2.0
1.0
0.0
HC-C/EBP Beta Tg
AD-C/EBP Beta Tg

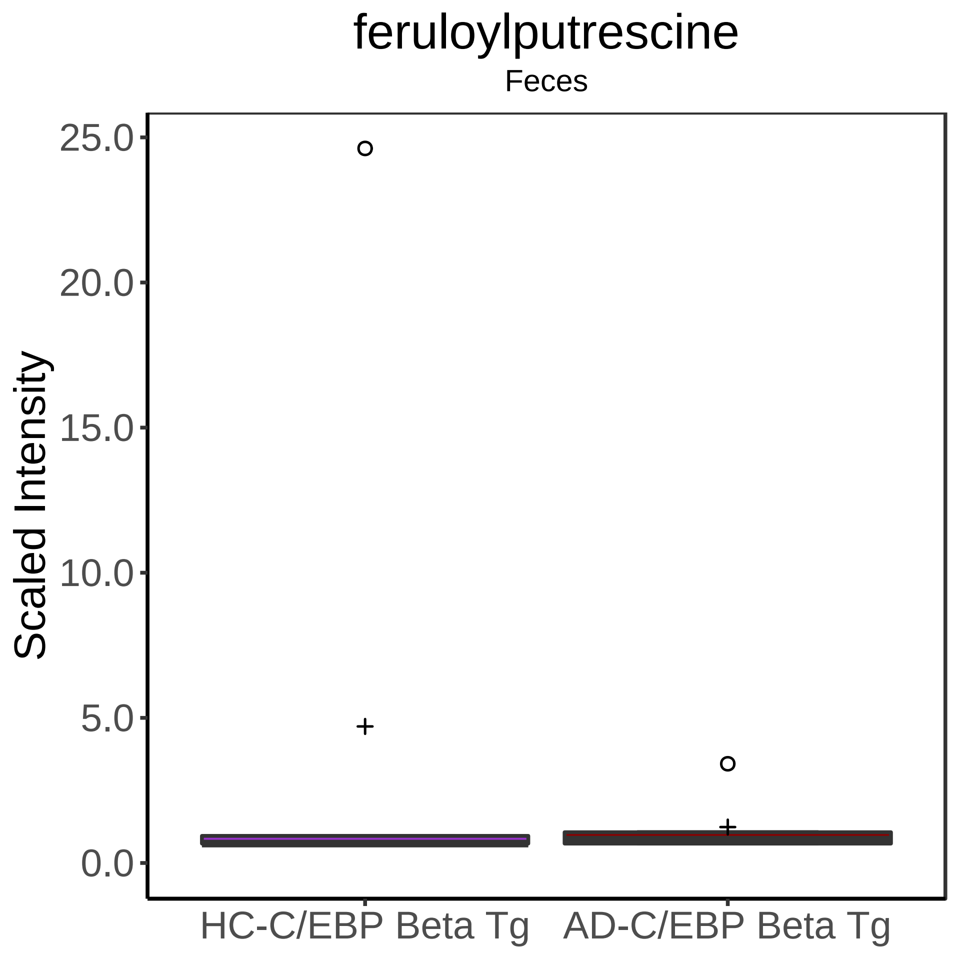
feruloylputrescine
Feces
Scaled Intensity
25.0
20.0
15.0
10.0
5.0
0.0
HC-C/EBP Beta Tg
AD-C/EBP Beta Tg

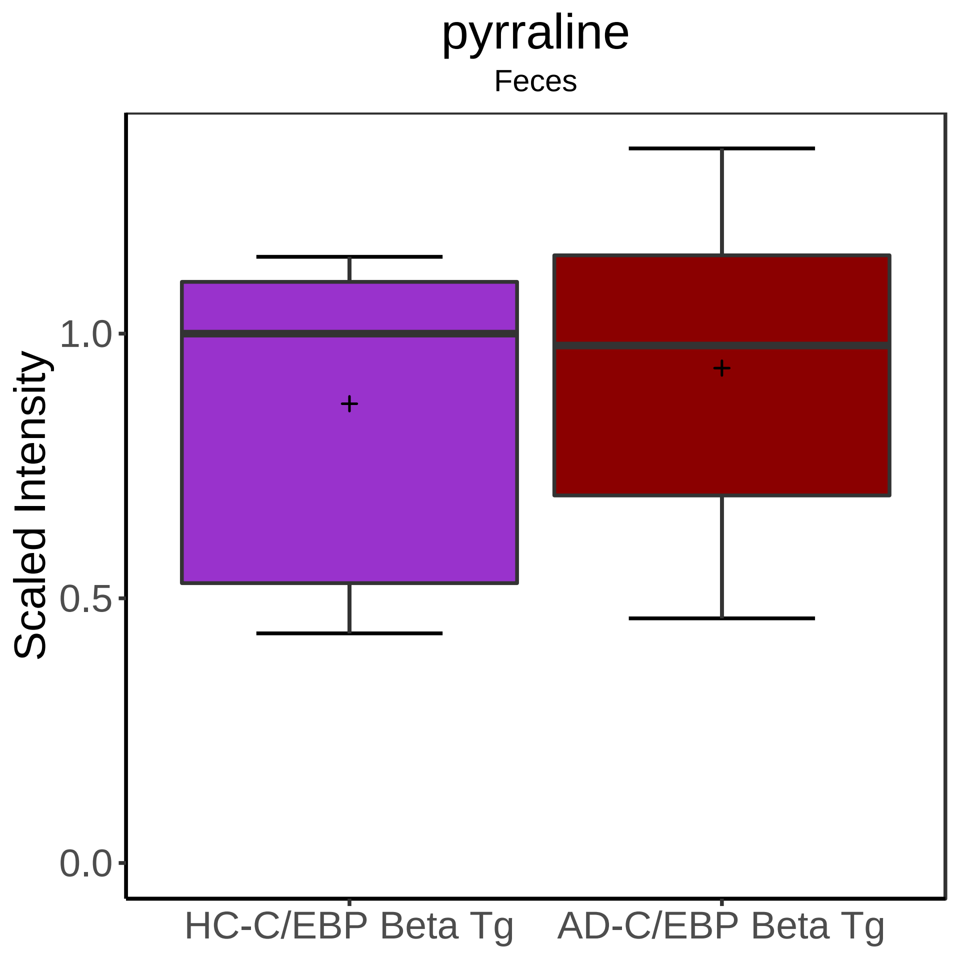

pyrraline
Feces
Scaled Intensity
1.0
0.5
0.0
HC-C/EBP Beta Tg
AD-C/EBP Beta Tg

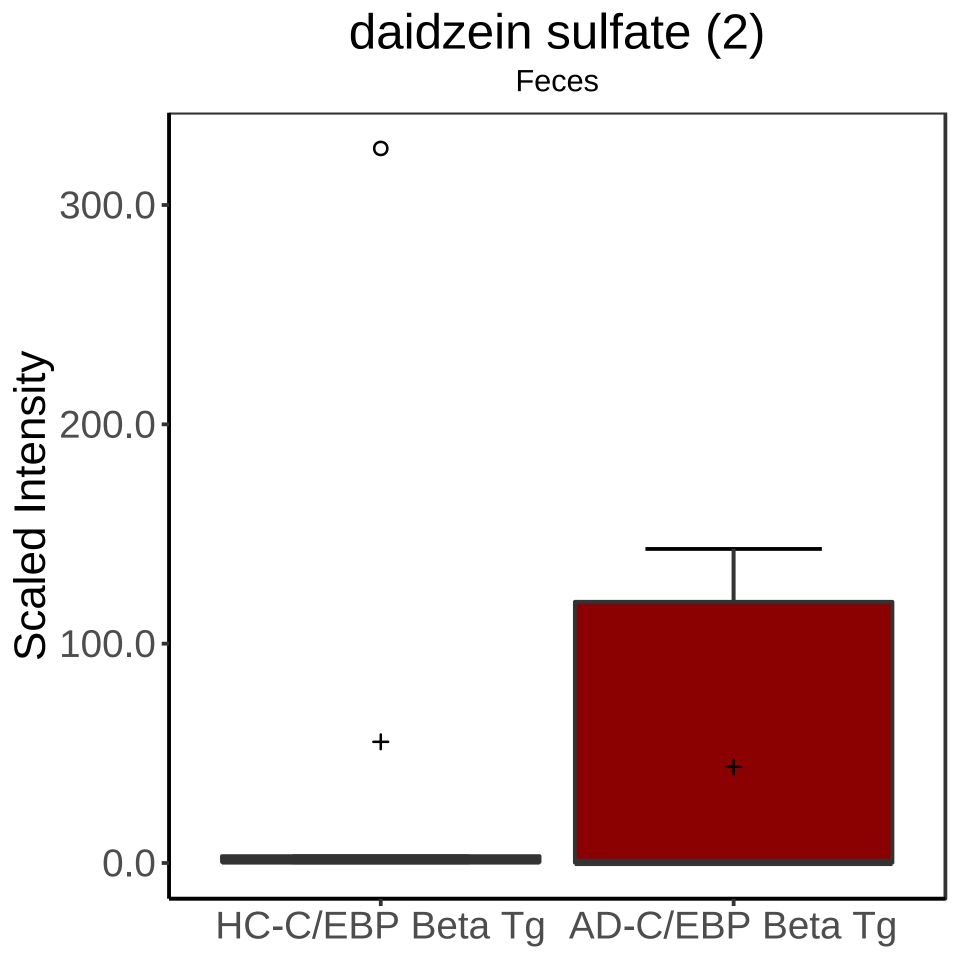
daidzein sulfate (2)
Feces
Scaled Intensity
300.0
200.0
100.0
0.0
HC-C/EBP Beta Tg
AD-C/EBP Beta Tg

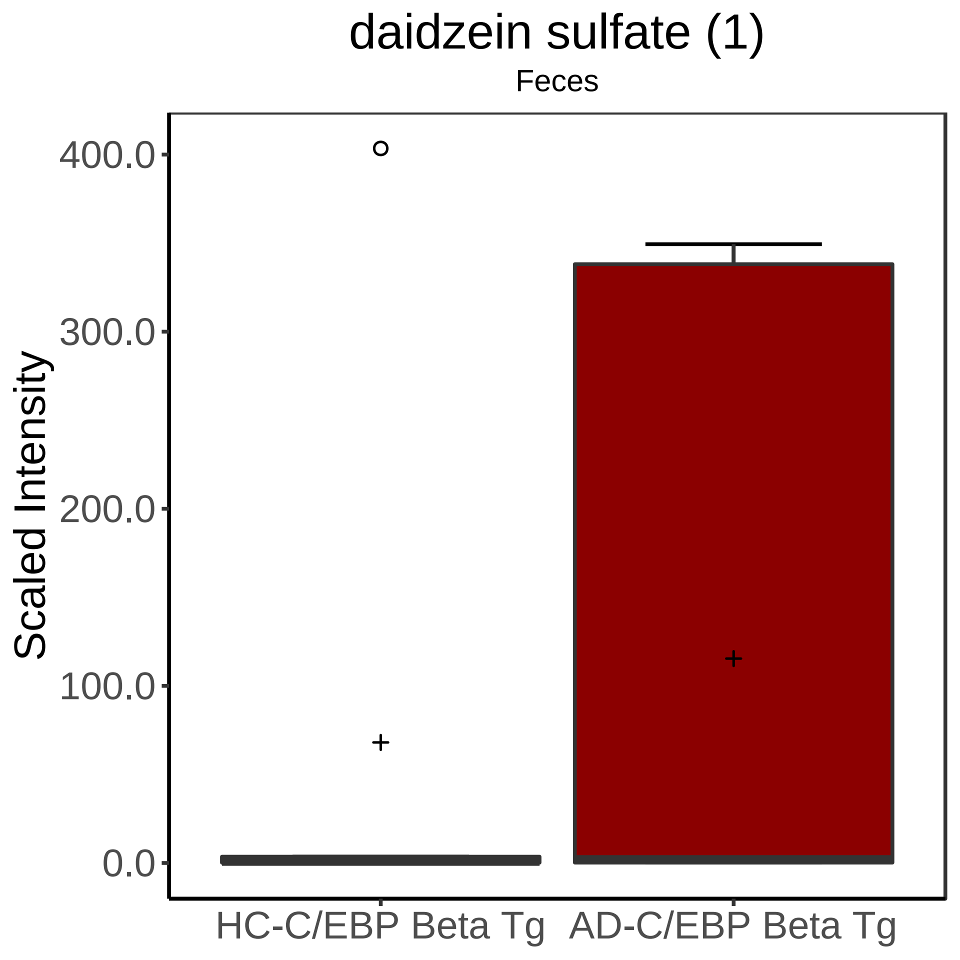

daidzein sulfate (1)
Feces
Scaled Intensity
400.0
300.0
200.0
100.0
0.0
HC-C/EBP Beta Tg
AD-C/EBP Beta Tg

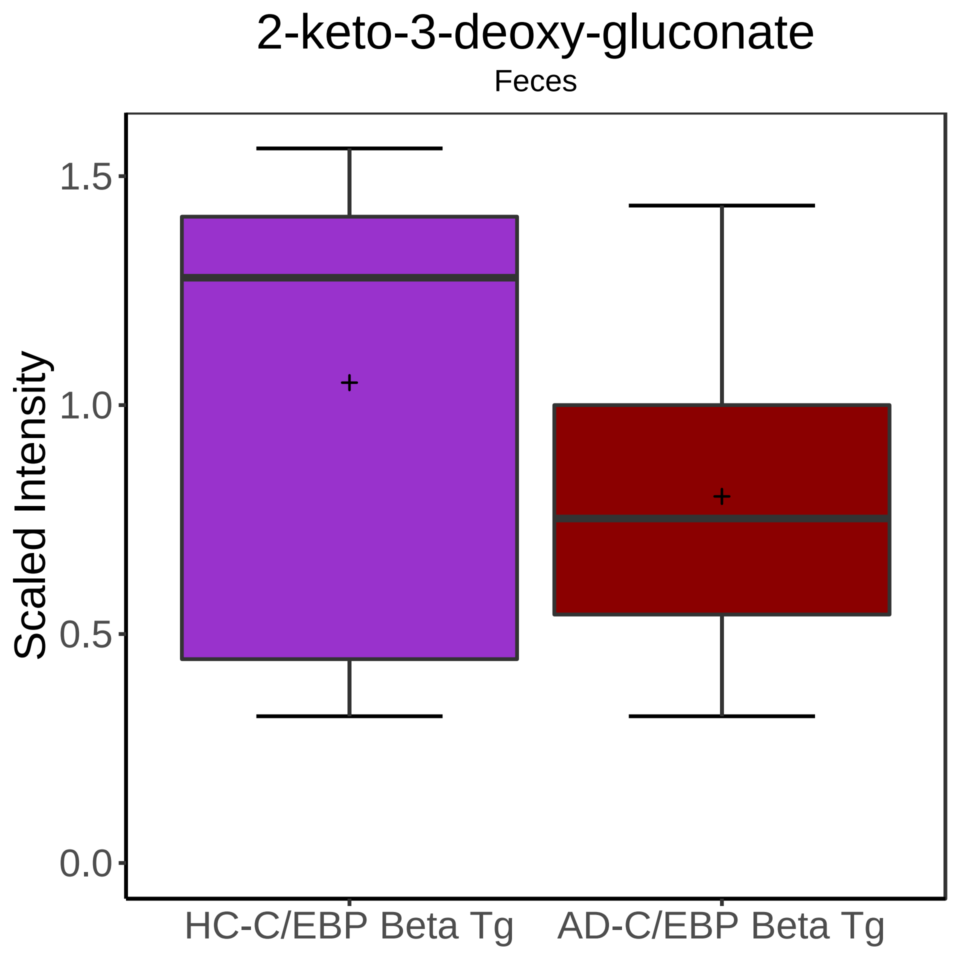
2-keto-3-deoxy-gluconate
Feces
Scaled Intensity
1.5
1.0
0.5
0.0
HC-C/EBP Beta Tg
AD-C/EBP Beta Tg

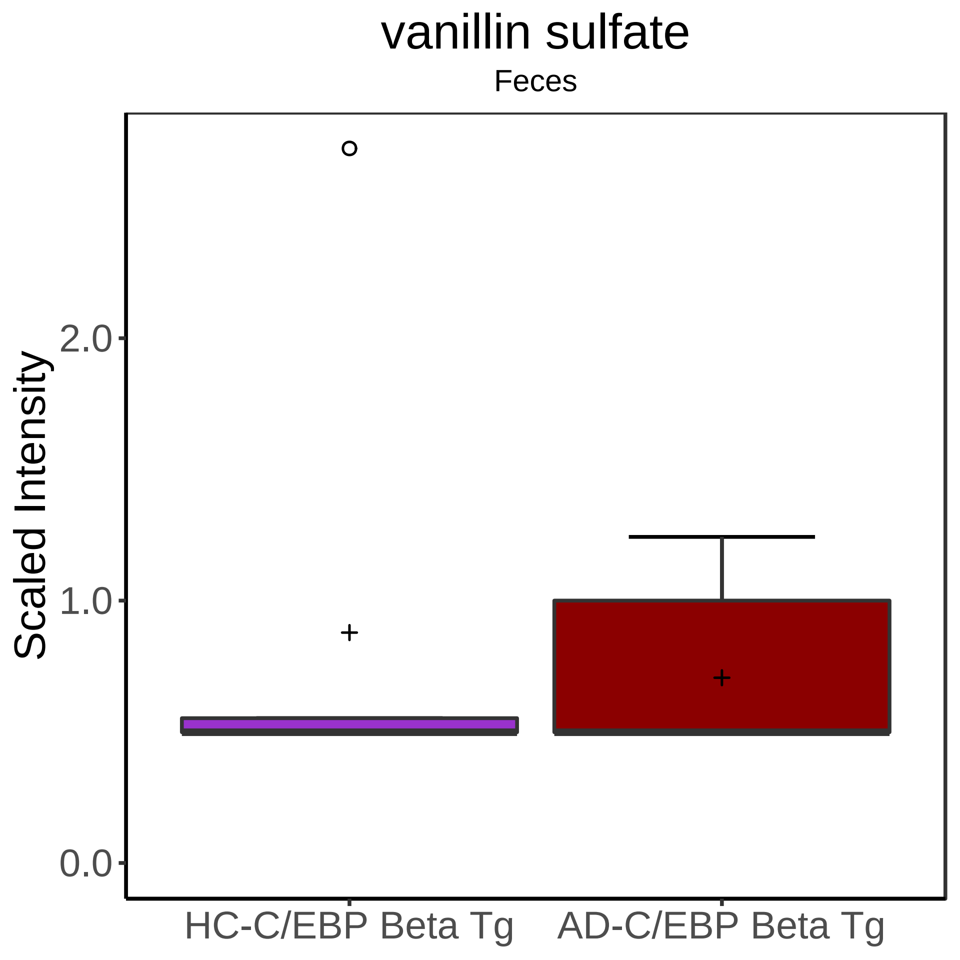

vanillin sulfate
Feces
Scaled Intensity
2.0
1.0
0.0
HC-C/EBP Beta Tg
AD-C/EBP Beta Tg

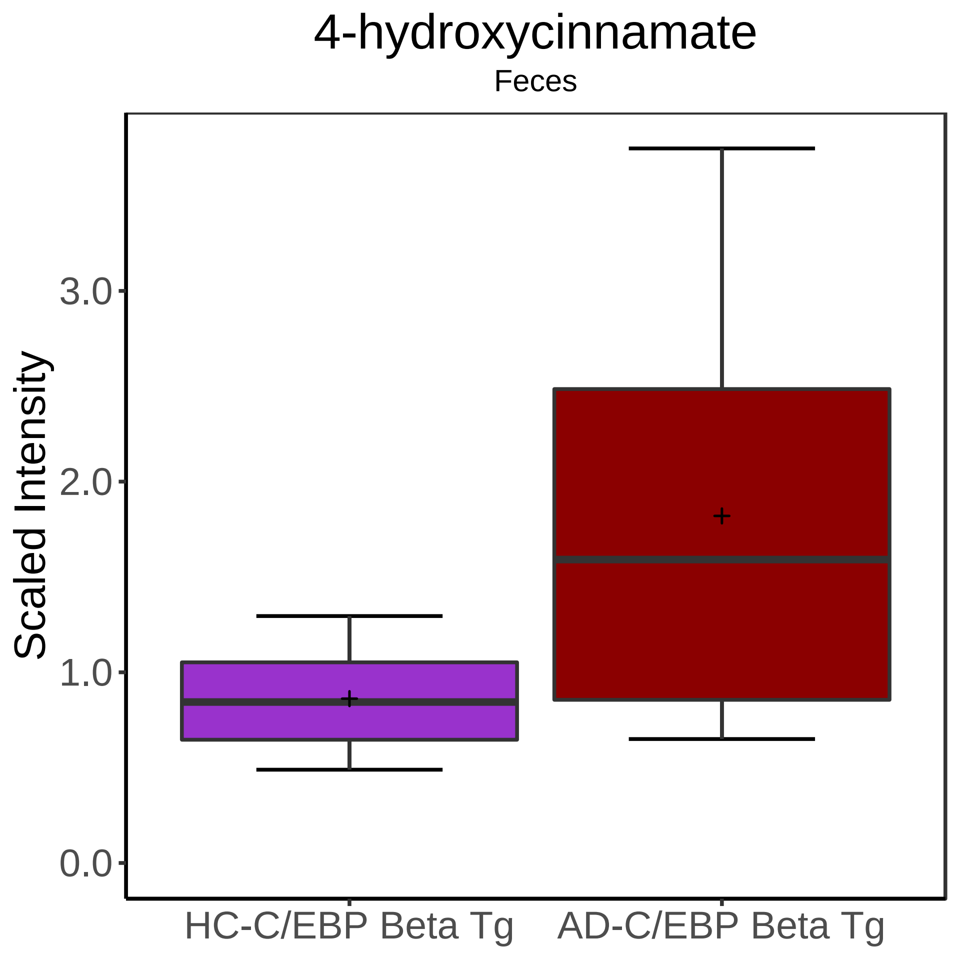

4-hydroxycinnamate
Feces
Scaled Intensity
3.0
2.0
1.0
0.0
HC-C/EBP Beta Tg
AD-C/EBP Beta Tg

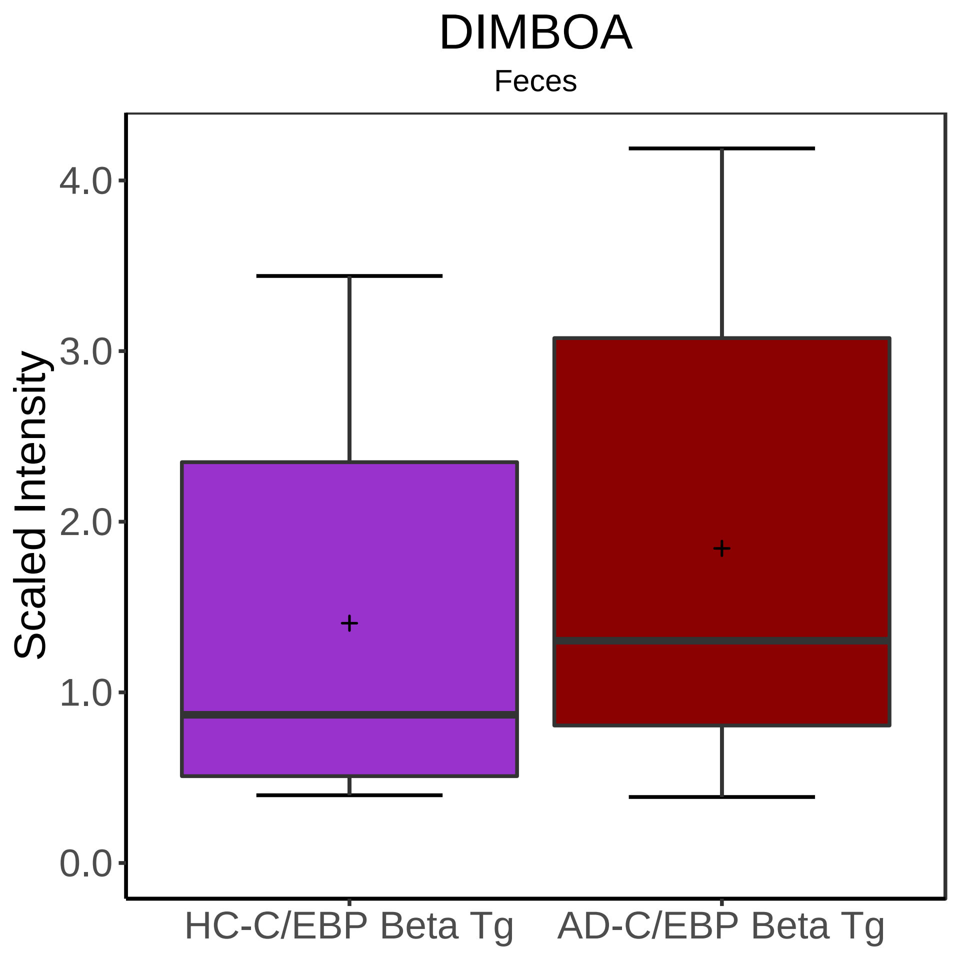

DIMBOA
Feces
Scaled Intensity
4.0
3.0
2.0
1.0
0.0
HC-C/EBP Beta Tg
AD-C/EBP Beta Tg

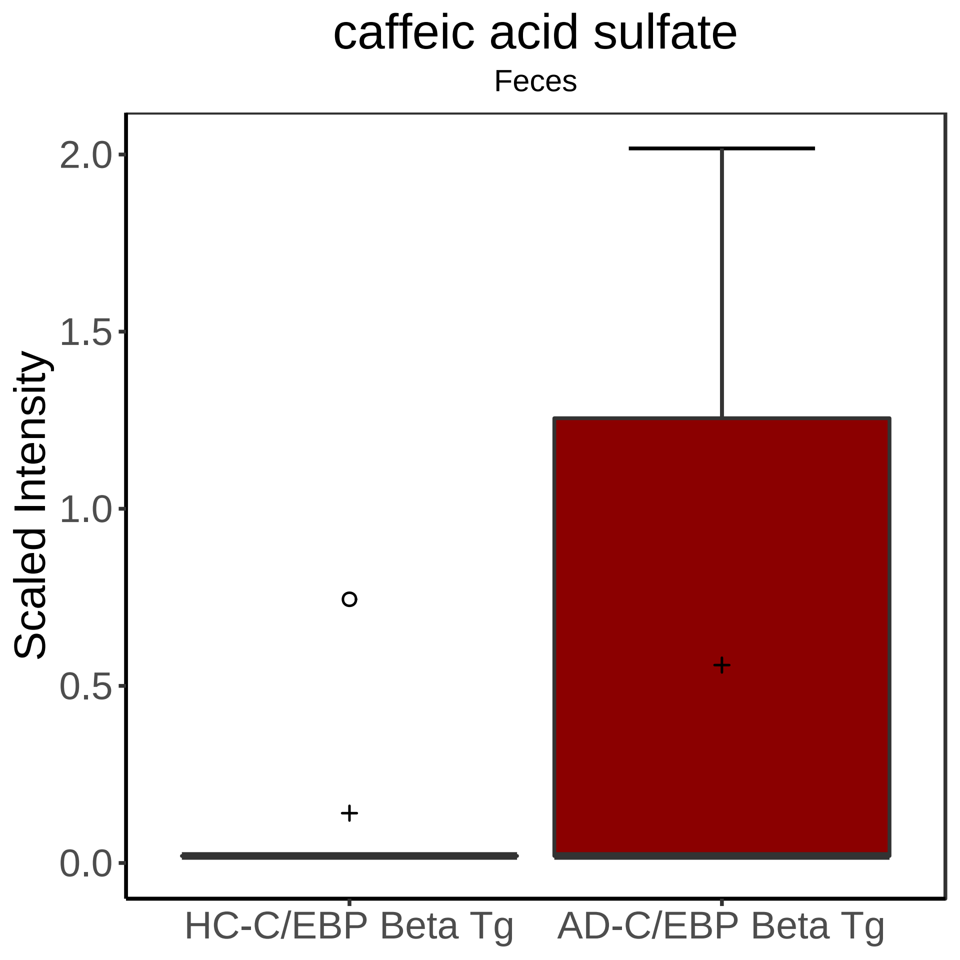

caffeic acid sulfate
Feces
Scaled Intensity
2.0
1.5
1.0
0.5
0.0
HC-C/EBP Beta Tg
AD-C/EBP Beta Tg

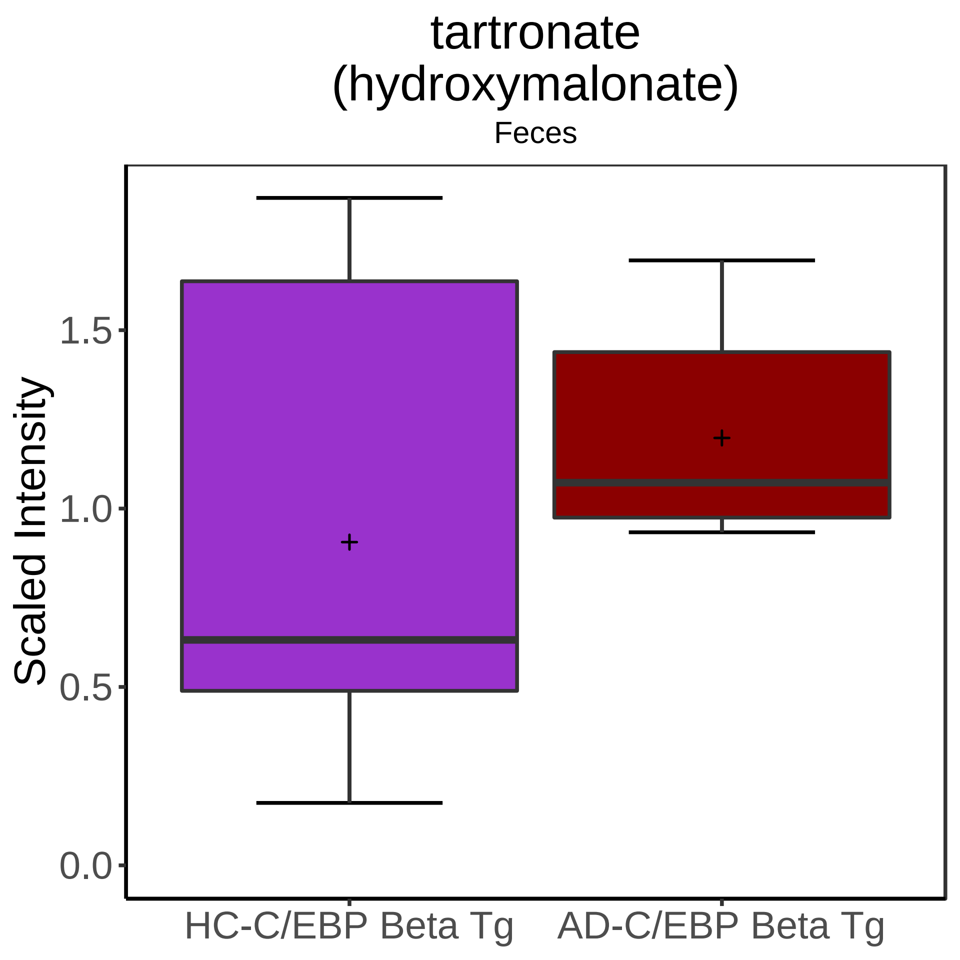
tartronate
(hydroxymalonate)
Feces
Scaled Intensity
1.5
1.0
0.5
0.0
HC-C/EBP Beta Tg
AD-C/EBP Beta Tg

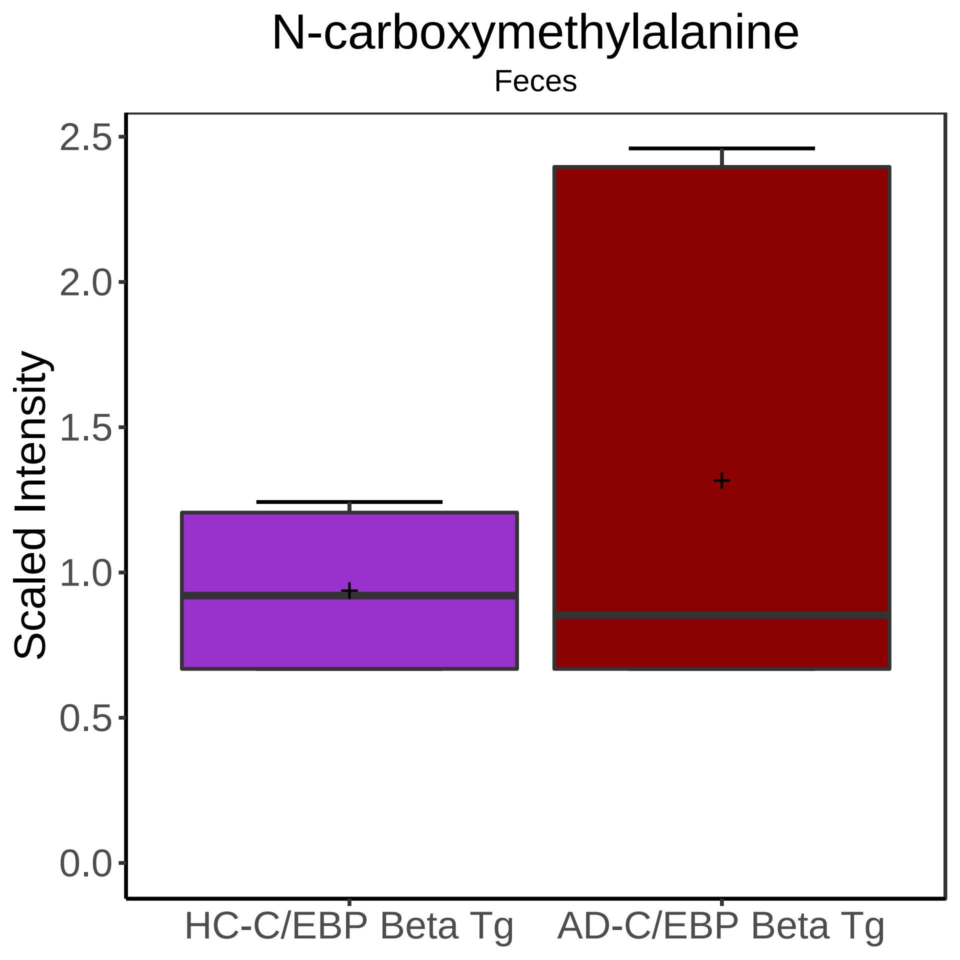
N-carboxymethylalanine
Feces
Scaled Intensity
2.5
2.0
1.5
1.0
0.5
0.0
HC-C/EBP Beta Tg
AD-C/EBP Beta Tg

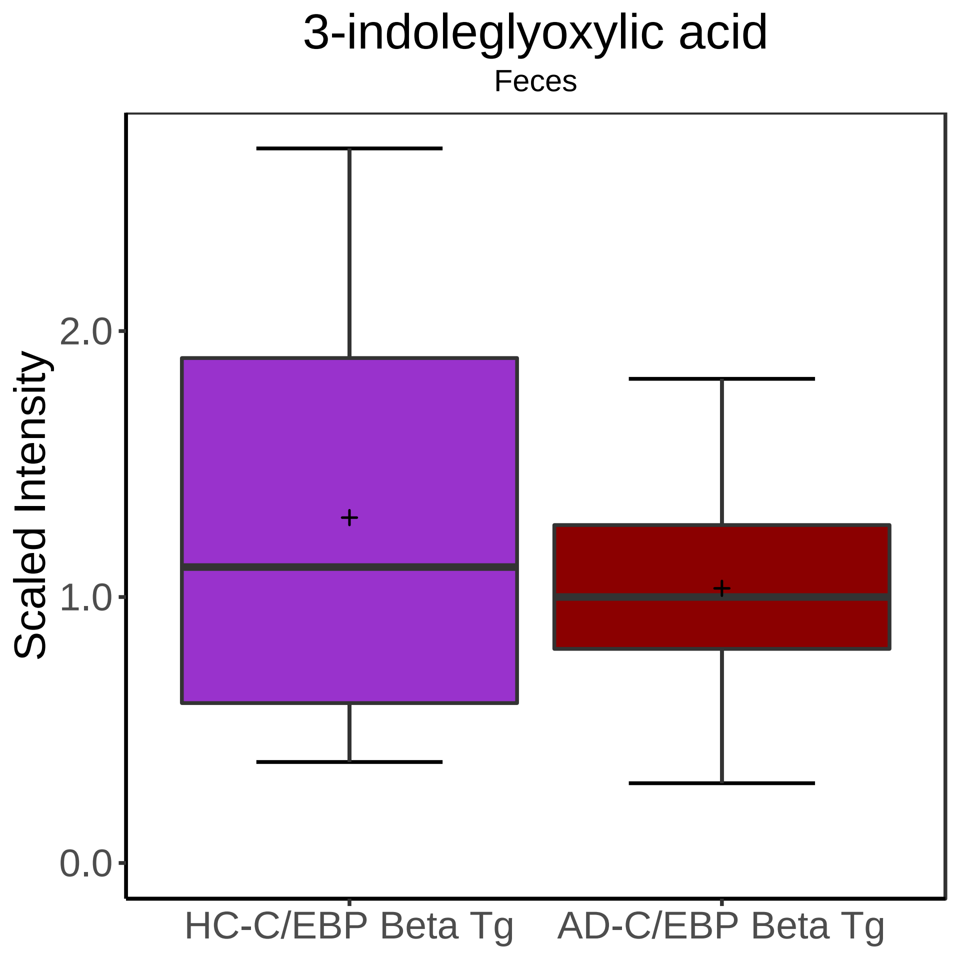
3-indoleglyoxylic acid
Feces
Scaled Intensity
2.0
1.0
0.0
HC-C/EBP Beta Tg
AD-C/EBP Beta Tg

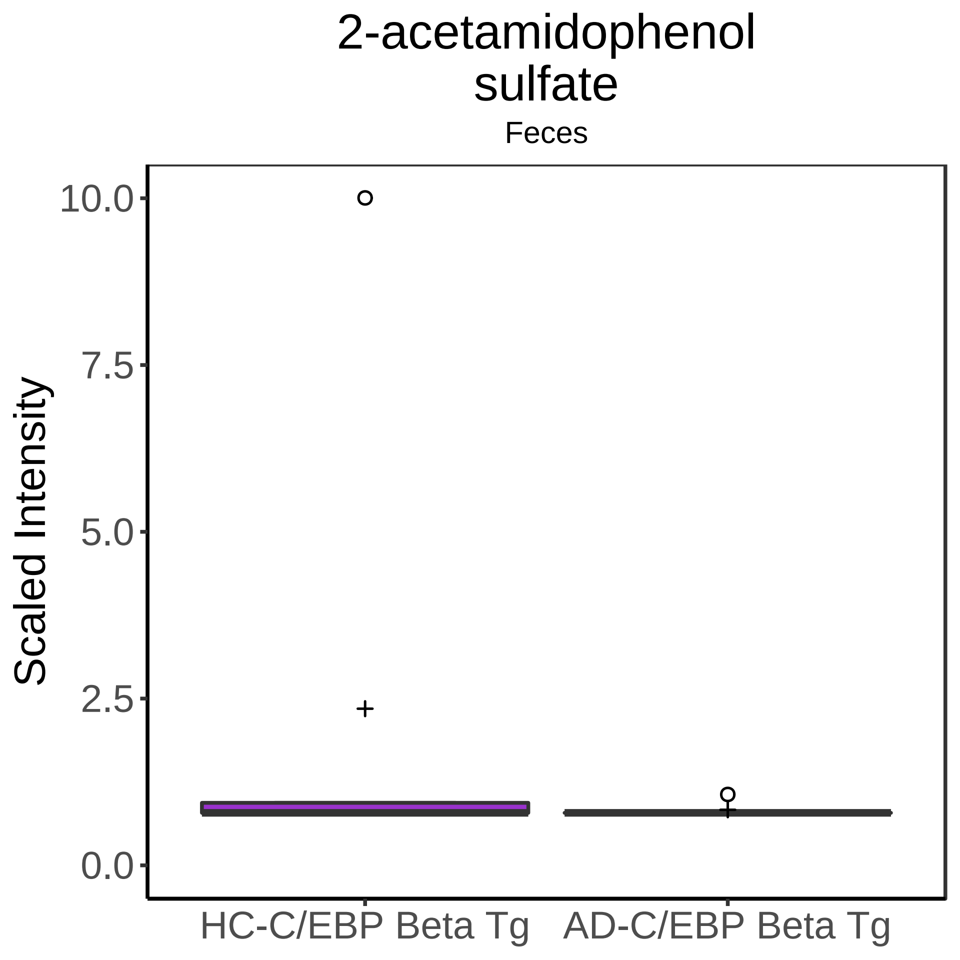
2-acetamidophenol
sulfate
Feces
Scaled Intensity
10.0
7.5
5.0
2.5
0.0
HC-C/EBP Beta Tg
AD-C/EBP Beta Tg

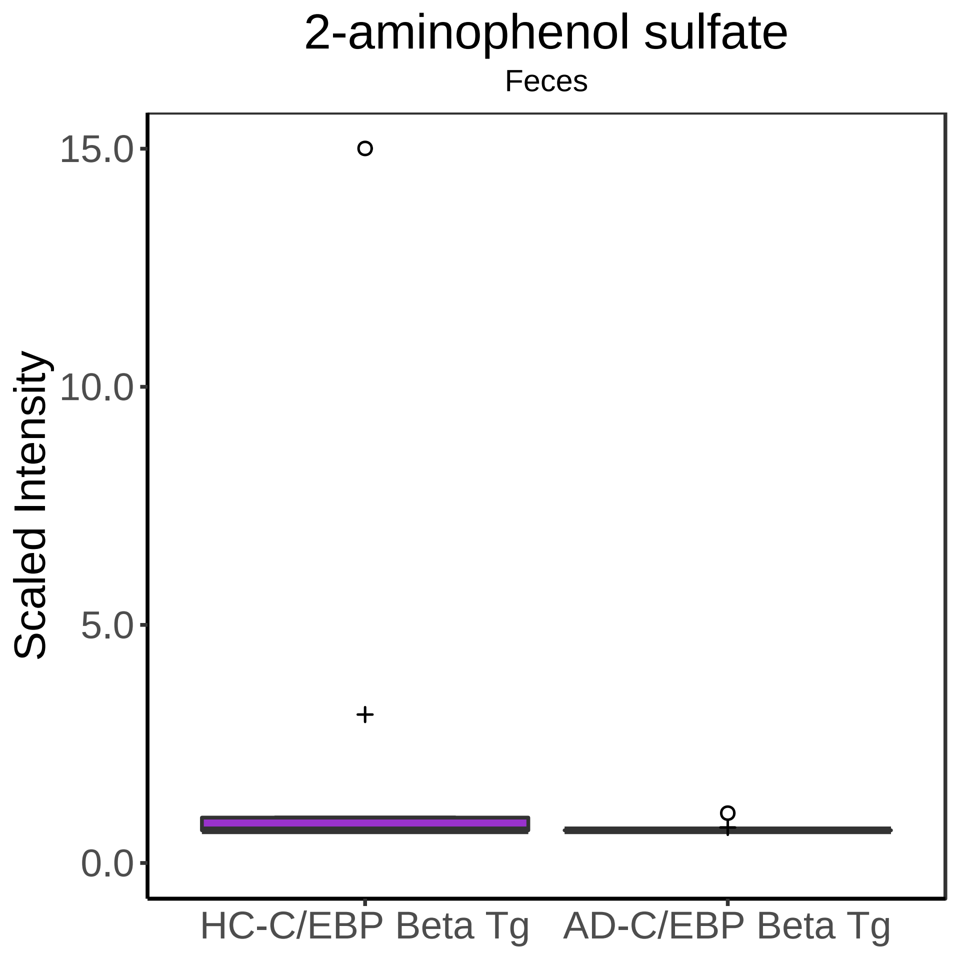
2-aminophenol sulfate
Feces
Scaled Intensity
15.0
10.0
5.0
0.0
HC-C/EBP Beta Tg
AD-C/EBP Beta Tg

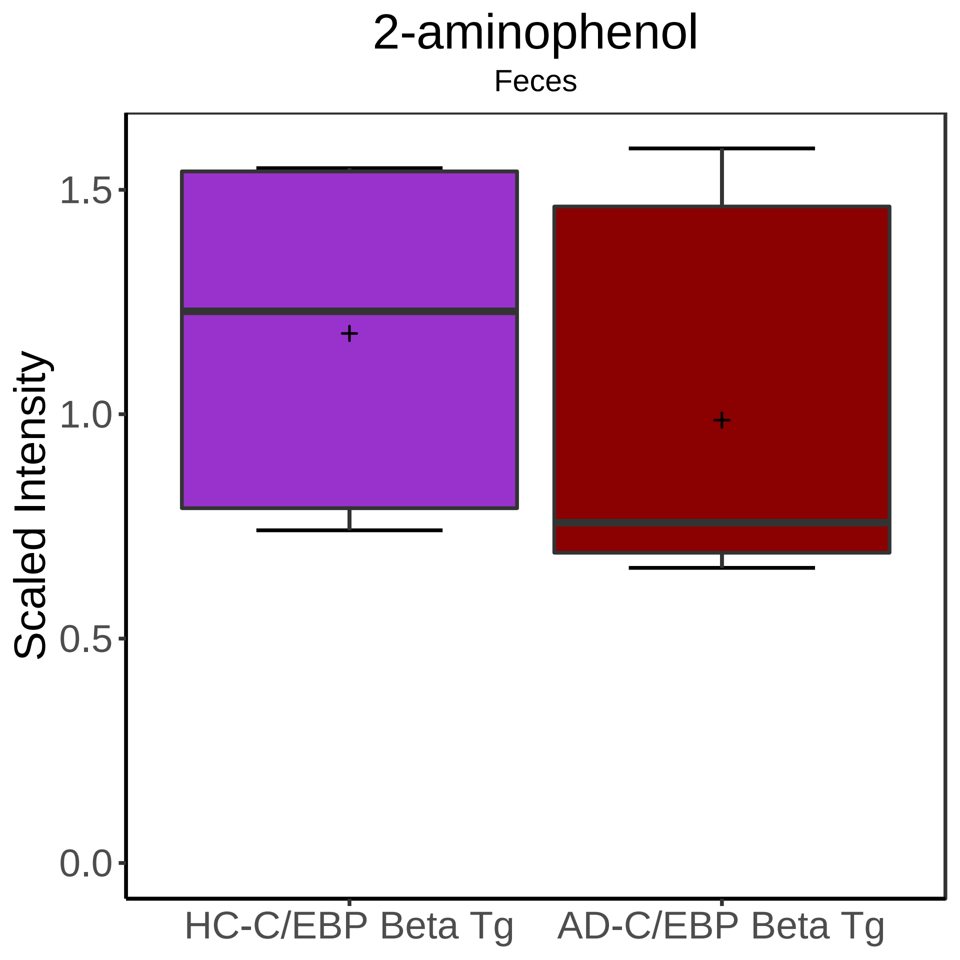

2-aminophenol
Feces
Scaled Intensity
1.5
1.0
0.5
0.0
HC-C/EBP Beta Tg
AD-C/EBP Beta Tg

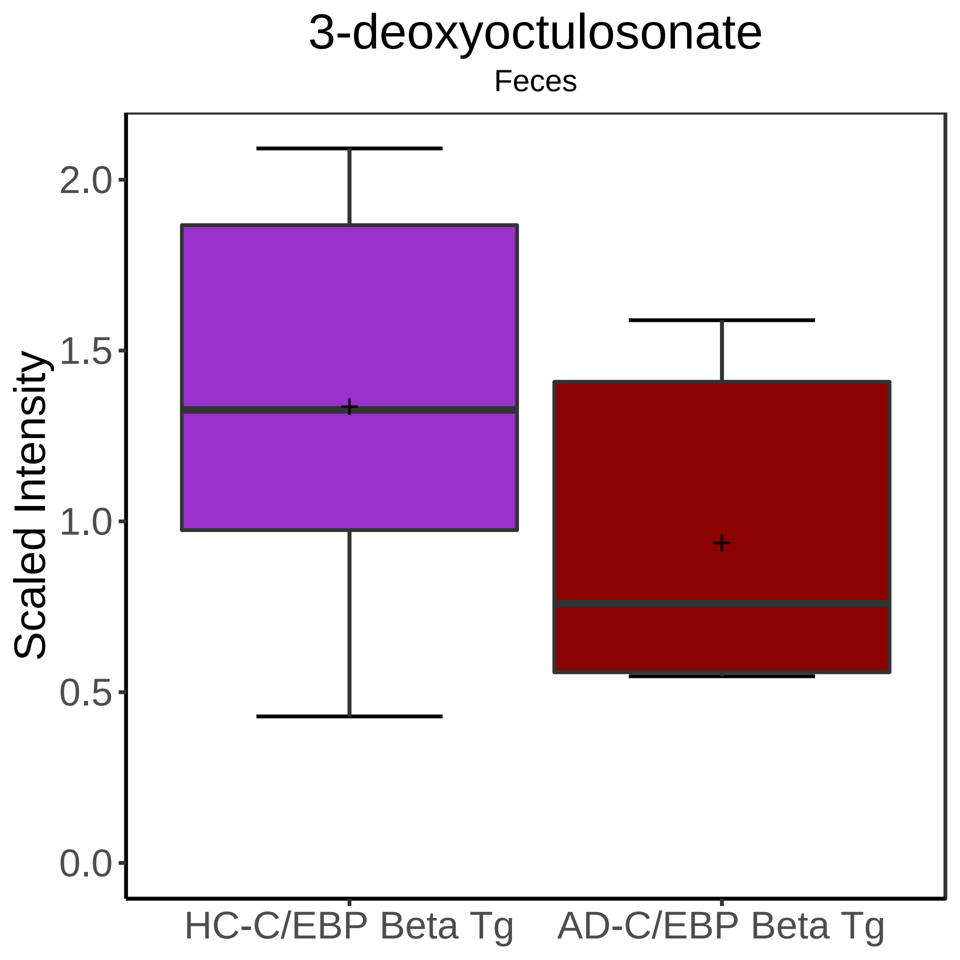
3-deoxyoctulosonate
Feces
Scaled Intensity
2.0
1.5
1.0
0.5
0.0
HC-C/EBP Beta Tg
AD-C/EBP Beta Tg

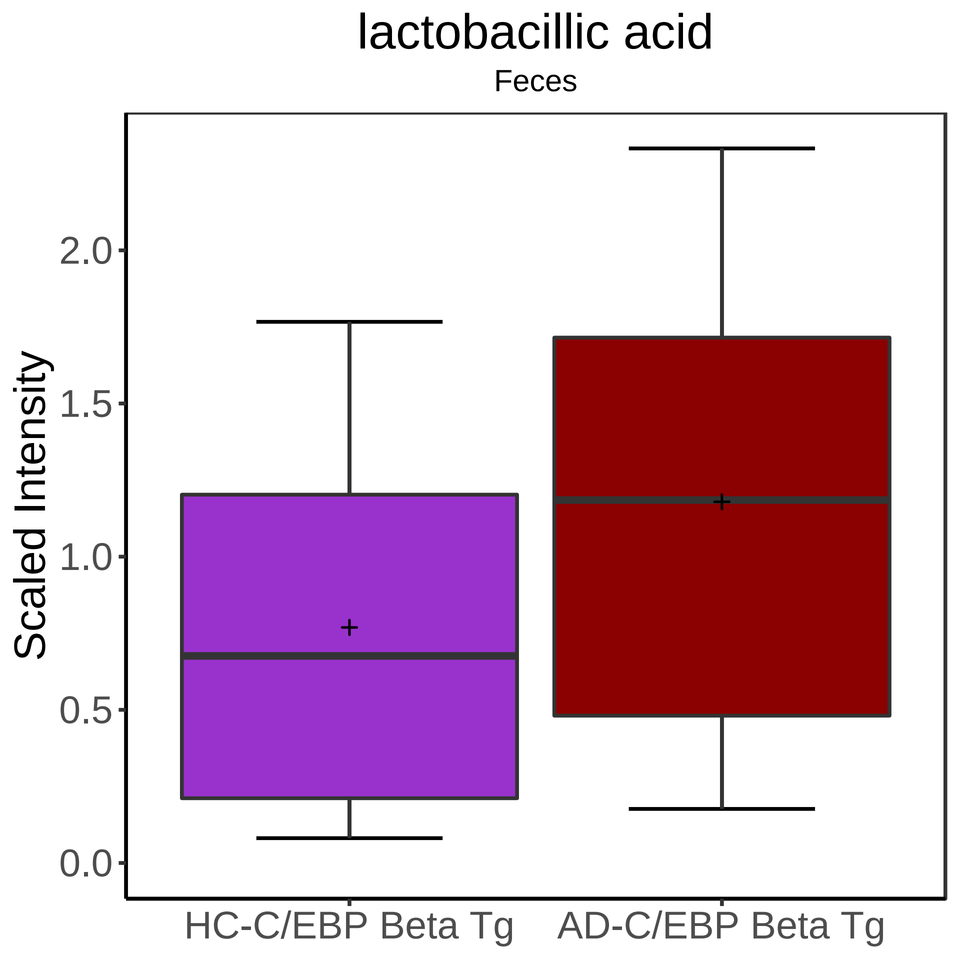
lactobacillic acid
Feces
Scaled Intensity
2.0
1.5
1.0
0.5
0.0
HC-C/EBP Beta Tg
AD-C/EBP Beta Tg

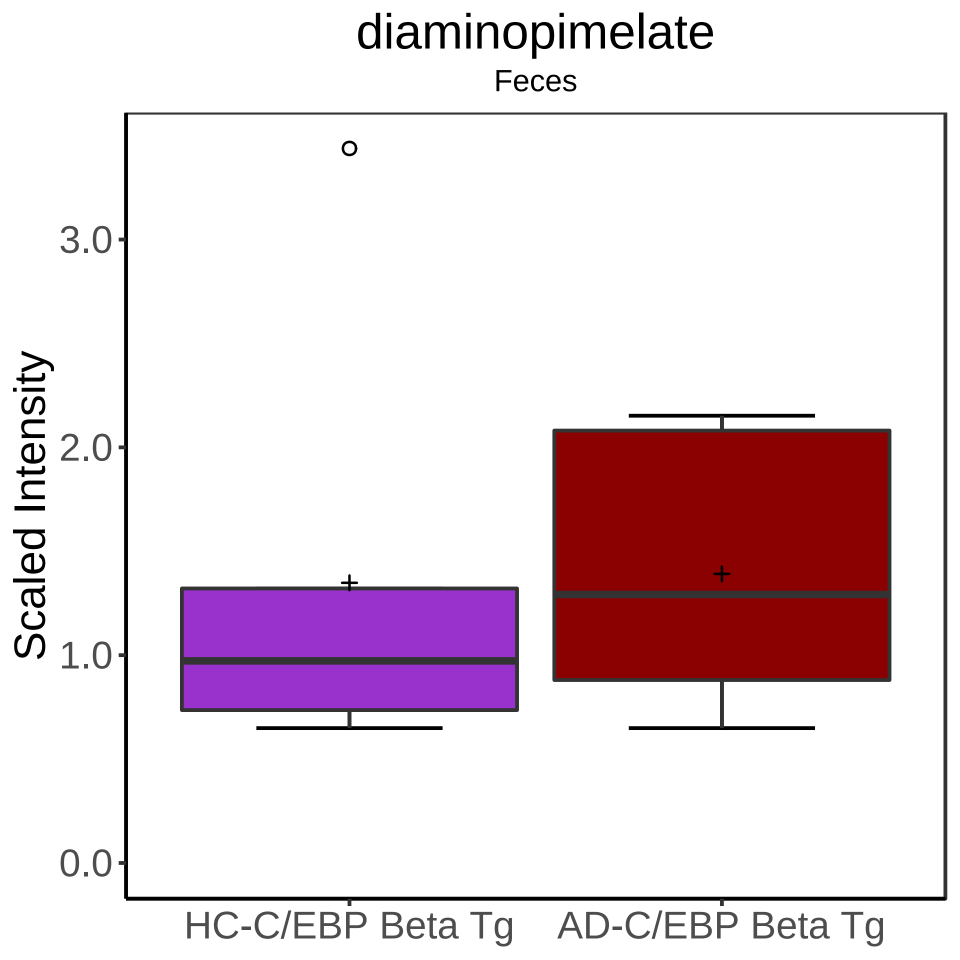

diaminopimelate
Feces
Scaled Intensity
3.0
2.0
1.0
0.0
HC-C/EBP Beta Tg
AD-C/EBP Beta Tg

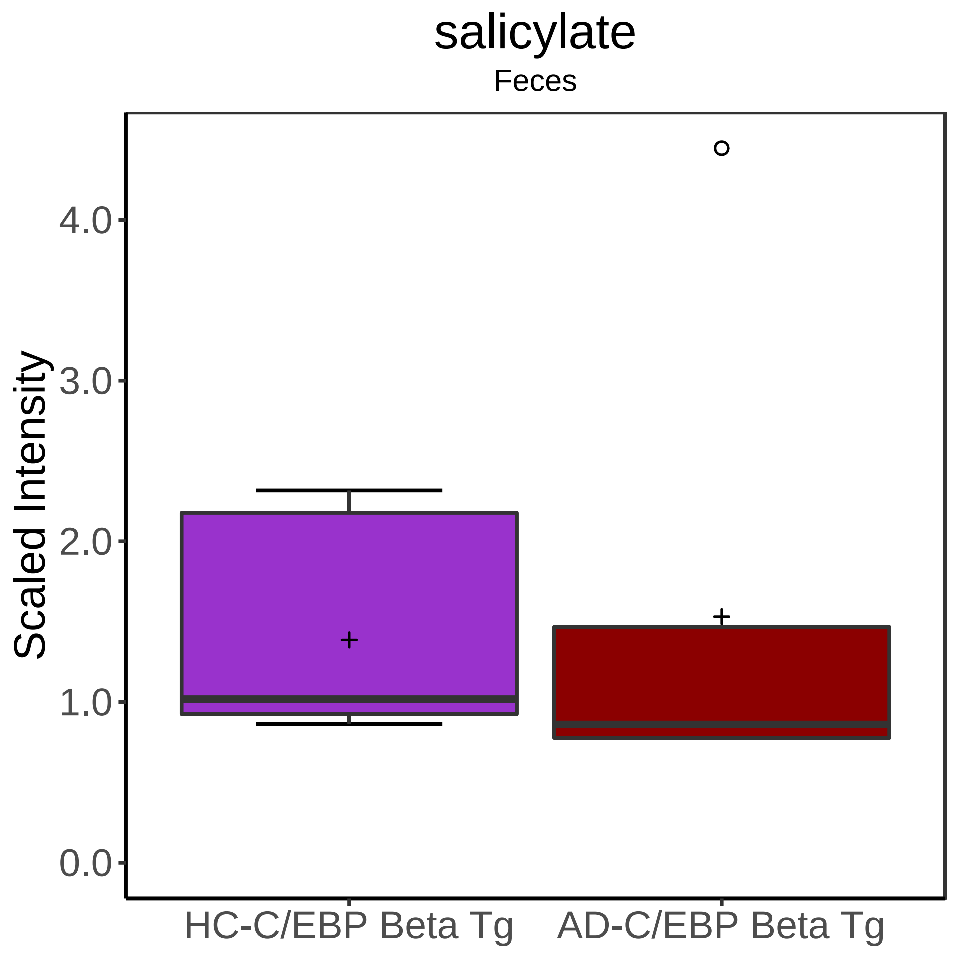

salicylate
Feces
Scaled Intensity
4.0
3.0
2.0
1.0
0.0
HC-C/EBP Beta Tg
AD-C/EBP Beta Tg

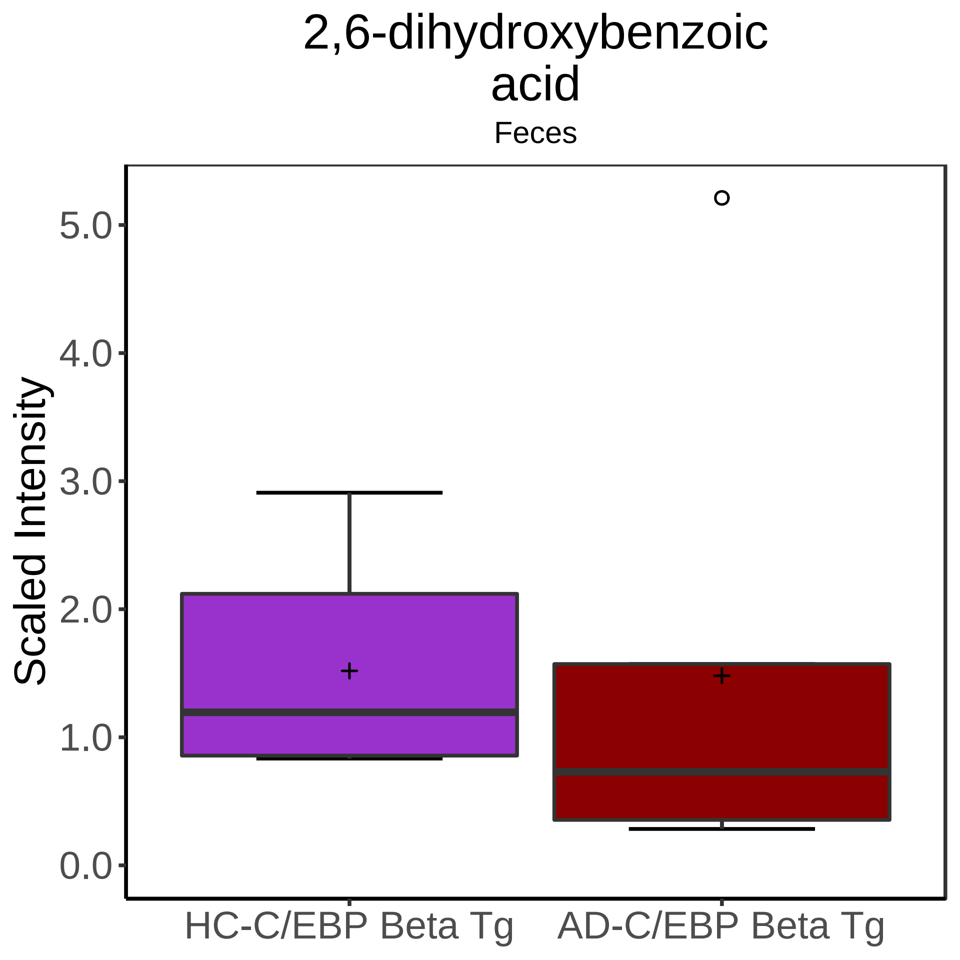
2,6-dihydroxybenzoic acid
Feces
Scaled Intensity
5.0
4.0
3.0
2.0
1.0
0.0
HC-C/EBP Beta Tg
AD-C/EBP Beta Tg

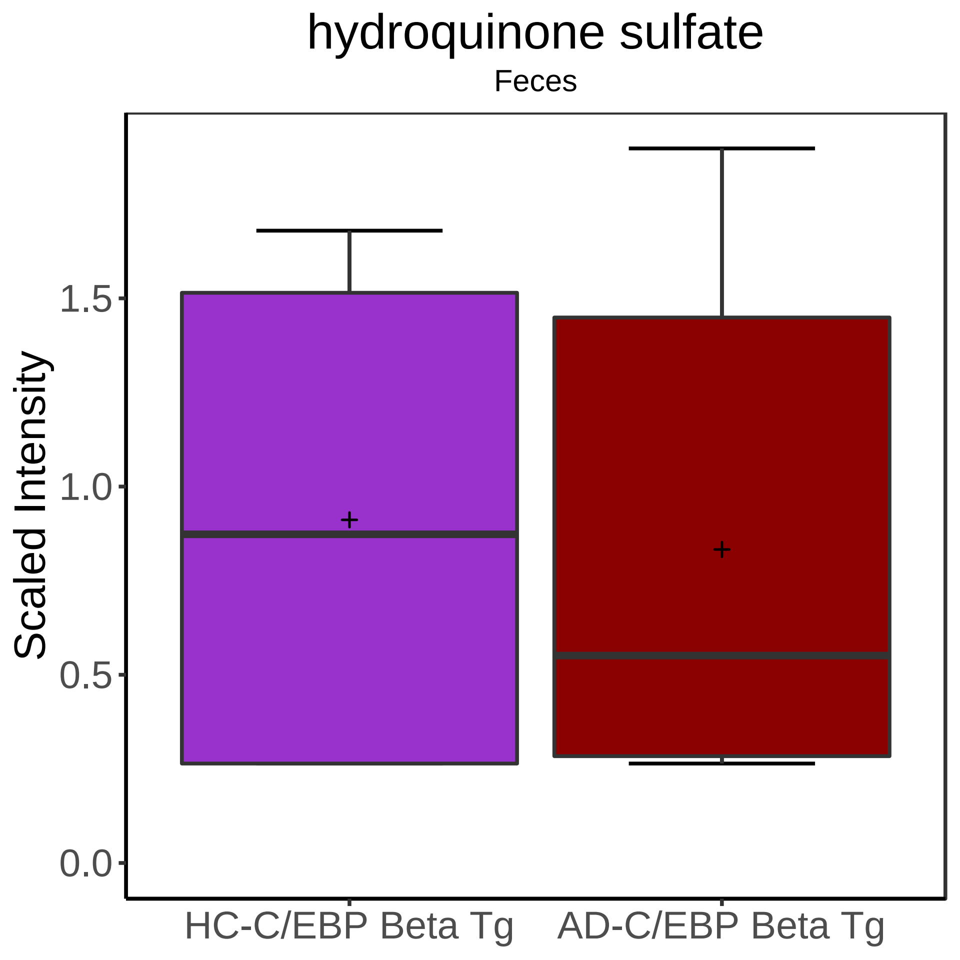

hydroquinone sulfate
Feces
Scaled Intensity
1.5
1.0
0.5
0.0
HC-C/EBP Beta Tg
AD-C/EBP Beta Tg

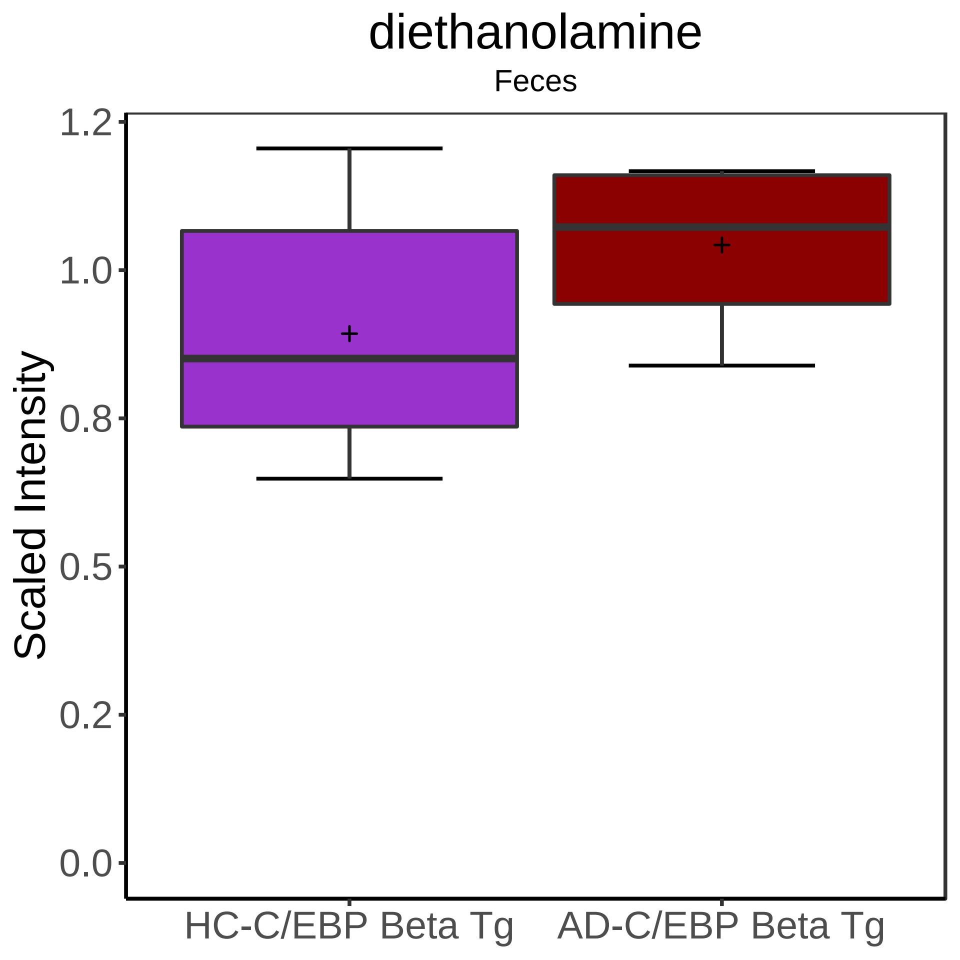

diethanolamine
Feces
Scaled Intensity
1.2
1.0
0.8
0.5
0.2
0.0
HC-C/EBP Beta Tg
AD-C/EBP Beta Tg

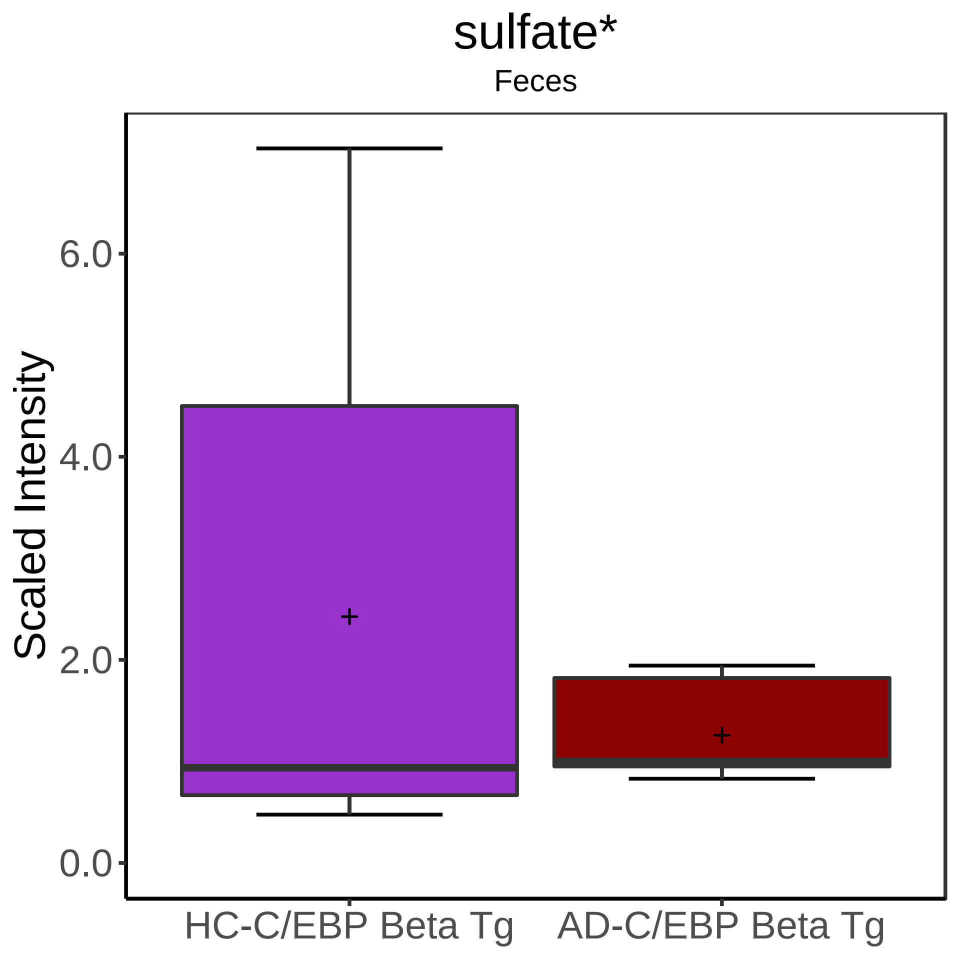
sulfate*
Feces
Scaled Intensity
6.0
4.0
2.0
0.0
HC-C/EBP Beta Tg
AD-C/EBP Beta Tg

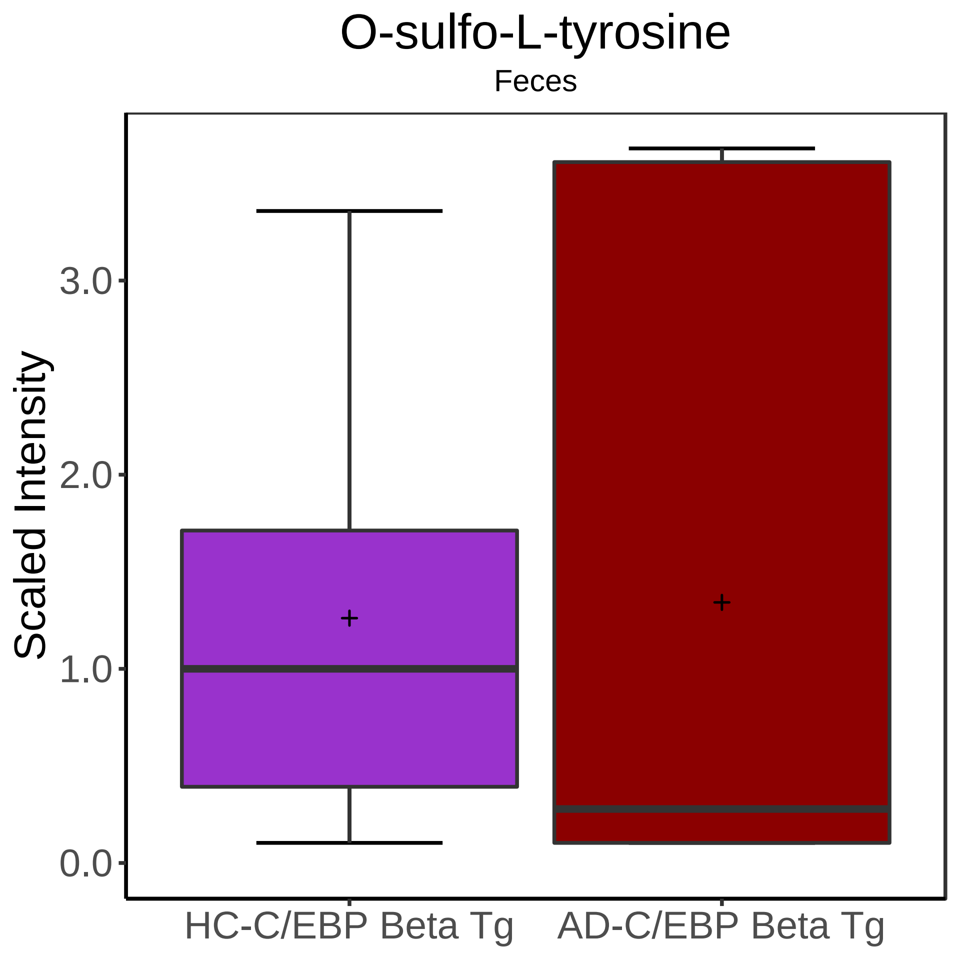

O-sulfo-L-tyrosine
Feces
Scaled Intensity
3.0
2.0
1.0
0.0
HC-C/EBP Beta Tg
AD-C/EBP Beta Tg

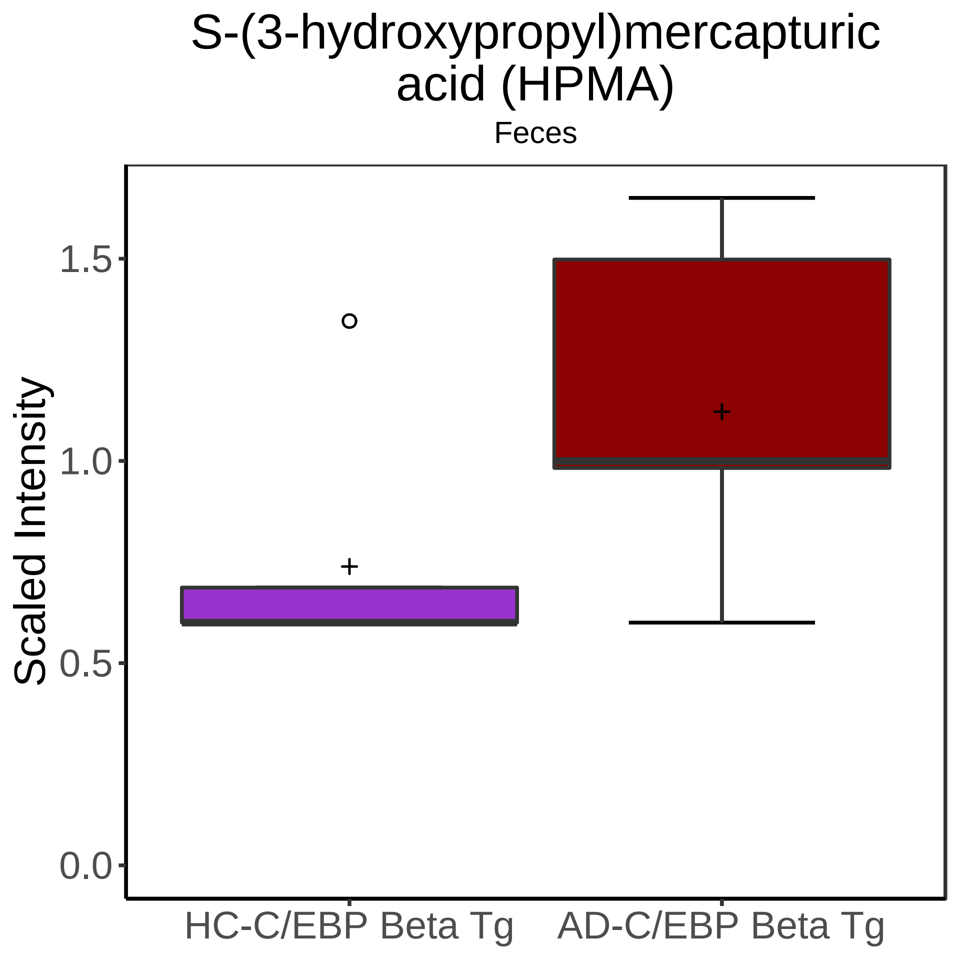

S-(3-hydroxypropyl)mercapturic acid (HPMA)
Feces
Scaled Intensity
1.5
1.0
0.5
0.0
HC-C/EBP Beta Tg
AD-C/EBP Beta Tg

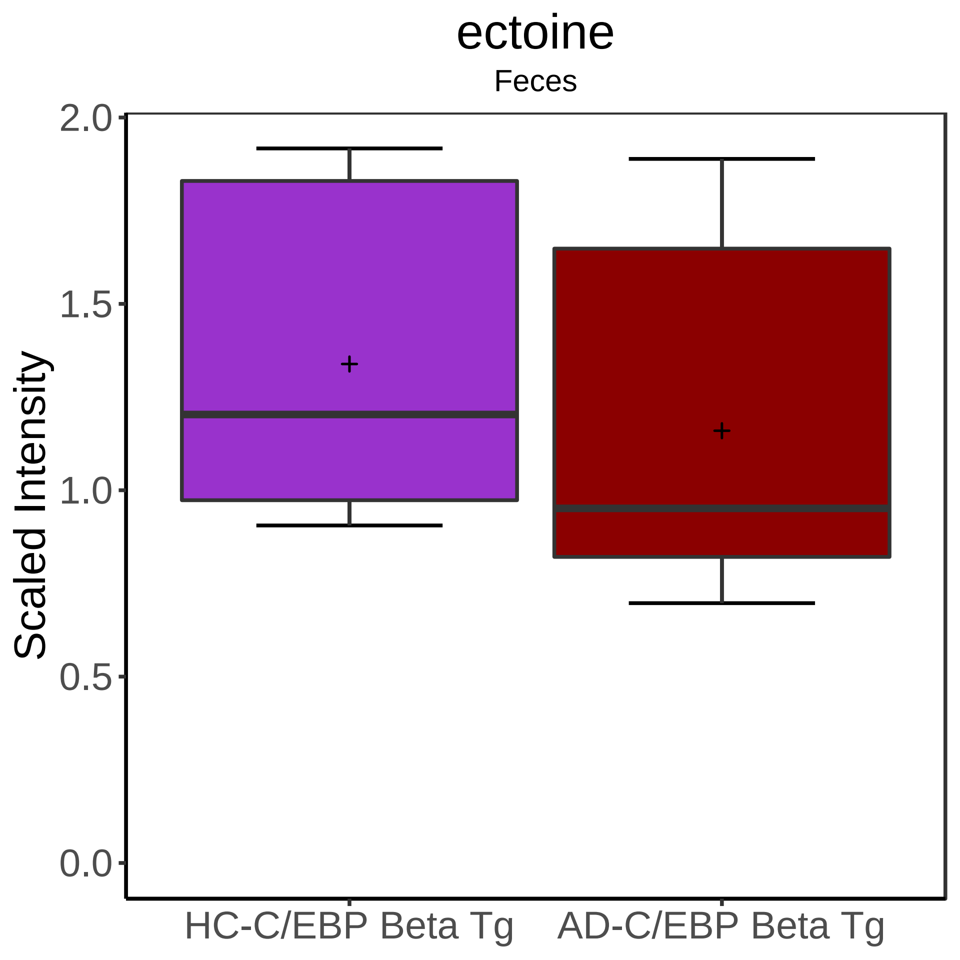
ectoine
Feces
Scaled Intensity
2.0
1.5
1.0
0.5
0.0
HC-C/EBP Beta Tg
AD-C/EBP Beta Tg

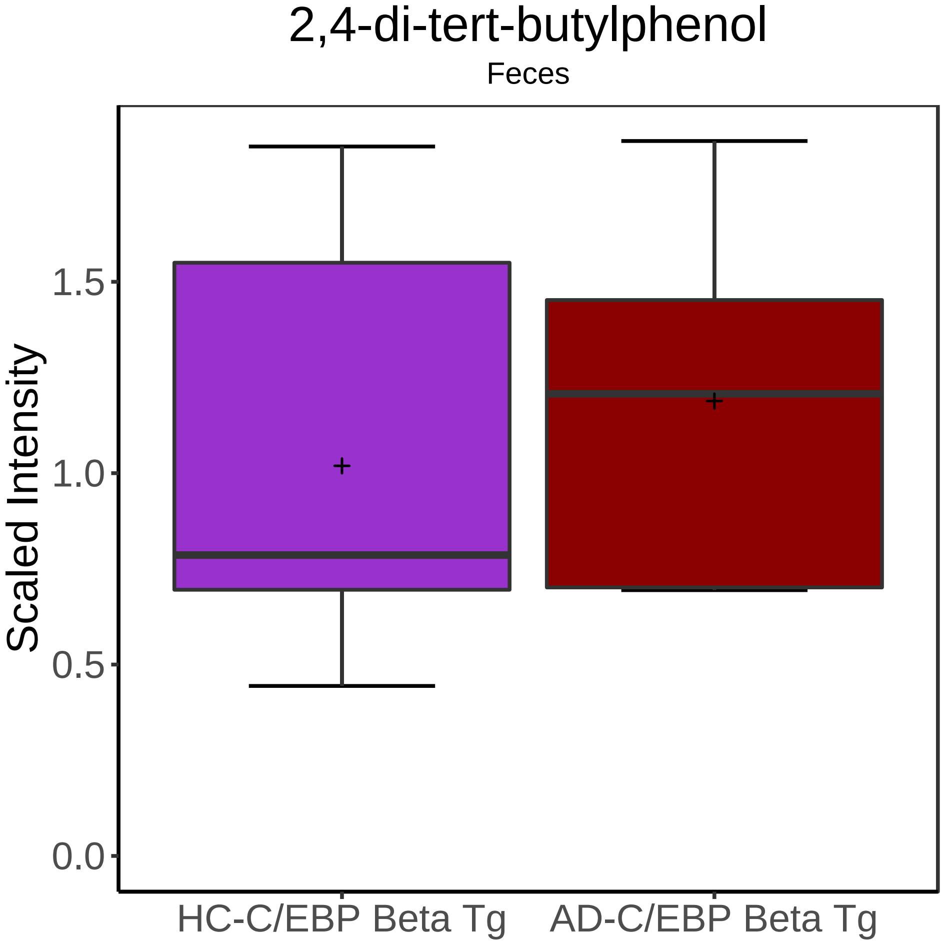
2,4-di-tert-butylphenol
Feces
Scaled Intensity
0.0
0.5
1.0
1.5
HC-C/EBP Beta Tg
AD-C/EBP Beta Tg

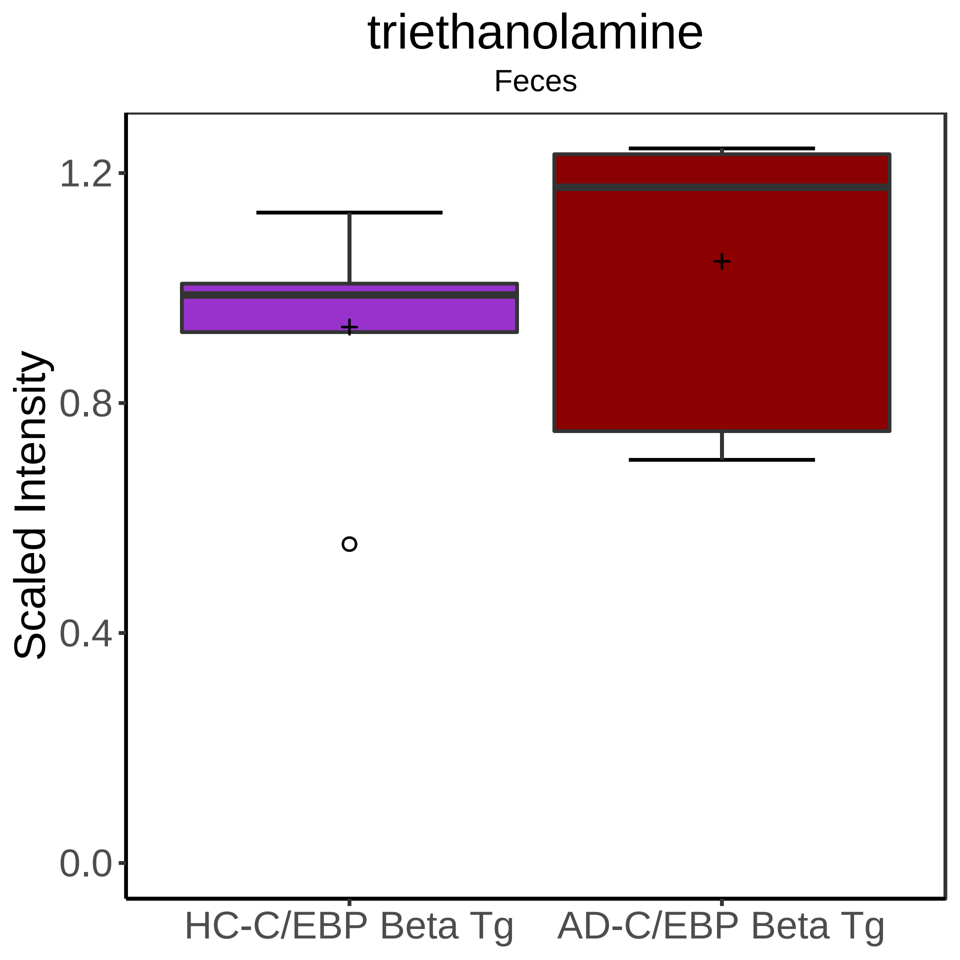
triethanolamine
Feces
Scaled Intensity
1.2
0.8
0.4
0.0
HC-C/EBP Beta Tg
AD-C/EBP Beta Tg

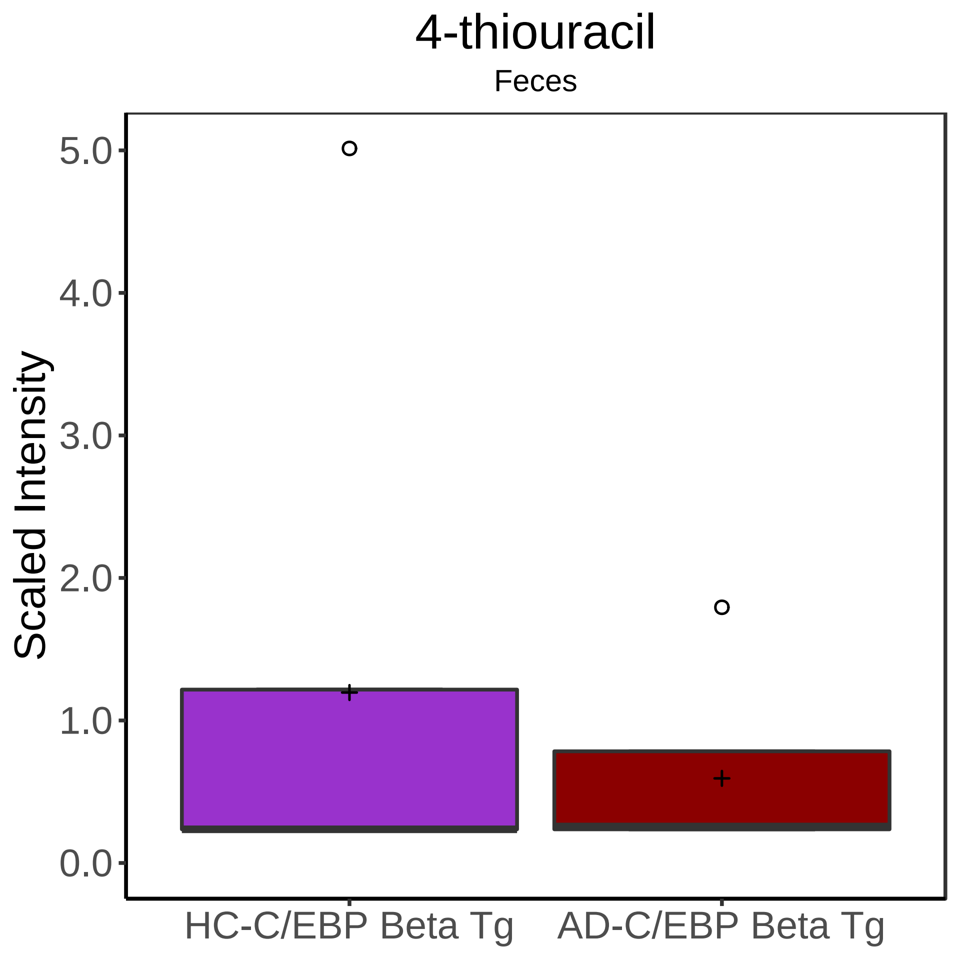
4-thiouracil
Feces
Scaled Intensity
5.0
4.0
3.0
2.0
1.0
0.0
HC-C/EBP Beta Tg
AD-C/EBP Beta Tg

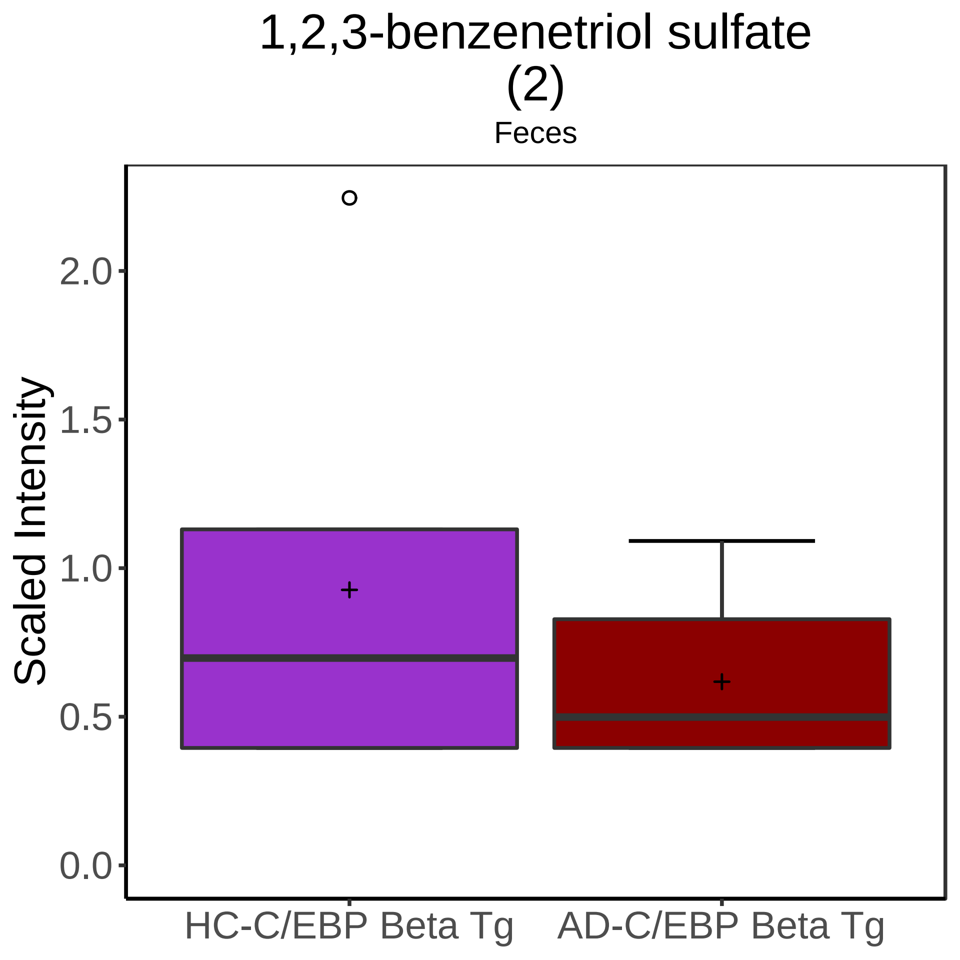
1,2,3-benzenetriol sulfate
(2)
Feces
Scaled Intensity
2.0
1.5
1.0
0.5
0.0
HC-C/EBP Beta Tg
AD-C/EBP Beta Tg

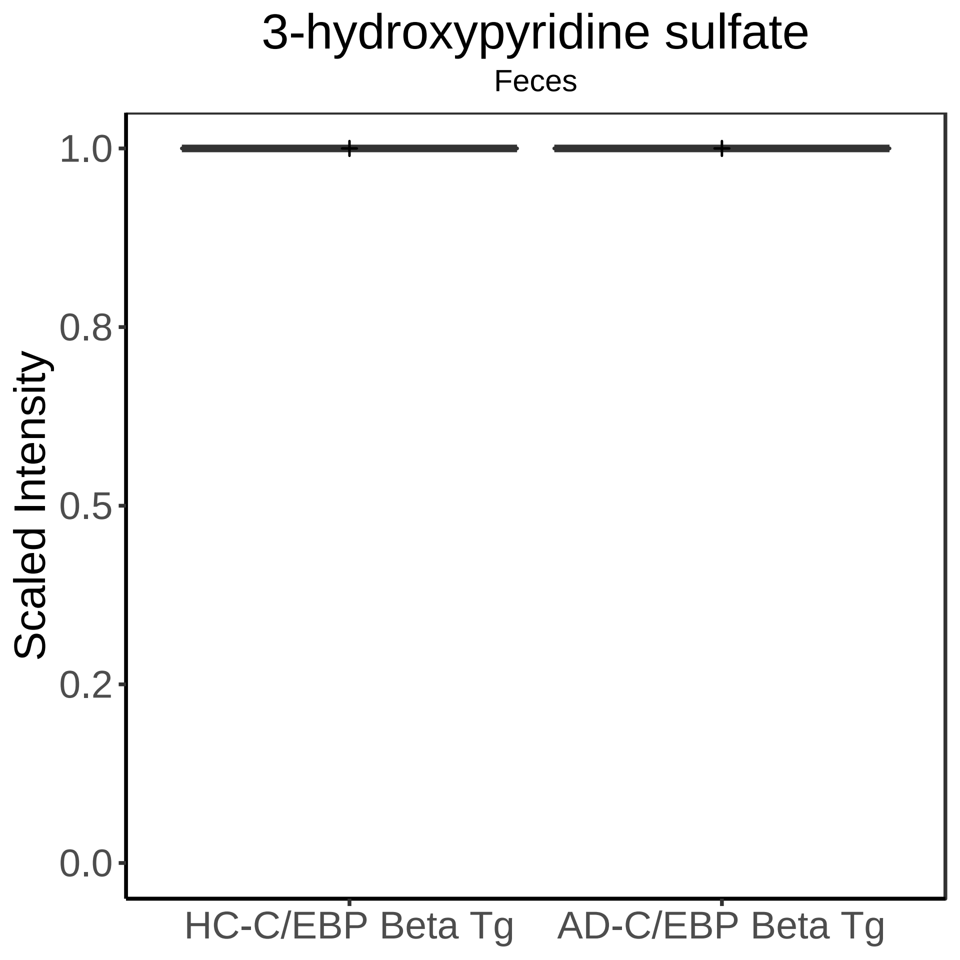
3-hydroxypyridine sulfate
Feces
Scaled Intensity
1.0
0.8
0.5
0.2
0.0
HC-C/EBP Beta Tg
AD-C/EBP Beta Tg

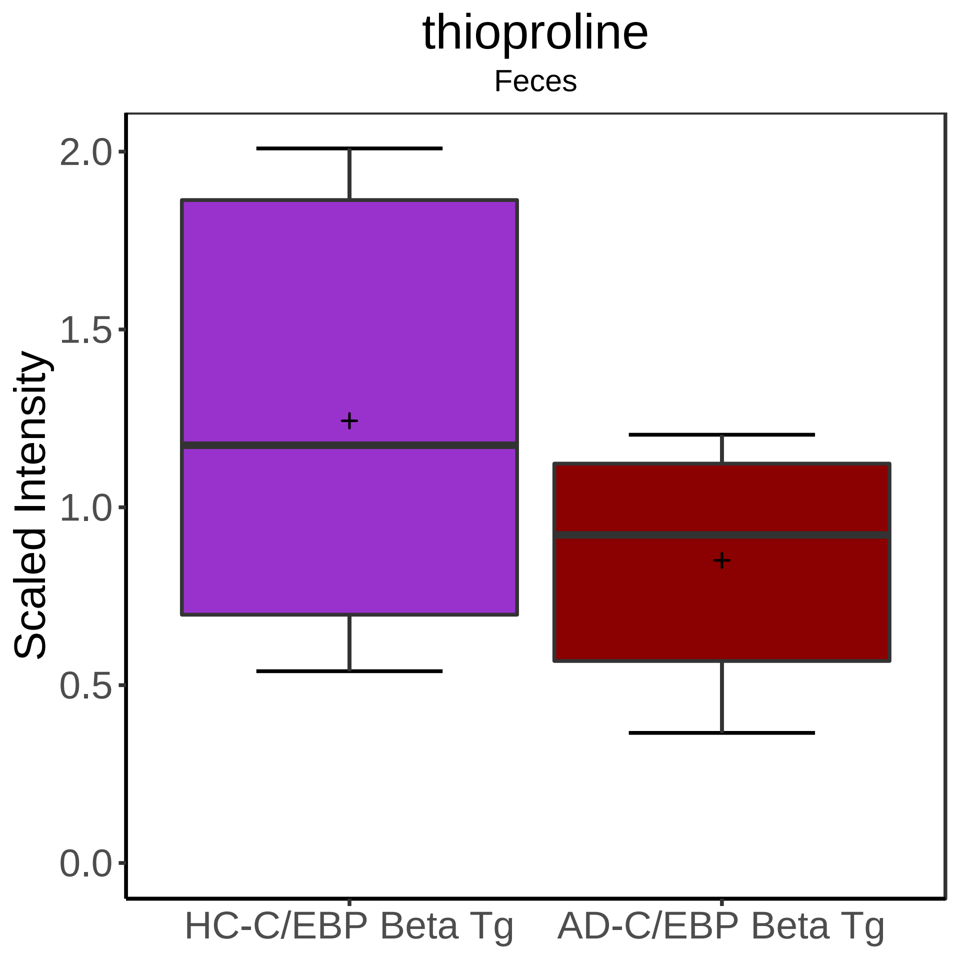
thioproline
Feces
Scaled Intensity
2.0
1.5
1.0
0.5
0.0
HC-C/EBP Beta Tg
AD-C/EBP Beta Tg

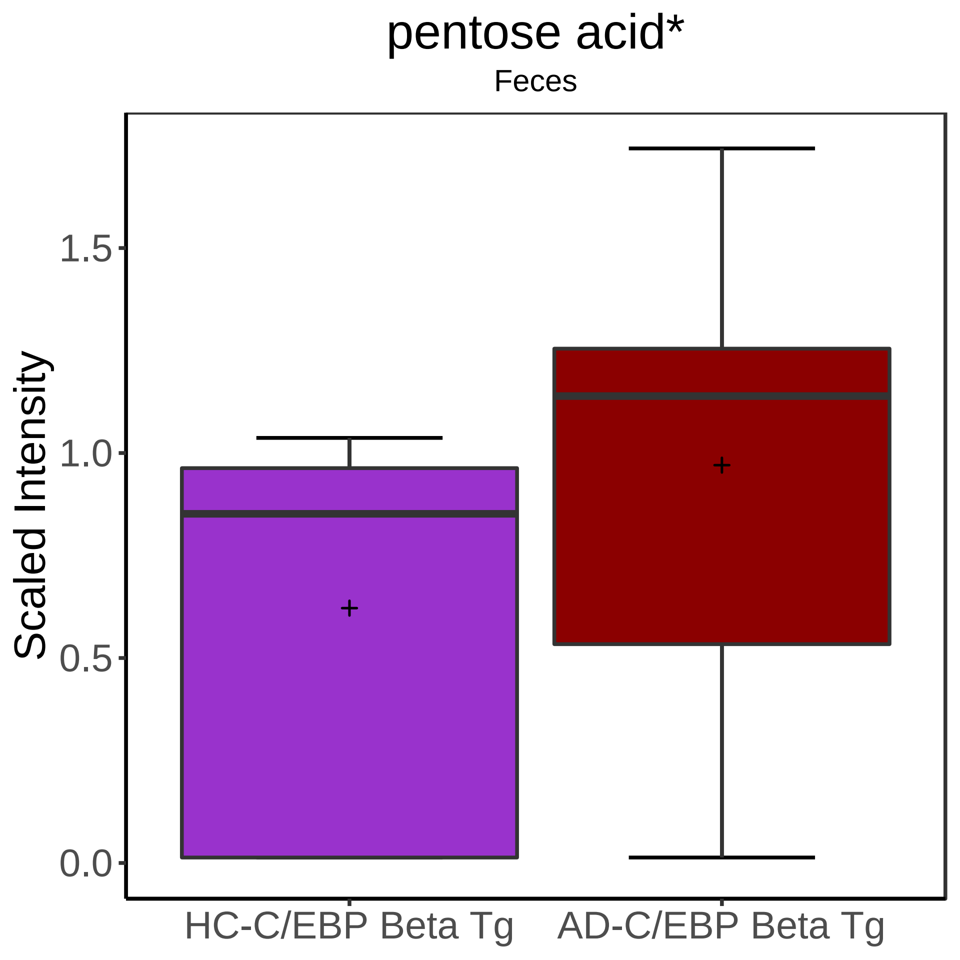

pentose acid*
Feces
Scaled Intensity
1.5
1.0
0.5
0.0
HC-C/EBP Beta Tg
AD-C/EBP Beta Tg

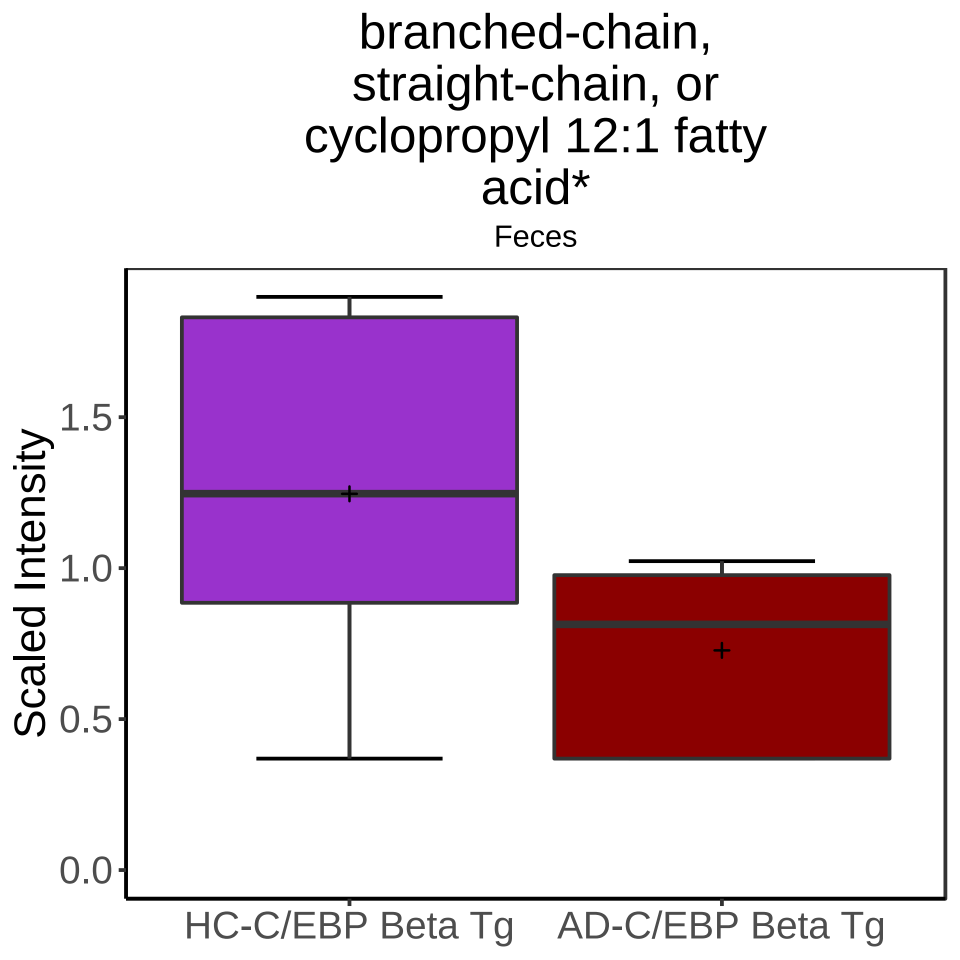

branched-chain,
straight-chain, or
cyclopropyl 12:1 fatty
acid*
Feces
Scaled Intensity
1.5
1.0
0.5
0.0
HC-C/EBP Beta Tg
AD-C/EBP Beta Tg

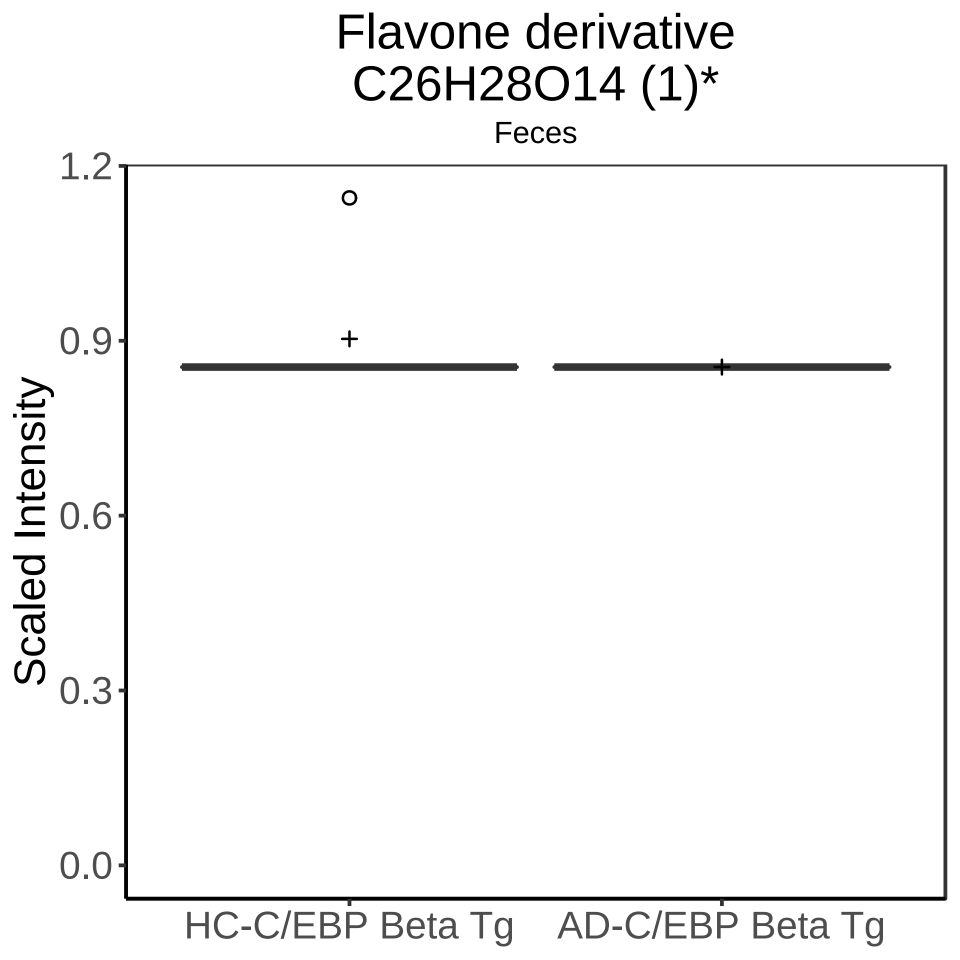

Flavone derivative
C26H28O14 (1)*
Feces
Scaled Intensity
1.2
0.9
0.6
0.3
0.0
HC-C/EBP Beta Tg
AD-C/EBP Beta Tg

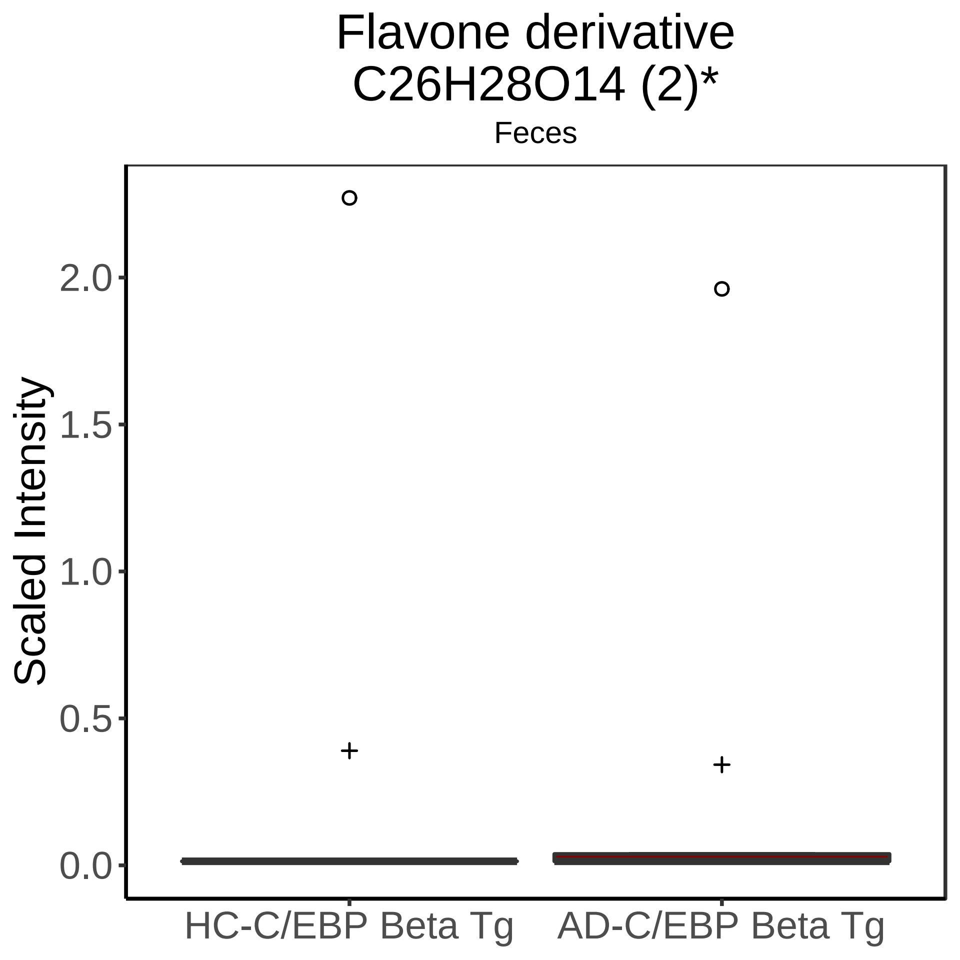
Flavone derivative
C26H28O14 (2)*
Feces
Scaled Intensity
0.0
0.5
1.0
1.5
2.0
HC-C/EBP Beta Tg
AD-C/EBP Beta Tg

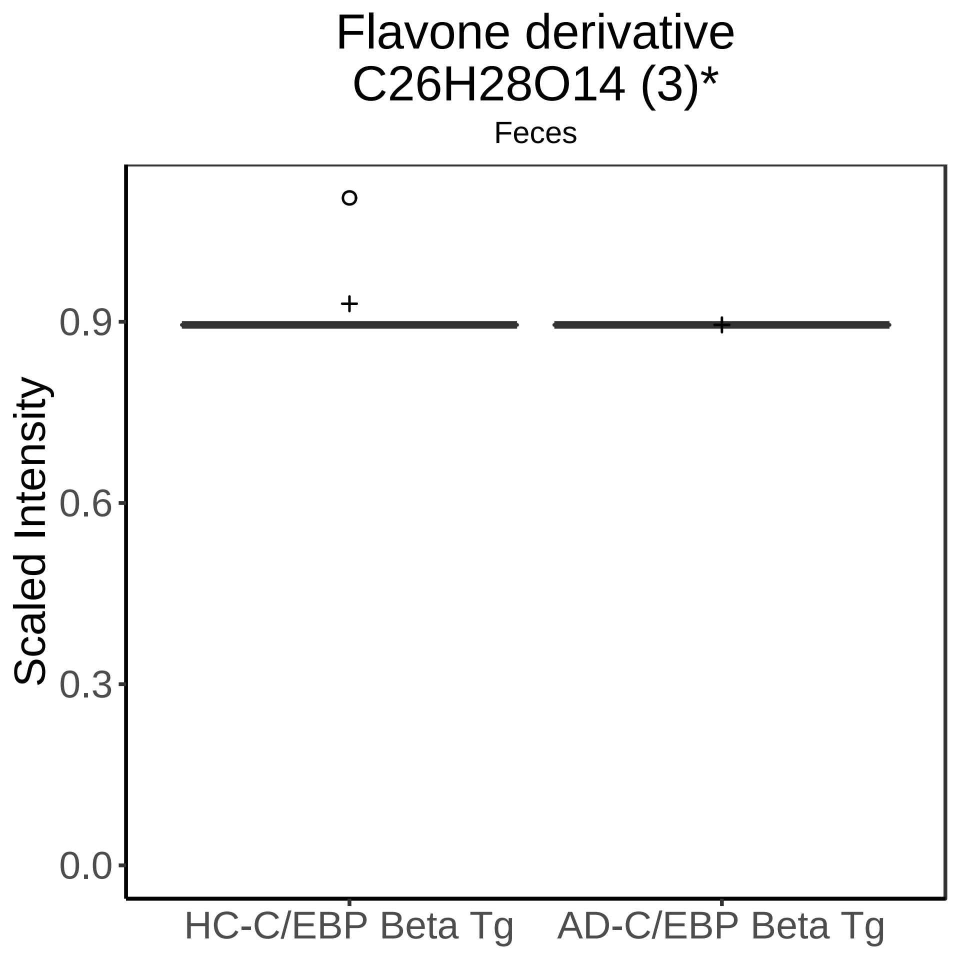
Flavone derivative
C26H28O14 (3)*
Feces
Scaled Intensity
0.0
0.3
0.6
0.9
HC-C/EBP Beta Tg
AD-C/EBP Beta Tg

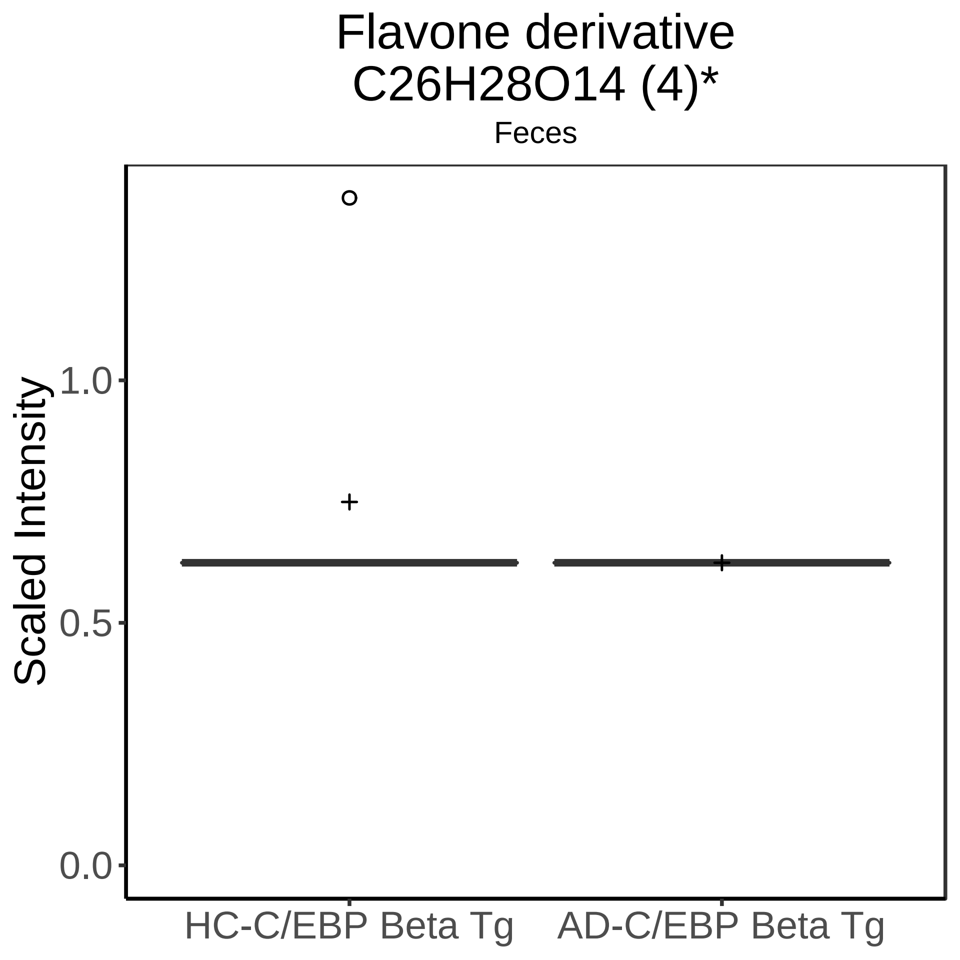
Flavone derivative
C26H28O14 (4)*
Feces
Scaled Intensity
1.0
0.5
0.0
HC-C/EBP Beta Tg
AD-C/EBP Beta Tg

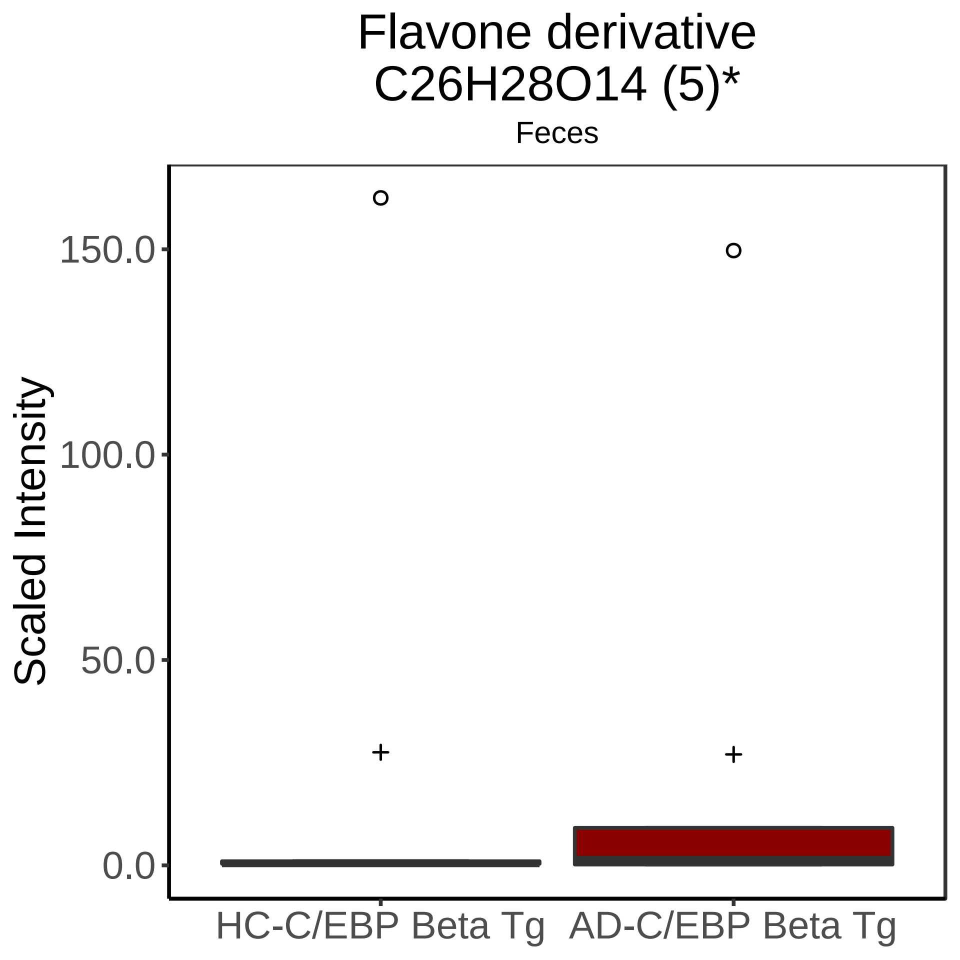

Flavone derivative
C26H28O14 (5)*
Feces
Scaled Intensity
150.0
100.0
50.0
0.0
HC-C/EBP Beta Tg
AD-C/EBP Beta Tg

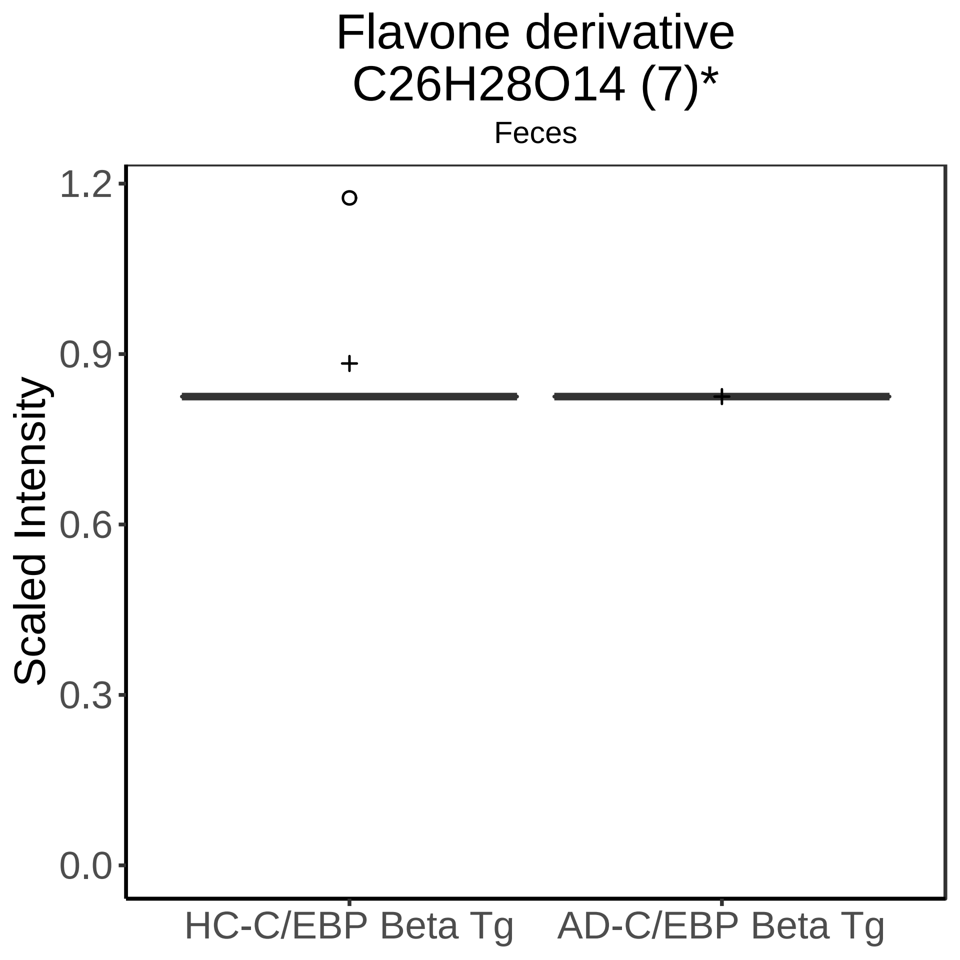
Flavone derivative
C26H28O14 (7)*
Feces
Scaled Intensity
1.2
0.9
0.6
0.3
0.0
HC-C/EBP Beta Tg
AD-C/EBP Beta Tg

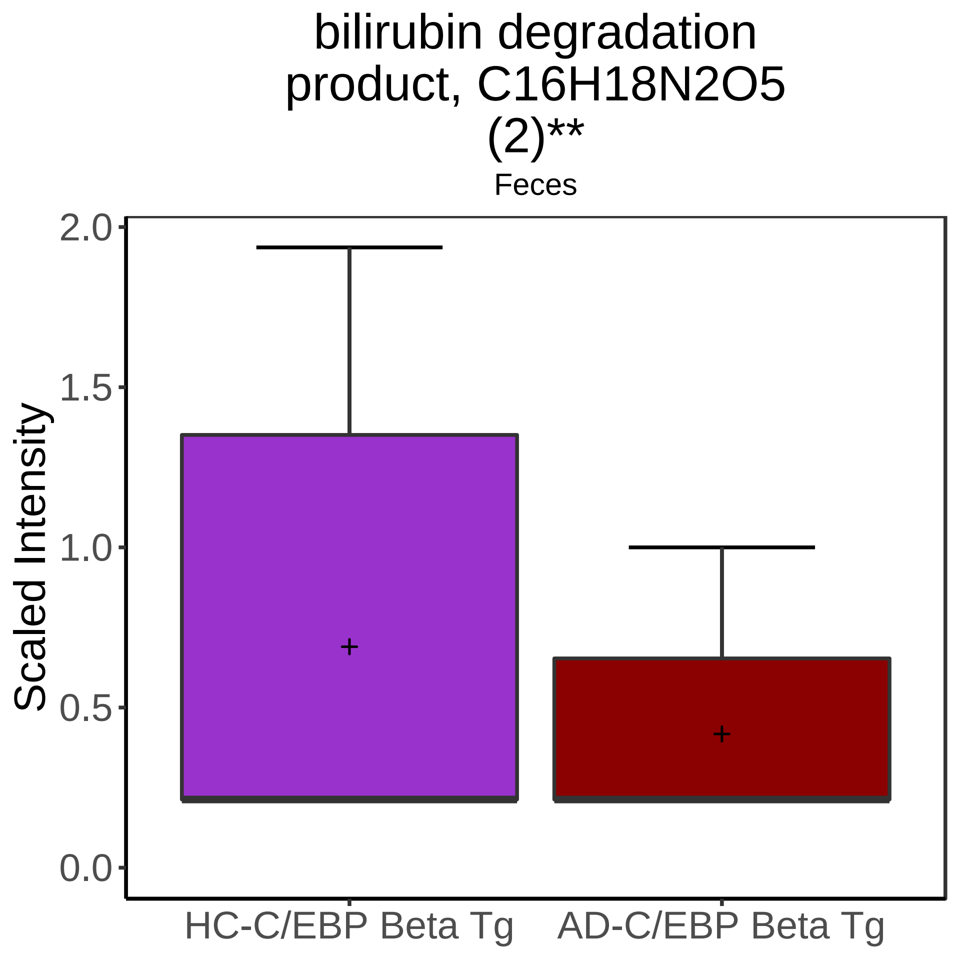
bilirubin degradation product, C16H18N2O5 (2)**
Feces
Scaled Intensity
2.0
1.5
1.0
0.5
0.0
HC-C/EBP Beta Tg
AD-C/EBP Beta Tg

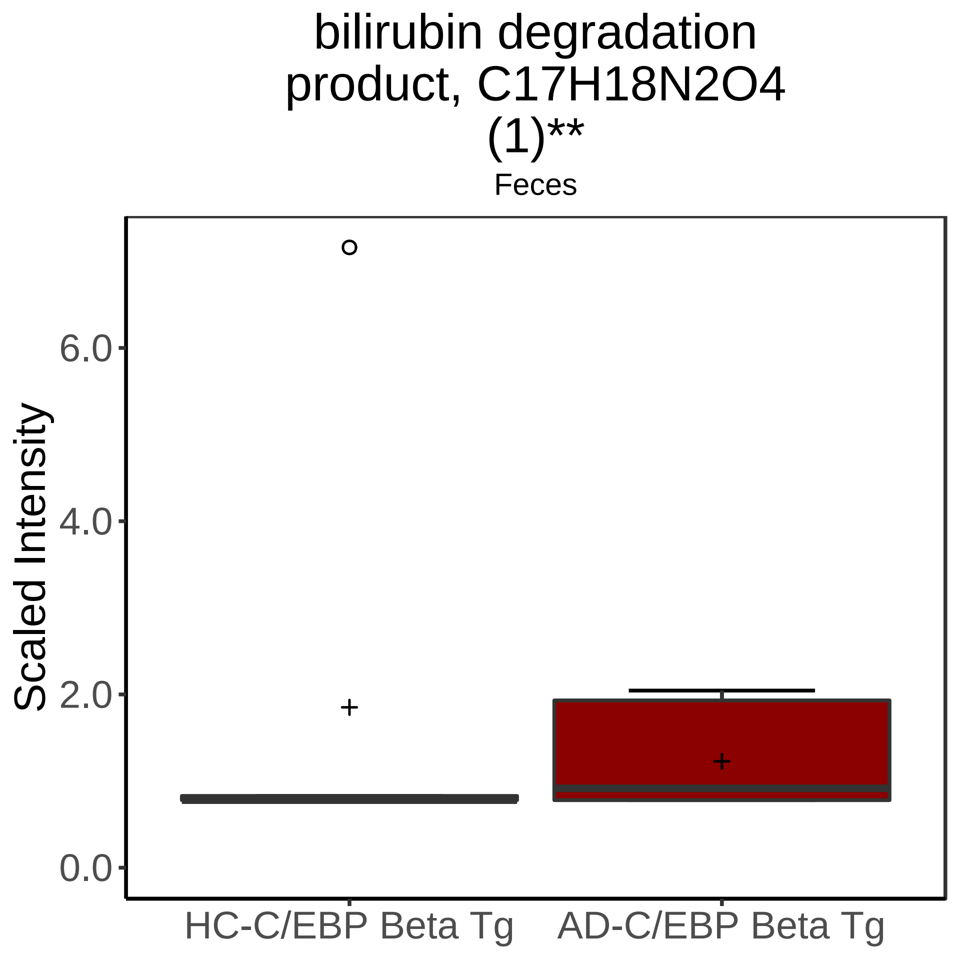
bilirubin degradation product, C17H18N2O4 (1)**
Feces
Scaled Intensity
6.0
4.0
2.0
0.0
HC-C/EBP Beta Tg
AD-C/EBP Beta Tg

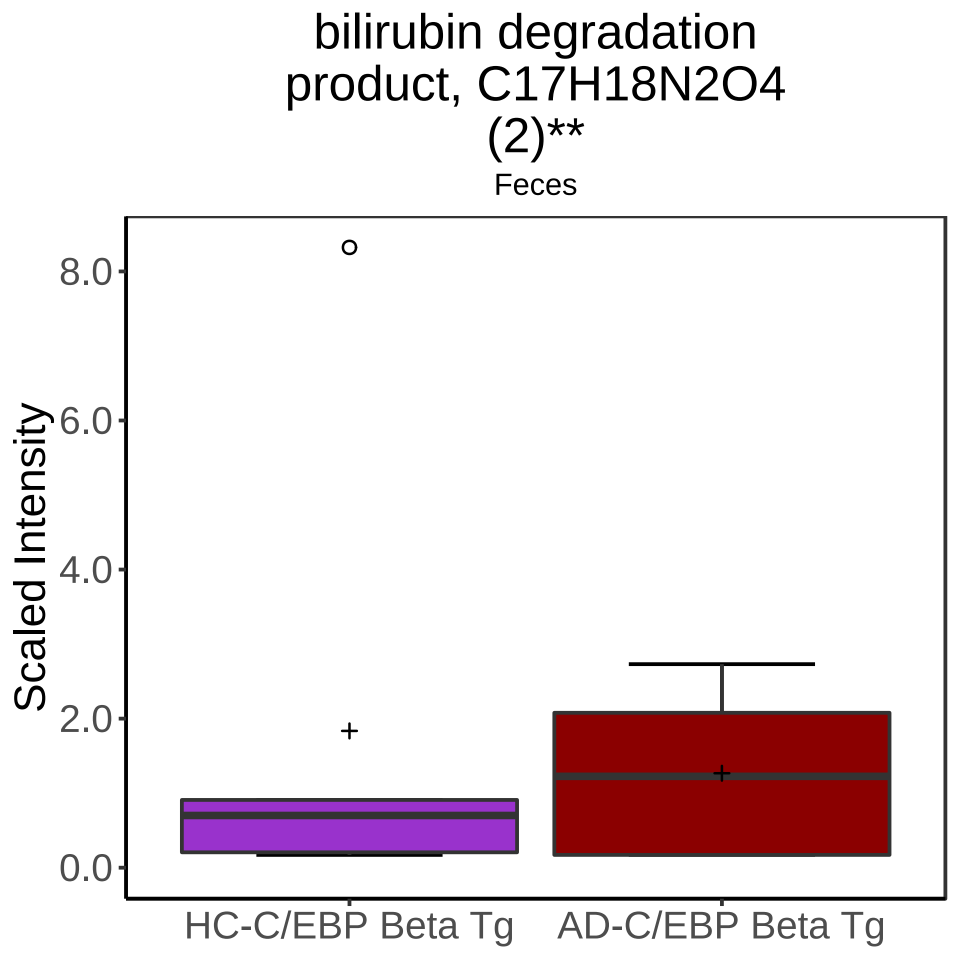

bilirubin degradation product, C17H18N2O4 (2)**
Feces
Scaled Intensity
8.0
6.0
4.0
2.0
0.0
HC-C/EBP Beta Tg
AD-C/EBP Beta Tg

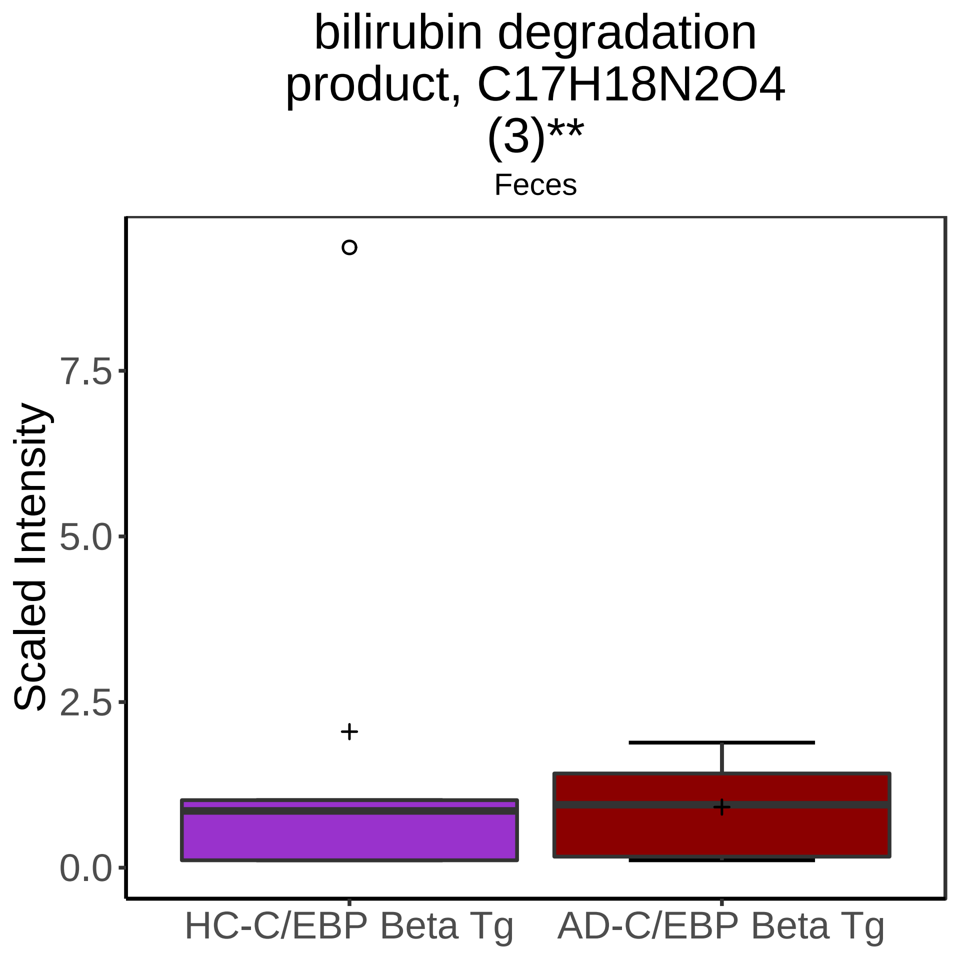

bilirubin degradation product, C17H18N2O4 (3)**
Feces
Scaled Intensity
7.5
5.0
2.5
0.0
HC-C/EBP Beta Tg
AD-C/EBP Beta Tg

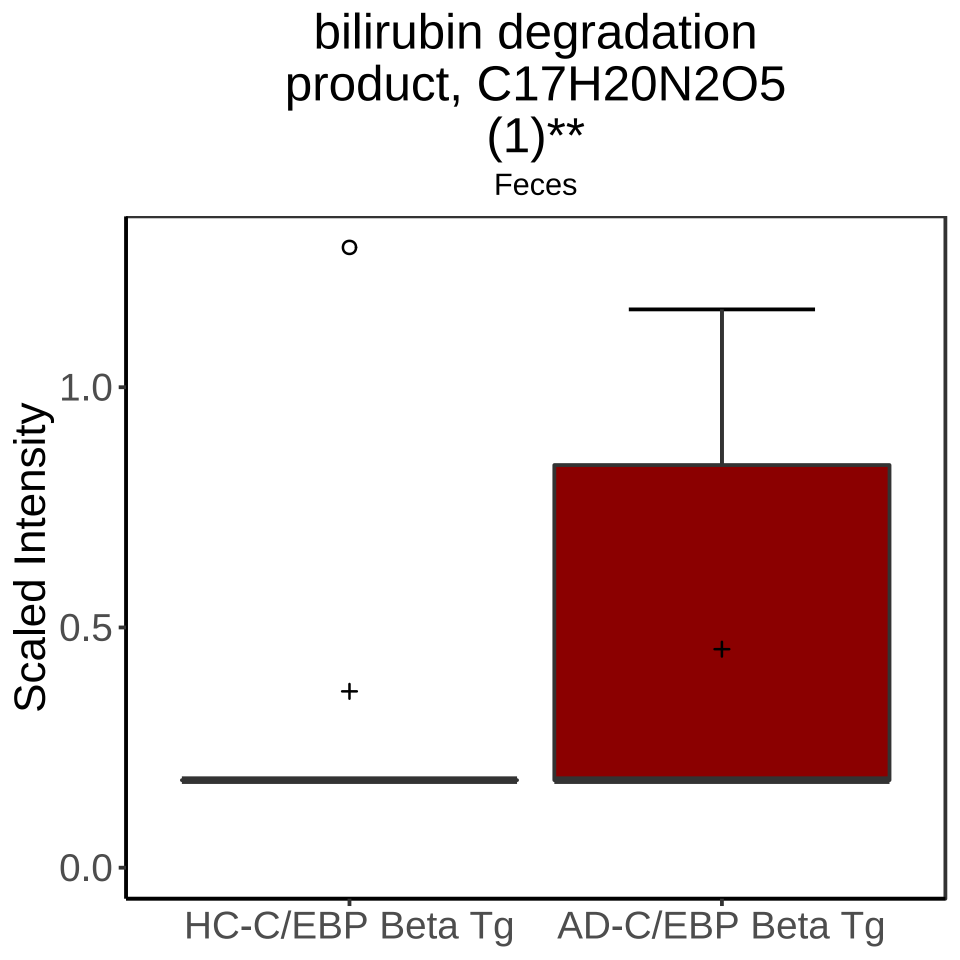
bilirubin degradation
product, C17H20N2O5
(1)**
Feces
Scaled Intensity
1.0
0.5
0.0
HC-C/EBP Beta Tg
AD-C/EBP Beta Tg

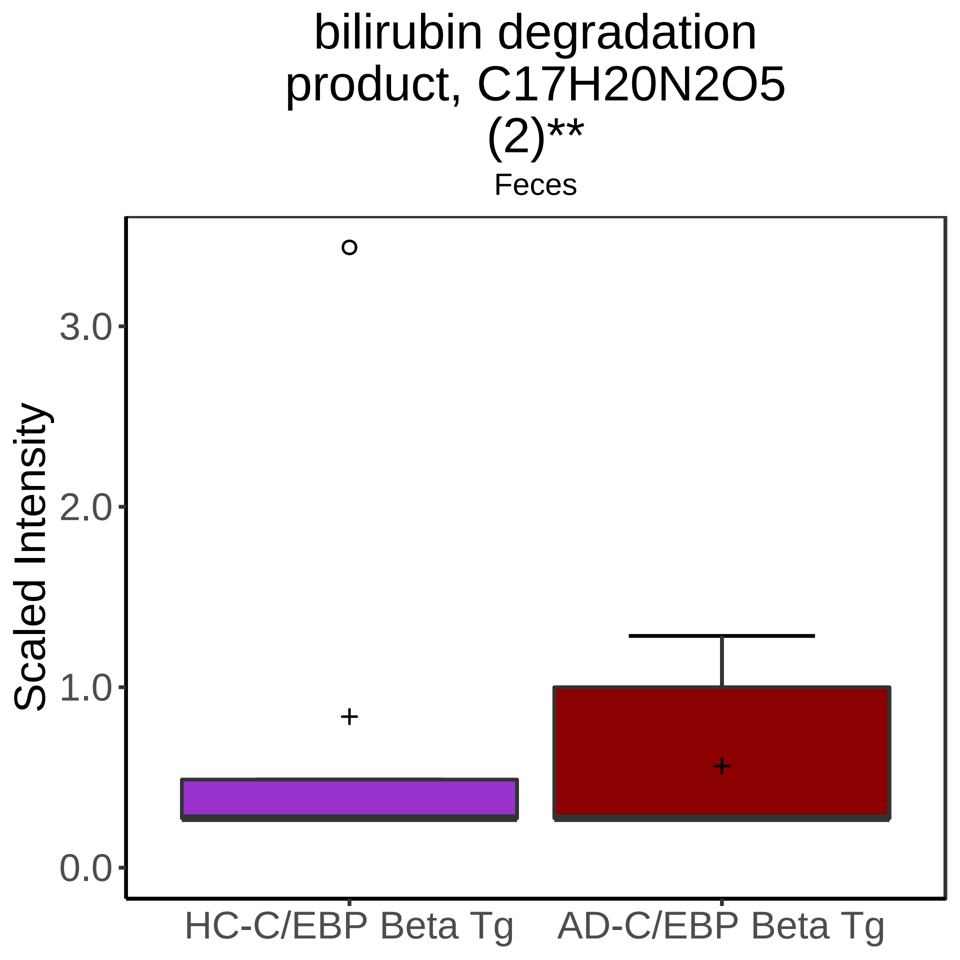
bilirubin degradation product, C17H20N2O5 (2)**
Feces
Scaled Intensity
3.0
2.0
1.0
0.0
HC-C/EBP Beta Tg
AD-C/EBP Beta Tg

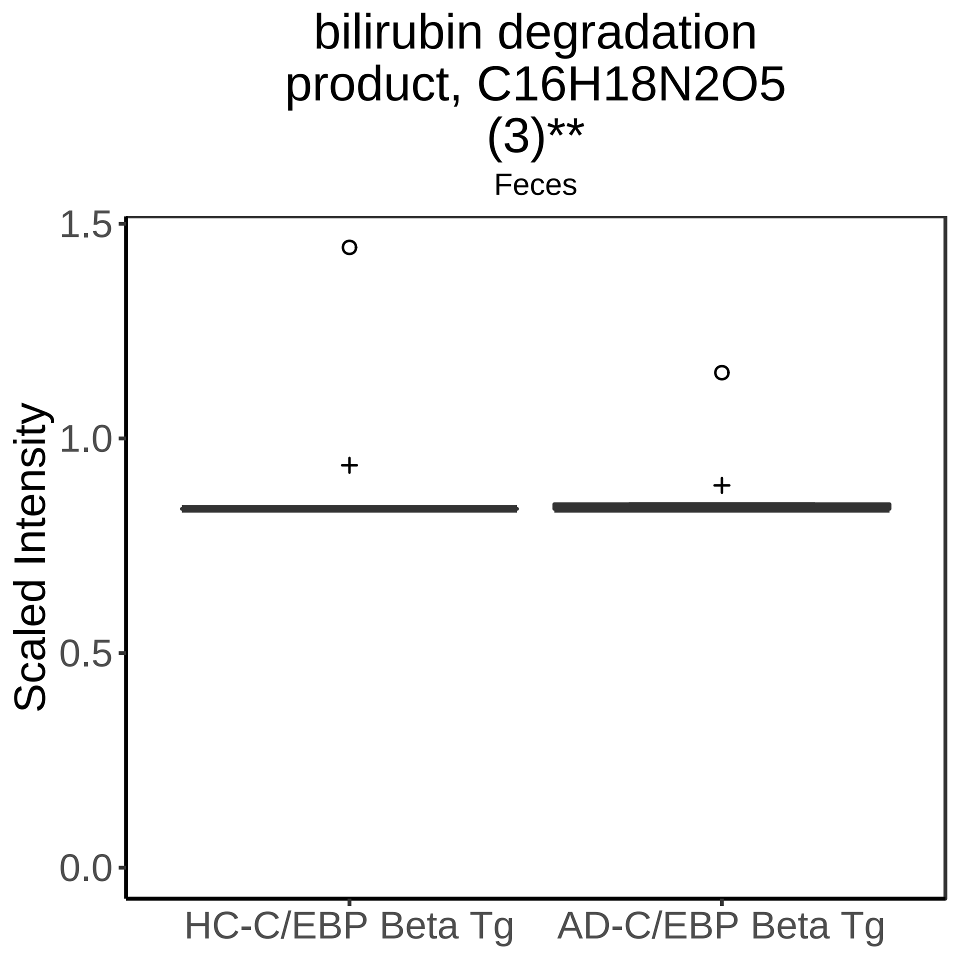

bilirubin degradation product, C16H18N2O5 (3)**
Feces
Scaled Intensity
1.5
1.0
0.5
0.0
HC-C/EBP Beta Tg
AD-C/EBP Beta Tg

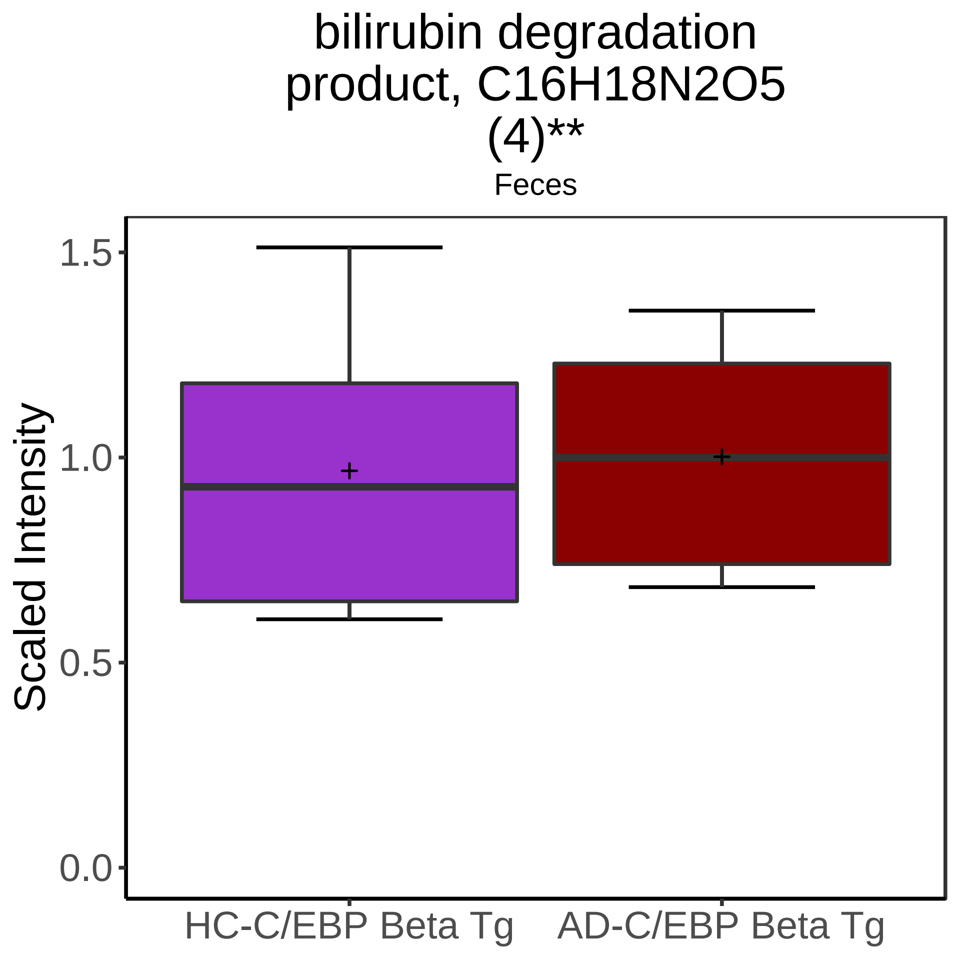
bilirubin degradation product, C16H18N2O5 (4)**
Feces
Scaled Intensity
0.0
0.5
1.0
1.5
HC-C/EBP Beta Tg
AD-C/EBP Beta Tg

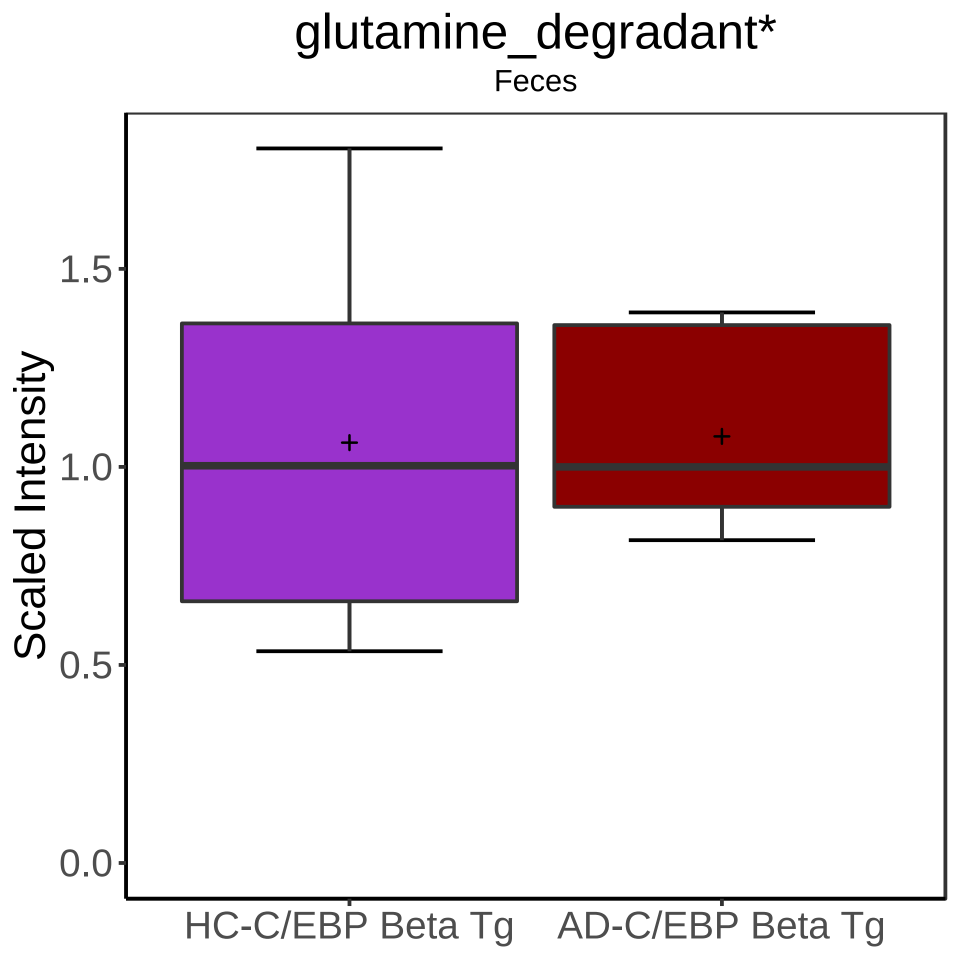

glutamine_degradant*
Feces
Scaled Intensity
1.5
1.0
0.5
0.0
HC-C/EBP Beta Tg
AD-C/EBP Beta Tg

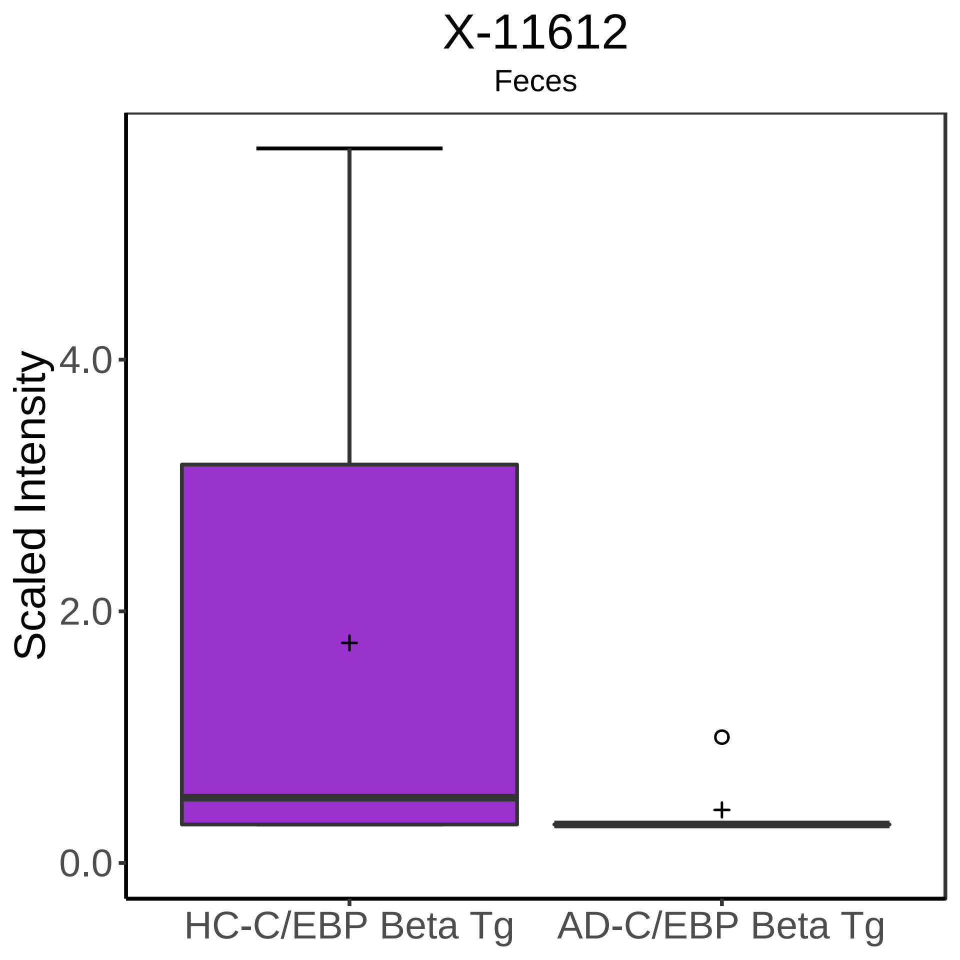
X-11612
Feces
Scaled Intensity
4.0
2.0
0.0
HC-C/EBP Beta Tg
AD-C/EBP Beta Tg

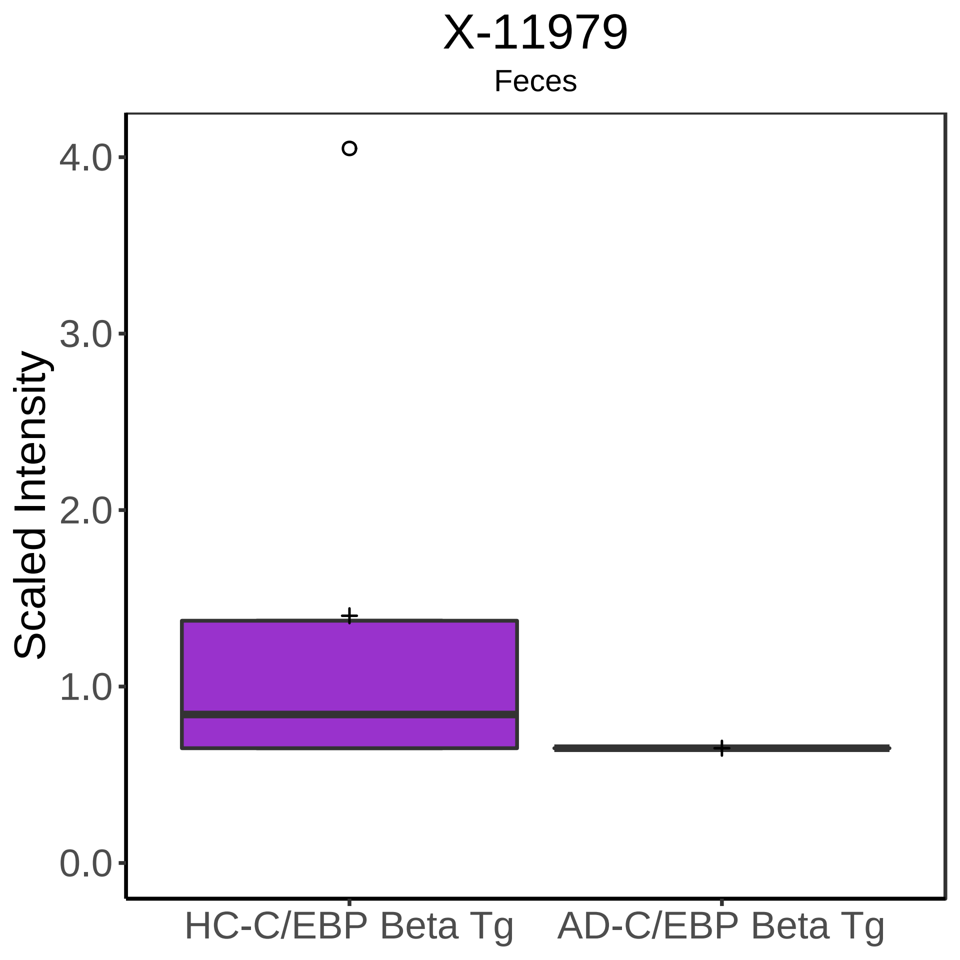
X-11979
Feces
Scaled Intensity
4.0
3.0
2.0
1.0
0.0
HC-C/EBP Beta Tg
AD-C/EBP Beta Tg

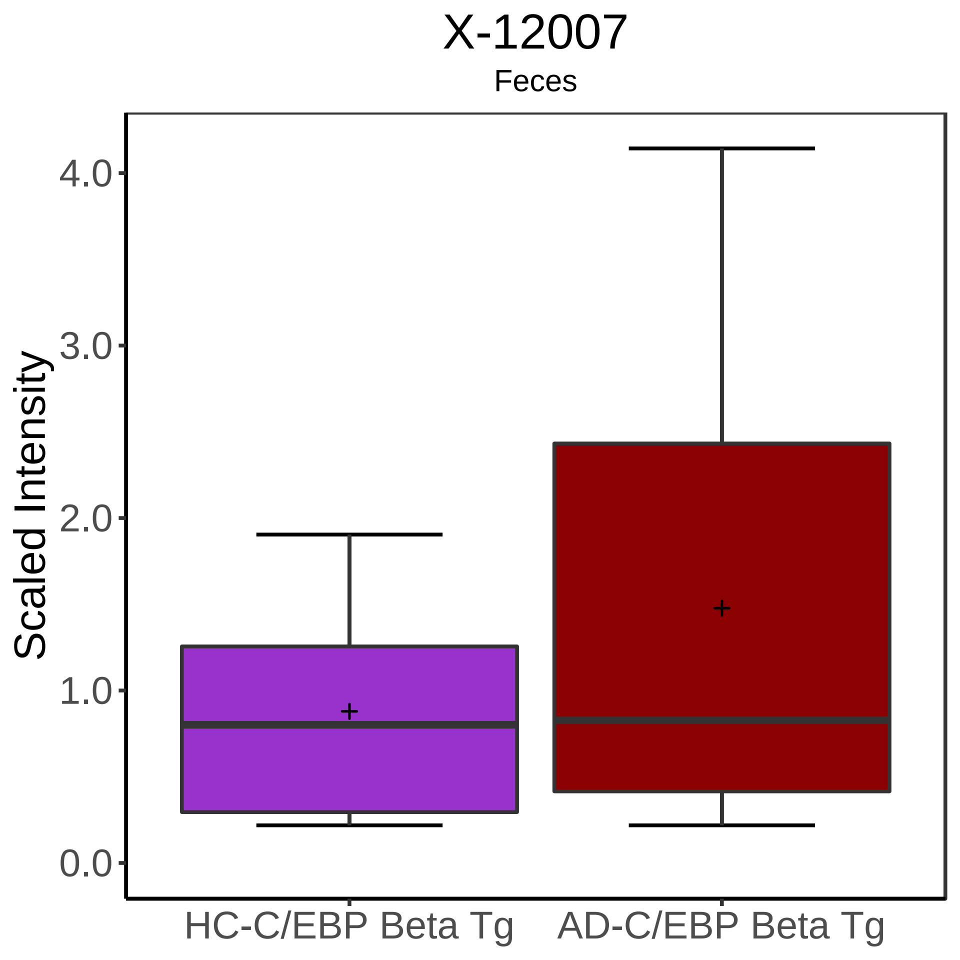

X-12007
Feces
Scaled Intensity
4.0
3.0
2.0
1.0
0.0
HC-C/EBP Beta Tg
AD-C/EBP Beta Tg

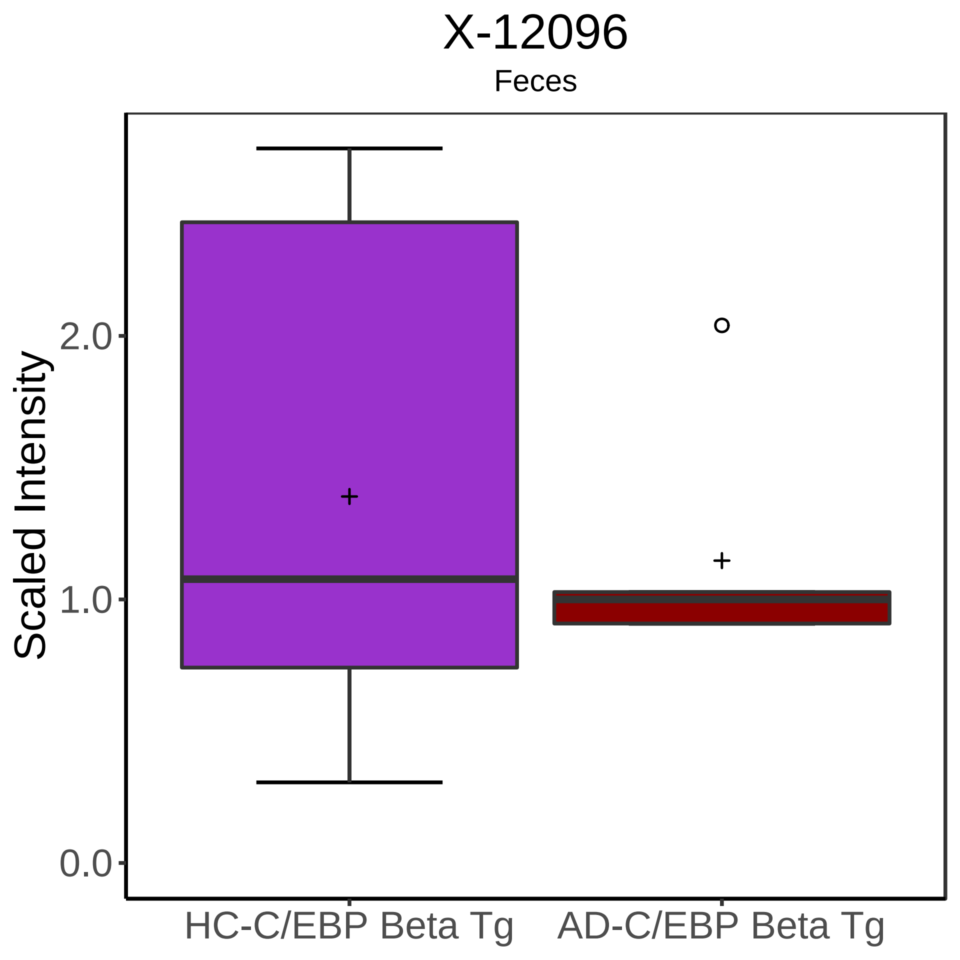
X-12096
Feces
Scaled Intensity
2.0
1.0
0.0
HC-C/EBP Beta Tg
AD-C/EBP Beta Tg

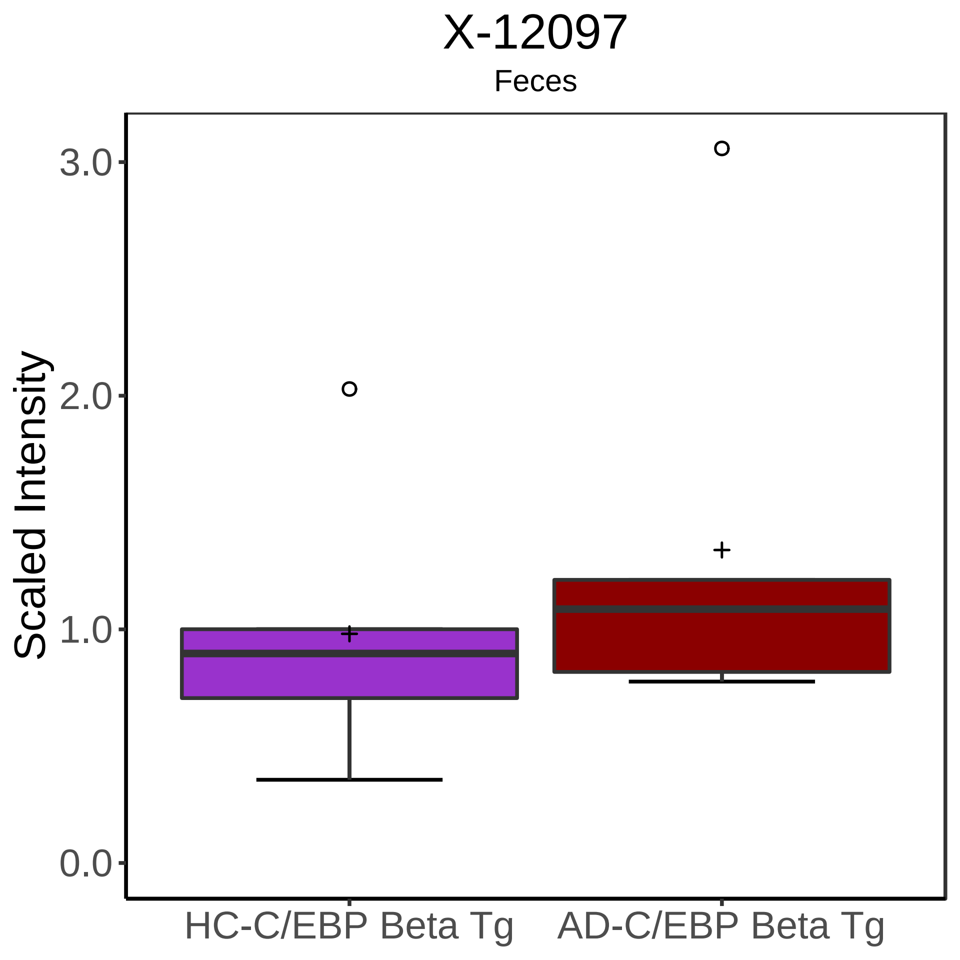
X-12097
Feces
Scaled Intensity
3.0
2.0
1.0
0.0
HC-C/EBP Beta Tg
AD-C/EBP Beta Tg

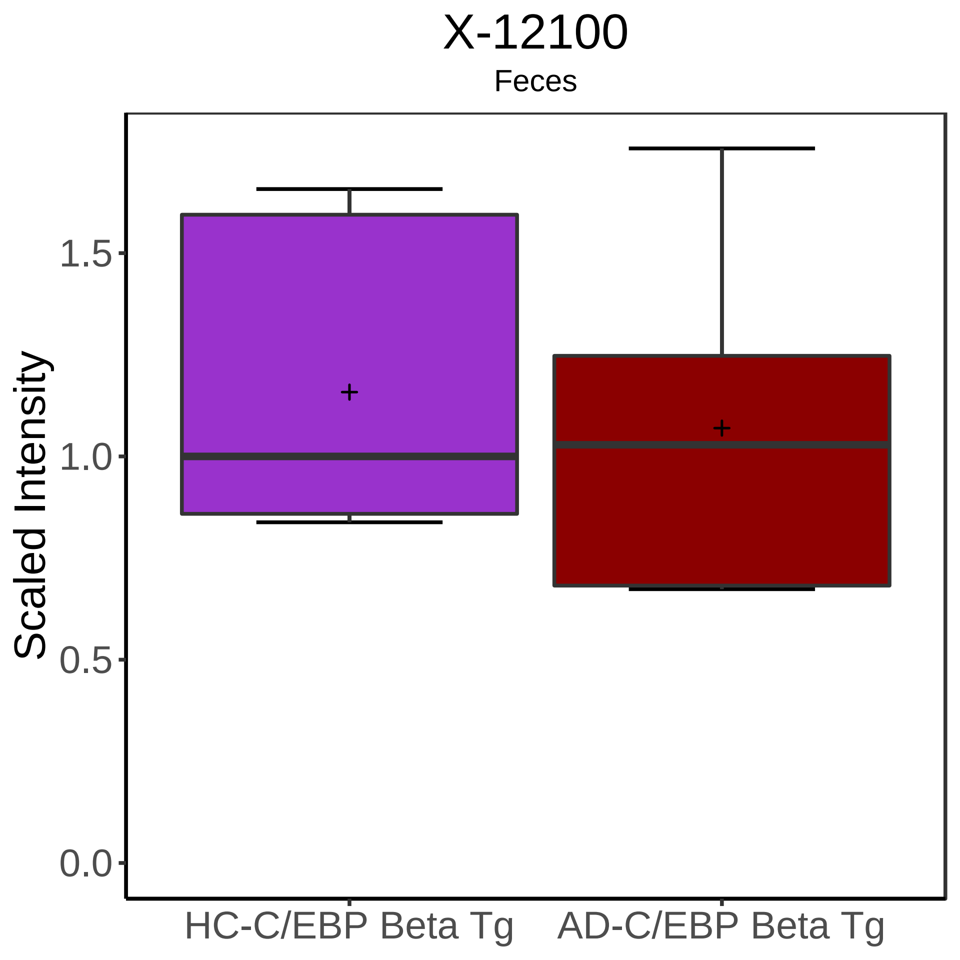
X-12100
Feces
Scaled Intensity
0.0
0.5
1.0
1.5
HC-C/EBP Beta Tg
AD-C/EBP Beta Tg

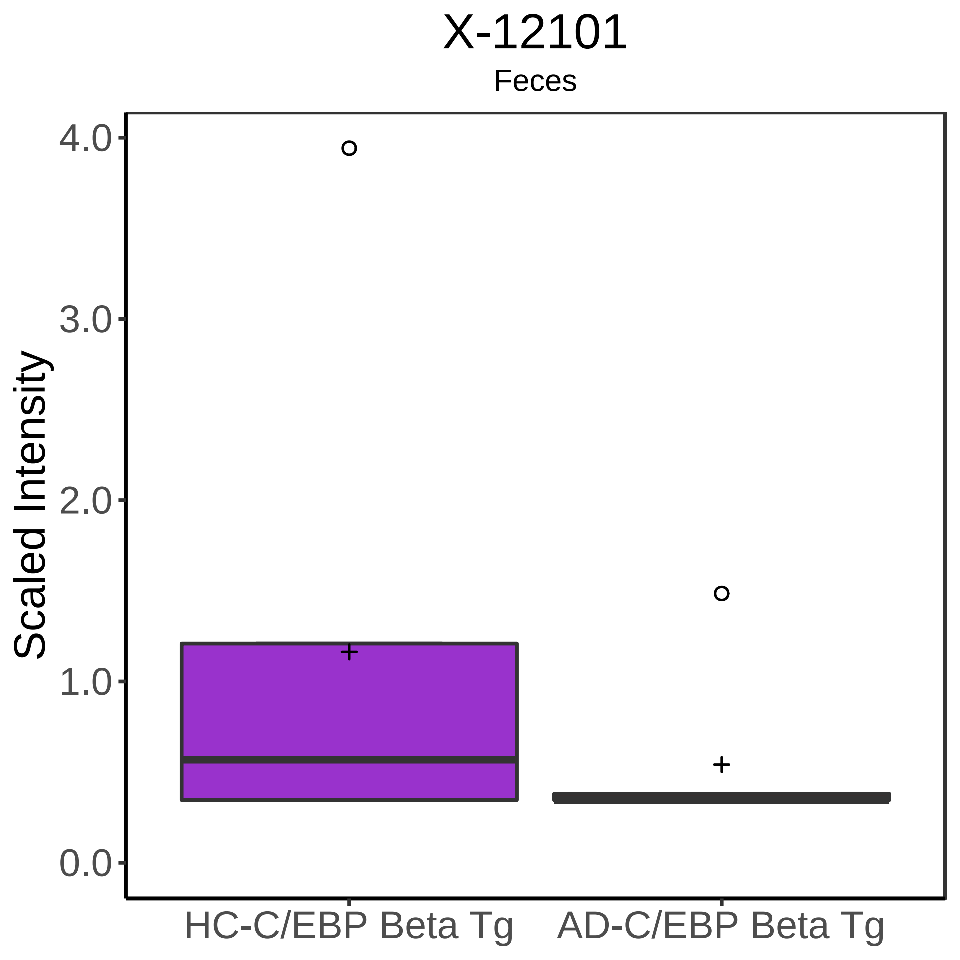
X-12101
Feces
Scaled Intensity
4.0
3.0
2.0
1.0
0.0
HC-C/EBP Beta Tg
AD-C/EBP Beta Tg

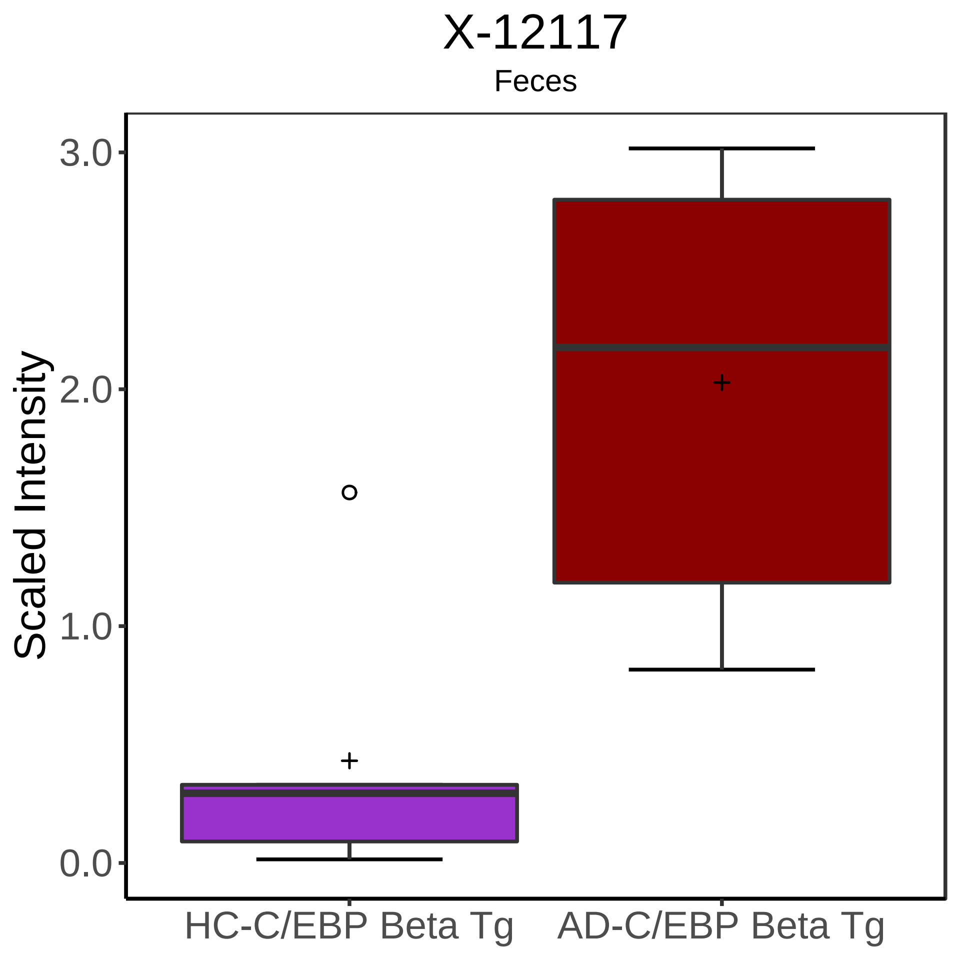
X-12117
Feces
Scaled Intensity
3.0
2.0
1.0
0.0
HC-C/EBP Beta Tg
AD-C/EBP Beta Tg

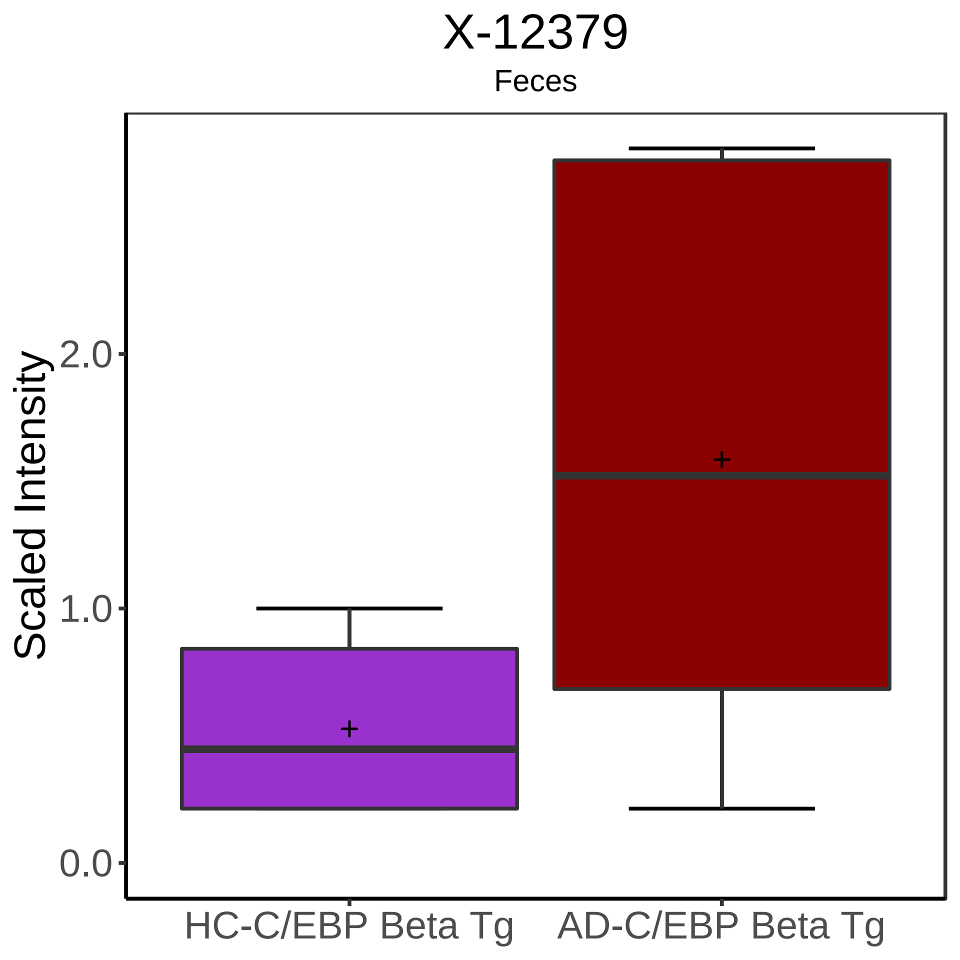
X-12379
Feces
Scaled Intensity
2.0
1.0
0.0
HC-C/EBP Beta Tg
AD-C/EBP Beta Tg

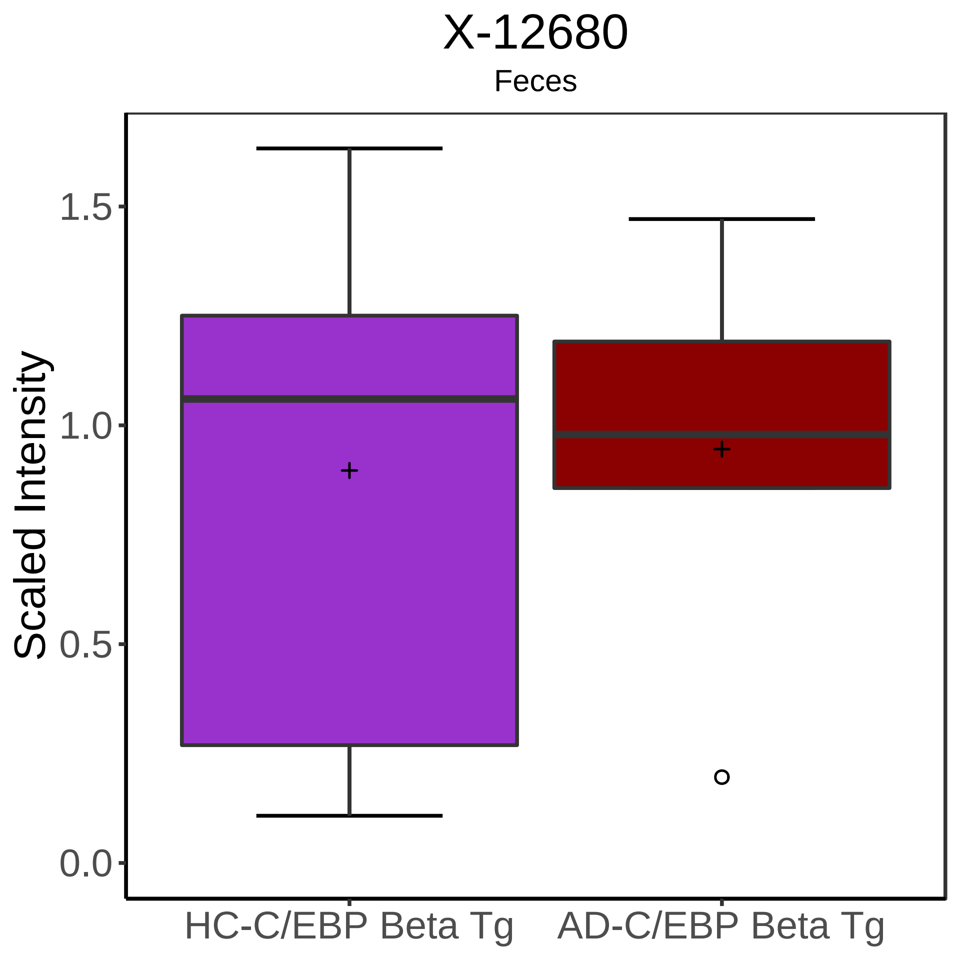
X-12680
Feces
Scaled Intensity
1.5
1.0
0.5
0.0
HC-C/EBP Beta Tg
AD-C/EBP Beta Tg

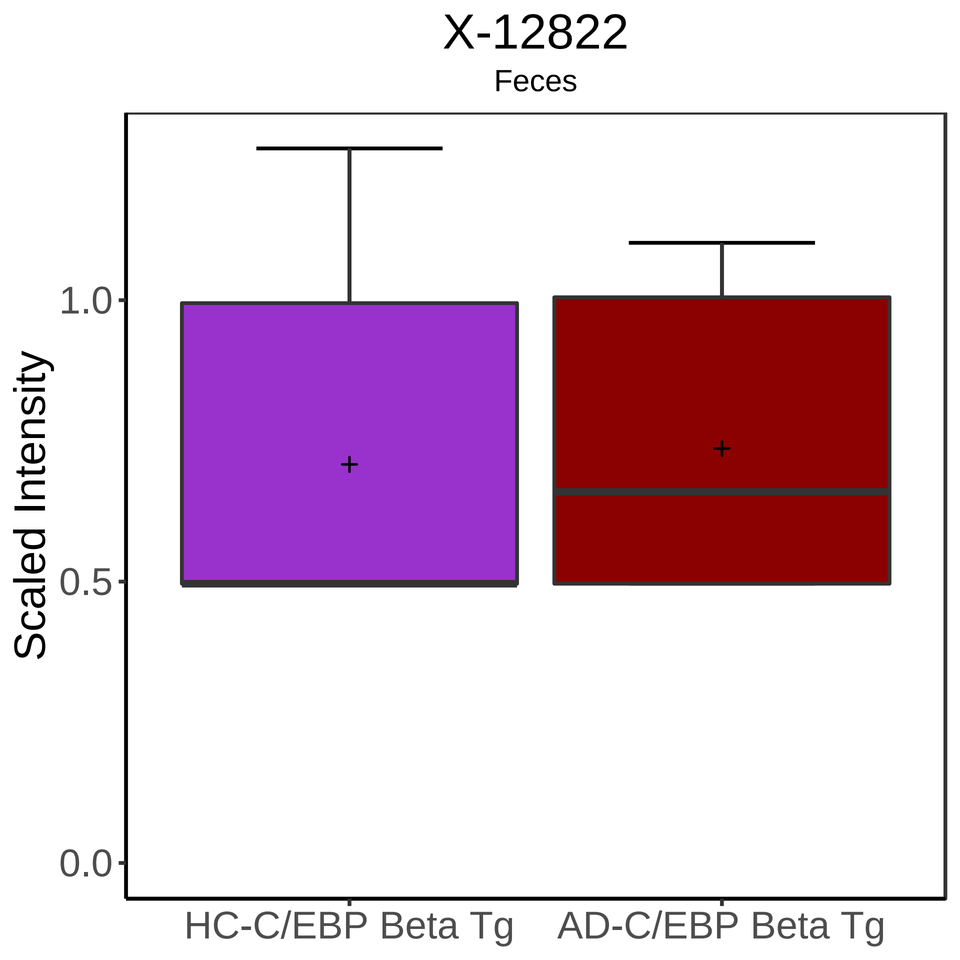
X-12822
Feces
Scaled Intensity
1.0
0.5
0.0
HC-C/EBP Beta Tg
AD-C/EBP Beta Tg

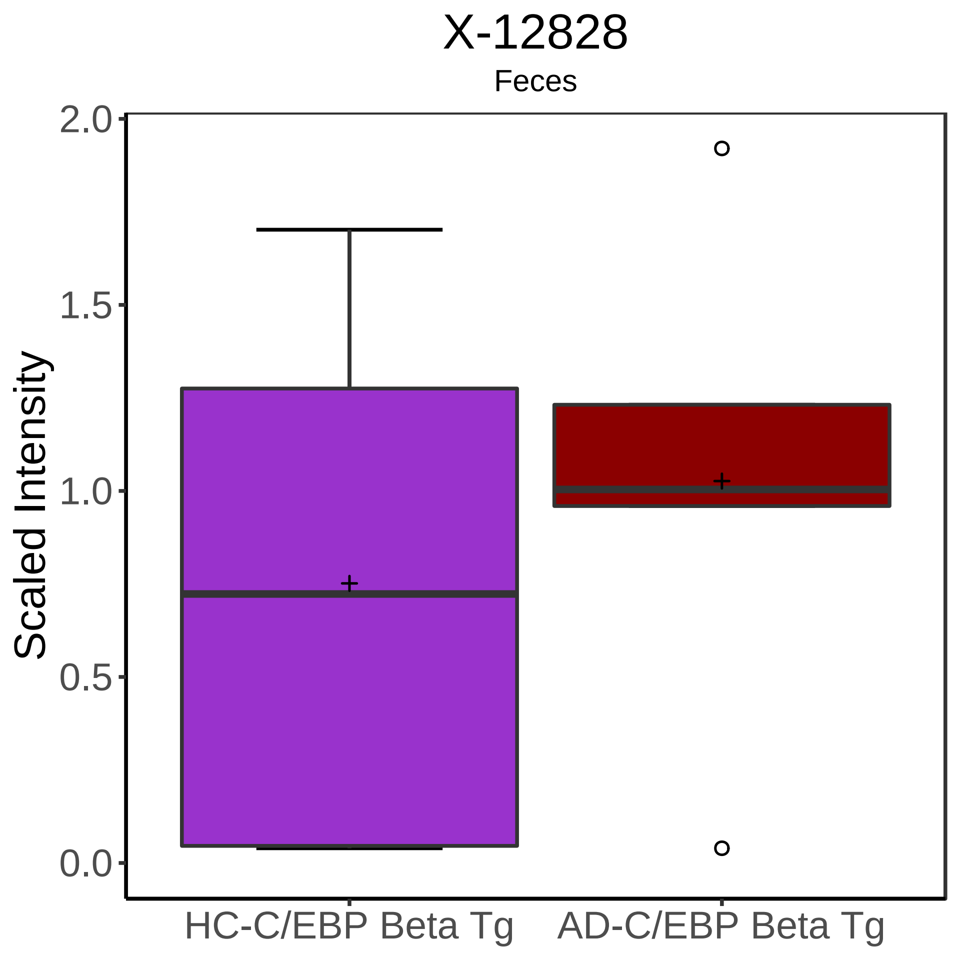

X-12828
Feces
Scaled Intensity
2.0
1.5
1.0
0.5
0.0
HC-C/EBP Beta Tg
AD-C/EBP Beta Tg

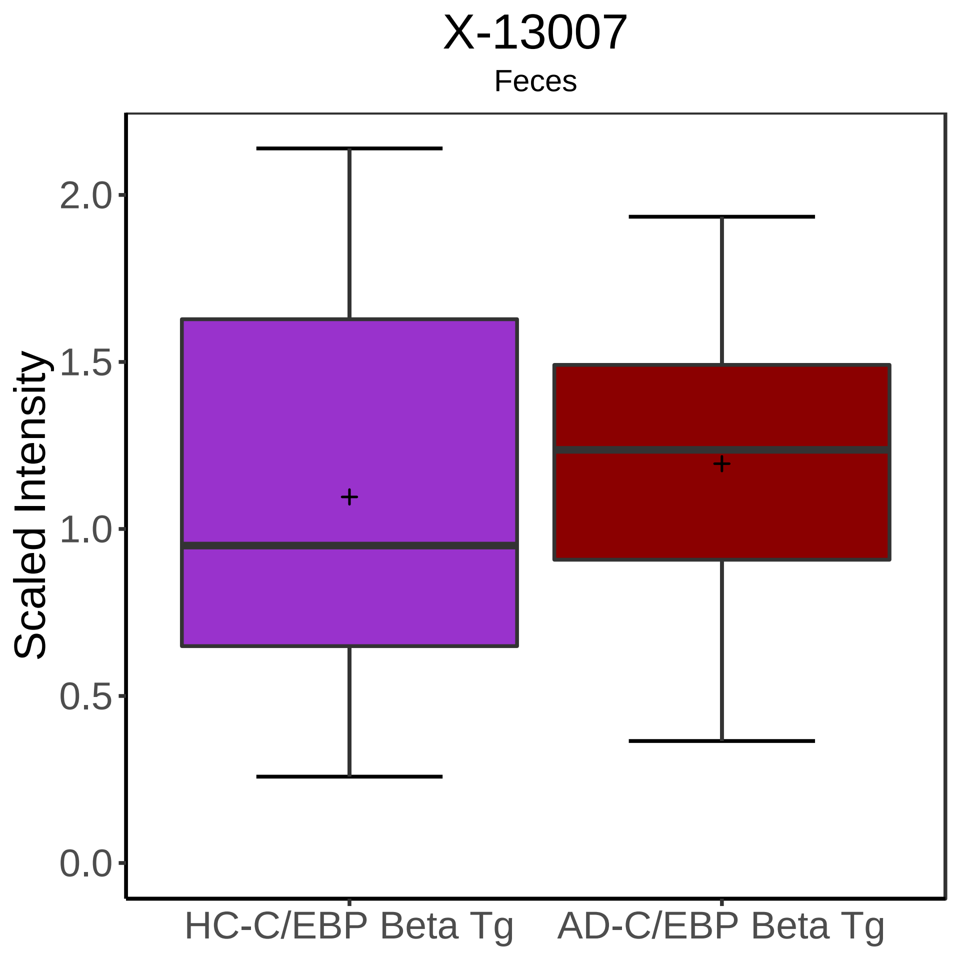
X-13007
Feces
Scaled Intensity
2.0
1.5
1.0
0.5
0.0
HC-C/EBP Beta Tg
AD-C/EBP Beta Tg

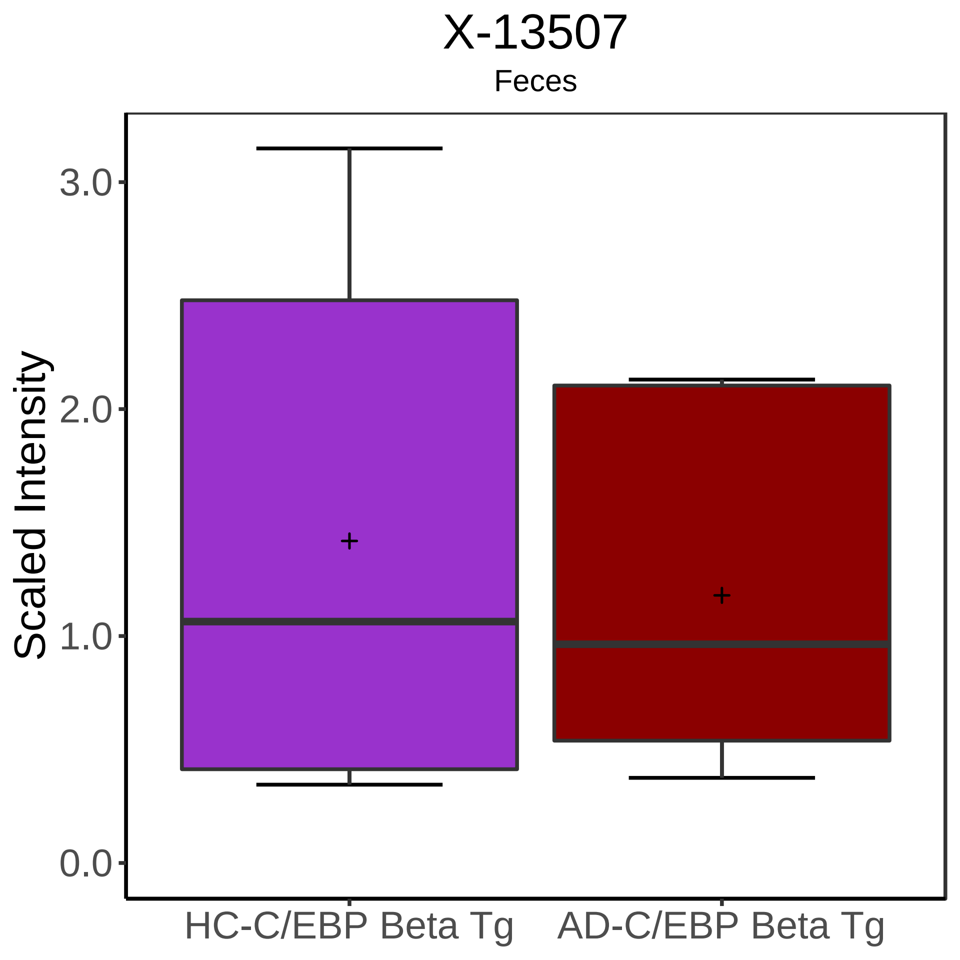

X-13507
Feces
Scaled Intensity
3.0
2.0
1.0
0.0
HC-C/EBP Beta Tg
AD-C/EBP Beta Tg

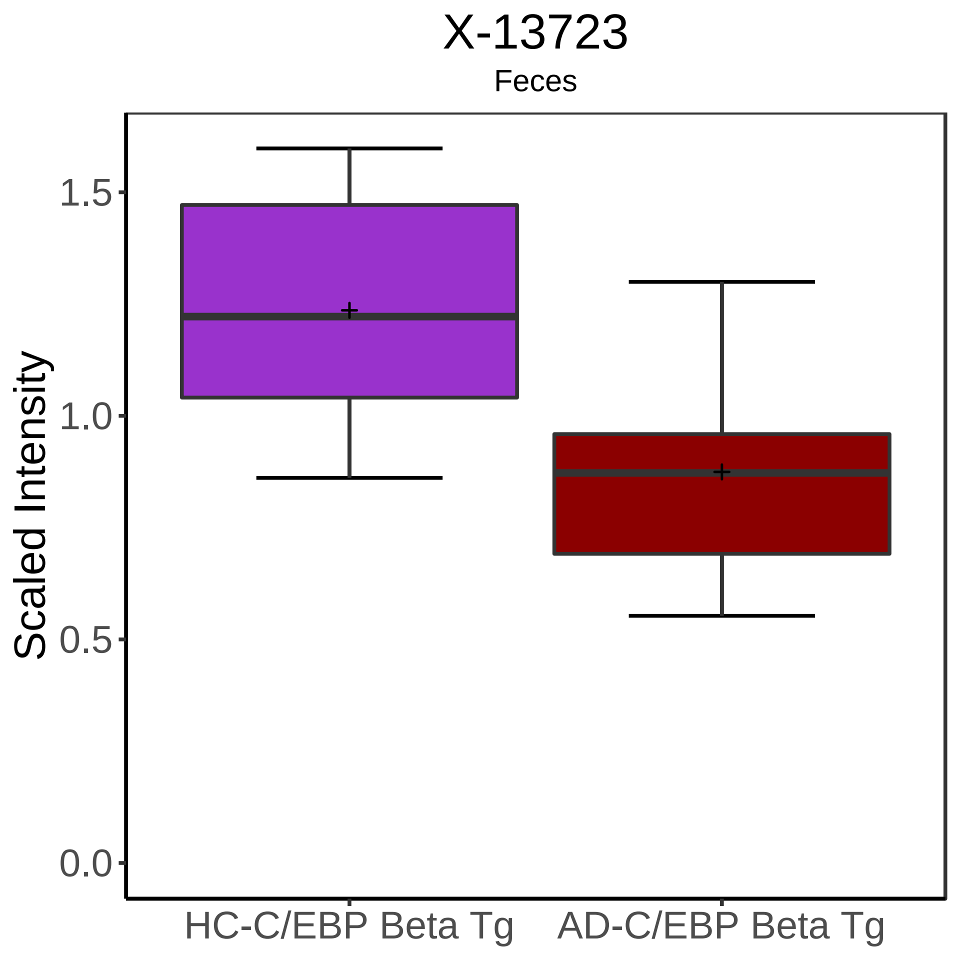
X-13723
Feces
Scaled Intensity
1.5
1.0
0.5
0.0
HC-C/EBP Beta Tg
AD-C/EBP Beta Tg

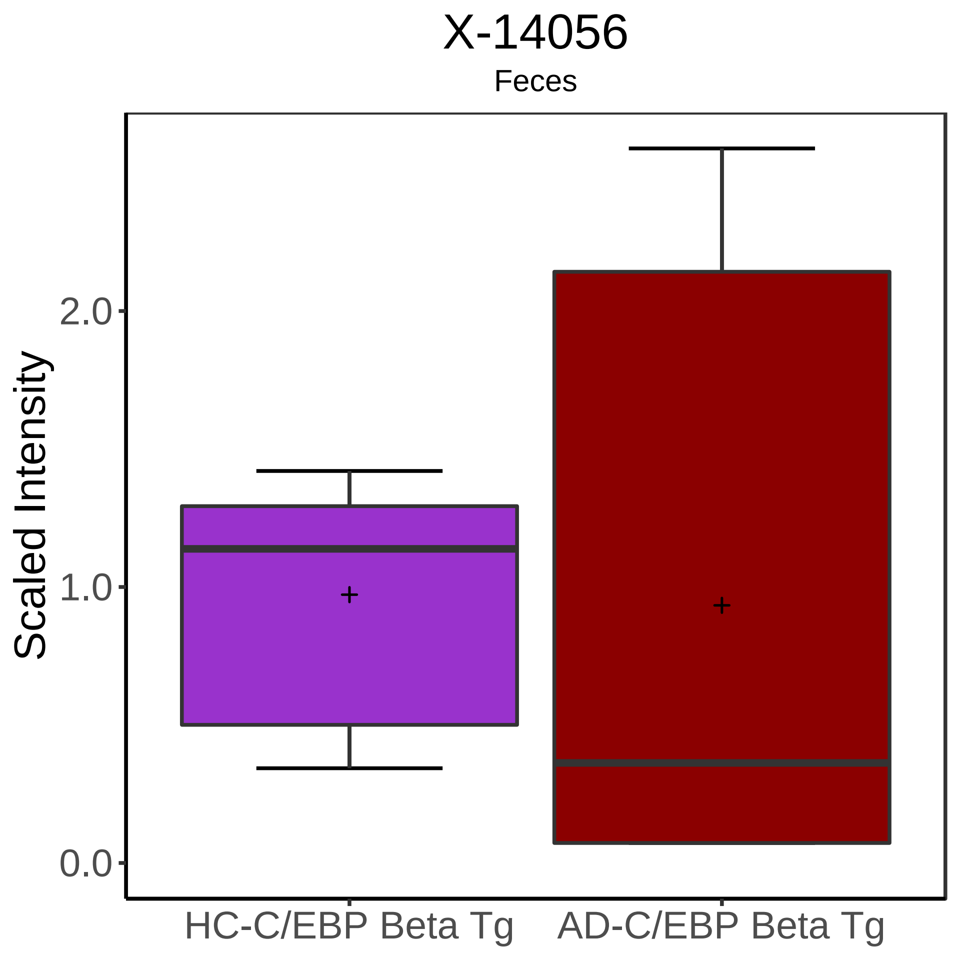
X-14056
Feces
Scaled Intensity
2.0
1.0
0.0
HC-C/EBP Beta Tg
AD-C/EBP Beta Tg

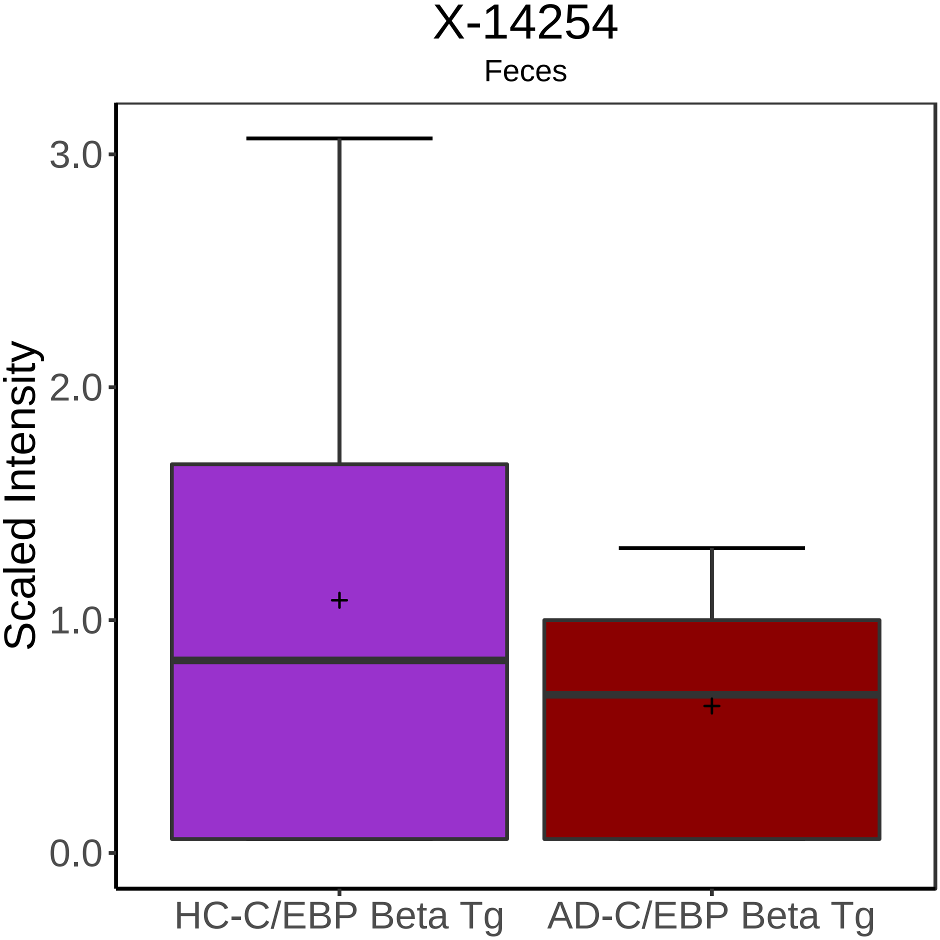
X-14254
Feces
Scaled Intensity
3.0
2.0
1.0
0.0
HC-C/EBP Beta Tg
AD-C/EBP Beta Tg

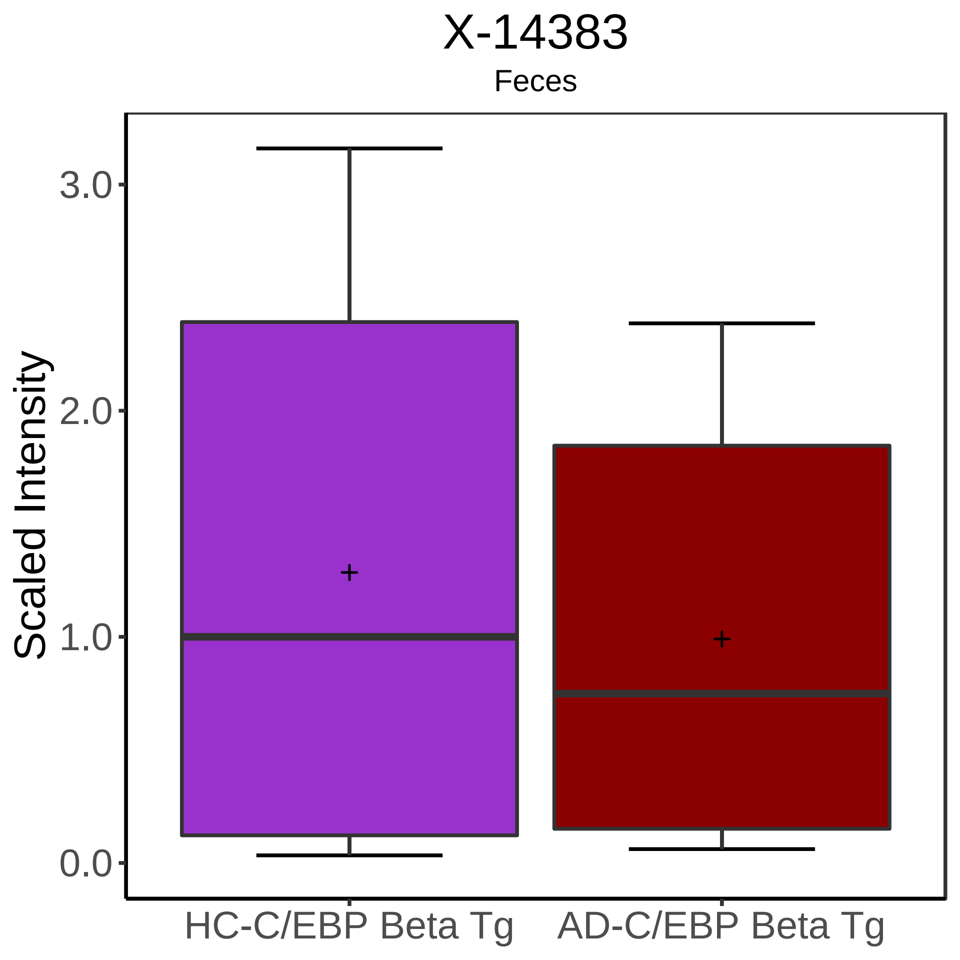

X-14383
Feces
Scaled Intensity
3.0
2.0
1.0
0.0
HC-C/EBP Beta Tg
AD-C/EBP Beta Tg

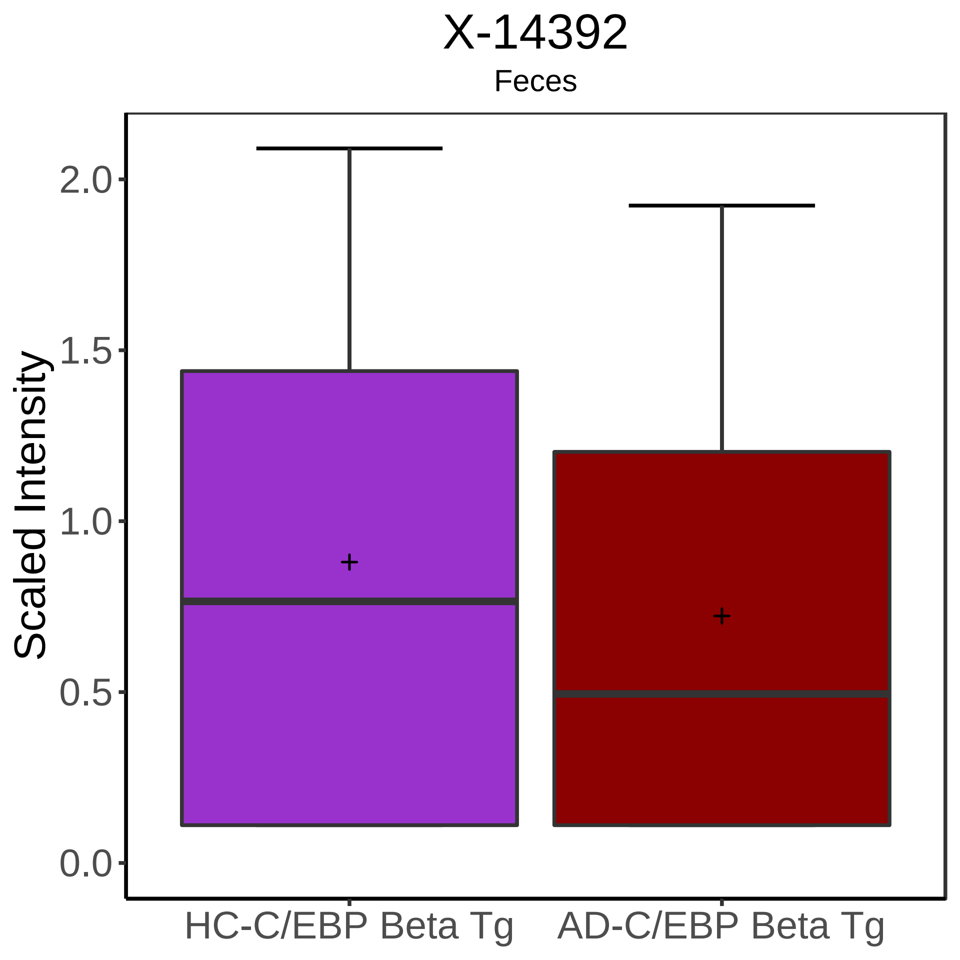

X-14392
Feces
Scaled Intensity
2.0
1.5
1.0
0.5
0.0
HC-C/EBP Beta Tg
AD-C/EBP Beta Tg

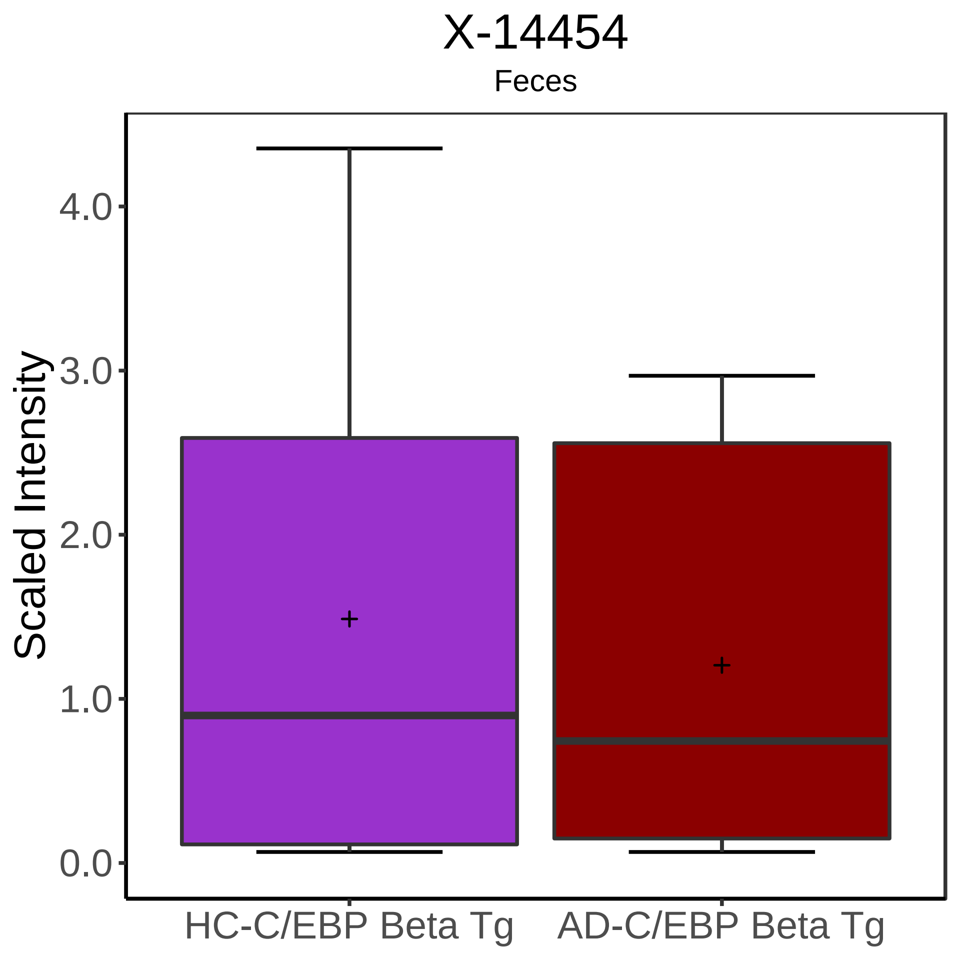
X-14454
Feces
Scaled Intensity
4.0
3.0
2.0
1.0
0.0
HC-C/EBP Beta Tg
AD-C/EBP Beta Tg

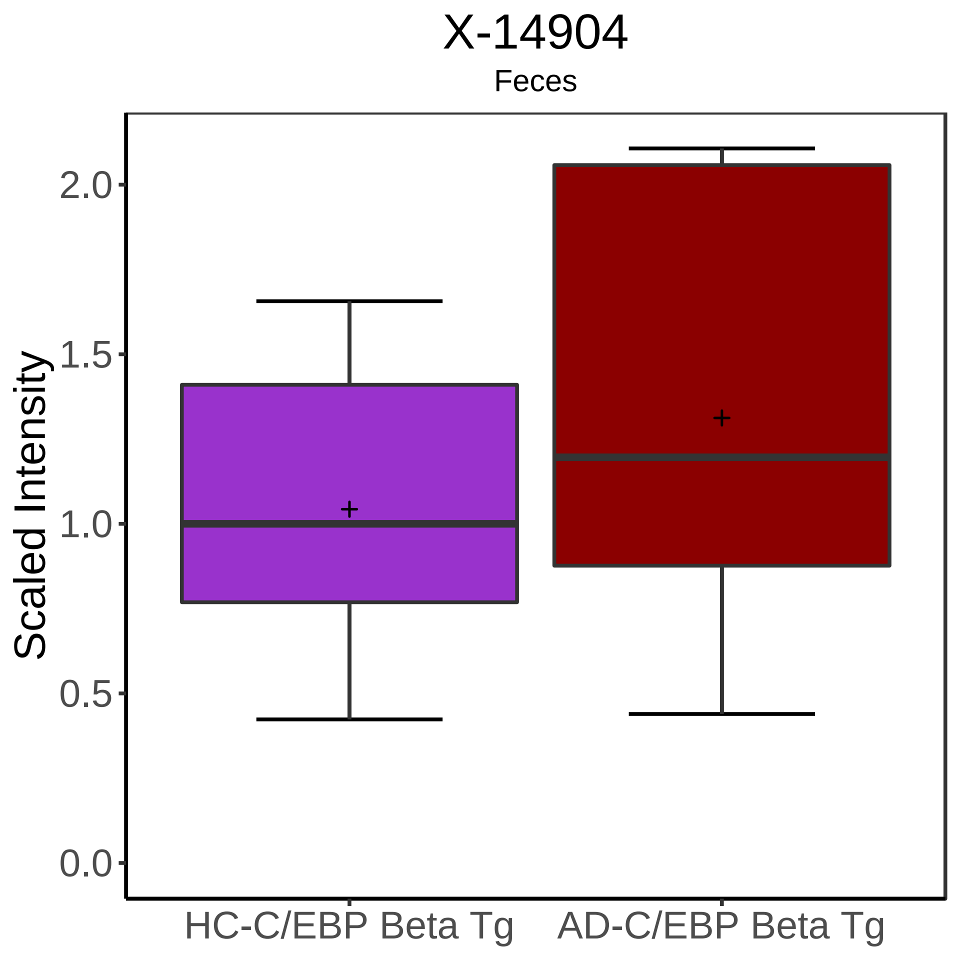
X-14904
Feces
Scaled Intensity
0.0
0.5
1.0
1.5
2.0
HC-C/EBP Beta Tg
AD-C/EBP Beta Tg

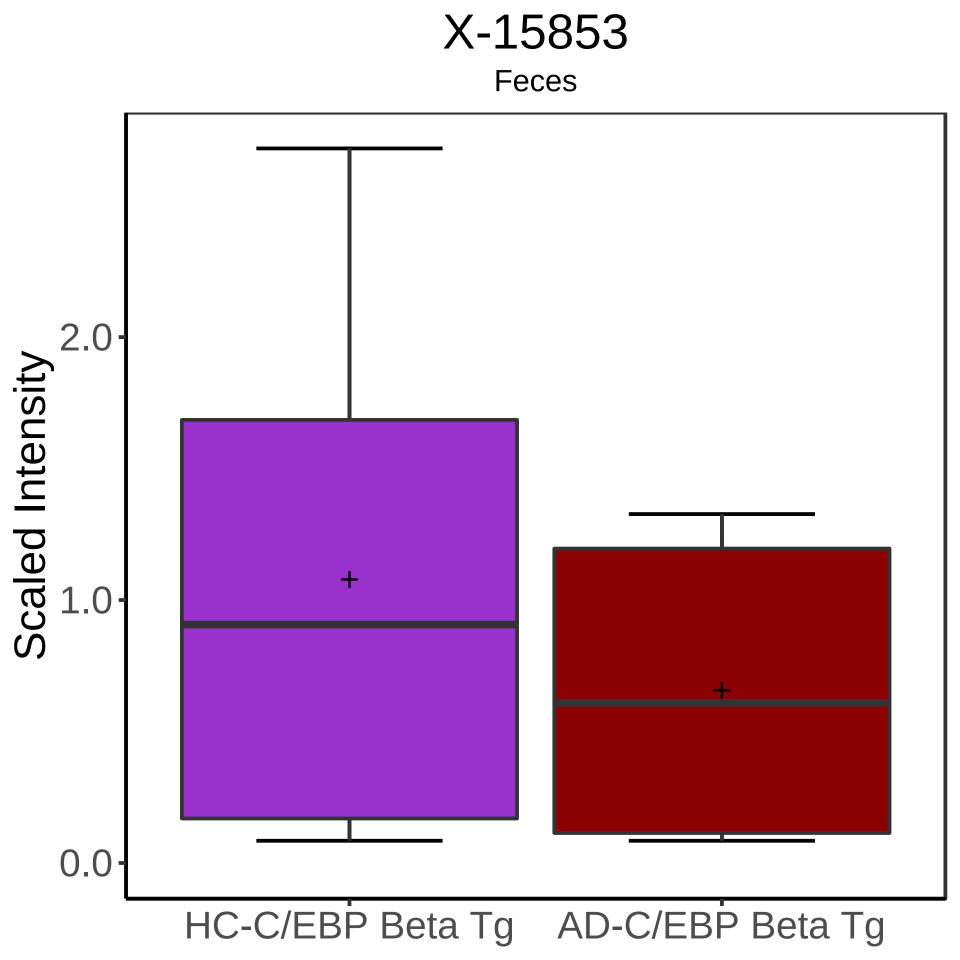
X-15853
Feces
Scaled Intensity
2.0
1.0
0.0
HC-C/EBP Beta Tg
AD-C/EBP Beta Tg

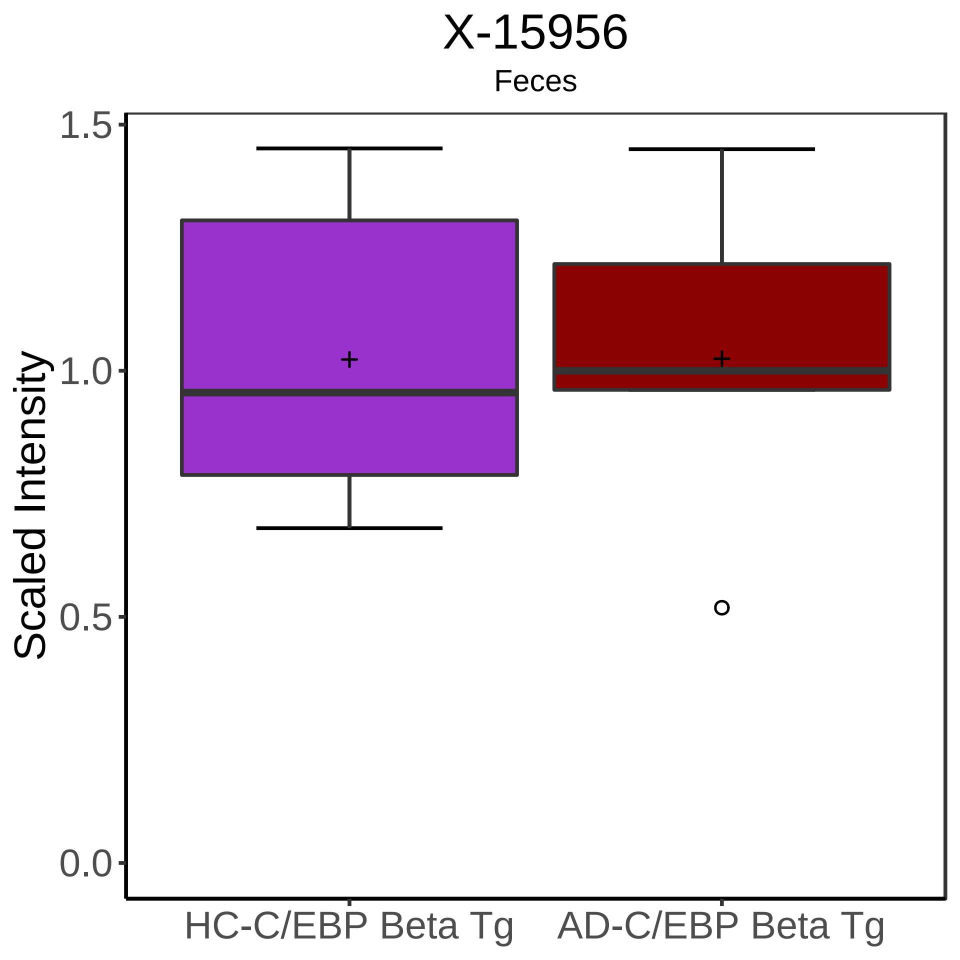
X-15956
Feces
Scaled Intensity
1.5
1.0
0.5
0.0
HC-C/EBP Beta Tg
AD-C/EBP Beta Tg

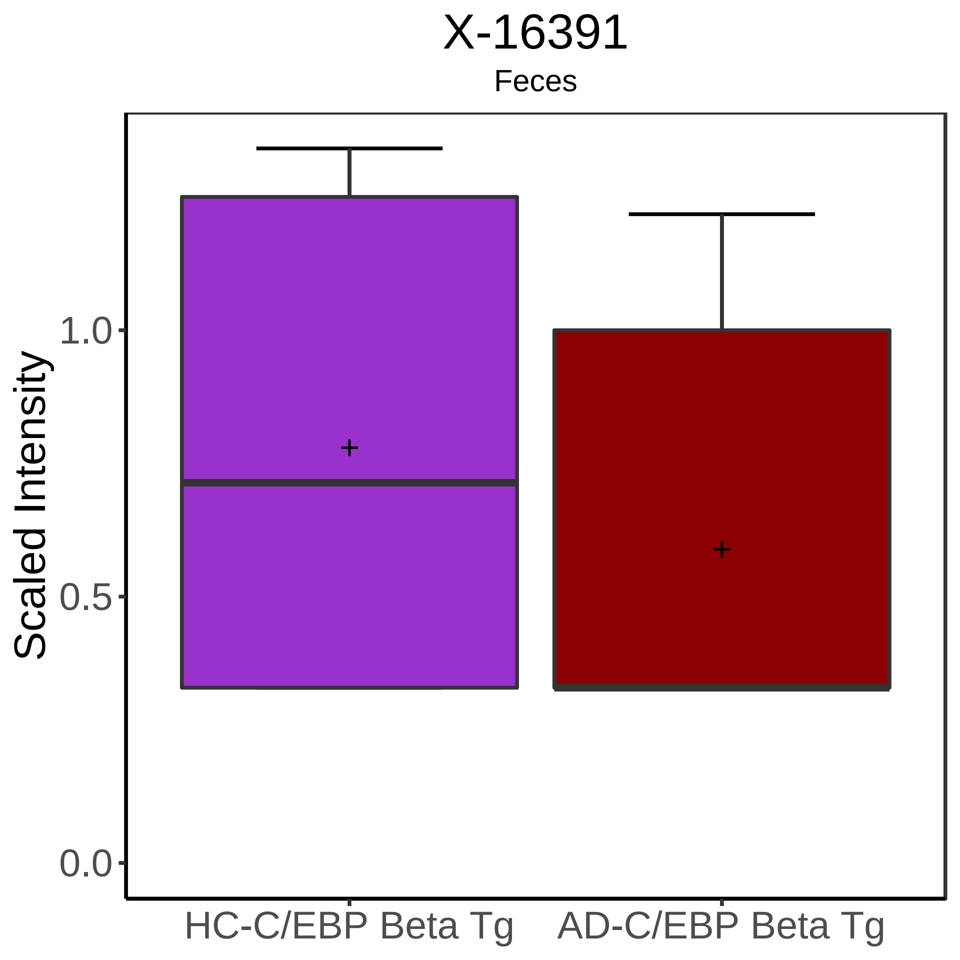
X-16391
Feces
Scaled Intensity
1.0
0.5
0.0
HC-C/EBP Beta Tg
AD-C/EBP Beta Tg

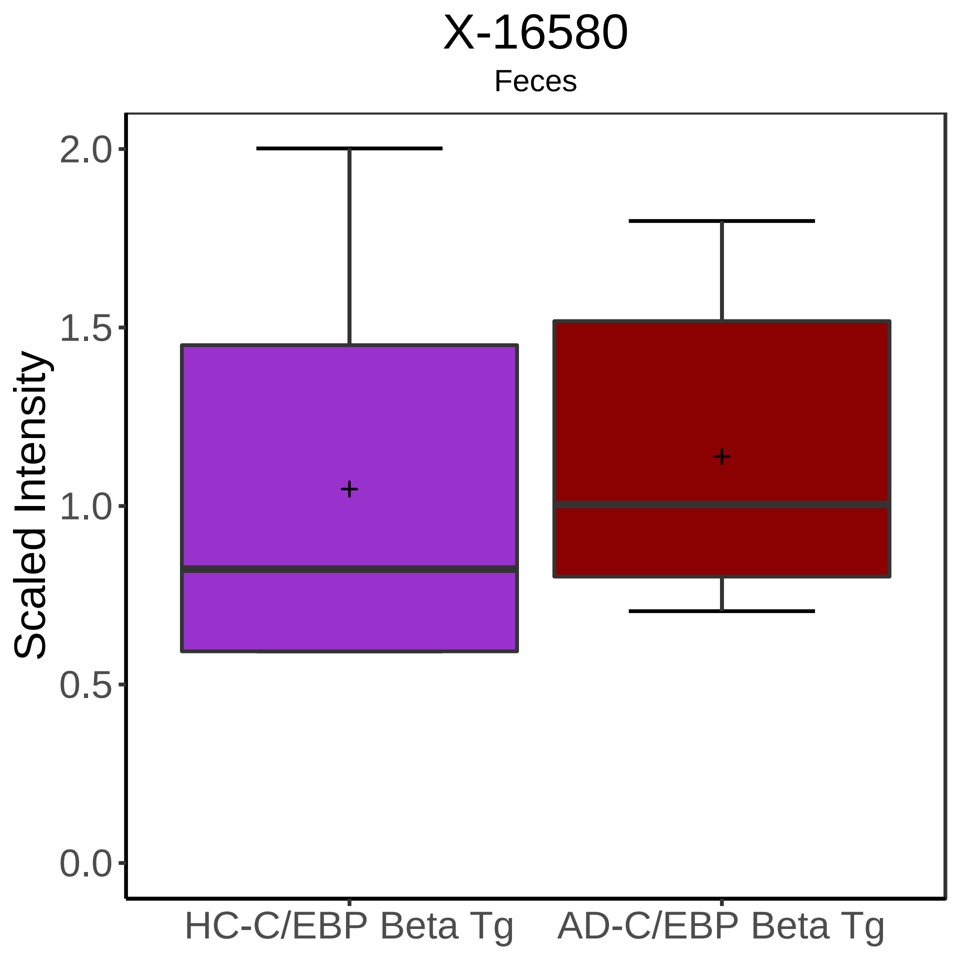

X-16580
Feces
Scaled Intensity
2.0
1.5
1.0
0.5
0.0
HC-C/EBP Beta Tg
AD-C/EBP Beta Tg

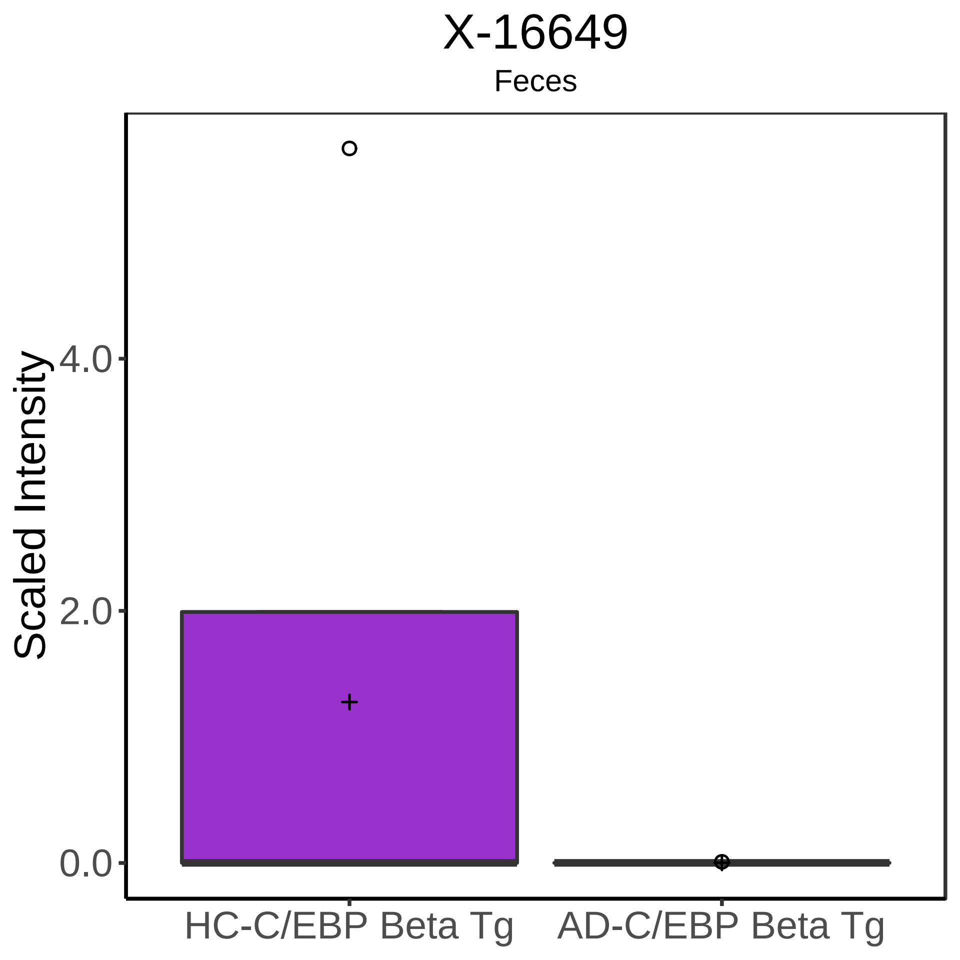

X-16649
Feces
Scaled Intensity
4.0
2.0
0.0
HC-C/EBP Beta Tg
AD-C/EBP Beta Tg

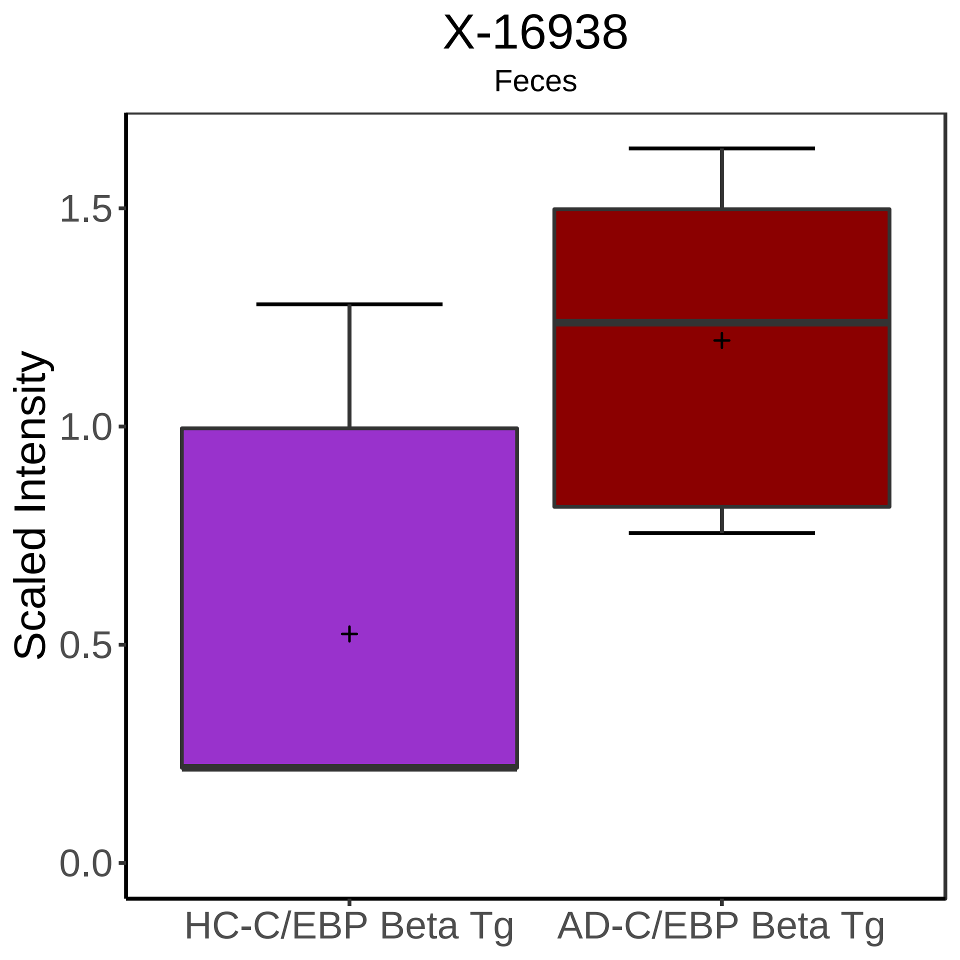
X-16938
Feces
Scaled Intensity
0.0
0.5
1.0
1.5
HC-C/EBP Beta Tg
AD-C/EBP Beta Tg

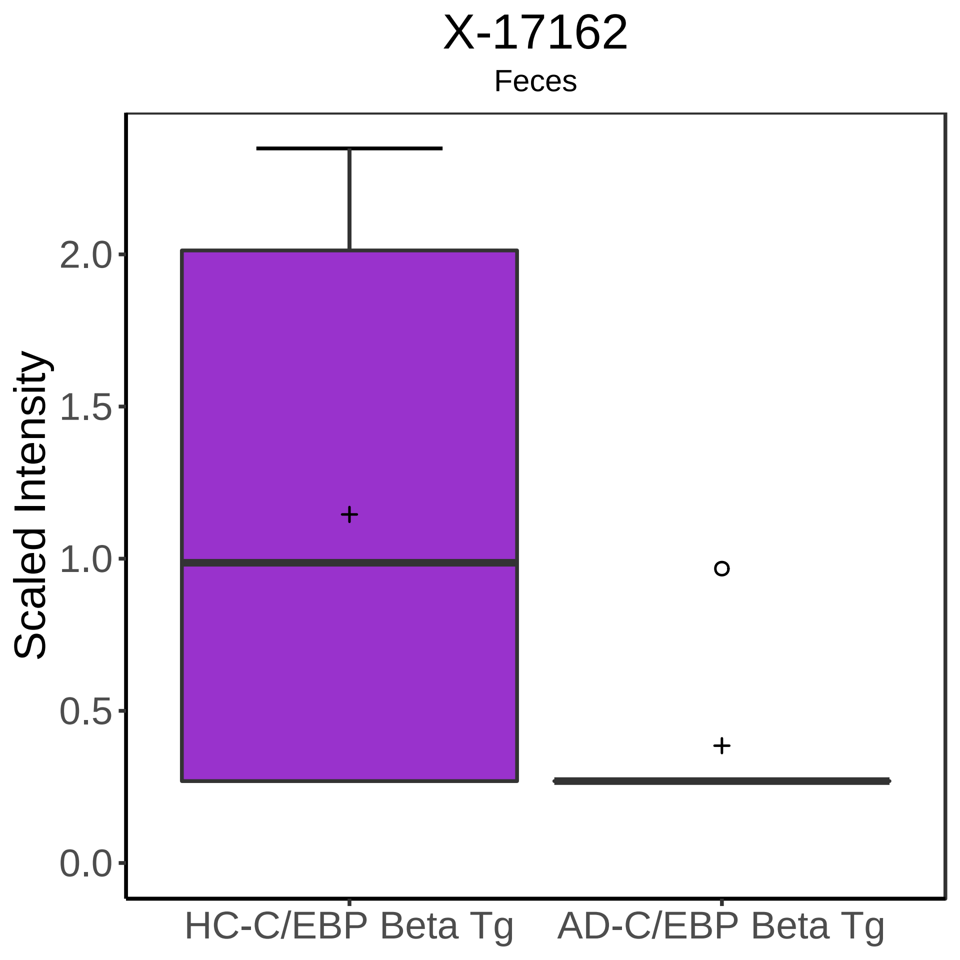

X-17162
Feces
Scaled Intensity
2.0
1.5
1.0
0.5
0.0
HC-C/EBP Beta Tg
AD-C/EBP Beta Tg

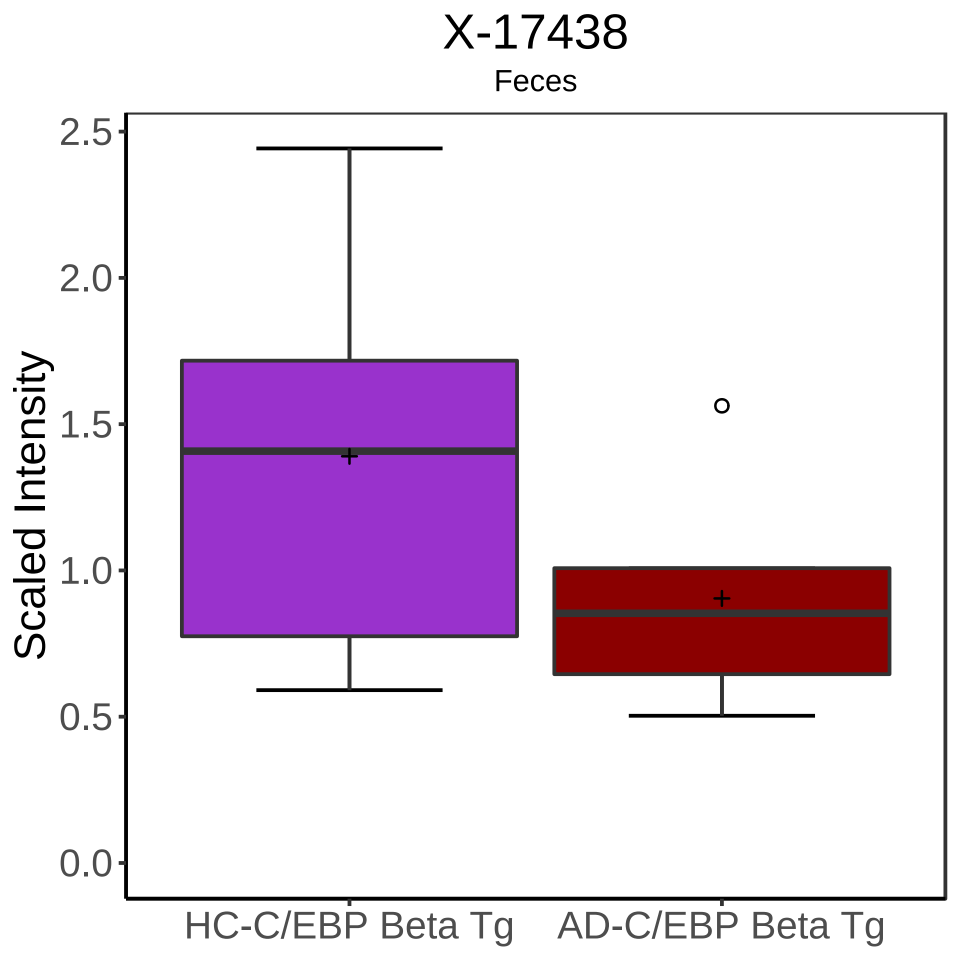

X-17438
Feces
Scaled Intensity
2.5
2.0
1.5
1.0
0.5
0.0
HC-C/EBP Beta Tg
AD-C/EBP Beta Tg

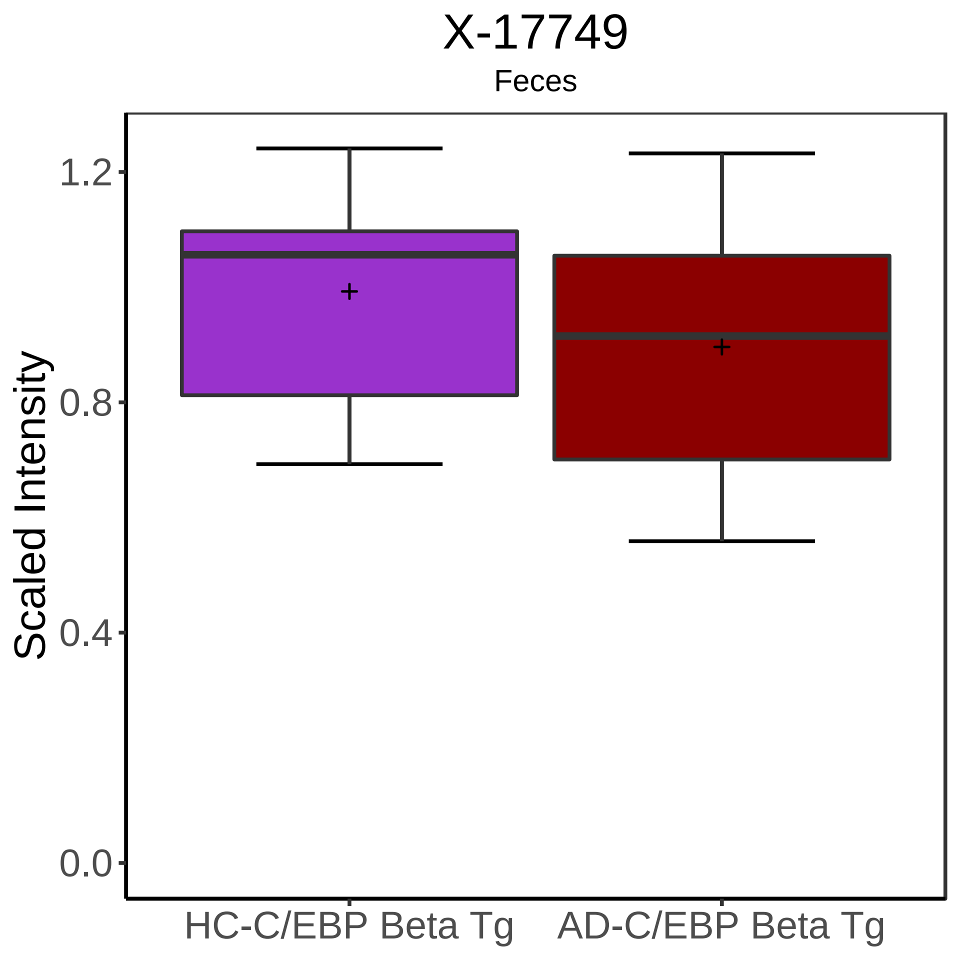

X-17749
Feces
Scaled Intensity
1.2
0.8
0.4
0.0
HC-C/EBP Beta Tg
AD-C/EBP Beta Tg

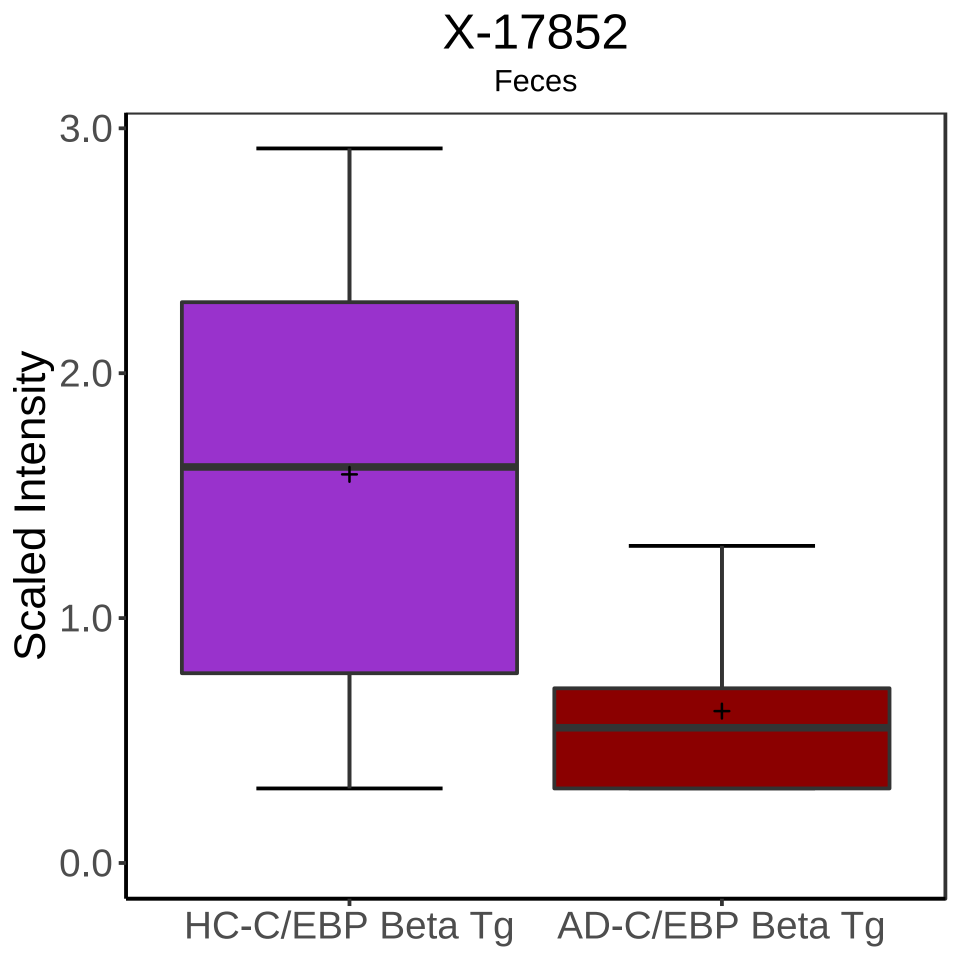

X-17852
Feces
Scaled Intensity
3.0
2.0
1.0
0.0
HC-C/EBP Beta Tg
AD-C/EBP Beta Tg

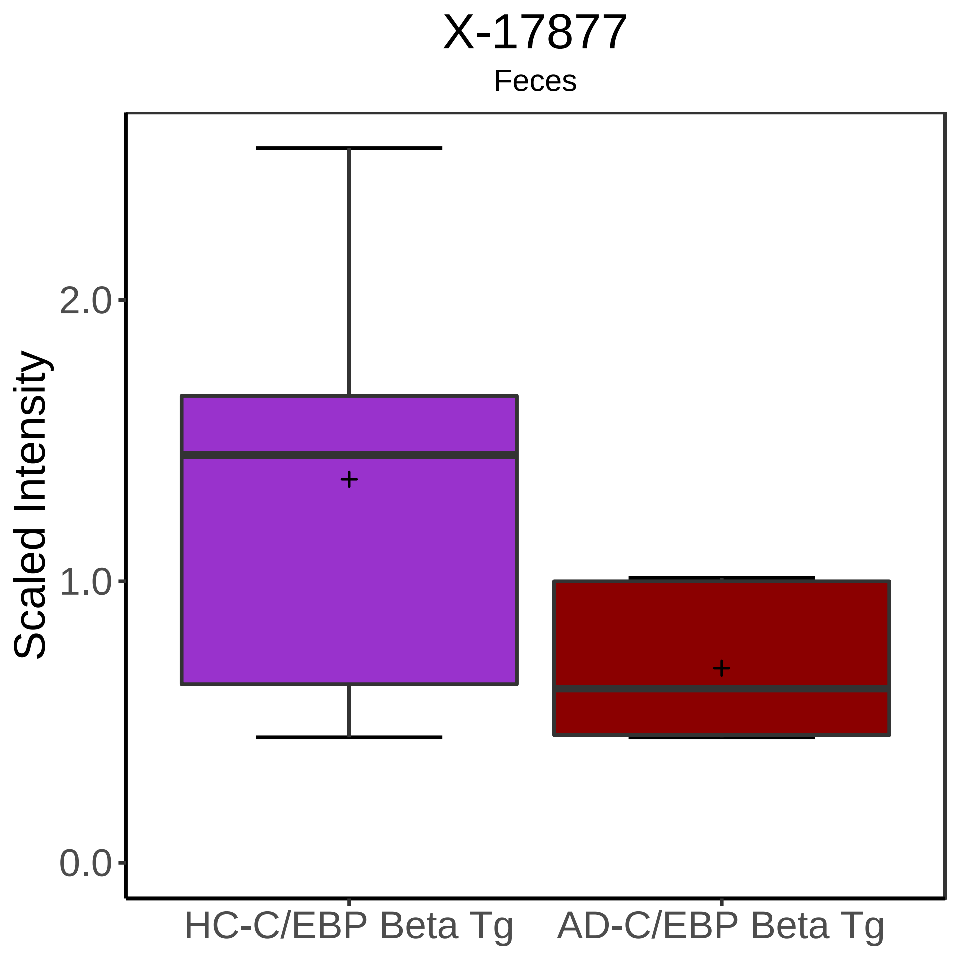
X-17877
Feces
Scaled Intensity
2.0
1.0
0.0
HC-C/EBP Beta Tg
AD-C/EBP Beta Tg

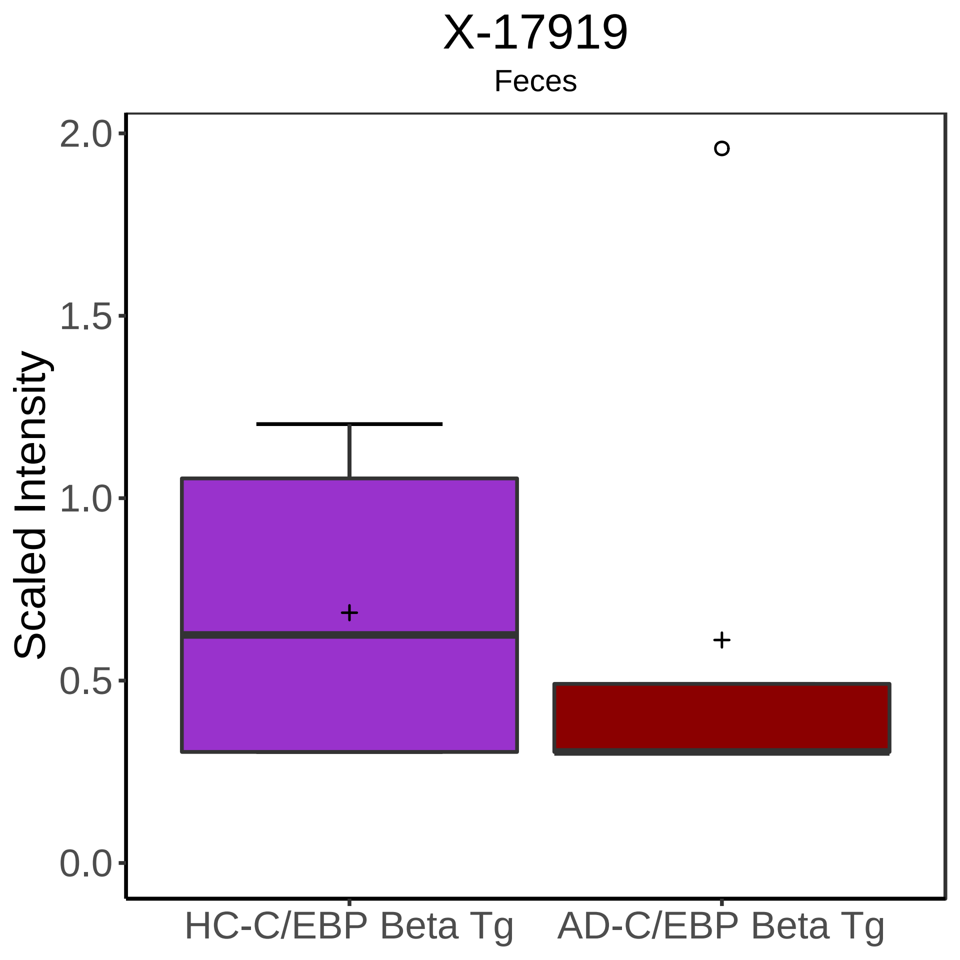
X-17919
Feces
Scaled Intensity
2.0
1.5
1.0
0.5
0.0
HC-C/EBP Beta Tg
AD-C/EBP Beta Tg

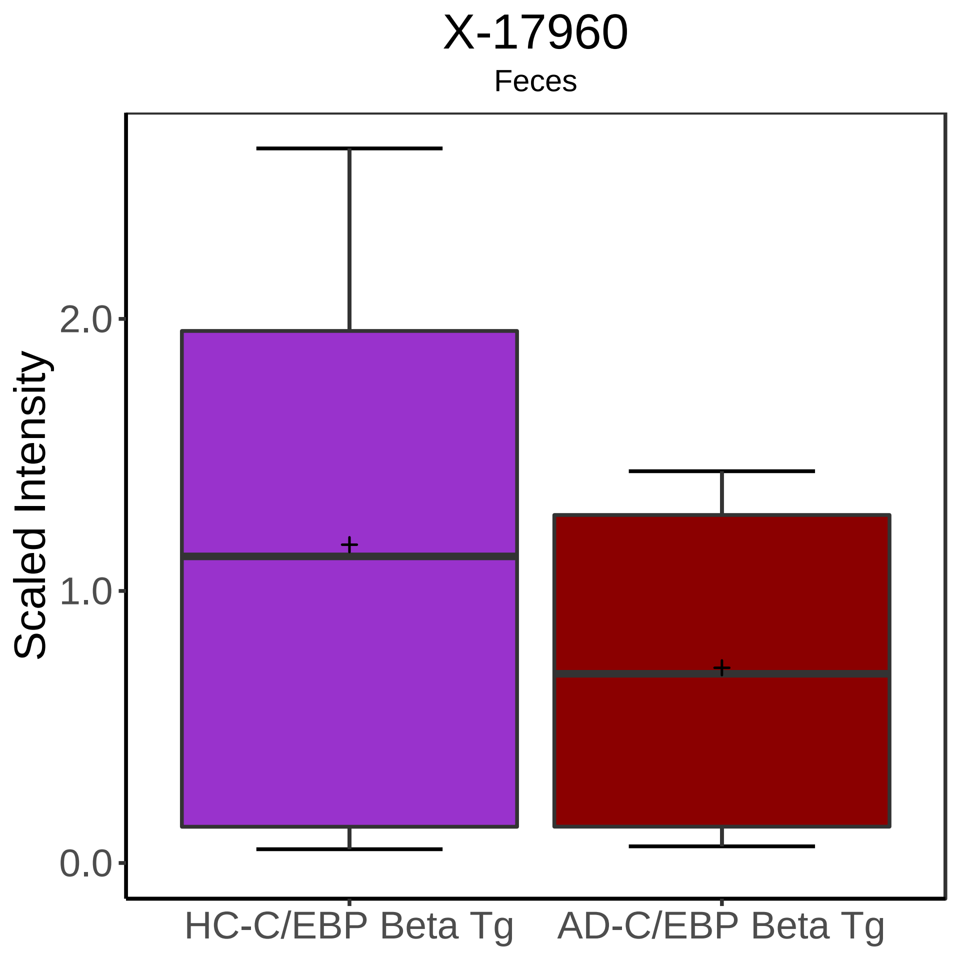
X-17960
Feces
Scaled Intensity
2.0
1.0
0.0
HC-C/EBP Beta Tg
AD-C/EBP Beta Tg

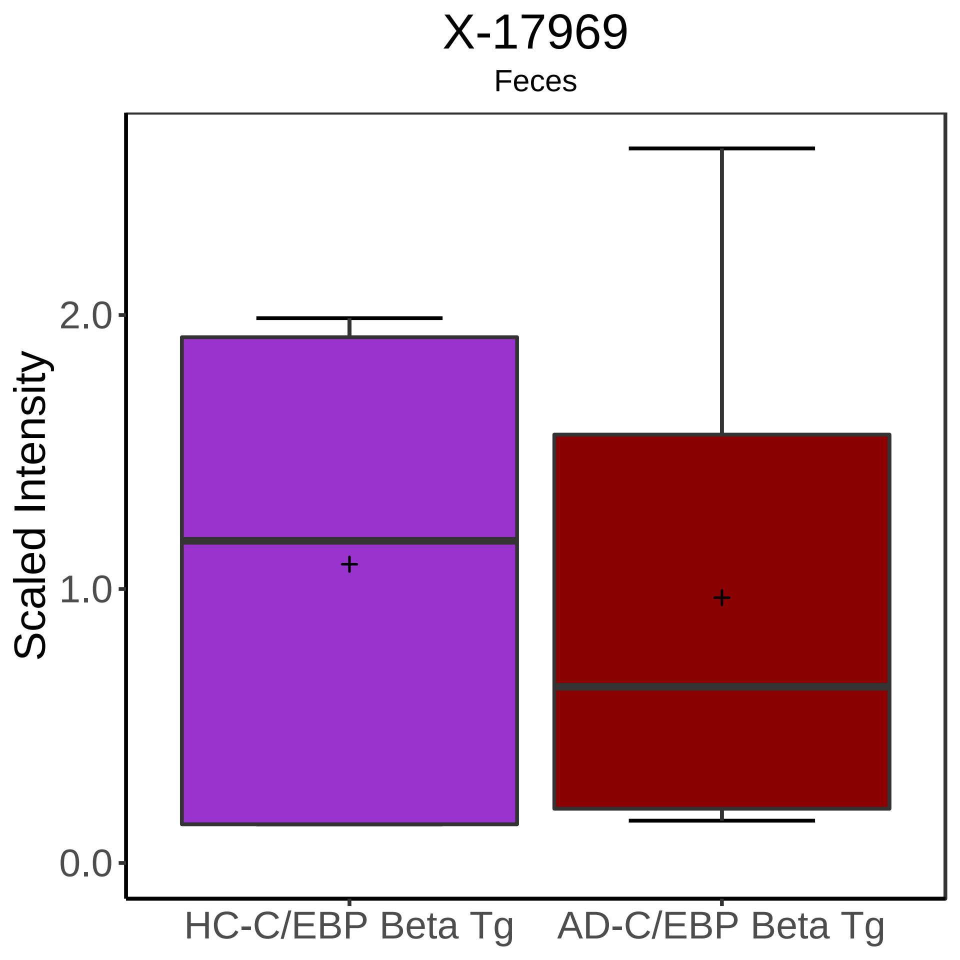
X-17969
Feces
Scaled Intensity
2.0
1.0
0.0
HC-C/EBP Beta Tg
AD-C/EBP Beta Tg

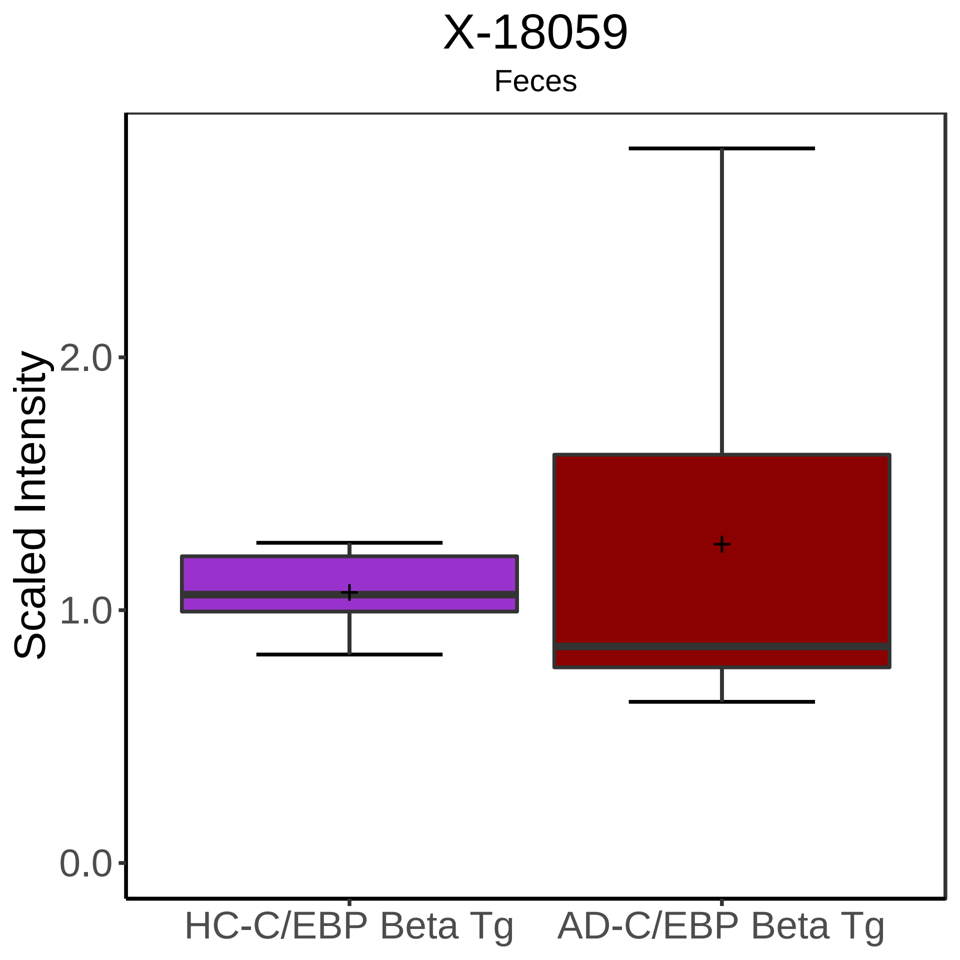

X-18059
Feces
Scaled Intensity
2.0
1.0
0.0
HC-C/EBP Beta Tg
AD-C/EBP Beta Tg

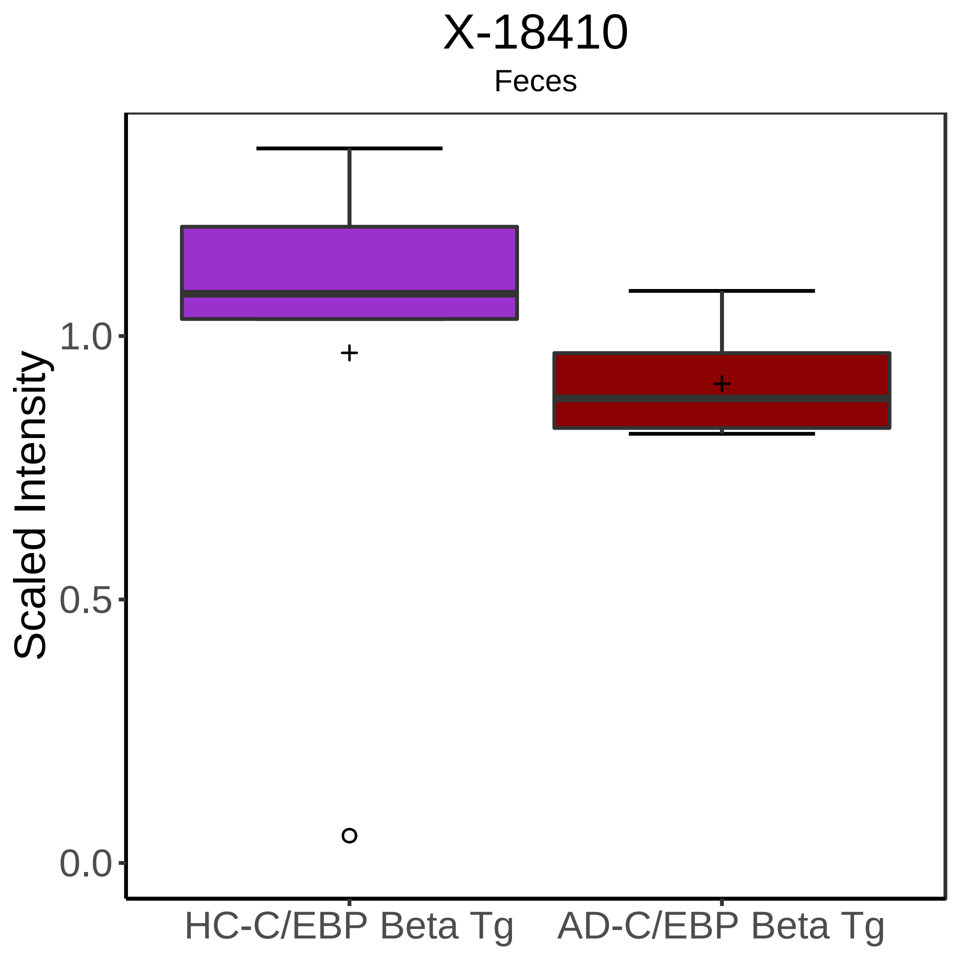
X-18410
Feces
Scaled Intensity
1.0
0.5
0.0
HC-C/EBP Beta Tg
AD-C/EBP Beta Tg

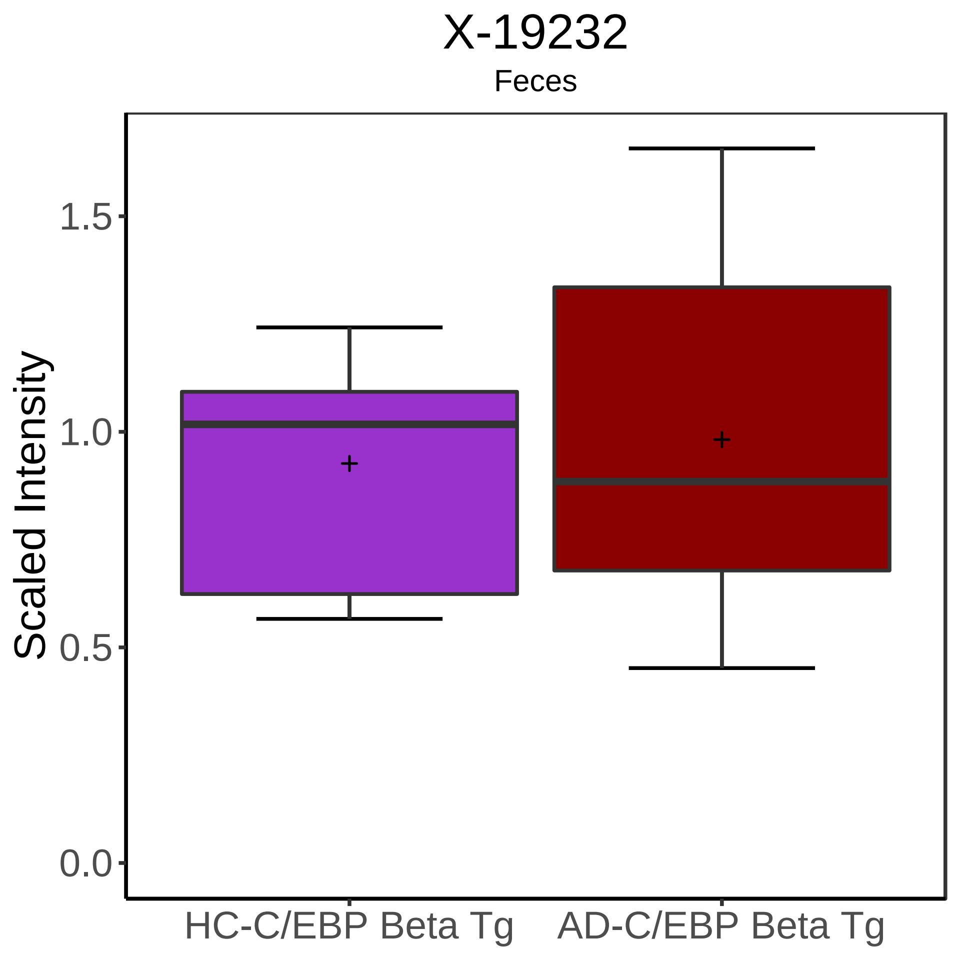
X-19232
Feces
Scaled Intensity
1.5
1.0
0.5
0.0
HC-C/EBP Beta Tg
AD-C/EBP Beta Tg

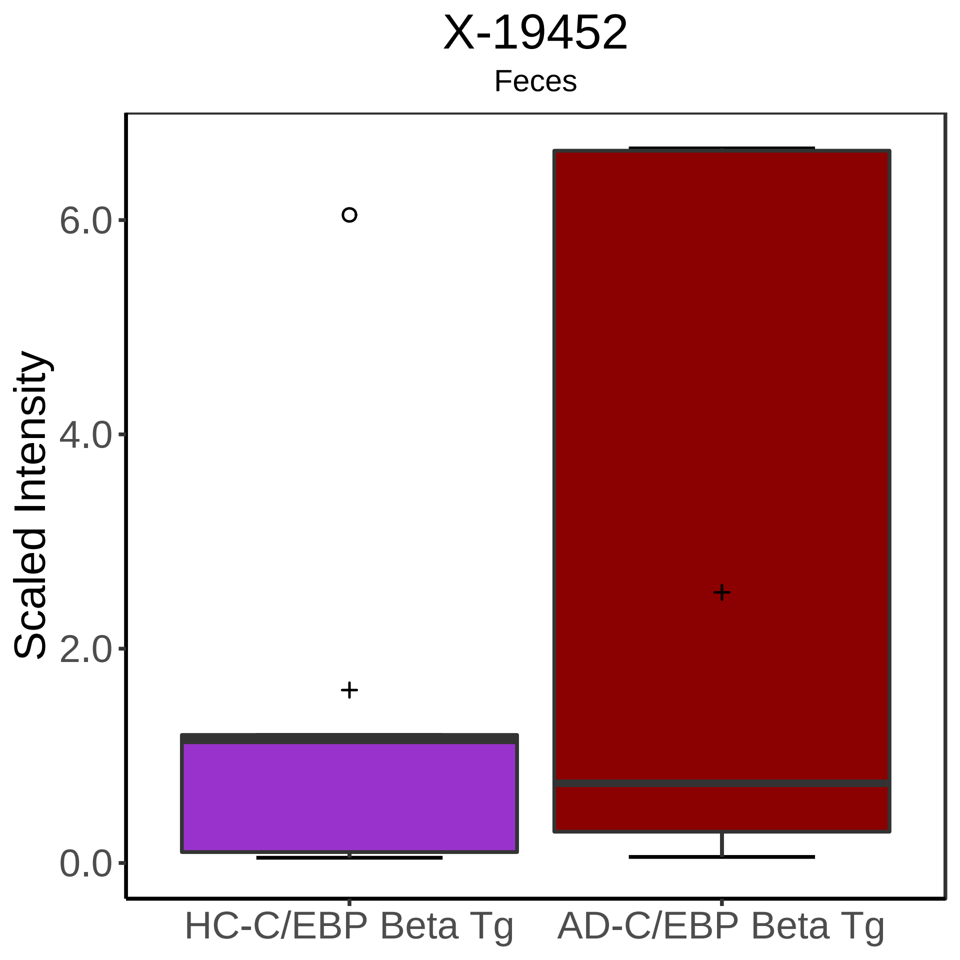
X-19452
Feces
Scaled Intensity
6.0
4.0
2.0
0.0
HC-C/EBP Beta Tg
AD-C/EBP Beta Tg

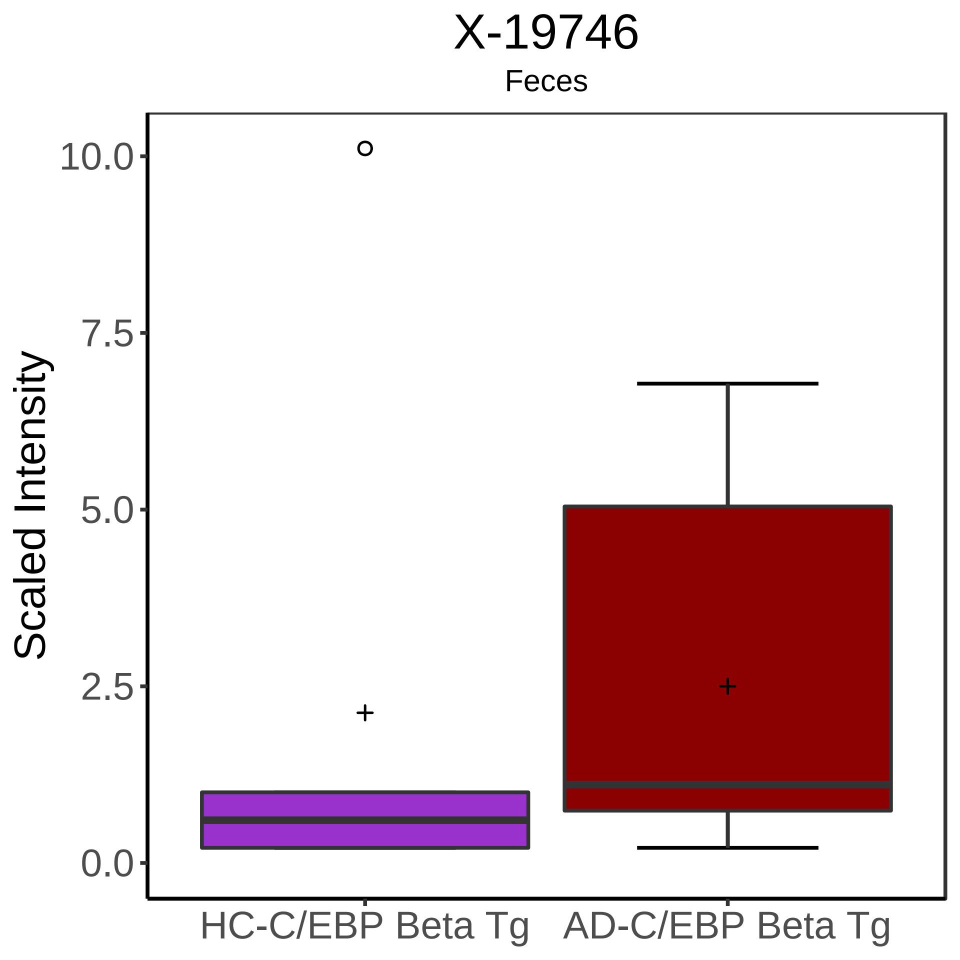
X-19746
Feces
Scaled Intensity
10.0
7.5
5.0
2.5
0.0
HC-C/EBP Beta Tg
AD-C/EBP Beta Tg

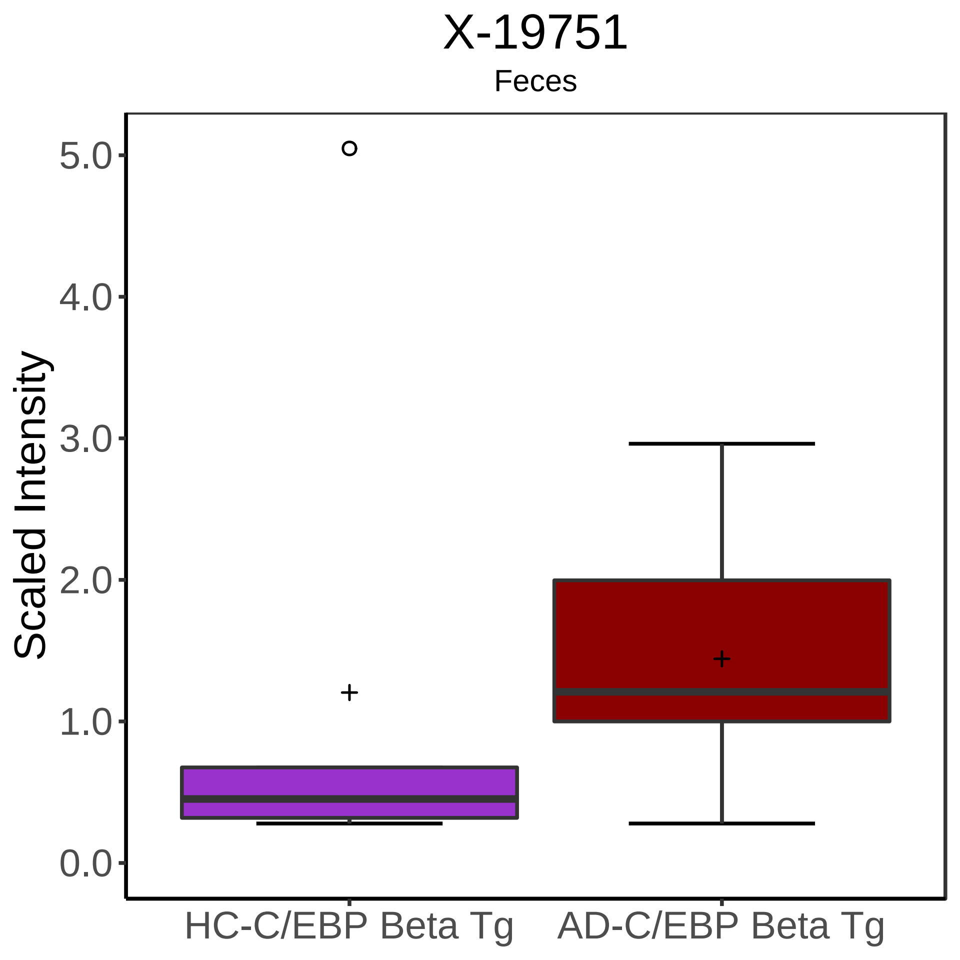
X-19751
Feces
Scaled Intensity
5.0
4.0
3.0
2.0
1.0
0.0
HC-C/EBP Beta Tg
AD-C/EBP Beta Tg

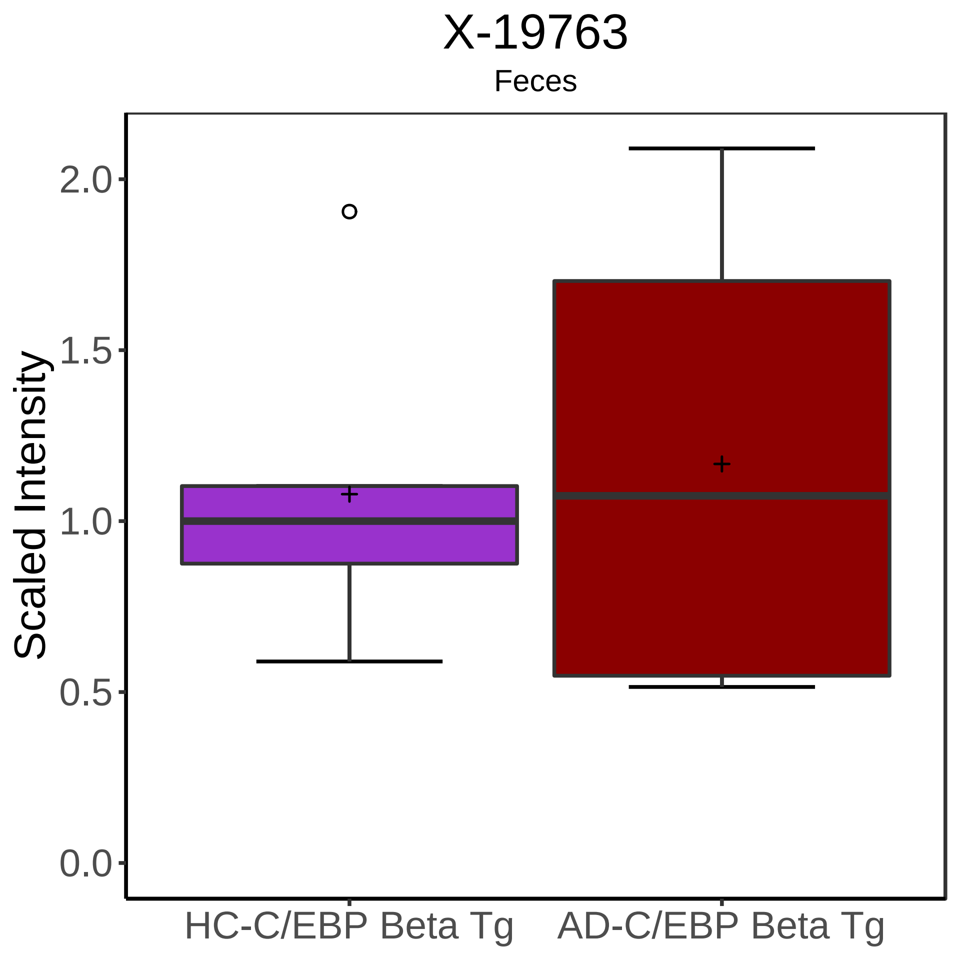
X-19763
Feces
Scaled Intensity
2.0
1.5
1.0
0.5
0.0
HC-C/EBP Beta Tg
AD-C/EBP Beta Tg

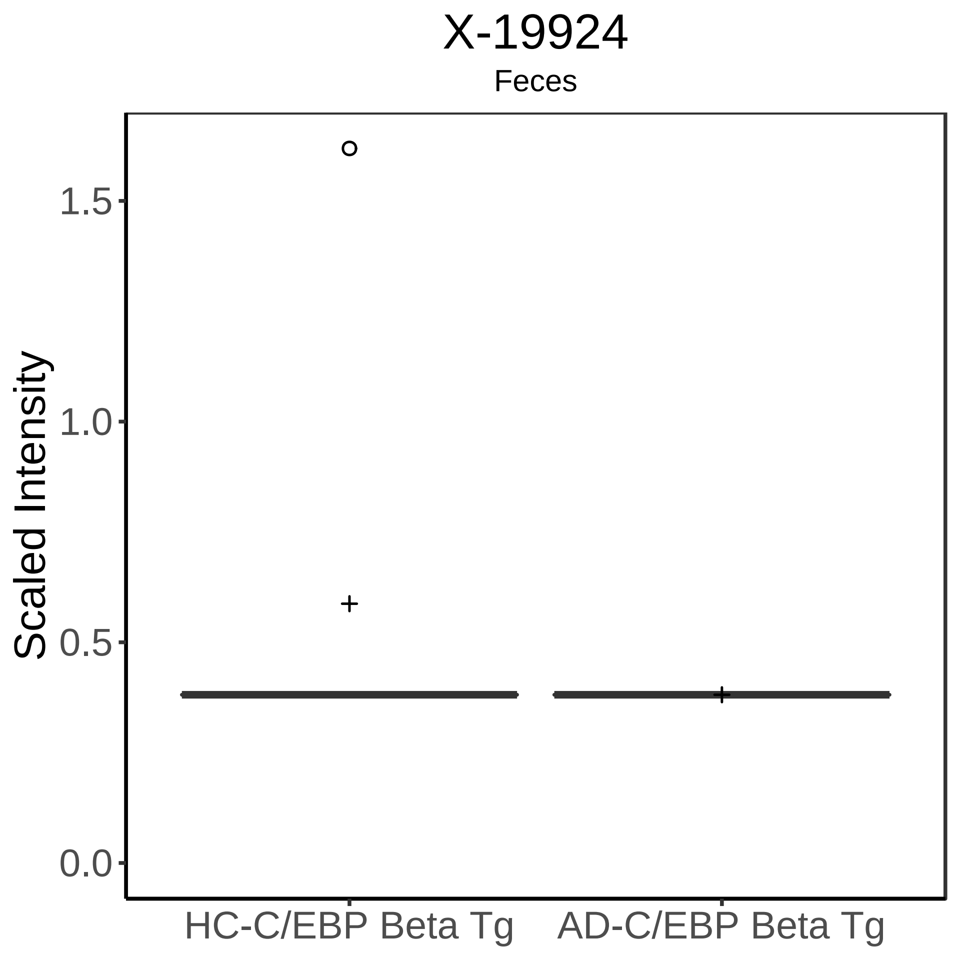

X-19924
Feces
Scaled Intensity
1.5
1.0
0.5
0.0
HC-C/EBP Beta Tg
AD-C/EBP Beta Tg

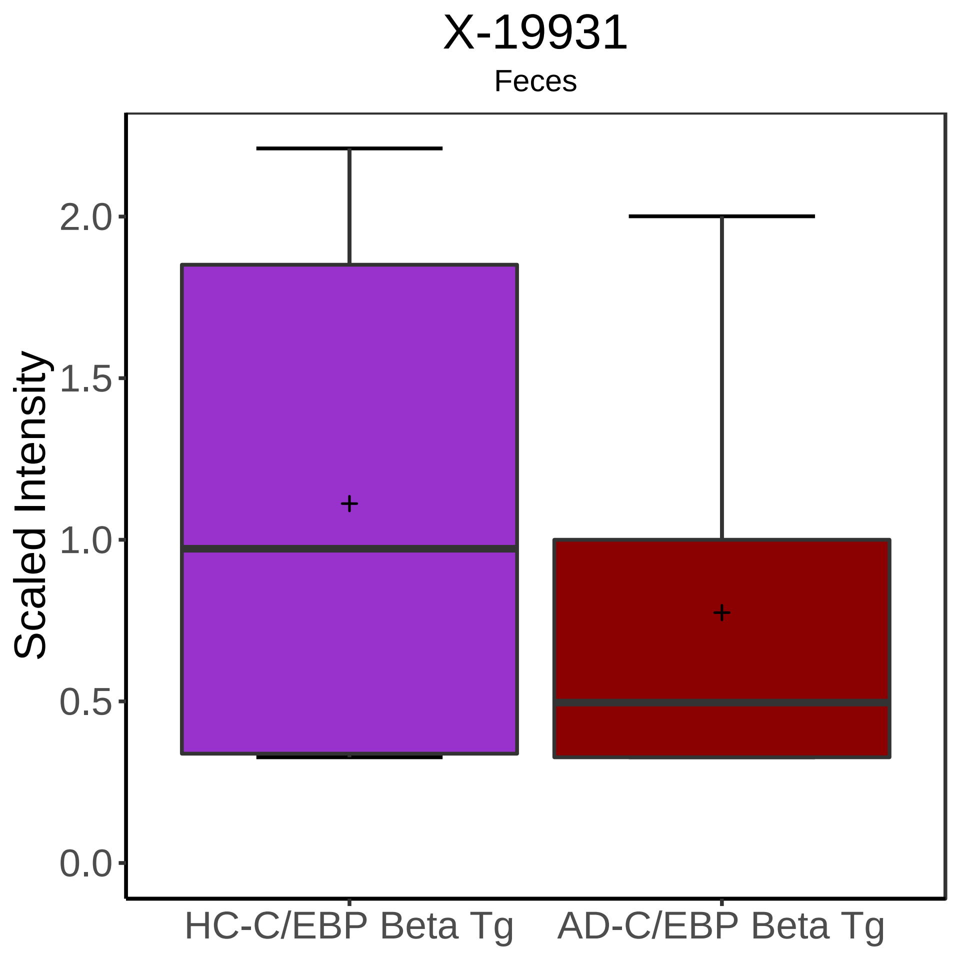
X-19931
Feces
Scaled Intensity
2.0
1.5
1.0
0.5
0.0
HC-C/EBP Beta Tg
AD-C/EBP Beta Tg

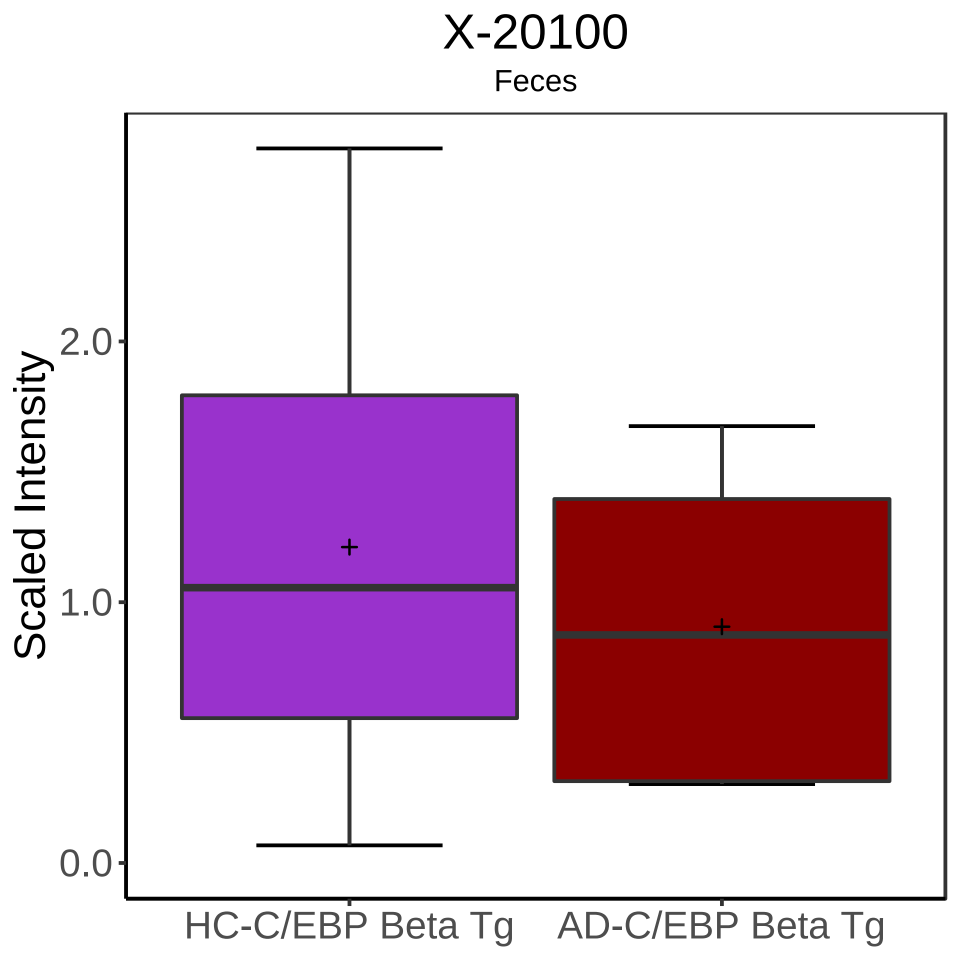
X-20100
Feces
Scaled Intensity
2.0
1.0
0.0
HC-C/EBP Beta Tg
AD-C/EBP Beta Tg

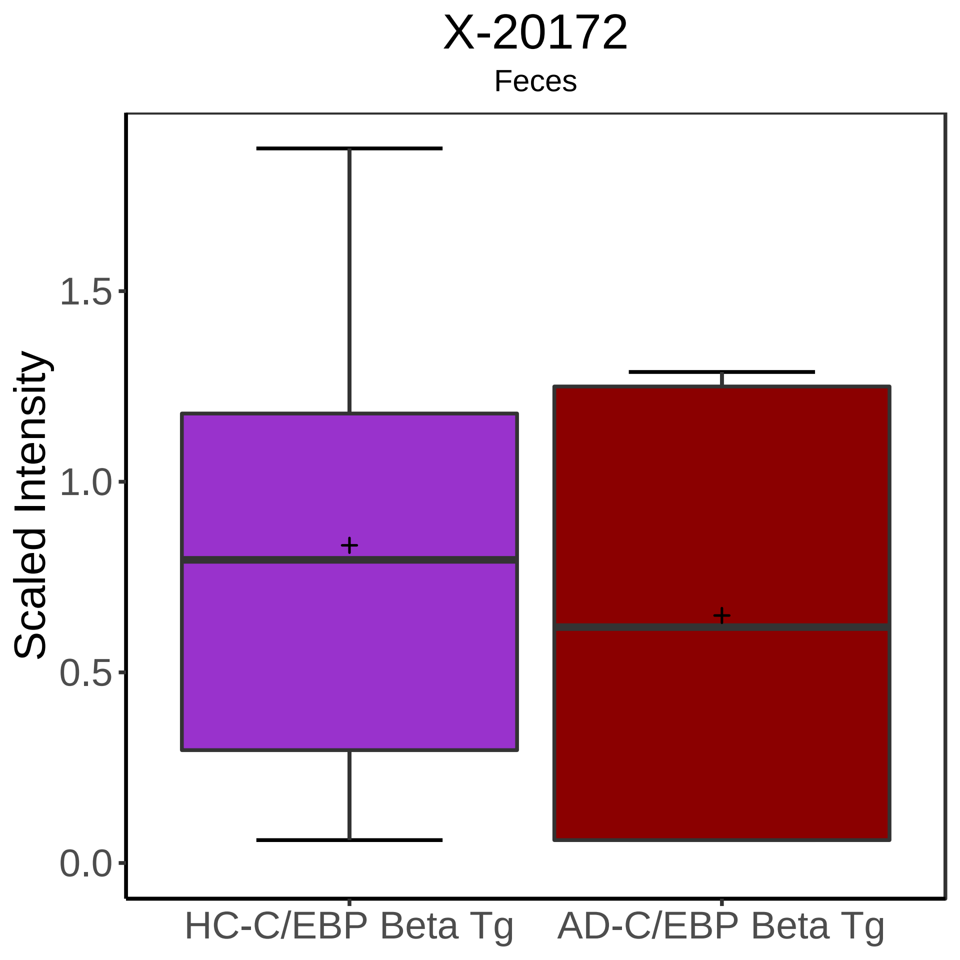

X-20172
Feces
Scaled Intensity
1.5
1.0
0.5
0.0
HC-C/EBP Beta Tg
AD-C/EBP Beta Tg

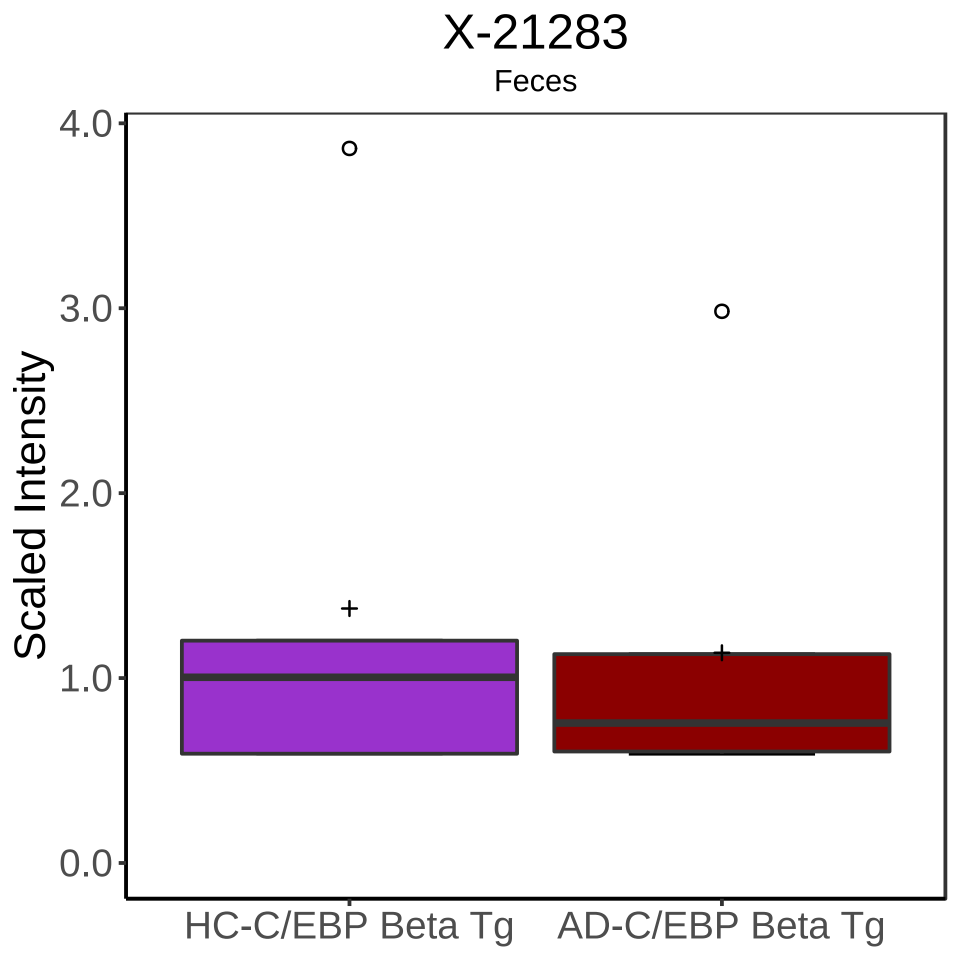

X-21283
Feces
Scaled Intensity
4.0
3.0
2.0
1.0
0.0
HC-C/EBP Beta Tg
AD-C/EBP Beta Tg

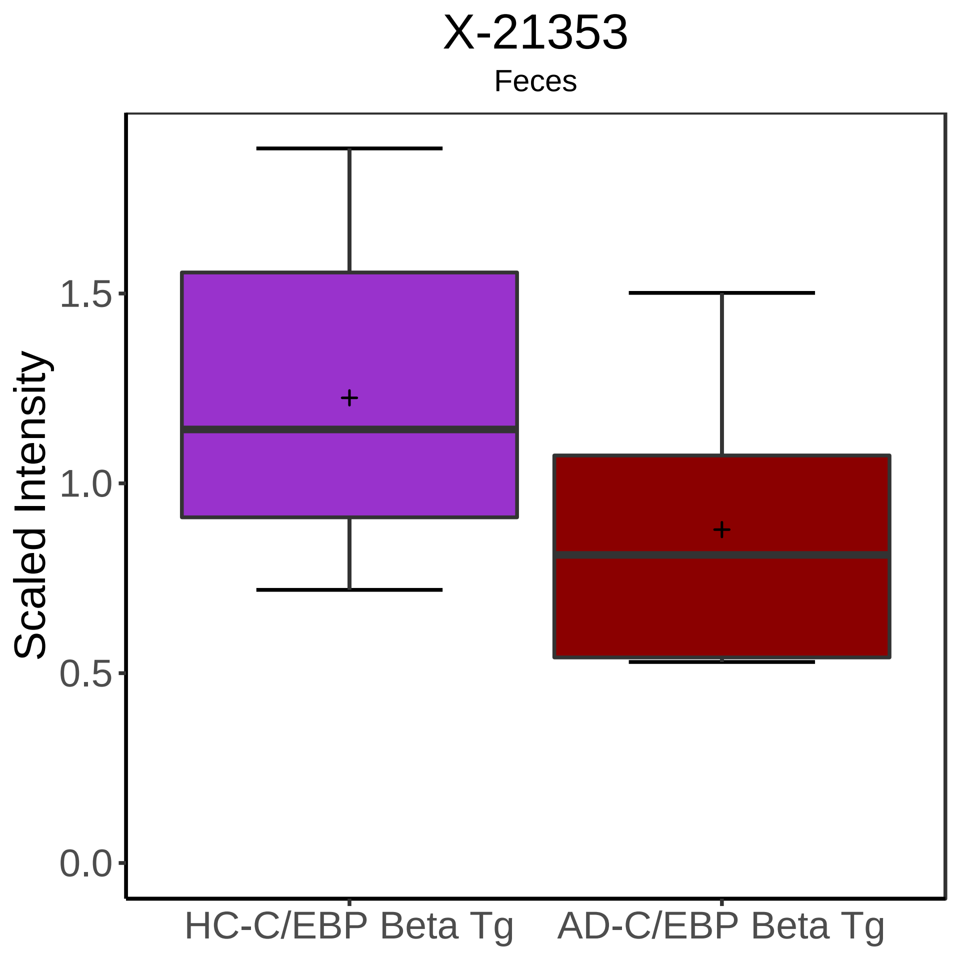
X-21353
Feces
Scaled Intensity
1.5
1.0
0.5
0.0
HC-C/EBP Beta Tg
AD-C/EBP Beta Tg

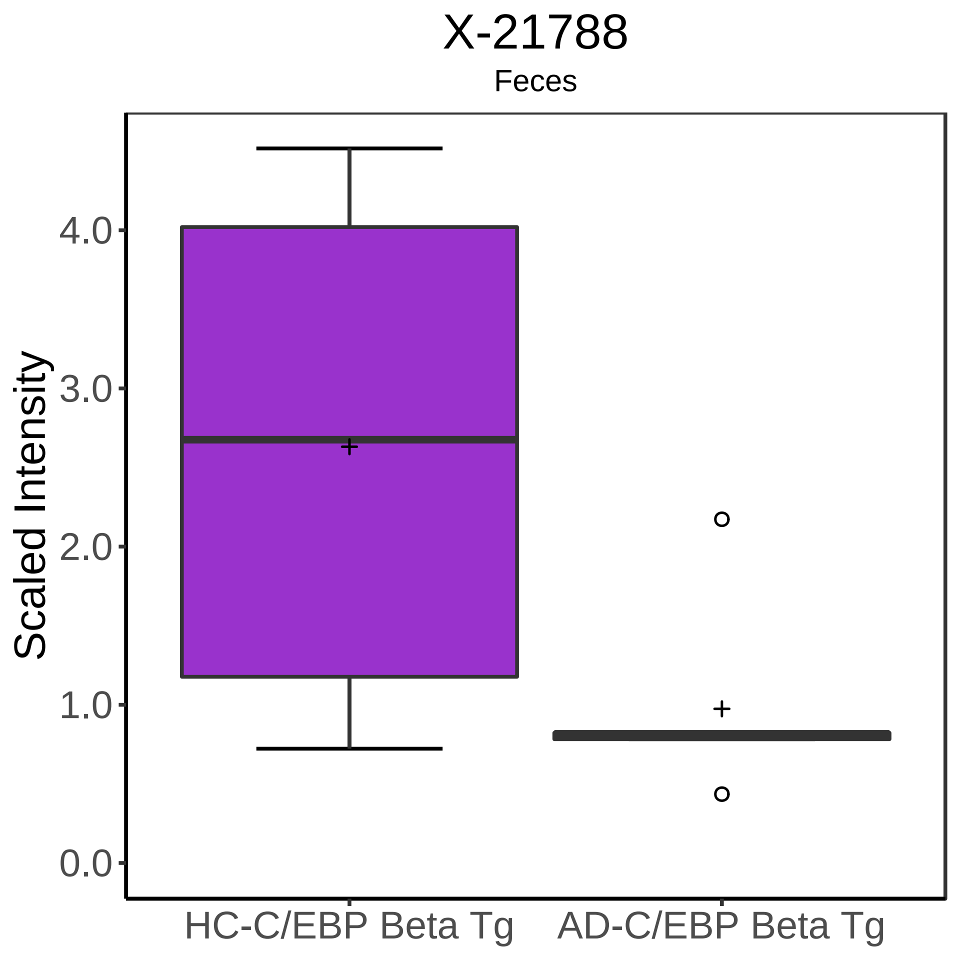
X-21788
Feces
Scaled Intensity
4.0
3.0
2.0
1.0
0.0
HC-C/EBP Beta Tg
AD-C/EBP Beta Tg

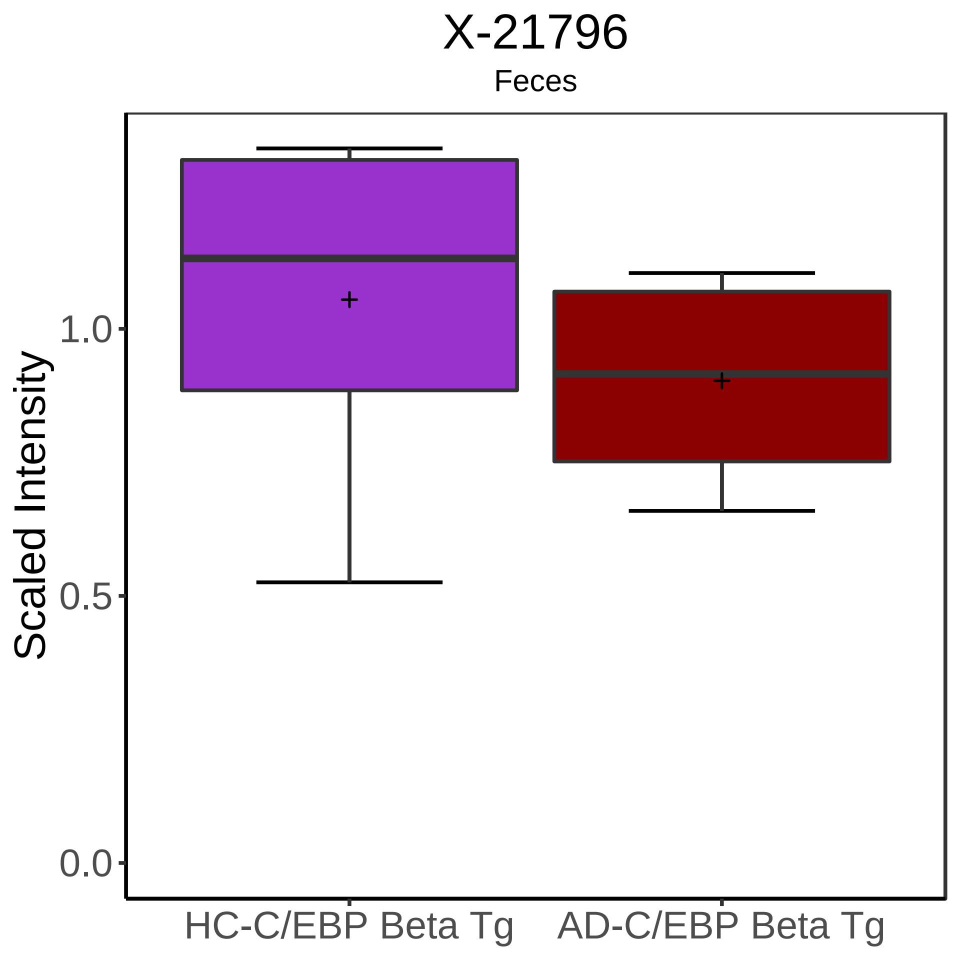
X-21796
Feces
Scaled Intensity
1.0
0.5
0.0
HC-C/EBP Beta Tg
AD-C/EBP Beta Tg

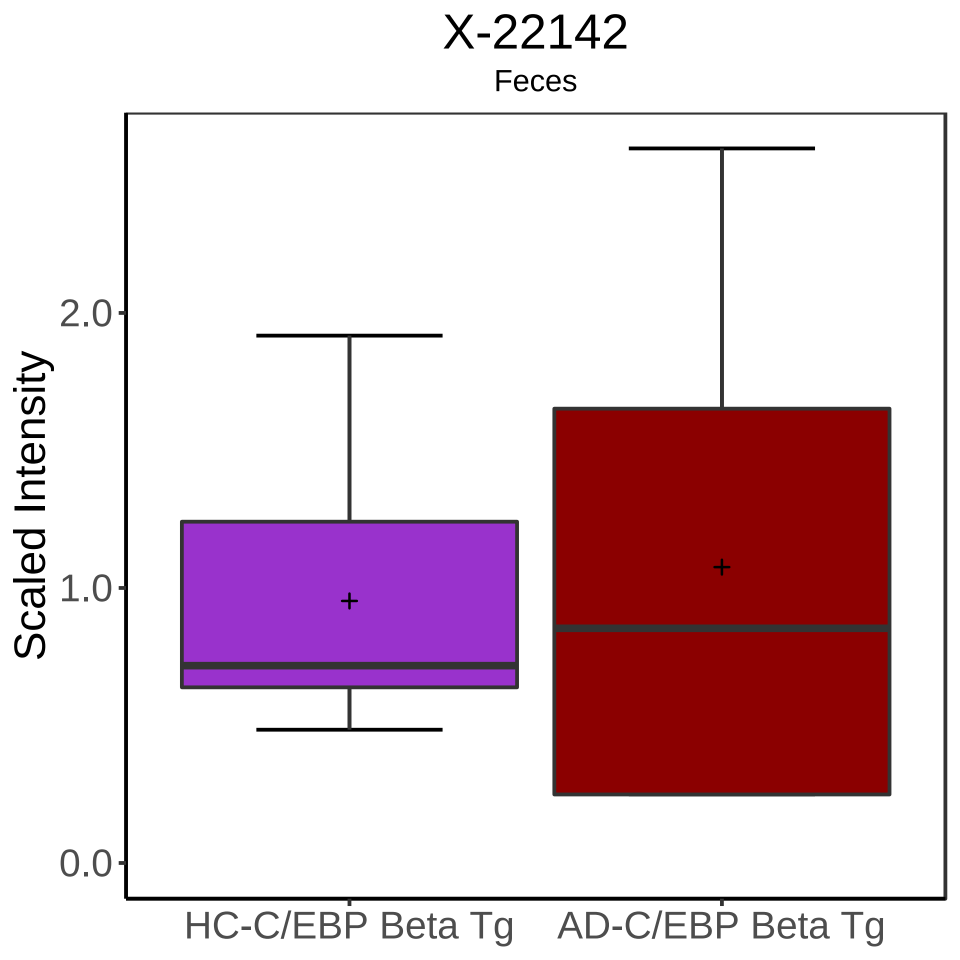

X-22142
Feces
Scaled Intensity
2.0
1.0
0.0
HC-C/EBP Beta Tg
AD-C/EBP Beta Tg

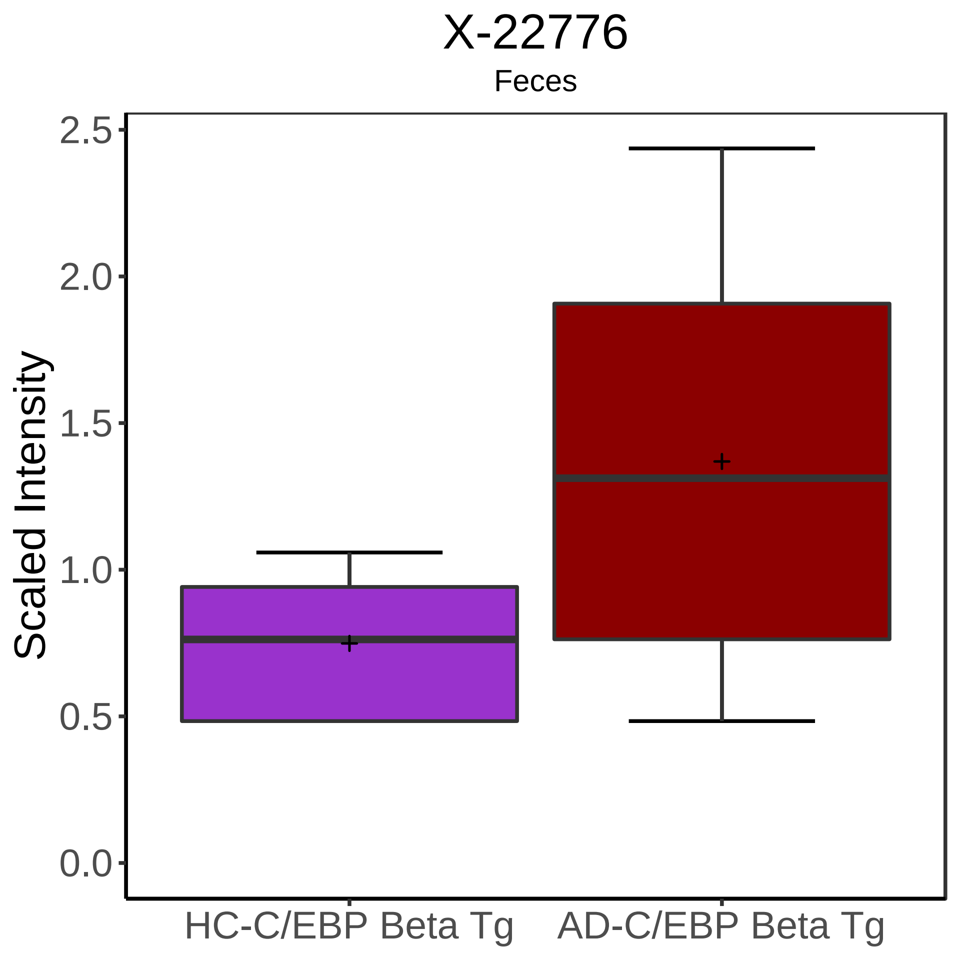

X-22776
Feces
Scaled Intensity
2.5
2.0
1.5
1.0
0.5
0.0
HC-C/EBP Beta Tg
AD-C/EBP Beta Tg

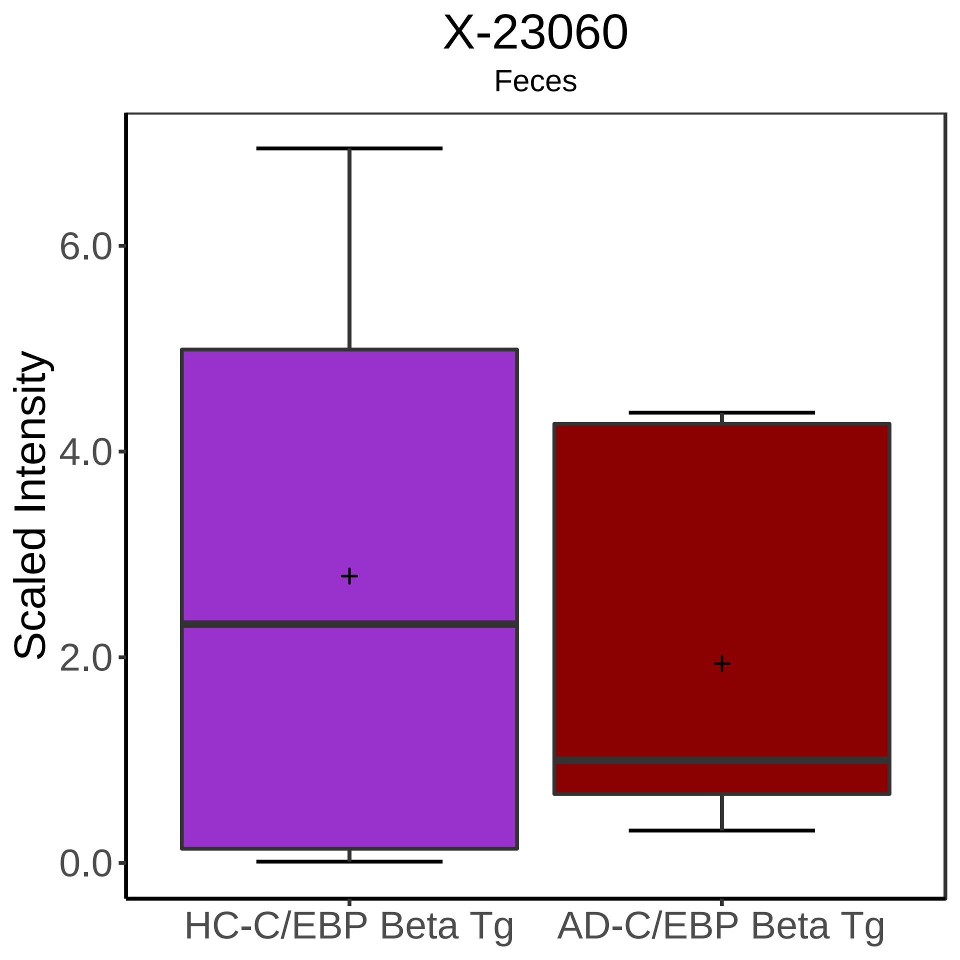

X-23060
Feces
Scaled Intensity
6.0
4.0
2.0
0.0
HC-C/EBP Beta Tg
AD-C/EBP Beta Tg

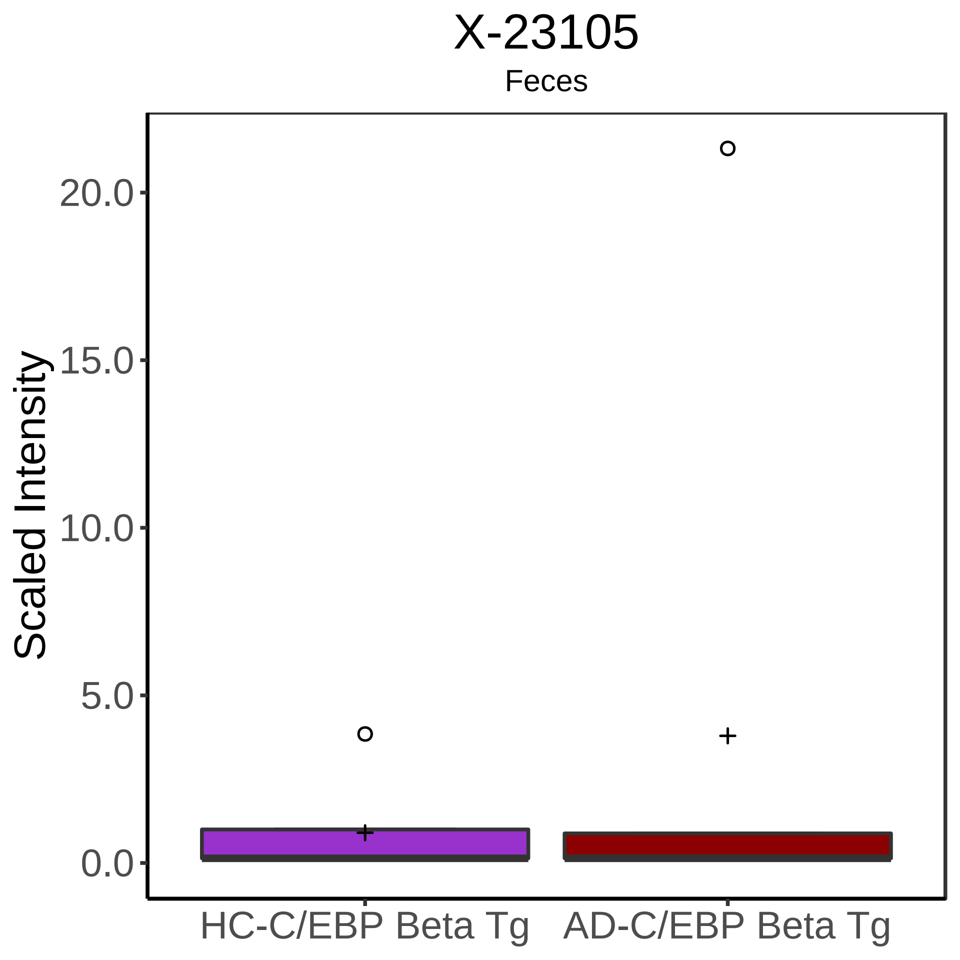
X-23105
Feces
Scaled Intensity
20.0
15.0
10.0
5.0
0.0
HC-C/EBP Beta Tg
AD-C/EBP Beta Tg

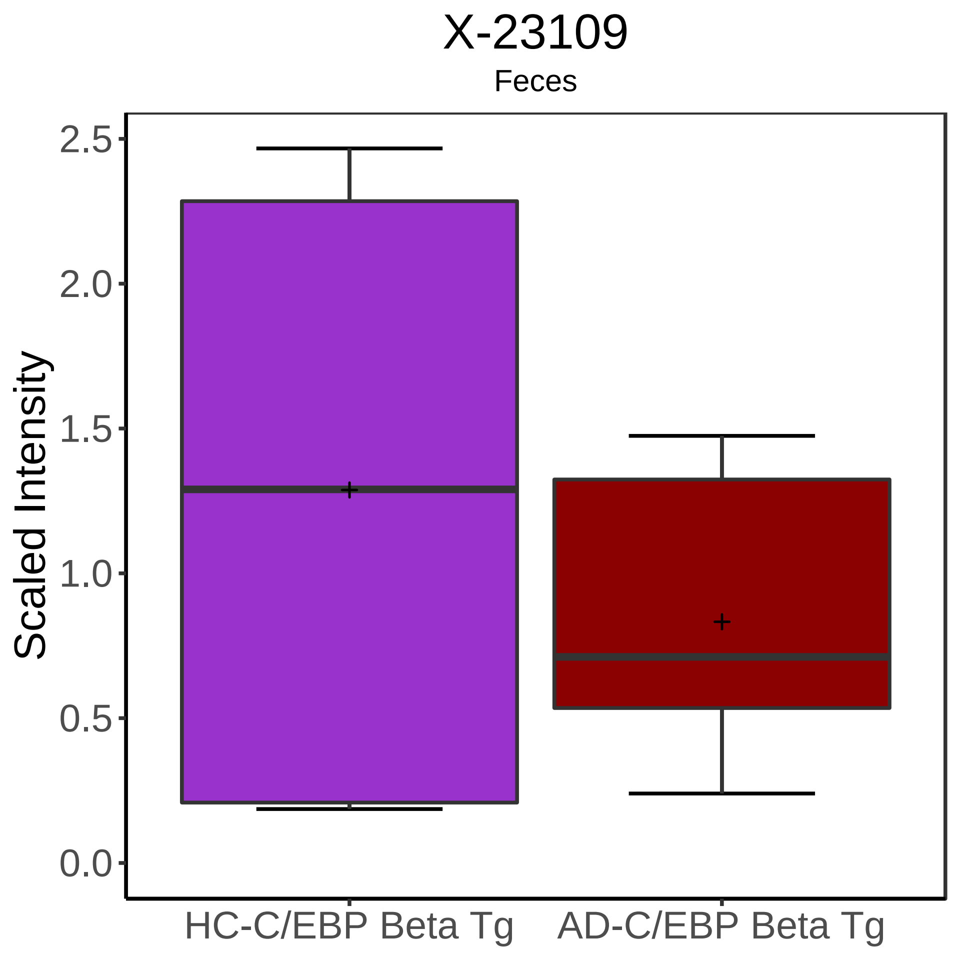

X-23109
Feces
Scaled Intensity
2.5
2.0
1.5
1.0
0.5
0.0
HC-C/EBP Beta Tg
AD-C/EBP Beta Tg

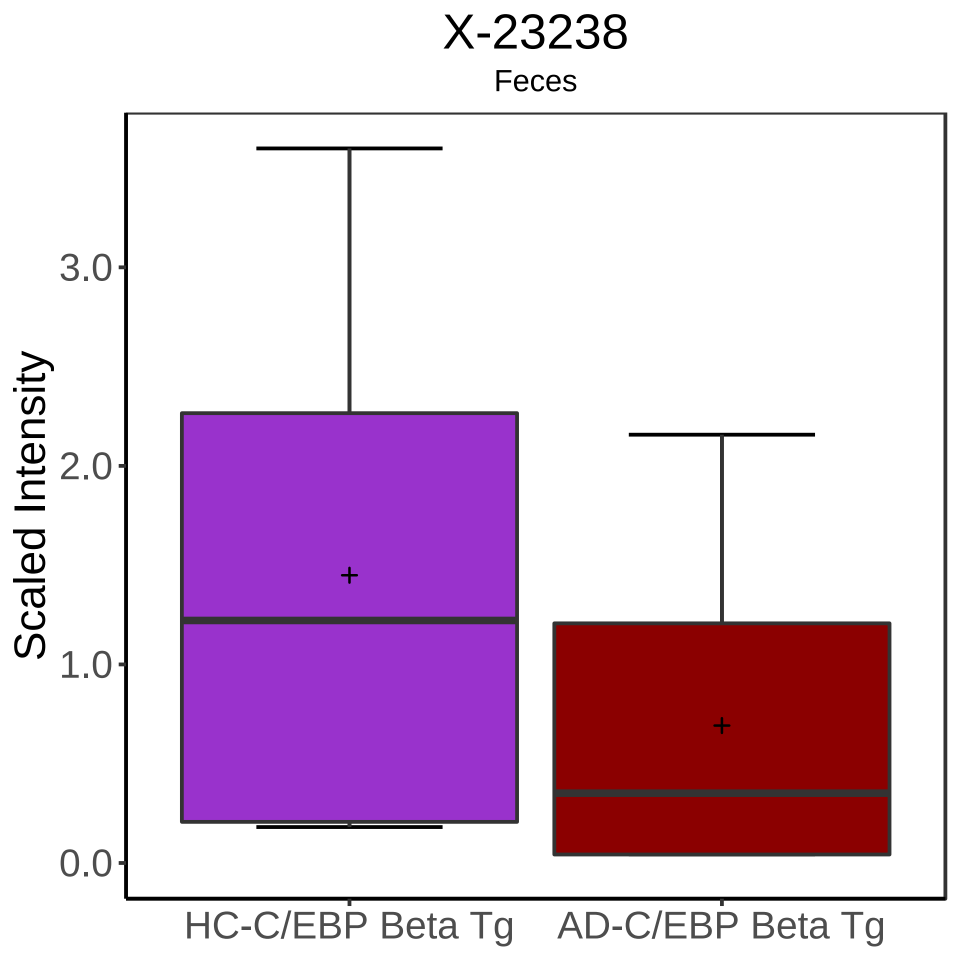

X-23238
Feces
Scaled Intensity
3.0
2.0
1.0
0.0
HC-C/EBP Beta Tg
AD-C/EBP Beta Tg

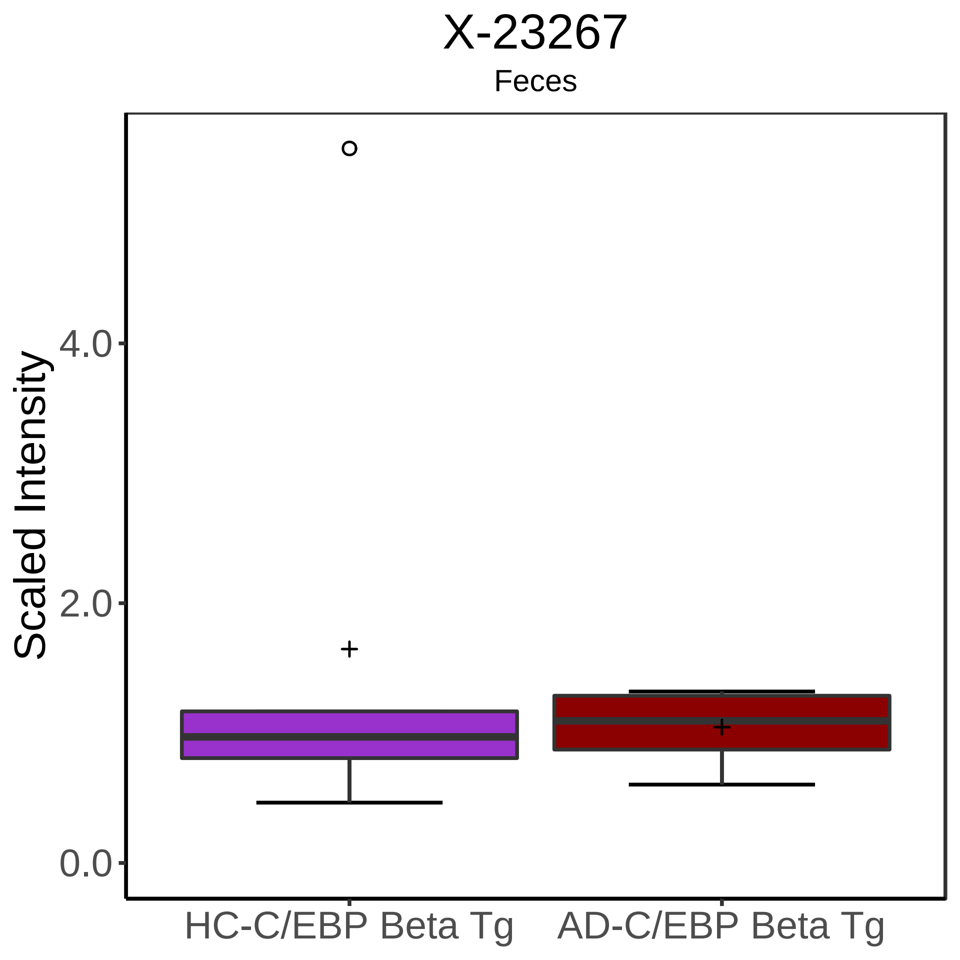

X-23267
Feces
Scaled Intensity
4.0
2.0
0.0
HC-C/EBP Beta Tg
AD-C/EBP Beta Tg

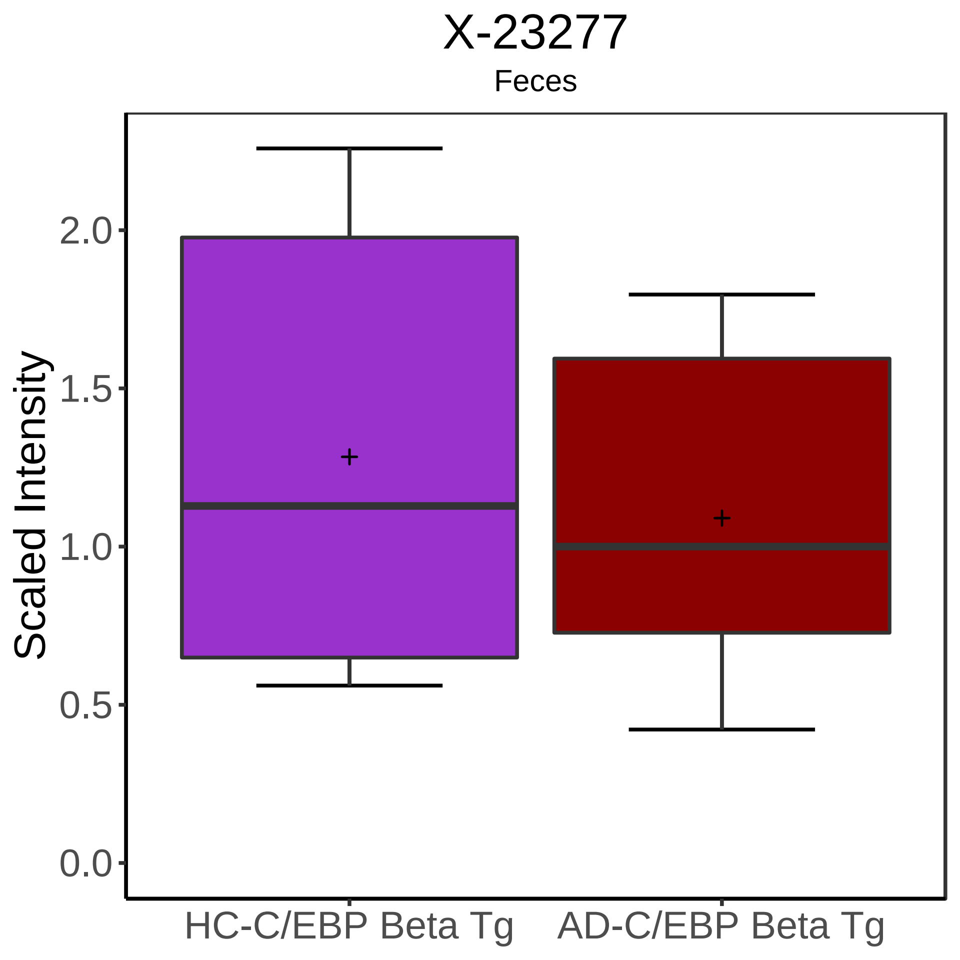

X-23277
Feces
Scaled Intensity
2.0
1.5
1.0
0.5
0.0
HC-C/EBP Beta Tg
AD-C/EBP Beta Tg

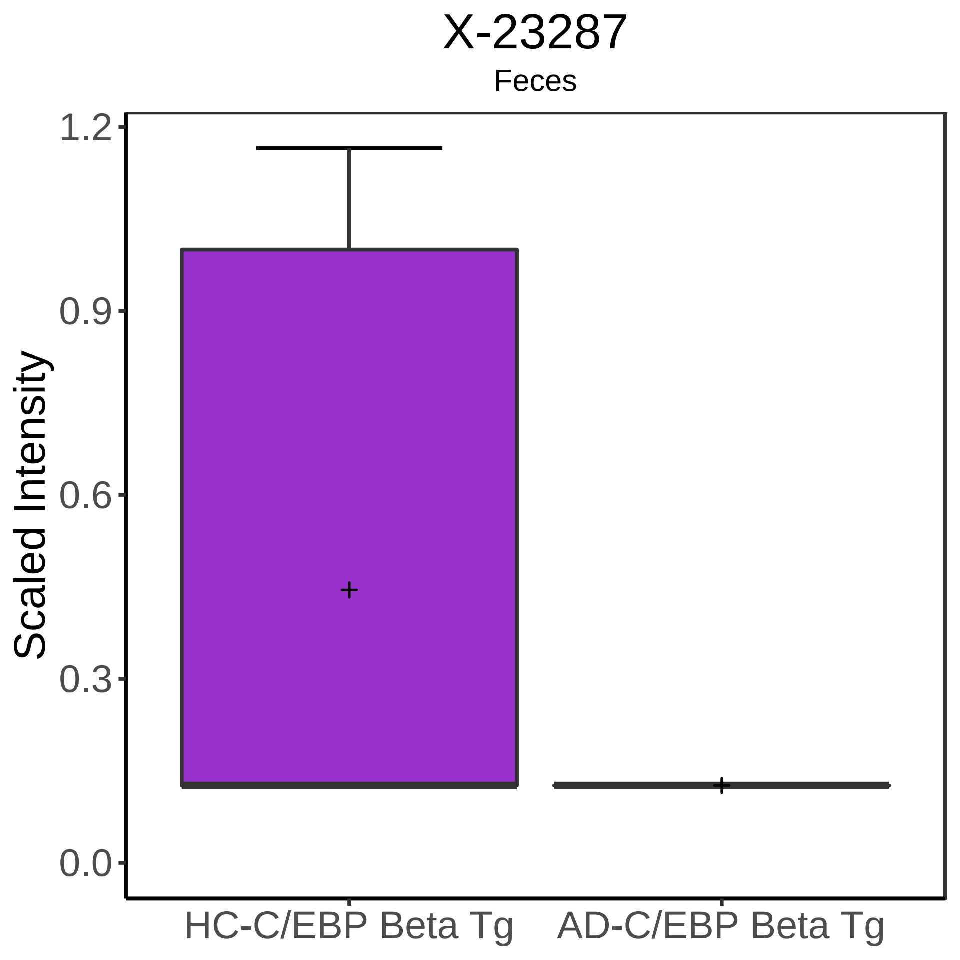
X-23287
Feces
Scaled Intensity
1.2
0.9
0.6
0.3
0.0
HC-C/EBP Beta Tg
AD-C/EBP Beta Tg

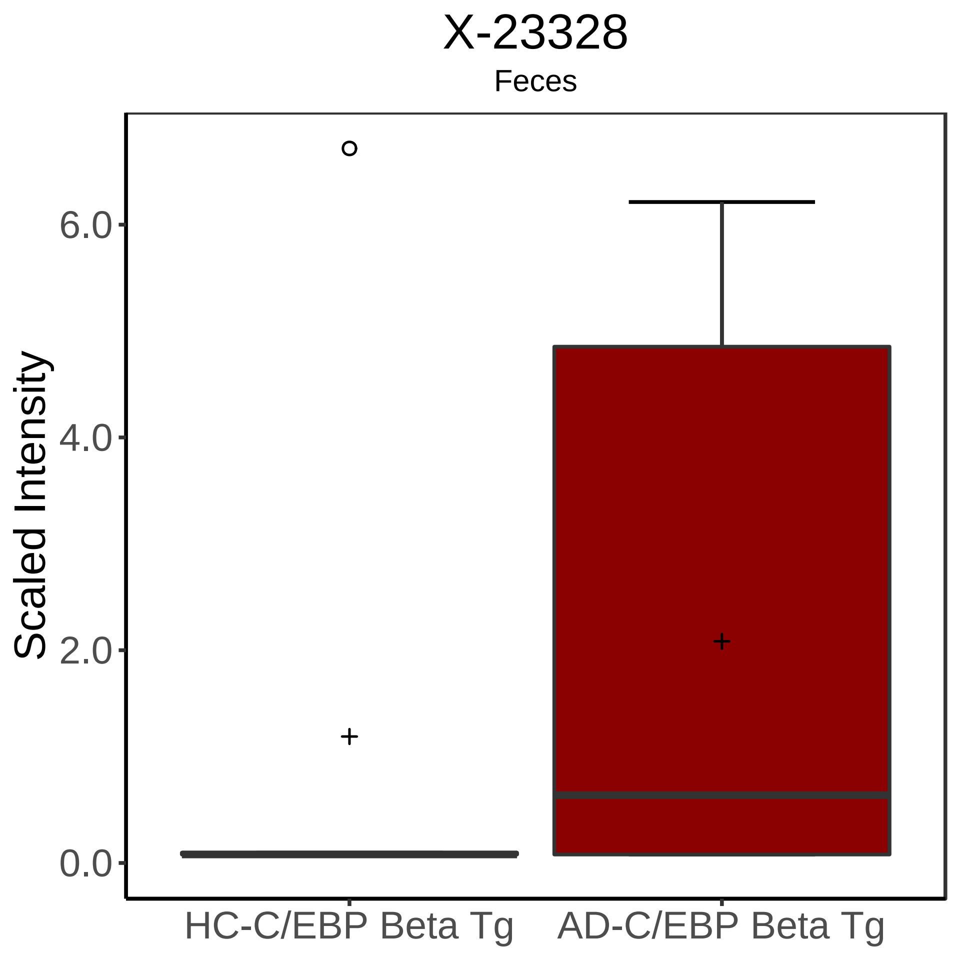

X-23328
Feces
Scaled Intensity
6.0
4.0
2.0
0.0
HC-C/EBP Beta Tg
AD-C/EBP Beta Tg

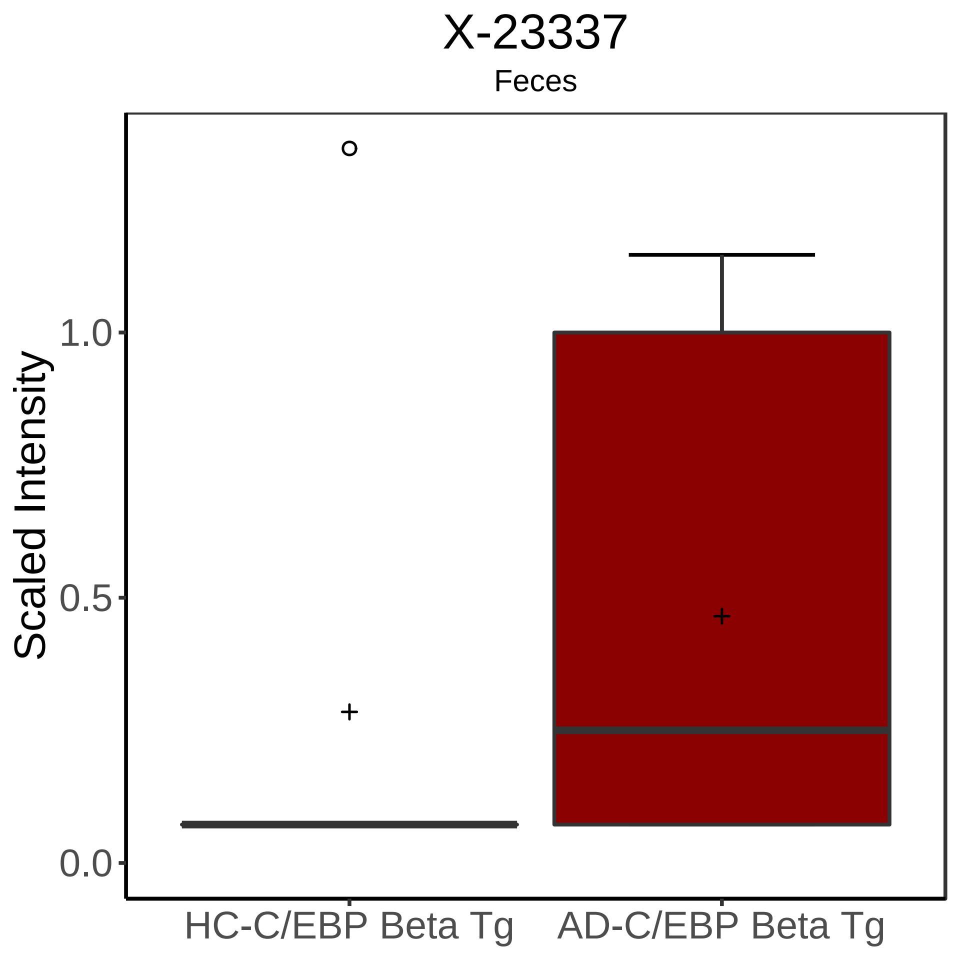
X-23337
Feces
Scaled Intensity
0.0
0.5
1.0
HC-C/EBP Beta Tg
AD-C/EBP Beta Tg

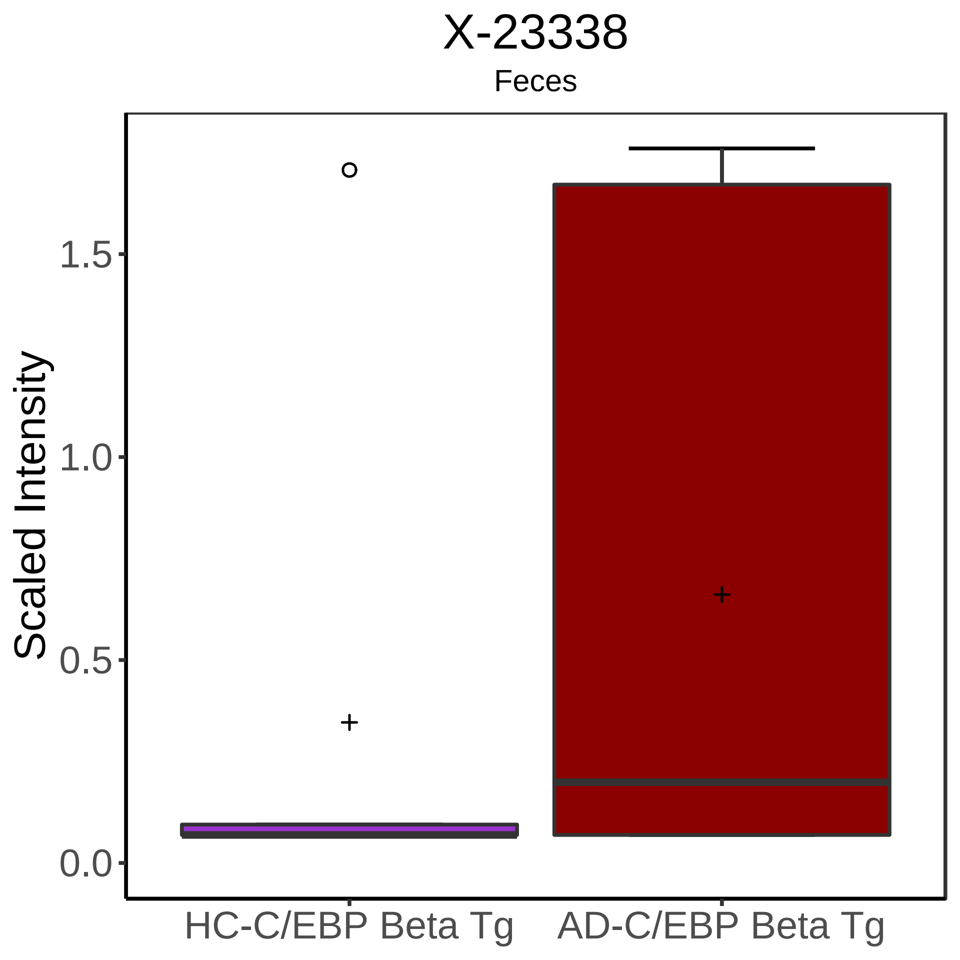

X-23338
Feces
Scaled Intensity
1.5
1.0
0.5
0.0
HC-C/EBP Beta Tg
AD-C/EBP Beta Tg

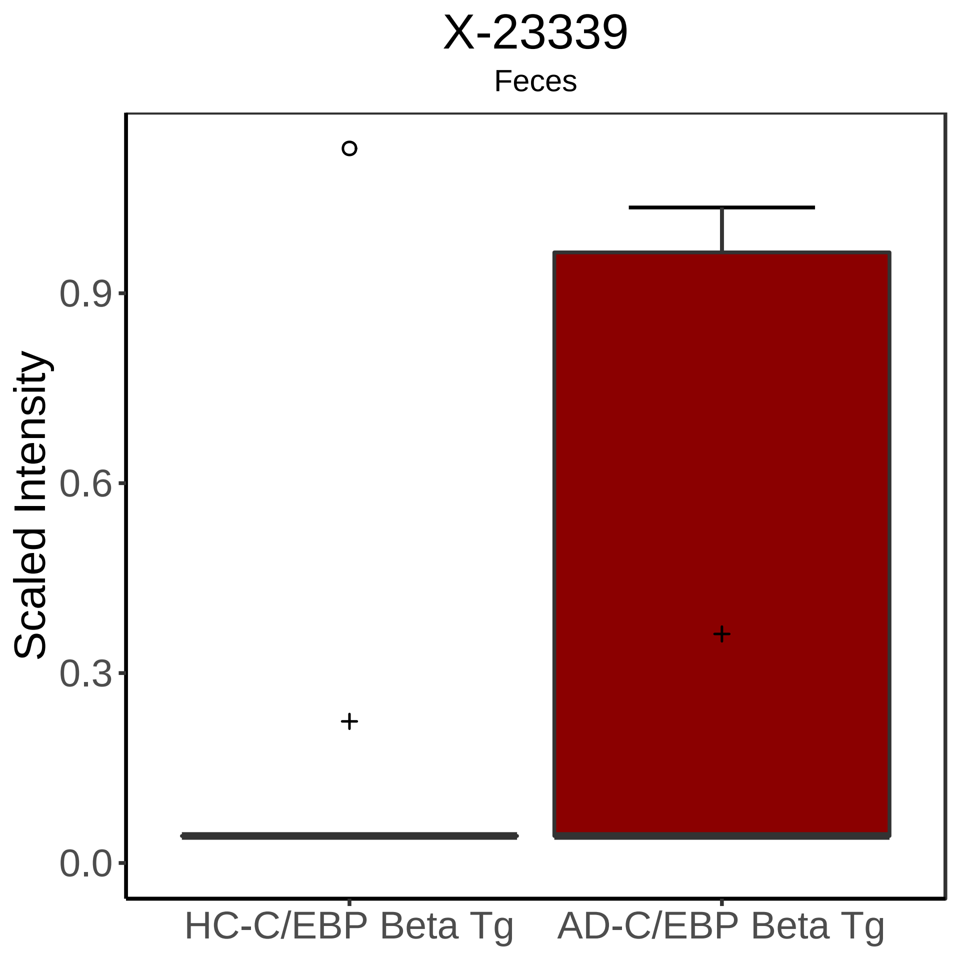
X-23339
Feces
Scaled Intensity
0.0
0.3
0.6
0.9
HC-C/EBP Beta Tg
AD-C/EBP Beta Tg

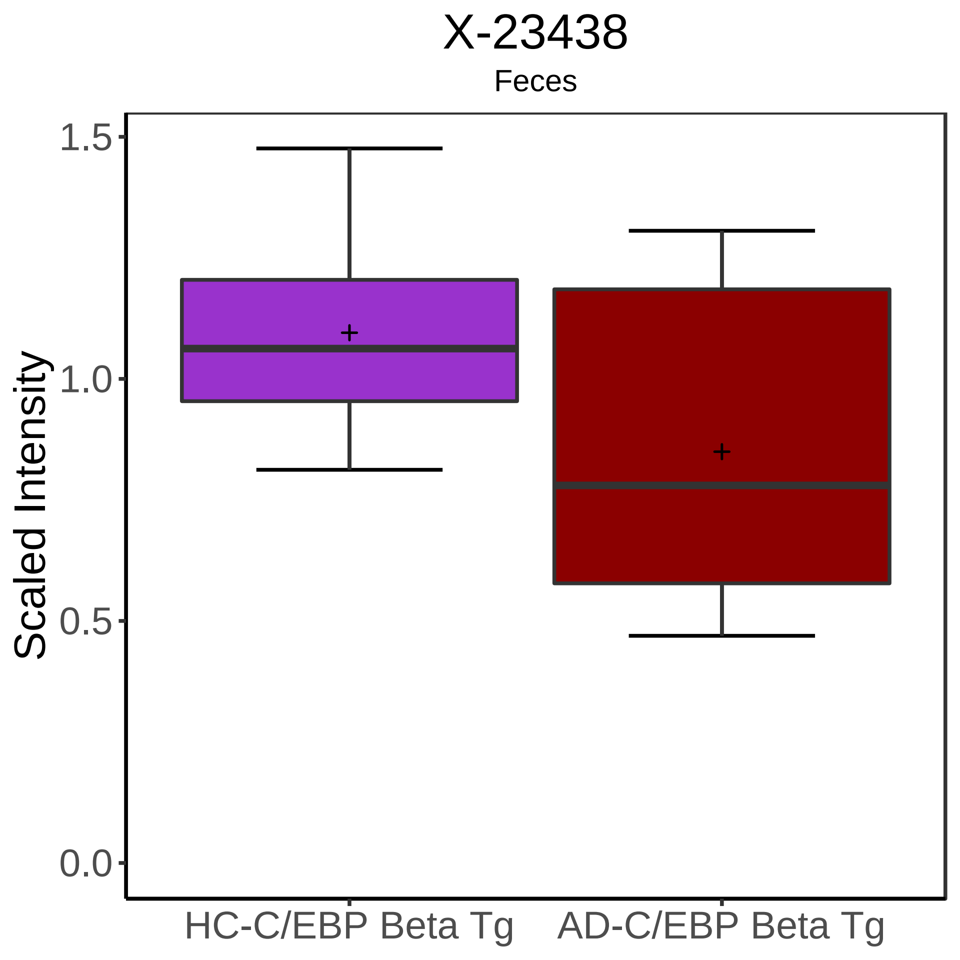
X-23438
Feces
Scaled Intensity
1.5
1.0
0.5
0.0
HC-C/EBP Beta Tg
AD-C/EBP Beta Tg

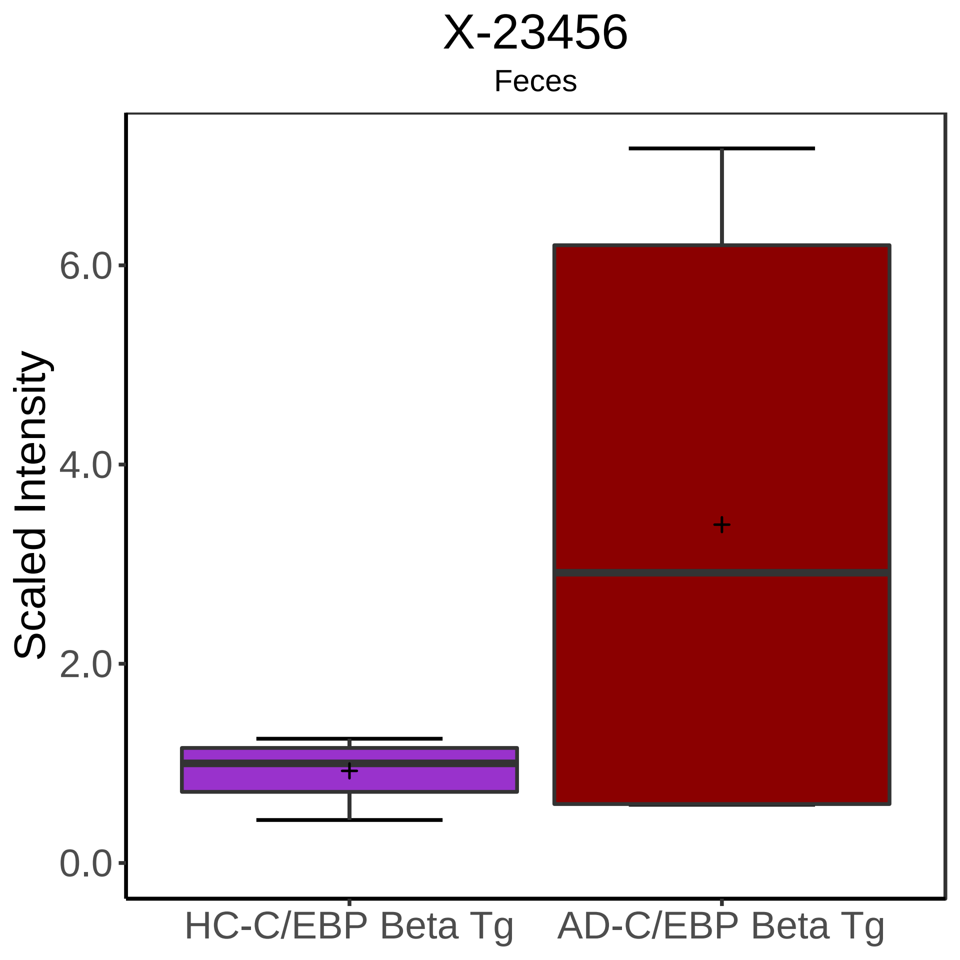

X-23456
Feces
Scaled Intensity
6.0
4.0
2.0
0.0
HC-C/EBP Beta Tg
AD-C/EBP Beta Tg

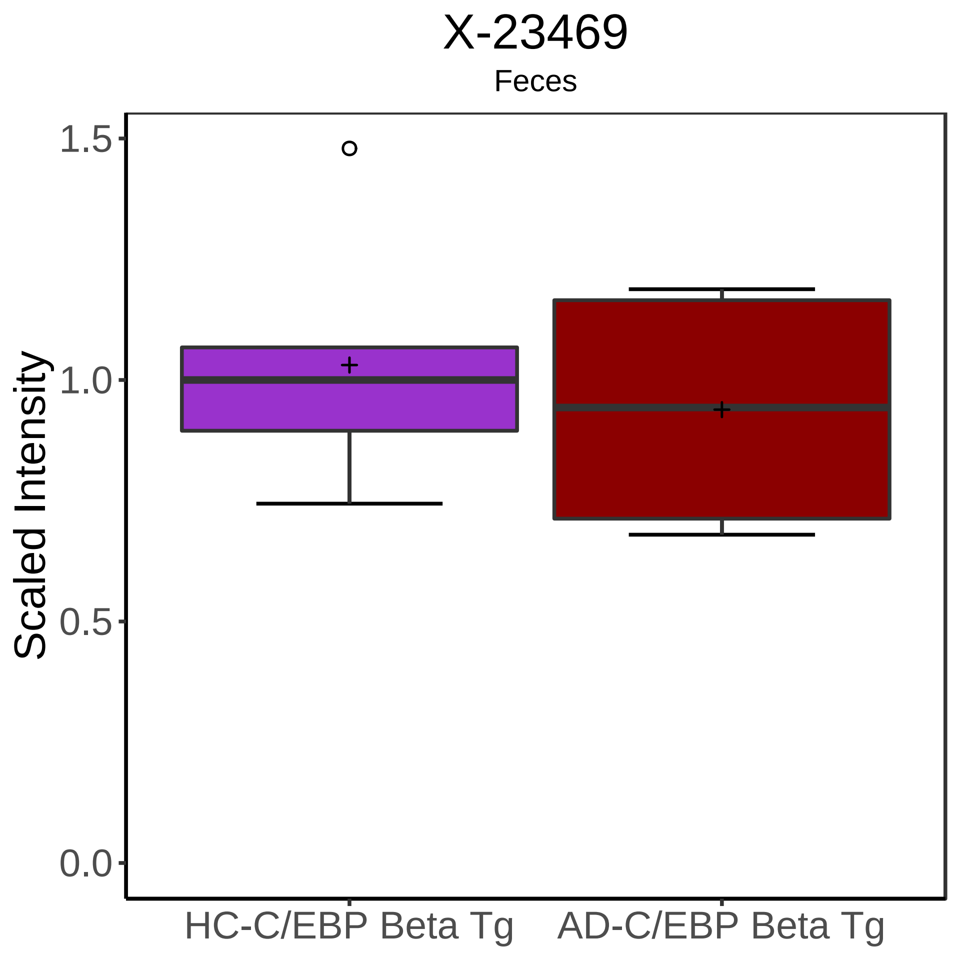
X-23469
Feces
Scaled Intensity
1.5
1.0
0.5
0.0
HC-C/EBP Beta Tg
AD-C/EBP Beta Tg

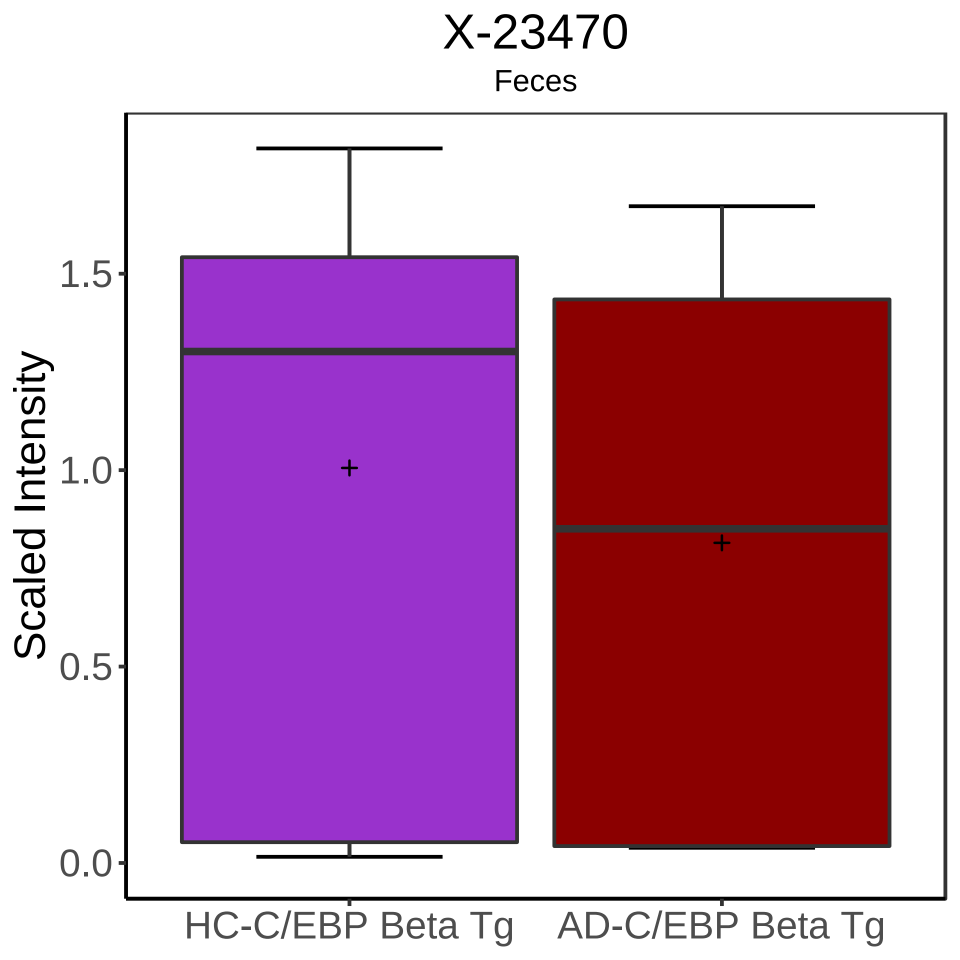
X-23470
Feces
Scaled Intensity
1.5
1.0
0.5
0.0
HC-C/EBP Beta Tg
AD-C/EBP Beta Tg

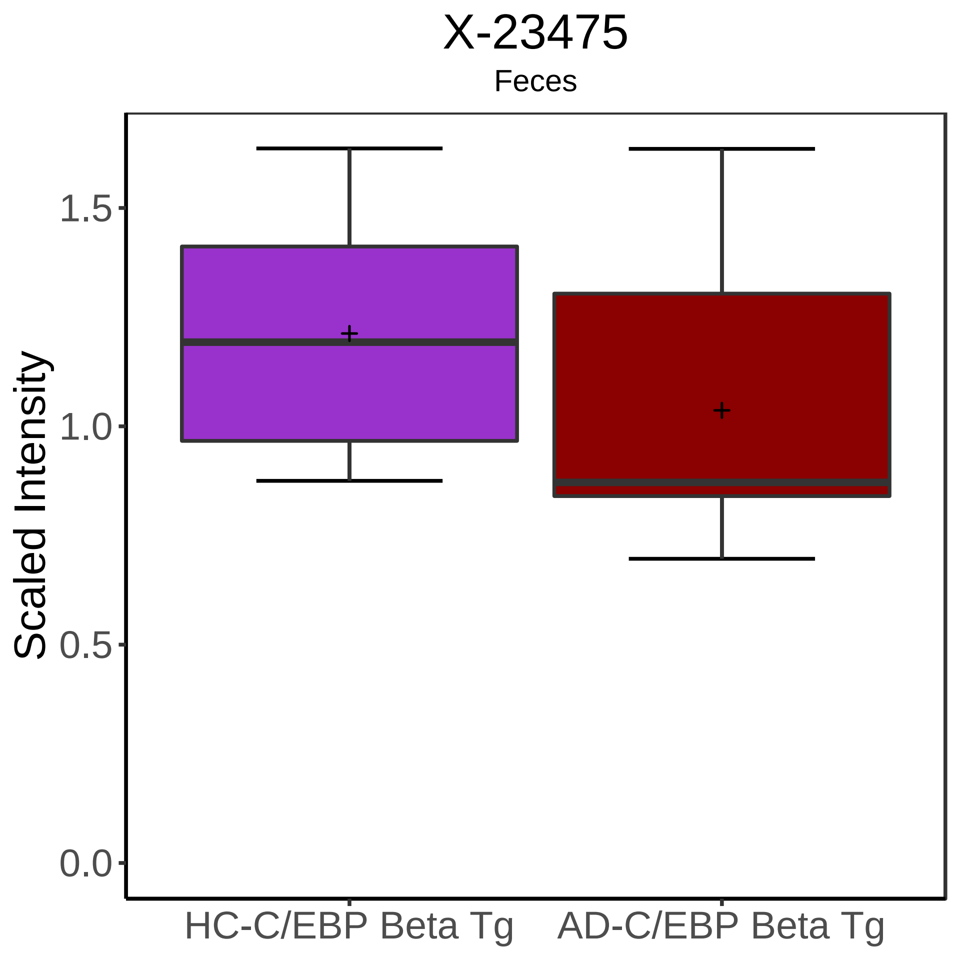

X-23475
Feces
Scaled Intensity
1.5
1.0
0.5
0.0
HC-C/EBP Beta Tg
AD-C/EBP Beta Tg

# X-23479

Feces

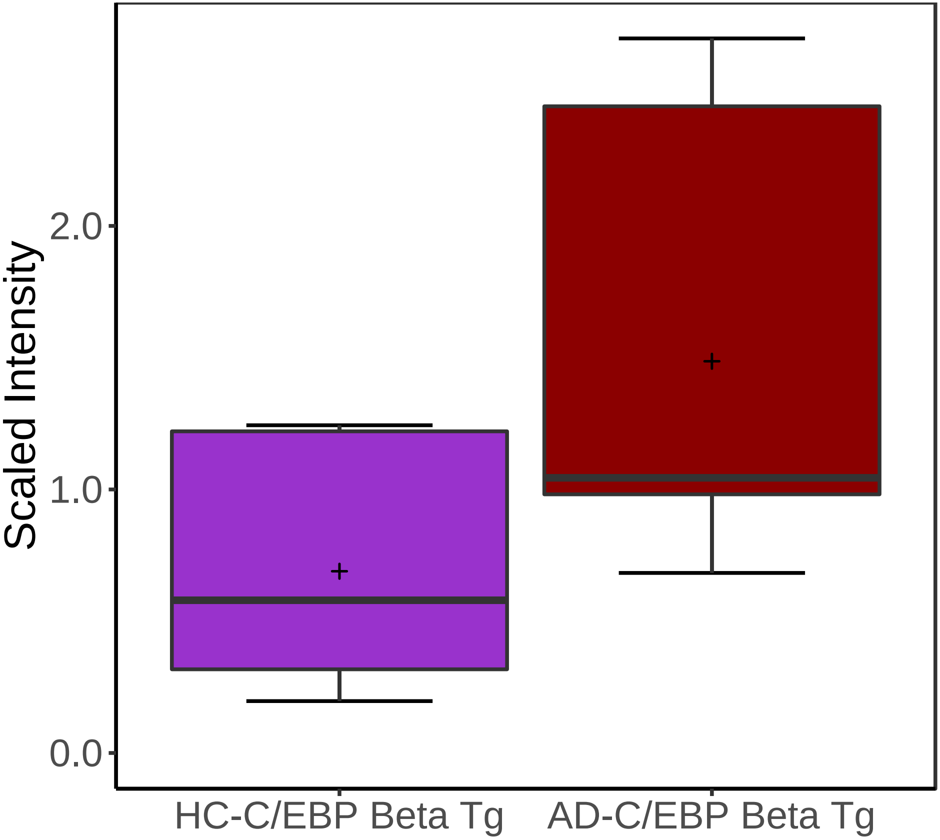

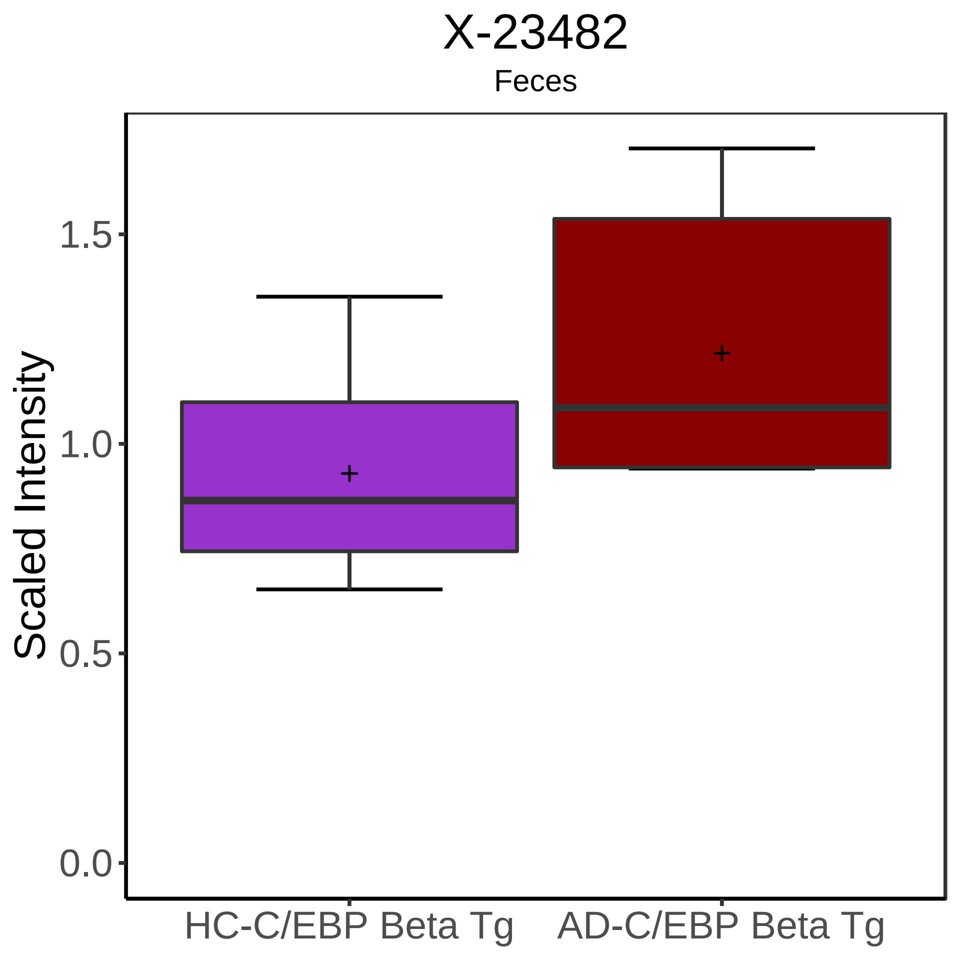
X-23482
Feces
Scaled Intensity
0.0
0.5
1.0
1.5
HC-C/EBP Beta Tg
AD-C/EBP Beta Tg

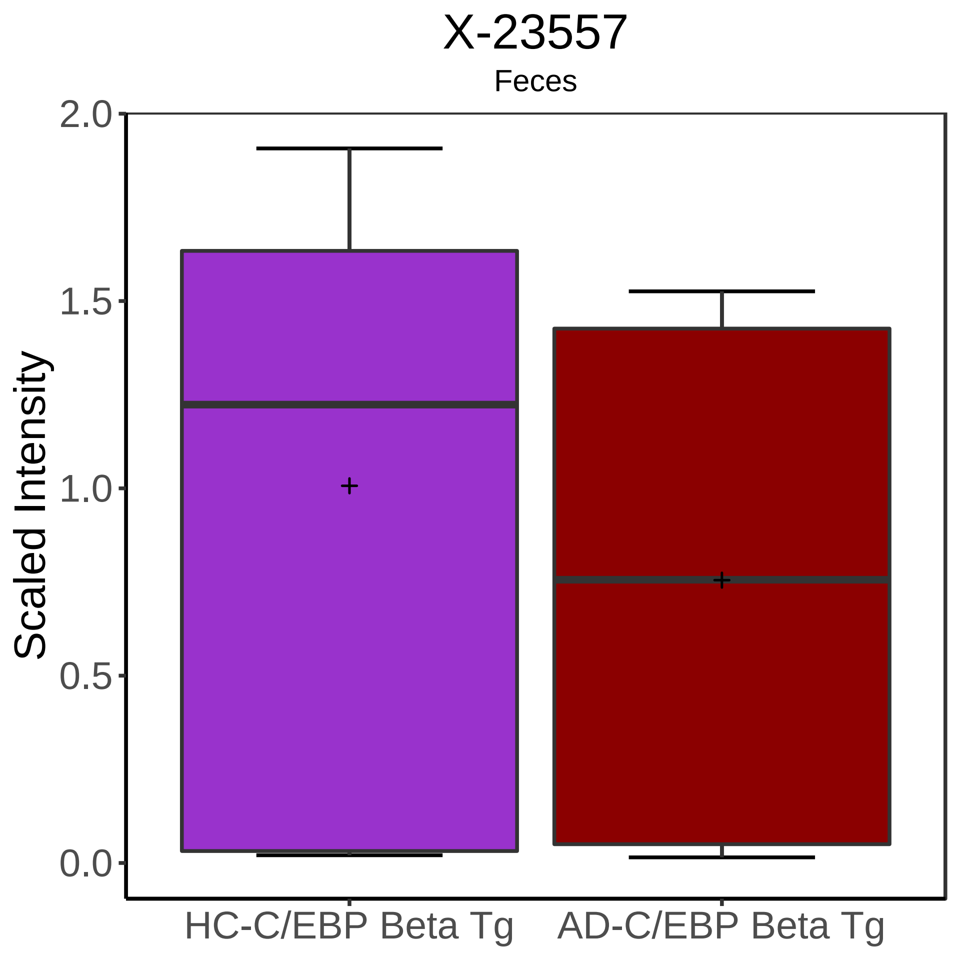
X-23557
Feces
Scaled Intensity
2.0
1.5
1.0
0.5
0.0
HC-C/EBP Beta Tg
AD-C/EBP Beta Tg

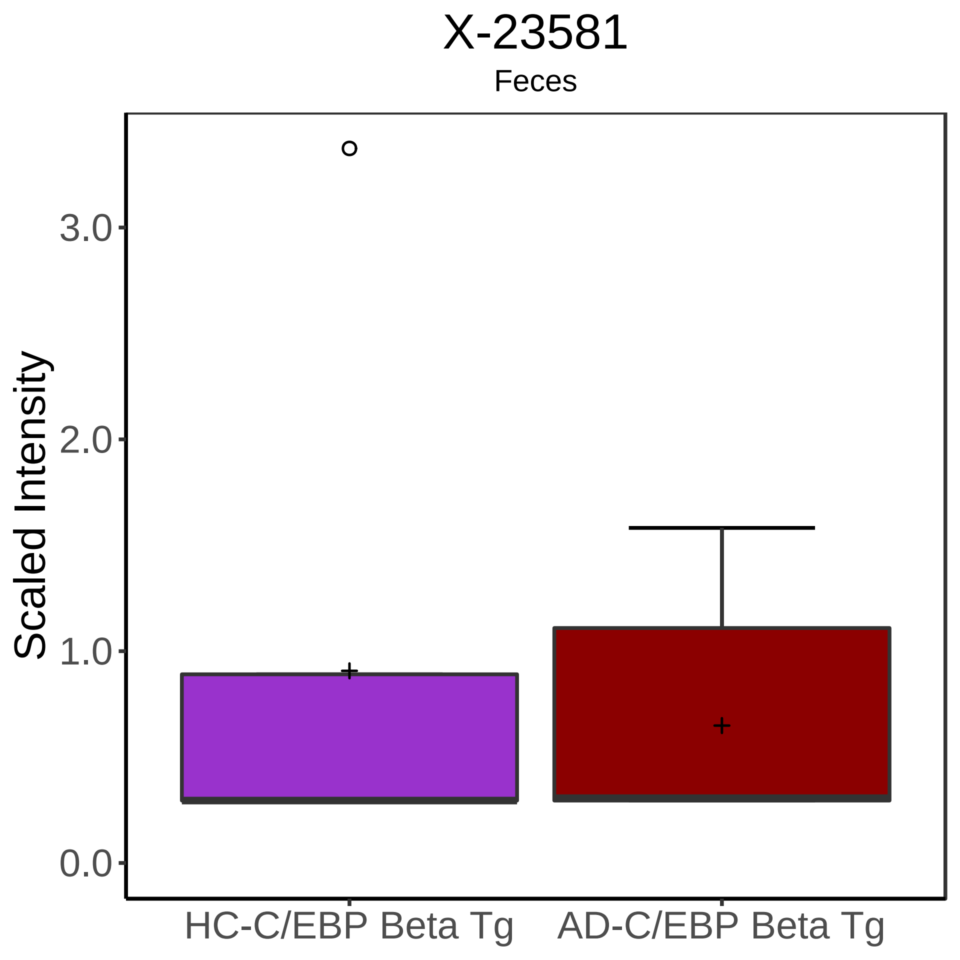
X-23581
Feces
Scaled Intensity
3.0
2.0
1.0
0.0
HC-C/EBP Beta Tg
AD-C/EBP Beta Tg

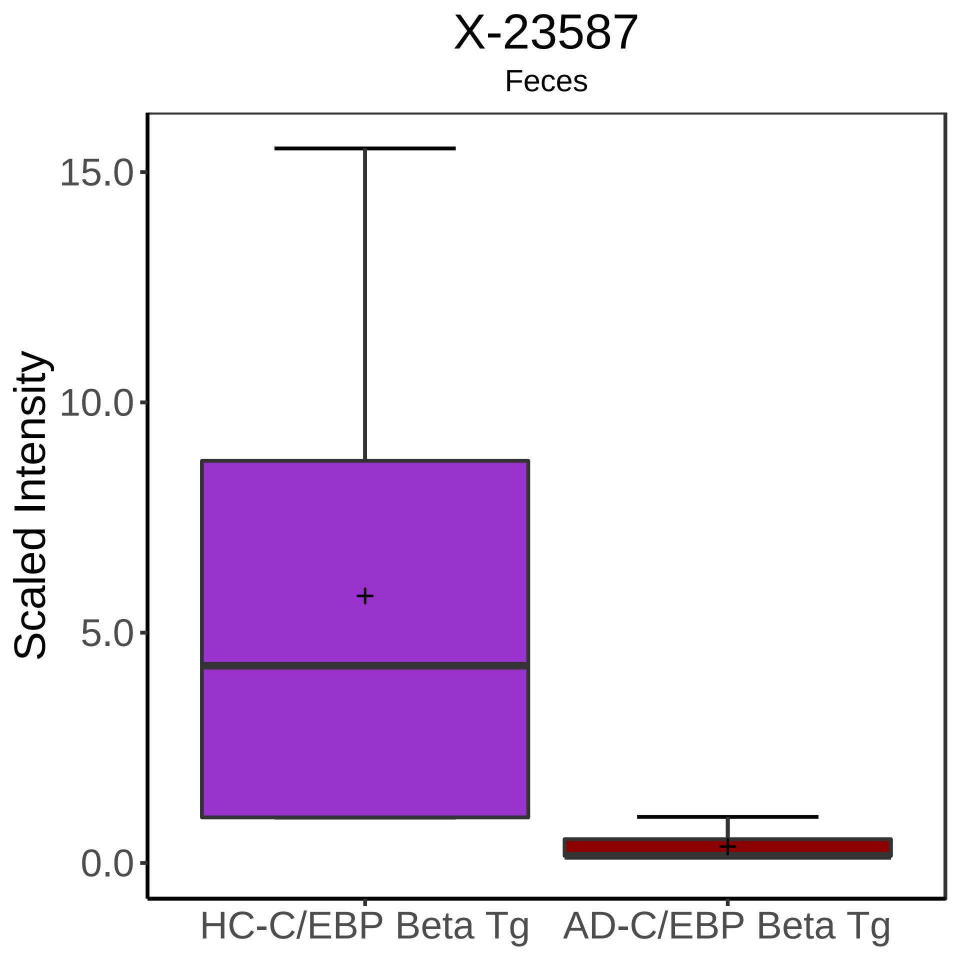

X-23587
Feces
Scaled Intensity
15.0
10.0
5.0
0.0
HC-C/EBP Beta Tg
AD-C/EBP Beta Tg

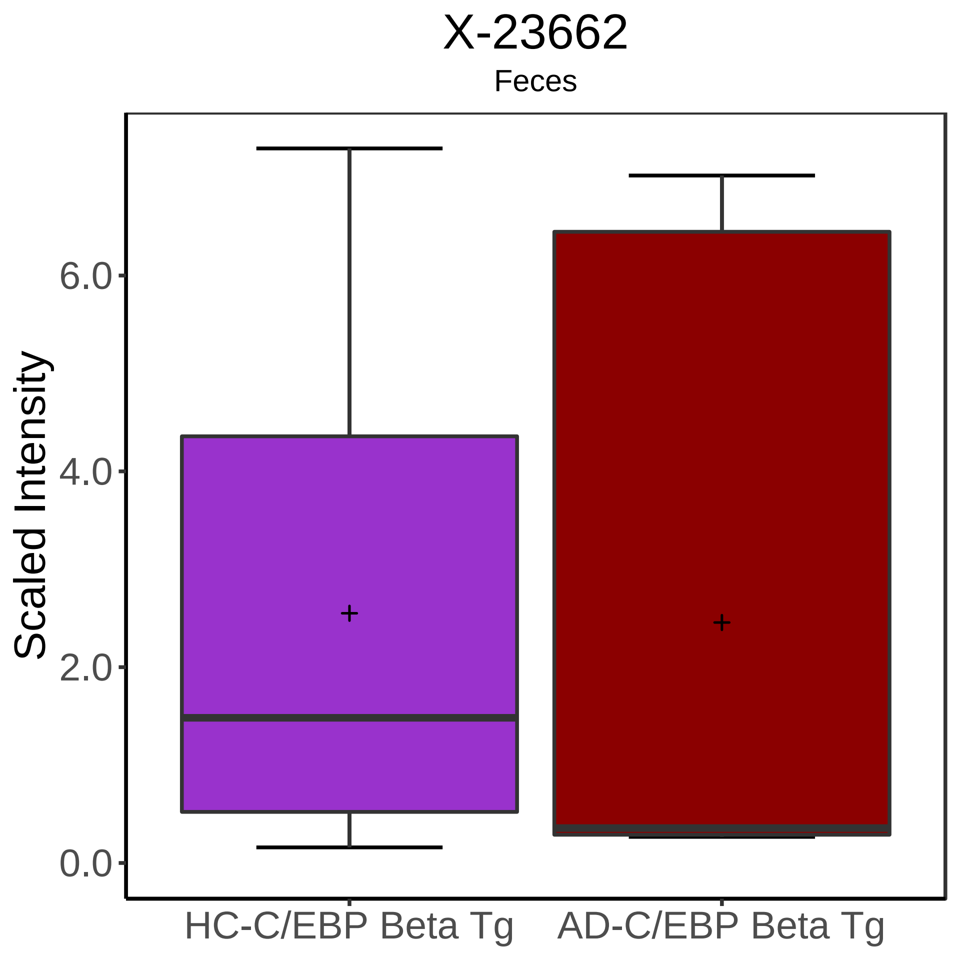
X-23662
Feces
Scaled Intensity
6.0
4.0
2.0
0.0
HC-C/EBP Beta Tg
AD-C/EBP Beta Tg

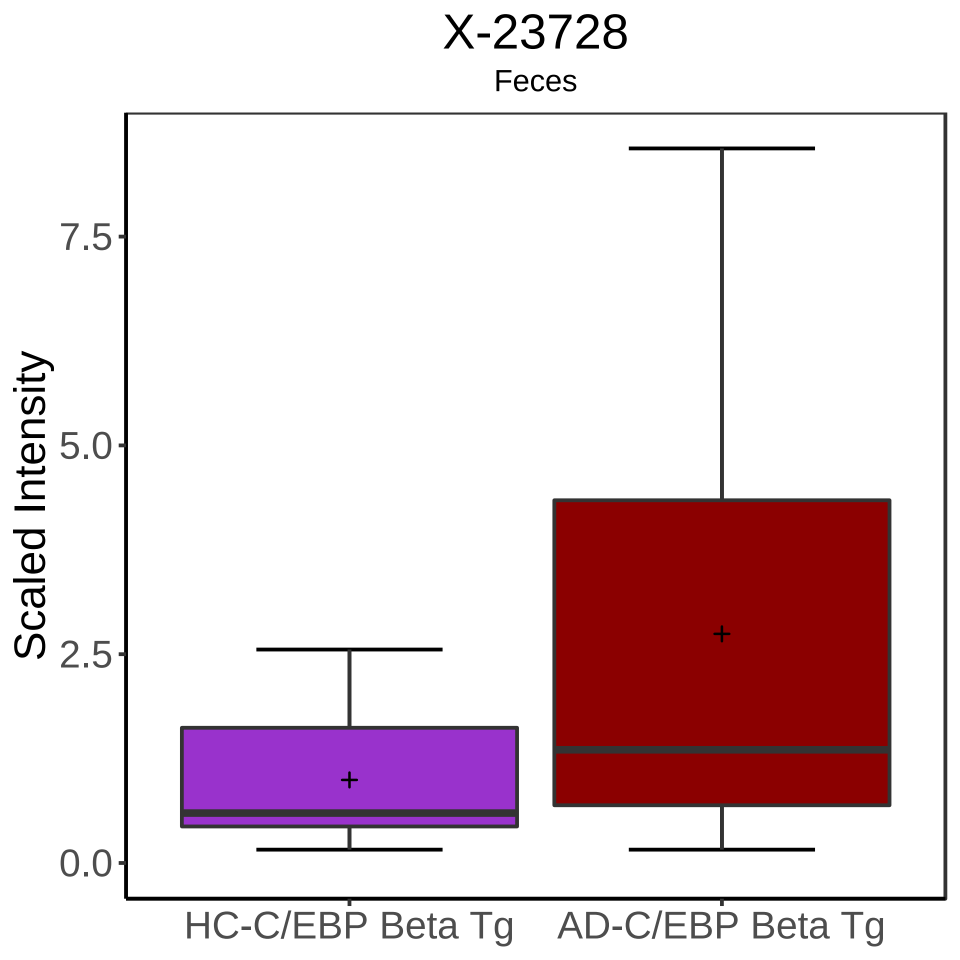
X-23728
Feces
Scaled Intensity
7.5
5.0
2.5
0.0
HC-C/EBP Beta Tg
AD-C/EBP Beta Tg

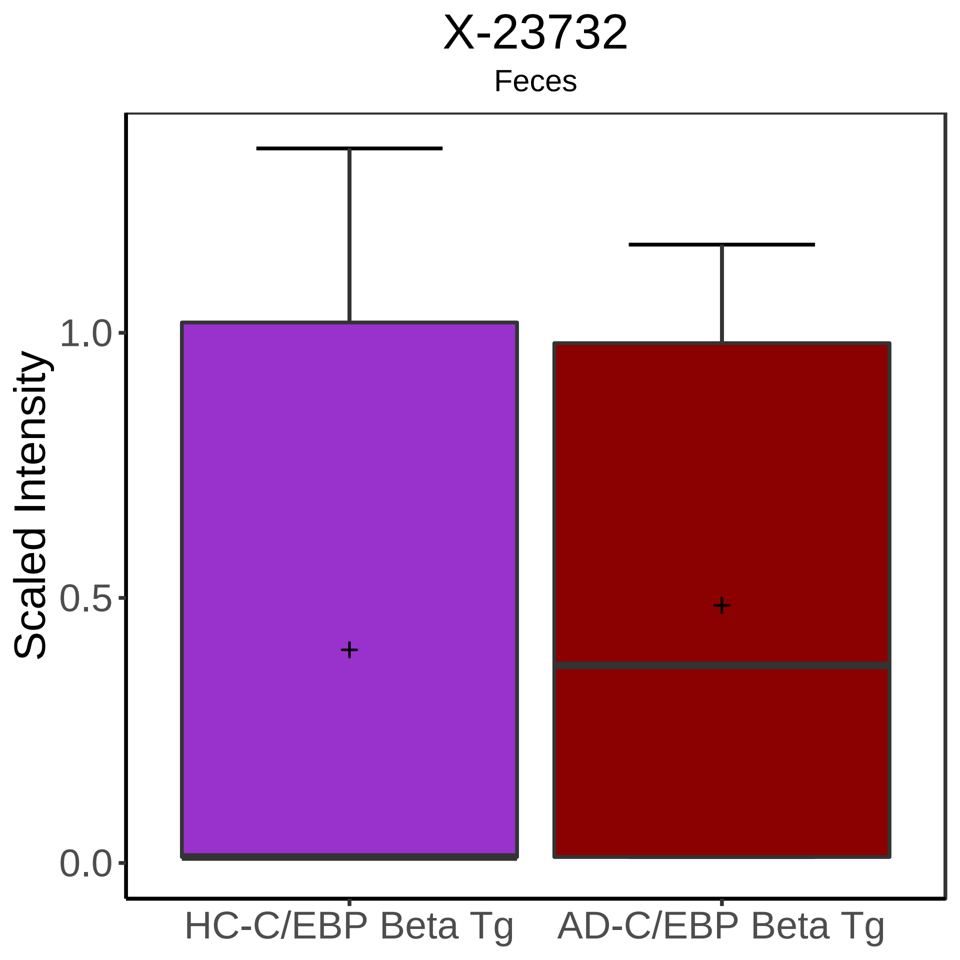
X-23732
Feces
Scaled Intensity
1.0
0.5
0.0
HC-C/EBP Beta Tg
AD-C/EBP Beta Tg

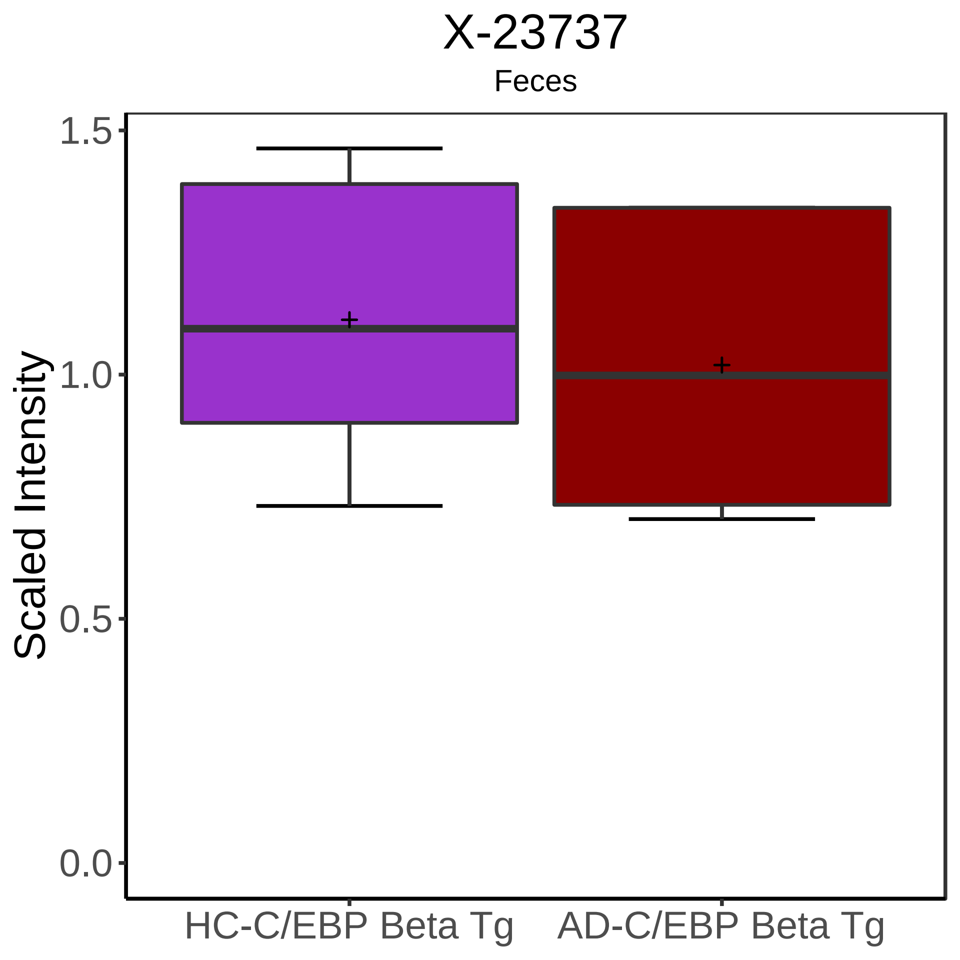

X-23737
Feces
Scaled Intensity
1.5
1.0
0.5
0.0
HC-C/EBP Beta Tg
AD-C/EBP Beta Tg

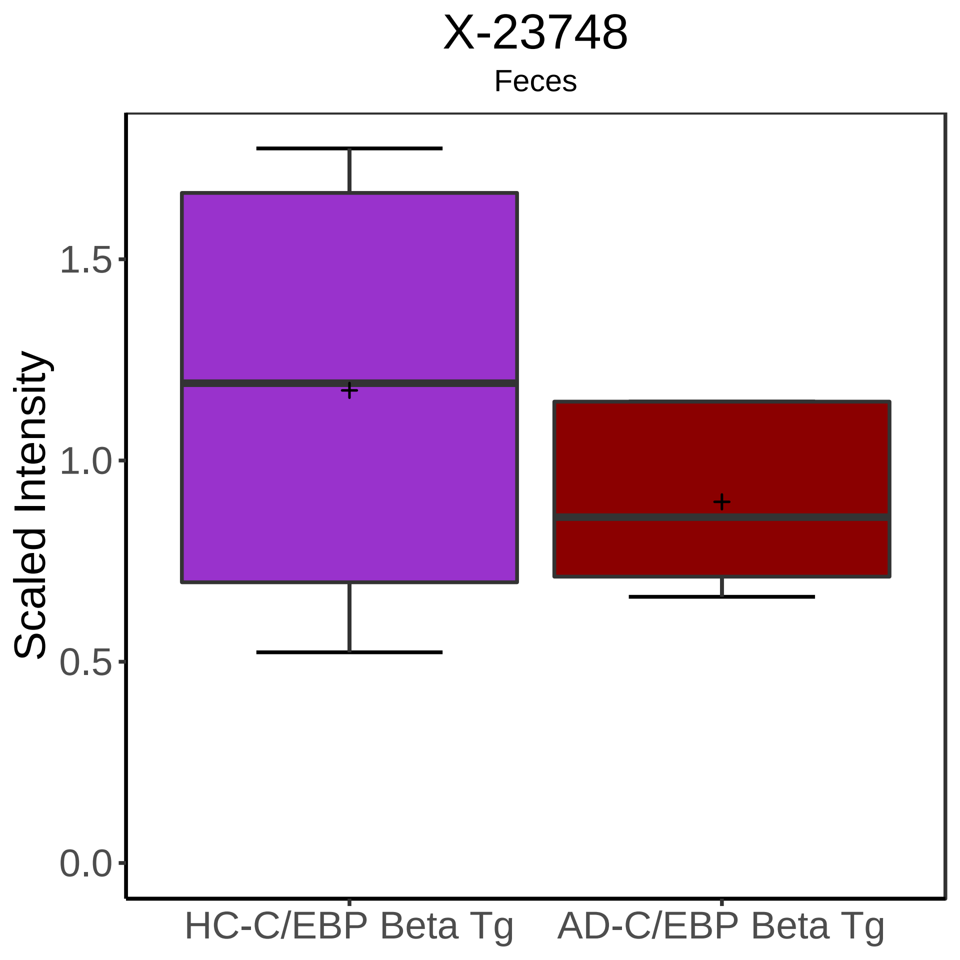
X-23748
Feces
Scaled Intensity
1.5
1.0
0.5
0.0
HC-C/EBP Beta Tg
AD-C/EBP Beta Tg

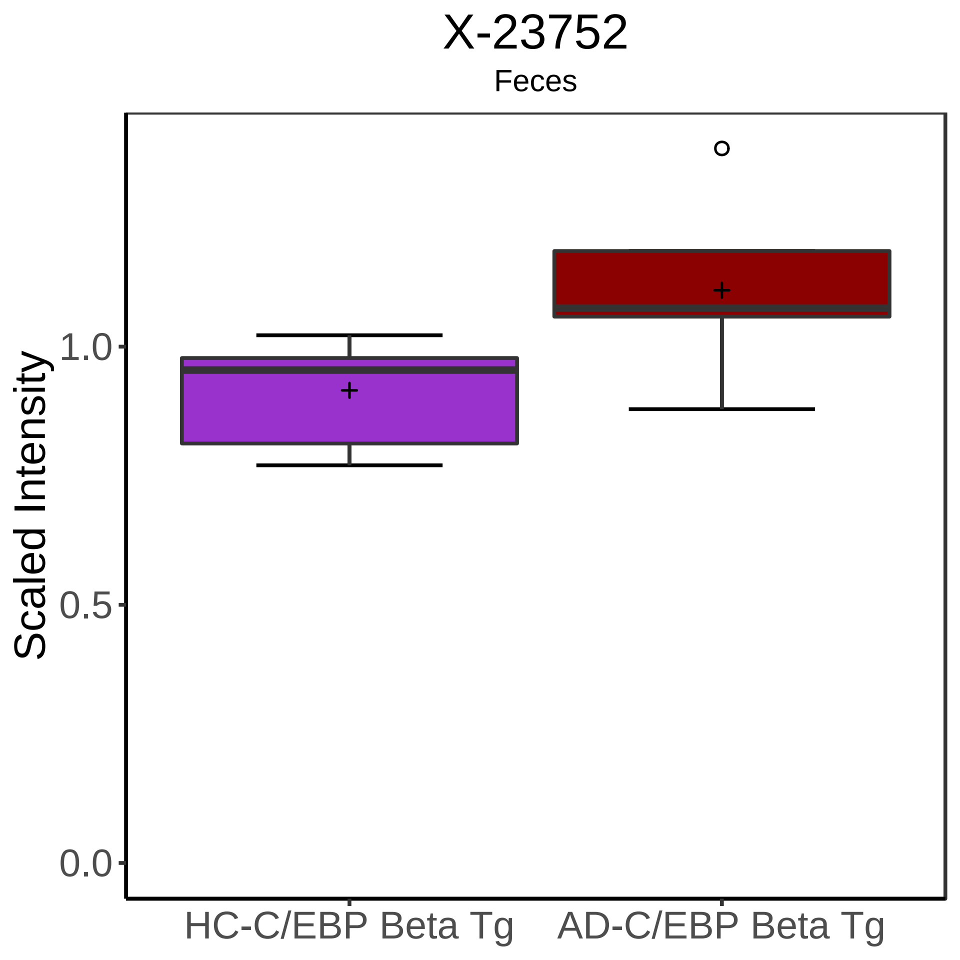
X-23752
Feces
Scaled Intensity
1.0
0.5
0.0
HC-C/EBP Beta Tg
AD-C/EBP Beta Tg

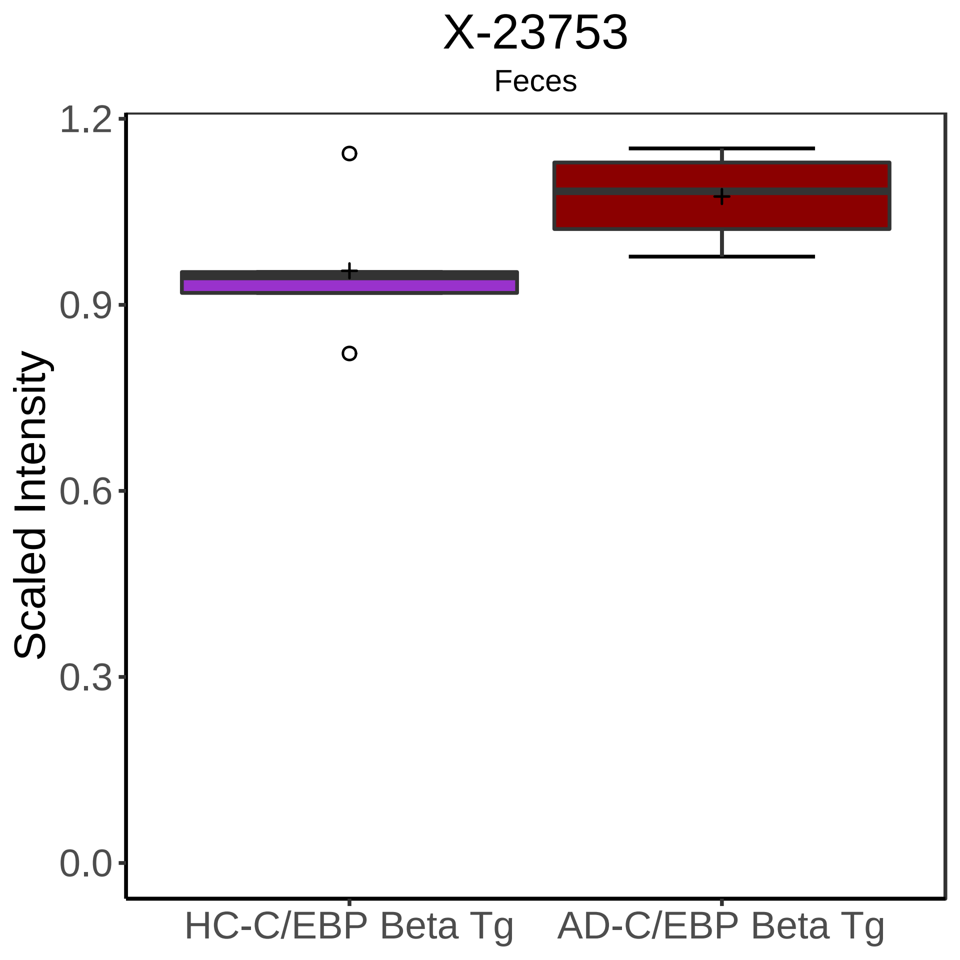
X-23753
Feces
Scaled Intensity
1.2
0.9
0.6
0.3
0.0
HC-C/EBP Beta Tg
AD-C/EBP Beta Tg

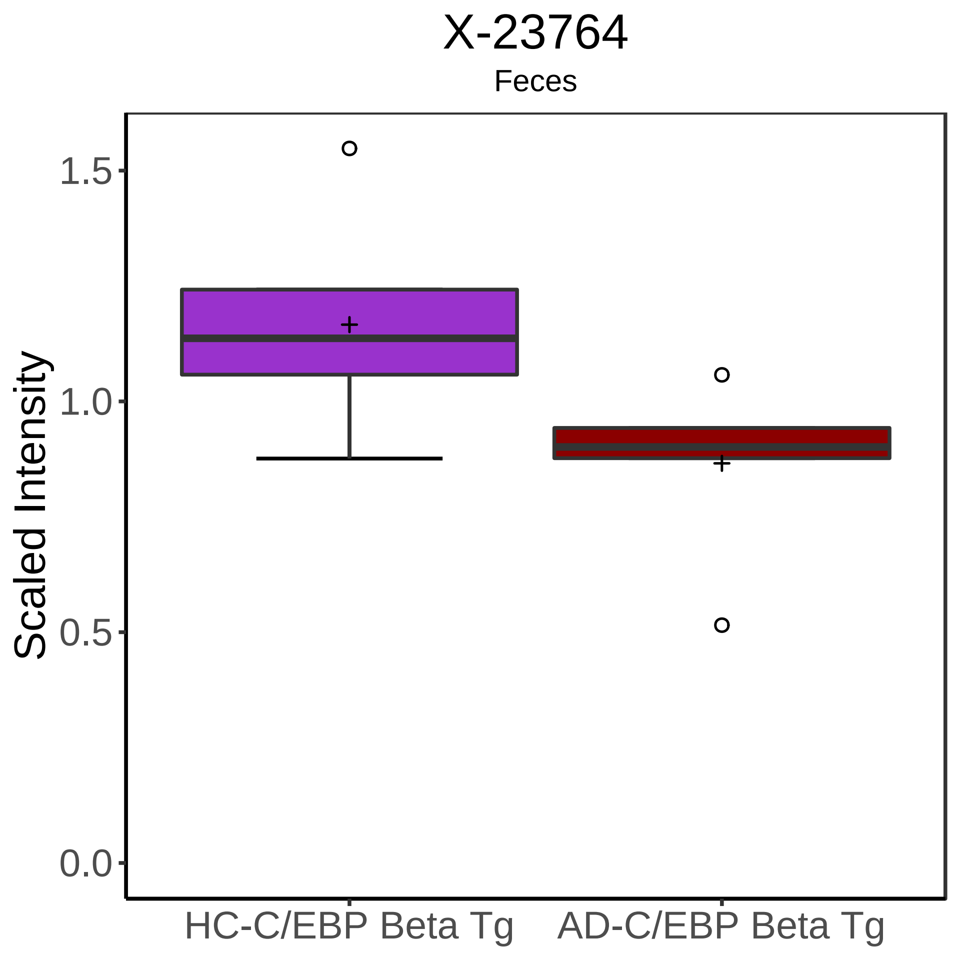
X-23764
Feces
Scaled Intensity
1.5
1.0
0.5
0.0
HC-C/EBP Beta Tg
AD-C/EBP Beta Tg

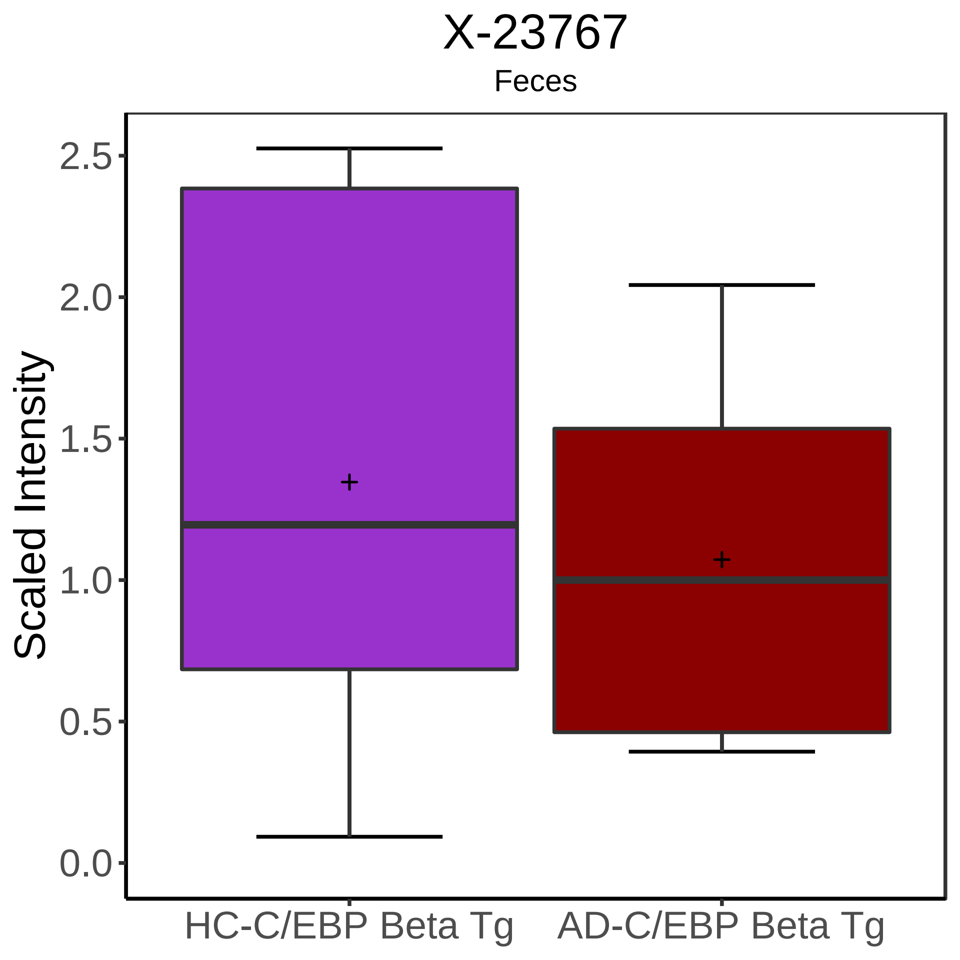

X-23767
Feces
Scaled Intensity
2.5
2.0
1.5
1.0
0.5
0.0
HC-C/EBP Beta Tg
AD-C/EBP Beta Tg

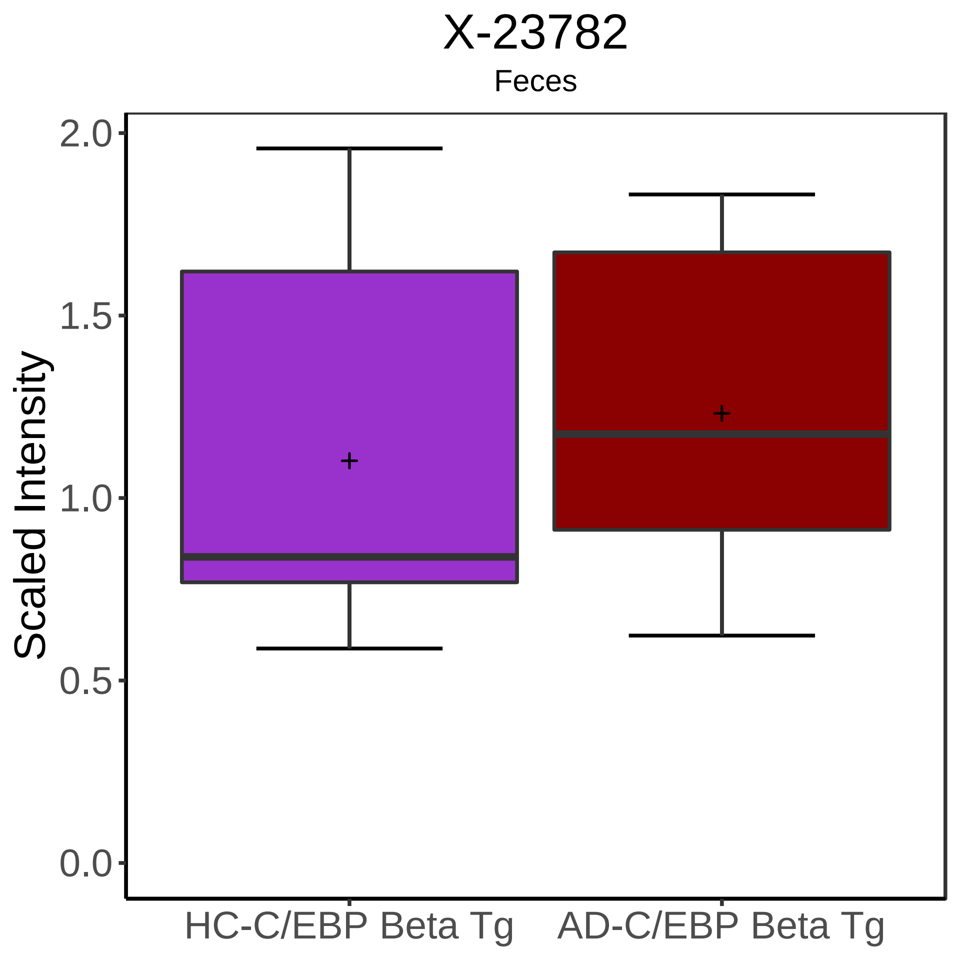
X-23782
Feces
Scaled Intensity
2.0
1.5
1.0
0.5
0.0
HC-C/EBP Beta Tg
AD-C/EBP Beta Tg

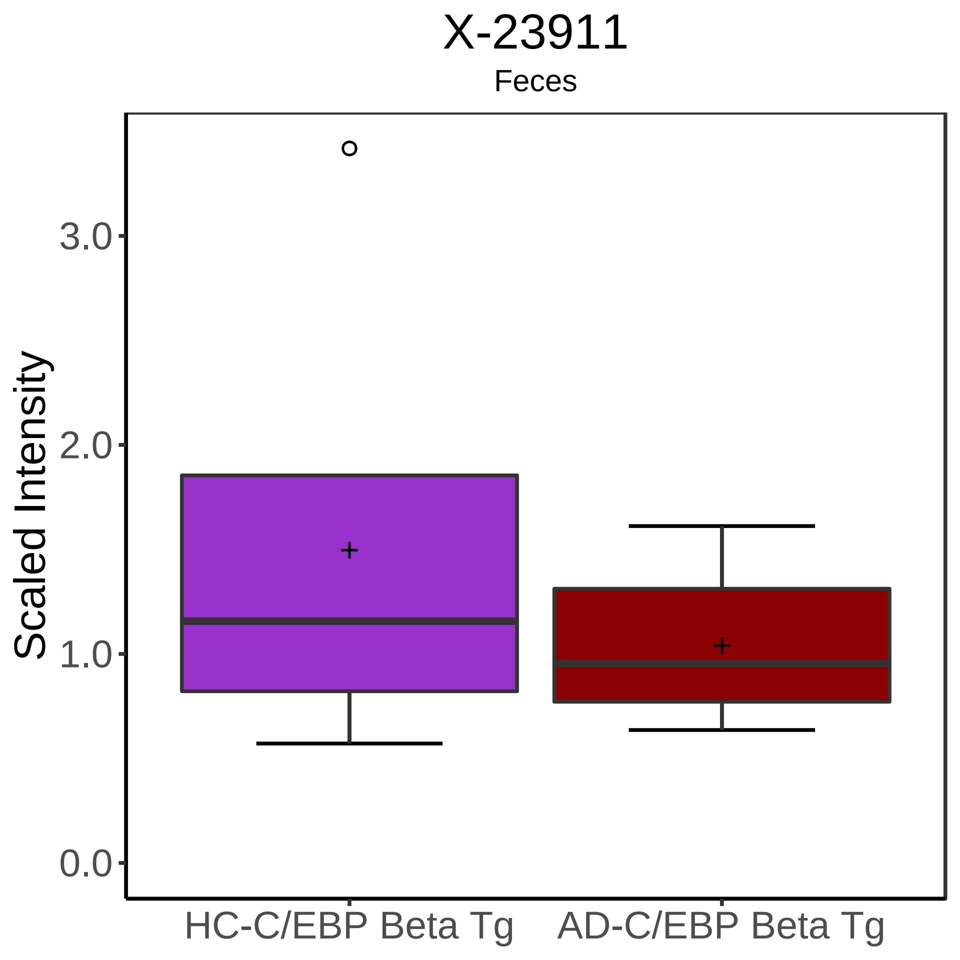
X-23911
Feces
Scaled Intensity
3.0
2.0
1.0
0.0
HC-C/EBP Beta Tg
AD-C/EBP Beta Tg

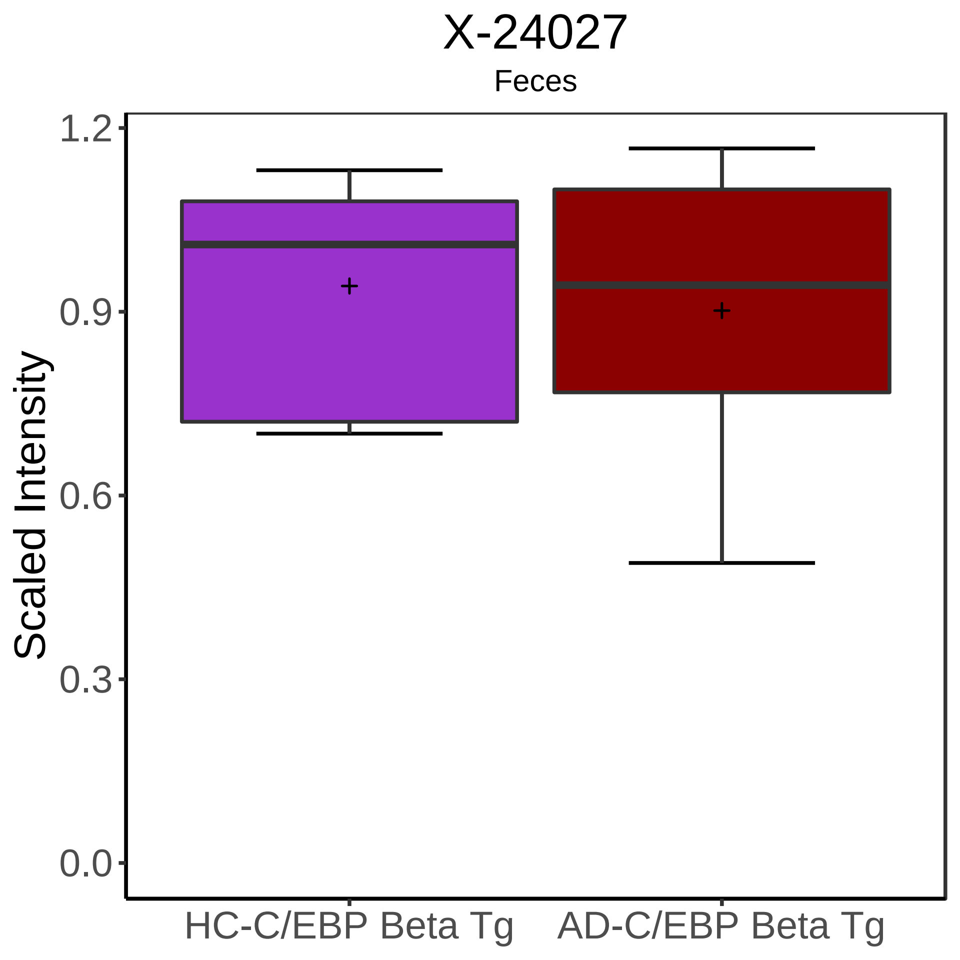
X-24027
Feces
Scaled Intensity
1.2
0.9
0.6
0.3
0.0
HC-C/EBP Beta Tg
AD-C/EBP Beta Tg

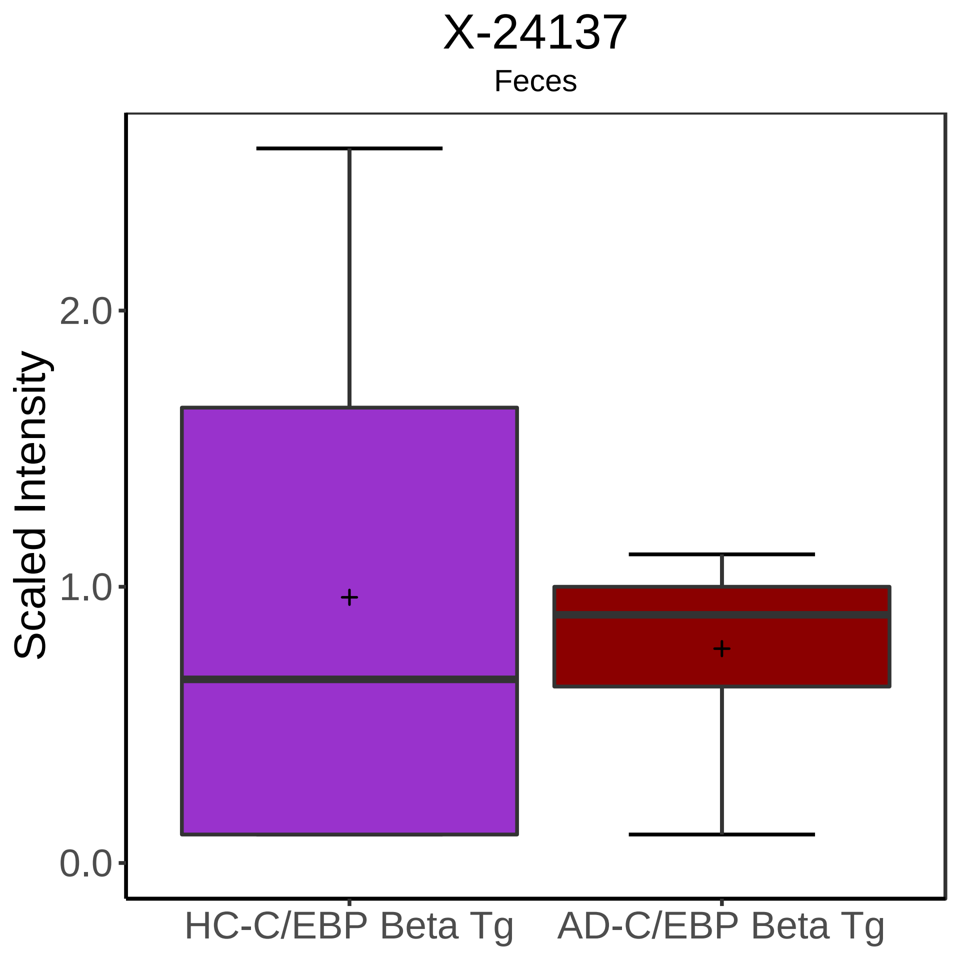
X-24137
Feces
Scaled Intensity
2.0
1.0
0.0
HC-C/EBP Beta Tg
AD-C/EBP Beta Tg

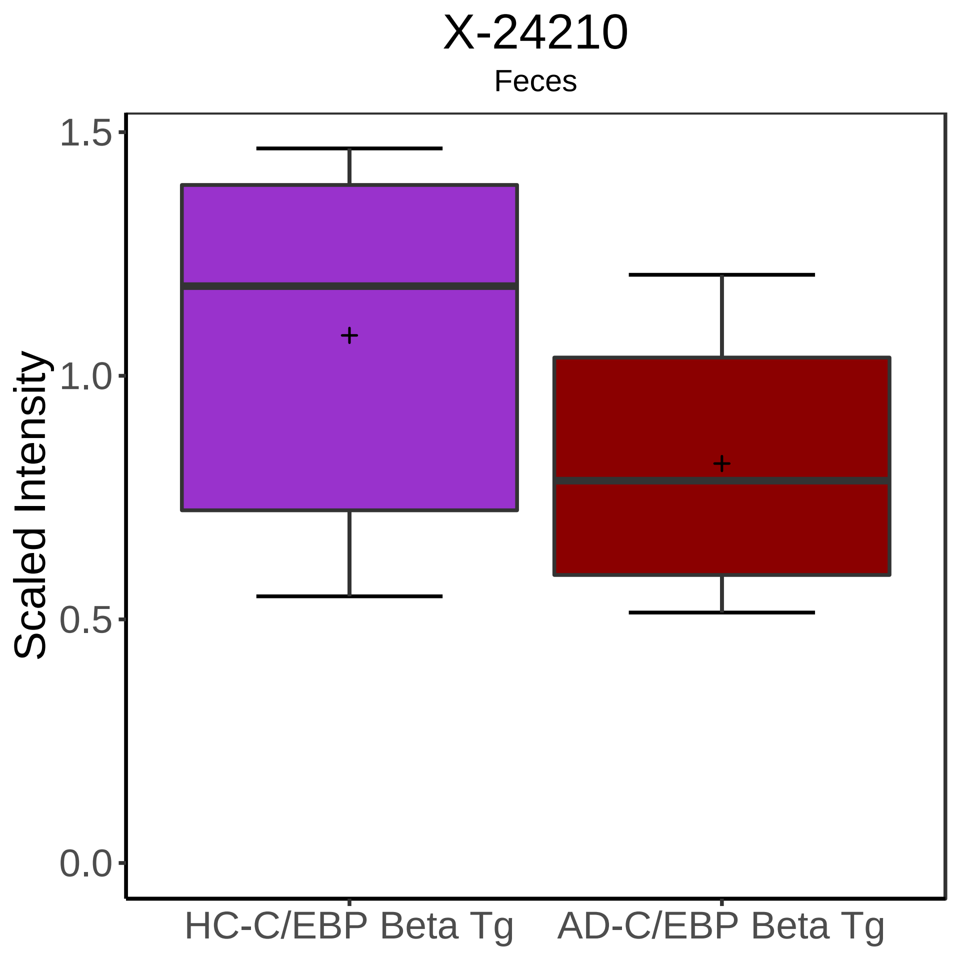
X-24210
Feces
Scaled Intensity
1.5
1.0
0.5
0.0
HC-C/EBP Beta Tg
AD-C/EBP Beta Tg

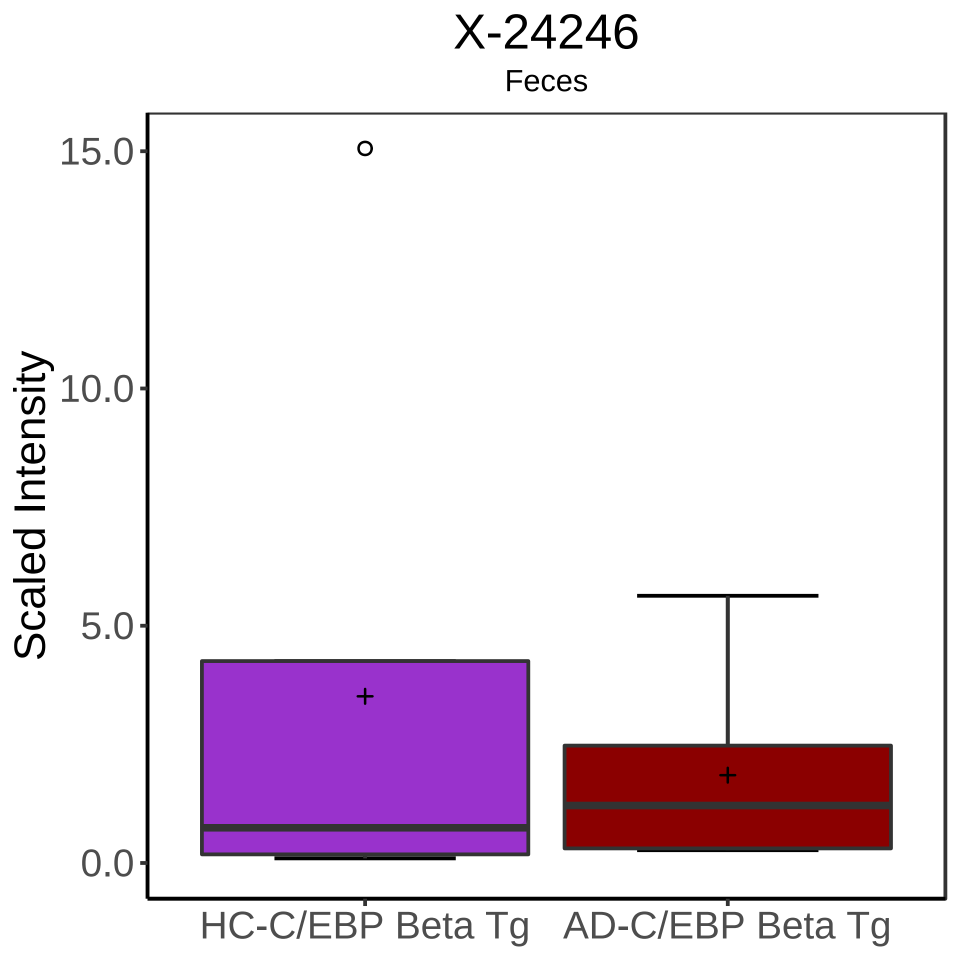
X-24246
Feces
Scaled Intensity
15.0
10.0
5.0
0.0
HC-C/EBP Beta Tg
AD-C/EBP Beta Tg

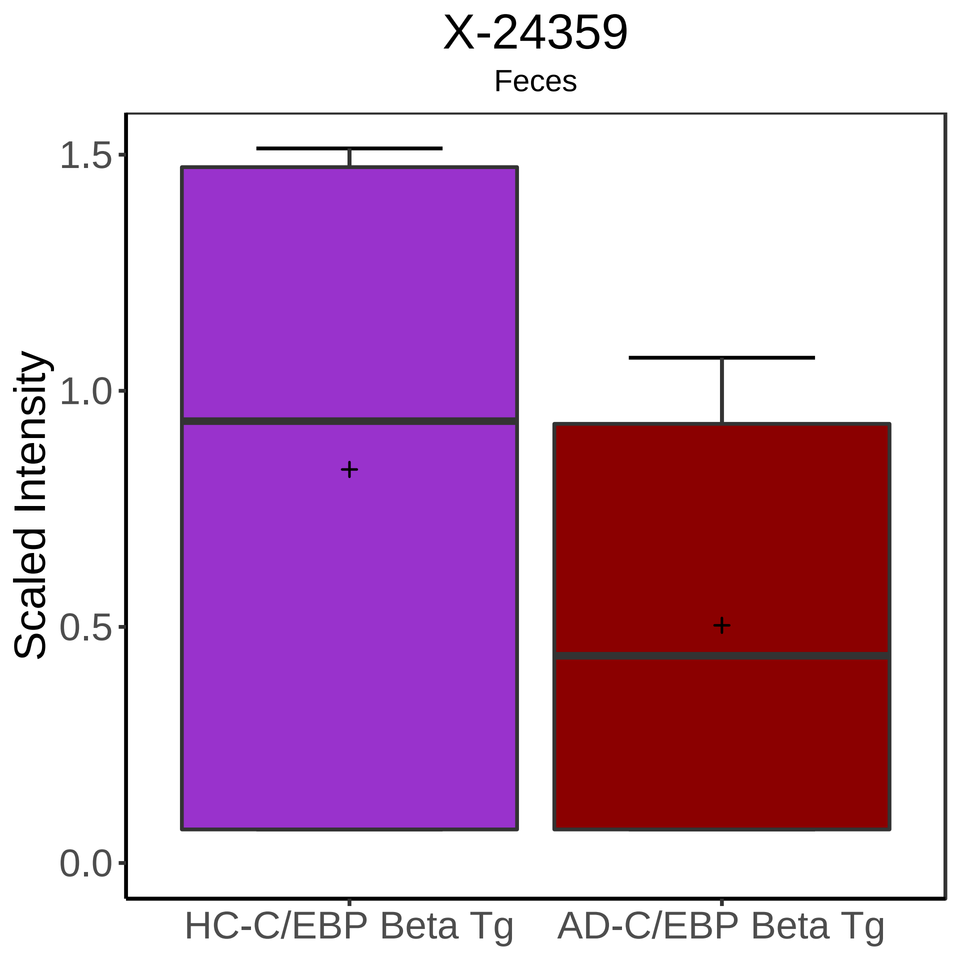

X-24359
Feces
Scaled Intensity
1.5
1.0
0.5
0.0
HC-C/EBP Beta Tg
AD-C/EBP Beta Tg

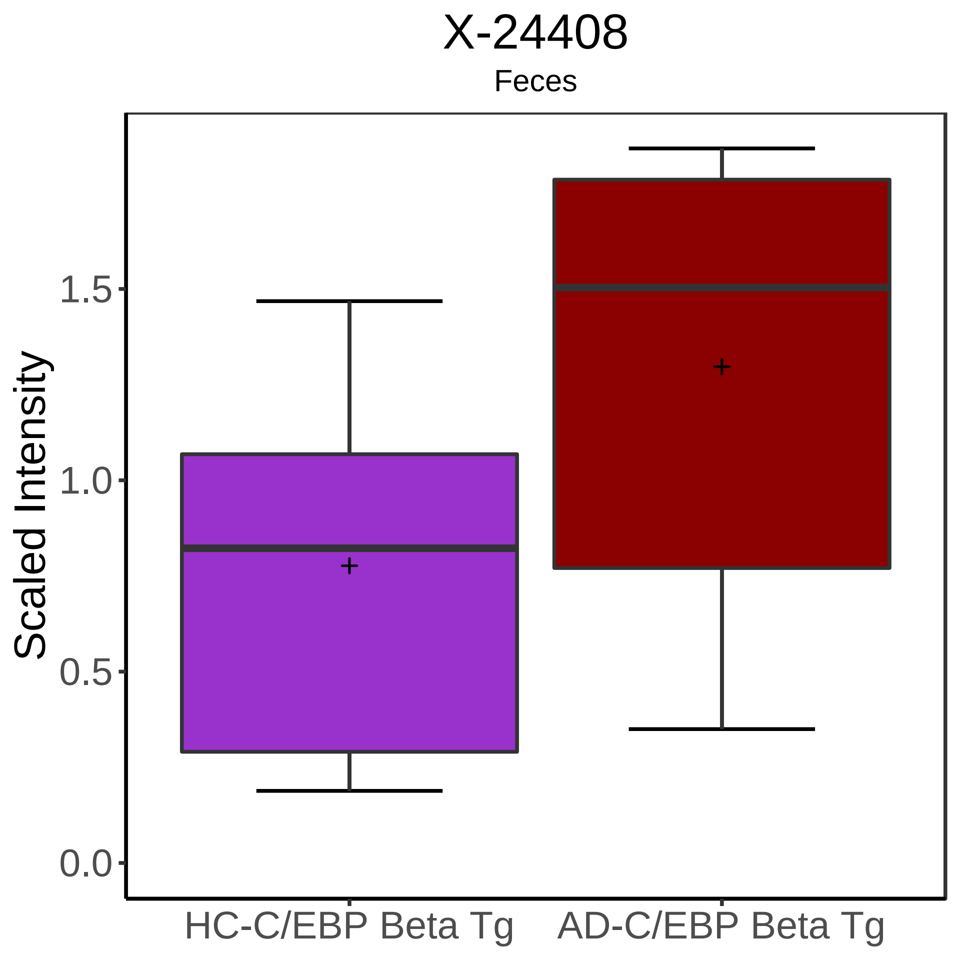

X-24408
Feces
Scaled Intensity
1.5
1.0
0.5
0.0
HC-C/EBP Beta Tg
AD-C/EBP Beta Tg

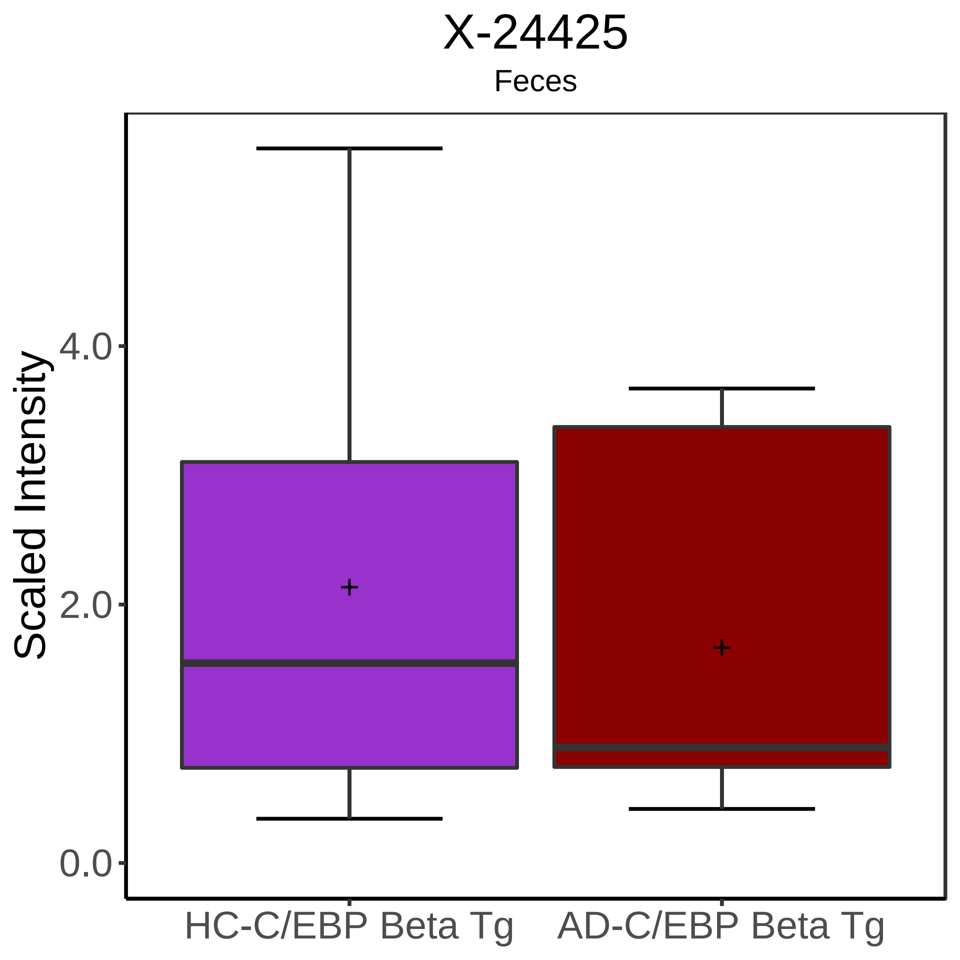
X-24425
Feces
Scaled Intensity
4.0
2.0
0.0
HC-C/EBP Beta Tg
AD-C/EBP Beta Tg

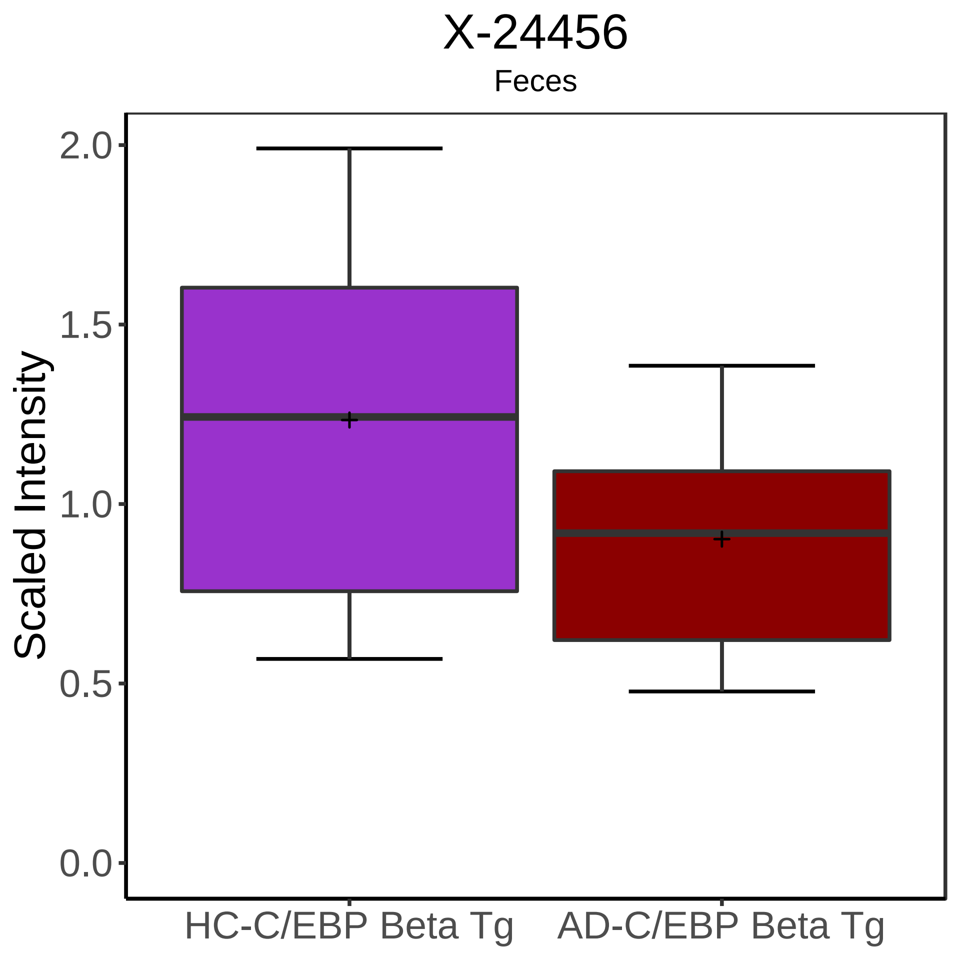
X-24456
Feces
Scaled Intensity
2.0
1.5
1.0
0.5
0.0
HC-C/EBP Beta Tg
AD-C/EBP Beta Tg

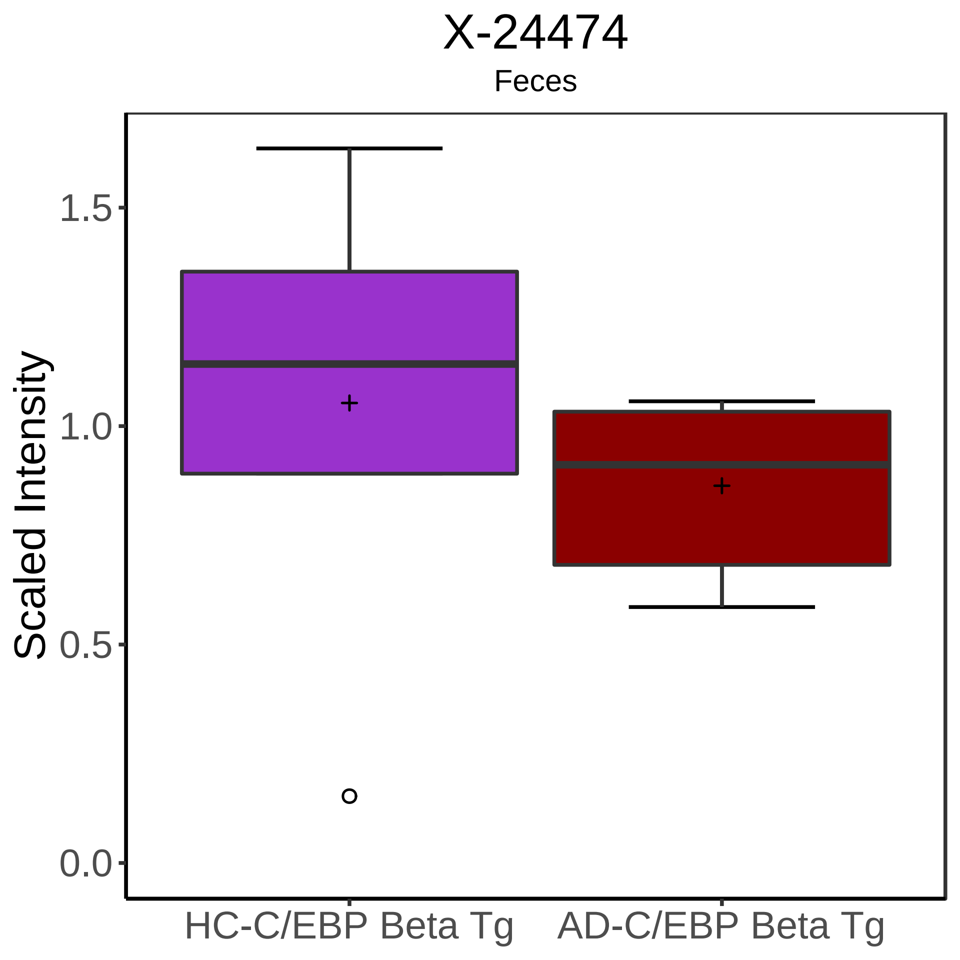
X-24474
Feces
Scaled Intensity
1.5
1.0
0.5
0.0
HC-C/EBP Beta Tg
AD-C/EBP Beta Tg

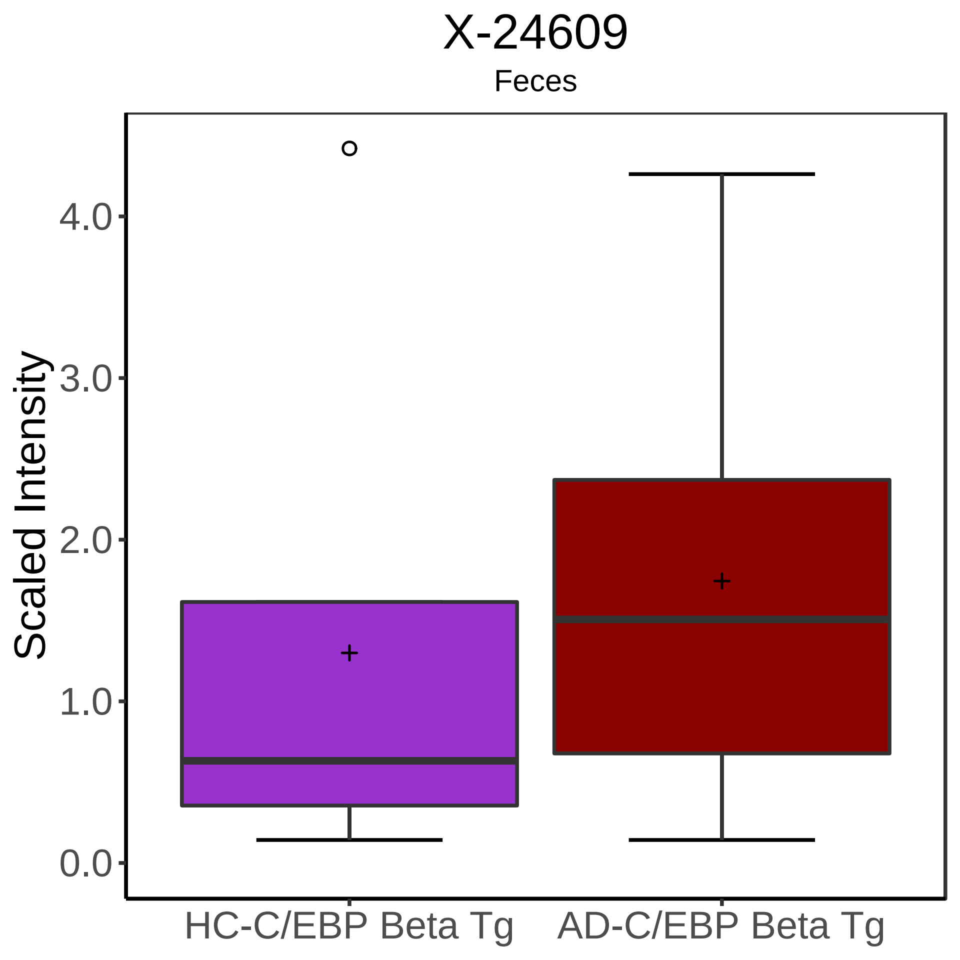

X-24609
Feces
Scaled Intensity
4.0
3.0
2.0
1.0
0.0
HC-C/EBP Beta Tg
AD-C/EBP Beta Tg

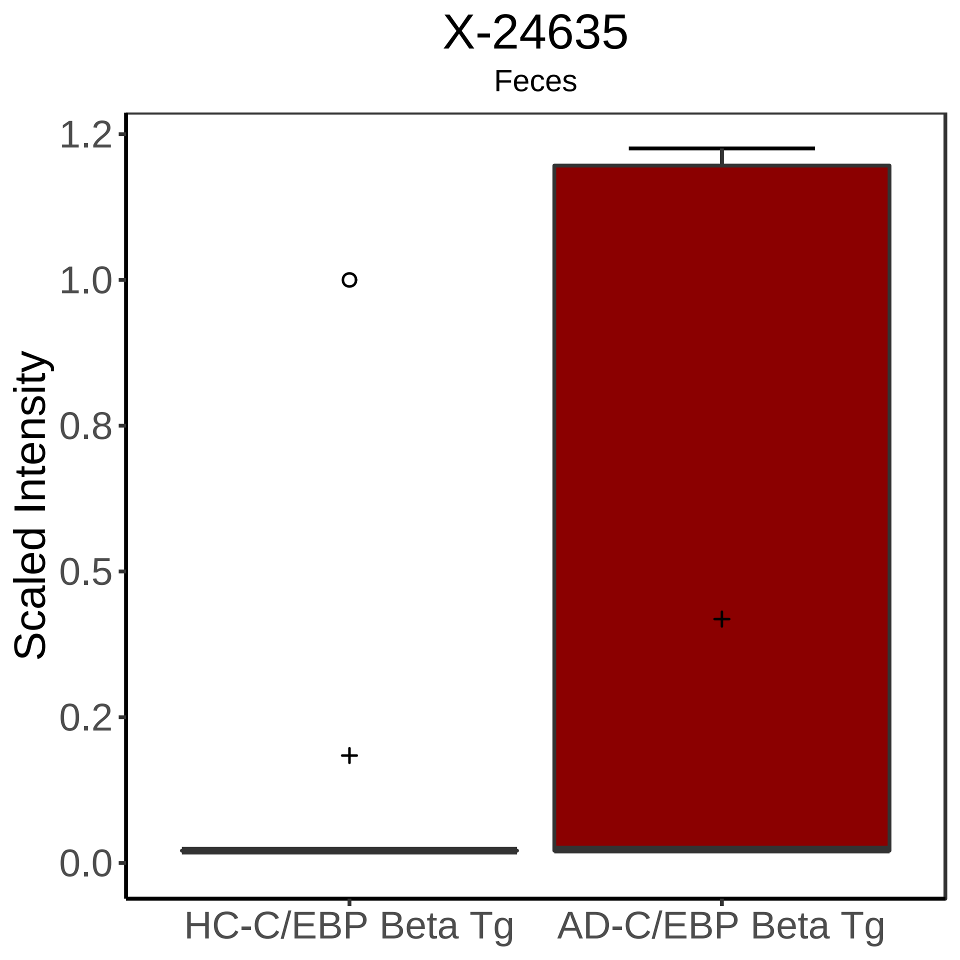

X-24635
Feces
Scaled Intensity
1.2
1.0
0.8
0.5
0.2
0.0
HC-C/EBP Beta Tg
AD-C/EBP Beta Tg

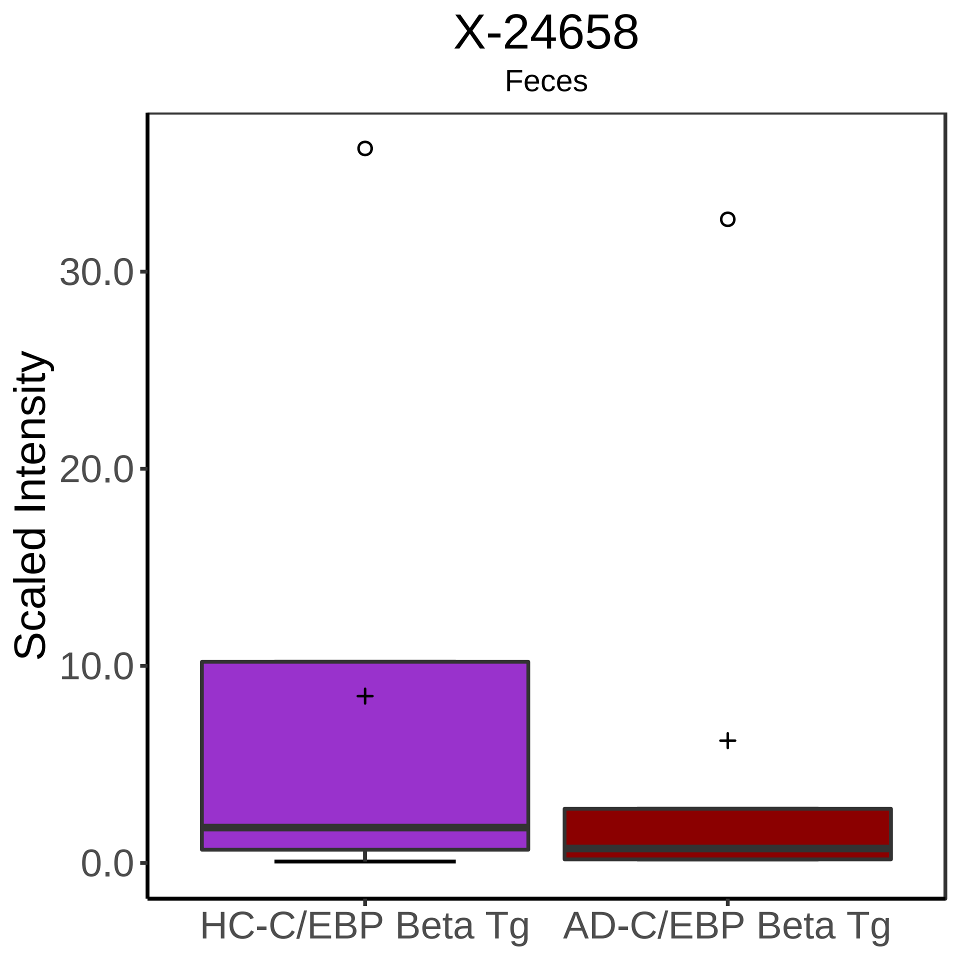
X-24658
Feces
Scaled Intensity
30.0
20.0
10.0
0.0
HC-C/EBP Beta Tg
AD-C/EBP Beta Tg

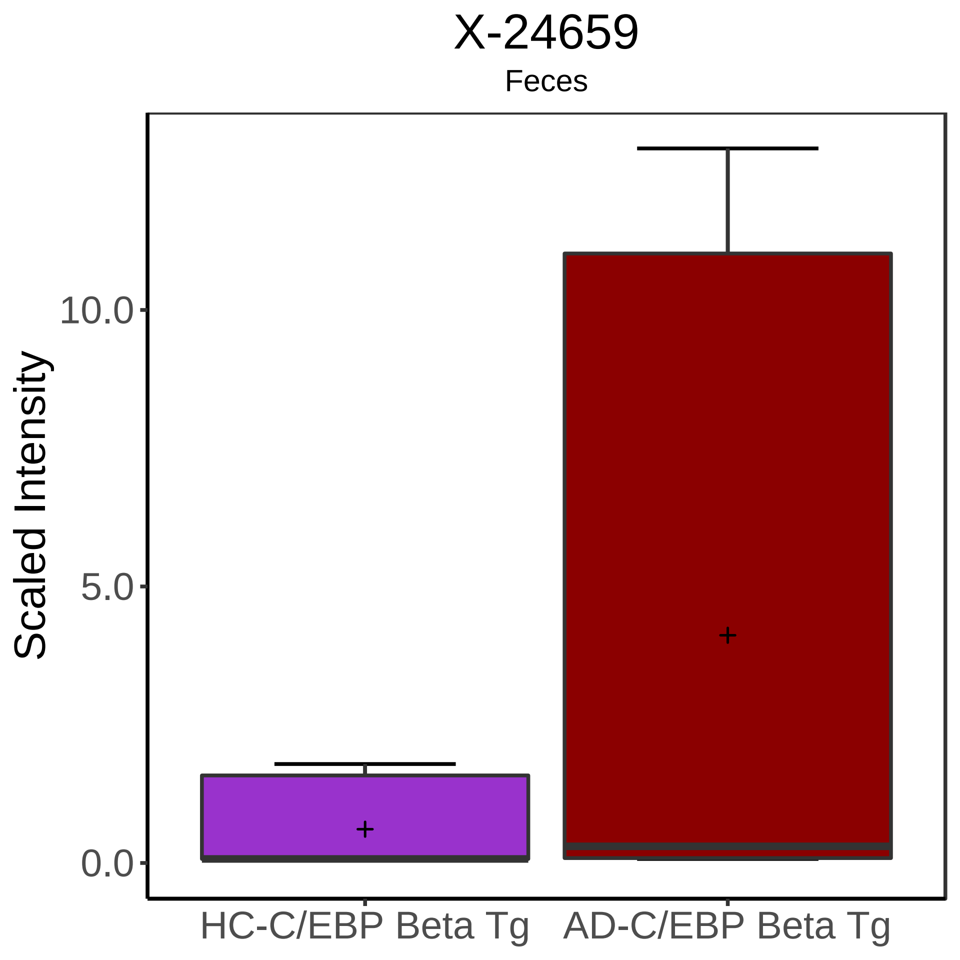
X-24659
Feces
Scaled Intensity
10.0
5.0
0.0
HC-C/EBP Beta Tg
AD-C/EBP Beta Tg

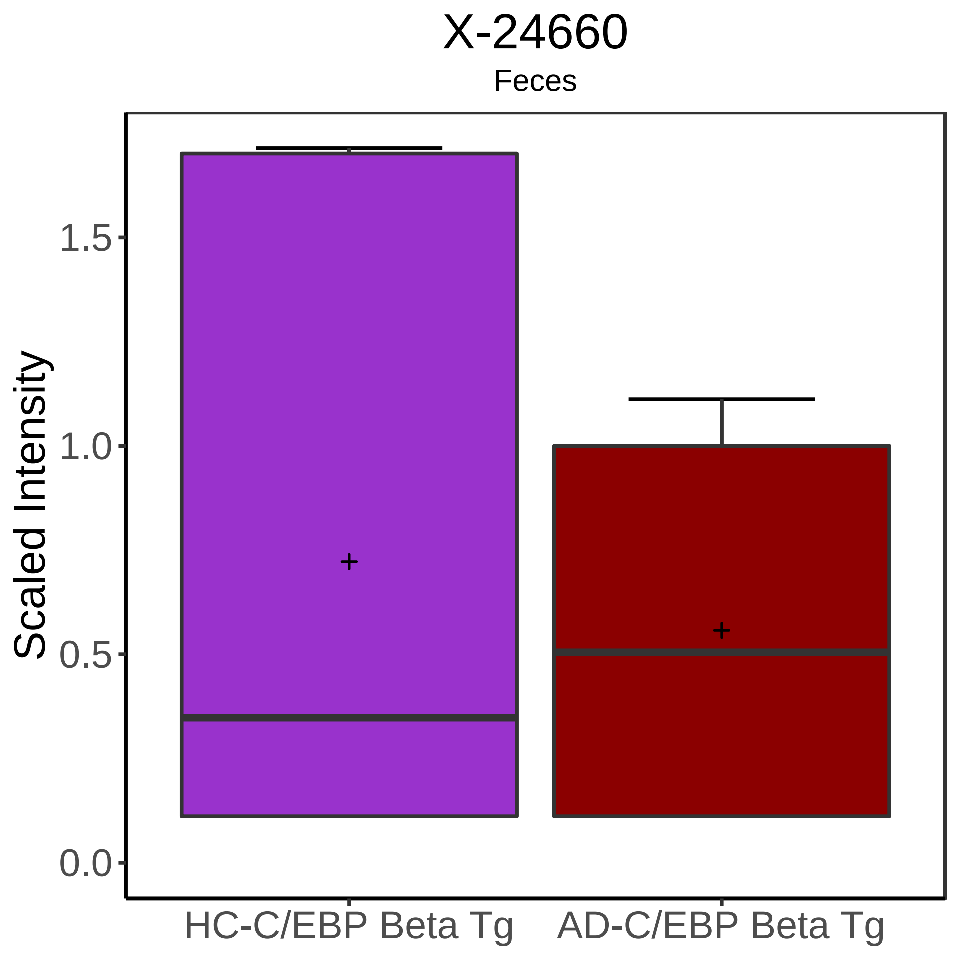
X-24660
Feces
Scaled Intensity
1.5
1.0
0.5
0.0
HC-C/EBP Beta Tg
AD-C/EBP Beta Tg

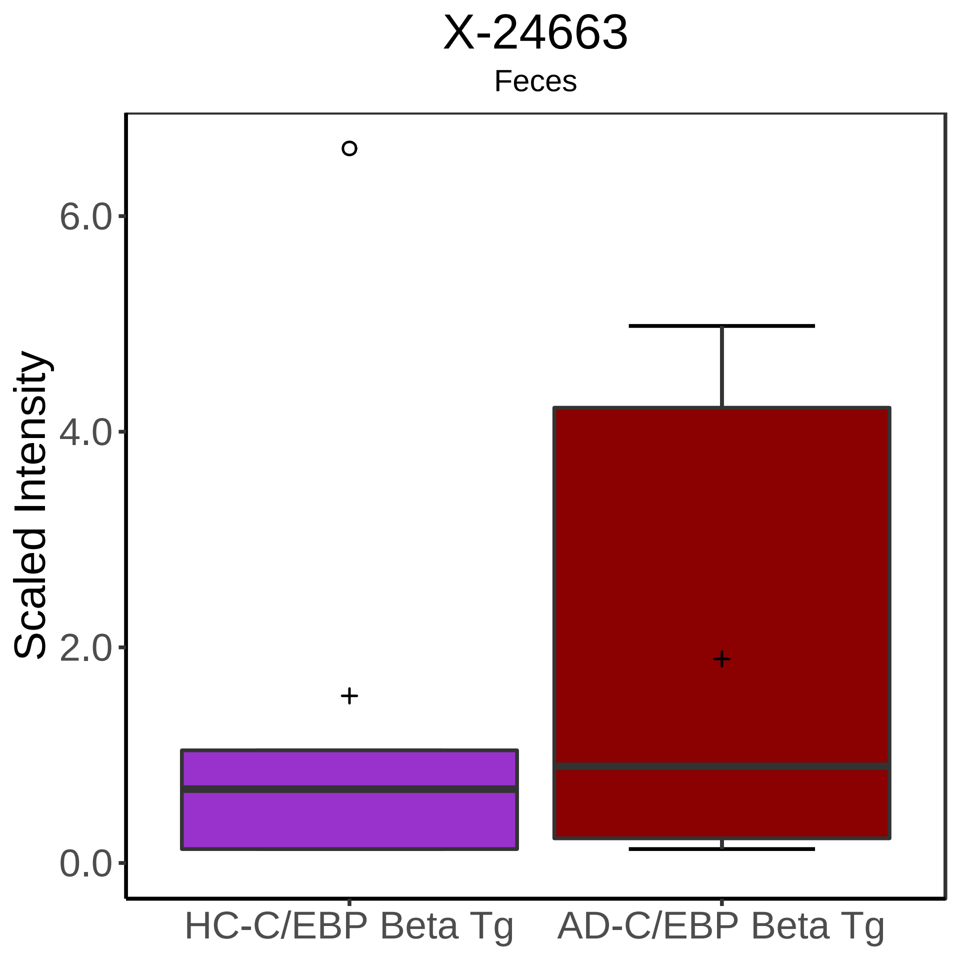
X-24663
Feces
Scaled Intensity
6.0
4.0
2.0
0.0
HC-C/EBP Beta Tg
AD-C/EBP Beta Tg

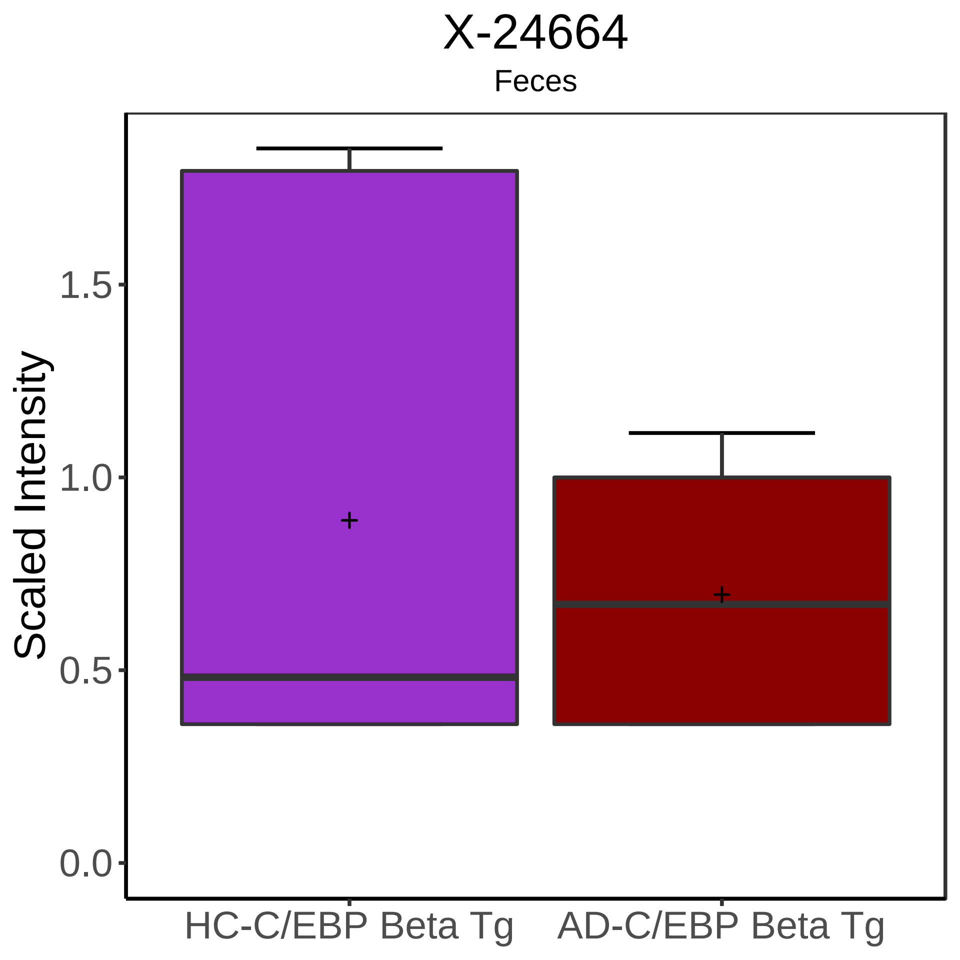

X-24664
Feces
Scaled Intensity
1.5
1.0
0.5
0.0
HC-C/EBP Beta Tg
AD-C/EBP Beta Tg

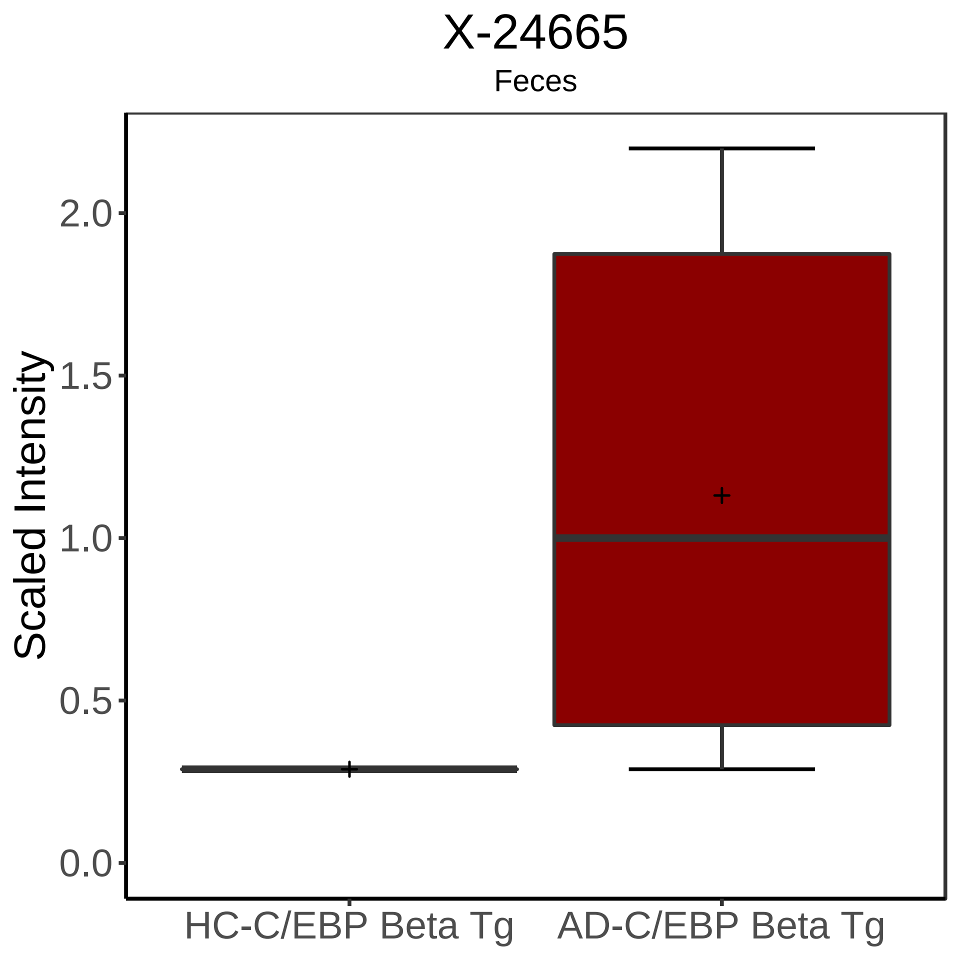
X-24665
Feces
Scaled Intensity
2.0
1.5
1.0
0.5
0.0
HC-C/EBP Beta Tg
AD-C/EBP Beta Tg

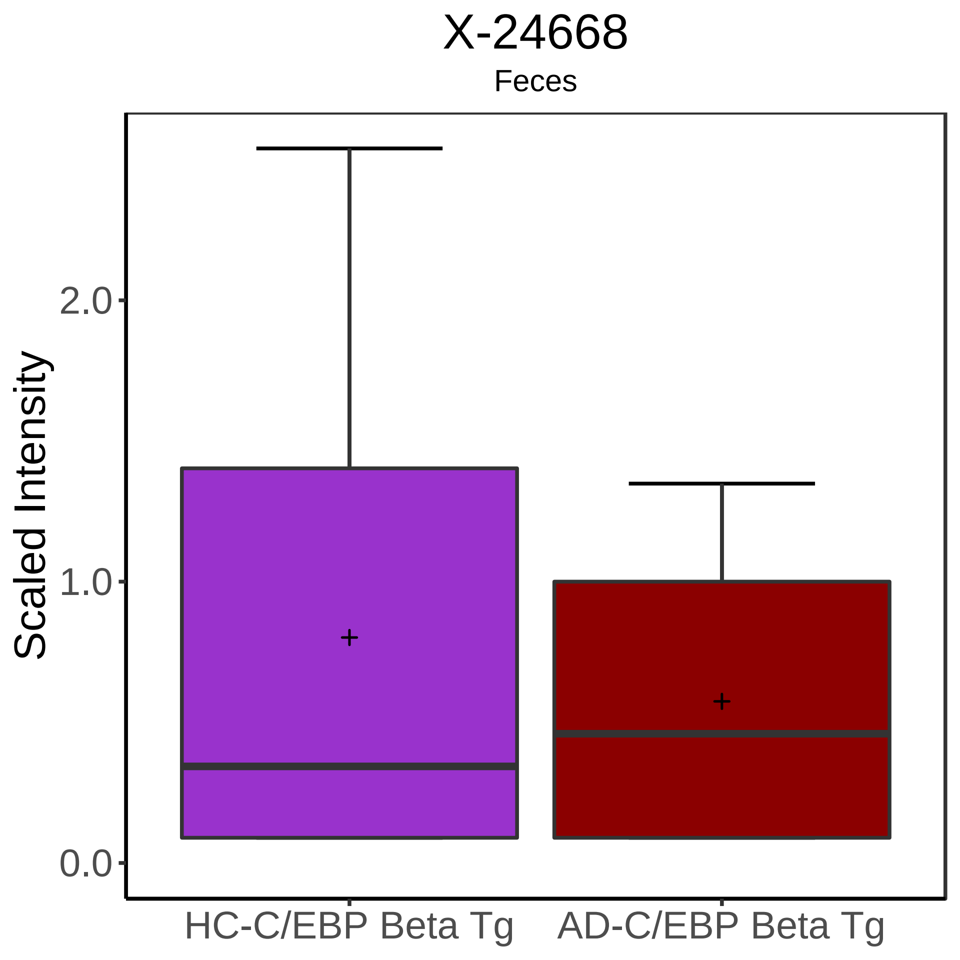

X-24668
Feces
Scaled Intensity
2.0
1.0
0.0
HC-C/EBP Beta Tg
AD-C/EBP Beta Tg

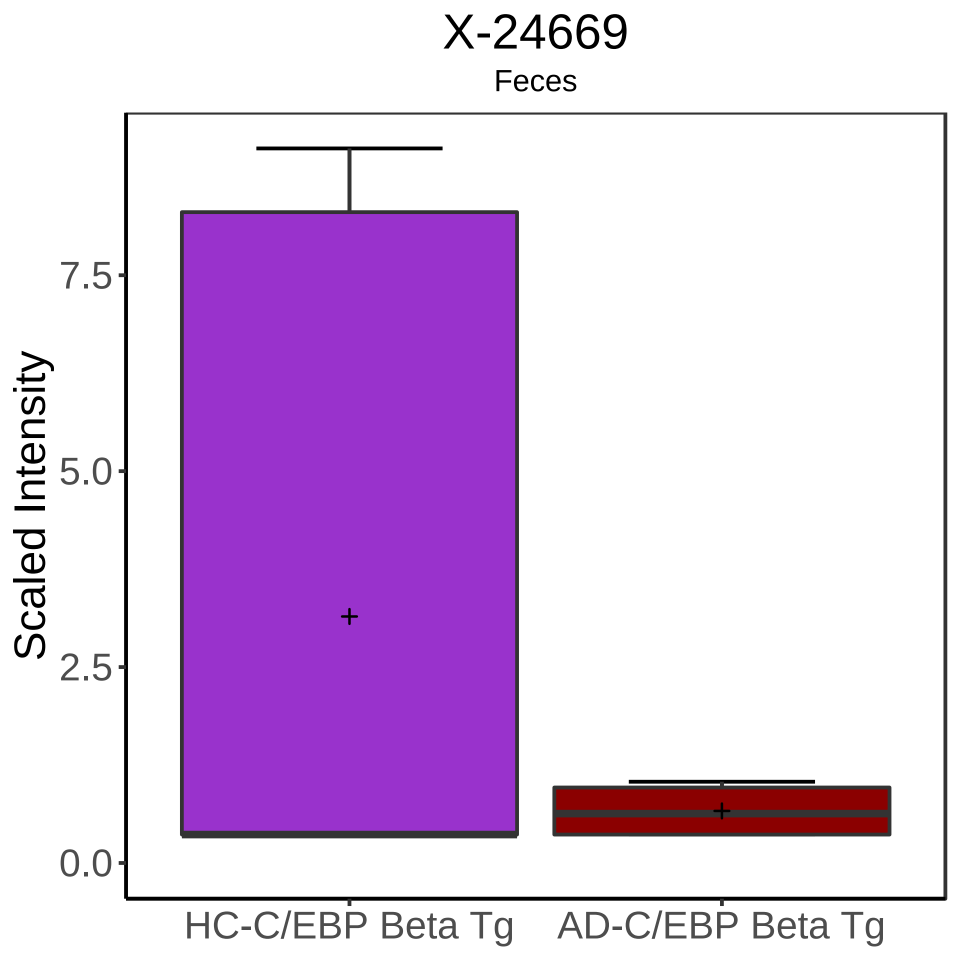
X-24669
Feces
Scaled Intensity
7.5
5.0
2.5
0.0
HC-C/EBP Beta Tg
AD-C/EBP Beta Tg

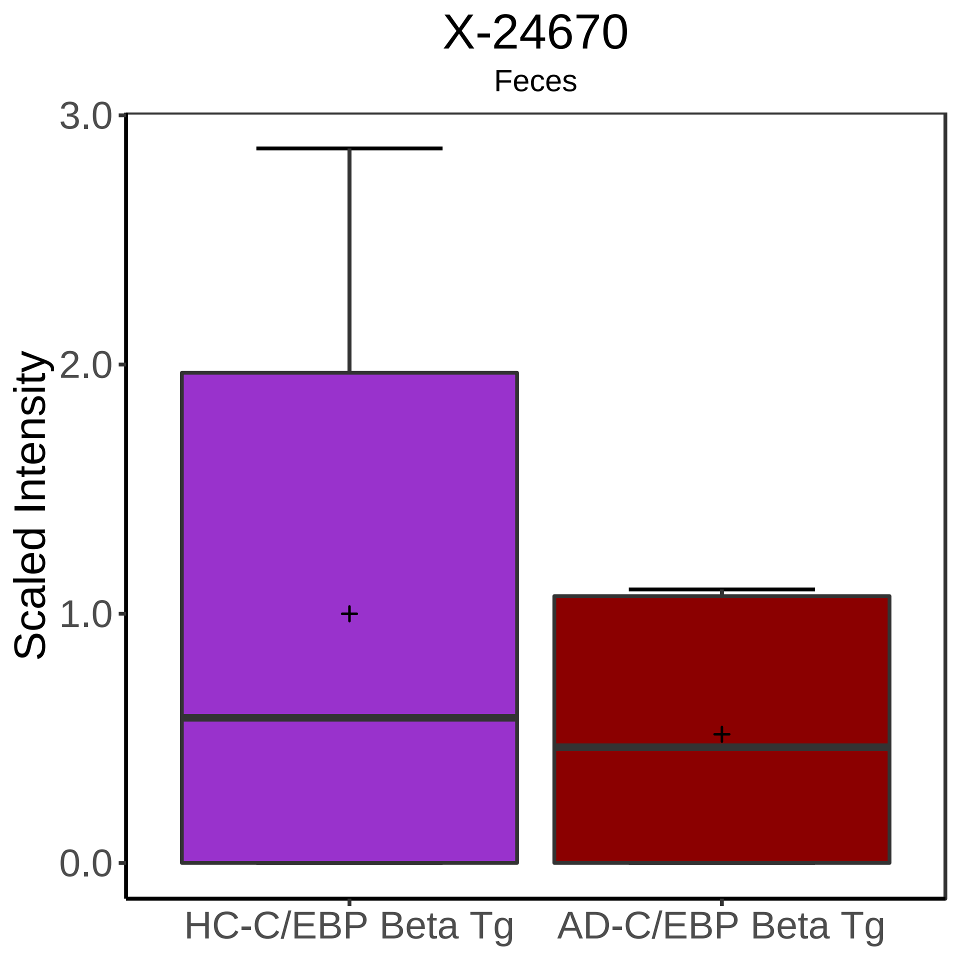
X-24670
Feces
Scaled Intensity
3.0
2.0
1.0
0.0
HC-C/EBP Beta Tg
AD-C/EBP Beta Tg

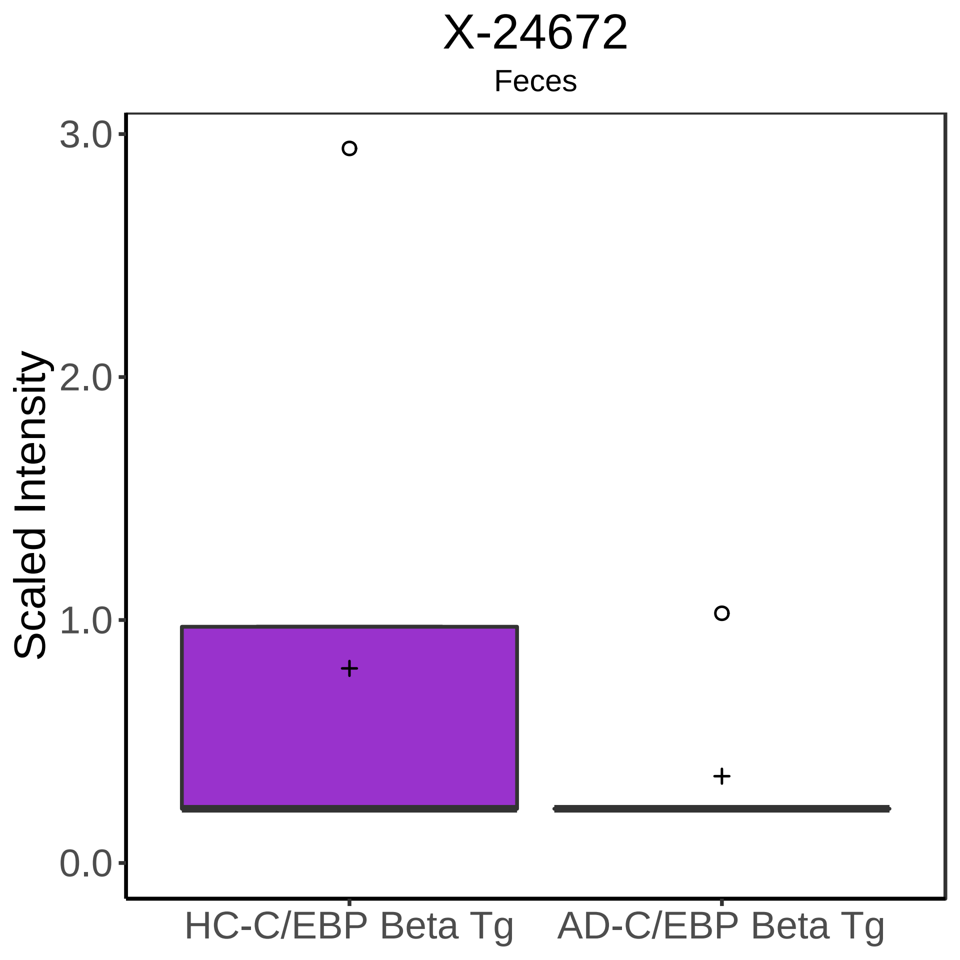
X-24672
Feces
Scaled Intensity
3.0
2.0
1.0
0.0
HC-C/EBP Beta Tg
AD-C/EBP Beta Tg

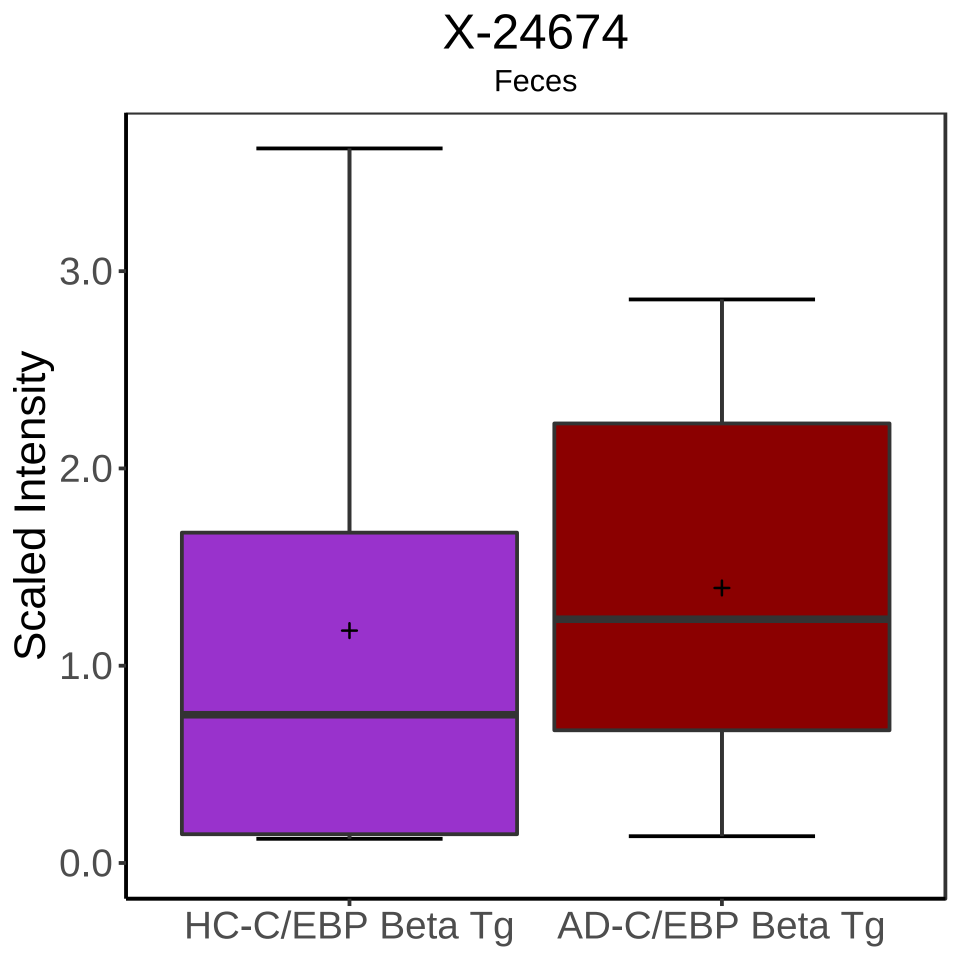
X-24674
Feces
Scaled Intensity
3.0
2.0
1.0
0.0
HC-C/EBP Beta Tg
AD-C/EBP Beta Tg

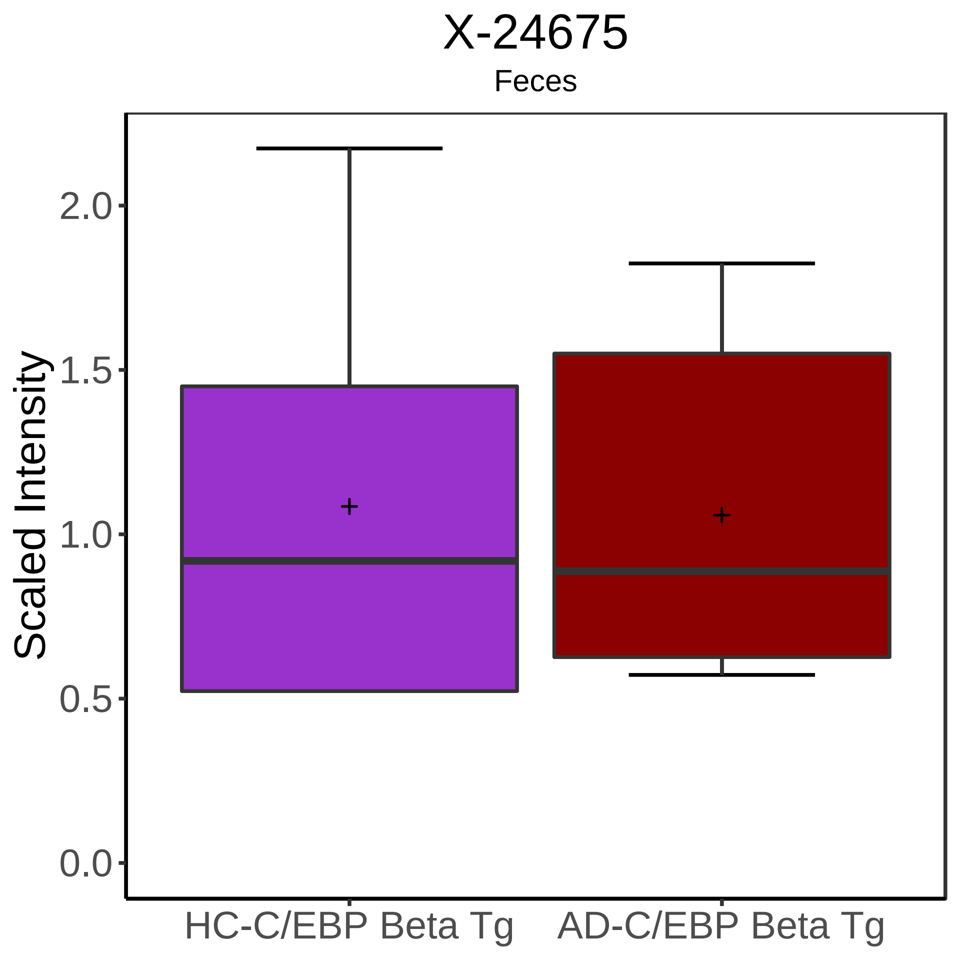
X-24675
Feces
Scaled Intensity
2.0
1.5
1.0
0.5
0.0
HC-C/EBP Beta Tg
AD-C/EBP Beta Tg

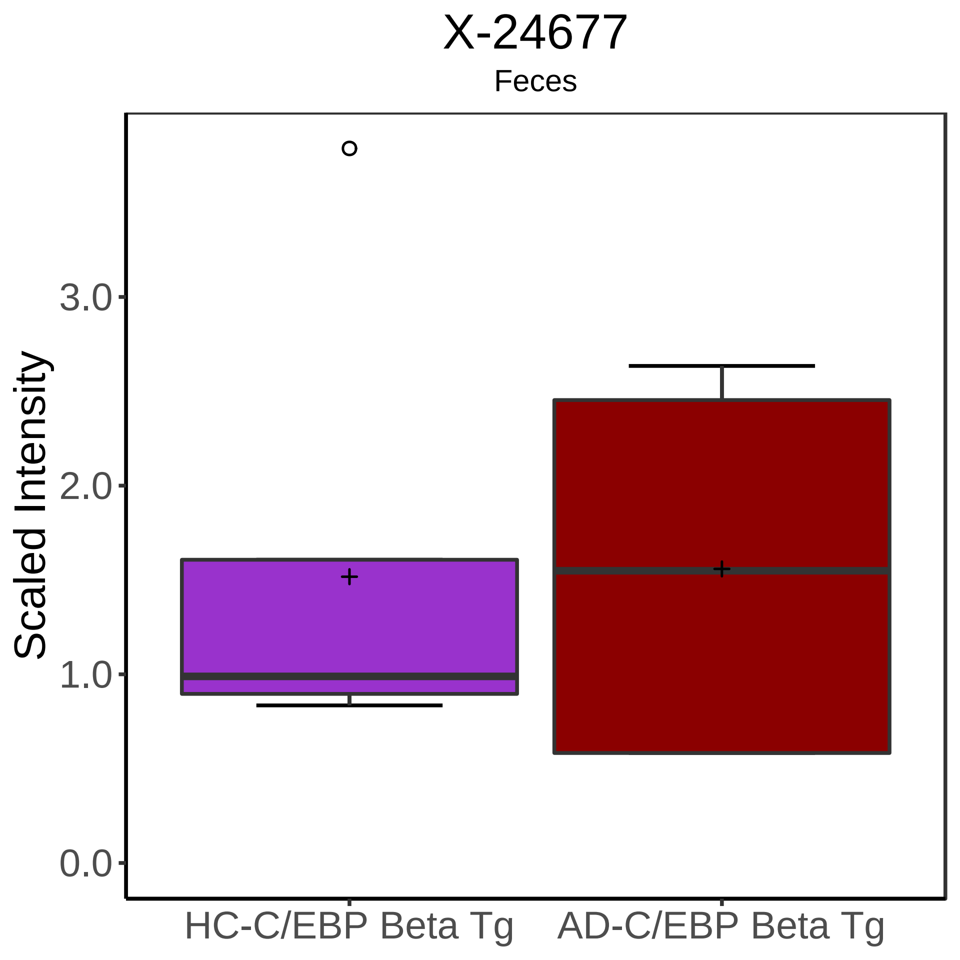
X-24677
Feces
Scaled Intensity
3.0
2.0
1.0
0.0
HC-C/EBP Beta Tg
AD-C/EBP Beta Tg

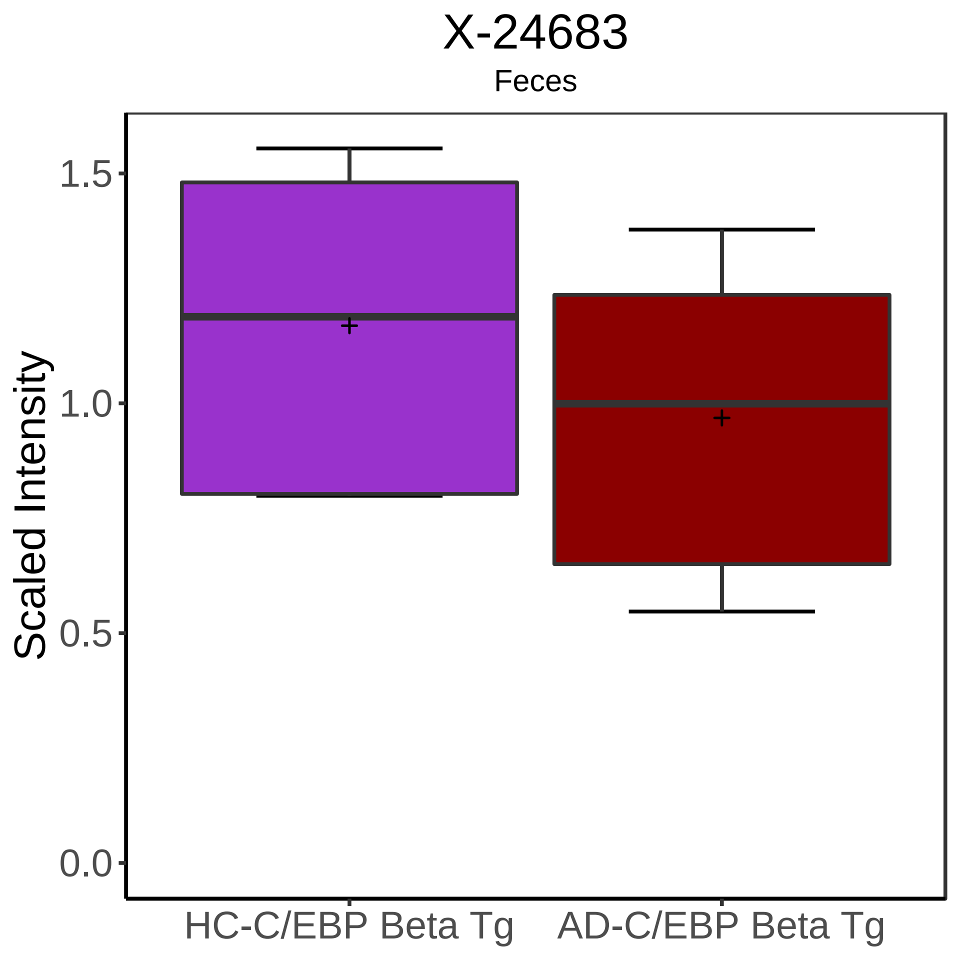

X-24683
Feces
Scaled Intensity
1.5
1.0
0.5
0.0
HC-C/EBP Beta Tg
AD-C/EBP Beta Tg

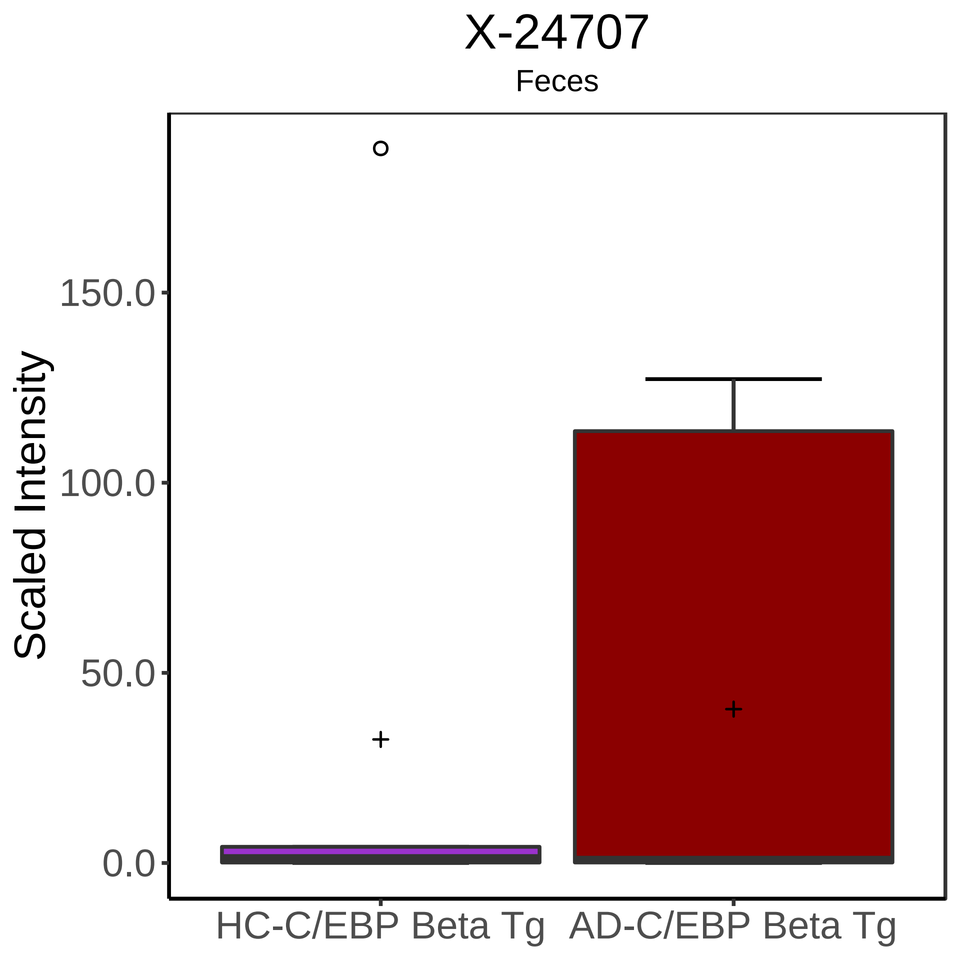

X-24707
Feces
Scaled Intensity
150.0
100.0
50.0
0.0
HC-C/EBP Beta Tg
AD-C/EBP Beta Tg

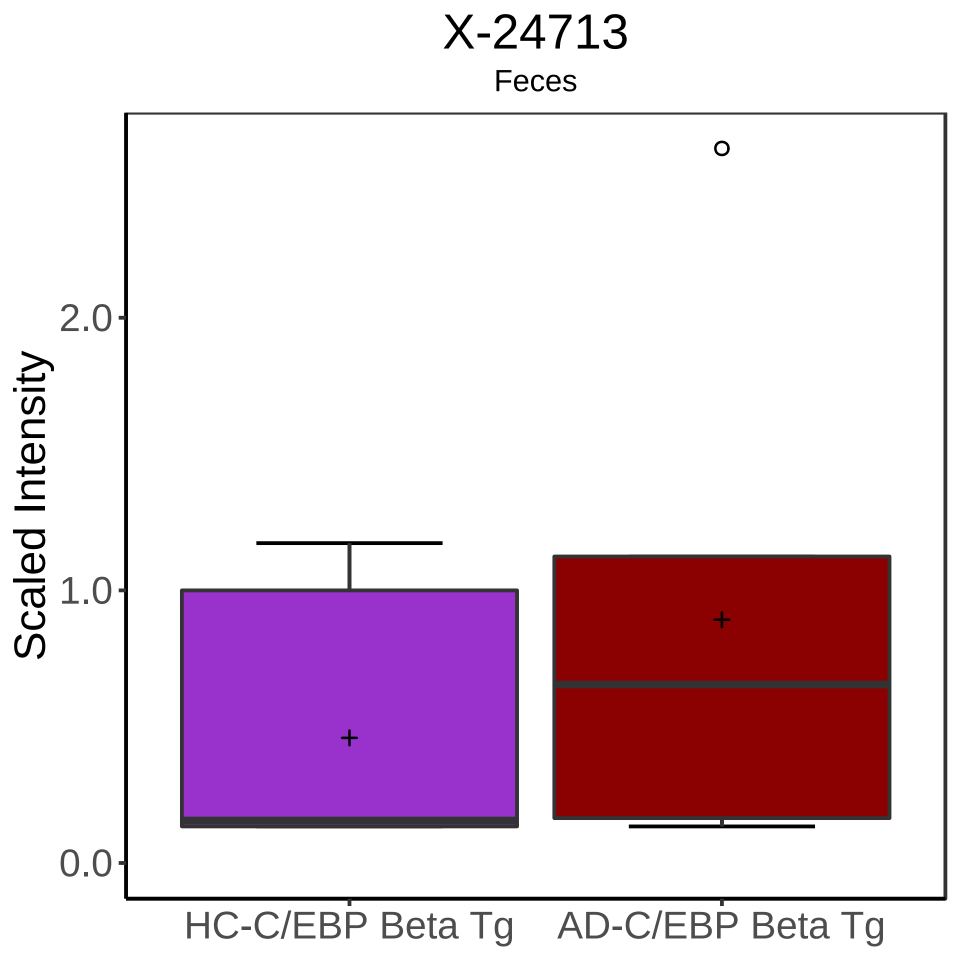
X-24713
Feces
Scaled Intensity
2.0
1.0
0.0
HC-C/EBP Beta Tg
AD-C/EBP Beta Tg

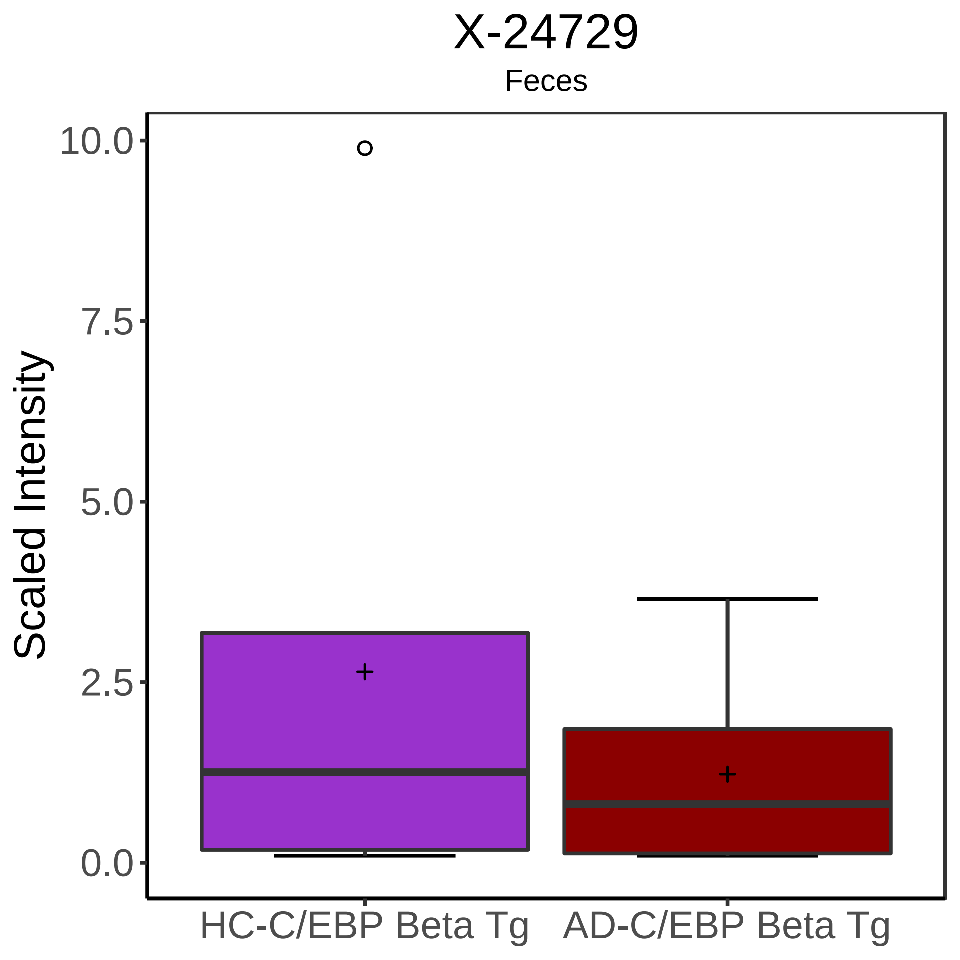

X-24729
Feces
Scaled Intensity
10.0
7.5
5.0
2.5
0.0
HC-C/EBP Beta Tg
AD-C/EBP Beta Tg

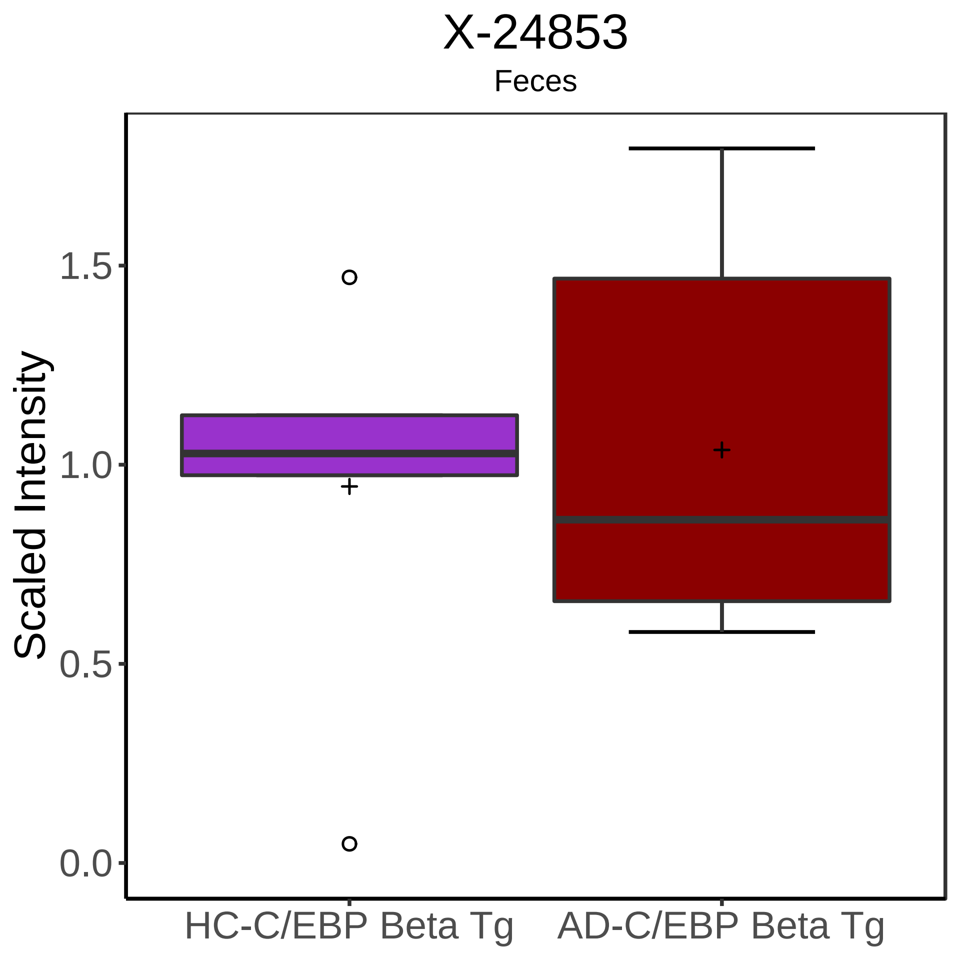

X-24853
Feces
Scaled Intensity
0.0
0.5
1.0
1.5
HC-C/EBP Beta Tg
AD-C/EBP Beta Tg

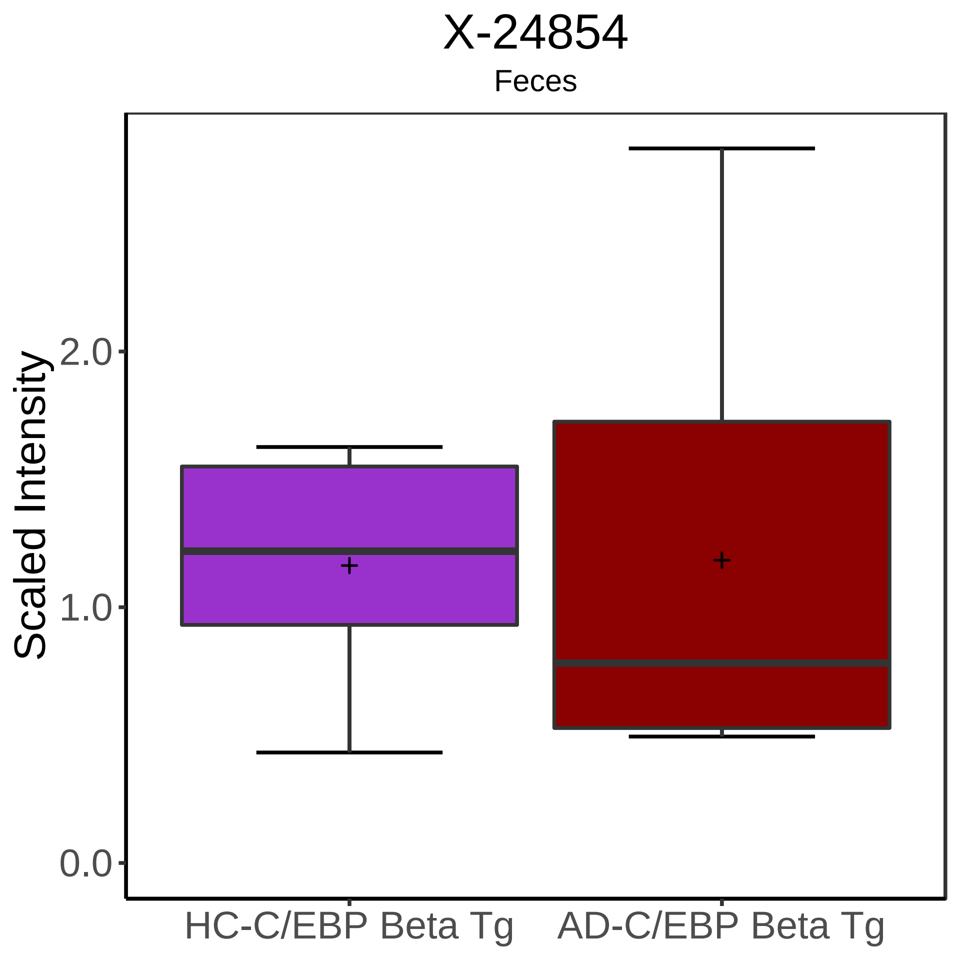
X-24854
Feces
Scaled Intensity
2.0
1.0
0.0
HC-C/EBP Beta Tg
AD-C/EBP Beta Tg

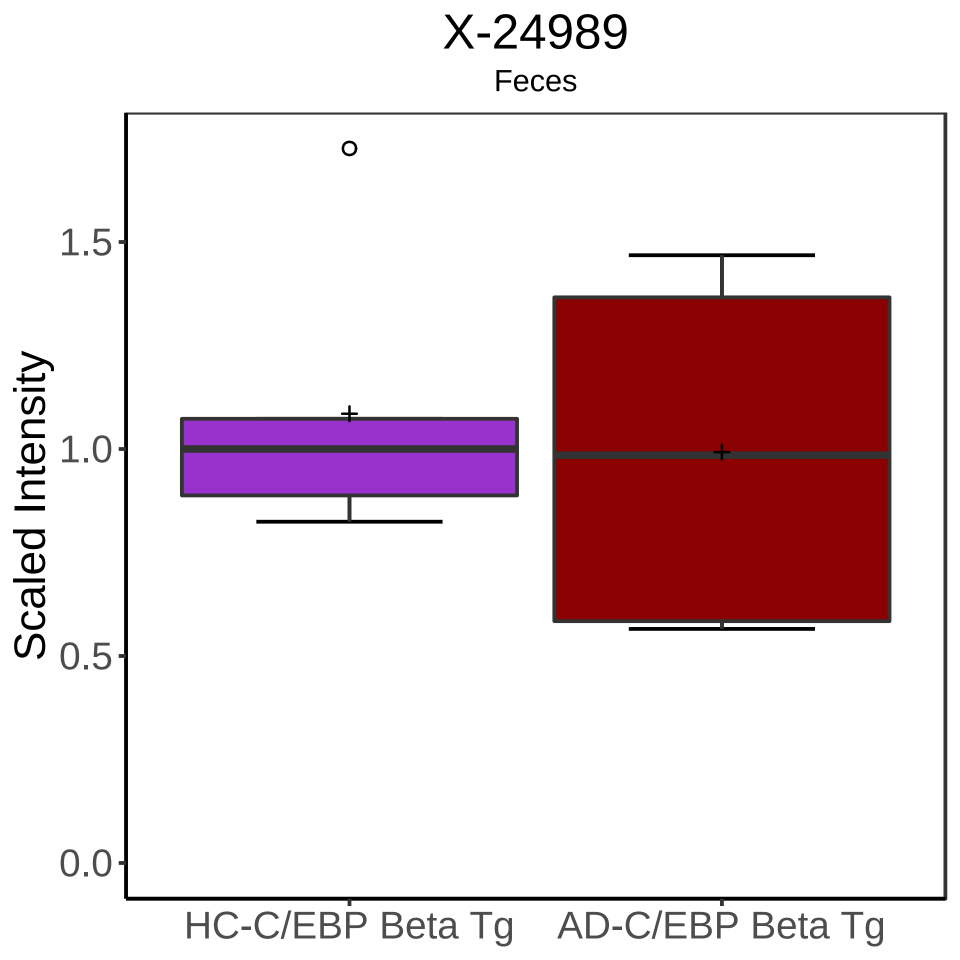

X-24989
Feces
Scaled Intensity
1.5
1.0
0.5
0.0
HC-C/EBP Beta Tg
AD-C/EBP Beta Tg

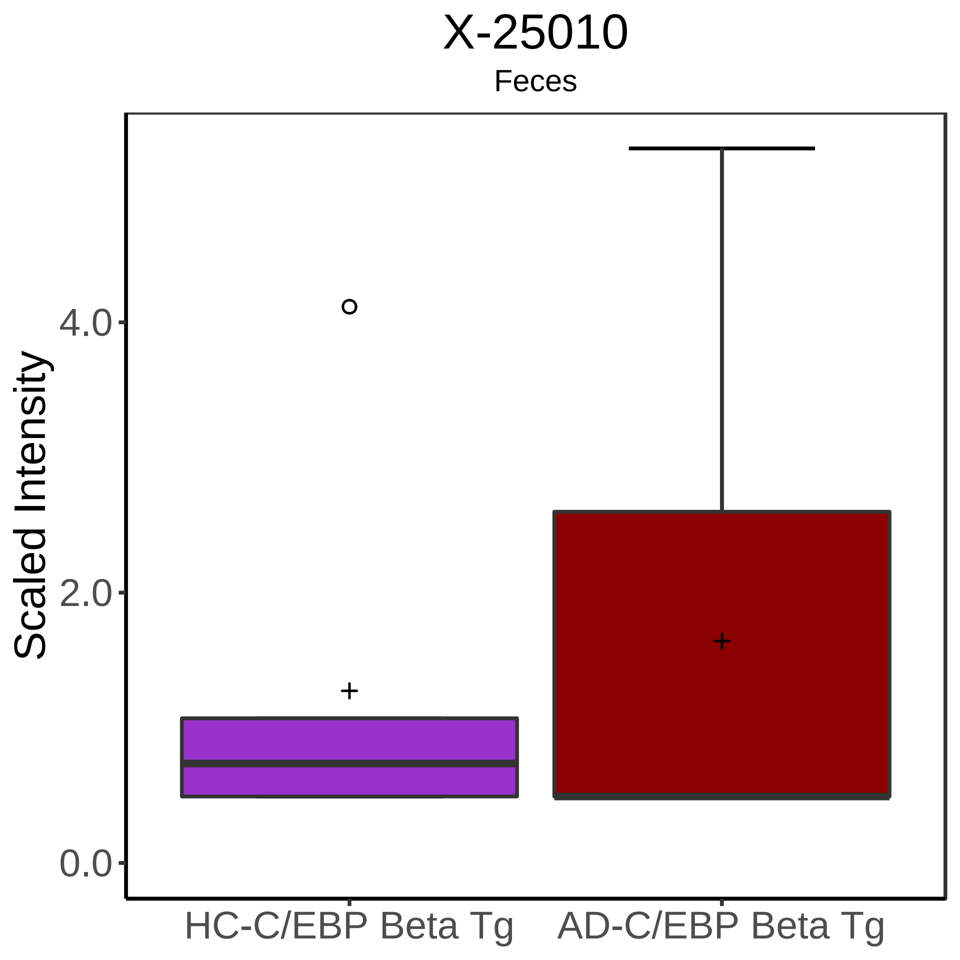
X-25010
Feces
Scaled Intensity
4.0
2.0
0.0
HC-C/EBP Beta Tg
AD-C/EBP Beta Tg

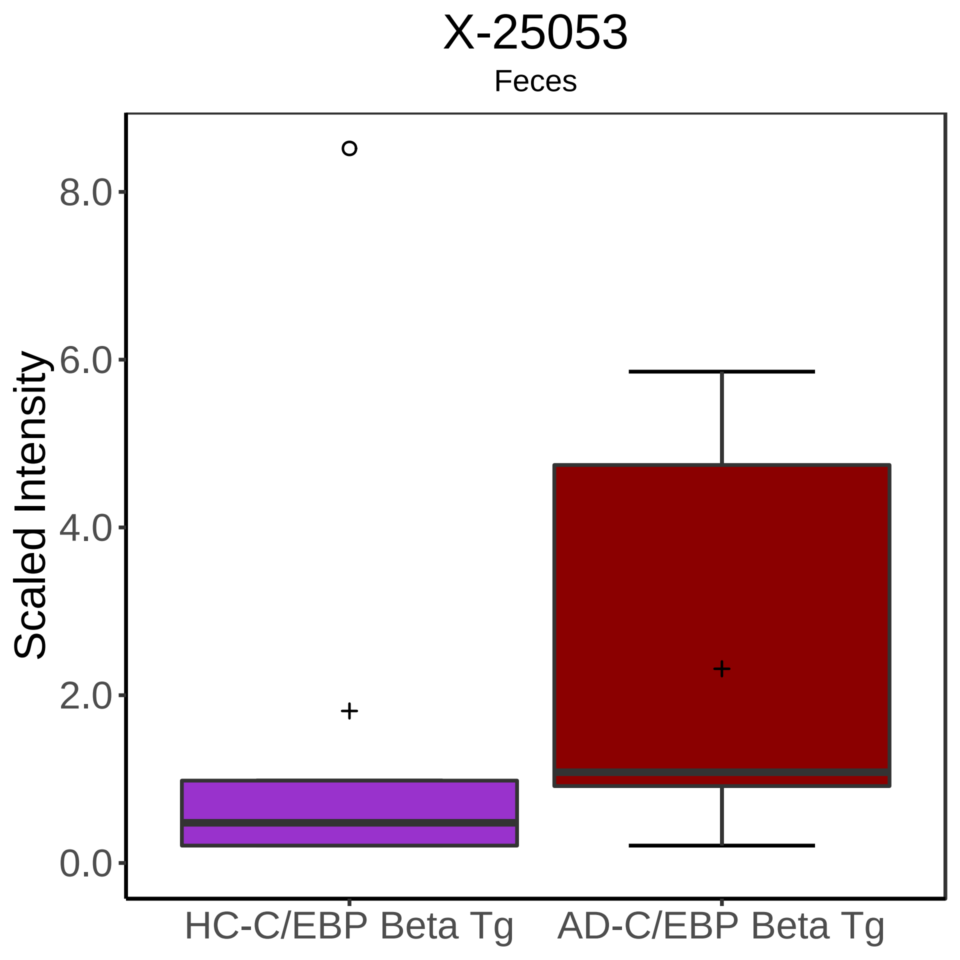

X-25053
Feces
Scaled Intensity
8.0
6.0
4.0
2.0
0.0
HC-C/EBP Beta Tg
AD-C/EBP Beta Tg

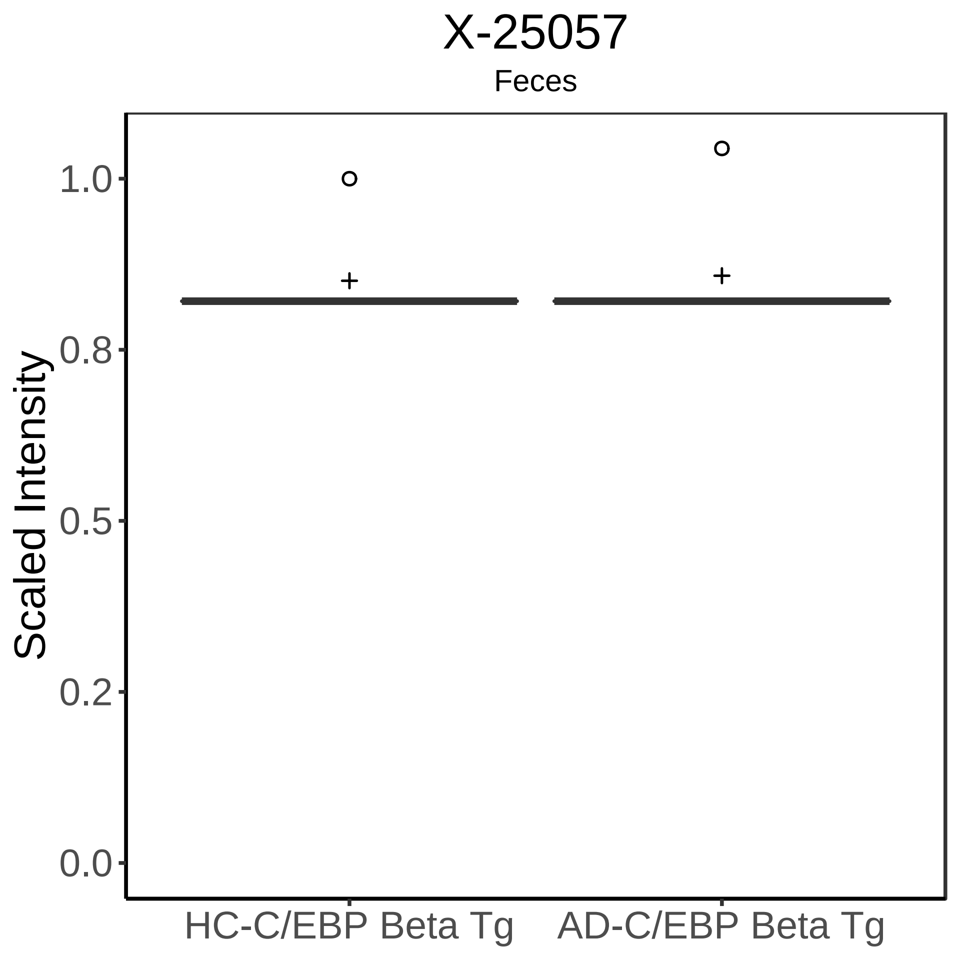

X-25057
Feces
Scaled Intensity
1.0
0.8
0.5
0.2
0.0
HC-C/EBP Beta Tg
AD-C/EBP Beta Tg

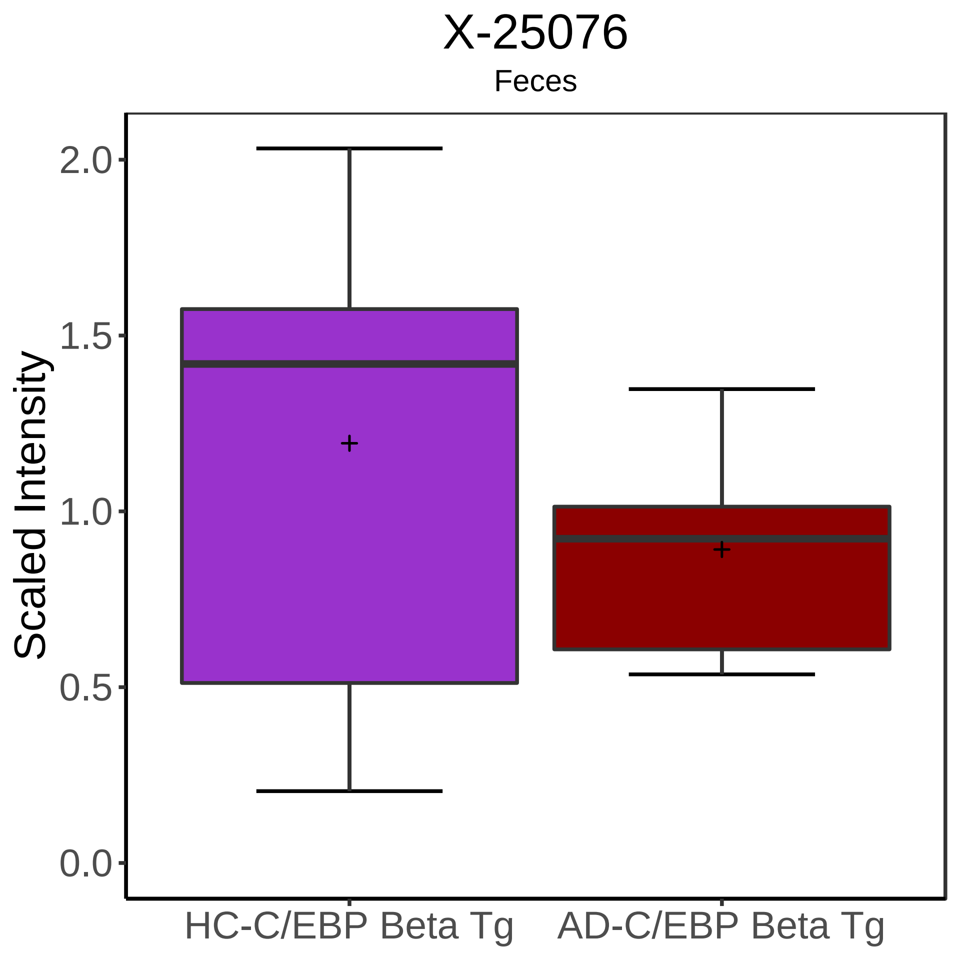
X-25076
Feces
Scaled Intensity
2.0
1.5
1.0
0.5
0.0
HC-C/EBP Beta Tg
AD-C/EBP Beta Tg

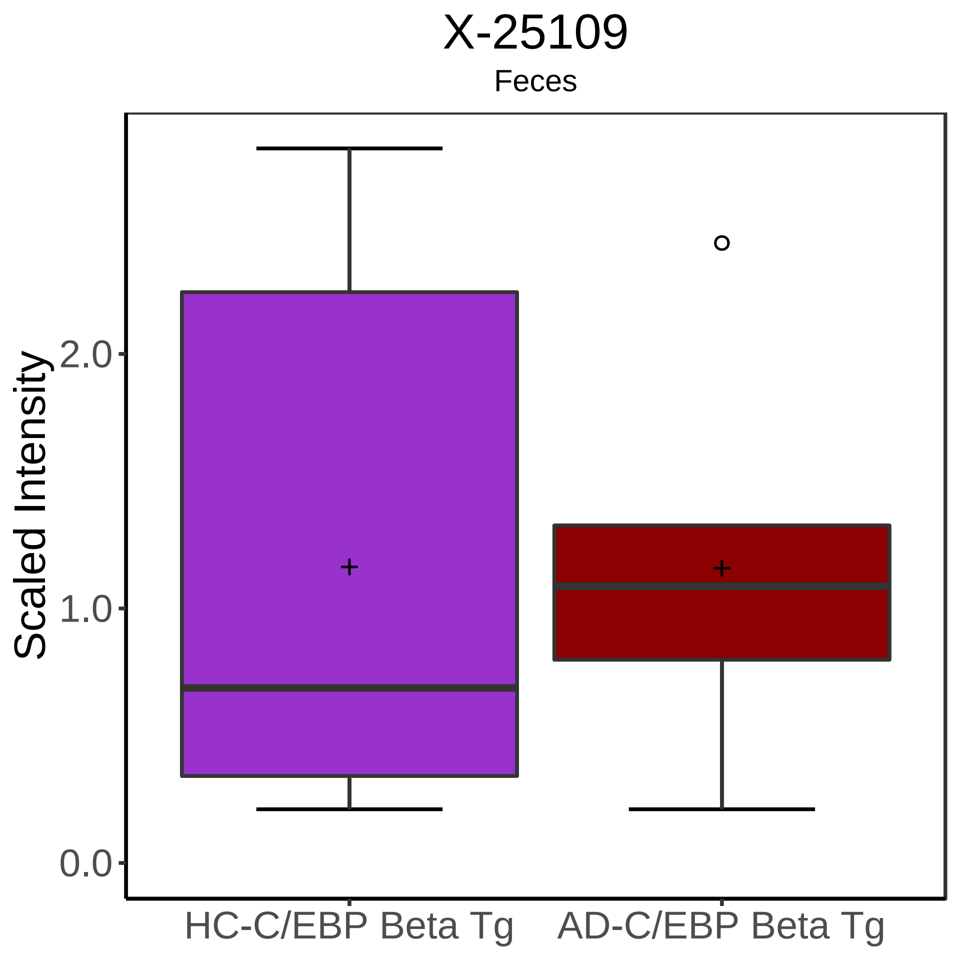
X-25109
Feces
Scaled Intensity
2.0
1.0
0.0
HC-C/EBP Beta Tg
AD-C/EBP Beta Tg

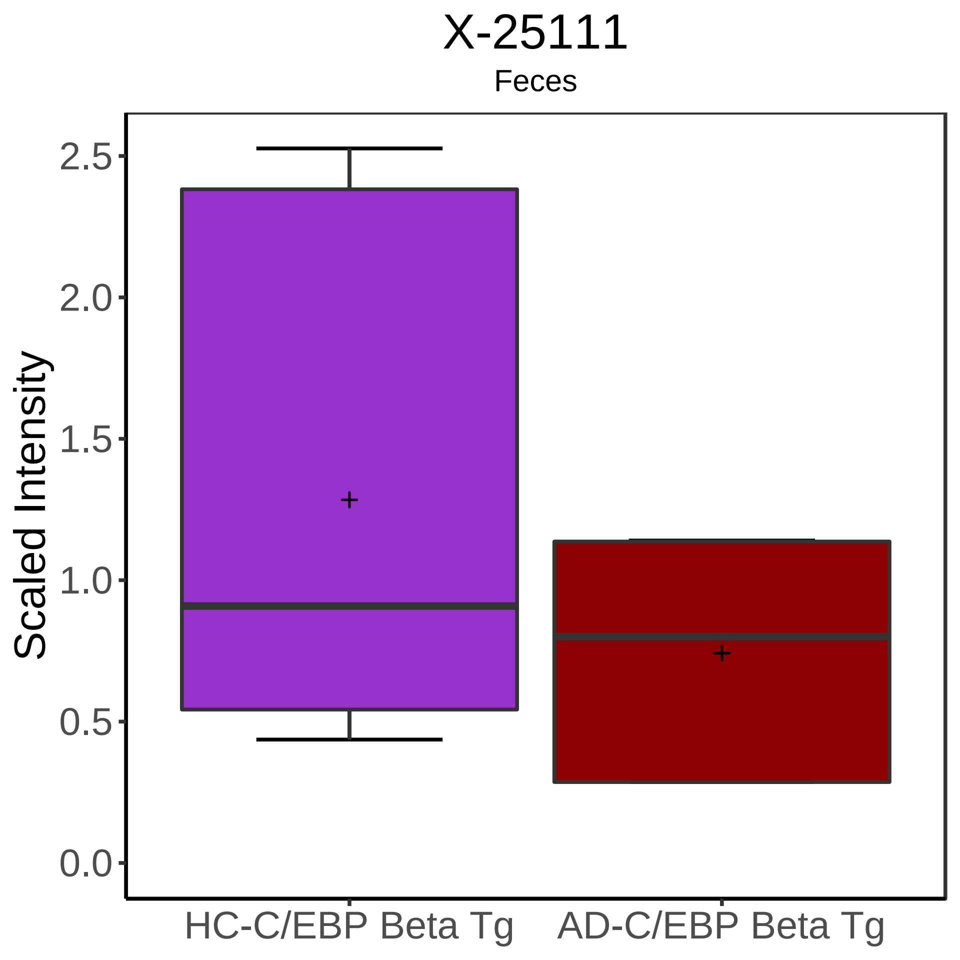
X-25111
Feces
Scaled Intensity
2.5
2.0
1.5
1.0
0.5
0.0
HC-C/EBP Beta Tg
AD-C/EBP Beta Tg

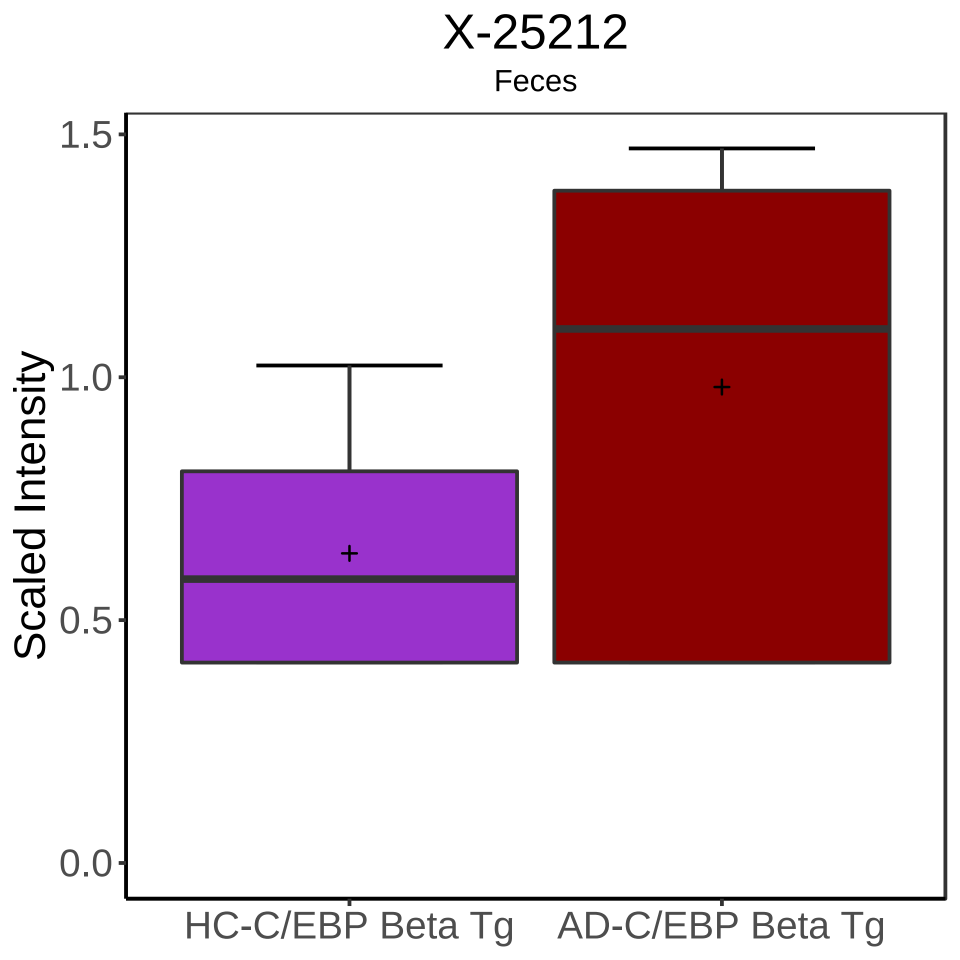
X-25212
Feces
Scaled Intensity
1.5
1.0
0.5
0.0
HC-C/EBP Beta Tg
AD-C/EBP Beta Tg

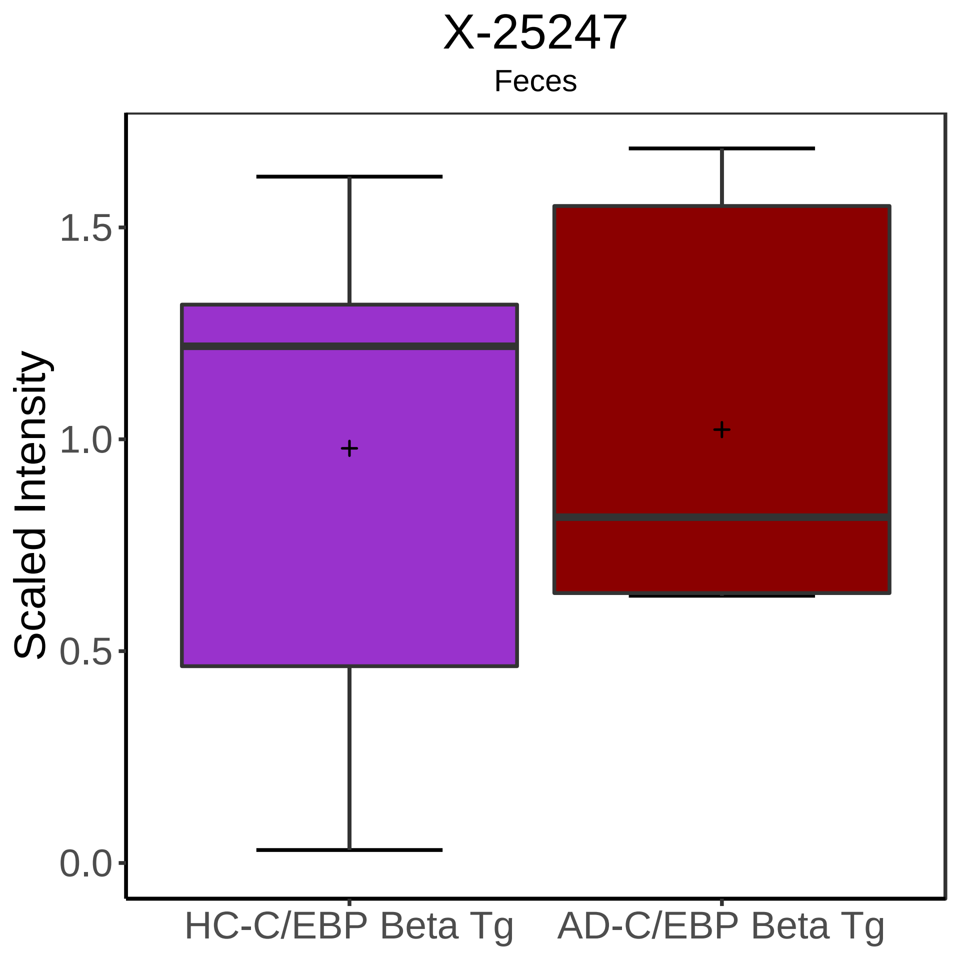

X-25247
Feces
Scaled Intensity
1.5
1.0
0.5
0.0
HC-C/EBP Beta Tg
AD-C/EBP Beta Tg

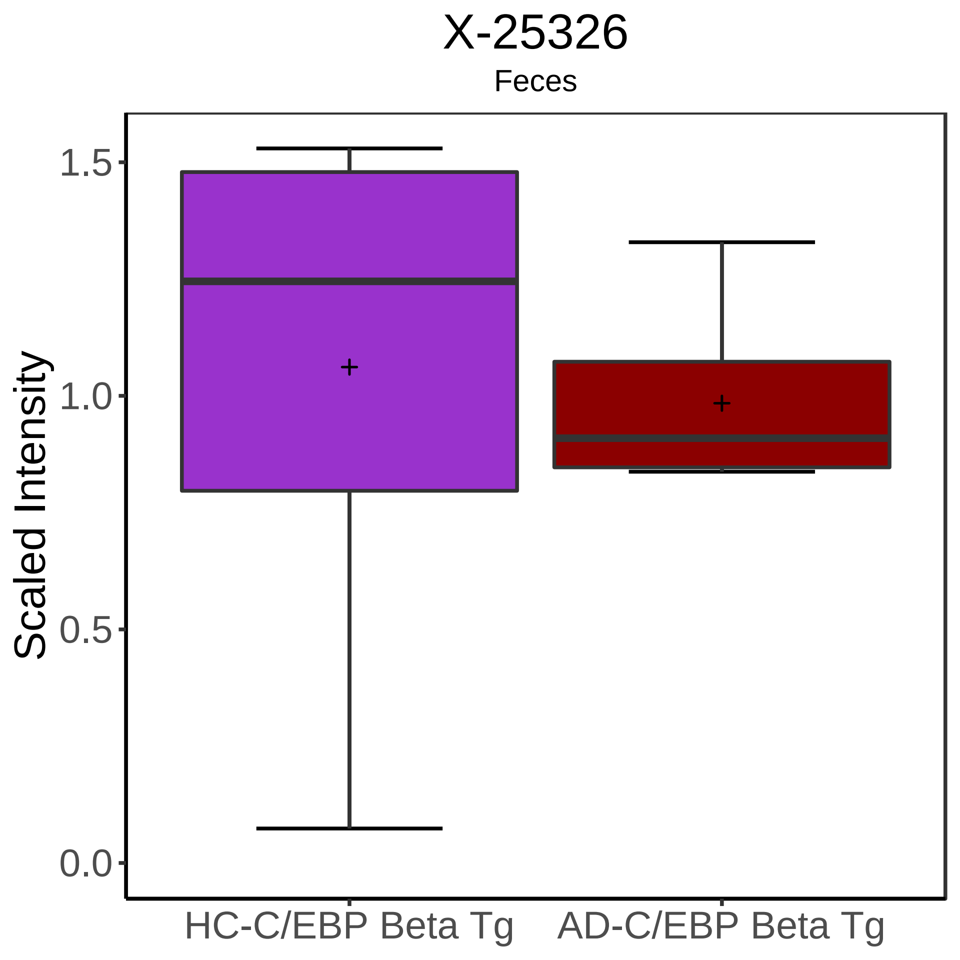

X-25326
Feces
Scaled Intensity
1.5
1.0
0.5
0.0
HC-C/EBP Beta Tg
AD-C/EBP Beta Tg

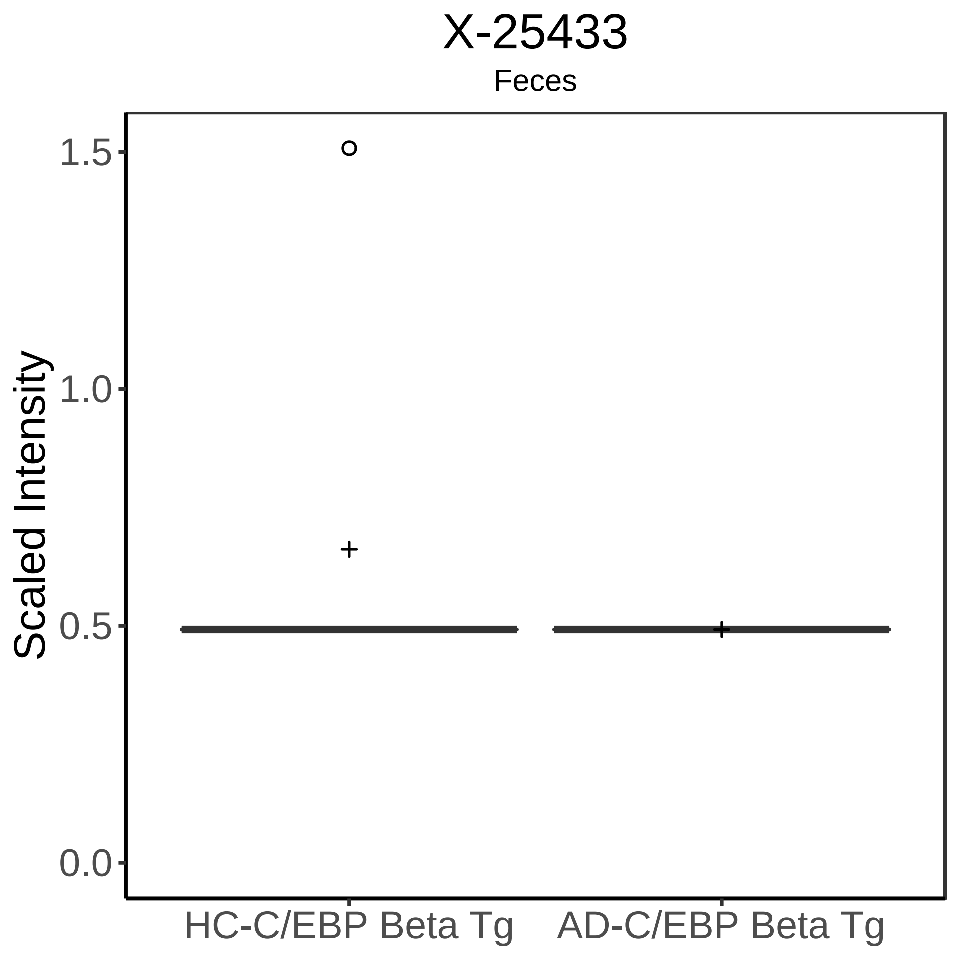
X-25433
Feces
Scaled Intensity
1.5
1.0
0.5
0.0
HC-C/EBP Beta Tg
AD-C/EBP Beta Tg

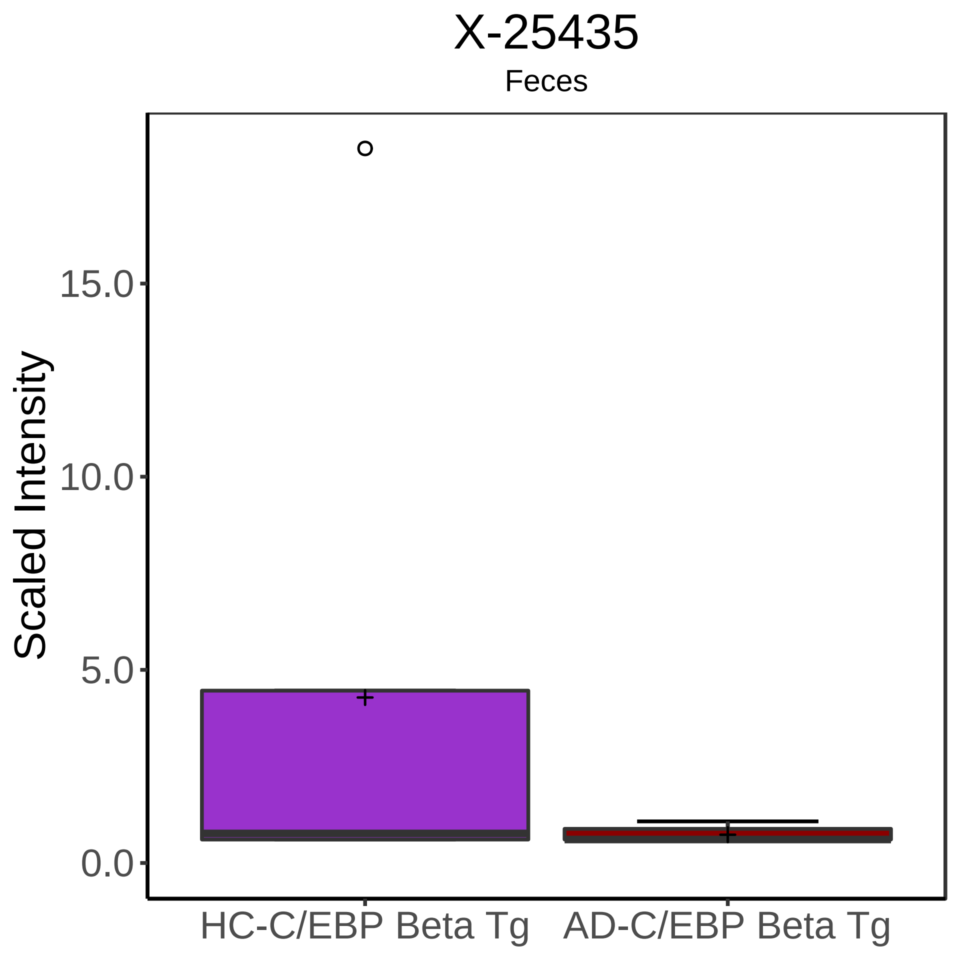
X-25435
Feces
Scaled Intensity
15.0
10.0
5.0
0.0
HC-C/EBP Beta Tg
AD-C/EBP Beta Tg

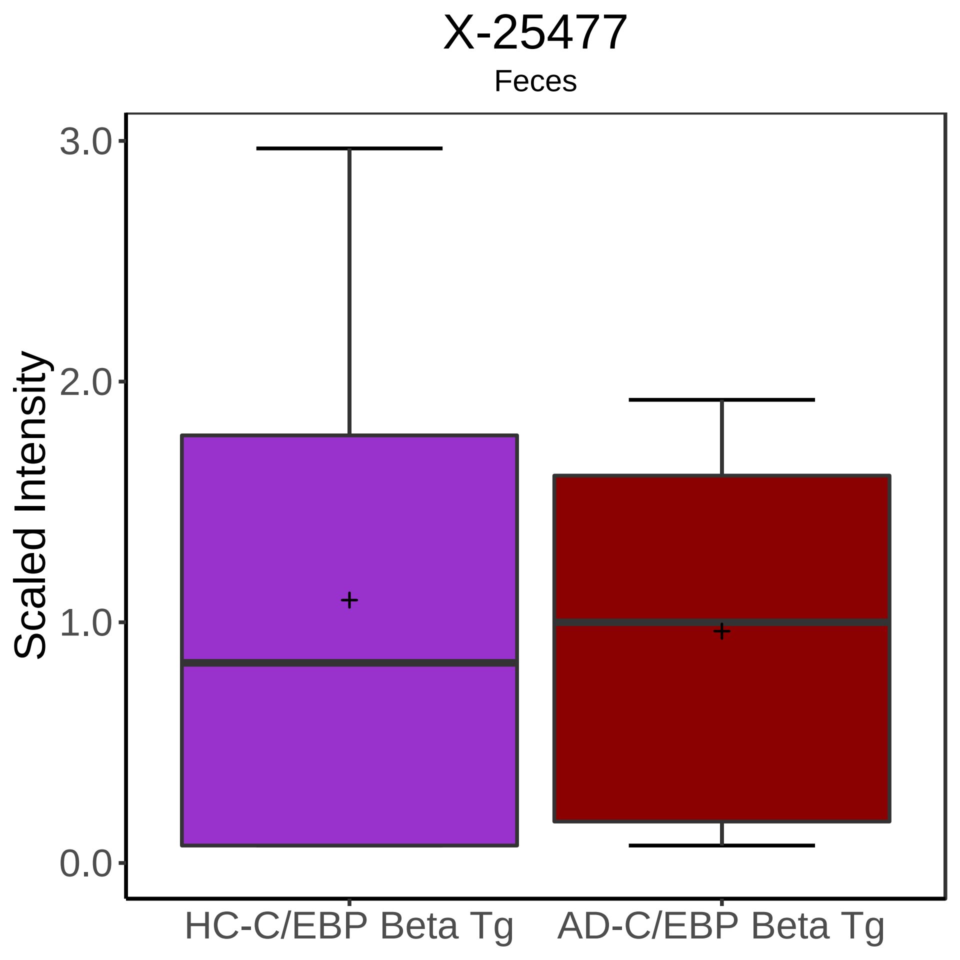
X-25477
Feces
Scaled Intensity
3.0
2.0
1.0
0.0
HC-C/EBP Beta Tg
AD-C/EBP Beta Tg

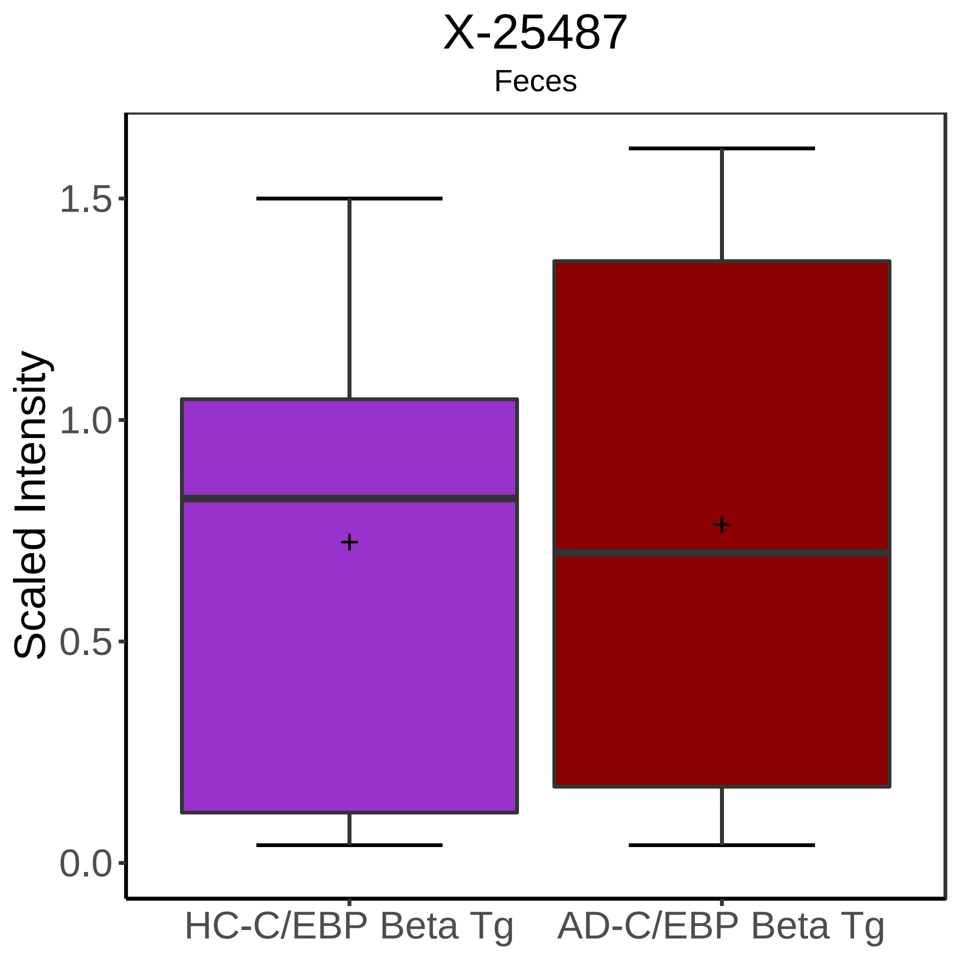
X-25487
Feces
Scaled Intensity
1.5
1.0
0.5
0.0
HC-C/EBP Beta Tg
AD-C/EBP Beta Tg

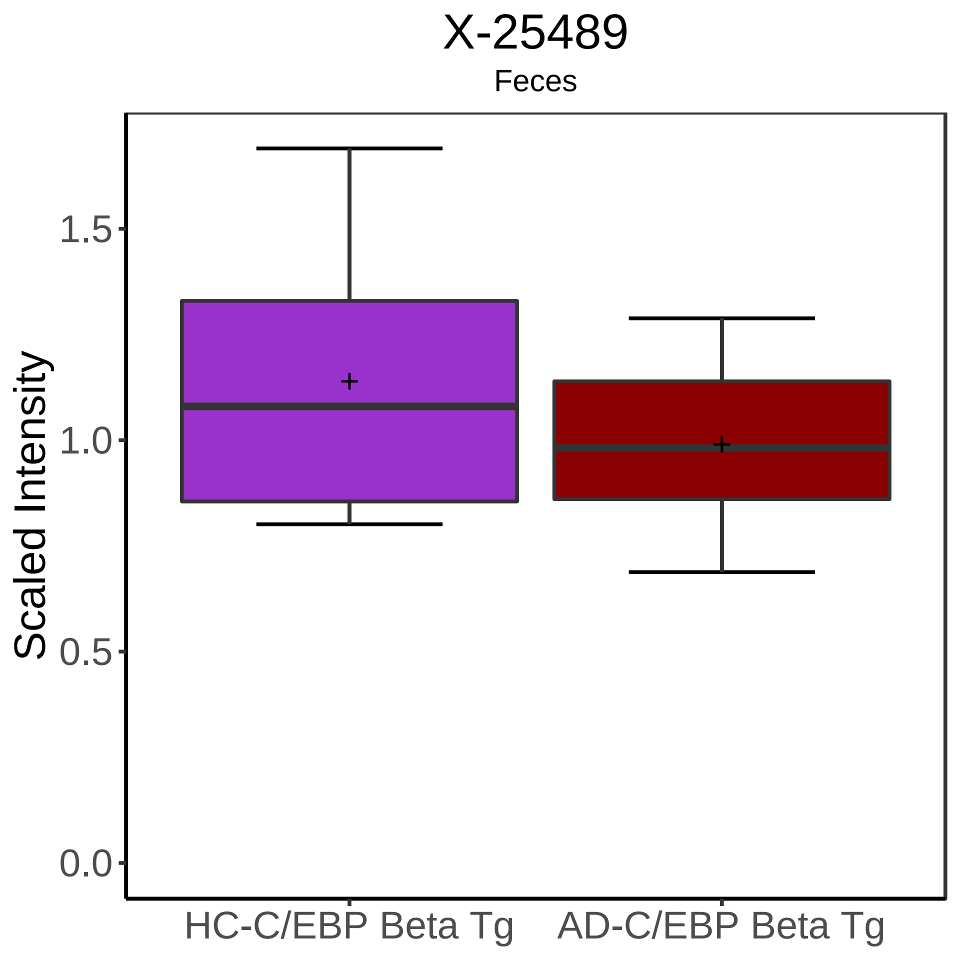
X-25489
Feces
Scaled Intensity
1.5
1.0
0.5
0.0
HC-C/EBP Beta Tg
AD-C/EBP Beta Tg

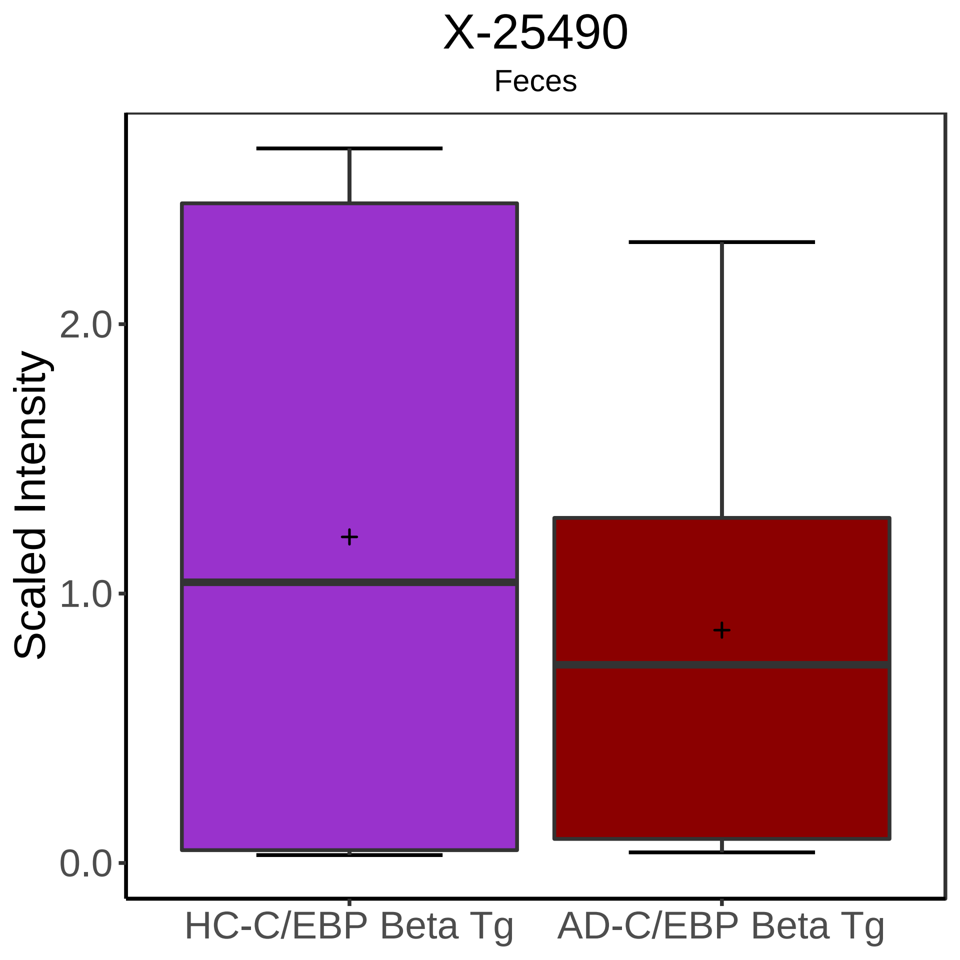

X-25490
Feces
Scaled Intensity
2.0
1.0
0.0
HC-C/EBP Beta Tg
AD-C/EBP Beta Tg

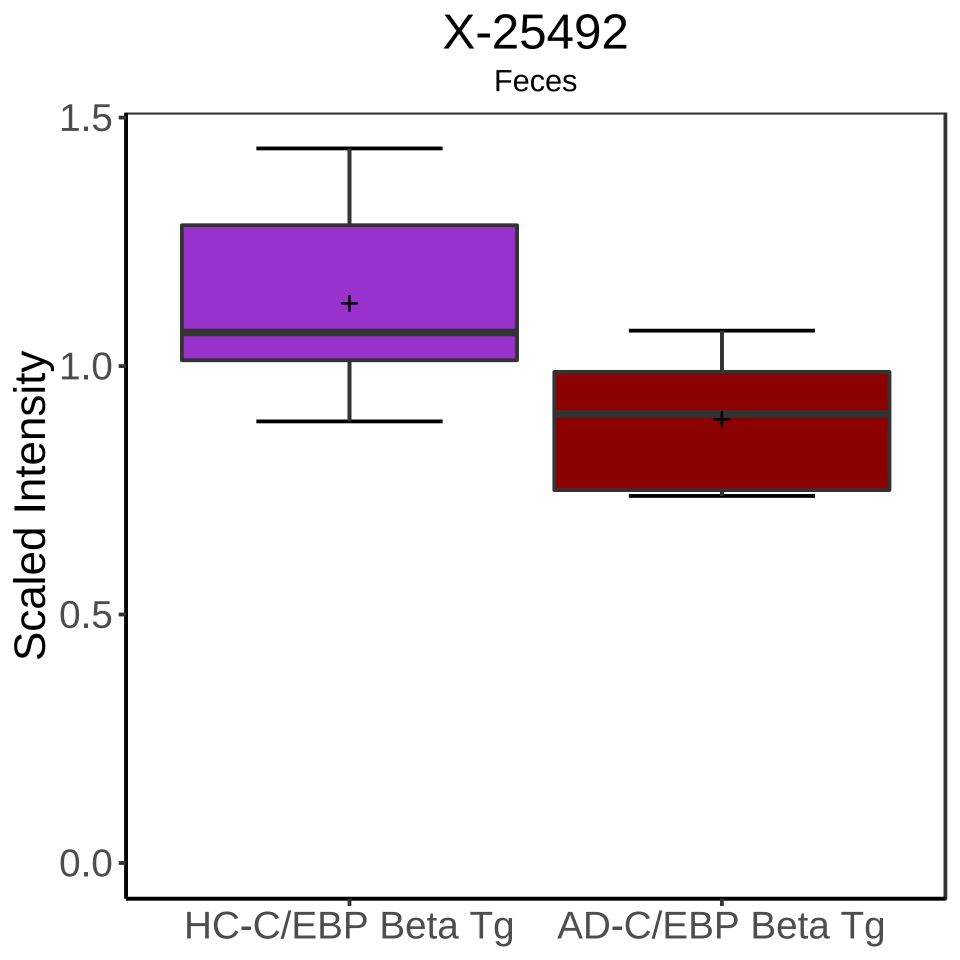
X-25492
Feces
Scaled Intensity
1.5
1.0
0.5
0.0
HC-C/EBP Beta Tg
AD-C/EBP Beta Tg

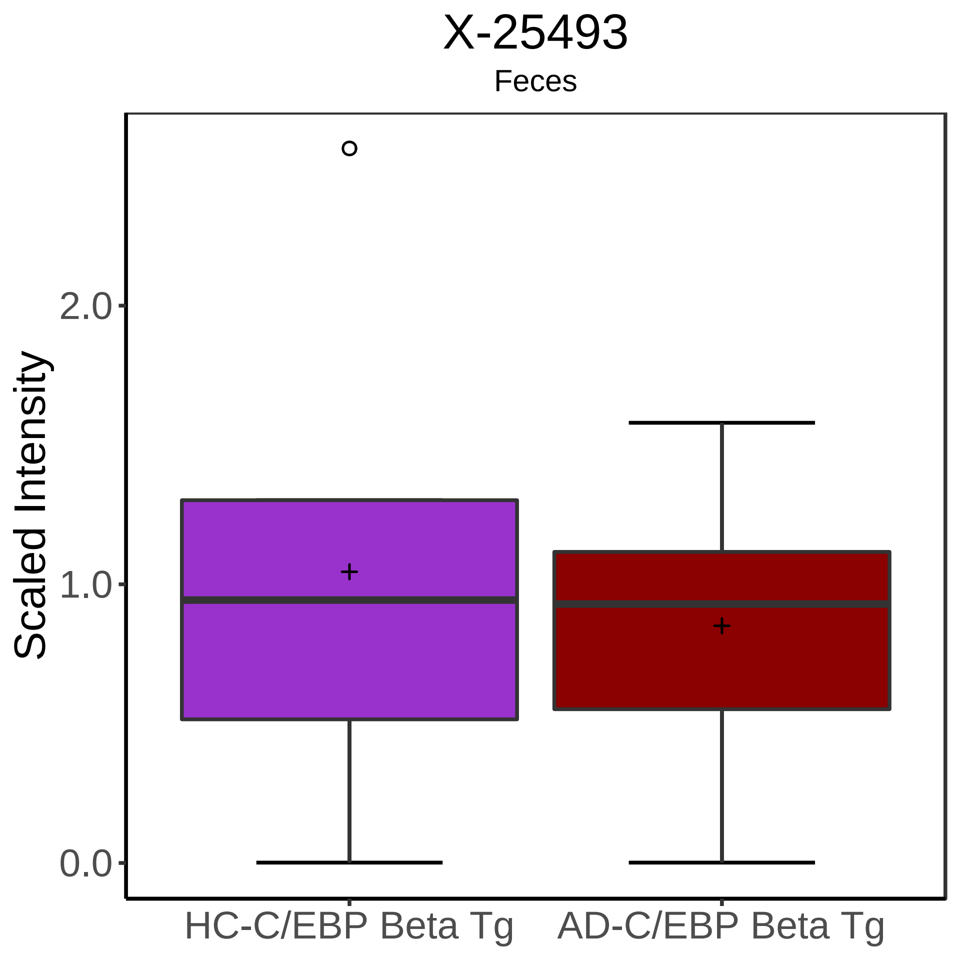
X-25493
Feces
Scaled Intensity
2.0
1.0
0.0
HC-C/EBP Beta Tg
AD-C/EBP Beta Tg

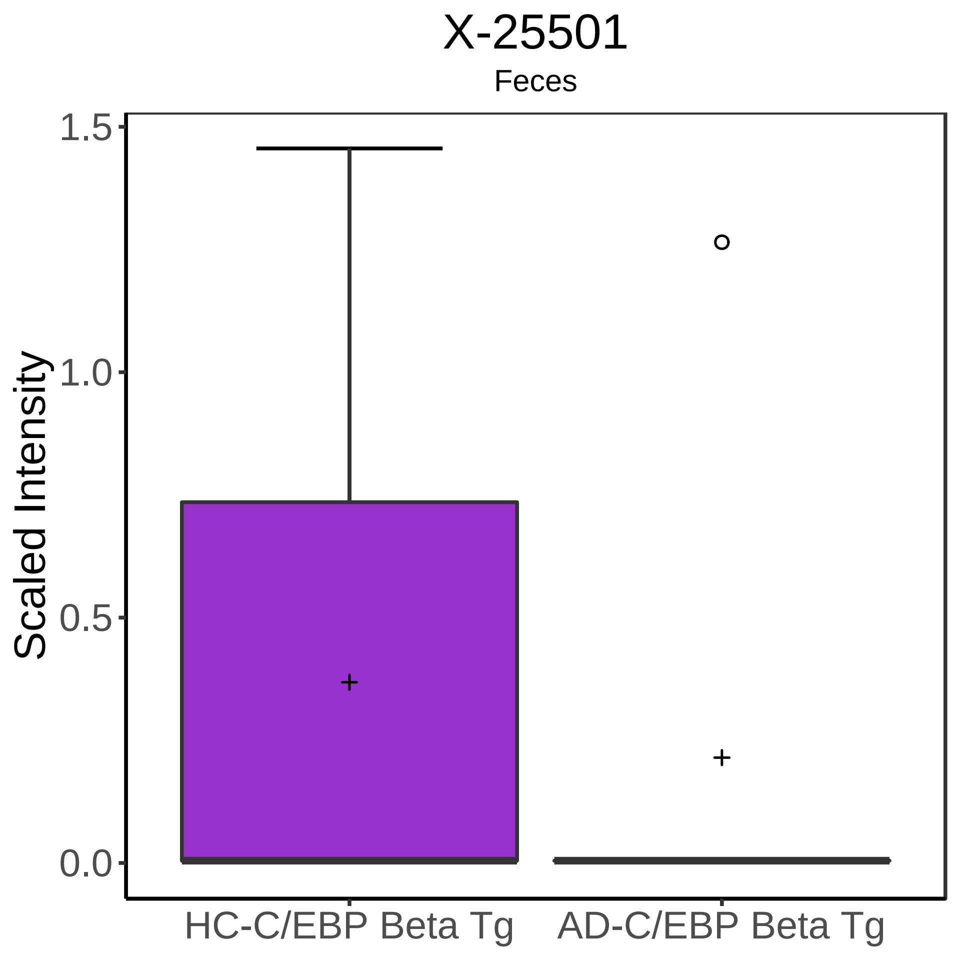
X-25501
Feces
Scaled Intensity
1.5
1.0
0.5
0.0
HC-C/EBP Beta Tg
AD-C/EBP Beta Tg

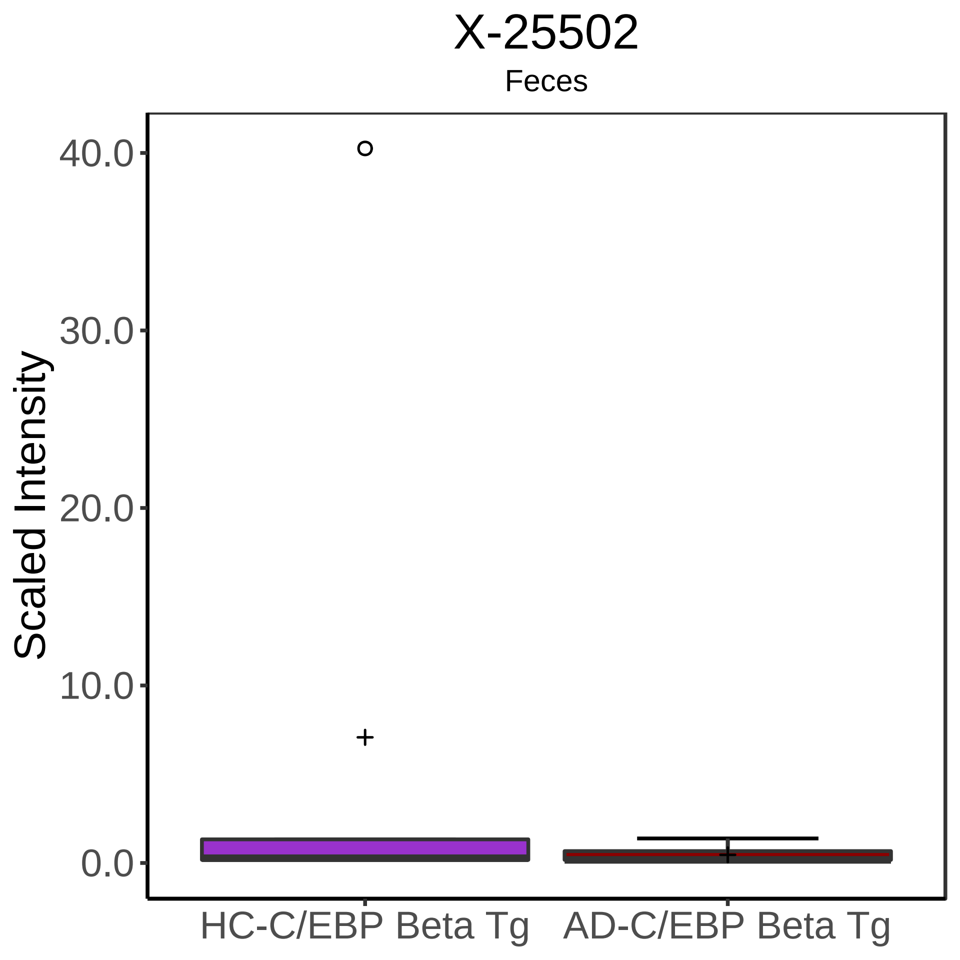

X-25502
Feces
Scaled Intensity
40.0
30.0
20.0
10.0
0.0
HC-C/EBP Beta Tg
AD-C/EBP Beta Tg

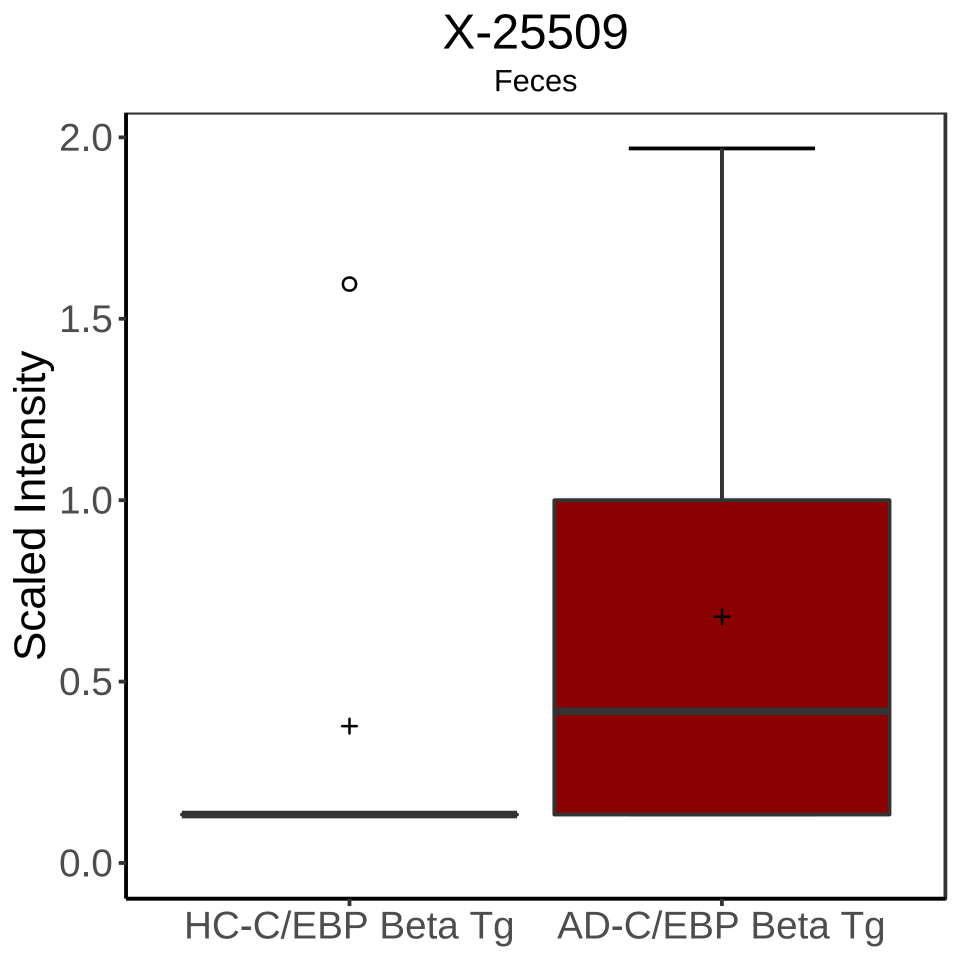

X-25509
Feces
Scaled Intensity
2.0
1.5
1.0
0.5
0.0
HC-C/EBP Beta Tg
AD-C/EBP Beta Tg

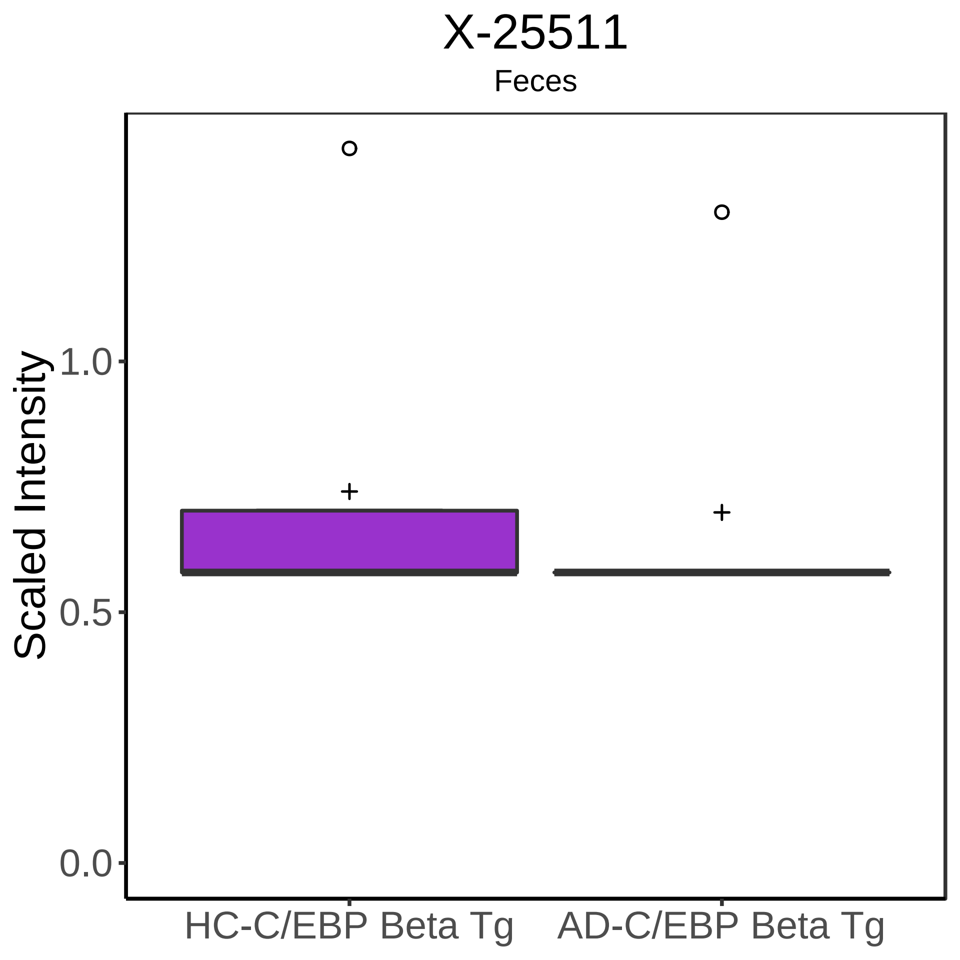
X-25511
Feces
Scaled Intensity
1.0
0.5
0.0
HC-C/EBP Beta Tg
AD-C/EBP Beta Tg

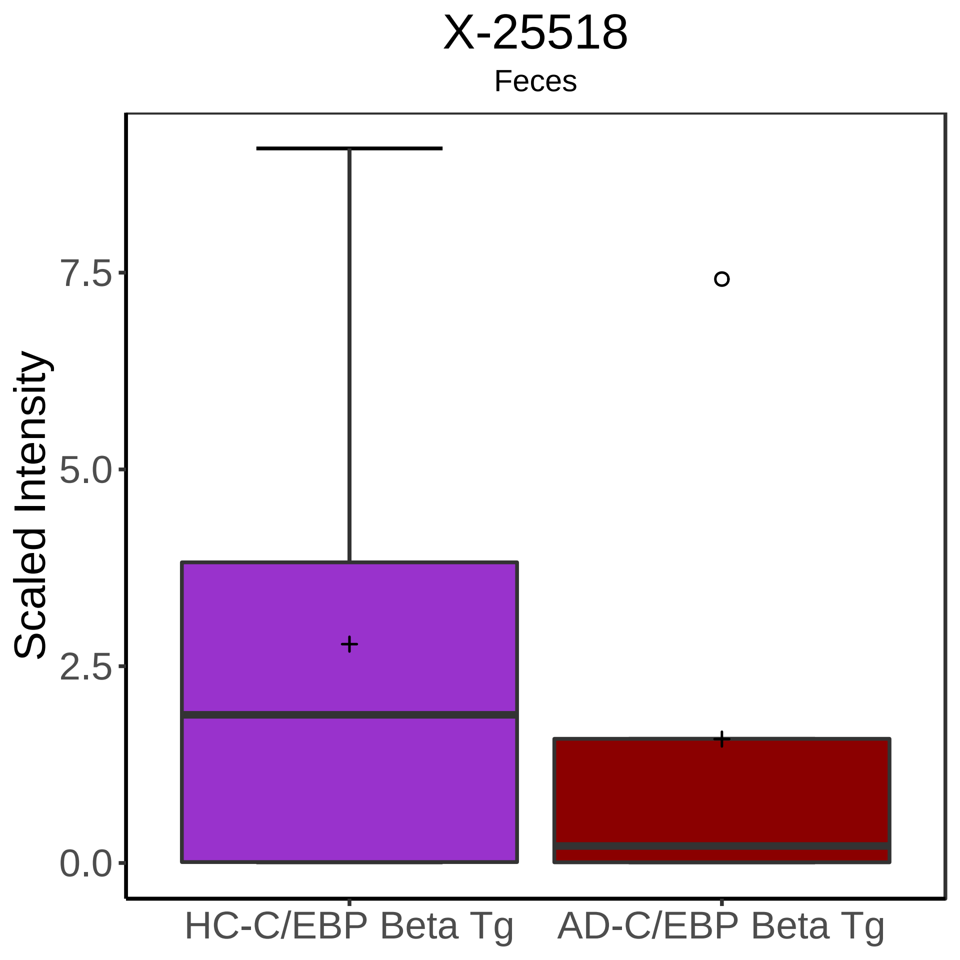

X-25518
Feces
Scaled Intensity
7.5
5.0
2.5
0.0
HC-C/EBP Beta Tg
AD-C/EBP Beta Tg

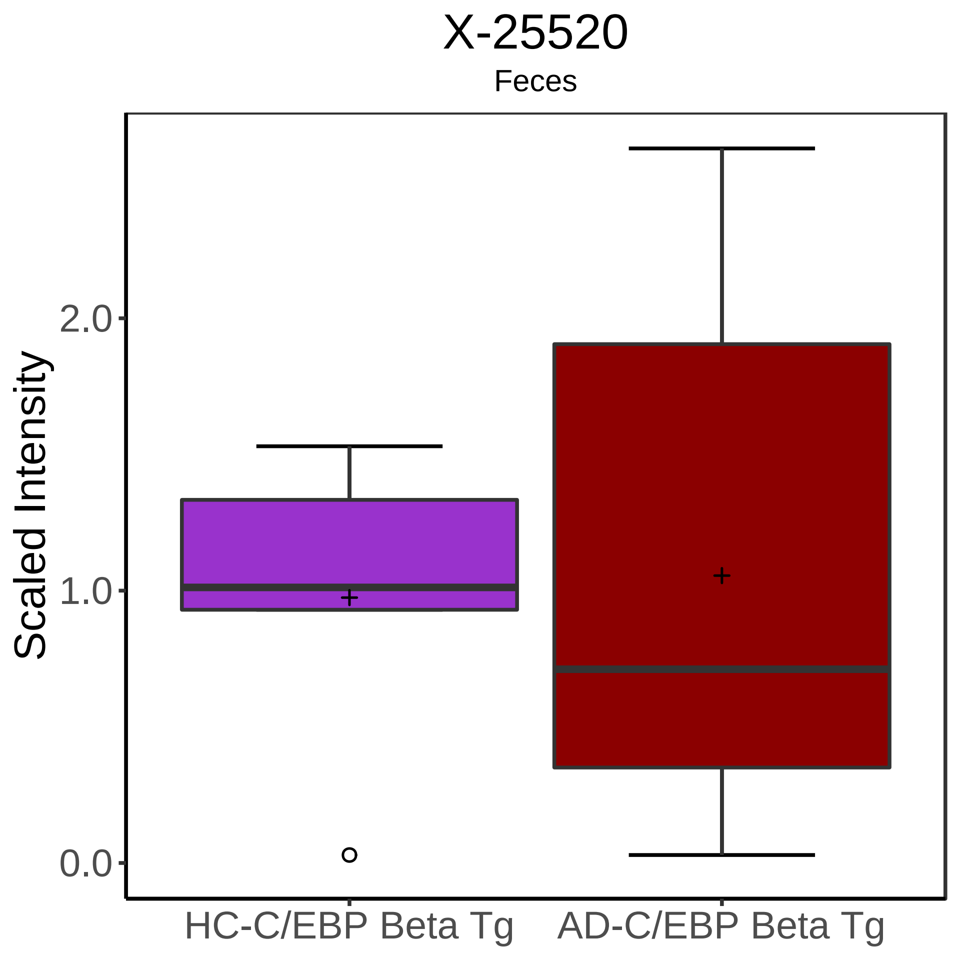
X-25520
Feces
Scaled Intensity
2.0
1.0
0.0
HC-C/EBP Beta Tg
AD-C/EBP Beta Tg

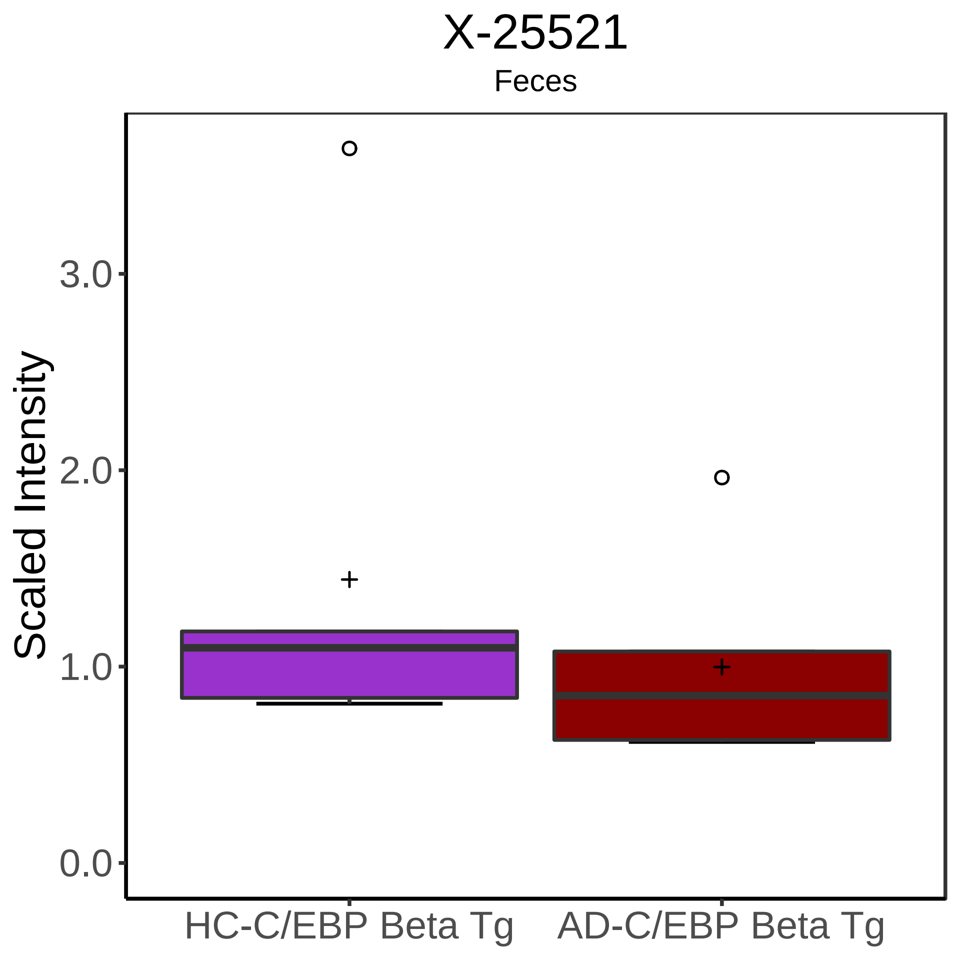

X-25521
Feces
Scaled Intensity
3.0
2.0
1.0
0.0
HC-C/EBP Beta Tg
AD-C/EBP Beta Tg

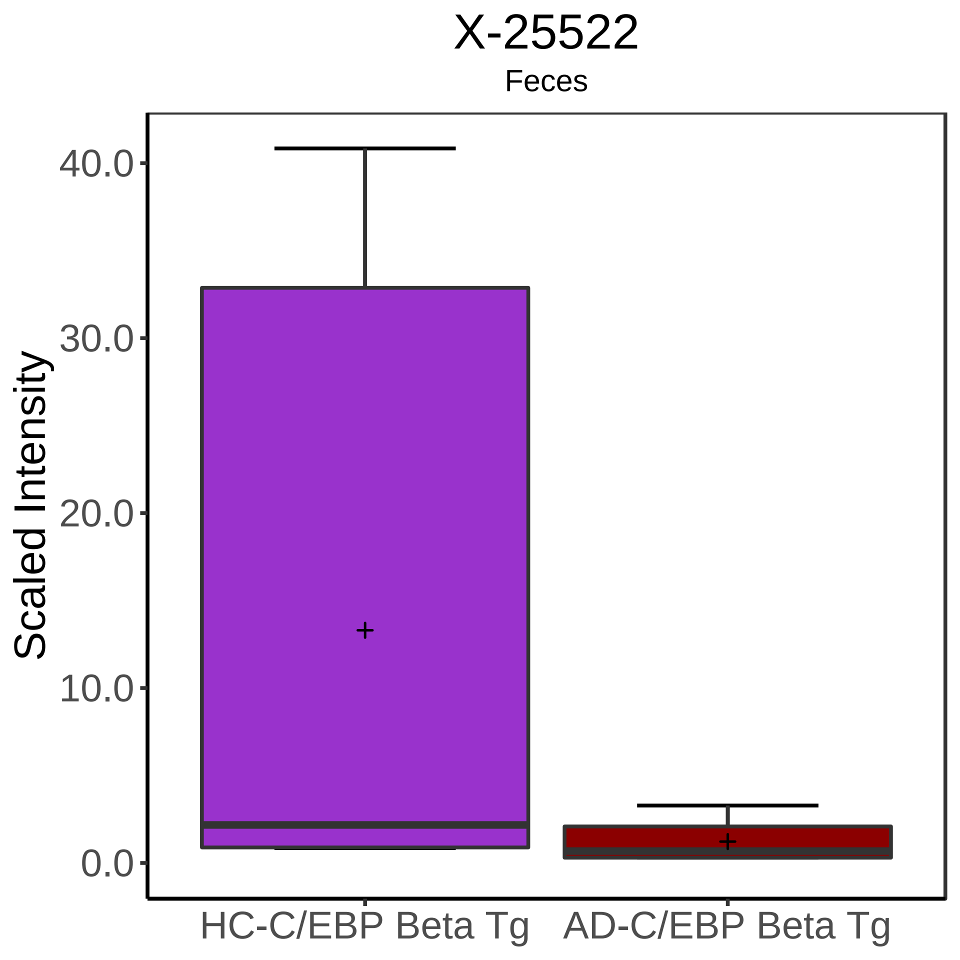
X-25522
Feces
Scaled Intensity
40.0
30.0
20.0
10.0
0.0
HC-C/EBP Beta Tg
AD-C/EBP Beta Tg

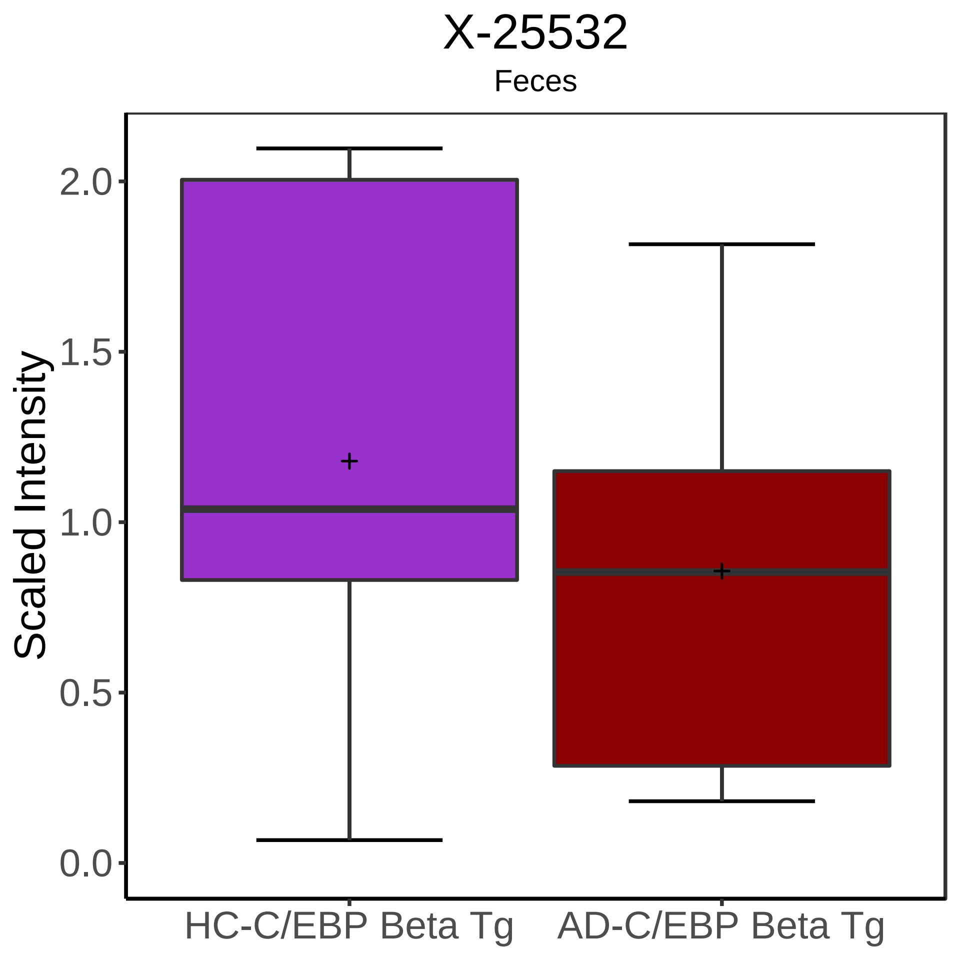
X-25532
Feces
Scaled Intensity
2.0
1.5
1.0
0.5
0.0
HC-C/EBP Beta Tg
AD-C/EBP Beta Tg

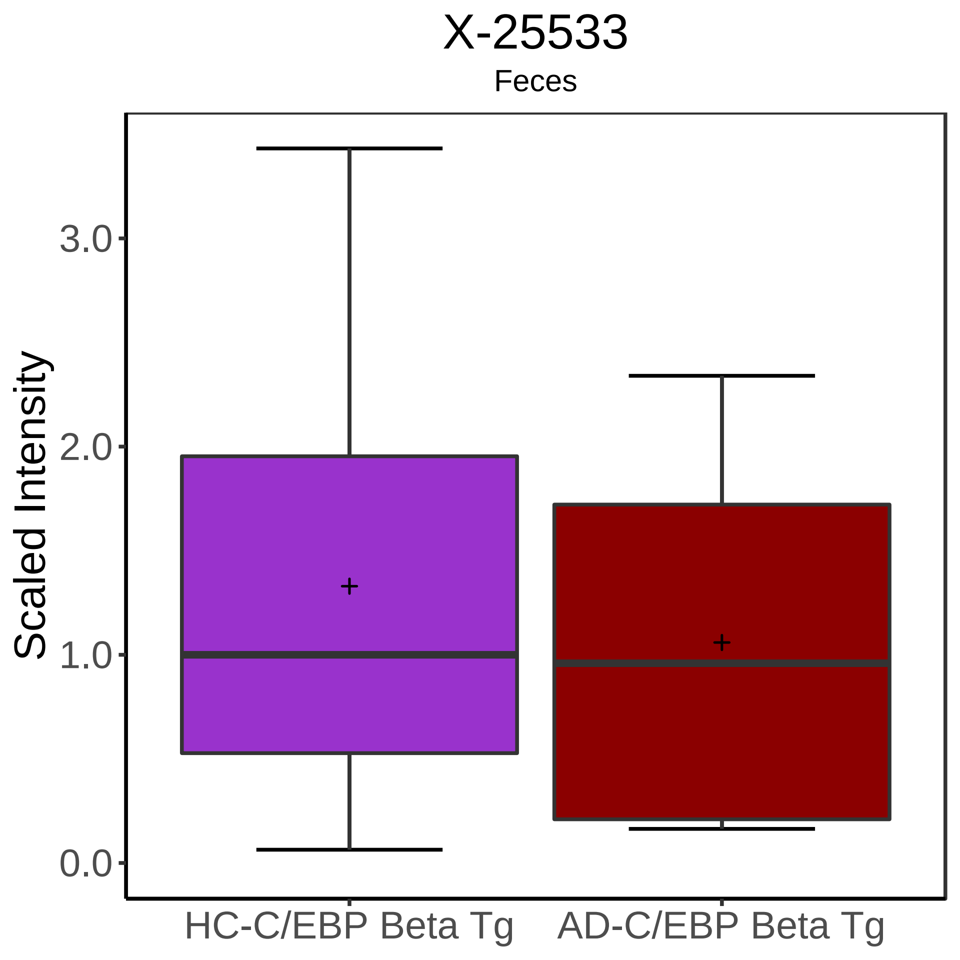
X-25533
Feces
Scaled Intensity
3.0
2.0
1.0
0.0
HC-C/EBP Beta Tg
AD-C/EBP Beta Tg

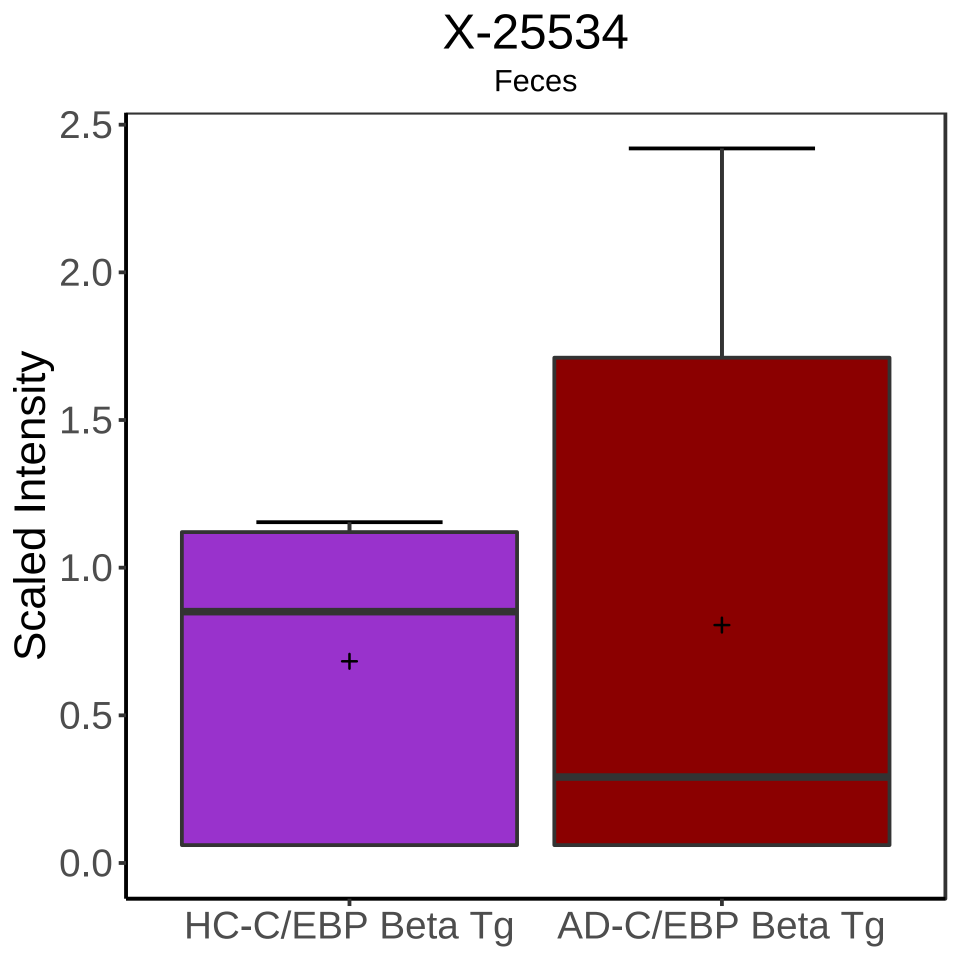
X-25534
Feces
Scaled Intensity
2.5
2.0
1.5
1.0
0.5
0.0
HC-C/EBP Beta Tg
AD-C/EBP Beta Tg

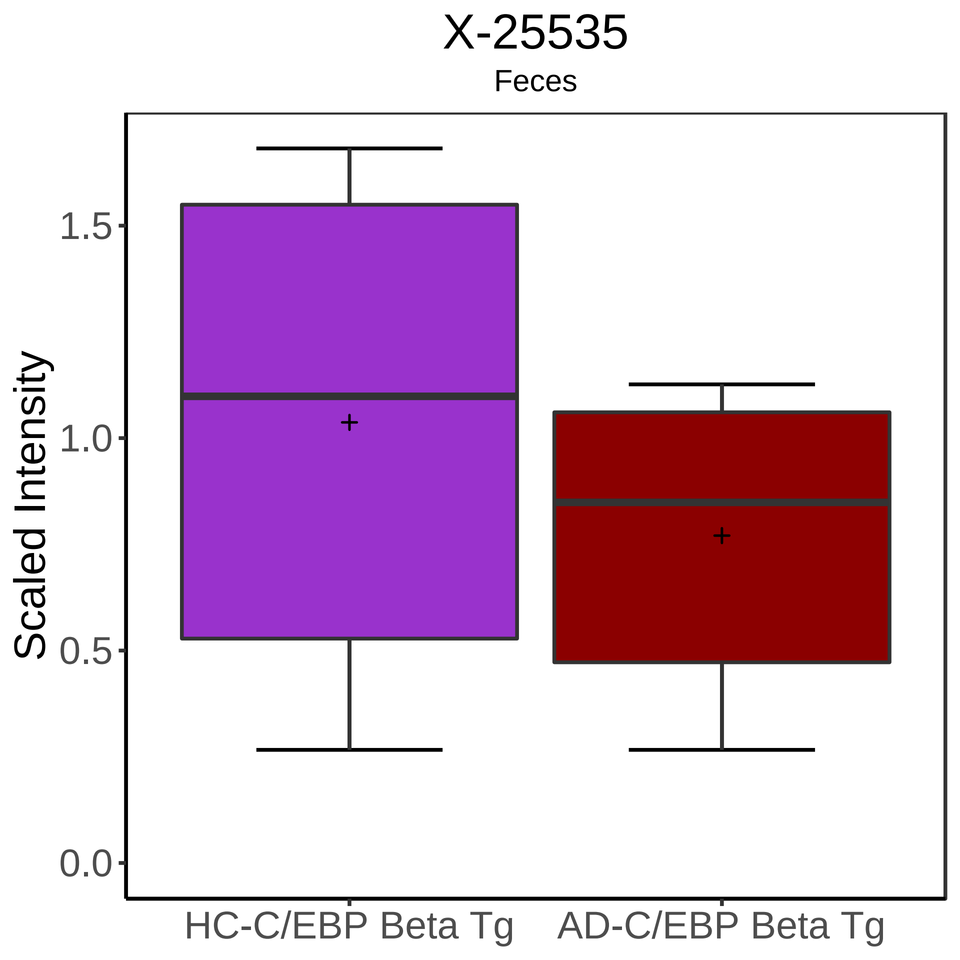
X-25535
Feces
Scaled Intensity
1.5
1.0
0.5
0.0
HC-C/EBP Beta Tg
AD-C/EBP Beta Tg

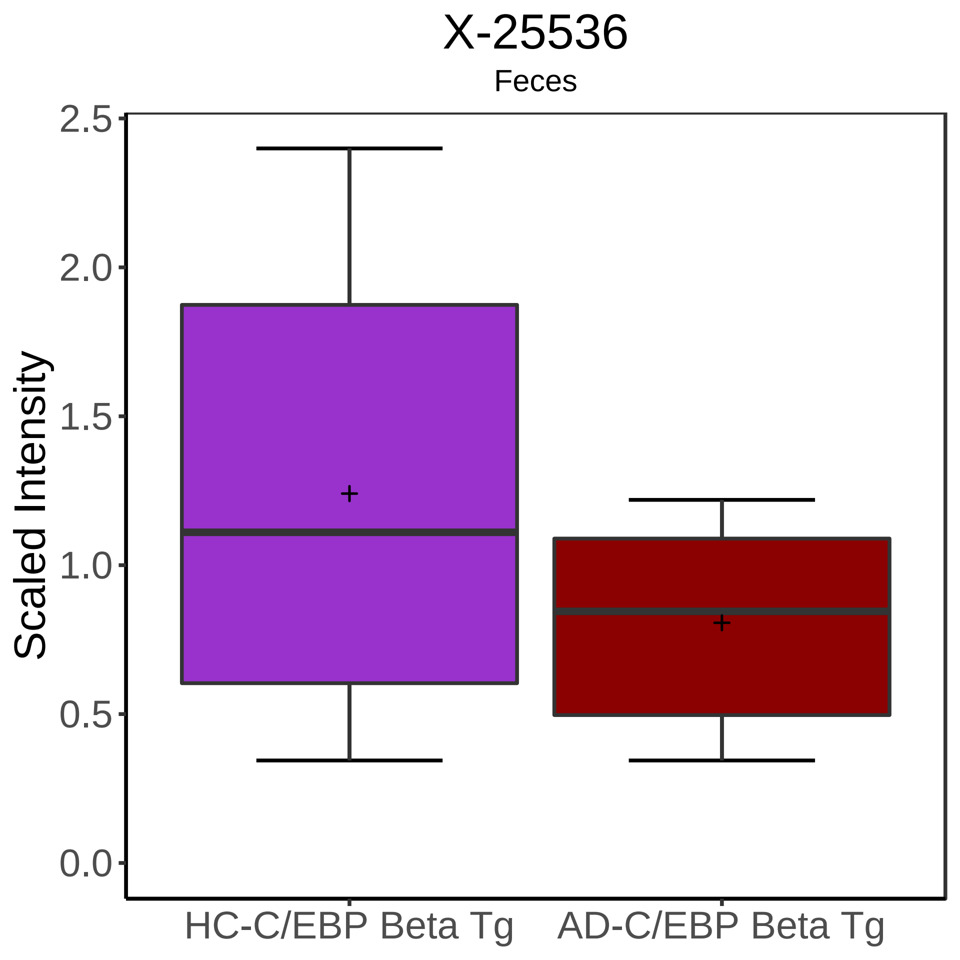
X-25536
Feces
Scaled Intensity
2.5
2.0
1.5
1.0
0.5
0.0
HC-C/EBP Beta Tg
AD-C/EBP Beta Tg

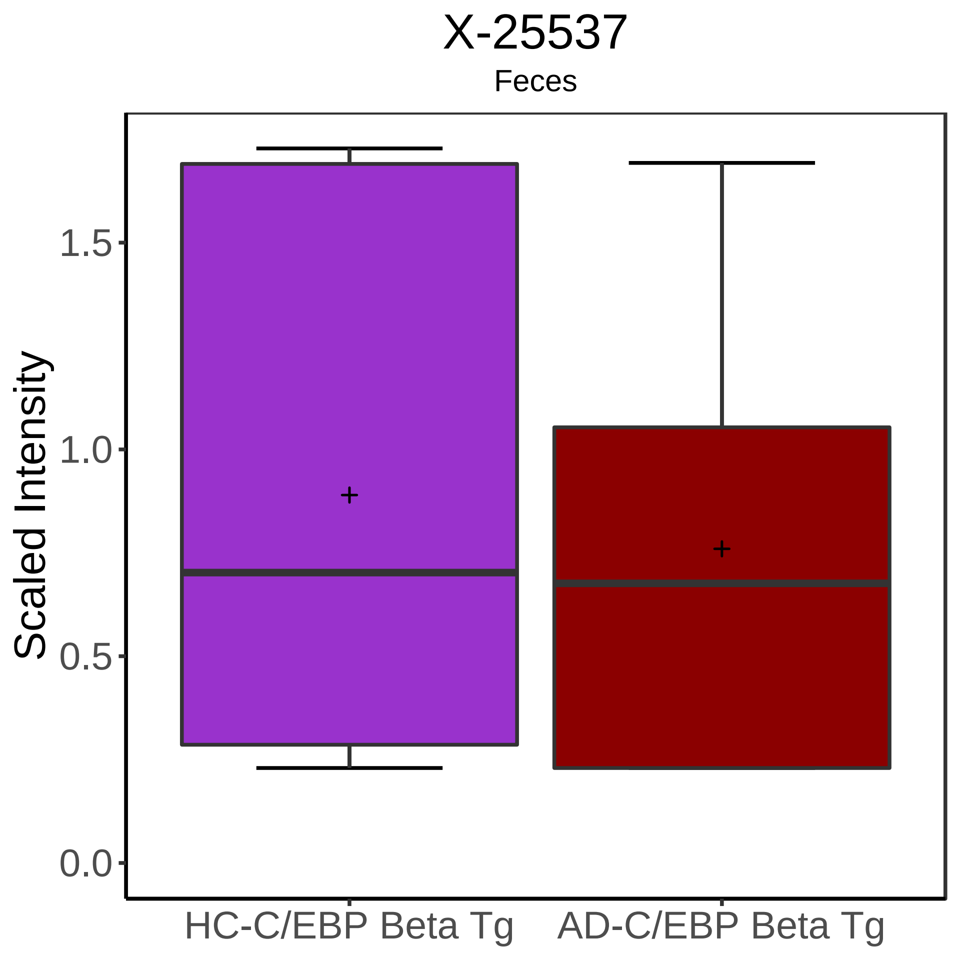
X-25537
Feces
Scaled Intensity
1.5
1.0
0.5
0.0
HC-C/EBP Beta Tg
AD-C/EBP Beta Tg

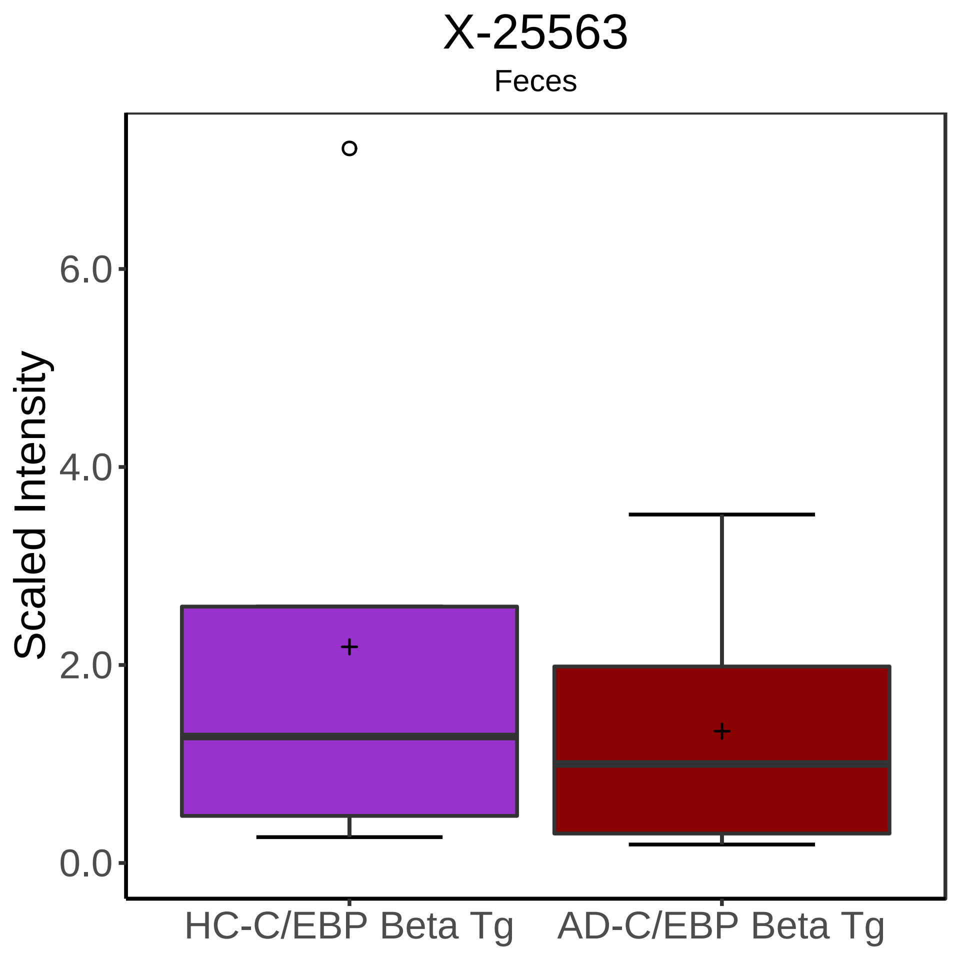
X-25563
Feces
Scaled Intensity
6.0
4.0
2.0
0.0
HC-C/EBP Beta Tg
AD-C/EBP Beta Tg

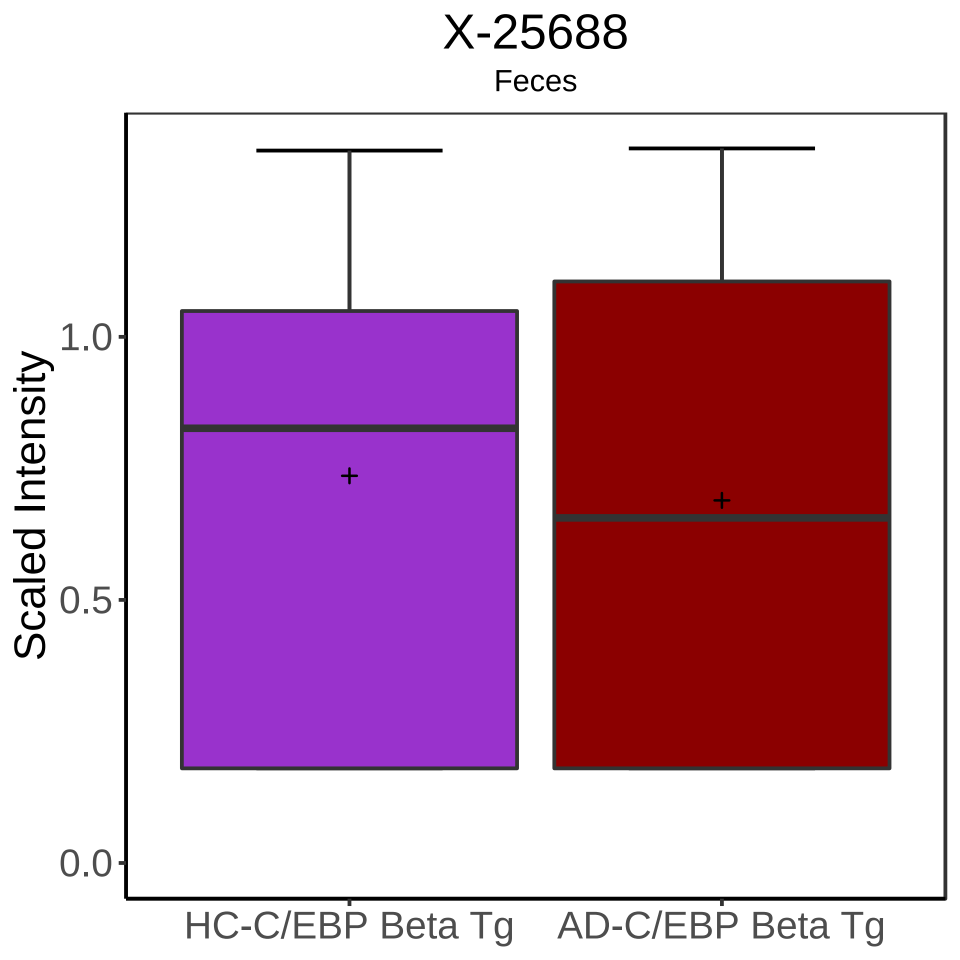
X-25688
Feces
Scaled Intensity
1.0
0.5
0.0
HC-C/EBP Beta Tg
AD-C/EBP Beta Tg

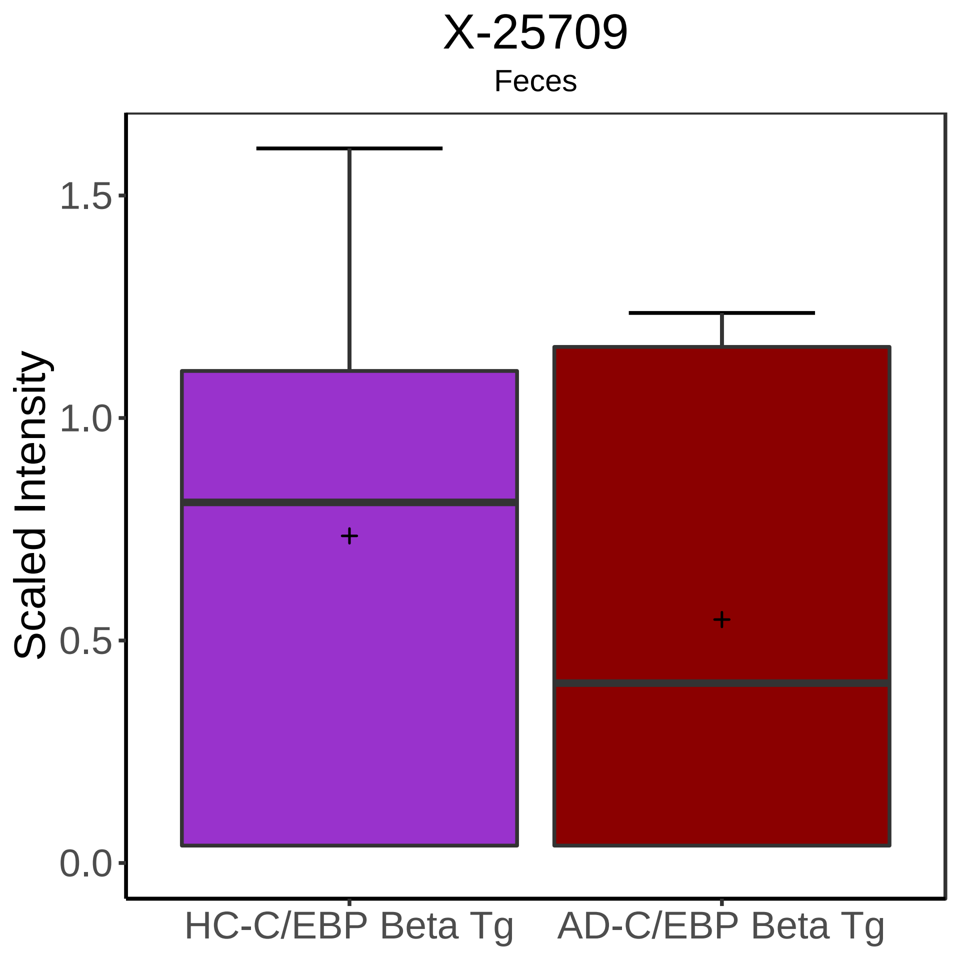

X-25709
Feces
Scaled Intensity
1.5
1.0
0.5
0.0
HC-C/EBP Beta Tg
AD-C/EBP Beta Tg

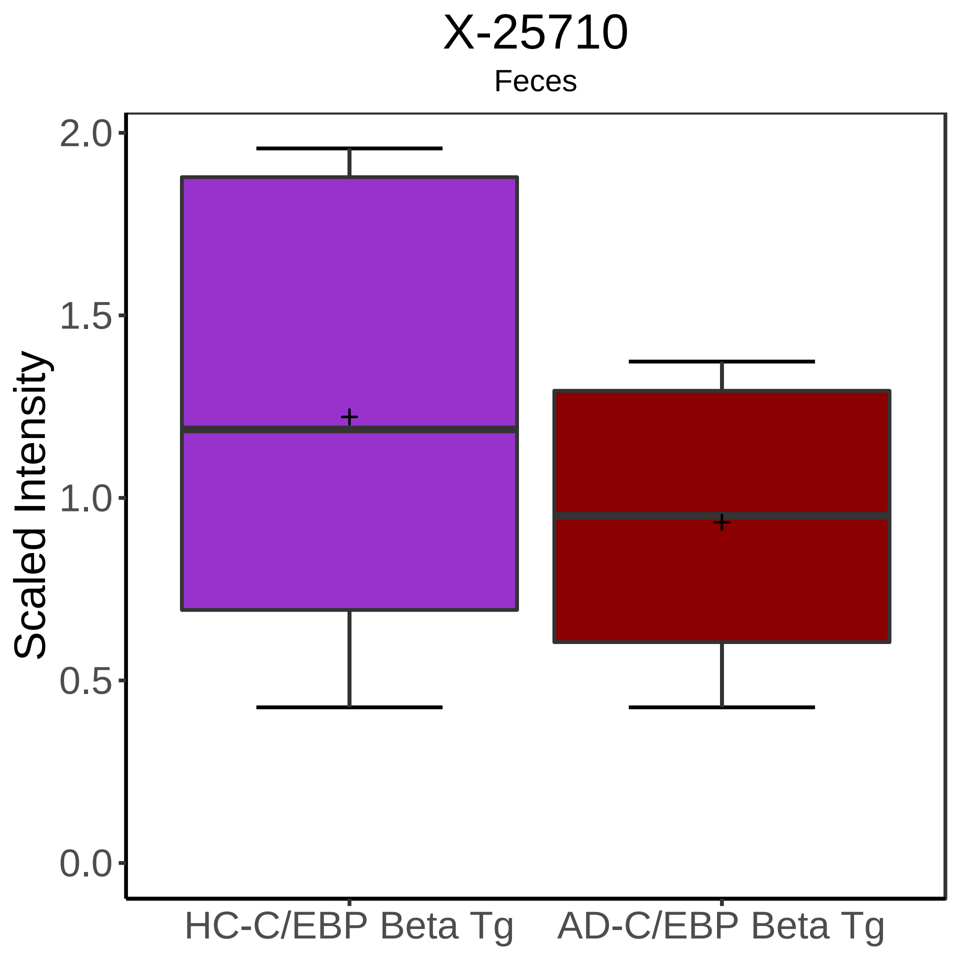
X-25710
Feces
Scaled Intensity
2.0
1.5
1.0
0.5
0.0
HC-C/EBP Beta Tg
AD-C/EBP Beta Tg

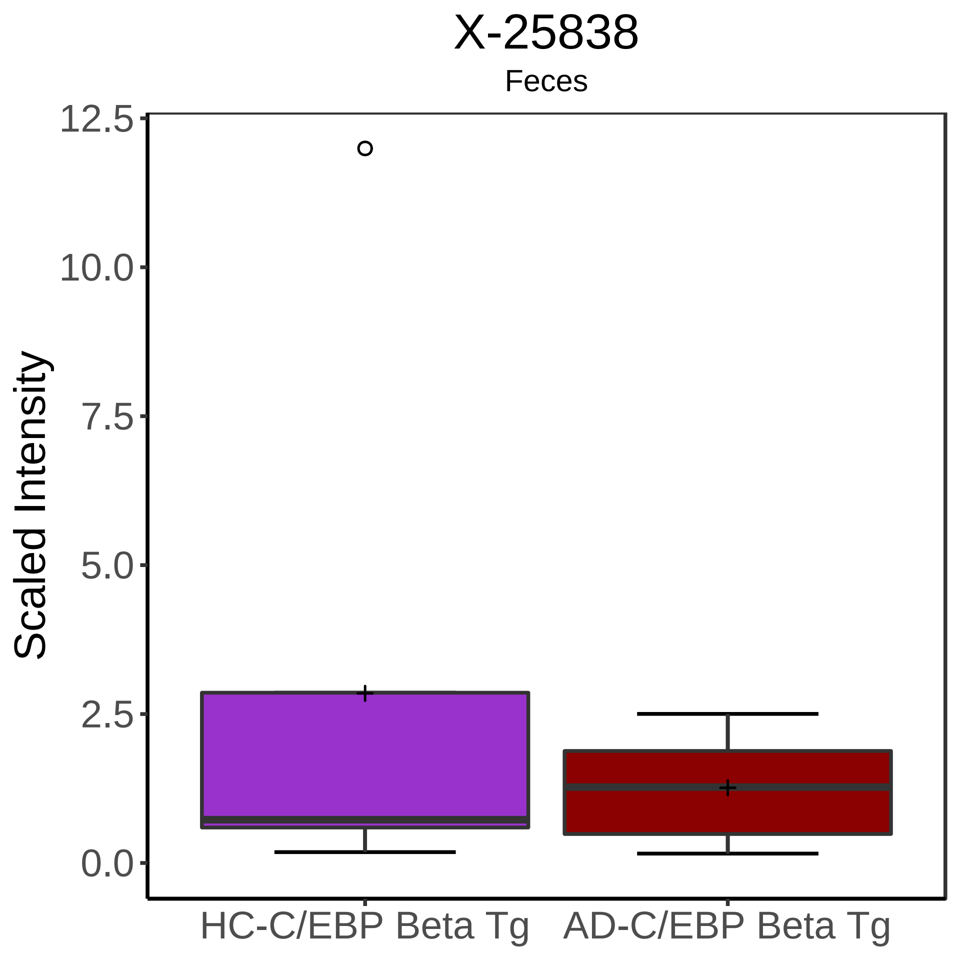
X-25838
Feces
Scaled Intensity
12.5
10.0
7.5
5.0
2.5
0.0
HC-C/EBP Beta Tg
AD-C/EBP Beta Tg

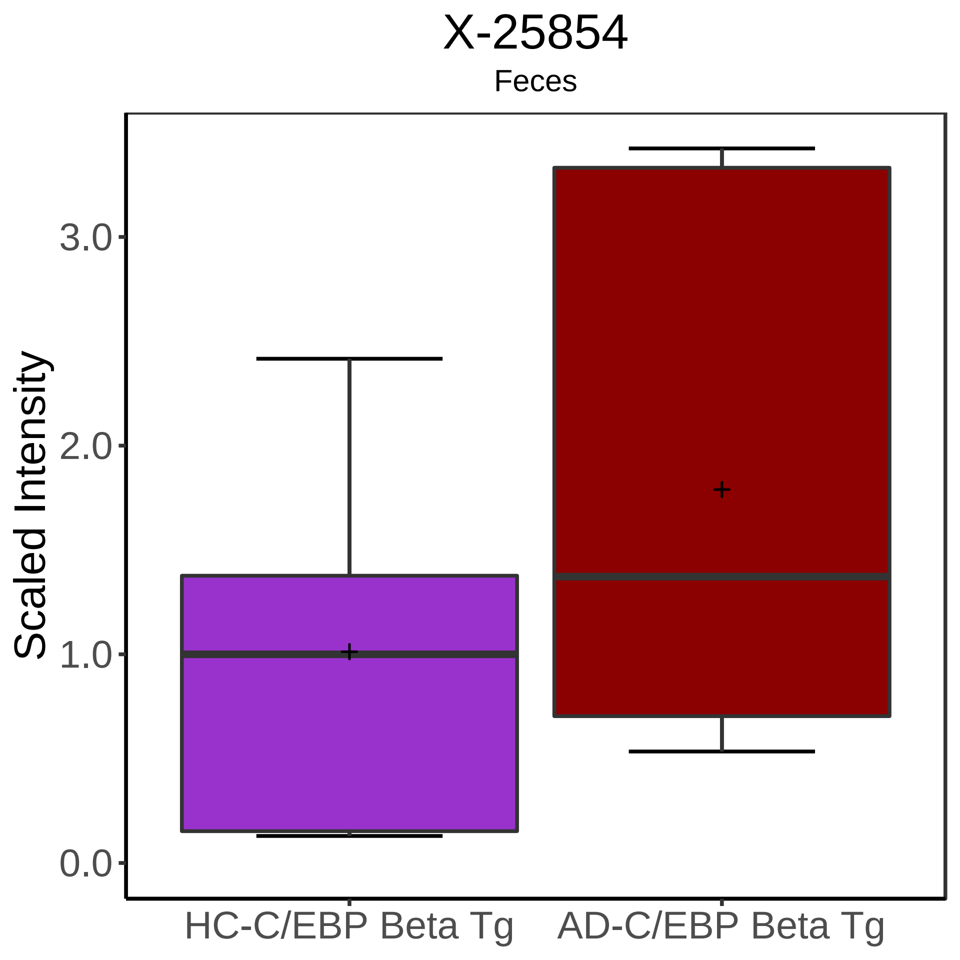

X-25854
Feces
Scaled Intensity
3.0
2.0
1.0
0.0
HC-C/EBP Beta Tg
AD-C/EBP Beta Tg

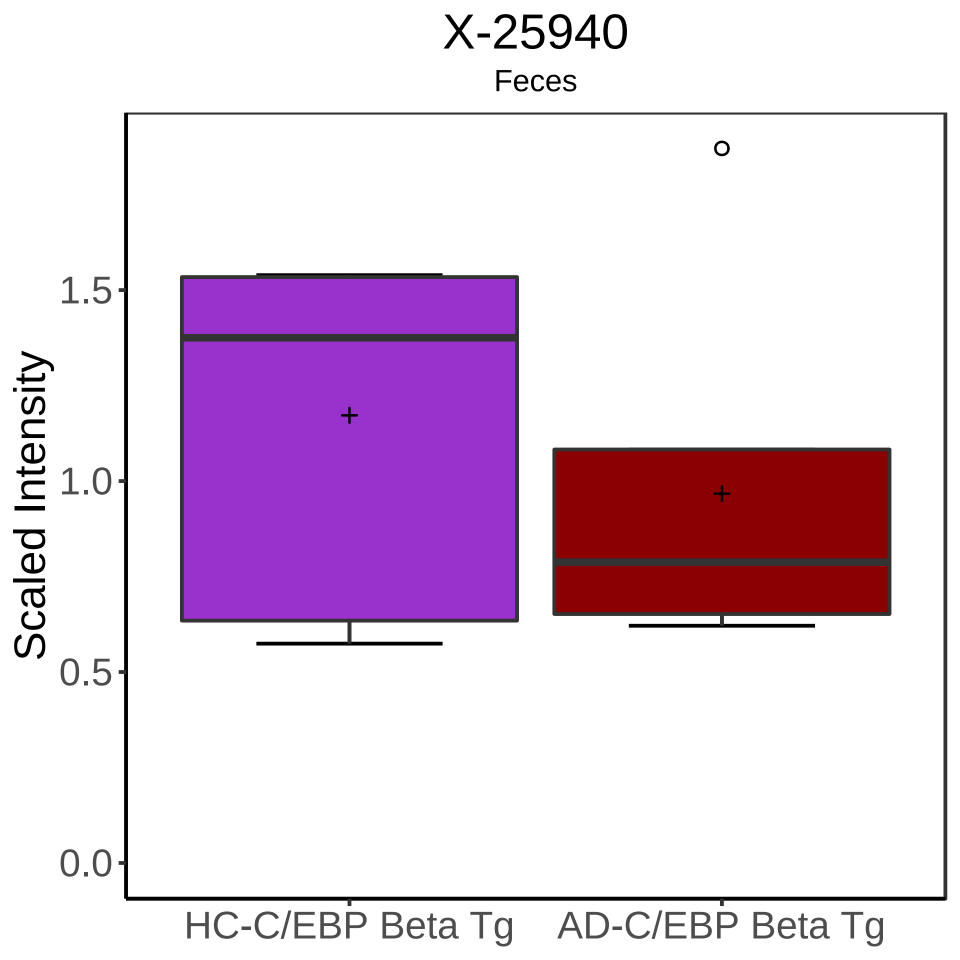
X-25940
Feces
Scaled Intensity
1.5
1.0
0.5
0.0
HC-C/EBP Beta Tg
AD-C/EBP Beta Tg

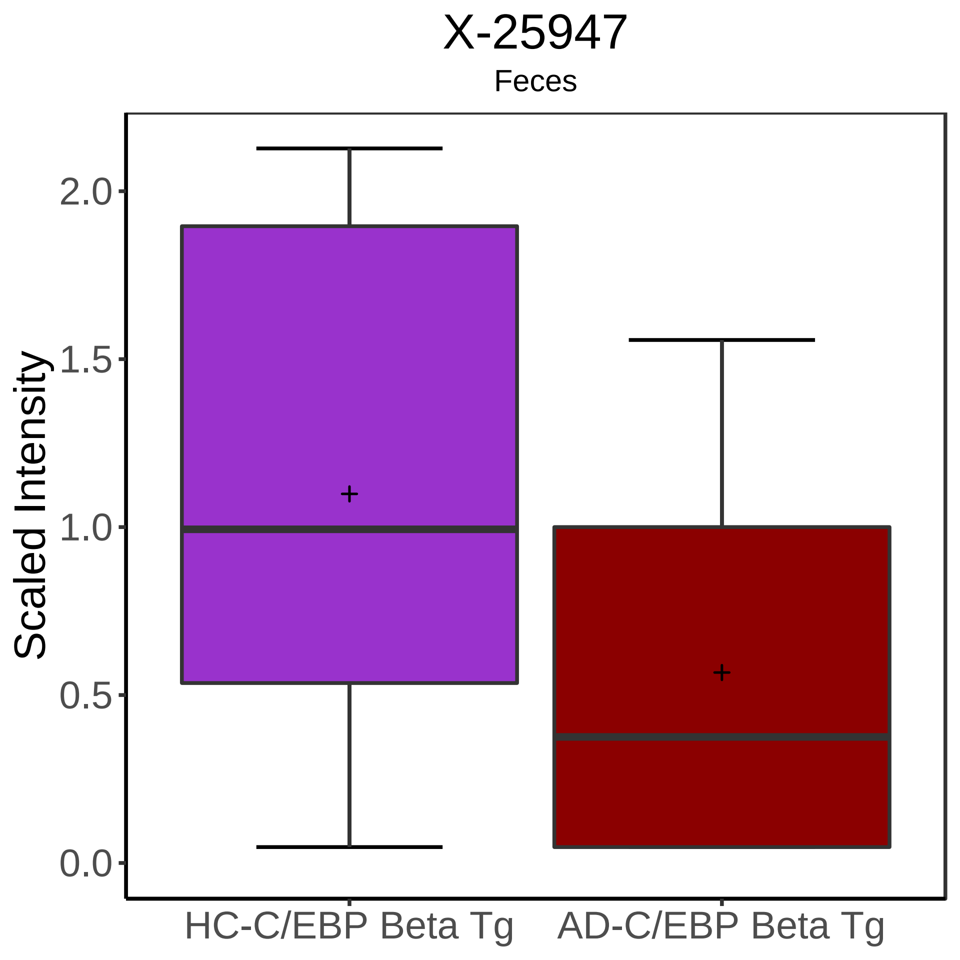
X-25947
Feces
Scaled Intensity
2.0
1.5
1.0
0.5
0.0
HC-C/EBP Beta Tg
AD-C/EBP Beta Tg

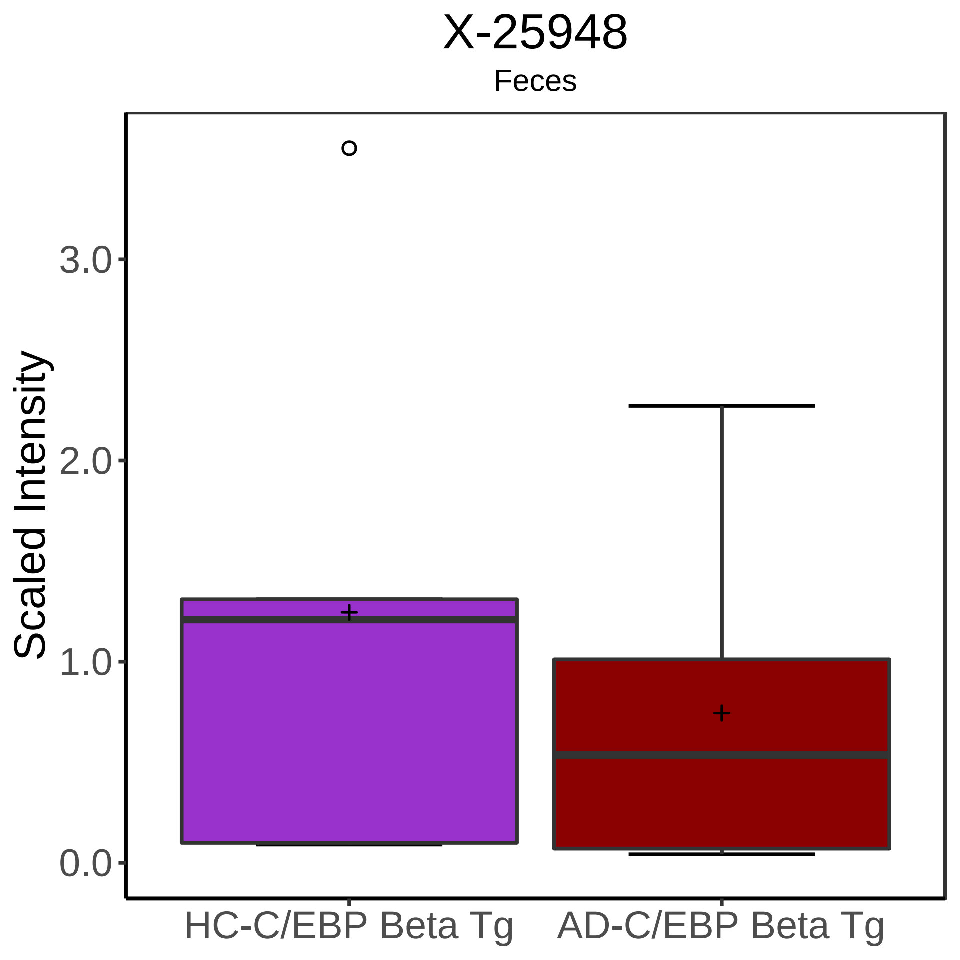

X-25948
Feces
Scaled Intensity
3.0
2.0
1.0
0.0
HC-C/EBP Beta Tg
AD-C/EBP Beta Tg

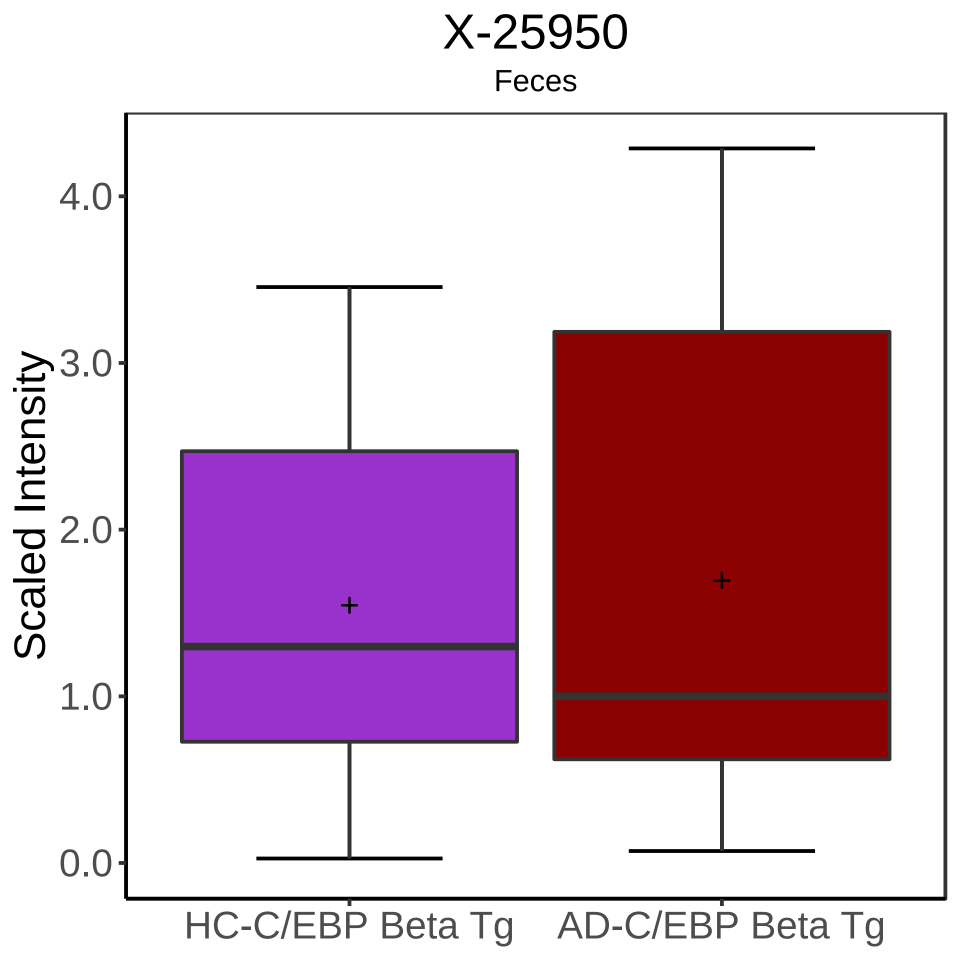
X-25950
Feces
Scaled Intensity
4.0
3.0
2.0
1.0
0.0
HC-C/EBP Beta Tg
AD-C/EBP Beta Tg

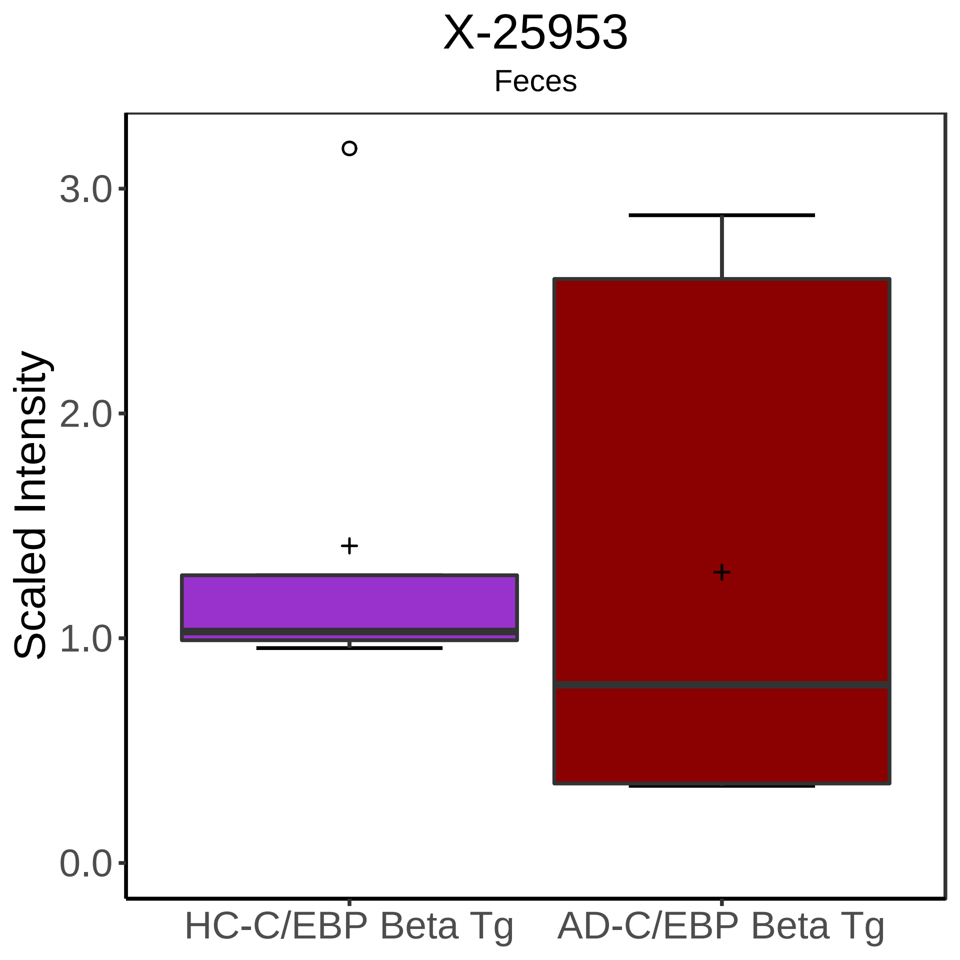
X-25953
Feces
Scaled Intensity
3.0
2.0
1.0
0.0
HC-C/EBP Beta Tg
AD-C/EBP Beta Tg

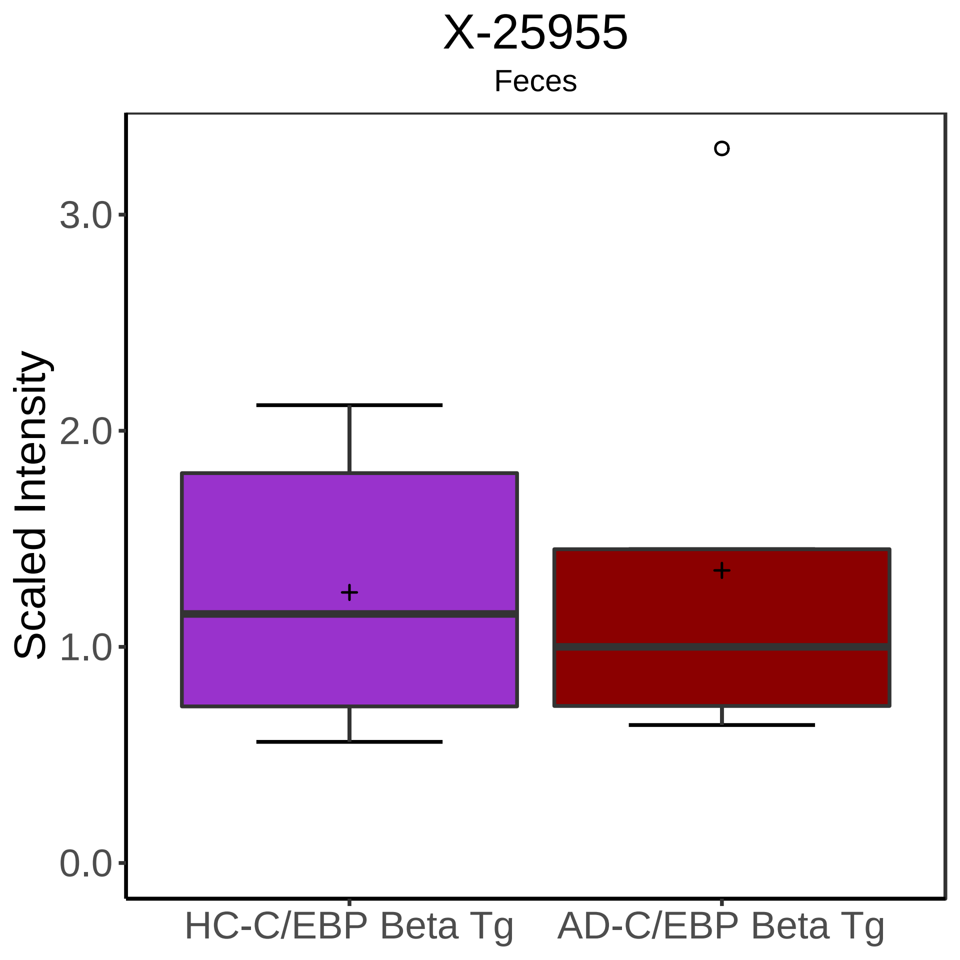

X-25955
Feces
Scaled Intensity
3.0
2.0
1.0
0.0
HC-C/EBP Beta Tg
AD-C/EBP Beta Tg

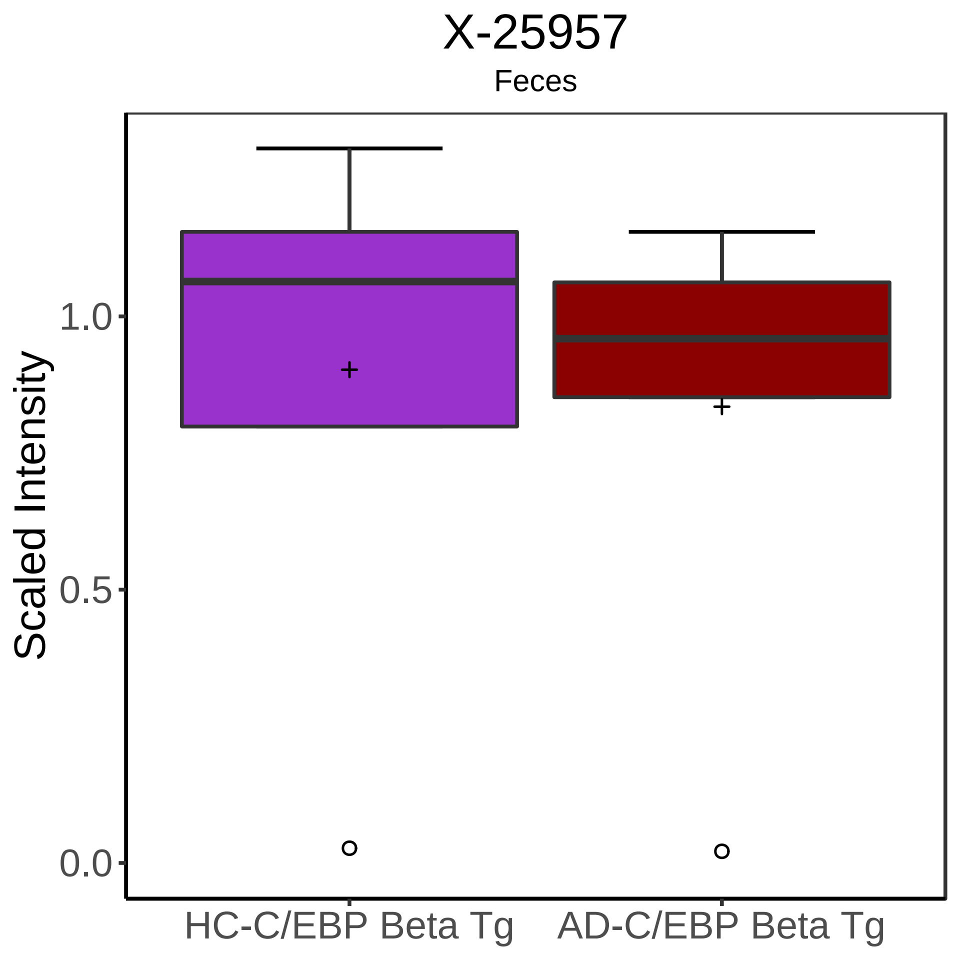
X-25957
Feces
Scaled Intensity
1.0
0.5
0.0
HC-C/EBP Beta Tg
AD-C/EBP Beta Tg

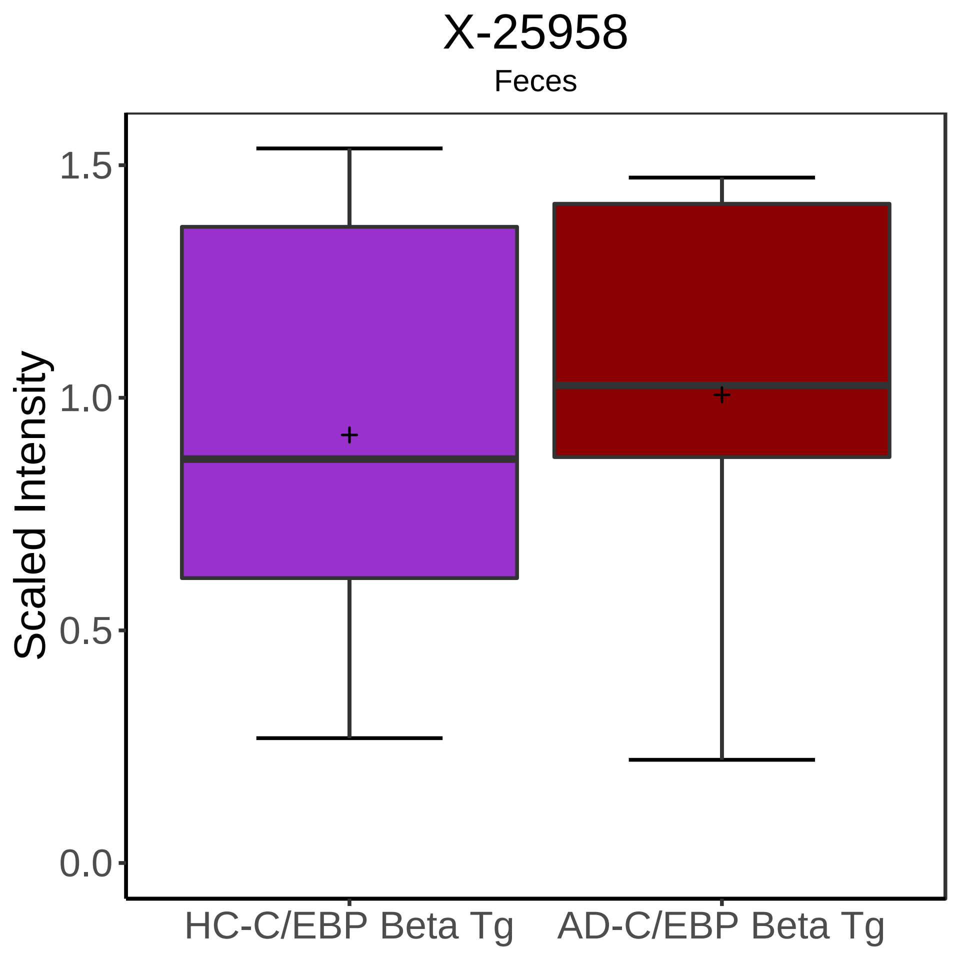
X-25958
Feces
Scaled Intensity
1.5
1.0
0.5
0.0
HC-C/EBP Beta Tg
AD-C/EBP Beta Tg

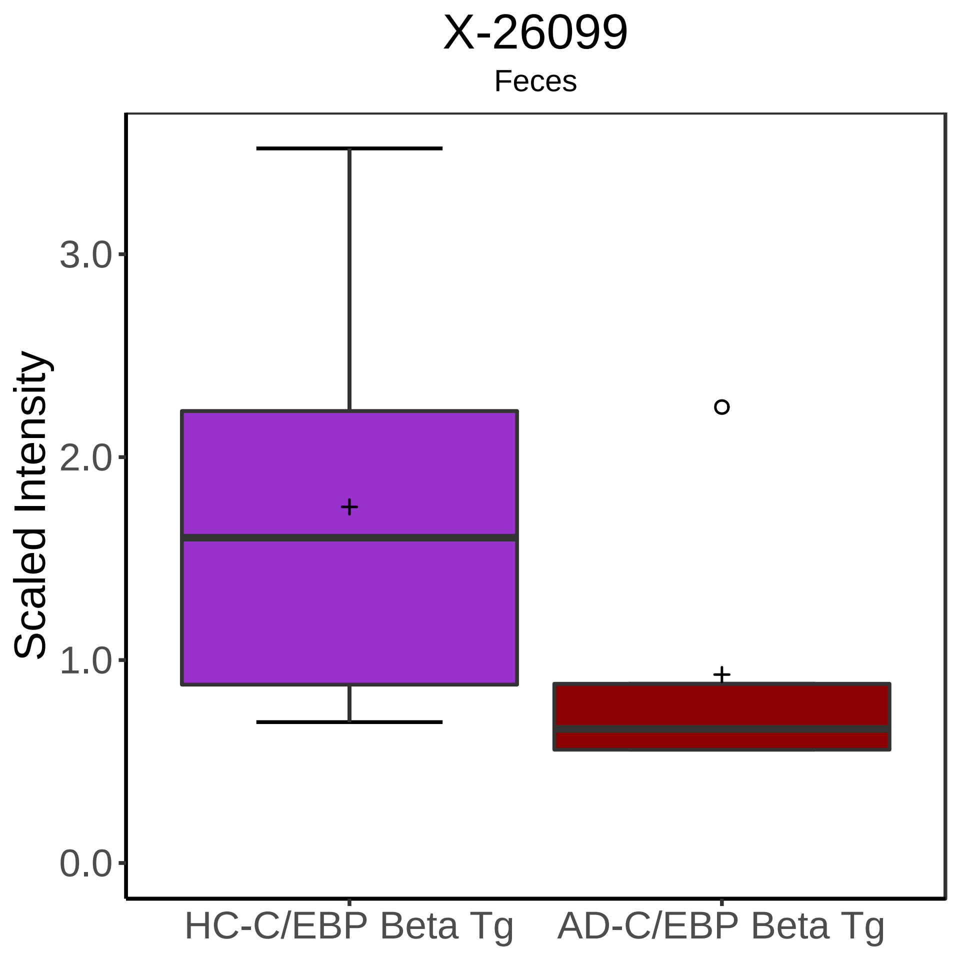
X-26099
Feces
Scaled Intensity
3.0
2.0
1.0
0.0
HC-C/EBP Beta Tg
AD-C/EBP Beta Tg

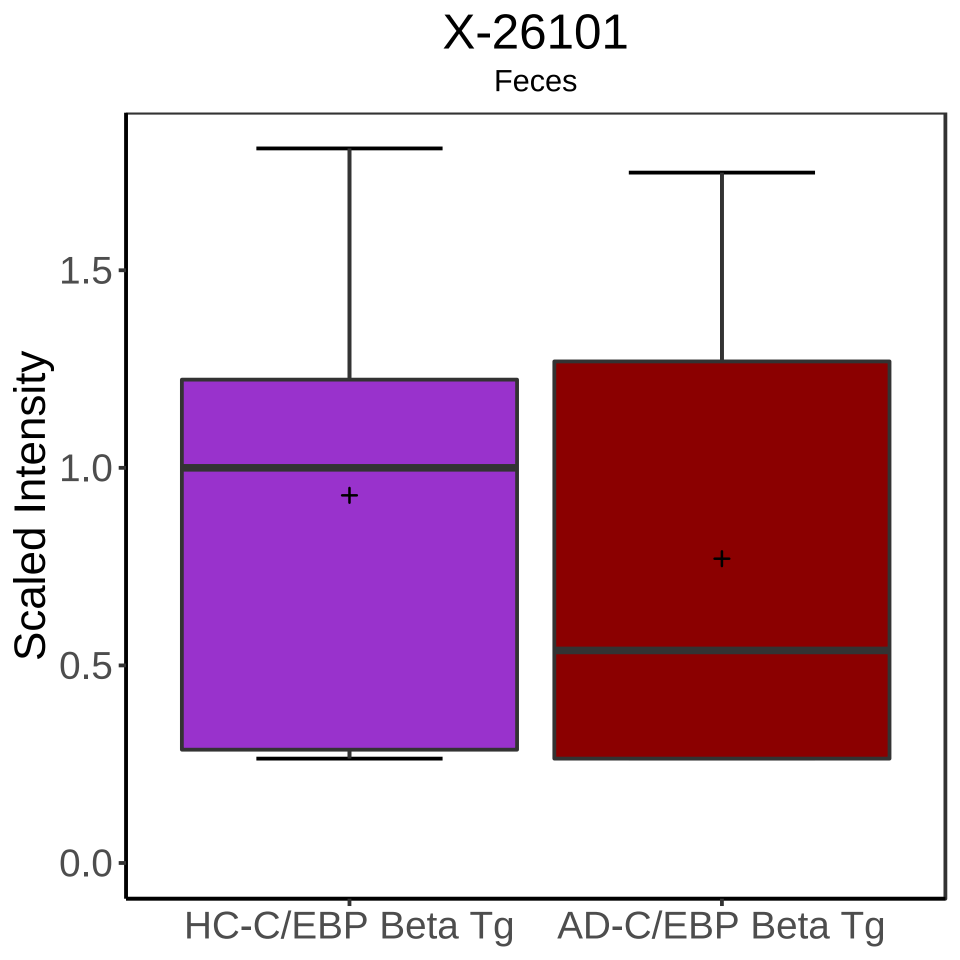
X-26101
Feces
Scaled Intensity
1.5
1.0
0.5
0.0
HC-C/EBP Beta Tg
AD-C/EBP Beta Tg

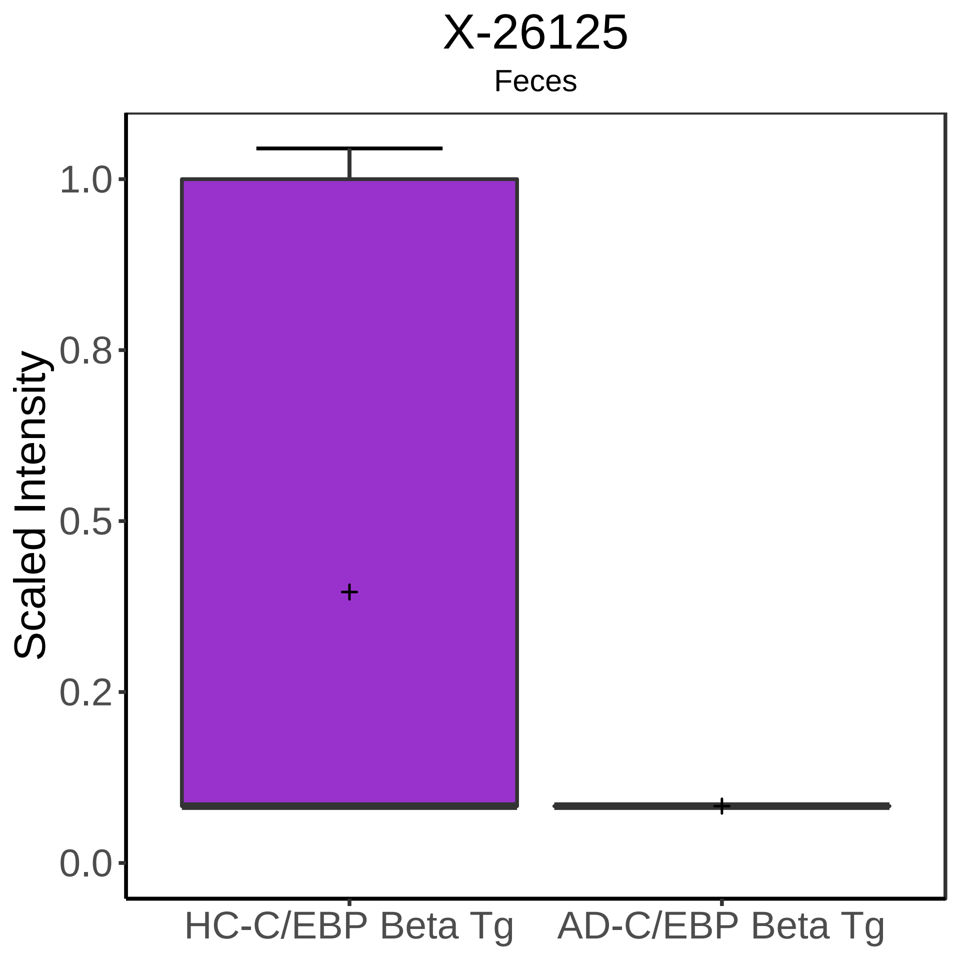
X-26125
Feces
Scaled Intensity
1.0
0.8
0.5
0.2
0.0
HC-C/EBP Beta Tg
AD-C/EBP Beta Tg

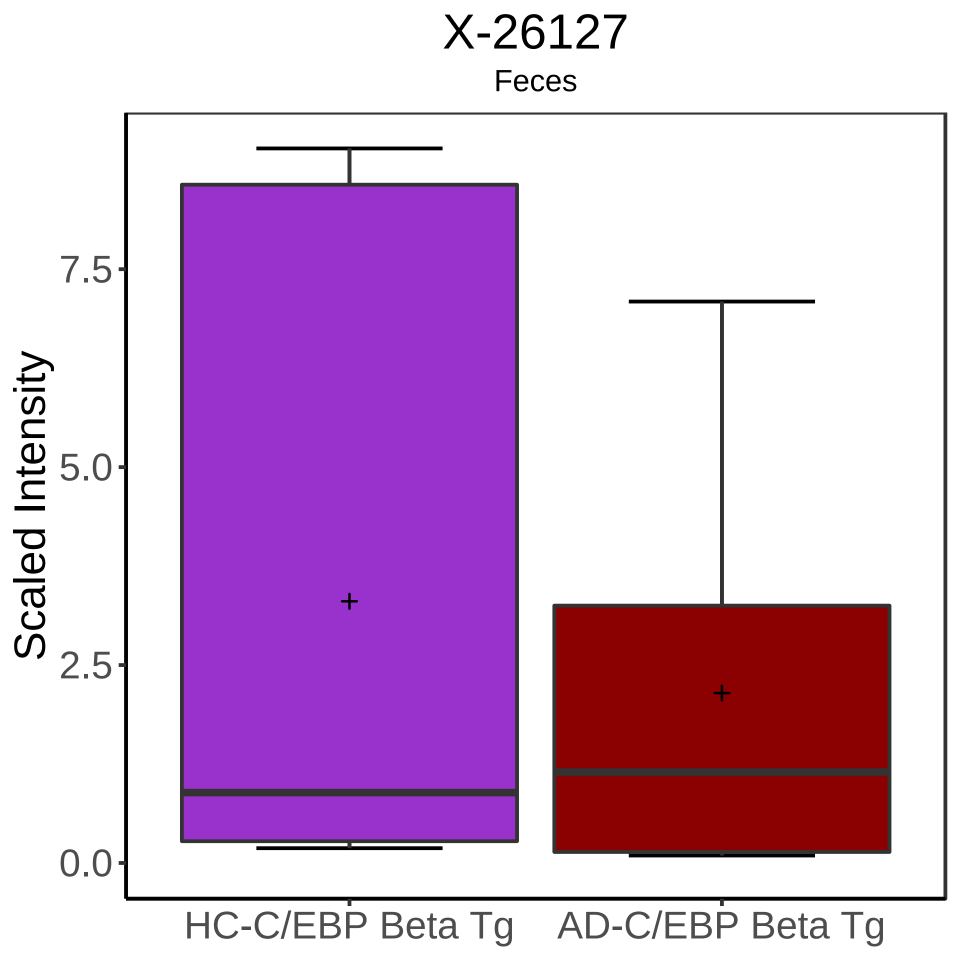
X-26127
Feces
Scaled Intensity
7.5
5.0
2.5
0.0
HC-C/EBP Beta Tg
AD-C/EBP Beta Tg
