# Supplementary material for: Bacteroides Fragilis in the gut microbiomes of Alzheimer’s disease activates microglia and triggers pathogenesis in neuronal C/EBPβ transgenic mice
Source: Nat Commun. 2023 Sep 6;14:5471. doi: 10.1038/s41467-023-41283-w (PMC10482867; doi:10.1038/s41467-023-41283-w)
Supplement: Supplementary file 4 — Supplementary Data 1 [file 41467_2023_41283_MOESM4_ESM.zip › EMOR-0201-20VW+/EMOR-0201-20VW+ BOX PLOTS (SERUM).PDF]

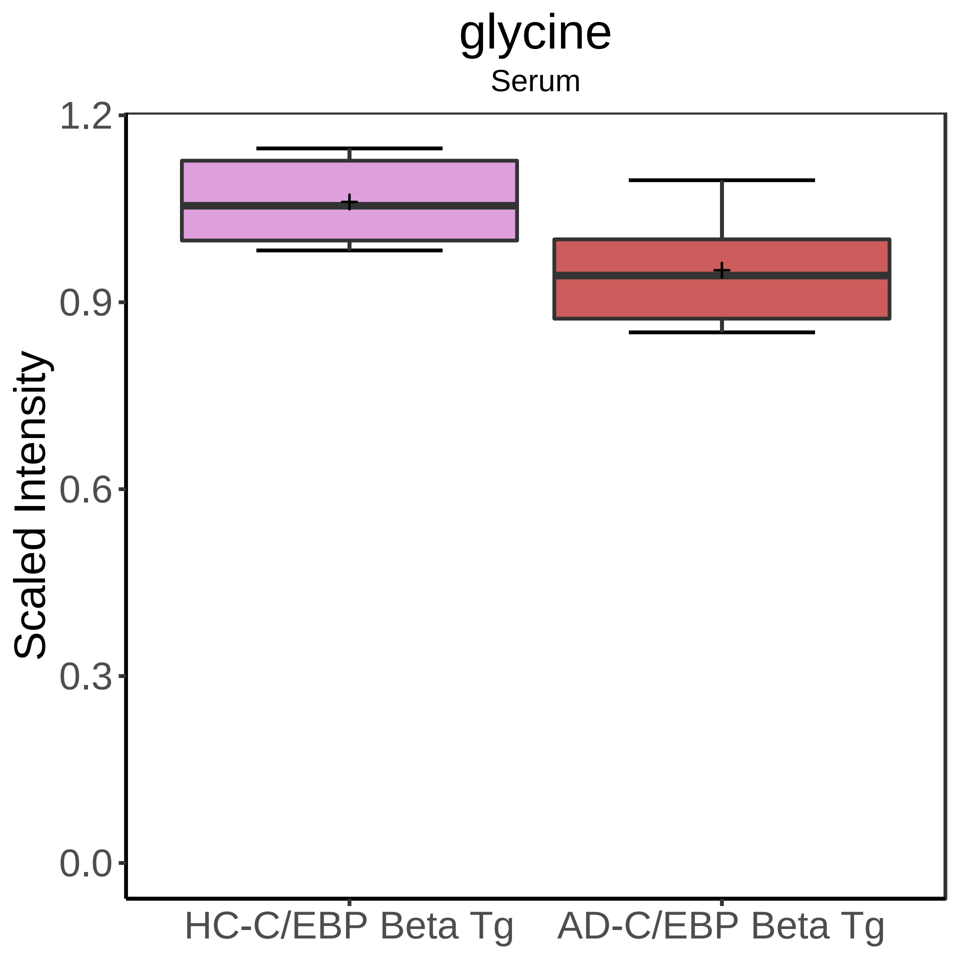
glycine
Serum
Scaled Intensity
1.2
0.9
0.6
0.3
0.0
HC-C/EBP Beta Tg
AD-C/EBP Beta Tg

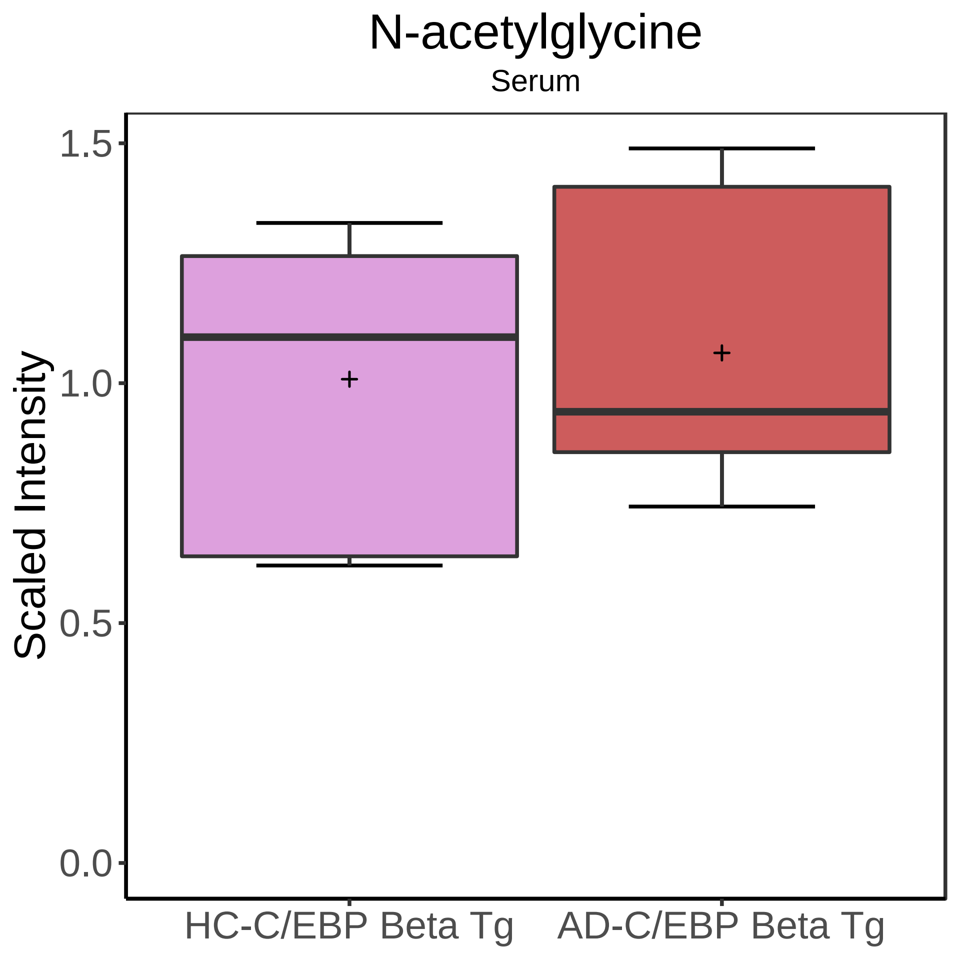

N-acetylglycine
Serum
Scaled Intensity
1.5
1.0
0.5
0.0
HC-C/EBP Beta Tg
AD-C/EBP Beta Tg

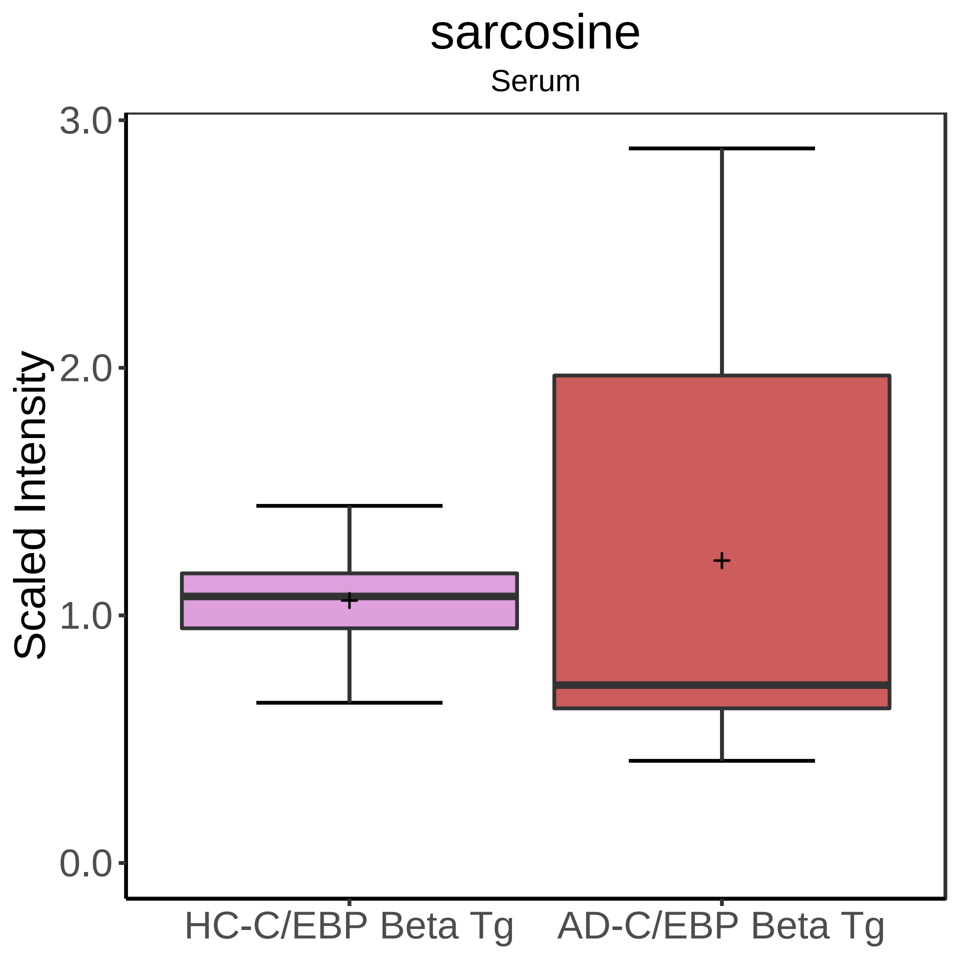
sarcosine
Serum
Scaled Intensity
3.0
2.0
1.0
0.0
HC-C/EBP Beta Tg
AD-C/EBP Beta Tg

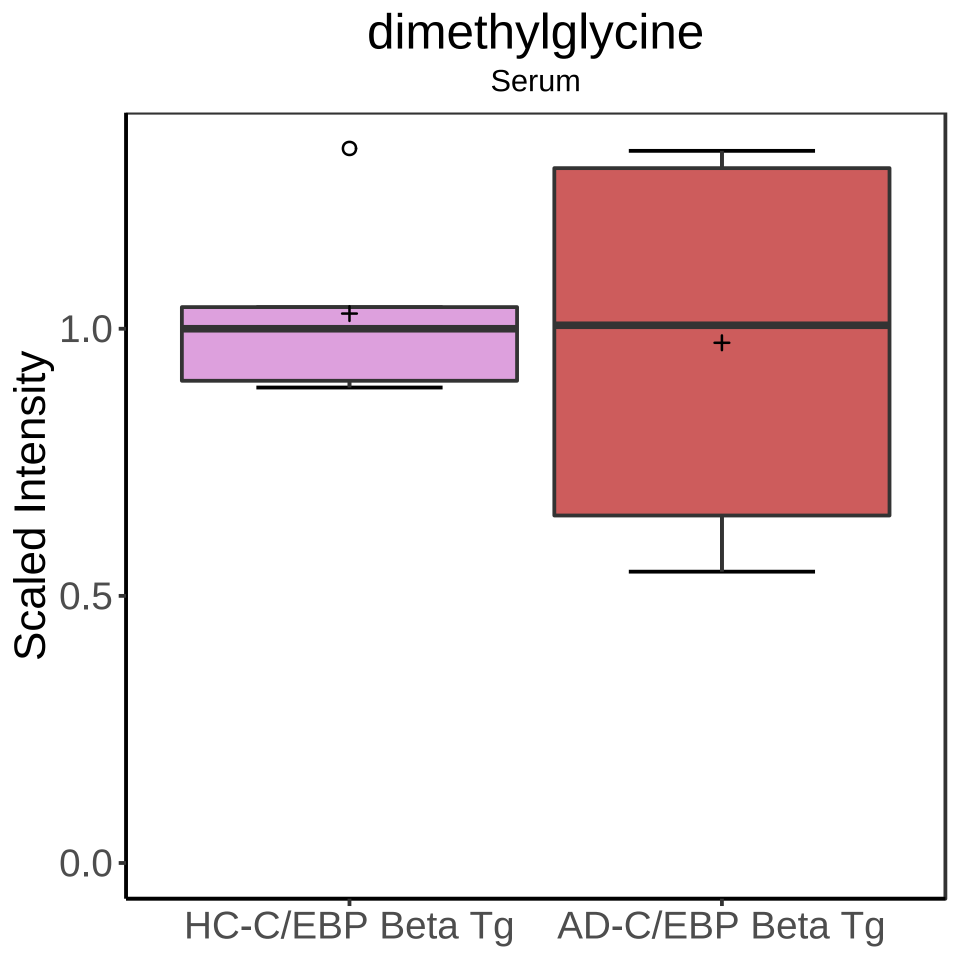
dimethylglycine
Serum
Scaled Intensity
1.0
0.5
0.0
HC-C/EBP Beta Tg
AD-C/EBP Beta Tg

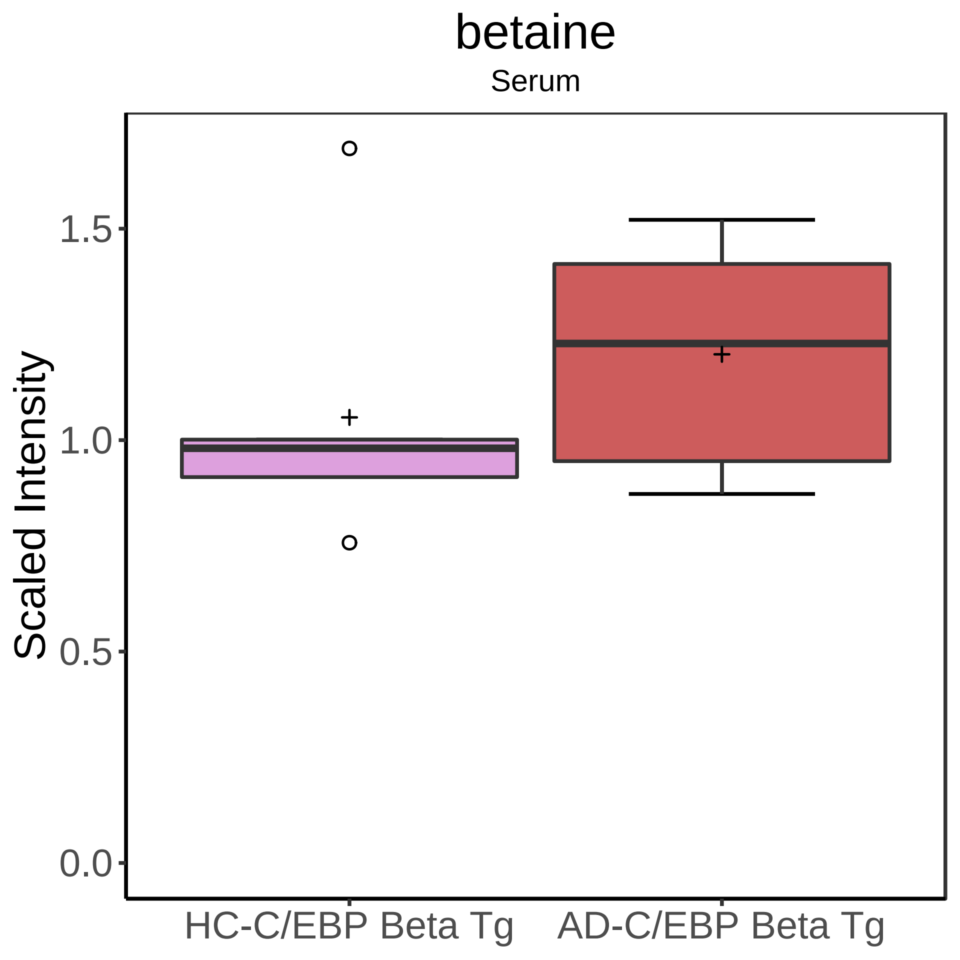
betaine
Serum
Scaled Intensity
1.5
1.0
0.5
0.0
HC-C/EBP Beta Tg
AD-C/EBP Beta Tg

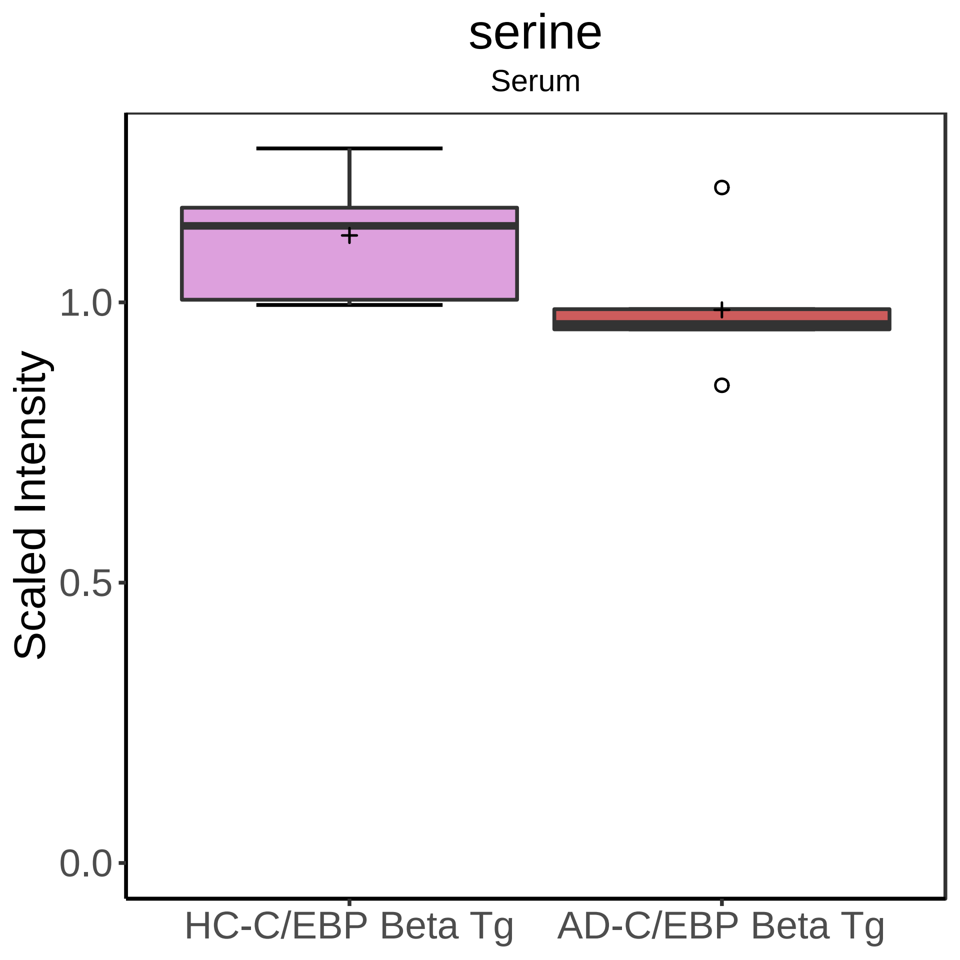
serine
Serum
Scaled Intensity
1.0
0.5
0.0
HC-C/EBP Beta Tg
AD-C/EBP Beta Tg

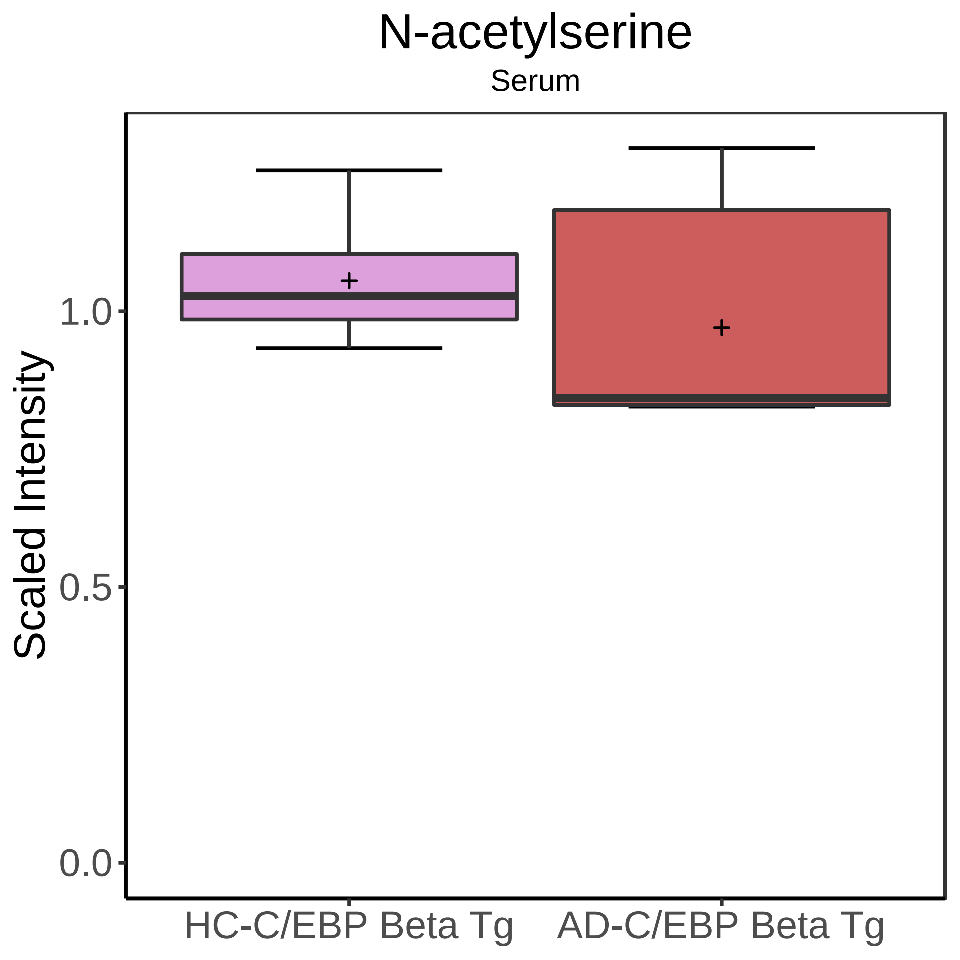
N-acetylserine
Serum
Scaled Intensity
1.0
0.5
0.0
HC-C/EBP Beta Tg
AD-C/EBP Beta Tg

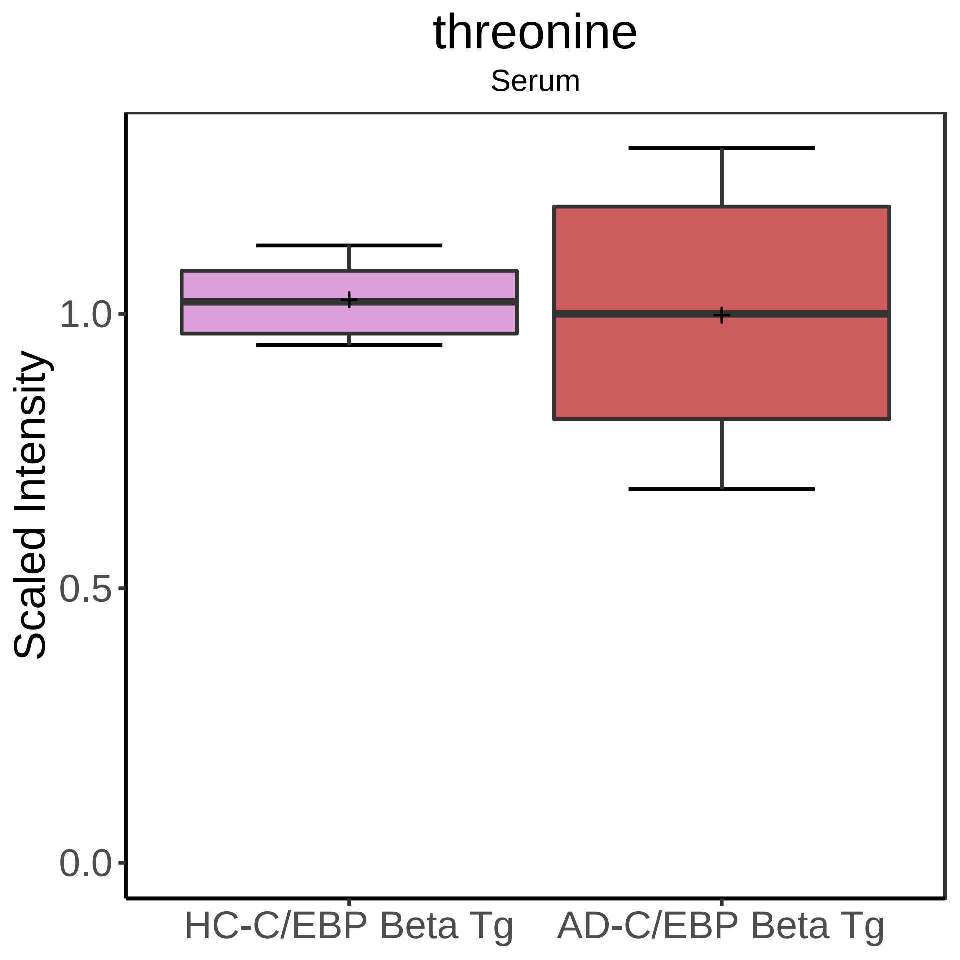
threonine
Serum
Scaled Intensity
1.0
0.5
0.0
HC-C/EBP Beta Tg
AD-C/EBP Beta Tg

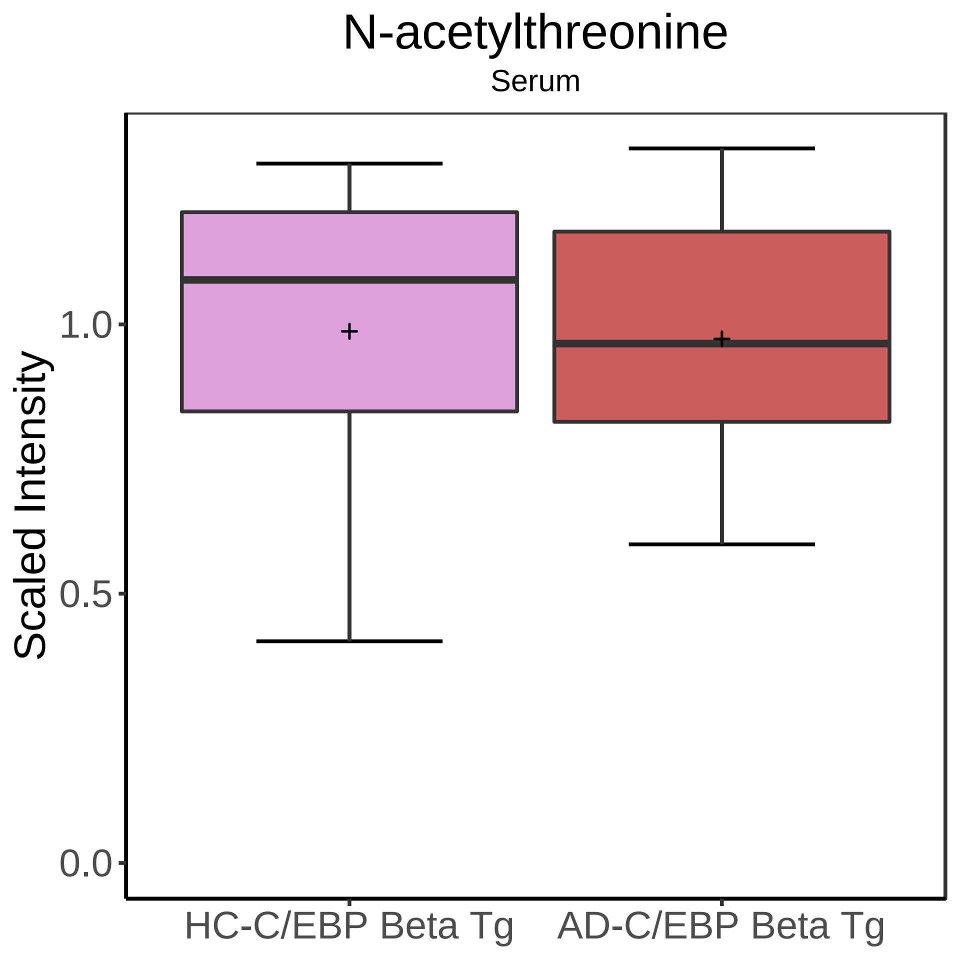
N-acetylthreonine
Serum
Scaled Intensity
1.0
0.5
0.0
HC-C/EBP Beta Tg
AD-C/EBP Beta Tg

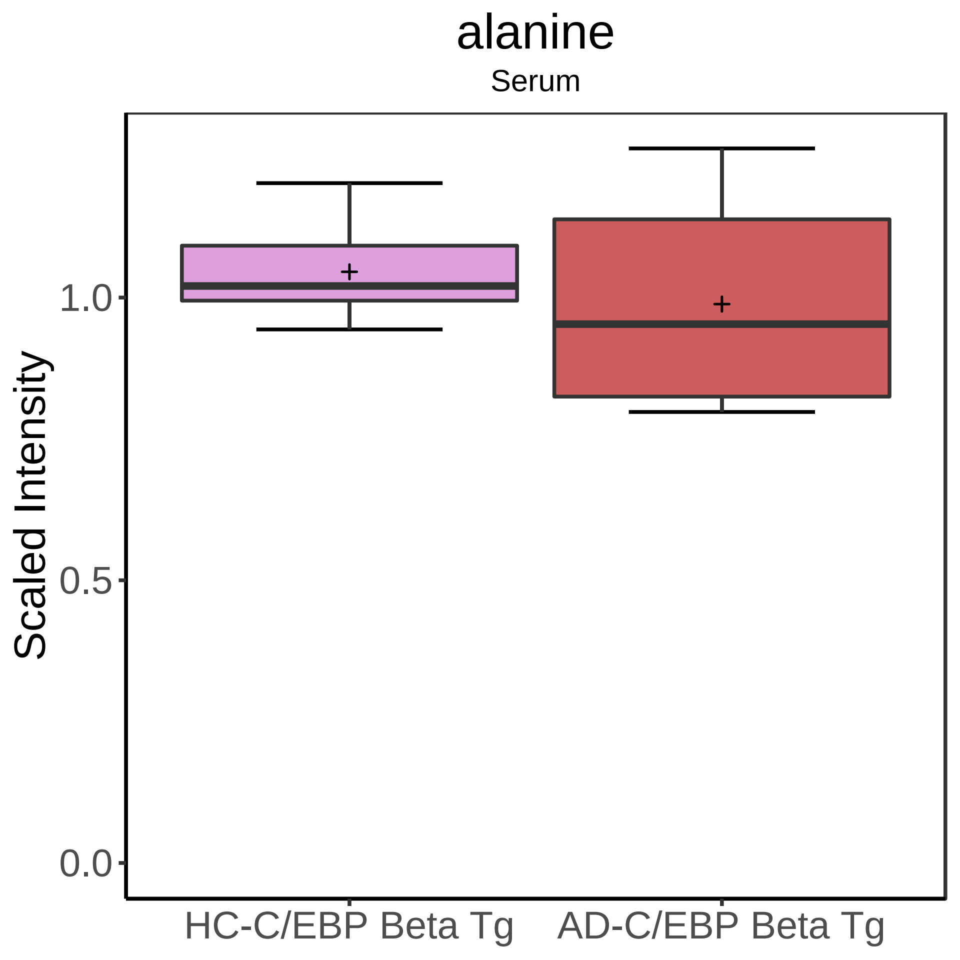
alanine
Serum
Scaled Intensity
1.0
0.5
0.0
HC-C/EBP Beta Tg
AD-C/EBP Beta Tg

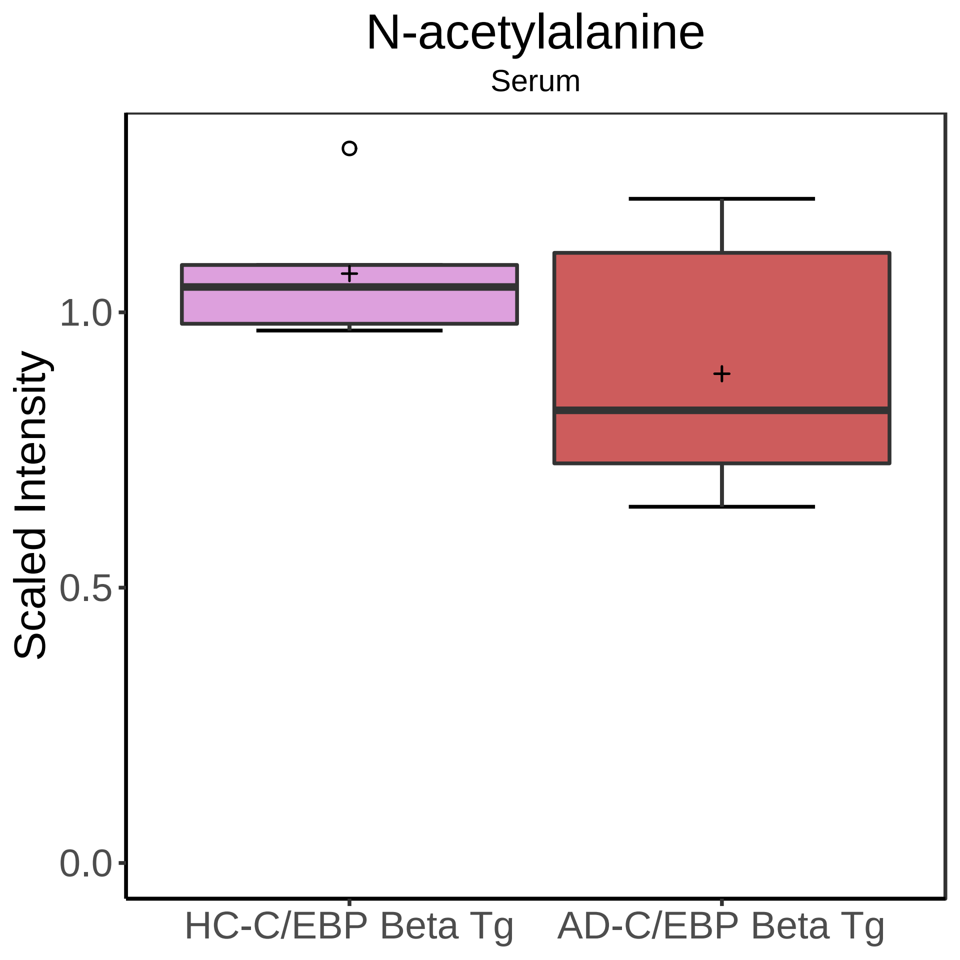
N-acetylalanine
Serum
Scaled Intensity
1.0
0.5
0.0
HC-C/EBP Beta Tg
AD-C/EBP Beta Tg

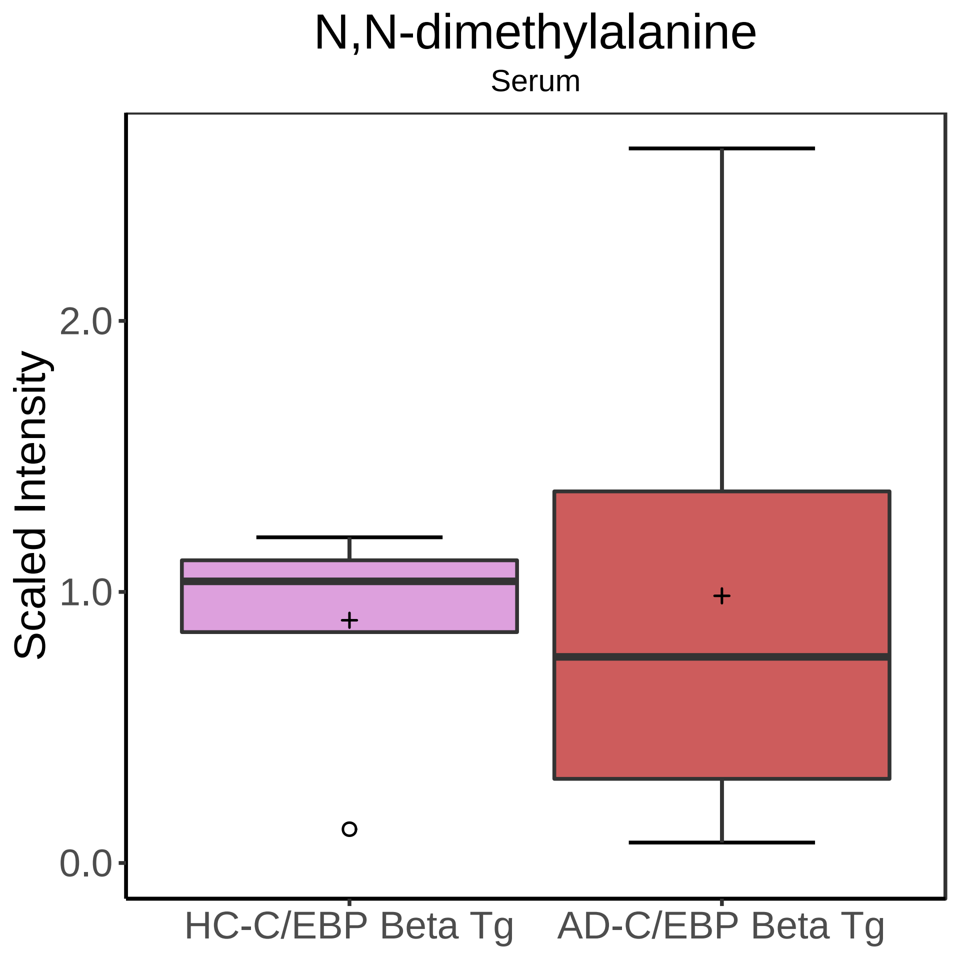
N,N-dimethylalanine
Serum
Scaled Intensity
2.0
1.0
0.0
HC-C/EBP Beta Tg
AD-C/EBP Beta Tg

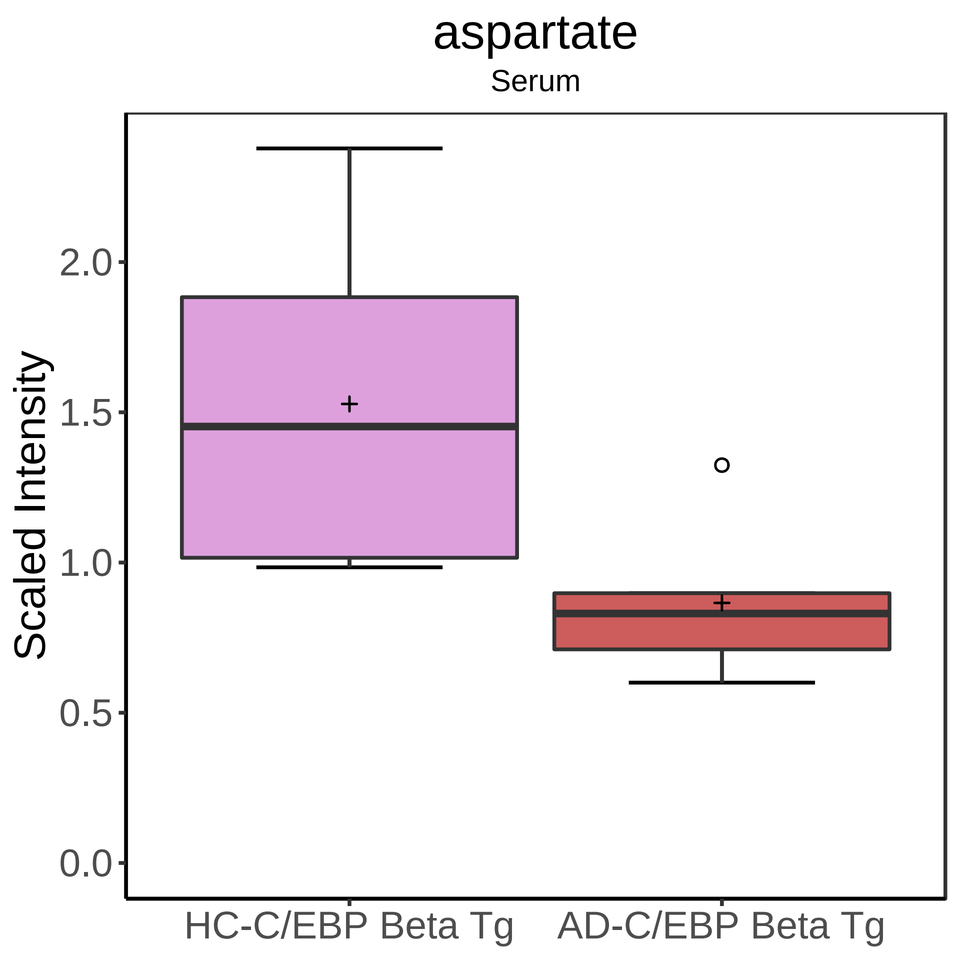
aspartate
Serum
Scaled Intensity
2.0
1.5
1.0
0.5
0.0
HC-C/EBP Beta Tg
AD-C/EBP Beta Tg

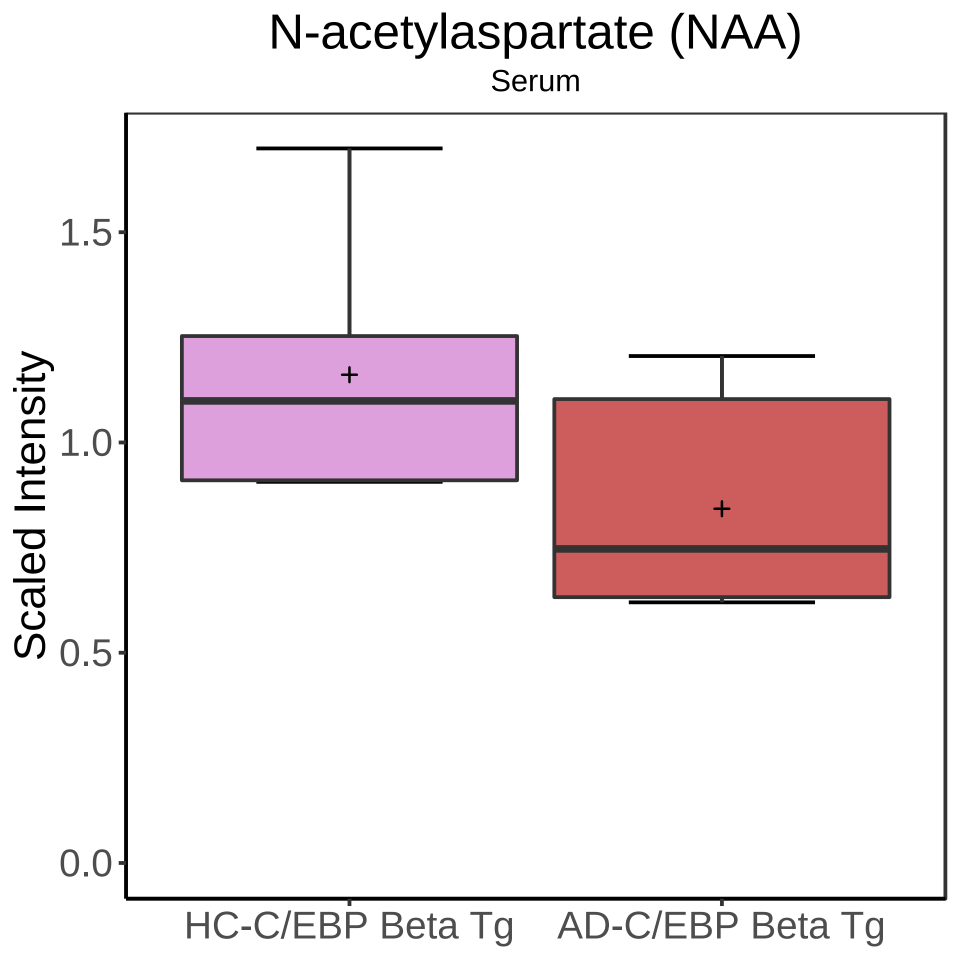
N-acetylaspartate (NAA)
Serum
Scaled Intensity
1.5
1.0
0.5
0.0
HC-C/EBP Beta Tg
AD-C/EBP Beta Tg

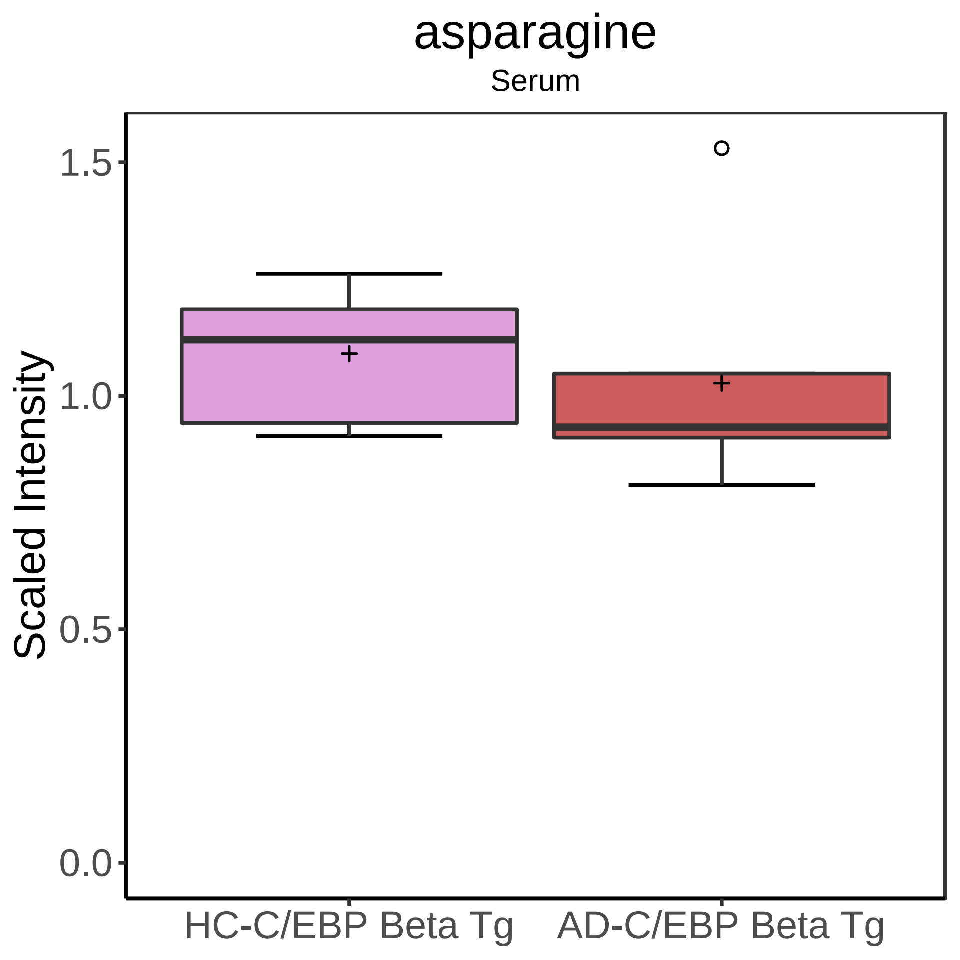
asparagine
Serum
Scaled Intensity
1.5
1.0
0.5
0.0
HC-C/EBP Beta Tg
AD-C/EBP Beta Tg

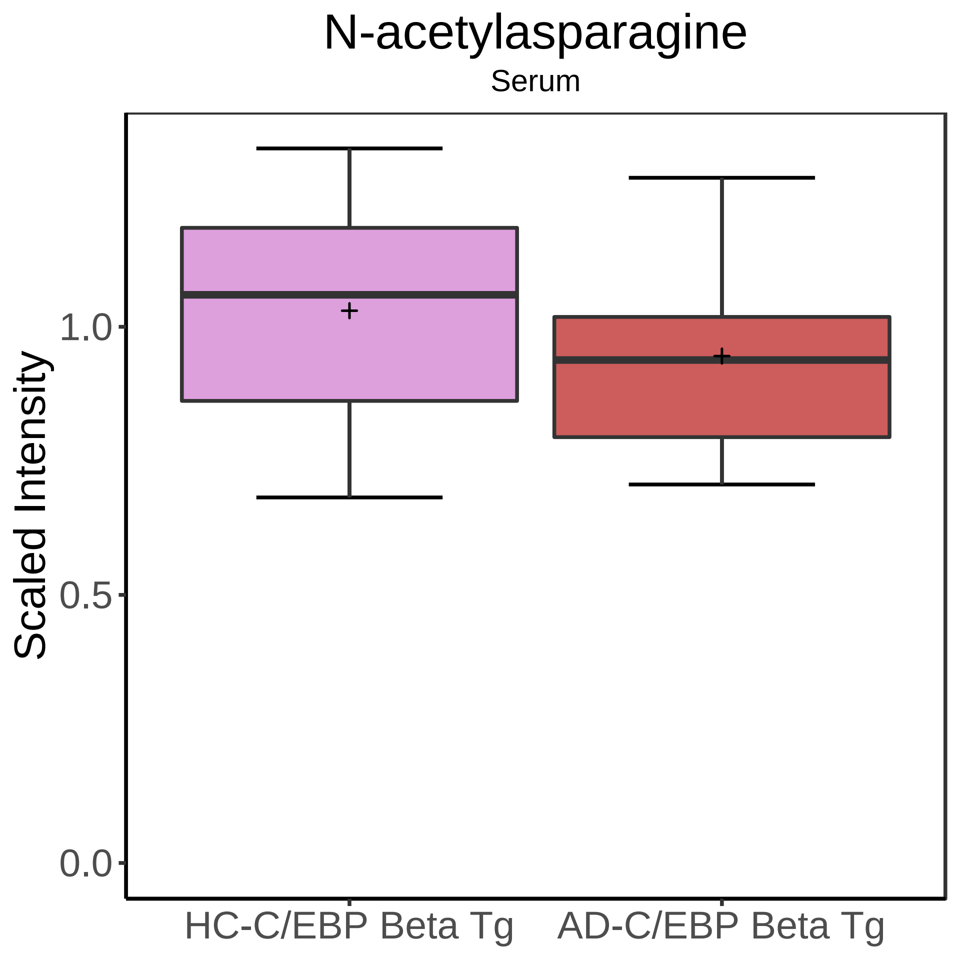
N-acetylasparagine
Serum
Scaled Intensity
1.0
0.5
0.0
HC-C/EBP Beta Tg
AD-C/EBP Beta Tg

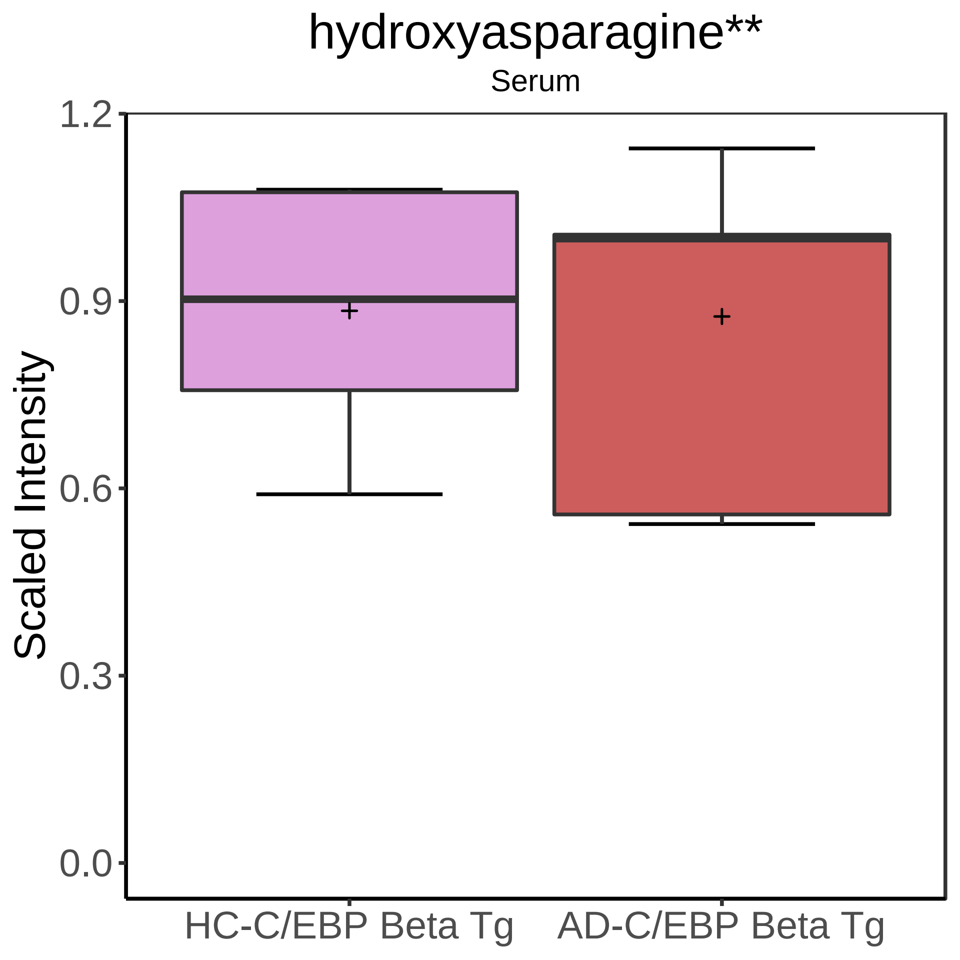
hydroxyasparagine**
Serum
Scaled Intensity
1.2
0.9
0.6
0.3
0.0
HC-C/EBP Beta Tg
AD-C/EBP Beta Tg

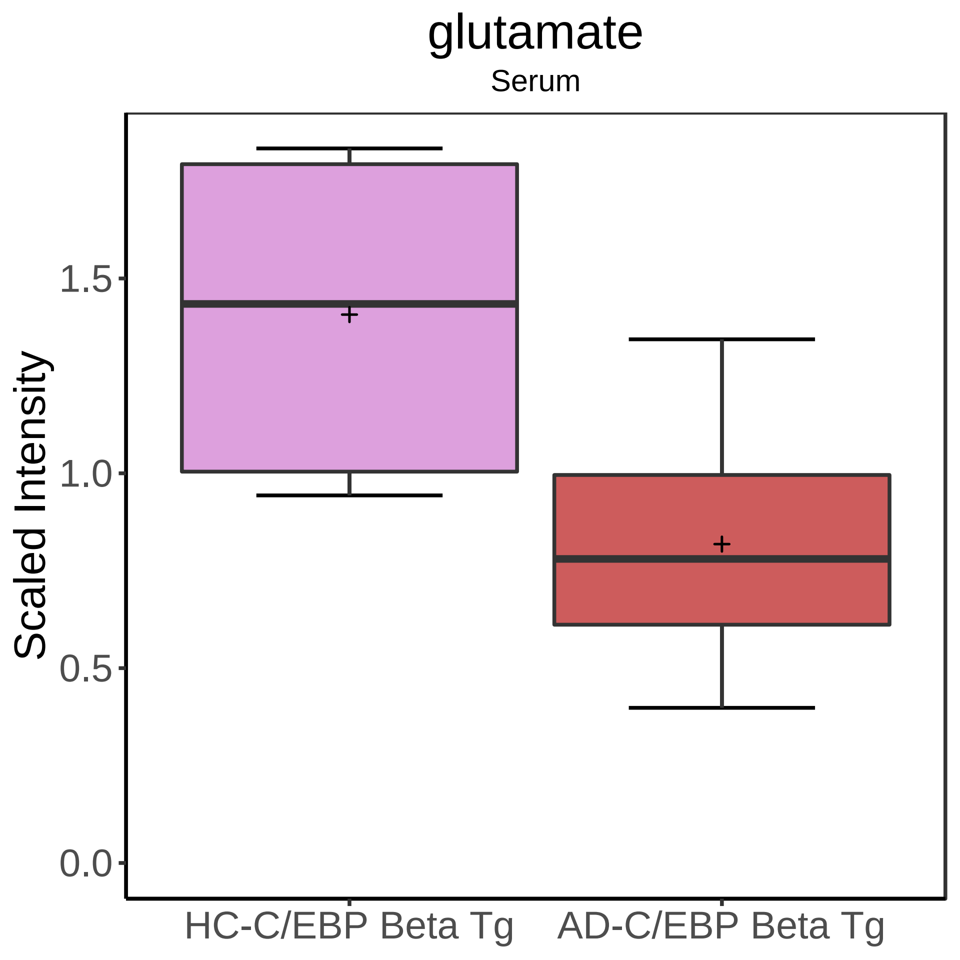

glutamate
Serum
Scaled Intensity
1.5
1.0
0.5
0.0
HC-C/EBP Beta Tg
AD-C/EBP Beta Tg

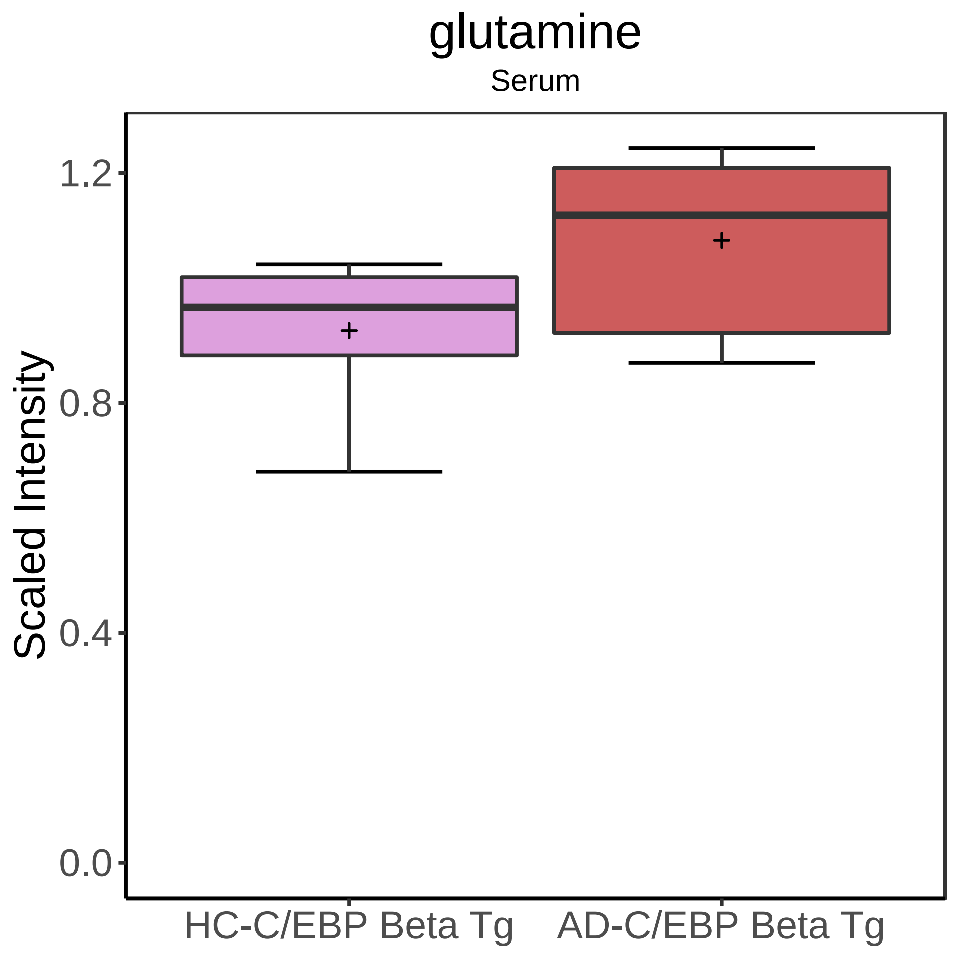
glutamine
Serum
Scaled Intensity
1.2
0.8
0.4
0.0
HC-C/EBP Beta Tg
AD-C/EBP Beta Tg

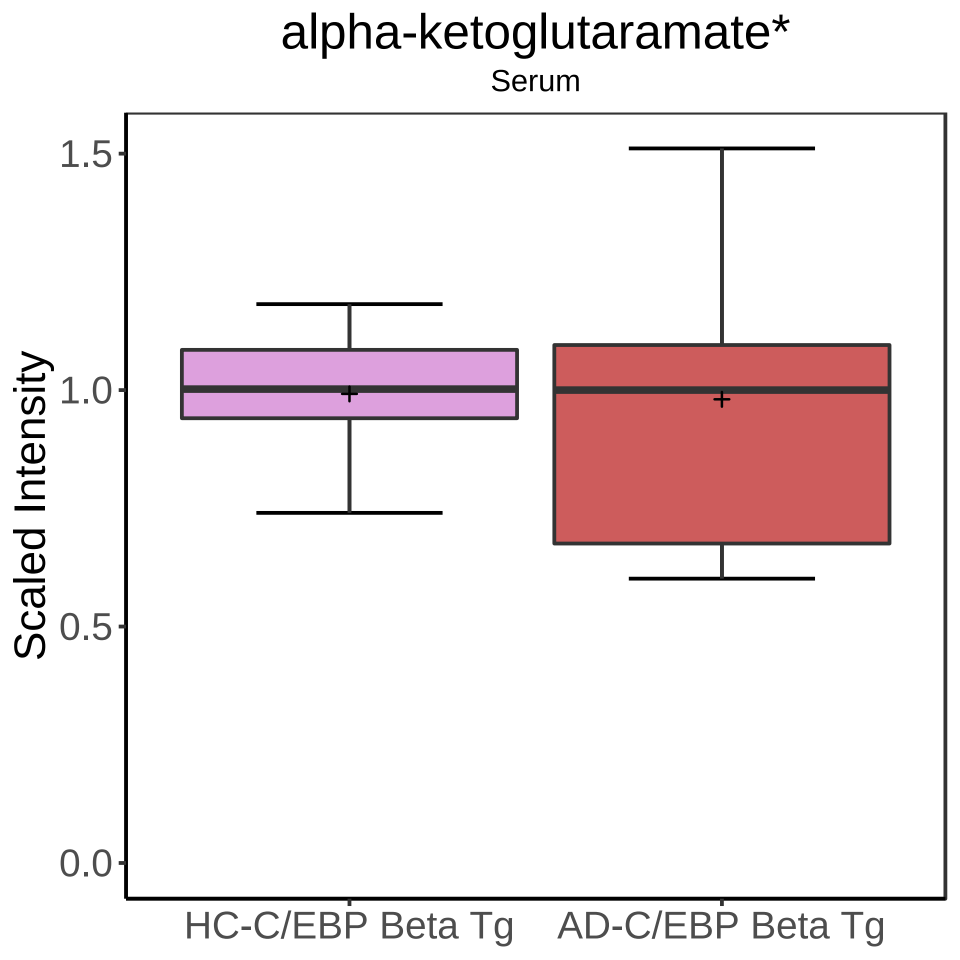
alpha-ketoglutaramate*
Serum
Scaled Intensity
1.5
1.0
0.5
0.0
HC-C/EBP Beta Tg
AD-C/EBP Beta Tg

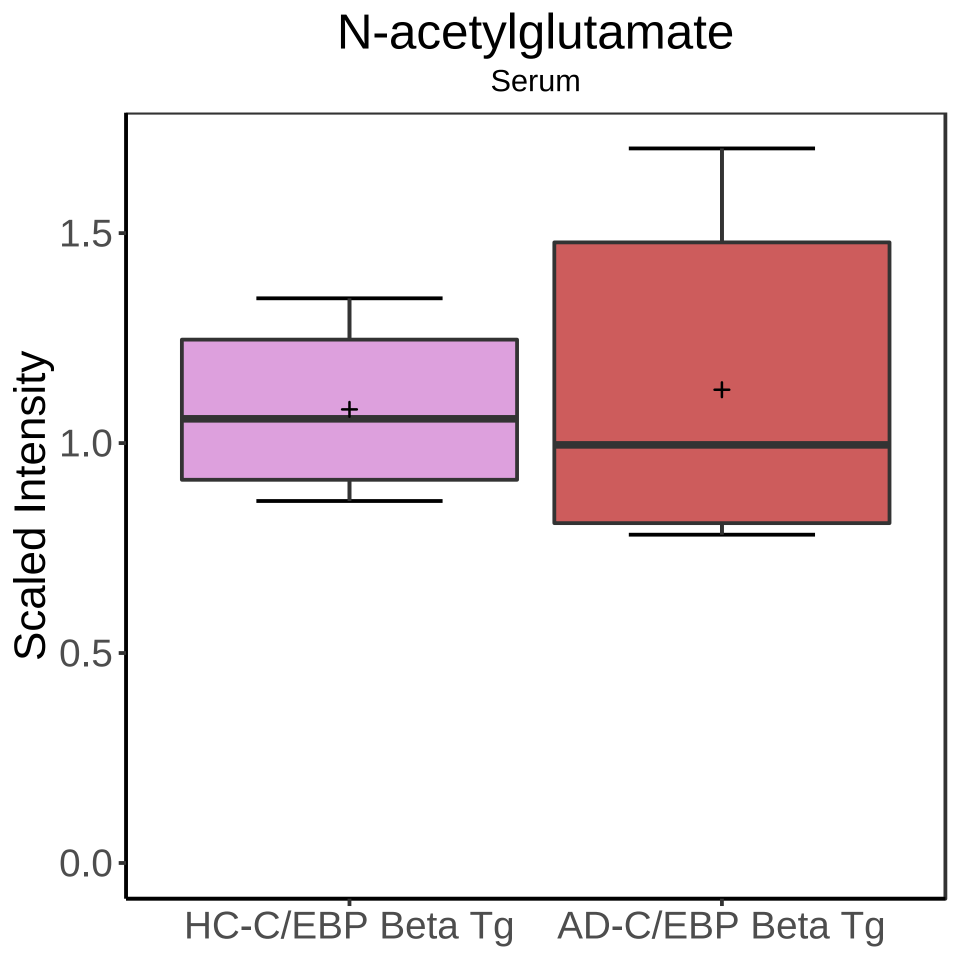
N-acetylglutamate
Serum
Scaled Intensity
1.5
1.0
0.5
0.0
HC-C/EBP Beta Tg
AD-C/EBP Beta Tg

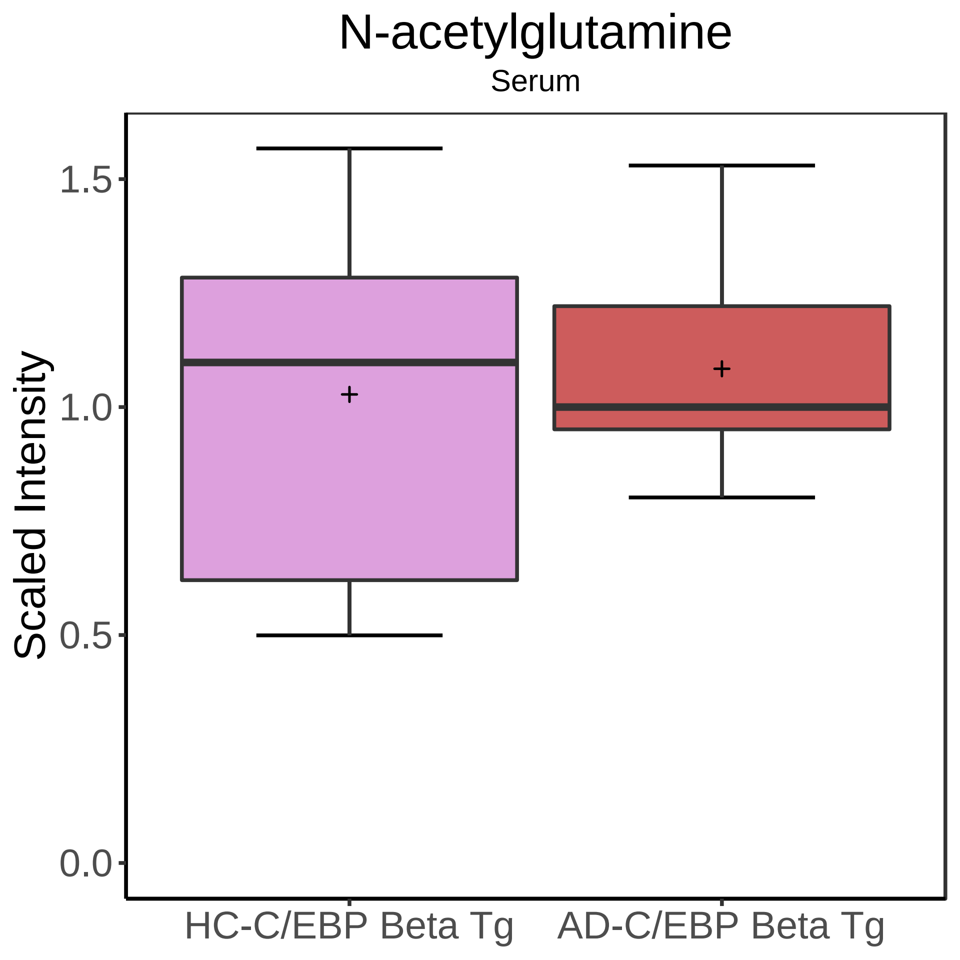
N-acetylglutamine
Serum
Scaled Intensity
1.5
1.0
0.5
0.0
HC-C/EBP Beta Tg
AD-C/EBP Beta Tg

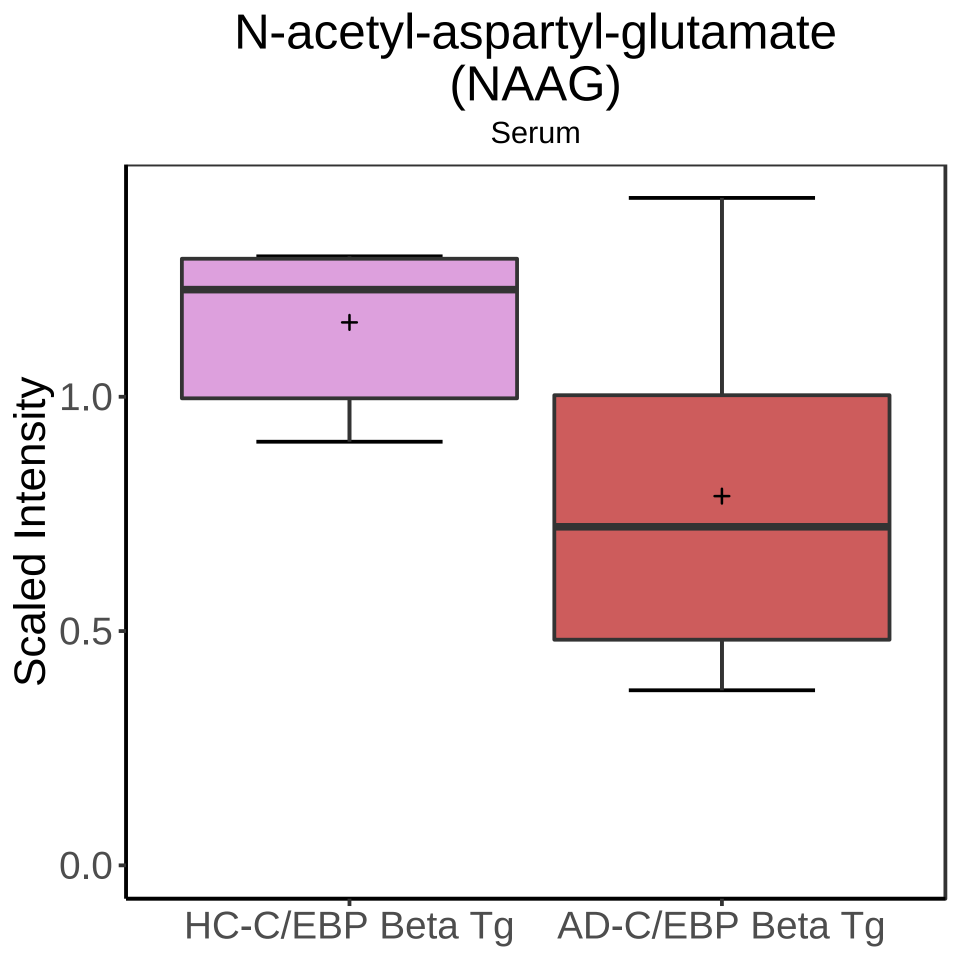
N-acetyl-aspartyl-glutamate
(NAAG)
Serum
Scaled Intensity
1.0
0.5
0.0
HC-C/EBP Beta Tg
AD-C/EBP Beta Tg

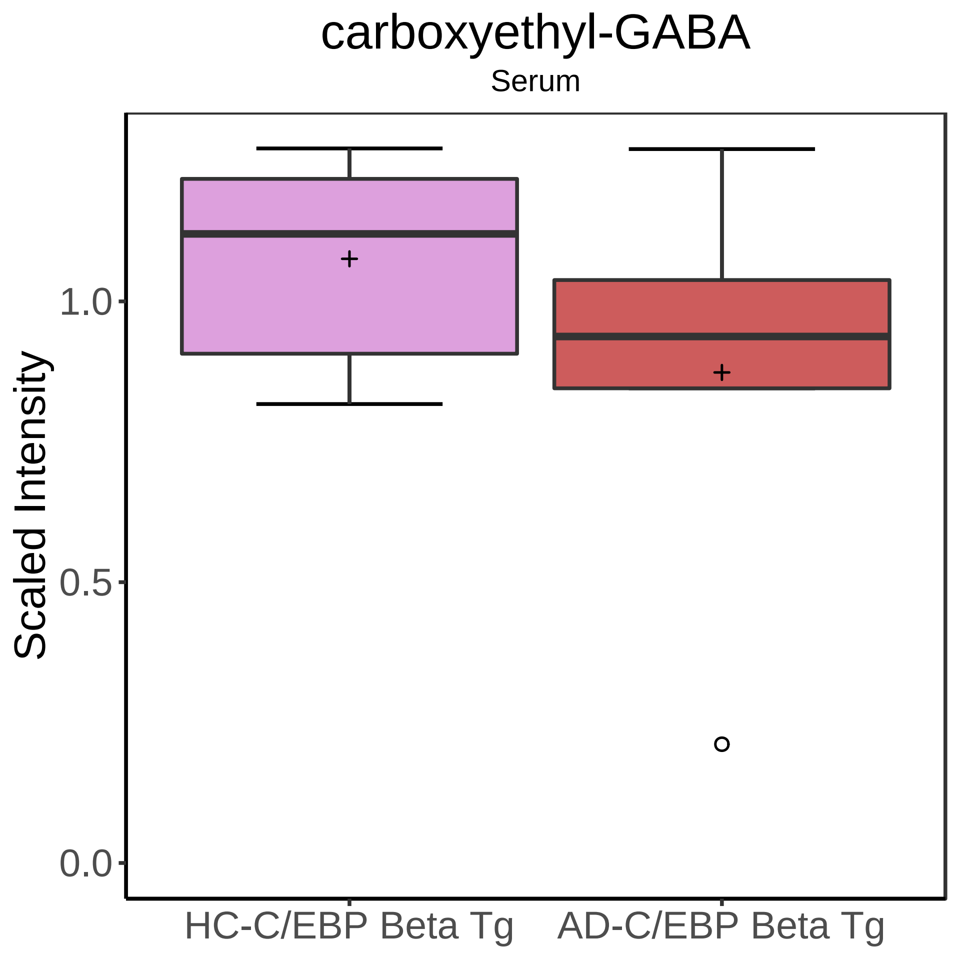

carboxyethyl-GABA
Serum
Scaled Intensity
1.0
0.5
0.0
HC-C/EBP Beta Tg
AD-C/EBP Beta Tg

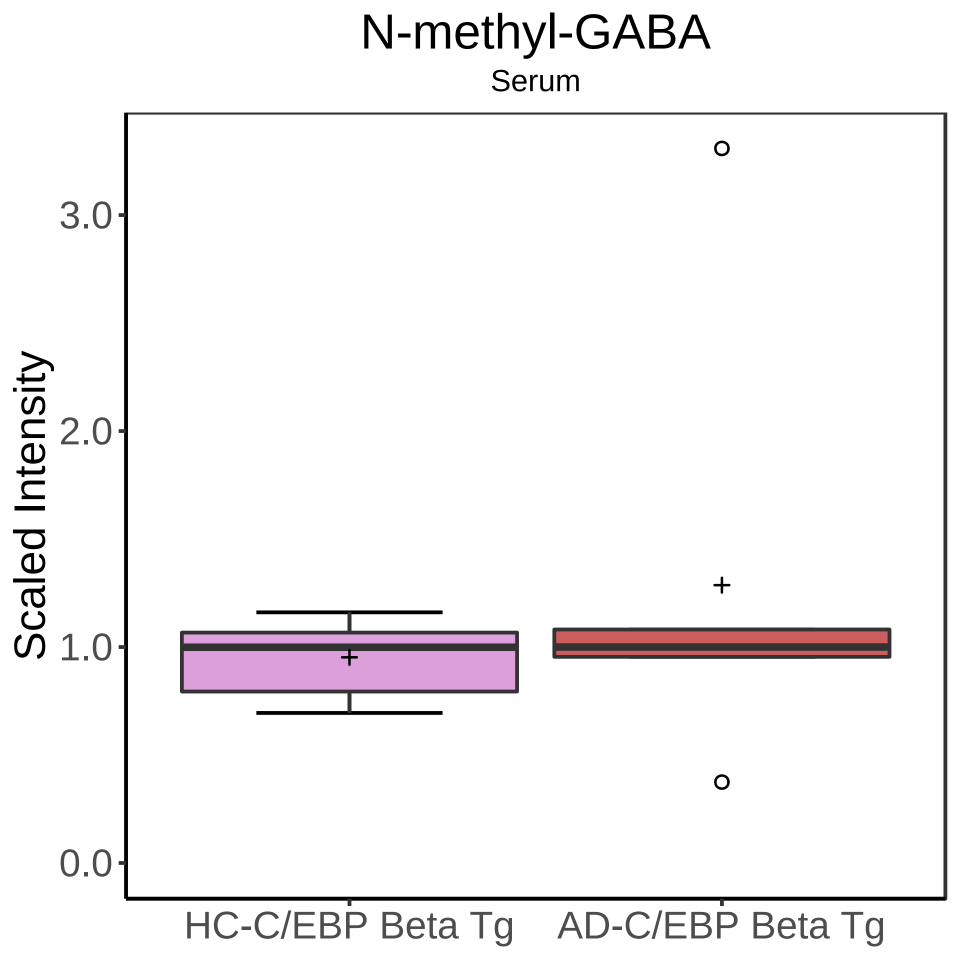
N-methyl-GABA
Serum
Scaled Intensity
3.0
2.0
1.0
0.0
HC-C/EBP Beta Tg
AD-C/EBP Beta Tg

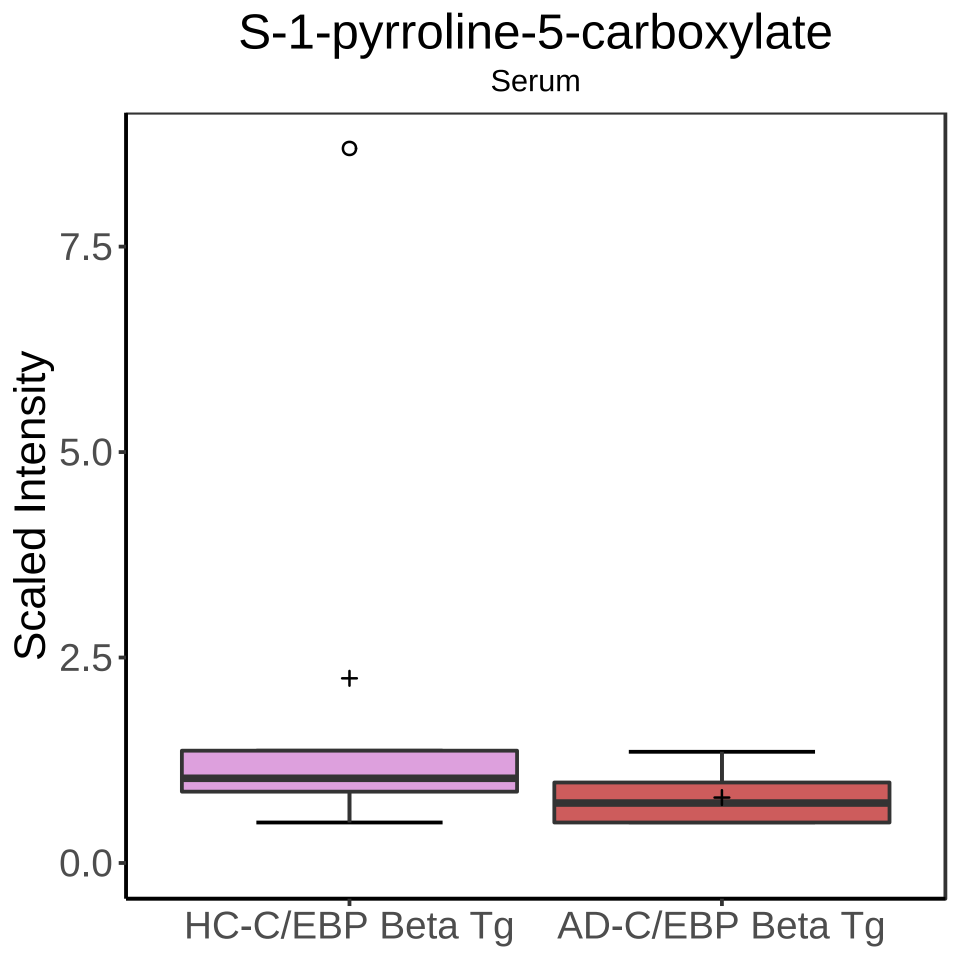
S-1-pyrroline-5-carboxylate
Serum
Scaled Intensity
7.5
5.0
2.5
0.0
HC-C/EBP Beta Tg
AD-C/EBP Beta Tg

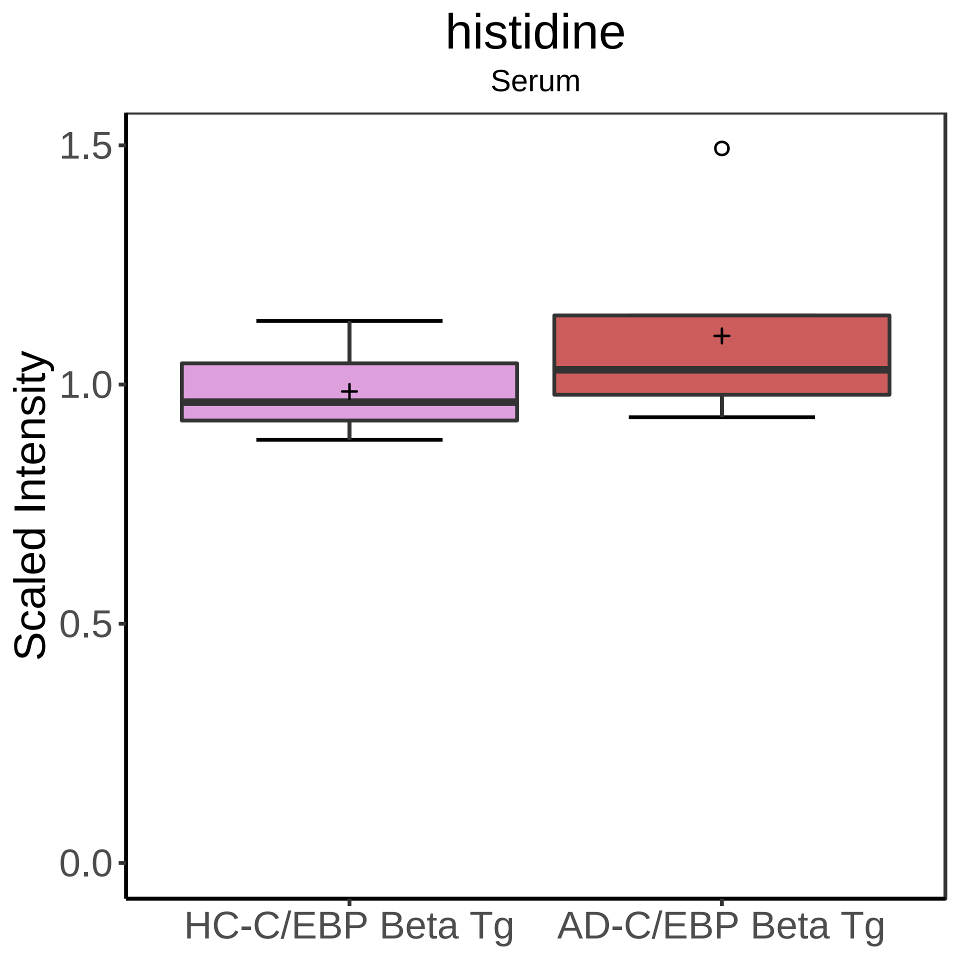
histidine
Serum
Scaled Intensity
1.5
1.0
0.5
0.0
HC-C/EBP Beta Tg
AD-C/EBP Beta Tg

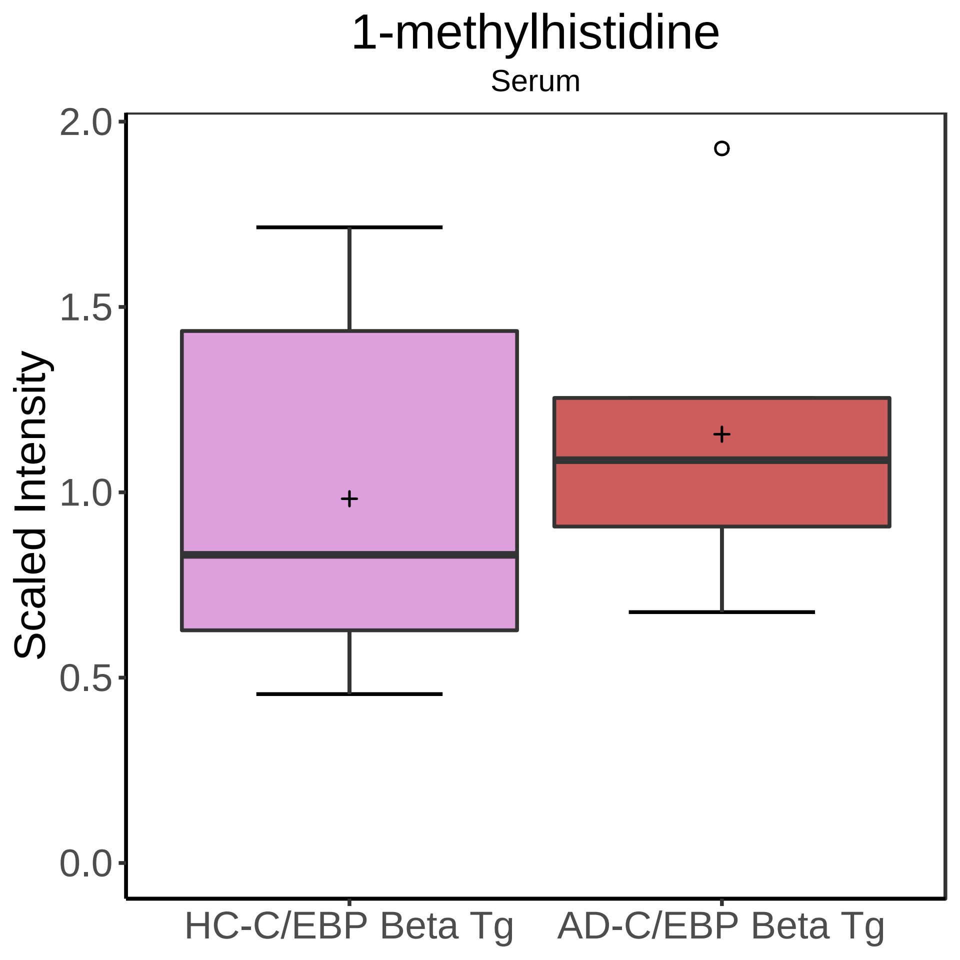
1-methylhistidine
Serum
Scaled Intensity
2.0
1.5
1.0
0.5
0.0
HC-C/EBP Beta Tg
AD-C/EBP Beta Tg

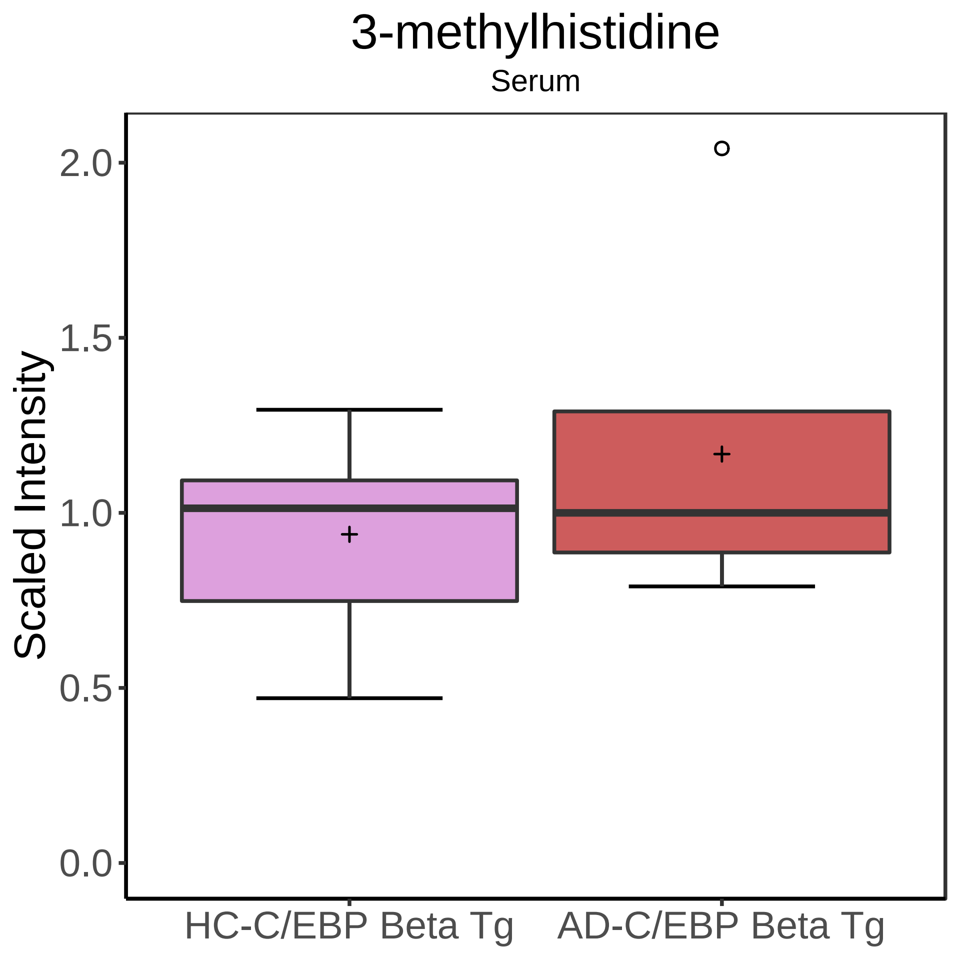
3-methylhistidine
Serum
Scaled Intensity
2.0
1.5
1.0
0.5
0.0
HC-C/EBP Beta Tg
AD-C/EBP Beta Tg

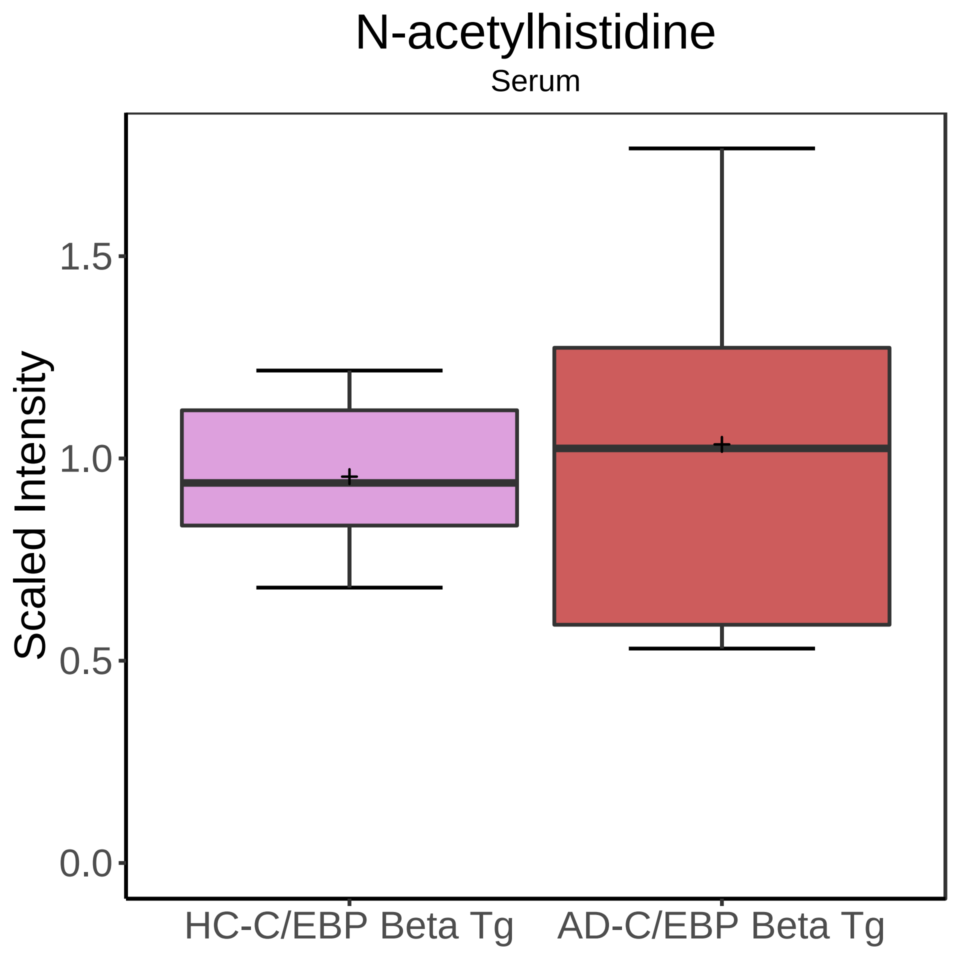

N-acetylhistidine
Serum
Scaled Intensity
1.5
1.0
0.5
0.0
HC-C/EBP Beta Tg
AD-C/EBP Beta Tg

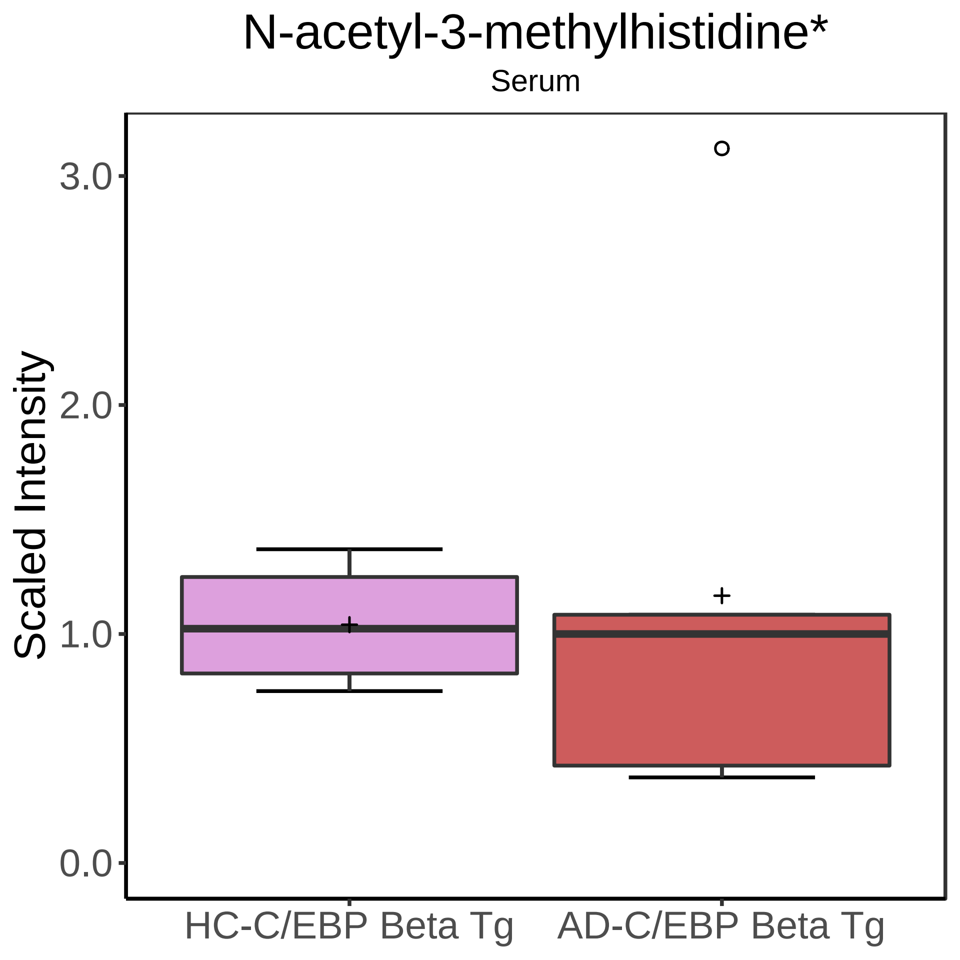
N-acetyl-3-methylhistidine*
Serum
Scaled Intensity
3.0
2.0
1.0
0.0
HC-C/EBP Beta Tg
AD-C/EBP Beta Tg

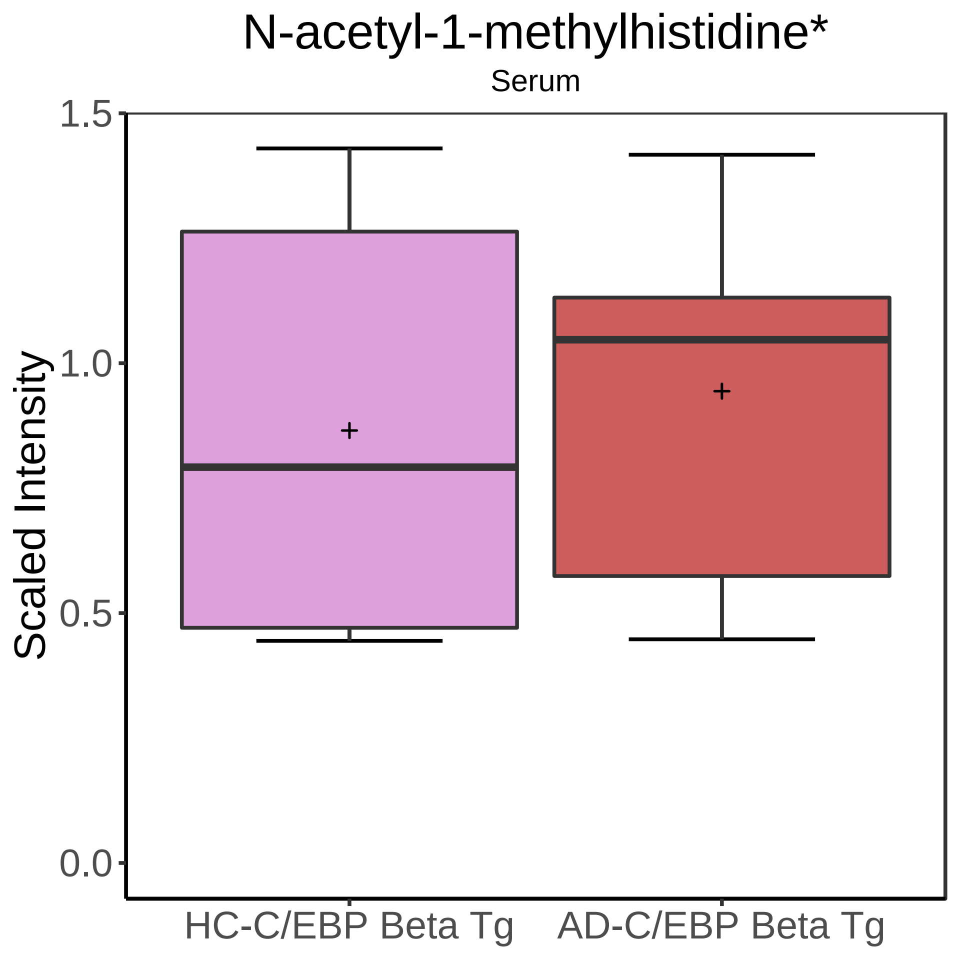

N-acetyl-1-methylhistidine*
Serum
Scaled Intensity
1.5
1.0
0.5
0.0
HC-C/EBP Beta Tg
AD-C/EBP Beta Tg

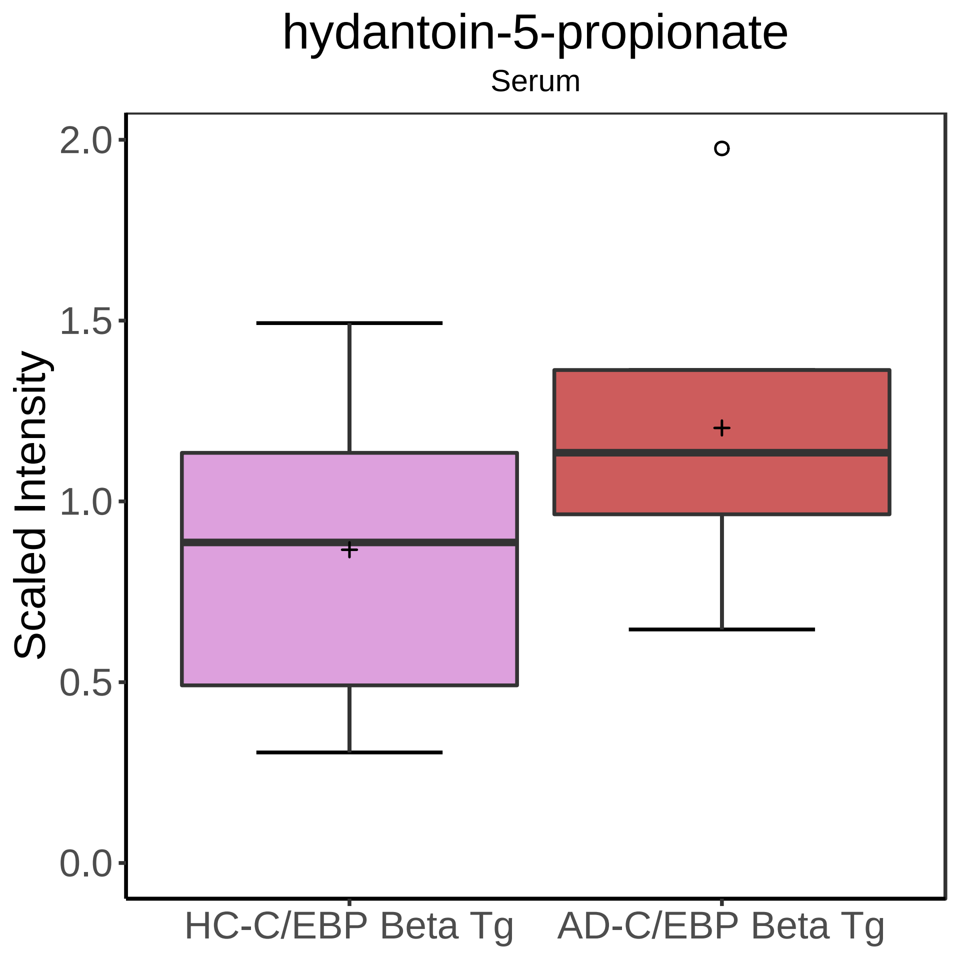
hydantoin-5-propionate
Serum
Scaled Intensity
2.0
1.5
1.0
0.5
0.0
HC-C/EBP Beta Tg
AD-C/EBP Beta Tg

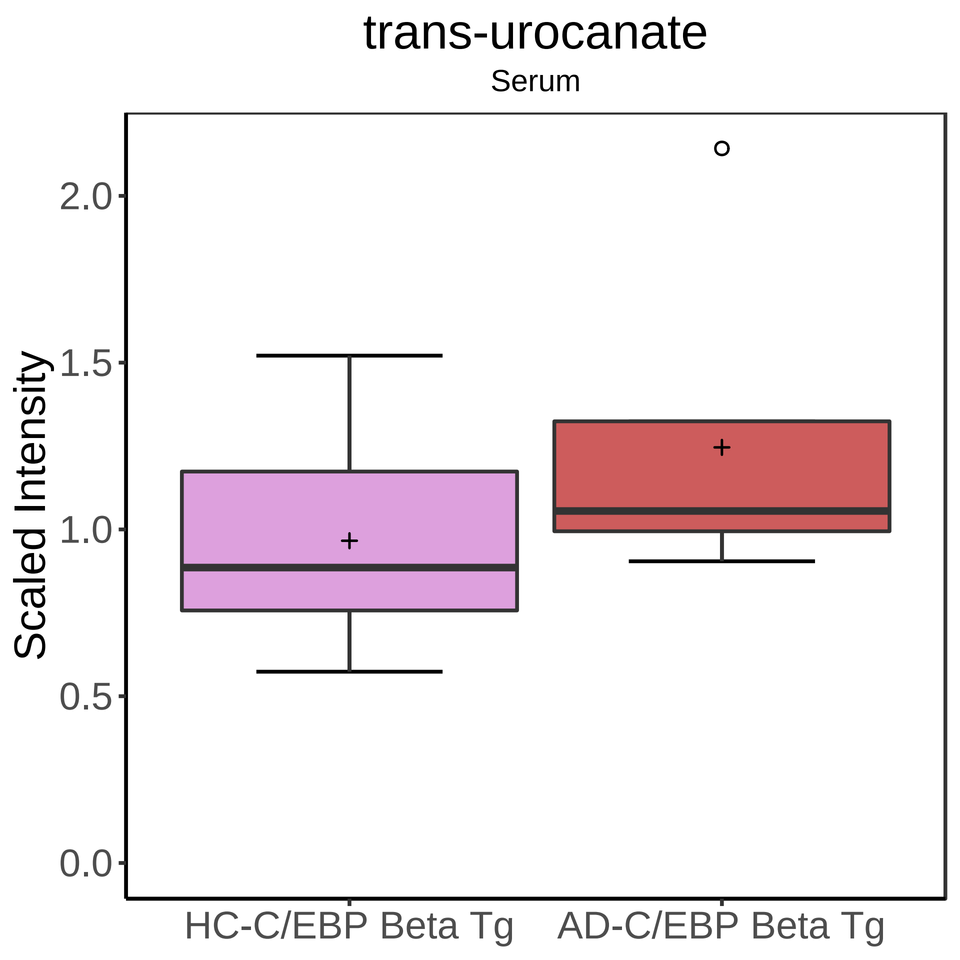
trans-urocanate
Serum
Scaled Intensity
0.0
0.5
1.0
1.5
2.0
HC-C/EBP Beta Tg
AD-C/EBP Beta Tg

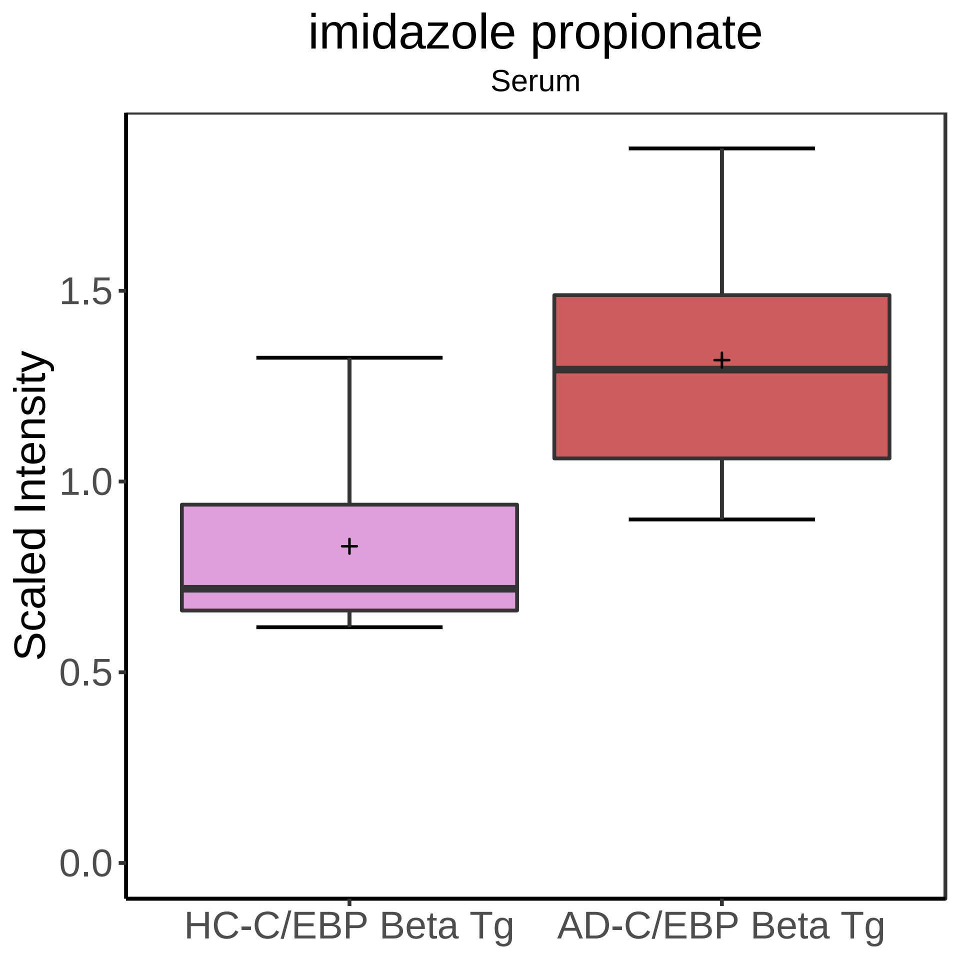
imidazole propionate
Serum
Scaled Intensity
1.5
1.0
0.5
0.0
HC-C/EBP Beta Tg
AD-C/EBP Beta Tg

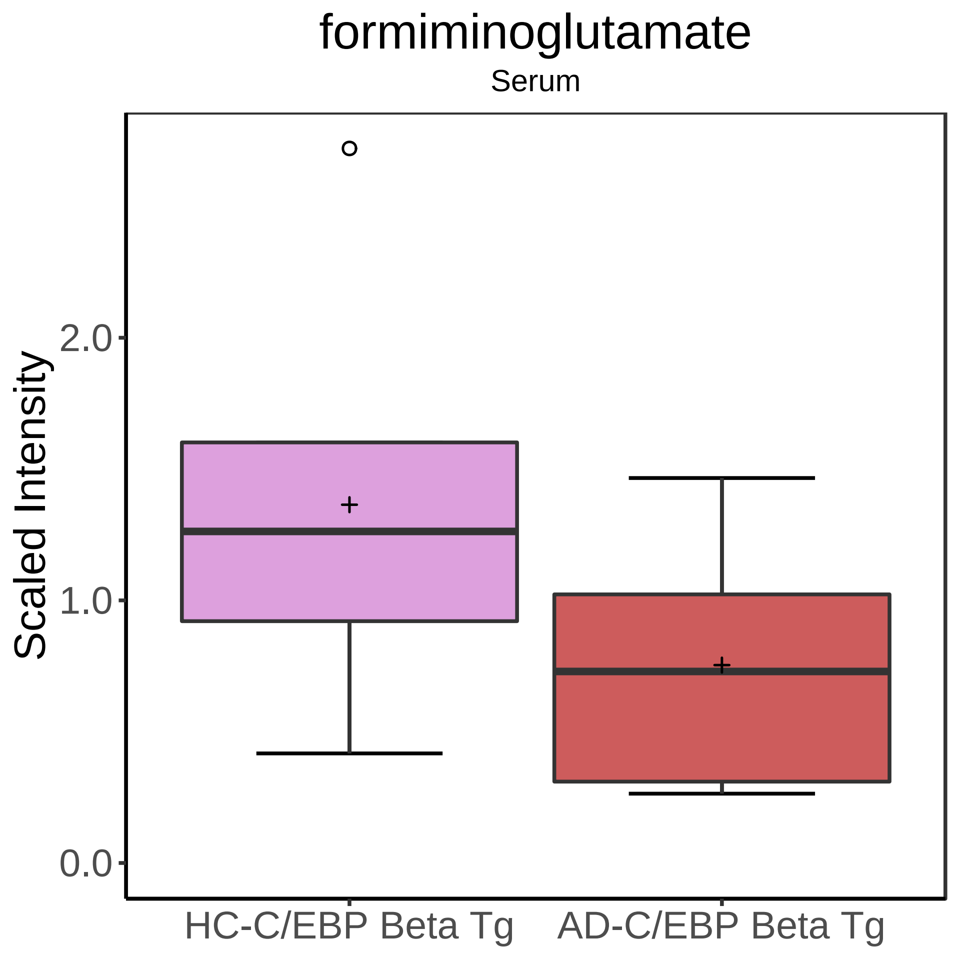
formiminoglutamate
Serum
Scaled Intensity
2.0
1.0
0.0
HC-C/EBP Beta Tg
AD-C/EBP Beta Tg

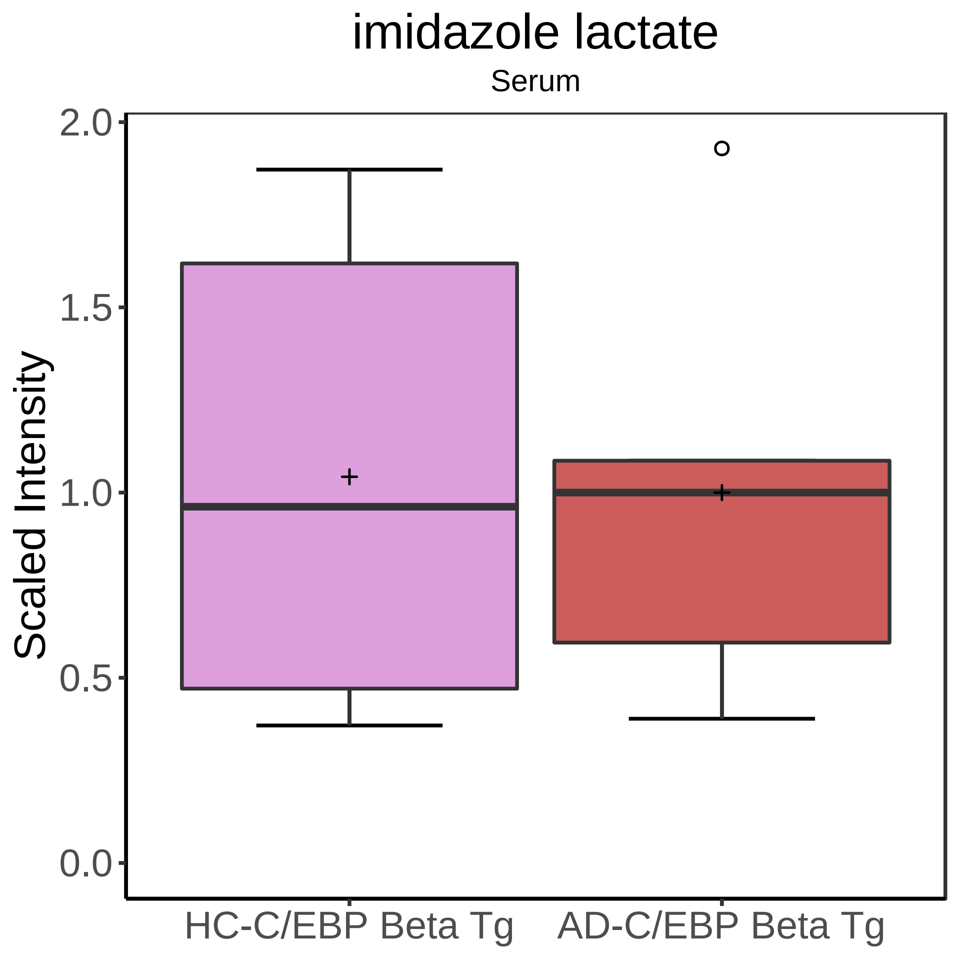
imidazole lactate
Serum
Scaled Intensity
2.0
1.5
1.0
0.5
0.0
HC-C/EBP Beta Tg
AD-C/EBP Beta Tg

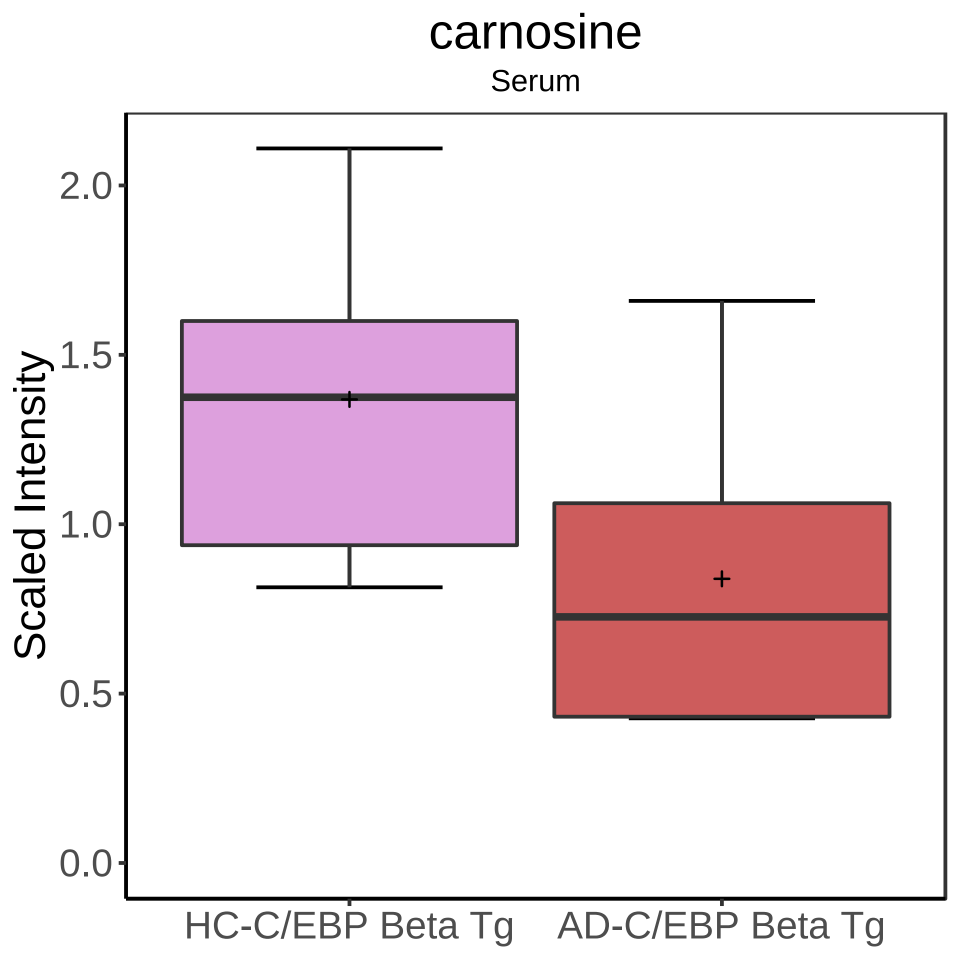
carnosine
Serum
Scaled Intensity
2.0
1.5
1.0
0.5
0.0
HC-C/EBP Beta Tg
AD-C/EBP Beta Tg

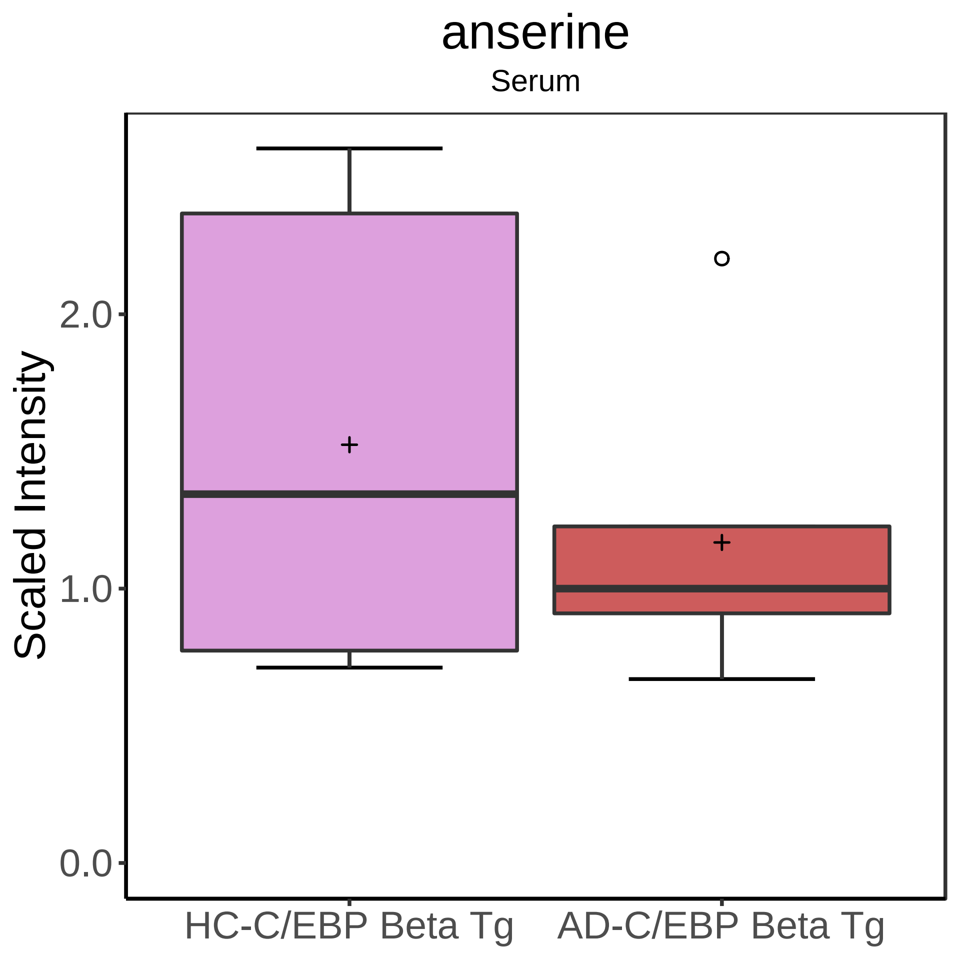

anserine
Serum
Scaled Intensity
2.0
1.0
0.0
HC-C/EBP Beta Tg
AD-C/EBP Beta Tg

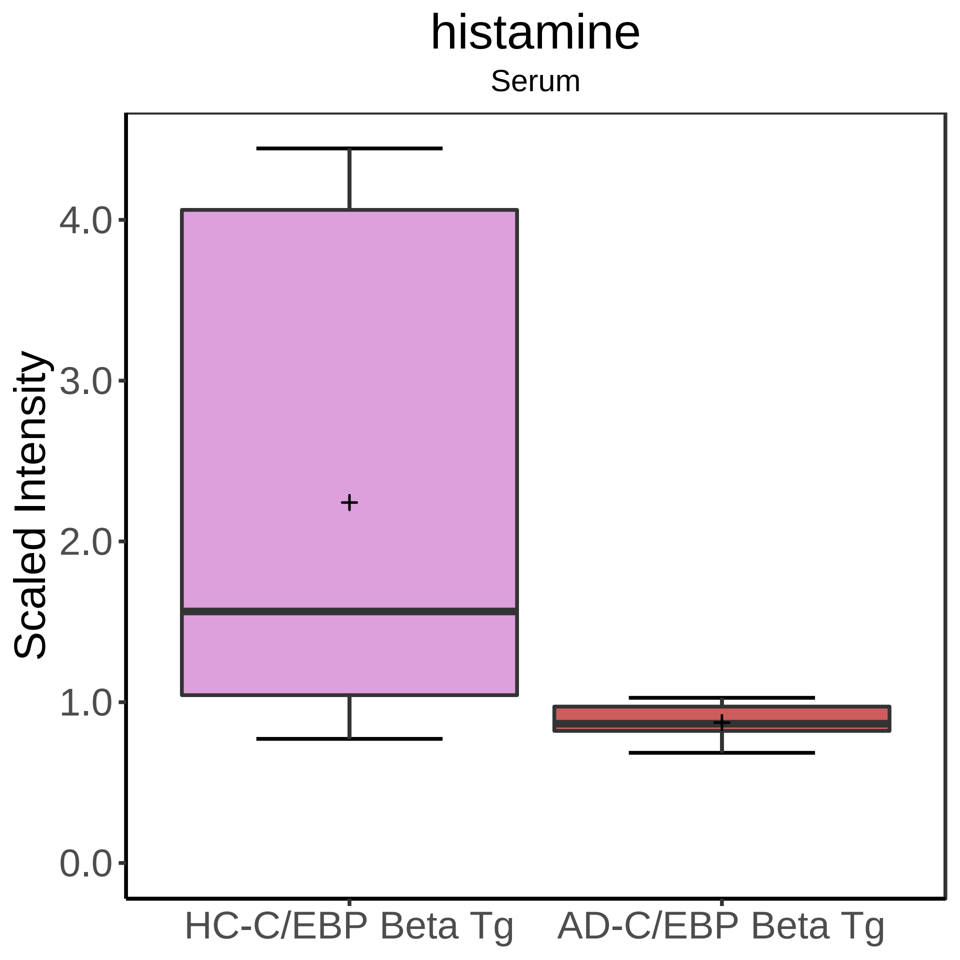

histamine
Serum
Scaled Intensity
4.0
3.0
2.0
1.0
0.0
HC-C/EBP Beta Tg
AD-C/EBP Beta Tg

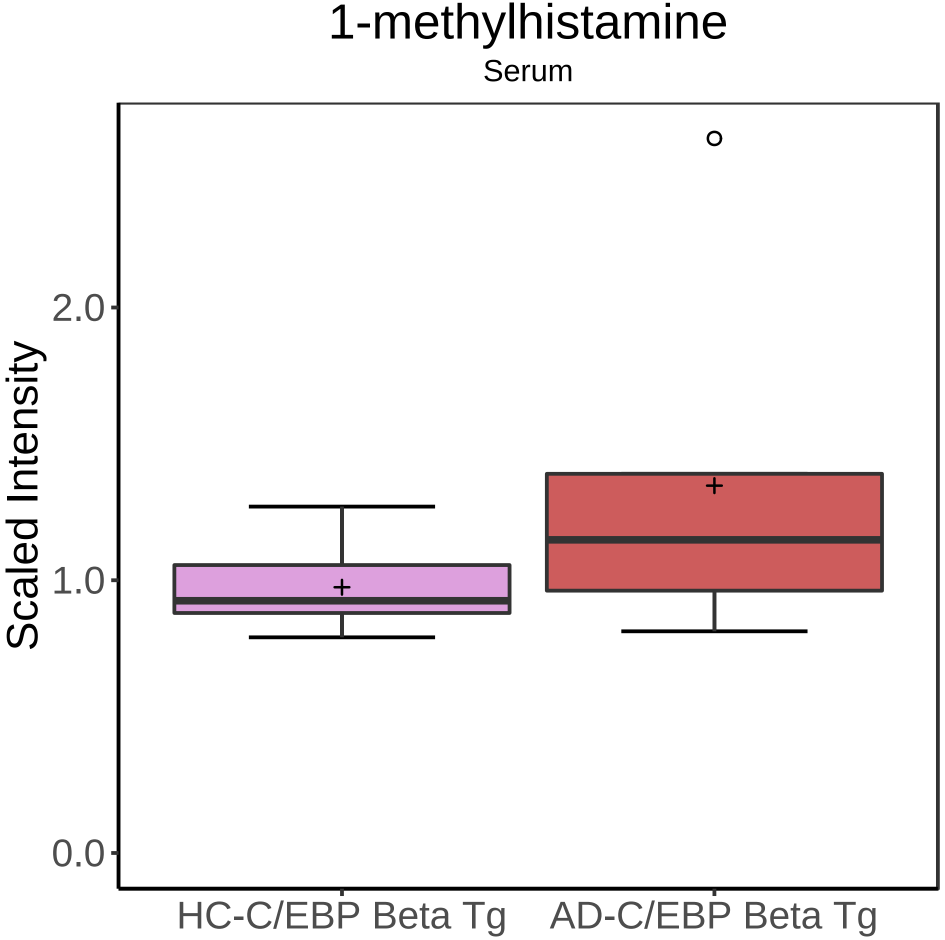
1-methylhistamine
Serum
Scaled Intensity
2.0
1.0
0.0
HC-C/EBP Beta Tg
AD-C/EBP Beta Tg

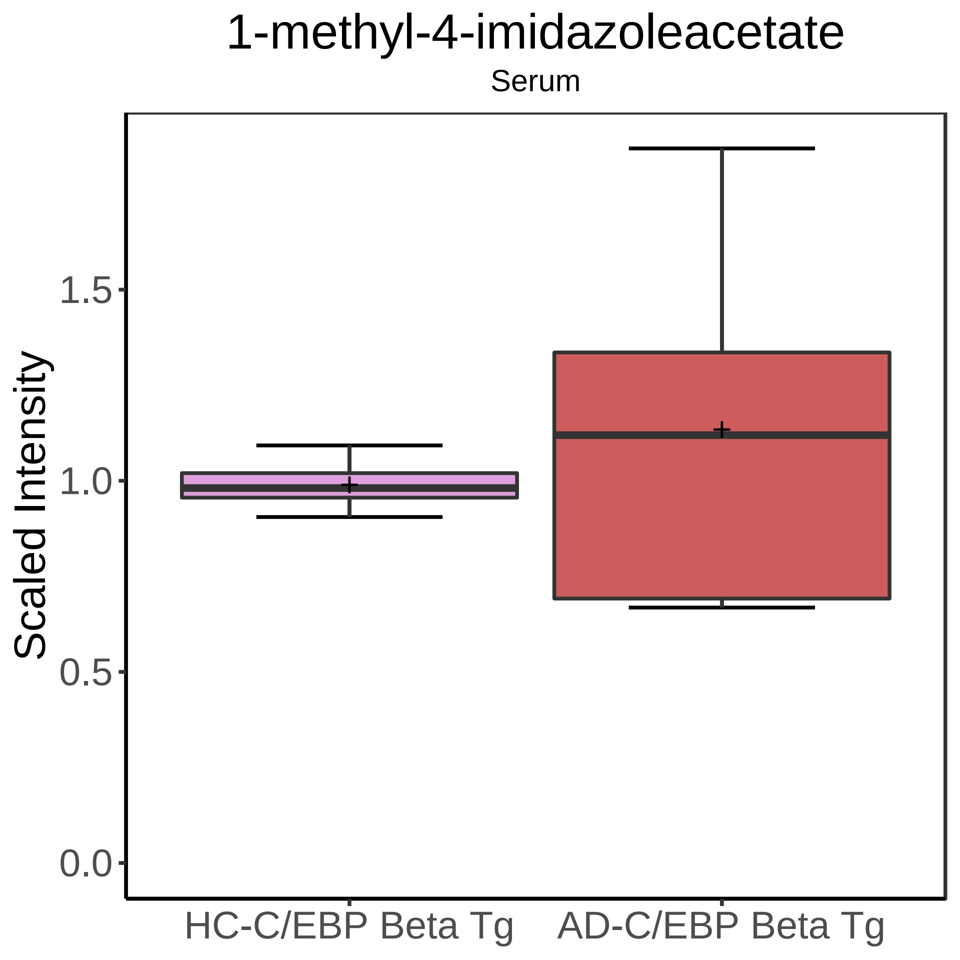
1-methyl-4-imidazoleacetate
Serum
Scaled Intensity
1.5
1.0
0.5
0.0
HC-C/EBP Beta Tg
AD-C/EBP Beta Tg

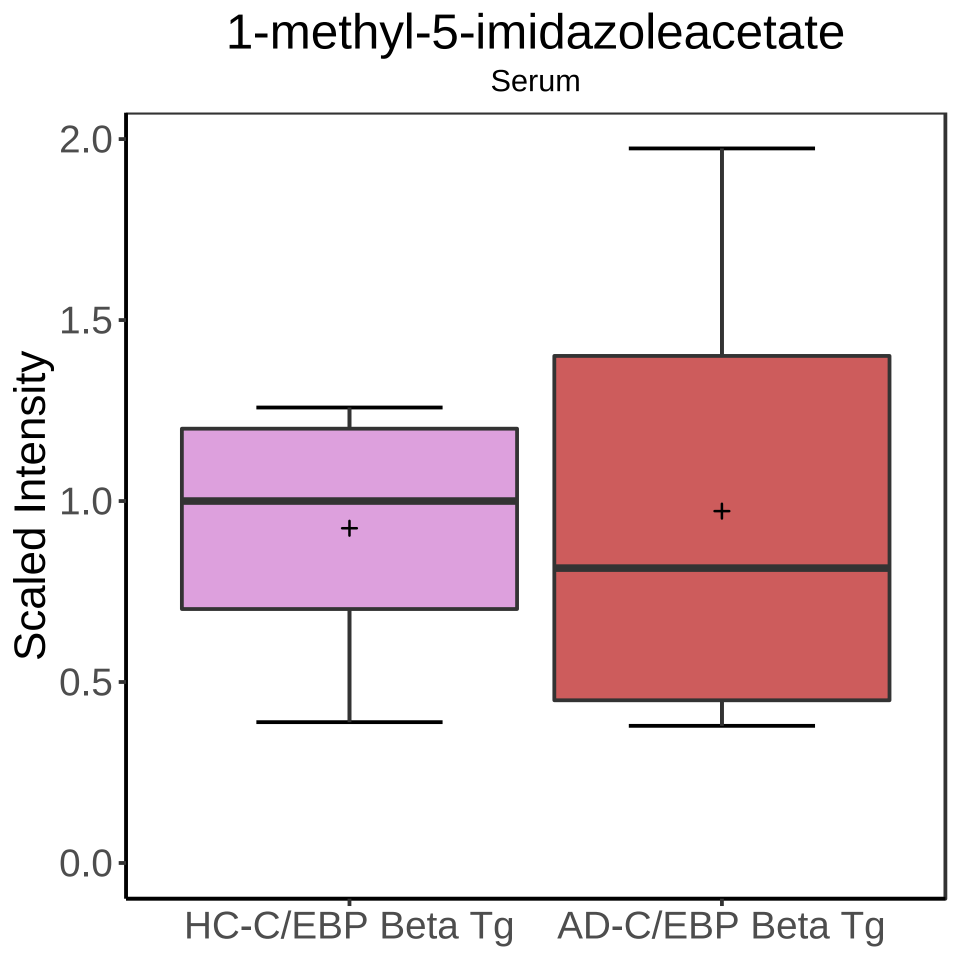

1-methyl-5-imidazoleacetate
Serum
Scaled Intensity
2.0
1.5
1.0
0.5
0.0
HC-C/EBP Beta Tg
AD-C/EBP Beta Tg

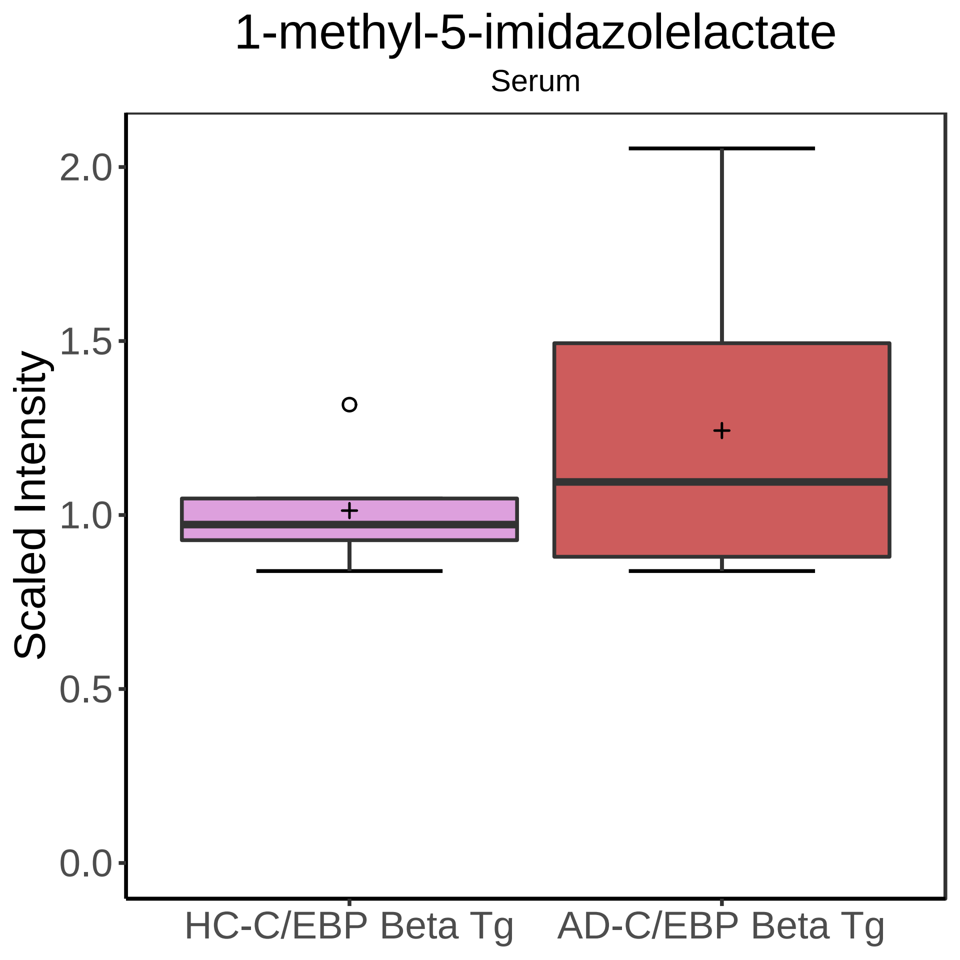
1-methyl-5-imidazolelactate
Serum
Scaled Intensity
2.0
1.5
1.0
0.5
0.0
HC-C/EBP Beta Tg
AD-C/EBP Beta Tg

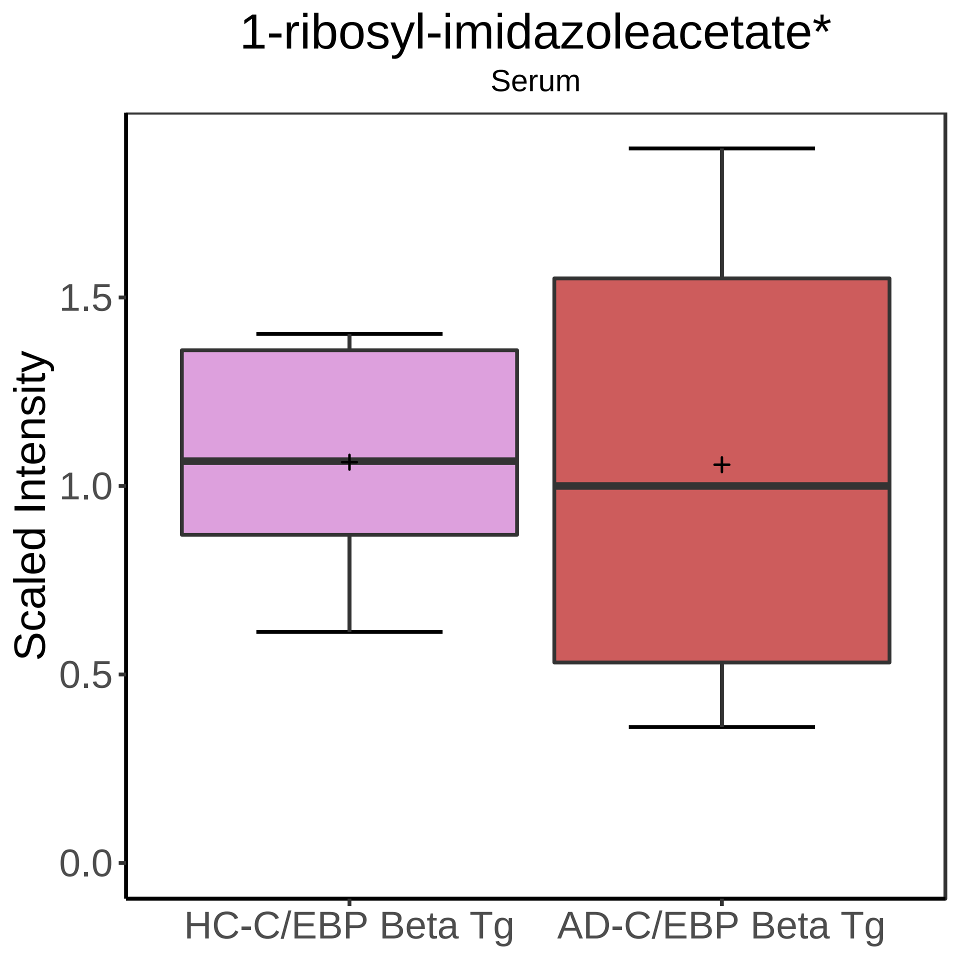
1-ribosyl-imidazoleacetate*
Serum
Scaled Intensity
0.0
0.5
1.0
1.5
HC-C/EBP Beta Tg
AD-C/EBP Beta Tg

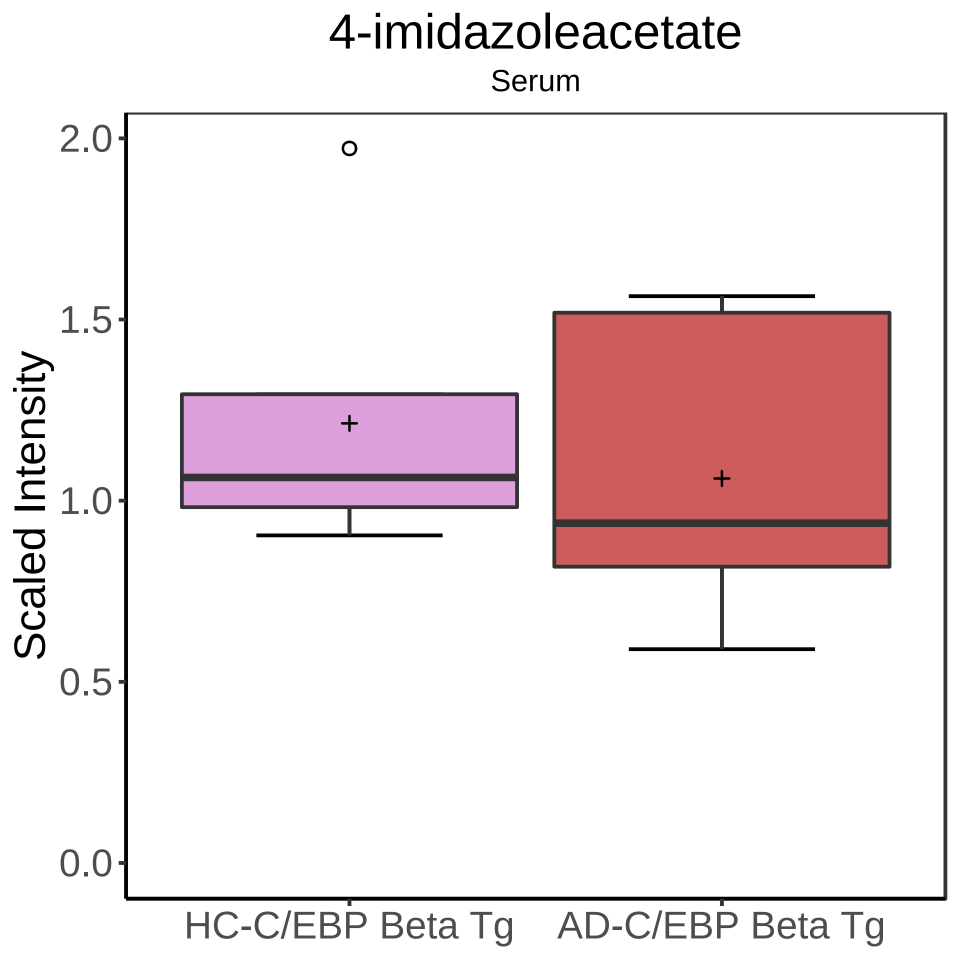
4-imidazoleacetate
Serum
Scaled Intensity
2.0
1.5
1.0
0.5
0.0
HC-C/EBP Beta Tg
AD-C/EBP Beta Tg

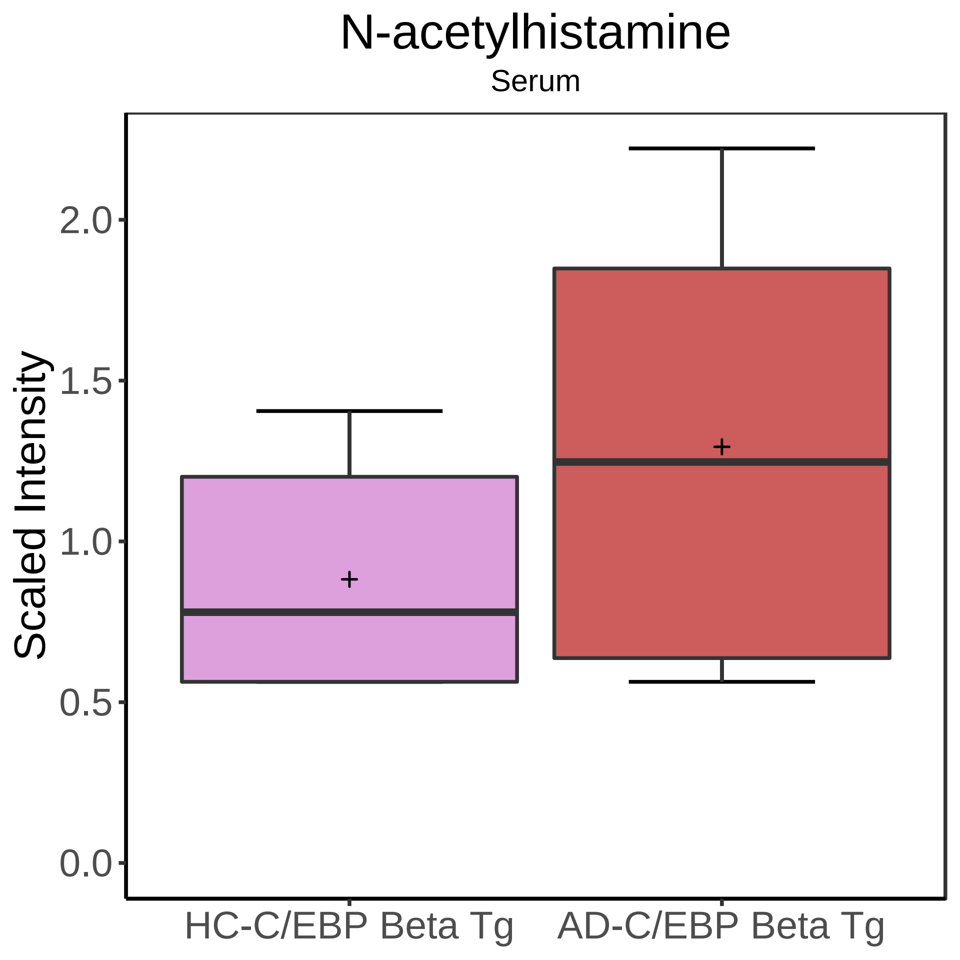
N-acetylhistamine
Serum
Scaled Intensity
0.0
0.5
1.0
1.5
2.0
HC-C/EBP Beta Tg
AD-C/EBP Beta Tg

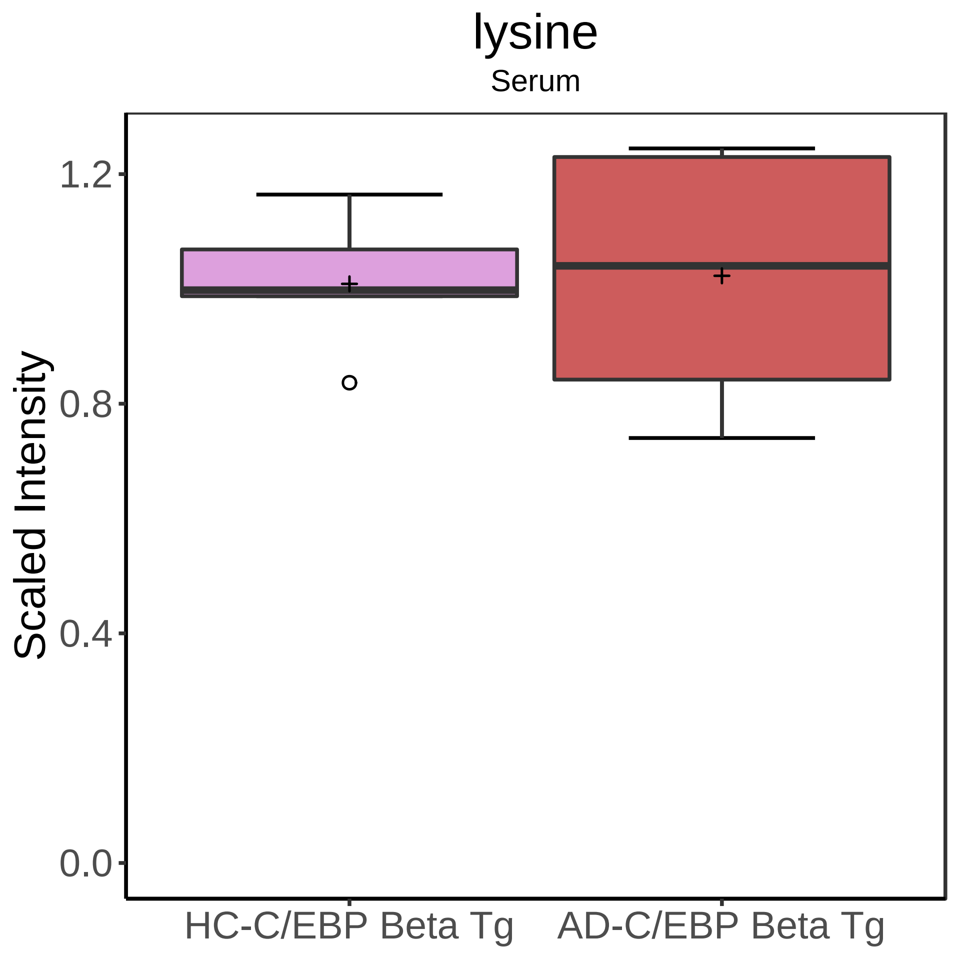
lysine
Serum
Scaled Intensity
1.2
0.8
0.4
0.0
HC-C/EBP Beta Tg
AD-C/EBP Beta Tg

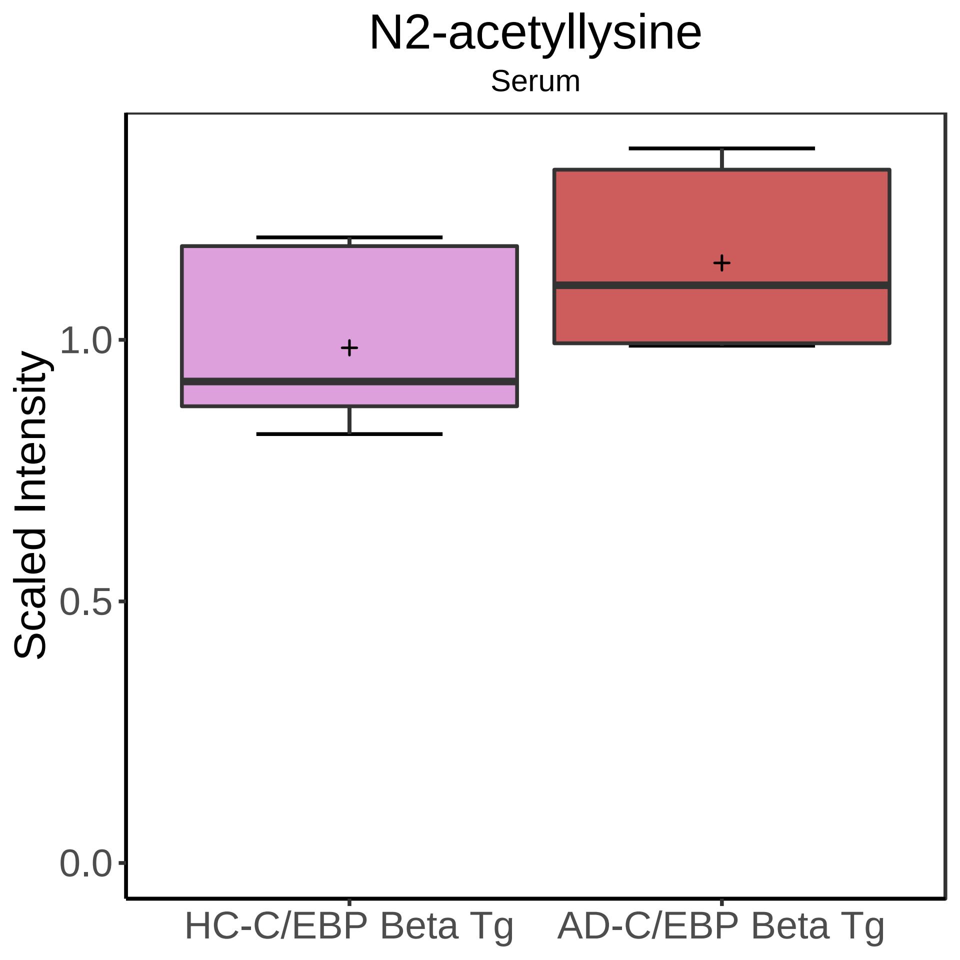

N2-acetyllysine
Serum
Scaled Intensity
1.0
0.5
0.0
HC-C/EBP Beta Tg
AD-C/EBP Beta Tg

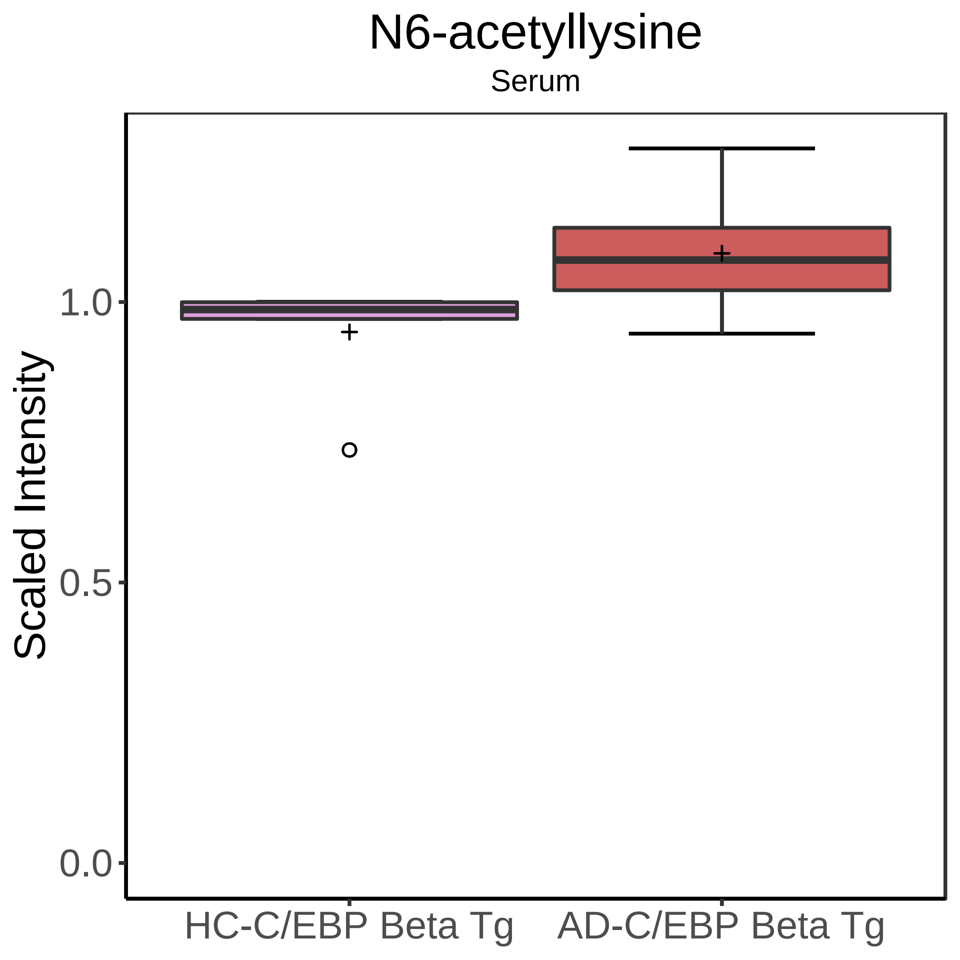
N6-acetyllysine
Serum
Scaled Intensity
1.0
0.5
0.0
HC-C/EBP Beta Tg
AD-C/EBP Beta Tg

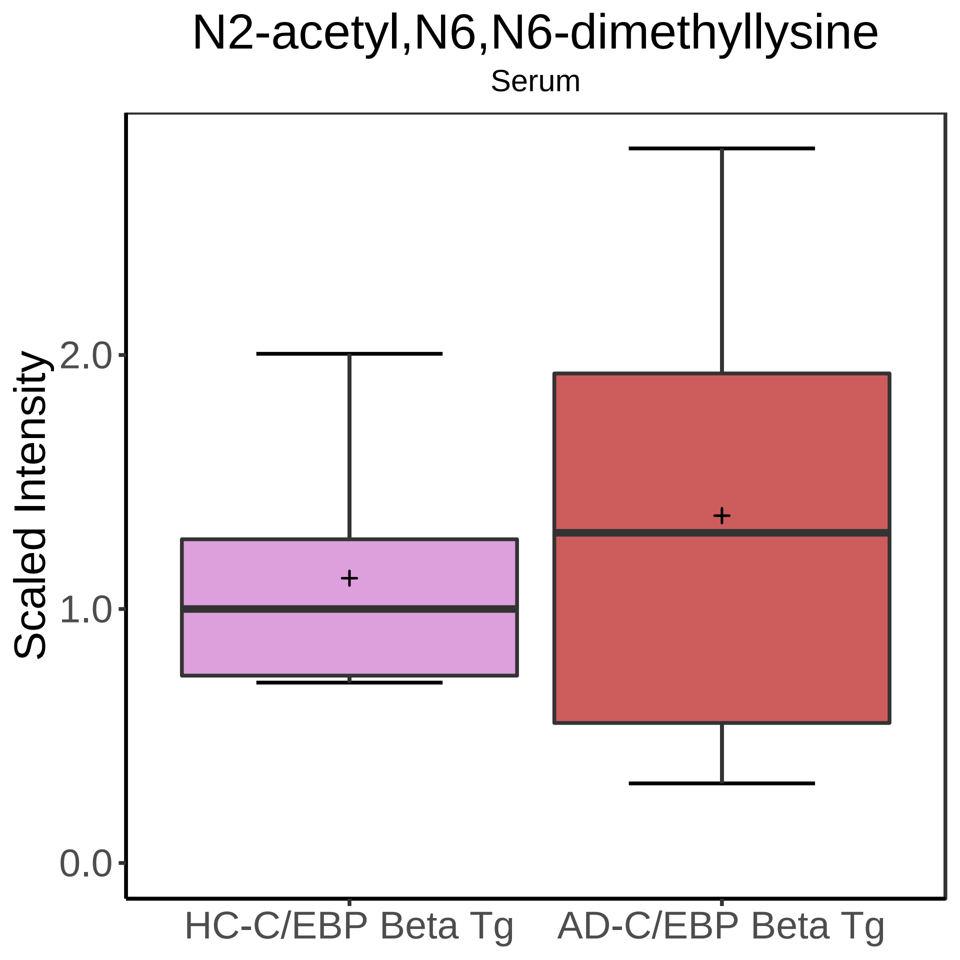
N2-acetyl,N6,N6-dimethyllysine
Serum
Scaled Intensity
2.0
1.0
0.0
HC-C/EBP Beta Tg
AD-C/EBP Beta Tg

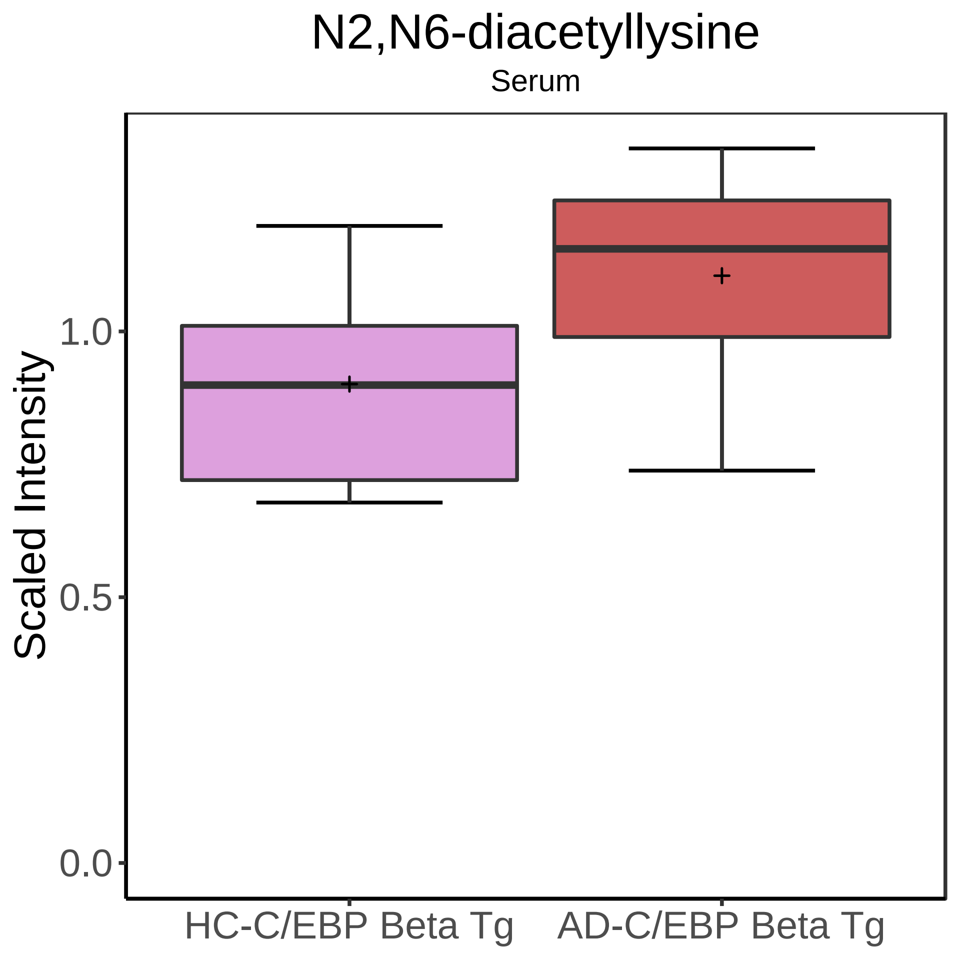

N2,N6-diacetyllysine
Serum
Scaled Intensity
1.0
0.5
0.0
HC-C/EBP Beta Tg
AD-C/EBP Beta Tg

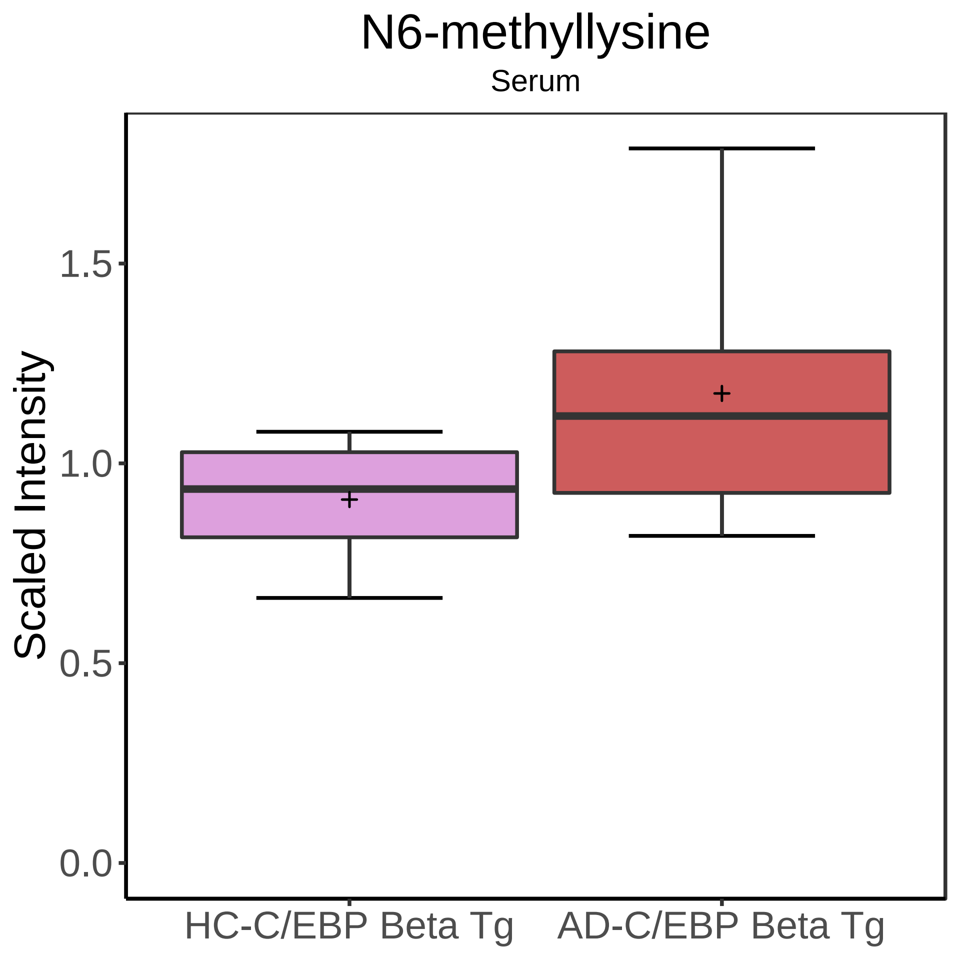
N6-methyllysine
Serum
Scaled Intensity
1.5
1.0
0.5
0.0
HC-C/EBP Beta Tg
AD-C/EBP Beta Tg

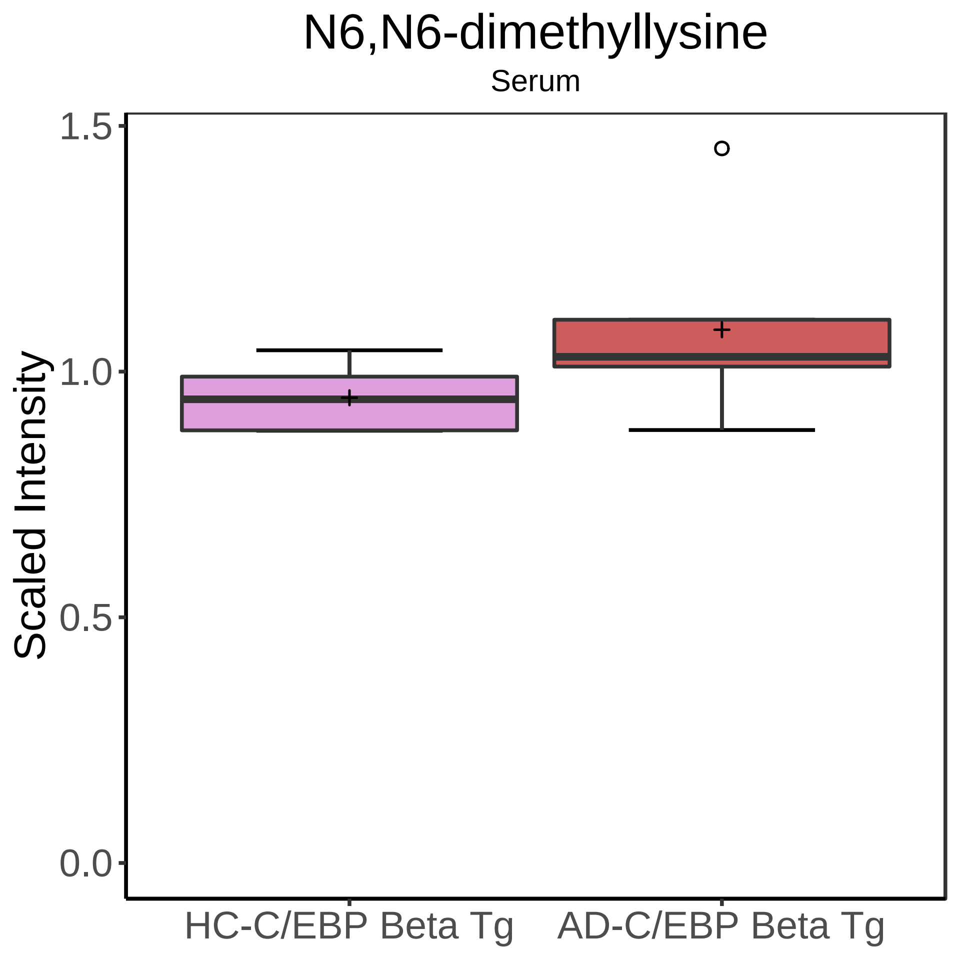
N6,N6-dimethyllysine
Serum
Scaled Intensity
1.5
1.0
0.5
0.0
HC-C/EBP Beta Tg
AD-C/EBP Beta Tg

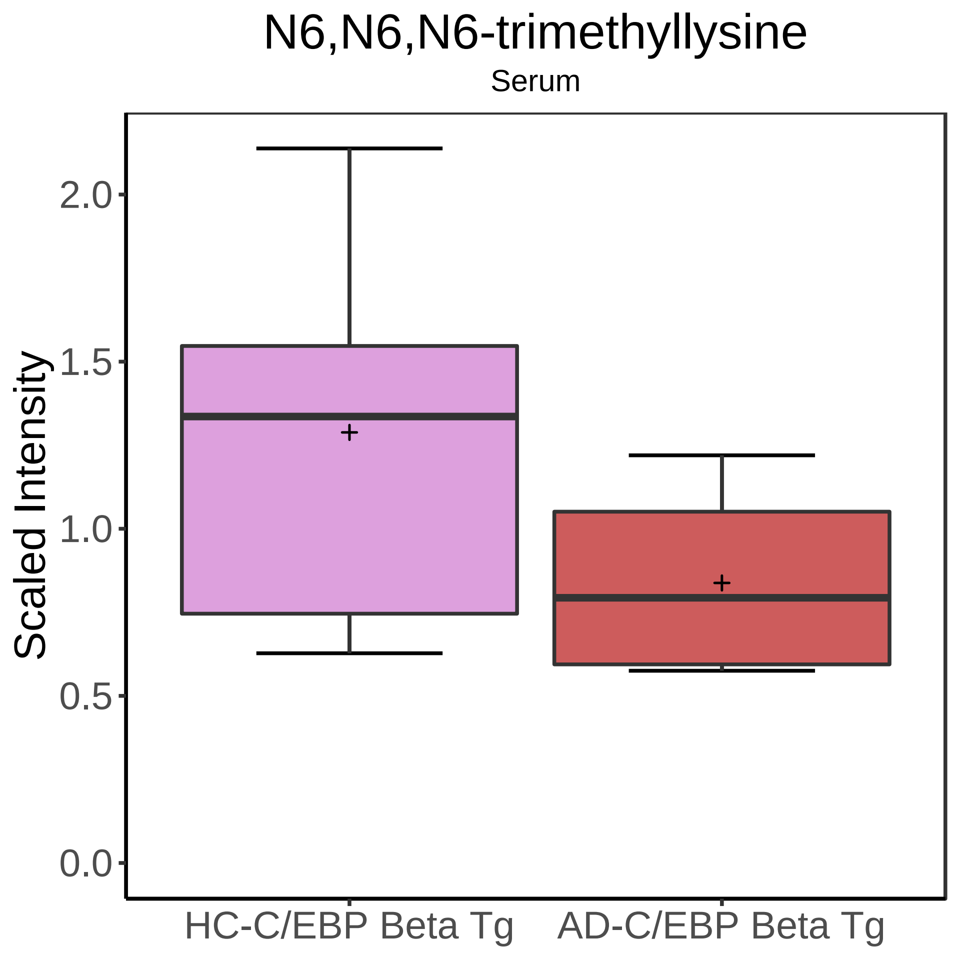
N6,N6,N6-trimethyllysine
Serum
Scaled Intensity
2.0
1.5
1.0
0.5
0.0
HC-C/EBP Beta Tg
AD-C/EBP Beta Tg

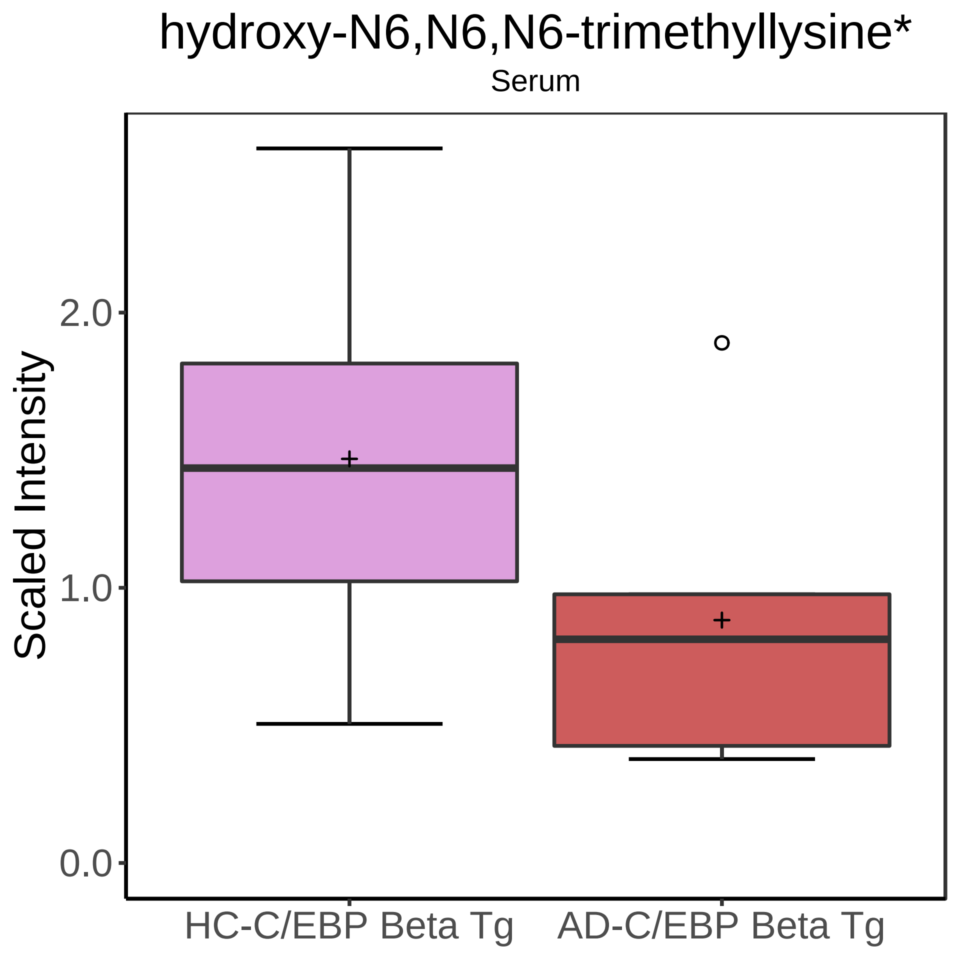
hydroxy-N6,N6,N6-trimethyllysine*
Serum
Scaled Intensity
2.0
1.0
0.0
HC-C/EBP Beta Tg
AD-C/EBP Beta Tg

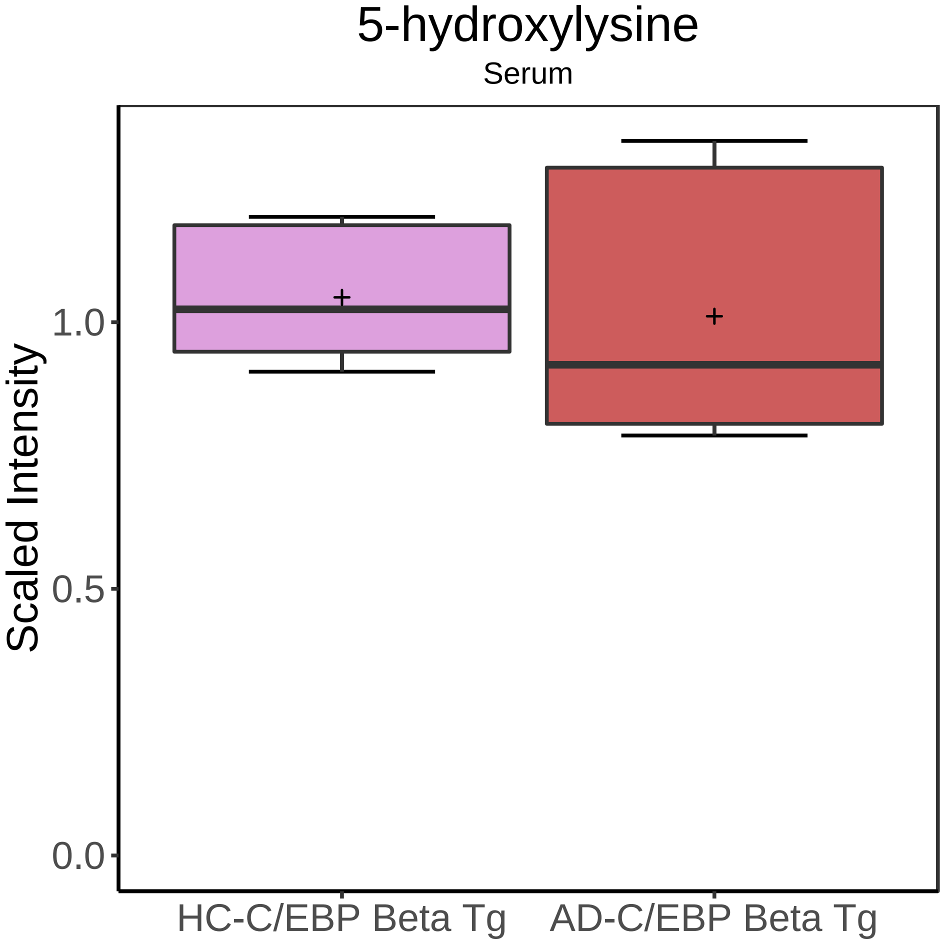
5-hydroxylysine
Serum
Scaled Intensity
1.0
0.5
0.0
HC-C/EBP Beta Tg
AD-C/EBP Beta Tg

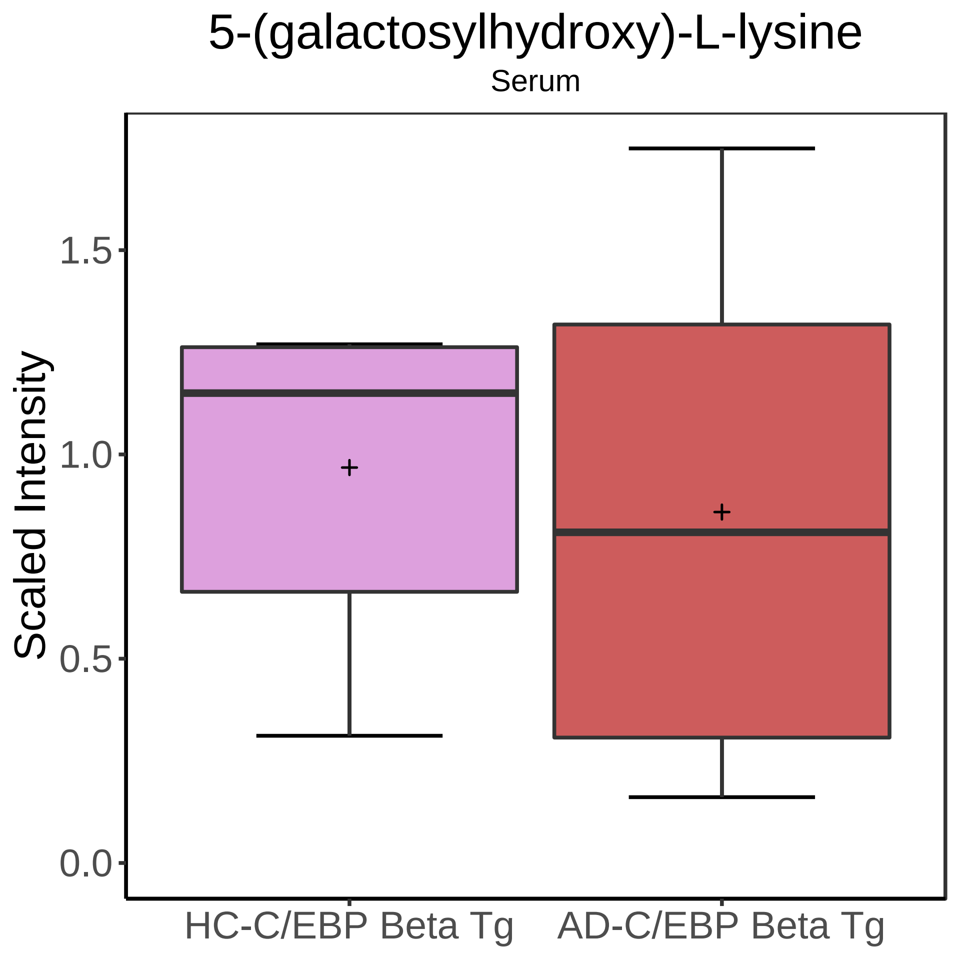
5-(galactosylhydroxy)-L-lysine
Serum
Scaled Intensity
1.5
1.0
0.5
0.0
HC-C/EBP Beta Tg
AD-C/EBP Beta Tg

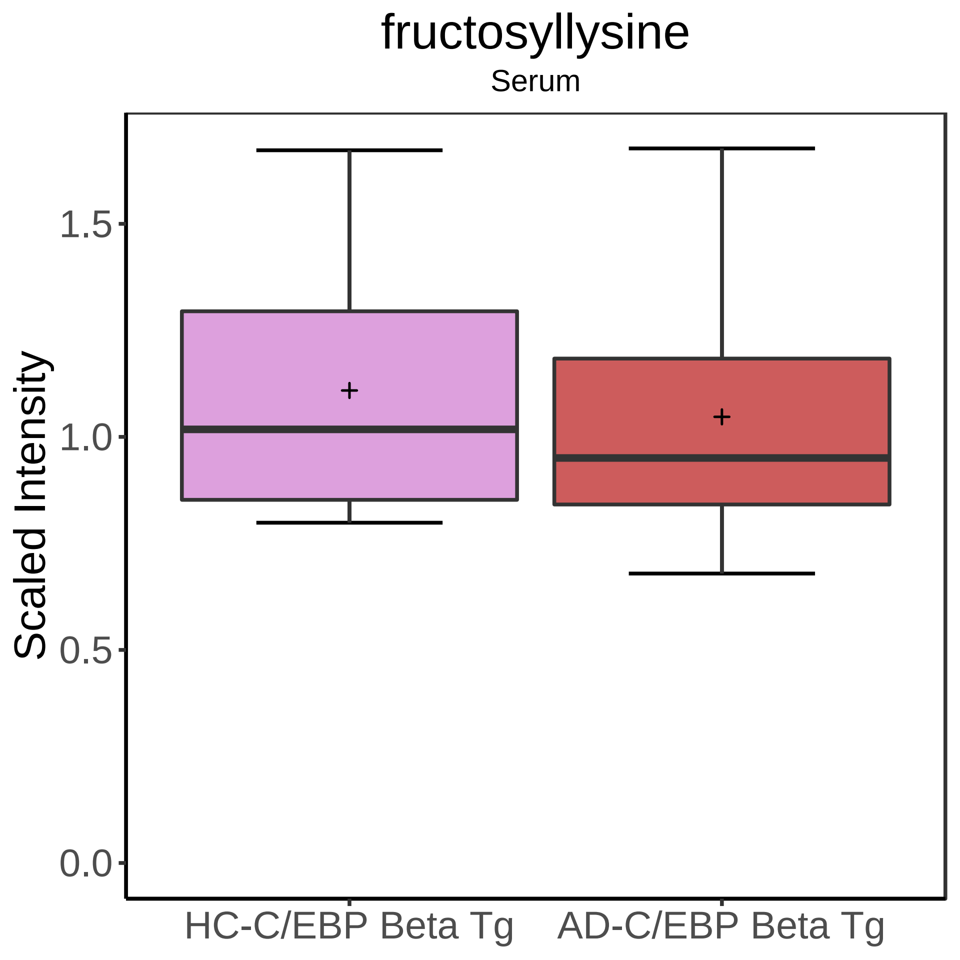
fructosyllysine
Serum
Scaled Intensity
1.5
1.0
0.5
0.0
HC-C/EBP Beta Tg
AD-C/EBP Beta Tg

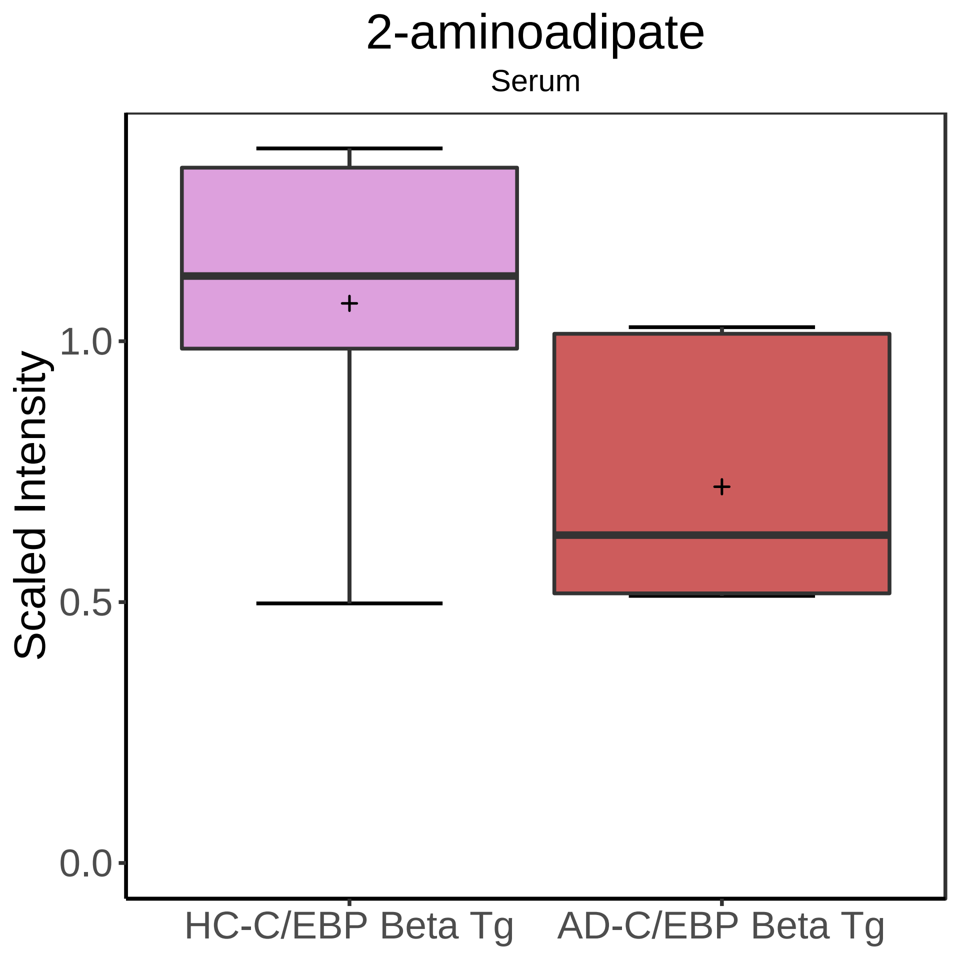

2-aminoadipate
Serum
Scaled Intensity
1.0
0.5
0.0
HC-C/EBP Beta Tg
AD-C/EBP Beta Tg

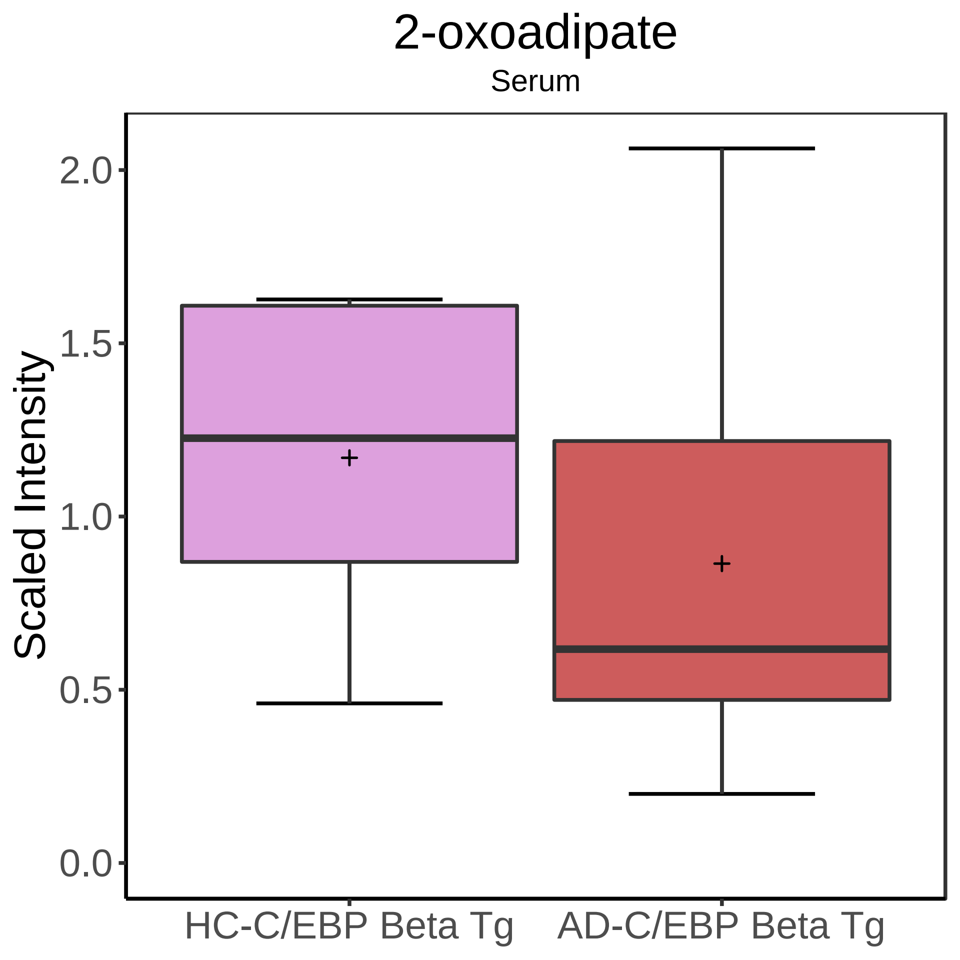
2-oxoadipate
Serum
Scaled Intensity
2.0
1.5
1.0
0.5
0.0
HC-C/EBP Beta Tg
AD-C/EBP Beta Tg

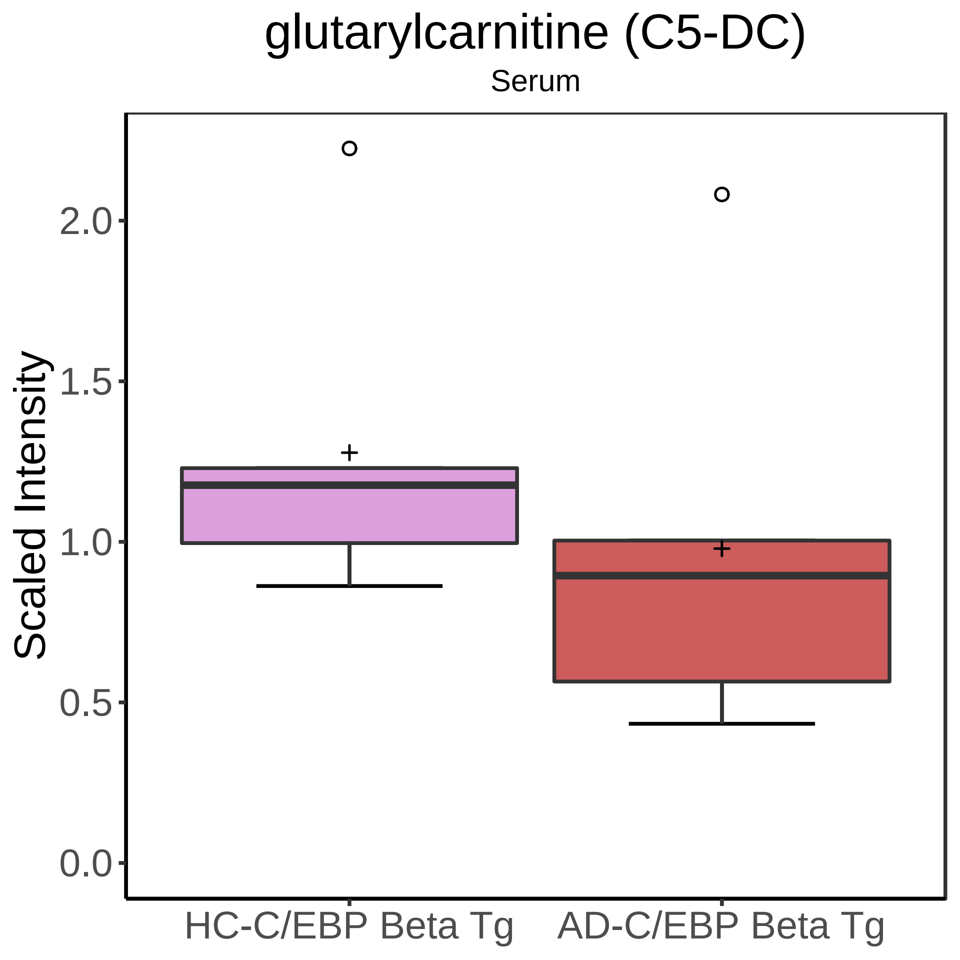
glutarylcarnitine (C5-DC)
Serum
Scaled Intensity
2.0
1.5
1.0
0.5
0.0
HC-C/EBP Beta Tg
AD-C/EBP Beta Tg

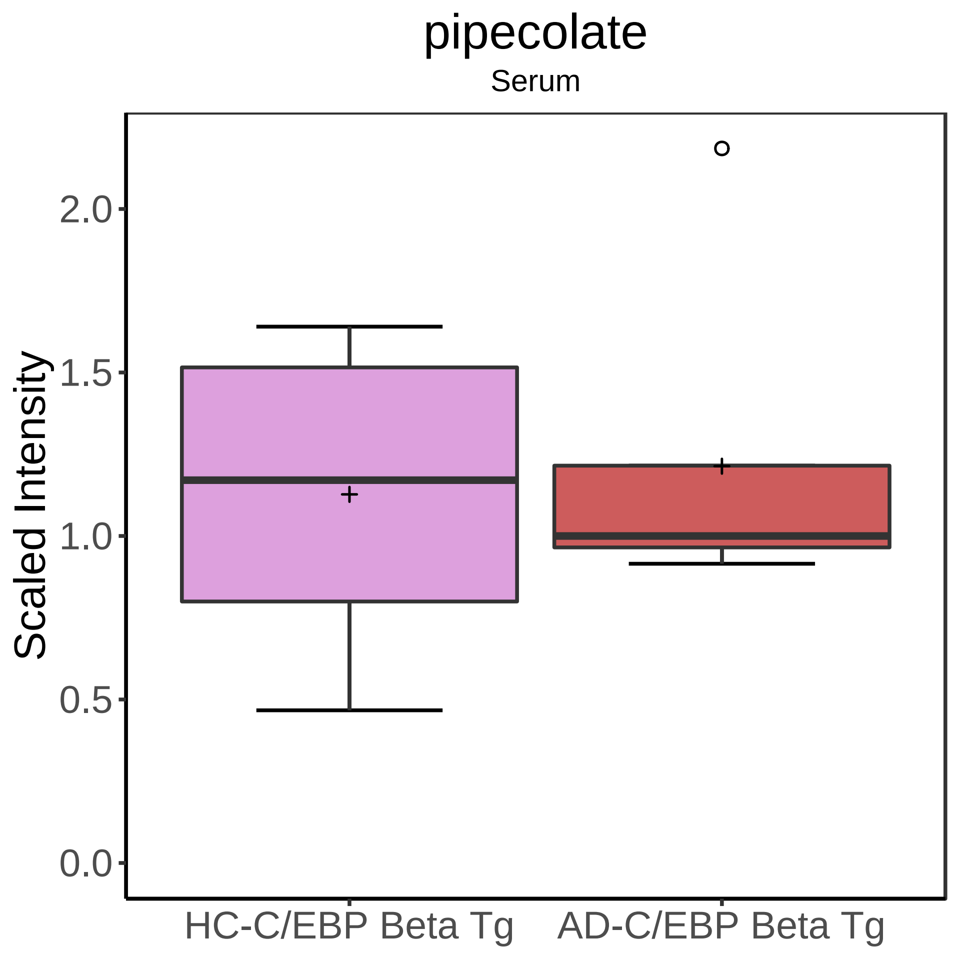
pipecolate
Serum
Scaled Intensity
2.0
1.5
1.0
0.5
0.0
HC-C/EBP Beta Tg
AD-C/EBP Beta Tg

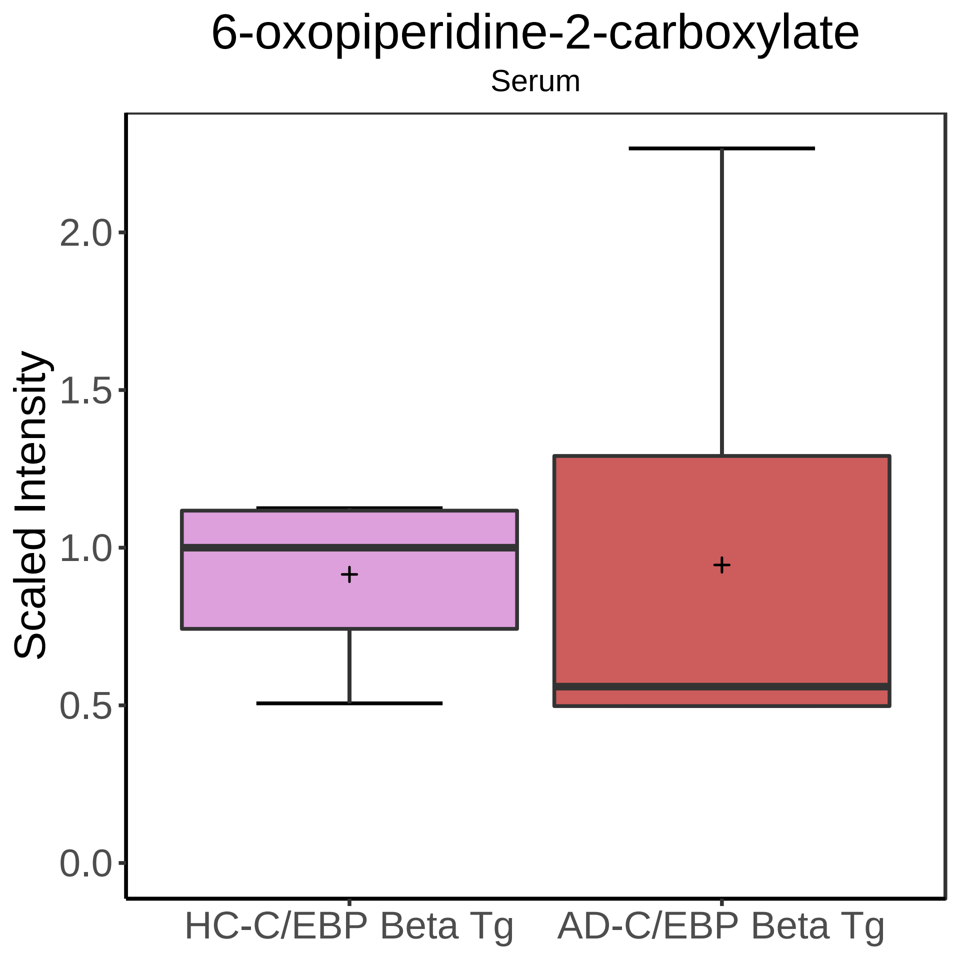
6-oxopiperidine-2-carboxylate
Serum
Scaled Intensity
2.0
1.5
1.0
0.5
0.0
HC-C/EBP Beta Tg
AD-C/EBP Beta Tg

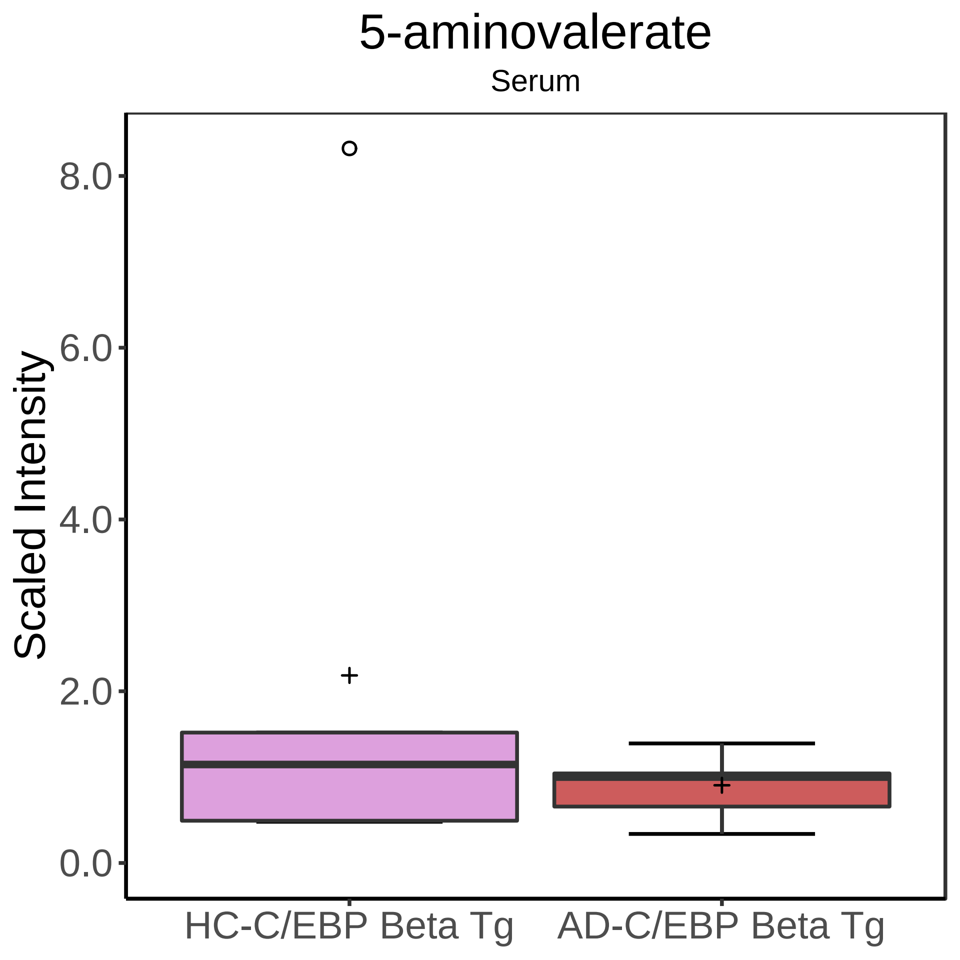
5-aminovalerate
Serum
Scaled Intensity
8.0
6.0
4.0
2.0
0.0
HC-C/EBP Beta Tg
AD-C/EBP Beta Tg

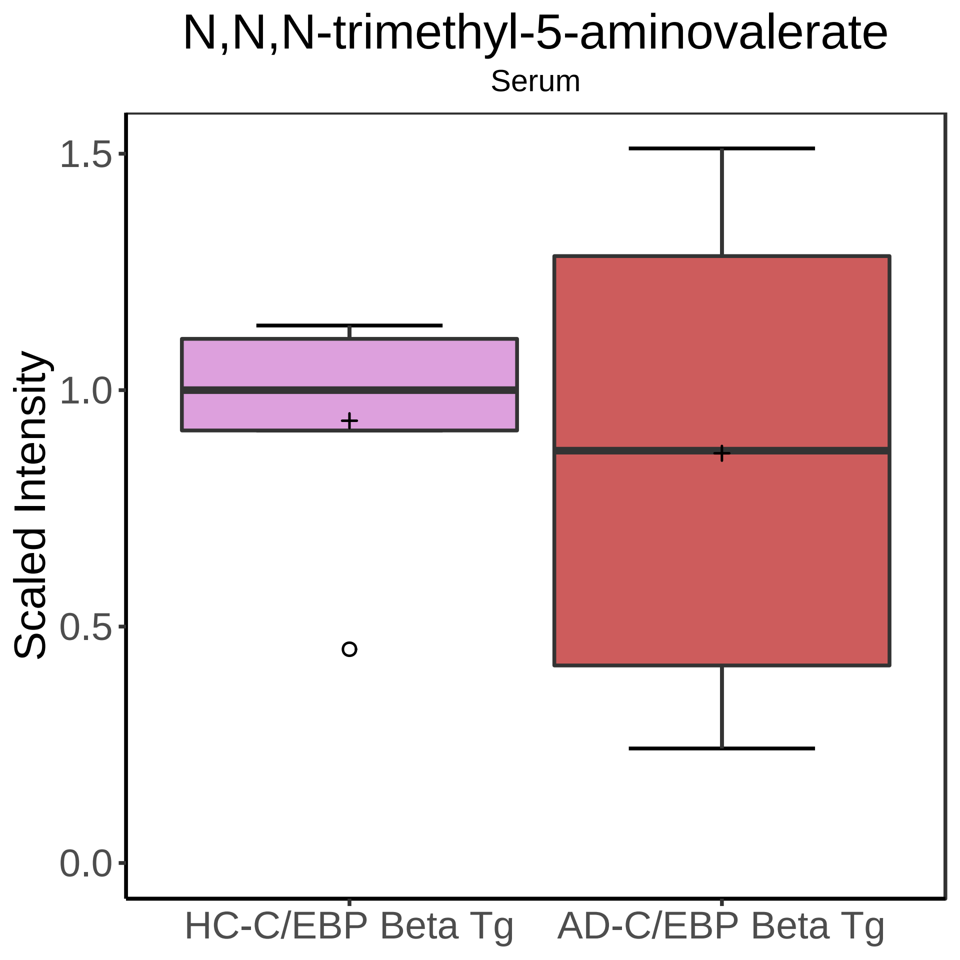

N,N,N-trimethyl-5-aminovalerate
Serum
Scaled Intensity
1.5
1.0
0.5
0.0
HC-C/EBP Beta Tg
AD-C/EBP Beta Tg

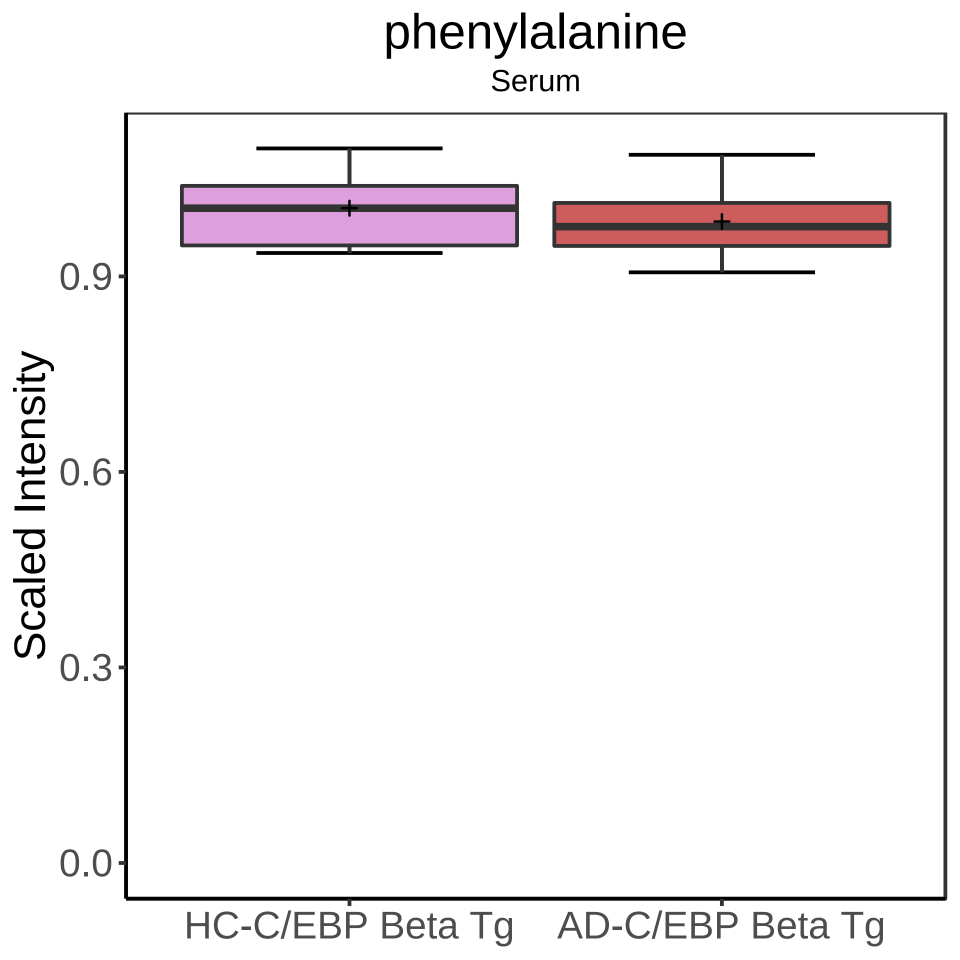
phenylalanine
Serum
Scaled Intensity
0.0
0.3
0.6
0.9
HC-C/EBP Beta Tg
AD-C/EBP Beta Tg

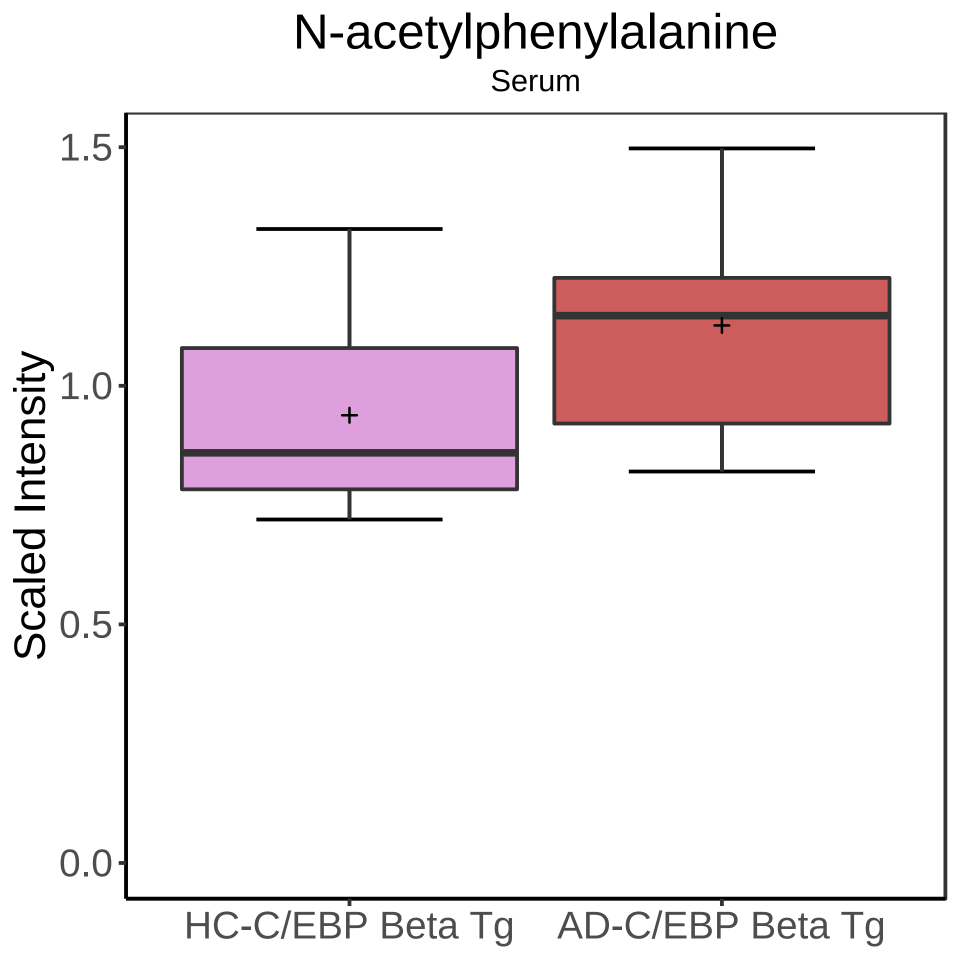
N-acetylphenylalanine
Serum
Scaled Intensity
1.5
1.0
0.5
0.0
HC-C/EBP Beta Tg
AD-C/EBP Beta Tg

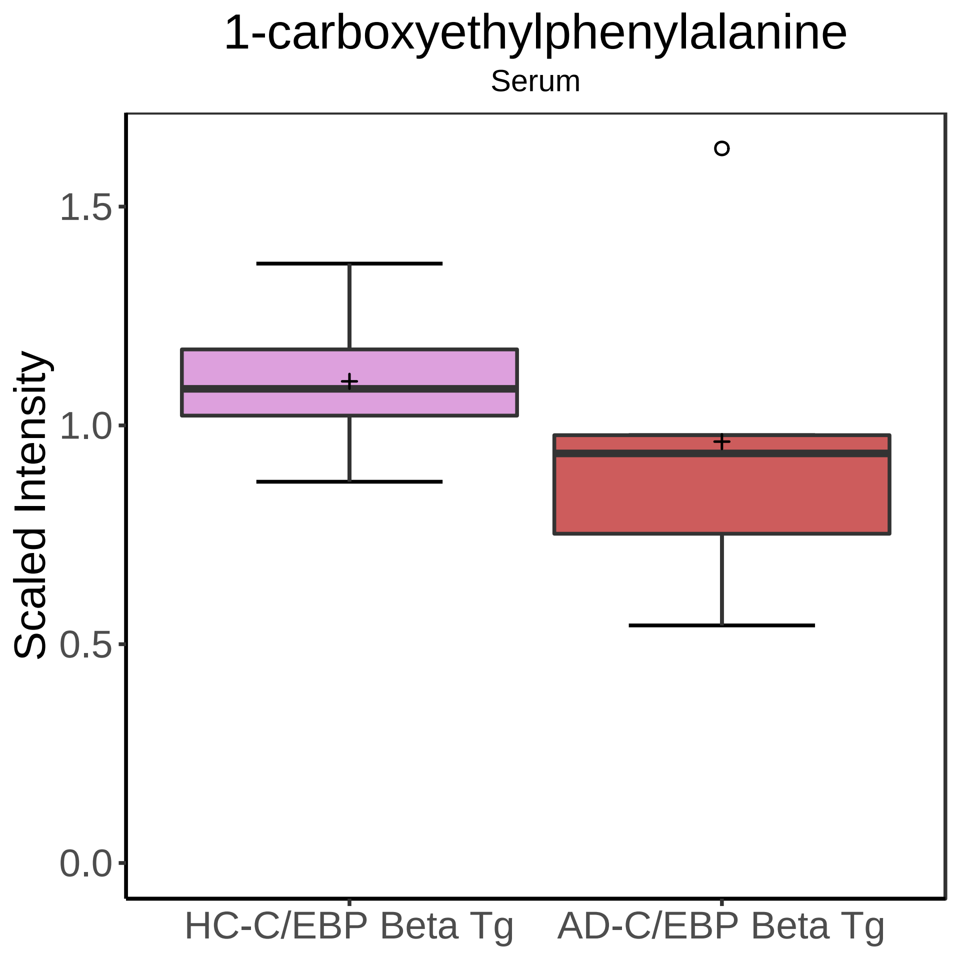
1-carboxyethylphenylalanine
Serum
Scaled Intensity
1.5
1.0
0.5
0.0
HC-C/EBP Beta Tg
AD-C/EBP Beta Tg

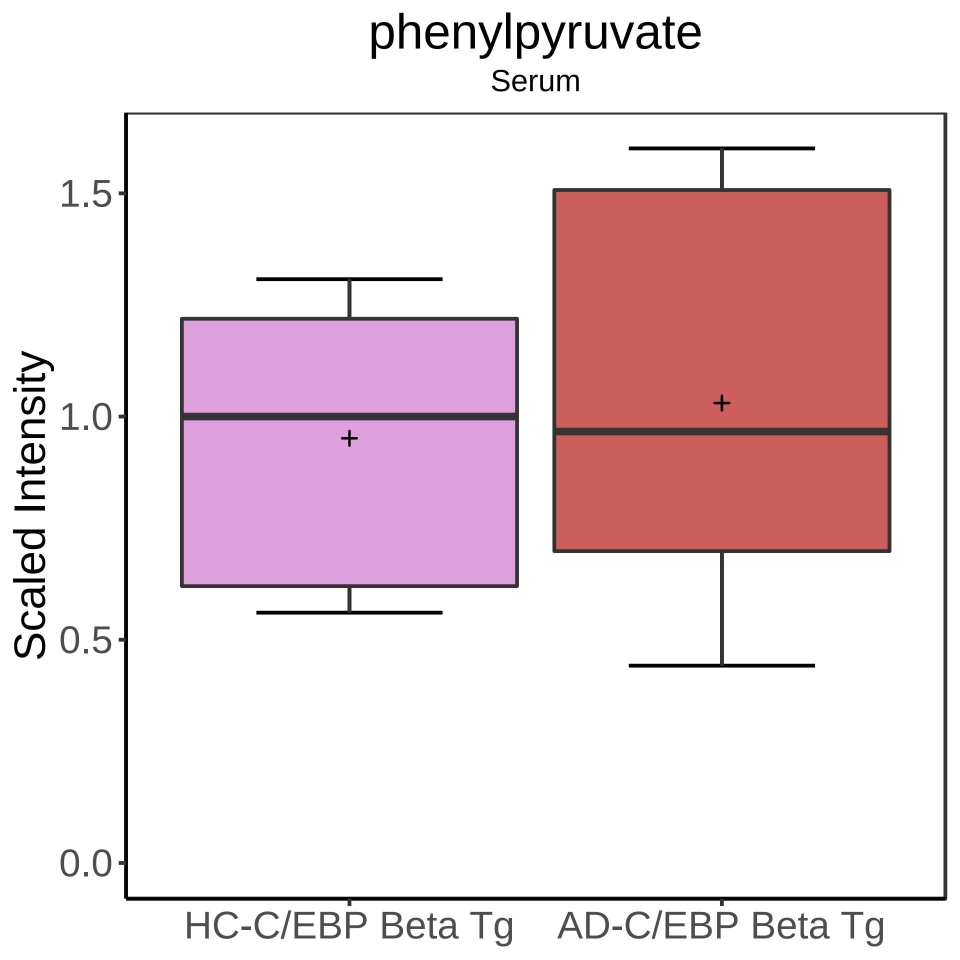
phenylpyruvate
Serum
Scaled Intensity
1.5
1.0
0.5
0.0
HC-C/EBP Beta Tg
AD-C/EBP Beta Tg

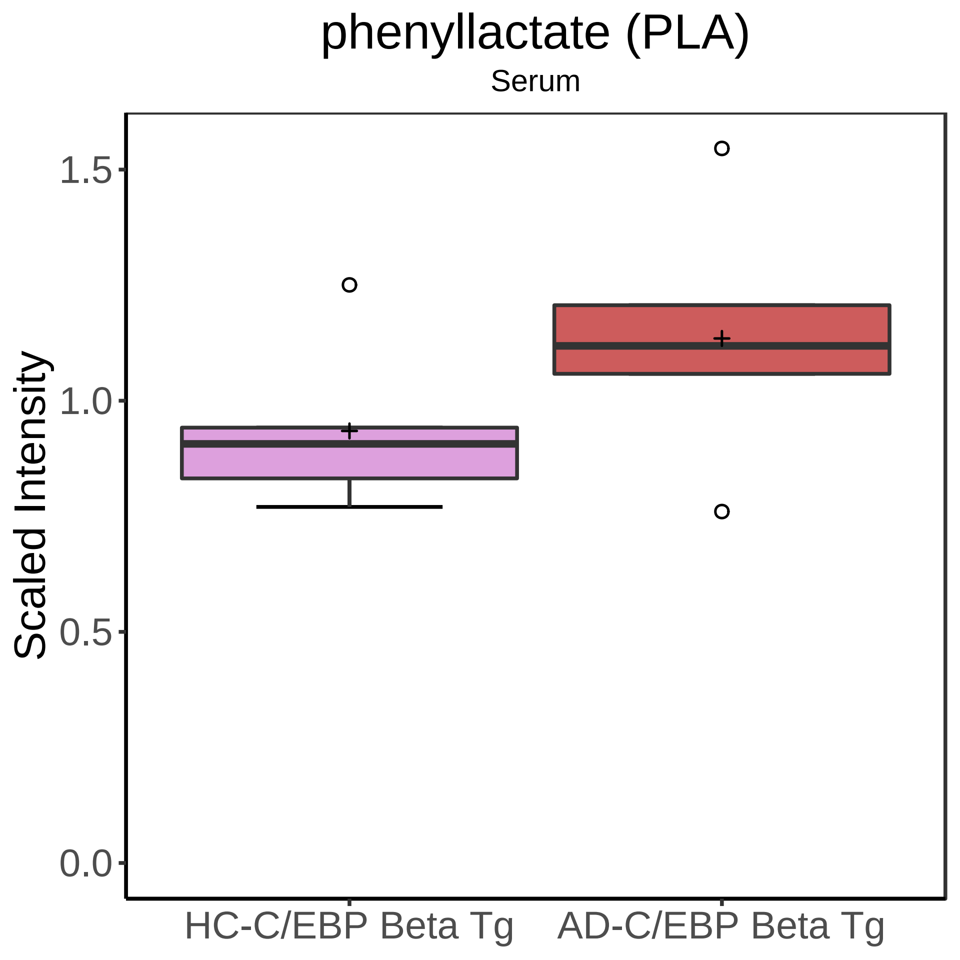
phenyllactate (PLA)
Serum
Scaled Intensity
1.5
1.0
0.5
0.0
HC-C/EBP Beta Tg
AD-C/EBP Beta Tg

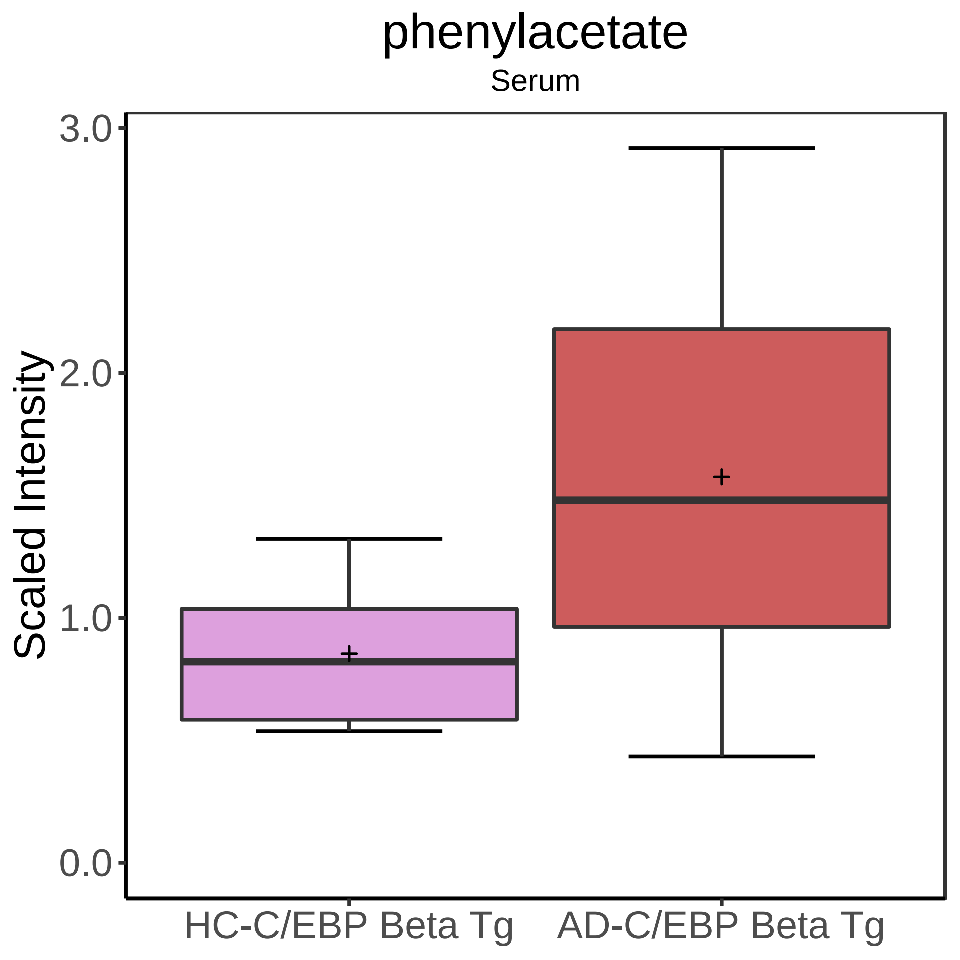
phenylacetate
Serum
Scaled Intensity
3.0
2.0
1.0
0.0
HC-C/EBP Beta Tg
AD-C/EBP Beta Tg

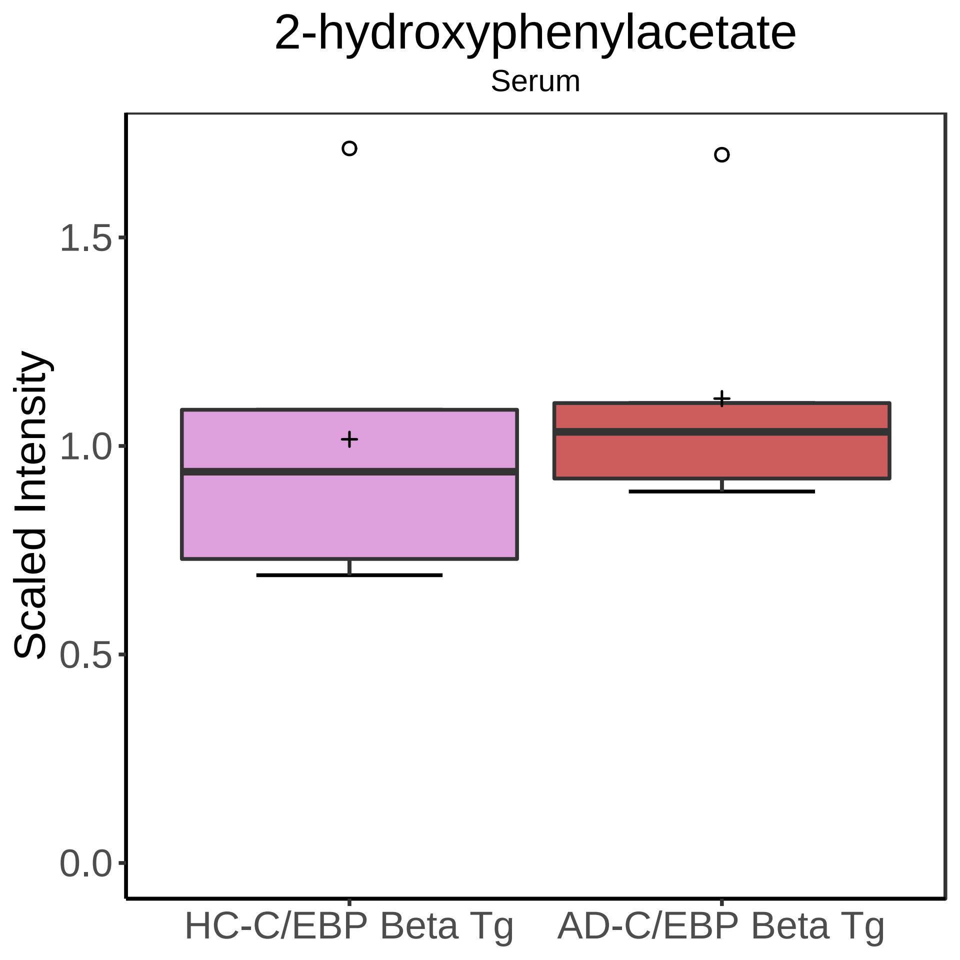
2-hydroxyphenylacetate
Serum
Scaled Intensity
0.0
0.5
1.0
1.5
HC-C/EBP Beta Tg
AD-C/EBP Beta Tg

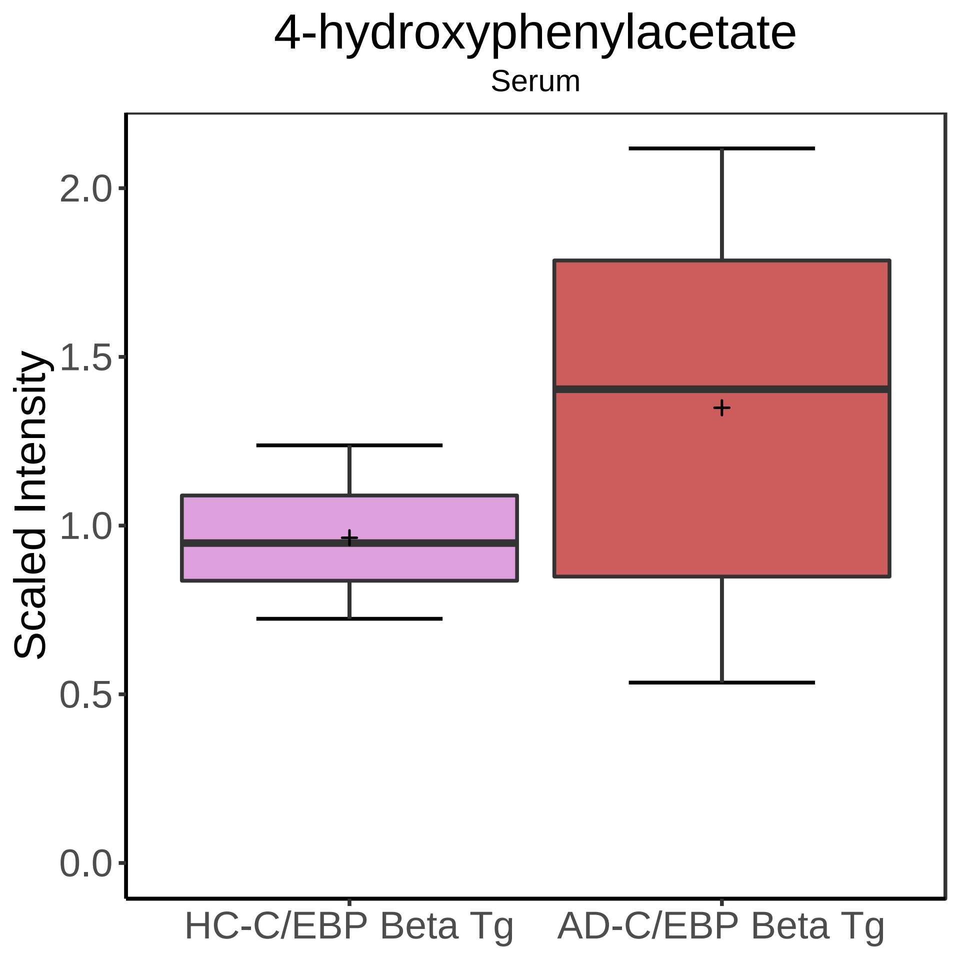
4-hydroxyphenylacetate
Serum
Scaled Intensity
2.0
1.5
1.0
0.5
0.0
HC-C/EBP Beta Tg
AD-C/EBP Beta Tg

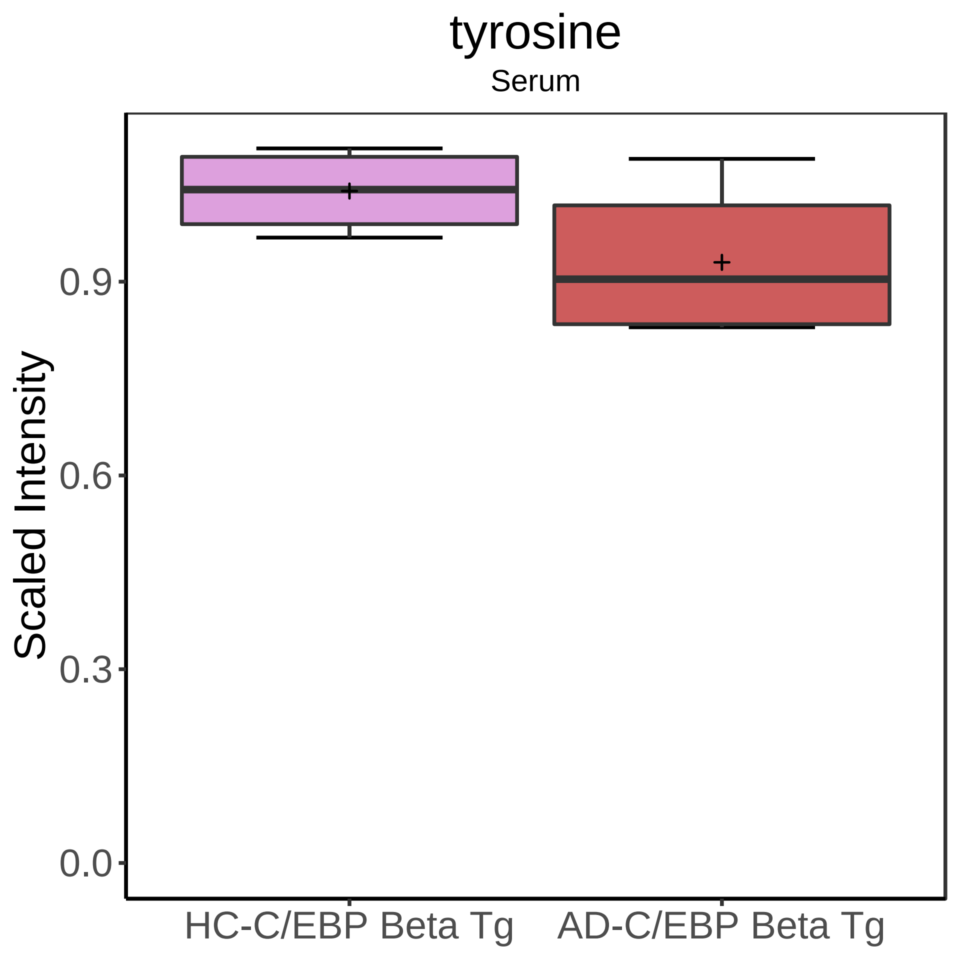
tyrosine
Serum
Scaled Intensity
0.0
0.3
0.6
0.9
HC-C/EBP Beta Tg
AD-C/EBP Beta Tg

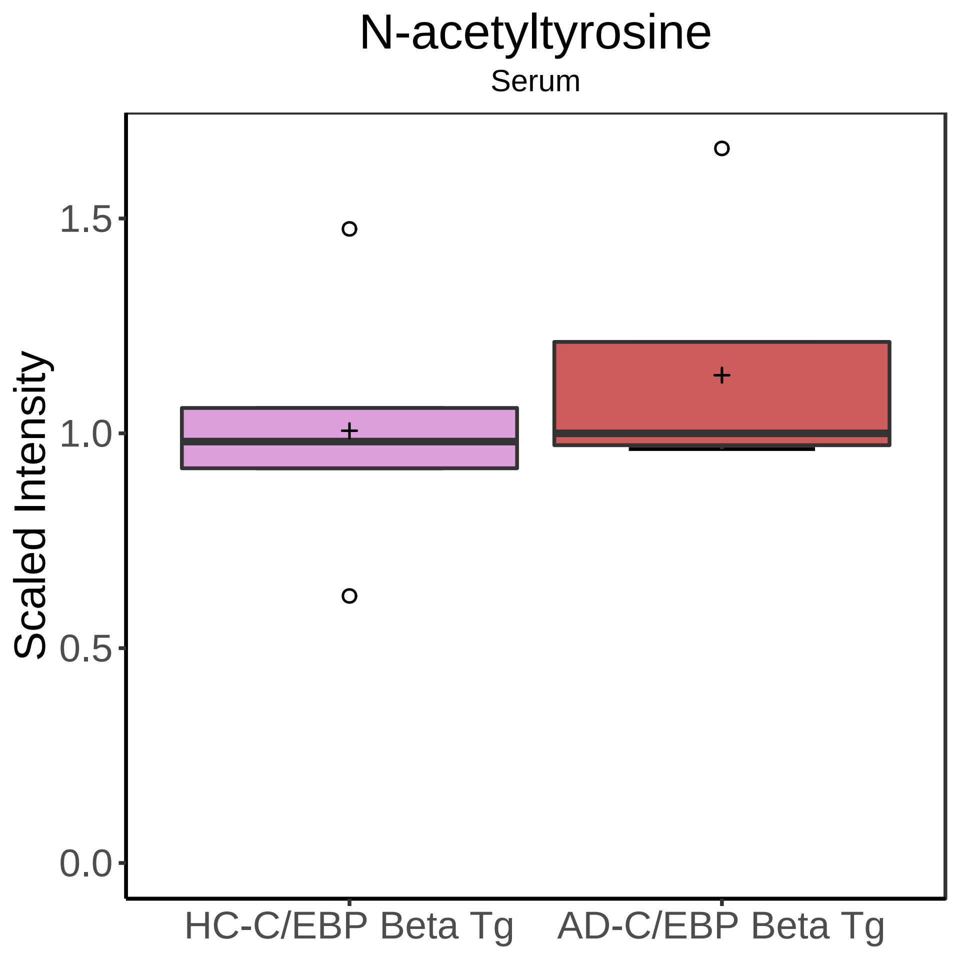
N-acetyltyrosine
Serum
Scaled Intensity
1.5
1.0
0.5
0.0
HC-C/EBP Beta Tg
AD-C/EBP Beta Tg

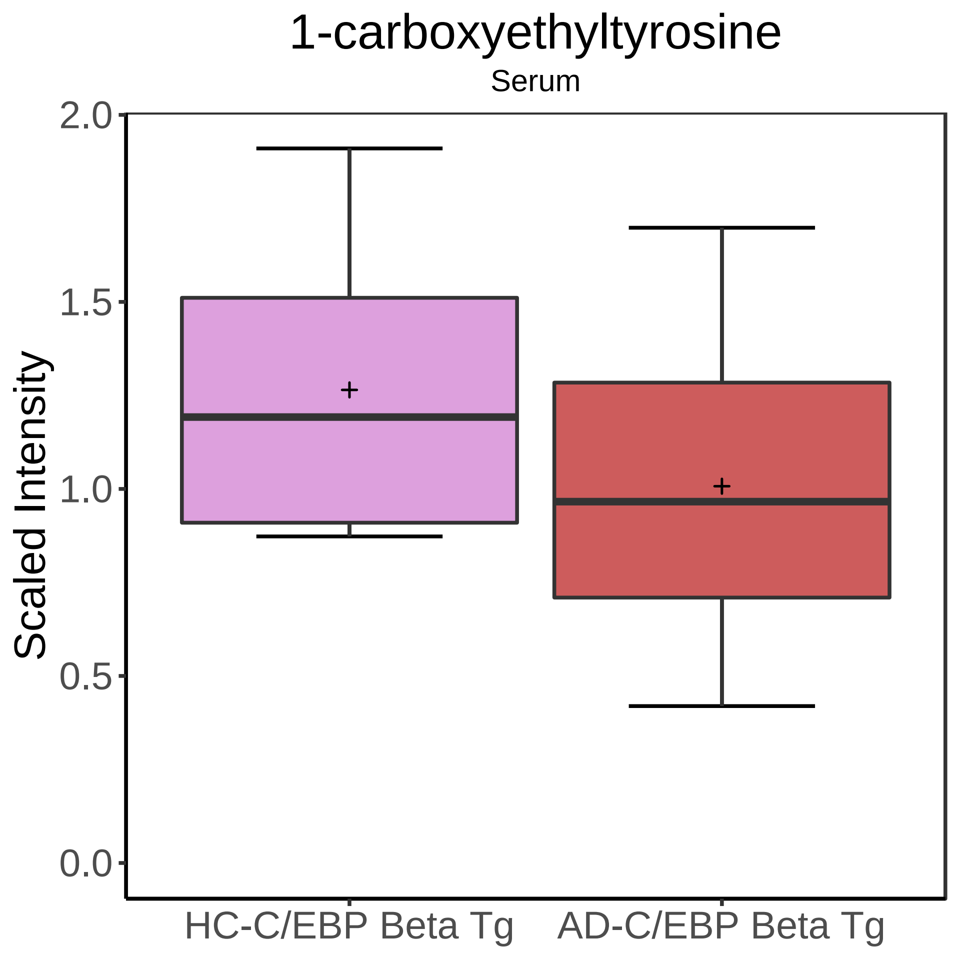

1-carboxyethyltyrosine
Serum
Scaled Intensity
2.0
1.5
1.0
0.5
0.0
HC-C/EBP Beta Tg
AD-C/EBP Beta Tg

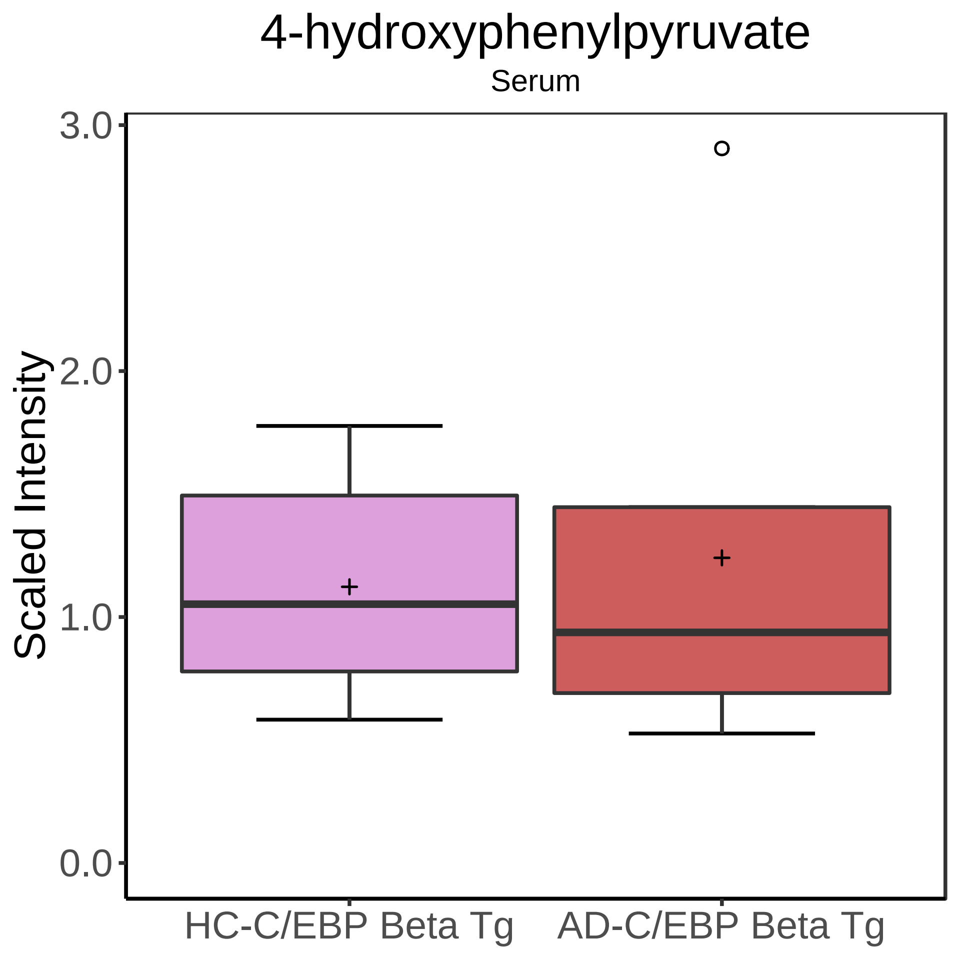
4-hydroxyphenylpyruvate
Serum
Scaled Intensity
3.0
2.0
1.0
0.0
HC-C/EBP Beta Tg
AD-C/EBP Beta Tg

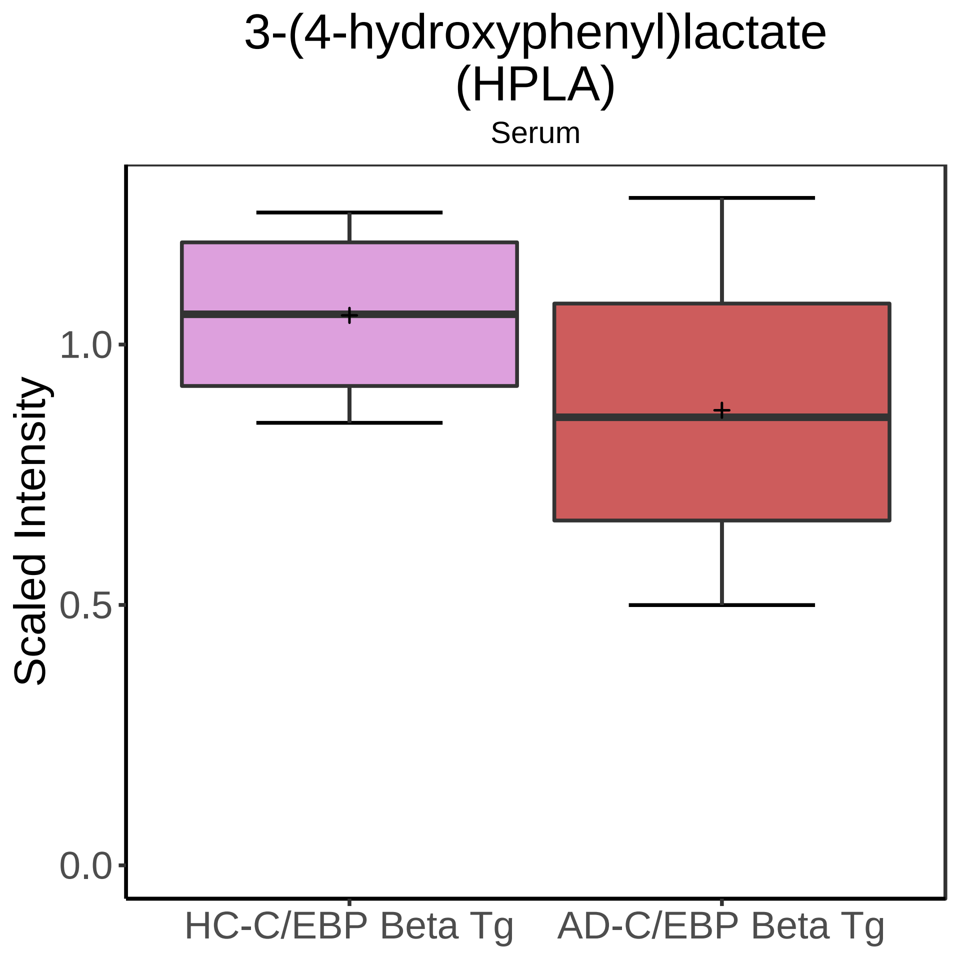
3-(4-hydroxyphenyl)lactate
(HPLA)
Serum
Scaled Intensity
1.0
0.5
0.0
HC-C/EBP Beta Tg
AD-C/EBP Beta Tg

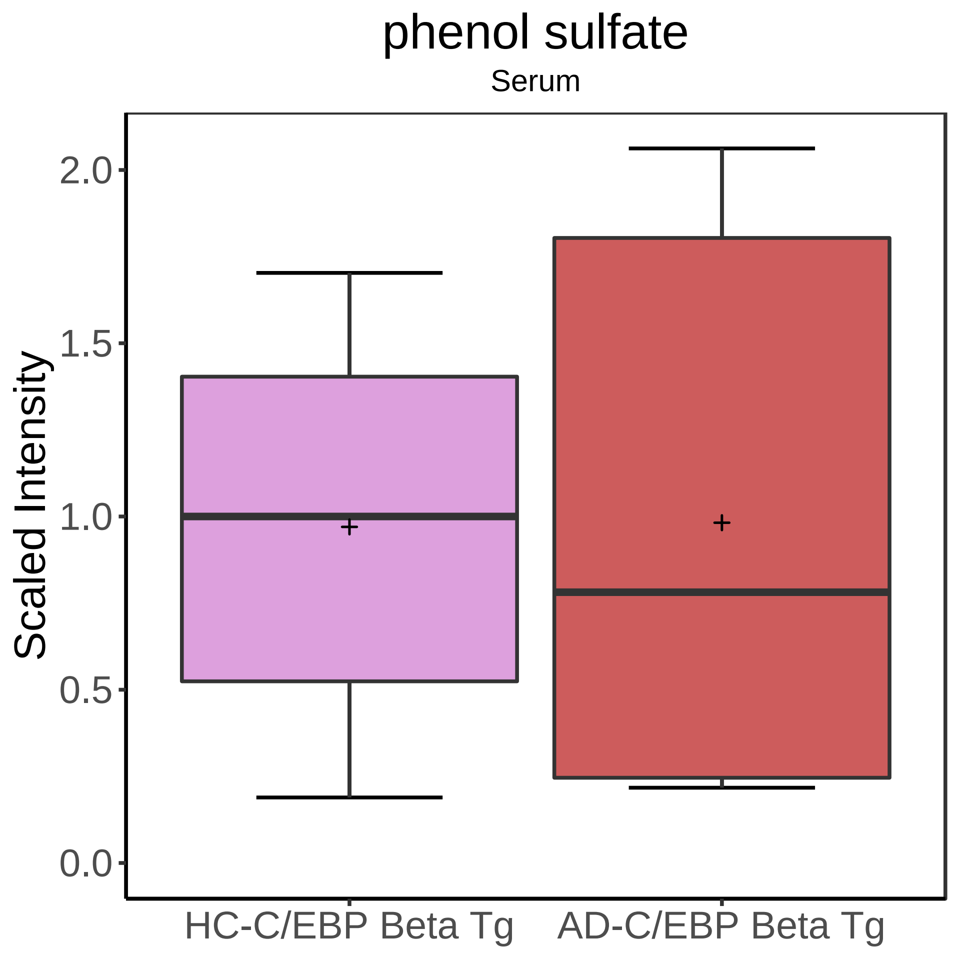
phenol sulfate
Serum
Scaled Intensity
2.0
1.5
1.0
0.5
0.0
HC-C/EBP Beta Tg
AD-C/EBP Beta Tg

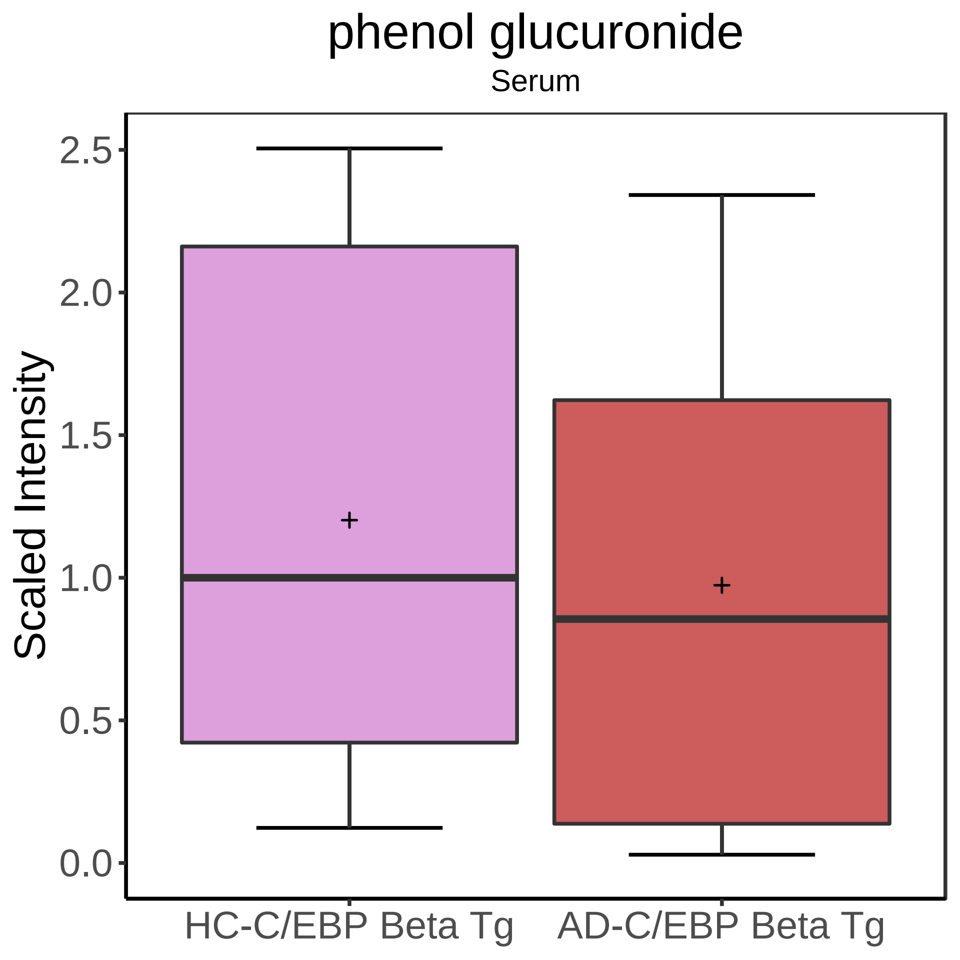
phenol glucuronide
Serum
Scaled Intensity
2.5
2.0
1.5
1.0
0.5
0.0
HC-C/EBP Beta Tg
AD-C/EBP Beta Tg

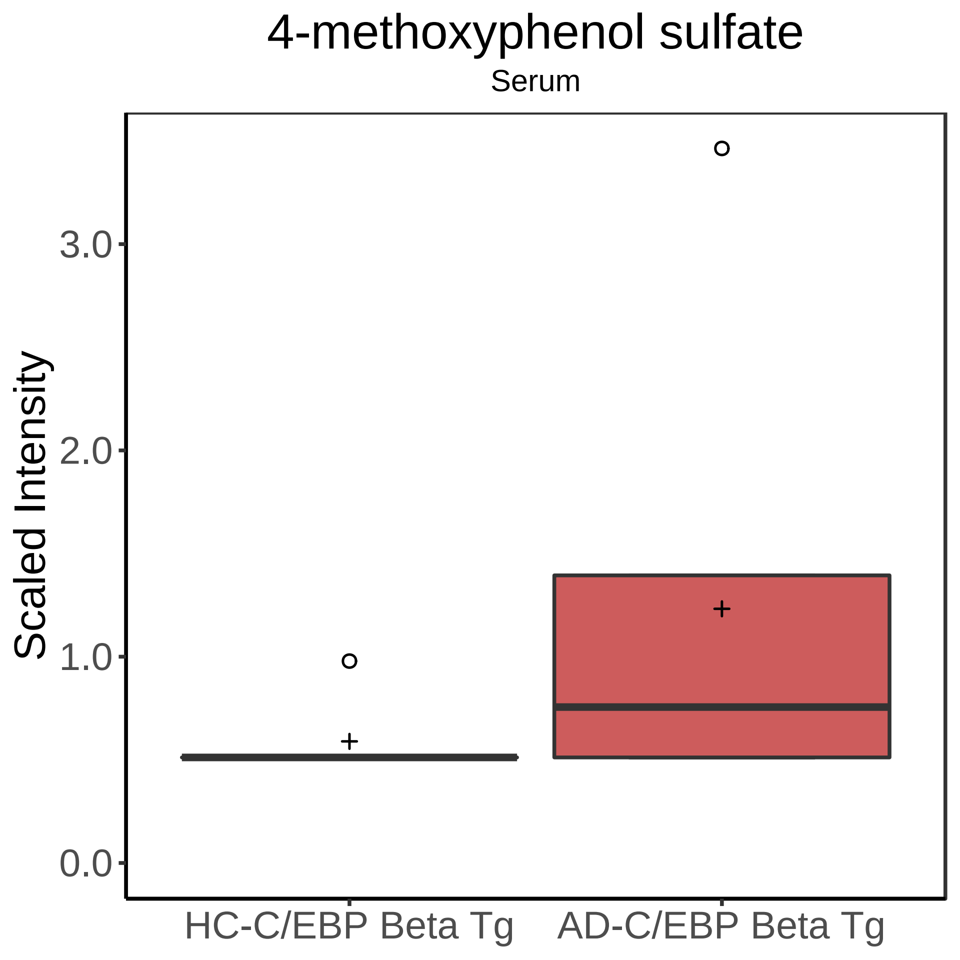
4-methoxyphenol sulfate
Serum
Scaled Intensity
3.0
2.0
1.0
0.0
HC-C/EBP Beta Tg
AD-C/EBP Beta Tg

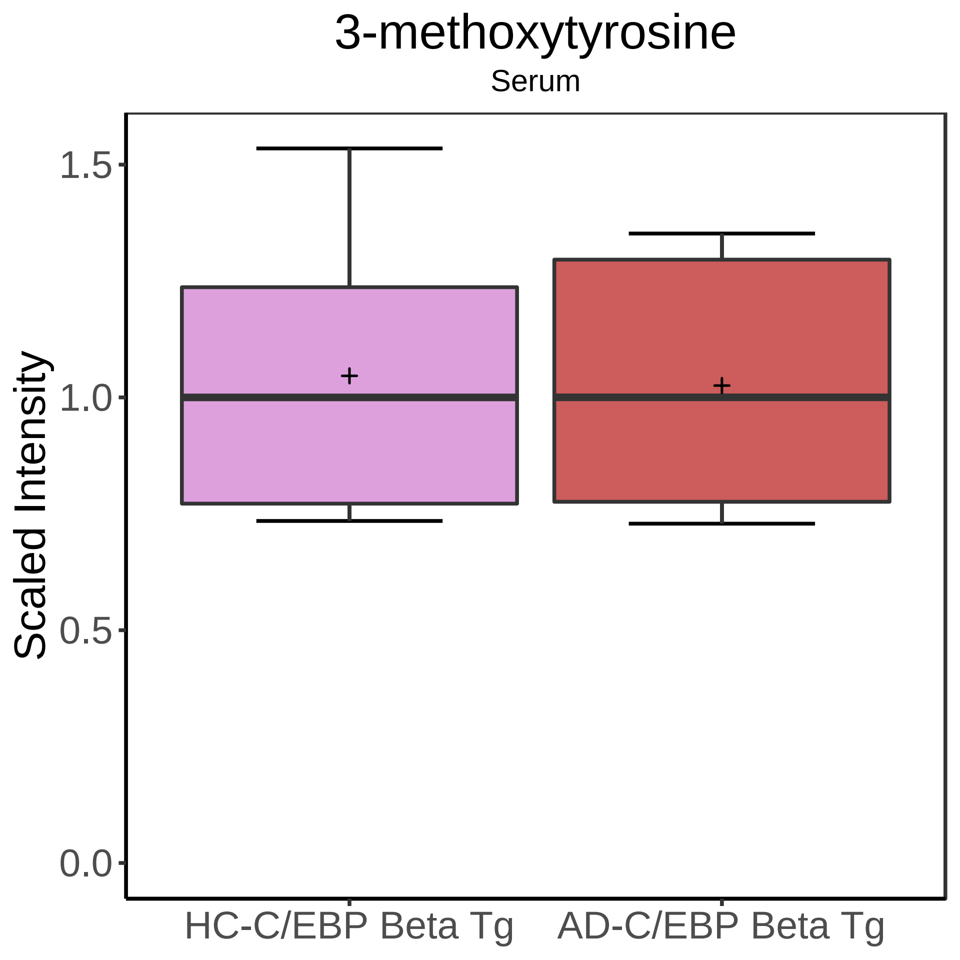

3-methoxytyrosine
Serum
Scaled Intensity
1.5
1.0
0.5
0.0
HC-C/EBP Beta Tg
AD-C/EBP Beta Tg

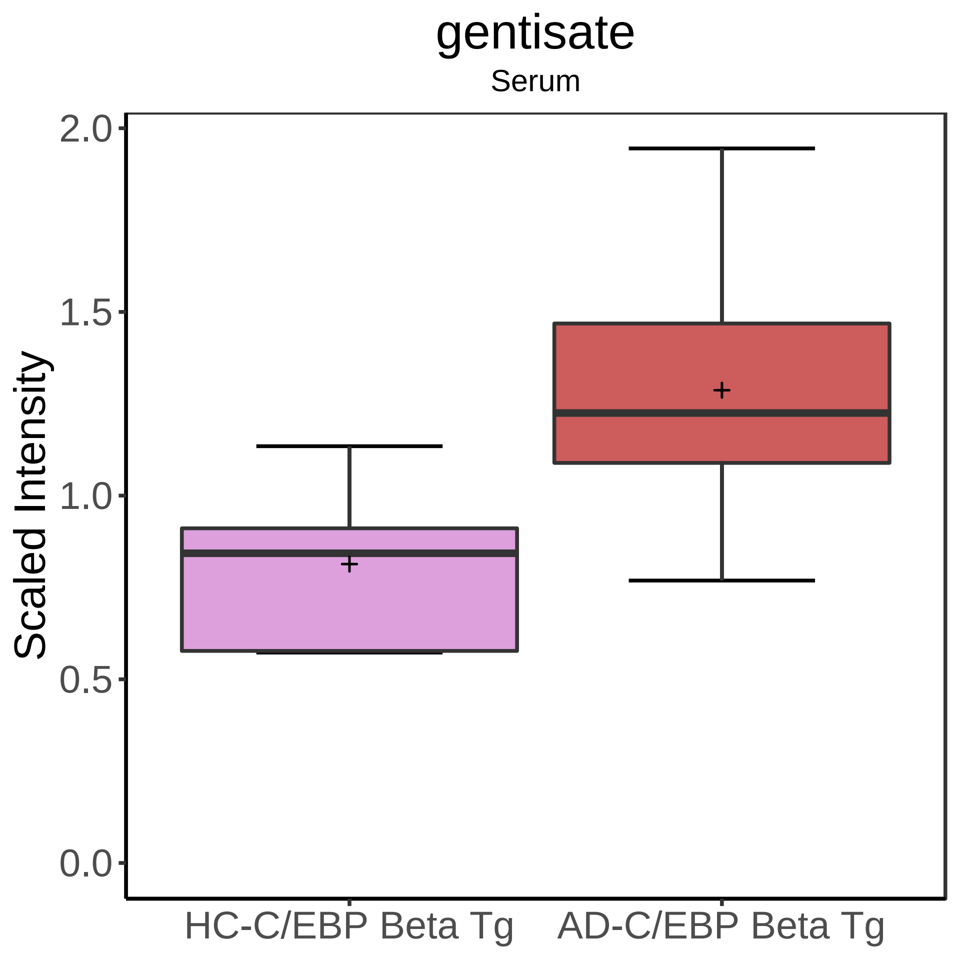
gentisate
Serum
Scaled Intensity
2.0
1.5
1.0
0.5
0.0
HC-C/EBP Beta Tg
AD-C/EBP Beta Tg

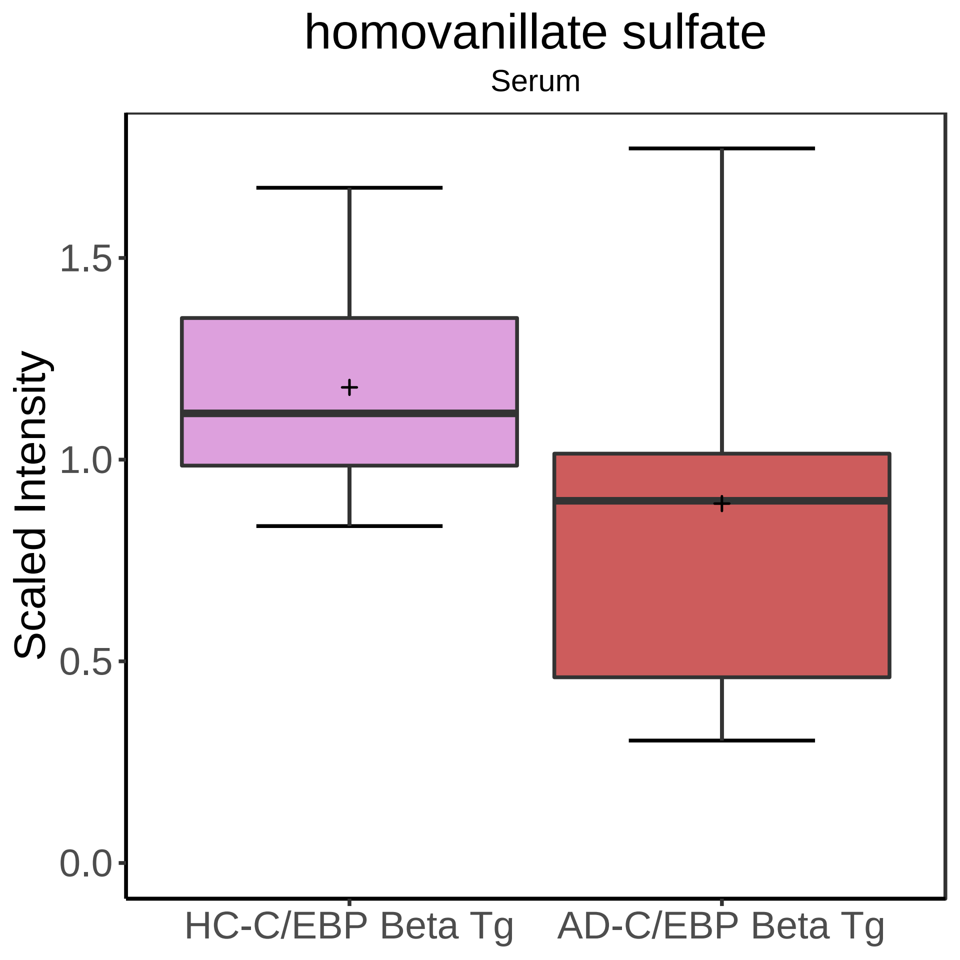
homovanillate sulfate
Serum
Scaled Intensity
1.5
1.0
0.5
0.0
HC-C/EBP Beta Tg
AD-C/EBP Beta Tg

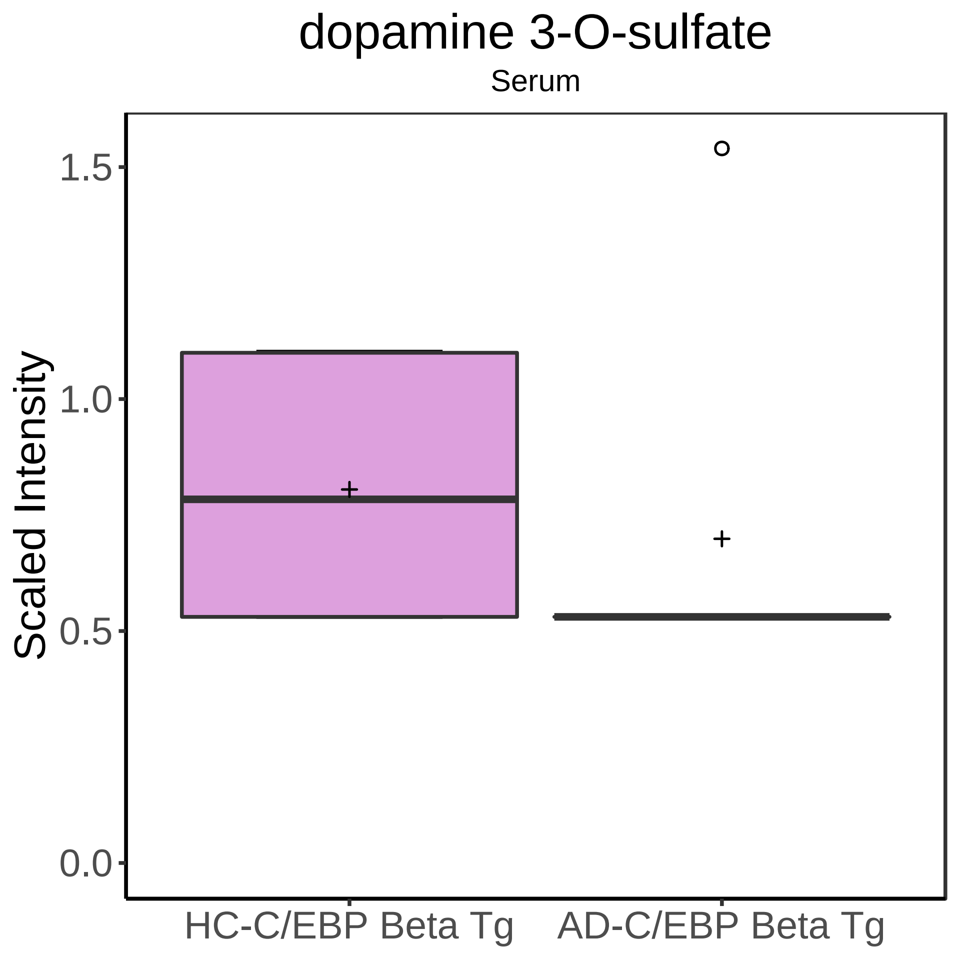
dopamine 3-O-sulfate
Serum
Scaled Intensity
1.5
1.0
0.5
0.0
HC-C/EBP Beta Tg
AD-C/EBP Beta Tg

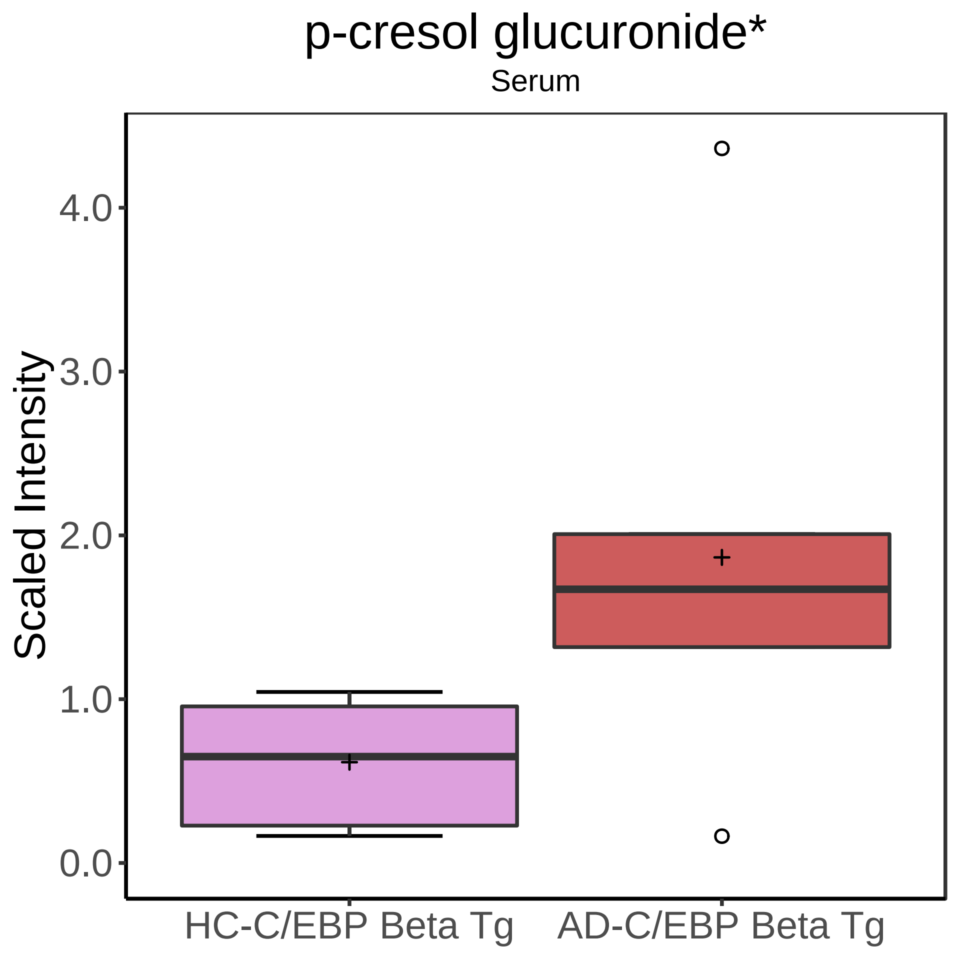
p-cresol glucuronide*
Serum
Scaled Intensity
4.0
3.0
2.0
1.0
0.0
HC-C/EBP Beta Tg
AD-C/EBP Beta Tg

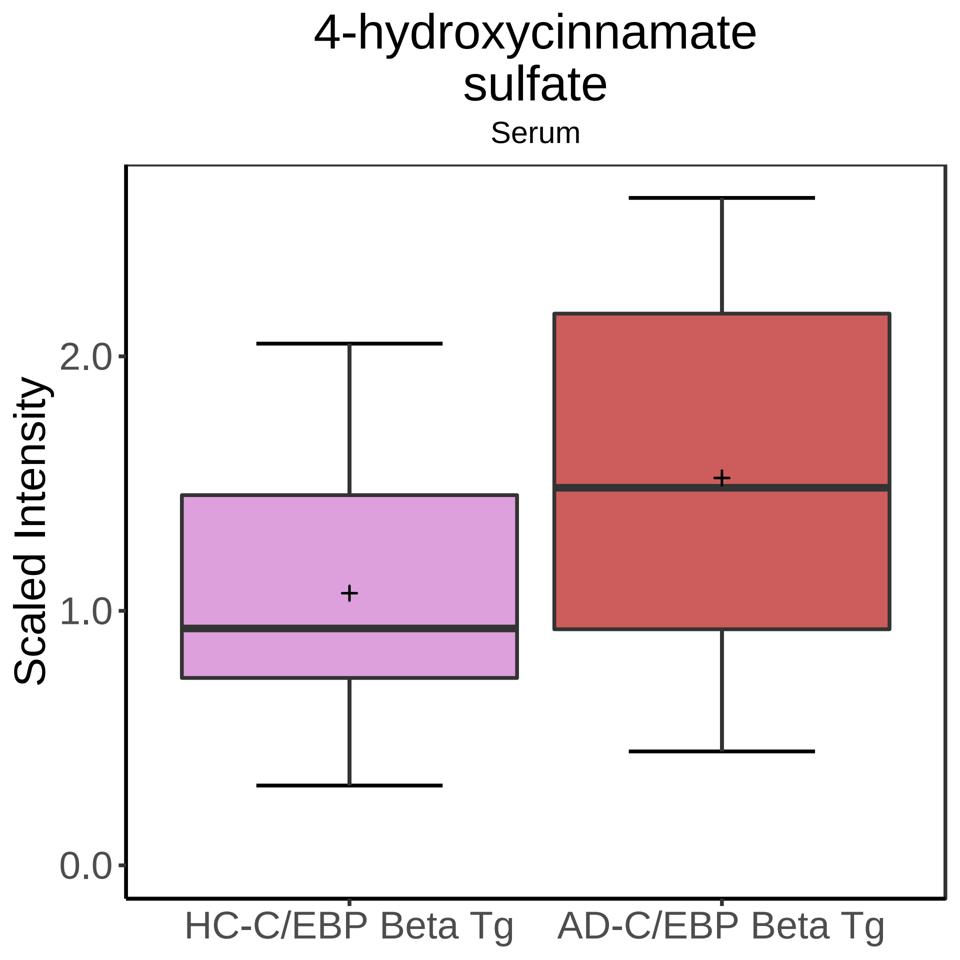
4-hydroxycinnamate sulfate
Serum
Scaled Intensity
2.0
1.0
0.0
HC-C/EBP Beta Tg
AD-C/EBP Beta Tg

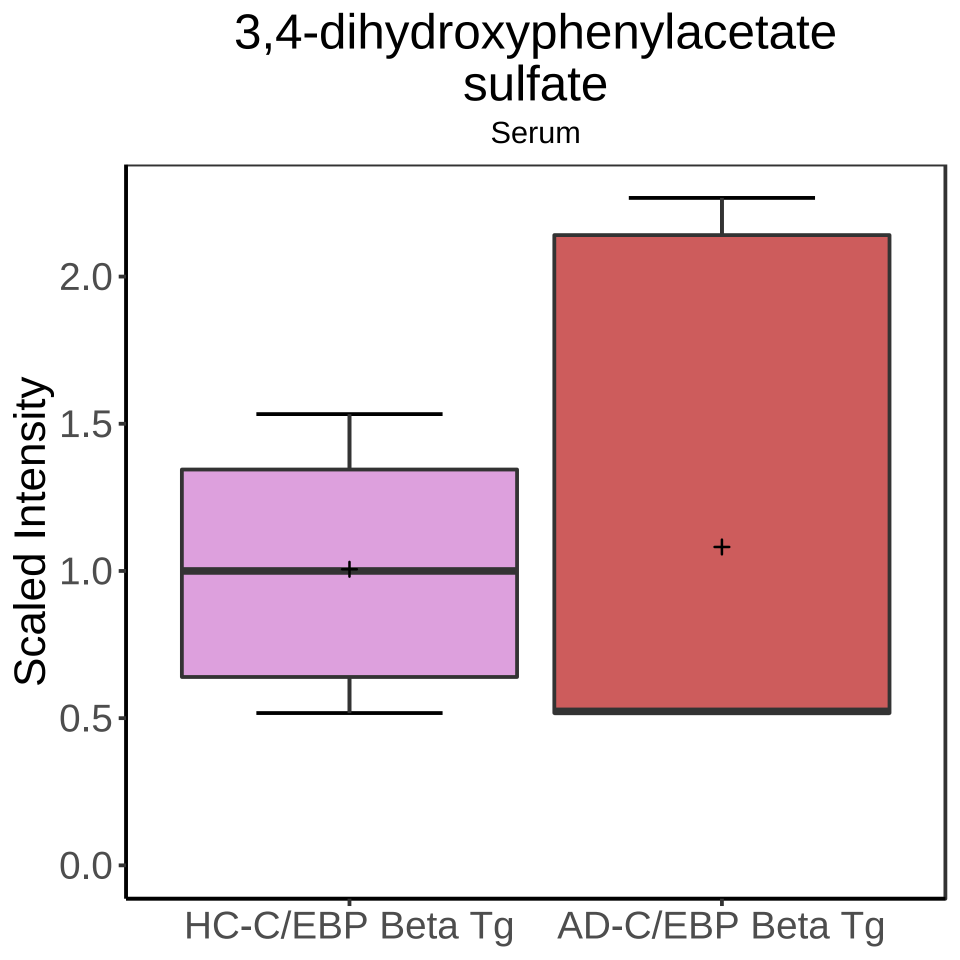
3,4-dihydroxyphenylacetate sulfate
Serum
Scaled Intensity
2.0
1.5
1.0
0.5
0.0
HC-C/EBP Beta Tg
AD-C/EBP Beta Tg

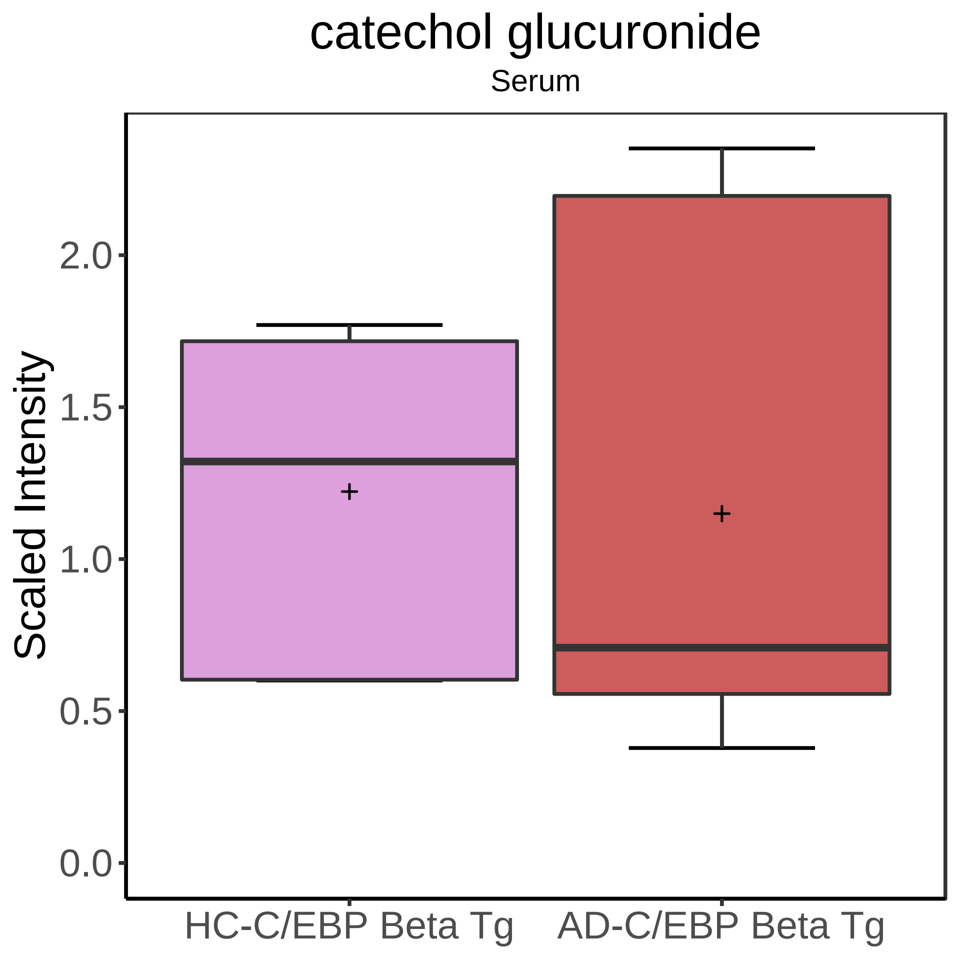
catechol glucuronide
Serum
Scaled Intensity
2.0
1.5
1.0
0.5
0.0
HC-C/EBP Beta Tg
AD-C/EBP Beta Tg

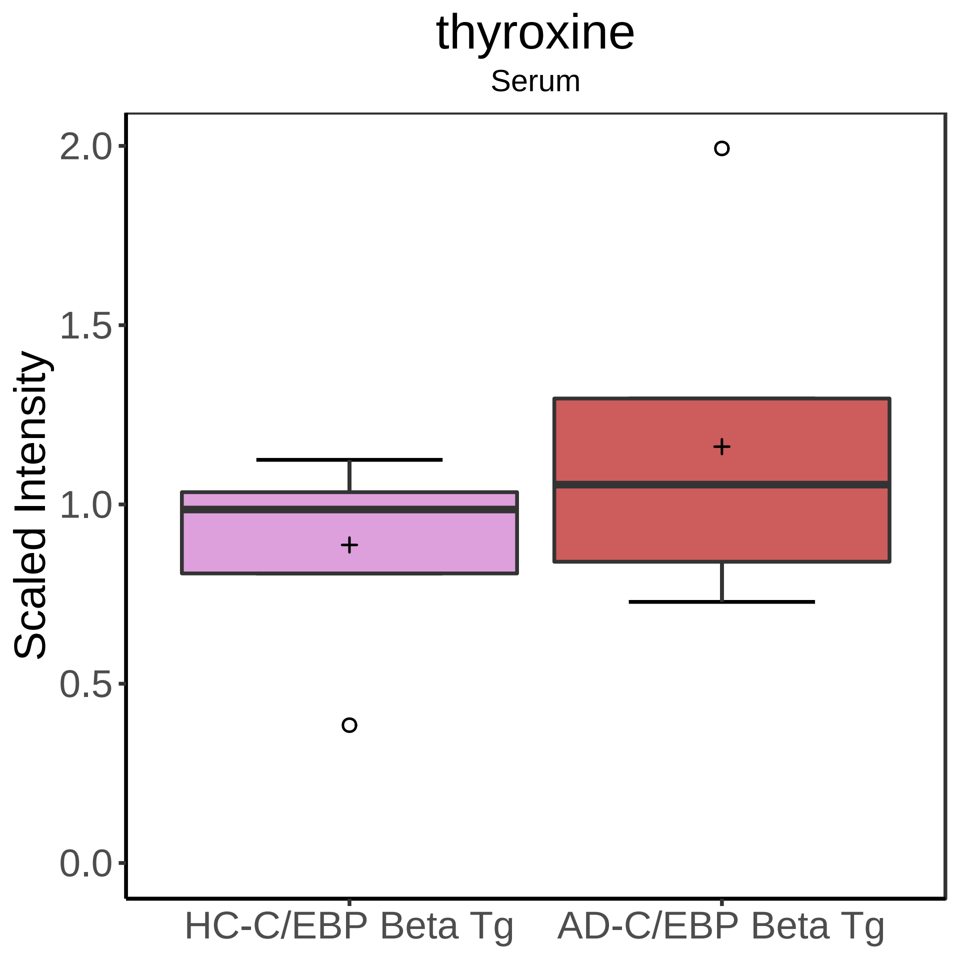
thyroxine
Serum
Scaled Intensity
2.0
1.5
1.0
0.5
0.0
HC-C/EBP Beta Tg
AD-C/EBP Beta Tg

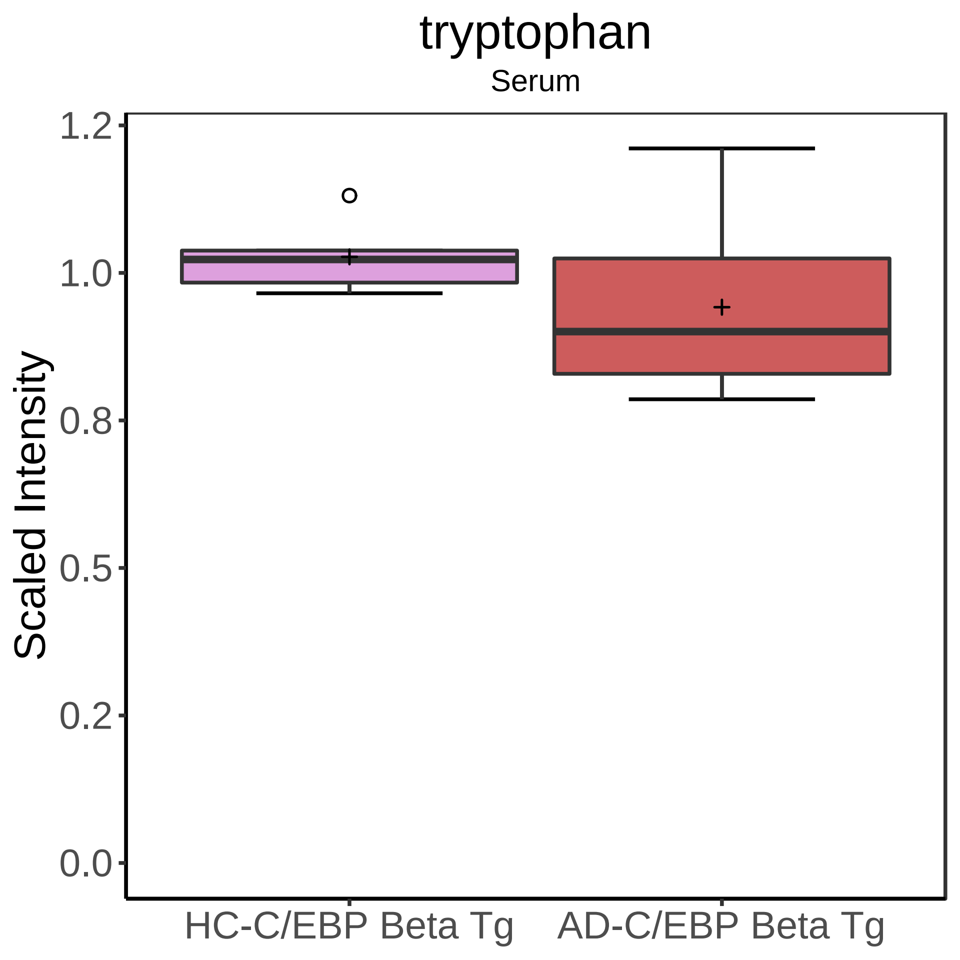
tryptophan
Serum
Scaled Intensity
1.2
1.0
0.8
0.5
0.2
0.0
HC-C/EBP Beta Tg
AD-C/EBP Beta Tg

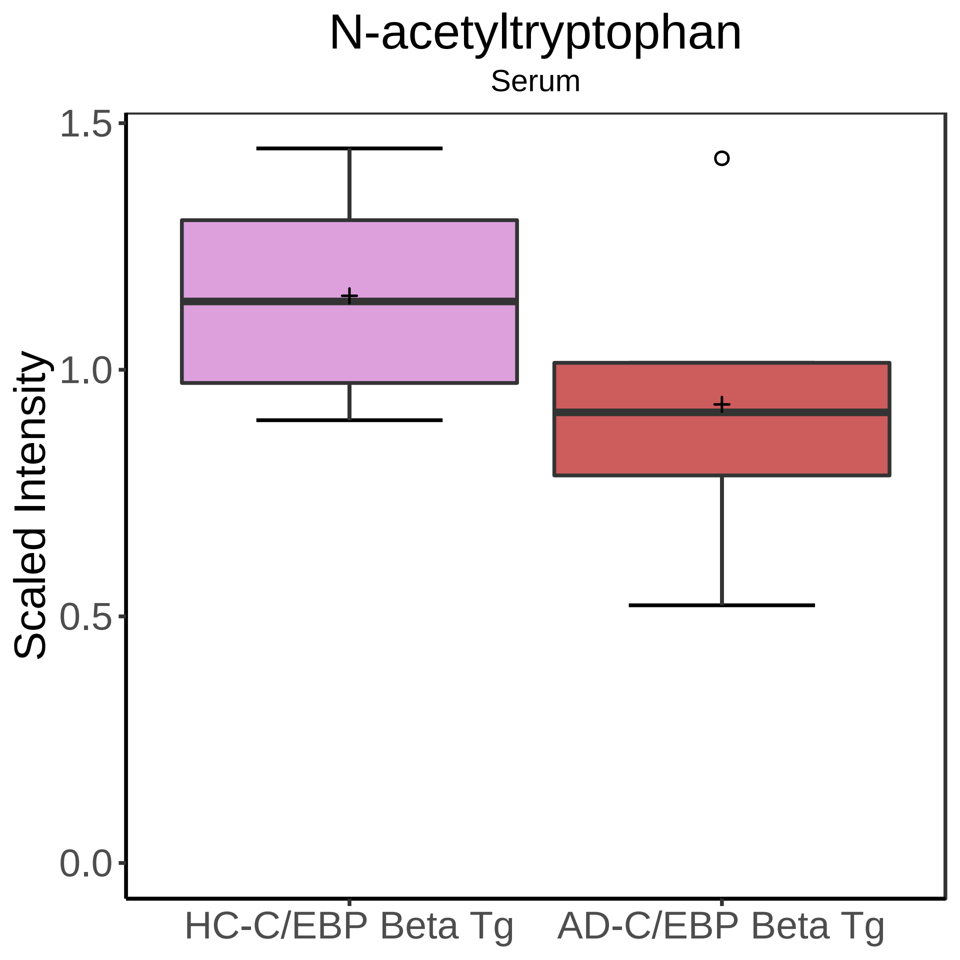
N-acetyltryptophan
Serum
Scaled Intensity
1.5
1.0
0.5
0.0
HC-C/EBP Beta Tg
AD-C/EBP Beta Tg

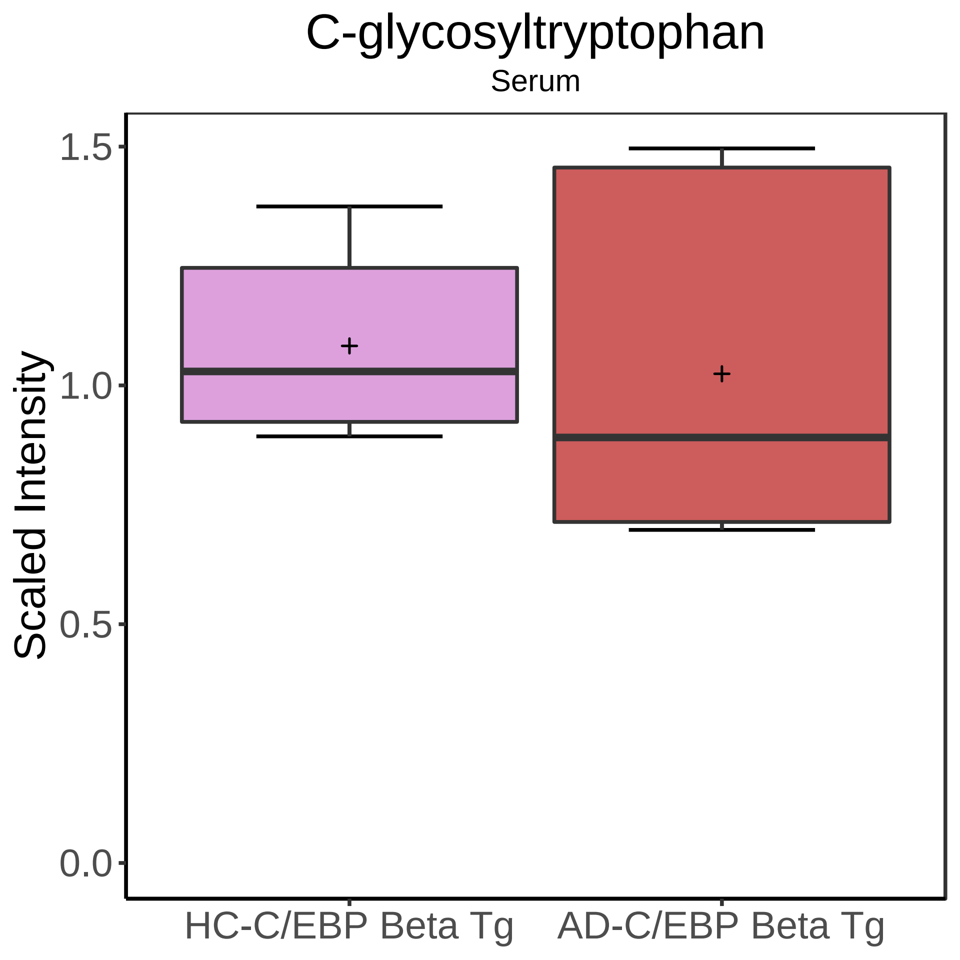
C-glycosyltryptophan
Serum
Scaled Intensity
1.5
1.0
0.5
0.0
HC-C/EBP Beta Tg
AD-C/EBP Beta Tg

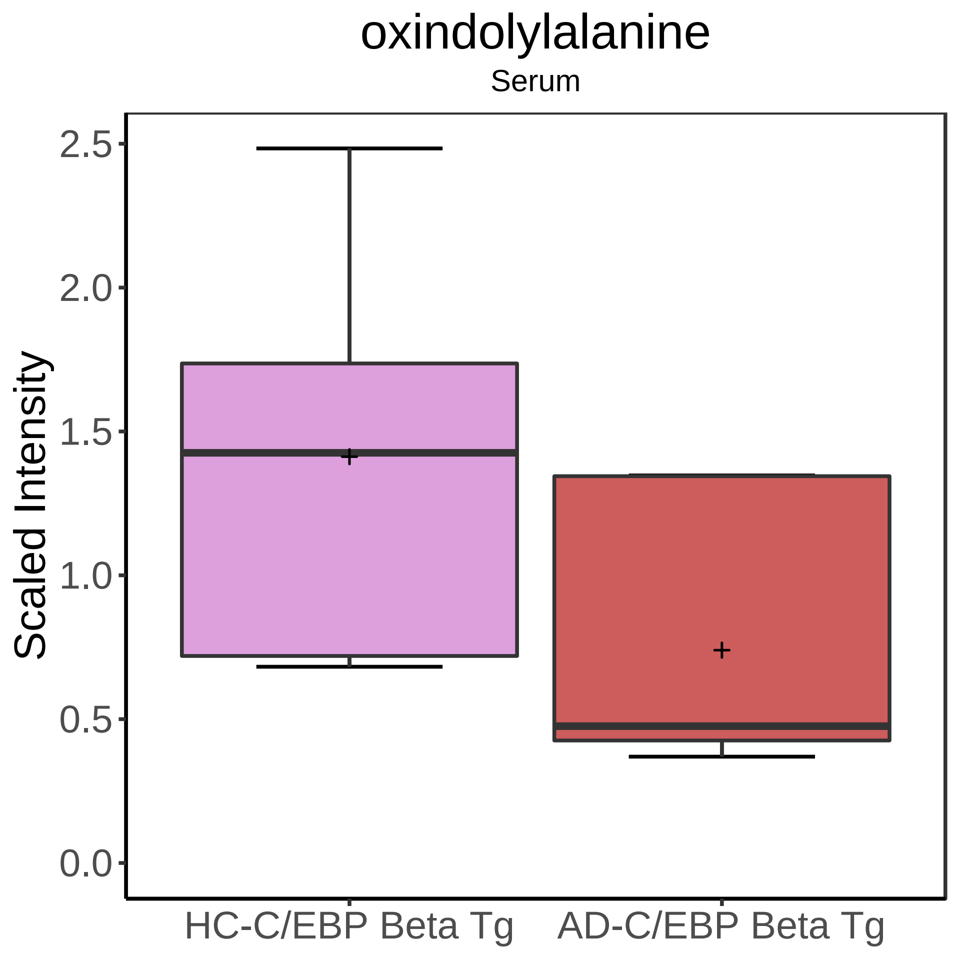

oxindolylalanine
Serum
Scaled Intensity
2.5
2.0
1.5
1.0
0.5
0.0
HC-C/EBP Beta Tg
AD-C/EBP Beta Tg

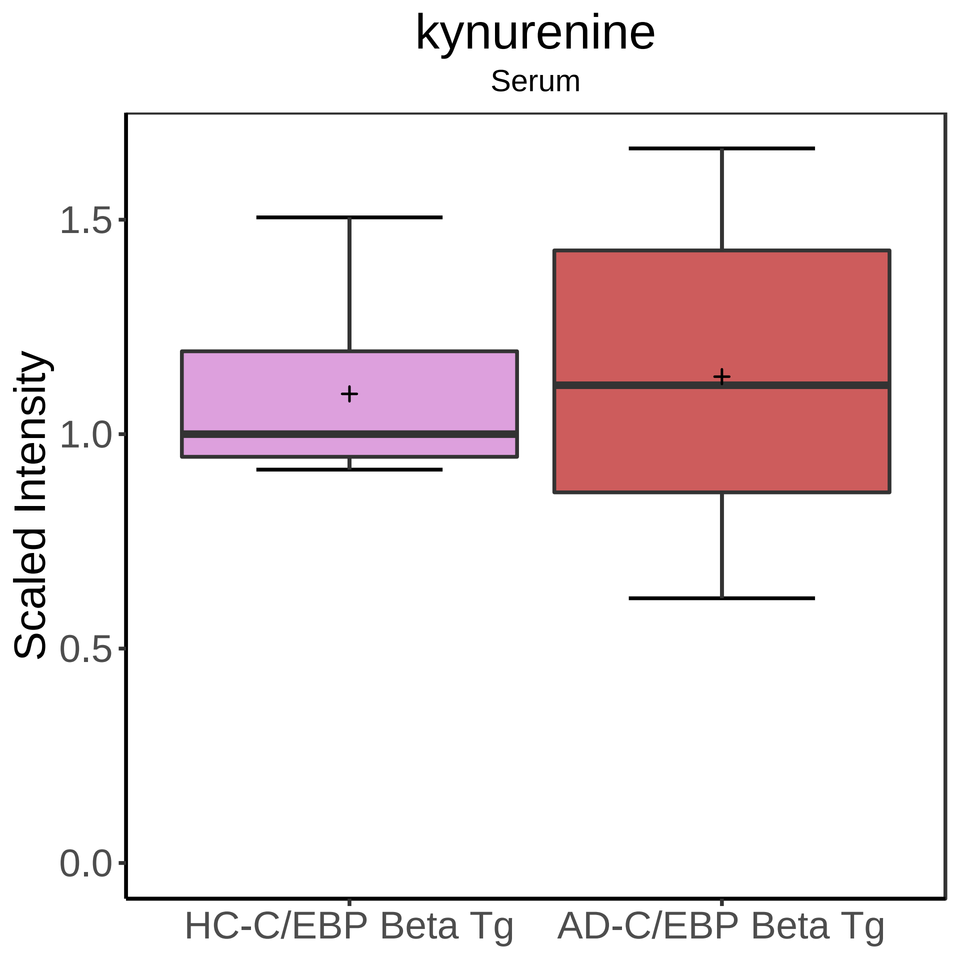

kynurenine
Serum
Scaled Intensity
1.5
1.0
0.5
0.0
HC-C/EBP Beta Tg
AD-C/EBP Beta Tg

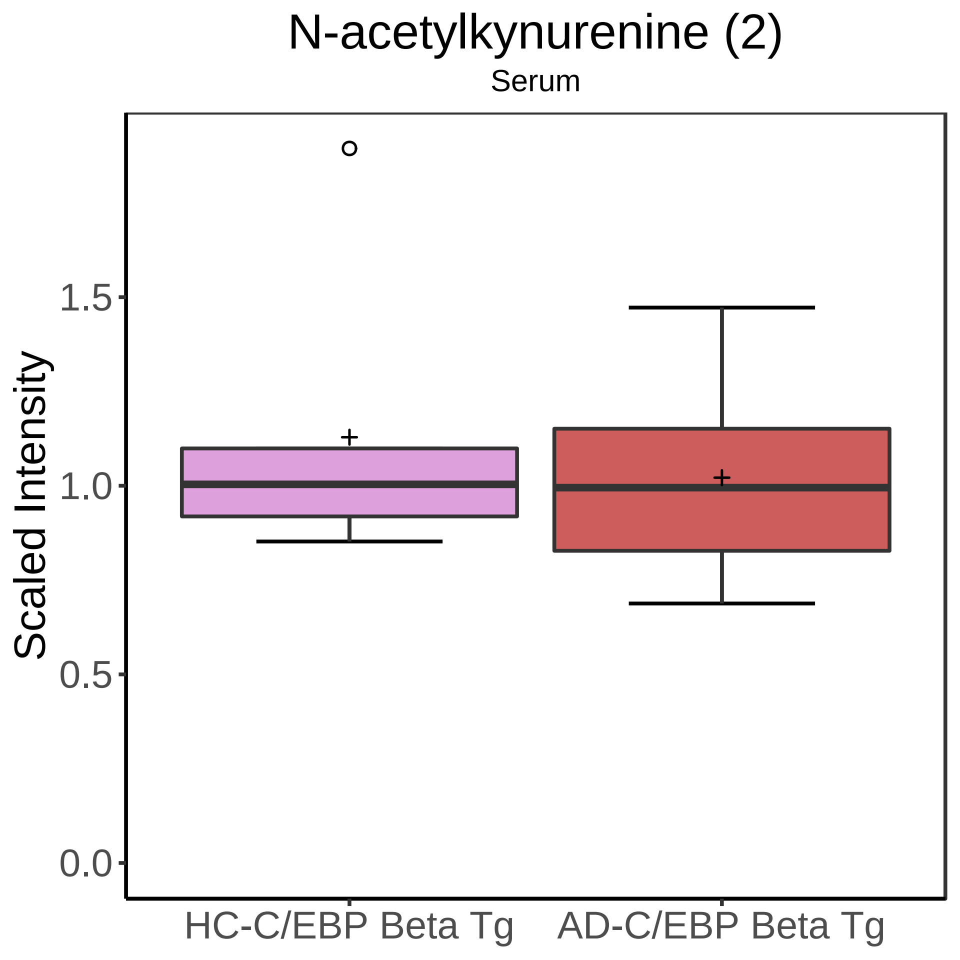

N-acetylkynurenine (2)
Serum
Scaled Intensity
1.5
1.0
0.5
0.0
HC-C/EBP Beta Tg
AD-C/EBP Beta Tg

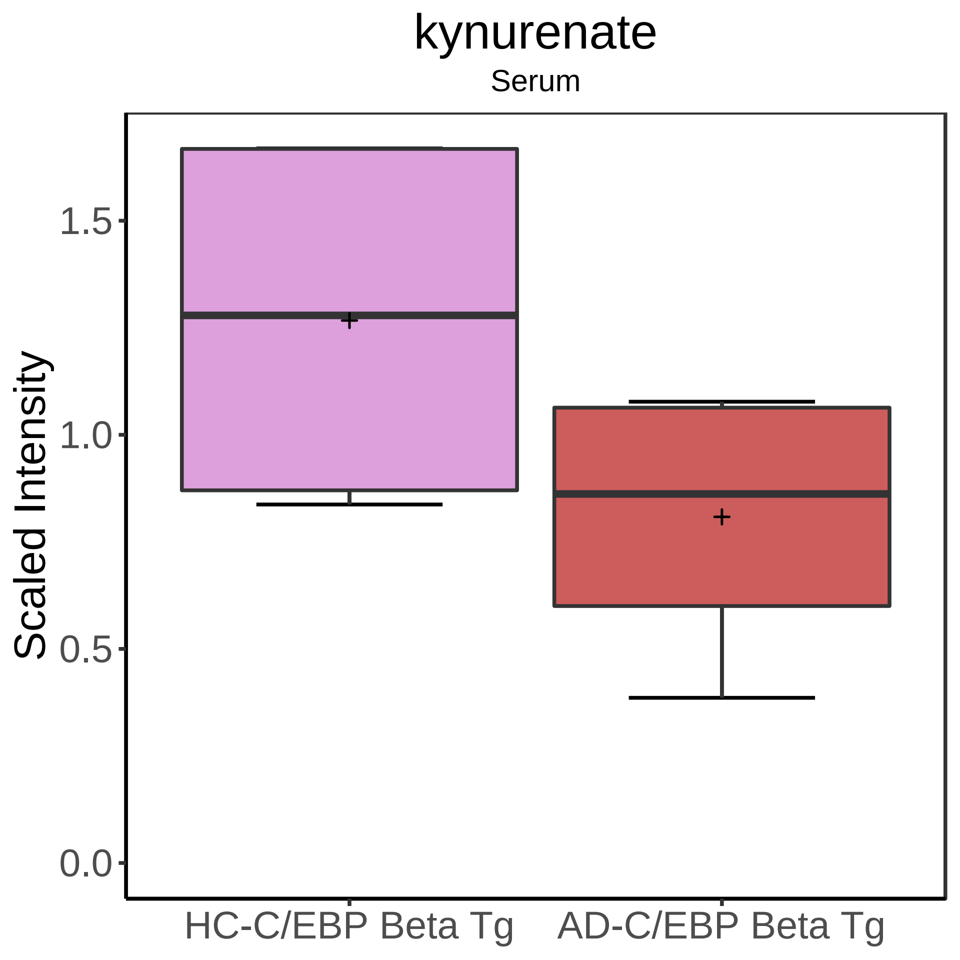
kynurenate
Serum
Scaled Intensity
1.5
1.0
0.5
0.0
HC-C/EBP Beta Tg
AD-C/EBP Beta Tg

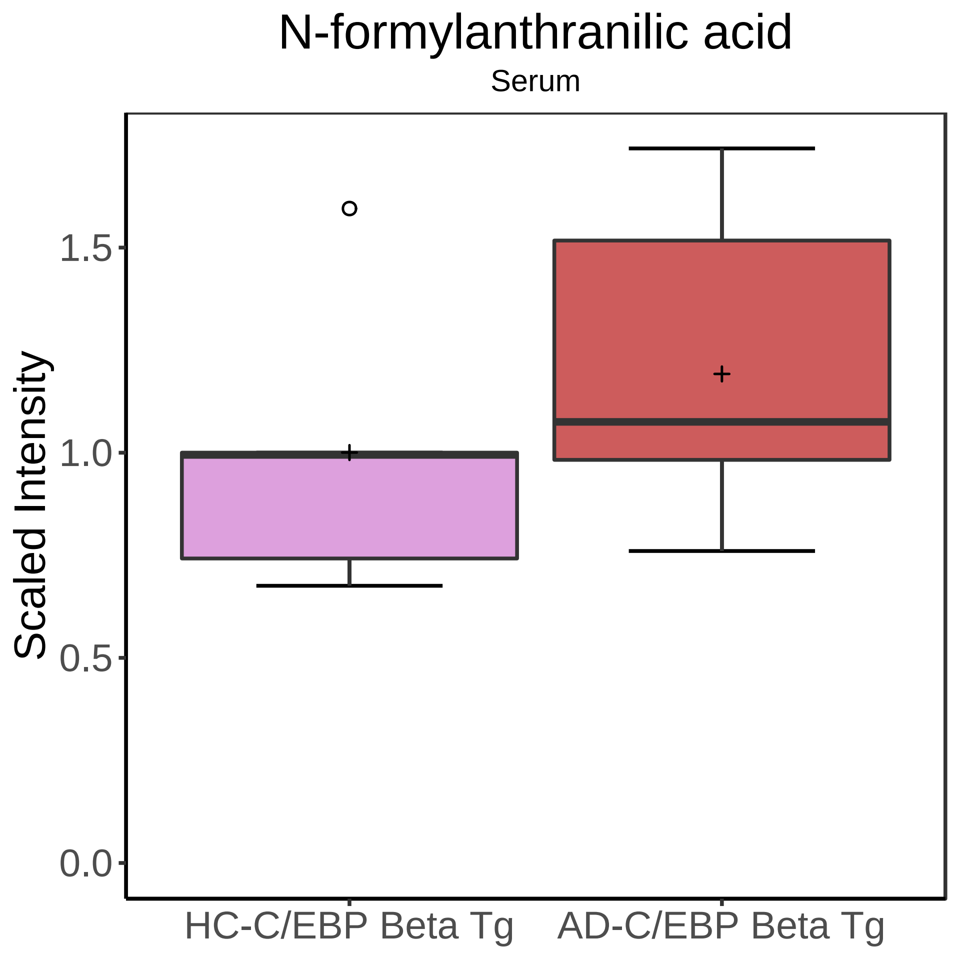

N-formylanthranilic acid
Serum
Scaled Intensity
1.5
1.0
0.5
0.0
HC-C/EBP Beta Tg
AD-C/EBP Beta Tg

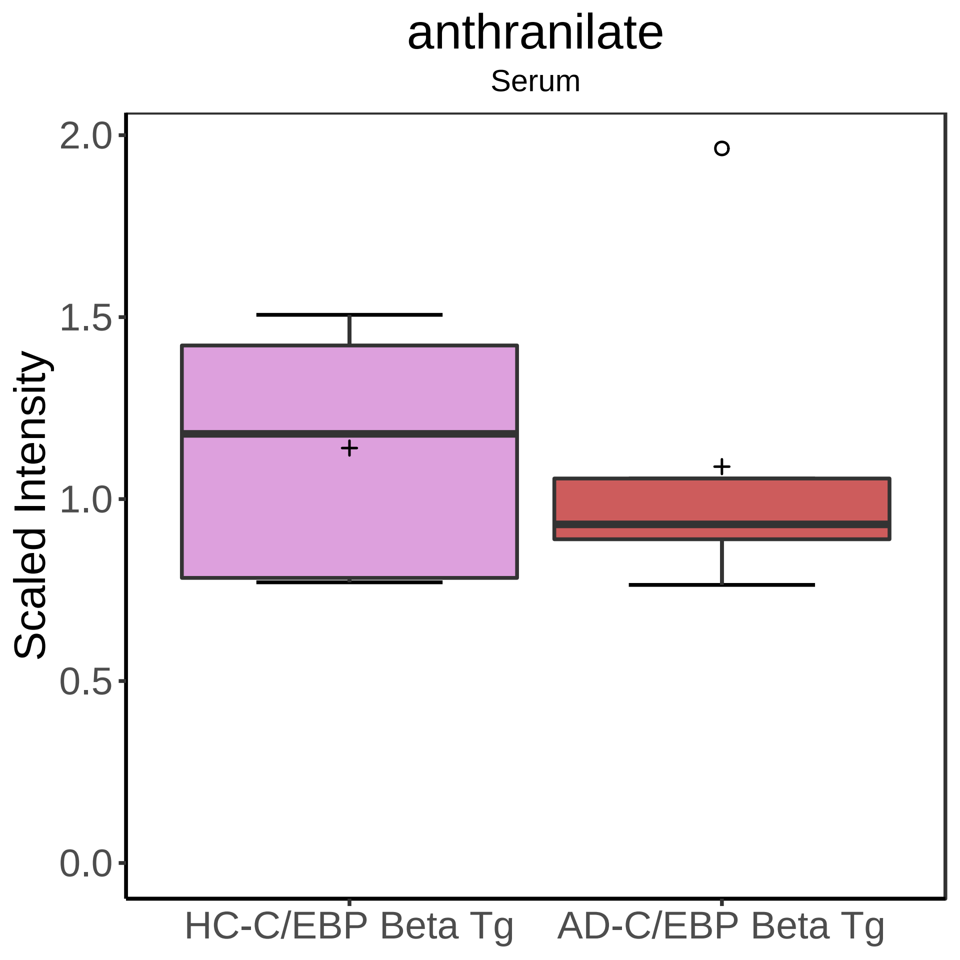
anthranilate
Serum
Scaled Intensity
2.0
1.5
1.0
0.5
0.0
HC-C/EBP Beta Tg
AD-C/EBP Beta Tg

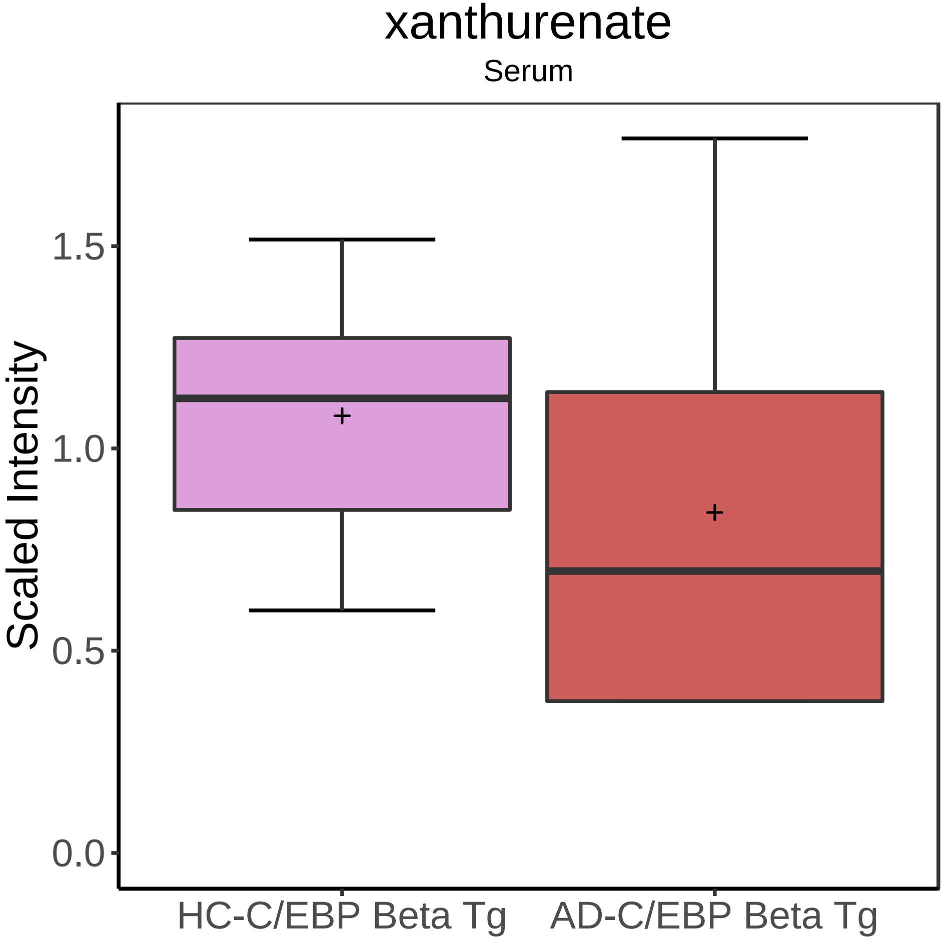
xanthurenate
Serum
Scaled Intensity
1.5
1.0
0.5
0.0
HC-C/EBP Beta Tg
AD-C/EBP Beta Tg

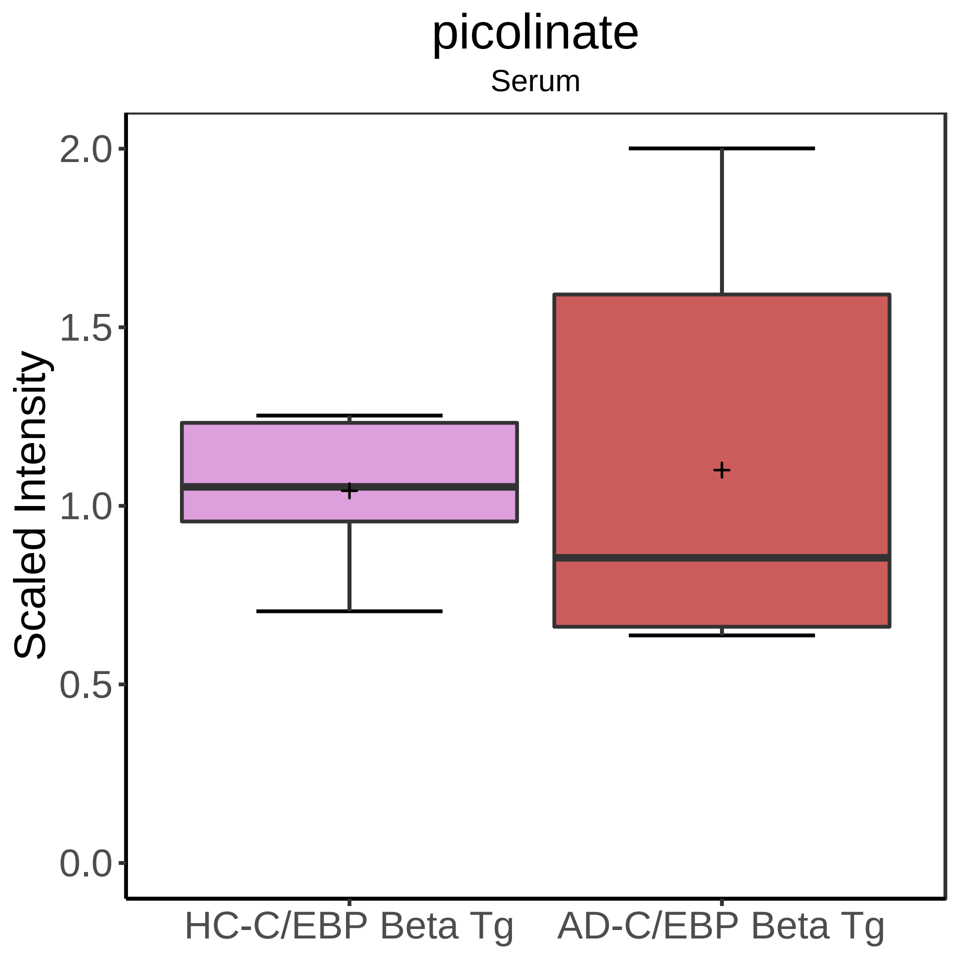
picolinate
Serum
Scaled Intensity
2.0
1.5
1.0
0.5
0.0
HC-C/EBP Beta Tg
AD-C/EBP Beta Tg

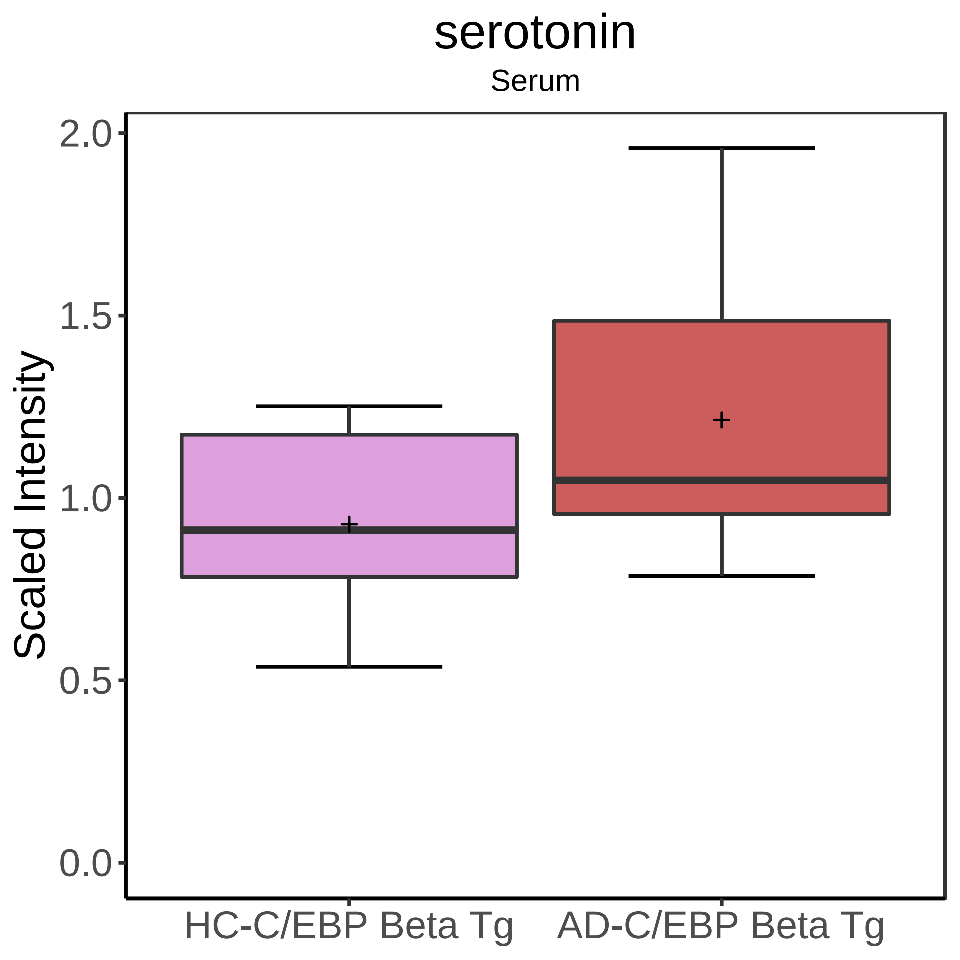
serotonin
Serum
Scaled Intensity
2.0
1.5
1.0
0.5
0.0
HC-C/EBP Beta Tg
AD-C/EBP Beta Tg

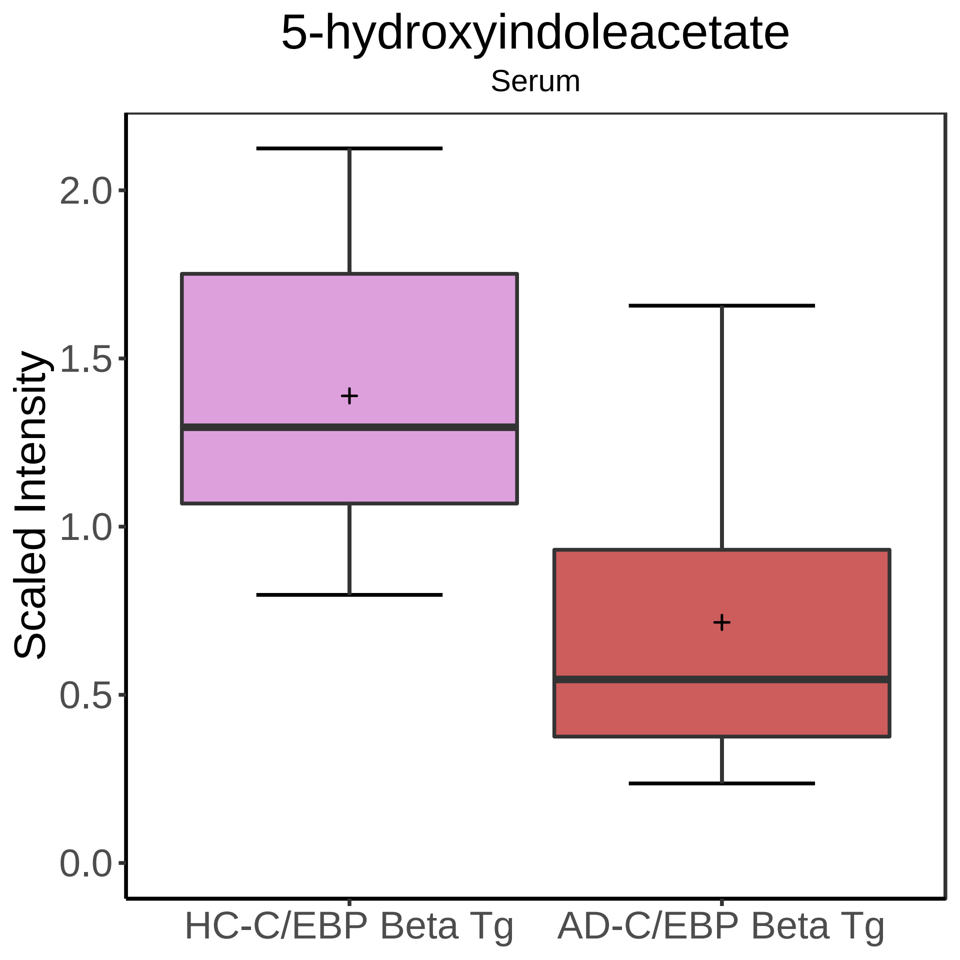
5-hydroxyindoleacetate
Serum
Scaled Intensity
2.0
1.5
1.0
0.5
0.0
HC-C/EBP Beta Tg
AD-C/EBP Beta Tg

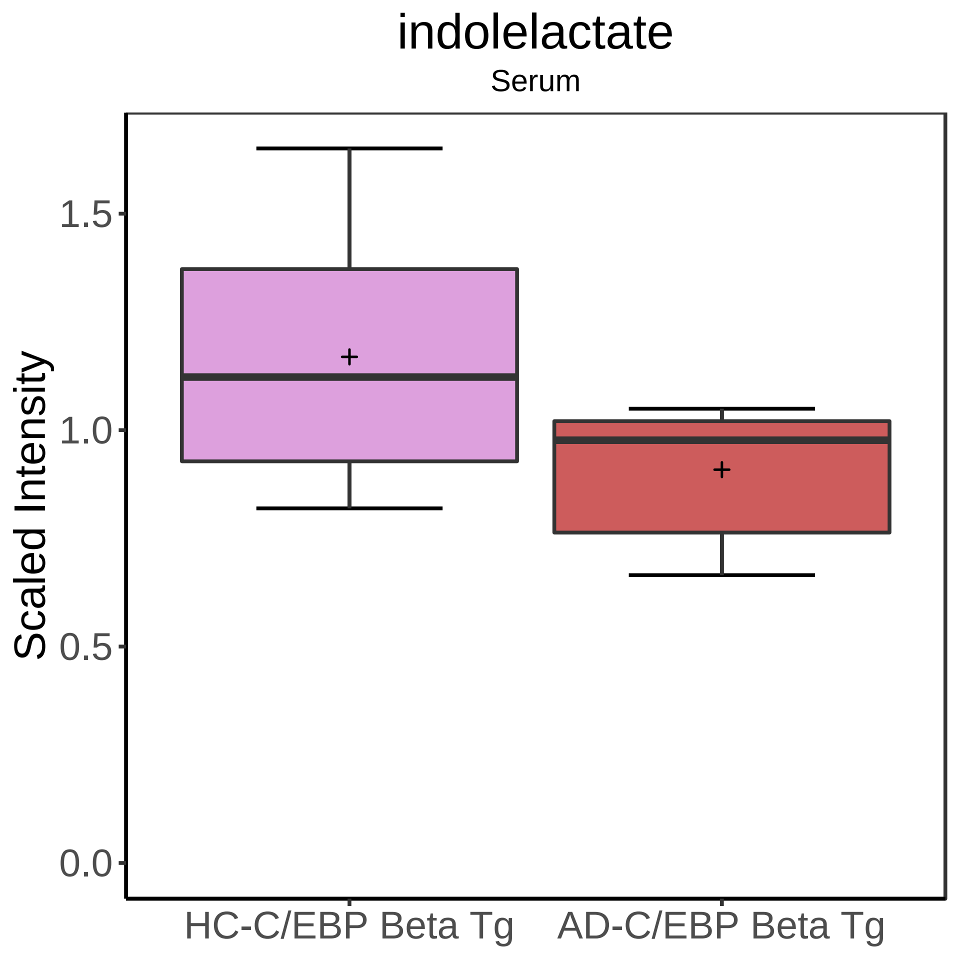
indolelactate
Serum
Scaled Intensity
1.5
1.0
0.5
0.0
HC-C/EBP Beta Tg
AD-C/EBP Beta Tg

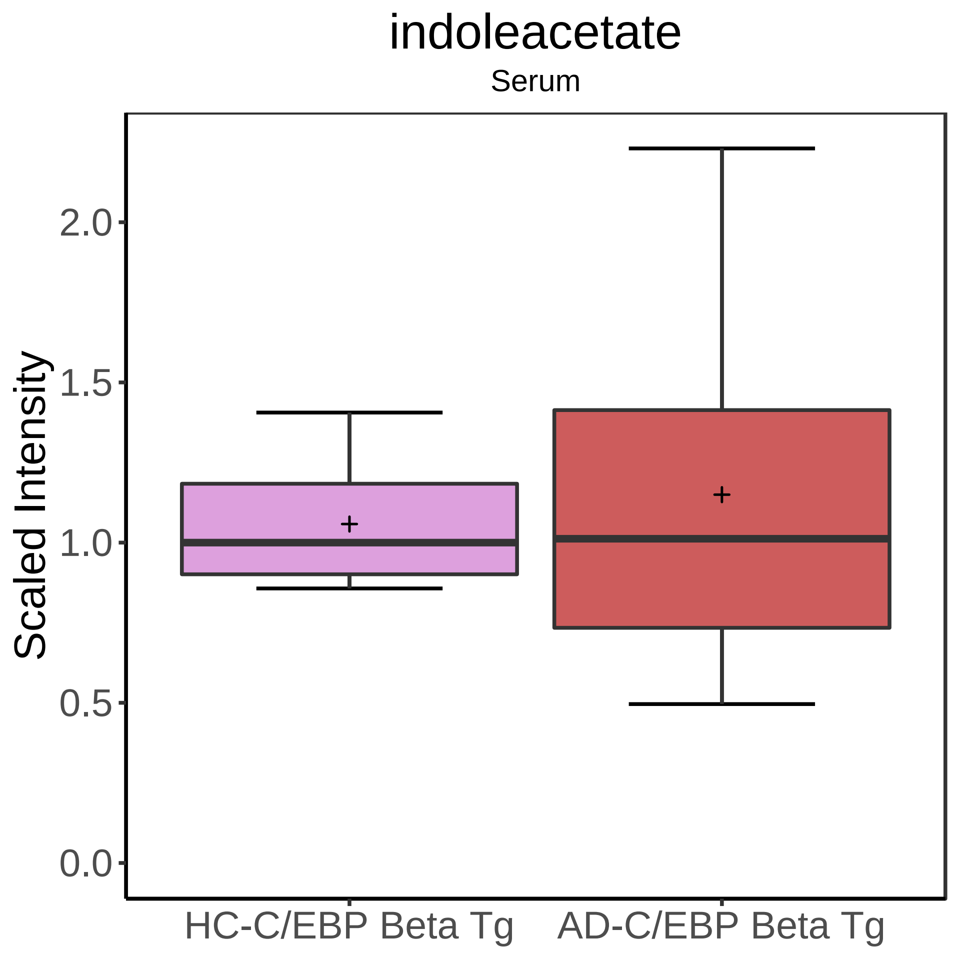
indoleacetate
Serum
Scaled Intensity
2.0
1.5
1.0
0.5
0.0
HC-C/EBP Beta Tg
AD-C/EBP Beta Tg

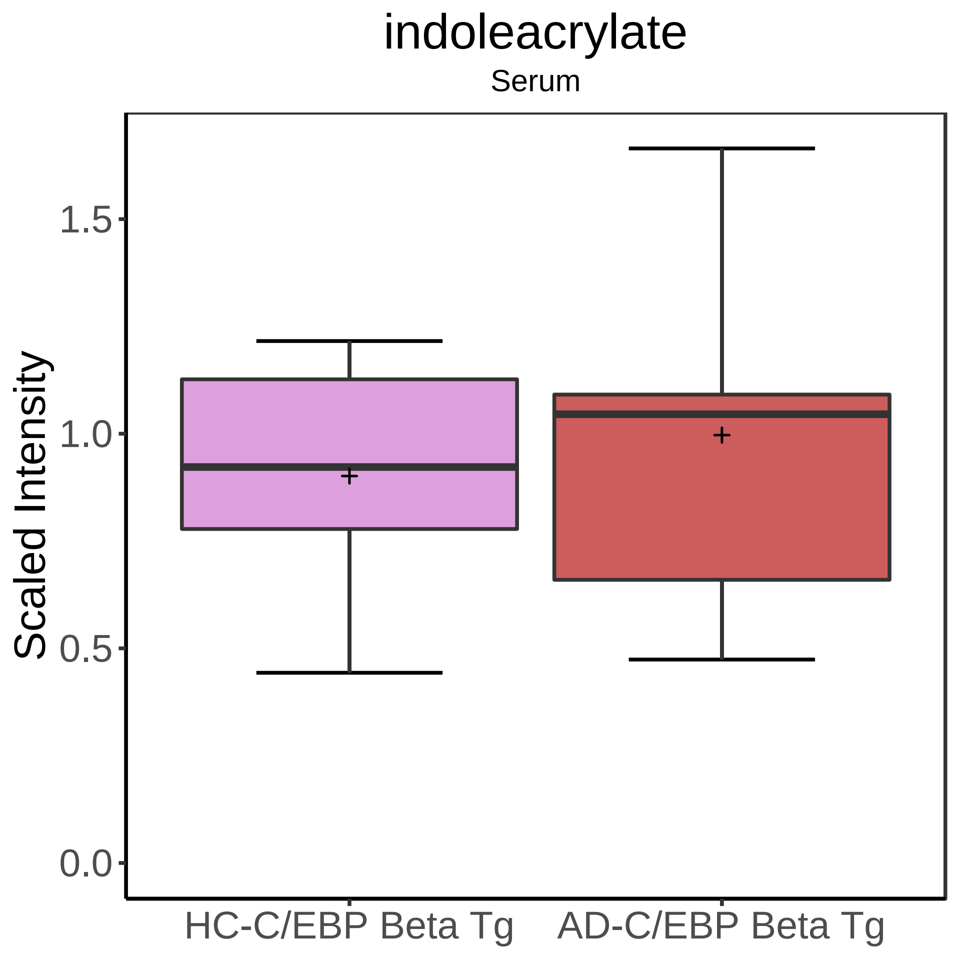
indoleacrylate
Serum
Scaled Intensity
1.5
1.0
0.5
0.0
HC-C/EBP Beta Tg
AD-C/EBP Beta Tg

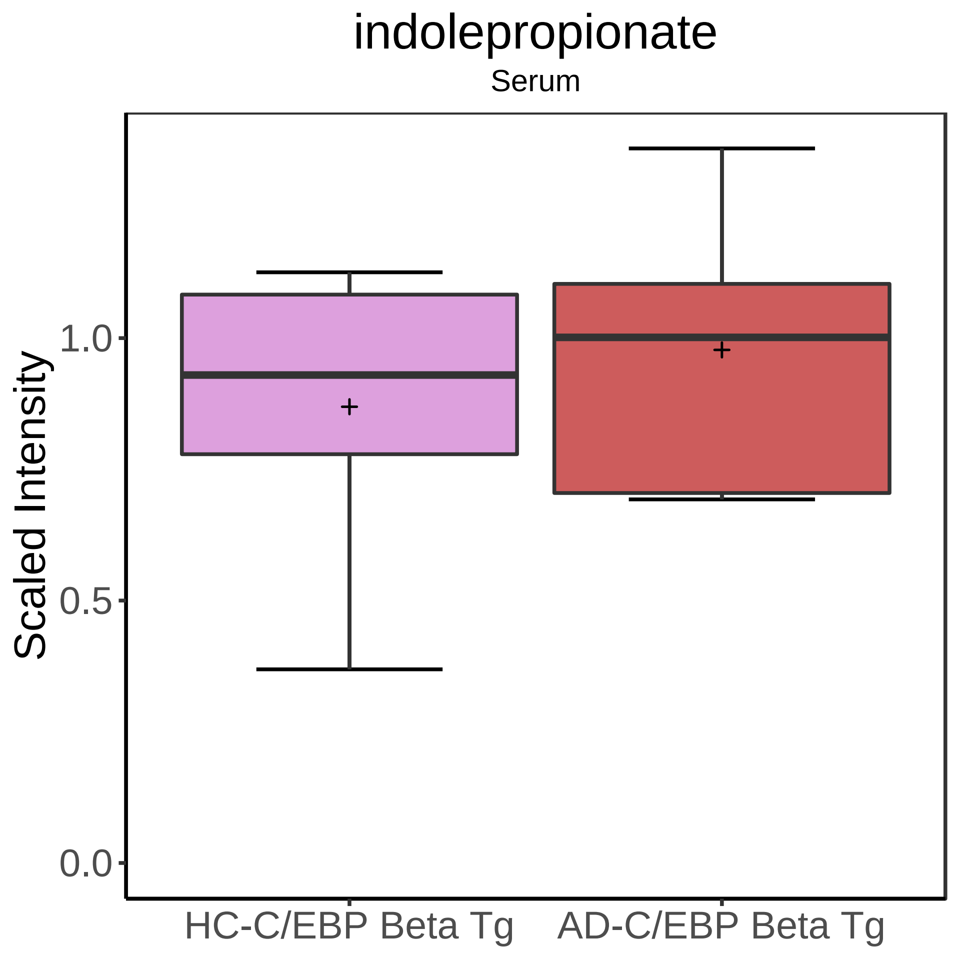

indolepropionate
Serum
Scaled Intensity
1.0
0.5
0.0
HC-C/EBP Beta Tg
AD-C/EBP Beta Tg

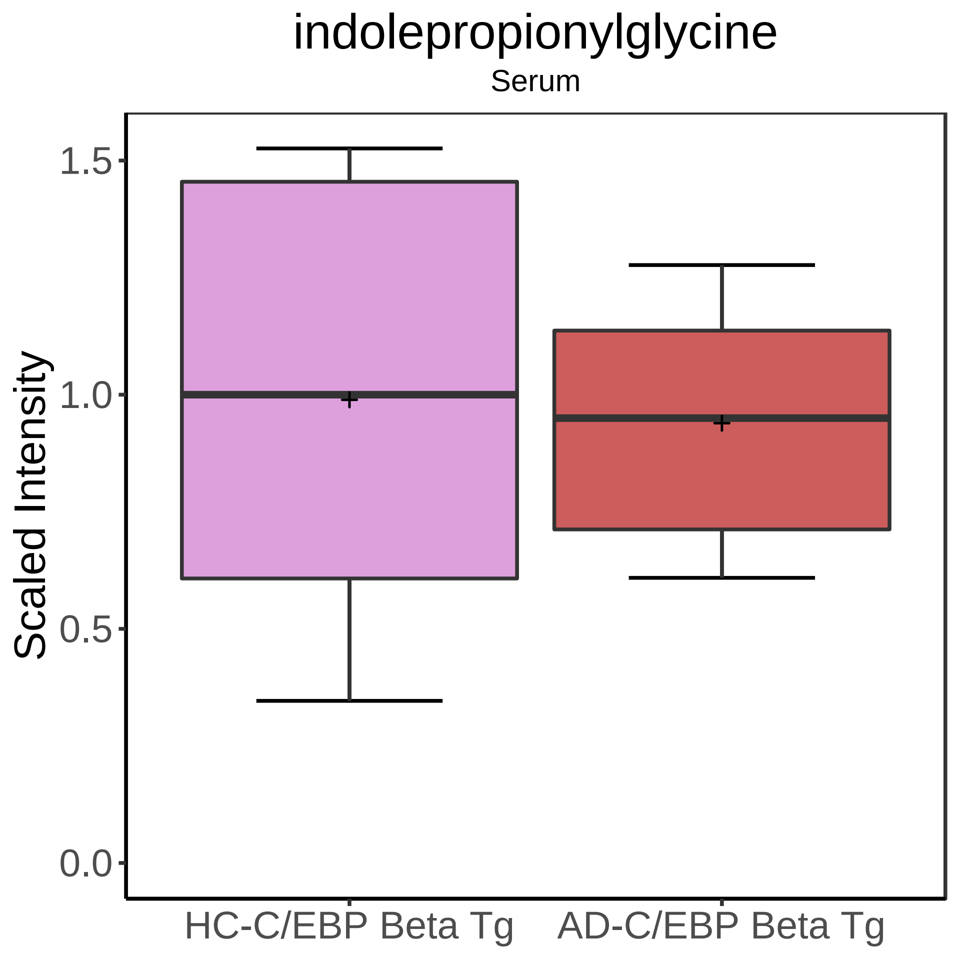
indolepropionylglycine
Serum
Scaled Intensity
1.5
1.0
0.5
0.0
HC-C/EBP Beta Tg
AD-C/EBP Beta Tg

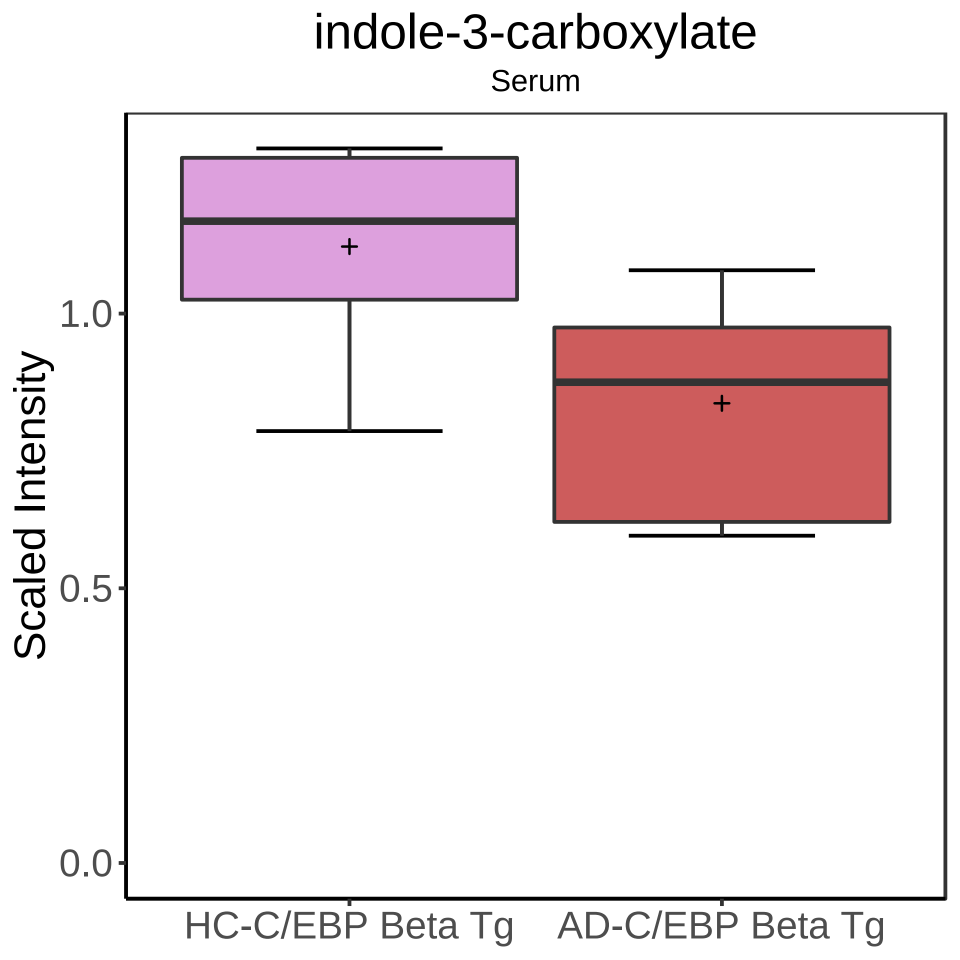
indole-3-carboxylate
Serum
Scaled Intensity
1.0
0.5
0.0
HC-C/EBP Beta Tg
AD-C/EBP Beta Tg

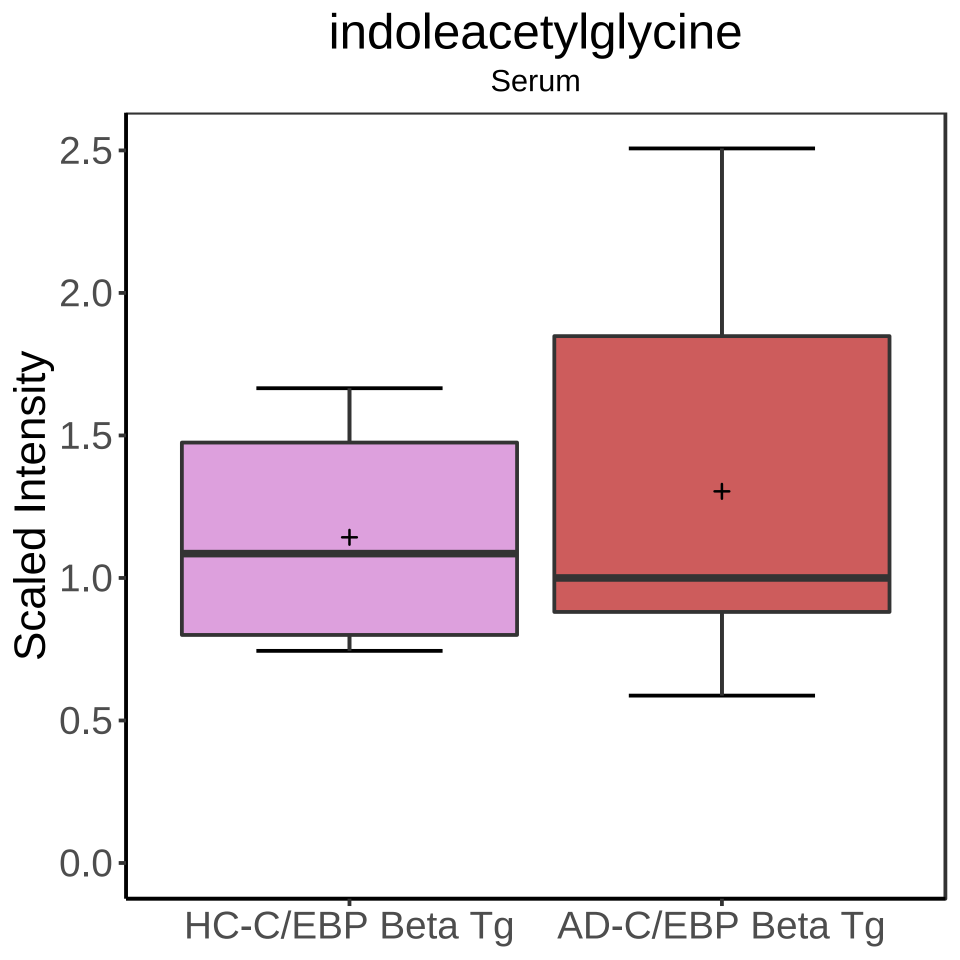
indoleacetylglycine
Serum
Scaled Intensity
2.5
2.0
1.5
1.0
0.5
0.0
HC-C/EBP Beta Tg
AD-C/EBP Beta Tg

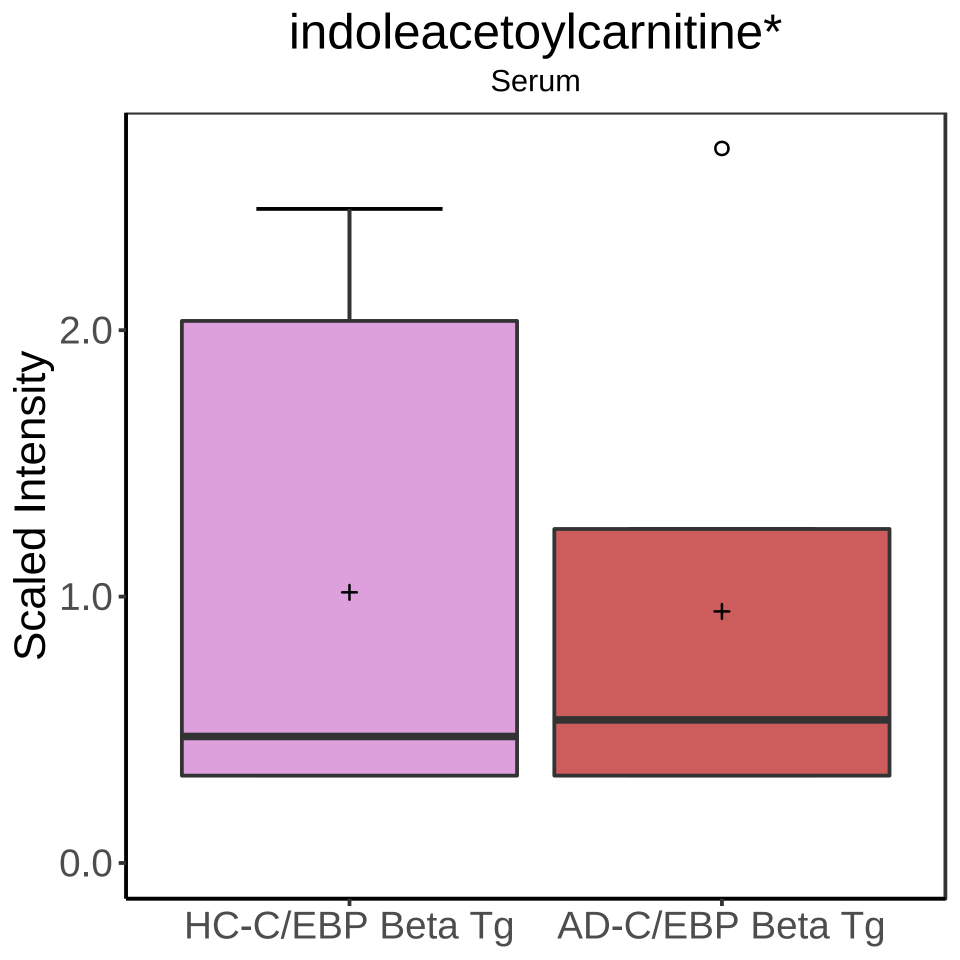
indoleacetoylcarnitine*
Serum
Scaled Intensity
2.0
1.0
0.0
HC-C/EBP Beta Tg
AD-C/EBP Beta Tg

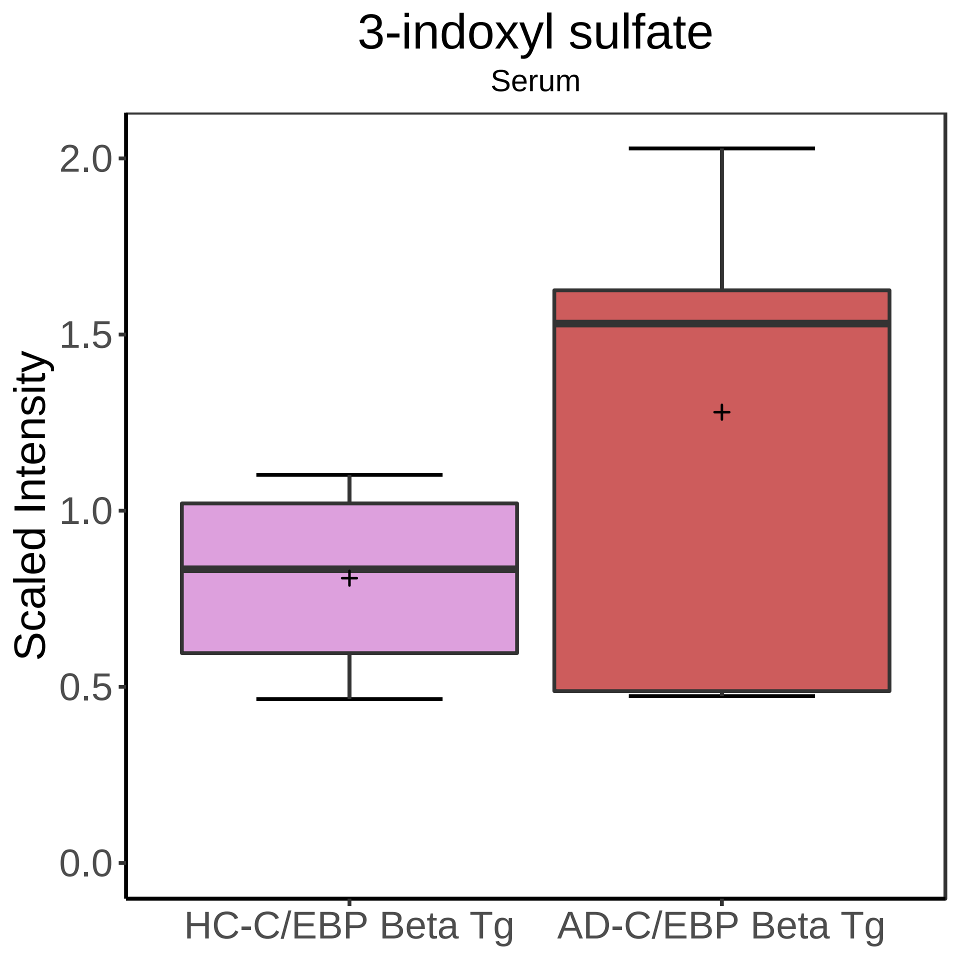
3-indoxyl sulfate
Serum
Scaled Intensity
2.0
1.5
1.0
0.5
0.0
HC-C/EBP Beta Tg
AD-C/EBP Beta Tg

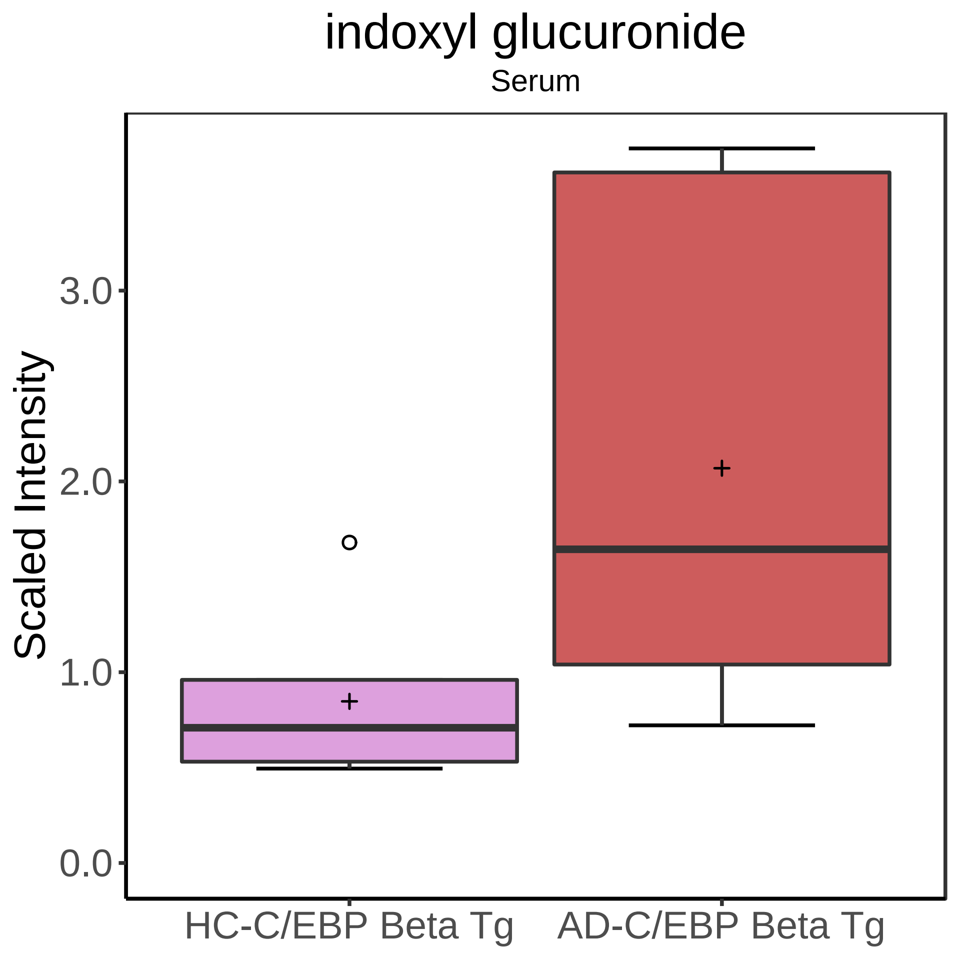
indoxyl glucuronide
Serum
Scaled Intensity
3.0
2.0
1.0
0.0
HC-C/EBP Beta Tg
AD-C/EBP Beta Tg

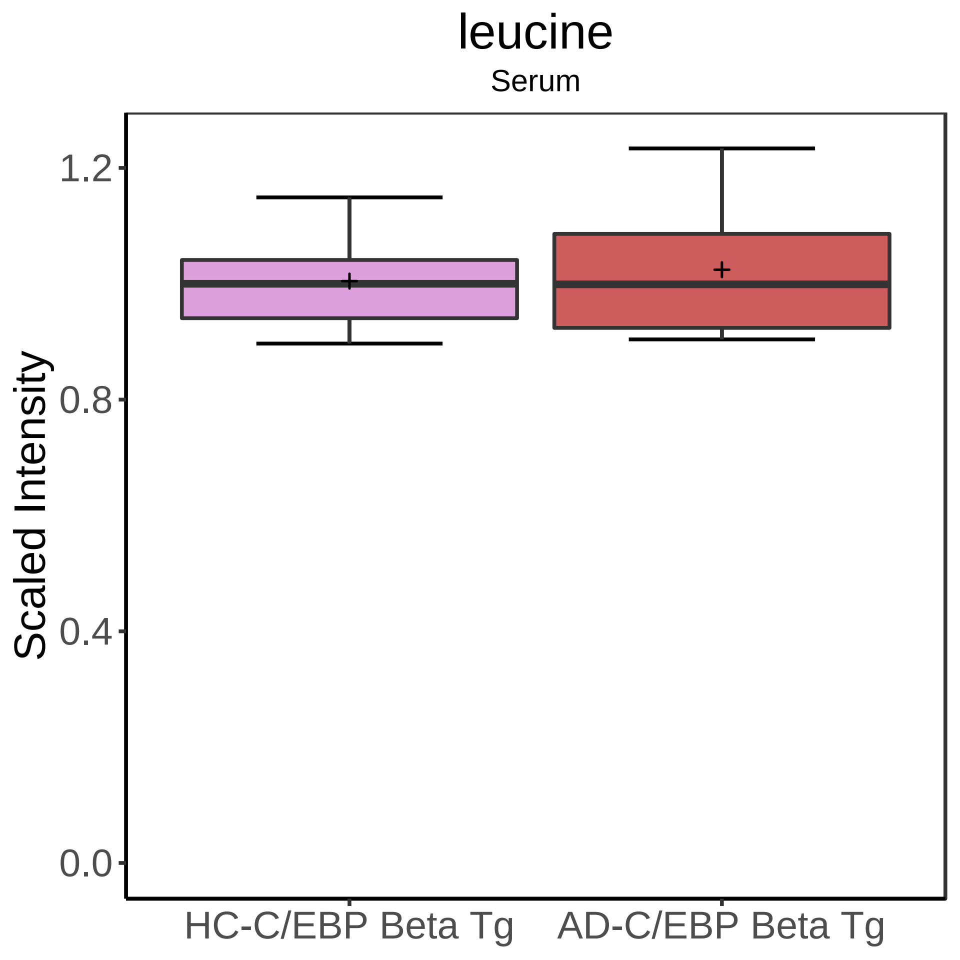
leucine
Serum
Scaled Intensity
1.2
0.8
0.4
0.0
HC-C/EBP Beta Tg
AD-C/EBP Beta Tg

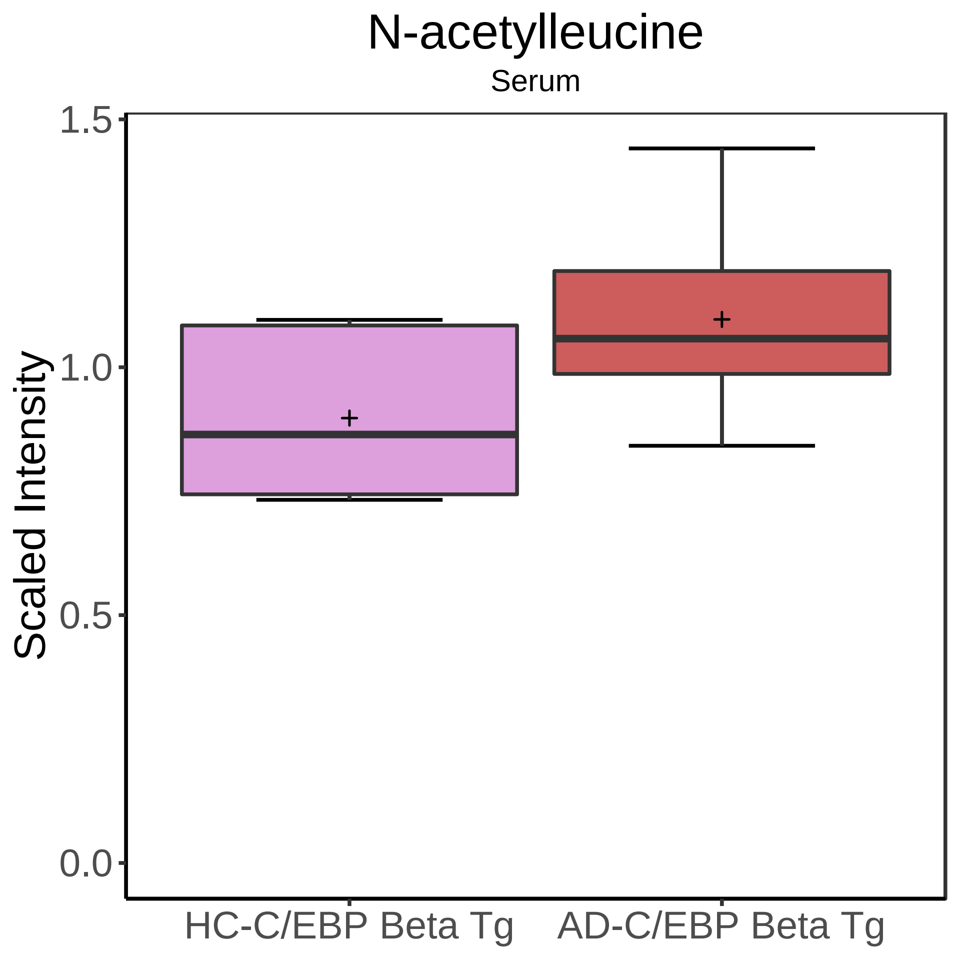
N-acetylleucine
Serum
Scaled Intensity
1.5
1.0
0.5
0.0
HC-C/EBP Beta Tg
AD-C/EBP Beta Tg

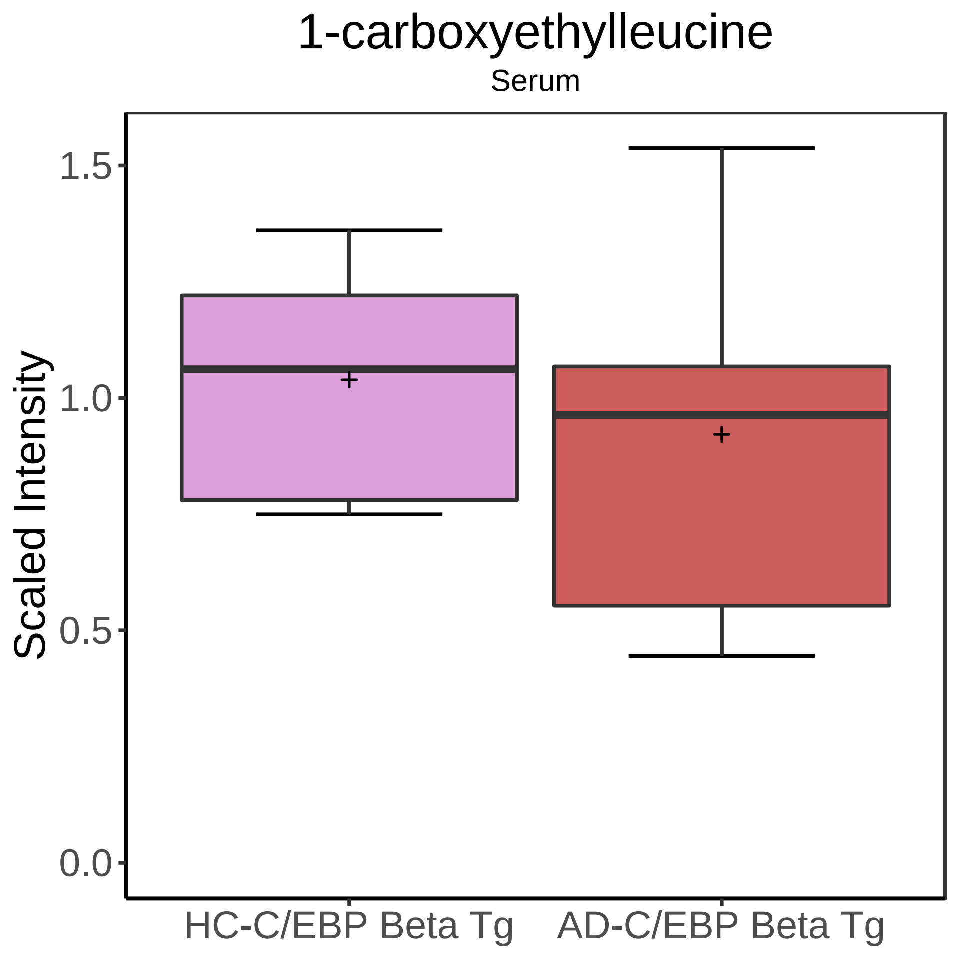

1-carboxyethylleucine
Serum
Scaled Intensity
1.5
1.0
0.5
0.0
HC-C/EBP Beta Tg
AD-C/EBP Beta Tg

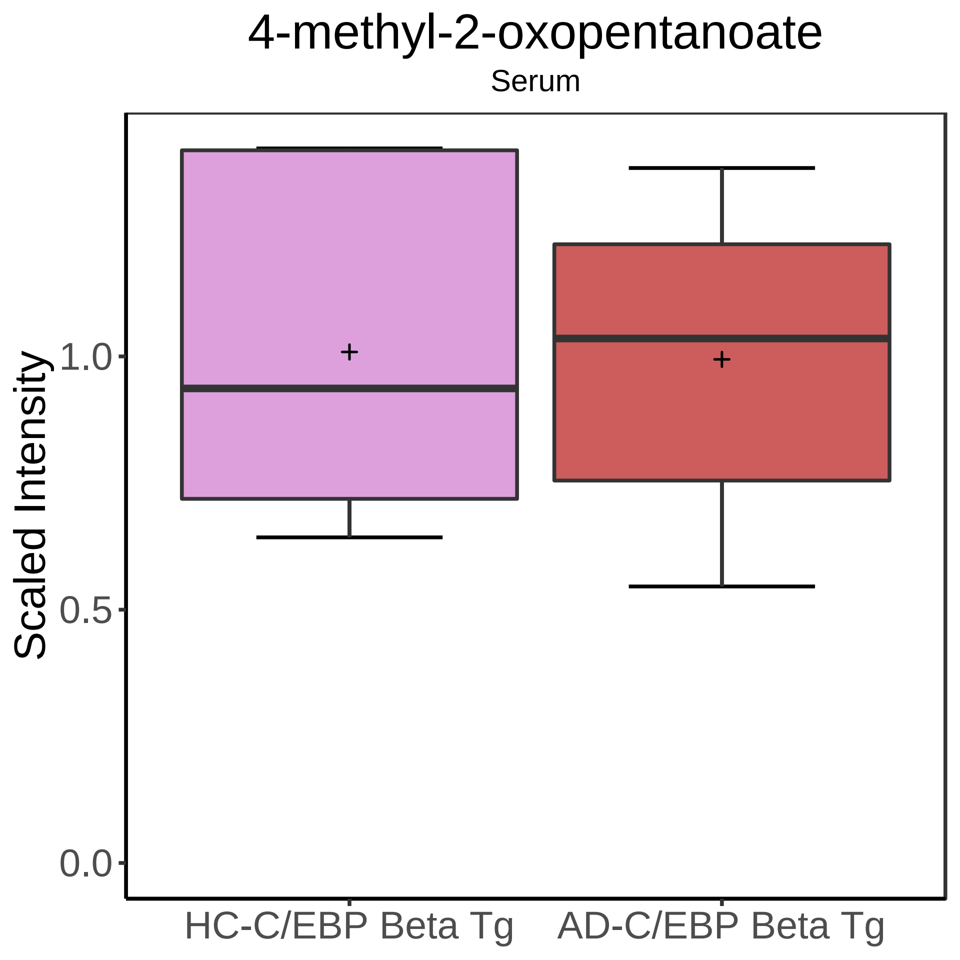
4-methyl-2-oxopentanoate
Serum
Scaled Intensity
1.0
0.5
0.0
HC-C/EBP Beta Tg
AD-C/EBP Beta Tg

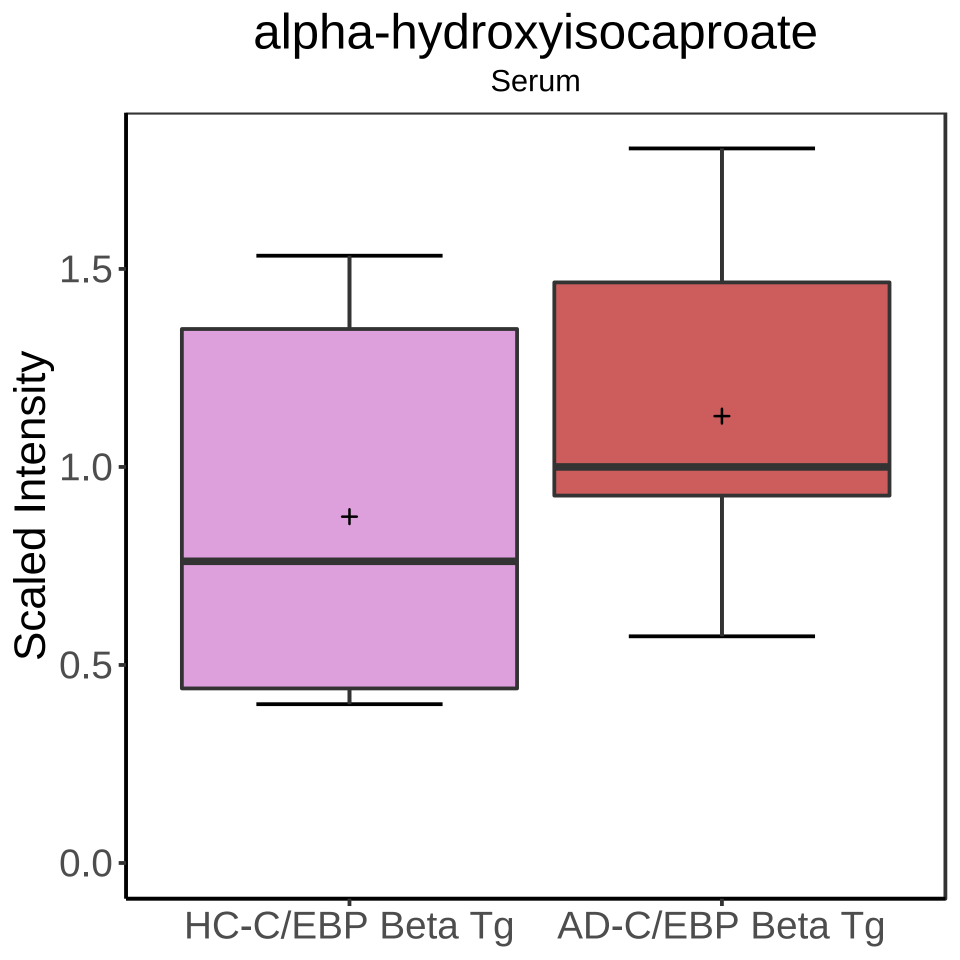
alpha-hydroxyisocaproate
Serum
Scaled Intensity
1.5
1.0
0.5
0.0
HC-C/EBP Beta Tg
AD-C/EBP Beta Tg

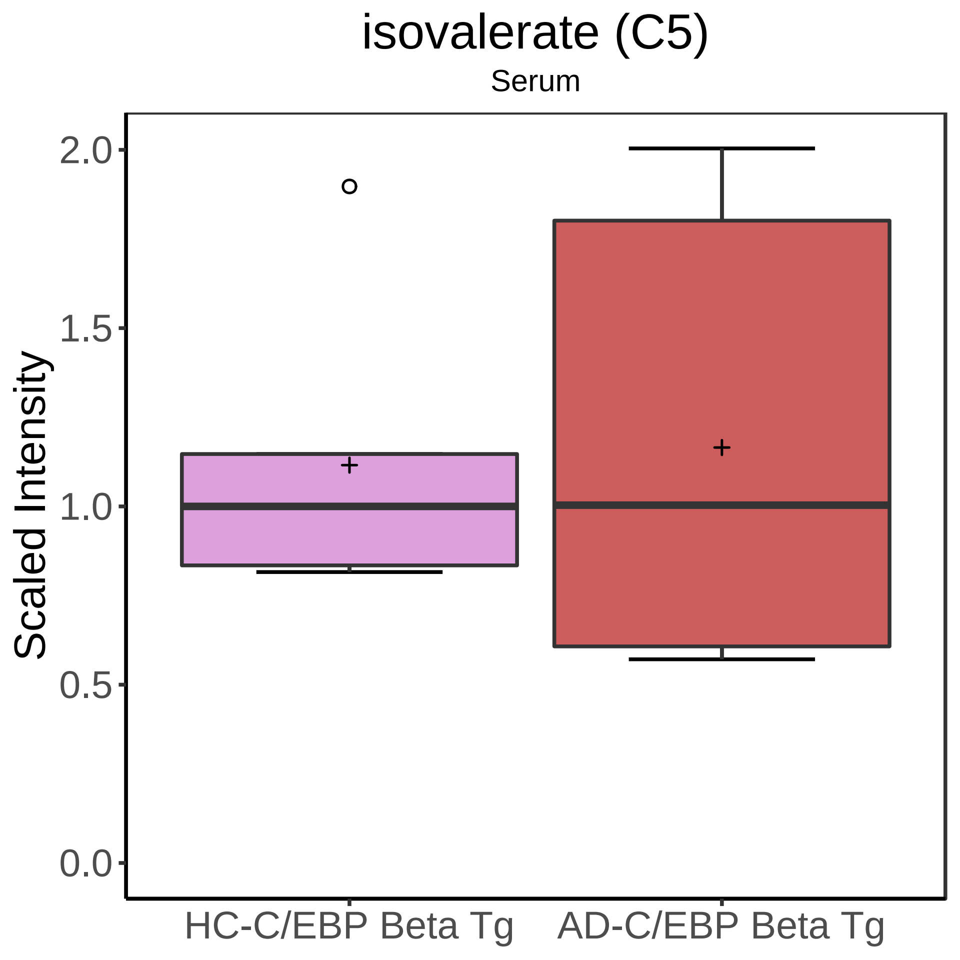
isovalerate (C5)
Serum
Scaled Intensity
2.0
1.5
1.0
0.5
0.0
HC-C/EBP Beta Tg
AD-C/EBP Beta Tg

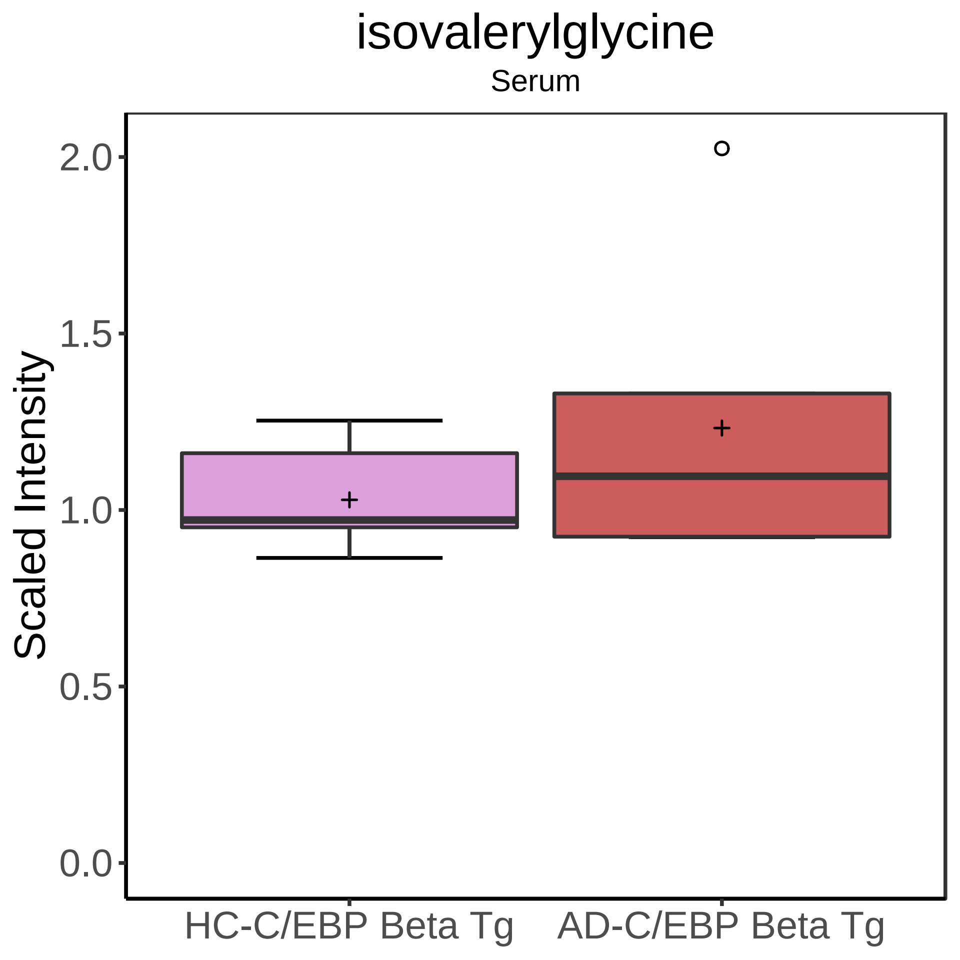

isovalerylglycine
Serum
Scaled Intensity
2.0
1.5
1.0
0.5
0.0
HC-C/EBP Beta Tg
AD-C/EBP Beta Tg

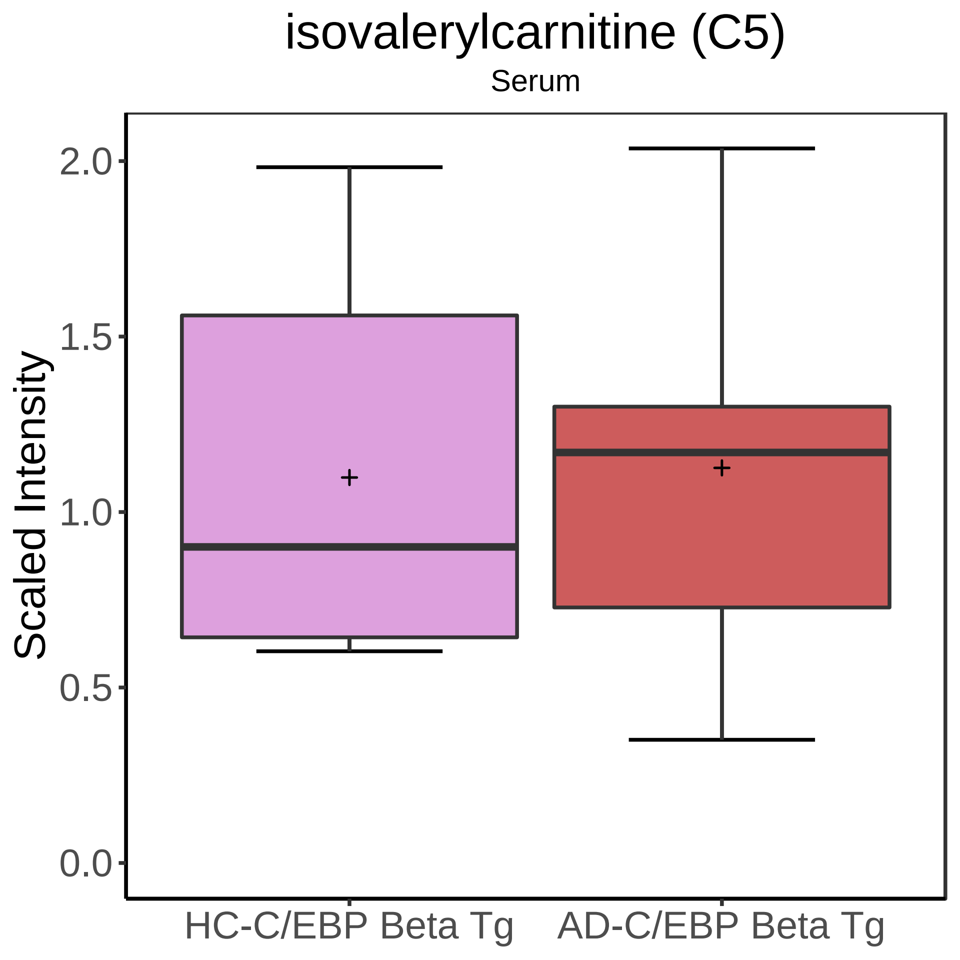
isovalerylcarnitine (C5)
Serum
Scaled Intensity
2.0
1.5
1.0
0.5
0.0
HC-C/EBP Beta Tg
AD-C/EBP Beta Tg

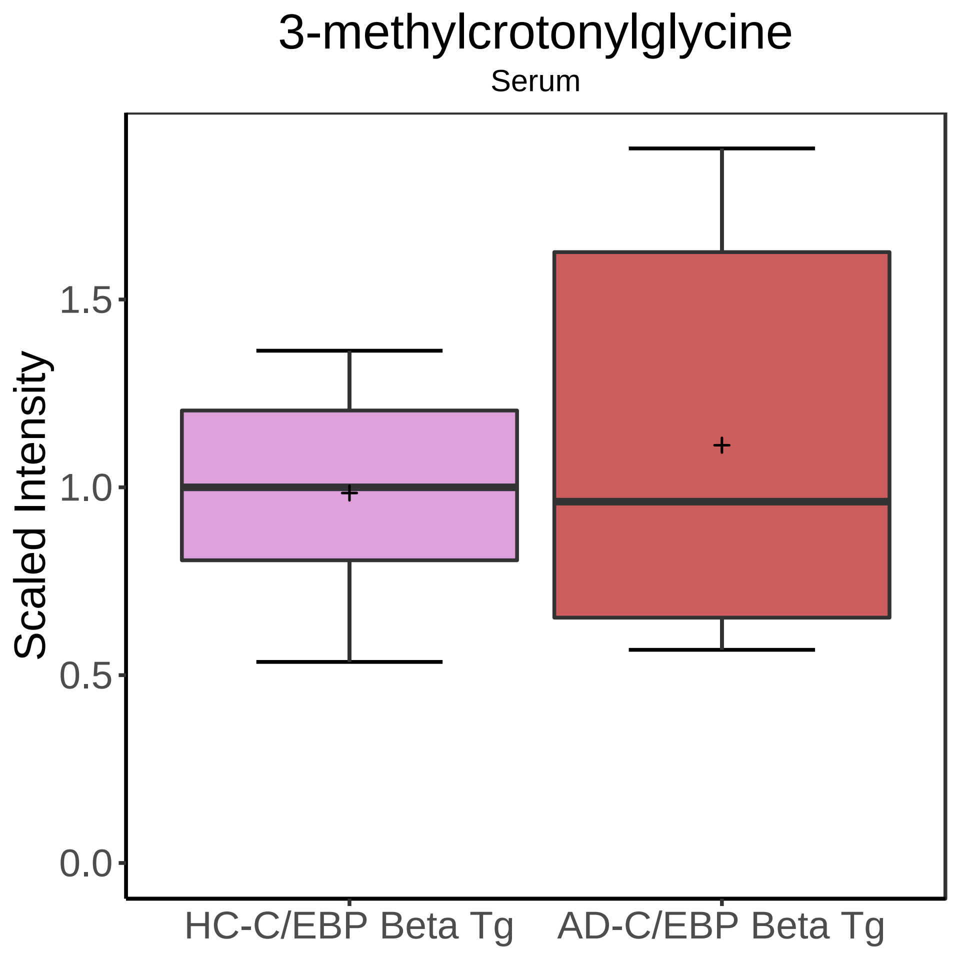
3-methylcrotonylglycine
Serum
Scaled Intensity
0.0
0.5
1.0
1.5
HC-C/EBP Beta Tg
AD-C/EBP Beta Tg

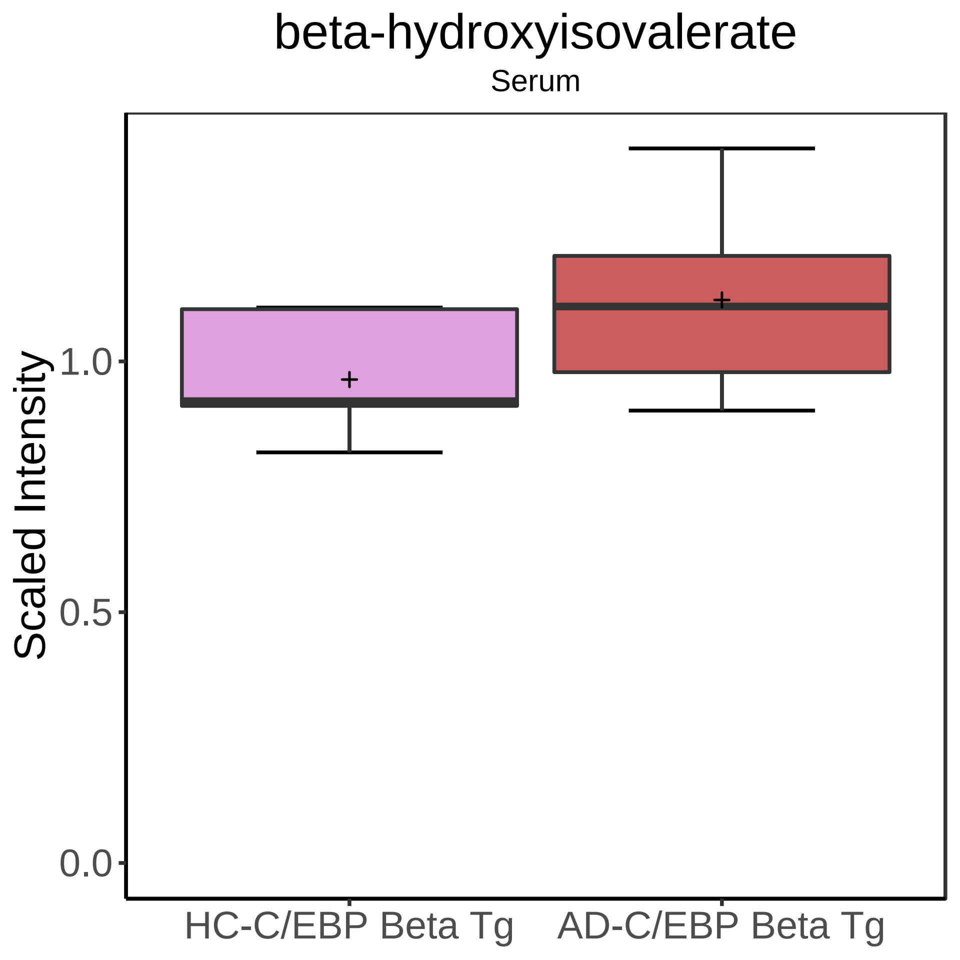
beta-hydroxyisovalerate
Serum
Scaled Intensity
1.0
0.5
0.0
HC-C/EBP Beta Tg
AD-C/EBP Beta Tg

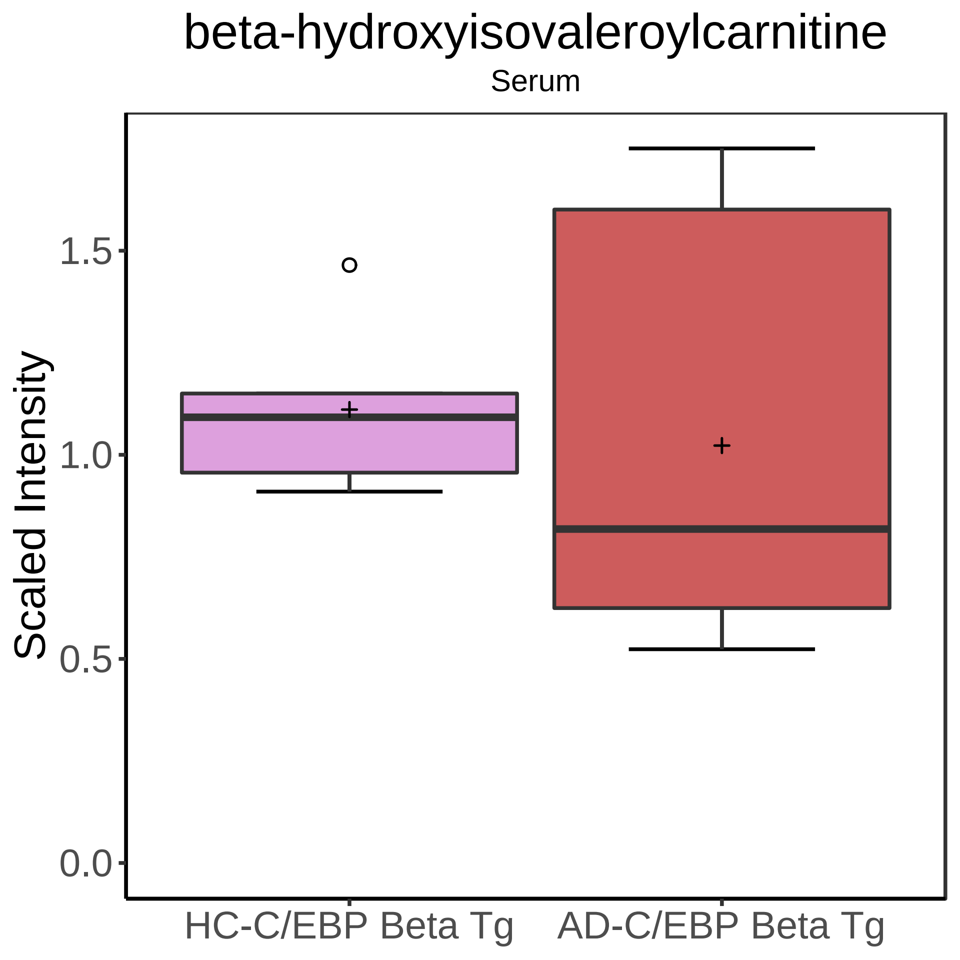
beta-hydroxyisovaleroylcarnitine
Serum
Scaled Intensity
1.5
1.0
0.5
0.0
HC-C/EBP Beta Tg
AD-C/EBP Beta Tg

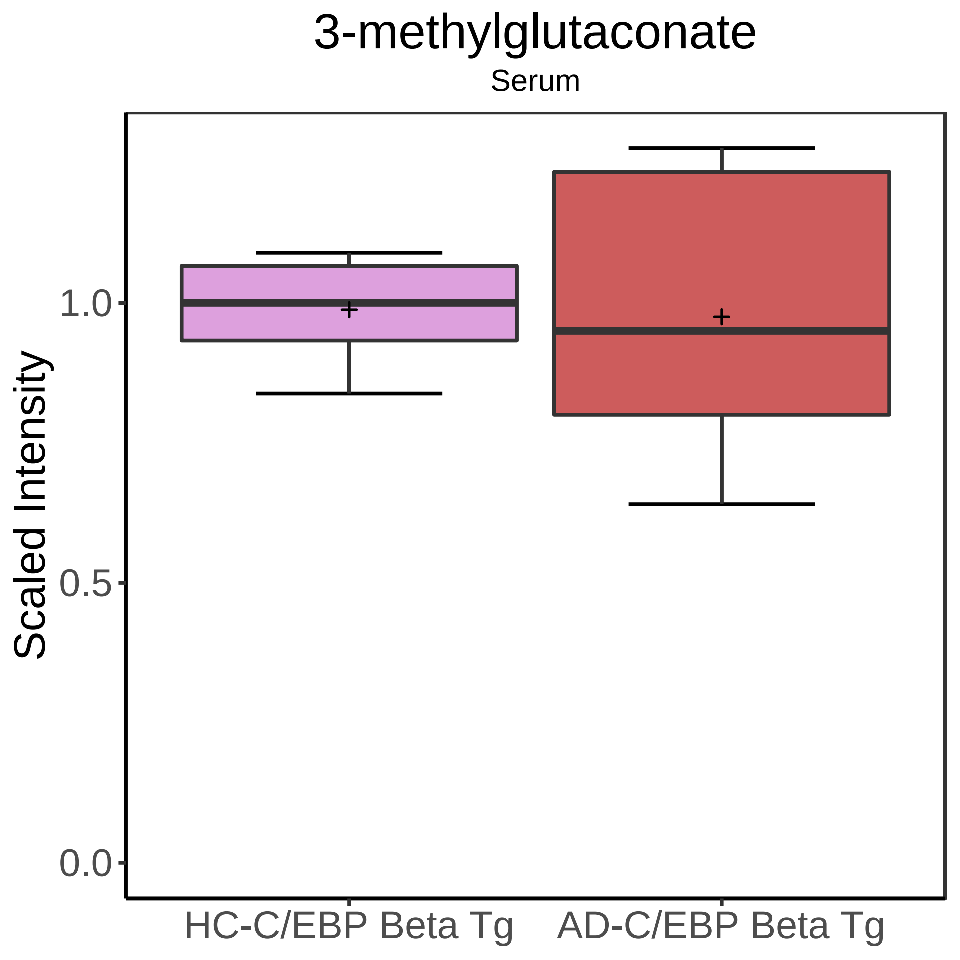
3-methylglutaconate
Serum
Scaled Intensity
1.0
0.5
0.0
HC-C/EBP Beta Tg
AD-C/EBP Beta Tg

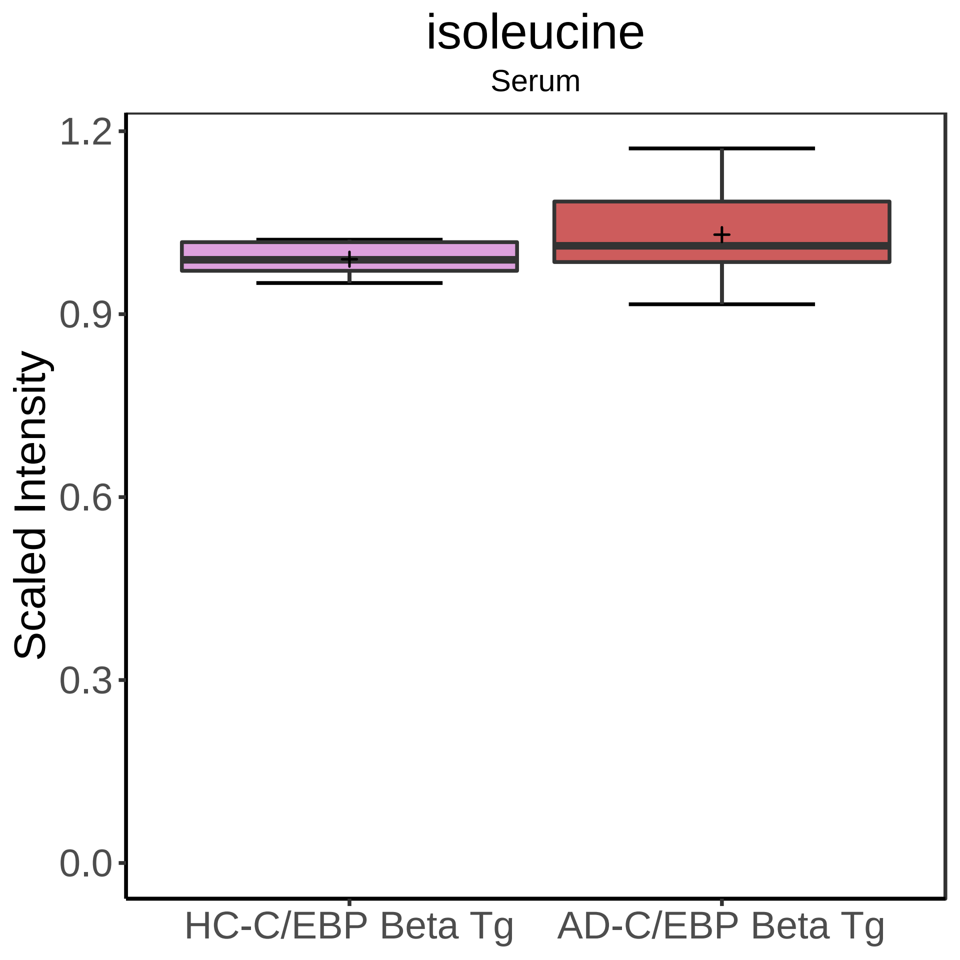

isoleucine
Serum
Scaled Intensity
1.2
0.9
0.6
0.3
0.0
HC-C/EBP Beta Tg
AD-C/EBP Beta Tg

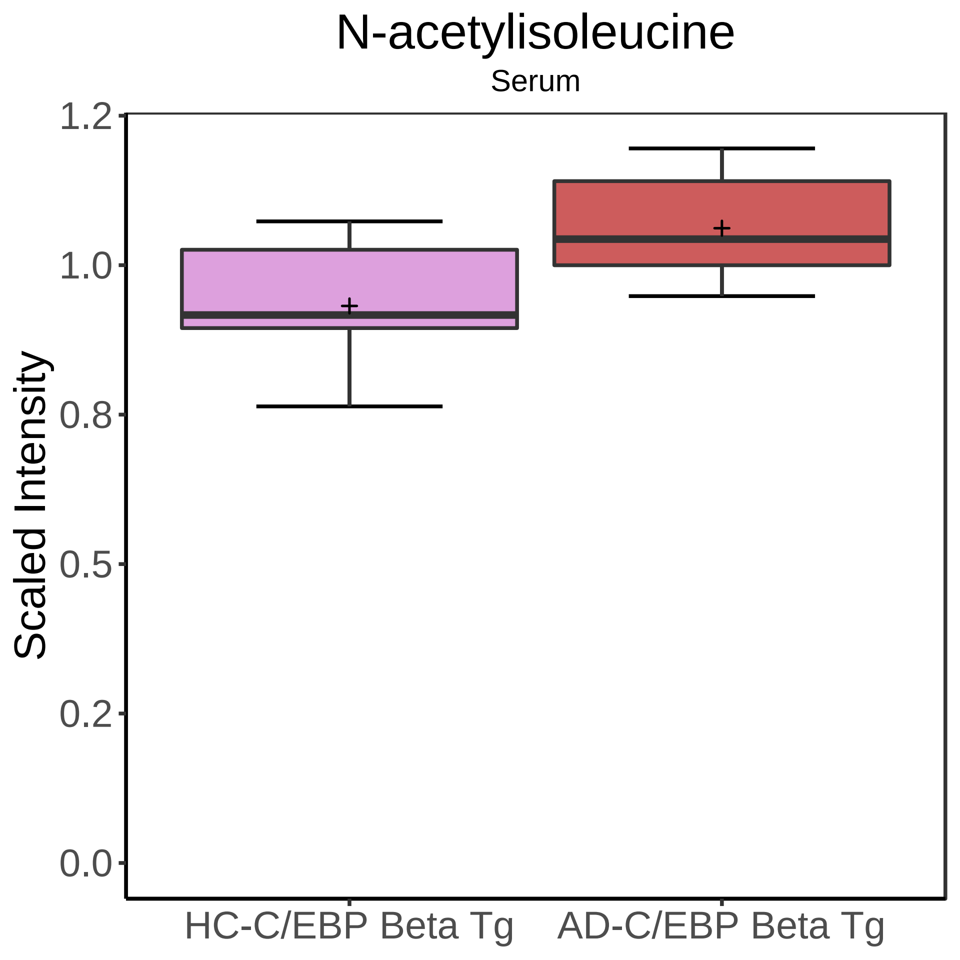
N-acetylisoleucine
Serum
Scaled Intensity
1.2
1.0
0.8
0.5
0.2
0.0
HC-C/EBP Beta Tg
AD-C/EBP Beta Tg

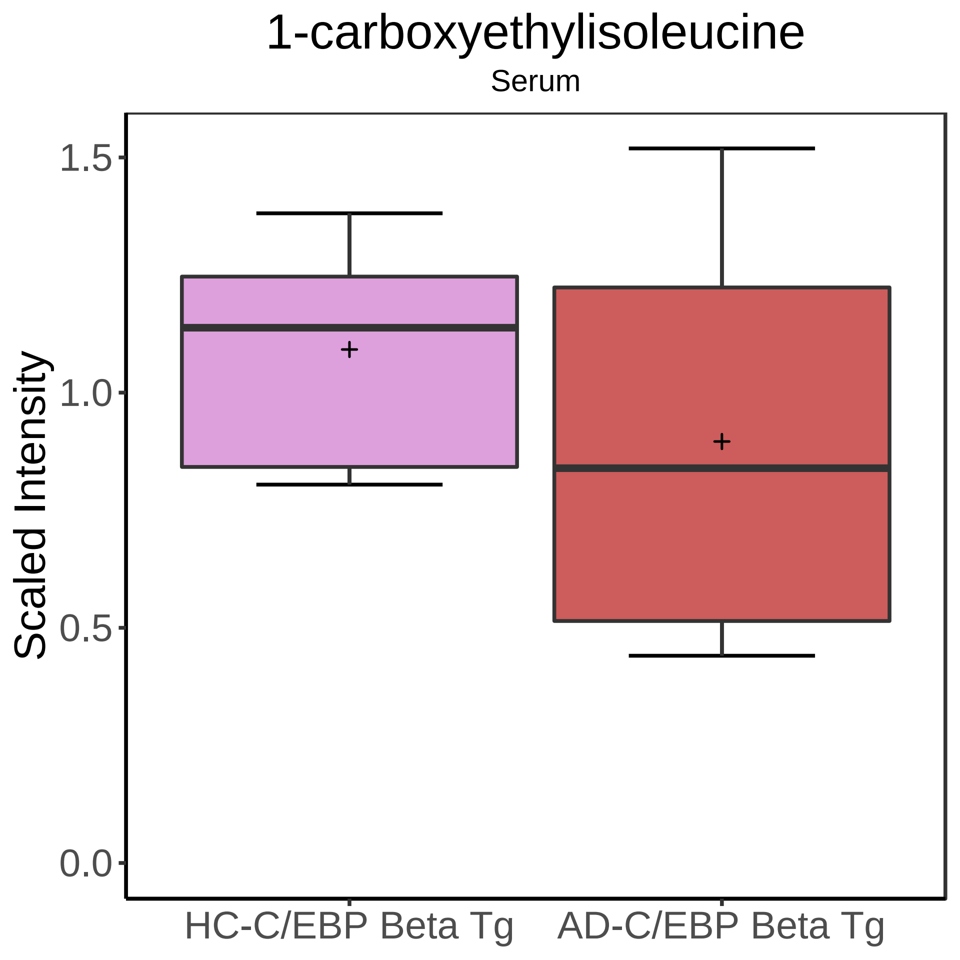
1-carboxyethylisoleucine
Serum
Scaled Intensity
1.5
1.0
0.5
0.0
HC-C/EBP Beta Tg
AD-C/EBP Beta Tg

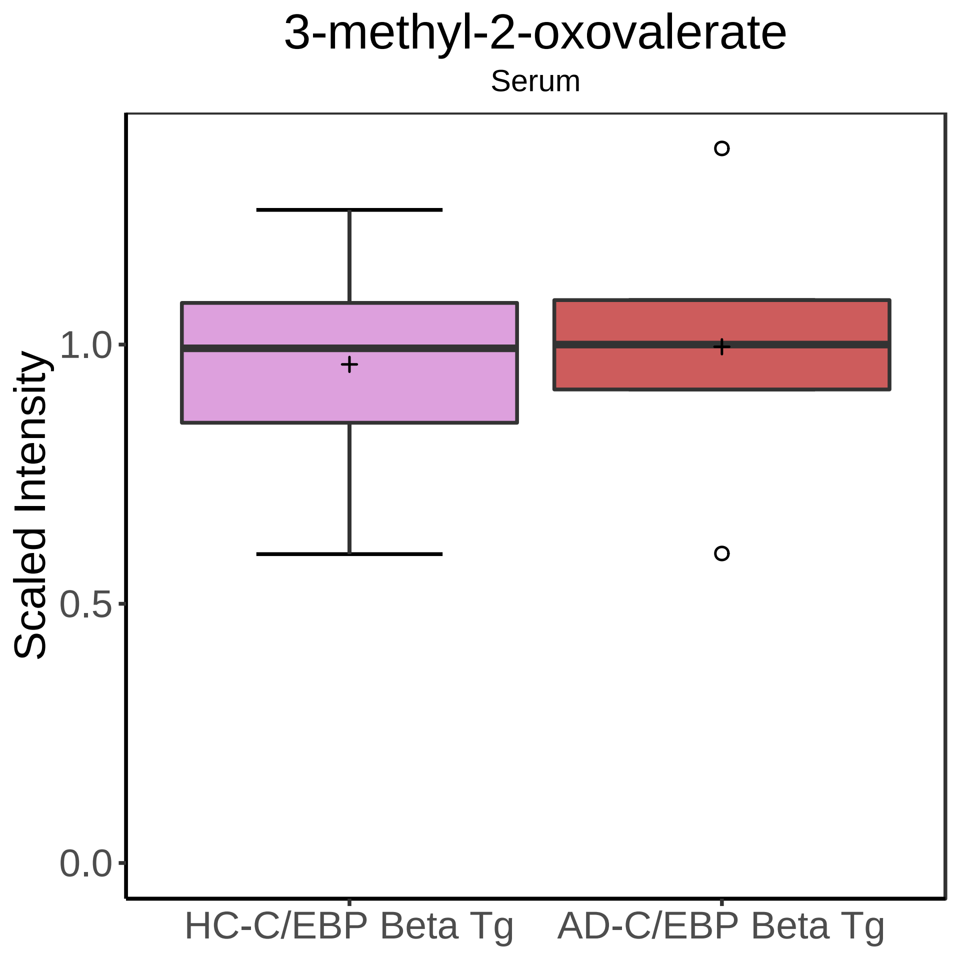
3-methyl-2-oxovalerate
Serum
Scaled Intensity
1.0
0.5
0.0
HC-C/EBP Beta Tg
AD-C/EBP Beta Tg

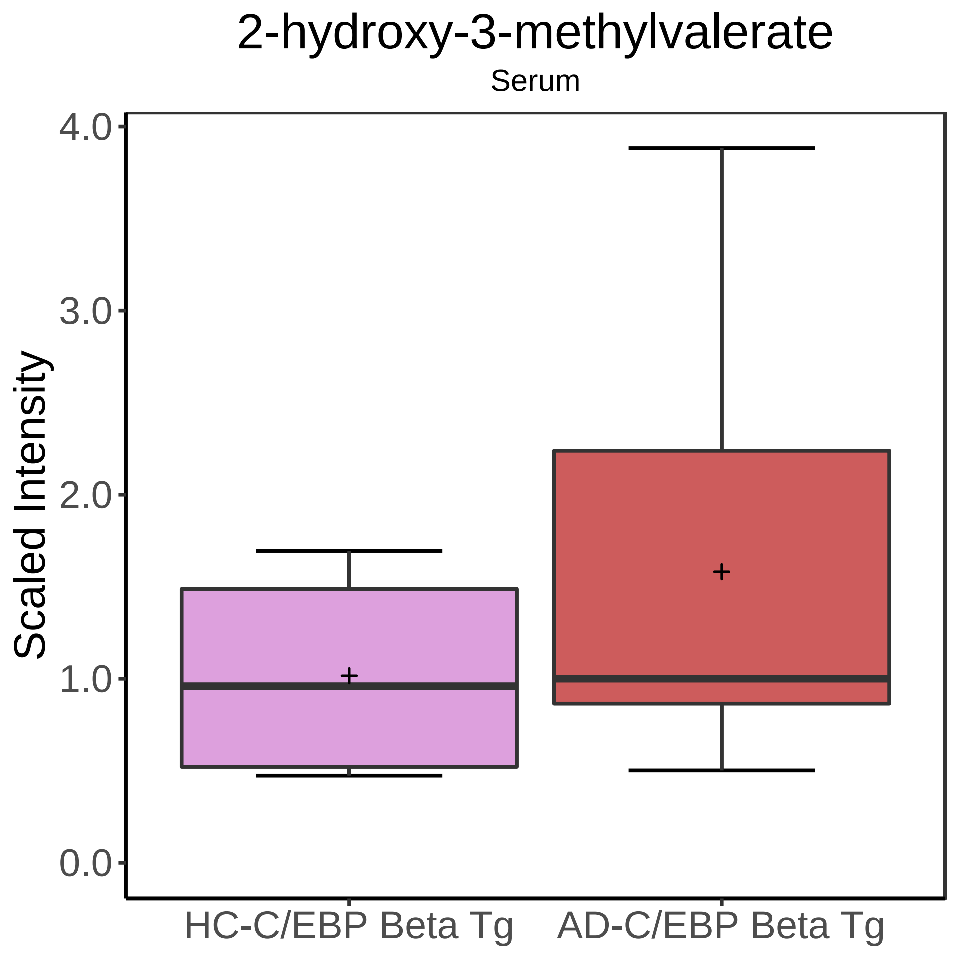
2-hydroxy-3-methylvalerate
Serum
Scaled Intensity
4.0
3.0
2.0
1.0
0.0
HC-C/EBP Beta Tg
AD-C/EBP Beta Tg

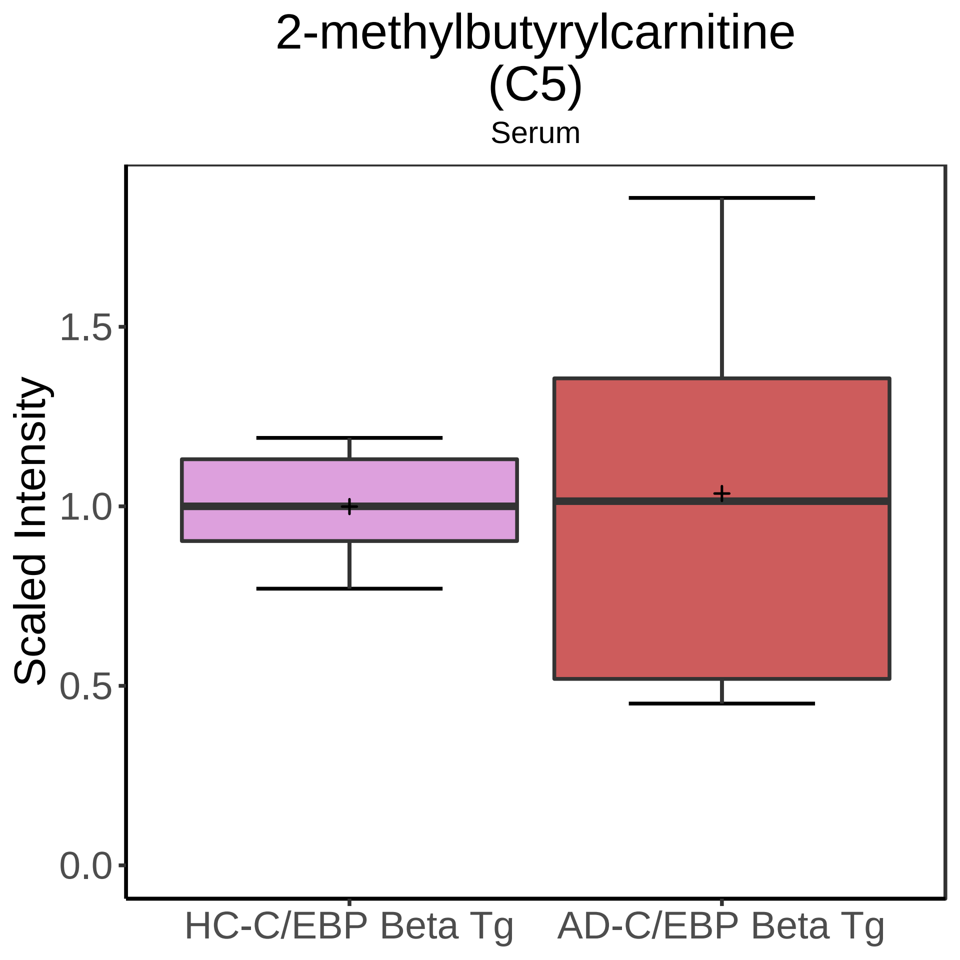
2-methylbutyrylcarnitine
(C5)
Serum
Scaled Intensity
1.5
1.0
0.5
0.0
HC-C/EBP Beta Tg
AD-C/EBP Beta Tg

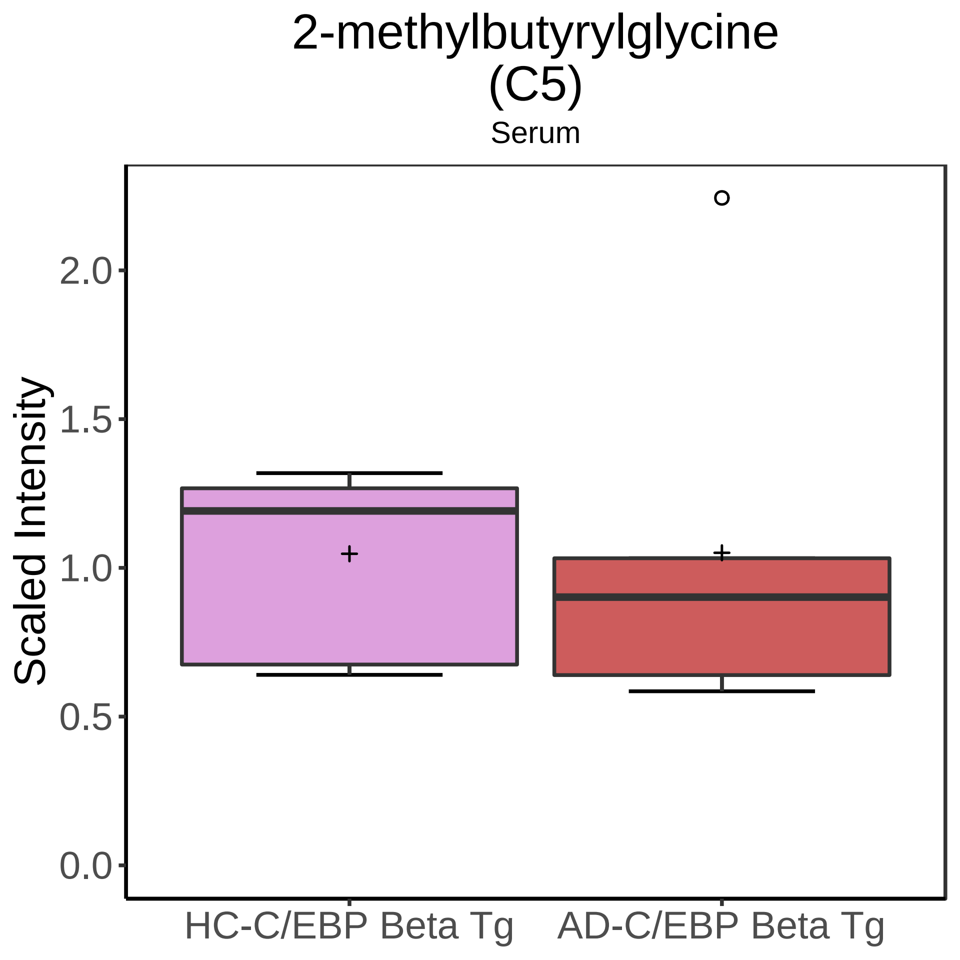
2-methylbutyrylglycine
(C5)
Serum
Scaled Intensity
2.0
1.5
1.0
0.5
0.0
HC-C/EBP Beta Tg
AD-C/EBP Beta Tg

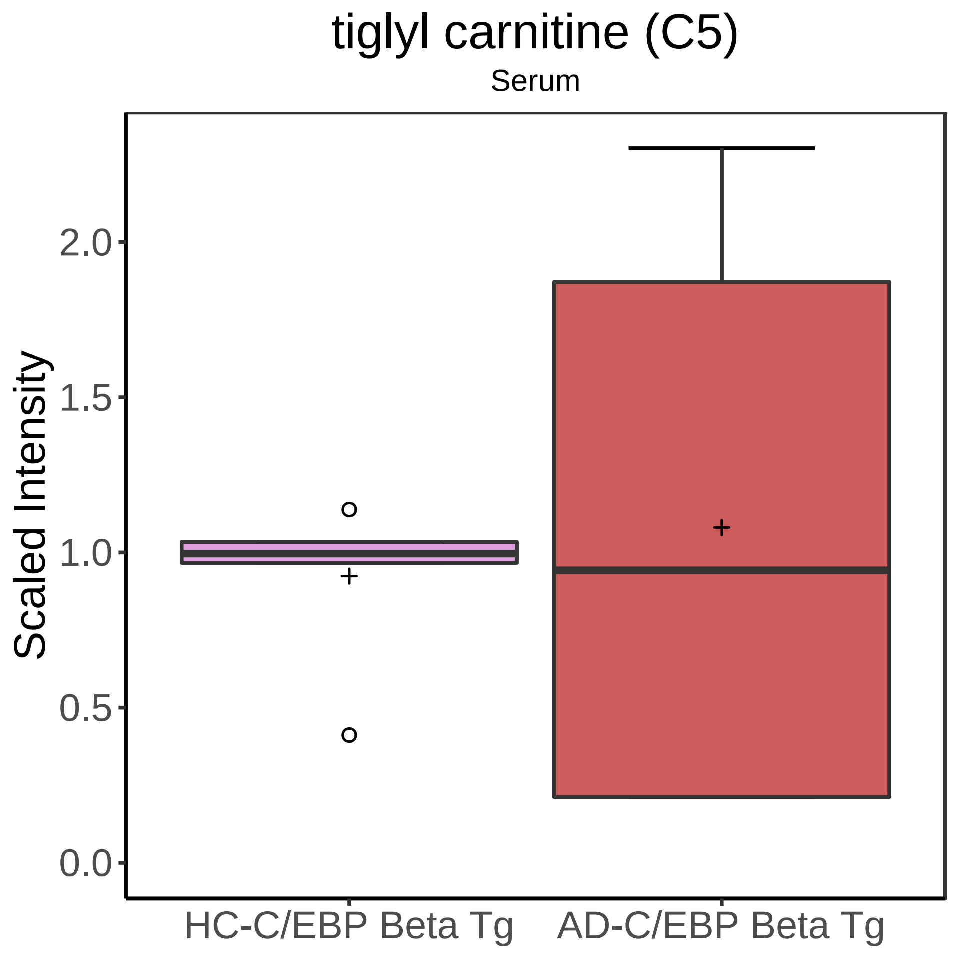
tiglyl carnitine (C5)
Serum
Scaled Intensity
2.0
1.5
1.0
0.5
0.0
HC-C/EBP Beta Tg
AD-C/EBP Beta Tg

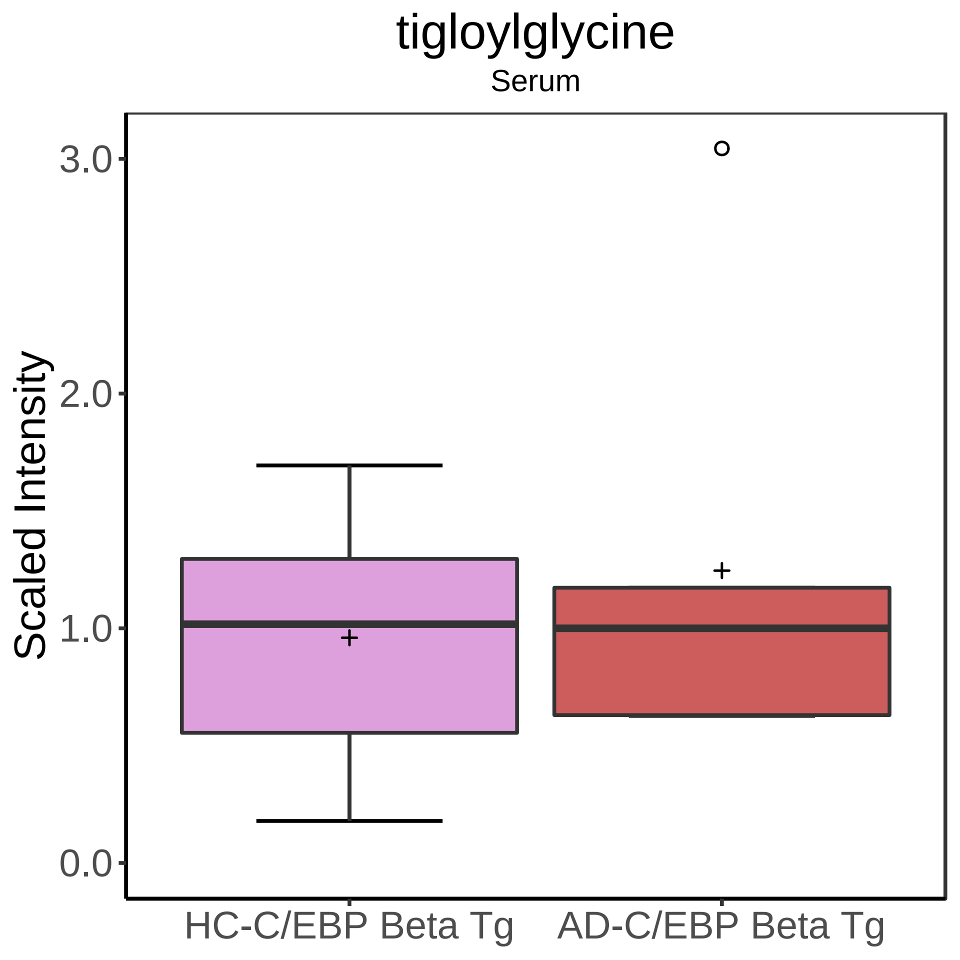
tigloylglycine
Serum
Scaled Intensity
3.0
2.0
1.0
0.0
HC-C/EBP Beta Tg
AD-C/EBP Beta Tg

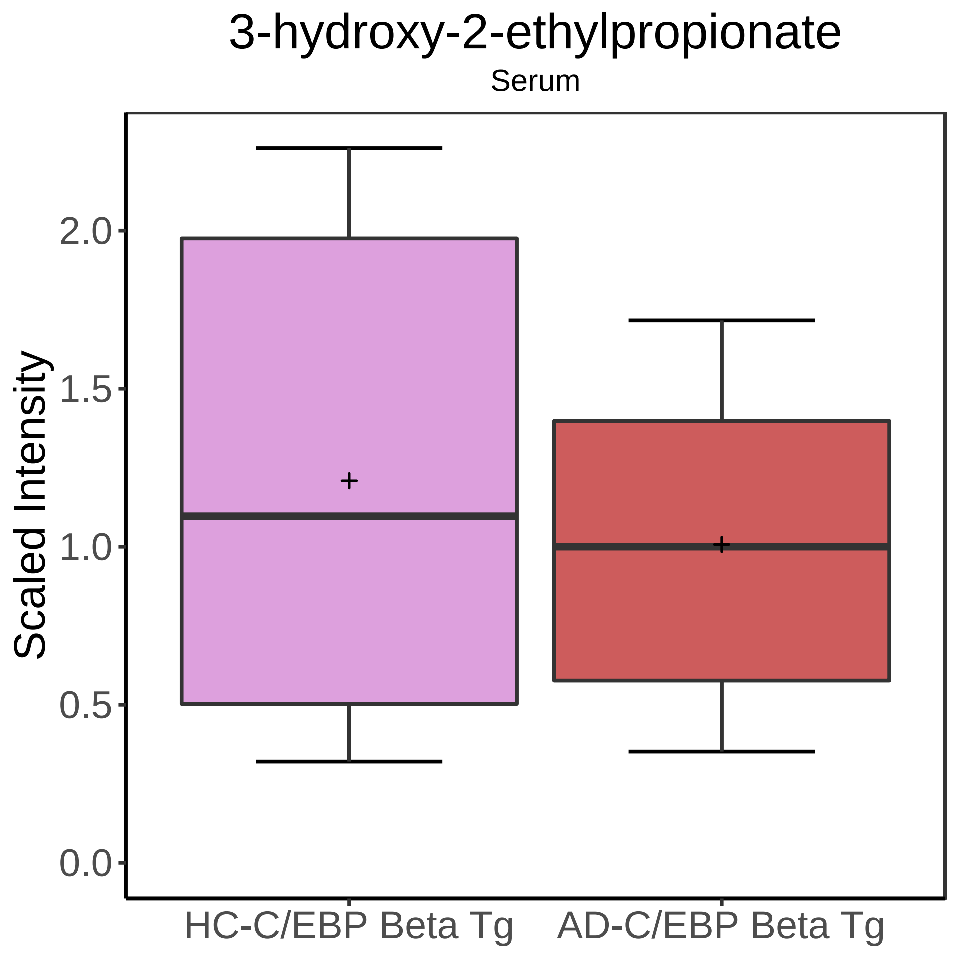
3-hydroxy-2-ethylpropionate
Serum
Scaled Intensity
2.0
1.5
1.0
0.5
0.0
HC-C/EBP Beta Tg
AD-C/EBP Beta Tg

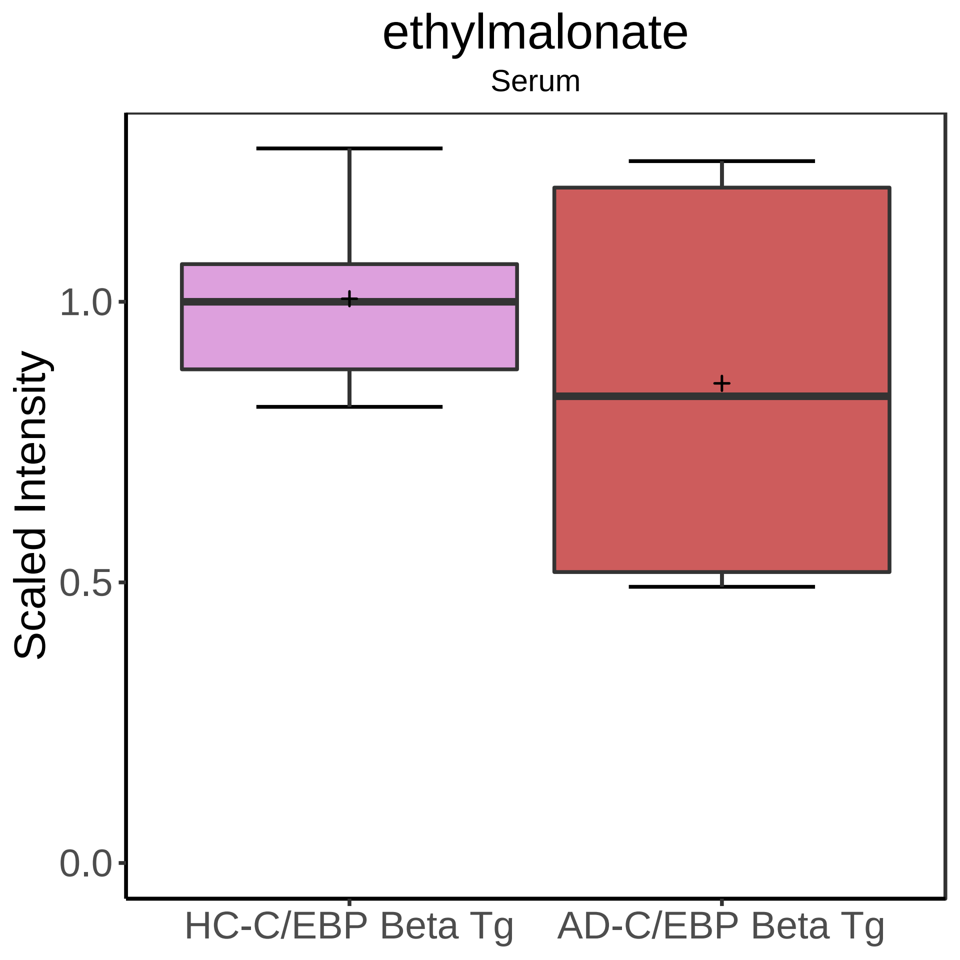
ethylmalonate
Serum
Scaled Intensity
1.0
0.5
0.0
HC-C/EBP Beta Tg
AD-C/EBP Beta Tg

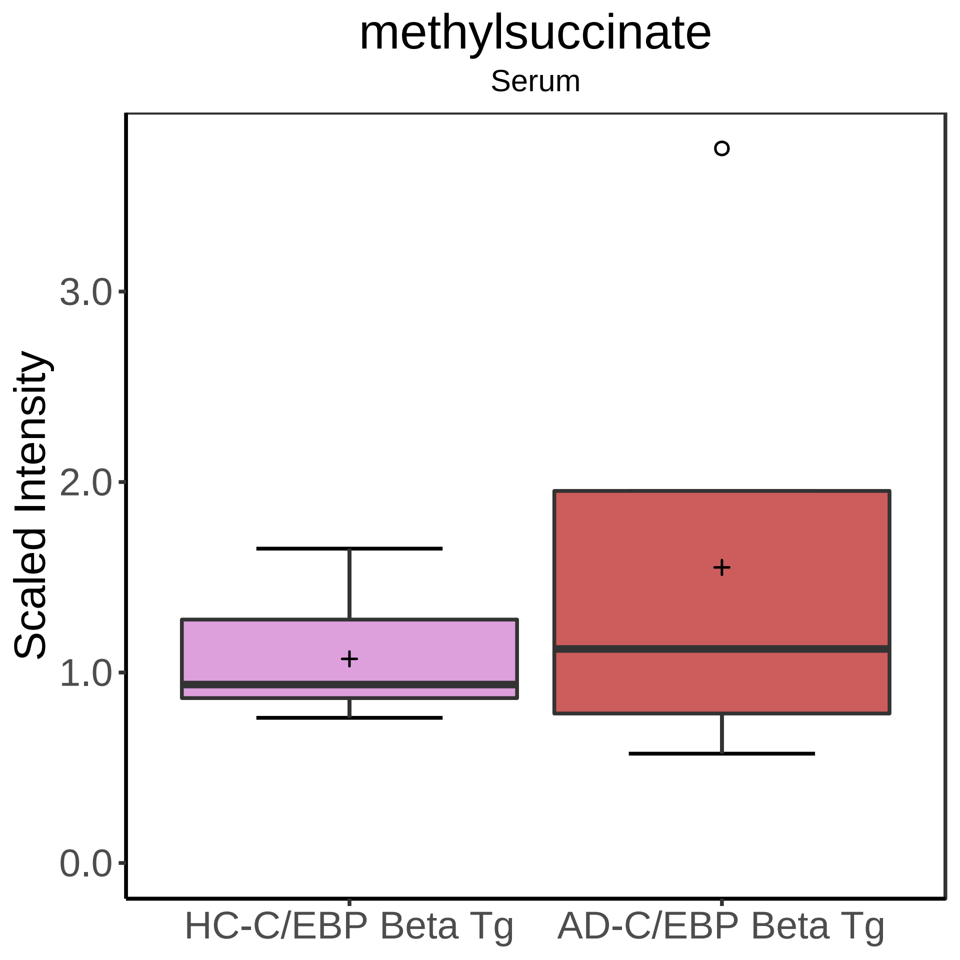
methylsuccinate
Serum
Scaled Intensity
0.0
1.0
2.0
3.0
HC-C/EBP Beta Tg
AD-C/EBP Beta Tg

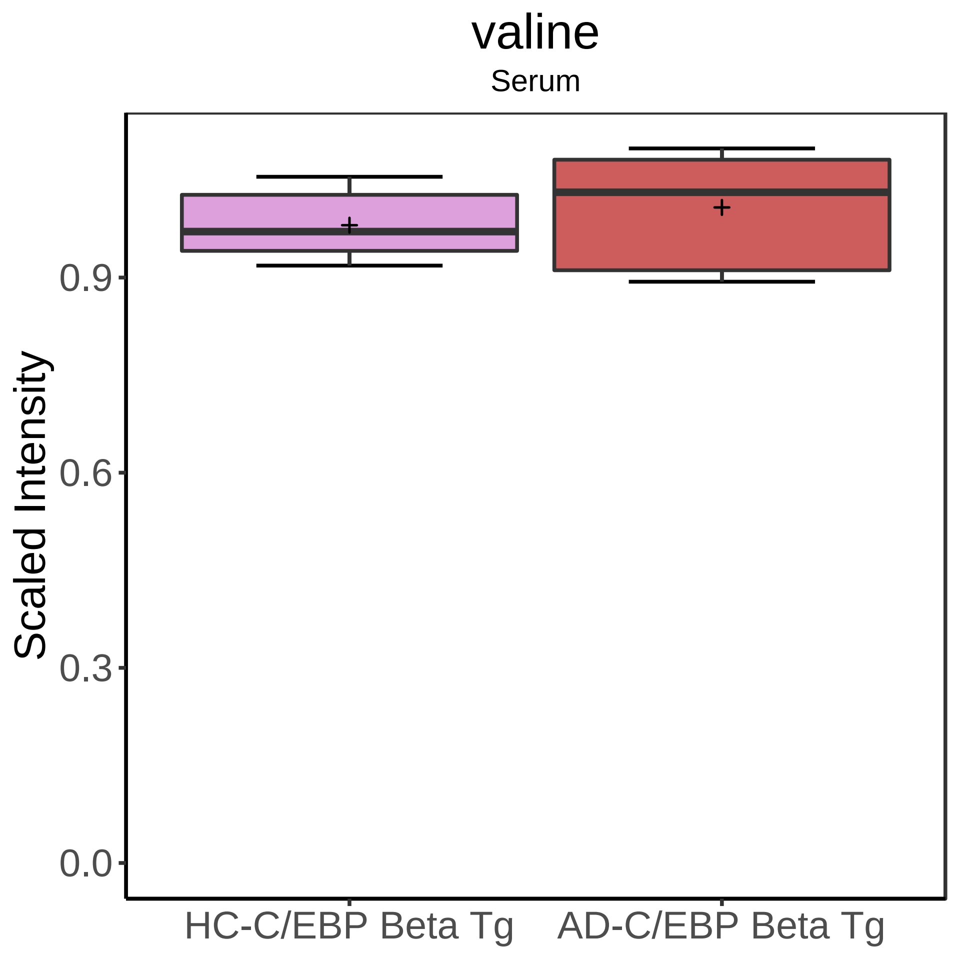
valine
Serum
Scaled Intensity
0.0
0.3
0.6
0.9
HC-C/EBP Beta Tg
AD-C/EBP Beta Tg

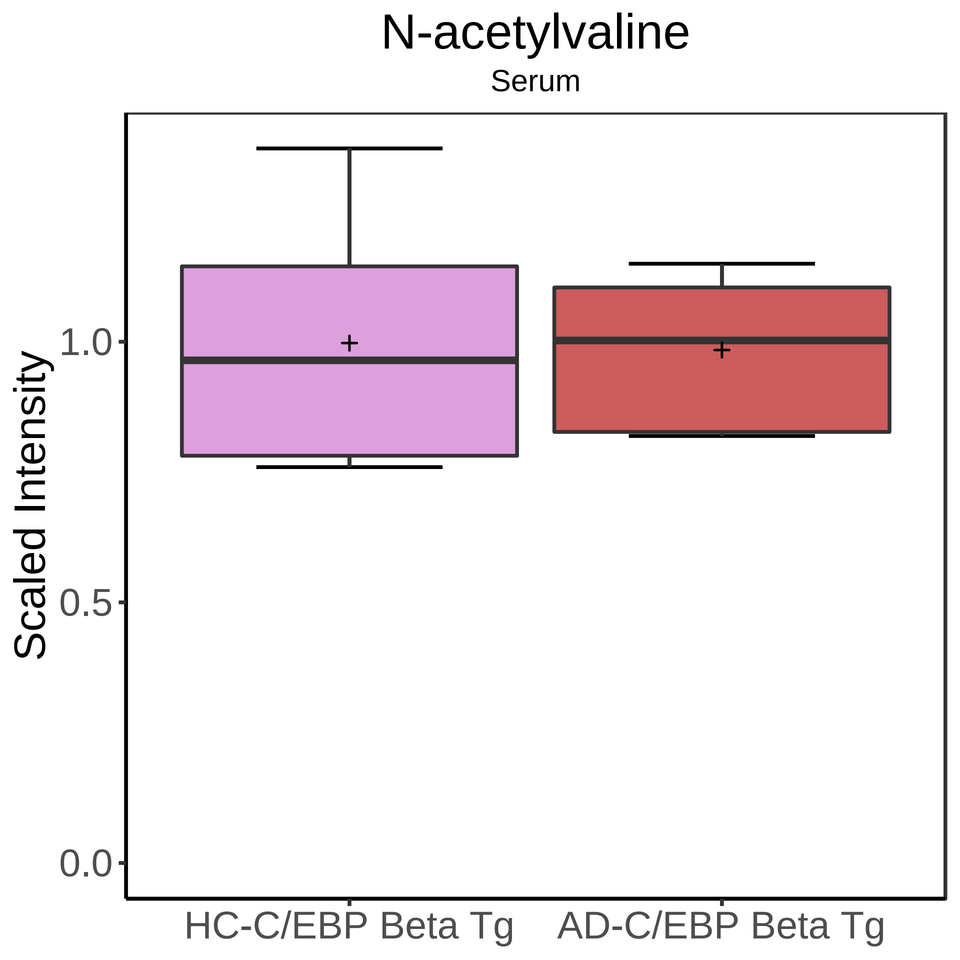
N-acetylvaline
Serum
Scaled Intensity
1.0
0.5
0.0
HC-C/EBP Beta Tg
AD-C/EBP Beta Tg

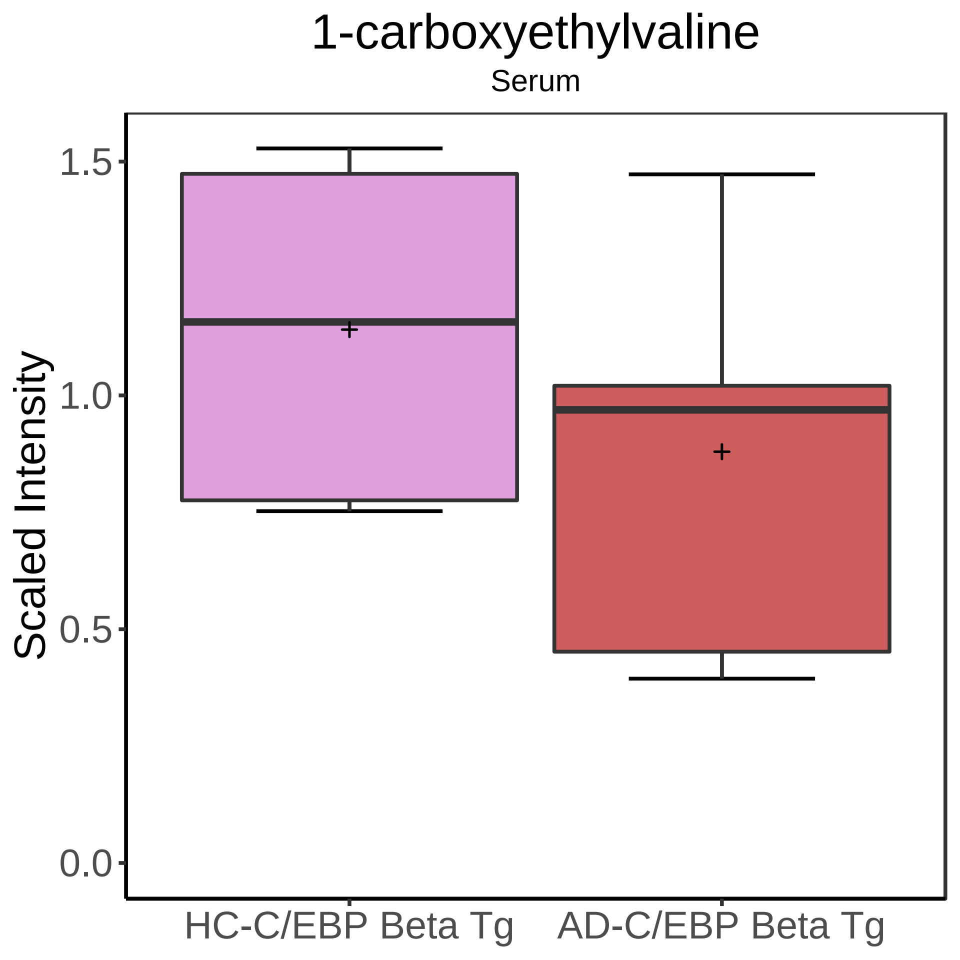
1-carboxyethylvaline
Serum
Scaled Intensity
1.5
1.0
0.5
0.0
HC-C/EBP Beta Tg
AD-C/EBP Beta Tg

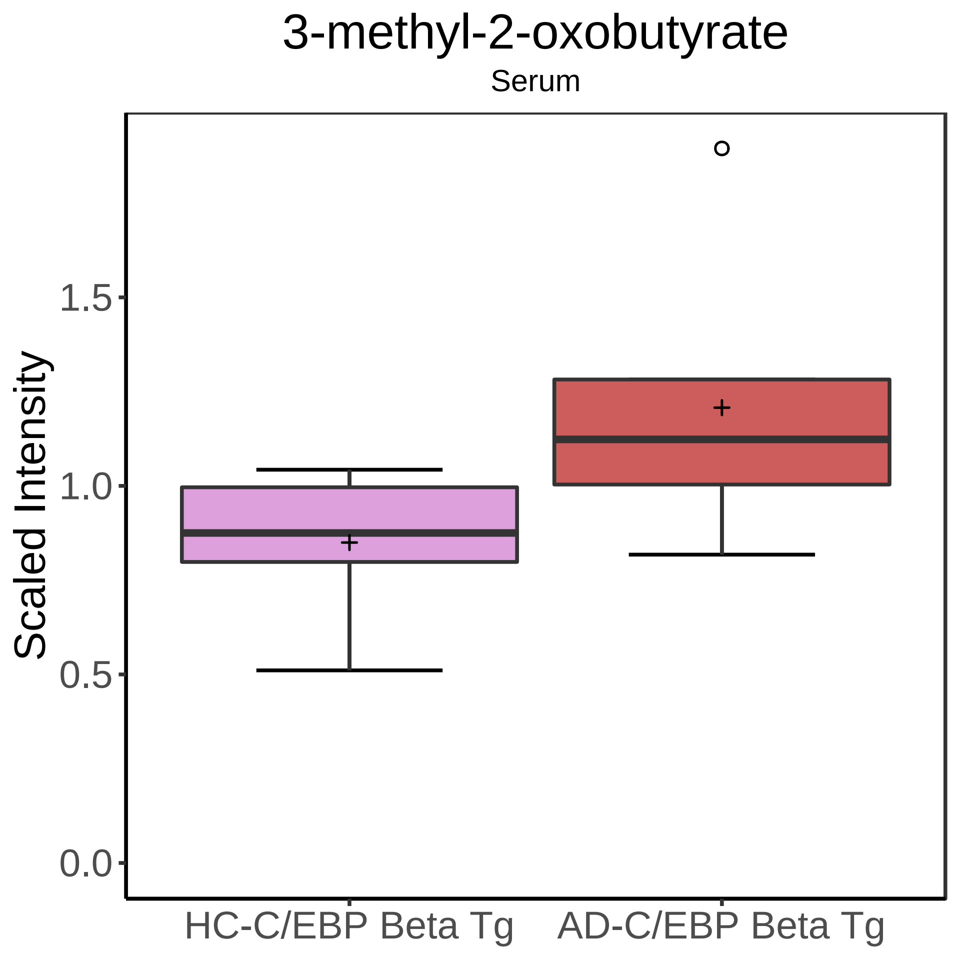

3-methyl-2-oxobutyrate
Serum
Scaled Intensity
0.0
0.5
1.0
1.5
HC-C/EBP Beta Tg
AD-C/EBP Beta Tg

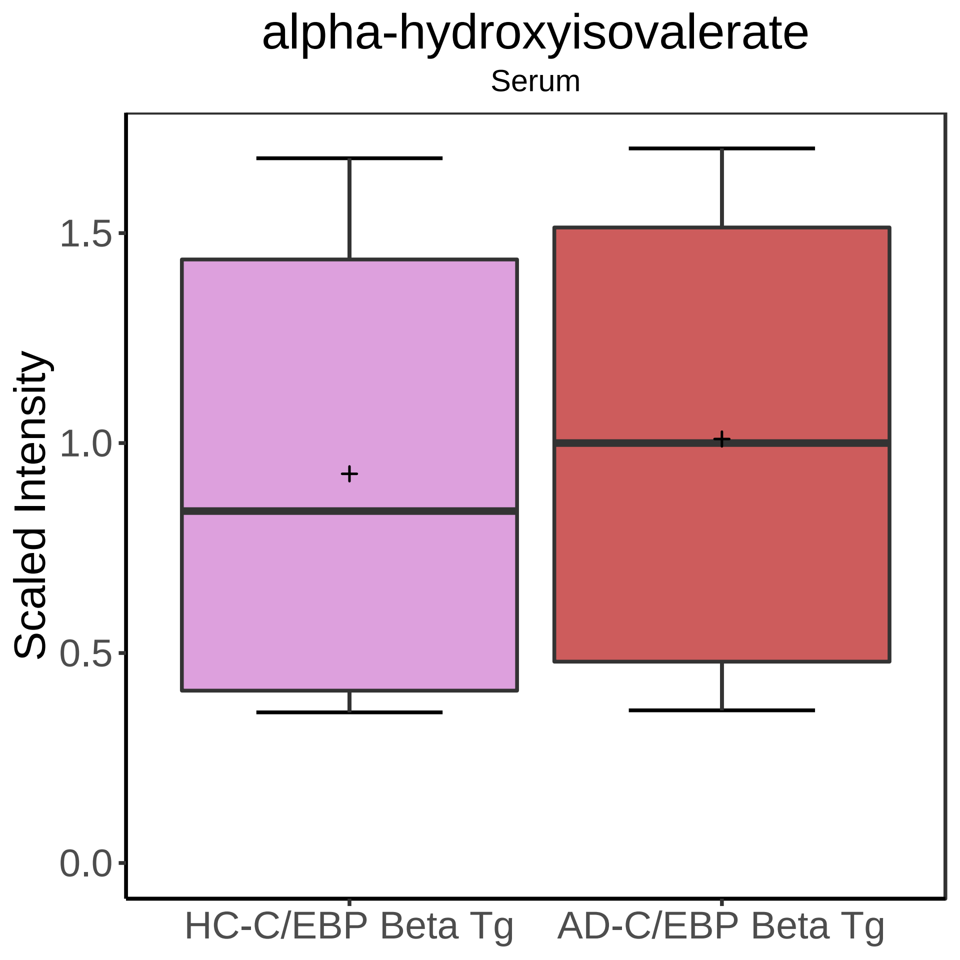
alpha-hydroxyisovalerate
Serum
Scaled Intensity
1.5
1.0
0.5
0.0
HC-C/EBP Beta Tg
AD-C/EBP Beta Tg

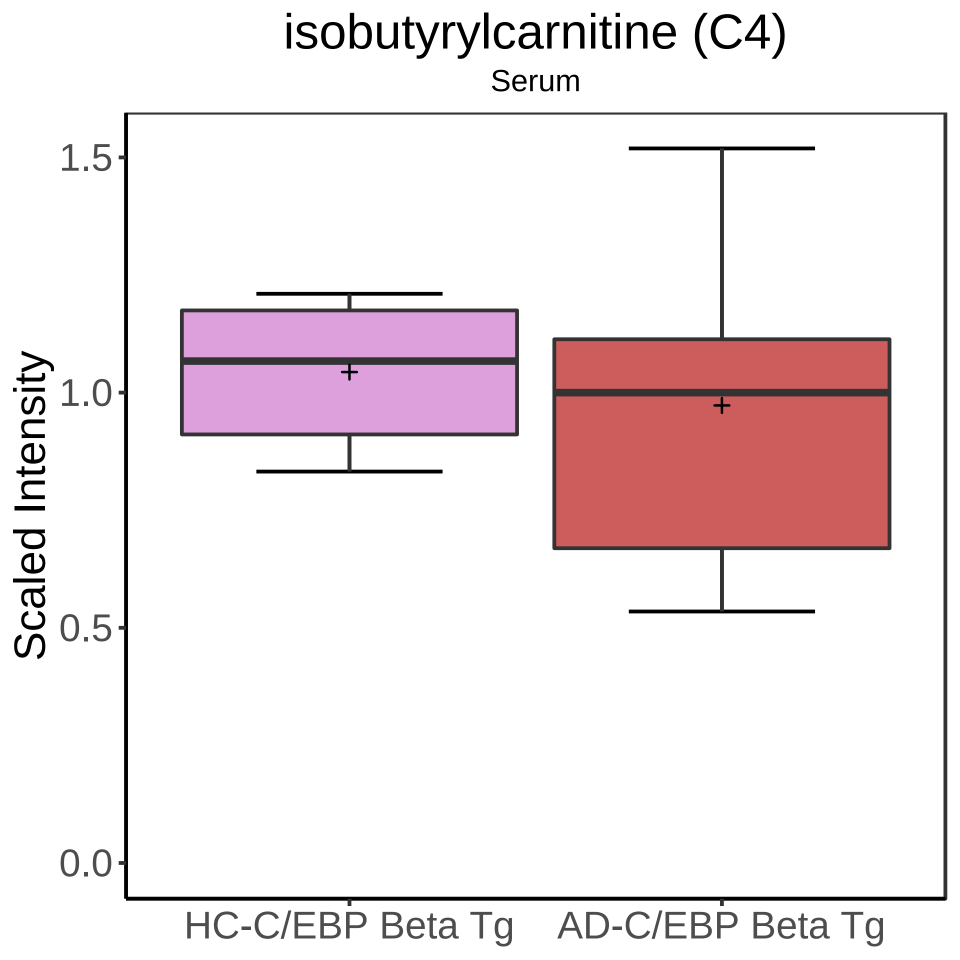
isobutyrylcarnitine (C4)
Serum
Scaled Intensity
1.5
1.0
0.5
0.0
HC-C/EBP Beta Tg
AD-C/EBP Beta Tg

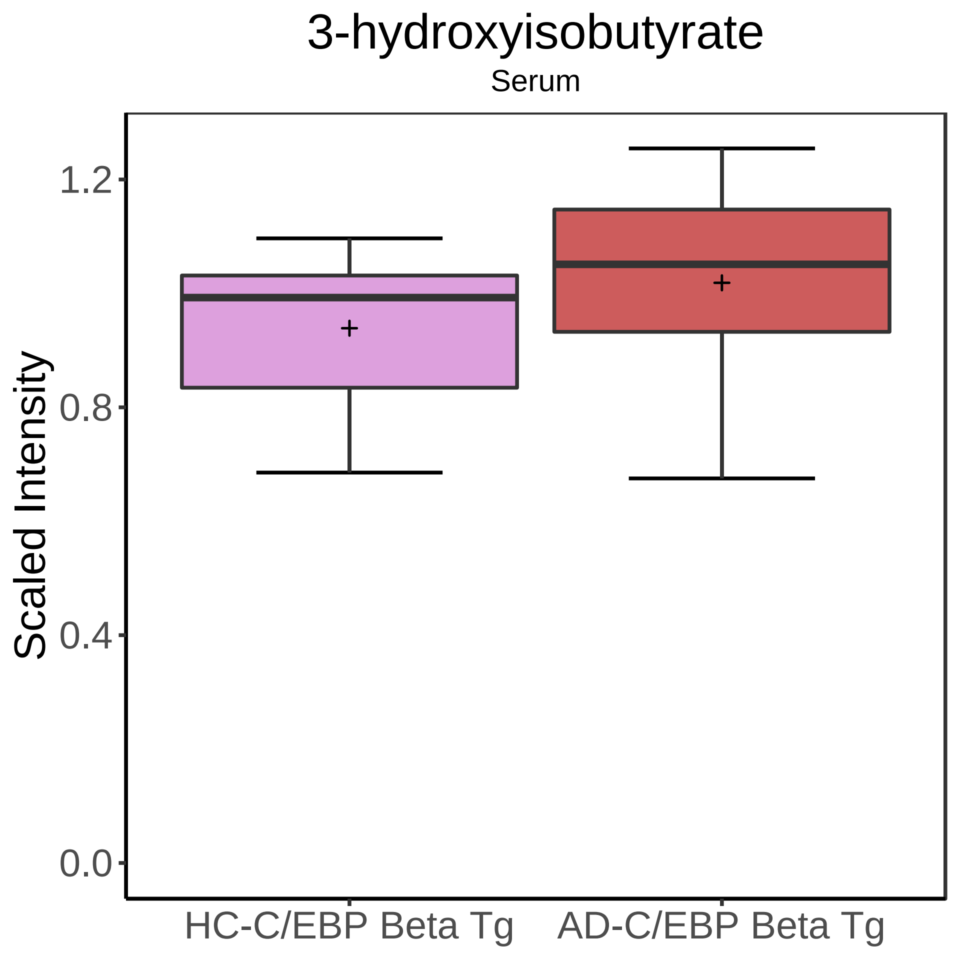

3-hydroxyisobutyrate
Serum
Scaled Intensity
1.2
0.8
0.4
0.0
HC-C/EBP Beta Tg
AD-C/EBP Beta Tg

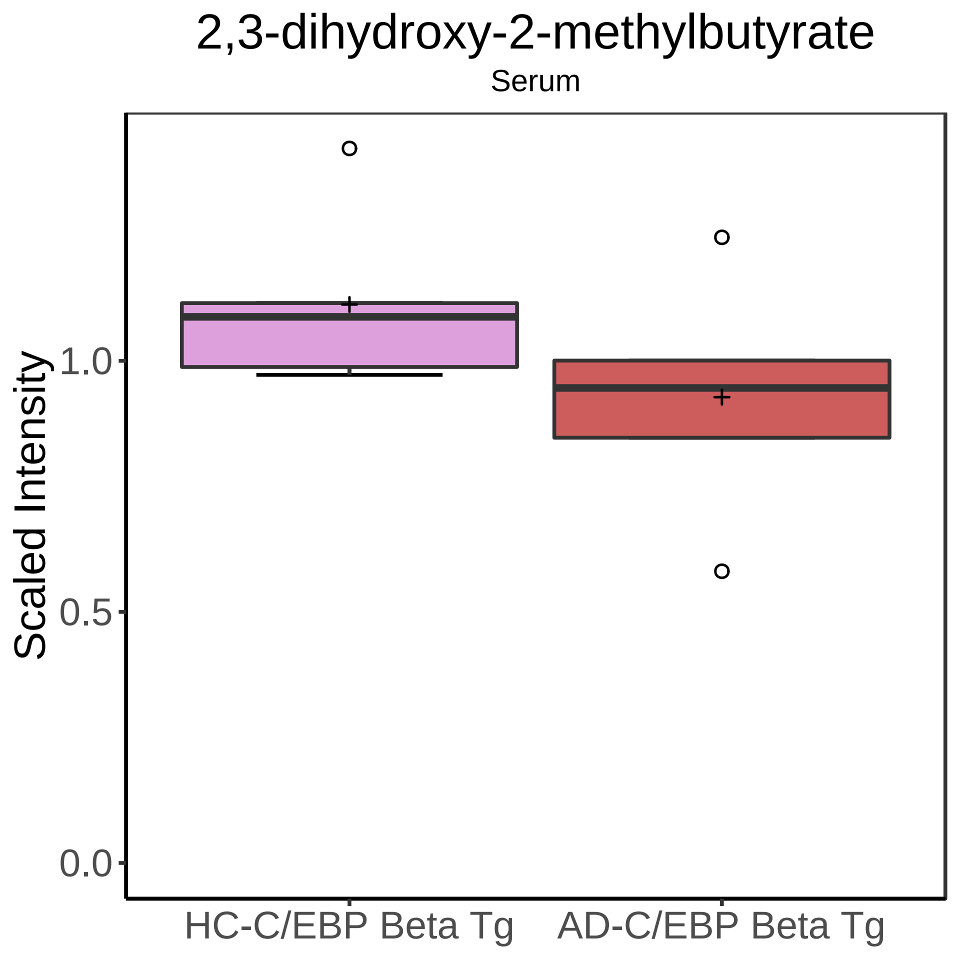
2,3-dihydroxy-2-methylbutyrate
Serum
Scaled Intensity
1.0
0.5
0.0
HC-C/EBP Beta Tg
AD-C/EBP Beta Tg

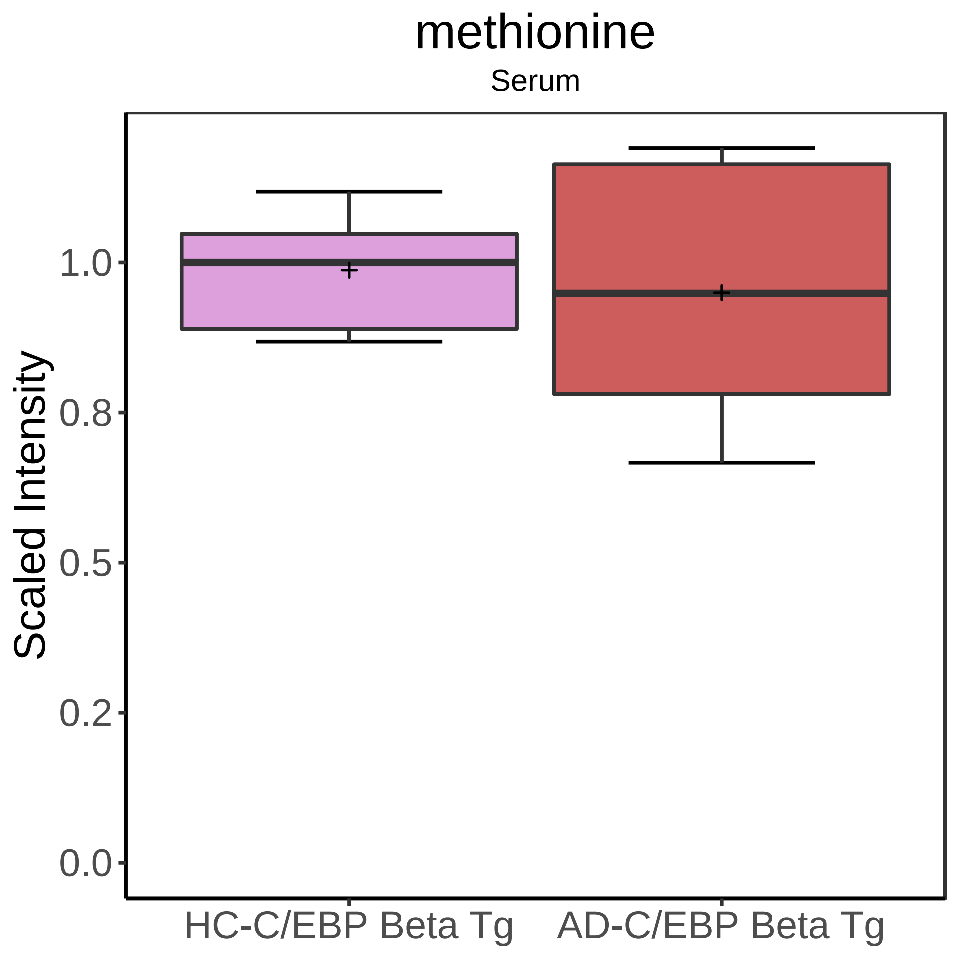
methionine
Serum
Scaled Intensity
1.0
0.8
0.5
0.2
0.0
HC-C/EBP Beta Tg
AD-C/EBP Beta Tg

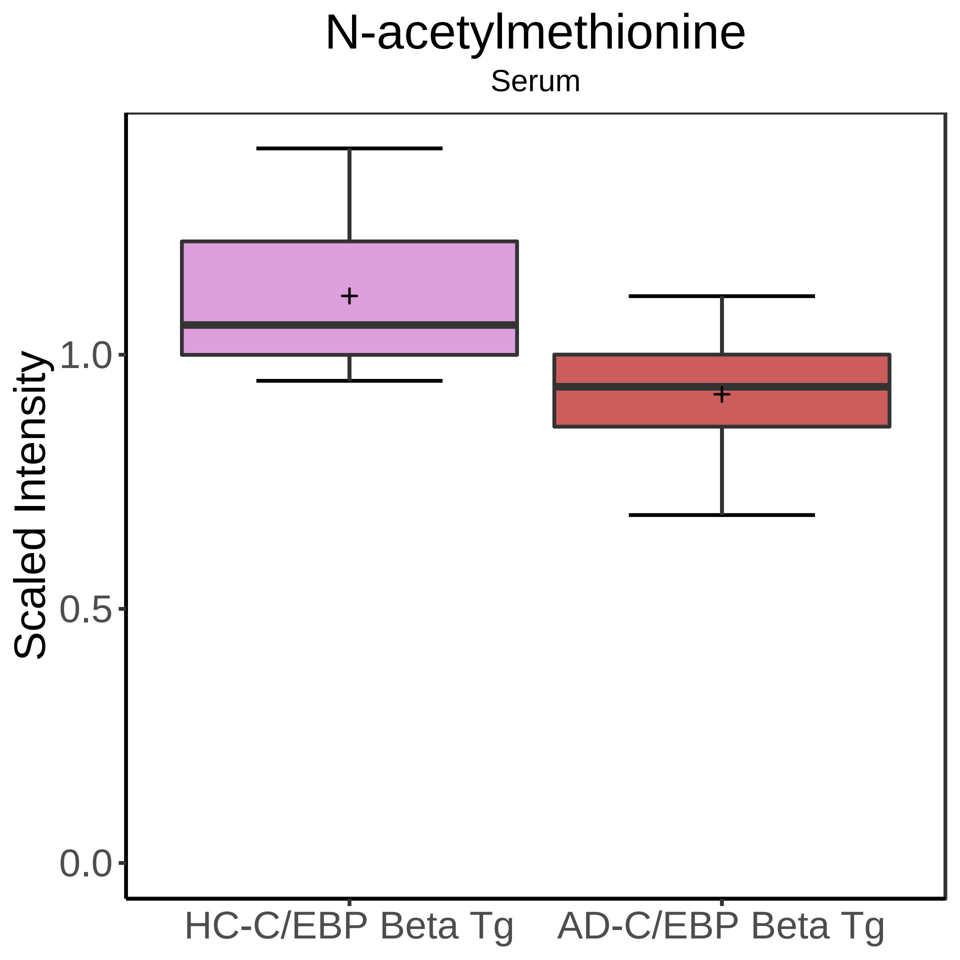
N-acetylmethionine
Serum
Scaled Intensity
1.0
0.5
0.0
HC-C/EBP Beta Tg
AD-C/EBP Beta Tg

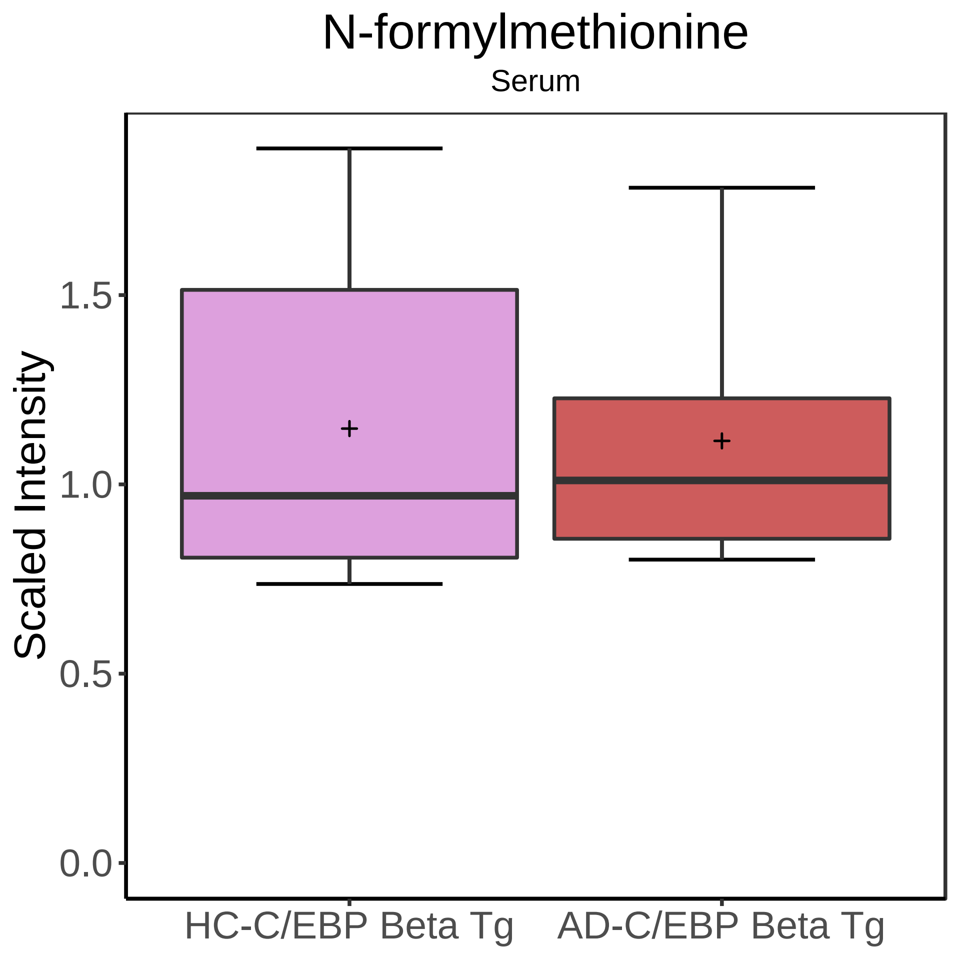

N-formylmethionine
Serum
Scaled Intensity
1.5
1.0
0.5
0.0
HC-C/EBP Beta Tg
AD-C/EBP Beta Tg

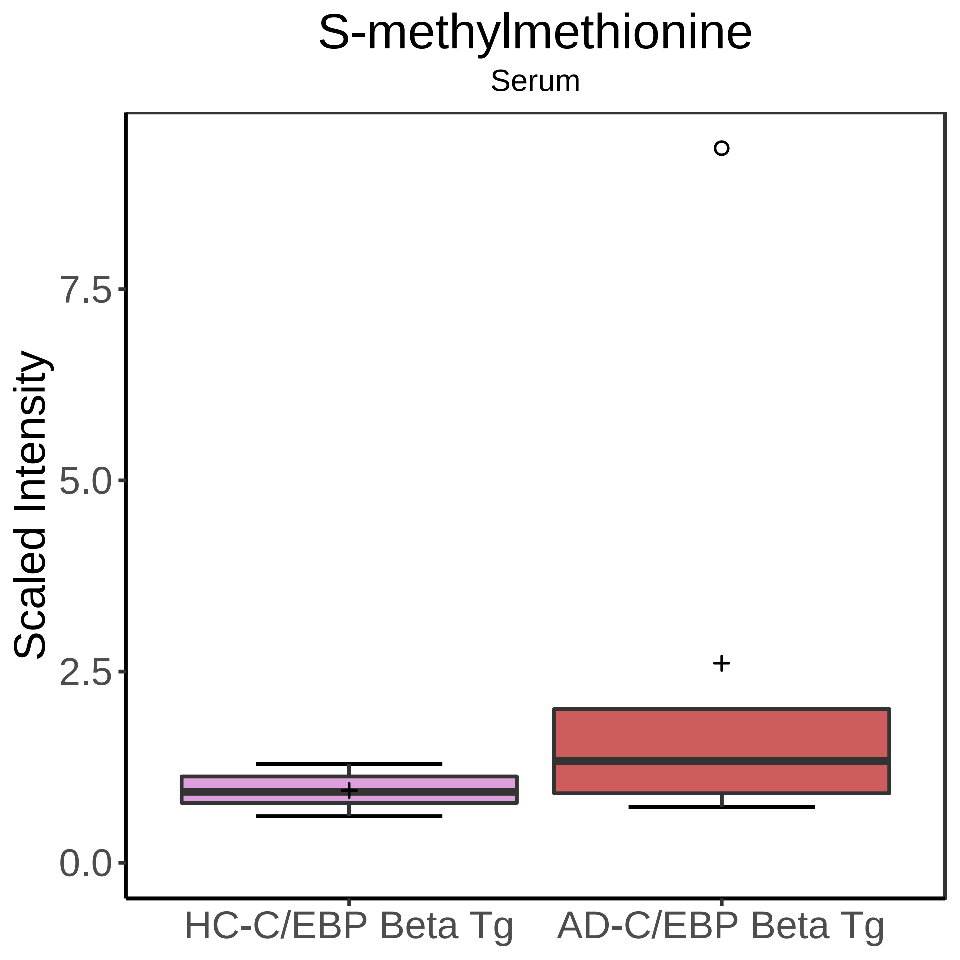
S-methylmethionine
Serum
Scaled Intensity
7.5
5.0
2.5
0.0
HC-C/EBP Beta Tg
AD-C/EBP Beta Tg

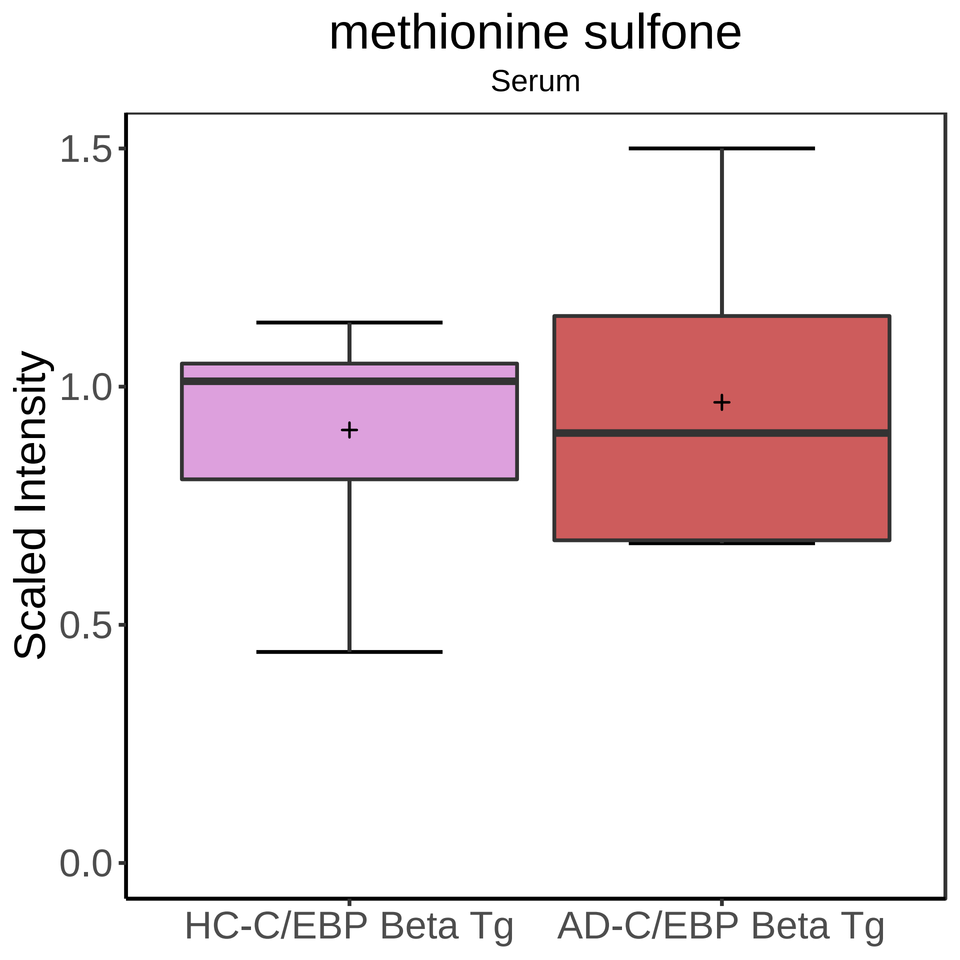
methionine sulfone
Serum
Scaled Intensity
1.5
1.0
0.5
0.0
HC-C/EBP Beta Tg
AD-C/EBP Beta Tg

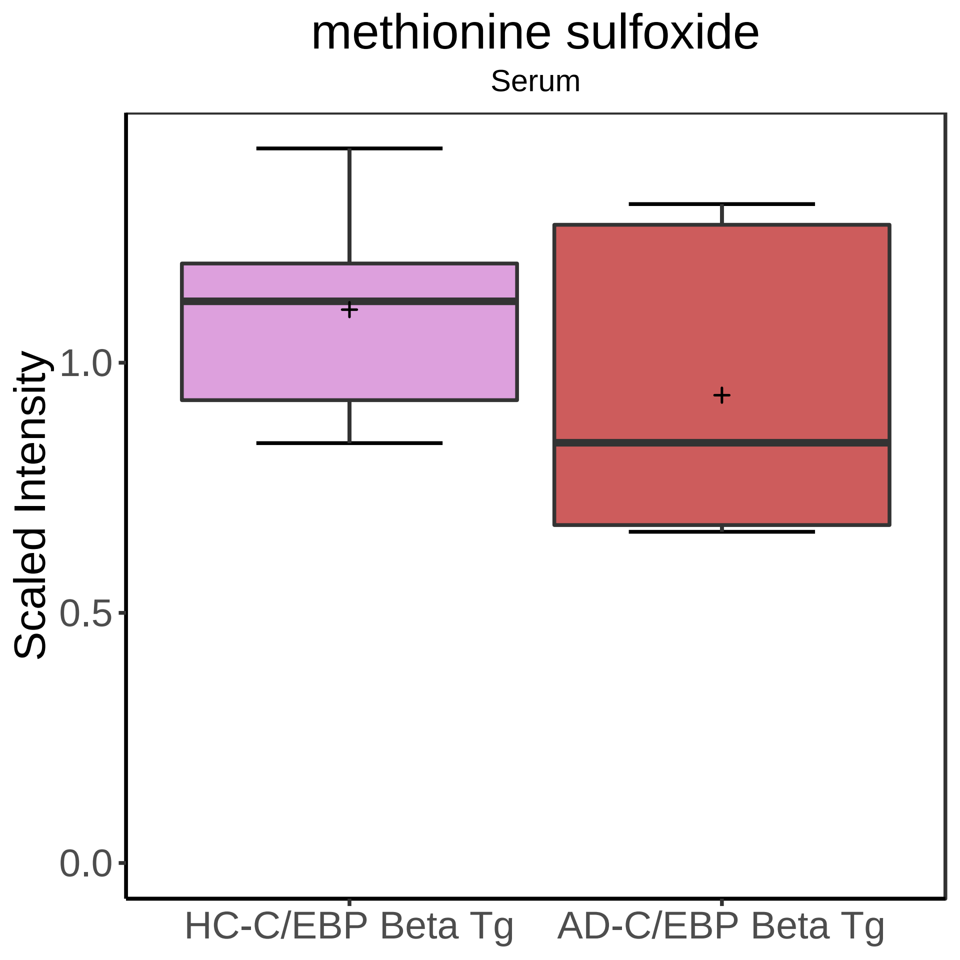
methionine sulfoxide
Serum
Scaled Intensity
1.0
0.5
0.0
HC-C/EBP Beta Tg
AD-C/EBP Beta Tg

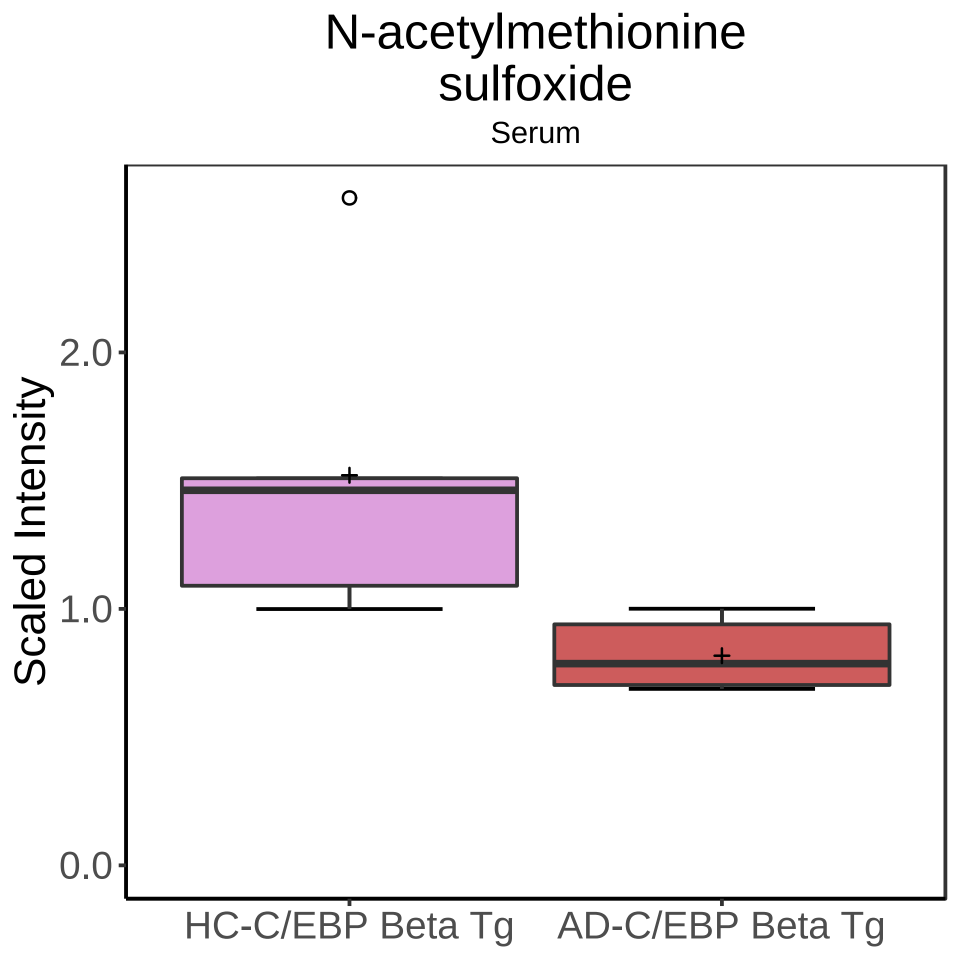

N-acetylmethionine
sulfoxide
Serum
Scaled Intensity
2.0
1.0
0.0
HC-C/EBP Beta Tg
AD-C/EBP Beta Tg

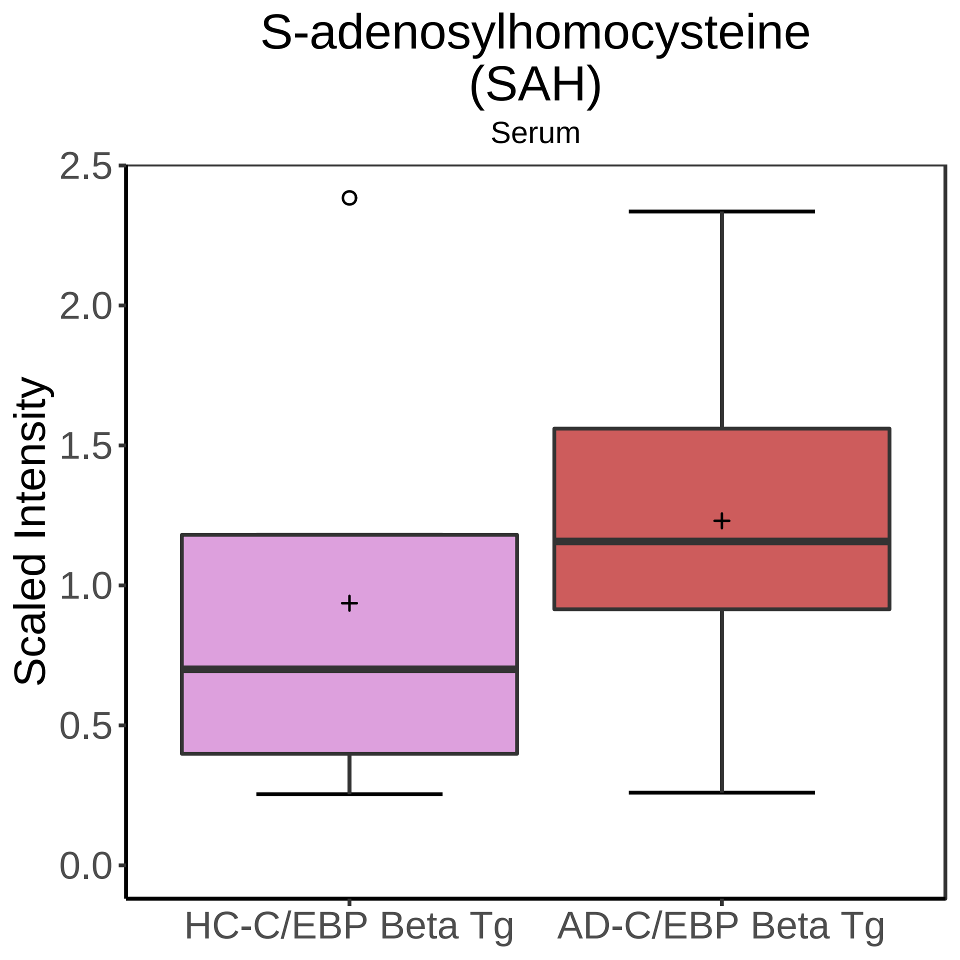
S-adenosylhomocysteine
(SAH)
Serum
Scaled Intensity
2.5
2.0
1.5
1.0
0.5
0.0
HC-C/EBP Beta Tg
AD-C/EBP Beta Tg

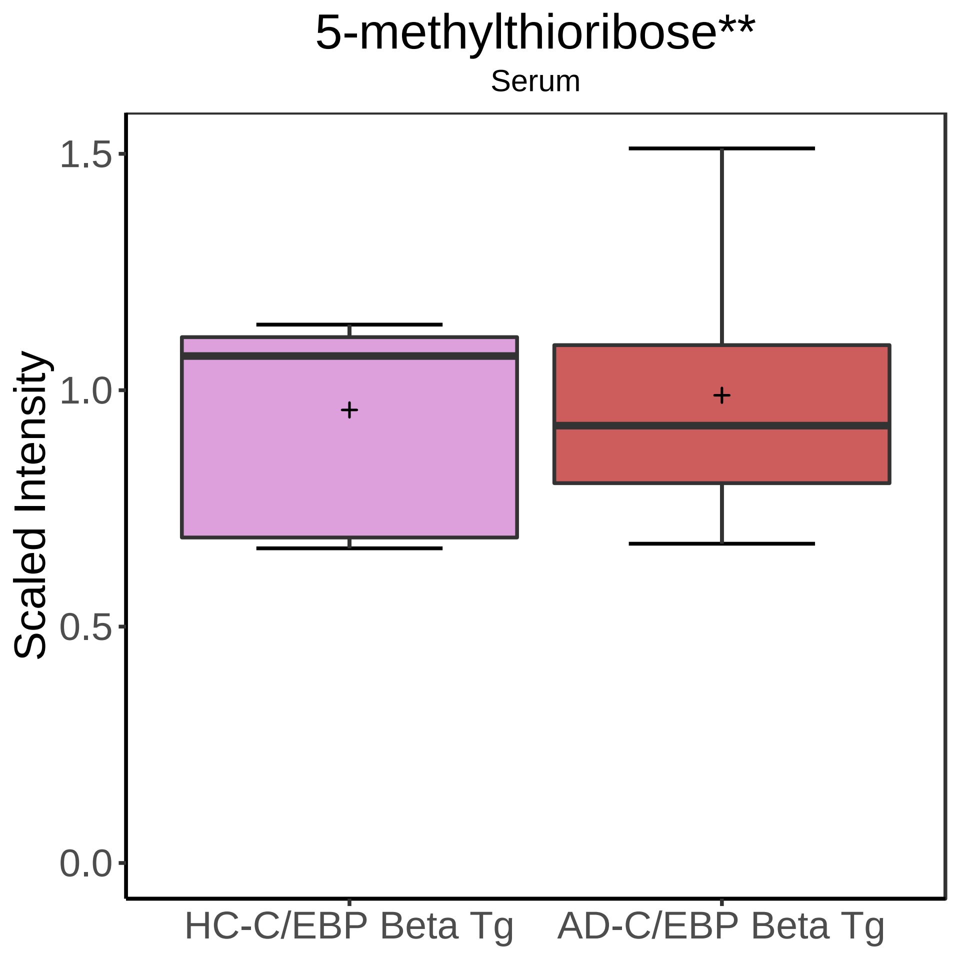
5-methylthioribose**
Serum
Scaled Intensity
1.5
1.0
0.5
0.0
HC-C/EBP Beta Tg
AD-C/EBP Beta Tg

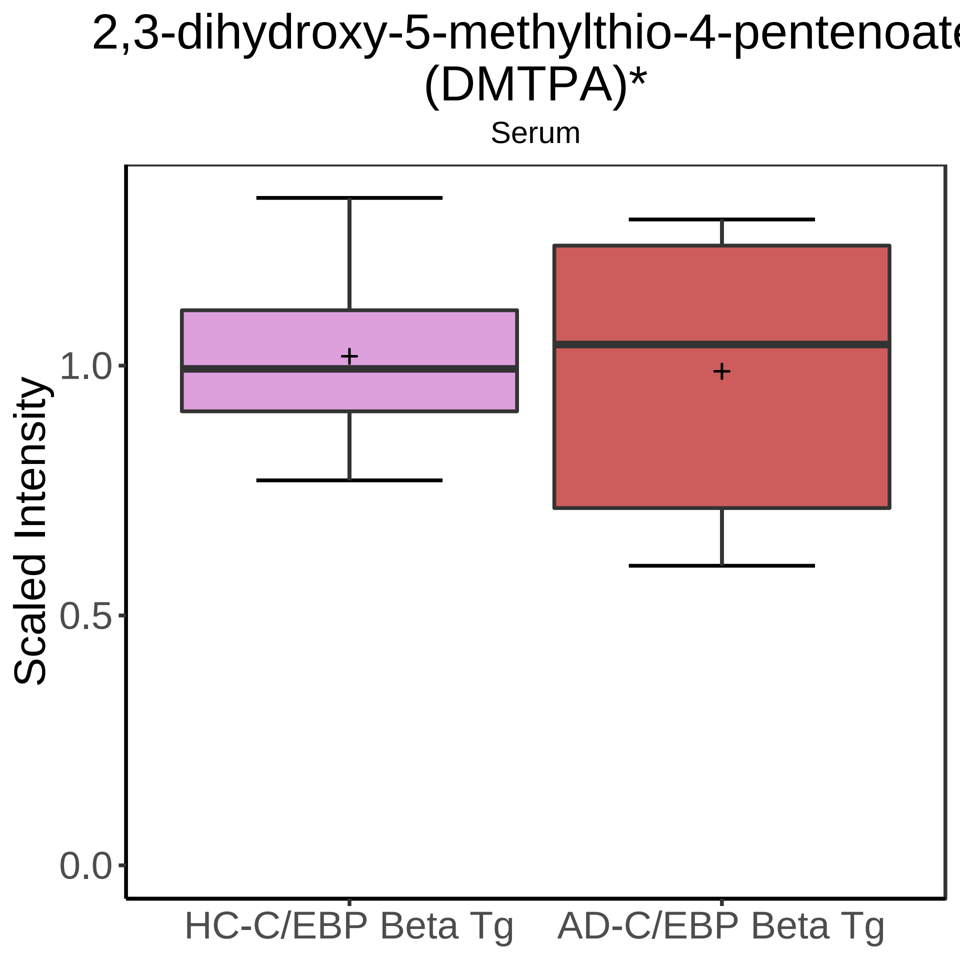

2,3-dihydroxy-5-methylthio-4-pentenoate
(DMTPA)*
Serum
Scaled Intensity
1.0
0.5
0.0
HC-C/EBP Beta Tg
AD-C/EBP Beta Tg

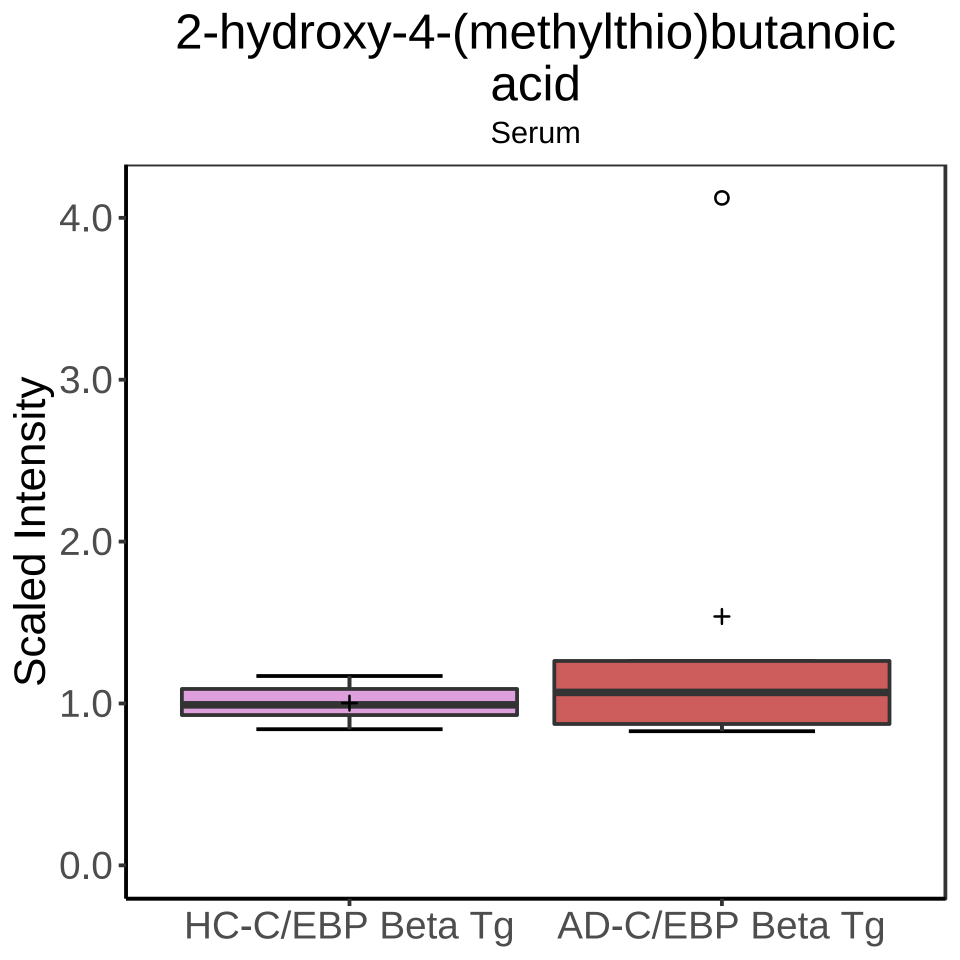

2-hydroxy-4-(methylthio)butanoic acid
Serum
Scaled Intensity
4.0
3.0
2.0
1.0
0.0
HC-C/EBP Beta Tg
AD-C/EBP Beta Tg

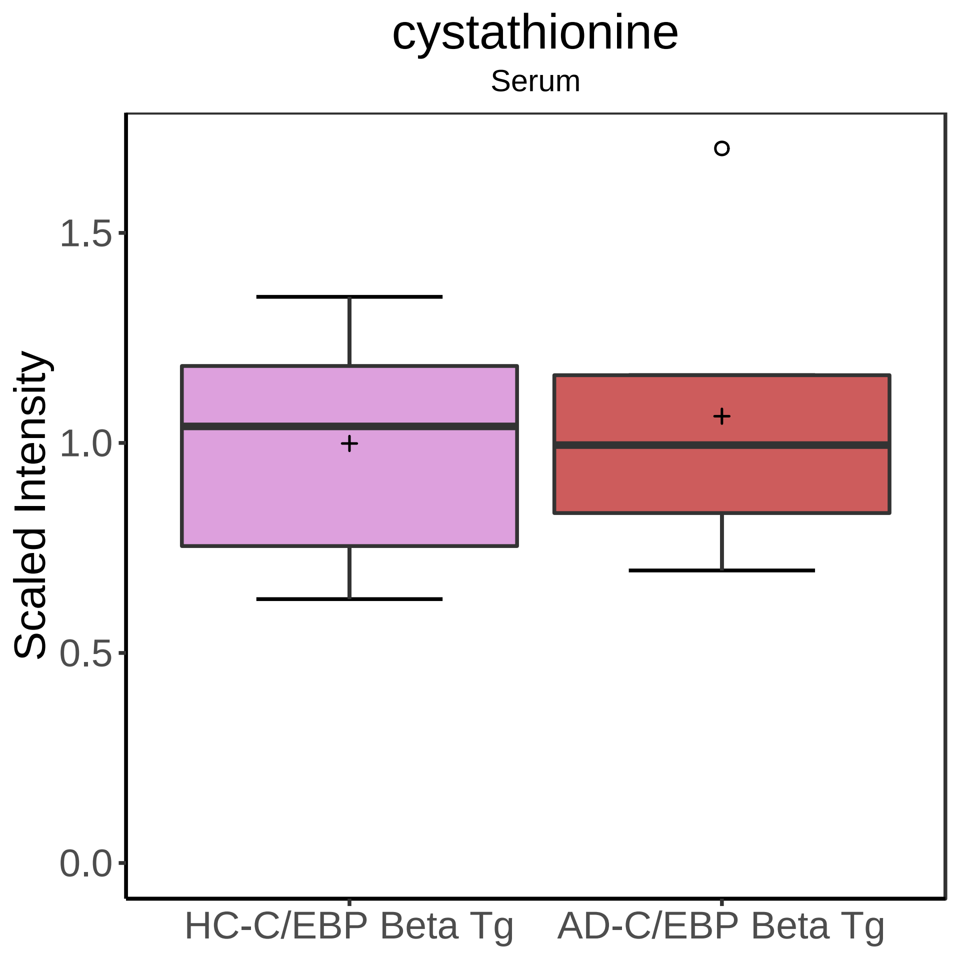
cystathionine
Serum
Scaled Intensity
1.5
1.0
0.5
0.0
HC-C/EBP Beta Tg
AD-C/EBP Beta Tg

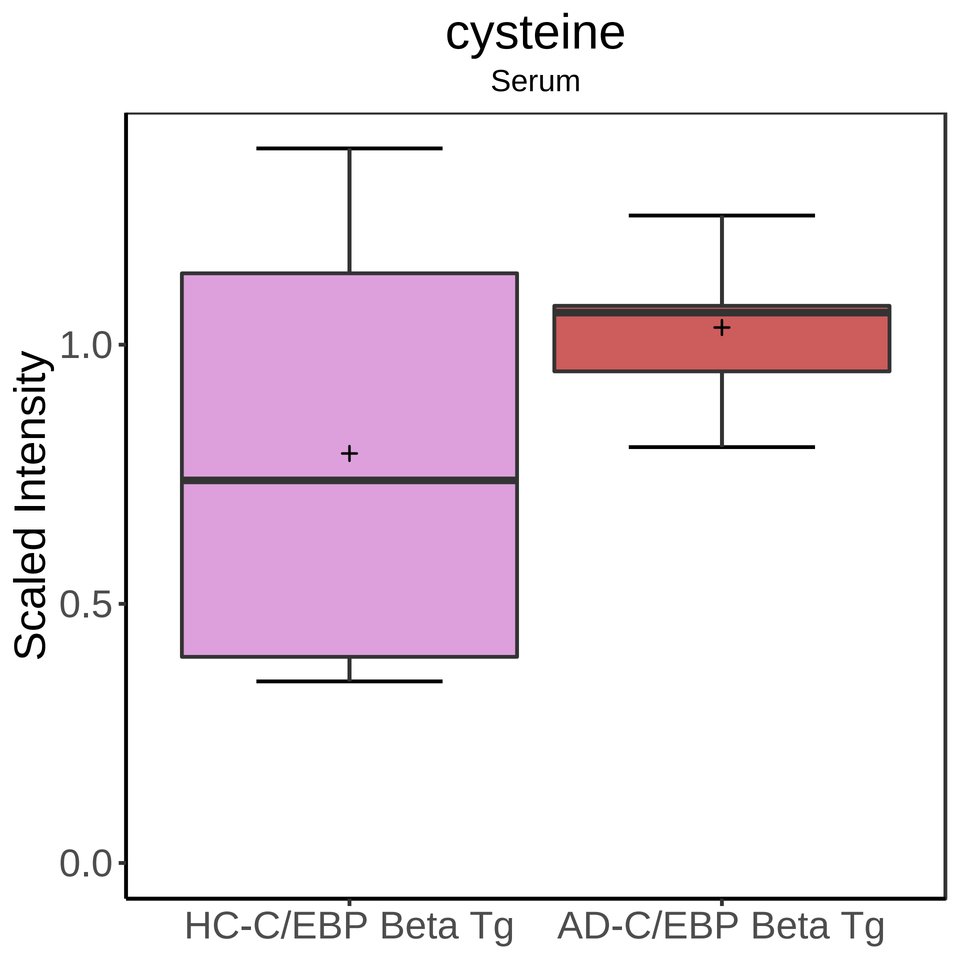
cysteine
Serum
Scaled Intensity
1.0
0.5
0.0
HC-C/EBP Beta Tg
AD-C/EBP Beta Tg

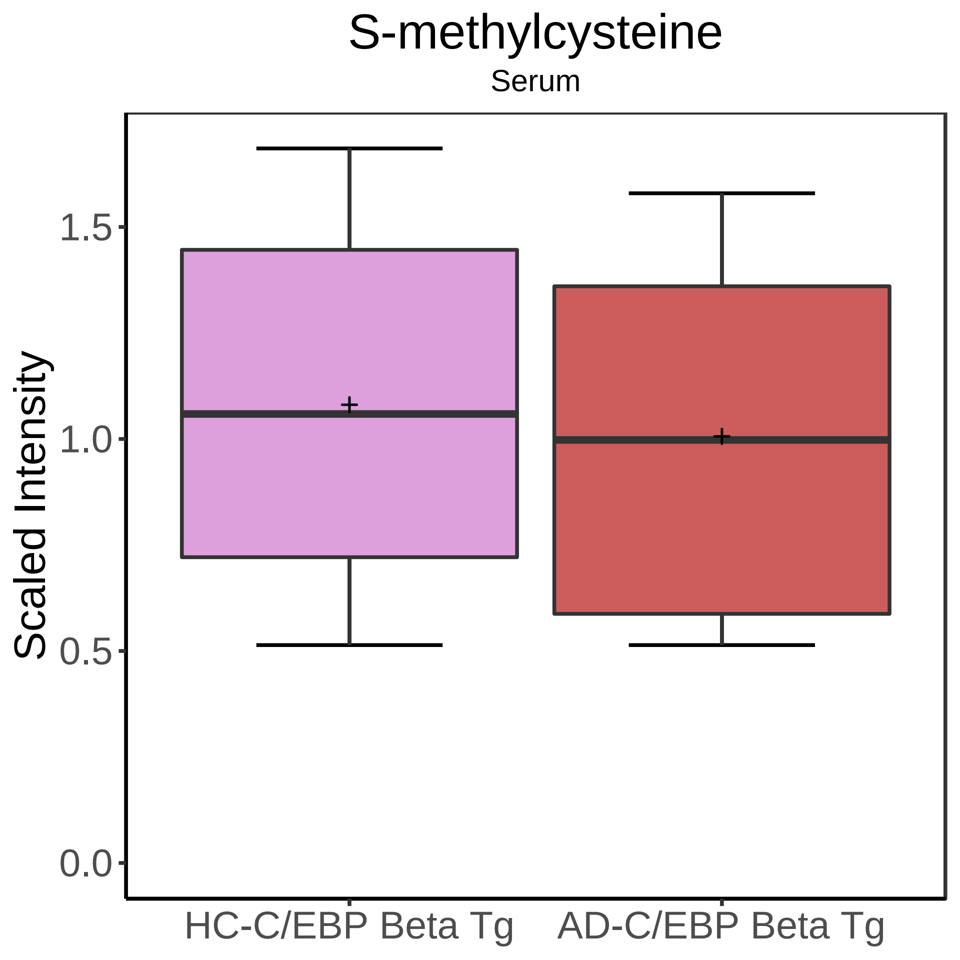

S-methylcysteine
Serum
Scaled Intensity
1.5
1.0
0.5
0.0
HC-C/EBP Beta Tg
AD-C/EBP Beta Tg

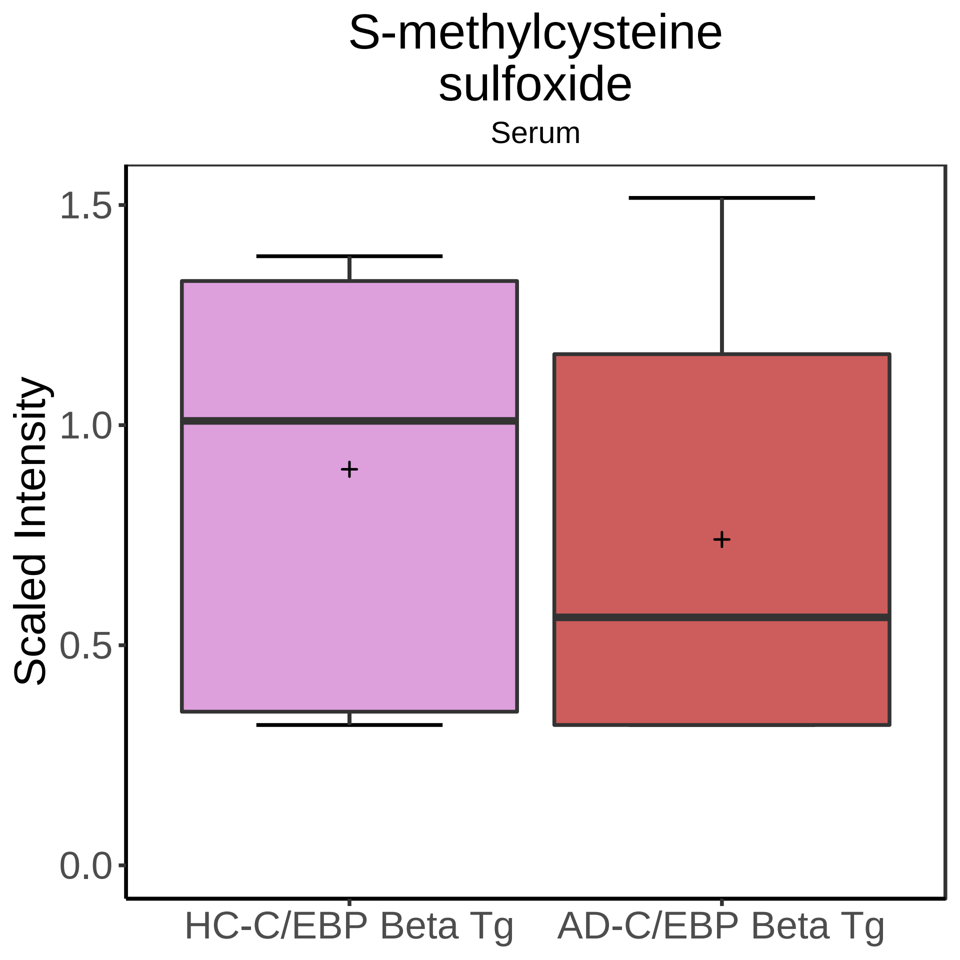
S-methylcysteine
sulfoxide
Serum
Scaled Intensity
1.5
1.0
0.5
0.0
HC-C/EBP Beta Tg
AD-C/EBP Beta Tg

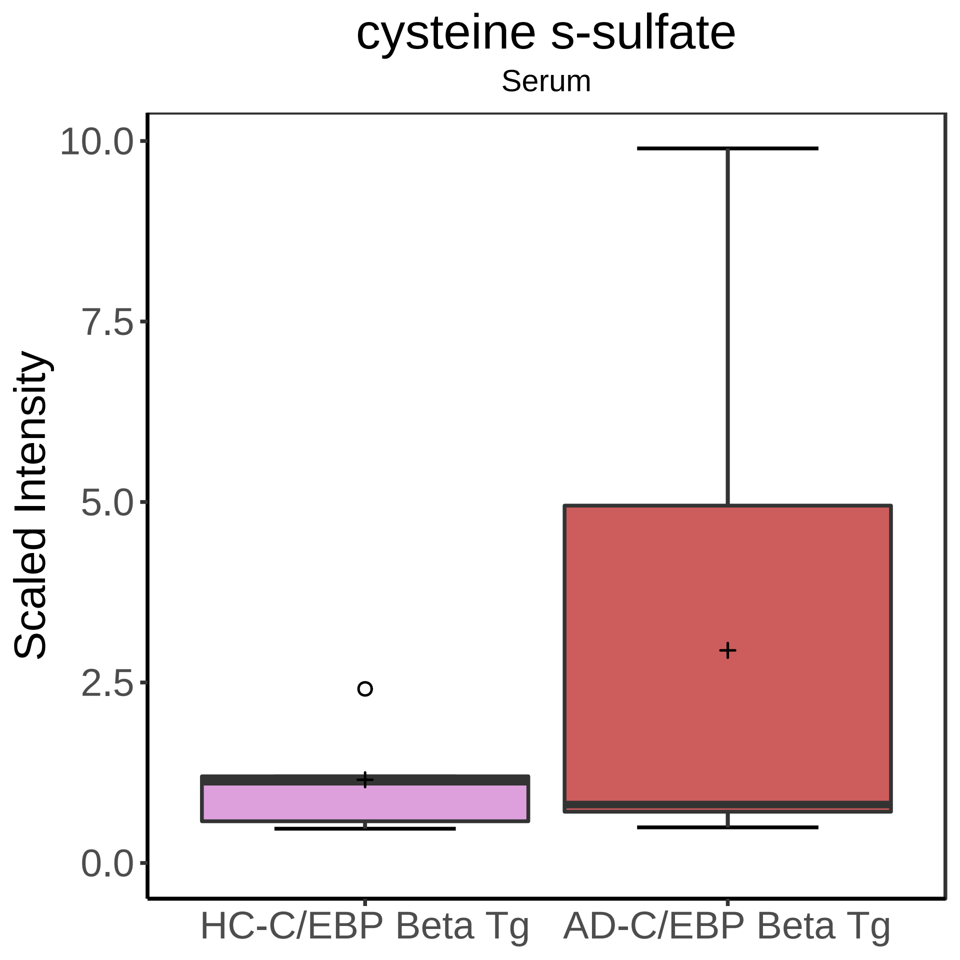
cysteine s-sulfate
Serum
Scaled Intensity
10.0
7.5
5.0
2.5
0.0
HC-C/EBP Beta Tg
AD-C/EBP Beta Tg

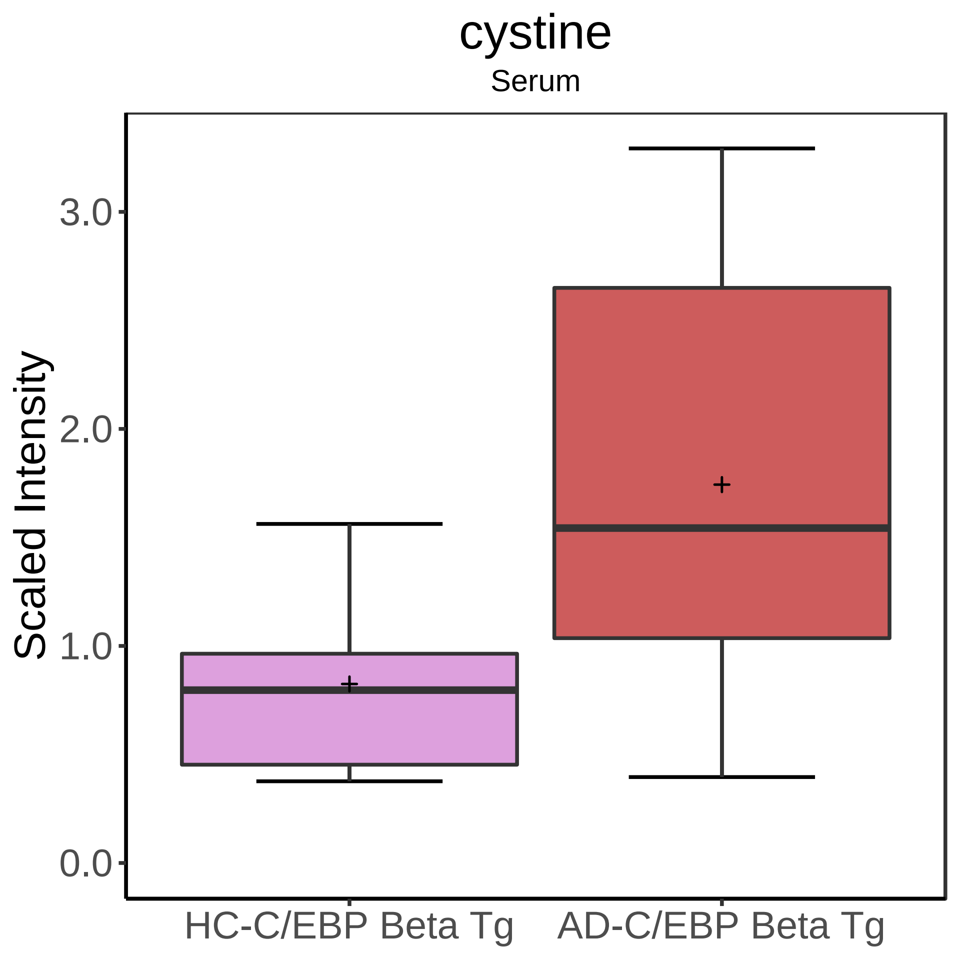
cystine
Serum
Scaled Intensity
3.0
2.0
1.0
0.0
HC-C/EBP Beta Tg
AD-C/EBP Beta Tg

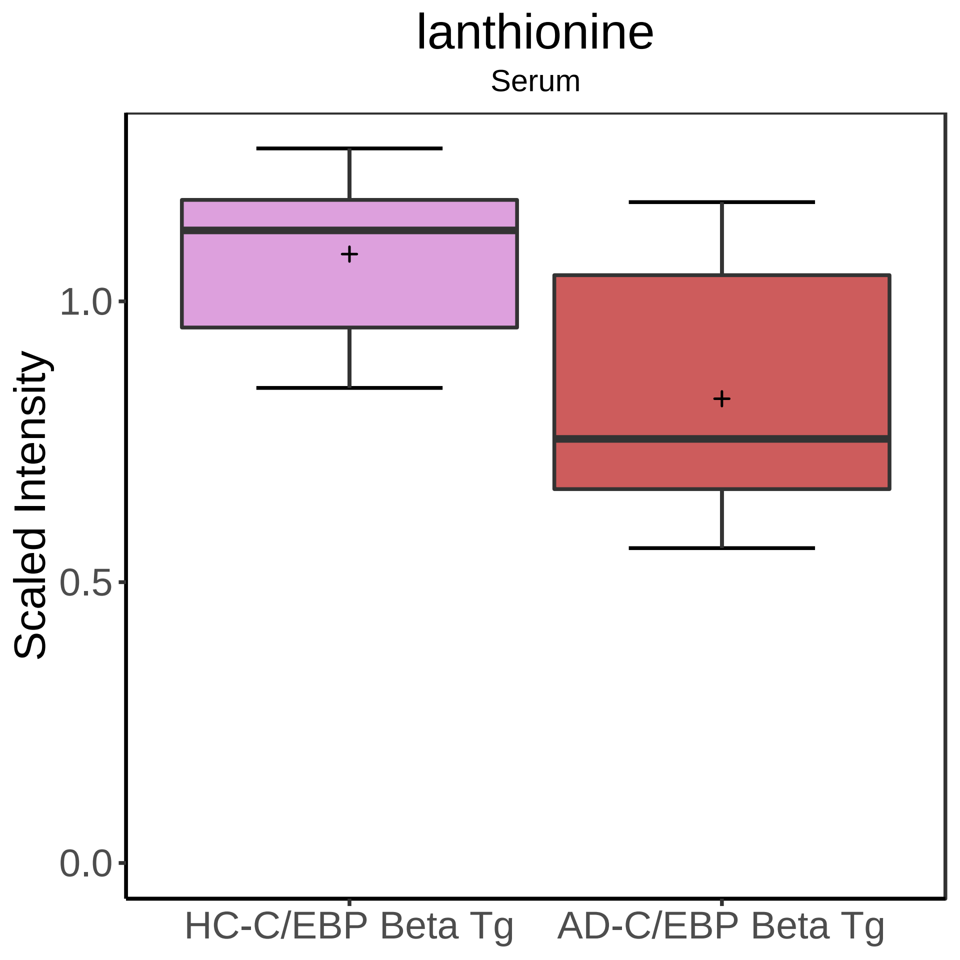
lanthionine
Serum
Scaled Intensity
1.0
0.5
0.0
HC-C/EBP Beta Tg
AD-C/EBP Beta Tg

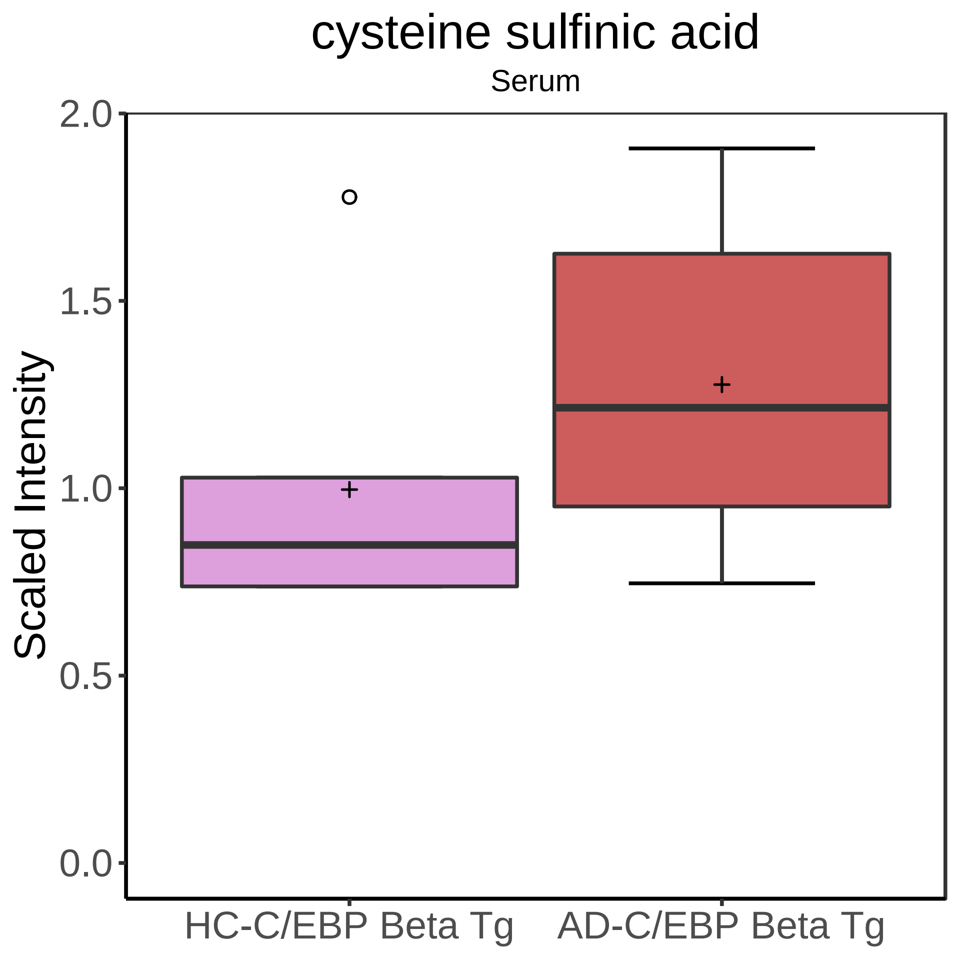

cysteine sulfinic acid
Serum
Scaled Intensity
2.0
1.5
1.0
0.5
0.0
HC-C/EBP Beta Tg
AD-C/EBP Beta Tg

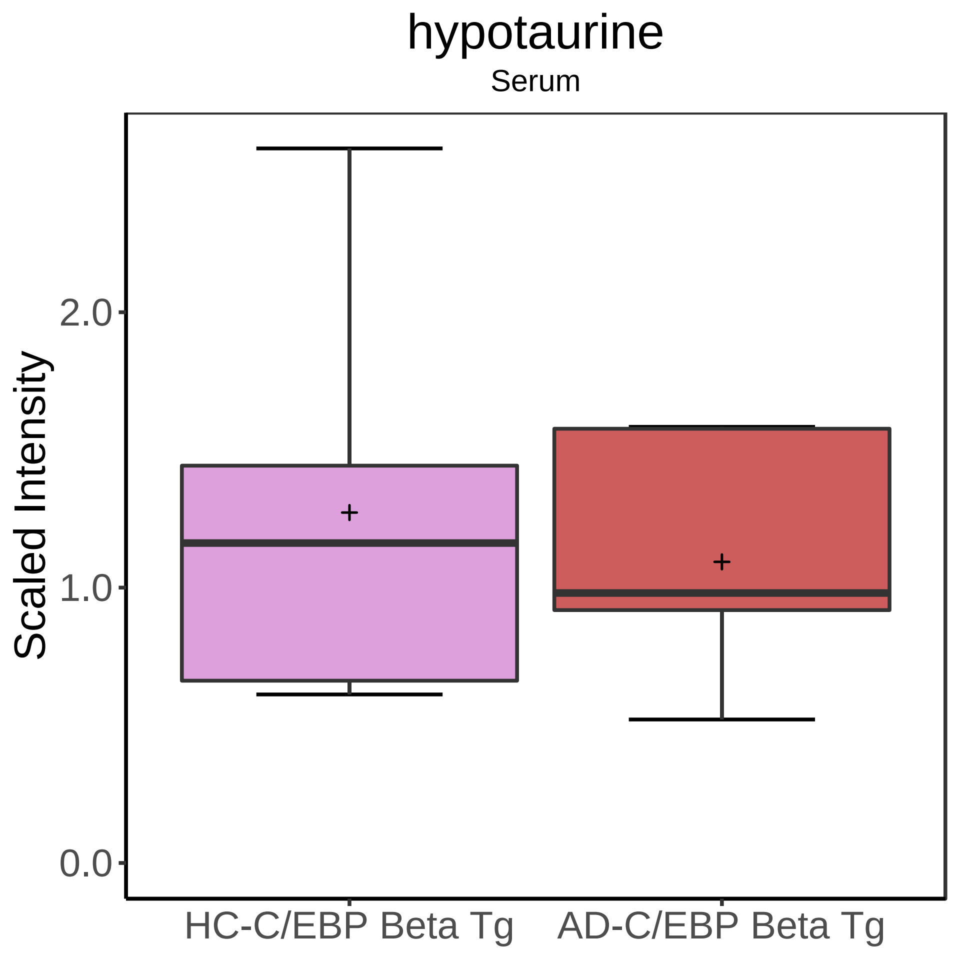
hypotaurine
Serum
Scaled Intensity
2.0
1.0
0.0
HC-C/EBP Beta Tg
AD-C/EBP Beta Tg

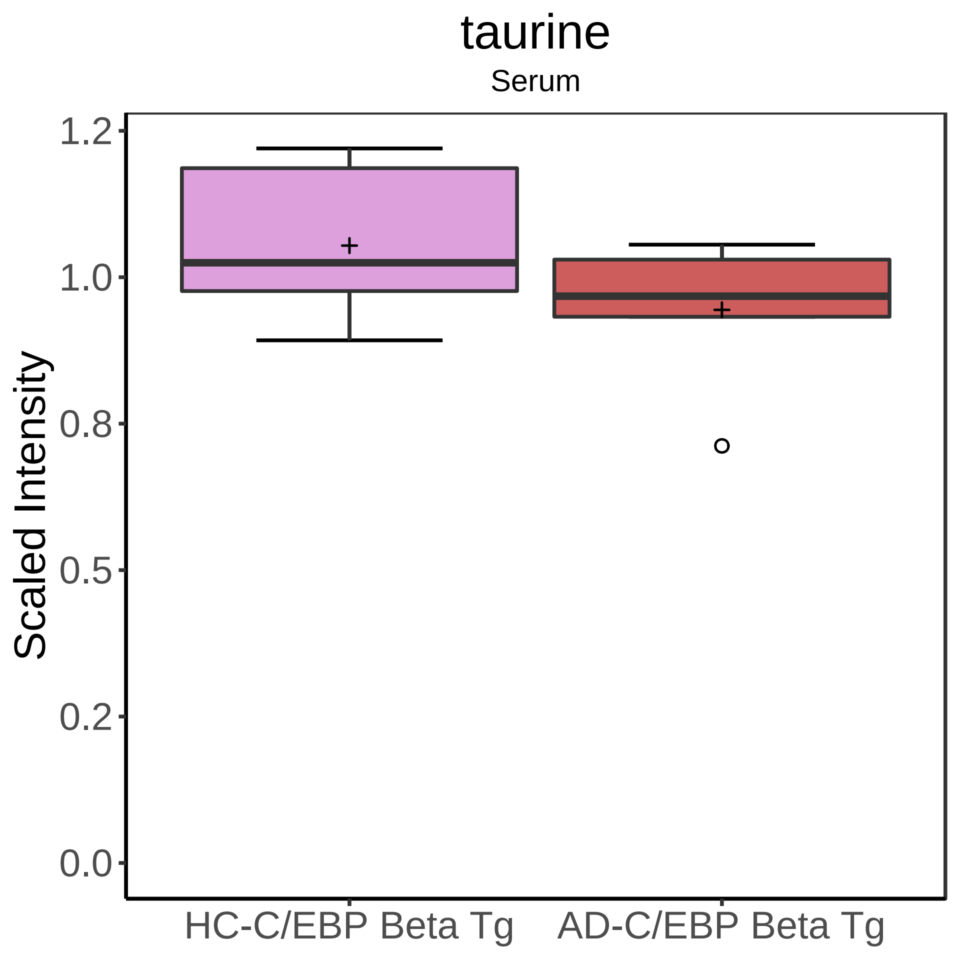
taurine
Serum
Scaled Intensity
1.2
1.0
0.8
0.5
0.2
0.0
HC-C/EBP Beta Tg
AD-C/EBP Beta Tg

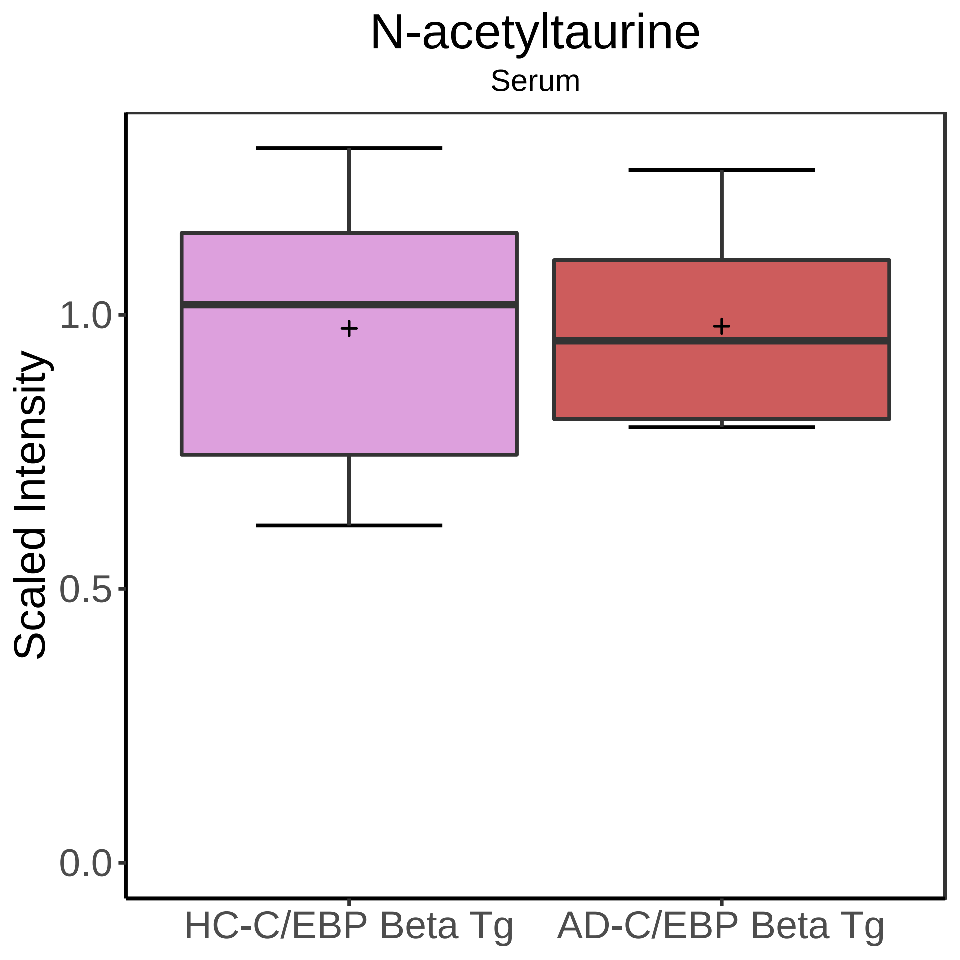
N-acetyltaurine
Serum
Scaled Intensity
1.0
0.5
0.0
HC-C/EBP Beta Tg
AD-C/EBP Beta Tg

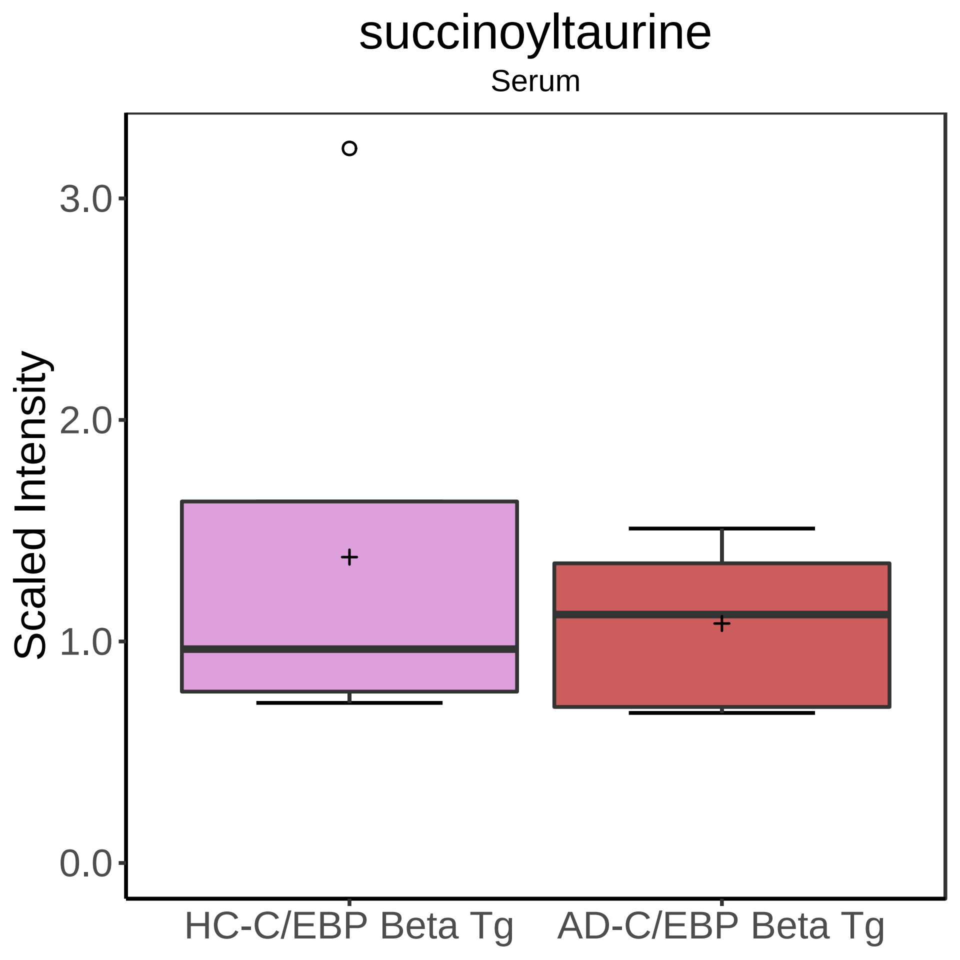

succinoyltaurine
Serum
Scaled Intensity
3.0
2.0
1.0
0.0
HC-C/EBP Beta Tg
AD-C/EBP Beta Tg

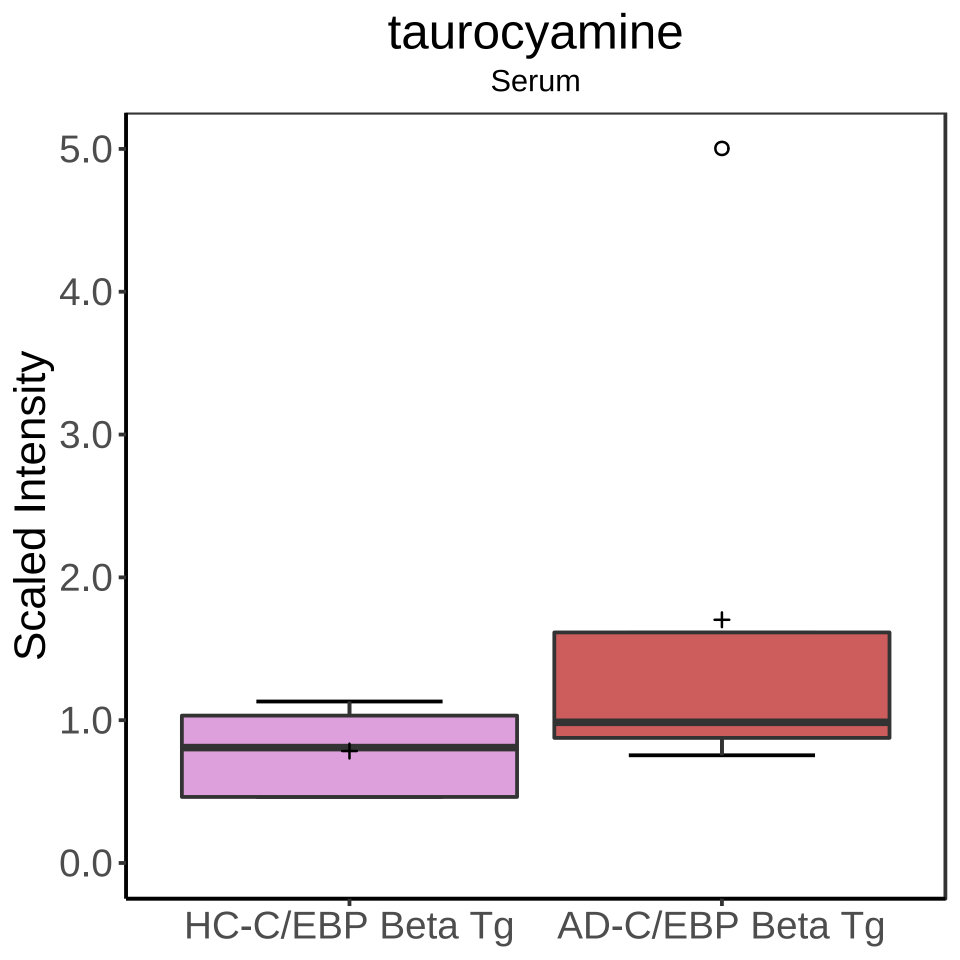

taurocyamine
Serum
Scaled Intensity
5.0
4.0
3.0
2.0
1.0
0.0
HC-C/EBP Beta Tg
AD-C/EBP Beta Tg

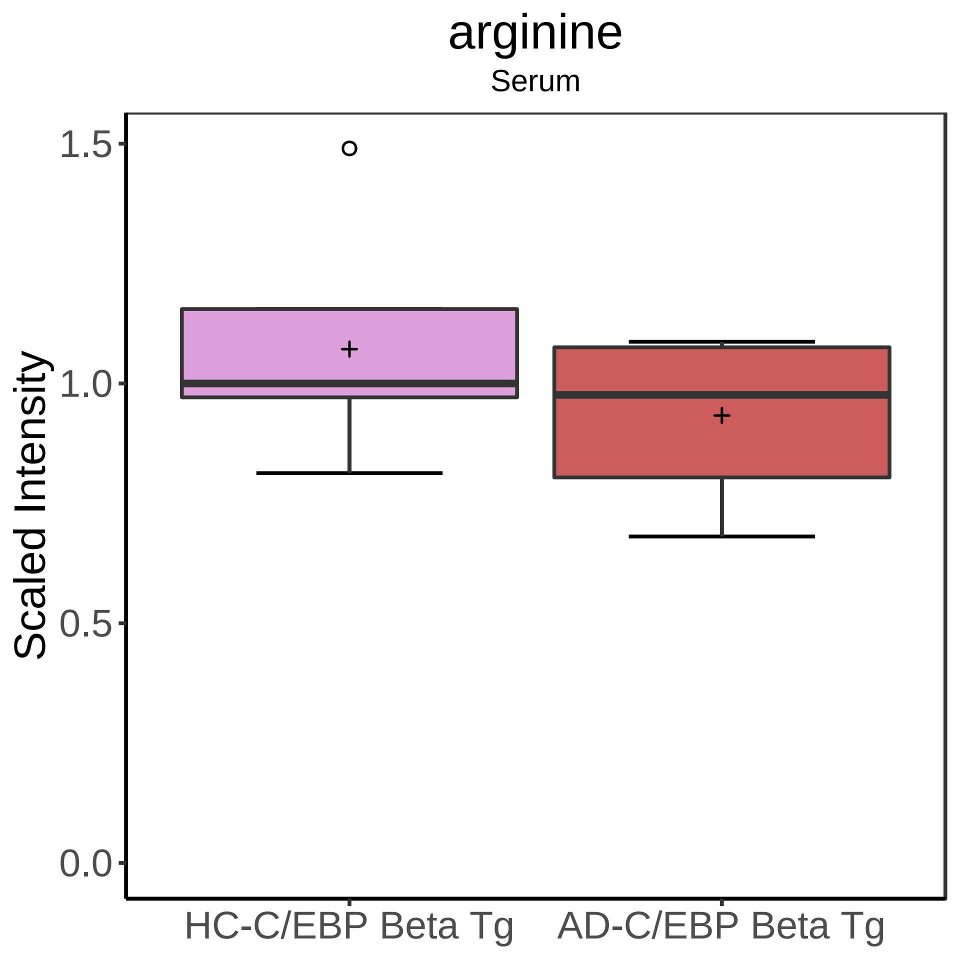

arginine
Serum
Scaled Intensity
1.5
1.0
0.5
0.0
HC-C/EBP Beta Tg
AD-C/EBP Beta Tg

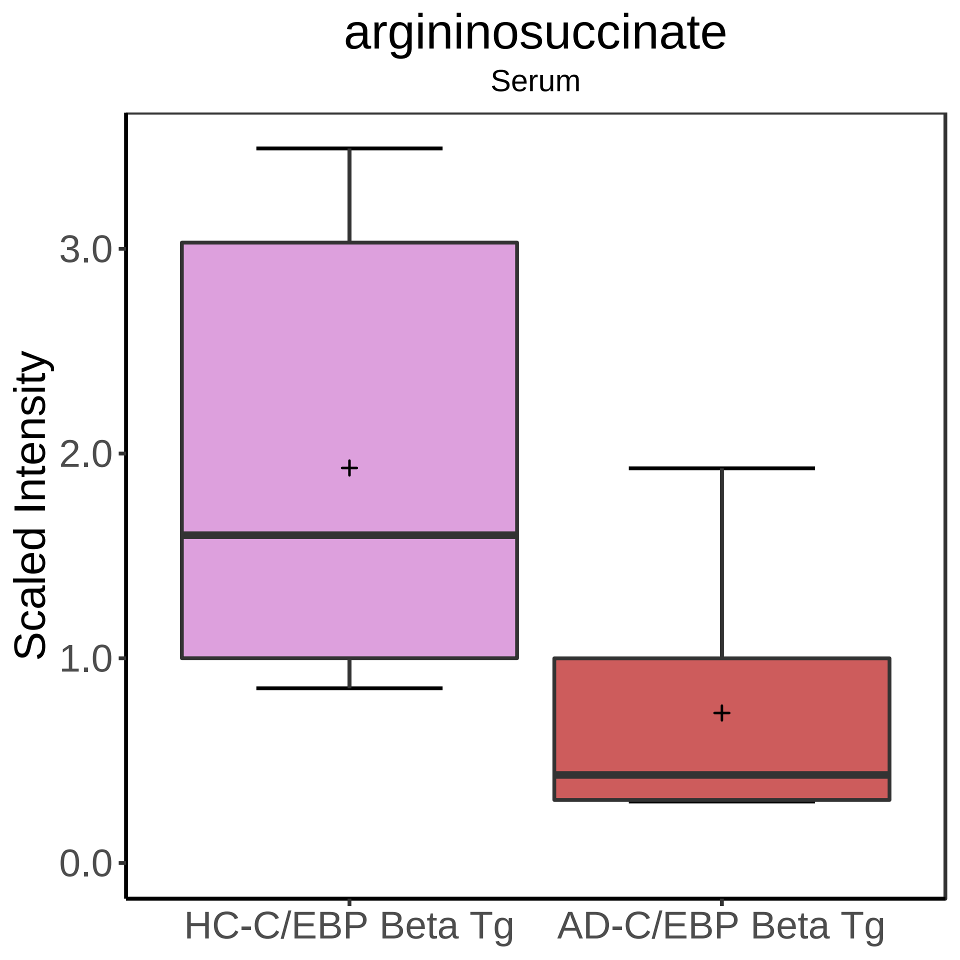
argininosuccinate
Serum
Scaled Intensity
3.0
2.0
1.0
0.0
HC-C/EBP Beta Tg
AD-C/EBP Beta Tg

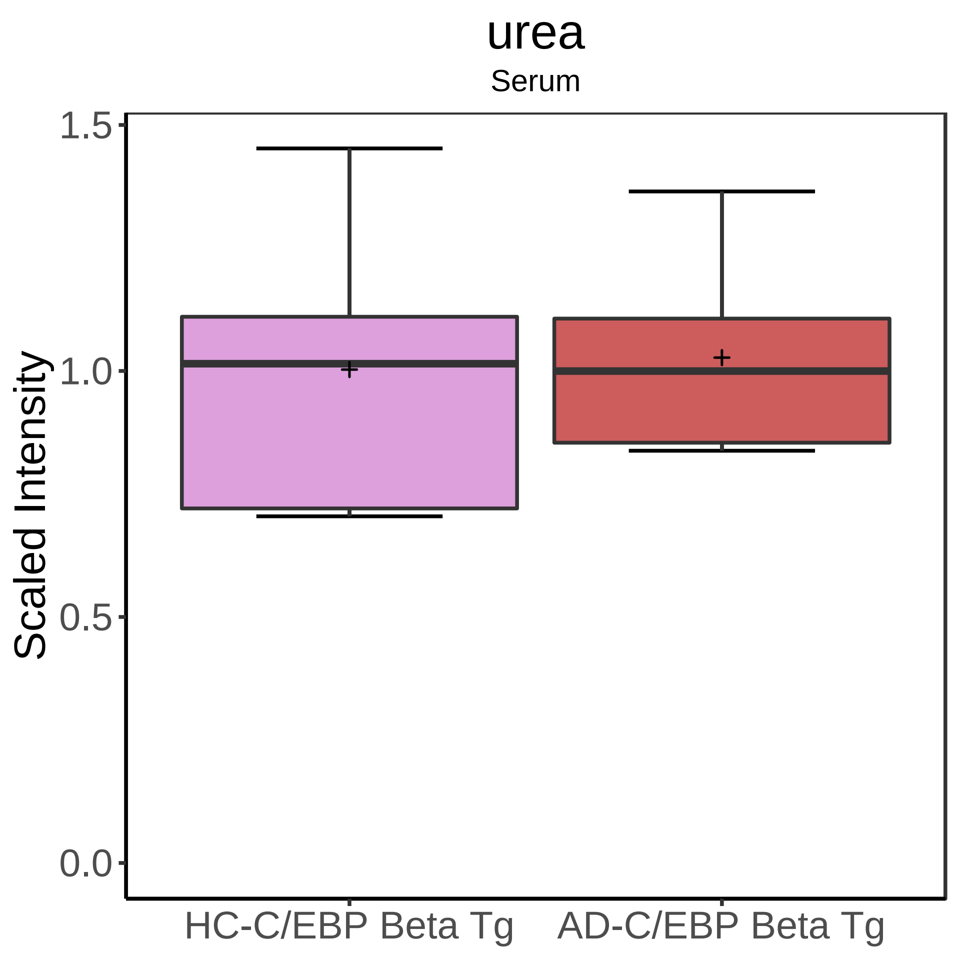
urea
Serum
Scaled Intensity
1.5
1.0
0.5
0.0
HC-C/EBP Beta Tg
AD-C/EBP Beta Tg

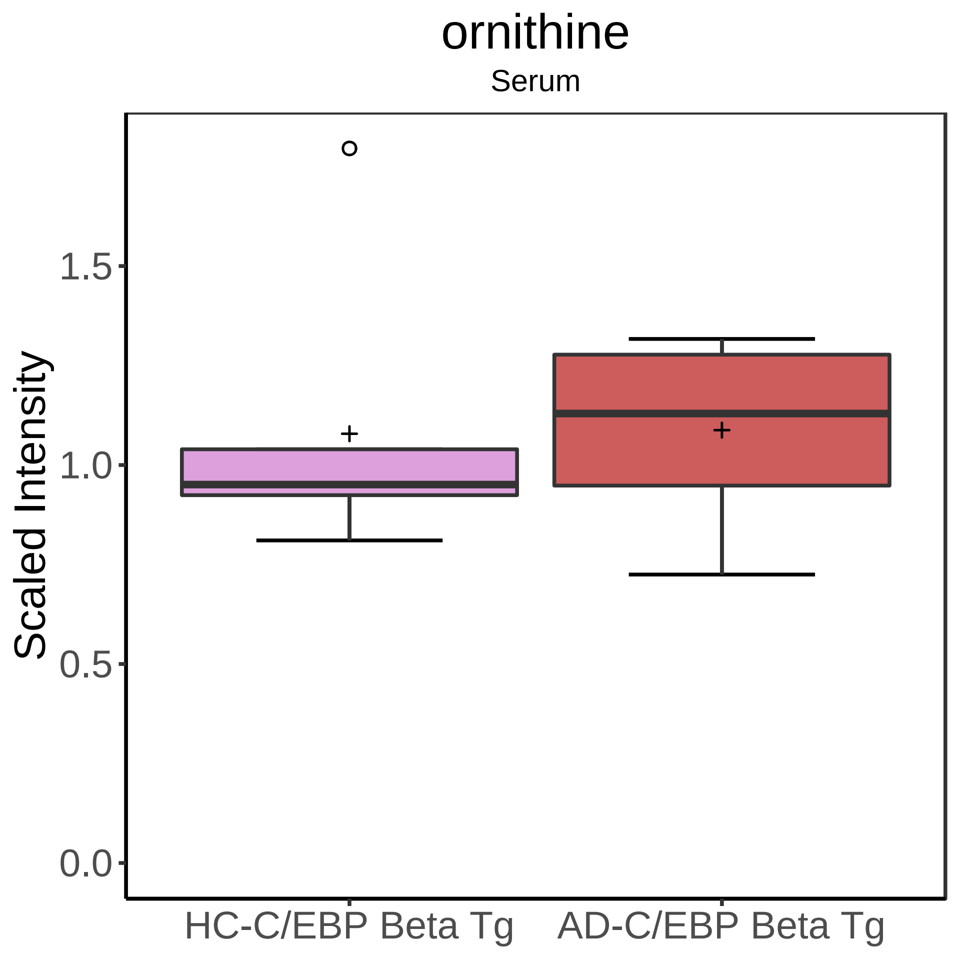
ornithine
Serum
Scaled Intensity
1.5
1.0
0.5
0.0
HC-C/EBP Beta Tg
AD-C/EBP Beta Tg

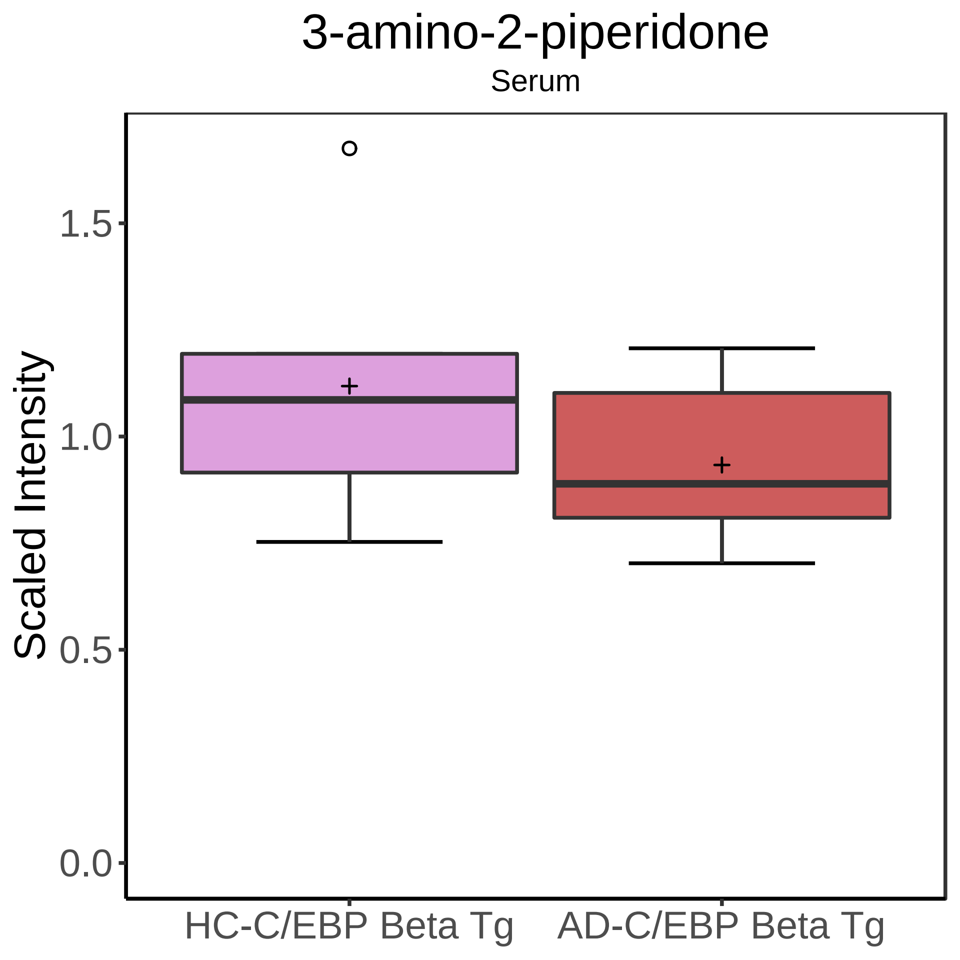
3-amino-2-piperidone
Serum
Scaled Intensity
1.5
1.0
0.5
0.0
HC-C/EBP Beta Tg
AD-C/EBP Beta Tg

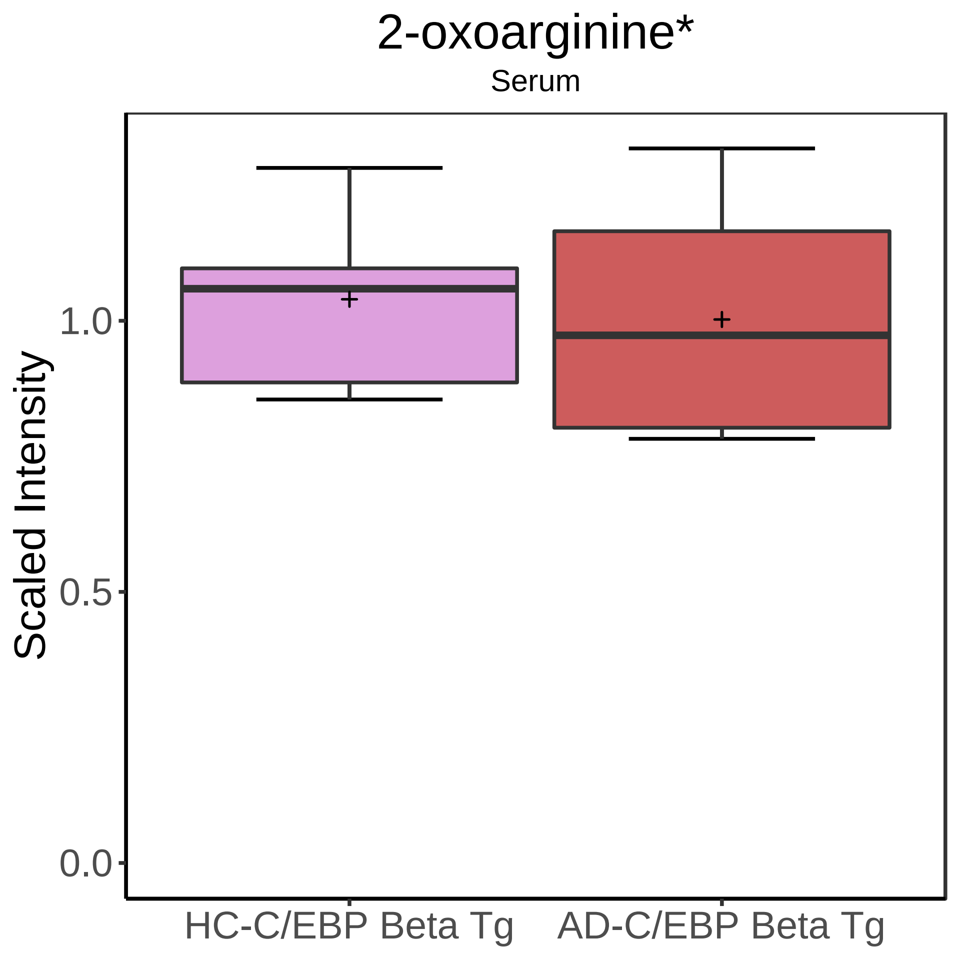
2-oxoarginine*
Serum
Scaled Intensity
1.0
0.5
0.0
HC-C/EBP Beta Tg
AD-C/EBP Beta Tg

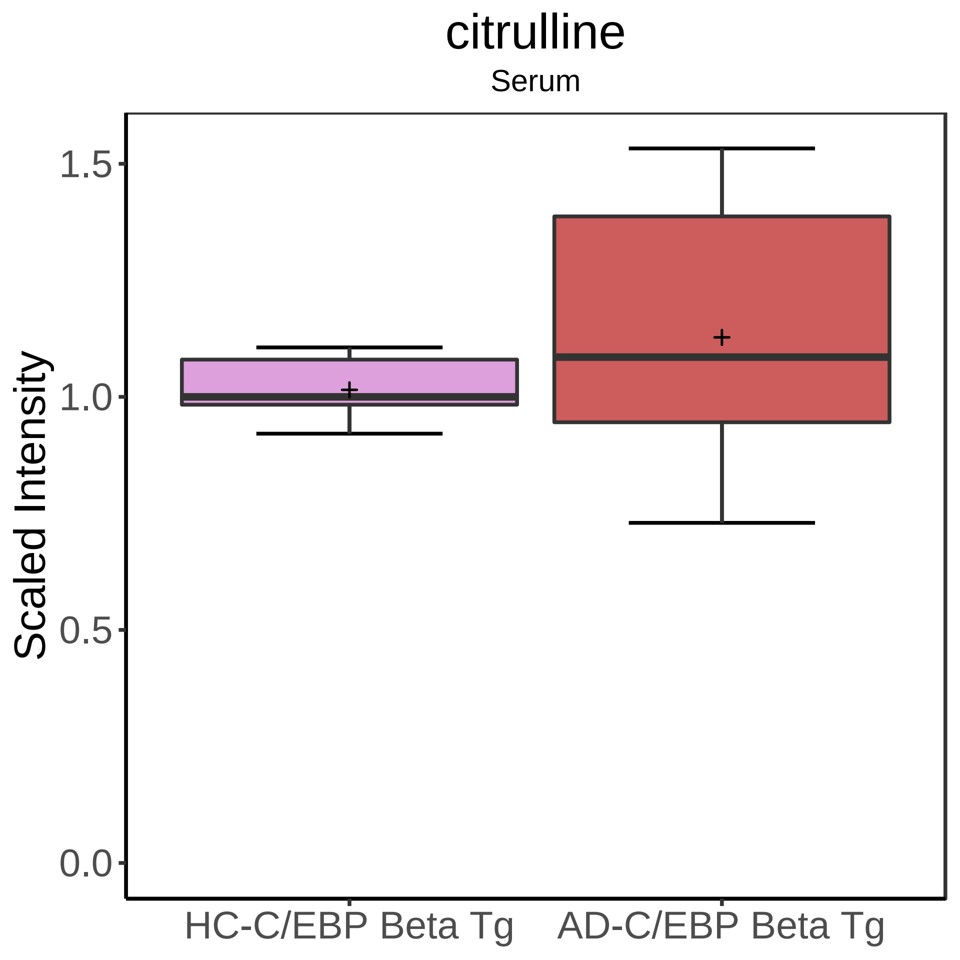

citrulline
Serum
Scaled Intensity
1.5
1.0
0.5
0.0
HC-C/EBP Beta Tg
AD-C/EBP Beta Tg

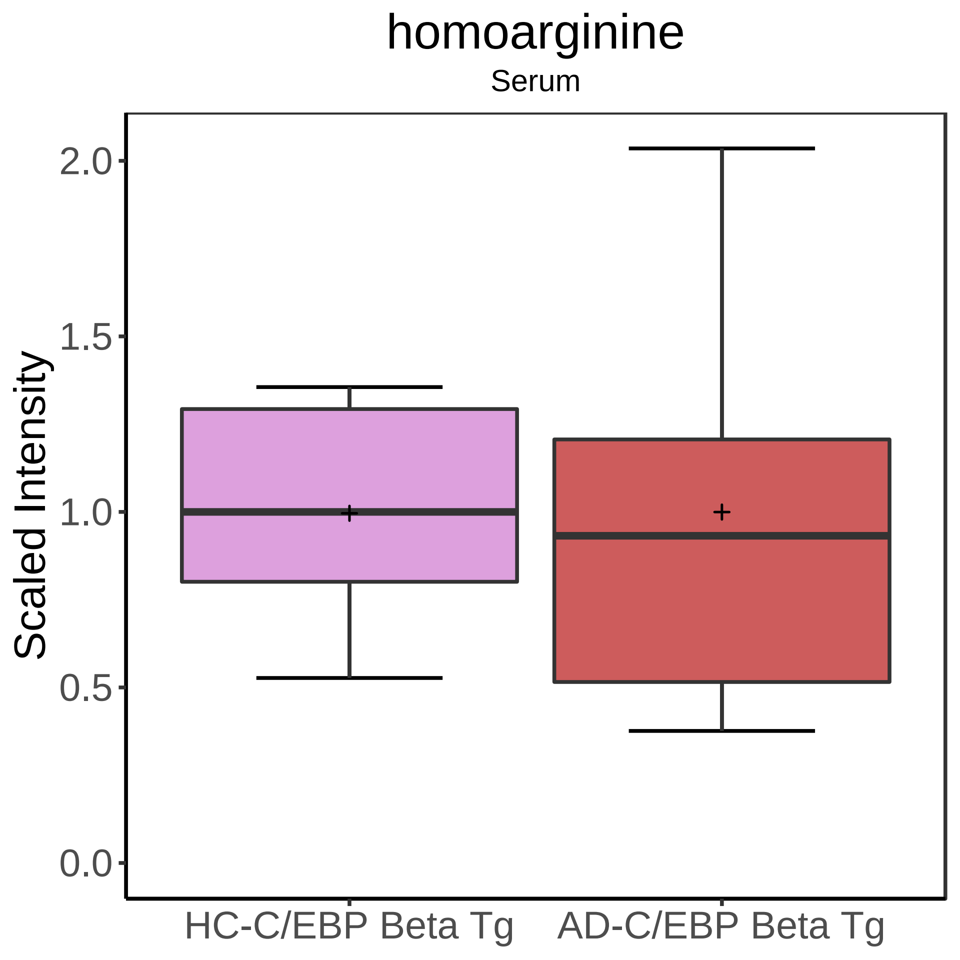
homoarginine
Serum
Scaled Intensity
2.0
1.5
1.0
0.5
0.0
HC-C/EBP Beta Tg
AD-C/EBP Beta Tg

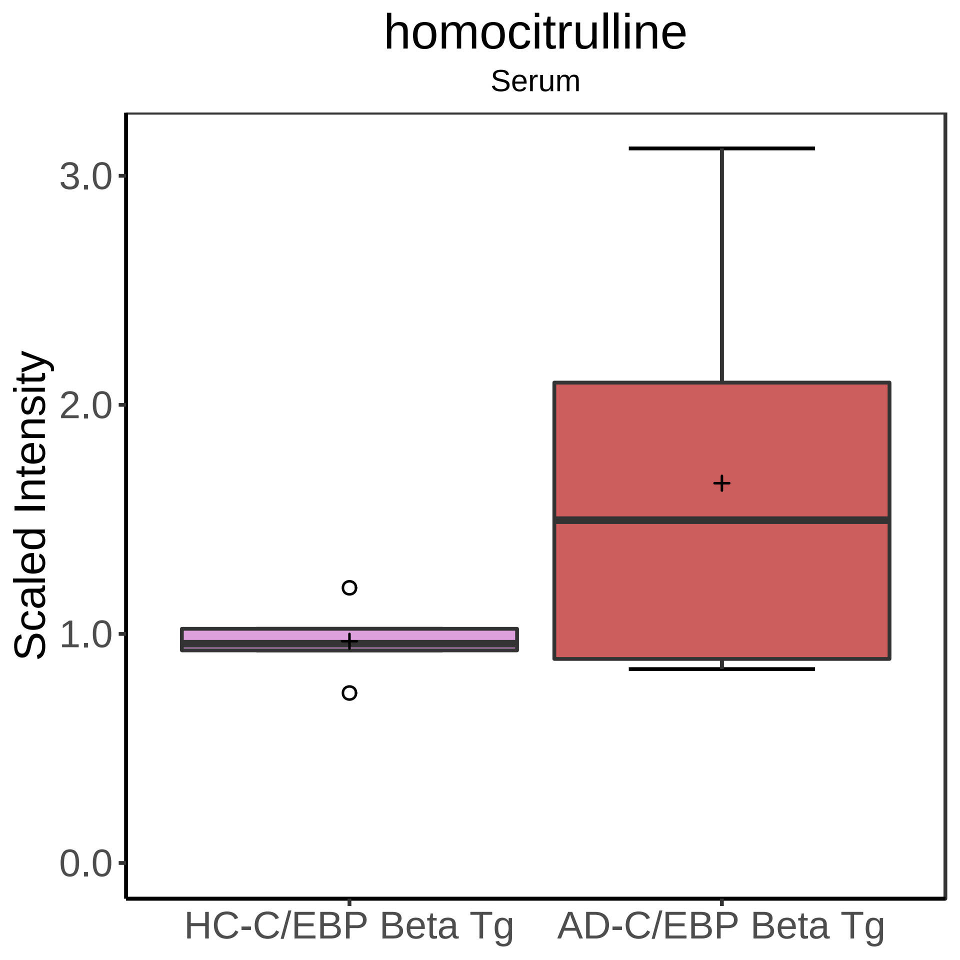
homocitrulline
Serum
Scaled Intensity
3.0
2.0
1.0
0.0
HC-C/EBP Beta Tg
AD-C/EBP Beta Tg

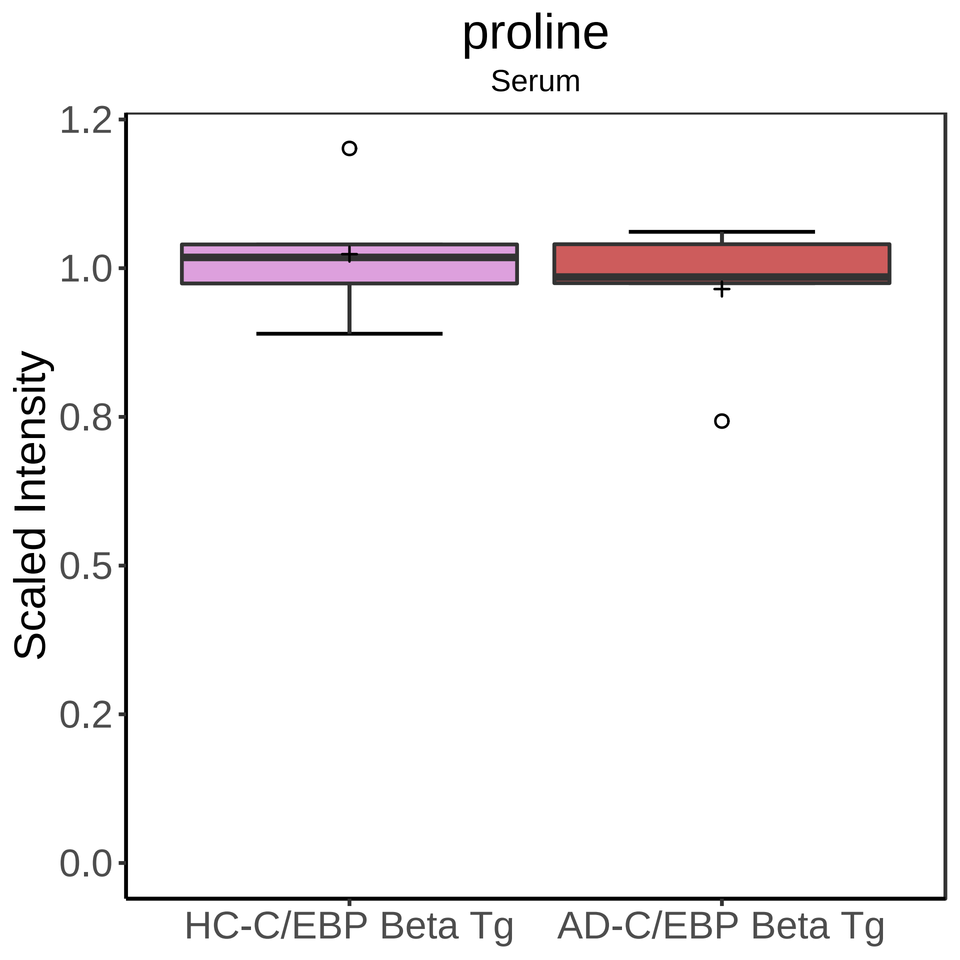
proline
Serum
Scaled Intensity
1.2
1.0
0.8
0.5
0.2
0.0
HC-C/EBP Beta Tg
AD-C/EBP Beta Tg

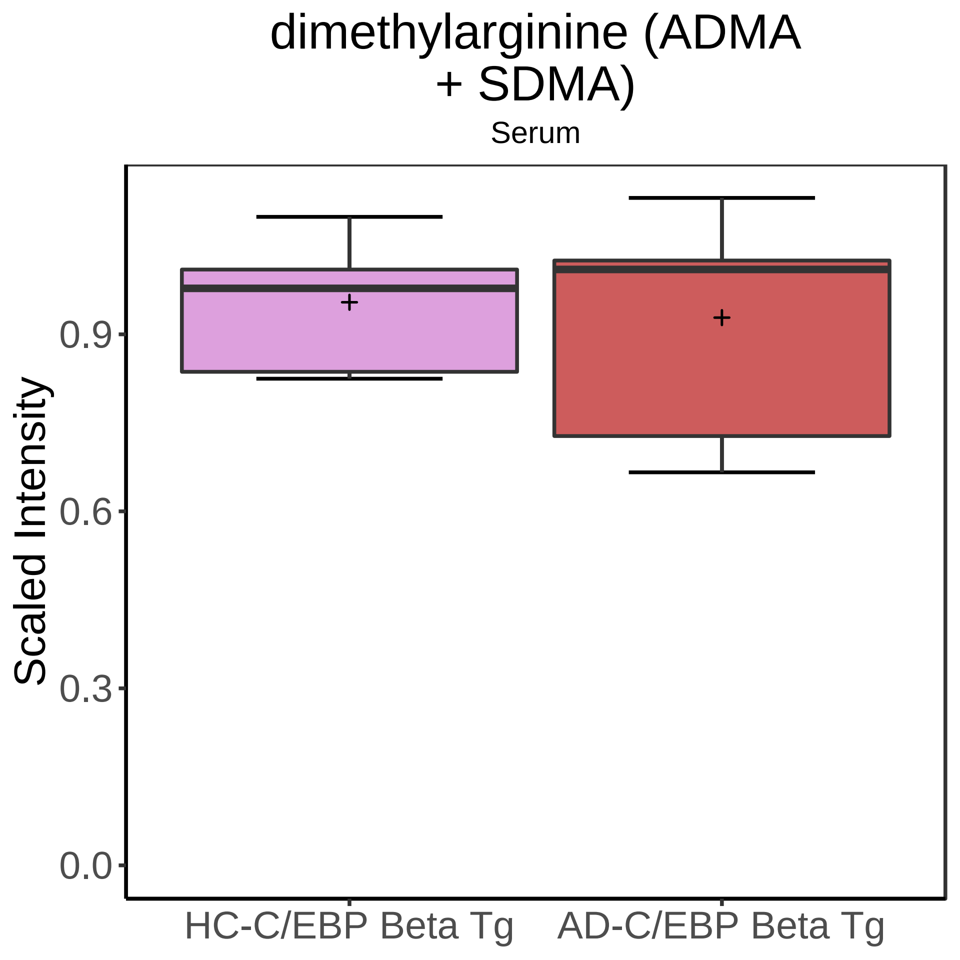
dimethylarginine (ADMA + SDMA)
Serum
Scaled Intensity
0.0
0.3
0.6
0.9
HC-C/EBP Beta Tg
AD-C/EBP Beta Tg

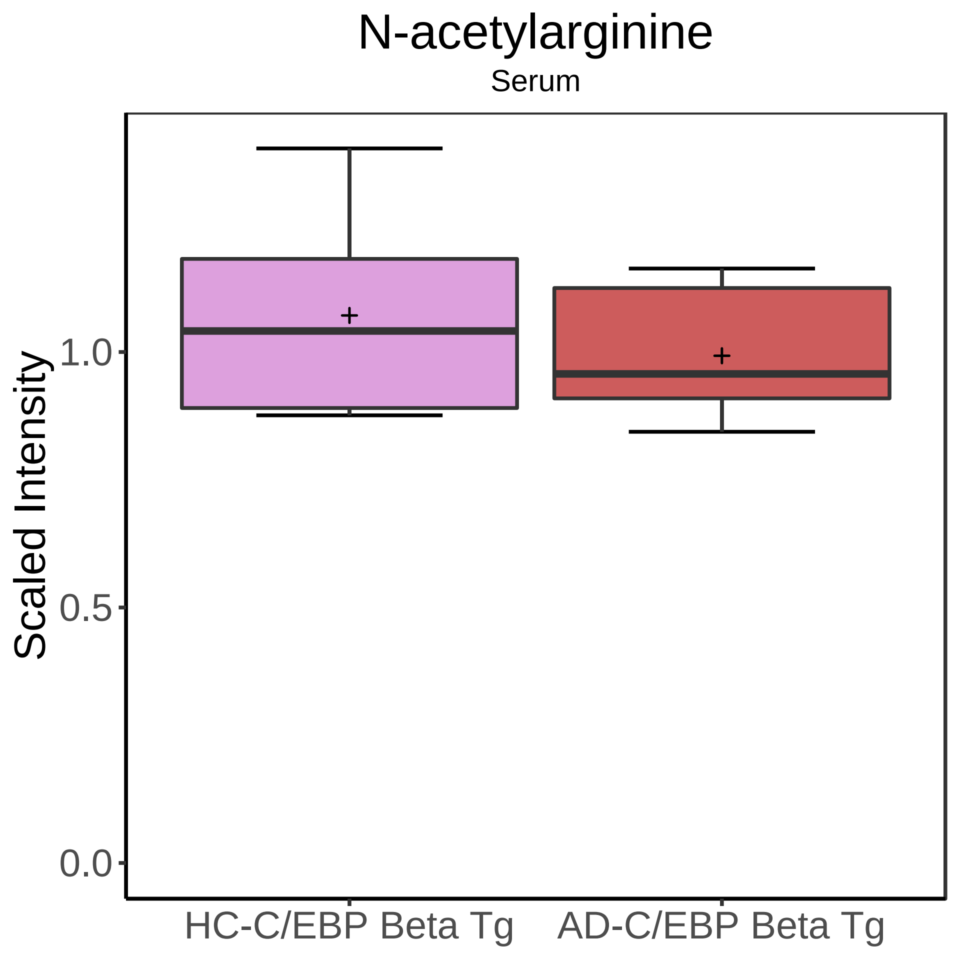
N-acetylarginine
Serum
Scaled Intensity
1.0
0.5
0.0
HC-C/EBP Beta Tg
AD-C/EBP Beta Tg

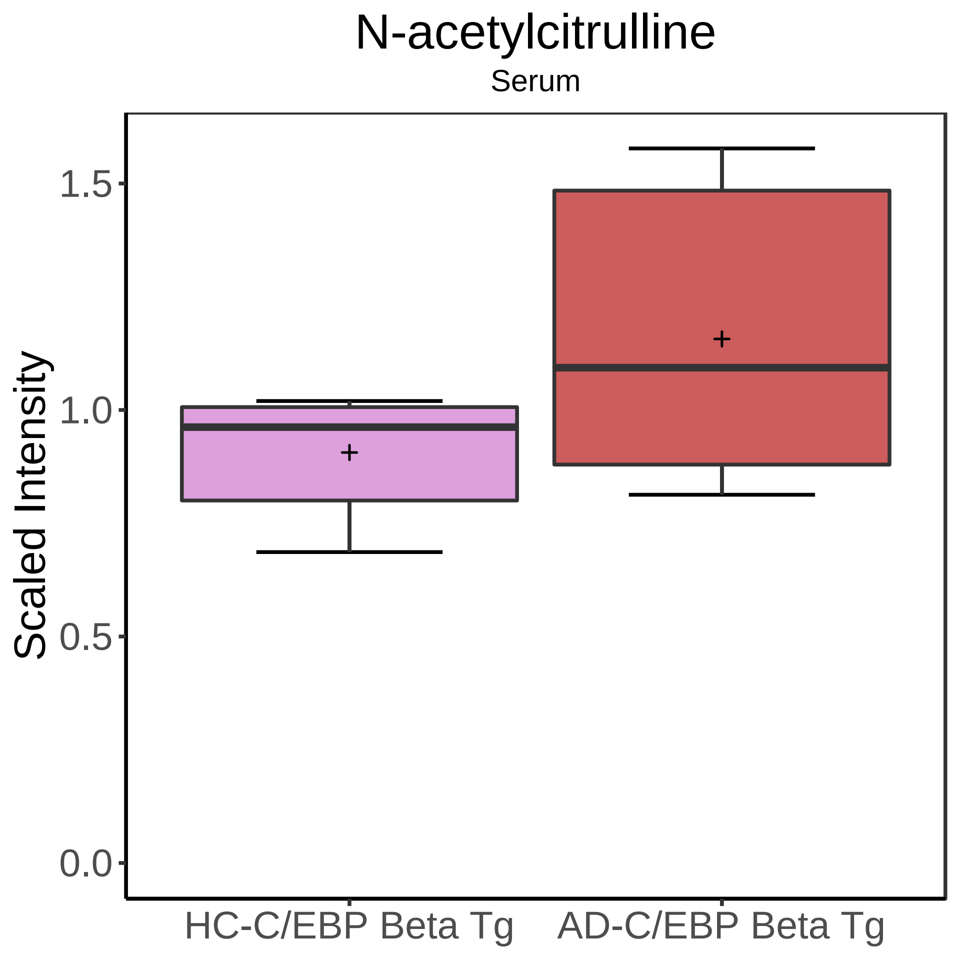
N-acetylcitrulline
Serum
Scaled Intensity
1.5
1.0
0.5
0.0
HC-C/EBP Beta Tg
AD-C/EBP Beta Tg

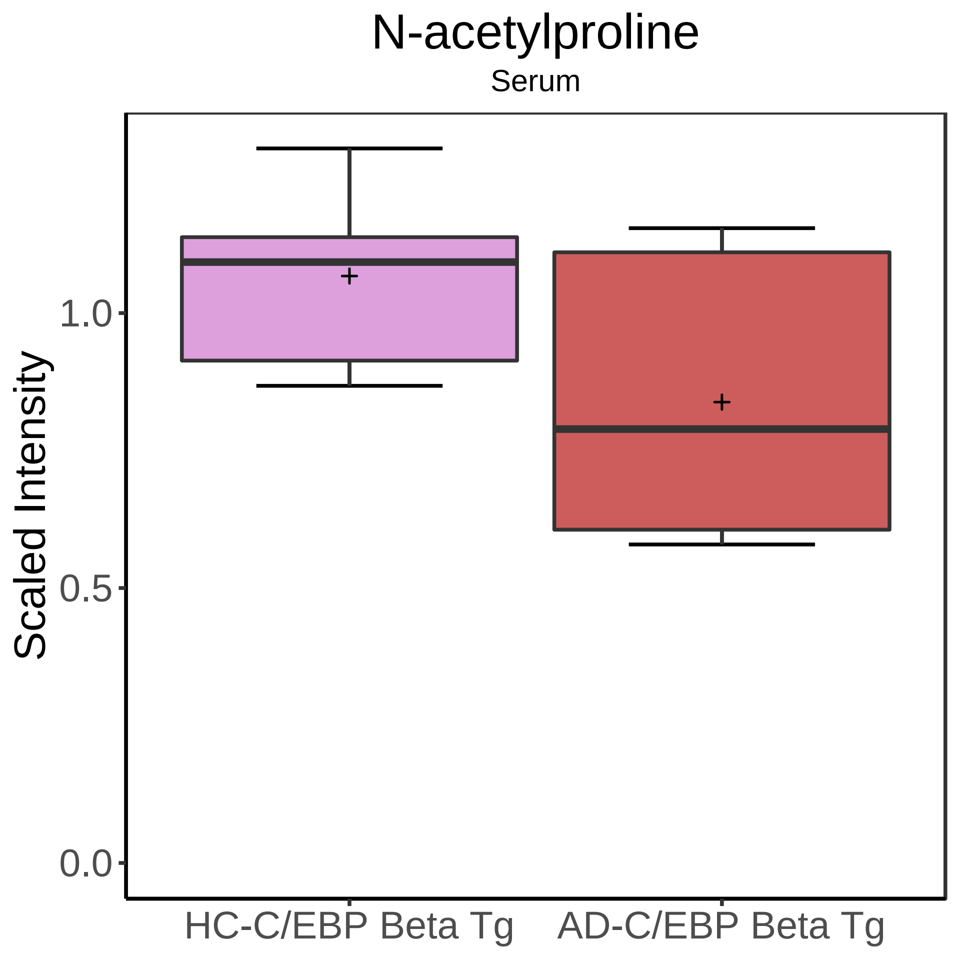
N-acetylproline
Serum
Scaled Intensity
1.0
0.5
0.0
HC-C/EBP Beta Tg
AD-C/EBP Beta Tg

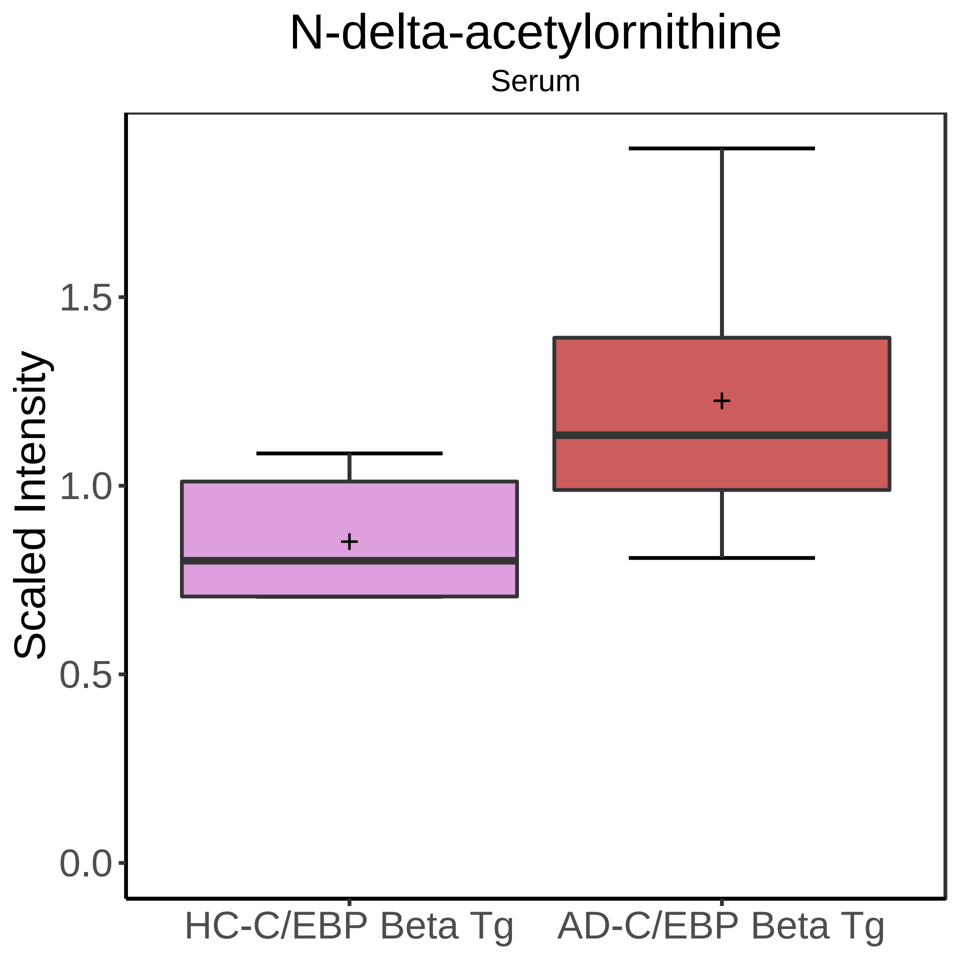
N-delta-acetylornithine
Serum
Scaled Intensity
1.5
1.0
0.5
0.0
HC-C/EBP Beta Tg
AD-C/EBP Beta Tg

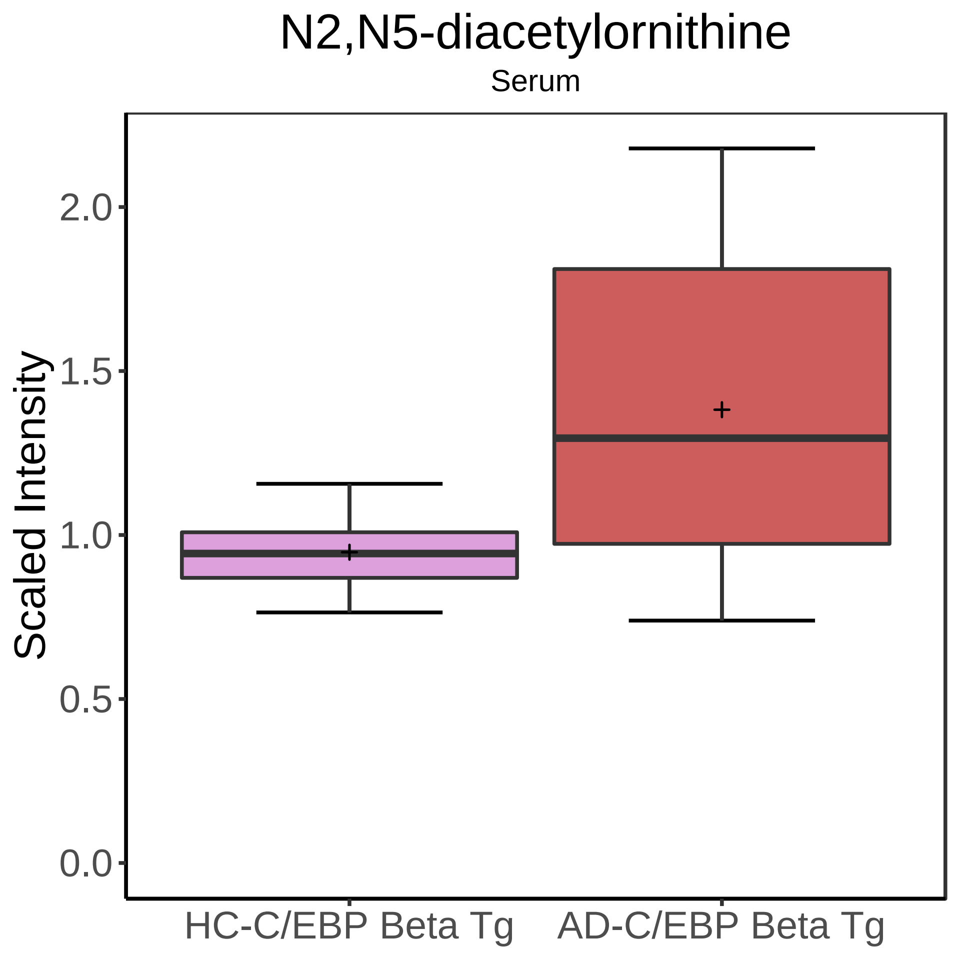
N2,N5-diacetylornithine
Serum
Scaled Intensity
2.0
1.5
1.0
0.5
0.0
HC-C/EBP Beta Tg
AD-C/EBP Beta Tg

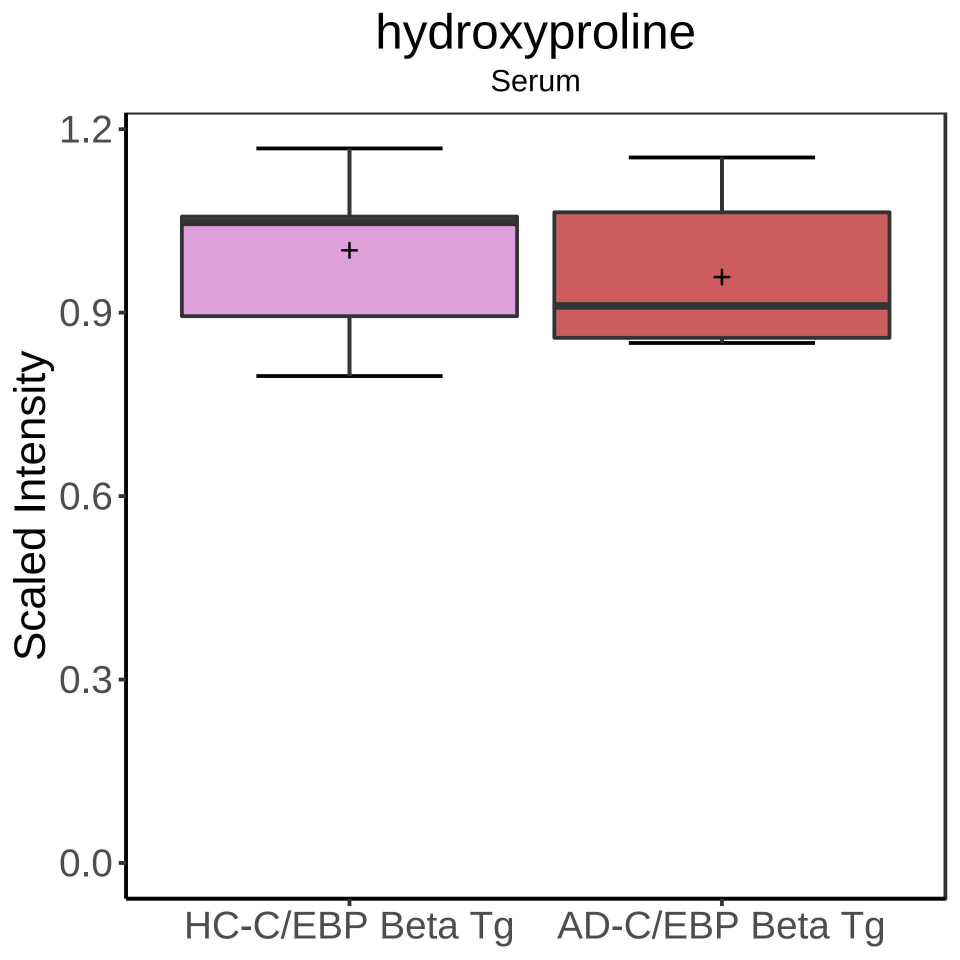
hydroxyproline
Serum
Scaled Intensity
1.2
0.9
0.6
0.3
0.0
HC-C/EBP Beta Tg
AD-C/EBP Beta Tg

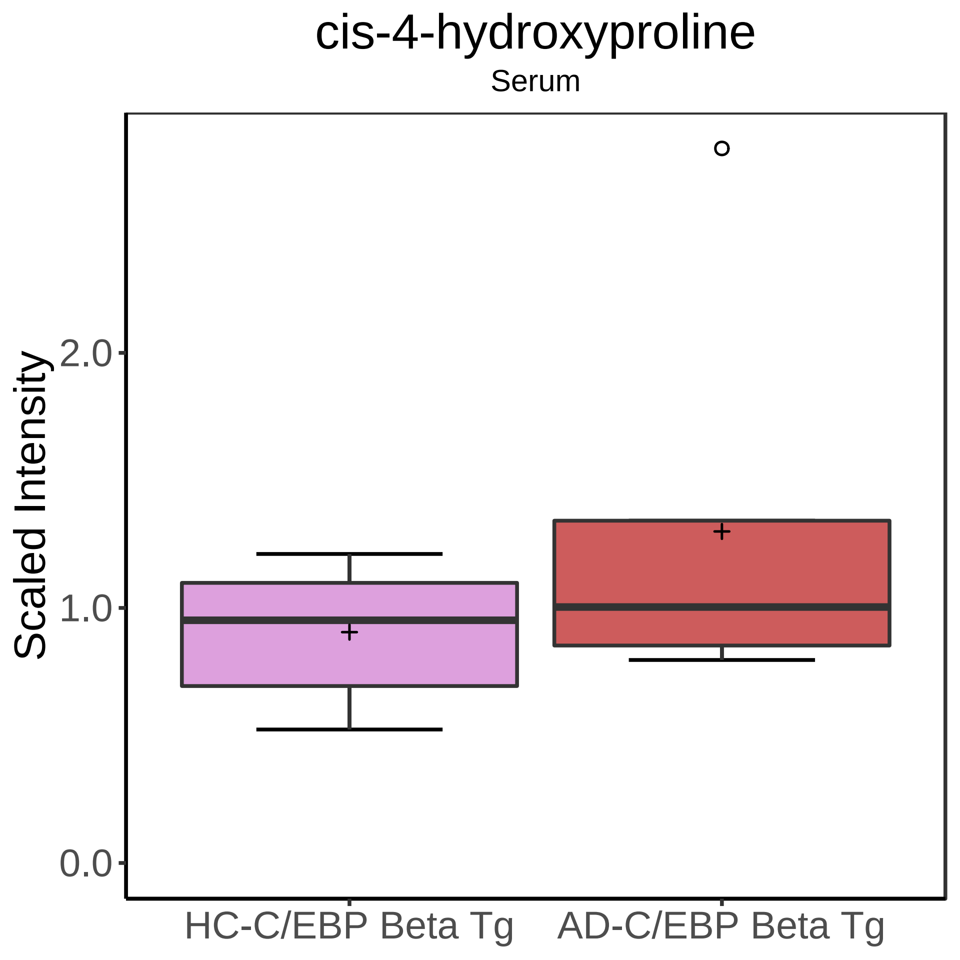
cis-4-hydroxyproline
Serum
Scaled Intensity
2.0
1.0
0.0
HC-C/EBP Beta Tg
AD-C/EBP Beta Tg

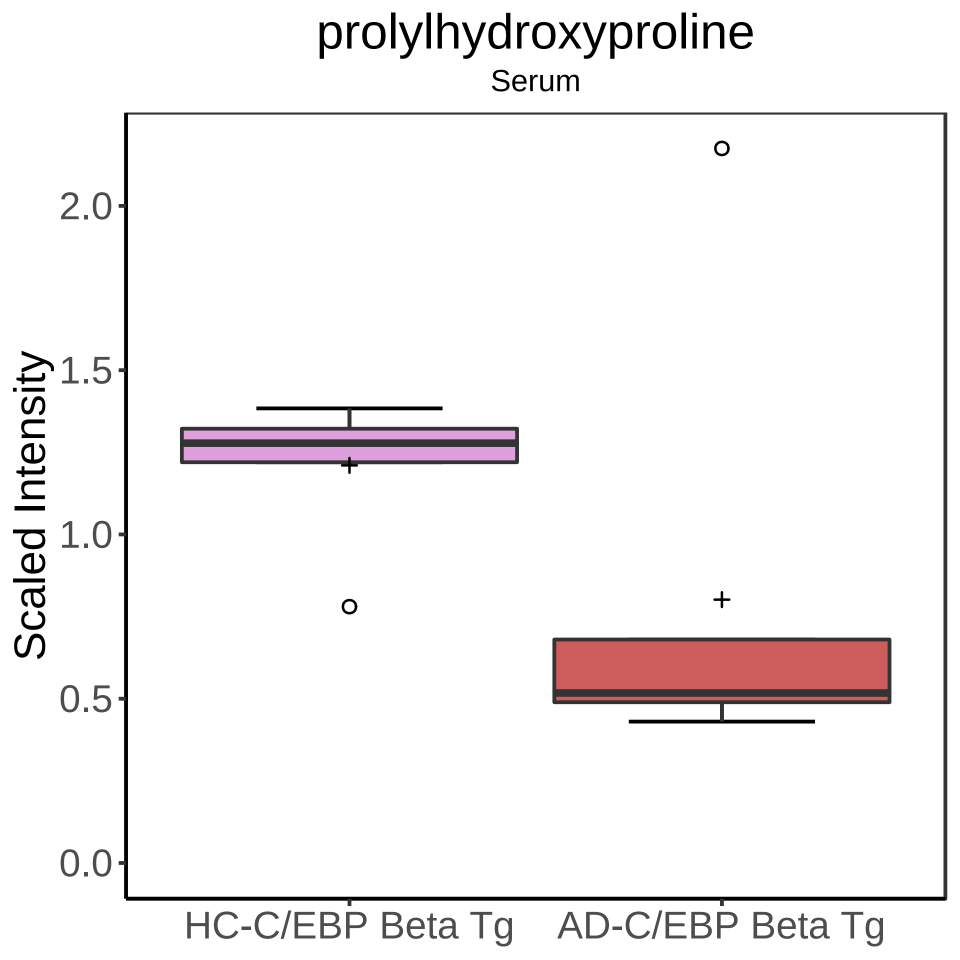

prolylhydroxyproline
Serum
Scaled Intensity
2.0
1.5
1.0
0.5
0.0
HC-C/EBP Beta Tg
AD-C/EBP Beta Tg

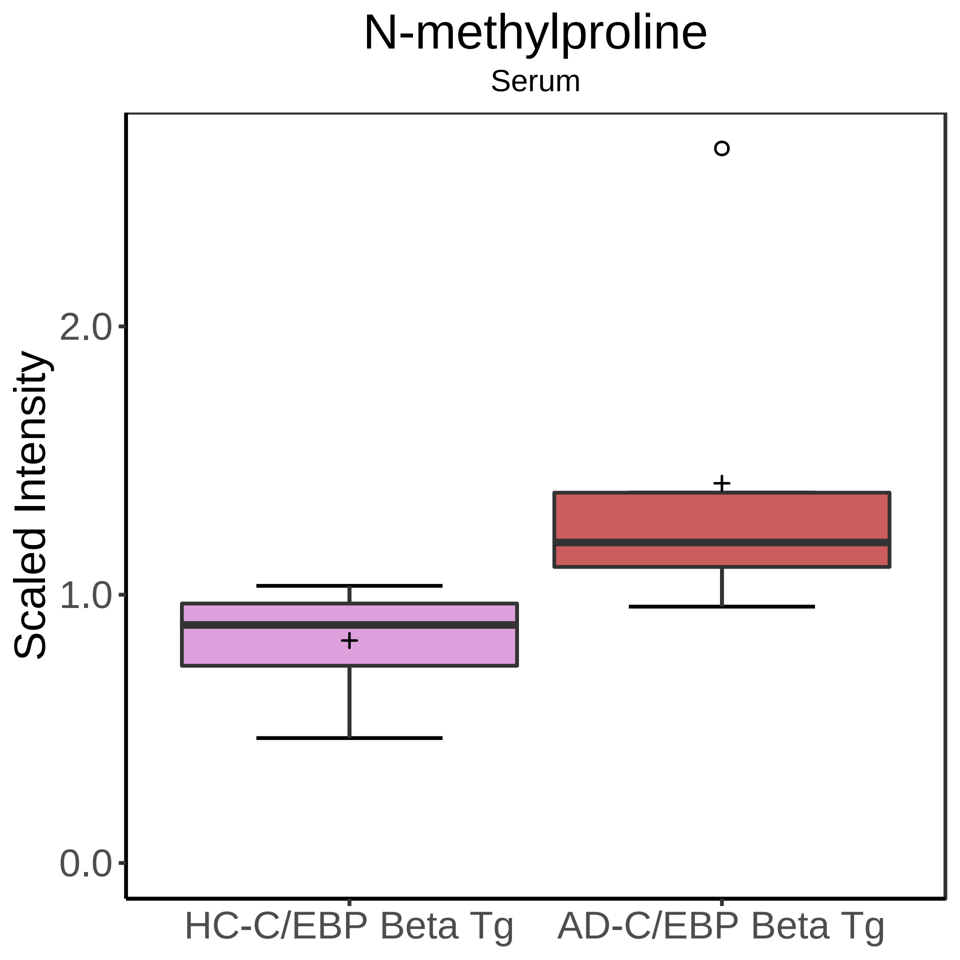
N-methylproline
Serum
Scaled Intensity
2.0
1.0
0.0
HC-C/EBP Beta Tg
AD-C/EBP Beta Tg

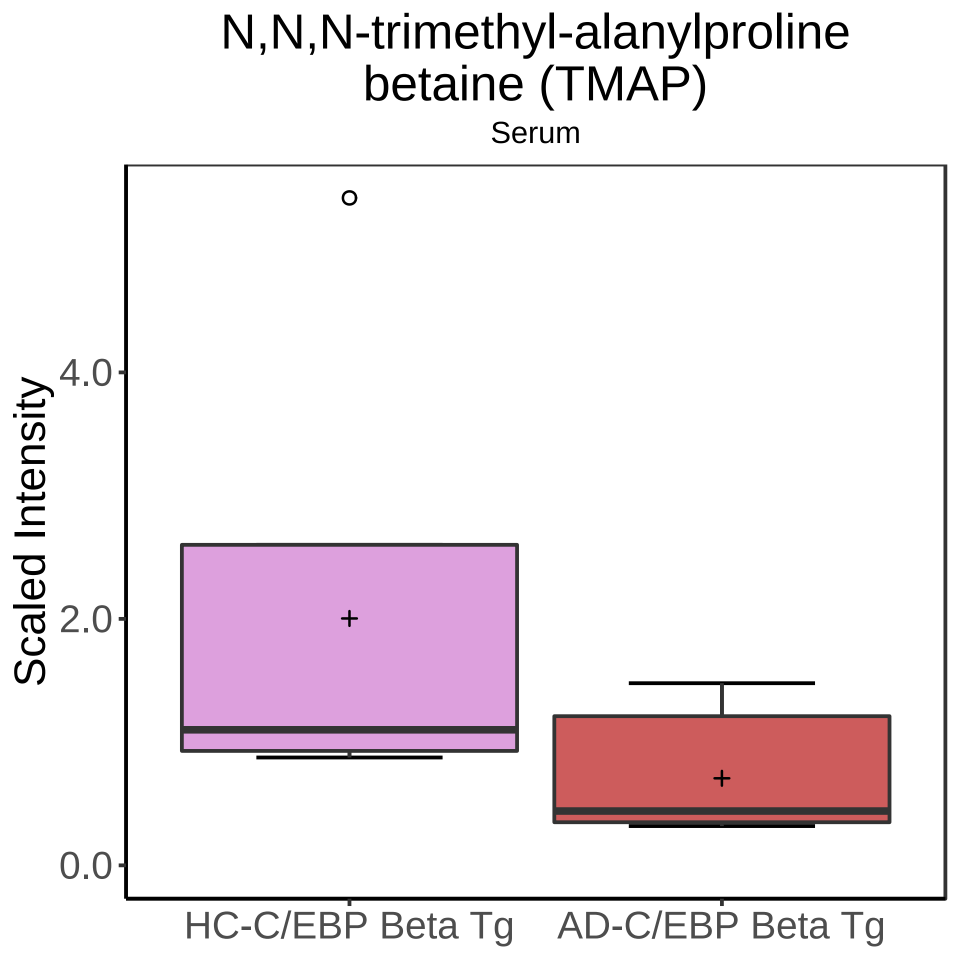
N,N,N-trimethyl-alanylproline
betaine (TMAP)
Serum
Scaled Intensity
4.0
2.0
0.0
HC-C/EBP Beta Tg
AD-C/EBP Beta Tg

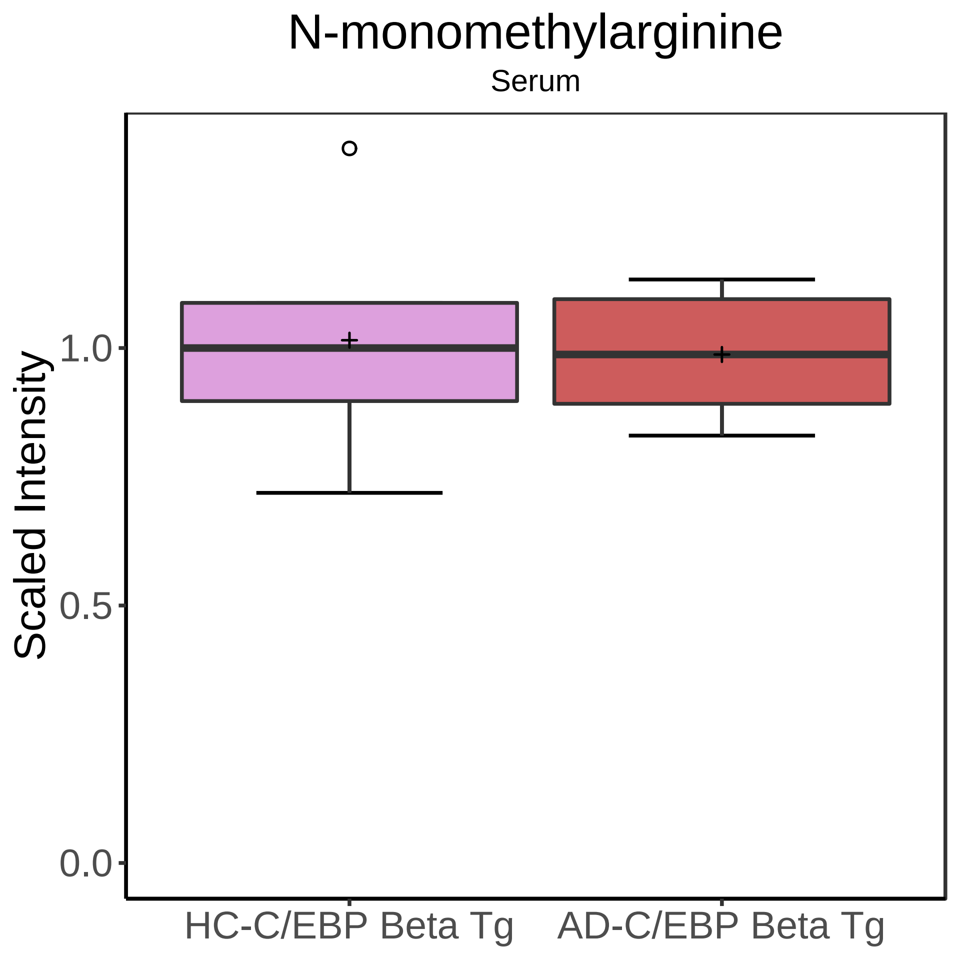

N-monomethylarginine
Serum
Scaled Intensity
1.0
0.5
0.0
HC-C/EBP Beta Tg
AD-C/EBP Beta Tg

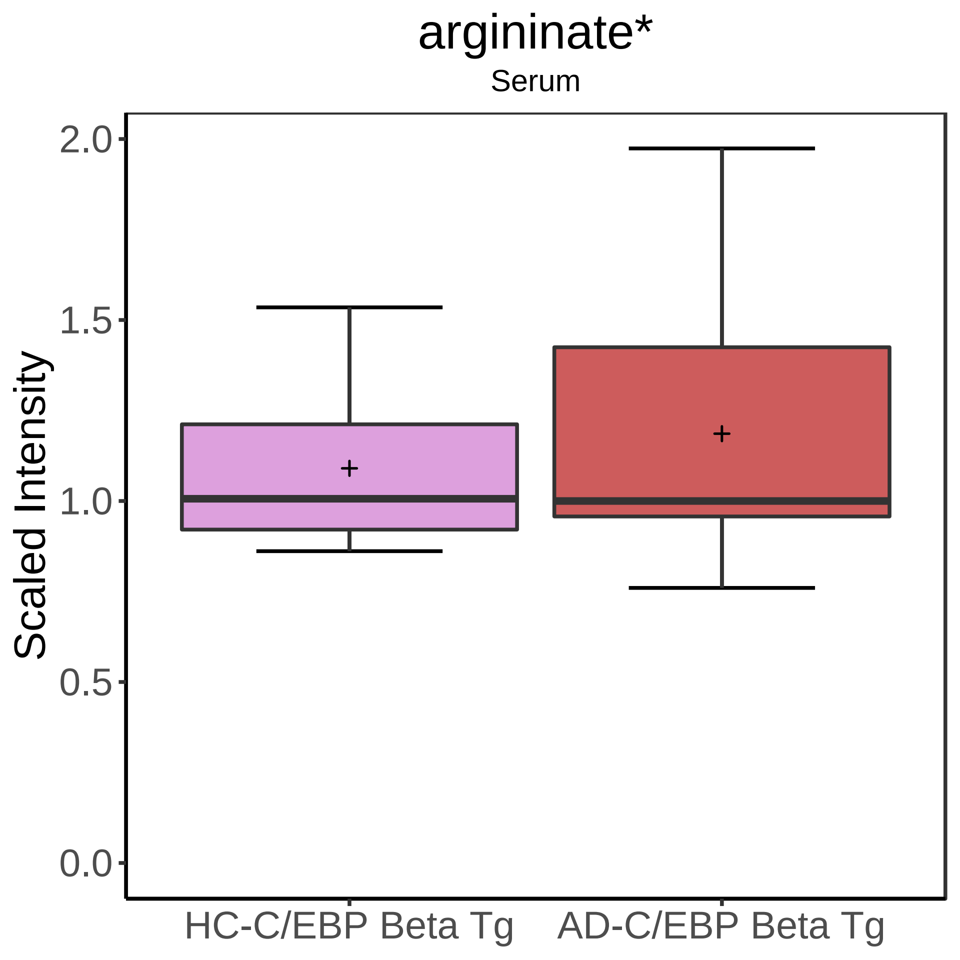
arginiate*
Serum
Scaled Intensity
2.0
1.5
1.0
0.5
0.0
HC-C/EBP Beta Tg
AD-C/EBP Beta Tg

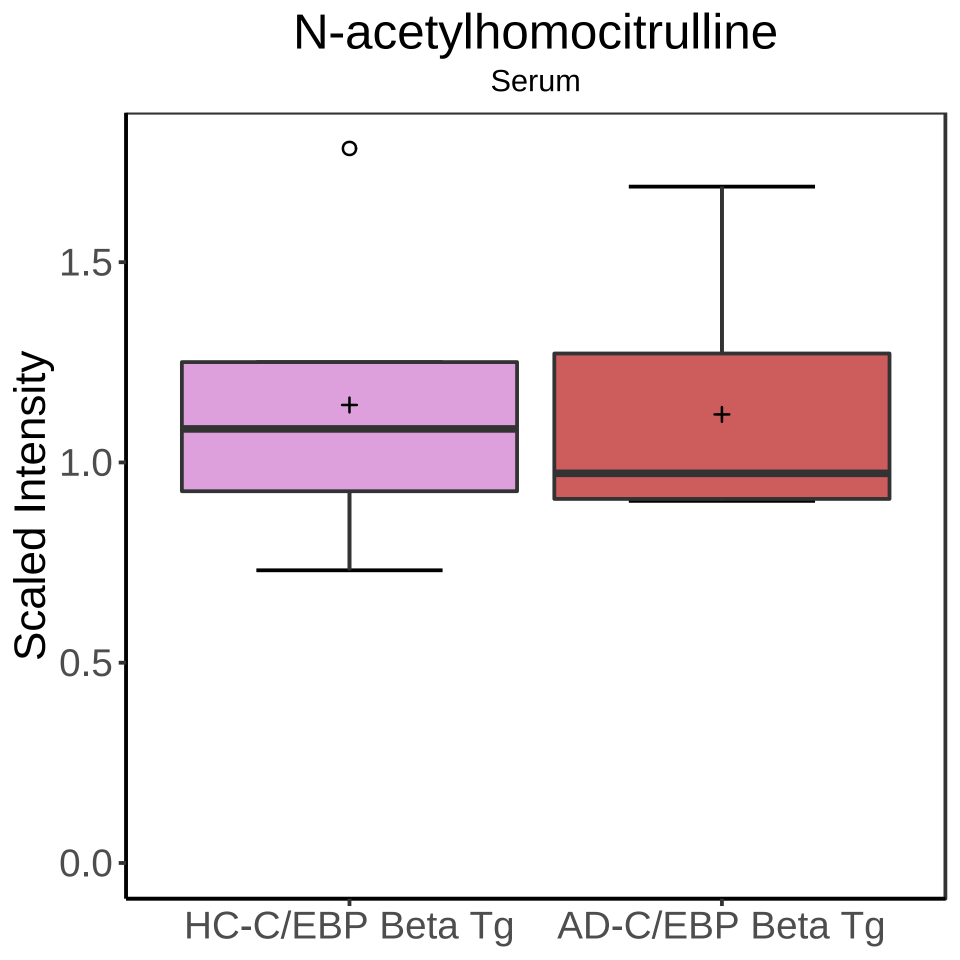
N-acetylhomocitrulline
Serum
Scaled Intensity
0.0
0.5
1.0
1.5
HC-C/EBP Beta Tg
AD-C/EBP Beta Tg

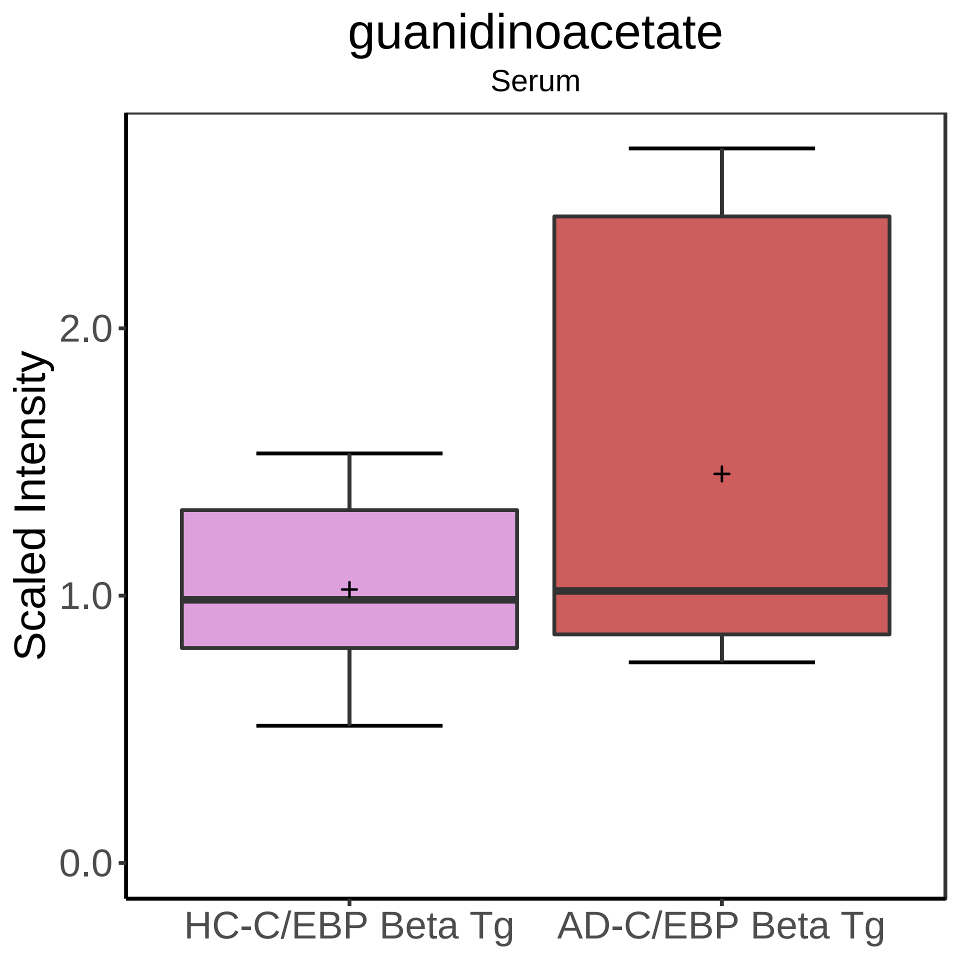

guanidinoacetate
Serum
Scaled Intensity
2.0
1.0
0.0
HC-C/EBP Beta Tg
AD-C/EBP Beta Tg

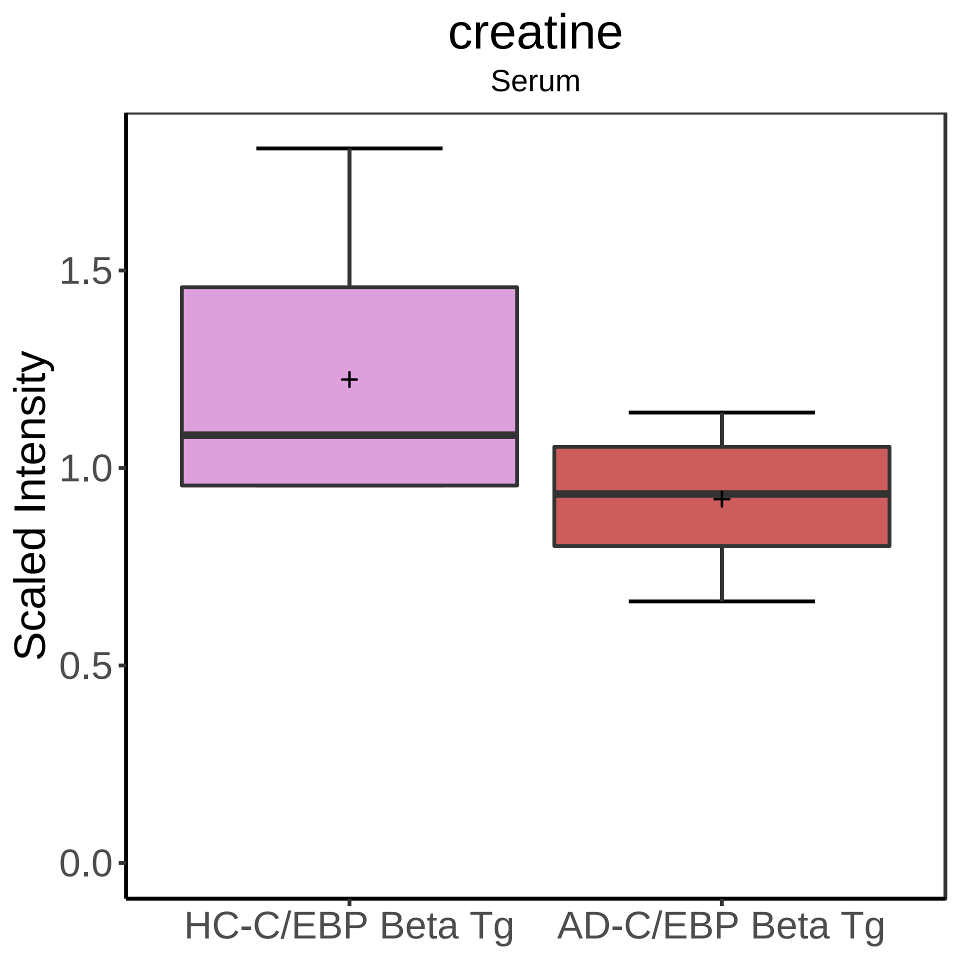
creatine
Serum
Scaled Intensity
1.5
1.0
0.5
0.0
HC-C/EBP Beta Tg
AD-C/EBP Beta Tg

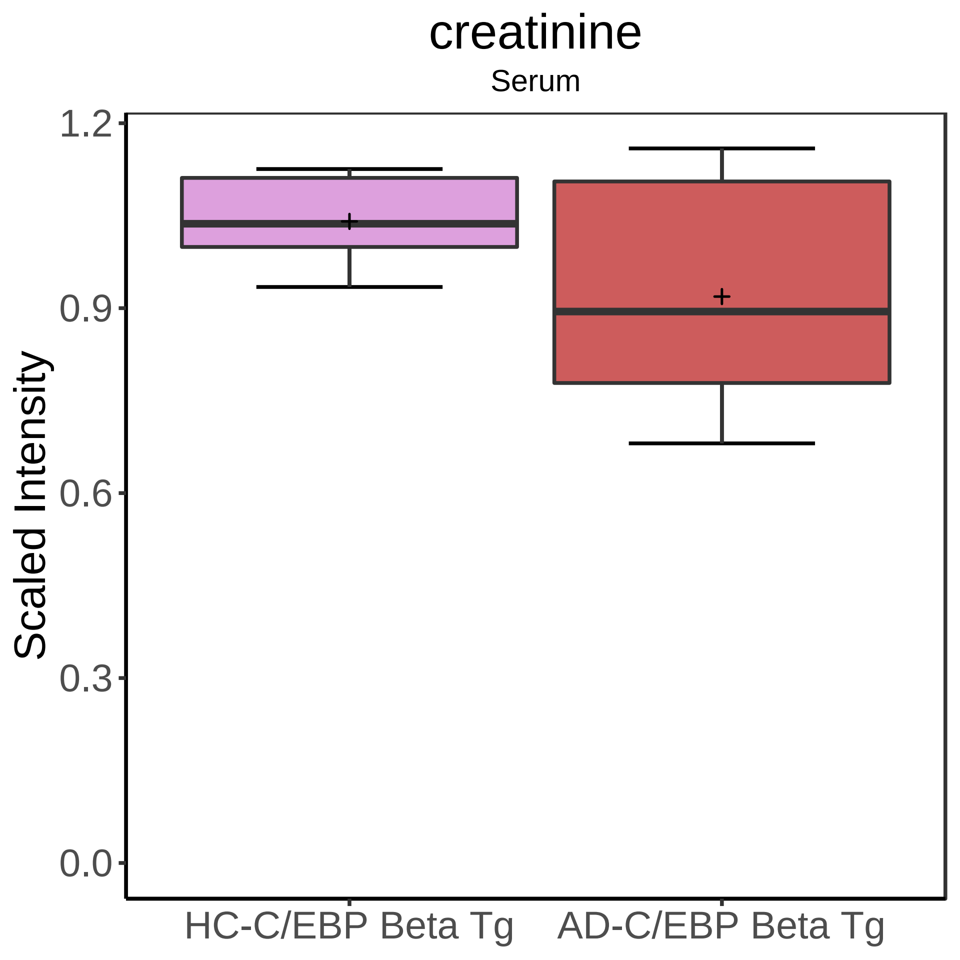
creatinine
Serum
Scaled Intensity
1.2
0.9
0.6
0.3
0.0
HC-C/EBP Beta Tg
AD-C/EBP Beta Tg

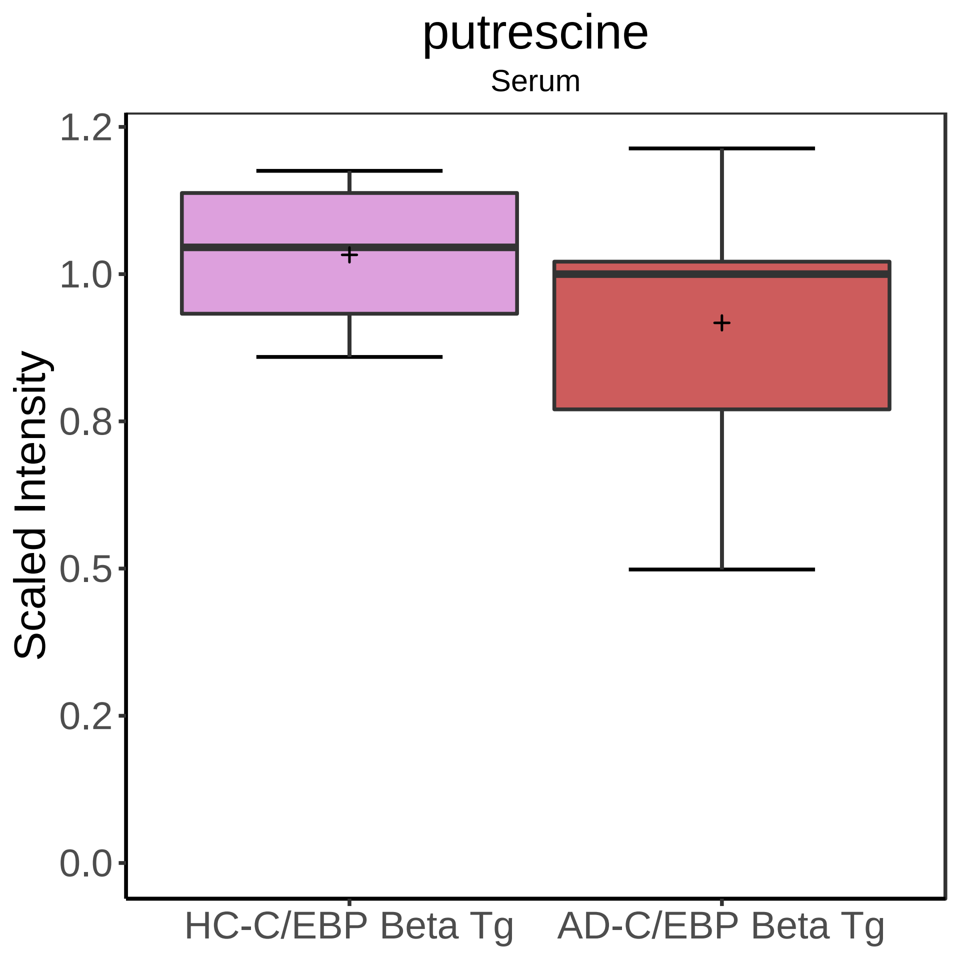

putrescine
Serum
Scaled Intensity
1.2
1.0
0.8
0.5
0.2
0.0
HC-C/EBP Beta Tg
AD-C/EBP Beta Tg

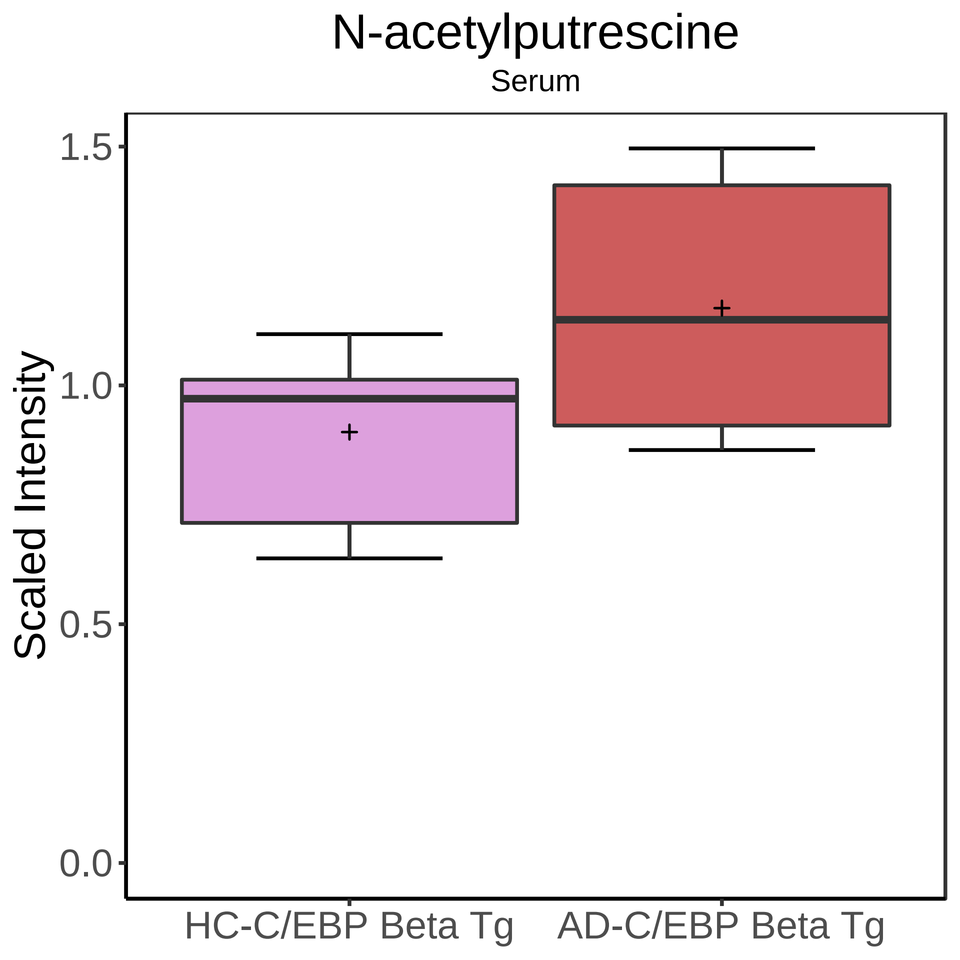
N-acetylputrescine
Serum
Scaled Intensity
1.5
1.0
0.5
0.0
HC-C/EBP Beta Tg
AD-C/EBP Beta Tg

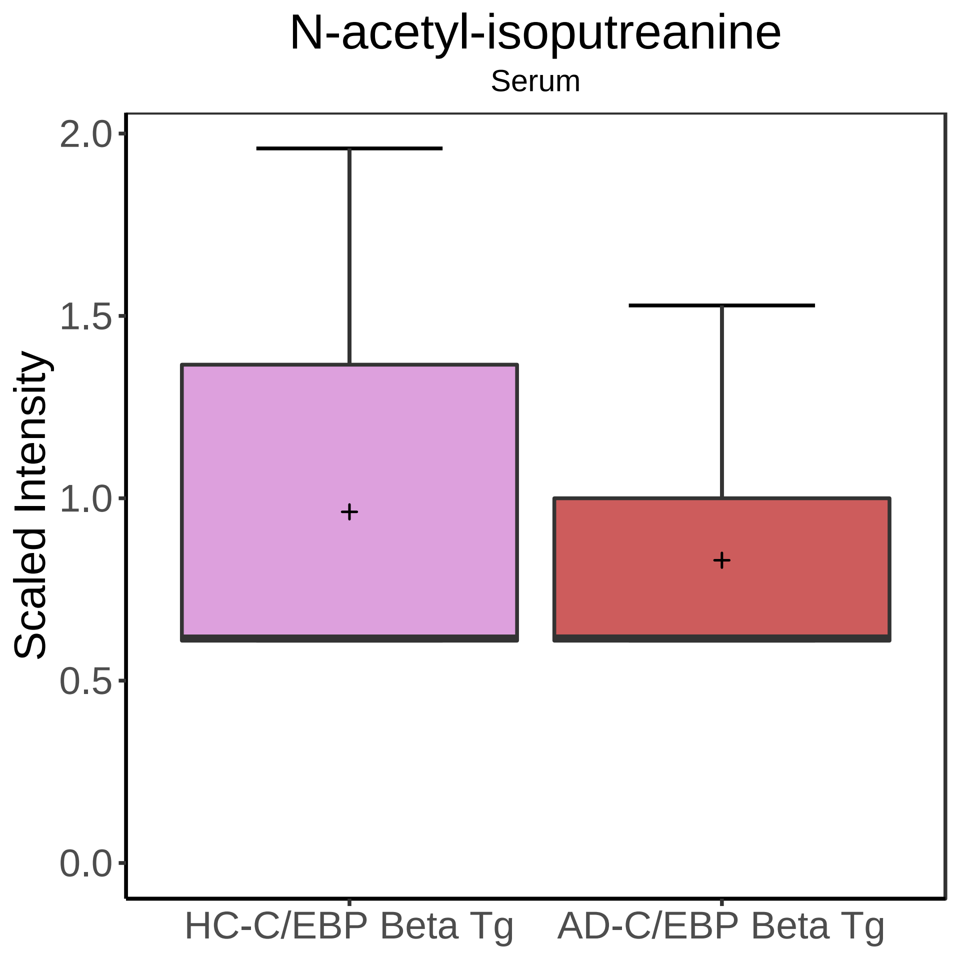
N-acetyl-isoputreanine
Serum
Scaled Intensity
2.0
1.5
1.0
0.5
0.0
HC-C/EBP Beta Tg
AD-C/EBP Beta Tg

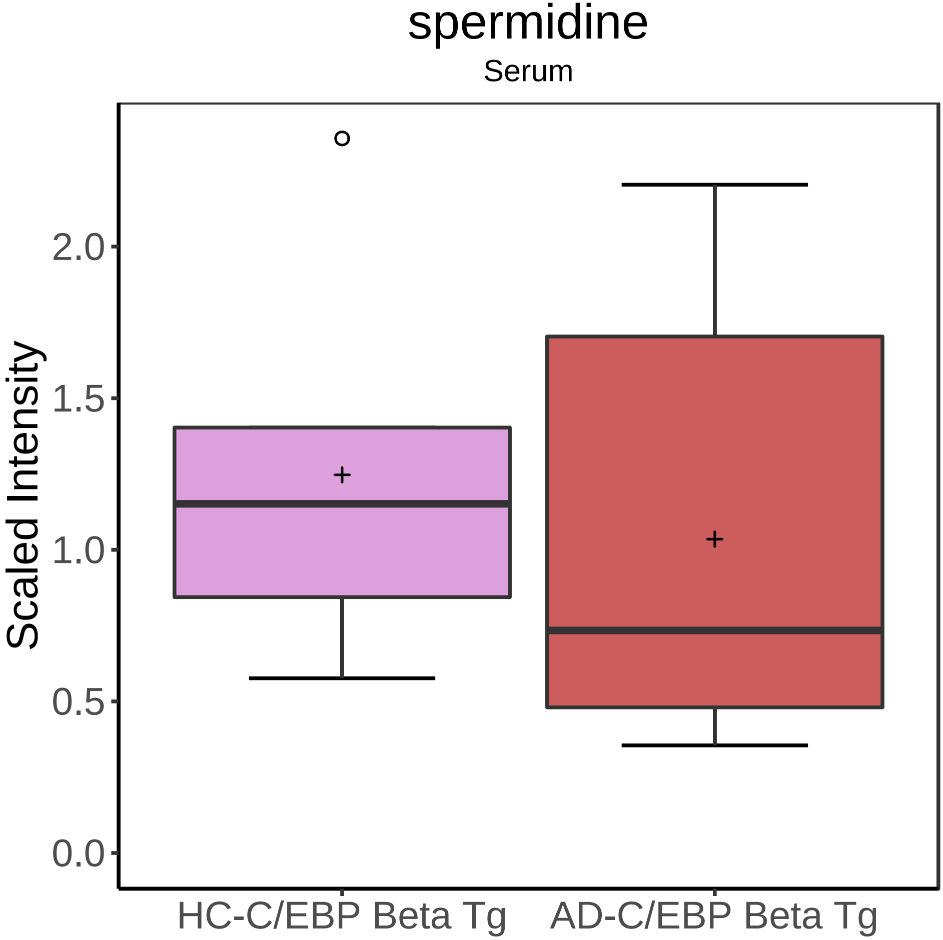
spermidine
Serum
Scaled Intensity
0.0
0.5
1.0
1.5
2.0
HC-C/EBP Beta Tg
AD-C/EBP Beta Tg

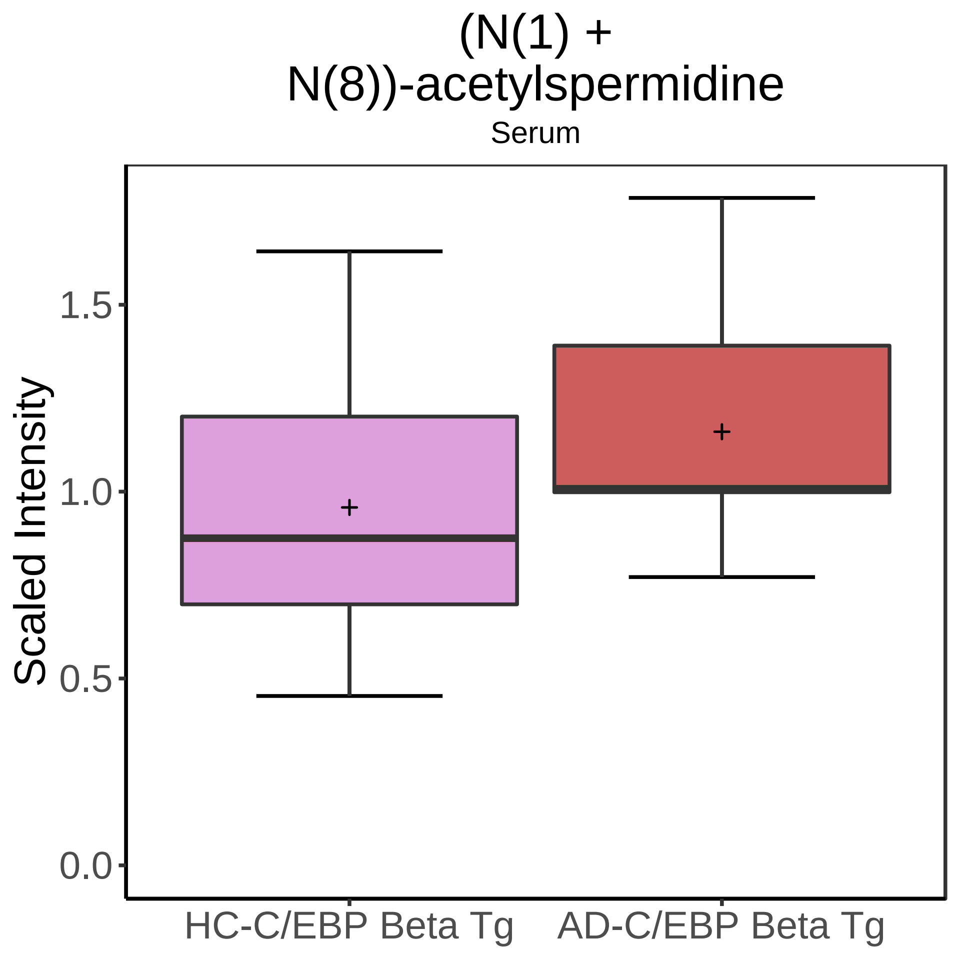
(N(1) +
N(8))-acetylspermidine
Serum
Scaled Intensity
0.0
0.5
1.0
1.5
HC-C/EBP Beta Tg
AD-C/EBP Beta Tg

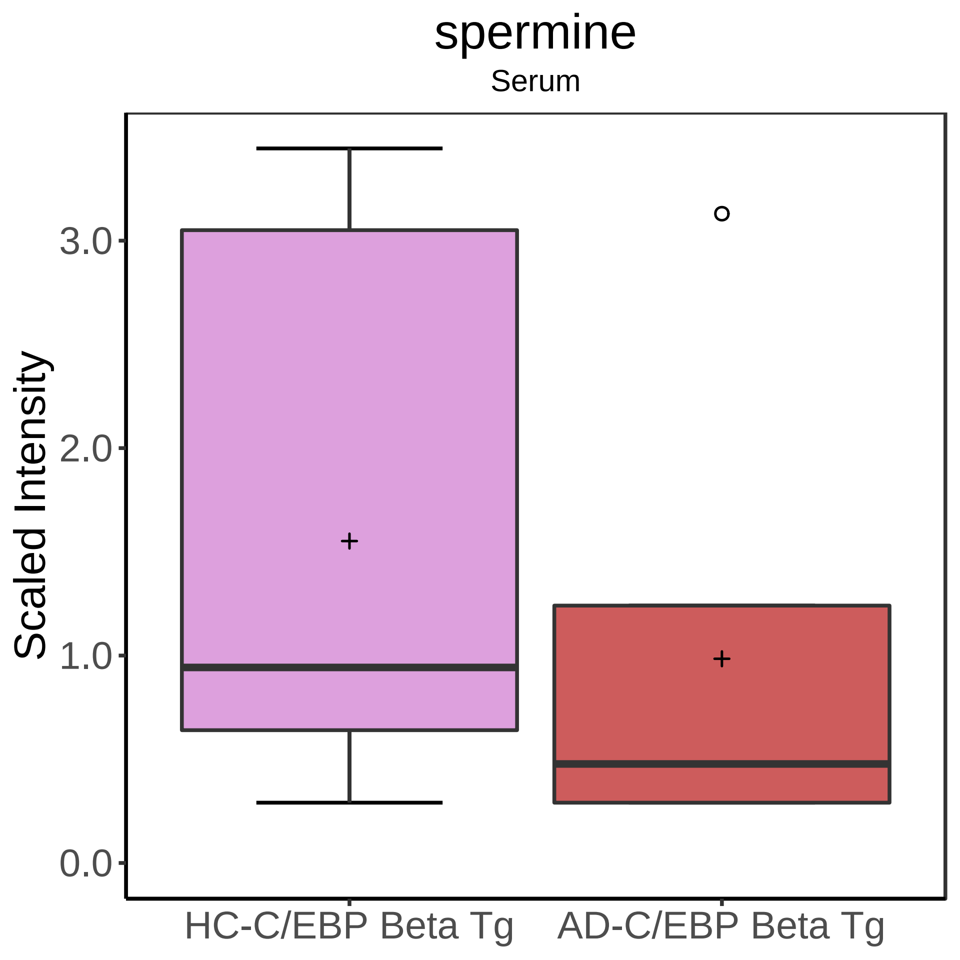
spermine
Serum
Scaled Intensity
3.0
2.0
1.0
0.0
HC-C/EBP Beta Tg
AD-C/EBP Beta Tg

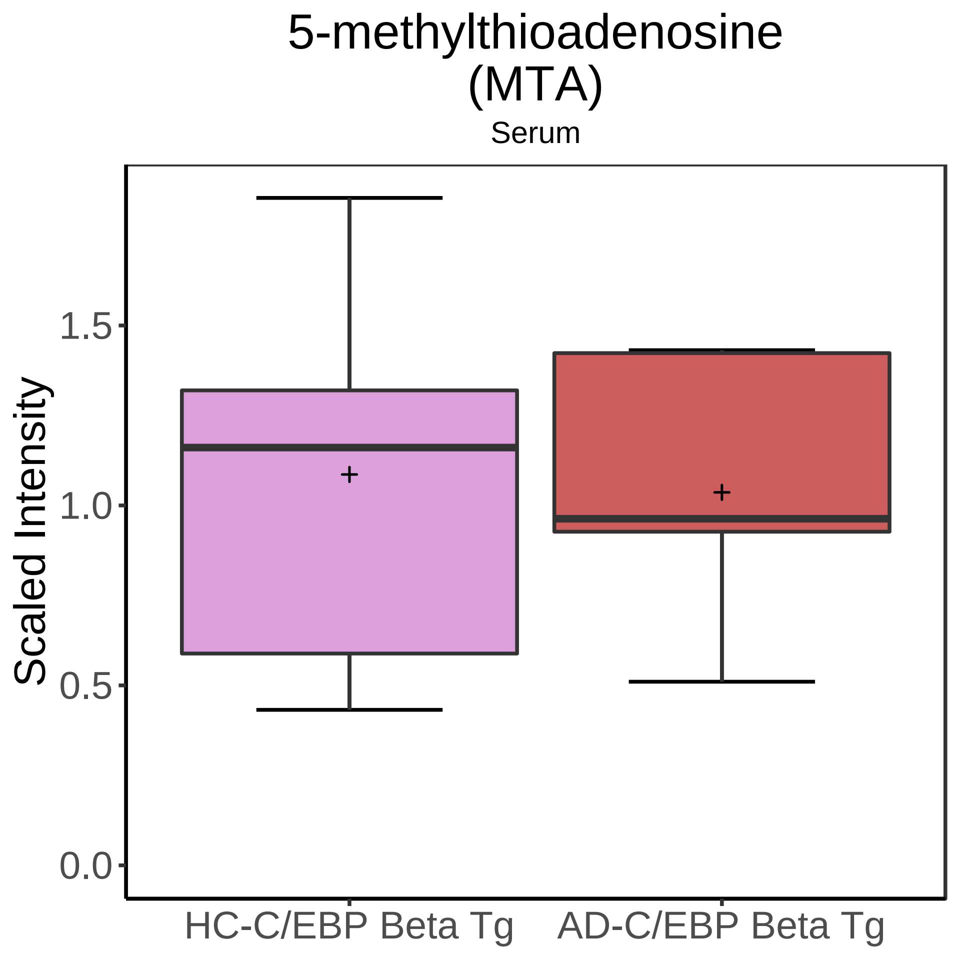
5-methylthioadenosine
(MTA)
Serum
Scaled Intensity
1.5
1.0
0.5
0.0
HC-C/EBP Beta Tg
AD-C/EBP Beta Tg

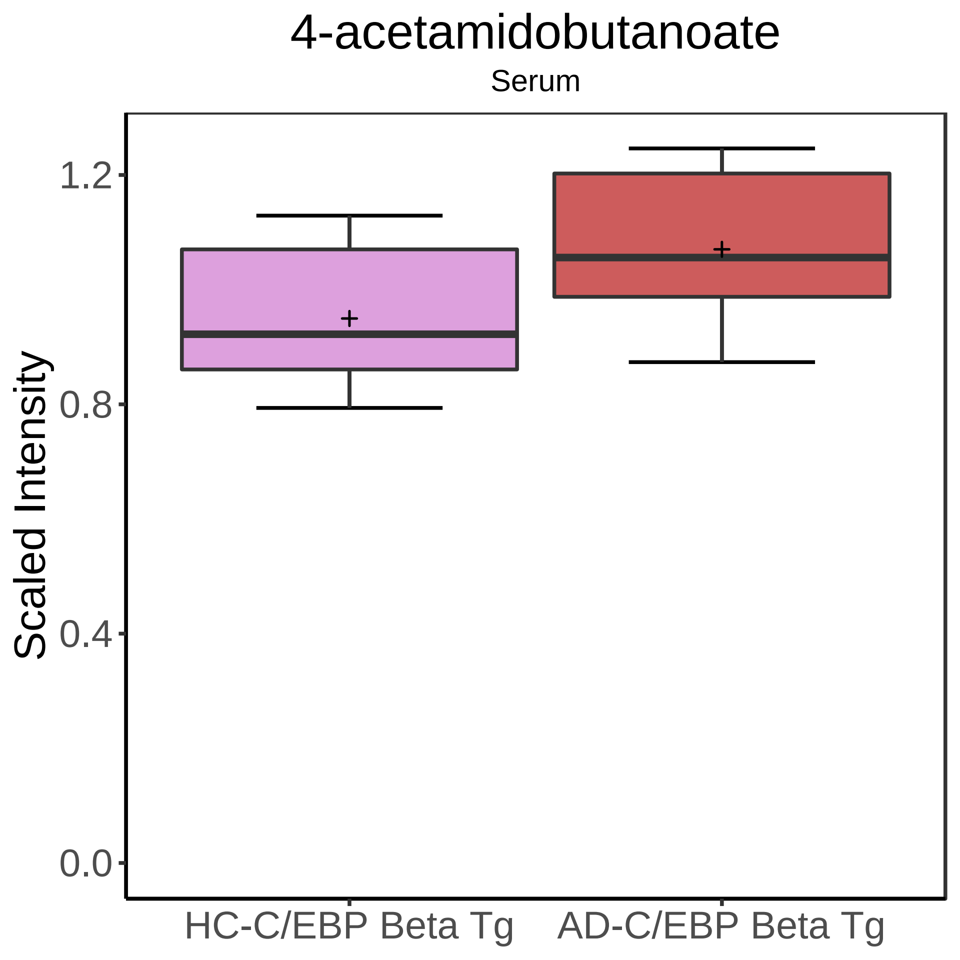

4-acetamidobutanoate
Serum
Scaled Intensity
1.2
0.8
0.4
0.0
HC-C/EBP Beta Tg
AD-C/EBP Beta Tg

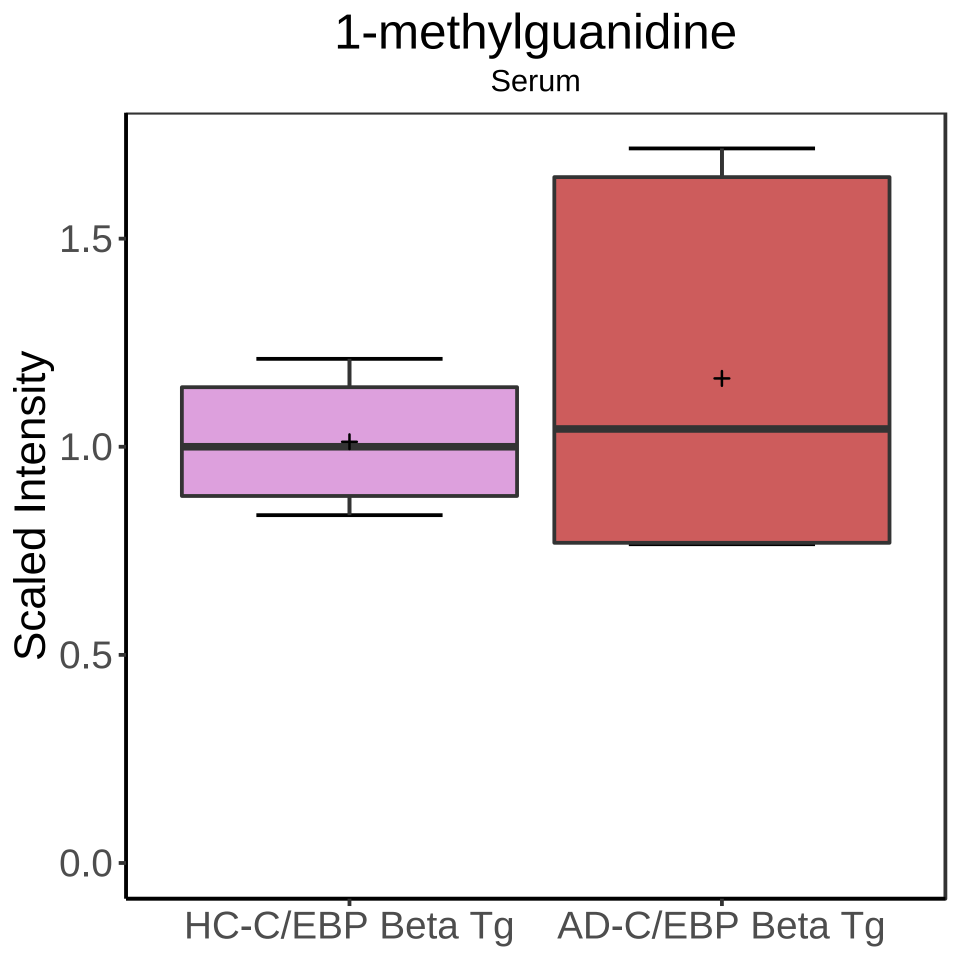
1-methylguanidine
Serum
Scaled Intensity
1.5
1.0
0.5
0.0
HC-C/EBP Beta Tg
AD-C/EBP Beta Tg

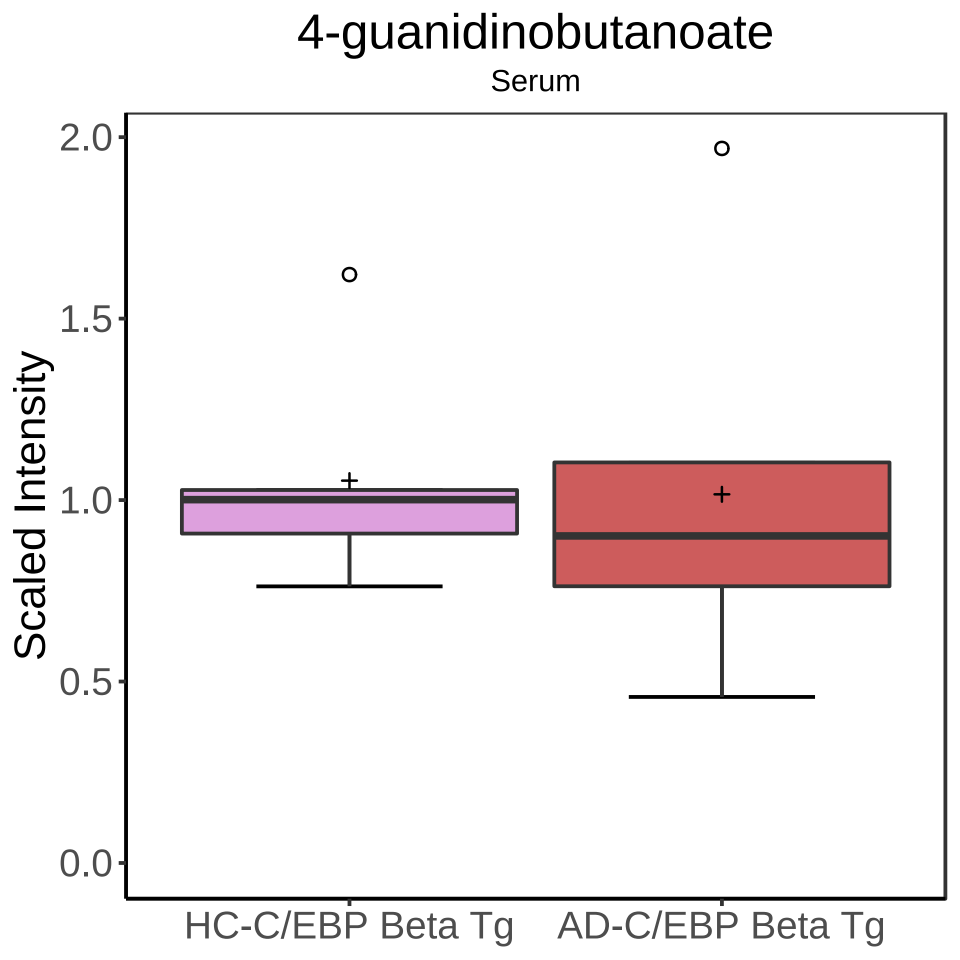
4-guanidinobutanoate
Serum
Scaled Intensity
2.0
1.5
1.0
0.5
0.0
HC-C/EBP Beta Tg
AD-C/EBP Beta Tg

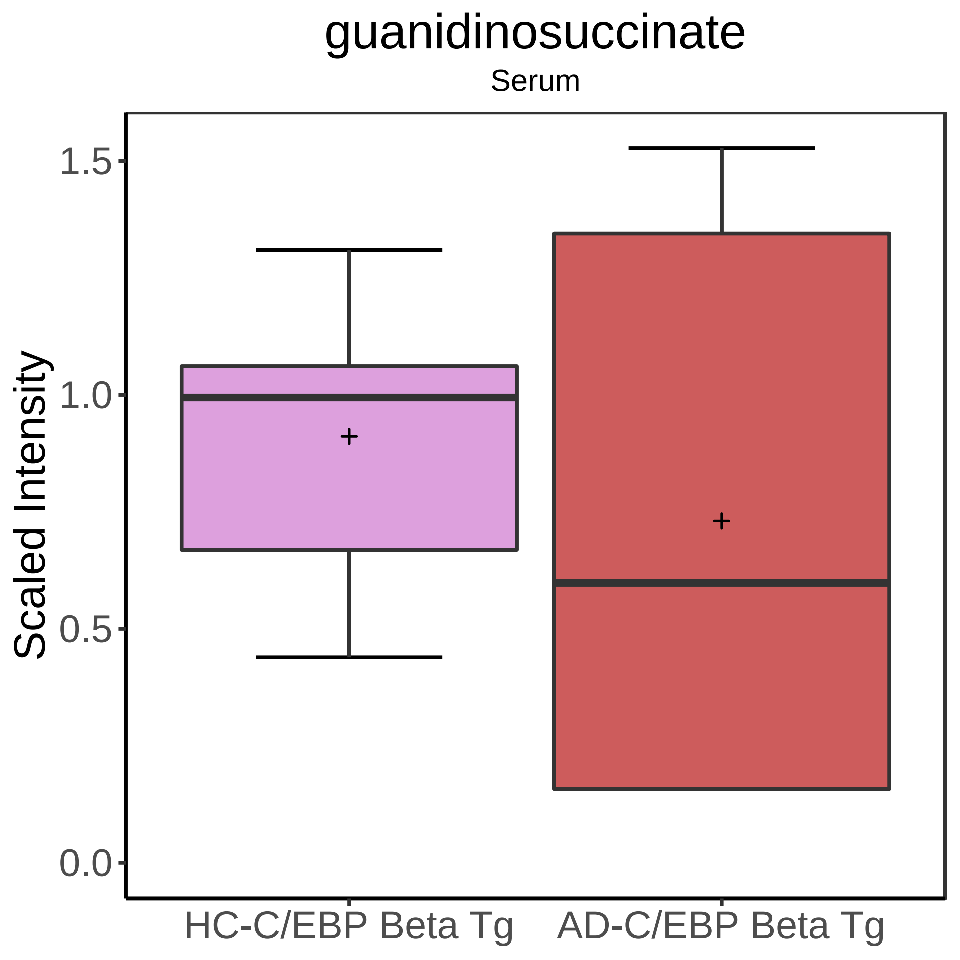
guanidinosuccinate
Serum
Scaled Intensity
1.5
1.0
0.5
0.0
HC-C/EBP Beta Tg
AD-C/EBP Beta Tg

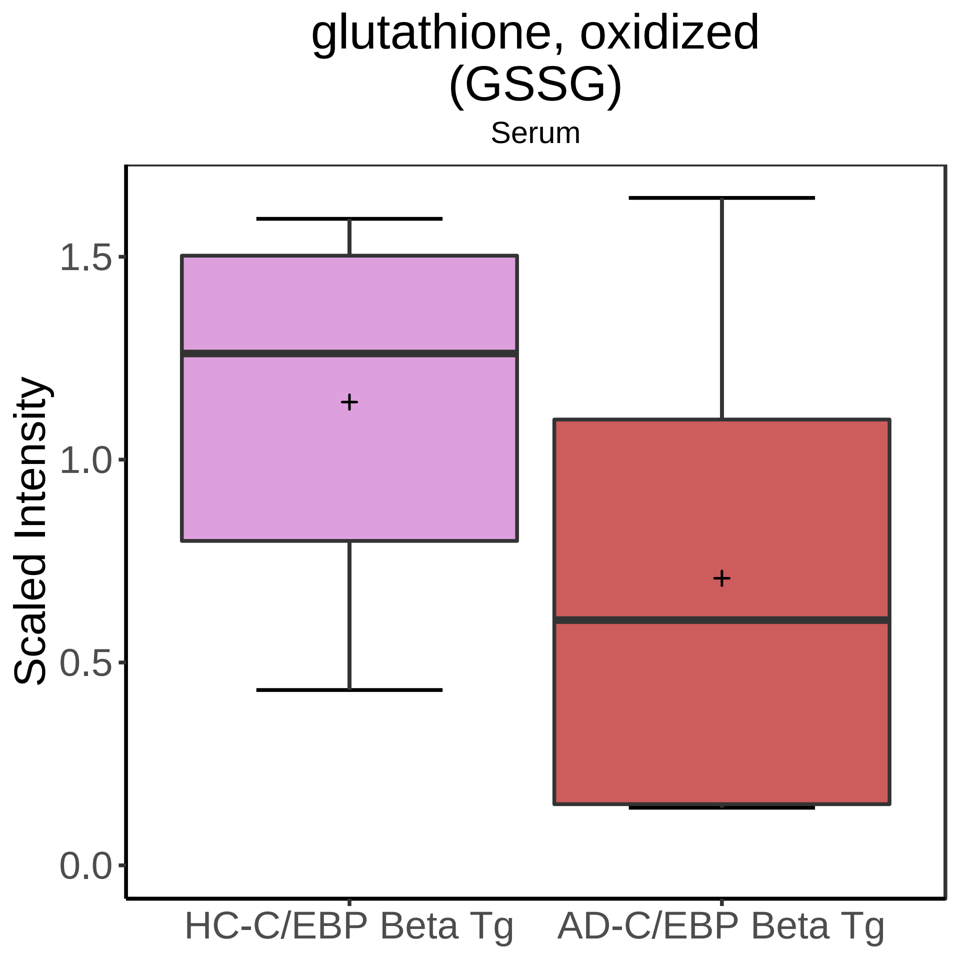

glutathione, oxidized
(GSSG)
Serum
Scaled Intensity
1.5
1.0
0.5
0.0
HC-C/EBP Beta Tg
AD-C/EBP Beta Tg

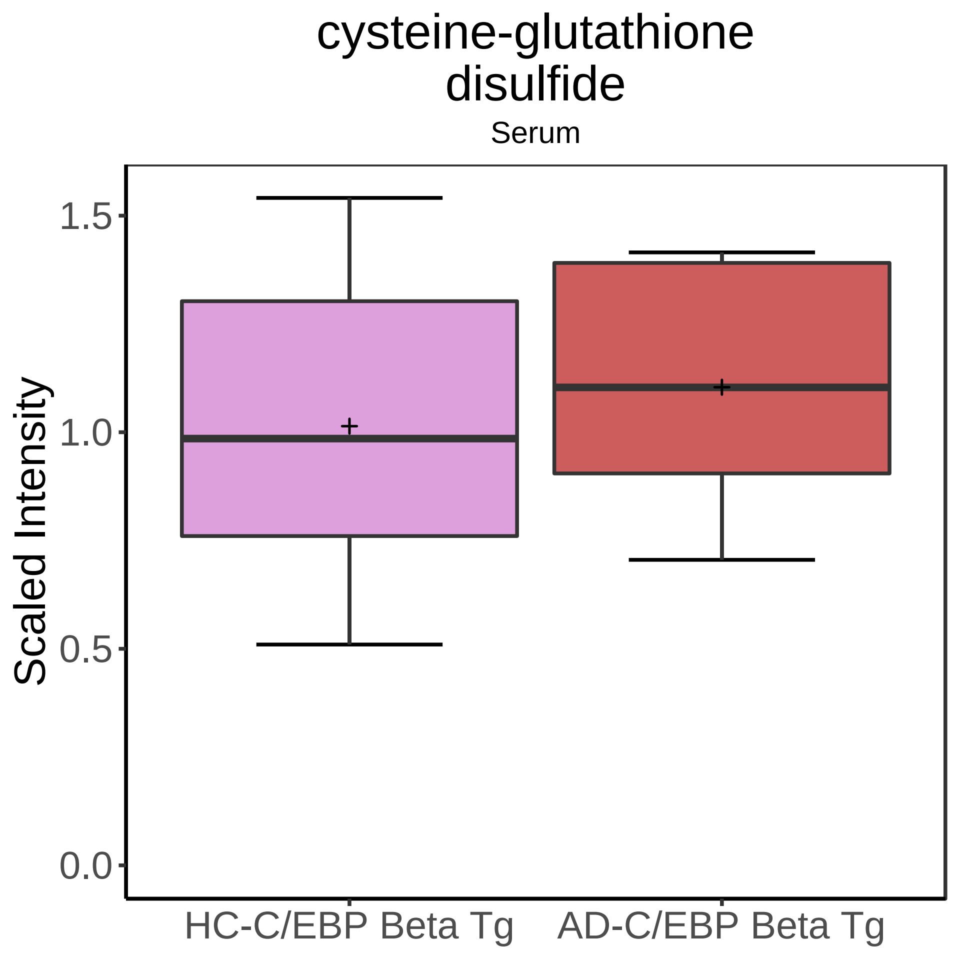
cysteine-glutathione
disulfide
Serum
Scaled Intensity
1.5
1.0
0.5
0.0
HC-C/EBP Beta Tg
AD-C/EBP Beta Tg

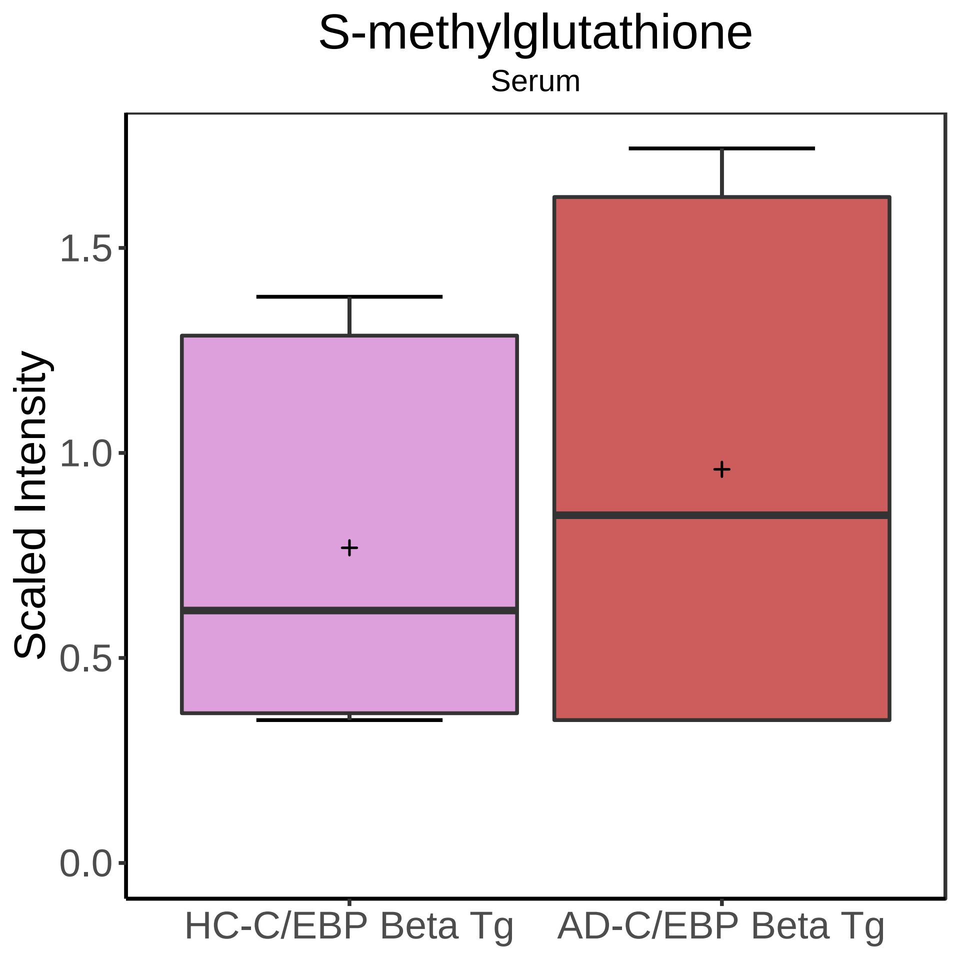
S-methylglutathione
Serum
Scaled Intensity
1.5
1.0
0.5
0.0
HC-C/EBP Beta Tg
AD-C/EBP Beta Tg

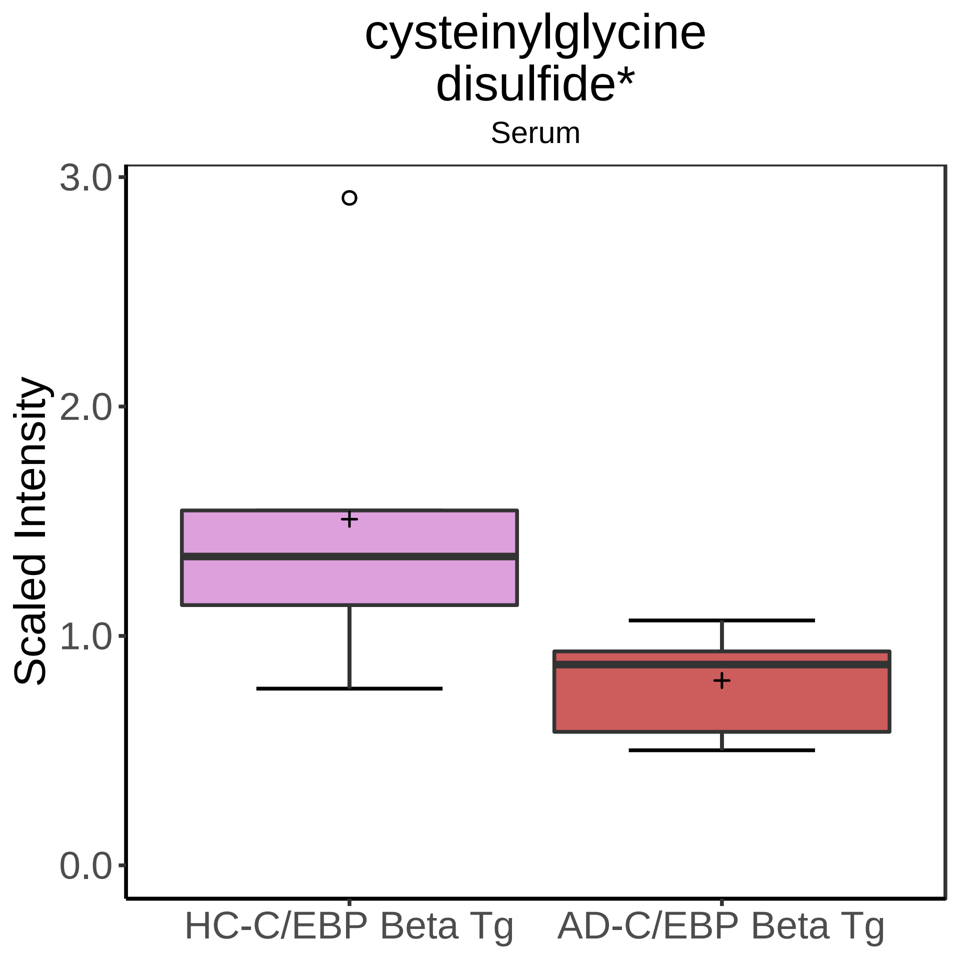
cysteinylglycine
disulfide*
Serum
Scaled Intensity
3.0
2.0
1.0
0.0
HC-C/EBP Beta Tg
AD-C/EBP Beta Tg

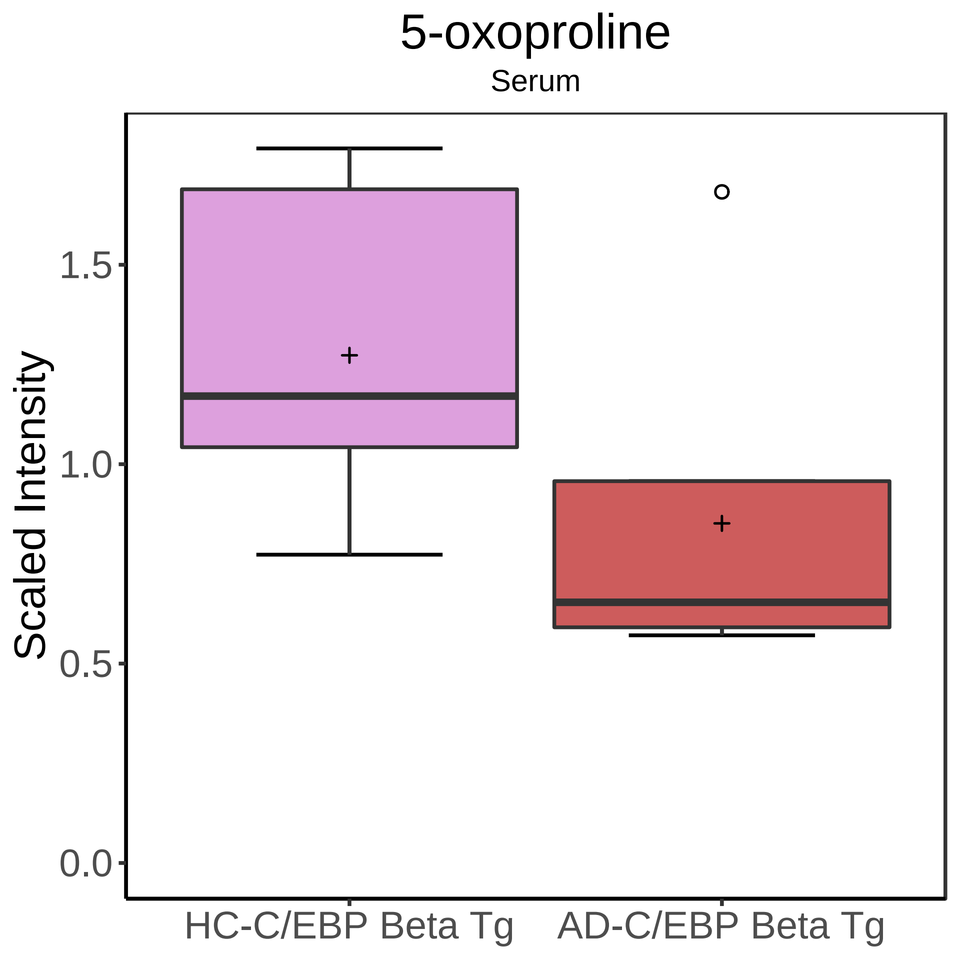

5-oxoproline
Serum
Scaled Intensity
0.0
0.5
1.0
1.5
HC-C/EBP Beta Tg
AD-C/EBP Beta Tg

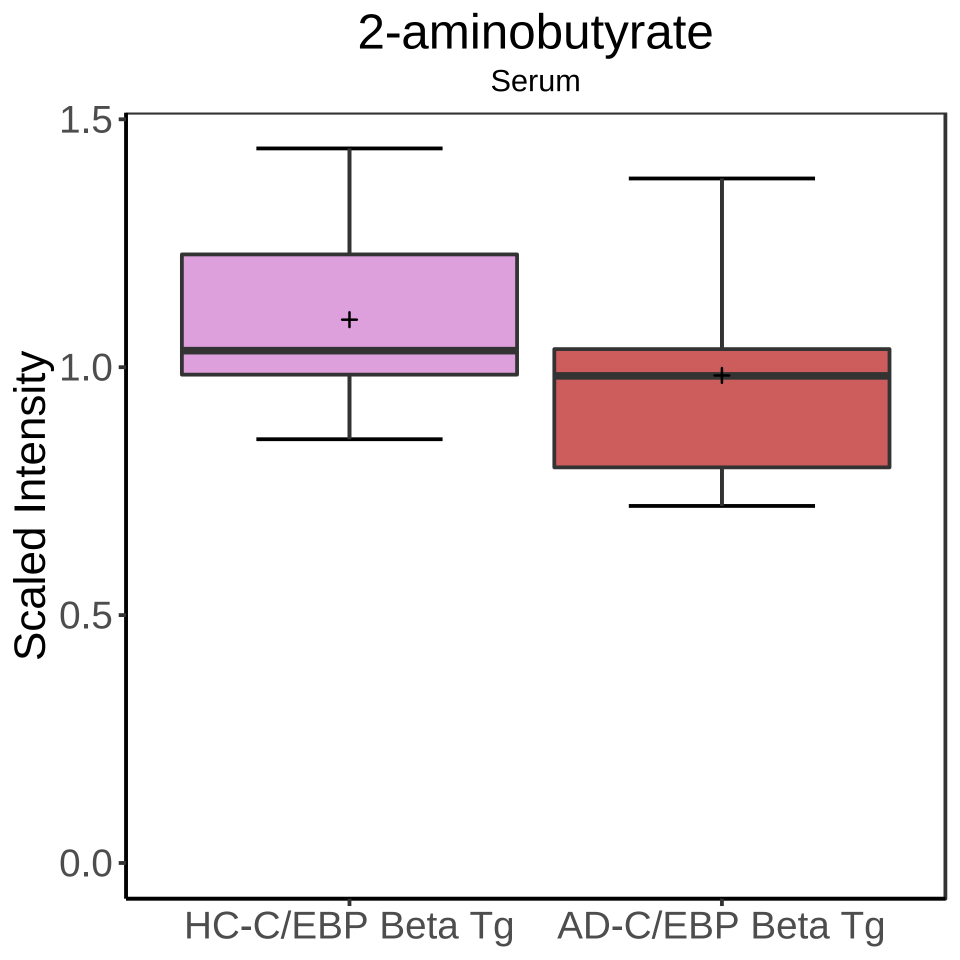

2-aminobutyrate
Serum
Scaled Intensity
1.5
1.0
0.5
0.0
HC-C/EBP Beta Tg
AD-C/EBP Beta Tg

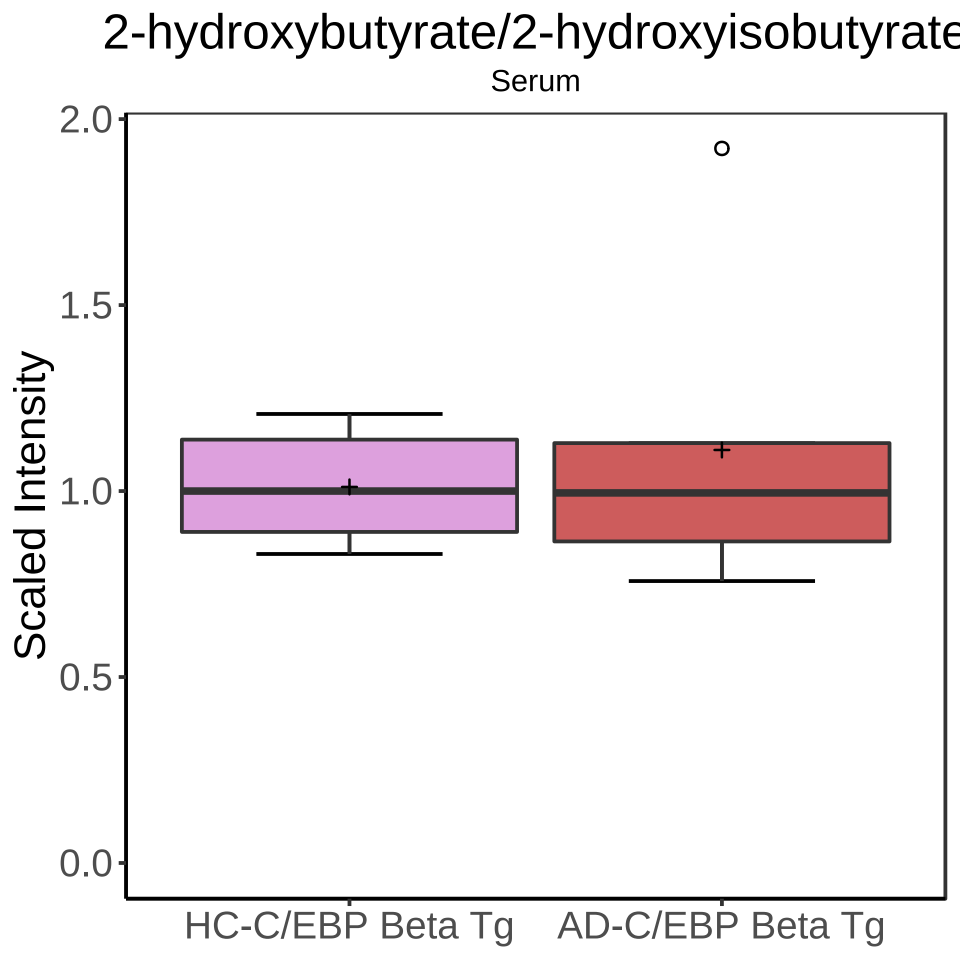
2-hydroxybutyrate/2-hydroxyisobutyrate
Serum
Scaled Intensity
2.0
1.5
1.0
0.5
0.0
HC-C/EBP Beta Tg
AD-C/EBP Beta Tg

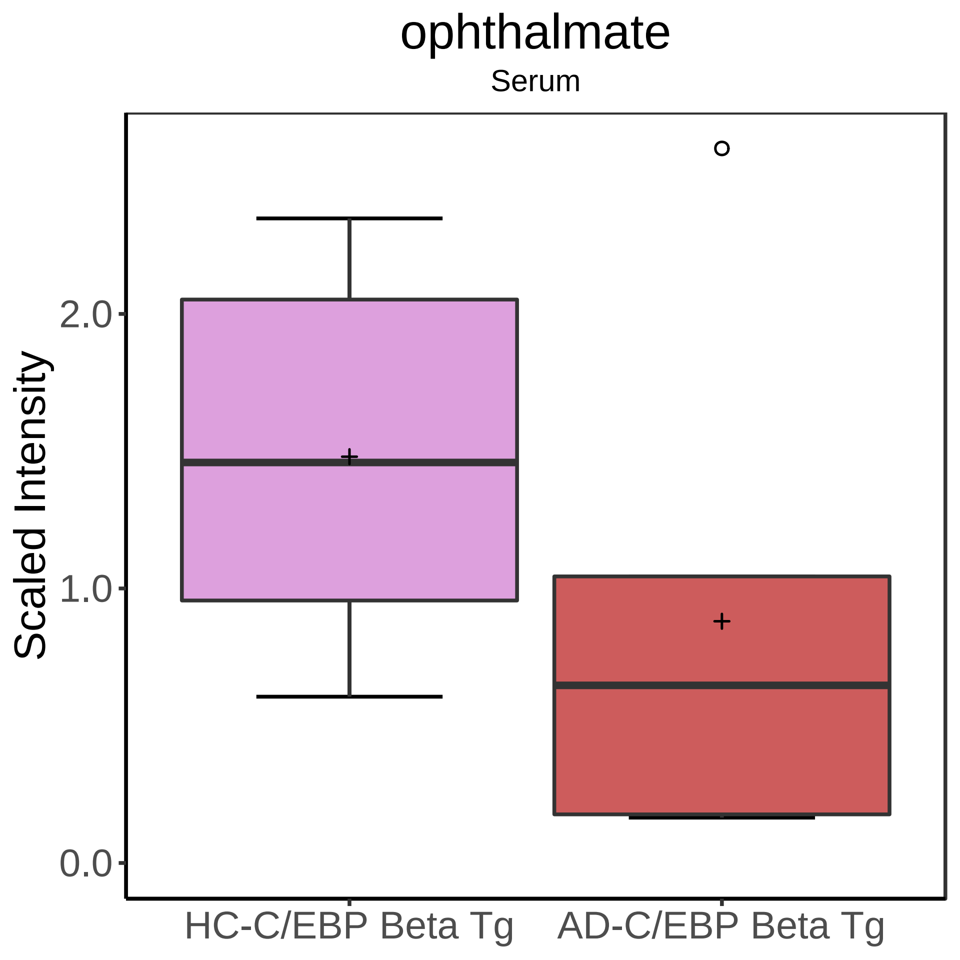
ophthalmate
Serum
Scaled Intensity
2.0
1.0
0.0
HC-C/EBP Beta Tg
AD-C/EBP Beta Tg

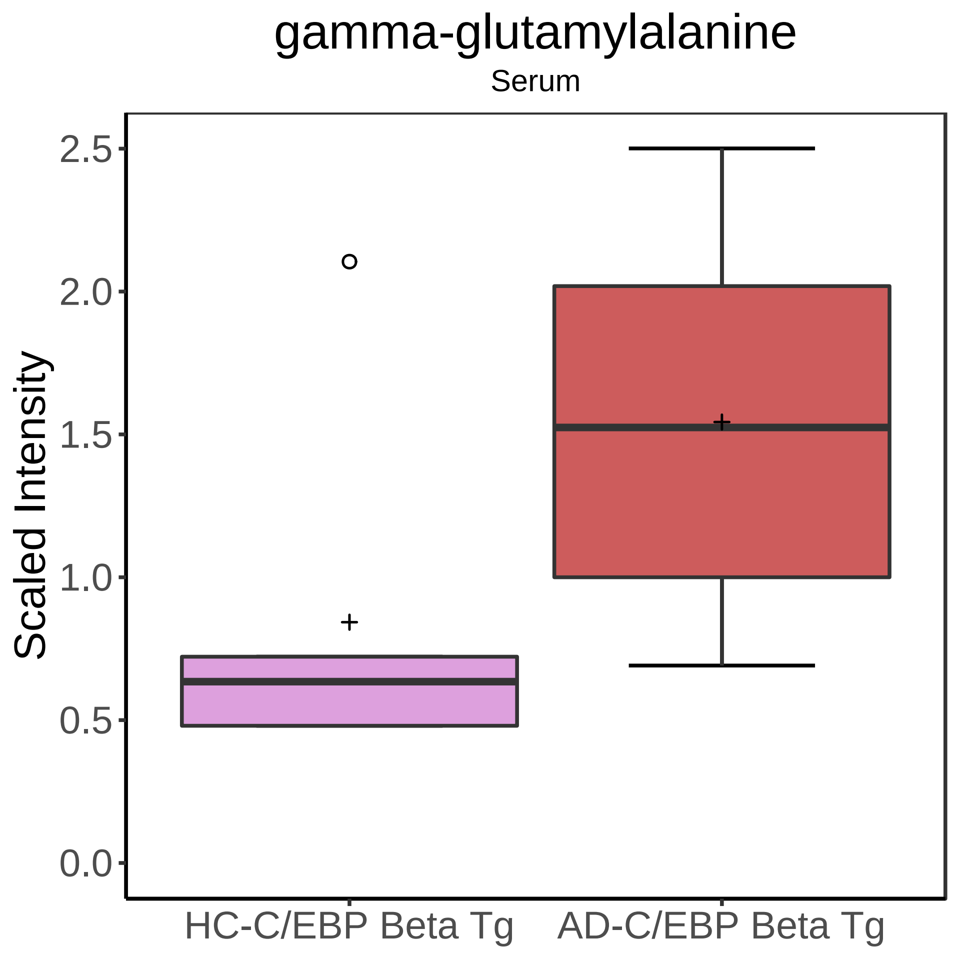
gamma-glutamylalanine
Serum
Scaled Intensity
2.5
2.0
1.5
1.0
0.5
0.0
HC-C/EBP Beta Tg
AD-C/EBP Beta Tg

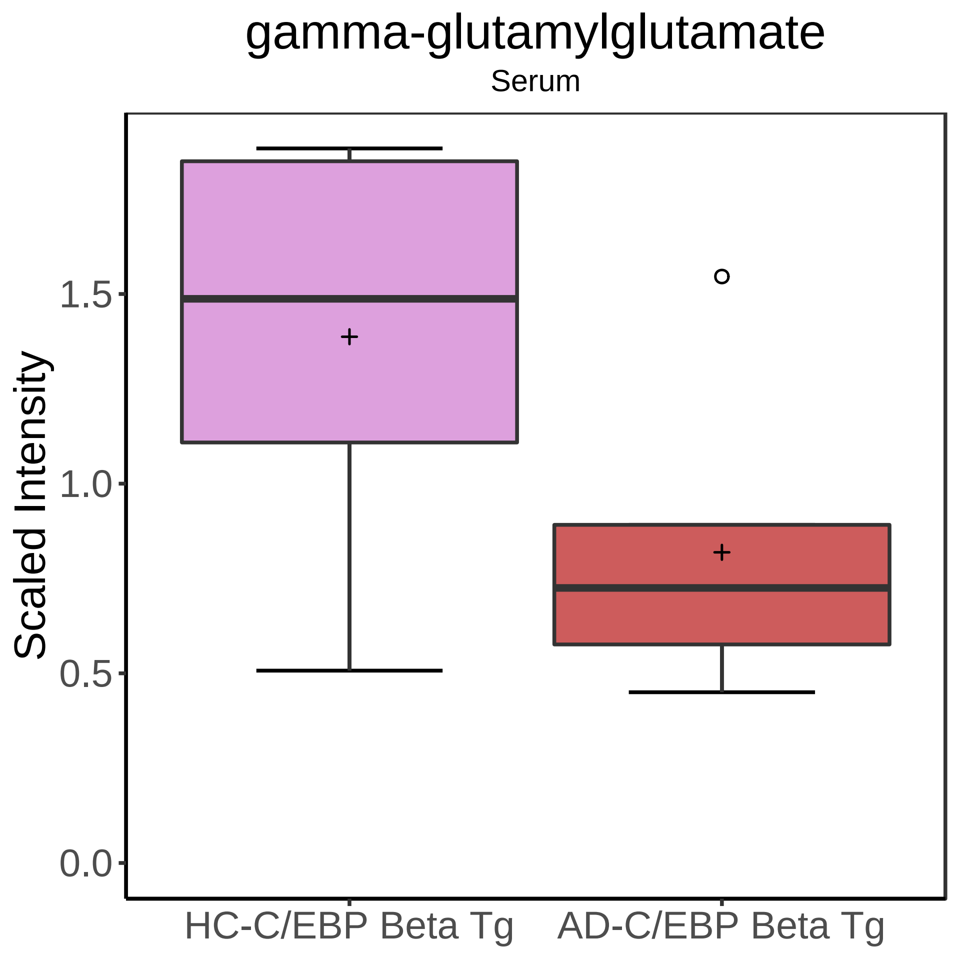
gamma-glutamylglutamate
Serum
Scaled Intensity
1.5
1.0
0.5
0.0
HC-C/EBP Beta Tg
AD-C/EBP Beta Tg

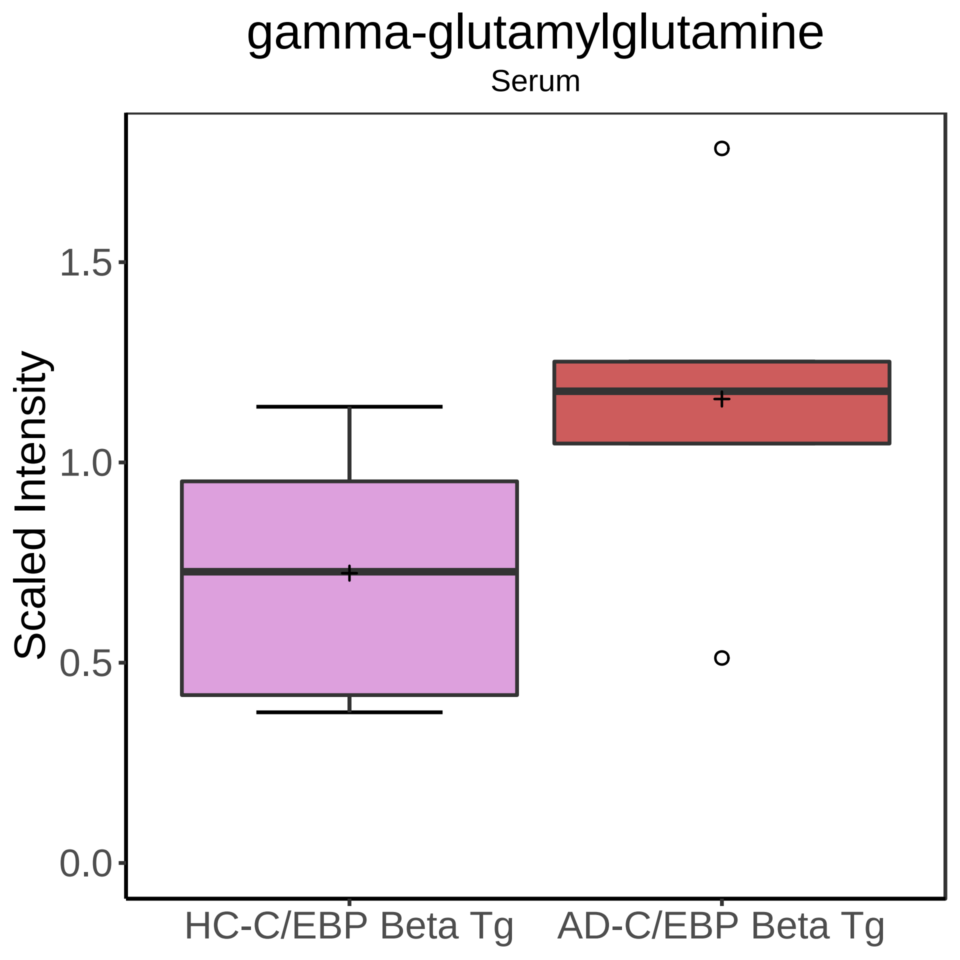
gamma-glutamylglutamine
Serum
Scaled Intensity
1.5
1.0
0.5
0.0
HC-C/EBP Beta Tg
AD-C/EBP Beta Tg

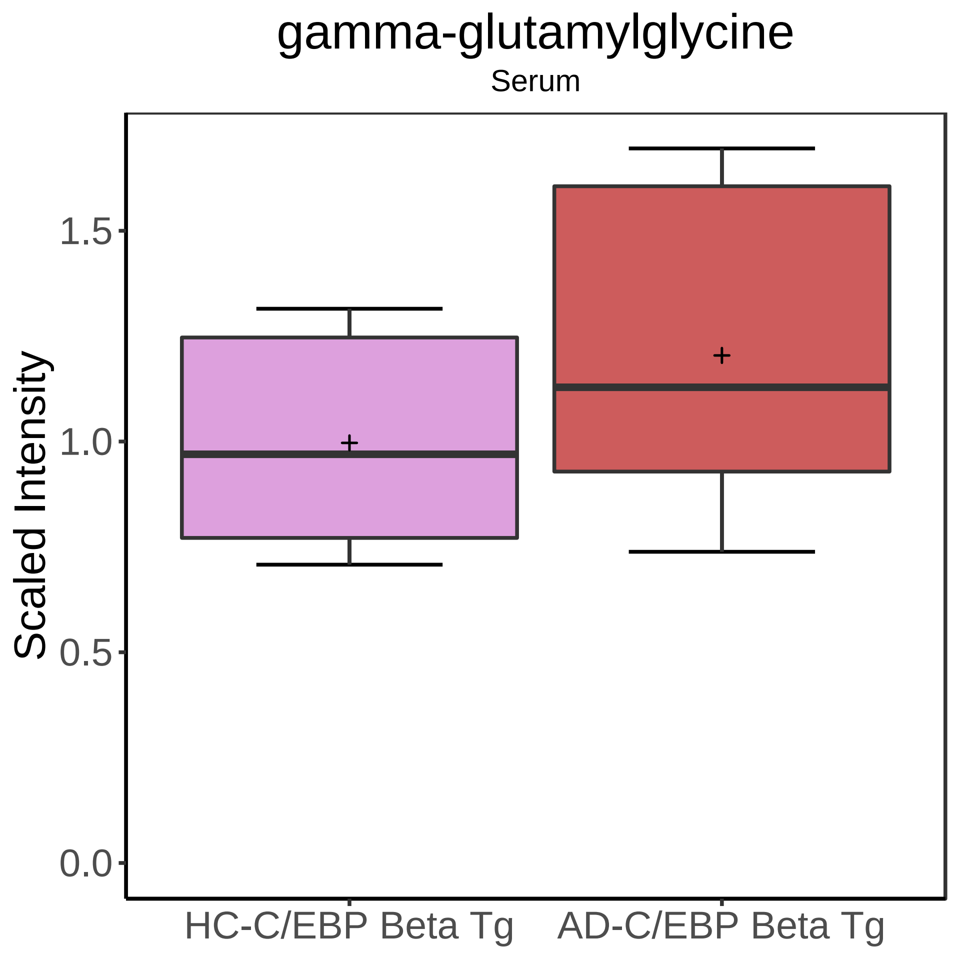
gamma-glutamylglycine
Serum
Scaled Intensity
0.0
0.5
1.0
1.5
HC-C/EBP Beta Tg
AD-C/EBP Beta Tg

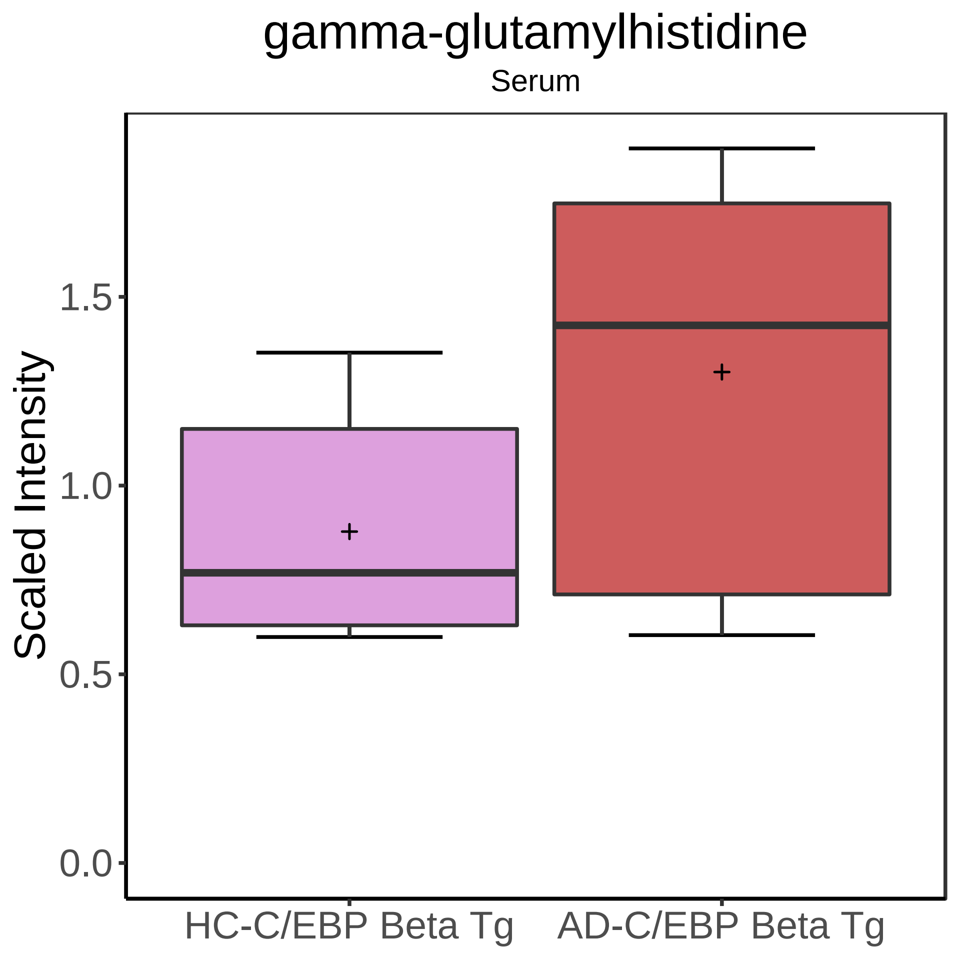
gamma-glutamylhistidine
Serum
Scaled Intensity
1.5
1.0
0.5
0.0
HC-C/EBP Beta Tg
AD-C/EBP Beta Tg

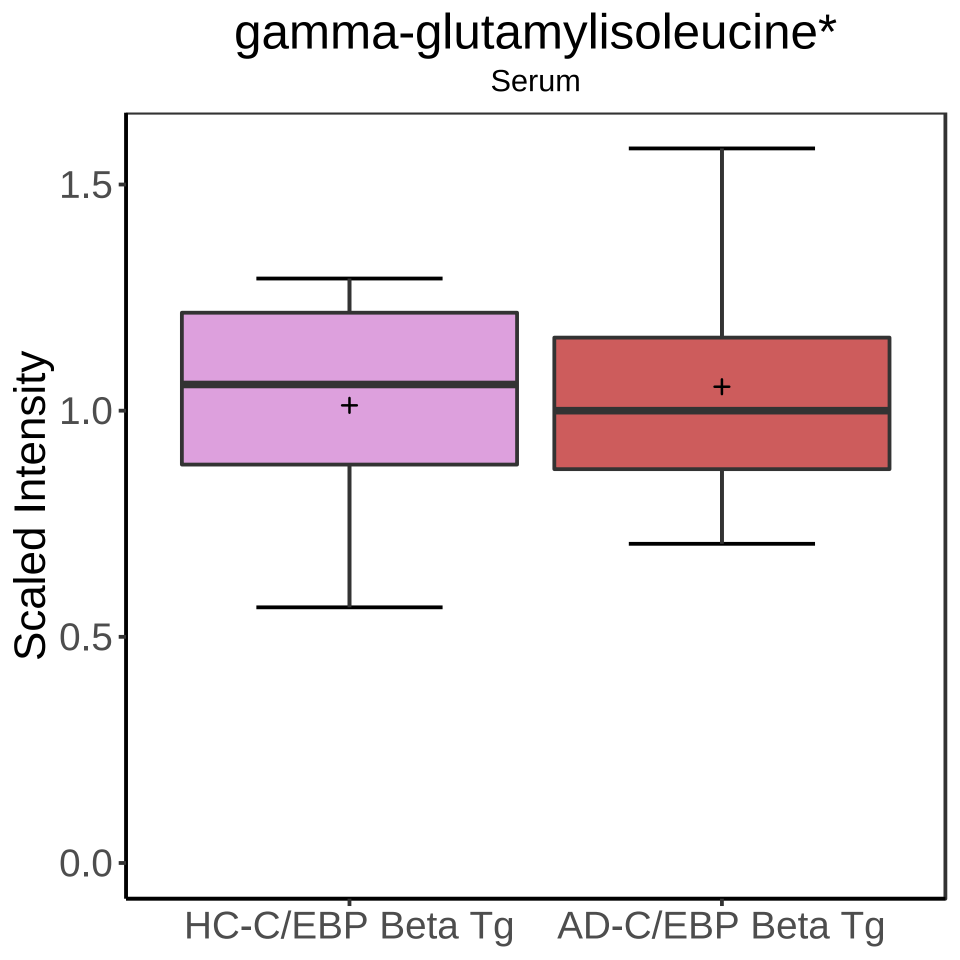
gamma-glutamylisoleucine*
Serum
Scaled Intensity
1.5
1.0
0.5
0.0
HC-C/EBP Beta Tg
AD-C/EBP Beta Tg

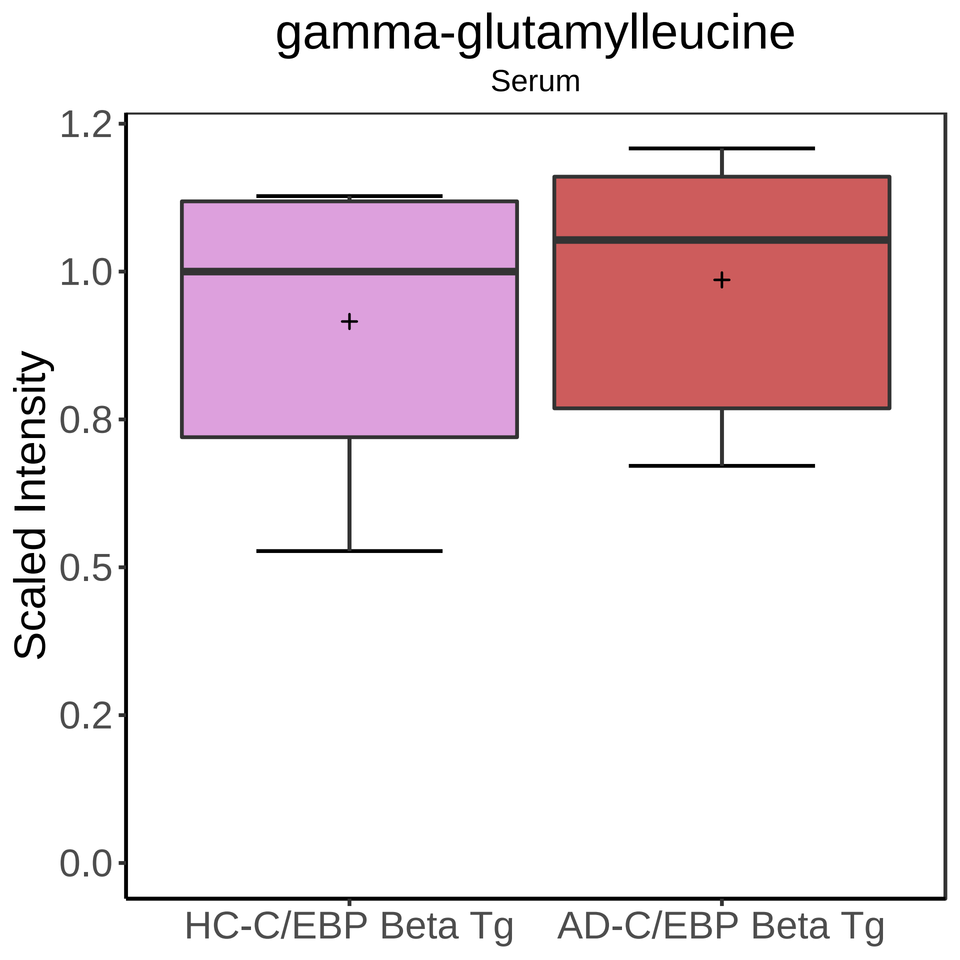
gamma-glutamylleucine
Serum
Scaled Intensity
1.2
1.0
0.8
0.5
0.2
0.0
HC-C/EBP Beta Tg
AD-C/EBP Beta Tg

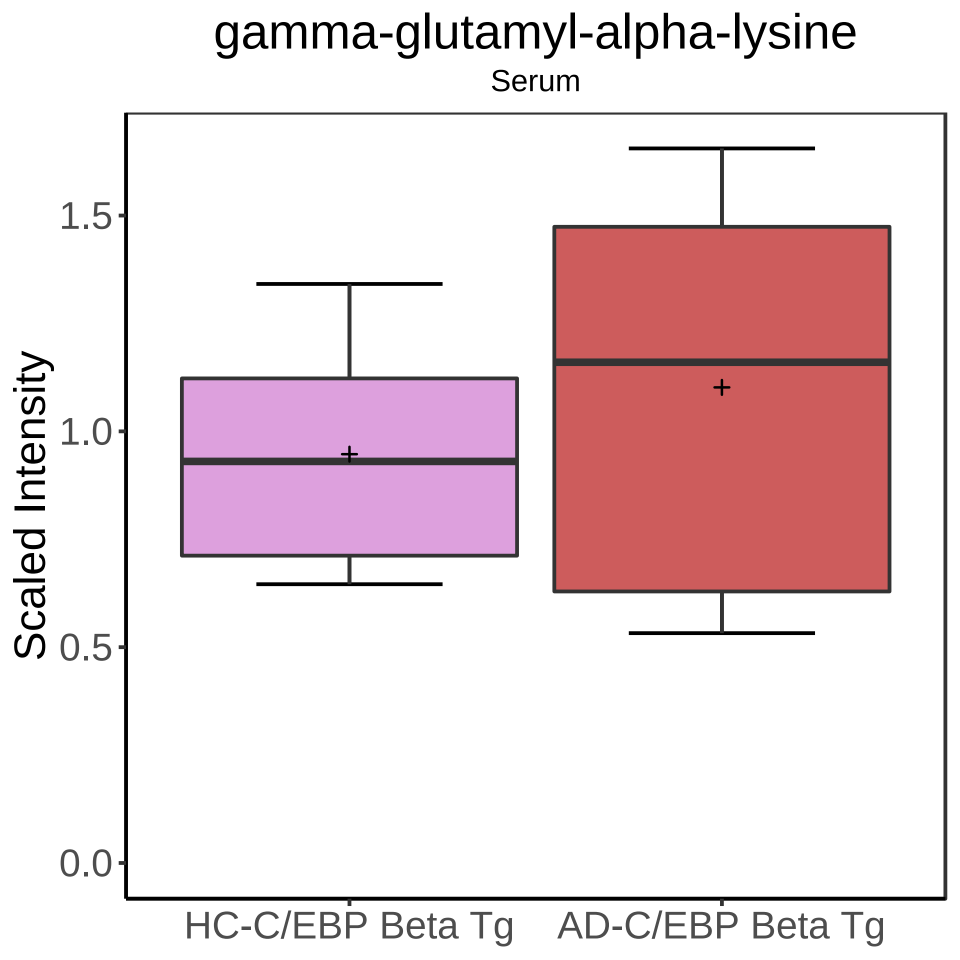
gamma-glutamyl-alpha-lysine
Serum
Scaled Intensity
1.5
1.0
0.5
0.0
HC-C/EBP Beta Tg
AD-C/EBP Beta Tg

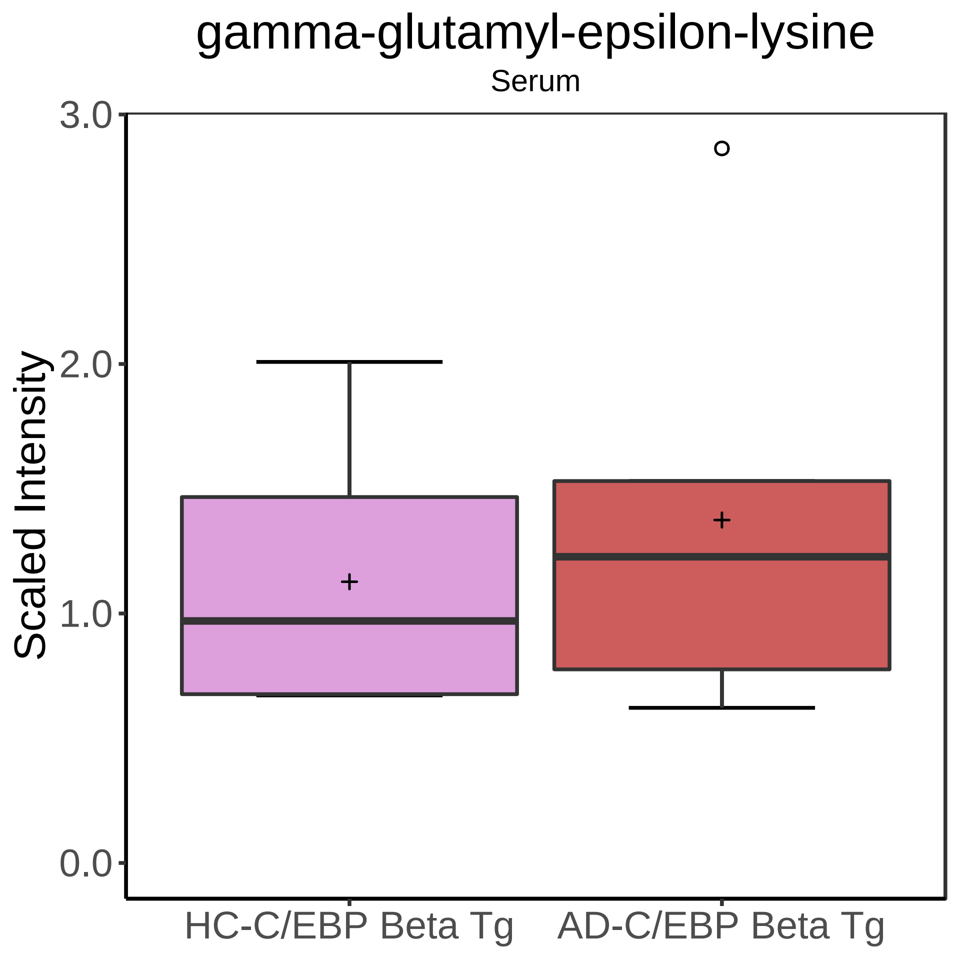
gamma-glutamyl-epsilon-lysine
Serum
Scaled Intensity
3.0
2.0
1.0
0.0
HC-C/EBP Beta Tg
AD-C/EBP Beta Tg

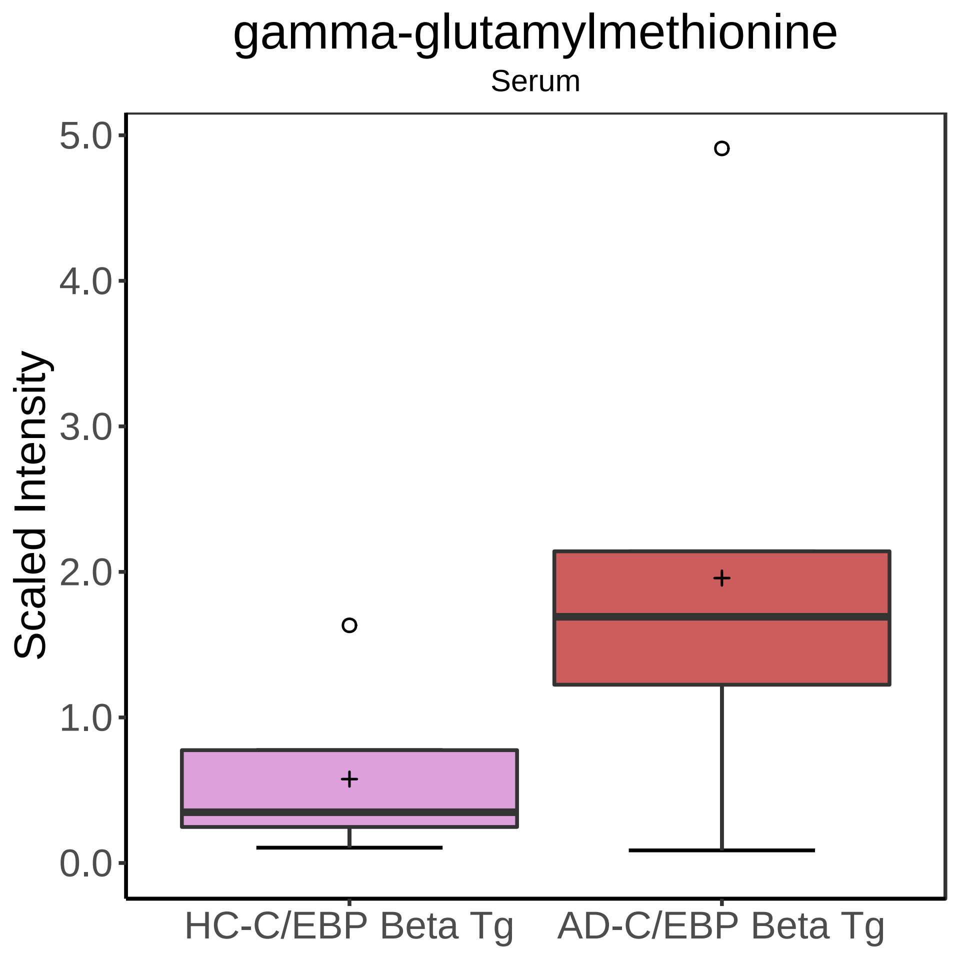
gamma-glutamylmethionine
Serum
Scaled Intensity
5.0
4.0
3.0
2.0
1.0
0.0
HC-C/EBP Beta Tg
AD-C/EBP Beta Tg

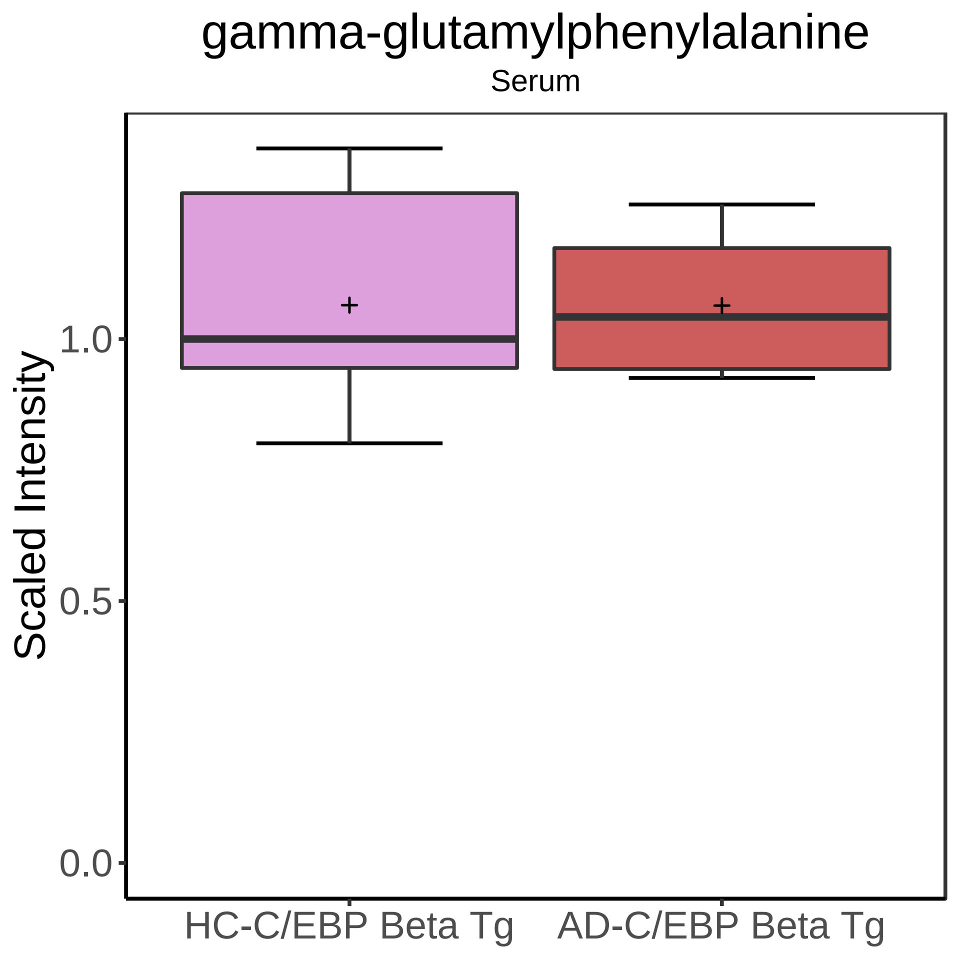
gamma-glutamylphenylalanine
Serum
Scaled Intensity
1.0
0.5
0.0
HC-C/EBP Beta Tg
AD-C/EBP Beta Tg

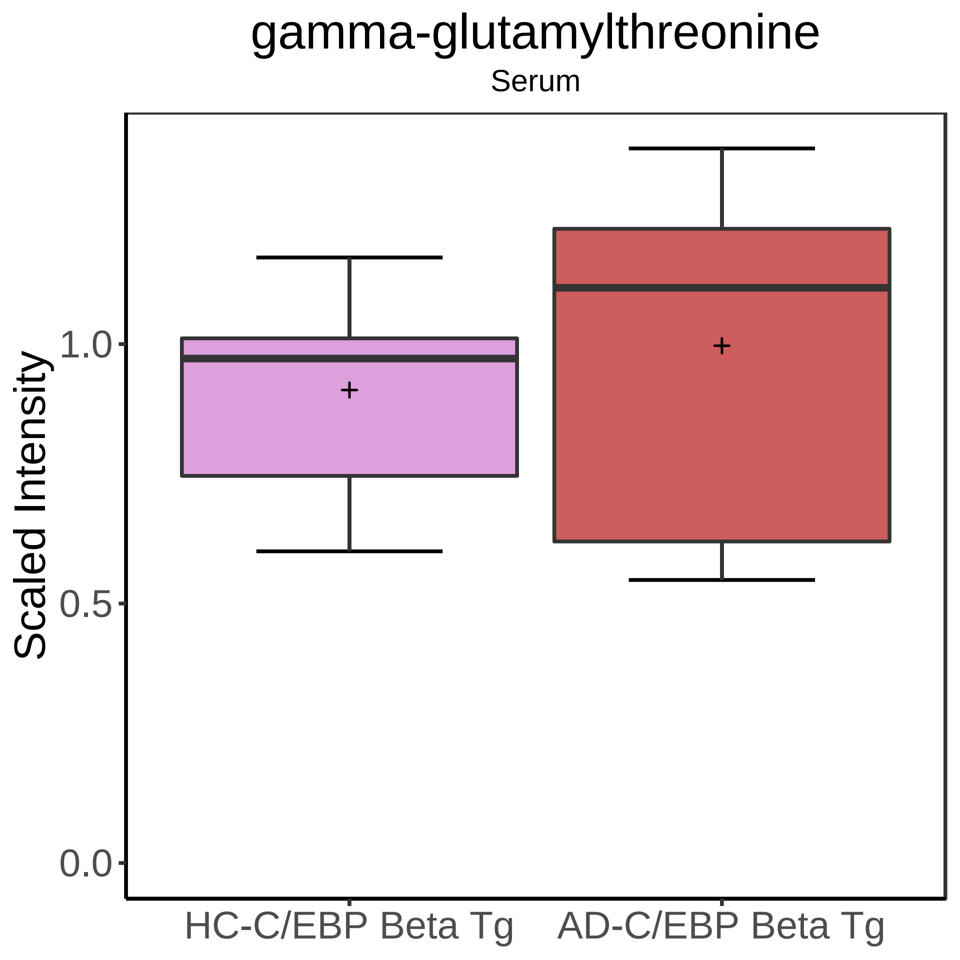

gamma-glutamylthreonine
Serum
Scaled Intensity
1.0
0.5
0.0
HC-C/EBP Beta Tg
AD-C/EBP Beta Tg

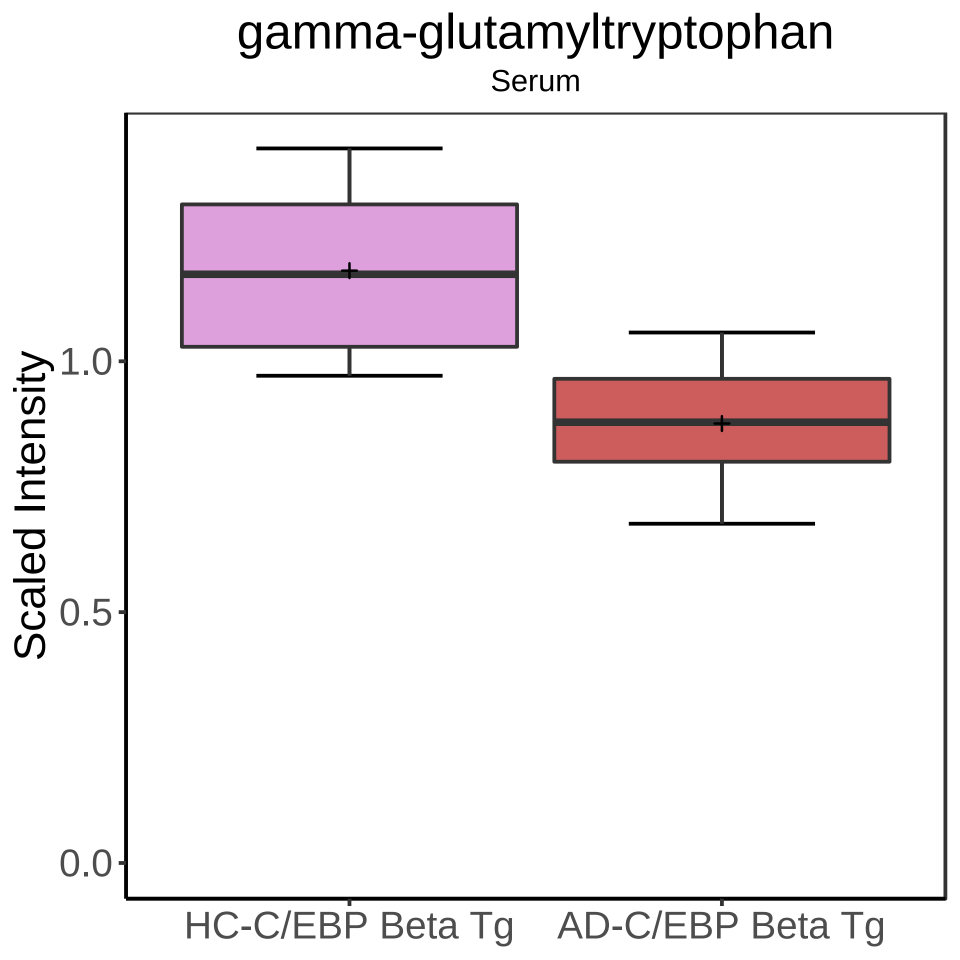

gamma-glutamyltryptophan
Serum
Scaled Intensity
1.0
0.5
0.0
HC-C/EBP Beta Tg
AD-C/EBP Beta Tg

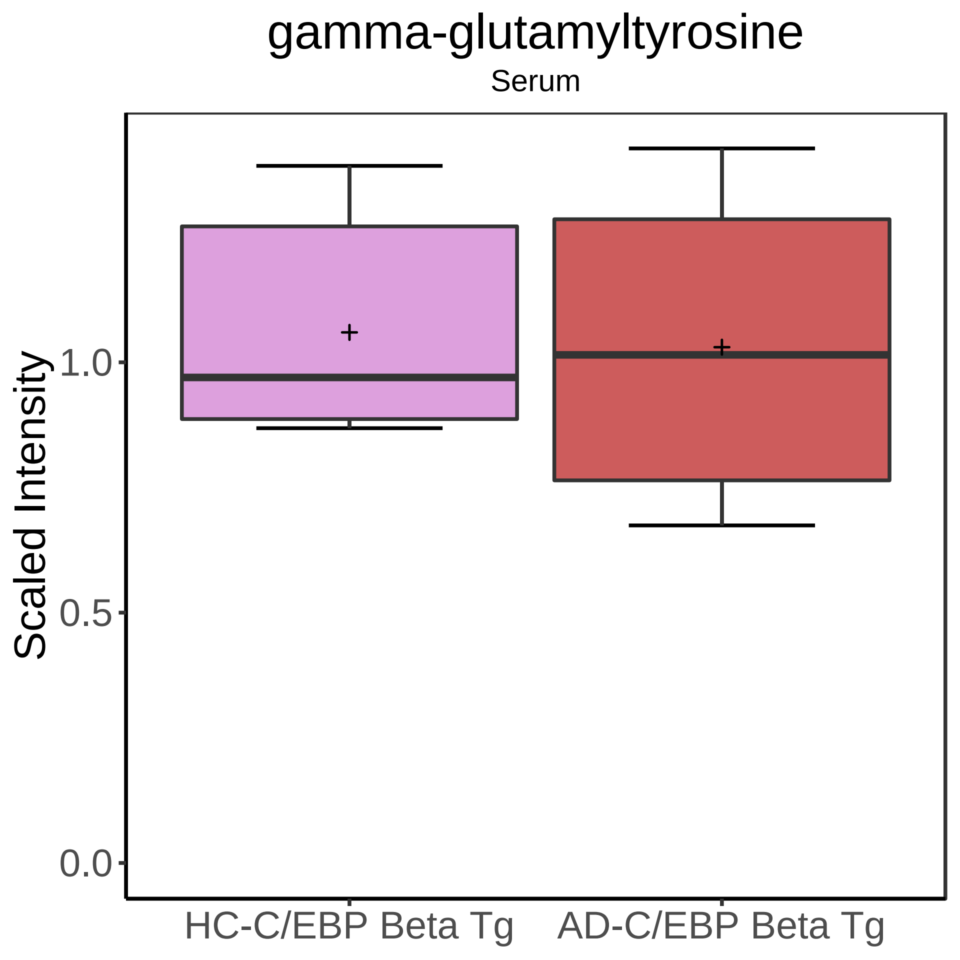
gamma-glutamyltyrosine
Serum
Scaled Intensity
1.0
0.5
0.0
HC-C/EBP Beta Tg
AD-C/EBP Beta Tg

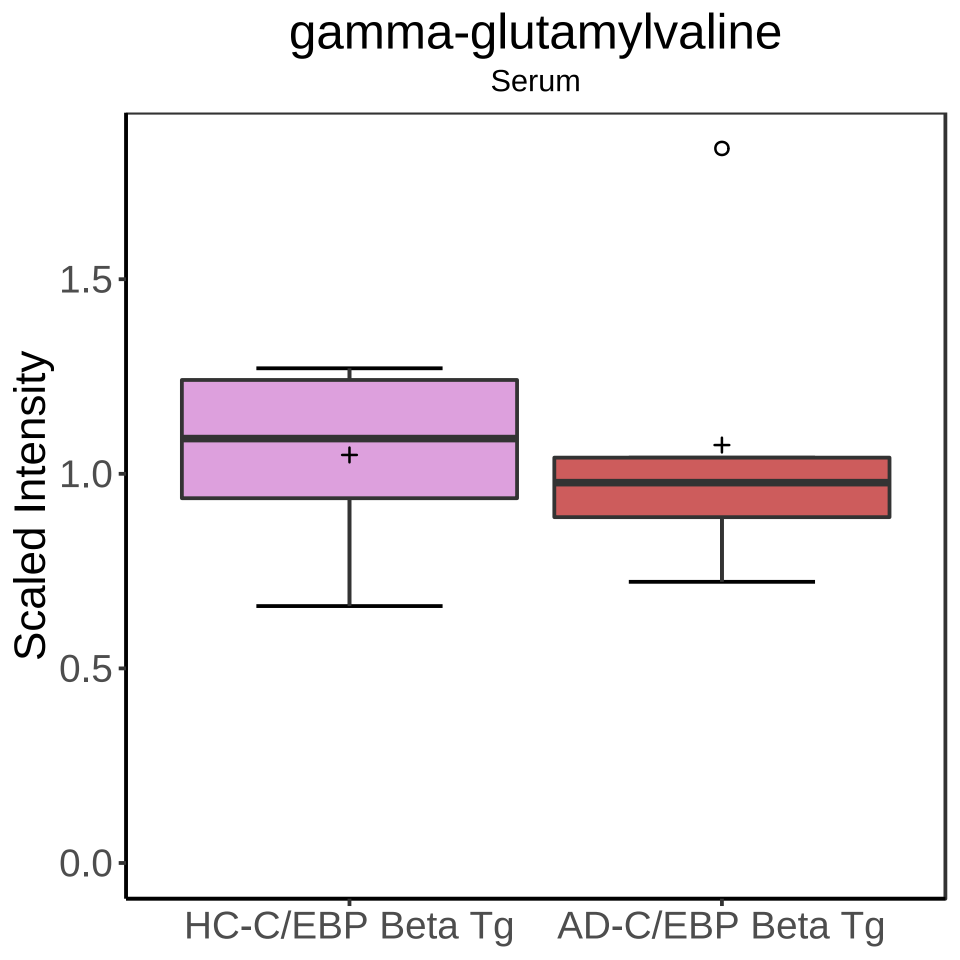
gamma-glutamylvaline
Serum
Scaled Intensity
0.0
0.5
1.0
1.5
HC-C/EBP Beta Tg
AD-C/EBP Beta Tg

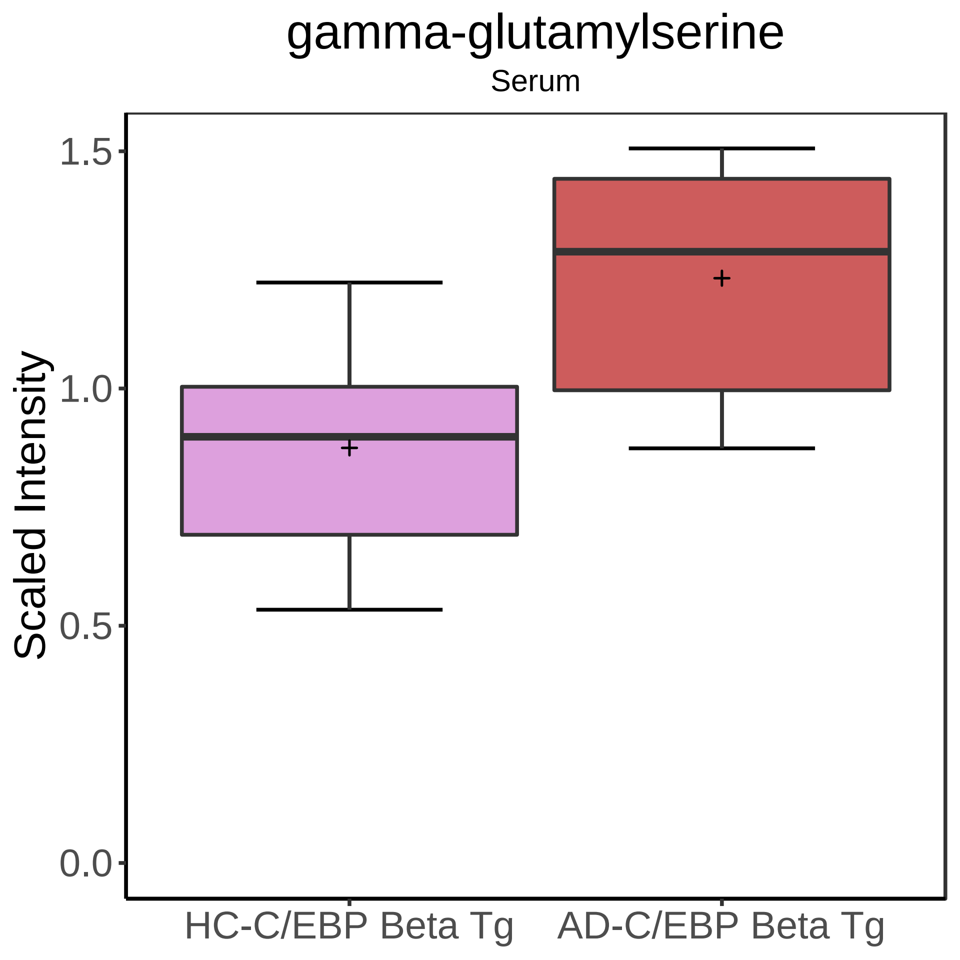
gamma-glutamylserine
Serum
Scaled Intensity
1.5
1.0
0.5
0.0
HC-C/EBP Beta Tg
AD-C/EBP Beta Tg

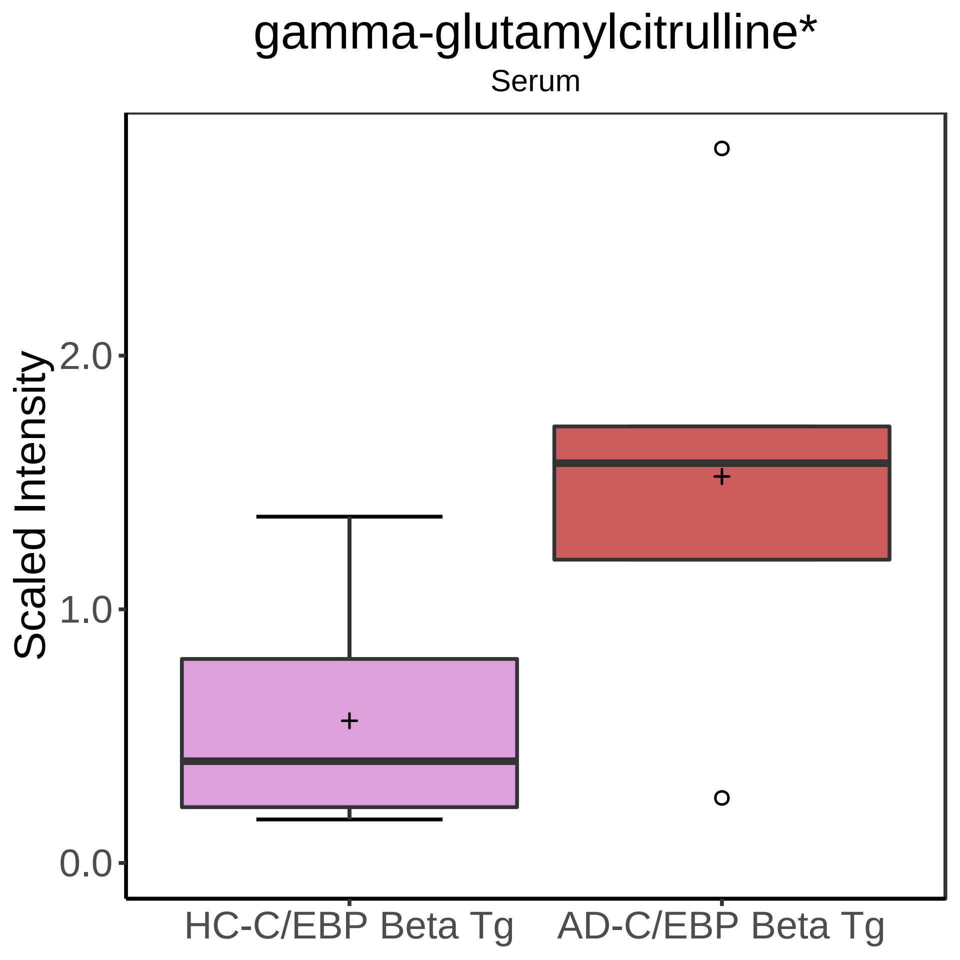
gamma-glutamylcitrulline*
Serum
Scaled Intensity
2.0
1.0
0.0
HC-C/EBP Beta Tg
AD-C/EBP Beta Tg

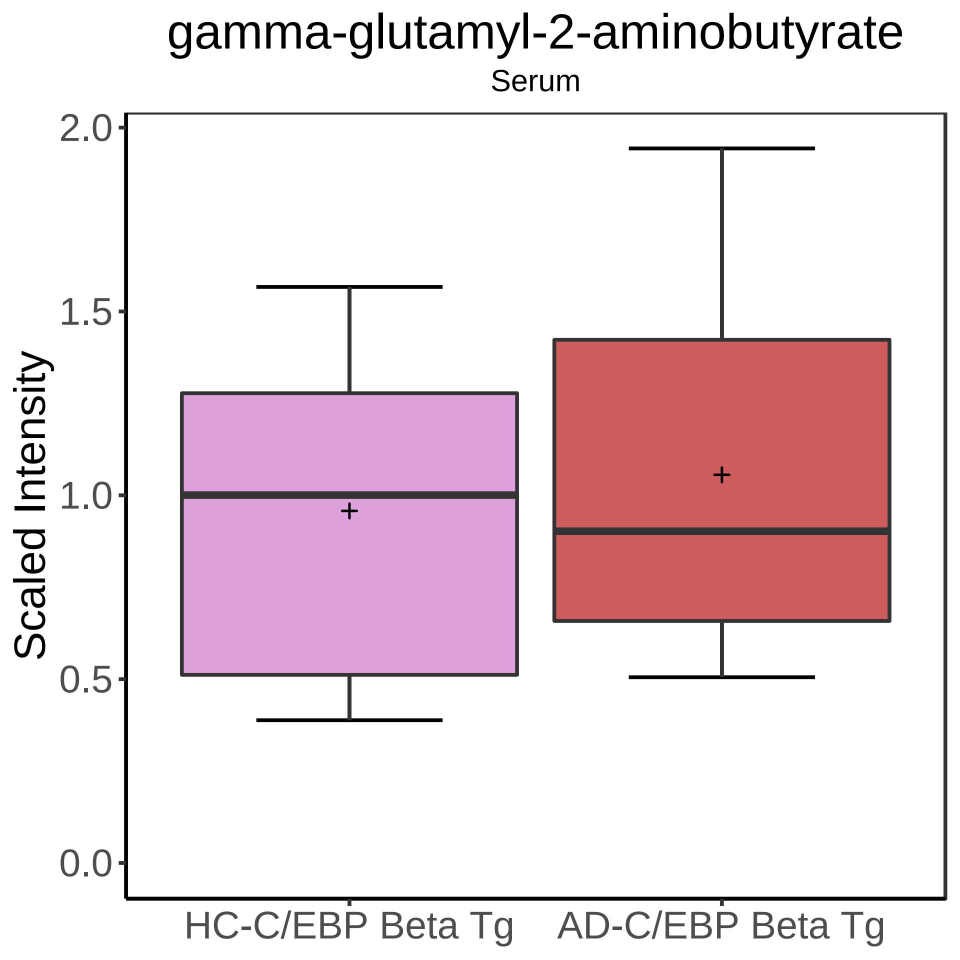
gamma-glutamyl-2-aminobutyrate
Serum
Scaled Intensity
2.0
1.5
1.0
0.5
0.0
HC-C/EBP Beta Tg
AD-C/EBP Beta Tg

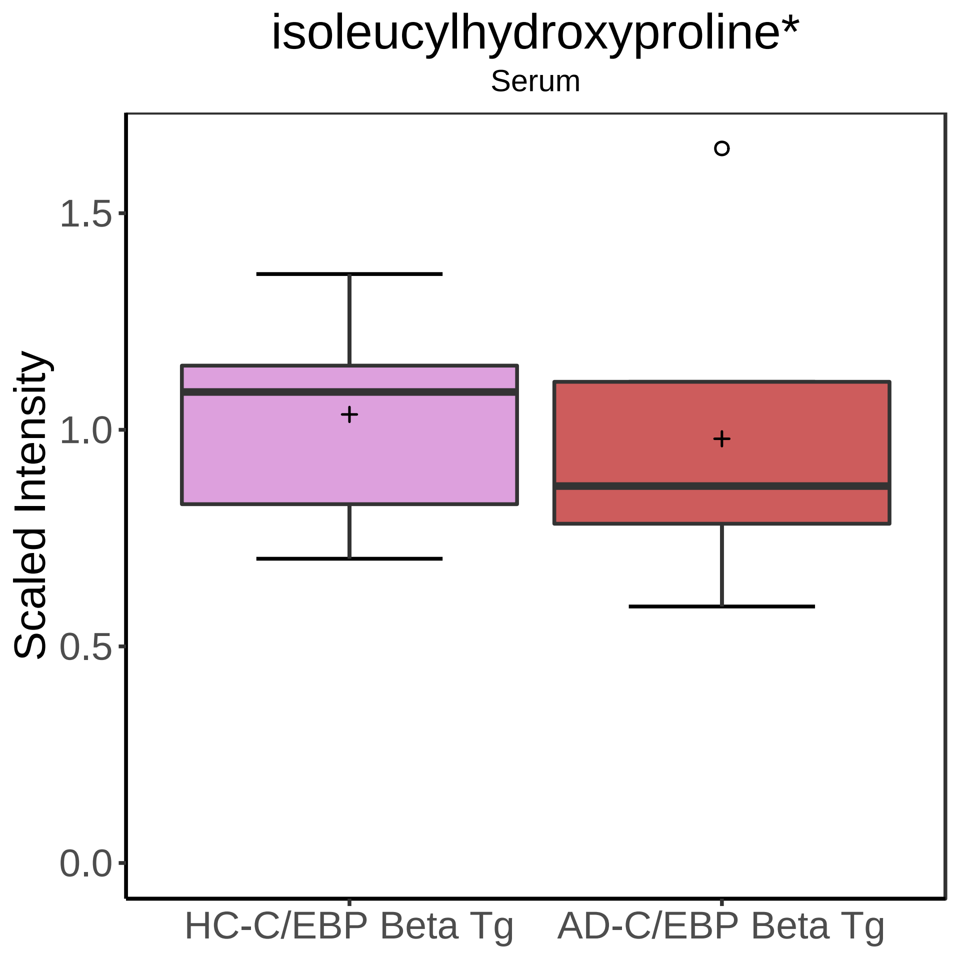

isoleucylhydroxyproline*
Serum
Scaled Intensity
1.5
1.0
0.5
0.0
HC-C/EBP Beta Tg
AD-C/EBP Beta Tg

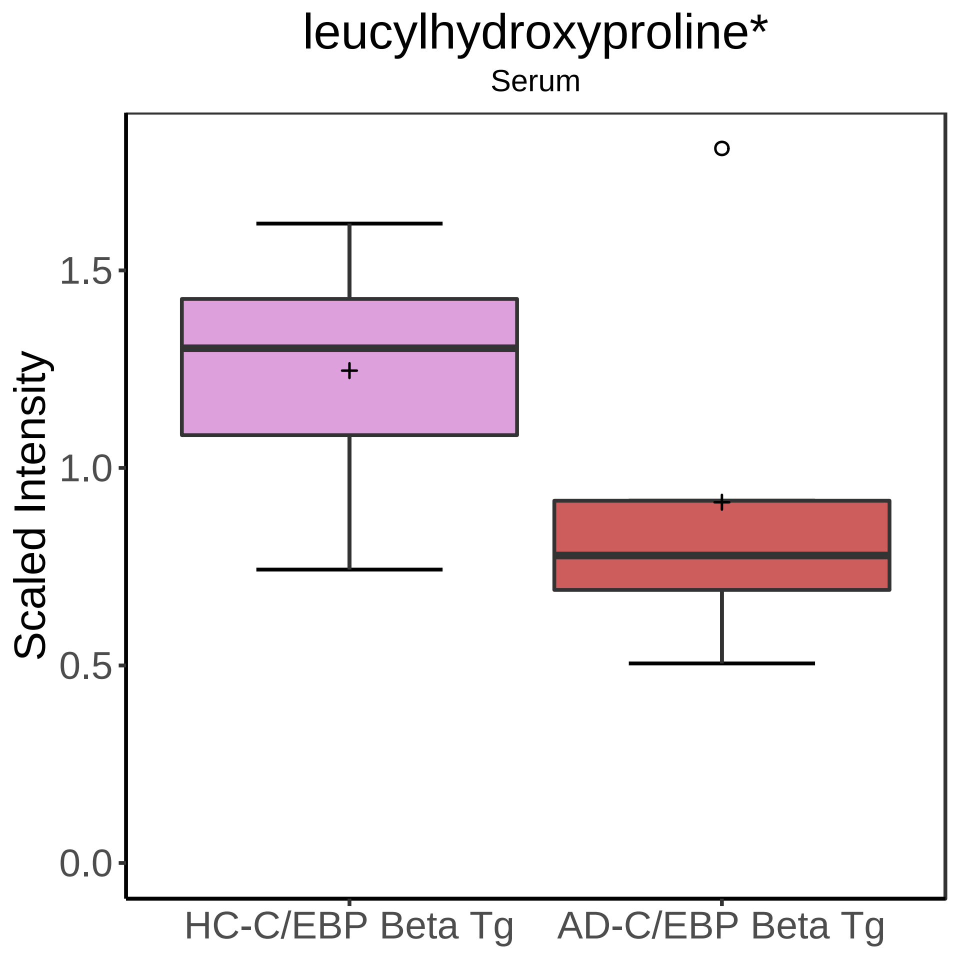
leucylhydroxyproline*
Serum
Scaled Intensity
1.5
1.0
0.5
0.0
HC-C/EBP Beta Tg
AD-C/EBP Beta Tg

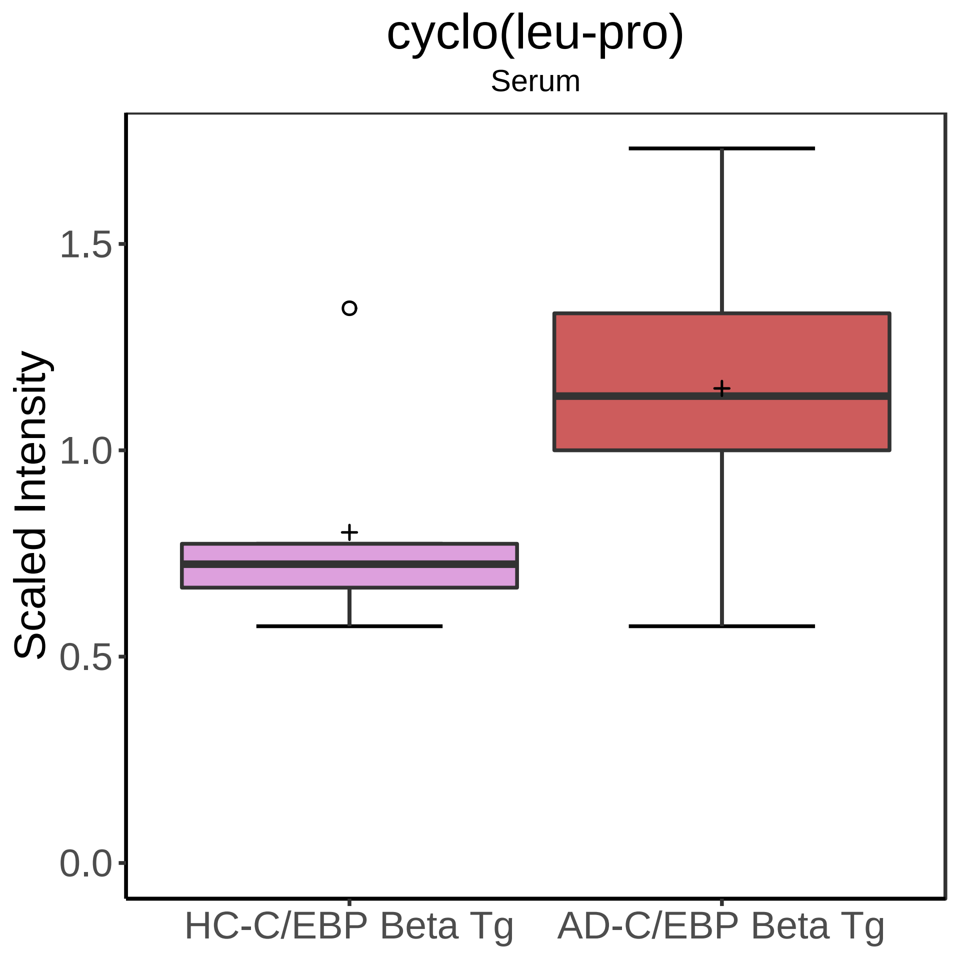
cyclo(leu-pro)
Serum
Scaled Intensity
0.0
0.5
1.0
1.5
HC-C/EBP Beta Tg
AD-C/EBP Beta Tg

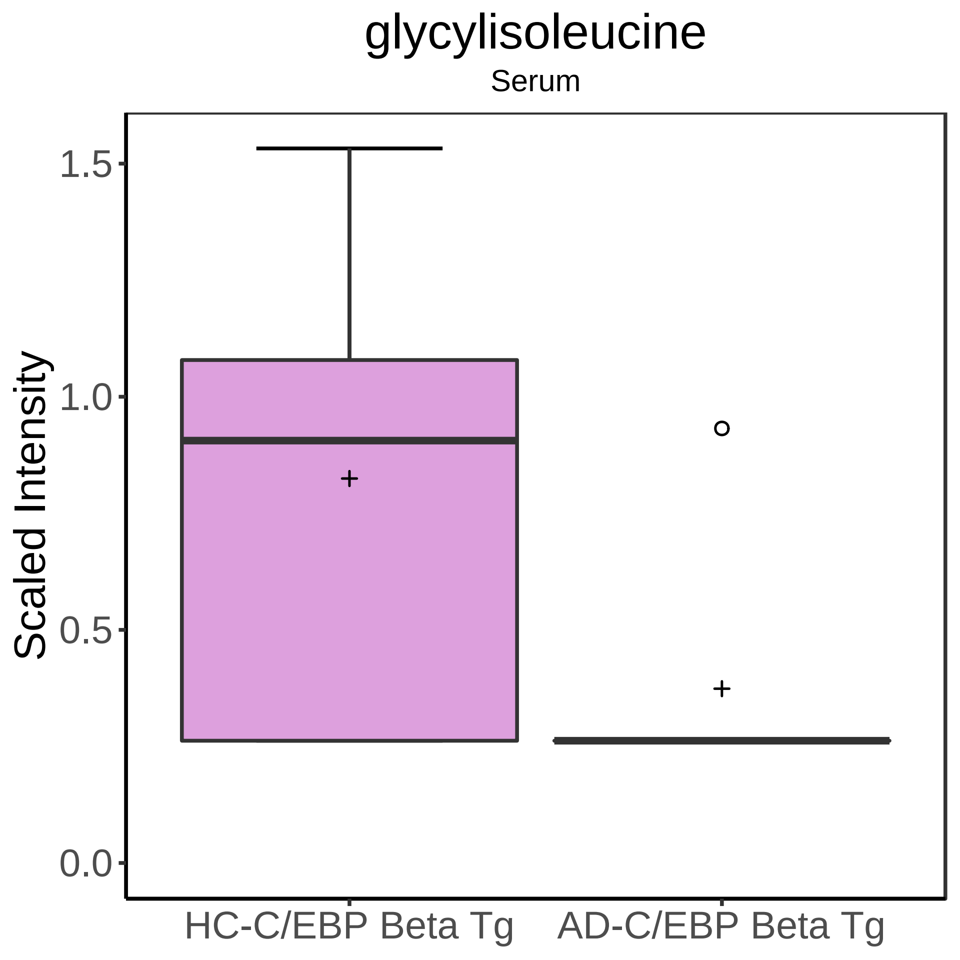
glycylisoleucine
Serum
Scaled Intensity
1.5
1.0
0.5
0.0
HC-C/EBP Beta Tg
AD-C/EBP Beta Tg

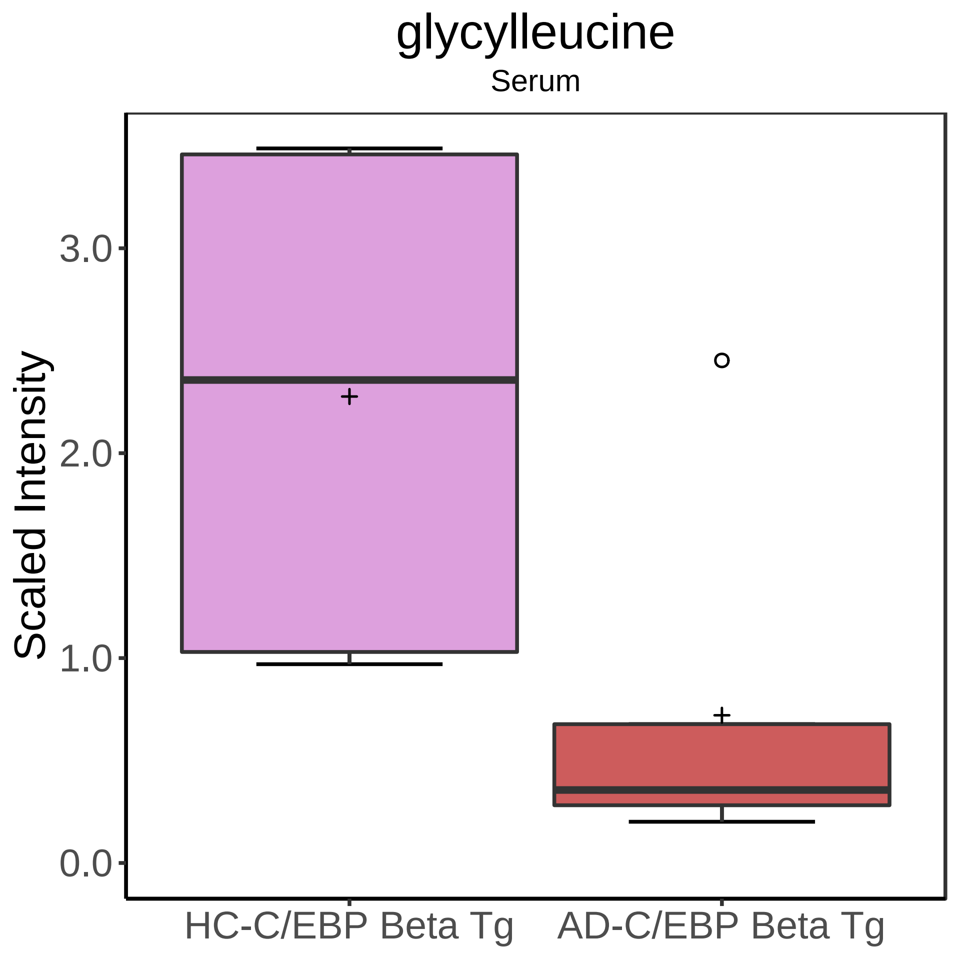
glycylleucine
Serum
Scaled Intensity
3.0
2.0
1.0
0.0
HC-C/EBP Beta Tg
AD-C/EBP Beta Tg

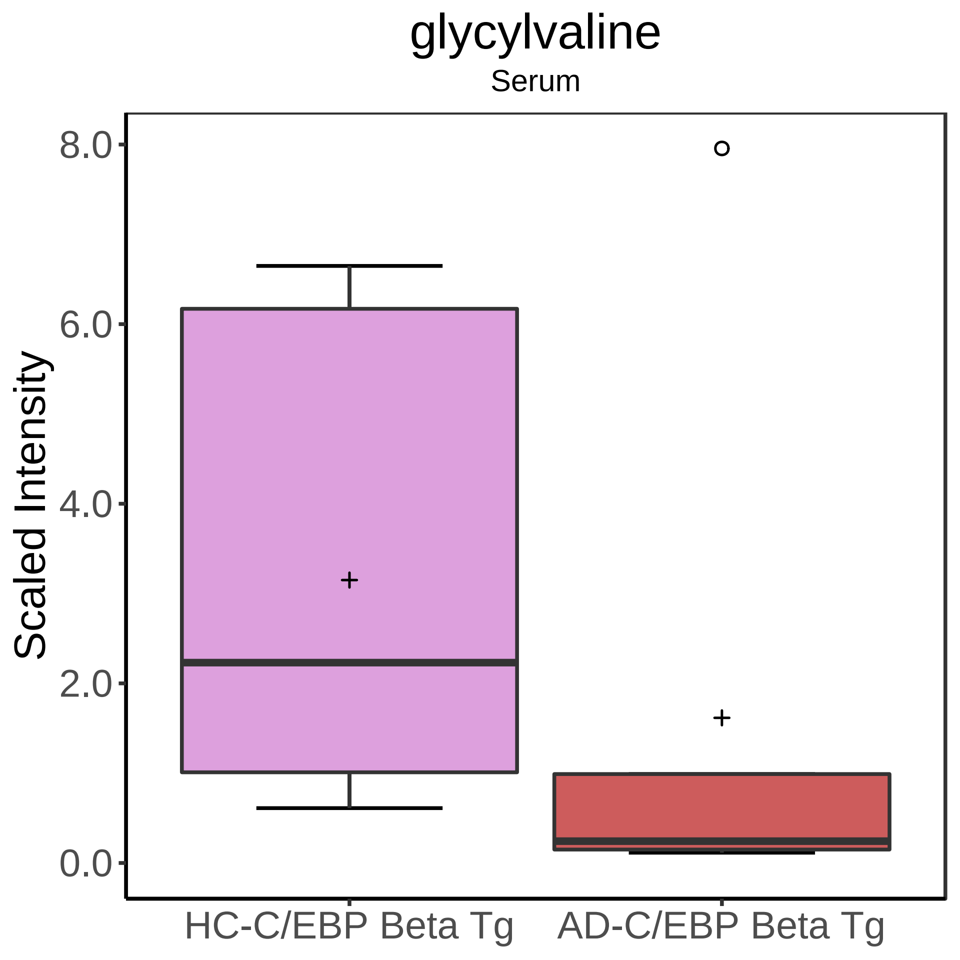

glycylvaline
Serum
Scaled Intensity
8.0
6.0
4.0
2.0
0.0
HC-C/EBP Beta Tg
AD-C/EBP Beta Tg

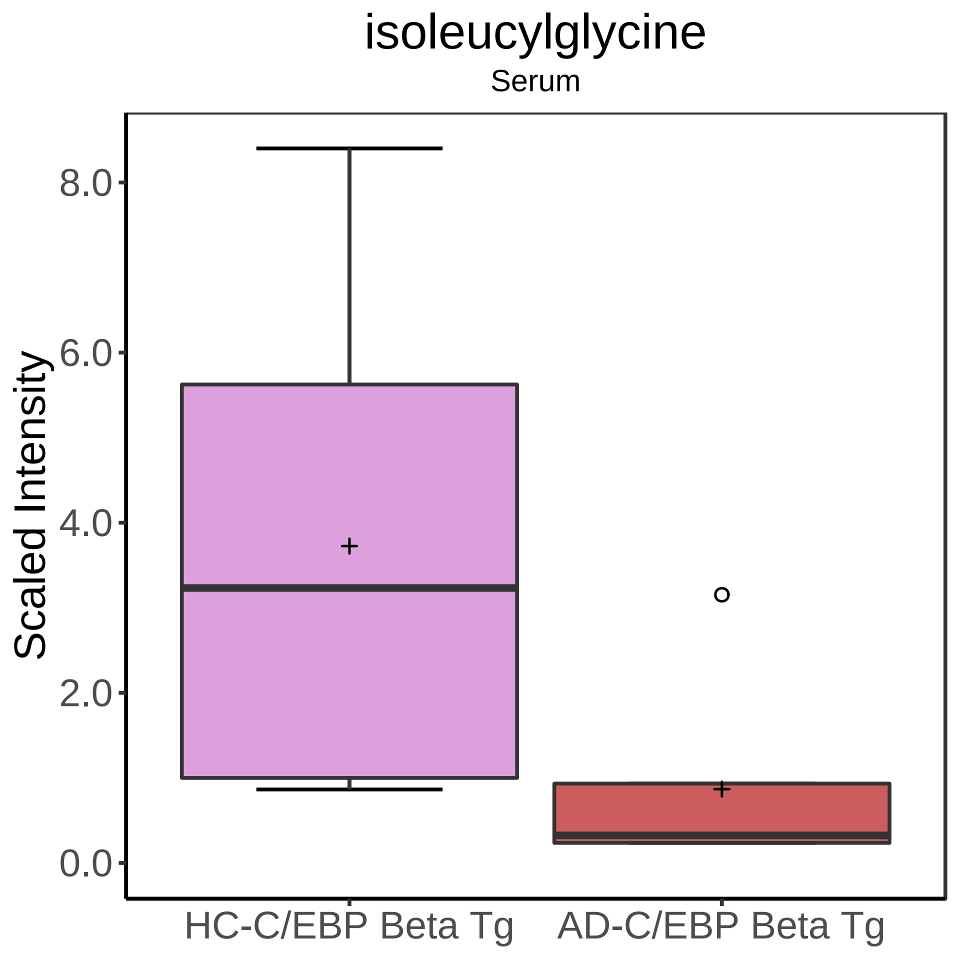
isoleucylglycine
Serum
Scaled Intensity
8.0
6.0
4.0
2.0
0.0
HC-C/EBP Beta Tg
AD-C/EBP Beta Tg

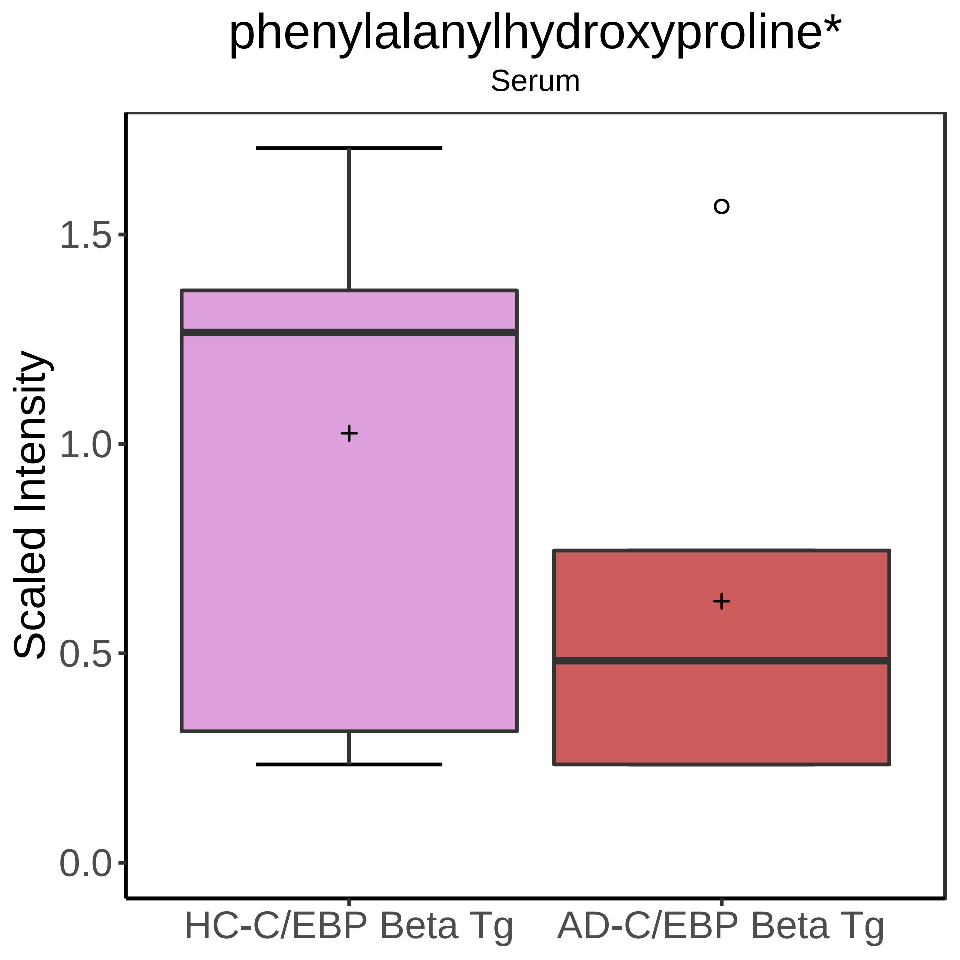

phenylalanylhydroxyproline*
Serum
Scaled Intensity
1.5
1.0
0.5
0.0
HC-C/EBP Beta Tg
AD-C/EBP Beta Tg

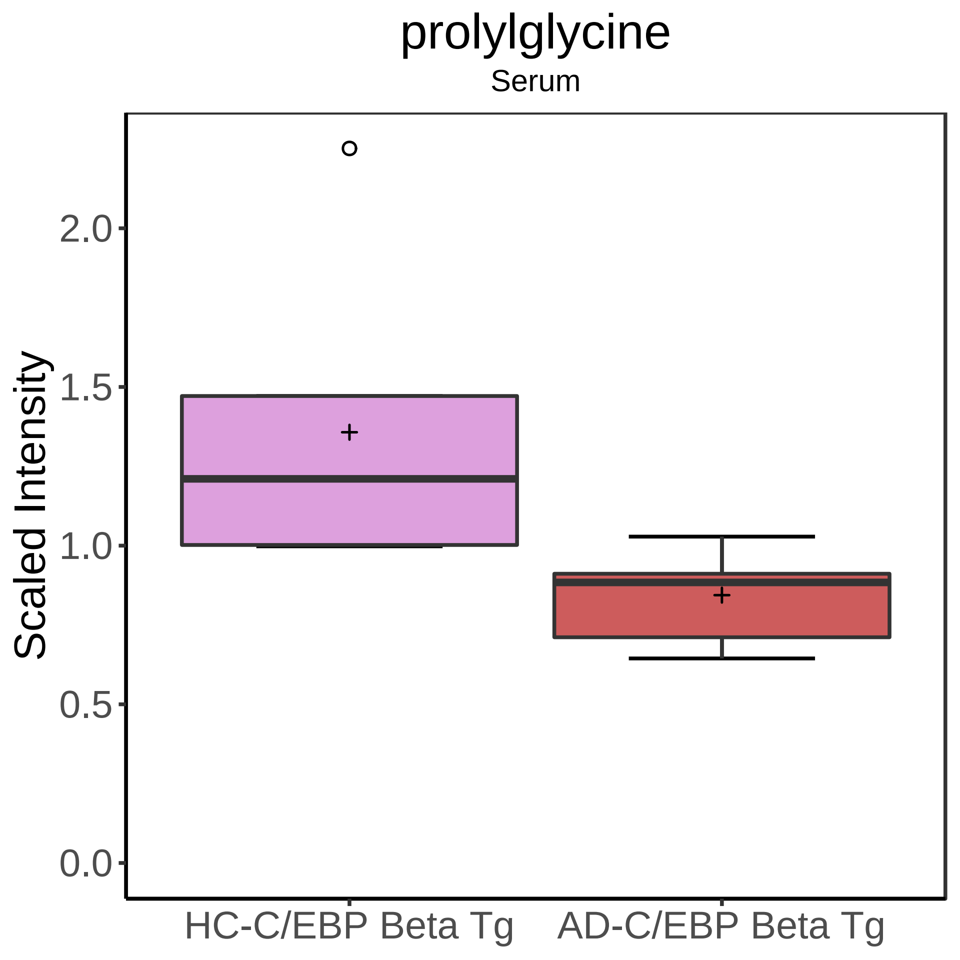

prolylglycine
Serum
Scaled Intensity
2.0
1.5
1.0
0.5
0.0
HC-C/EBP Beta Tg
AD-C/EBP Beta Tg

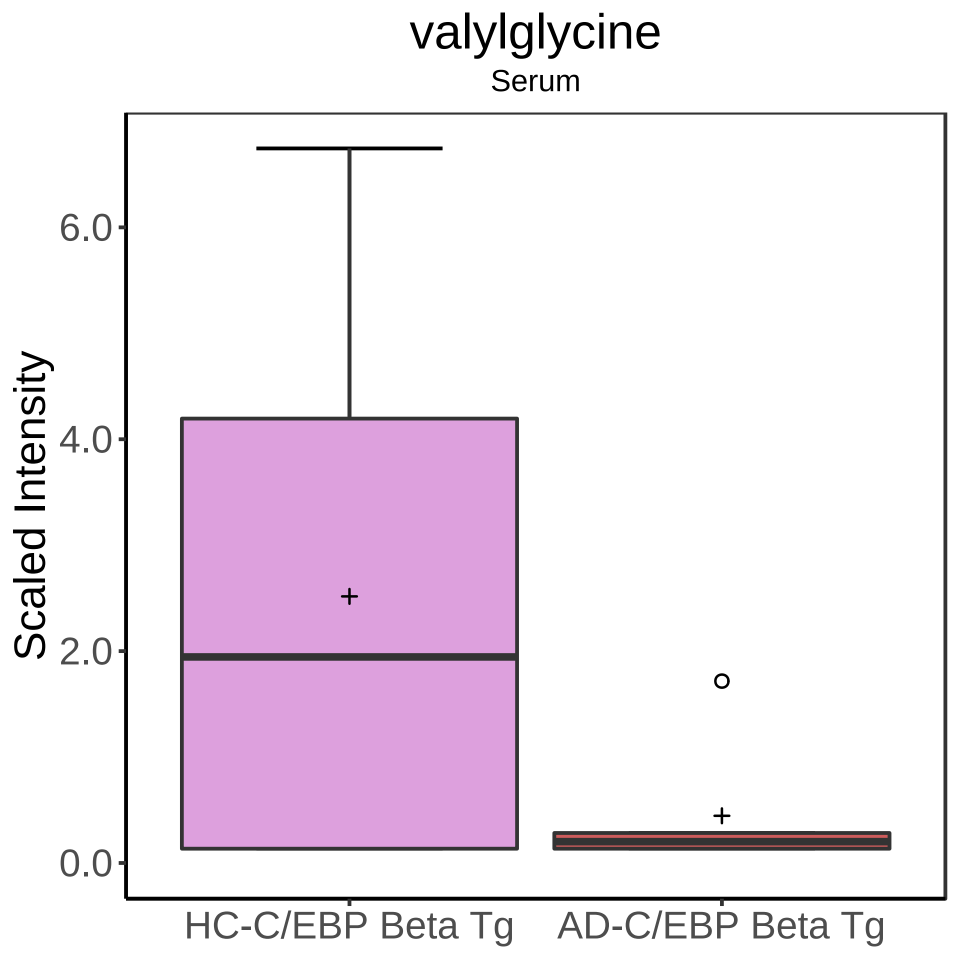
valylglycine
Serum
Scaled Intensity
6.0
4.0
2.0
0.0
HC-C/EBP Beta Tg
AD-C/EBP Beta Tg

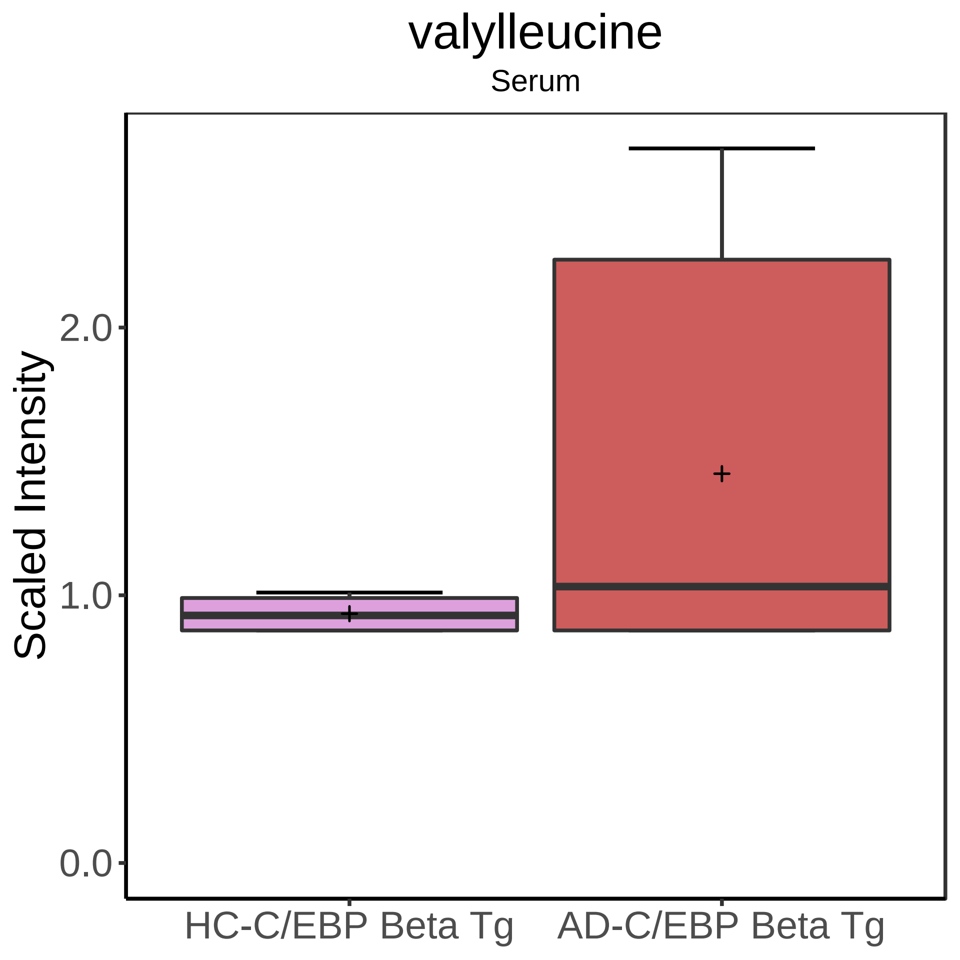
valylleucine
Serum
Scaled Intensity
2.0
1.0
0.0
HC-C/EBP Beta Tg
AD-C/EBP Beta Tg

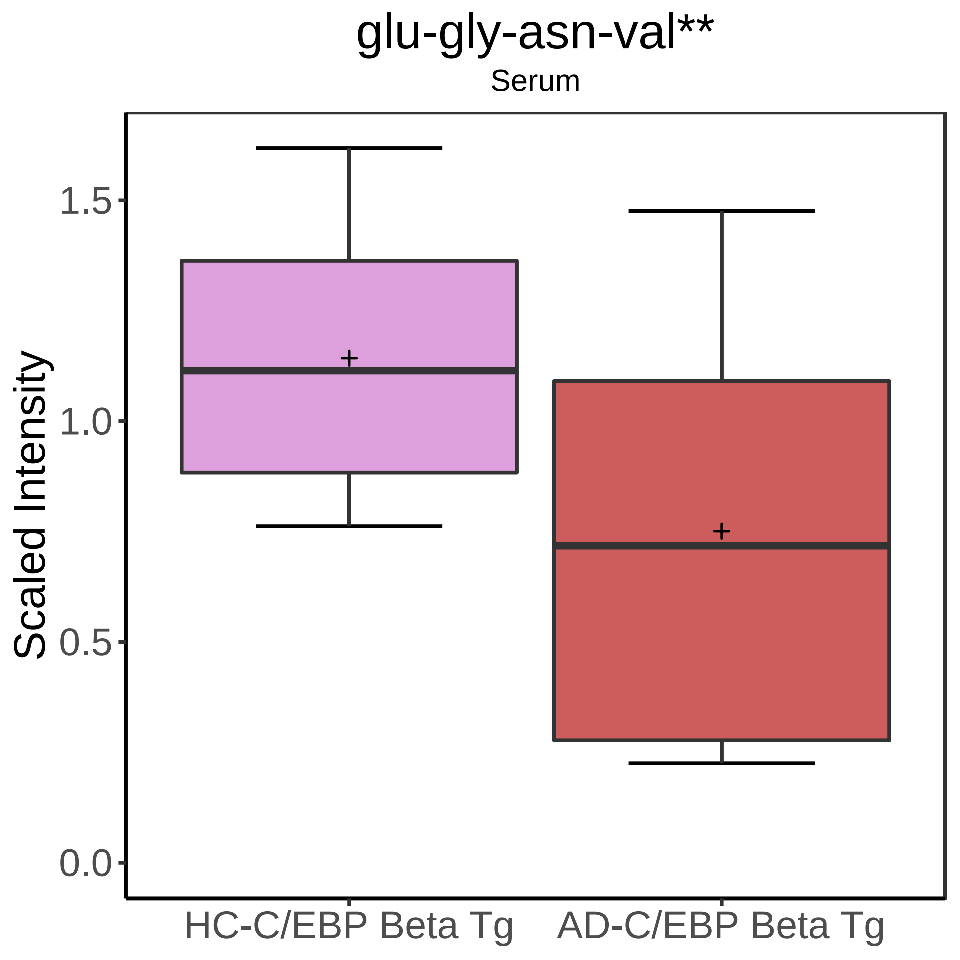
glu-gly-asn-val**
Serum
Scaled Intensity
1.5
1.0
0.5
0.0
HC-C/EBP Beta Tg
AD-C/EBP Beta Tg

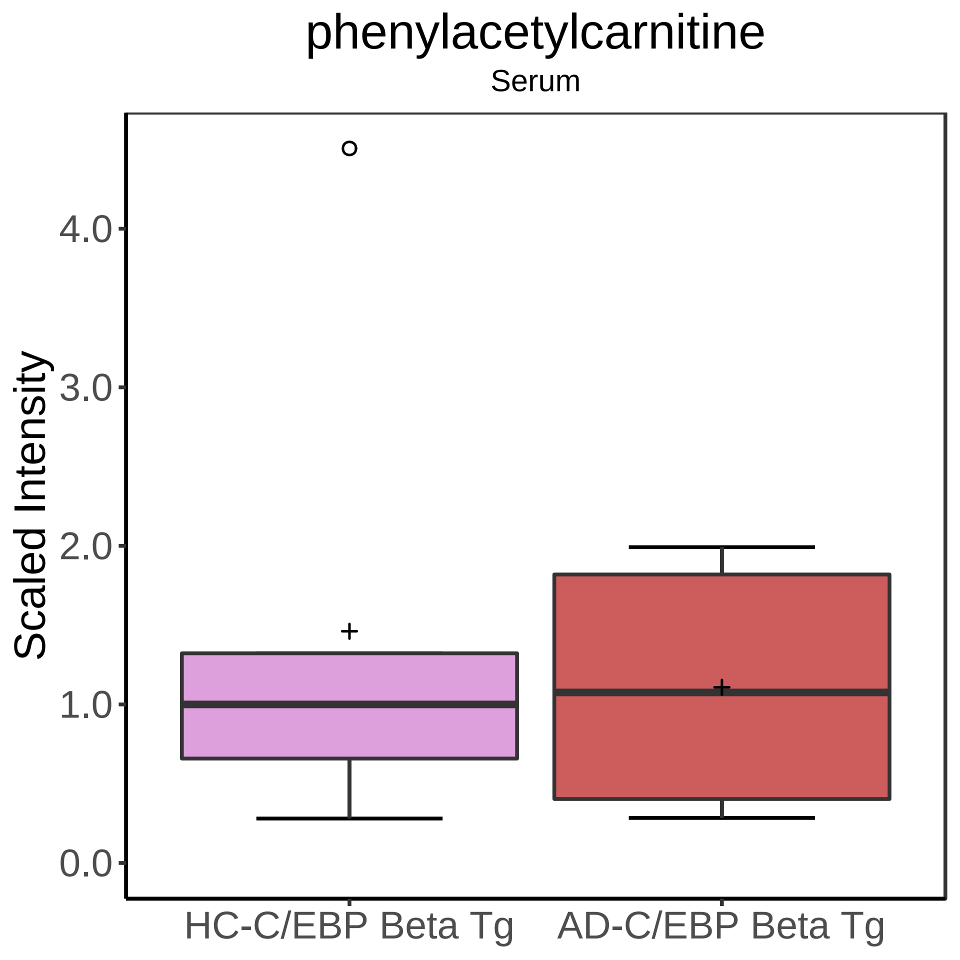
phenylacetylcarnitine
Serum
Scaled Intensity
4.0
3.0
2.0
1.0
0.0
HC-C/EBP Beta Tg
AD-C/EBP Beta Tg

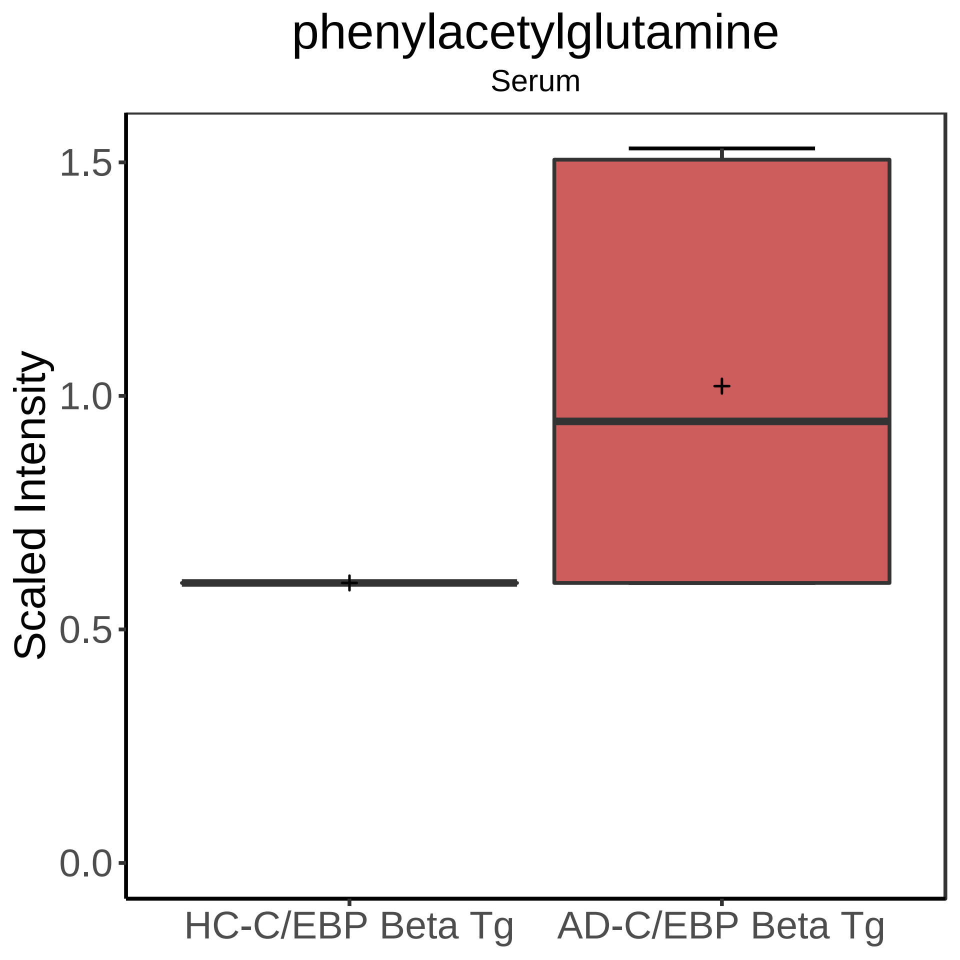
phenylacetylglutamine
Serum
Scaled Intensity
1.5
1.0
0.5
0.0
HC-C/EBP Beta Tg
AD-C/EBP Beta Tg

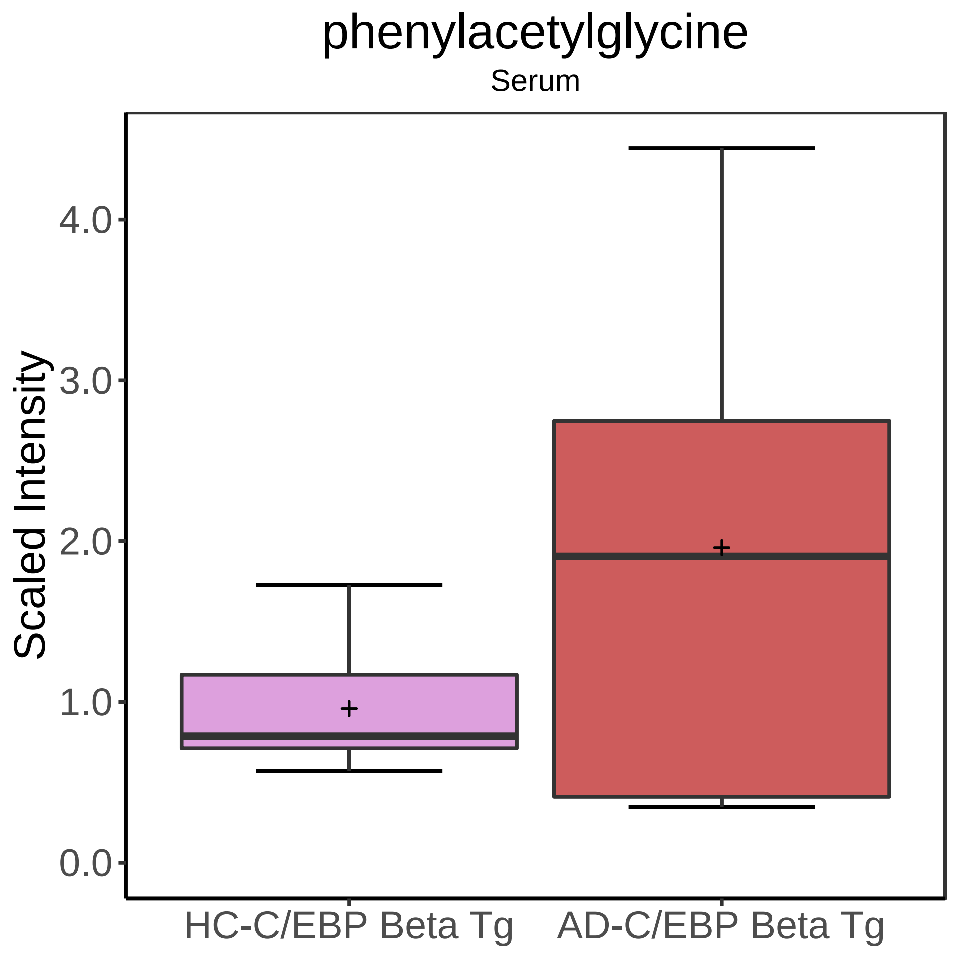
phenylacetylglycine
Serum
Scaled Intensity
4.0
3.0
2.0
1.0
0.0
HC-C/EBP Beta Tg
AD-C/EBP Beta Tg

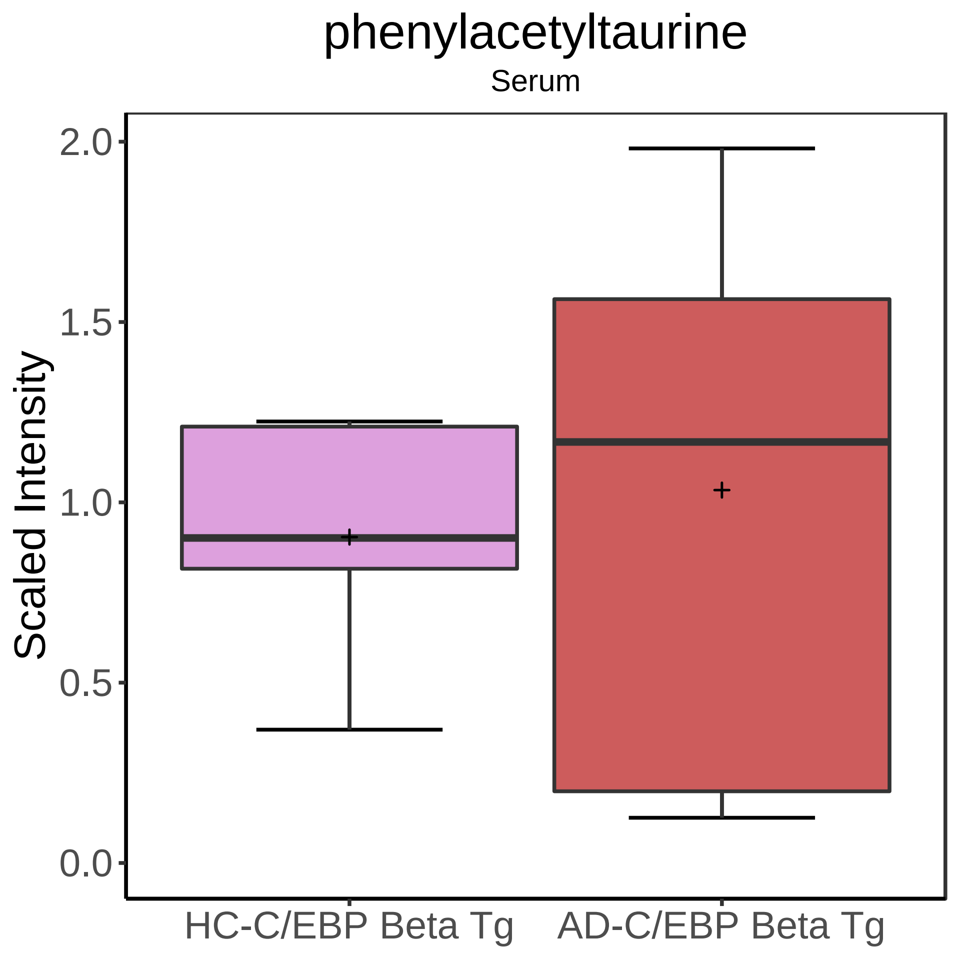
phenylacetyltaurine
Serum
Scaled Intensity
2.0
1.5
1.0
0.5
0.0
HC-C/EBP Beta Tg
AD-C/EBP Beta Tg

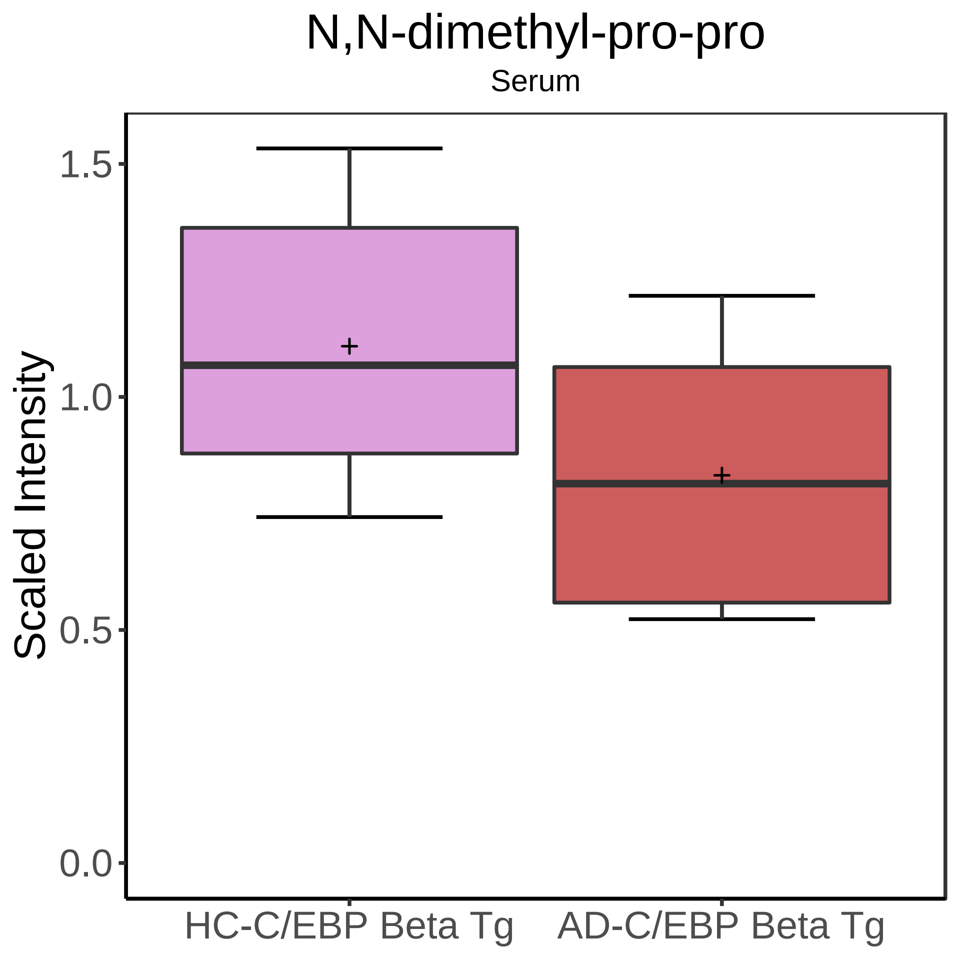
N,N-dimethyl-pro-pro
Serum
Scaled Intensity
1.5
1.0
0.5
0.0
HC-C/EBP Beta Tg
AD-C/EBP Beta Tg

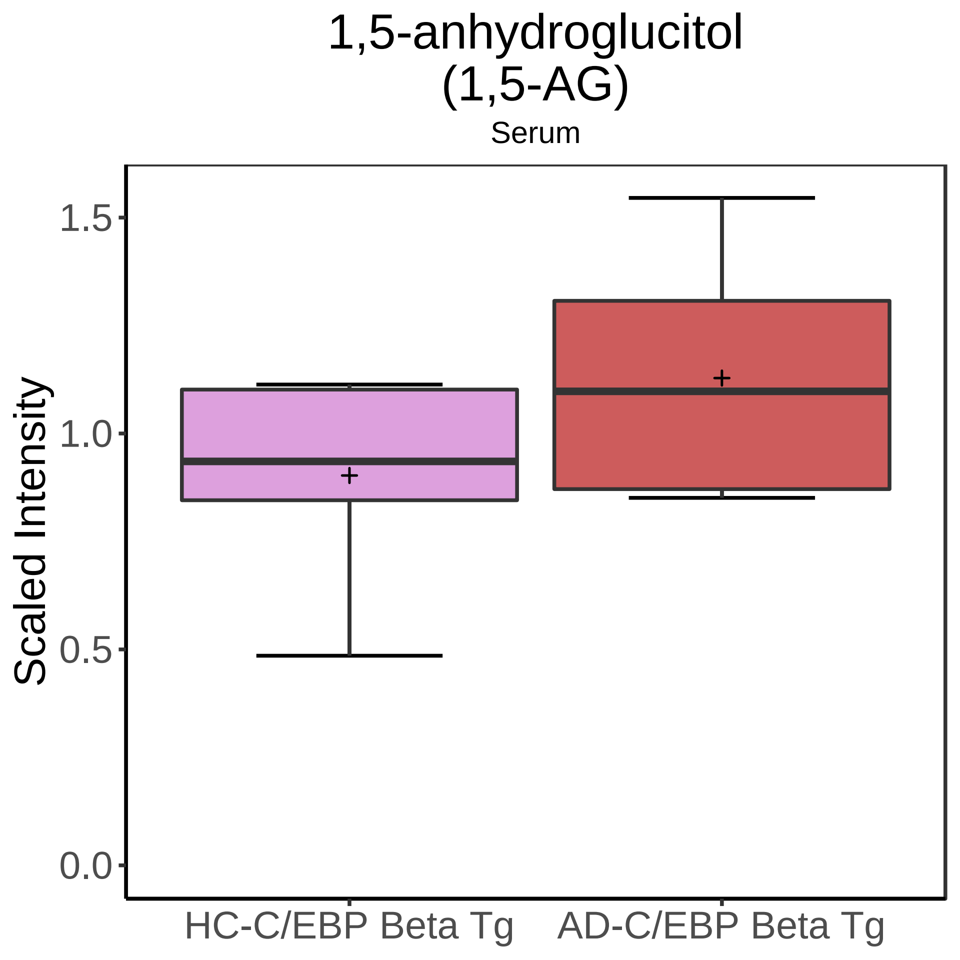
1,5-anhydroglucitol
(1,5-AG)
Serum
Scaled Intensity
1.5
1.0
0.5
0.0
HC-C/EBP Beta Tg
AD-C/EBP Beta Tg

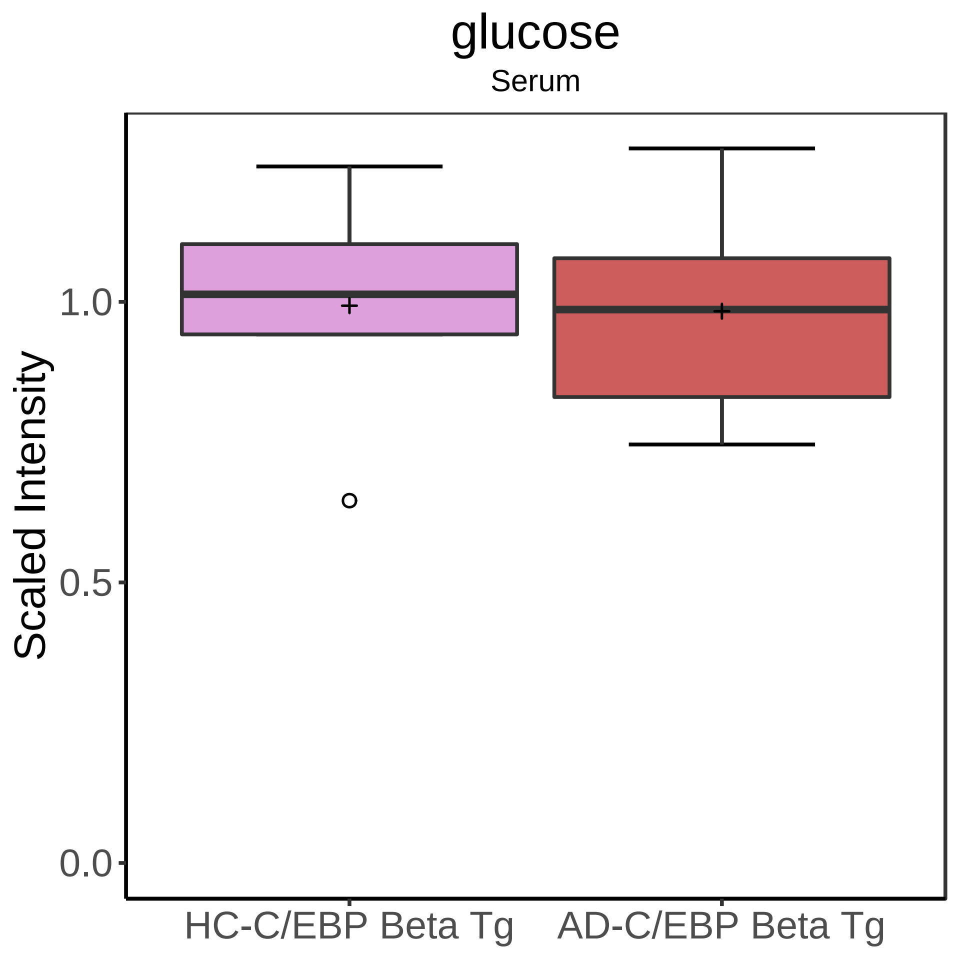
glucose
Serum
Scaled Intensity
1.0
0.5
0.0
HC-C/EBP Beta Tg
AD-C/EBP Beta Tg

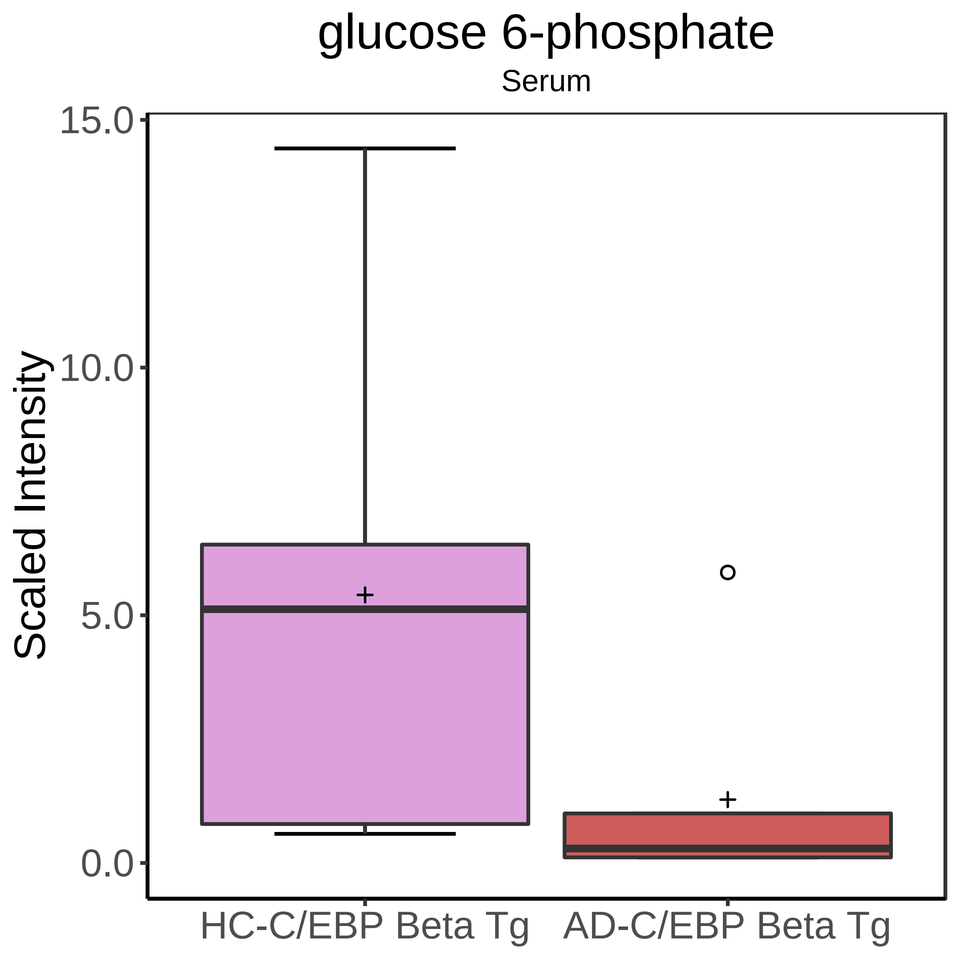
glucose 6-phosphate
Serum
Scaled Intensity
15.0
10.0
5.0
0.0
HC-C/EBP Beta Tg
AD-C/EBP Beta Tg

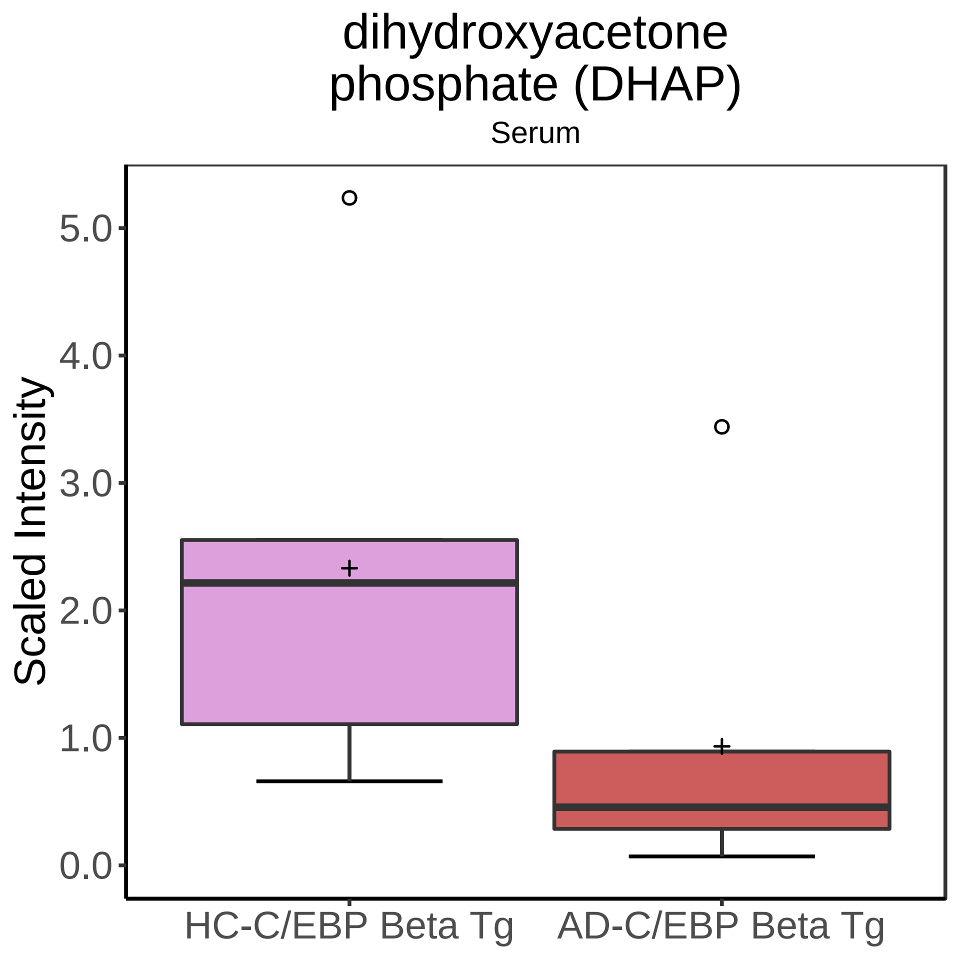
dihydroxyacetone
phosphate (DHAP)
Serum
Scaled Intensity
5.0
4.0
3.0
2.0
1.0
0.0
HC-C/EBP Beta Tg
AD-C/EBP Beta Tg

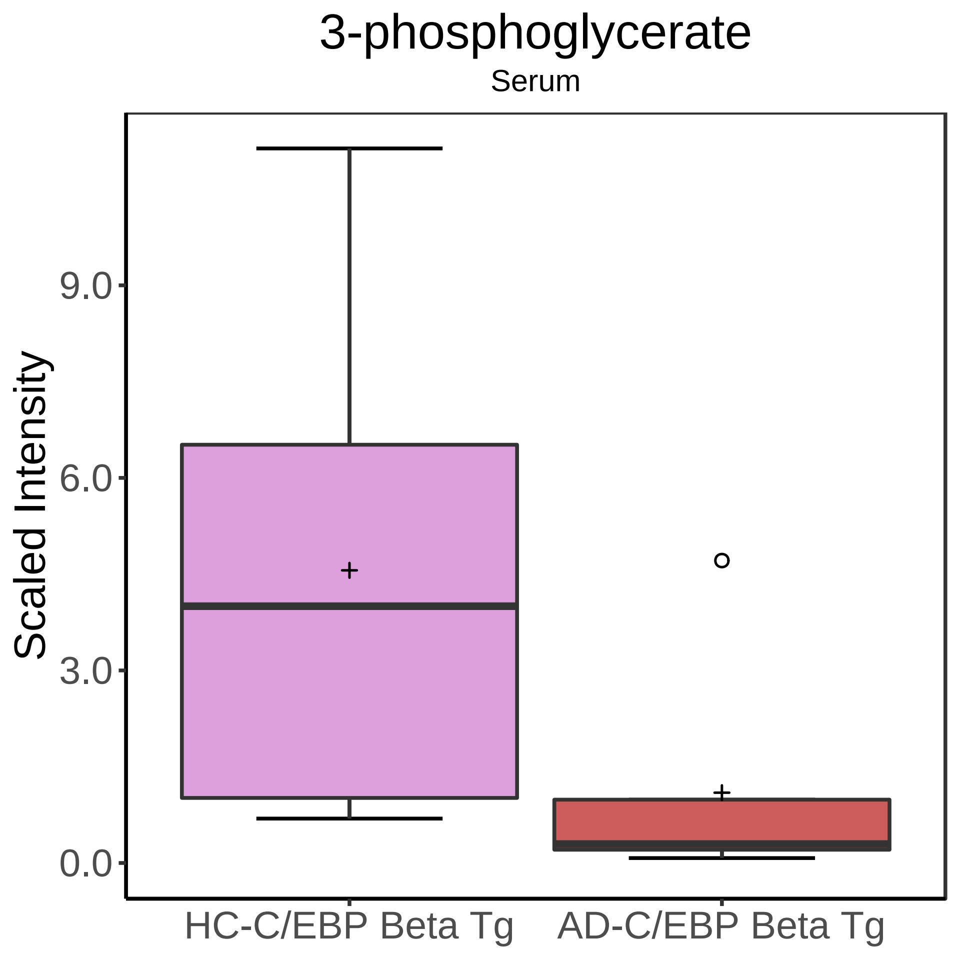
3-phosphoglycerate
Serum
Scaled Intensity
9.0
6.0
3.0
0.0
HC-C/EBP Beta Tg
AD-C/EBP Beta Tg

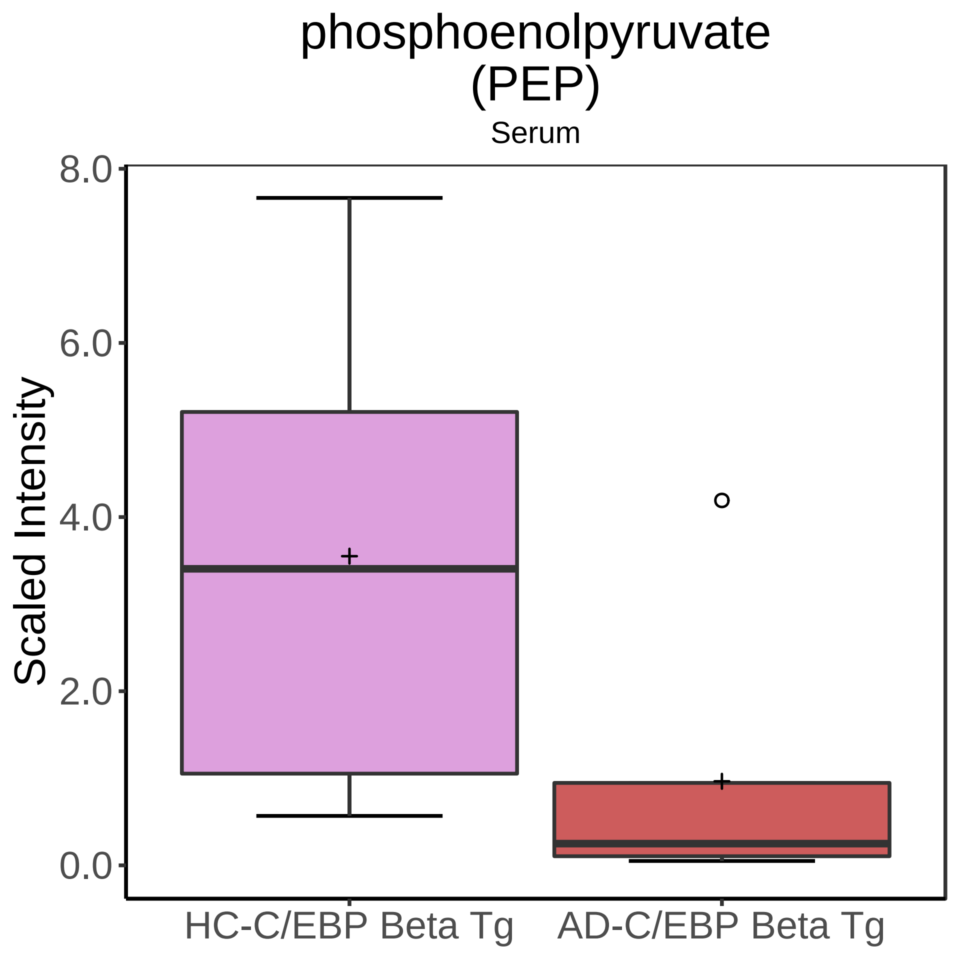
phosphoenolpyruvate
(PEP)
Serum
Scaled Intensity
8.0
6.0
4.0
2.0
0.0
HC-C/EBP Beta Tg
AD-C/EBP Beta Tg

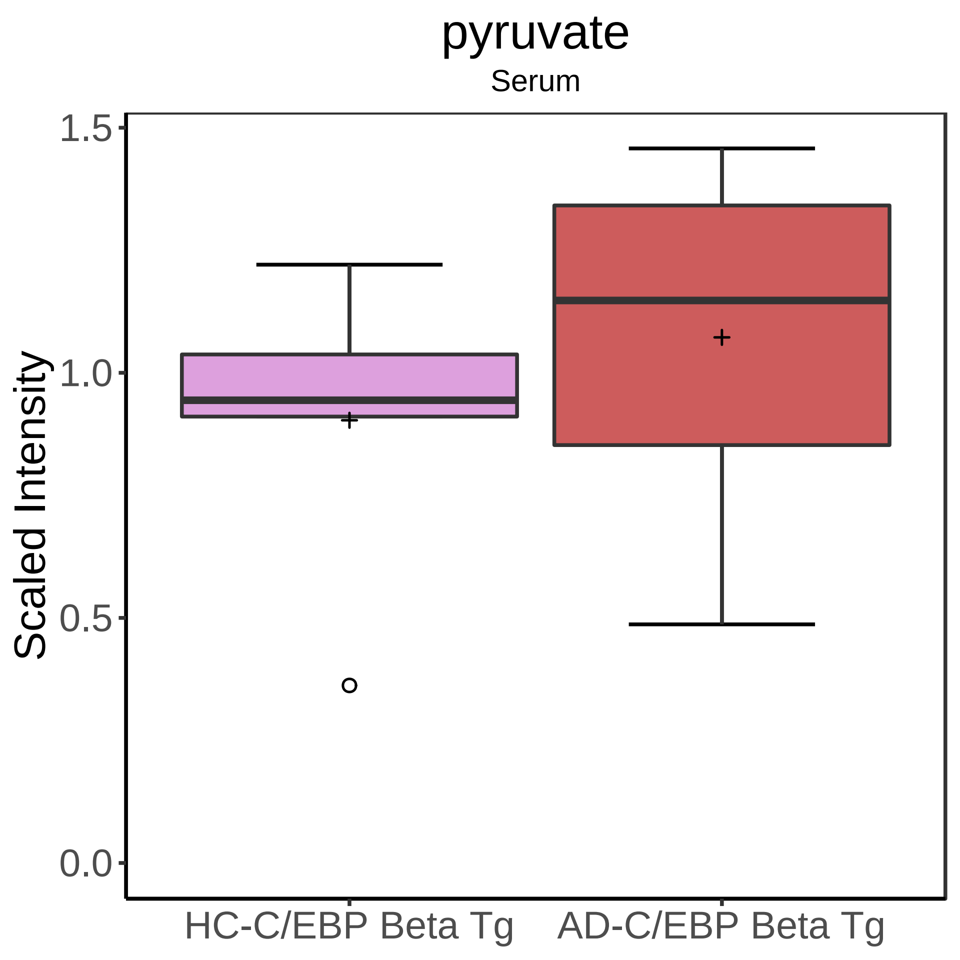
pyruvate
Serum
Scaled Intensity
1.5
1.0
0.5
0.0
HC-C/EBP Beta Tg
AD-C/EBP Beta Tg

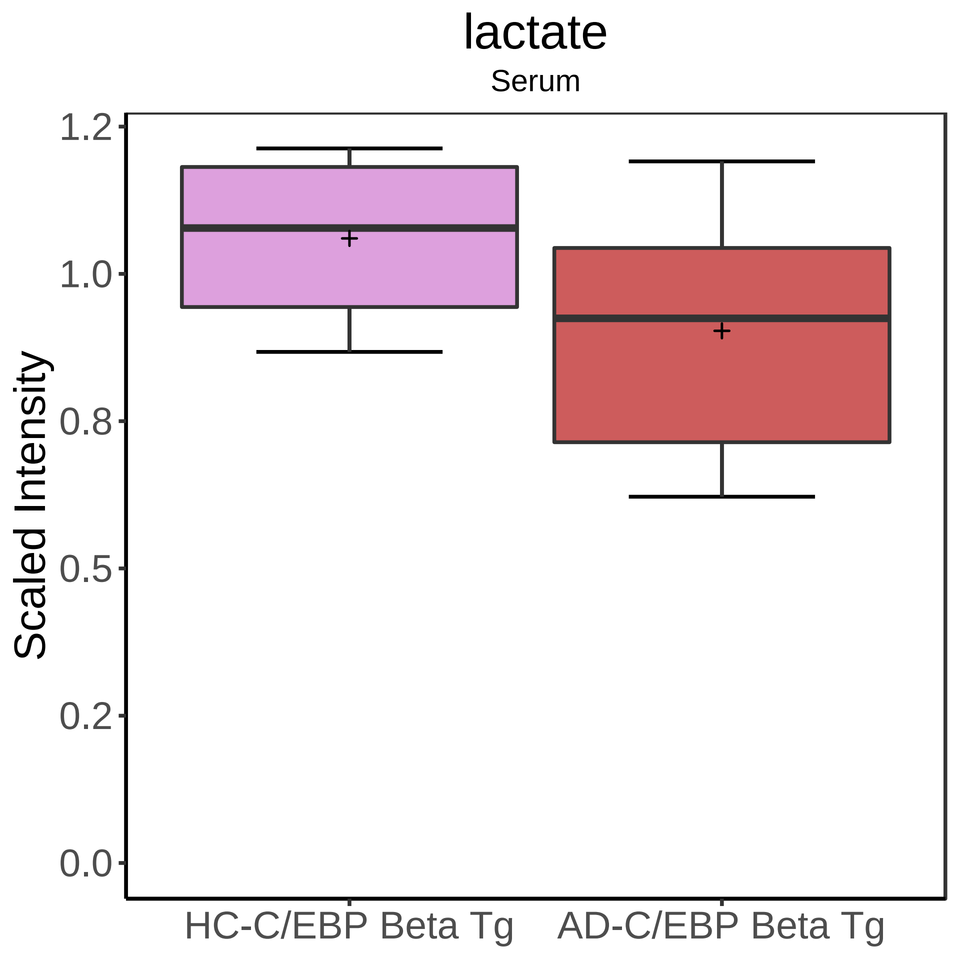
lactate
Serum
Scaled Intensity
1.2
1.0
0.8
0.5
0.2
0.0
HC-C/EBP Beta Tg
AD-C/EBP Beta Tg

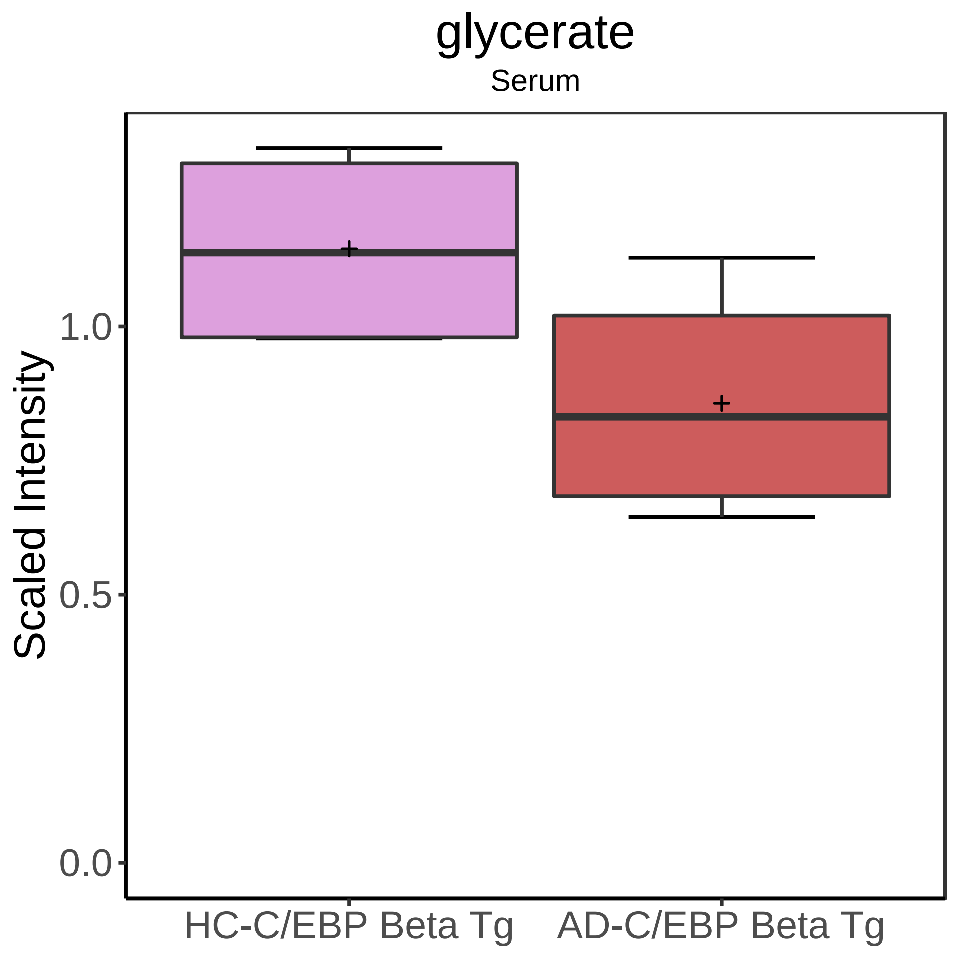
glycerate
Serum
Scaled Intensity
1.0
0.5
0.0
HC-C/EBP Beta Tg
AD-C/EBP Beta Tg

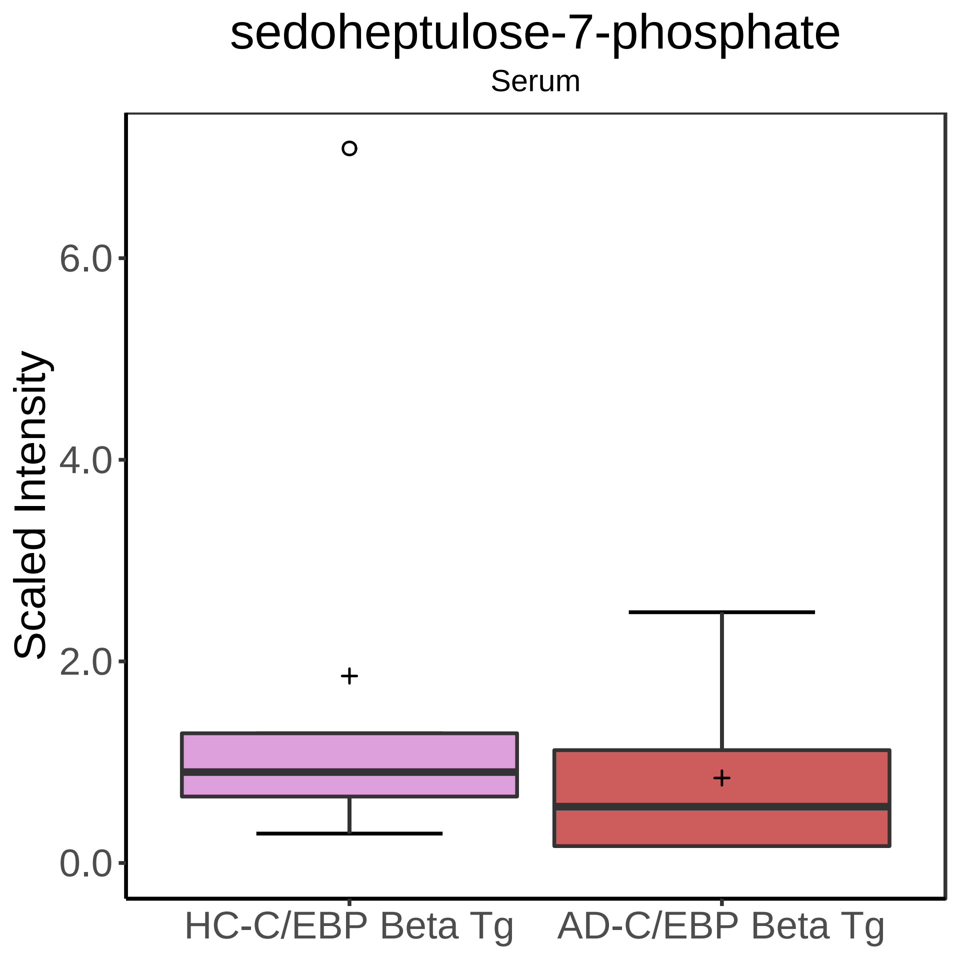
sedoheptulose-7-phosphate
Serum
Scaled Intensity
6.0
4.0
2.0
0.0
HC-C/EBP Beta Tg
AD-C/EBP Beta Tg

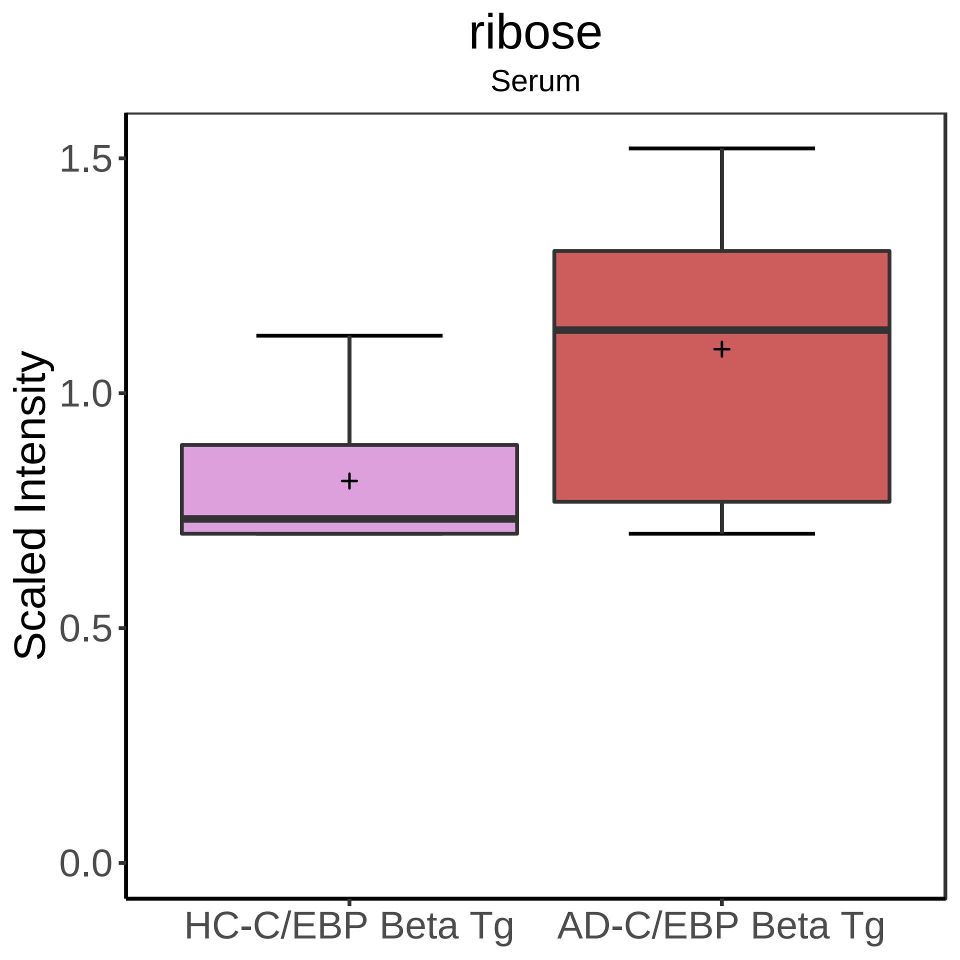
ribose
Serum
Scaled Intensity
1.5
1.0
0.5
0.0
HC-C/EBP Beta Tg
AD-C/EBP Beta Tg

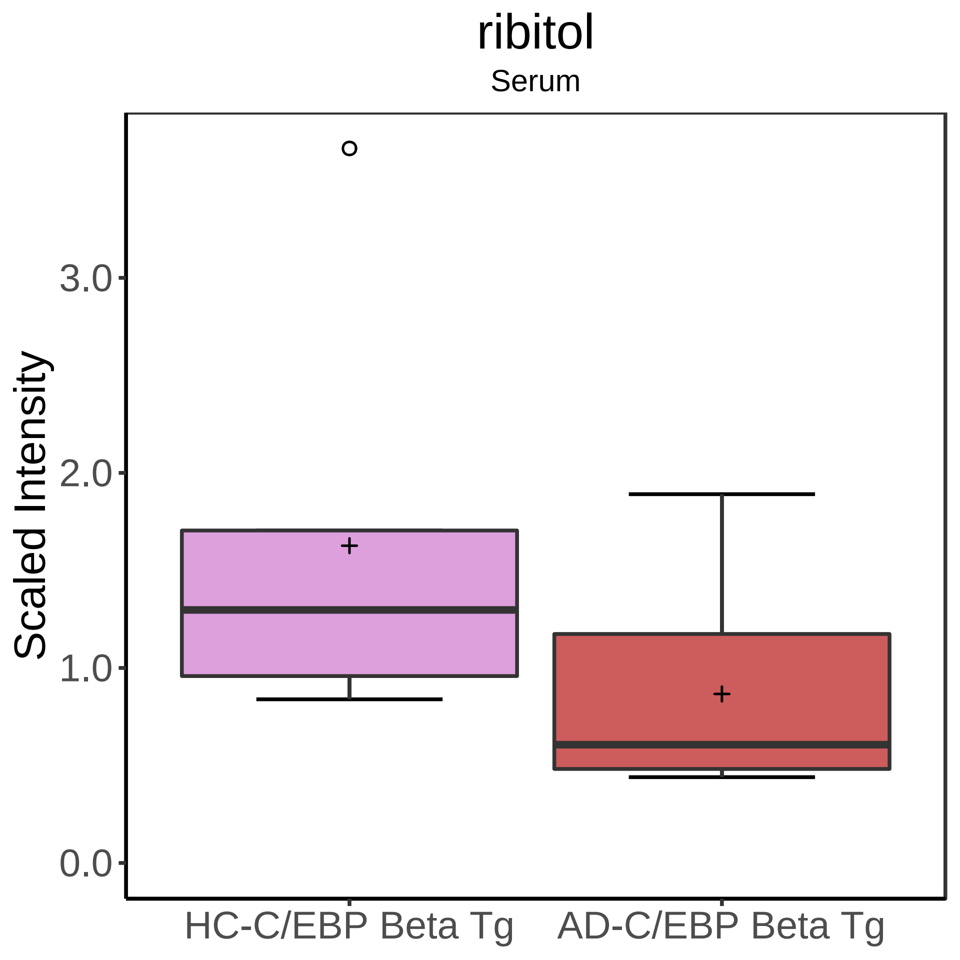
ribitol
Serum
Scaled Intensity
3.0
2.0
1.0
0.0
HC-C/EBP Beta Tg
AD-C/EBP Beta Tg

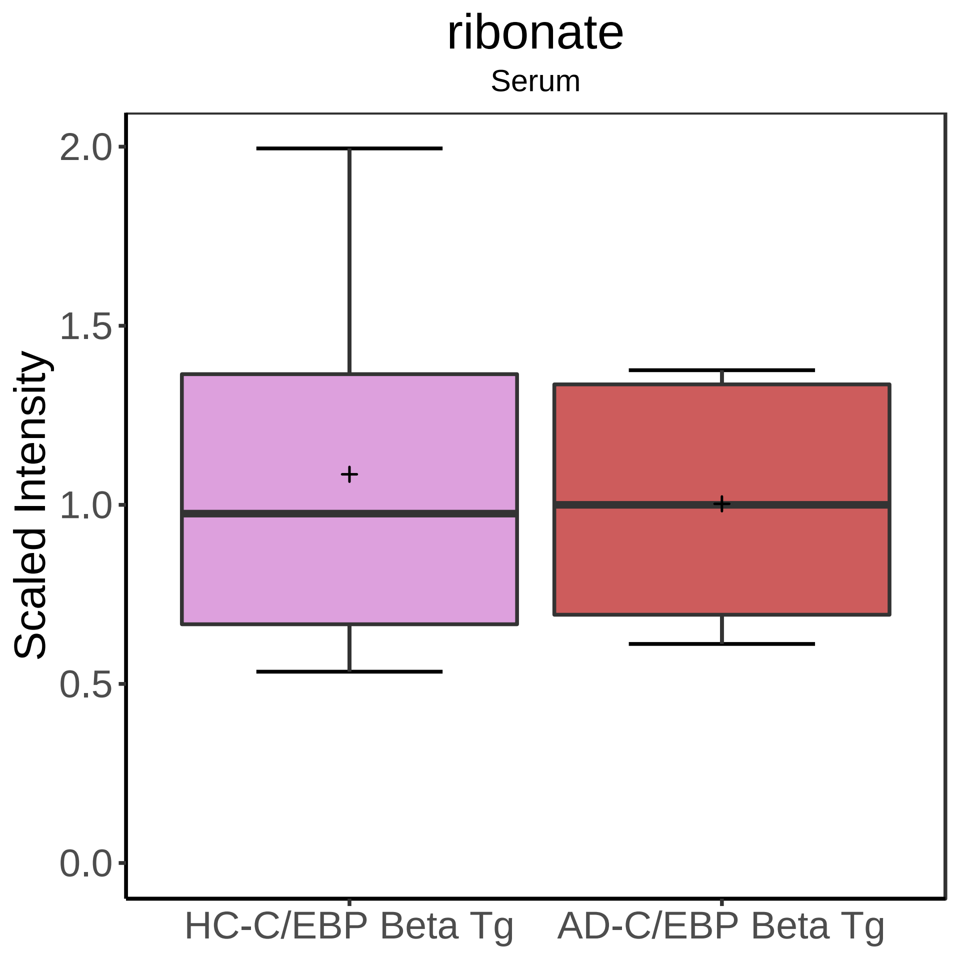
ribonate
Serum
Scaled Intensity
2.0
1.5
1.0
0.5
0.0
HC-C/EBP Beta Tg
AD-C/EBP Beta Tg

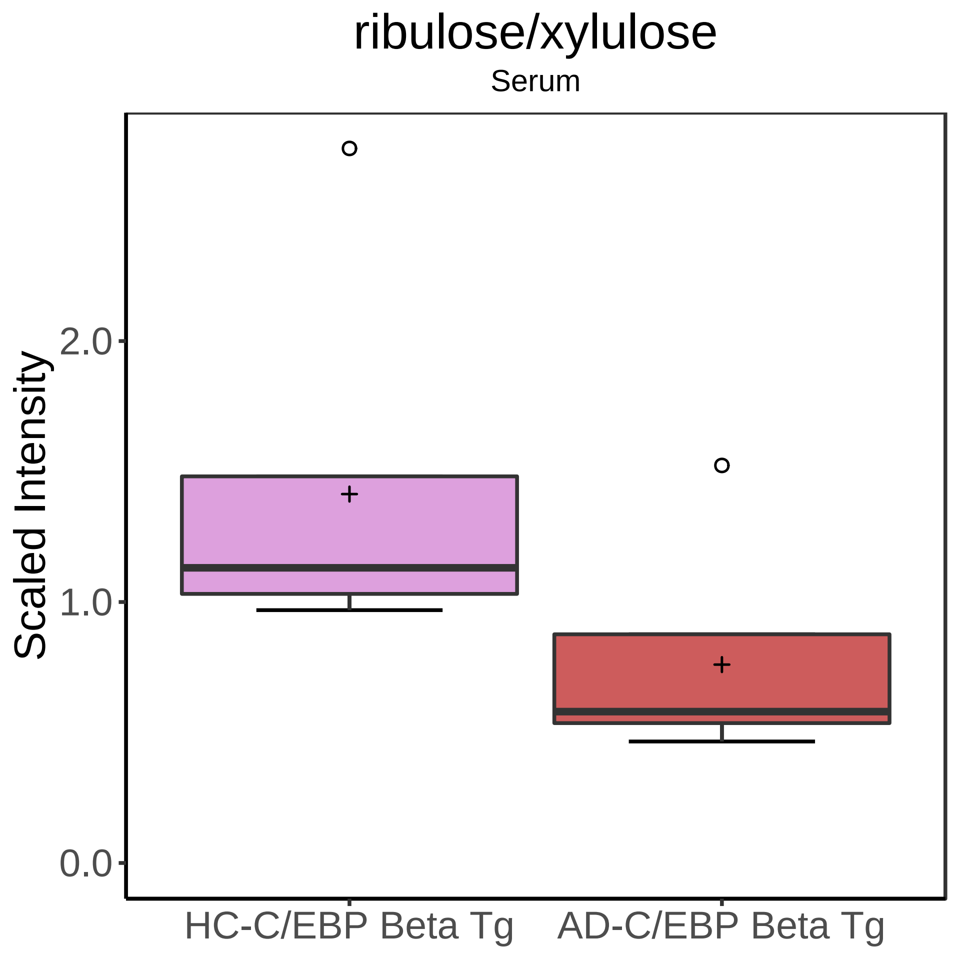
ribulose/xylulose
Serum
Scaled Intensity
2.0
1.0
0.0
HC-C/EBP Beta Tg
AD-C/EBP Beta Tg

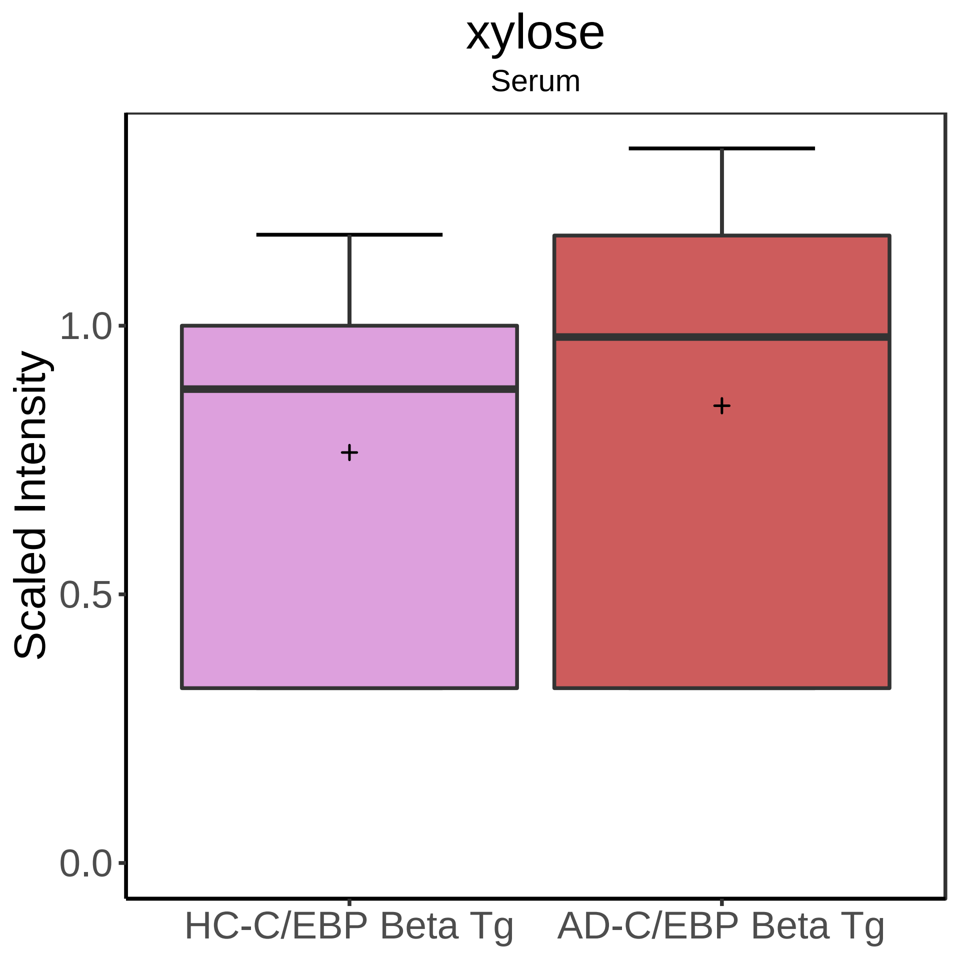
xylose
Serum
Scaled Intensity
1.0
0.5
0.0
HC-C/EBP Beta Tg
AD-C/EBP Beta Tg

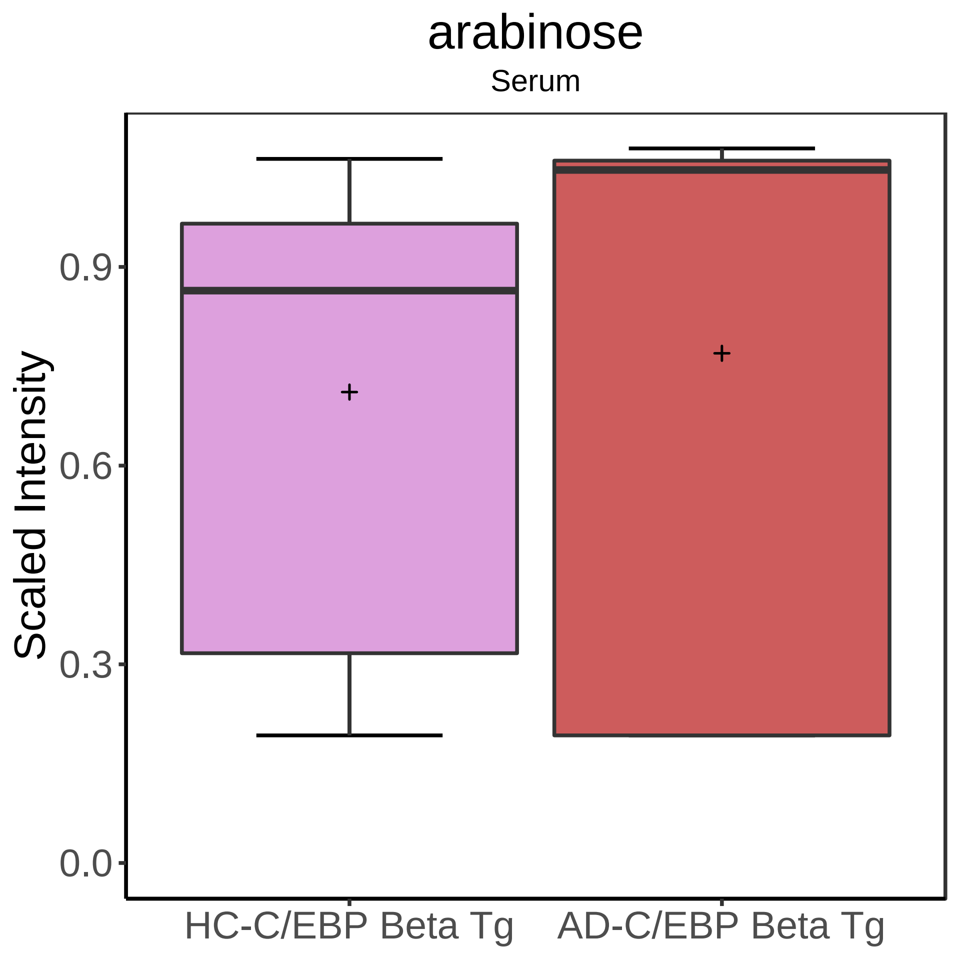
arabinose
Serum
Scaled Intensity
0.9
0.6
0.3
0.0
HC-C/EBP Beta Tg
AD-C/EBP Beta Tg

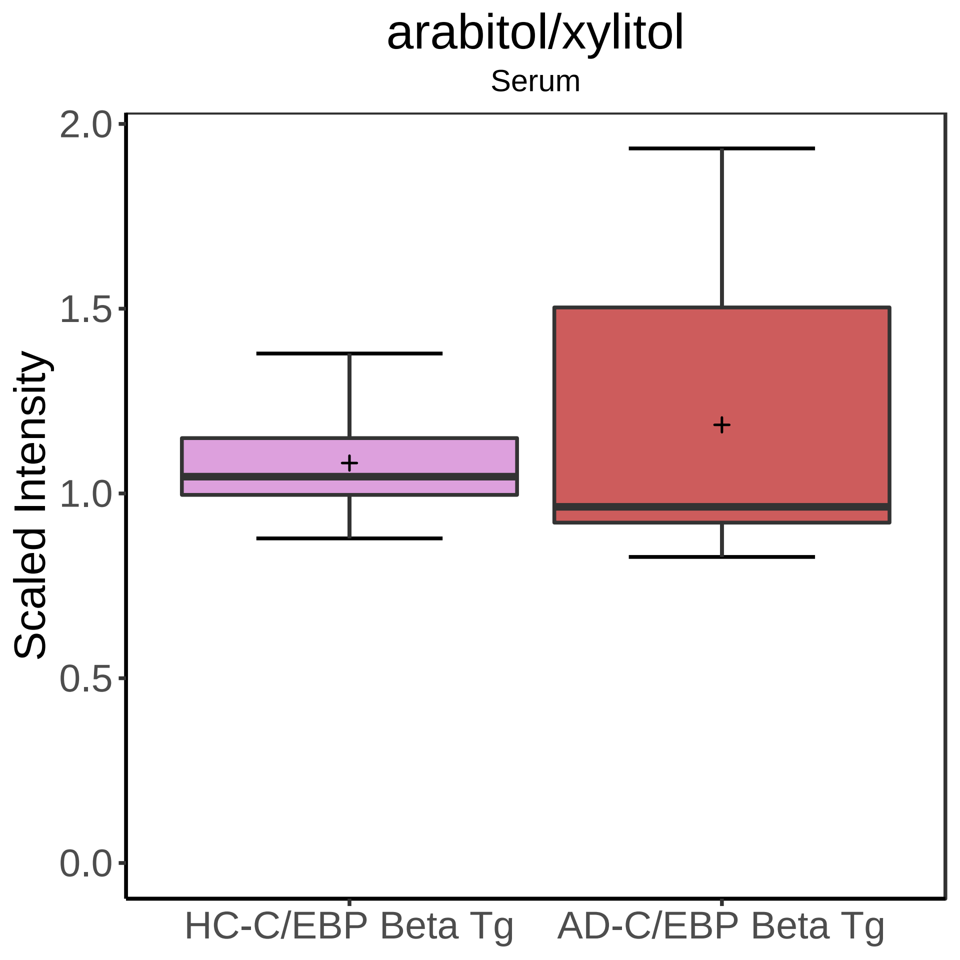
arabitol/xylitol
Serum
Scaled Intensity
2.0
1.5
1.0
0.5
0.0
HC-C/EBP Beta Tg
AD-C/EBP Beta Tg

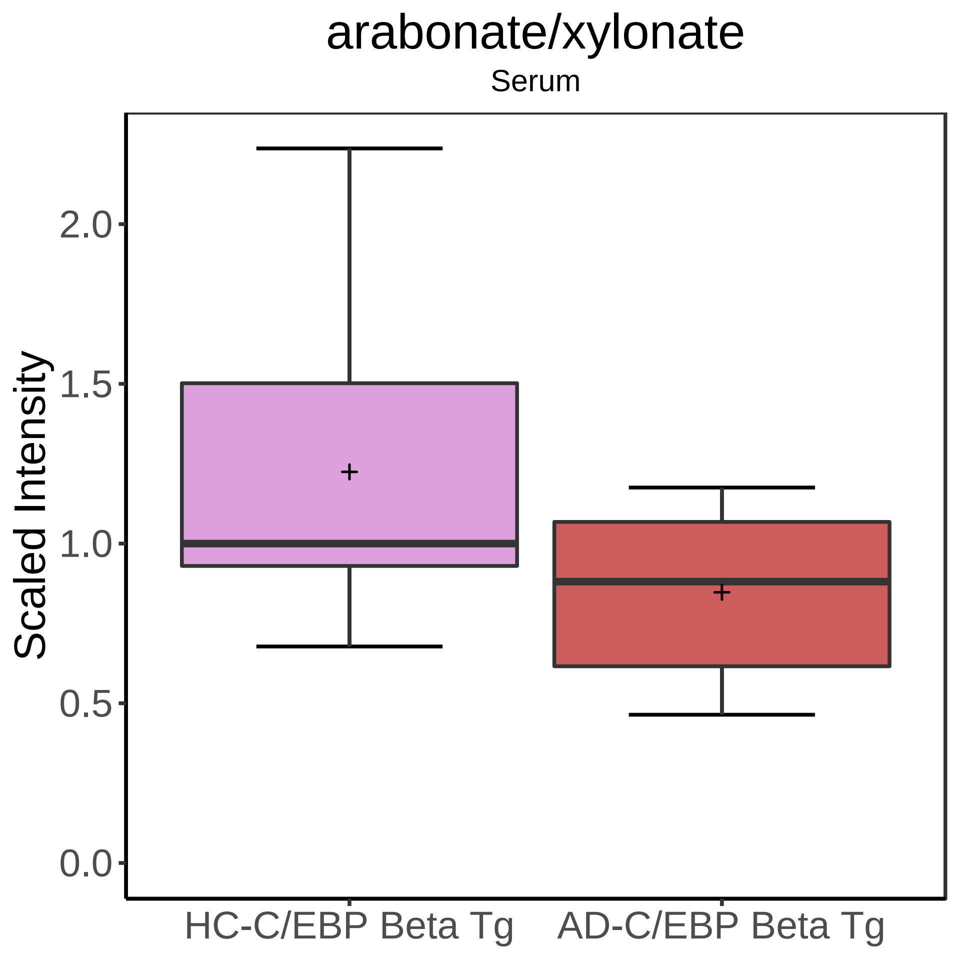
arabonate/xylonate
Serum
Scaled Intensity
2.0
1.5
1.0
0.5
0.0
HC-C/EBP Beta Tg
AD-C/EBP Beta Tg

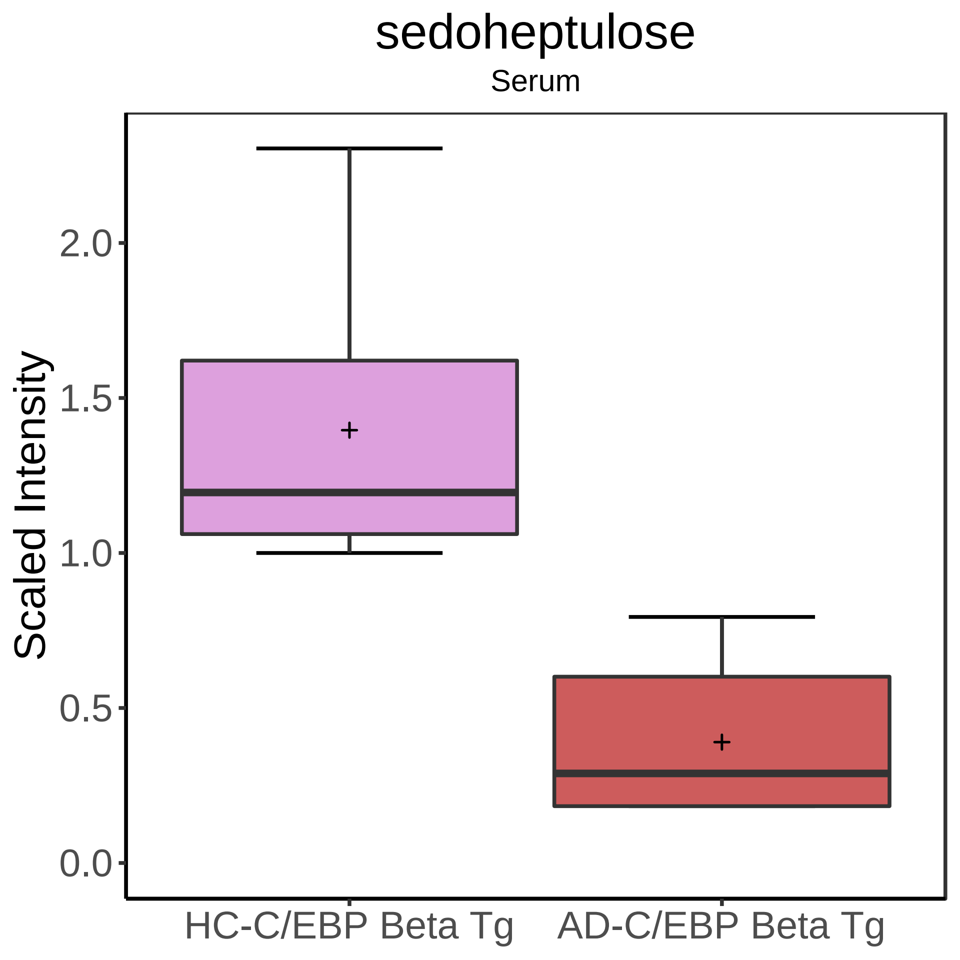
sedoheptulose
Serum
Scaled Intensity
2.0
1.5
1.0
0.5
0.0
HC-C/EBP Beta Tg
AD-C/EBP Beta Tg

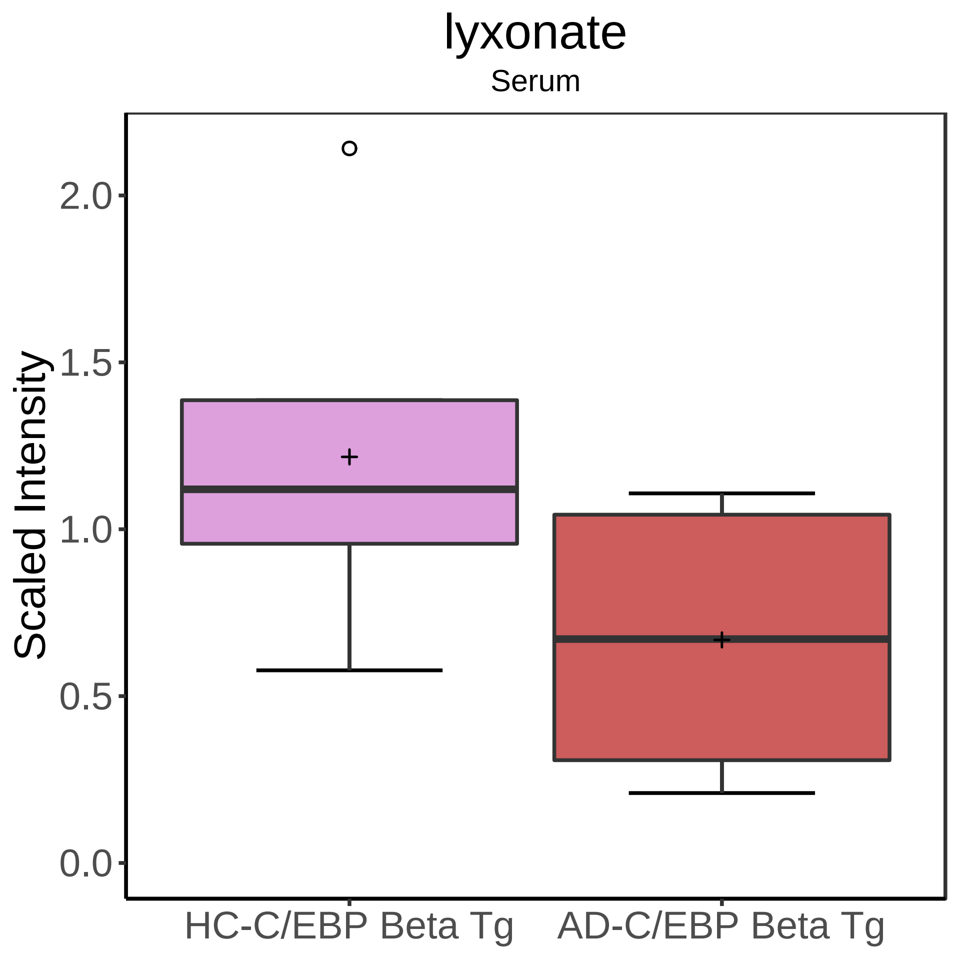
lyxonate
Serum
Scaled Intensity
2.0
1.5
1.0
0.5
0.0
HC-C/EBP Beta Tg
AD-C/EBP Beta Tg

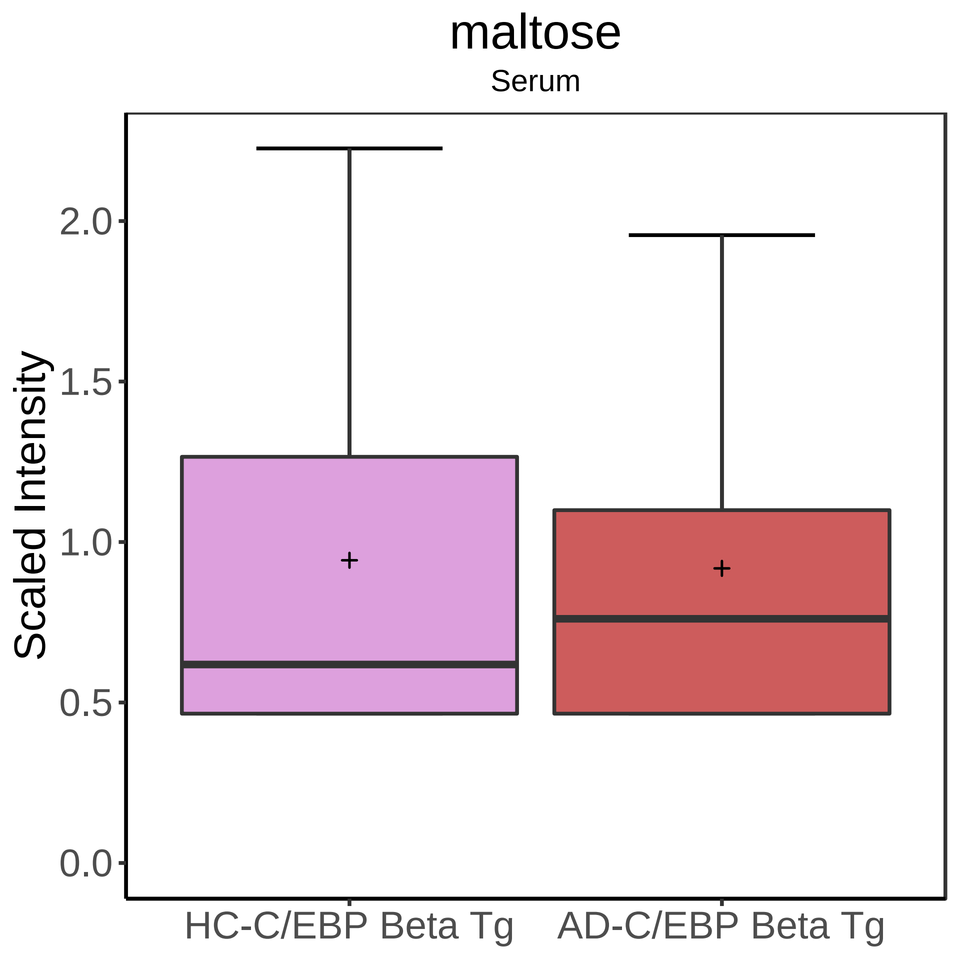
maltose
Serum
Scaled Intensity
2.0
1.5
1.0
0.5
0.0
HC-C/EBP Beta Tg
AD-C/EBP Beta Tg

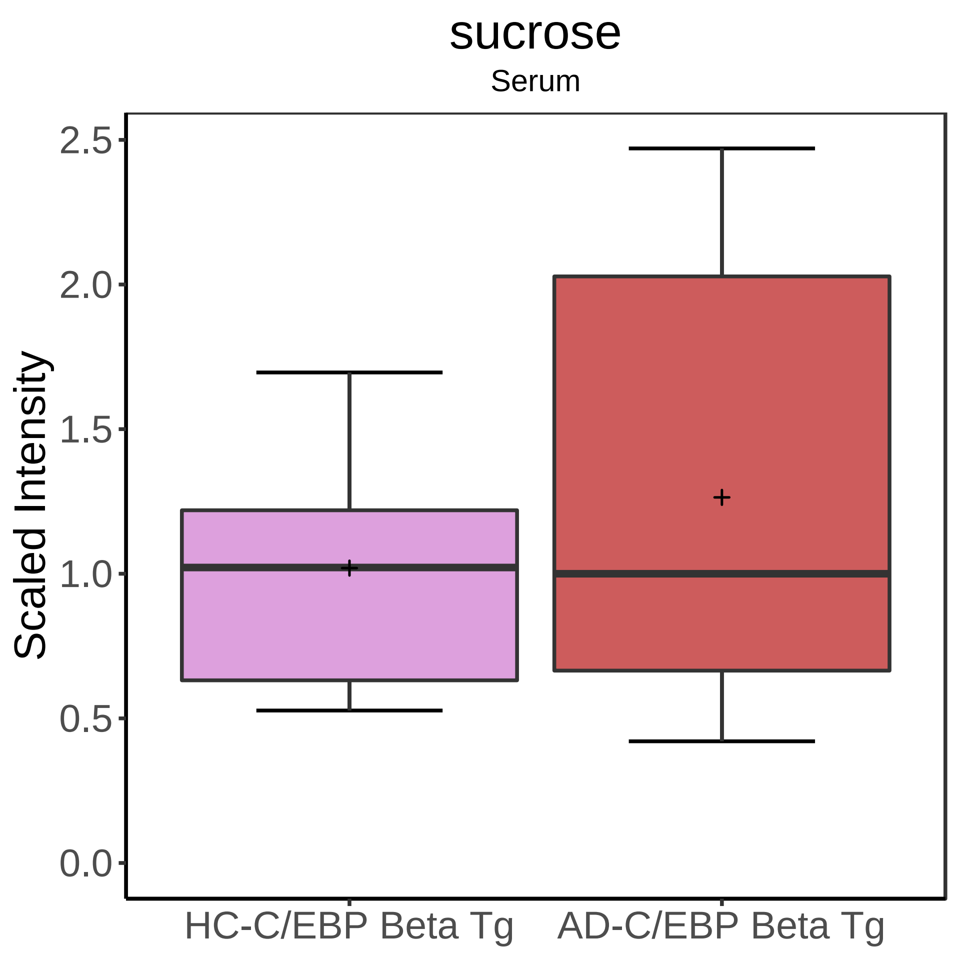
sucrose
Serum
Scaled Intensity
2.5
2.0
1.5
1.0
0.5
0.0
HC-C/EBP Beta Tg
AD-C/EBP Beta Tg

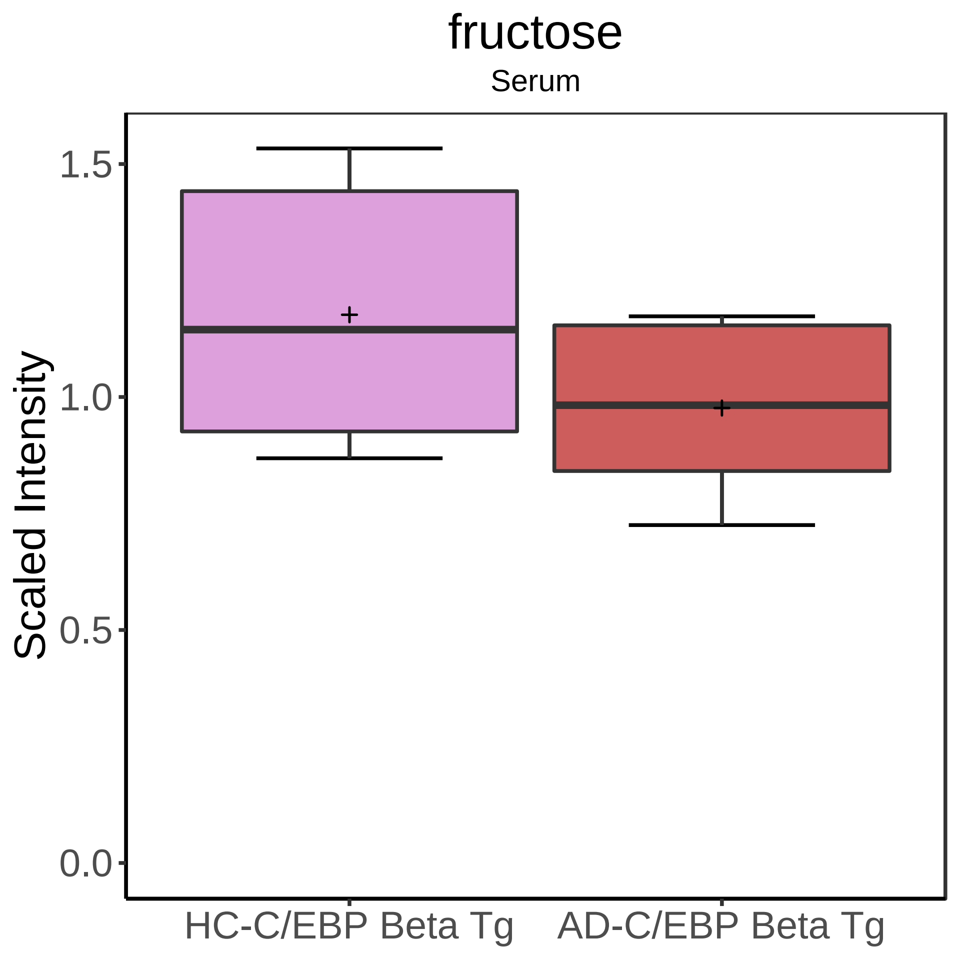

fructose
Serum
Scaled Intensity
1.5
1.0
0.5
0.0
HC-C/EBP Beta Tg
AD-C/EBP Beta Tg

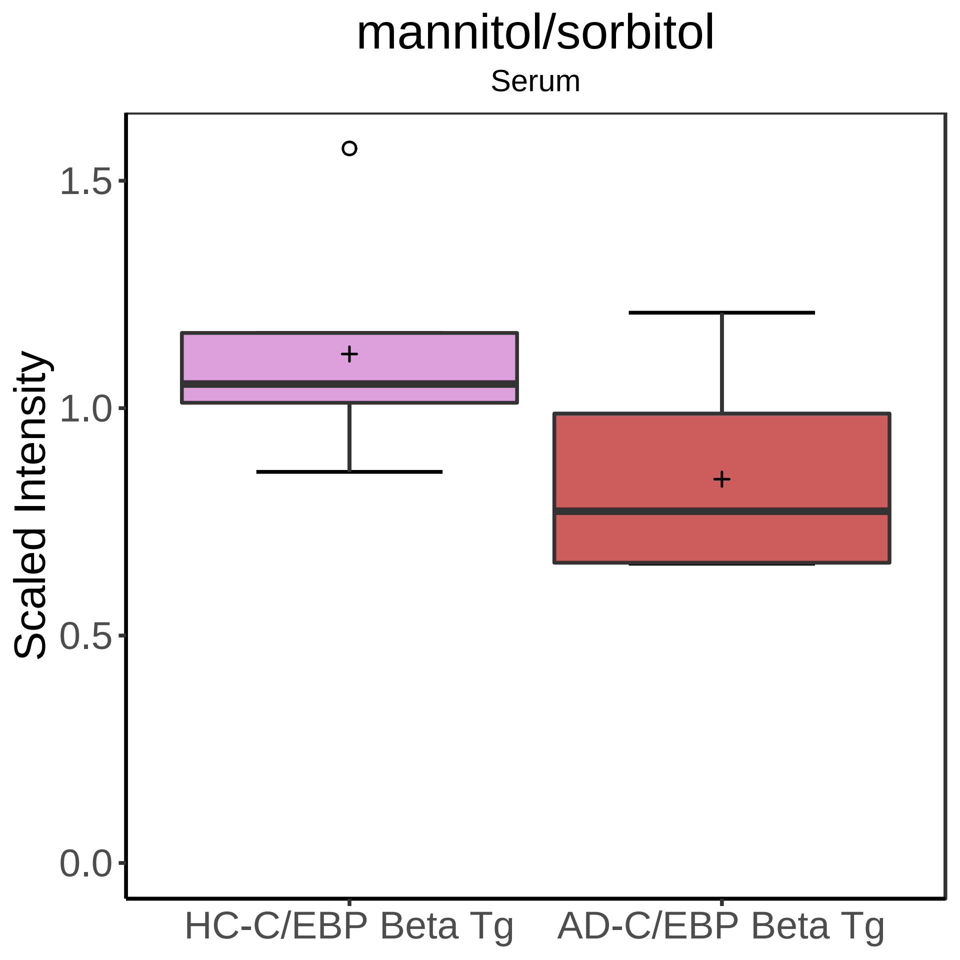
mannitol/sorbitol
Serum
Scaled Intensity
1.5
1.0
0.5
0.0
HC-C/EBP Beta Tg
AD-C/EBP Beta Tg

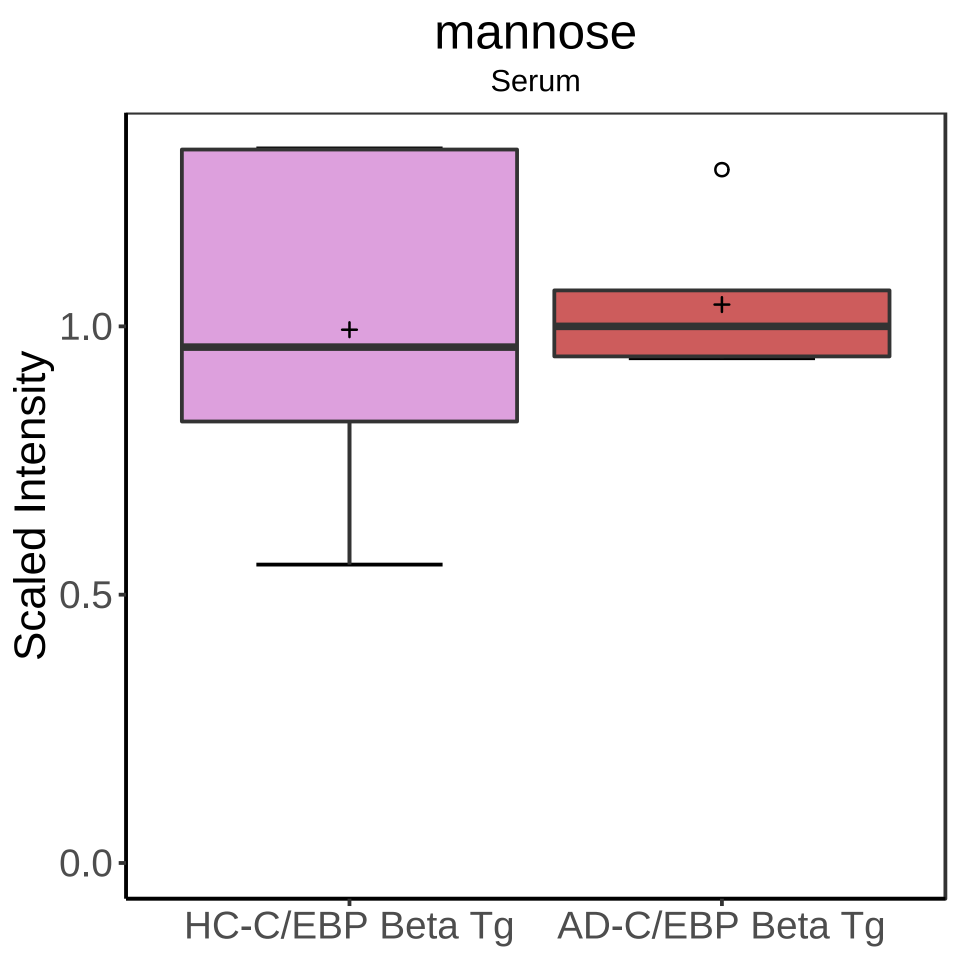
mannose
Serum
Scaled Intensity
1.0
0.5
0.0
HC-C/EBP Beta Tg
AD-C/EBP Beta Tg

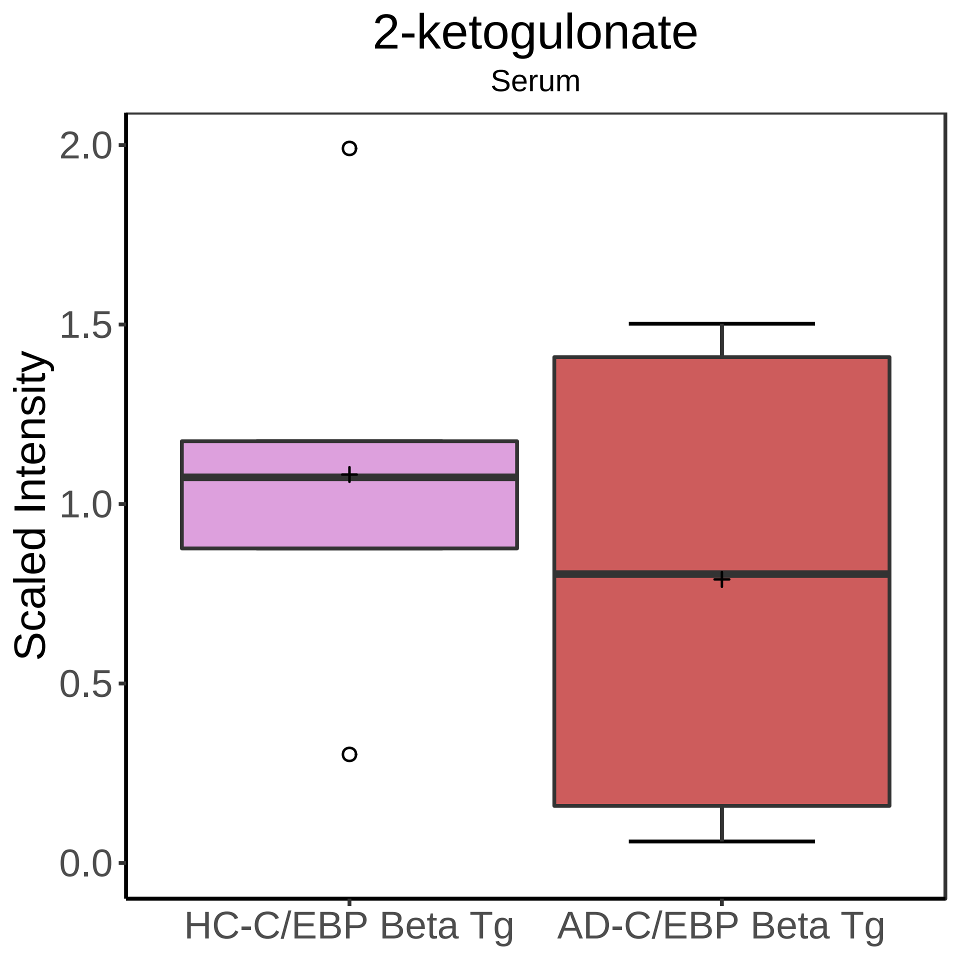
2-ketogulonate
Serum
Scaled Intensity
2.0
1.5
1.0
0.5
0.0
HC-C/EBP Beta Tg
AD-C/EBP Beta Tg

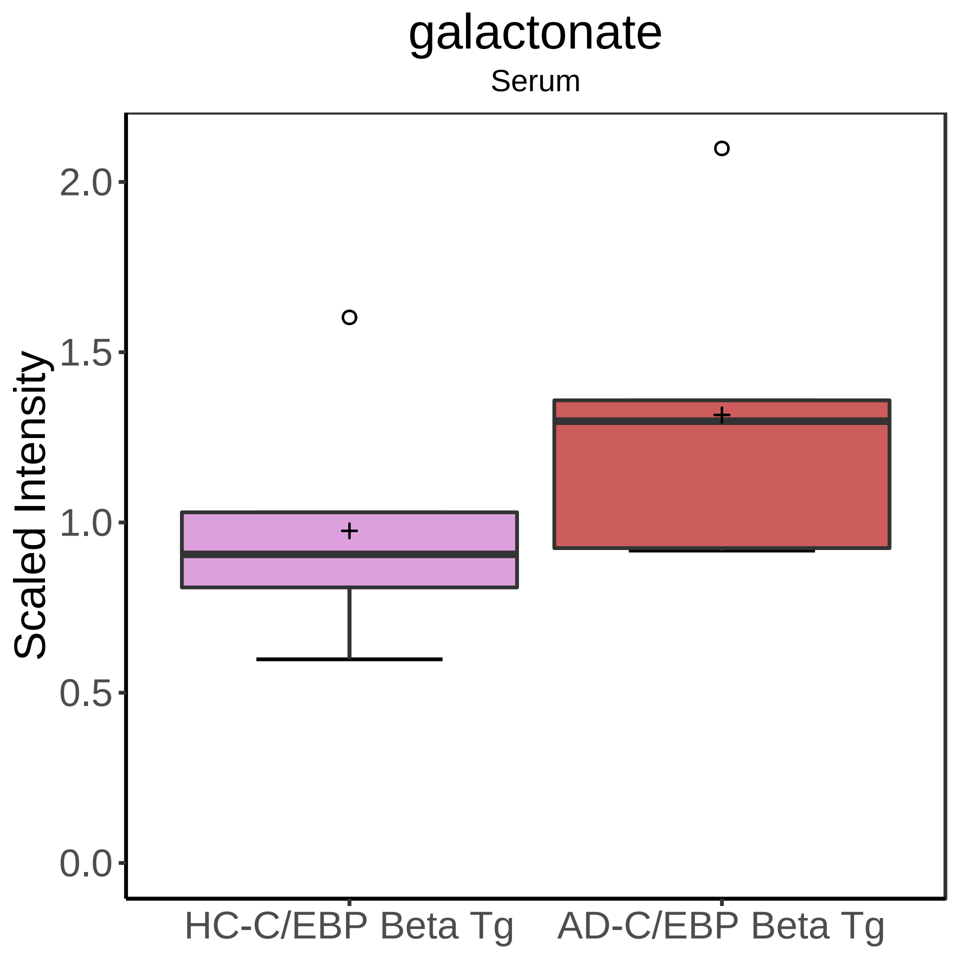
galactonate
Serum
Scaled Intensity
2.0
1.5
1.0
0.5
0.0
HC-C/EBP Beta Tg
AD-C/EBP Beta Tg

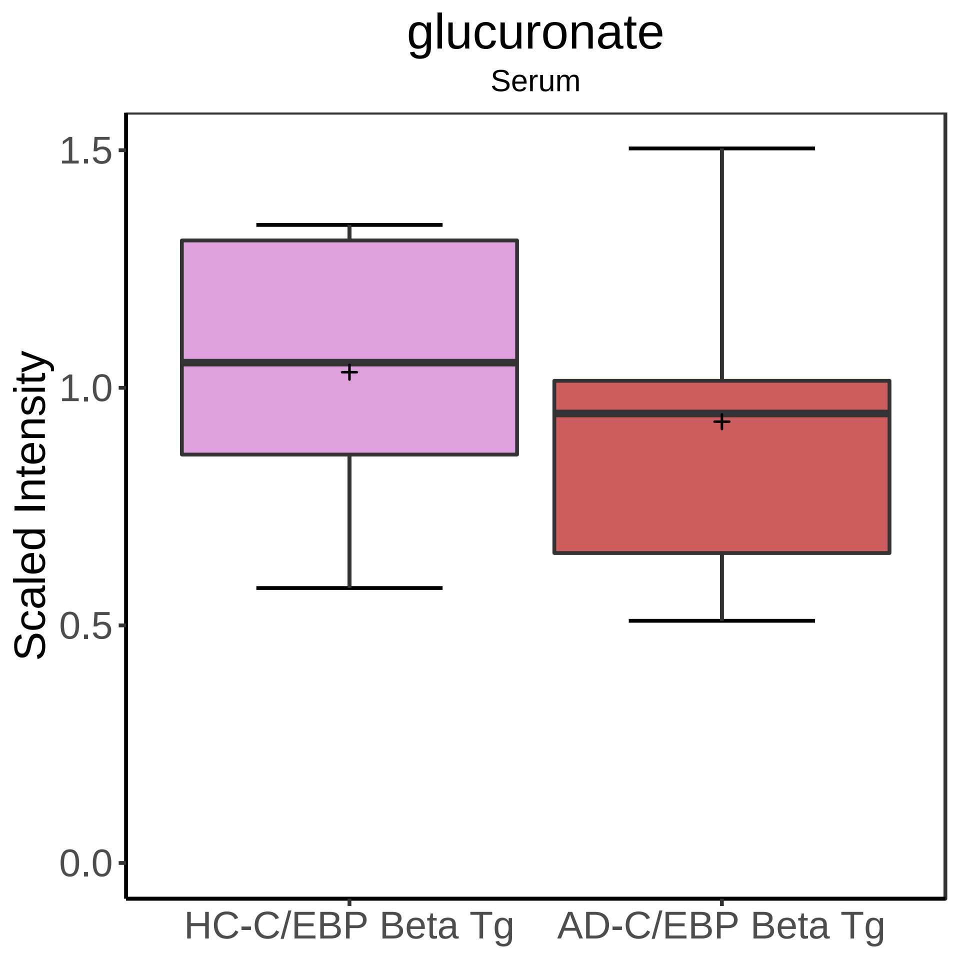
glucuronate
Serum
Scaled Intensity
1.5
1.0
0.5
0.0
HC-C/EBP Beta Tg
AD-C/EBP Beta Tg

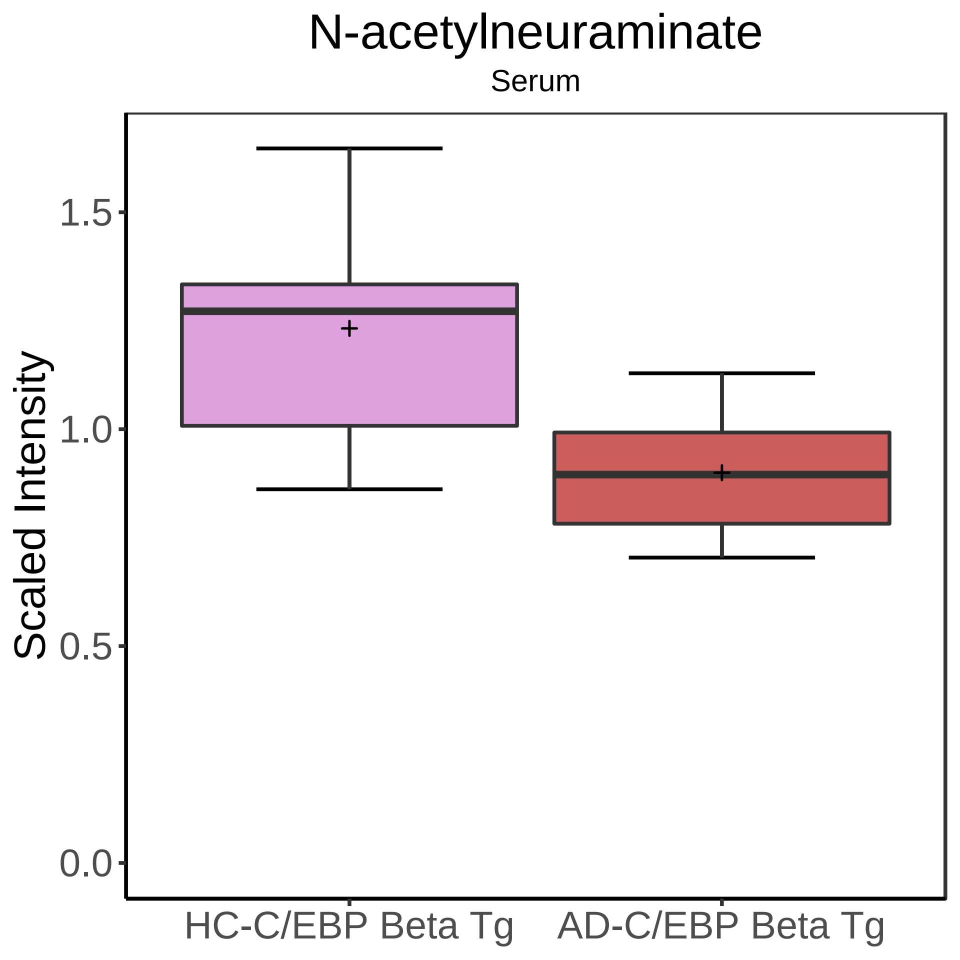

N-acetylneuraminate
Serum
Scaled Intensity
1.5
1.0
0.5
0.0
HC-C/EBP Beta Tg
AD-C/EBP Beta Tg

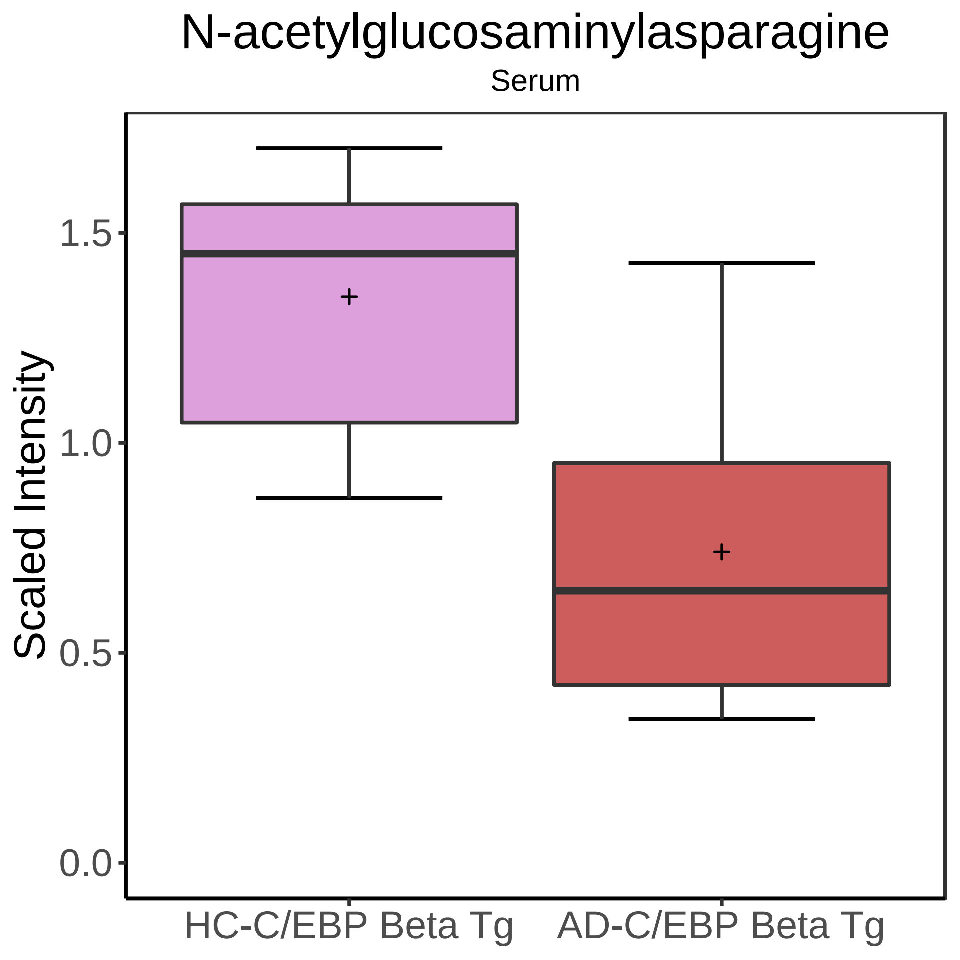
N-acetylglucosaminylasparagine
Serum
Scaled Intensity
0.0
0.5
1.0
1.5
HC-C/EBP Beta Tg
AD-C/EBP Beta Tg

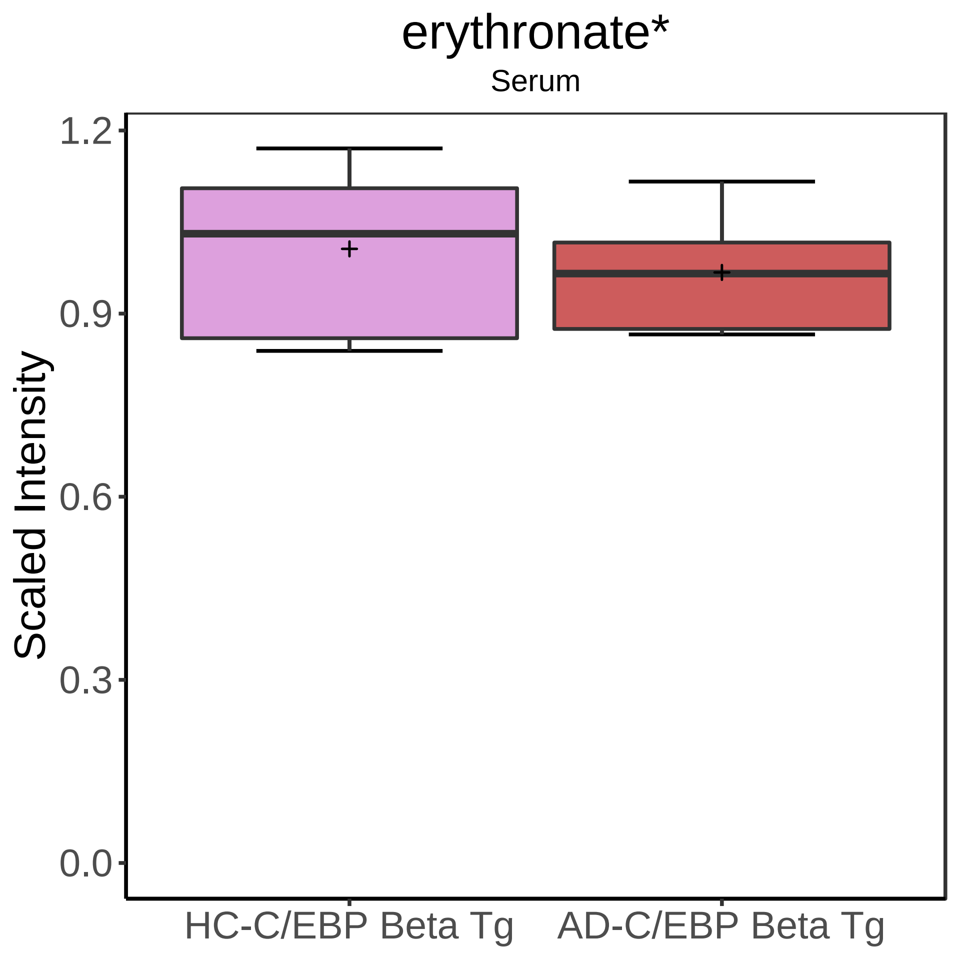
erythronate*
Serum
Scaled Intensity
1.2
0.9
0.6
0.3
0.0
HC-C/EBP Beta Tg
AD-C/EBP Beta Tg

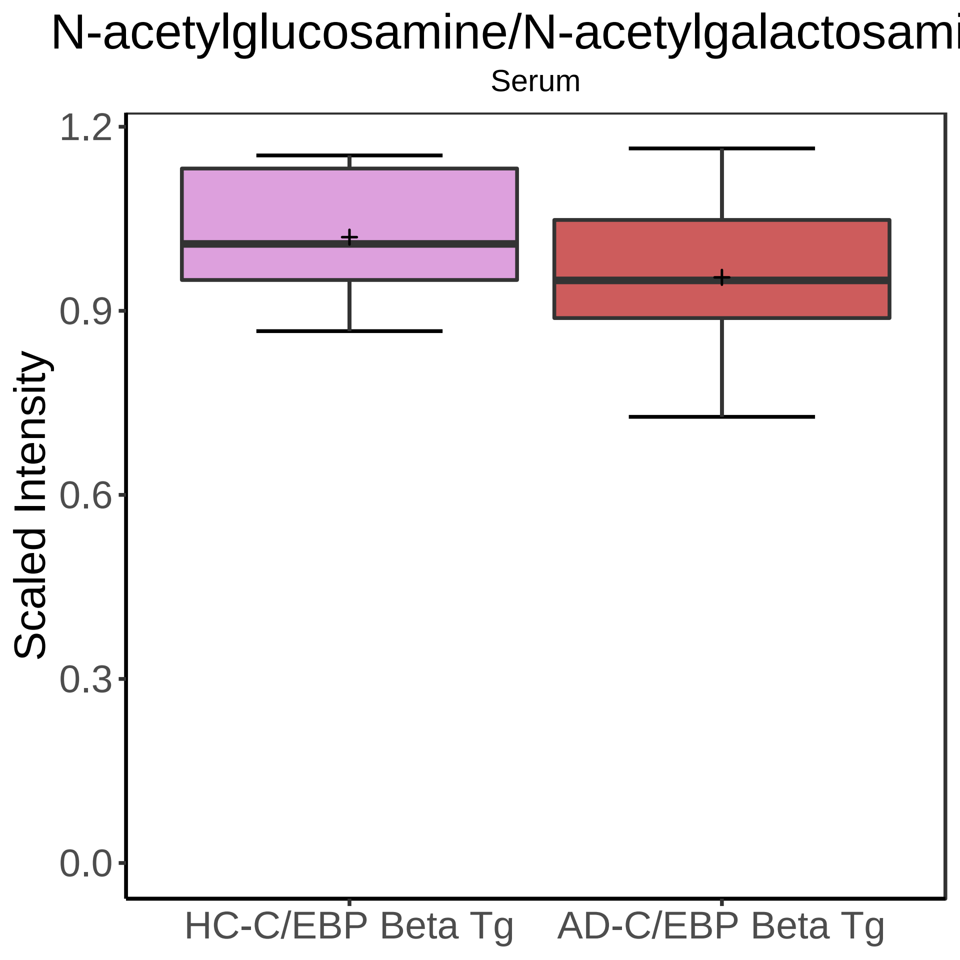
N-acetylglucosamine/N-acetylgalactosami
Serum
Scaled Intensity
1.2
0.9
0.6
0.3
0.0
HC-C/EBP Beta Tg
AD-C/EBP Beta Tg

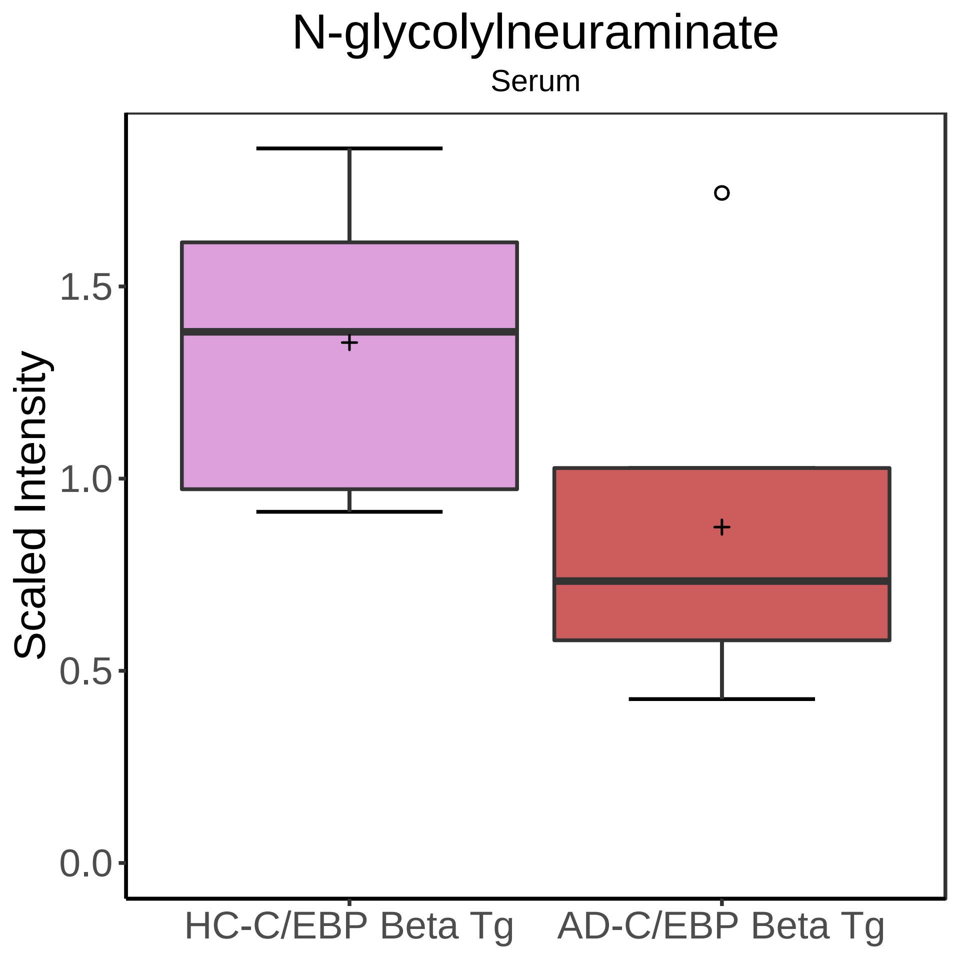
N-glycolylneuraminate
Serum
Scaled Intensity
1.5
1.0
0.5
0.0
HC-C/EBP Beta Tg
AD-C/EBP Beta Tg

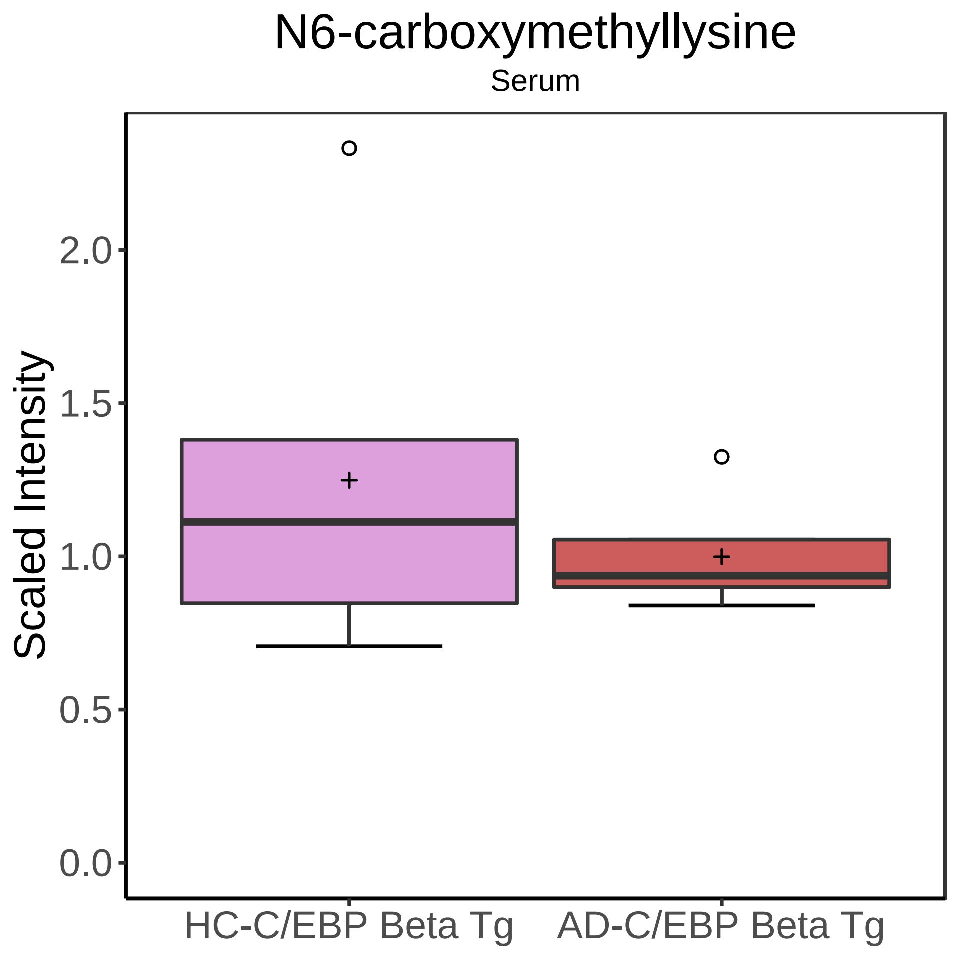
N6-carboxymethyllysine
Serum
Scaled Intensity
2.0
1.5
1.0
0.5
0.0
HC-C/EBP Beta Tg
AD-C/EBP Beta Tg

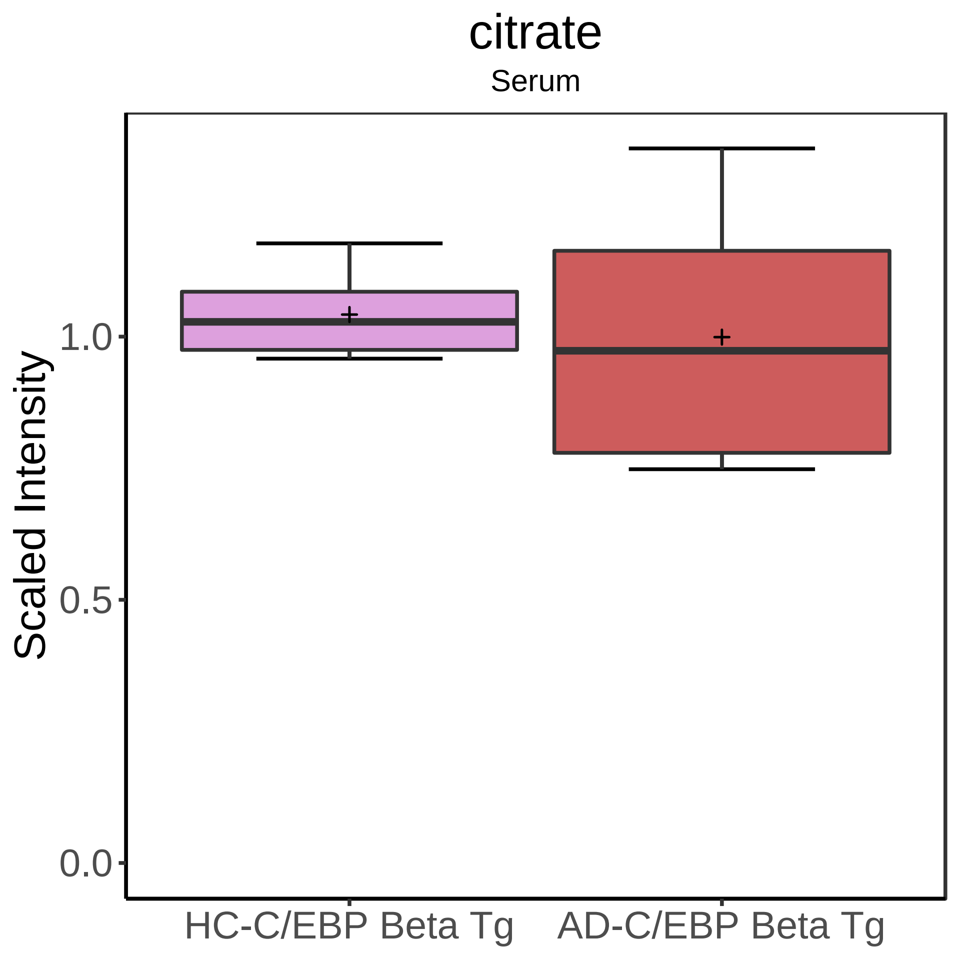
citrate
Serum
Scaled Intensity
1.0
0.5
0.0
HC-C/EBP Beta Tg
AD-C/EBP Beta Tg

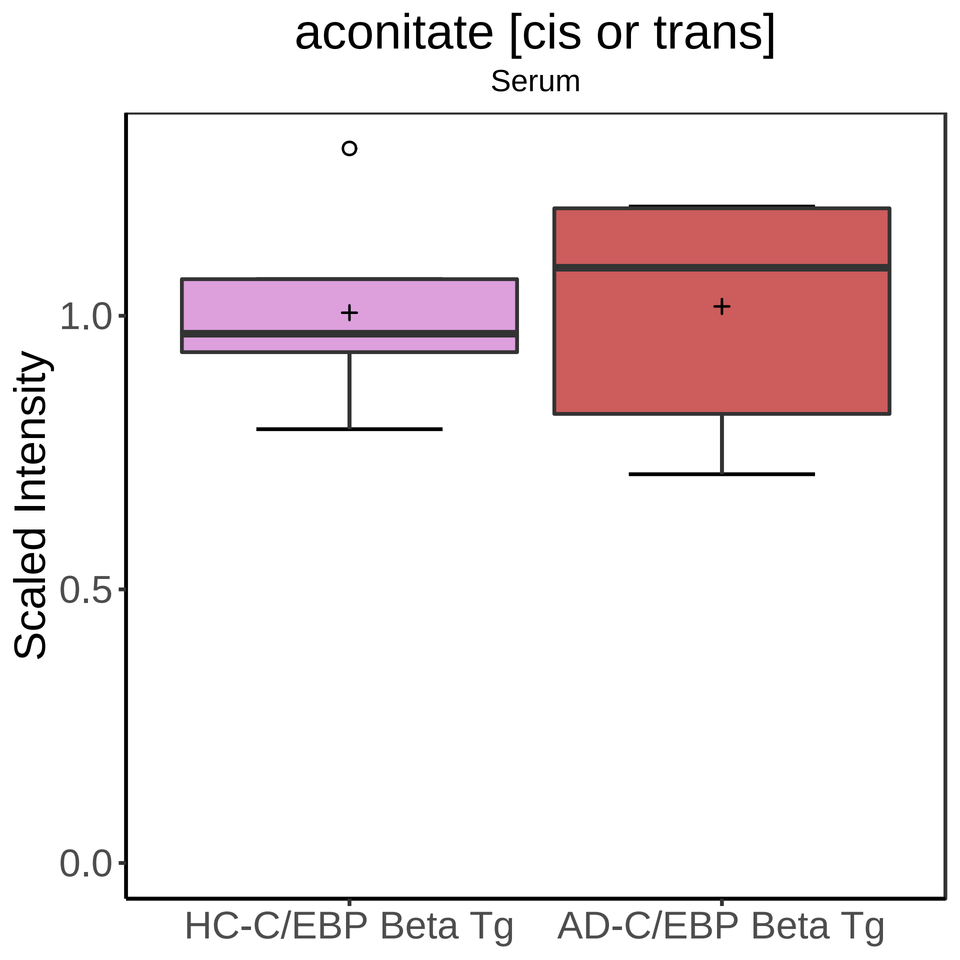
aconitate [cis or trans]
Serum
Scaled Intensity
1.0
0.5
0.0
HC-C/EBP Beta Tg
AD-C/EBP Beta Tg

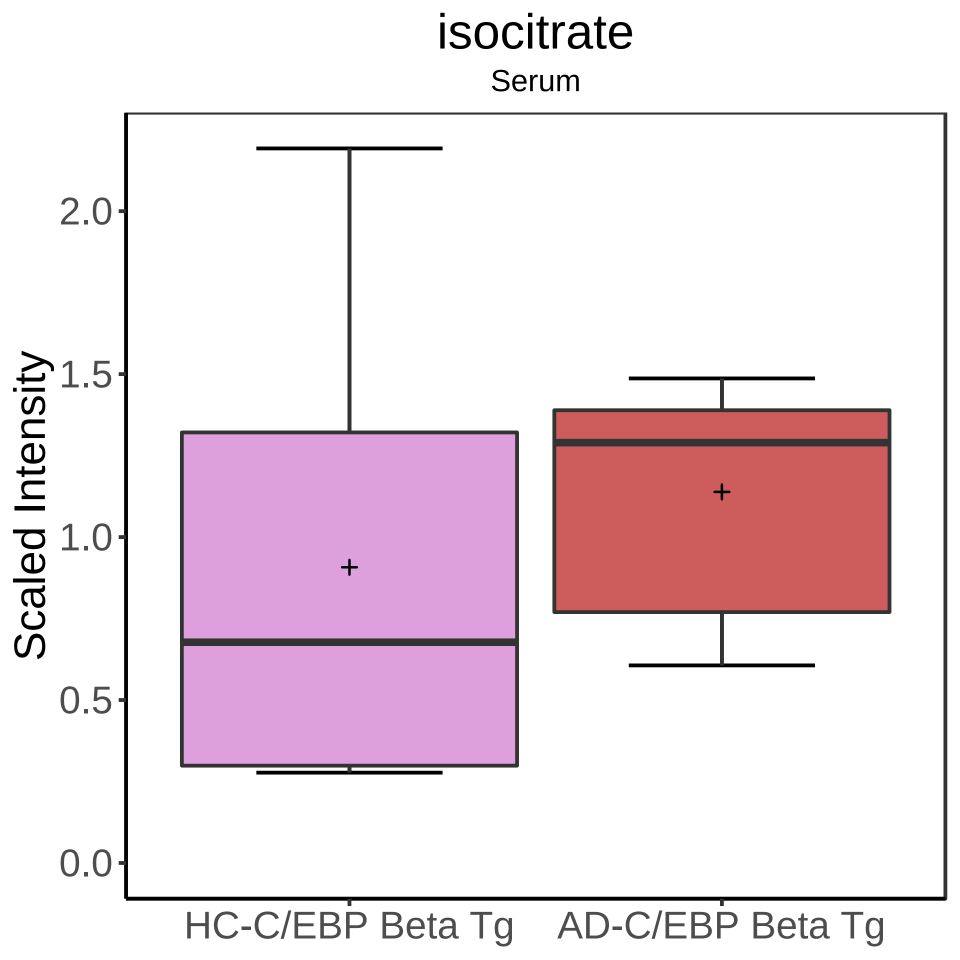
isocitrate
Serum
Scaled Intensity
2.0
1.5
1.0
0.5
0.0
HC-C/EBP Beta Tg
AD-C/EBP Beta Tg

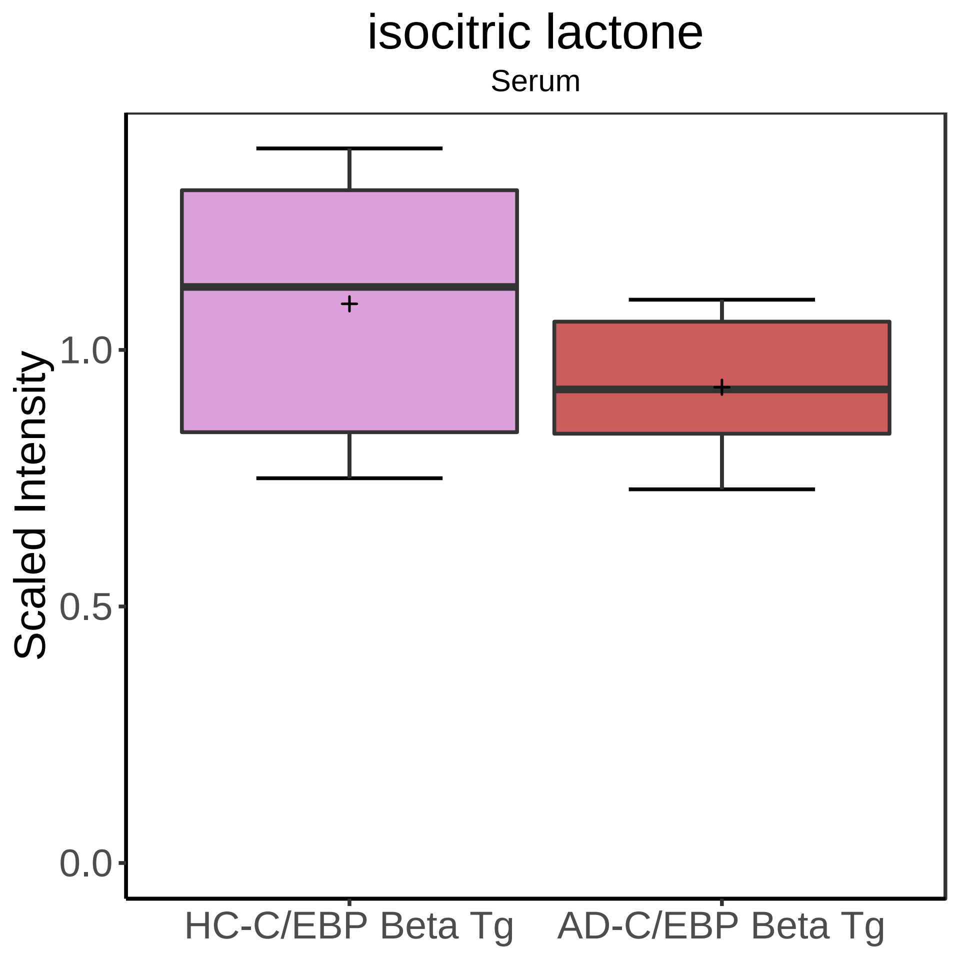
isocitric lactone
Serum
Scaled Intensity
1.0
0.5
0.0
HC-C/EBP Beta Tg
AD-C/EBP Beta Tg

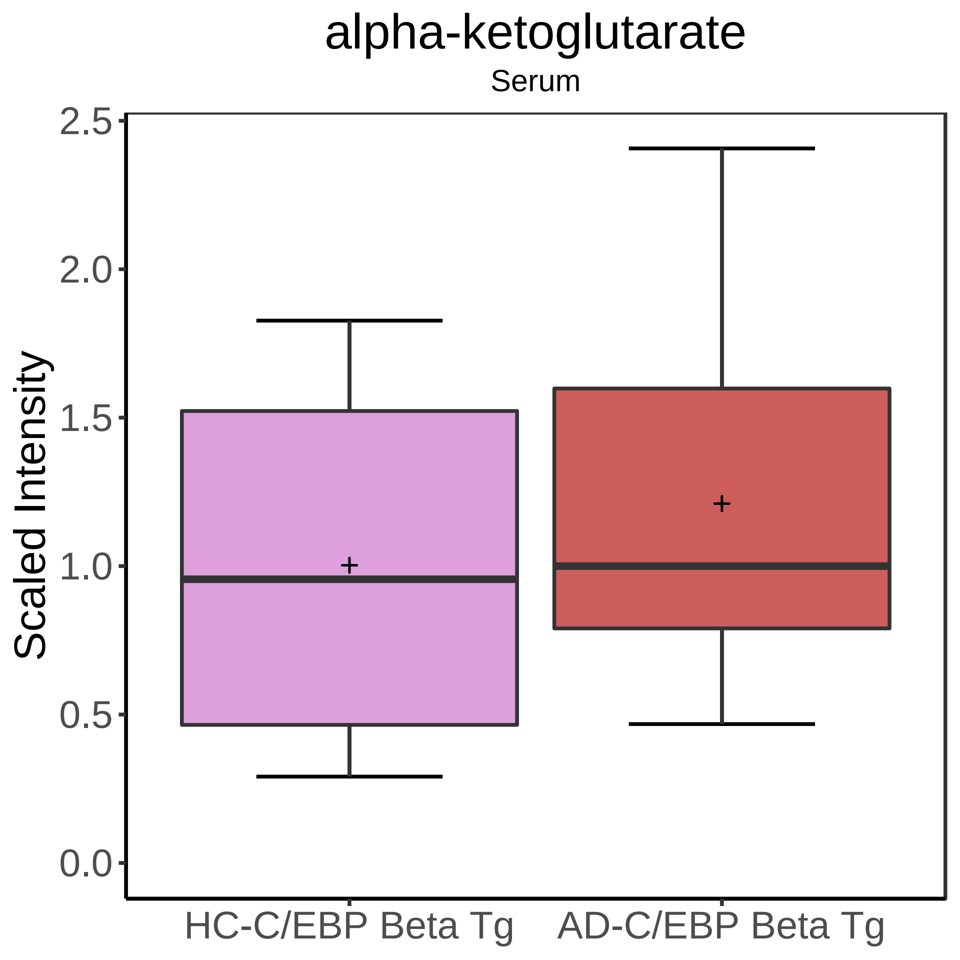
alpha-ketoglutarate
Serum
Scaled Intensity
0.0
0.5
1.0
1.5
2.0
2.5
HC-C/EBP Beta Tg
AD-C/EBP Beta Tg

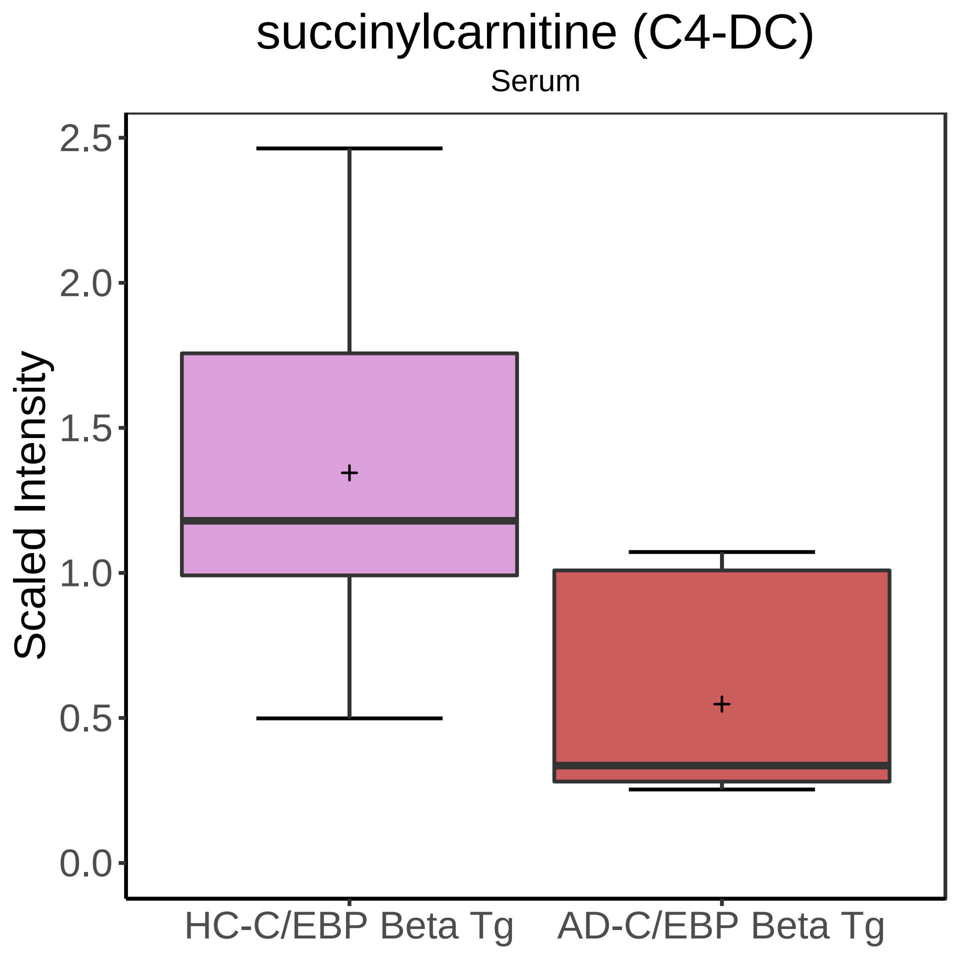
succinylcarnitine (C4-DC)
Serum
Scaled Intensity
2.5
2.0
1.5
1.0
0.5
0.0
HC-C/EBP Beta Tg
AD-C/EBP Beta Tg

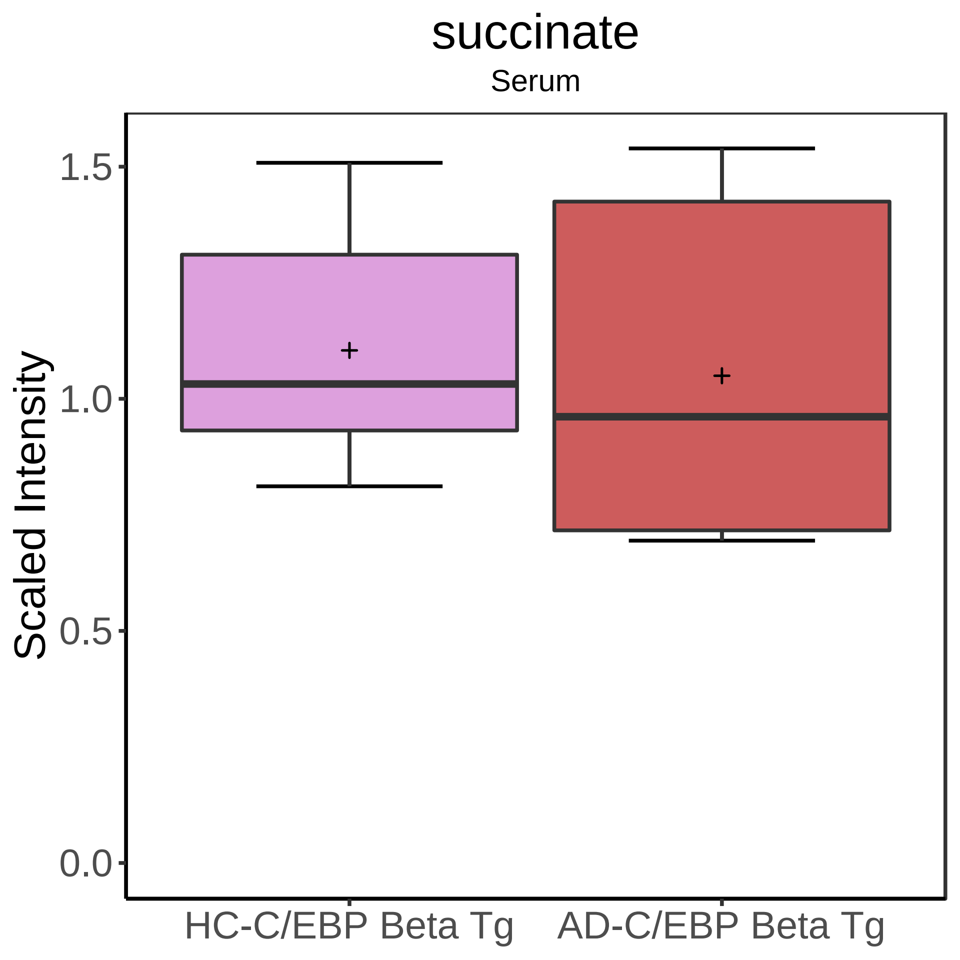
succinate
Serum
Scaled Intensity
1.5
1.0
0.5
0.0
HC-C/EBP Beta Tg
AD-C/EBP Beta Tg

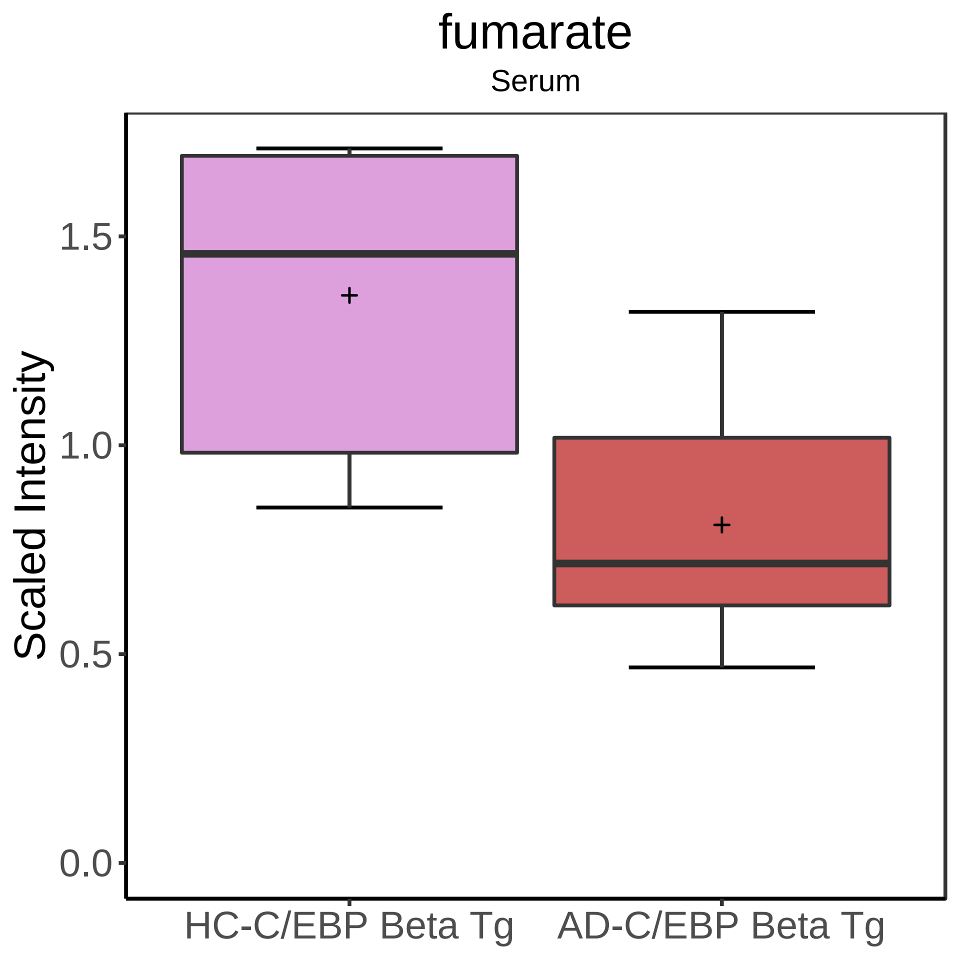

fumarate
Serum
Scaled Intensity
1.5
1.0
0.5
0.0
HC-C/EBP Beta Tg
AD-C/EBP Beta Tg

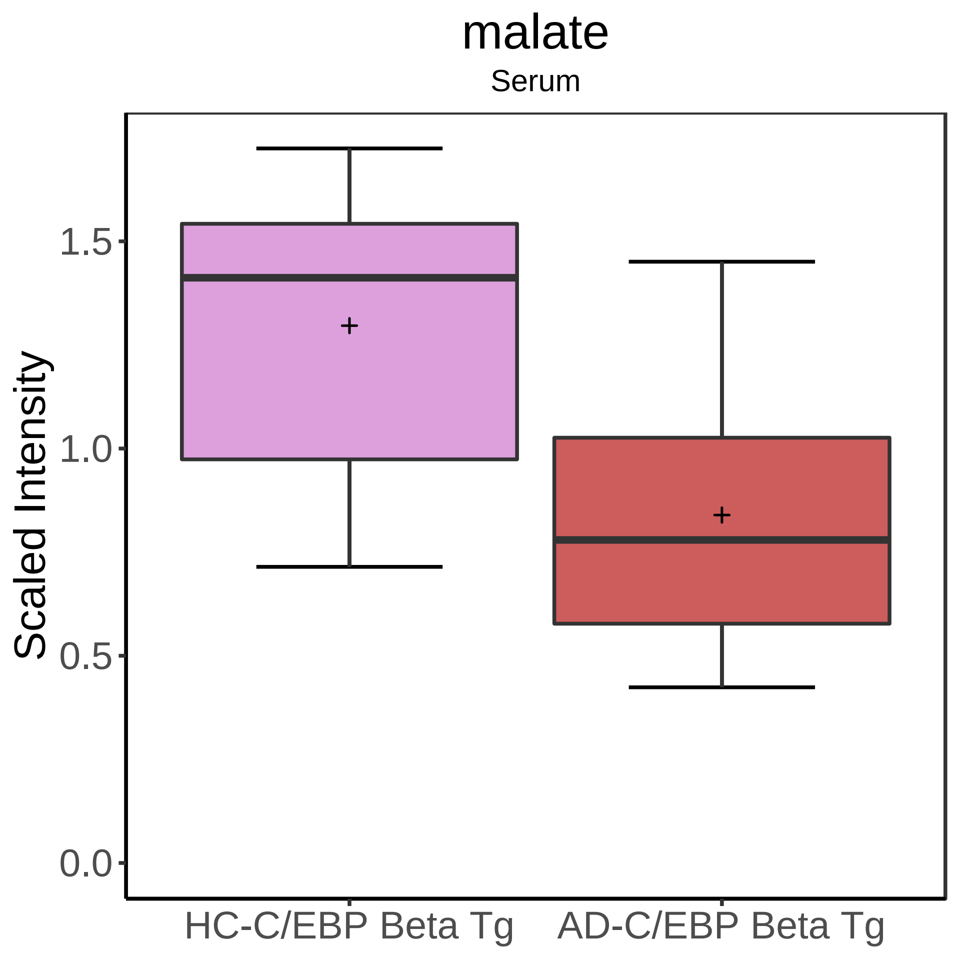
malate
Serum
Scaled Intensity
1.5
1.0
0.5
0.0
HC-C/EBP Beta Tg
AD-C/EBP Beta Tg

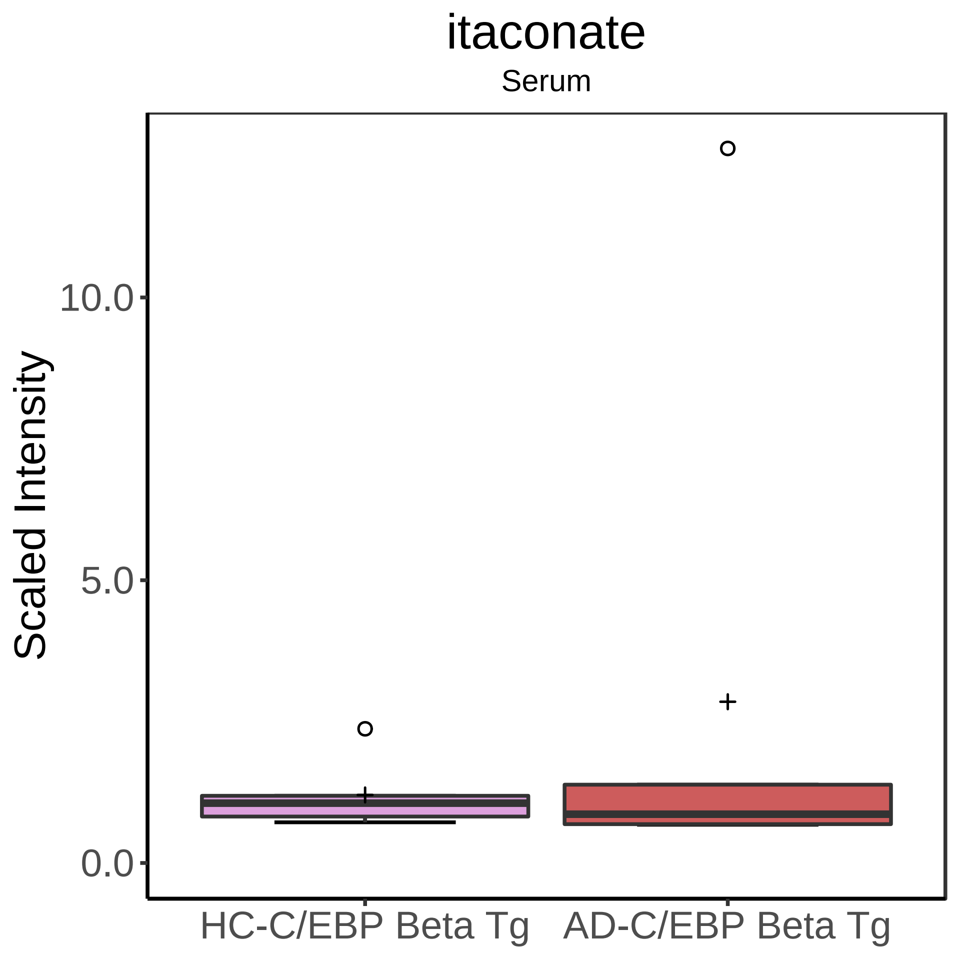
itaconate
Serum
Scaled Intensity
10.0
5.0
0.0
HC-C/EBP Beta Tg
AD-C/EBP Beta Tg

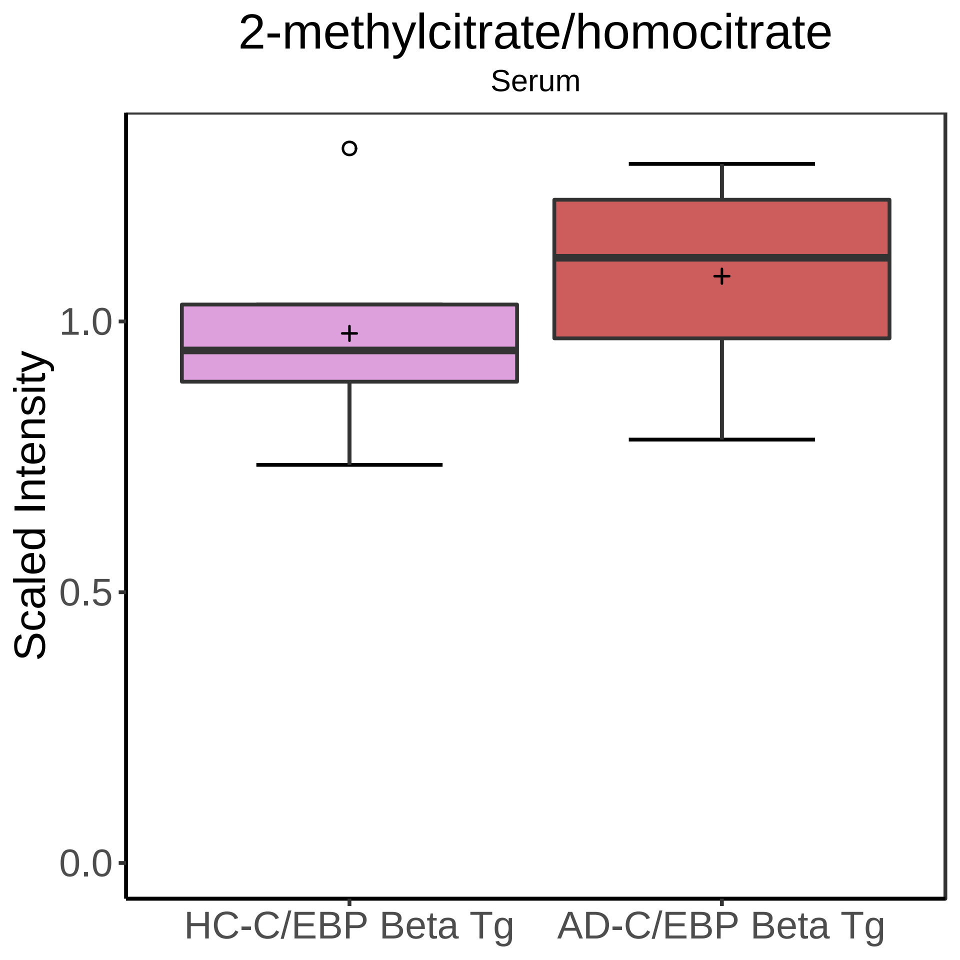
2-methylcitrate/homocitrate
Serum
Scaled Intensity
1.0
0.5
0.0
HC-C/EBP Beta Tg
AD-C/EBP Beta Tg

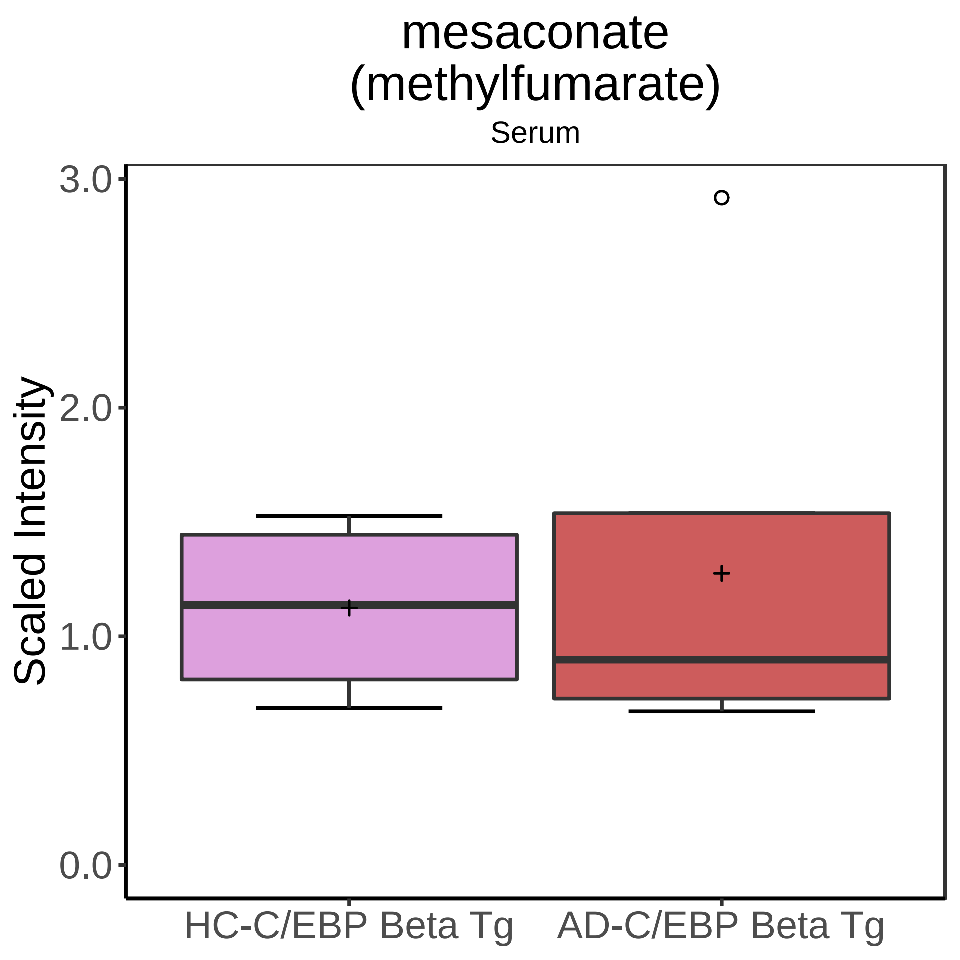
mesaconate
(methylfumarate)
Serum
Scaled Intensity
3.0
2.0
1.0
0.0
HC-C/EBP Beta Tg
AD-C/EBP Beta Tg

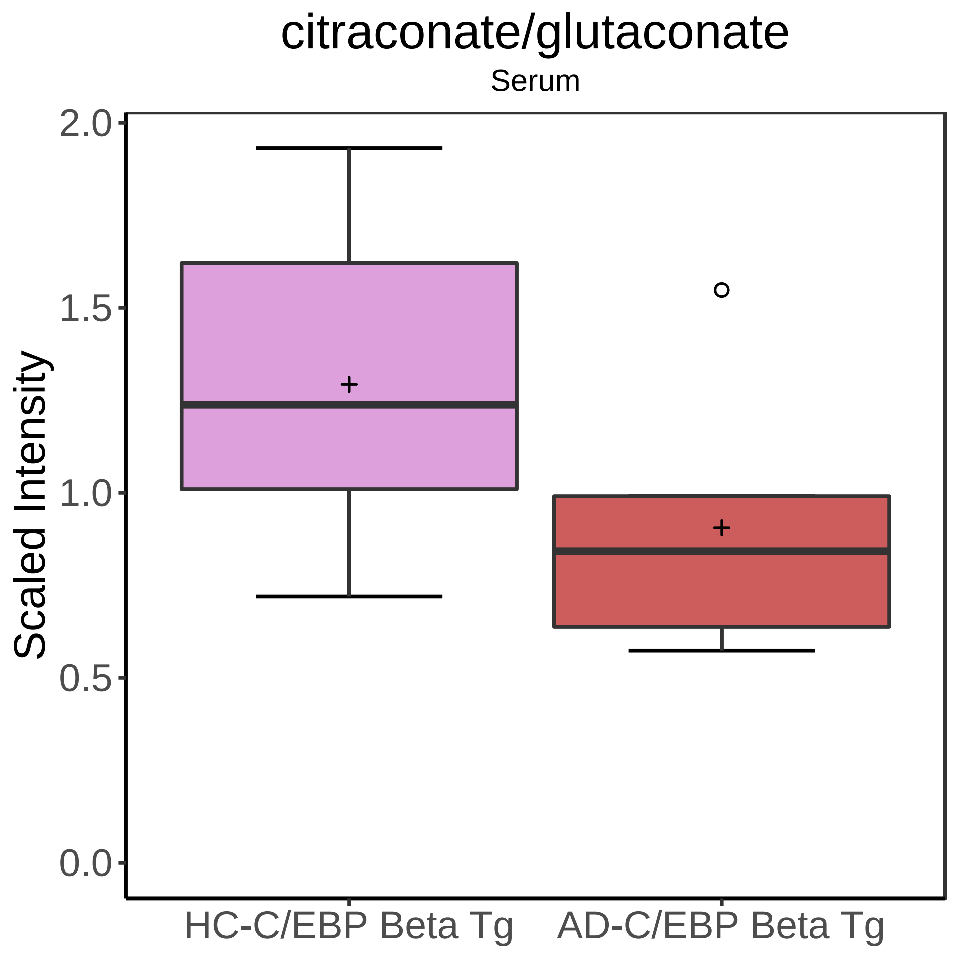
citraconate/glutaconate
Serum
Scaled Intensity
2.0
1.5
1.0
0.5
0.0
HC-C/EBP Beta Tg
AD-C/EBP Beta Tg

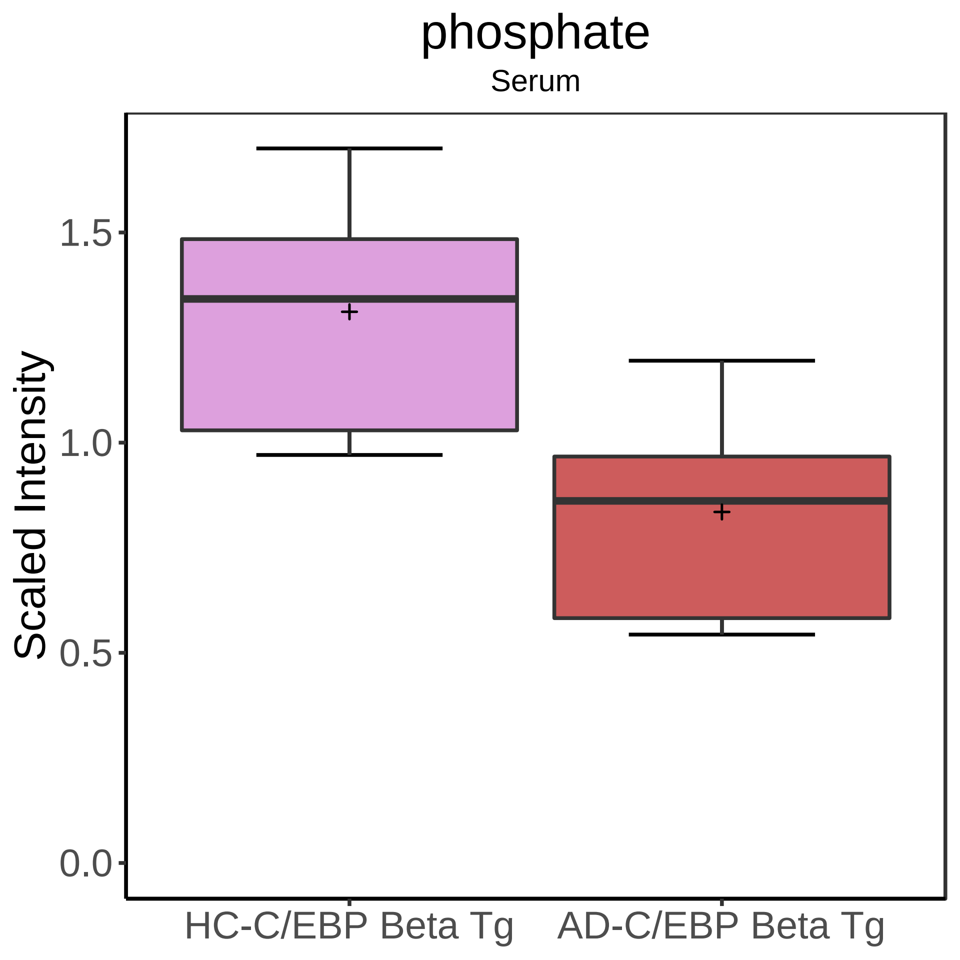
phosphate
Serum
Scaled Intensity
1.5
1.0
0.5
0.0
HC-C/EBP Beta Tg
AD-C/EBP Beta Tg

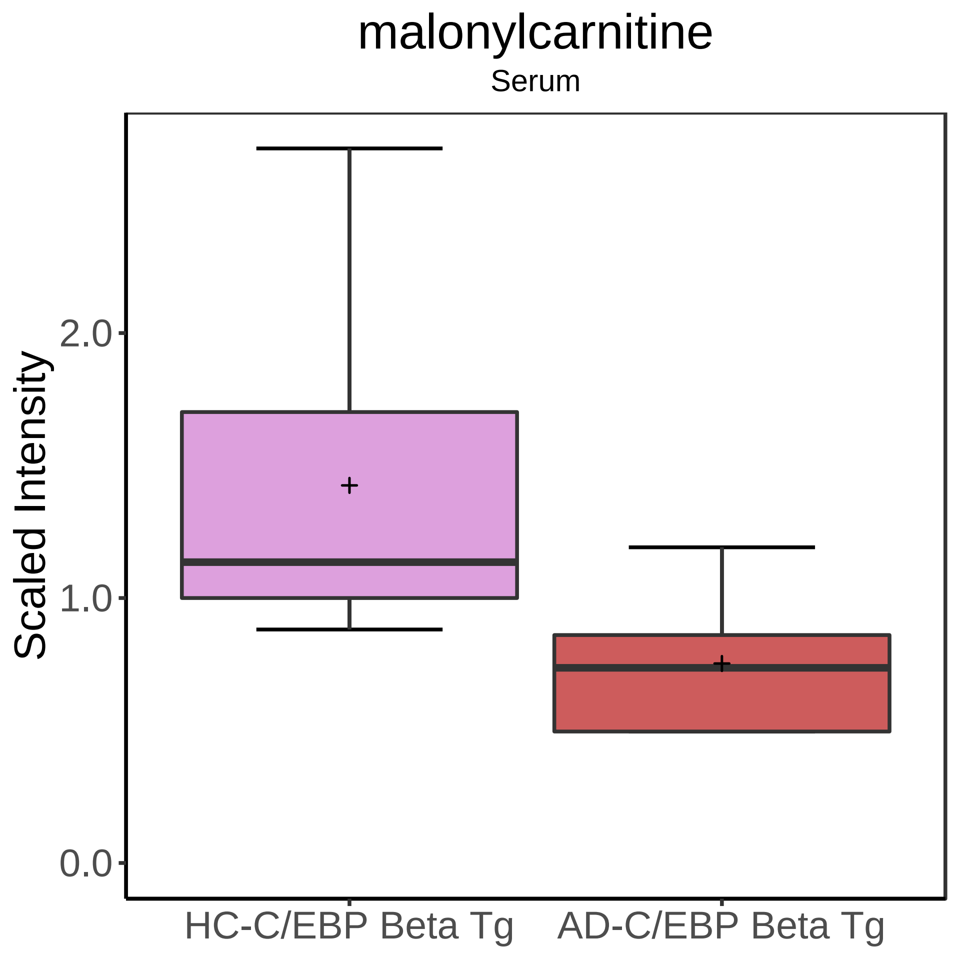
malonylcarnitine
Serum
Scaled Intensity
2.0
1.0
0.0
HC-C/EBP Beta Tg
AD-C/EBP Beta Tg

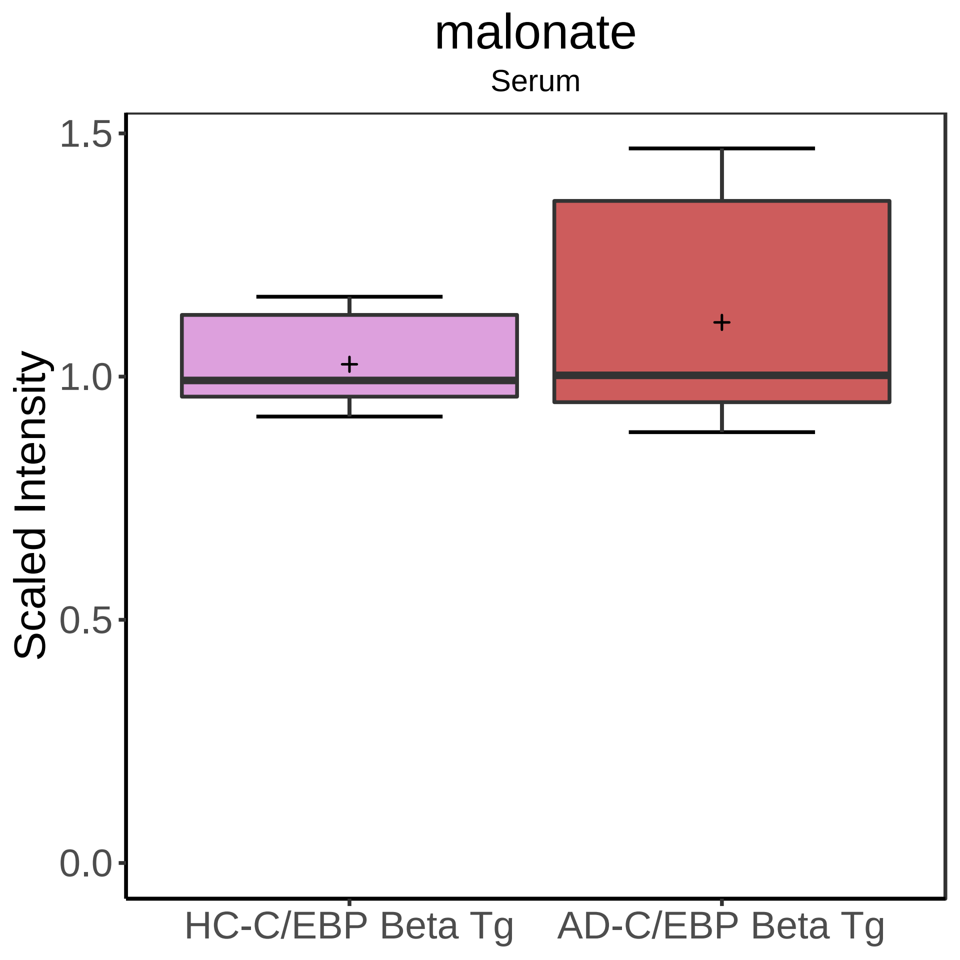
malonate
Serum
Scaled Intensity
1.5
1.0
0.5
0.0
HC-C/EBP Beta Tg
AD-C/EBP Beta Tg

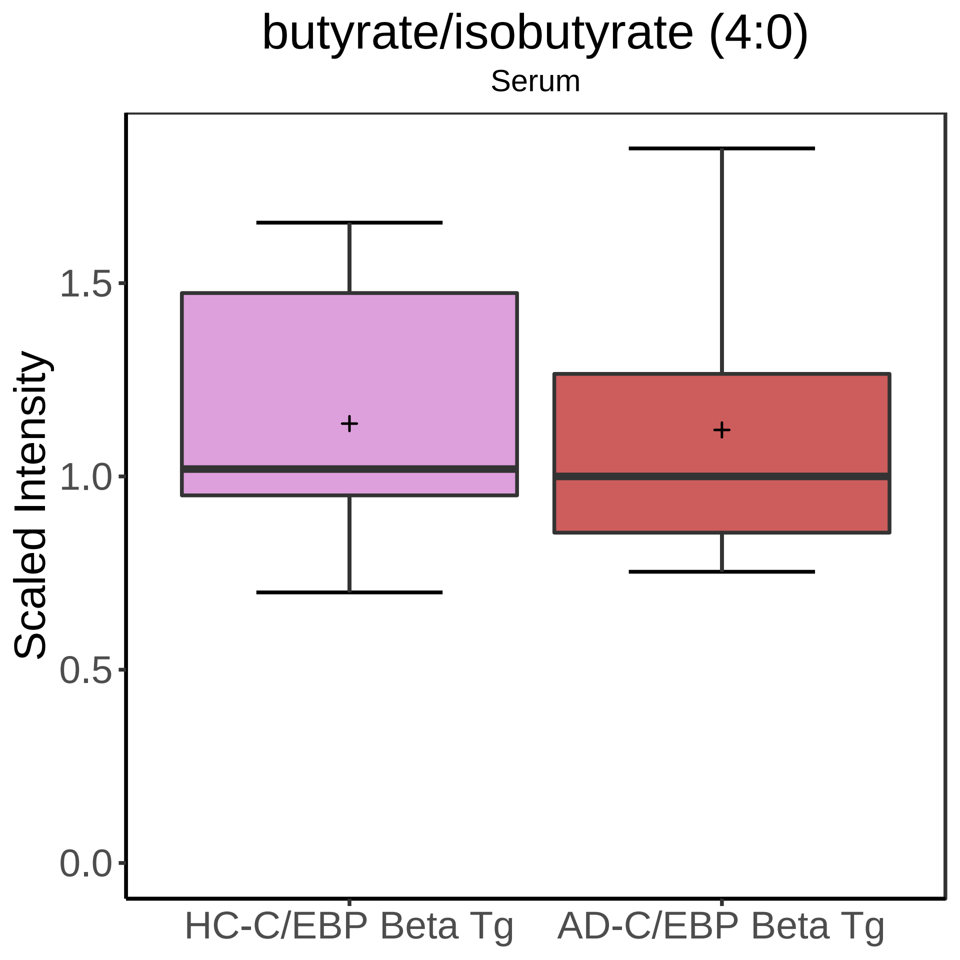
butyrate/isobutyrate (4:0)
Serum
Scaled Intensity
1.5
1.0
0.5
0.0
HC-C/EBP Beta Tg
AD-C/EBP Beta Tg

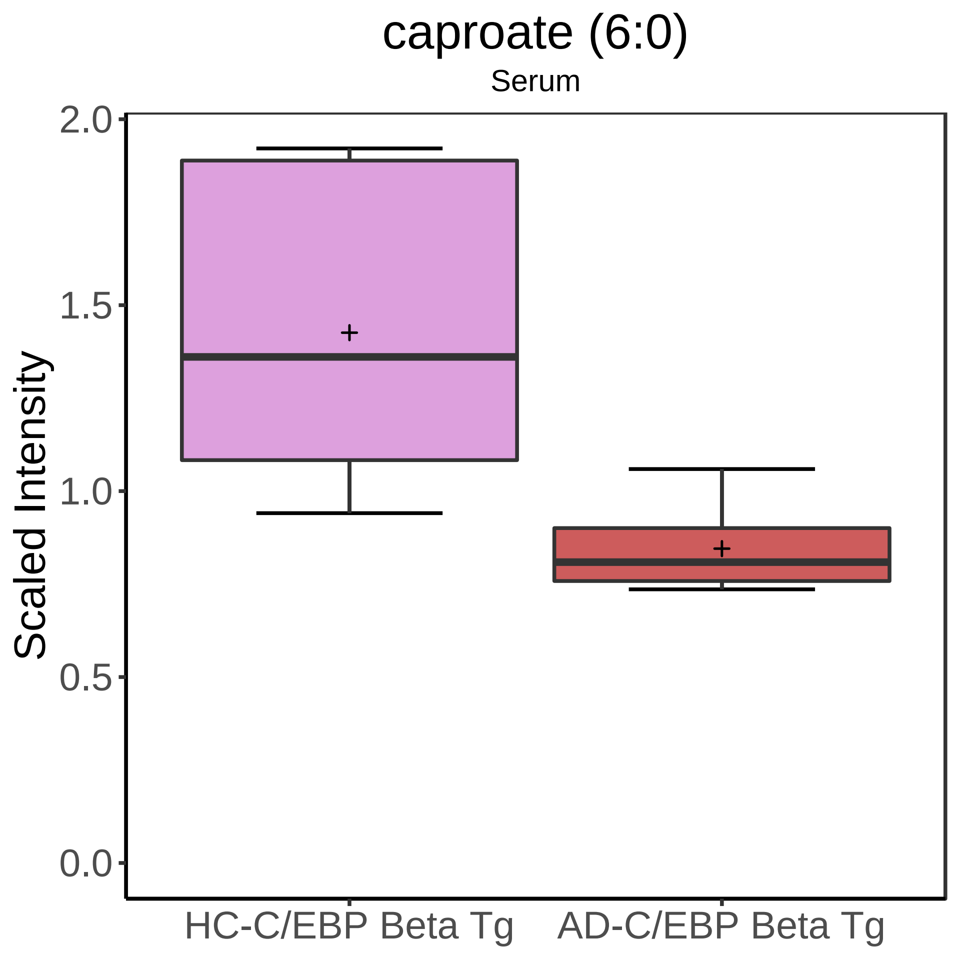
caproate (6:0)
Serum
Scaled Intensity
2.0
1.5
1.0
0.5
0.0
HC-C/EBP Beta Tg
AD-C/EBP Beta Tg

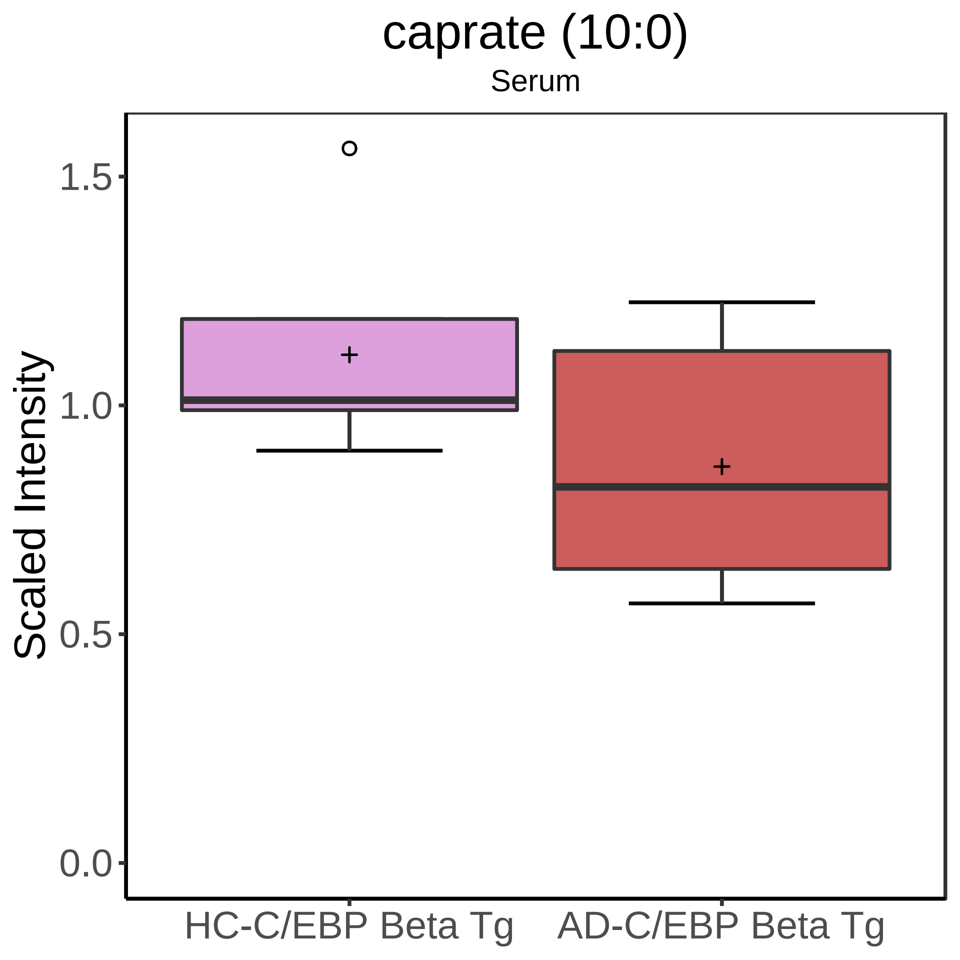
caprate (10:0)
Serum
Scaled Intensity
1.5
1.0
0.5
0.0
HC-C/EBP Beta Tg
AD-C/EBP Beta Tg

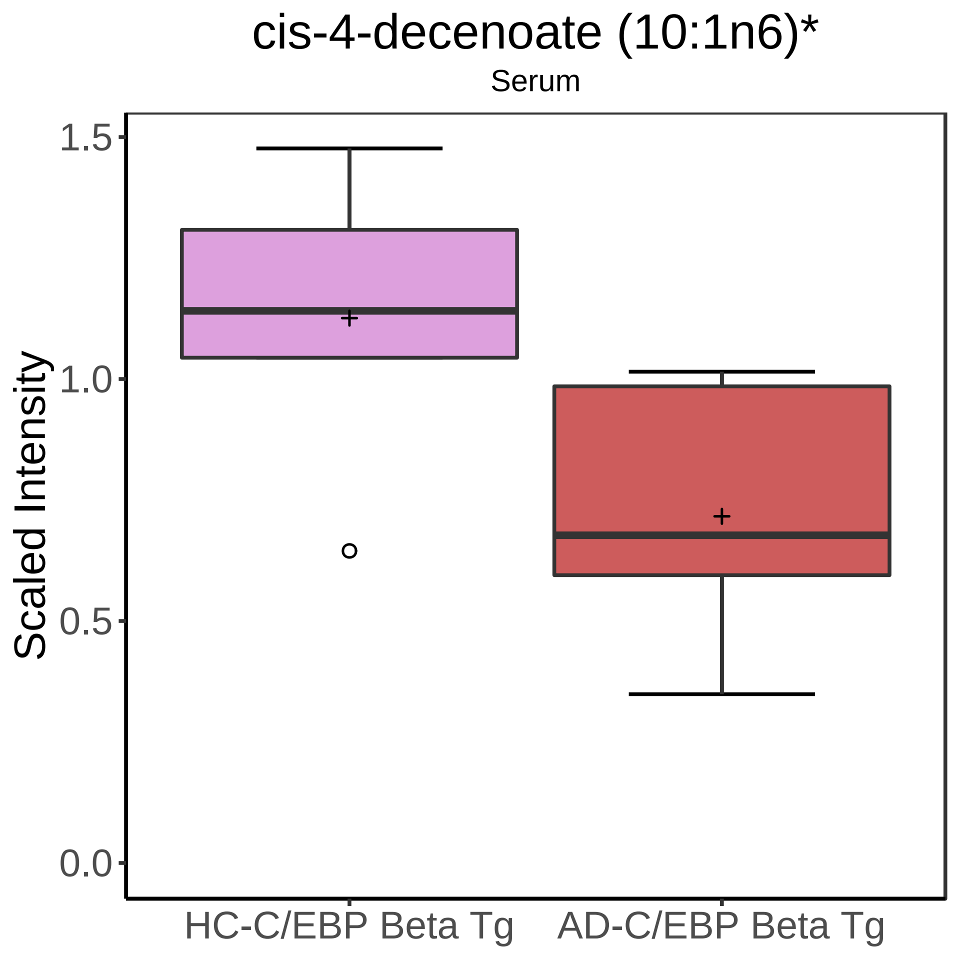
cis-4-decenoate (10:1n6)*
Serum
Scaled Intensity
1.5
1.0
0.5
0.0
HC-C/EBP Beta Tg
AD-C/EBP Beta Tg

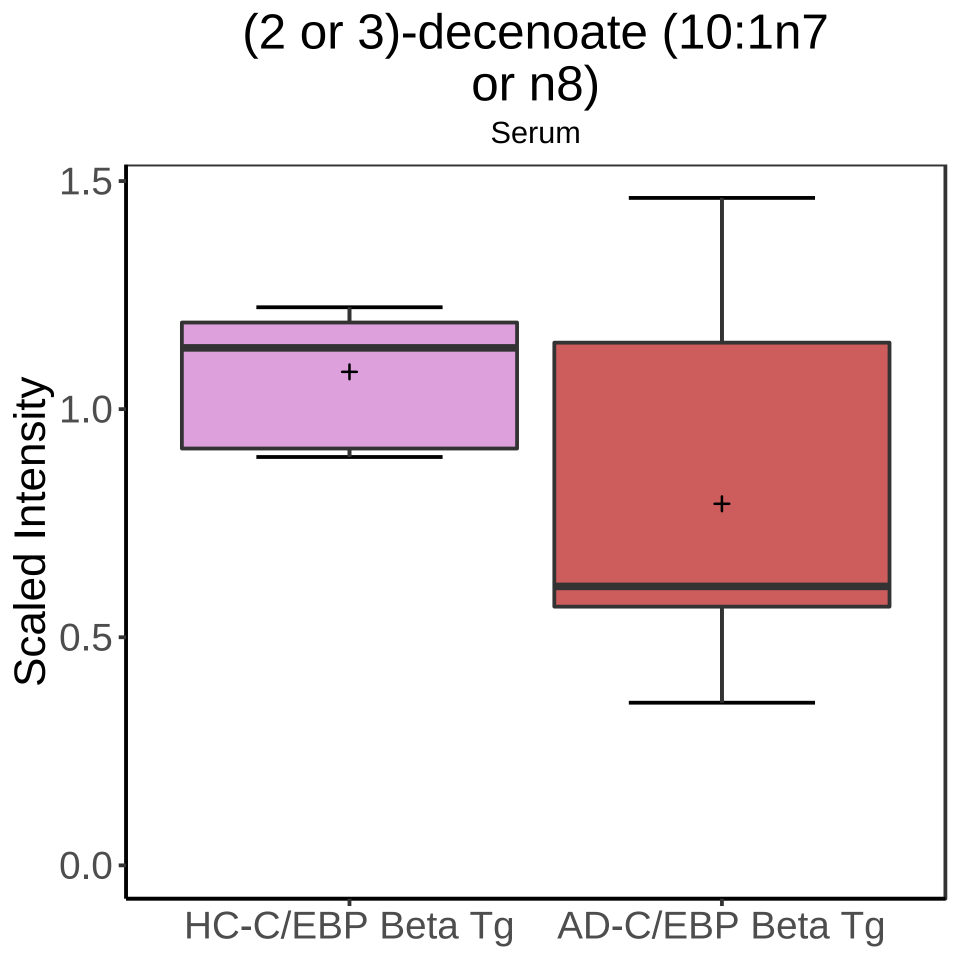

(2 or 3)-decenoate (10:1n7 or n8)
Serum
Scaled Intensity
1.5
1.0
0.5
0.0
HC-C/EBP Beta Tg
AD-C/EBP Beta Tg

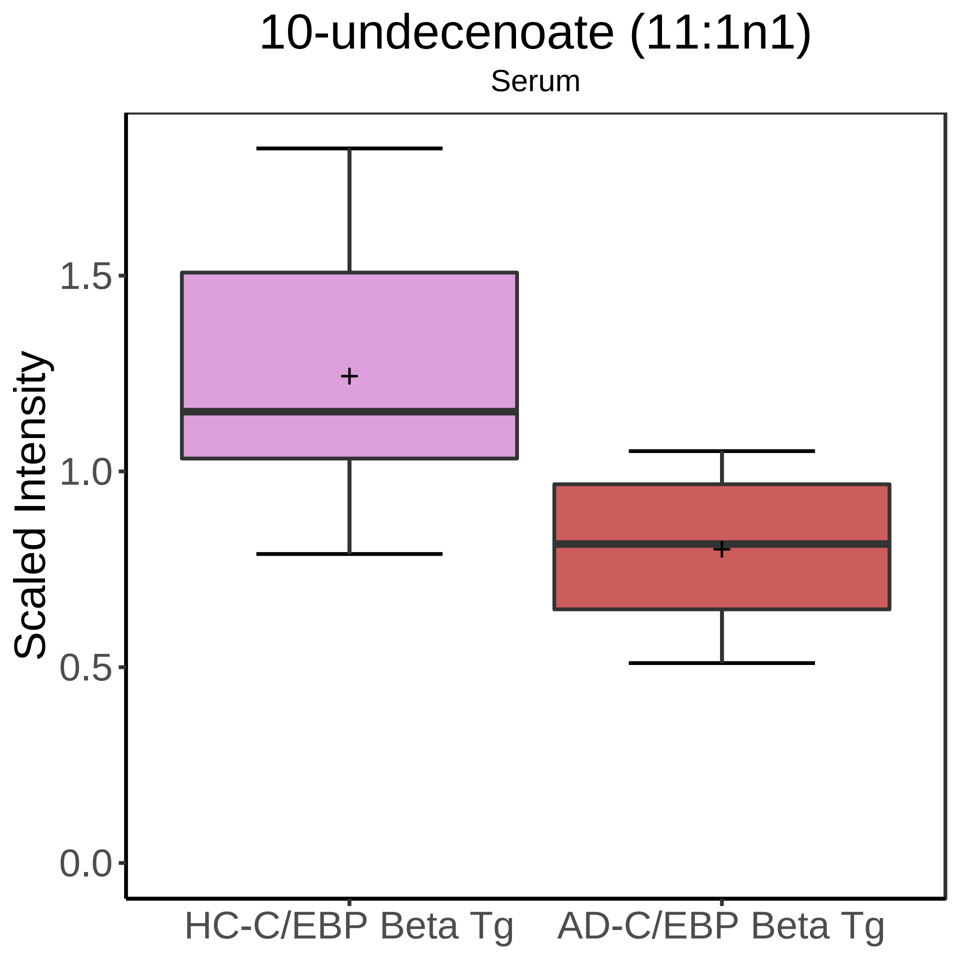
10-undecenoate (11:1n1)
Serum
Scaled Intensity
1.5
1.0
0.5
0.0
HC-C/EBP Beta Tg
AD-C/EBP Beta Tg

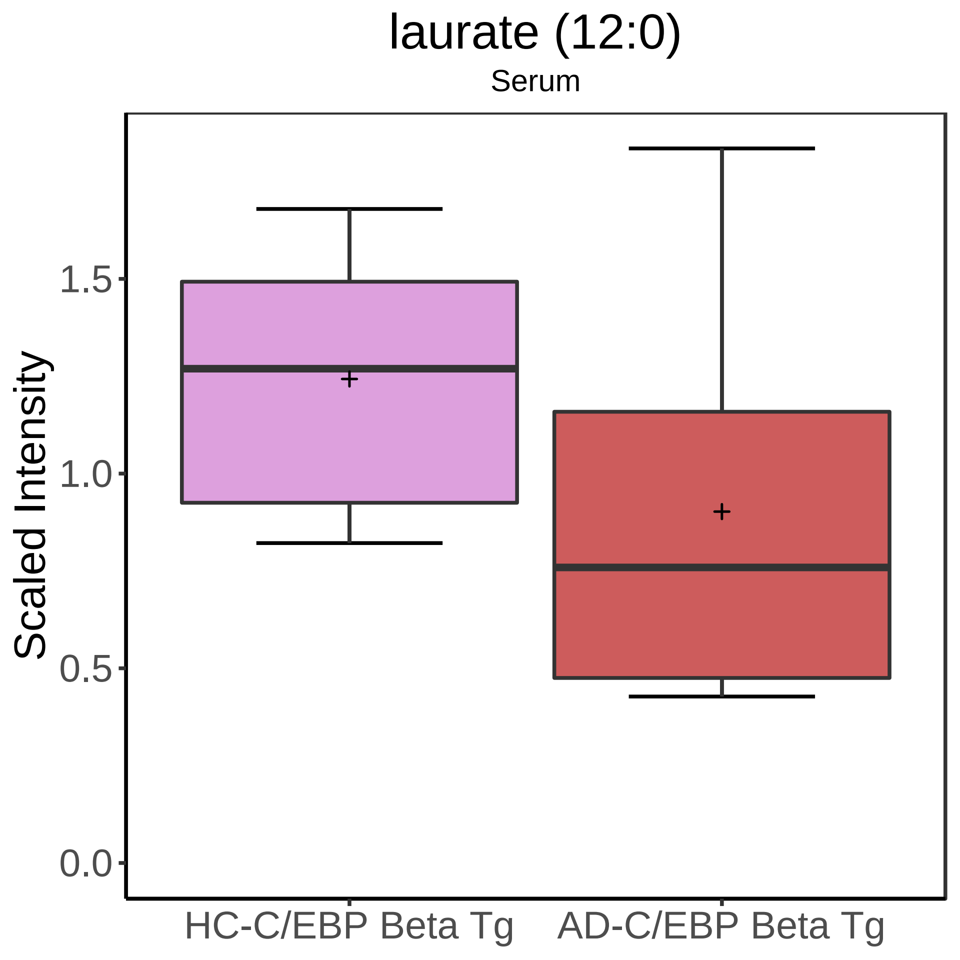
laurate (12:0)
Serum
Scaled Intensity
1.5
1.0
0.5
0.0
HC-C/EBP Beta Tg
AD-C/EBP Beta Tg

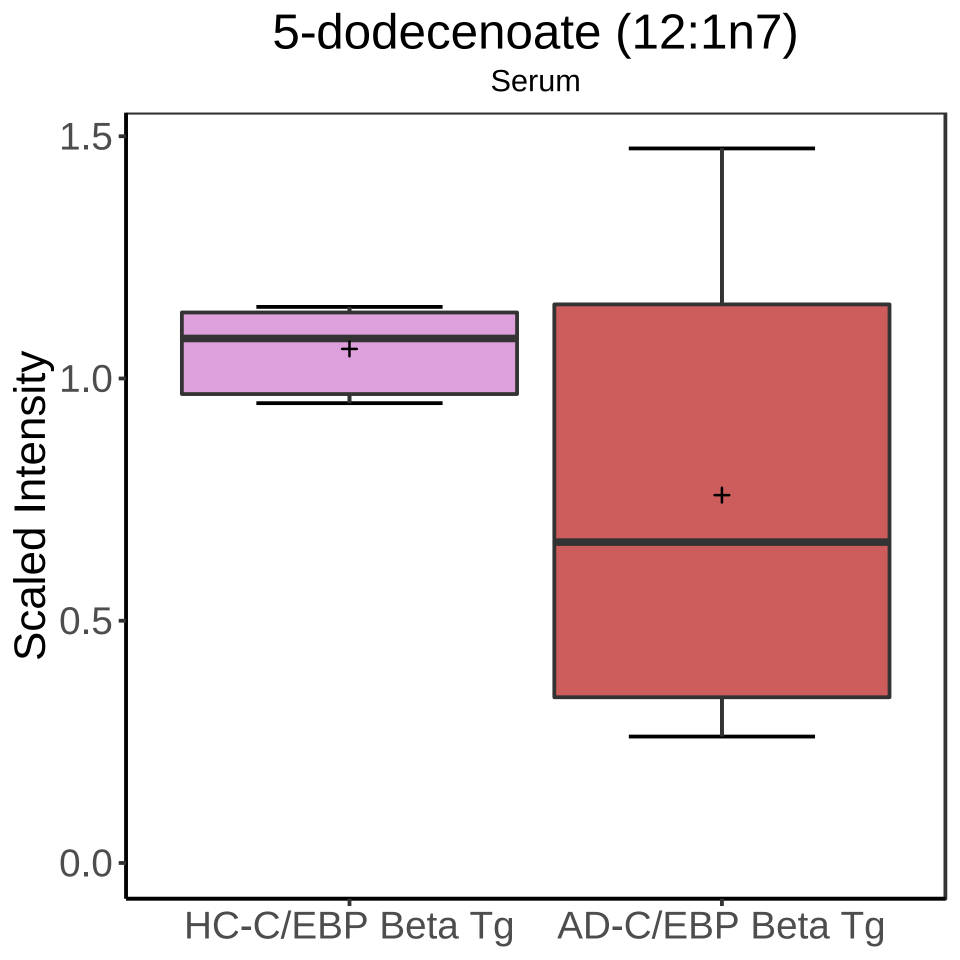

5-dodecenoate (12:1n7)
Serum
Scaled Intensity
1.5
1.0
0.5
0.0
HC-C/EBP Beta Tg
AD-C/EBP Beta Tg

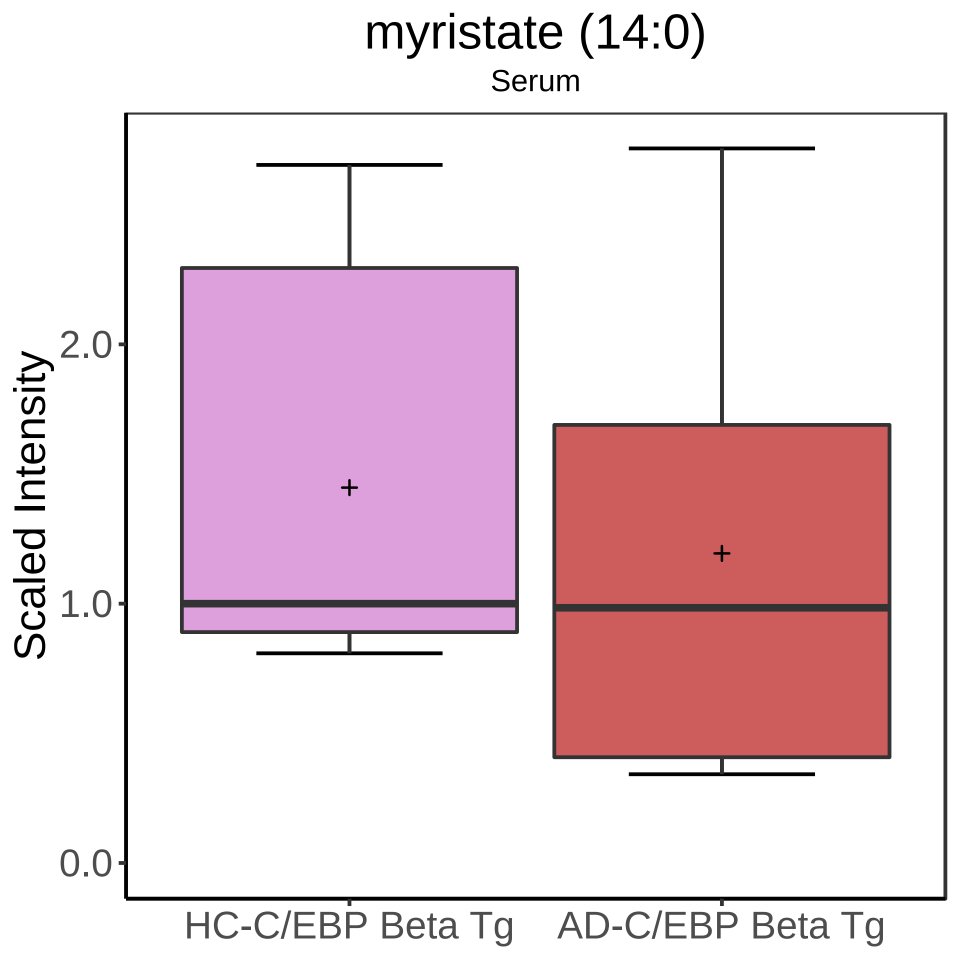
myristate (14:0)
Serum
Scaled Intensity
2.0
1.0
0.0
HC-C/EBP Beta Tg
AD-C/EBP Beta Tg

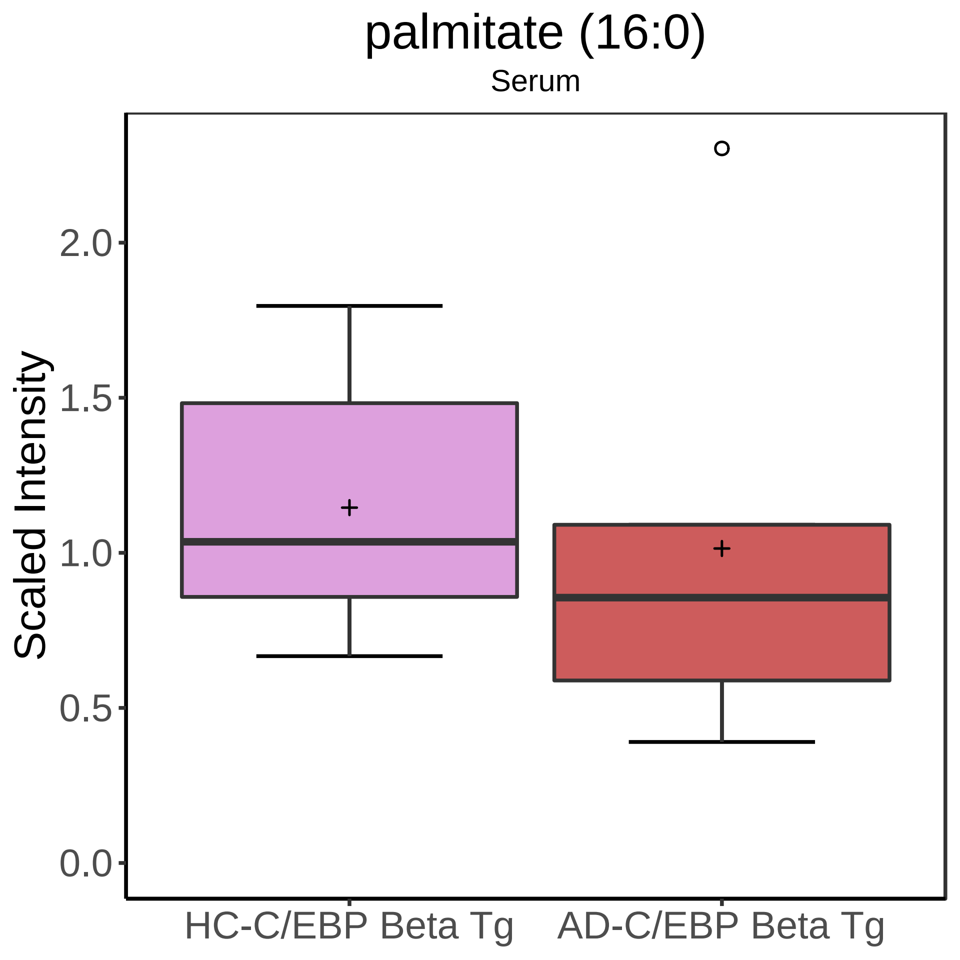
palmitate (16:0)
Serum
Scaled Intensity
2.0
1.5
1.0
0.5
0.0
HC-C/EBP Beta Tg
AD-C/EBP Beta Tg

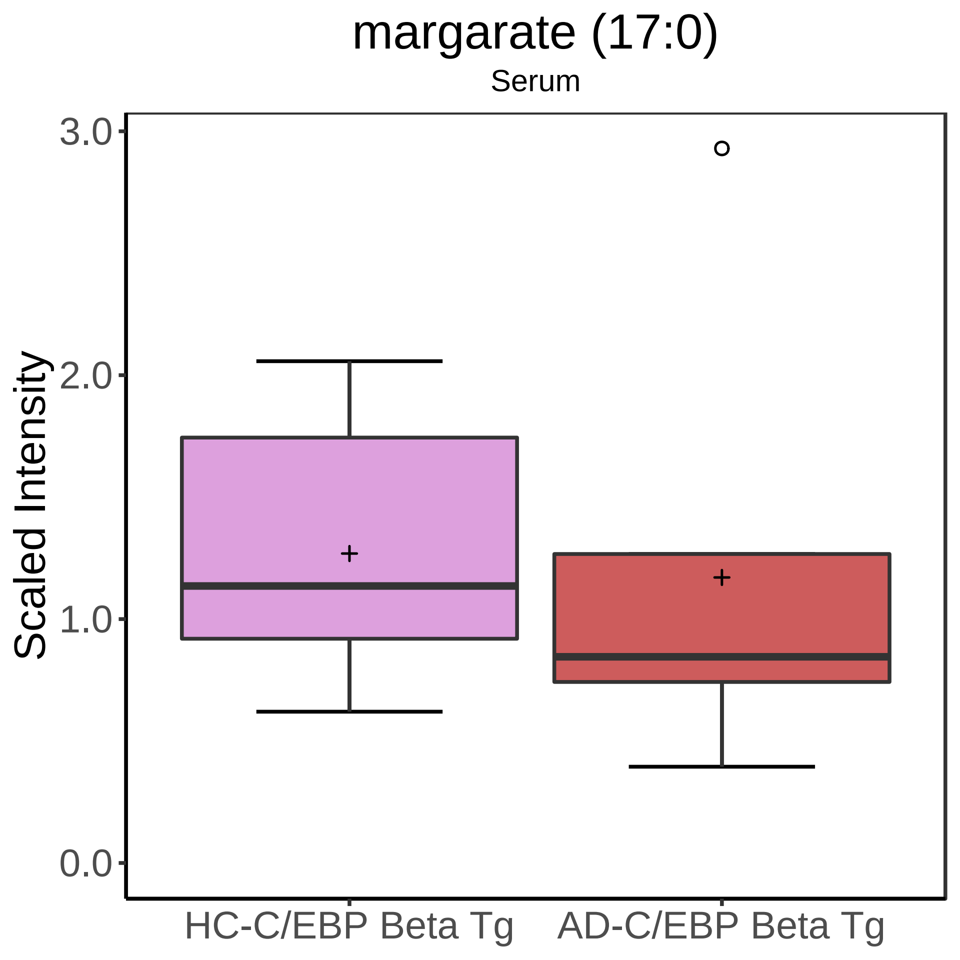
margarate (17:0)
Serum
Scaled Intensity
3.0
2.0
1.0
0.0
HC-C/EBP Beta Tg
AD-C/EBP Beta Tg

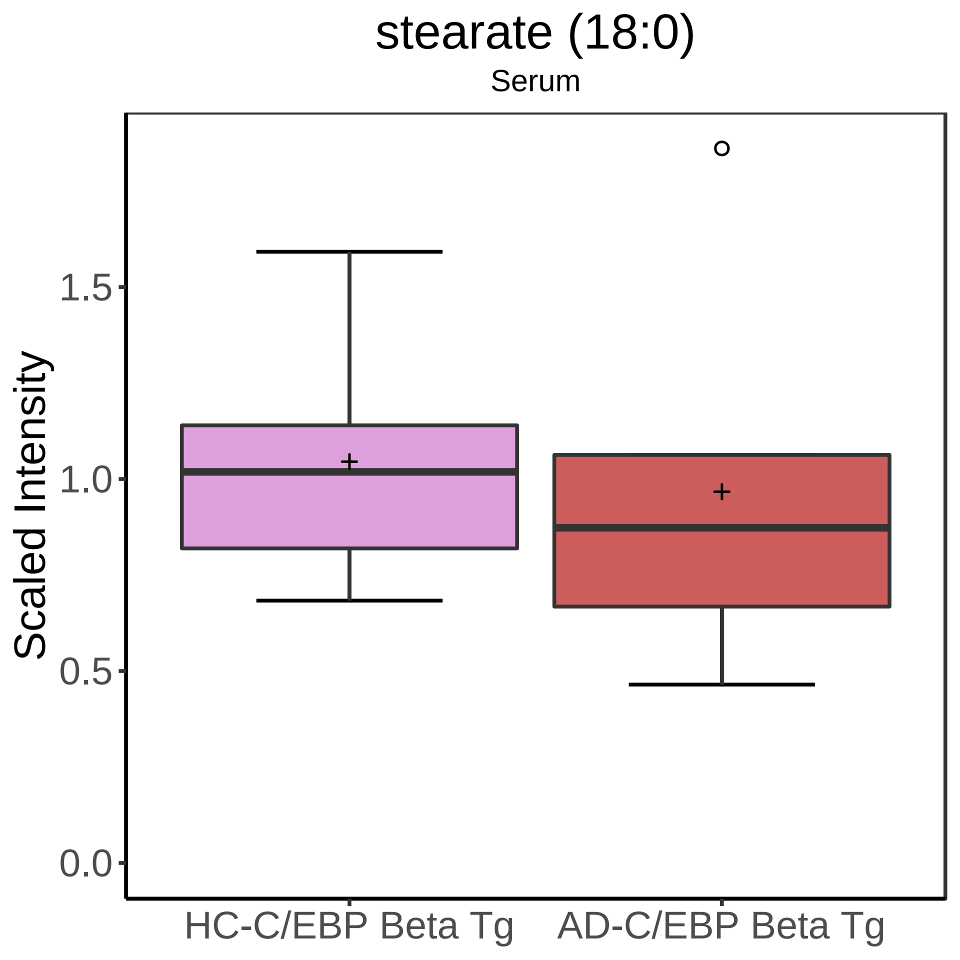
stearate (18:0)
Serum
Scaled Intensity
1.5
1.0
0.5
0.0
HC-C/EBP Beta Tg
AD-C/EBP Beta Tg

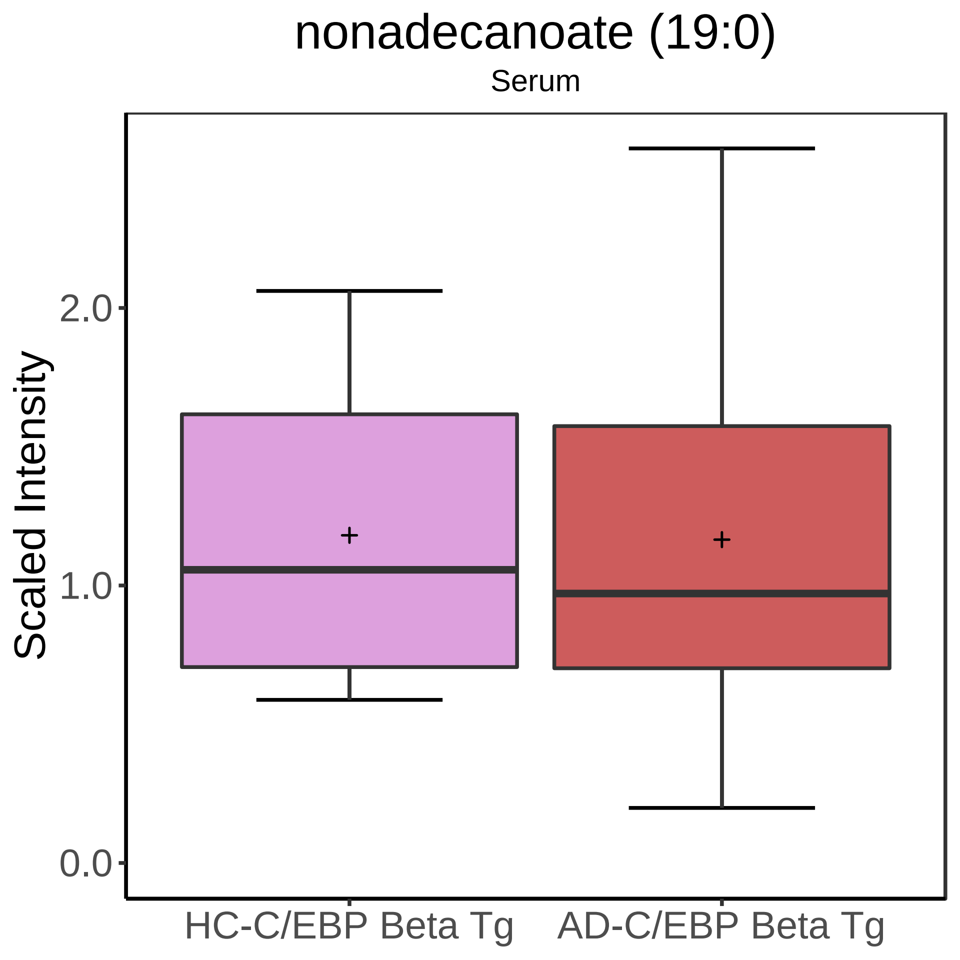
nonadecanoate (19:0)
Serum
Scaled Intensity
2.0
1.0
0.0
HC-C/EBP Beta Tg
AD-C/EBP Beta Tg

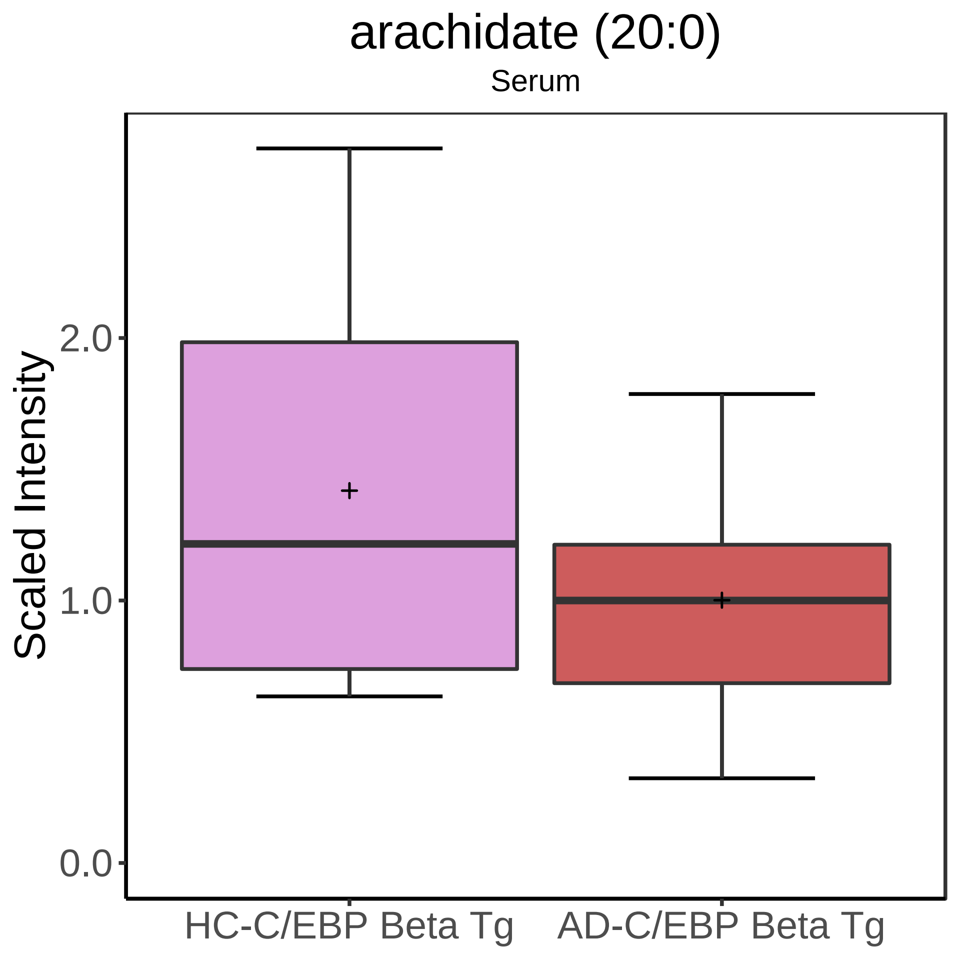
arachidate (20:0)
Serum
Scaled Intensity
2.0
1.0
0.0
HC-C/EBP Beta Tg
AD-C/EBP Beta Tg

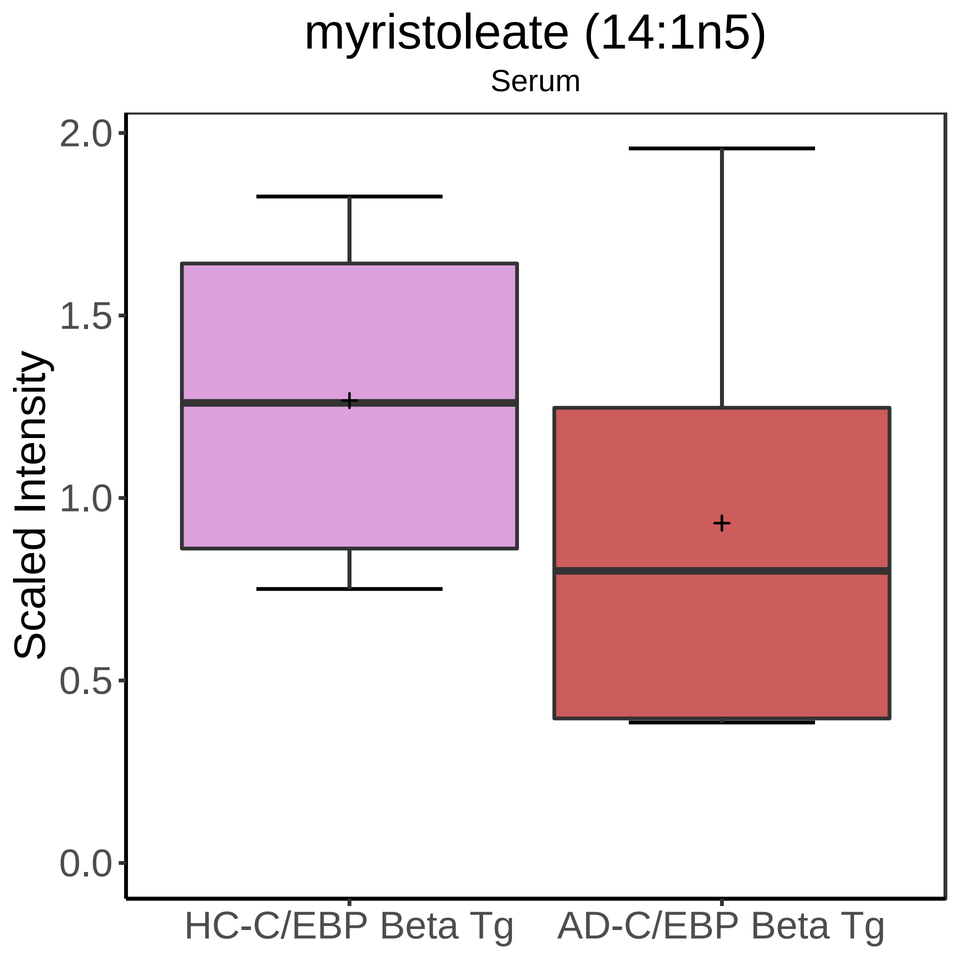
myristoleate (14:1n5)
Serum
Scaled Intensity
2.0
1.5
1.0
0.5
0.0
HC-C/EBP Beta Tg
AD-C/EBP Beta Tg

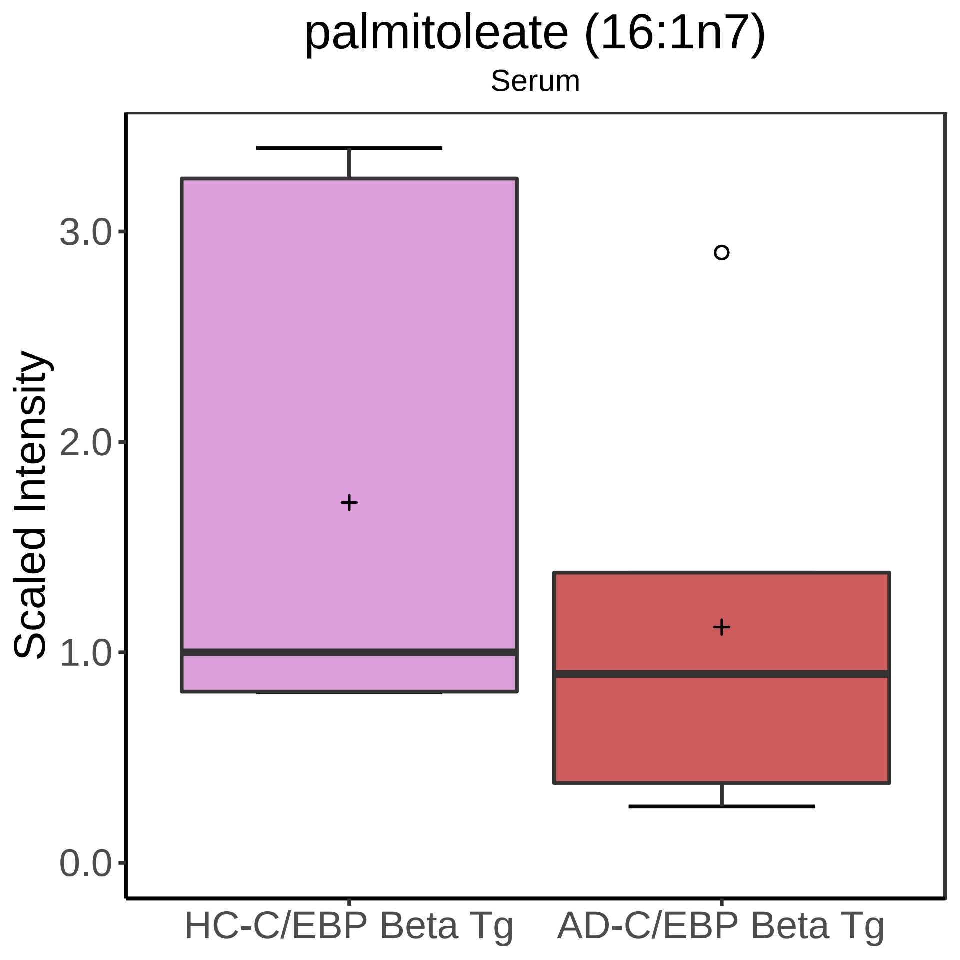

palmitoleate (16:1n7)
Serum
Scaled Intensity
3.0
2.0
1.0
0.0
HC-C/EBP Beta Tg
AD-C/EBP Beta Tg

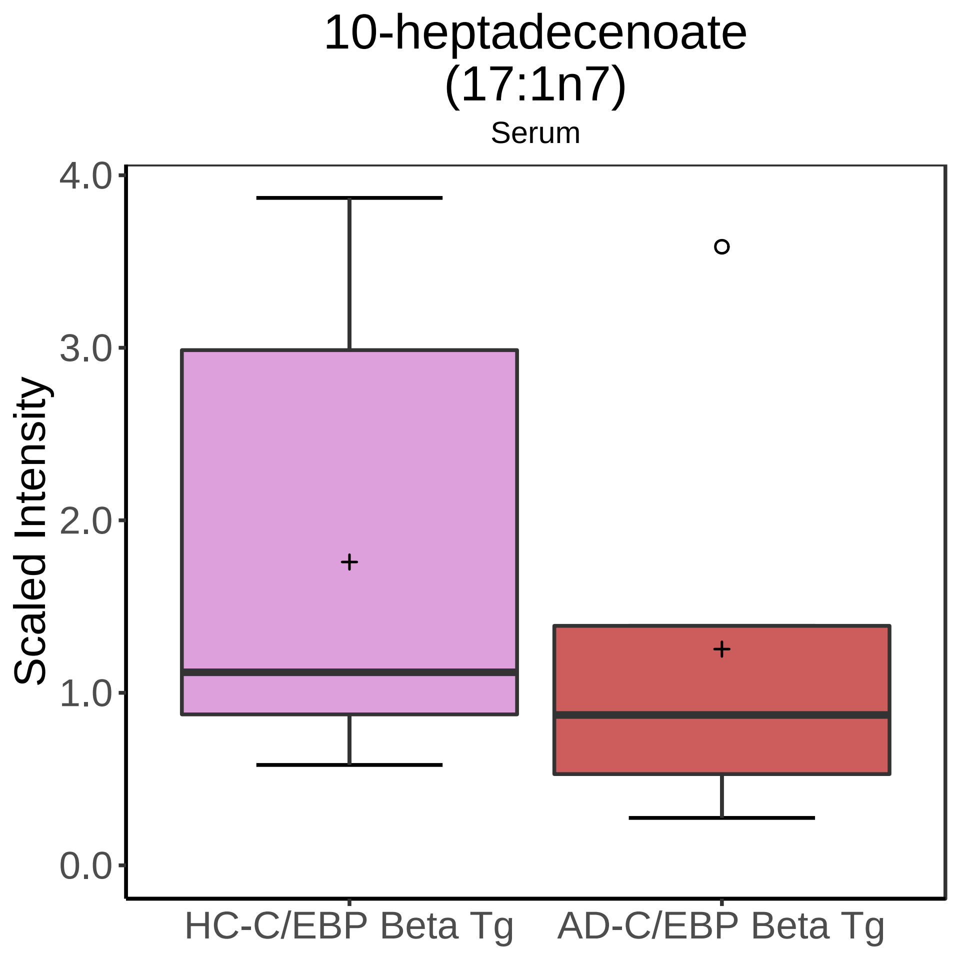
10-heptadecenoate
(17:1n7)
Serum
Scaled Intensity
4.0
3.0
2.0
1.0
0.0
HC-C/EBP Beta Tg
AD-C/EBP Beta Tg

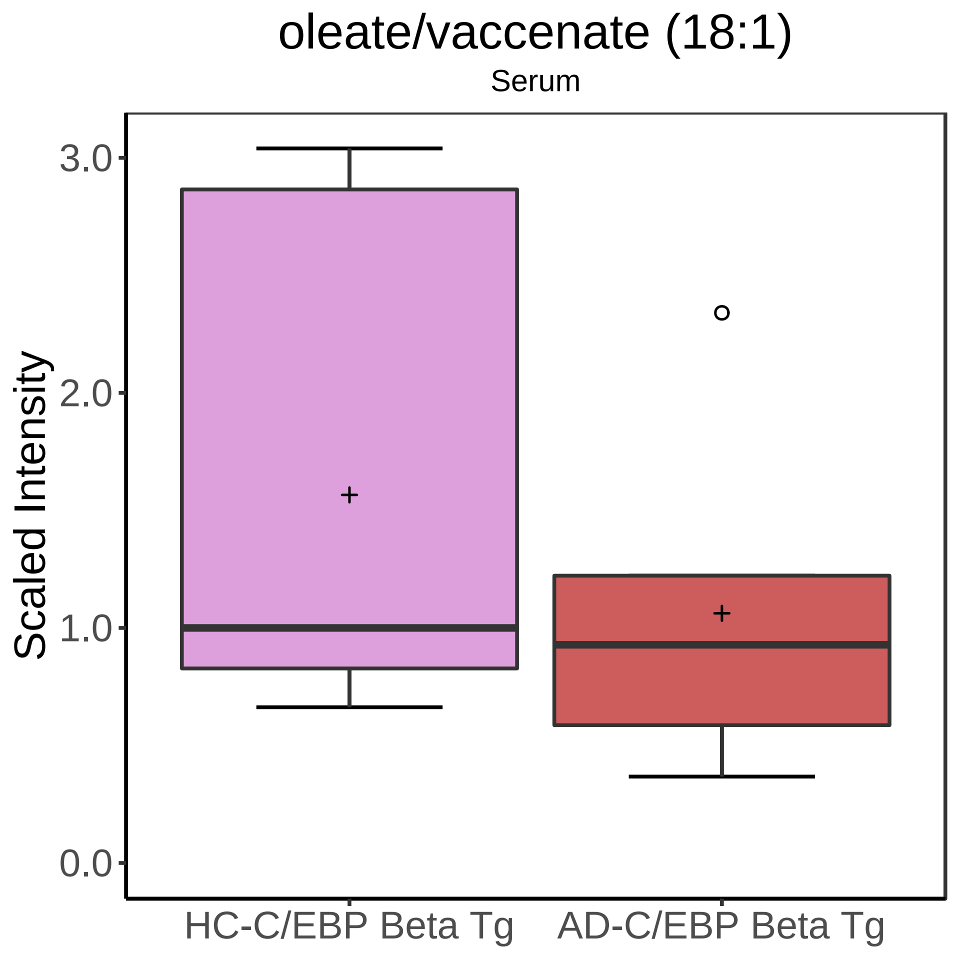
oleate/vaccenate (18:1)
Serum
Scaled Intensity
3.0
2.0
1.0
0.0
HC-C/EBP Beta Tg
AD-C/EBP Beta Tg

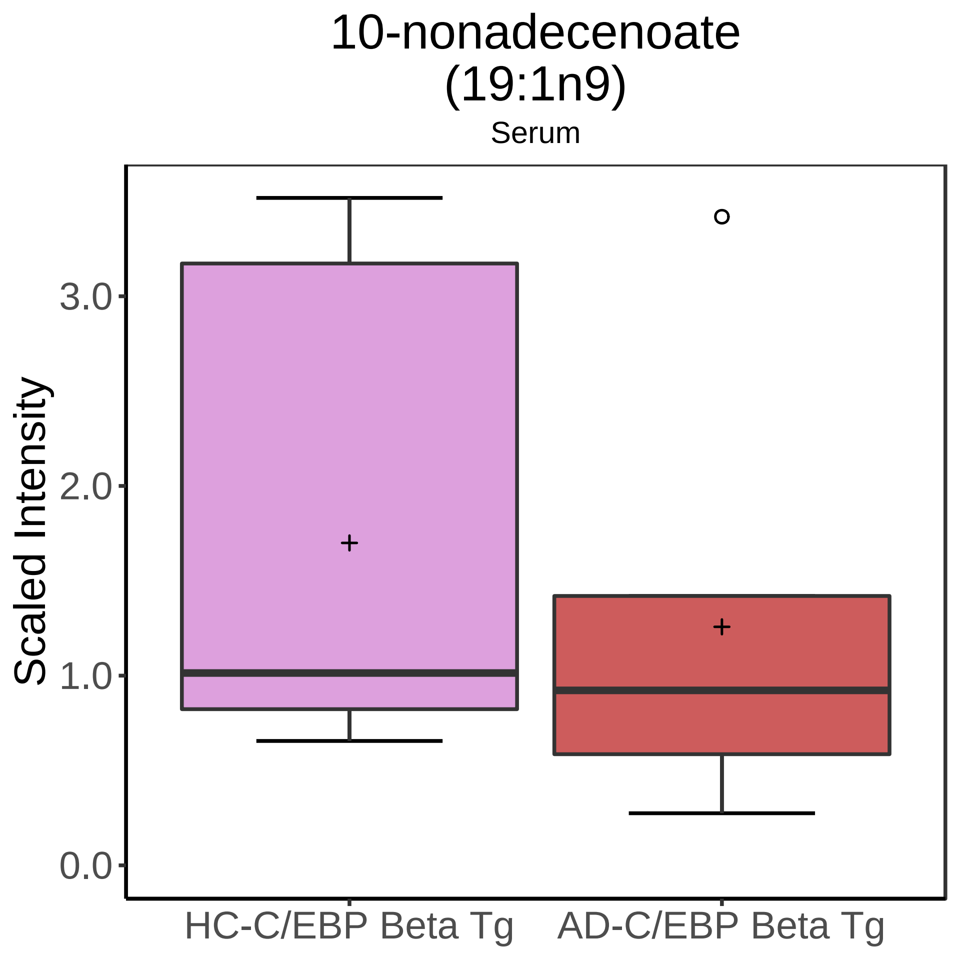
10-nonadecenoate
(19:1n9)
Serum
Scaled Intensity
3.0
2.0
1.0
0.0
HC-C/EBP Beta Tg
AD-C/EBP Beta Tg

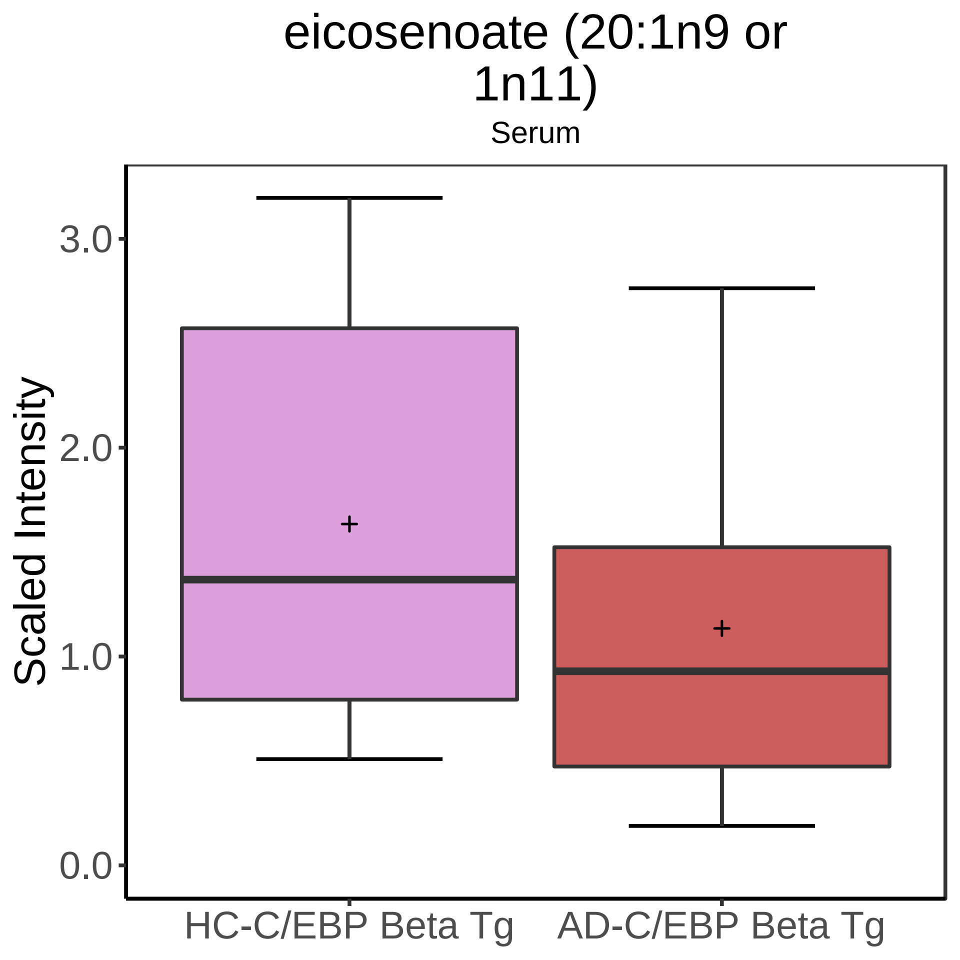
eicosenoate (20:1n9 or 1n11)
Serum
Scaled Intensity
3.0
2.0
1.0
0.0
HC-C/EBP Beta Tg
AD-C/EBP Beta Tg

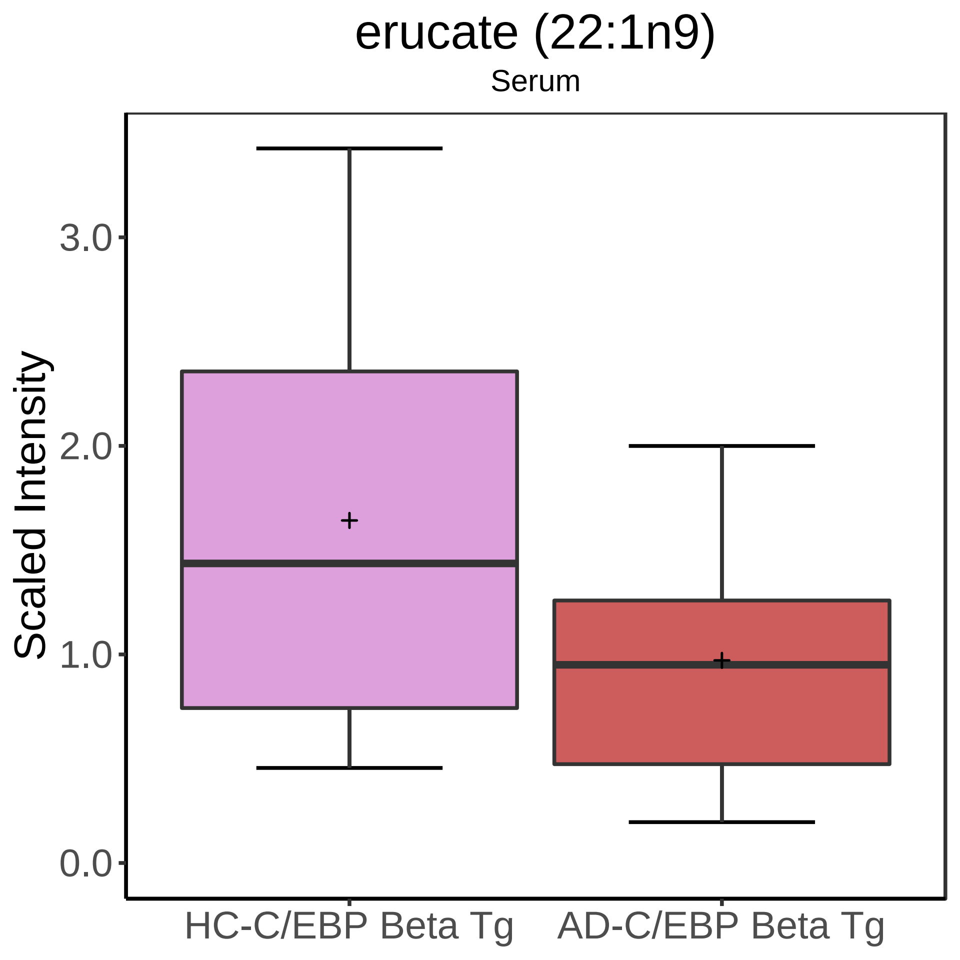
erucate (22:1n9)
Serum
Scaled Intensity
3.0
2.0
1.0
0.0
HC-C/EBP Beta Tg
AD-C/EBP Beta Tg

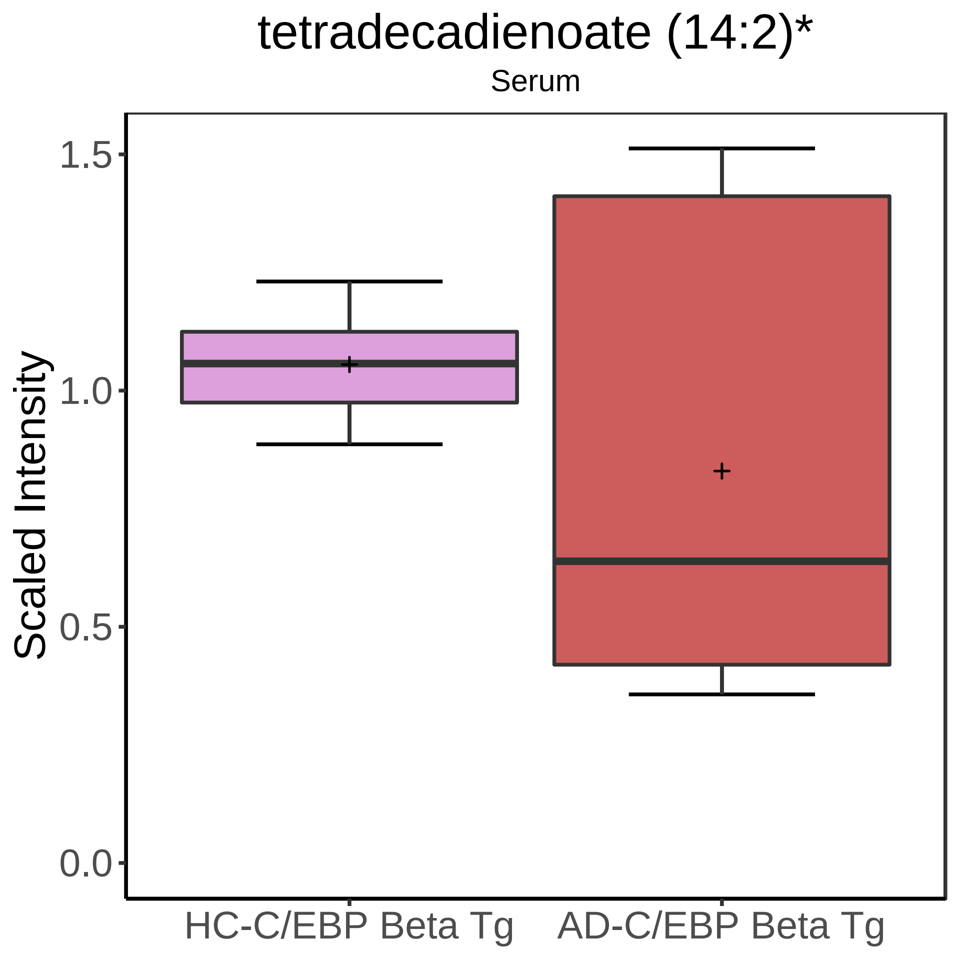
tetradecadienoate (14:2)*
Serum
Scaled Intensity
1.5
1.0
0.5
0.0
HC-C/EBP Beta Tg
AD-C/EBP Beta Tg

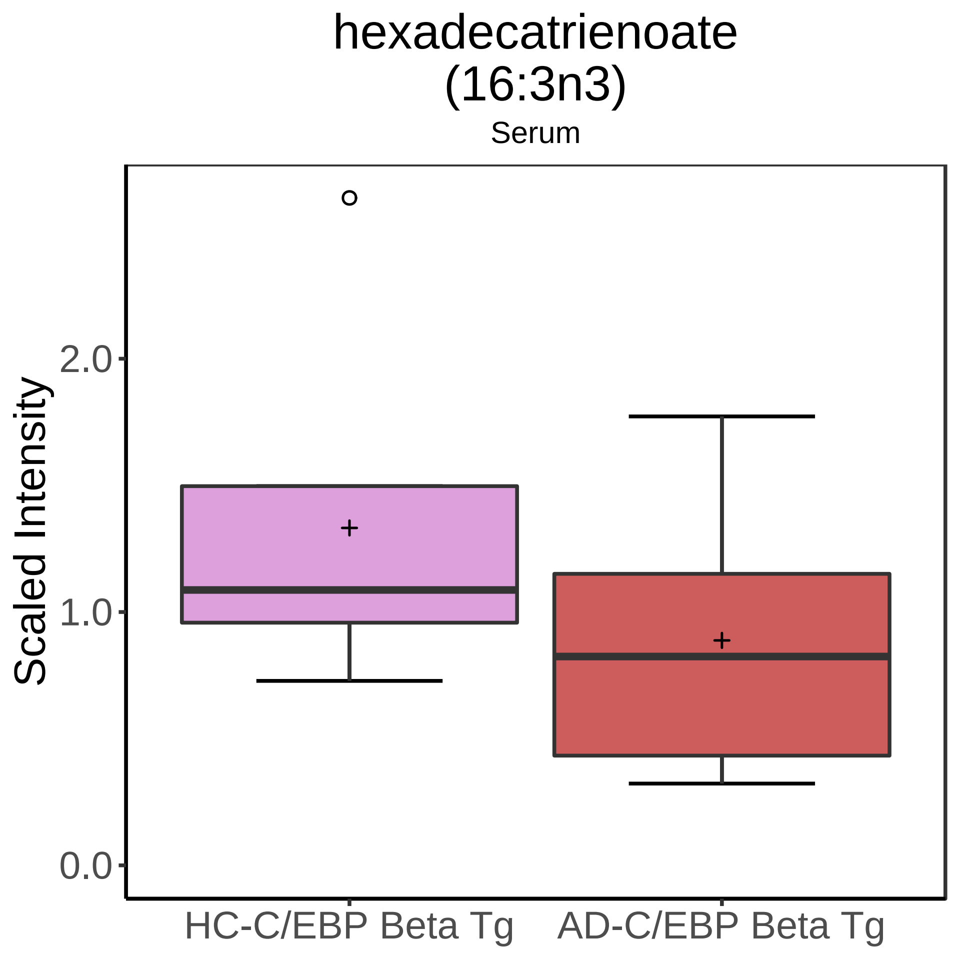
hexadecatrienoate
(16:3n3)
Serum
Scaled Intensity
2.0
1.0
0.0
HC-C/EBP Beta Tg
AD-C/EBP Beta Tg

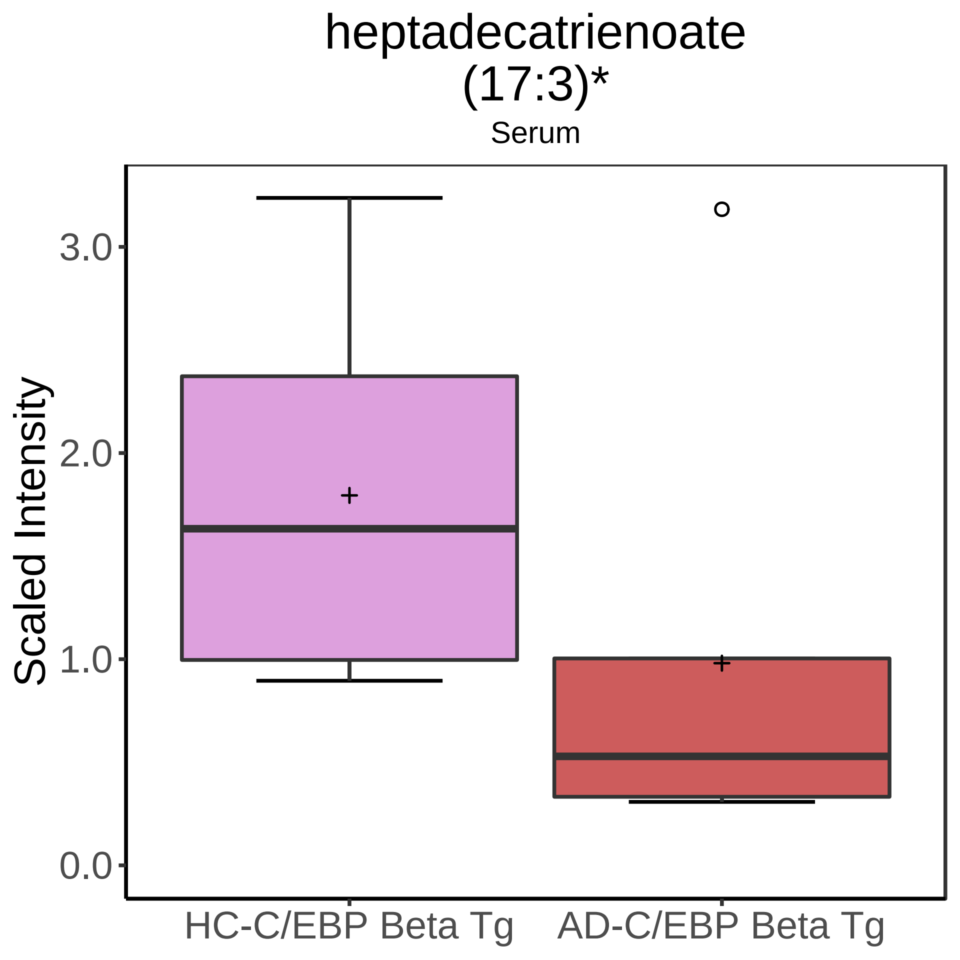
heptadecatrienoate
(17:3)*
Serum
Scaled Intensity
3.0
2.0
1.0
0.0
HC-C/EBP Beta Tg
AD-C/EBP Beta Tg

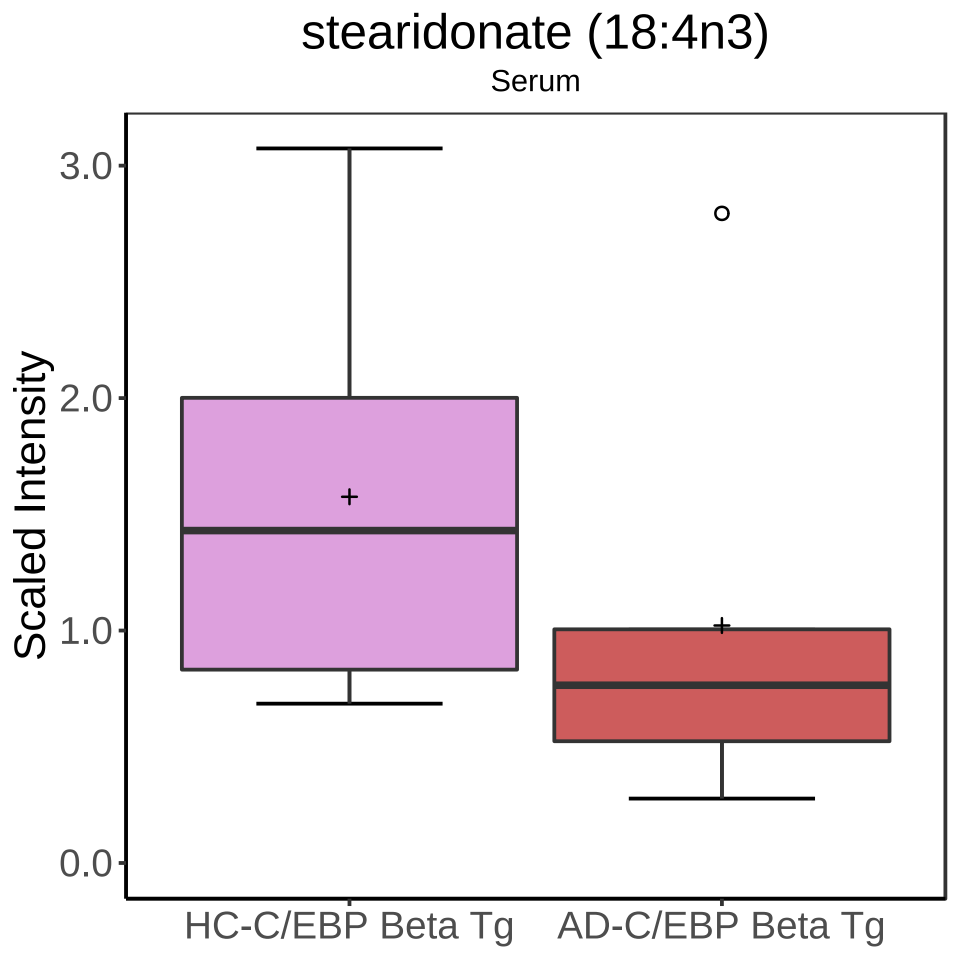

stearidonate (18:4n3)
Serum
Scaled Intensity
3.0
2.0
1.0
0.0
HC-C/EBP Beta Tg
AD-C/EBP Beta Tg

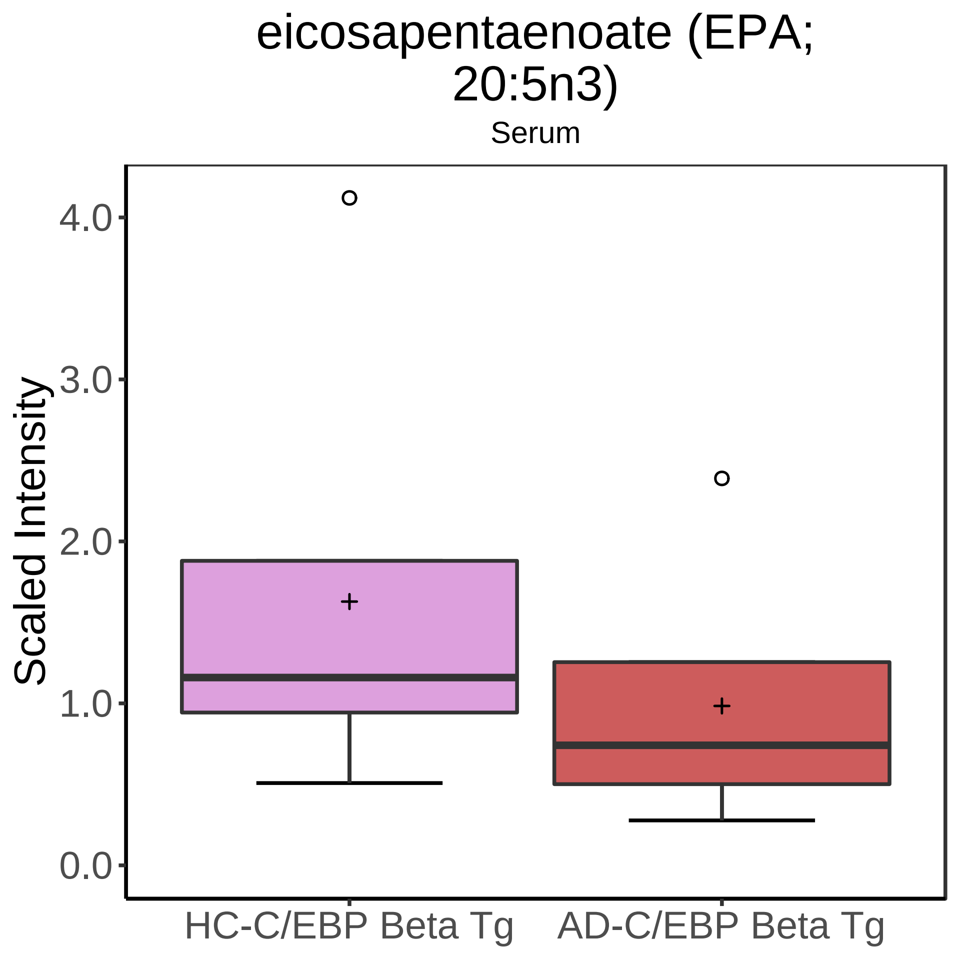
eicosapentaenoate (EPA; 20:5n3)
Serum
Scaled Intensity
4.0
3.0
2.0
1.0
0.0
HC-C/EBP Beta Tg
AD-C/EBP Beta Tg

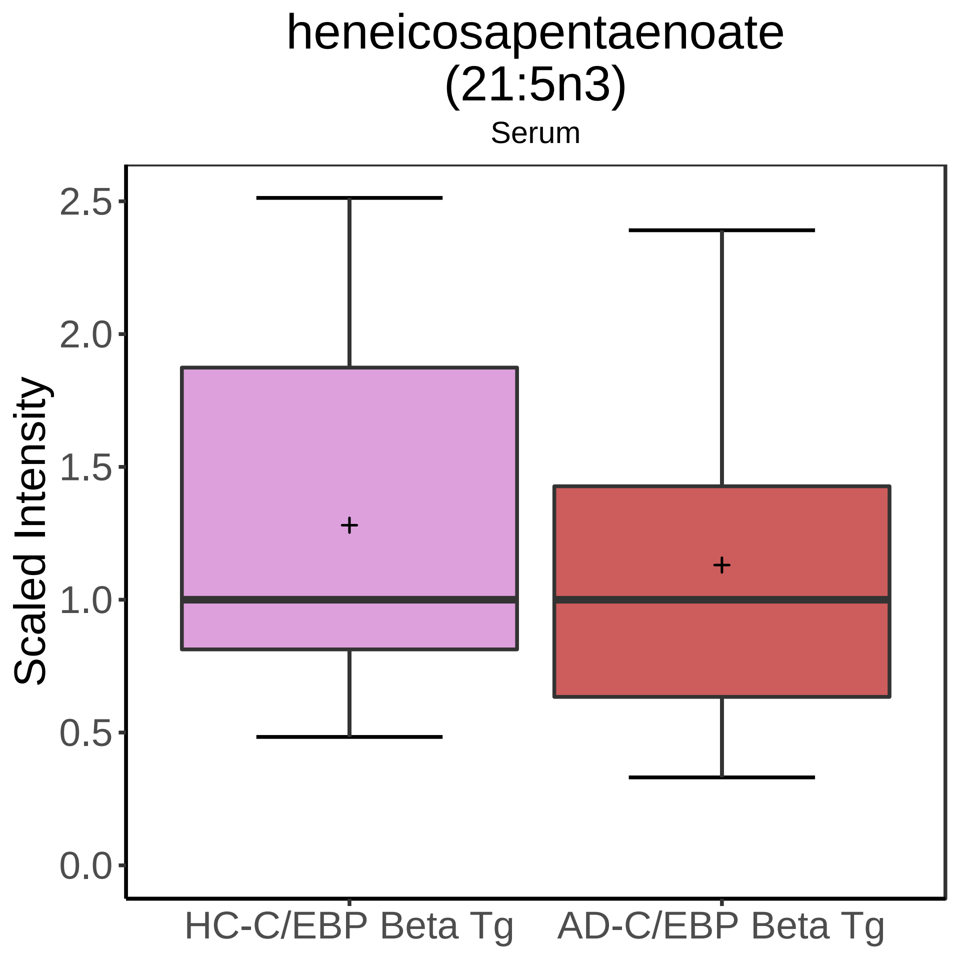
heneicosapentaenoate
(21:5n3)
Serum
Scaled Intensity
2.5
2.0
1.5
1.0
0.5
0.0
HC-C/EBP Beta Tg
AD-C/EBP Beta Tg

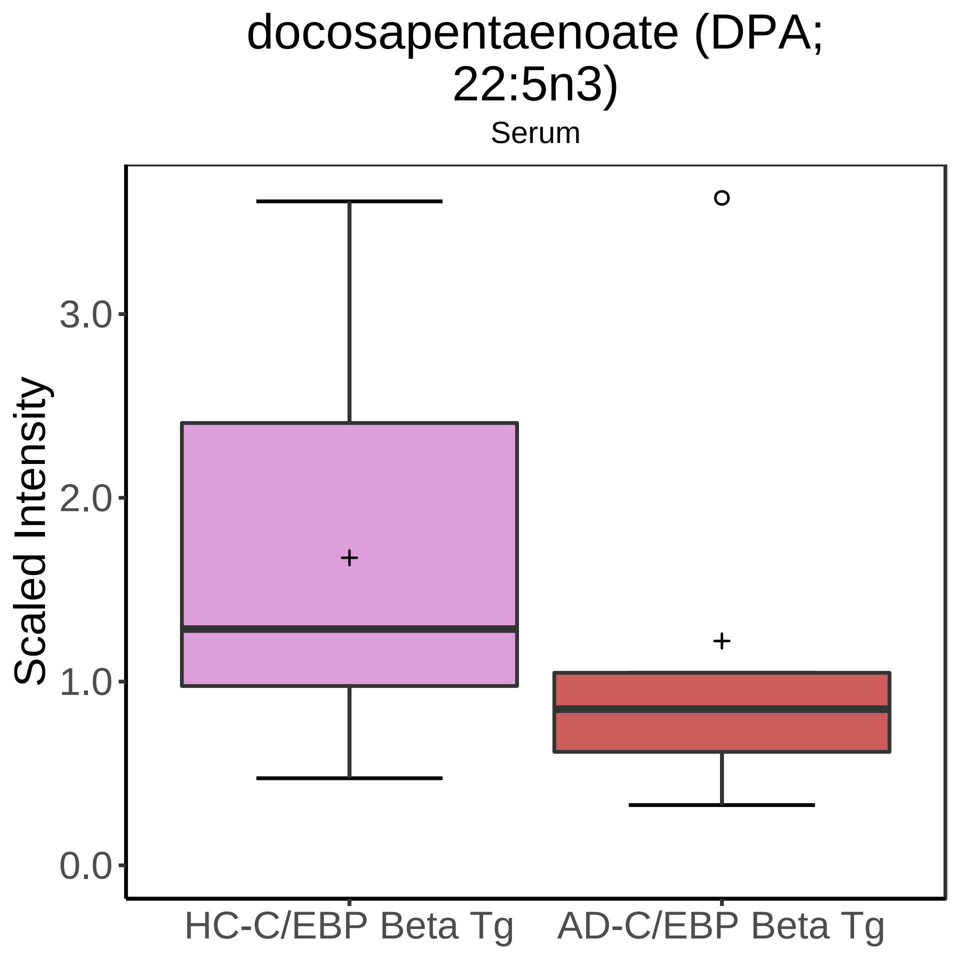
docosapentaenoate (DPA; 22:5n3)
Serum
Scaled Intensity
3.0
2.0
1.0
0.0
HC-C/EBP Beta Tg
AD-C/EBP Beta Tg

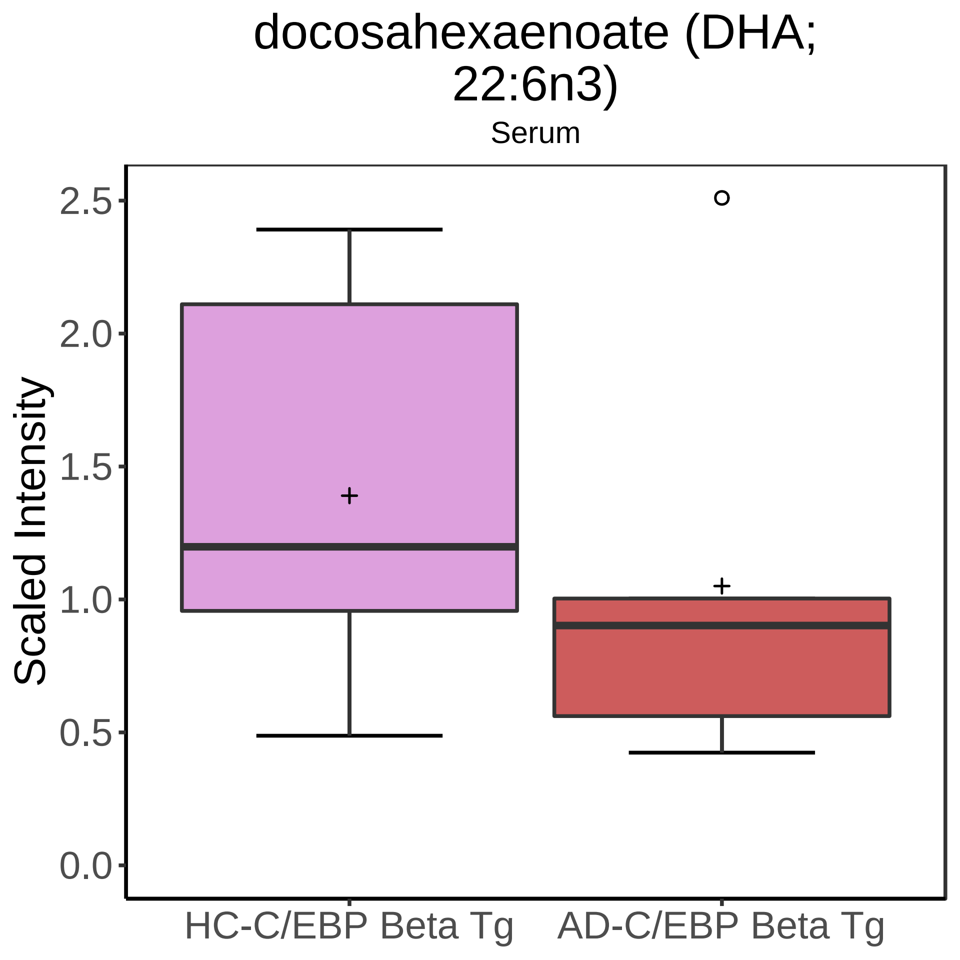
docosahexaenoate (DHA;
22:6n3)
Serum
Scaled Intensity
2.5
2.0
1.5
1.0
0.5
0.0
HC-C/EBP Beta Tg
AD-C/EBP Beta Tg

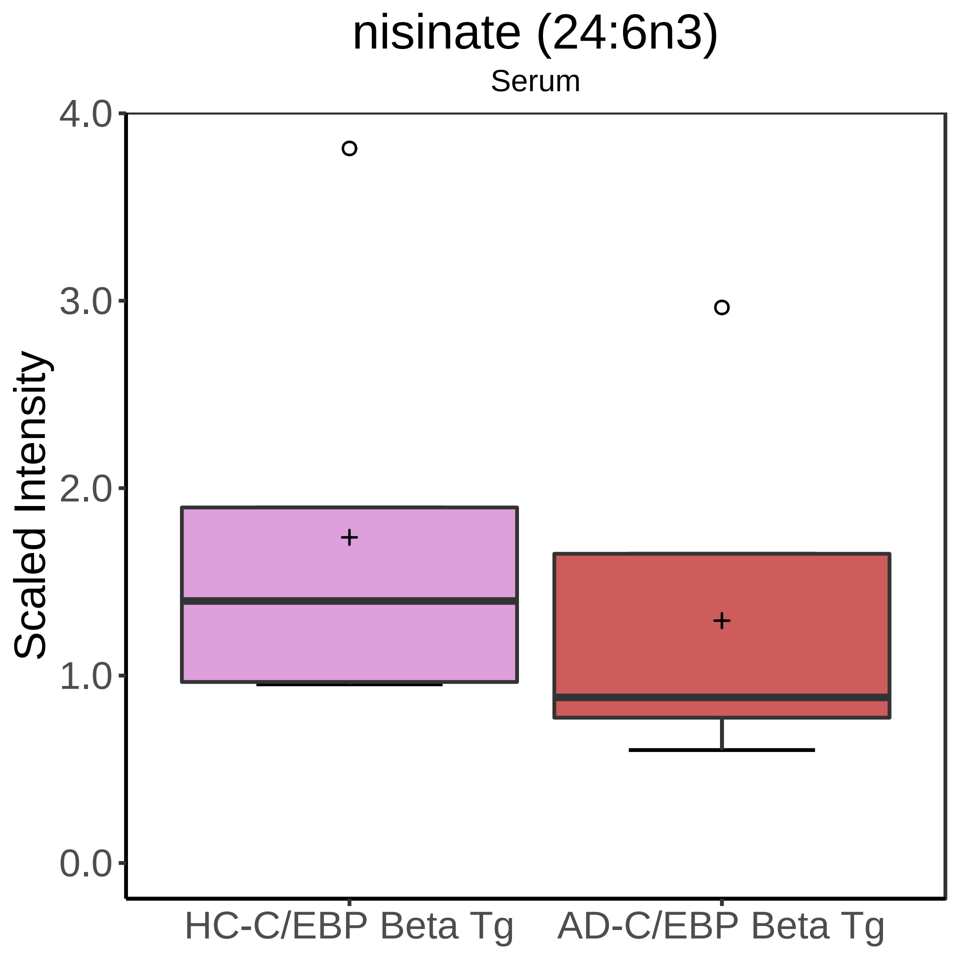
nisinate (24:6n3)
Serum
Scaled Intensity
4.0
3.0
2.0
1.0
0.0
HC-C/EBP Beta Tg
AD-C/EBP Beta Tg

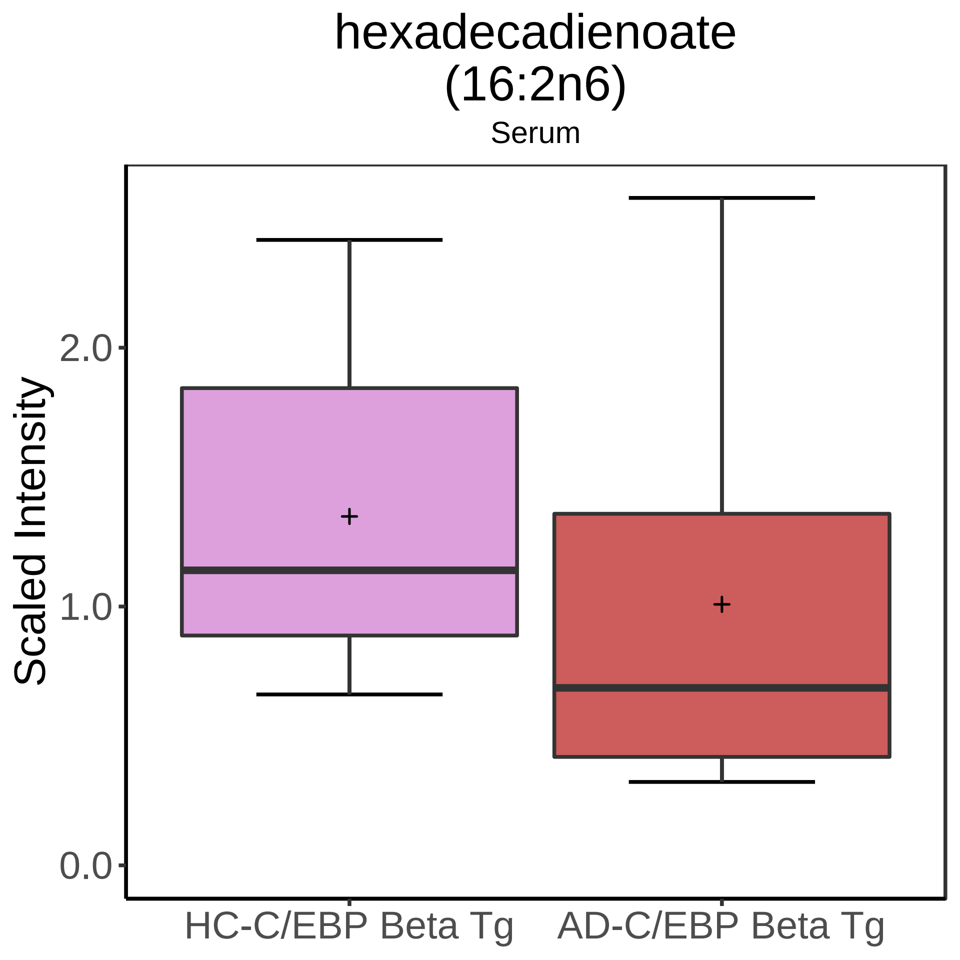
hexadecadienoate
(16:2n6)
Serum
Scaled Intensity
2.0
1.0
0.0
HC-C/EBP Beta Tg
AD-C/EBP Beta Tg

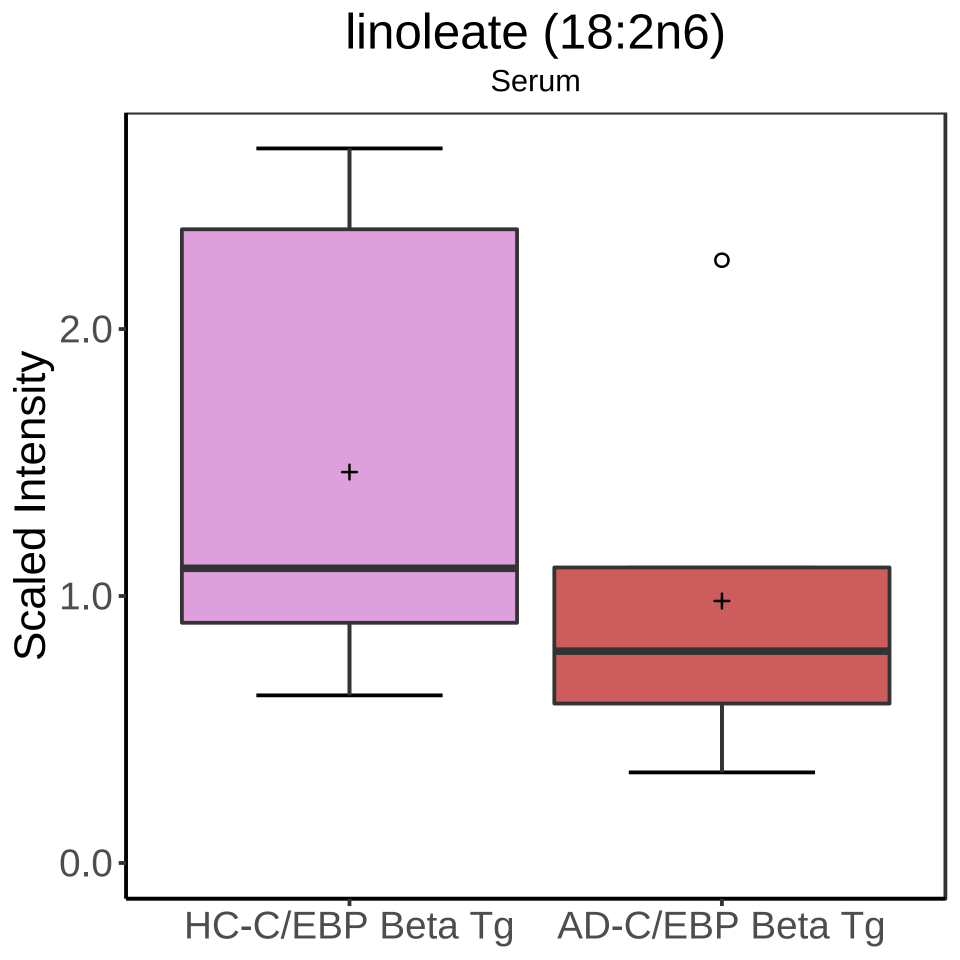
linoleate (18:2n6)
Serum
Scaled Intensity
2.0
1.0
0.0
HC-C/EBP Beta Tg
AD-C/EBP Beta Tg

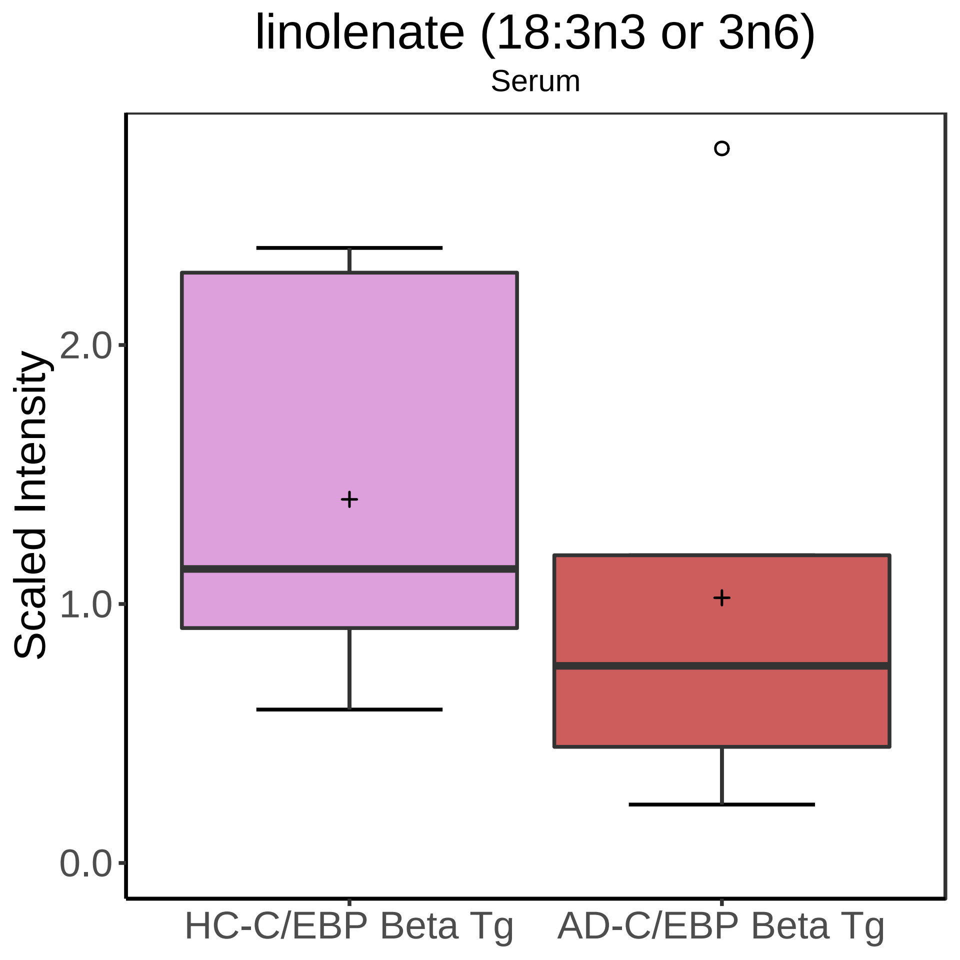

linolenate (18:3n3 or 3n6)
Serum
Scaled Intensity
2.0
1.0
0.0
HC-C/EBP Beta Tg
AD-C/EBP Beta Tg

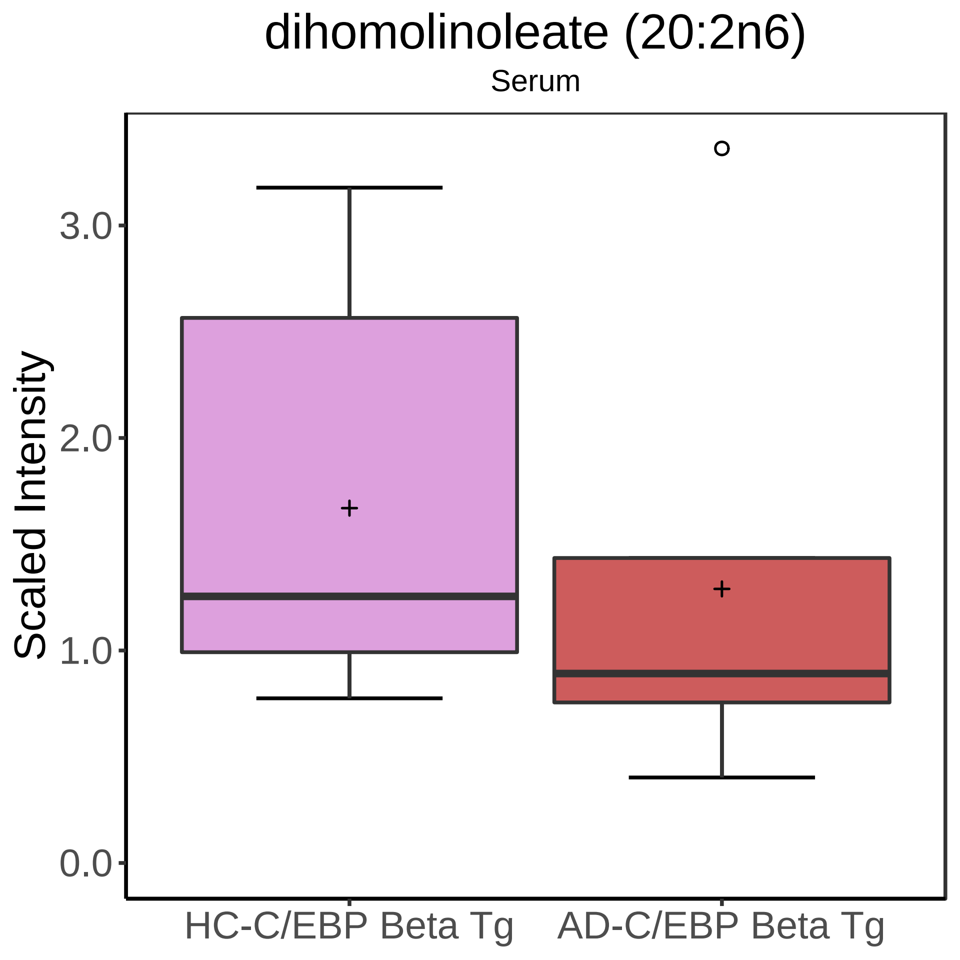
dihomolinoleate (20:2n6)
Serum
Scaled Intensity
3.0
2.0
1.0
0.0
HC-C/EBP Beta Tg
AD-C/EBP Beta Tg

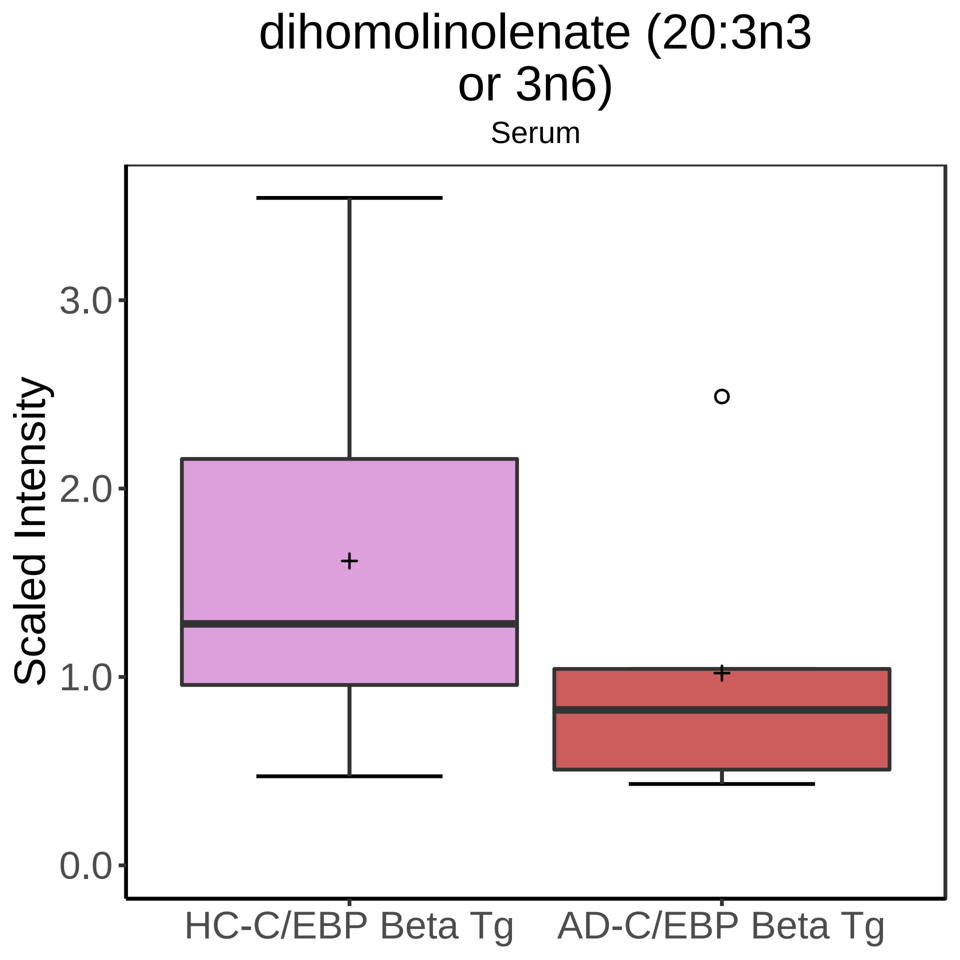
dihomolinolenate (20:3n3 or 3n6)
Serum
Scaled Intensity
3.0
2.0
1.0
0.0
HC-C/EBP Beta Tg
AD-C/EBP Beta Tg

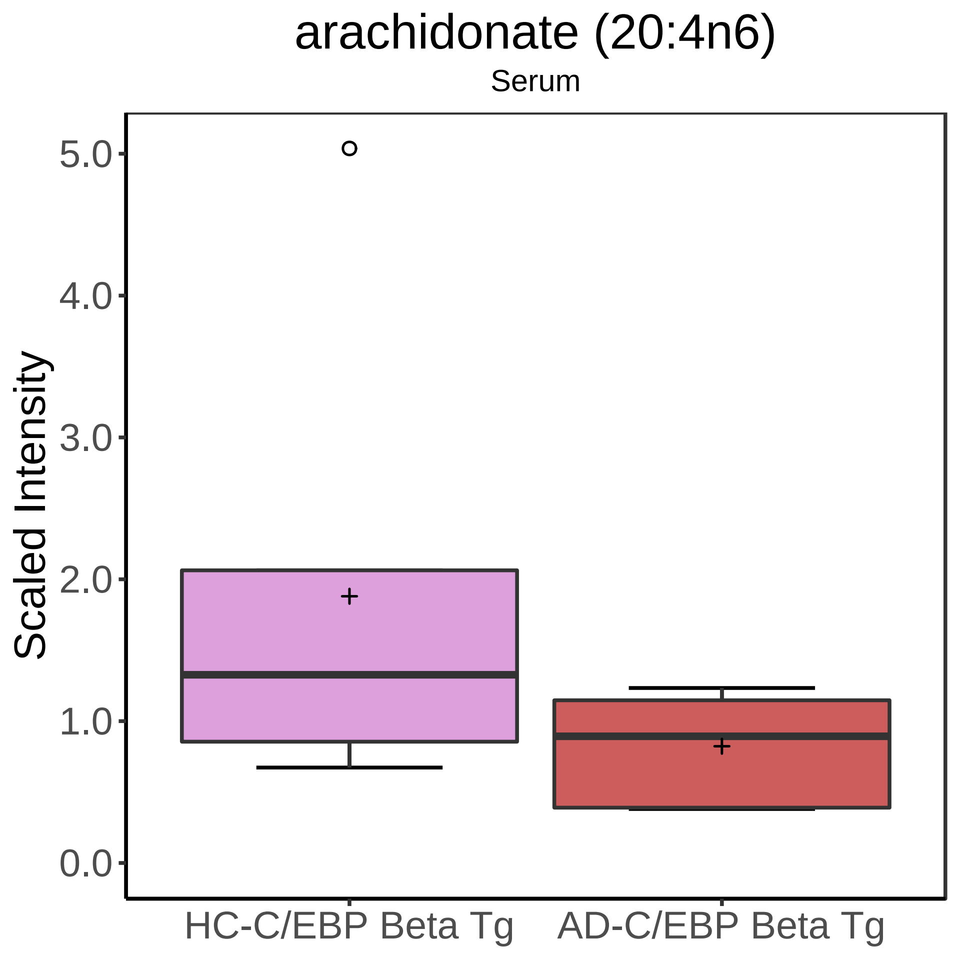

arachidonate (20:4n6)
Serum
Scaled Intensity
5.0
4.0
3.0
2.0
1.0
0.0
HC-C/EBP Beta Tg
AD-C/EBP Beta Tg

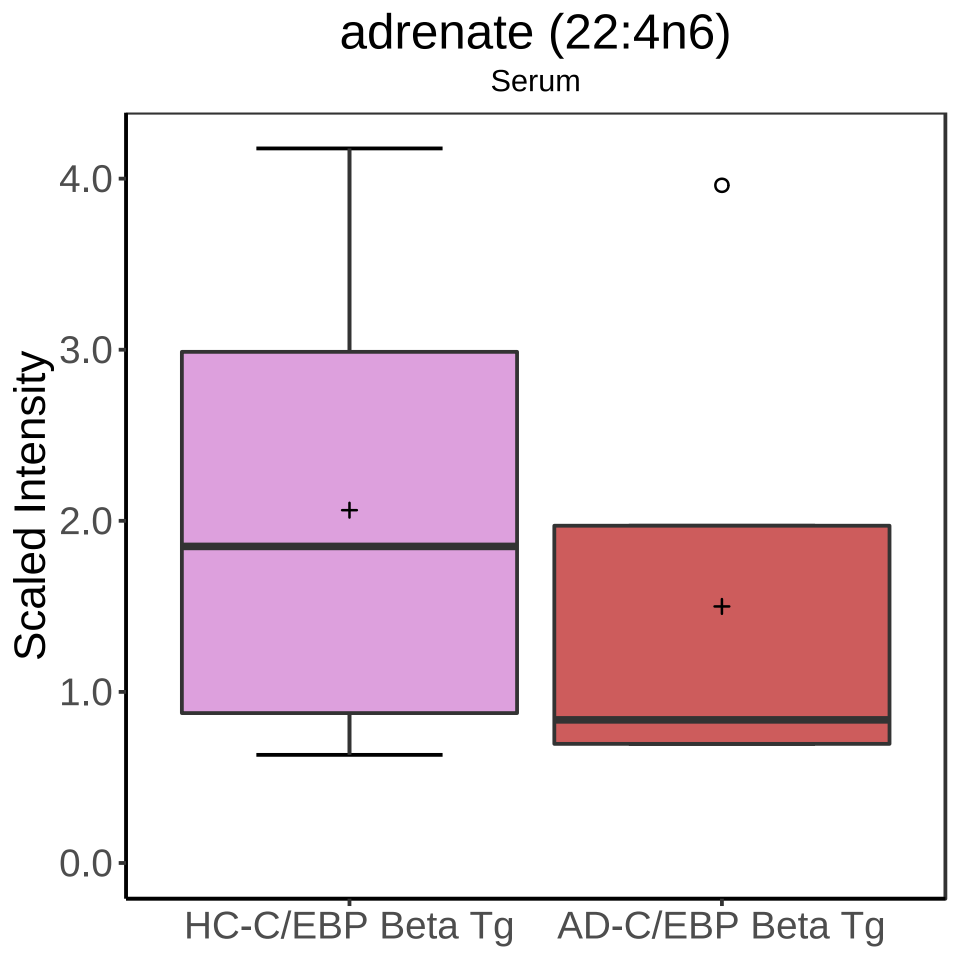

adrenate (22:4n6)
Serum
Scaled Intensity
4.0
3.0
2.0
1.0
0.0
HC-C/EBP Beta Tg
AD-C/EBP Beta Tg

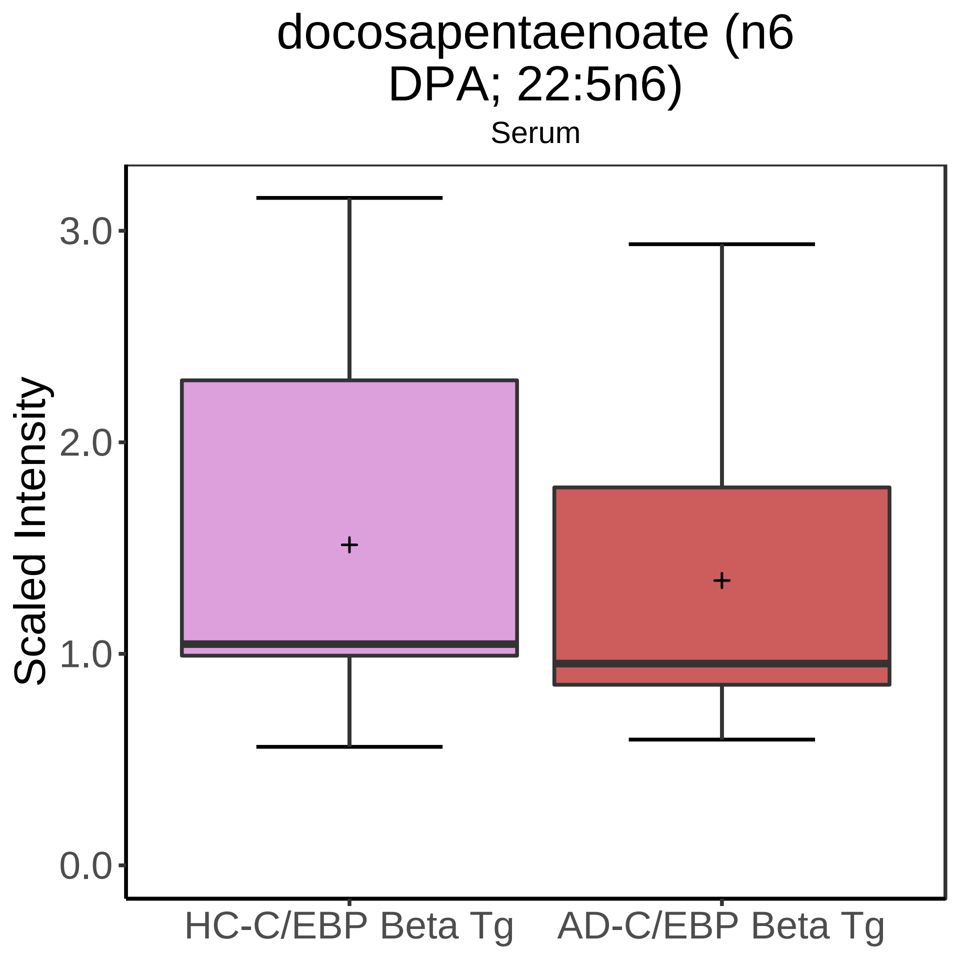

docosapentaenoate (n6
DPA; 22:5n6)
Serum
Scaled Intensity
3.0
2.0
1.0
0.0
HC-C/EBP Beta Tg
AD-C/EBP Beta Tg

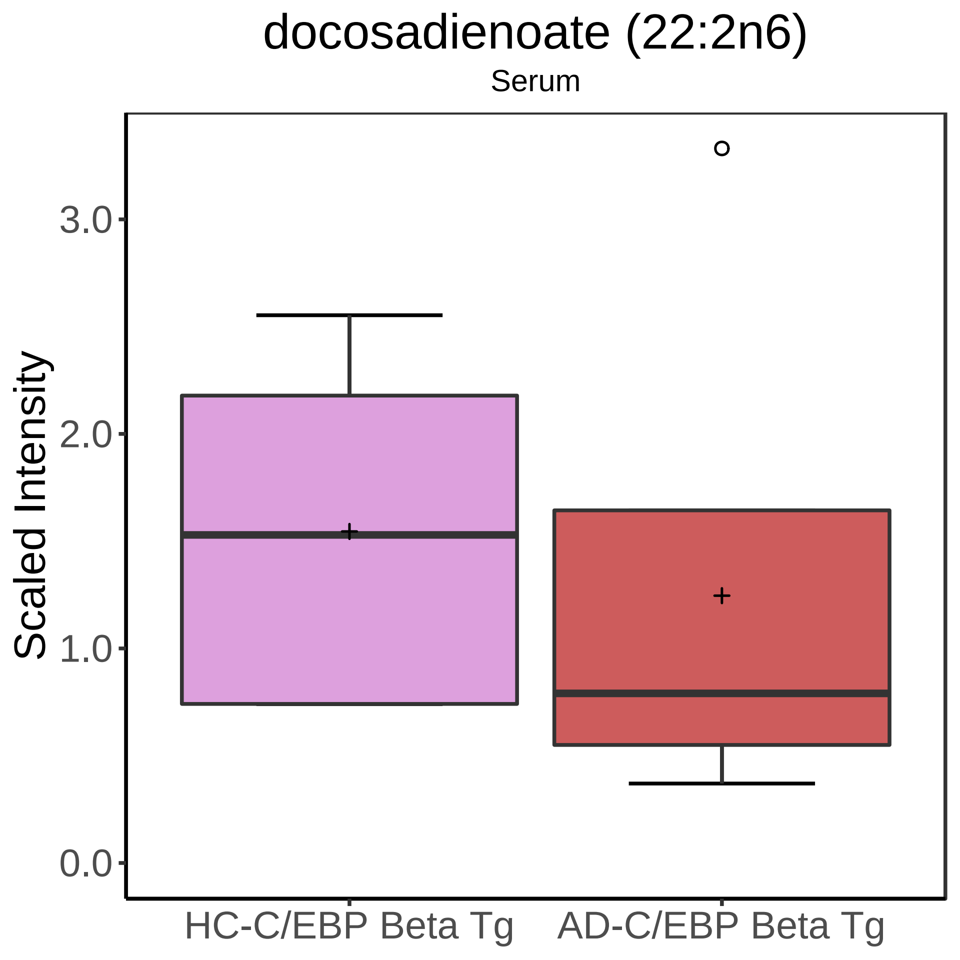
docosadienoate (22:2n6)
Serum
Scaled Intensity
3.0
2.0
1.0
0.0
HC-C/EBP Beta Tg
AD-C/EBP Beta Tg

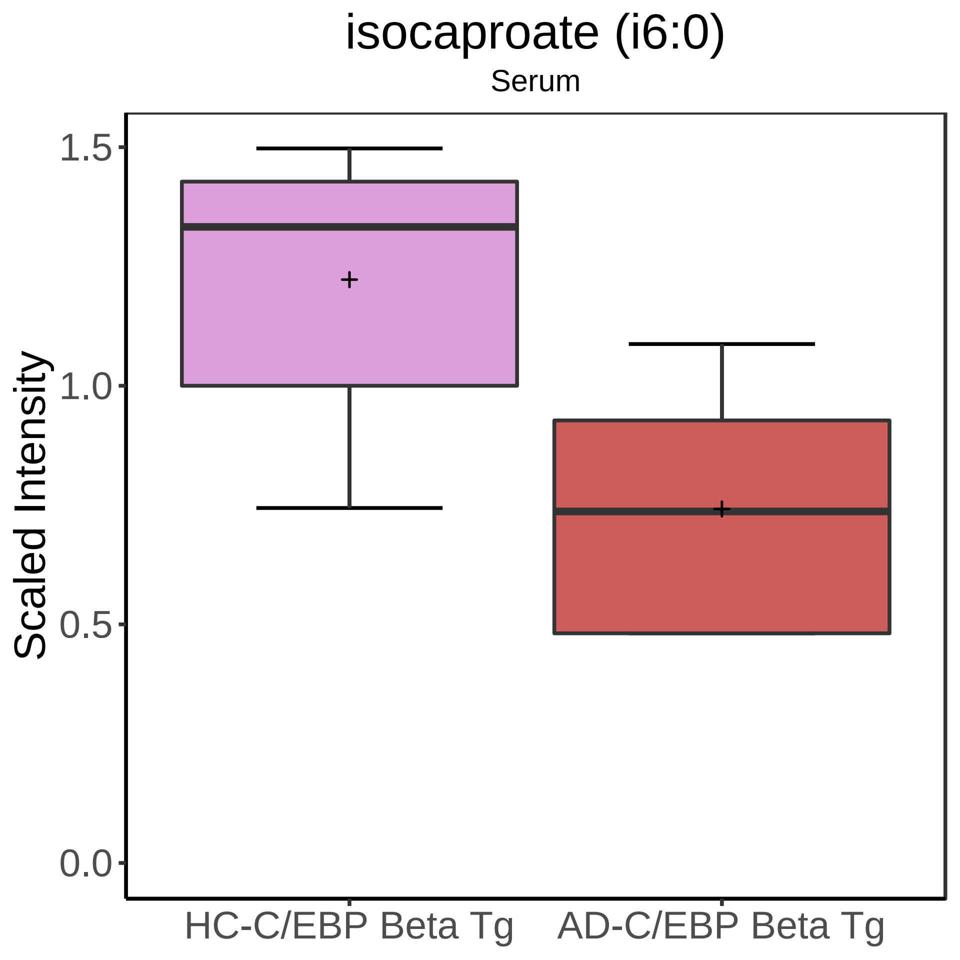

isocaproate (i6:0)
Serum
Scaled Intensity
1.5
1.0
0.5
0.0
HC-C/EBP Beta Tg
AD-C/EBP Beta Tg

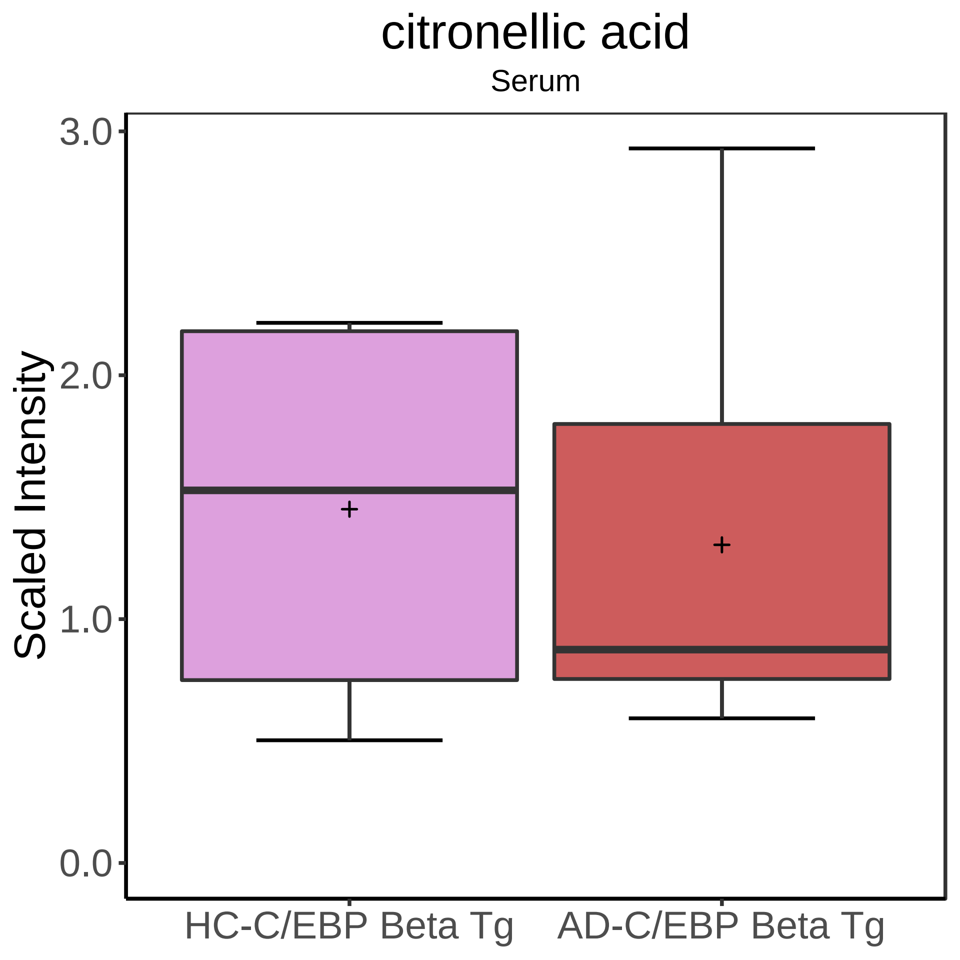
citronellic acid
Serum
Scaled Intensity
3.0
2.0
1.0
0.0
HC-C/EBP Beta Tg
AD-C/EBP Beta Tg

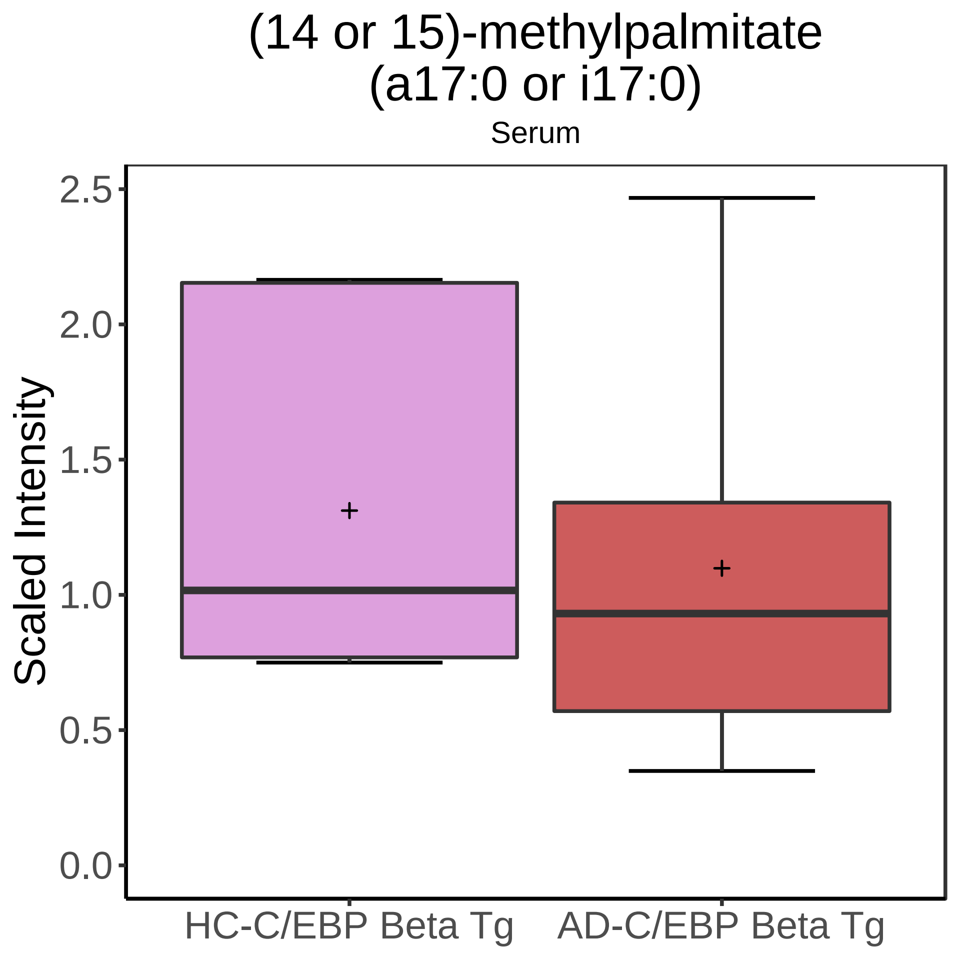

(14 or 15)-methylpalmitate
(a17:0 or i17:0)
Serum
Scaled Intensity
2.5
2.0
1.5
1.0
0.5
0.0
HC-C/EBP Beta Tg
AD-C/EBP Beta Tg

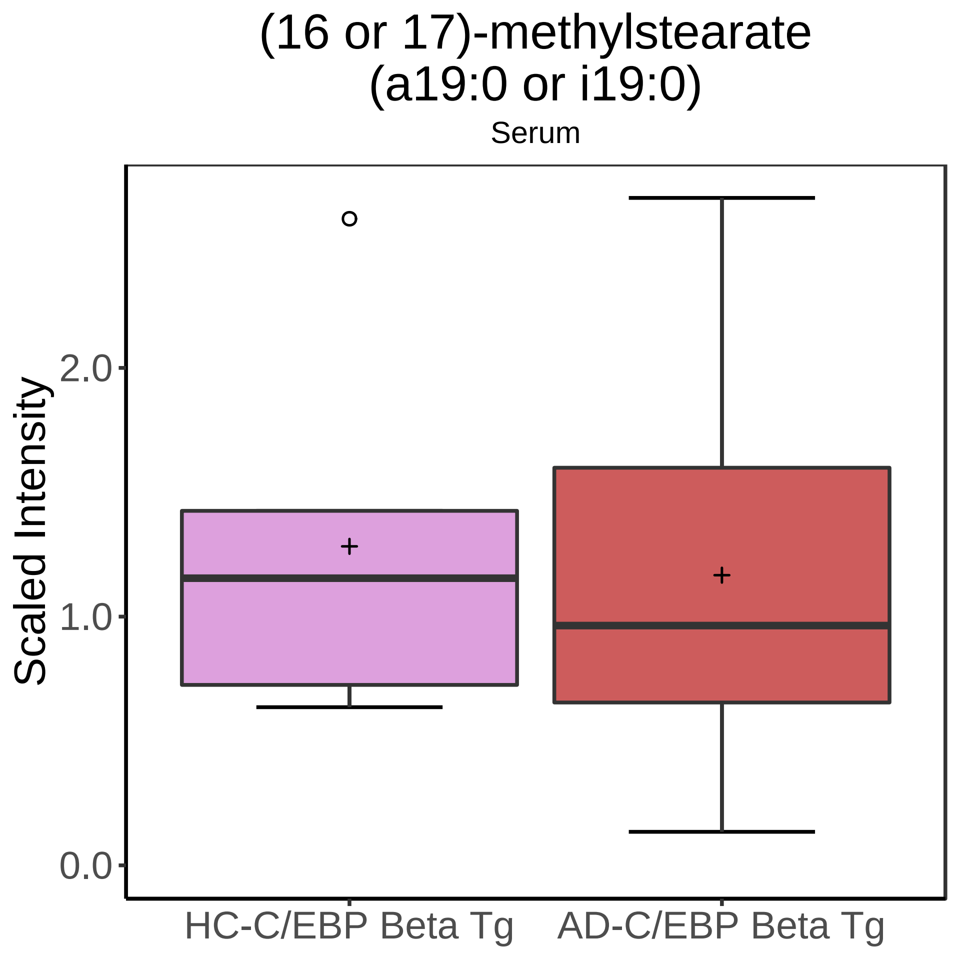
(16 or 17)-methylstearate
(a19:0 or i19:0)
Serum
Scaled Intensity
2.0
1.0
0.0
HC-C/EBP Beta Tg
AD-C/EBP Beta Tg

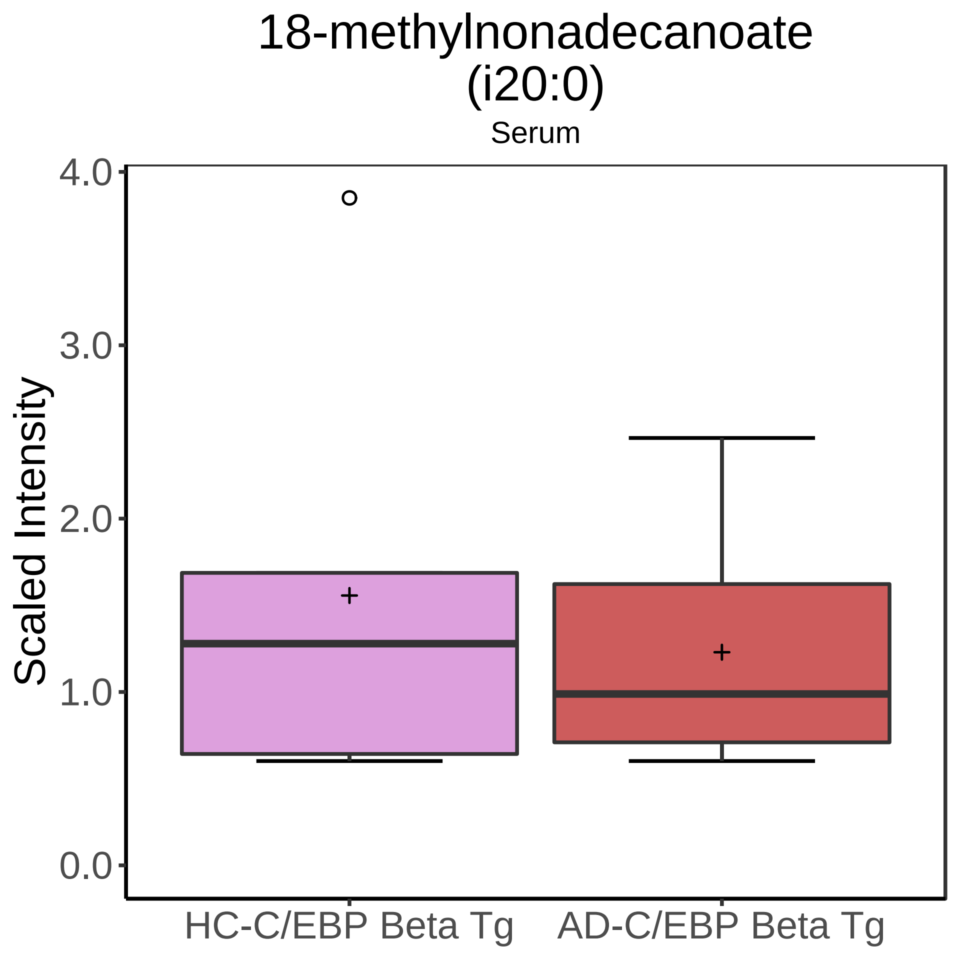
18-methylnonadecanoate
(i20:0)
Serum
Scaled Intensity
4.0
3.0
2.0
1.0
0.0
HC-C/EBP Beta Tg
AD-C/EBP Beta Tg

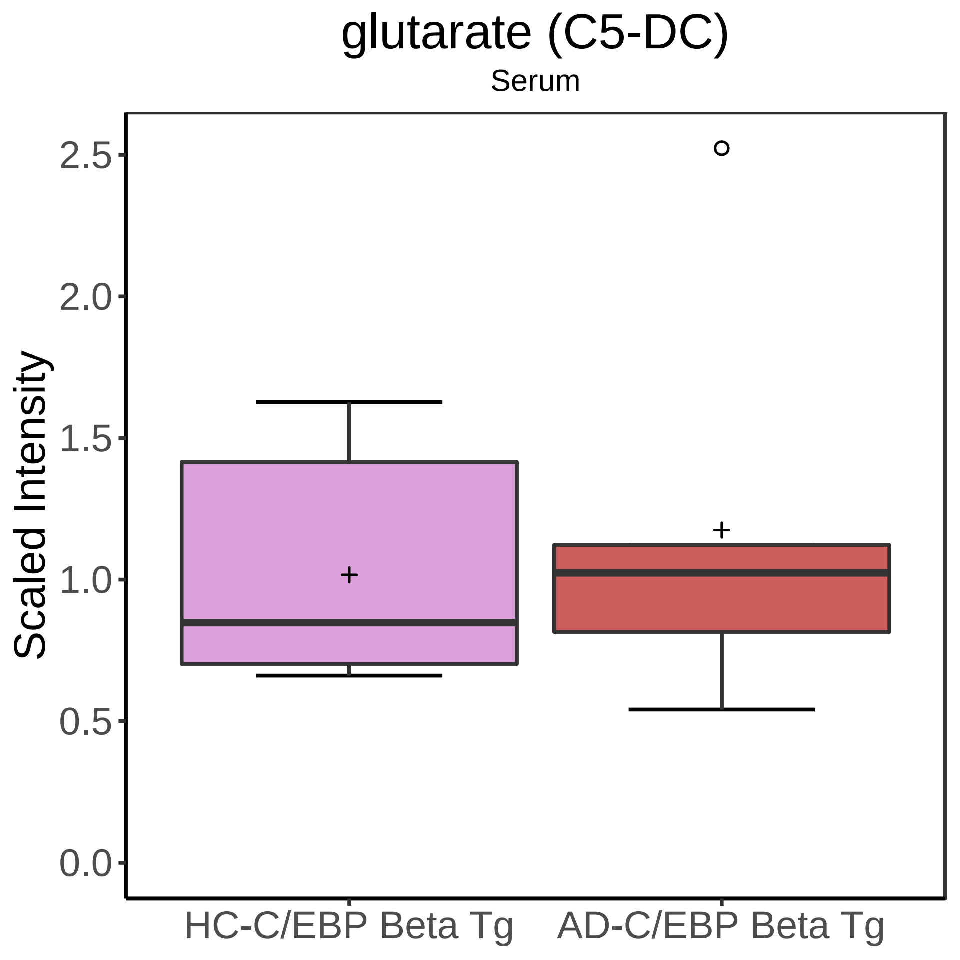
glutarate (C5-DC)
Serum
Scaled Intensity
2.5
2.0
1.5
1.0
0.5
0.0
HC-C/EBP Beta Tg
AD-C/EBP Beta Tg

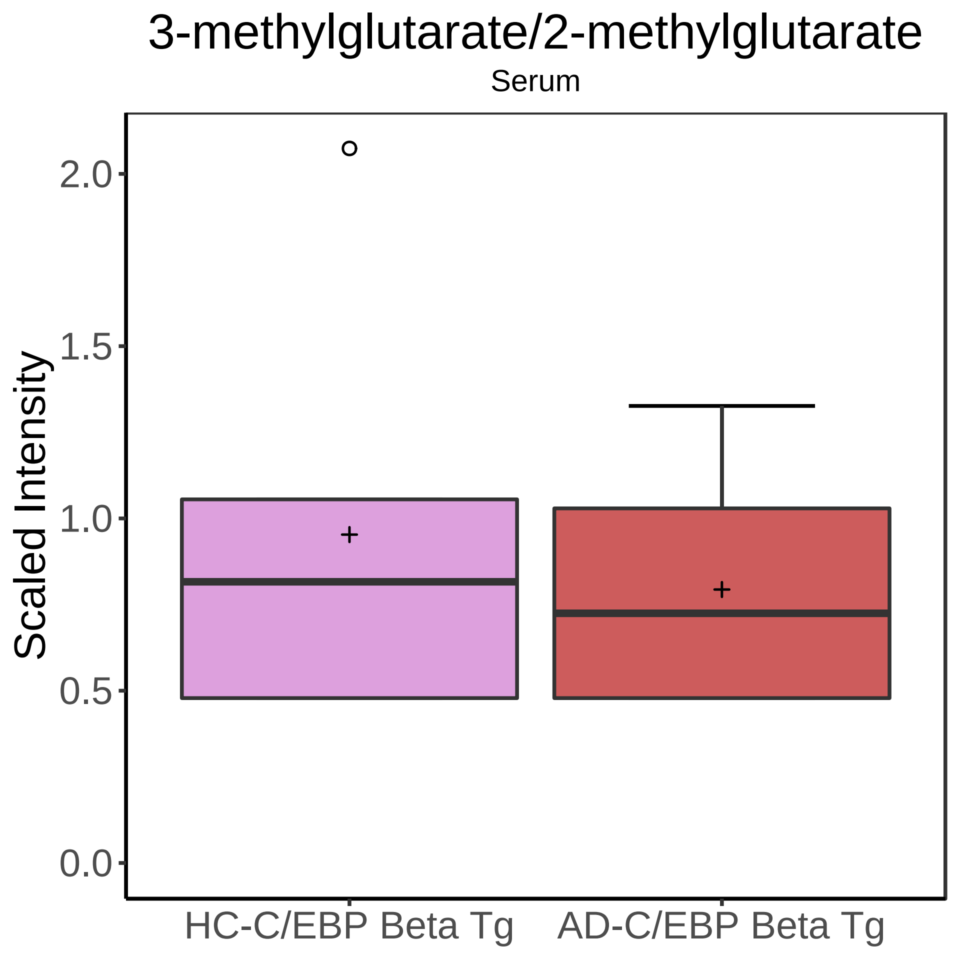
3-methylglutarate/2-methylglutarate
Serum
Scaled Intensity
2.0
1.5
1.0
0.5
0.0
HC-C/EBP Beta Tg
AD-C/EBP Beta Tg

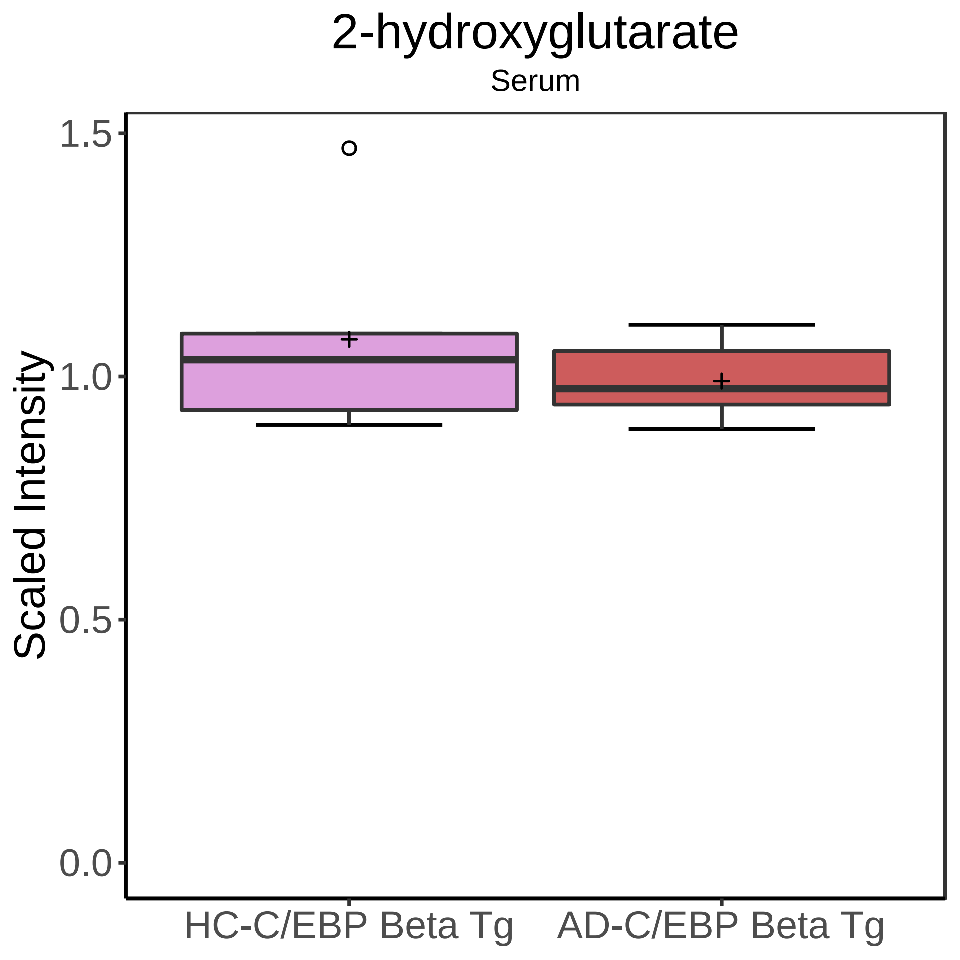
2-hydroxyglutarate
Serum
Scaled Intensity
1.5
1.0
0.5
0.0
HC-C/EBP Beta Tg
AD-C/EBP Beta Tg

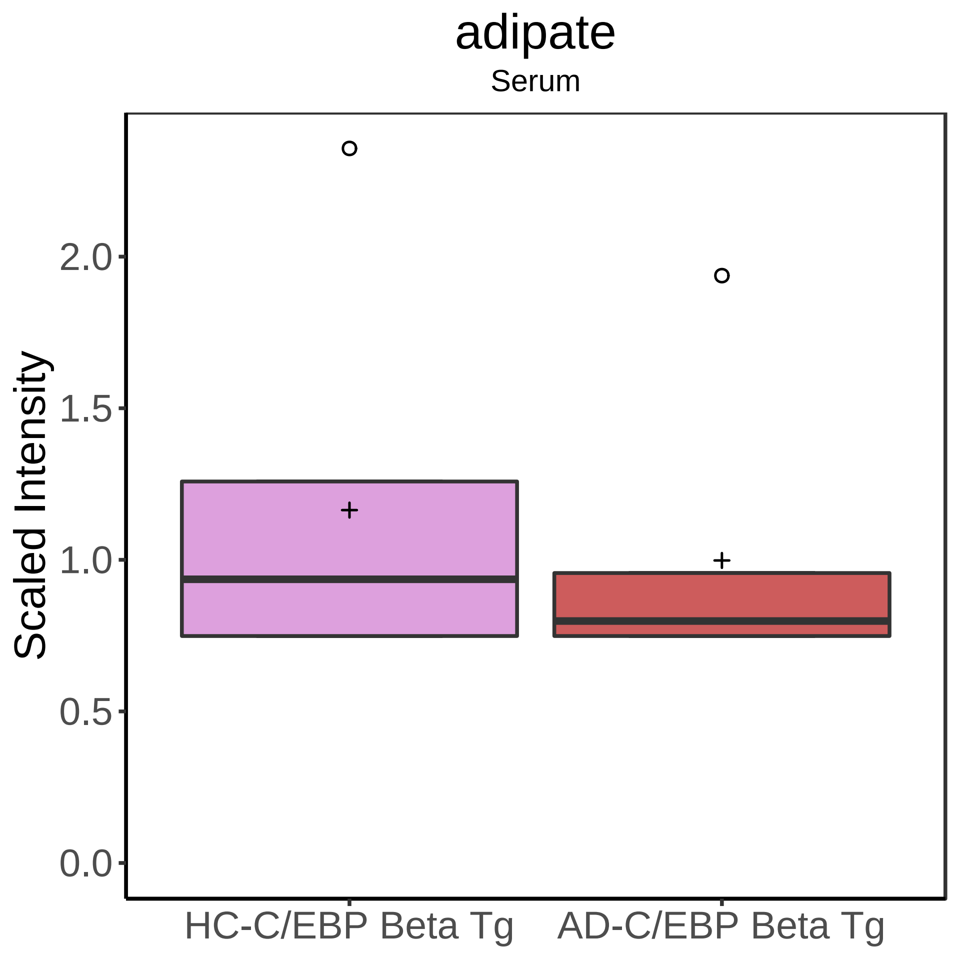
adipate
Serum
Scaled Intensity
2.0
1.5
1.0
0.5
0.0
HC-C/EBP Beta Tg
AD-C/EBP Beta Tg

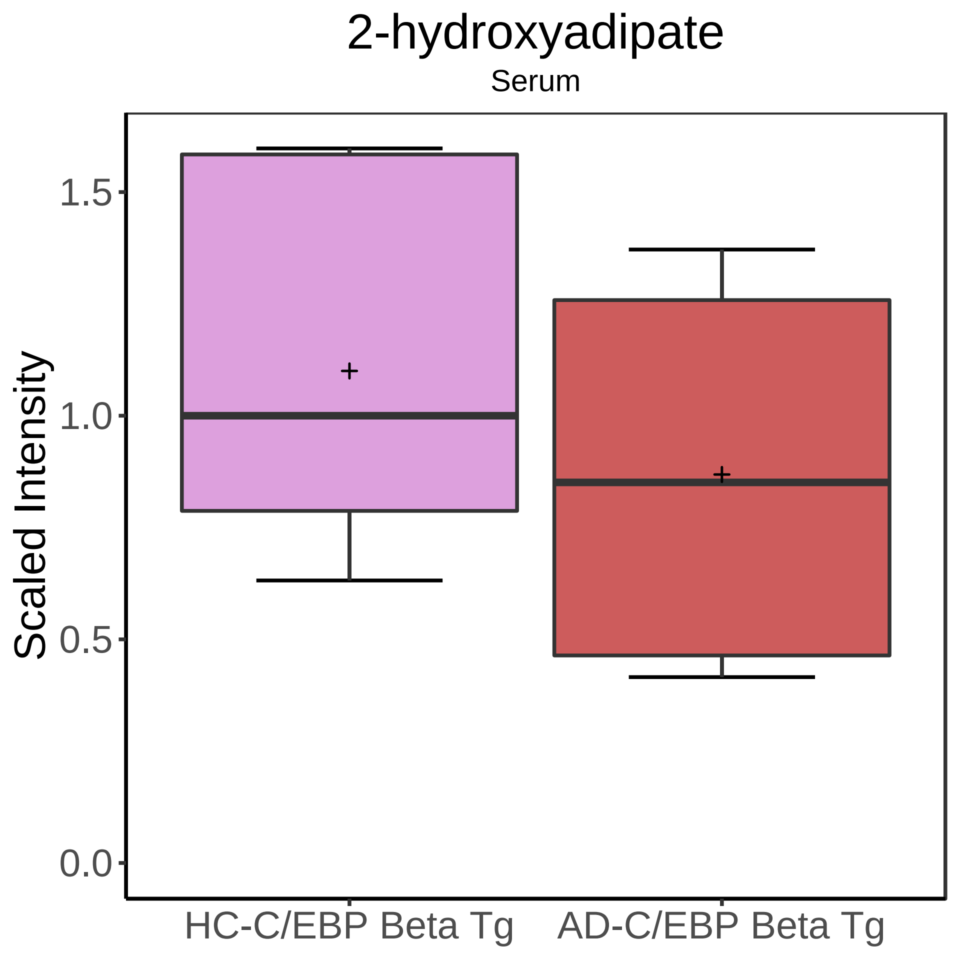
2-hydroxyadipate
Serum
Scaled Intensity
1.5
1.0
0.5
0.0
HC-C/EBP Beta Tg
AD-C/EBP Beta Tg

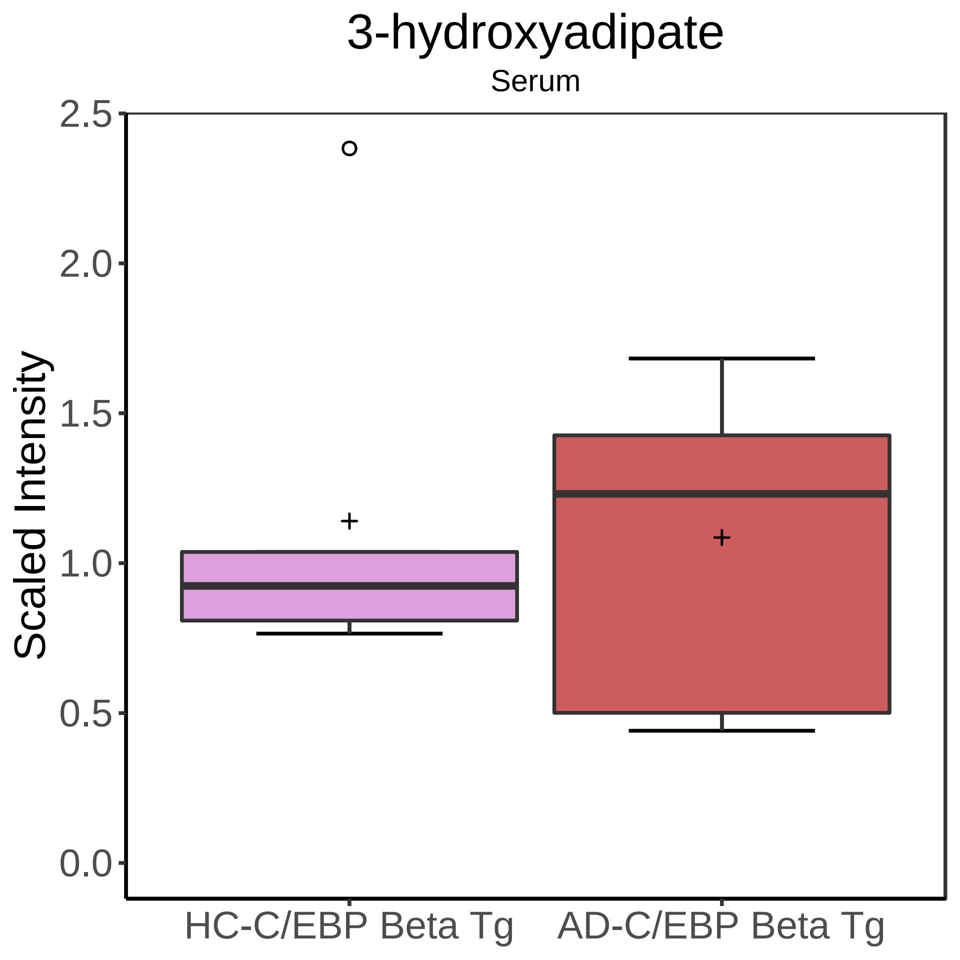
3-hydroxyadipate
Serum
Scaled Intensity
2.5
2.0
1.5
1.0
0.5
0.0
HC-C/EBP Beta Tg
AD-C/EBP Beta Tg

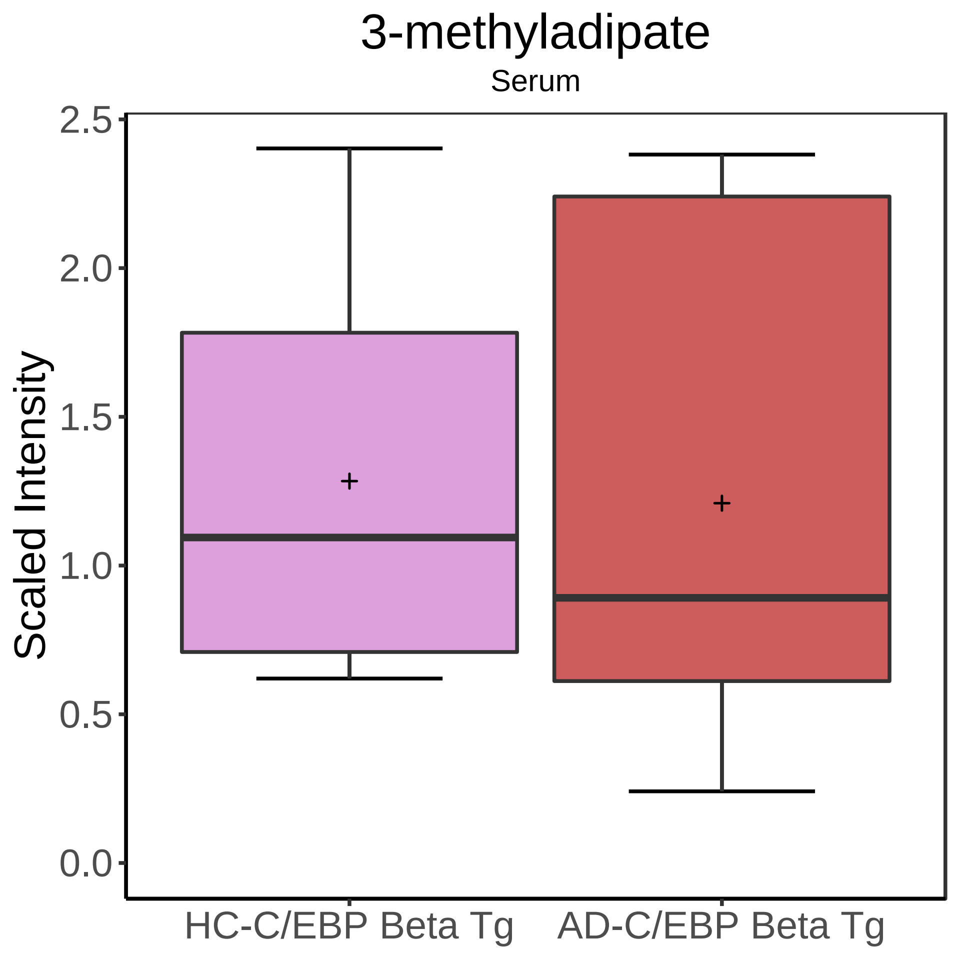
3-methyladipate
Serum
Scaled Intensity
2.5
2.0
1.5
1.0
0.5
0.0
HC-C/EBP Beta Tg
AD-C/EBP Beta Tg

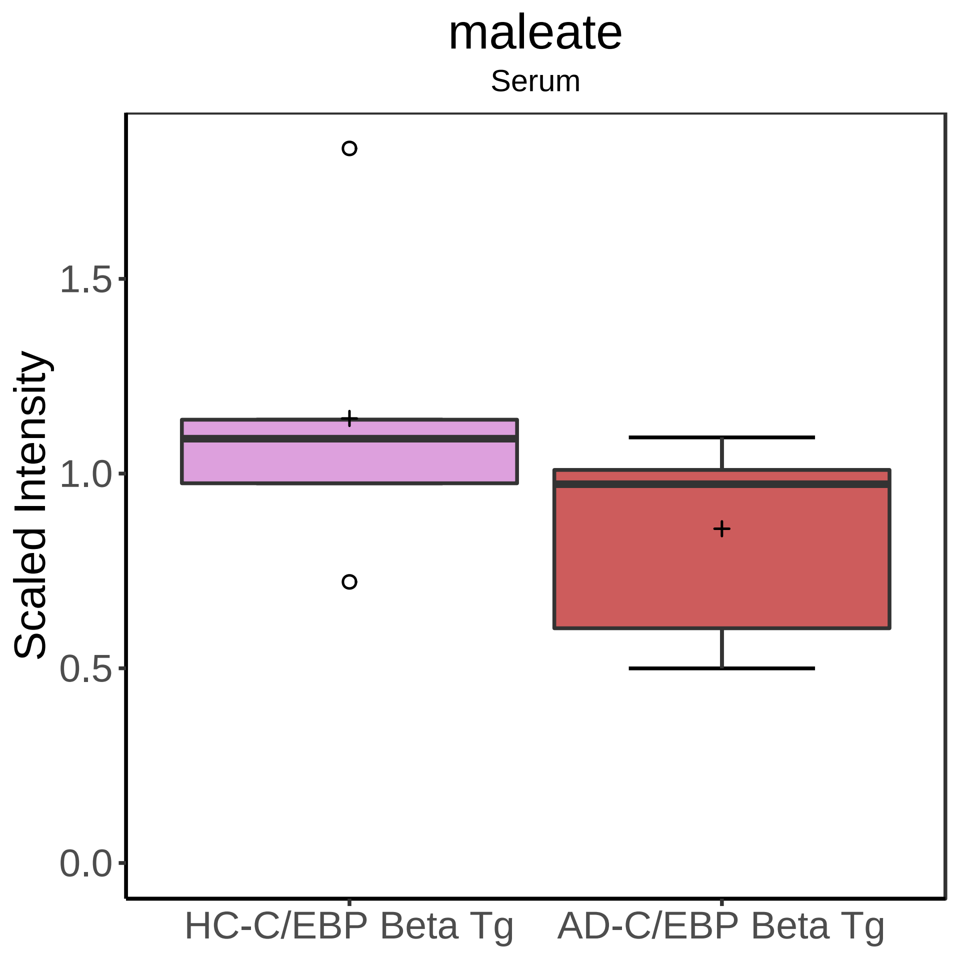
maleate
Serum
Scaled Intensity
1.5
1.0
0.5
0.0
HC-C/EBP Beta Tg
AD-C/EBP Beta Tg

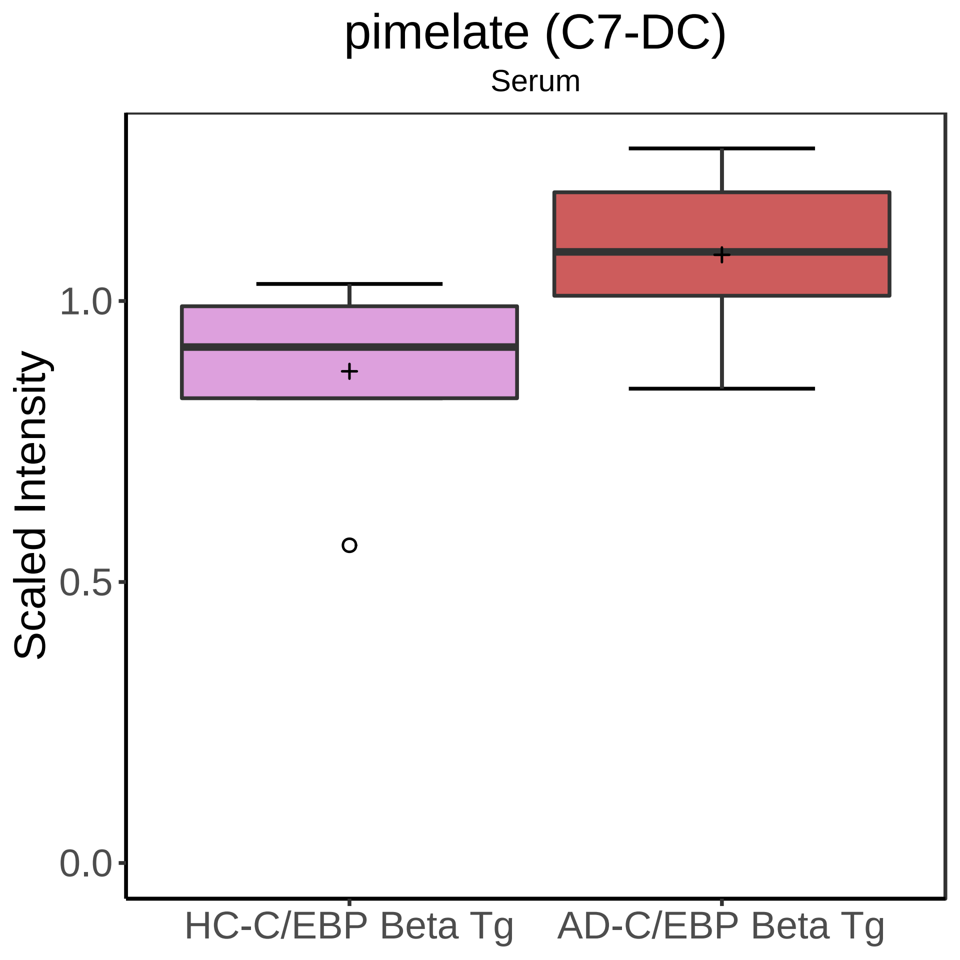
pimelate (C7-DC)
Serum
Scaled Intensity
1.0
0.5
0.0
HC-C/EBP Beta Tg
AD-C/EBP Beta Tg

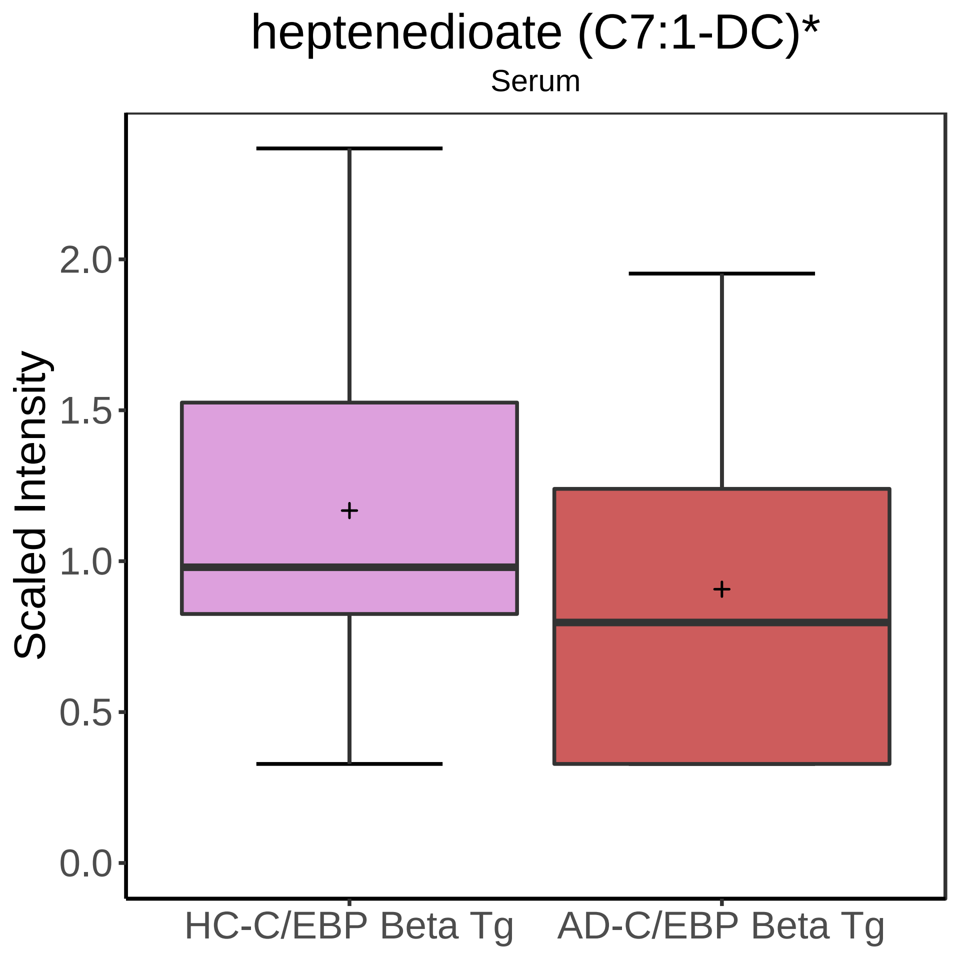
heptenedioate (C7:1-DC)*
Serum
Scaled Intensity
0.0
0.5
1.0
1.5
2.0
HC-C/EBP Beta Tg
AD-C/EBP Beta Tg

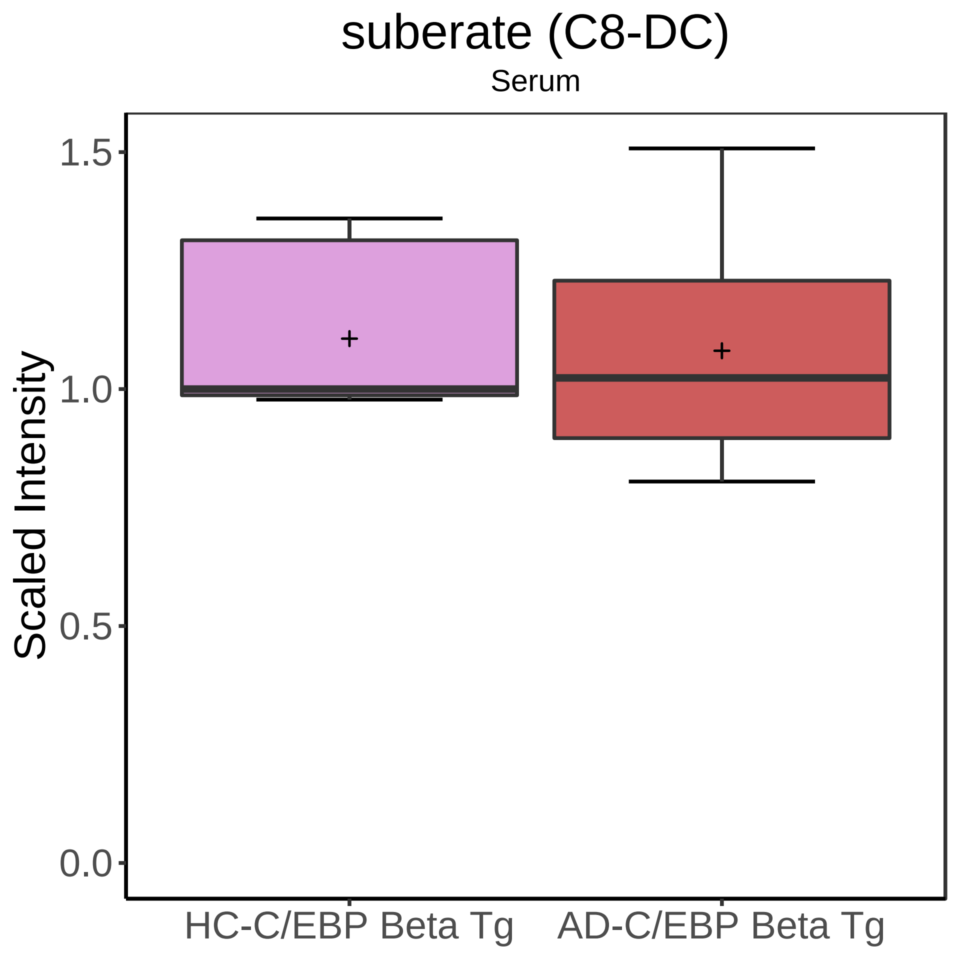
suberate (C8-DC)
Serum
Scaled Intensity
1.5
1.0
0.5
0.0
HC-C/EBP Beta Tg
AD-C/EBP Beta Tg

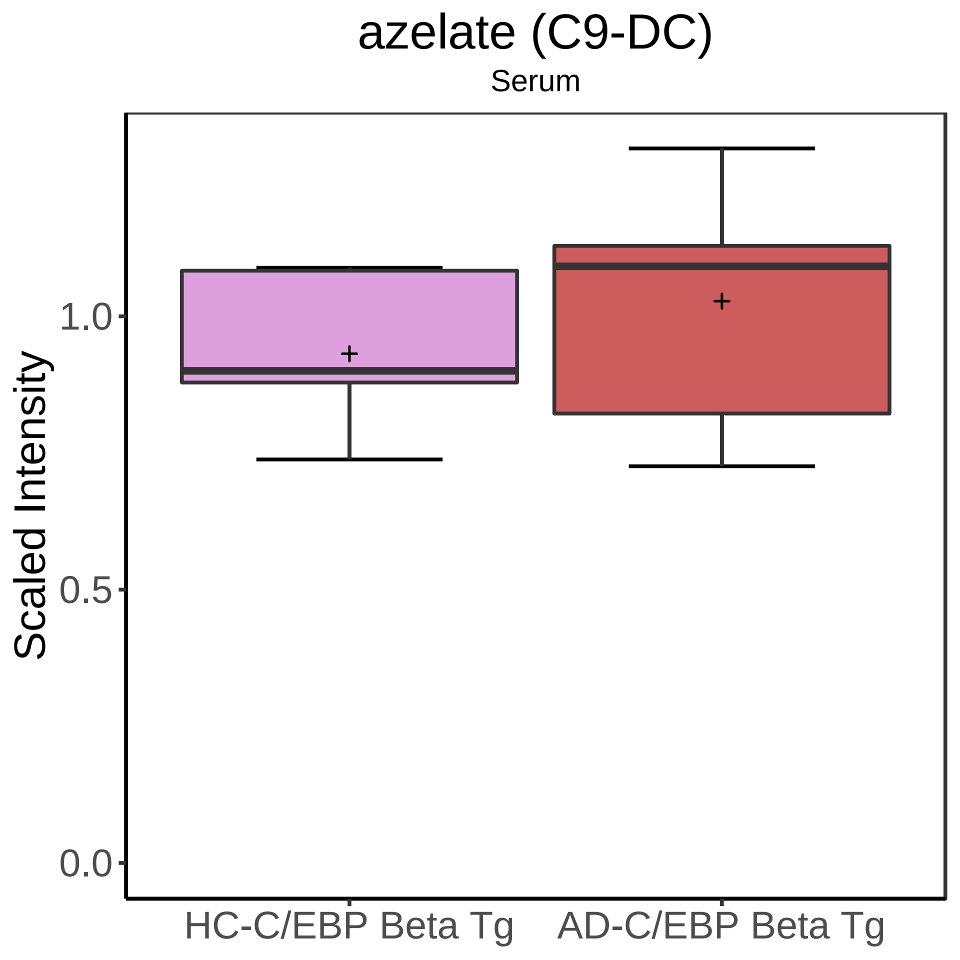
azelate (C9-DC)
Serum
Scaled Intensity
1.0
0.5
0.0
HC-C/EBP Beta Tg
AD-C/EBP Beta Tg

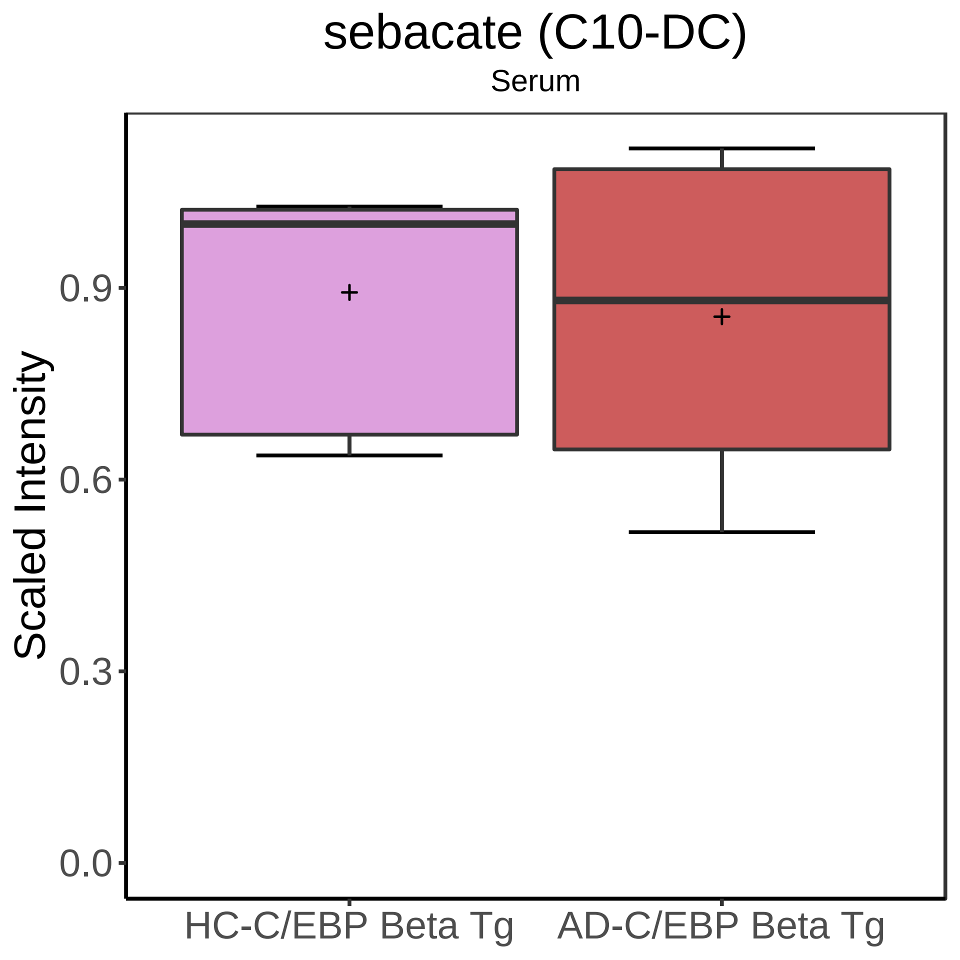
sebacate (C10-DC)
Serum
Scaled Intensity
0.9
0.6
0.3
0.0
HC-C/EBP Beta Tg
AD-C/EBP Beta Tg

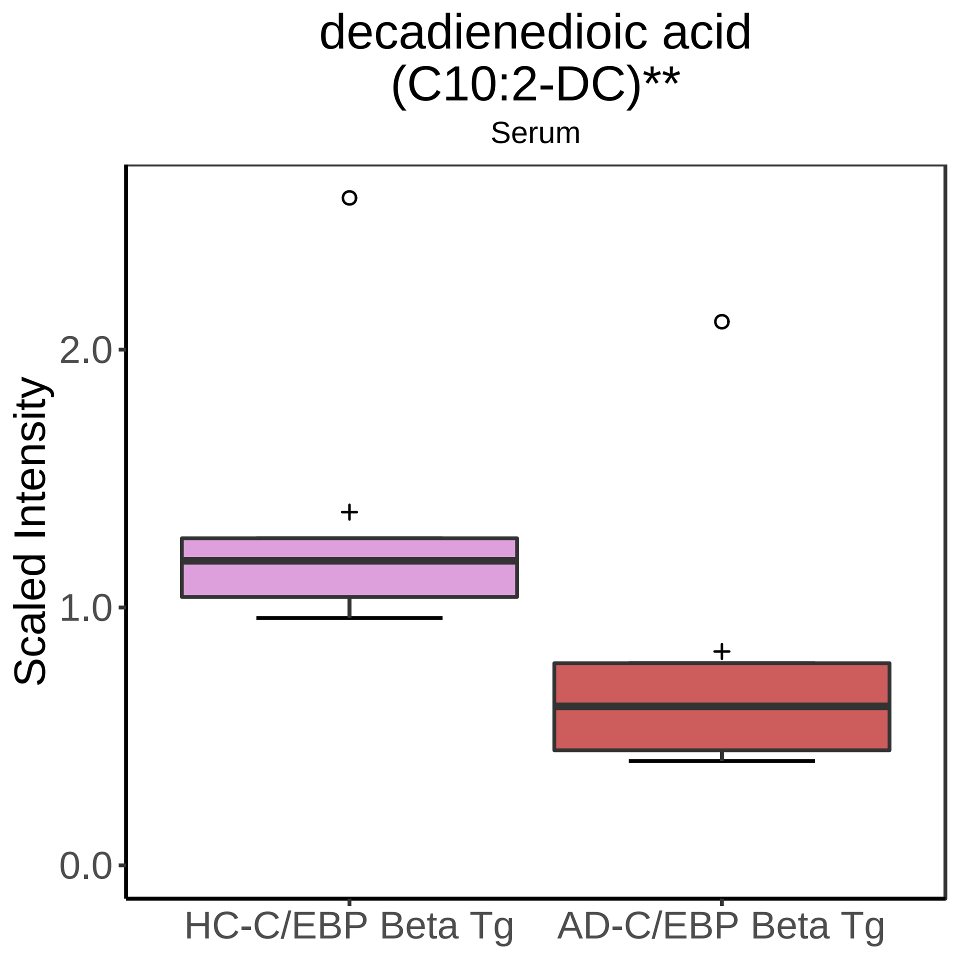
decadienedioic acid
(C10:2-DC)**
Serum
Scaled Intensity
2.0
1.0
0.0
HC-C/EBP Beta Tg
AD-C/EBP Beta Tg

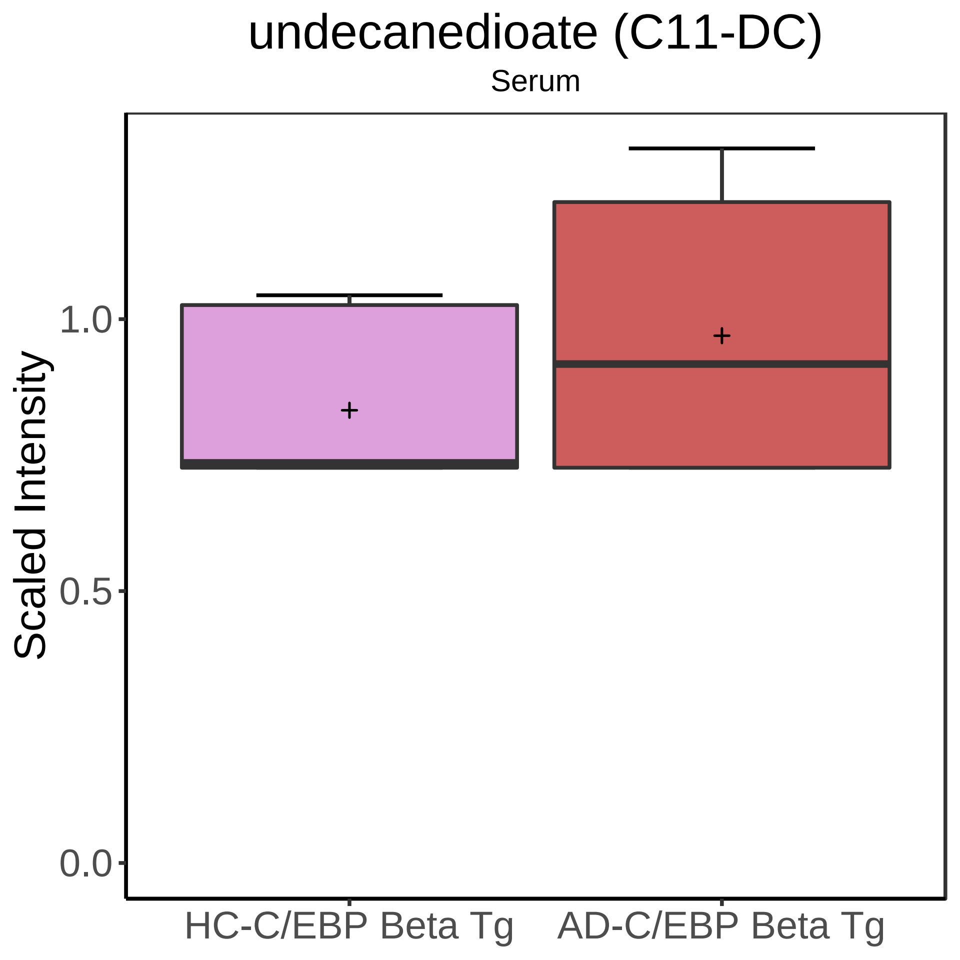
undecanedioate (C11-DC)
Serum
Scaled Intensity
1.0
0.5
0.0
HC-C/EBP Beta Tg
AD-C/EBP Beta Tg

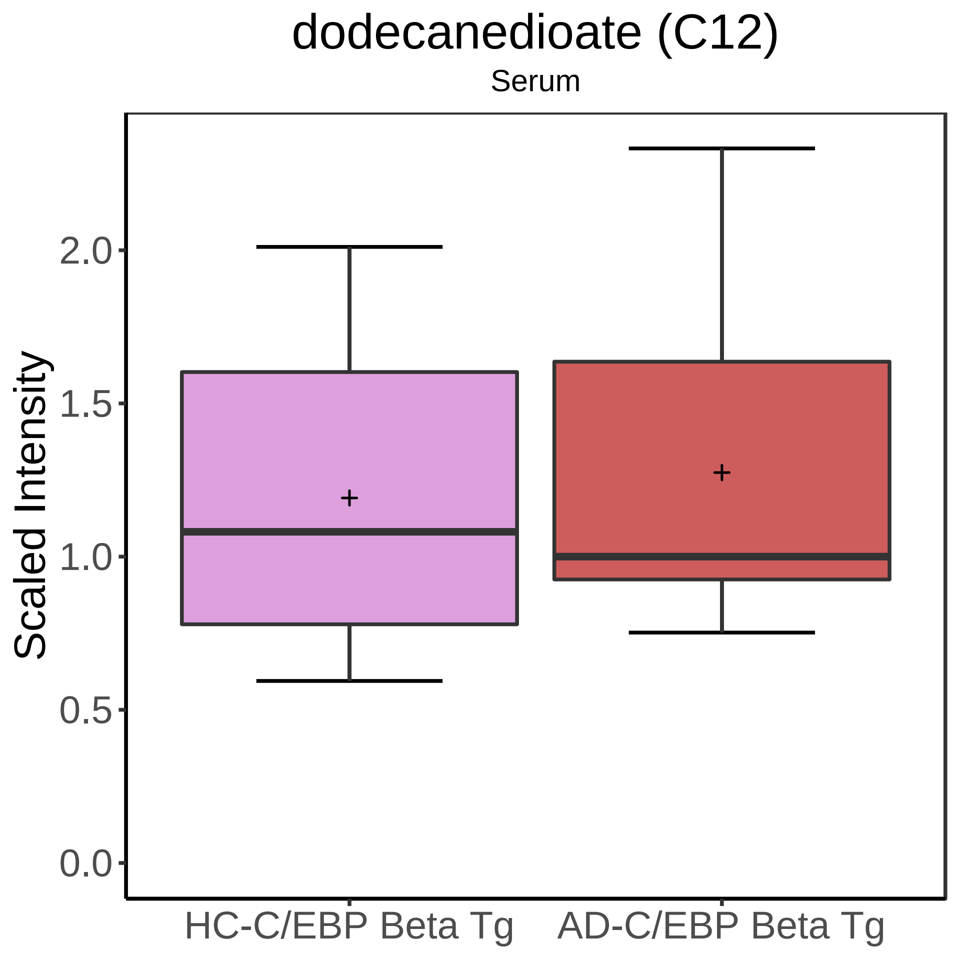
dodecanedioate (C12)
Serum
Scaled Intensity
2.0
1.5
1.0
0.5
0.0
HC-C/EBP Beta Tg
AD-C/EBP Beta Tg

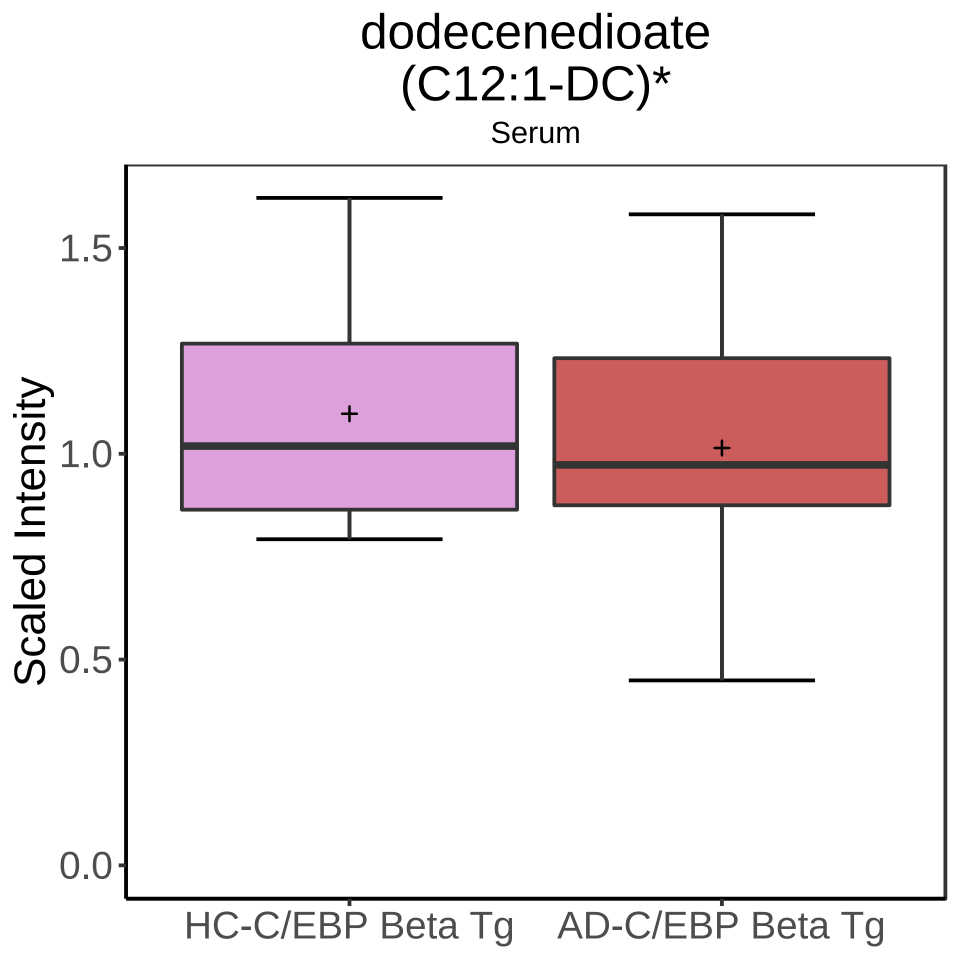
dodecenedioate
(C12:1-DC)*
Serum
Scaled Intensity
0.0
0.5
1.0
1.5
HC-C/EBP Beta Tg
AD-C/EBP Beta Tg

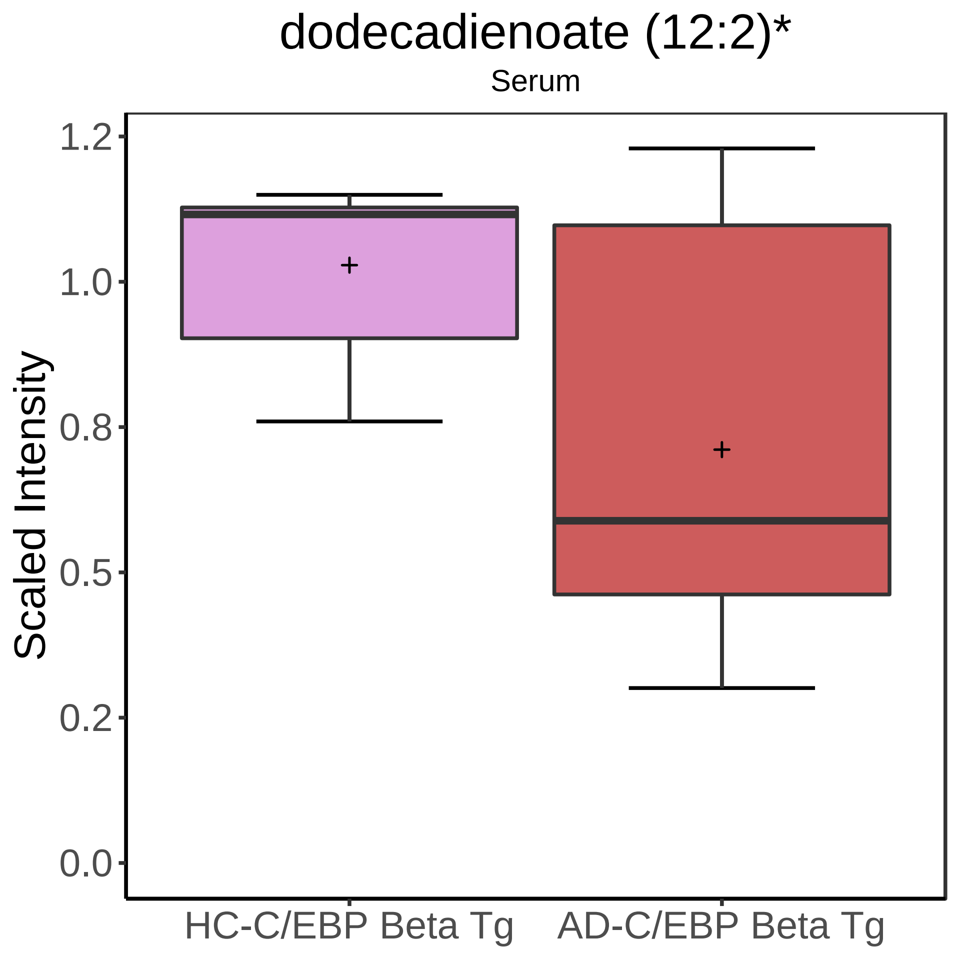

dodecadienoate (12:2)*
Serum
Scaled Intensity
1.2
1.0
0.8
0.5
0.2
0.0
HC-C/EBP Beta Tg
AD-C/EBP Beta Tg

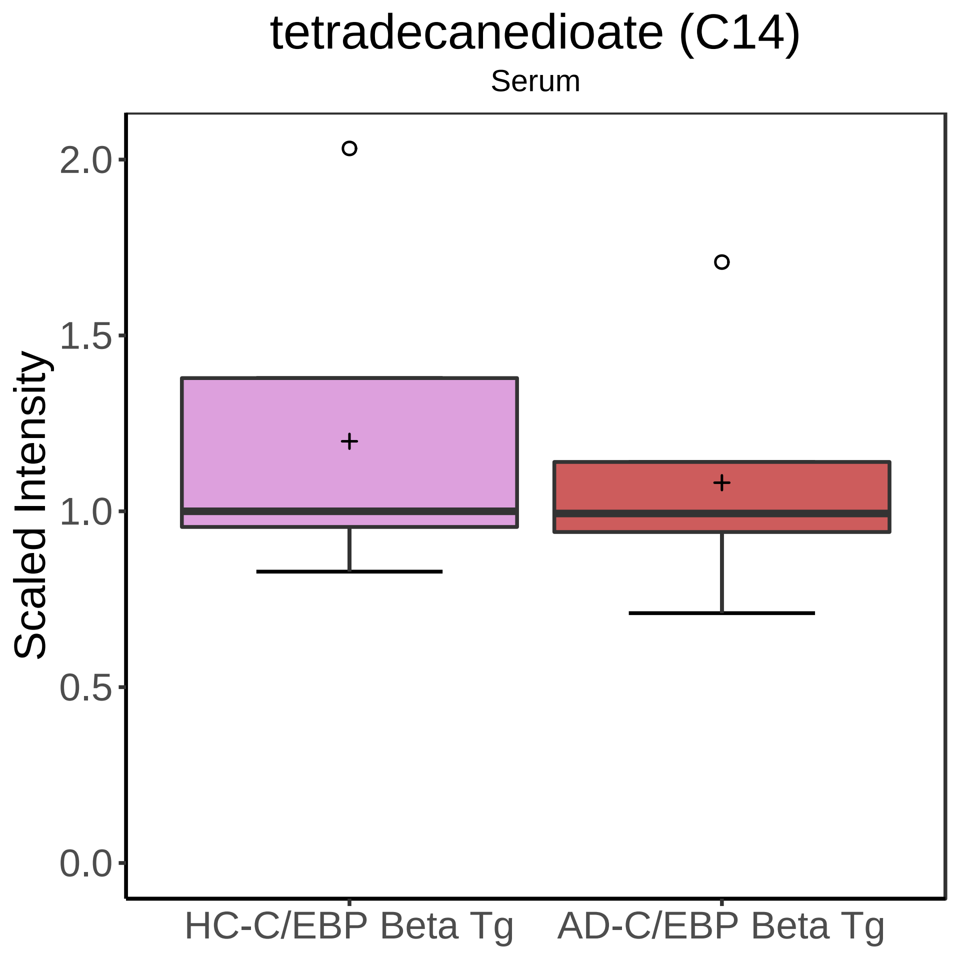
tetradecanedioate (C14)
Serum
Scaled Intensity
2.0
1.5
1.0
0.5
0.0
HC-C/EBP Beta Tg
AD-C/EBP Beta Tg

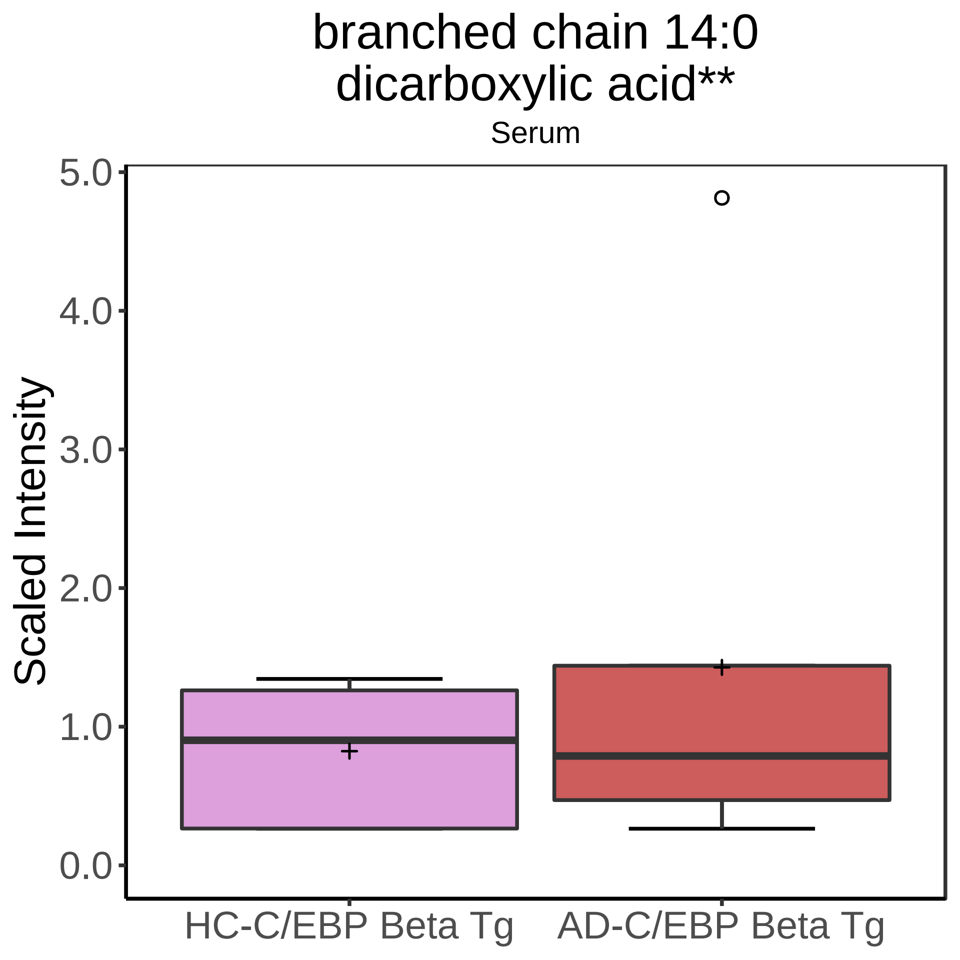
branched chain 14:0
dicarboxylic acid**
Serum
Scaled Intensity
5.0
4.0
3.0
2.0
1.0
0.0
HC-C/EBP Beta Tg
AD-C/EBP Beta Tg

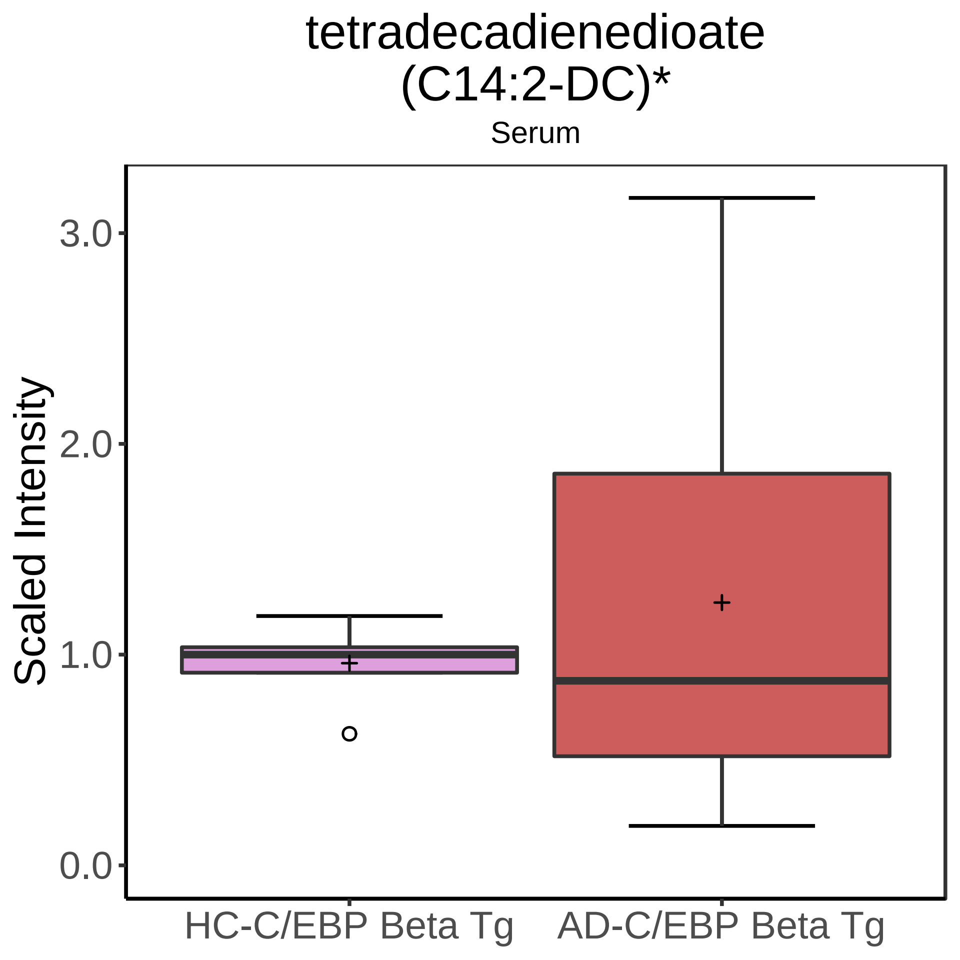

tetradecadienedioate
(C14:2-DC)*
Serum
Scaled Intensity
3.0
2.0
1.0
0.0
HC-C/EBP Beta Tg
AD-C/EBP Beta Tg

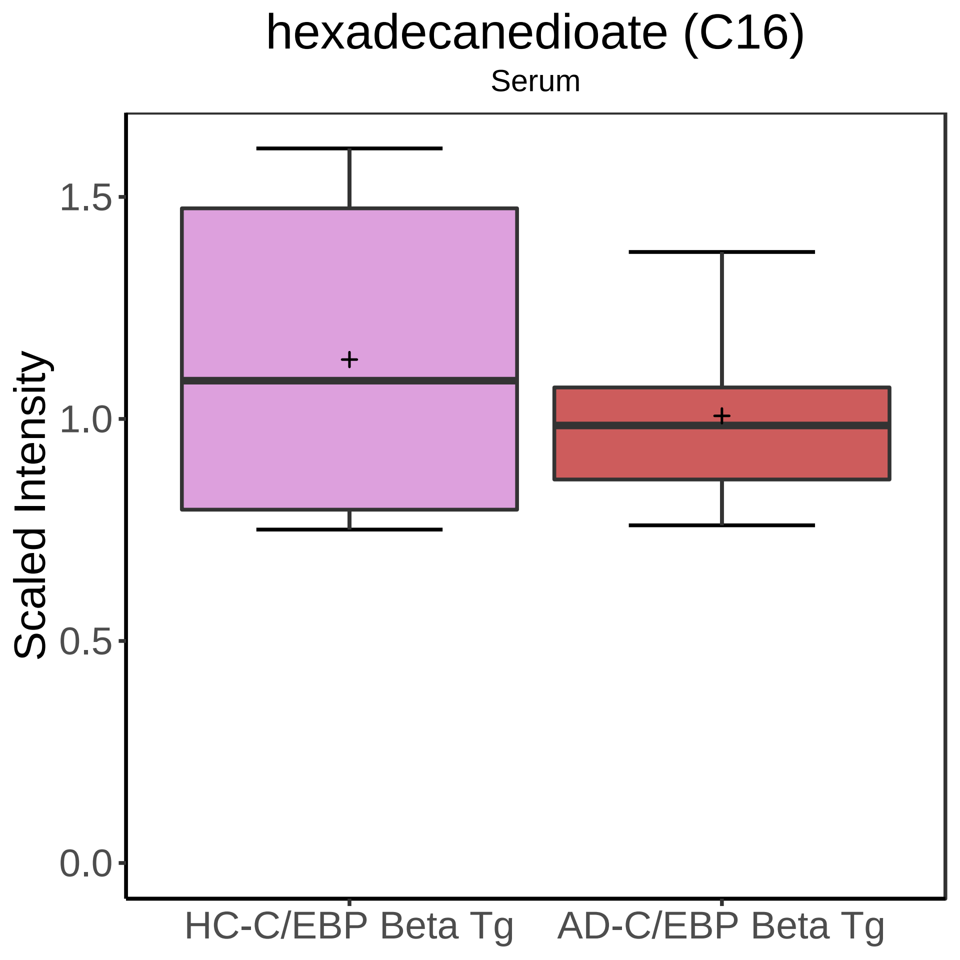
hexadecanedioate (C16)
Serum
Scaled Intensity
1.5
1.0
0.5
0.0
HC-C/EBP Beta Tg
AD-C/EBP Beta Tg

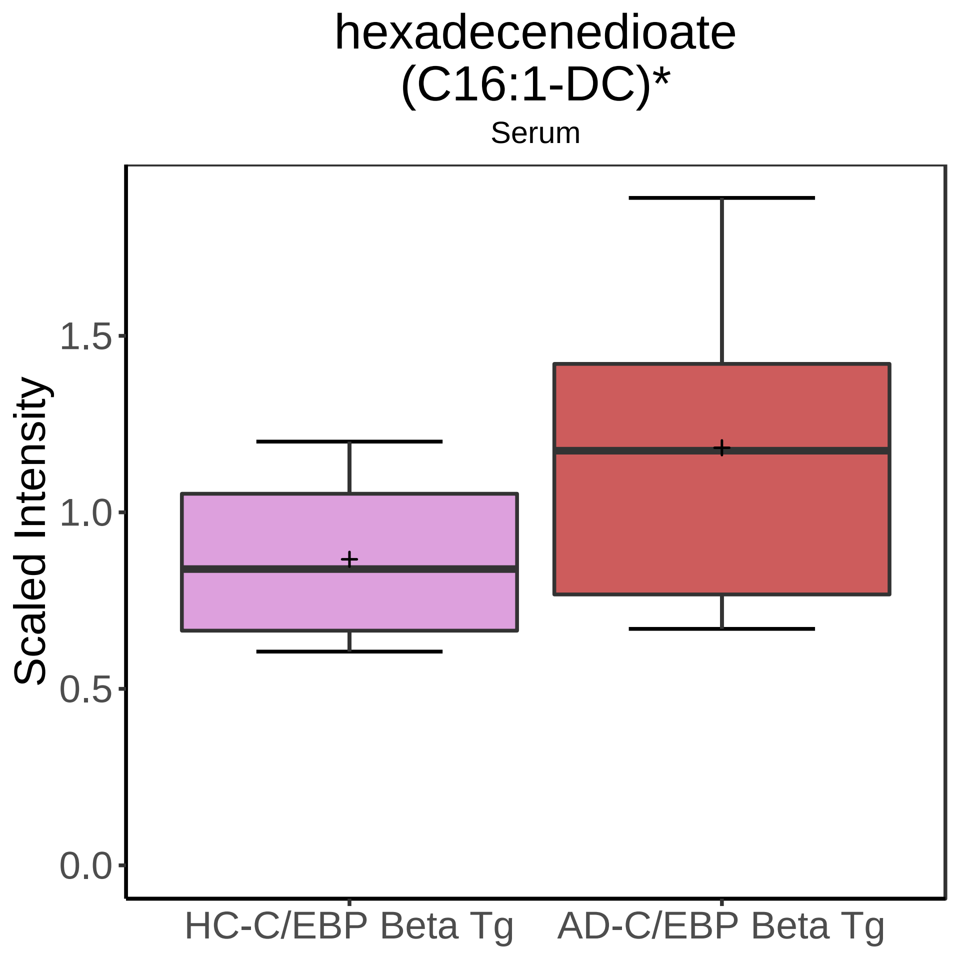

hexadecenedioate
(C16:1-DC)*
Serum
Scaled Intensity
1.5
1.0
0.5
0.0
HC-C/EBP Beta Tg
AD-C/EBP Beta Tg

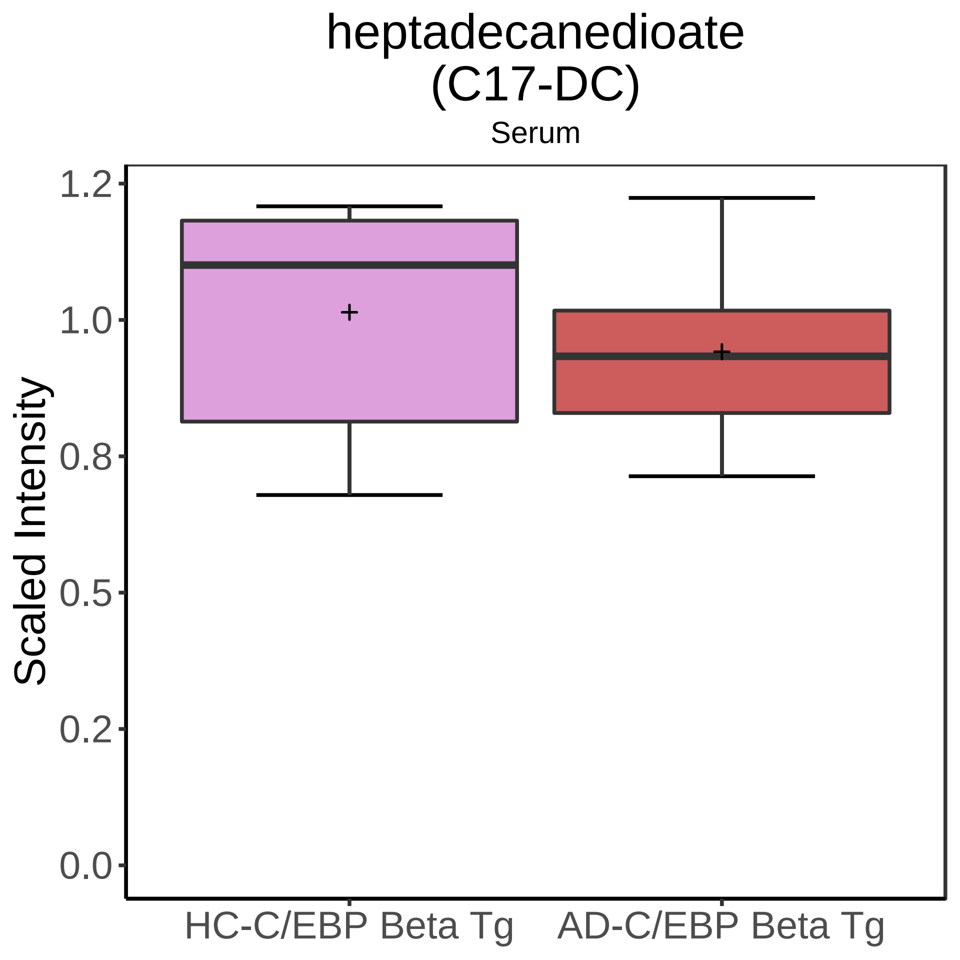
heptadecanedioate
(C17-DC)
Serum
Scaled Intensity
1.2
1.0
0.8
0.5
0.2
0.0
HC-C/EBP Beta Tg
AD-C/EBP Beta Tg

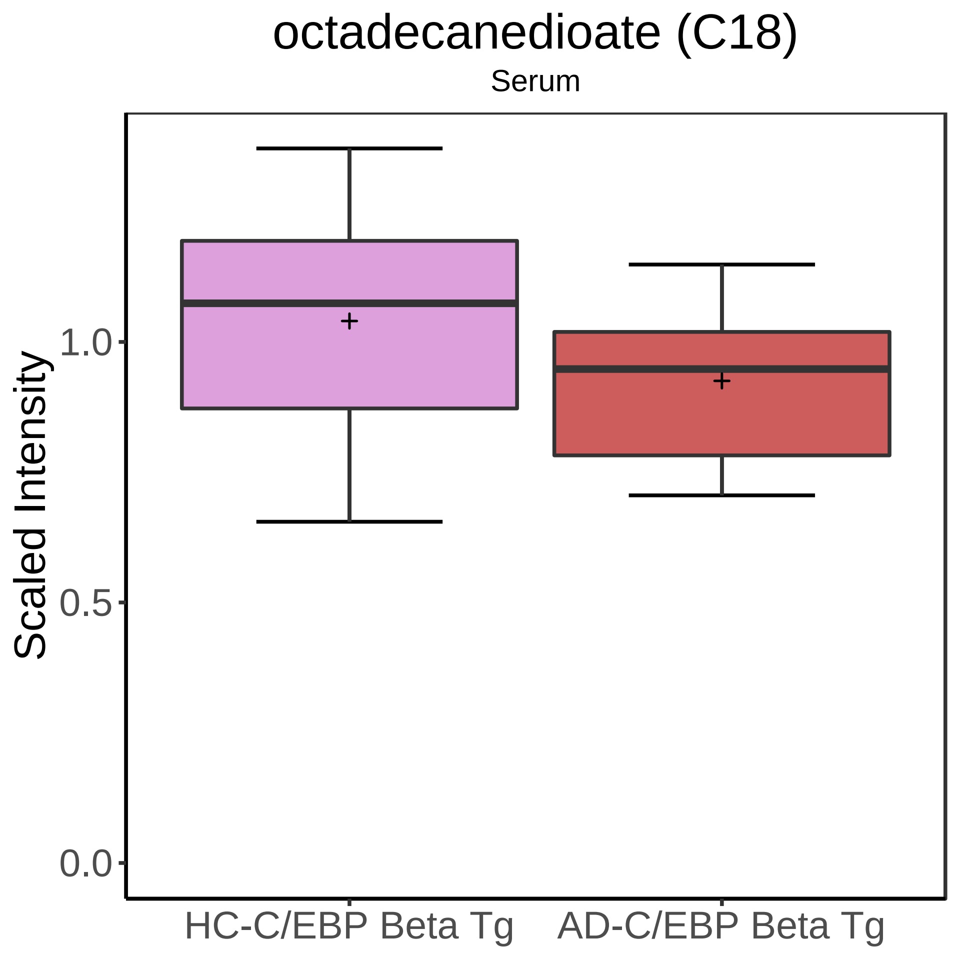
octadecanedioate (C18)
Serum
Scaled Intensity
1.0
0.5
0.0
HC-C/EBP Beta Tg
AD-C/EBP Beta Tg

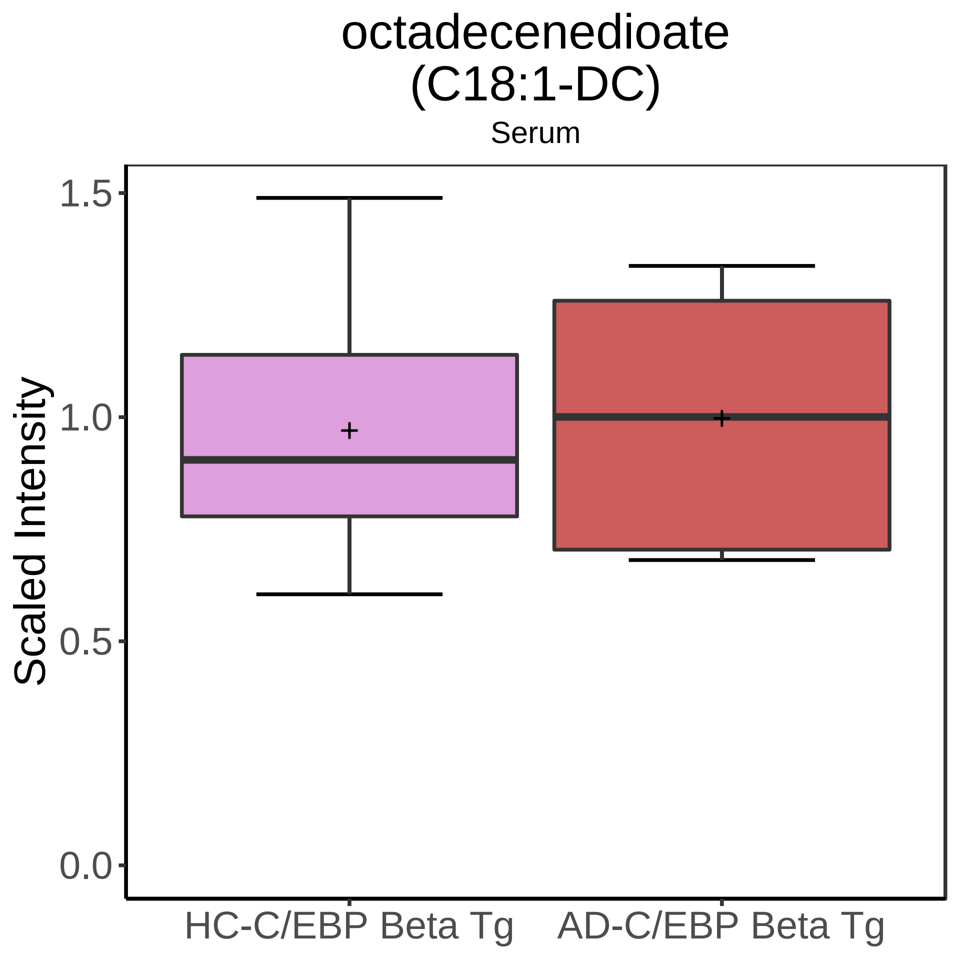
octadecenedioate
(C18:1-DC)
Serum
Scaled Intensity
1.5
1.0
0.5
0.0
HC-C/EBP Beta Tg
AD-C/EBP Beta Tg

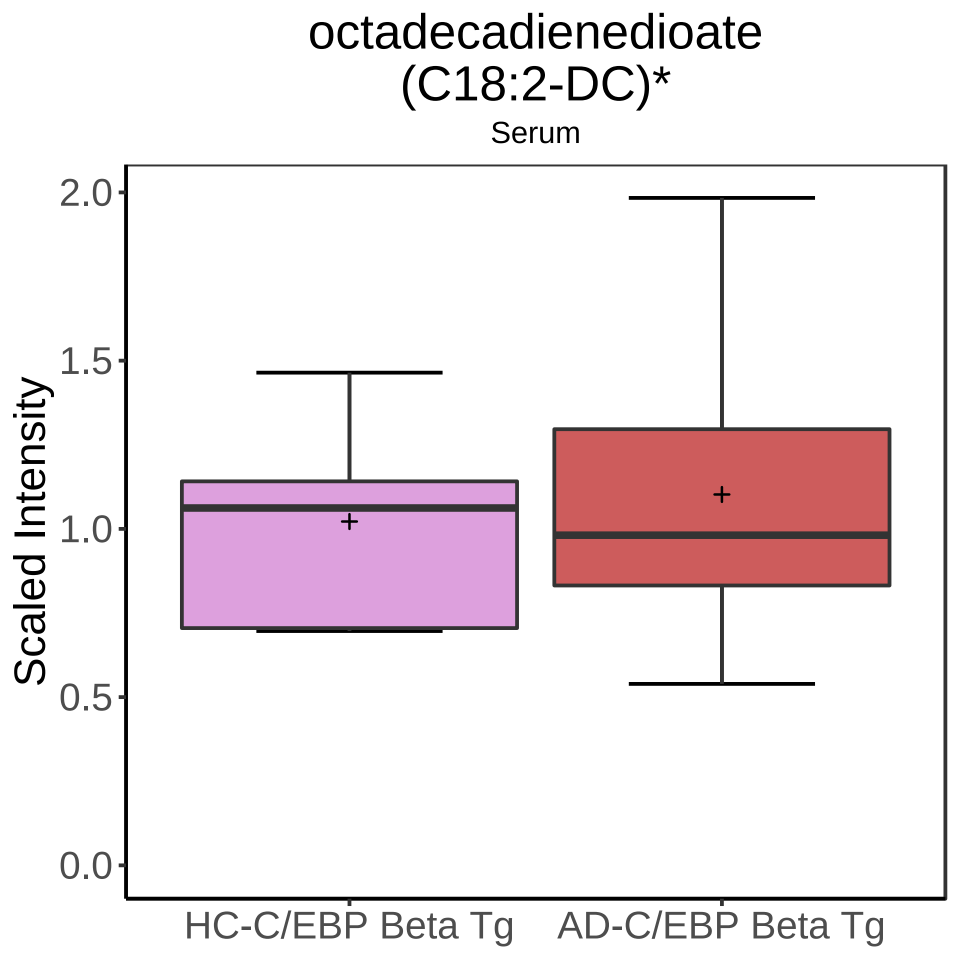
octadecadienedioate
(C18:2-DC)*
Serum
Scaled Intensity
2.0
1.5
1.0
0.5
0.0
HC-C/EBP Beta Tg
AD-C/EBP Beta Tg

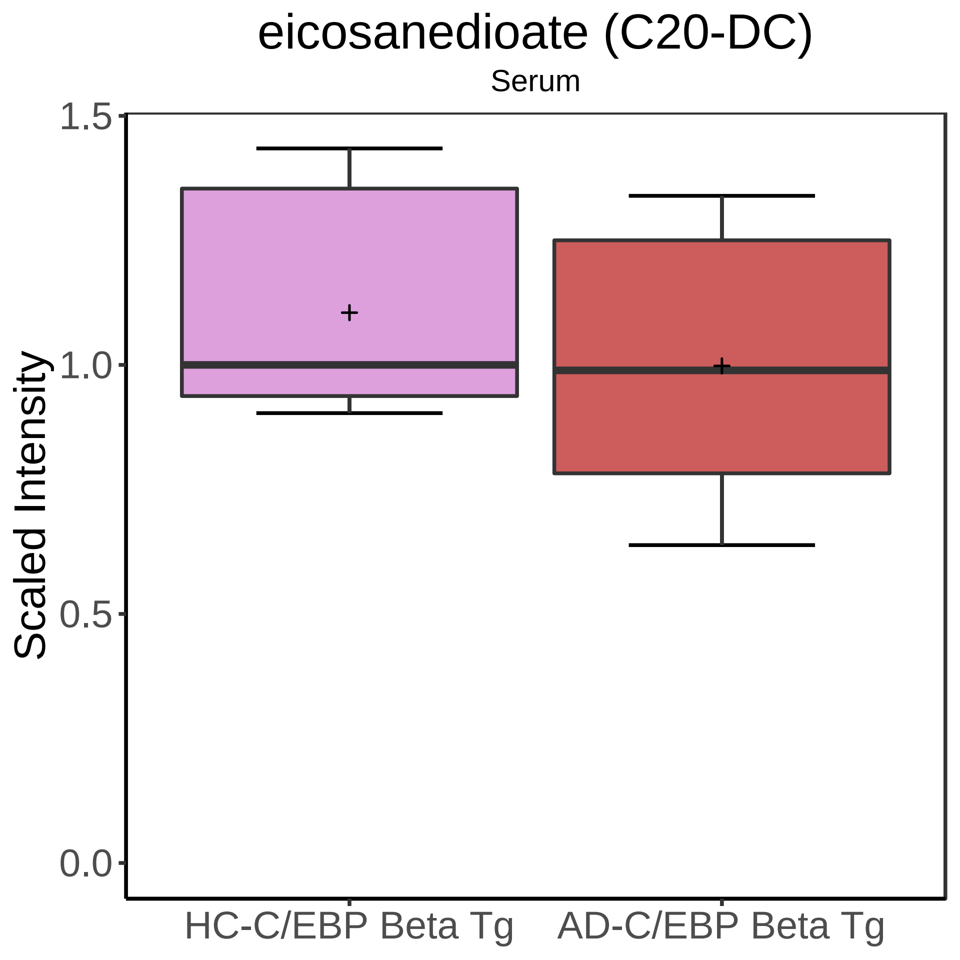
eicosanedioate (C20-DC)
Serum
Scaled Intensity
1.5
1.0
0.5
0.0
HC-C/EBP Beta Tg
AD-C/EBP Beta Tg

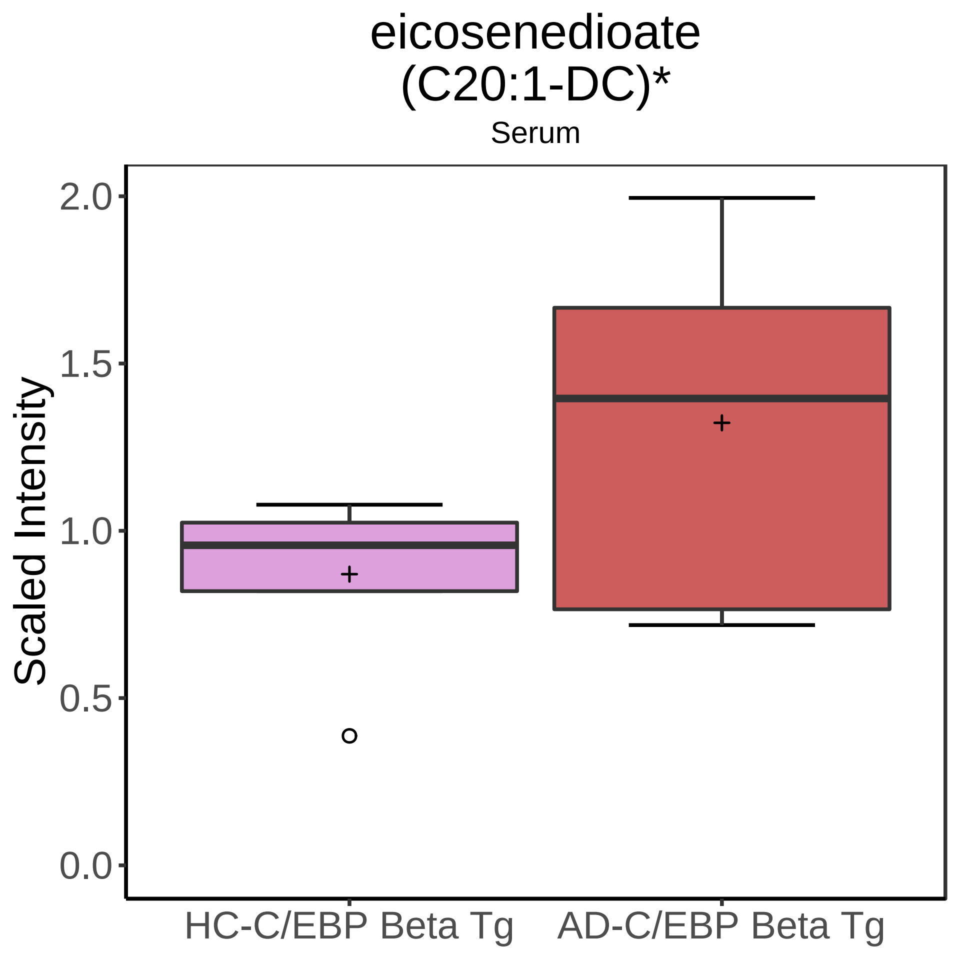
eicosenedioate
(C20:1-DC)*
Serum
Scaled Intensity
2.0
1.5
1.0
0.5
0.0
HC-C/EBP Beta Tg
AD-C/EBP Beta Tg

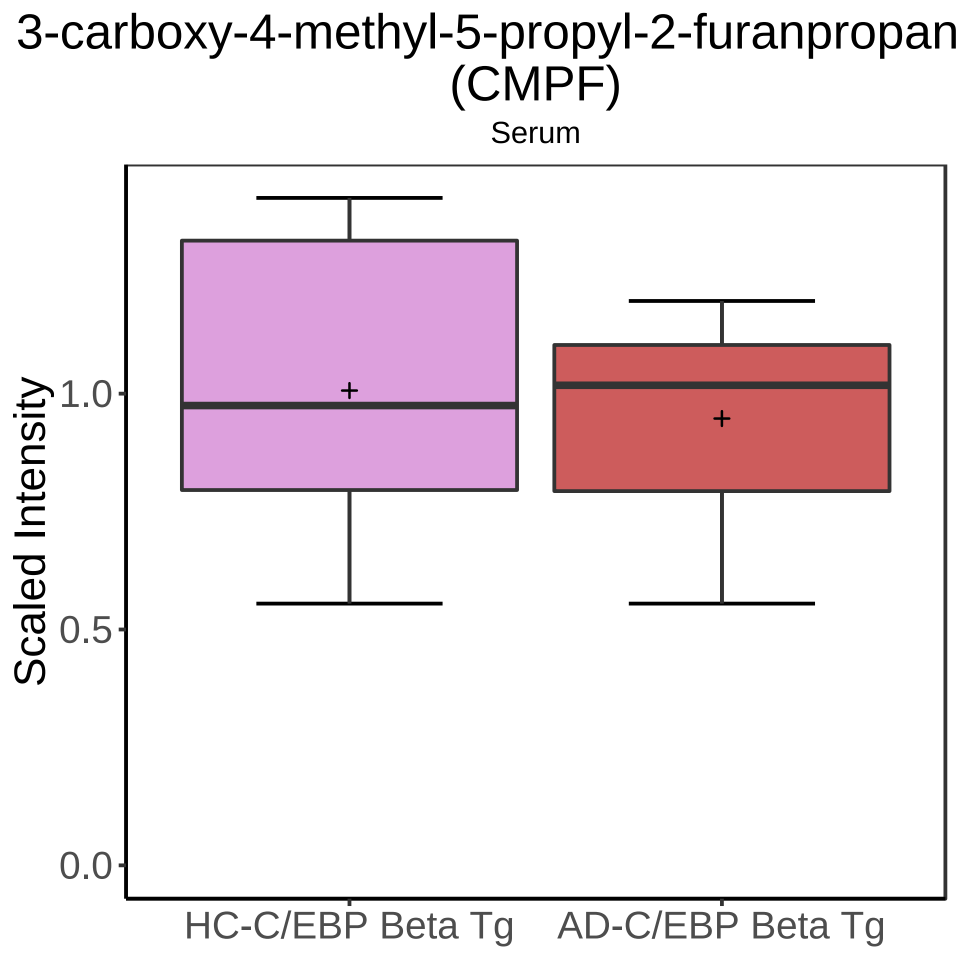
3-carboxy-4-methyl-5-propyl-2-furanpropan
(CMPF)
Serum
Scaled Intensity
1.0
0.5
0.0
HC-C/EBP Beta Tg
AD-C/EBP Beta Tg

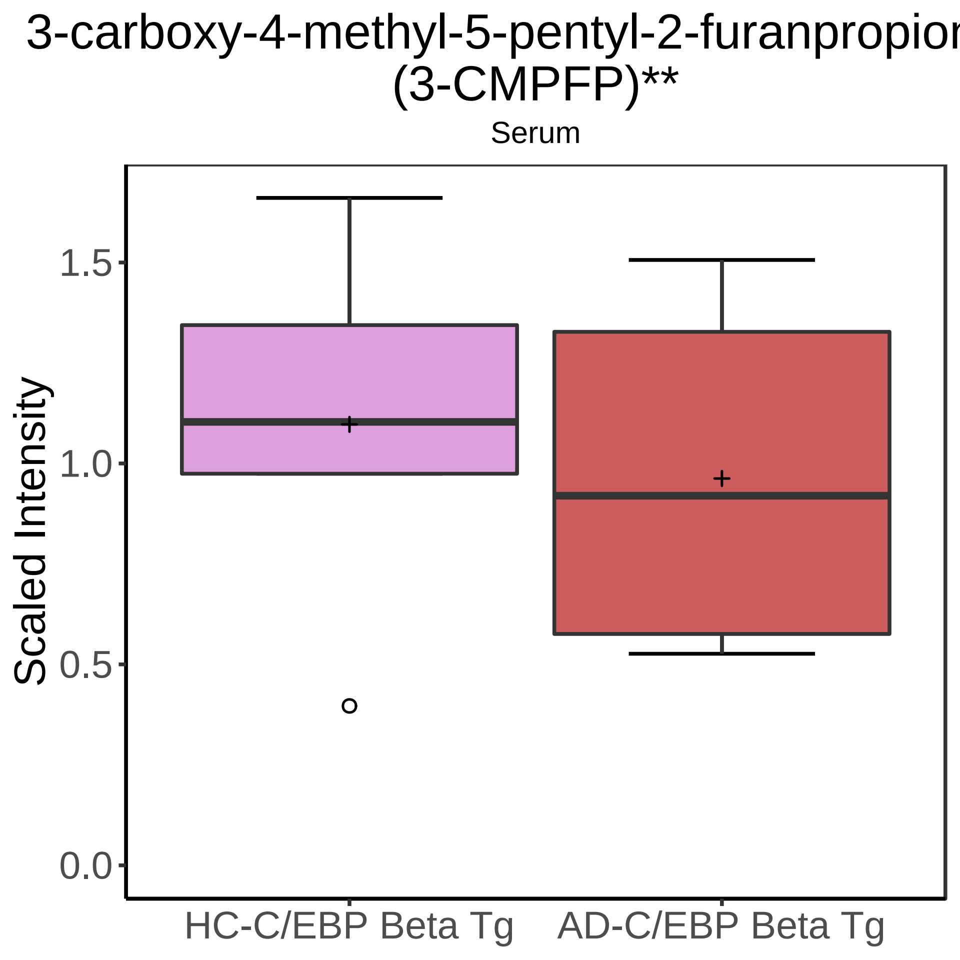
3-carboxy-4-methyl-5-pentyl-2-furanpropion
(3-CMPFP)**
Serum
Scaled Intensity
1.5
1.0
0.5
0.0
HC-C/EBP Beta Tg
AD-C/EBP Beta Tg

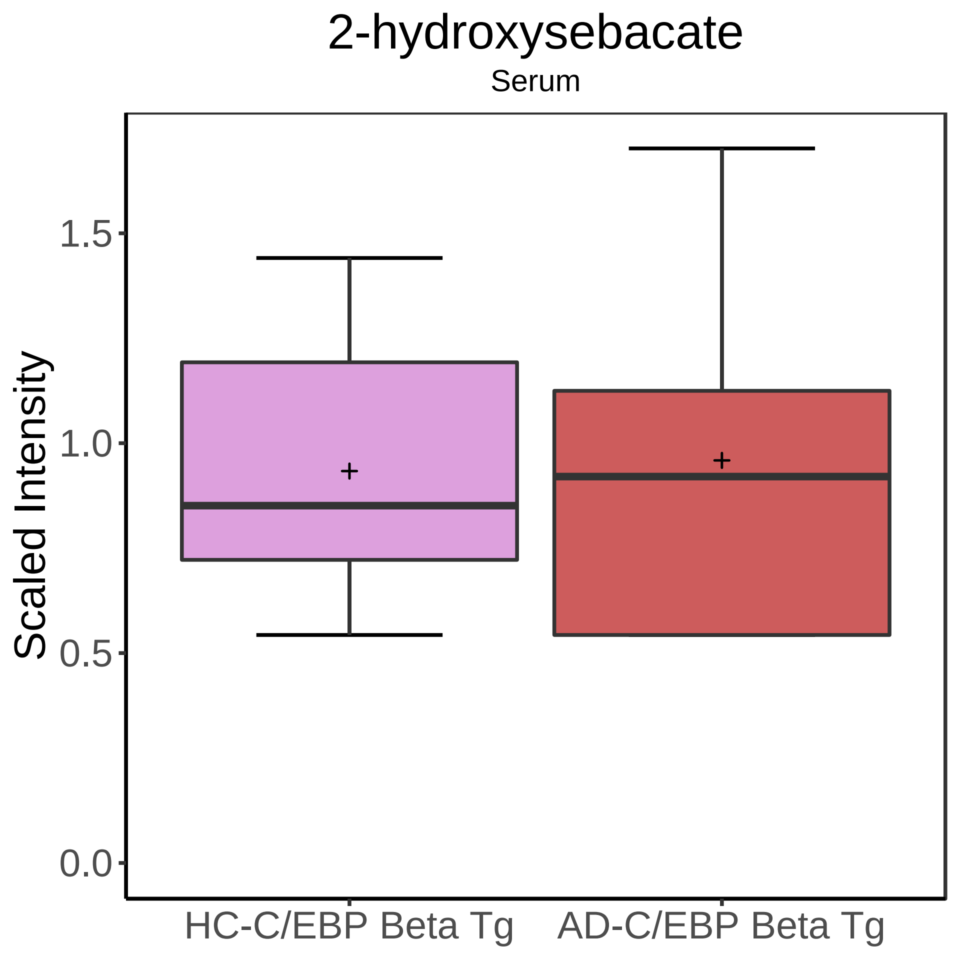
2-hydroxysebacate
Serum
Scaled Intensity
1.5
1.0
0.5
0.0
HC-C/EBP Beta Tg
AD-C/EBP Beta Tg

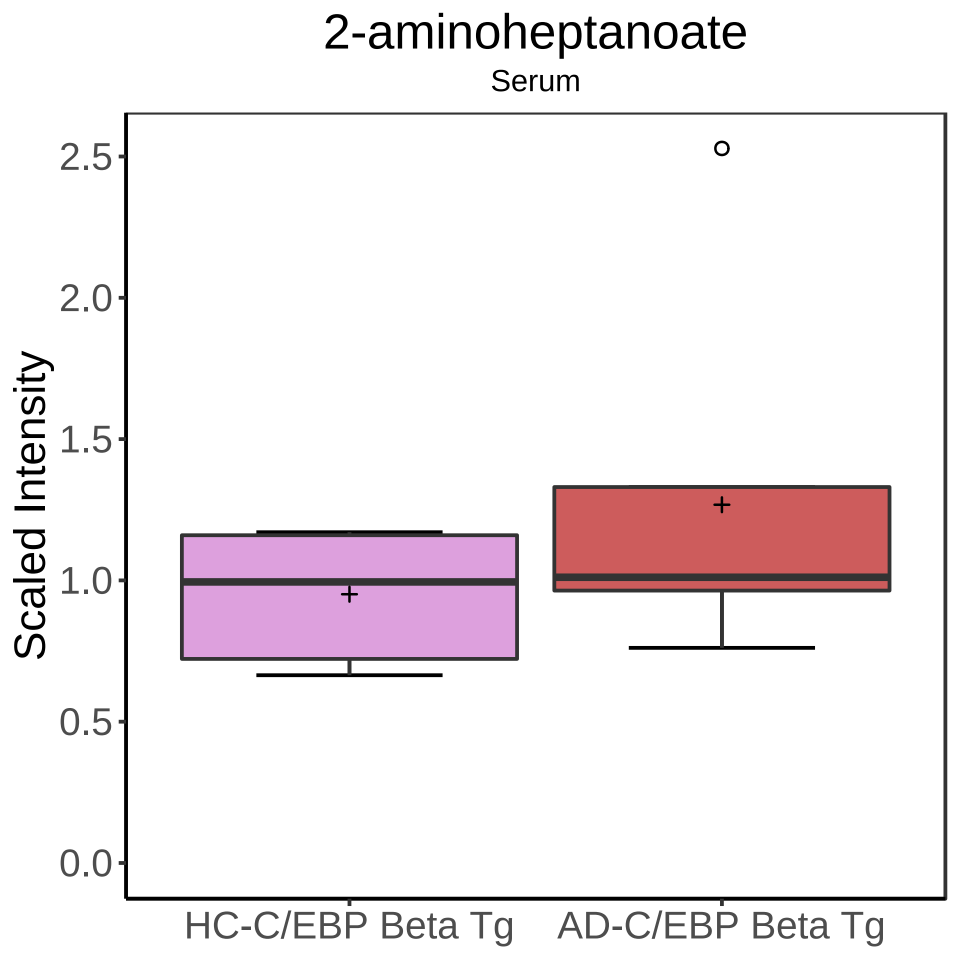
2-aminoheptanoate
Serum
Scaled Intensity
2.5
2.0
1.5
1.0
0.5
0.0
HC-C/EBP Beta Tg
AD-C/EBP Beta Tg

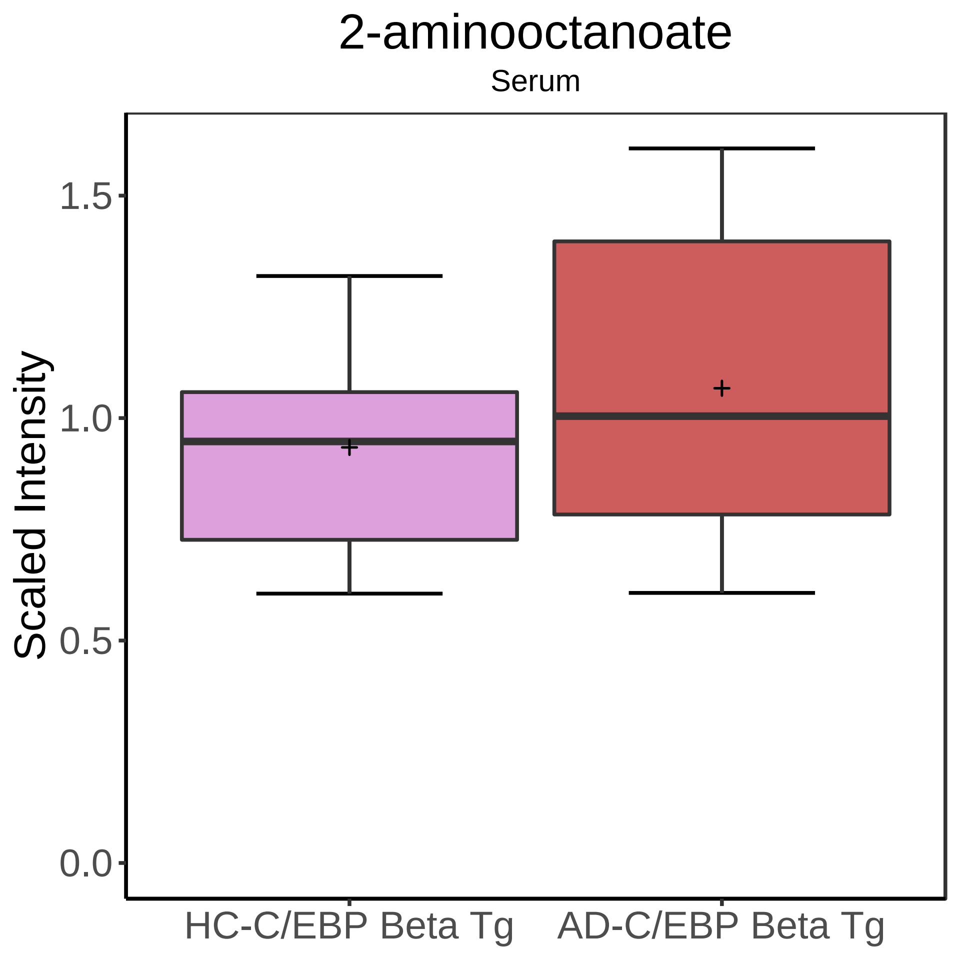

2-aminooctanoate
Serum
Scaled Intensity
1.5
1.0
0.5
0.0
HC-C/EBP Beta Tg
AD-C/EBP Beta Tg

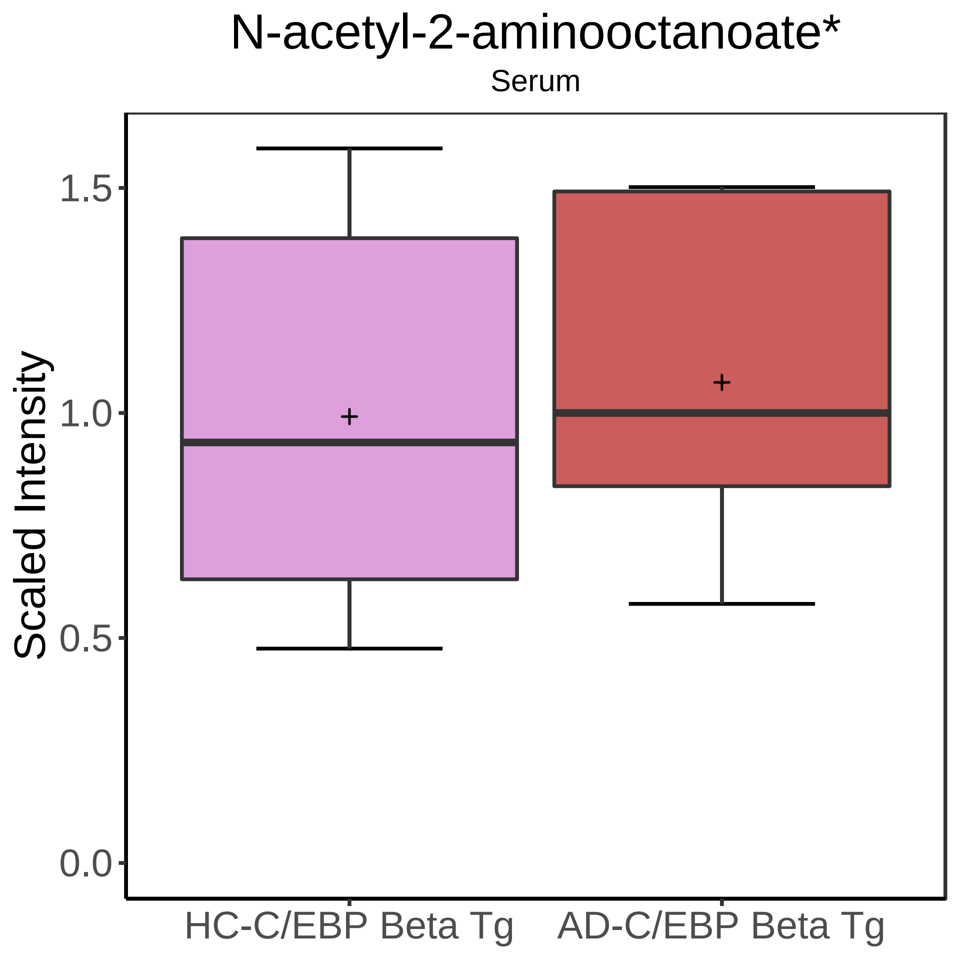
N-acetyl-2-aminooctanoate*
Serum
Scaled Intensity
1.5
1.0
0.5
0.0
HC-C/EBP Beta Tg
AD-C/EBP Beta Tg

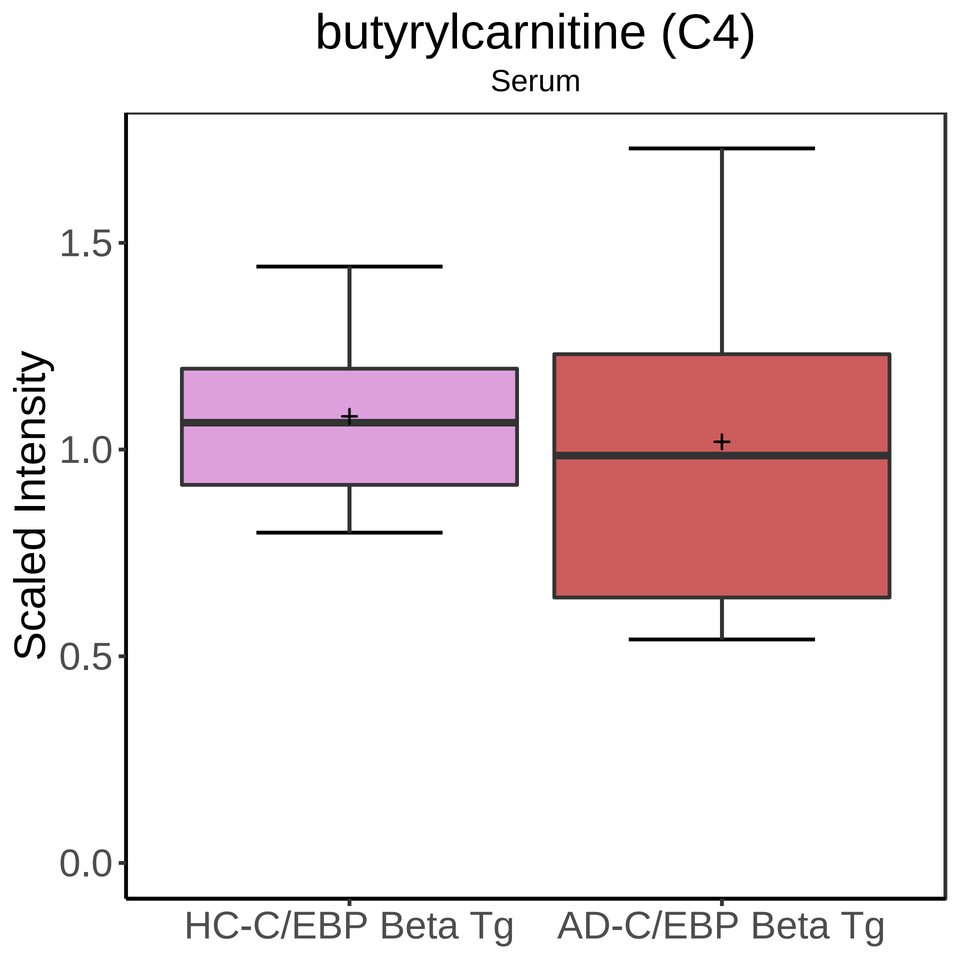

butyrylcarnitine (C4)
Serum
Scaled Intensity
0.0
0.5
1.0
1.5
HC-C/EBP Beta Tg
AD-C/EBP Beta Tg

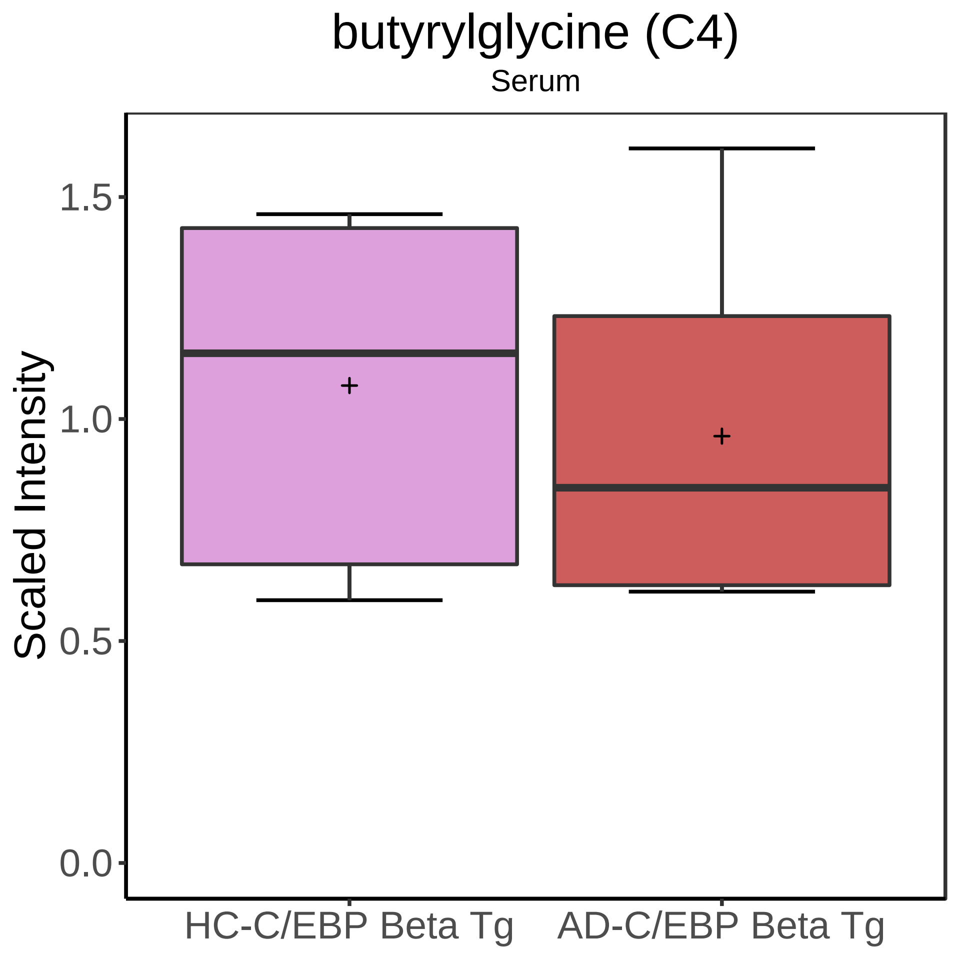
butyrylglycine (C4)
Serum
Scaled Intensity
1.5
1.0
0.5
0.0
HC-C/EBP Beta Tg
AD-C/EBP Beta Tg

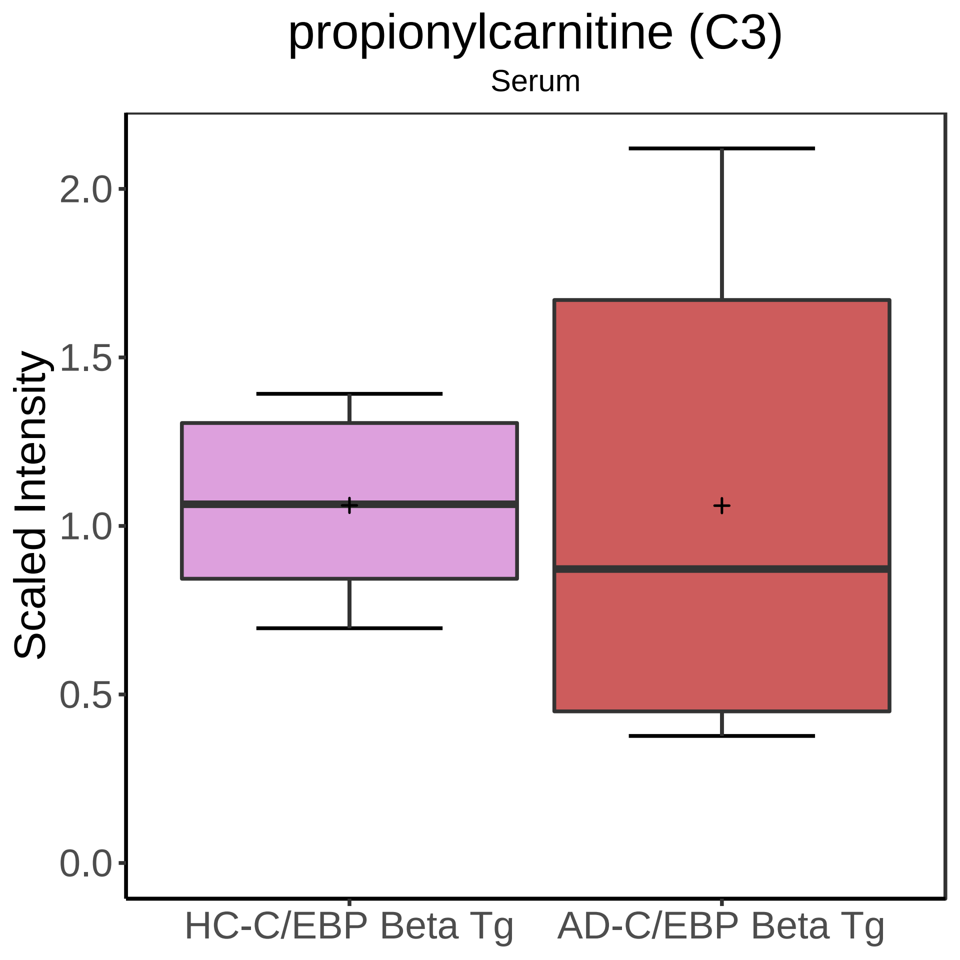
propionylcarnitine (C3)
Serum
Scaled Intensity
2.0
1.5
1.0
0.5
0.0
HC-C/EBP Beta Tg
AD-C/EBP Beta Tg

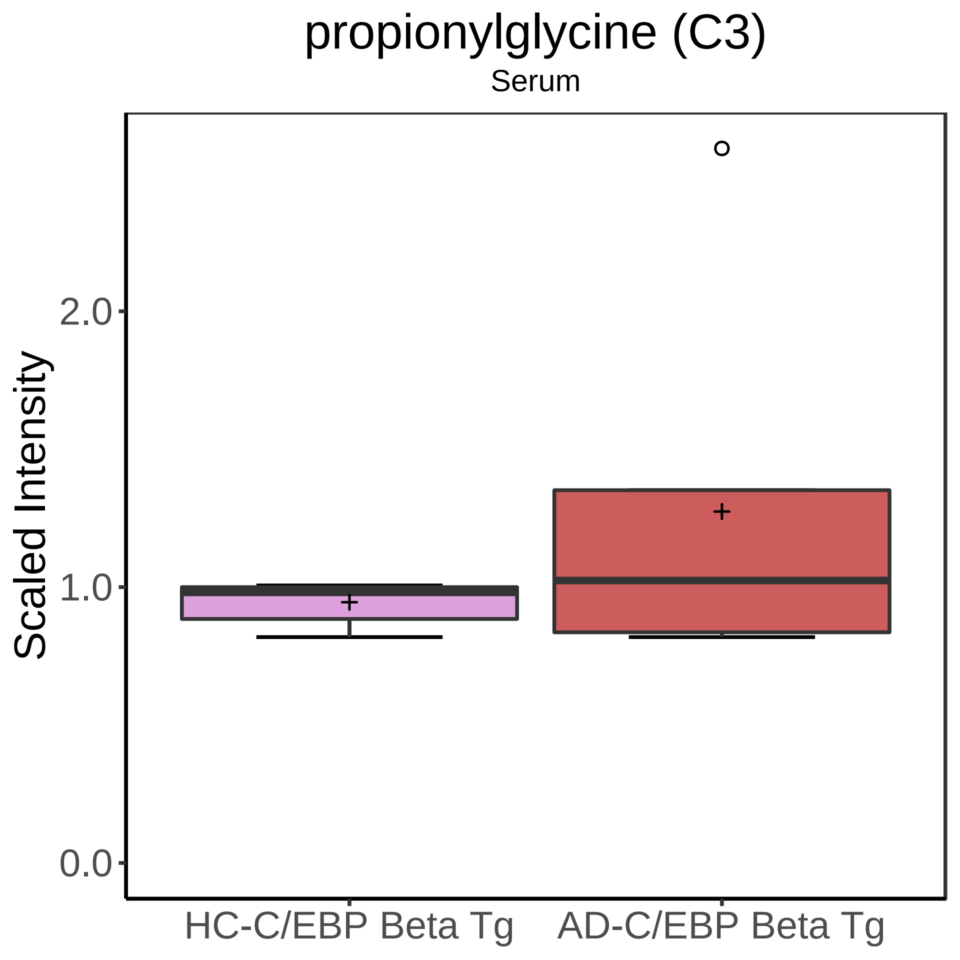
propionylglycine (C3)
Serum
Scaled Intensity
2.0
1.0
0.0
HC-C/EBP Beta Tg
AD-C/EBP Beta Tg

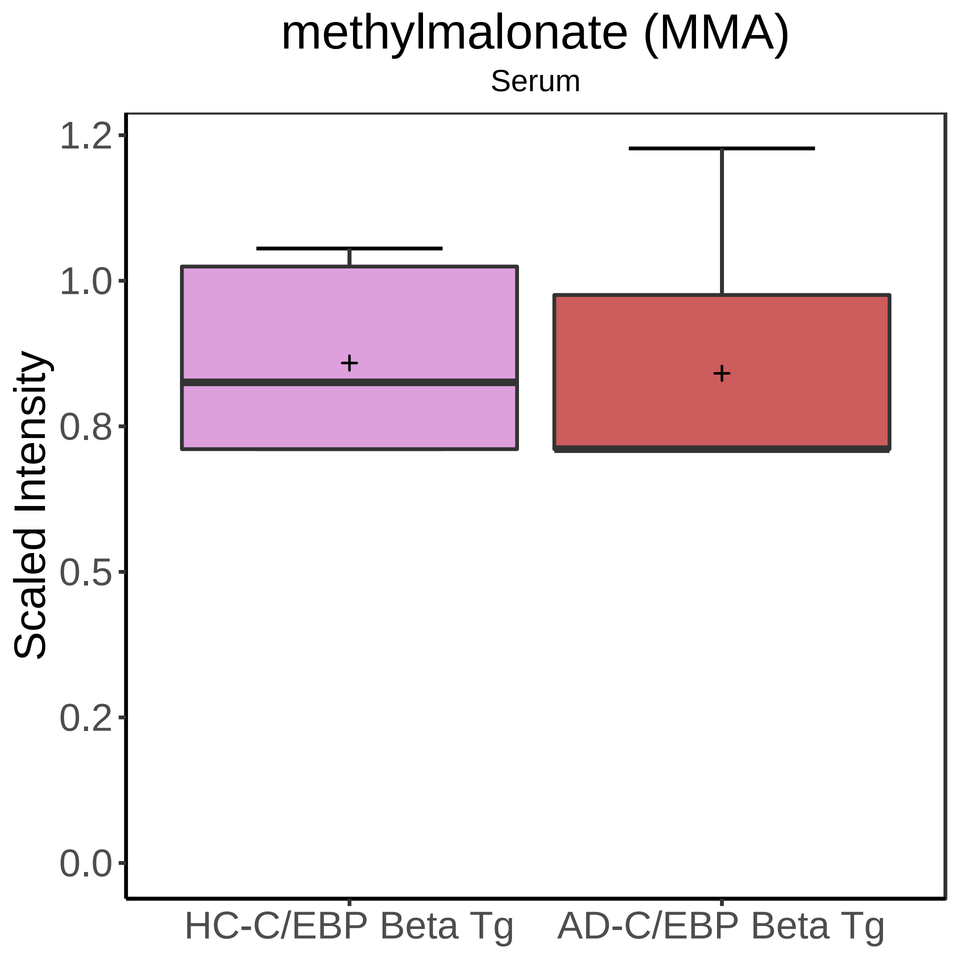
methylmalonate (MMA)
Serum
Scaled Intensity
1.2
1.0
0.8
0.5
0.2
0.0
HC-C/EBP Beta Tg
AD-C/EBP Beta Tg

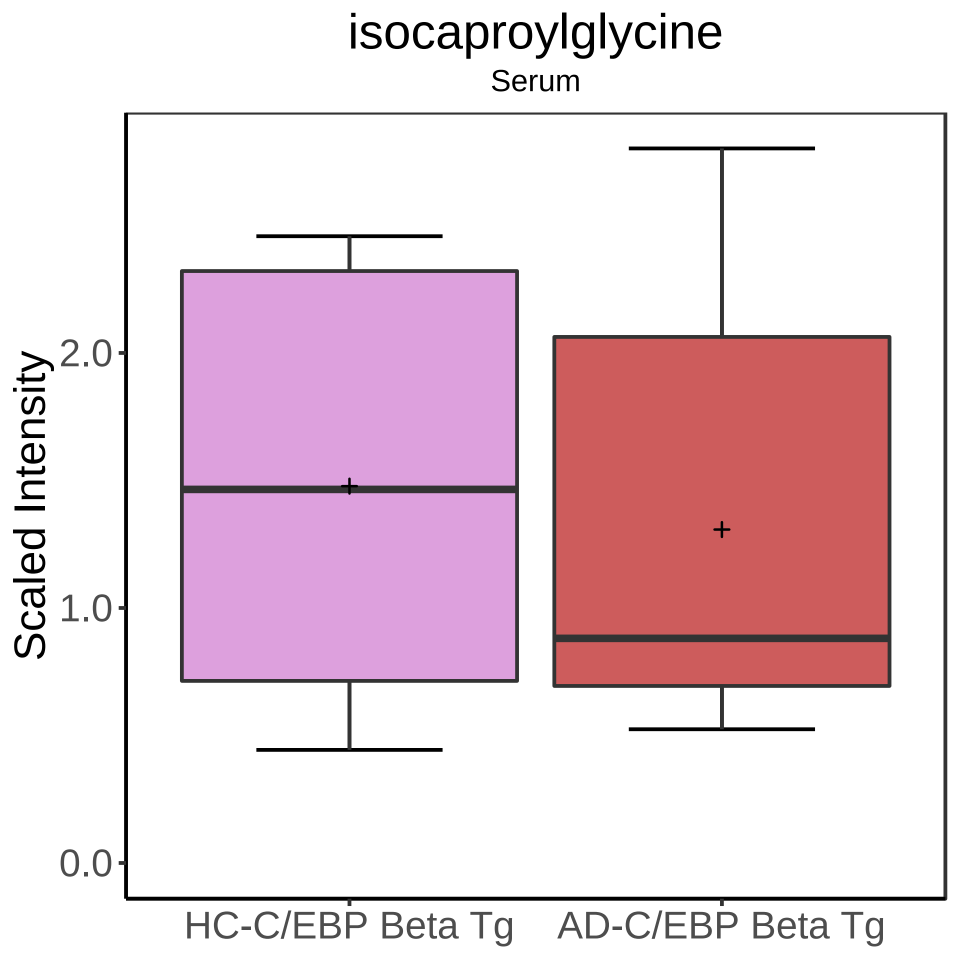

isocaproylglycine
Serum
Scaled Intensity
2.0
1.0
0.0
HC-C/EBP Beta Tg
AD-C/EBP Beta Tg

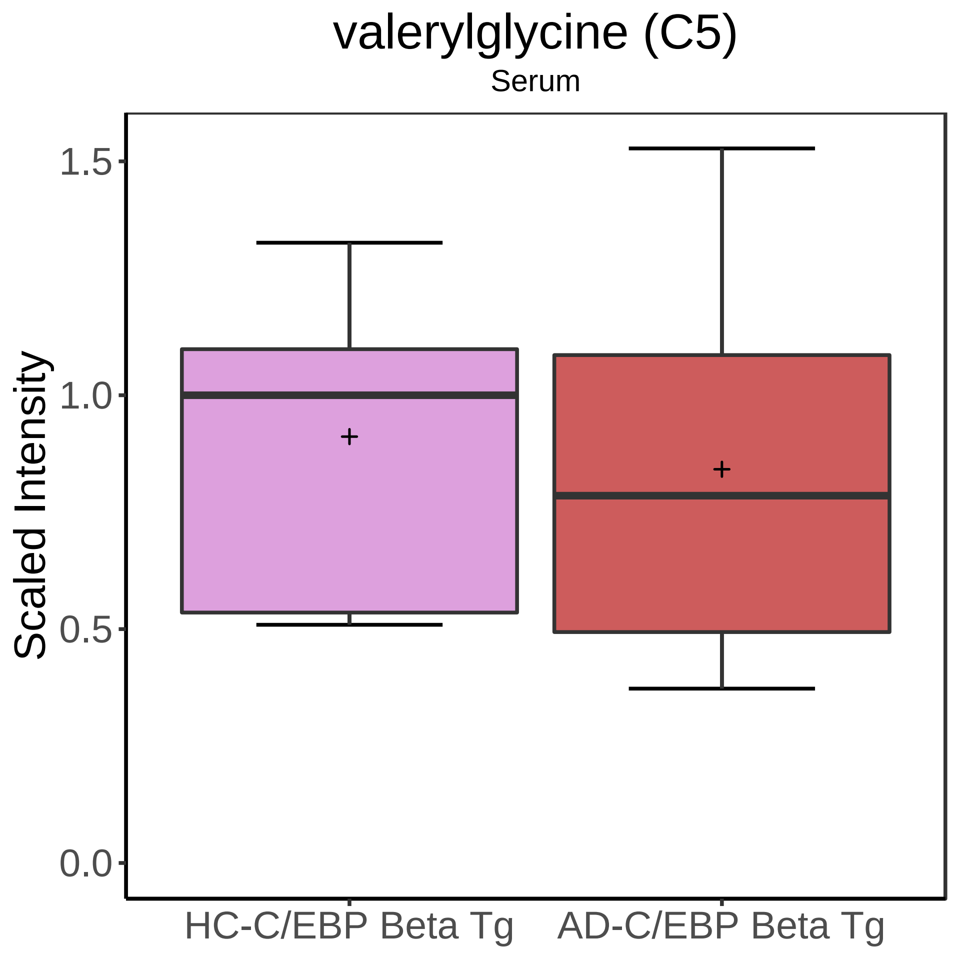
valerylglycine (C5)
Serum
Scaled Intensity
1.5
1.0
0.5
0.0
HC-C/EBP Beta Tg
AD-C/EBP Beta Tg

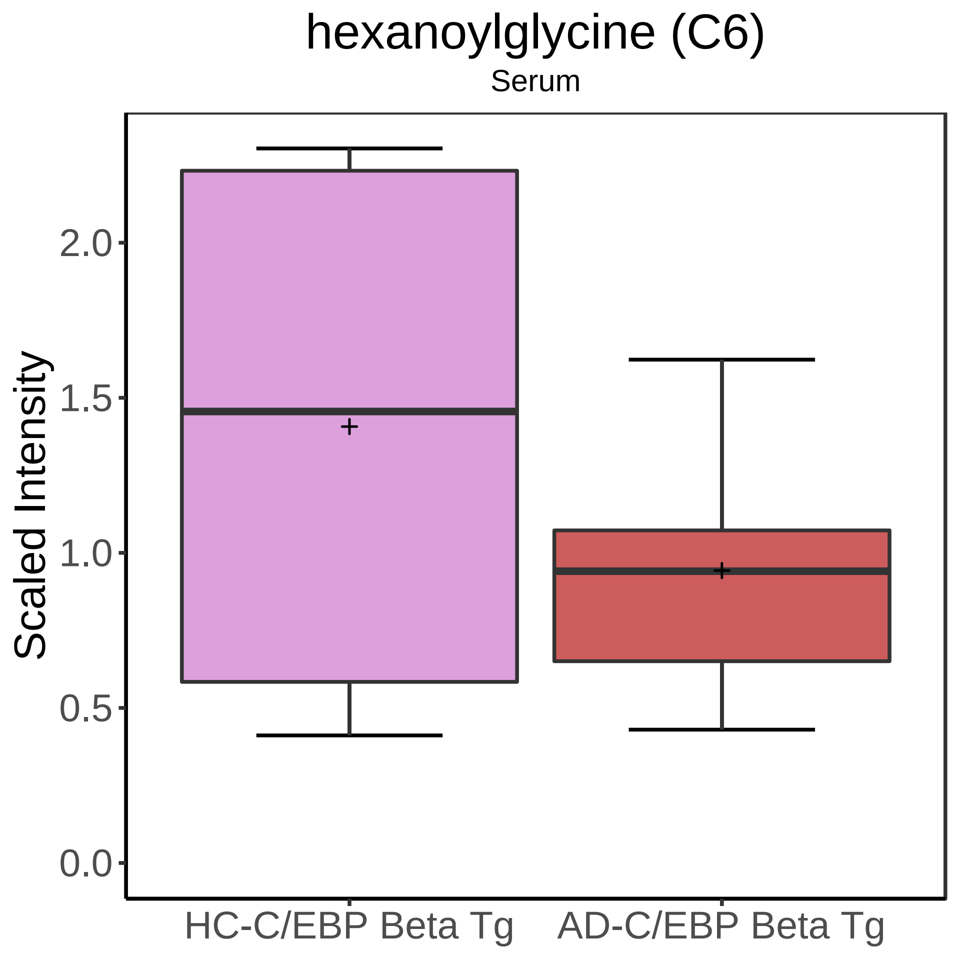
hexanoylglycine (C6)
Serum
Scaled Intensity
2.0
1.5
1.0
0.5
0.0
HC-C/EBP Beta Tg
AD-C/EBP Beta Tg

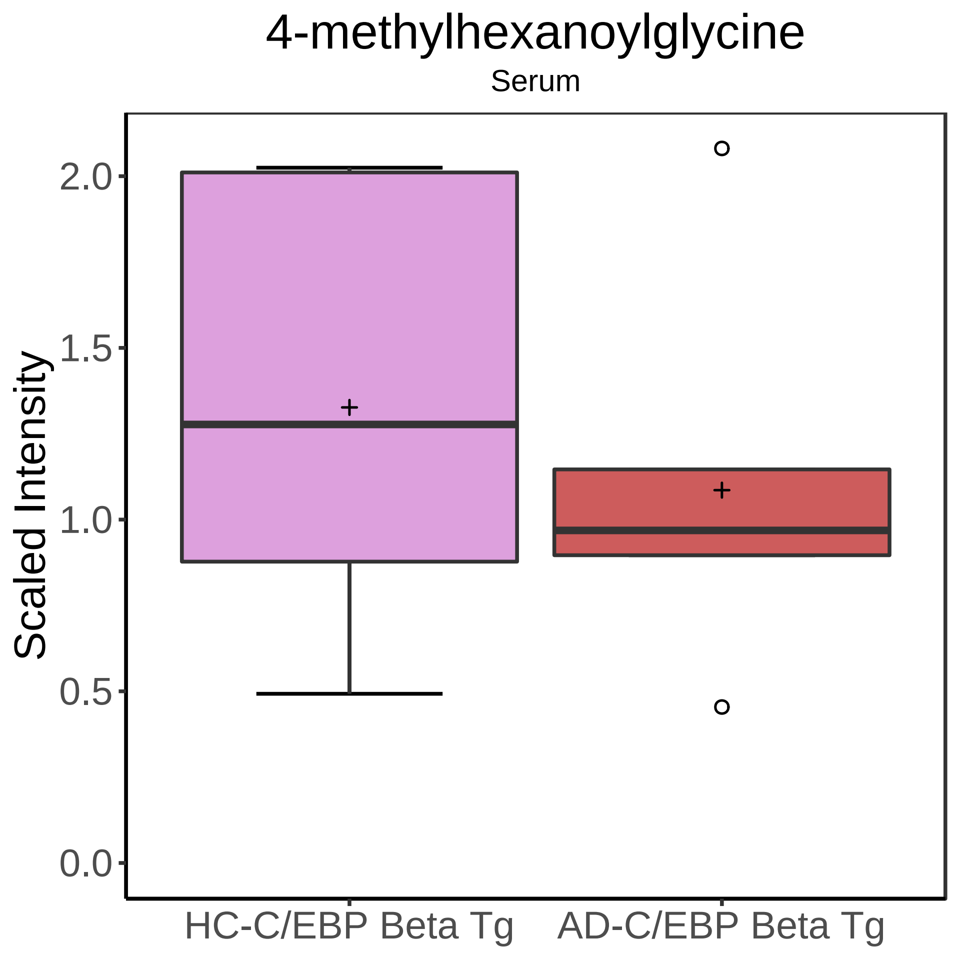

4-methylhexanoylglycine
Serum
Scaled Intensity
0.0
0.5
1.0
1.5
2.0
HC-C/EBP Beta Tg
AD-C/EBP Beta Tg

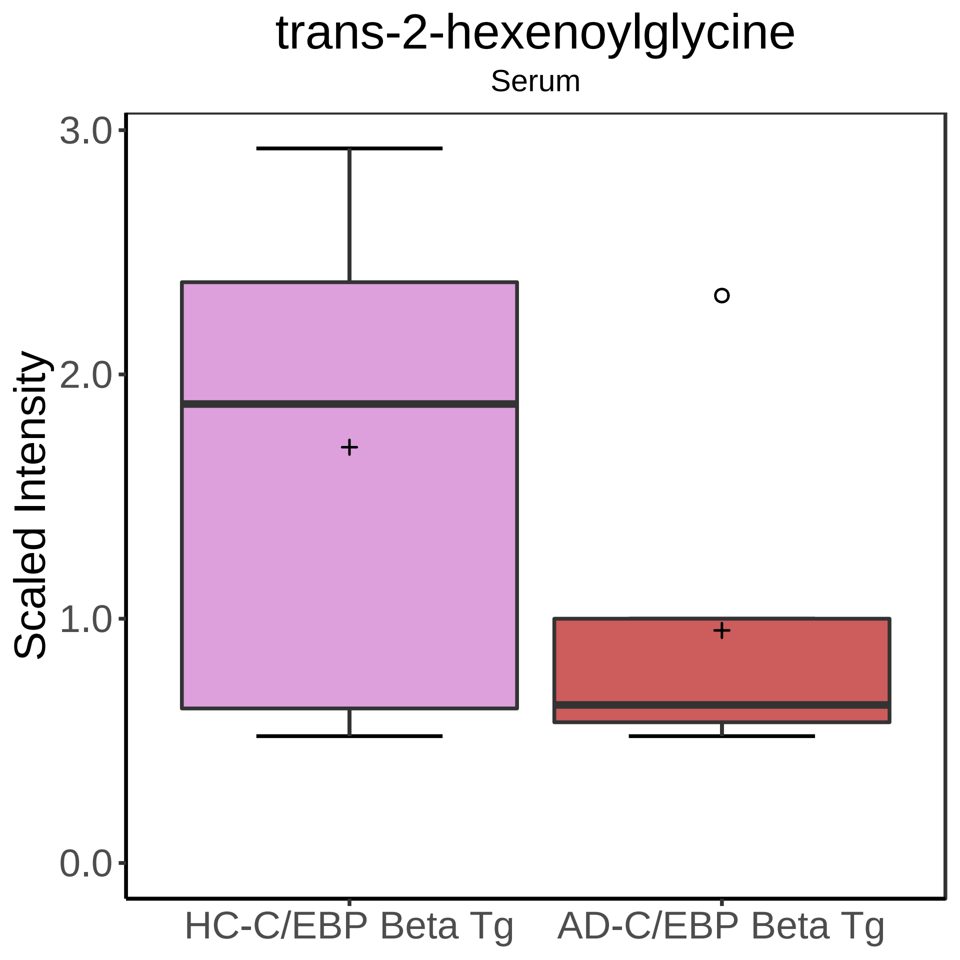
trans-2-hexenoylglycine
Serum
Scaled Intensity
3.0
2.0
1.0
0.0
HC-C/EBP Beta Tg
AD-C/EBP Beta Tg

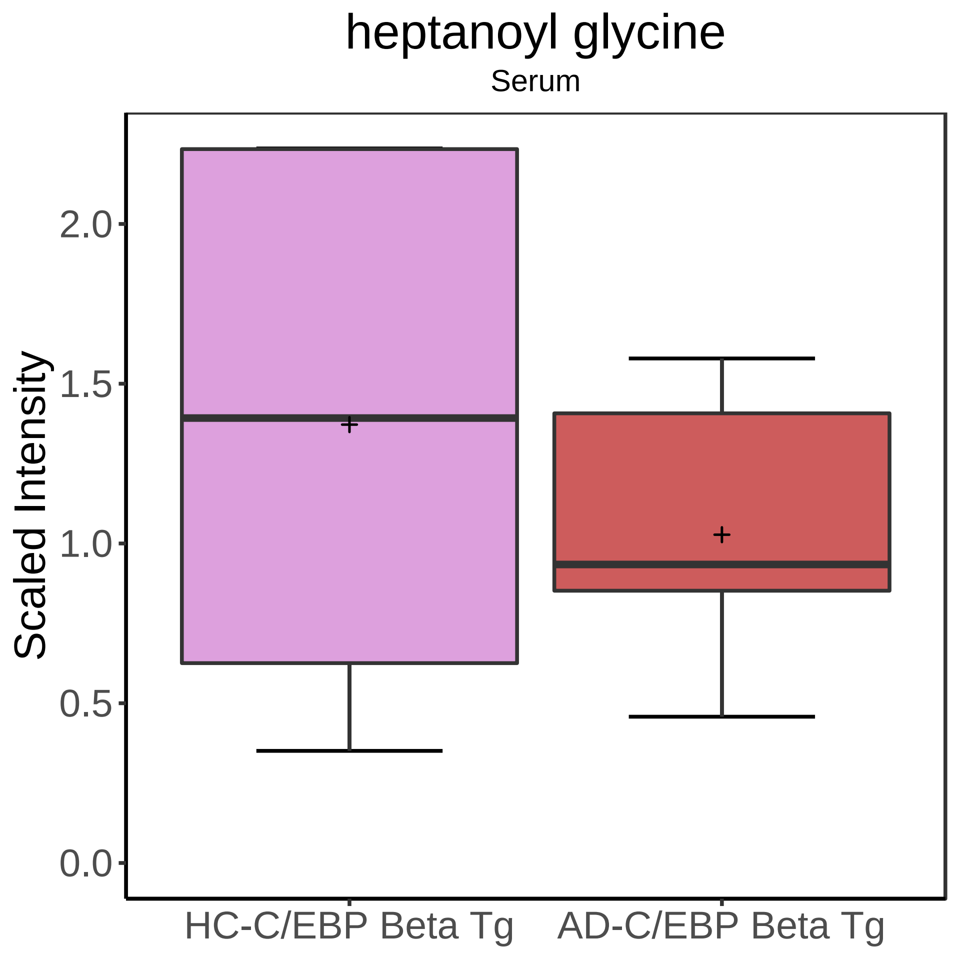
heptanoyl glycine
Serum
Scaled Intensity
2.0
1.5
1.0
0.5
0.0
HC-C/EBP Beta Tg
AD-C/EBP Beta Tg

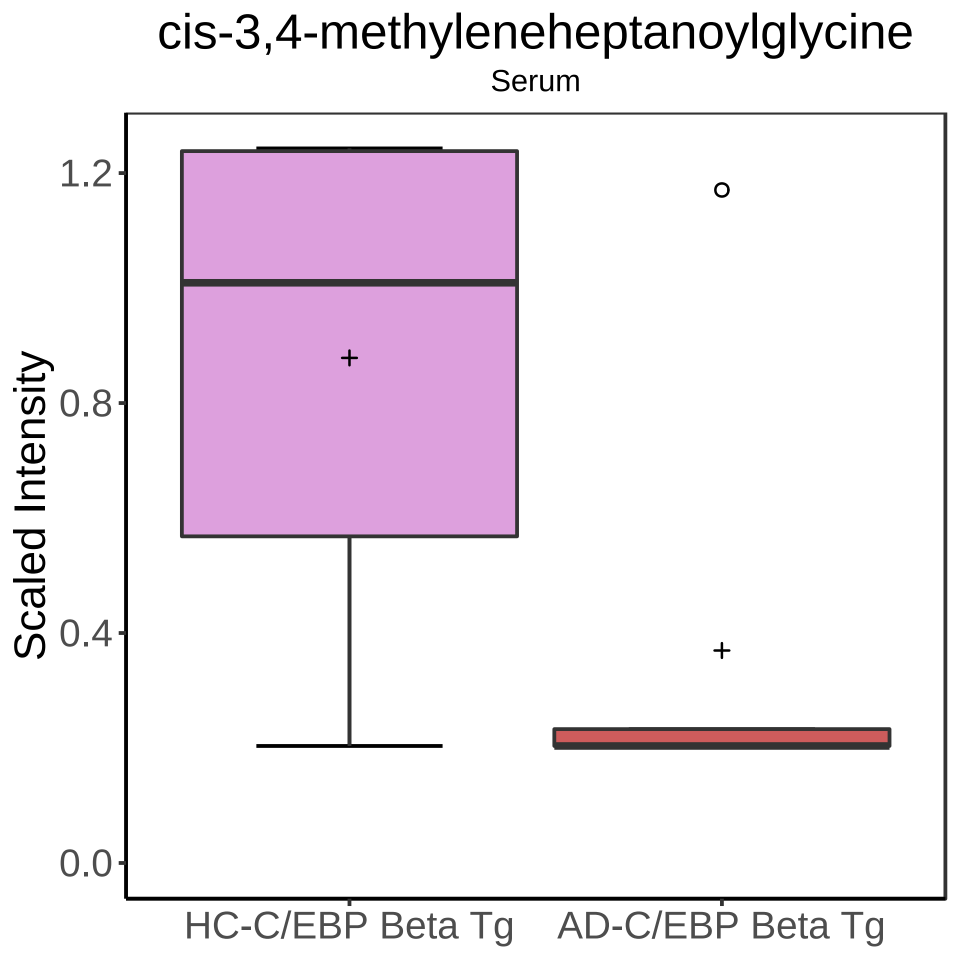
cis-3,4-methyleneheptanoylglycine
Serum
Scaled Intensity
1.2
0.8
0.4
0.0
HC-C/EBP Beta Tg
AD-C/EBP Beta Tg

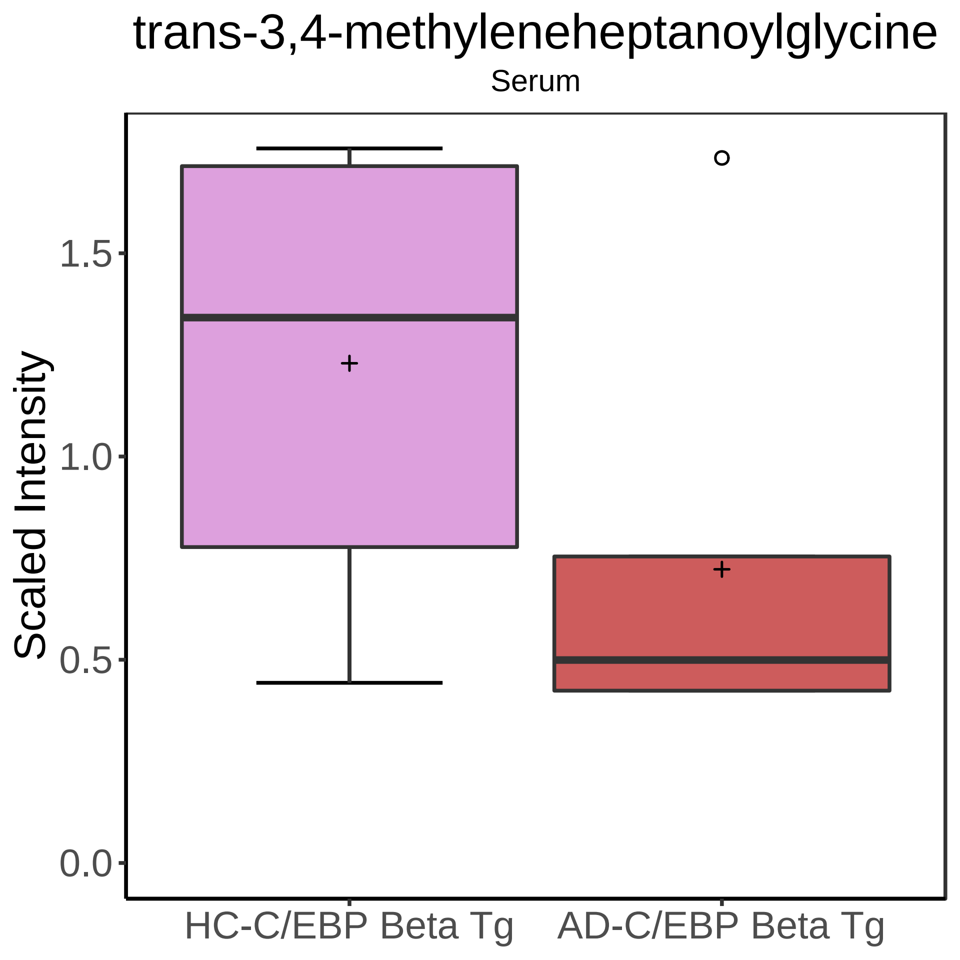

trans-3,4-methyleneheptanoylglycine
Serum
Scaled Intensity
1.5
1.0
0.5
0.0
HC-C/EBP Beta Tg
AD-C/EBP Beta Tg

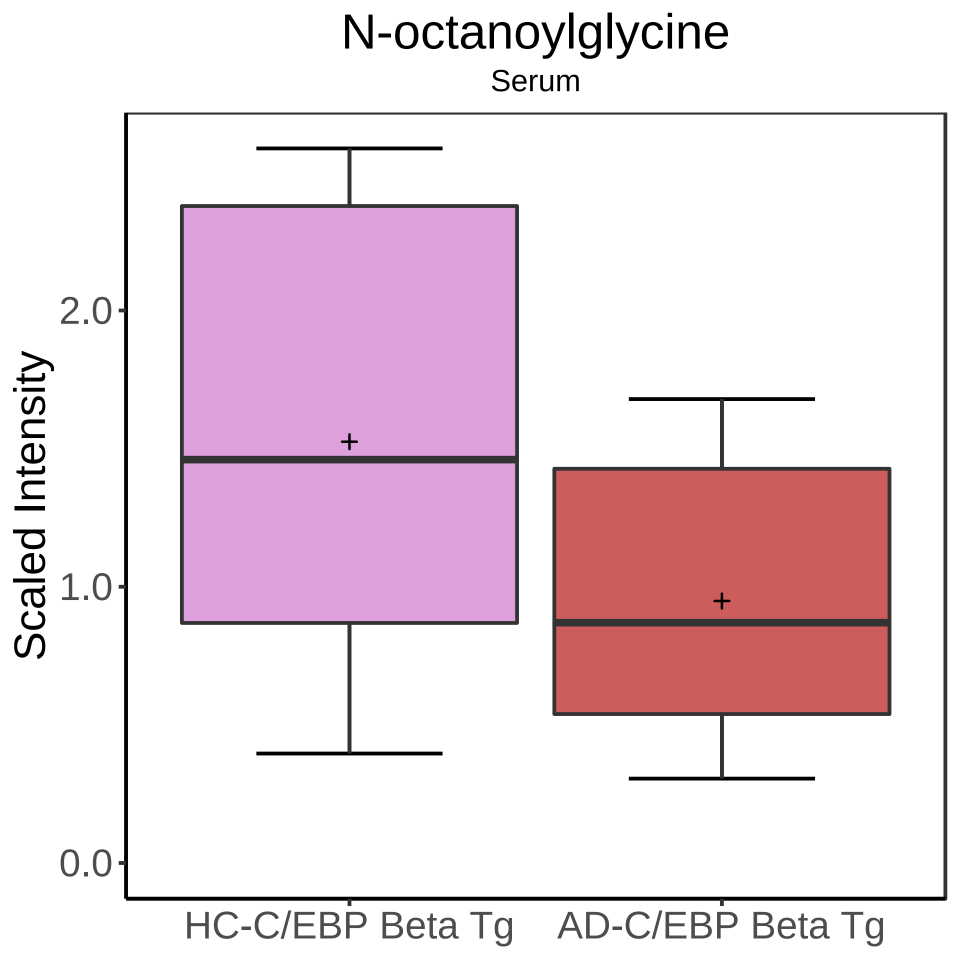
N-octanoylglycine
Serum
Scaled Intensity
2.0
1.0
0.0
HC-C/EBP Beta Tg
AD-C/EBP Beta Tg

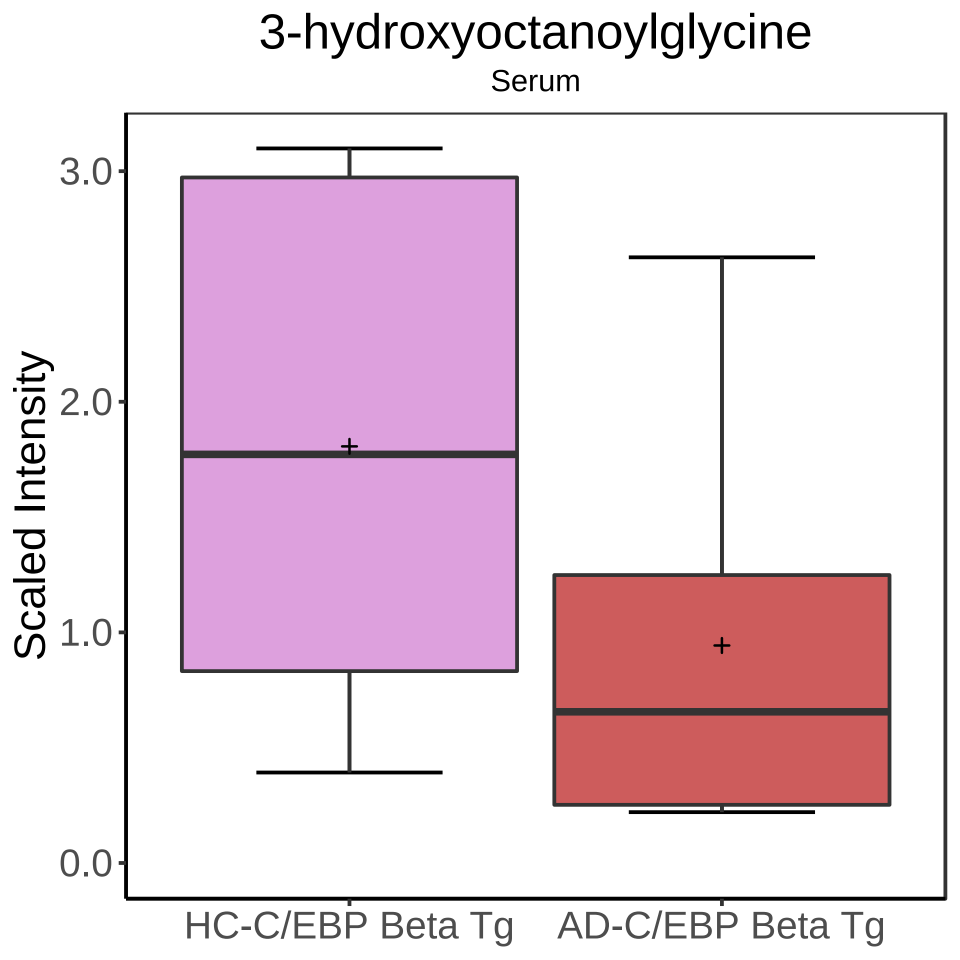
3-hydroxyoctanoylglycine
Serum
Scaled Intensity
3.0
2.0
1.0
0.0
HC-C/EBP Beta Tg
AD-C/EBP Beta Tg

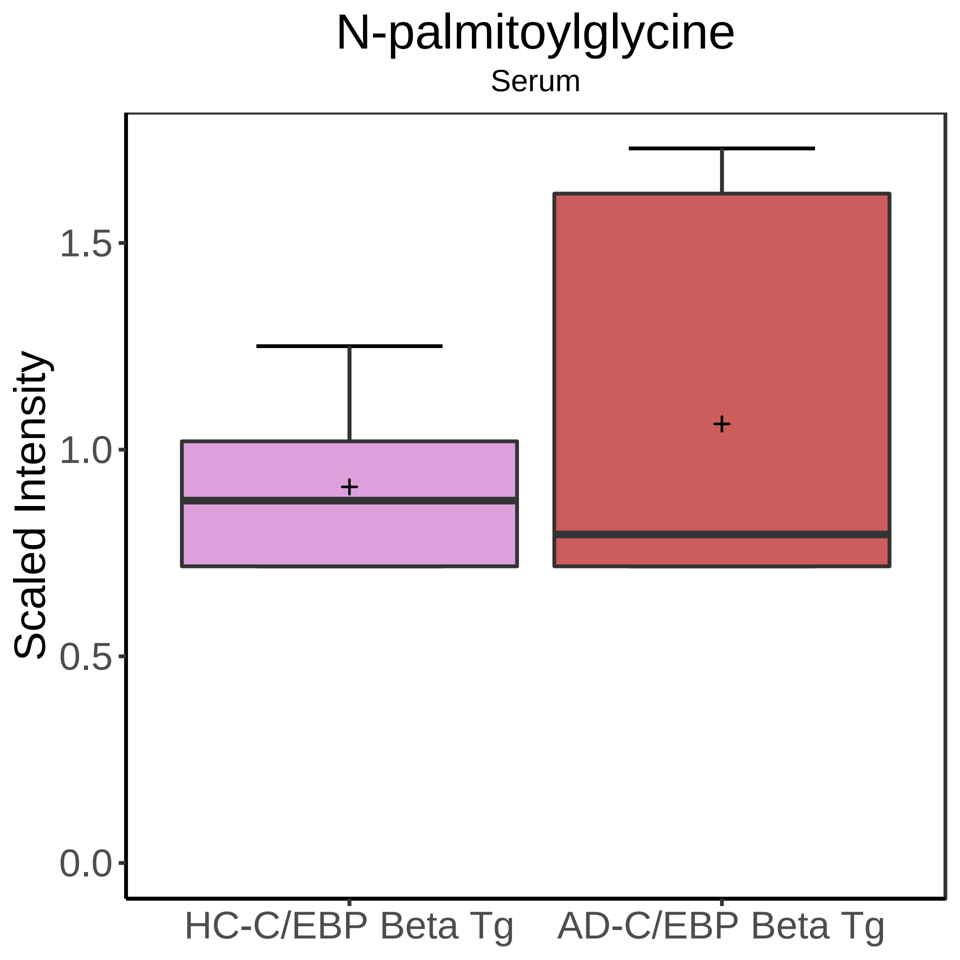
N-palmitoylglycine
Serum
Scaled Intensity
0.0
0.5
1.0
1.5
HC-C/EBP Beta Tg
AD-C/EBP Beta Tg

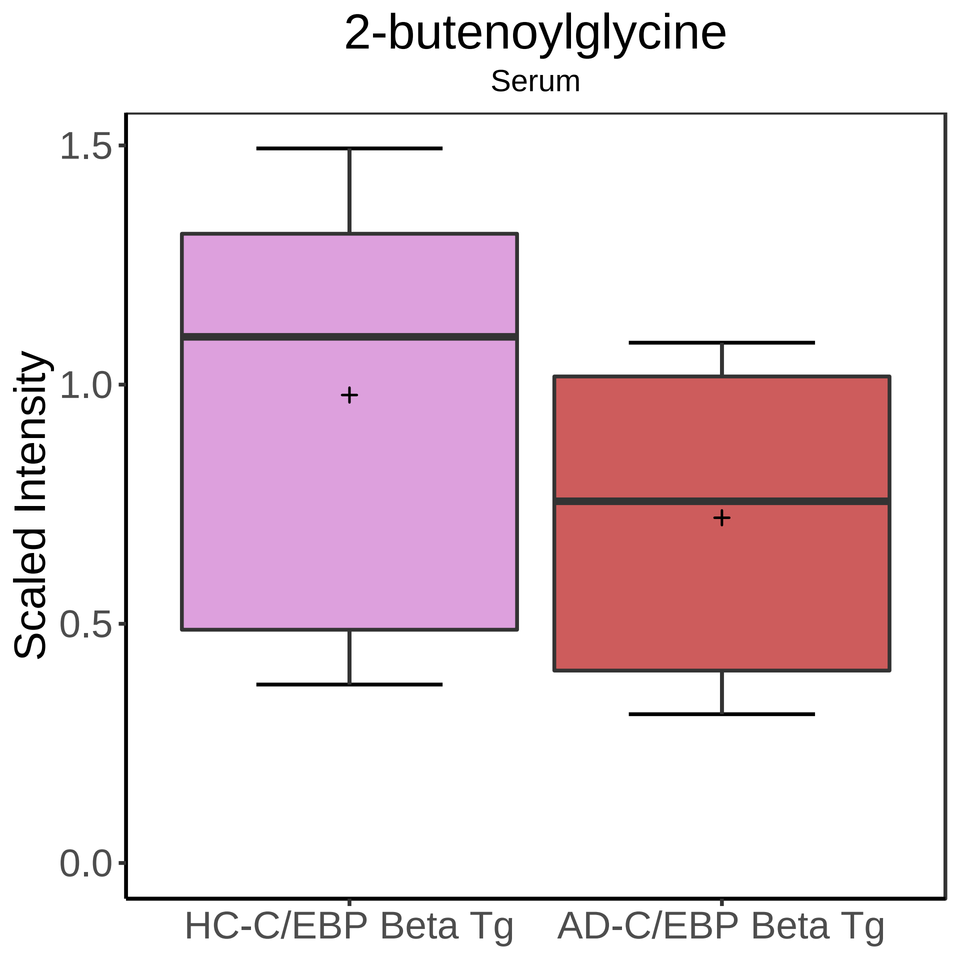
2-butenoylglycine
Serum
Scaled Intensity
1.5
1.0
0.5
0.0
HC-C/EBP Beta Tg
AD-C/EBP Beta Tg

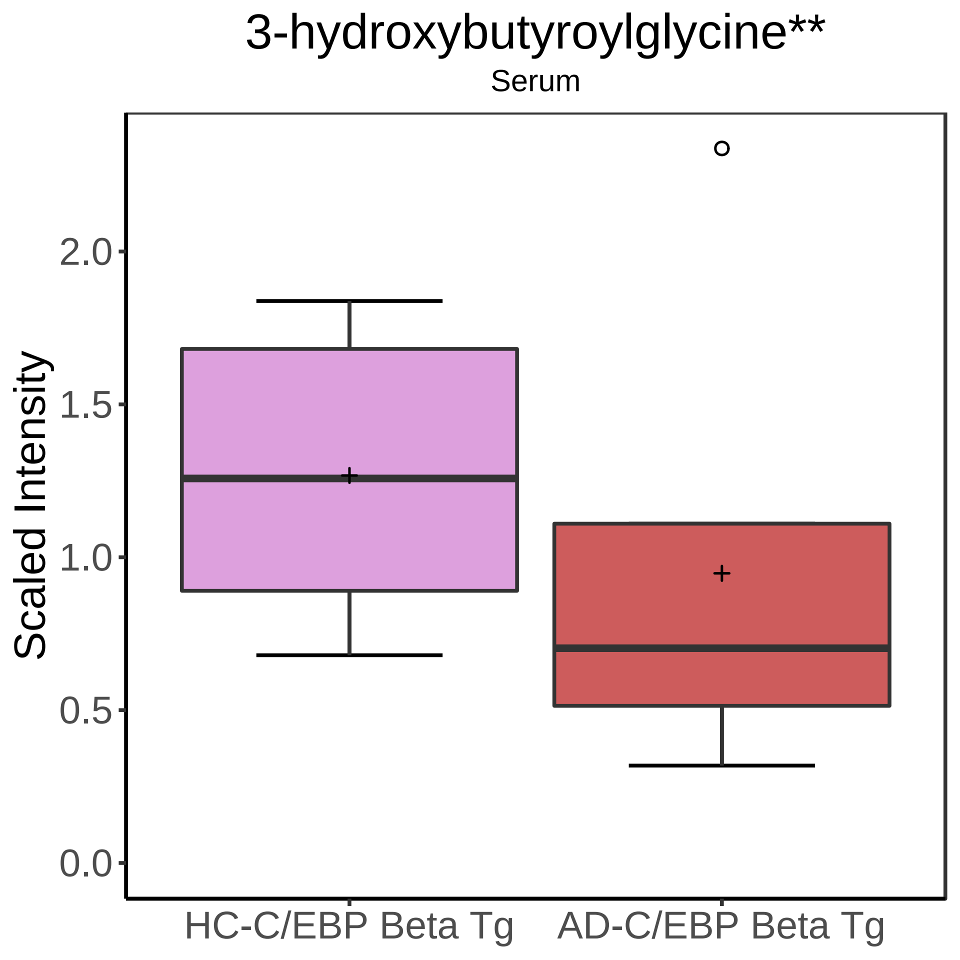
3-hydroxybutyroylglycine**
Serum
Scaled Intensity
2.0
1.5
1.0
0.5
0.0
HC-C/EBP Beta Tg
AD-C/EBP Beta Tg

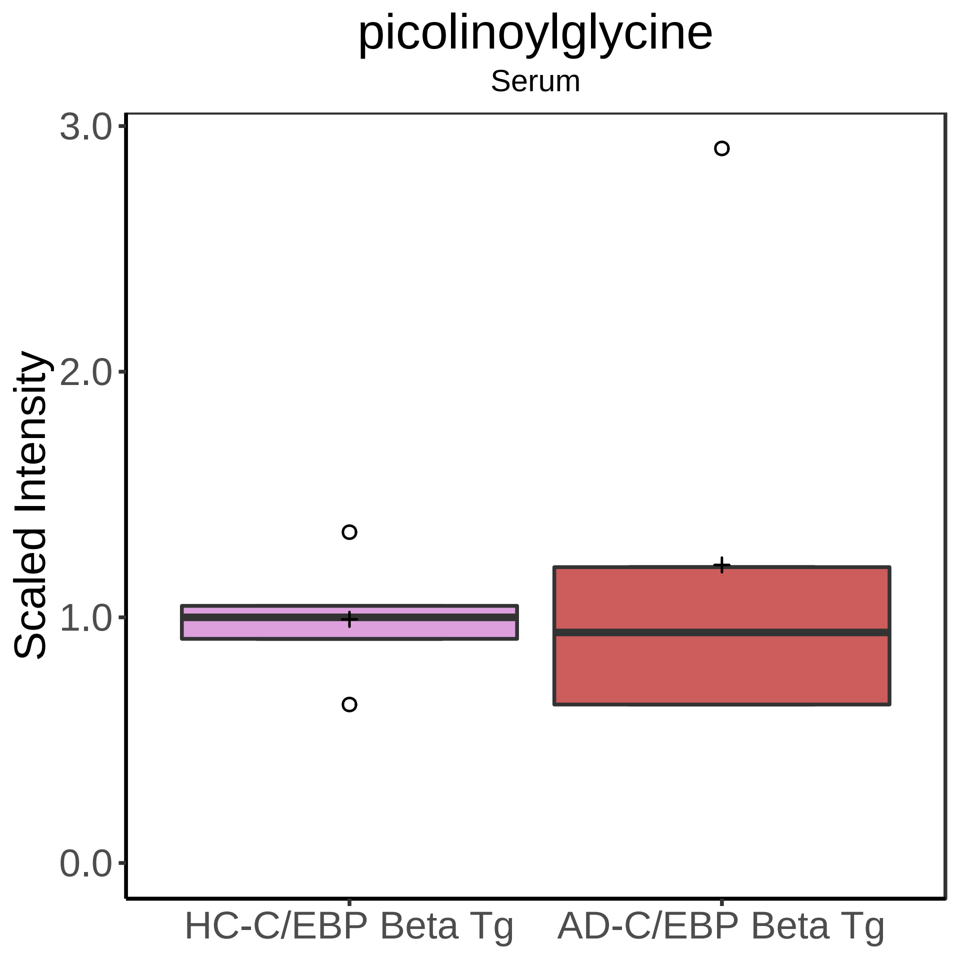

picolinoylglycine
Serum
Scaled Intensity
3.0
2.0
1.0
0.0
HC-C/EBP Beta Tg
AD-C/EBP Beta Tg

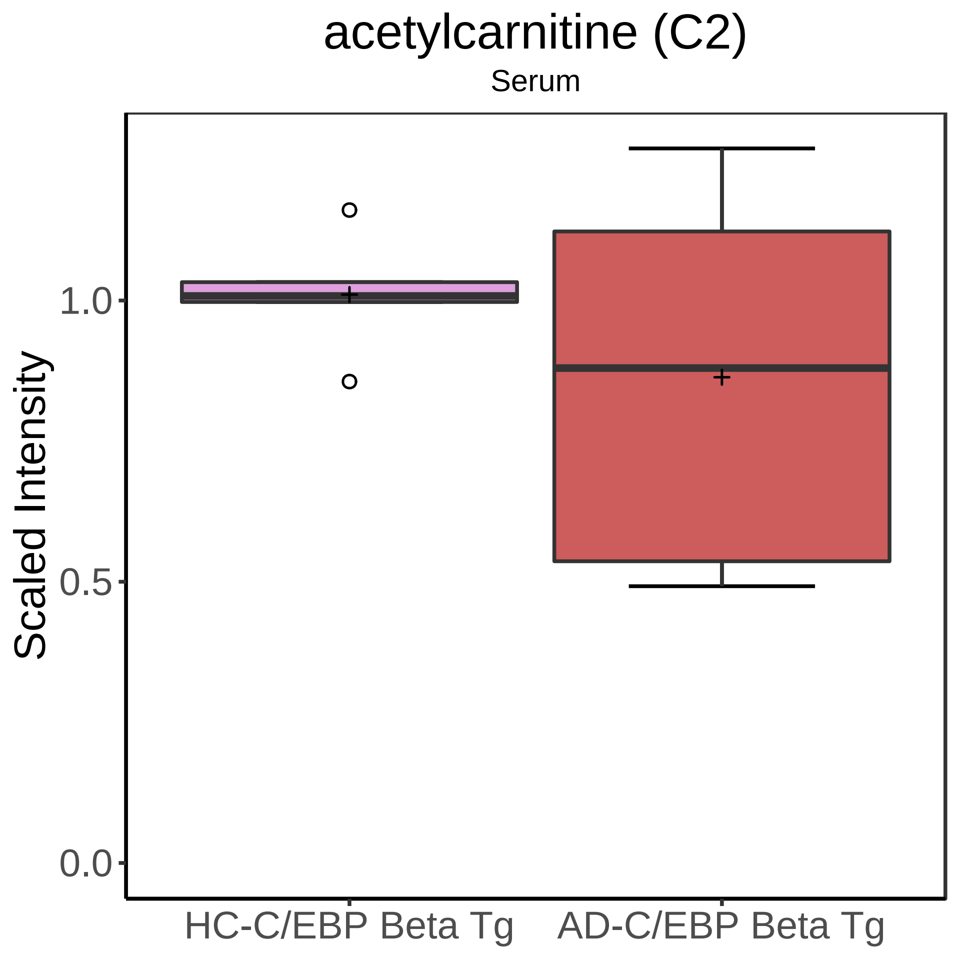
acetylcarnitine (C2)
Serum
Scaled Intensity
1.0
0.5
0.0
HC-C/EBP Beta Tg
AD-C/EBP Beta Tg

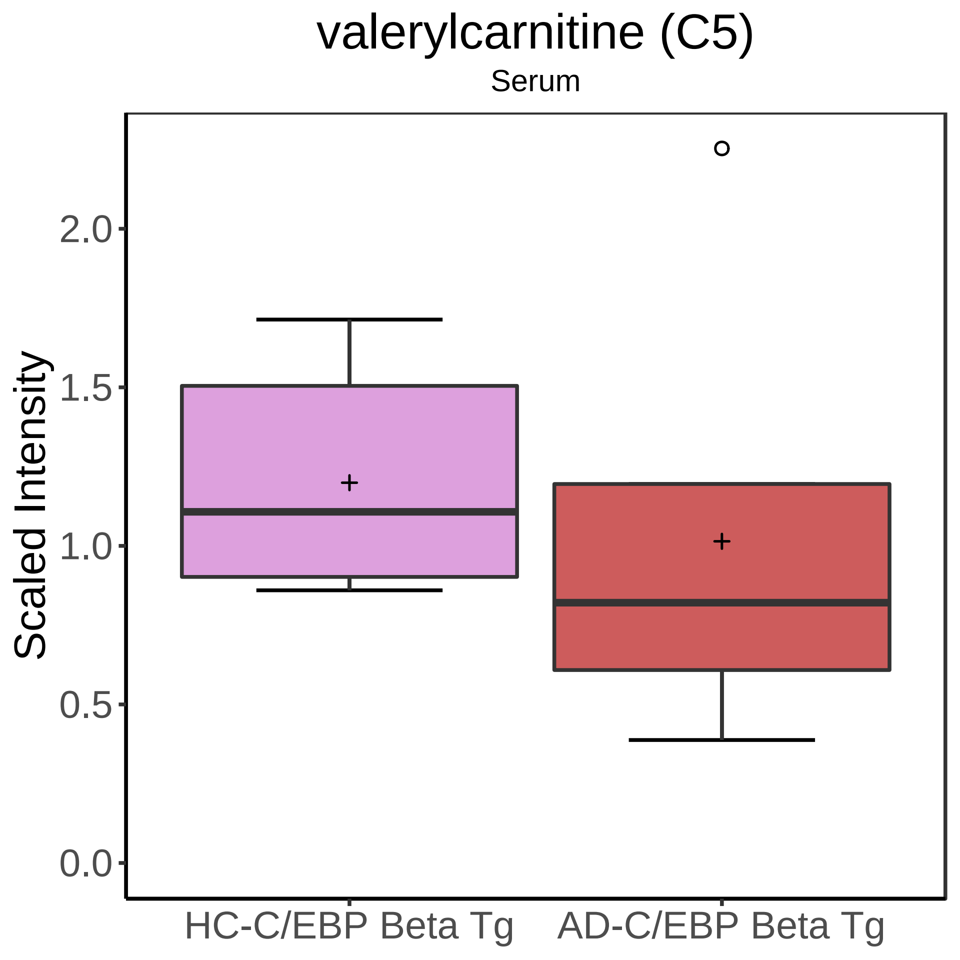
valerylcarnitine (C5)
Serum
Scaled Intensity
2.0
1.5
1.0
0.5
0.0
HC-C/EBP Beta Tg
AD-C/EBP Beta Tg

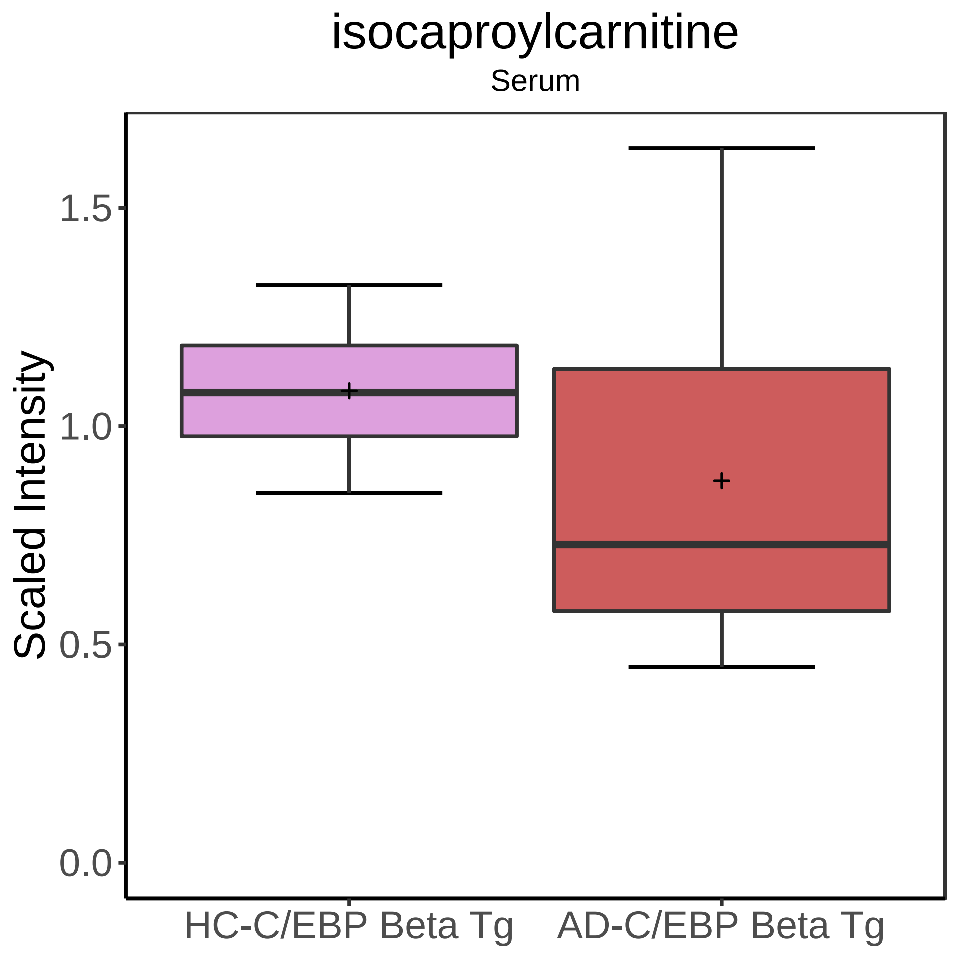
isocaproylcarnitine
Serum
Scaled Intensity
1.5
1.0
0.5
0.0
HC-C/EBP Beta Tg
AD-C/EBP Beta Tg

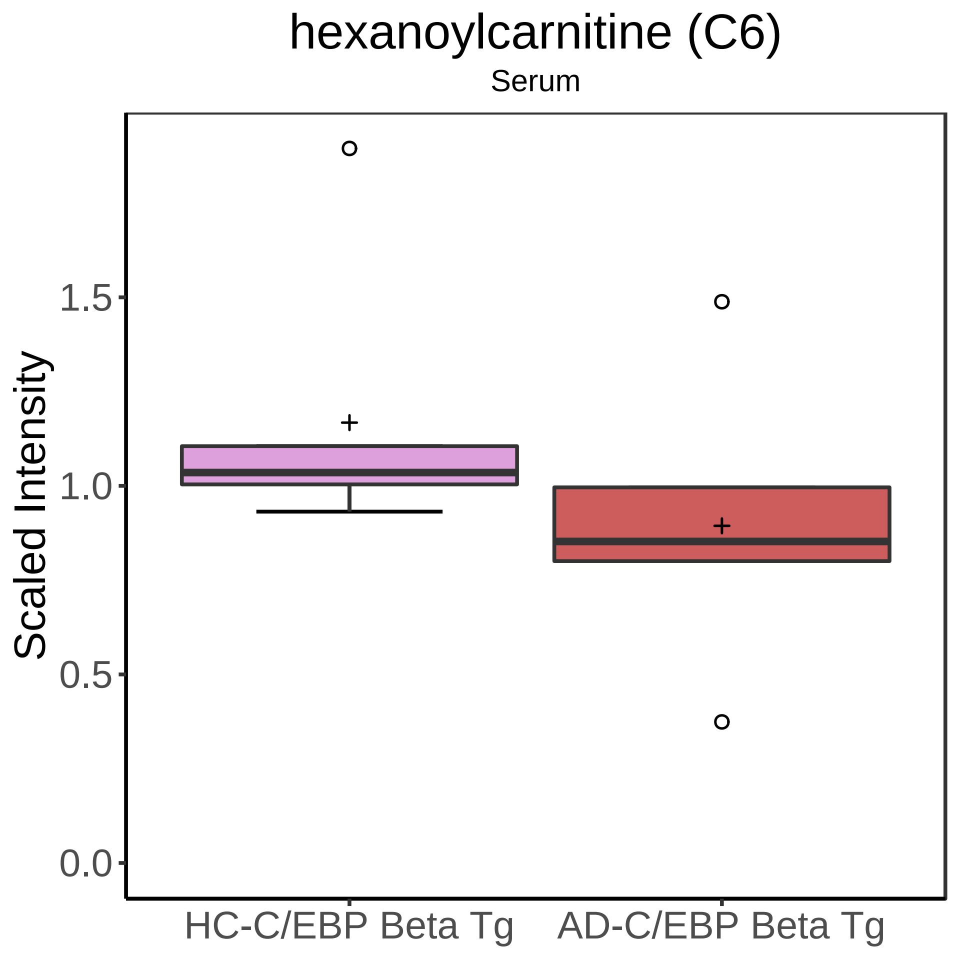
hexanoylcarnitine (C6)
Serum
Scaled Intensity
1.5
1.0
0.5
0.0
HC-C/EBP Beta Tg
AD-C/EBP Beta Tg

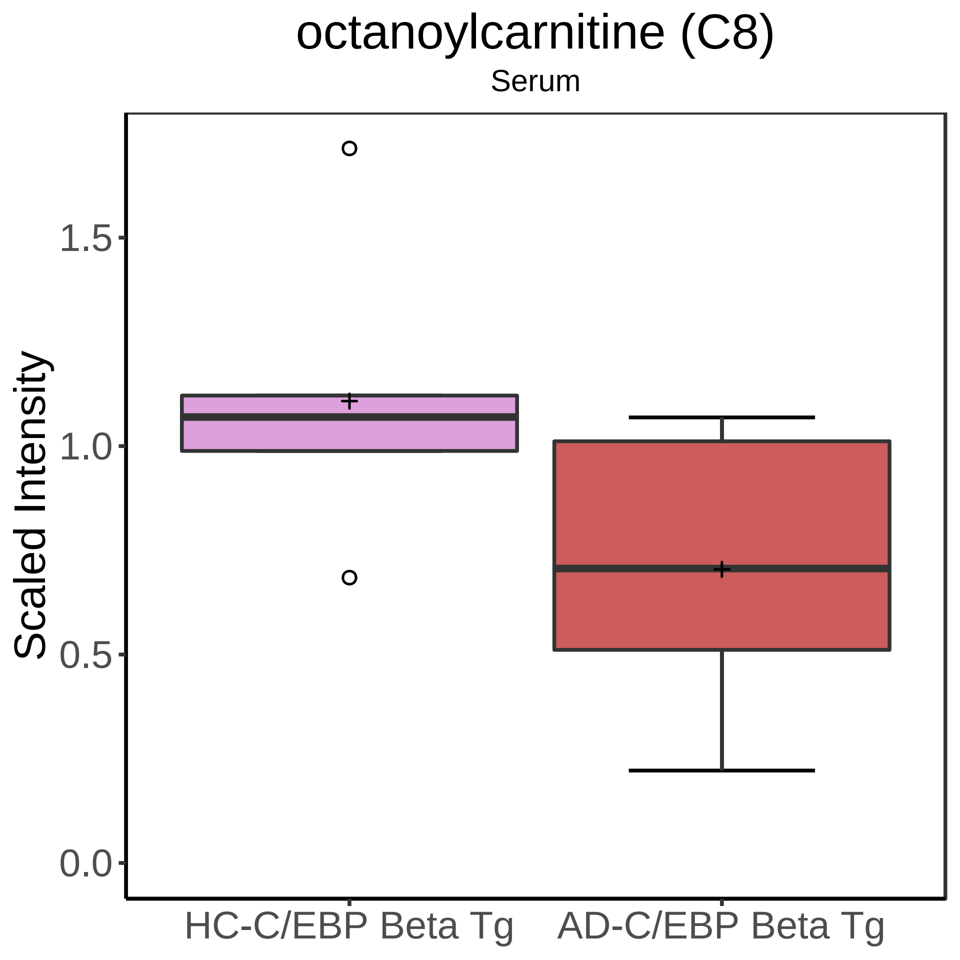
octanoylcarnitine (C8)
Serum
Scaled Intensity
1.5
1.0
0.5
0.0
HC-C/EBP Beta Tg
AD-C/EBP Beta Tg

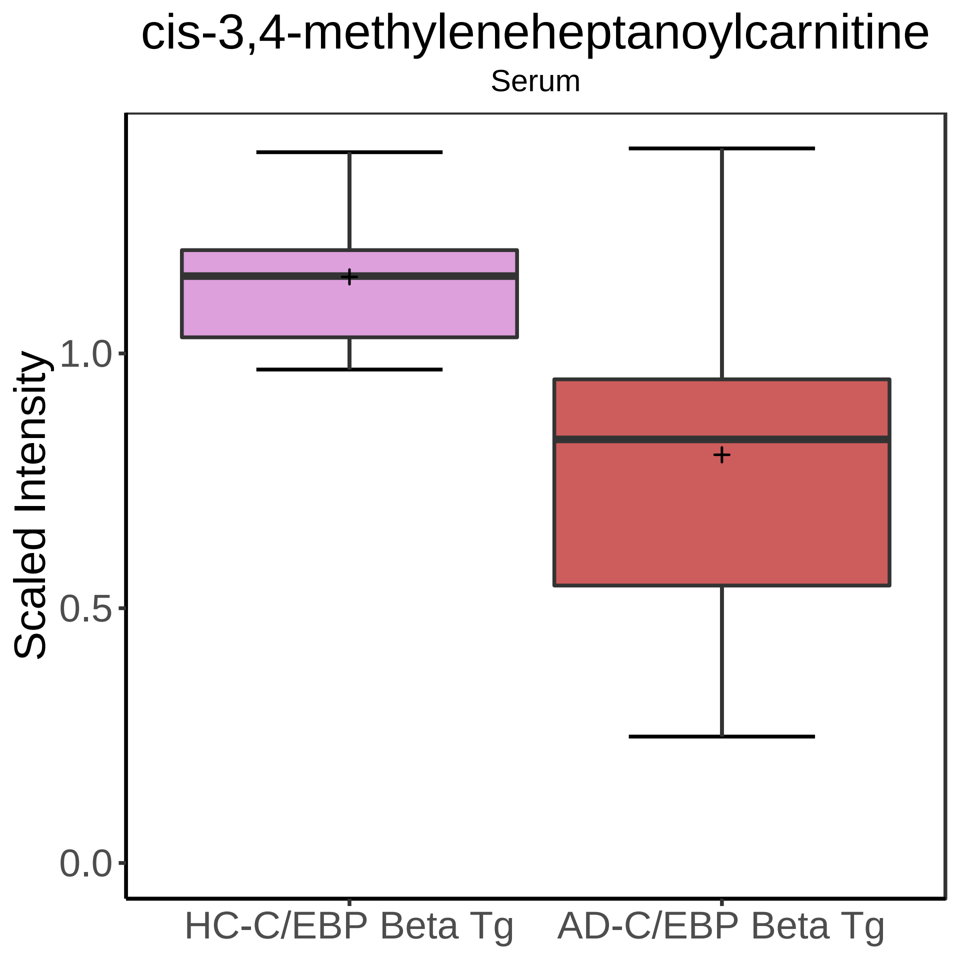
cis-3,4-methyleneheptanoylcarnitine
Serum
Scaled Intensity
1.0
0.5
0.0
HC-C/EBP Beta Tg
AD-C/EBP Beta Tg

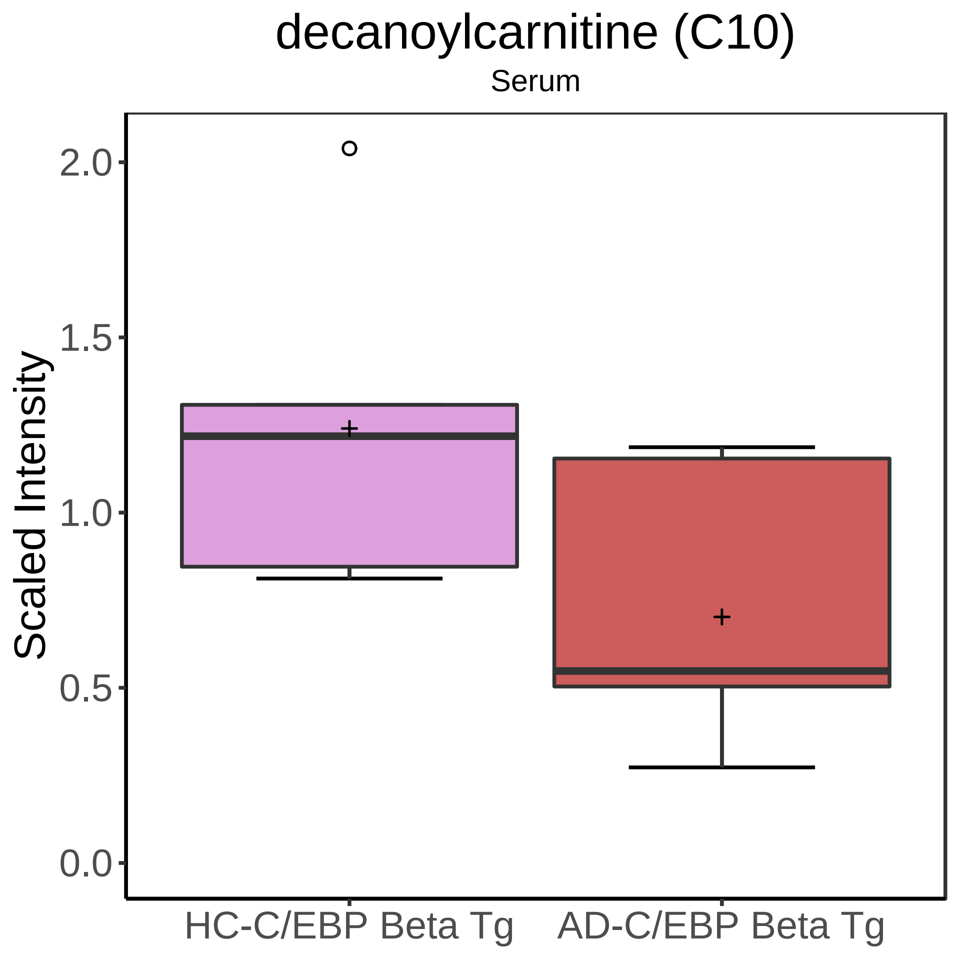
decanoylcarnitine (C10)
Serum
Scaled Intensity
2.0
1.5
1.0
0.5
0.0
HC-C/EBP Beta Tg
AD-C/EBP Beta Tg

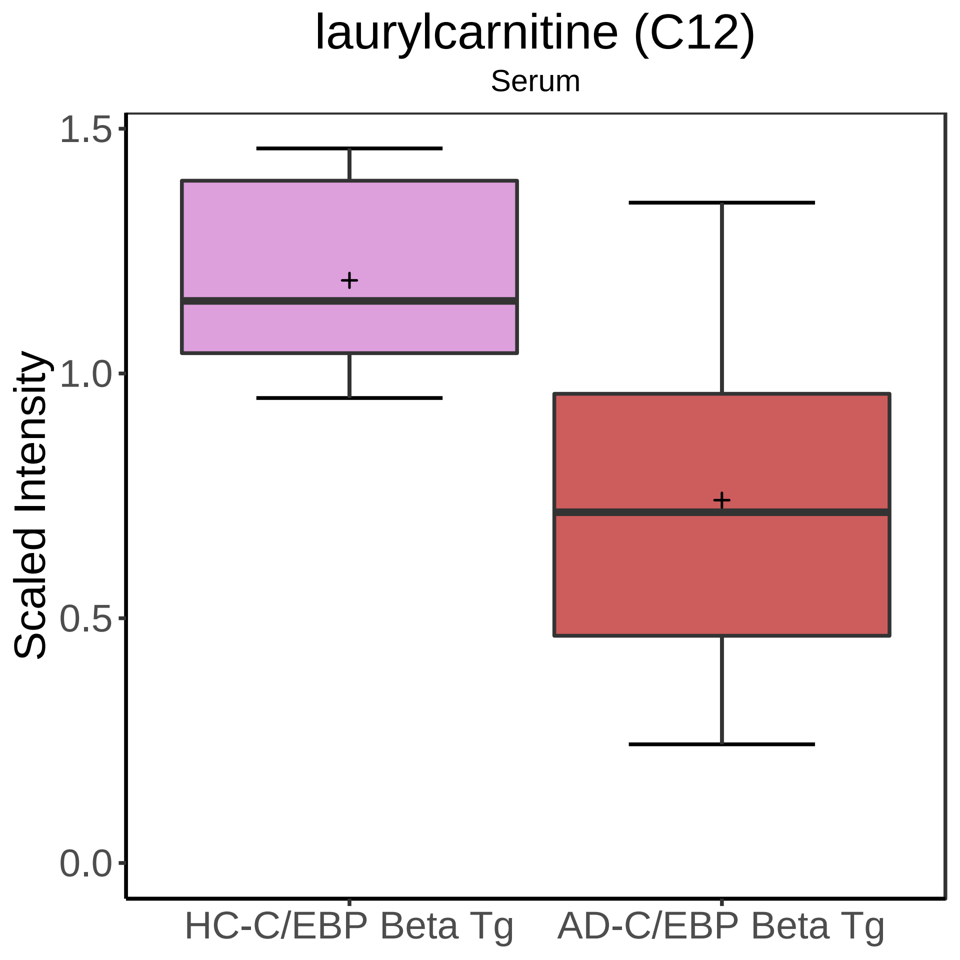
laurylcarnitine (C12)
Serum
Scaled Intensity
1.5
1.0
0.5
0.0
HC-C/EBP Beta Tg
AD-C/EBP Beta Tg

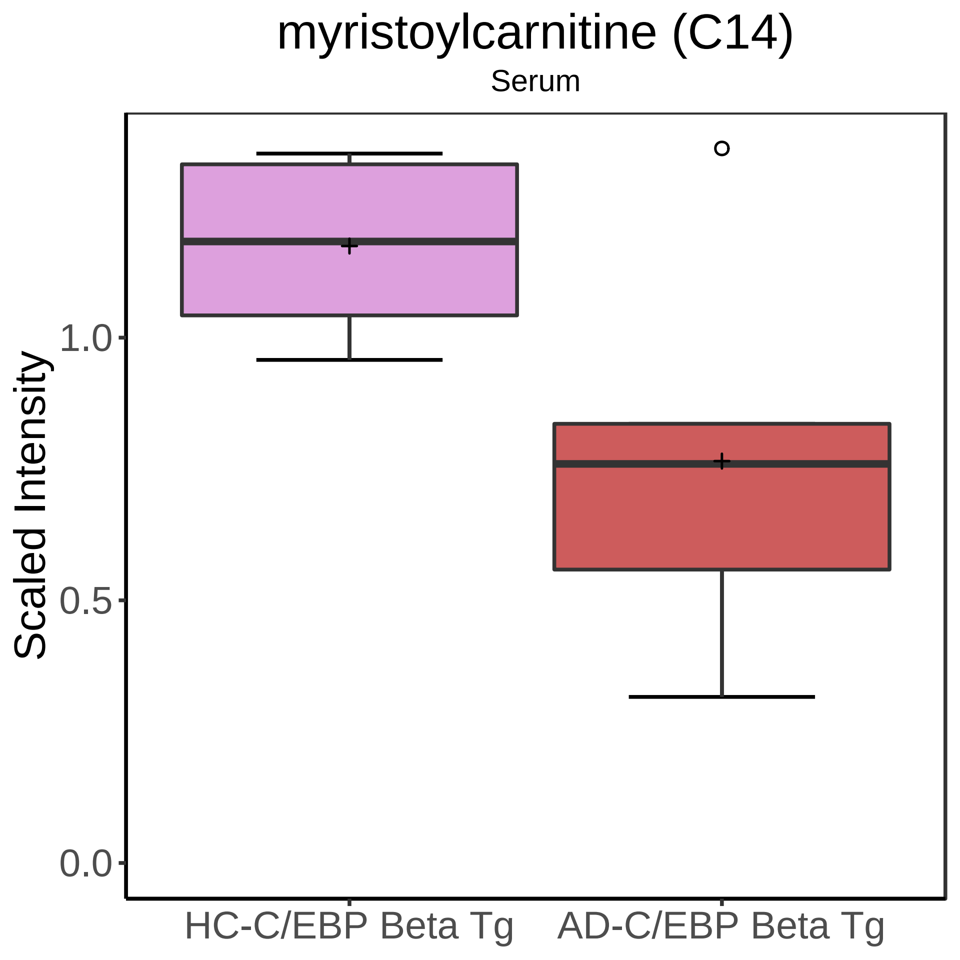

myristoylcarnitine (C14)
Serum
Scaled Intensity
1.0
0.5
0.0
HC-C/EBP Beta Tg
AD-C/EBP Beta Tg

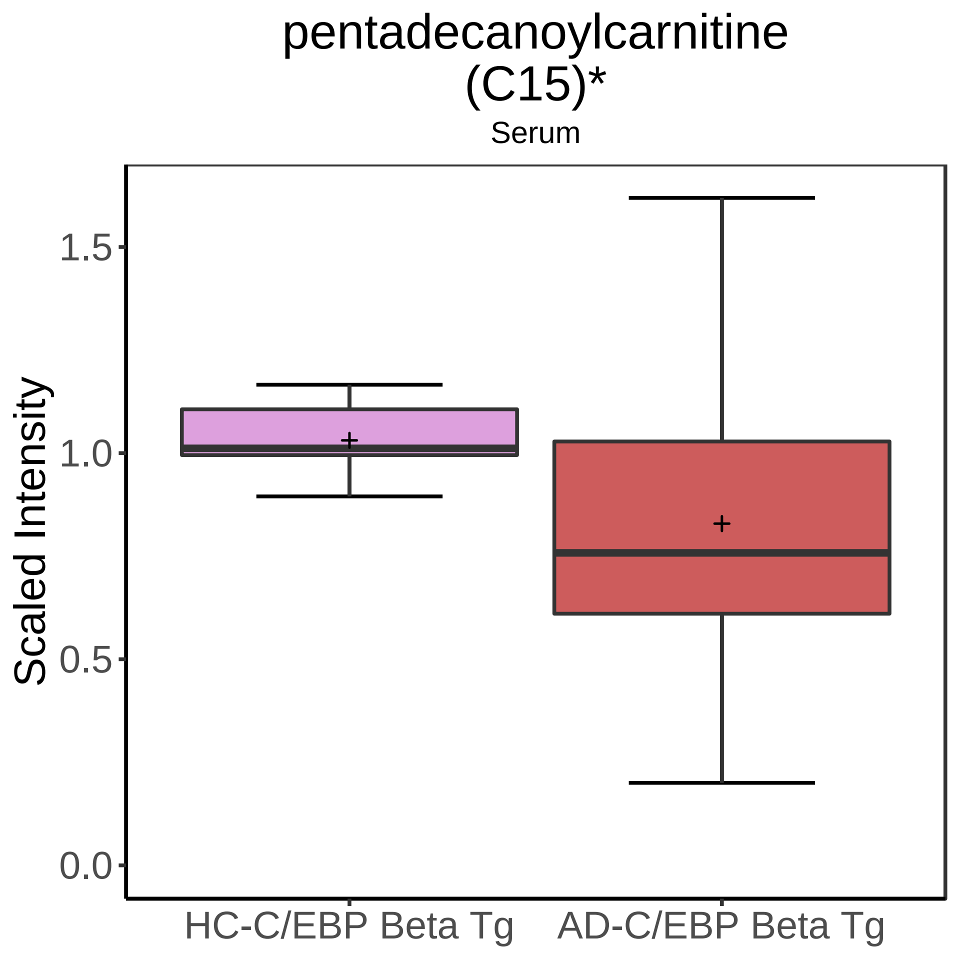
pentadecanoylcarnitine
(C15)*
Serum
Scaled Intensity
1.5
1.0
0.5
0.0
HC-C/EBP Beta Tg
AD-C/EBP Beta Tg

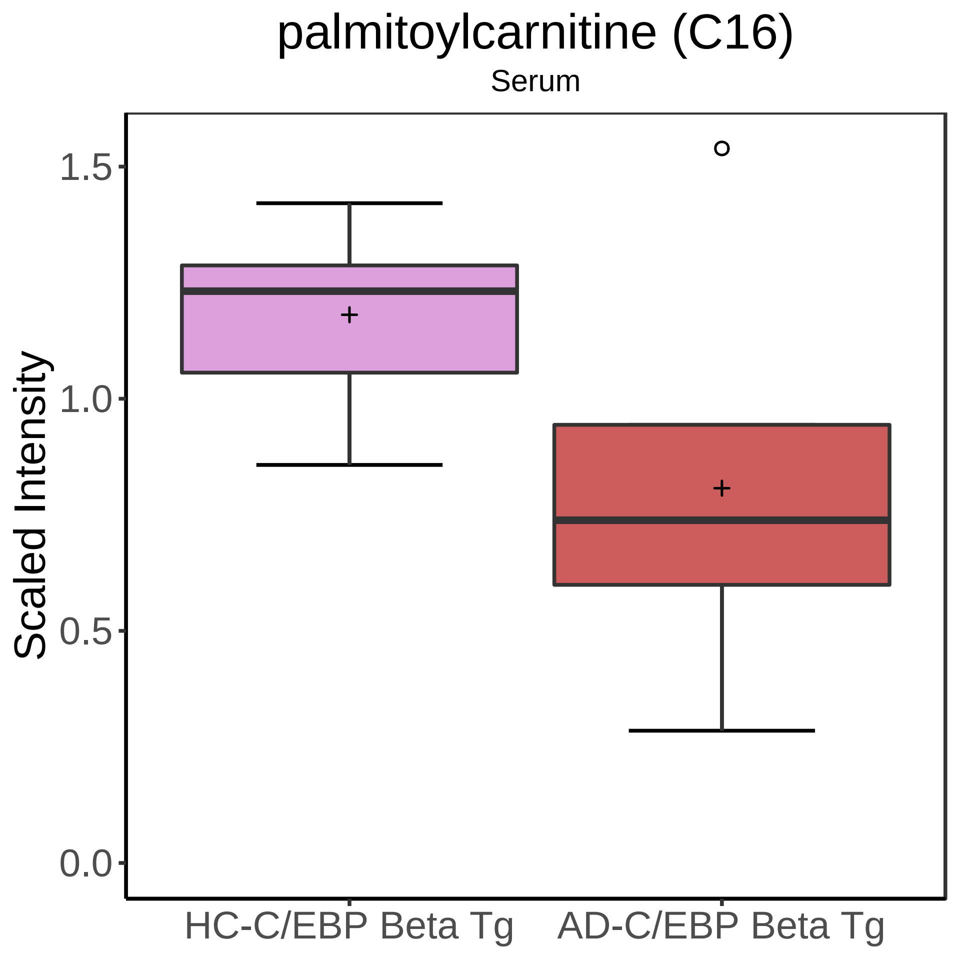
palmitoylcarnitine (C16)
Serum
Scaled Intensity
1.5
1.0
0.5
0.0
HC-C/EBP Beta Tg
AD-C/EBP Beta Tg

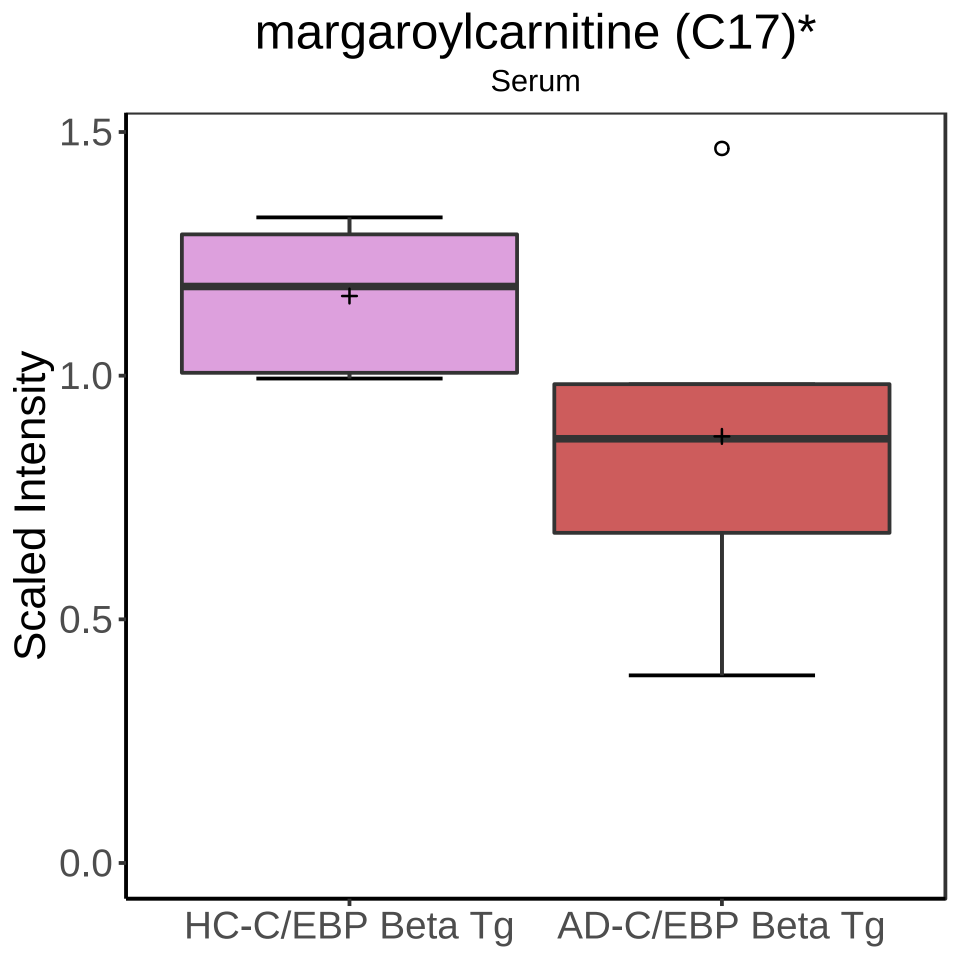

margaroylcarnitine (C17)*
Serum
Scaled Intensity
1.5
1.0
0.5
0.0
HC-C/EBP Beta Tg
AD-C/EBP Beta Tg

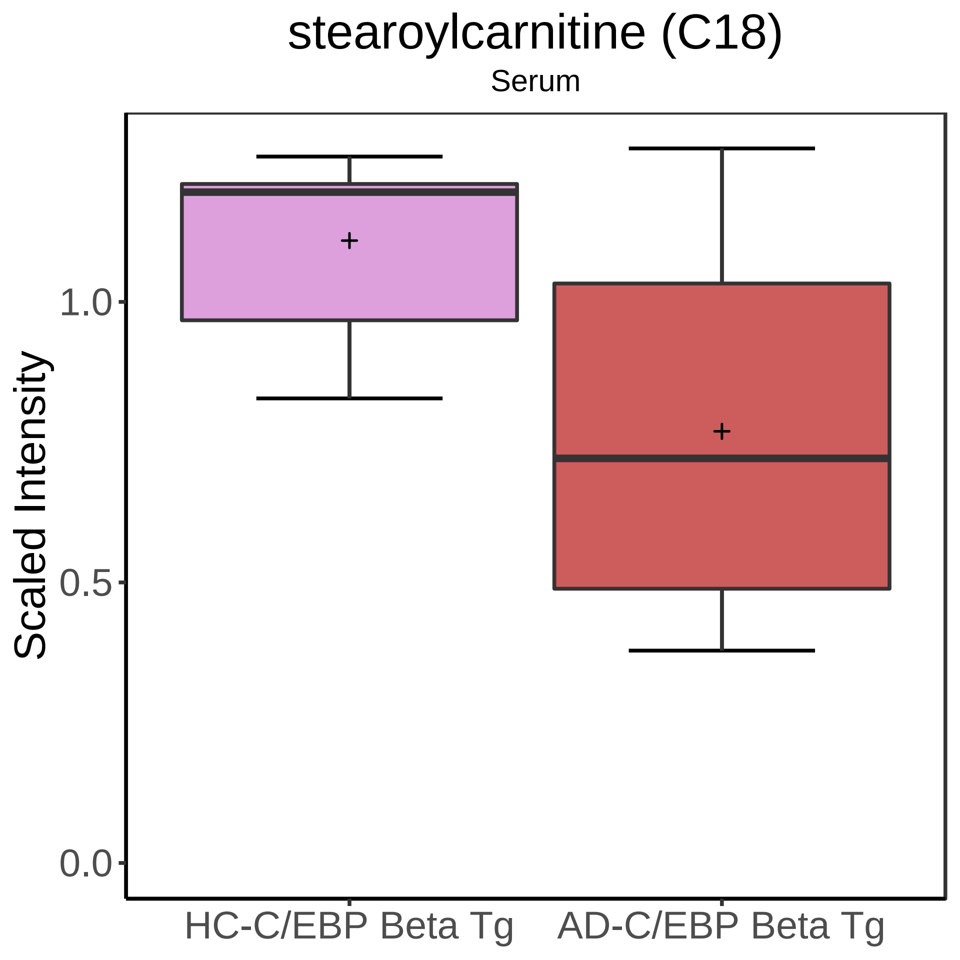

stearoylcarnitine (C18)
Serum
Scaled Intensity
1.0
0.5
0.0
HC-C/EBP Beta Tg
AD-C/EBP Beta Tg

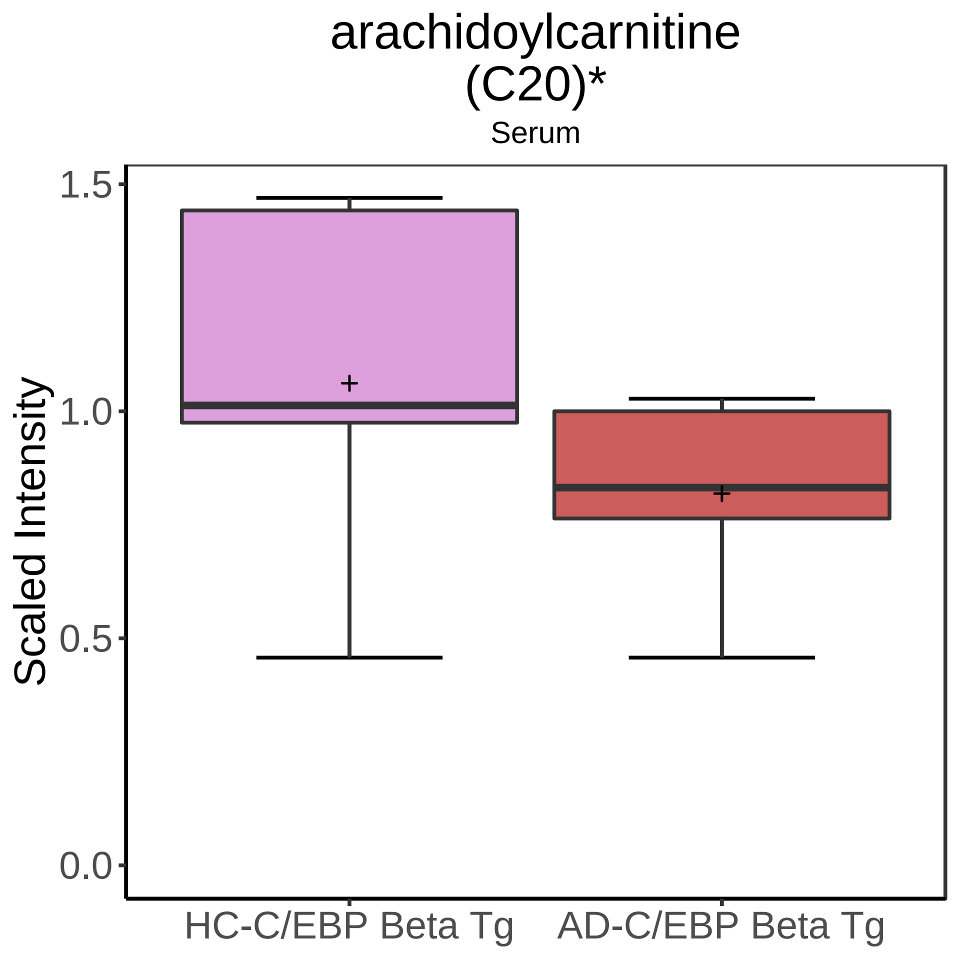
arachidoylcarnitine
(C20)*
Serum
Scaled Intensity
1.5
1.0
0.5
0.0
HC-C/EBP Beta Tg
AD-C/EBP Beta Tg

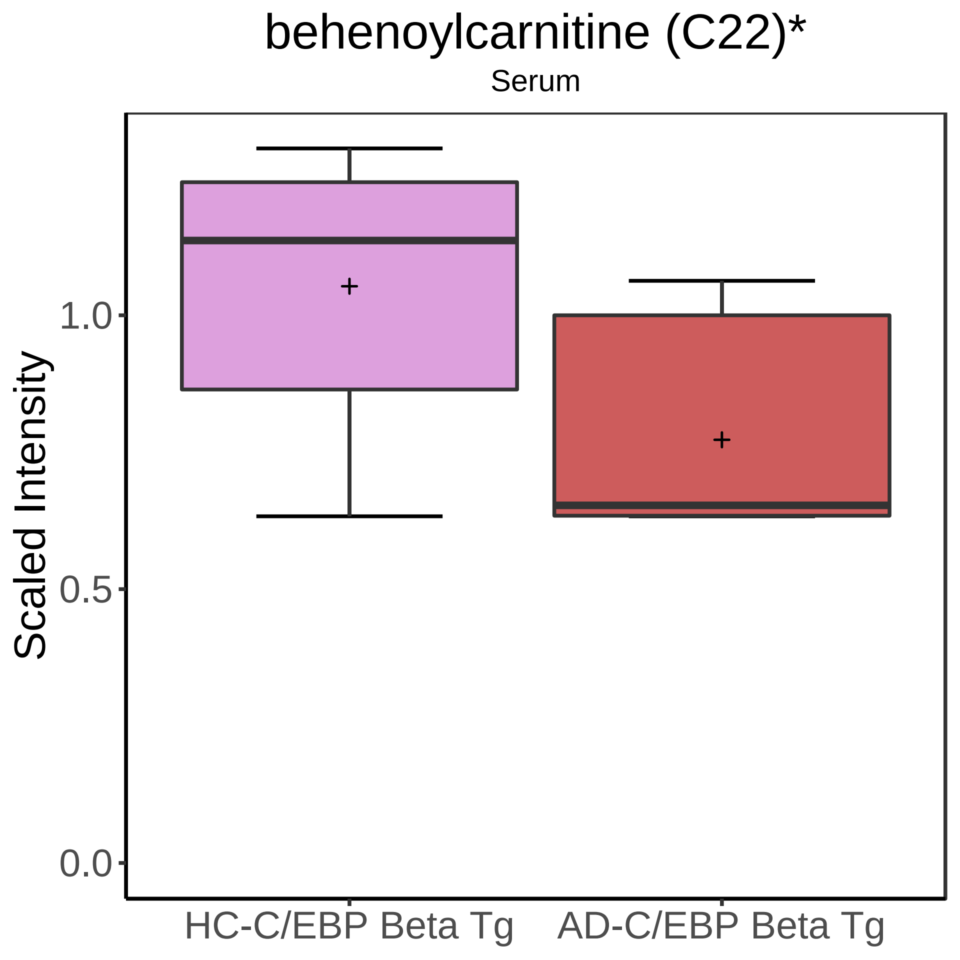
behenoylcarnitine (C22)*
Serum
Scaled Intensity
1.0
0.5
0.0
HC-C/EBP Beta Tg
AD-C/EBP Beta Tg

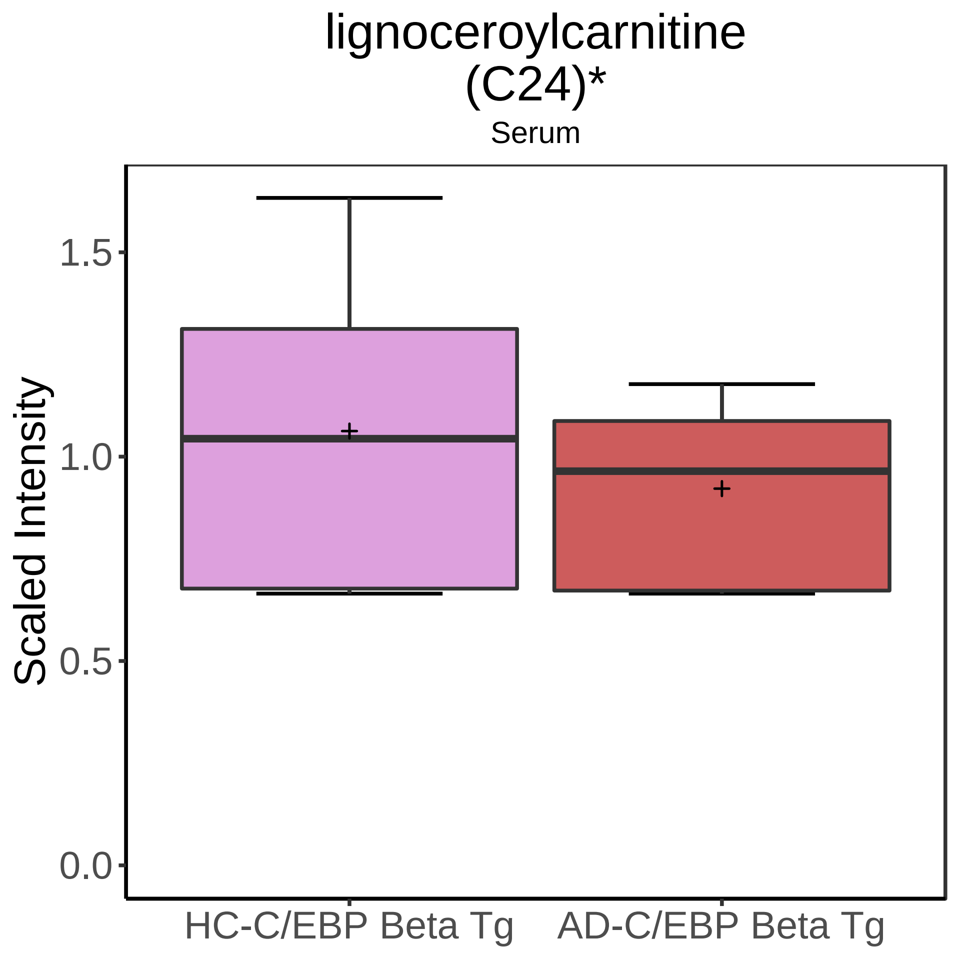
lignoceroylcarnitine
(C24)*
Serum
Scaled Intensity
1.5
1.0
0.5
0.0
HC-C/EBP Beta Tg
AD-C/EBP Beta Tg

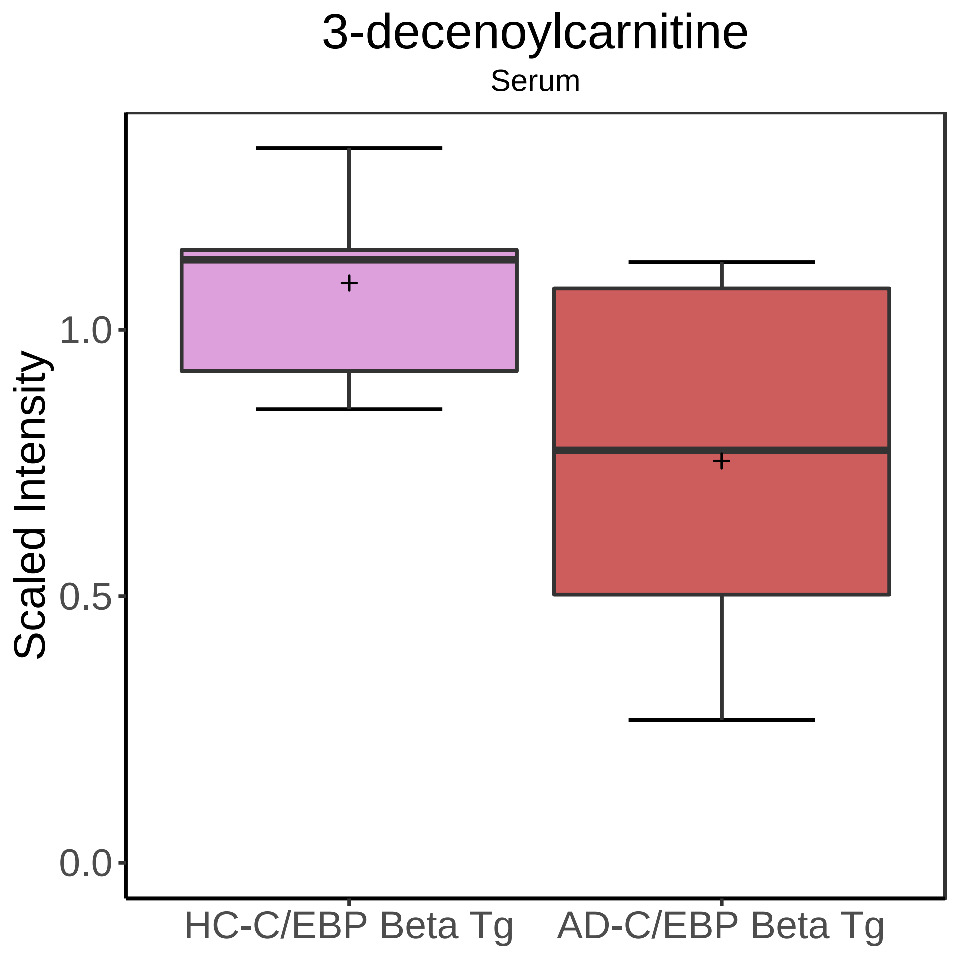
3-decenoylcarnitine
Serum
Scaled Intensity
1.0
0.5
0.0
HC-C/EBP Beta Tg
AD-C/EBP Beta Tg

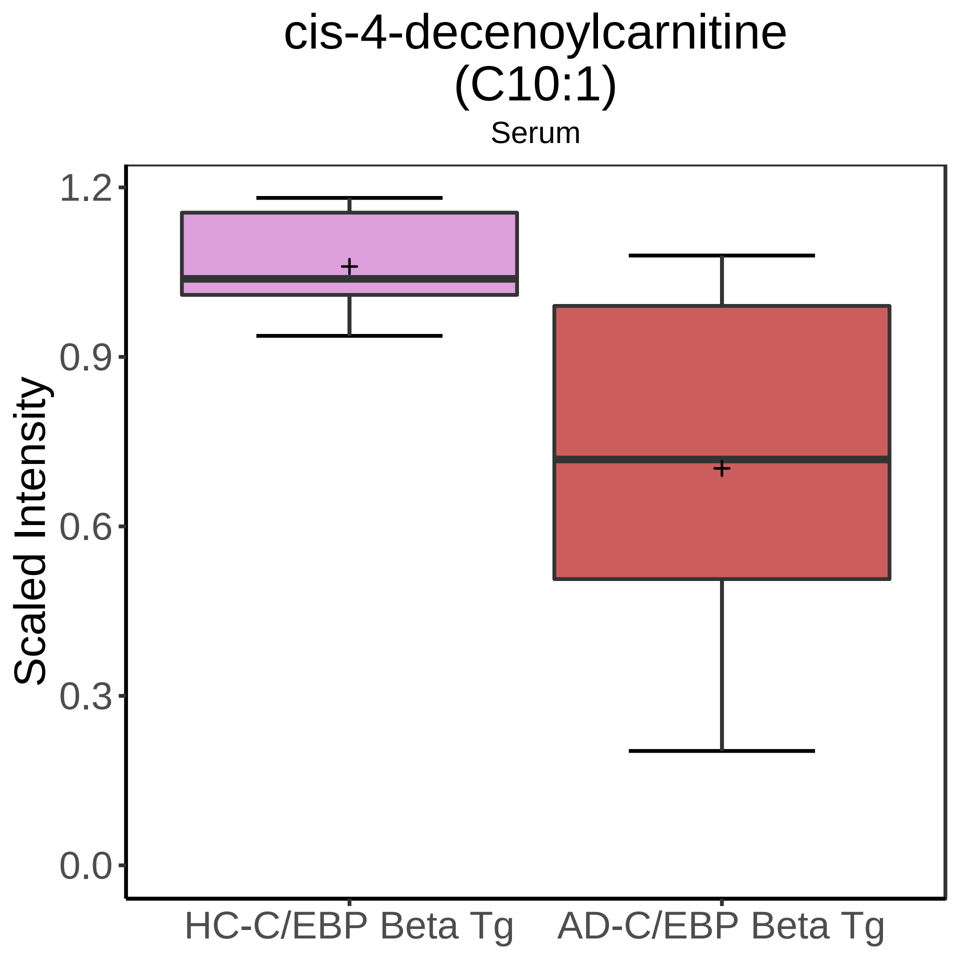

cis-4-decenoylcarnitine
(C10:1)
Serum
Scaled Intensity
1.2
0.9
0.6
0.3
0.0
HC-C/EBP Beta Tg
AD-C/EBP Beta Tg

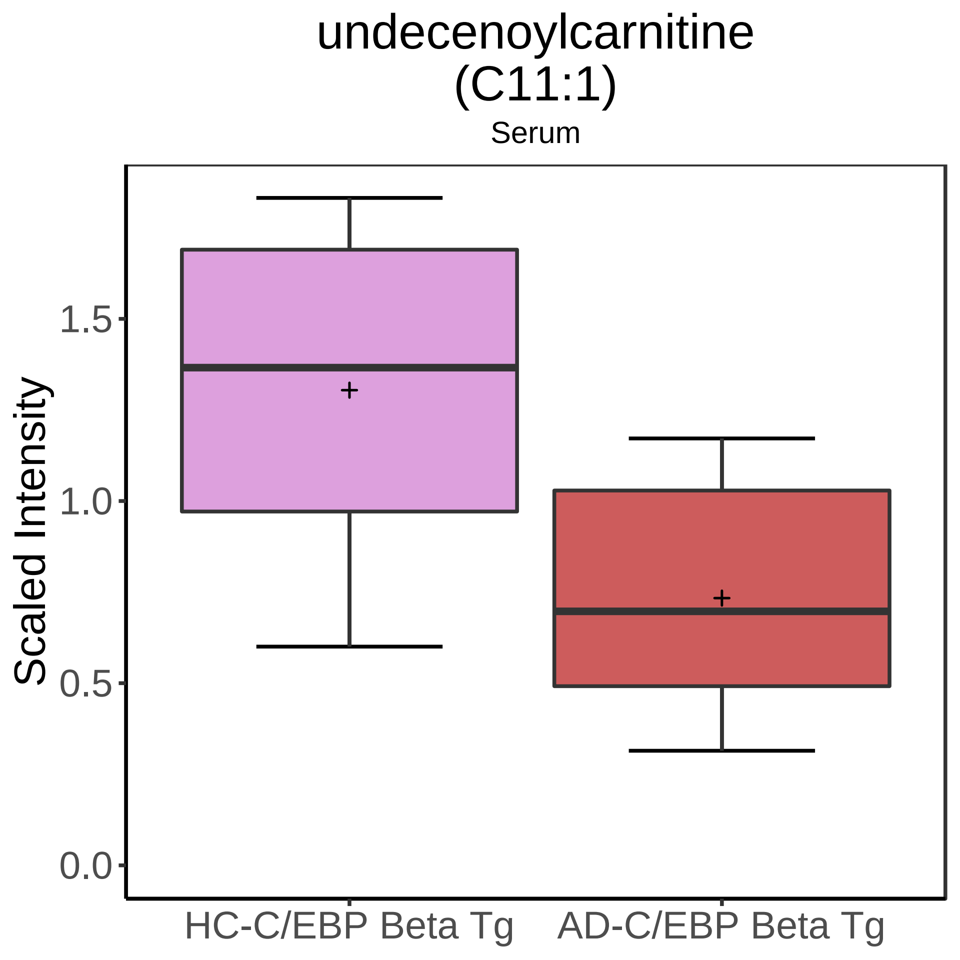
undecenoylcarnitine
(C11:1)
Serum
Scaled Intensity
0.0
0.5
1.0
1.5
HC-C/EBP Beta Tg
AD-C/EBP Beta Tg

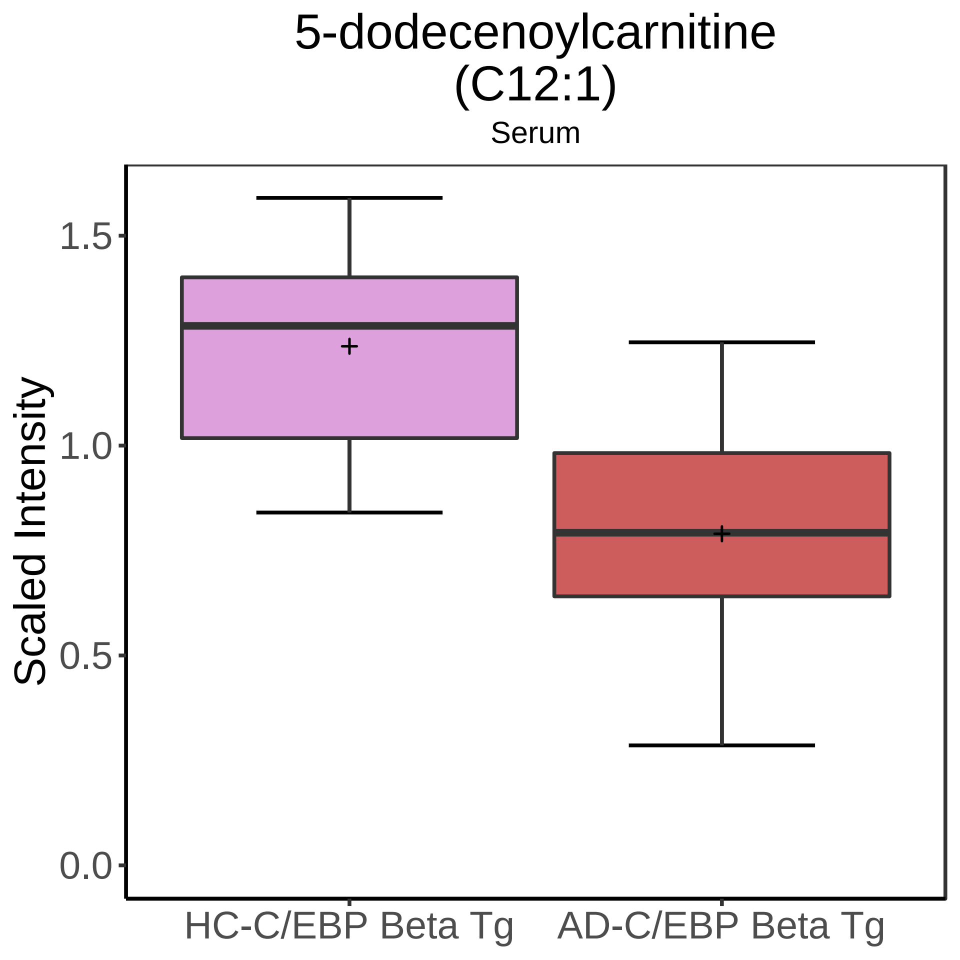
5-dodecenoylcarnitine
(C12:1)
Serum
Scaled Intensity
1.5
1.0
0.5
0.0
HC-C/EBP Beta Tg
AD-C/EBP Beta Tg

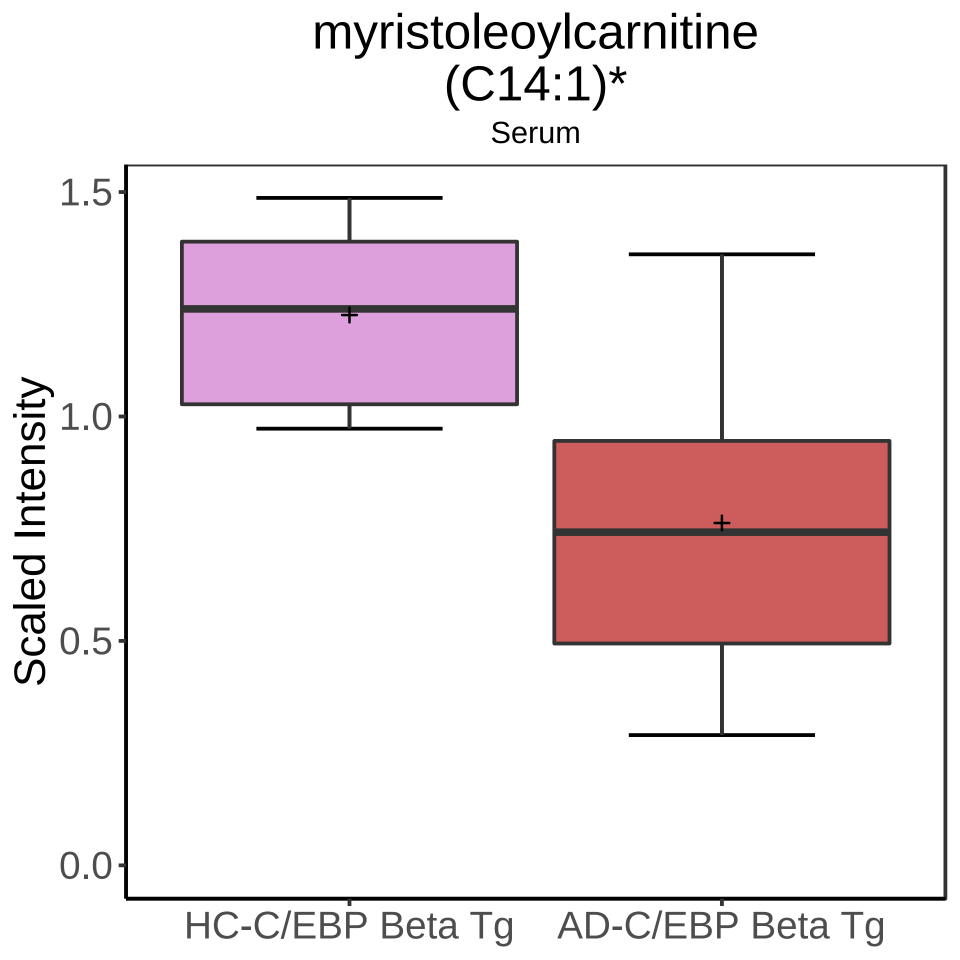
myristoleoylcarnitine
(C14:1)*
Serum
Scaled Intensity
1.5
1.0
0.5
0.0
HC-C/EBP Beta Tg
AD-C/EBP Beta Tg

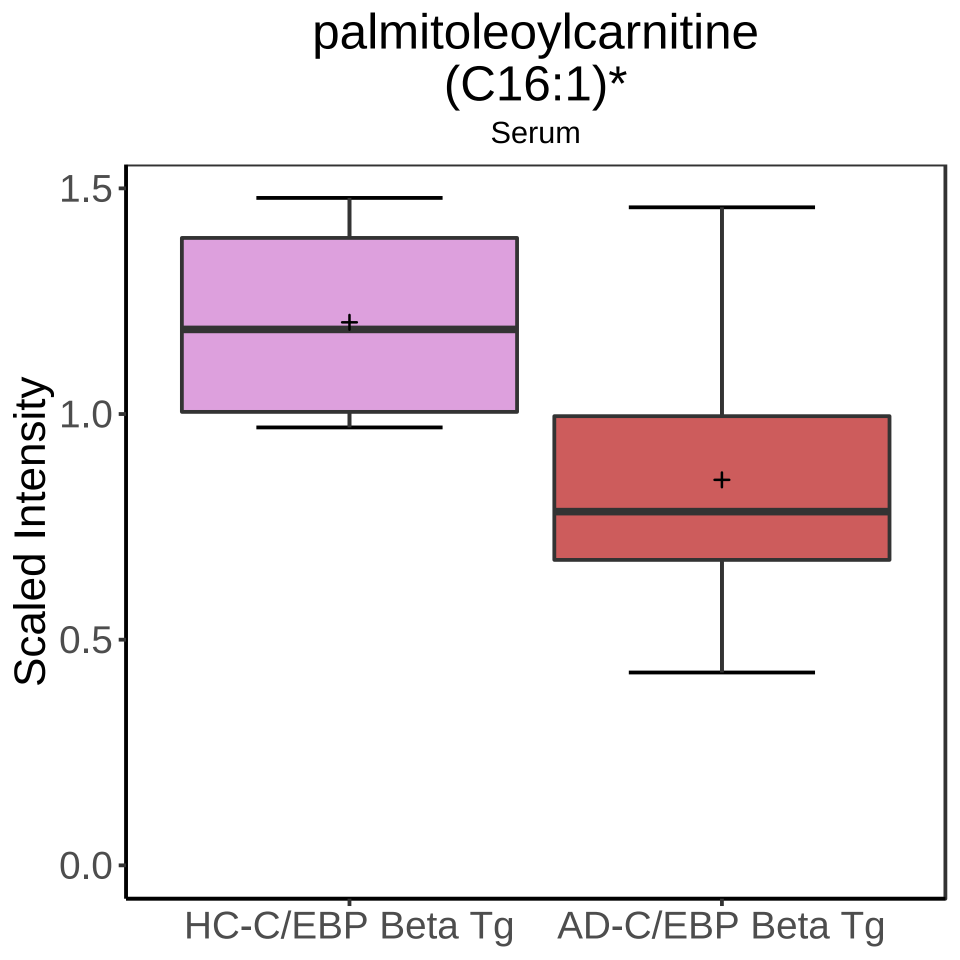

palmitoleoylcarnitine (C16:1)*
Serum
Scaled Intensity
1.5
1.0
0.5
0.0
HC-C/EBP Beta Tg
AD-C/EBP Beta Tg

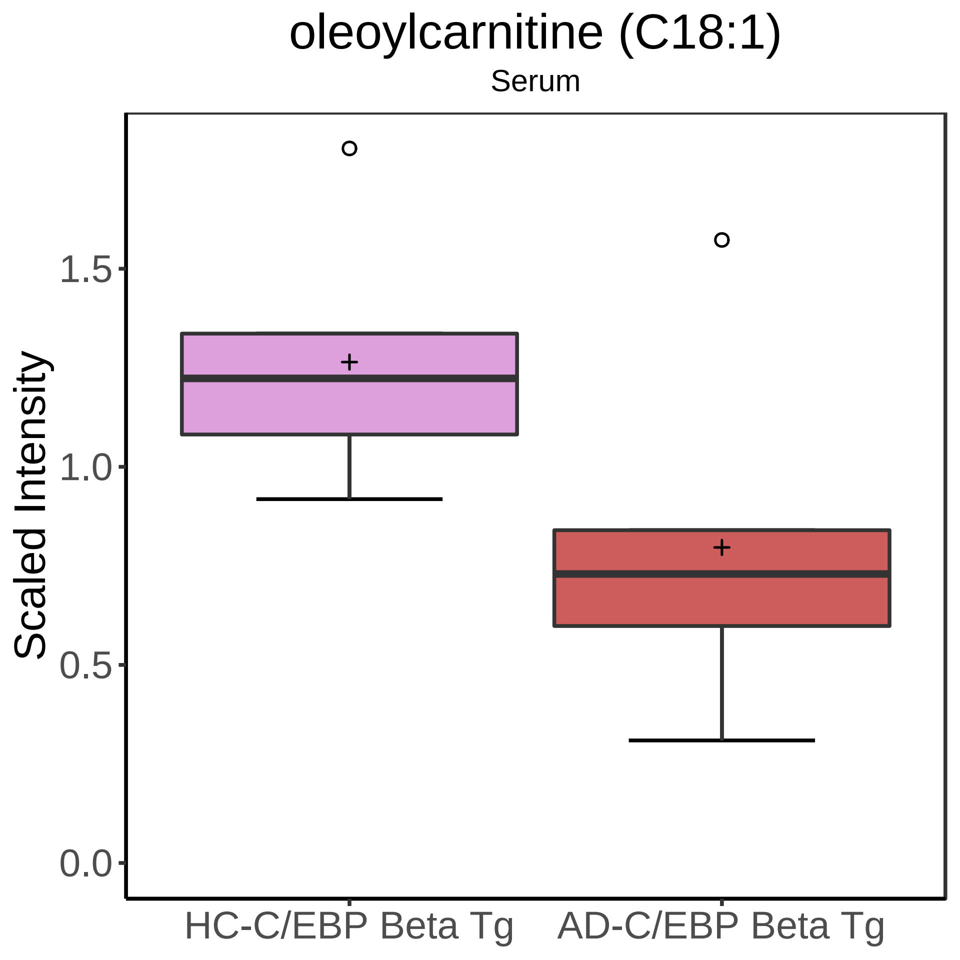
oleoylcarnitine (C18:1)
Serum
Scaled Intensity
1.5
1.0
0.5
0.0
HC-C/EBP Beta Tg
AD-C/EBP Beta Tg

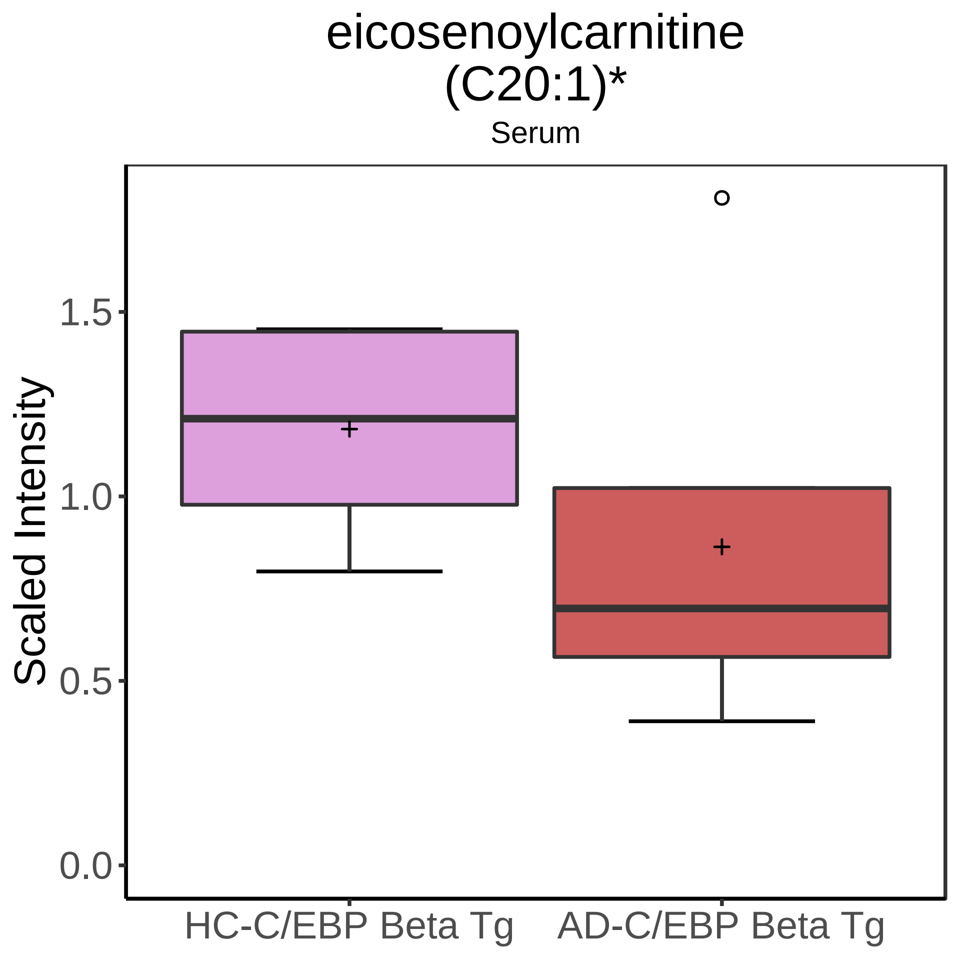

eicosenoylcarnitine
(C20:1)*
Serum
Scaled Intensity
1.5
1.0
0.5
0.0
HC-C/EBP Beta Tg
AD-C/EBP Beta Tg

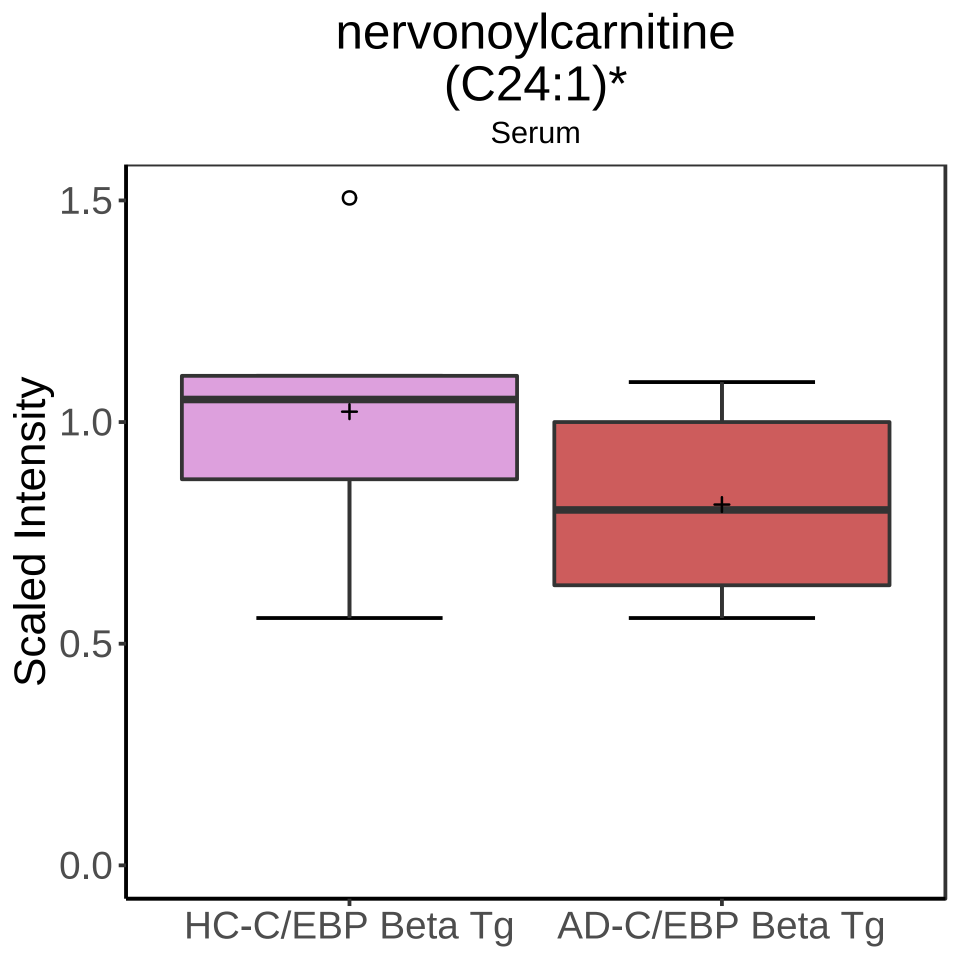

nervonoylcarnitine
(C24:1)*
Serum
Scaled Intensity
1.5
1.0
0.5
0.0
HC-C/EBP Beta Tg
AD-C/EBP Beta Tg

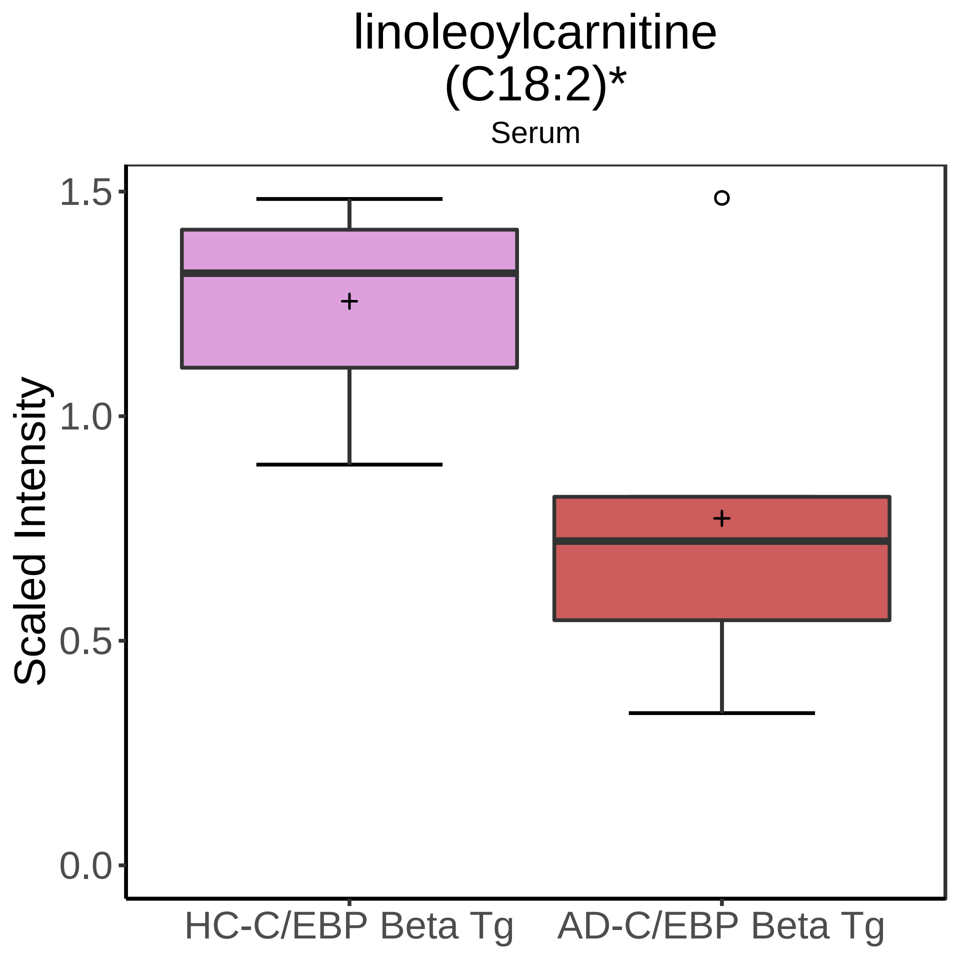
linoleoylcarnitine
(C18:2)*
Serum
Scaled Intensity
1.5
1.0
0.5
0.0
HC-C/EBP Beta Tg
AD-C/EBP Beta Tg

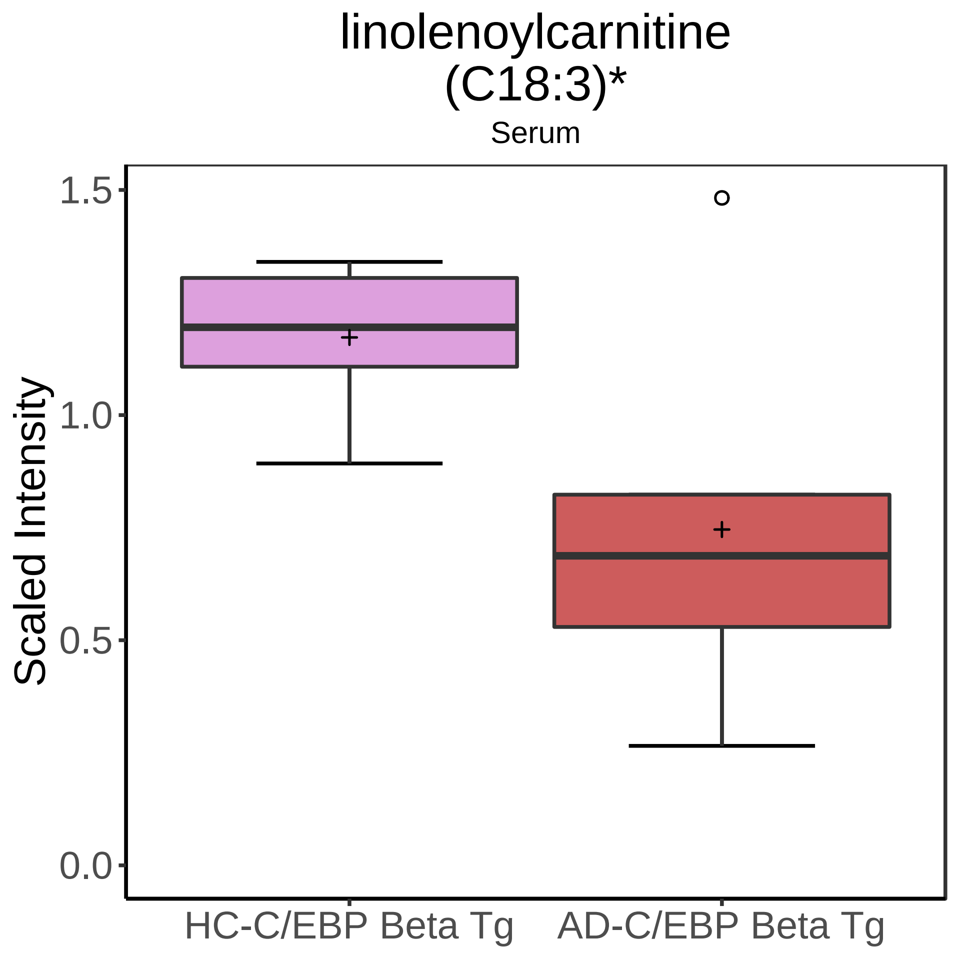
linolenoylcarnitine
(C18:3)*
Serum
Scaled Intensity
1.5
1.0
0.5
0.0
HC-C/EBP Beta Tg
AD-C/EBP Beta Tg

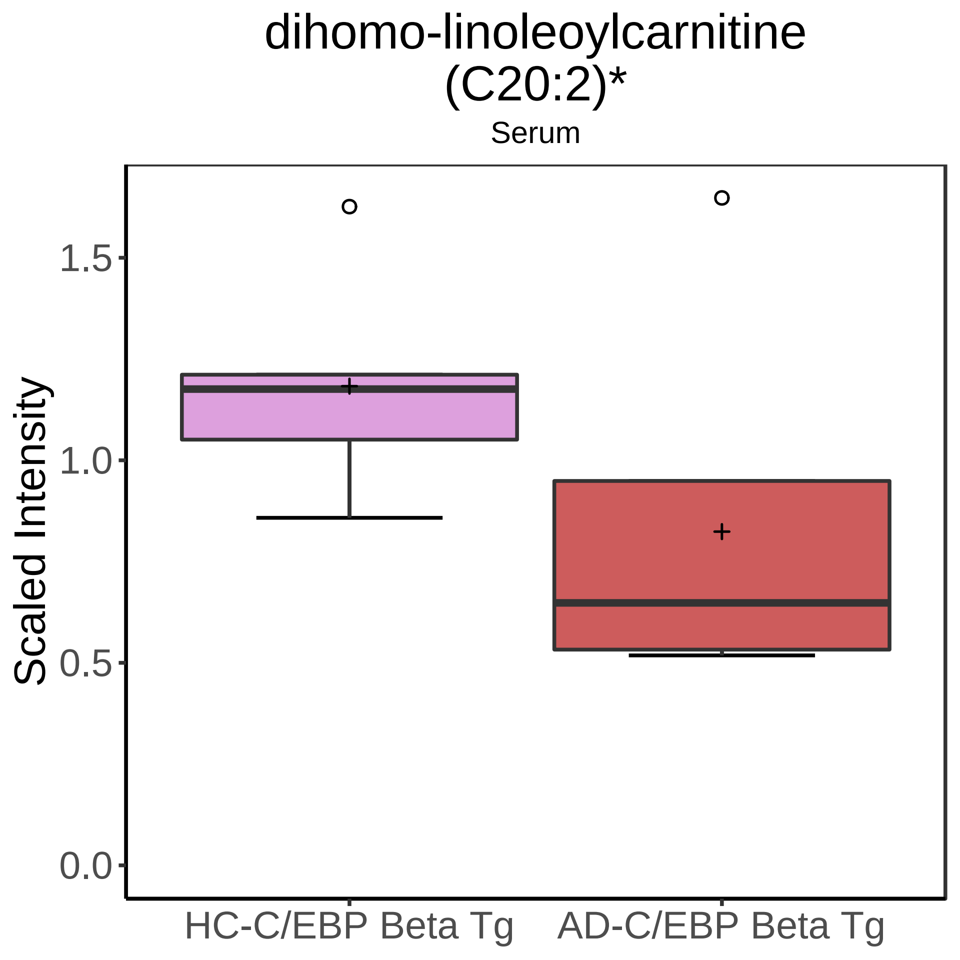
dihomo-linoleoylcarnitine
(C20:2)*
Serum
Scaled Intensity
1.5
1.0
0.5
0.0
HC-C/EBP Beta Tg
AD-C/EBP Beta Tg

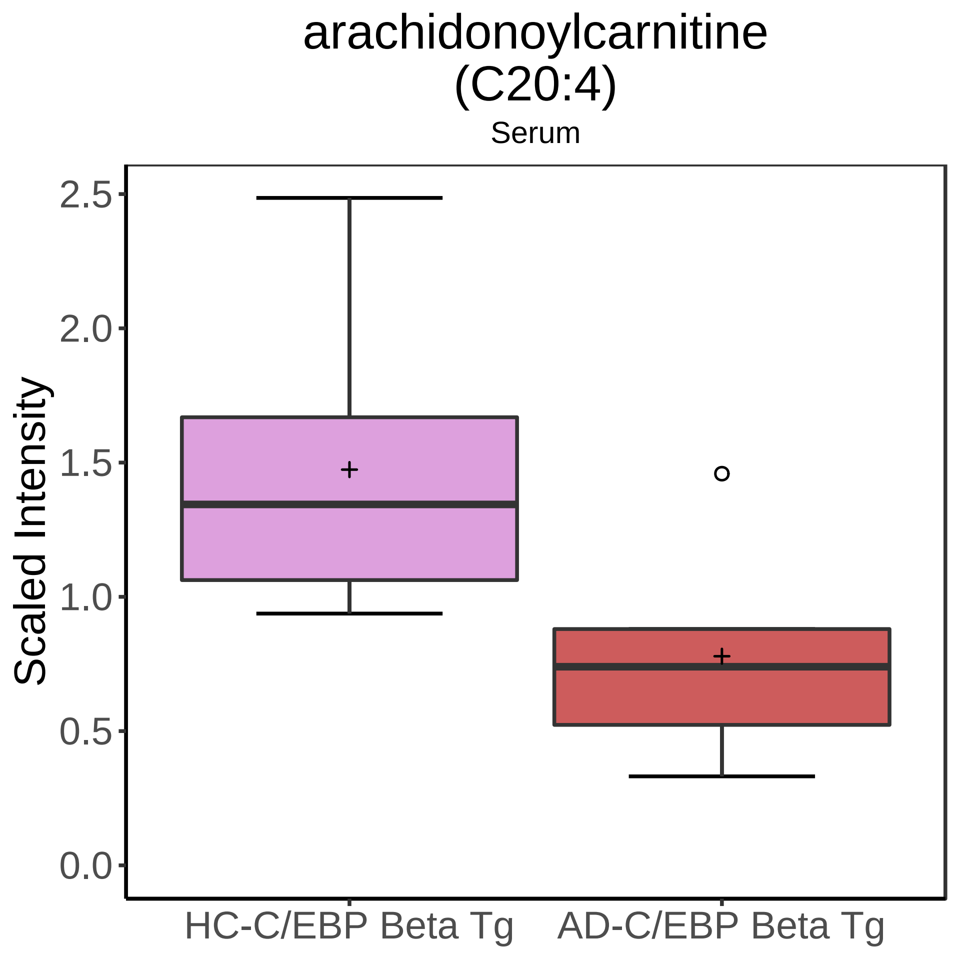
arachidonoylcarnitine
(C20:4)
Serum
Scaled Intensity
2.5
2.0
1.5
1.0
0.5
0.0
HC-C/EBP Beta Tg
AD-C/EBP Beta Tg

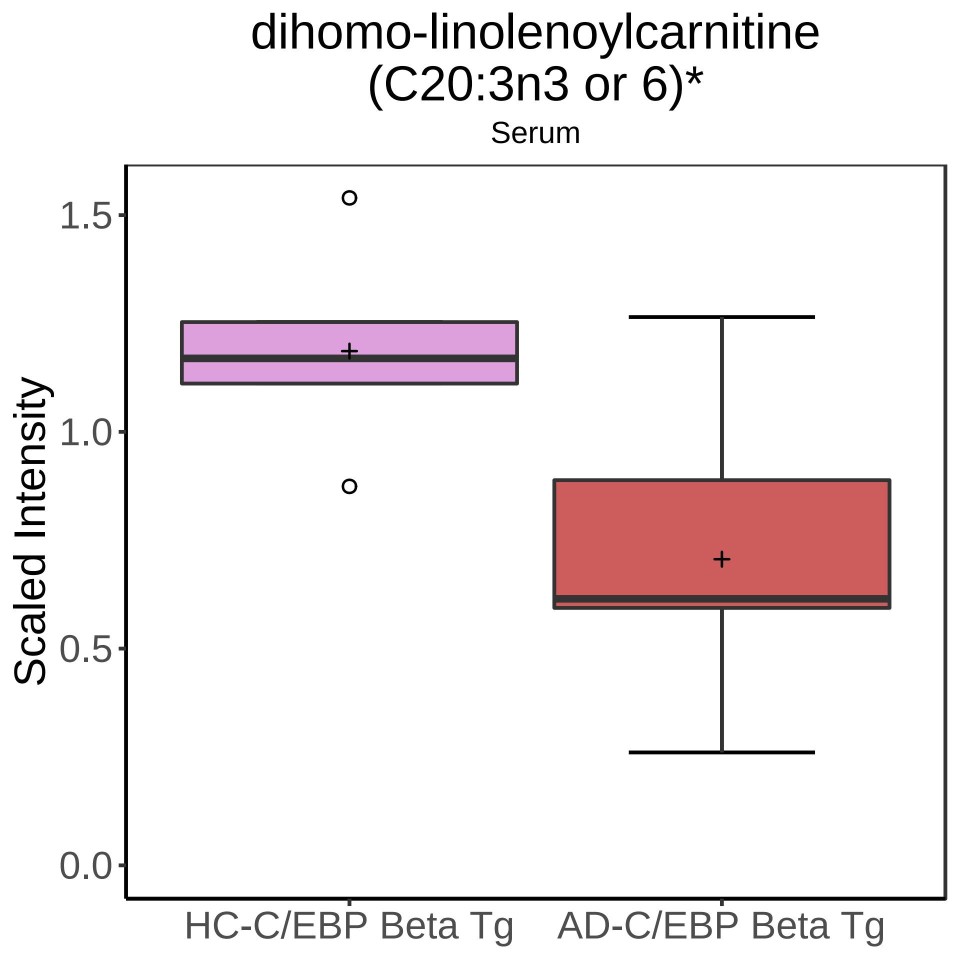
dihomo-linolenoylcarnitine
(C20:3n3 or 6)*
Serum
Scaled Intensity
1.5
1.0
0.5
0.0
HC-C/EBP Beta Tg
AD-C/EBP Beta Tg

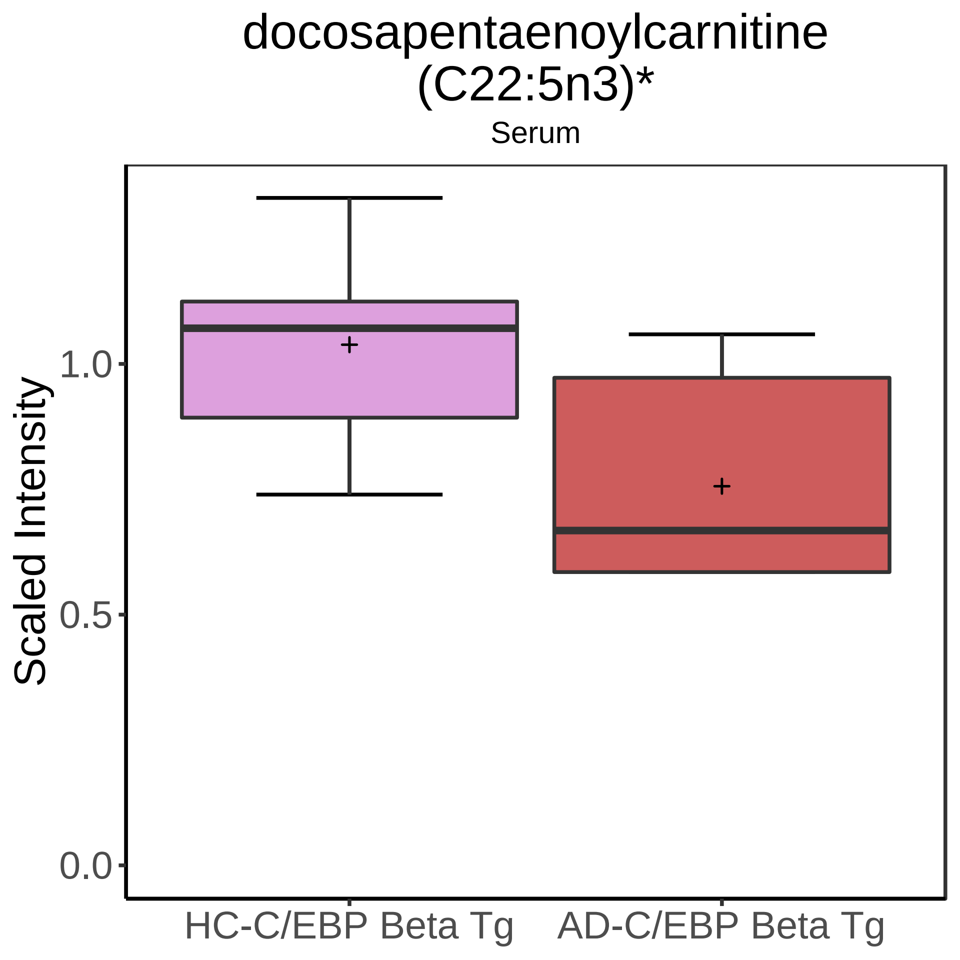
docosapentaenoylcarnitine
(C22:5n3)*
Serum
Scaled Intensity
1.0
0.5
0.0
HC-C/EBP Beta Tg
AD-C/EBP Beta Tg

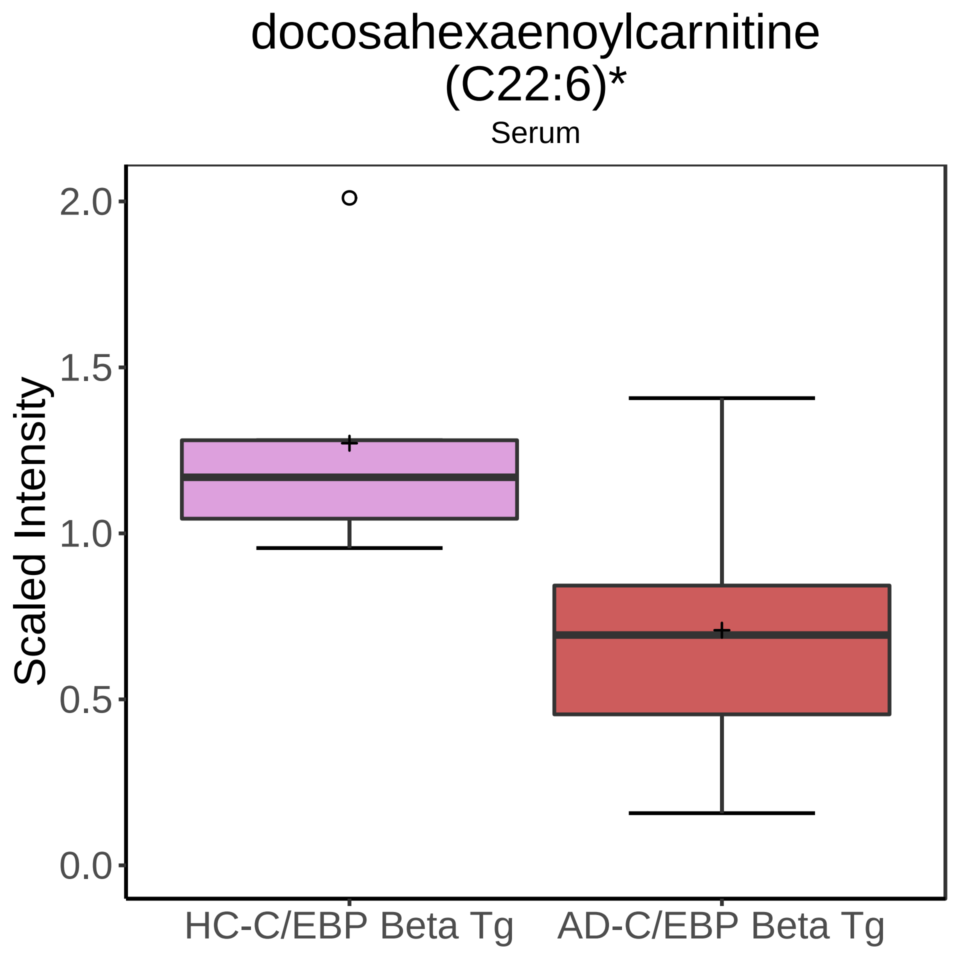
docosahexaenoylcarnitine
(C22:6)*
Serum
Scaled Intensity
2.0
1.5
1.0
0.5
0.0
HC-C/EBP Beta Tg
AD-C/EBP Beta Tg

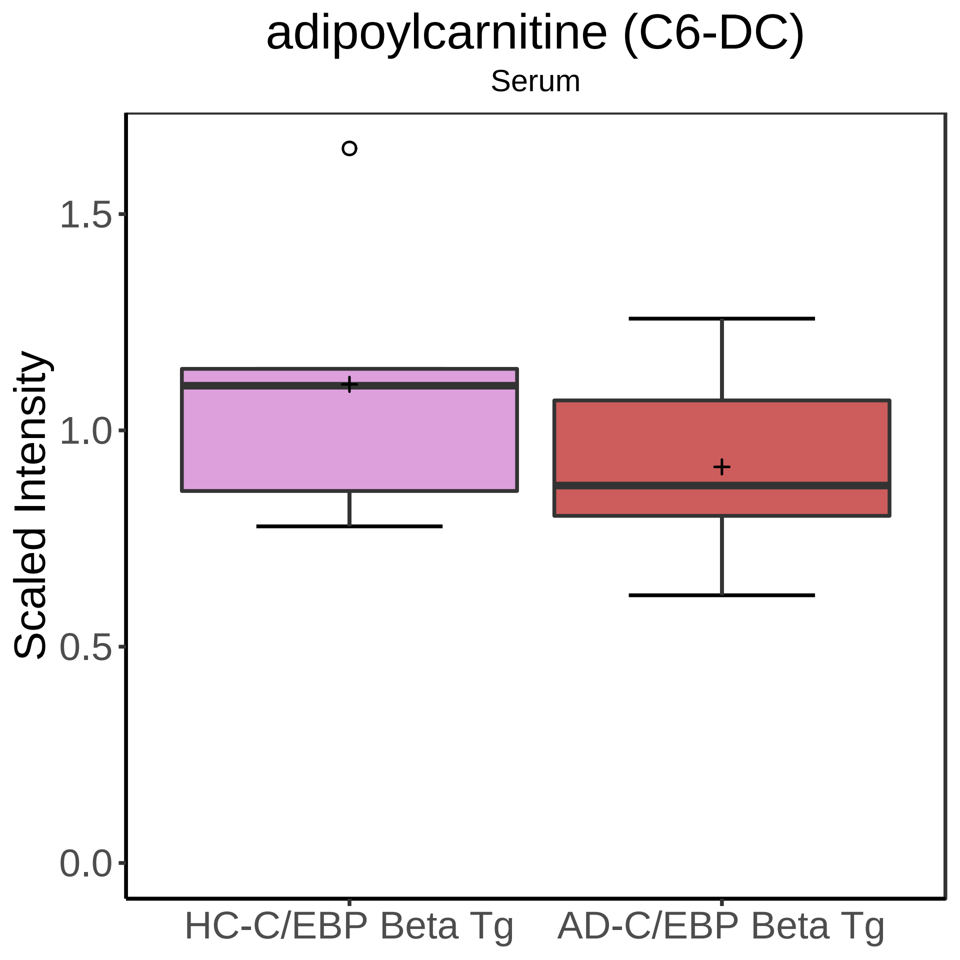
adipoylcarnitine (C6-DC)
Serum
Scaled Intensity
1.5
1.0
0.5
0.0
HC-C/EBP Beta Tg
AD-C/EBP Beta Tg

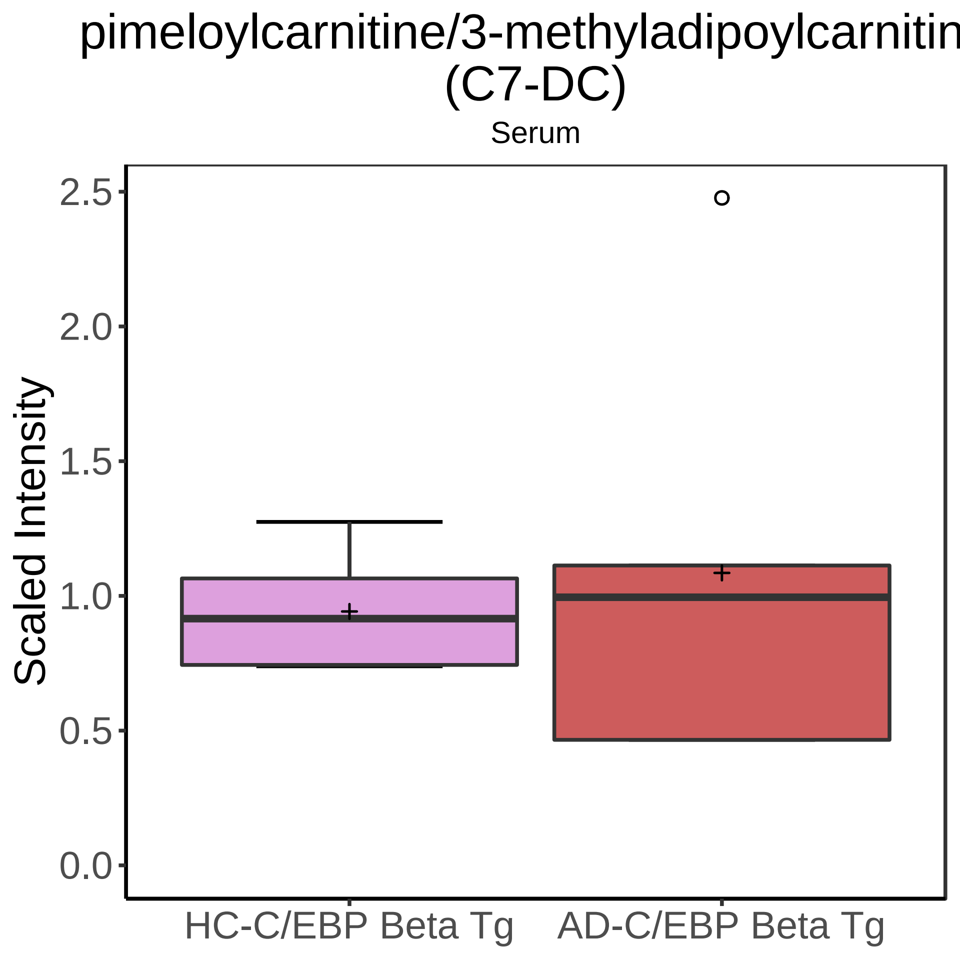

pimeloylcarnitine/3-methyladipoylcarnitin
(C7-DC)
Serum
Scaled Intensity
2.5
2.0
1.5
1.0
0.5
0.0
HC-C/EBP Beta Tg
AD-C/EBP Beta Tg

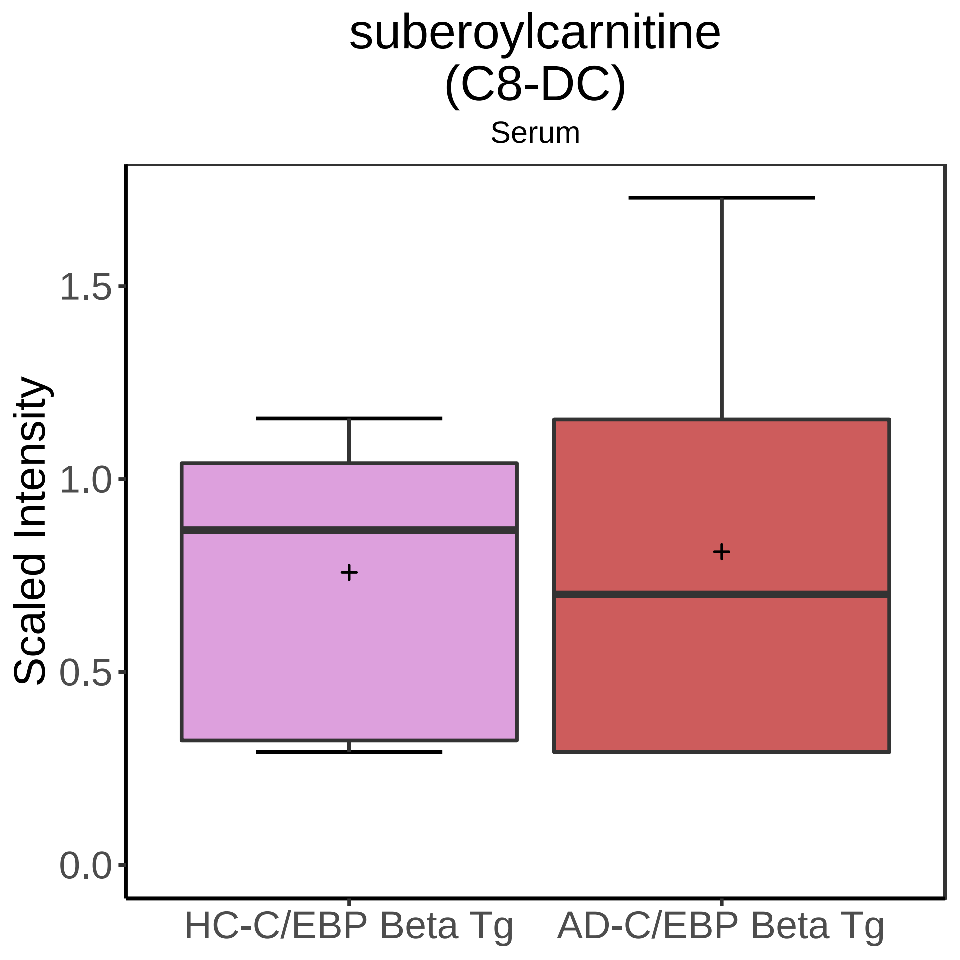
suberoylcarnitine
(C8-DC)
Serum
Scaled Intensity
0.0
0.5
1.0
1.5
HC-C/EBP Beta Tg
AD-C/EBP Beta Tg

octadecanedioylcarnitine (C18-DC)*

Serum

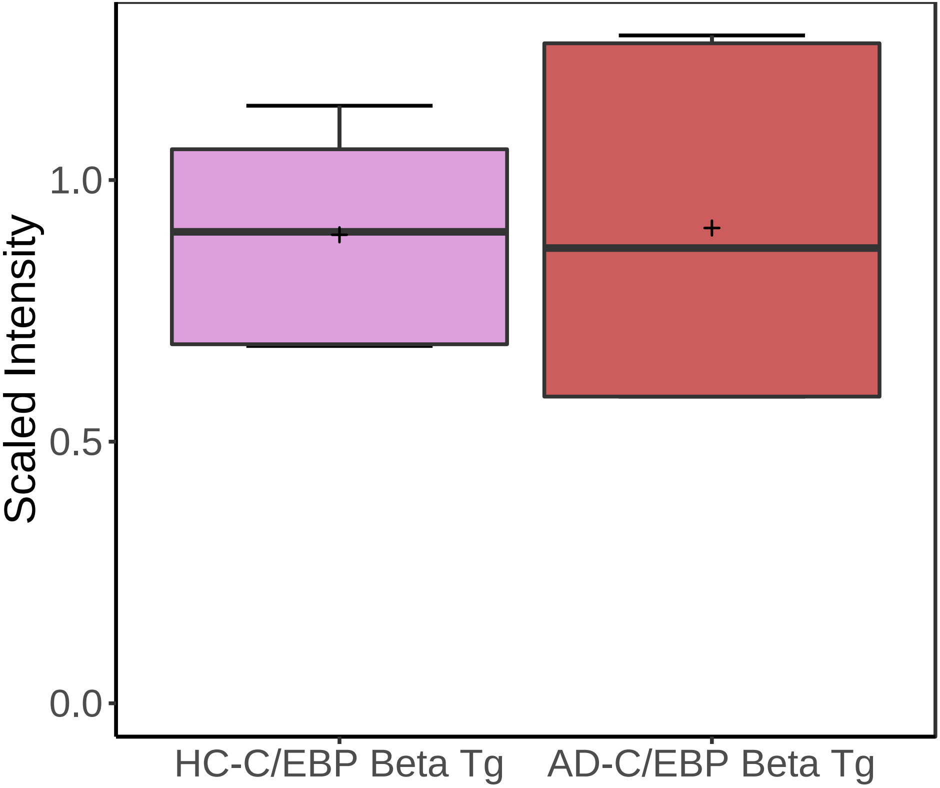

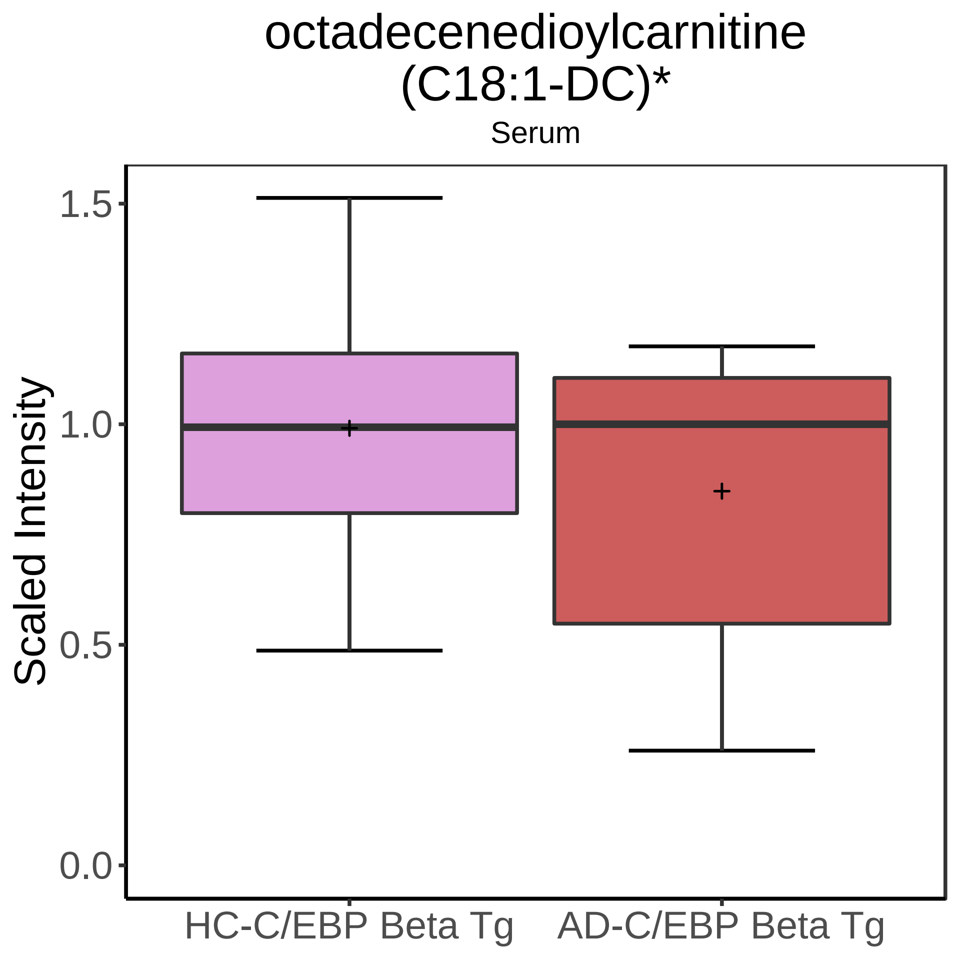
octadecenedioylcarnitine
(C18:1-DC)*
Serum
Scaled Intensity
1.5
1.0
0.5
0.0
HC-C/EBP Beta Tg
AD-C/EBP Beta Tg

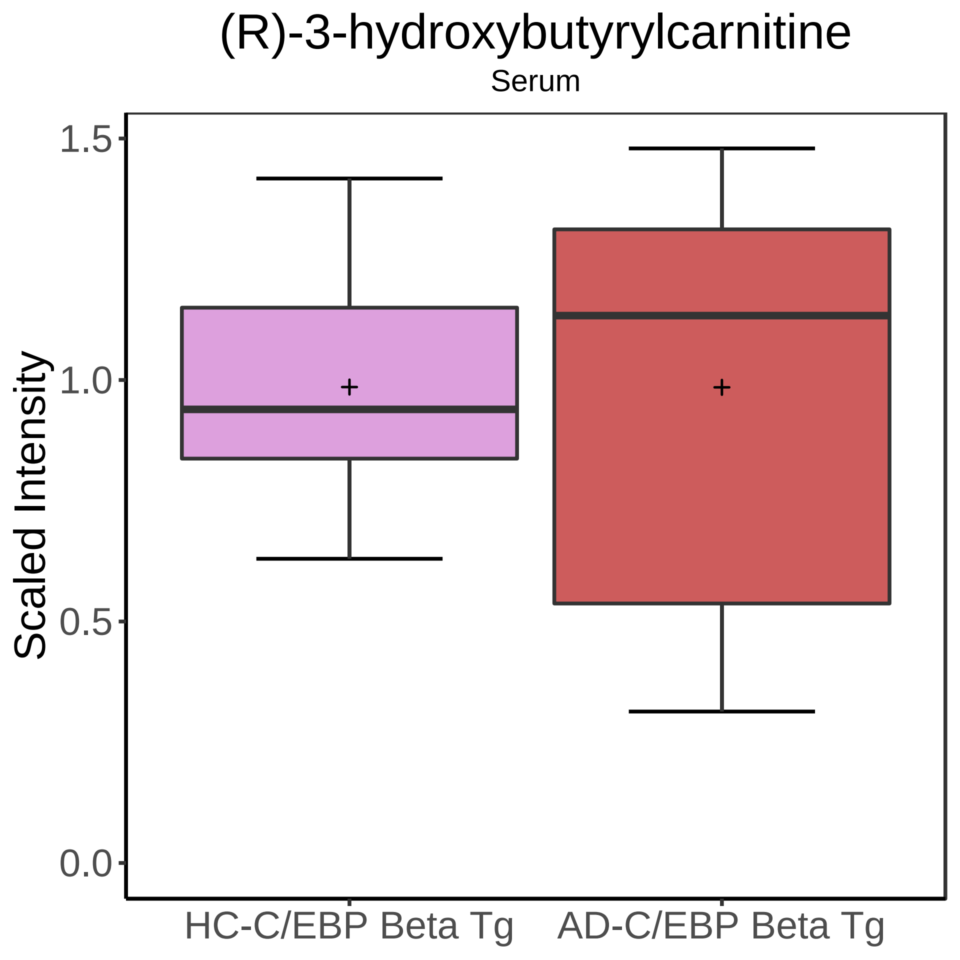
(R)-3-hydroxybutyrylcarnitine
Serum
Scaled Intensity
1.5
1.0
0.5
0.0
HC-C/EBP Beta Tg
AD-C/EBP Beta Tg

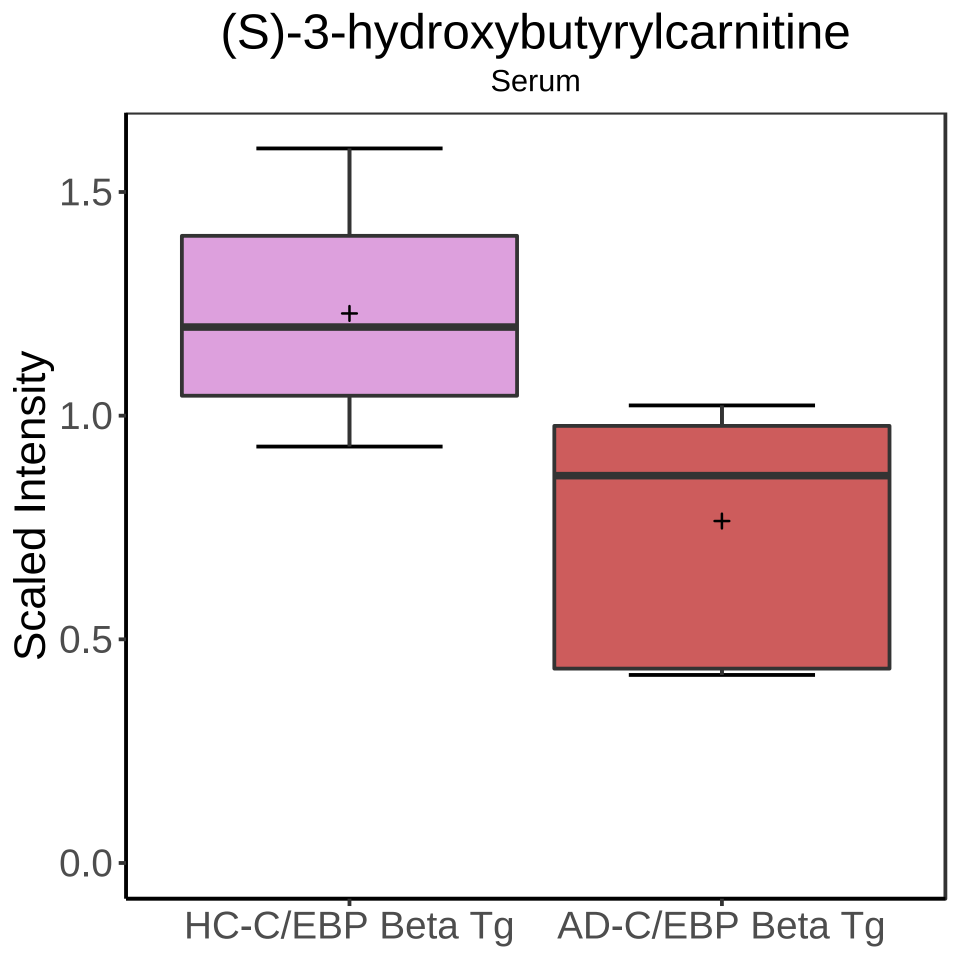
(S)-3-hydroxybutyrylcarnitine
Serum
Scaled Intensity
1.5
1.0
0.5
0.0
HC-C/EBP Beta Tg
AD-C/EBP Beta Tg

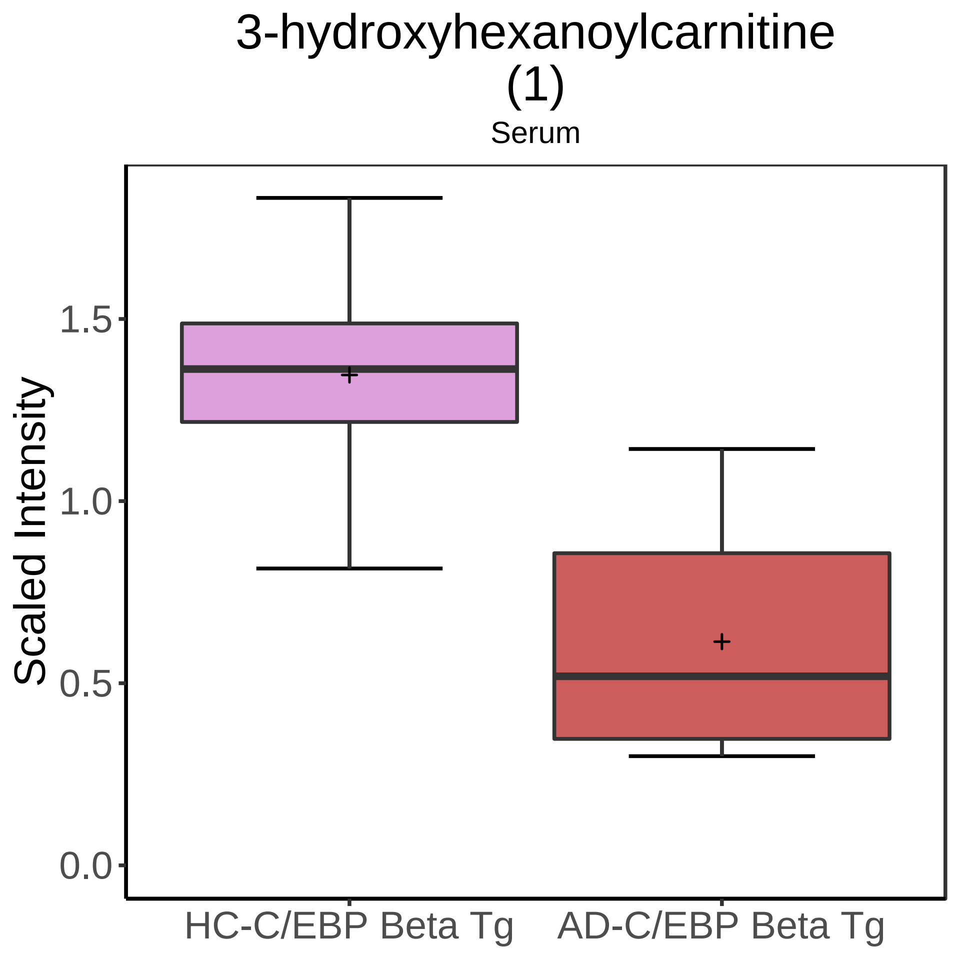
3-hydroxyhexanoylcarnitine (1)
Serum
Scaled Intensity
0.0
0.5
1.0
1.5
HC-C/EBP Beta Tg
AD-C/EBP Beta Tg

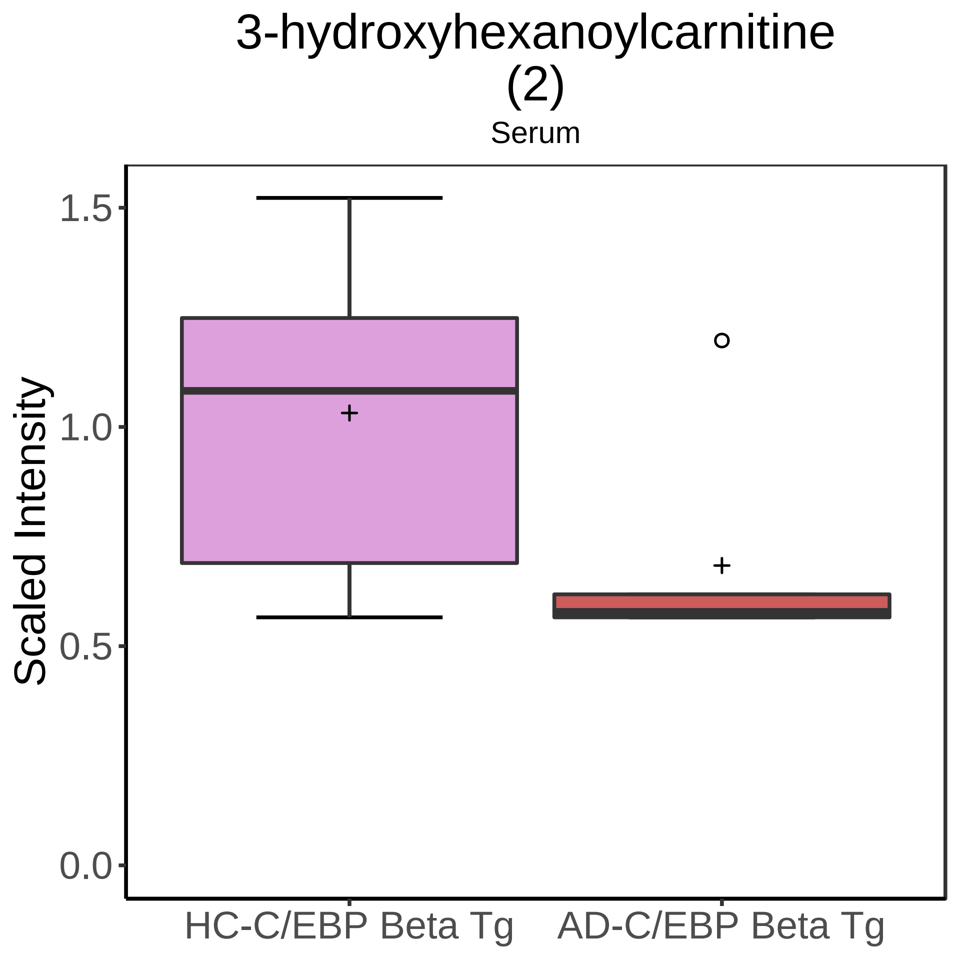

3-hydroxyhexanoylcarnitine (2)
Serum
Scaled Intensity
1.5
1.0
0.5
0.0
HC-C/EBP Beta Tg
AD-C/EBP Beta Tg

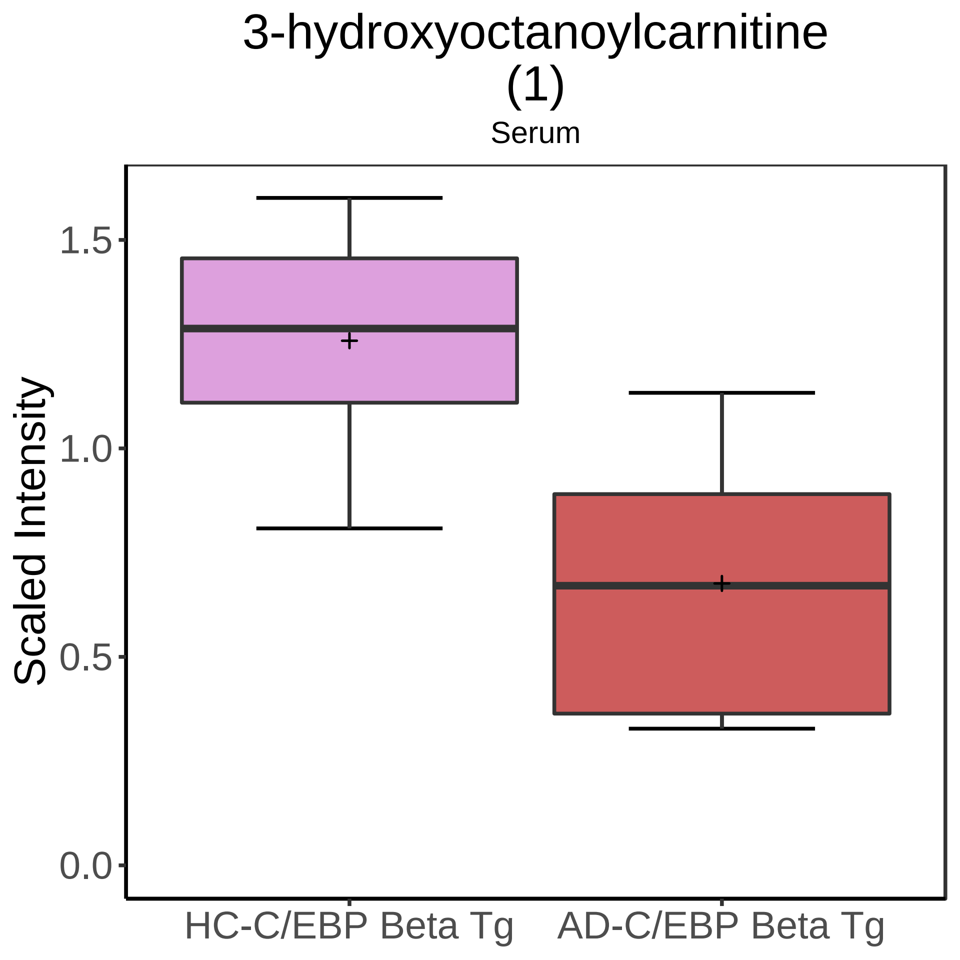
3-hydroxyoctanoylcarnitine
(1)
Serum
Scaled Intensity
1.5
1.0
0.5
0.0
HC-C/EBP Beta Tg
AD-C/EBP Beta Tg

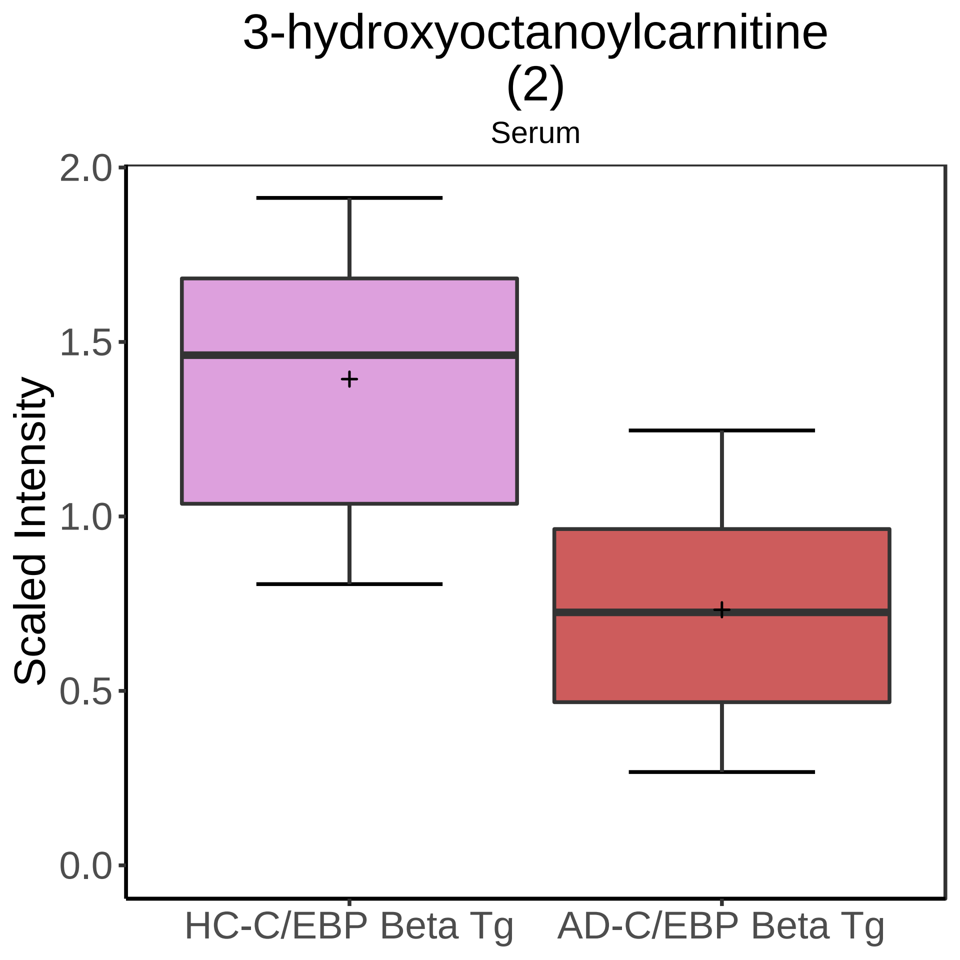
3-hydroxyoctanoylcarnitine (2)
Serum
Scaled Intensity
2.0
1.5
1.0
0.5
0.0
HC-C/EBP Beta Tg
AD-C/EBP Beta Tg

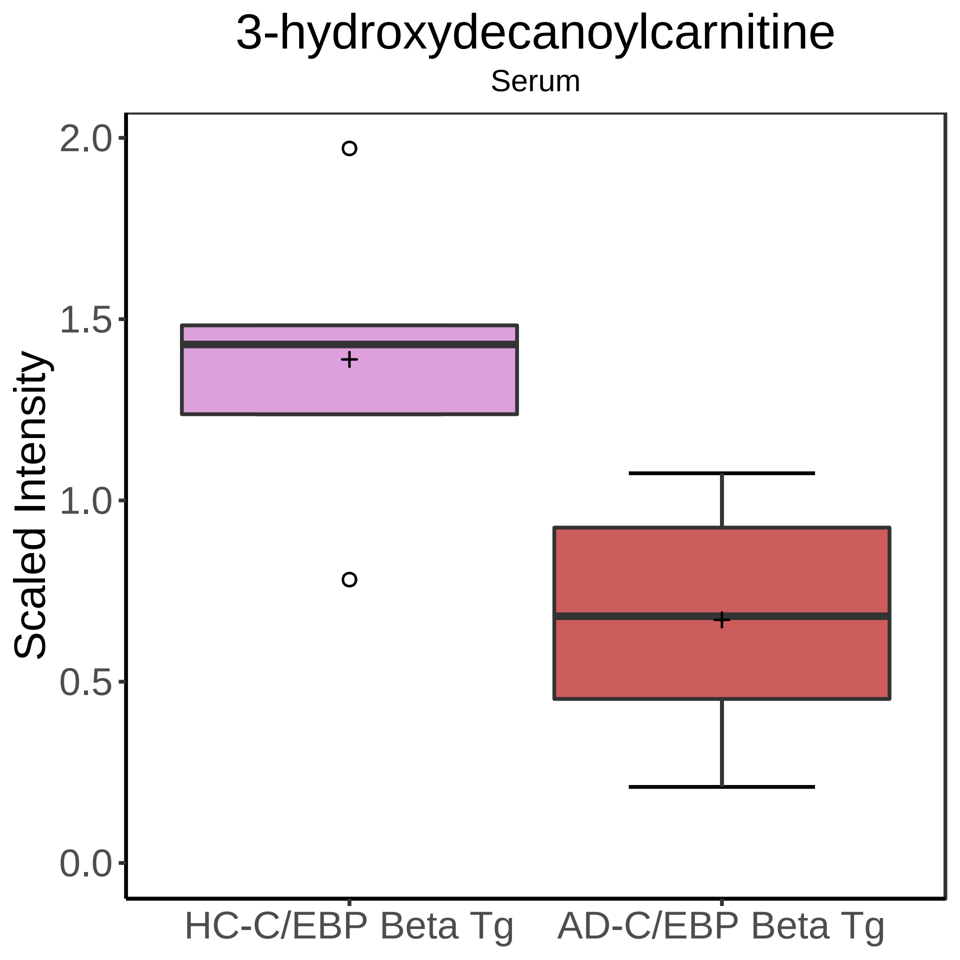
3-hydroxydecanoylcarnitine
Serum
Scaled Intensity
2.0
1.5
1.0
0.5
0.0
HC-C/EBP Beta Tg
AD-C/EBP Beta Tg

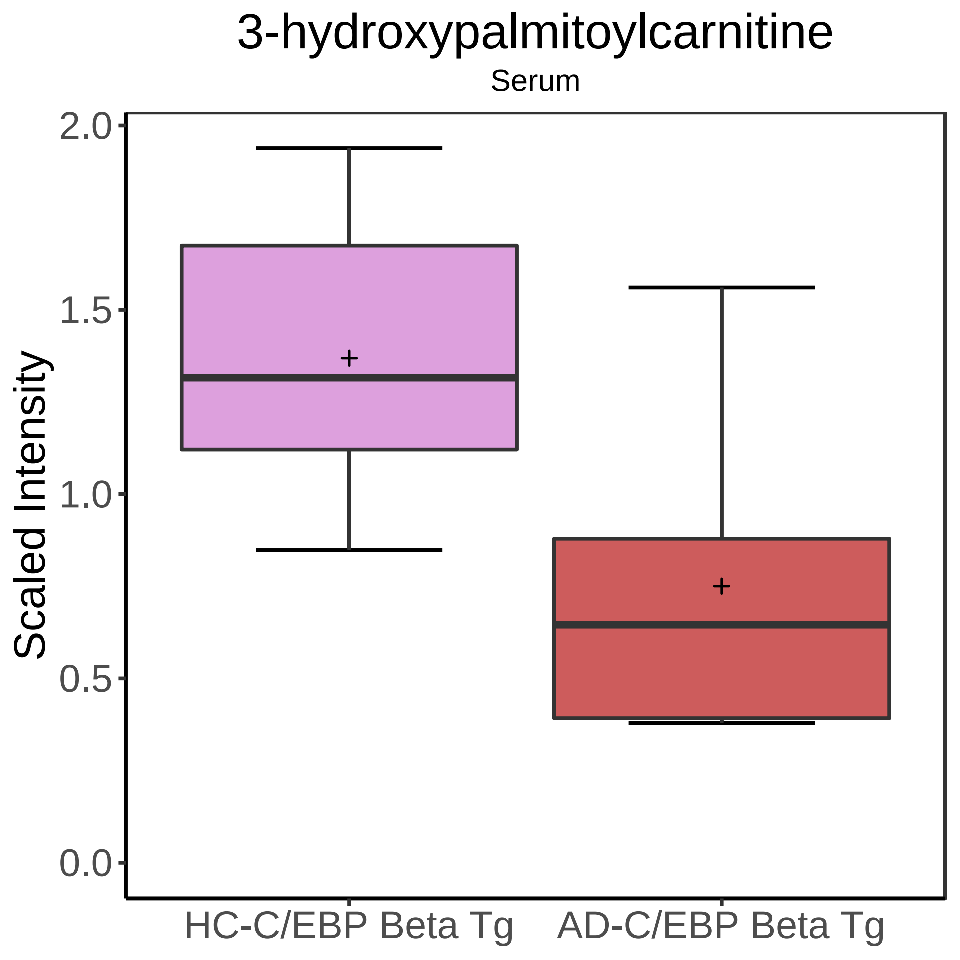
3-hydroxypalmitoylcarnitine
Serum
Scaled Intensity
2.0
1.5
1.0
0.5
0.0
HC-C/EBP Beta Tg
AD-C/EBP Beta Tg

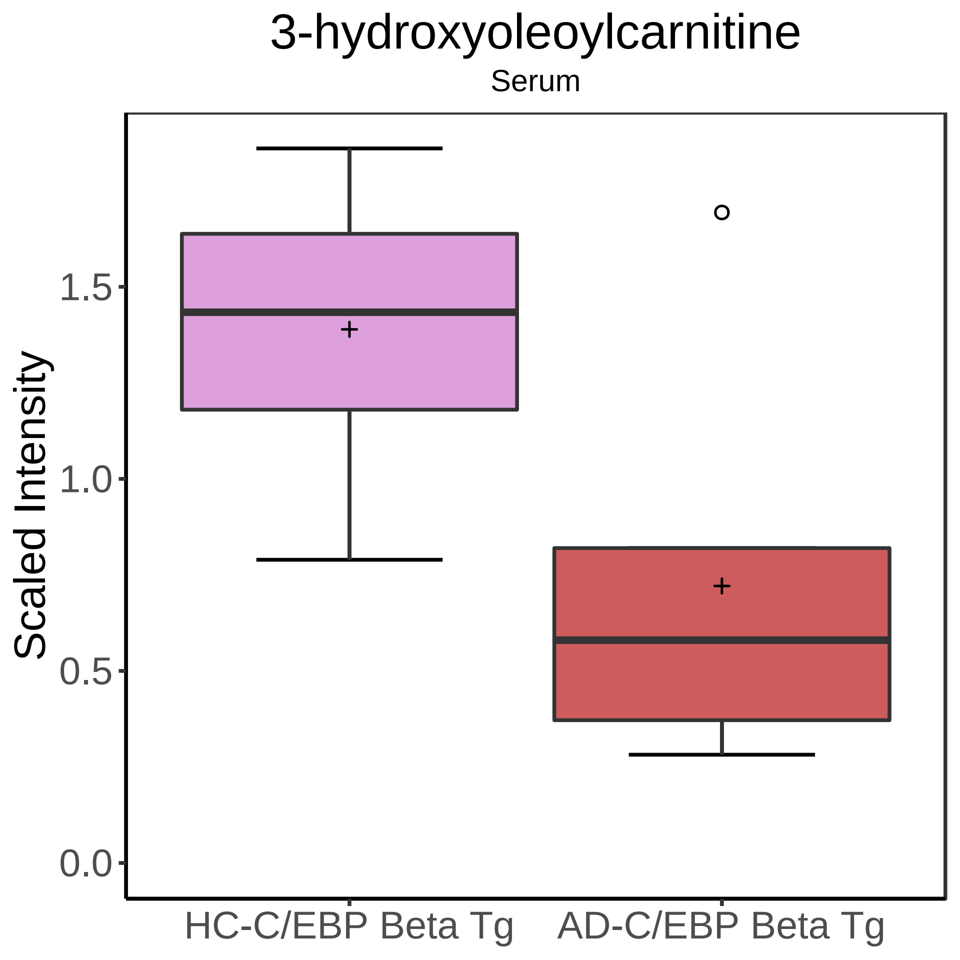

3-hydroxyoleoylcarnitine
Serum
Scaled Intensity
0.0
0.5
1.0
1.5
HC-C/EBP Beta Tg
AD-C/EBP Beta Tg

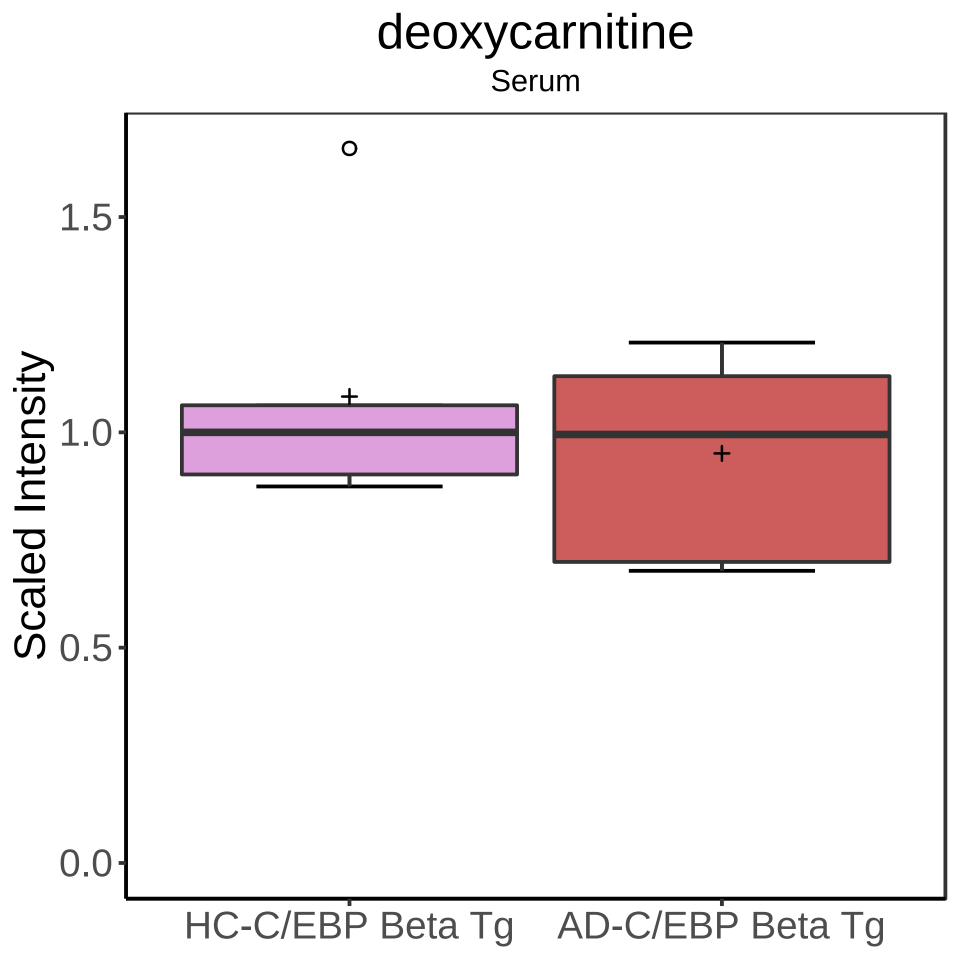
deoxycarnitine
Serum
Scaled Intensity
1.5
1.0
0.5
0.0
HC-C/EBP Beta Tg
AD-C/EBP Beta Tg

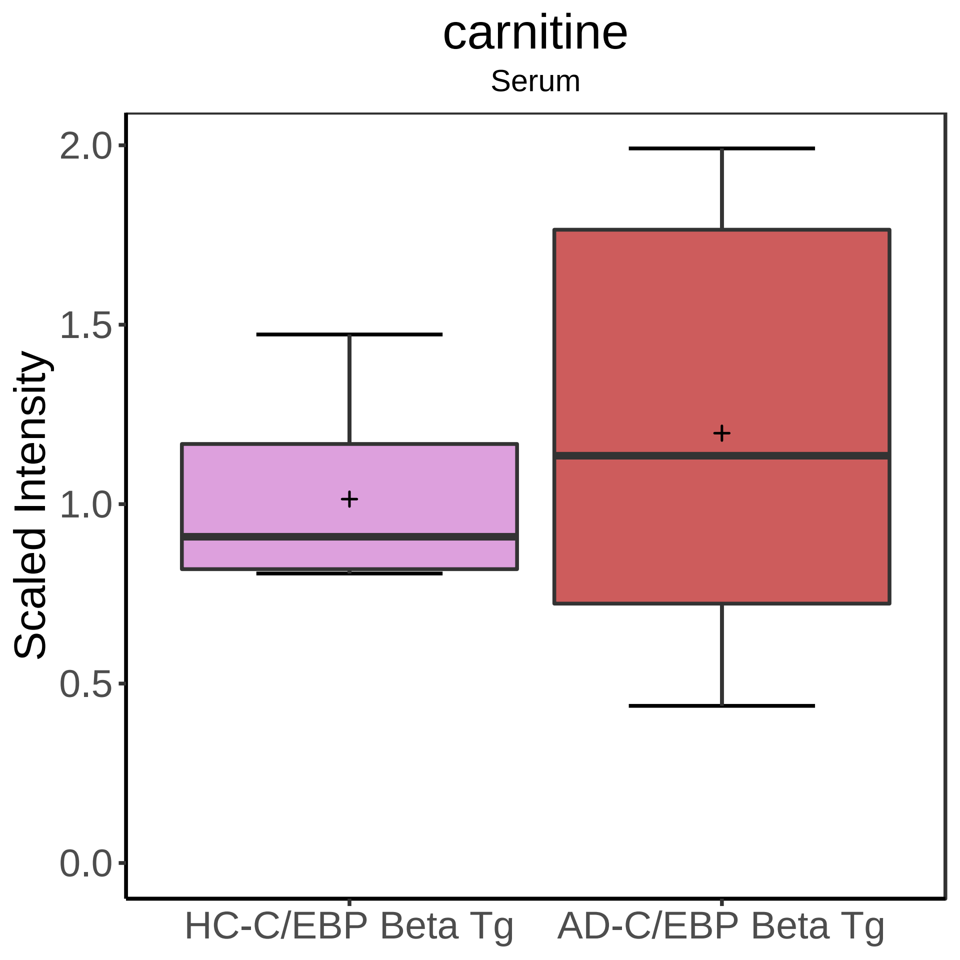
carnitine
Serum
Scaled Intensity
2.0
1.5
1.0
0.5
0.0
HC-C/EBP Beta Tg
AD-C/EBP Beta Tg

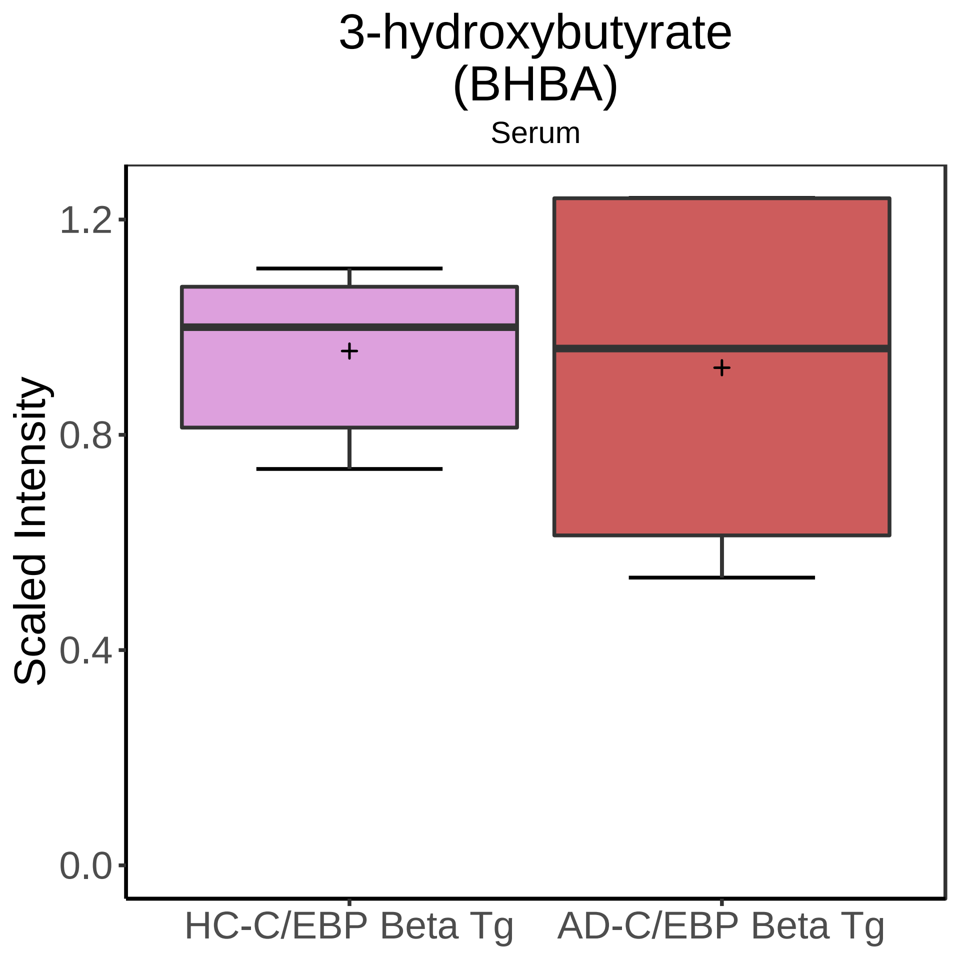
3-hydroxybutyrate
(BHBA)
Serum
Scaled Intensity
1.2
0.8
0.4
0.0
HC-C/EBP Beta Tg
AD-C/EBP Beta Tg

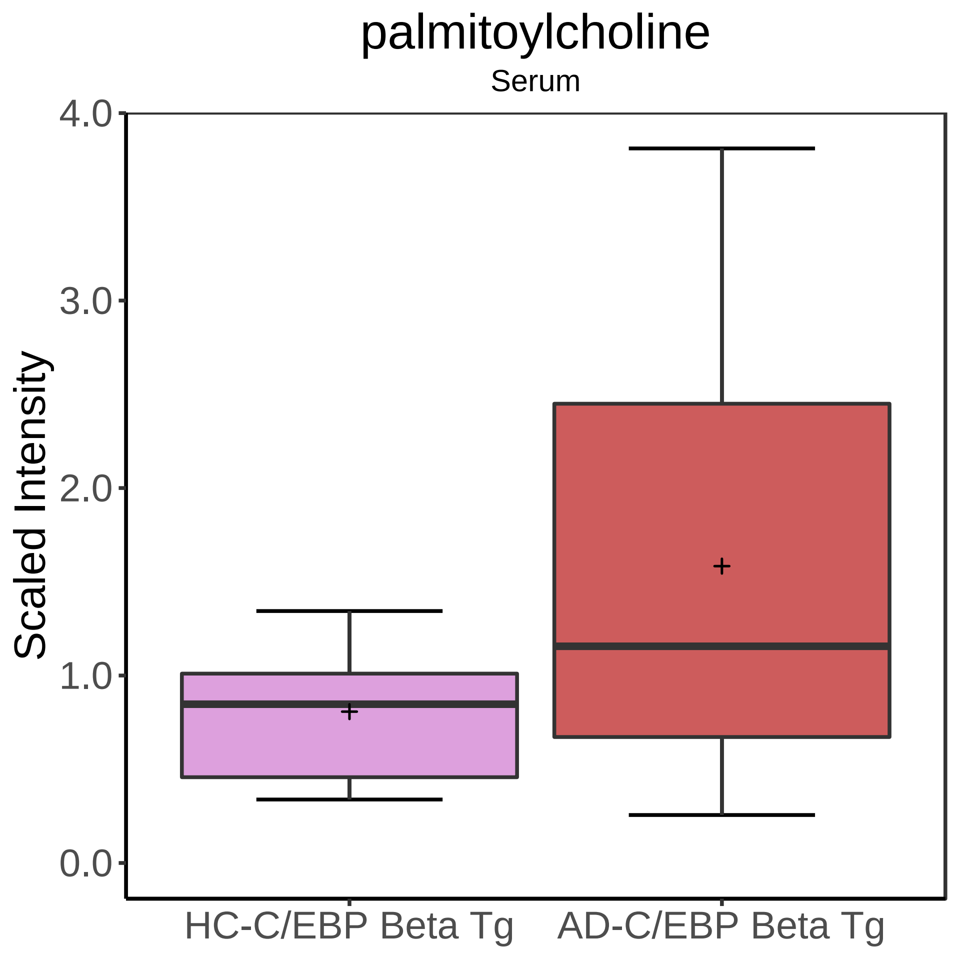
palmitoylcholine
Serum
Scaled Intensity
4.0
3.0
2.0
1.0
0.0
HC-C/EBP Beta Tg
AD-C/EBP Beta Tg

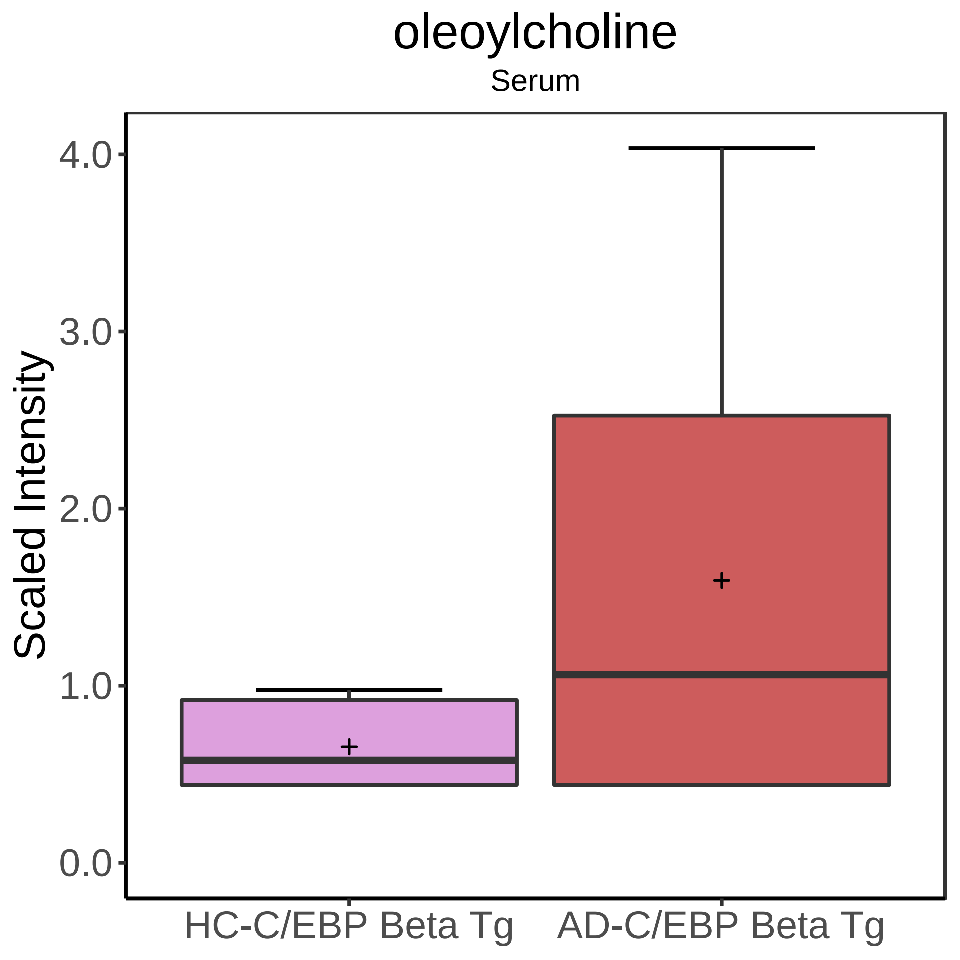
oleoylcholine
Serum
Scaled Intensity
4.0
3.0
2.0
1.0
0.0
HC-C/EBP Beta Tg
AD-C/EBP Beta Tg

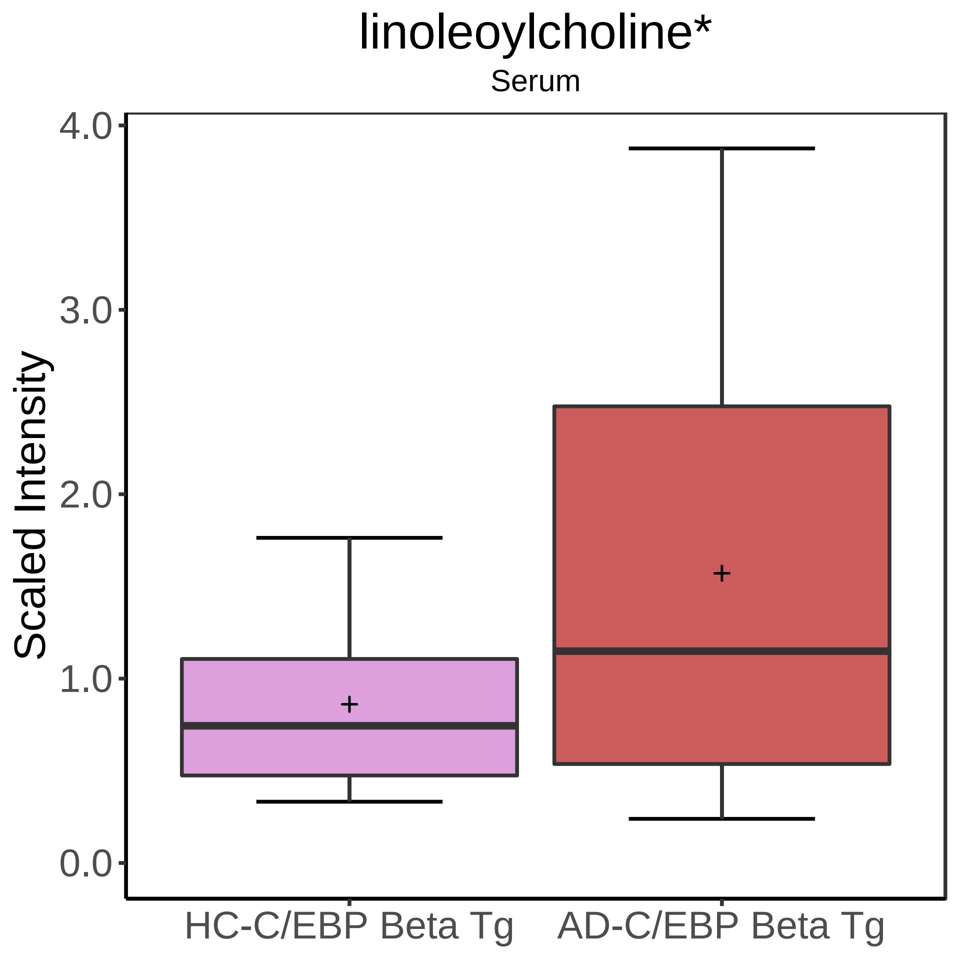
linoleoylcholine*
Serum
Scaled Intensity
4.0
3.0
2.0
1.0
0.0
HC-C/EBP Beta Tg
AD-C/EBP Beta Tg

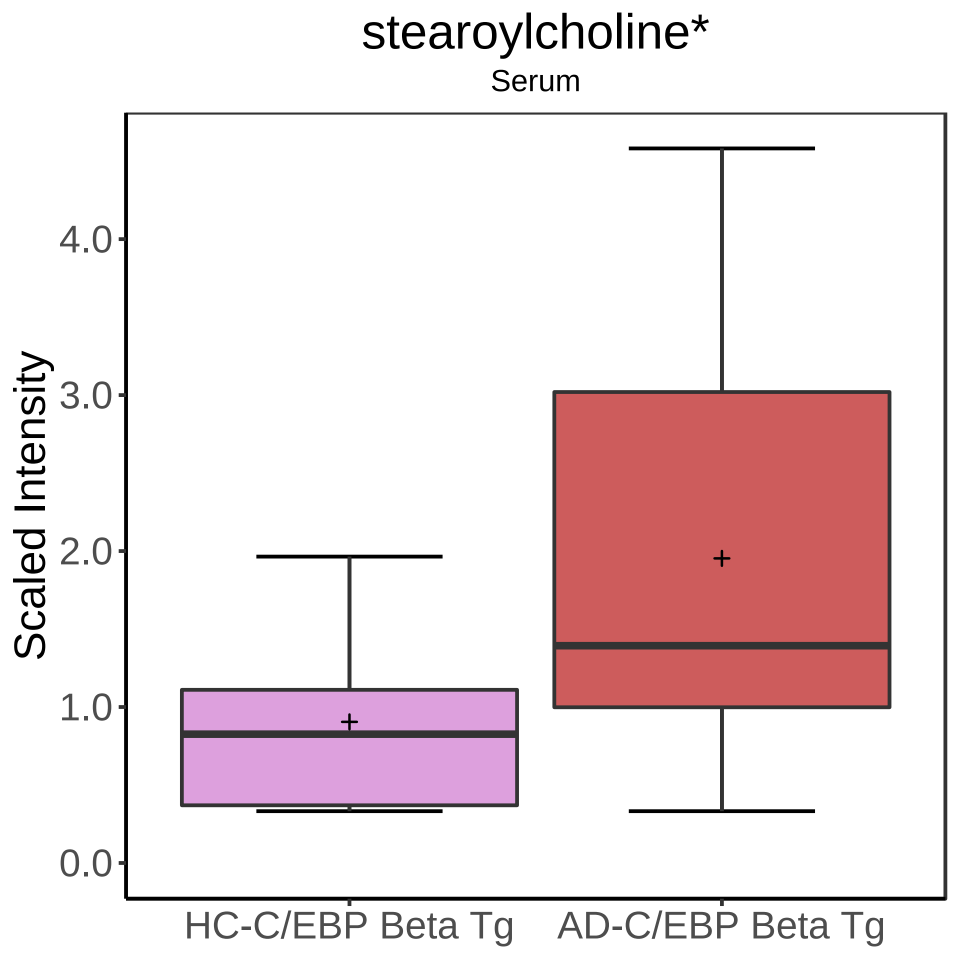
stearoylcholine*
Serum
Scaled Intensity
4.0
3.0
2.0
1.0
0.0
HC-C/EBP Beta Tg
AD-C/EBP Beta Tg

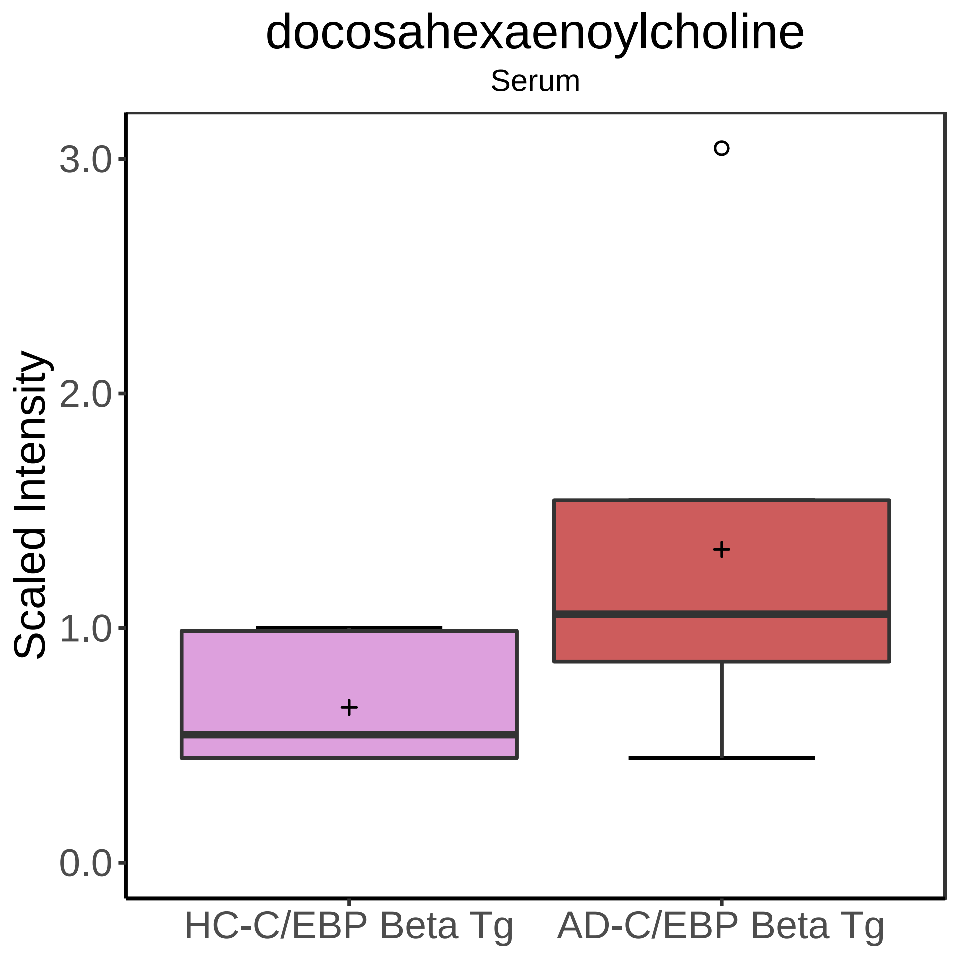
docosahexaenoylcholine
Serum
Scaled Intensity
3.0
2.0
1.0
0.0
HC-C/EBP Beta Tg
AD-C/EBP Beta Tg

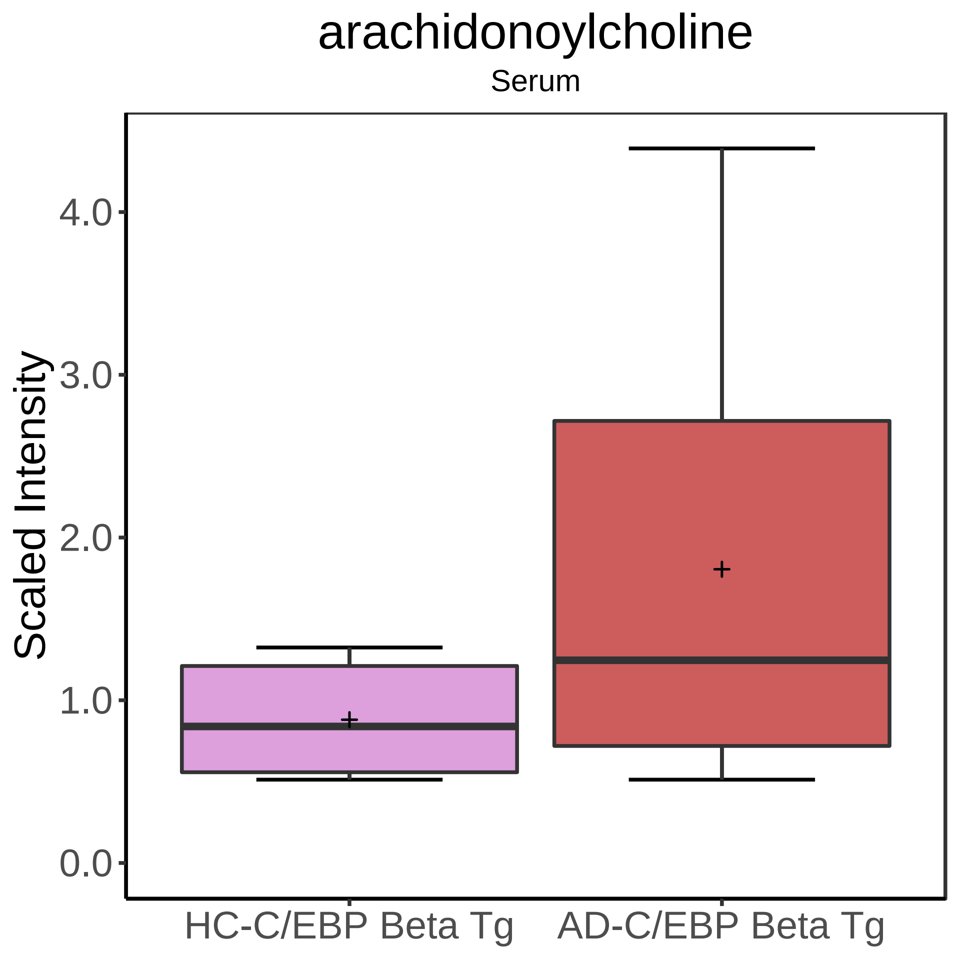
arachidonoylcholine
Serum
Scaled Intensity
4.0
3.0
2.0
1.0
0.0
HC-C/EBP Beta Tg
AD-C/EBP Beta Tg

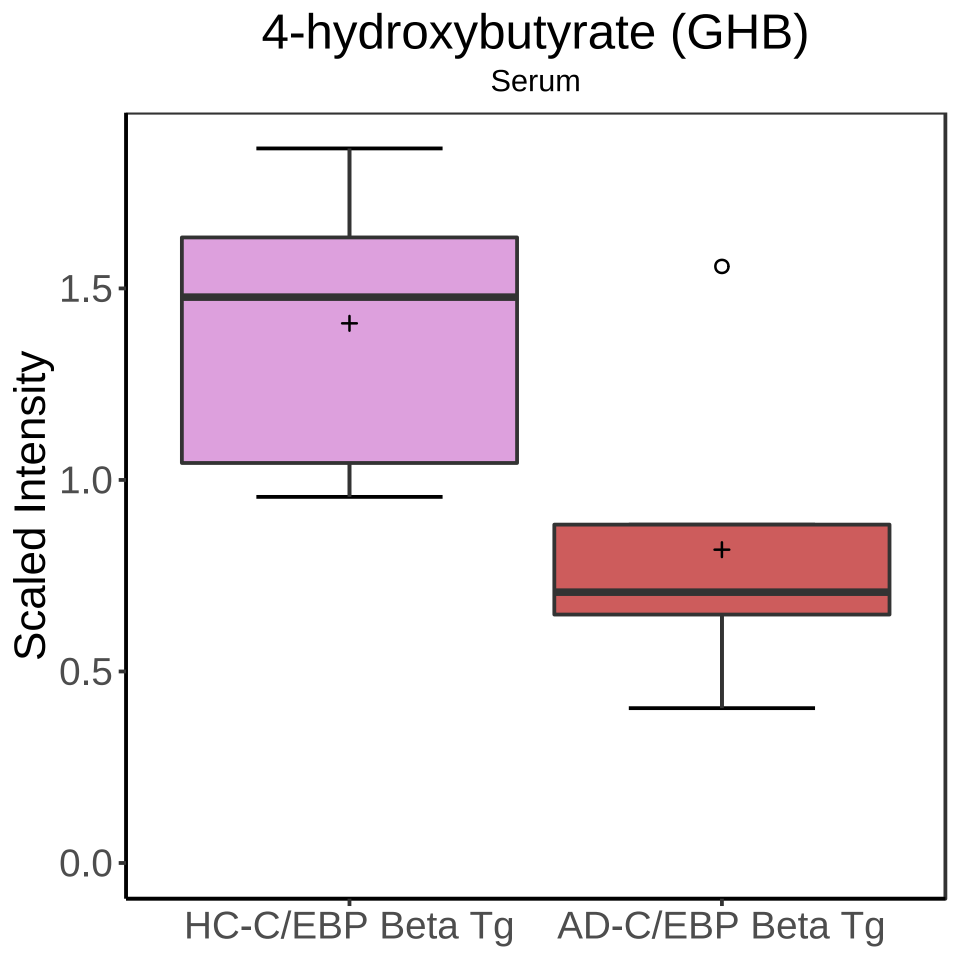
4-hydroxybutyrate (GHB)
Serum
Scaled Intensity
1.5
1.0
0.5
0.0
HC-C/EBP Beta Tg
AD-C/EBP Beta Tg

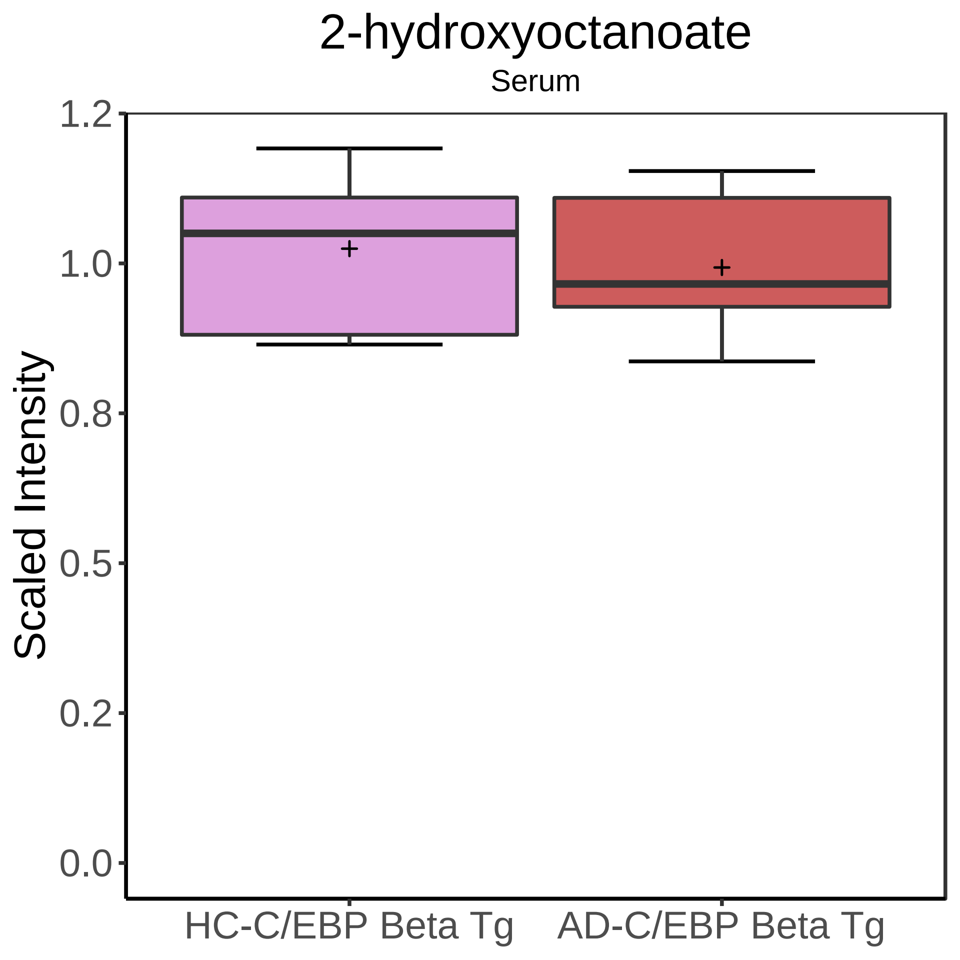
2-hydroxyoctanoate
Serum
Scaled Intensity
1.2
1.0
0.8
0.5
0.2
0.0
HC-C/EBP Beta Tg
AD-C/EBP Beta Tg

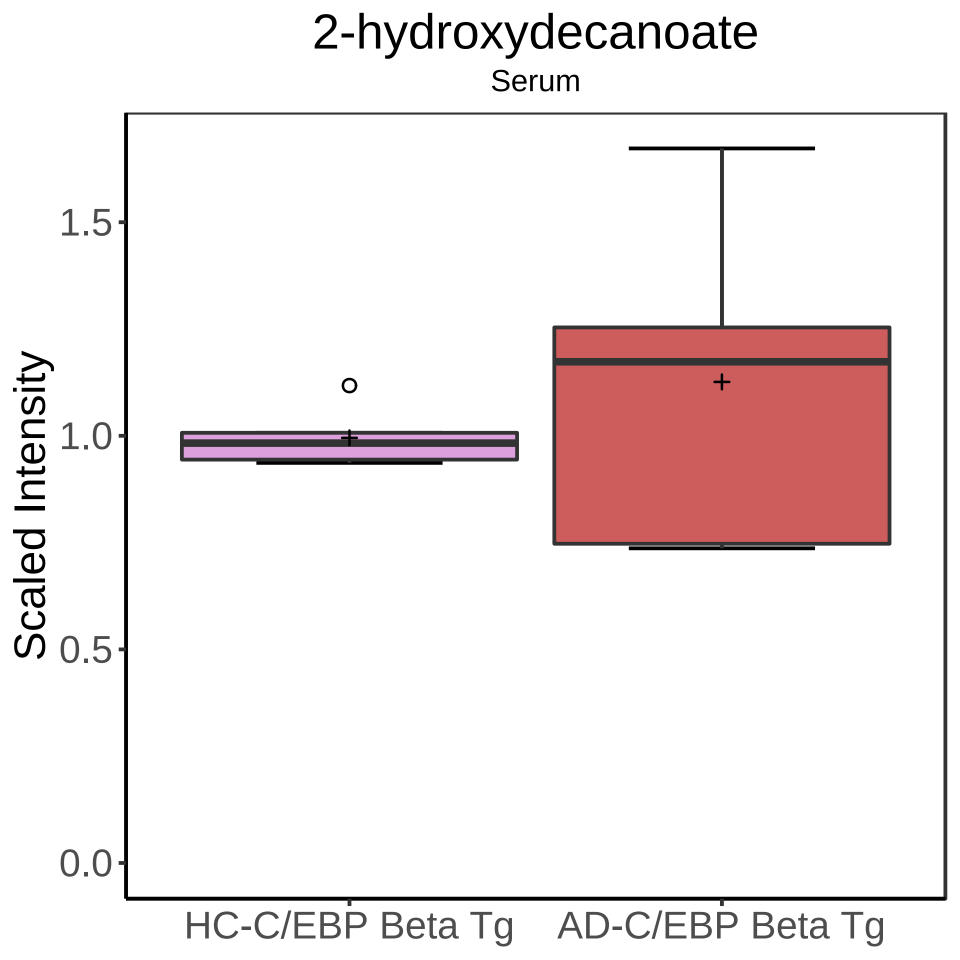
2-hydroxydecanoate
Serum
Scaled Intensity
0.0
0.5
1.0
1.5
HC-C/EBP Beta Tg
AD-C/EBP Beta Tg

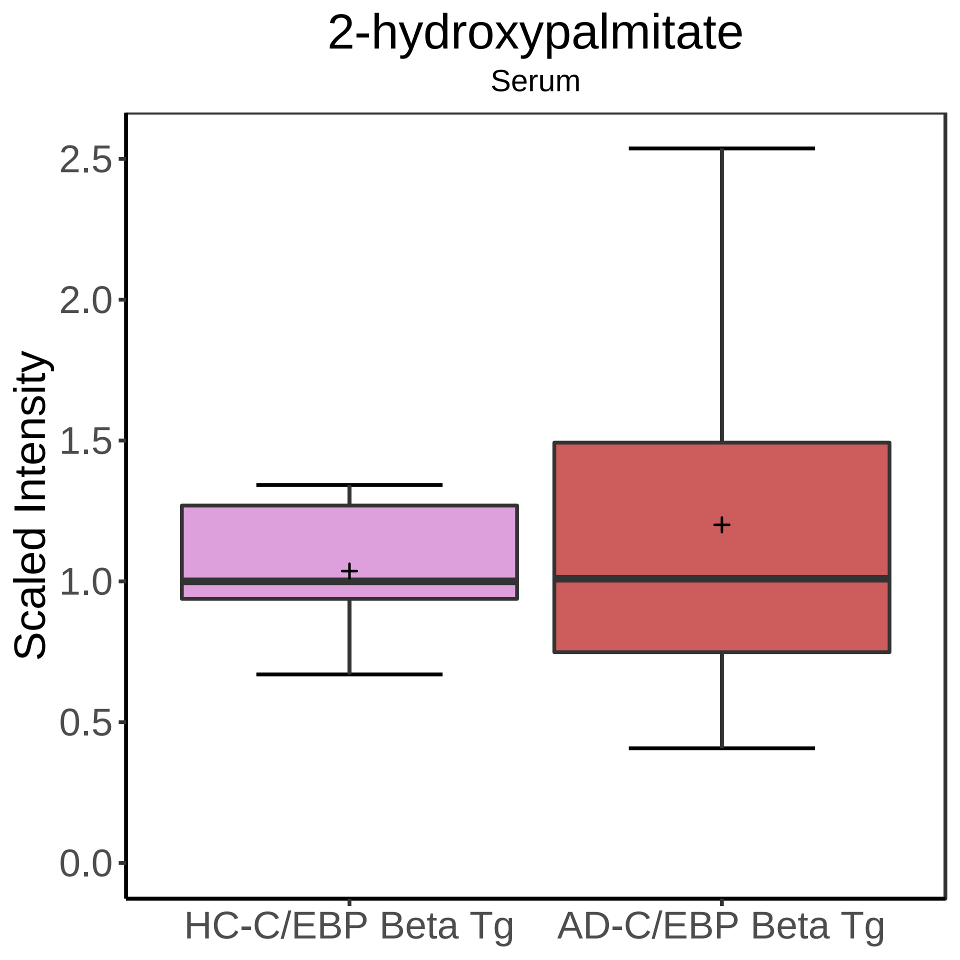
2-hydroxypalmitate
Serum
Scaled Intensity
2.5
2.0
1.5
1.0
0.5
0.0
HC-C/EBP Beta Tg
AD-C/EBP Beta Tg

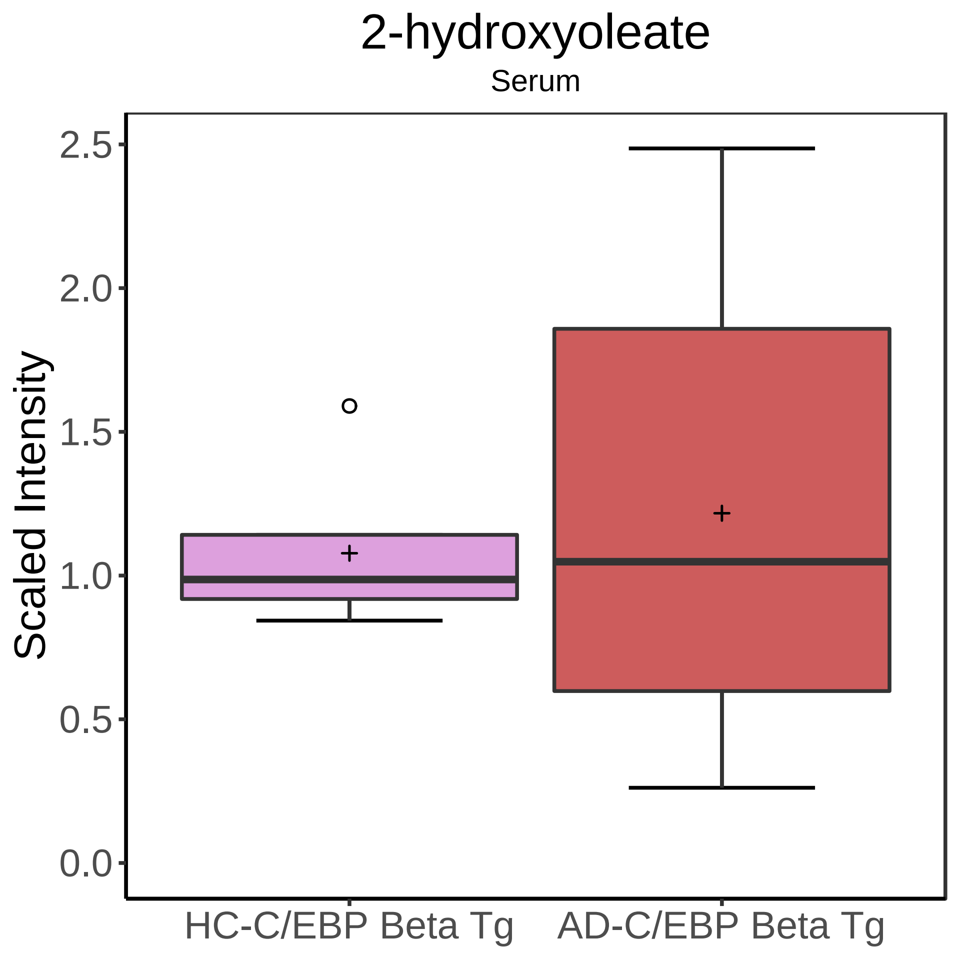
2-hydroxyoleate
Serum
Scaled Intensity
2.5
2.0
1.5
1.0
0.5
0.0
HC-C/EBP Beta Tg
AD-C/EBP Beta Tg

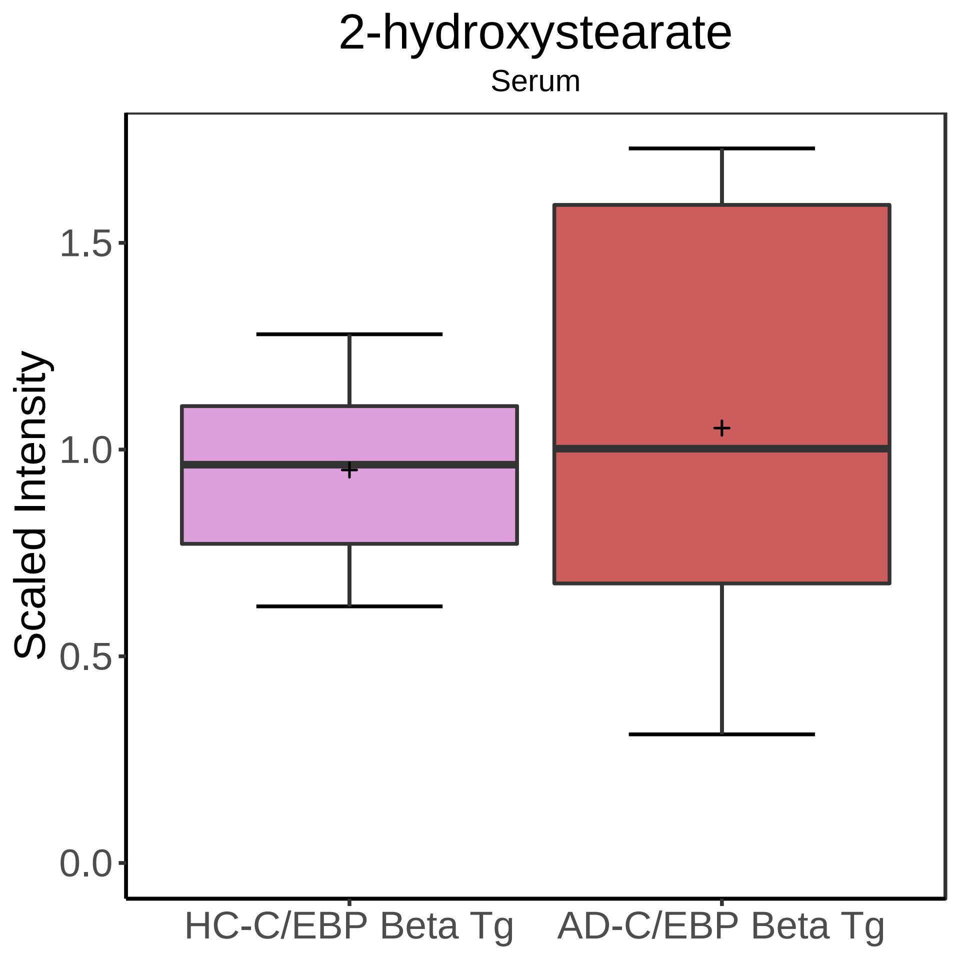
2-hydroxystearate
Serum
Scaled Intensity
0.0
0.5
1.0
1.5
HC-C/EBP Beta Tg
AD-C/EBP Beta Tg

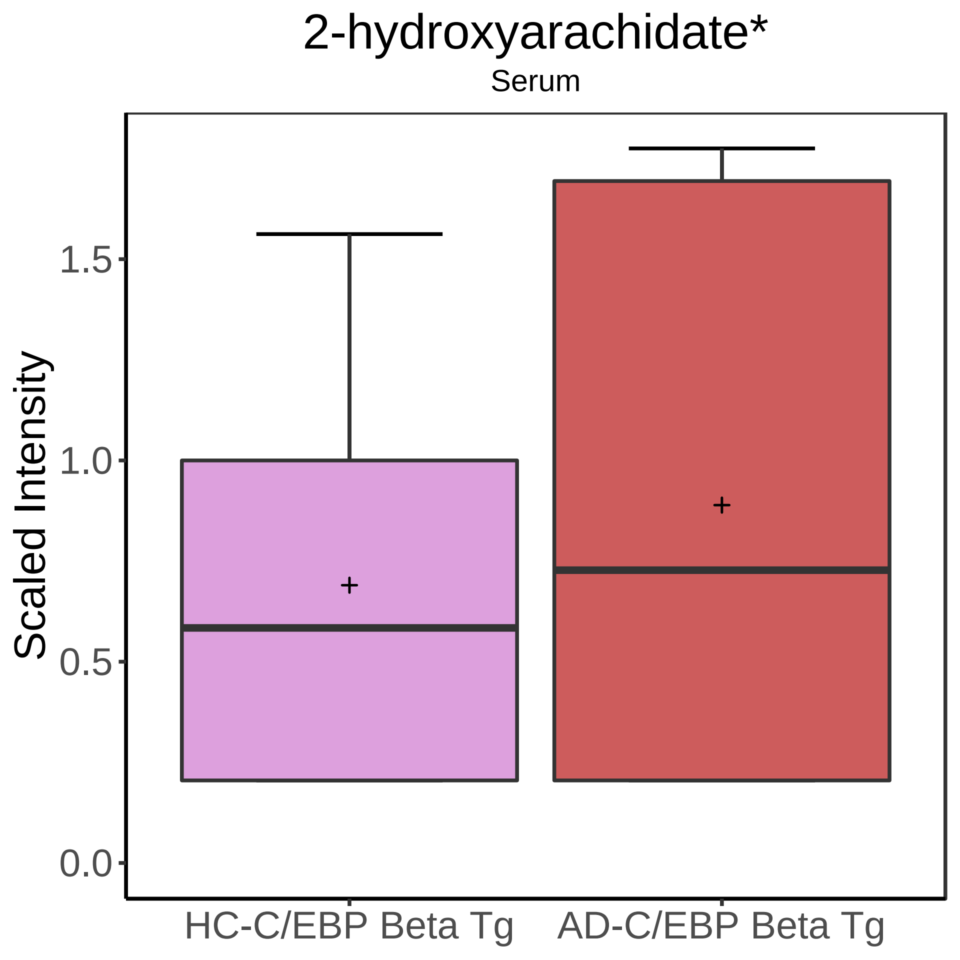
2-hydroxyarachidate*
Serum
Scaled Intensity
0.0
0.5
1.0
1.5
HC-C/EBP Beta Tg
AD-C/EBP Beta Tg

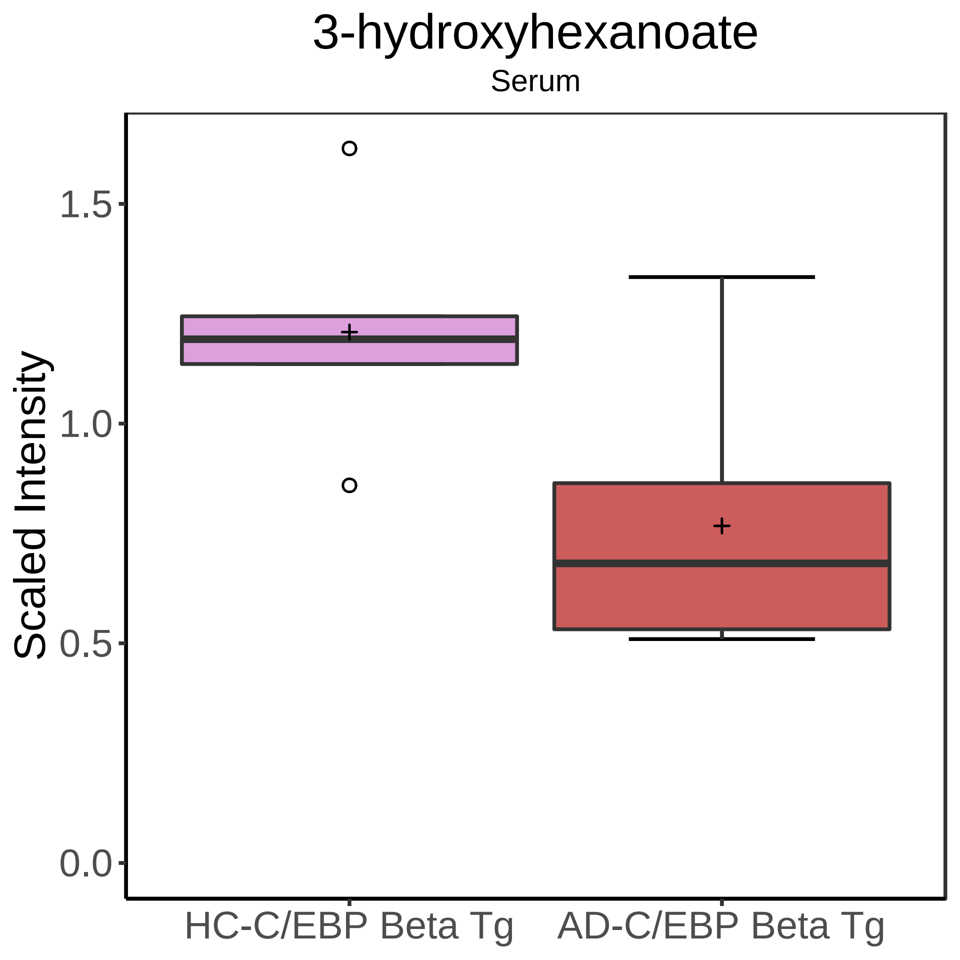
3-hydroxyhexanoate
Serum
Scaled Intensity
1.5
1.0
0.5
0.0
HC-C/EBP Beta Tg
AD-C/EBP Beta Tg

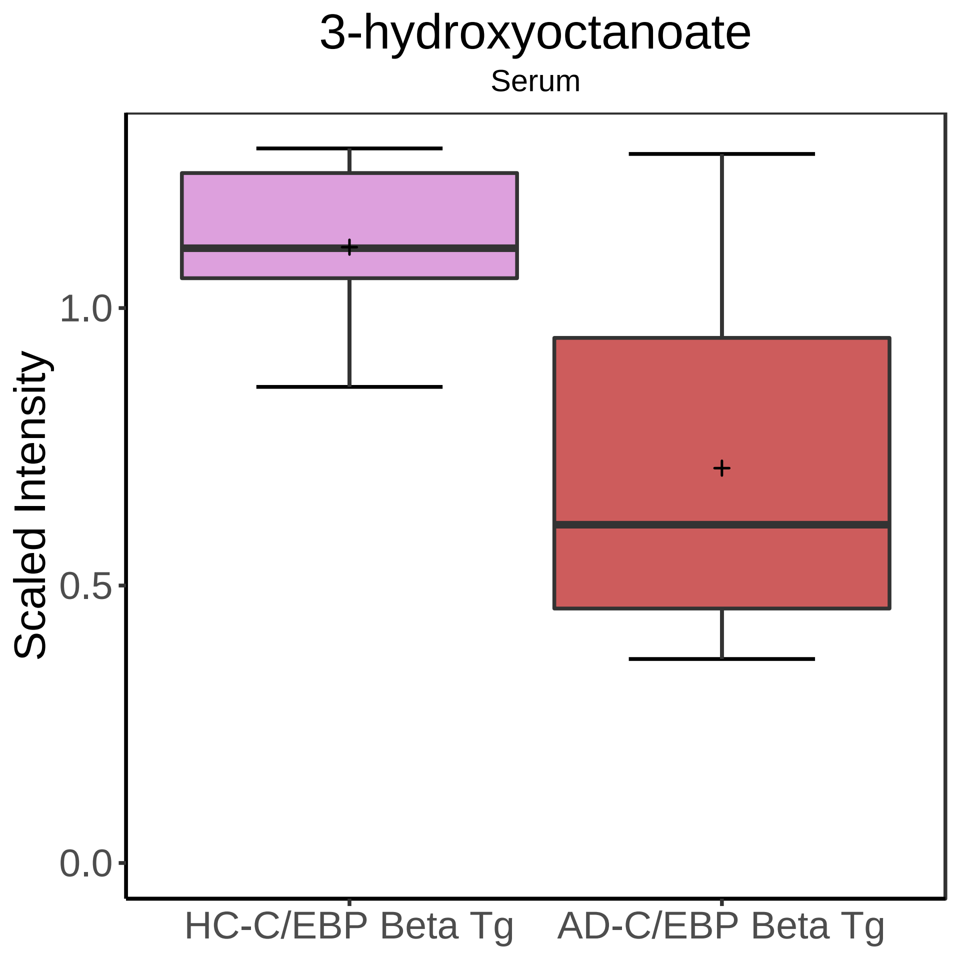
3-hydroxyoctanoate
Serum
Scaled Intensity
1.0
0.5
0.0
HC-C/EBP Beta Tg
AD-C/EBP Beta Tg

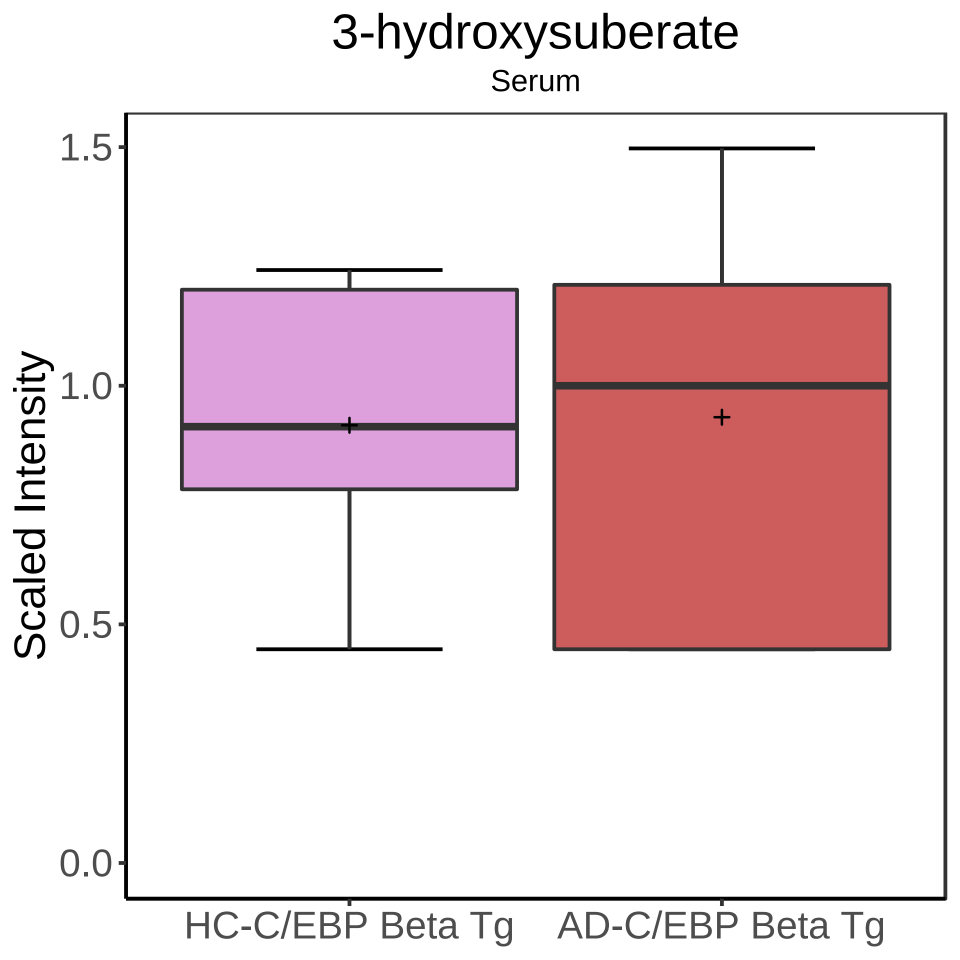
3-hydroxysuberate
Serum
Scaled Intensity
1.5
1.0
0.5
0.0
HC-C/EBP Beta Tg
AD-C/EBP Beta Tg

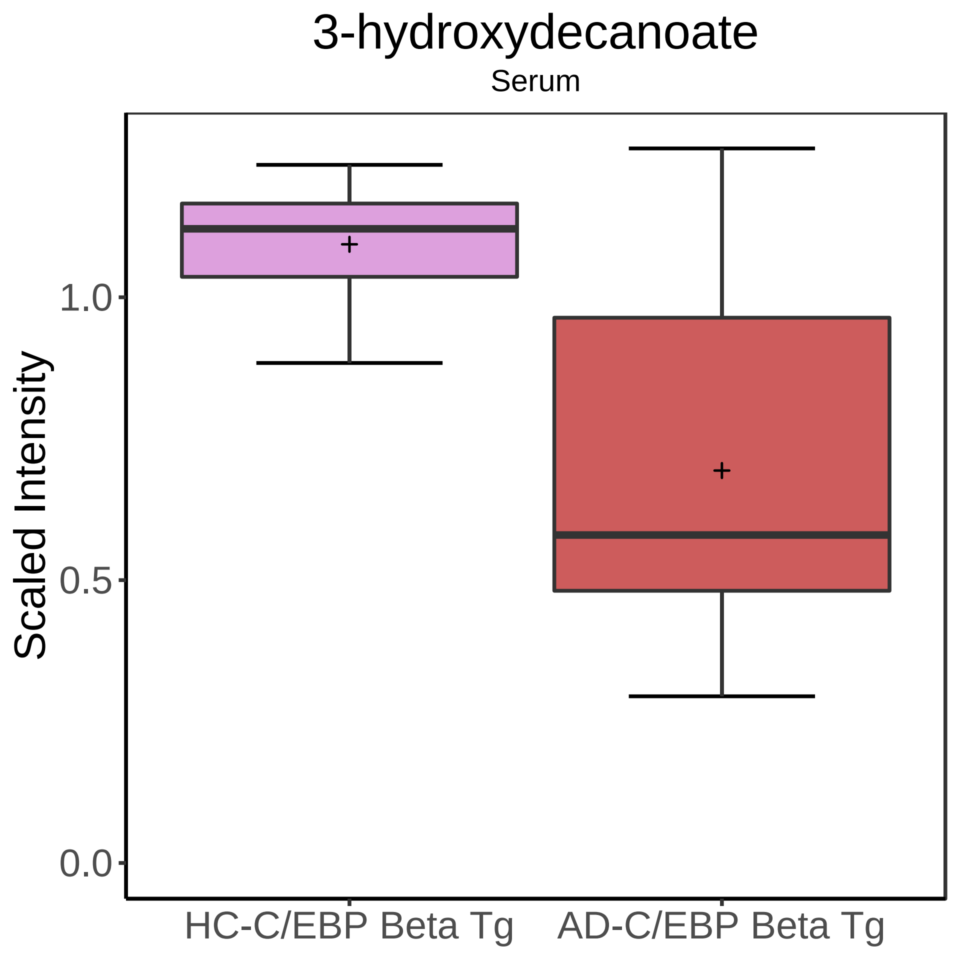
3-hydroxydecanoate
Serum
Scaled Intensity
1.0
0.5
0.0
HC-C/EBP Beta Tg
AD-C/EBP Beta Tg

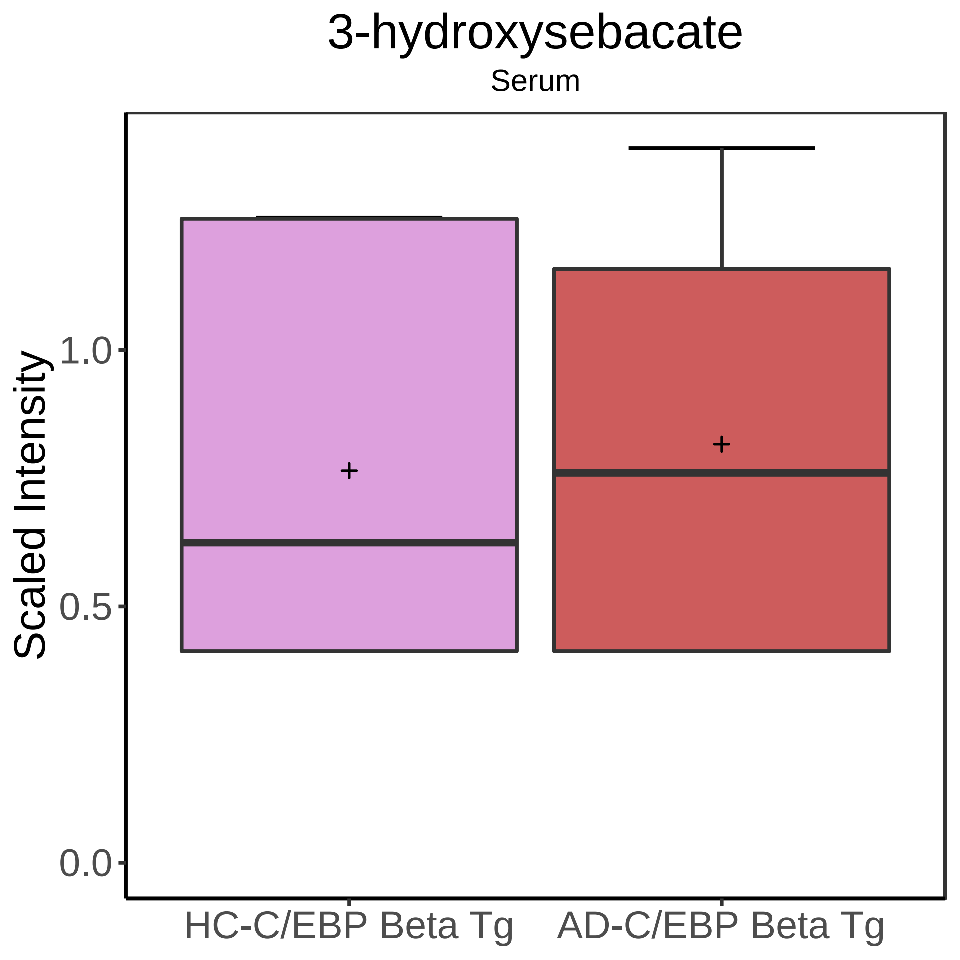
3-hydroxysebacate
Serum
Scaled Intensity
1.0
0.5
0.0
HC-C/EBP Beta Tg
AD-C/EBP Beta Tg

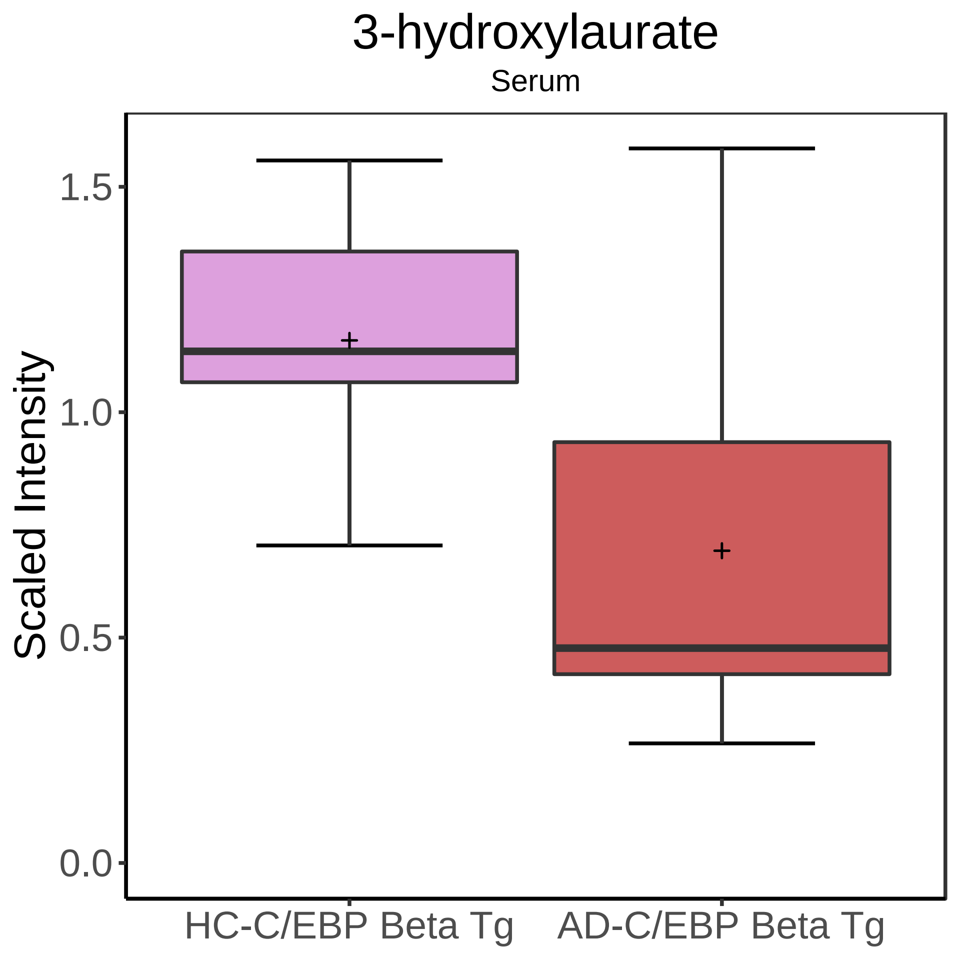
3-hydroxylaurate
Serum
Scaled Intensity
1.5
1.0
0.5
0.0
HC-C/EBP Beta Tg
AD-C/EBP Beta Tg

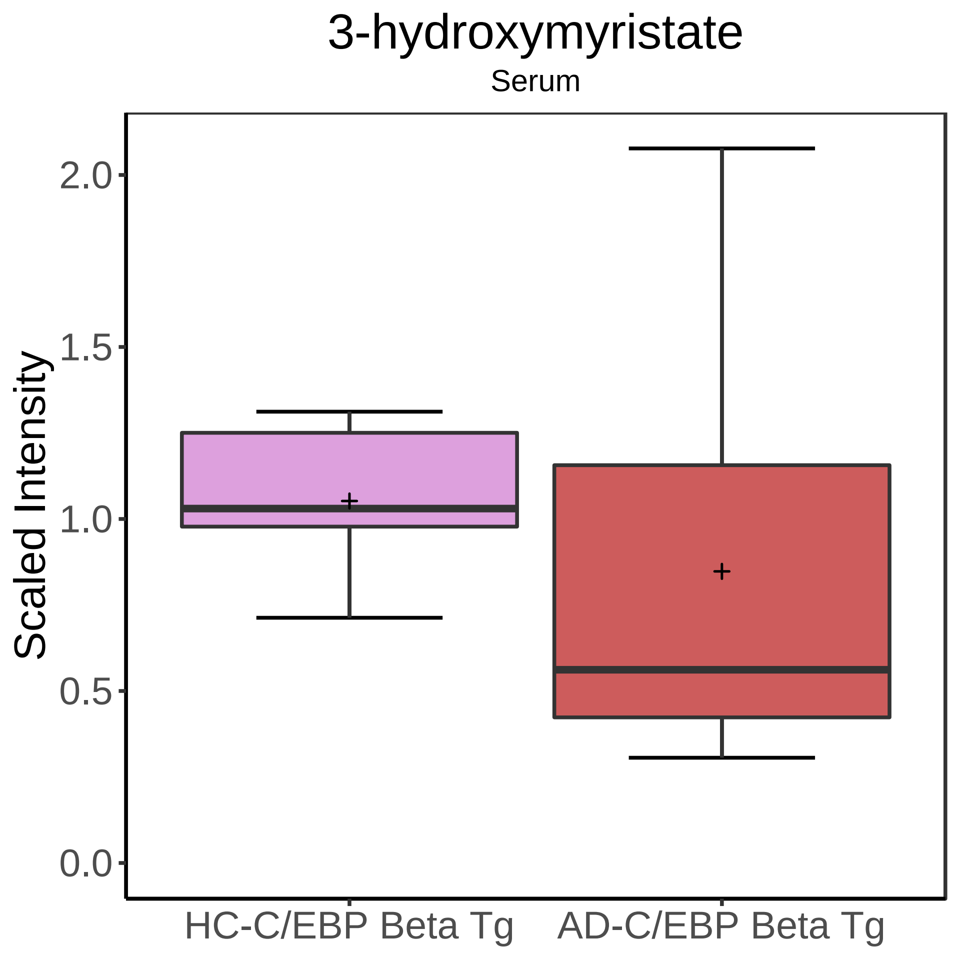
3-hydroxymyristate
Serum
Scaled Intensity
2.0
1.5
1.0
0.5
0.0
HC-C/EBP Beta Tg
AD-C/EBP Beta Tg

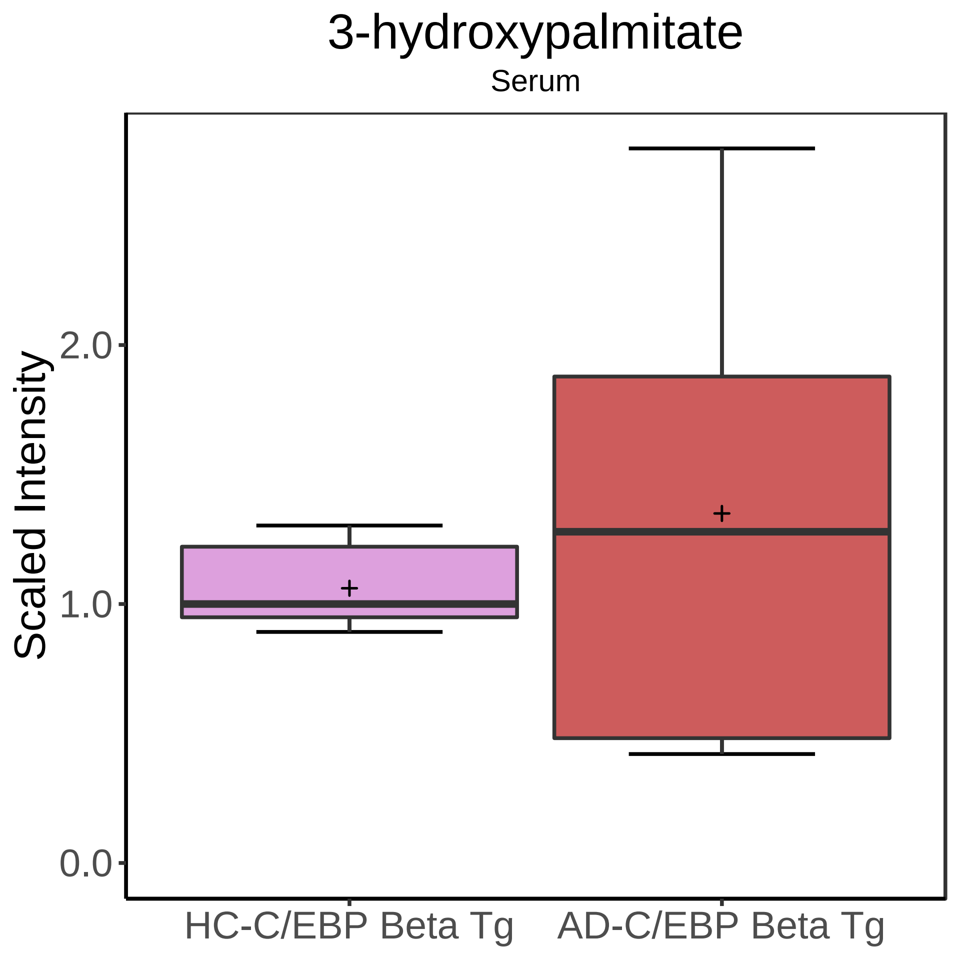
3-hydroxypalmitate
Serum
Scaled Intensity
2.0
1.0
0.0
HC-C/EBP Beta Tg
AD-C/EBP Beta Tg

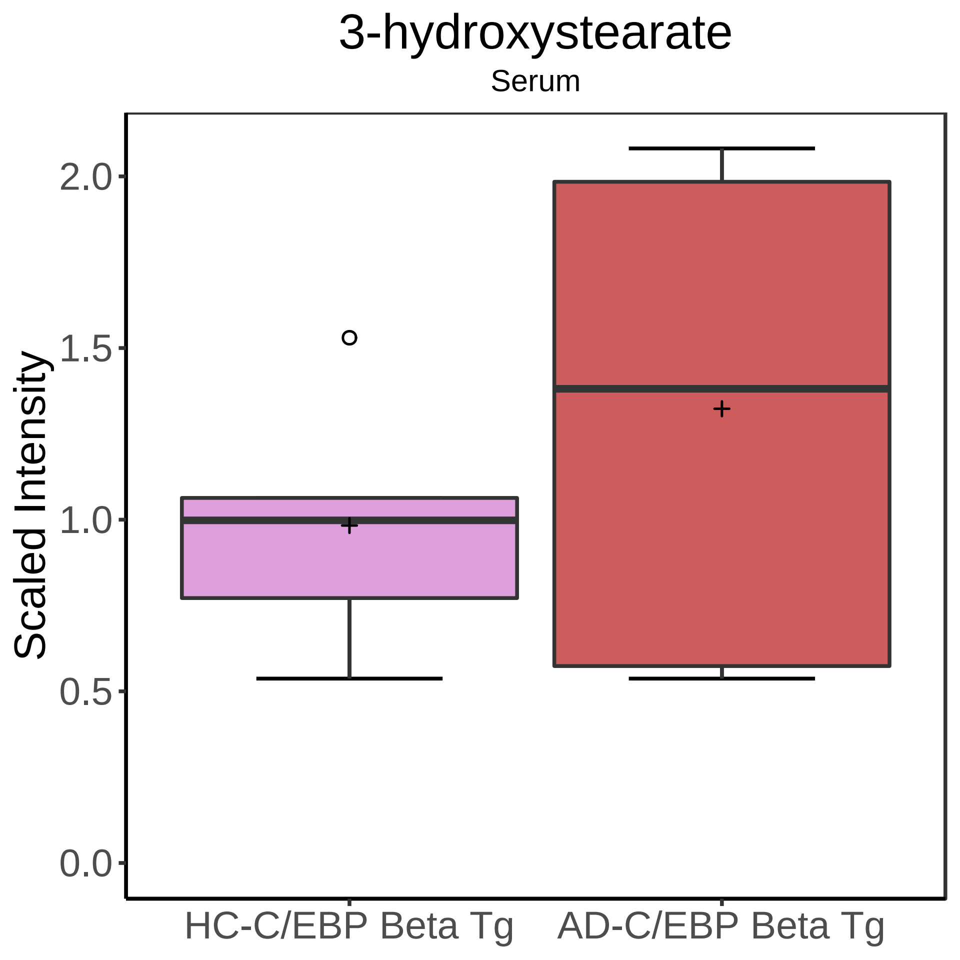
3-hydroxystearate
Serum
Scaled Intensity
2.0
1.5
1.0
0.5
0.0
HC-C/EBP Beta Tg
AD-C/EBP Beta Tg

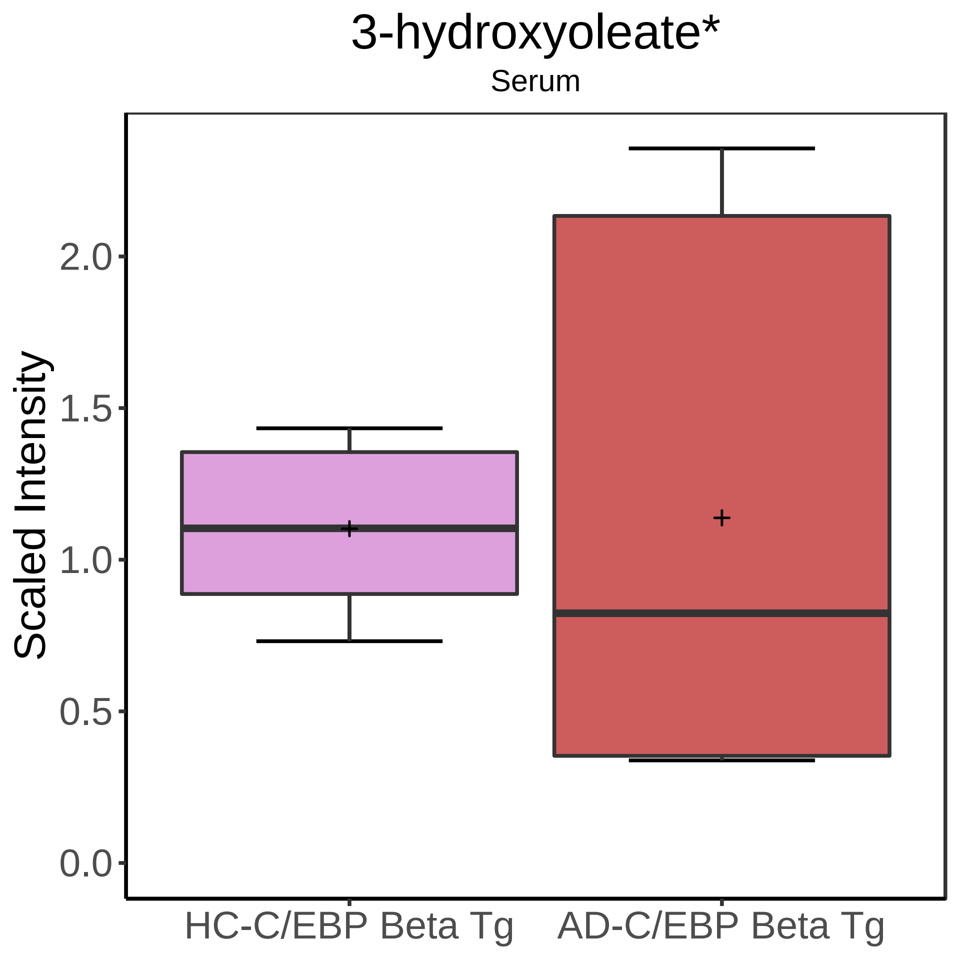
3-hydroxyoleate*
Serum
Scaled Intensity
2.0
1.5
1.0
0.5
0.0
HC-C/EBP Beta Tg
AD-C/EBP Beta Tg

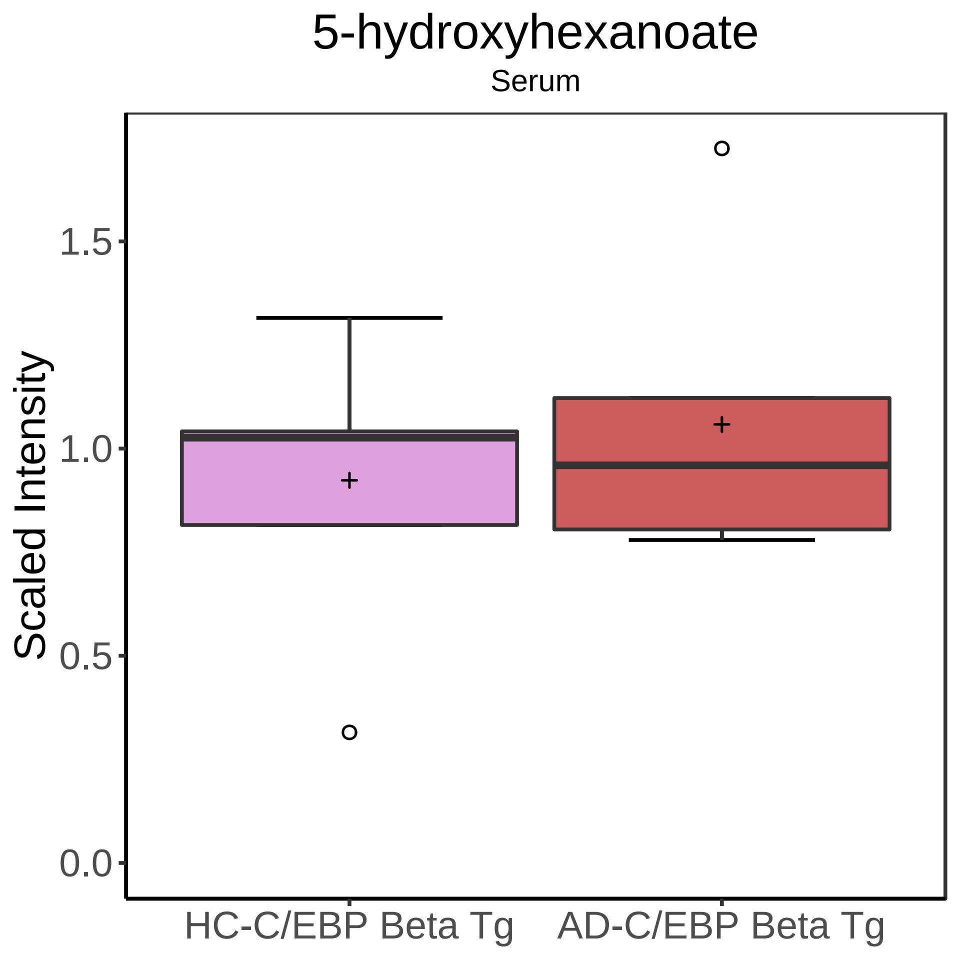
5-hydroxyhexanoate
Serum
Scaled Intensity
0.0
0.5
1.0
1.5
HC-C/EBP Beta Tg
AD-C/EBP Beta Tg

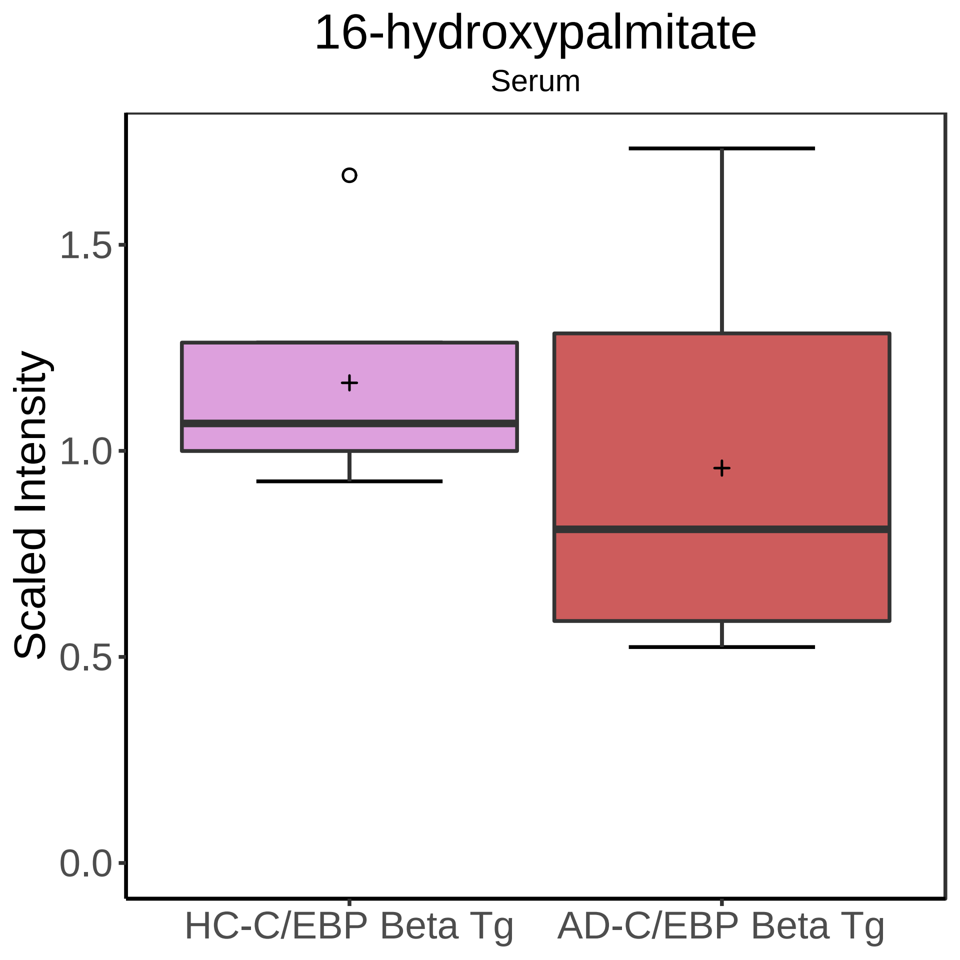
16-hydroxypalmitate
Serum
Scaled Intensity
1.5
1.0
0.5
0.0
HC-C/EBP Beta Tg
AD-C/EBP Beta Tg

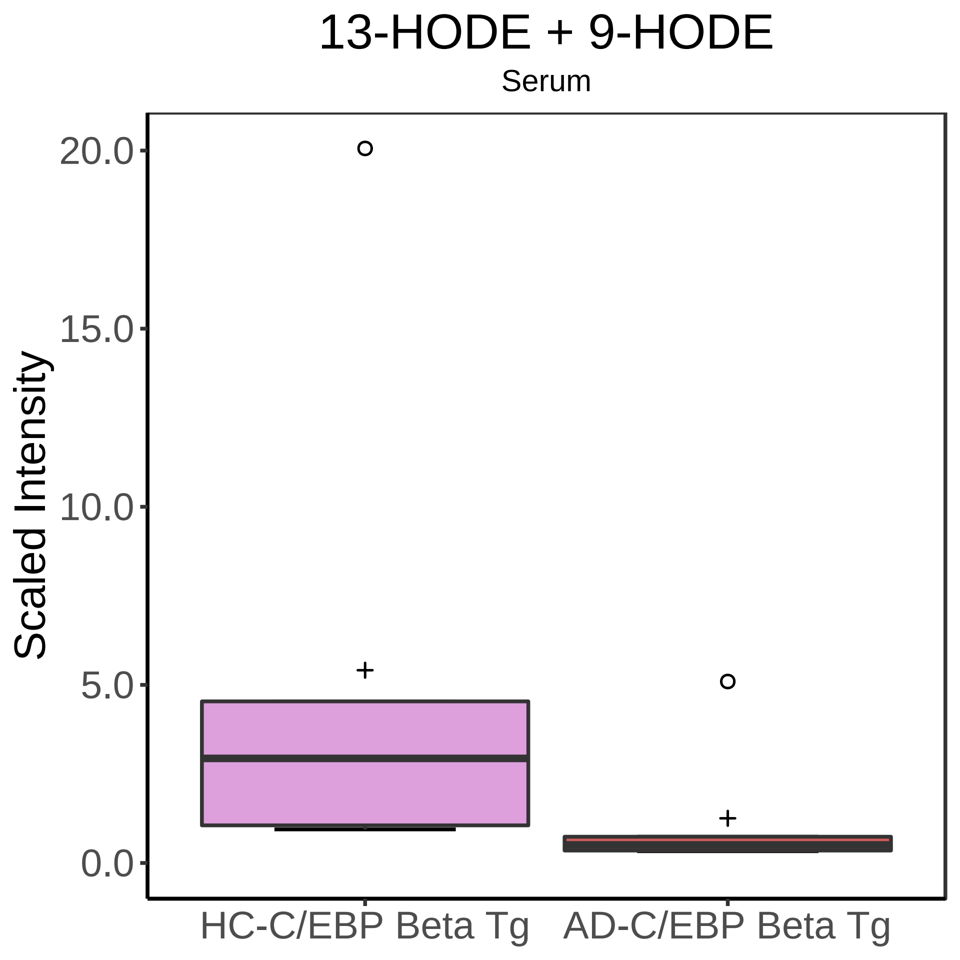

13-HODE + 9-HODE
Serum
Scaled Intensity
20.0
15.0
10.0
5.0
0.0
HC-C/EBP Beta Tg
AD-C/EBP Beta Tg

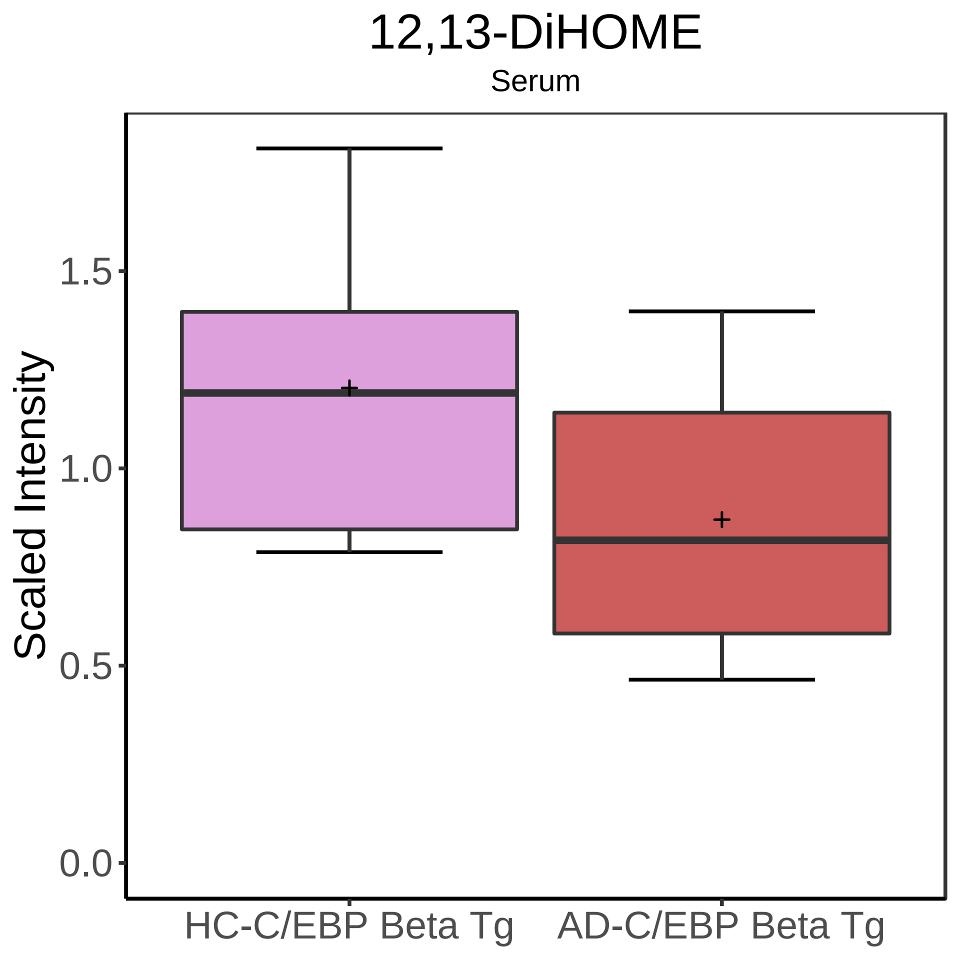
12,13-DiHOME
Serum
Scaled Intensity
1.5
1.0
0.5
0.0
HC-C/EBP Beta Tg
AD-C/EBP Beta Tg

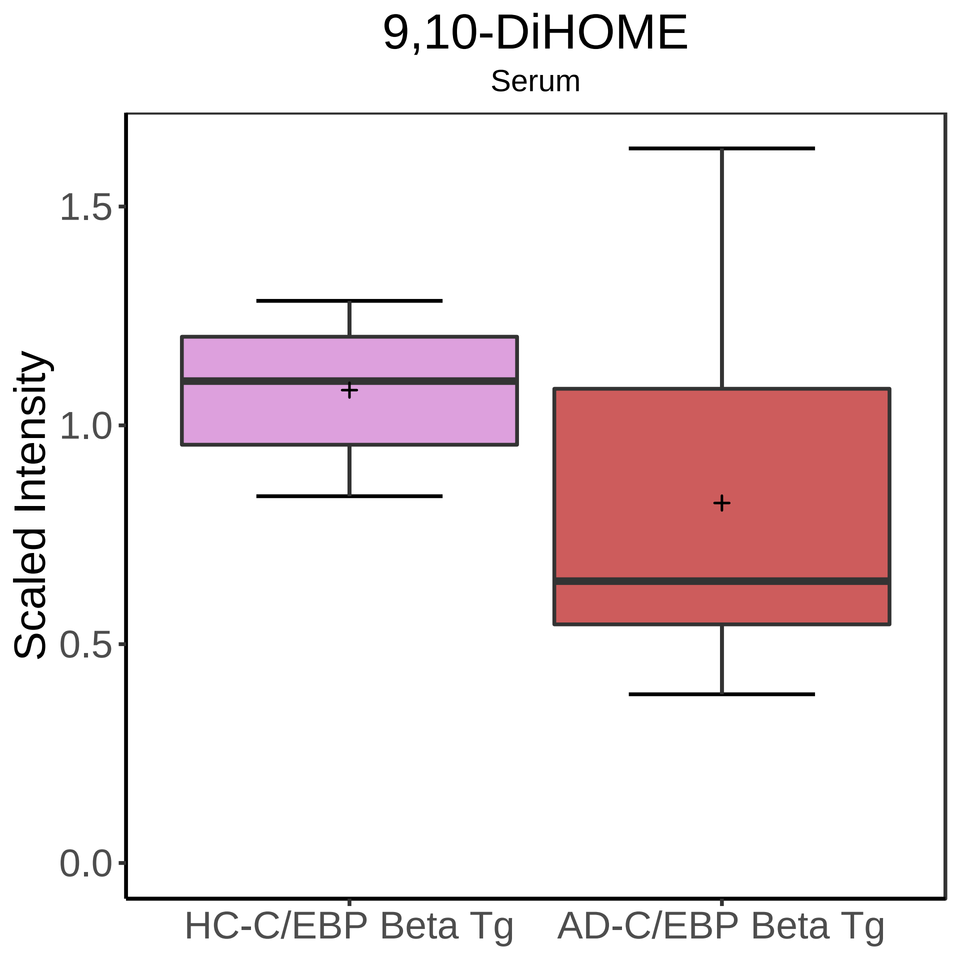
9,10-DiHOME
Serum
Scaled Intensity
1.5
1.0
0.5
0.0
HC-C/EBP Beta Tg
AD-C/EBP Beta Tg

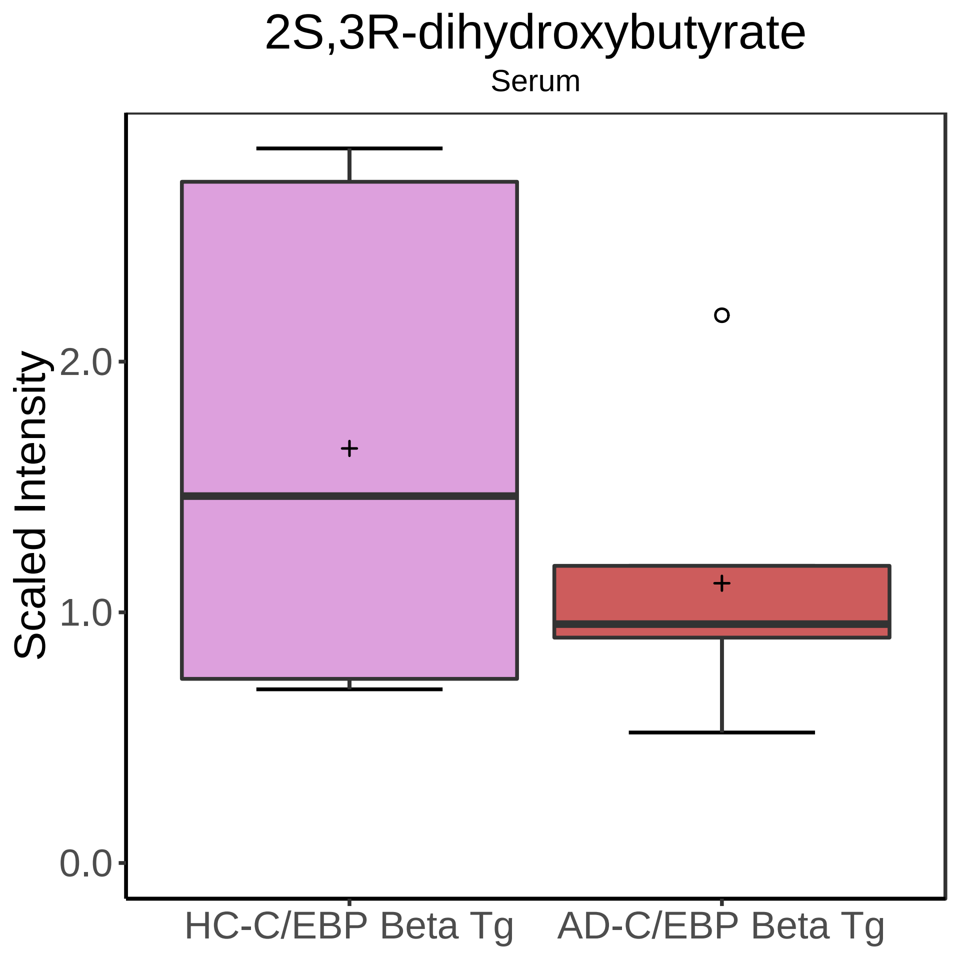
2S,3R-dihydroxybutyrate
Serum
Scaled Intensity
2.0
1.0
0.0
HC-C/EBP Beta Tg
AD-C/EBP Beta Tg

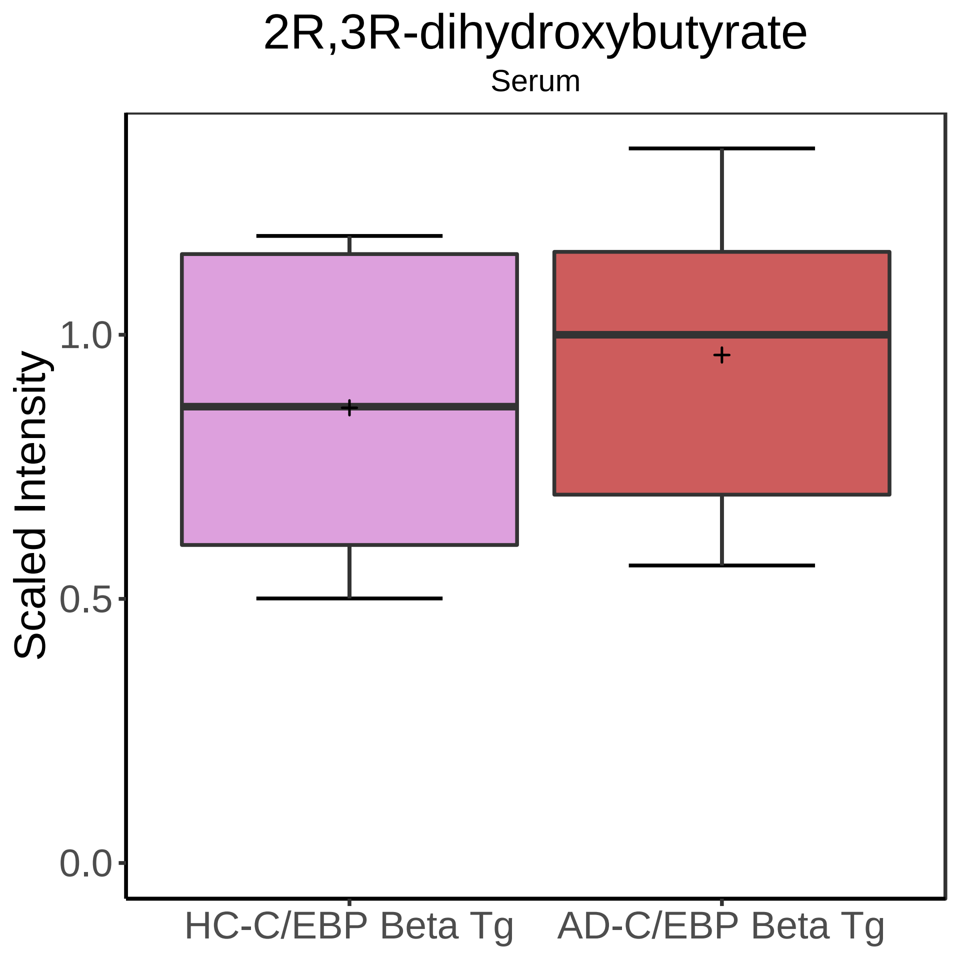
2R,3R-dihydroxybutyrate
Serum
Scaled Intensity
1.0
0.5
0.0
HC-C/EBP Beta Tg
AD-C/EBP Beta Tg

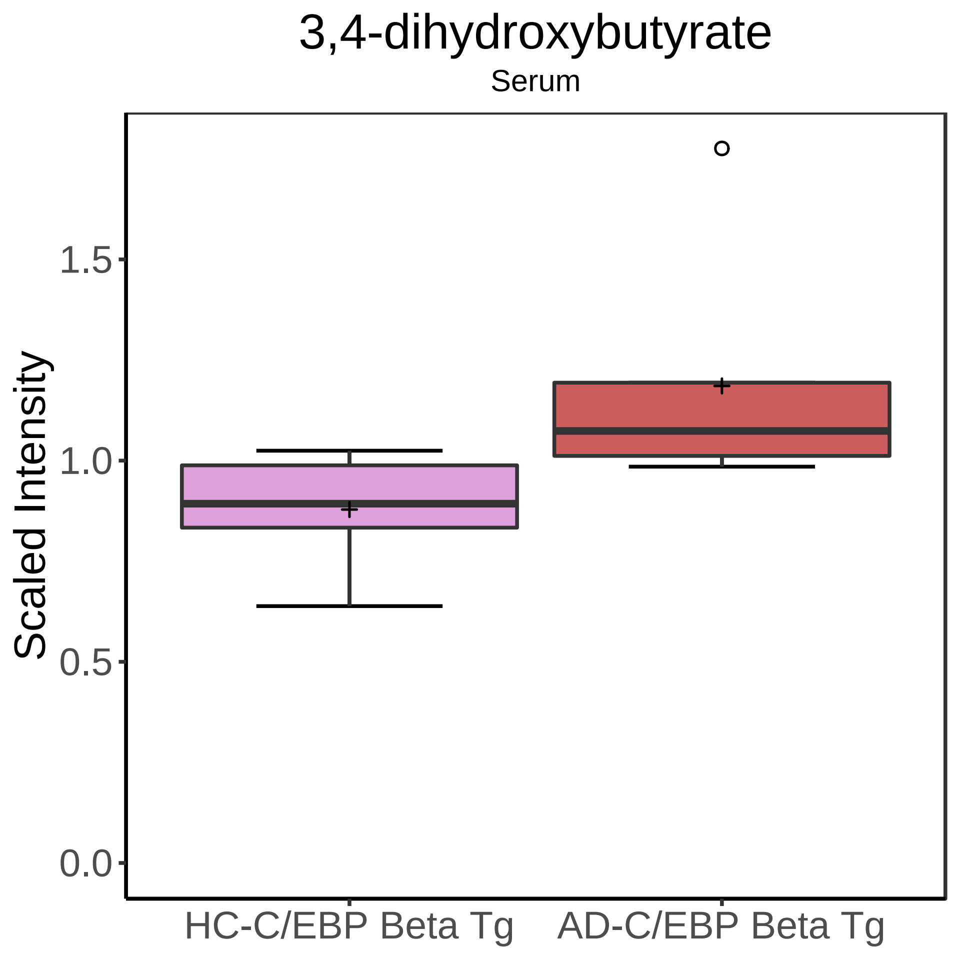
3,4-dihydroxybutyrate
Serum
Scaled Intensity
0.0
0.5
1.0
1.5
HC-C/EBP Beta Tg
AD-C/EBP Beta Tg

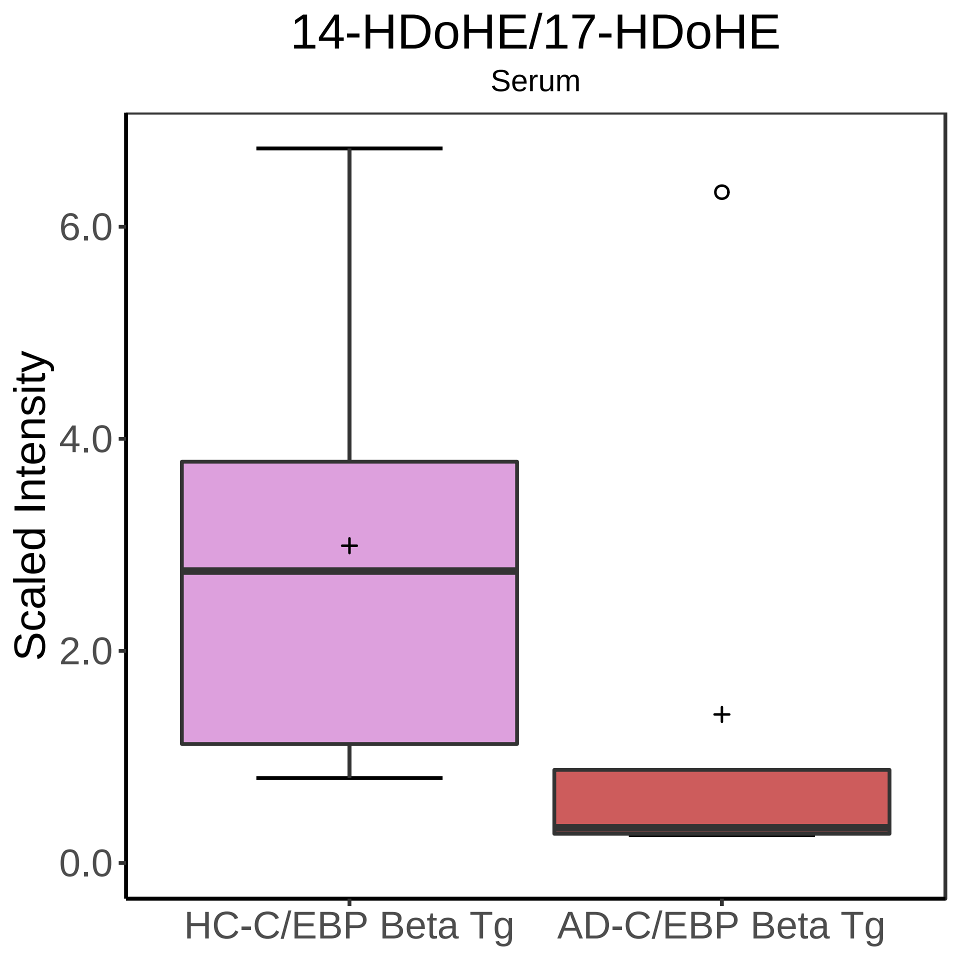
14-HDoHE/17-HDoHE
Serum
Scaled Intensity
6.0
4.0
2.0
0.0
HC-C/EBP Beta Tg
AD-C/EBP Beta Tg

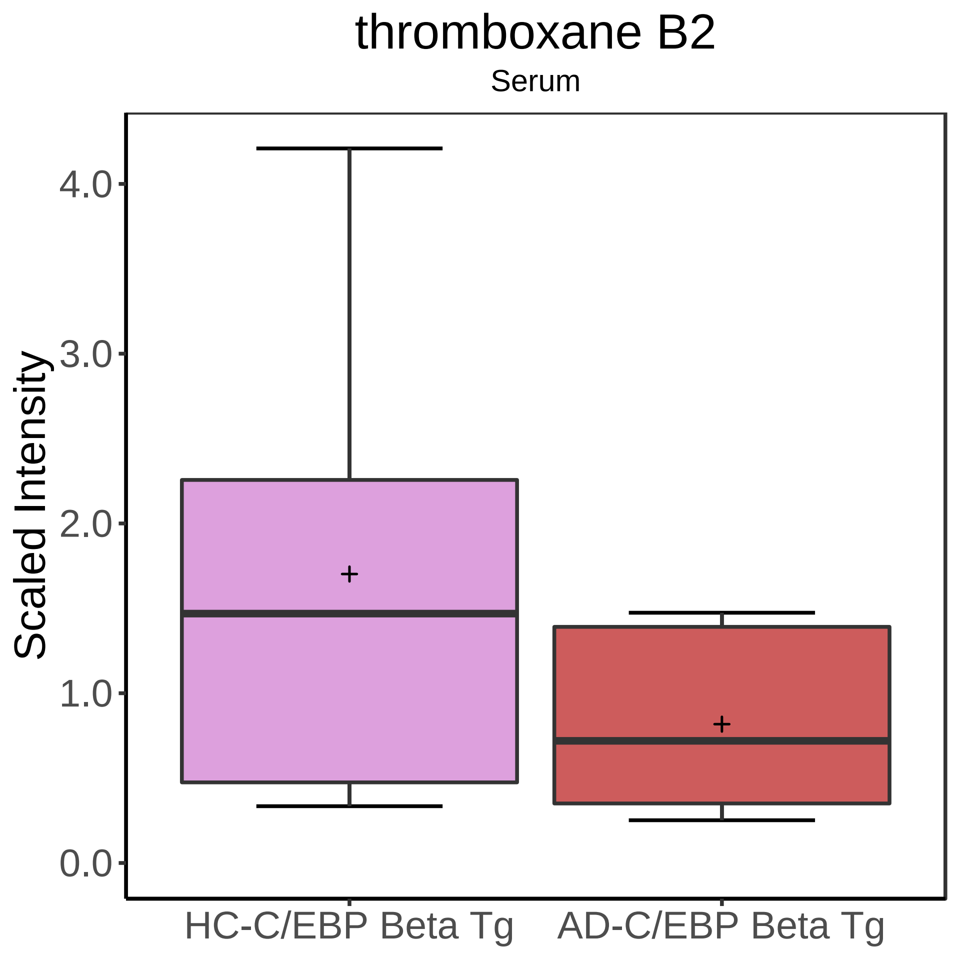
thromboxane B2
Serum
Scaled Intensity
4.0
3.0
2.0
1.0
0.0
HC-C/EBP Beta Tg
AD-C/EBP Beta Tg

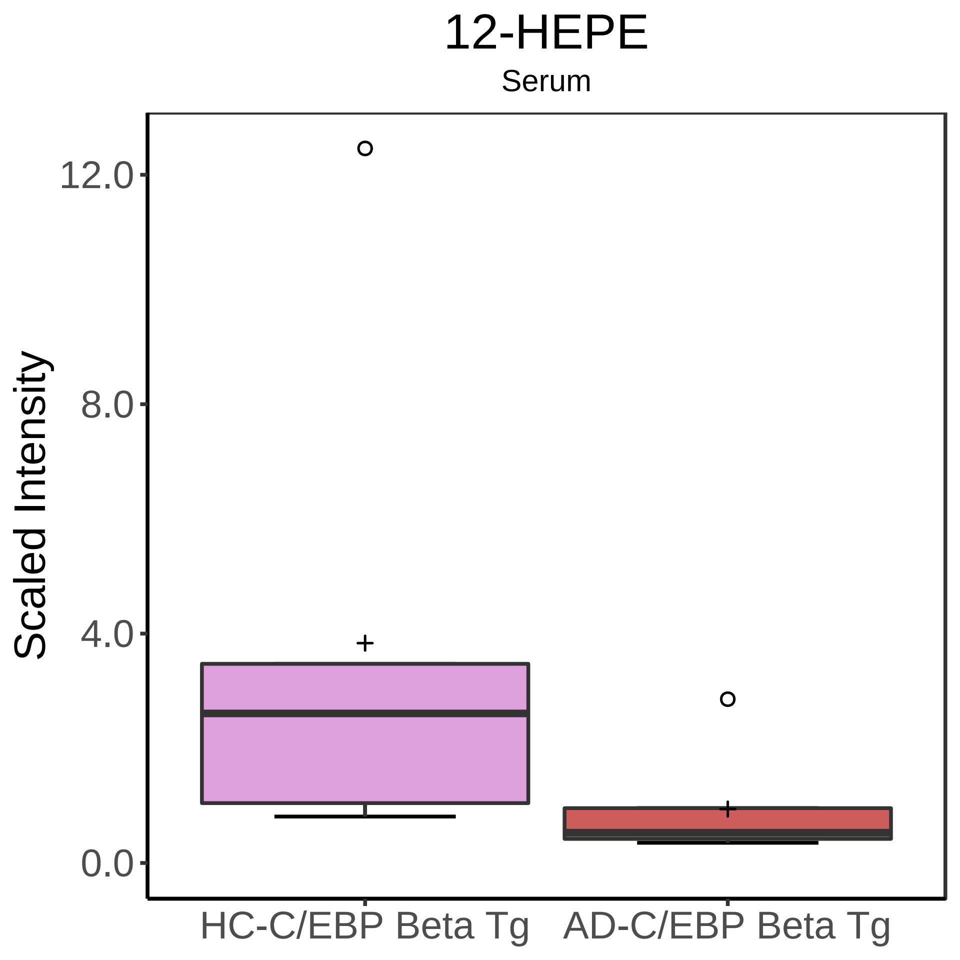
12-HEPE
Serum
Scaled Intensity
12.0
8.0
4.0
0.0
HC-C/EBP Beta Tg
AD-C/EBP Beta Tg

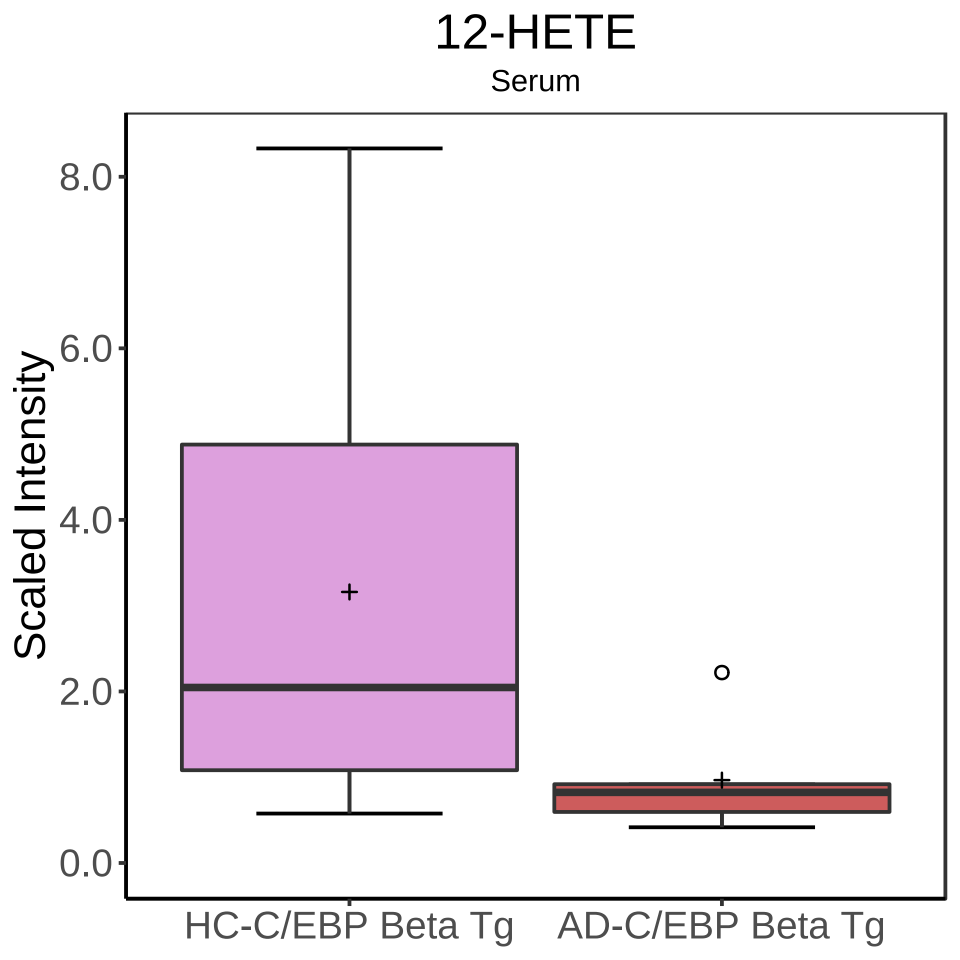
12-HETE
Serum
Scaled Intensity
8.0
6.0
4.0
2.0
0.0
HC-C/EBP Beta Tg
AD-C/EBP Beta Tg

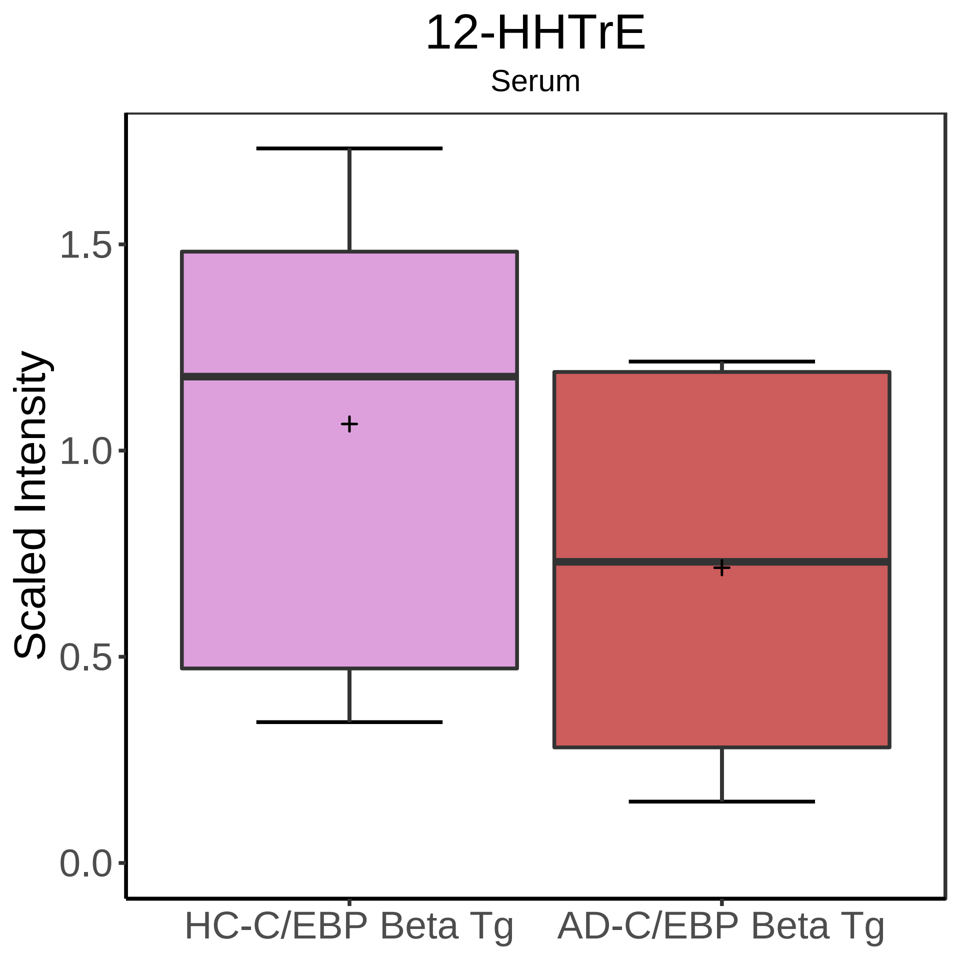

12-HHTrE
Serum
Scaled Intensity
1.5
1.0
0.5
0.0
HC-C/EBP Beta Tg
AD-C/EBP Beta Tg

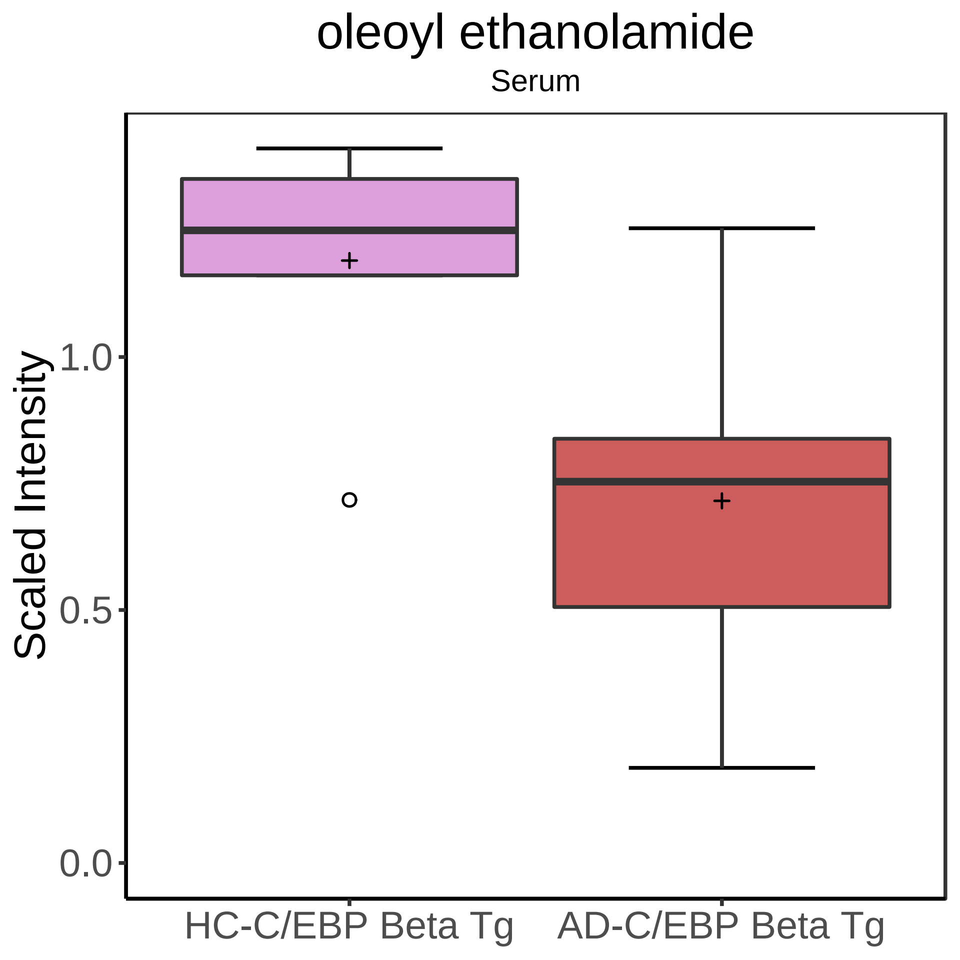

oleoyl ethanolamide
Serum
Scaled Intensity
1.0
0.5
0.0
HC-C/EBP Beta Tg
AD-C/EBP Beta Tg

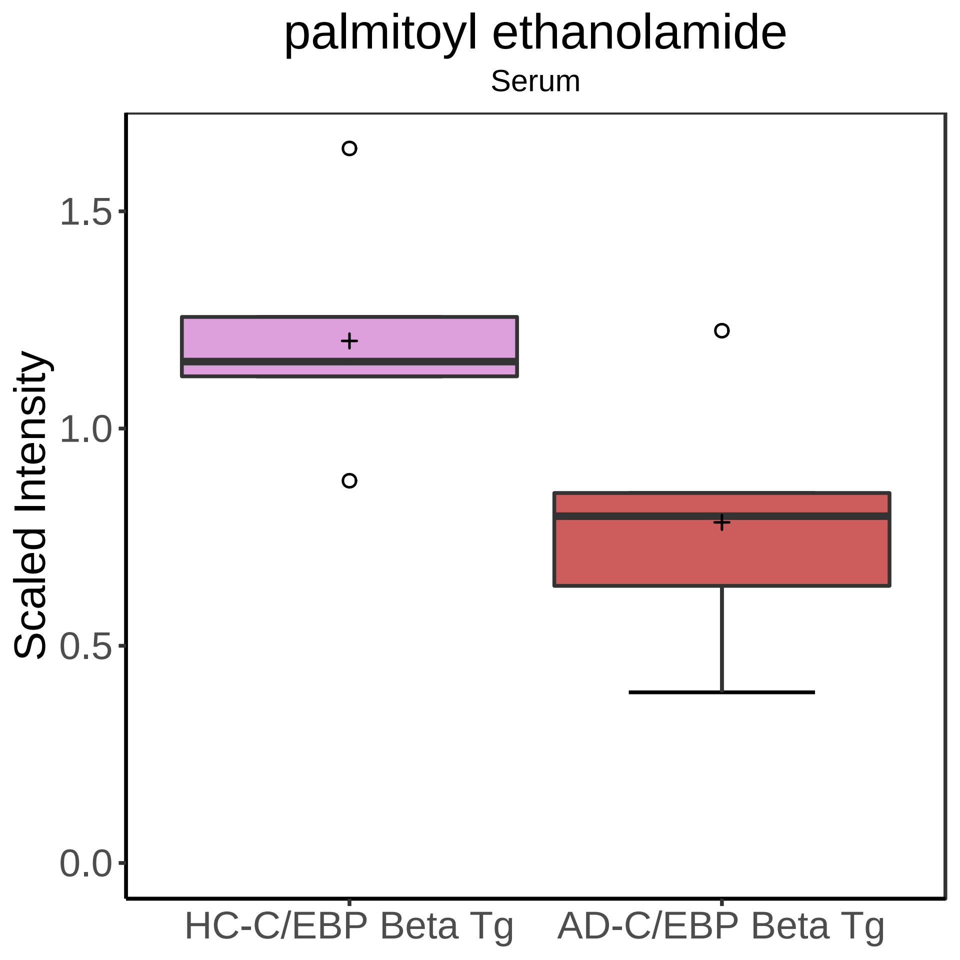
palmitoyl ethanolamide
Serum
Scaled Intensity
0.0
0.5
1.0
1.5
HC-C/EBP Beta Tg
AD-C/EBP Beta Tg

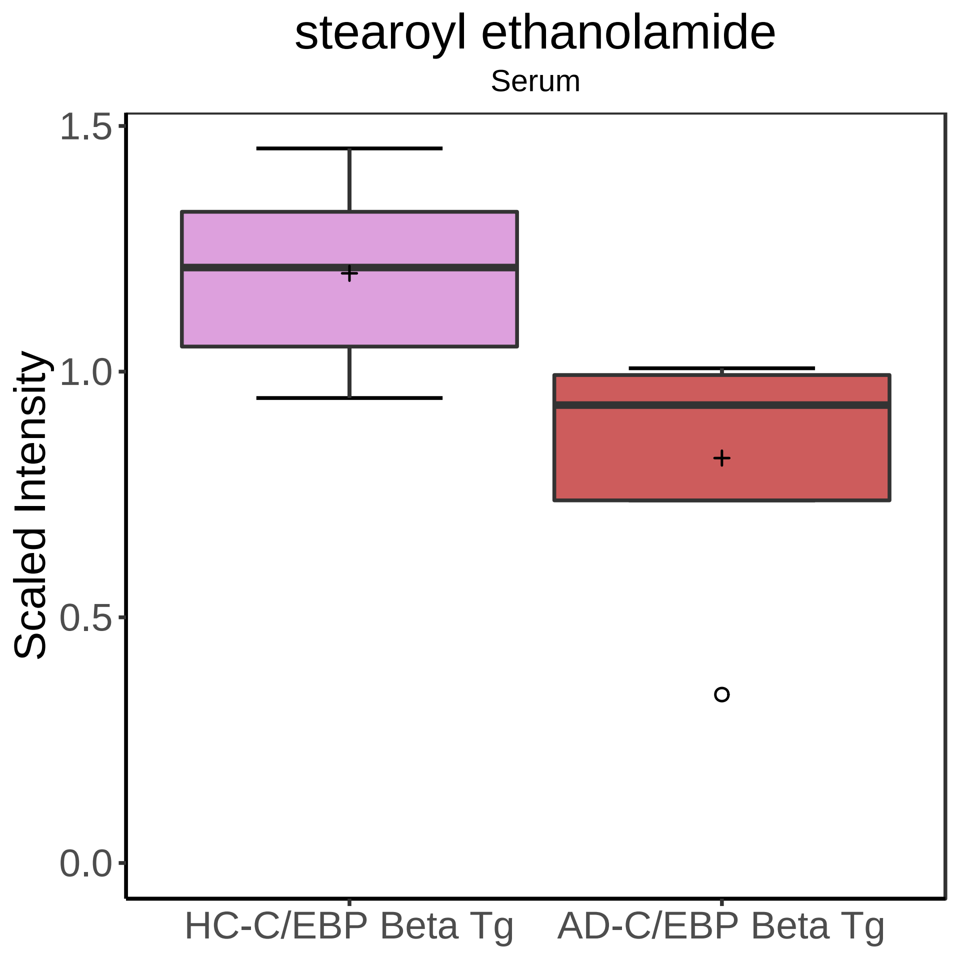
stearoyl ethanolamide
Serum
Scaled Intensity
1.5
1.0
0.5
0.0
HC-C/EBP Beta Tg
AD-C/EBP Beta Tg

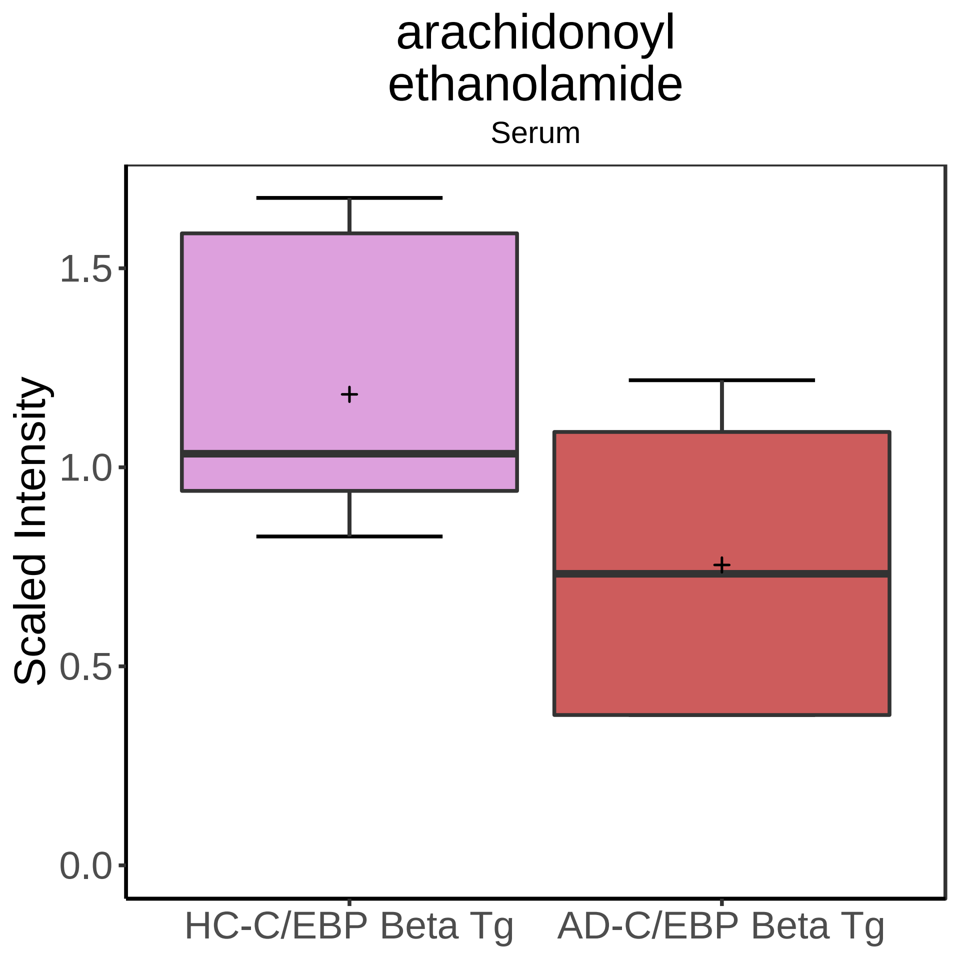

arachidonoyl
ethanolamide
Serum
Scaled Intensity
1.5
1.0
0.5
0.0
HC-C/EBP Beta Tg
AD-C/EBP Beta Tg

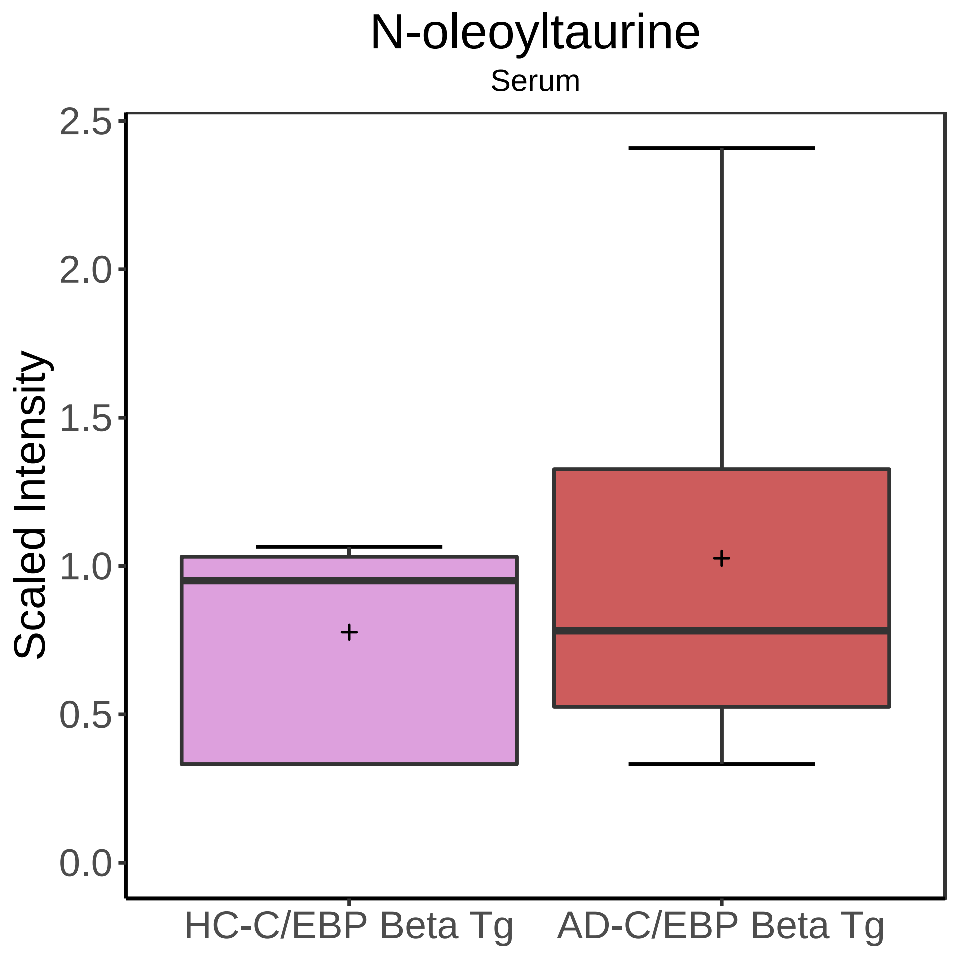
N-oleoyltaurine
Serum
Scaled Intensity
2.5
2.0
1.5
1.0
0.5
0.0
HC-C/EBP Beta Tg
AD-C/EBP Beta Tg

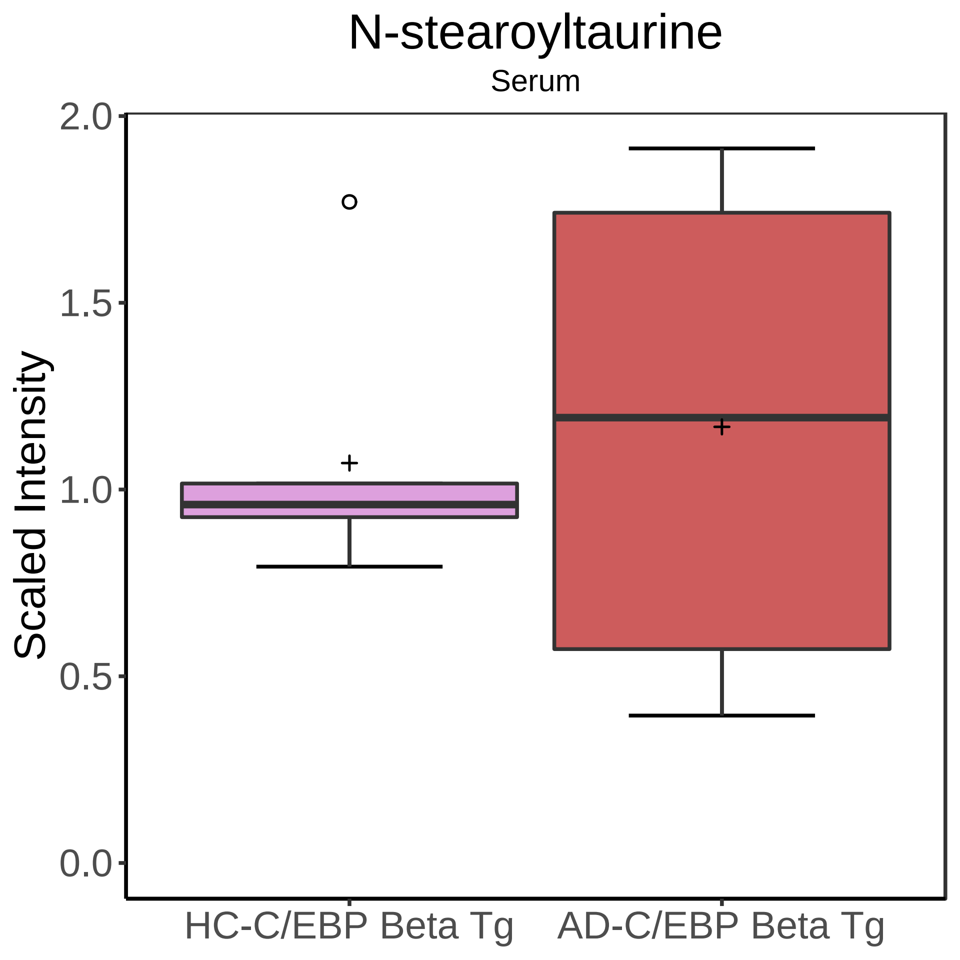
N-stearoyltaurine
Serum
Scaled Intensity
2.0
1.5
1.0
0.5
0.0
HC-C/EBP Beta Tg
AD-C/EBP Beta Tg

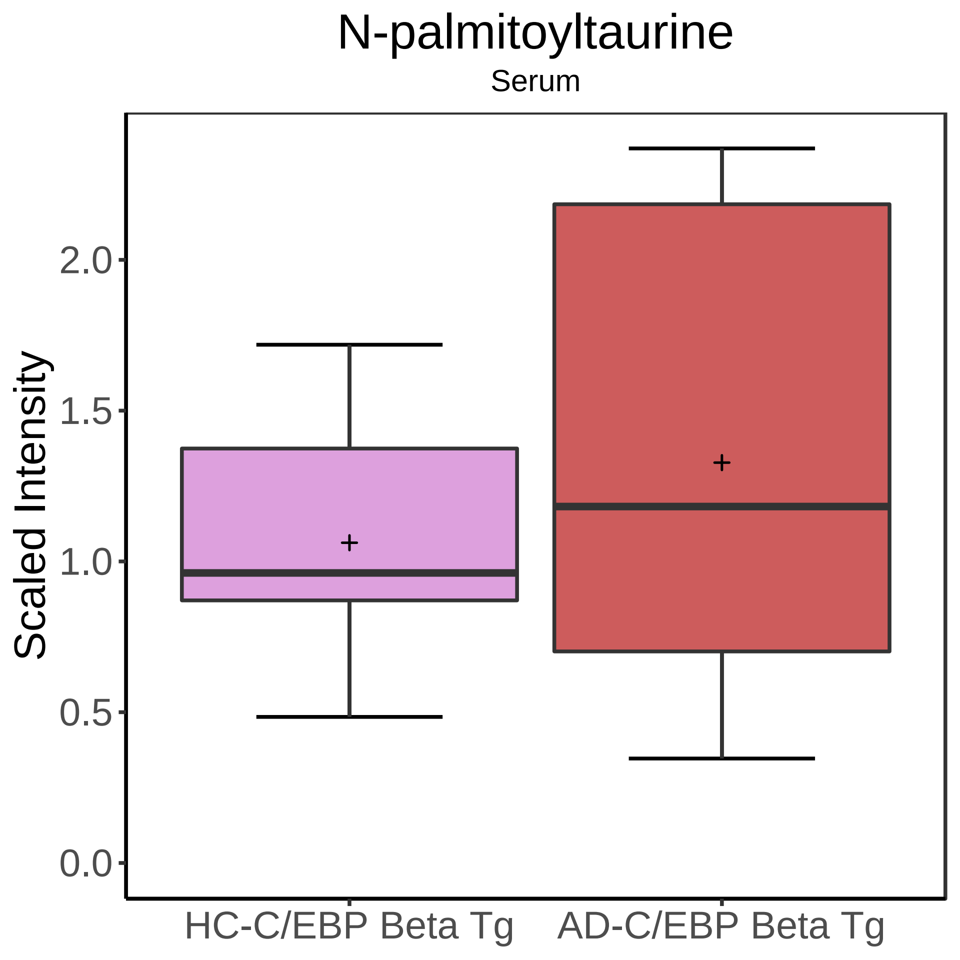

N-palmitoyltaurine
Serum
Scaled Intensity
2.0
1.5
1.0
0.5
0.0
HC-C/EBP Beta Tg
AD-C/EBP Beta Tg

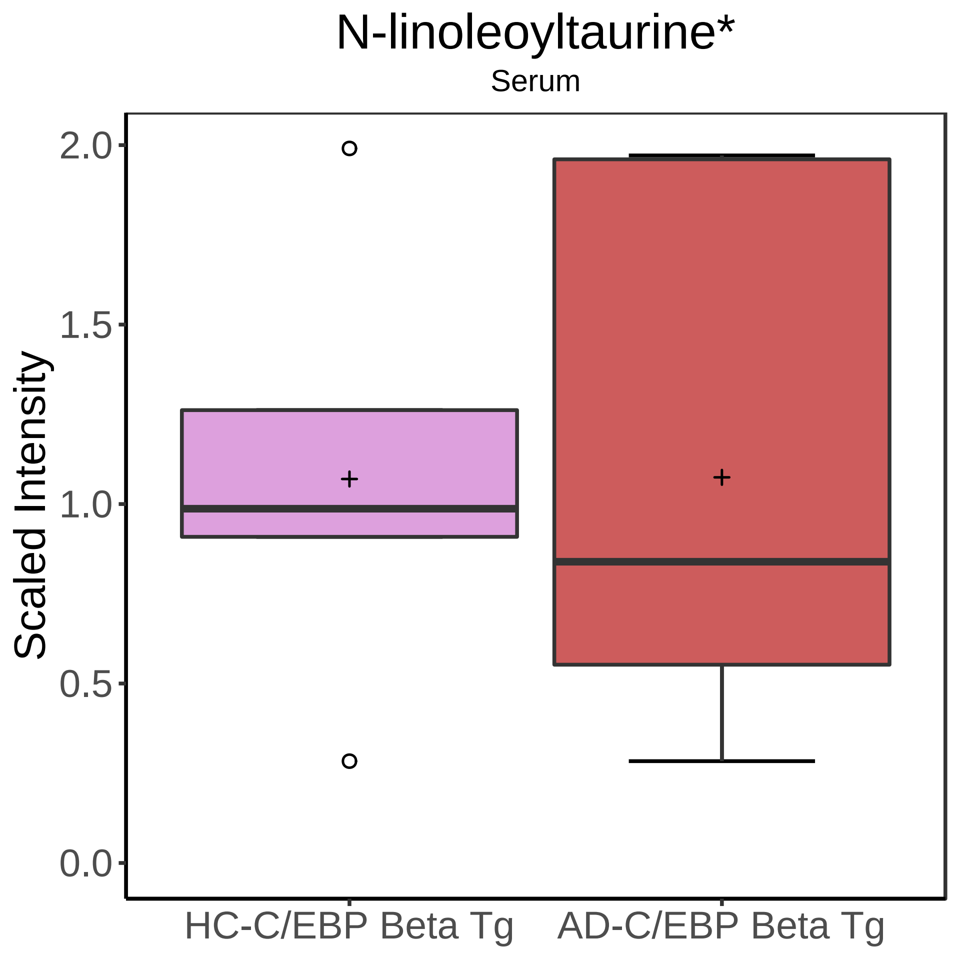
N-linoleoyltaurine*
Serum
Scaled Intensity
2.0
1.5
1.0
0.5
0.0
HC-C/EBP Beta Tg
AD-C/EBP Beta Tg

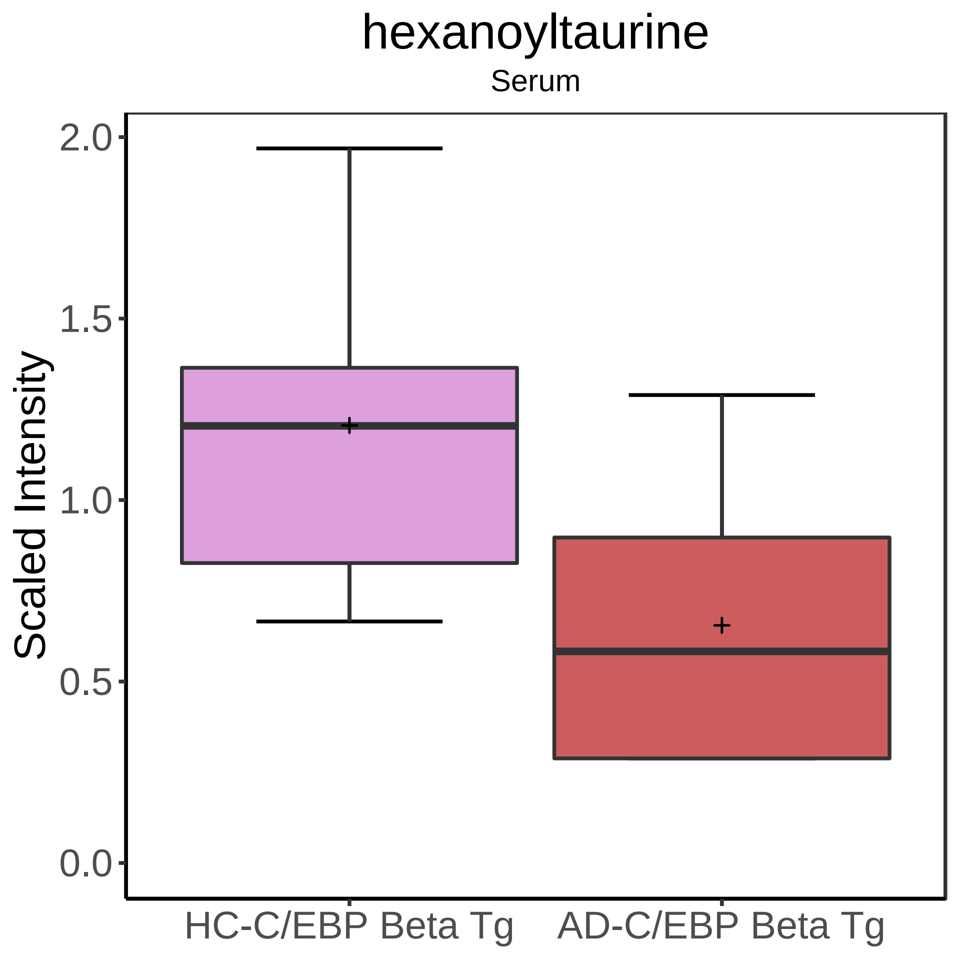
hexanoyltaurine
Serum
Scaled Intensity
2.0
1.5
1.0
0.5
0.0
HC-C/EBP Beta Tg
AD-C/EBP Beta Tg

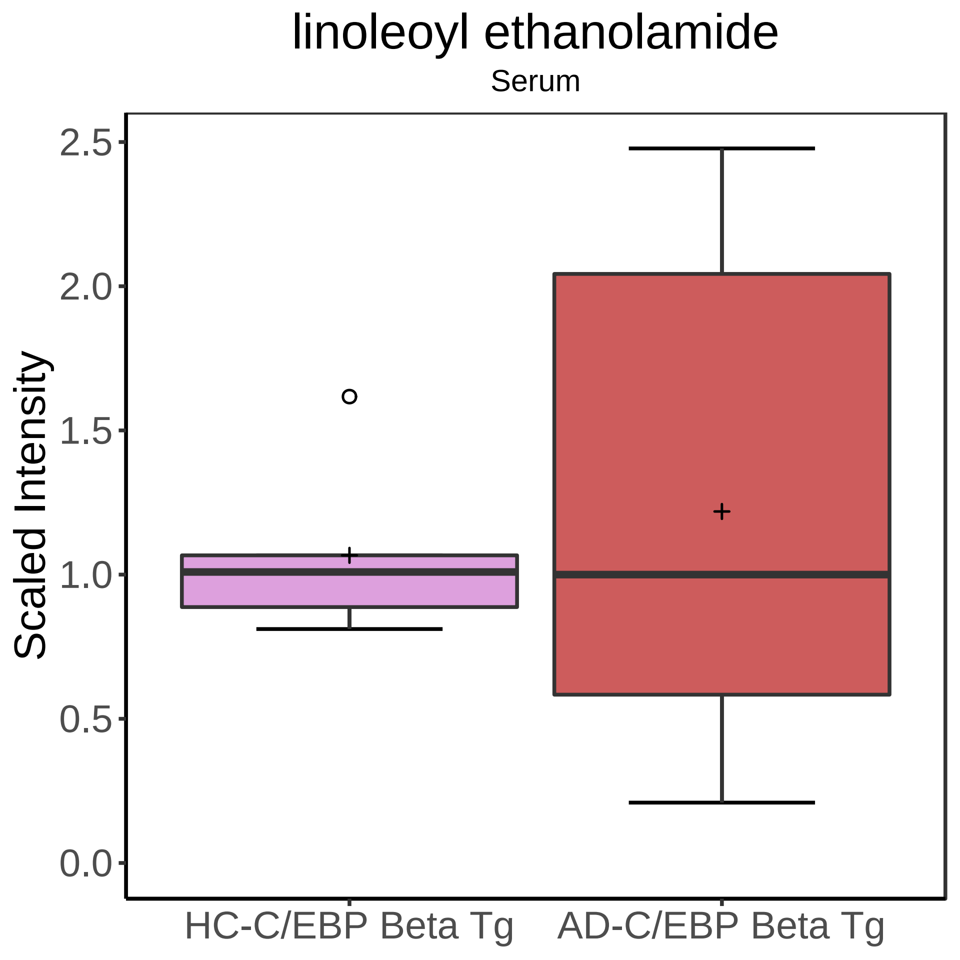

linoleoyl ethanolamide
Serum
Scaled Intensity
2.5
2.0
1.5
1.0
0.5
0.0
HC-C/EBP Beta Tg
AD-C/EBP Beta Tg

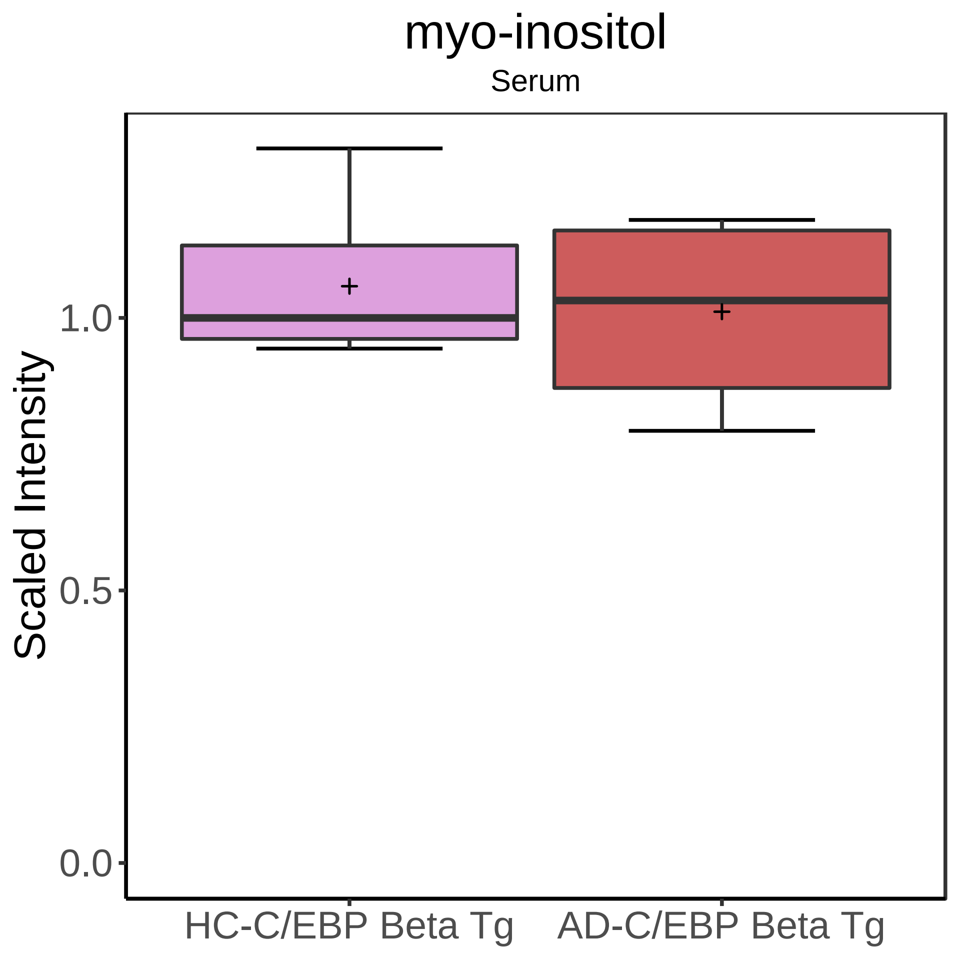
myo-inositol
Serum
Scaled Intensity
1.0
0.5
0.0
HC-C/EBP Beta Tg
AD-C/EBP Beta Tg

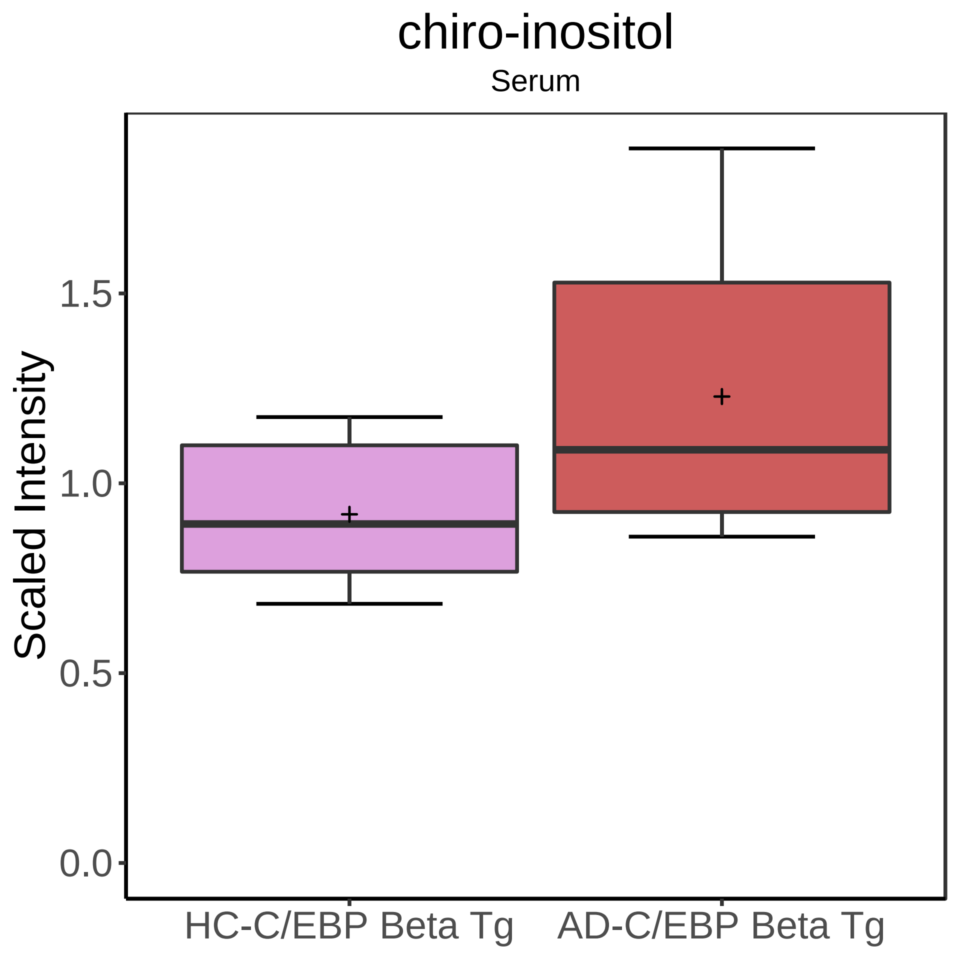
chiro-inositol
Serum
Scaled Intensity
1.5
1.0
0.5
0.0
HC-C/EBP Beta Tg
AD-C/EBP Beta Tg

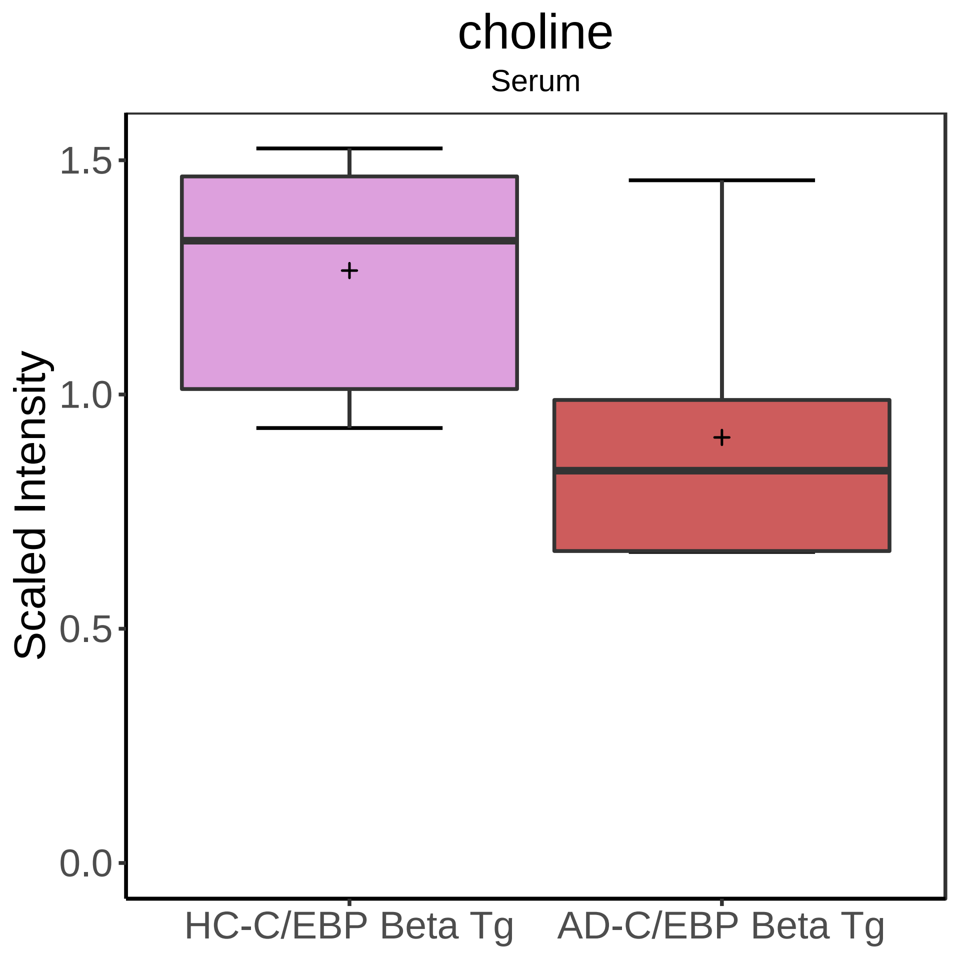

choline
Serum
Scaled Intensity
1.5
1.0
0.5
0.0
HC-C/EBP Beta Tg
AD-C/EBP Beta Tg

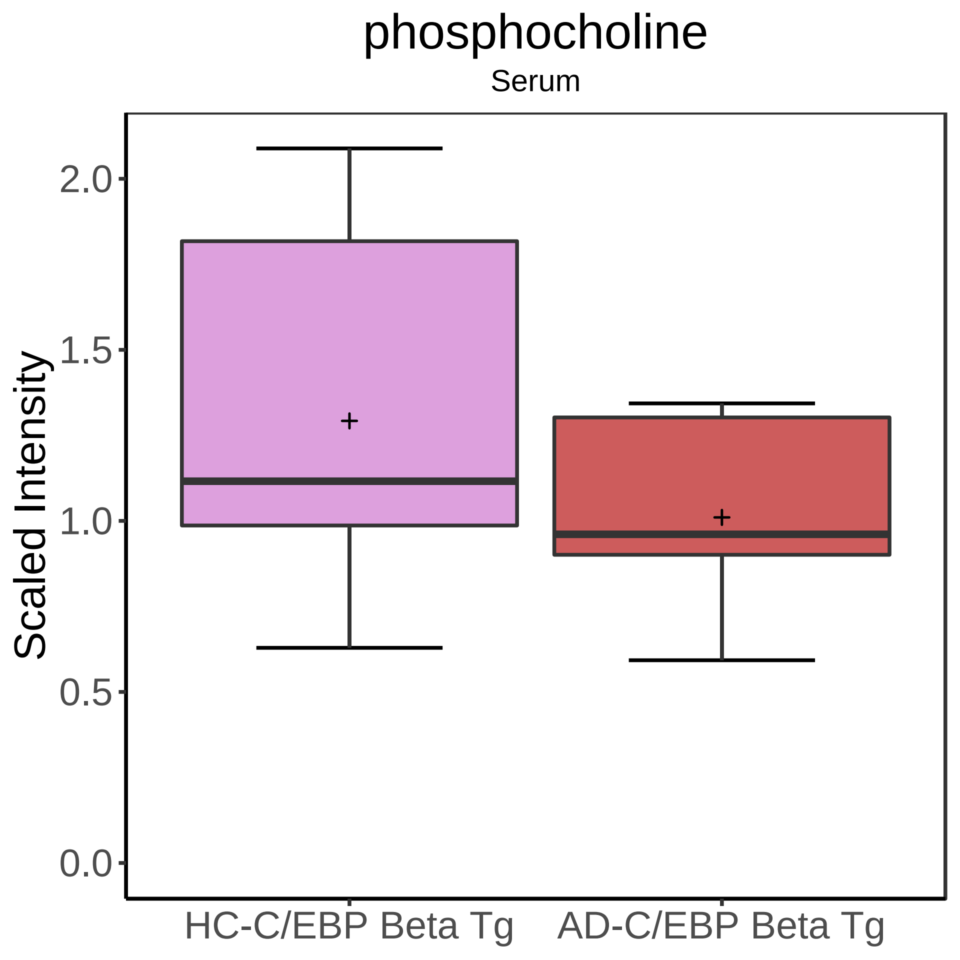
phosphocholine
Serum
Scaled Intensity
2.0
1.5
1.0
0.5
0.0
HC-C/EBP Beta Tg
AD-C/EBP Beta Tg

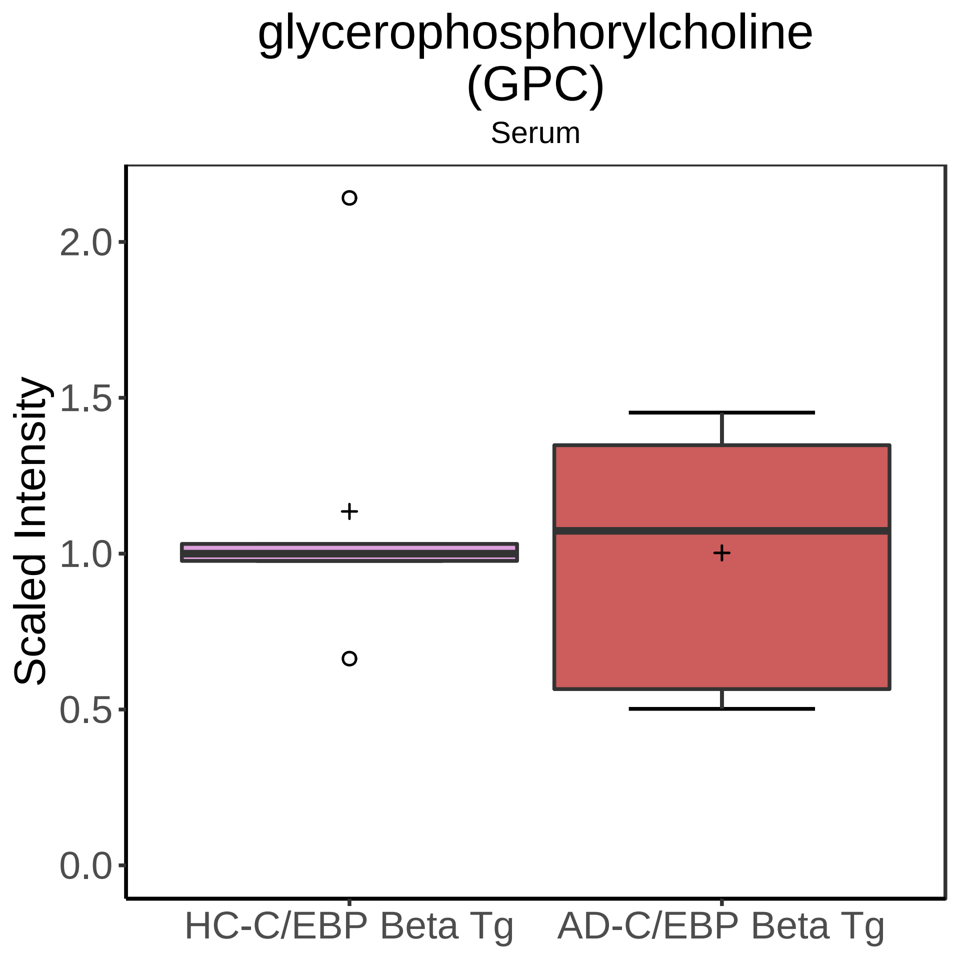

glycerophosphorylcholine
(GPC)
Serum
Scaled Intensity
2.0
1.5
1.0
0.5
0.0
HC-C/EBP Beta Tg
AD-C/EBP Beta Tg

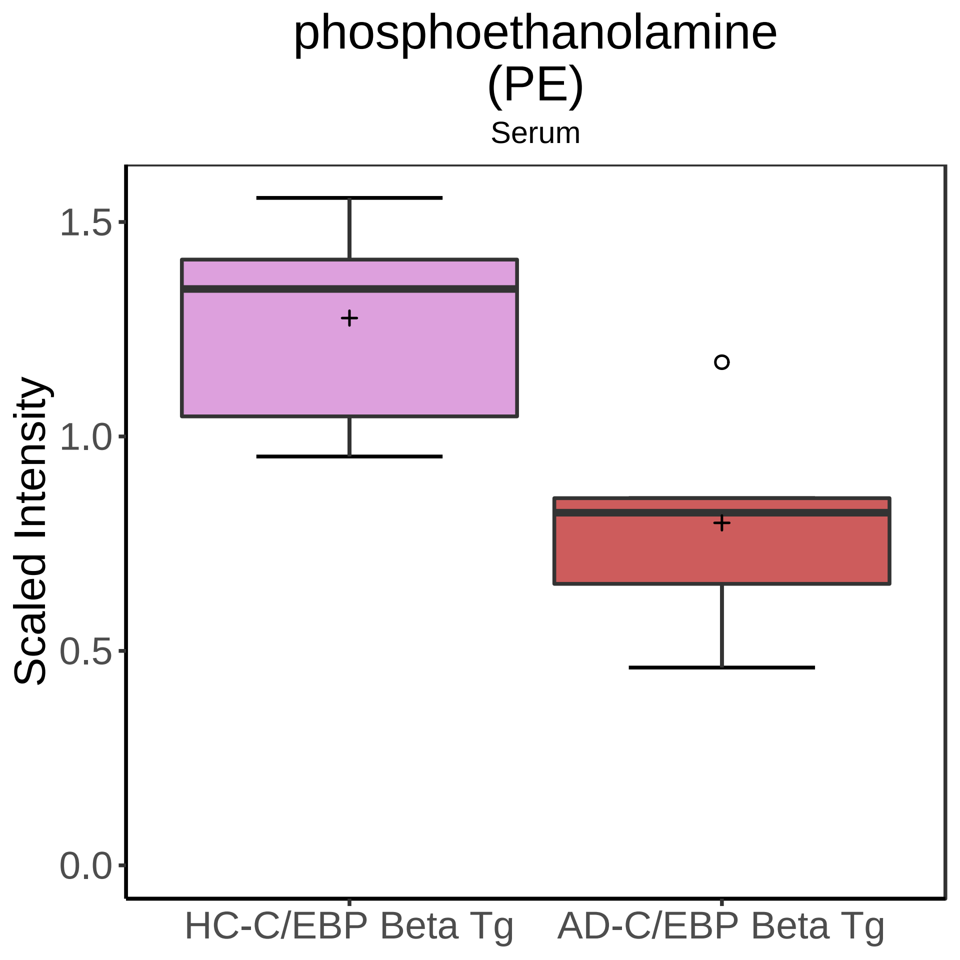

phosphoethanolamine
(PE)
Serum
Scaled Intensity
1.5
1.0
0.5
0.0
HC-C/EBP Beta Tg
AD-C/EBP Beta Tg

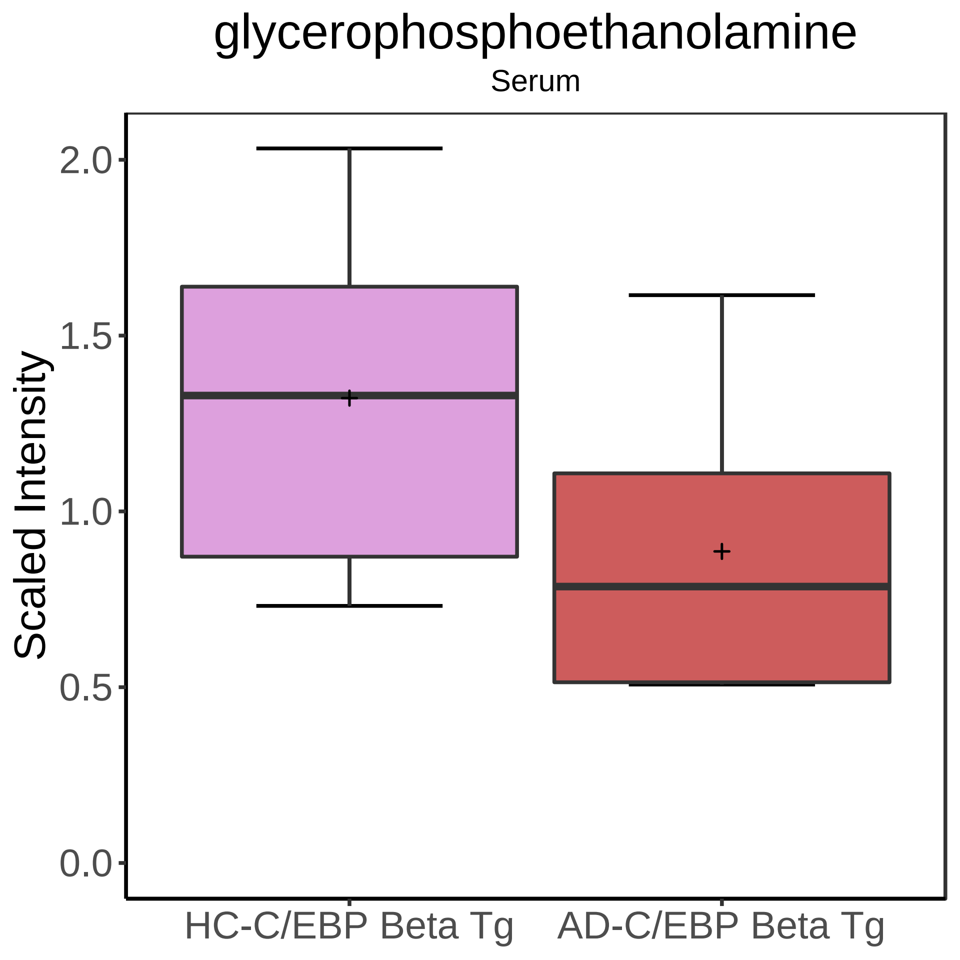
glycerophosphoethanolamine
Serum
Scaled Intensity
2.0
1.5
1.0
0.5
0.0
HC-C/EBP Beta Tg
AD-C/EBP Beta Tg

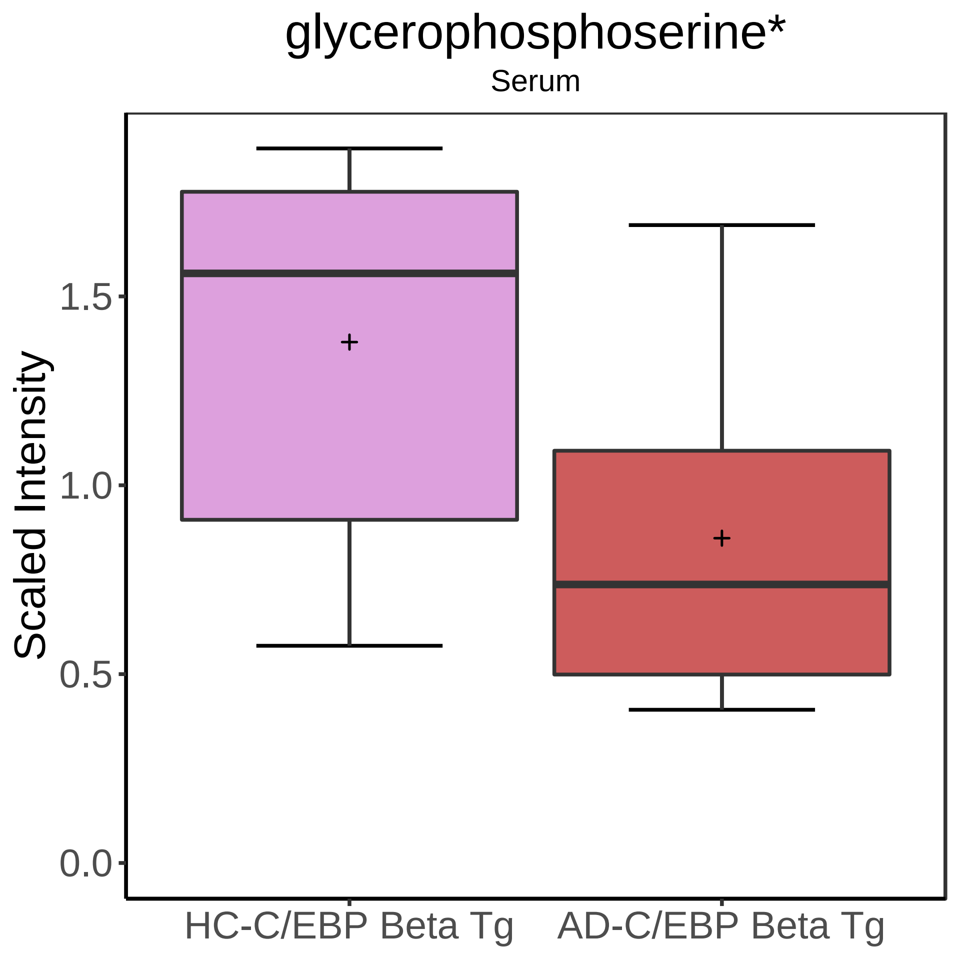
glycerophosphoserine*
Serum
Scaled Intensity
1.5
1.0
0.5
0.0
HC-C/EBP Beta Tg
AD-C/EBP Beta Tg

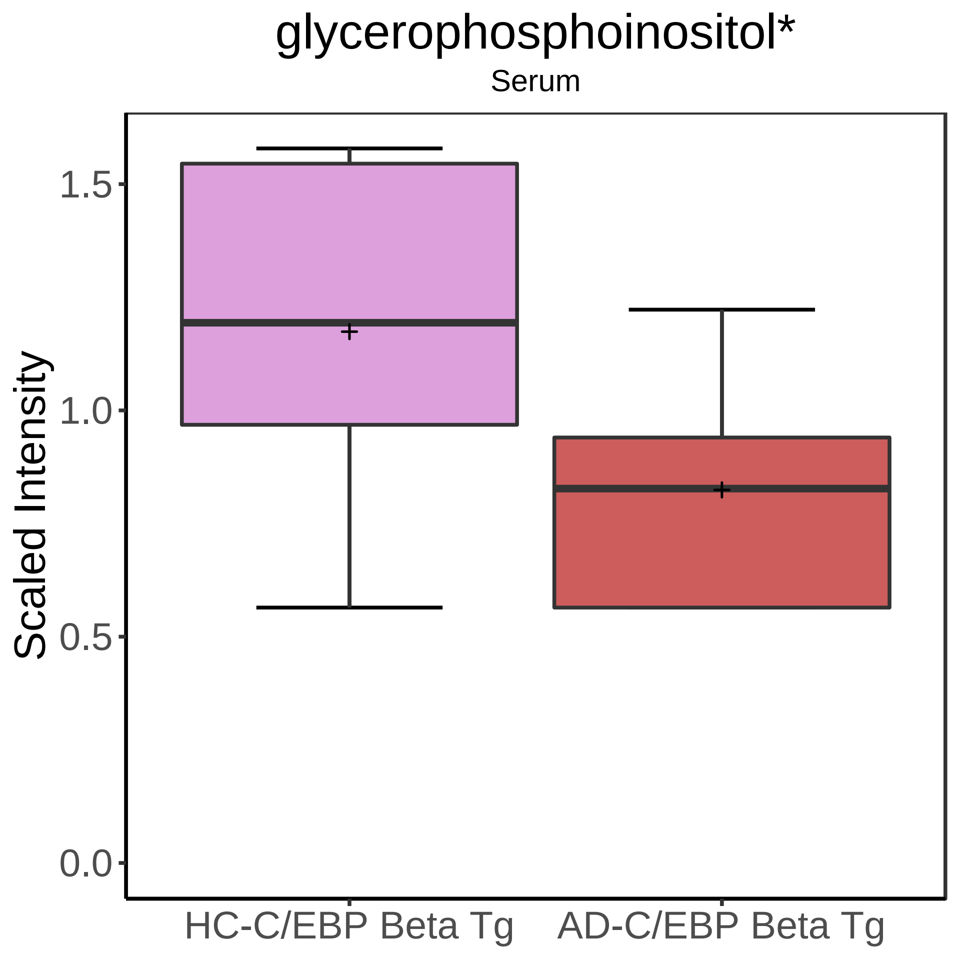
glycerophosphoinositol*
Serum
Scaled Intensity
1.5
1.0
0.5
0.0
HC-C/EBP Beta Tg
AD-C/EBP Beta Tg

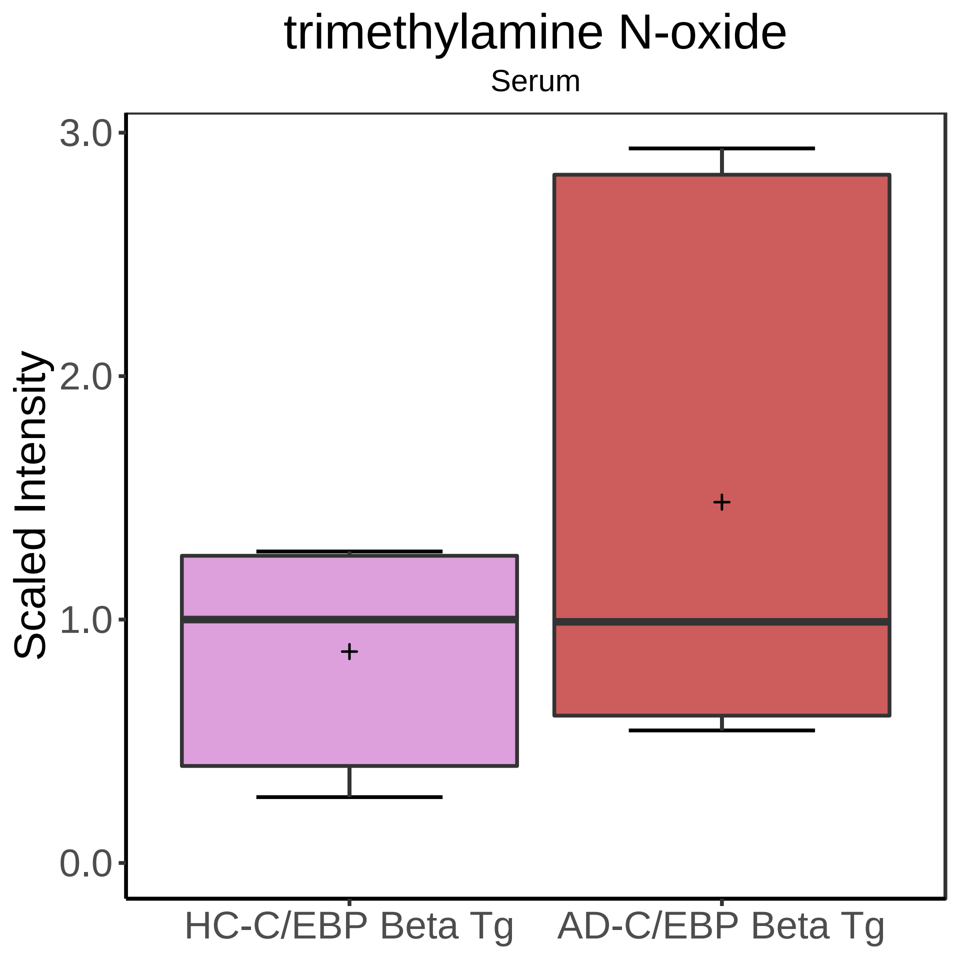

trimethylamine N-oxide
Serum
Scaled Intensity
3.0
2.0
1.0
0.0
HC-C/EBP Beta Tg
AD-C/EBP Beta Tg

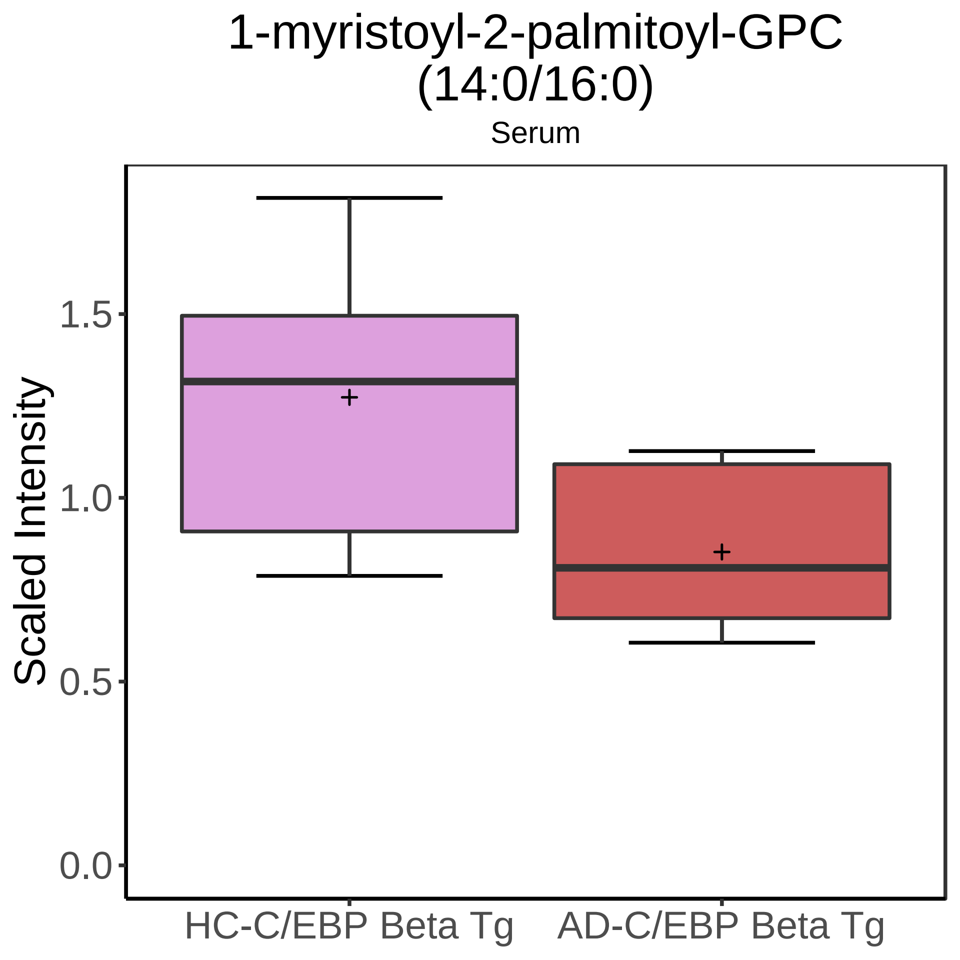
1-myristoyl-2-palmitoyl-GPC
(14:0/16:0)
Serum
Scaled Intensity
1.5
1.0
0.5
0.0
HC-C/EBP Beta Tg
AD-C/EBP Beta Tg

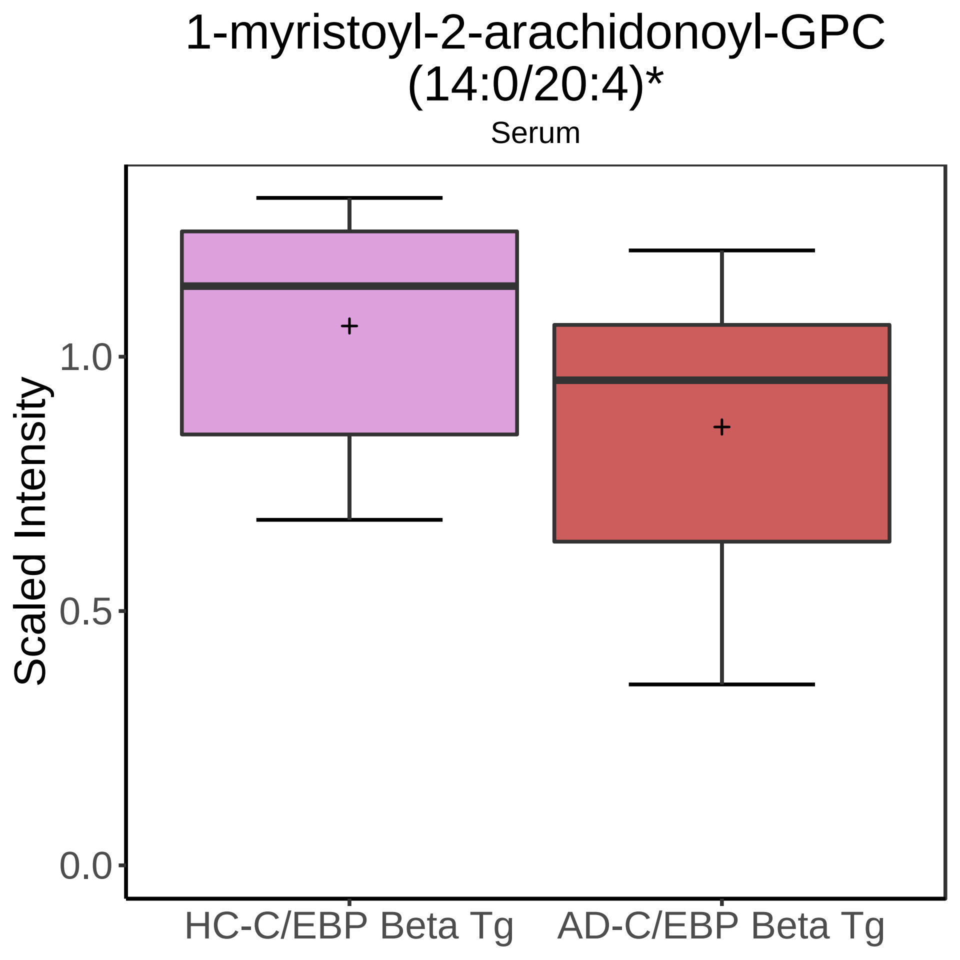
1-myristoyl-2-arachidonoyl-GPC
(14:0/20:4)*
Serum
Scaled Intensity
1.0
0.5
0.0
HC-C/EBP Beta Tg
AD-C/EBP Beta Tg

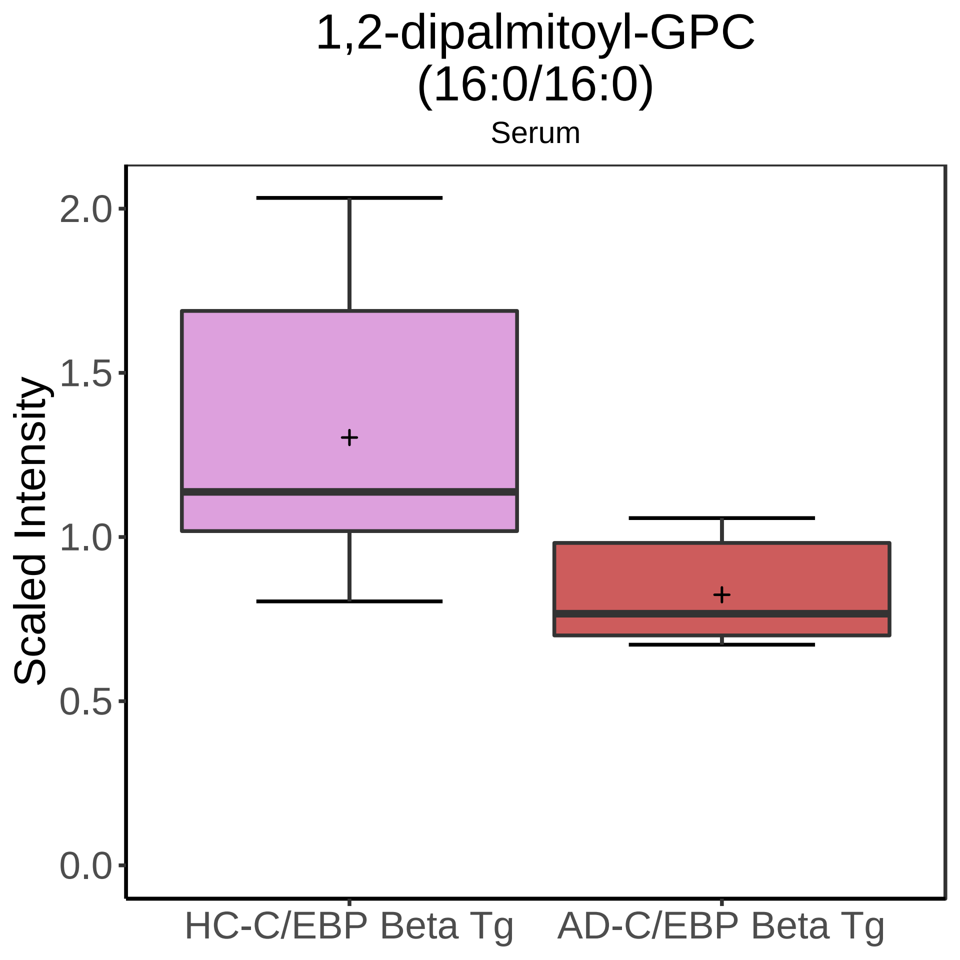
1,2-dipalmitoyl-GPC
(16:0/16:0)
Serum
Scaled Intensity
2.0
1.5
1.0
0.5
0.0
HC-C/EBP Beta Tg
AD-C/EBP Beta Tg

1-palmitoyl-2-palmitoleoyl-GPC (16:0/16:1)*

Serum

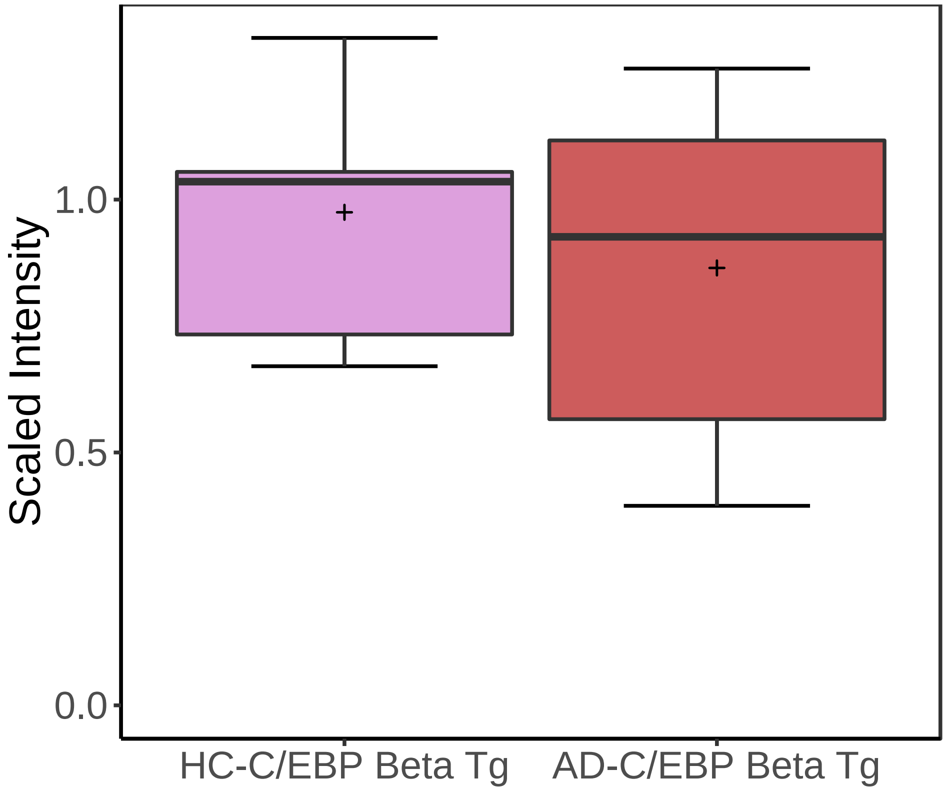

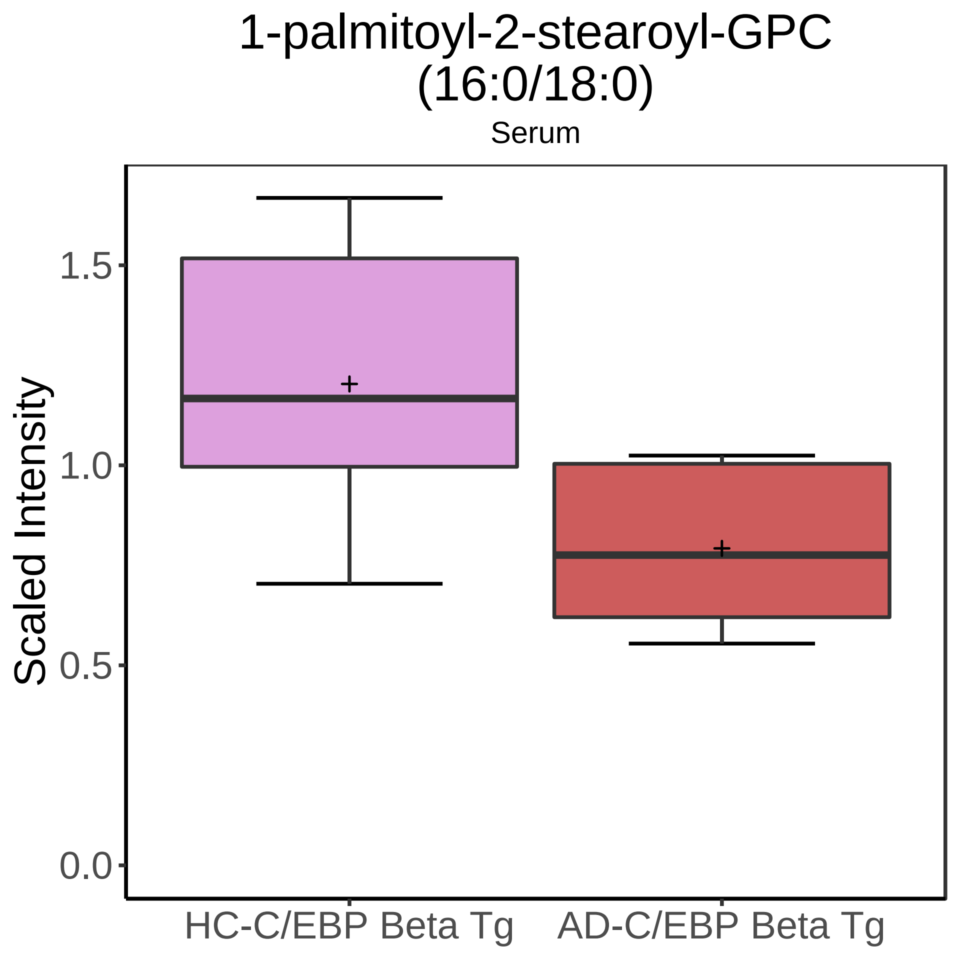

1-palmitoyl-2-stearoyl-GPC
(16:0/18:0)
Serum
Scaled Intensity
1.5
1.0
0.5
0.0
HC-C/EBP Beta Tg
AD-C/EBP Beta Tg

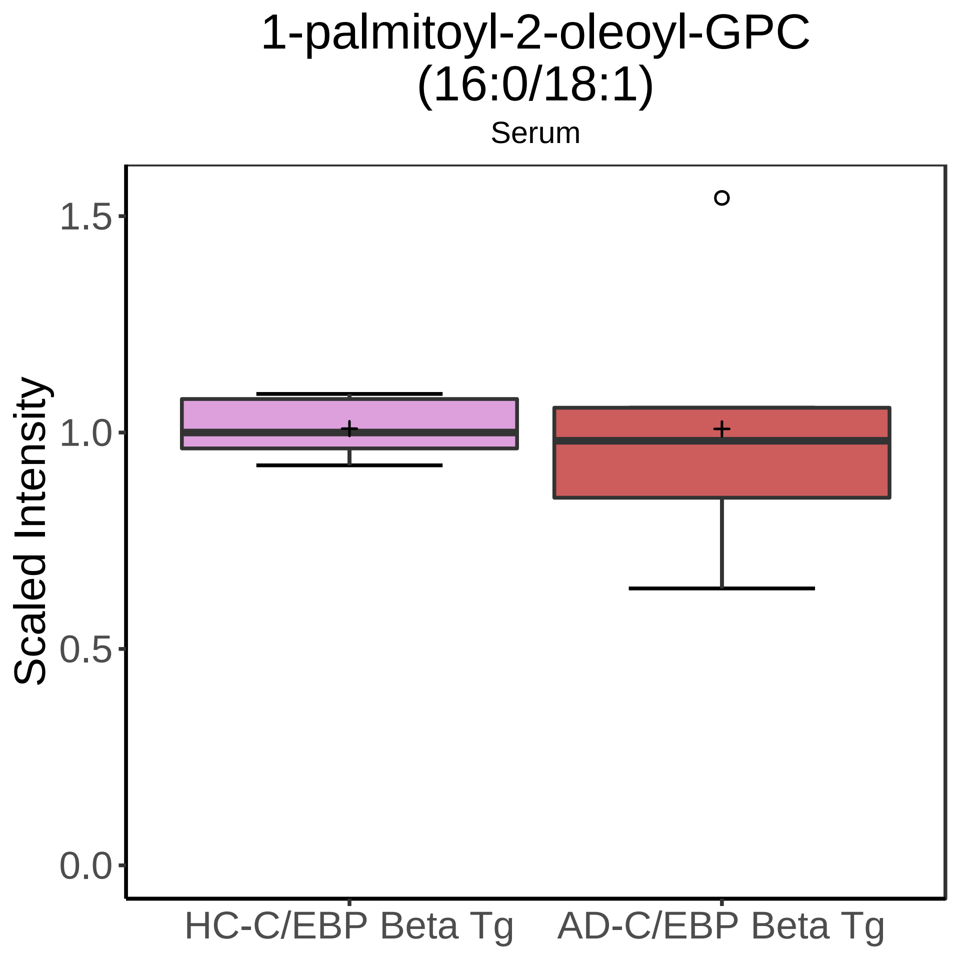
1-palmitoyl-2-oleoyl-GPC
(16:0/18:1)
Serum
Scaled Intensity
1.5
1.0
0.5
0.0
HC-C/EBP Beta Tg
AD-C/EBP Beta Tg

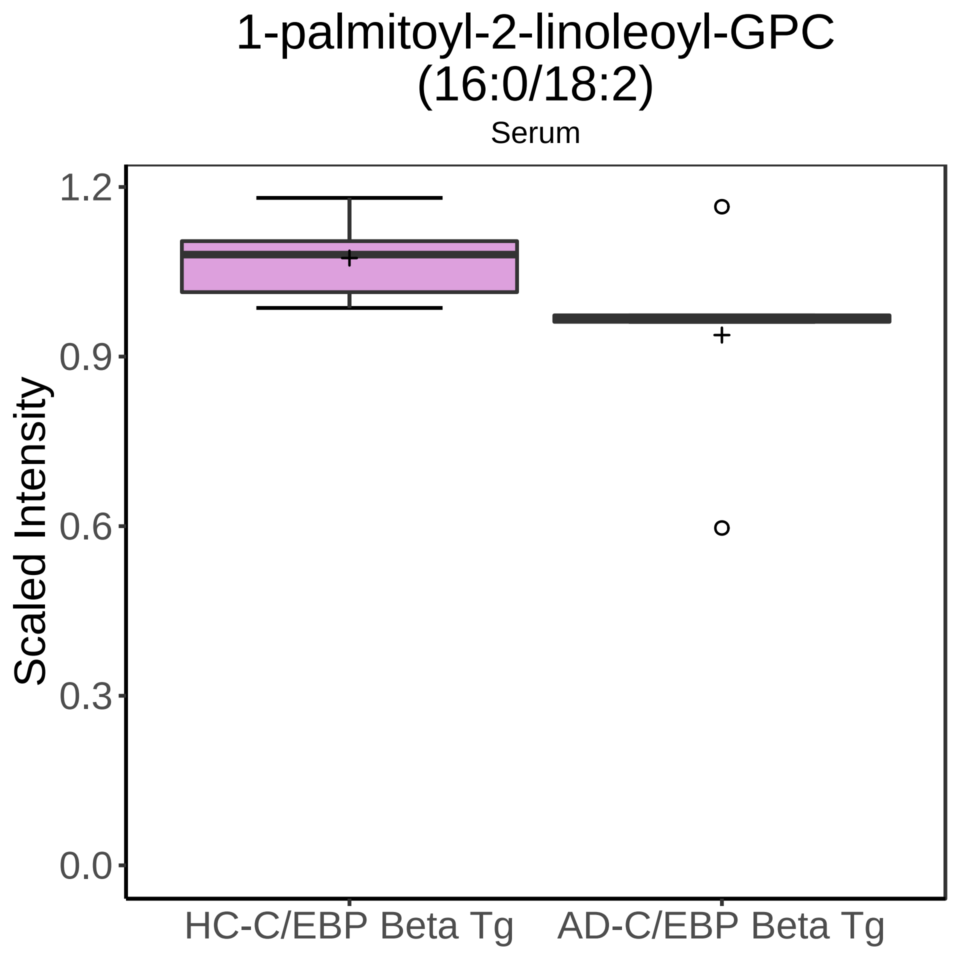
1-palmitoyl-2-linoleoyl-GPC
(16:0/18:2)
Serum
Scaled Intensity
1.2
0.9
0.6
0.3
0.0
HC-C/EBP Beta Tg
AD-C/EBP Beta Tg

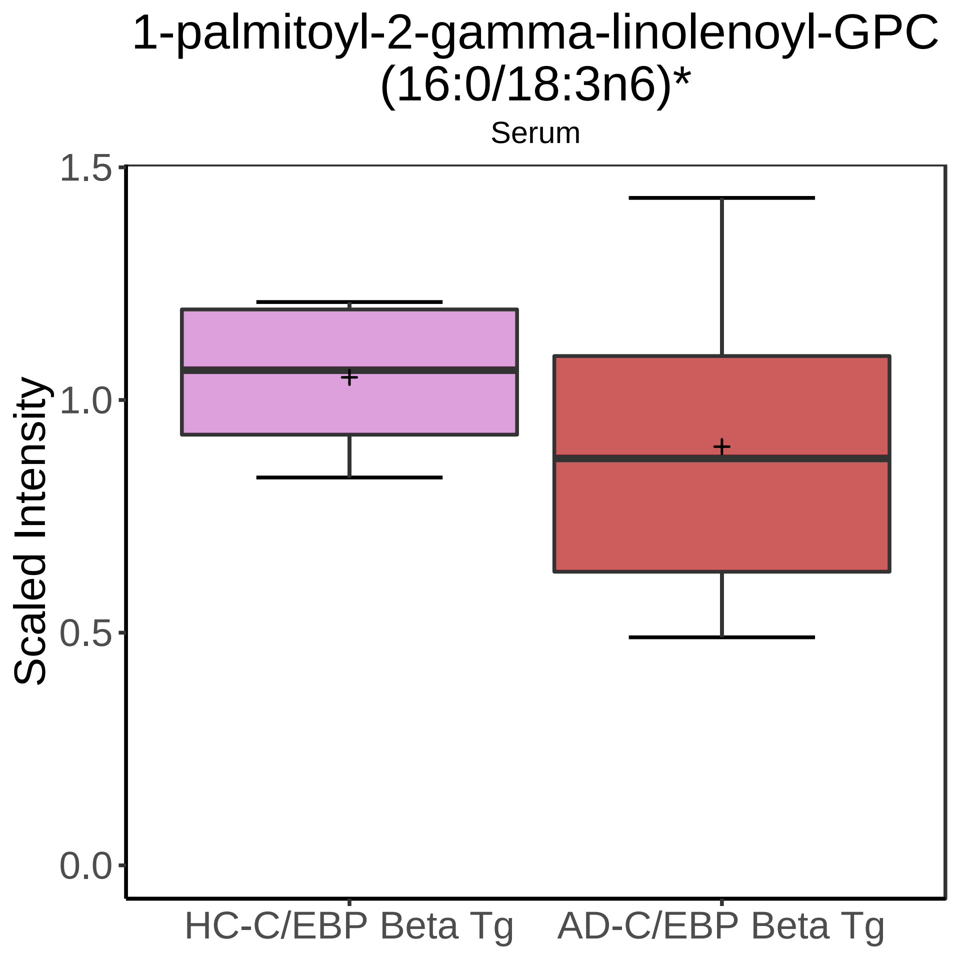

1-palmitoyl-2-gamma-linolenoyl-GPC
(16:0/18:3n6)*
Serum
Scaled Intensity
1.5
1.0
0.5
0.0
HC-C/EBP Beta Tg
AD-C/EBP Beta Tg

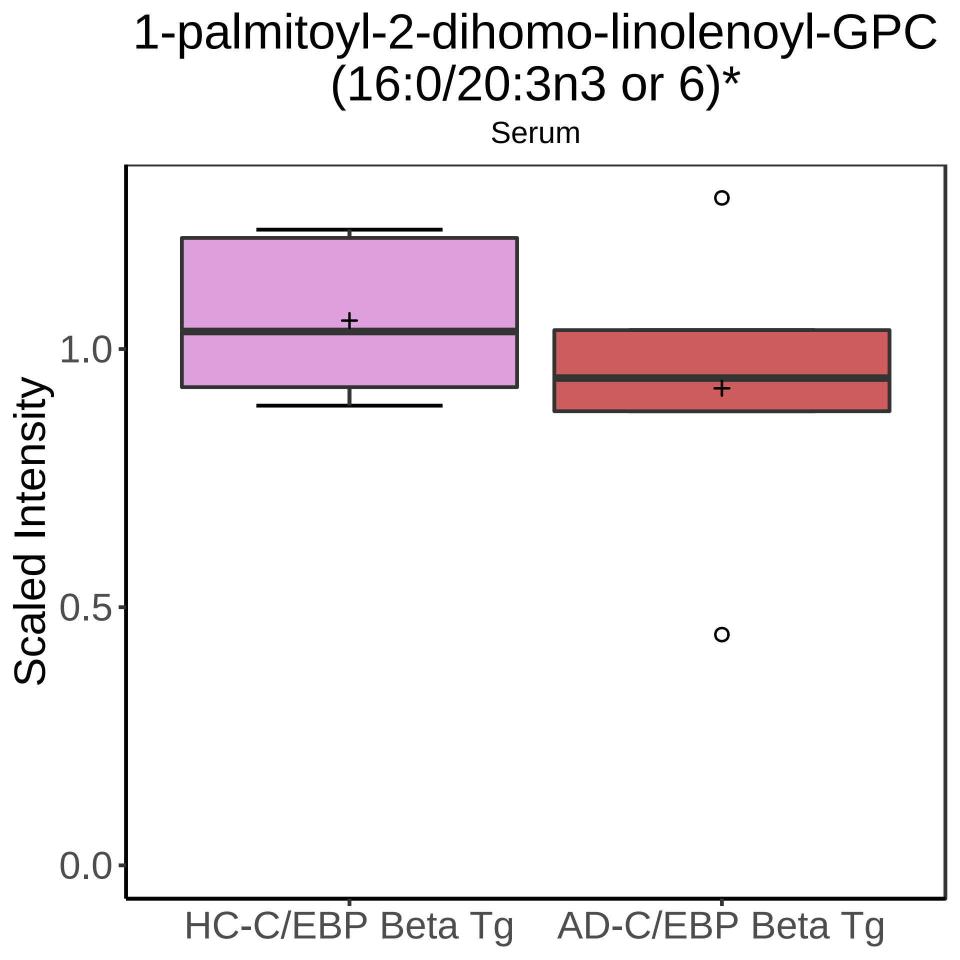
1-palmitoyl-2-dihomo-linolenoyl-GPC
(16:0/20:3n3 or 6)*
Serum
Scaled Intensity
1.0
0.5
0.0
HC-C/EBP Beta Tg
AD-C/EBP Beta Tg

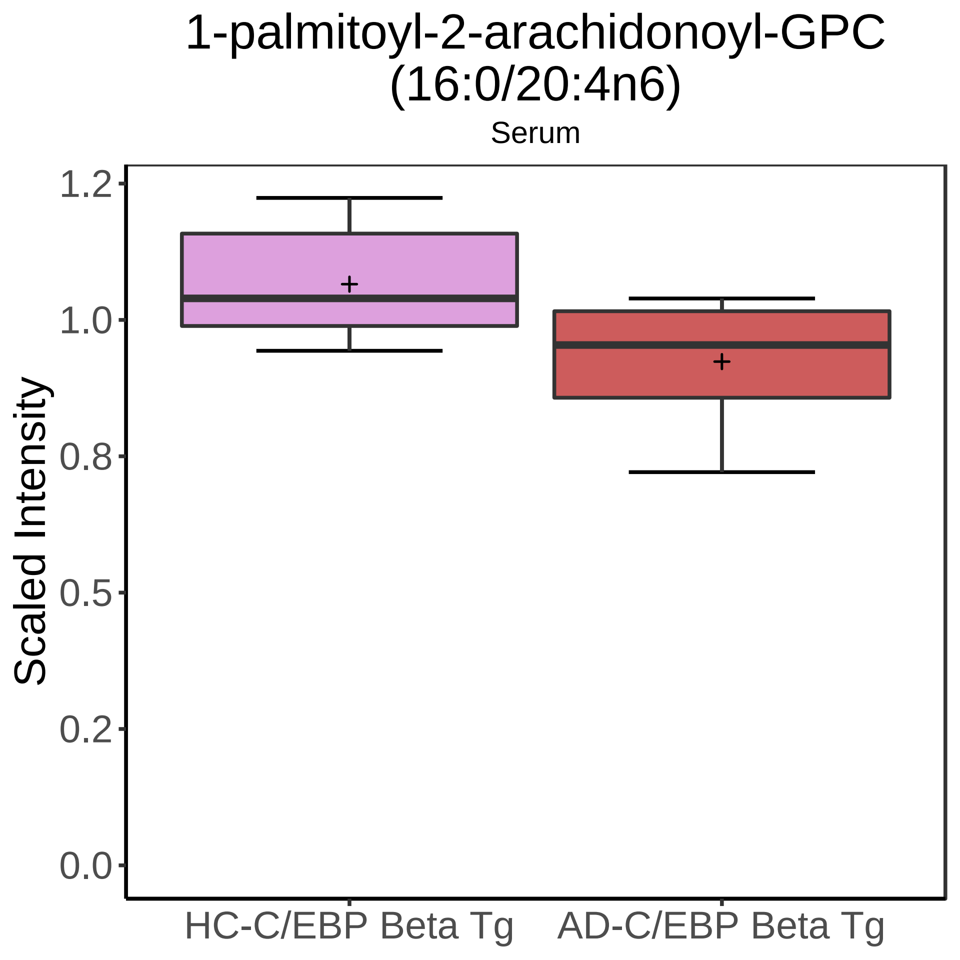
1-palmitoyl-2-arachidonoyl-GPC
(16:0/20:4n6)
Serum
Scaled Intensity
1.2
1.0
0.8
0.5
0.2
0.0
HC-C/EBP Beta Tg
AD-C/EBP Beta Tg

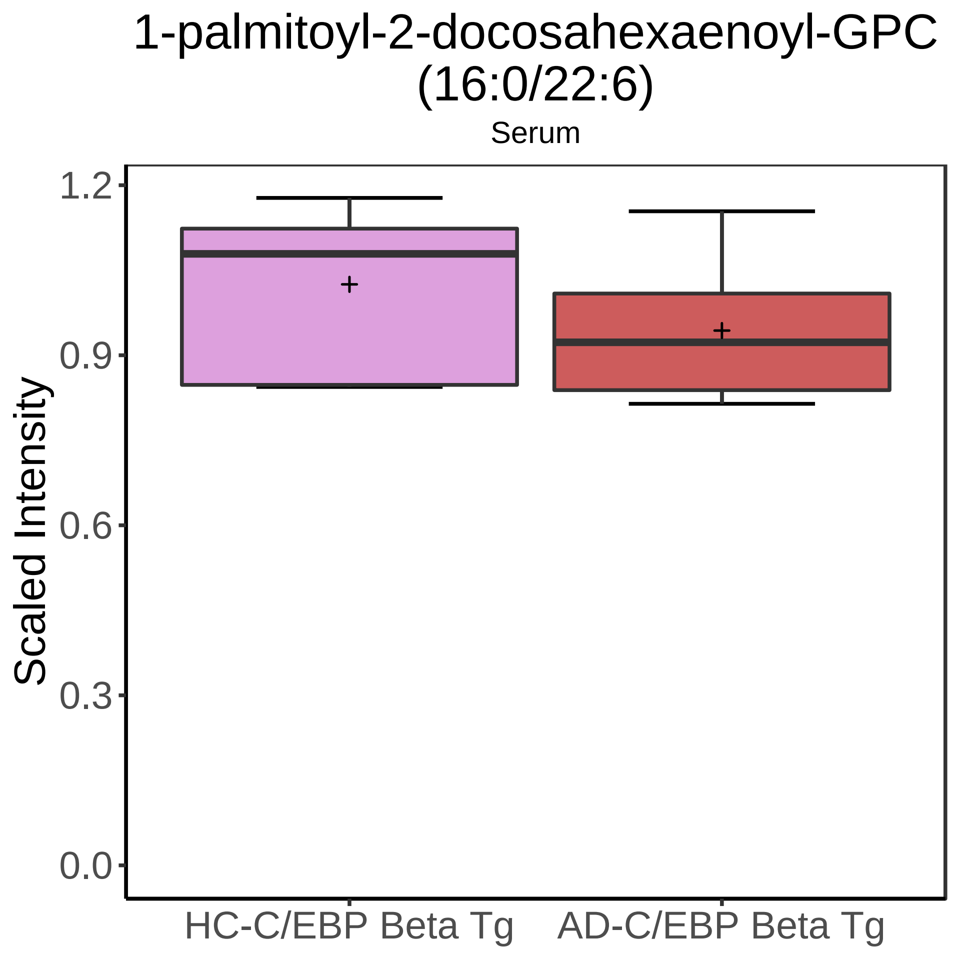
1-palmitoyl-2-docosahexaenoyl-GPC (16:0/22:6)
Serum
Scaled Intensity
1.2
0.9
0.6
0.3
0.0
HC-C/EBP Beta Tg
AD-C/EBP Beta Tg

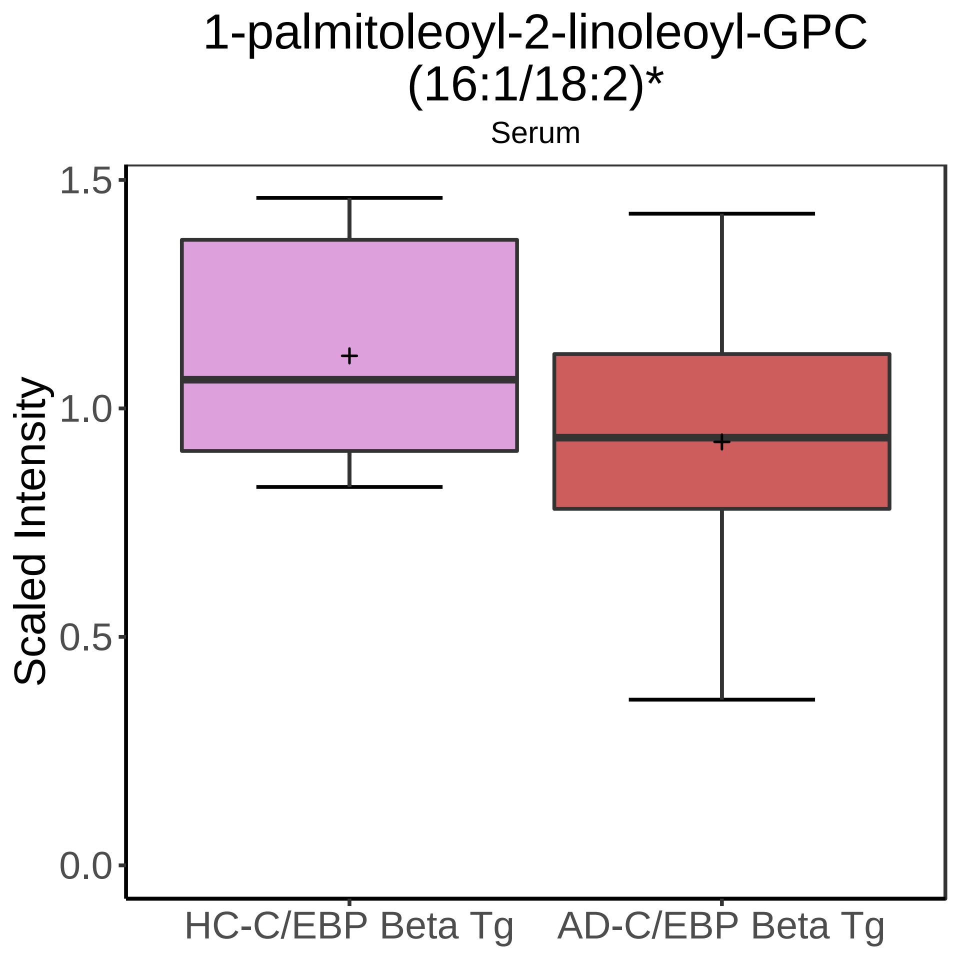

1-palmitoleoyl-2-linoleoyl-GPC
(16:1/18:2)*
Serum
Scaled Intensity
1.5
1.0
0.5
0.0
HC-C/EBP Beta Tg
AD-C/EBP Beta Tg

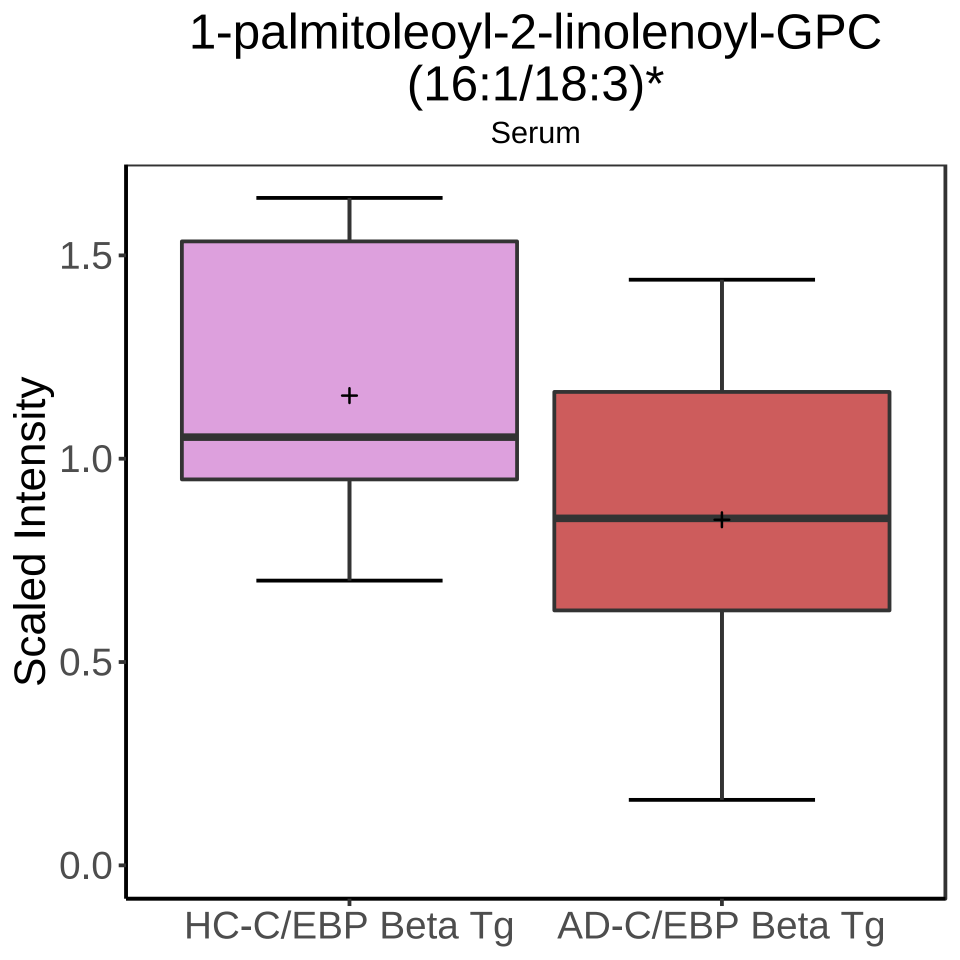

1-palmitoleoyl-2-linolenoyl-GPC
(16:1/18:3)*
Serum
Scaled Intensity
1.5
1.0
0.5
0.0
HC-C/EBP Beta Tg
AD-C/EBP Beta Tg

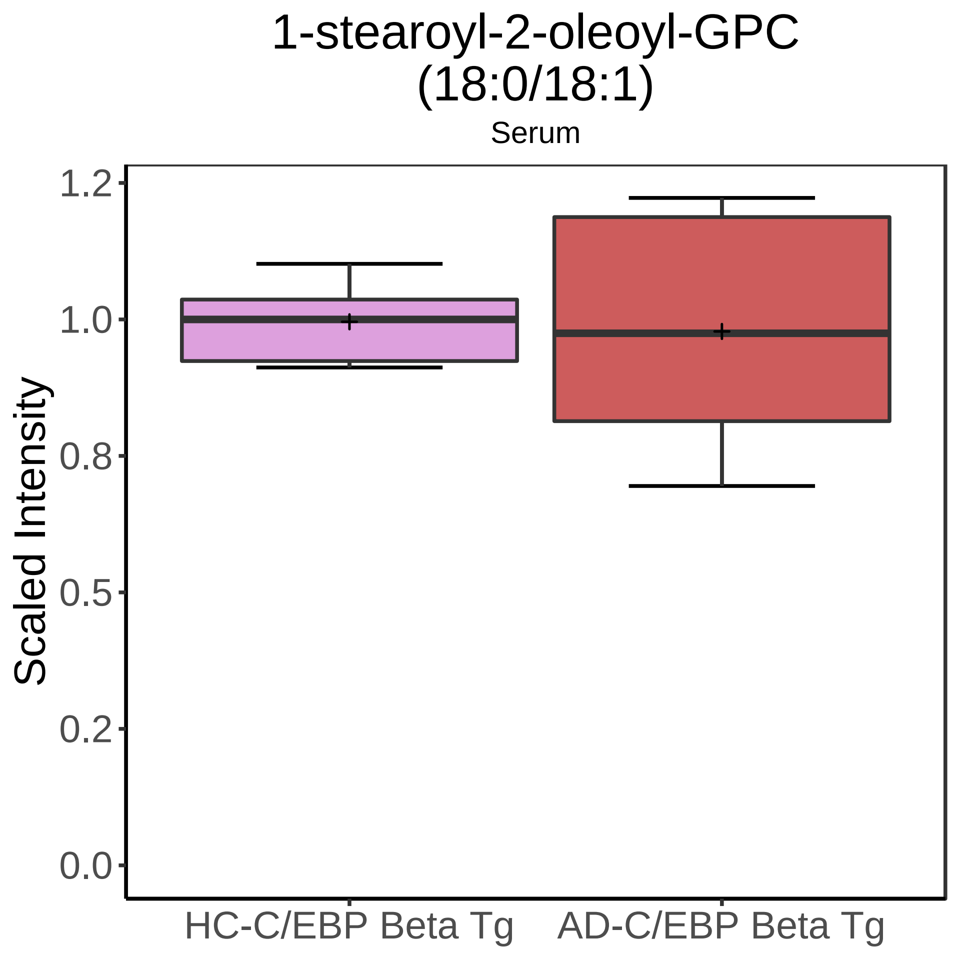
1-stearoyl-2-oleoyl-GPC
(18:0/18:1)
Serum
Scaled Intensity
1.2
1.0
0.8
0.5
0.2
0.0
HC-C/EBP Beta Tg
AD-C/EBP Beta Tg

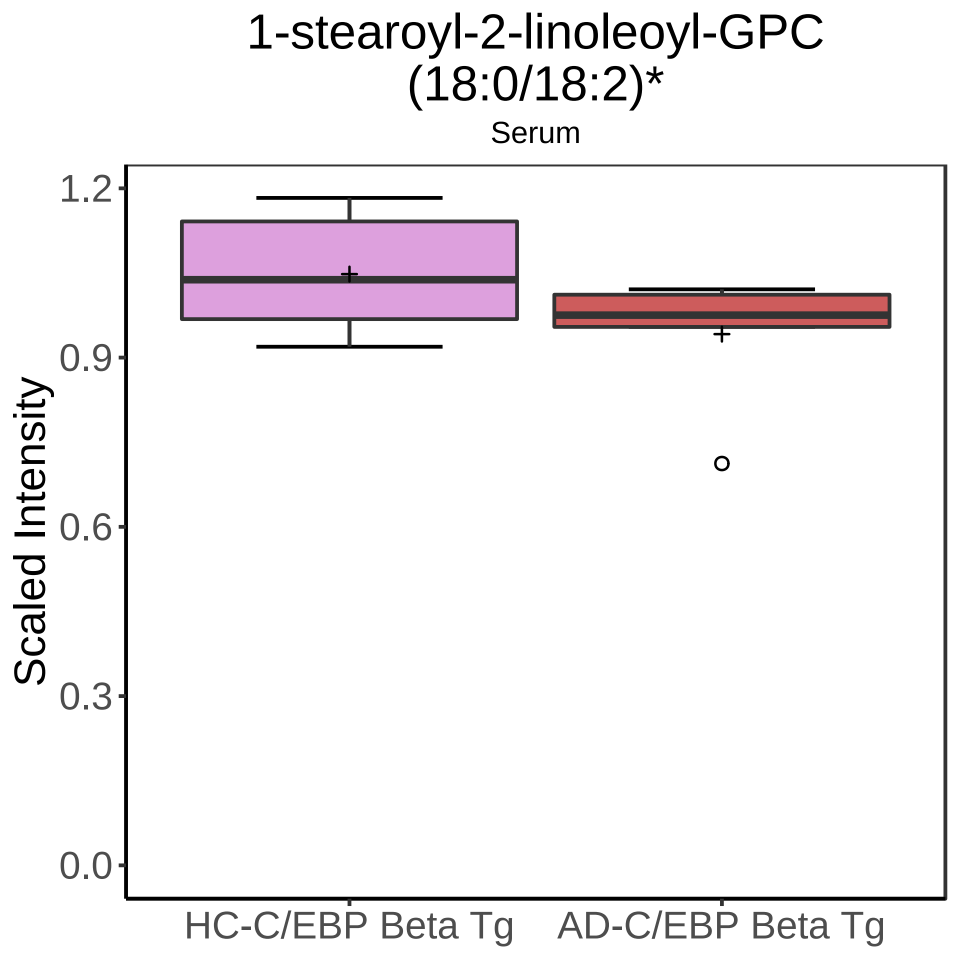
1-stearoyl-2-linoleoyl-GPC
(18:0/18:2)*
Serum
Scaled Intensity
1.2
0.9
0.6
0.3
0.0
HC-C/EBP Beta Tg
AD-C/EBP Beta Tg

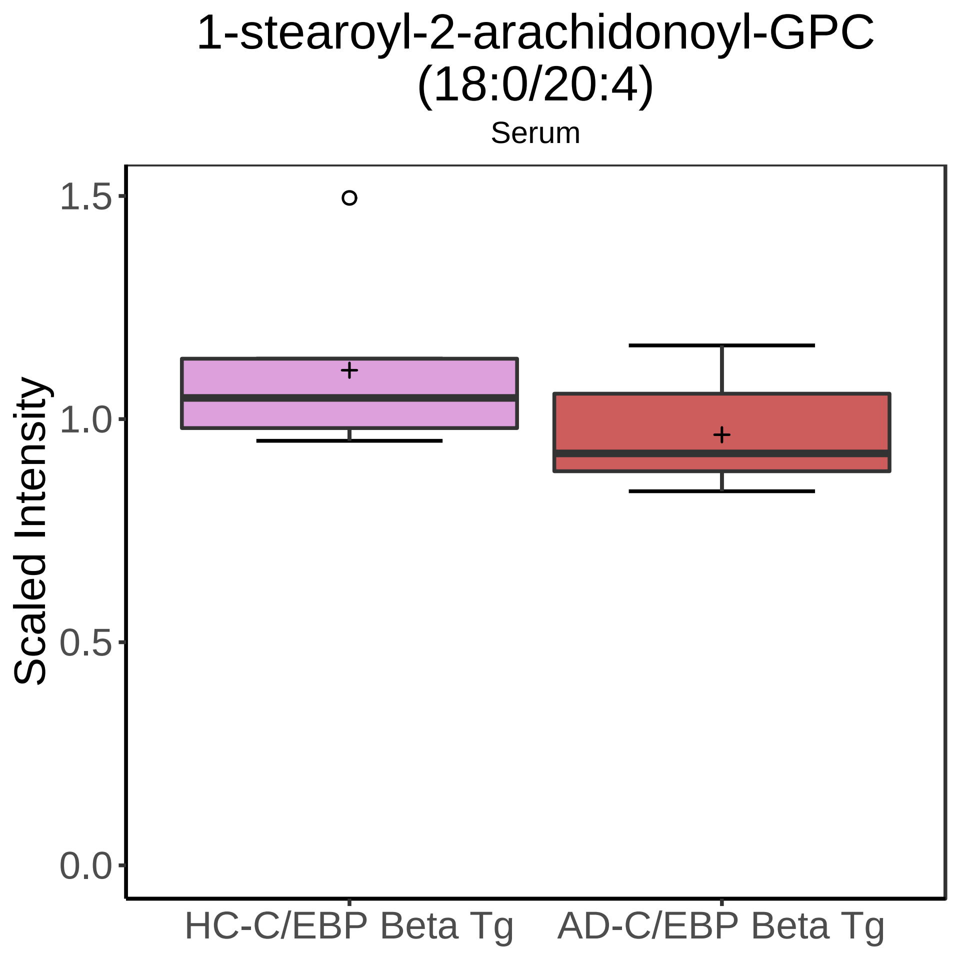
1-stearoyl-2-arachidonoyl-GPC
(18:0/20:4)
Serum
Scaled Intensity
1.5
1.0
0.5
0.0
HC-C/EBP Beta Tg
AD-C/EBP Beta Tg

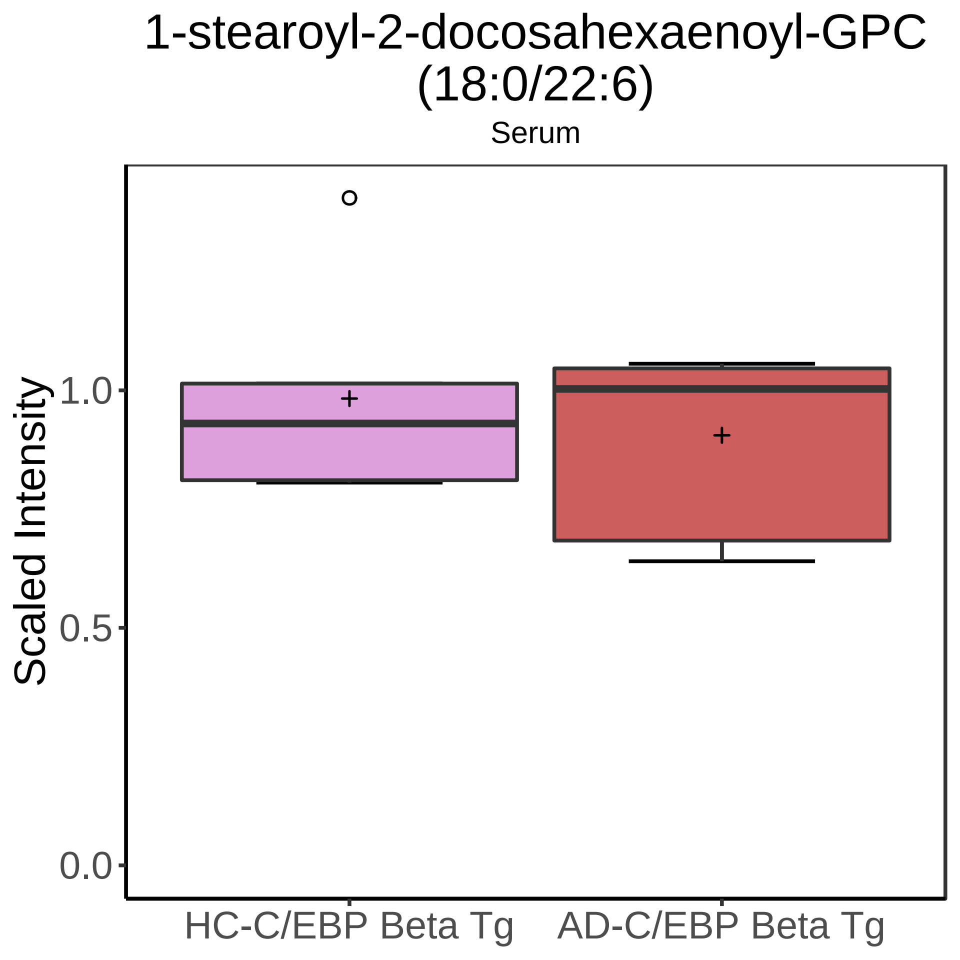

1-stearoyl-2-docosahexaenoyl-GPC
(18:0/22:6)
Serum
Scaled Intensity
1.0
0.5
0.0
HC-C/EBP Beta Tg
AD-C/EBP Beta Tg

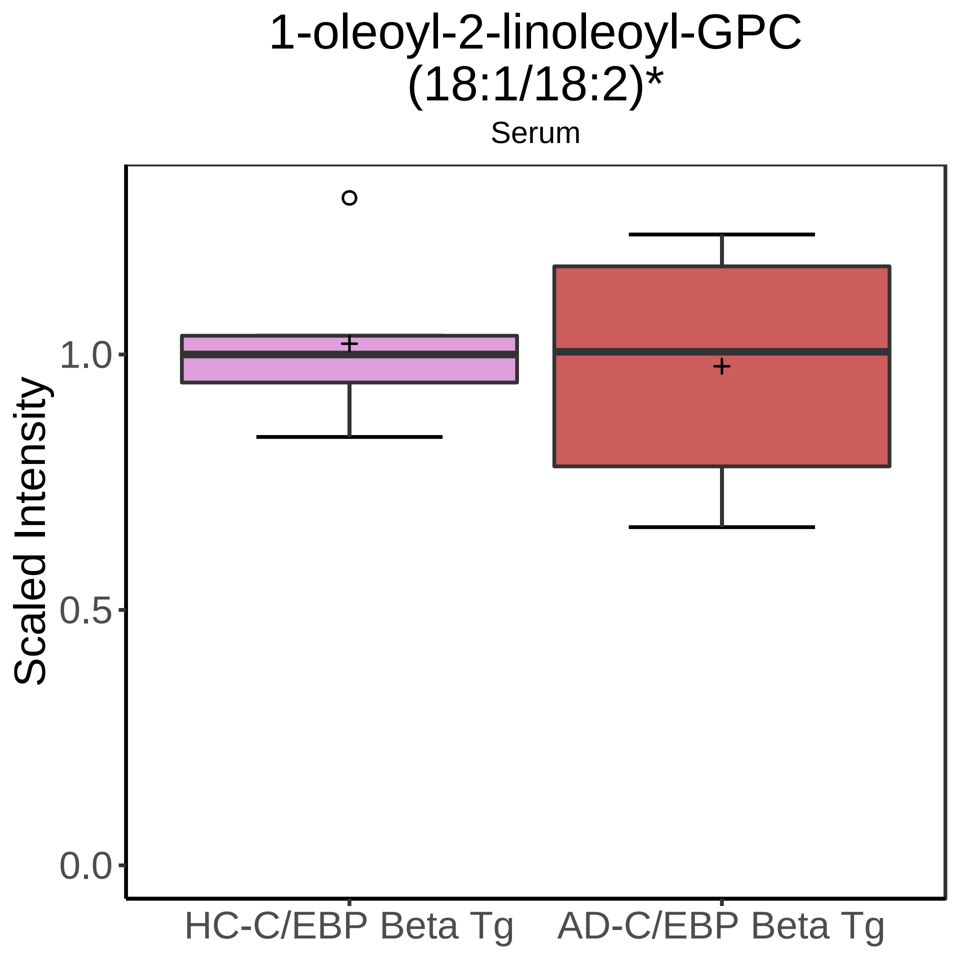
1-oleoyl-2-linoleoyl-GPC (18:1/18:2)*
Serum
Scaled Intensity
1.0
0.5
0.0
HC-C/EBP Beta Tg
AD-C/EBP Beta Tg

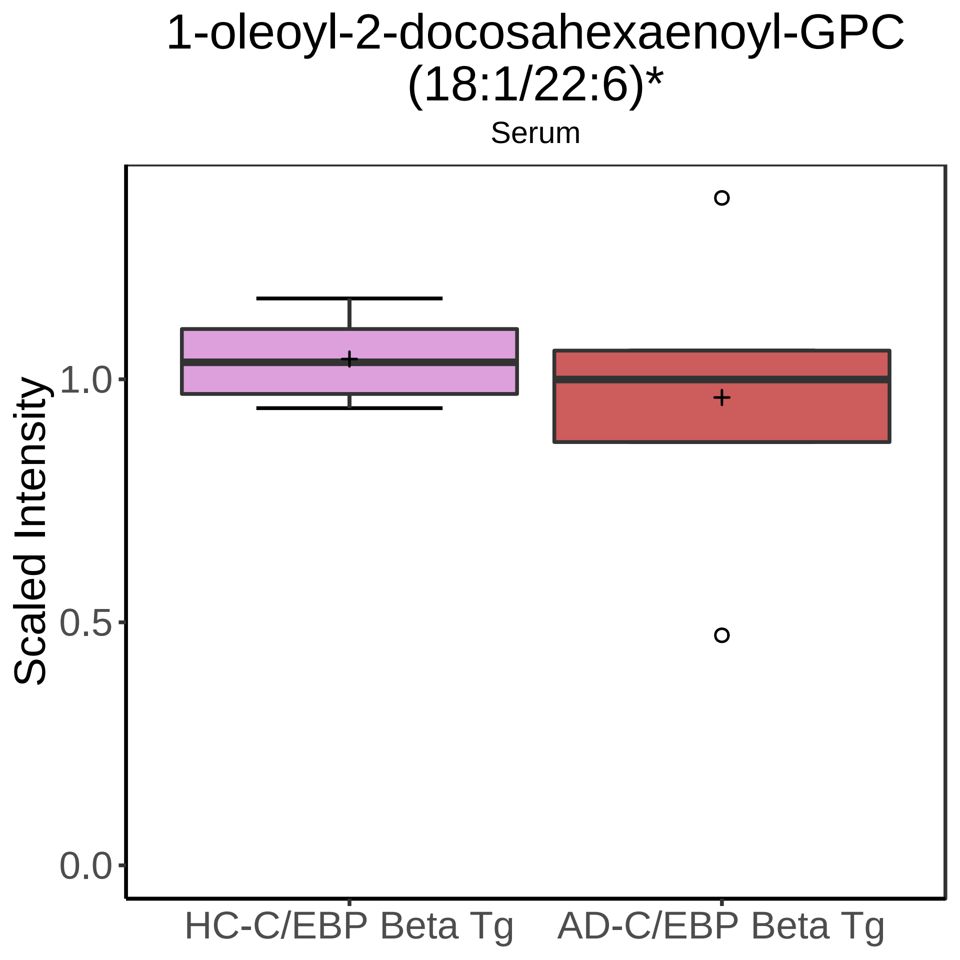
1-oleoyl-2-docosahexaenoyl-GPC
(18:1/22:6)*
Serum
Scaled Intensity
1.0
0.5
0.0
HC-C/EBP Beta Tg
AD-C/EBP Beta Tg

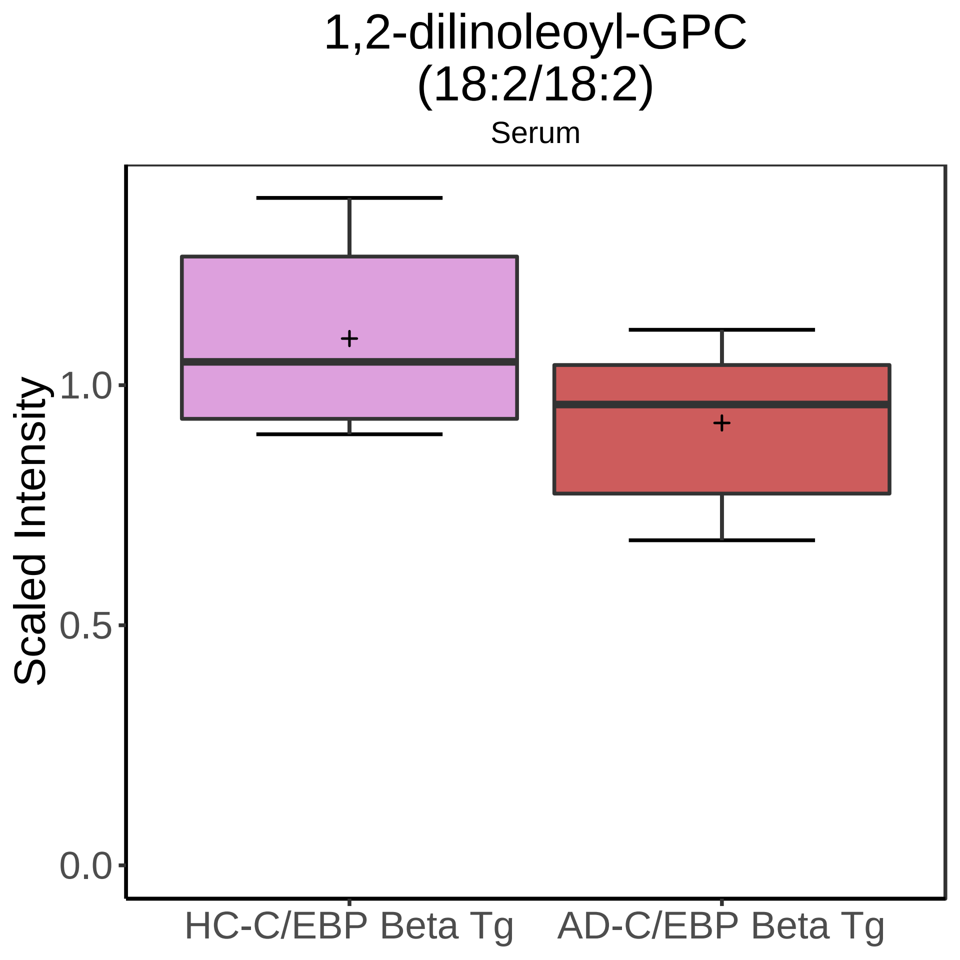
1,2-dilinoleoyl-GPC
(18:2/18:2)
Serum
Scaled Intensity
1.0
0.5
0.0
HC-C/EBP Beta Tg
AD-C/EBP Beta Tg

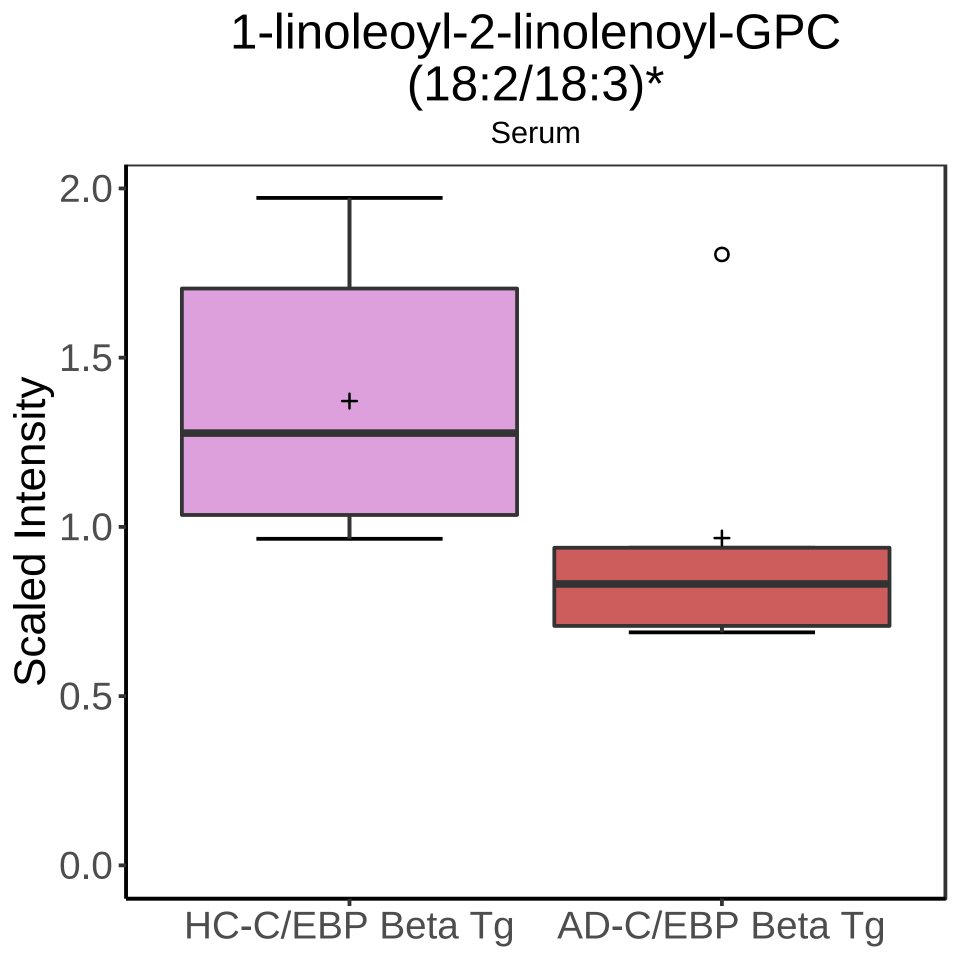
1-linoleoyl-2-linolenoyl-GPC (18:2/18:3)*
Serum
Scaled Intensity
2.0
1.5
1.0
0.5
0.0
HC-C/EBP Beta Tg
AD-C/EBP Beta Tg

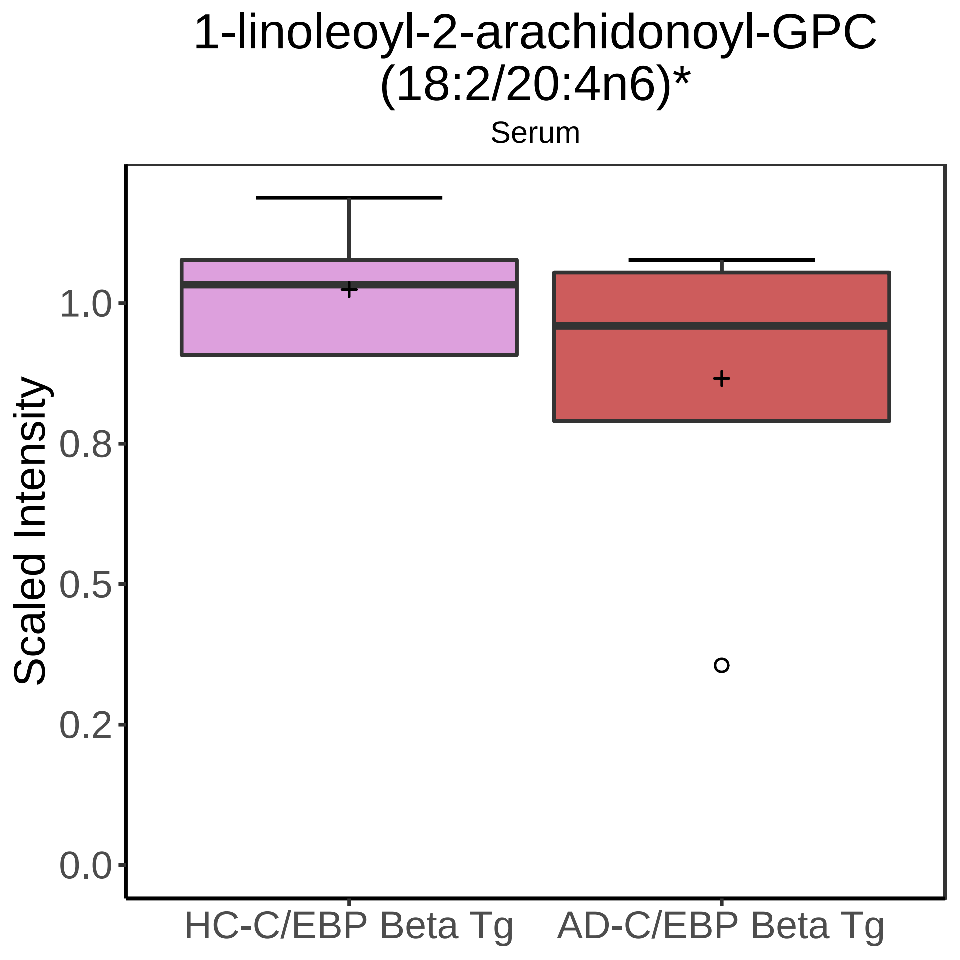
1-linoleoyl-2-arachidonoyl-GPC
(18:2/20:4n6)*
Serum
Scaled Intensity
1.0
0.8
0.5
0.2
0.0
HC-C/EBP Beta Tg
AD-C/EBP Beta Tg

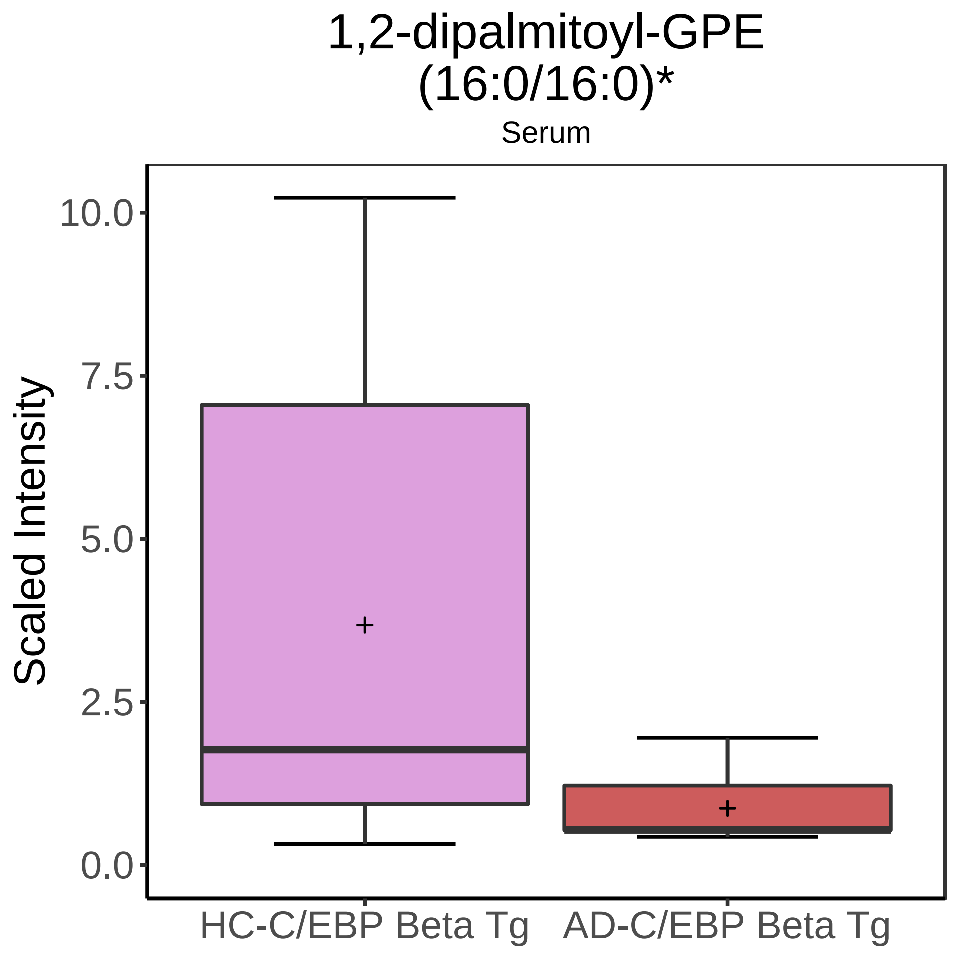
1,2-dipalmitoyl-GPE
(16:0/16:0)*
Serum
Scaled Intensity
10.0
7.5
5.0
2.5
0.0
HC-C/EBP Beta Tg
AD-C/EBP Beta Tg

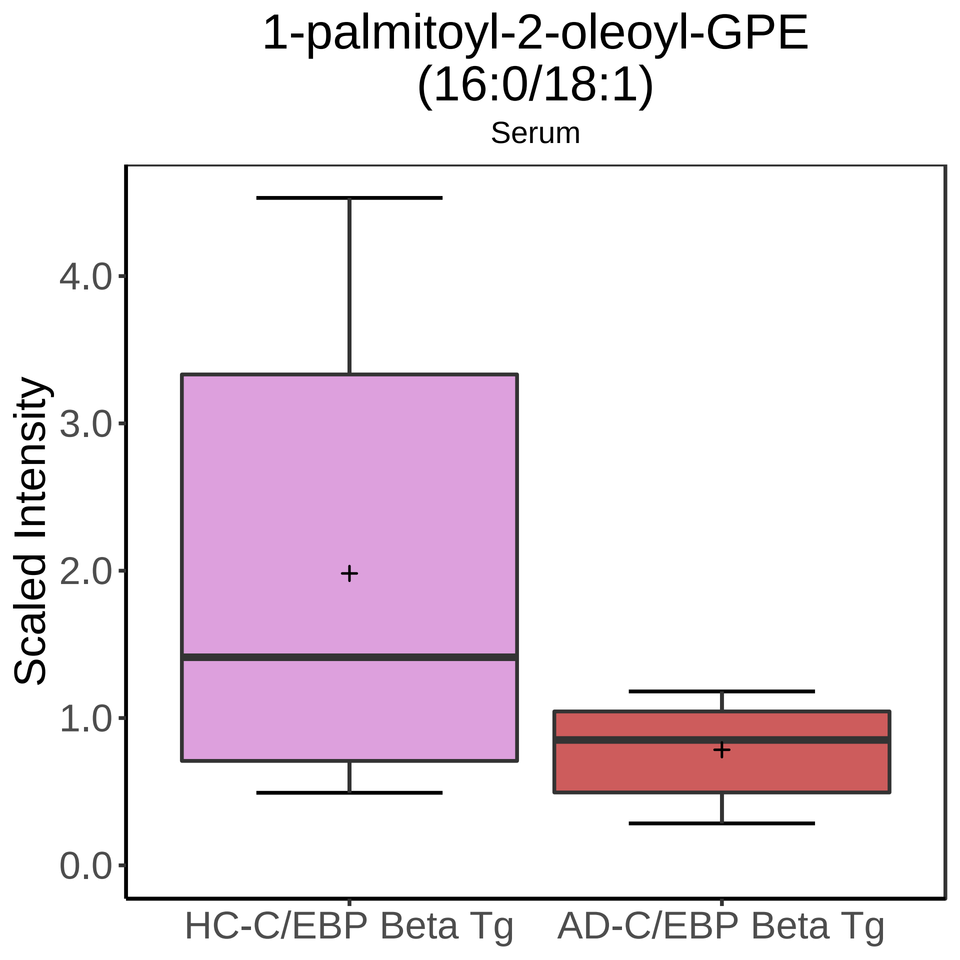

1-palmitoyl-2-oleoyl-GPE
(16:0/18:1)
Serum
Scaled Intensity
4.0
3.0
2.0
1.0
0.0
HC-C/EBP Beta Tg
AD-C/EBP Beta Tg

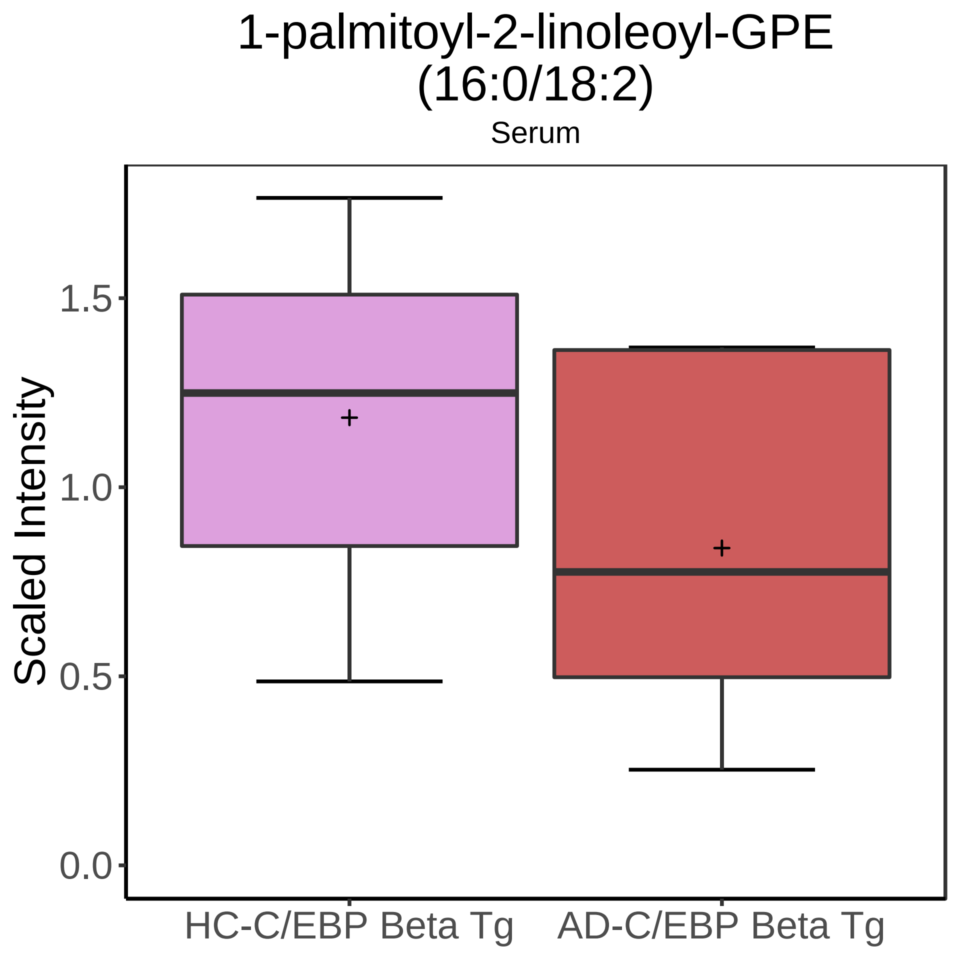
1-palmitoyl-2-linoleoyl-GPE
(16:0/18:2)
Serum
Scaled Intensity
1.5
1.0
0.5
0.0
HC-C/EBP Beta Tg
AD-C/EBP Beta Tg

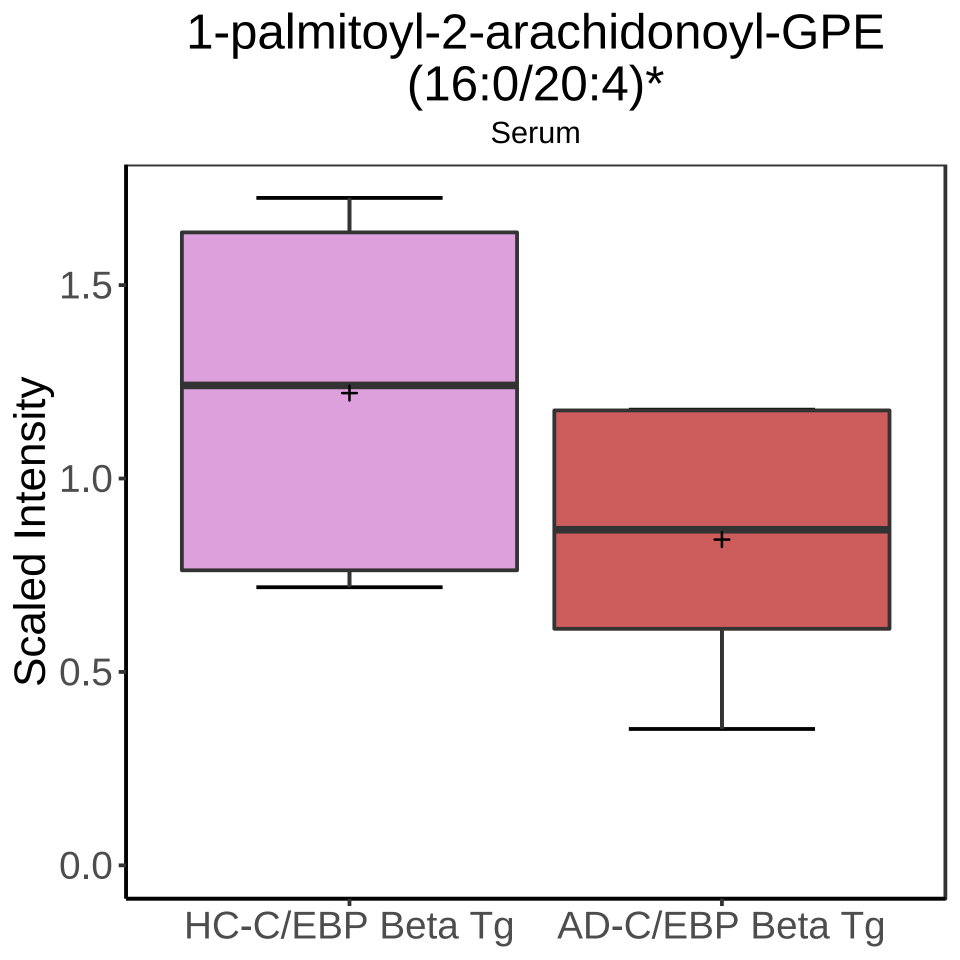
1-palmitoyl-2-arachidonoyl-GPE
(16:0/20:4)*
Serum
Scaled Intensity
1.5
1.0
0.5
0.0
HC-C/EBP Beta Tg
AD-C/EBP Beta Tg

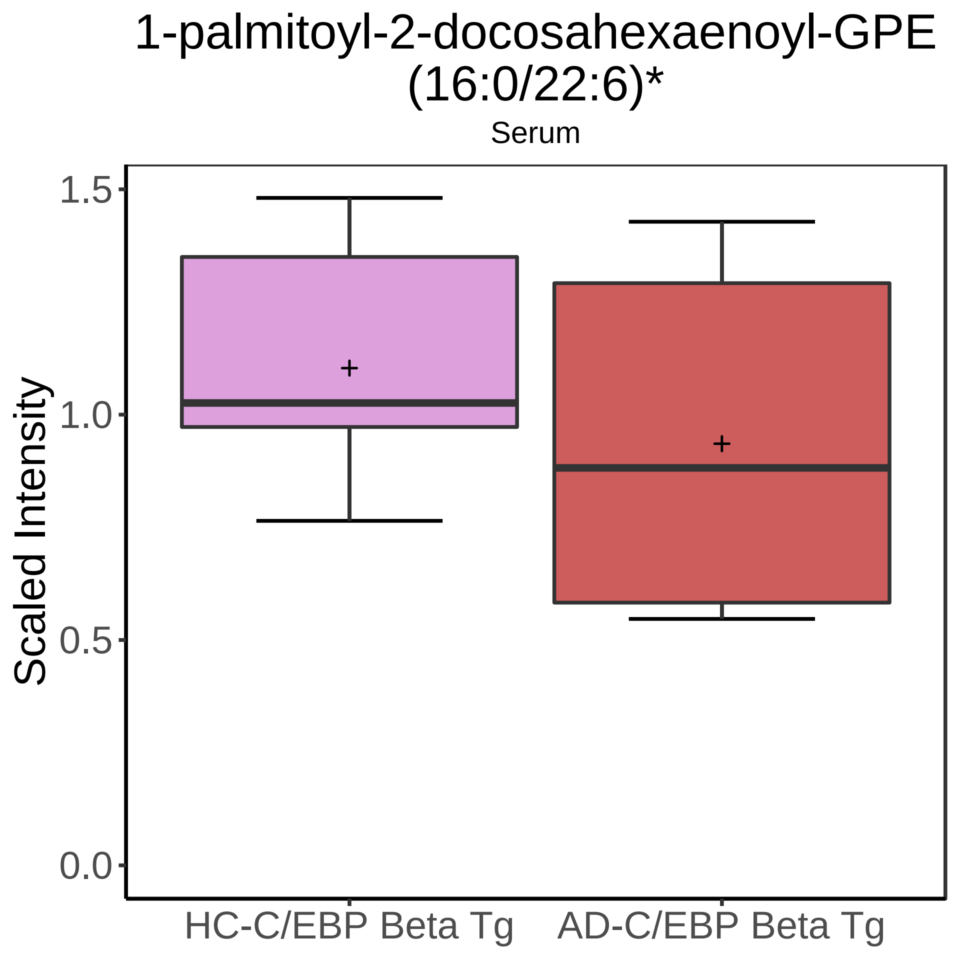
1-palmitoyl-2-docosahexaenoyl-GPE
(16:0/22:6)*
Serum
Scaled Intensity
1.5
1.0
0.5
0.0
HC-C/EBP Beta Tg
AD-C/EBP Beta Tg

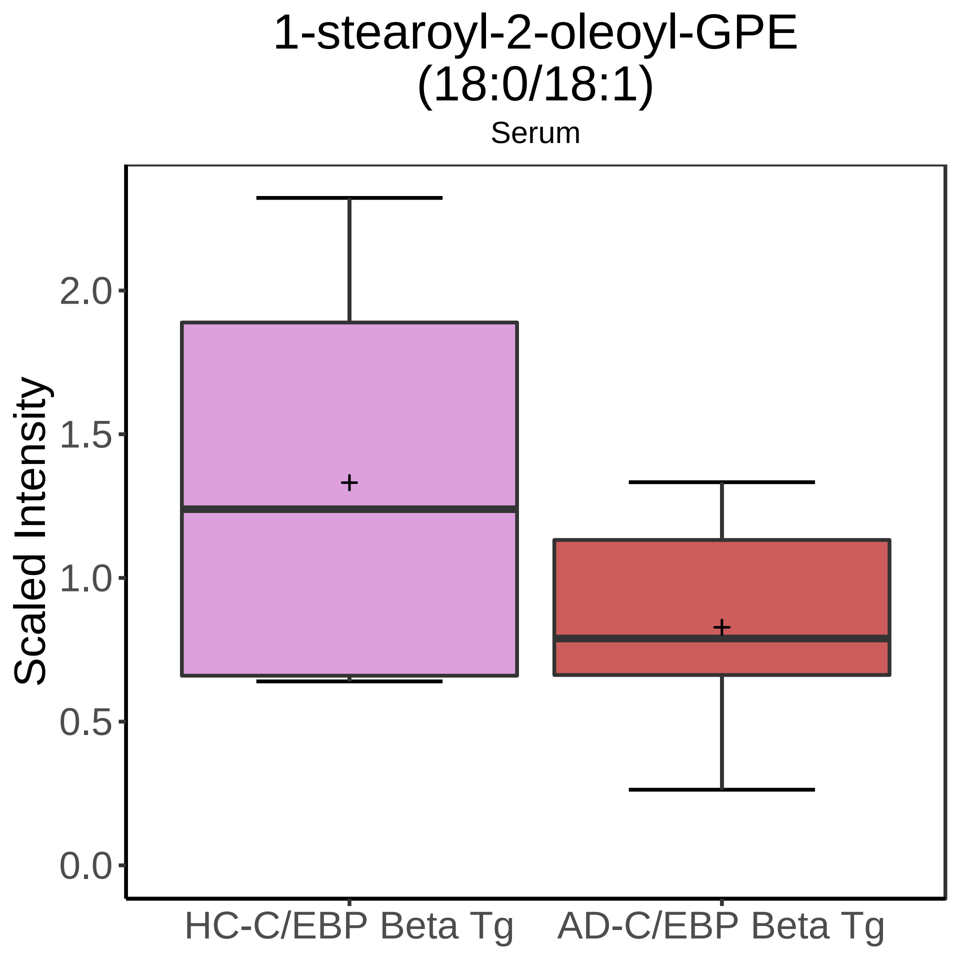
1-stearoyl-2-oleoyl-GPE
(18:0/18:1)
Serum
Scaled Intensity
2.0
1.5
1.0
0.5
0.0
HC-C/EBP Beta Tg
AD-C/EBP Beta Tg

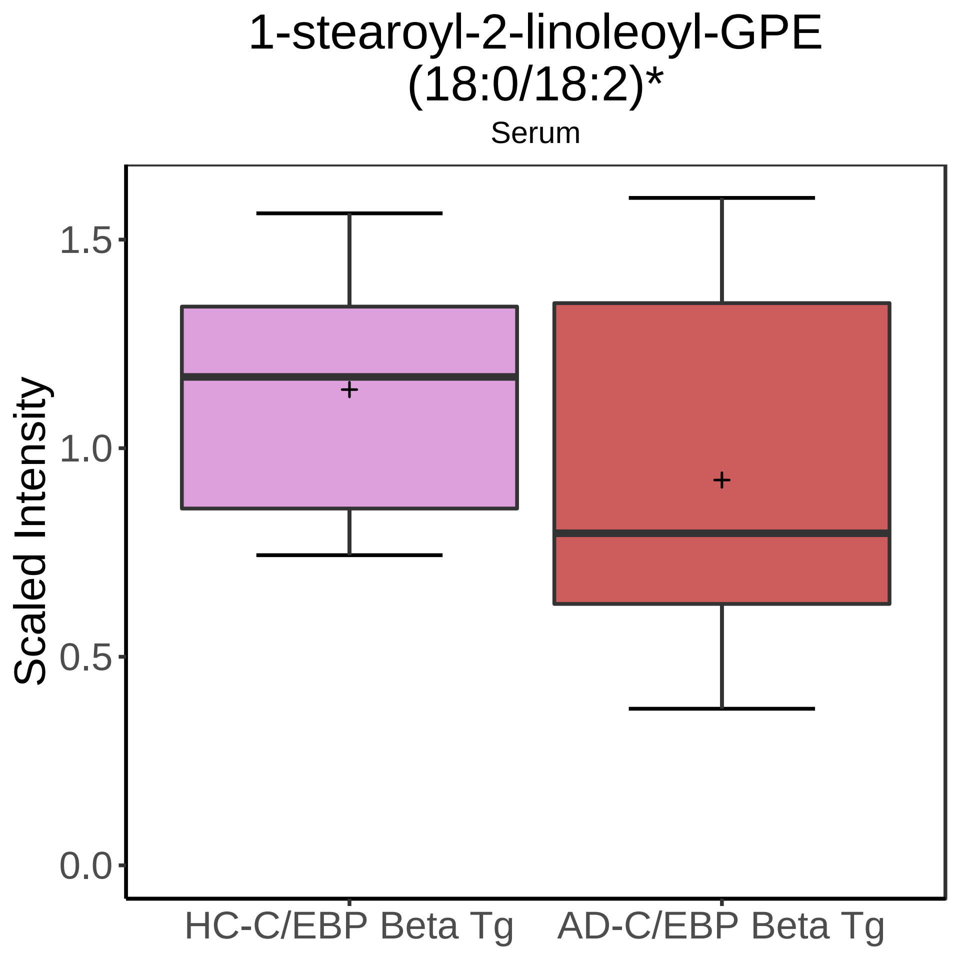
1-stearoyl-2-linoleoyl-GPE
(18:0/18:2)*
Serum
Scaled Intensity
1.5
1.0
0.5
0.0
HC-C/EBP Beta Tg
AD-C/EBP Beta Tg

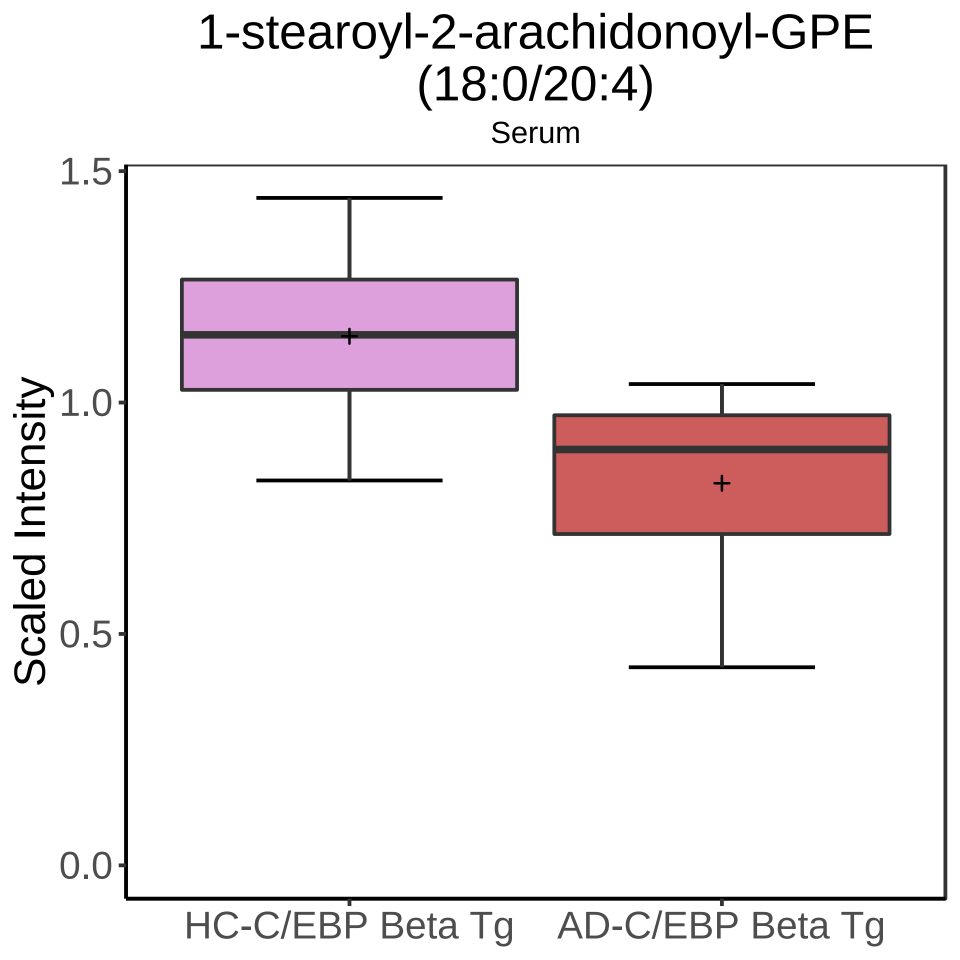
1-stearoyl-2-arachidonoyl-GPE
(18:0/20:4)
Serum
Scaled Intensity
1.5
1.0
0.5
0.0
HC-C/EBP Beta Tg
AD-C/EBP Beta Tg

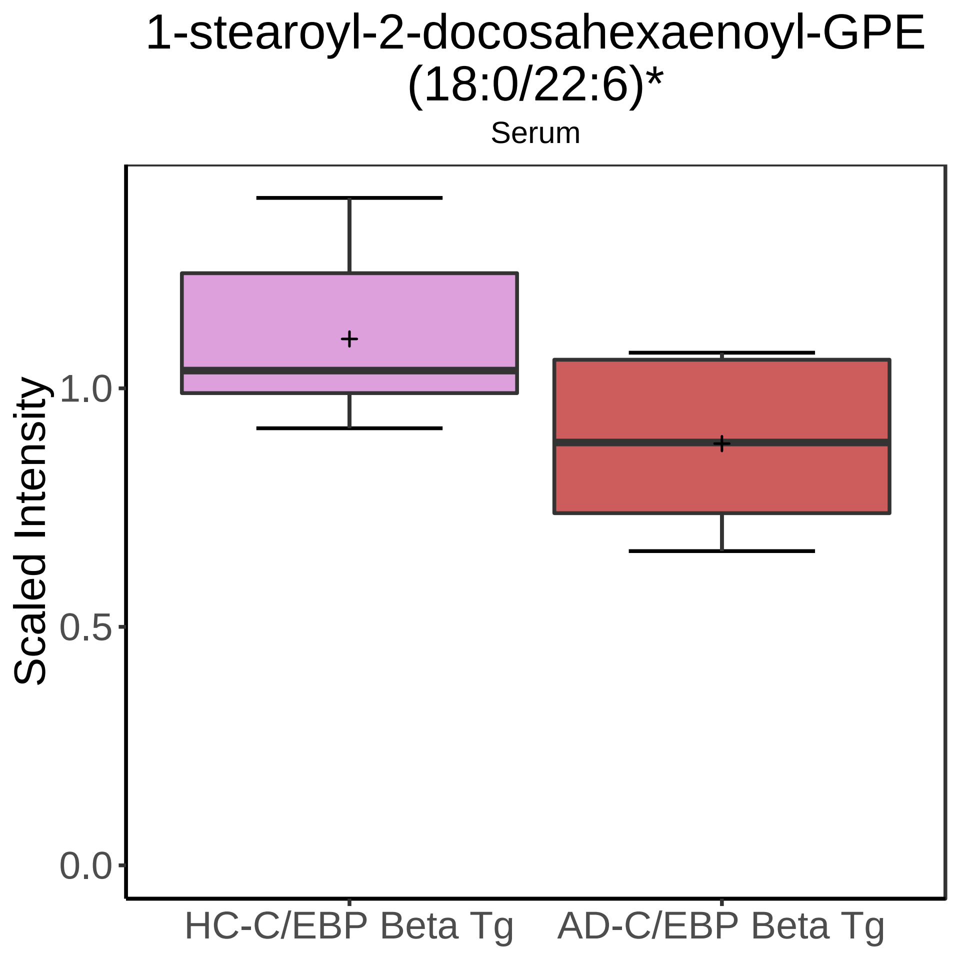
1-stearoyl-2-docosahexaenoyl-GPE
(18:0/22:6)*
Serum
Scaled Intensity
1.0
0.5
0.0
HC-C/EBP Beta Tg
AD-C/EBP Beta Tg

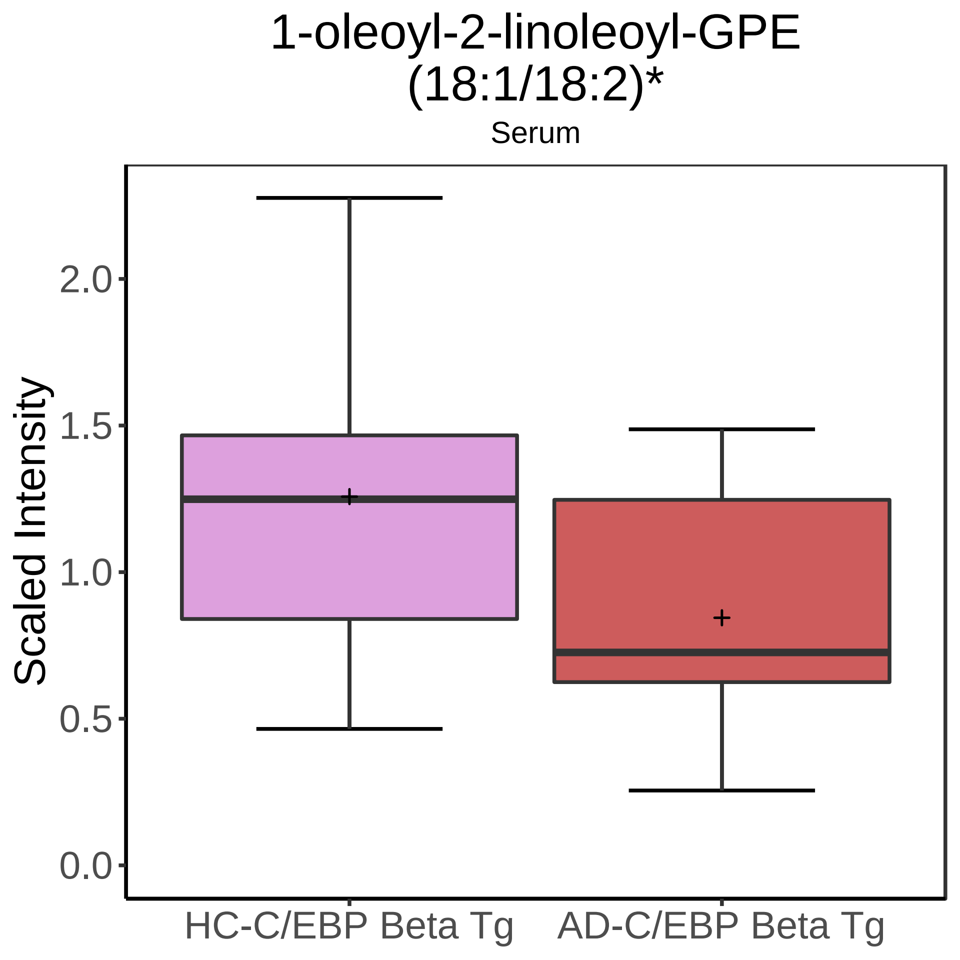
1-oleoyl-2-linoleoyl-GPE
(18:1/18:2)*
Serum
Scaled Intensity
2.0
1.5
1.0
0.5
0.0
HC-C/EBP Beta Tg
AD-C/EBP Beta Tg

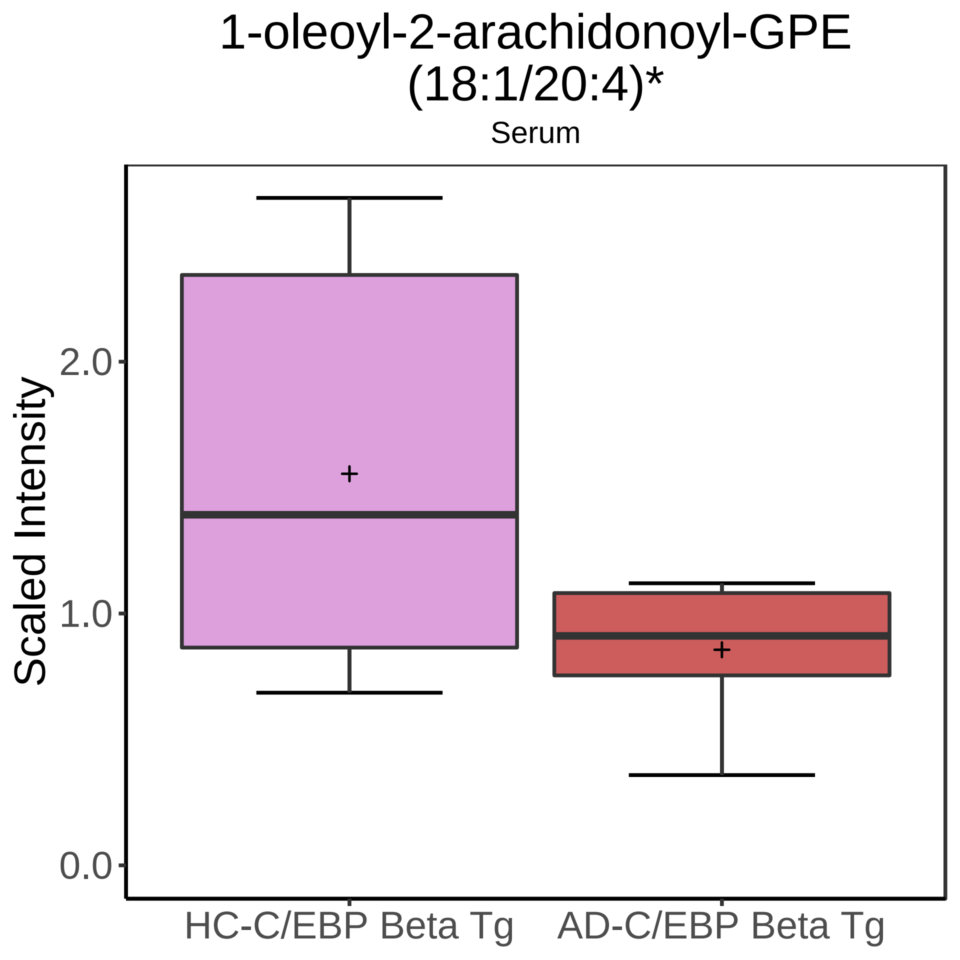

1-oleoyl-2-arachidonoyl-GPE (18:1/20:4)*
Serum
Scaled Intensity
2.0
1.0
0.0
HC-C/EBP Beta Tg
AD-C/EBP Beta Tg

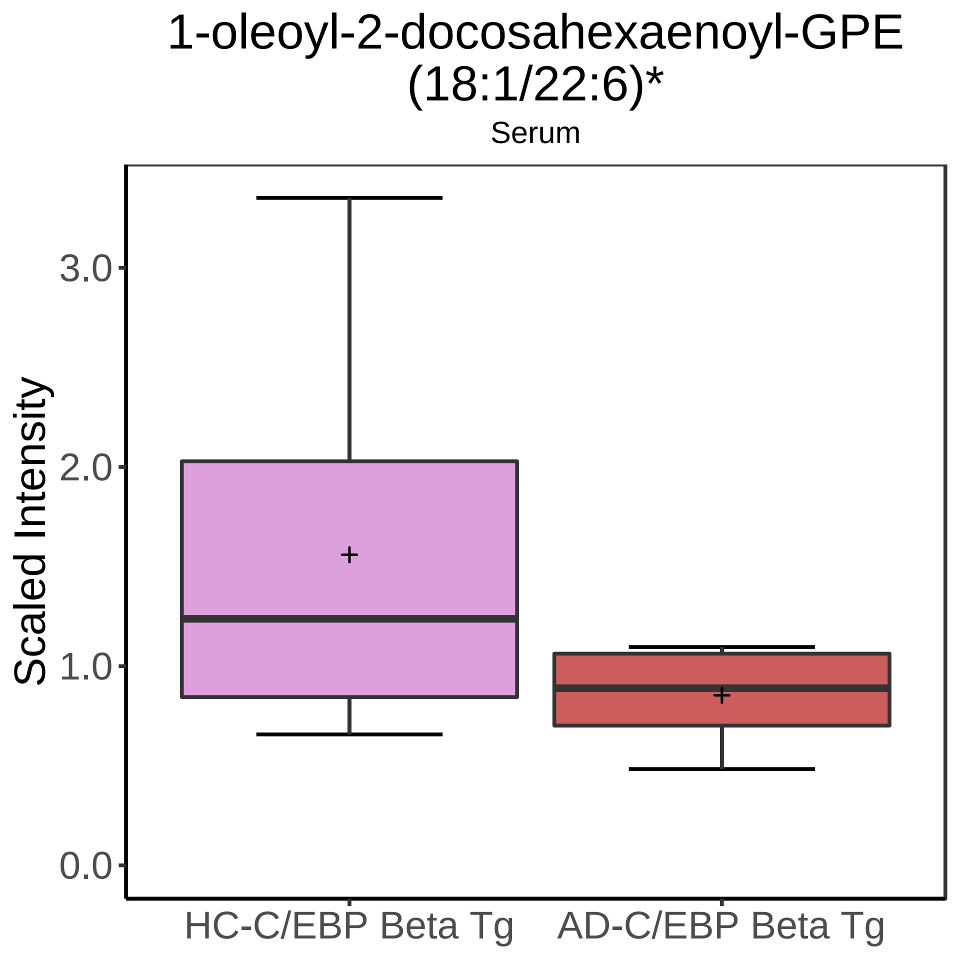

1-oleoyl-2-docosahexaenoyl-GPE
(18:1/22:6)*
Serum
Scaled Intensity
3.0
2.0
1.0
0.0
HC-C/EBP Beta Tg
AD-C/EBP Beta Tg

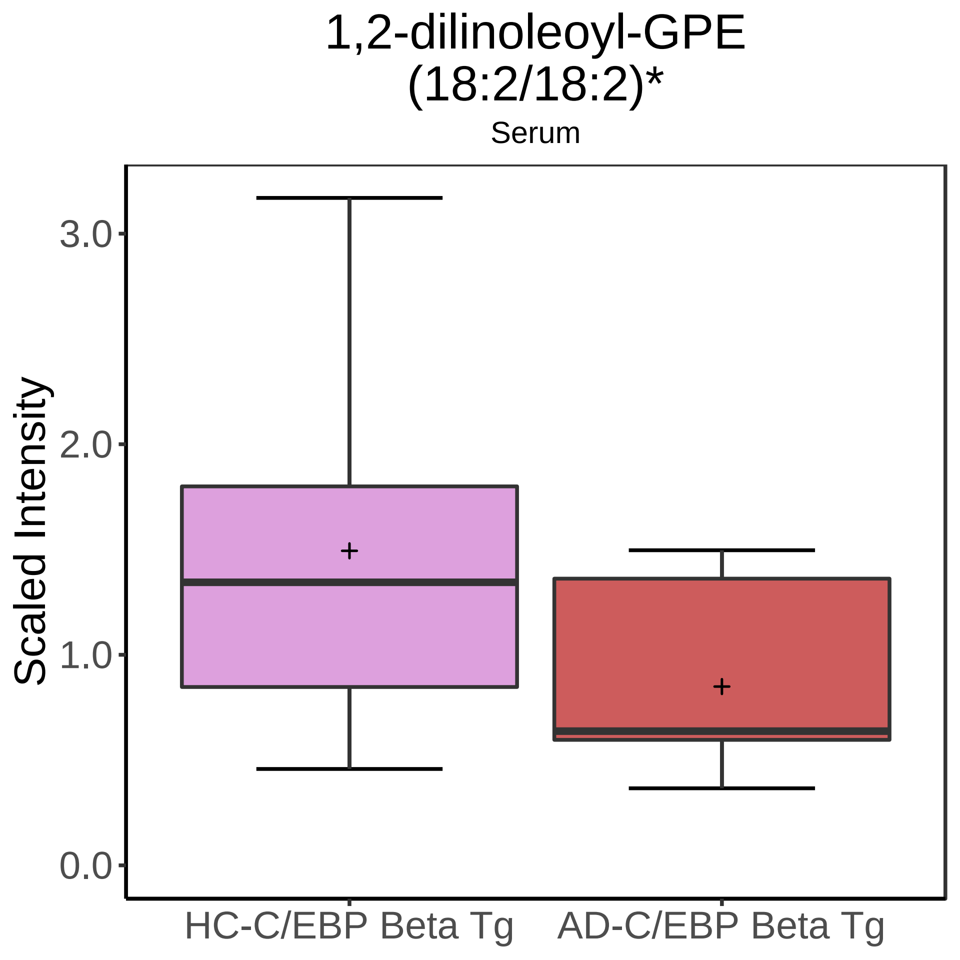

1,2-dilinoleoyl-GPE
(18:2/18:2)*
Serum
Scaled Intensity
3.0
2.0
1.0
0.0
HC-C/EBP Beta Tg
AD-C/EBP Beta Tg

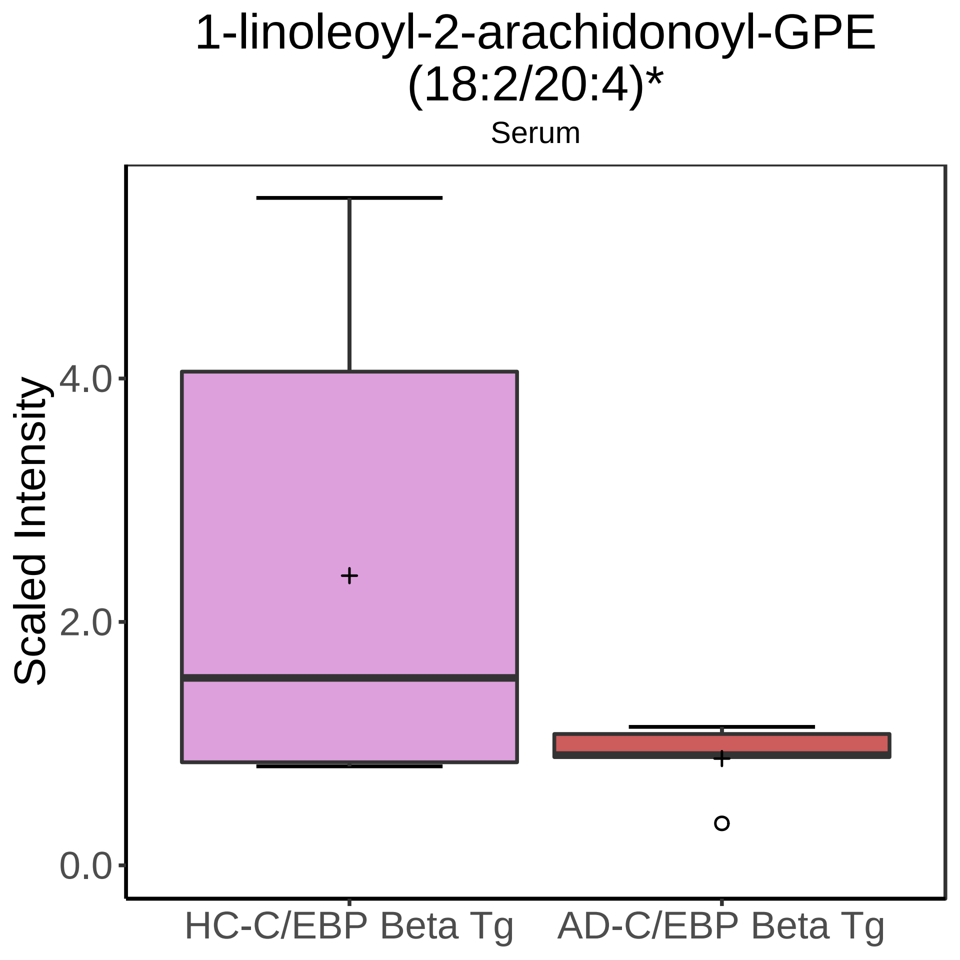
1-linoleoyl-2-arachidonoyl-GPE
(18:2/20:4)*
Serum
Scaled Intensity
4.0
2.0
0.0
HC-C/EBP Beta Tg
AD-C/EBP Beta Tg

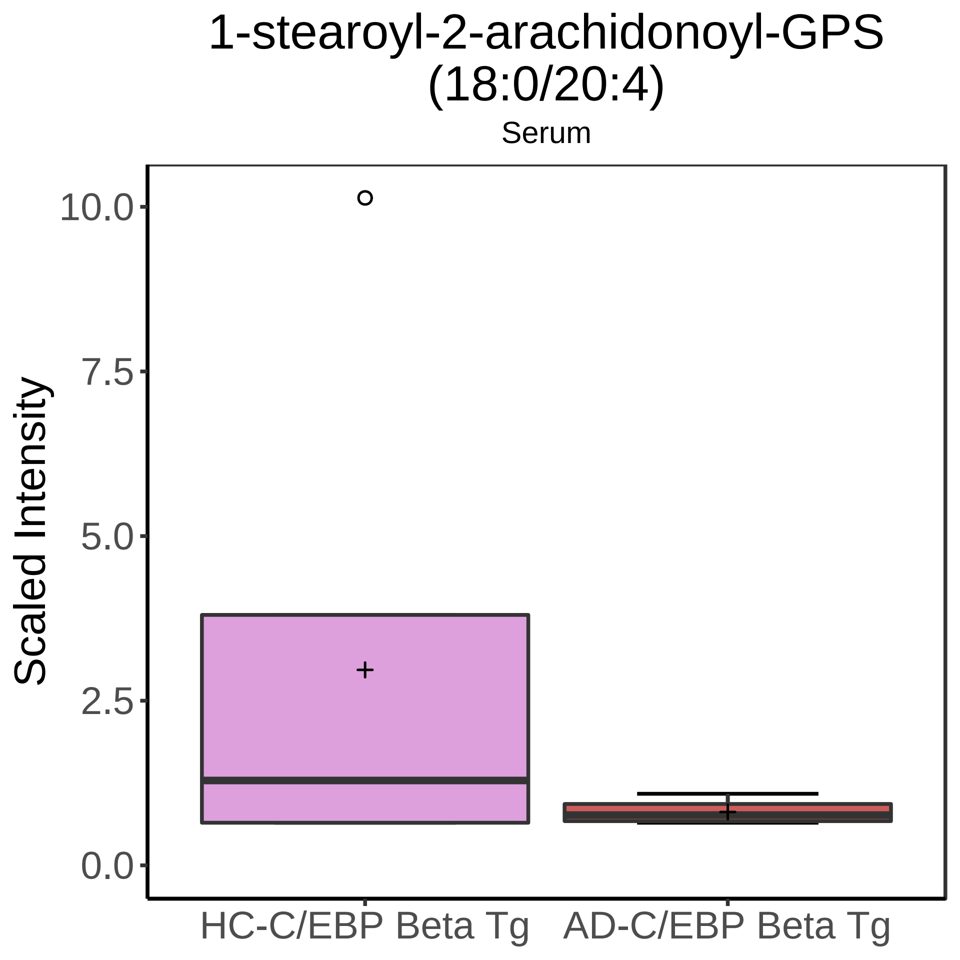
1-stearoyl-2-arachidonoyl-GPS
(18:0/20:4)
Serum
Scaled Intensity
10.0
7.5
5.0
2.5
0.0
HC-C/EBP Beta Tg
AD-C/EBP Beta Tg

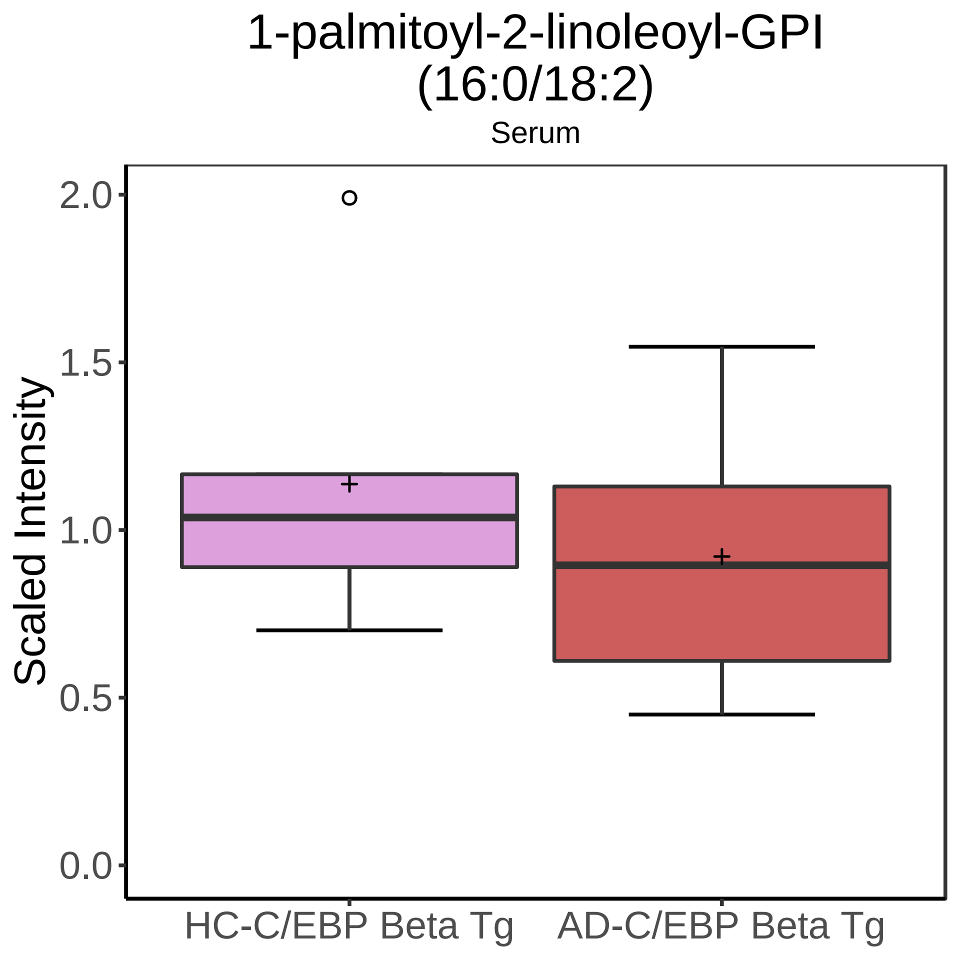
1-palmitoyl-2-linoleoyl-GPI
(16:0/18:2)
Serum
Scaled Intensity
2.0
1.5
1.0
0.5
0.0
HC-C/EBP Beta Tg
AD-C/EBP Beta Tg

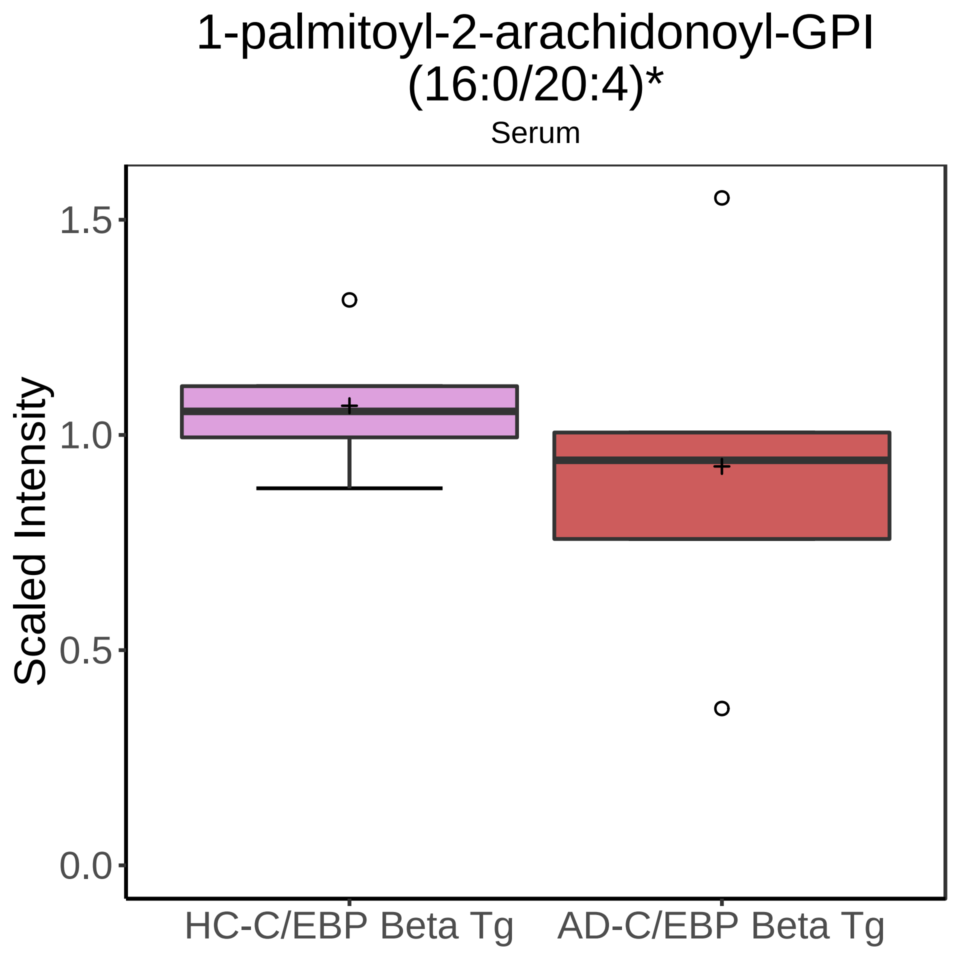
1-palmitoyl-2-arachidonoyl-GPI
(16:0/20:4)*
Serum
Scaled Intensity
1.5
1.0
0.5
0.0
HC-C/EBP Beta Tg
AD-C/EBP Beta Tg

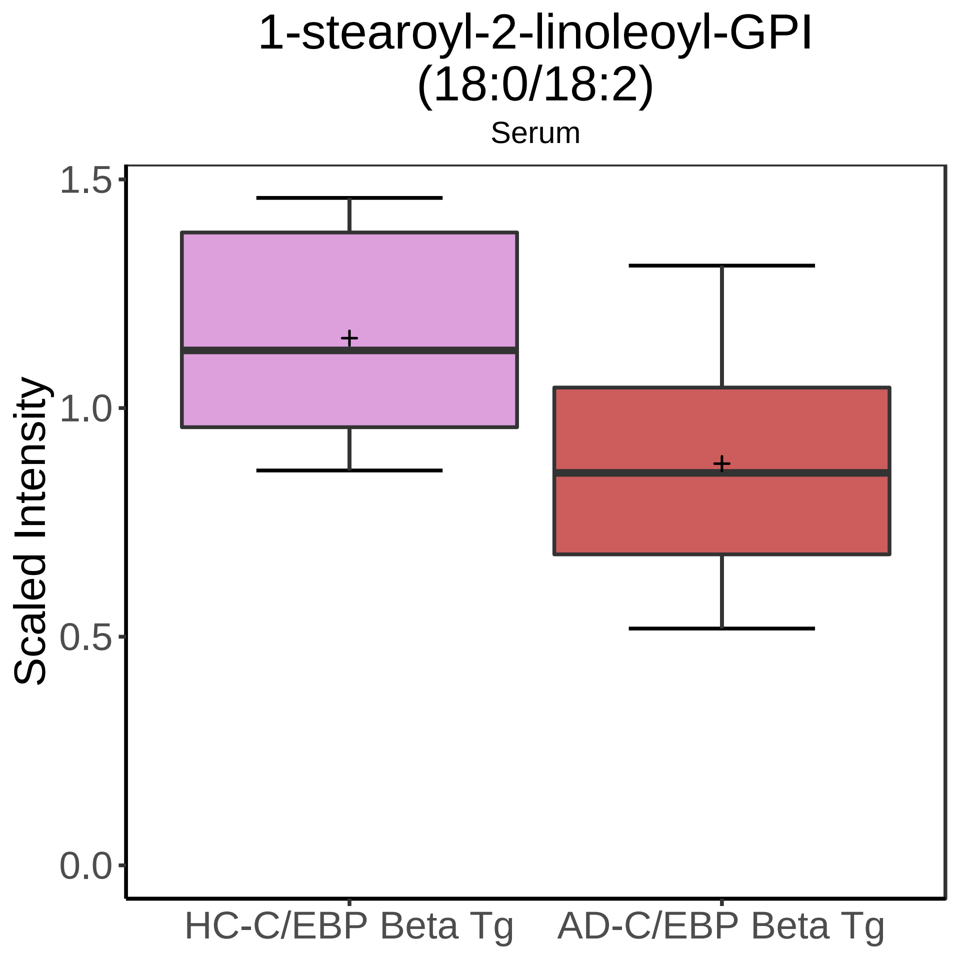
1-stearoyl-2-linoleoyl-GPI
(18:0/18:2)
Serum
Scaled Intensity
1.5
1.0
0.5
0.0
HC-C/EBP Beta Tg
AD-C/EBP Beta Tg

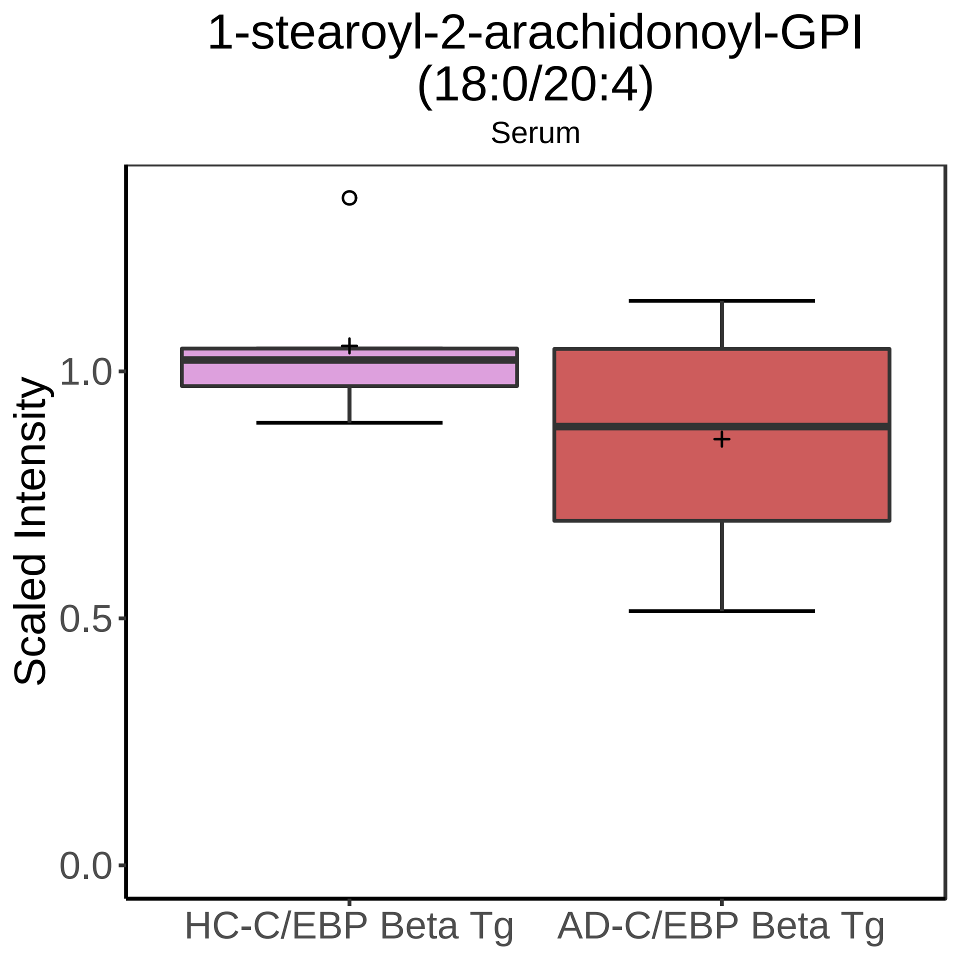
1-stearoyl-2-arachidonoyl-GPI
(18:0/20:4)
Serum
Scaled Intensity
1.0
0.5
0.0
HC-C/EBP Beta Tg
AD-C/EBP Beta Tg

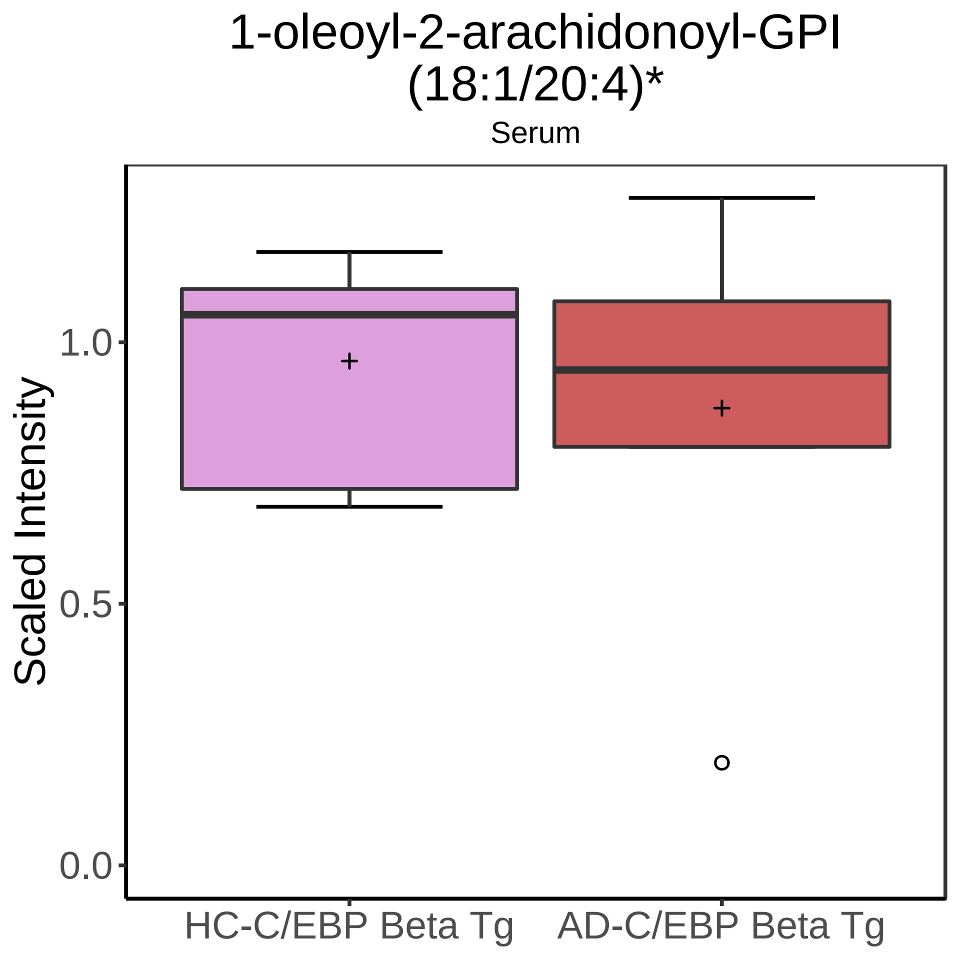
1-oleoyl-2-arachidonoyl-GPI
(18:1/20:4)*
Serum
Scaled Intensity
1.0
0.5
0.0
HC-C/EBP Beta Tg
AD-C/EBP Beta Tg

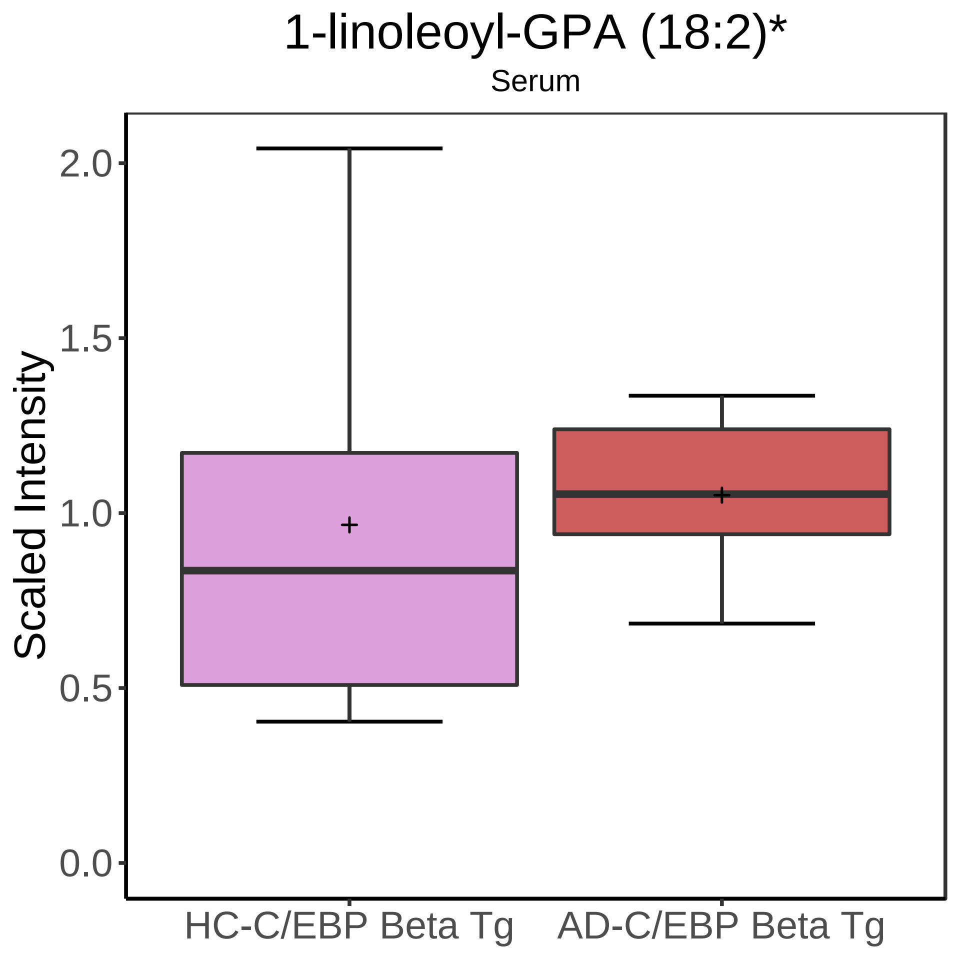
1-linoleoyl-GPA (18:2)*
Serum
Scaled Intensity
2.0
1.5
1.0
0.5
0.0
HC-C/EBP Beta Tg
AD-C/EBP Beta Tg

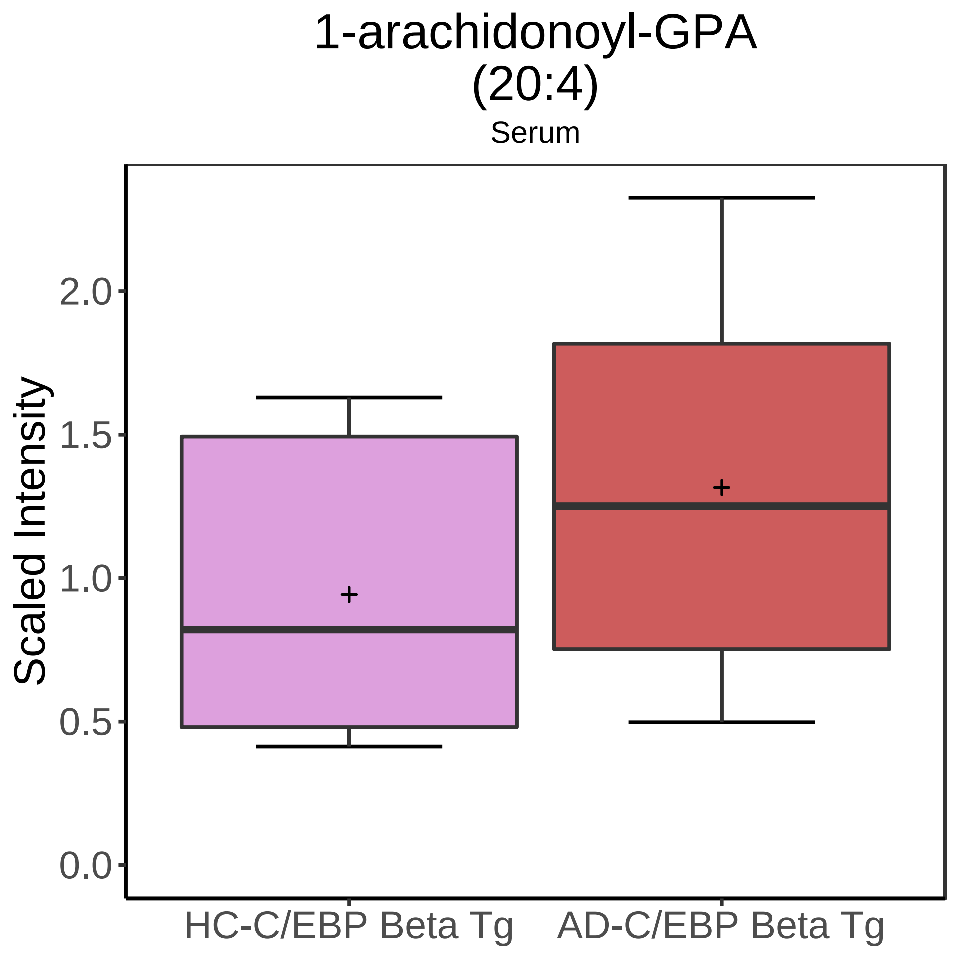
1-arachidonoyl-GPA
(20:4)
Serum
Scaled Intensity
2.0
1.5
1.0
0.5
0.0
HC-C/EBP Beta Tg
AD-C/EBP Beta Tg

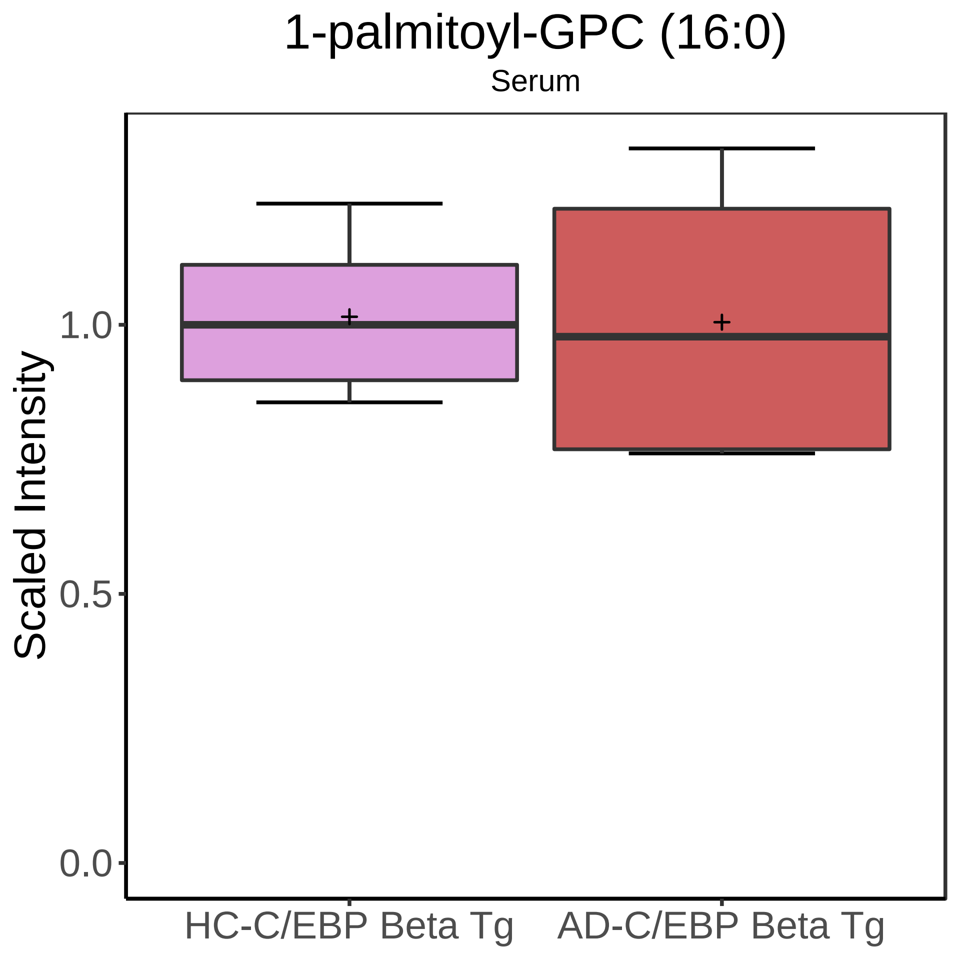
1-palmitoyl-GPC (16:0)
Serum
Scaled Intensity
1.0
0.5
0.0
HC-C/EBP Beta Tg
AD-C/EBP Beta Tg

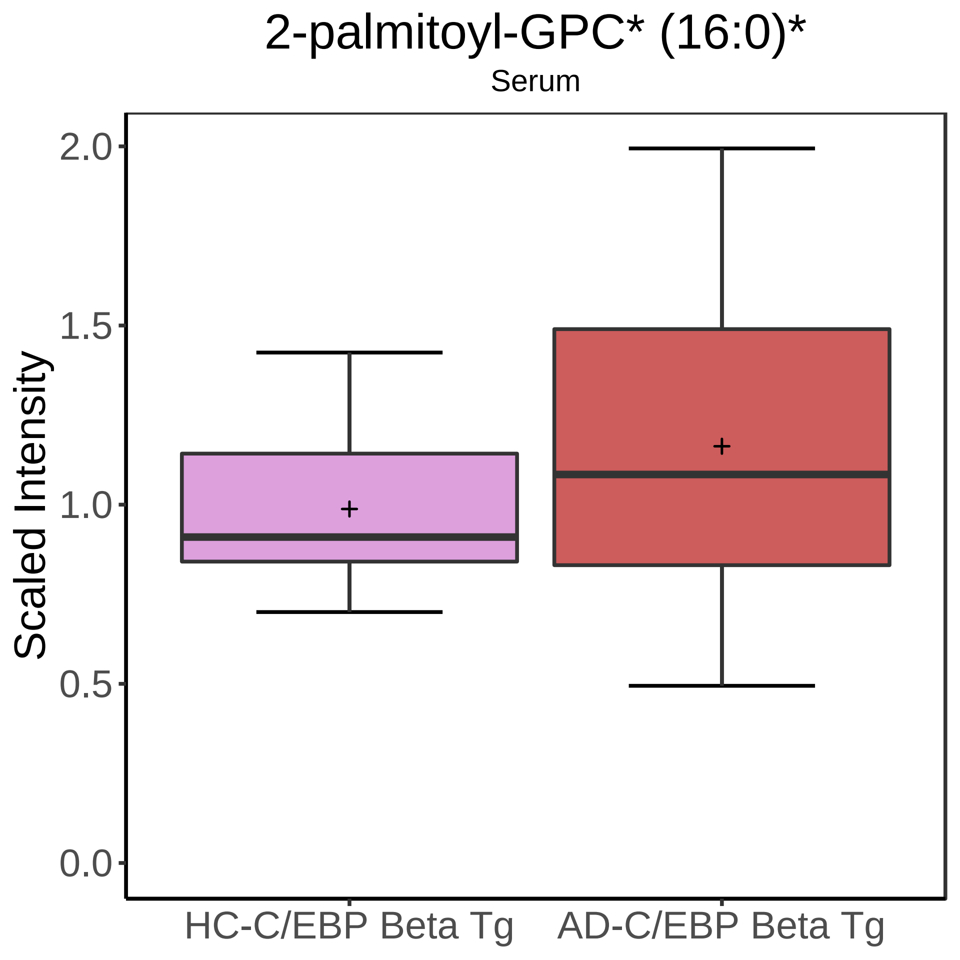
2-palmitoyl-GPC* (16:0)*
Serum
Scaled Intensity
2.0
1.5
1.0
0.5
0.0
HC-C/EBP Beta Tg
AD-C/EBP Beta Tg

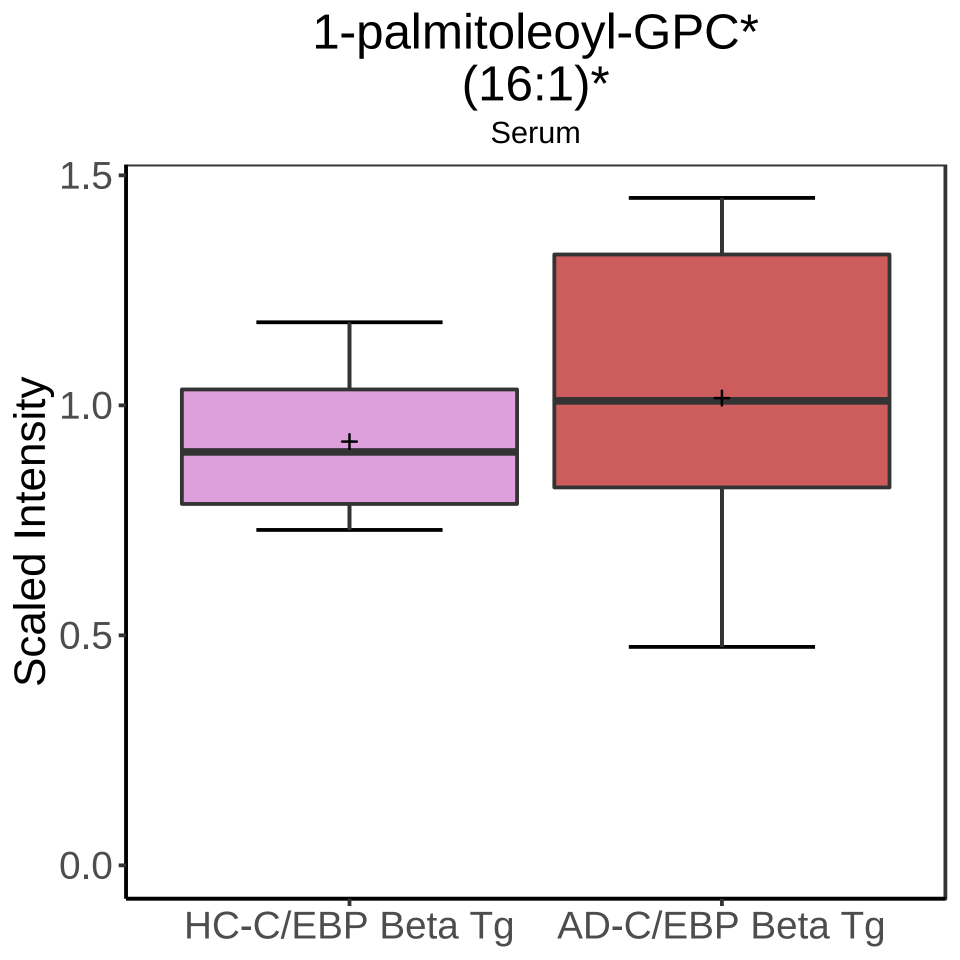
1-palmitoleoyl-GPC*
(16:1)*
Serum
Scaled Intensity
1.5
1.0
0.5
0.0
HC-C/EBP Beta Tg
AD-C/EBP Beta Tg

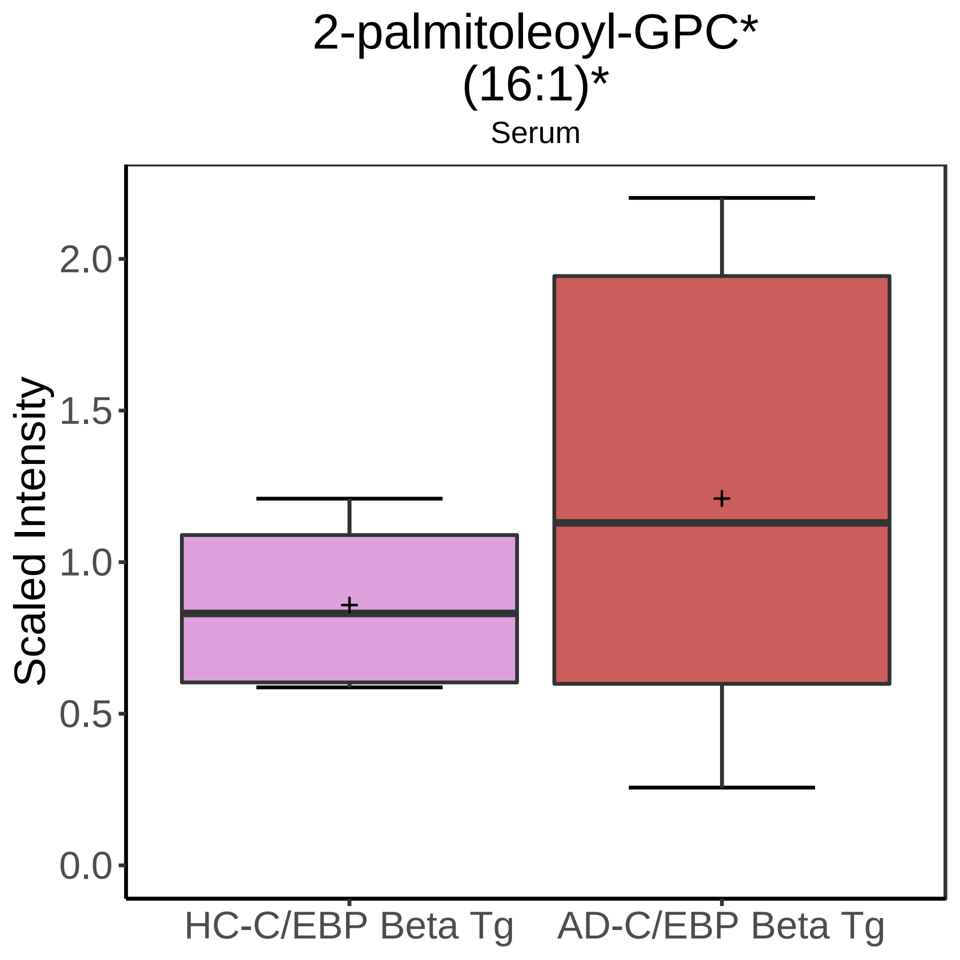
2-palmitoleoyl-GPC*
(16:1)*
Serum
Scaled Intensity
2.0
1.5
1.0
0.5
0.0
HC-C/EBP Beta Tg
AD-C/EBP Beta Tg

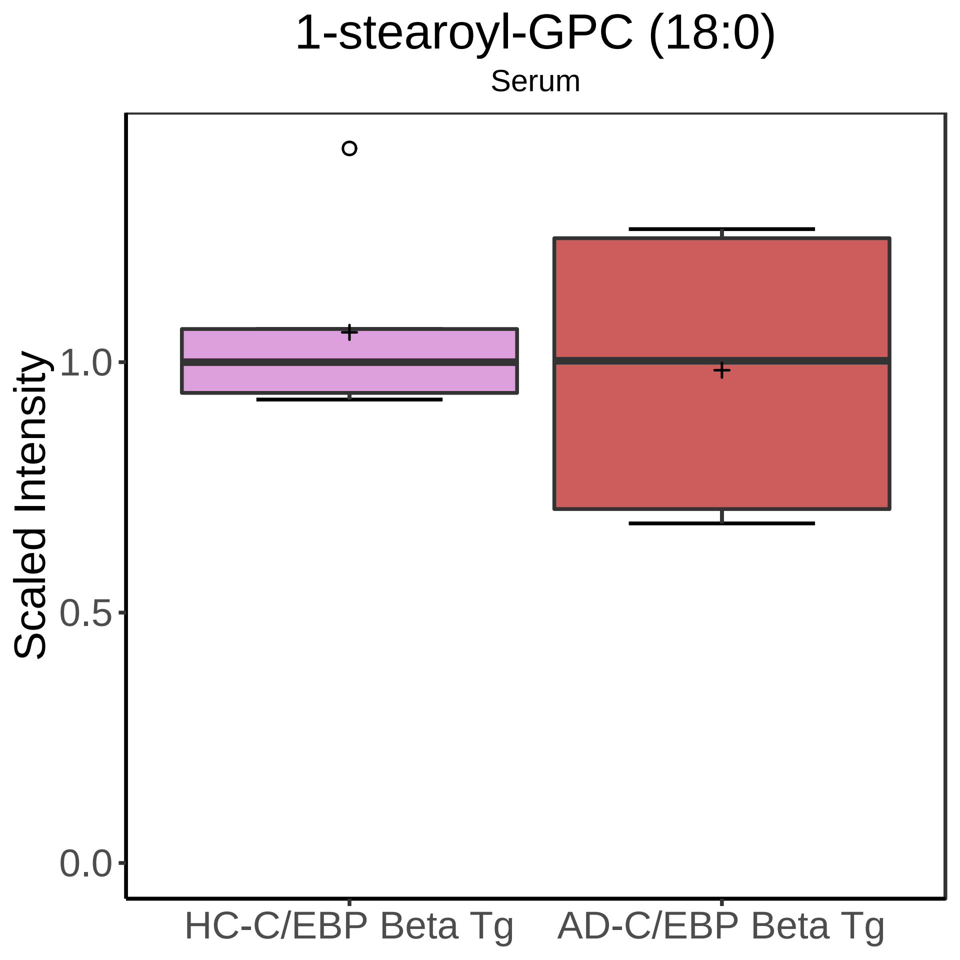
1-stearoyl-GPC (18:0)
Serum
Scaled Intensity
1.0
0.5
0.0
HC-C/EBP Beta Tg
AD-C/EBP Beta Tg

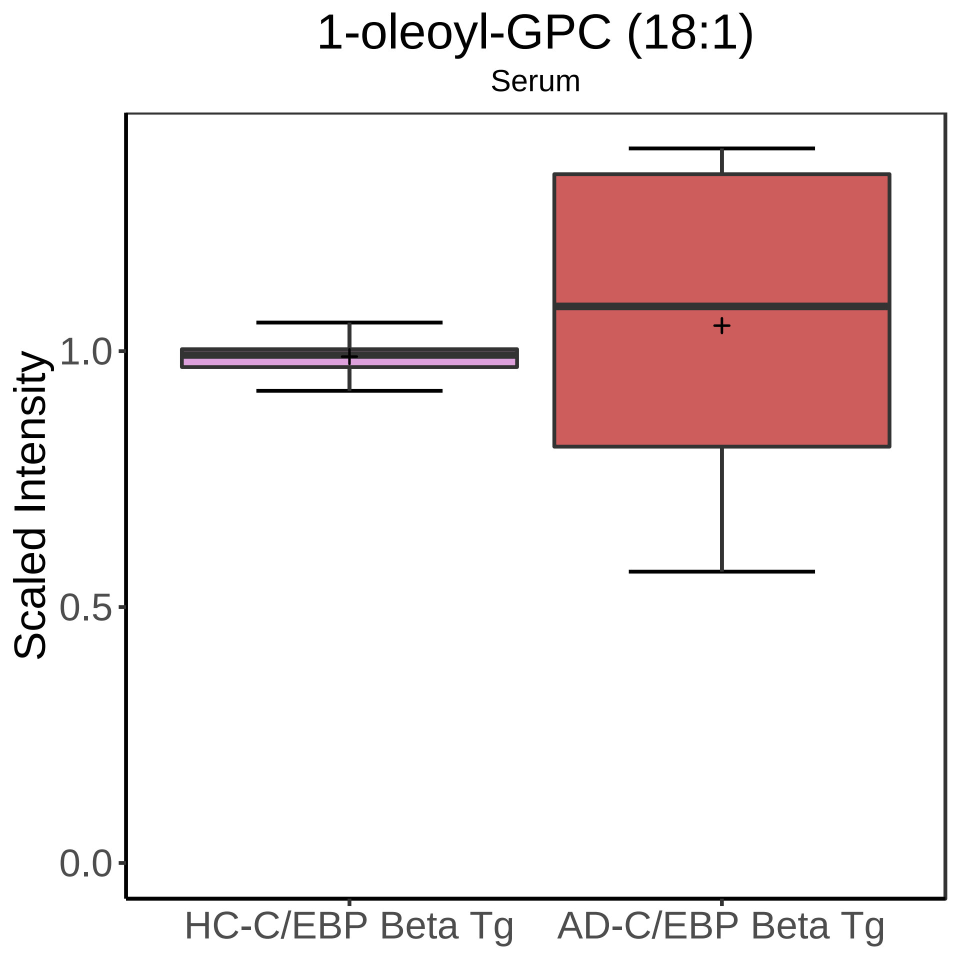
1-oleoyl-GPC (18:1)
Serum
Scaled Intensity
1.0
0.5
0.0
HC-C/EBP Beta Tg
AD-C/EBP Beta Tg

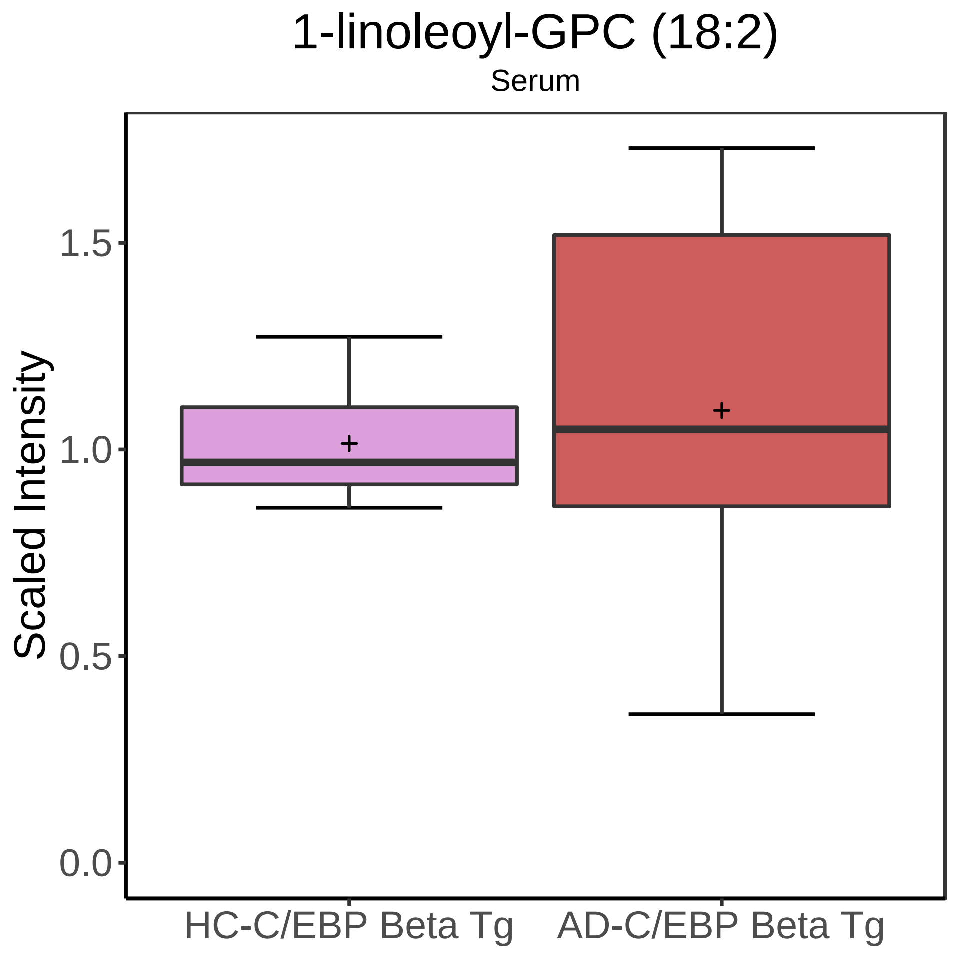

1-linoleoyl-GPC (18:2)
Serum
Scaled Intensity
1.5
1.0
0.5
0.0
HC-C/EBP Beta Tg
AD-C/EBP Beta Tg

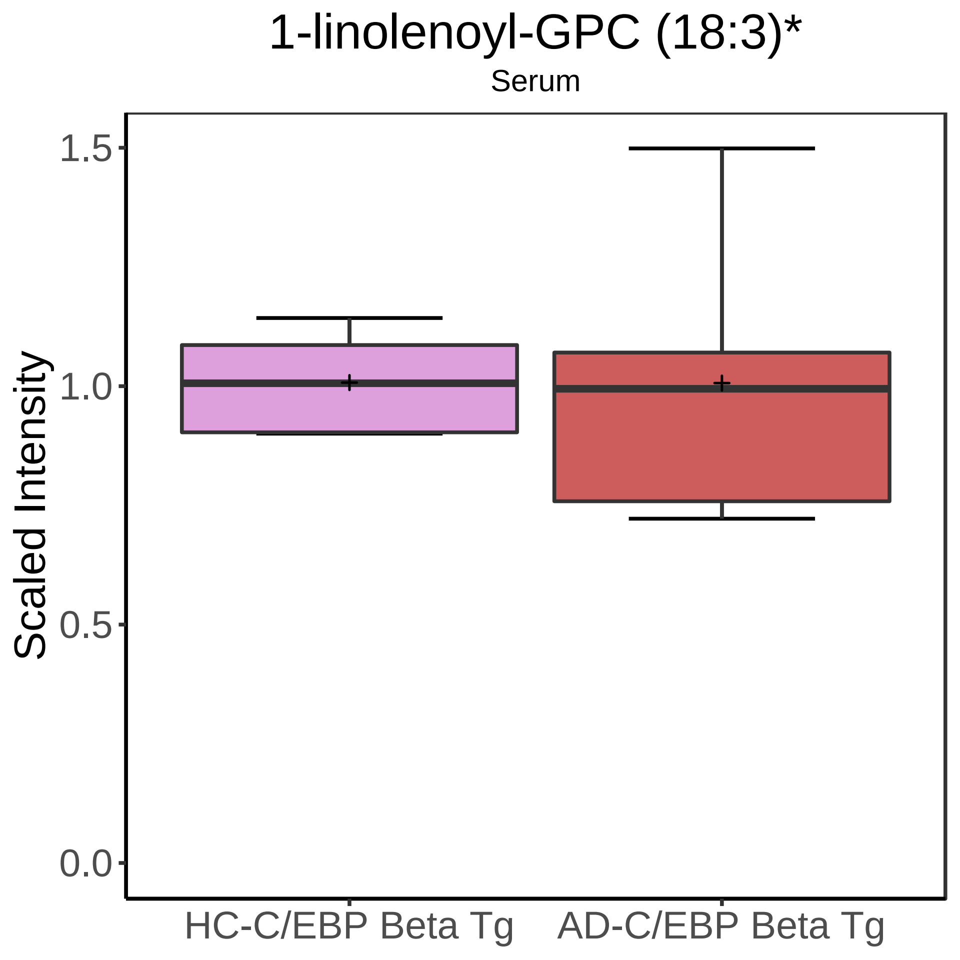
1-linolenoyl-GPC (18:3)*
Serum
Scaled Intensity
1.5
1.0
0.5
0.0
HC-C/EBP Beta Tg
AD-C/EBP Beta Tg

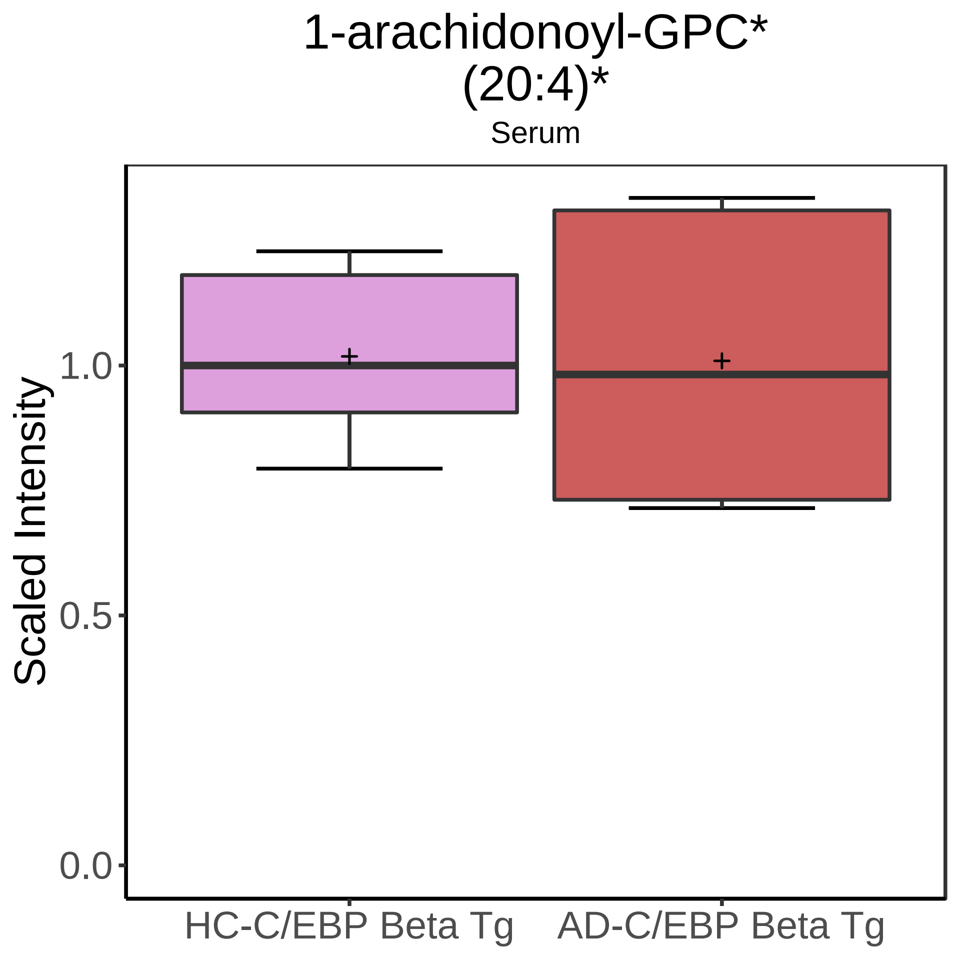

1-arachidonoyl-GPC*
(20:4)*
Serum
Scaled Intensity
1.0
0.5
0.0
HC-C/EBP Beta Tg
AD-C/EBP Beta Tg

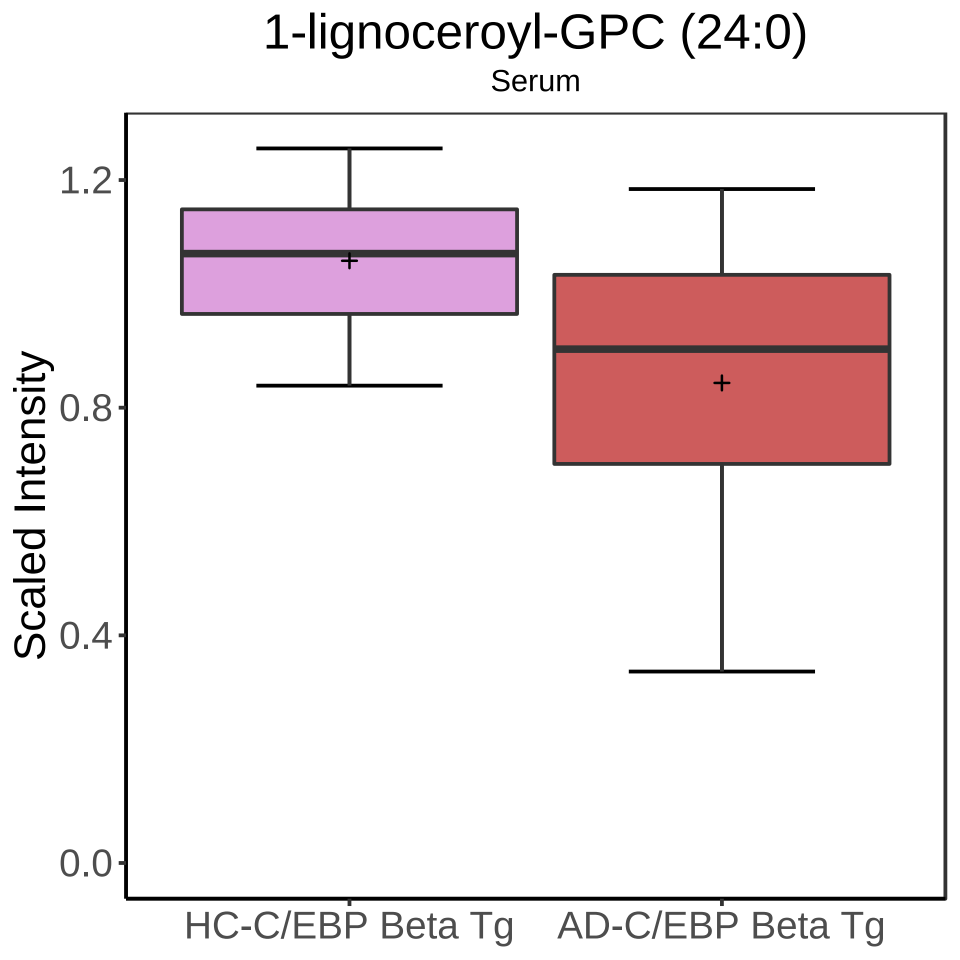
1-lignoceroyl-GPC (24:0)
Serum
Scaled Intensity
1.2
0.8
0.4
0.0
HC-C/EBP Beta Tg
AD-C/EBP Beta Tg

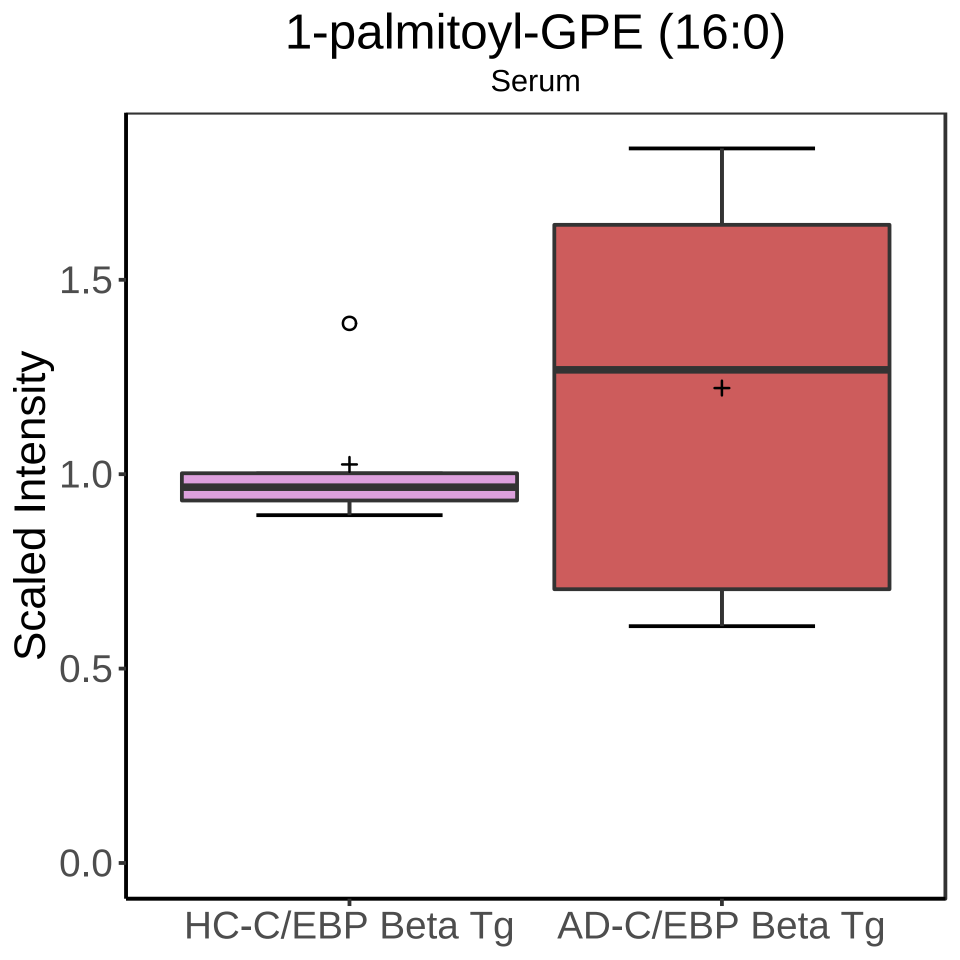
1-palmitoyl-GPE (16:0)
Serum
Scaled Intensity
1.5
1.0
0.5
0.0
HC-C/EBP Beta Tg
AD-C/EBP Beta Tg

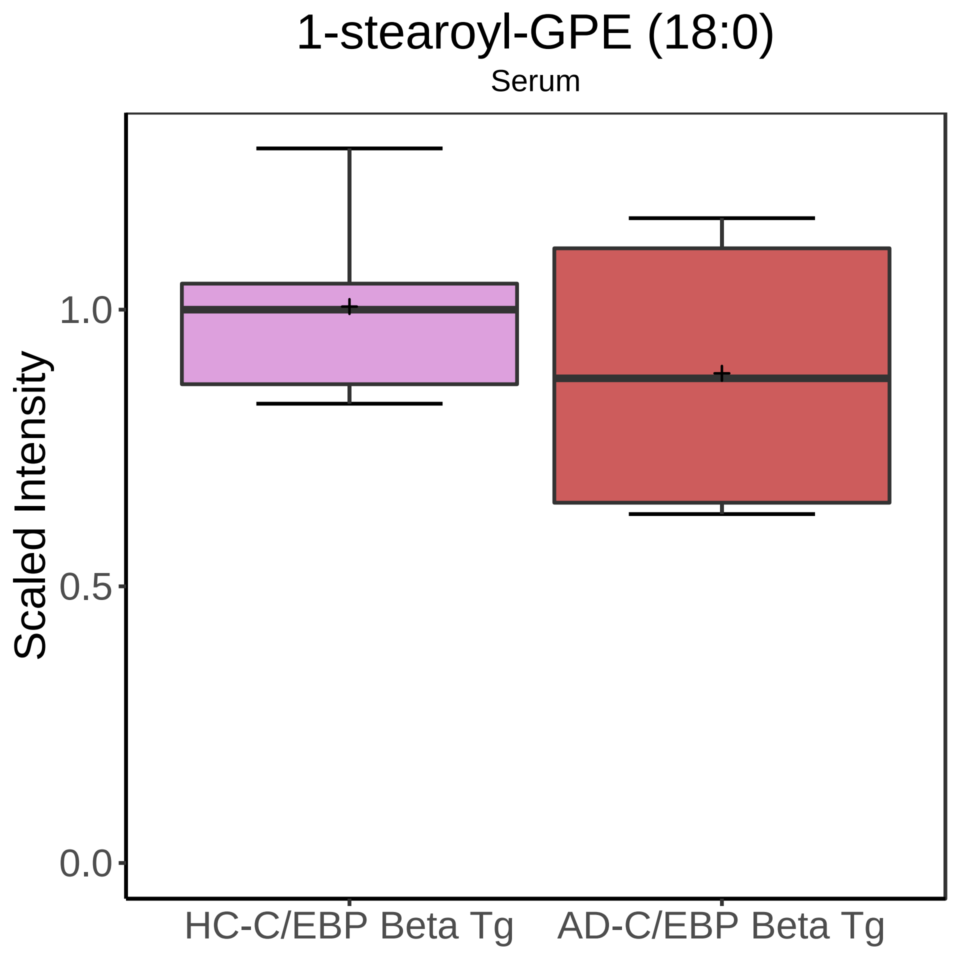
1-stearoyl-GPE (18:0)
Serum
Scaled Intensity
1.0
0.5
0.0
HC-C/EBP Beta Tg
AD-C/EBP Beta Tg

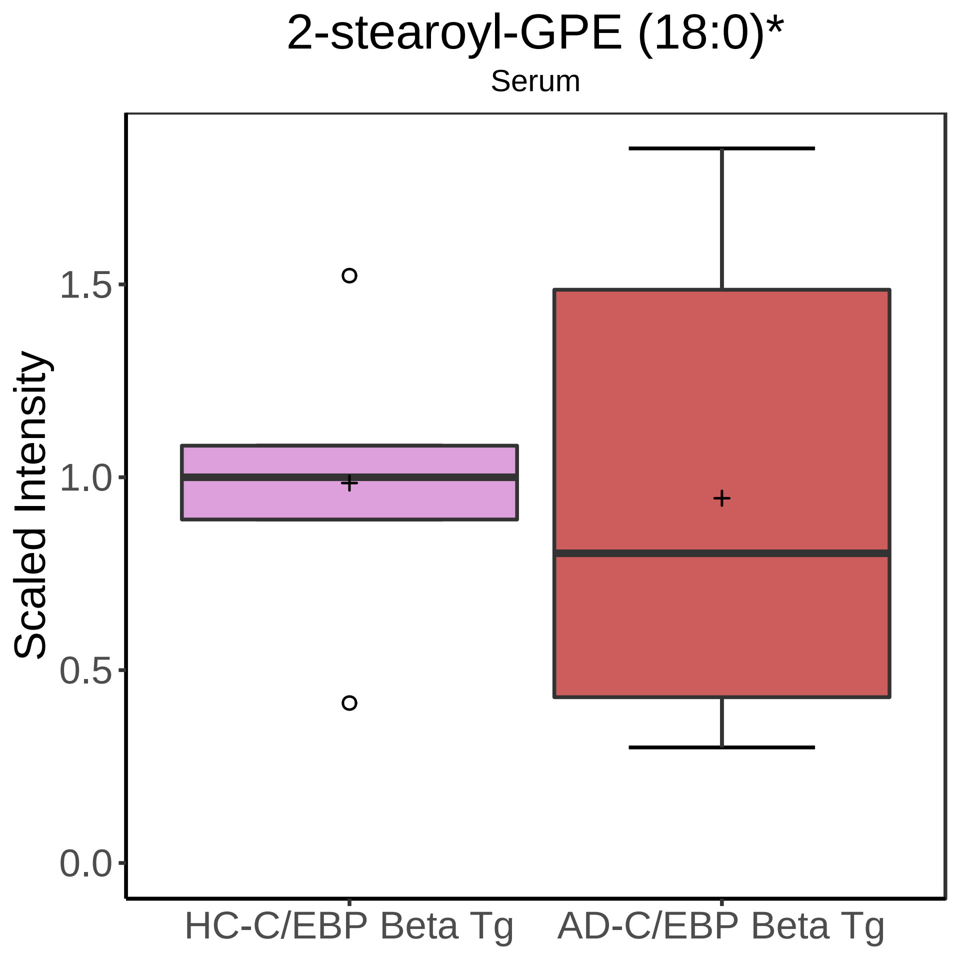
2-stearoyl-GPE (18:0)*
Serum
Scaled Intensity
1.5
1.0
0.5
0.0
HC-C/EBP Beta Tg
AD-C/EBP Beta Tg

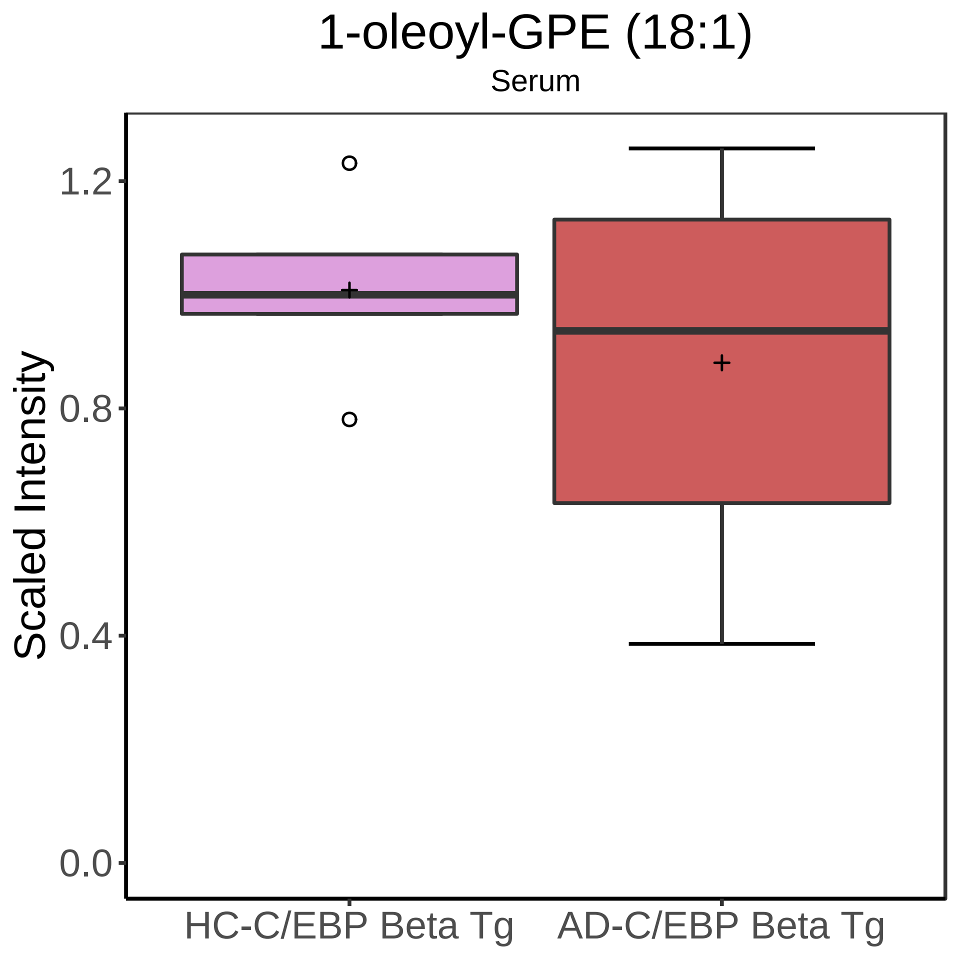
1-oleoyl-GPE (18:1)
Serum
Scaled Intensity
1.2
0.8
0.4
0.0
HC-C/EBP Beta Tg
AD-C/EBP Beta Tg

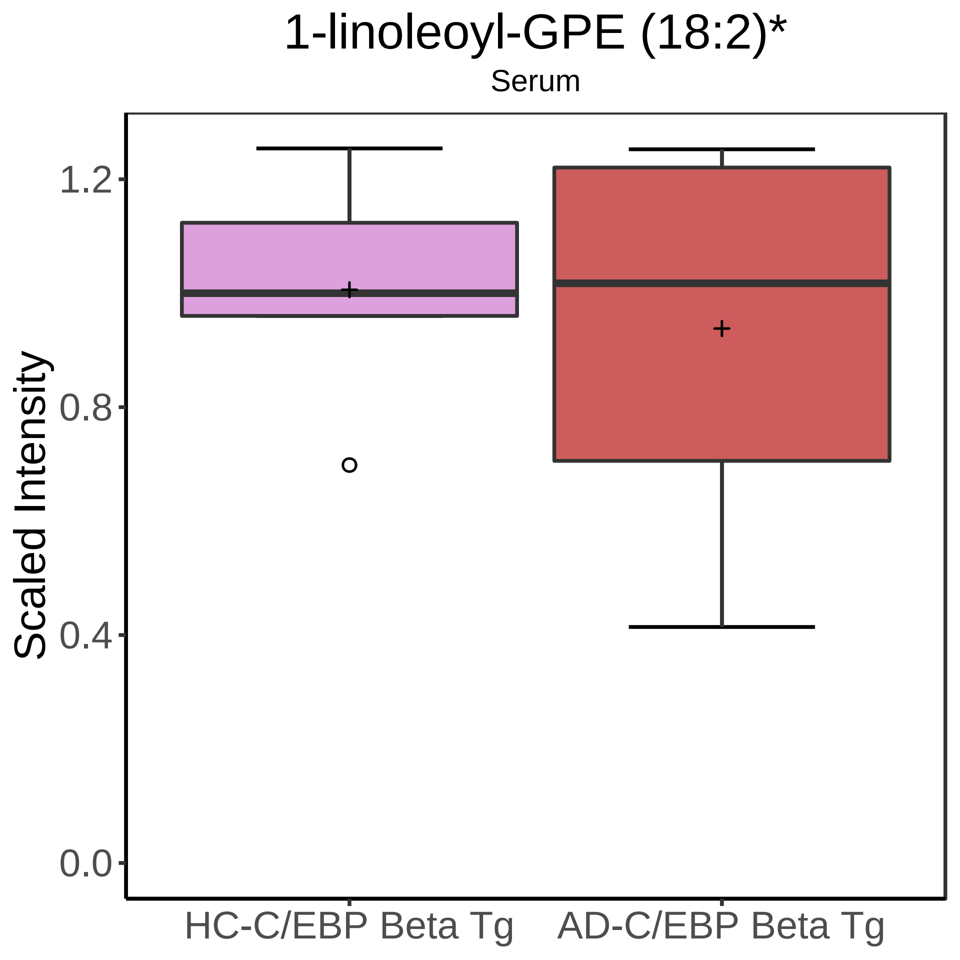
1-linoleoyl-GPE (18:2)*
Serum
Scaled Intensity
1.2
0.8
0.4
0.0
HC-C/EBP Beta Tg
AD-C/EBP Beta Tg

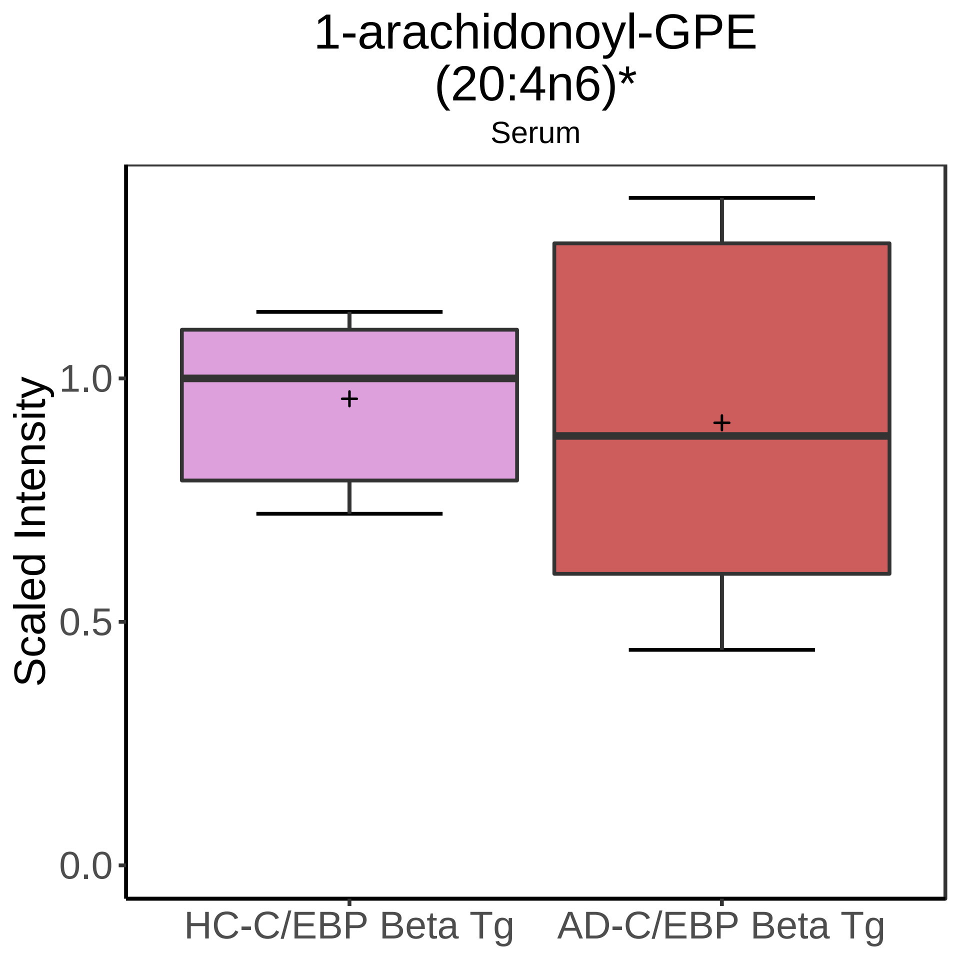

1-arachidonoyl-GPE
(20:4n6)*
Serum
Scaled Intensity
1.0
0.5
0.0
HC-C/EBP Beta Tg
AD-C/EBP Beta Tg

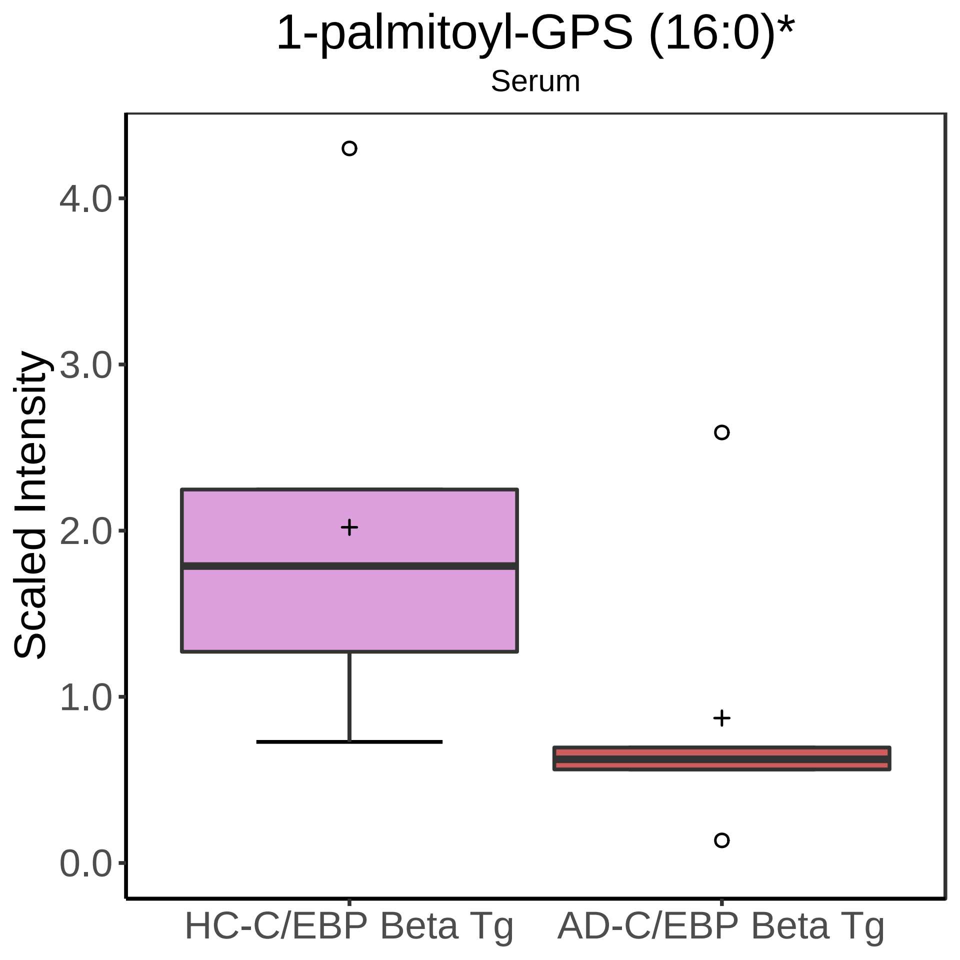

1-palmitoyl-GPS (16:0)*
Serum
Scaled Intensity
4.0
3.0
2.0
1.0
0.0
HC-C/EBP Beta Tg
AD-C/EBP Beta Tg

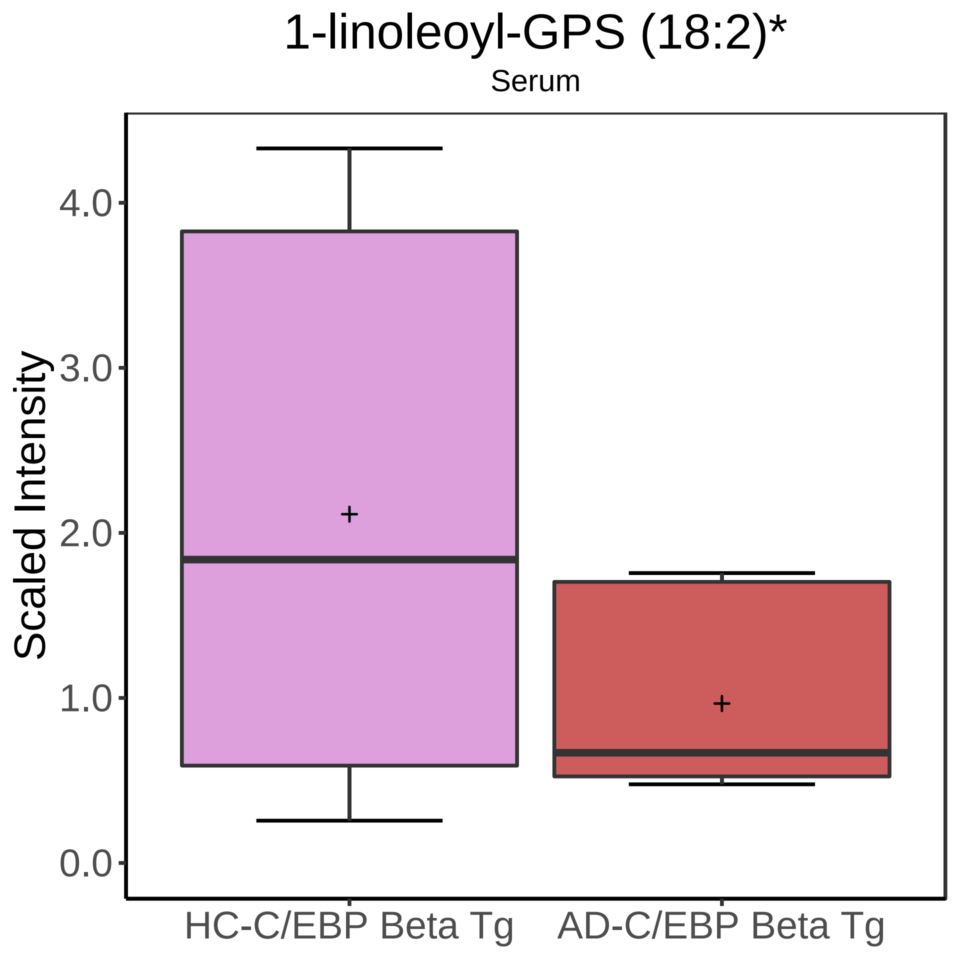
1-linoleoyl-GPS (18:2)*
Serum
Scaled Intensity
0.0
1.0
2.0
3.0
4.0
HC-C/EBP Beta Tg
AD-C/EBP Beta Tg

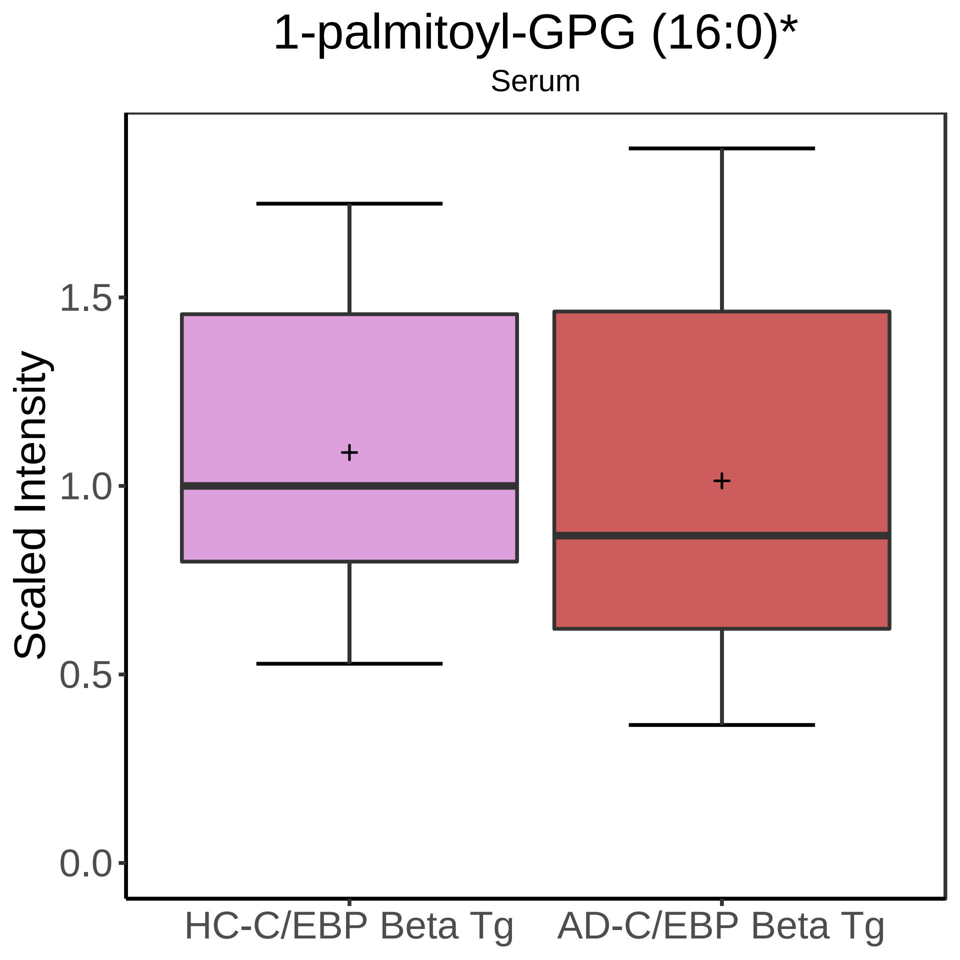
1-palmitoyl-GPG (16:0)*
Serum
Scaled Intensity
1.5
1.0
0.5
0.0
HC-C/EBP Beta Tg
AD-C/EBP Beta Tg

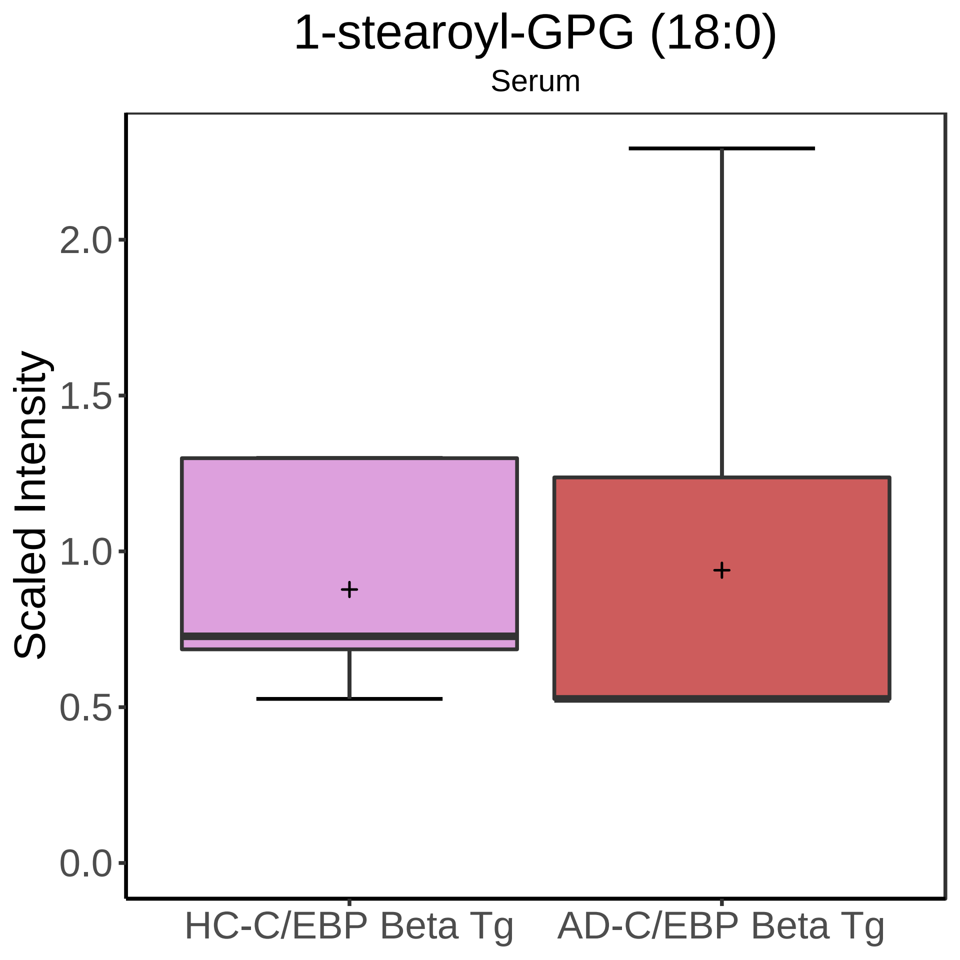
1-stearoyl-GPG (18:0)
Serum
Scaled Intensity
2.0
1.5
1.0
0.5
0.0
HC-C/EBP Beta Tg
AD-C/EBP Beta Tg

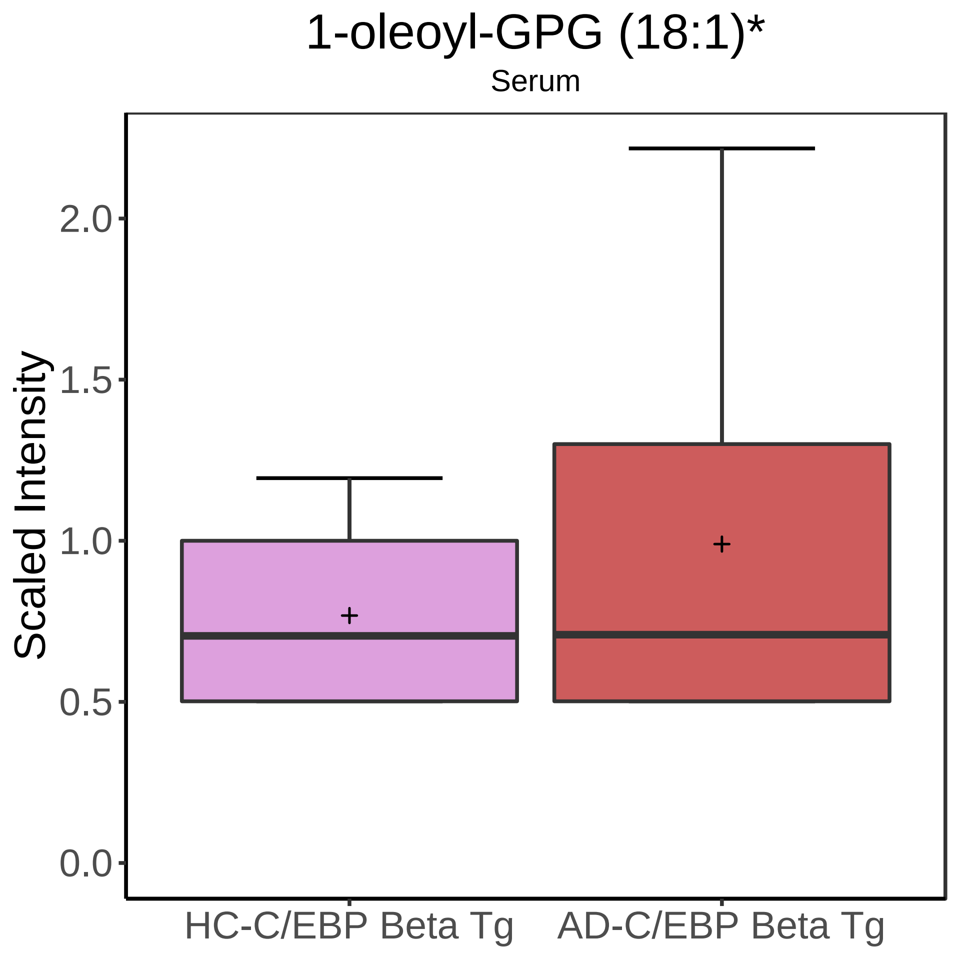
1-oleoyl-GPG (18:1)*
Serum
Scaled Intensity
2.0
1.5
1.0
0.5
0.0
HC-C/EBP Beta Tg
AD-C/EBP Beta Tg

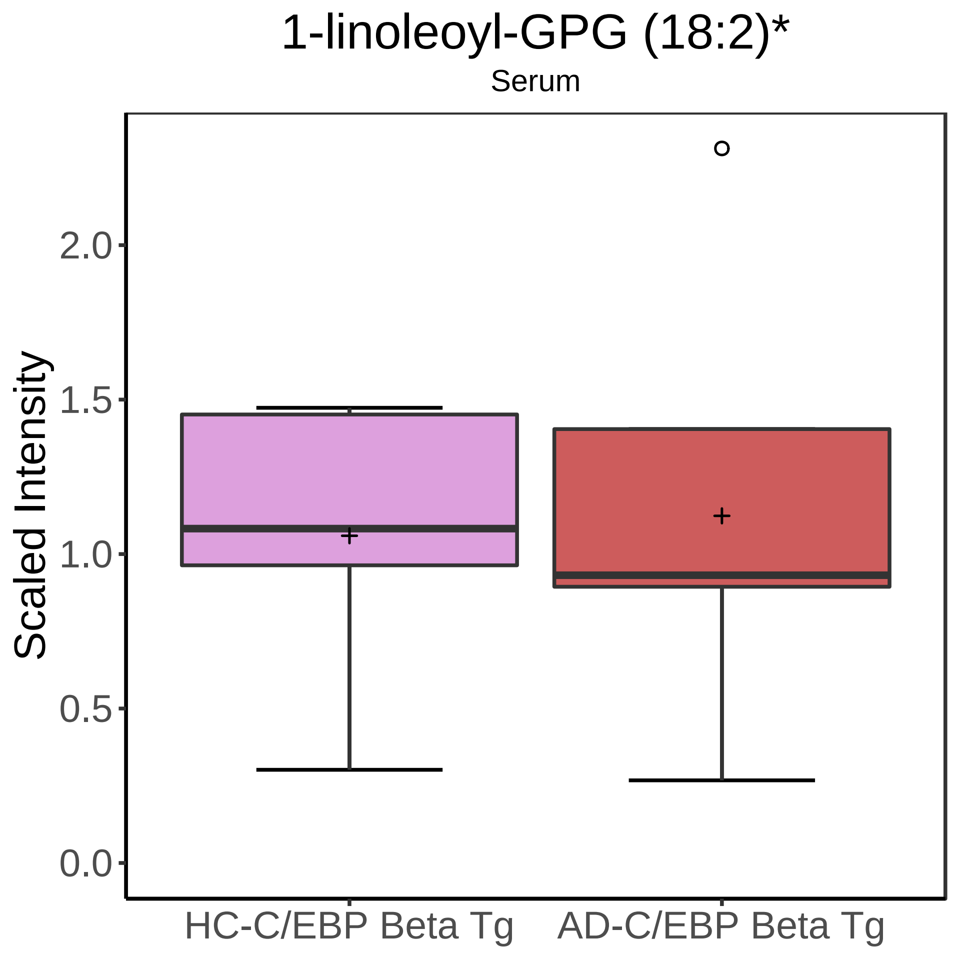
1-linoleoyl-GPG (18:2)*
Serum
Scaled Intensity
2.0
1.5
1.0
0.5
0.0
HC-C/EBP Beta Tg
AD-C/EBP Beta Tg

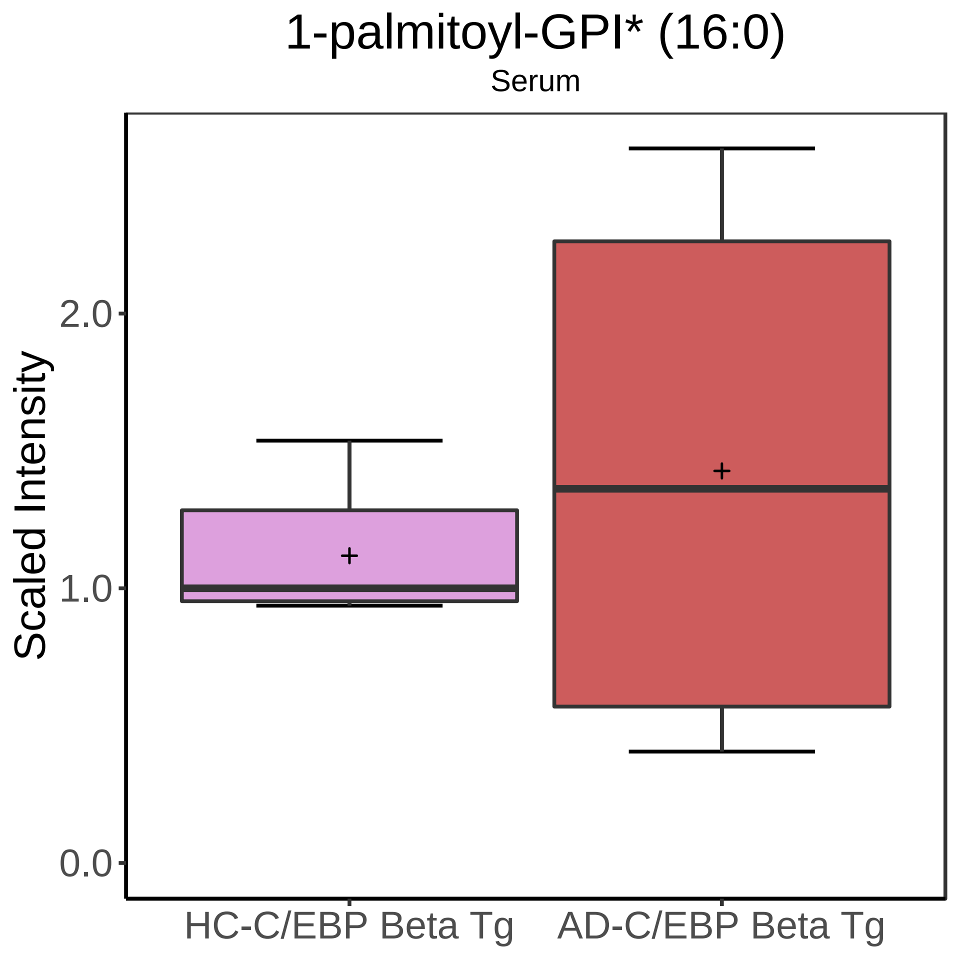

1-palmitoyl-GPI* (16:0)
Serum
Scaled Intensity
2.0
1.0
0.0
HC-C/EBP Beta Tg
AD-C/EBP Beta Tg

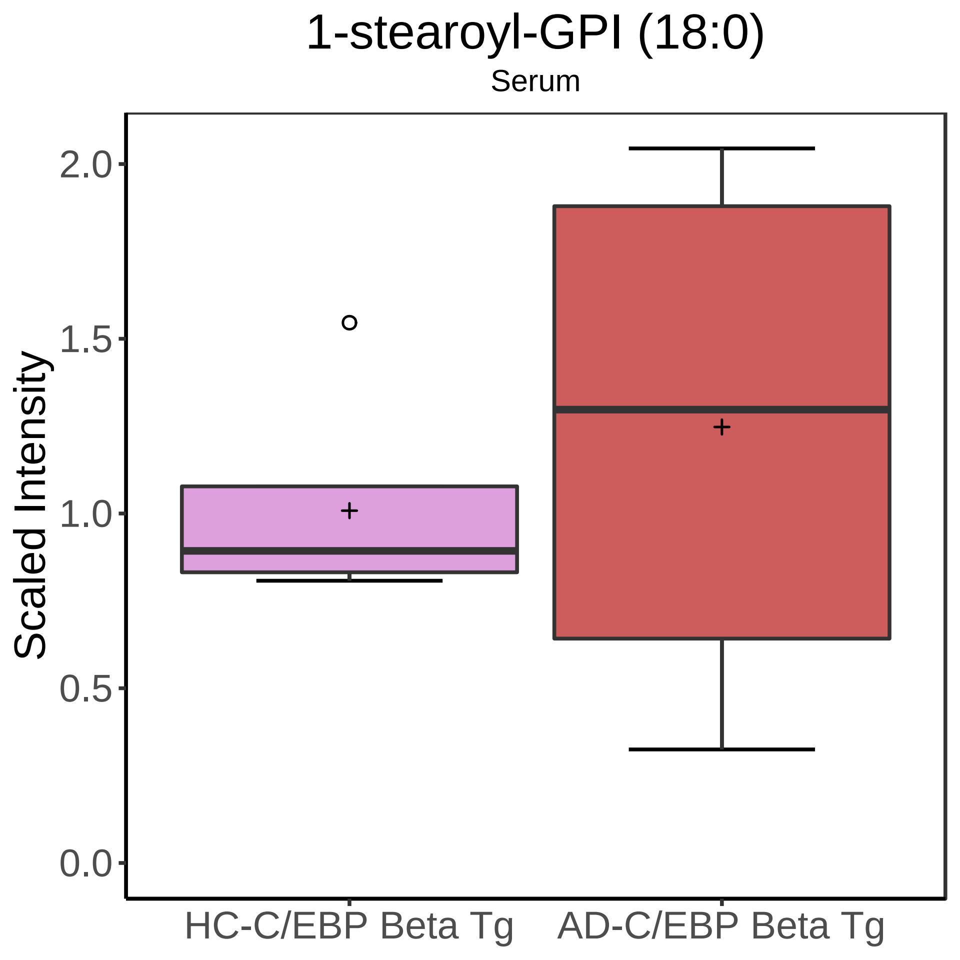

1-stearoyl-GPI (18:0)
Serum
Scaled Intensity
2.0
1.5
1.0
0.5
0.0
HC-C/EBP Beta Tg
AD-C/EBP Beta Tg

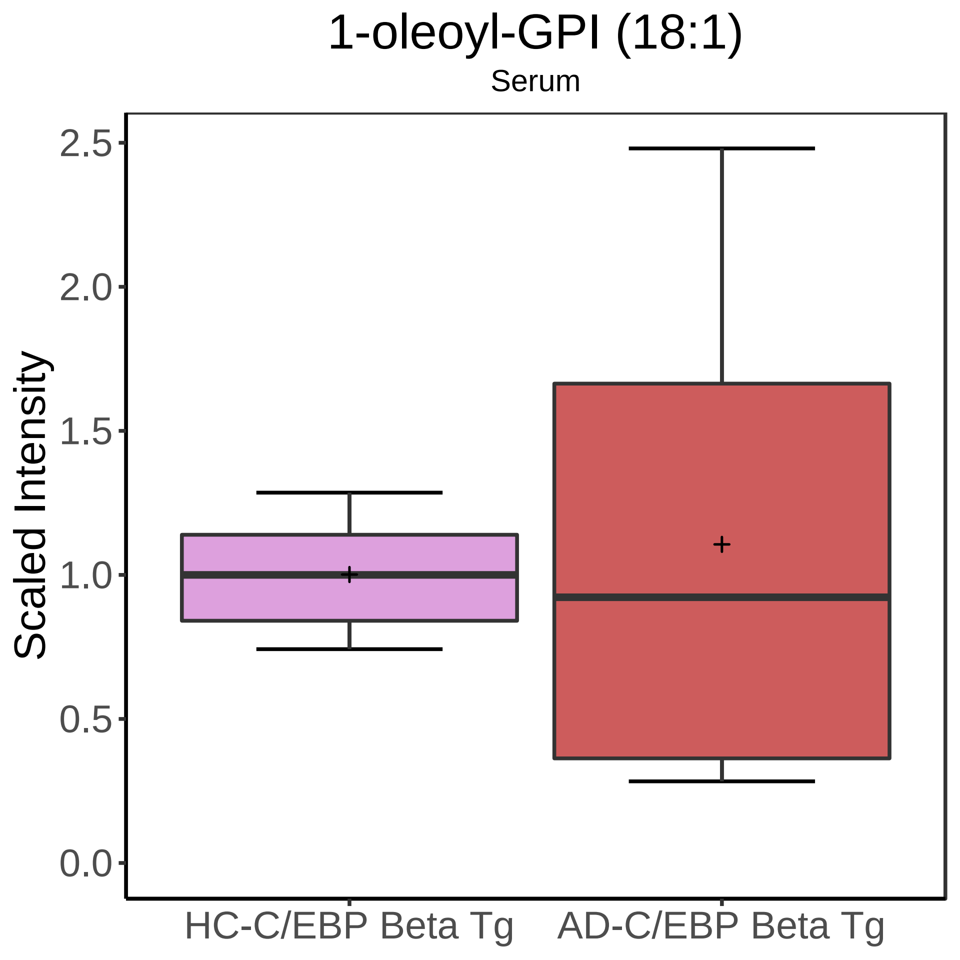
1-oleoyl-GPI (18:1)
Serum
Scaled Intensity
2.5
2.0
1.5
1.0
0.5
0.0
HC-C/EBP Beta Tg
AD-C/EBP Beta Tg

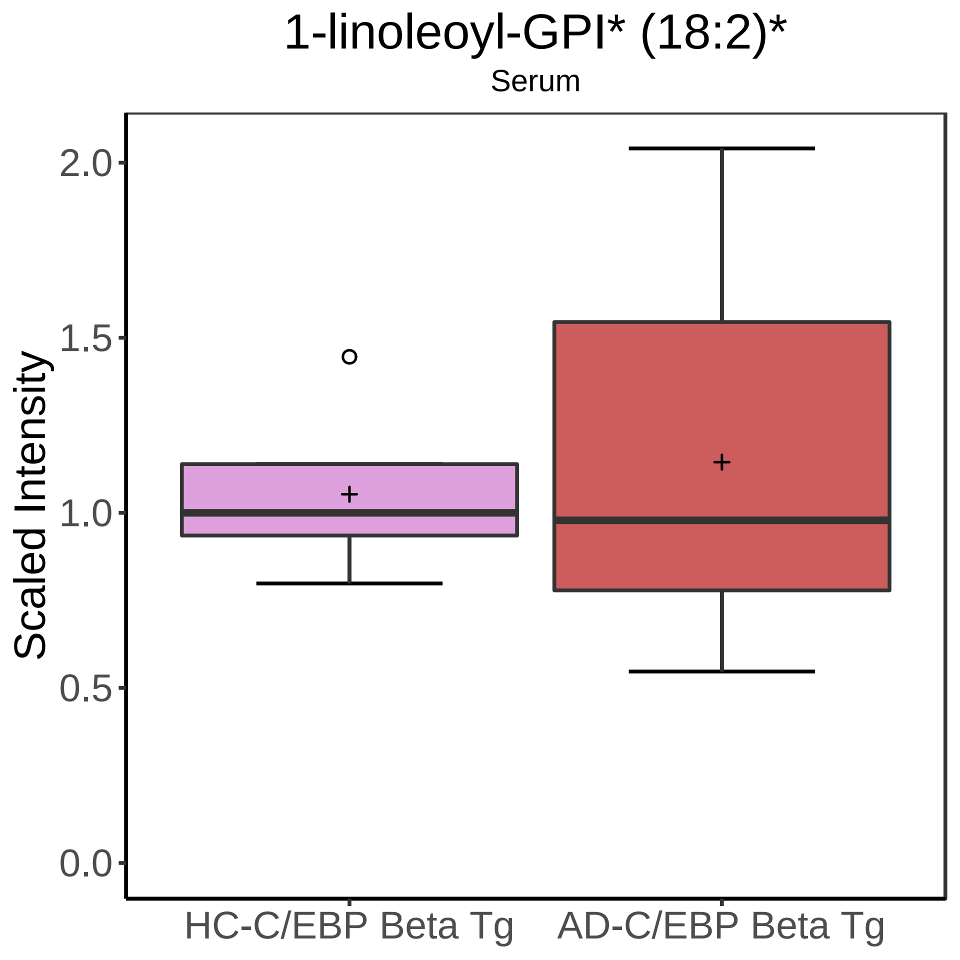
1-linoleoyl-GPI* (18:2)*
Serum
Scaled Intensity
2.0
1.5
1.0
0.5
0.0
HC-C/EBP Beta Tg
AD-C/EBP Beta Tg

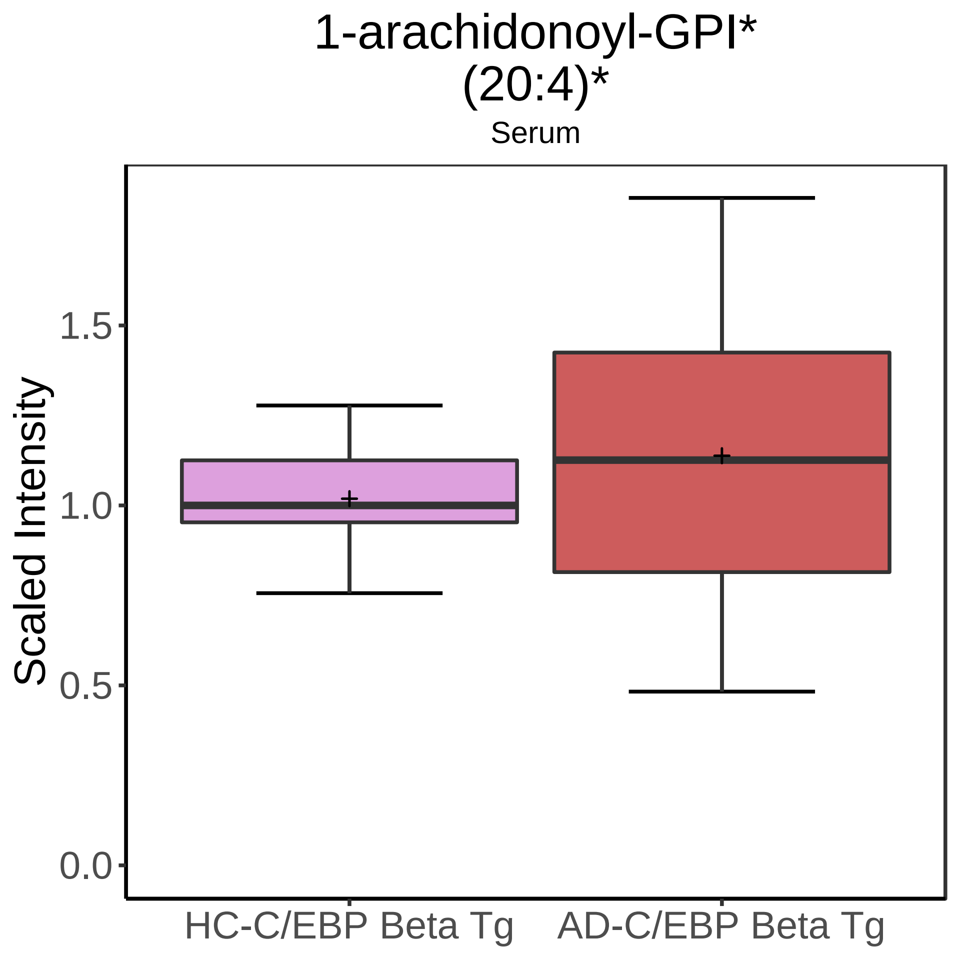

1-arachidonoyl-GPI*
(20:4)*
Serum
Scaled Intensity
1.5
1.0
0.5
0.0
HC-C/EBP Beta Tg
AD-C/EBP Beta Tg

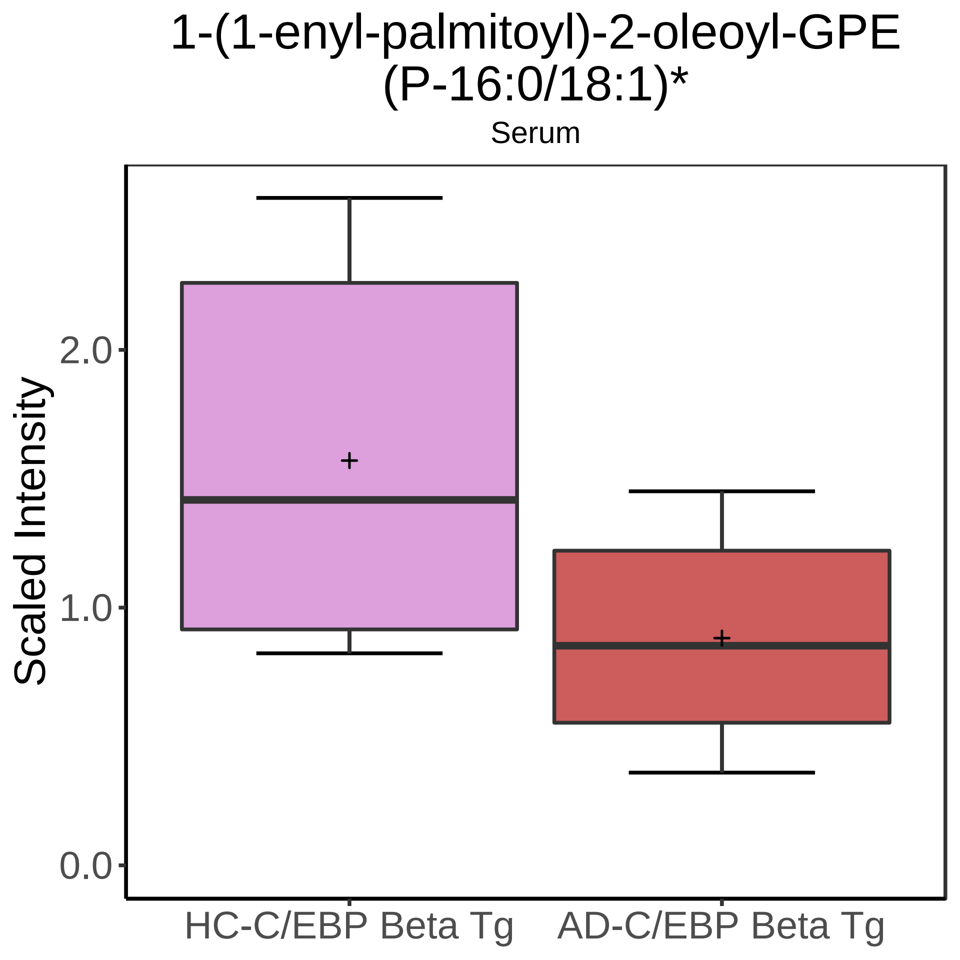
1-(1-enyl-palmitoyl)-2-oleoyl-GPE
(P-16:0/18:1)*
Serum
Scaled Intensity
2.0
1.0
0.0
HC-C/EBP Beta Tg
AD-C/EBP Beta Tg

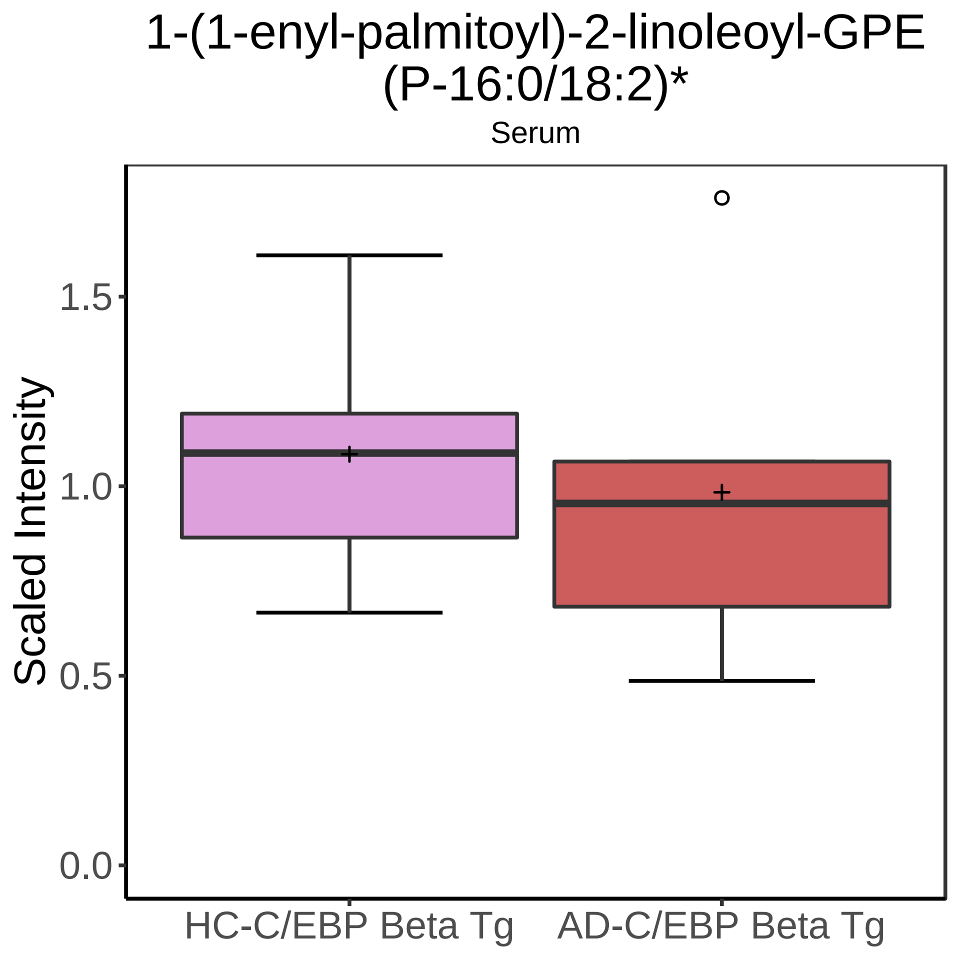
1-(1-enyl-palmitoyl)-2-linoleoyl-GPE
(P-16:0/18:2)*
Serum
Scaled Intensity
1.5
1.0
0.5
0.0
HC-C/EBP Beta Tg
AD-C/EBP Beta Tg

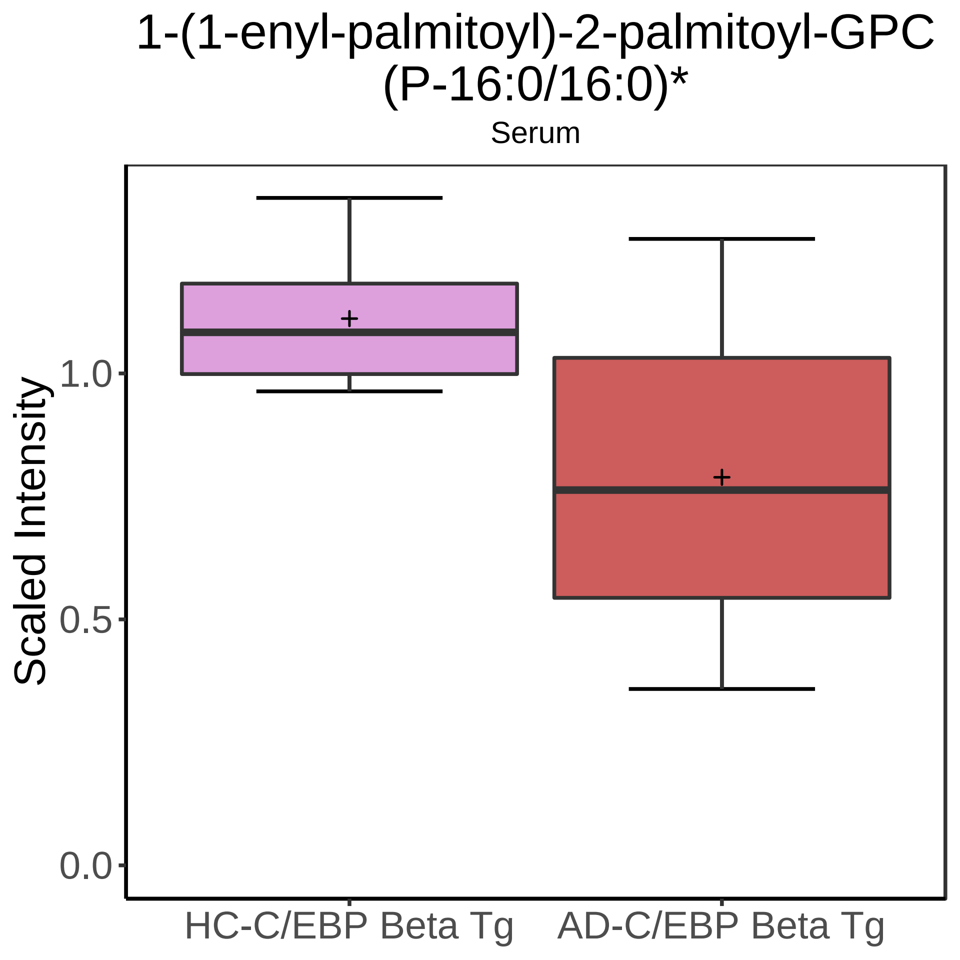
1-(1-enyl-palmitoyl)-2-palmitoyl-GPC
(P-16:0/16:0)*
Serum
Scaled Intensity
1.0
0.5
0.0
HC-C/EBP Beta Tg
AD-C/EBP Beta Tg

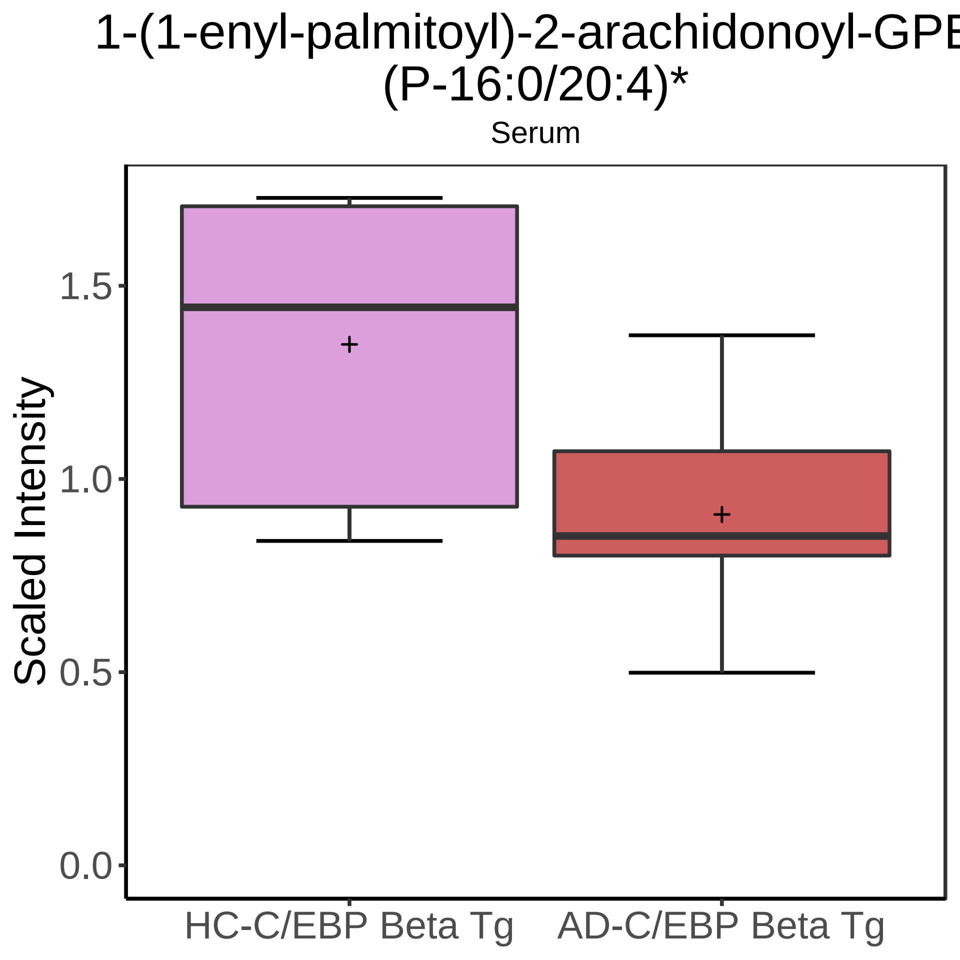
1-(1-enyl-palmitoyl)-2-arachidonoyl-GPE
(P-16:0/20:4)*
Serum
Scaled Intensity
1.5
1.0
0.5
0.0
HC-C/EBP Beta Tg
AD-C/EBP Beta Tg

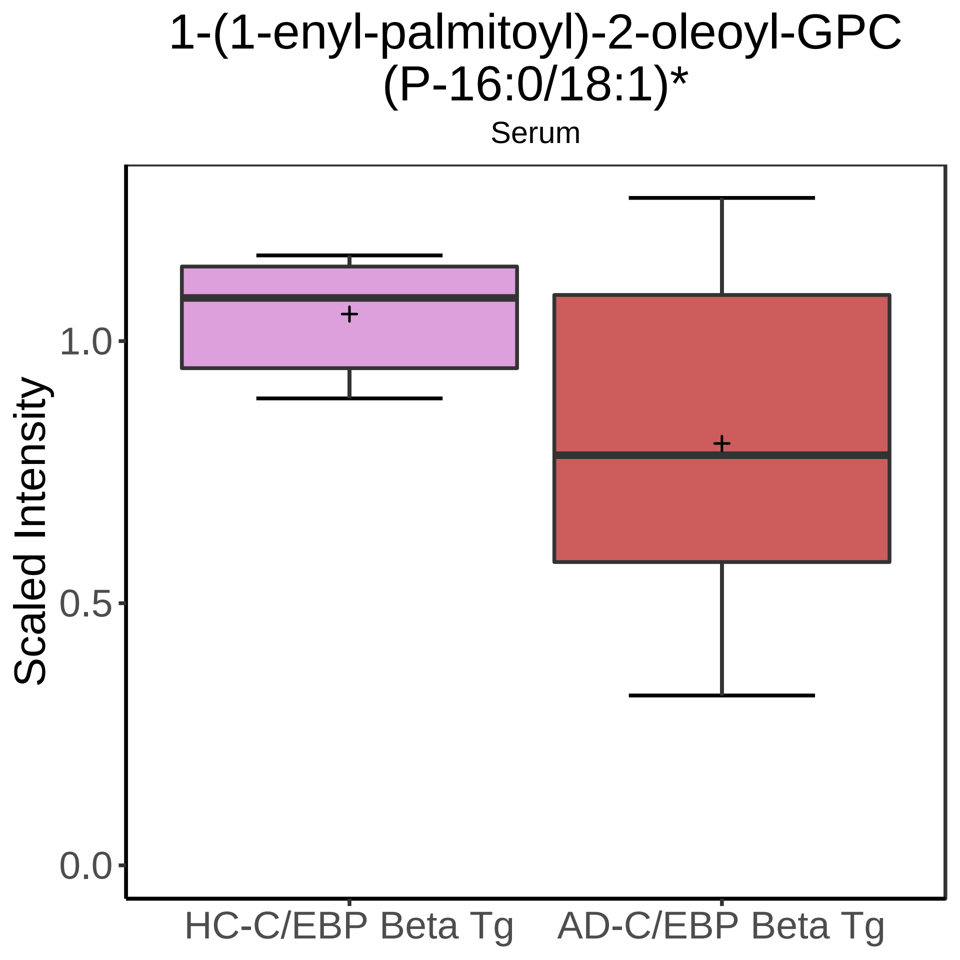

1-(1-enyl-palmitoyl)-2-oleoyl-GPC
(P-16:0/18:1)*
Serum
Scaled Intensity
1.0
0.5
0.0
HC-C/EBP Beta Tg
AD-C/EBP Beta Tg

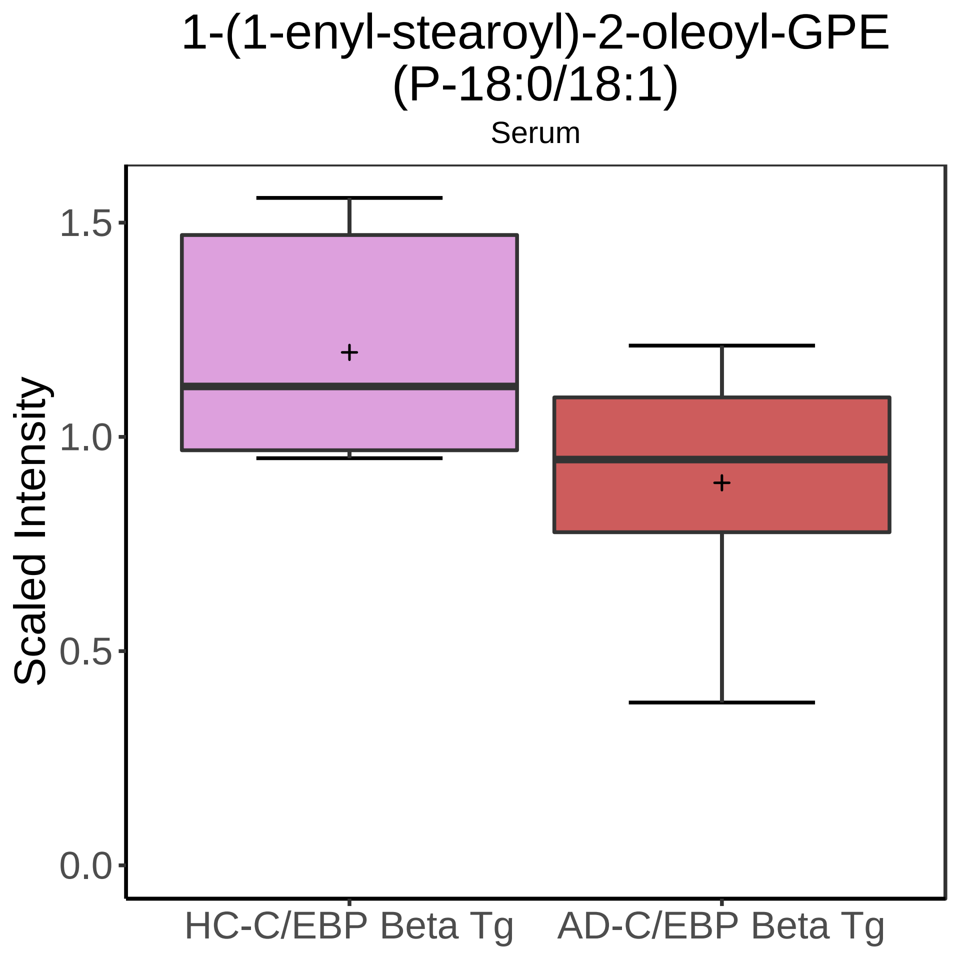
1-(1-enyl-stearoyl)-2-oleoyl-GPE
(P-18:0/18:1)
Serum
Scaled Intensity
1.5
1.0
0.5
0.0
HC-C/EBP Beta Tg
AD-C/EBP Beta Tg

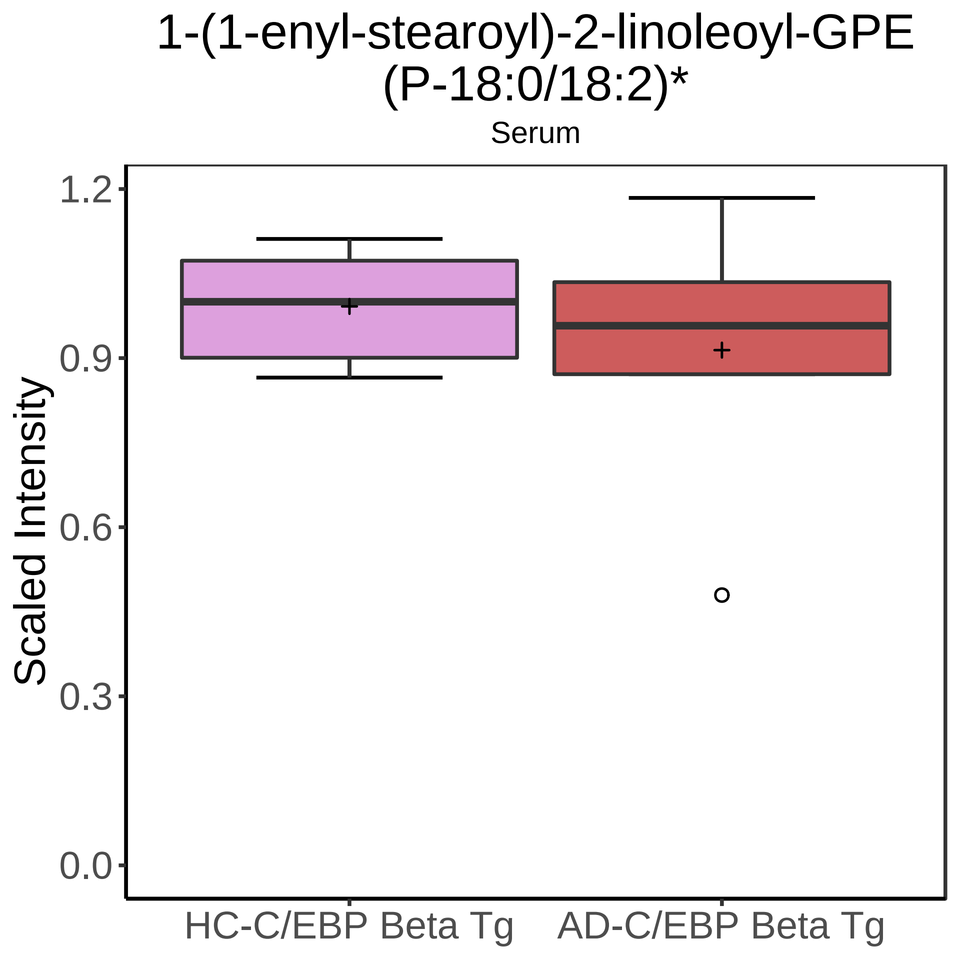

1-(1-enyl-stearoyl)-2-linoleoyl-GPE
(P-18:0/18:2)*
Serum
Scaled Intensity
1.2
0.9
0.6
0.3
0.0
HC-C/EBP Beta Tg
AD-C/EBP Beta Tg

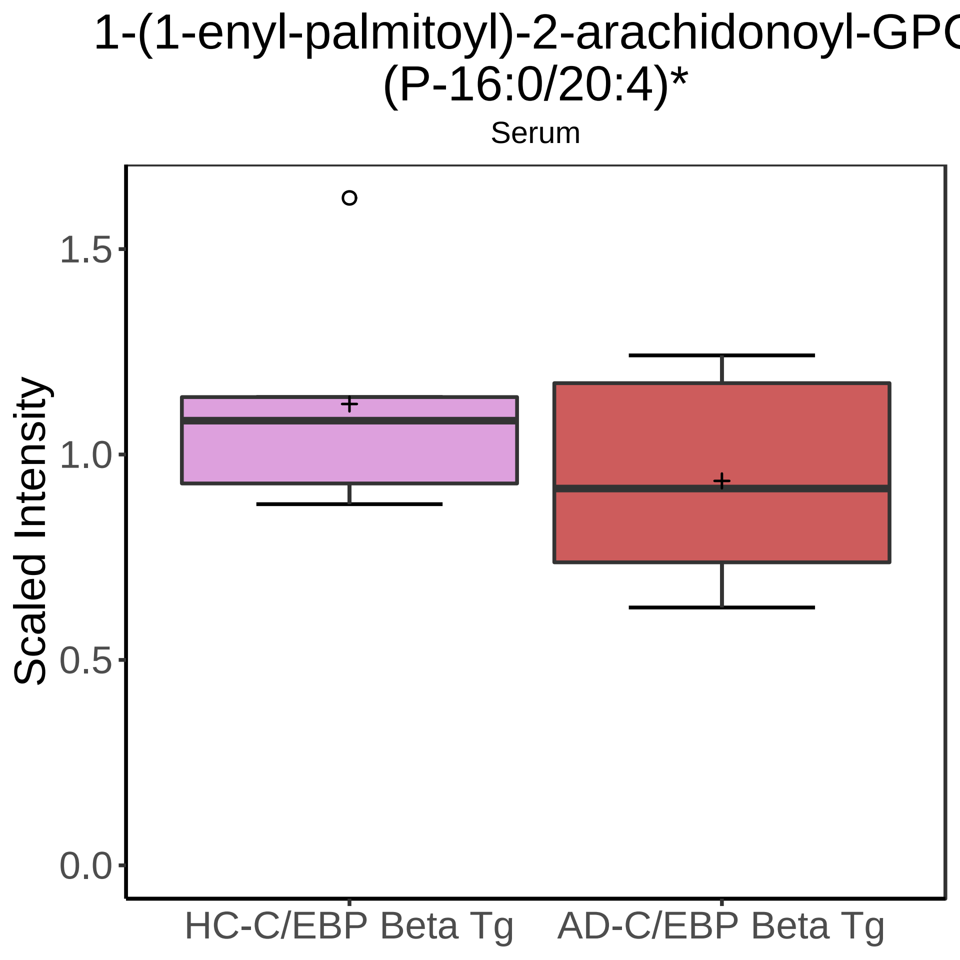

1-(1-enyl-palmitoyl)-2-arachidonoyl-GPC
(P-16:0/20:4)*
Serum
Scaled Intensity
1.5
1.0
0.5
0.0
HC-C/EBP Beta Tg
AD-C/EBP Beta Tg

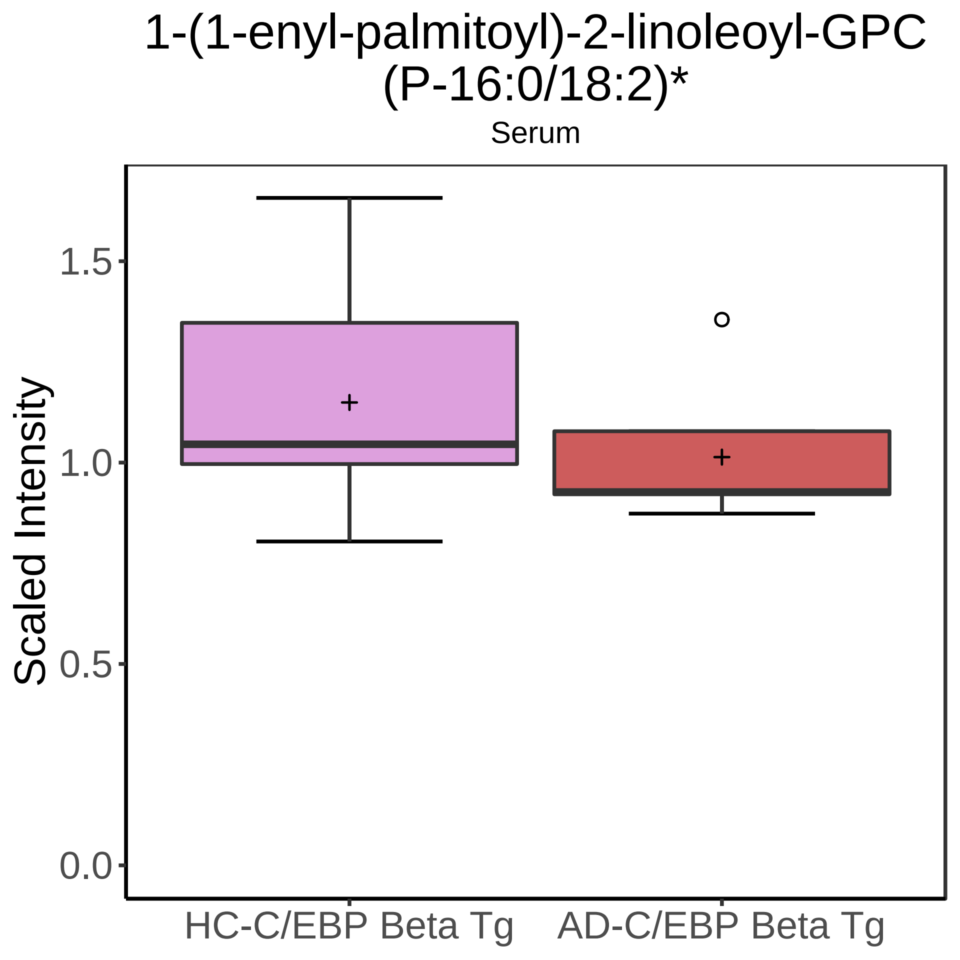

1-(1-enyl-palmitoyl)-2-linoleoyl-GPC
(P-16:0/18:2)*
Serum
Scaled Intensity
0.0
0.5
1.0
1.5
HC-C/EBP Beta Tg
AD-C/EBP Beta Tg

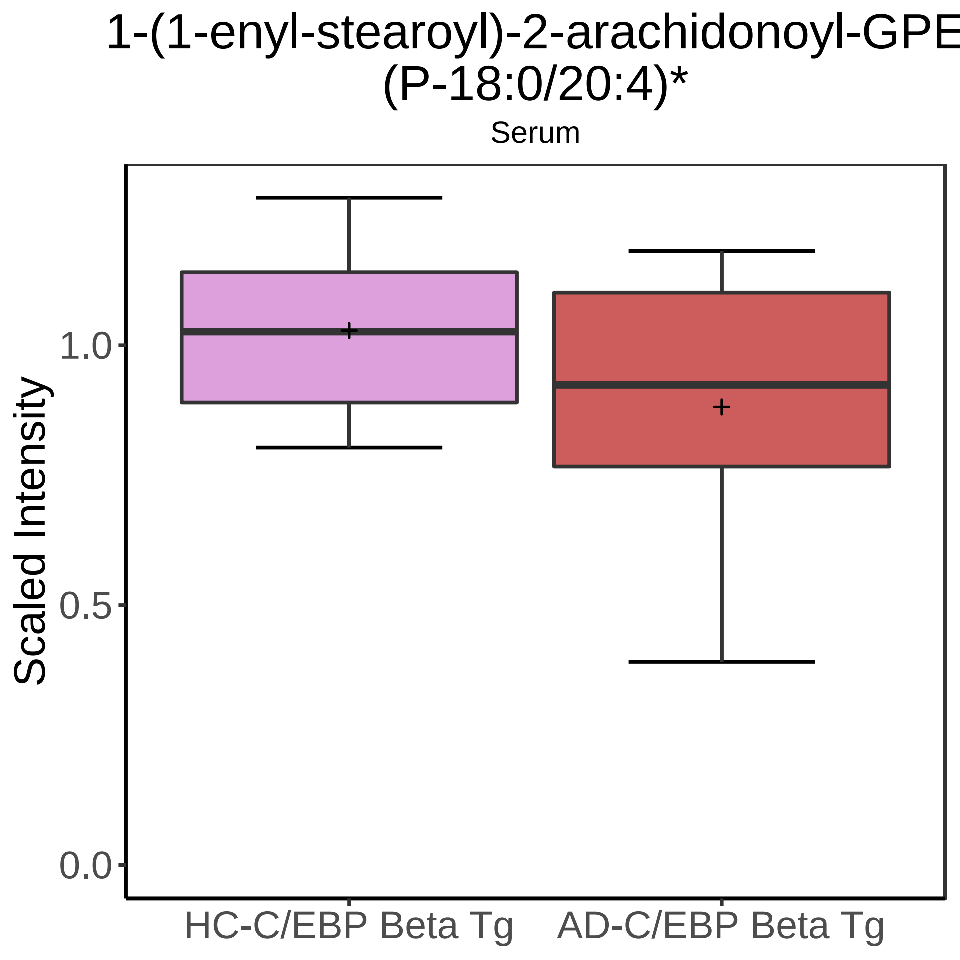
1-(1-enyl-stearoyl)-2-arachidonoyl-GPE
(P-18:0/20:4)*
Serum
Scaled Intensity
1.0
0.5
0.0
HC-C/EBP Beta Tg
AD-C/EBP Beta Tg

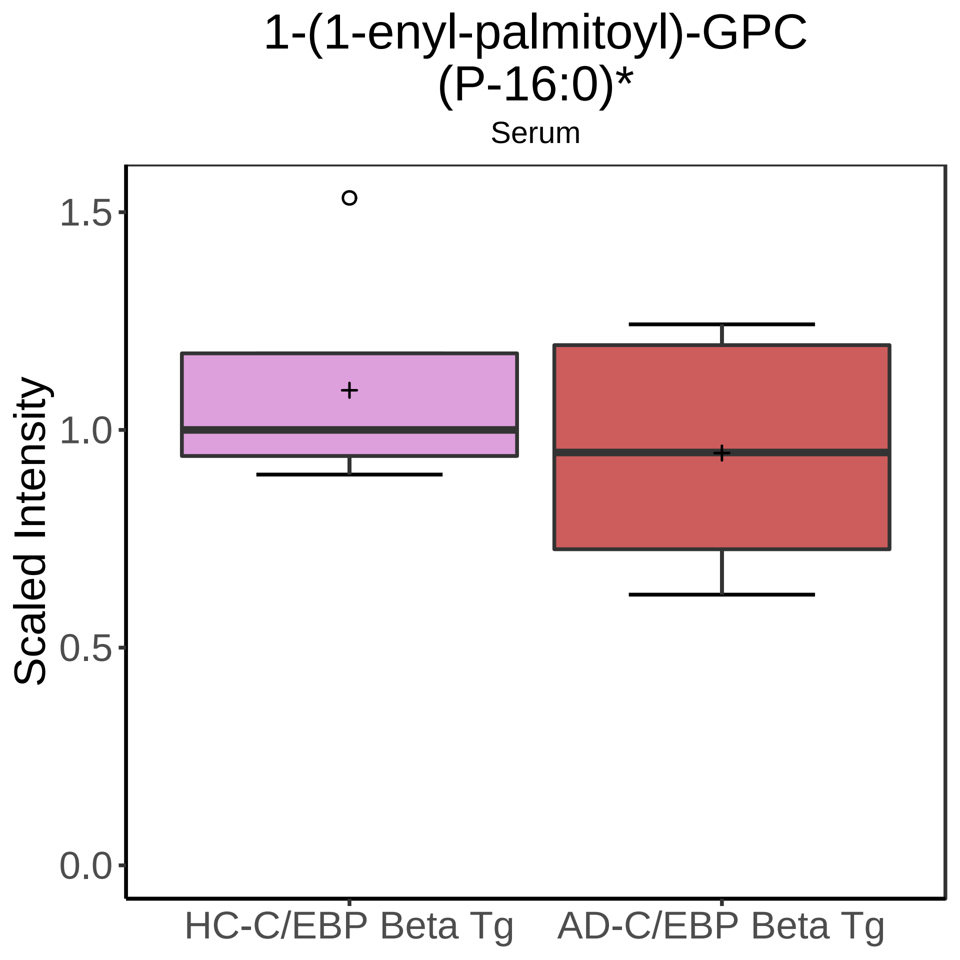
1-(1-enyl-palmitoyl)-GPC
(P-16:0)*
Serum
Scaled Intensity
1.5
1.0
0.5
0.0
HC-C/EBP Beta Tg
AD-C/EBP Beta Tg

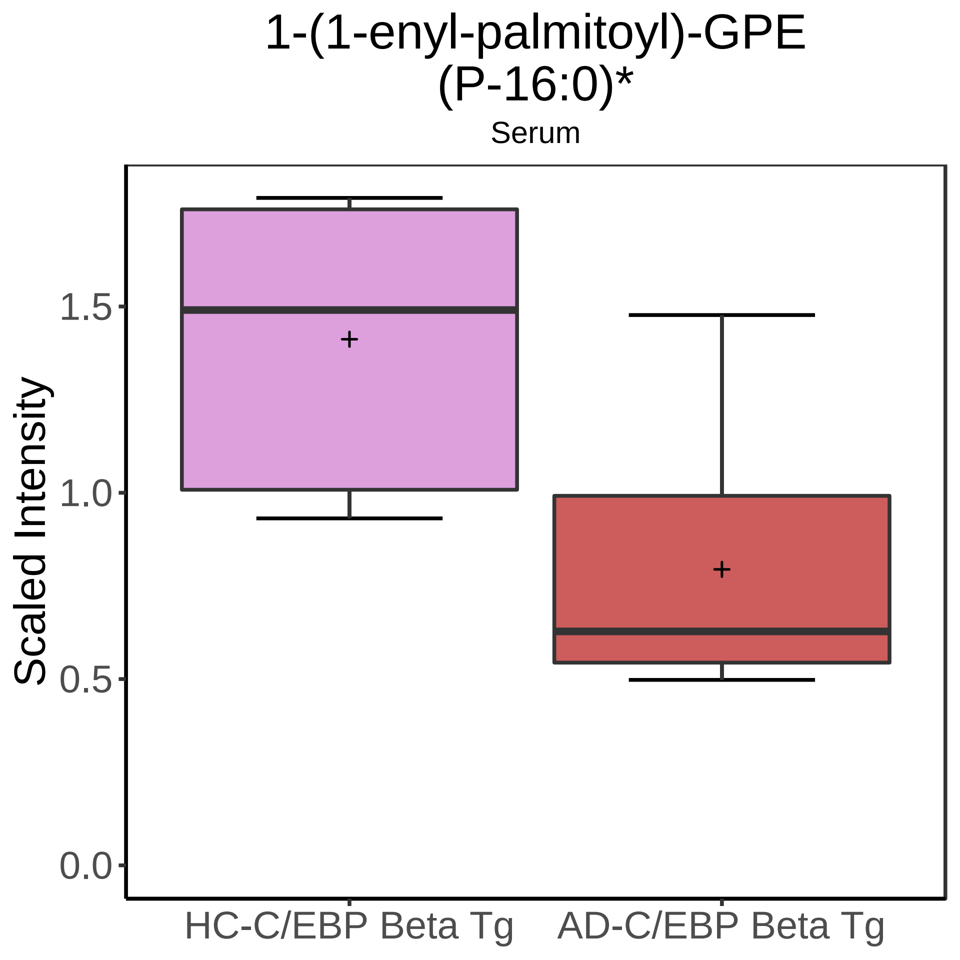
1-(1-enyl-palmitoyl)-GPE
(P-16:0)*
Serum
Scaled Intensity
0.0
0.5
1.0
1.5
HC-C/EBP Beta Tg
AD-C/EBP Beta Tg

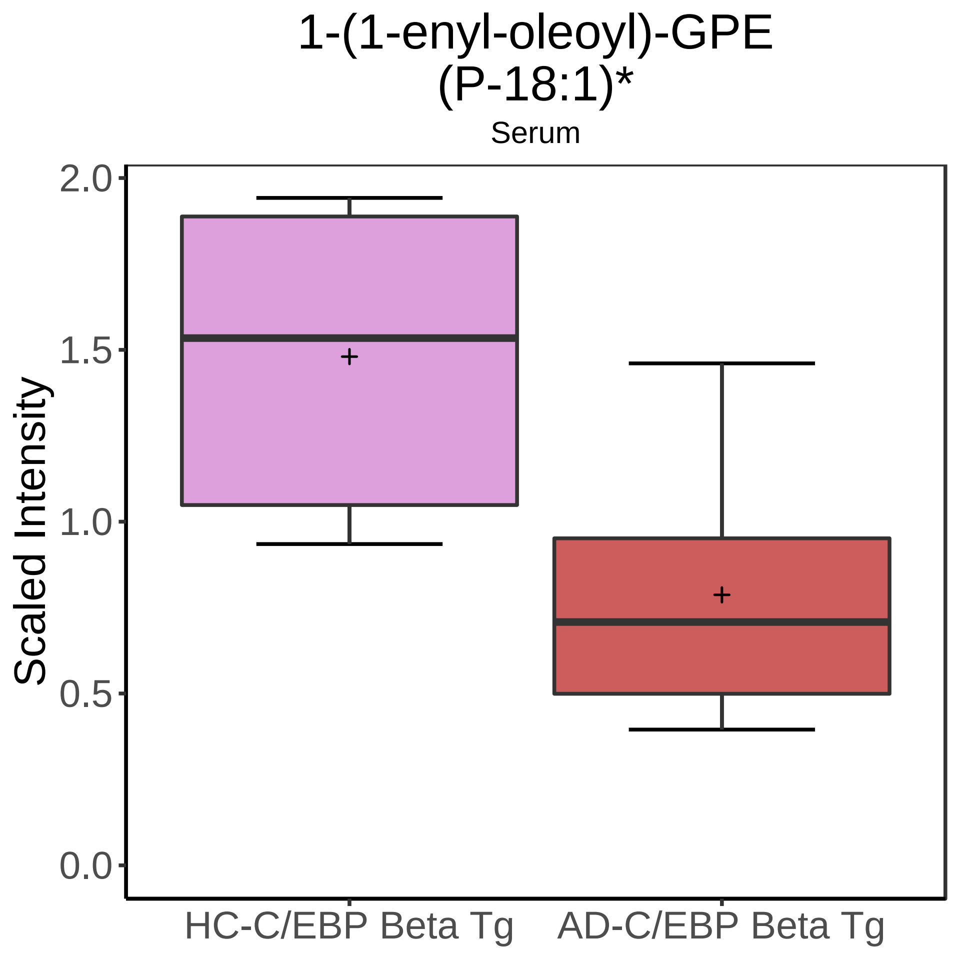
1-(1-enyl-oleoyl)-GPE
(P-18:1)*
Serum
Scaled Intensity
2.0
1.5
1.0
0.5
0.0
HC-C/EBP Beta Tg
AD-C/EBP Beta Tg

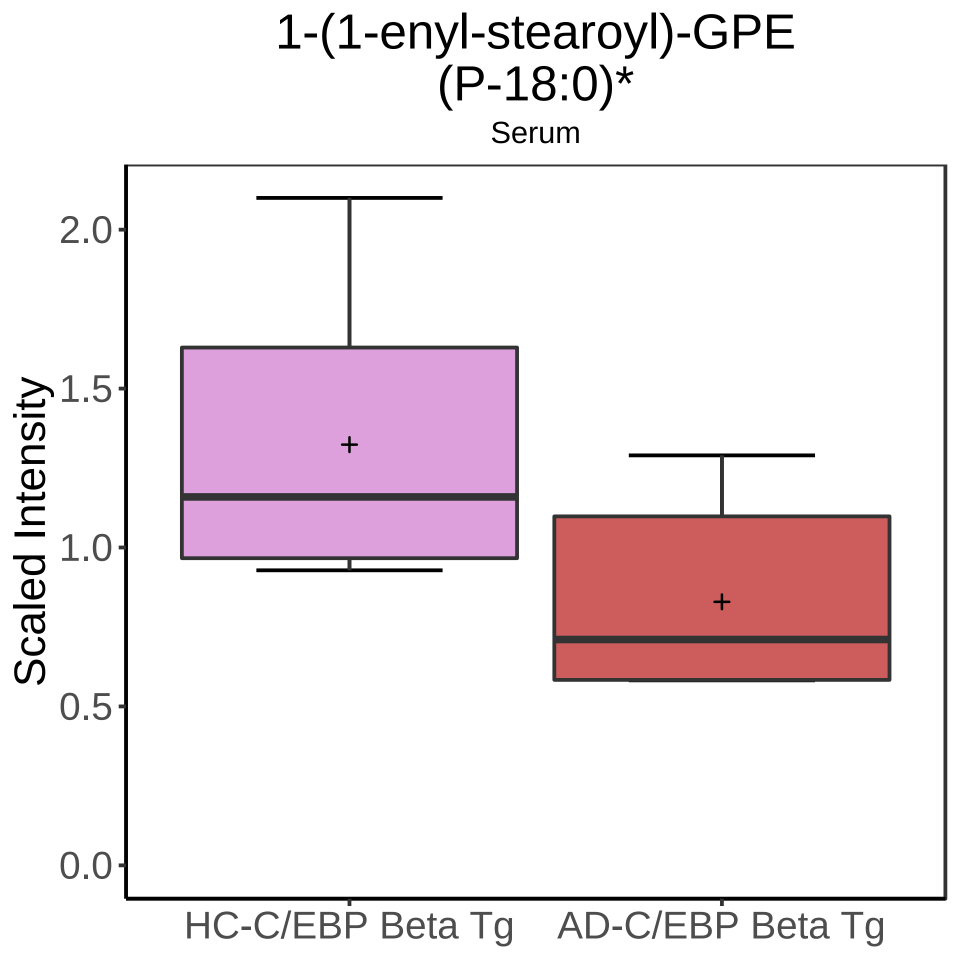
1-(1-enyl-stearoyl)-GPE
(P-18:0)*
Serum
Scaled Intensity
2.0
1.5
1.0
0.5
0.0
HC-C/EBP Beta Tg
AD-C/EBP Beta Tg

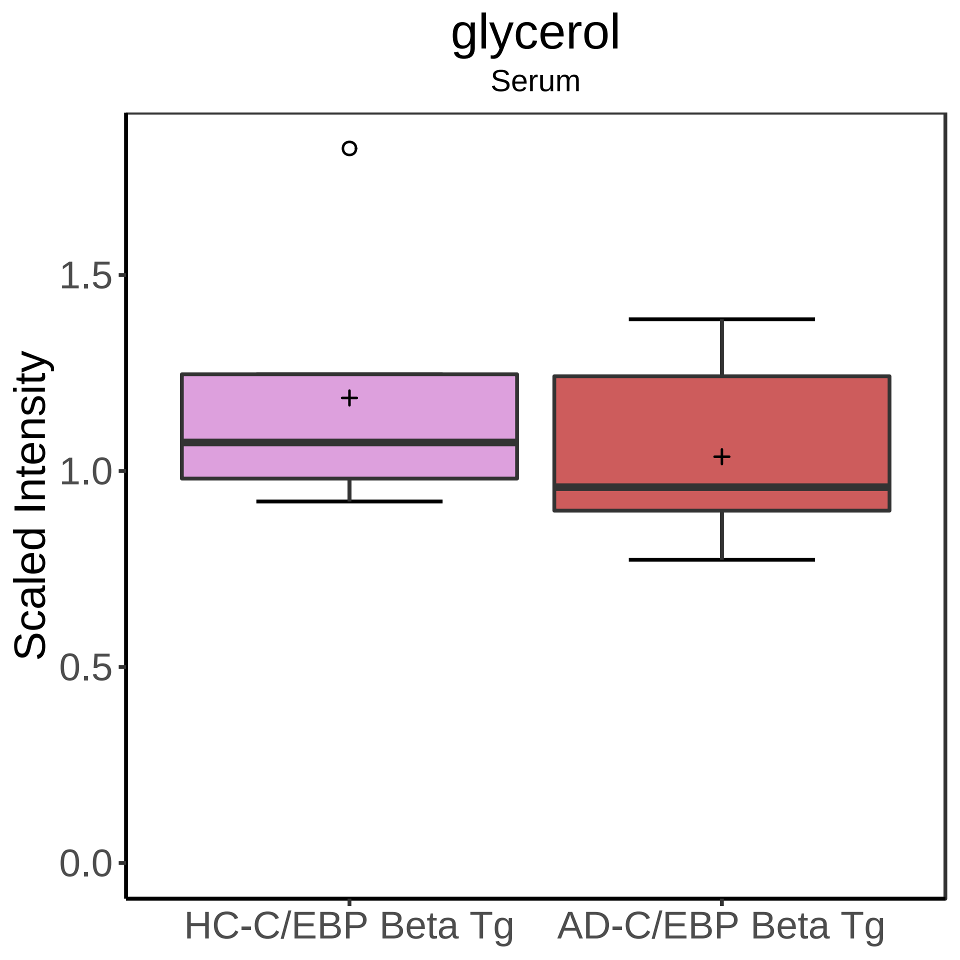
glycerol
Serum
Scaled Intensity
1.5
1.0
0.5
0.0
HC-C/EBP Beta Tg
AD-C/EBP Beta Tg

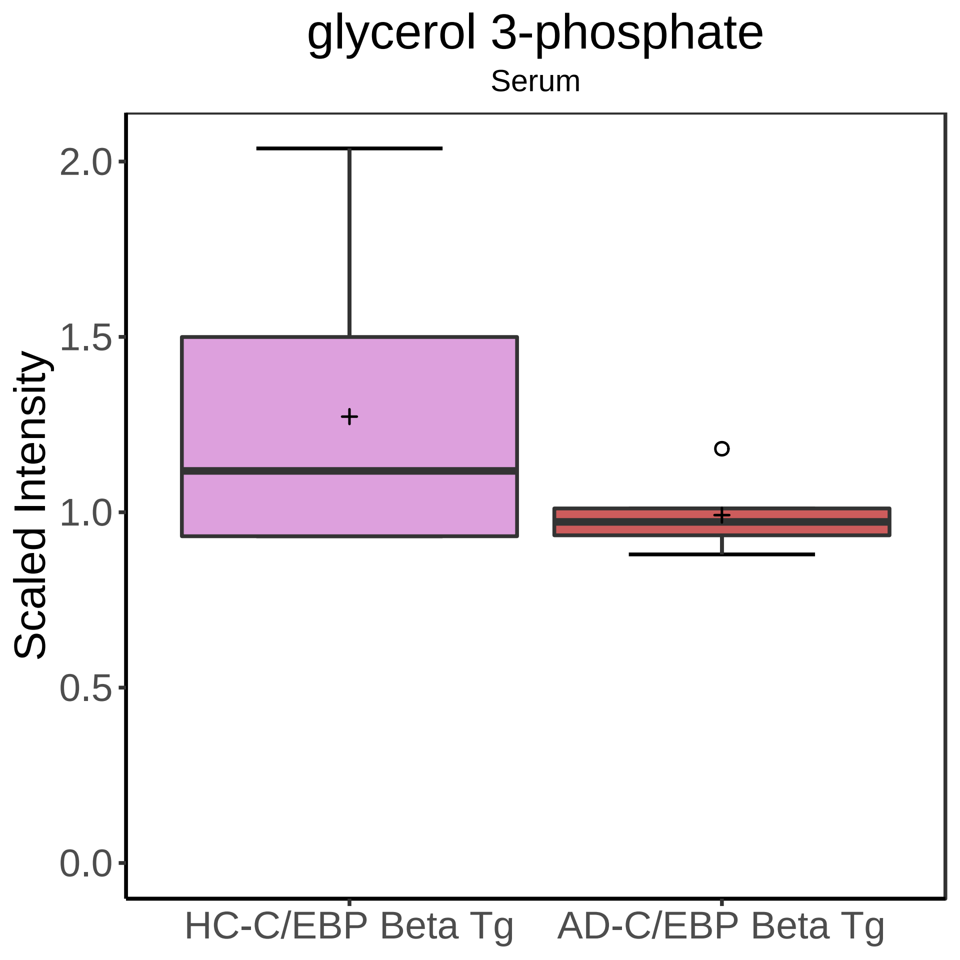
glycerol 3-phosphate
Serum
Scaled Intensity
2.0
1.5
1.0
0.5
0.0
HC-C/EBP Beta Tg
AD-C/EBP Beta Tg

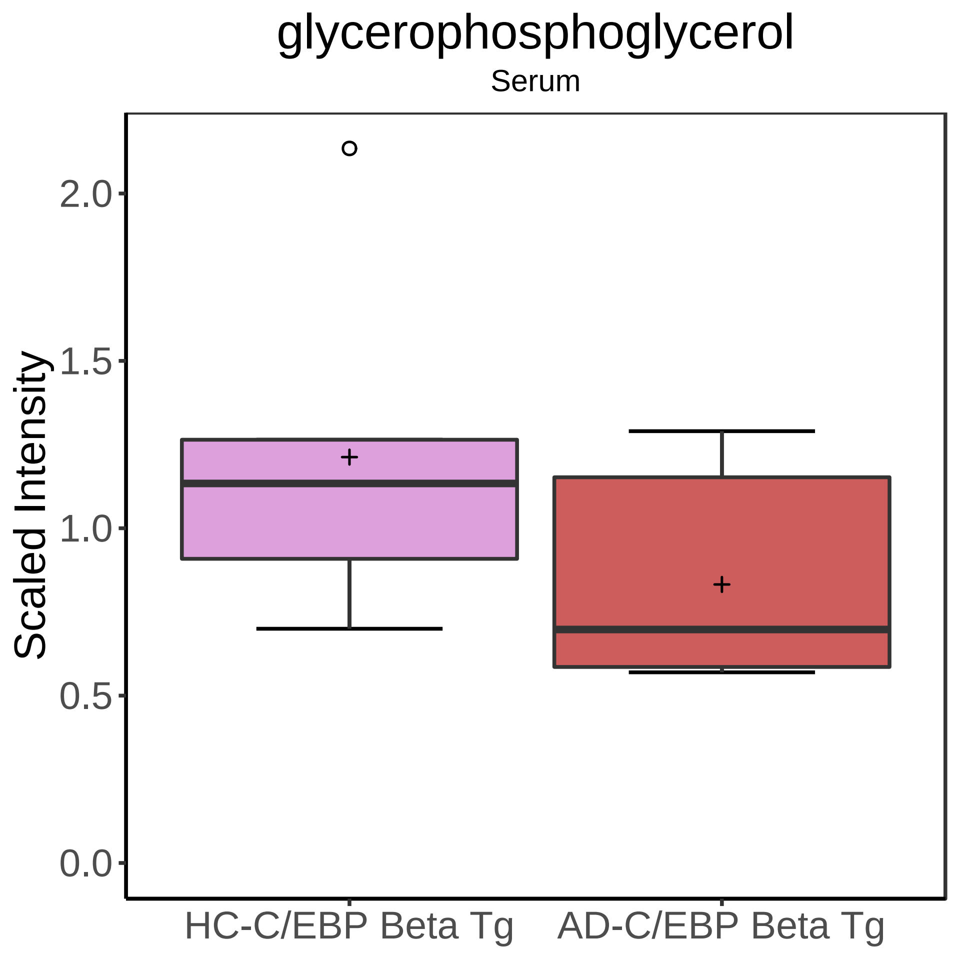

glycerophosphoglycerol
Serum
Scaled Intensity
2.0
1.5
1.0
0.5
0.0
HC-C/EBP Beta Tg
AD-C/EBP Beta Tg

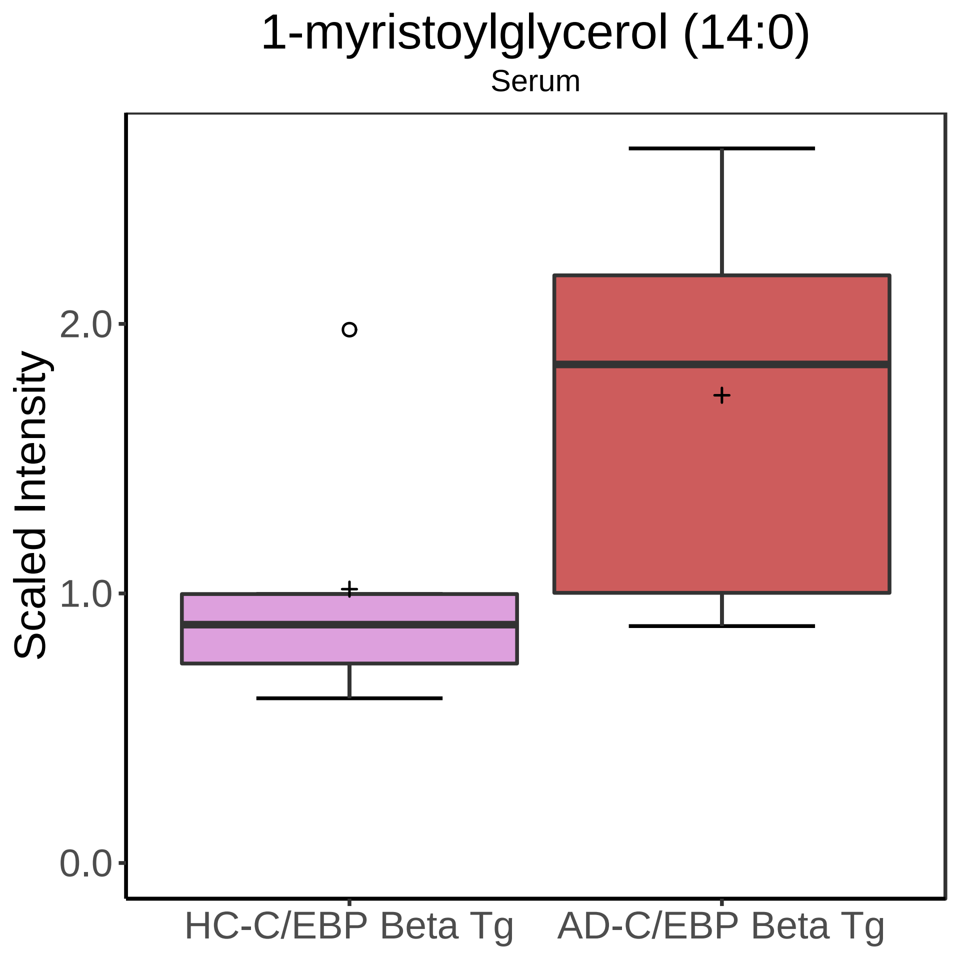
1-myristoylglycerol (14:0)
Serum
Scaled Intensity
2.0
1.0
0.0
HC-C/EBP Beta Tg
AD-C/EBP Beta Tg

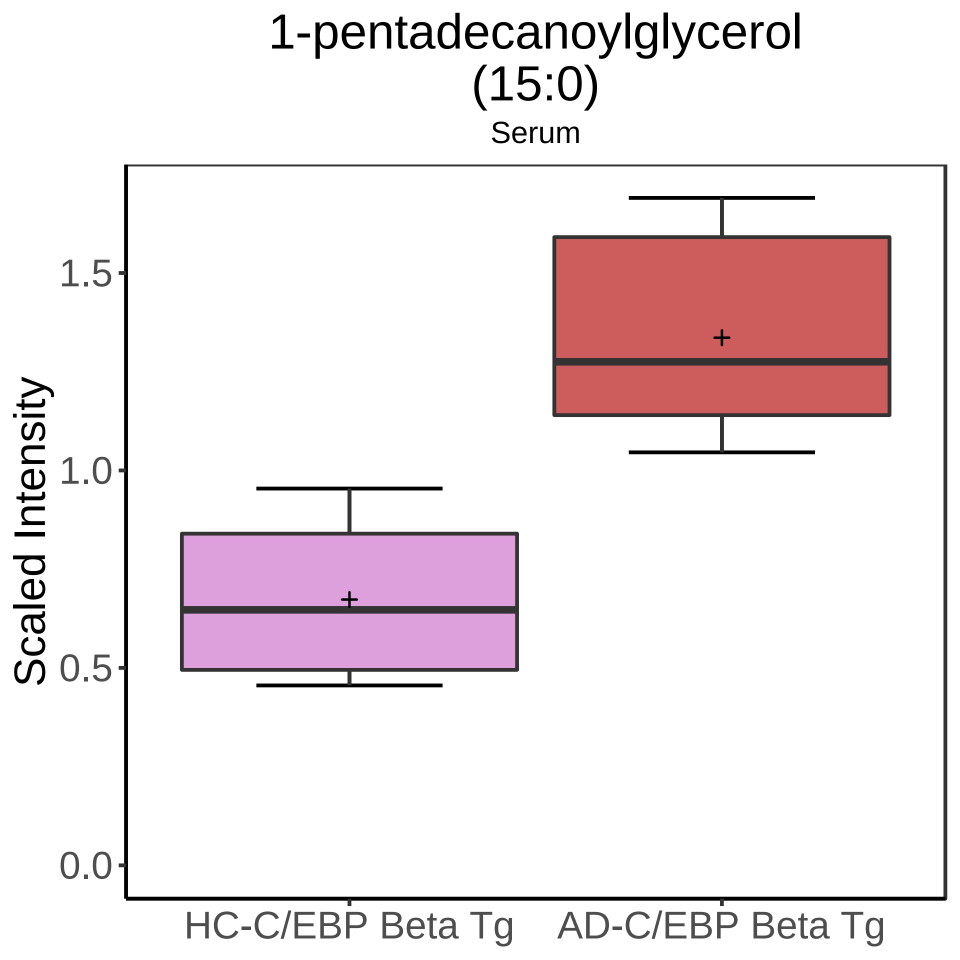
1-pentadecanoylglycerol
(15:0)
Serum
Scaled Intensity
0.0
0.5
1.0
1.5
HC-C/EBP Beta Tg
AD-C/EBP Beta Tg

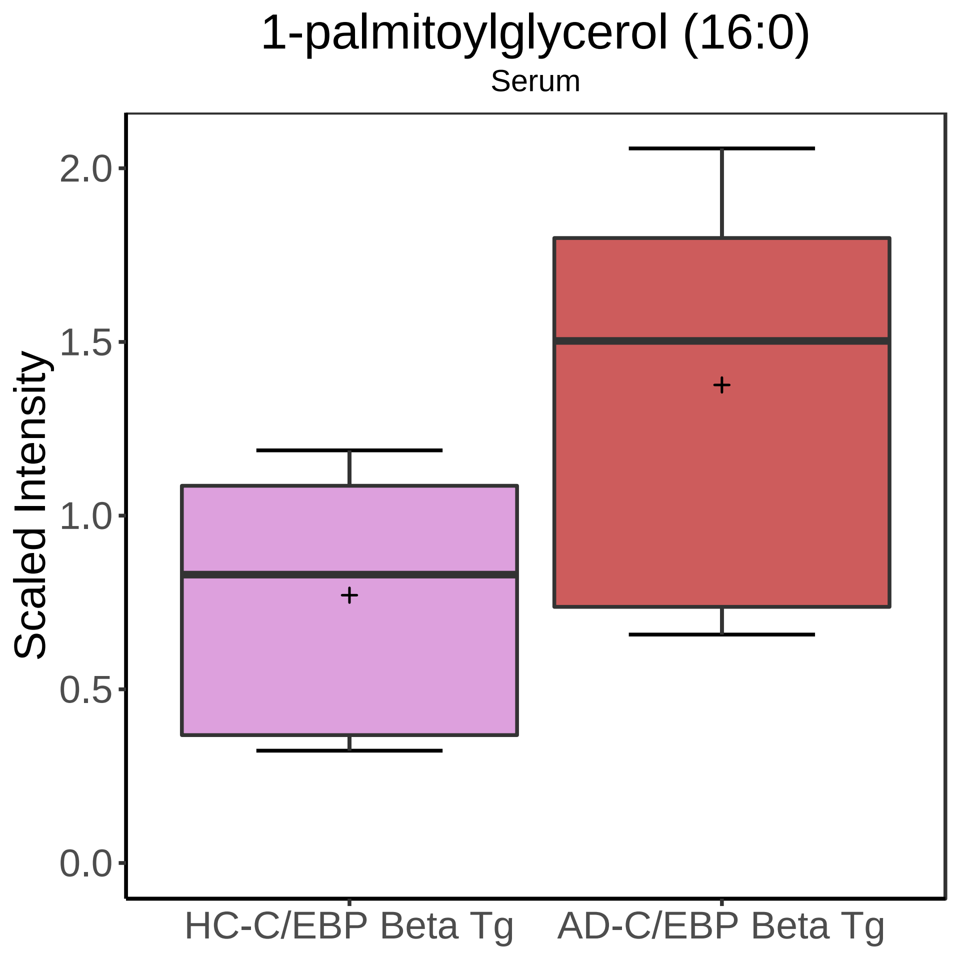
1-palmitoylglycerol (16:0)
Serum
Scaled Intensity
2.0
1.5
1.0
0.5
0.0
HC-C/EBP Beta Tg
AD-C/EBP Beta Tg

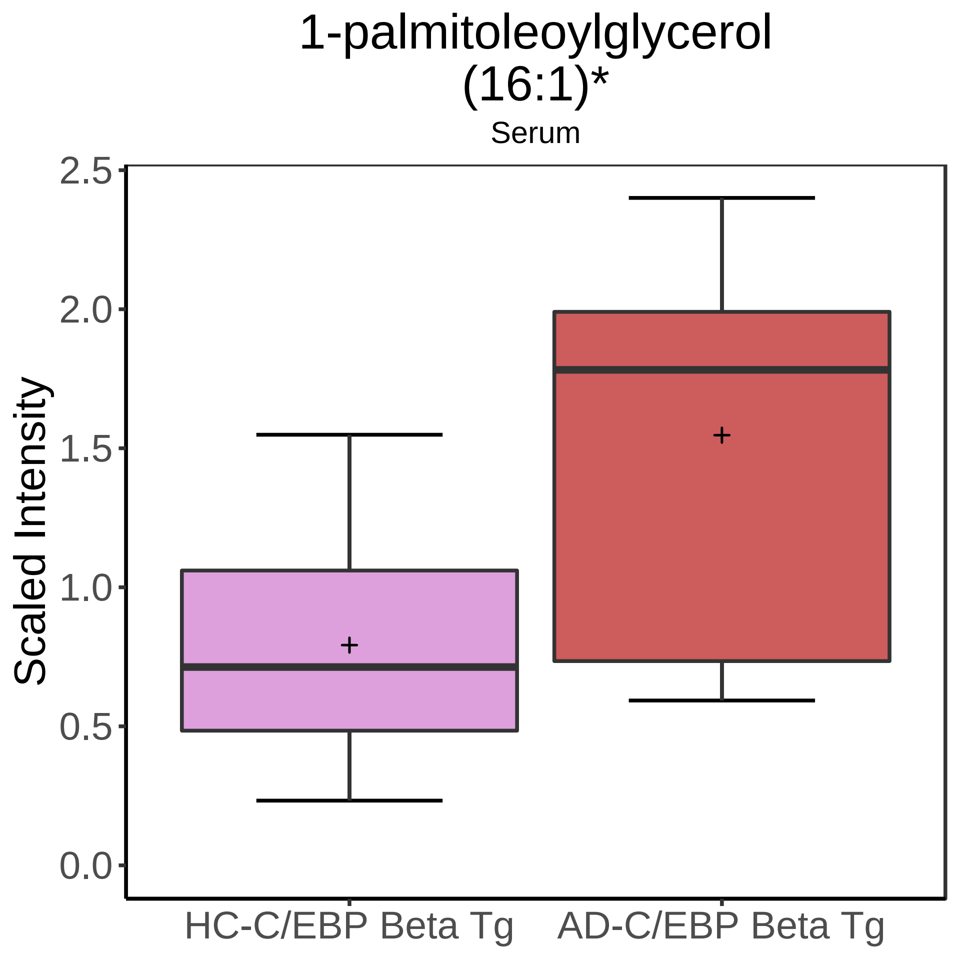

1-palmitoleoylglycerol
(16:1)*
Serum
Scaled Intensity
2.5
2.0
1.5
1.0
0.5
0.0
HC-C/EBP Beta Tg
AD-C/EBP Beta Tg

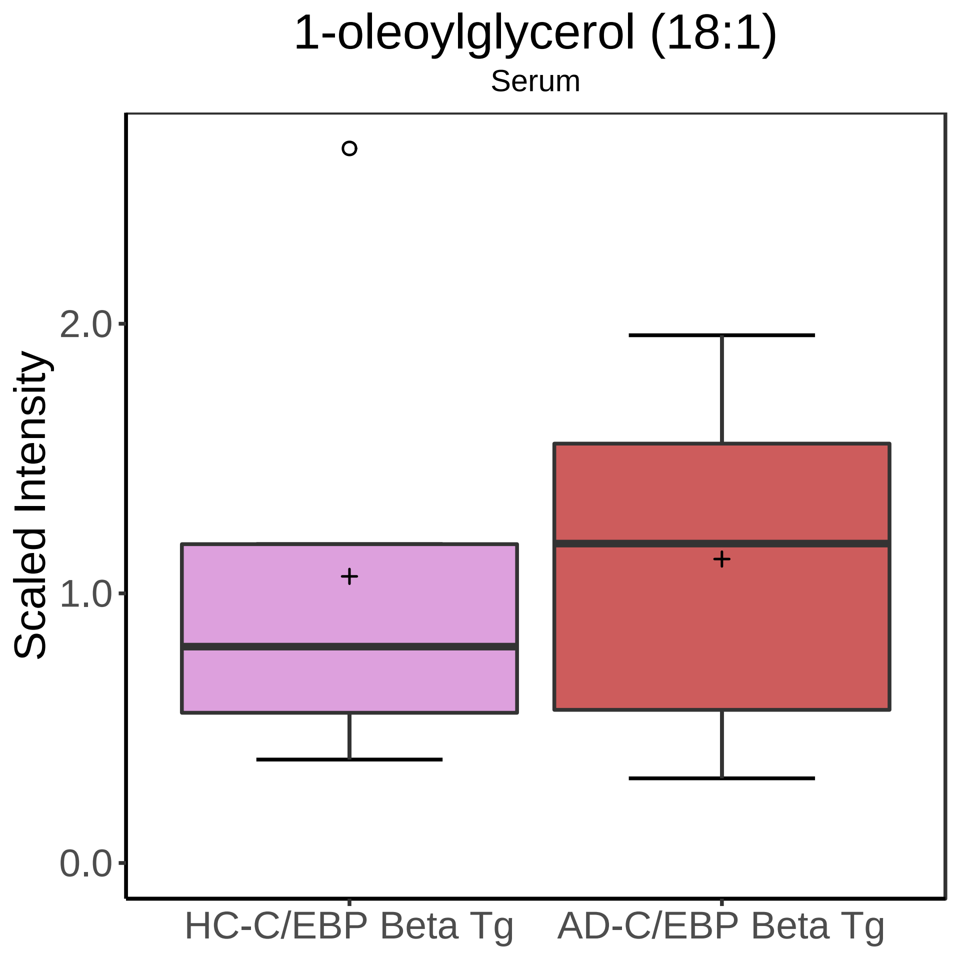

1-oleoylglycerol (18:1)
Serum
Scaled Intensity
2.0
1.0
0.0
HC-C/EBP Beta Tg
AD-C/EBP Beta Tg

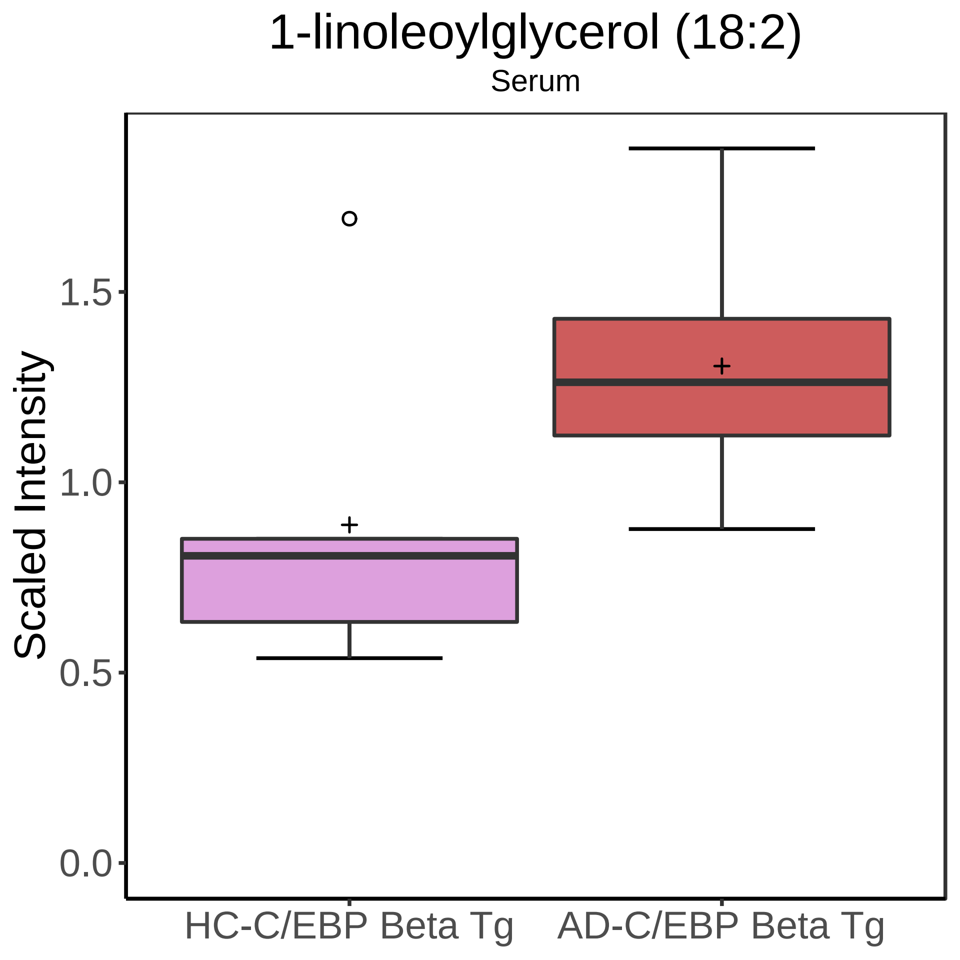
1-linoleoylglycerol (18:2)
Serum
Scaled Intensity
1.5
1.0
0.5
0.0
HC-C/EBP Beta Tg
AD-C/EBP Beta Tg

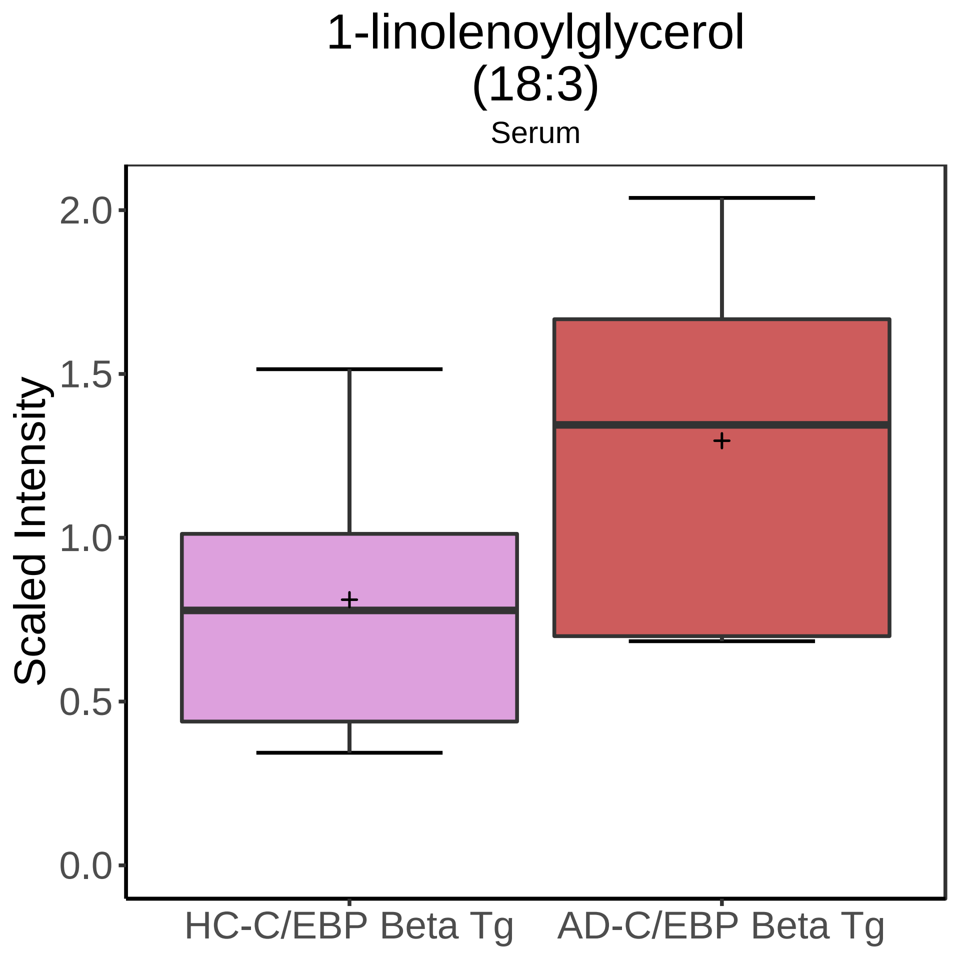
1-linolenoylglycerol
(18:3)
Serum
Scaled Intensity
2.0
1.5
1.0
0.5
0.0
HC-C/EBP Beta Tg
AD-C/EBP Beta Tg

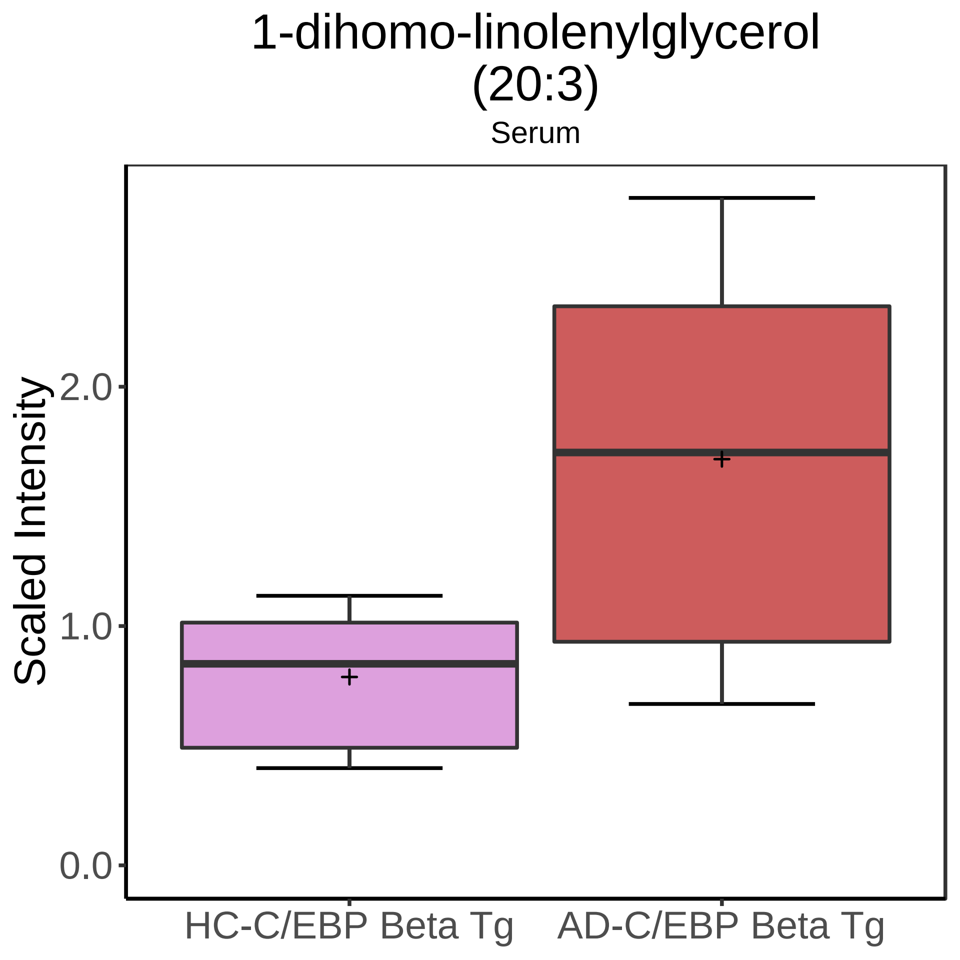
1-dihomo-linolenylglycerol (20:3)
Serum
Scaled Intensity
2.0
1.0
0.0
HC-C/EBP Beta Tg
AD-C/EBP Beta Tg

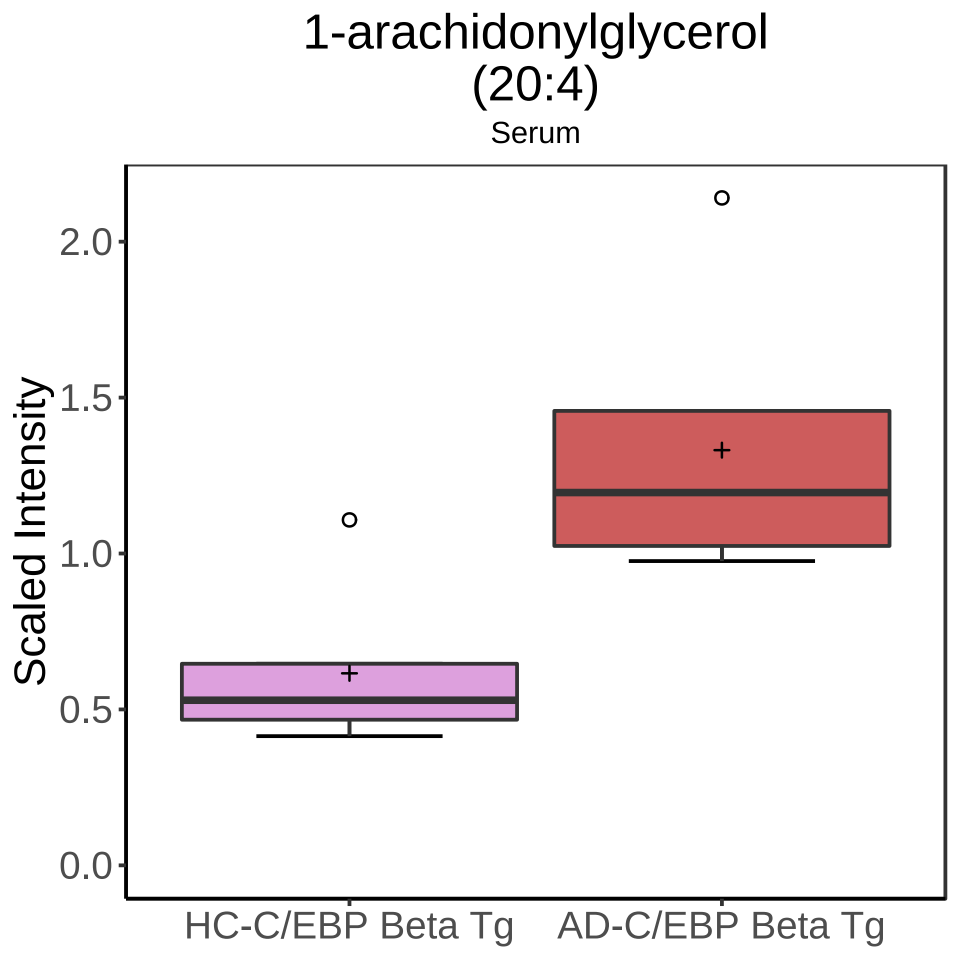
1-arachidonylglycerol
(20:4)
Serum
Scaled Intensity
2.0
1.5
1.0
0.5
0.0
HC-C/EBP Beta Tg
AD-C/EBP Beta Tg

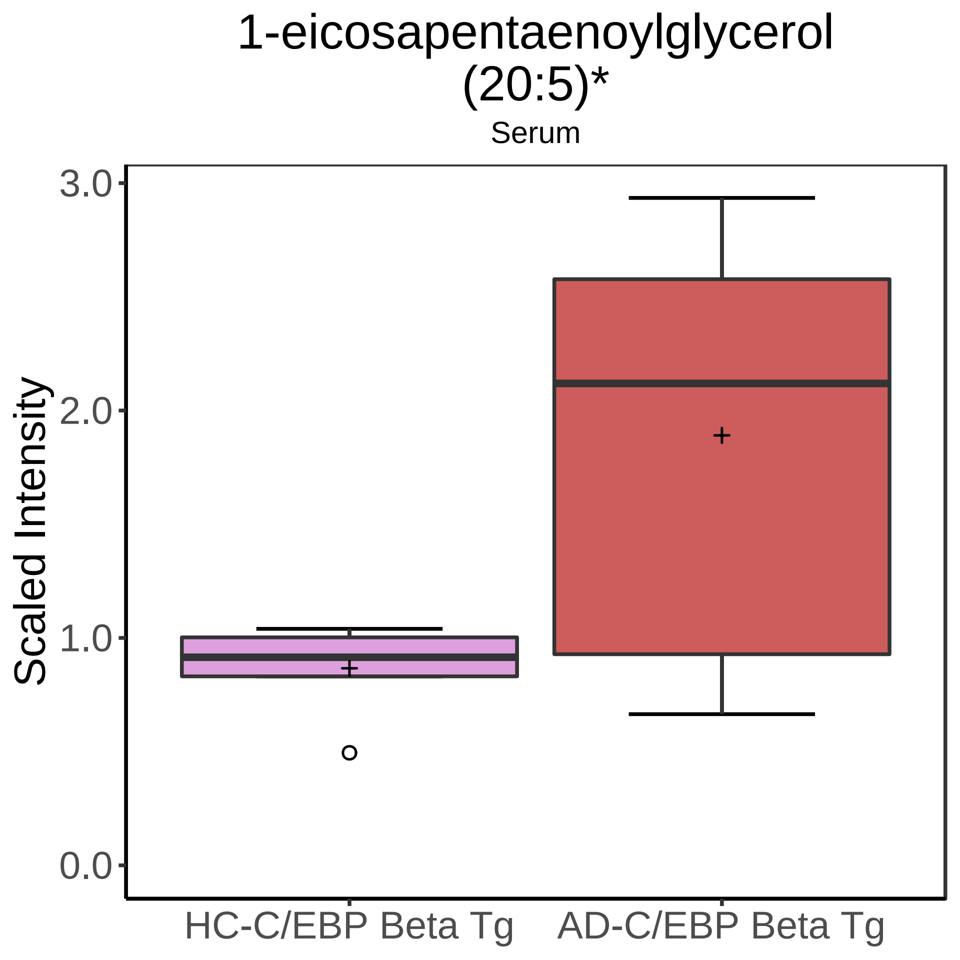

1-eicosapentaenoylglycerol
(20:5)*
Serum
Scaled Intensity
3.0
2.0
1.0
0.0
HC-C/EBP Beta Tg
AD-C/EBP Beta Tg

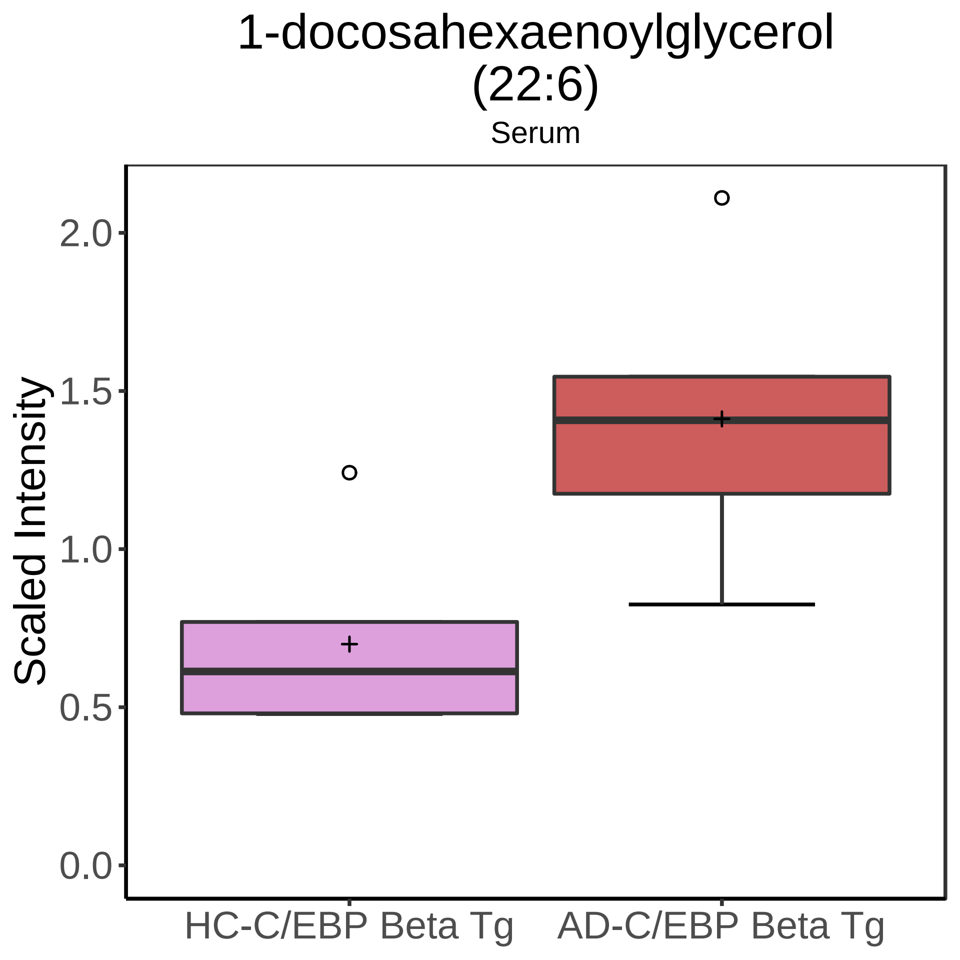
1-docosahexaenoylglycerol (22:6)
Serum
Scaled Intensity
2.0
1.5
1.0
0.5
0.0
HC-C/EBP Beta Tg
AD-C/EBP Beta Tg

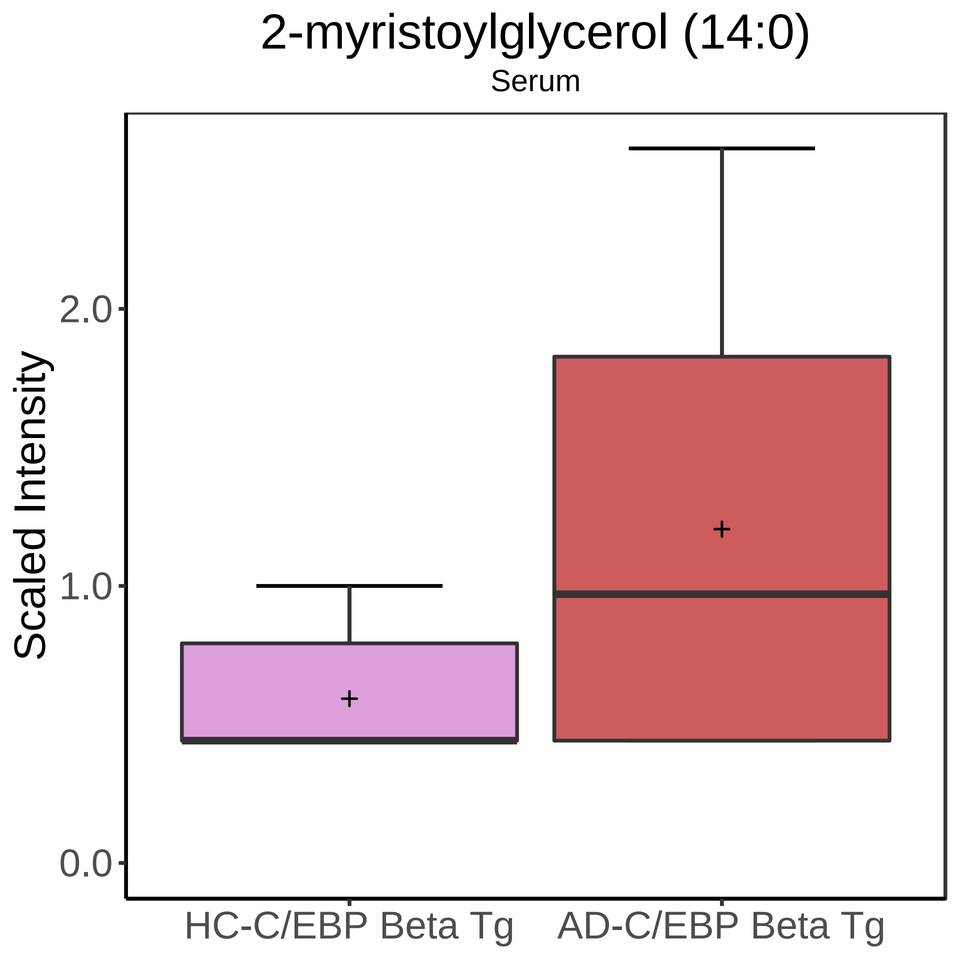
2-myristoylglycerol (14:0)
Serum
Scaled Intensity
2.0
1.0
0.0
HC-C/EBP Beta Tg
AD-C/EBP Beta Tg

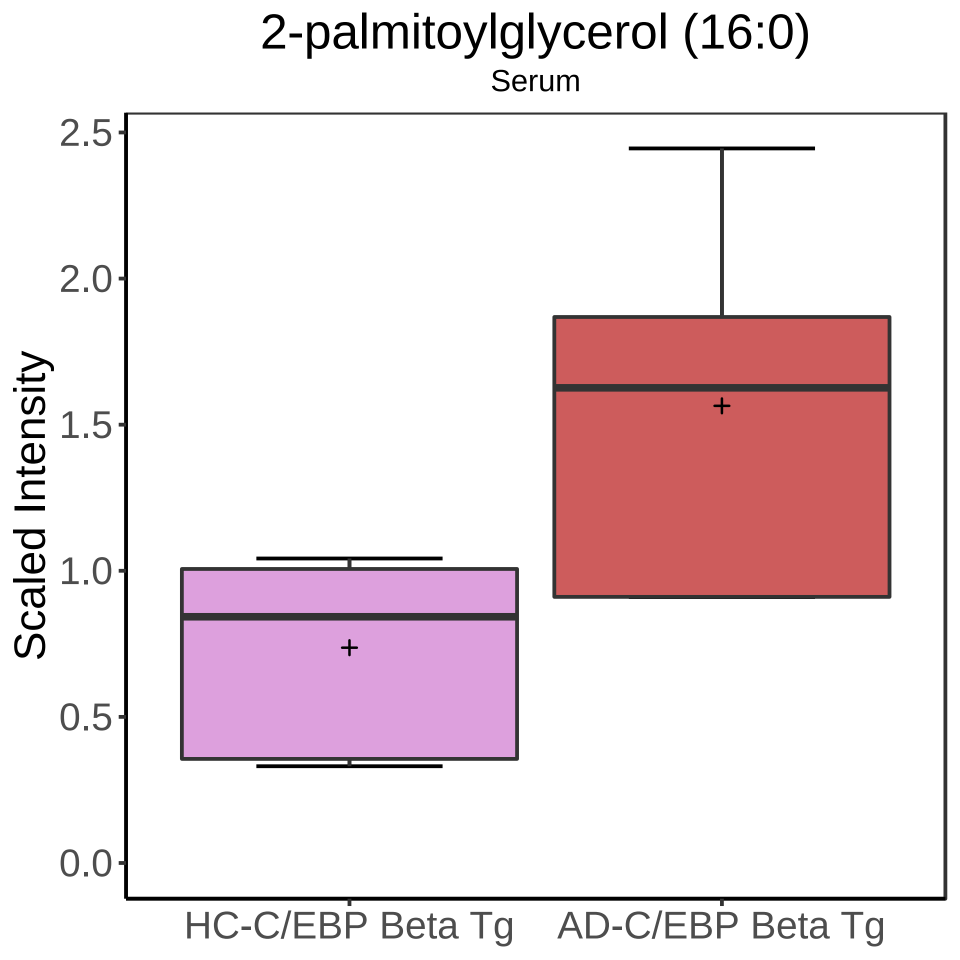

2-palmitoylglycerol (16:0)
Serum
Scaled Intensity
0.0
0.5
1.0
1.5
2.0
2.5
HC-C/EBP Beta Tg
AD-C/EBP Beta Tg

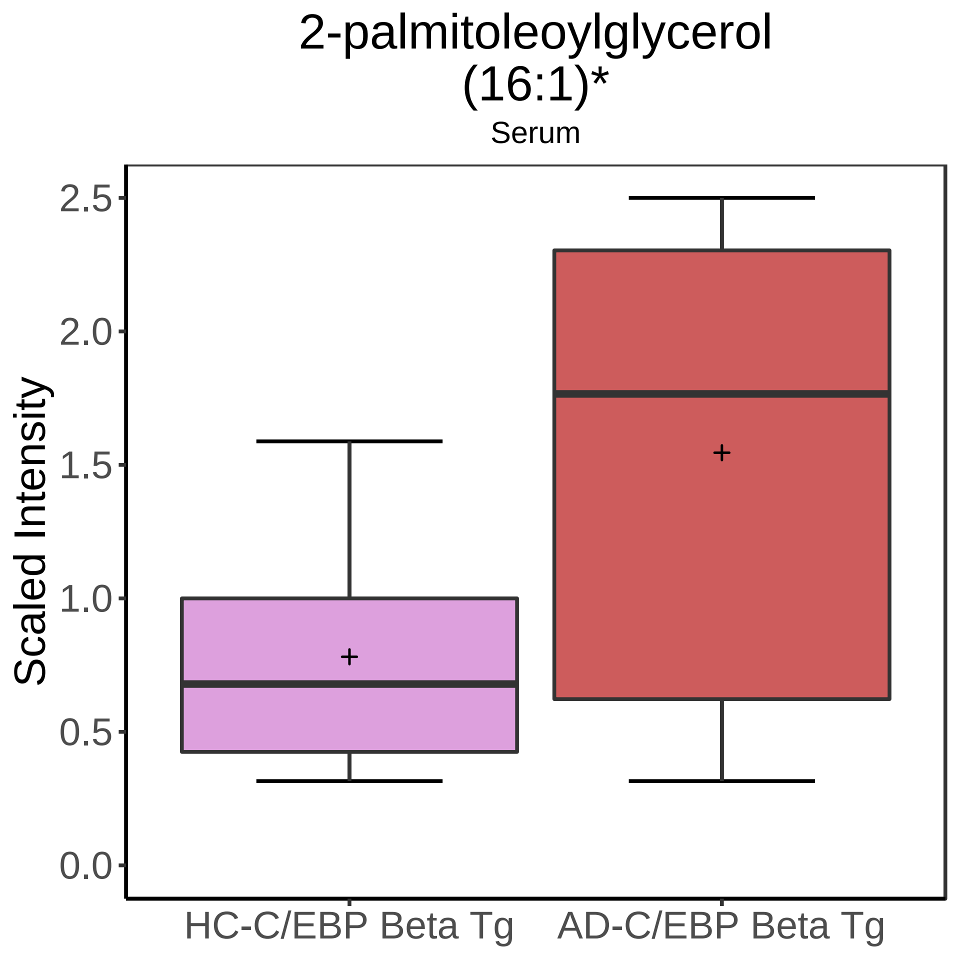
2-palmitoleoylglycerol
(16:1)*
Serum
Scaled Intensity
2.5
2.0
1.5
1.0
0.5
0.0
HC-C/EBP Beta Tg
AD-C/EBP Beta Tg

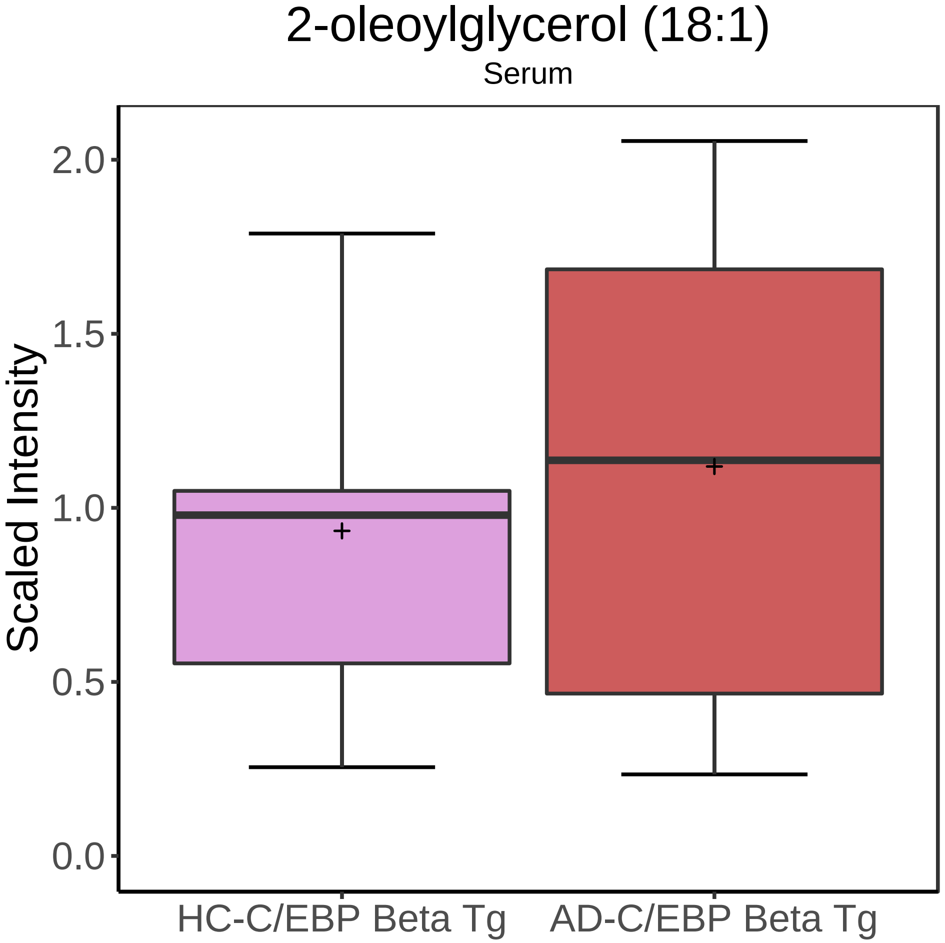
2-oleoylglycerol (18:1)
Serum
Scaled Intensity
0.0
0.5
1.0
1.5
2.0
HC-C/EBP Beta Tg
AD-C/EBP Beta Tg

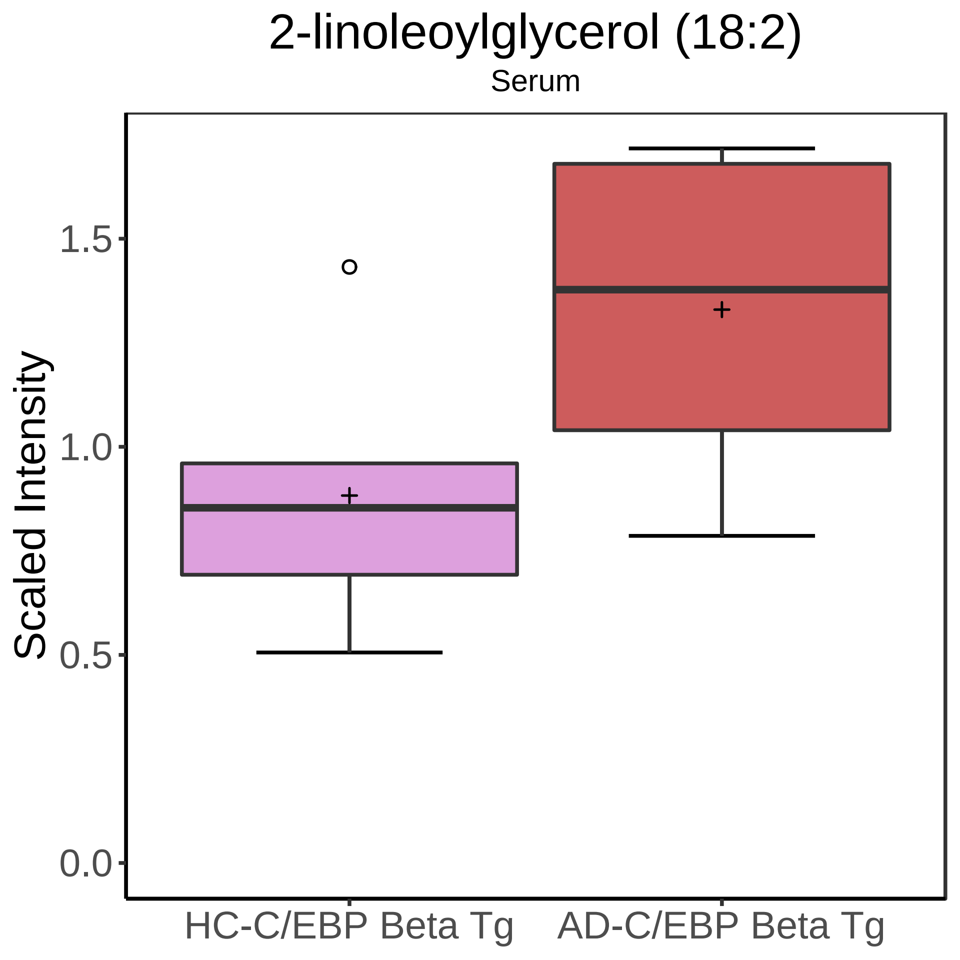

2-linoleoylglycerol (18:2)
Serum
Scaled Intensity
1.5
1.0
0.5
0.0
HC-C/EBP Beta Tg
AD-C/EBP Beta Tg

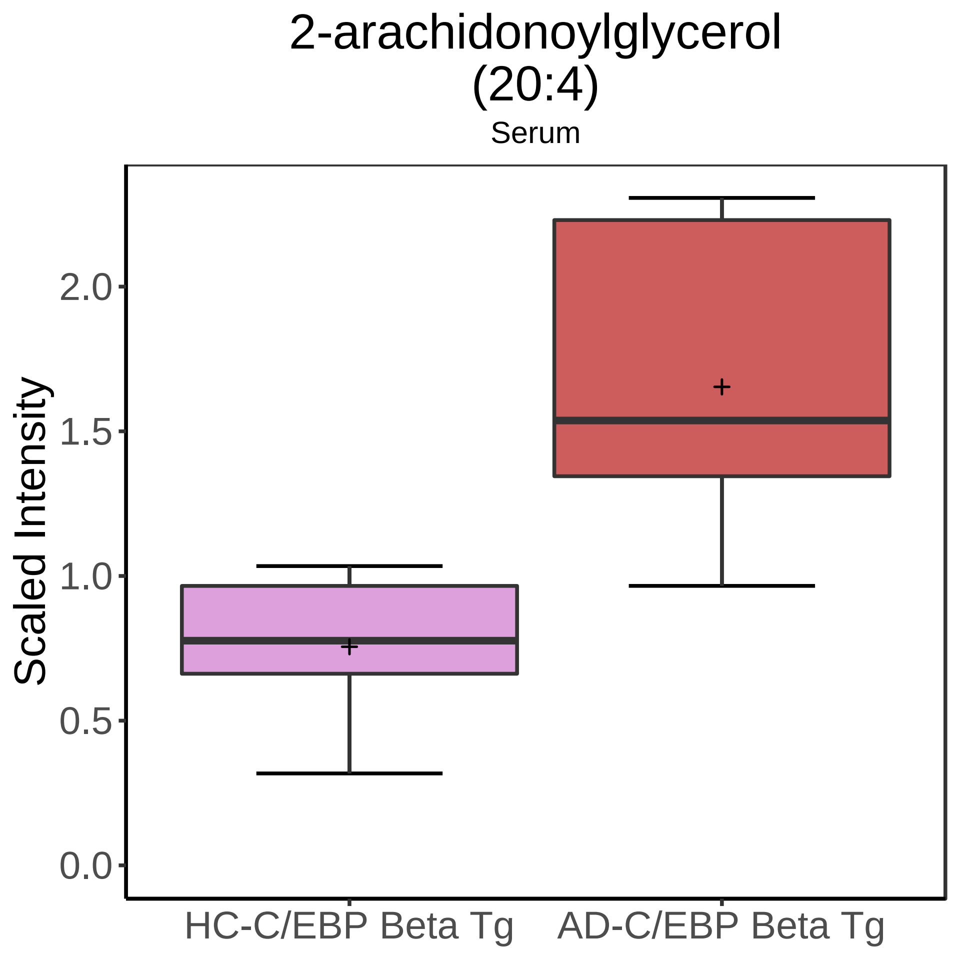
2-arachidonoylglycerol
(20:4)
Serum
Scaled Intensity
0.0
0.5
1.0
1.5
2.0
HC-C/EBP Beta Tg
AD-C/EBP Beta Tg

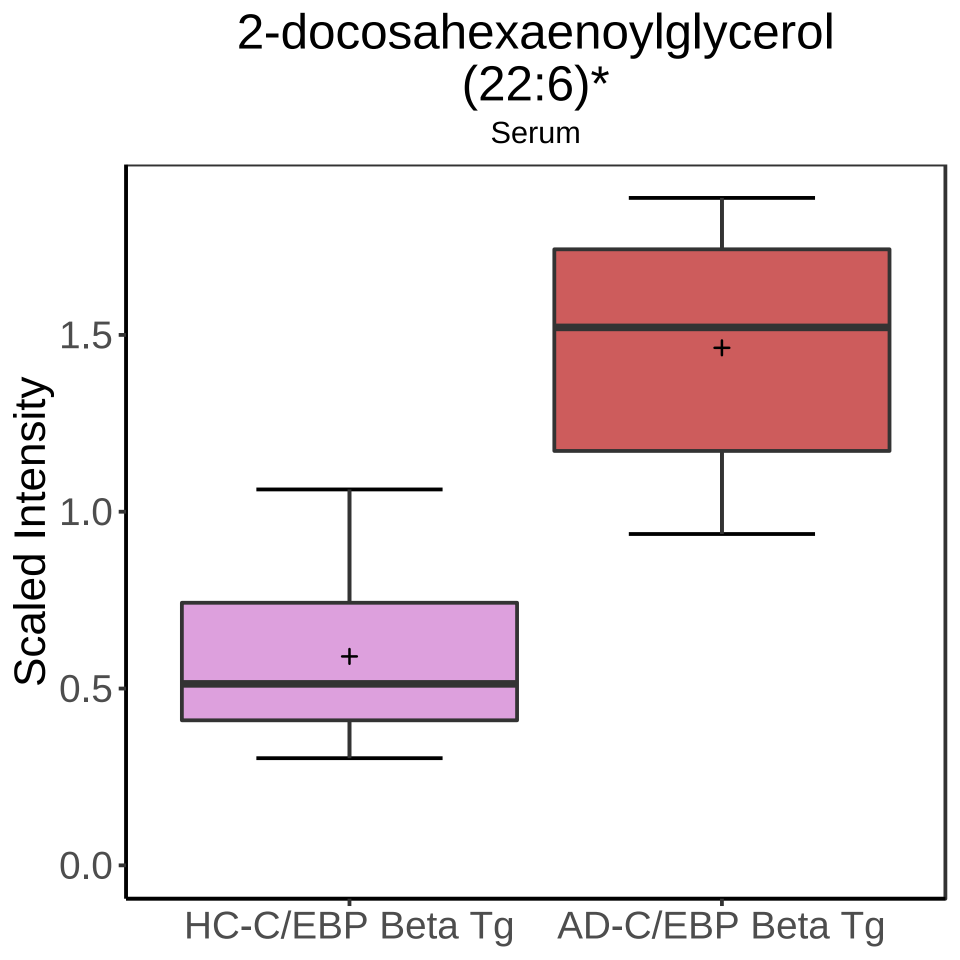
2-docosahexaenoylglycerol
(22:6)*
Serum
Scaled Intensity
0.0
0.5
1.0
1.5
HC-C/EBP Beta Tg
AD-C/EBP Beta Tg

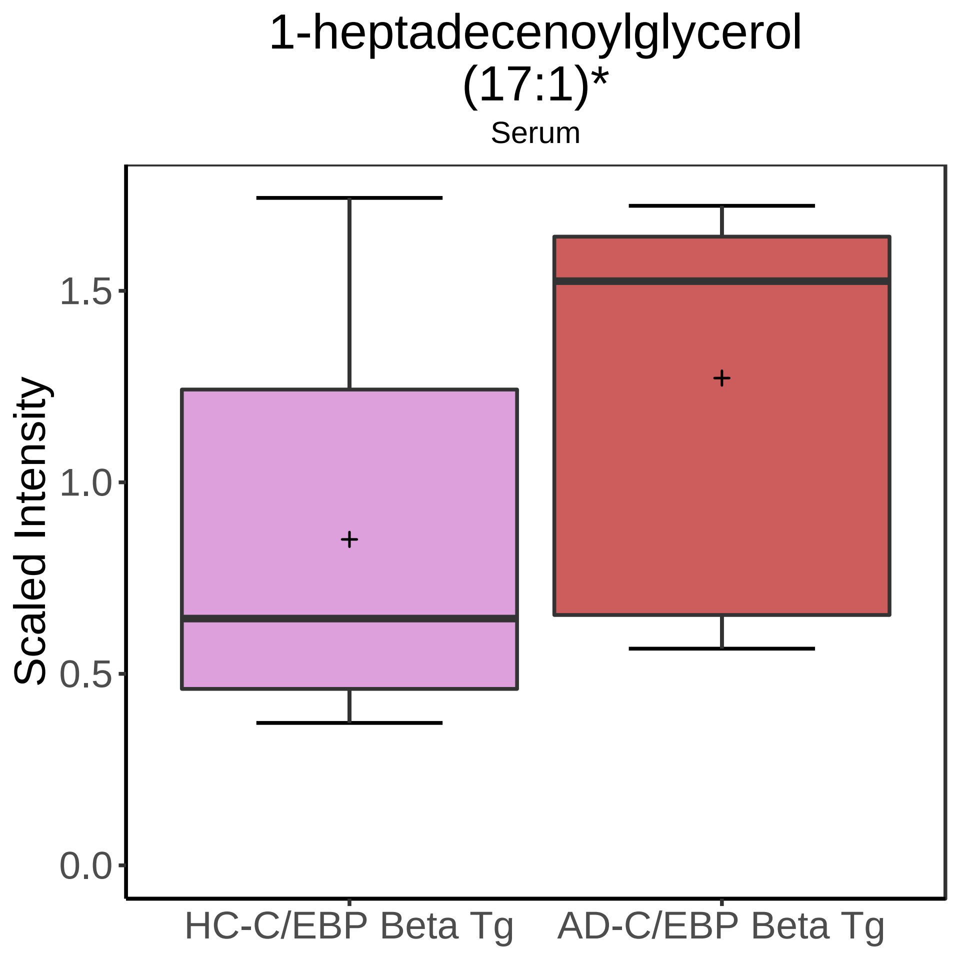
1-heptadecenoylglycerol
(17:1)*
Serum
Scaled Intensity
1.5
1.0
0.5
0.0
HC-C/EBP Beta Tg
AD-C/EBP Beta Tg

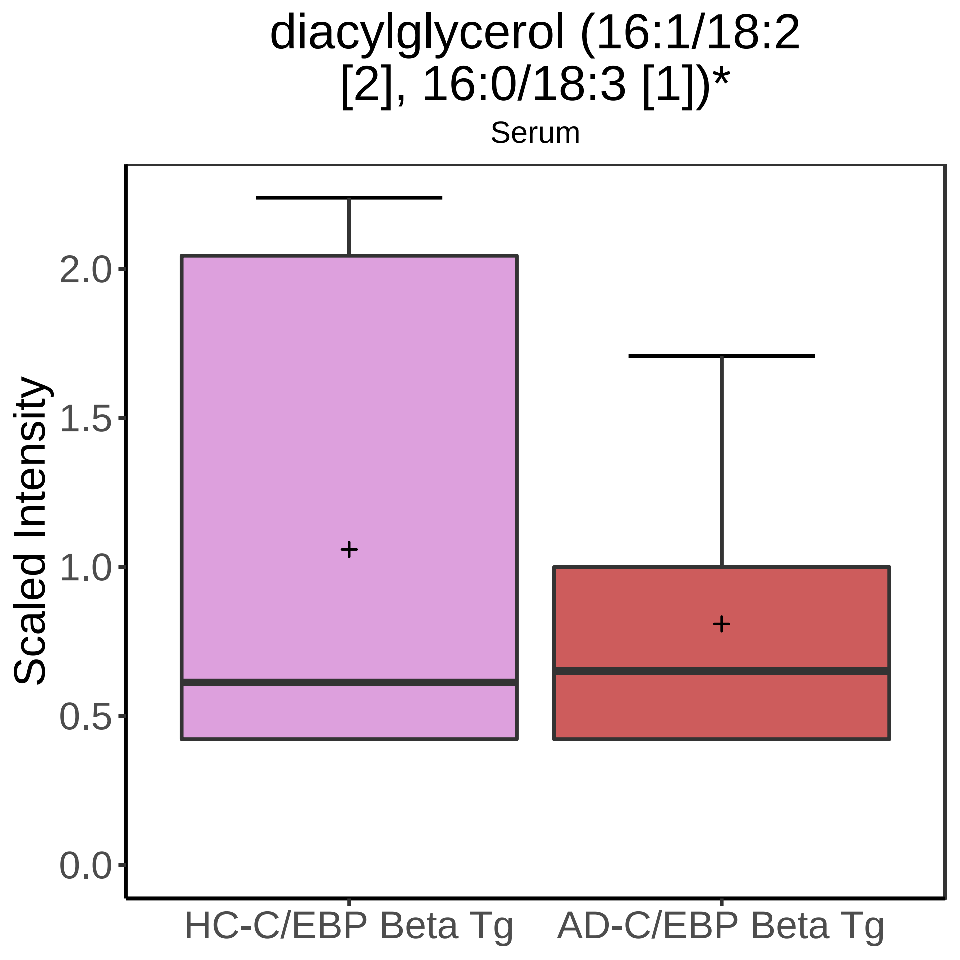
diacylglycerol (16:1/18:2
[2], 16:0/18:3 [1])*
Serum
Scaled Intensity
2.0
1.5
1.0
0.5
0.0
HC-C/EBP Beta Tg
AD-C/EBP Beta Tg

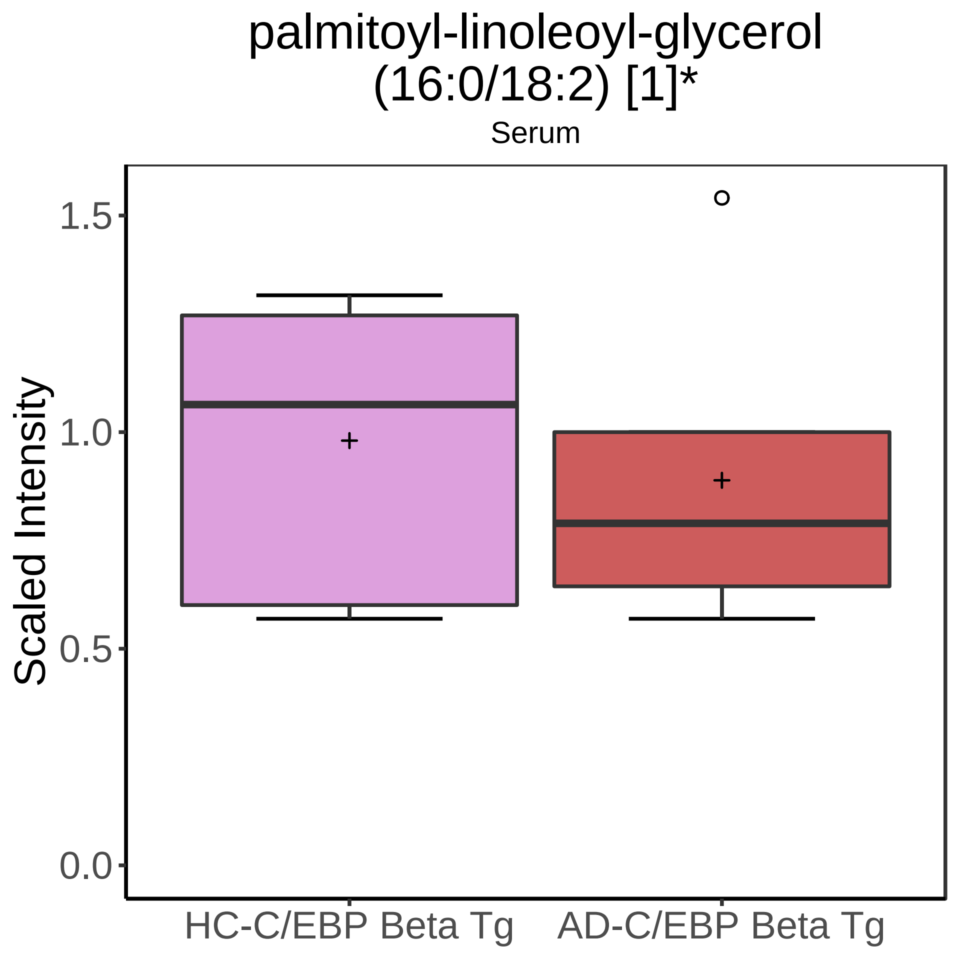
palmitoyl-linoleoyl-glycerol
(16:0/18:2) [1]*
Serum
Scaled Intensity
1.5
1.0
0.5
0.0
HC-C/EBP Beta Tg
AD-C/EBP Beta Tg

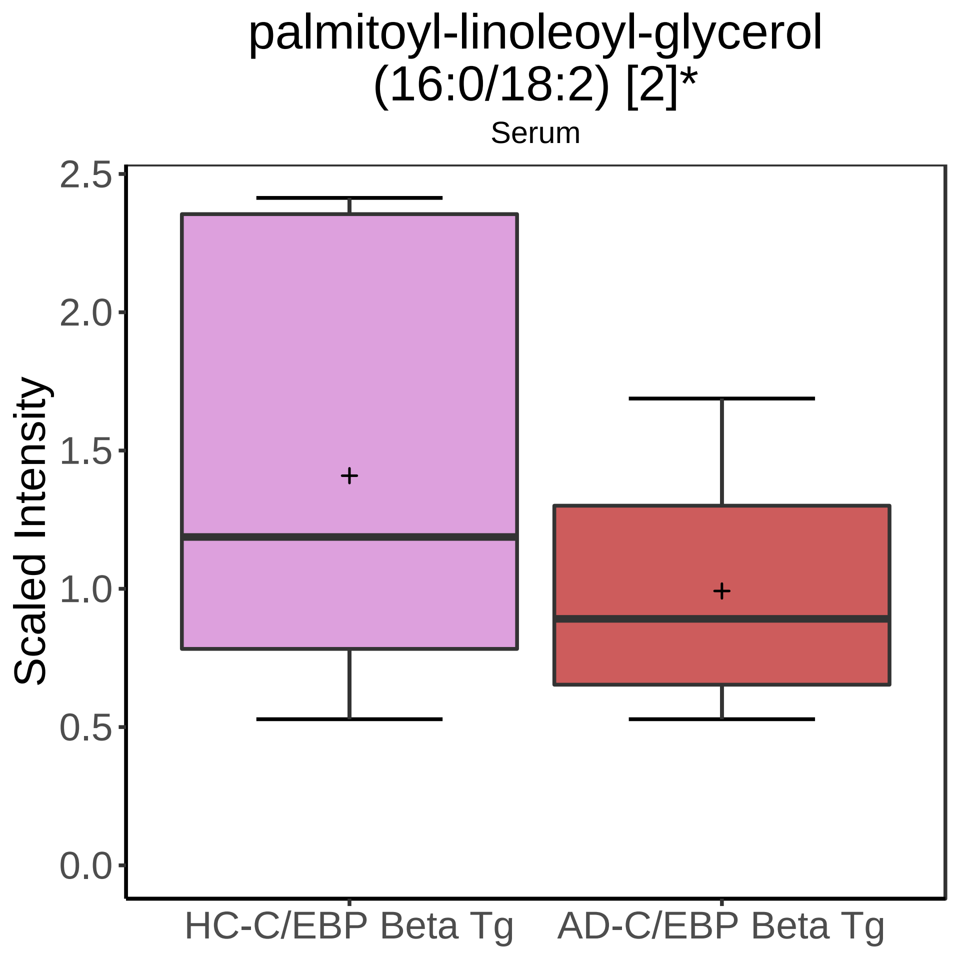

palmitoyl-linoleoyl-glycerol (16:0/18:2) [2]*
Serum
Scaled Intensity
0.0
0.5
1.0
1.5
2.0
2.5
HC-C/EBP Beta Tg
AD-C/EBP Beta Tg

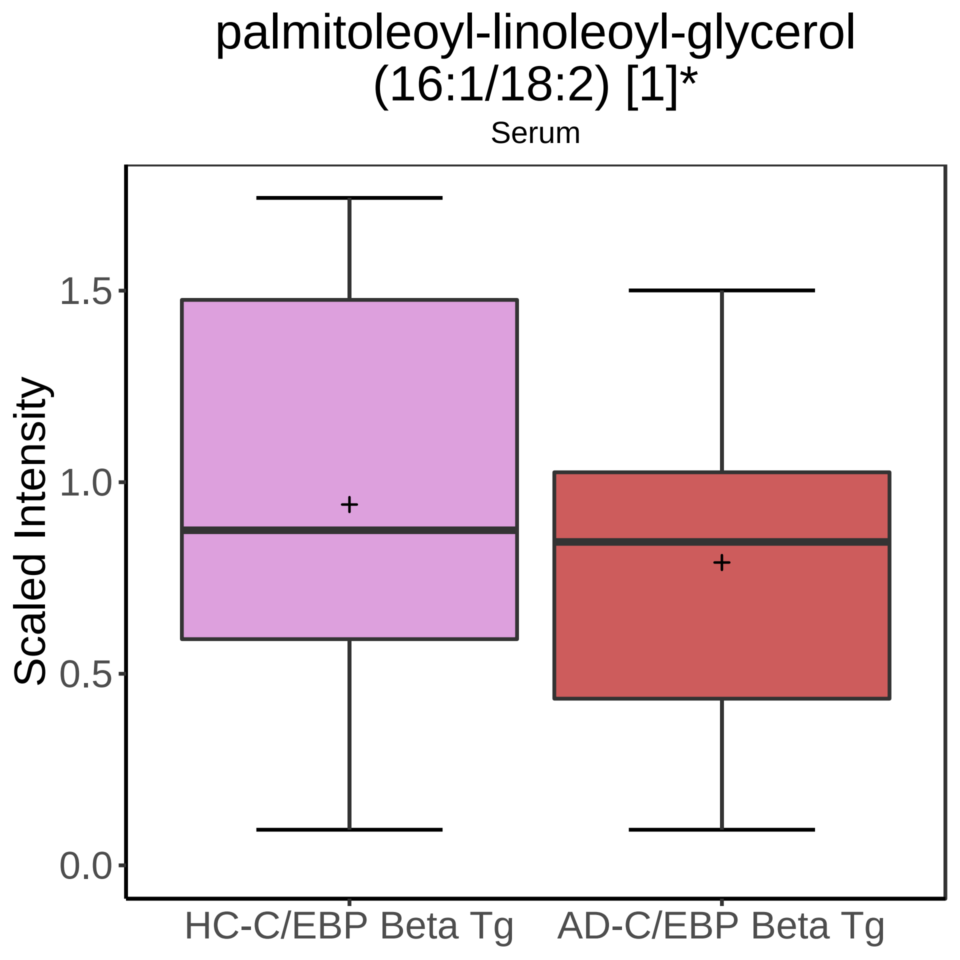
palmitoleoyl-linoleoyl-glycerol
(16:1/18:2) [1]*
Serum
Scaled Intensity
1.5
1.0
0.5
0.0
HC-C/EBP Beta Tg
AD-C/EBP Beta Tg

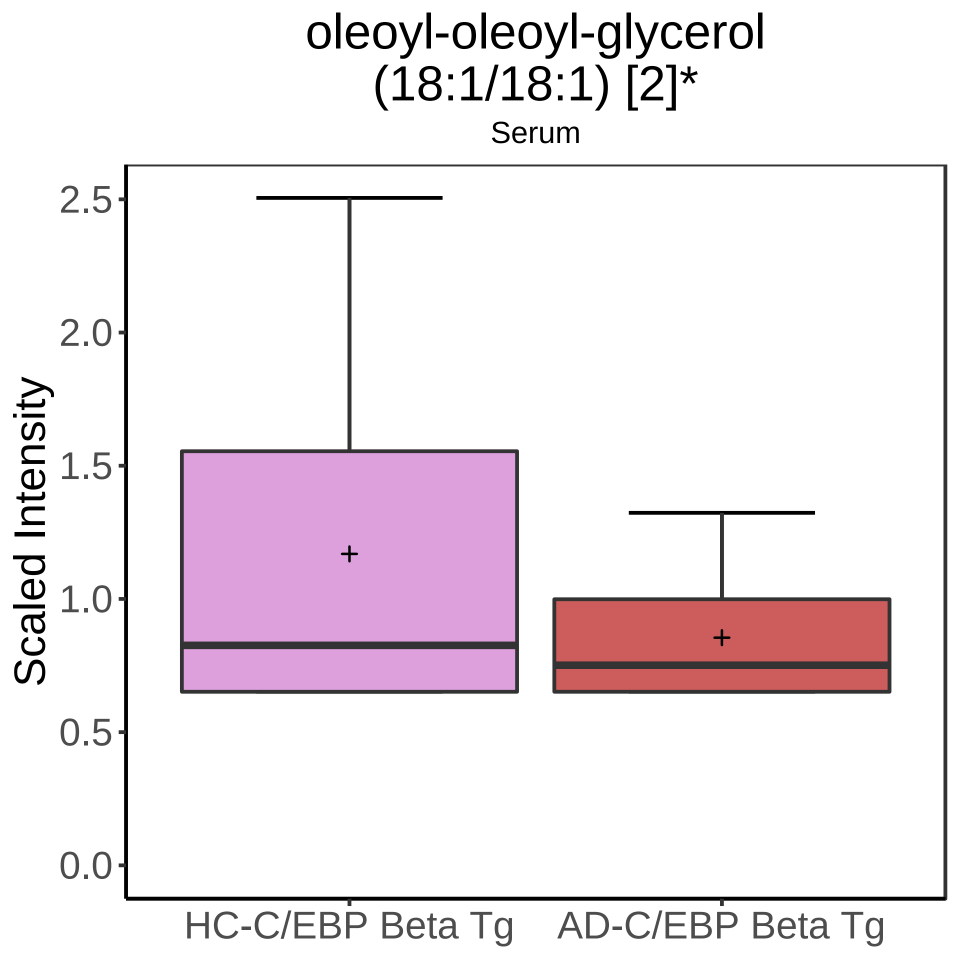

oleoyl-oleoyl-glycerol
(18:1/18:1) [2]*
Serum
Scaled Intensity
2.5
2.0
1.5
1.0
0.5
0.0
HC-C/EBP Beta Tg
AD-C/EBP Beta Tg

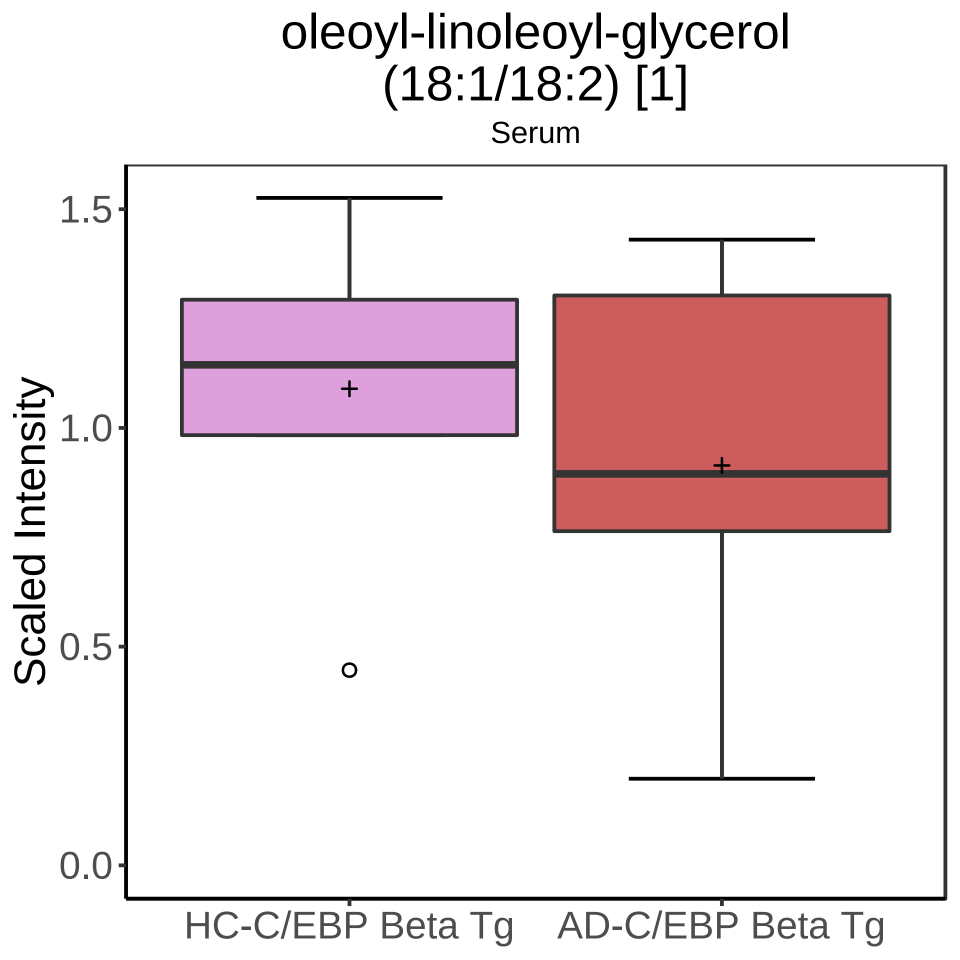
oleoyl-linoleoyl-glycerol
(18:1/18:2) [1]
Serum
Scaled Intensity
1.5
1.0
0.5
0.0
HC-C/EBP Beta Tg
AD-C/EBP Beta Tg

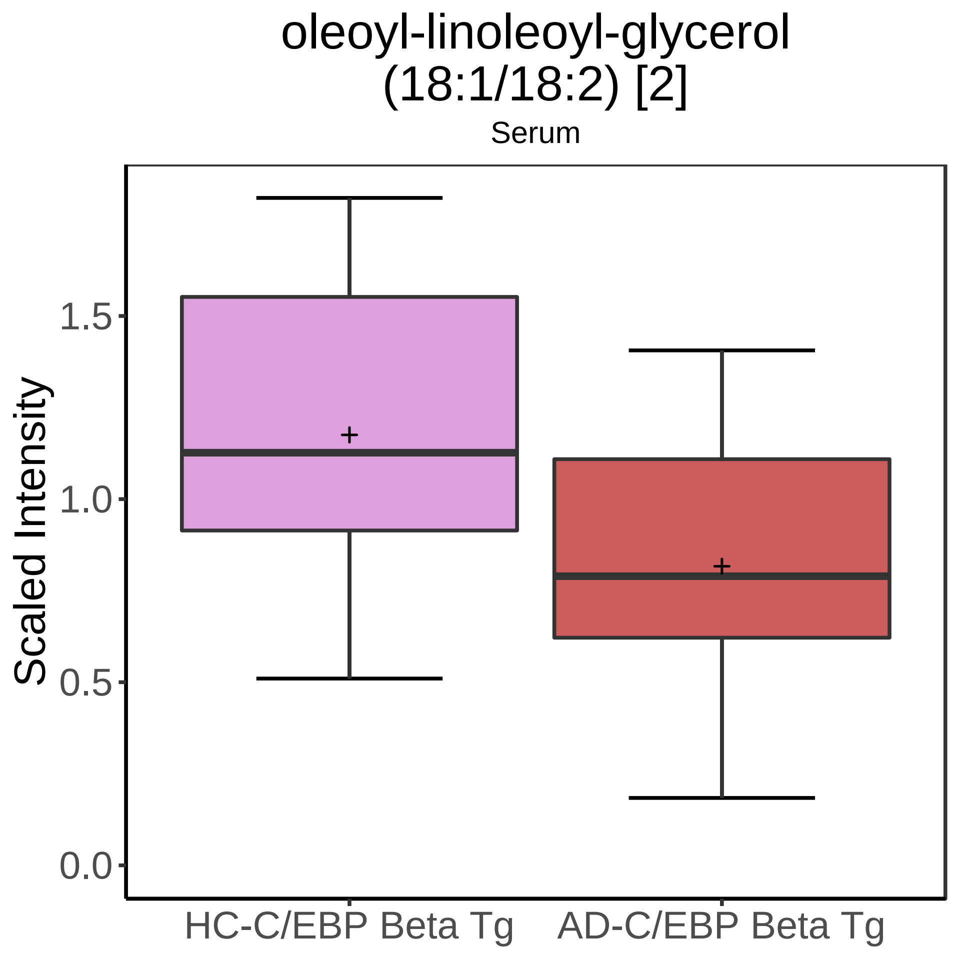
oleoyl-linoleoyl-glycerol
(18:1/18:2) [2]
Serum
Scaled Intensity
1.5
1.0
0.5
0.0
HC-C/EBP Beta Tg
AD-C/EBP Beta Tg

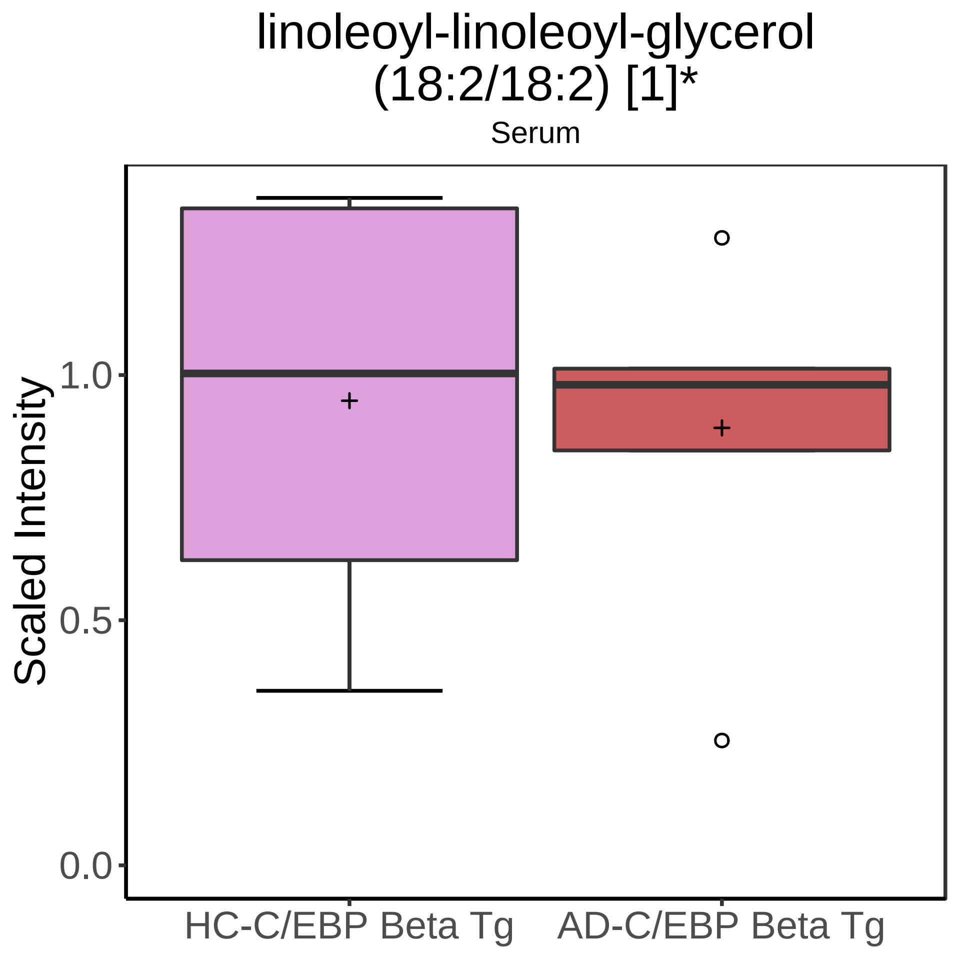

linoleoyl-linoleoyl-glycerol
(18:2/18:2) [1]*
Serum
Scaled Intensity
1.0
0.5
0.0
HC-C/EBP Beta Tg
AD-C/EBP Beta Tg

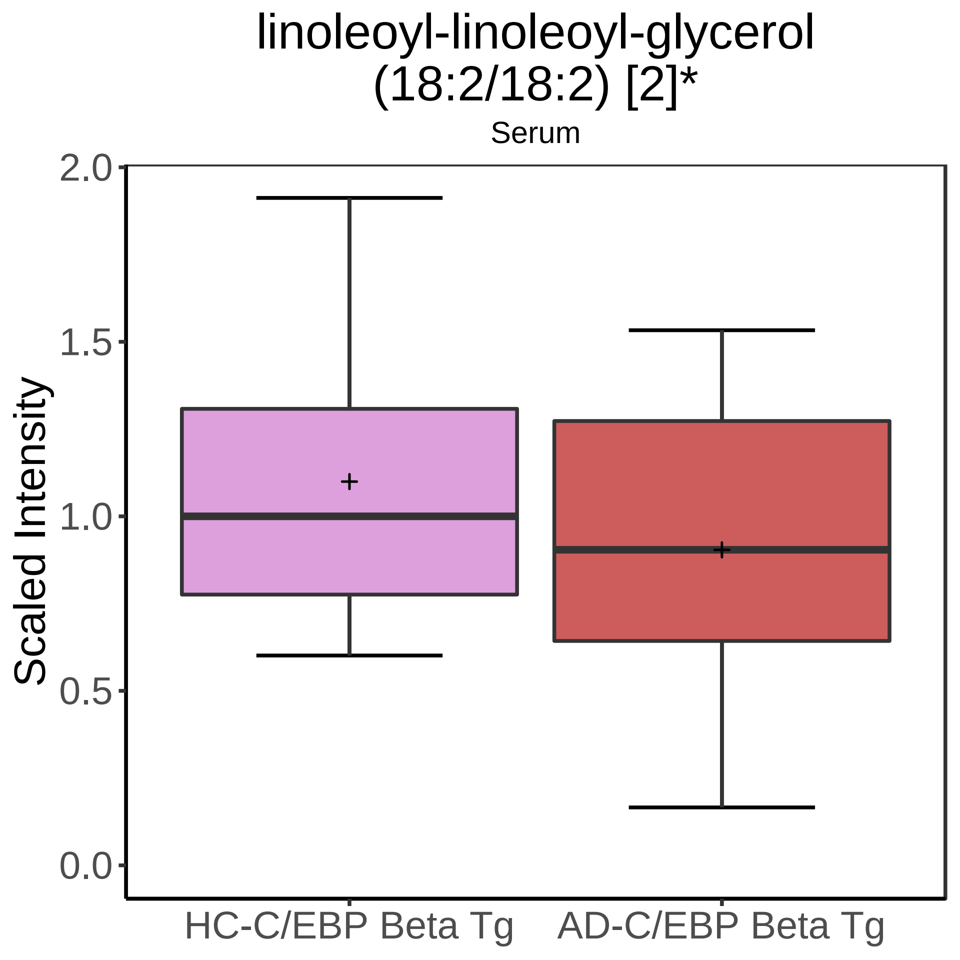
linoleoyl-linoleoyl-glycerol
(18:2/18:2) [2]*
Serum
Scaled Intensity
2.0
1.5
1.0
0.5
0.0
HC-C/EBP Beta Tg
AD-C/EBP Beta Tg

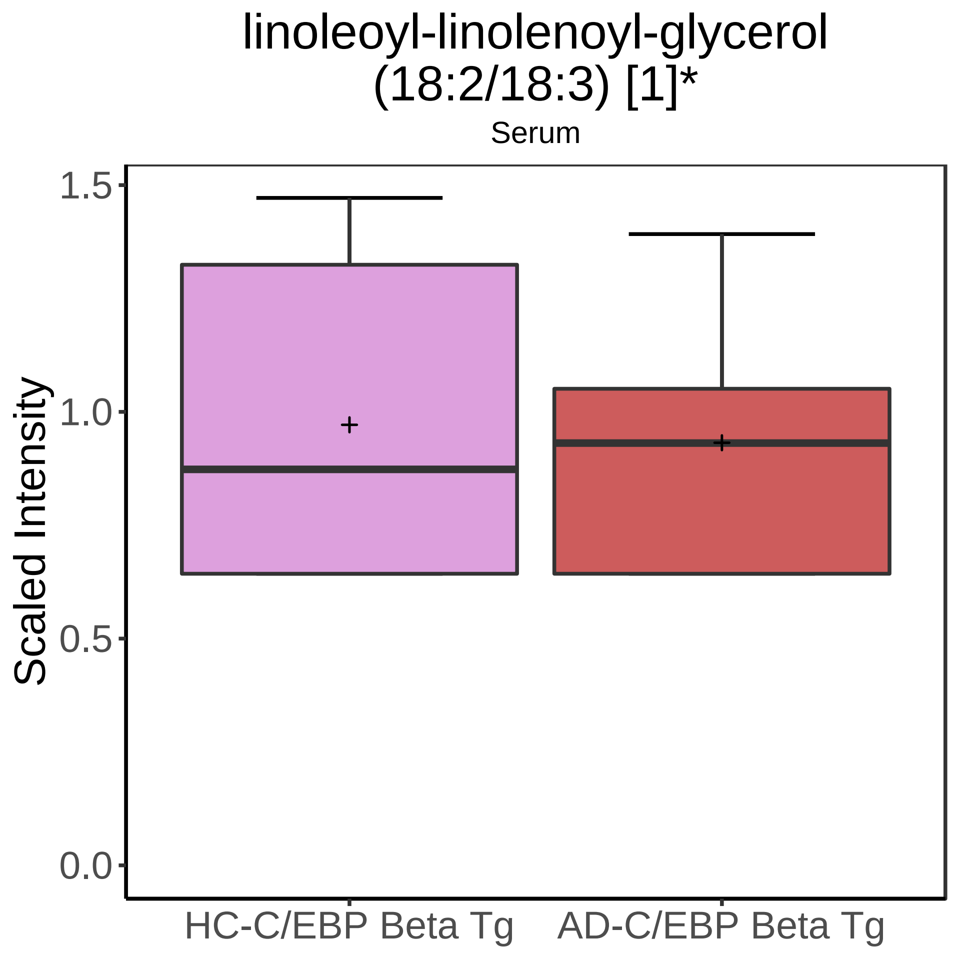
linoleoyl-linolenoyl-glycerol
(18:2/18:3) [1]*
Serum
Scaled Intensity
1.5
1.0
0.5
0.0
HC-C/EBP Beta Tg
AD-C/EBP Beta Tg

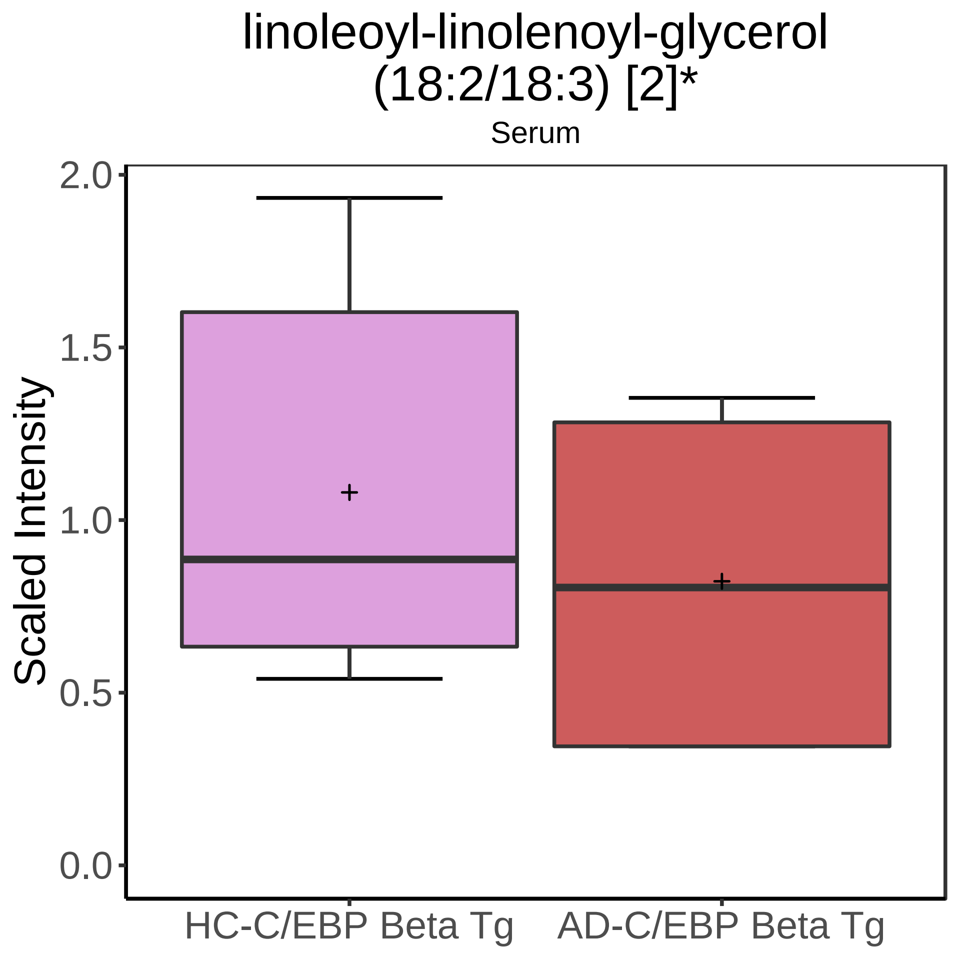

linoleoyl-linolenoyl-glycerol
(18:2/18:3) [2]*
Serum
Scaled Intensity
2.0
1.5
1.0
0.5
0.0
HC-C/EBP Beta Tg
AD-C/EBP Beta Tg

oleoyl-arachidonoyl-glycerol (18:1/20:4) [2]*

Serum

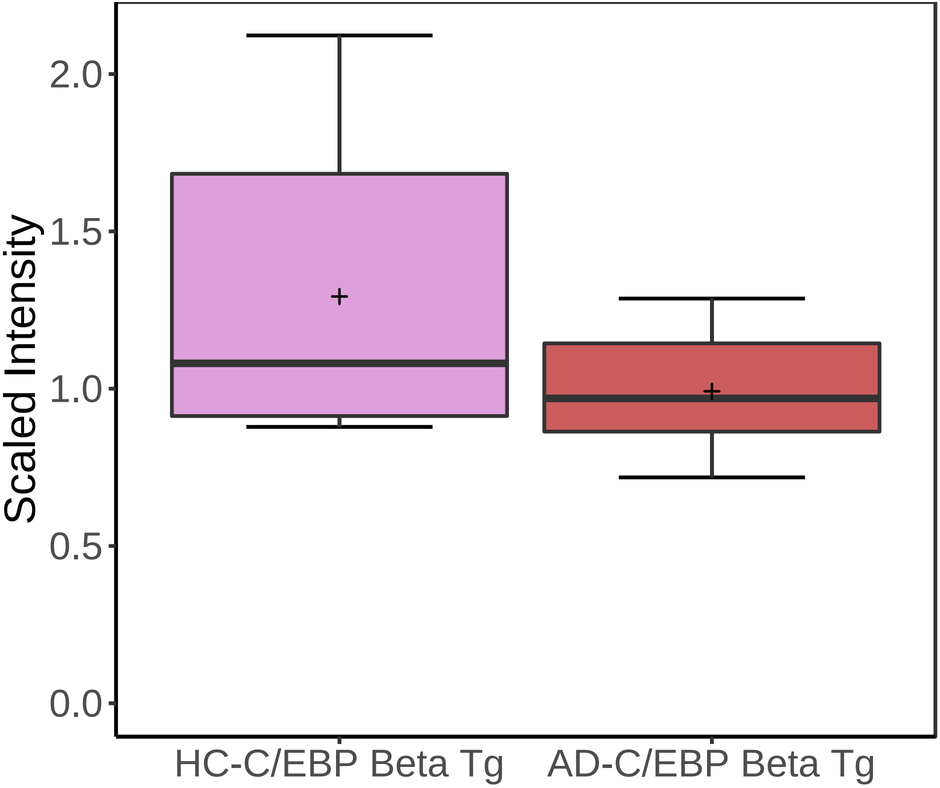

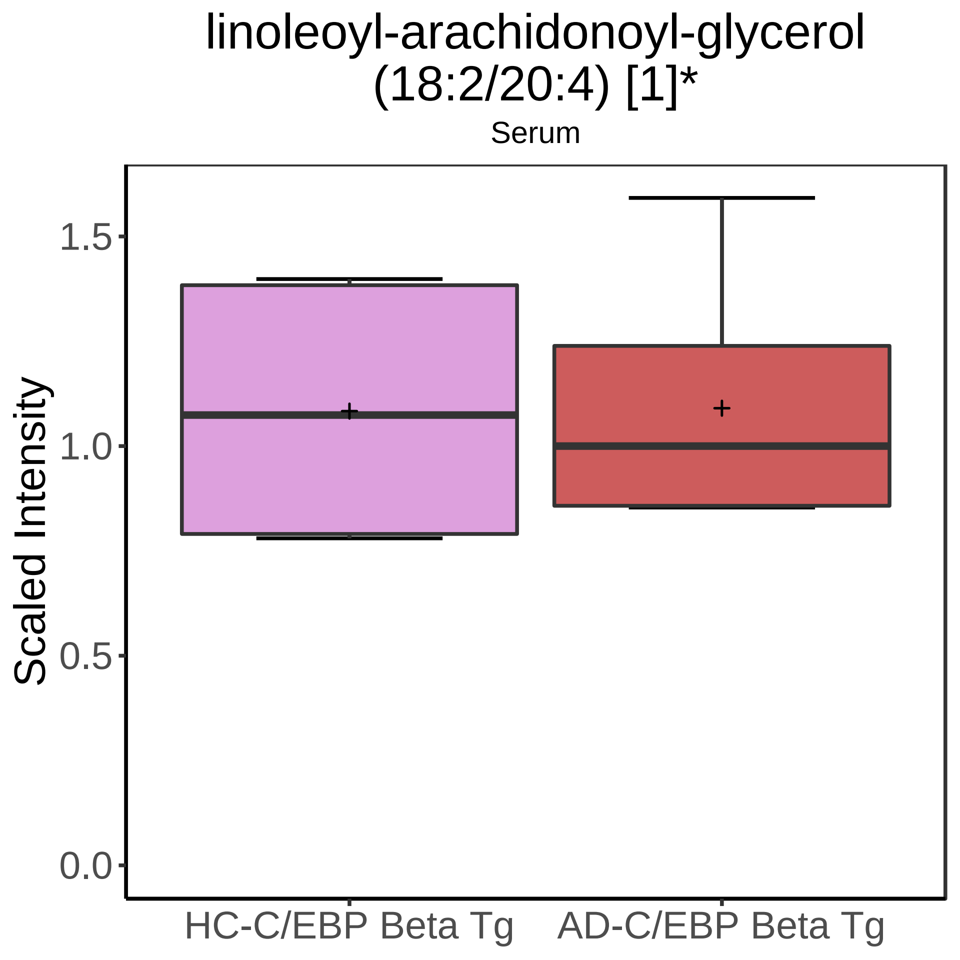
linoleoyl-arachidonoyl-glycerol (18:2/20:4) [1]*
Serum
Scaled Intensity
1.5
1.0
0.5
0.0
HC-C/EBP Beta Tg
AD-C/EBP Beta Tg

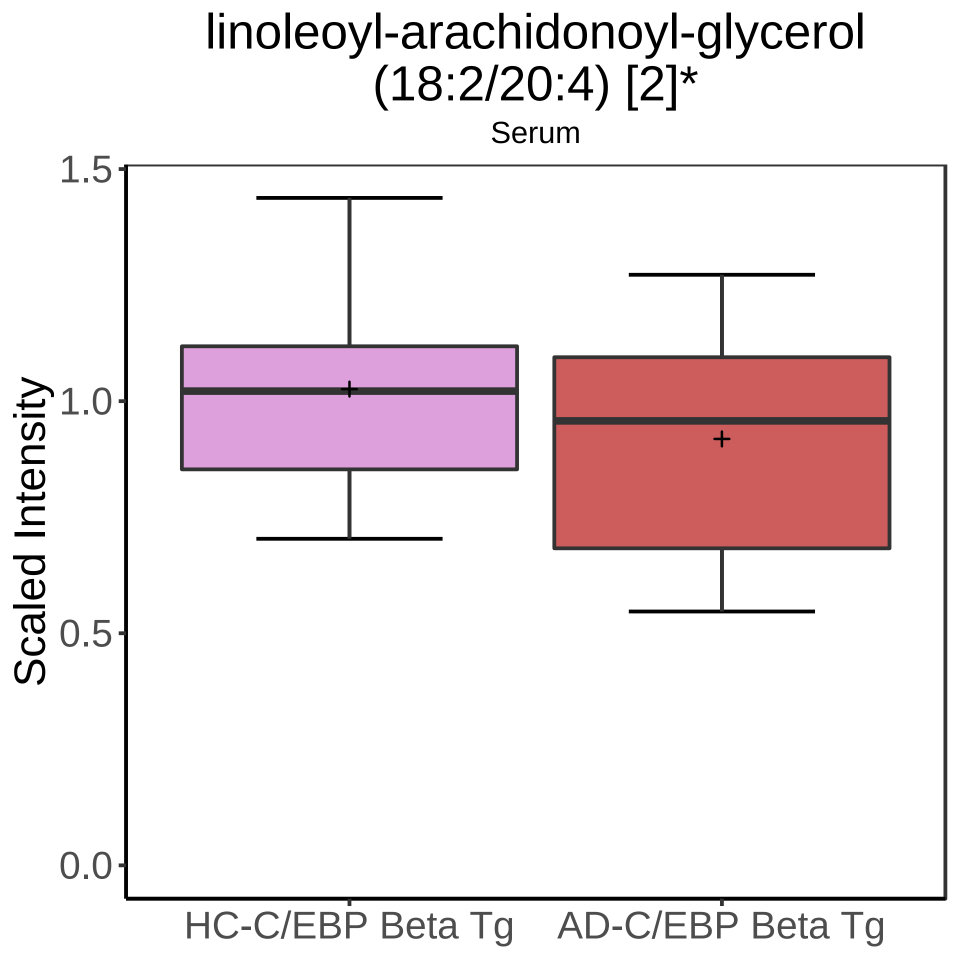
linoleoyl-arachidonoyl-glycerol
(18:2/20:4) [2]*
Serum
Scaled Intensity
1.5
1.0
0.5
0.0
HC-C/EBP Beta Tg
AD-C/EBP Beta Tg

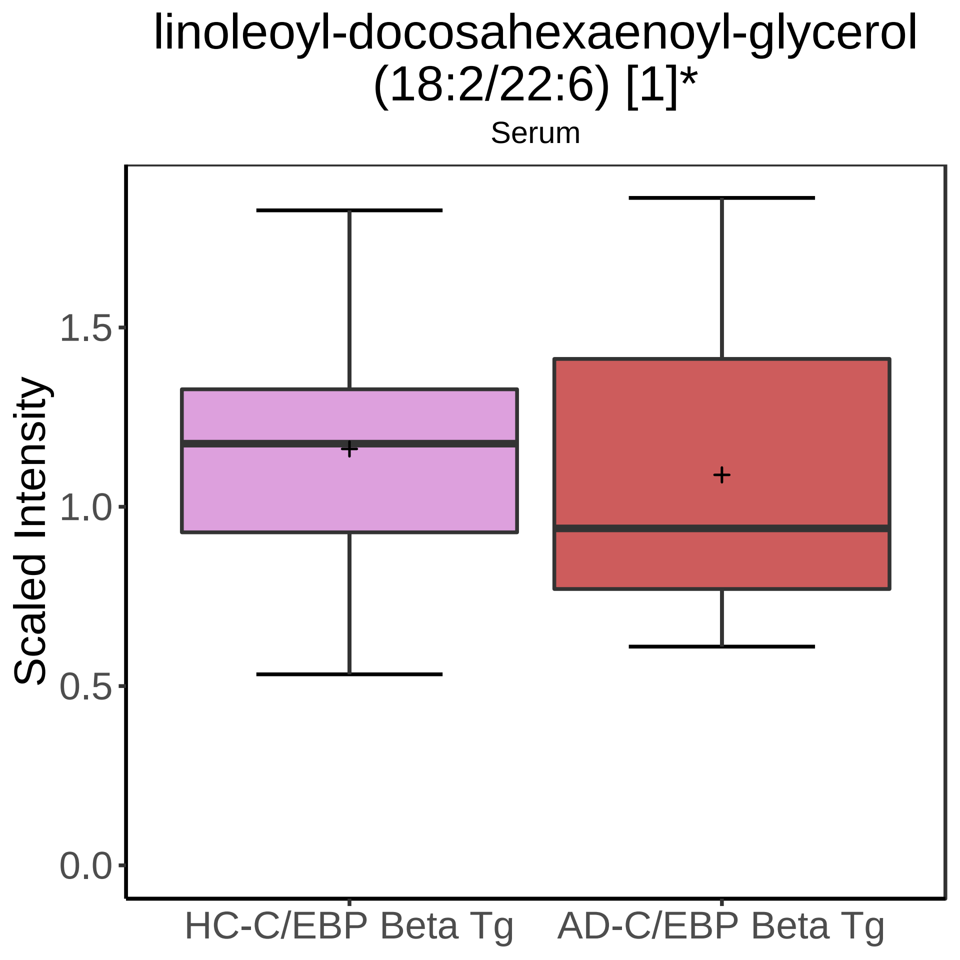
linoleoyl-docosahexaenoyl-glycerol (18:2/22:6) [1]*
Serum
Scaled Intensity
0.0
0.5
1.0
1.5
HC-C/EBP Beta Tg
AD-C/EBP Beta Tg

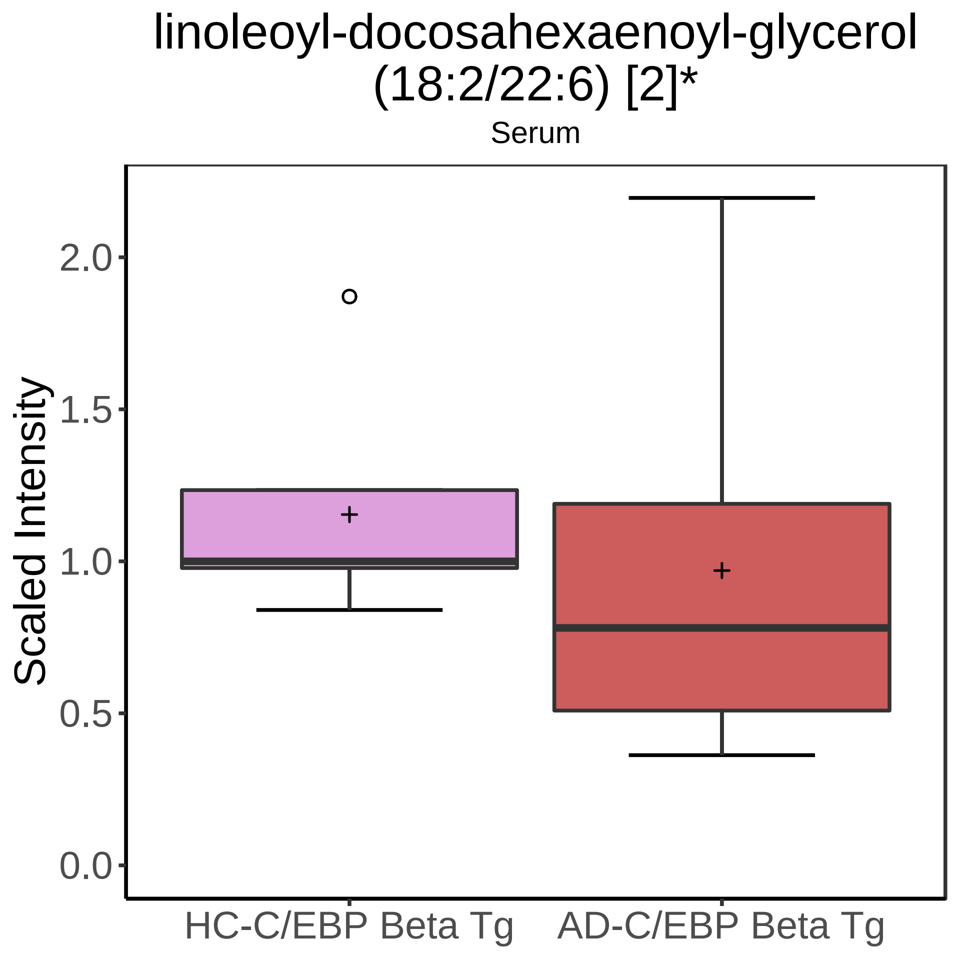

linoleoyl-docosahexaenoyl-glycerol
(18:2/22:6) [2]*
Serum
Scaled Intensity
2.0
1.5
1.0
0.5
0.0
HC-C/EBP Beta Tg
AD-C/EBP Beta Tg

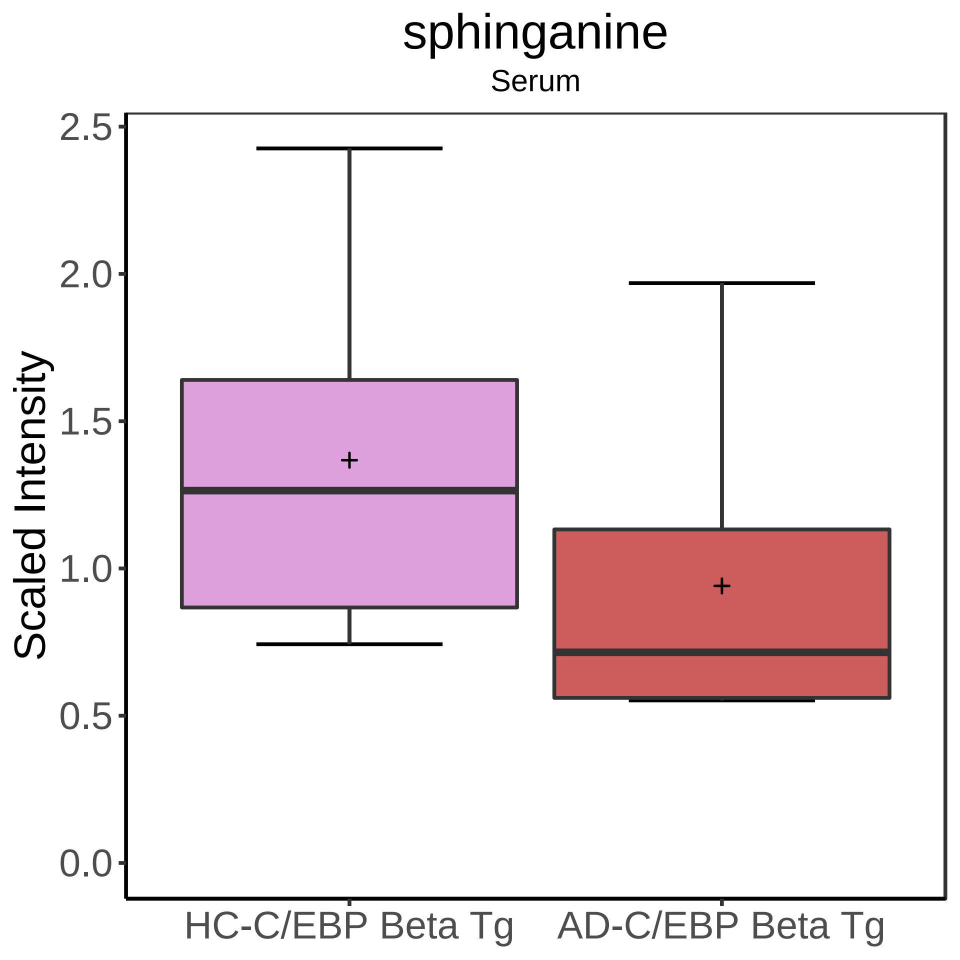
sphinganine
Serum
Scaled Intensity
2.5
2.0
1.5
1.0
0.5
0.0
HC-C/EBP Beta Tg
AD-C/EBP Beta Tg

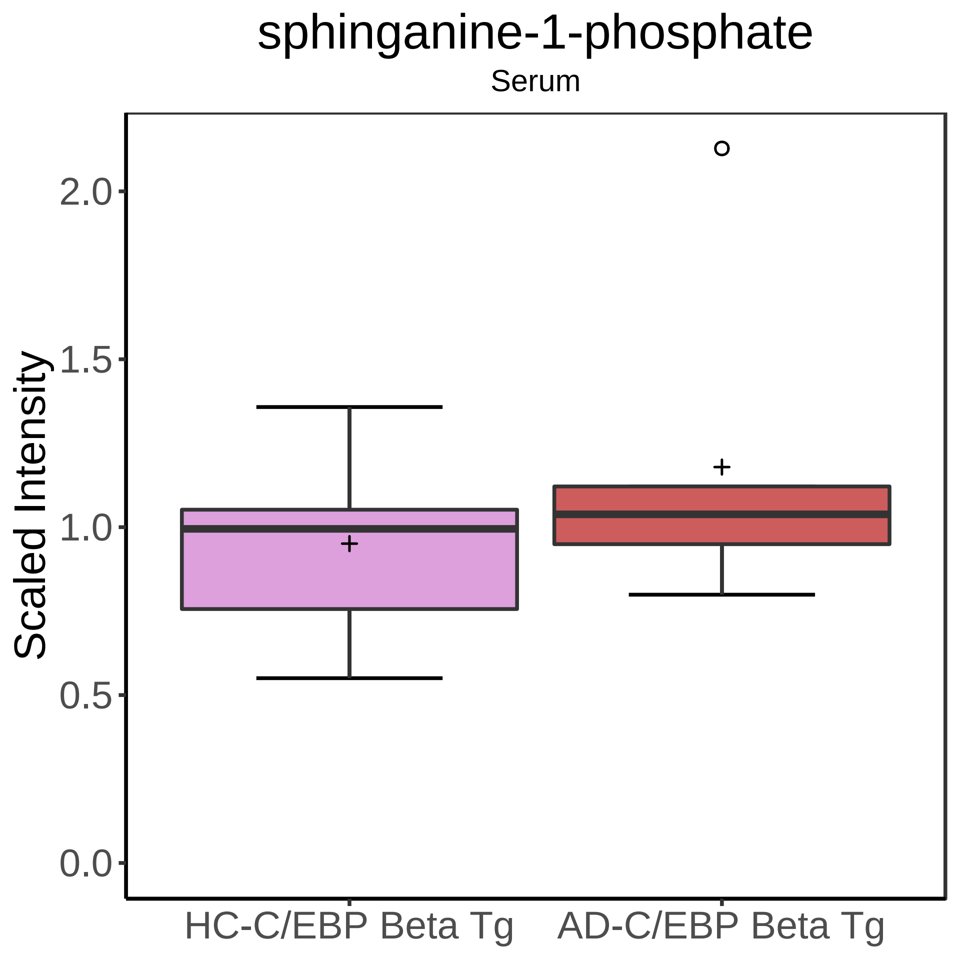

sphinganine-1-phosphate
Serum
Scaled Intensity
2.0
1.5
1.0
0.5
0.0
HC-C/EBP Beta Tg
AD-C/EBP Beta Tg

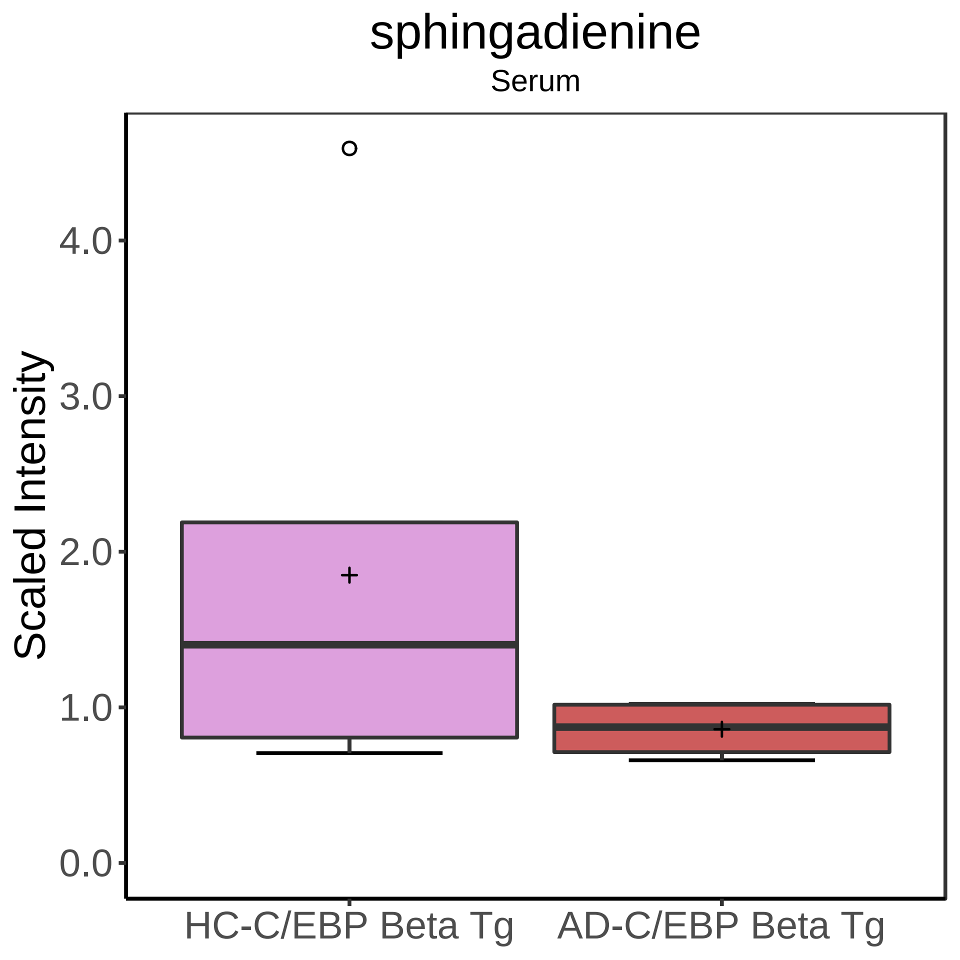
sphingadienine
Serum
Scaled Intensity
0.0
1.0
2.0
3.0
4.0
HC-C/EBP Beta Tg
AD-C/EBP Beta Tg

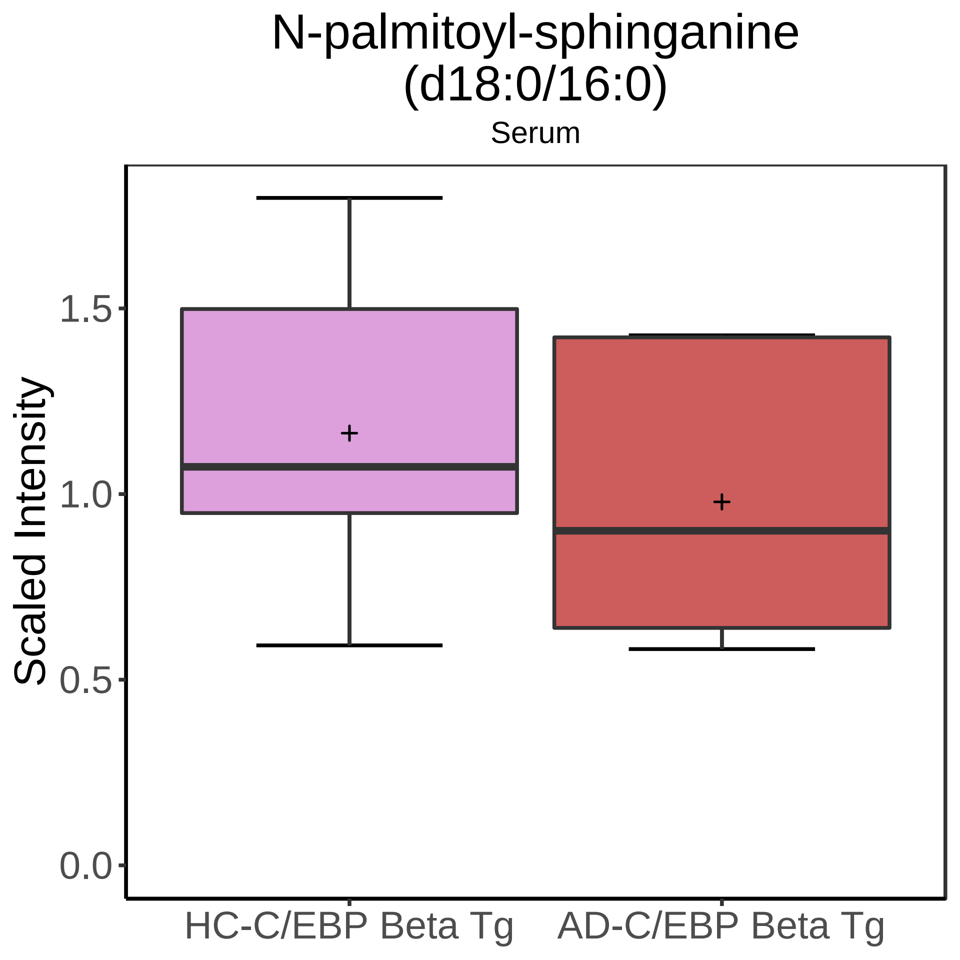

N-palmitoyl-sphinganine
(d18:0/16:0)
Serum
Scaled Intensity
1.5
1.0
0.5
0.0
HC-C/EBP Beta Tg
AD-C/EBP Beta Tg

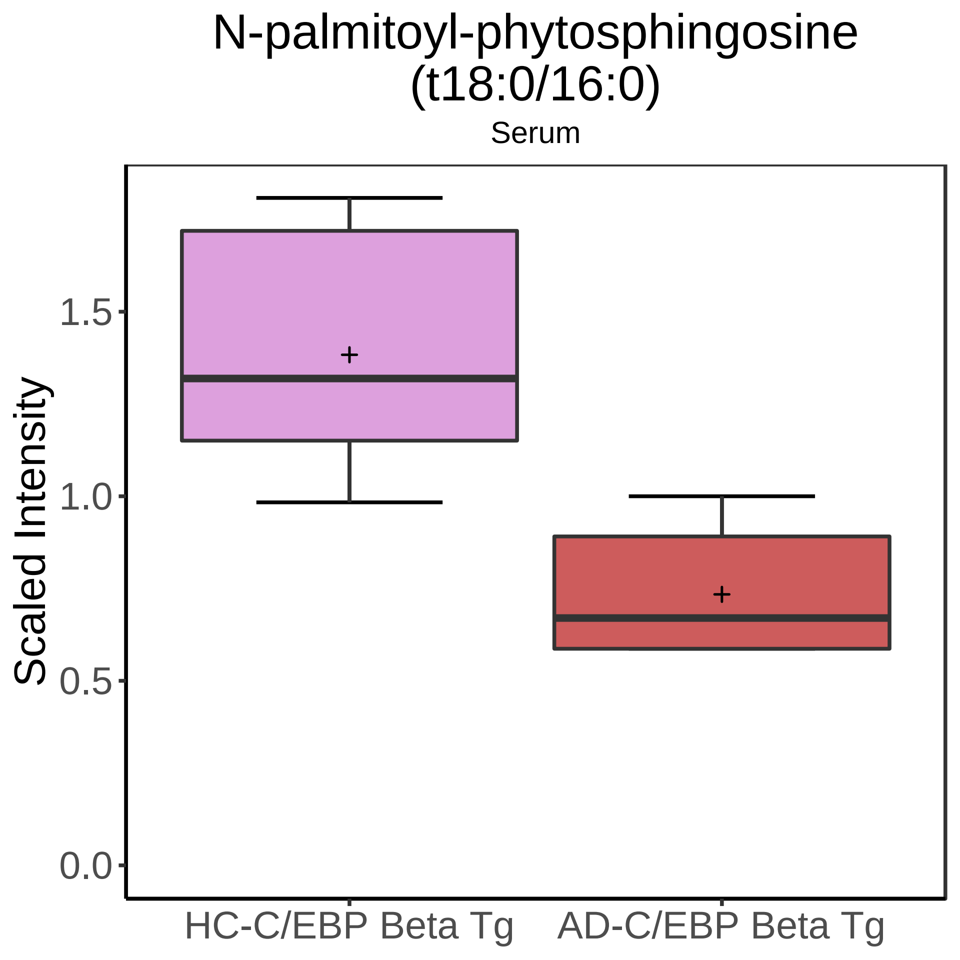

N-palmitoyl-phytosphingosine (t18:0/16:0)
Serum
Scaled Intensity
1.5
1.0
0.5
0.0
HC-C/EBP Beta Tg
AD-C/EBP Beta Tg

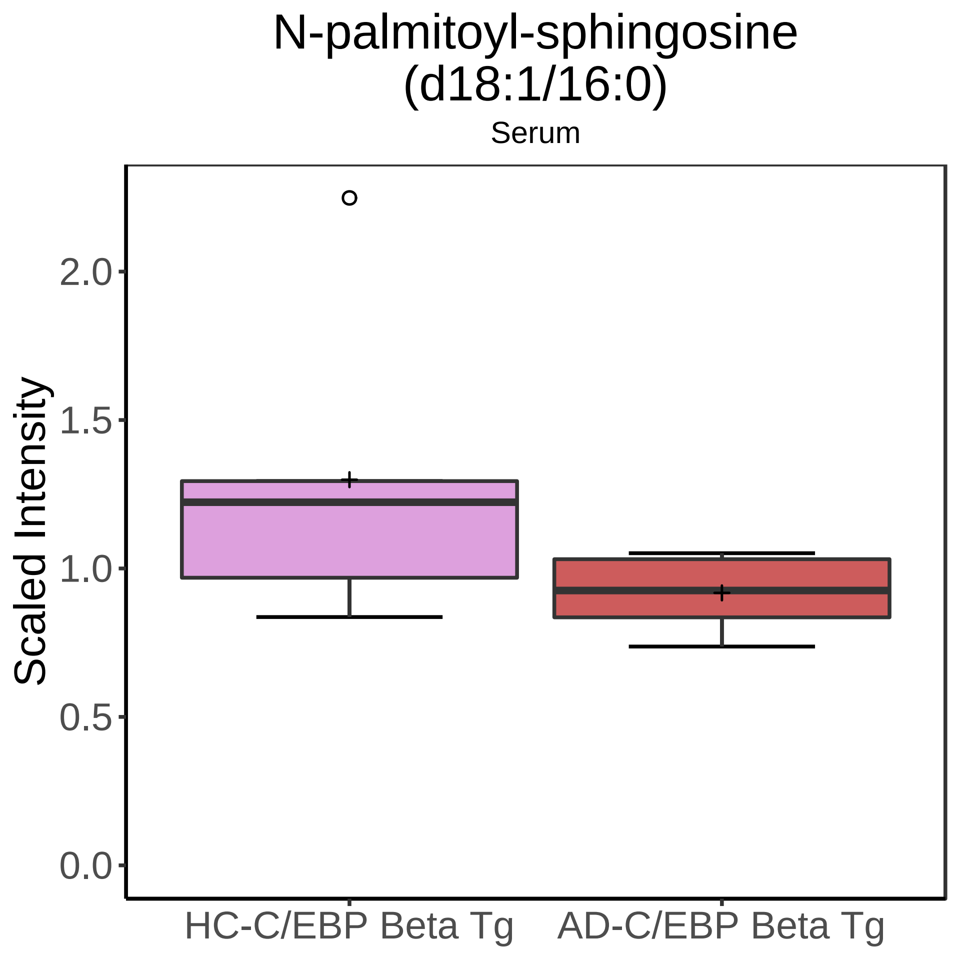
N-palmitoyl-sphingosine
(d18:1/16:0)
Serum
Scaled Intensity
2.0
1.5
1.0
0.5
0.0
HC-C/EBP Beta Tg
AD-C/EBP Beta Tg

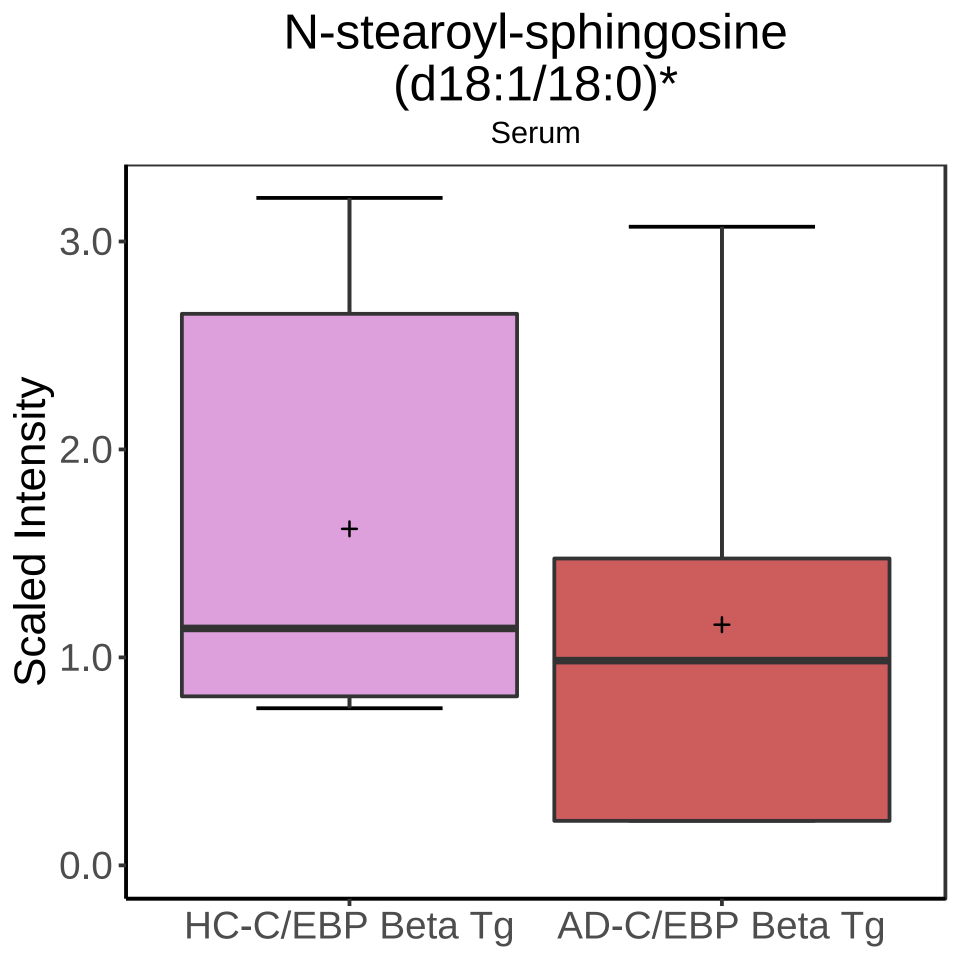
N-stearoyl-sphingosine
(d18:1/18:0)*
Serum
Scaled Intensity
3.0
2.0
1.0
0.0
HC-C/EBP Beta Tg
AD-C/EBP Beta Tg

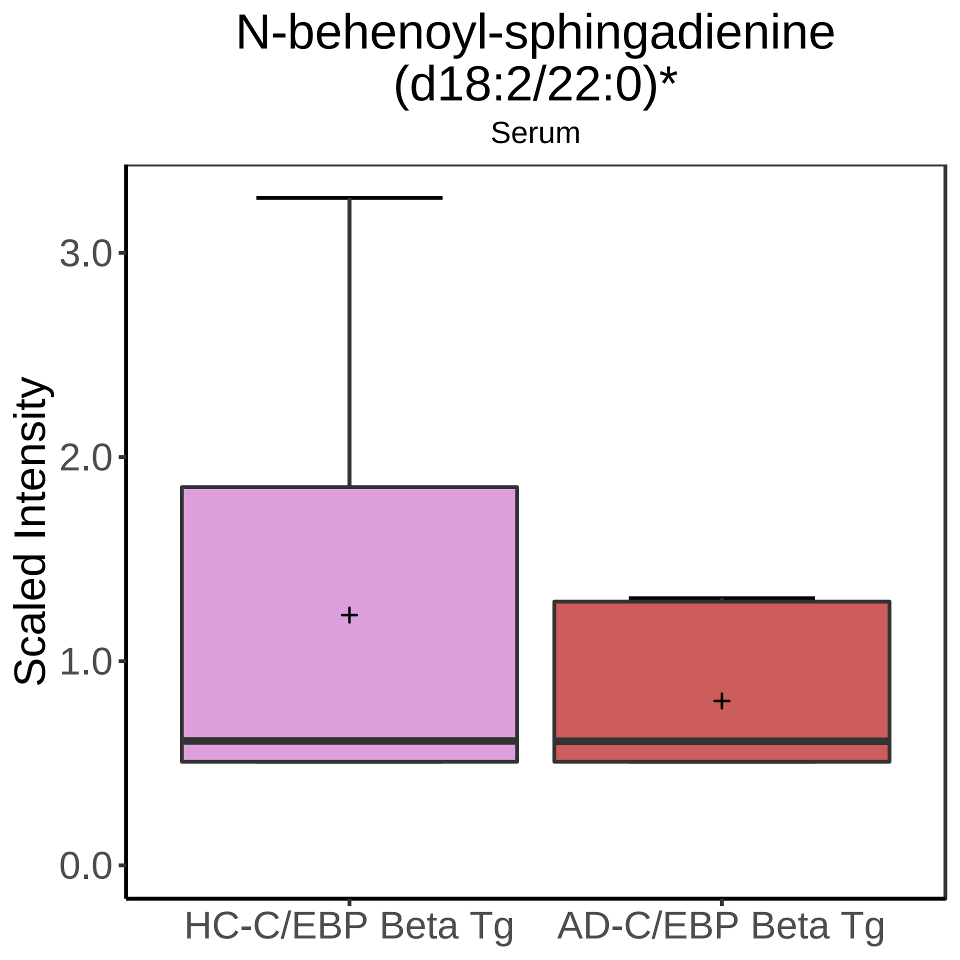
N-behenoyl-sphingadienine
(d18:2/22:0)*
Serum
Scaled Intensity
3.0
2.0
1.0
0.0
HC-C/EBP Beta Tg
AD-C/EBP Beta Tg

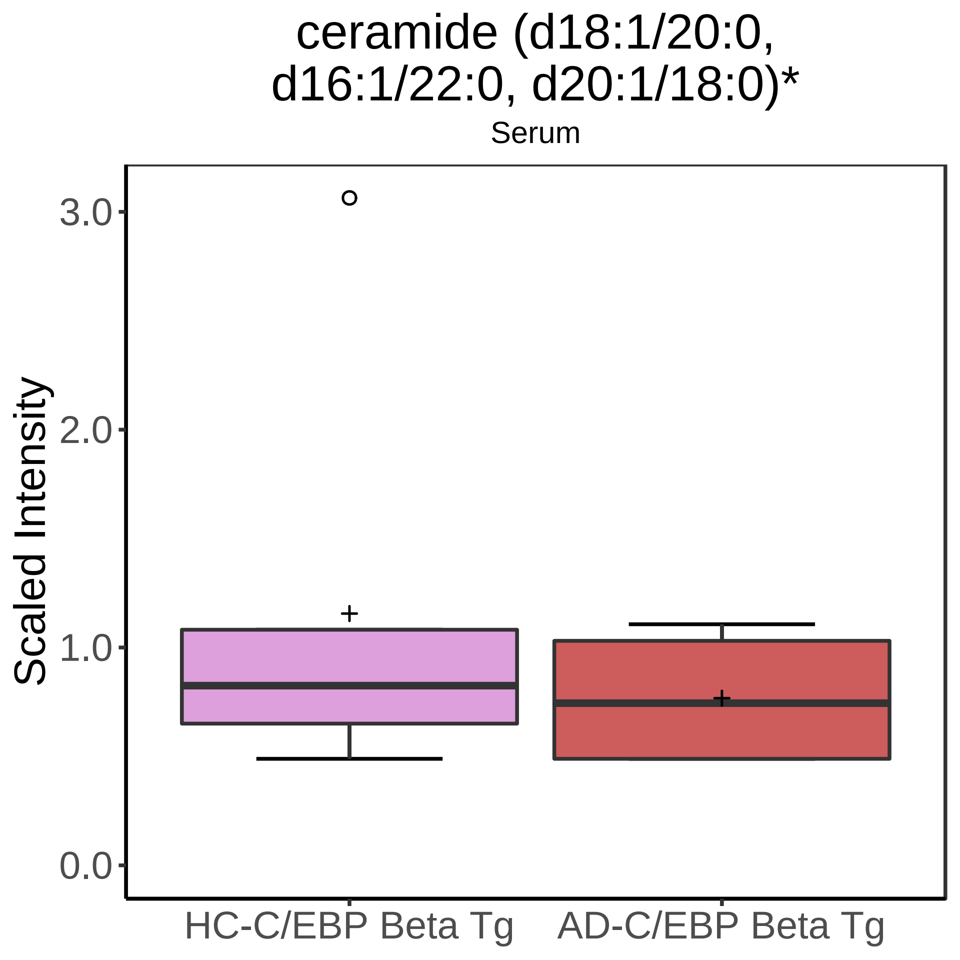
ceramide (d18:1/20:0,
d16:1/22:0, d20:1/18:0)*
Serum
Scaled Intensity
3.0
2.0
1.0
0.0
HC-C/EBP Beta Tg
AD-C/EBP Beta Tg

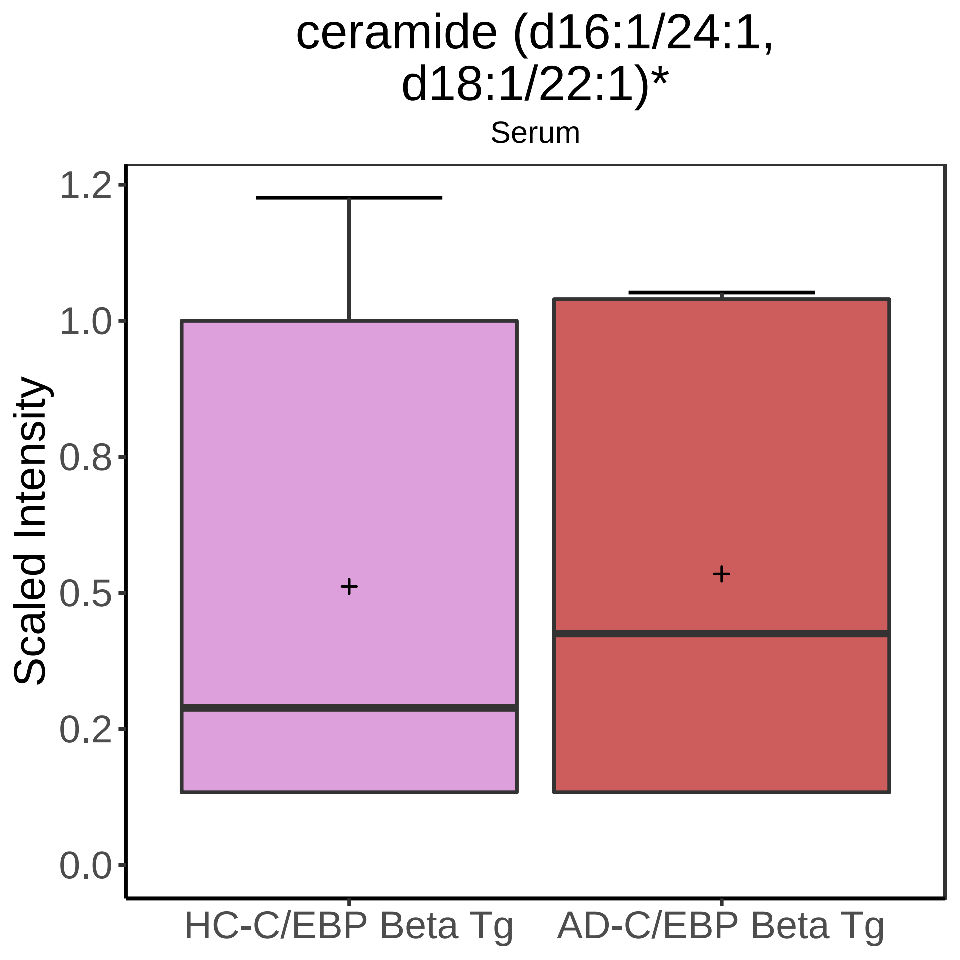
ceramide (d16:1/24:1, d18:1/22:1)*
Serum
Scaled Intensity
1.2
1.0
0.8
0.5
0.2
0.0
HC-C/EBP Beta Tg
AD-C/EBP Beta Tg

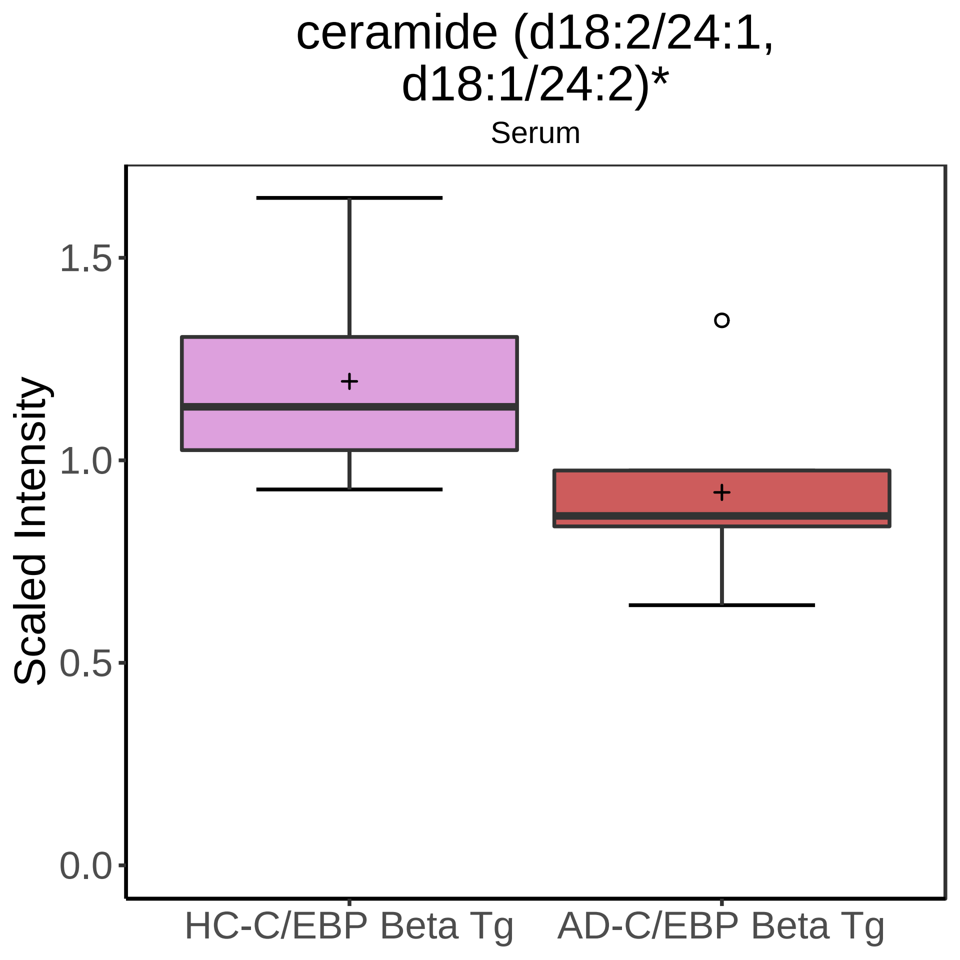
ceramide (d18:2/24:1, d18:1/24:2)*
Serum
Scaled Intensity
1.5
1.0
0.5
0.0
HC-C/EBP Beta Tg
AD-C/EBP Beta Tg

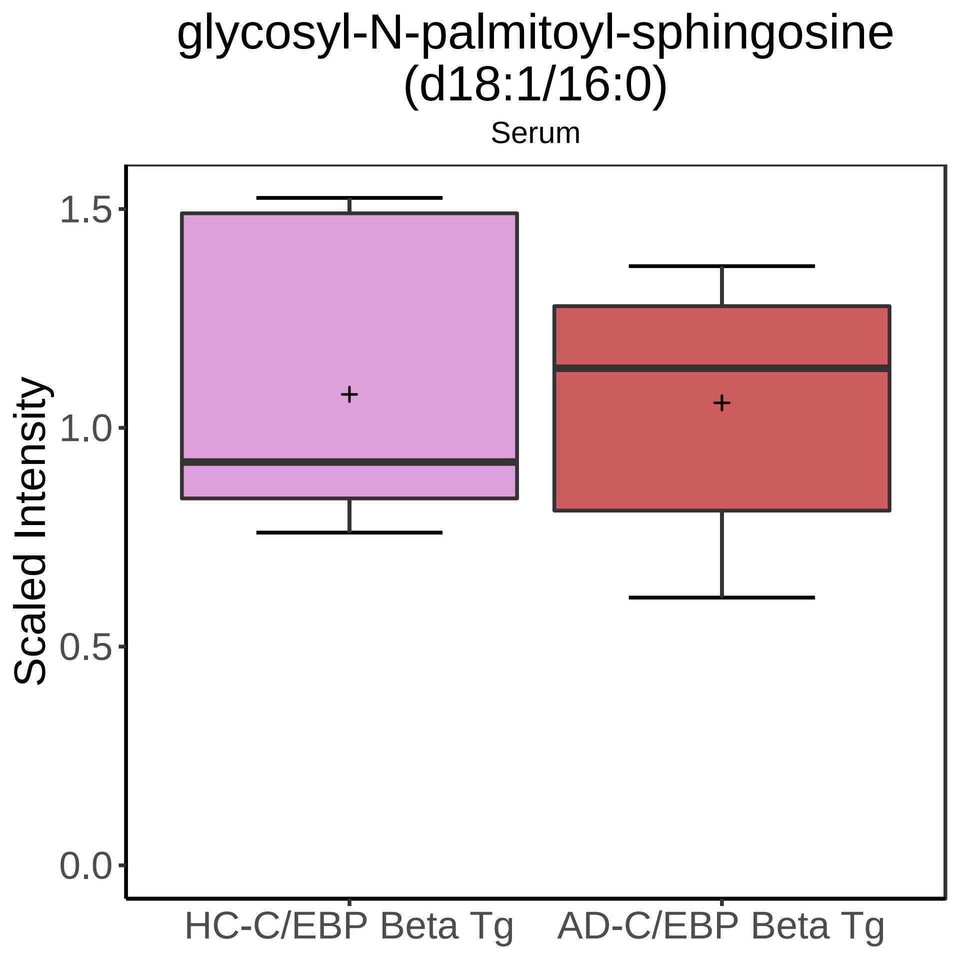
glycosyl-N-palmitoyl-sphingosine
(d18:1/16:0)
Serum
Scaled Intensity
1.5
1.0
0.5
0.0
HC-C/EBP Beta Tg
AD-C/EBP Beta Tg

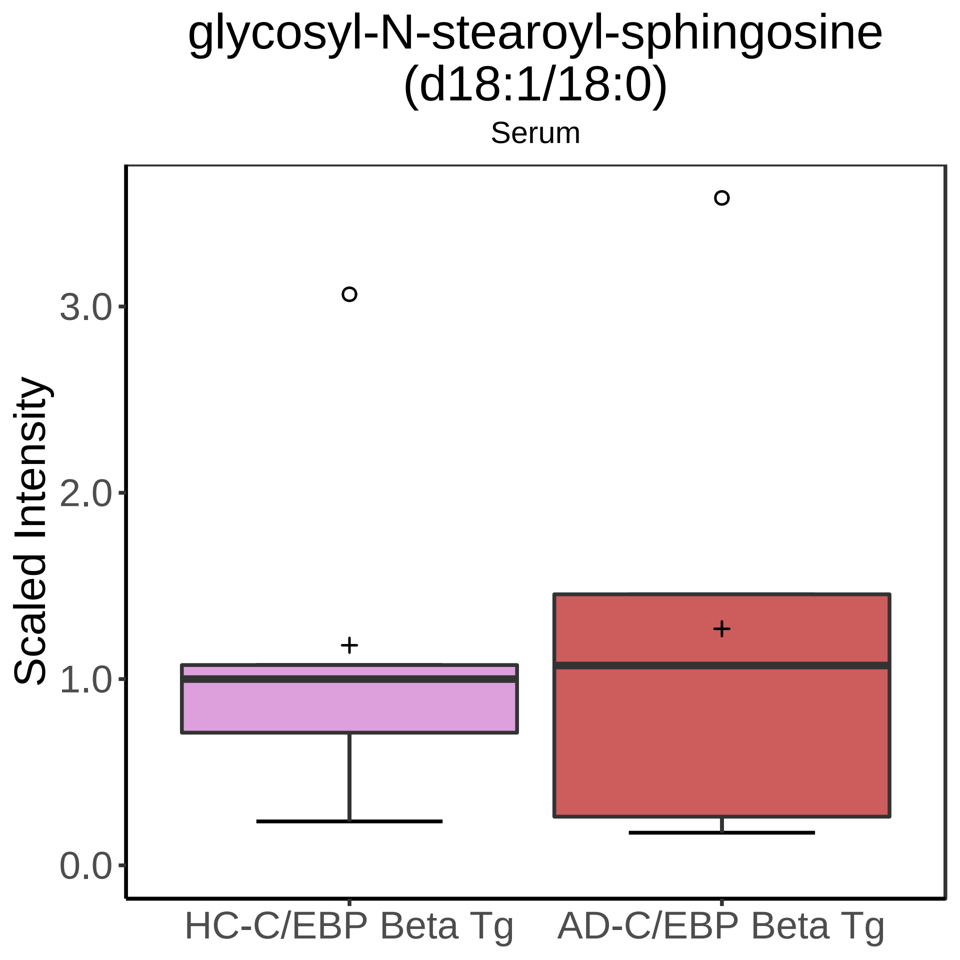

glycosyl-N-stearoyl-sphingosine
(d18:1/18:0)
Serum
Scaled Intensity
3.0
2.0
1.0
0.0
HC-C/EBP Beta Tg
AD-C/EBP Beta Tg

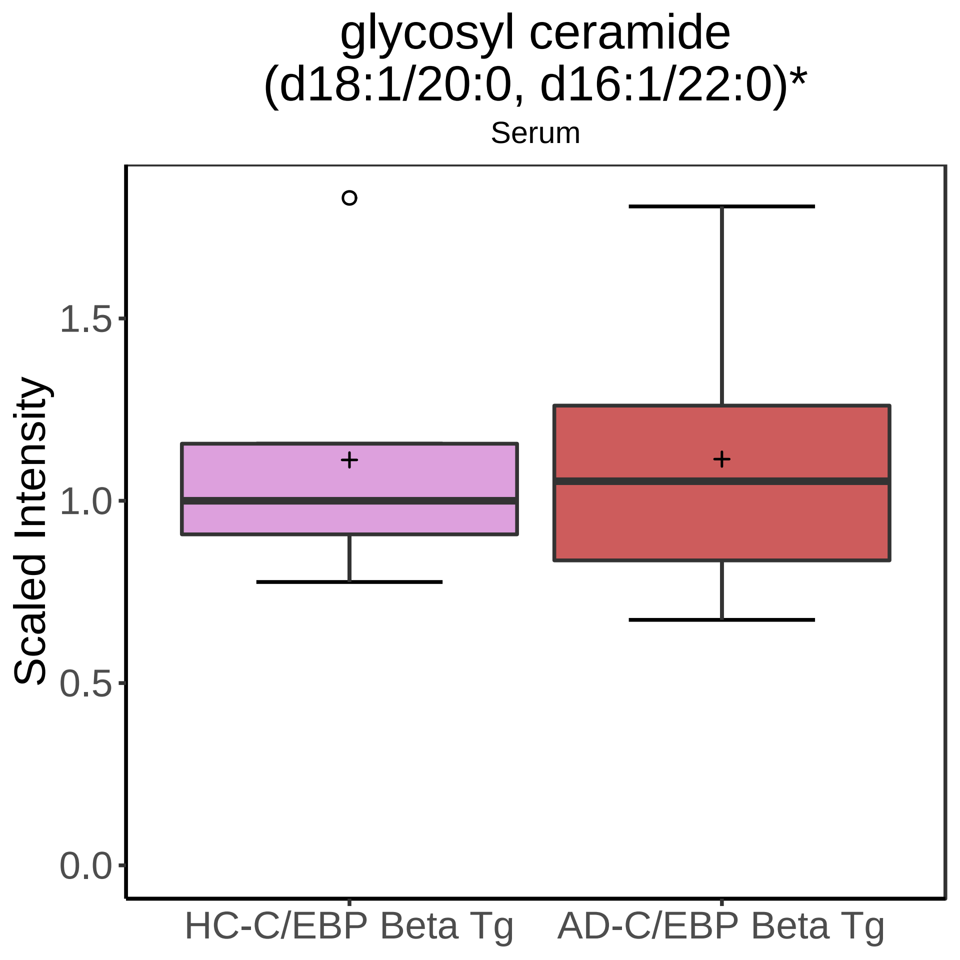
glycosyl ceramide
(d18:1/20:0, d16:1/22:0)*
Serum
Scaled Intensity
0.0
0.5
1.0
1.5
HC-C/EBP Beta Tg
AD-C/EBP Beta Tg

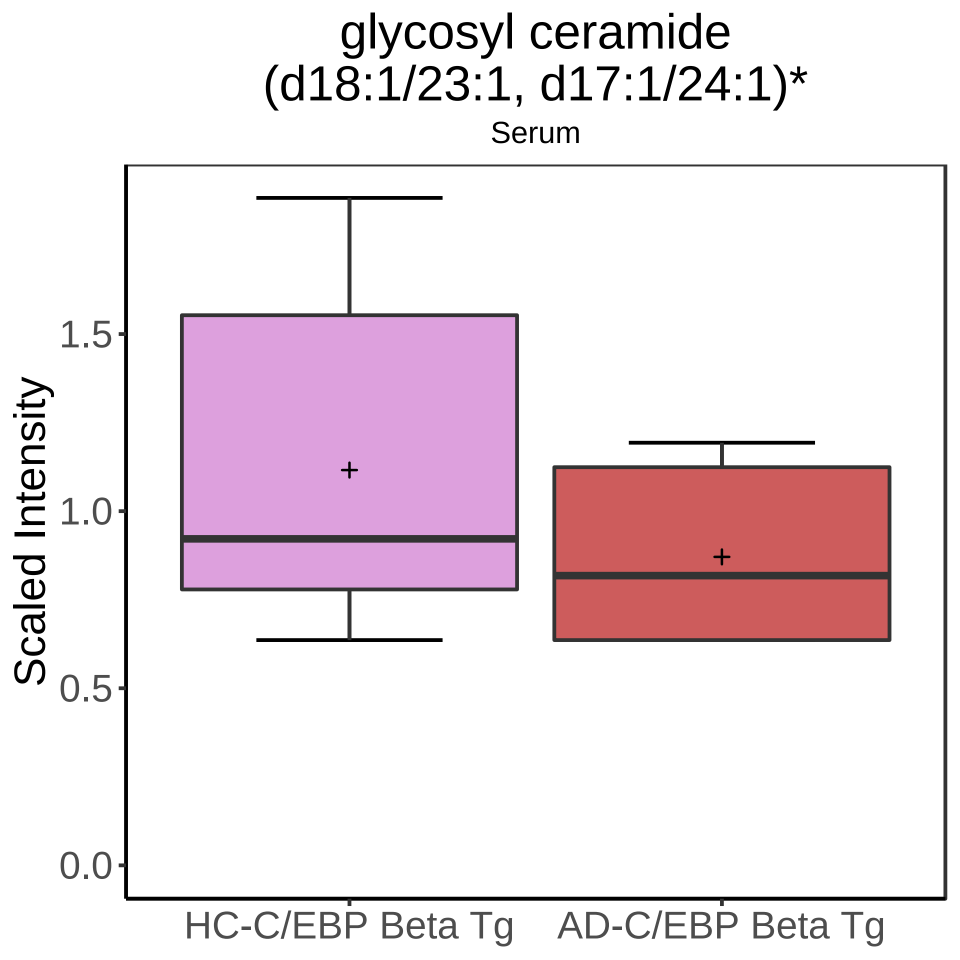
glycosyl ceramide
(d18:1/23:1, d17:1/24:1)*
Serum
Scaled Intensity
0.0
0.5
1.0
1.5
HC-C/EBP Beta Tg
AD-C/EBP Beta Tg

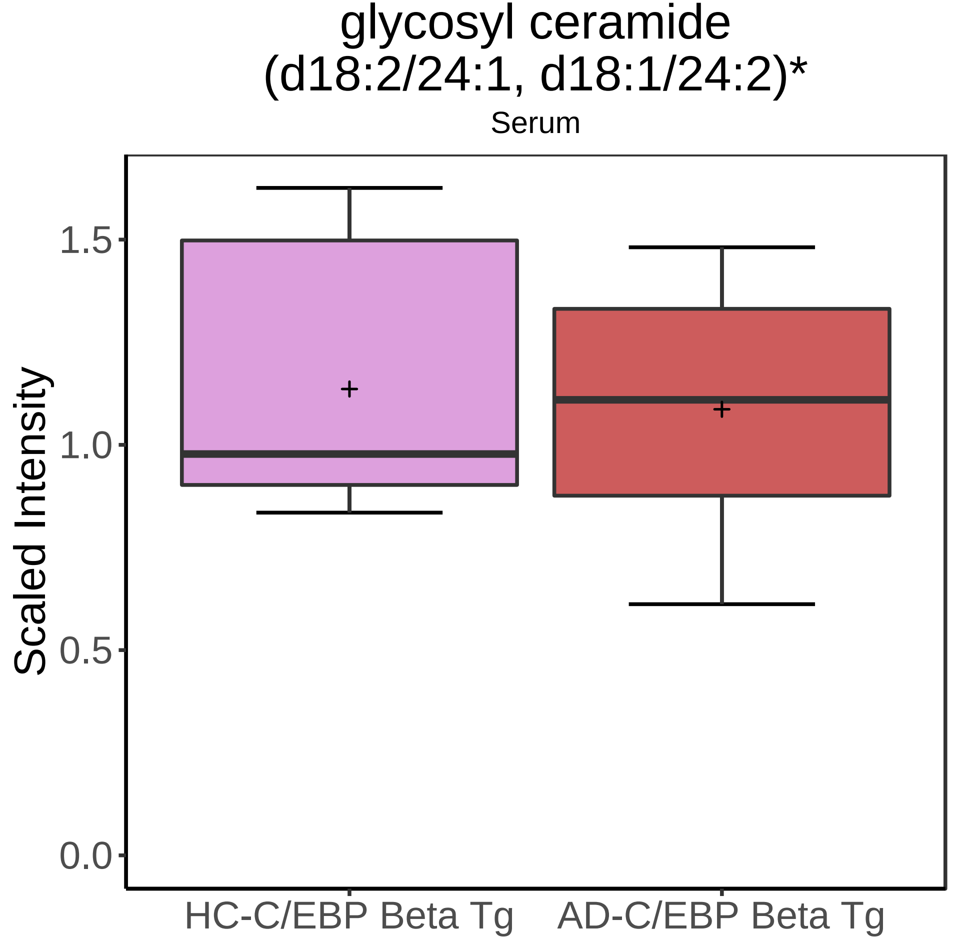

glycosyl ceramide
(d18:2/24:1, d18:1/24:2)*
Serum
Scaled Intensity
1.5
1.0
0.5
0.0
HC-C/EBP Beta Tg
AD-C/EBP Beta Tg

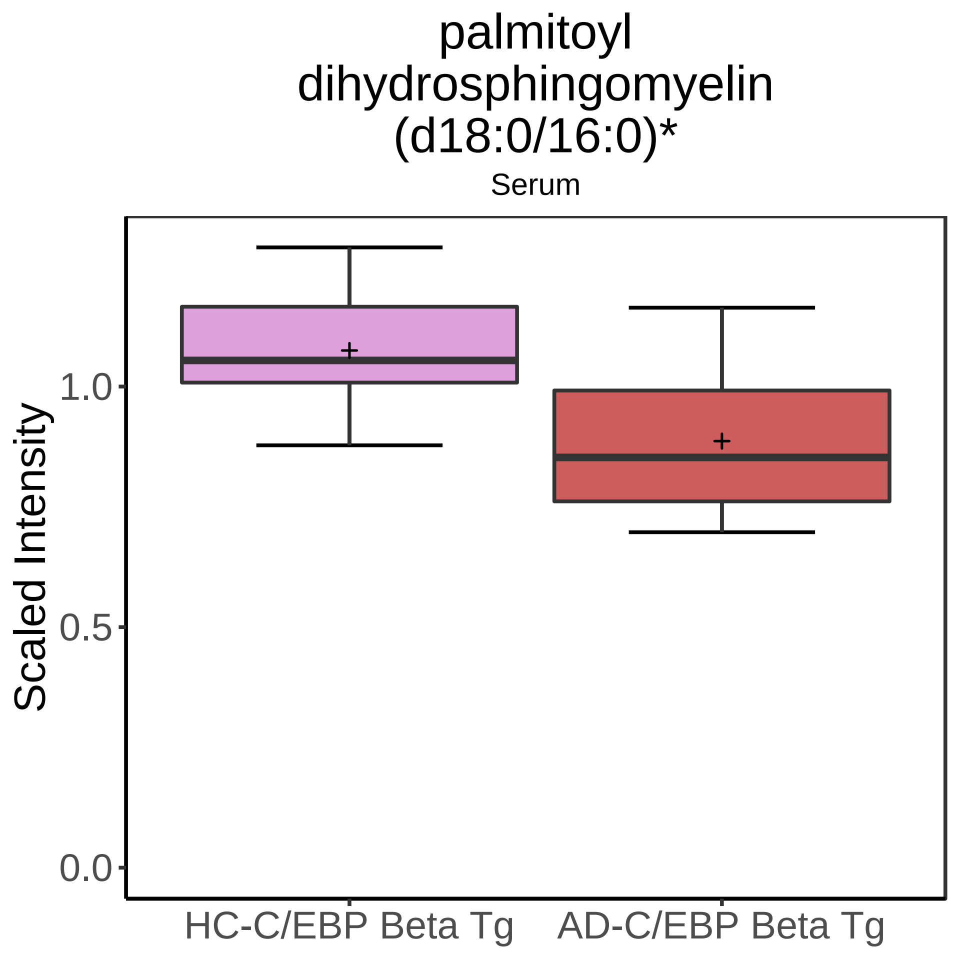
palmitoyl
dihydrosphingomyelin
(d18:0/16:0)*
Serum
Scaled Intensity
1.0
0.5
0.0
HC-C/EBP Beta Tg
AD-C/EBP Beta Tg

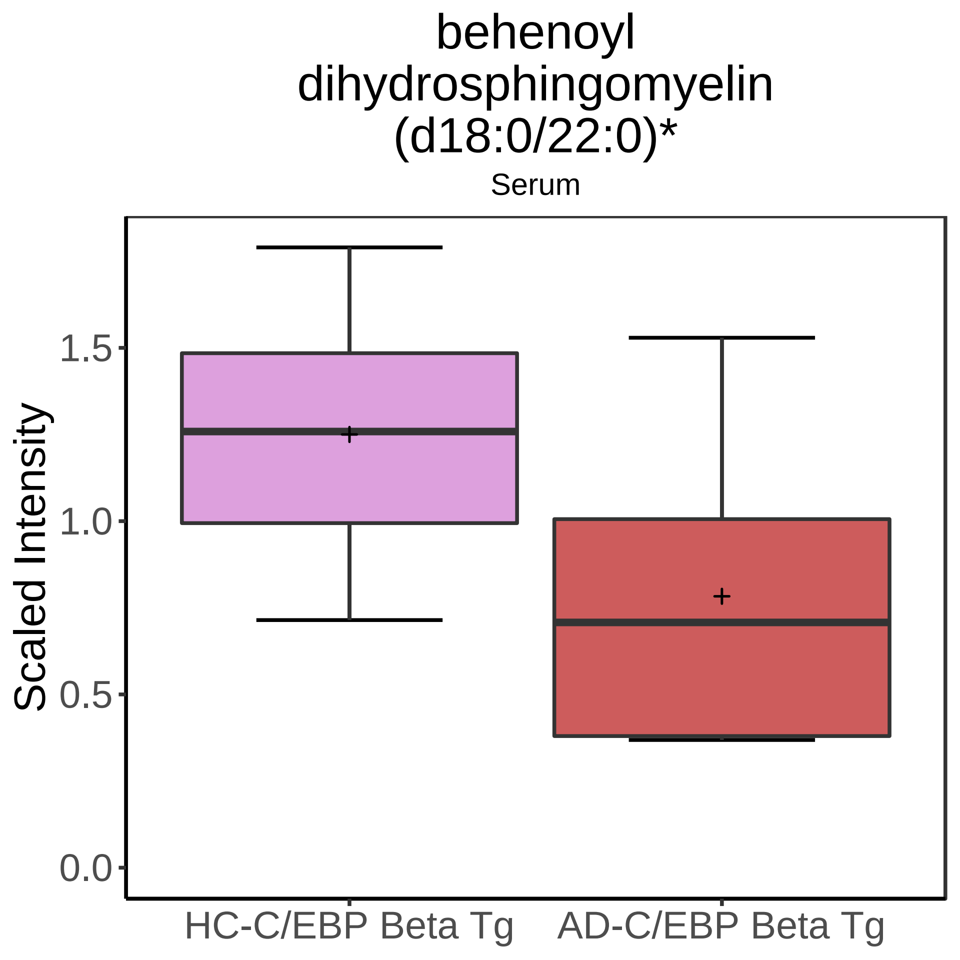
behenoyl dihydrosphingomyelin (d18:0/22:0)*
Serum
Scaled Intensity
0.0
0.5
1.0
1.5
HC-C/EBP Beta Tg
AD-C/EBP Beta Tg

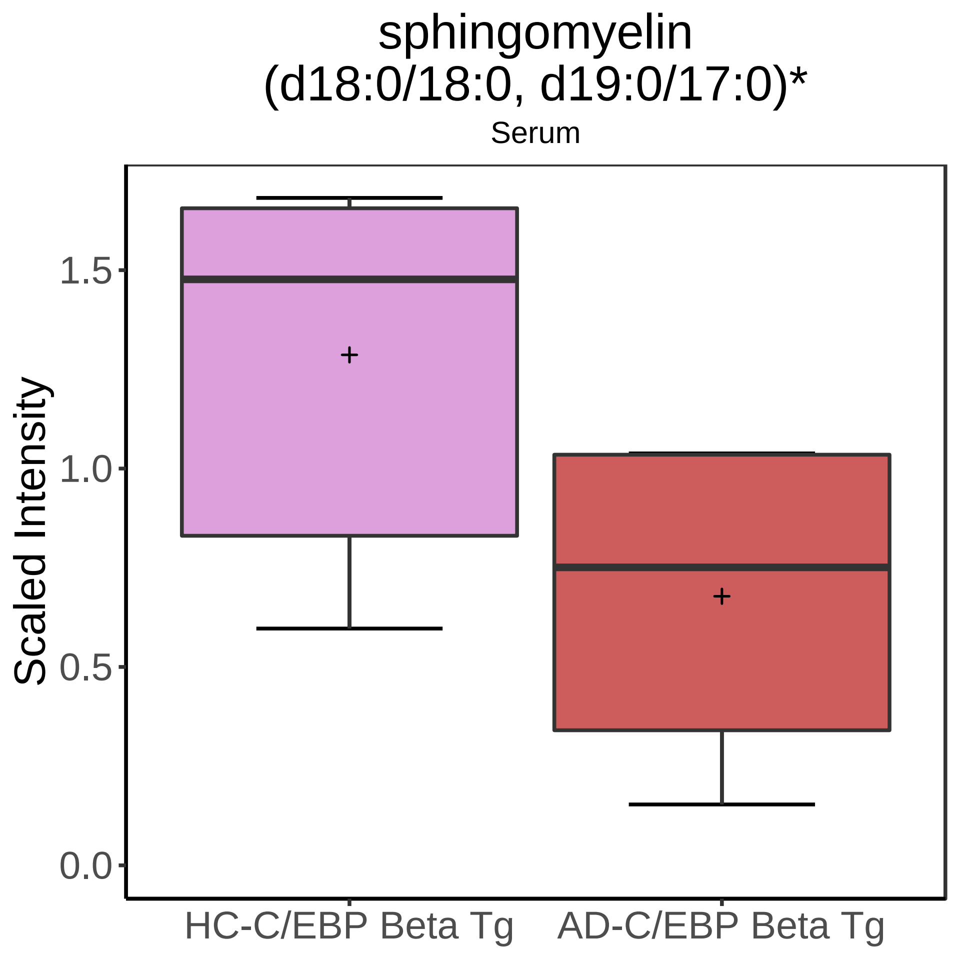
sphingomyelin
(d18:0/18:0, d19:0/17:0)*
Serum
Scaled Intensity
1.5
1.0
0.5
0.0
HC-C/EBP Beta Tg
AD-C/EBP Beta Tg

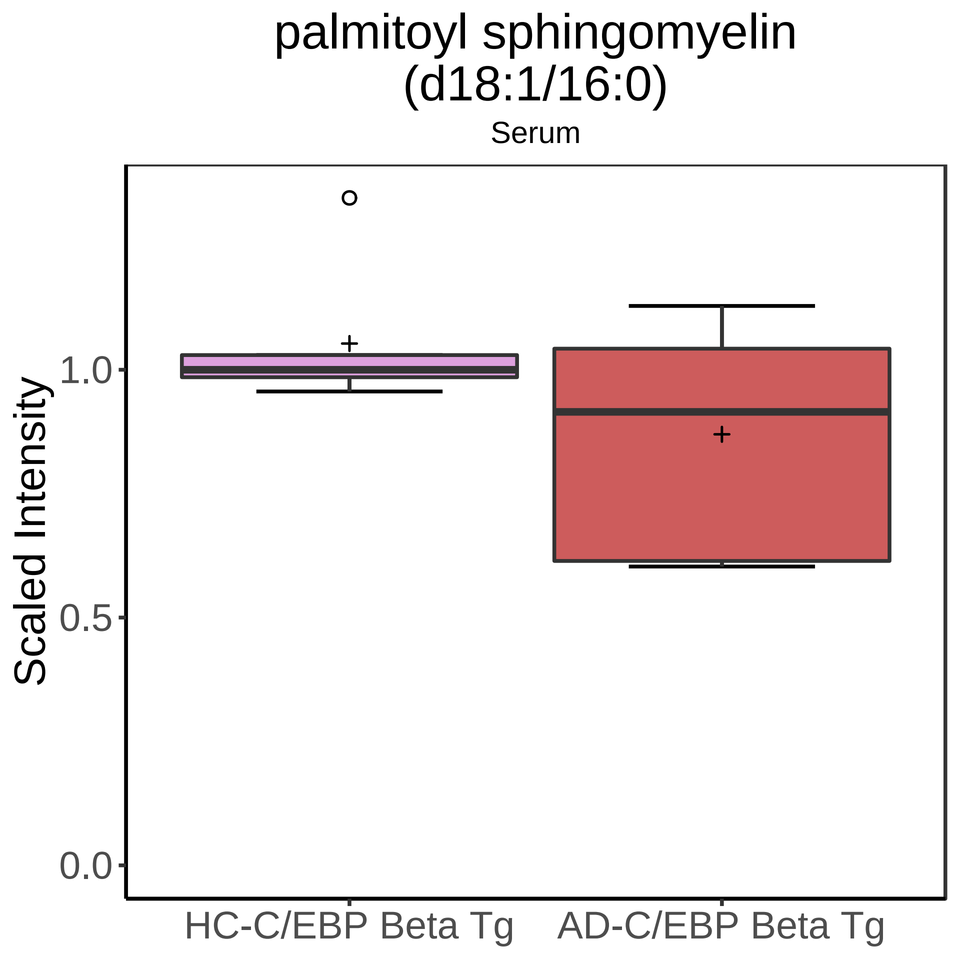
palmitoyl sphingomyelin
(d18:1/16:0)
Serum
Scaled Intensity
1.0
0.5
0.0
HC-C/EBP Beta Tg
AD-C/EBP Beta Tg

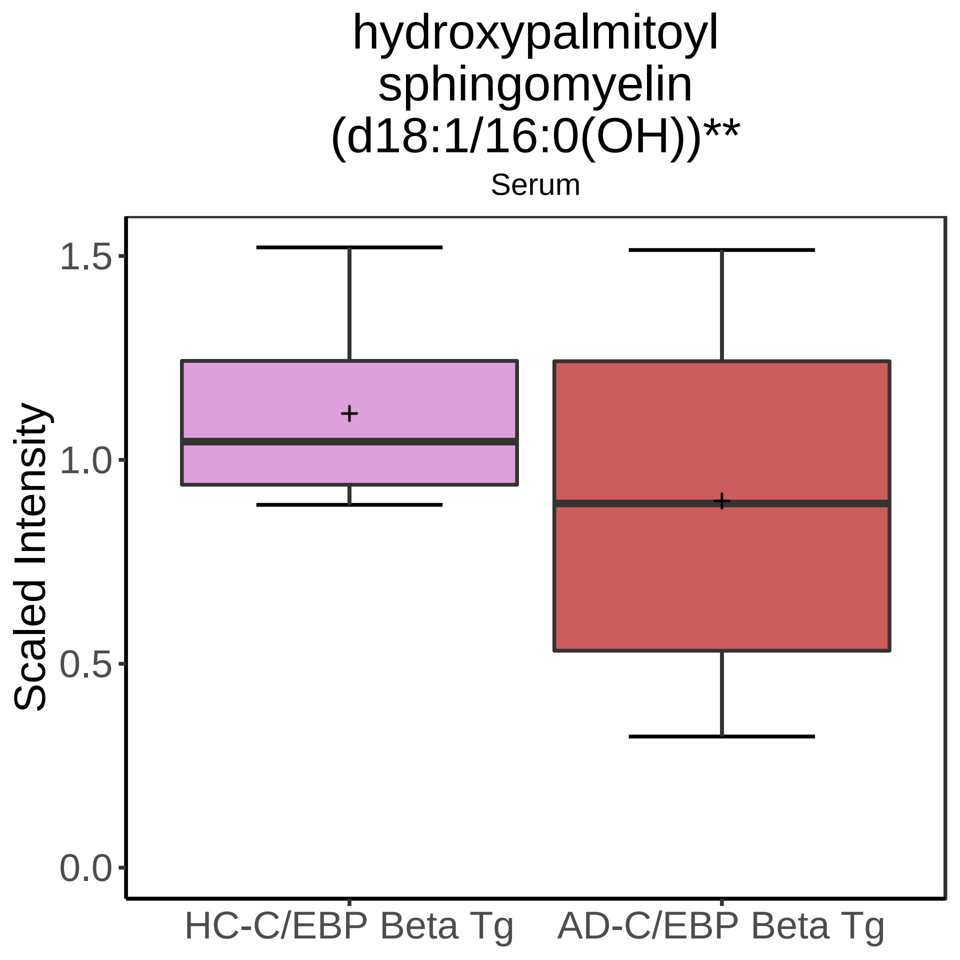
hydroxypalmitoyl sphingomyelin (d18:1/16:0(OH))**
Serum
Scaled Intensity
1.5
1.0
0.5
0.0
HC-C/EBP Beta Tg
AD-C/EBP Beta Tg

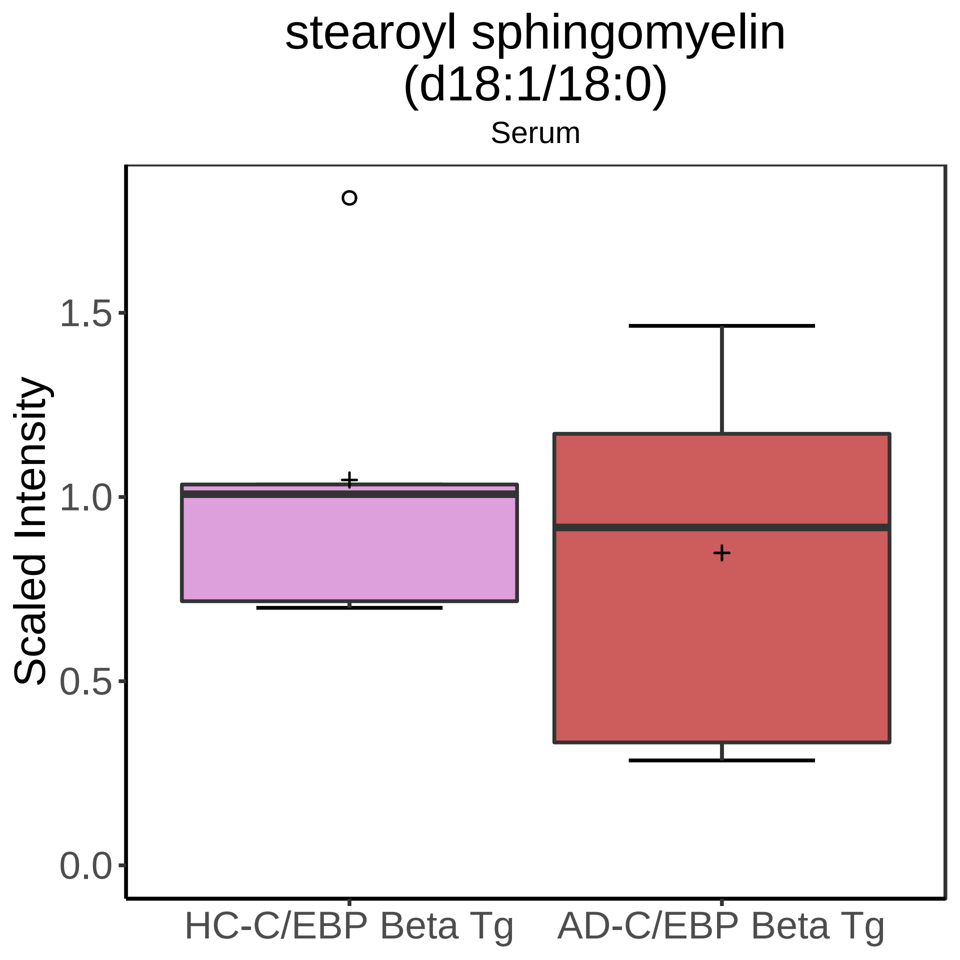
stearoyl sphingomyelin
(d18:1/18:0)
Serum
Scaled Intensity
1.5
1.0
0.5
0.0
HC-C/EBP Beta Tg
AD-C/EBP Beta Tg

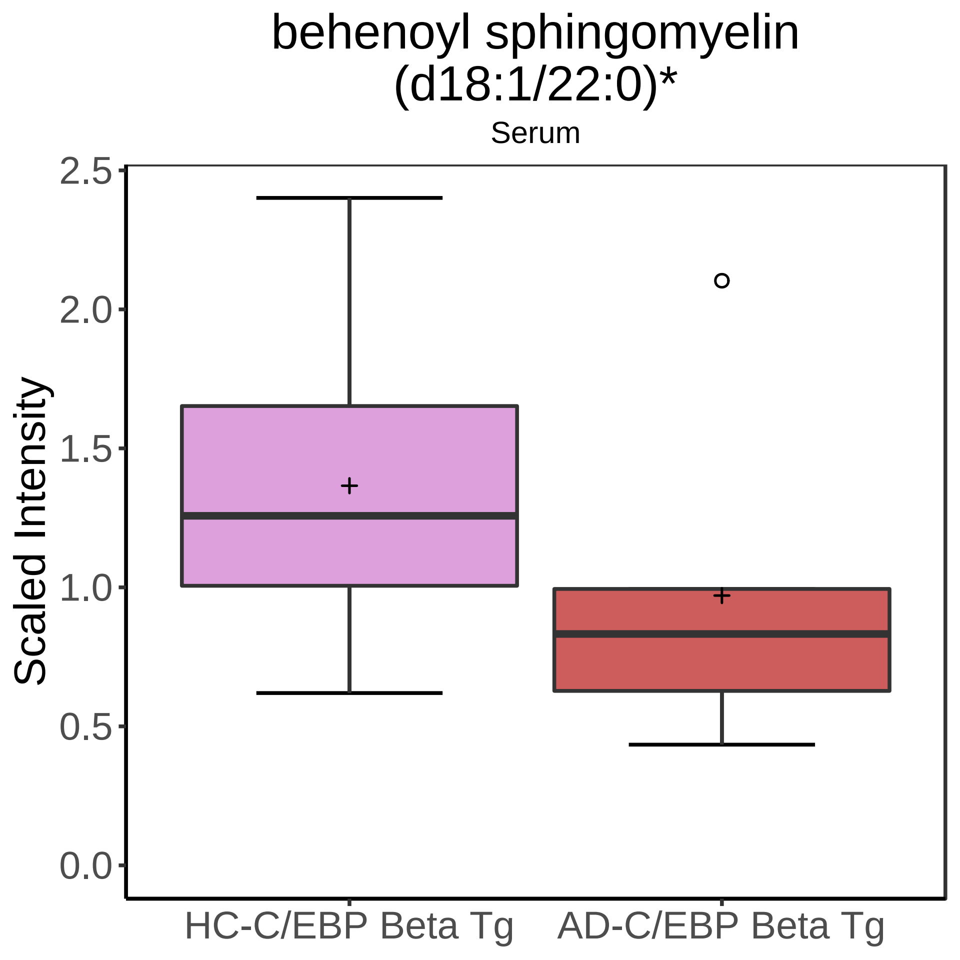
behenoyl sphingomyelin
(d18:1/22:0)*
Serum
Scaled Intensity
2.5
2.0
1.5
1.0
0.5
0.0
HC-C/EBP Beta Tg
AD-C/EBP Beta Tg

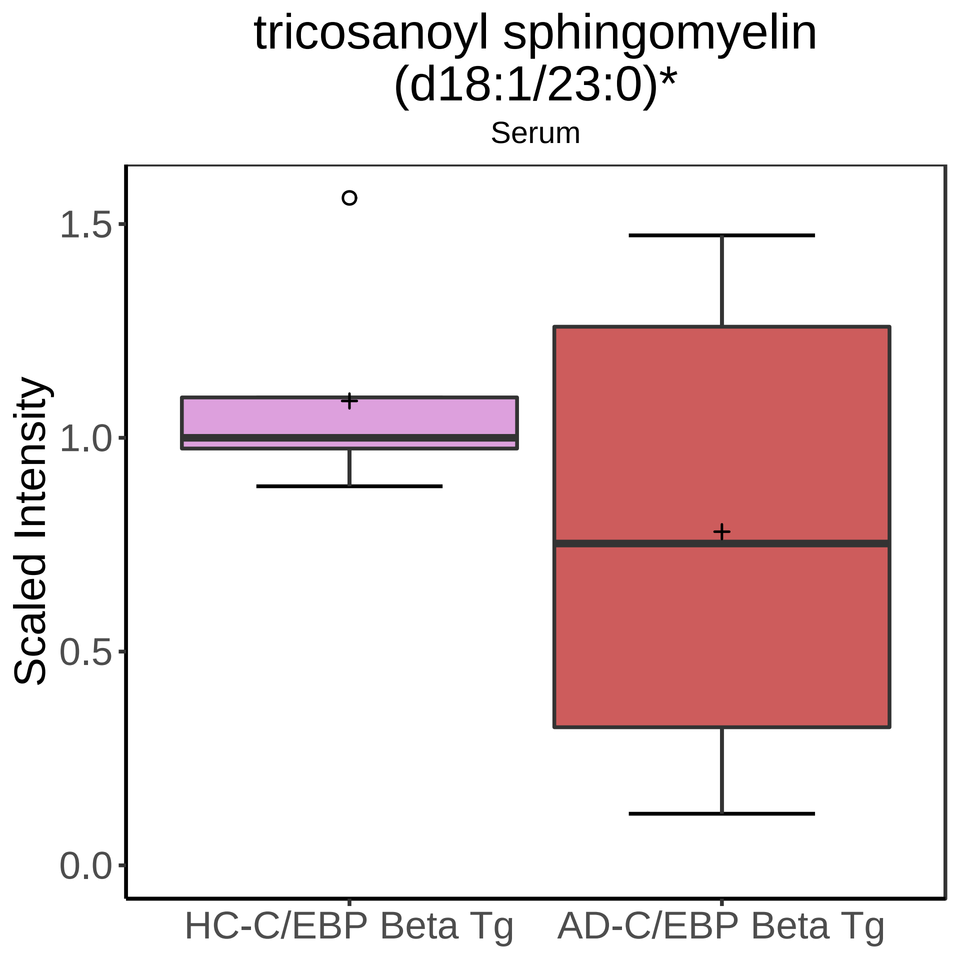
tricosanoyl sphingomyelin
(d18:1/23:0)*
Serum
Scaled Intensity
1.5
1.0
0.5
0.0
HC-C/EBP Beta Tg
AD-C/EBP Beta Tg

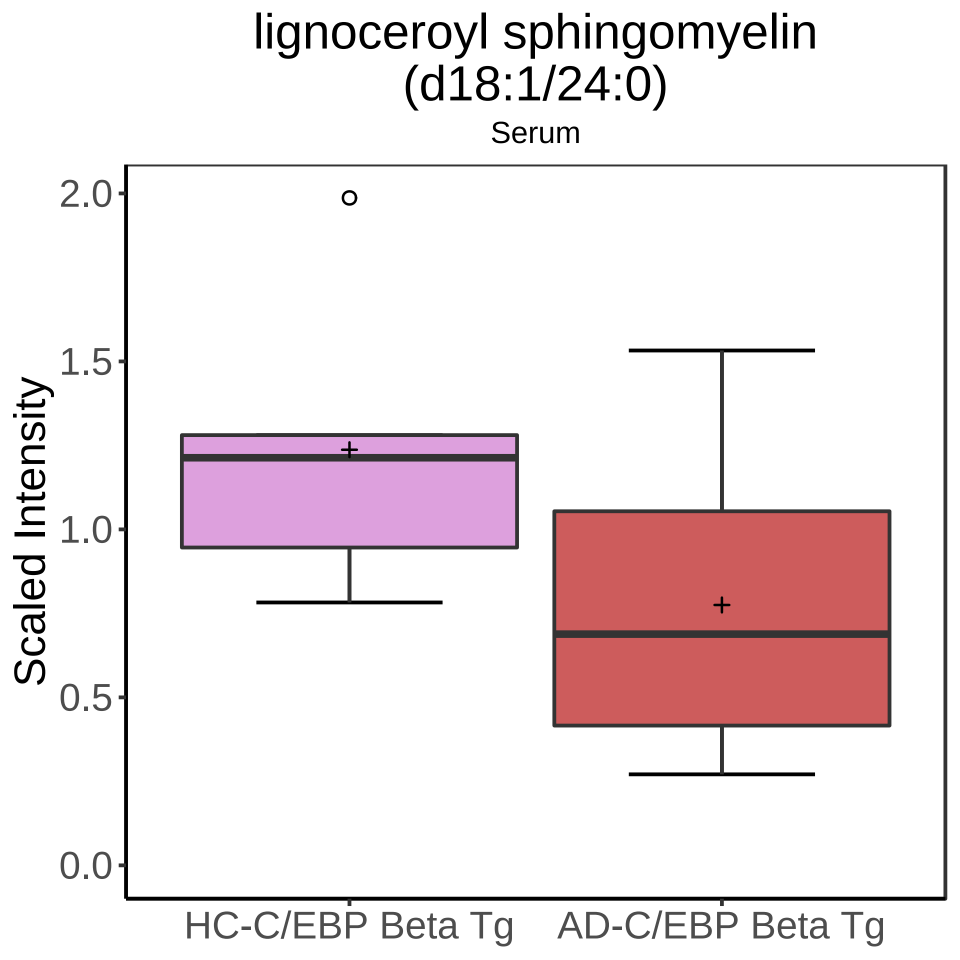
lignoceroyl sphingomyelin
(d18:1/24:0)
Serum
Scaled Intensity
2.0
1.5
1.0
0.5
0.0
HC-C/EBP Beta Tg
AD-C/EBP Beta Tg

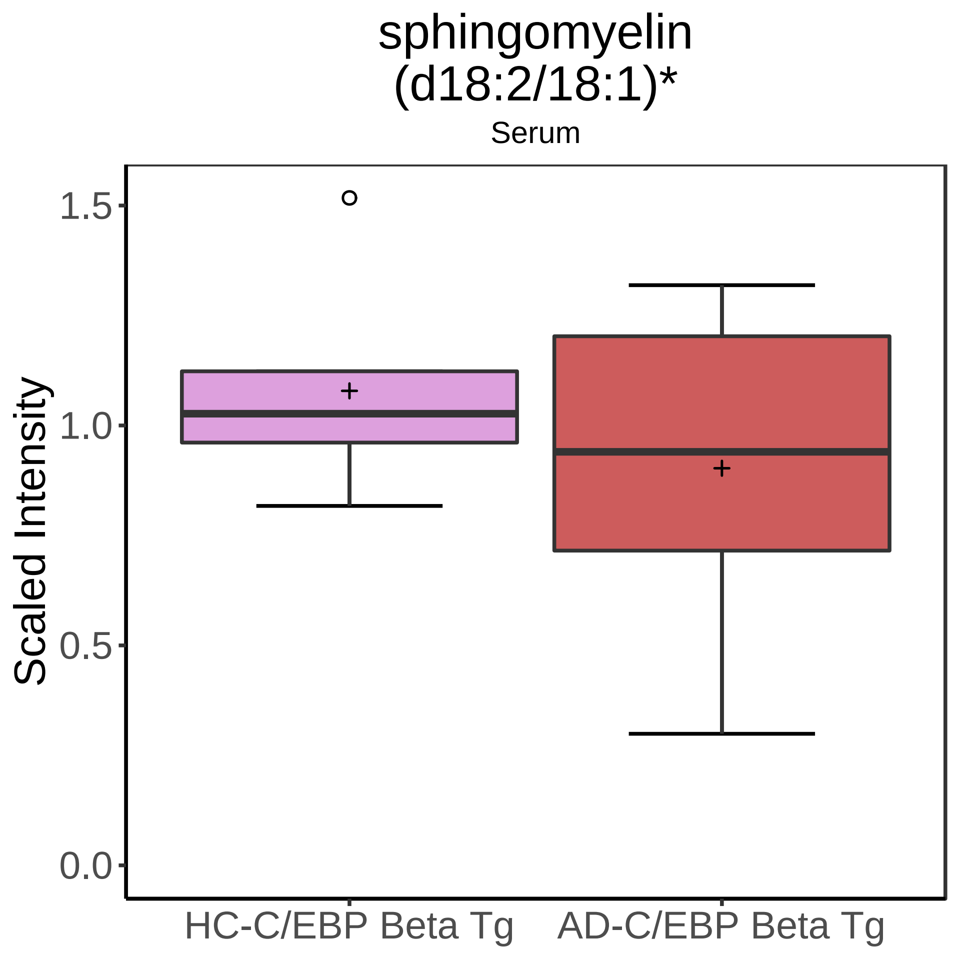
sphingomyelin
(d18:2/18:1)*
Serum
Scaled Intensity
1.5
1.0
0.5
0.0
HC-C/EBP Beta Tg
AD-C/EBP Beta Tg

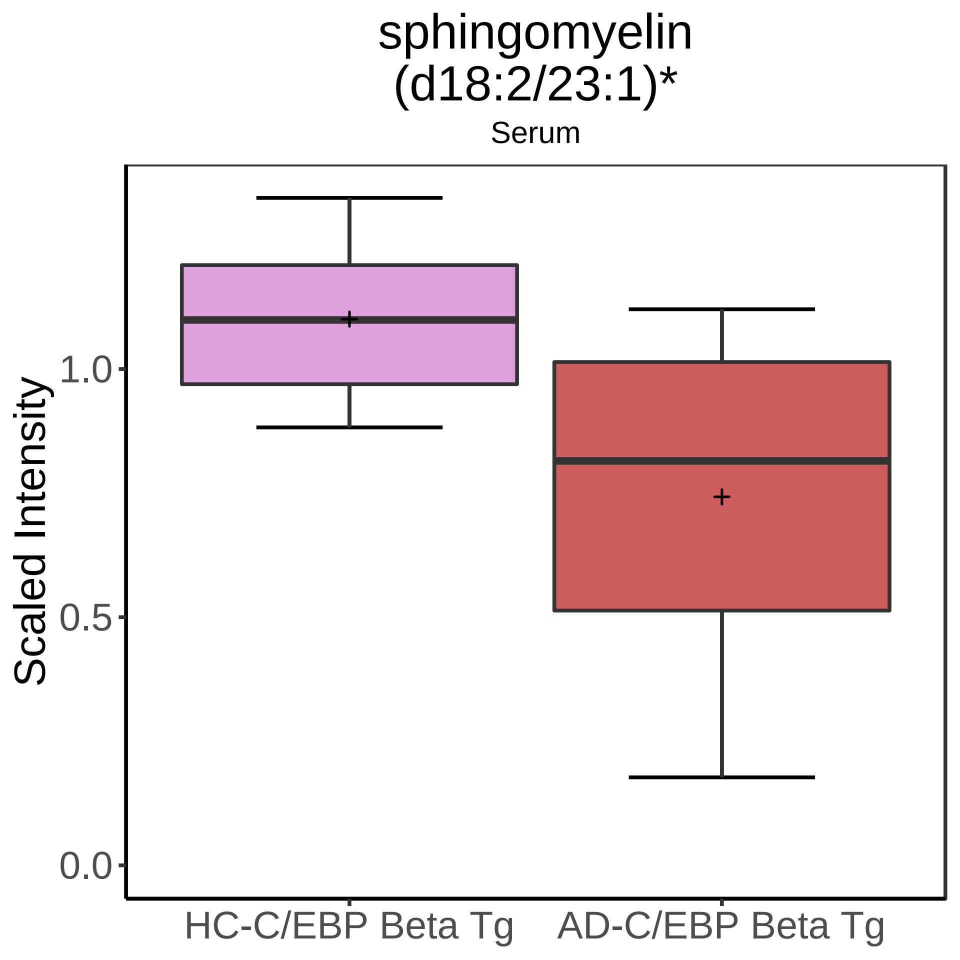
sphingomyelin
(d18:2/23:1)*
Serum
Scaled Intensity
1.0
0.5
0.0
HC-C/EBP Beta Tg
AD-C/EBP Beta Tg

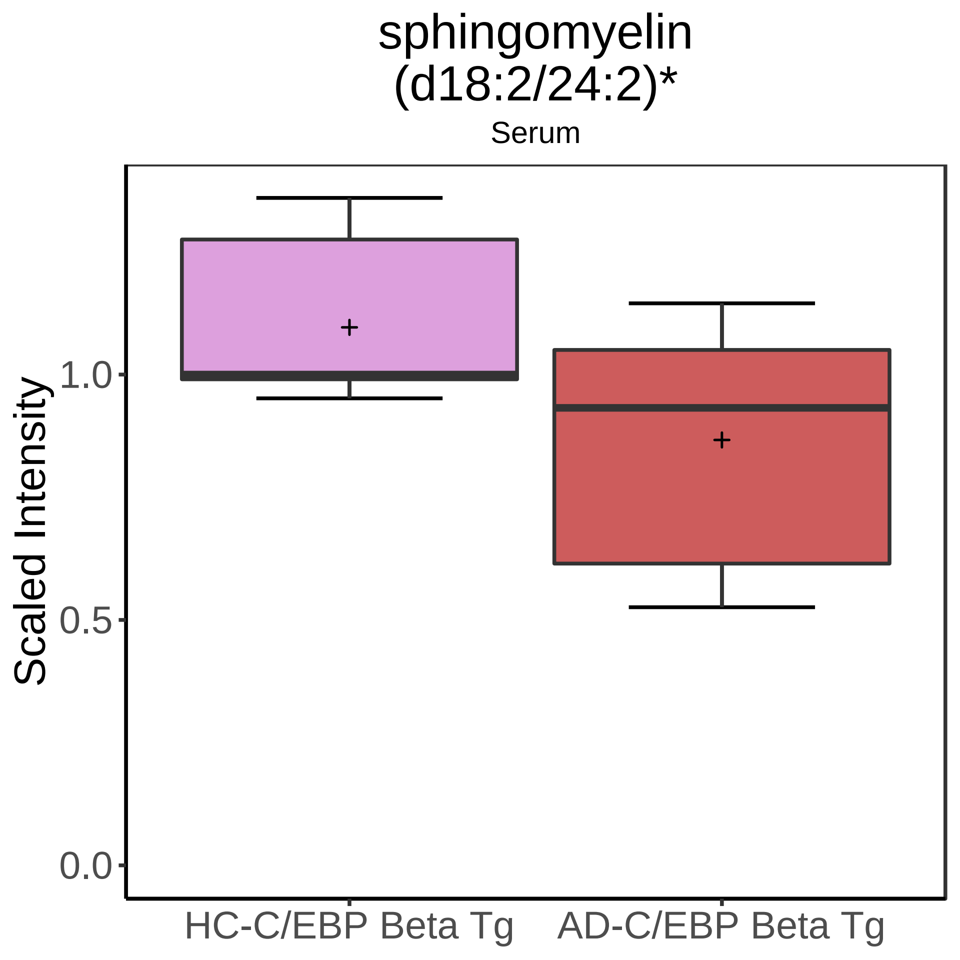
sphingomyelin
(d18:2/24:2)*
Serum
Scaled Intensity
1.0
0.5
0.0
HC-C/EBP Beta Tg
AD-C/EBP Beta Tg

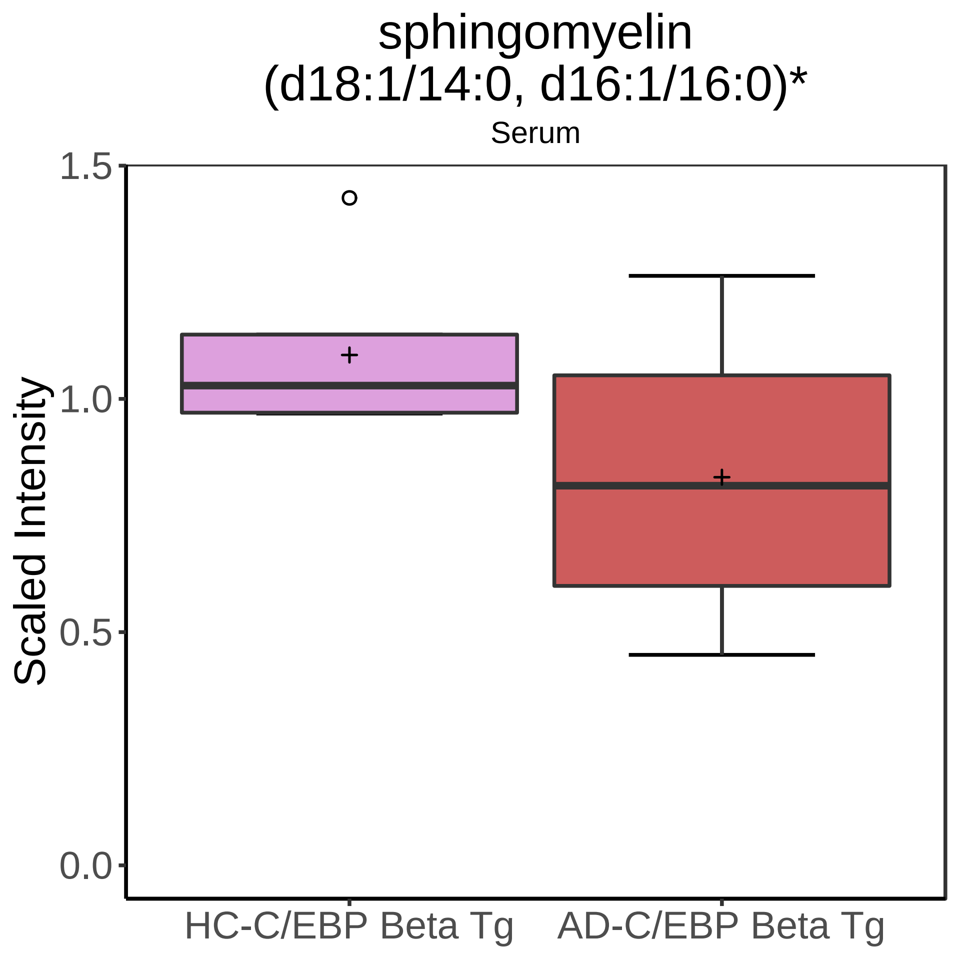
sphingomyelin
(d18:1/14:0, d16:1/16:0)*
Serum
Scaled Intensity
1.5
1.0
0.5
0.0
HC-C/EBP Beta Tg
AD-C/EBP Beta Tg

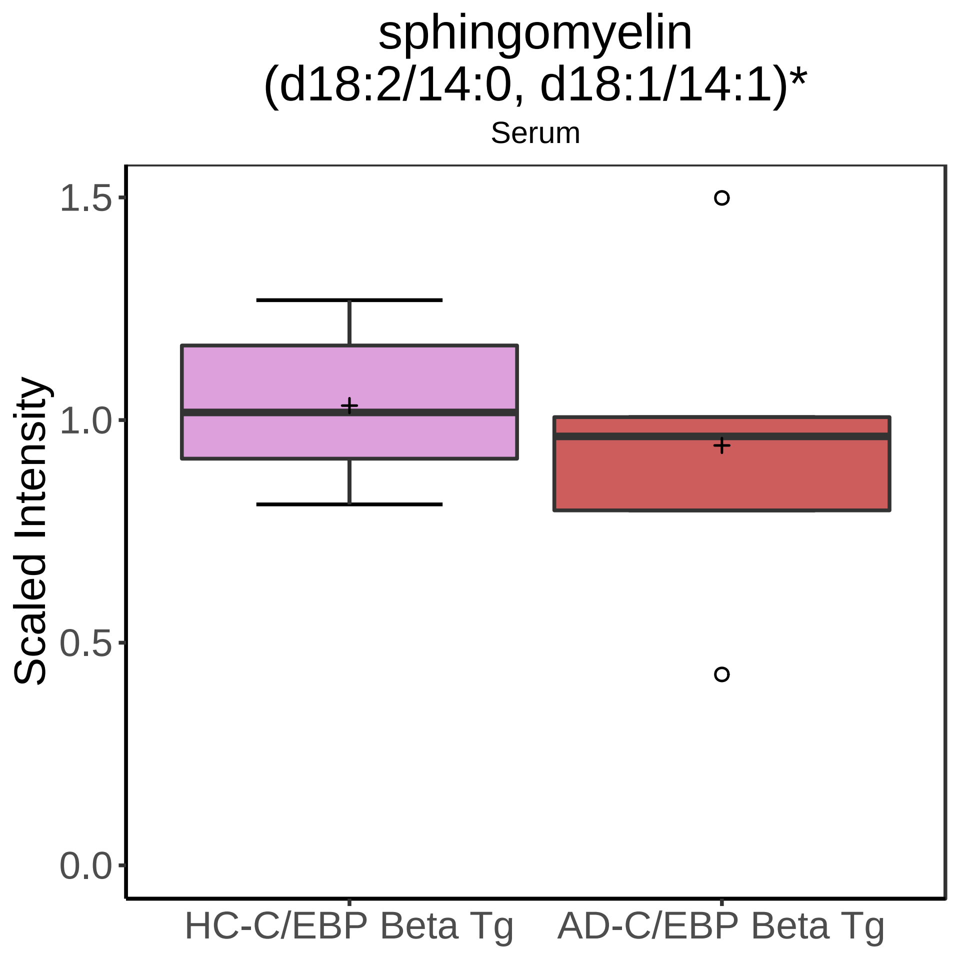
sphingomyelin
(d18:2/14:0, d18:1/14:1)*
Serum
Scaled Intensity
1.5
1.0
0.5
0.0
HC-C/EBP Beta Tg
AD-C/EBP Beta Tg

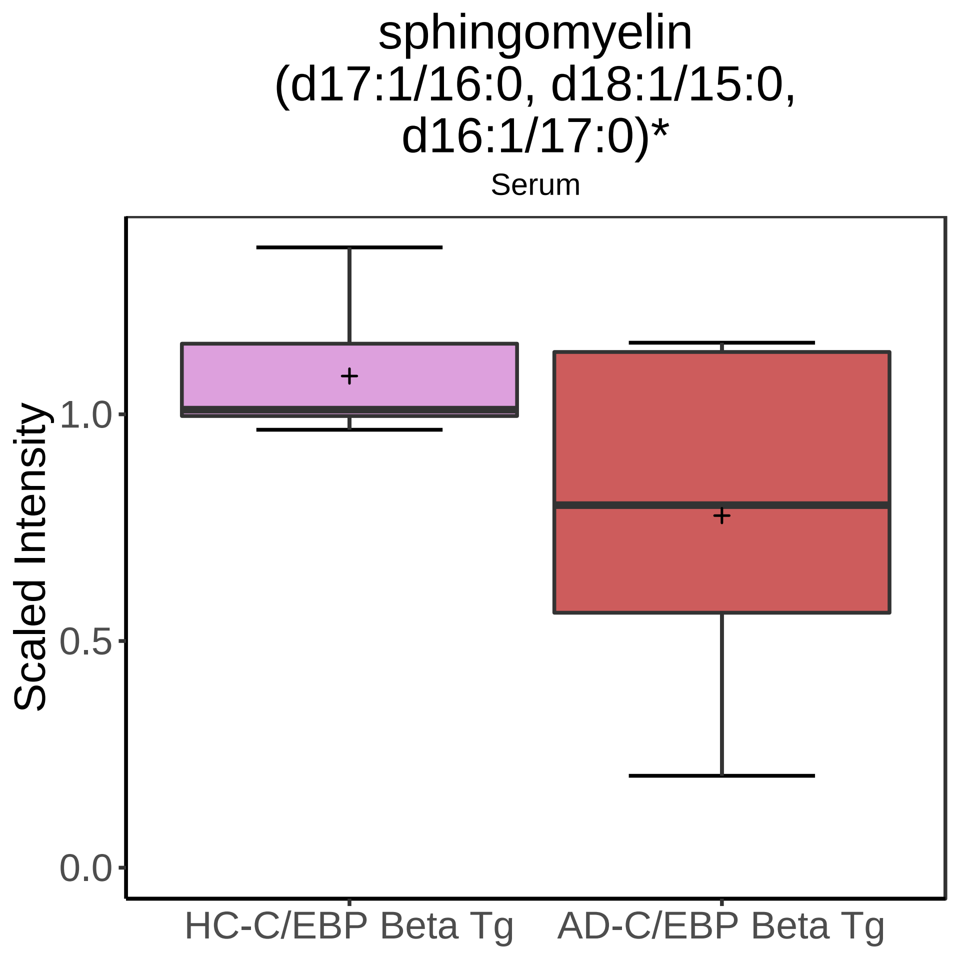
sphingomyelin
(d17:1/16:0, d18:1/15:0,
d16:1/17:0)*
Serum
Scaled Intensity
1.0
0.5
0.0
HC-C/EBP Beta Tg
AD-C/EBP Beta Tg

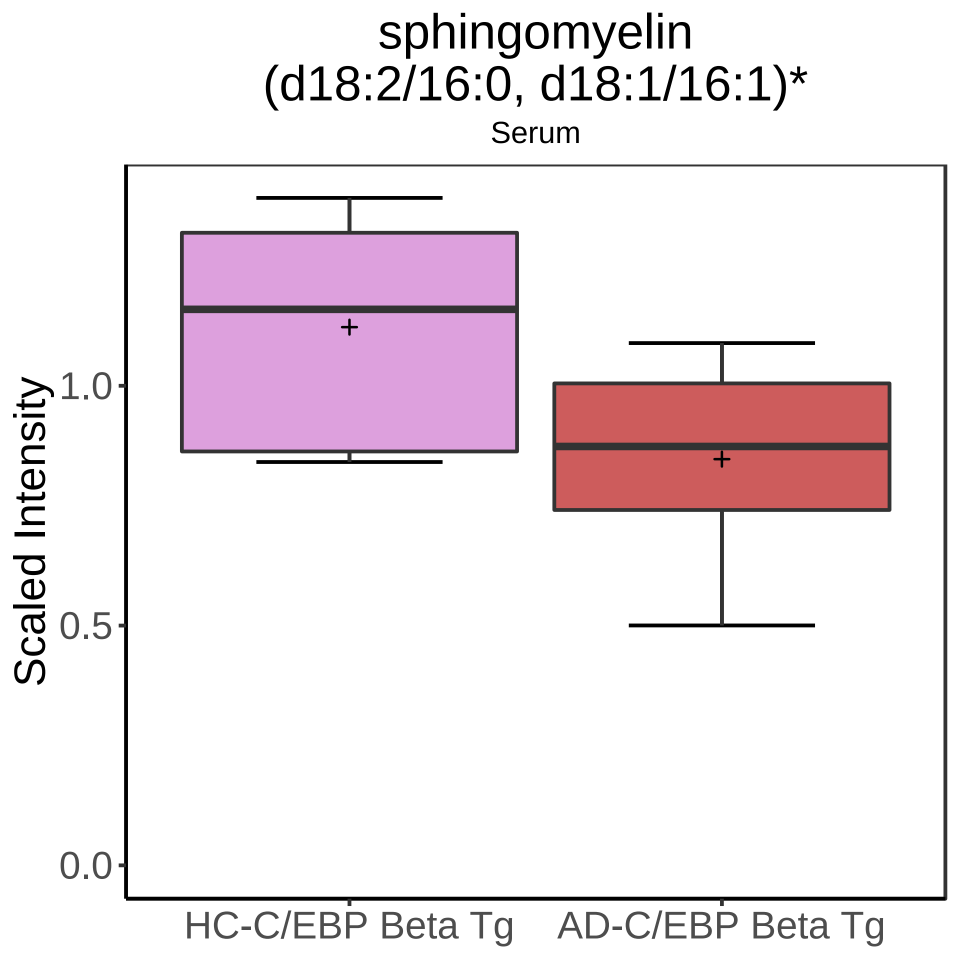
sphingomyelin
(d18:2/16:0, d18:1/16:1)*
Serum
Scaled Intensity
1.0
0.5
0.0
HC-C/EBP Beta Tg
AD-C/EBP Beta Tg

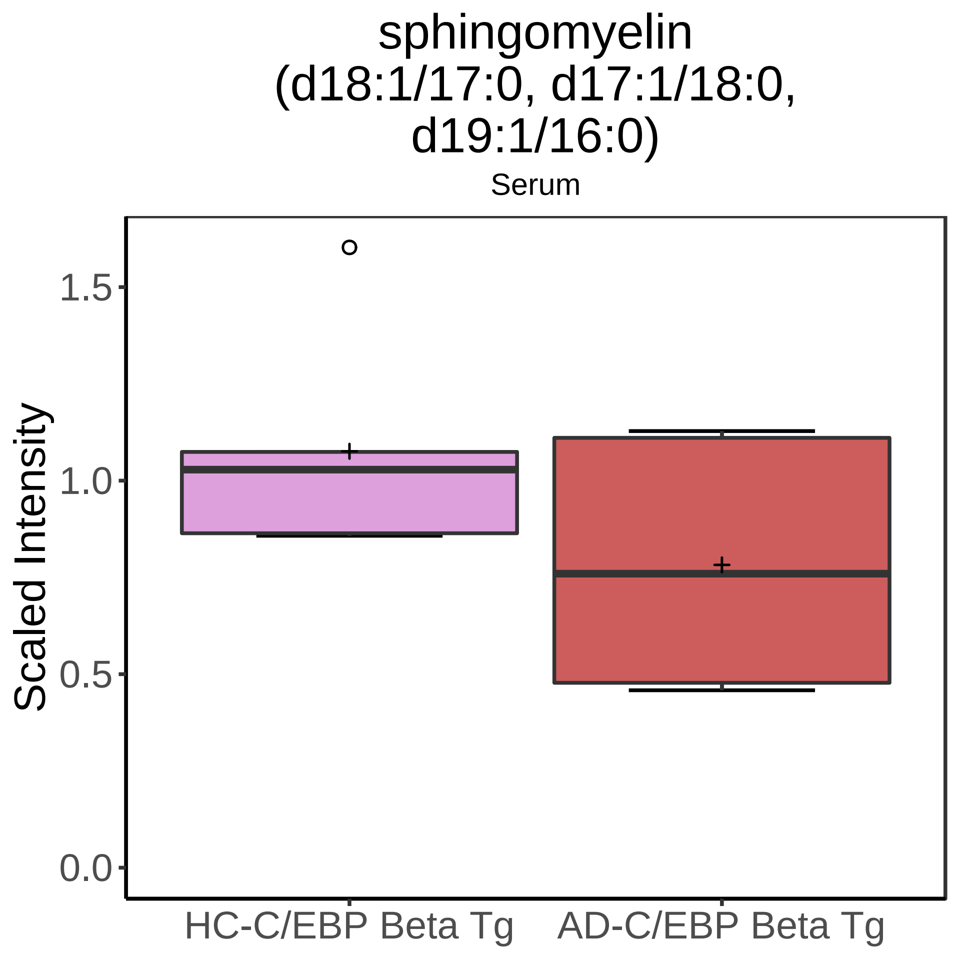

sphingomyelin
(d18:1/17:0, d17:1/18:0,
d19:1/16:0)
Serum
Scaled Intensity
1.5
1.0
0.5
0.0
HC-C/EBP Beta Tg
AD-C/EBP Beta Tg

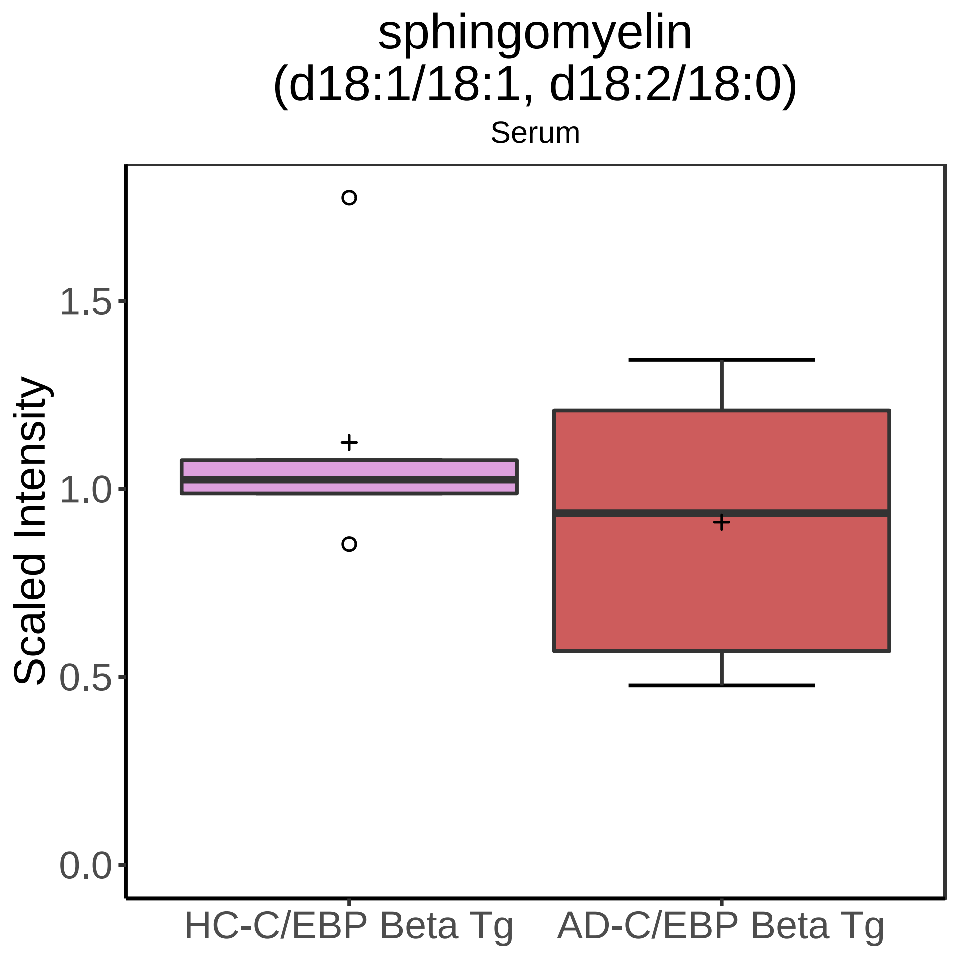
sphingomyelin
(d18:1/18:1, d18:2/18:0)
Serum
Scaled Intensity
1.5
1.0
0.5
0.0
HC-C/EBP Beta Tg
AD-C/EBP Beta Tg

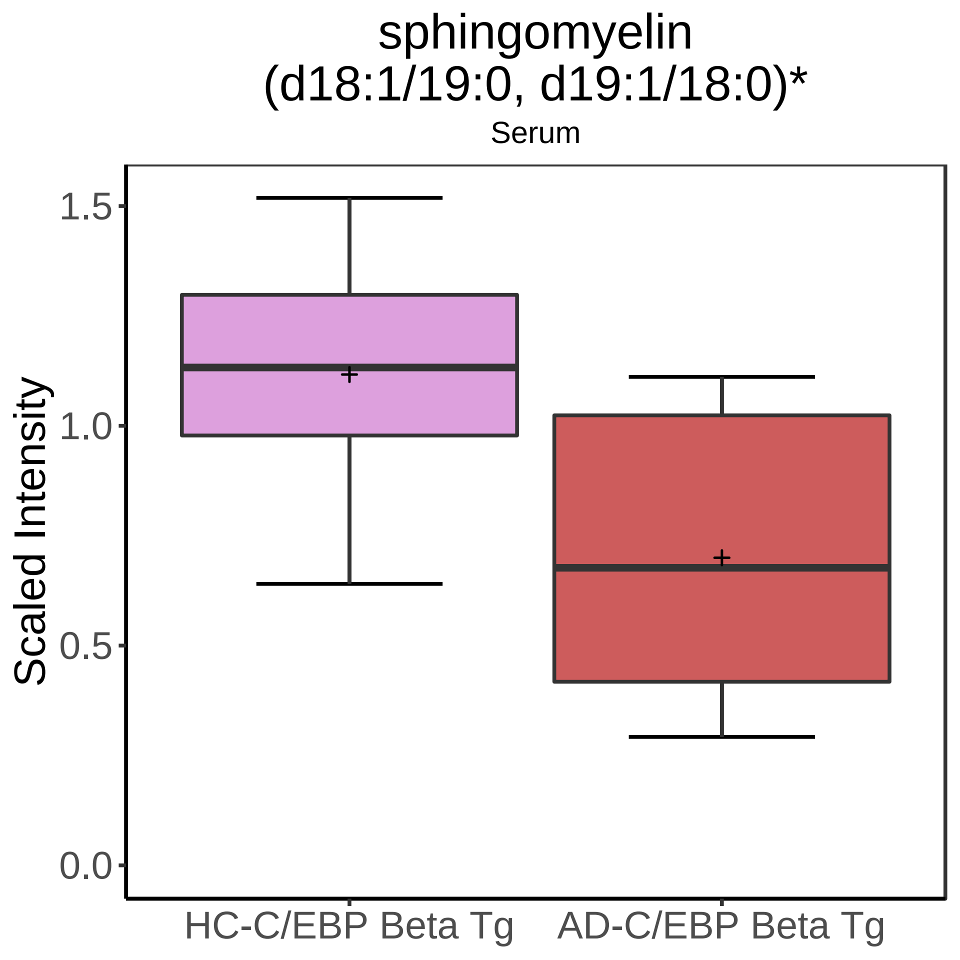

sphingomyelin
(d18:1/19:0, d19:1/18:0)*
Serum
Scaled Intensity
1.5
1.0
0.5
0.0
HC-C/EBP Beta Tg
AD-C/EBP Beta Tg

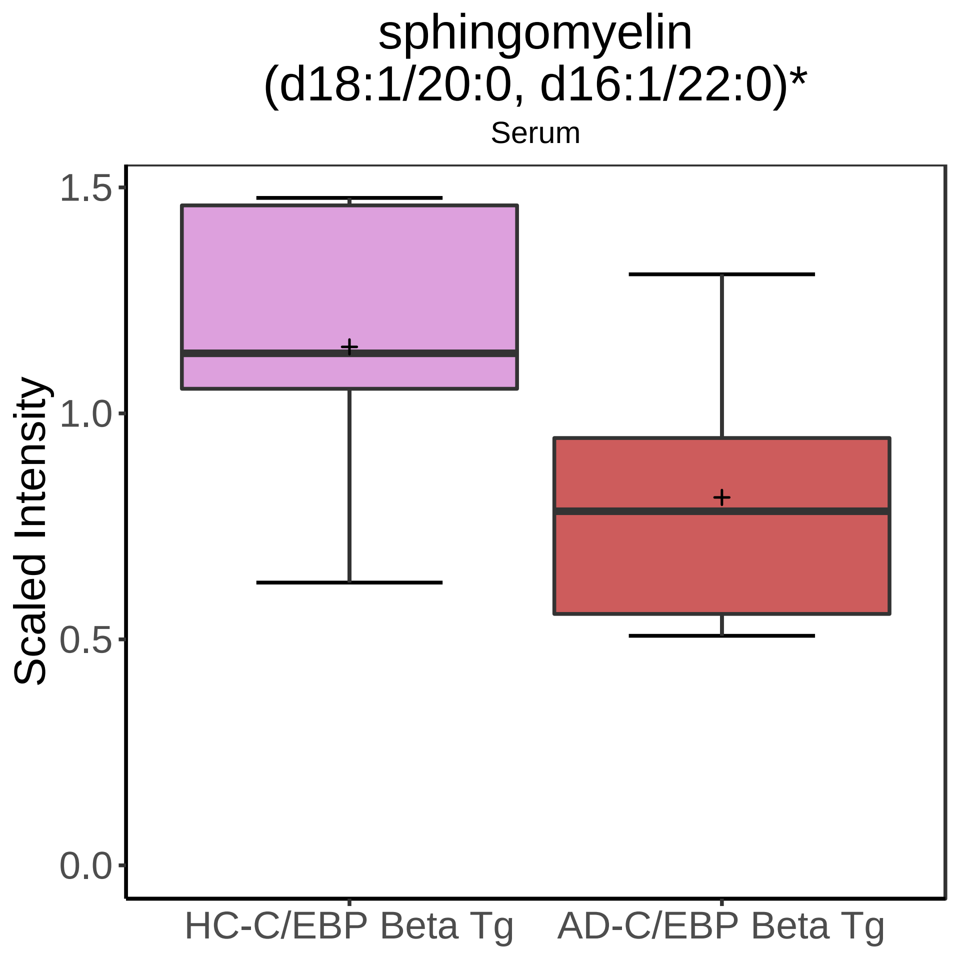

sphingomyelin
(d18:1/20:0, d16:1/22:0)*
Serum
Scaled Intensity
1.5
1.0
0.5
0.0
HC-C/EBP Beta Tg
AD-C/EBP Beta Tg

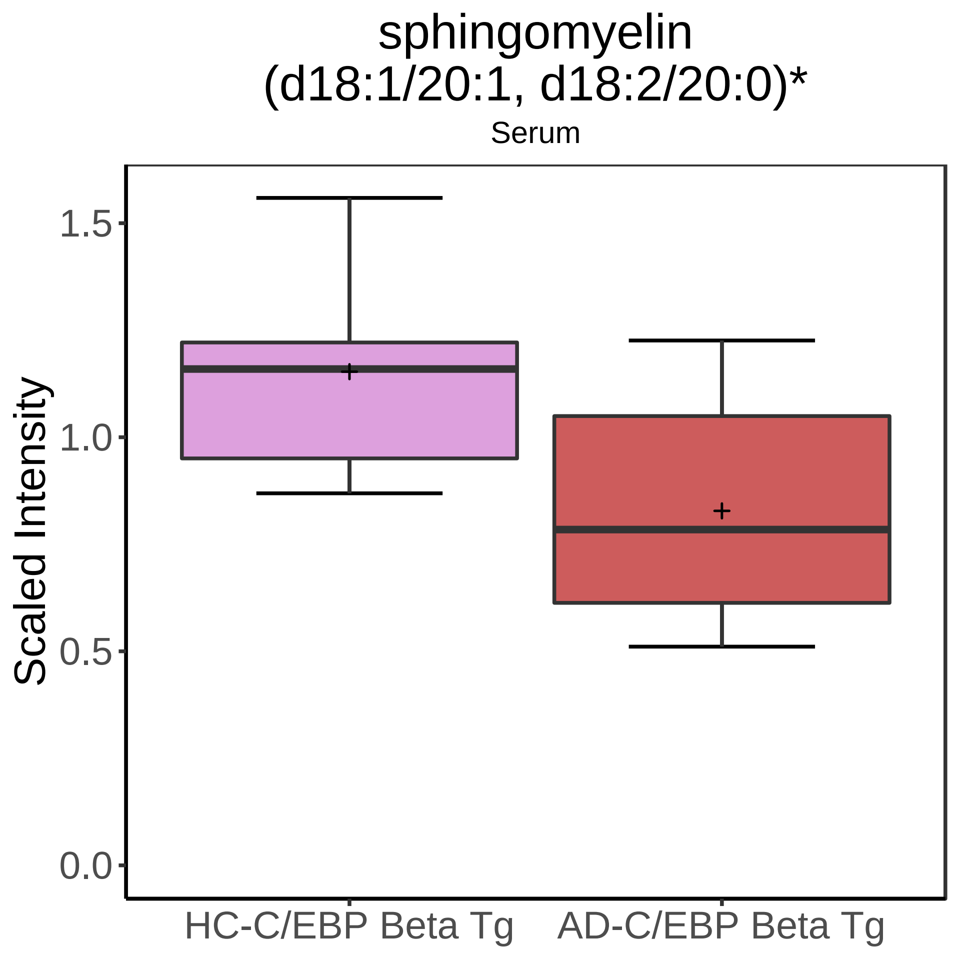
sphingomyelin
(d18:1/20:1, d18:2/20:0)*
Serum
Scaled Intensity
1.5
1.0
0.5
0.0
HC-C/EBP Beta Tg
AD-C/EBP Beta Tg

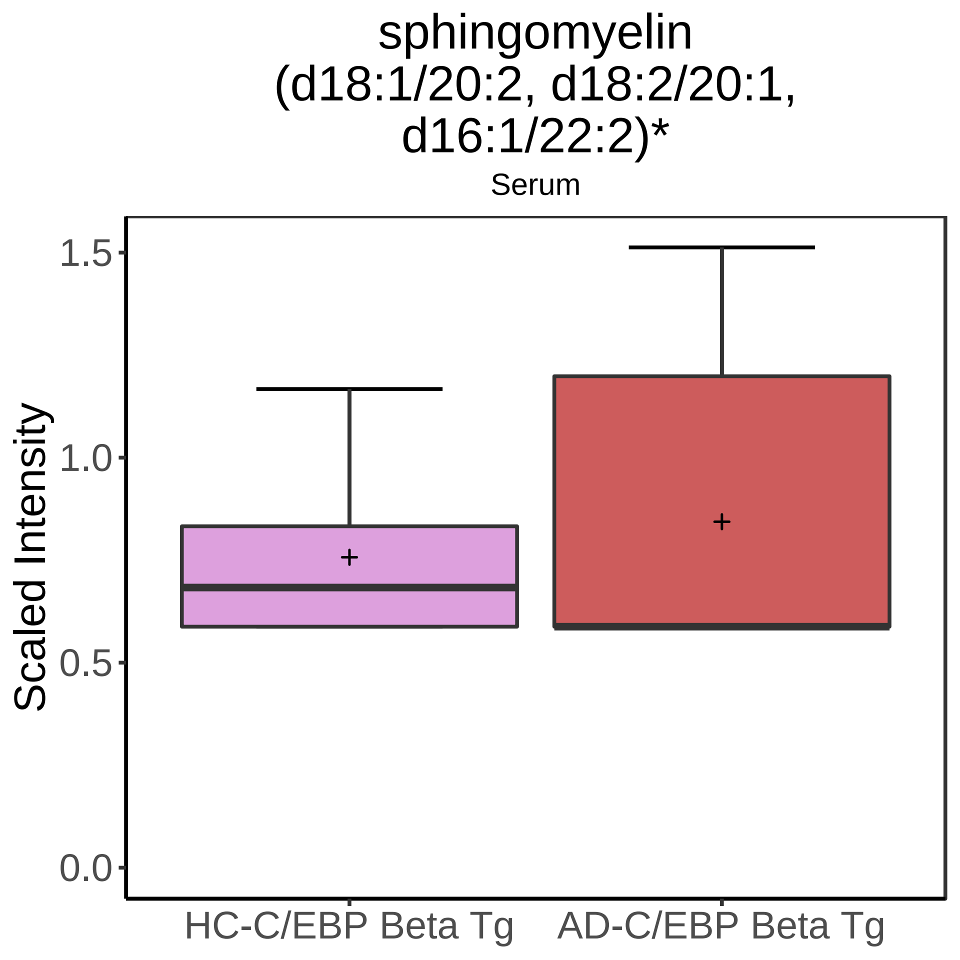
sphingomyelin
(d18:1/20:2, d18:2/20:1,
d16:1/22:2)*
Serum
Scaled Intensity
1.5
1.0
0.5
0.0
HC-C/EBP Beta Tg
AD-C/EBP Beta Tg

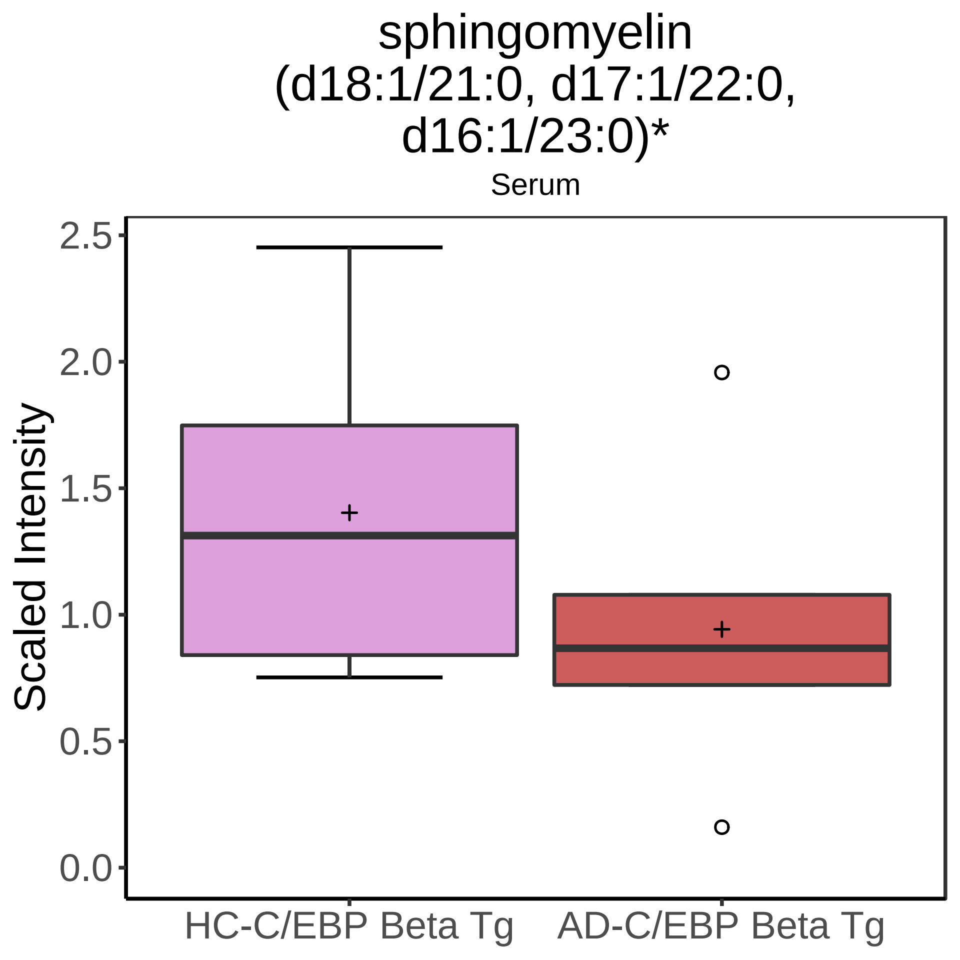
sphingomyelin
(d18:1/21:0, d17:1/22:0,
d16:1/23:0)*
Serum
Scaled Intensity
2.5
2.0
1.5
1.0
0.5
0.0
HC-C/EBP Beta Tg
AD-C/EBP Beta Tg

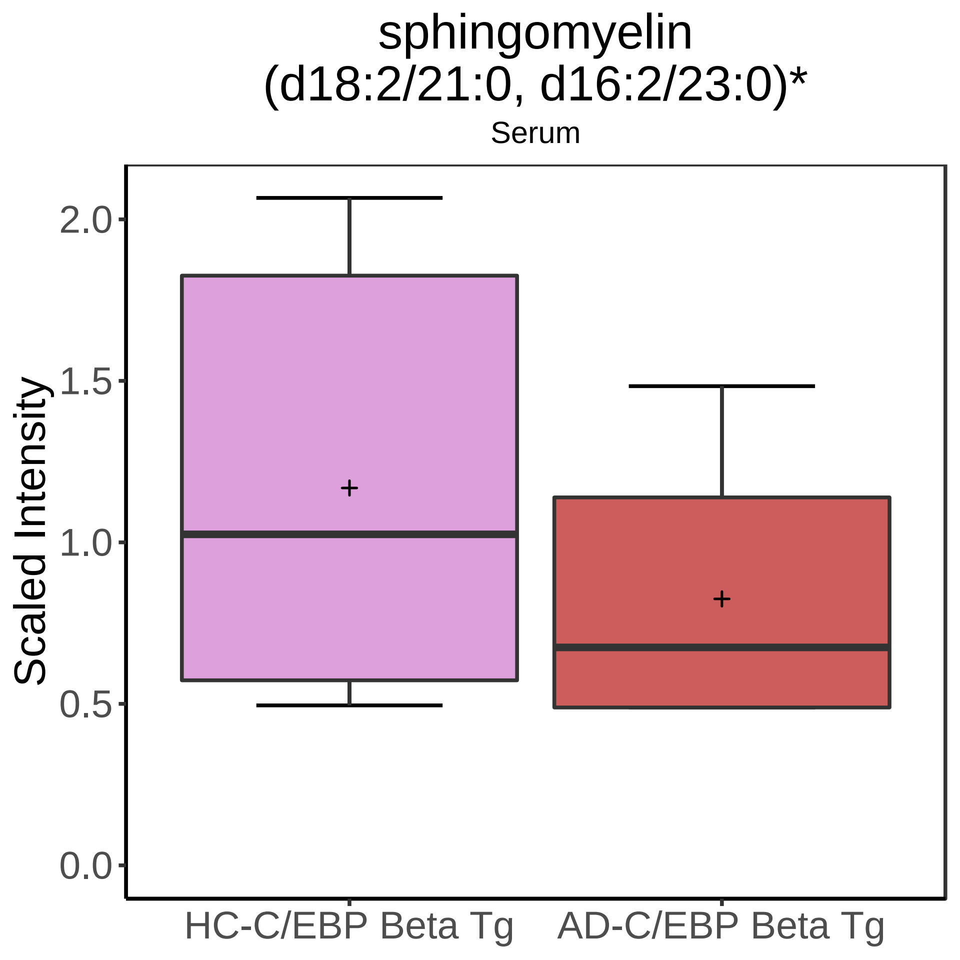

sphingomyelin
(d18:2/21:0, d16:2/23:0)*
Serum
Scaled Intensity
2.0
1.5
1.0
0.5
0.0
HC-C/EBP Beta Tg
AD-C/EBP Beta Tg

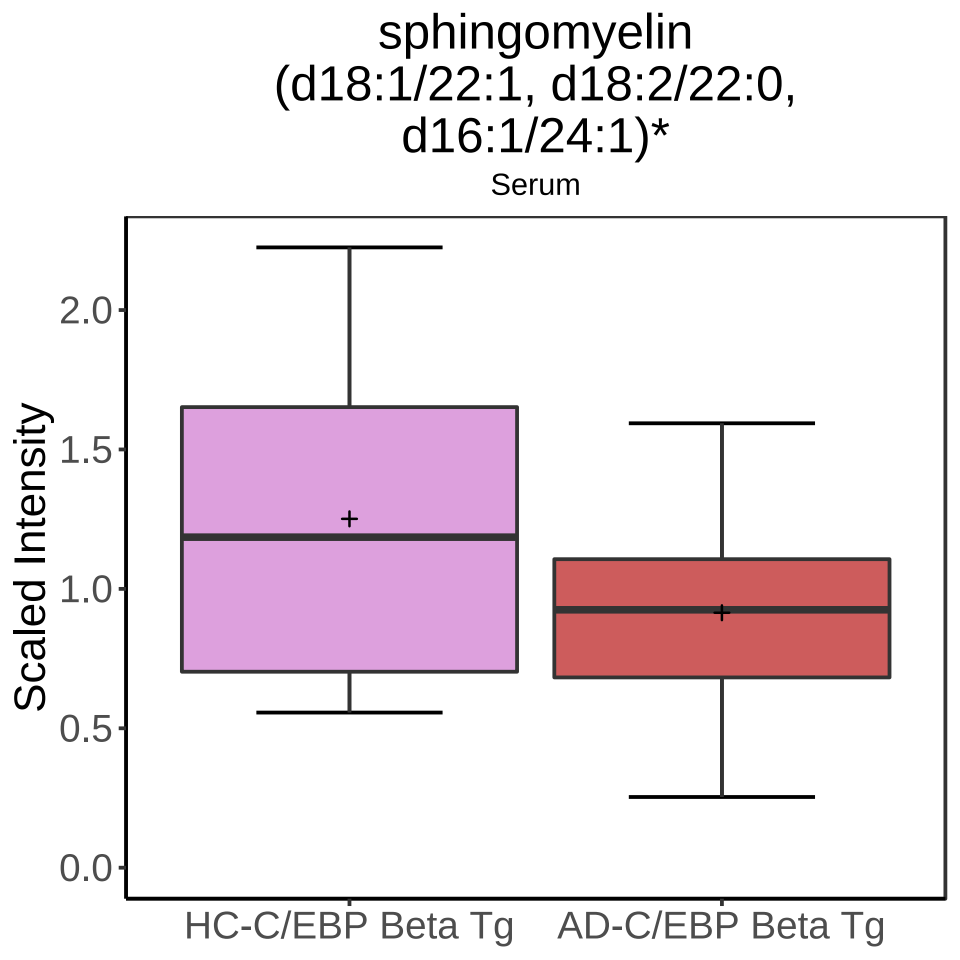
sphingomyelin
(d18:1/22:1, d18:2/22:0,
d16:1/24:1)*
Serum
Scaled Intensity
2.0
1.5
1.0
0.5
0.0
HC-C/EBP Beta Tg
AD-C/EBP Beta Tg

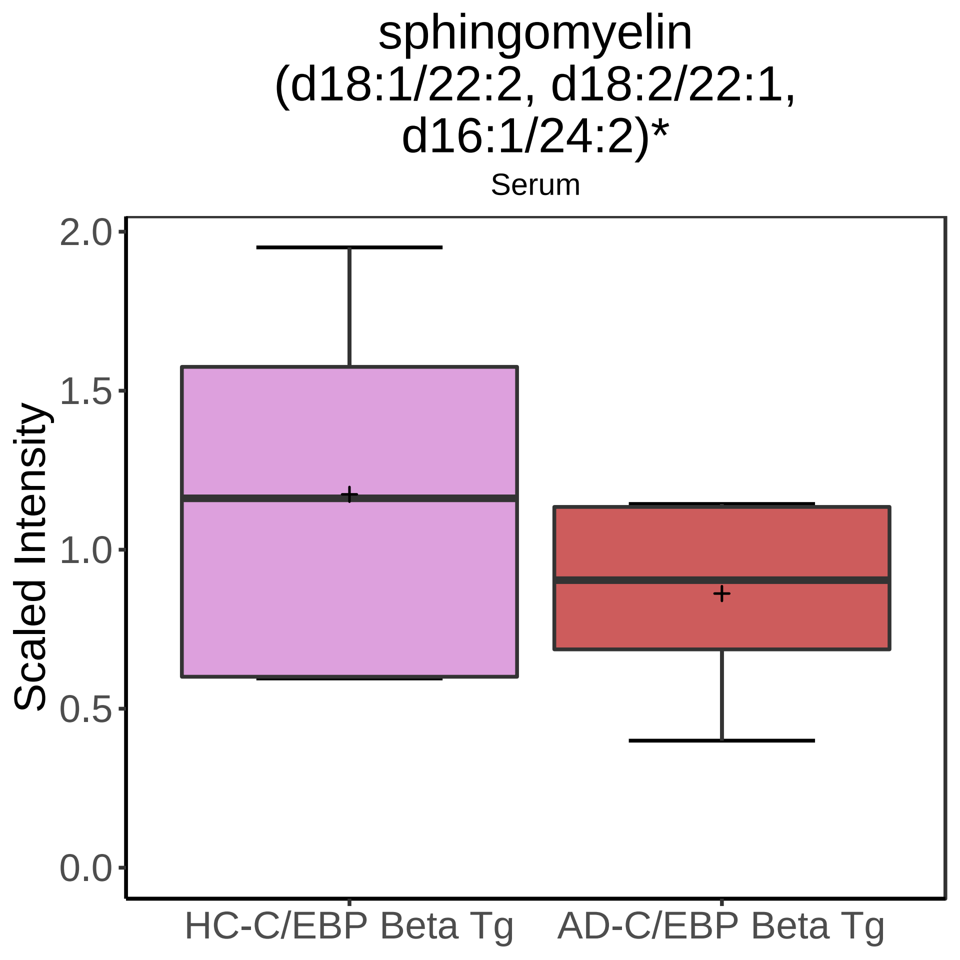

sphingomyelin
(d18:1/22:2, d18:2/22:1,
d16:1/24:2)*
Serum
Scaled Intensity
2.0
1.5
1.0
0.5
0.0
HC-C/EBP Beta Tg
AD-C/EBP Beta Tg

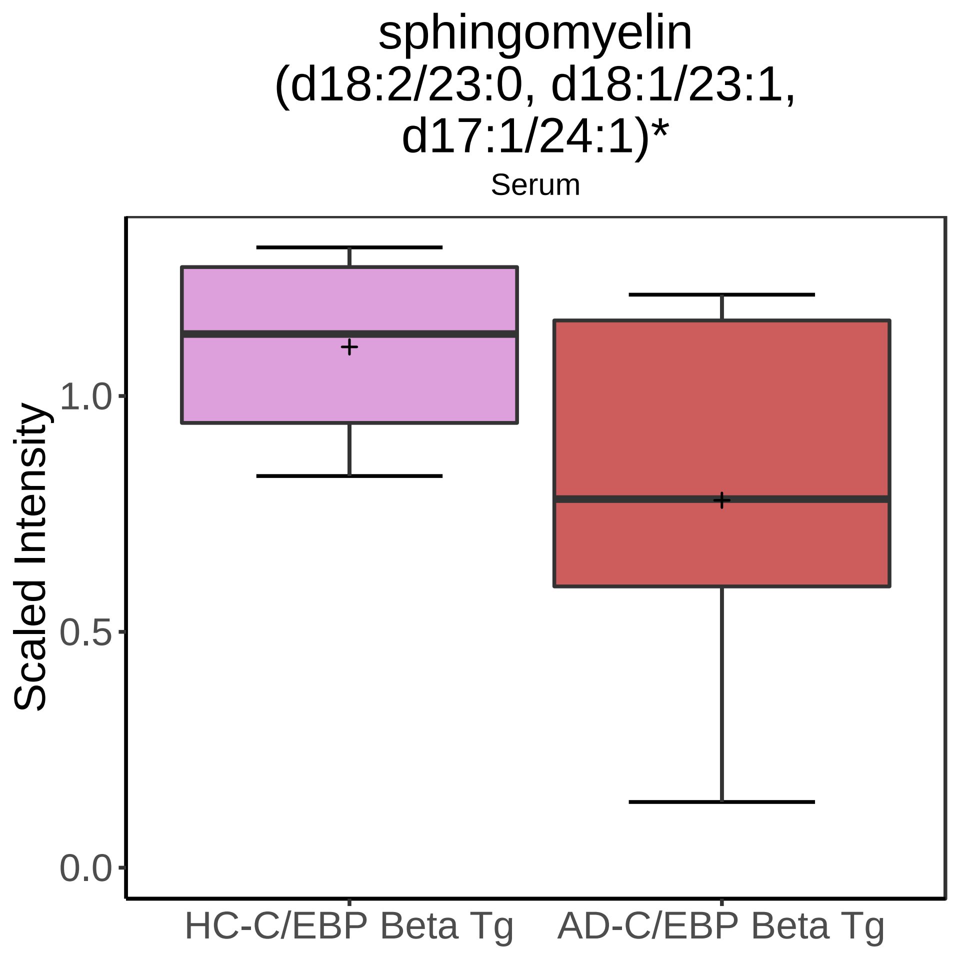
sphingomyelin
(d18:2/23:0, d18:1/23:1,
d17:1/24:1)*
Serum
Scaled Intensity
1.0
0.5
0.0
HC-C/EBP Beta Tg
AD-C/EBP Beta Tg

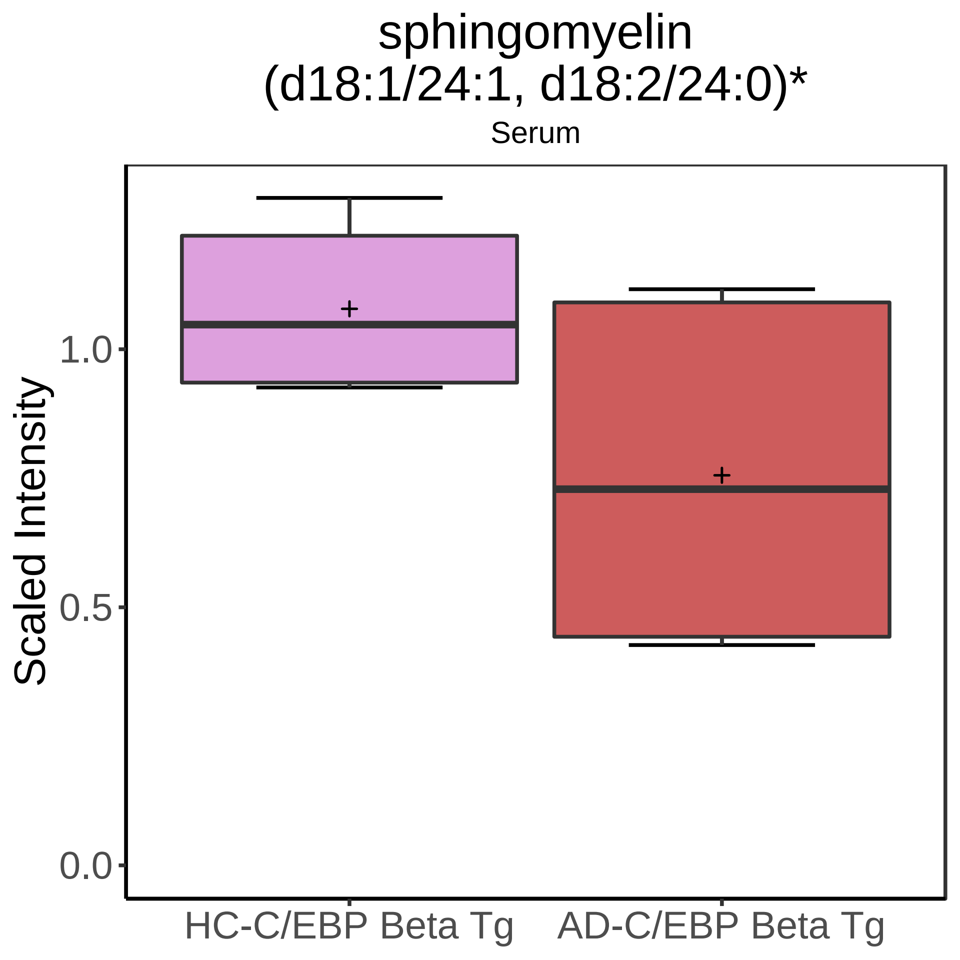
sphingomyelin
(d18:1/24:1, d18:2/24:0)*
Serum
Scaled Intensity
1.0
0.5
0.0
HC-C/EBP Beta Tg
AD-C/EBP Beta Tg

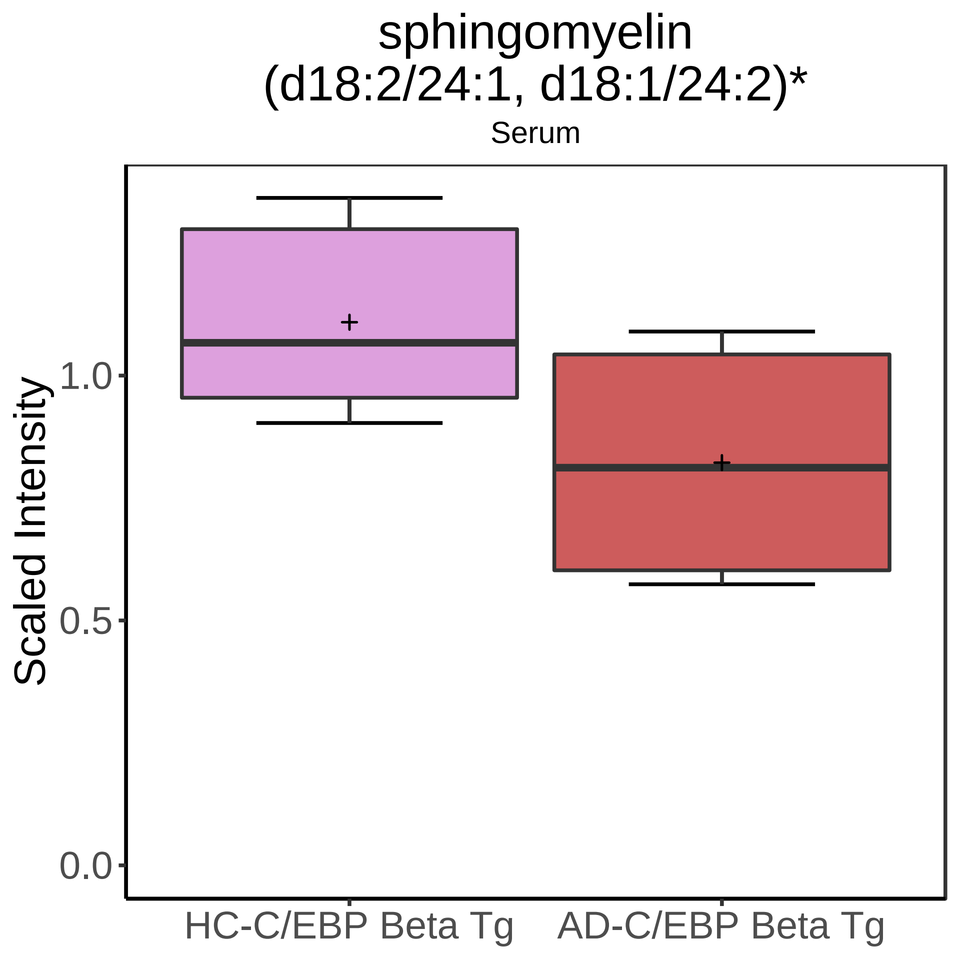
sphingomyelin
(d18:2/24:1, d18:1/24:2)*
Serum
Scaled Intensity
1.0
0.5
0.0
HC-C/EBP Beta Tg
AD-C/EBP Beta Tg

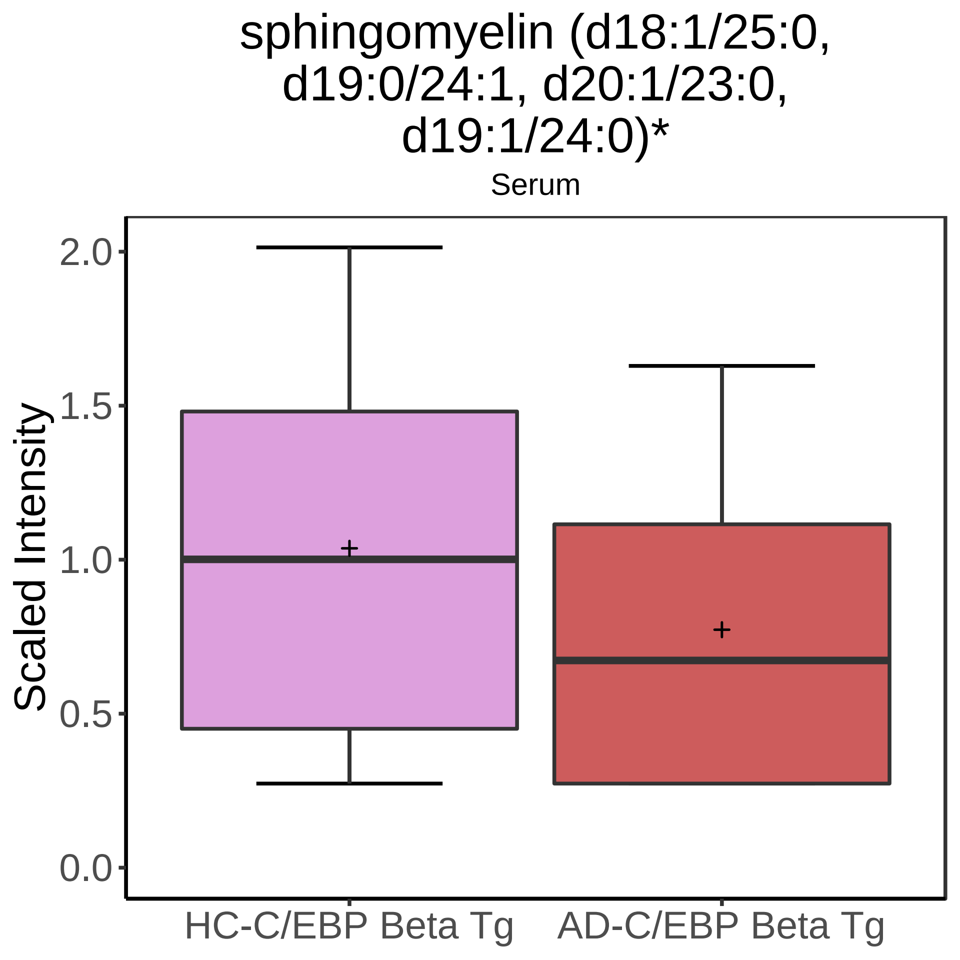
sphingomyelin (d18:1/25:0, d19:0/24:1, d20:1/23:0, d19:1/24:0)*
Serum
Scaled Intensity
2.0
1.5
1.0
0.5
0.0
HC-C/EBP Beta Tg
AD-C/EBP Beta Tg

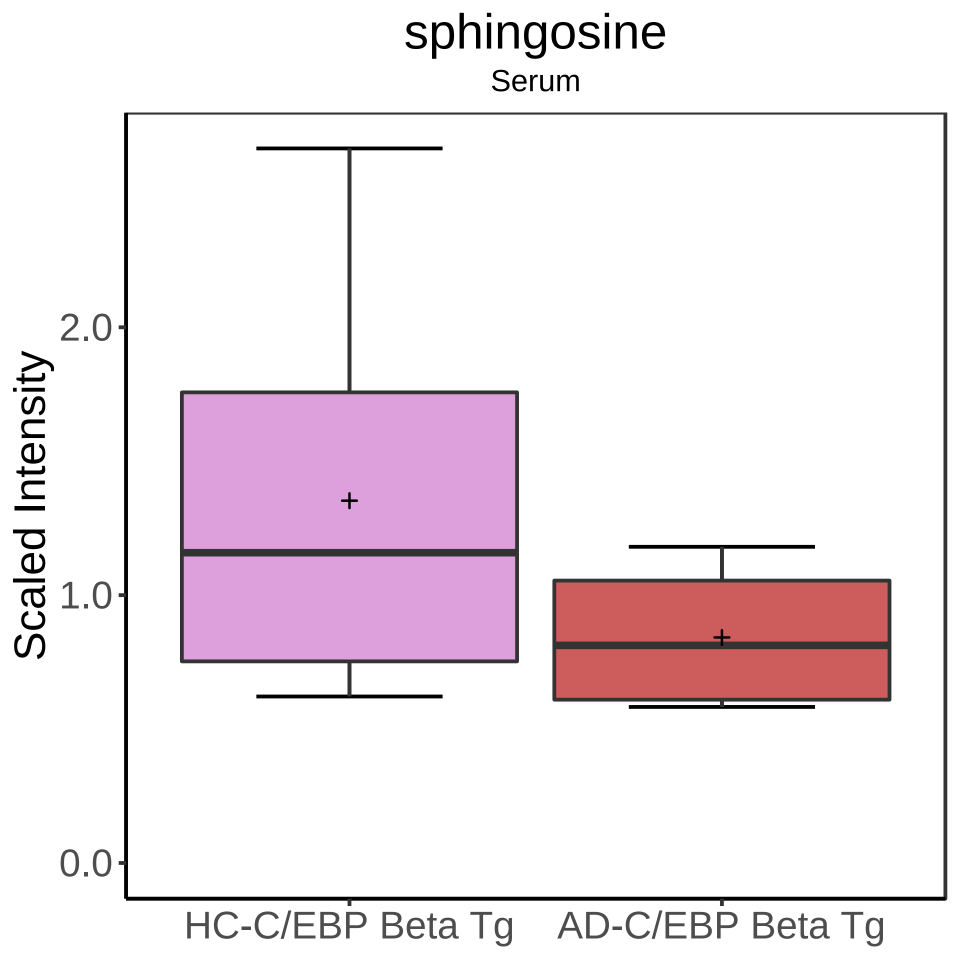
sphingosine
Serum
Scaled Intensity
2.0
1.0
0.0
HC-C/EBP Beta Tg
AD-C/EBP Beta Tg

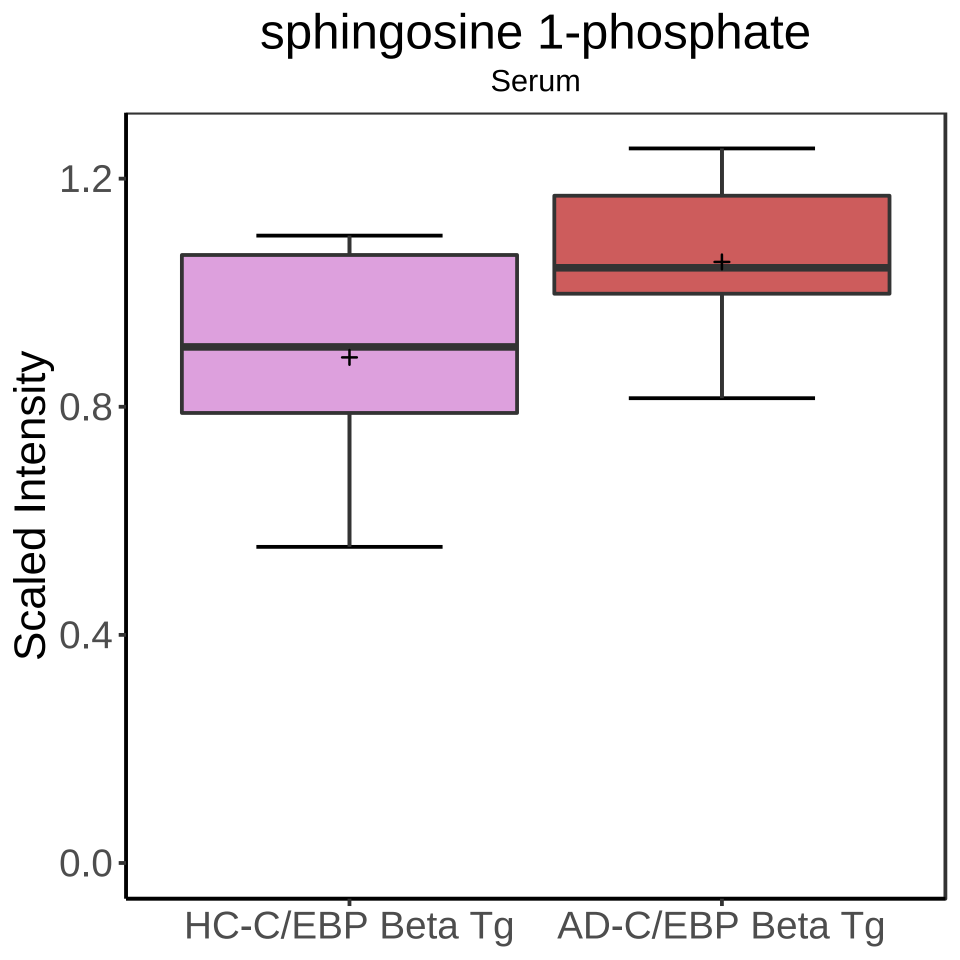
sphingosine 1-phosphate
Serum
Scaled Intensity
1.2
0.8
0.4
0.0
HC-C/EBP Beta Tg
AD-C/EBP Beta Tg

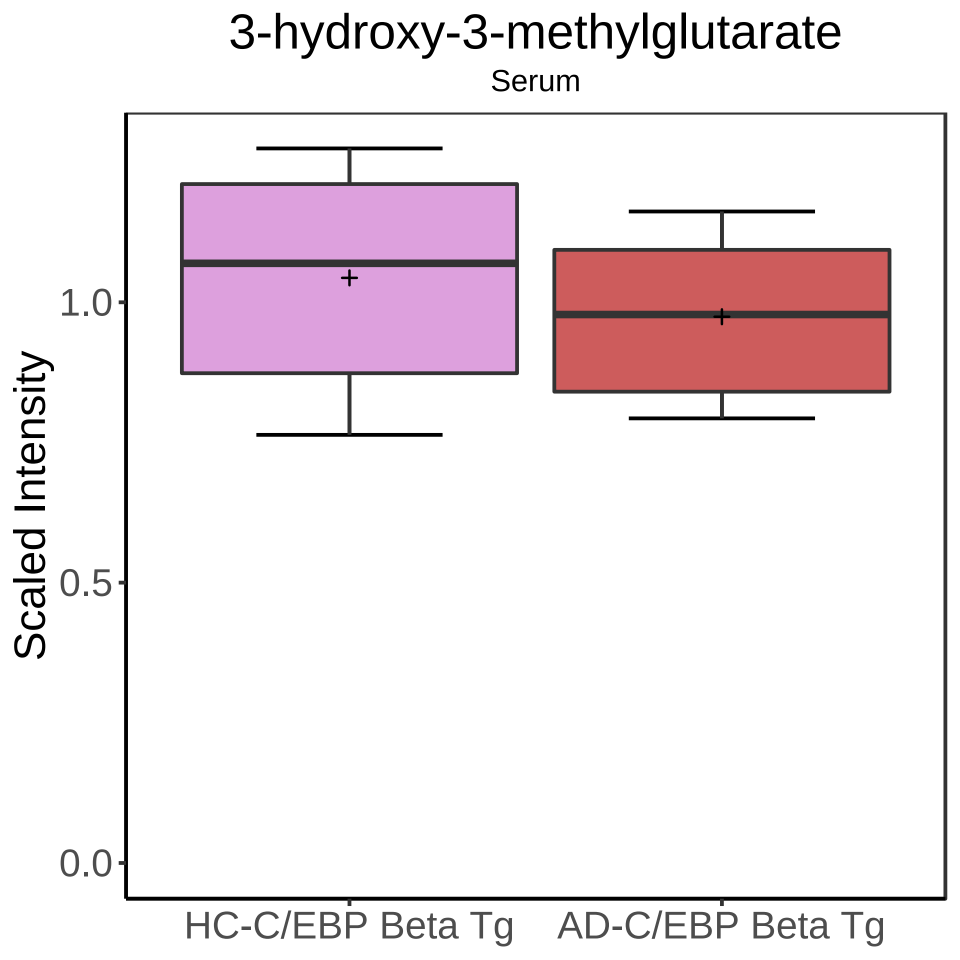
3-hydroxy-3-methylglutarate
Serum
Scaled Intensity
1.0
0.5
0.0
HC-C/EBP Beta Tg
AD-C/EBP Beta Tg

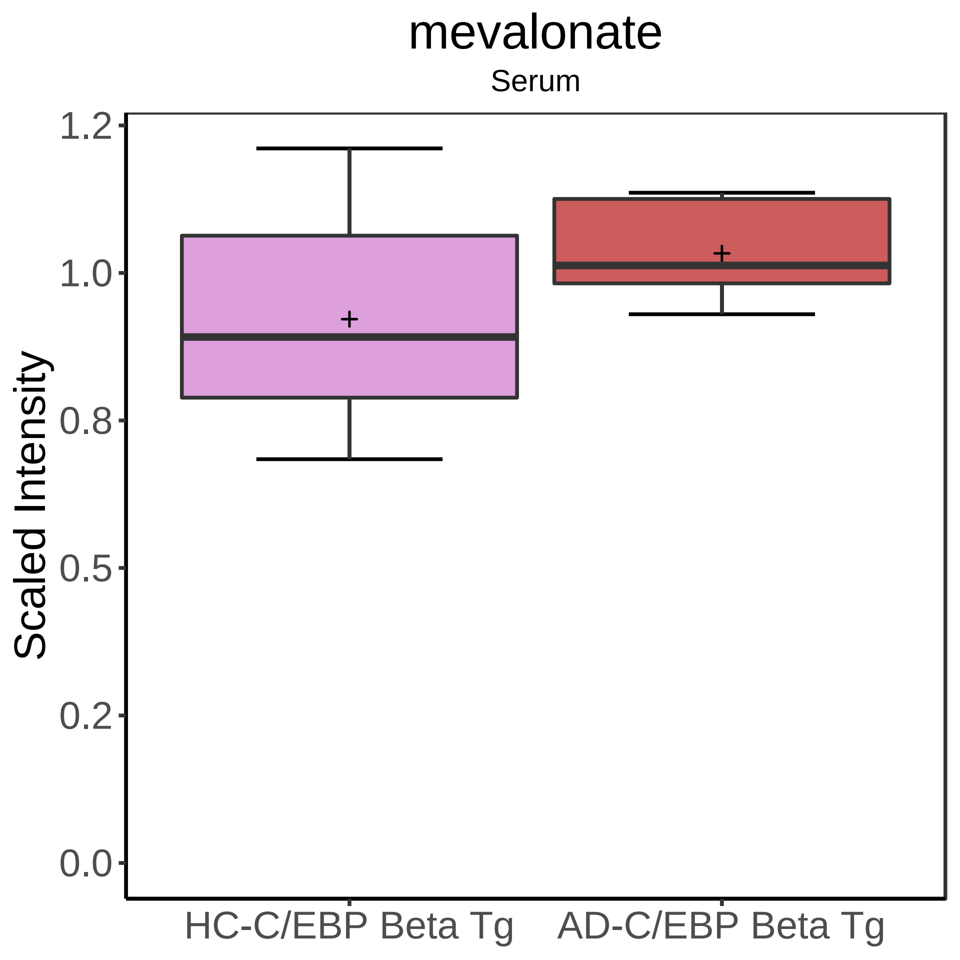

mevalonate
Serum
Scaled Intensity
1.2
1.0
0.8
0.5
0.2
0.0
HC-C/EBP Beta Tg
AD-C/EBP Beta Tg

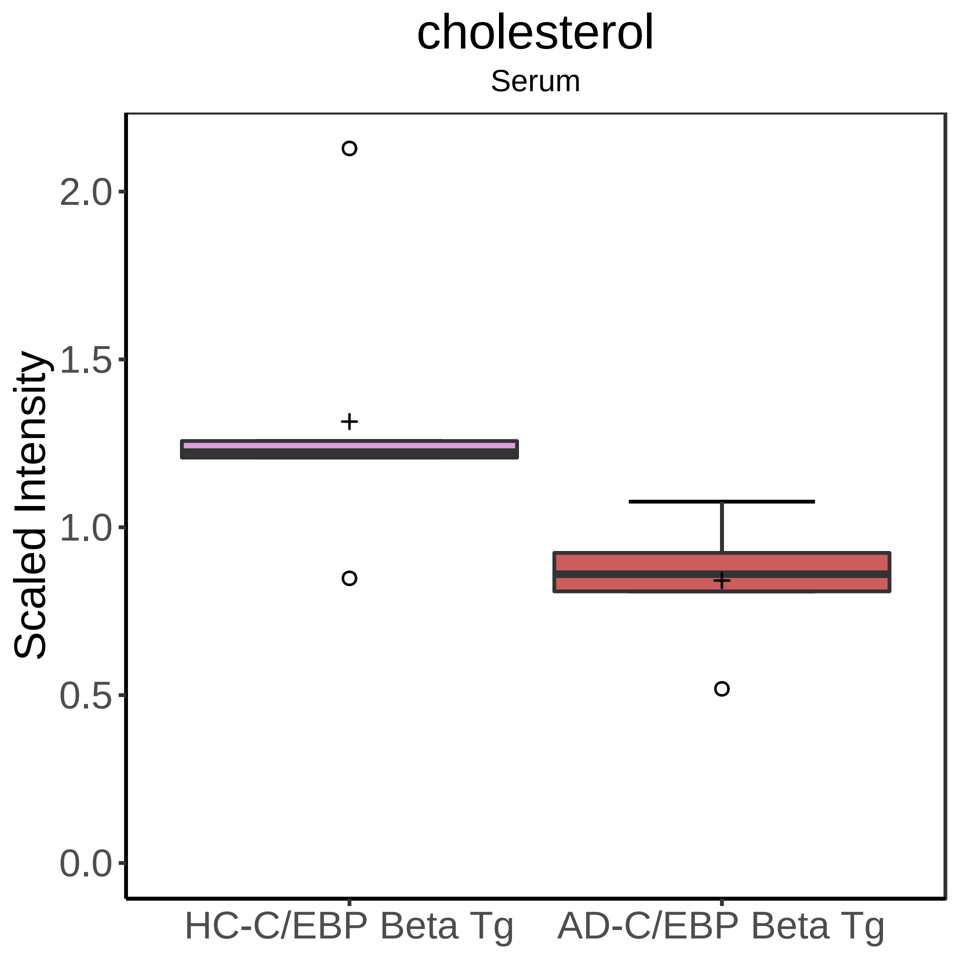
cholesterol
Serum
Scaled Intensity
2.0
1.5
1.0
0.5
0.0
HC-C/EBP Beta Tg
AD-C/EBP Beta Tg

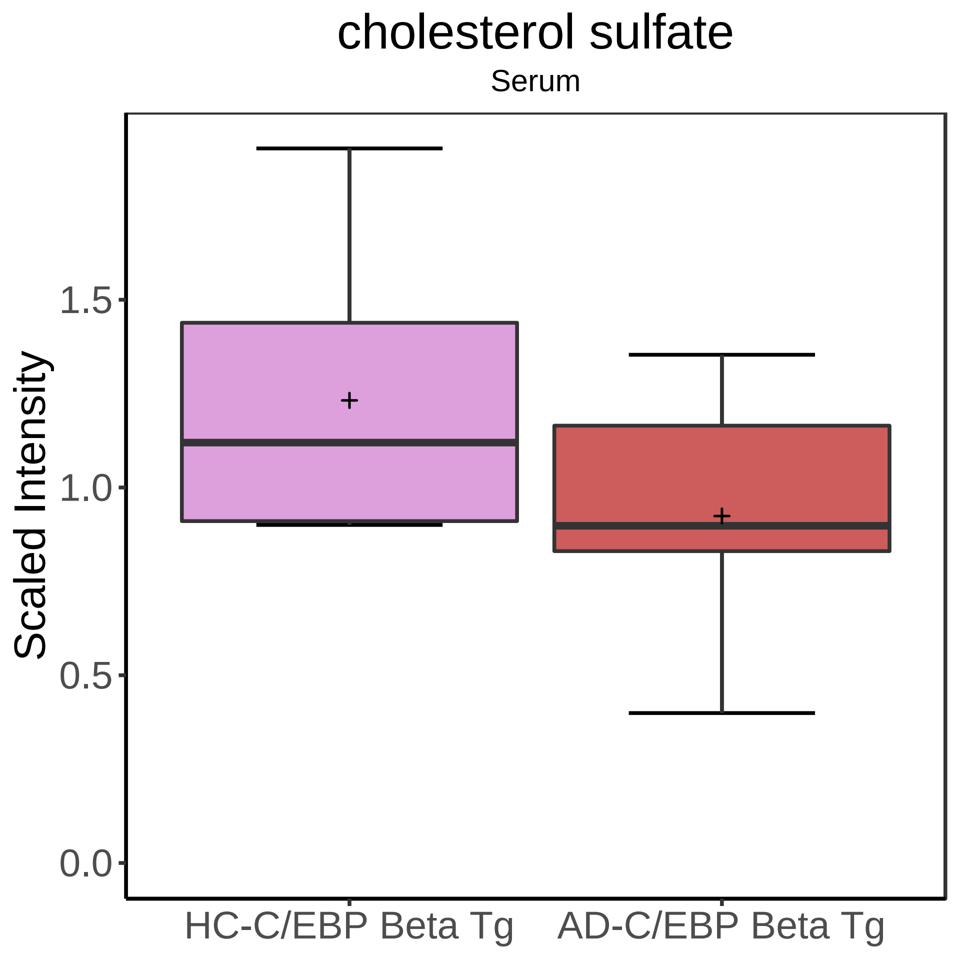
cholesterol sulfate
Serum
Scaled Intensity
1.5
1.0
0.5
0.0
HC-C/EBP Beta Tg
AD-C/EBP Beta Tg

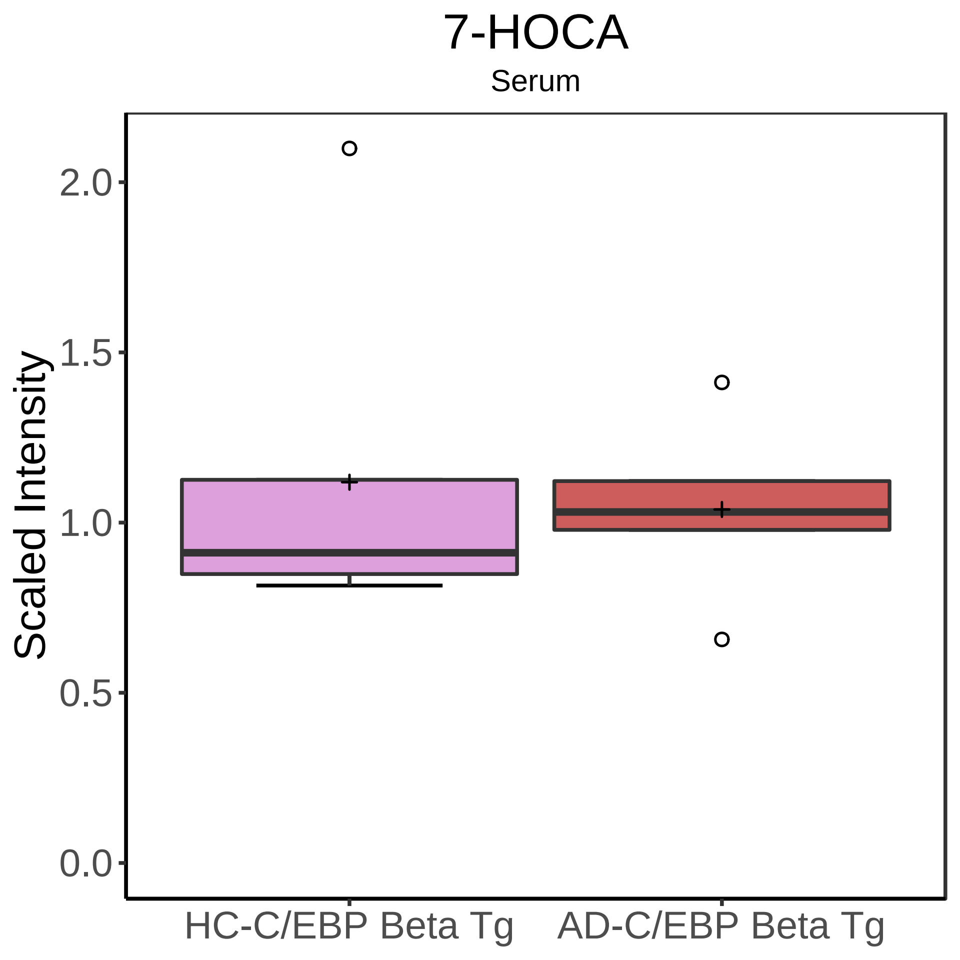

7-HOCA
Serum
Scaled Intensity
2.0
1.5
1.0
0.5
0.0
HC-C/EBP Beta Tg
AD-C/EBP Beta Tg

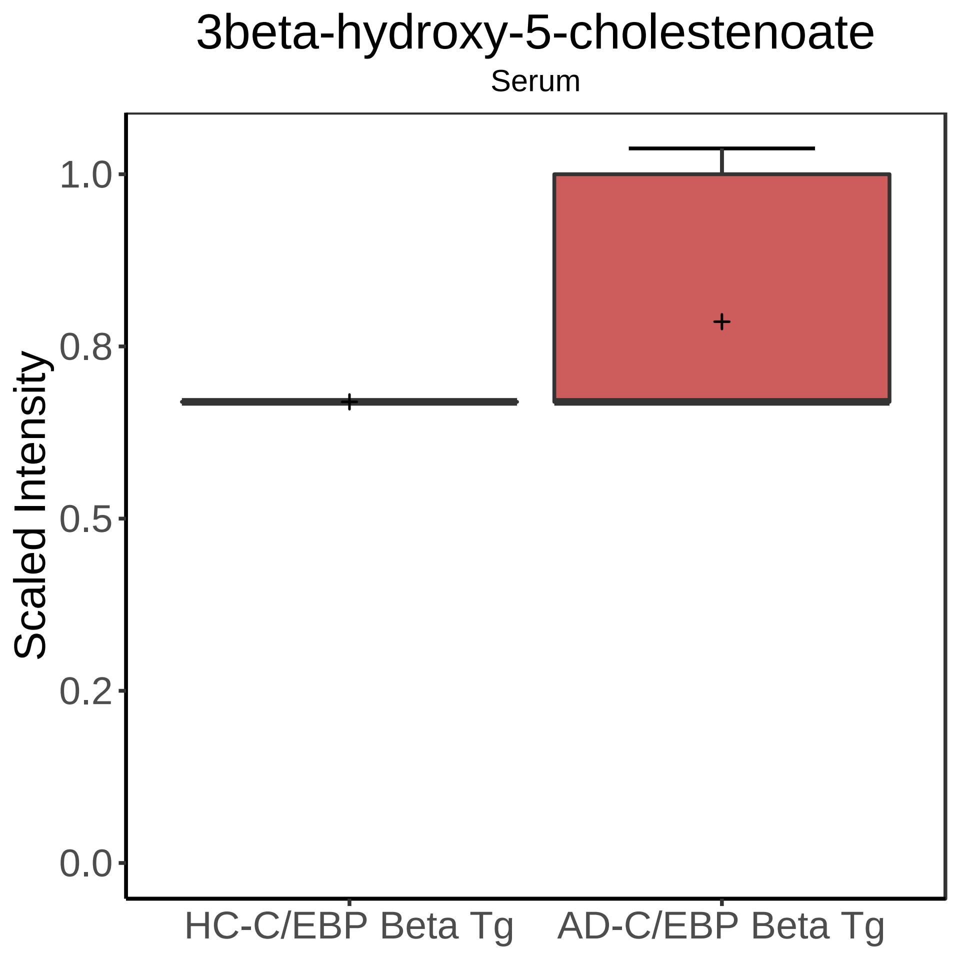
3beta-hydroxy-5-cholestenoate
Serum
Scaled Intensity
1.0
0.8
0.5
0.2
0.0
HC-C/EBP Beta Tg
AD-C/EBP Beta Tg

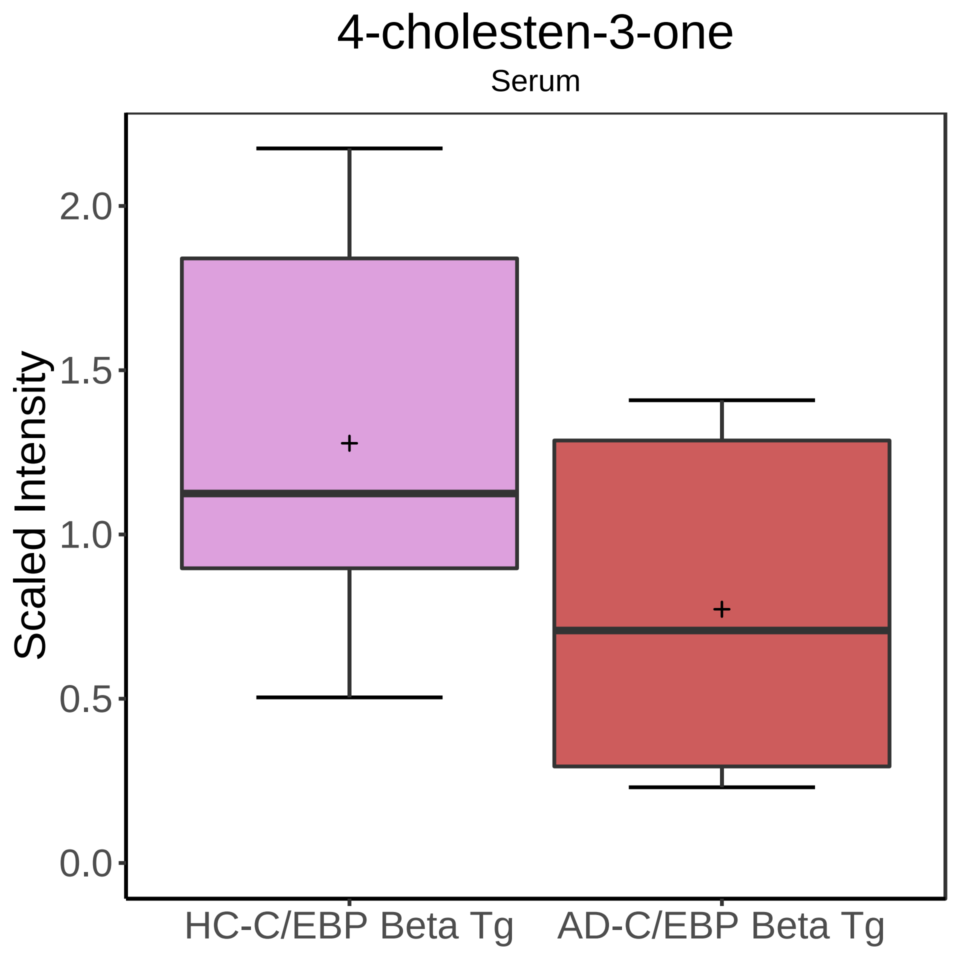
4-cholesten-3-one
Serum
Scaled Intensity
2.0
1.5
1.0
0.5
0.0
HC-C/EBP Beta Tg
AD-C/EBP Beta Tg

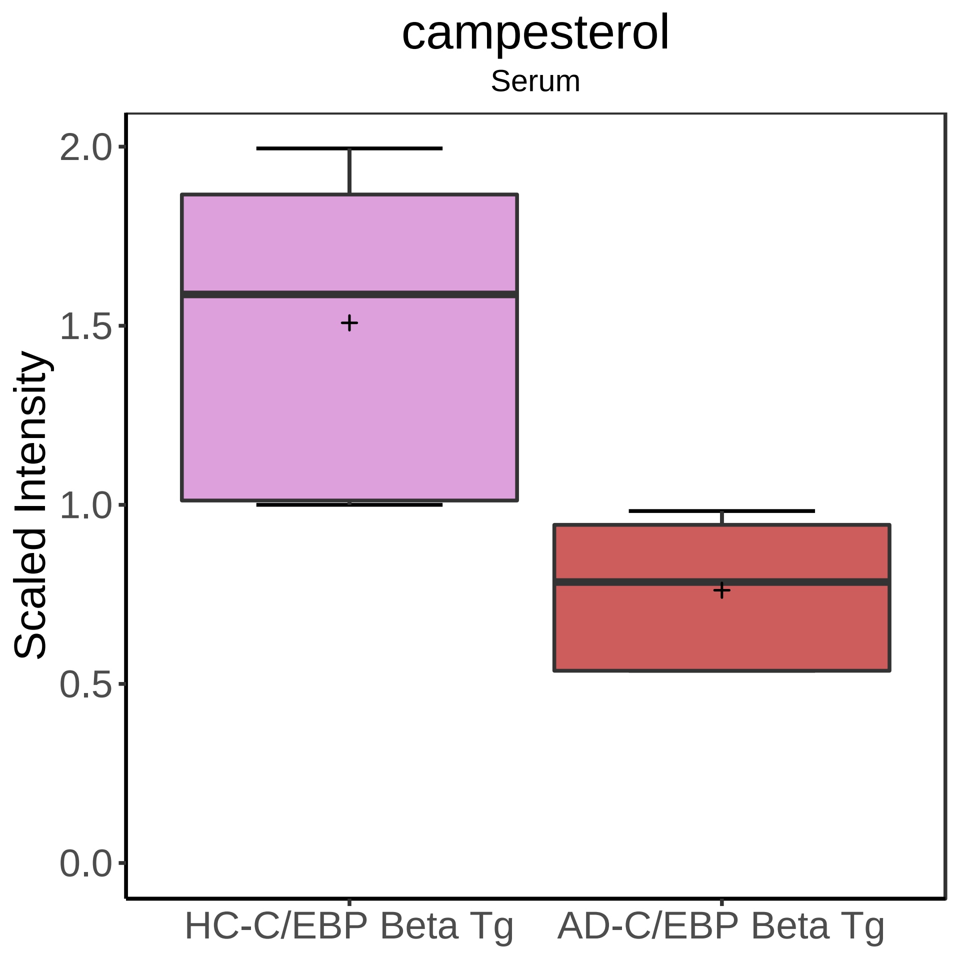
campesterol
Serum
Scaled Intensity
2.0
1.5
1.0
0.5
0.0
HC-C/EBP Beta Tg
AD-C/EBP Beta Tg

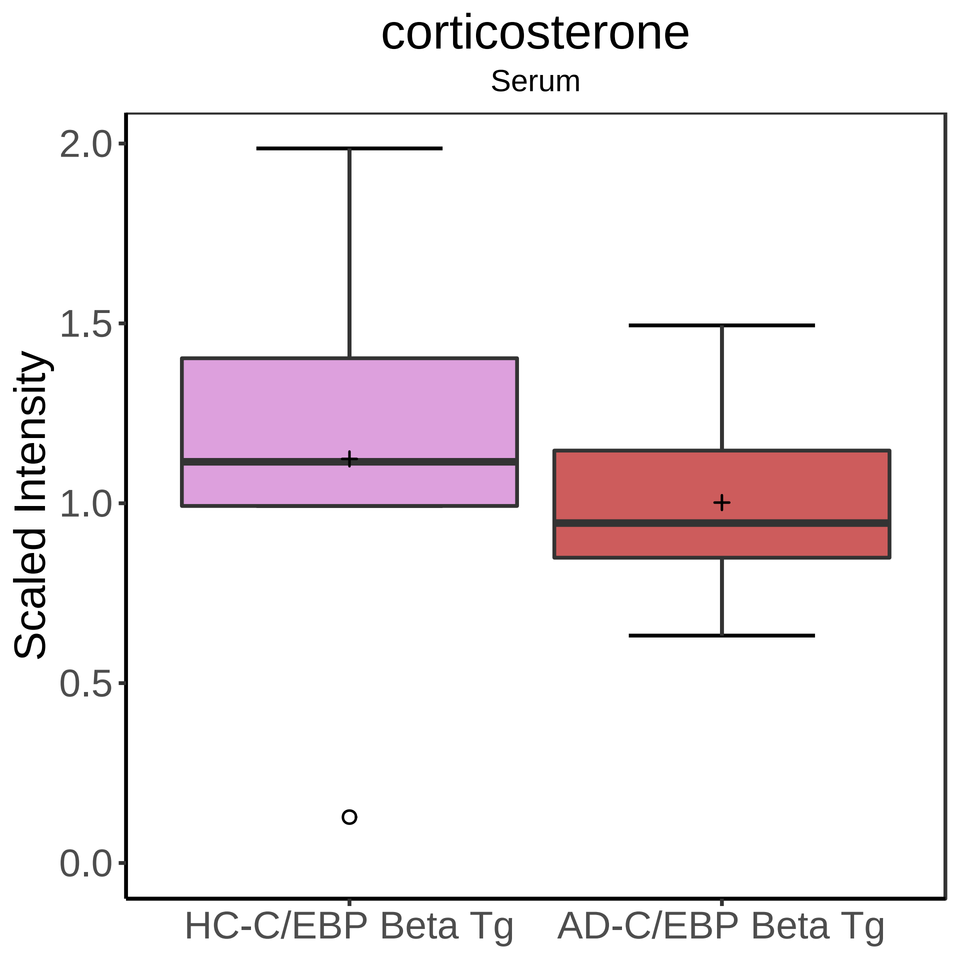
corticosterone
Serum
Scaled Intensity
2.0
1.5
1.0
0.5
0.0
HC-C/EBP Beta Tg
AD-C/EBP Beta Tg

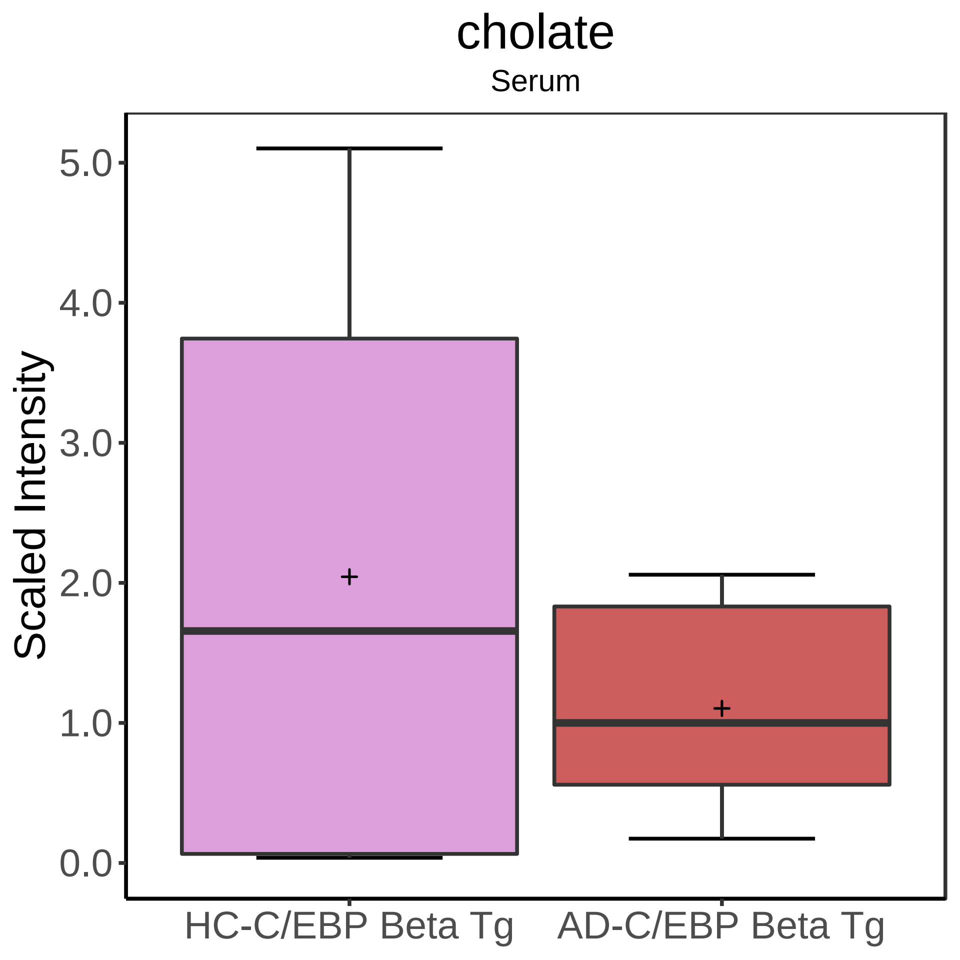
cholate
Serum
Scaled Intensity
5.0
4.0
3.0
2.0
1.0
0.0
HC-C/EBP Beta Tg
AD-C/EBP Beta Tg

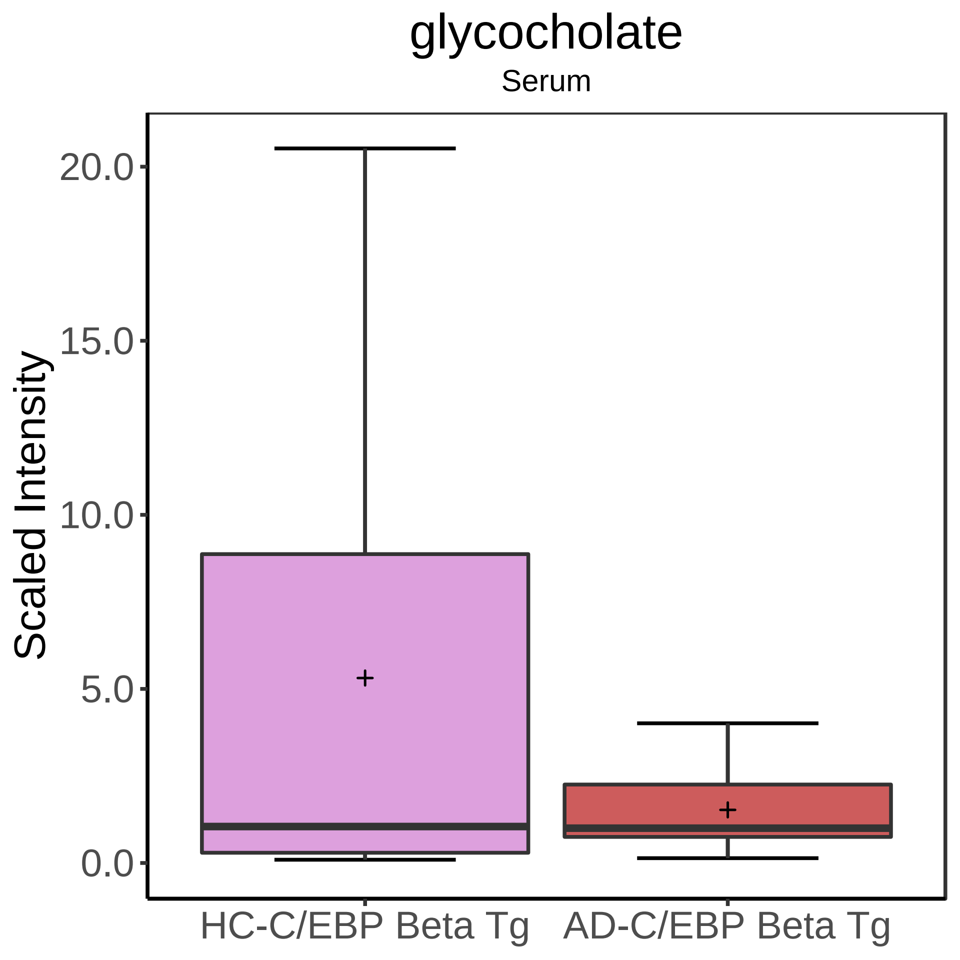
glycocholate
Serum
Scaled Intensity
20.0
15.0
10.0
5.0
0.0
HC-C/EBP Beta Tg
AD-C/EBP Beta Tg

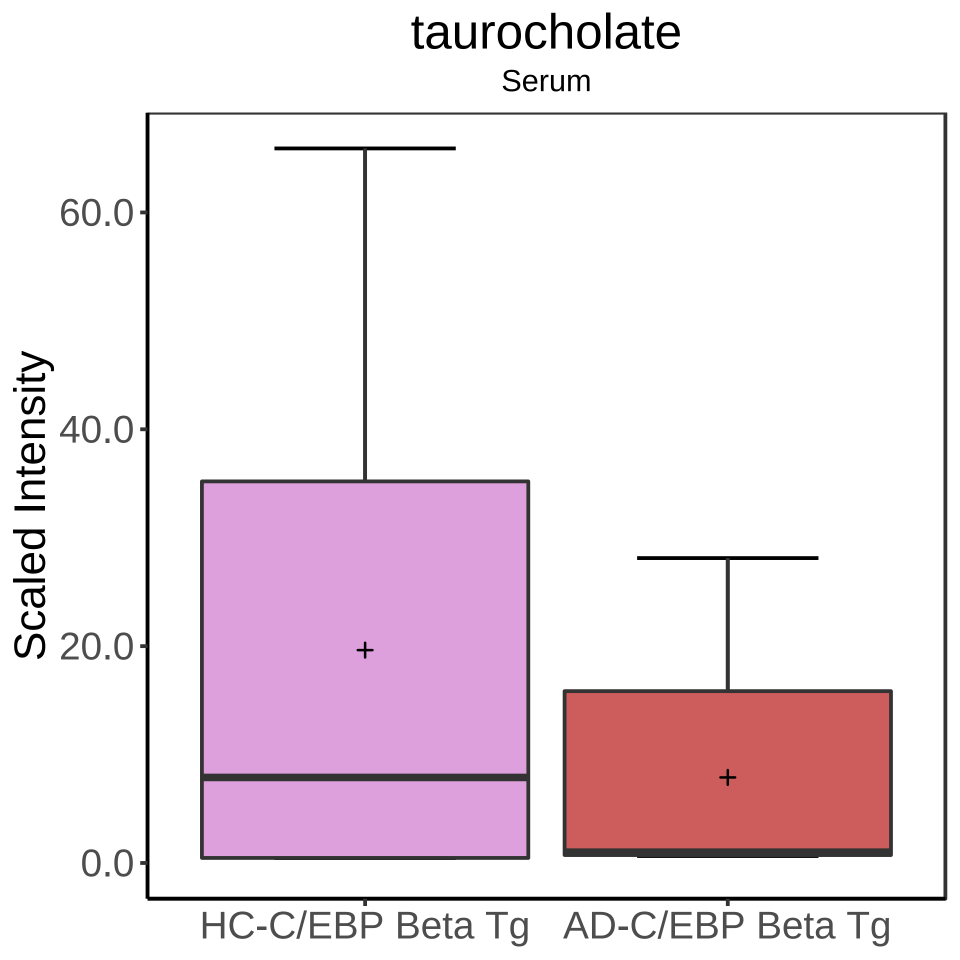
taurocholate
Serum
Scaled Intensity
60.0
40.0
20.0
0.0
HC-C/EBP Beta Tg
AD-C/EBP Beta Tg

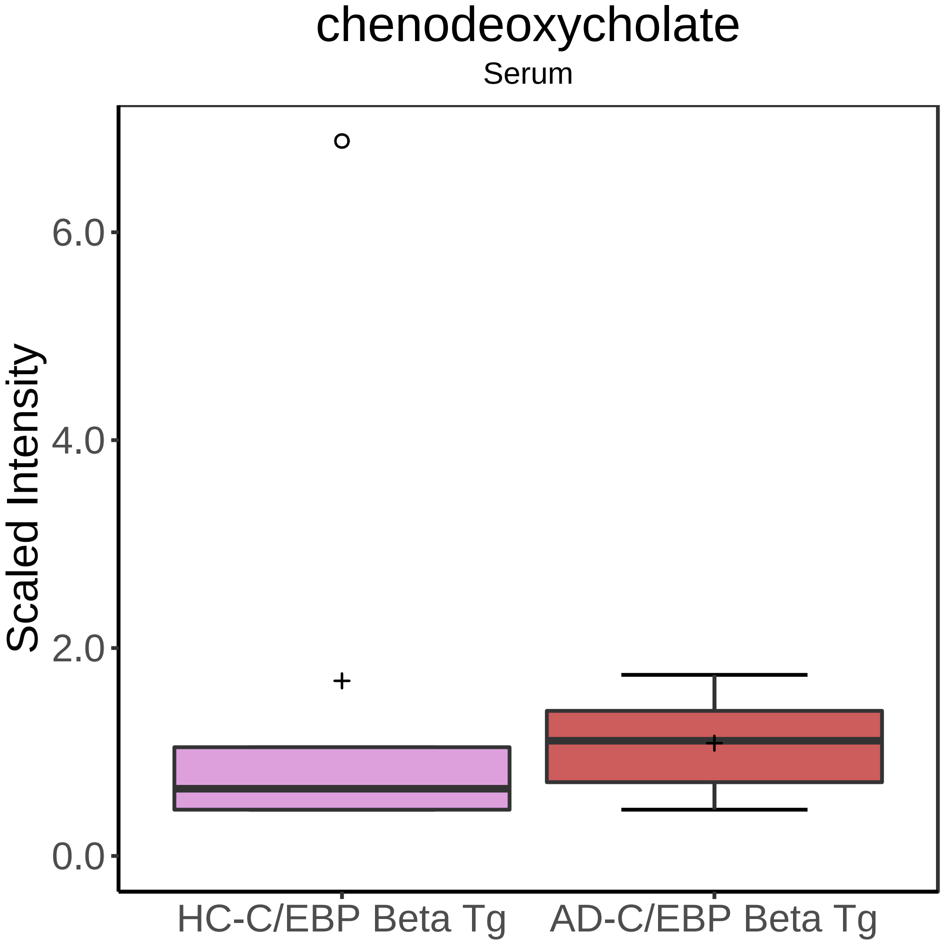
chenodeoxycholate
Serum
Scaled Intensity
6.0
4.0
2.0
0.0
HC-C/EBP Beta Tg
AD-C/EBP Beta Tg

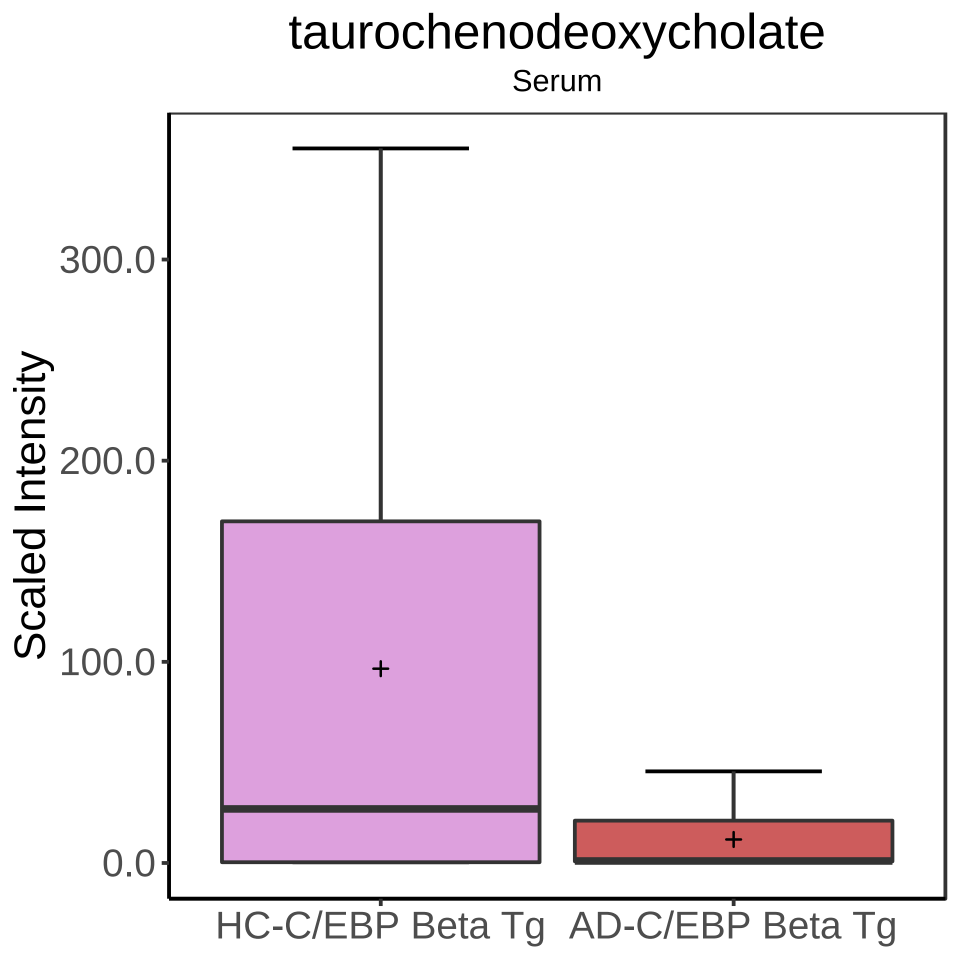
taurochenodeoxycholate
Serum
Scaled Intensity
300.0
200.0
100.0
0.0
HC-C/EBP Beta Tg
AD-C/EBP Beta Tg

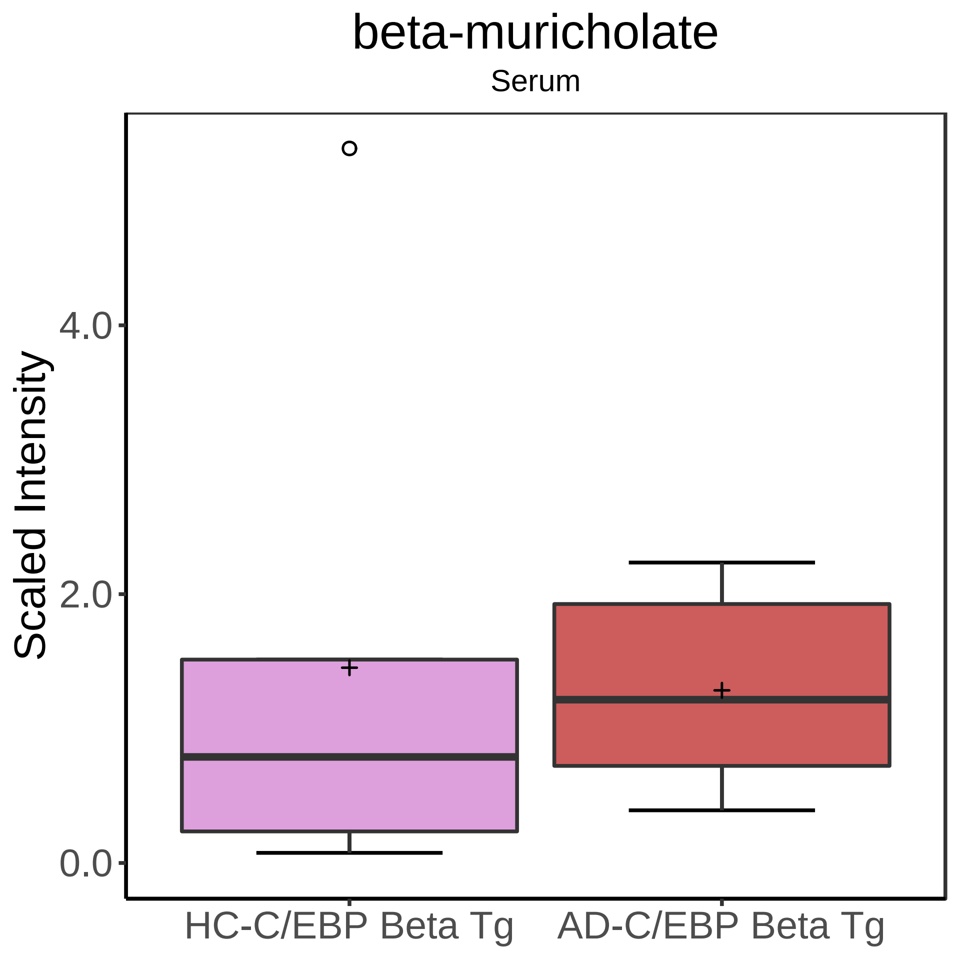

beta-muricholate
Serum
Scaled Intensity
4.0
2.0
0.0
HC-C/EBP Beta Tg
AD-C/EBP Beta Tg

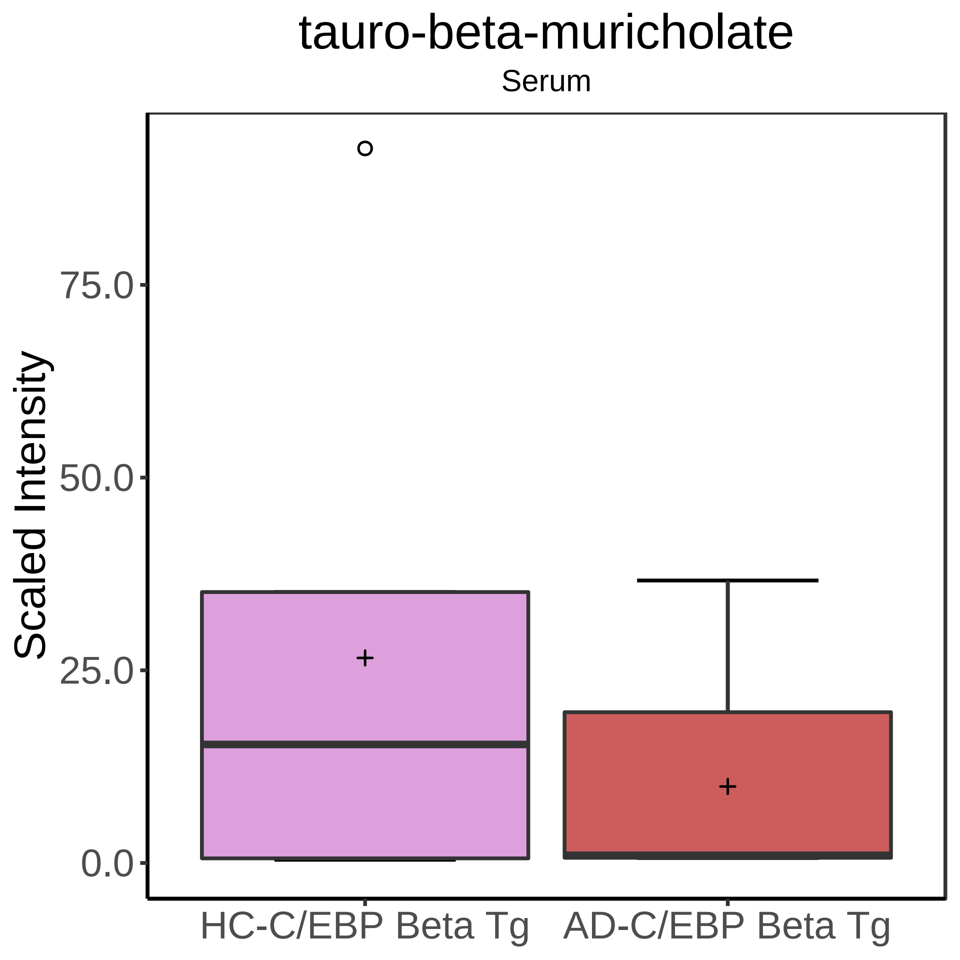

tauro-beta-muricholate
Serum
Scaled Intensity
75.0
50.0
25.0
0.0
HC-C/EBP Beta Tg
AD-C/EBP Beta Tg

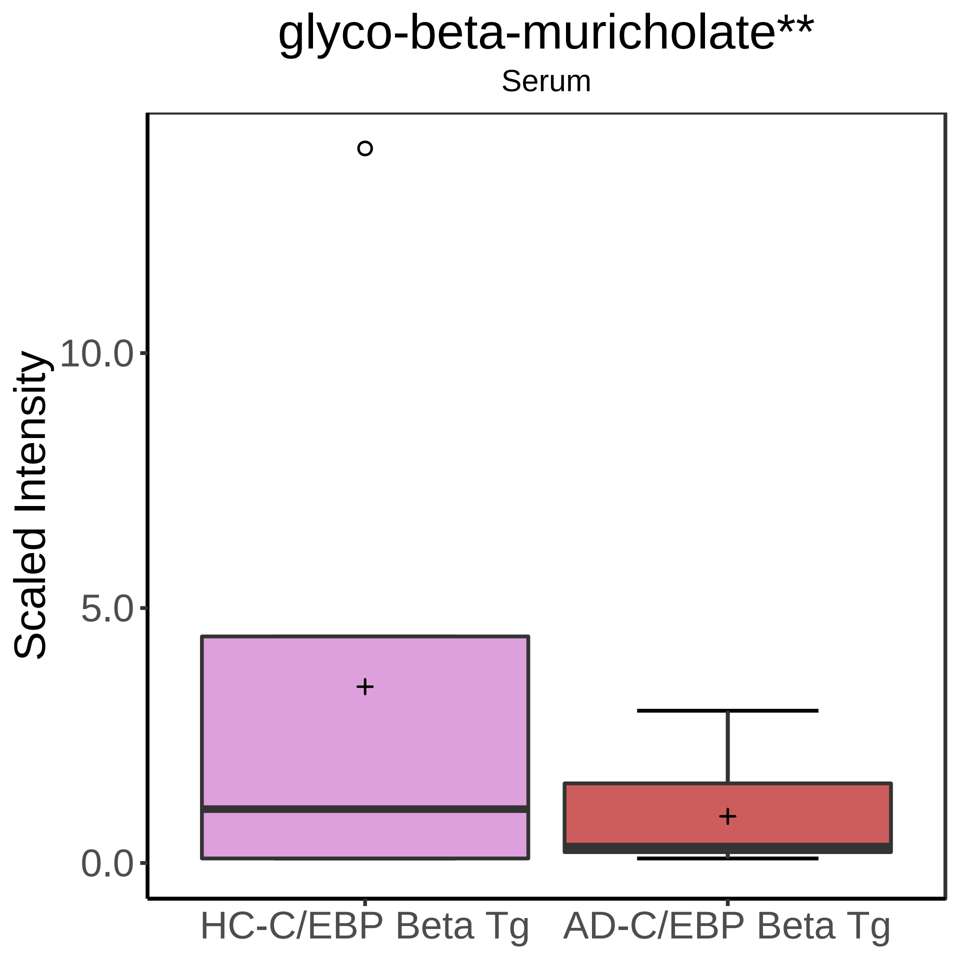

glyco-beta-muricholate**
Serum
Scaled Intensity
10.0
5.0
0.0
HC-C/EBP Beta Tg
AD-C/EBP Beta Tg

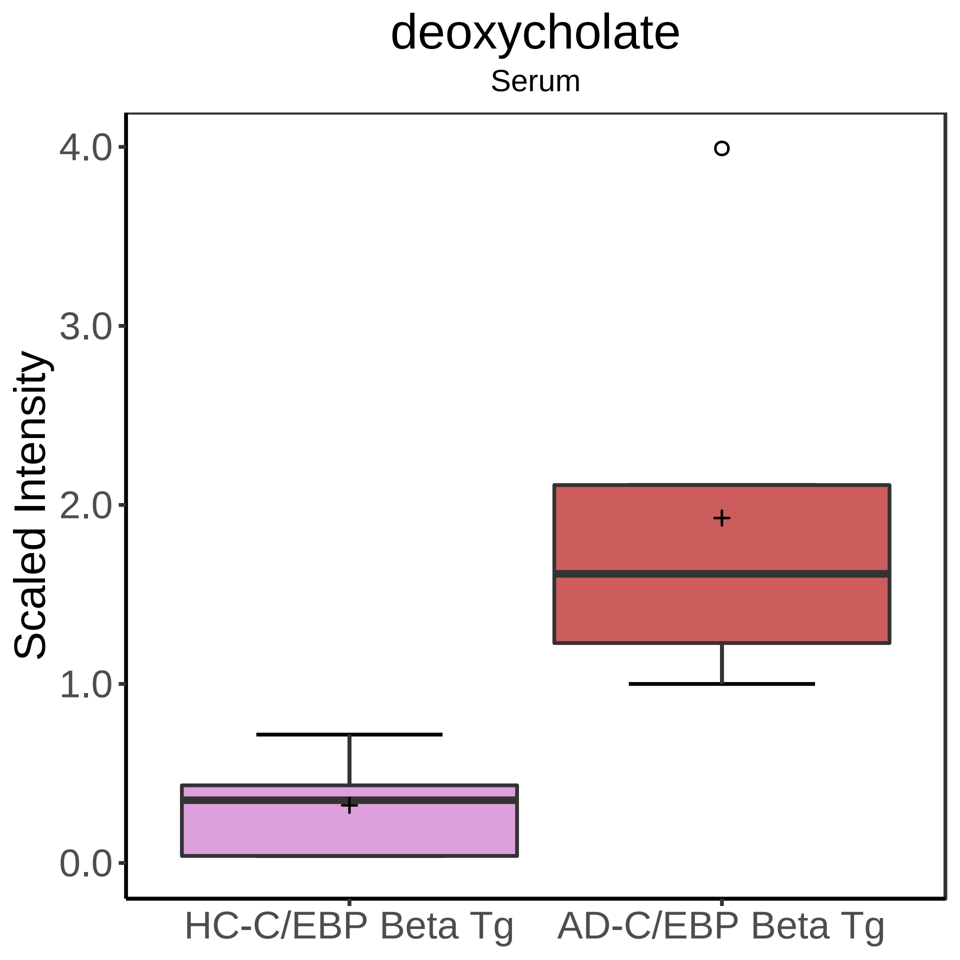
deoxycholate
Serum
Scaled Intensity
4.0
3.0
2.0
1.0
0.0
HC-C/EBP Beta Tg
AD-C/EBP Beta Tg

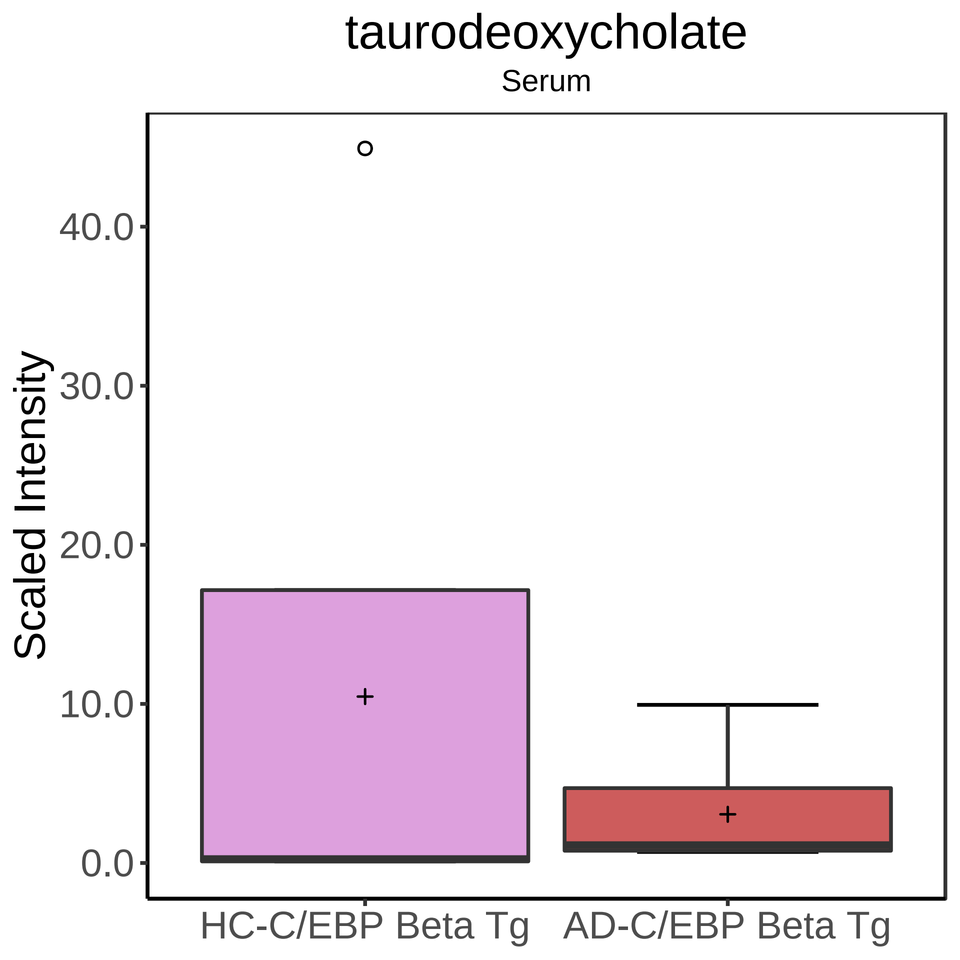
taurodeoxycholate
Serum
Scaled Intensity
40.0
30.0
20.0
10.0
0.0
HC-C/EBP Beta Tg
AD-C/EBP Beta Tg

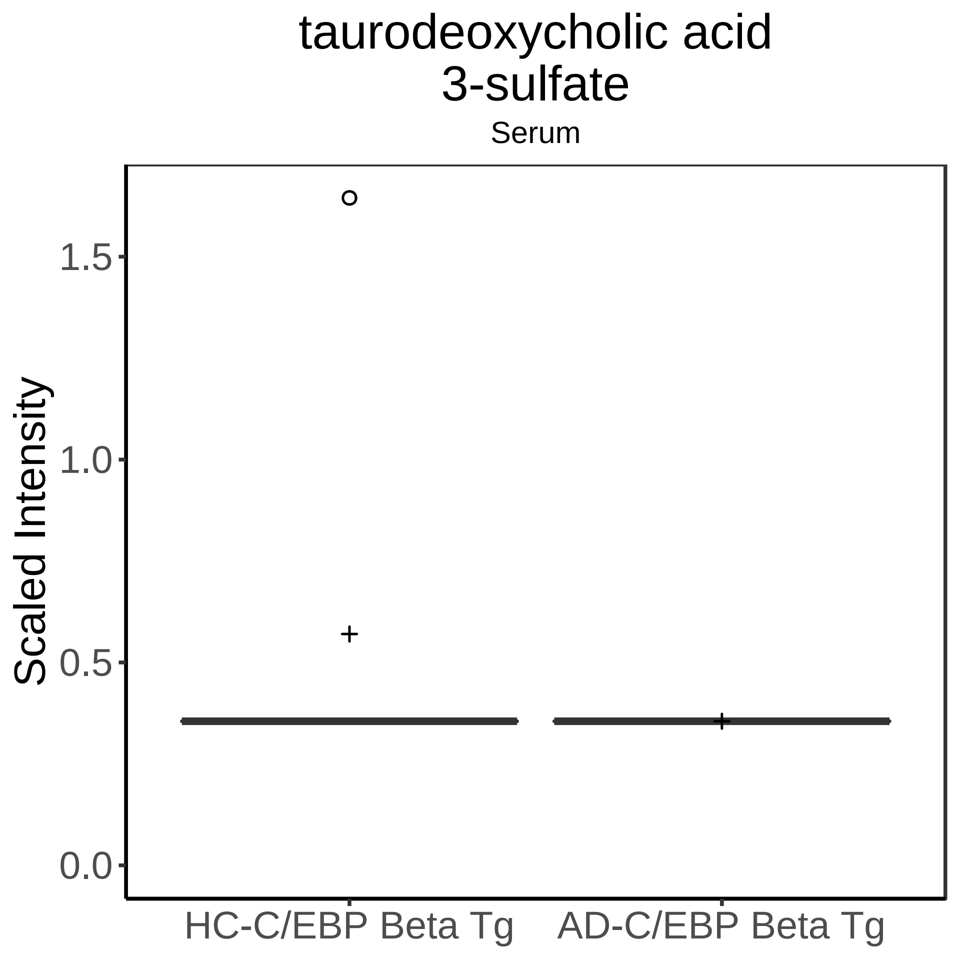
taurodeoxycholic acid
3-sulfate
Serum
Scaled Intensity
1.5
1.0
0.5
0.0
HC-C/EBP Beta Tg
AD-C/EBP Beta Tg

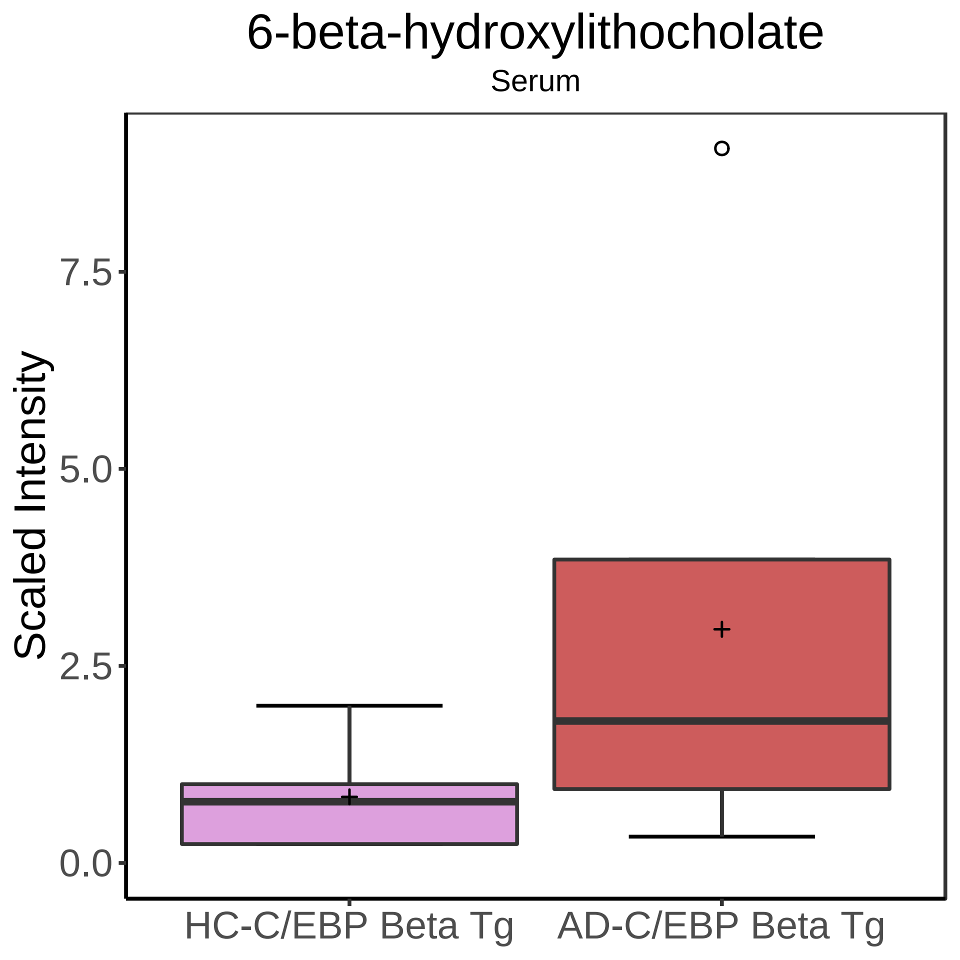
6-beta-hydroxylithocholate
Serum
Scaled Intensity
7.5
5.0
2.5
0.0
HC-C/EBP Beta Tg
AD-C/EBP Beta Tg

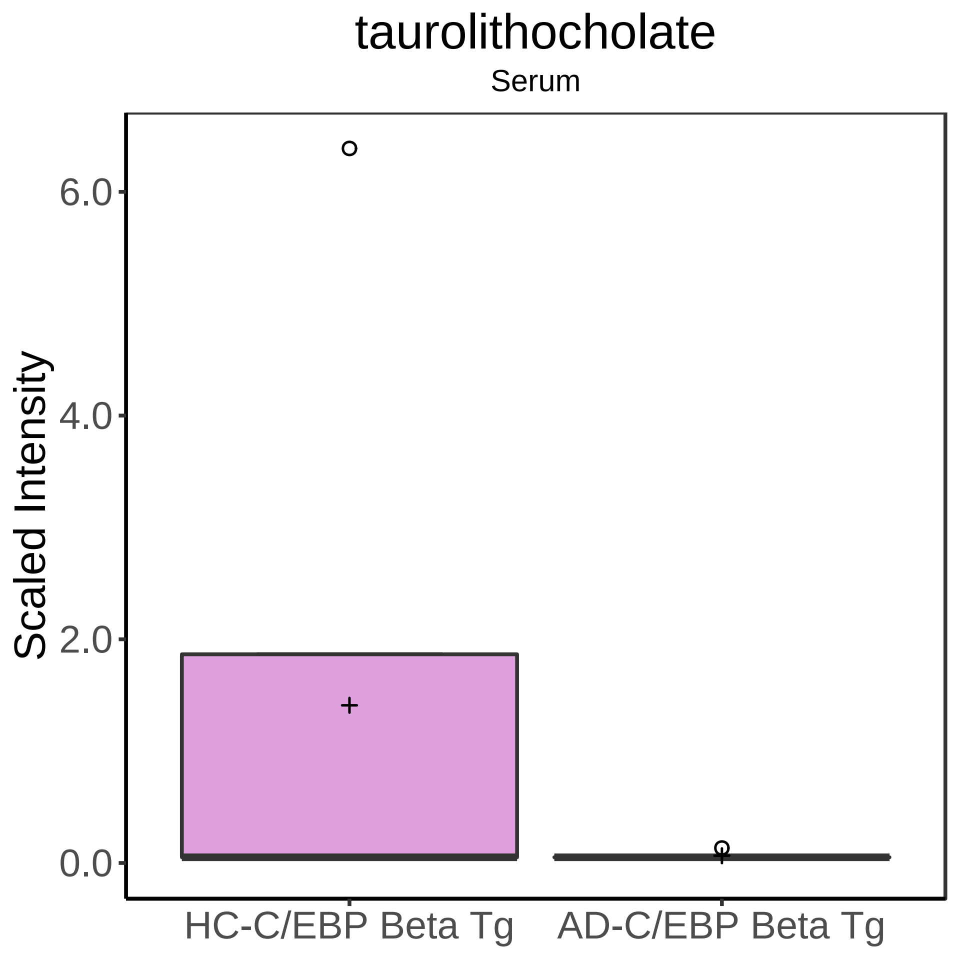
taurolithocholate
Serum
Scaled Intensity
6.0
4.0
2.0
0.0
HC-C/EBP Beta Tg
AD-C/EBP Beta Tg

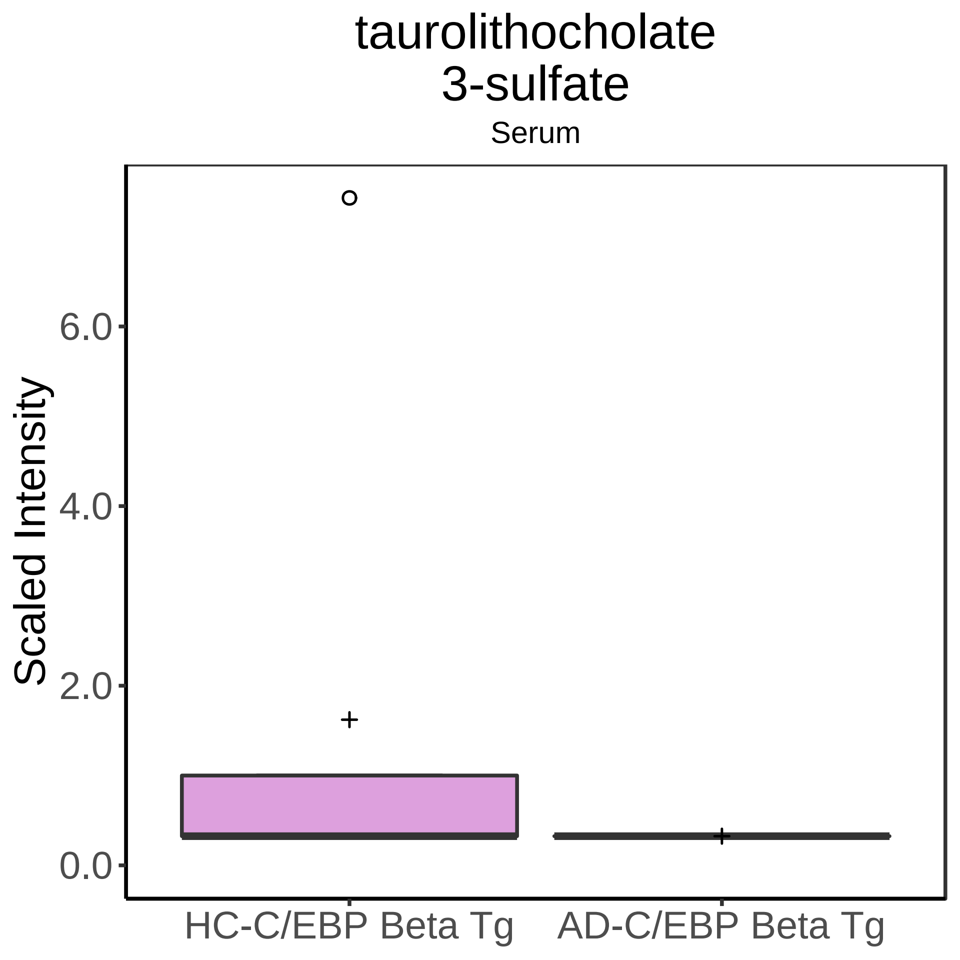
taurolithocholate
3-sulfate
Serum
Scaled Intensity
6.0
4.0
2.0
0.0
HC-C/EBP Beta Tg
AD-C/EBP Beta Tg

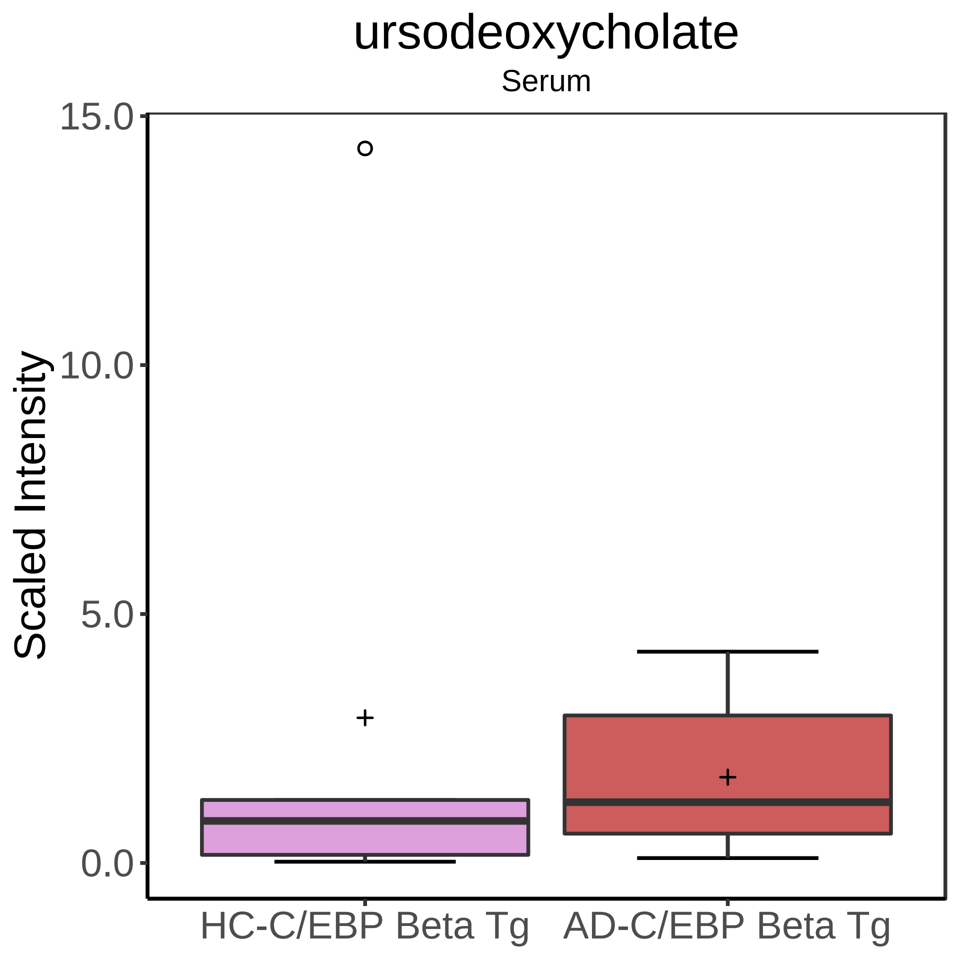
ursodeoxycholate
Serum
Scaled Intensity
15.0
10.0
5.0
0.0
HC-C/EBP Beta Tg
AD-C/EBP Beta Tg

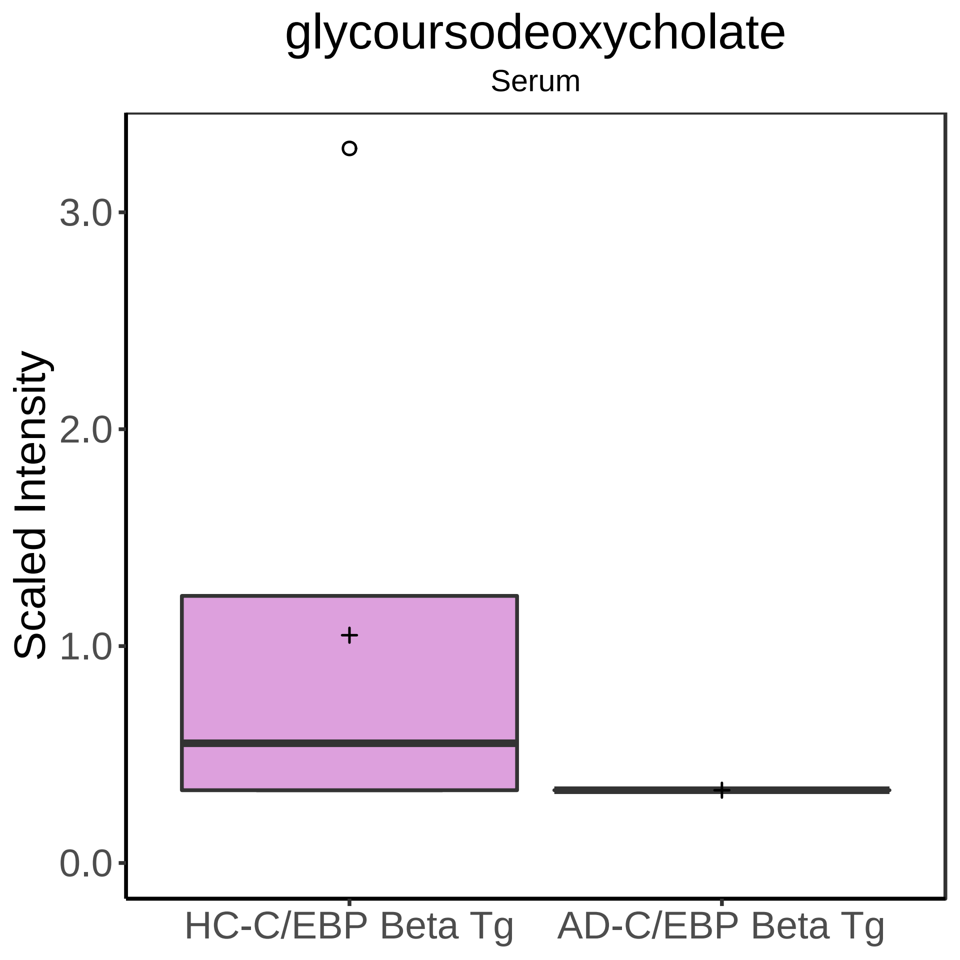
glycoursodeoxycholate
Serum
Scaled Intensity
3.0
2.0
1.0
0.0
HC-C/EBP Beta Tg
AD-C/EBP Beta Tg

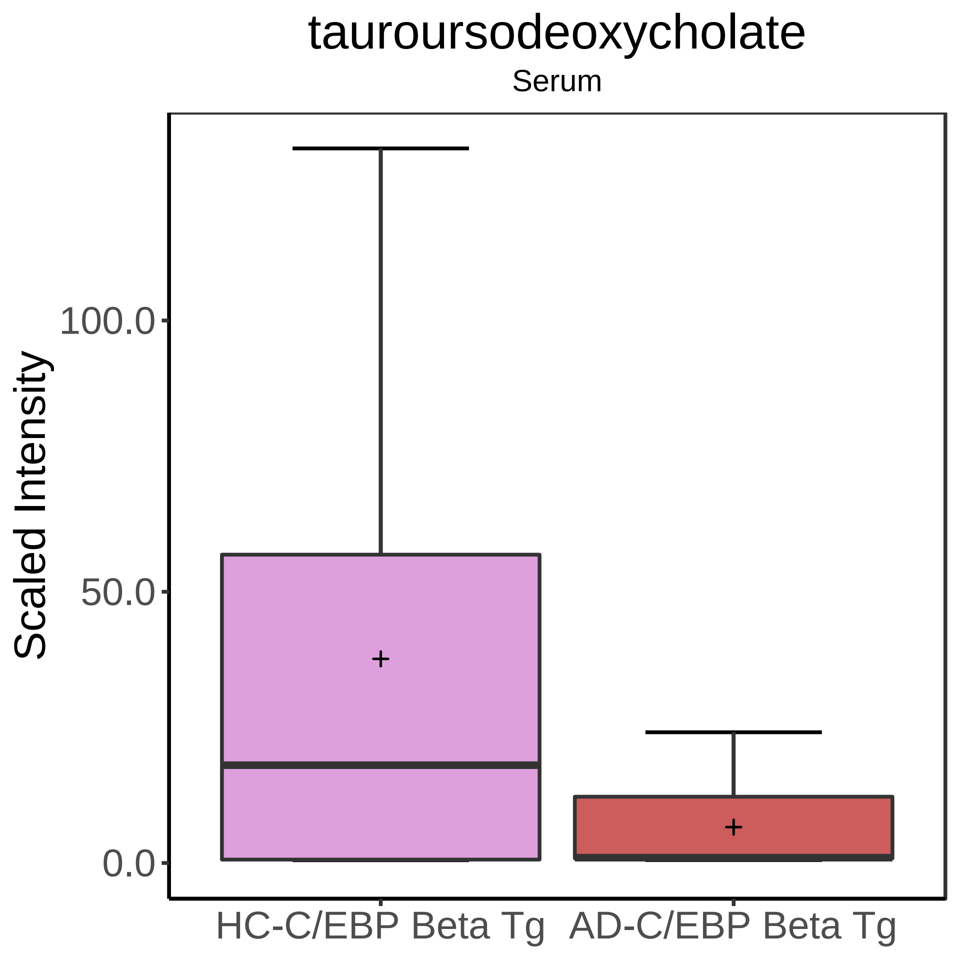
tauroursodeoxycholate
Serum
Scaled Intensity
100.0
50.0
0.0
HC-C/EBP Beta Tg
AD-C/EBP Beta Tg

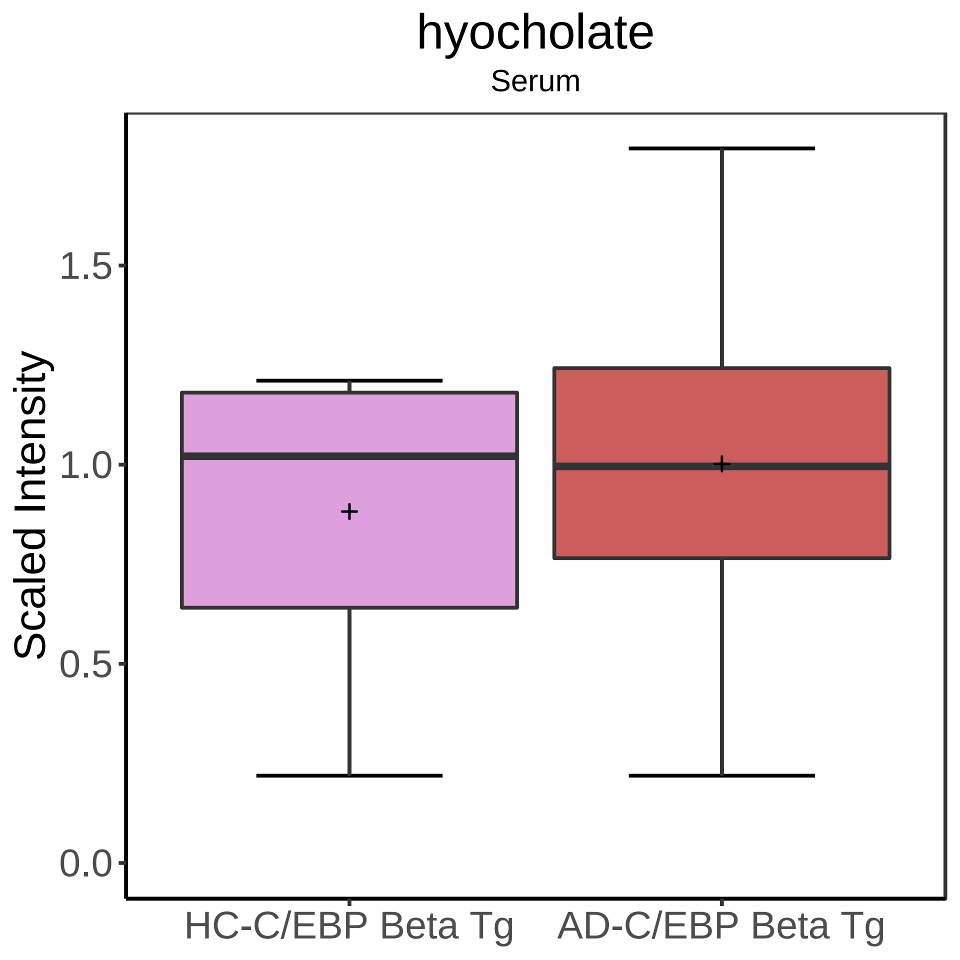
hyocholate
Serum
Scaled Intensity
0.0
0.5
1.0
1.5
HC-C/EBP Beta Tg
AD-C/EBP Beta Tg

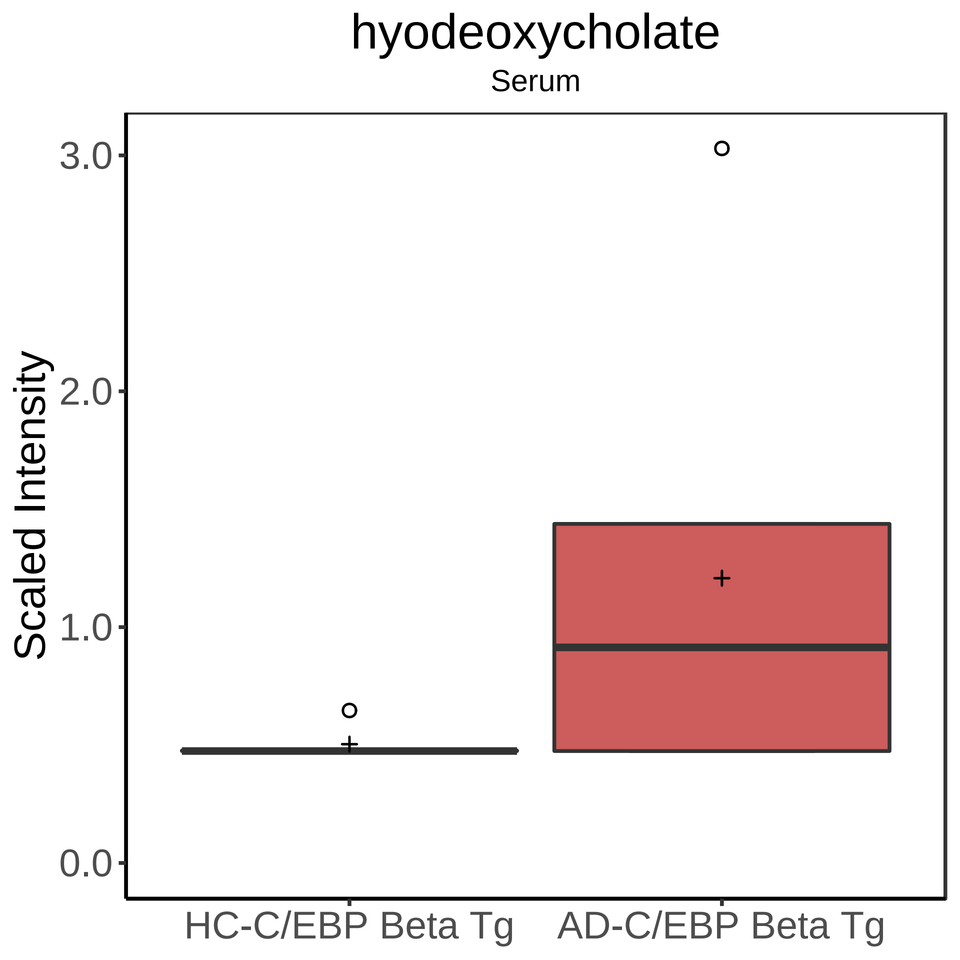
hyodeoxycholate
Serum
Scaled Intensity
3.0
2.0
1.0
0.0
HC-C/EBP Beta Tg
AD-C/EBP Beta Tg

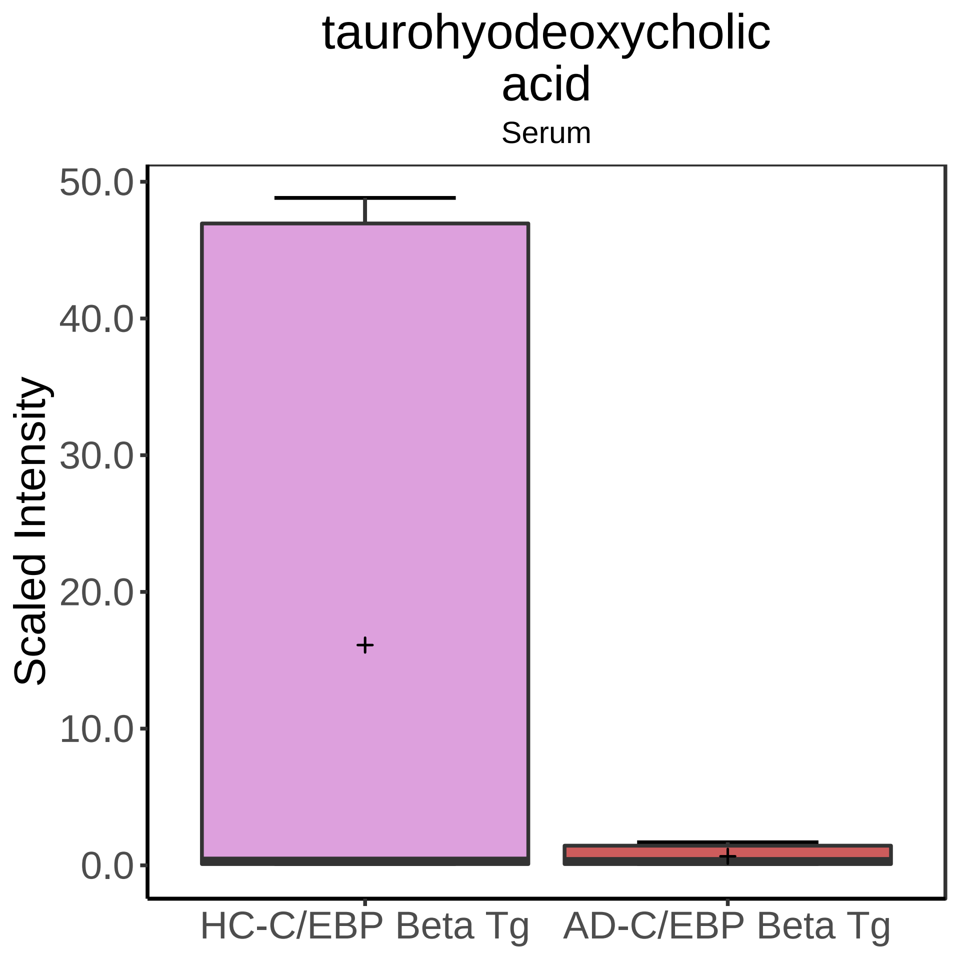

taurohyodeoxycholic acid
Serum
Scaled Intensity
50.0
40.0
30.0
20.0
10.0
0.0
HC-C/EBP Beta Tg
AD-C/EBP Beta Tg

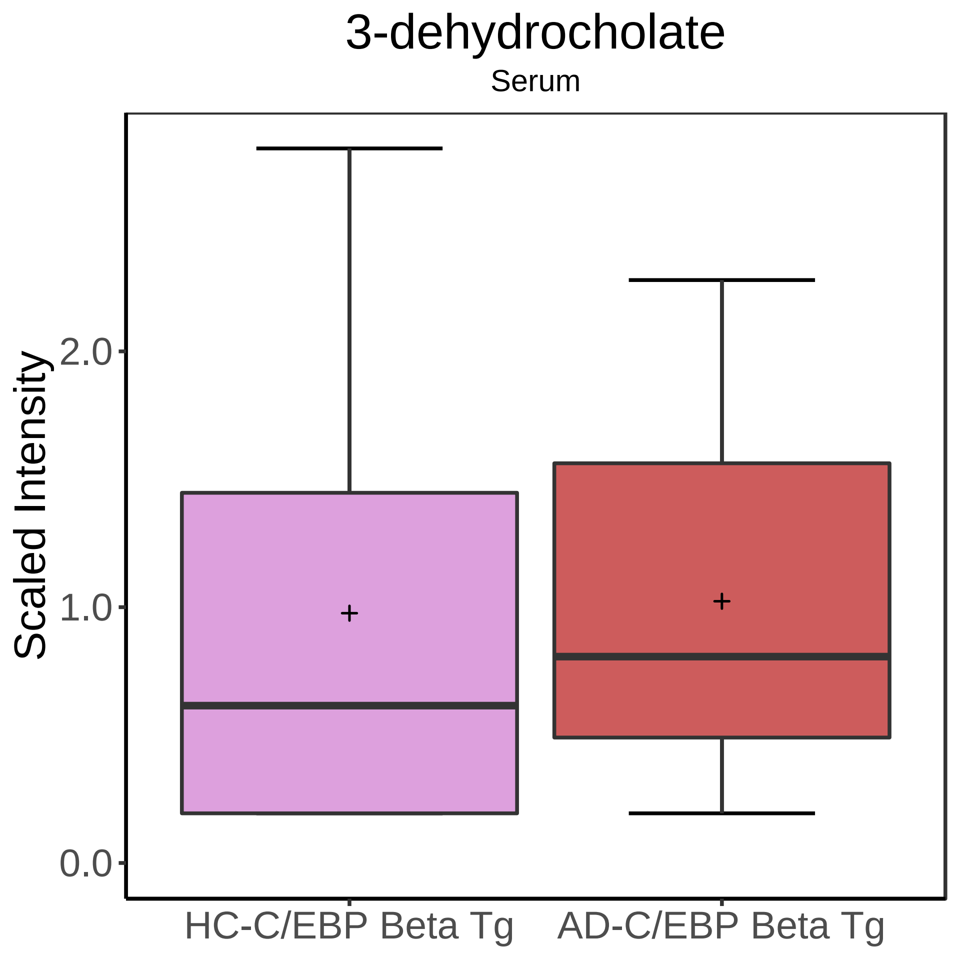
3-dehydrocholate
Serum
Scaled Intensity
2.0
1.0
0.0
HC-C/EBP Beta Tg
AD-C/EBP Beta Tg

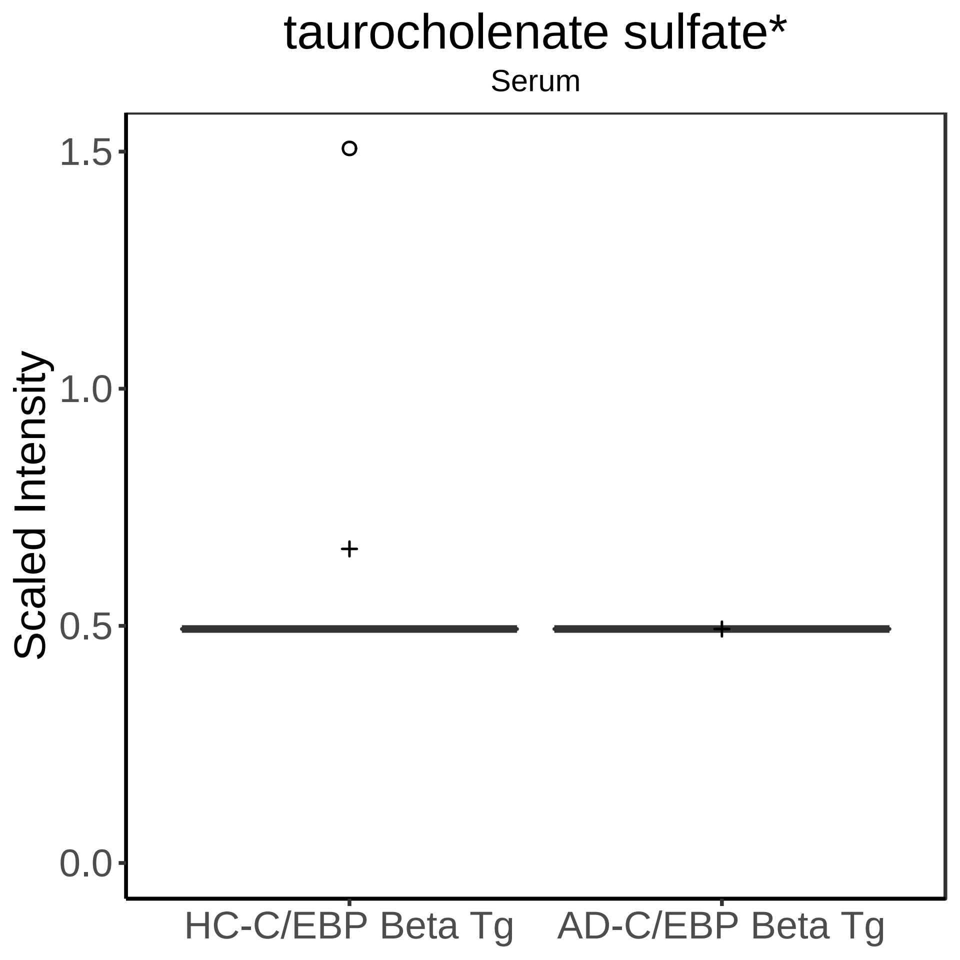
taurocholenate sulfate*
Serum
Scaled Intensity
1.5
1.0
0.5
0.0
HC-C/EBP Beta Tg
AD-C/EBP Beta Tg

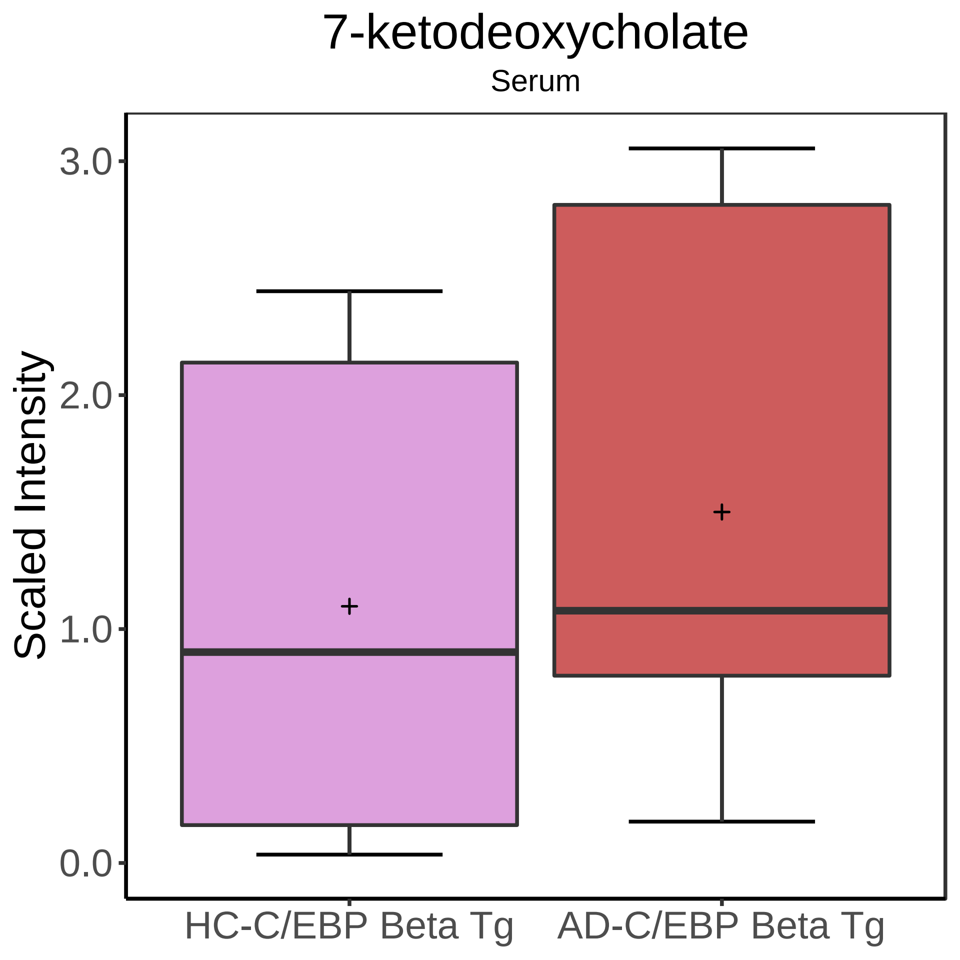
7-ketodeoxycholate
Serum
Scaled Intensity
3.0
2.0
1.0
0.0
HC-C/EBP Beta Tg
AD-C/EBP Beta Tg

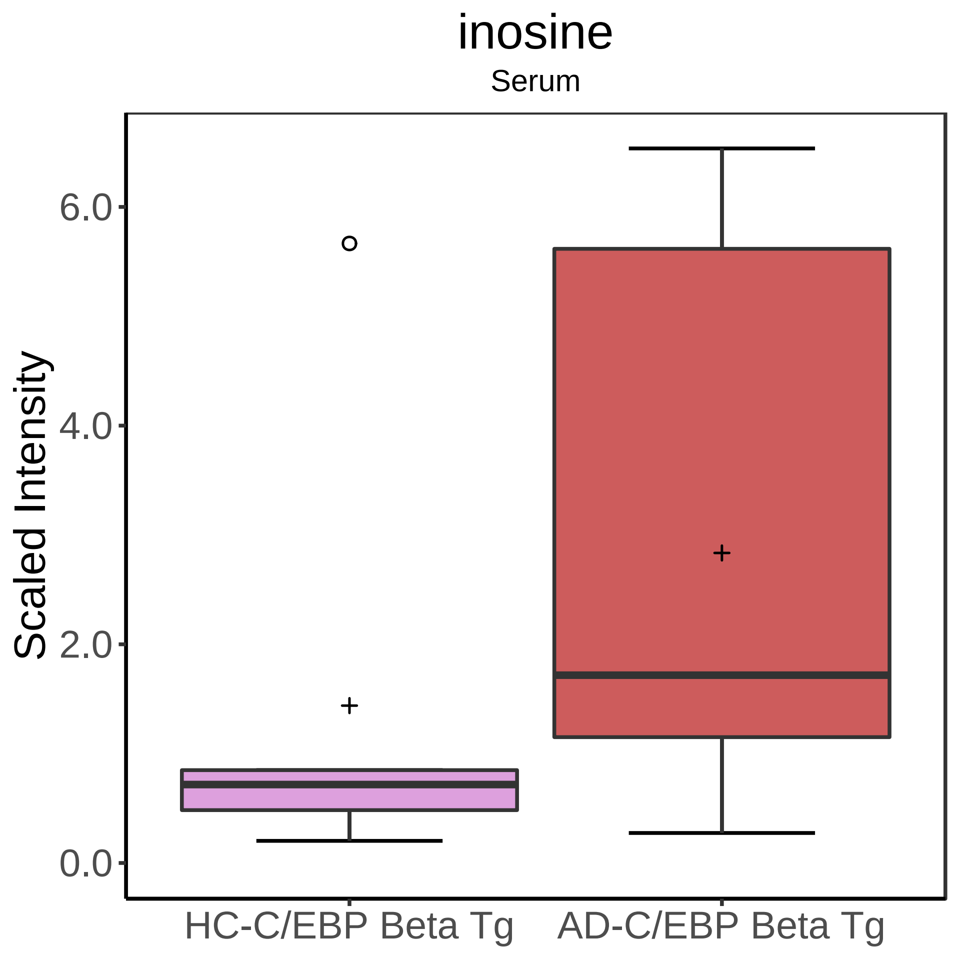

inosine
Serum
Scaled Intensity
6.0
4.0
2.0
0.0
HC-C/EBP Beta Tg
AD-C/EBP Beta Tg

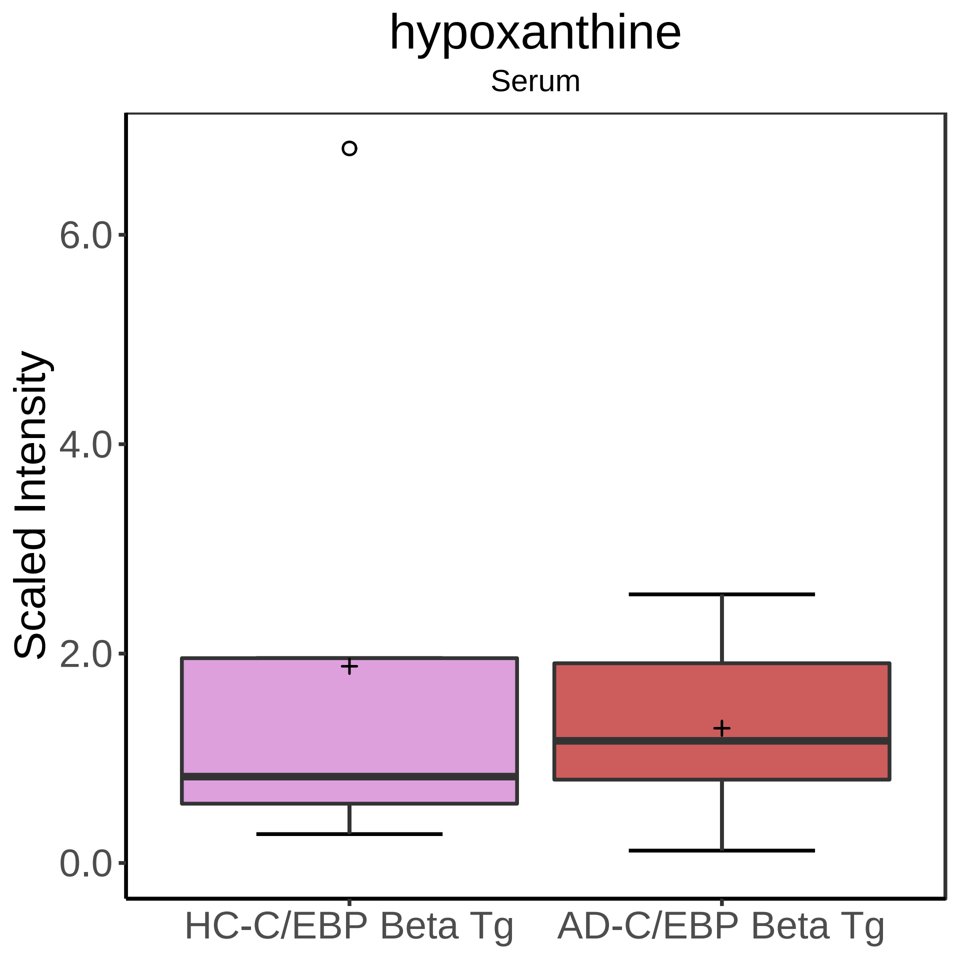
hypoxanthine
Serum
Scaled Intensity
6.0
4.0
2.0
0.0
HC-C/EBP Beta Tg
AD-C/EBP Beta Tg

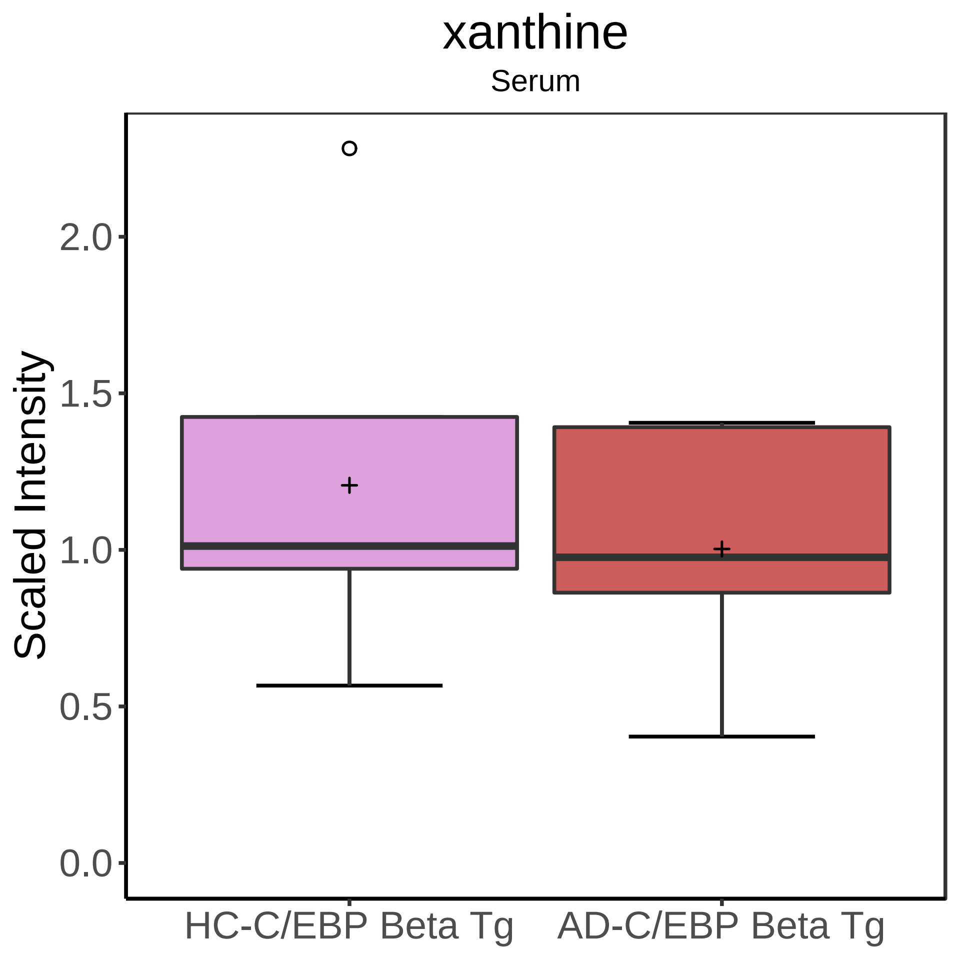
xanthine
Serum
Scaled Intensity
2.0
1.5
1.0
0.5
0.0
HC-C/EBP Beta Tg
AD-C/EBP Beta Tg

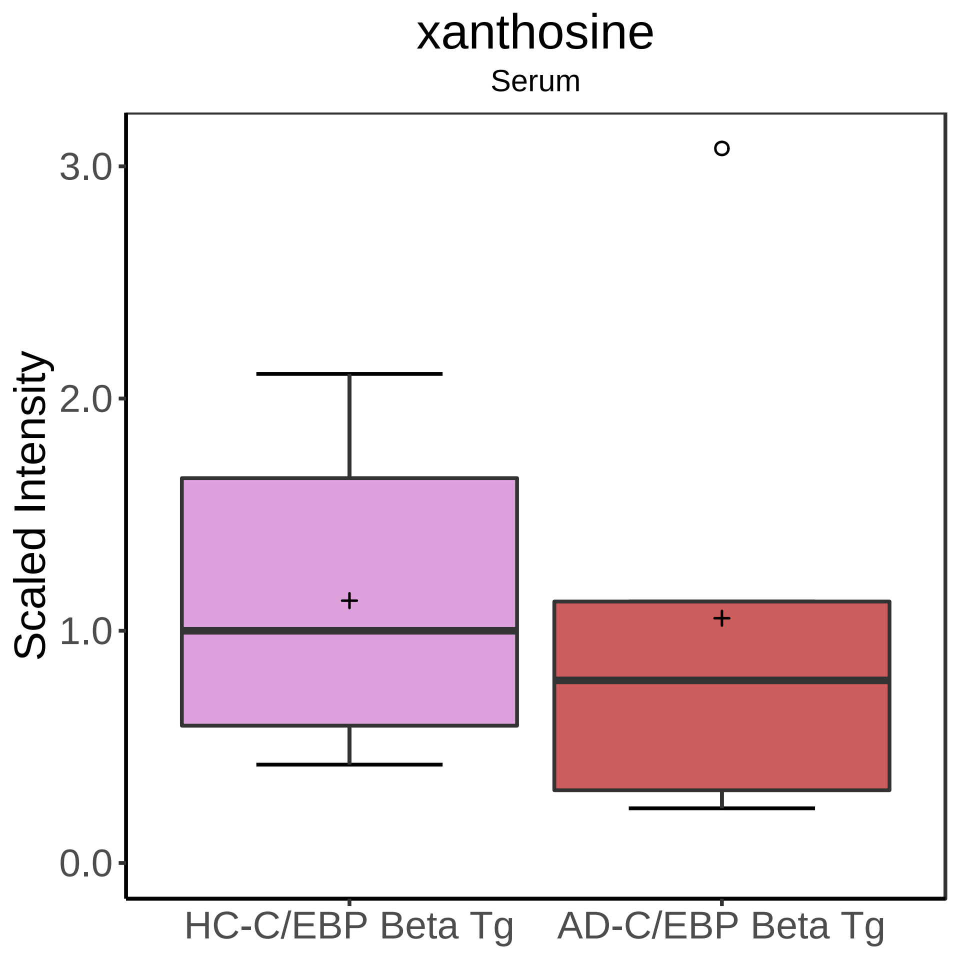
xanthosine
Serum
Scaled Intensity
3.0
2.0
1.0
0.0
HC-C/EBP Beta Tg
AD-C/EBP Beta Tg

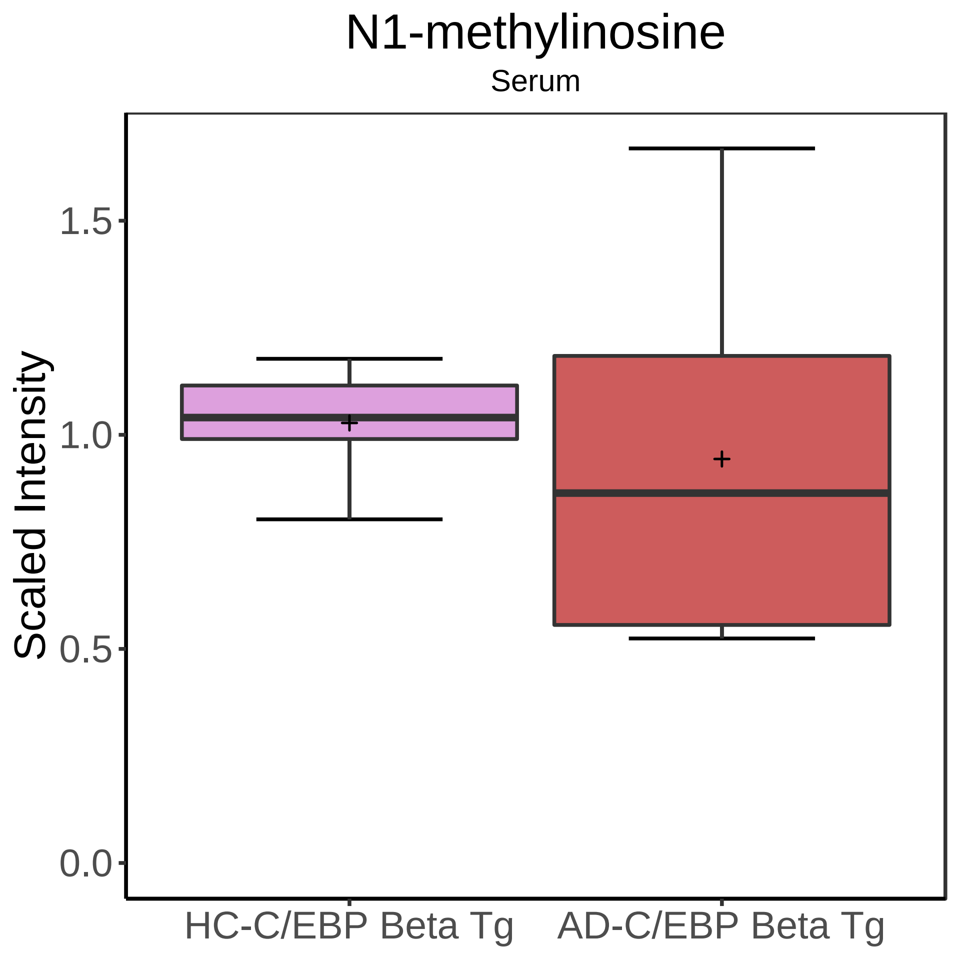
N1-methylinosine
Serum
Scaled Intensity
1.5
1.0
0.5
0.0
HC-C/EBP Beta Tg
AD-C/EBP Beta Tg

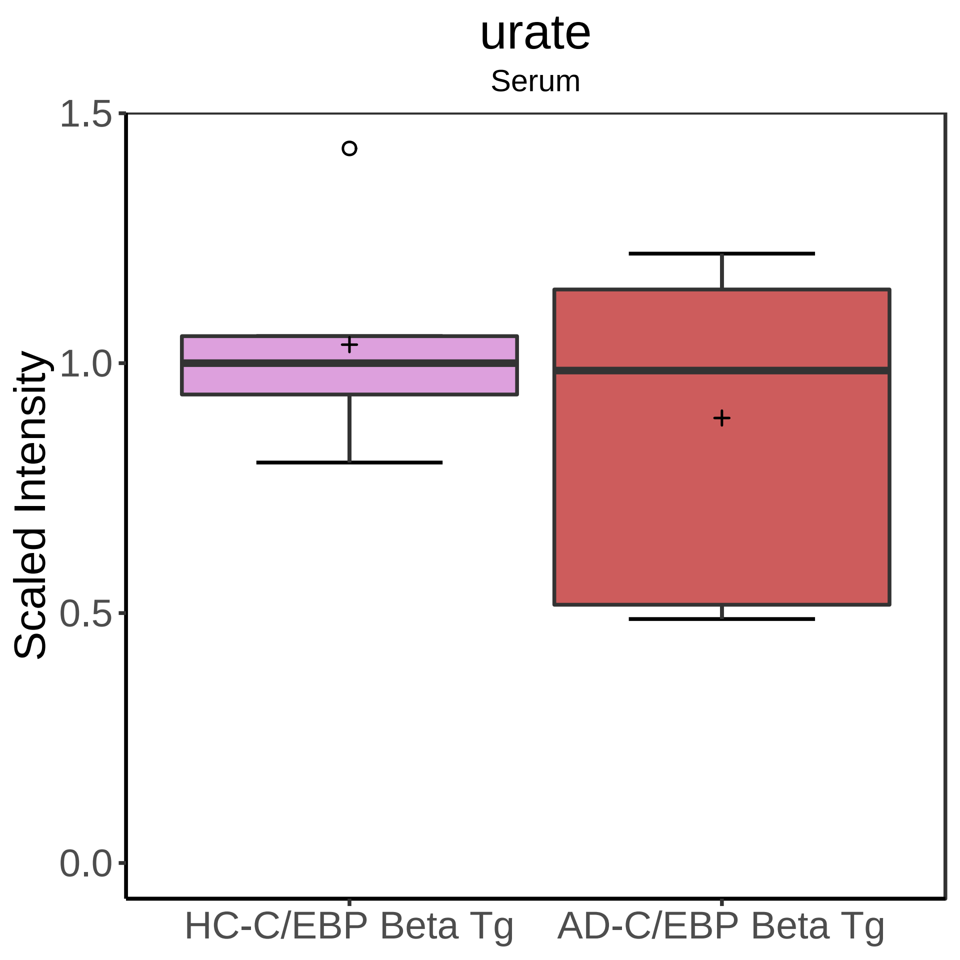

urate
Serum
Scaled Intensity
1.5
1.0
0.5
0.0
HC-C/EBP Beta Tg
AD-C/EBP Beta Tg

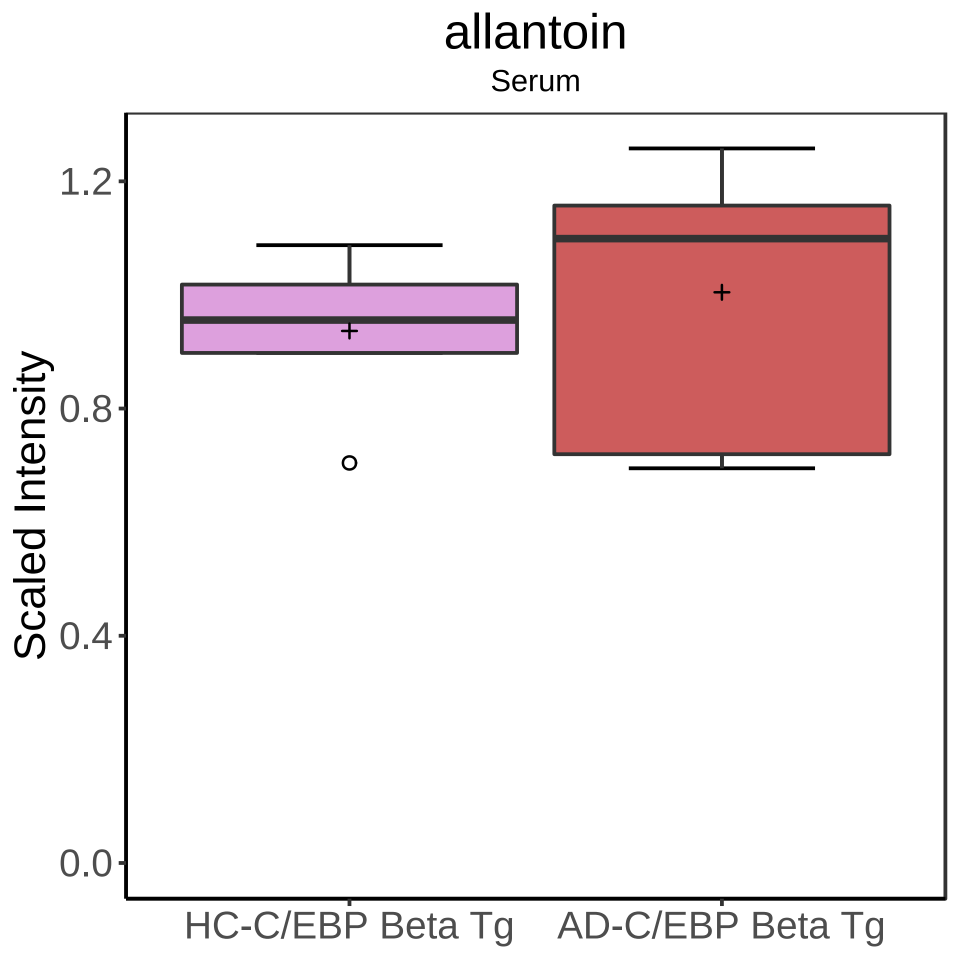
allantoin
Serum
Scaled Intensity
1.2
0.8
0.4
0.0
HC-C/EBP Beta Tg
AD-C/EBP Beta Tg

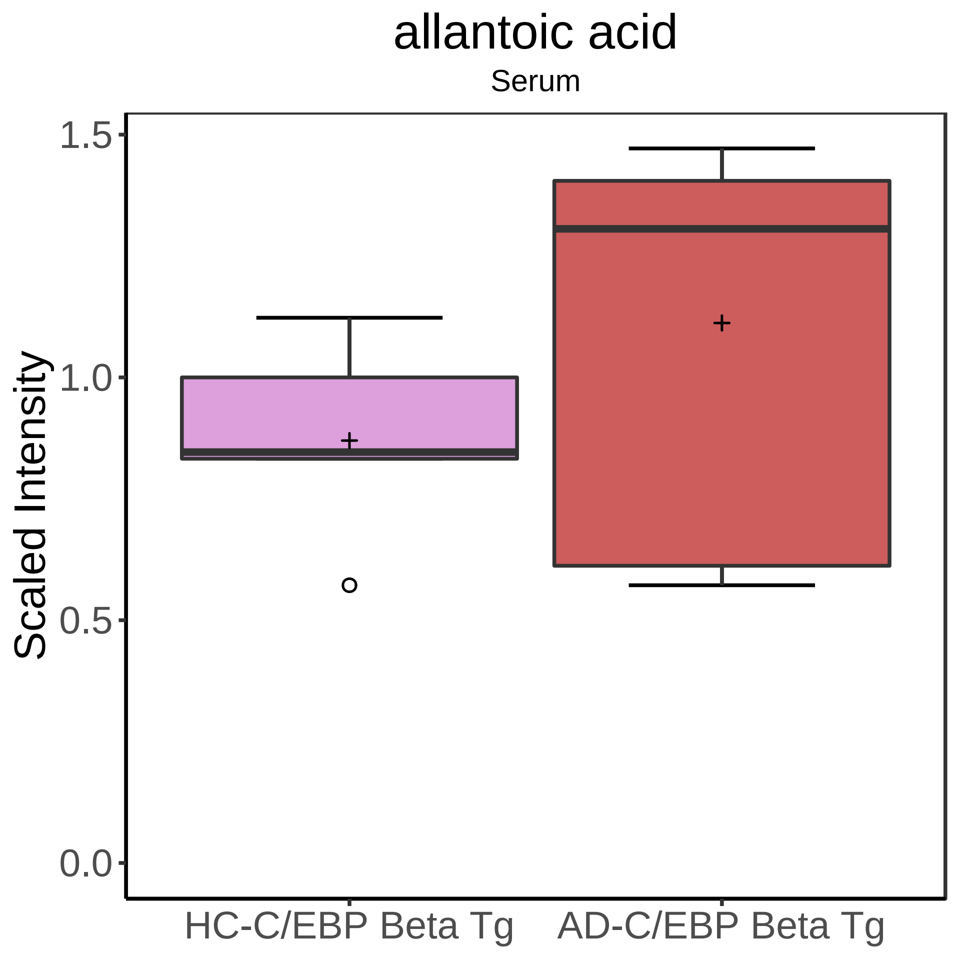
allantoic acid
Serum
Scaled Intensity
1.5
1.0
0.5
0.0
HC-C/EBP Beta Tg
AD-C/EBP Beta Tg

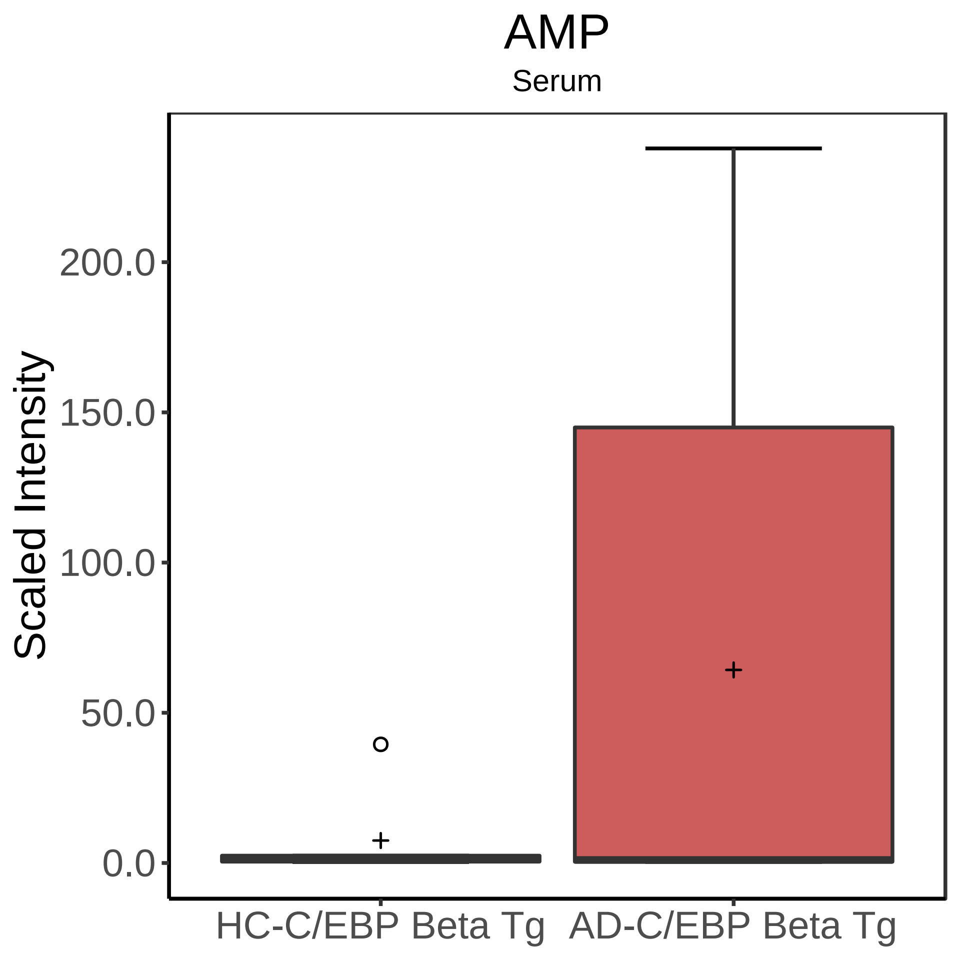
AMP
Serum
Scaled Intensity
200.0
150.0
100.0
50.0
0.0
HC-C/EBP Beta Tg
AD-C/EBP Beta Tg

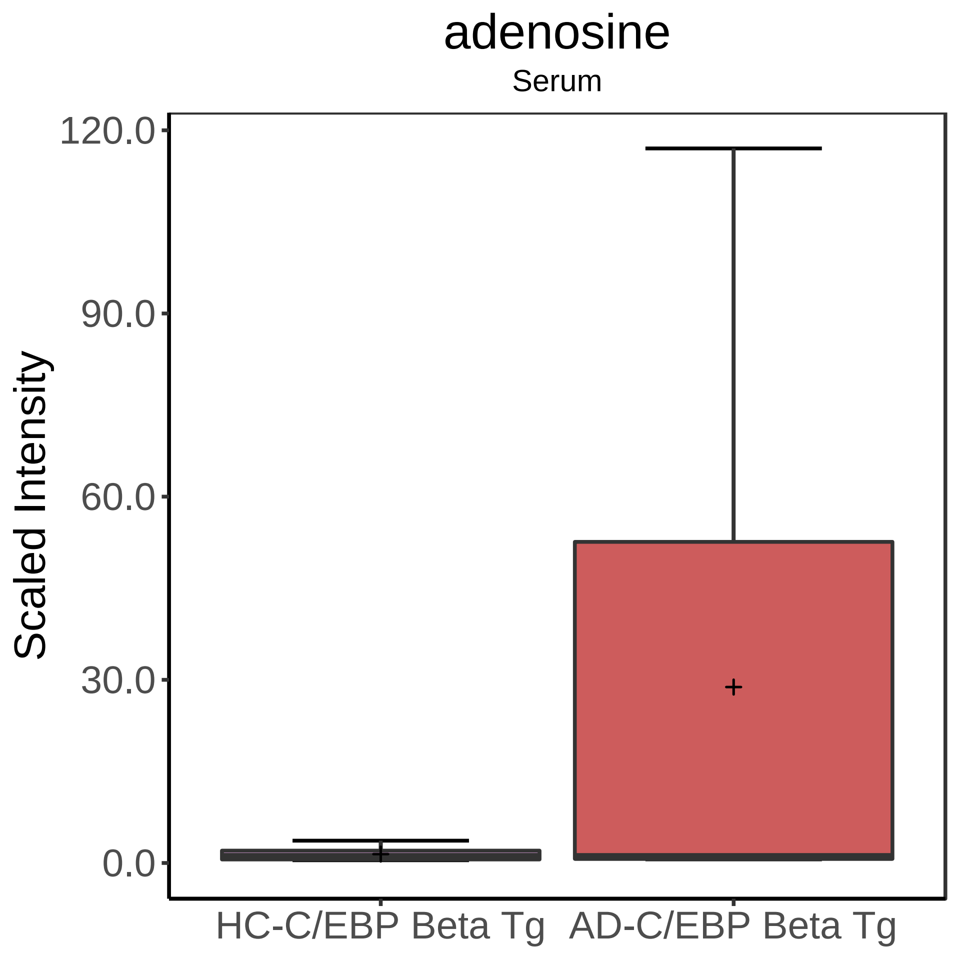

adenosine
Serum
Scaled Intensity
120.0
90.0
60.0
30.0
0.0
HC-C/EBP Beta Tg
AD-C/EBP Beta Tg

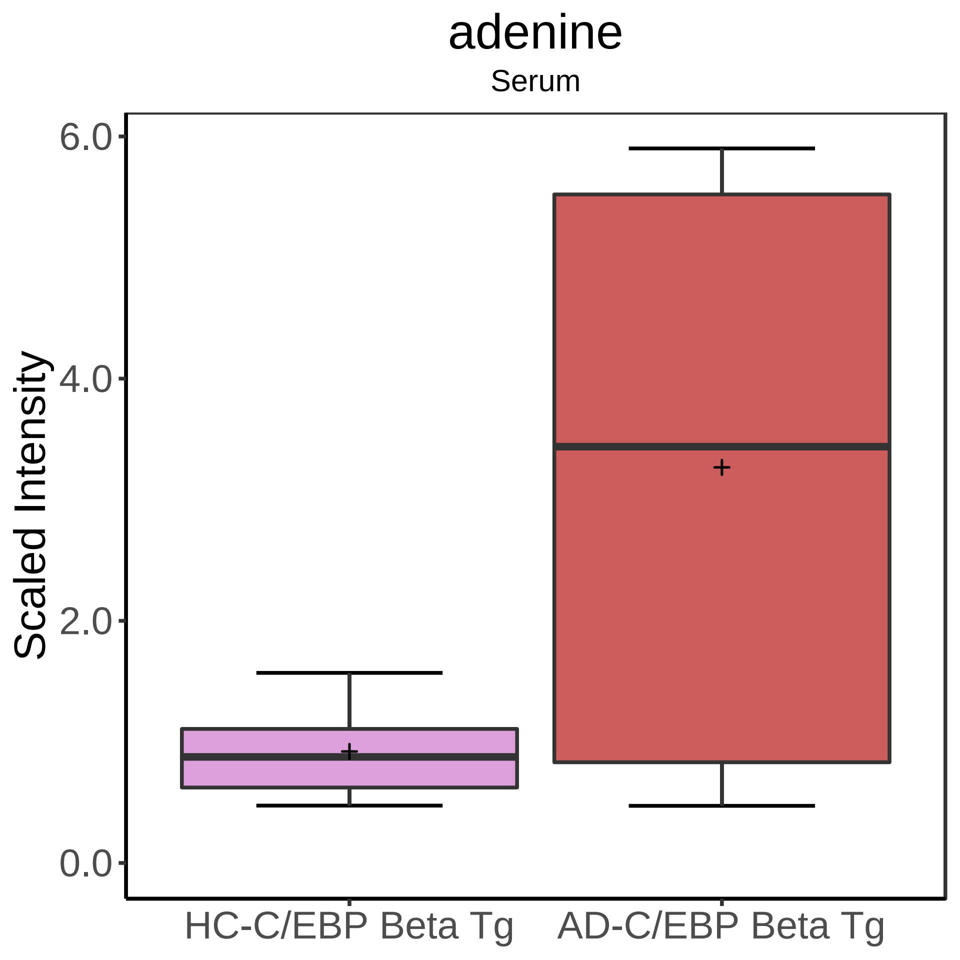
adenine
Serum
Scaled Intensity
6.0
4.0
2.0
0.0
HC-C/EBP Beta Tg
AD-C/EBP Beta Tg

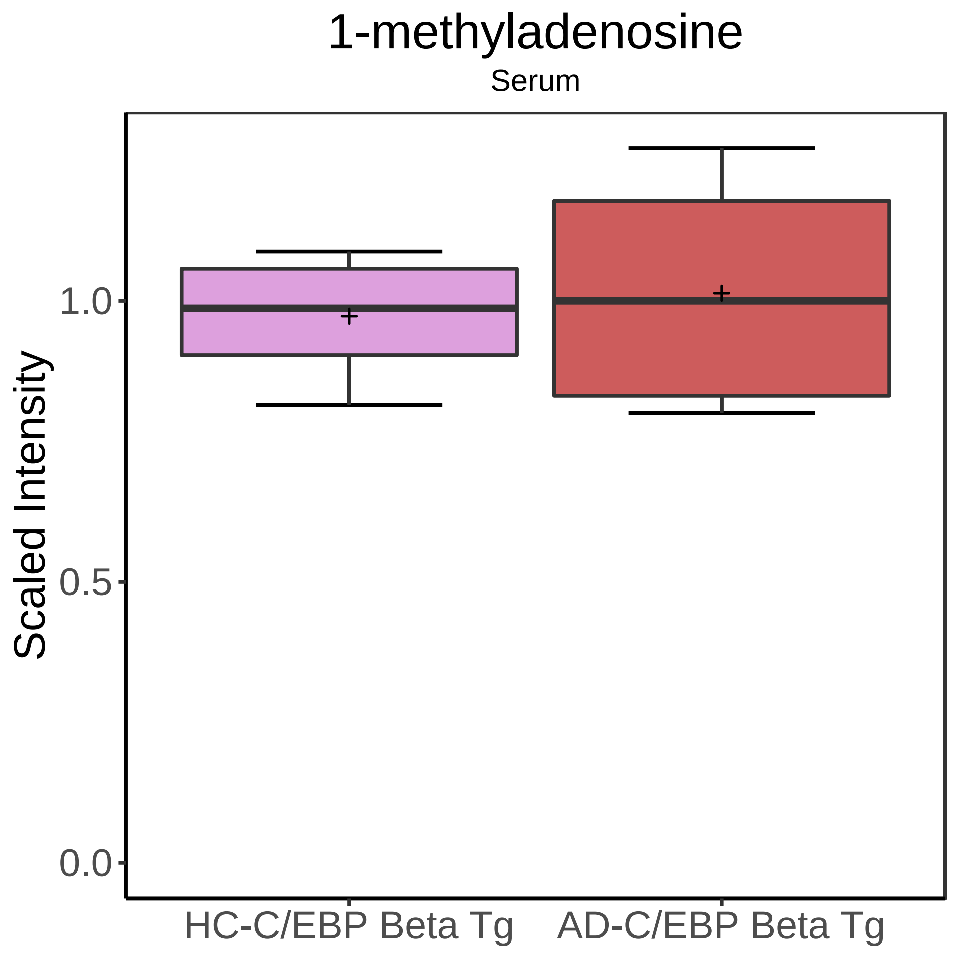
1-methyladenosine
Serum
Scaled Intensity
1.0
0.5
0.0
HC-C/EBP Beta Tg
AD-C/EBP Beta Tg

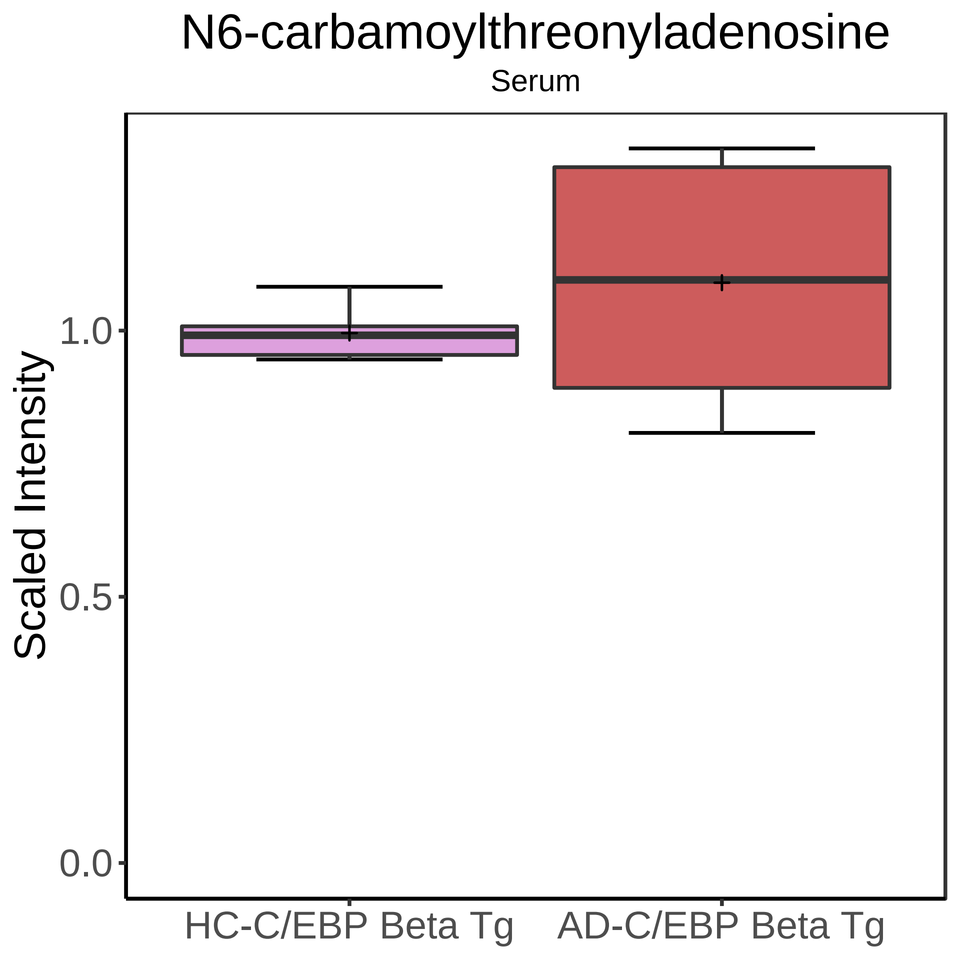
N6-carbamoylthreonyladenosine
Serum
Scaled Intensity
1.0
0.5
0.0
HC-C/EBP Beta Tg
AD-C/EBP Beta Tg

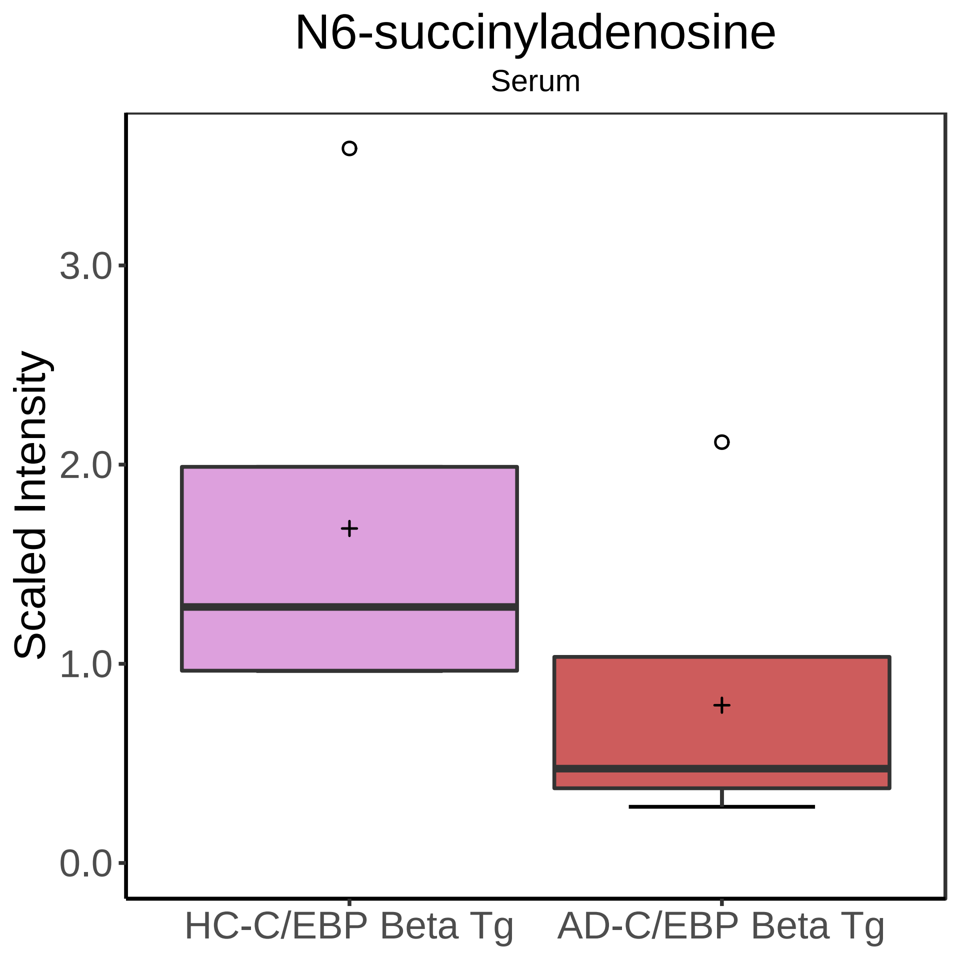

N6-succinyladenosine
Serum
Scaled Intensity
3.0
2.0
1.0
0.0
HC-C/EBP Beta Tg
AD-C/EBP Beta Tg

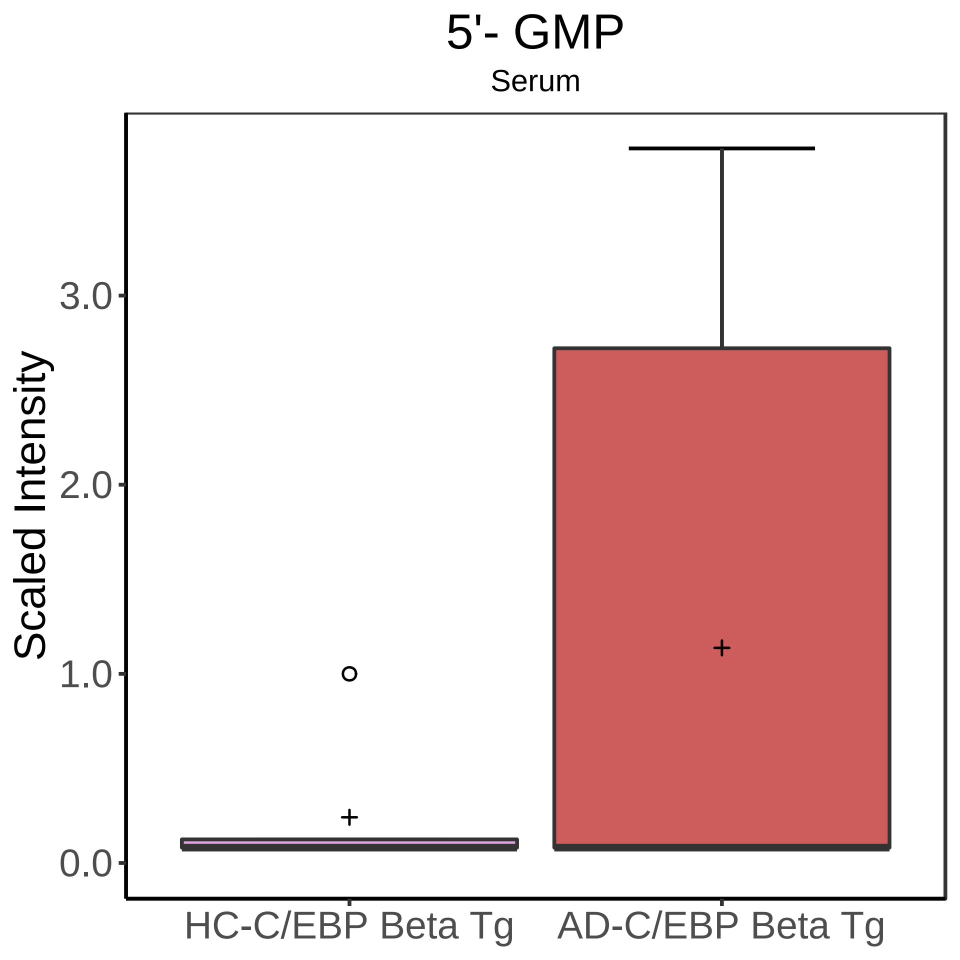
5'- GMP
Serum
Scaled Intensity
3.0
2.0
1.0
0.0
HC-C/EBP Beta Tg
AD-C/EBP Beta Tg

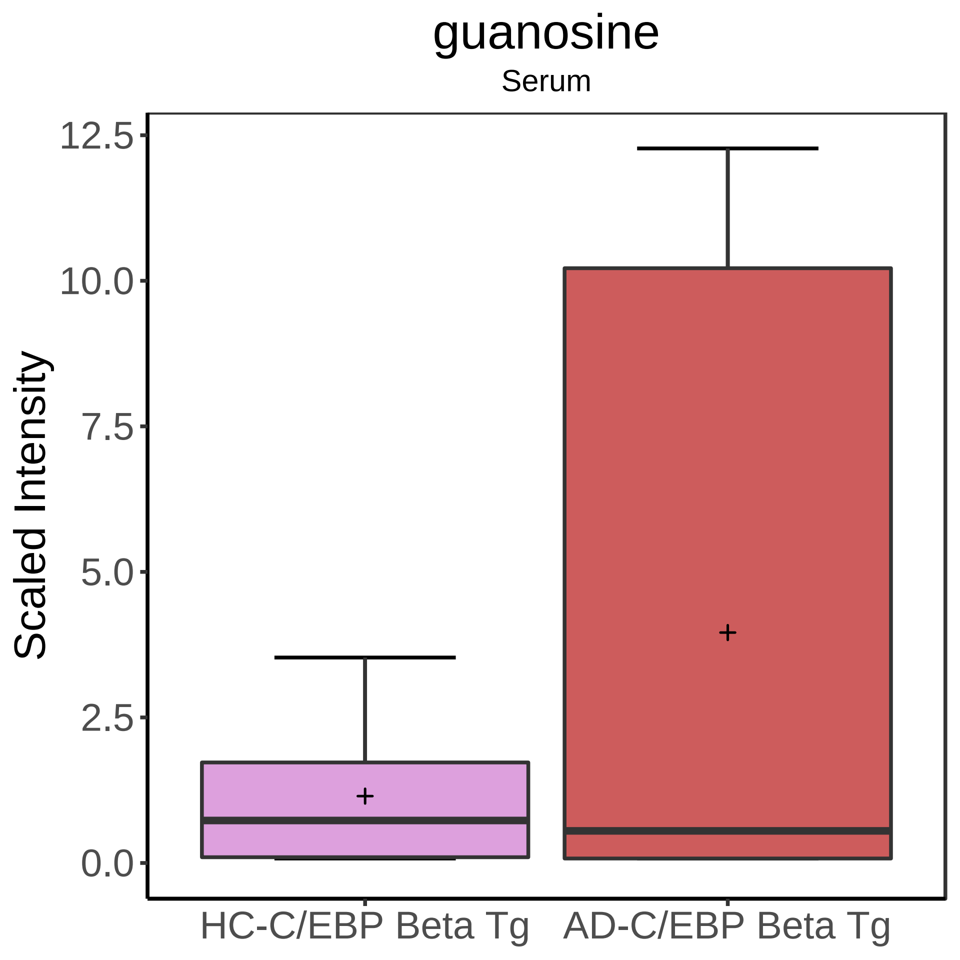
guanosine
Serum
Scaled Intensity
12.5
10.0
7.5
5.0
2.5
0.0
HC-C/EBP Beta Tg
AD-C/EBP Beta Tg

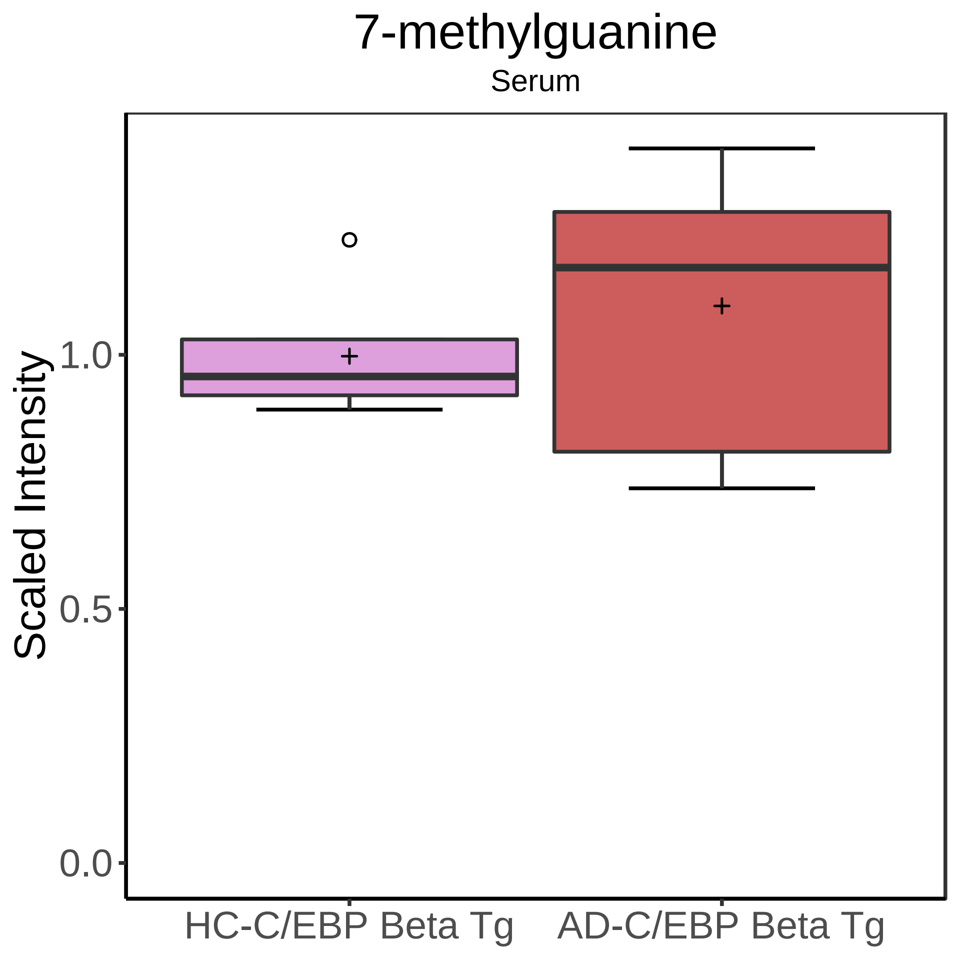
7-methylguanine
Serum
Scaled Intensity
1.0
0.5
0.0
HC-C/EBP Beta Tg
AD-C/EBP Beta Tg

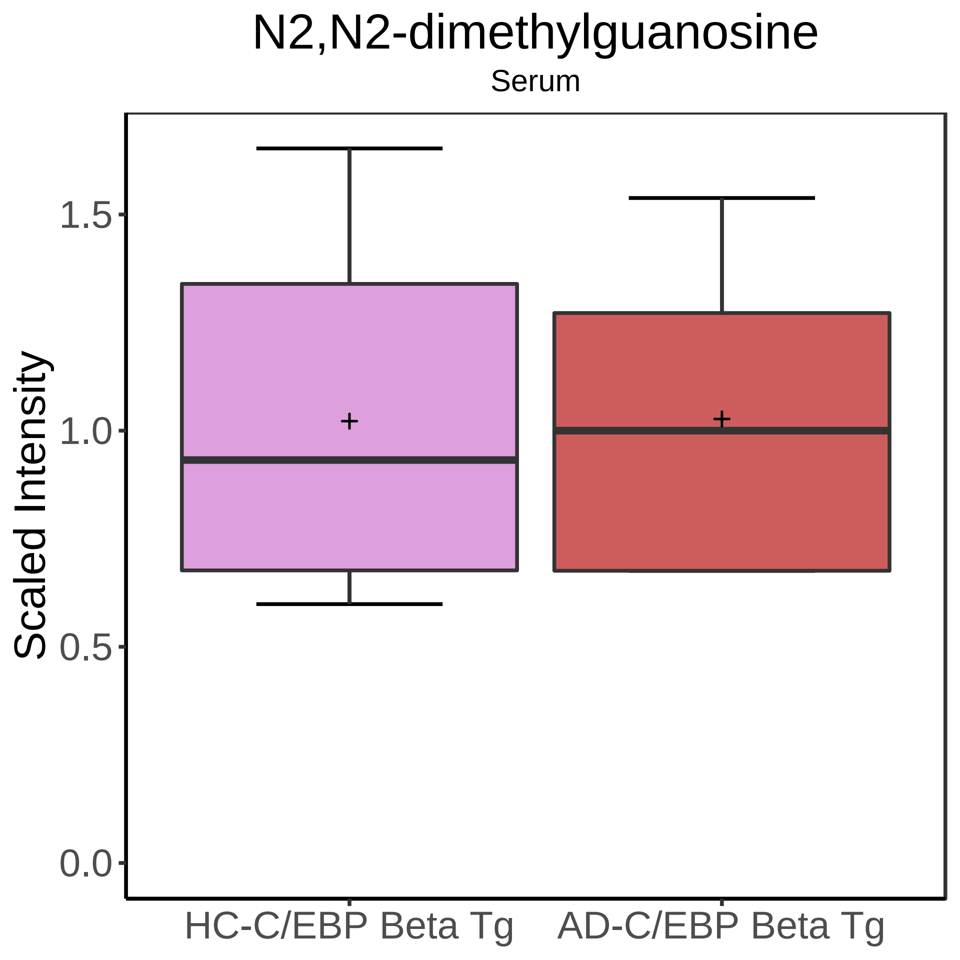
N2,N2-dimethylguanosine
Serum
Scaled Intensity
1.5
1.0
0.5
0.0
HC-C/EBP Beta Tg
AD-C/EBP Beta Tg

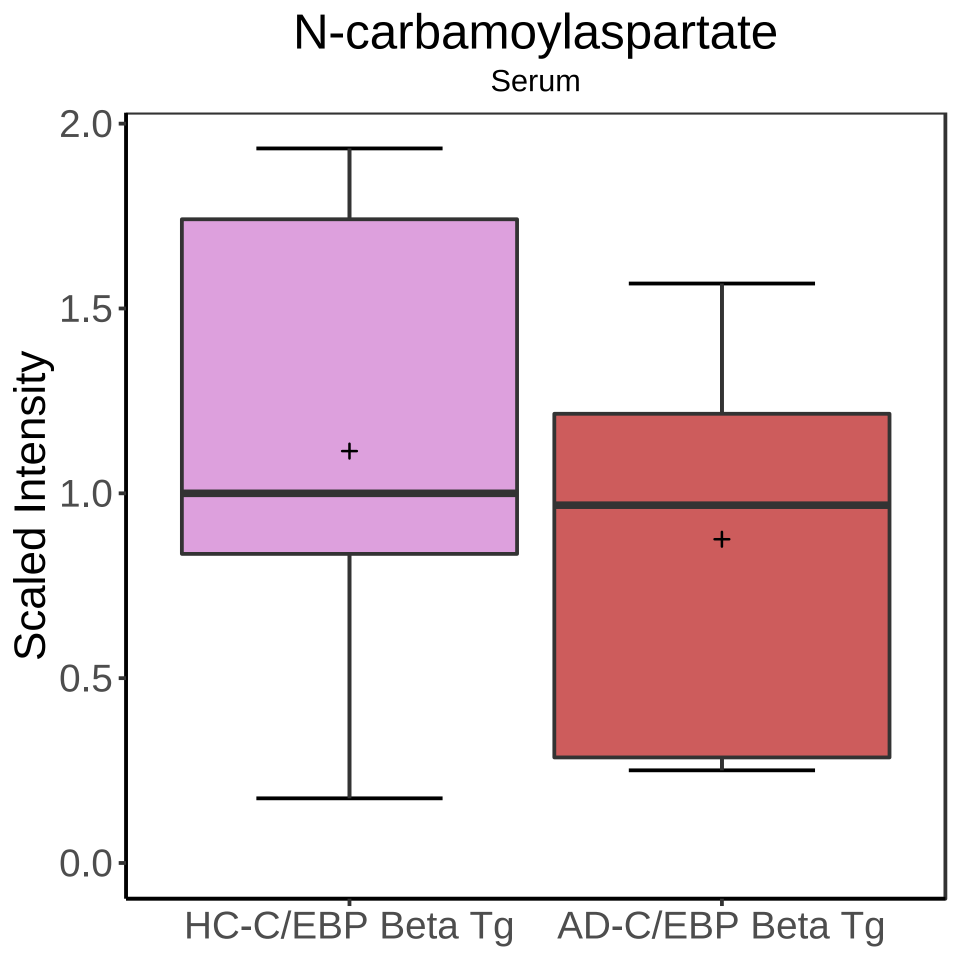
N-carbamoylaspartate
Serum
Scaled Intensity
2.0
1.5
1.0
0.5
0.0
HC-C/EBP Beta Tg
AD-C/EBP Beta Tg

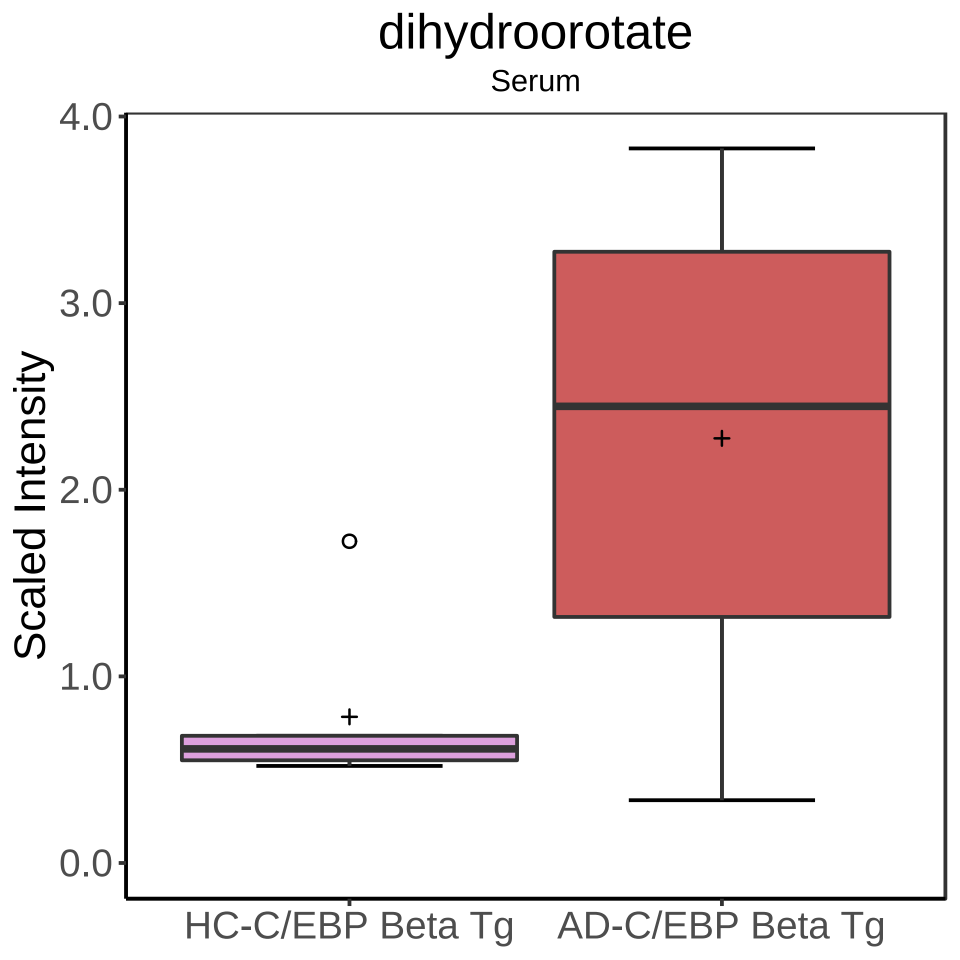

dihydroorotate
Serum
Scaled Intensity
4.0
3.0
2.0
1.0
0.0
HC-C/EBP Beta Tg
AD-C/EBP Beta Tg

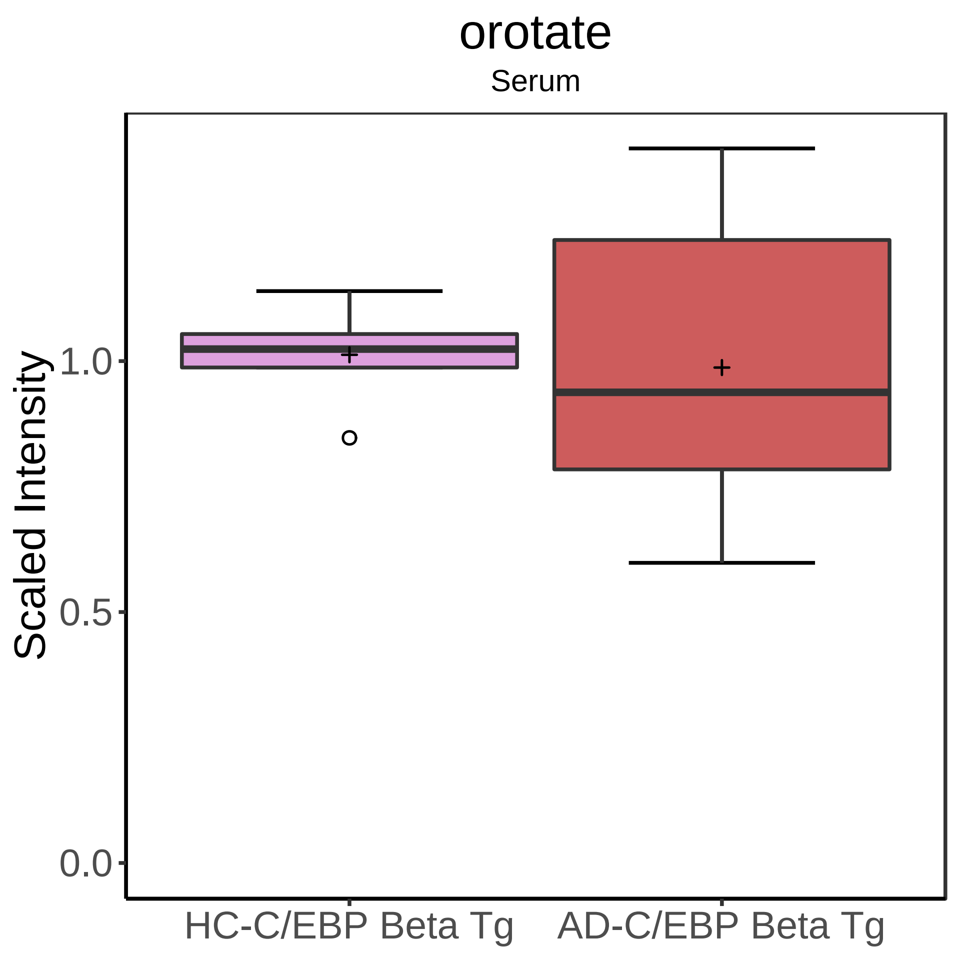
orotate
Serum
Scaled Intensity
1.0
0.5
0.0
HC-C/EBP Beta Tg
AD-C/EBP Beta Tg

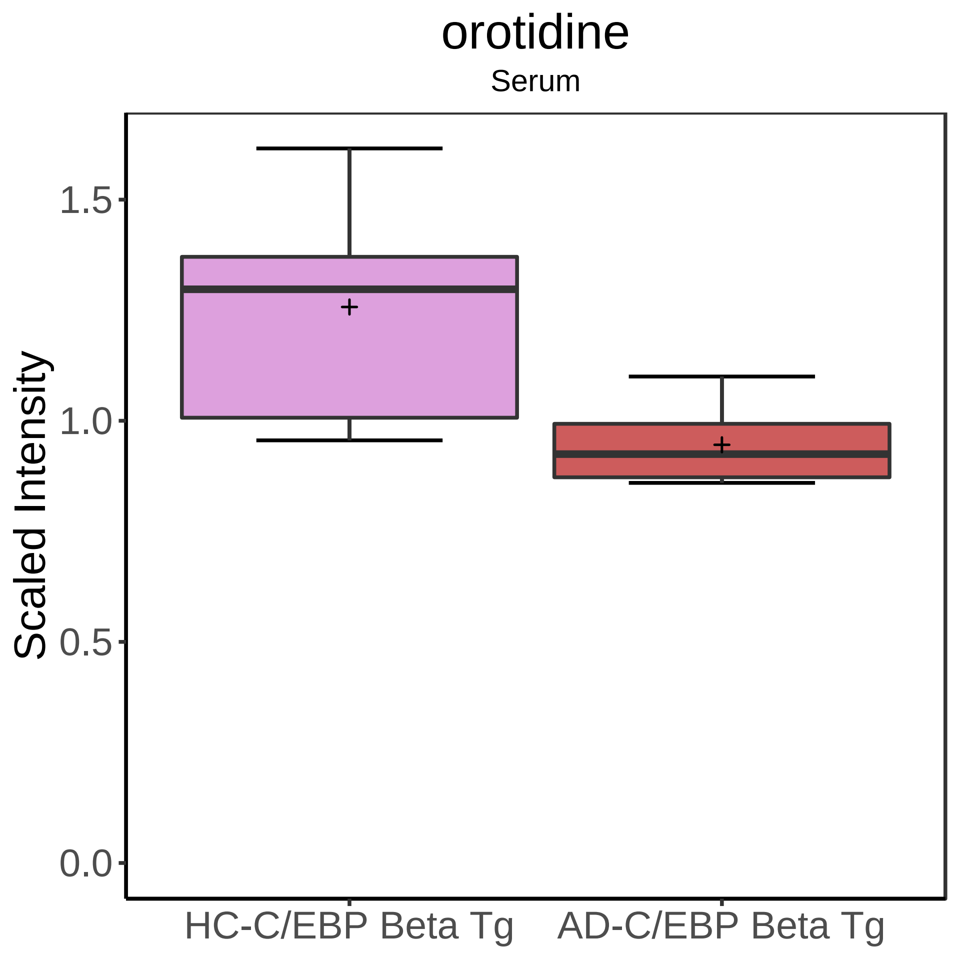
orotidine
Serum
Scaled Intensity
1.5
1.0
0.5
0.0
HC-C/EBP Beta Tg
AD-C/EBP Beta Tg

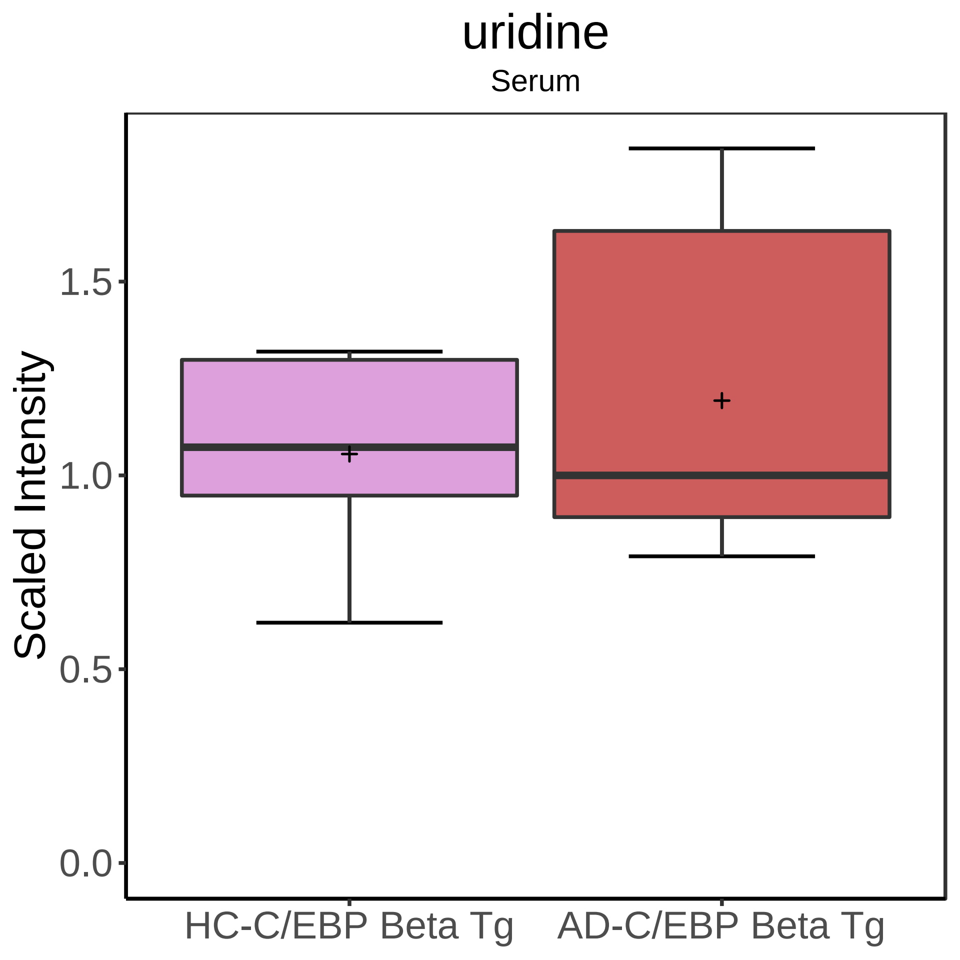
uridine
Serum
Scaled Intensity
0.0
0.5
1.0
1.5
HC-C/EBP Beta Tg
AD-C/EBP Beta Tg

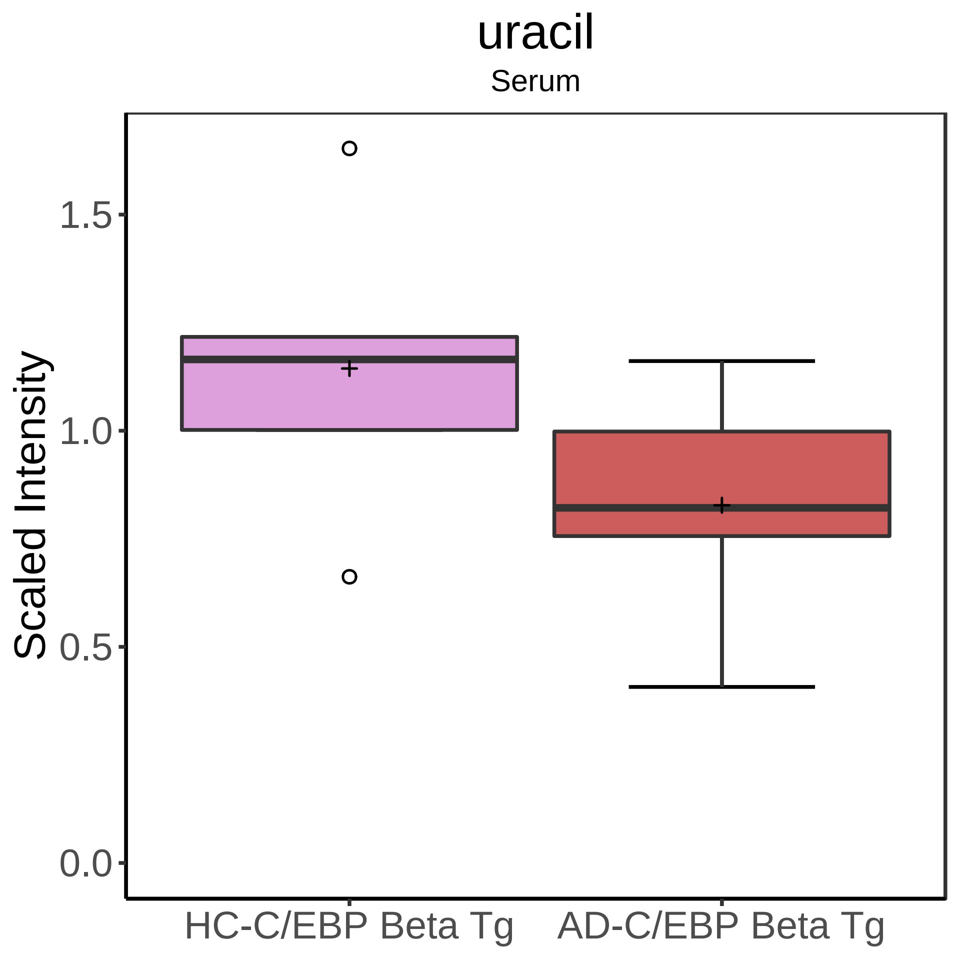
uracil
Serum
Scaled Intensity
1.5
1.0
0.5
0.0
HC-C/EBP Beta Tg
AD-C/EBP Beta Tg

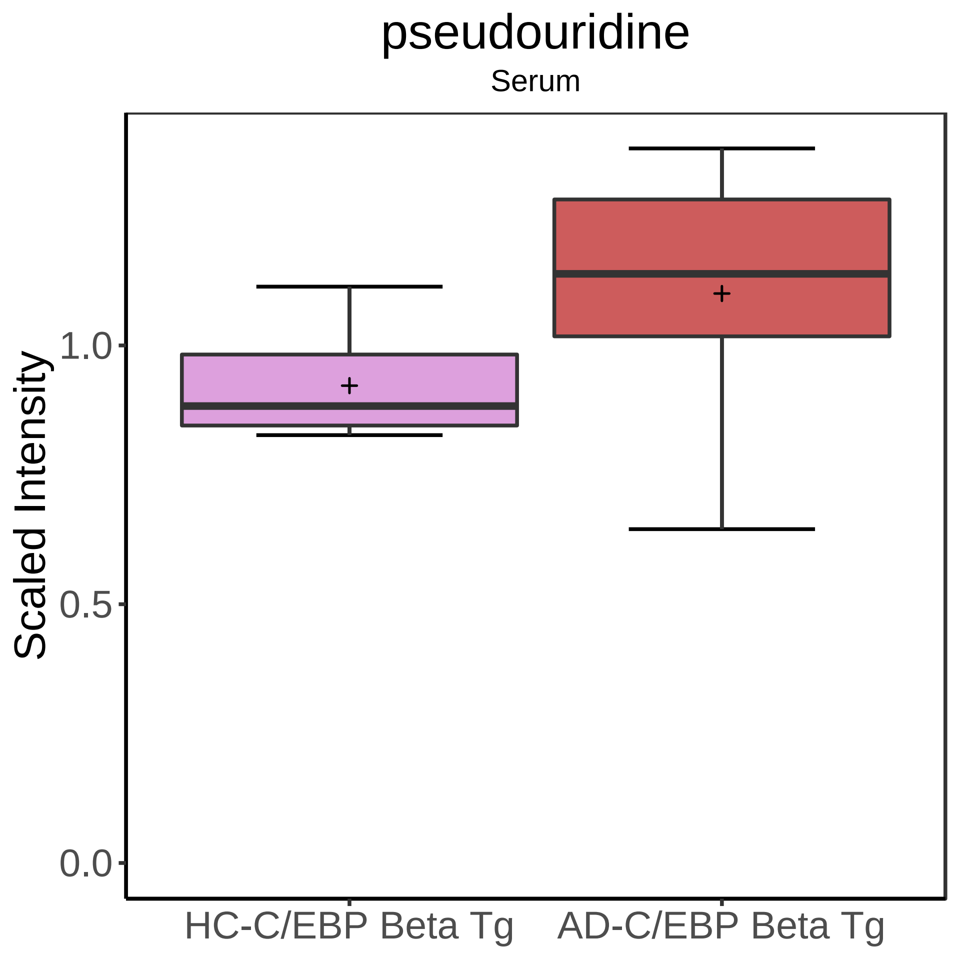
pseudouridine
Serum
Scaled Intensity
1.0
0.5
0.0
HC-C/EBP Beta Tg
AD-C/EBP Beta Tg

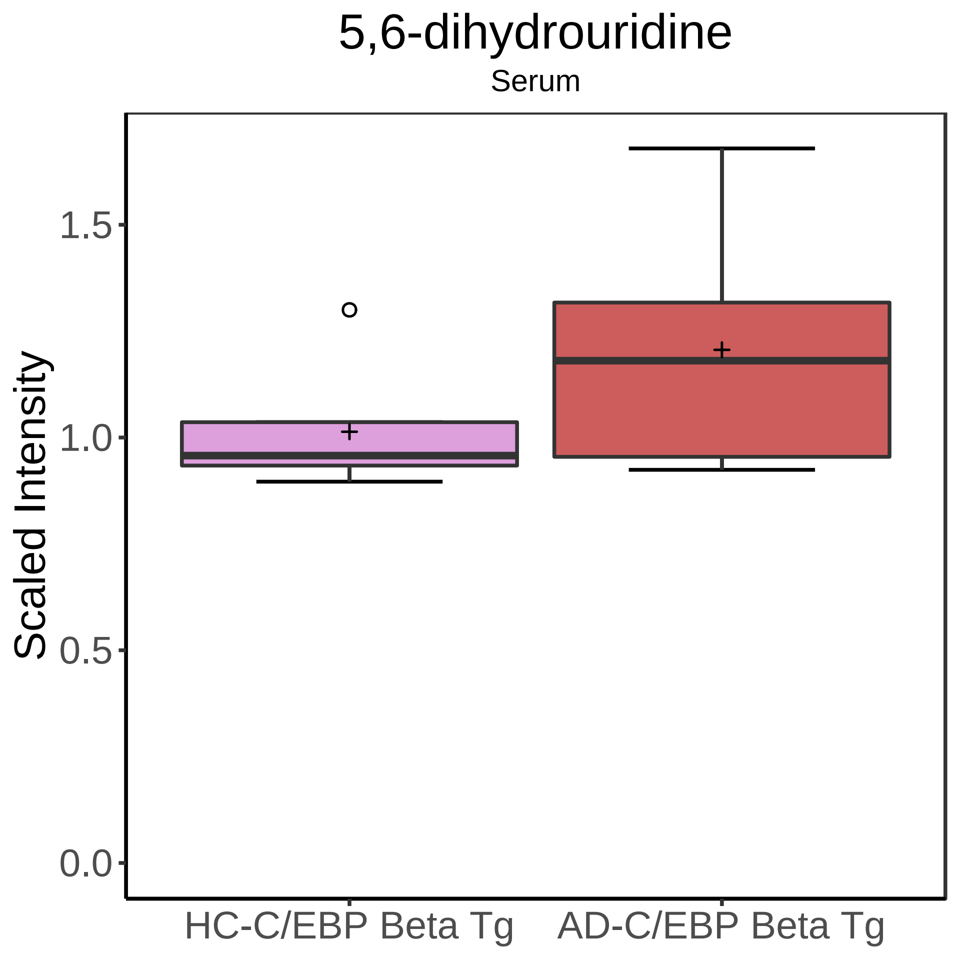
5,6-dihydrouridine
Serum
Scaled Intensity
1.5
1.0
0.5
0.0
HC-C/EBP Beta Tg
AD-C/EBP Beta Tg

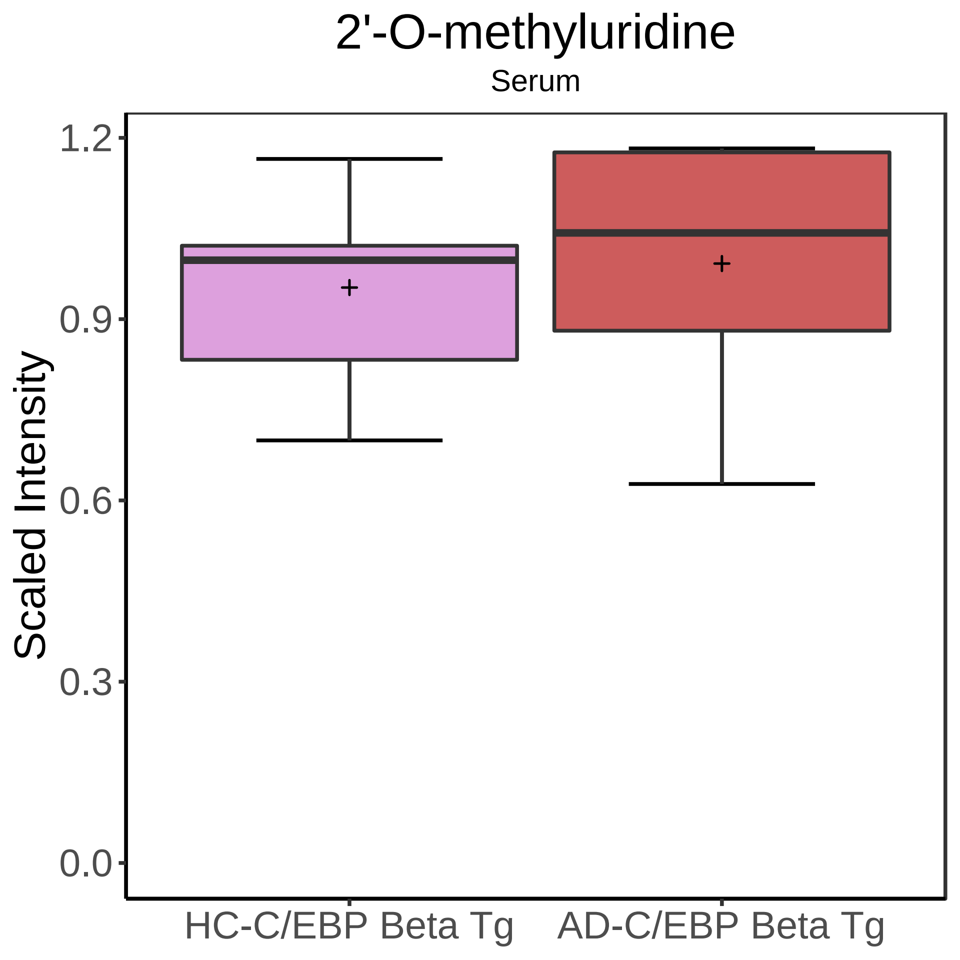
2'-O-methyluridine
Serum
Scaled Intensity
1.2
0.9
0.6
0.3
0.0
HC-C/EBP Beta Tg
AD-C/EBP Beta Tg

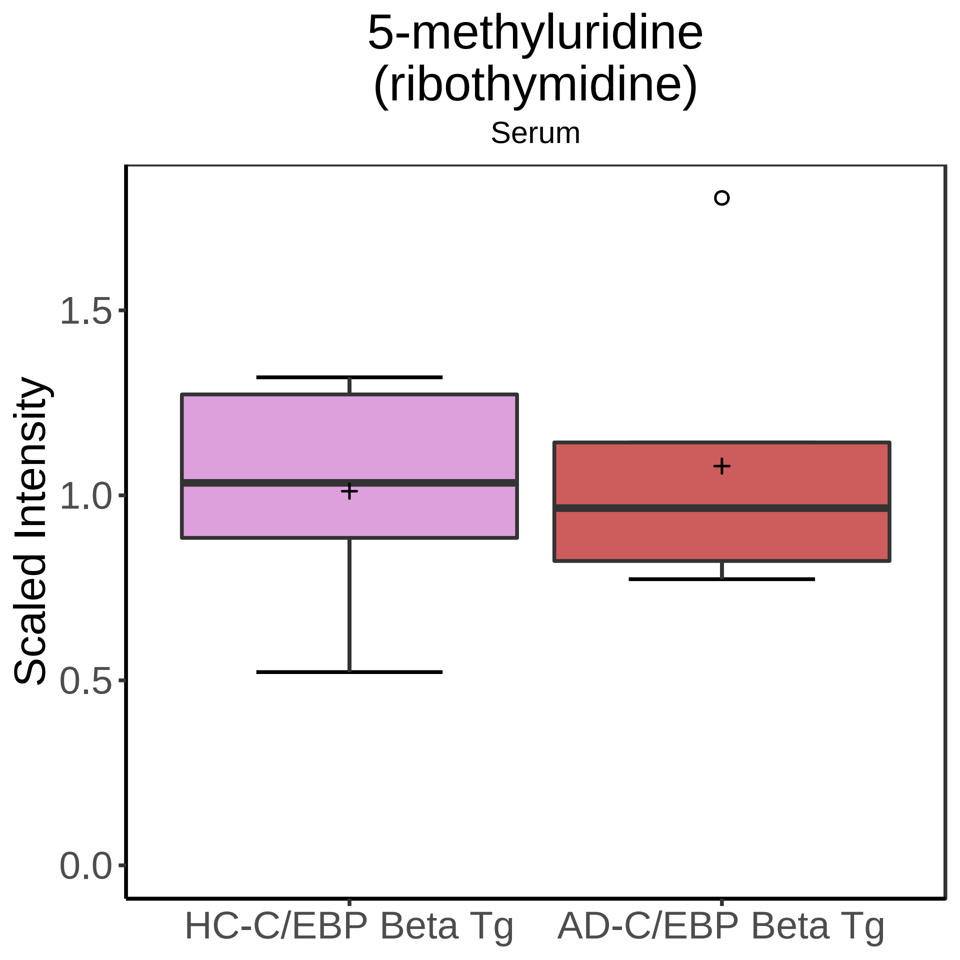
5-methyluridine
(ribothymidine)
Serum
Scaled Intensity
1.5
1.0
0.5
0.0
HC-C/EBP Beta Tg
AD-C/EBP Beta Tg

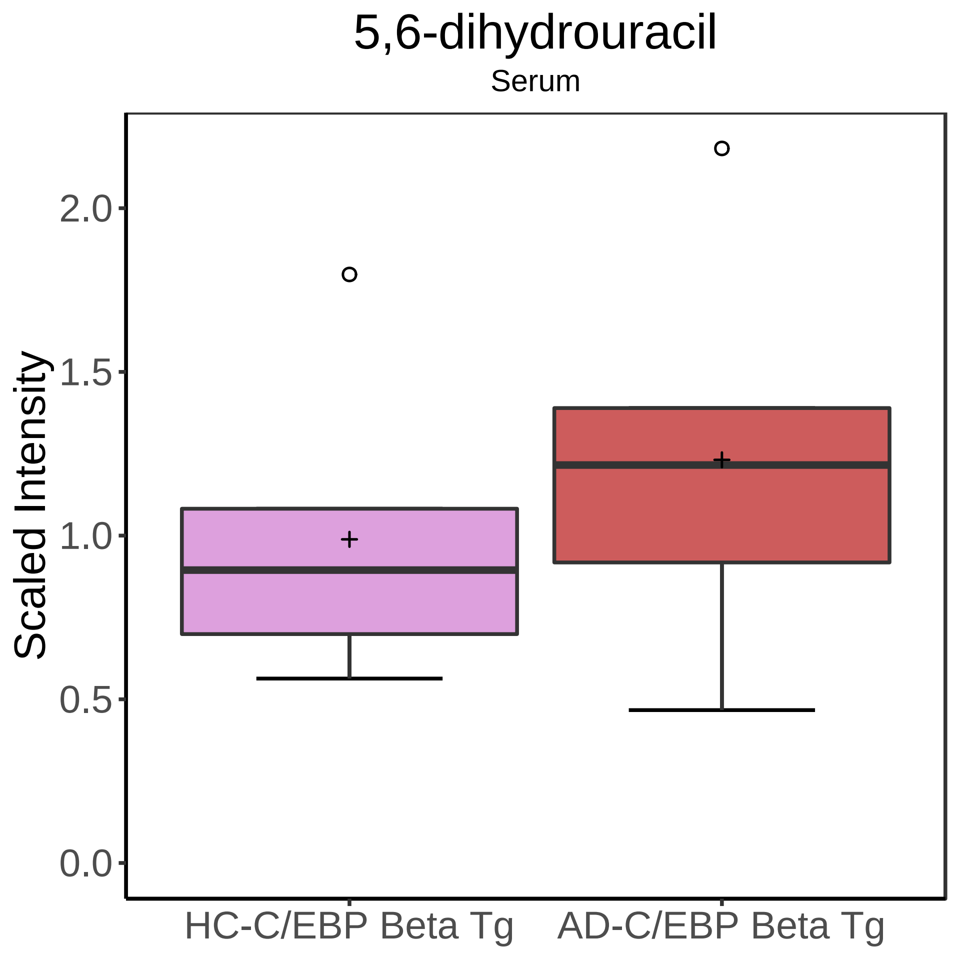
5,6-dihydrouracil
Serum
Scaled Intensity
2.0
1.5
1.0
0.5
0.0
HC-C/EBP Beta Tg
AD-C/EBP Beta Tg

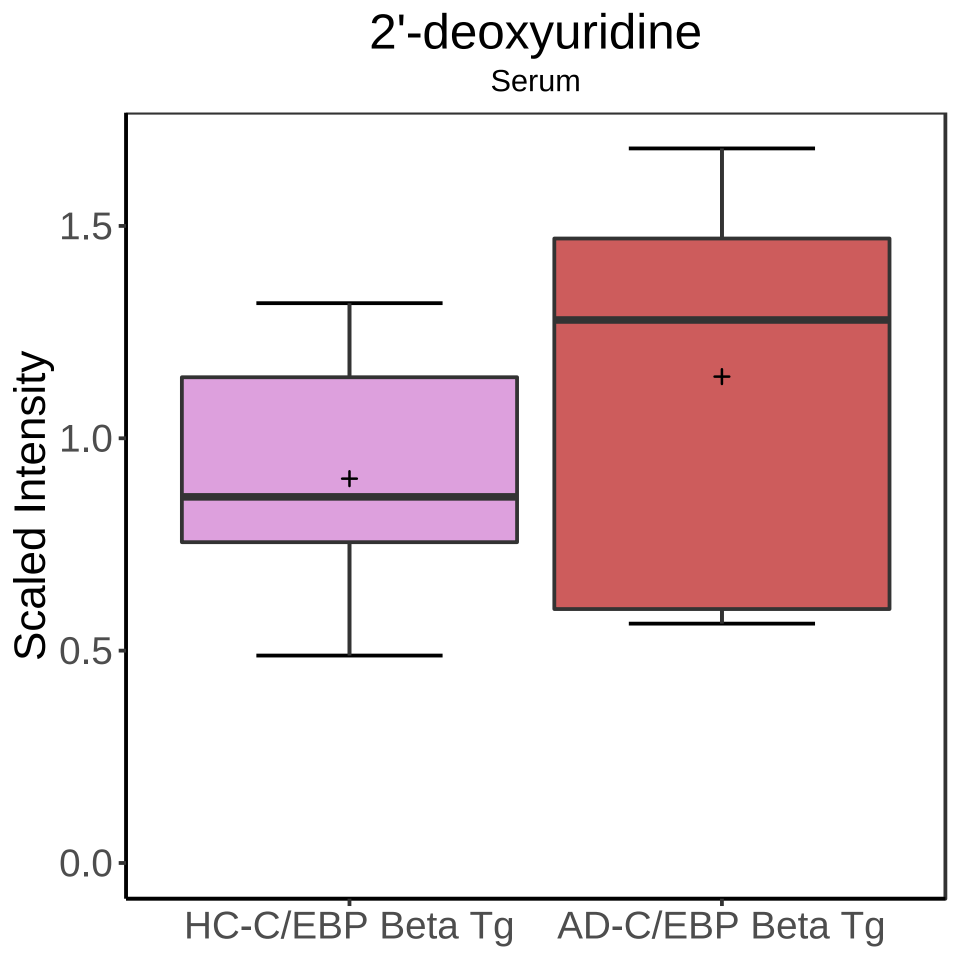
2'-deoxyuridine
Serum
Scaled Intensity
1.5
1.0
0.5
0.0
HC-C/EBP Beta Tg
AD-C/EBP Beta Tg

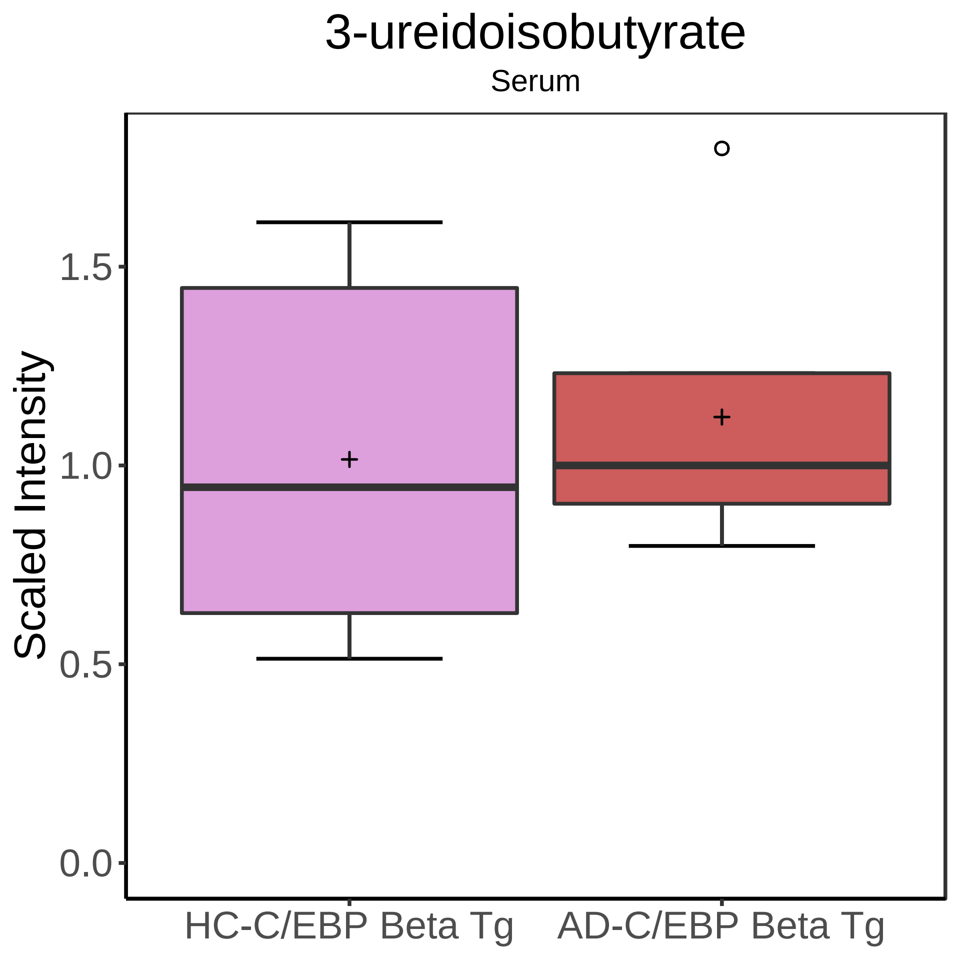
3-ureidoisobutyrate
Serum
Scaled Intensity
1.5
1.0
0.5
0.0
HC-C/EBP Beta Tg
AD-C/EBP Beta Tg

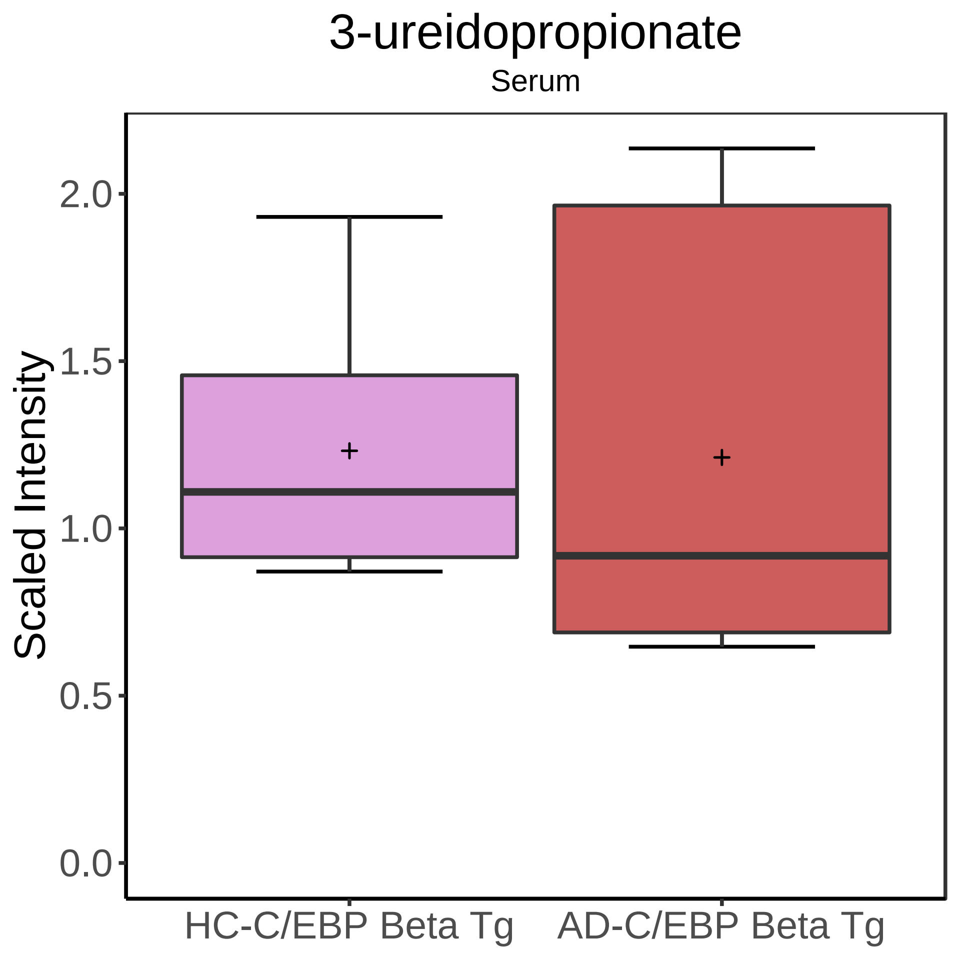
3-ureidopropionate
Serum
Scaled Intensity
2.0
1.5
1.0
0.5
0.0
HC-C/EBP Beta Tg
AD-C/EBP Beta Tg

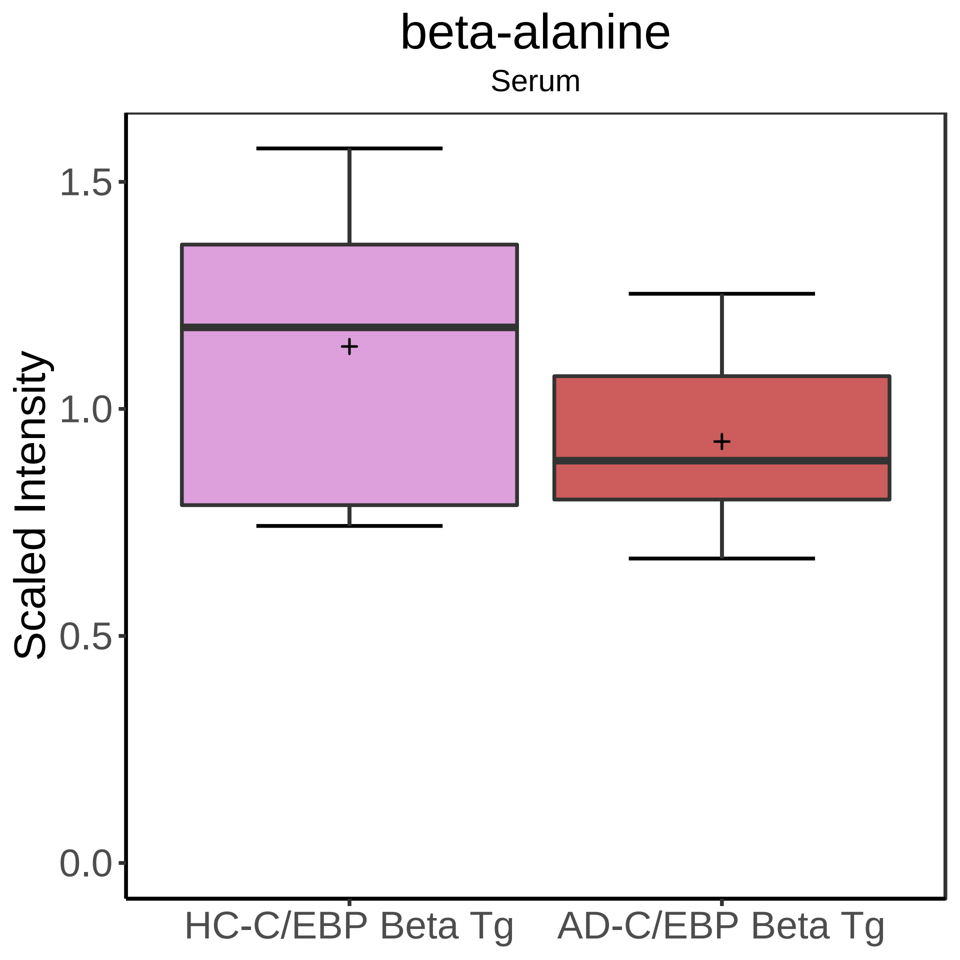
beta-alanine
Serum
Scaled Intensity
1.5
1.0
0.5
0.0
HC-C/EBP Beta Tg
AD-C/EBP Beta Tg

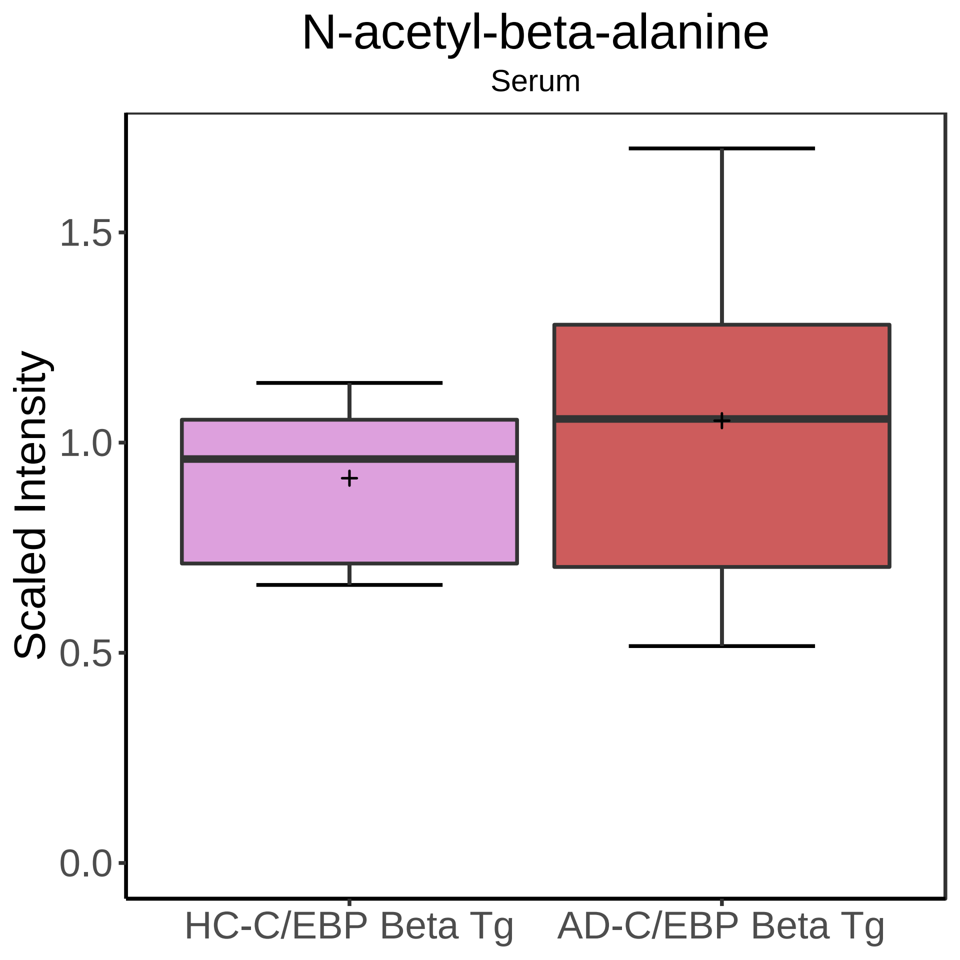
N-acetyl-beta-alanine
Serum
Scaled Intensity
1.5
1.0
0.5
0.0
HC-C/EBP Beta Tg
AD-C/EBP Beta Tg

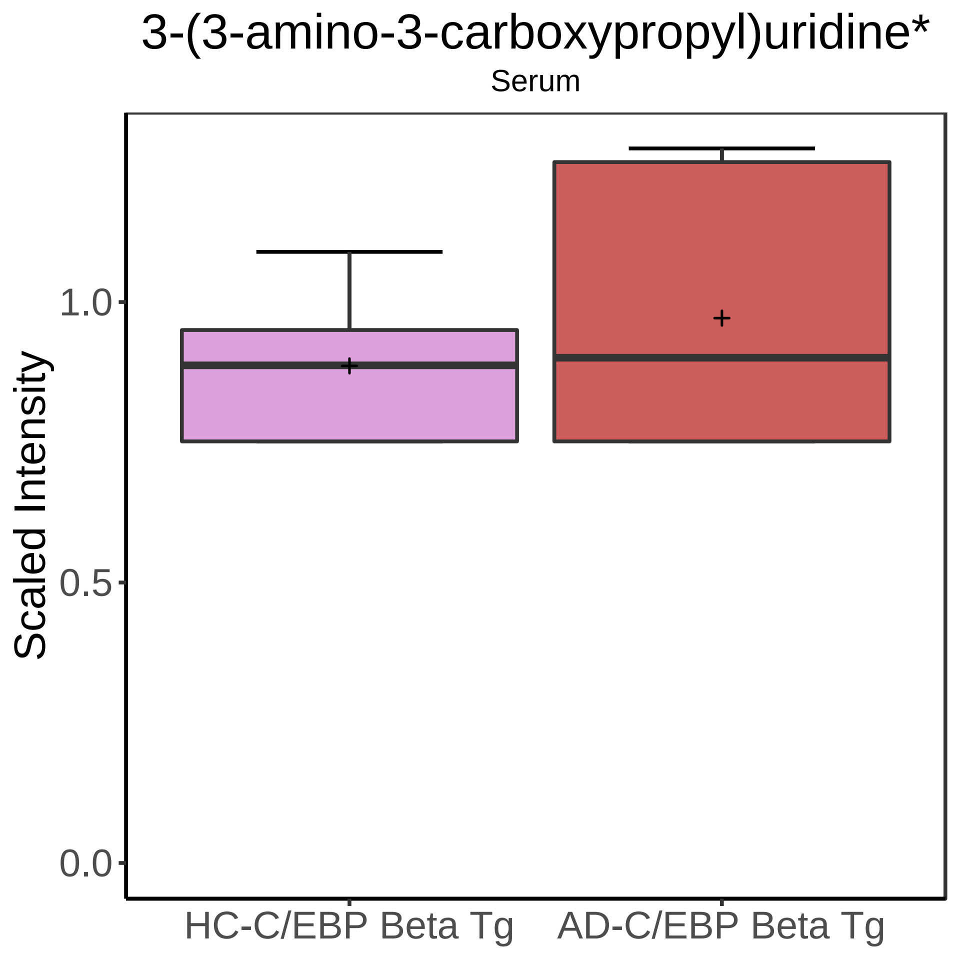

3-(3-amino-3-carboxypropyl)uridine*
Serum
Scaled Intensity
1.0
0.5
0.0
HC-C/EBP Beta Tg
AD-C/EBP Beta Tg

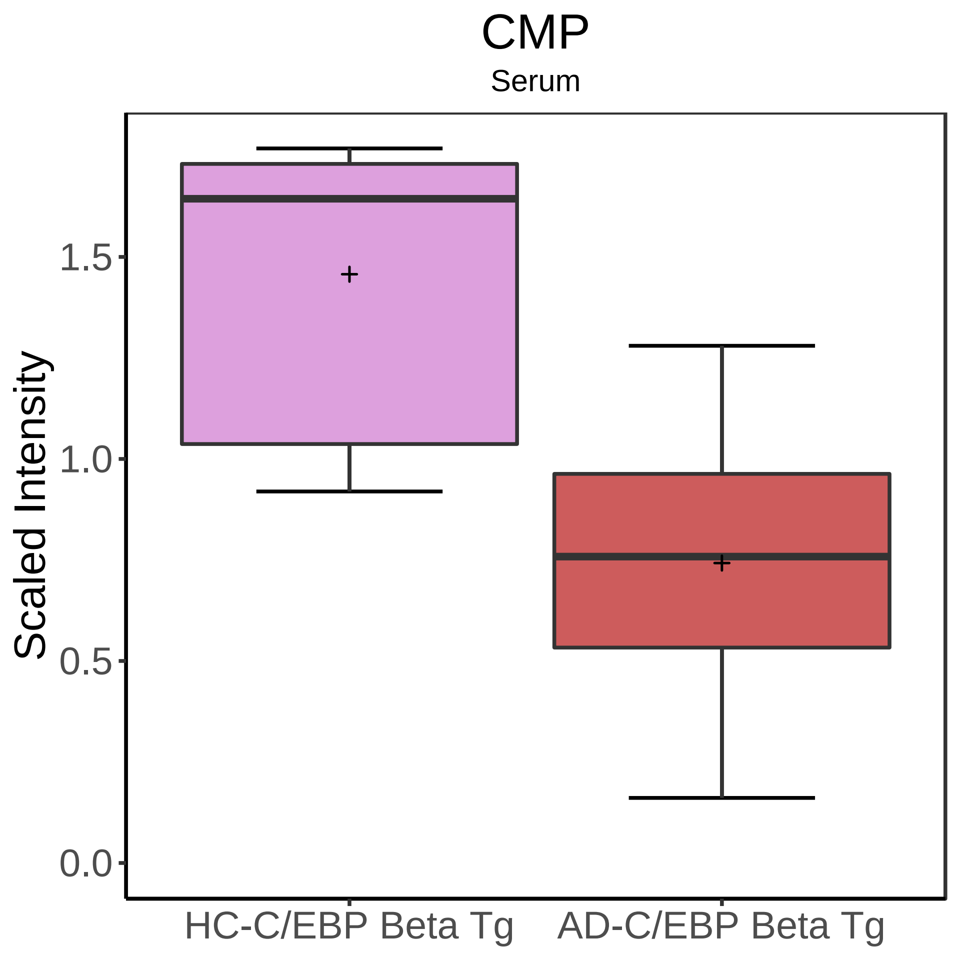
CMP
Serum
Scaled Intensity
1.5
1.0
0.5
0.0
HC-C/EBP Beta Tg
AD-C/EBP Beta Tg

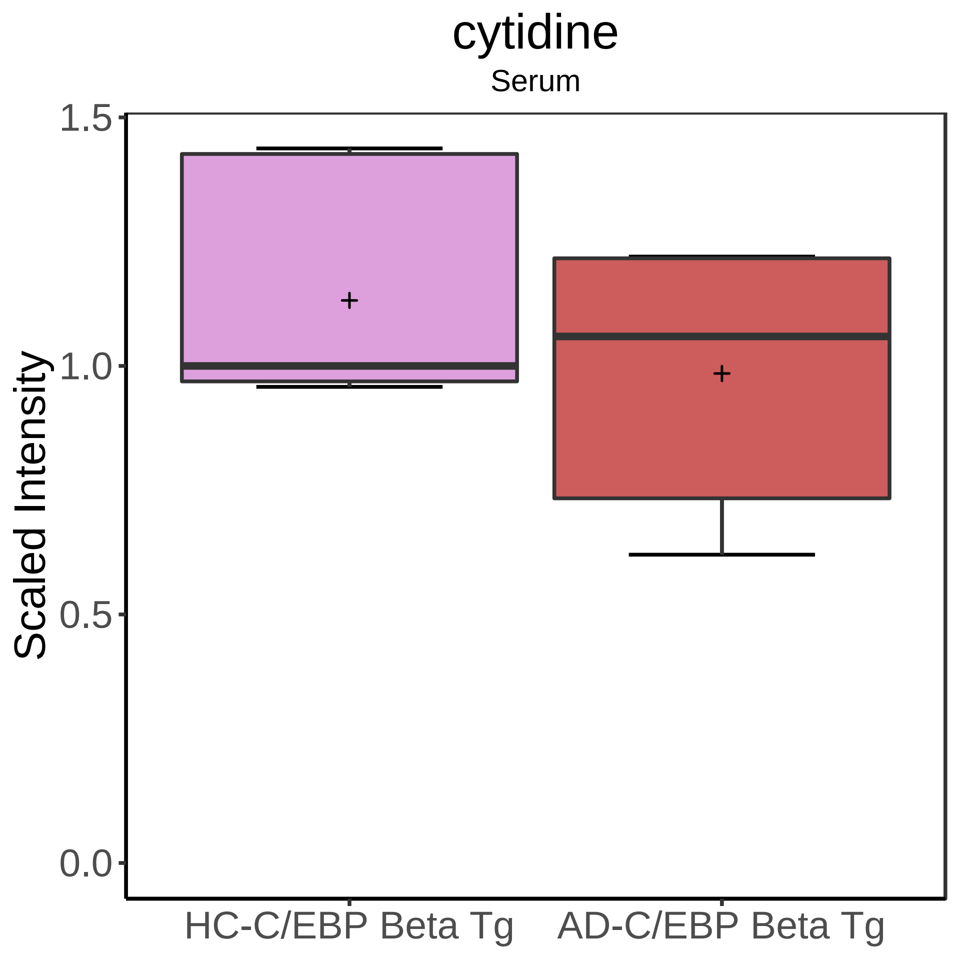
cytidine
Serum
Scaled Intensity
1.5
1.0
0.5
0.0
HC-C/EBP Beta Tg
AD-C/EBP Beta Tg

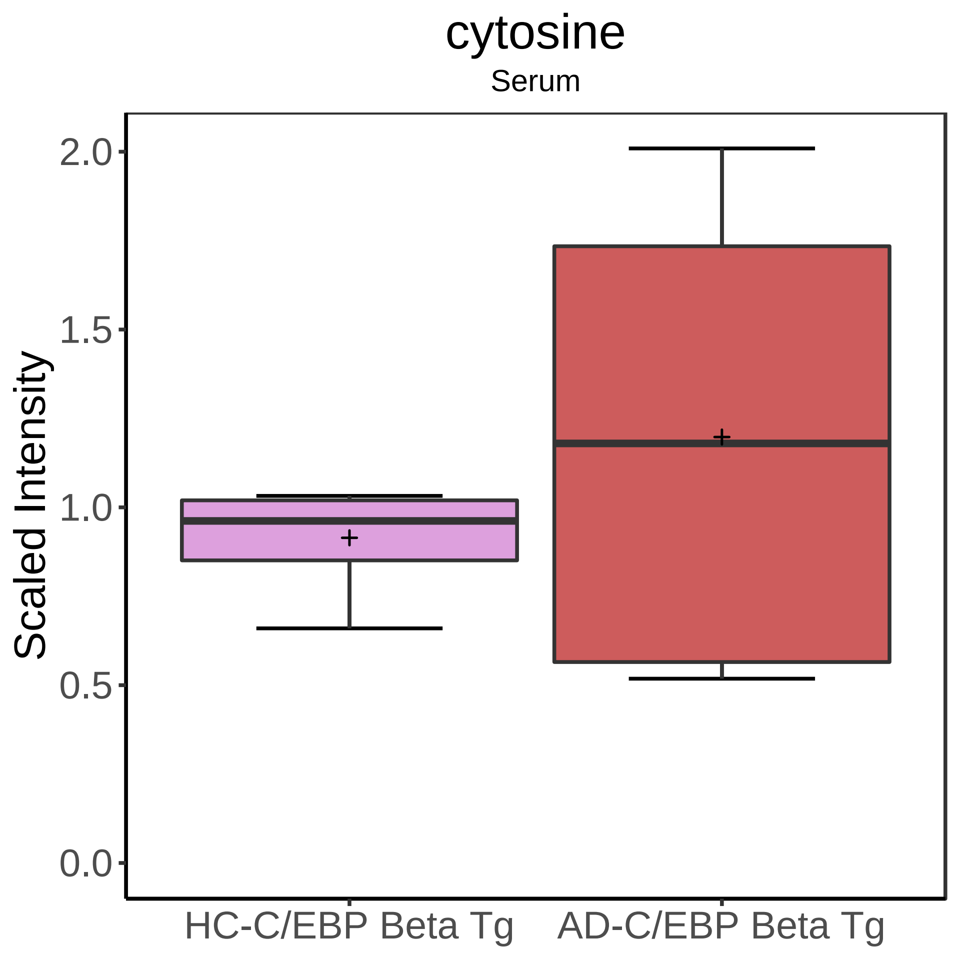
cytosine
Serum
Scaled Intensity
2.0
1.5
1.0
0.5
0.0
HC-C/EBP Beta Tg
AD-C/EBP Beta Tg

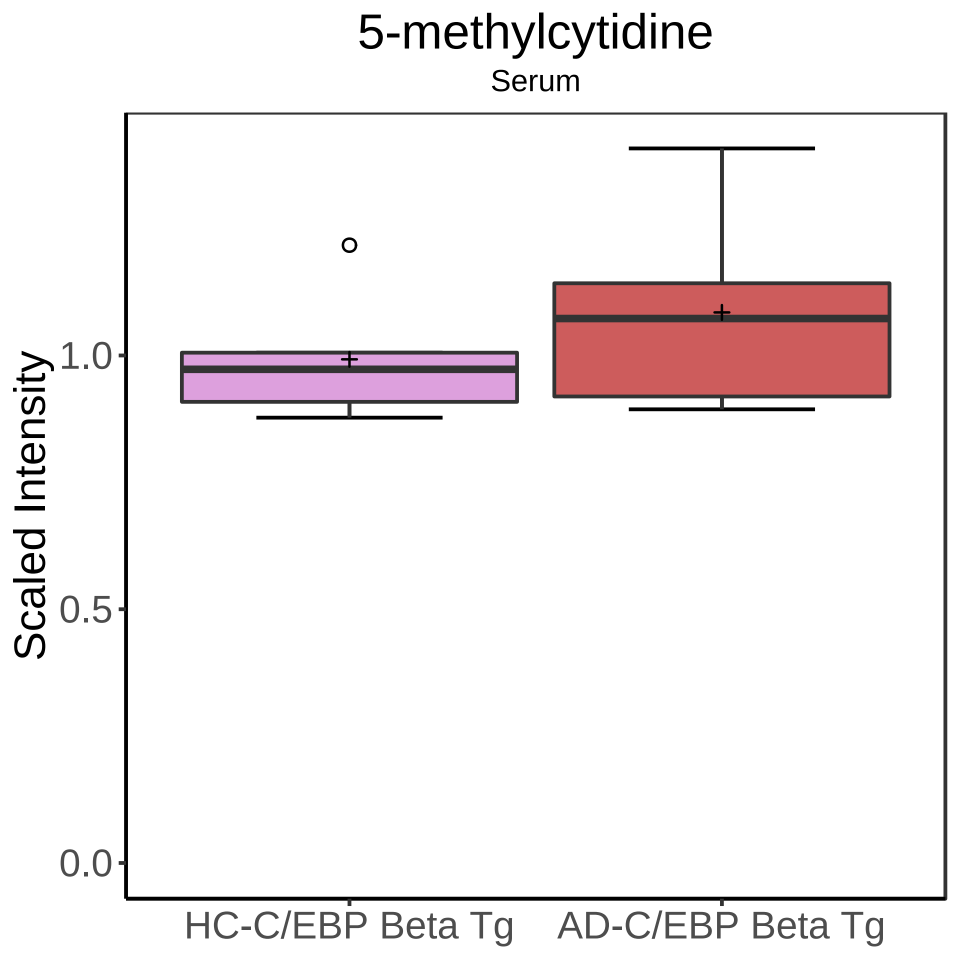
5-methylcytidine
Serum
Scaled Intensity
1.0
0.5
0.0
HC-C/EBP Beta Tg
AD-C/EBP Beta Tg

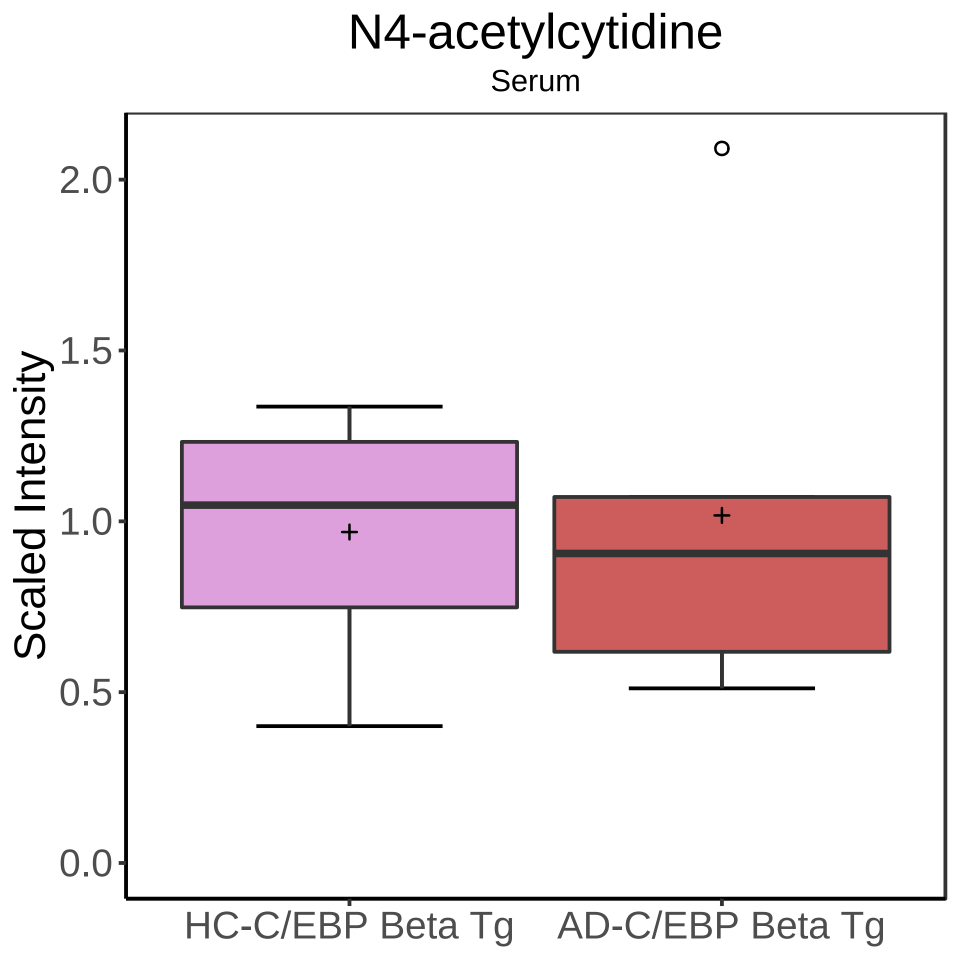

N4-acetylcytidine
Serum
Scaled Intensity
2.0
1.5
1.0
0.5
0.0
HC-C/EBP Beta Tg
AD-C/EBP Beta Tg

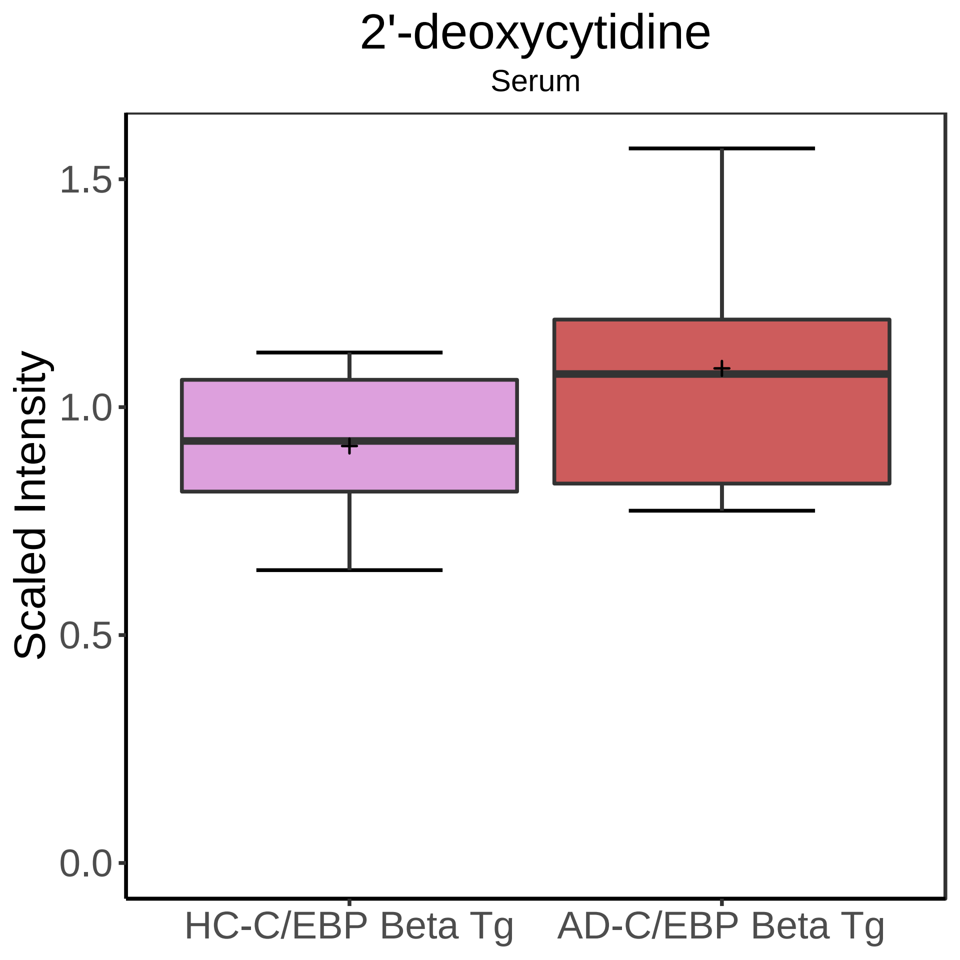
2'-deoxycytidine
Serum
Scaled Intensity
0.0
0.5
1.0
1.5
HC-C/EBP Beta Tg
AD-C/EBP Beta Tg

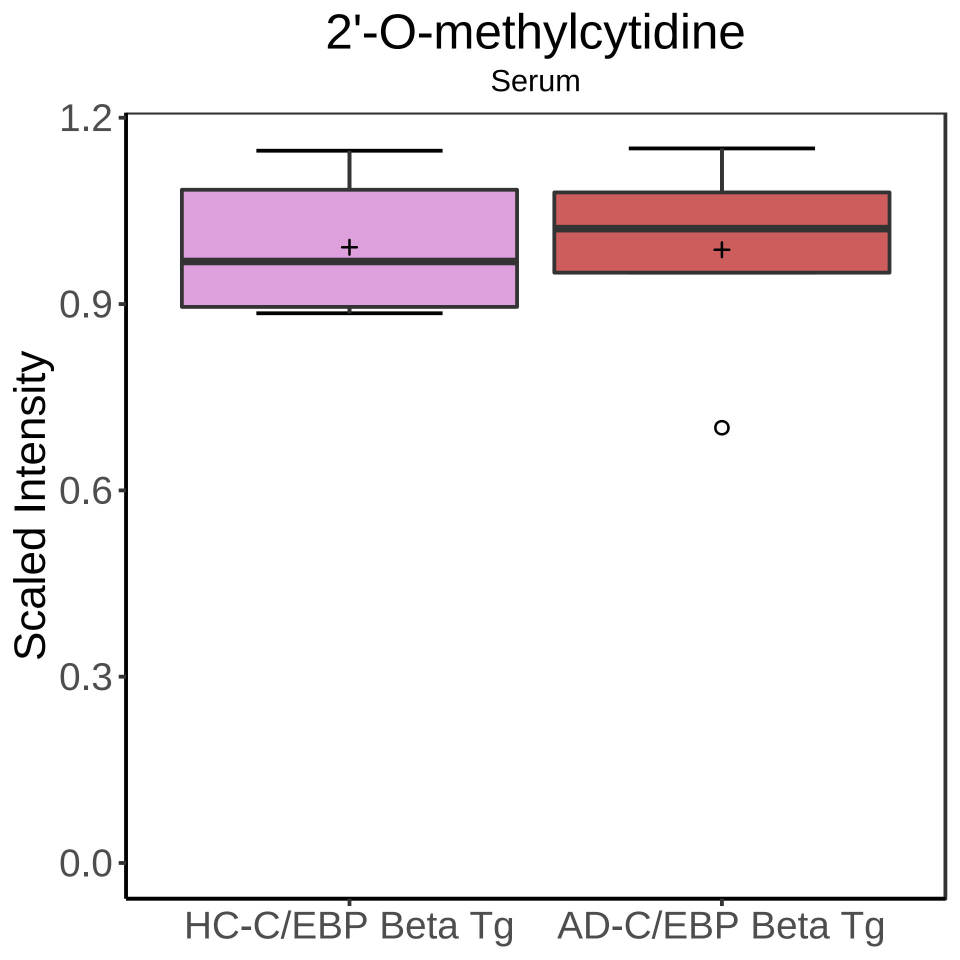
2'-O-methylcytidine
Serum
Scaled Intensity
1.2
0.9
0.6
0.3
0.0
HC-C/EBP Beta Tg
AD-C/EBP Beta Tg

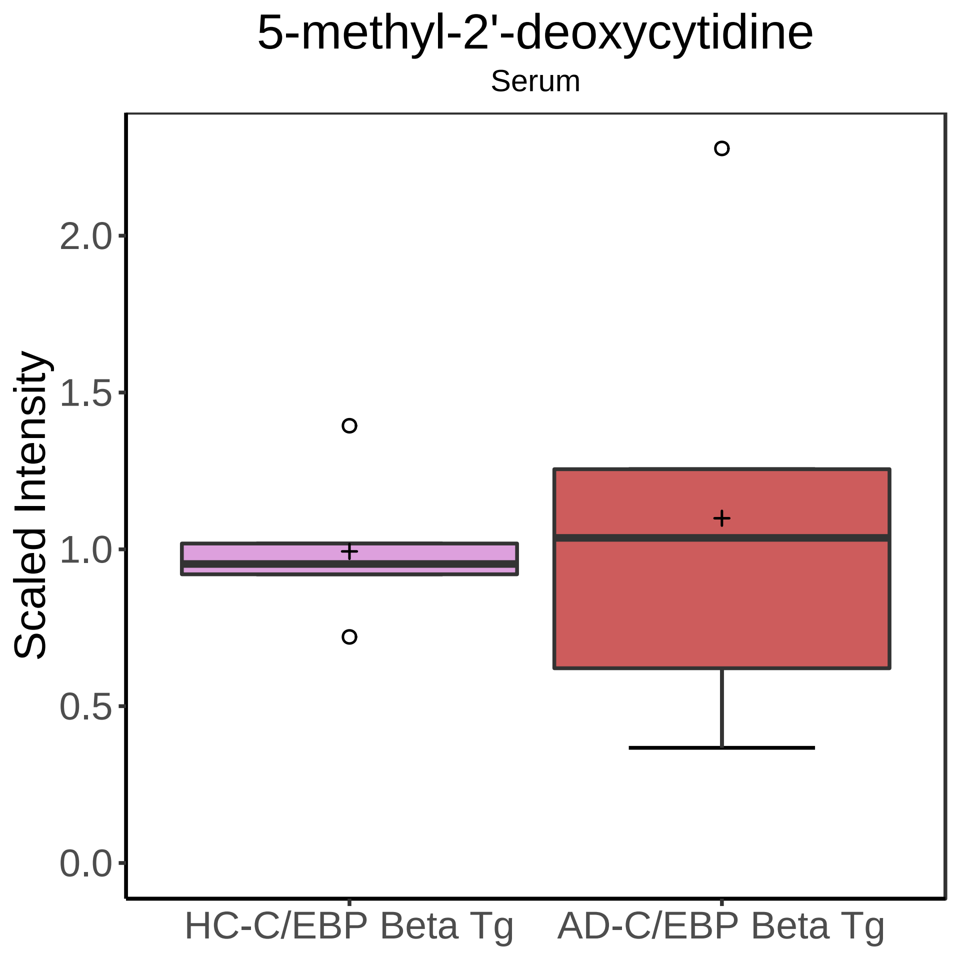
5-methyl-2'-deoxycytidine
Serum
Scaled Intensity
2.0
1.5
1.0
0.5
0.0
HC-C/EBP Beta Tg
AD-C/EBP Beta Tg

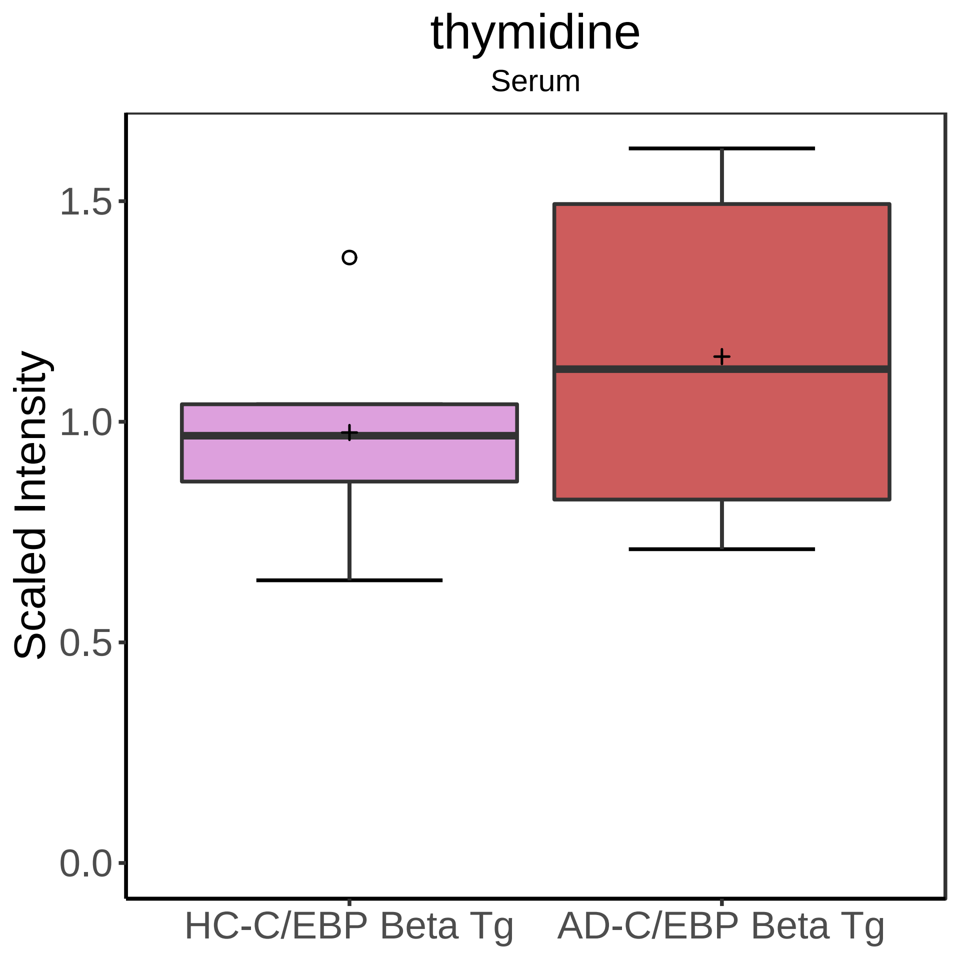

thymidine
Serum
Scaled Intensity
1.5
1.0
0.5
0.0
HC-C/EBP Beta Tg
AD-C/EBP Beta Tg

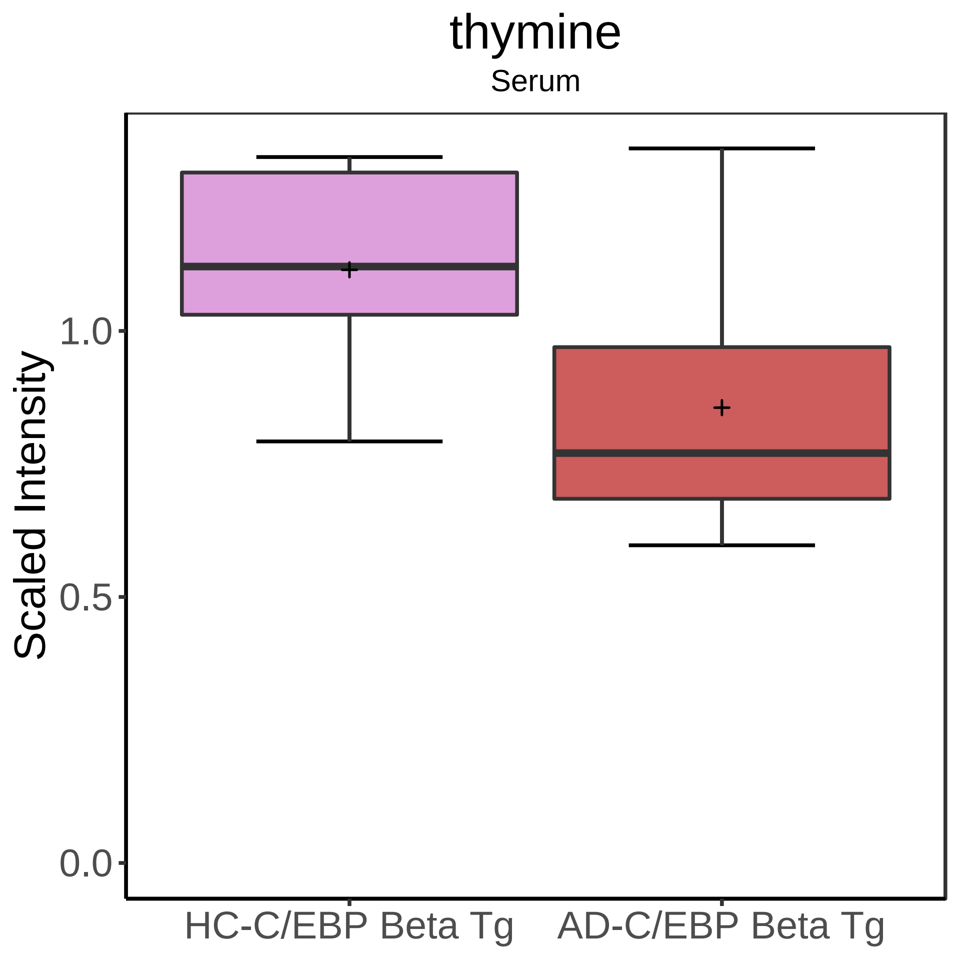
thymine
Serum
Scaled Intensity
1.0
0.5
0.0
HC-C/EBP Beta Tg
AD-C/EBP Beta Tg

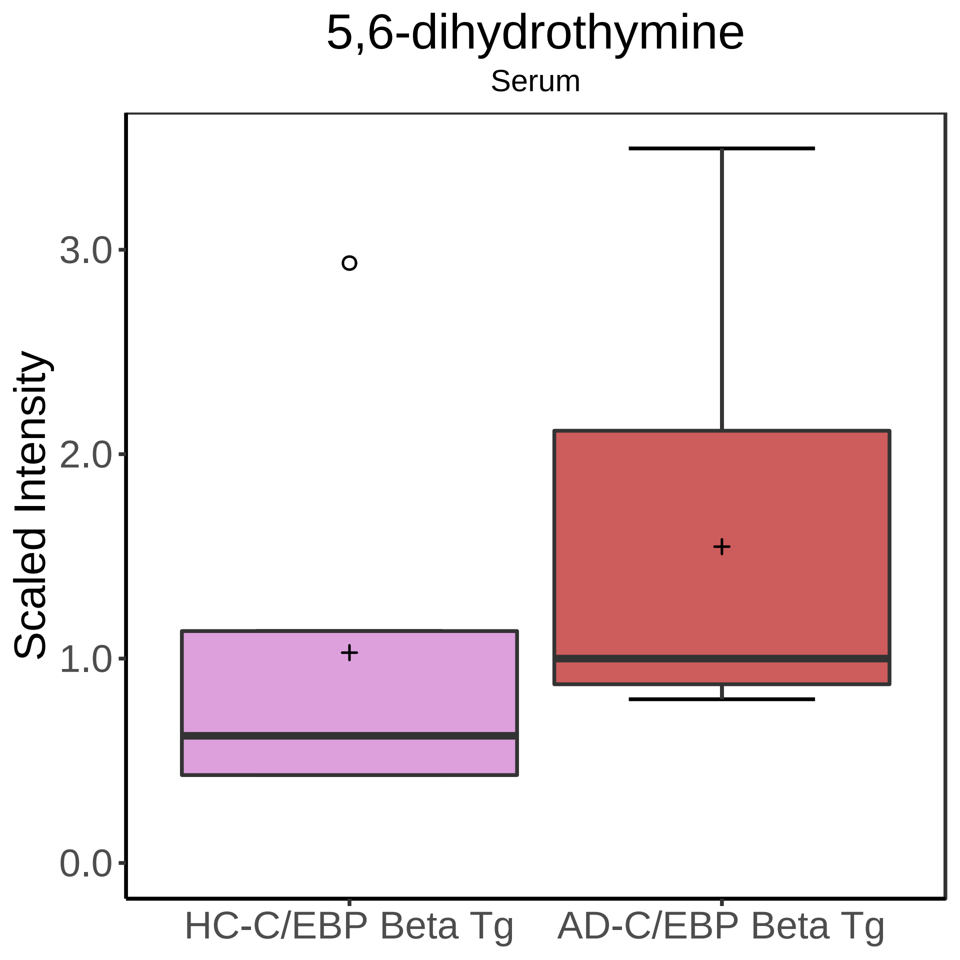
5,6-dihydrothymine
Serum
Scaled Intensity
3.0
2.0
1.0
0.0
HC-C/EBP Beta Tg
AD-C/EBP Beta Tg

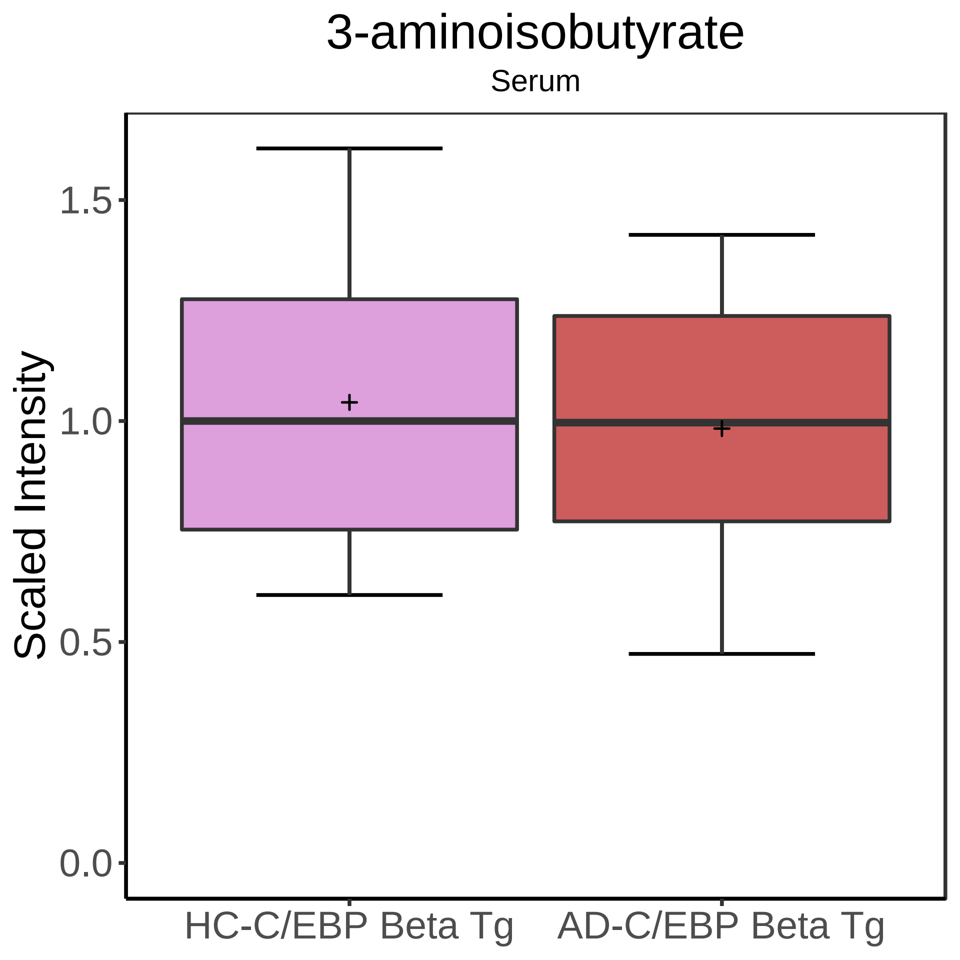

3-aminoisobutyrate
Serum
Scaled Intensity
1.5
1.0
0.5
0.0
HC-C/EBP Beta Tg
AD-C/EBP Beta Tg

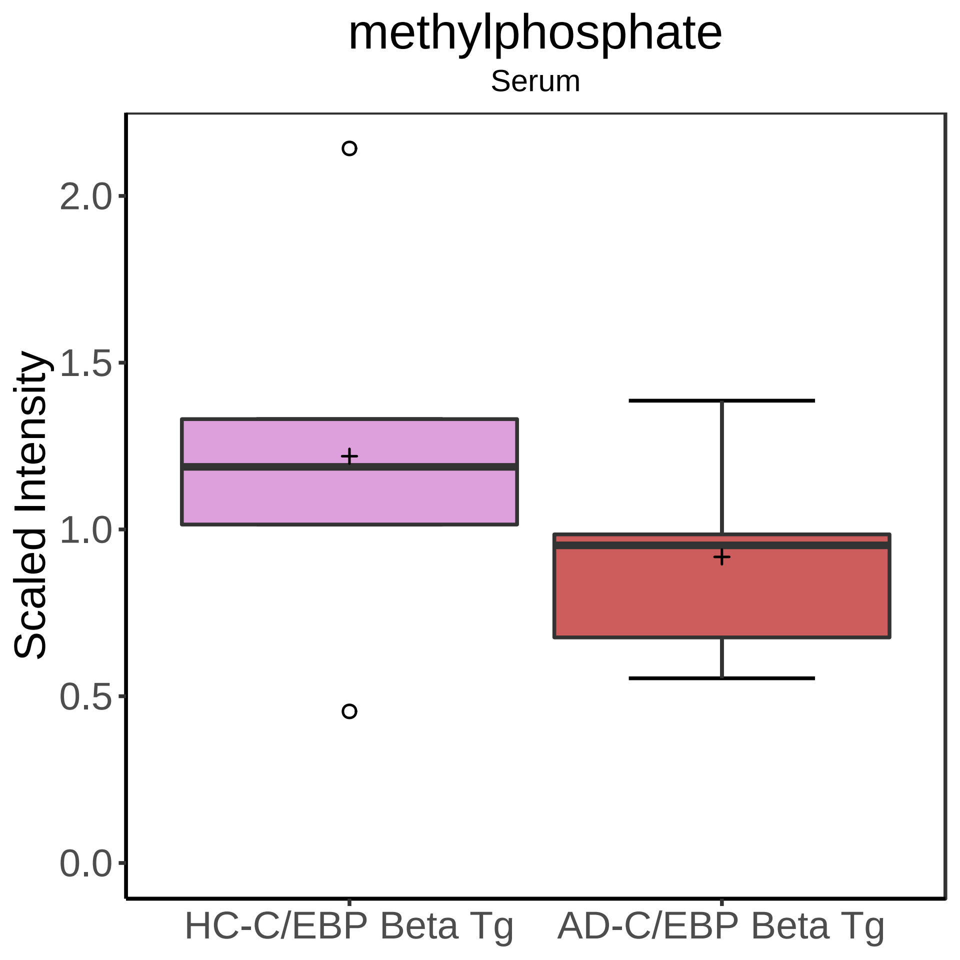
methylphosphate
Serum
Scaled Intensity
2.0
1.5
1.0
0.5
0.0
HC-C/EBP Beta Tg
AD-C/EBP Beta Tg

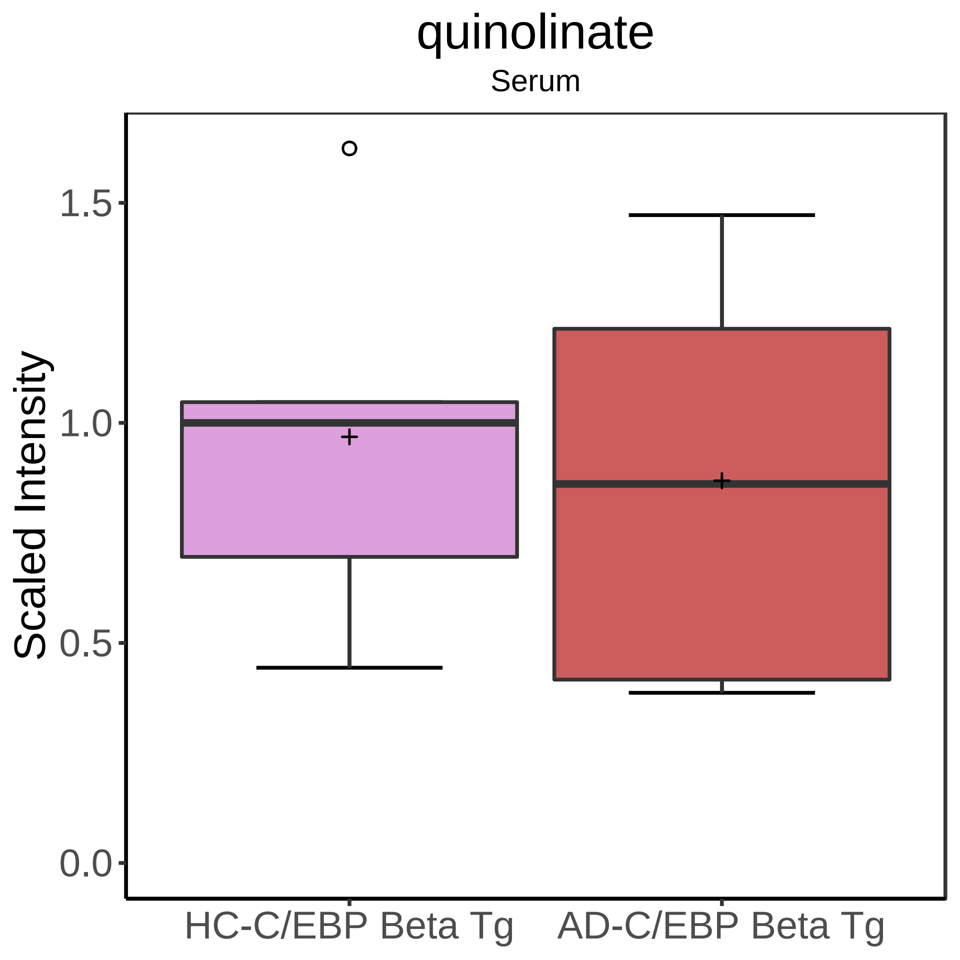
quinolinate
Serum
Scaled Intensity
1.5
1.0
0.5
0.0
HC-C/EBP Beta Tg
AD-C/EBP Beta Tg

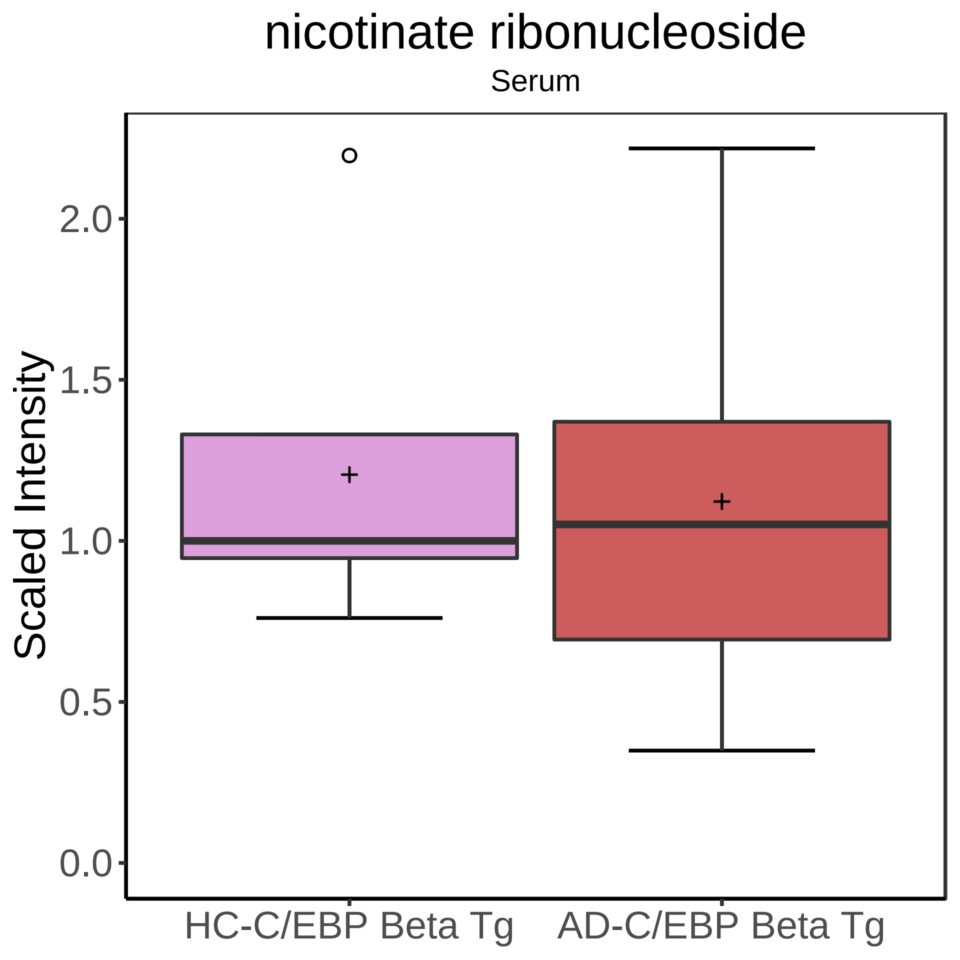
nicotinate ribonucleoside
Serum
Scaled Intensity
0.0
0.5
1.0
1.5
2.0
HC-C/EBP Beta Tg
AD-C/EBP Beta Tg

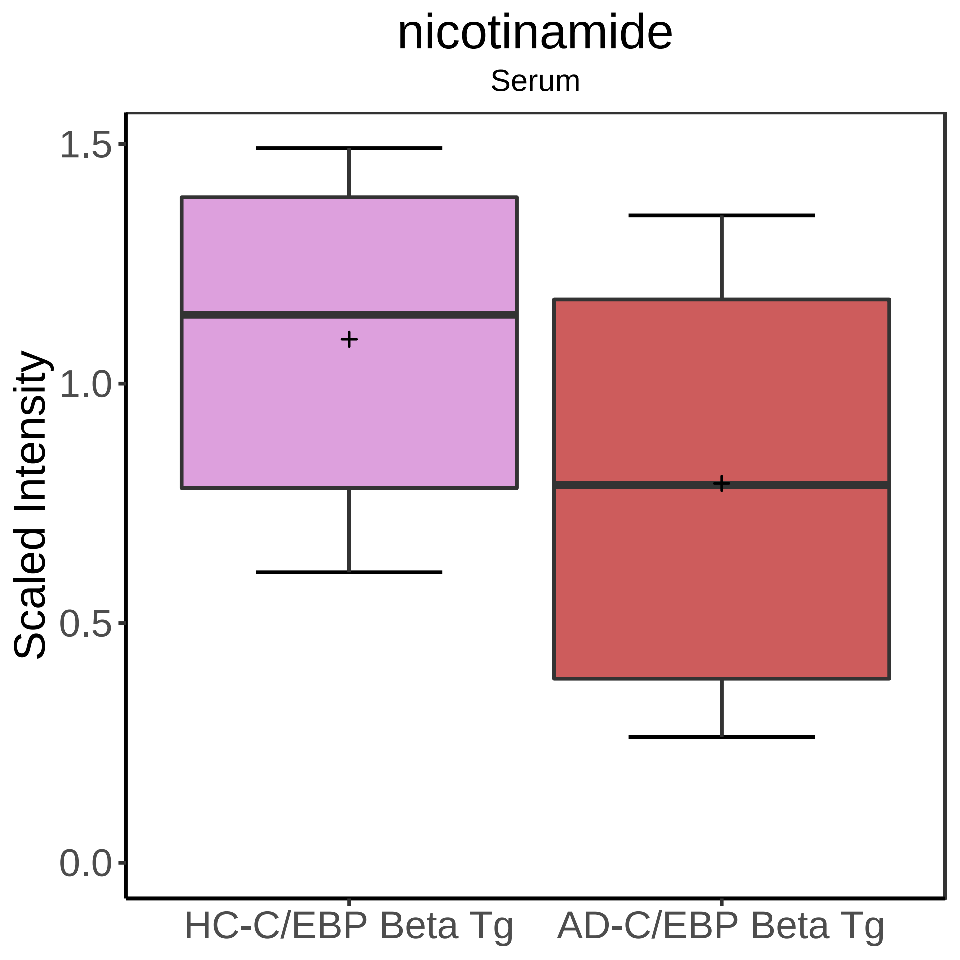
nicotinamide
Serum
Scaled Intensity
1.5
1.0
0.5
0.0
HC-C/EBP Beta Tg
AD-C/EBP Beta Tg

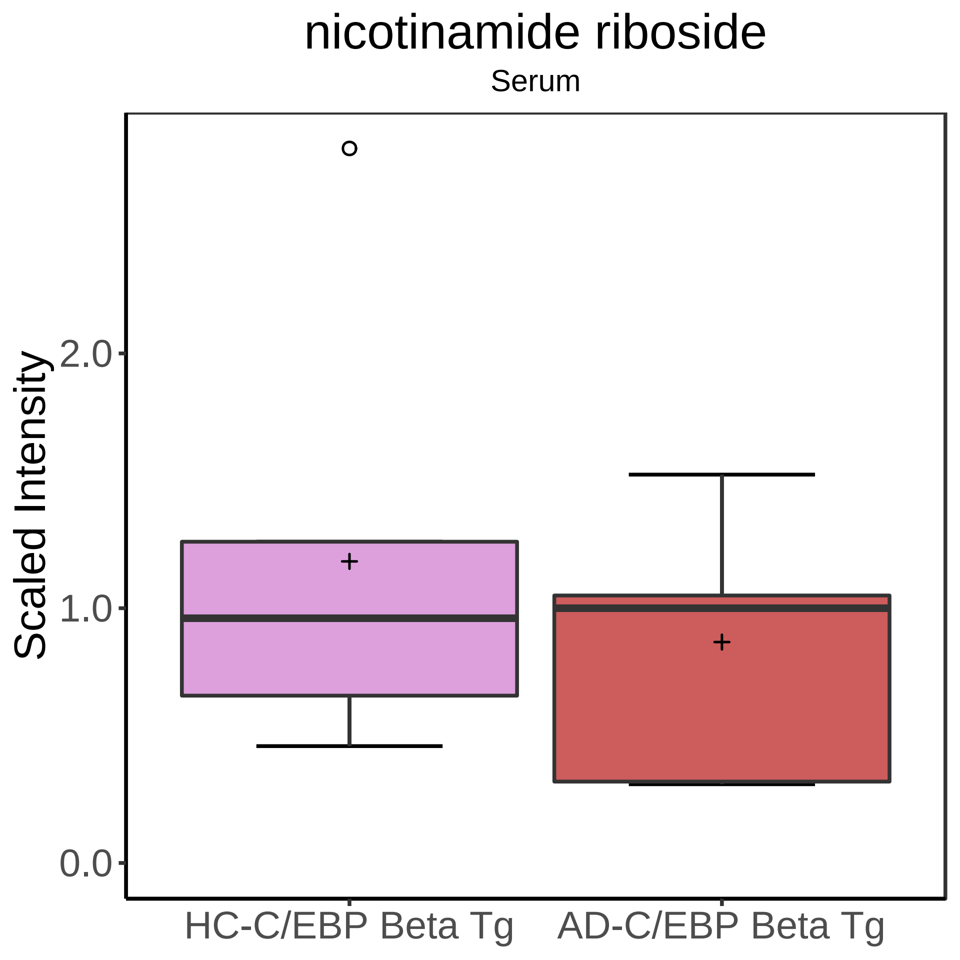
nicotinamide riboside
Serum
Scaled Intensity
2.0
1.0
0.0
HC-C/EBP Beta Tg
AD-C/EBP Beta Tg

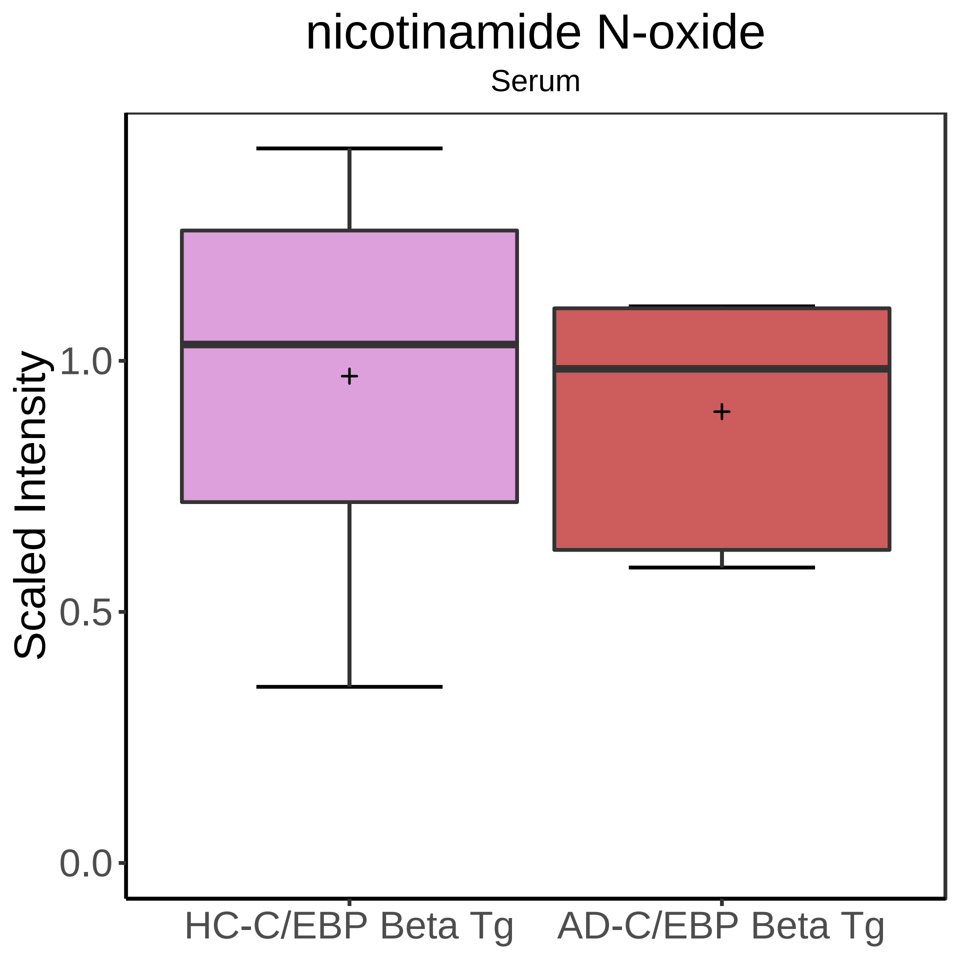
nicotinamide N-oxide
Serum
Scaled Intensity
1.0
0.5
0.0
HC-C/EBP Beta Tg
AD-C/EBP Beta Tg

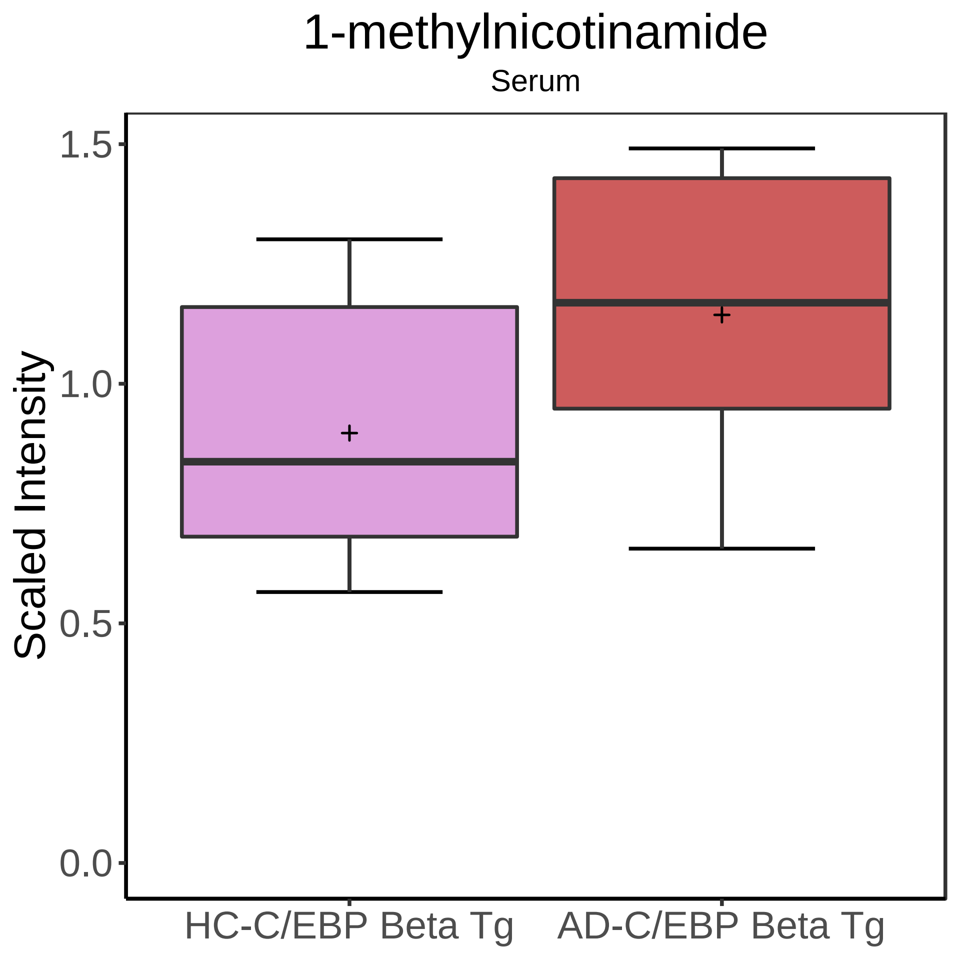

1-methylnicotinamide
Serum
Scaled Intensity
1.5
1.0
0.5
0.0
HC-C/EBP Beta Tg
AD-C/EBP Beta Tg

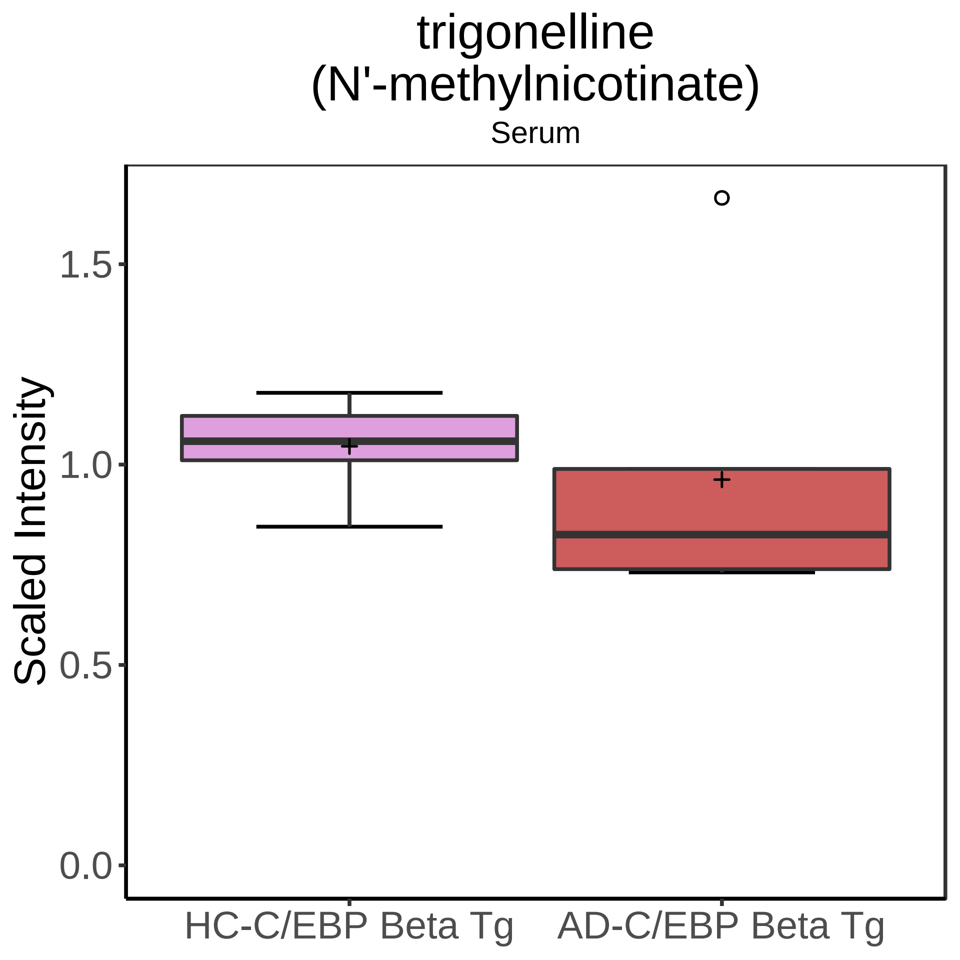
trigonelline
(N'-methylnicotinate)
Serum
Scaled Intensity
1.5
1.0
0.5
0.0
HC-C/EBP Beta Tg
AD-C/EBP Beta Tg

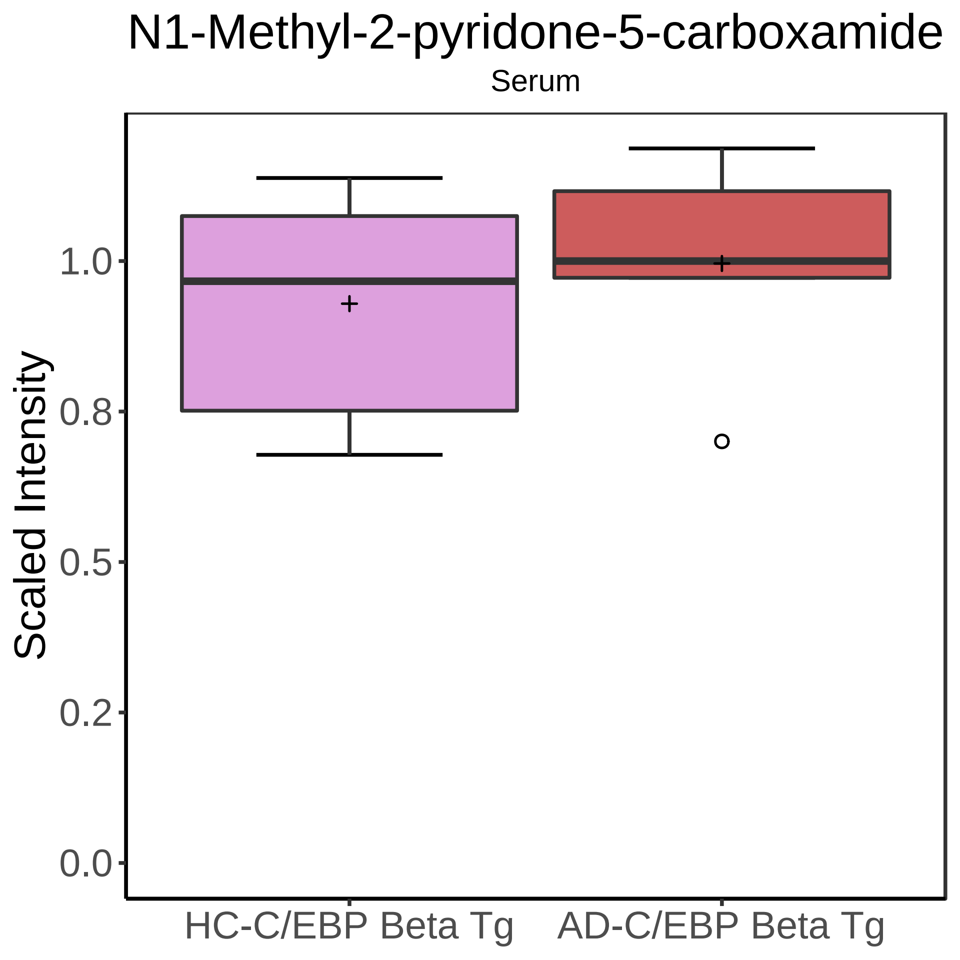
N1-Methyl-2-pyridone-5-carboxamide
Serum
Scaled Intensity
1.0
0.8
0.5
0.2
0.0
HC-C/EBP Beta Tg
AD-C/EBP Beta Tg

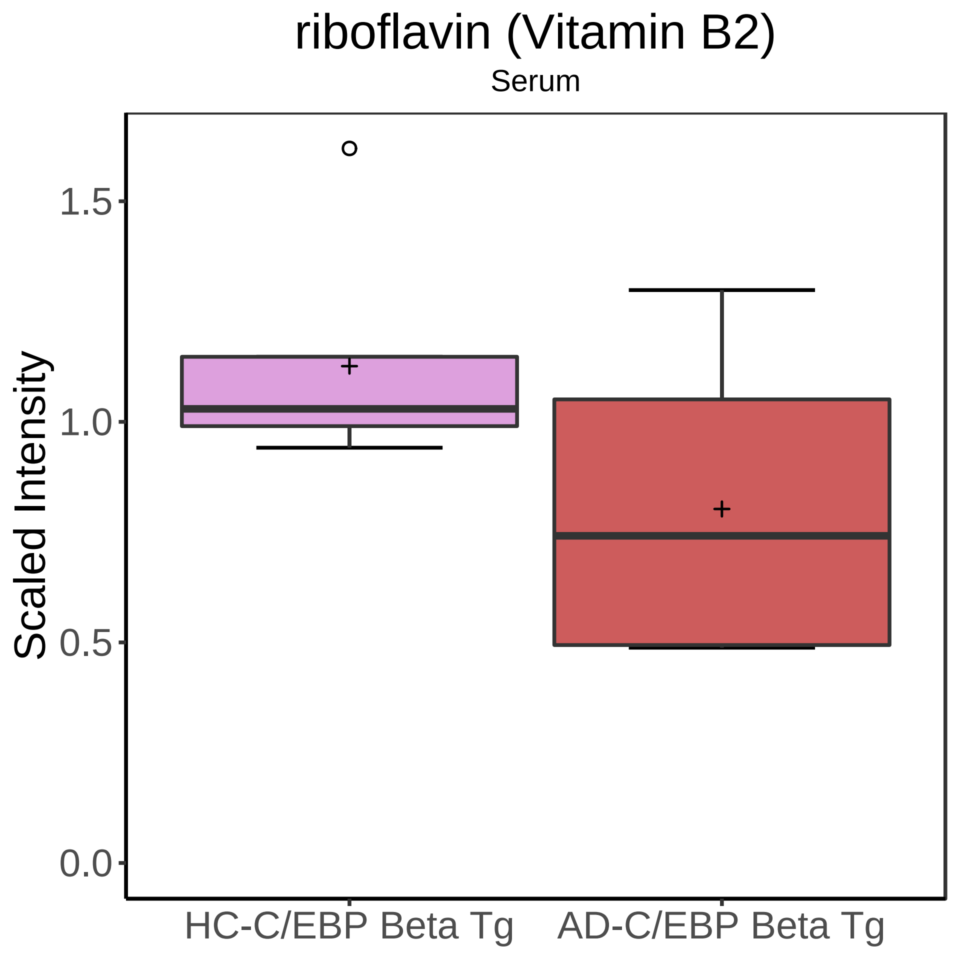

riboflavin (Vitamin B2)
Serum
Scaled Intensity
1.5
1.0
0.5
0.0
HC-C/EBP Beta Tg
AD-C/EBP Beta Tg

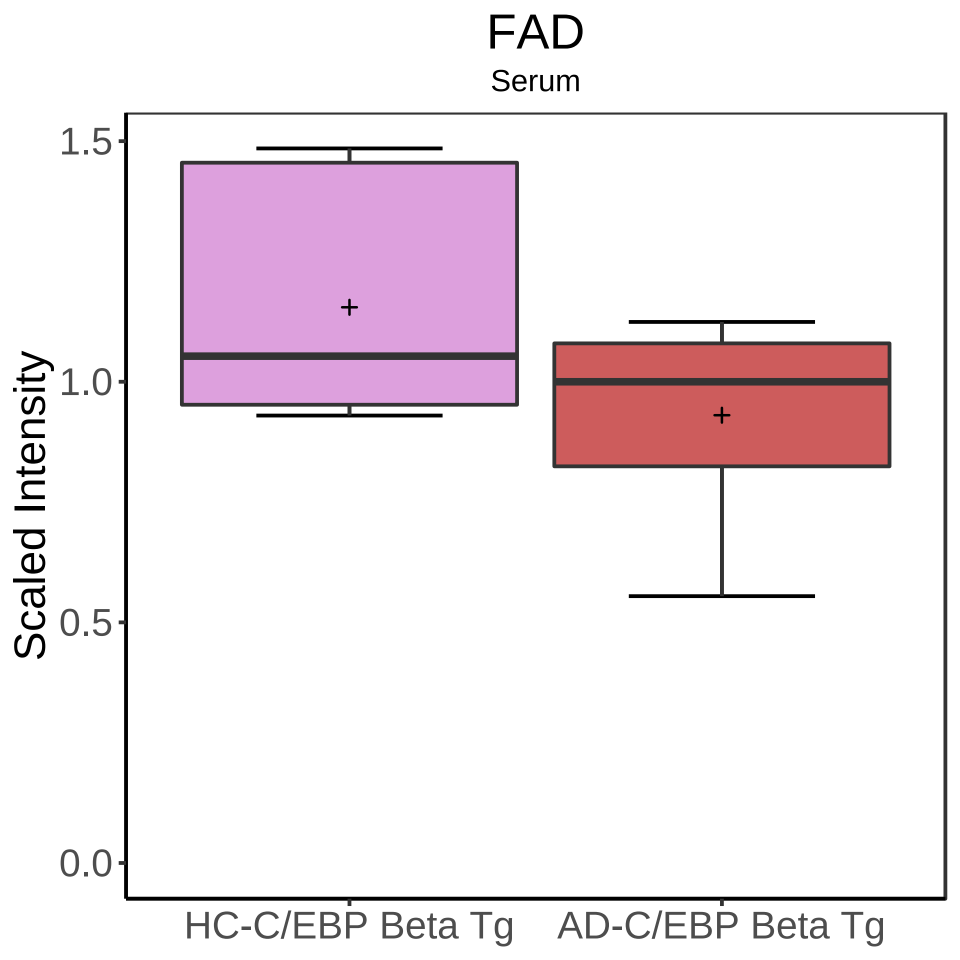
FAD
Serum
Scaled Intensity
1.5
1.0
0.5
0.0
HC-C/EBP Beta Tg
AD-C/EBP Beta Tg

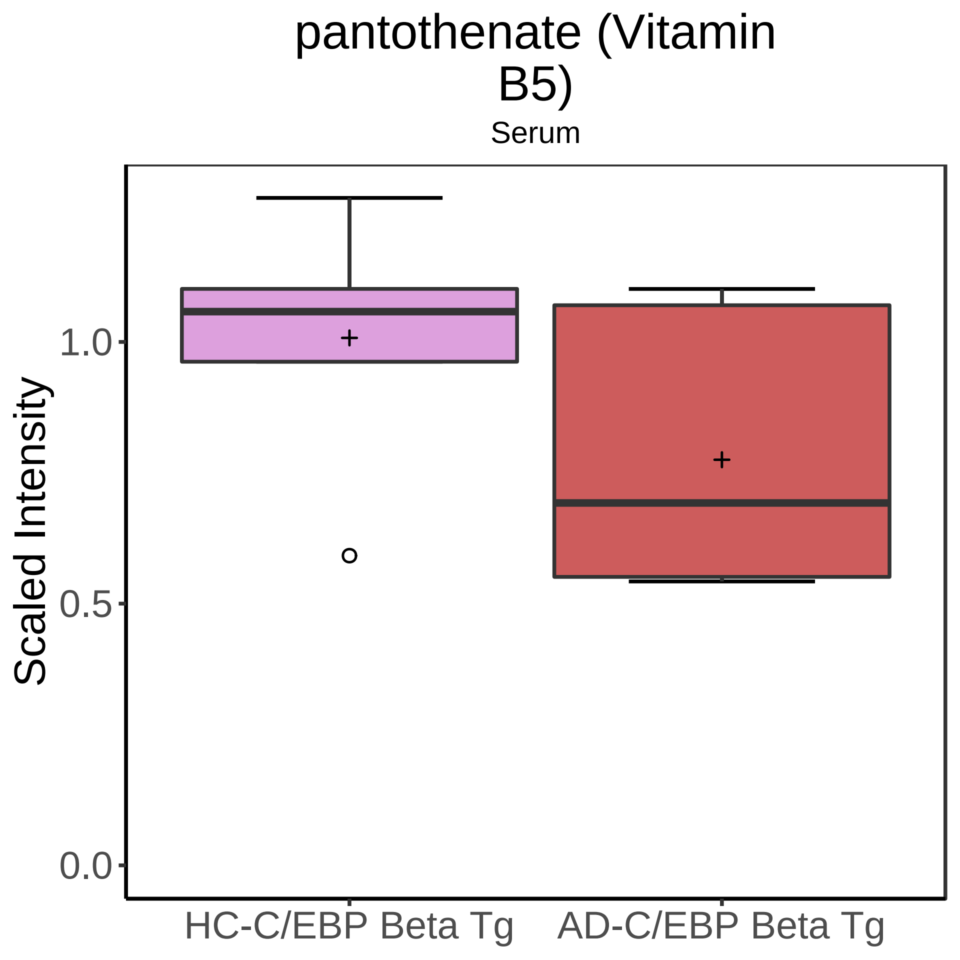
pantothenate (Vitamin B5)
Serum
Scaled Intensity
1.0
0.5
0.0
HC-C/EBP Beta Tg
AD-C/EBP Beta Tg

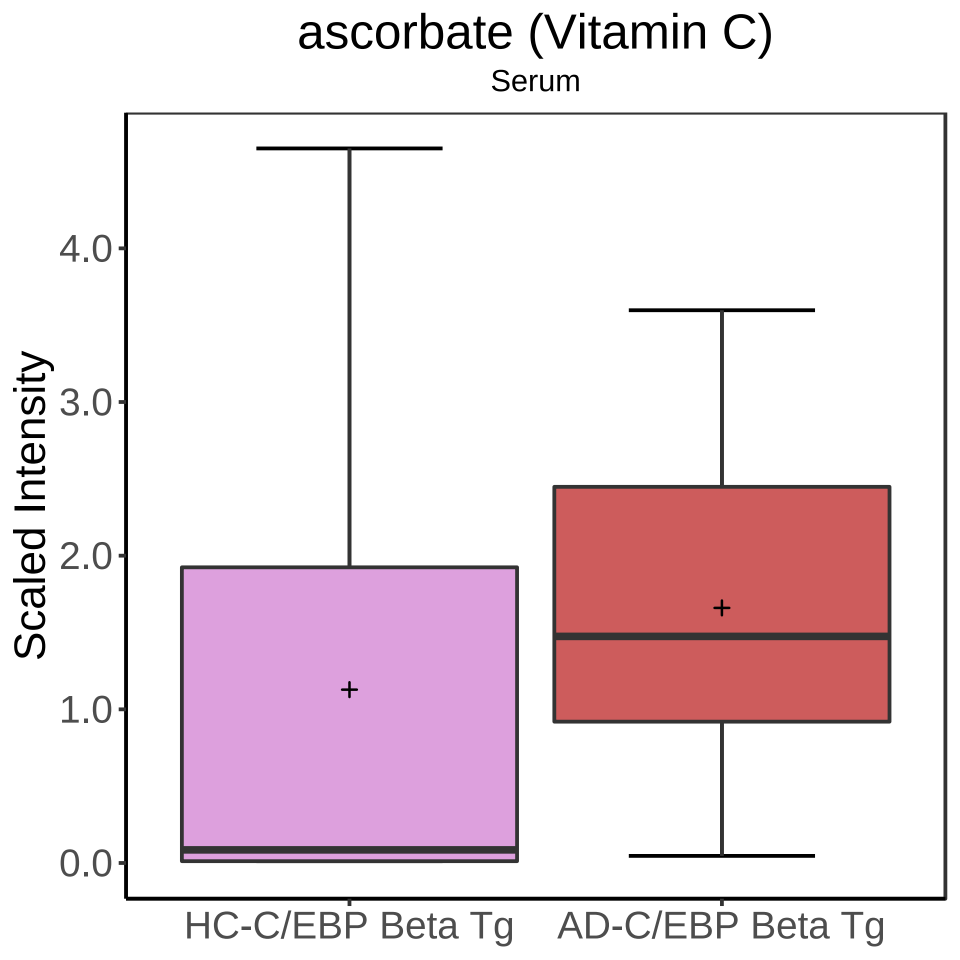

ascorbate (Vitamin C)
Serum
Scaled Intensity
4.0
3.0
2.0
1.0
0.0
HC-C/EBP Beta Tg
AD-C/EBP Beta Tg

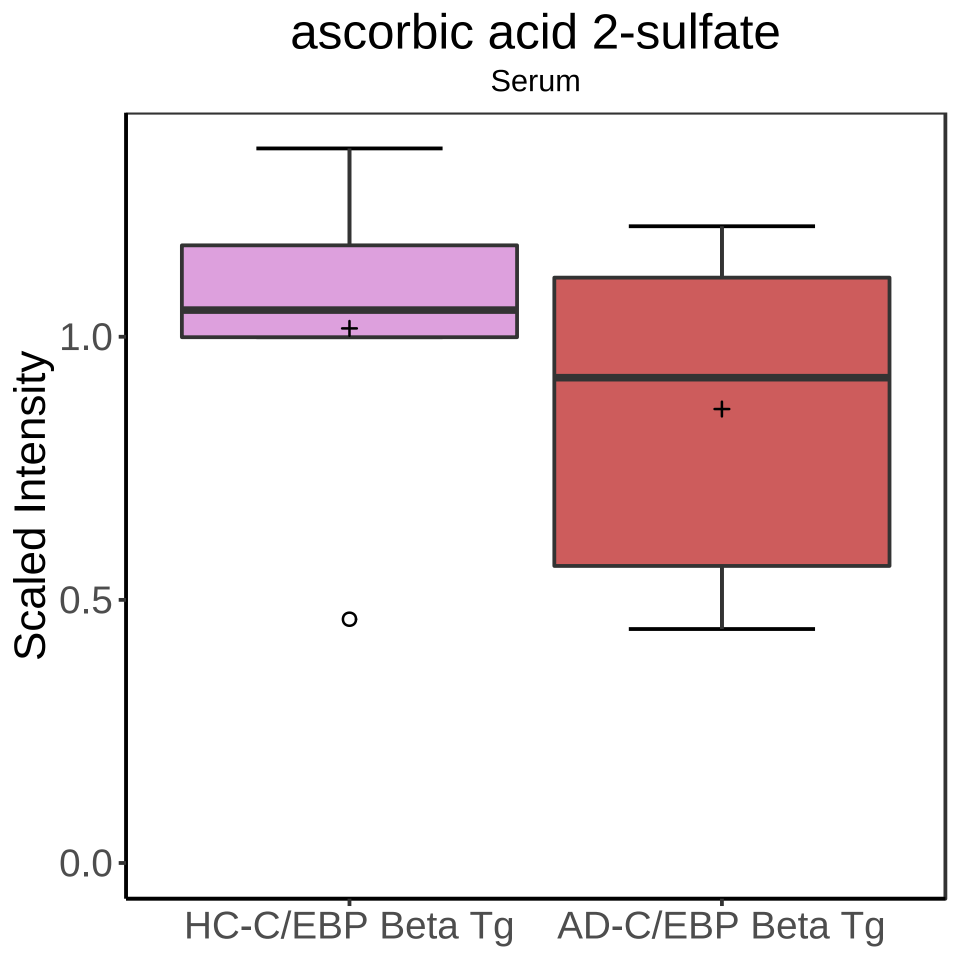
ascorbic acid 2-sulfate
Serum
Scaled Intensity
1.0
0.5
0.0
HC-C/EBP Beta Tg
AD-C/EBP Beta Tg

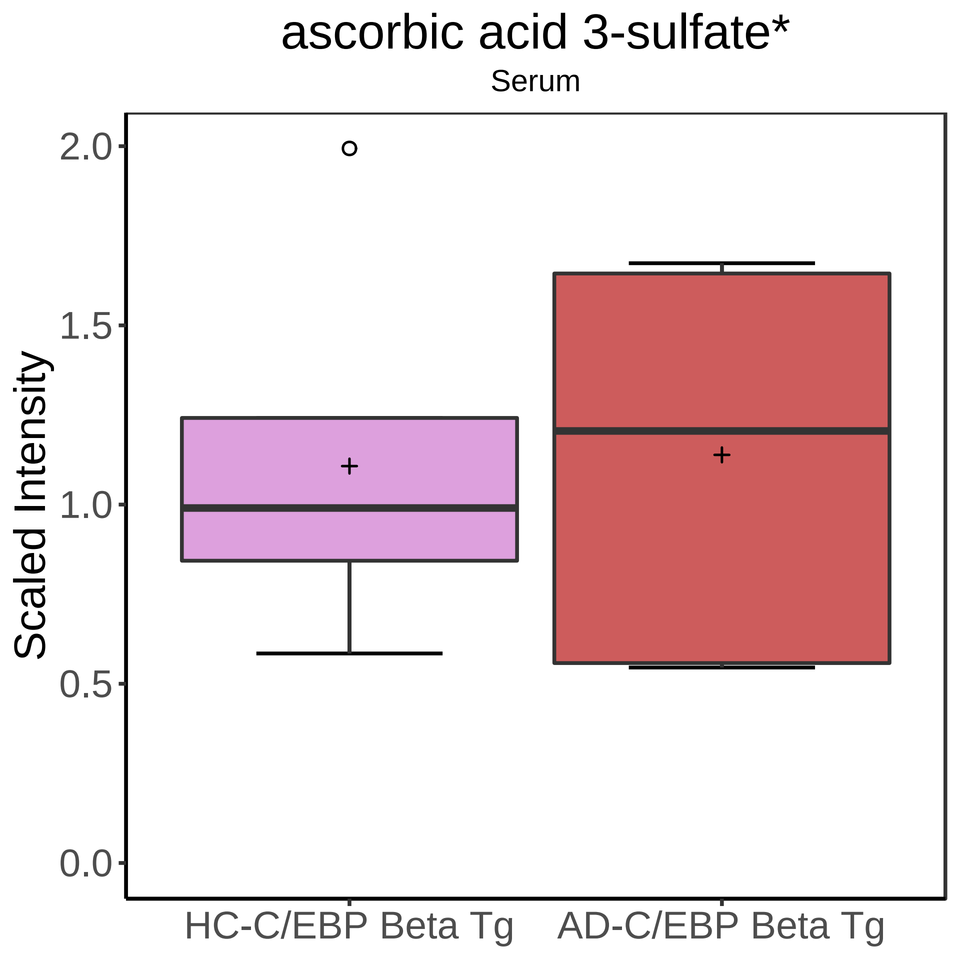
ascorbic acid 3-sulfate*
Serum
Scaled Intensity
2.0
1.5
1.0
0.5
0.0
HC-C/EBP Beta Tg
AD-C/EBP Beta Tg

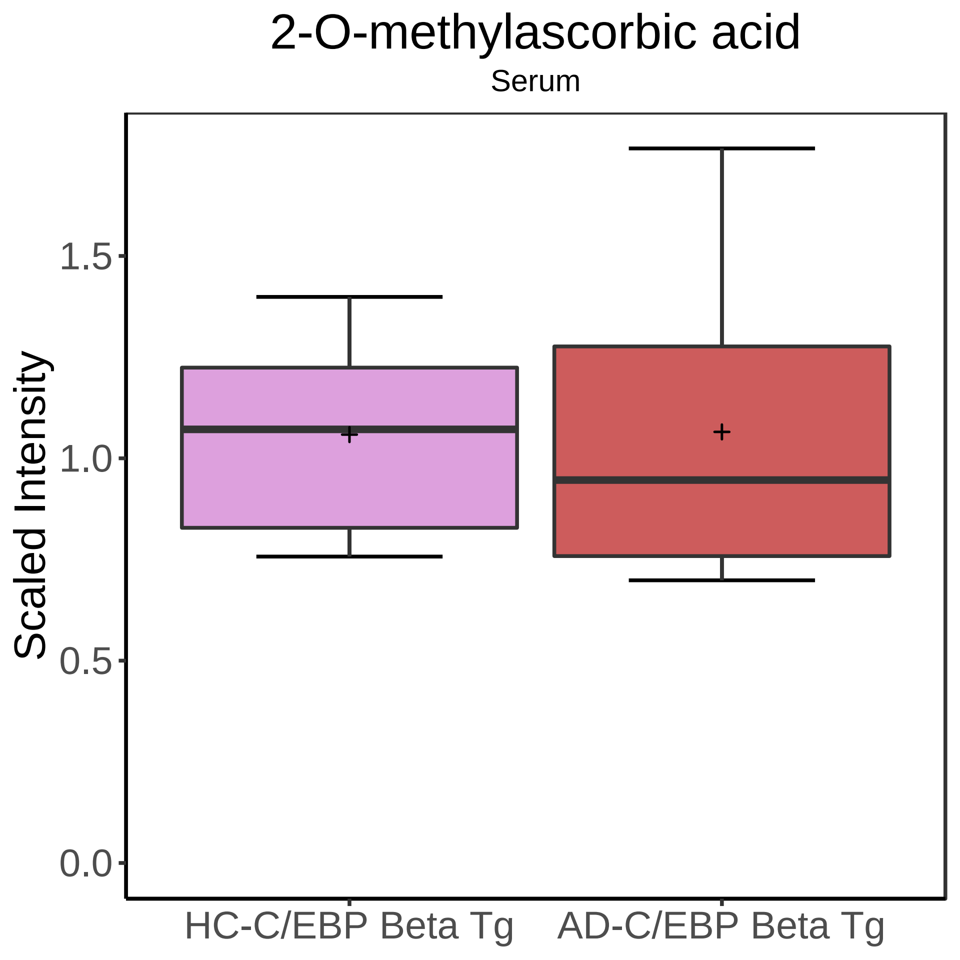
2-O-methylascorbic acid
Serum
Scaled Intensity
0.0
0.5
1.0
1.5
HC-C/EBP Beta Tg
AD-C/EBP Beta Tg

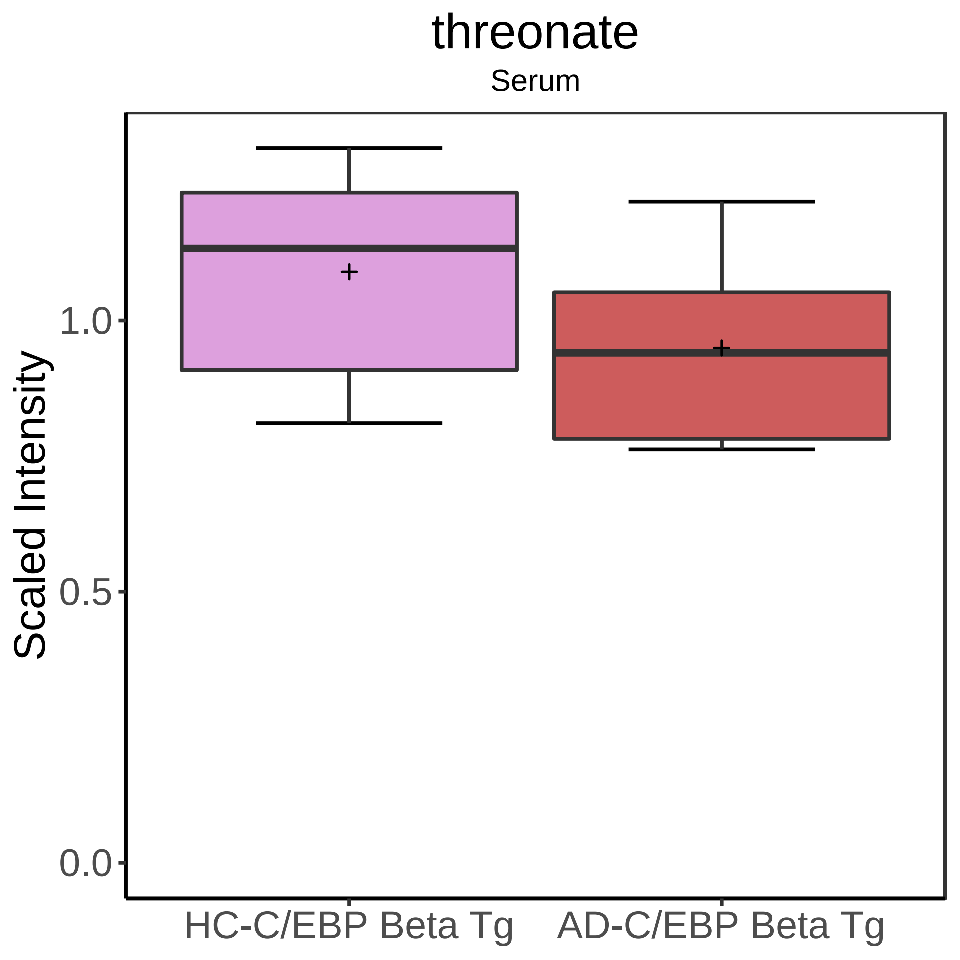
threonate
Serum
Scaled Intensity
1.0
0.5
0.0
HC-C/EBP Beta Tg
AD-C/EBP Beta Tg

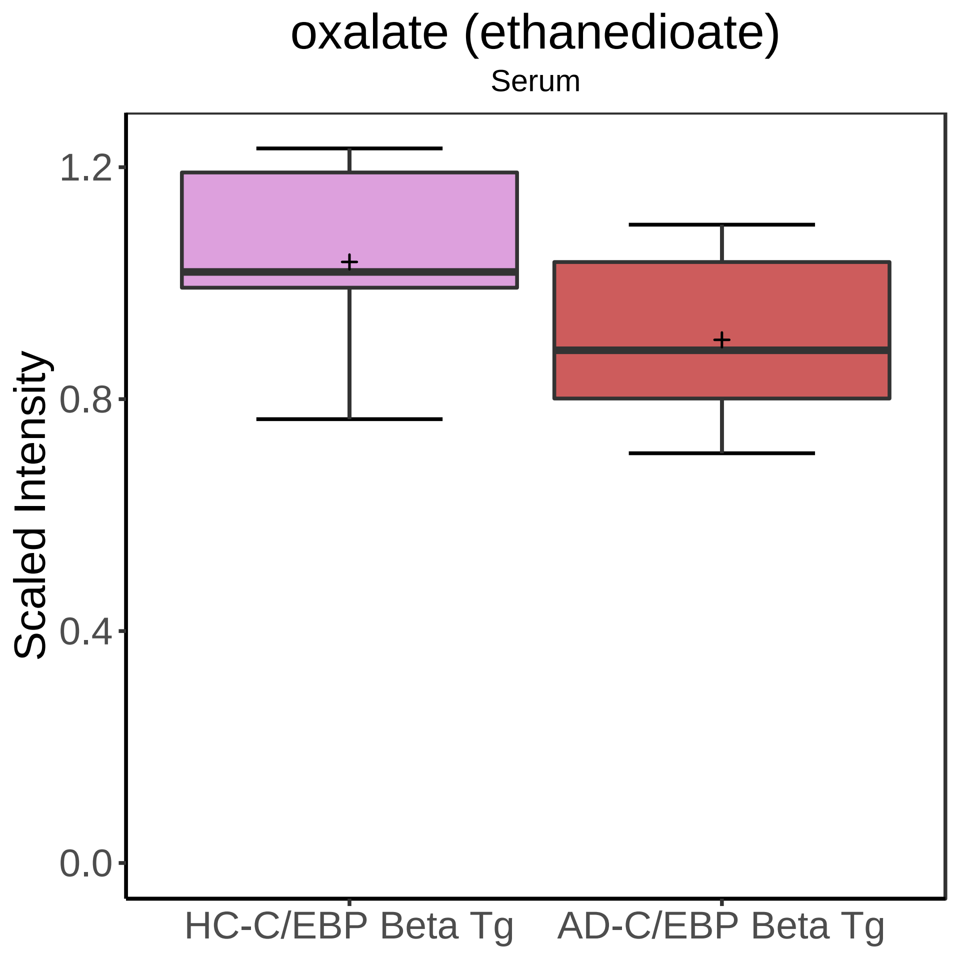
oxalate (ethanedioate)
Serum
Scaled Intensity
1.2
0.8
0.4
0.0
HC-C/EBP Beta Tg
AD-C/EBP Beta Tg

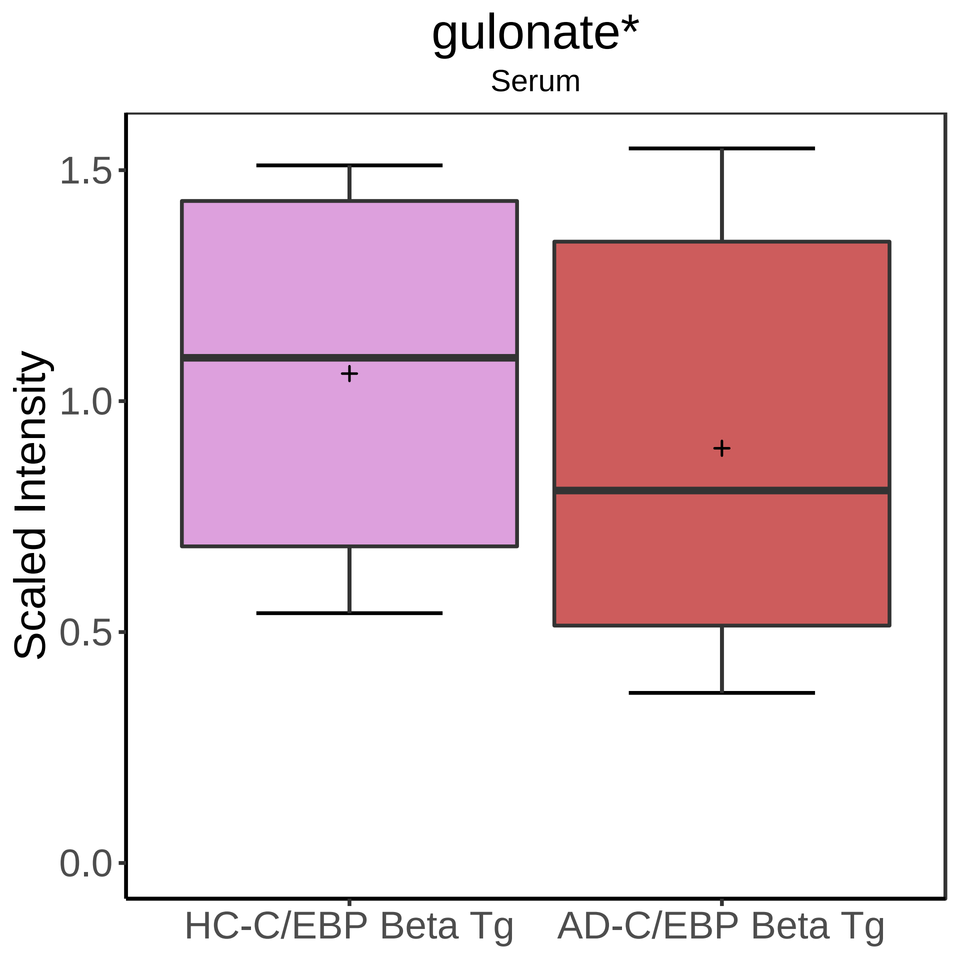

gulonate*
Serum
Scaled Intensity
1.5
1.0
0.5
0.0
HC-C/EBP Beta Tg
AD-C/EBP Beta Tg

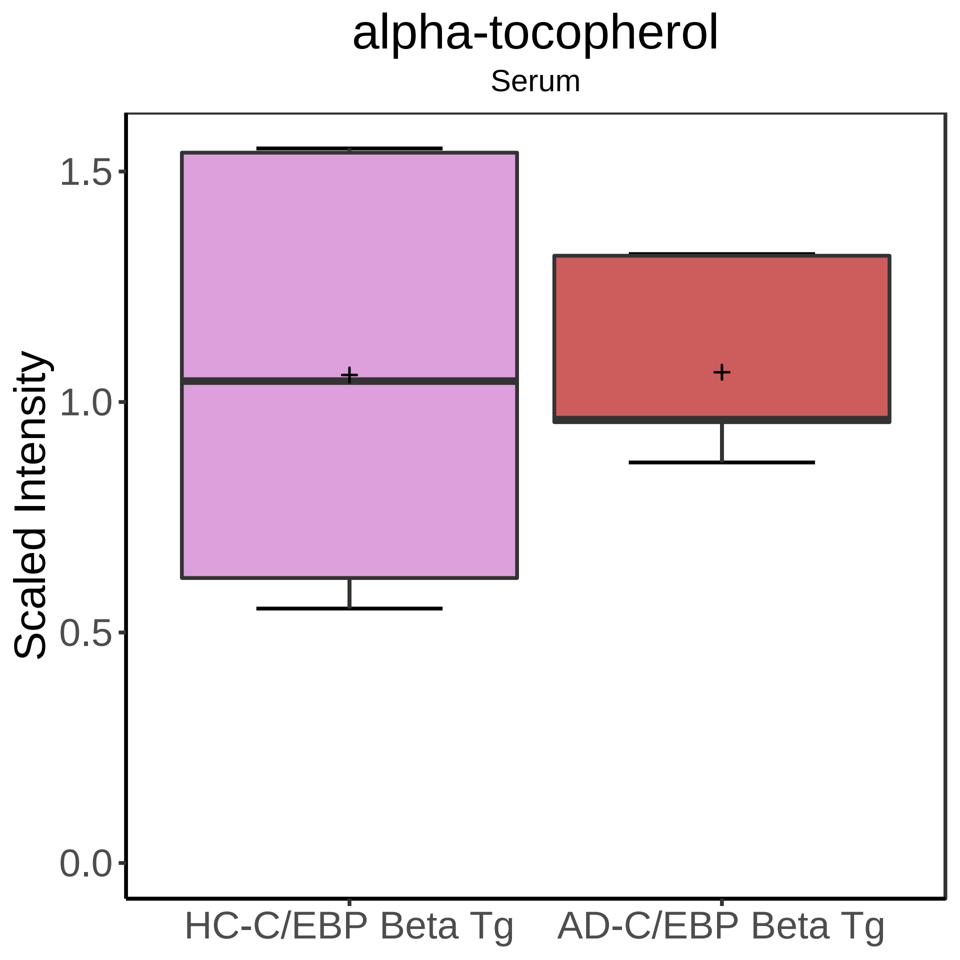
alpha-tocopherol
Serum
Scaled Intensity
0.0
0.5
1.0
1.5
HC-C/EBP Beta Tg
AD-C/EBP Beta Tg

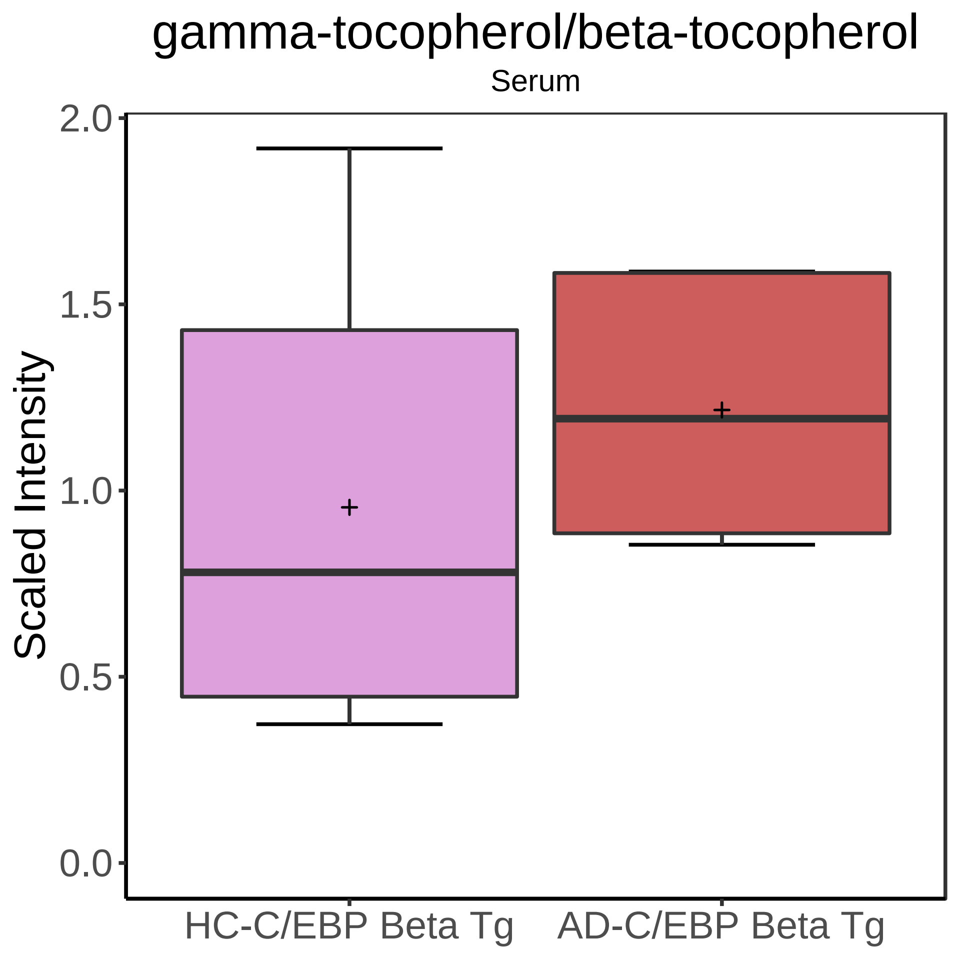
gamma-tocopherol/beta-tocopherol
Serum
Scaled Intensity
2.0
1.5
1.0
0.5
0.0
HC-C/EBP Beta Tg
AD-C/EBP Beta Tg

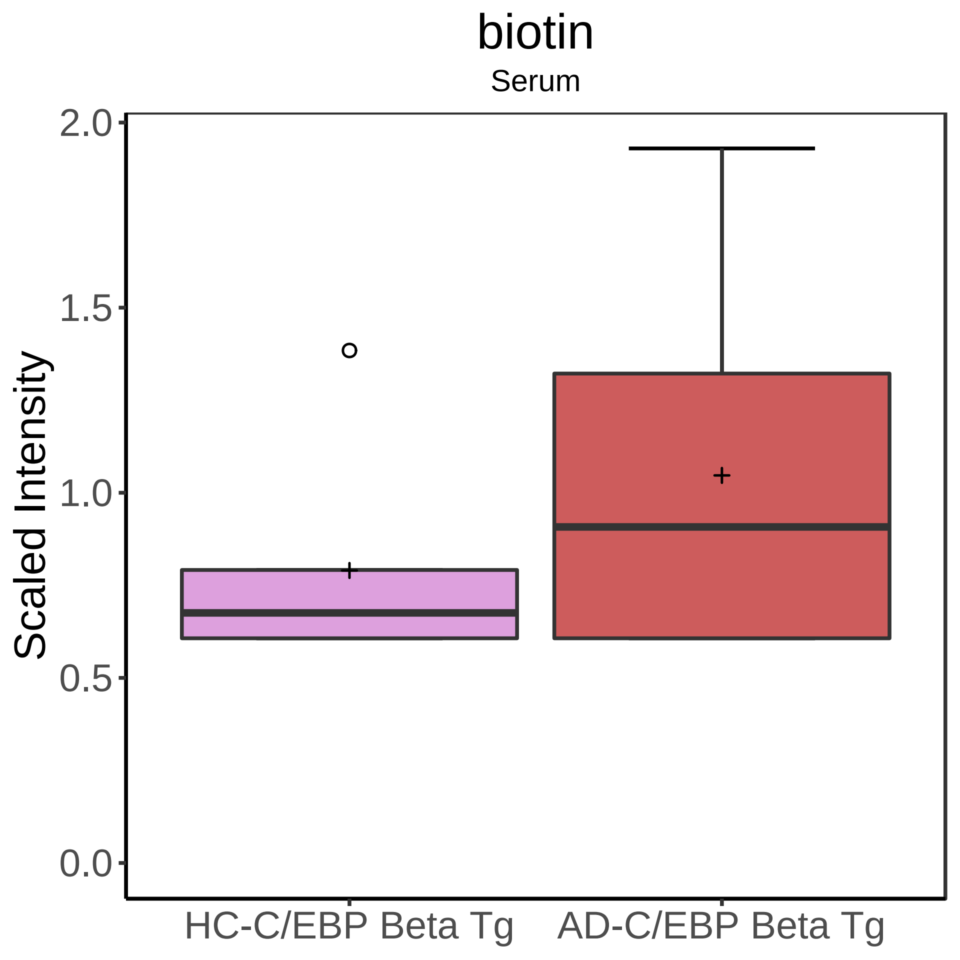
biotin
Serum
Scaled Intensity
2.0
1.5
1.0
0.5
0.0
HC-C/EBP Beta Tg
AD-C/EBP Beta Tg

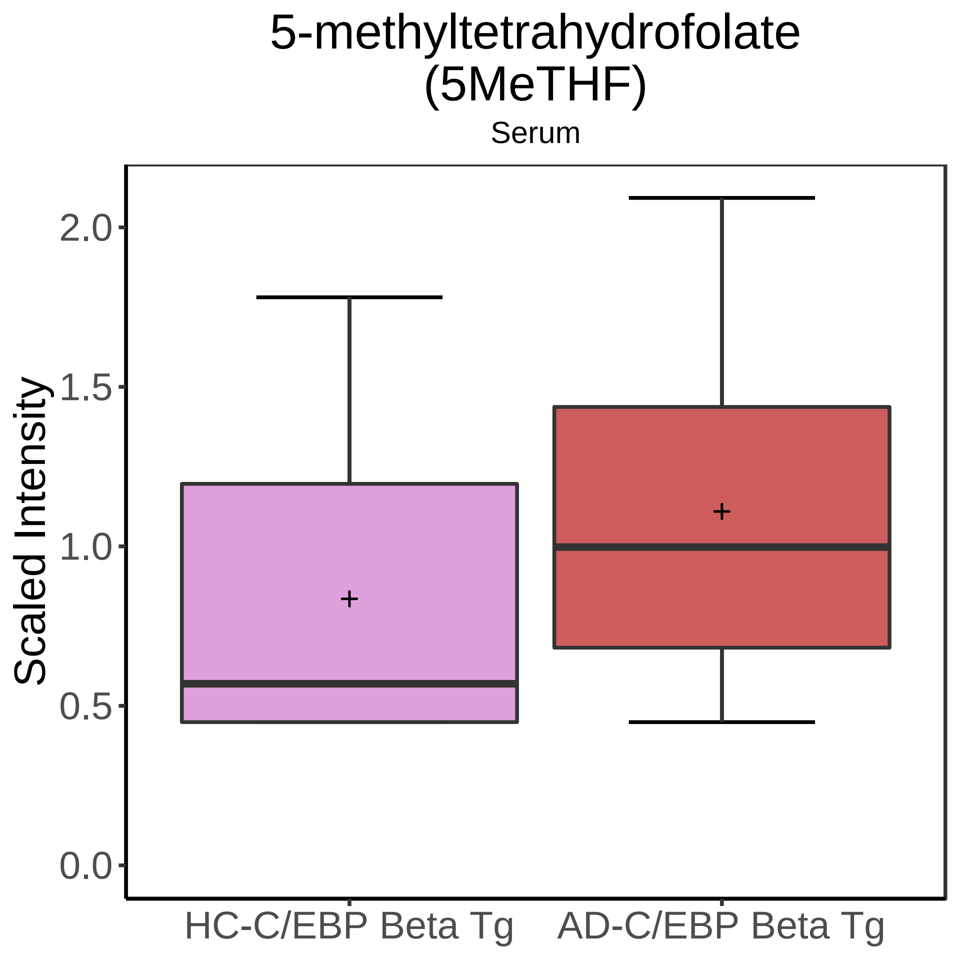

5-methyltetrahydrofolate
(5MeTHF)
Serum
Scaled Intensity
2.0
1.5
1.0
0.5
0.0
HC-C/EBP Beta Tg
AD-C/EBP Beta Tg

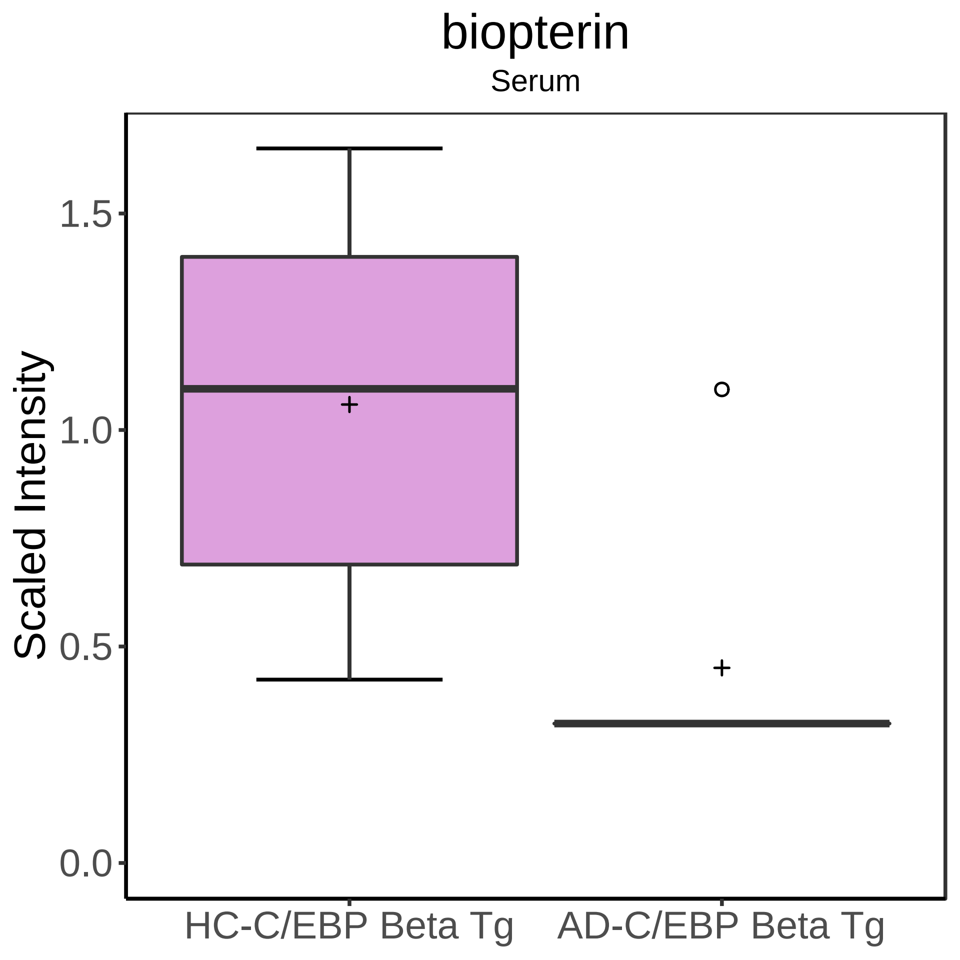

biopterin
Serum
Scaled Intensity
1.5
1.0
0.5
0.0
HC-C/EBP Beta Tg
AD-C/EBP Beta Tg

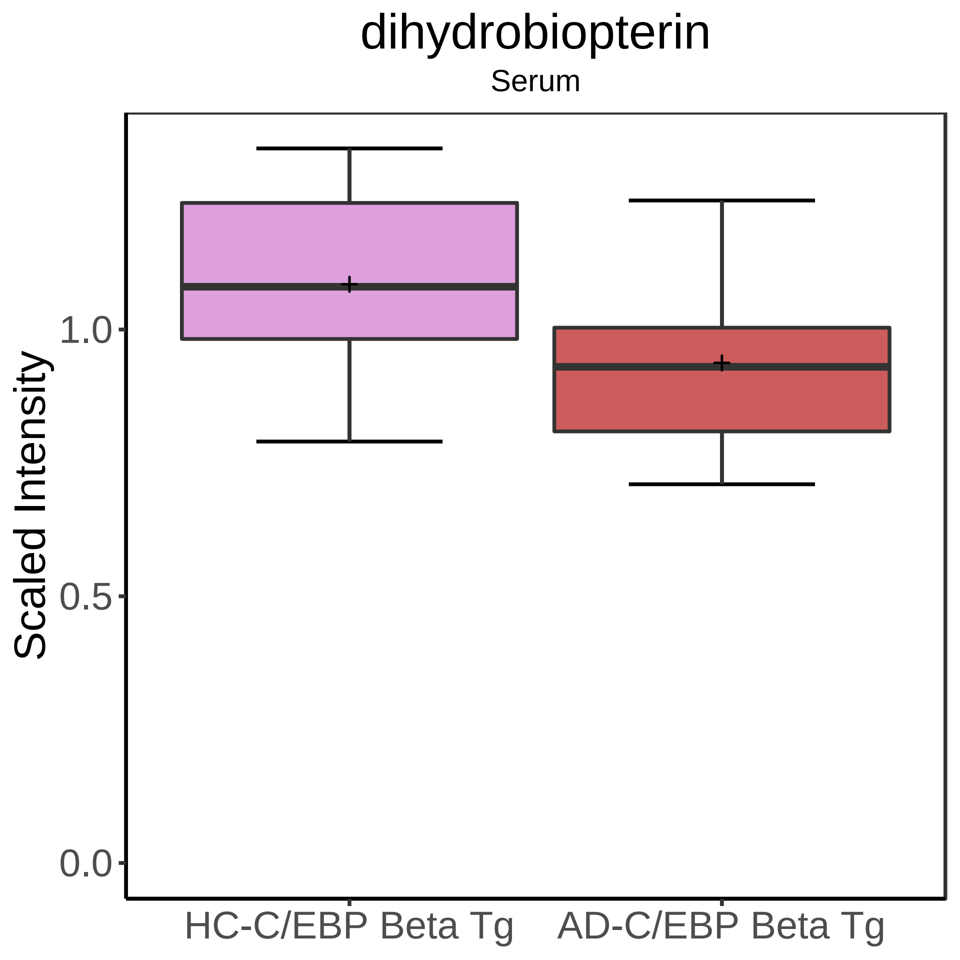
dihydrobiopterin
Serum
Scaled Intensity
1.0
0.5
0.0
HC-C/EBP Beta Tg
AD-C/EBP Beta Tg

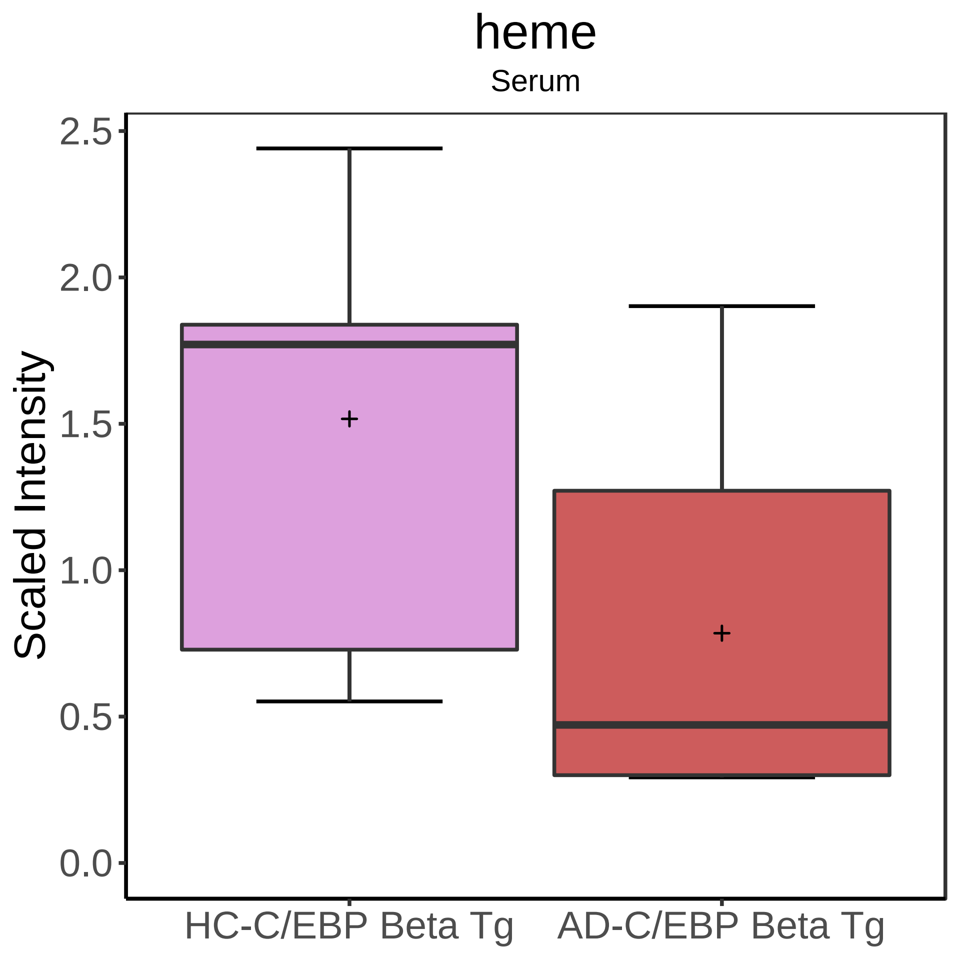

heme
Serum
Scaled Intensity
2.5
2.0
1.5
1.0
0.5
0.0
HC-C/EBP Beta Tg
AD-C/EBP Beta Tg

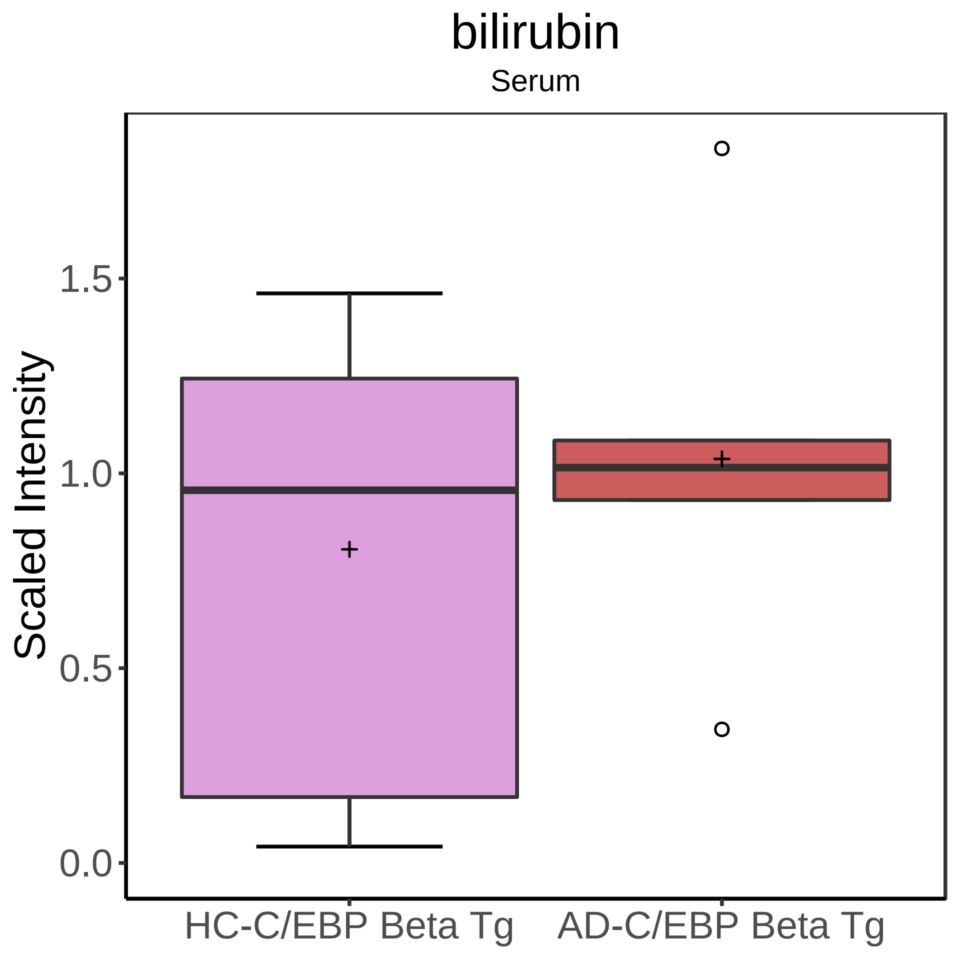
bilirubin
Serum
Scaled Intensity
1.5
1.0
0.5
0.0
HC-C/EBP Beta Tg
AD-C/EBP Beta Tg

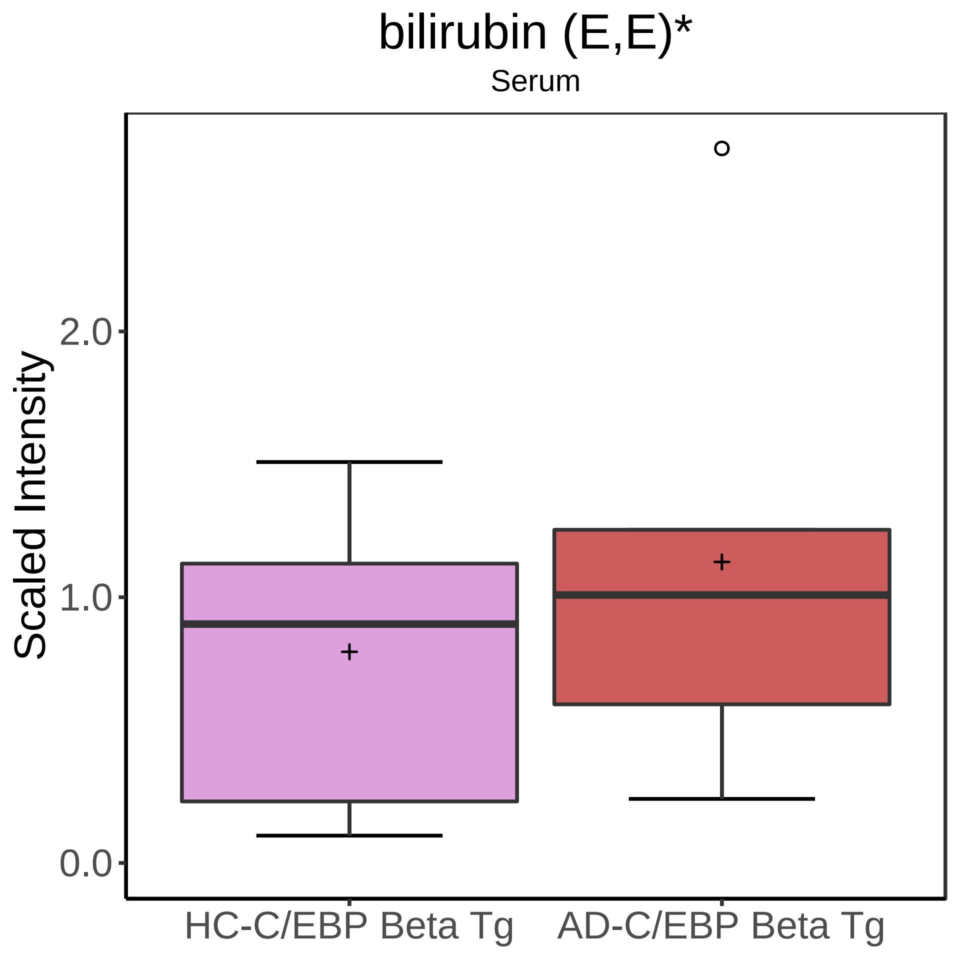
bilirubin (E,E)*
Serum
Scaled Intensity
2.0
1.0
0.0
HC-C/EBP Beta Tg
AD-C/EBP Beta Tg

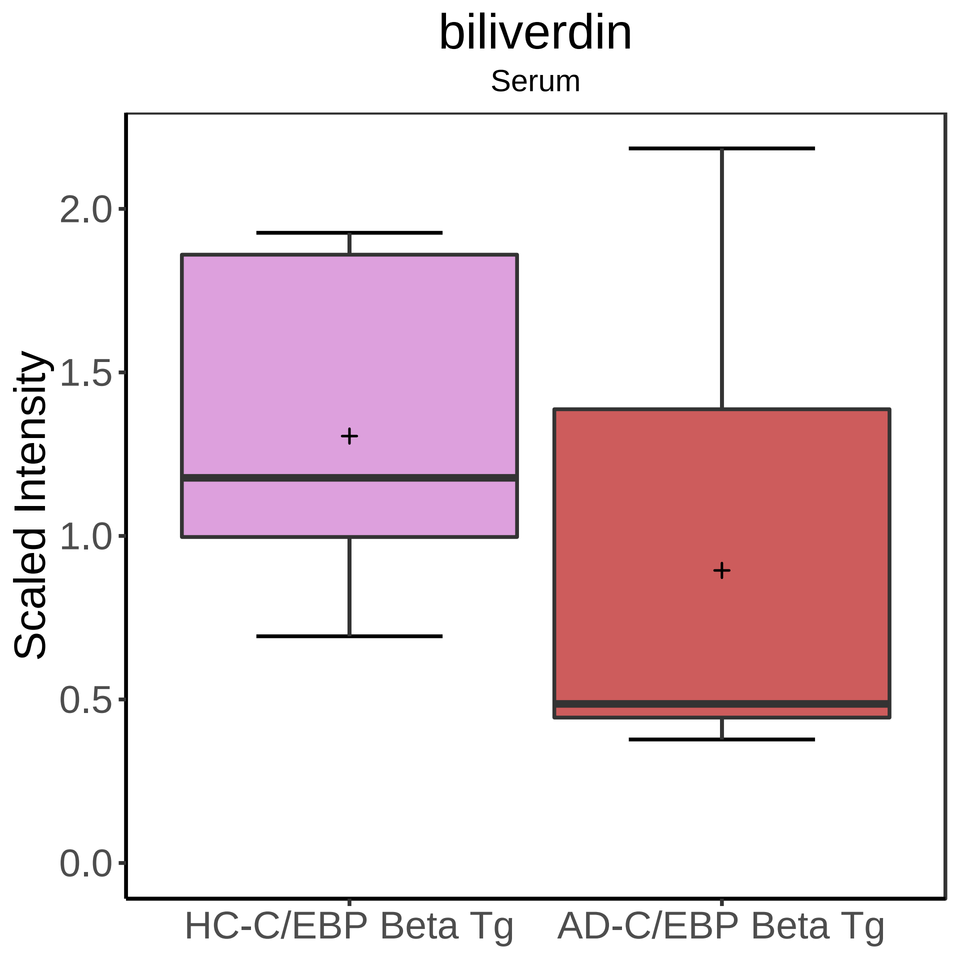
biliverdin
Serum
Scaled Intensity
2.0
1.5
1.0
0.5
0.0
HC-C/EBP Beta Tg
AD-C/EBP Beta Tg

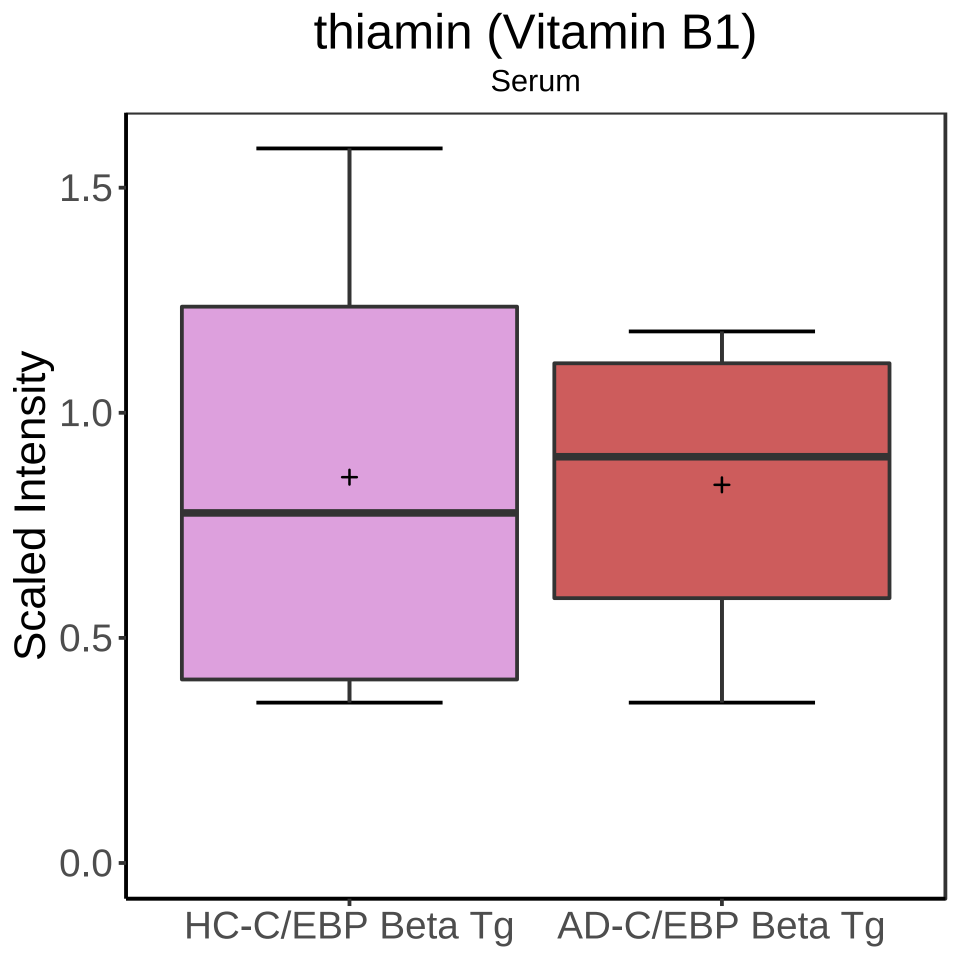
thiamin (Vitamin B1)
Serum
Scaled Intensity
1.5
1.0
0.5
0.0
HC-C/EBP Beta Tg
AD-C/EBP Beta Tg

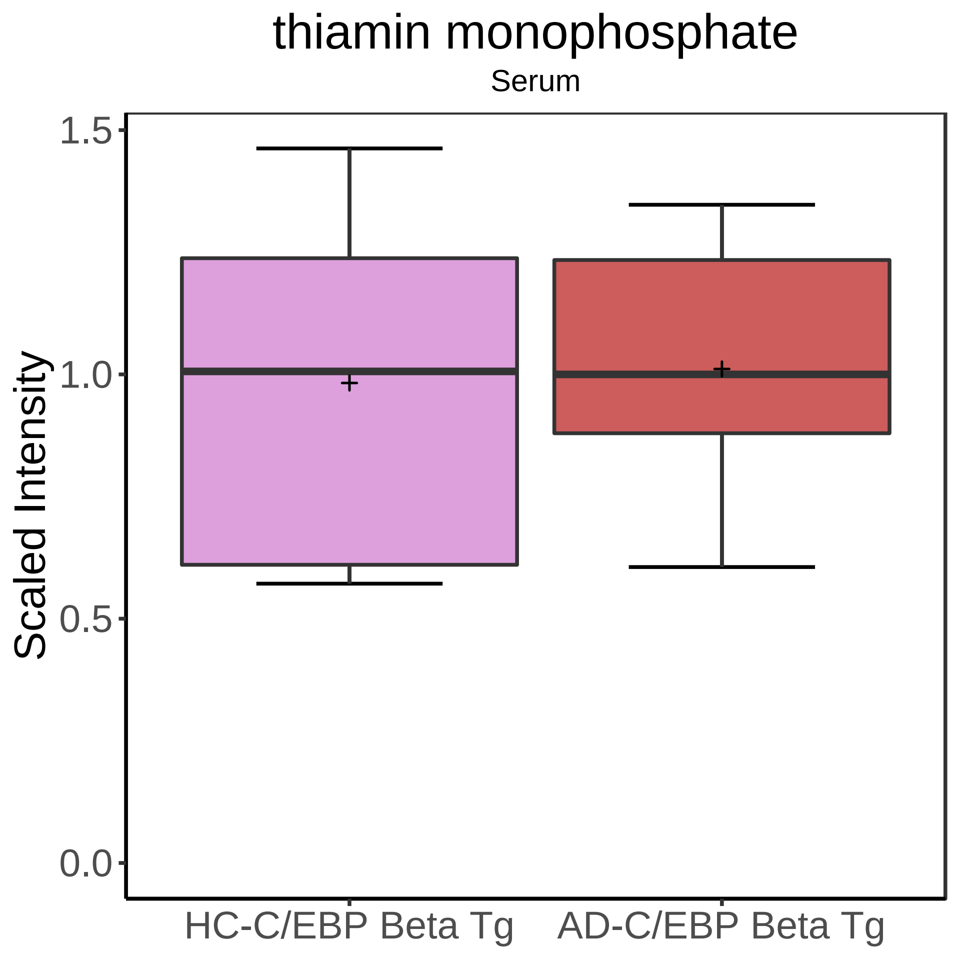
thiamin monophosphate
Serum
Scaled Intensity
1.5
1.0
0.5
0.0
HC-C/EBP Beta Tg
AD-C/EBP Beta Tg

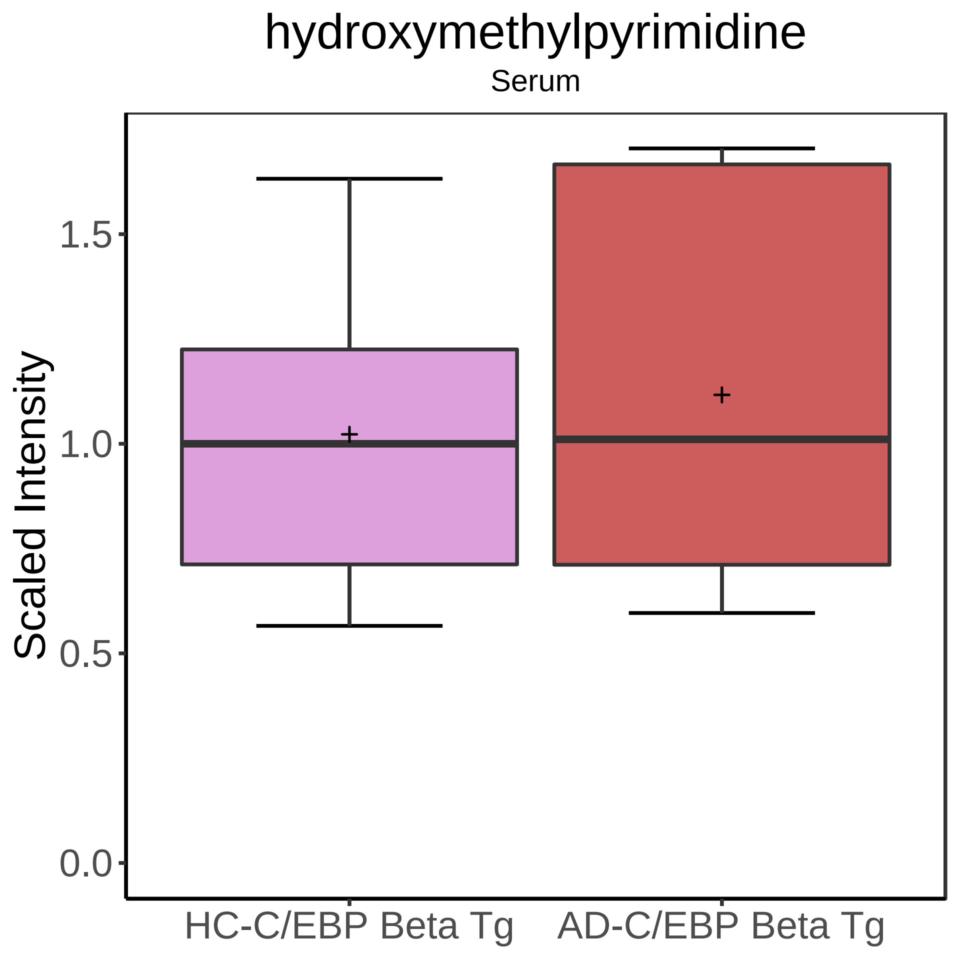

hydroxymethylpyrimidine
Serum
Scaled Intensity
1.5
1.0
0.5
0.0
HC-C/EBP Beta Tg
AD-C/EBP Beta Tg

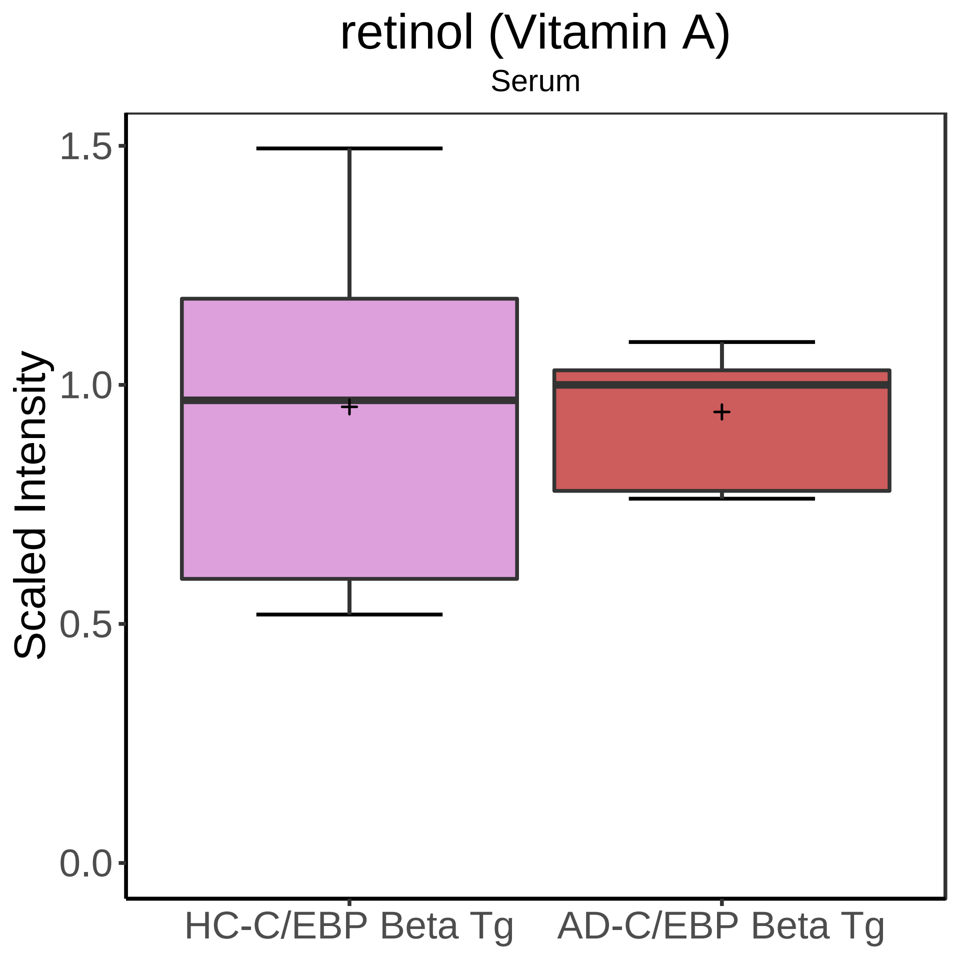
retinol (Vitamin A)
Serum
Scaled Intensity
1.5
1.0
0.5
0.0
HC-C/EBP Beta Tg
AD-C/EBP Beta Tg

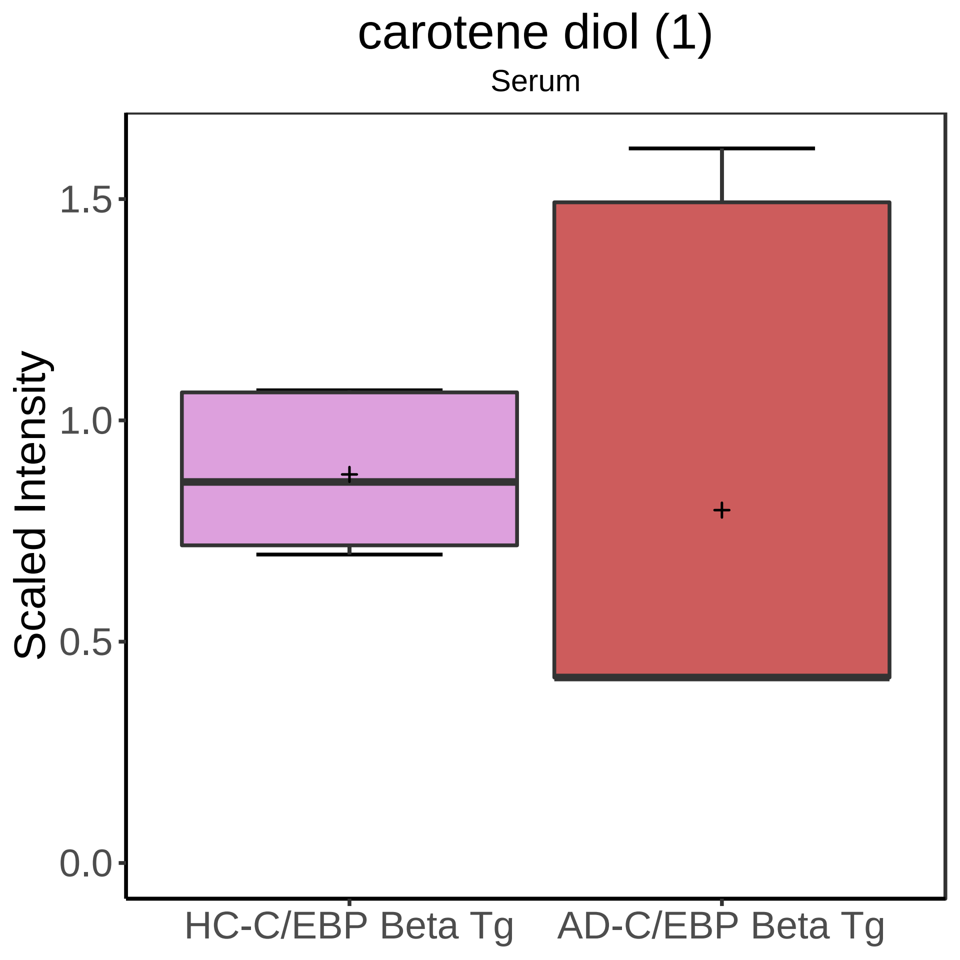
carotene diol (1)
Serum
Scaled Intensity
1.5
1.0
0.5
0.0
HC-C/EBP Beta Tg
AD-C/EBP Beta Tg

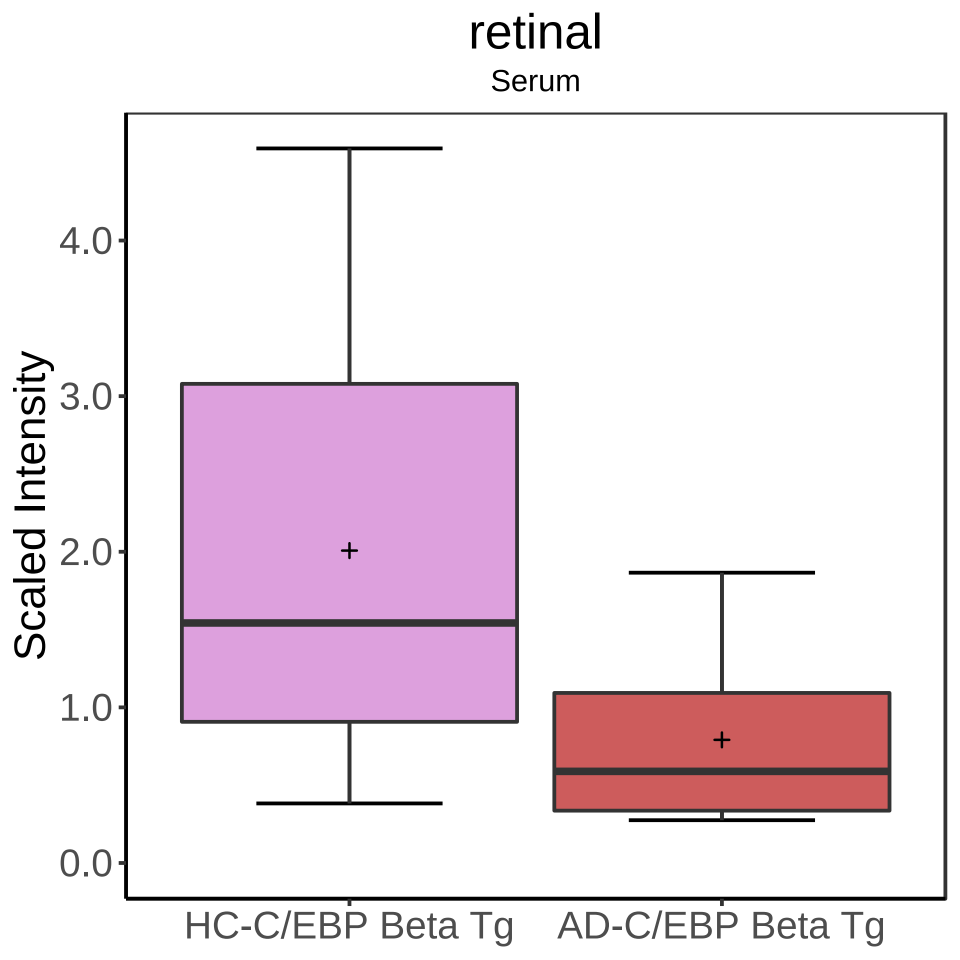

retinal
Serum
Scaled Intensity
4.0
3.0
2.0
1.0
0.0
HC-C/EBP Beta Tg
AD-C/EBP Beta Tg

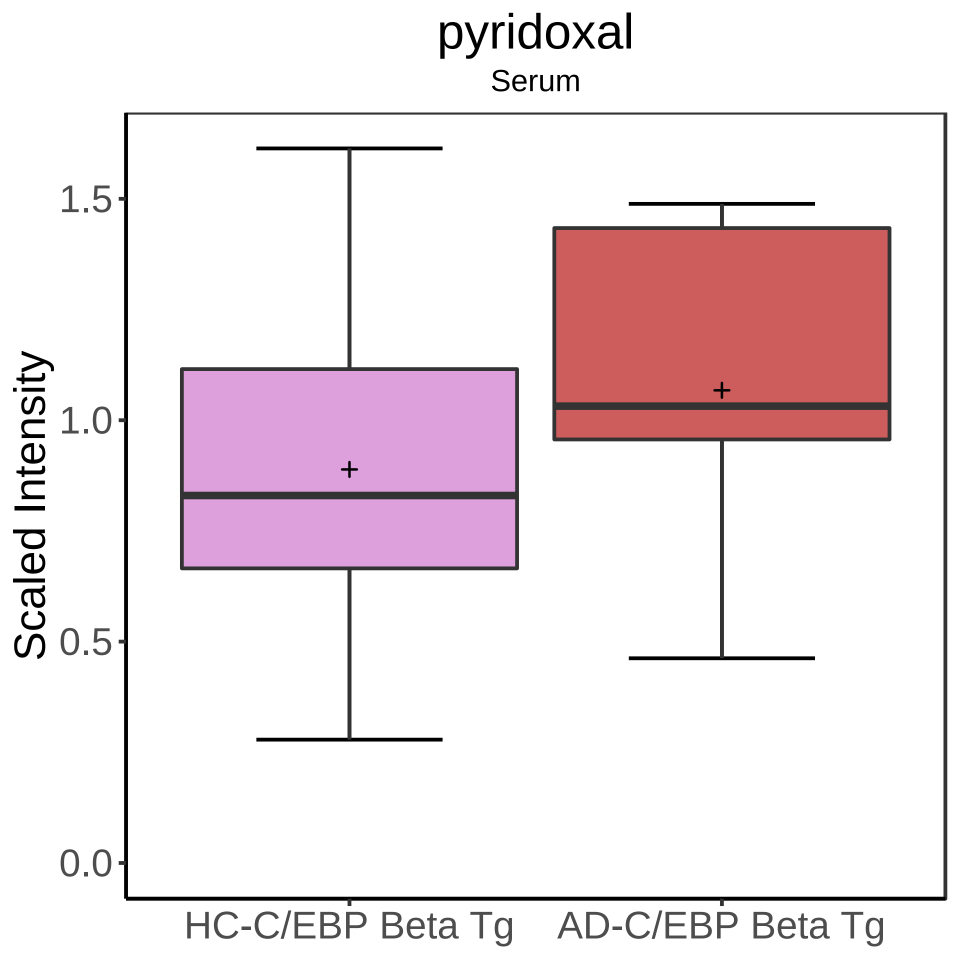
pyridoxal
Serum
Scaled Intensity
1.5
1.0
0.5
0.0
HC-C/EBP Beta Tg
AD-C/EBP Beta Tg

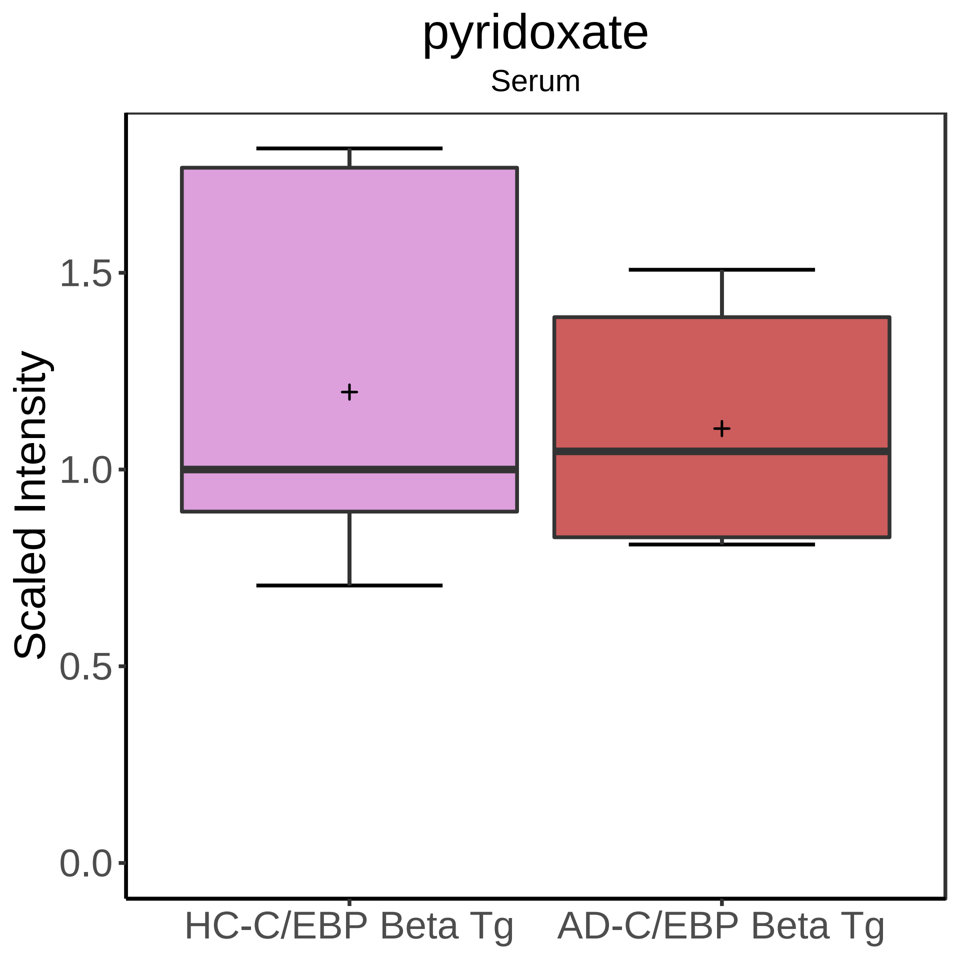
pyridoxate
Serum
Scaled Intensity
1.5
1.0
0.5
0.0
HC-C/EBP Beta Tg
AD-C/EBP Beta Tg

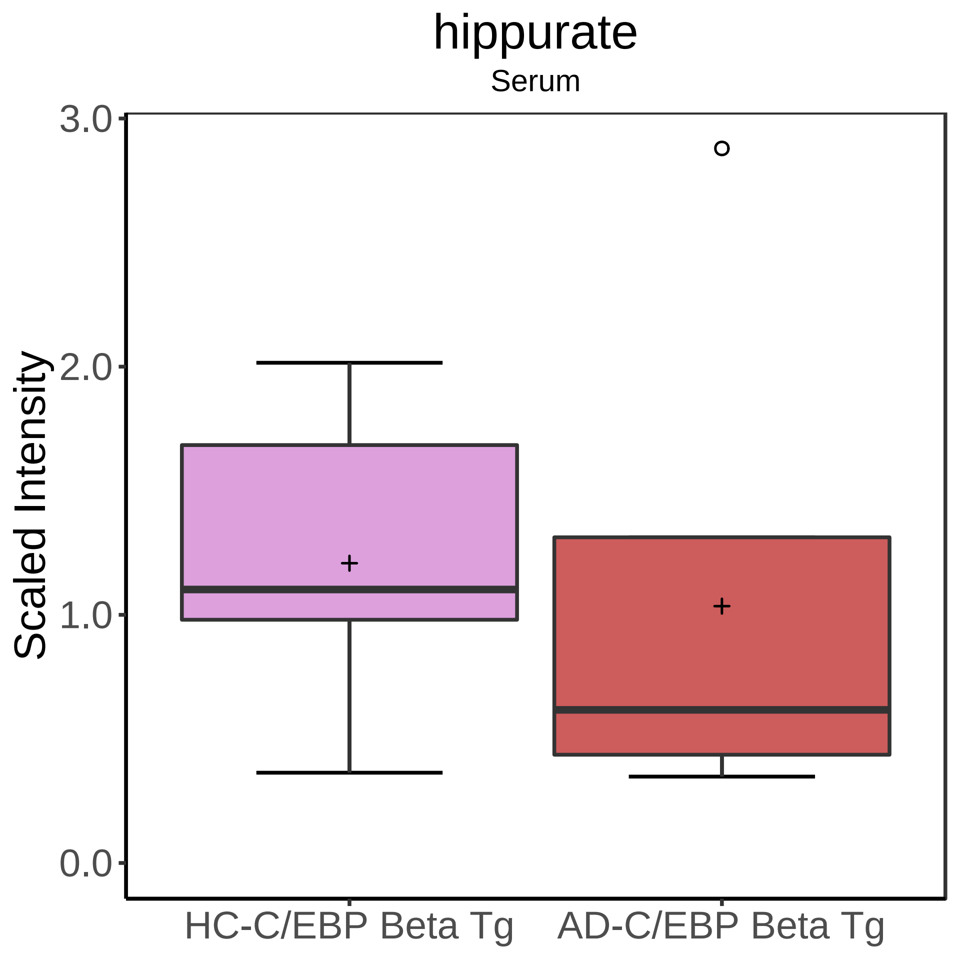
hippurate
Serum
Scaled Intensity
3.0
2.0
1.0
0.0
HC-C/EBP Beta Tg
AD-C/EBP Beta Tg

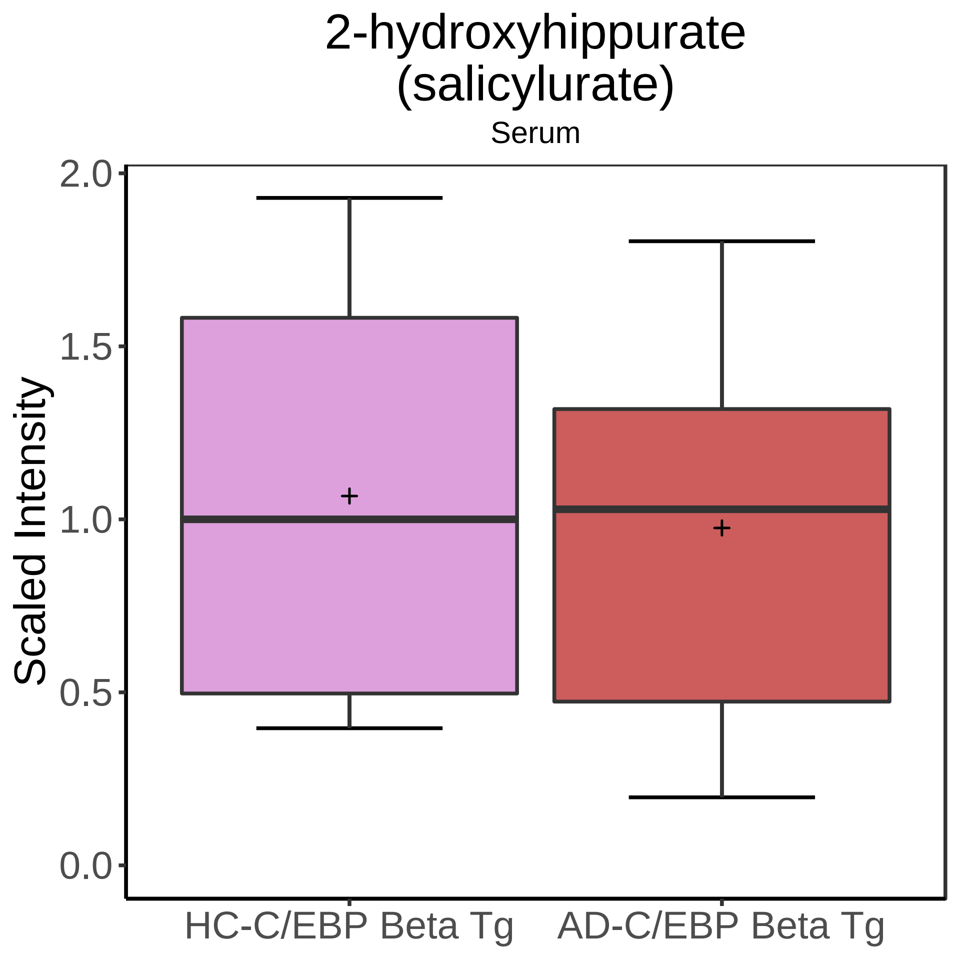

2-hydroxyhippurate
(salicylurate)
Serum
Scaled Intensity
2.0
1.5
1.0
0.5
0.0
HC-C/EBP Beta Tg
AD-C/EBP Beta Tg

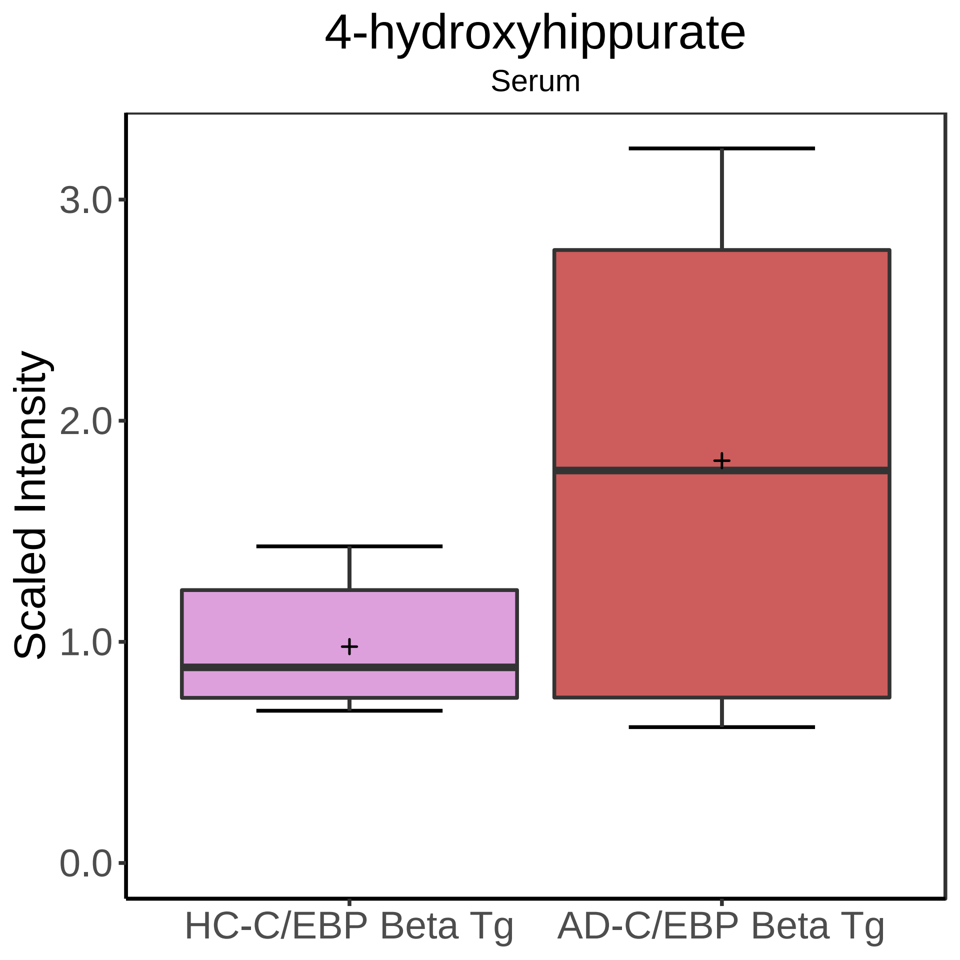

4-hydroxyhippurate
Serum
Scaled Intensity
3.0
2.0
1.0
0.0
HC-C/EBP Beta Tg
AD-C/EBP Beta Tg

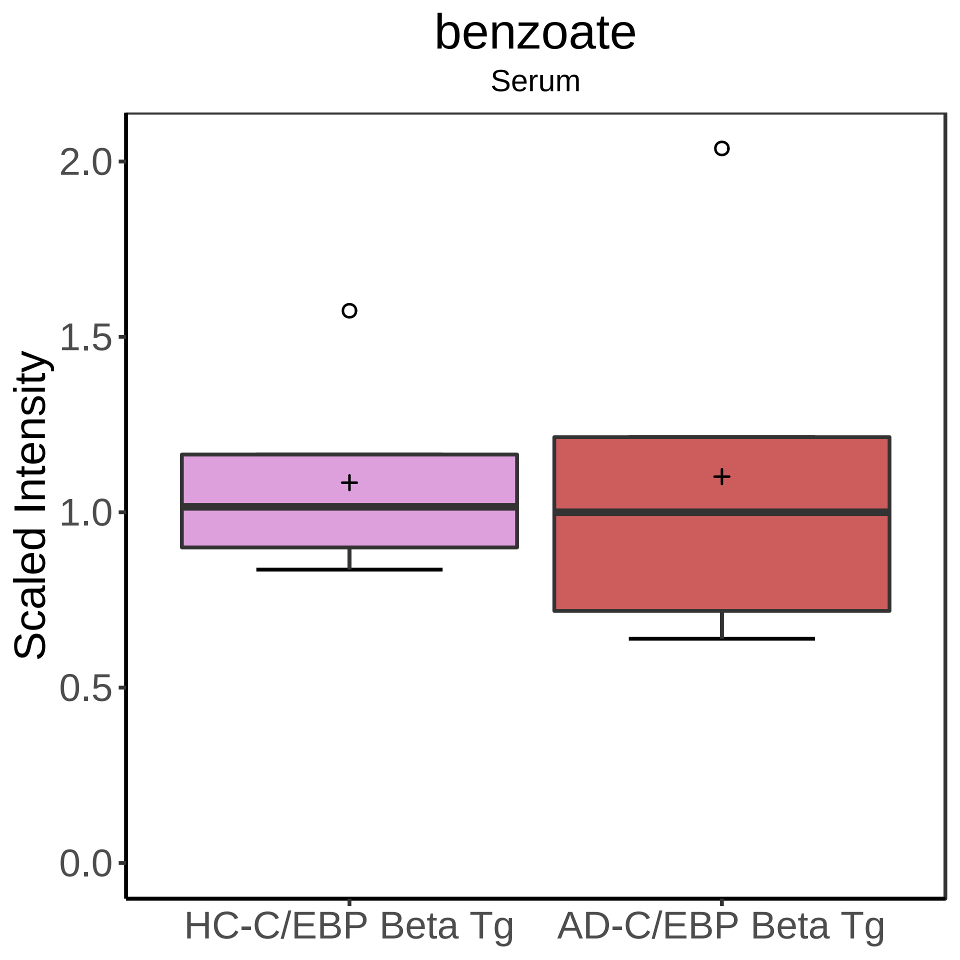
benzoate
Serum
Scaled Intensity
2.0
1.5
1.0
0.5
0.0
HC-C/EBP Beta Tg
AD-C/EBP Beta Tg

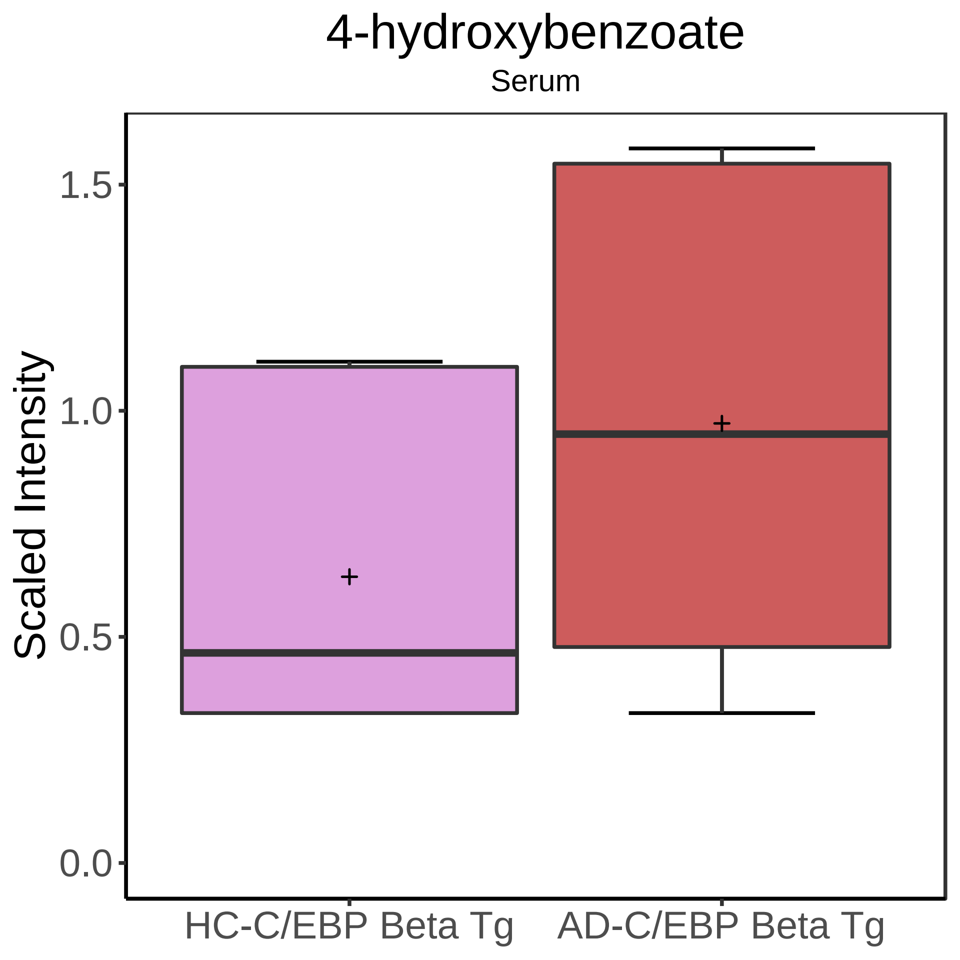
4-hydroxybenzoate
Serum
Scaled Intensity
1.5
1.0
0.5
0.0
HC-C/EBP Beta Tg
AD-C/EBP Beta Tg

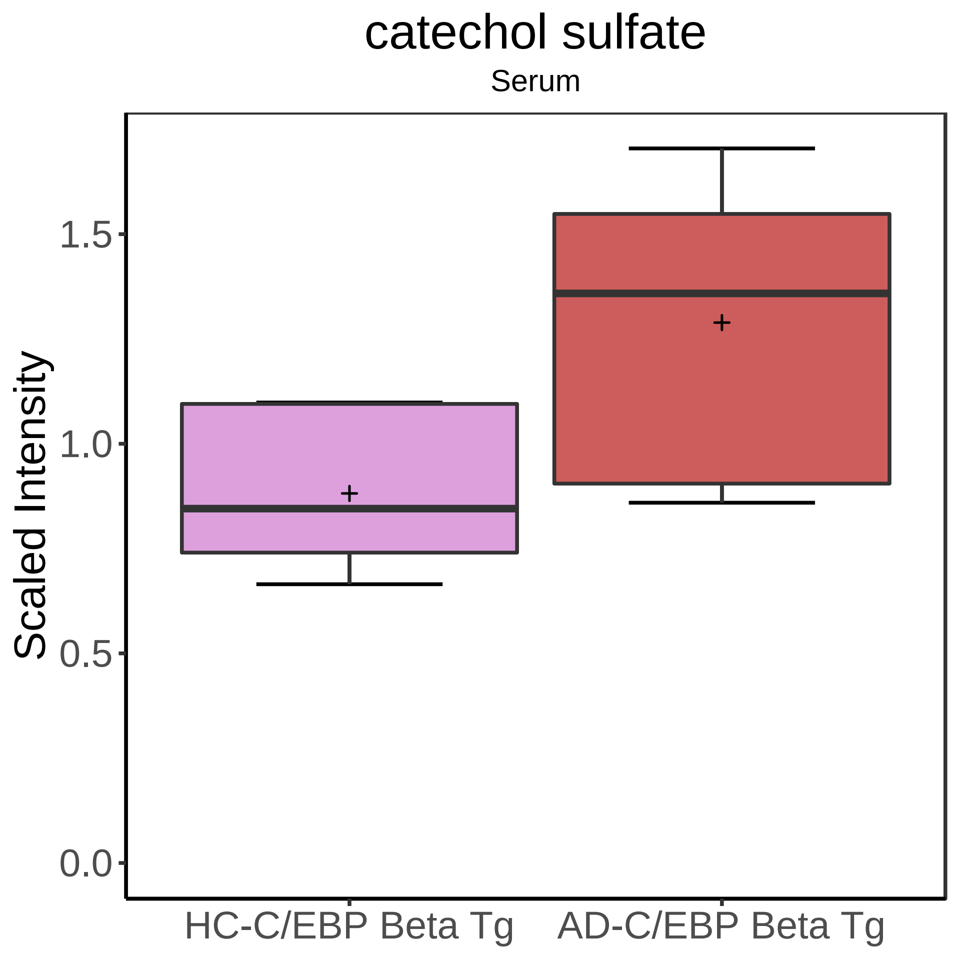

catechol sulfate
Serum
Scaled Intensity
1.5
1.0
0.5
0.0
HC-C/EBP Beta Tg
AD-C/EBP Beta Tg

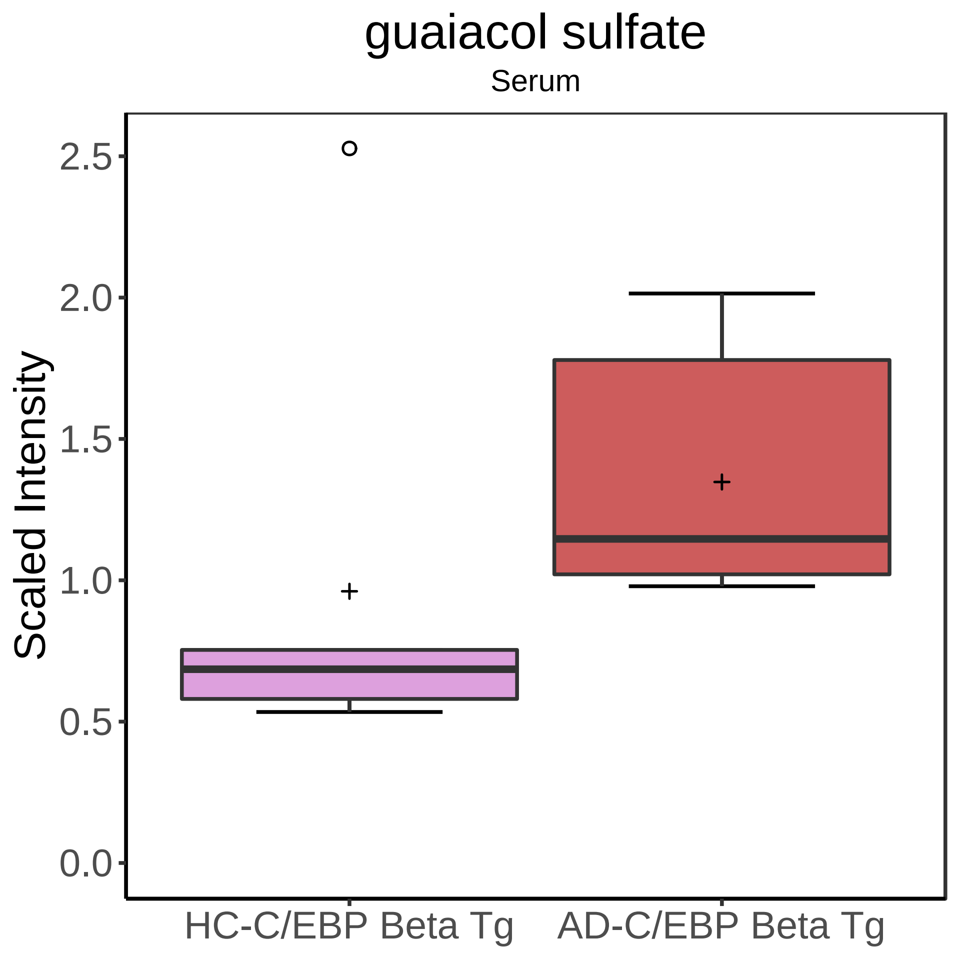

guaiacol sulfate
Serum
Scaled Intensity
2.5
2.0
1.5
1.0
0.5
0.0
HC-C/EBP Beta Tg
AD-C/EBP Beta Tg

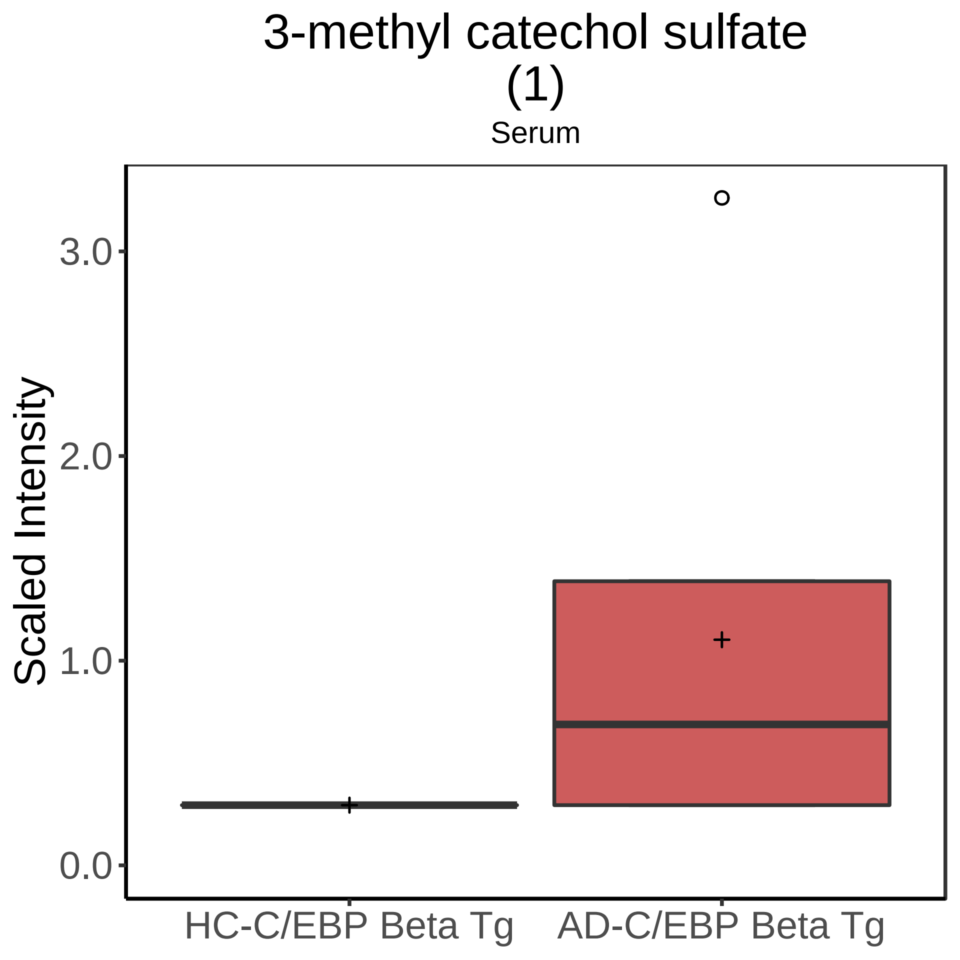
3-methyl catechol sulfate
(1)
Serum
Scaled Intensity
0.0
1.0
2.0
3.0
HC-C/EBP Beta Tg
AD-C/EBP Beta Tg

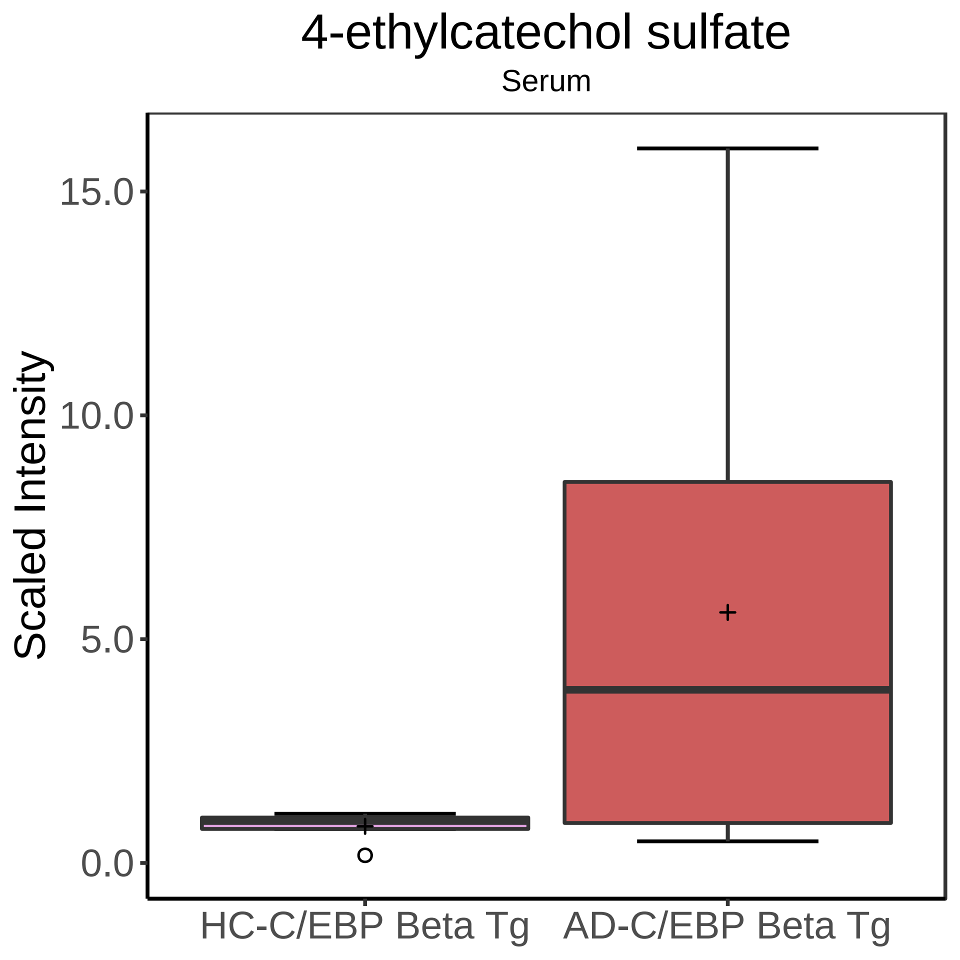
4-ethylcatechol sulfate
Serum
Scaled Intensity
15.0
10.0
5.0
0.0
HC-C/EBP Beta Tg
AD-C/EBP Beta Tg

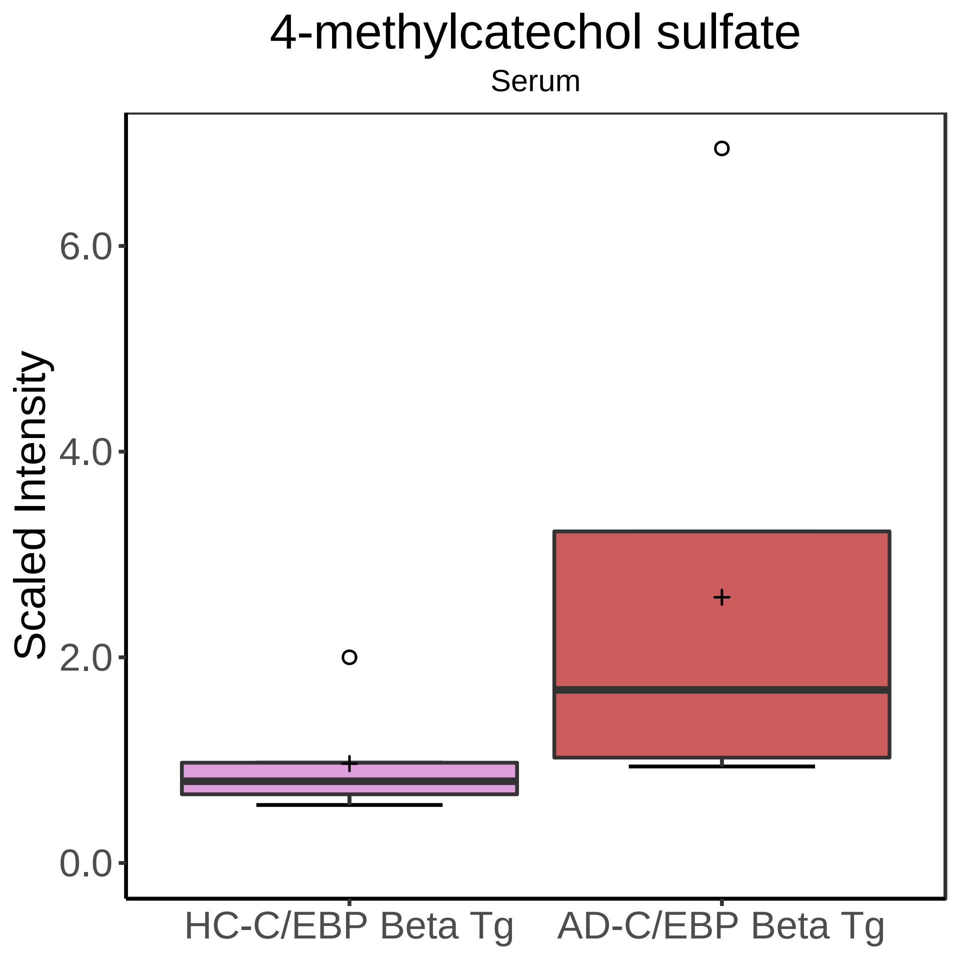
4-methylcatechol sulfate
Serum
Scaled Intensity
6.0
4.0
2.0
0.0
HC-C/EBP Beta Tg
AD-C/EBP Beta Tg

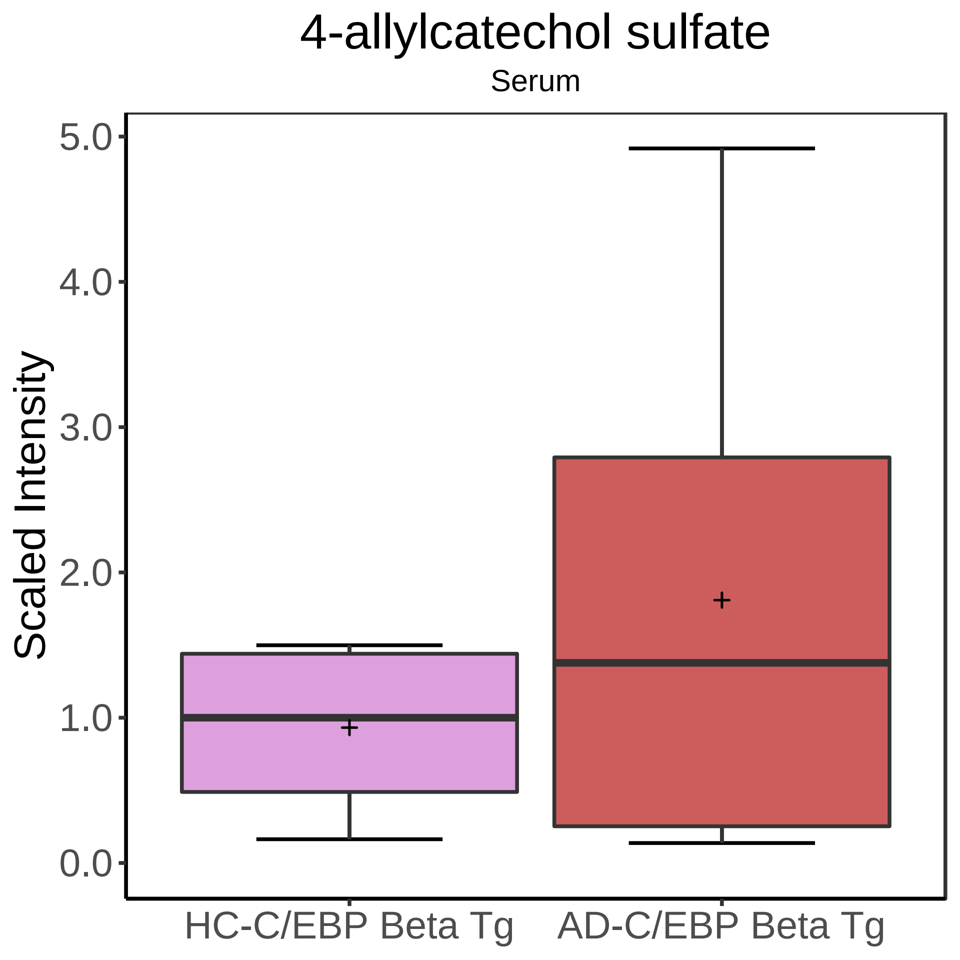

4-allylcatechol sulfate
Serum
Scaled Intensity
5.0
4.0
3.0
2.0
1.0
0.0
HC-C/EBP Beta Tg
AD-C/EBP Beta Tg

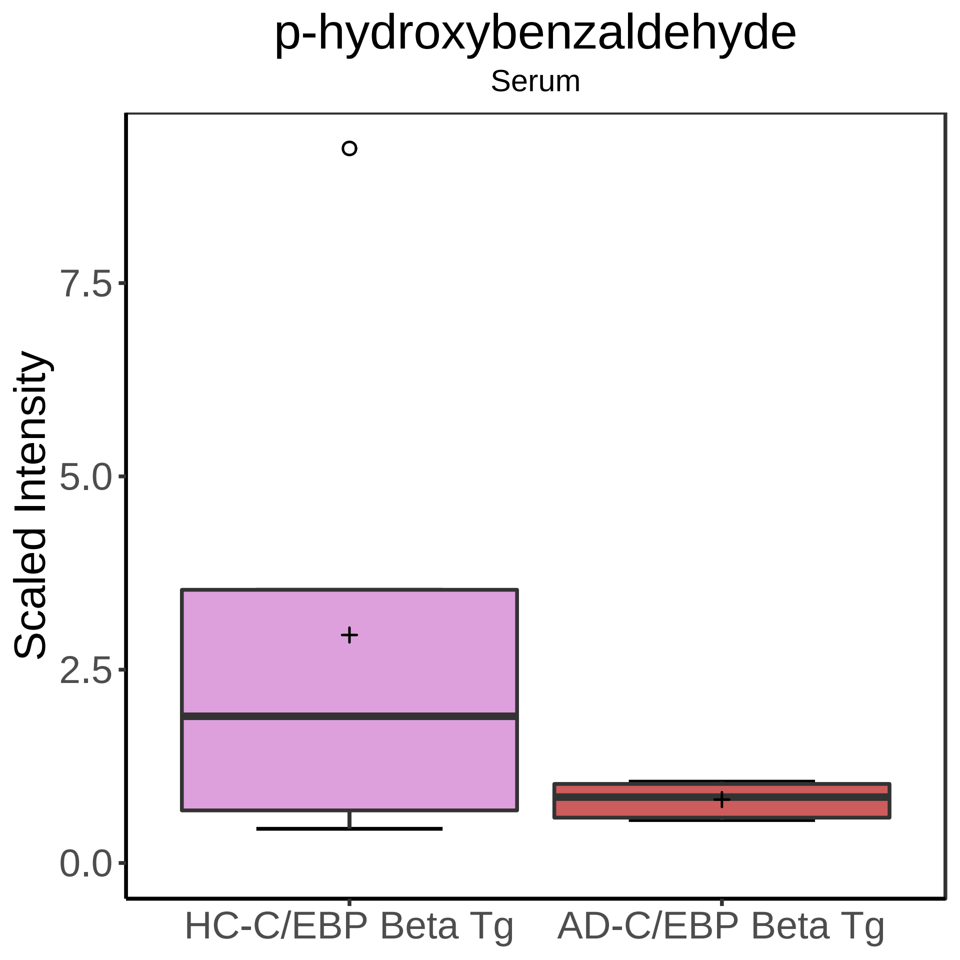
p-hydroxybenzaldehyde
Serum
Scaled Intensity
7.5
5.0
2.5
0.0
HC-C/EBP Beta Tg
AD-C/EBP Beta Tg

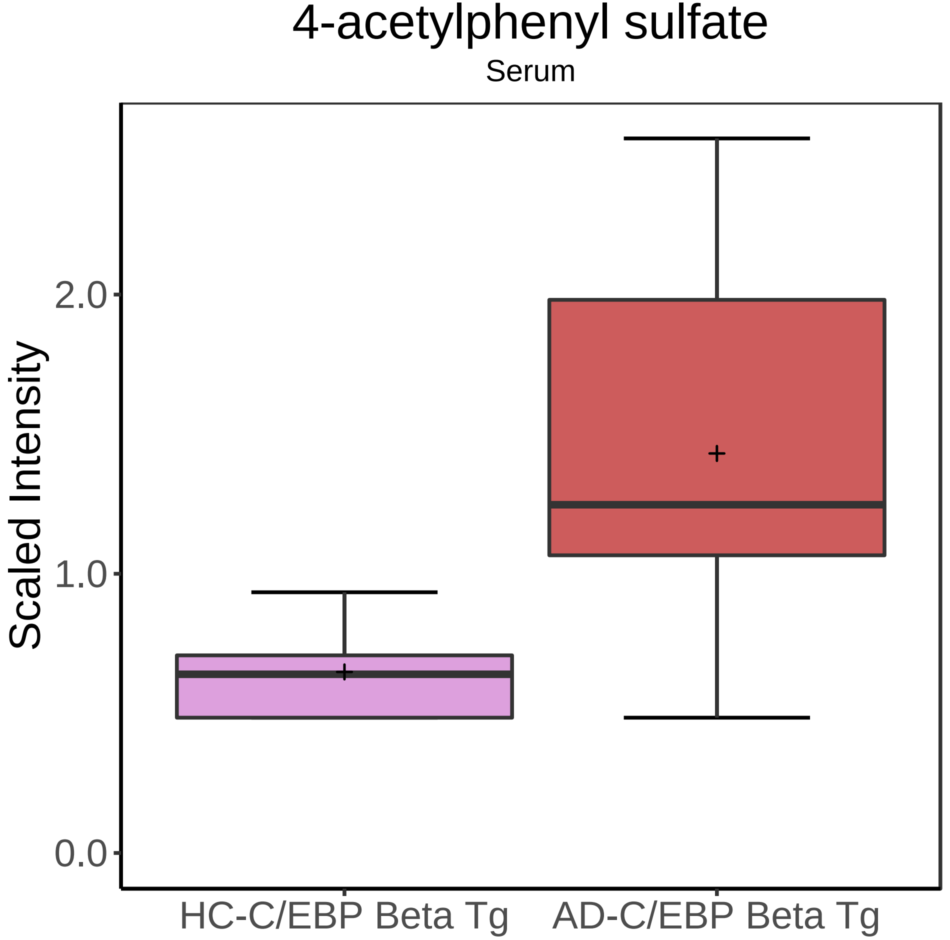
4-acetylphenyl sulfate
Serum
Scaled Intensity
2.0
1.0
0.0
HC-C/EBP Beta Tg
AD-C/EBP Beta Tg

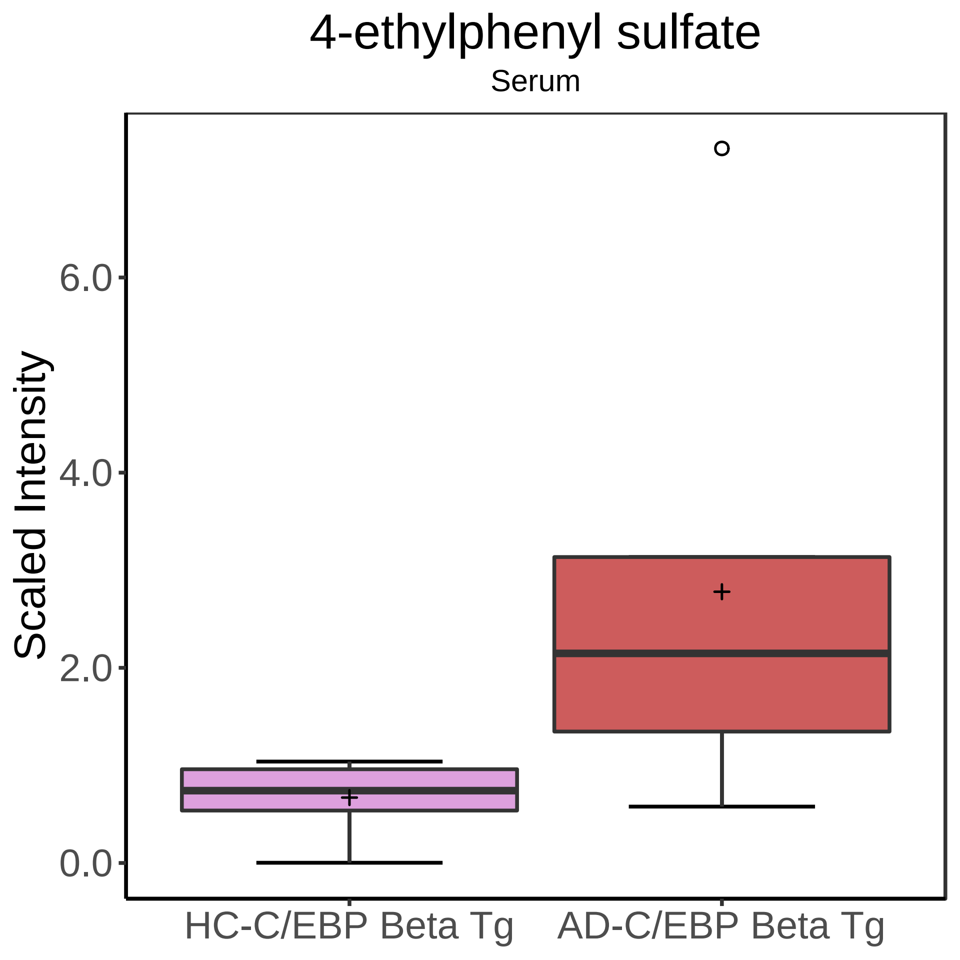
4-ethylphenyl sulfate
Serum
Scaled Intensity
6.0
4.0
2.0
0.0
HC-C/EBP Beta Tg
AD-C/EBP Beta Tg

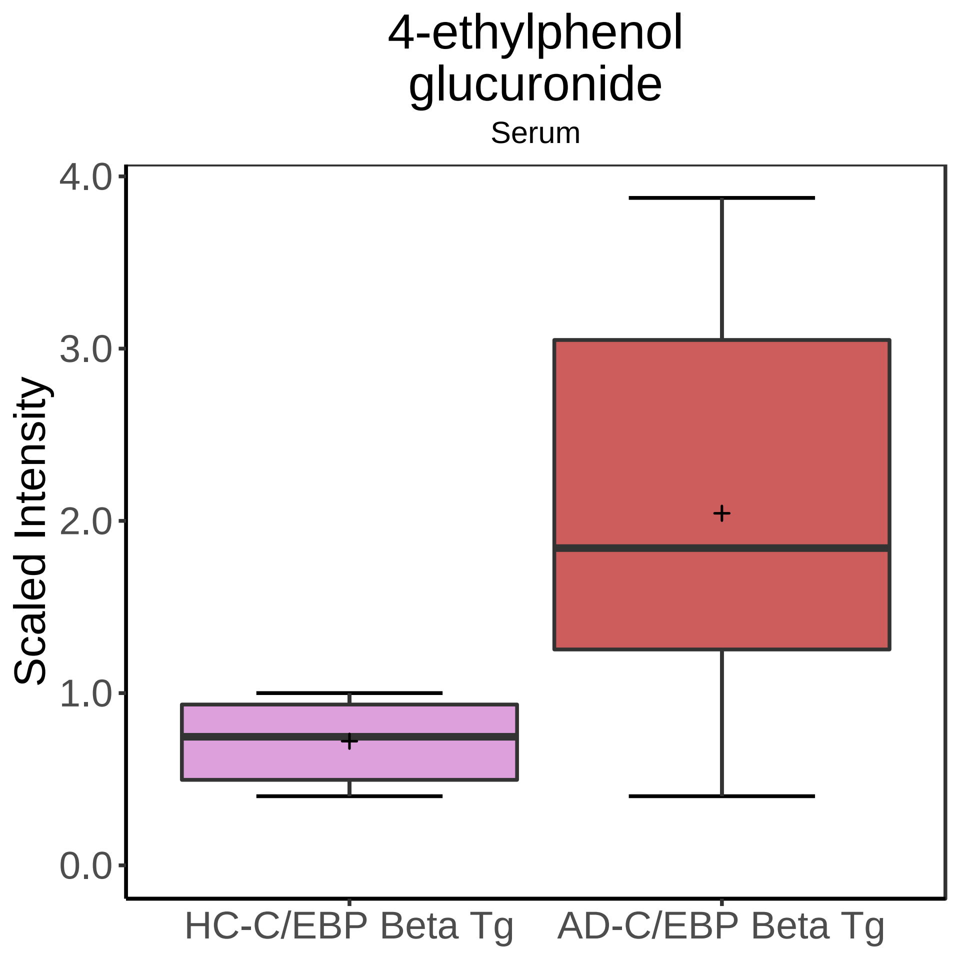
4-ethylphenol
glucuronide
Serum
Scaled Intensity
4.0
3.0
2.0
1.0
0.0
HC-C/EBP Beta Tg
AD-C/EBP Beta Tg

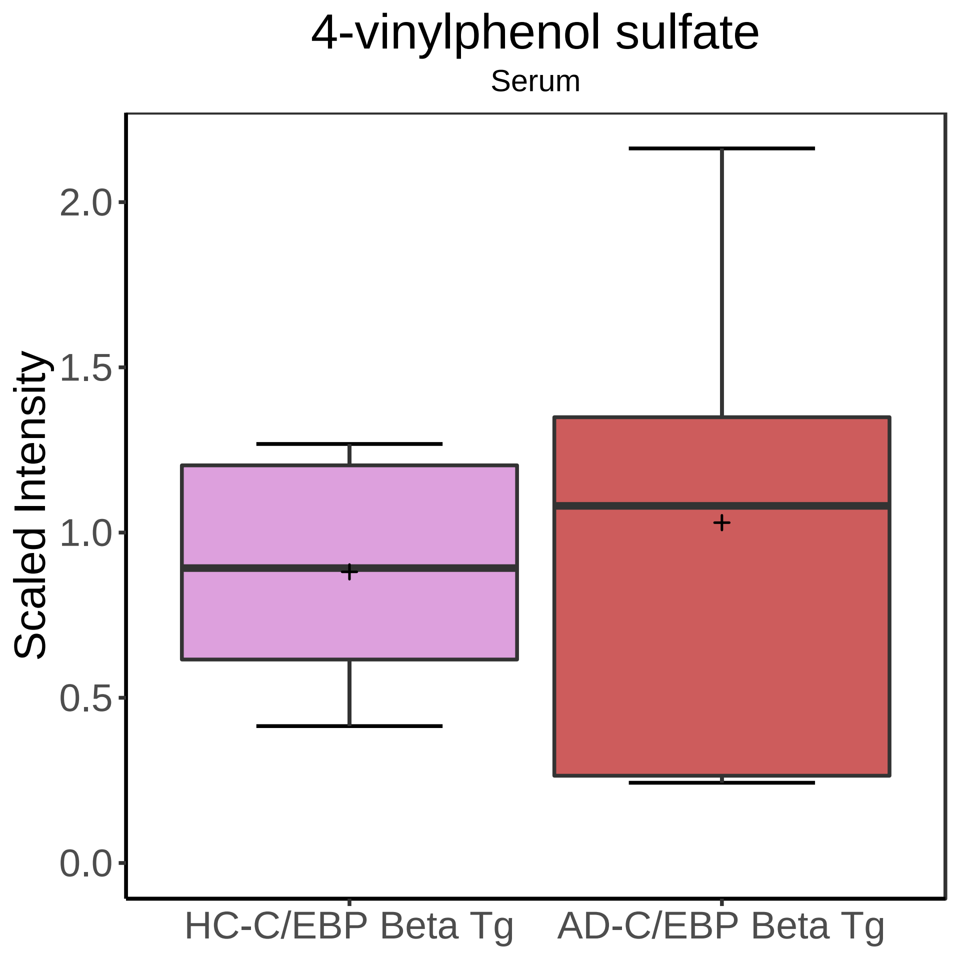
4-vinylphenol sulfate
Serum
Scaled Intensity
2.0
1.5
1.0
0.5
0.0
HC-C/EBP Beta Tg
AD-C/EBP Beta Tg

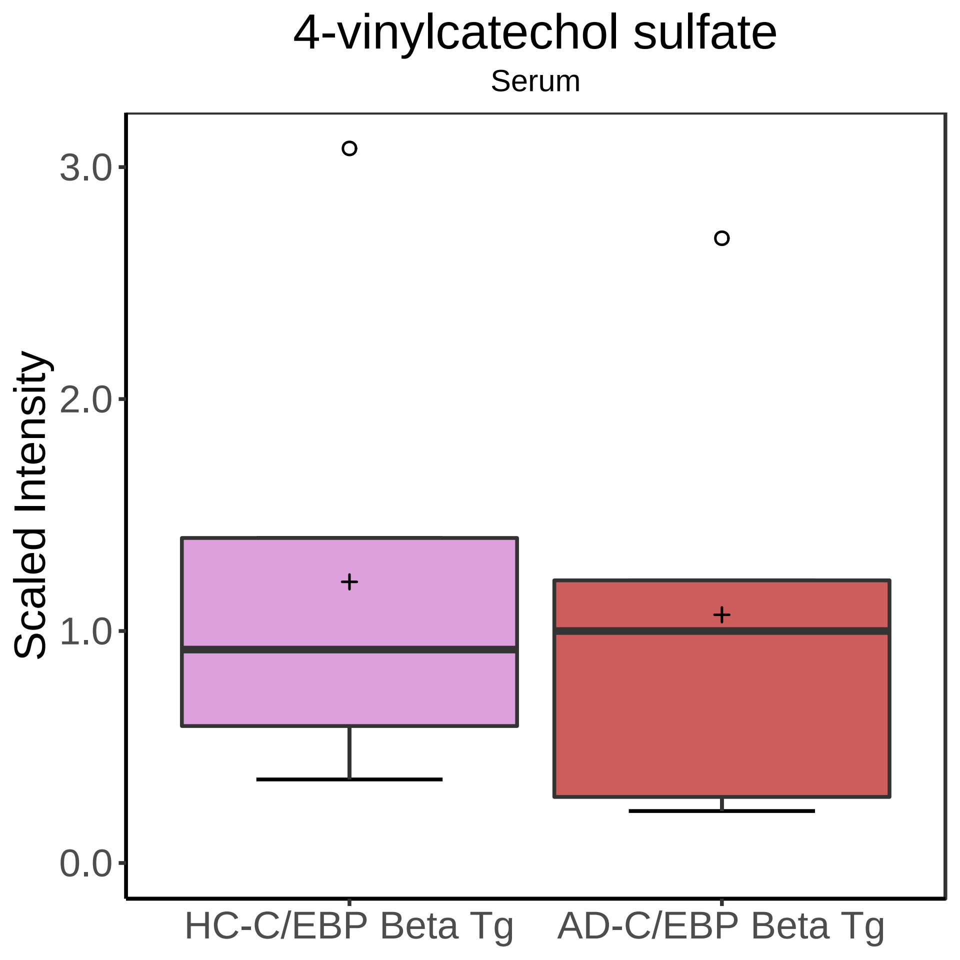

4-vinylcatechol sulfate
Serum
Scaled Intensity
3.0
2.0
1.0
0.0
HC-C/EBP Beta Tg
AD-C/EBP Beta Tg

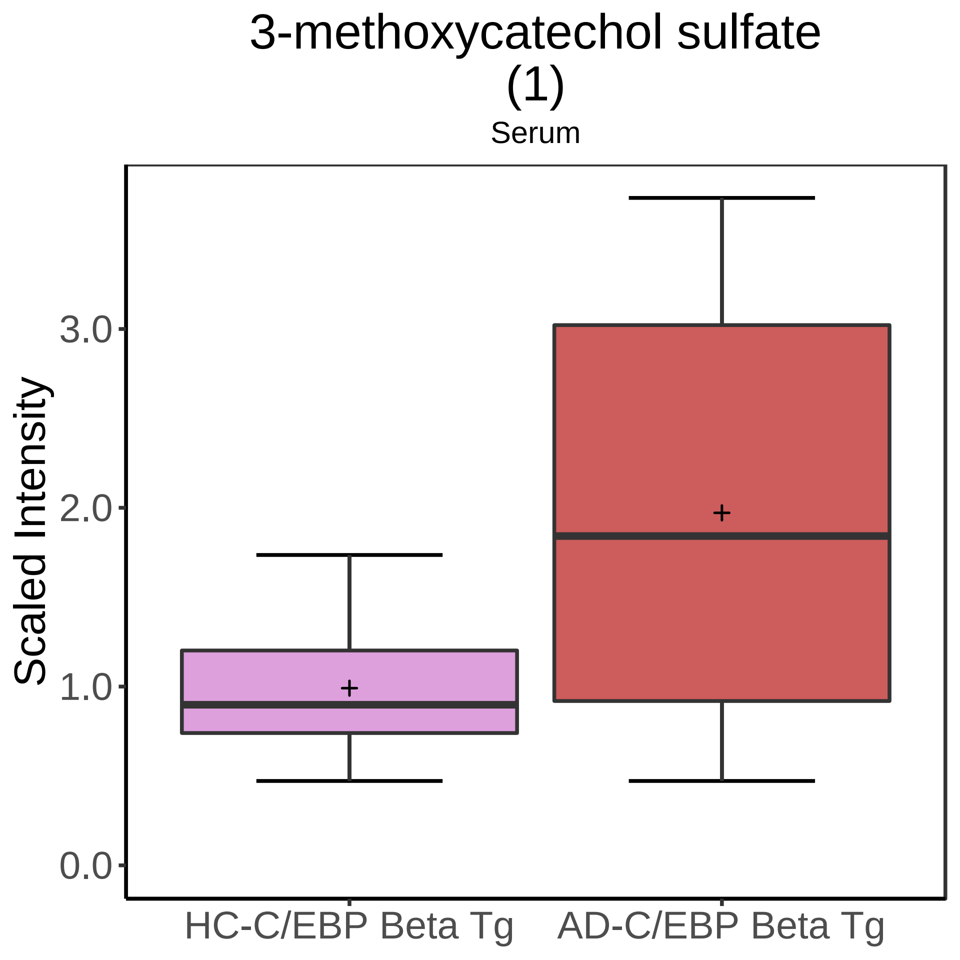
3-methoxycatechol sulfate
(1)
Serum
Scaled Intensity
3.0
2.0
1.0
0.0
HC-C/EBP Beta Tg
AD-C/EBP Beta Tg

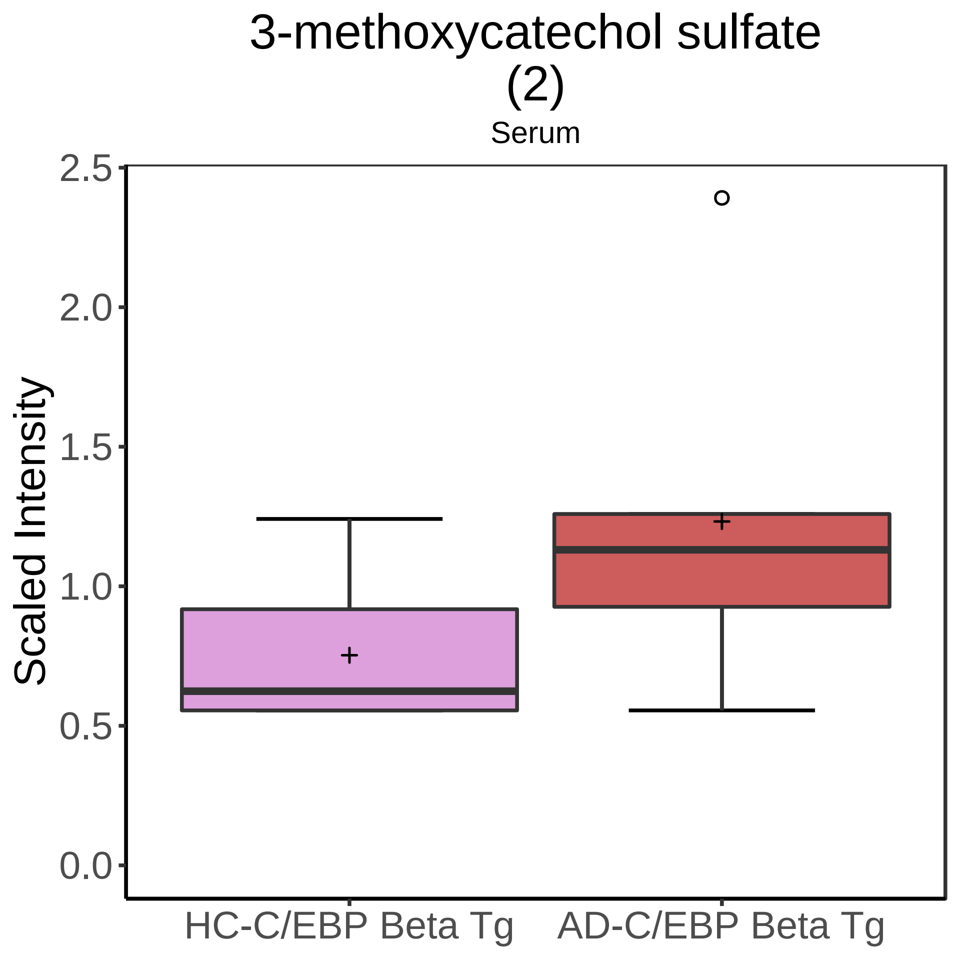
3-methoxycatechol sulfate
(2)
Serum
Scaled Intensity
2.5
2.0
1.5
1.0
0.5
0.0
HC-C/EBP Beta Tg
AD-C/EBP Beta Tg

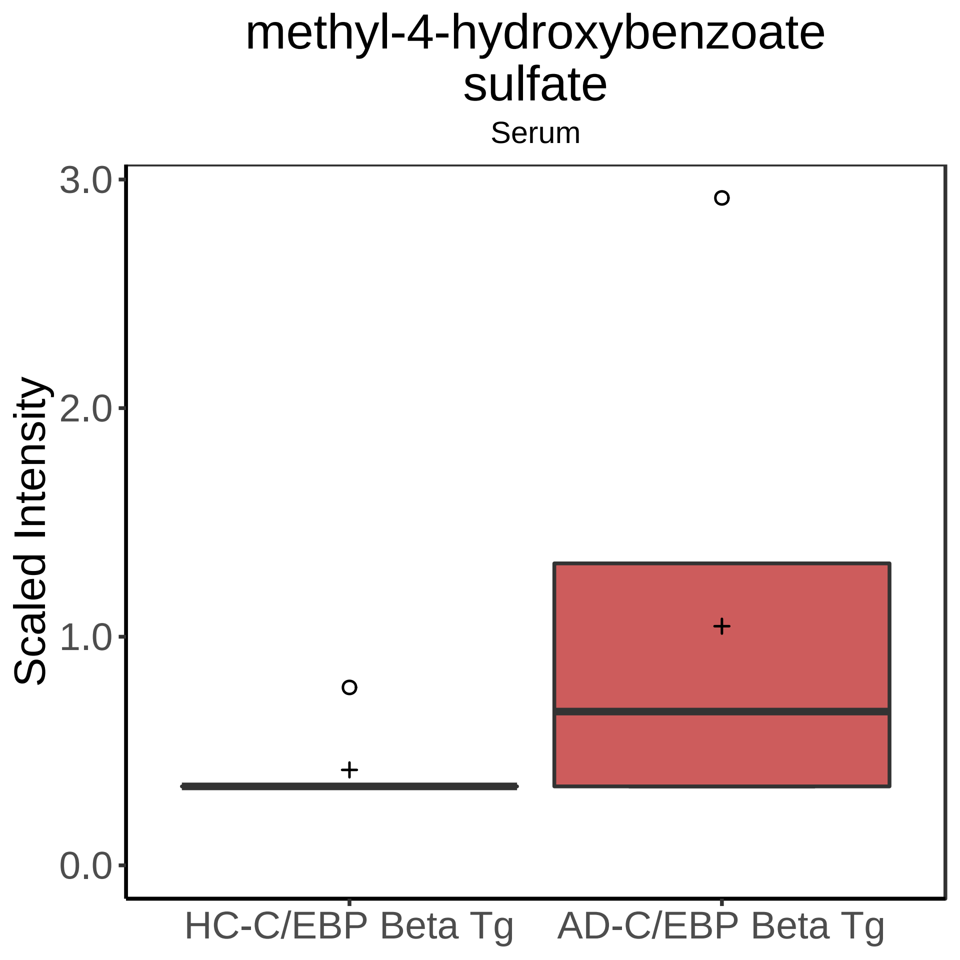
methyl-4-hydroxybenzoate sulfate
Serum
Scaled Intensity
3.0
2.0
1.0
0.0
HC-C/EBP Beta Tg
AD-C/EBP Beta Tg

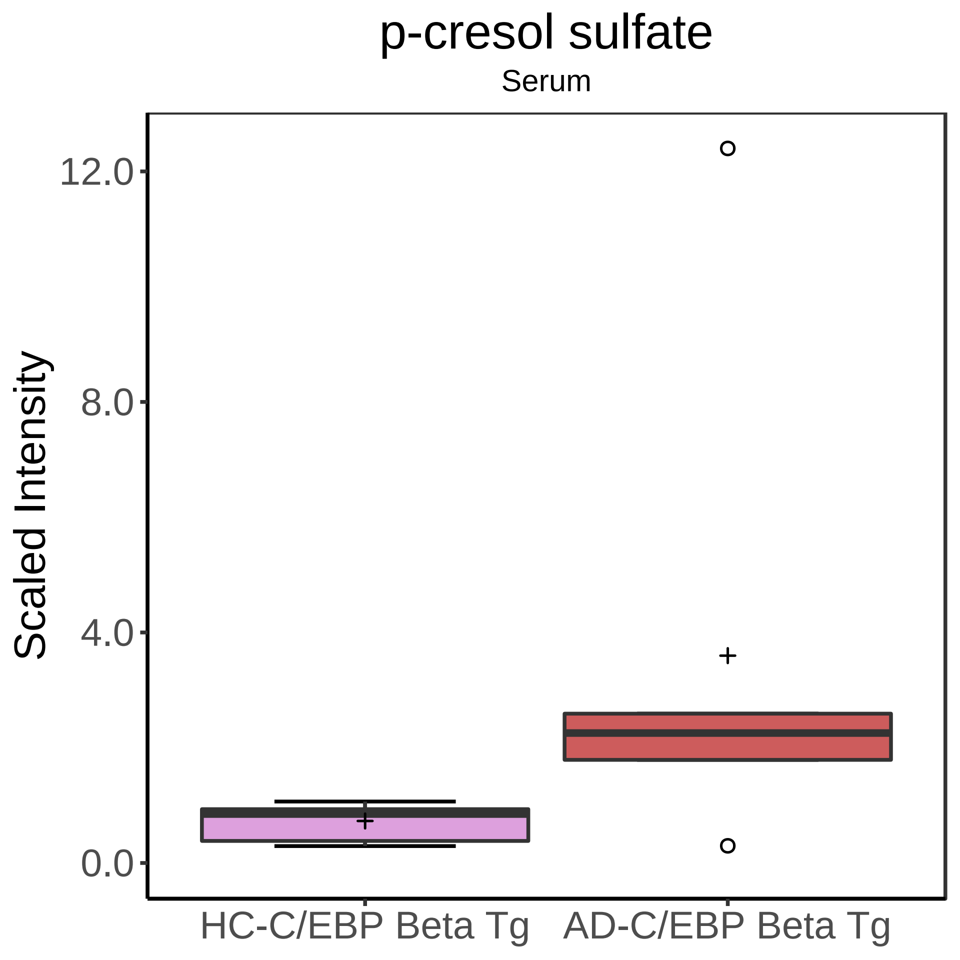
p-cresol sulfate
Serum
Scaled Intensity
12.0
8.0
4.0
0.0
HC-C/EBP Beta Tg
AD-C/EBP Beta Tg

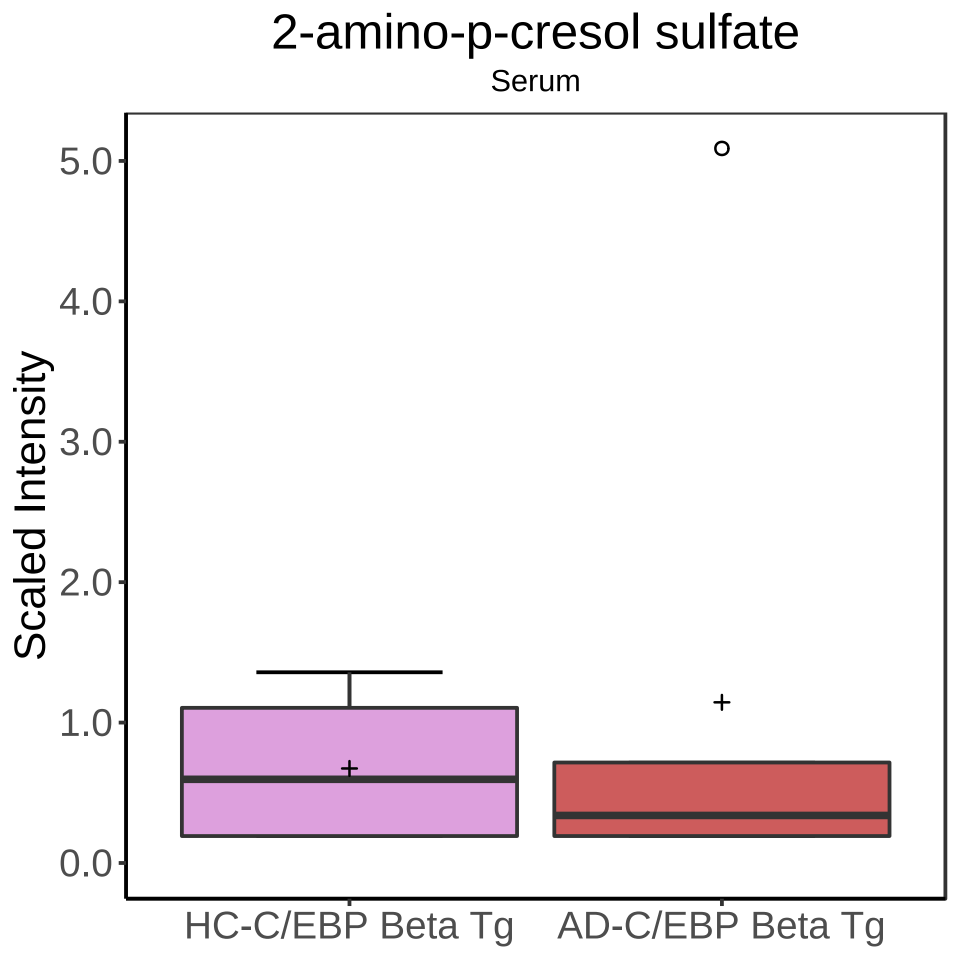

2-amino-p-cresol sulfate
Serum
Scaled Intensity
5.0
4.0
3.0
2.0
1.0
0.0
HC-C/EBP Beta Tg
AD-C/EBP Beta Tg

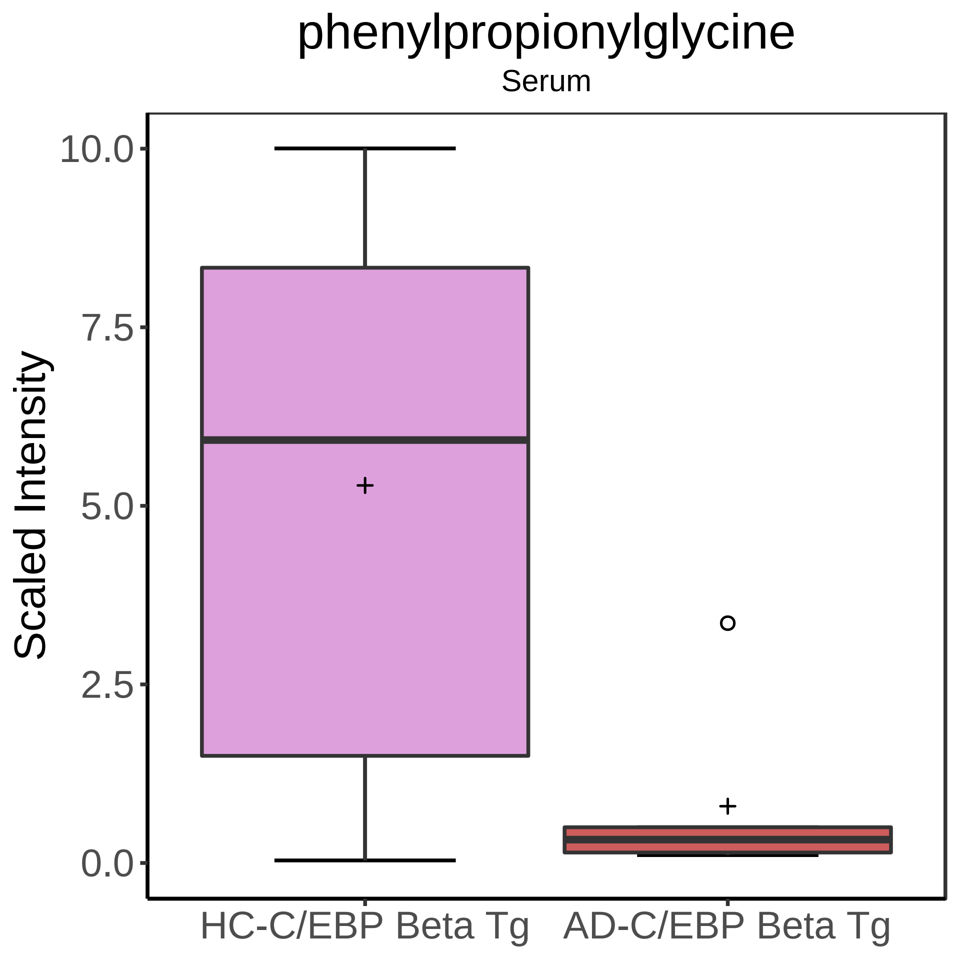
phenylpropionylglycine
Serum
Scaled Intensity
10.0
7.5
5.0
2.5
0.0
HC-C/EBP Beta Tg
AD-C/EBP Beta Tg

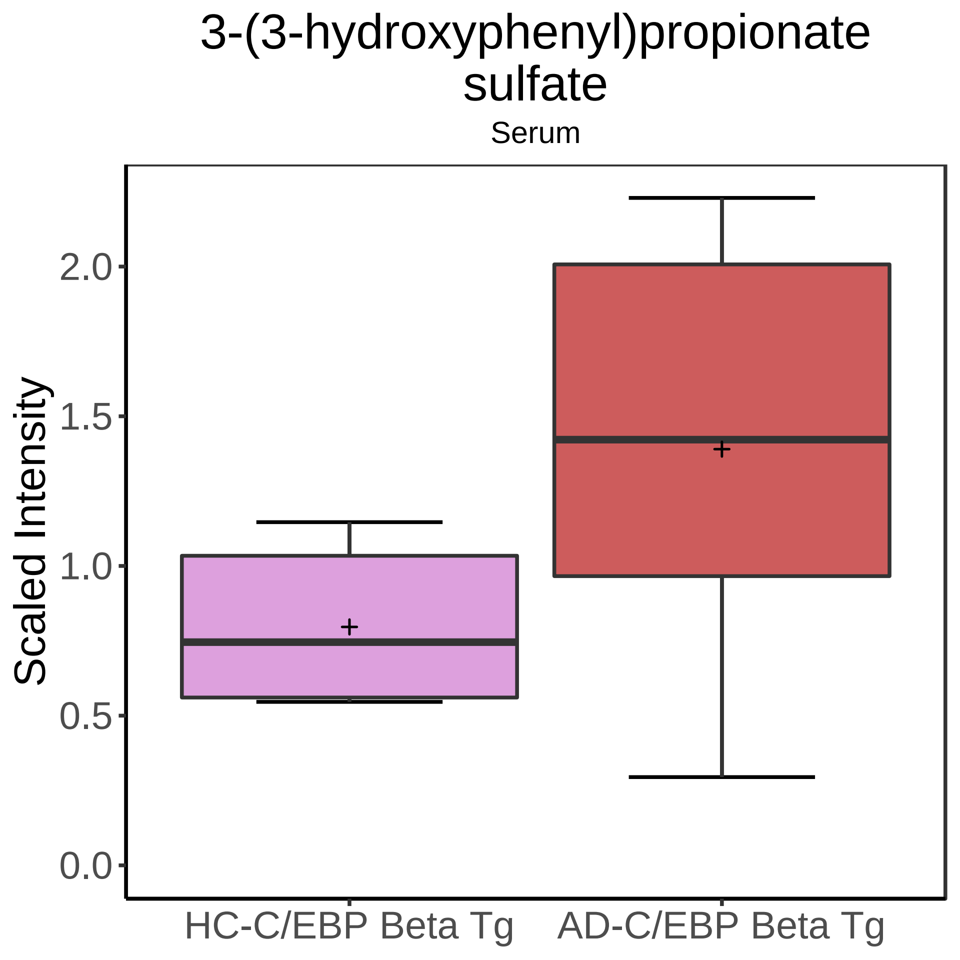
3-(3-hydroxyphenyl)propionate sulfate
Serum
Scaled Intensity
2.0
1.5
1.0
0.5
0.0
HC-C/EBP Beta Tg
AD-C/EBP Beta Tg

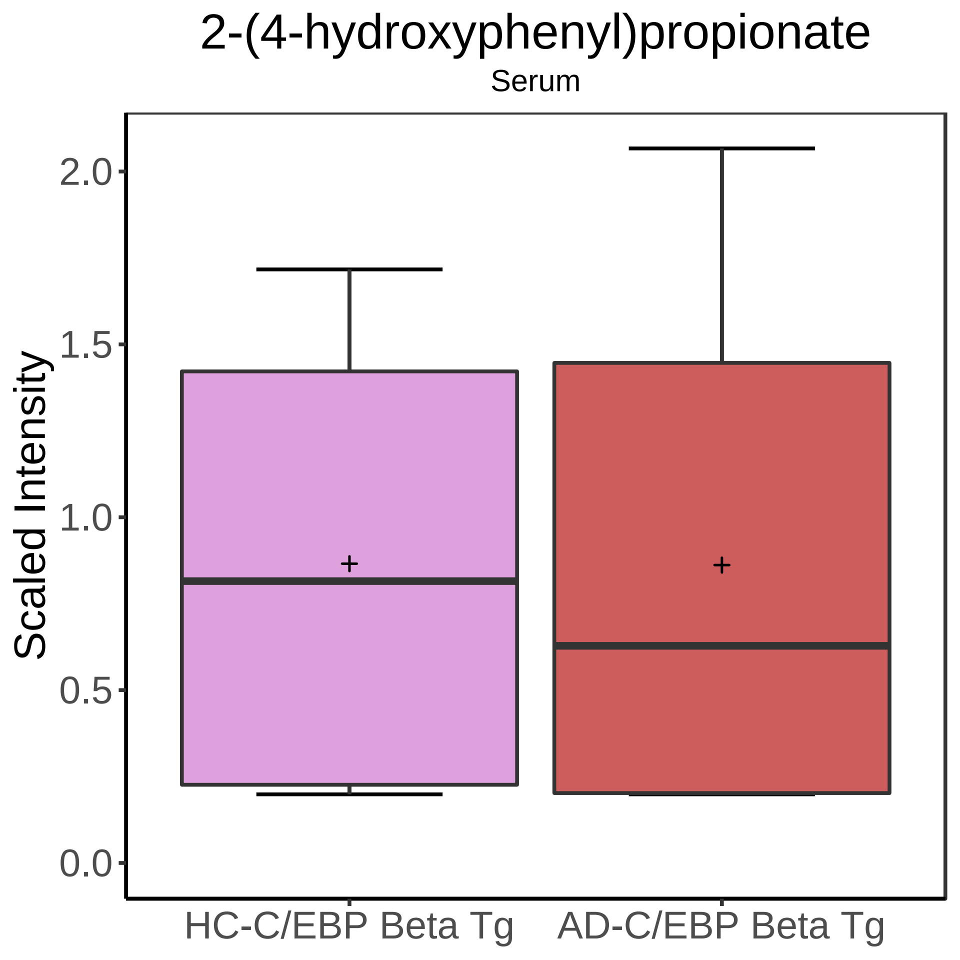
2-(4-hydroxyphenyl)propionate
Serum
Scaled Intensity
0.0
0.5
1.0
1.5
2.0
HC-C/EBP Beta Tg
AD-C/EBP Beta Tg

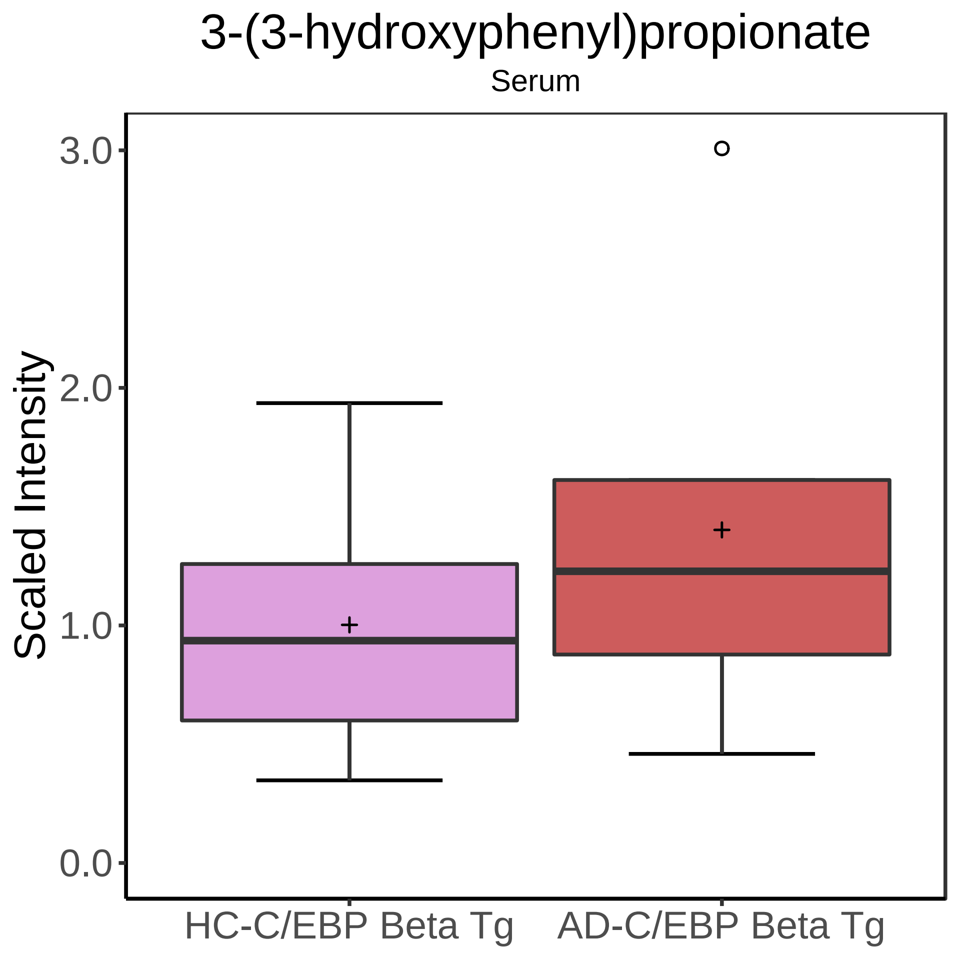
3-(3-hydroxyphenyl)propionate
Serum
Scaled Intensity
3.0
2.0
1.0
0.0
HC-C/EBP Beta Tg
AD-C/EBP Beta Tg

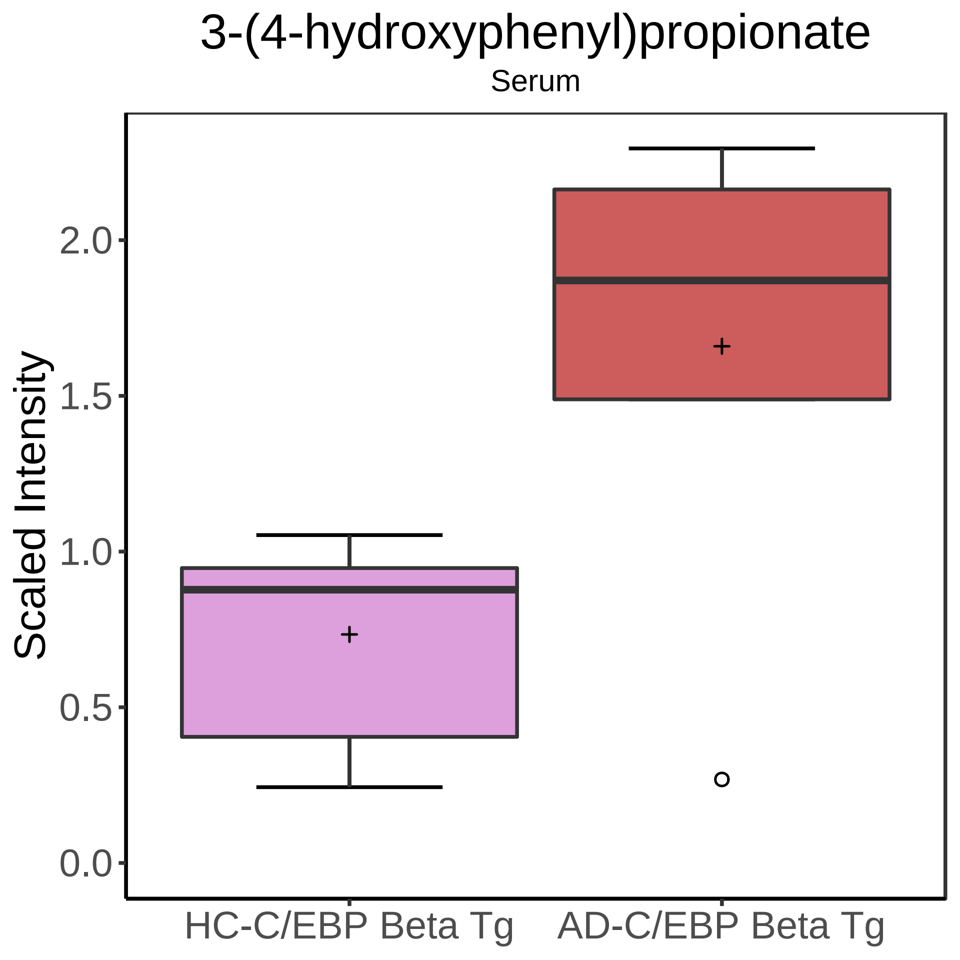
3-(4-hydroxyphenyl)propionate
Serum
Scaled Intensity
2.0
1.5
1.0
0.5
0.0
HC-C/EBP Beta Tg
AD-C/EBP Beta Tg

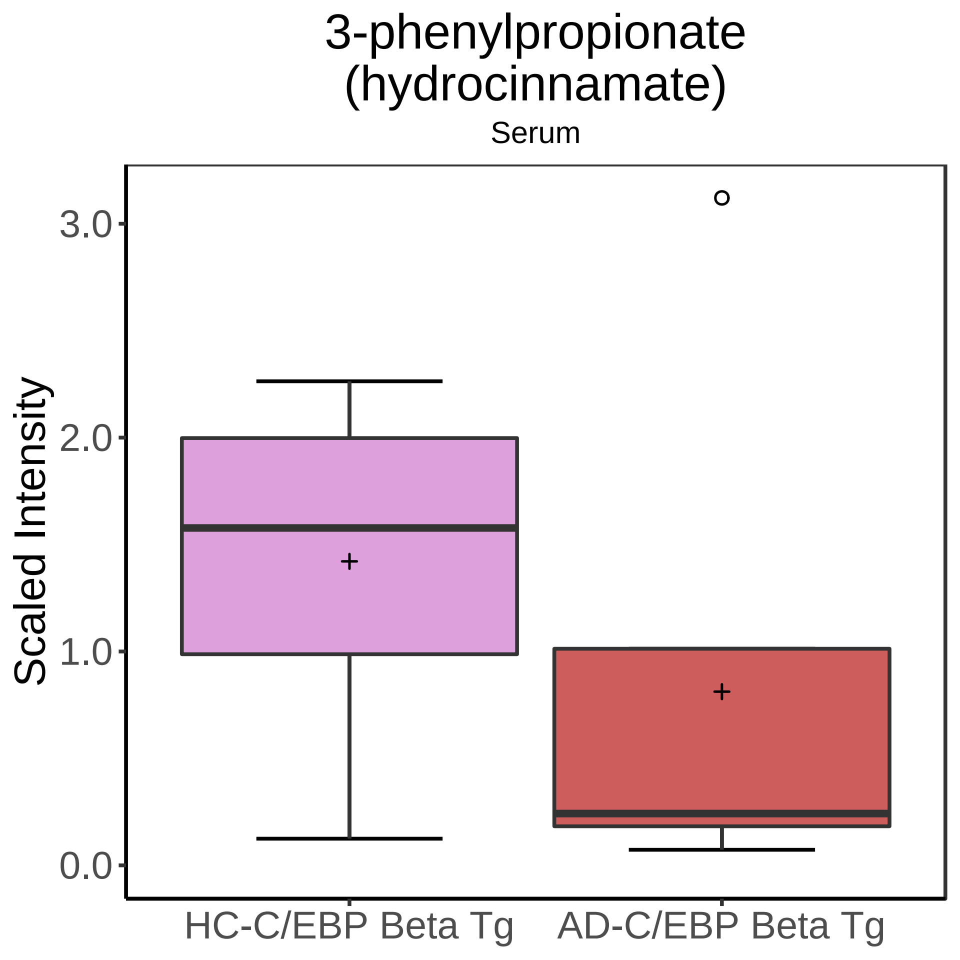
3-phenylpropionate
(hydrocinnamate)
Serum
Scaled Intensity
3.0
2.0
1.0
0.0
HC-C/EBP Beta Tg
AD-C/EBP Beta Tg

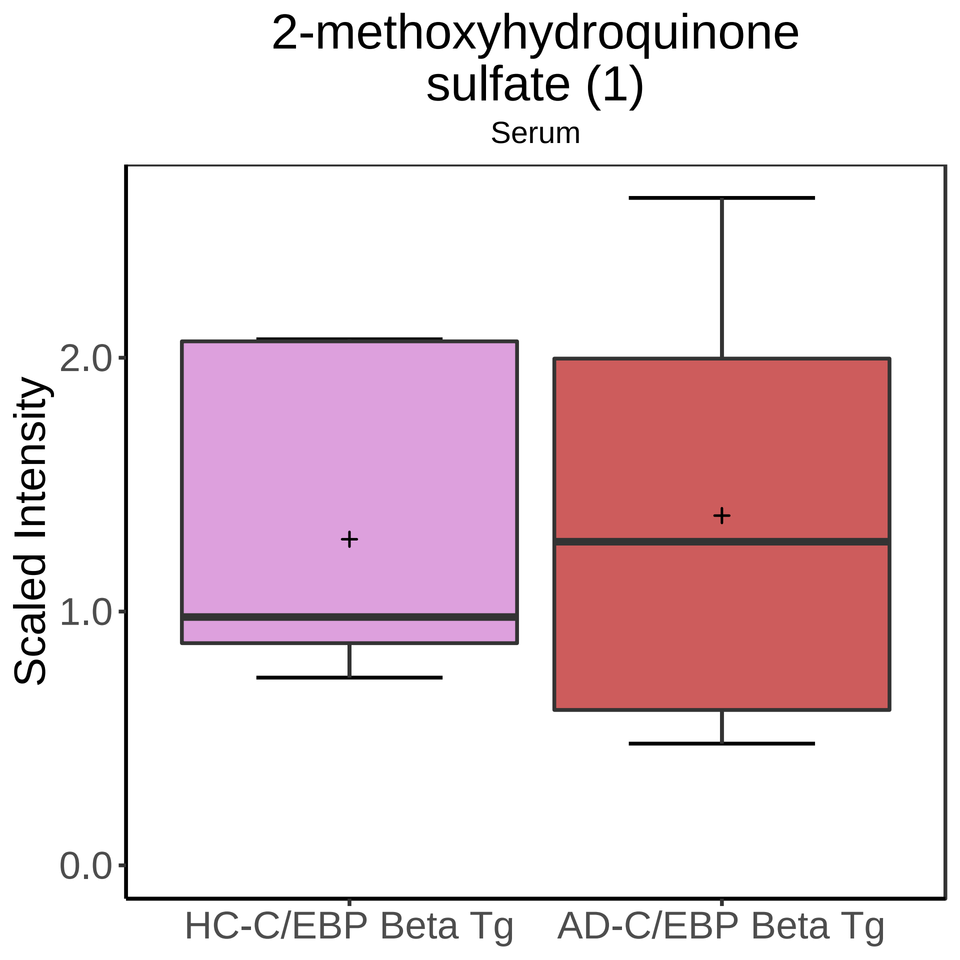
2-methoxyhydroquinone
sulfate (1)
Serum
Scaled Intensity
2.0
1.0
0.0
HC-C/EBP Beta Tg
AD-C/EBP Beta Tg

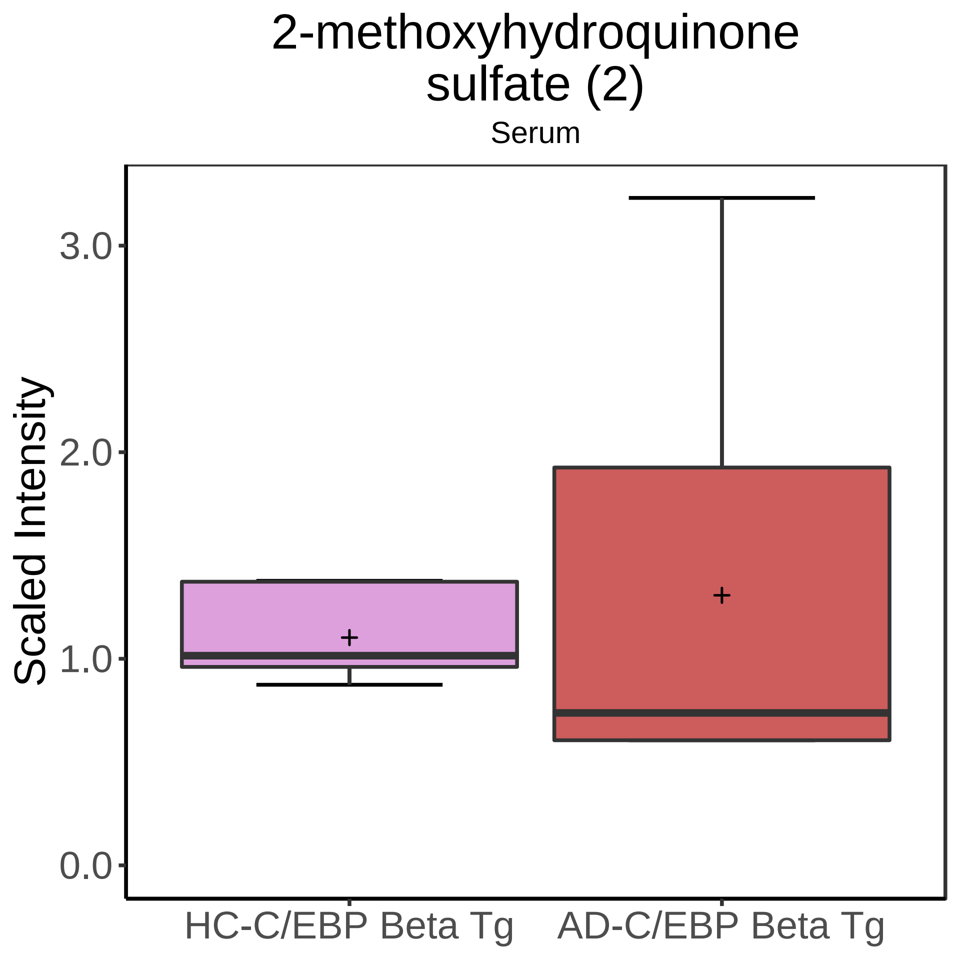
2-methoxyhydroquinone
sulfate (2)
Serum
Scaled Intensity
3.0
2.0
1.0
0.0
HC-C/EBP Beta Tg
AD-C/EBP Beta Tg

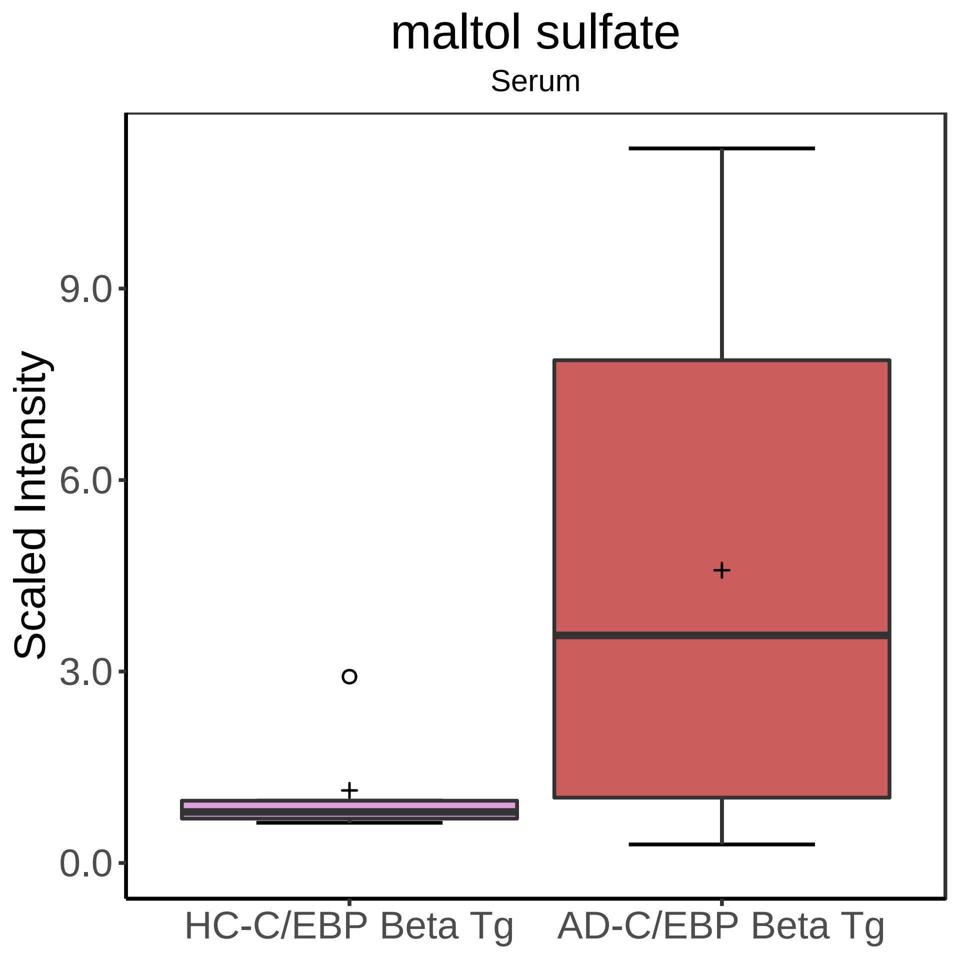

maltol sulfate
Serum
Scaled Intensity
9.0
6.0
3.0
0.0
HC-C/EBP Beta Tg
AD-C/EBP Beta Tg

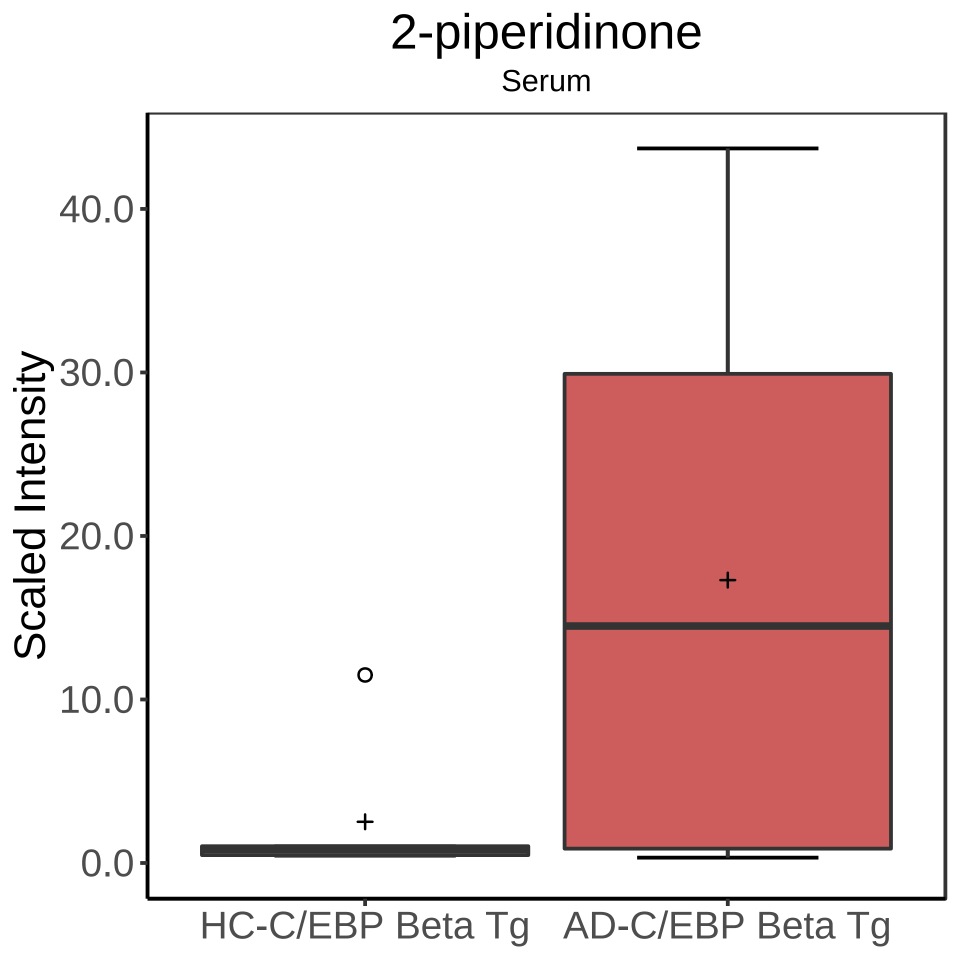

2-piperidinone
Serum
Scaled Intensity
40.0
30.0
20.0
10.0
0.0
HC-C/EBP Beta Tg
AD-C/EBP Beta Tg

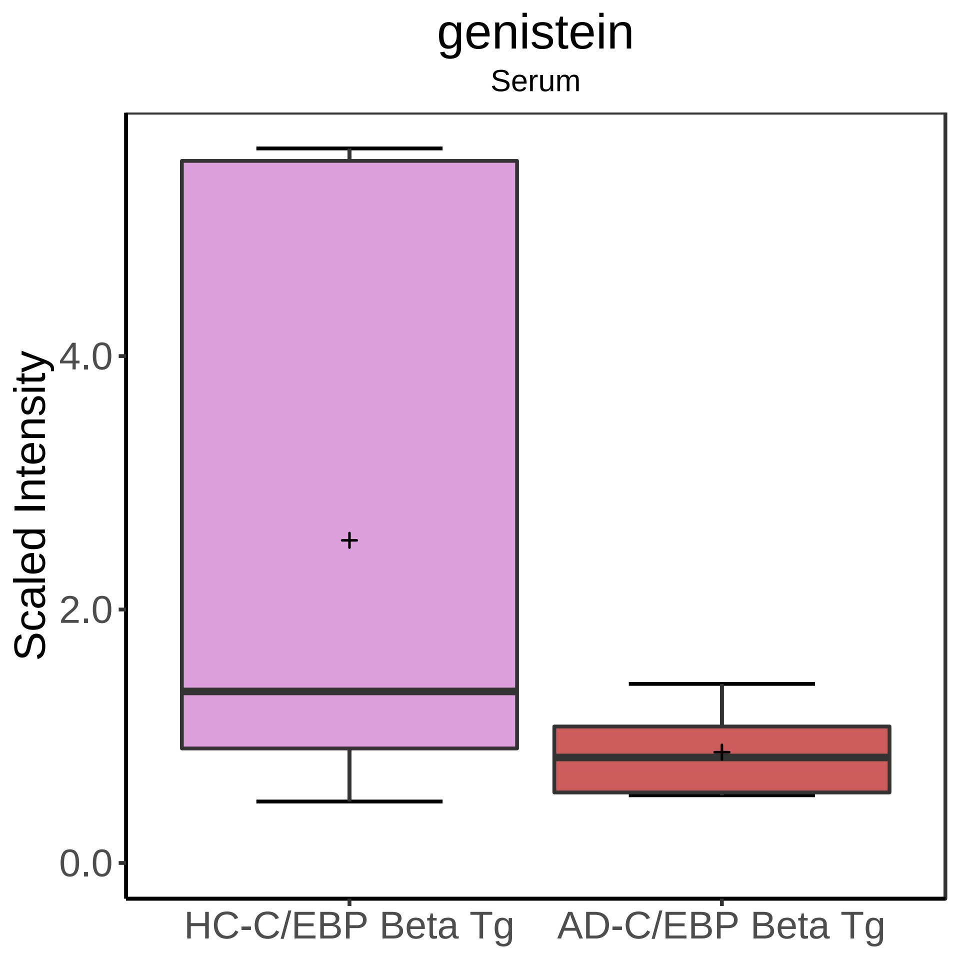
genistein
Serum
Scaled Intensity
4.0
2.0
0.0
HC-C/EBP Beta Tg
AD-C/EBP Beta Tg

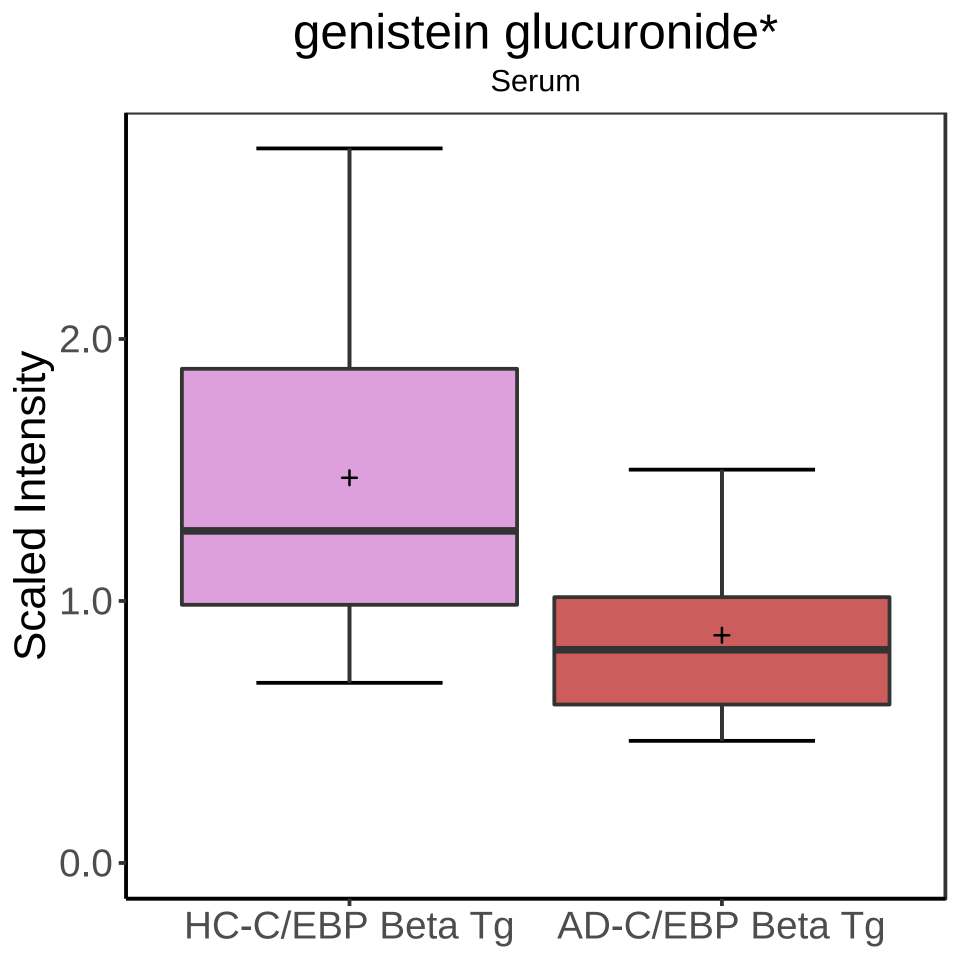
genistein glucuronide*
Serum
Scaled Intensity
2.0
1.0
0.0
HC-C/EBP Beta Tg
AD-C/EBP Beta Tg

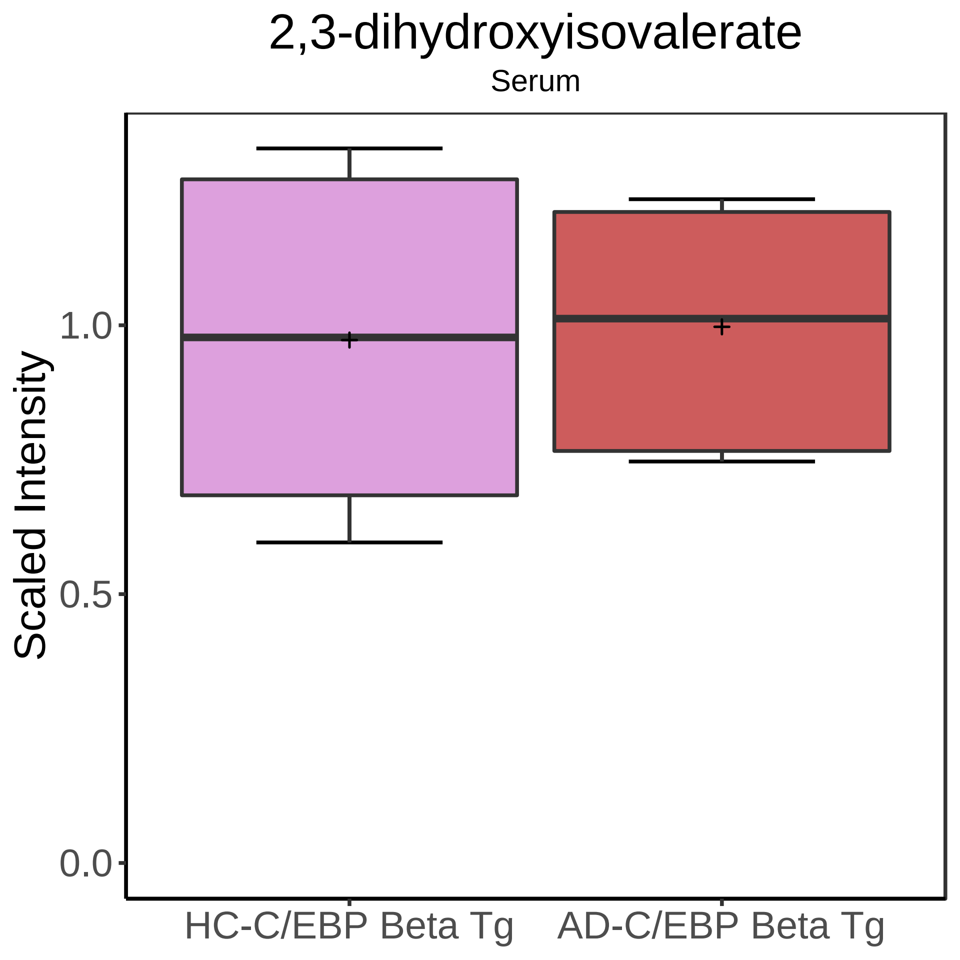

2,3-dihydroxyisovalerate
Serum
Scaled Intensity
1.0
0.5
0.0
HC-C/EBP Beta Tg
AD-C/EBP Beta Tg

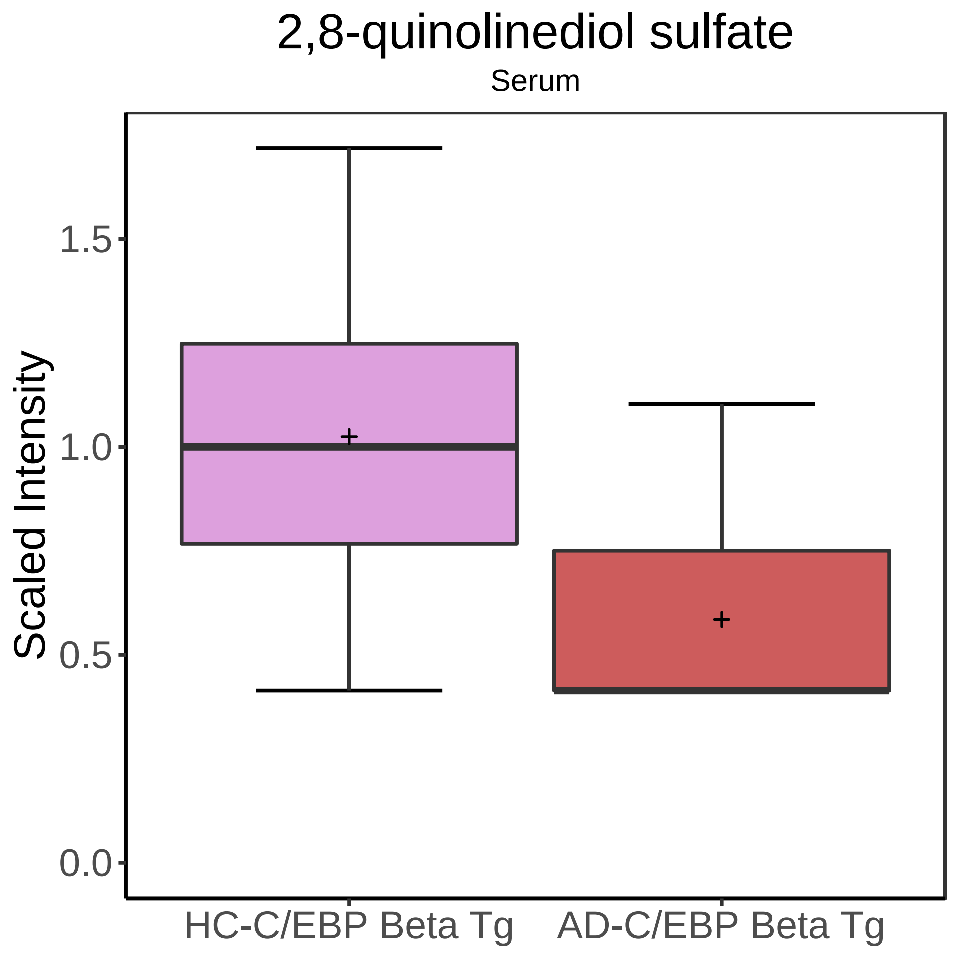
2,8-quinolinediol sulfate
Serum
Scaled Intensity
1.5
1.0
0.5
0.0
HC-C/EBP Beta Tg
AD-C/EBP Beta Tg

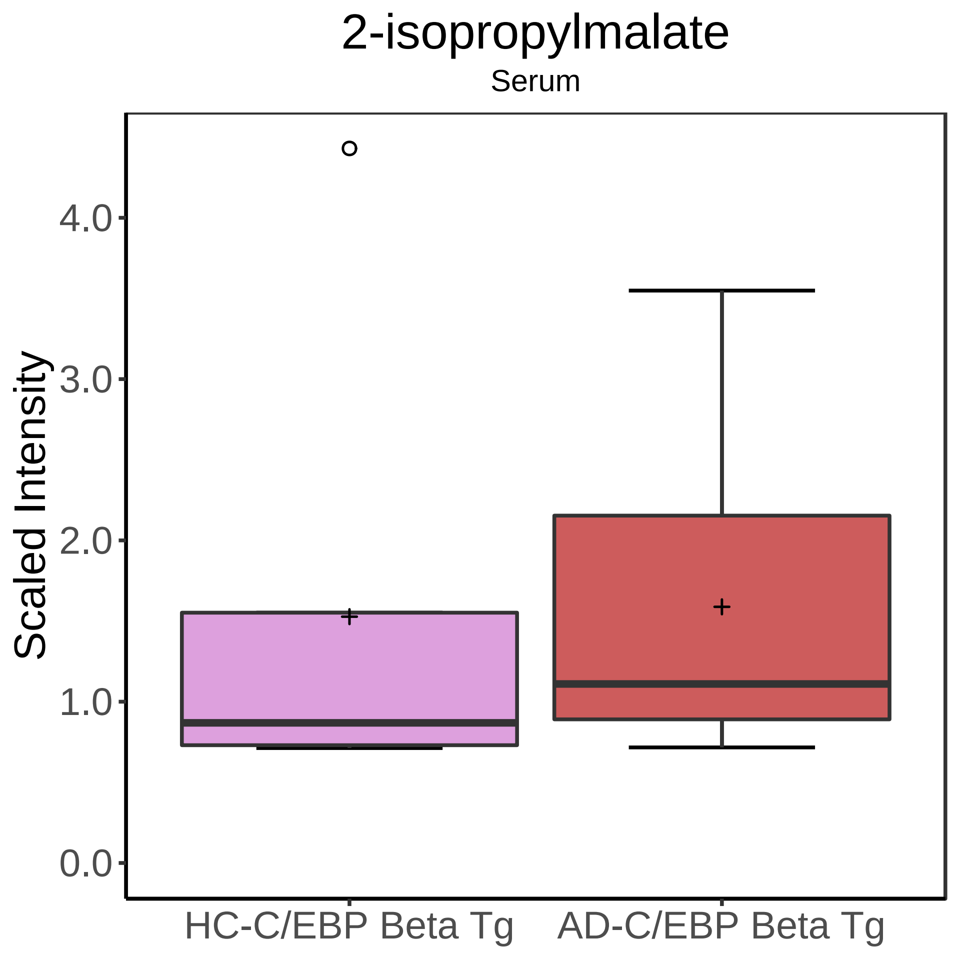
2-isopropylmalate
Serum
Scaled Intensity
4.0
3.0
2.0
1.0
0.0
HC-C/EBP Beta Tg
AD-C/EBP Beta Tg

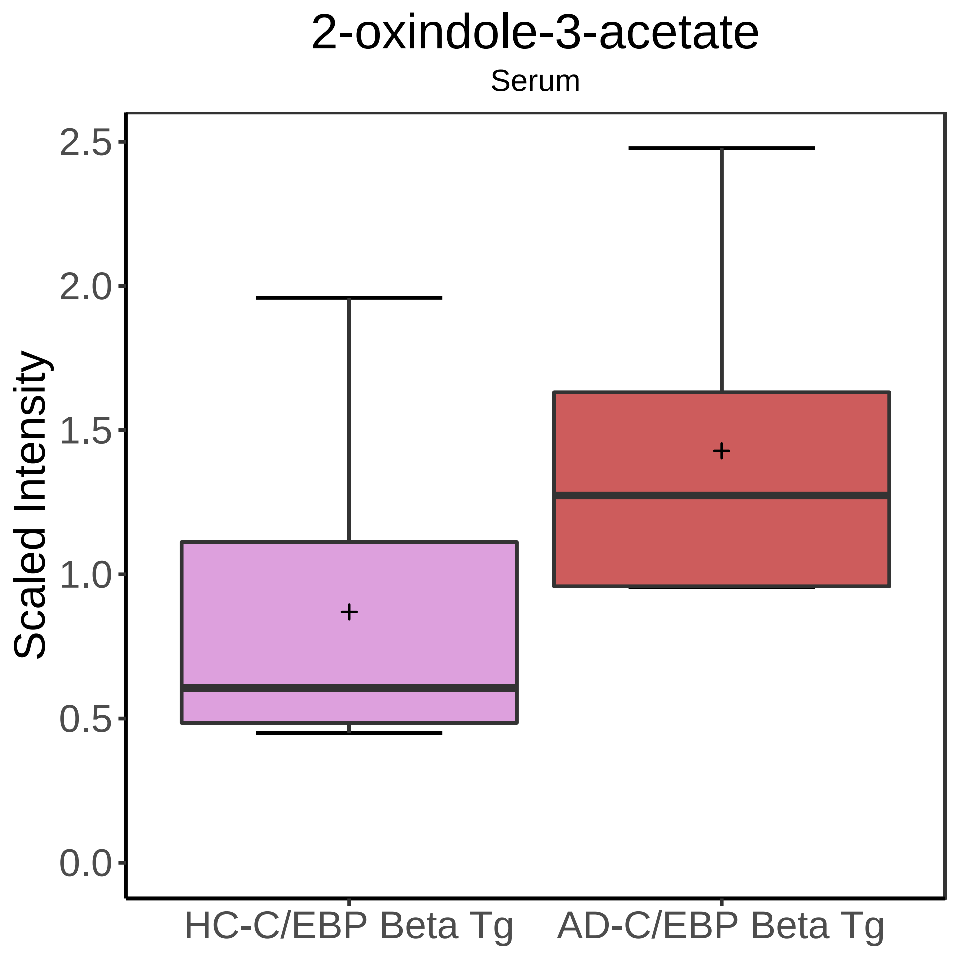
2-oxindole-3-acetate
Serum
Scaled Intensity
2.5
2.0
1.5
1.0
0.5
0.0
HC-C/EBP Beta Tg
AD-C/EBP Beta Tg

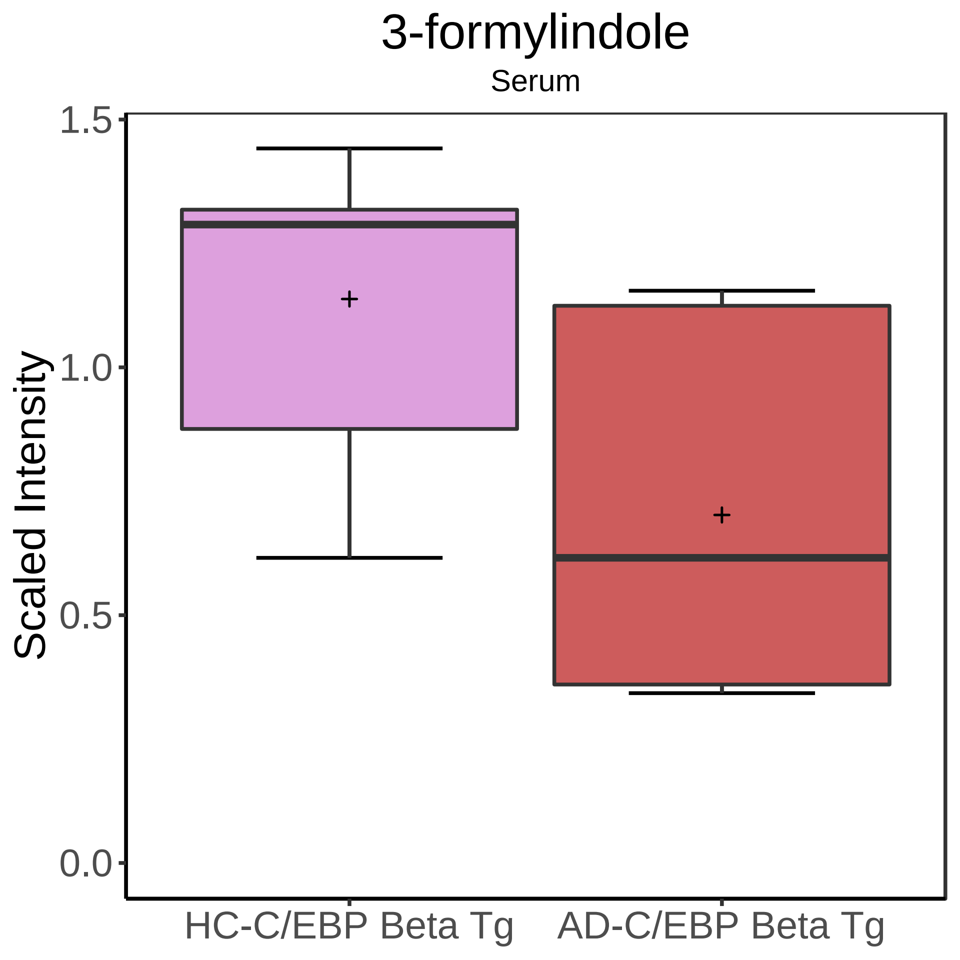

3-formylindole
Serum
Scaled Intensity
1.5
1.0
0.5
0.0
HC-C/EBP Beta Tg
AD-C/EBP Beta Tg

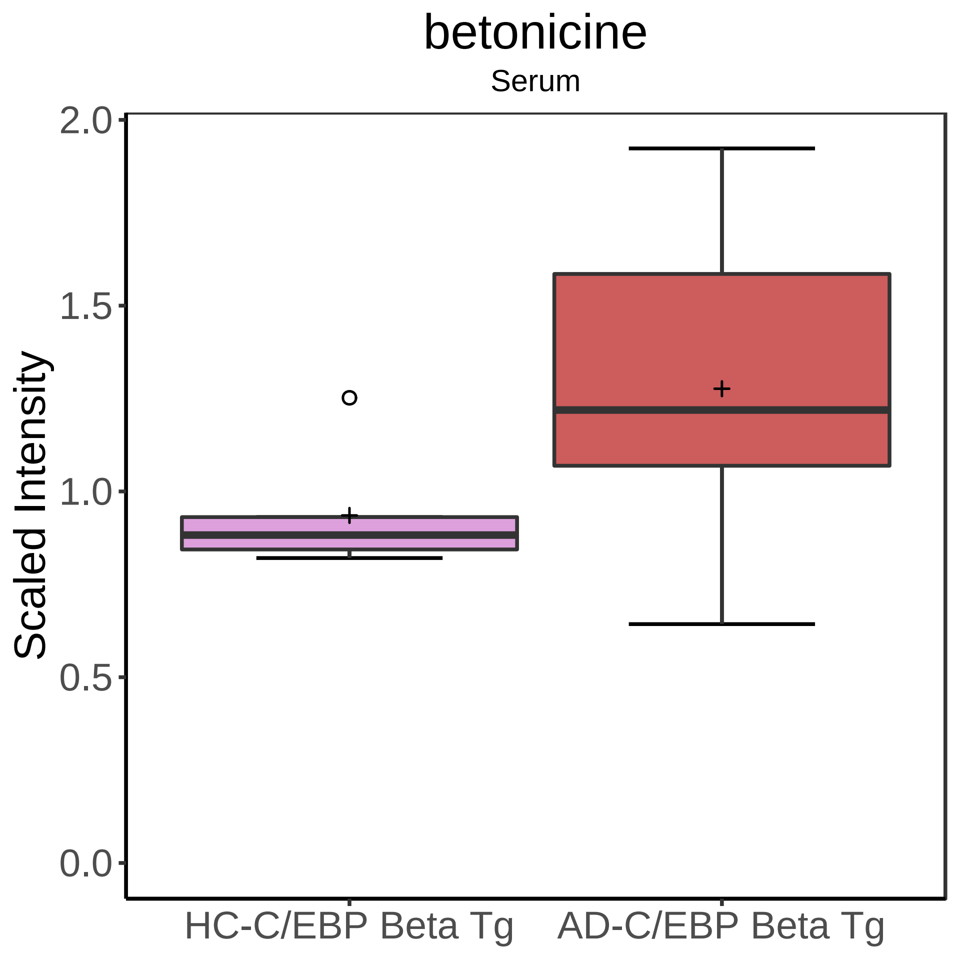
betonicine
Serum
Scaled Intensity
2.0
1.5
1.0
0.5
0.0
HC-C/EBP Beta Tg
AD-C/EBP Beta Tg

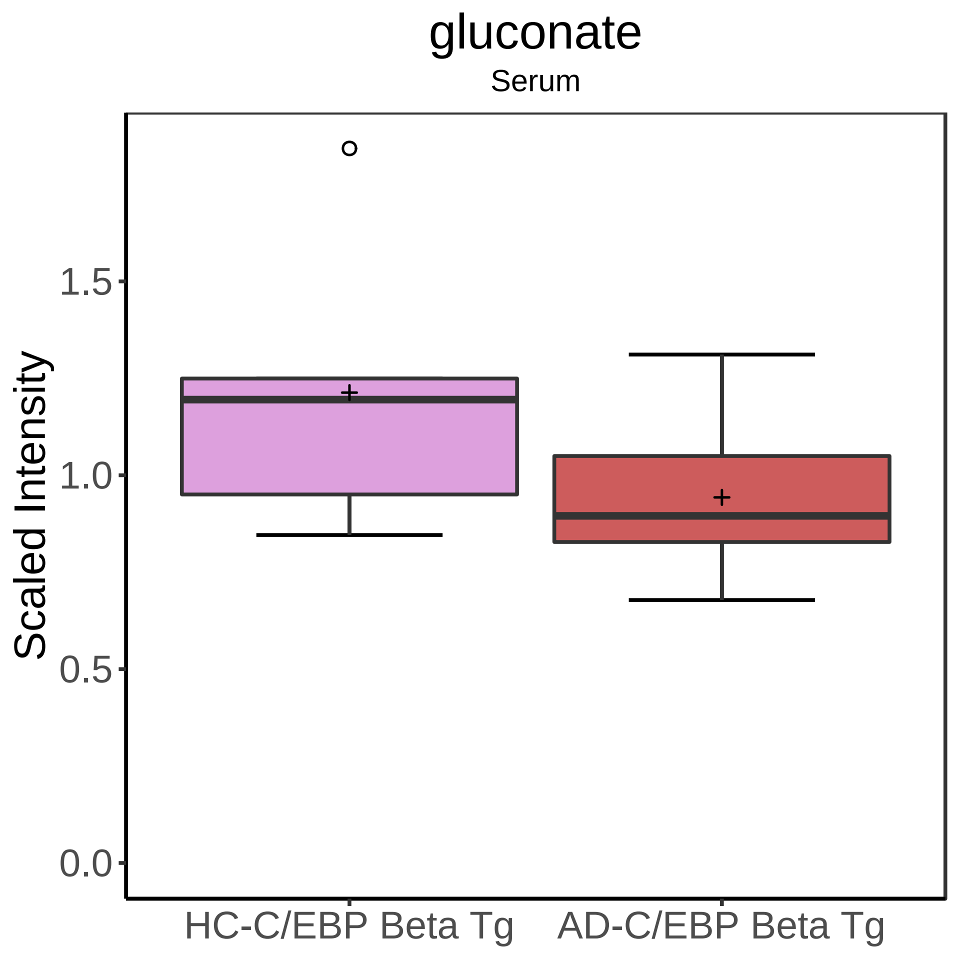
gluconate
Serum
Scaled Intensity
0.0
0.5
1.0
1.5
HC-C/EBP Beta Tg
AD-C/EBP Beta Tg

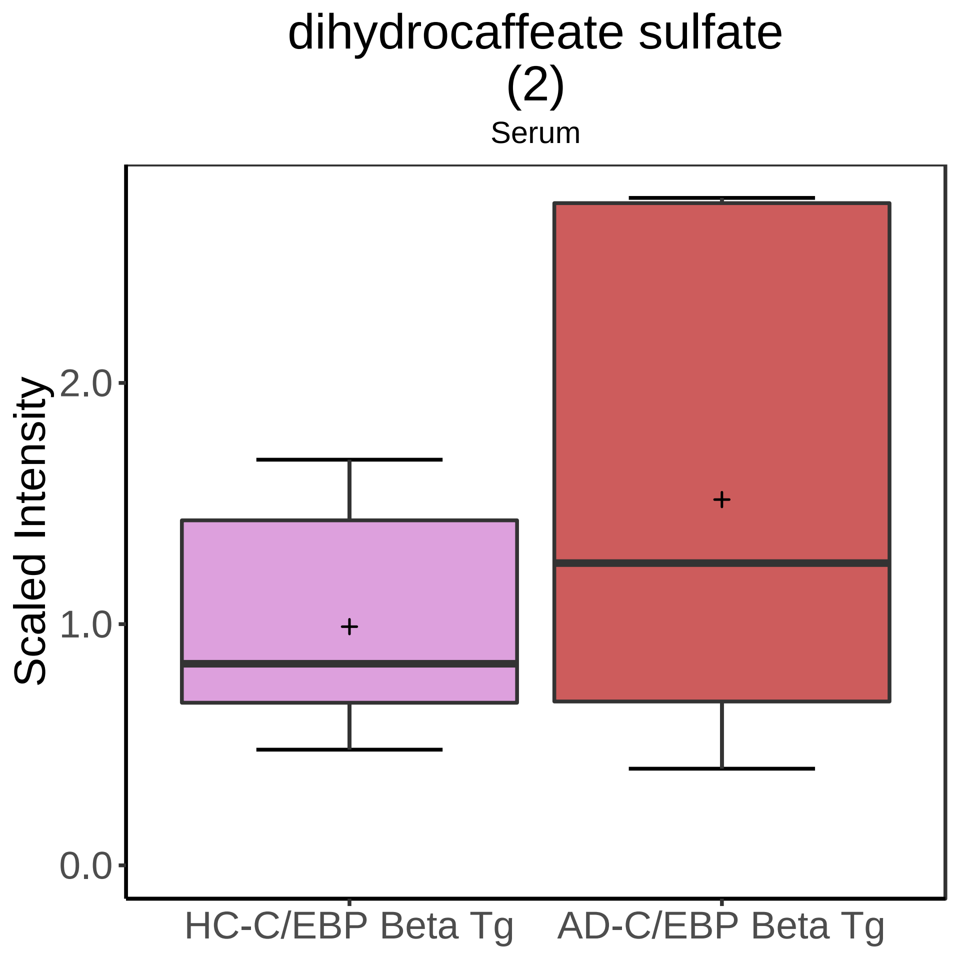
dihydrocaffeate sulfate
(2)
Serum
Scaled Intensity
2.0
1.0
0.0
HC-C/EBP Beta Tg
AD-C/EBP Beta Tg

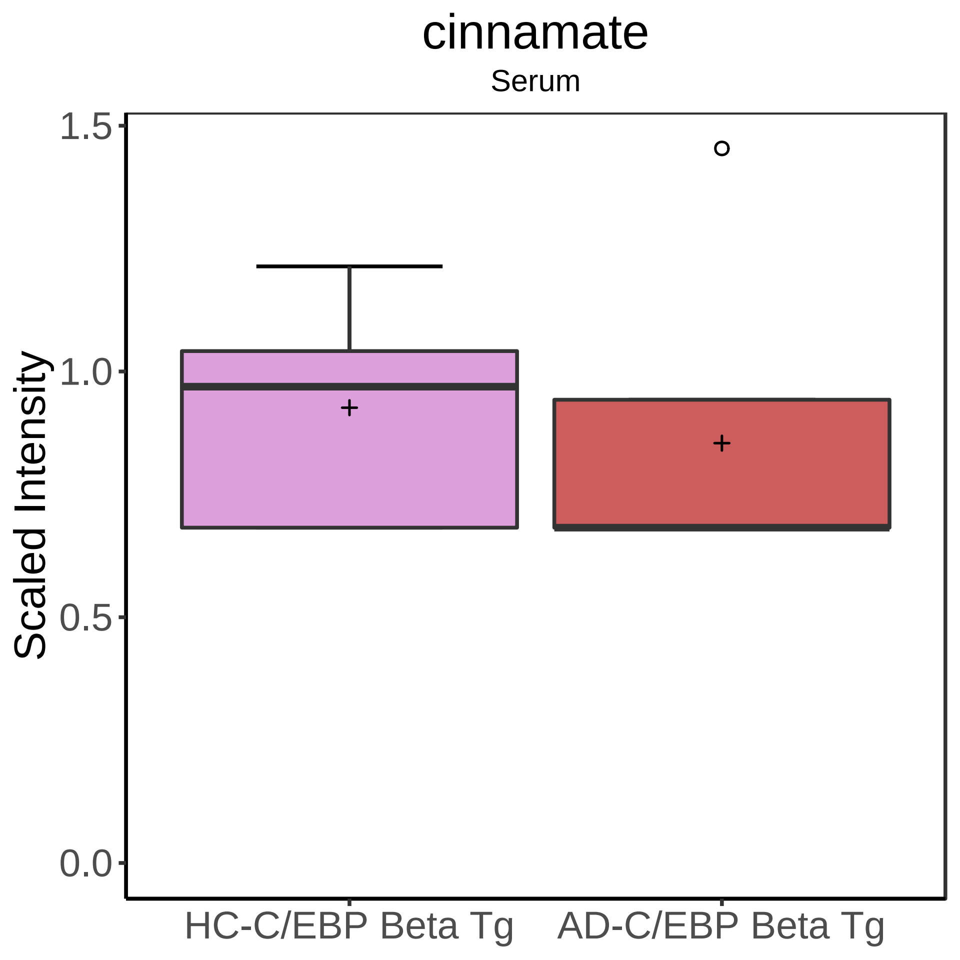
cinnamate
Serum
Scaled Intensity
1.5
1.0
0.5
0.0
HC-C/EBP Beta Tg
AD-C/EBP Beta Tg

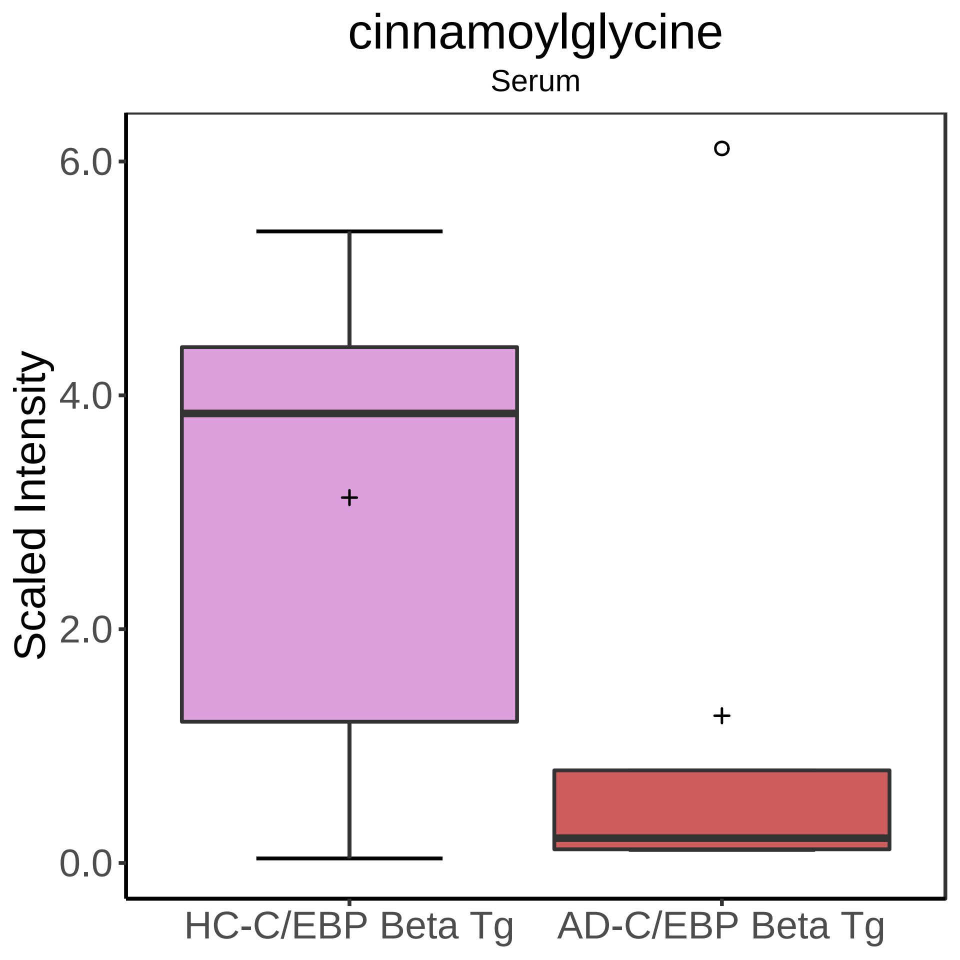
cinnamoylglycine
Serum
Scaled Intensity
6.0
4.0
2.0
0.0
HC-C/EBP Beta Tg
AD-C/EBP Beta Tg

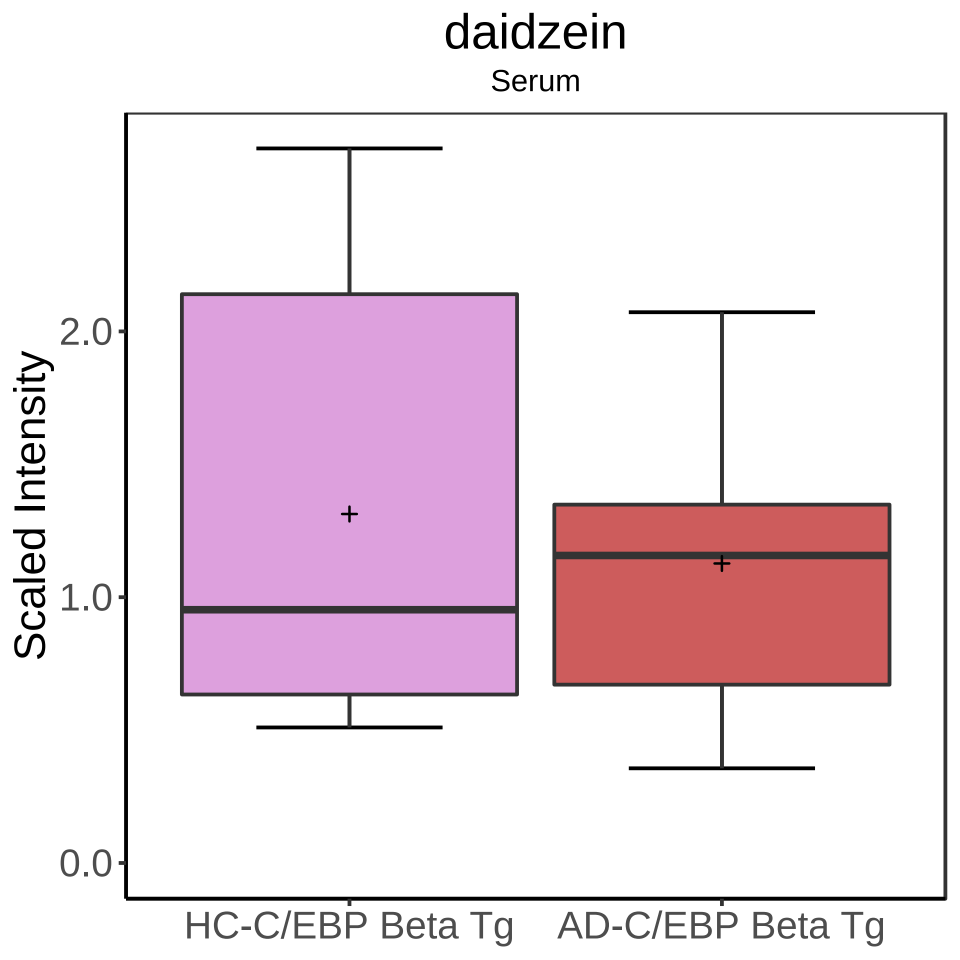

daidzein
Serum
Scaled Intensity
2.0
1.0
0.0
HC-C/EBP Beta Tg
AD-C/EBP Beta Tg

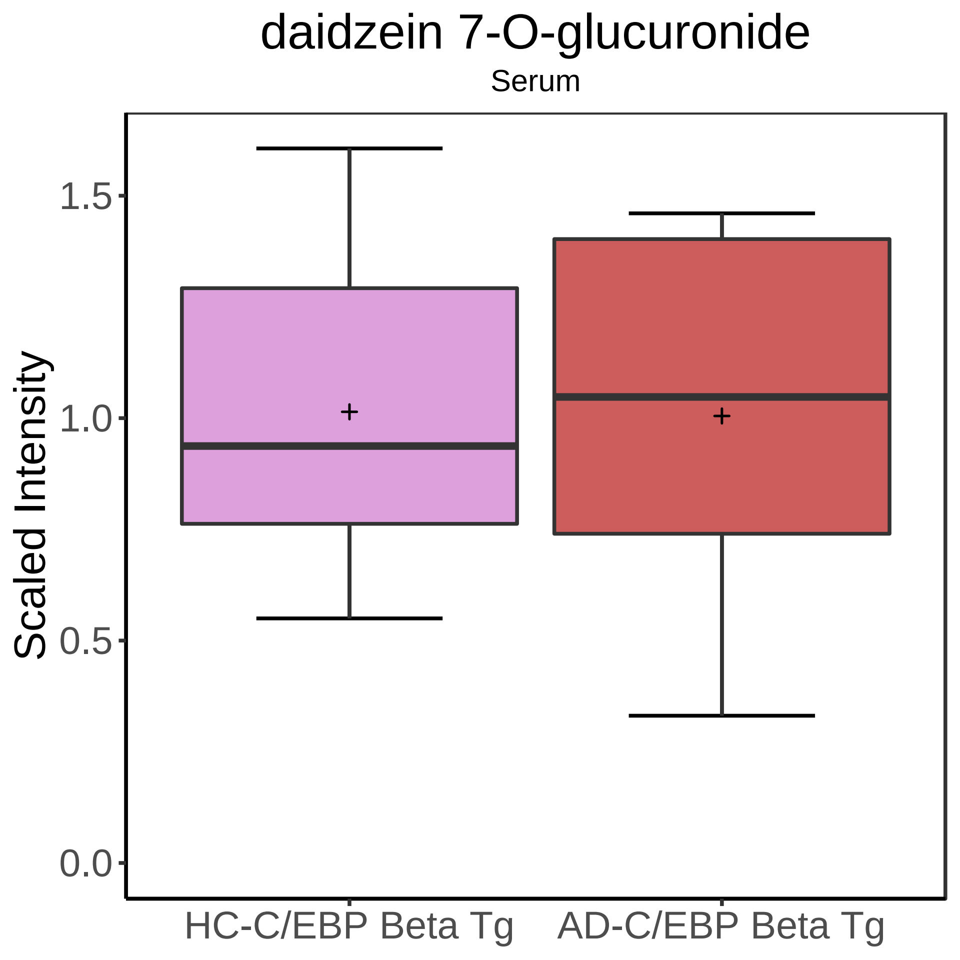
daidzein 7-O-glucuronide
Serum
Scaled Intensity
1.5
1.0
0.5
0.0
HC-C/EBP Beta Tg
AD-C/EBP Beta Tg

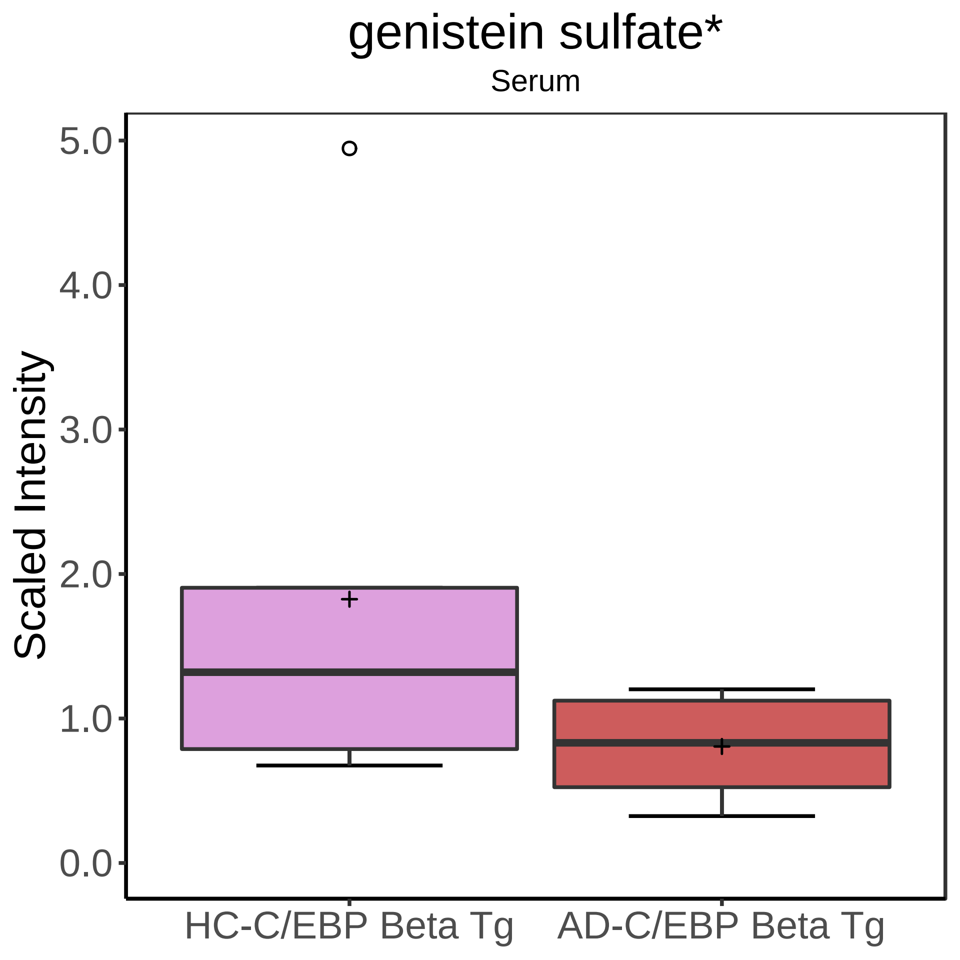

genistein sulfate*
Serum
Scaled Intensity
5.0
4.0
3.0
2.0
1.0
0.0
HC-C/EBP Beta Tg
AD-C/EBP Beta Tg

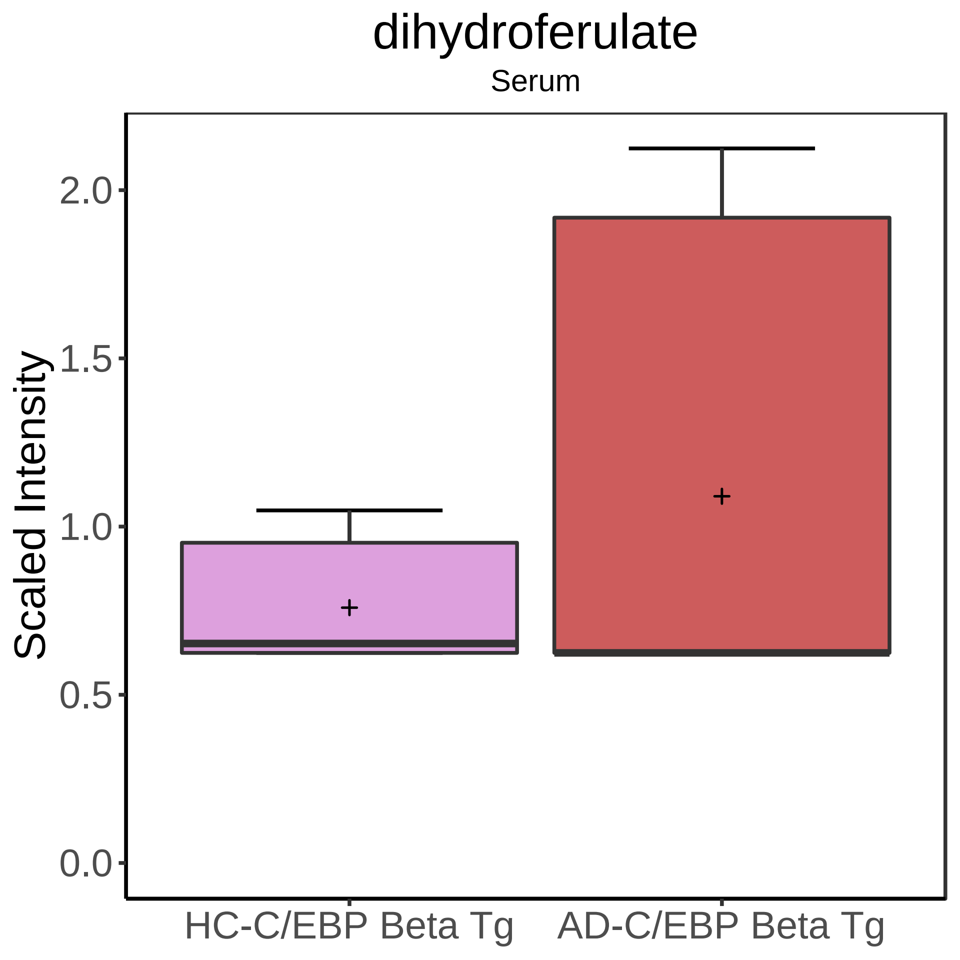
dihydroferulate
Serum
Scaled Intensity
2.0
1.5
1.0
0.5
0.0
HC-C/EBP Beta Tg
AD-C/EBP Beta Tg

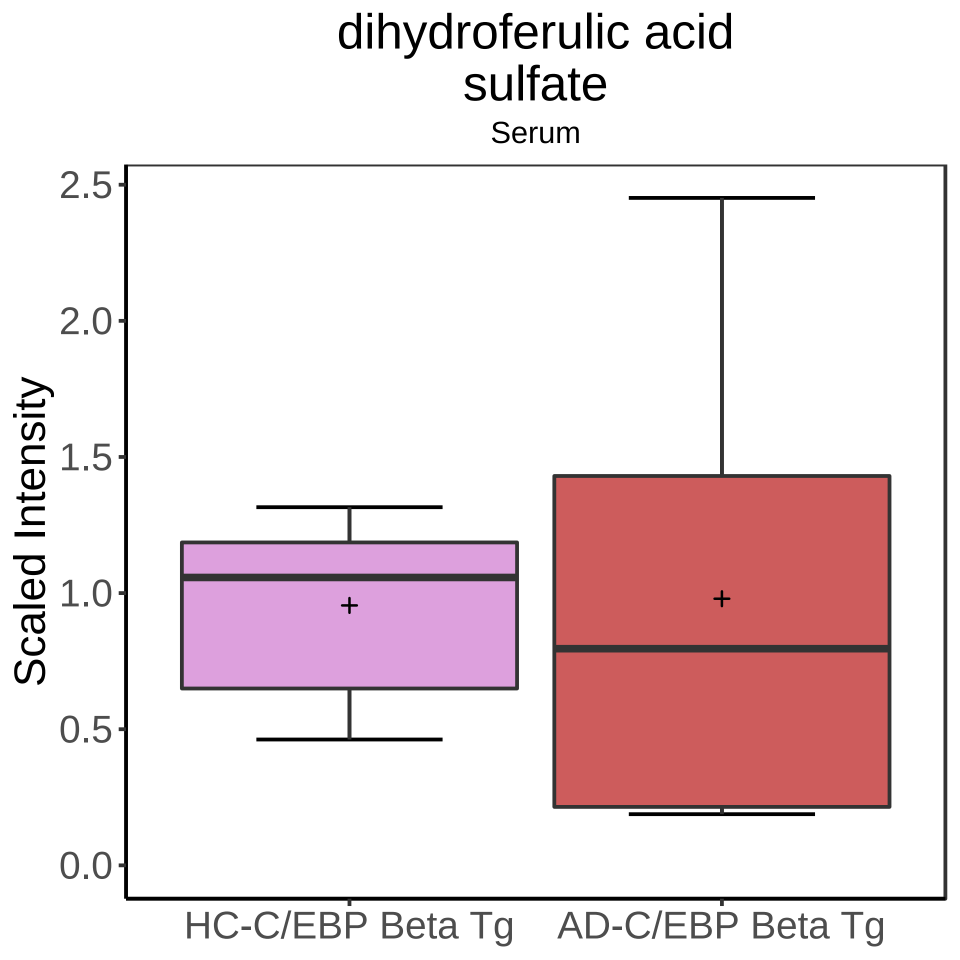
dihydroferulic acid
sulfate
Serum
Scaled Intensity
0.0
0.5
1.0
1.5
2.0
2.5
HC-C/EBP Beta Tg
AD-C/EBP Beta Tg

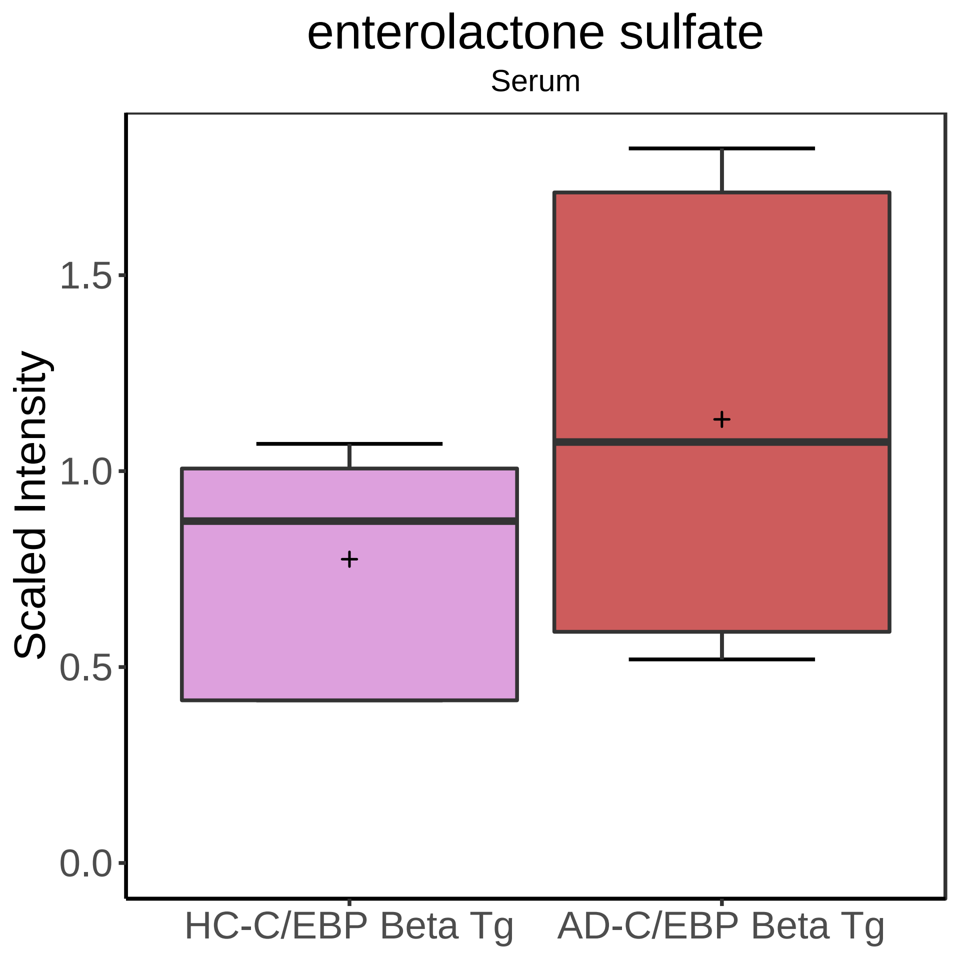
enterolactone sulfate
Serum
Scaled Intensity
1.5
1.0
0.5
0.0
HC-C/EBP Beta Tg
AD-C/EBP Beta Tg

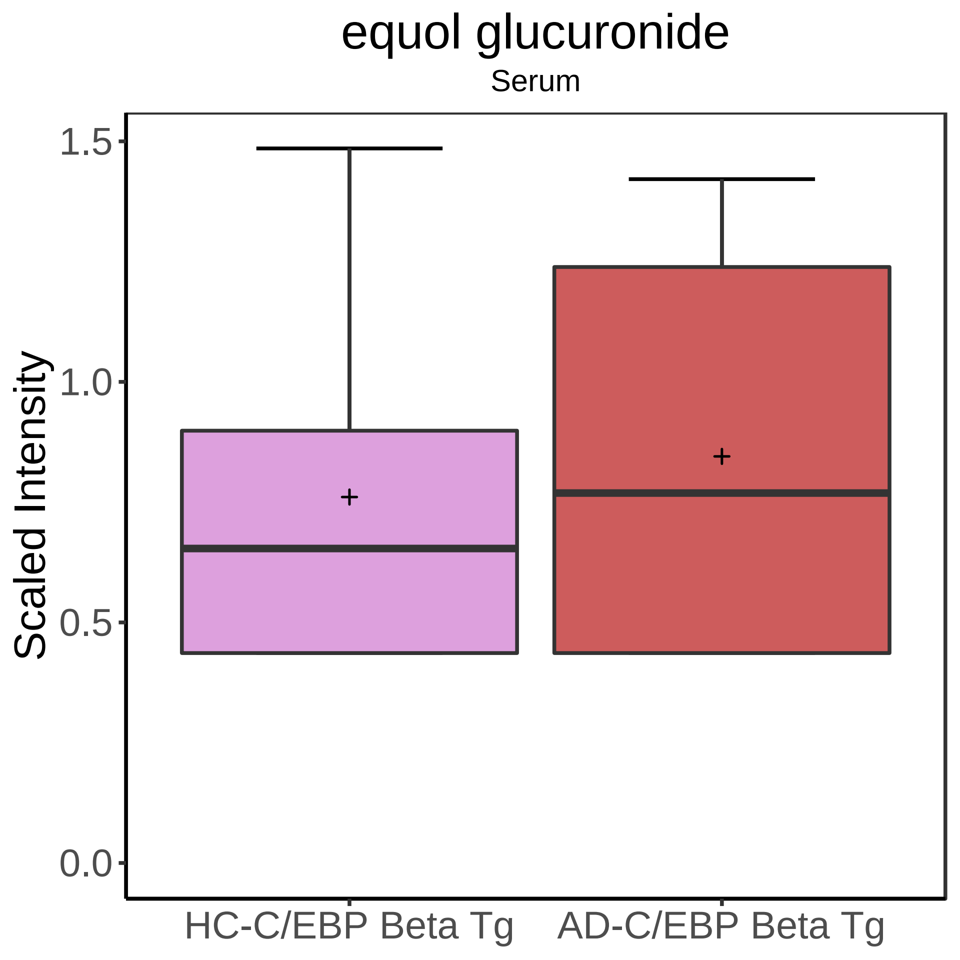
equol glucuronide
Serum
Scaled Intensity
1.5
1.0
0.5
0.0
HC-C/EBP Beta Tg
AD-C/EBP Beta Tg

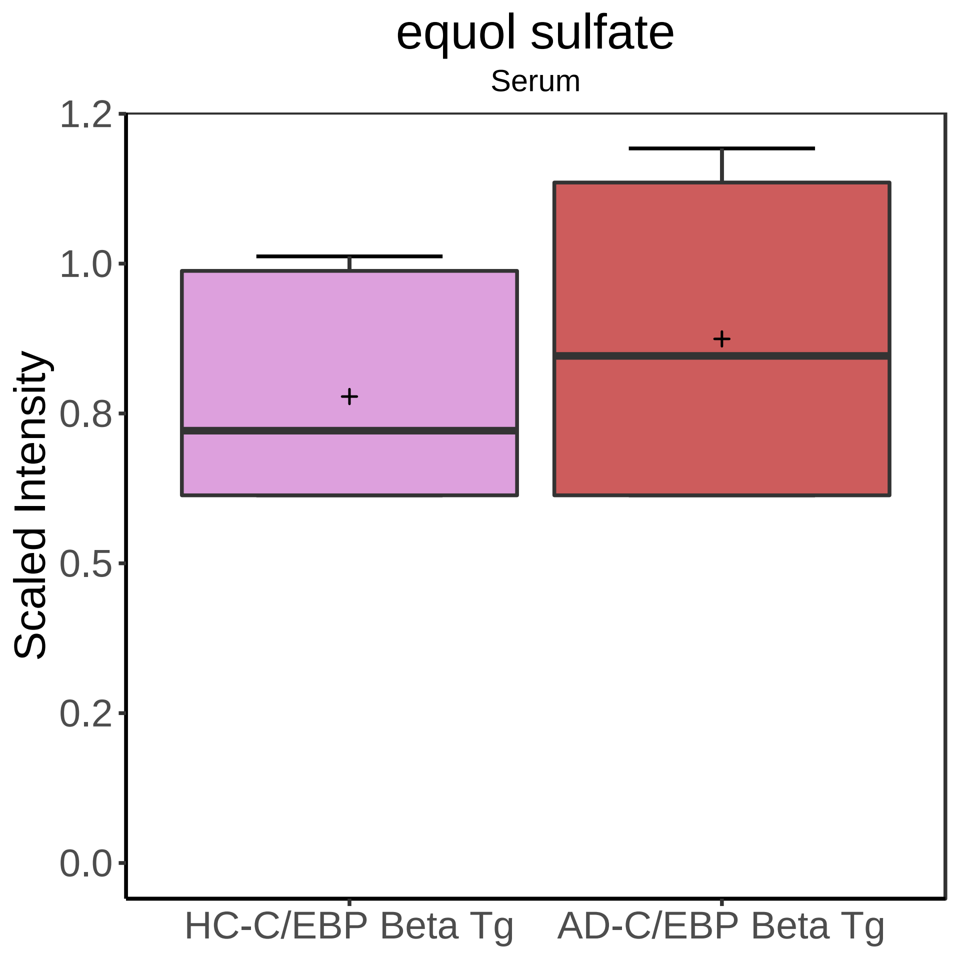
equol sulfate
Serum
Scaled Intensity
1.2
1.0
0.8
0.5
0.2
0.0
HC-C/EBP Beta Tg
AD-C/EBP Beta Tg

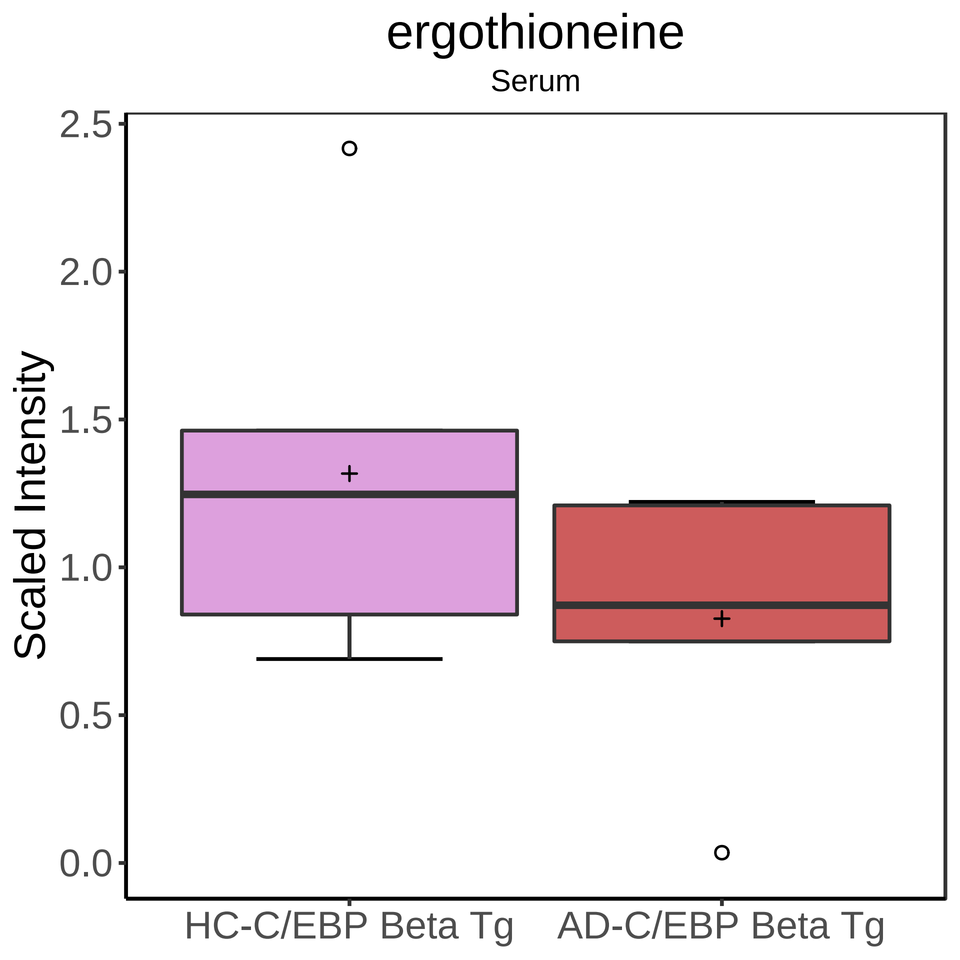
ergothioneine
Serum
Scaled Intensity
2.5
2.0
1.5
1.0
0.5
0.0
HC-C/EBP Beta Tg
AD-C/EBP Beta Tg

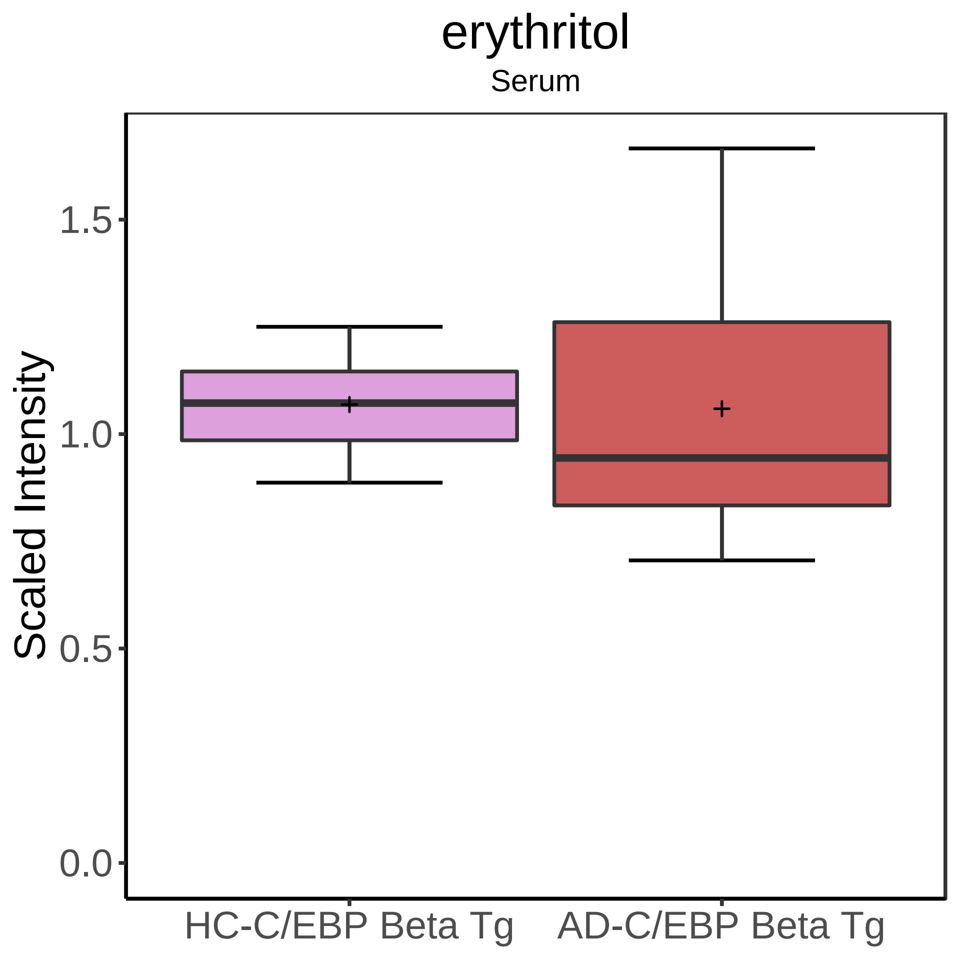
erythritol
Serum
Scaled Intensity
1.5
1.0
0.5
0.0
HC-C/EBP Beta Tg
AD-C/EBP Beta Tg

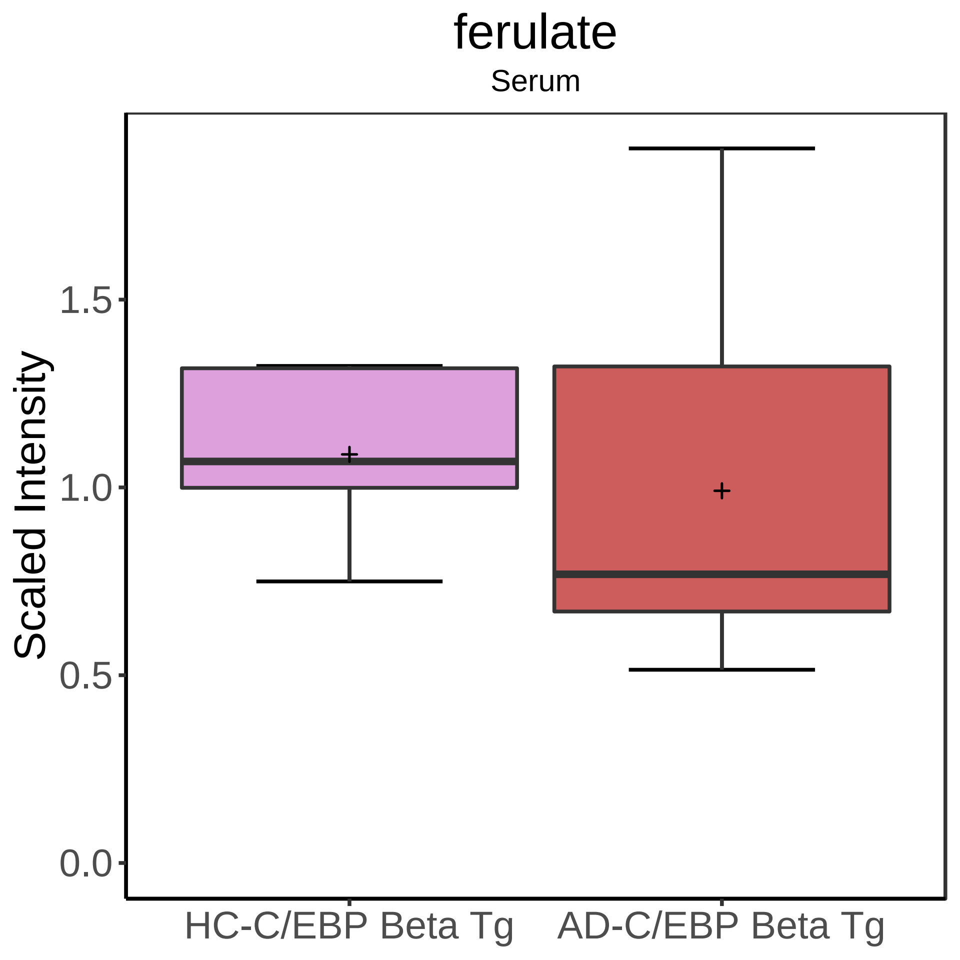
ferulate
Serum
Scaled Intensity
1.5
1.0
0.5
0.0
HC-C/EBP Beta Tg
AD-C/EBP Beta Tg

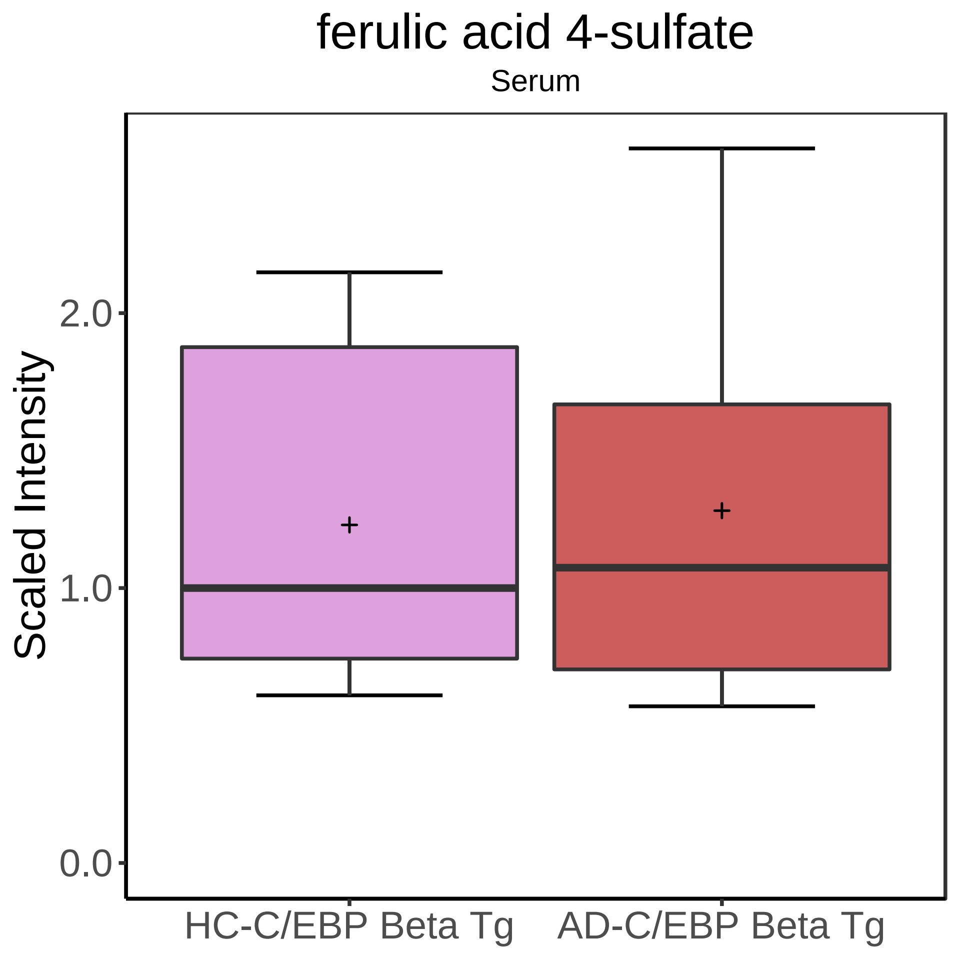

ferulic acid 4-sulfate
Serum
Scaled Intensity
2.0
1.0
0.0
HC-C/EBP Beta Tg
AD-C/EBP Beta Tg

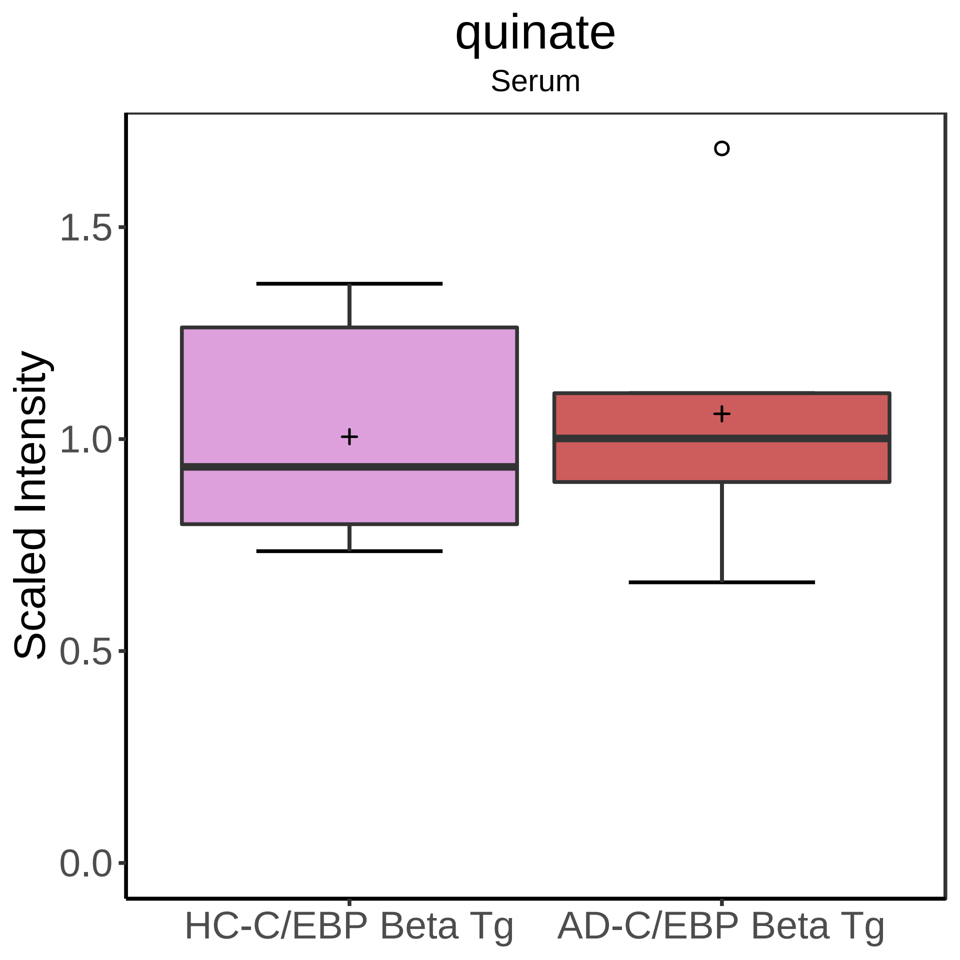
quinate
Serum
Scaled Intensity
1.5
1.0
0.5
0.0
HC-C/EBP Beta Tg
AD-C/EBP Beta Tg

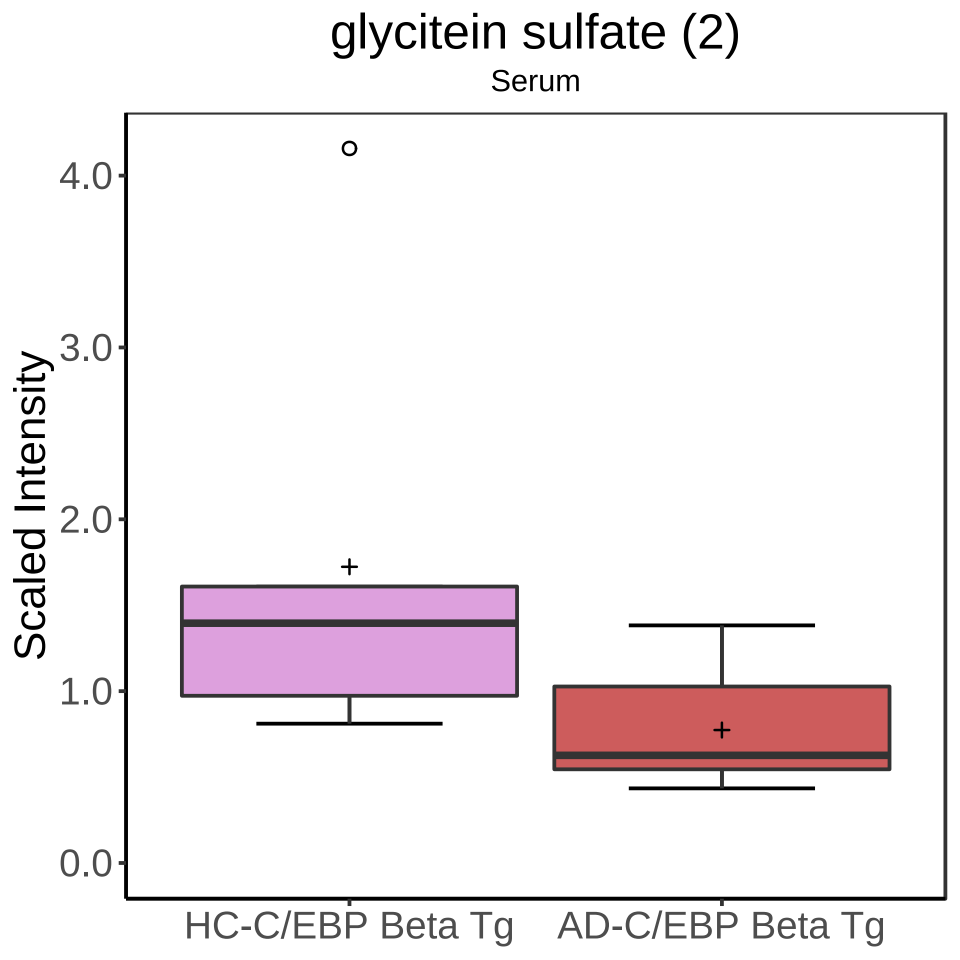
glycitein sulfate (2)
Serum
Scaled Intensity
4.0
3.0
2.0
1.0
0.0
HC-C/EBP Beta Tg
AD-C/EBP Beta Tg

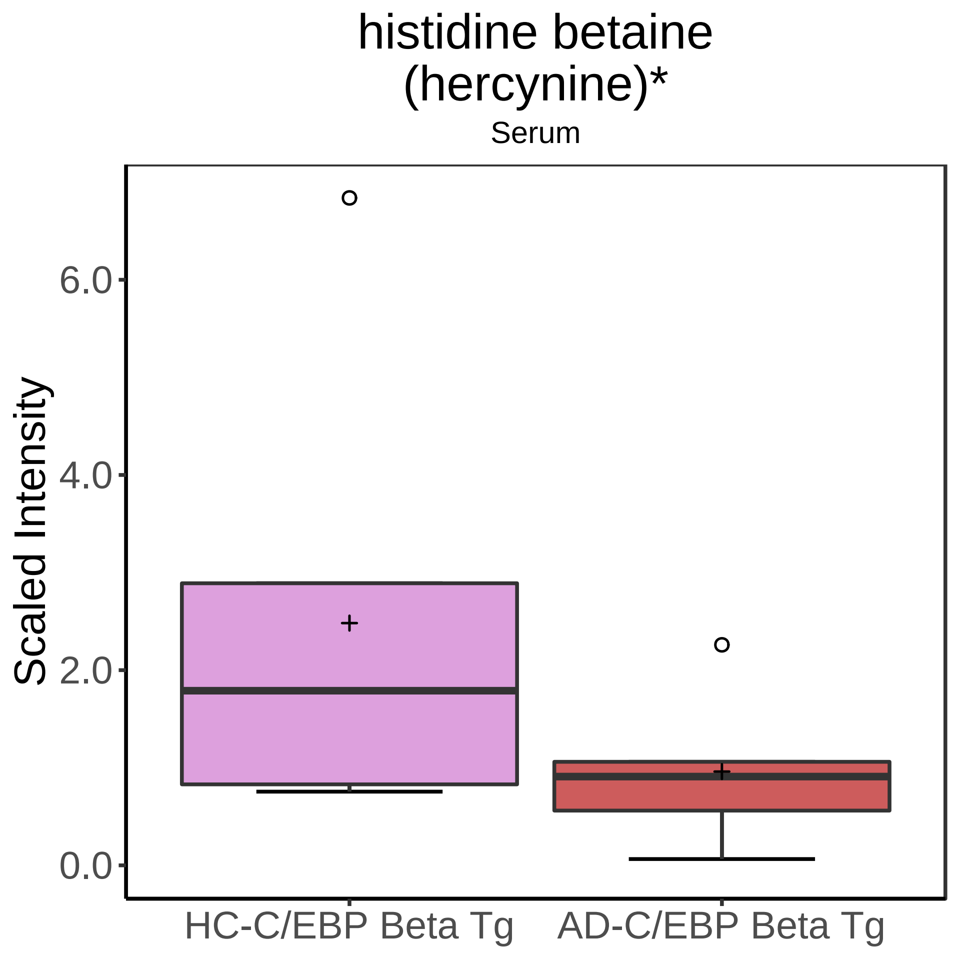

histidine betaine
(hercynine)*
Serum
Scaled Intensity
6.0
4.0
2.0
0.0
HC-C/EBP Beta Tg
AD-C/EBP Beta Tg

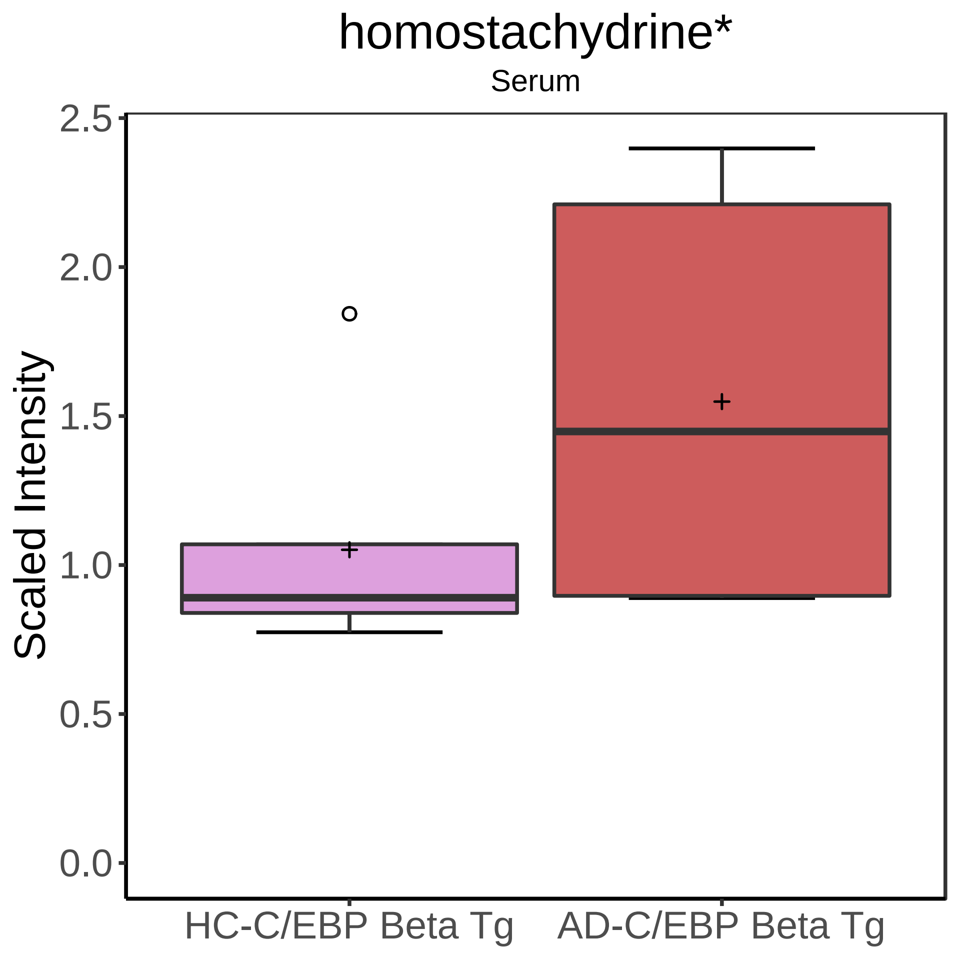
homostachydrine*
Serum
Scaled Intensity
2.5
2.0
1.5
1.0
0.5
0.0
HC-C/EBP Beta Tg
AD-C/EBP Beta Tg

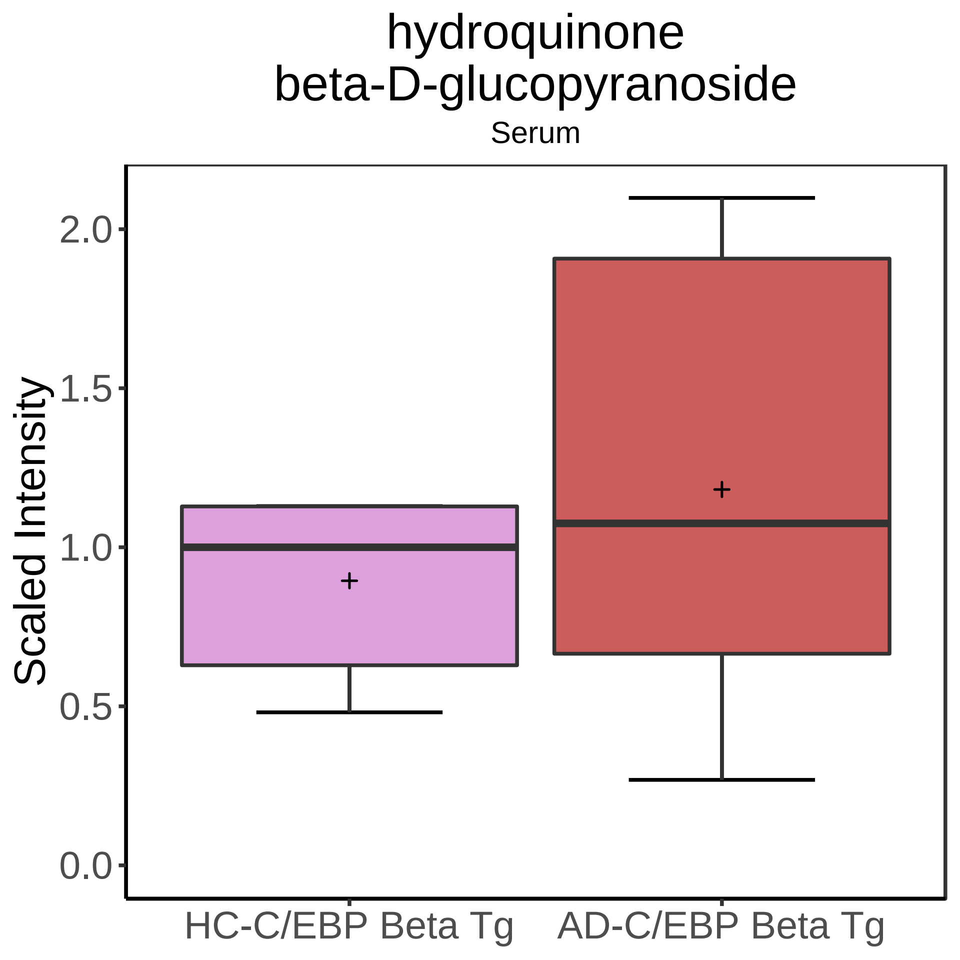
hydroquinone
beta-D-glucopyranoside
Serum
Scaled Intensity
2.0
1.5
1.0
0.5
0.0
HC-C/EBP Beta Tg
AD-C/EBP Beta Tg

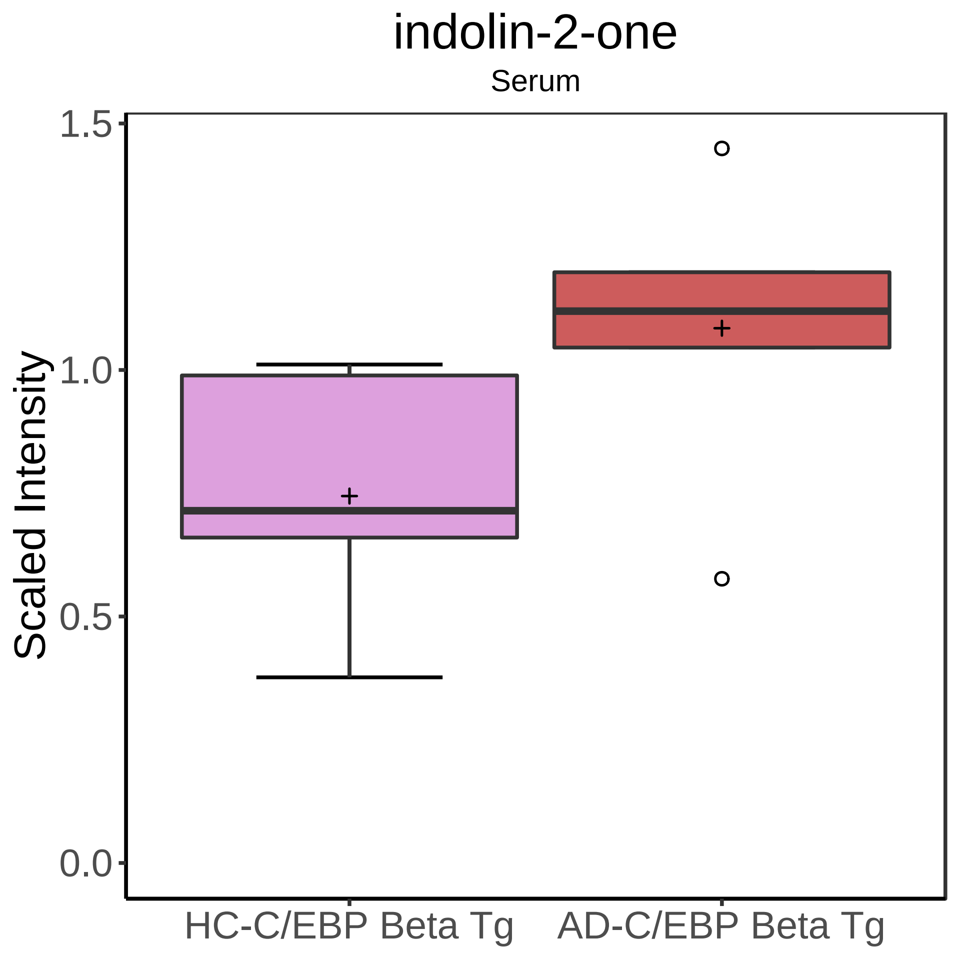
indolin-2-one
Serum
Scaled Intensity
1.5
1.0
0.5
0.0
HC-C/EBP Beta Tg
AD-C/EBP Beta Tg

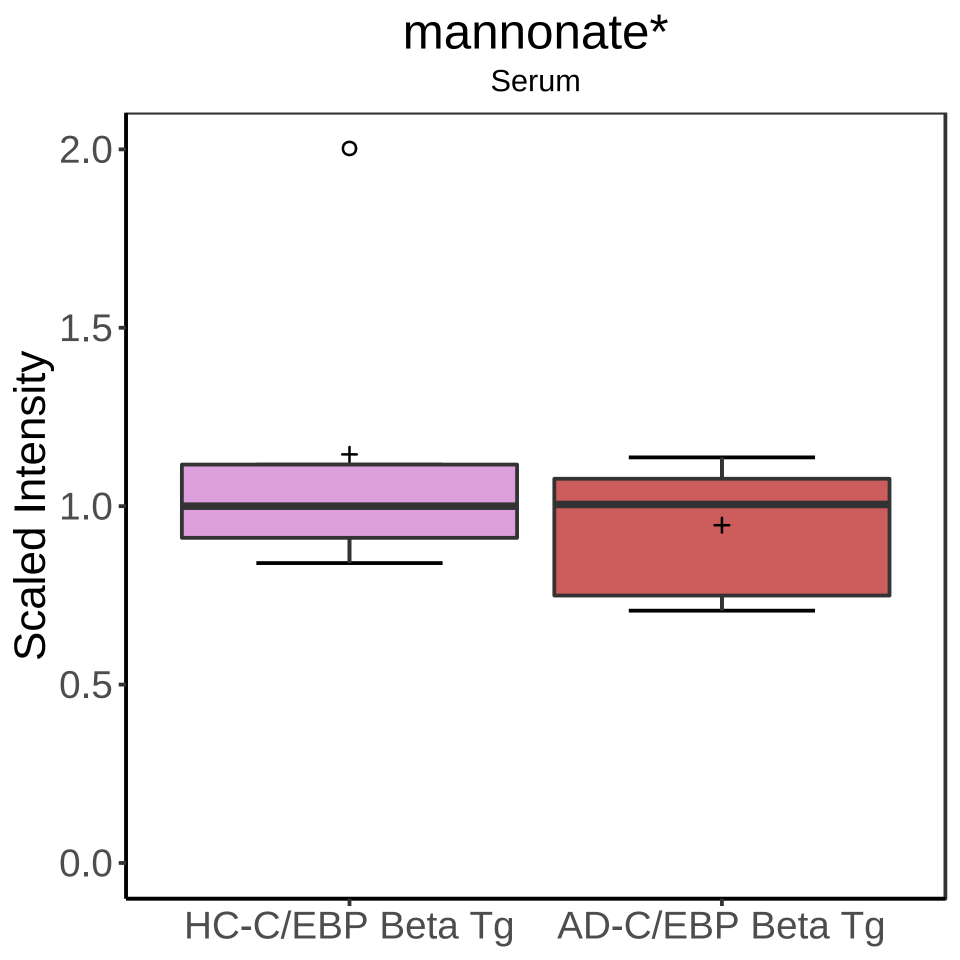
mannonate*
Serum
Scaled Intensity
2.0
1.5
1.0
0.5
0.0
HC-C/EBP Beta Tg
AD-C/EBP Beta Tg

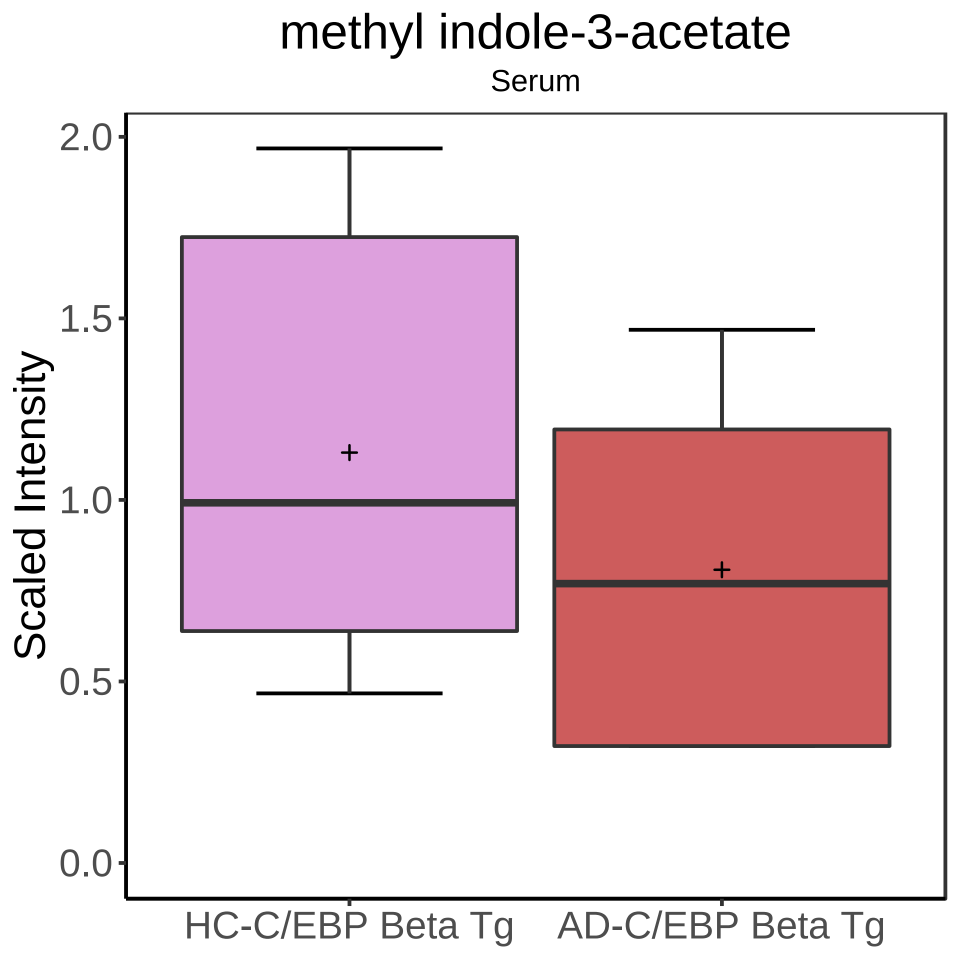

methyl indole-3-acetate
Serum
Scaled Intensity
2.0
1.5
1.0
0.5
0.0
HC-C/EBP Beta Tg
AD-C/EBP Beta Tg

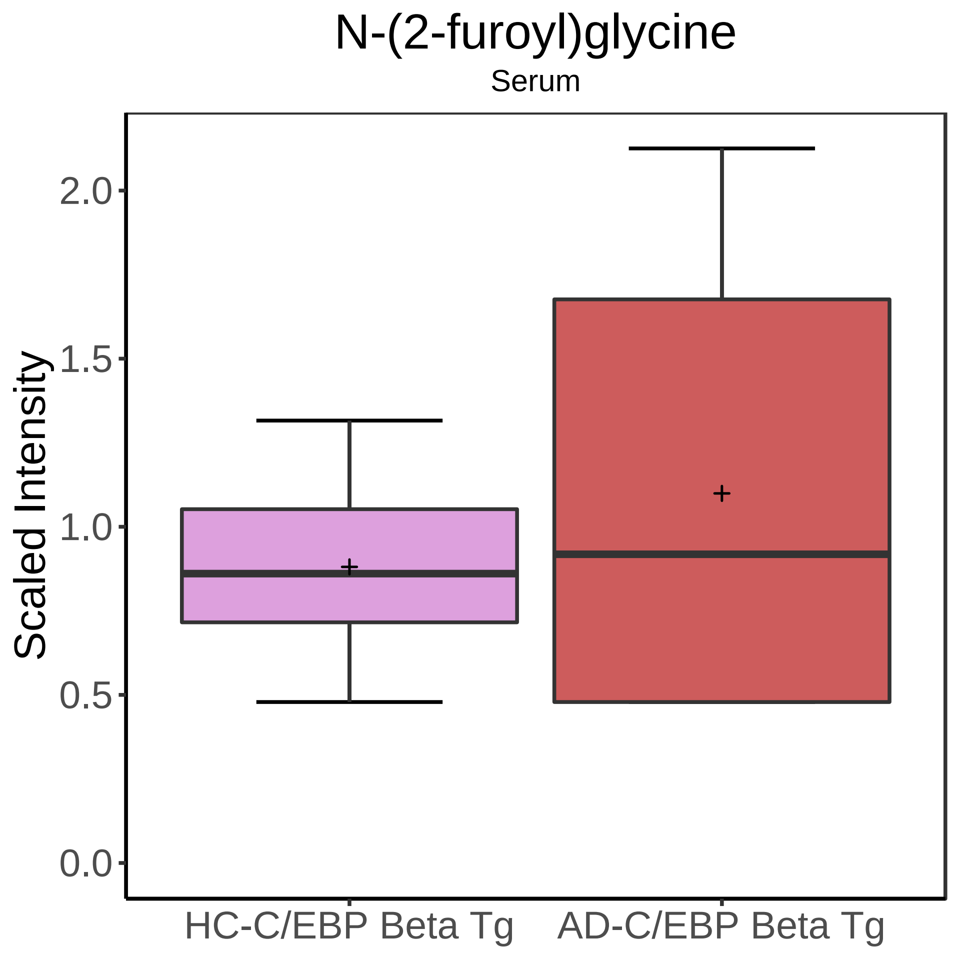
N-(2-furoyl)glycine
Serum
Scaled Intensity
2.0
1.5
1.0
0.5
0.0
HC-C/EBP Beta Tg
AD-C/EBP Beta Tg

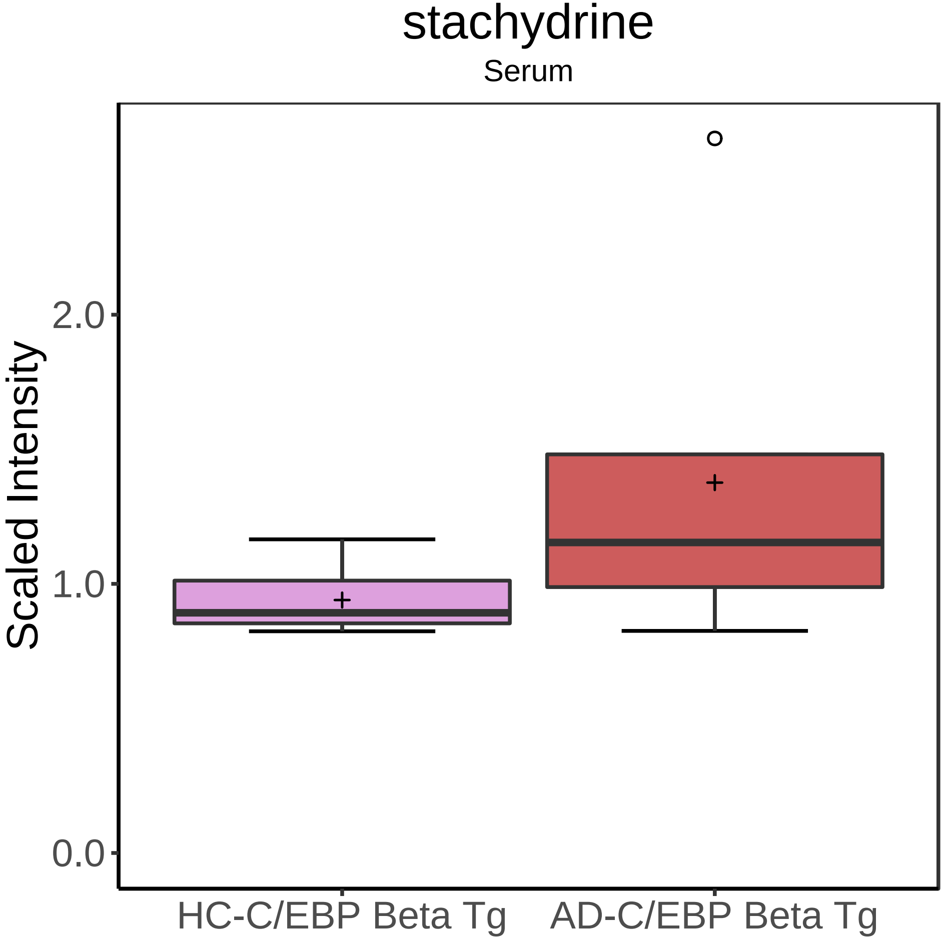

stachydrine
Serum
Scaled Intensity
2.0
1.0
0.0
HC-C/EBP Beta Tg
AD-C/EBP Beta Tg

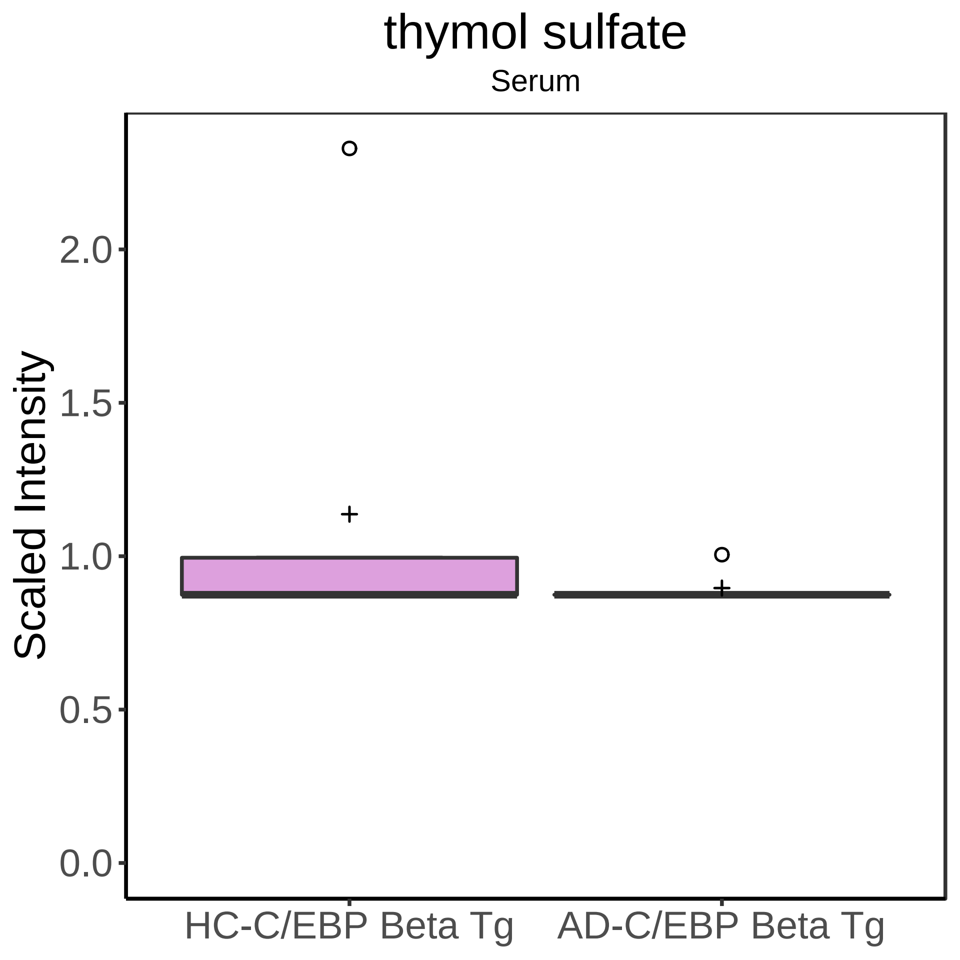
thymol sulfate
Serum
Scaled Intensity
2.0
1.5
1.0
0.5
0.0
HC-C/EBP Beta Tg
AD-C/EBP Beta Tg

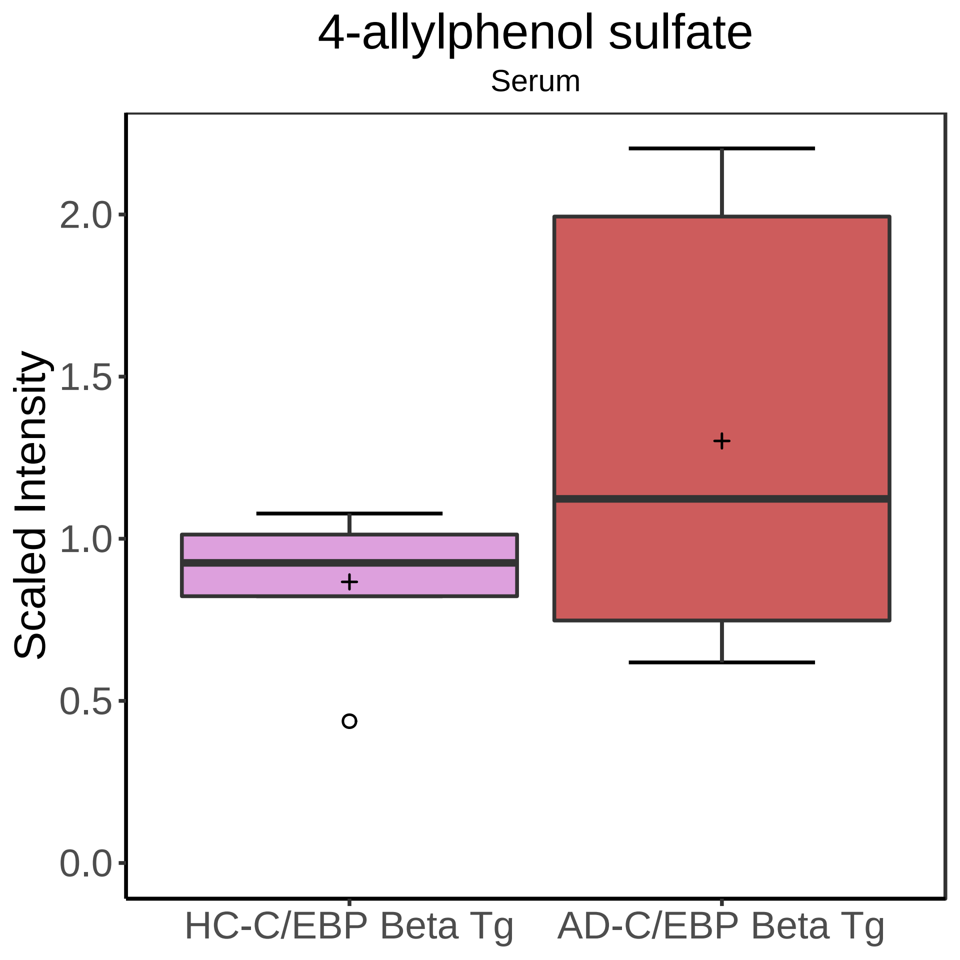

4-allylphenol sulfate
Serum
Scaled Intensity
2.0
1.5
1.0
0.5
0.0
HC-C/EBP Beta Tg
AD-C/EBP Beta Tg

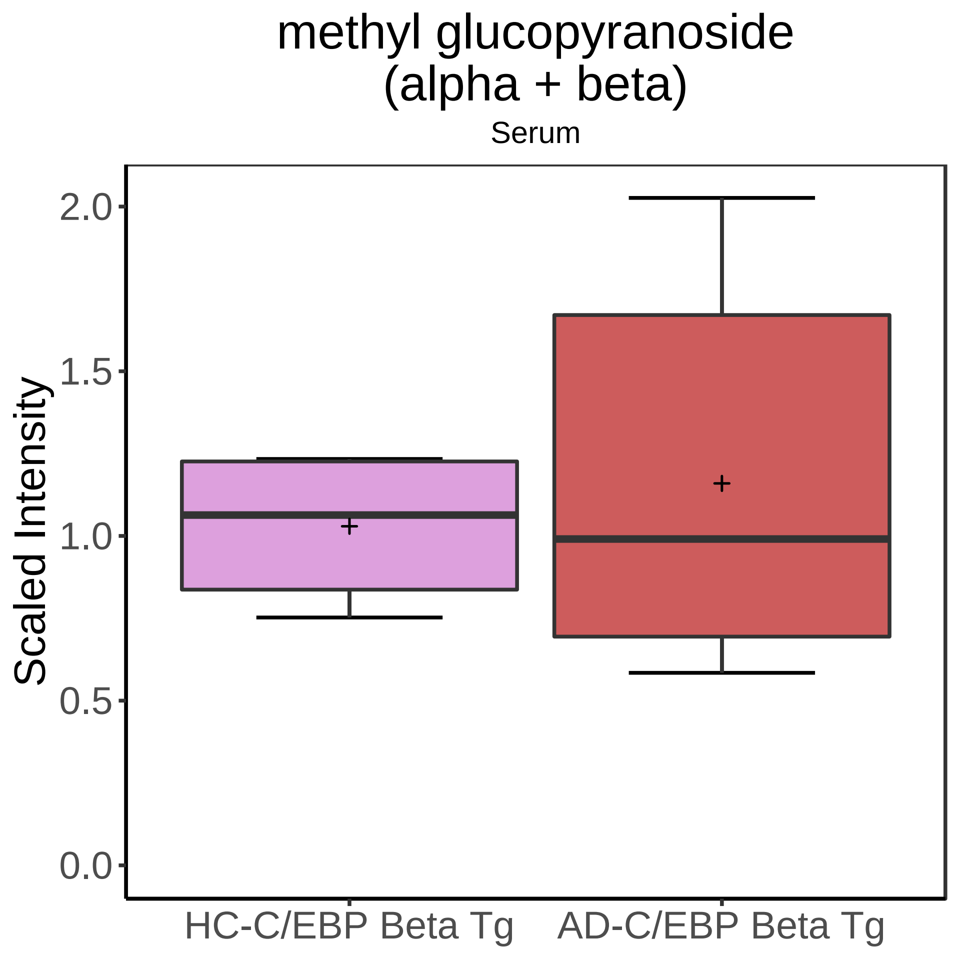
methyl glucopyranoside
(alpha + beta)
Serum
Scaled Intensity
2.0
1.5
1.0
0.5
0.0
HC-C/EBP Beta Tg
AD-C/EBP Beta Tg

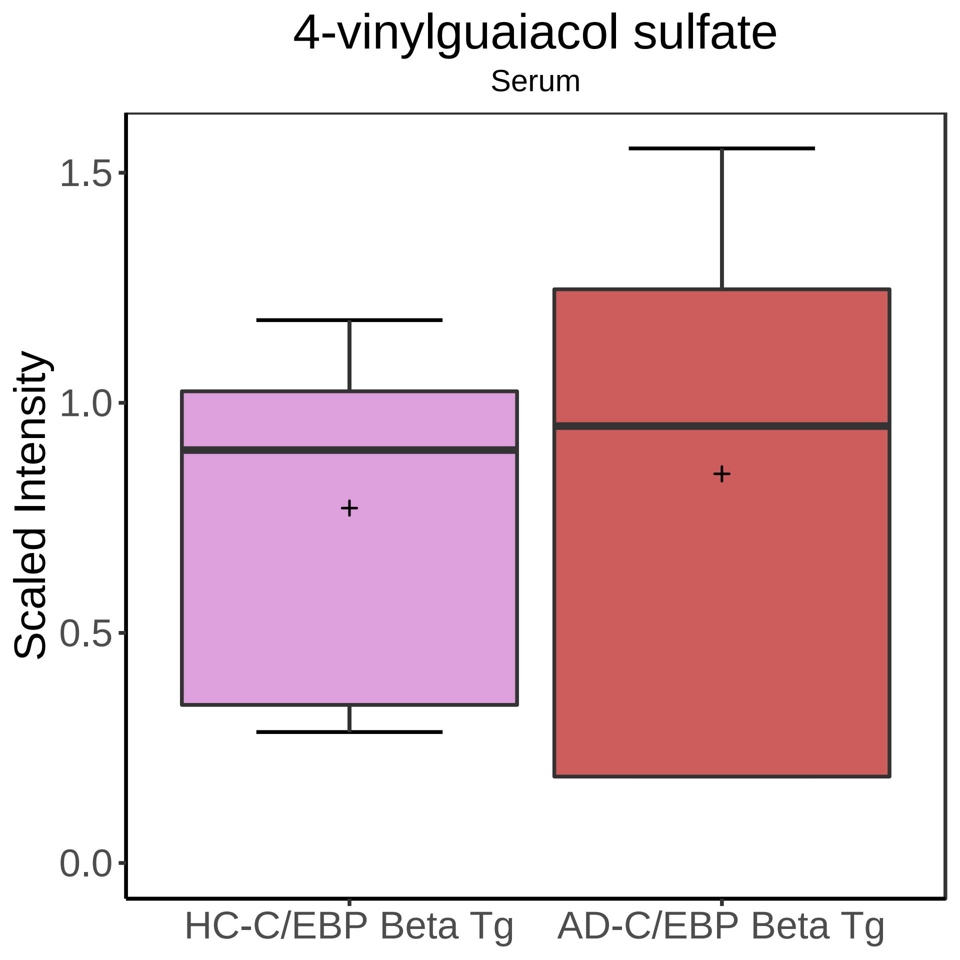
4-vinylguaiacol sulfate
Serum
Scaled Intensity
0.0
0.5
1.0
1.5
HC-C/EBP Beta Tg
AD-C/EBP Beta Tg

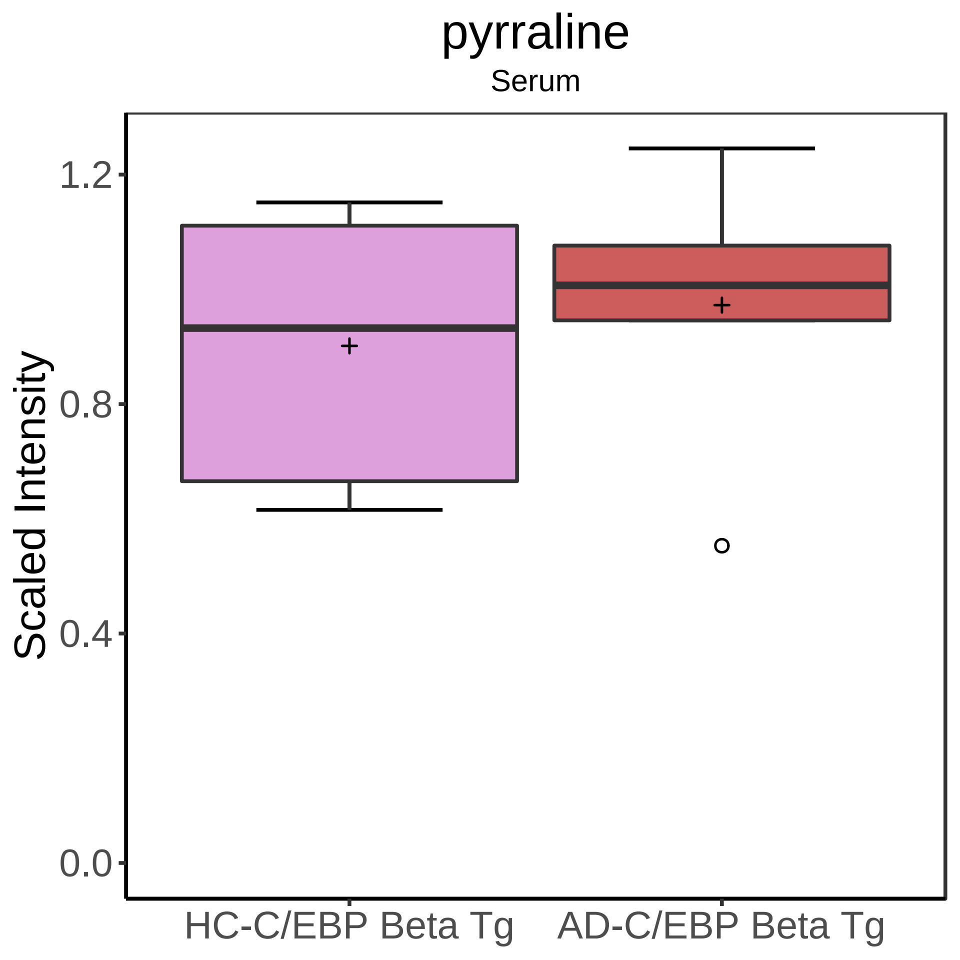
pyrraline
Serum
Scaled Intensity
1.2
0.8
0.4
0.0
HC-C/EBP Beta Tg
AD-C/EBP Beta Tg

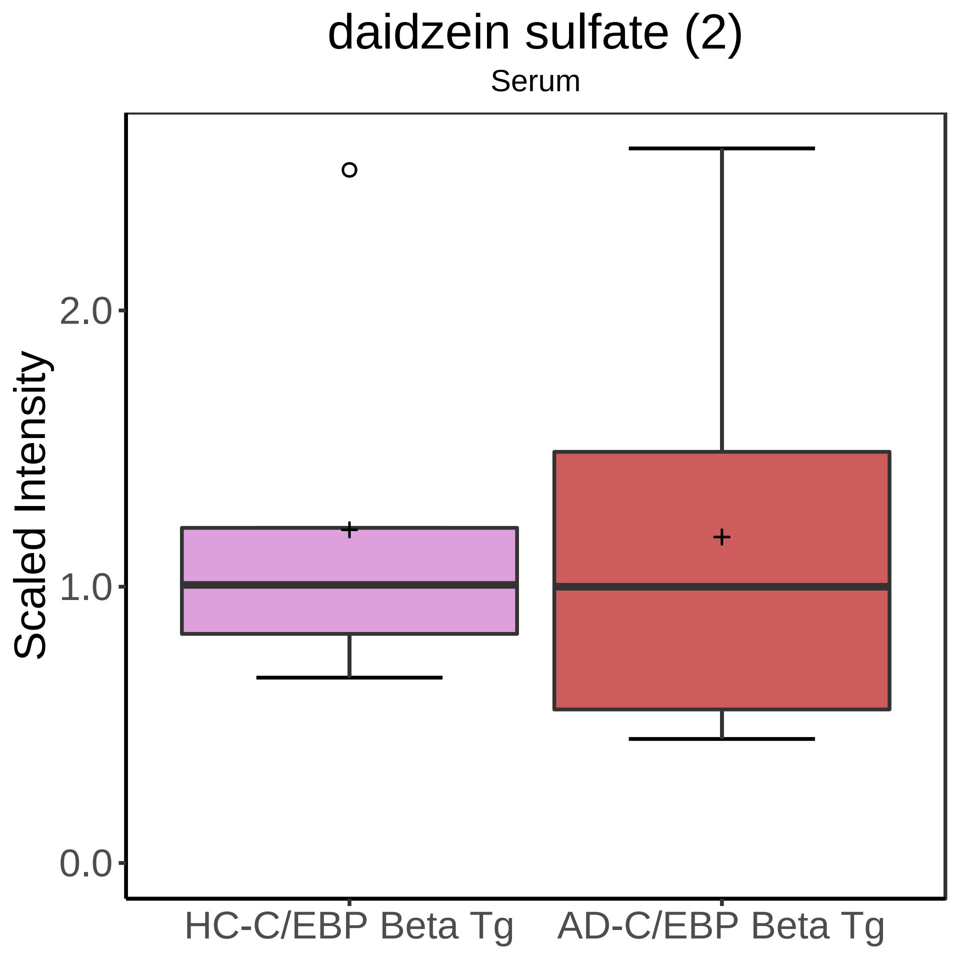
daidzein sulfate (2)
Serum
Scaled Intensity
2.0
1.0
0.0
HC-C/EBP Beta Tg
AD-C/EBP Beta Tg

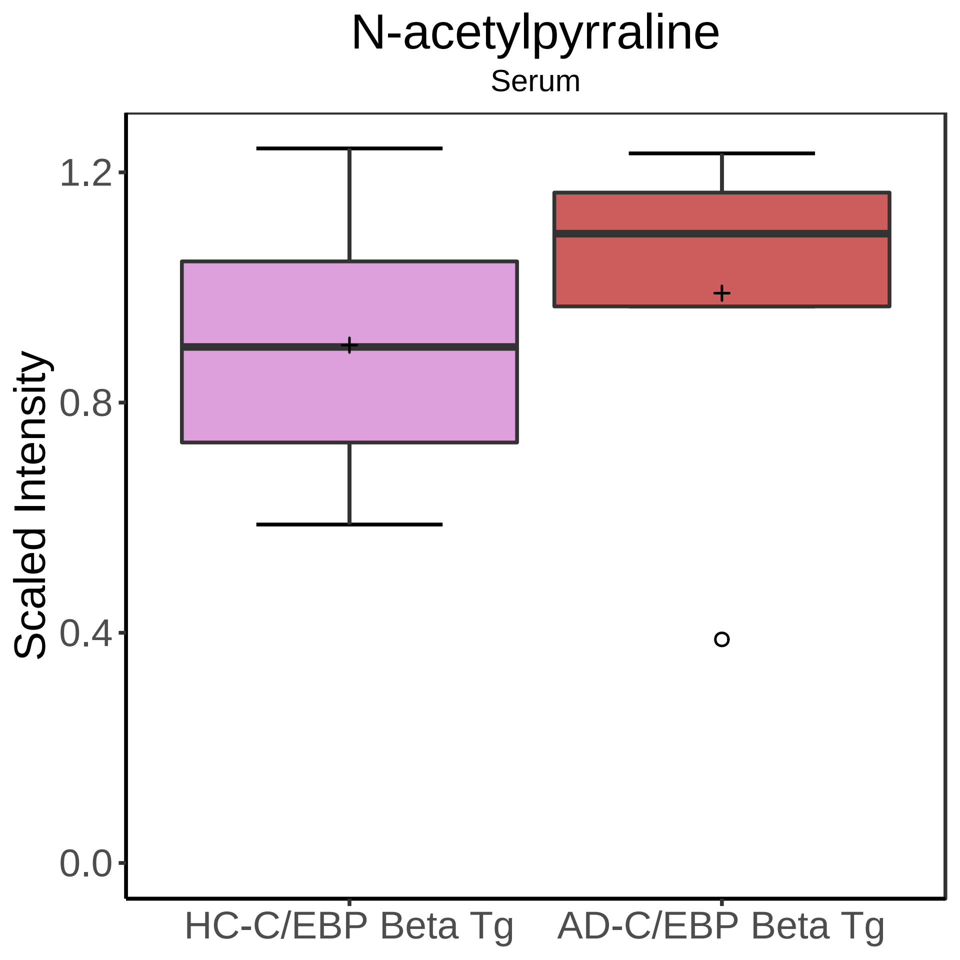

N-acetylpyrraline
Serum
Scaled Intensity
1.2
0.8
0.4
0.0
HC-C/EBP Beta Tg
AD-C/EBP Beta Tg

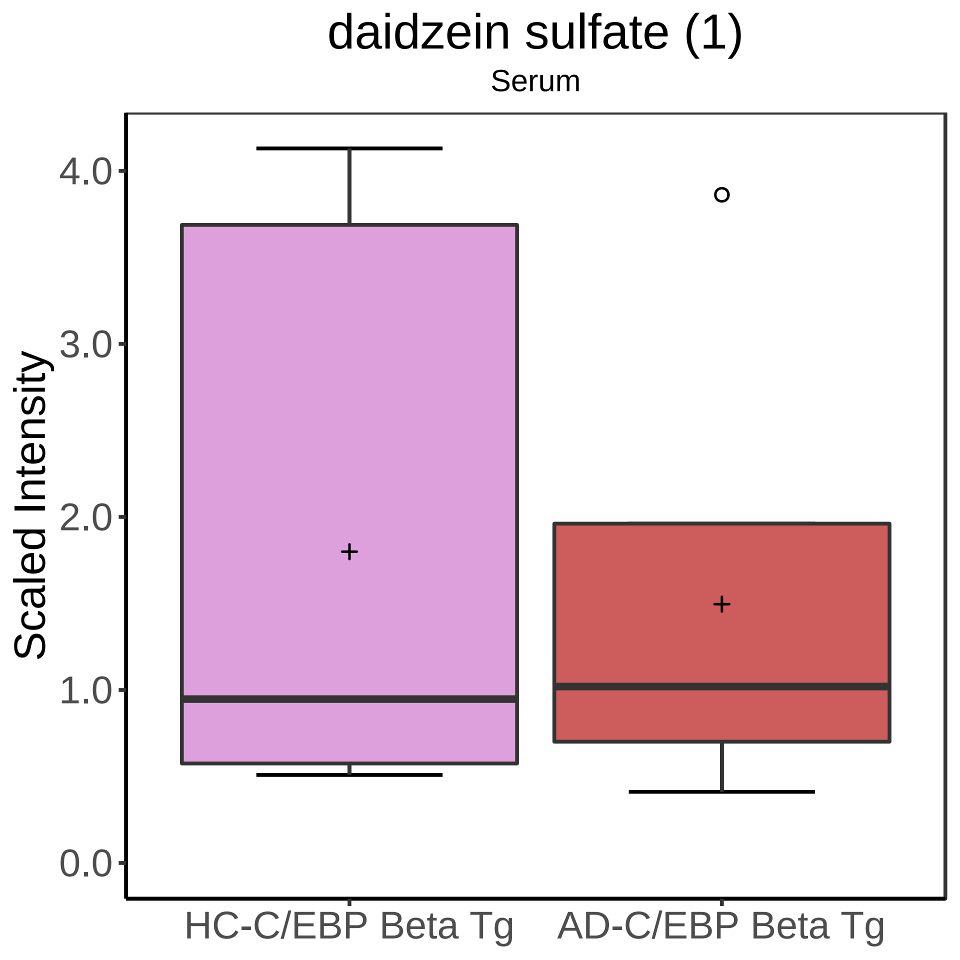
daidzein sulfate (1)
Serum
Scaled Intensity
4.0
3.0
2.0
1.0
0.0
HC-C/EBP Beta Tg
AD-C/EBP Beta Tg

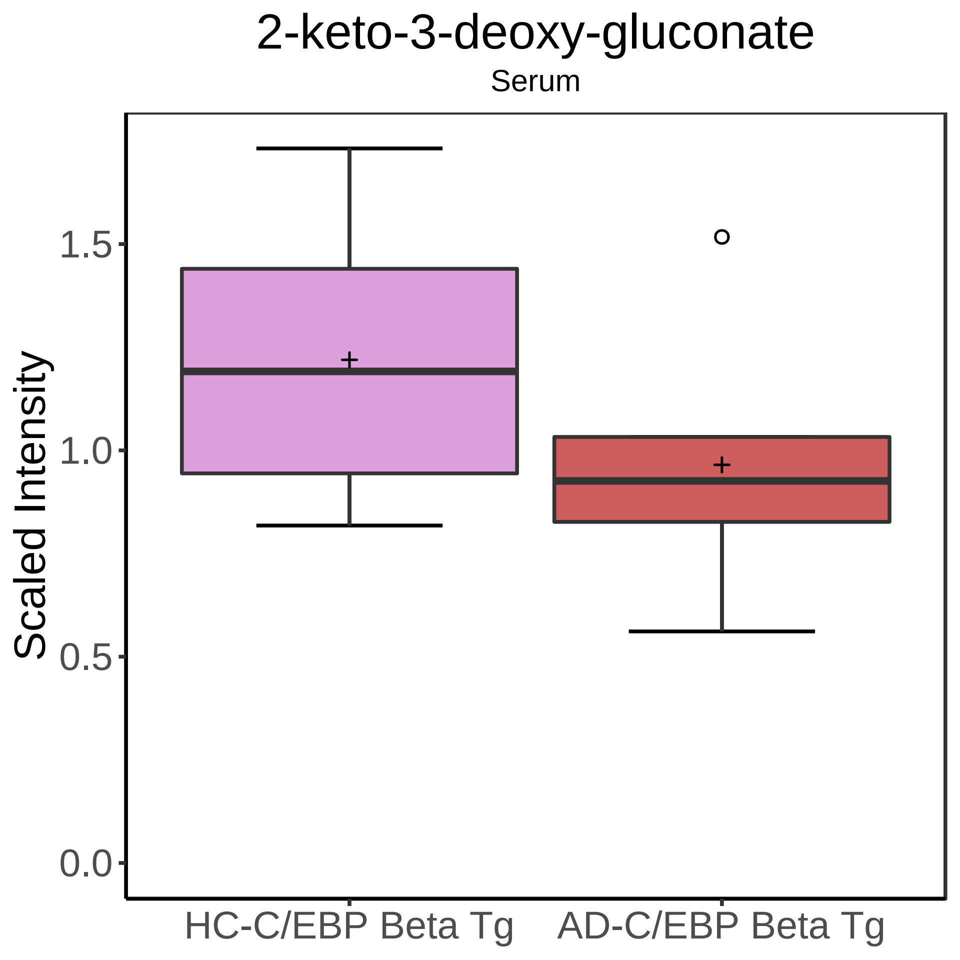
2-keto-3-deoxy-gluconate
Serum
Scaled Intensity
1.5
1.0
0.5
0.0
HC-C/EBP Beta Tg
AD-C/EBP Beta Tg

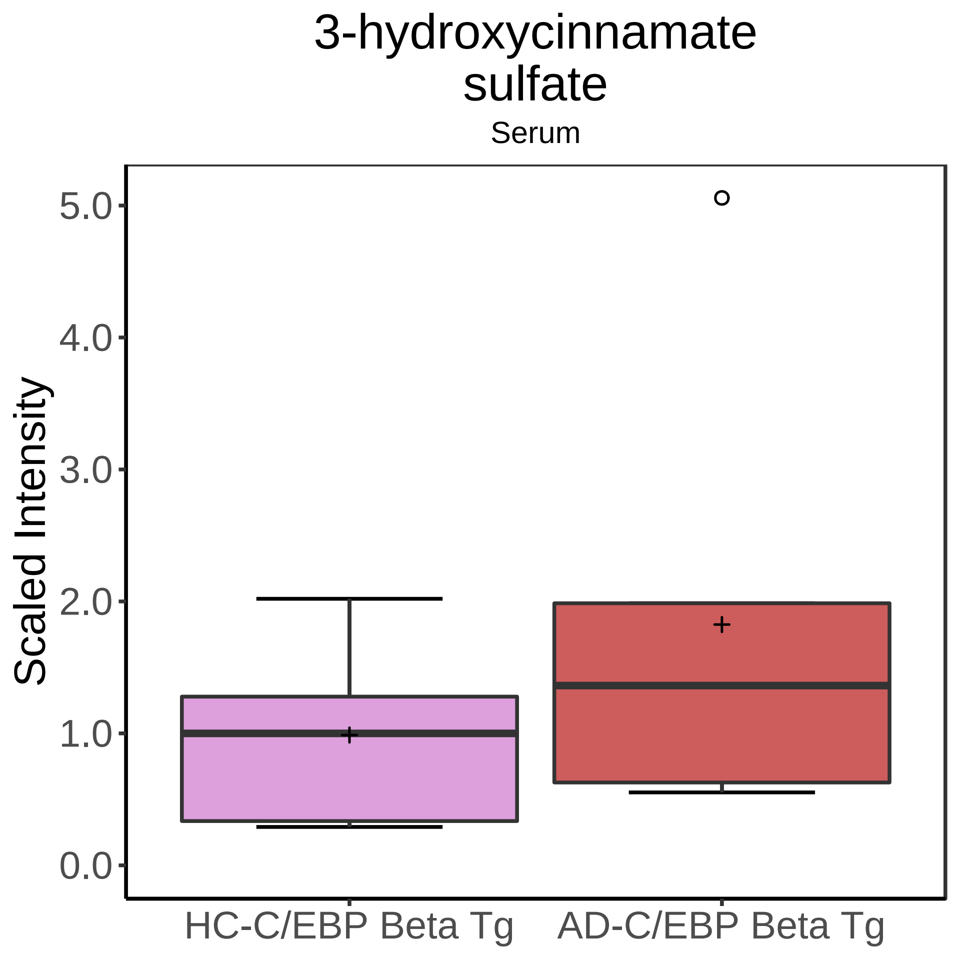
3-hydroxycinnamate sulfate
Serum
Scaled Intensity
5.0
4.0
3.0
2.0
1.0
0.0
HC-C/EBP Beta Tg
AD-C/EBP Beta Tg

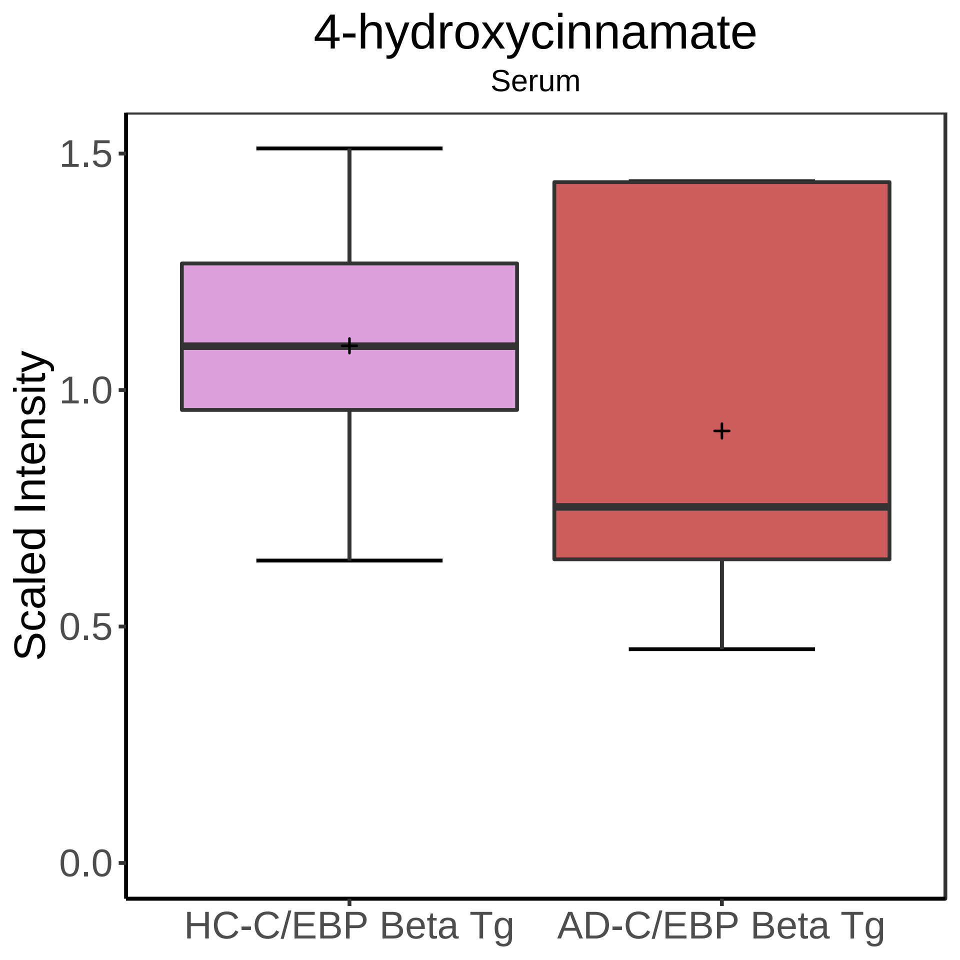
4-hydroxycinnamate
Serum
Scaled Intensity
1.5
1.0
0.5
0.0
HC-C/EBP Beta Tg
AD-C/EBP Beta Tg

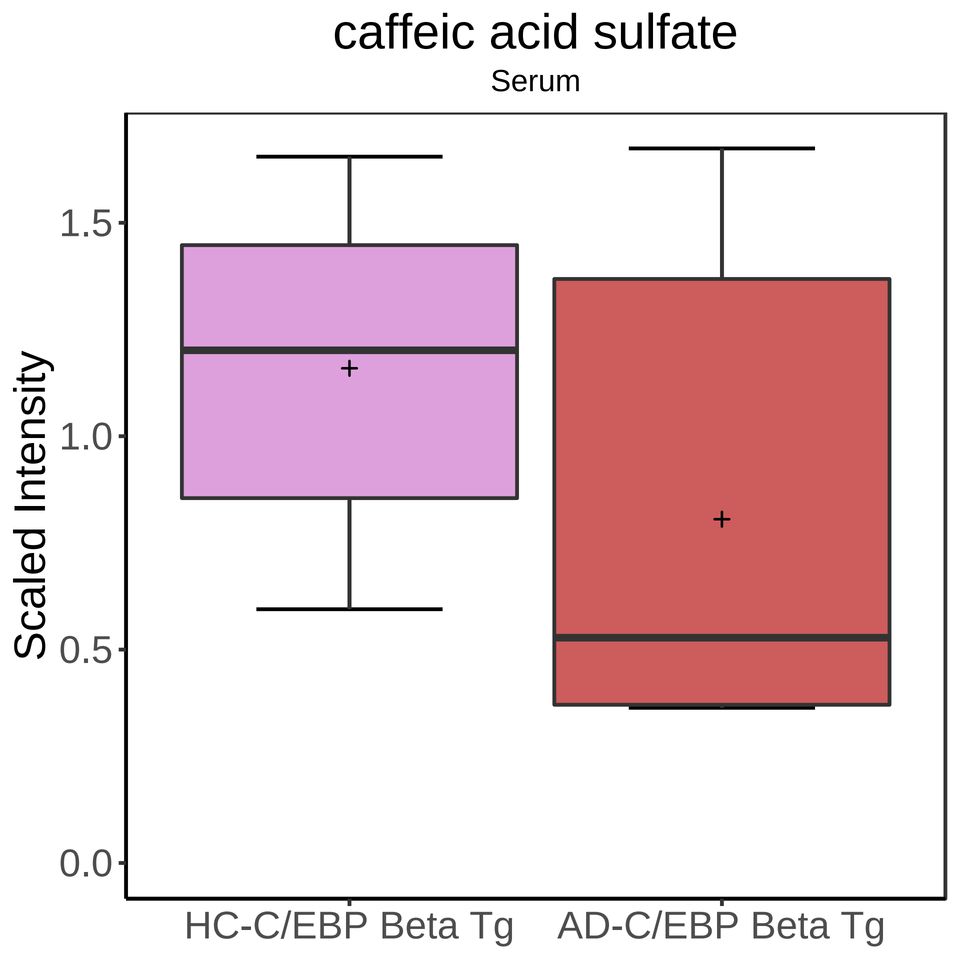
caffeic acid sulfate
Serum
Scaled Intensity
1.5
1.0
0.5
0.0
HC-C/EBP Beta Tg
AD-C/EBP Beta Tg

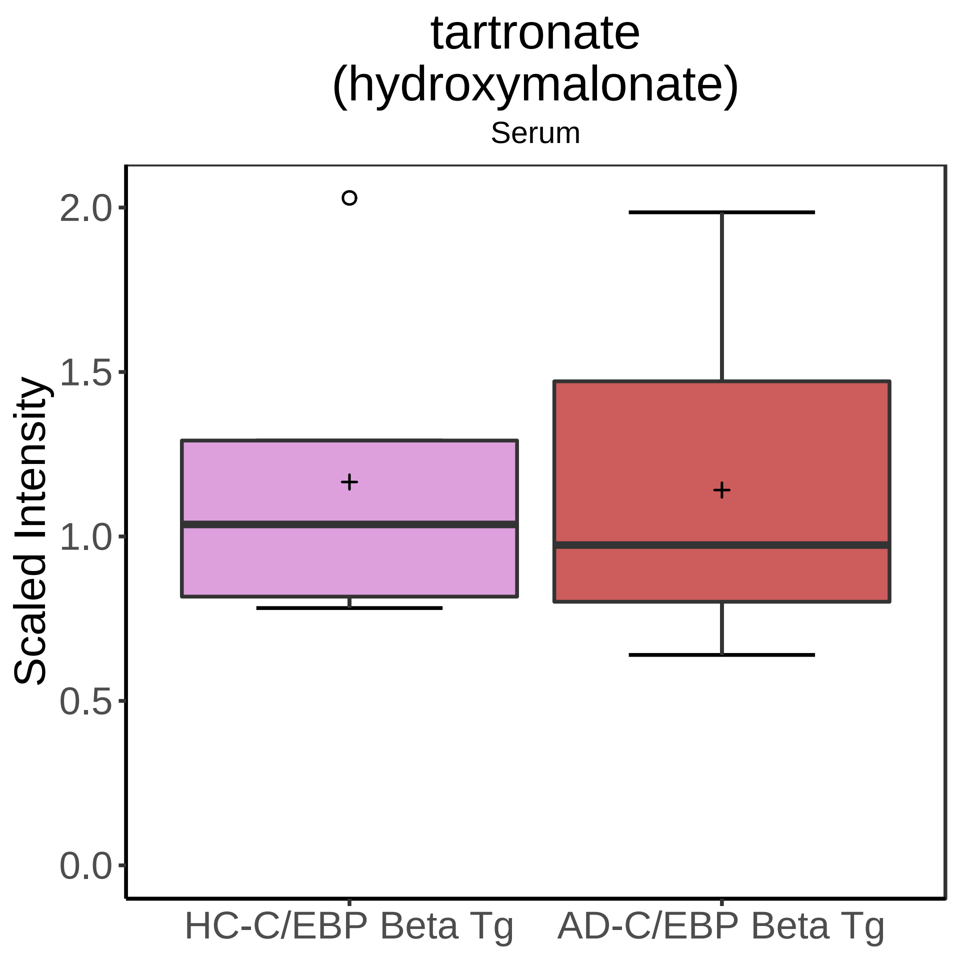

tartronate
(hydroxymalonate)
Serum
Scaled Intensity
2.0
1.5
1.0
0.5
0.0
HC-C/EBP Beta Tg
AD-C/EBP Beta Tg

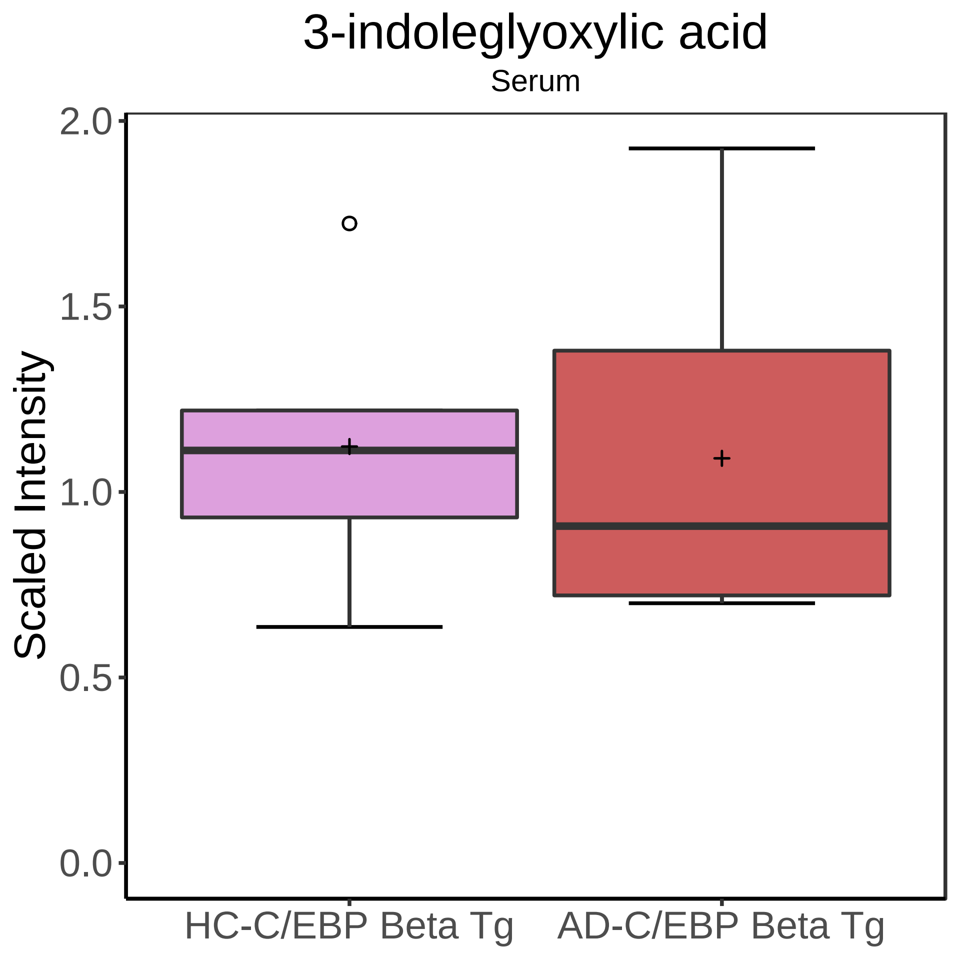

3-indoleglyoxylic acid
Serum
Scaled Intensity
2.0
1.5
1.0
0.5
0.0
HC-C/EBP Beta Tg
AD-C/EBP Beta Tg

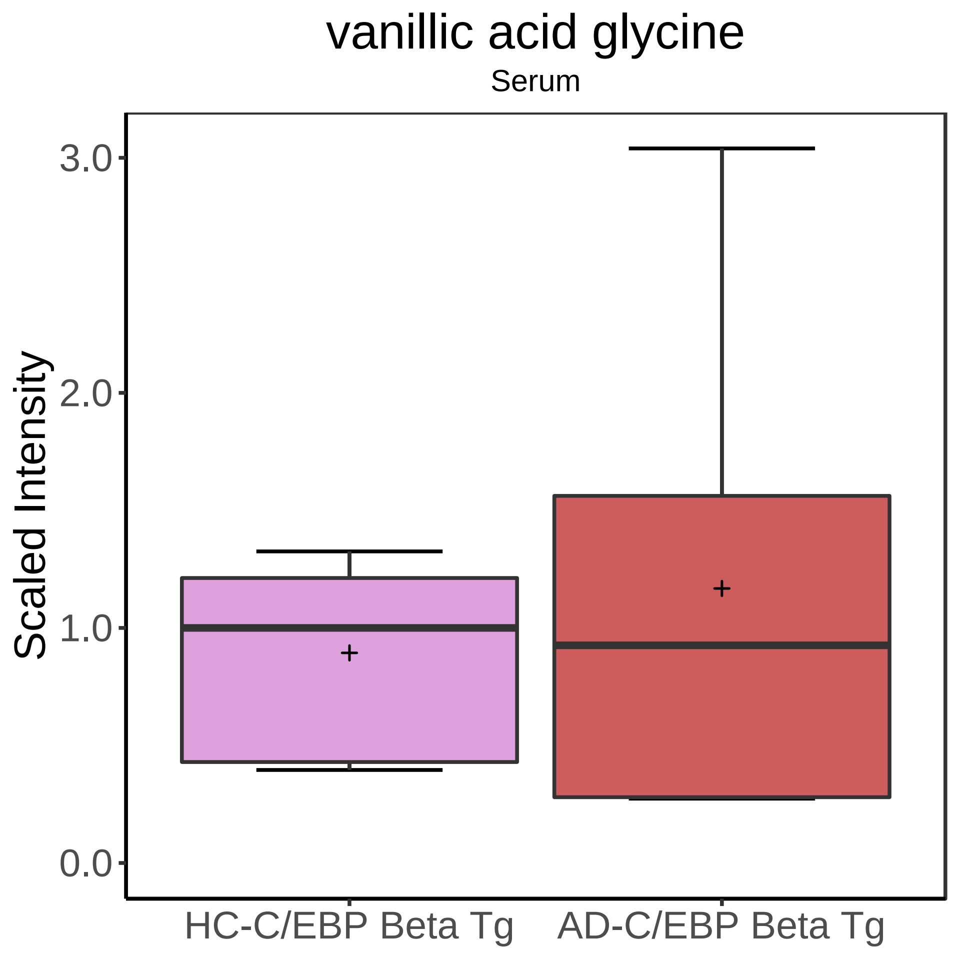
vanillic acid glycine
Serum
Scaled Intensity
3.0
2.0
1.0
0.0
HC-C/EBP Beta Tg
AD-C/EBP Beta Tg

4-vinylguaiacol glucuronide

Serum

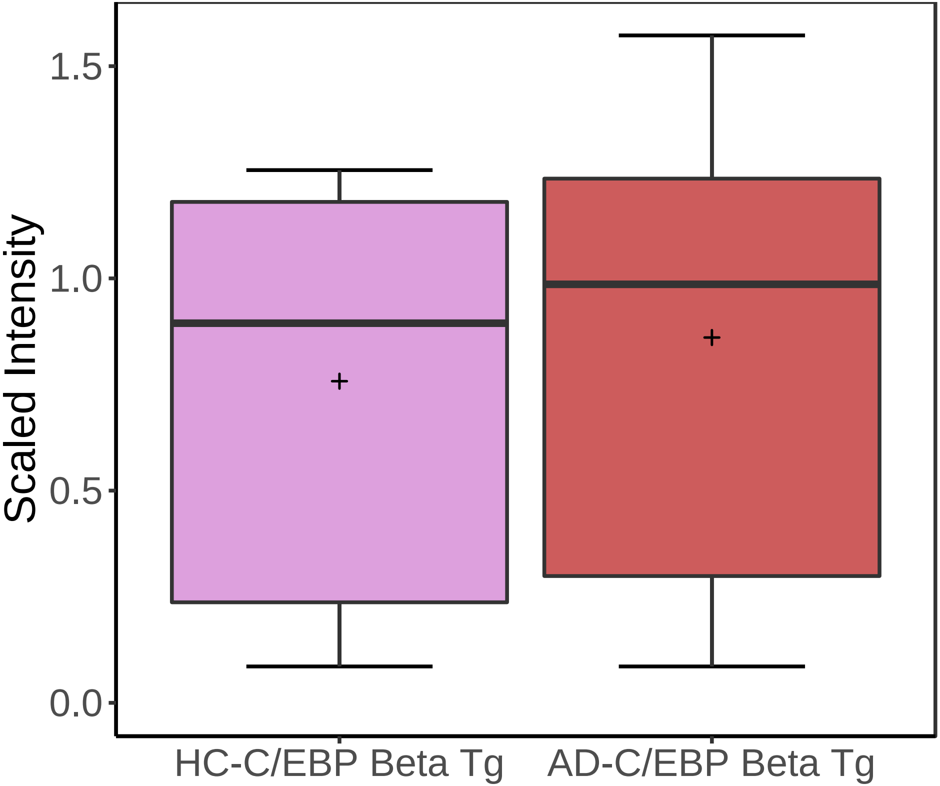

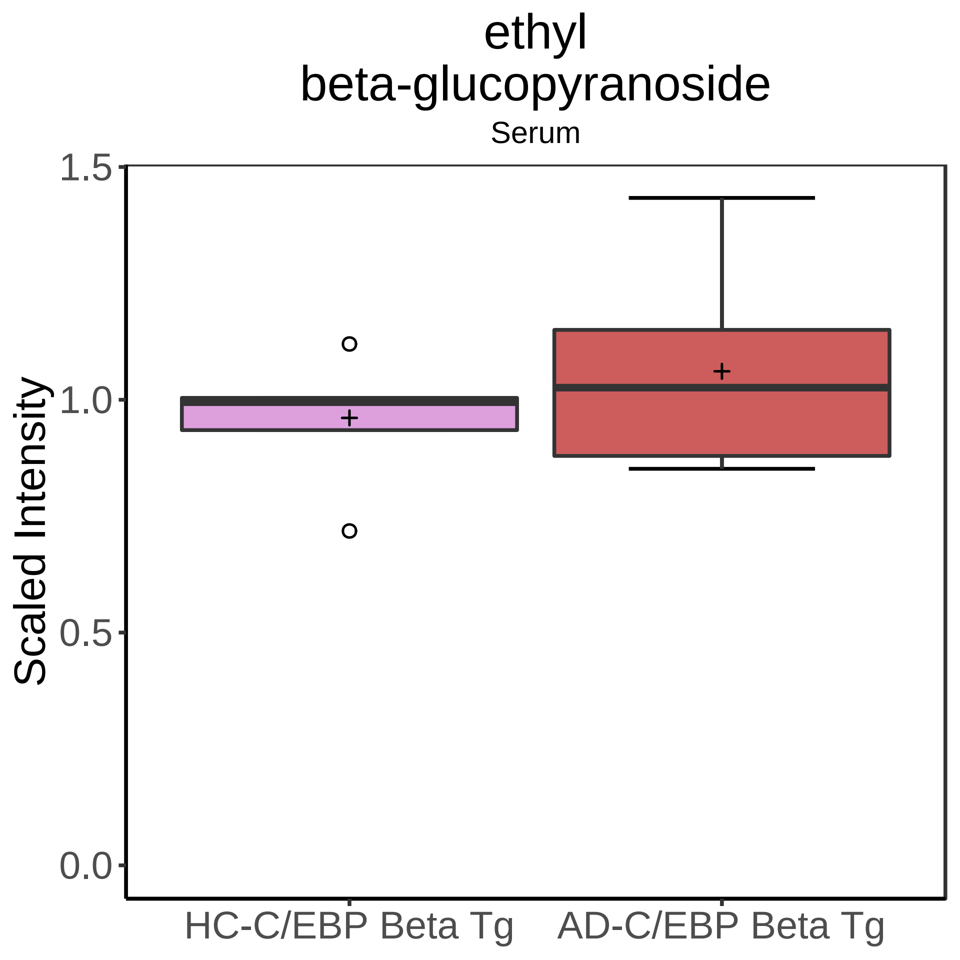
ethyl
beta-glucopyranoside
Serum
Scaled Intensity
1.5
1.0
0.5
0.0
HC-C/EBP Beta Tg
AD-C/EBP Beta Tg

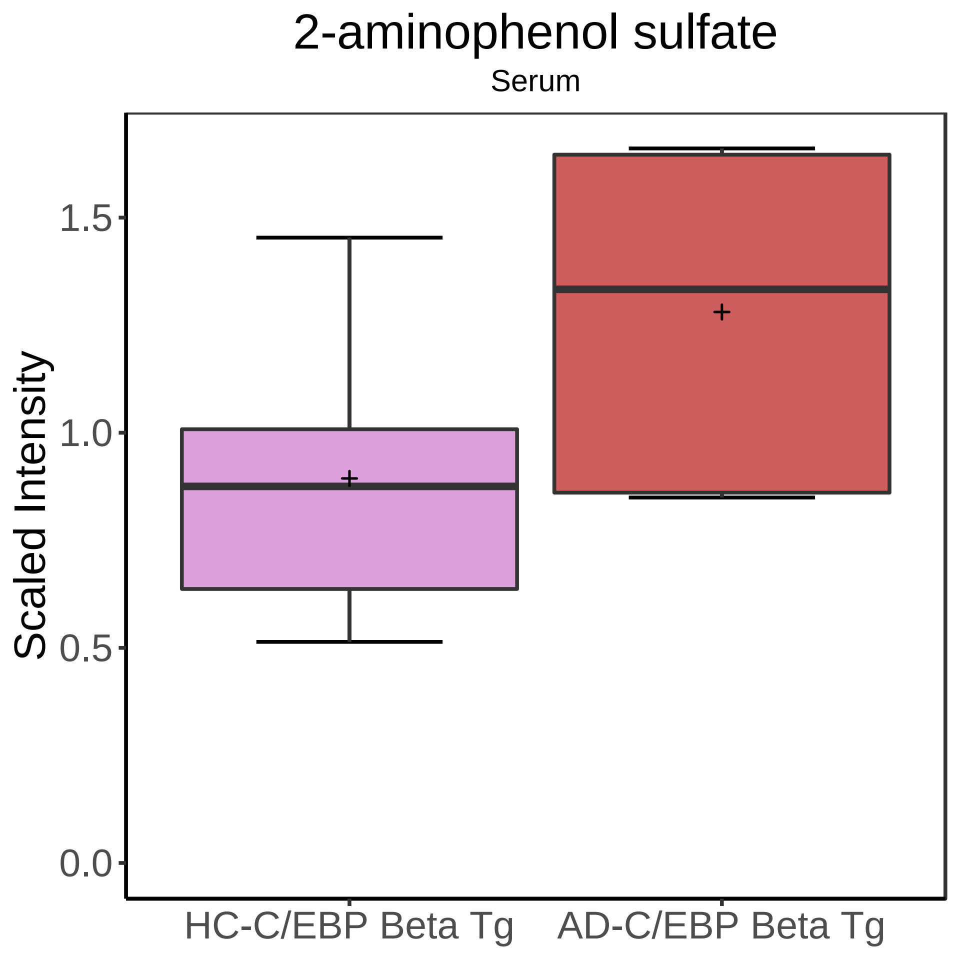

2-aminophenol sulfate
Serum
Scaled Intensity
1.5
1.0
0.5
0.0
HC-C/EBP Beta Tg
AD-C/EBP Beta Tg

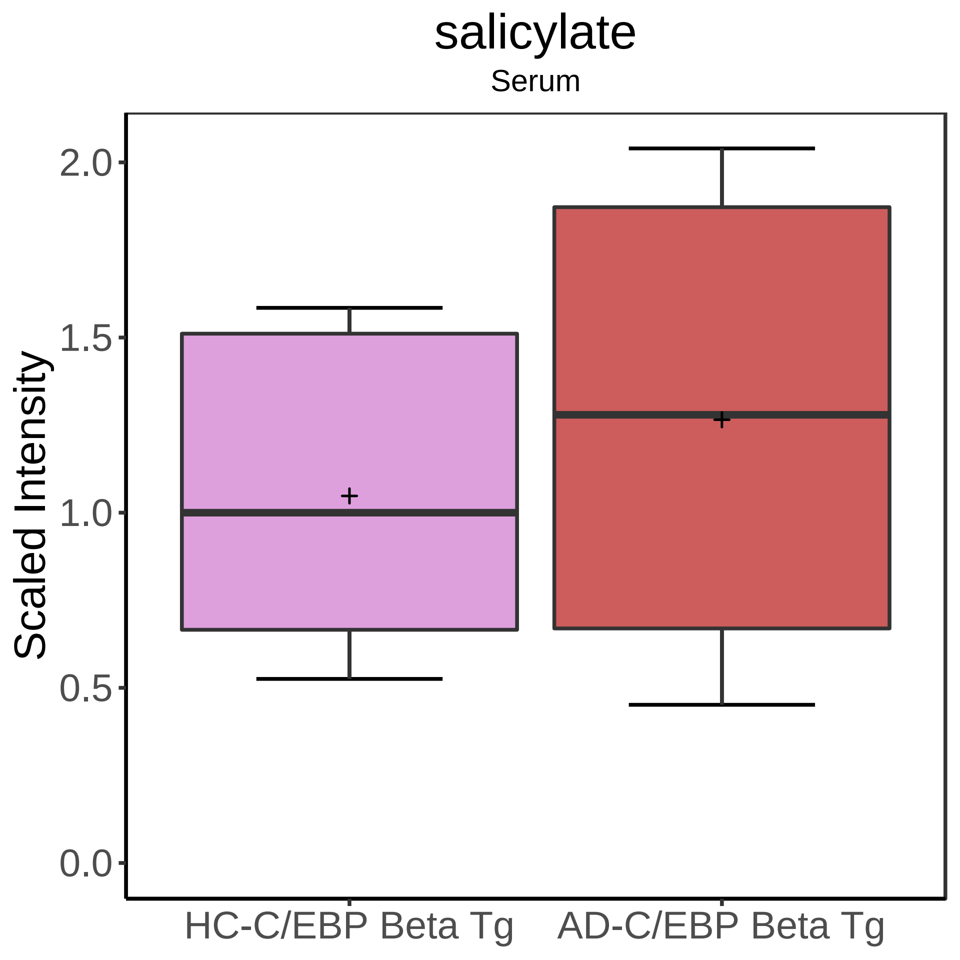
salicylate
Serum
Scaled Intensity
2.0
1.5
1.0
0.5
0.0
HC-C/EBP Beta Tg
AD-C/EBP Beta Tg

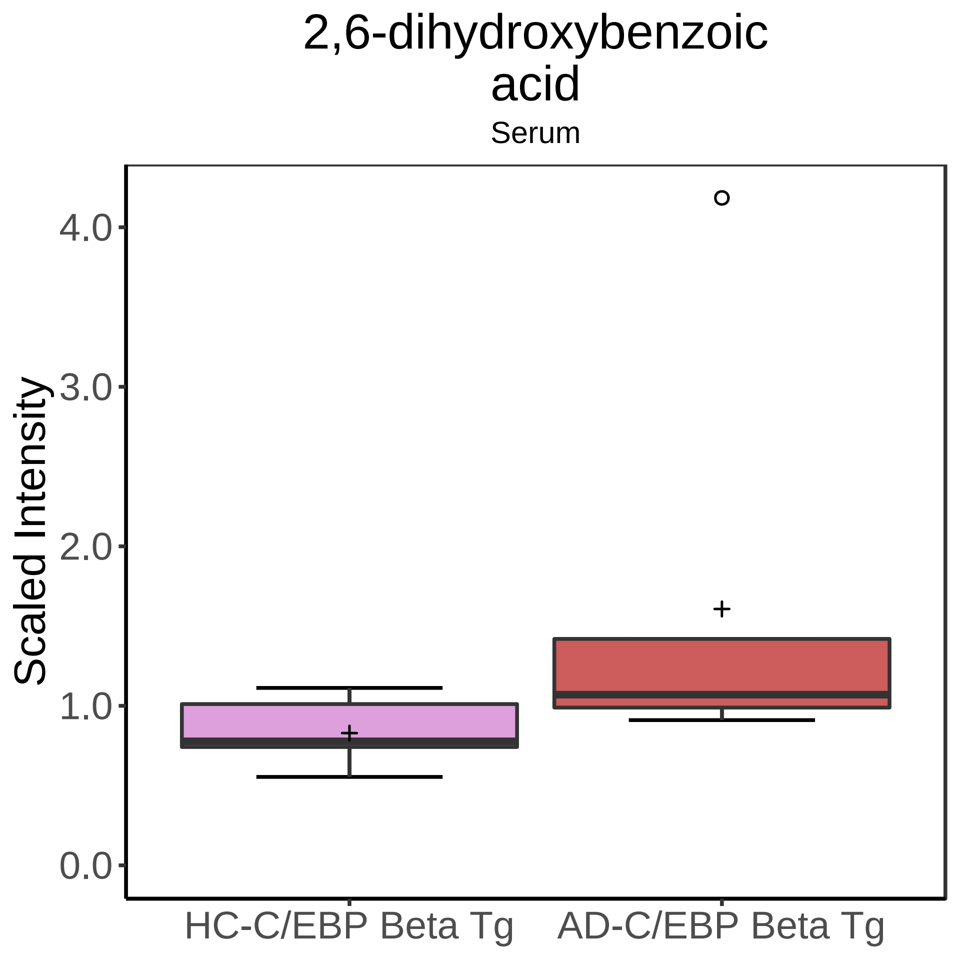
2,6-dihydroxybenzoic acid
Serum
Scaled Intensity
4.0
3.0
2.0
1.0
0.0
HC-C/EBP Beta Tg
AD-C/EBP Beta Tg

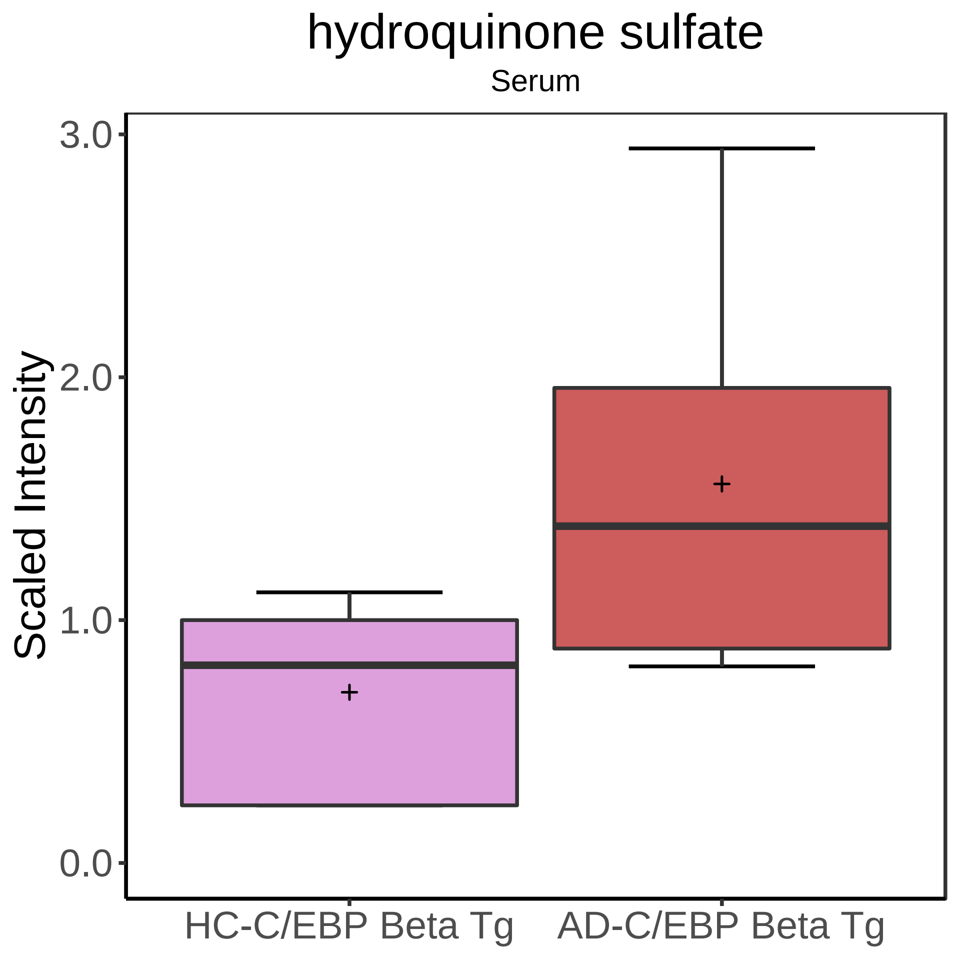
hydroquinone sulfate
Serum
Scaled Intensity
3.0
2.0
1.0
0.0
HC-C/EBP Beta Tg
AD-C/EBP Beta Tg

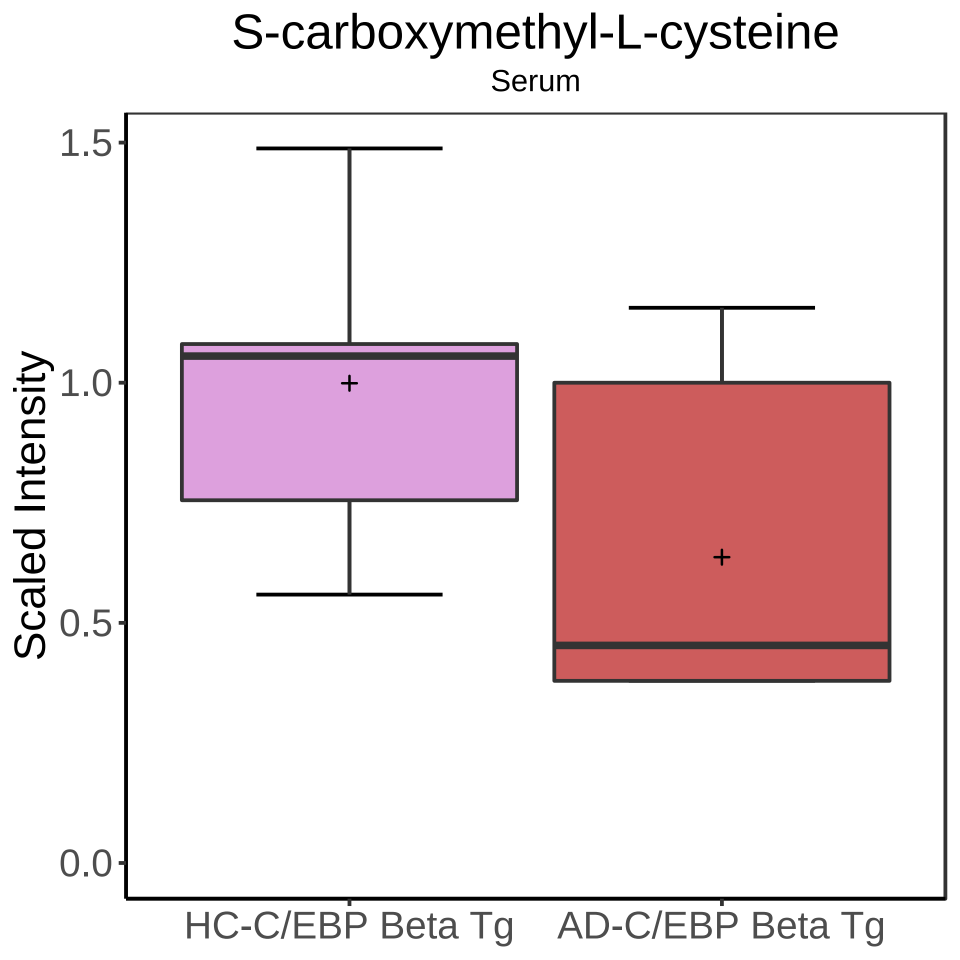
S-carboxymethyl-L-cysteine
Serum
Scaled Intensity
1.5
1.0
0.5
0.0
HC-C/EBP Beta Tg
AD-C/EBP Beta Tg

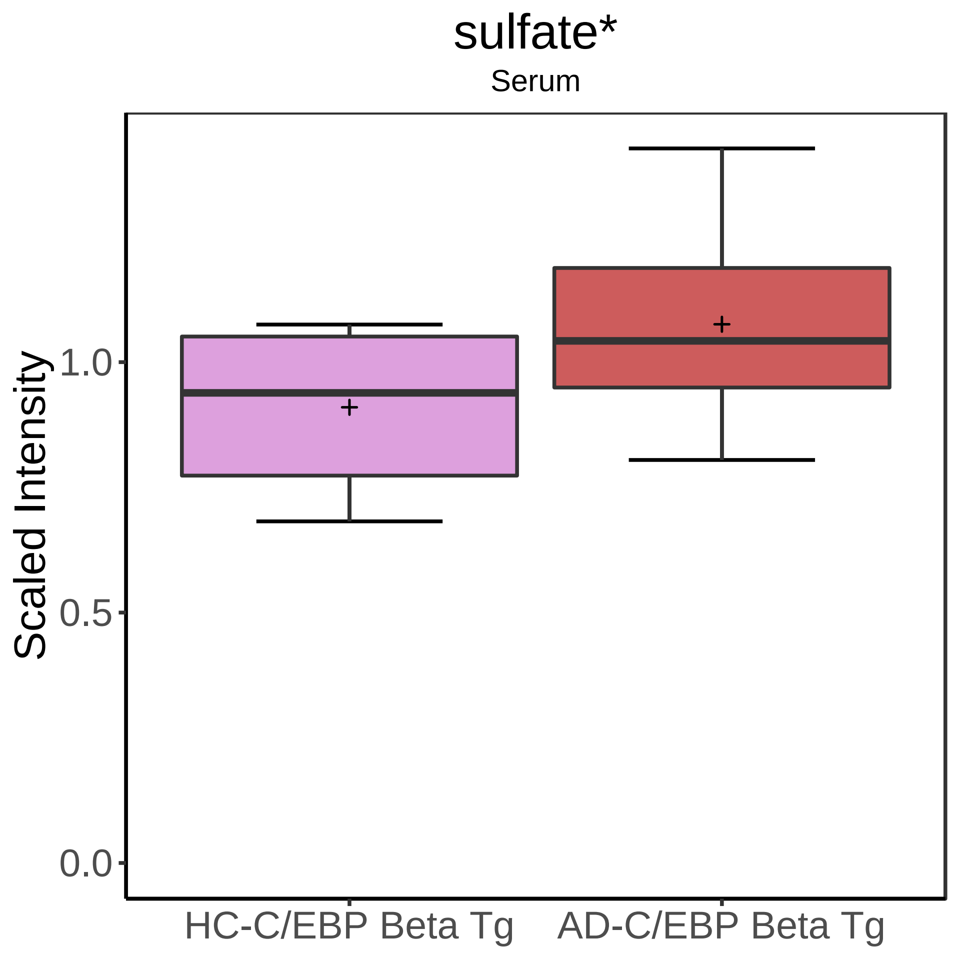
sulfate*
Serum
Scaled Intensity
1.0
0.5
0.0
HC-C/EBP Beta Tg
AD-C/EBP Beta Tg

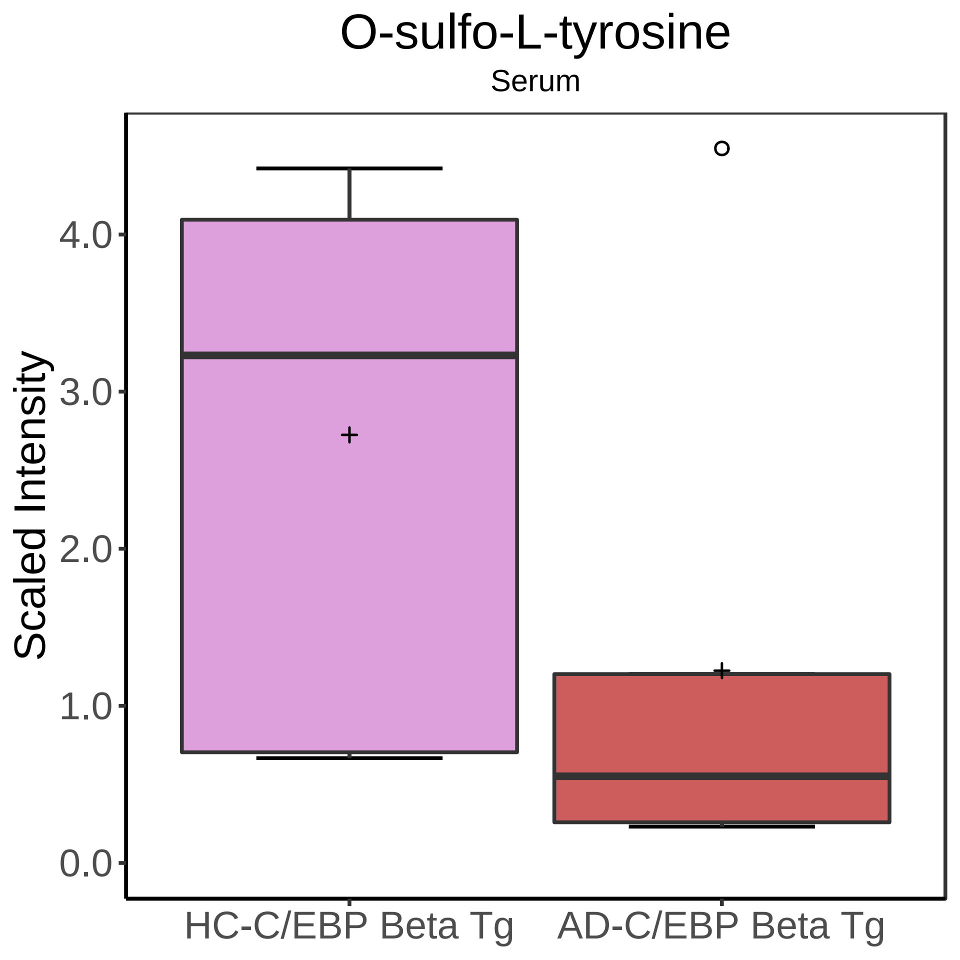
O-sulfo-L-tyrosine
Serum
Scaled Intensity
4.0
3.0
2.0
1.0
0.0
HC-C/EBP Beta Tg
AD-C/EBP Beta Tg

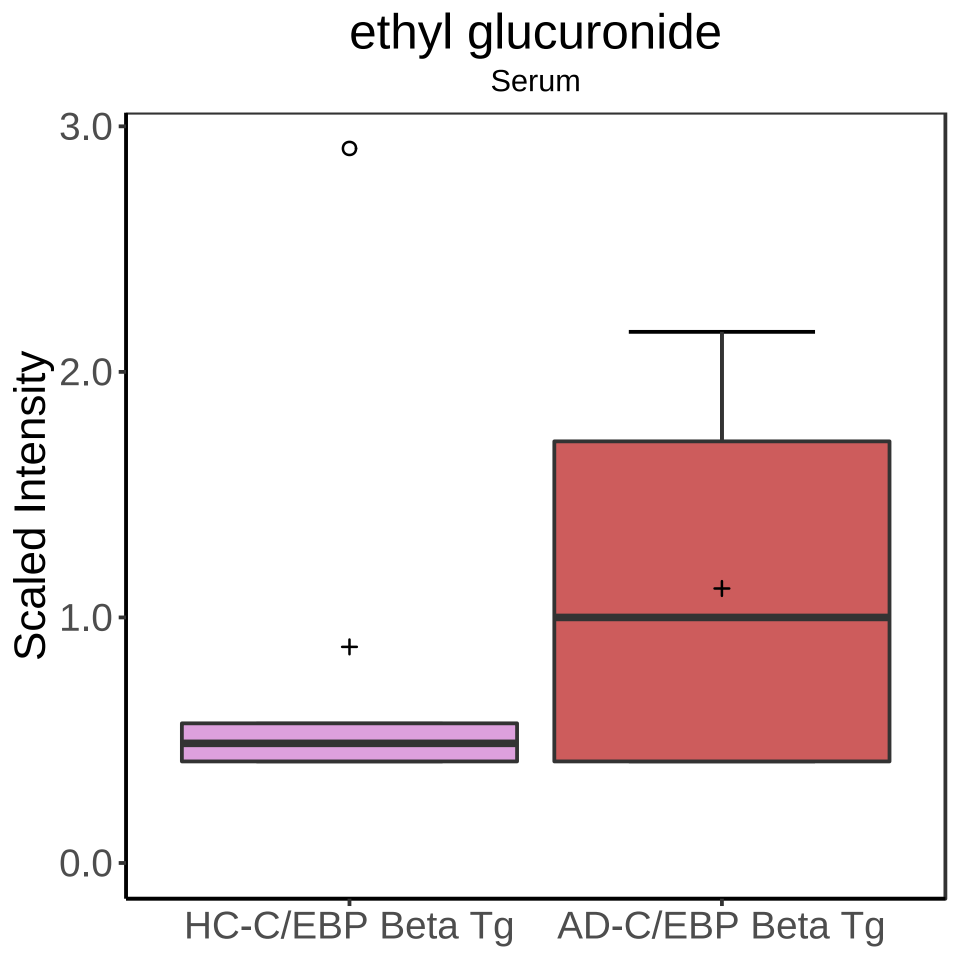
ethyl glucuronide
Serum
Scaled Intensity
3.0
2.0
1.0
0.0
HC-C/EBP Beta Tg
AD-C/EBP Beta Tg

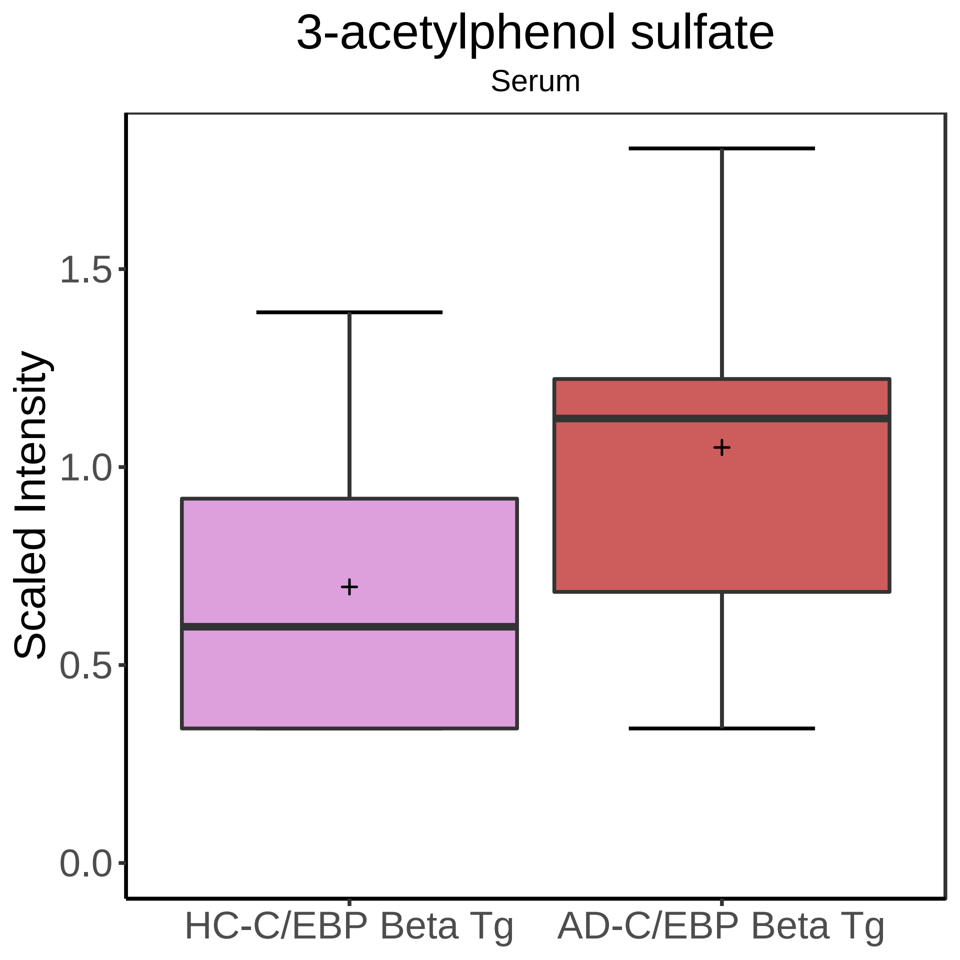
3-acetylphenol sulfate
Serum
Scaled Intensity
1.5
1.0
0.5
0.0
HC-C/EBP Beta Tg
AD-C/EBP Beta Tg

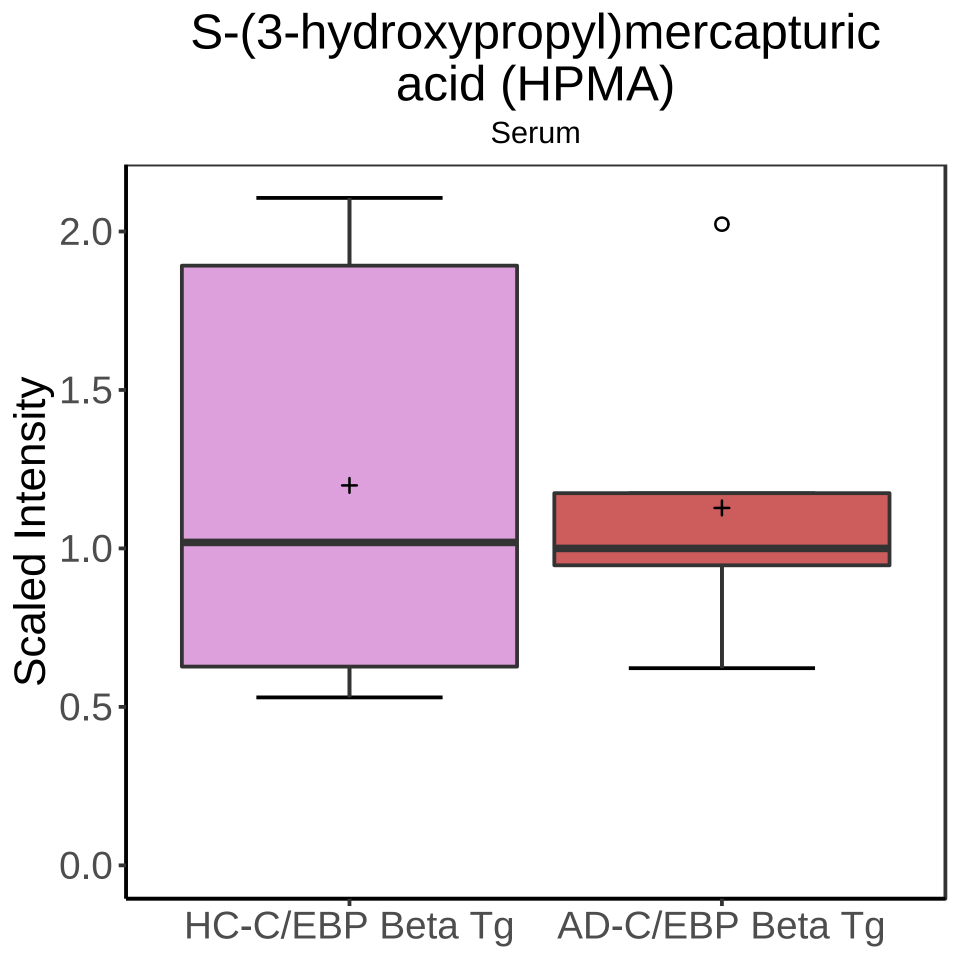
S-(3-hydroxypropyl)mercapturic acid (HPMA)
Serum
Scaled Intensity
2.0
1.5
1.0
0.5
0.0
HC-C/EBP Beta Tg
AD-C/EBP Beta Tg

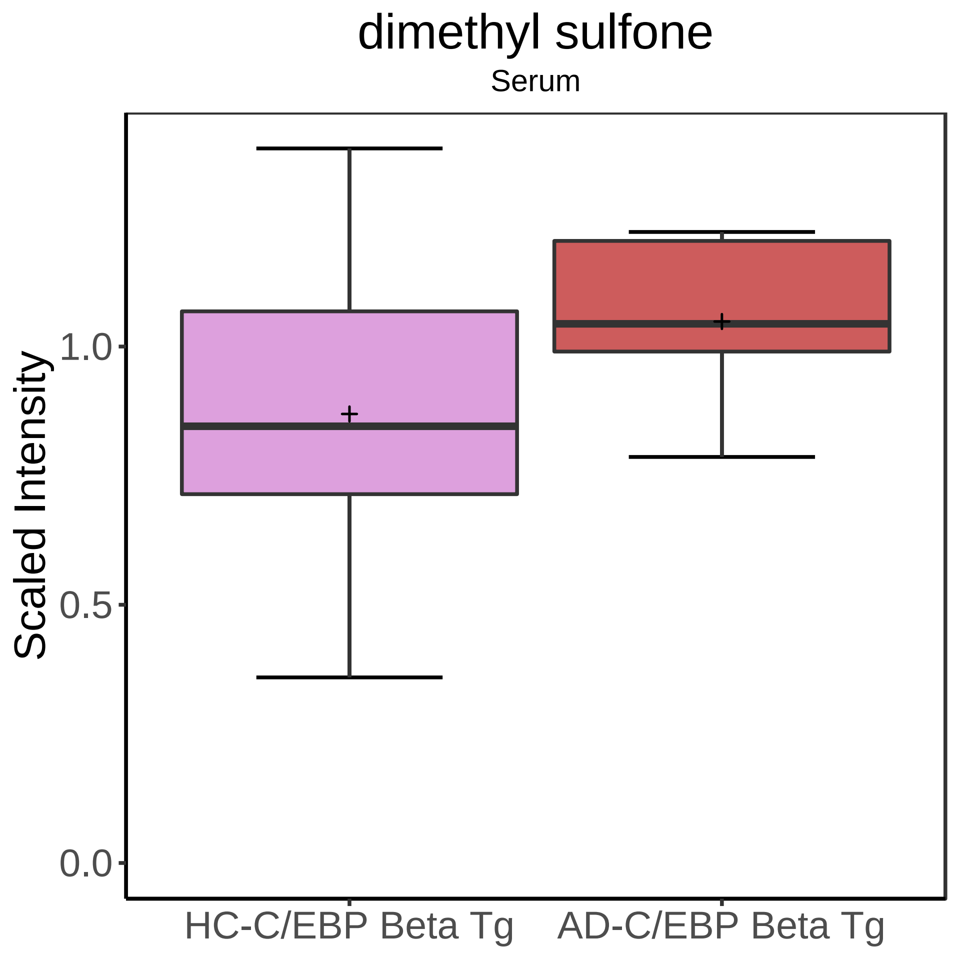
dimethyl sulfone
Serum
Scaled Intensity
1.0
0.5
0.0
HC-C/EBP Beta Tg
AD-C/EBP Beta Tg

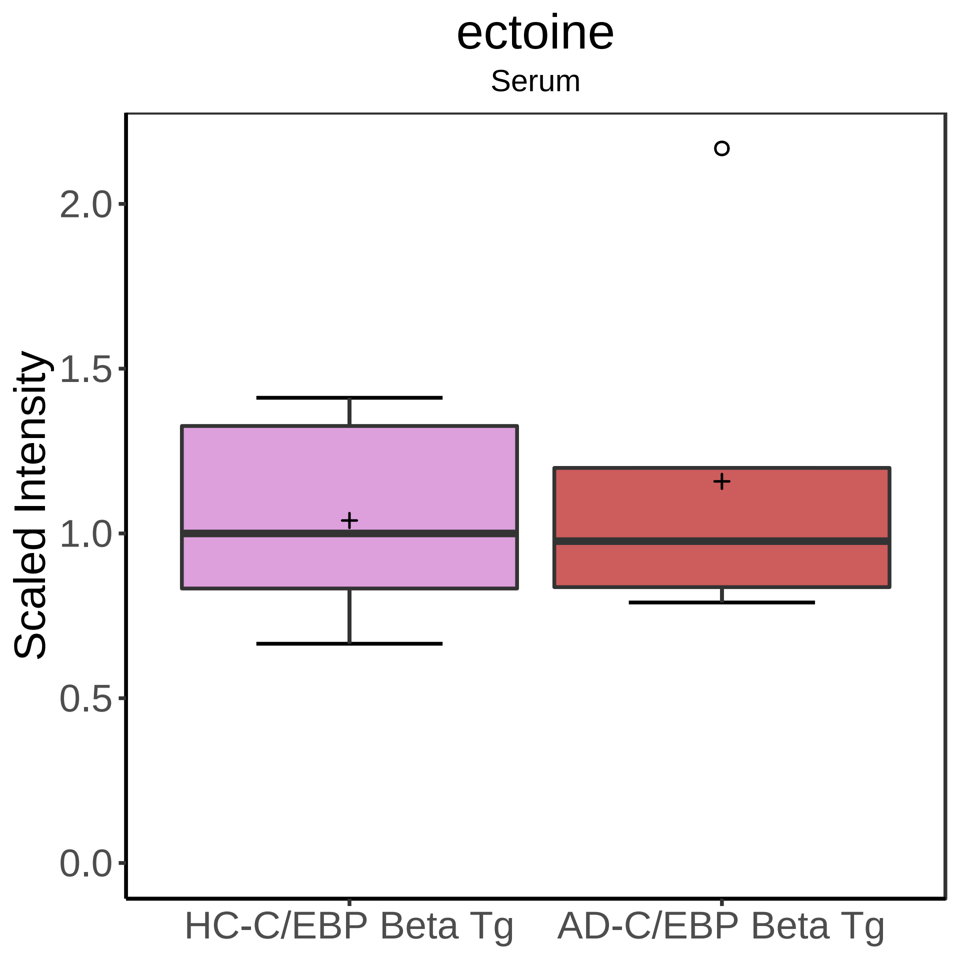
ectoine
Serum
Scaled Intensity
2.0
1.5
1.0
0.5
0.0
HC-C/EBP Beta Tg
AD-C/EBP Beta Tg

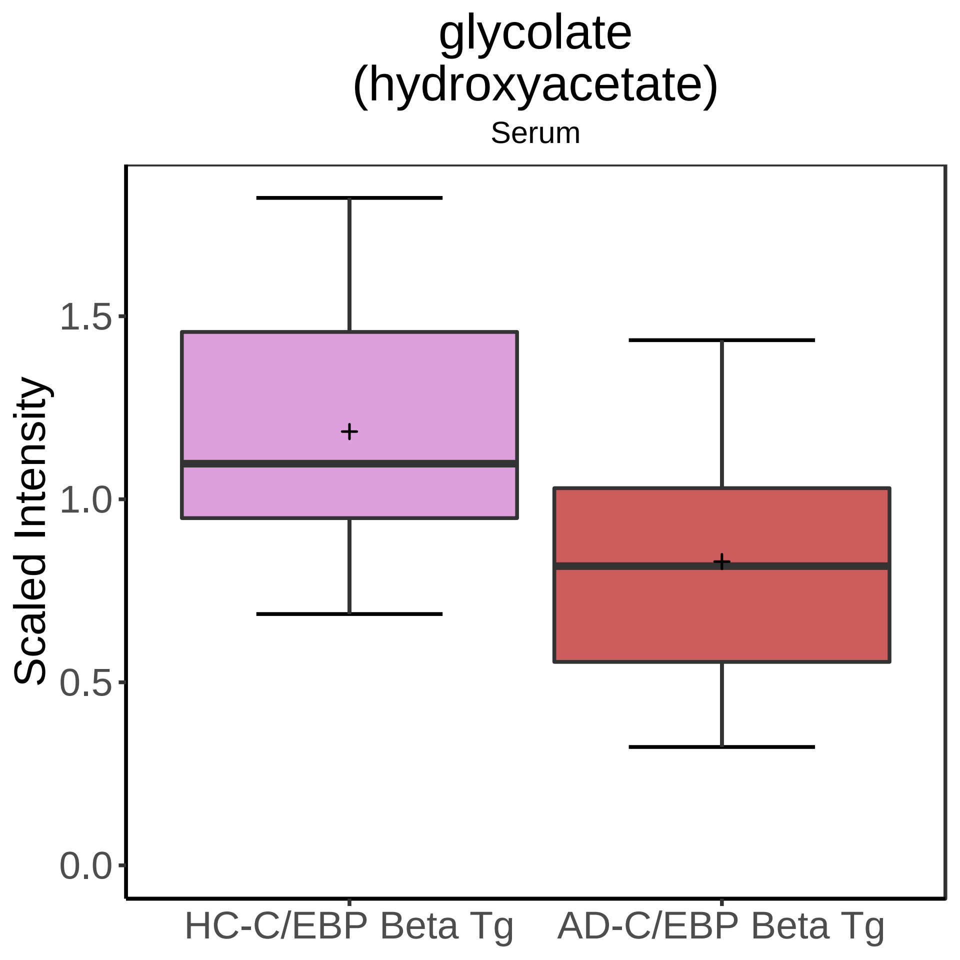

glycolate
(hydroxyacetate)
Serum
Scaled Intensity
0.0
0.5
1.0
1.5
HC-C/EBP Beta Tg
AD-C/EBP Beta Tg

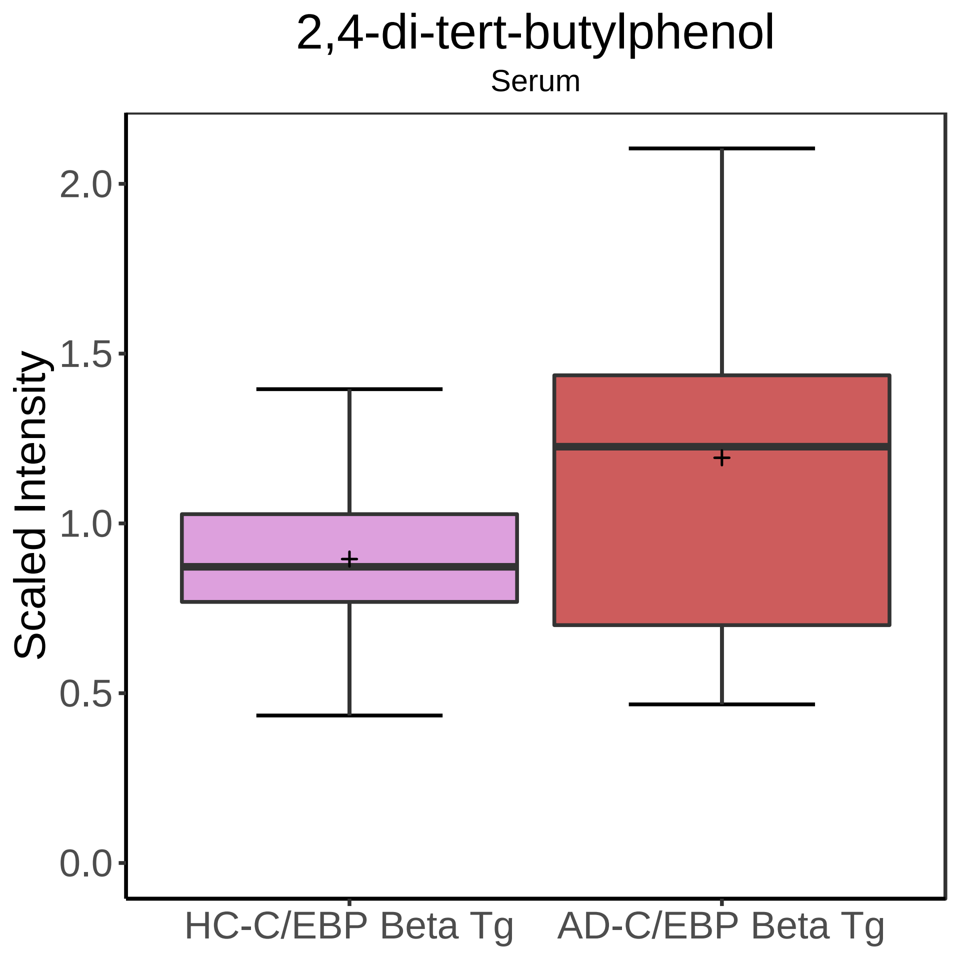
2,4-di-tert-butylphenol
Serum
Scaled Intensity
2.0
1.5
1.0
0.5
0.0
HC-C/EBP Beta Tg
AD-C/EBP Beta Tg

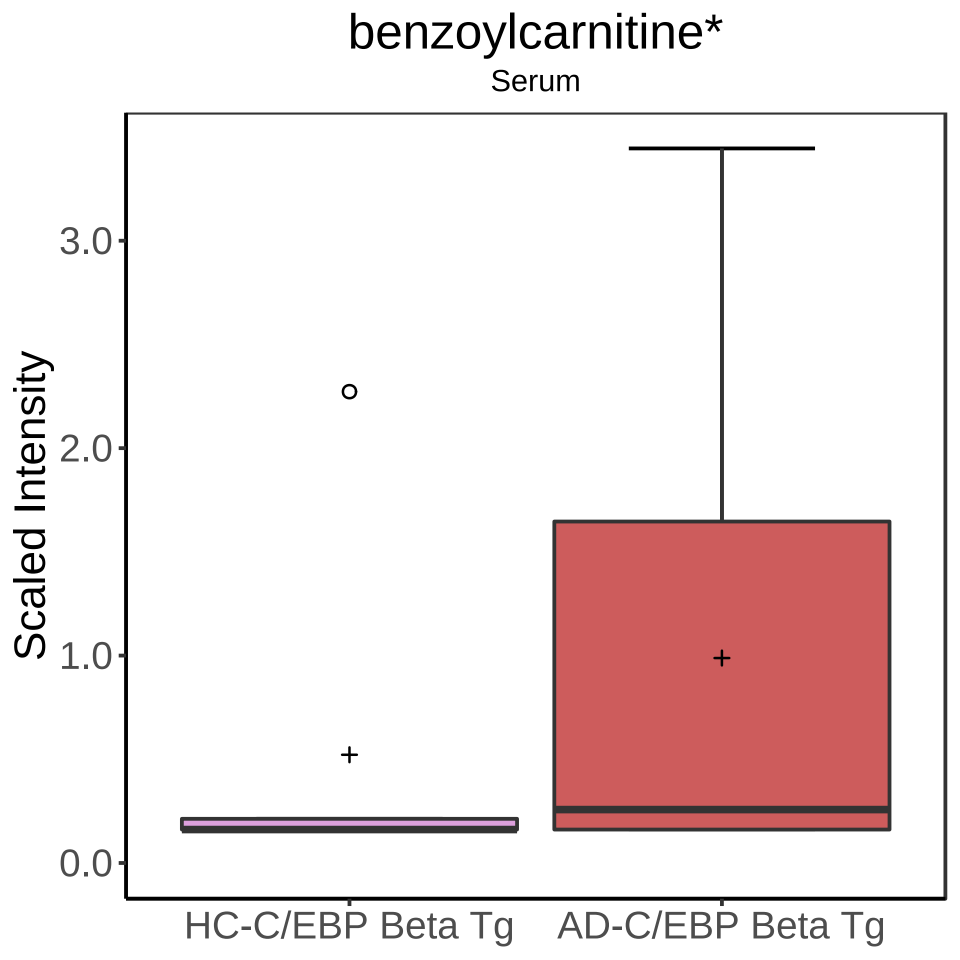

benzoylcarnitine*
Serum
Scaled Intensity
3.0
2.0
1.0
0.0
HC-C/EBP Beta Tg
AD-C/EBP Beta Tg

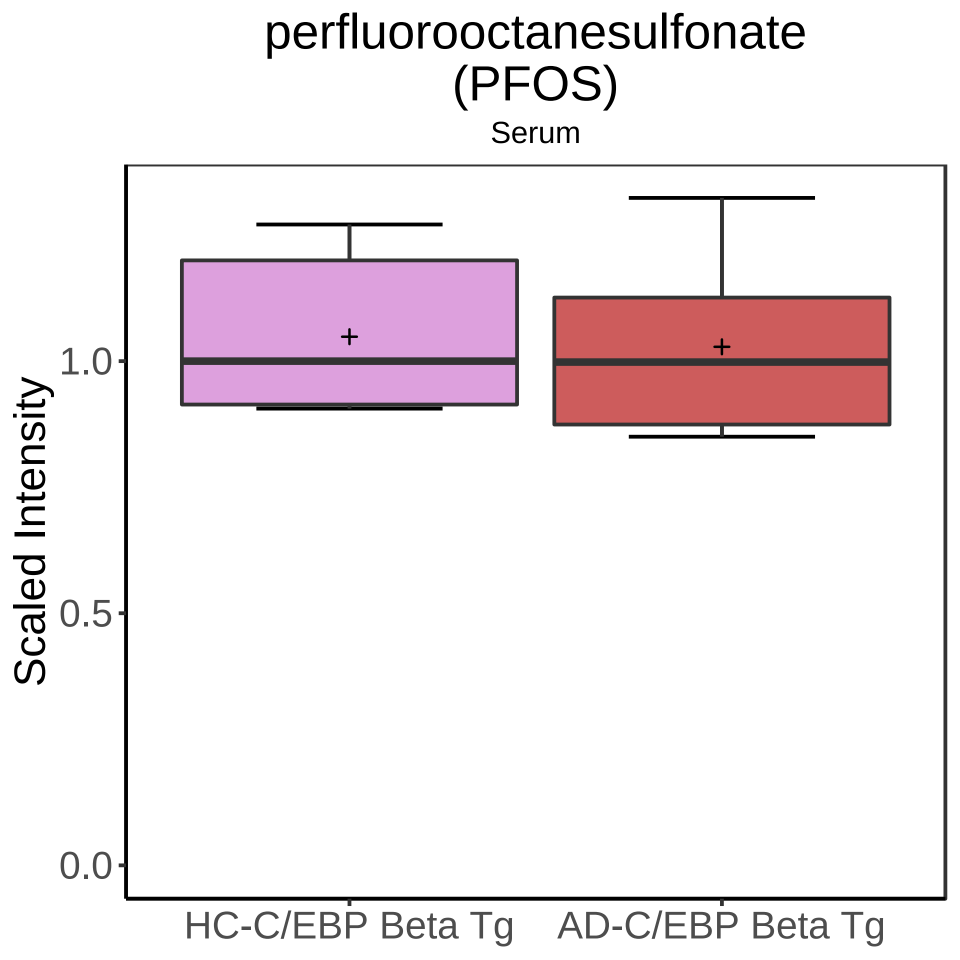
perfluorooctanesulfonate
(PFOS)
Serum
Scaled Intensity
1.0
0.5
0.0
HC-C/EBP Beta Tg
AD-C/EBP Beta Tg

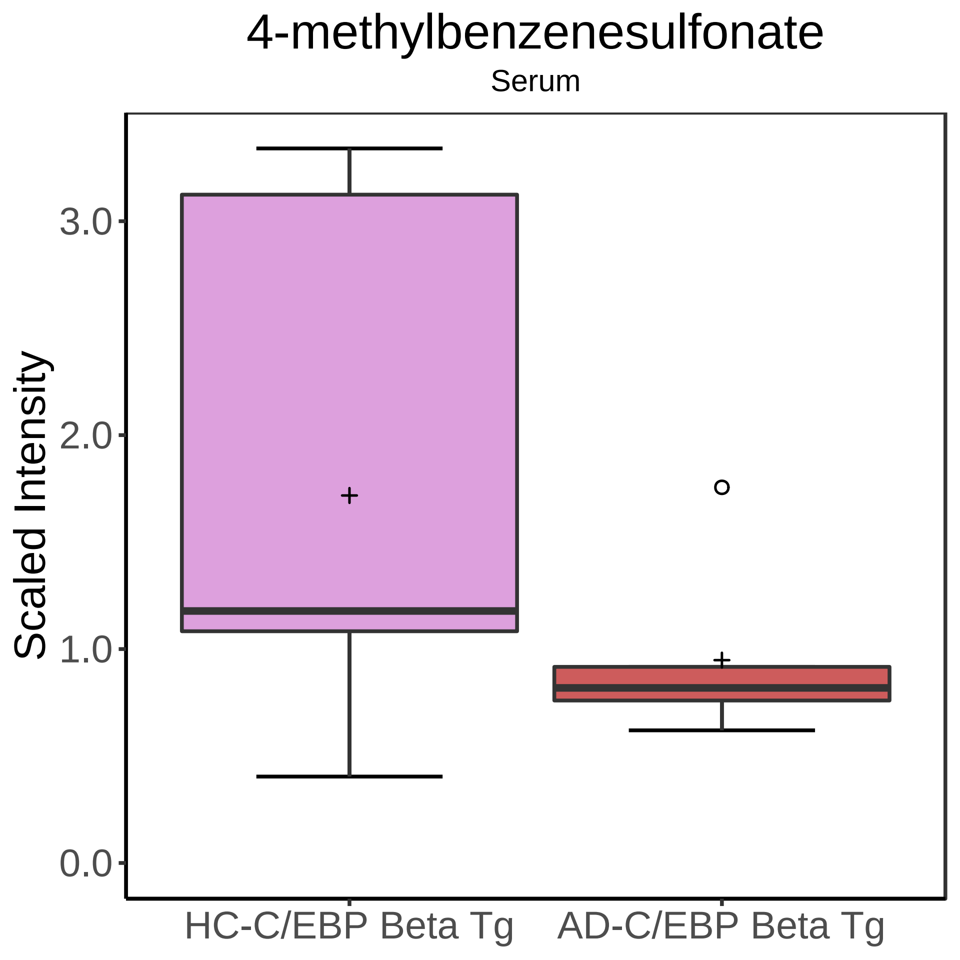
4-methylbenzenesulfonate
Serum
Scaled Intensity
3.0
2.0
1.0
0.0
HC-C/EBP Beta Tg
AD-C/EBP Beta Tg

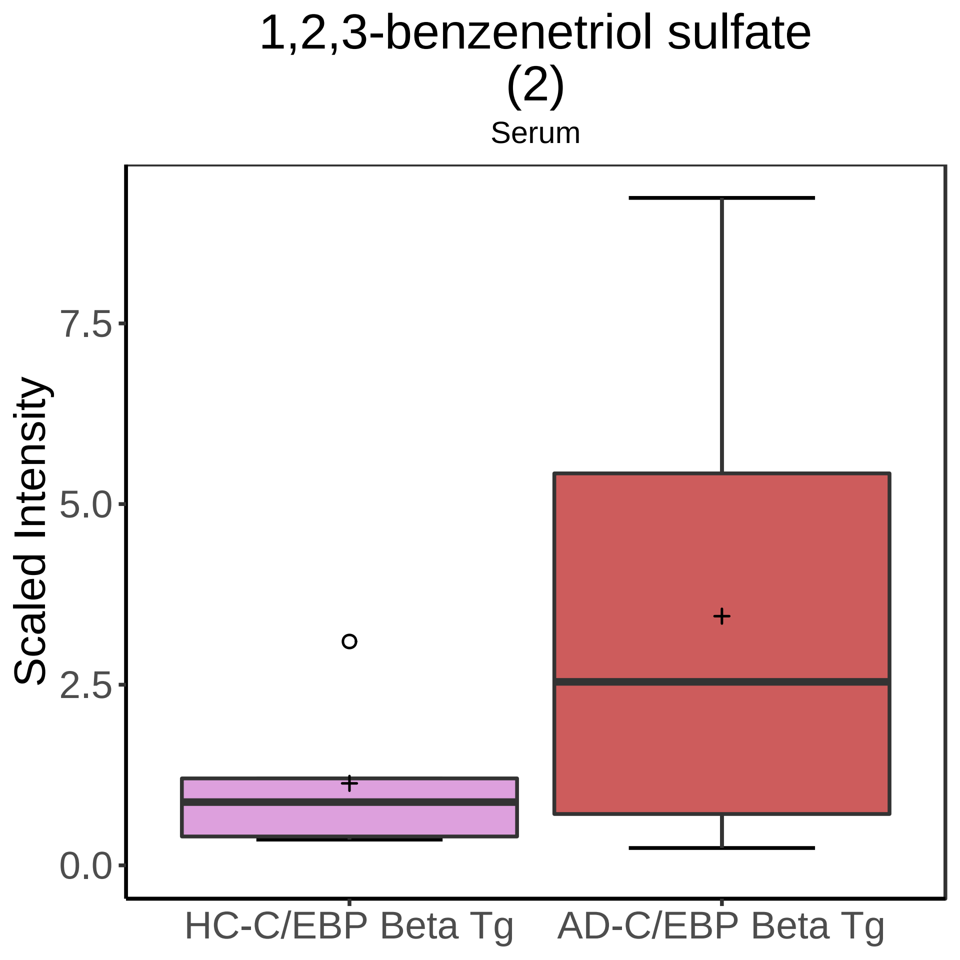
1,2,3-benzenetriol sulfate
(2)
Serum
Scaled Intensity
7.5
5.0
2.5
0.0
HC-C/EBP Beta Tg
AD-C/EBP Beta Tg

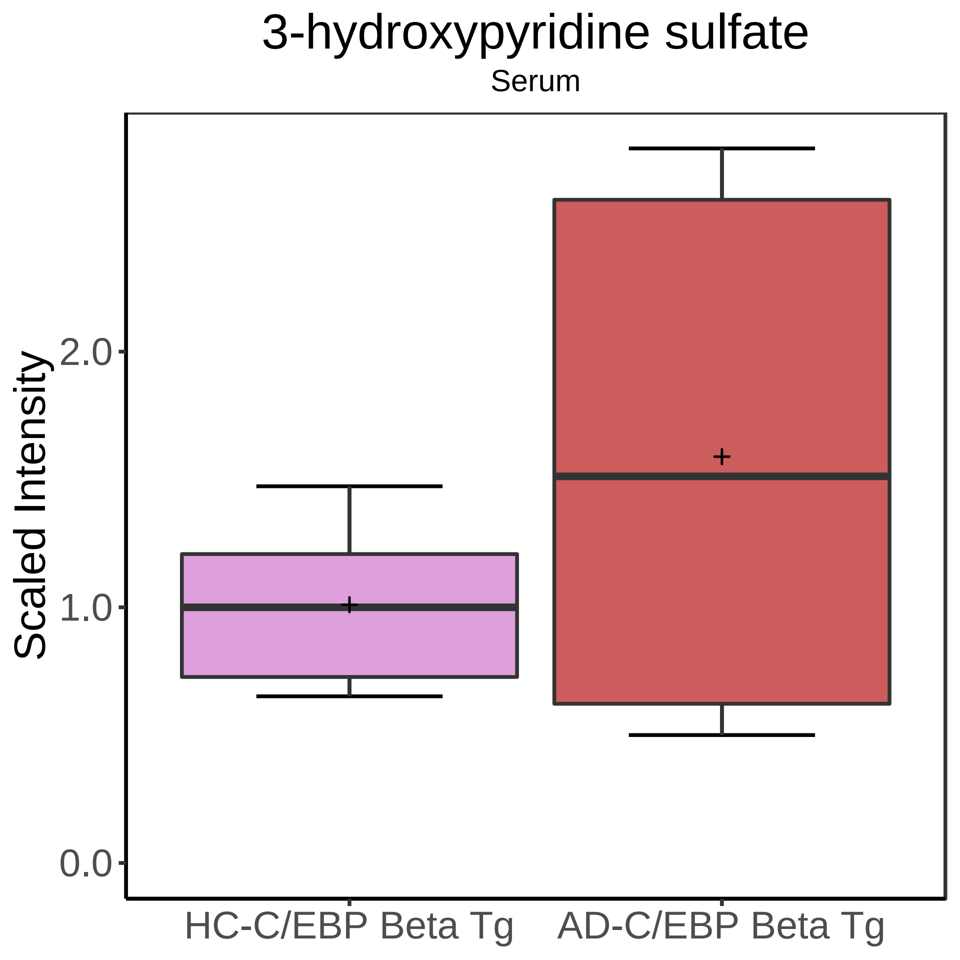
3-hydroxypyridine sulfate
Serum
Scaled Intensity
2.0
1.0
0.0
HC-C/EBP Beta Tg
AD-C/EBP Beta Tg

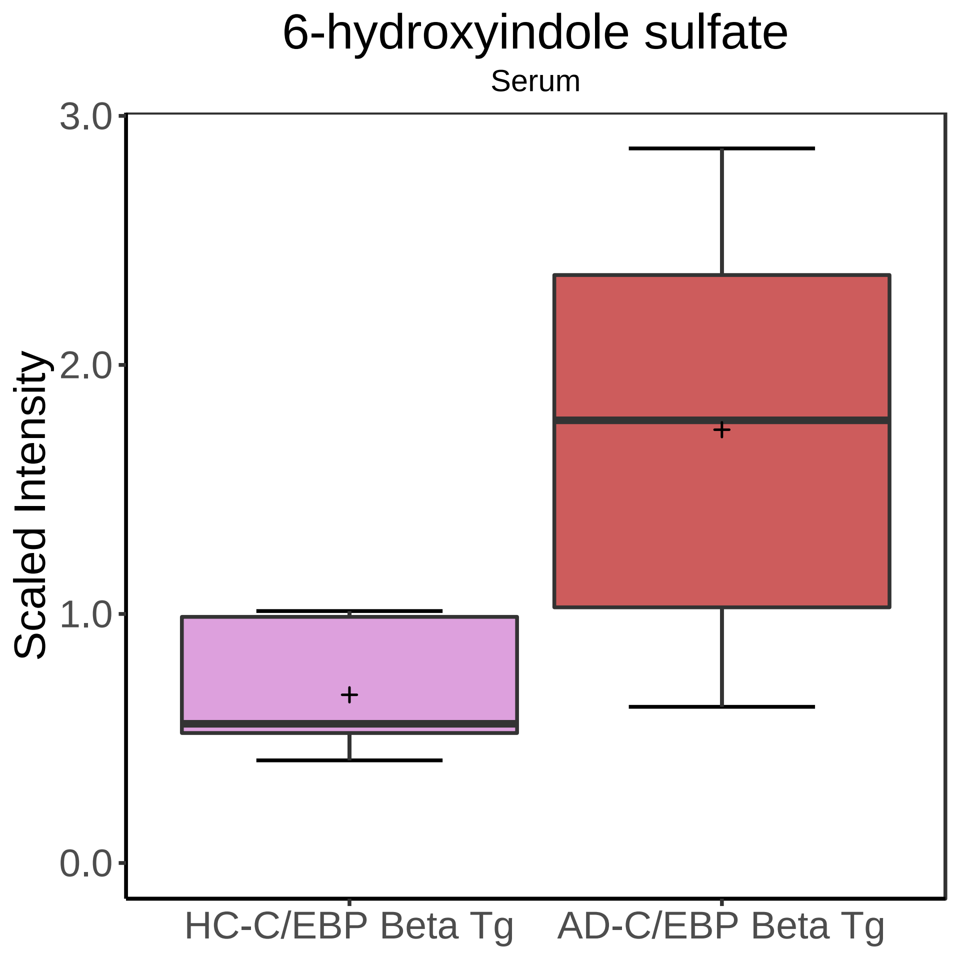
6-hydroxyindole sulfate
Serum
Scaled Intensity
3.0
2.0
1.0
0.0
HC-C/EBP Beta Tg
AD-C/EBP Beta Tg

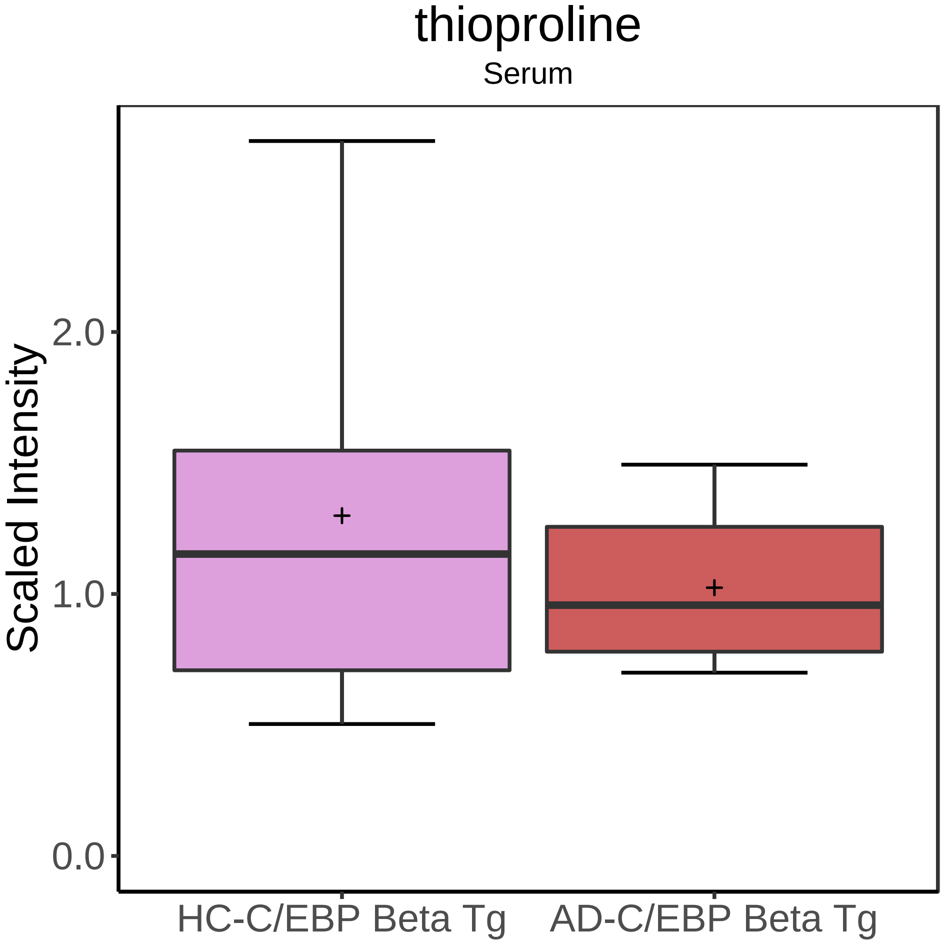
thioproline
Serum
Scaled Intensity
2.0
1.0
0.0
HC-C/EBP Beta Tg
AD-C/EBP Beta Tg

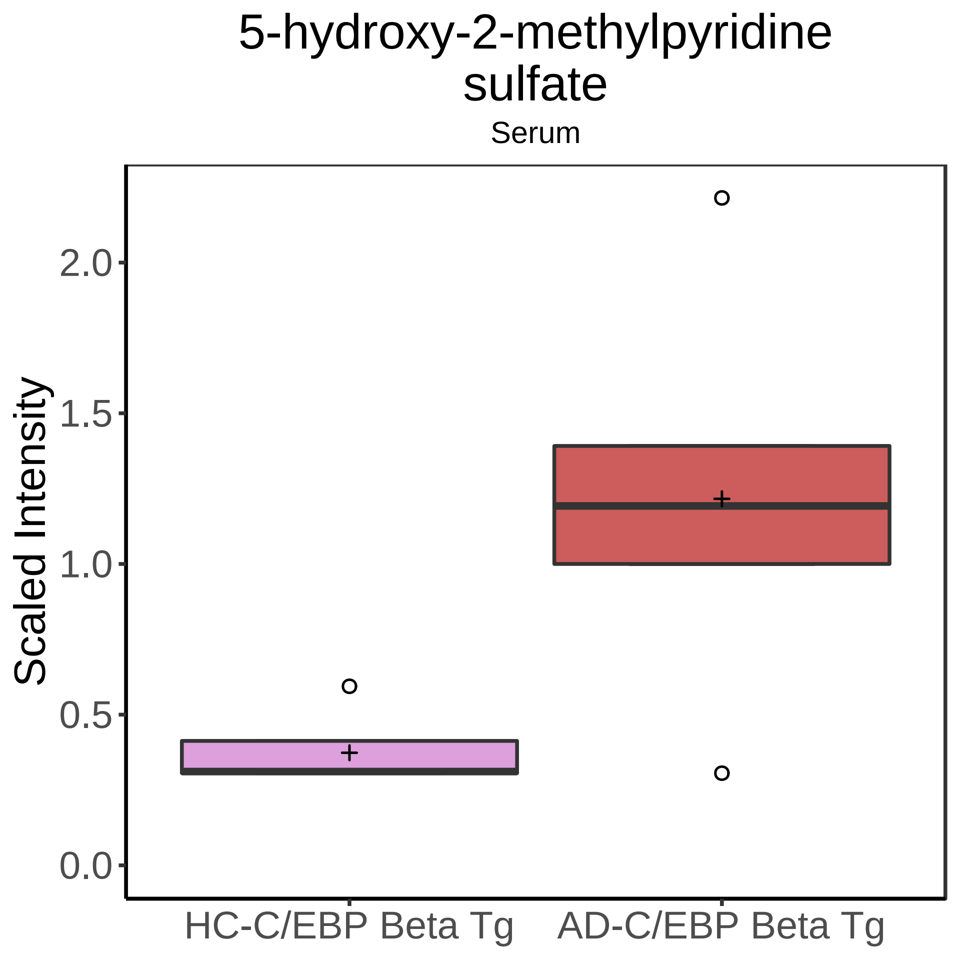
5-hydroxy-2-methylpyridine sulfate
Serum
Scaled Intensity
2.0
1.5
1.0
0.5
0.0
HC-C/EBP Beta Tg
AD-C/EBP Beta Tg

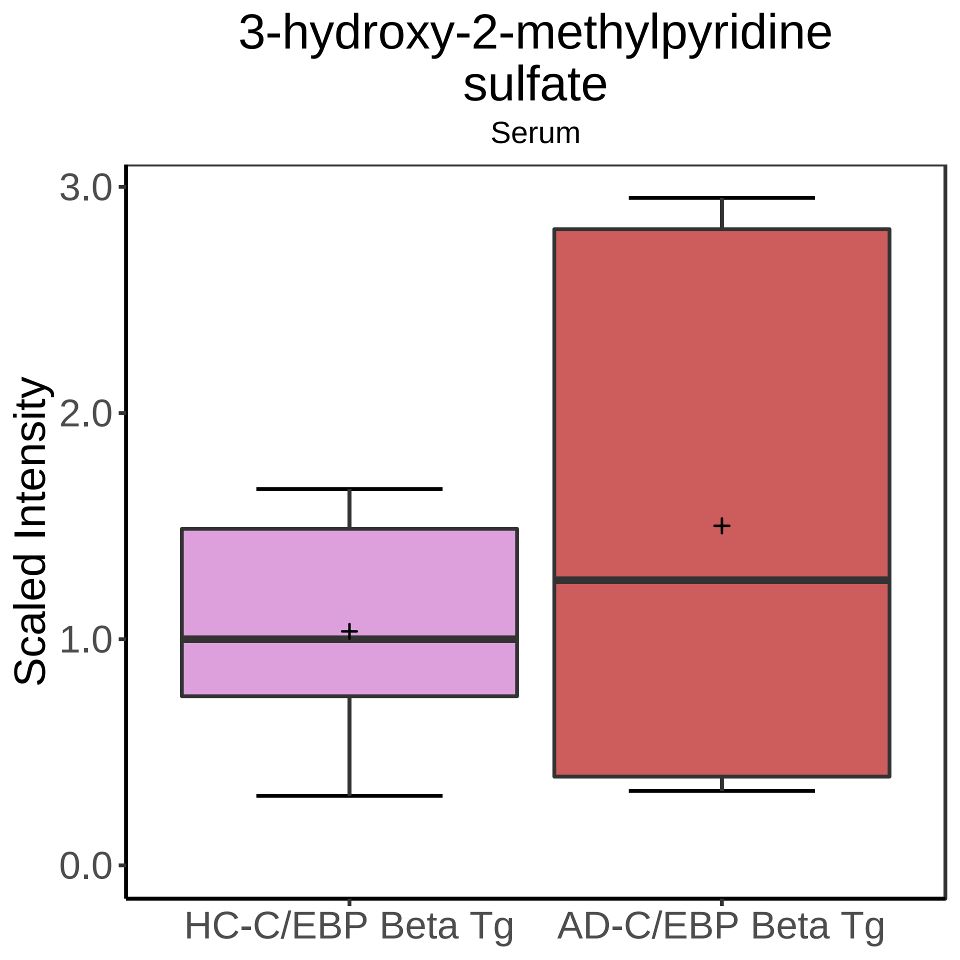
3-hydroxy-2-methylpyridine sulfate
Serum
Scaled Intensity
3.0
2.0
1.0
0.0
HC-C/EBP Beta Tg
AD-C/EBP Beta Tg

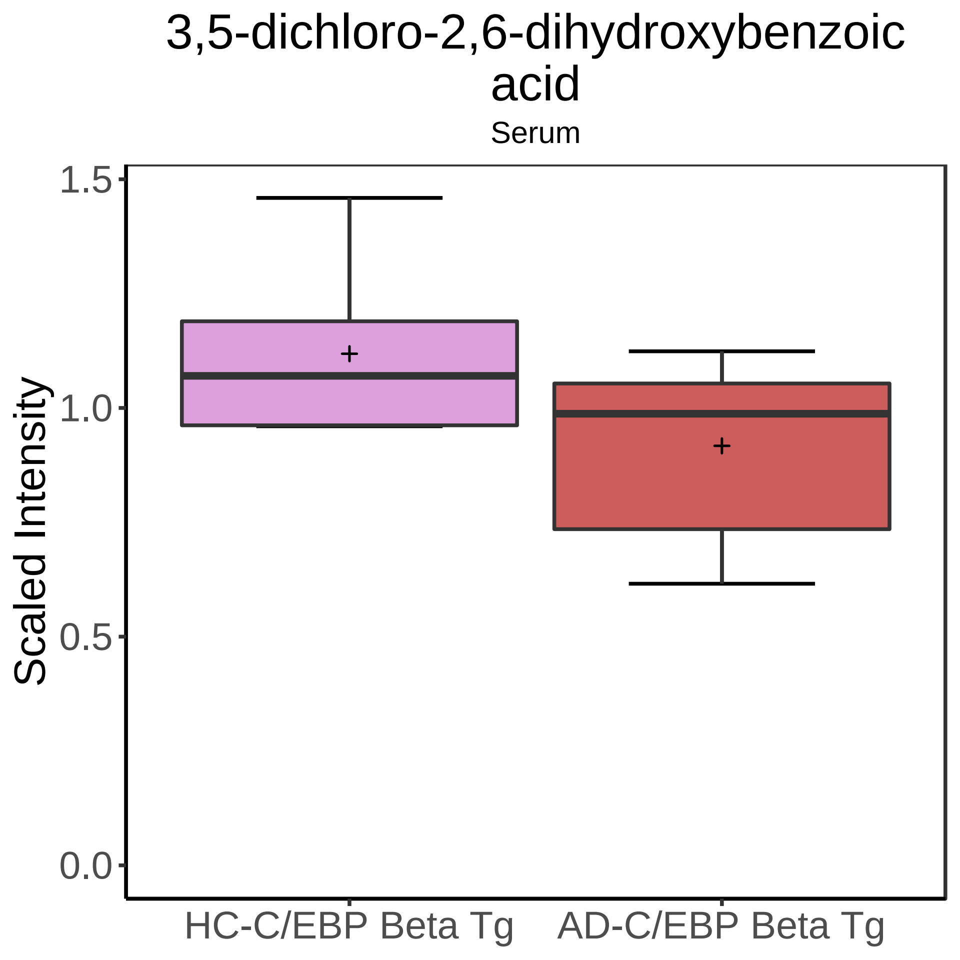
3,5-dichloro-2,6-dihydroxybenzoic acid
Serum
Scaled Intensity
1.5
1.0
0.5
0.0
HC-C/EBP Beta Tg
AD-C/EBP Beta Tg

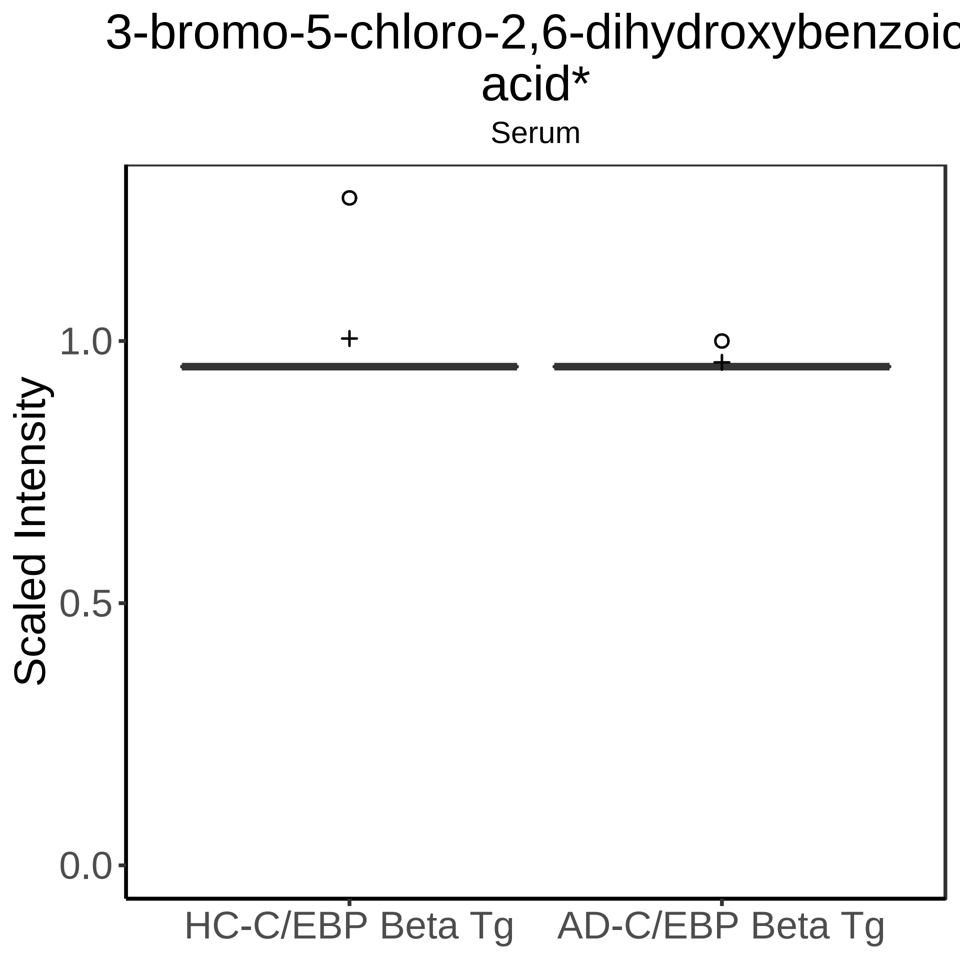

3-bromo-5-chloro-2,6-dihydroxybenzoic
acid*
Serum
Scaled Intensity
1.0
0.5
0.0
HC-C/EBP Beta Tg
AD-C/EBP Beta Tg

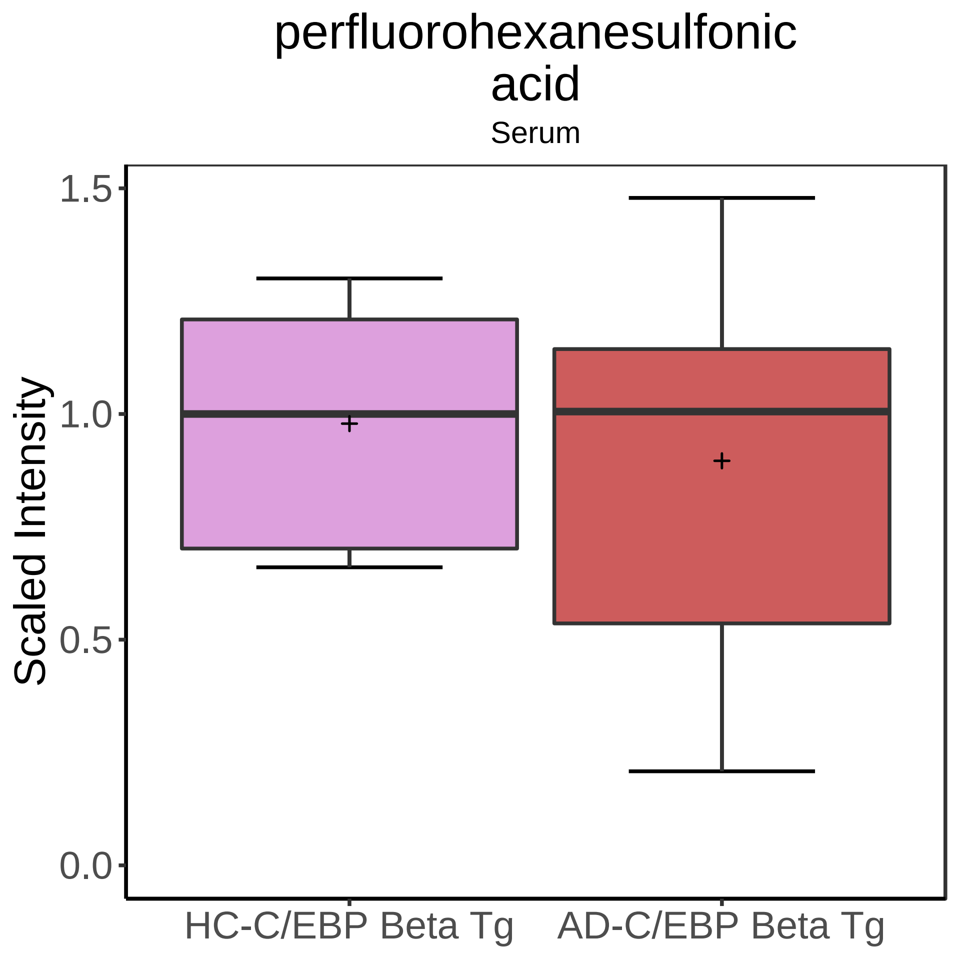
perfluorohexanesulfonic acid
Serum
Scaled Intensity
1.5
1.0
0.5
0.0
HC-C/EBP Beta Tg
AD-C/EBP Beta Tg

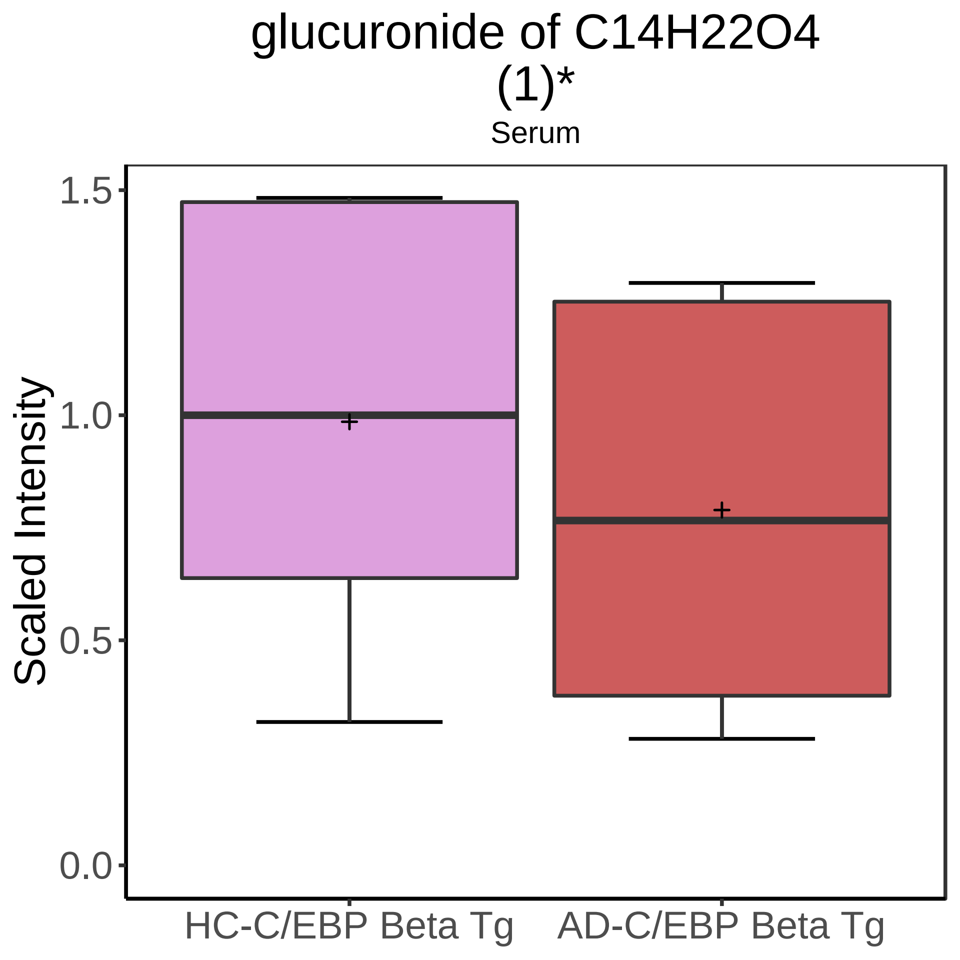
glucuronide of C14H22O4
(1)*
Serum
Scaled Intensity
1.5
1.0
0.5
0.0
HC-C/EBP Beta Tg
AD-C/EBP Beta Tg

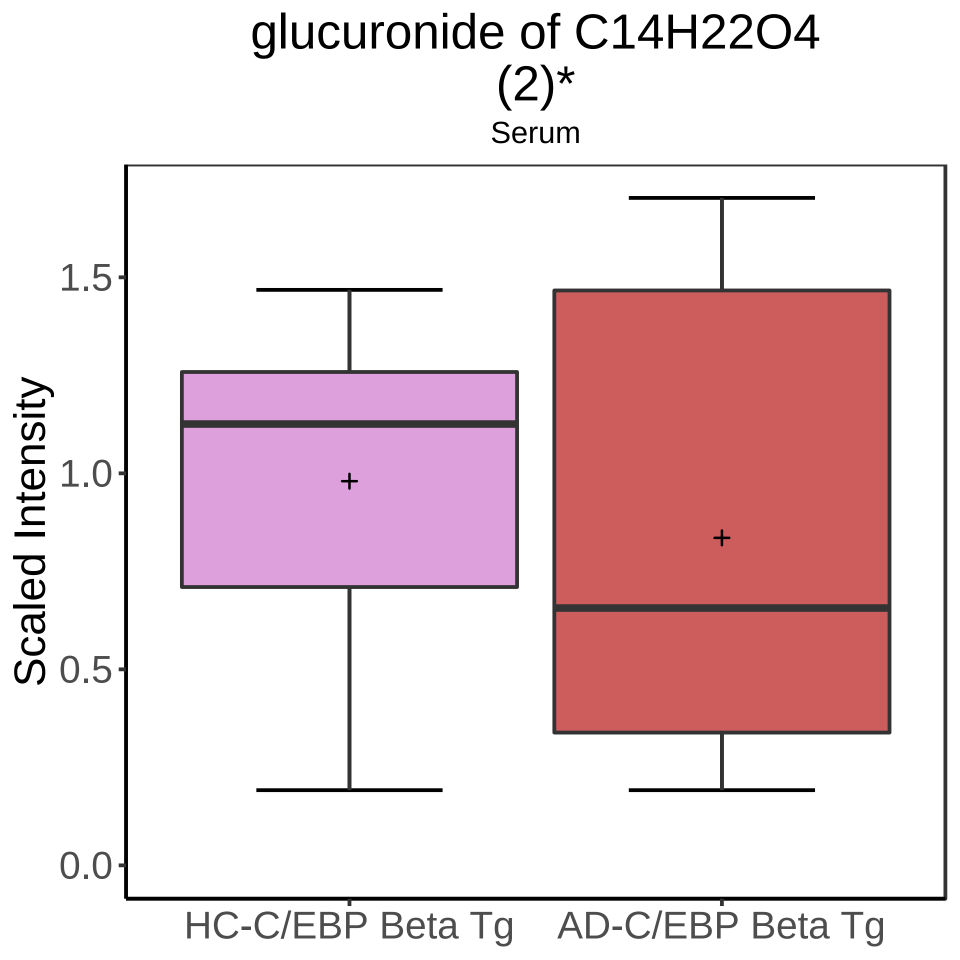
glucuronide of C14H22O4
(2)*
Serum
Scaled Intensity
1.5
1.0
0.5
0.0
HC-C/EBP Beta Tg
AD-C/EBP Beta Tg

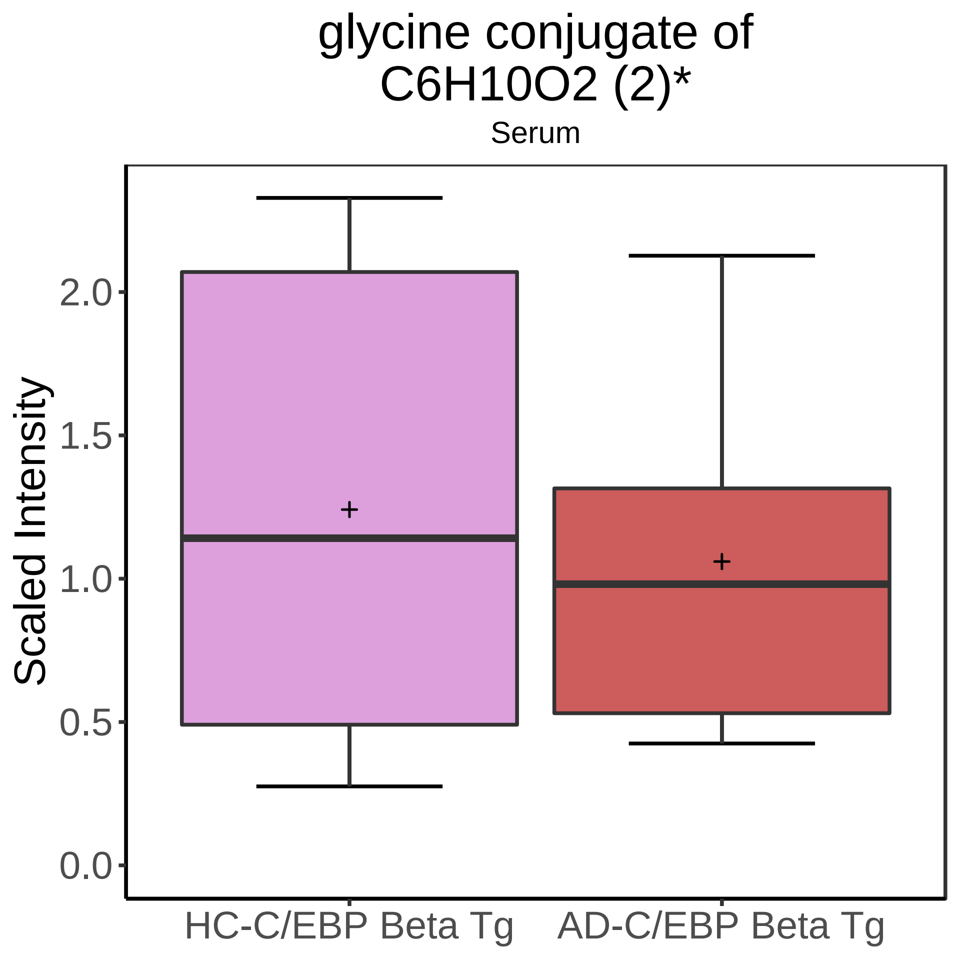
glycine conjugate of
C6H10O2 (2)*
Serum
Scaled Intensity
2.0
1.5
1.0
0.5
0.0
HC-C/EBP Beta Tg
AD-C/EBP Beta Tg

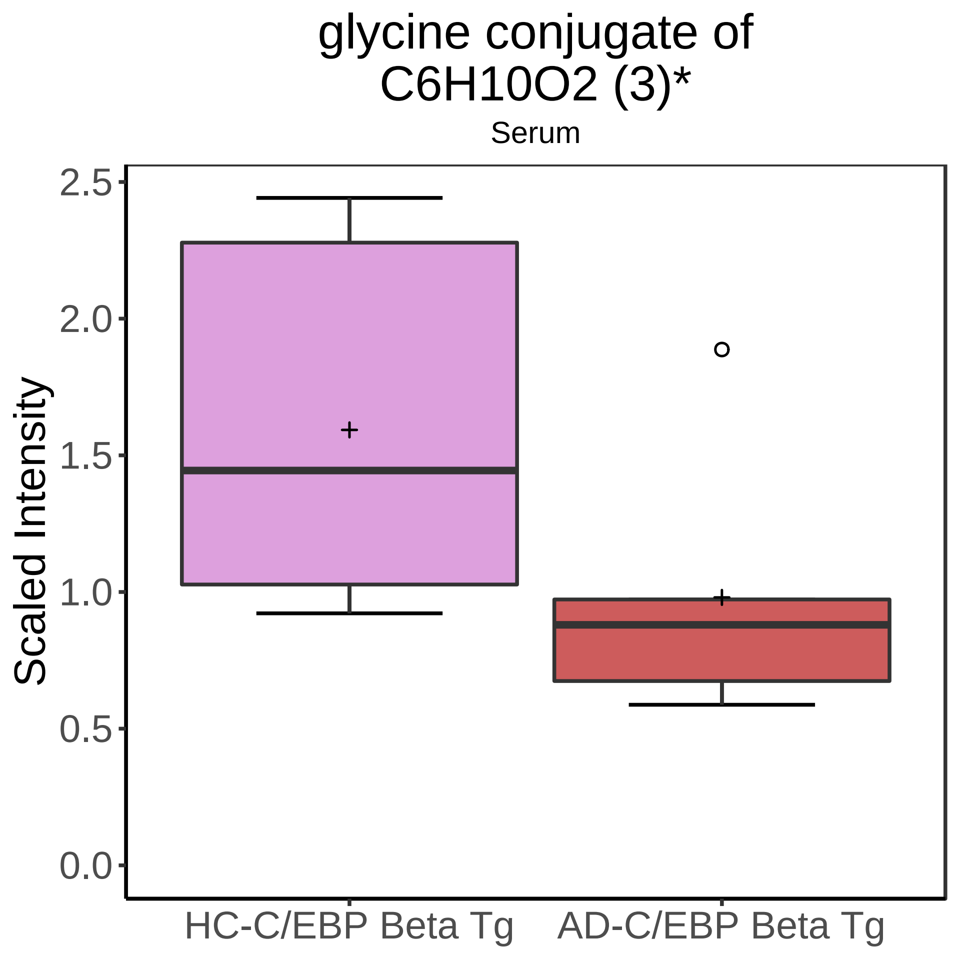

glycine conjugate of
C6H10O2 (3)*
Serum
Scaled Intensity
2.5
2.0
1.5
1.0
0.5
0.0
HC-C/EBP Beta Tg
AD-C/EBP Beta Tg

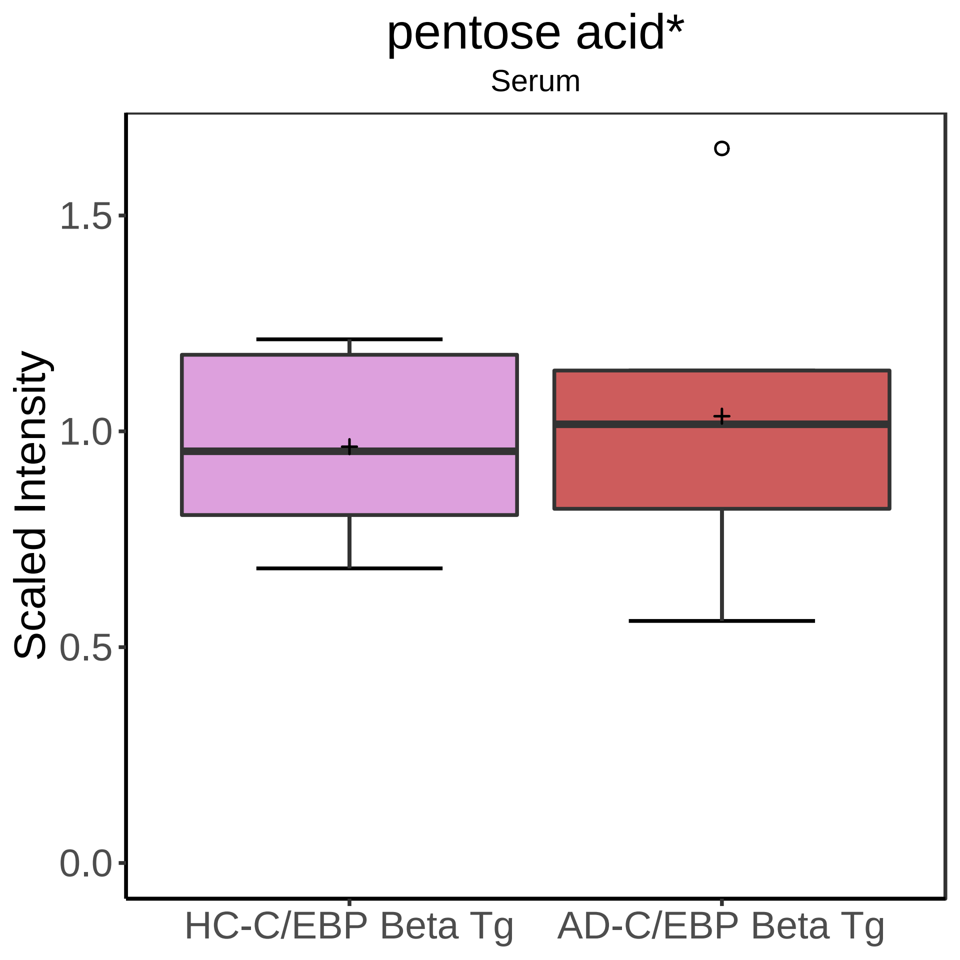

pentose acid*
Serum
Scaled Intensity
1.5
1.0
0.5
0.0
HC-C/EBP Beta Tg
AD-C/EBP Beta Tg

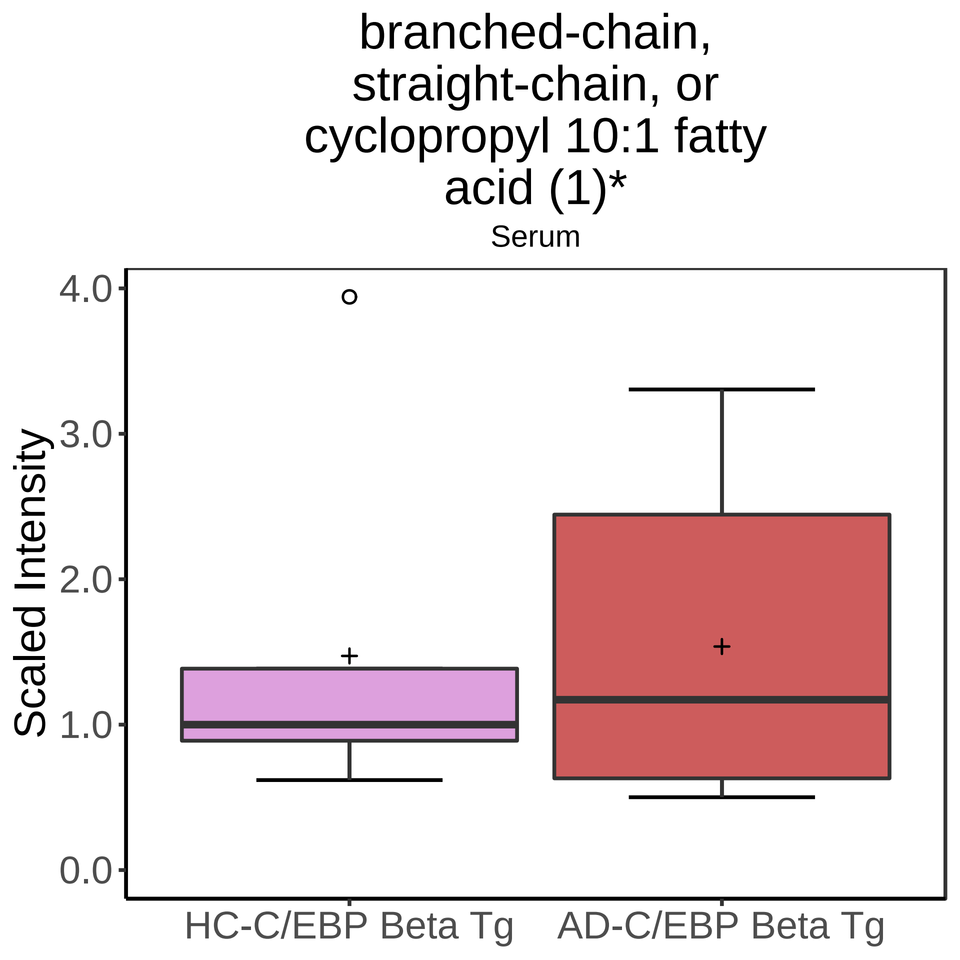
branched-chain,
straight-chain, or
cyclopropyl 10:1 fatty
acid (1)*
Serum
Scaled Intensity
4.0
3.0
2.0
1.0
0.0
HC-C/EBP Beta Tg
AD-C/EBP Beta Tg

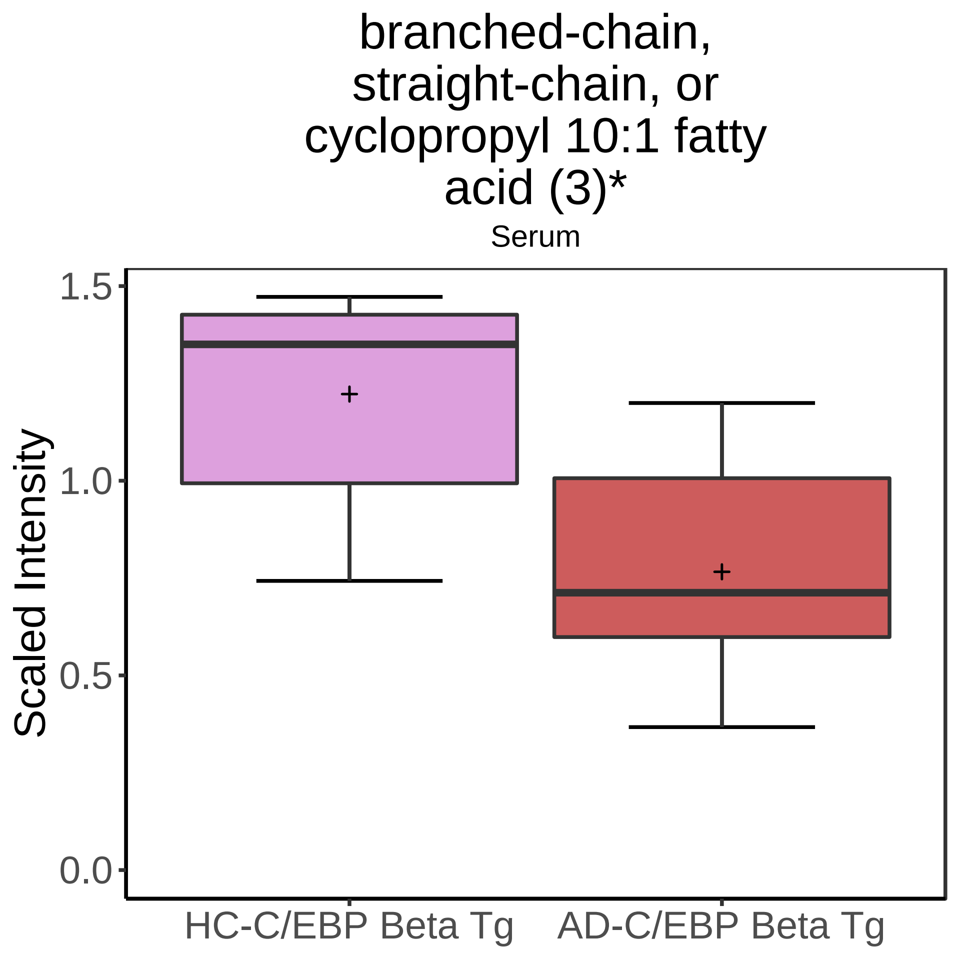
branched-chain,
straight-chain, or
cyclopropyl 10:1 fatty
acid (3)*
Serum
Scaled Intensity
1.5
1.0
0.5
0.0
HC-C/EBP Beta Tg
AD-C/EBP Beta Tg

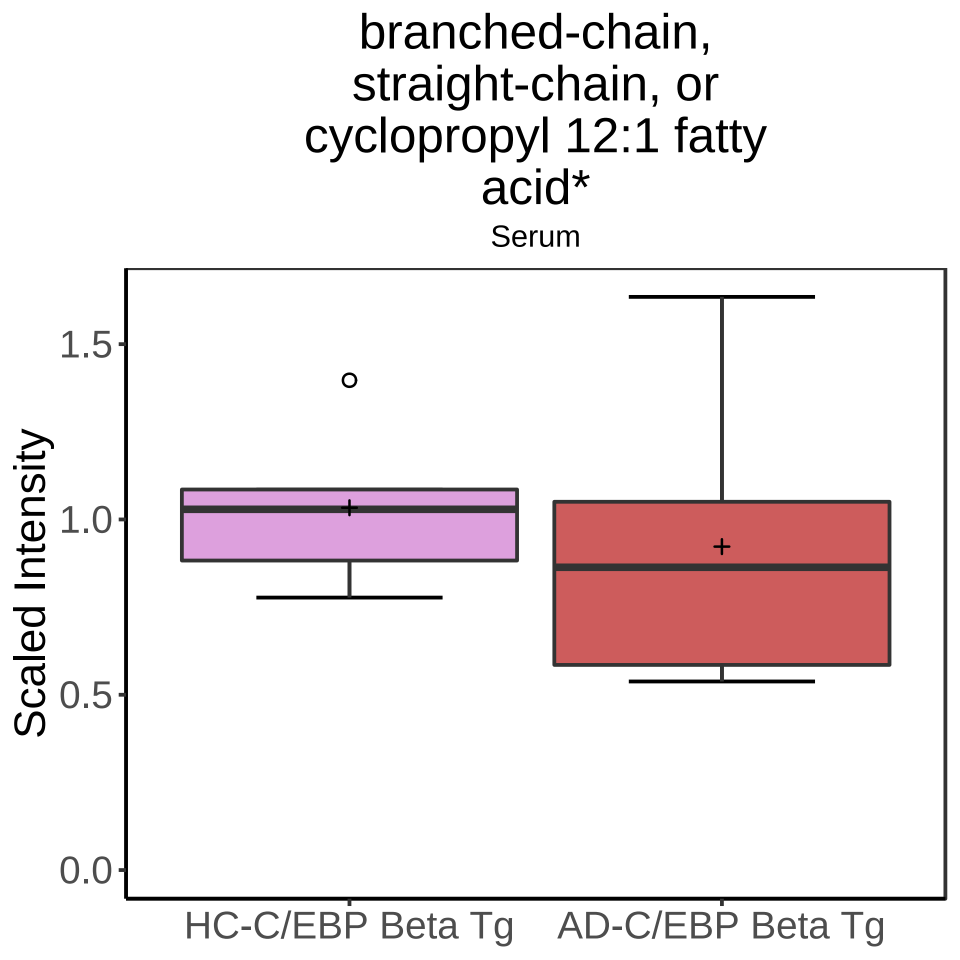
branched-chain, straight-chain, or cyclopropyl 12:1 fatty acid*
Serum
Scaled Intensity
1.5
1.0
0.5
0.0
HC-C/EBP Beta Tg
AD-C/EBP Beta Tg

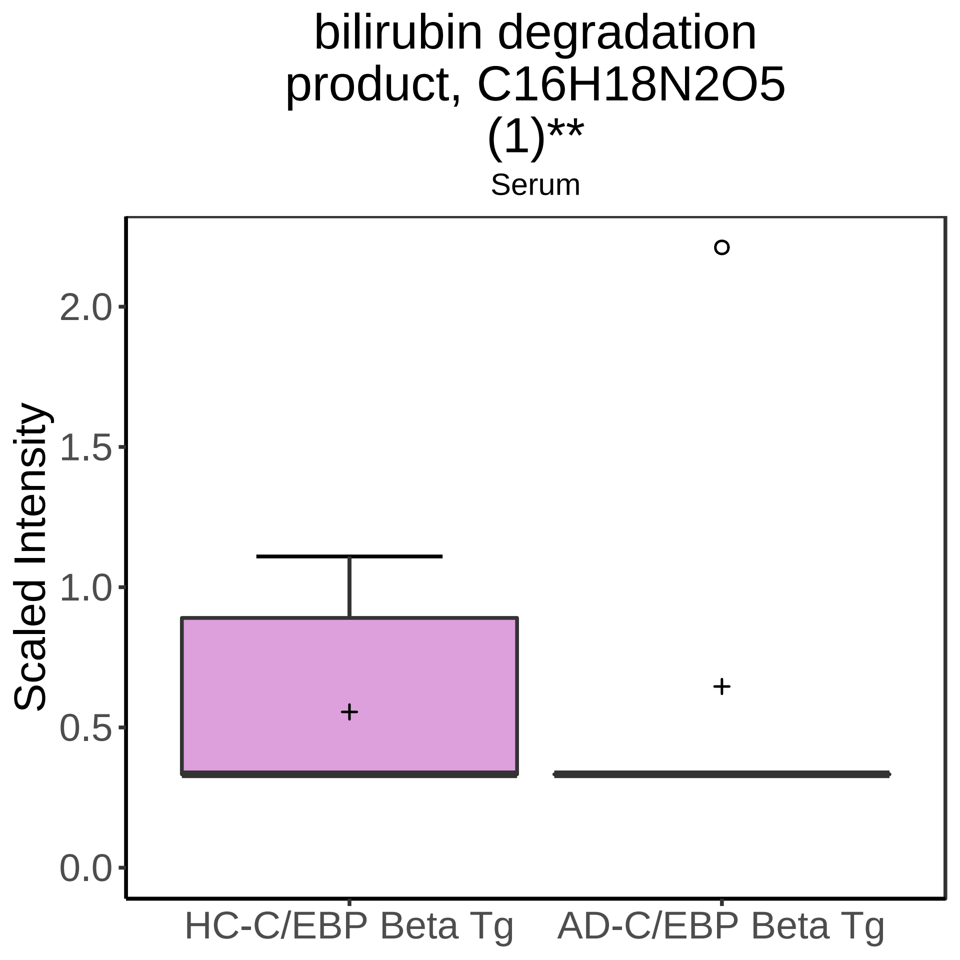
bilirubin degradation product, C16H18N2O5 (1)**
Serum
Scaled Intensity
2.0
1.5
1.0
0.5
0.0
HC-C/EBP Beta Tg
AD-C/EBP Beta Tg

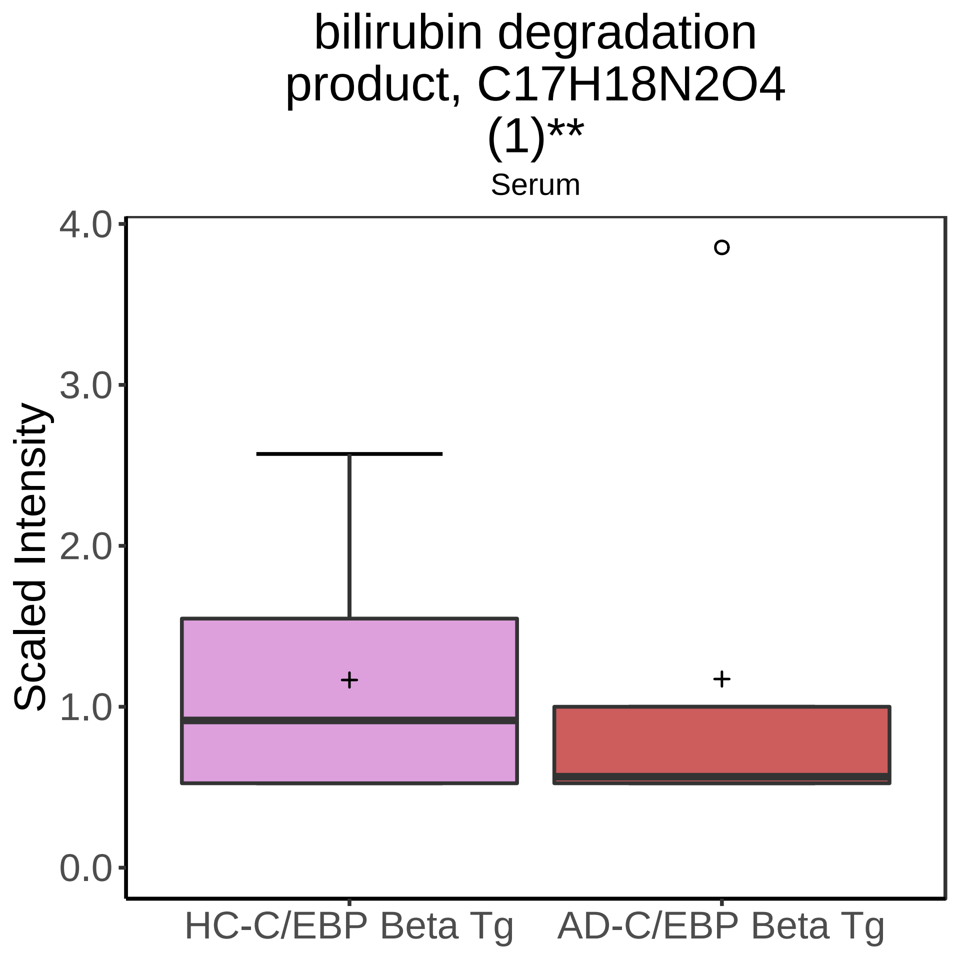

bilirubin degradation product, C17H18N2O4 (1)**
Serum
Scaled Intensity
4.0
3.0
2.0
1.0
0.0
HC-C/EBP Beta Tg
AD-C/EBP Beta Tg

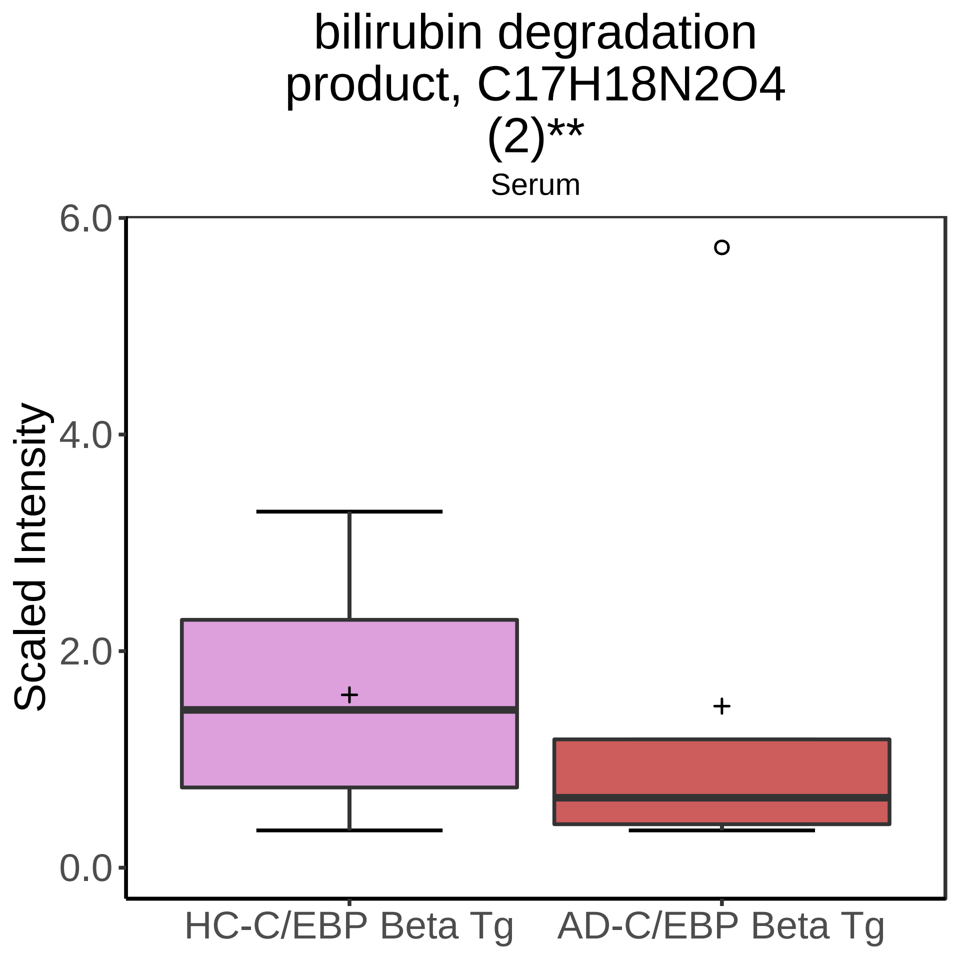

bilirubin degradation product, C17H18N2O4 (2)**
Serum
Scaled Intensity
6.0
4.0
2.0
0.0
HC-C/EBP Beta Tg
AD-C/EBP Beta Tg

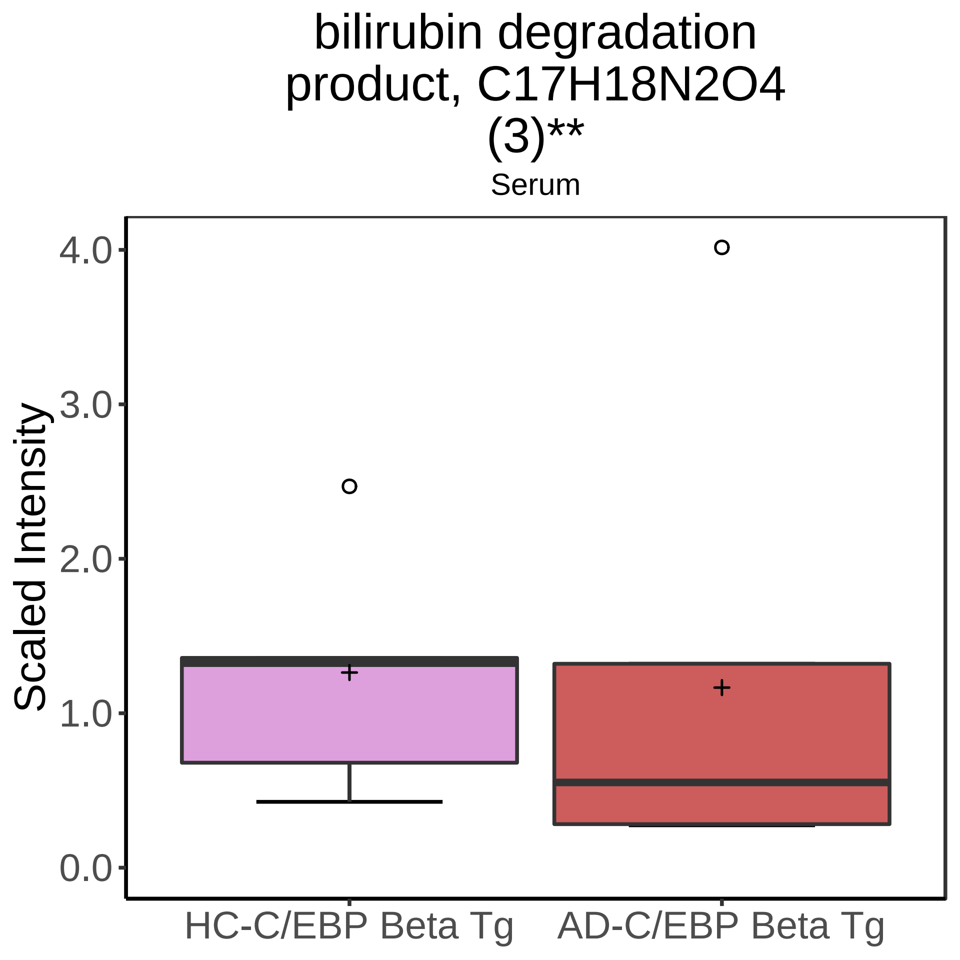
bilirubin degradation product, C17H18N2O4 (3)**
Serum
Scaled Intensity
4.0
3.0
2.0
1.0
0.0
HC-C/EBP Beta Tg
AD-C/EBP Beta Tg

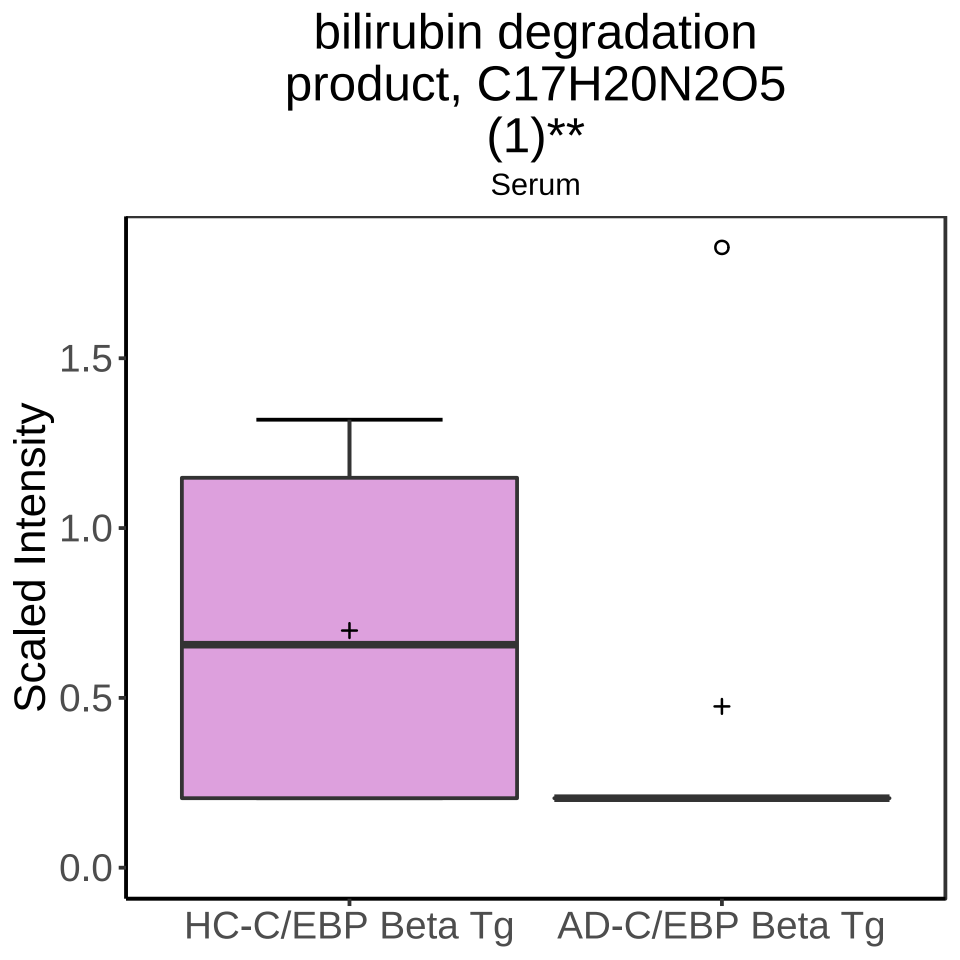
bilirubin degradation product, C17H20N2O5 (1)**
Serum
Scaled Intensity
0.0
0.5
1.0
1.5
HC-C/EBP Beta Tg
AD-C/EBP Beta Tg

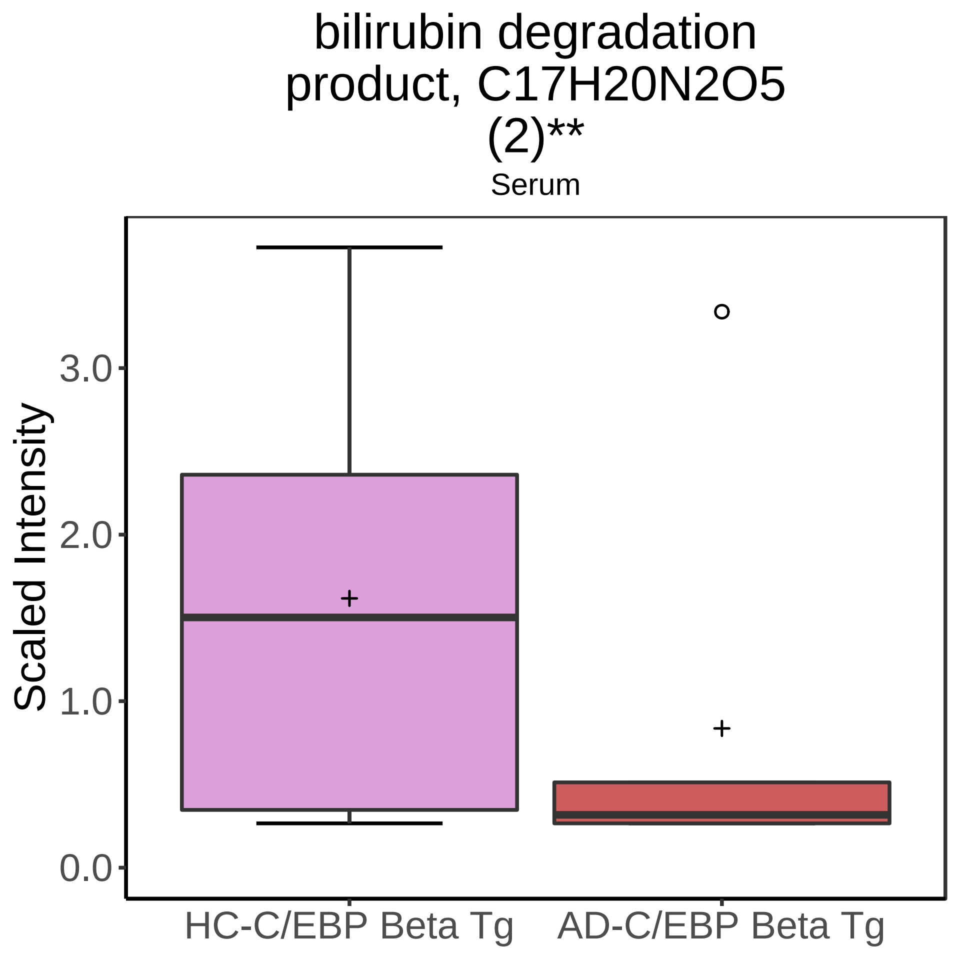

bilirubin degradation product, C17H20N2O5 (2)**
Serum
Scaled Intensity
3.0
2.0
1.0
0.0
HC-C/EBP Beta Tg
AD-C/EBP Beta Tg

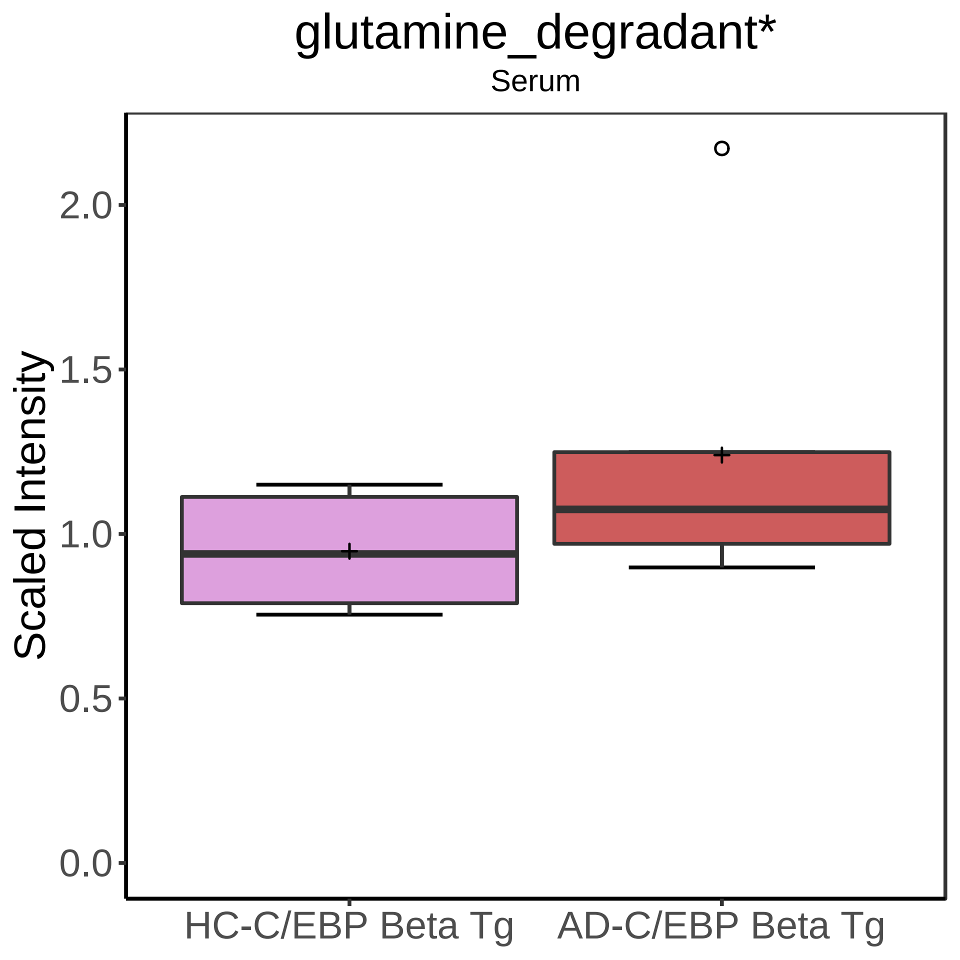

glutamine_degradant*
Serum
Scaled Intensity
2.0
1.5
1.0
0.5
0.0
HC-C/EBP Beta Tg
AD-C/EBP Beta Tg

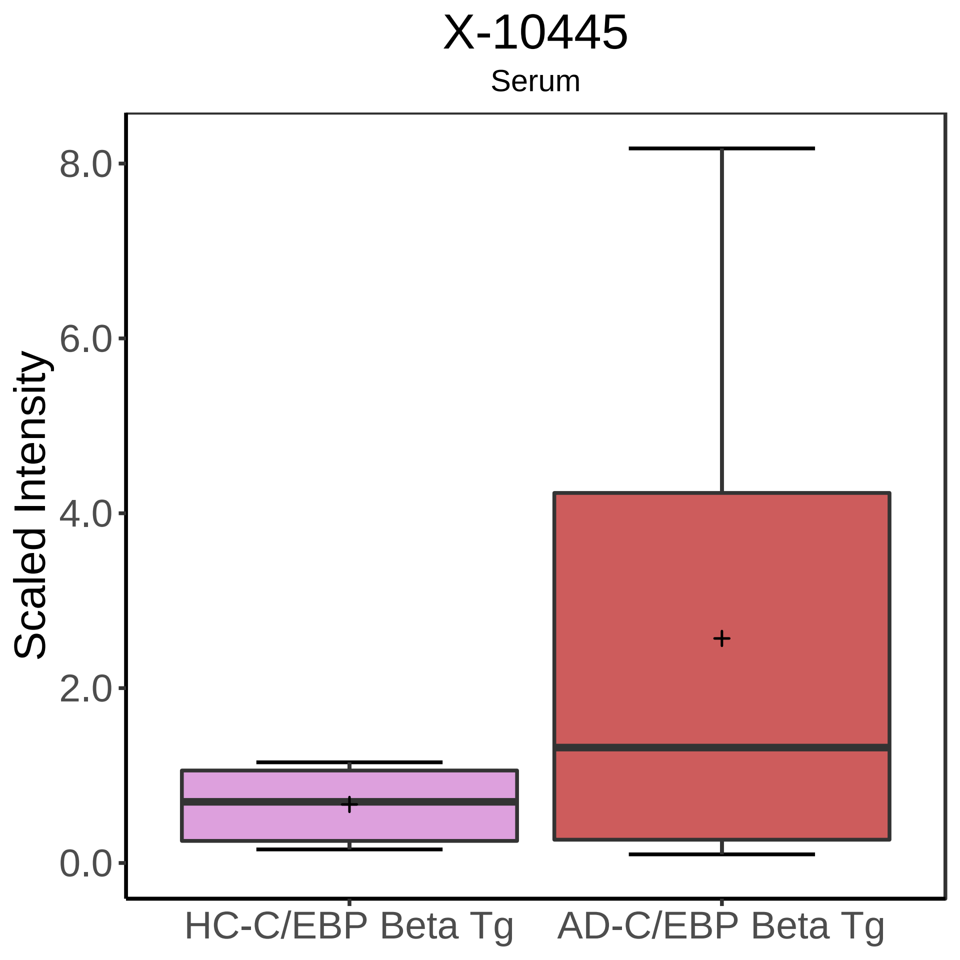
X-10445
Serum
Scaled Intensity
8.0
6.0
4.0
2.0
0.0
HC-C/EBP Beta Tg
AD-C/EBP Beta Tg

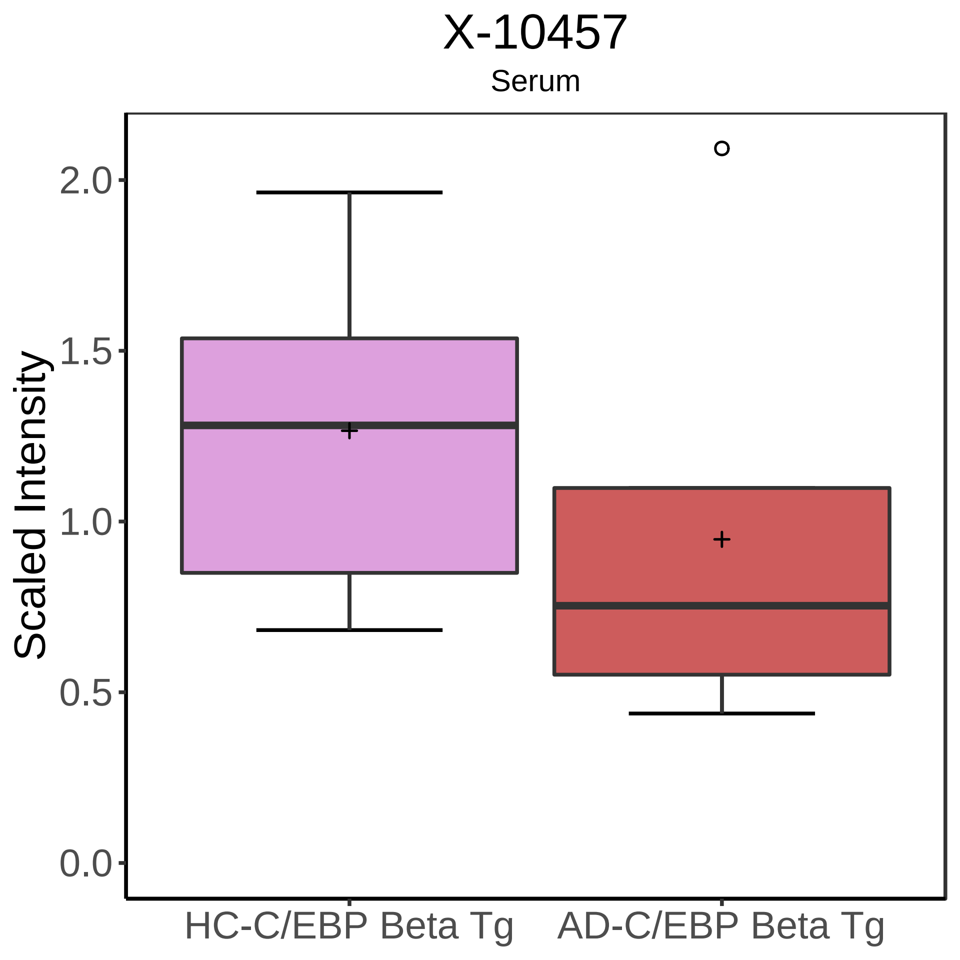
X-10457
Serum
Scaled Intensity
2.0
1.5
1.0
0.5
0.0
HC-C/EBP Beta Tg
AD-C/EBP Beta Tg

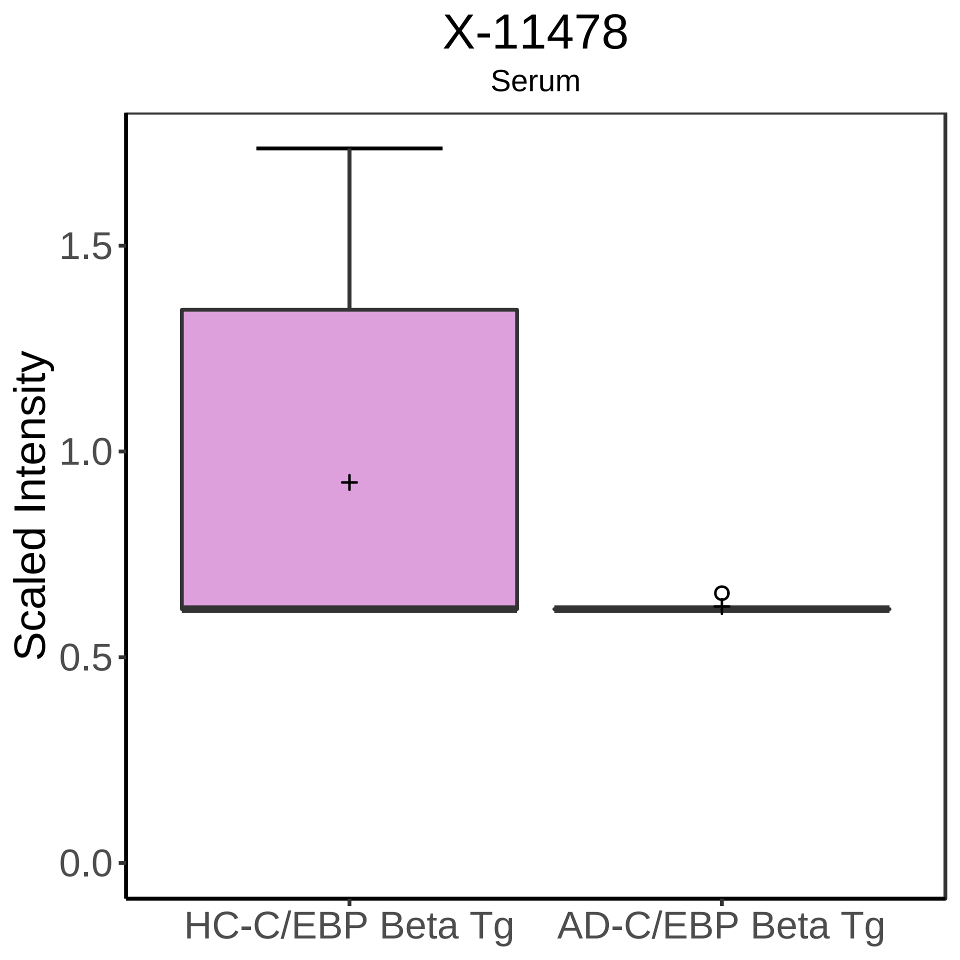
X-11478
Serum
Scaled Intensity
1.5
1.0
0.5
0.0
HC-C/EBP Beta Tg
AD-C/EBP Beta Tg

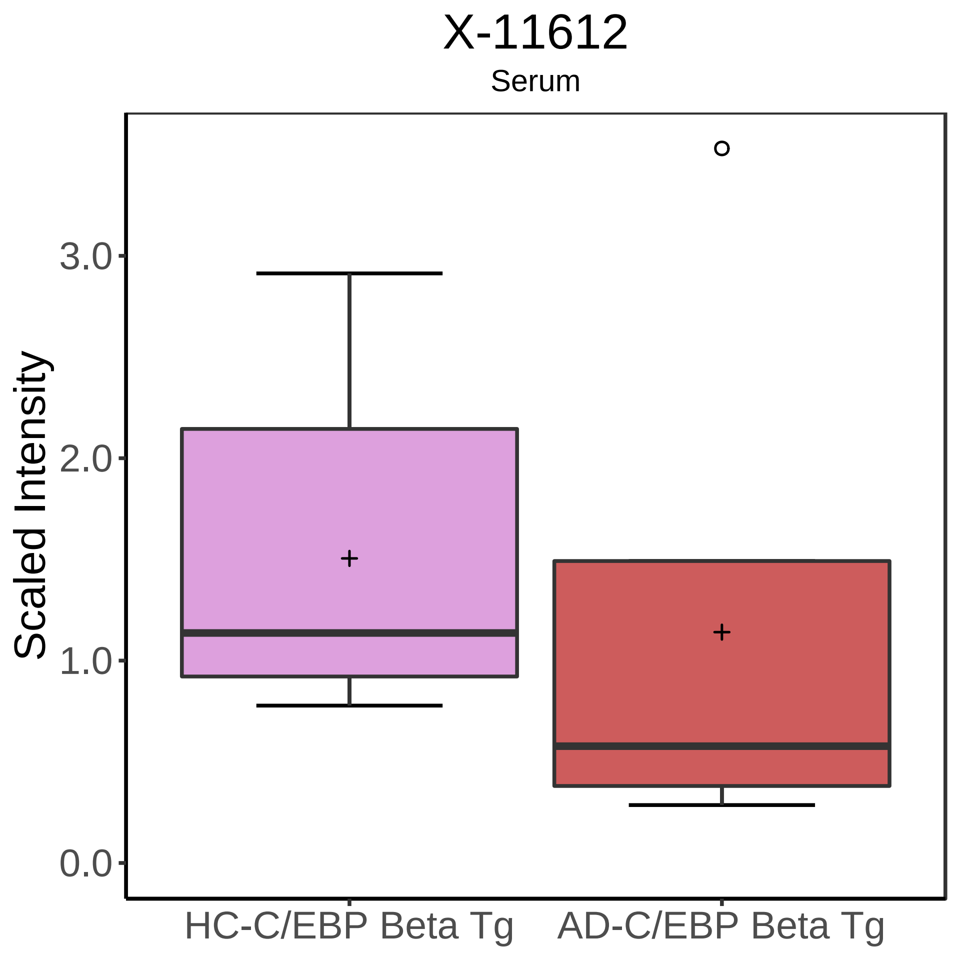
X-11612
Serum
Scaled Intensity
3.0
2.0
1.0
0.0
HC-C/EBP Beta Tg
AD-C/EBP Beta Tg

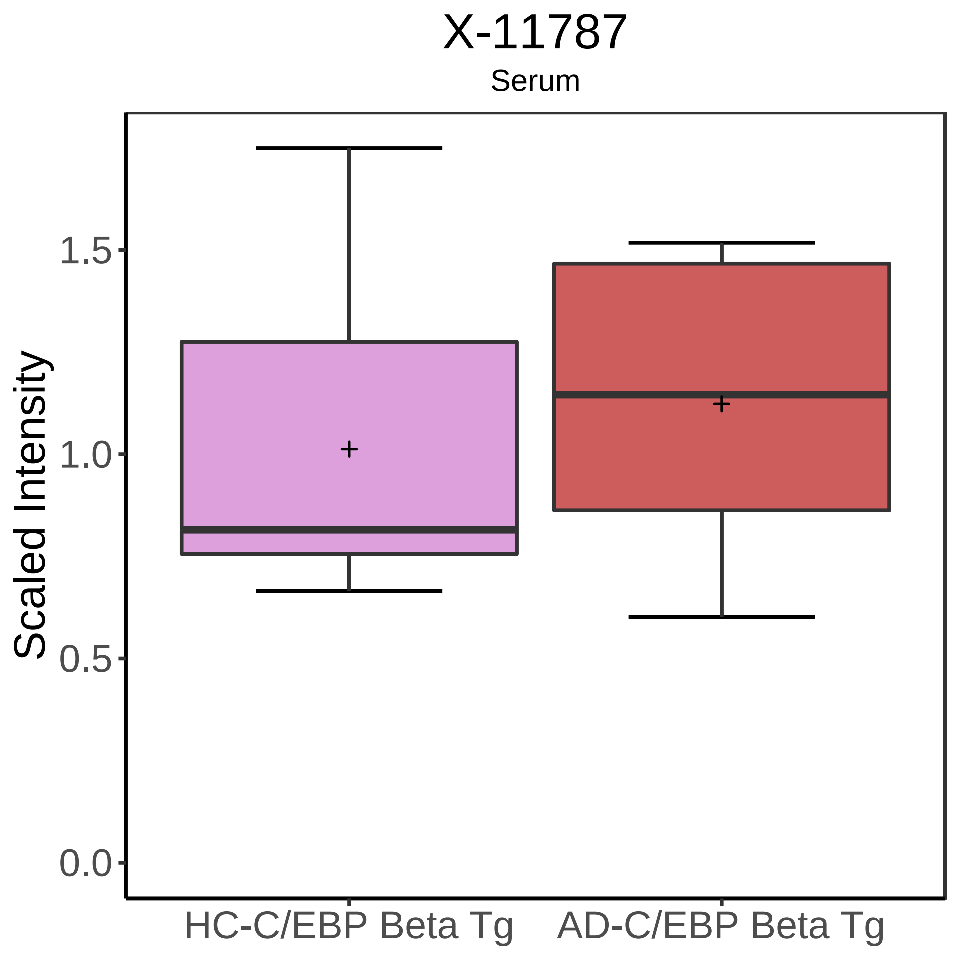
X-11787
Serum
Scaled Intensity
1.5
1.0
0.5
0.0
HC-C/EBP Beta Tg
AD-C/EBP Beta Tg

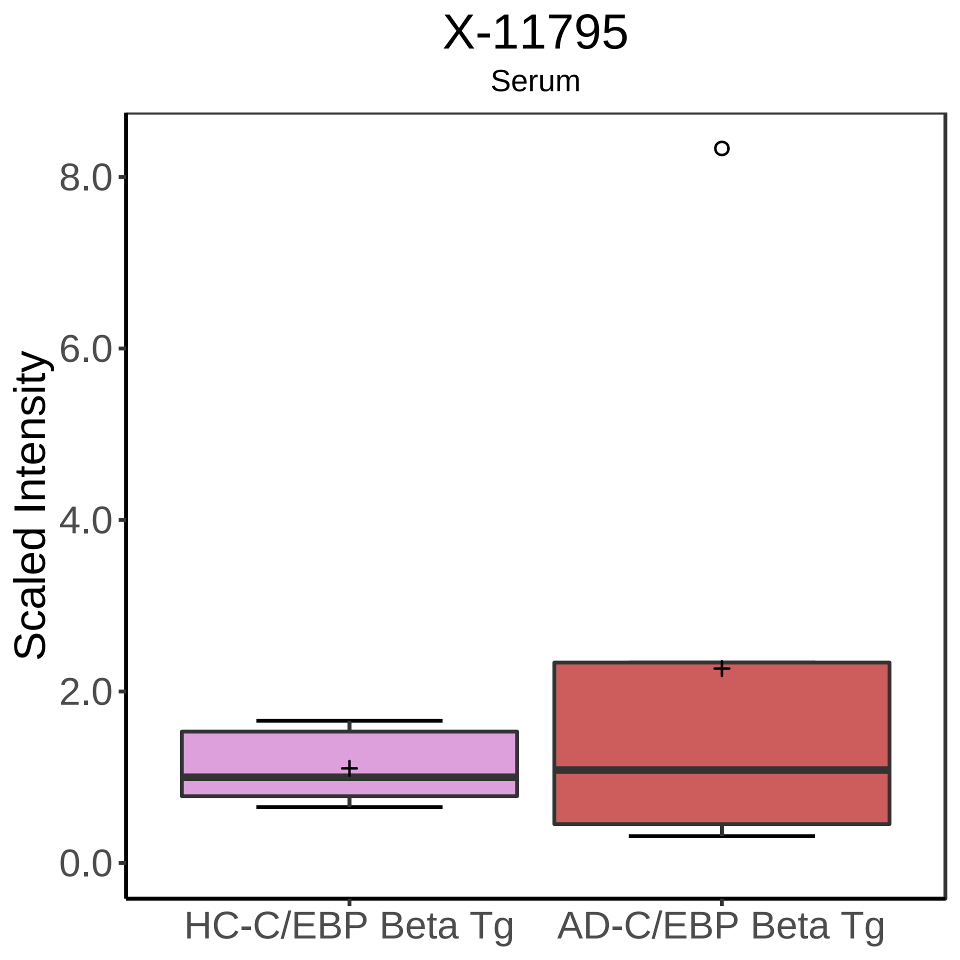
X-11795
Serum
Scaled Intensity
8.0
6.0
4.0
2.0
0.0
HC-C/EBP Beta Tg
AD-C/EBP Beta Tg

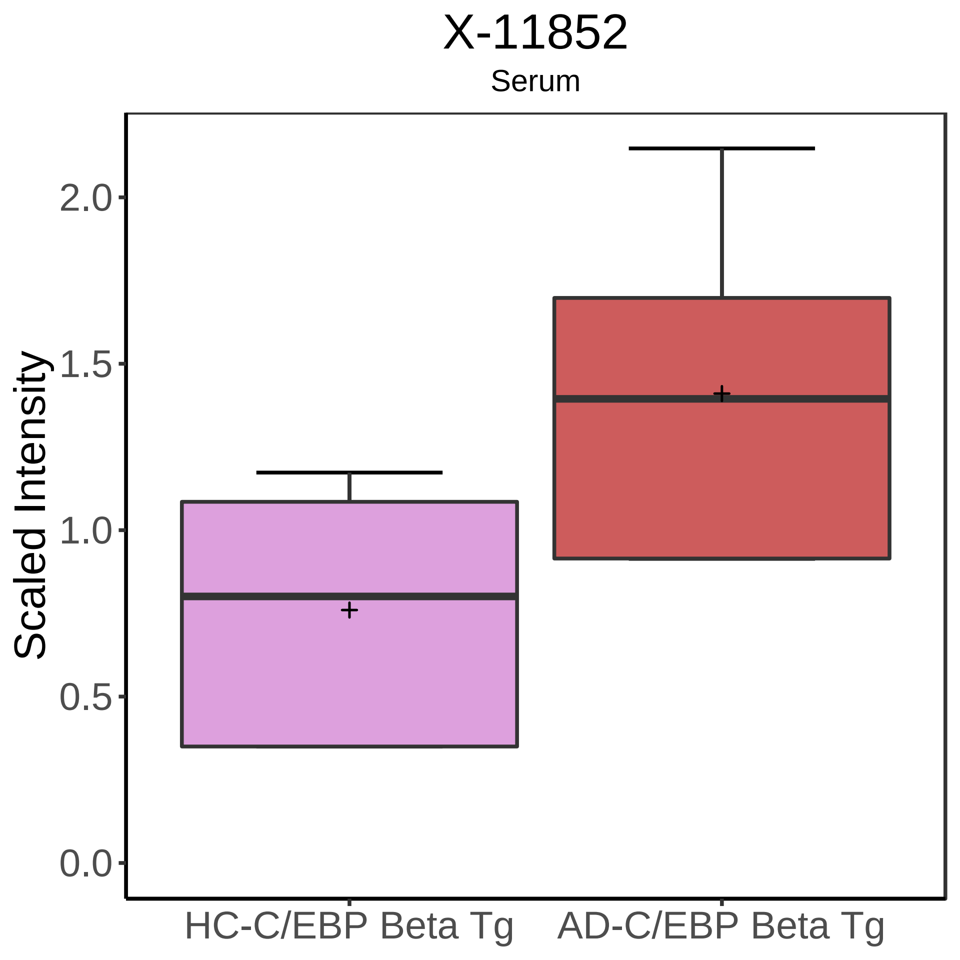
X-11852
Serum
Scaled Intensity
2.0
1.5
1.0
0.5
0.0
HC-C/EBP Beta Tg
AD-C/EBP Beta Tg

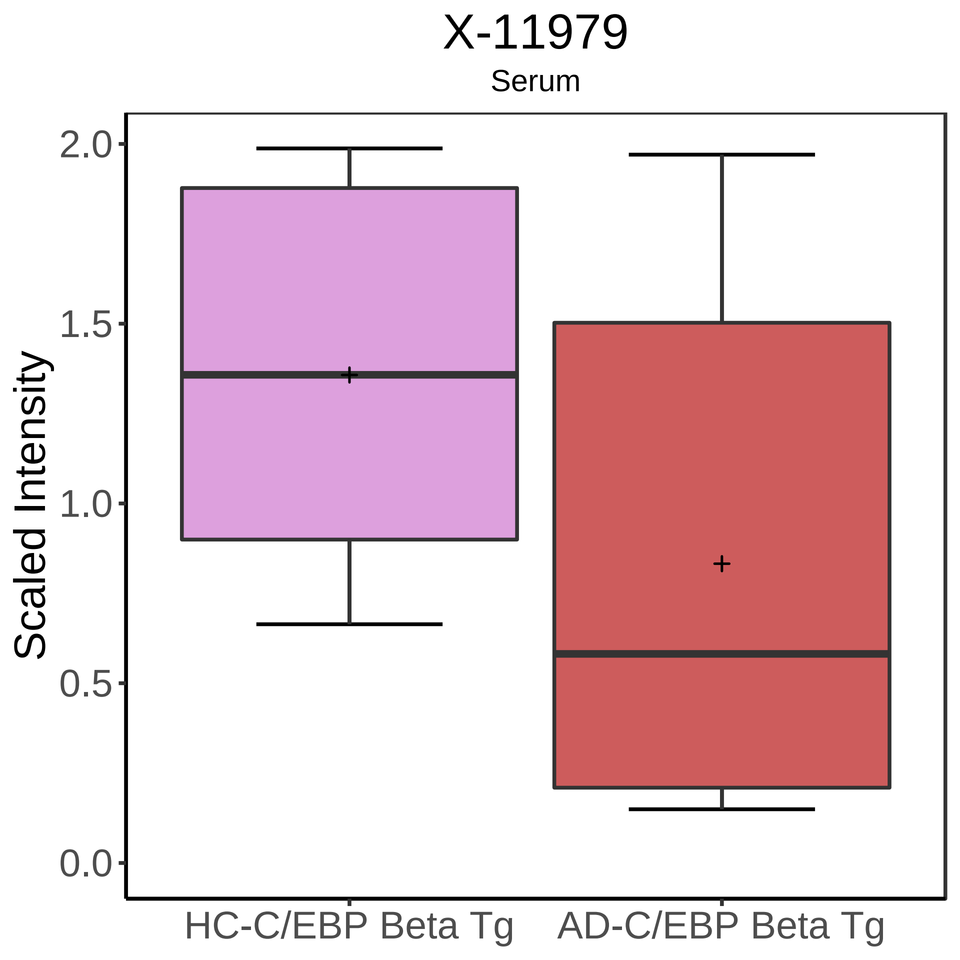
X-11979
Serum
Scaled Intensity
2.0
1.5
1.0
0.5
0.0
HC-C/EBP Beta Tg
AD-C/EBP Beta Tg

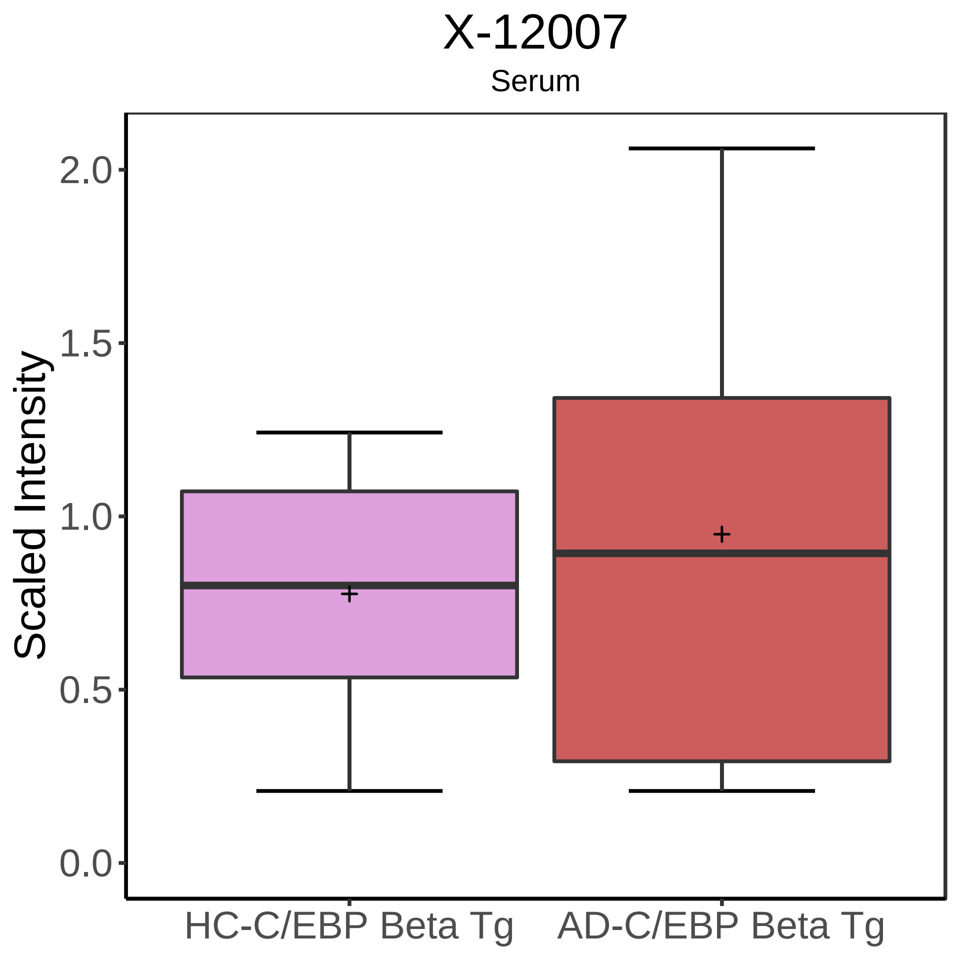
X-12007
Serum
Scaled Intensity
2.0
1.5
1.0
0.5
0.0
HC-C/EBP Beta Tg
AD-C/EBP Beta Tg

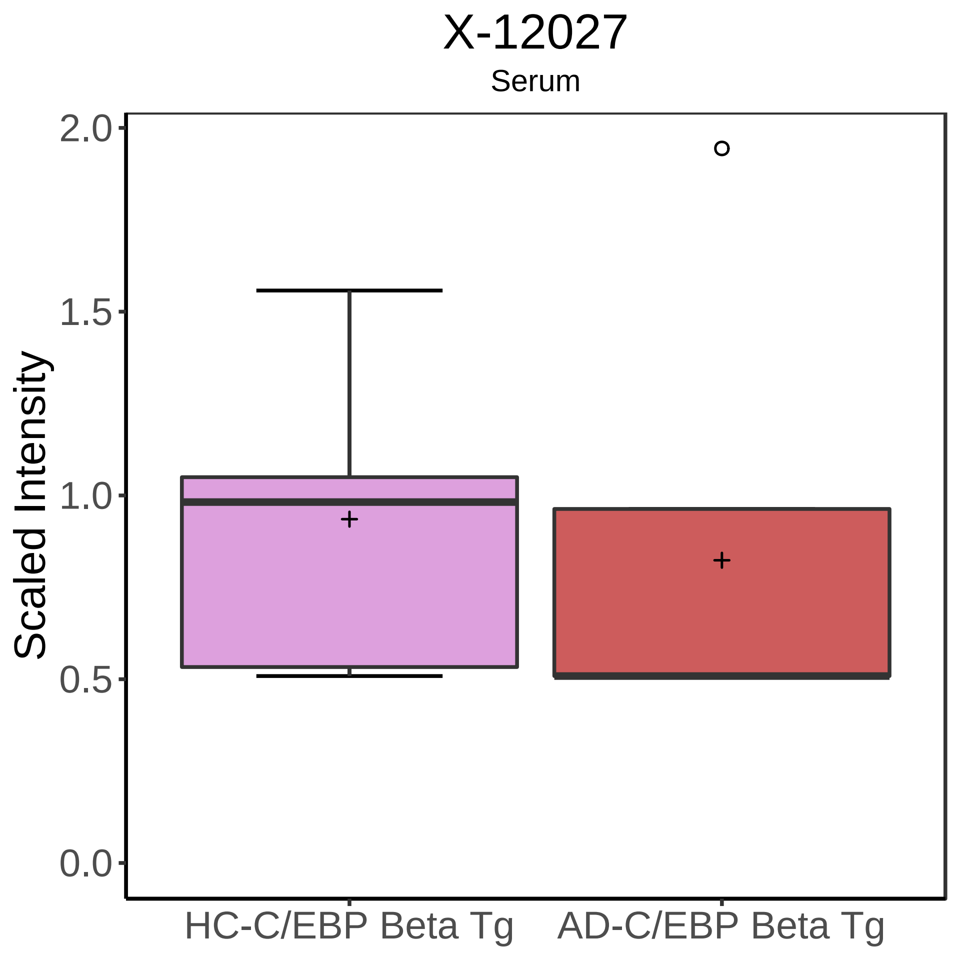
X-12027
Serum
Scaled Intensity
2.0
1.5
1.0
0.5
0.0
HC-C/EBP Beta Tg
AD-C/EBP Beta Tg

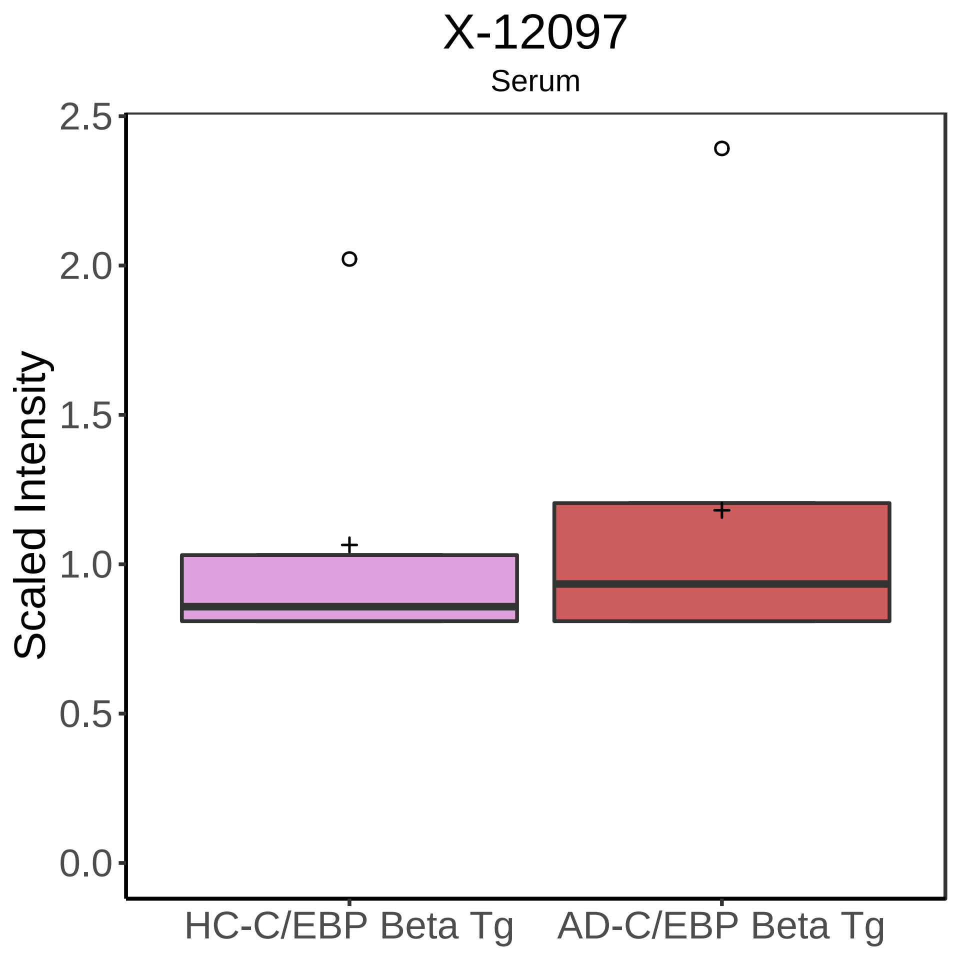

X-12097
Serum
Scaled Intensity
2.5
2.0
1.5
1.0
0.5
0.0
HC-C/EBP Beta Tg
AD-C/EBP Beta Tg

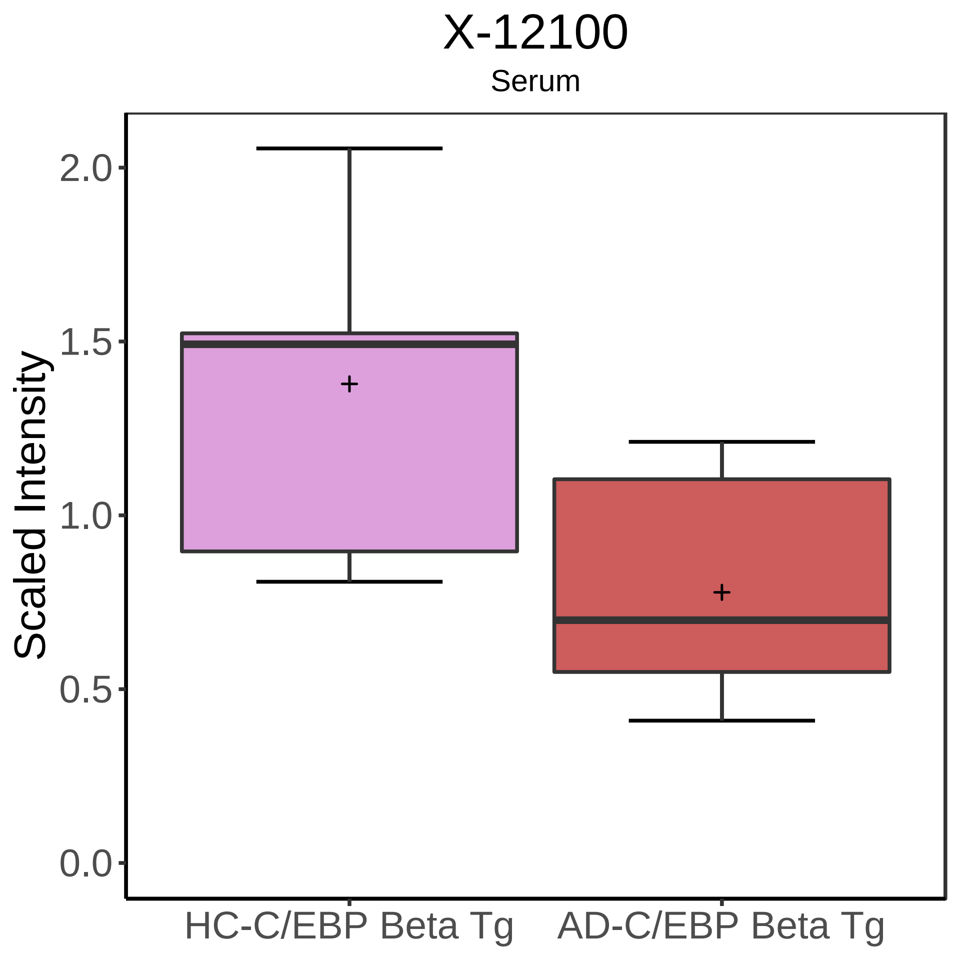
X-12100
Serum
Scaled Intensity
2.0
1.5
1.0
0.5
0.0
HC-C/EBP Beta Tg
AD-C/EBP Beta Tg

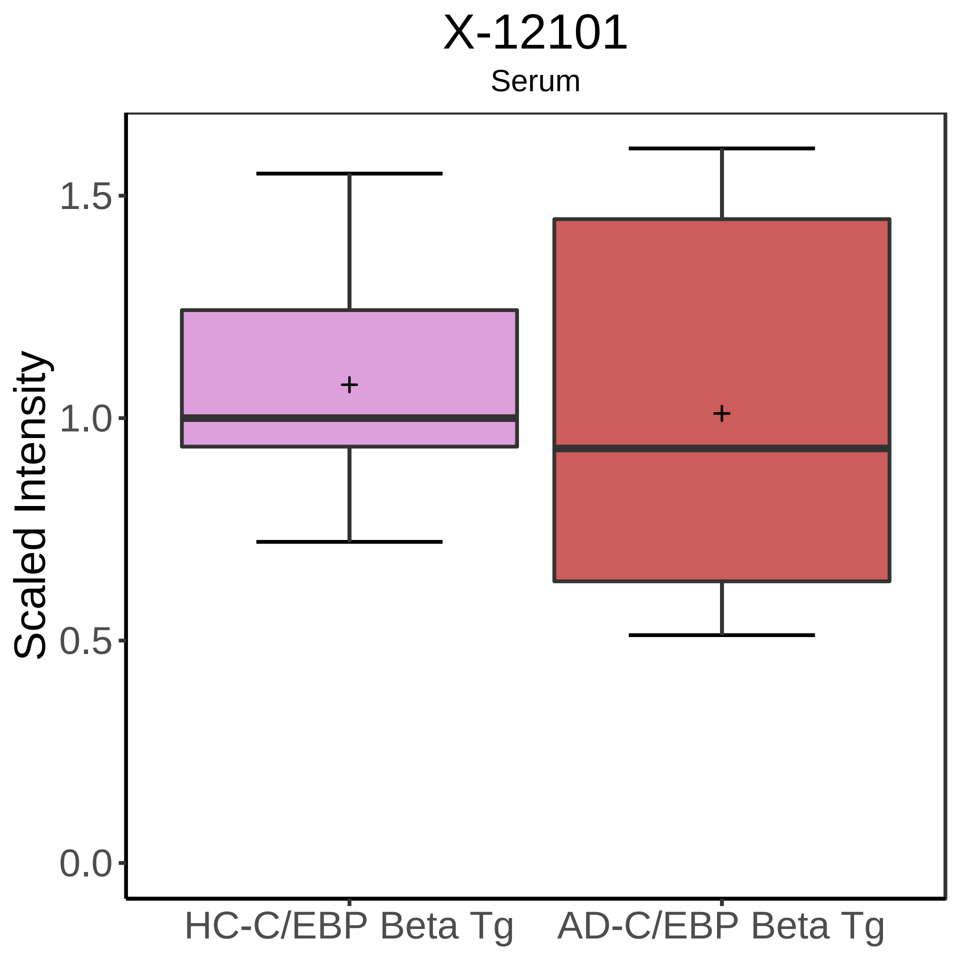
X-12101
Serum
Scaled Intensity
1.5
1.0
0.5
0.0
HC-C/EBP Beta Tg
AD-C/EBP Beta Tg

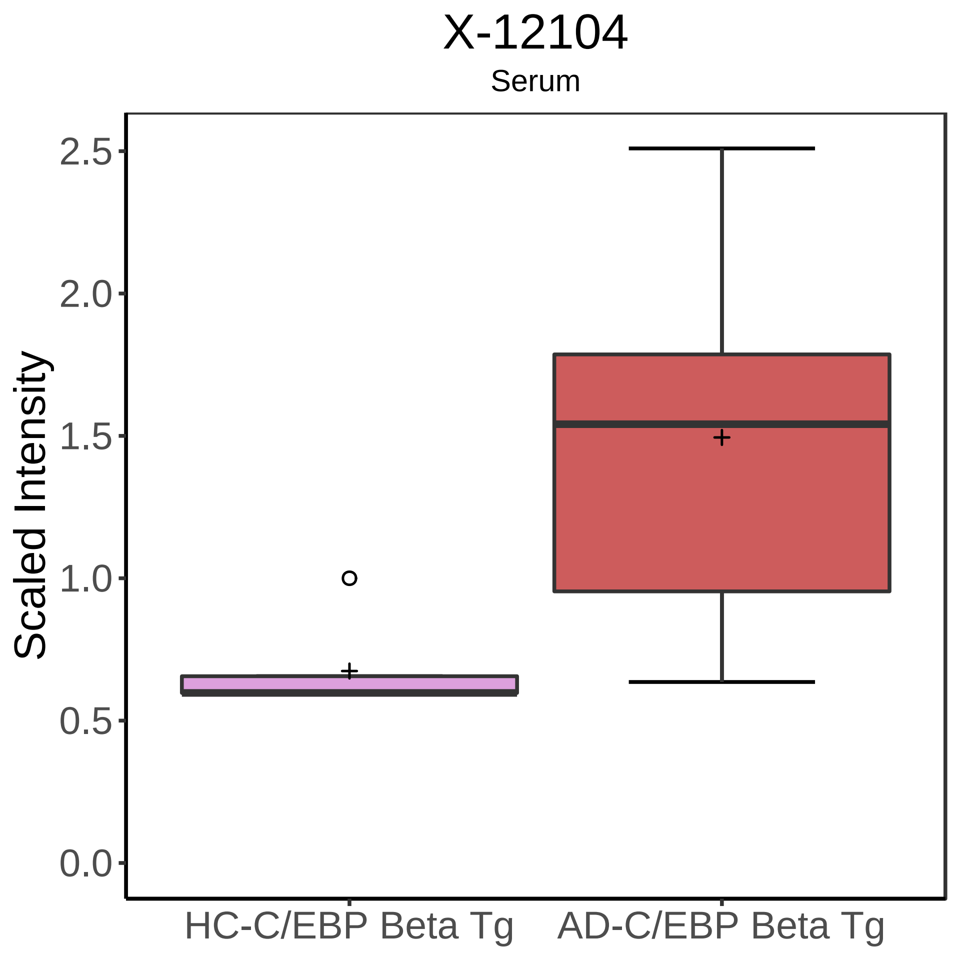
X-12104
Serum
Scaled Intensity
2.5
2.0
1.5
1.0
0.5
0.0
HC-C/EBP Beta Tg
AD-C/EBP Beta Tg

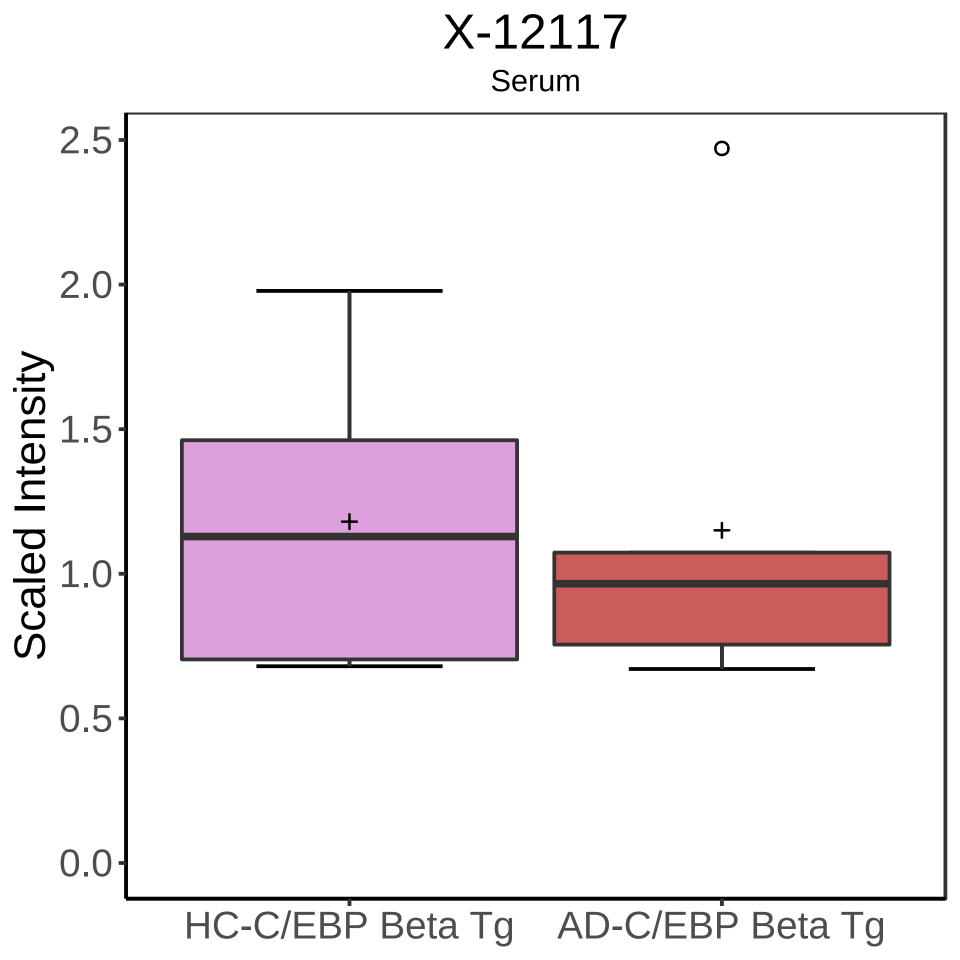
X-12117
Serum
Scaled Intensity
2.5
2.0
1.5
1.0
0.5
0.0
HC-C/EBP Beta Tg
AD-C/EBP Beta Tg

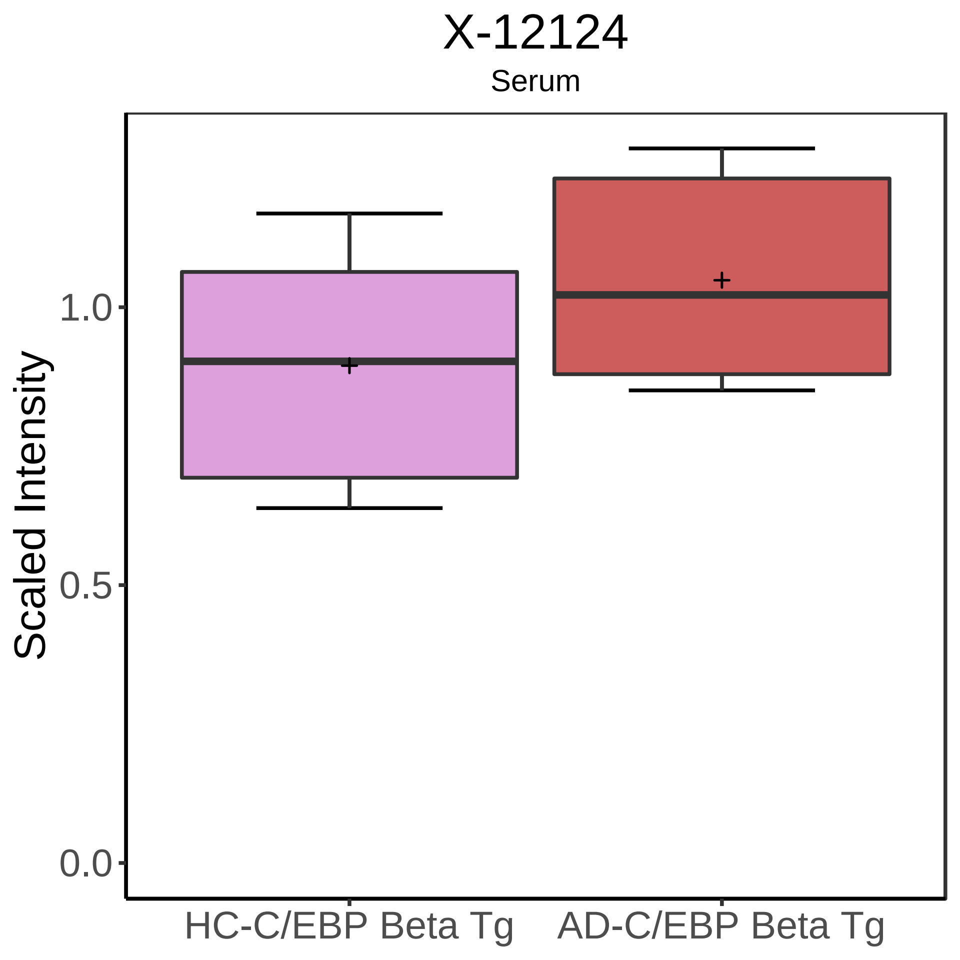
X-12124
Serum
Scaled Intensity
1.0
0.5
0.0
HC-C/EBP Beta Tg
AD-C/EBP Beta Tg

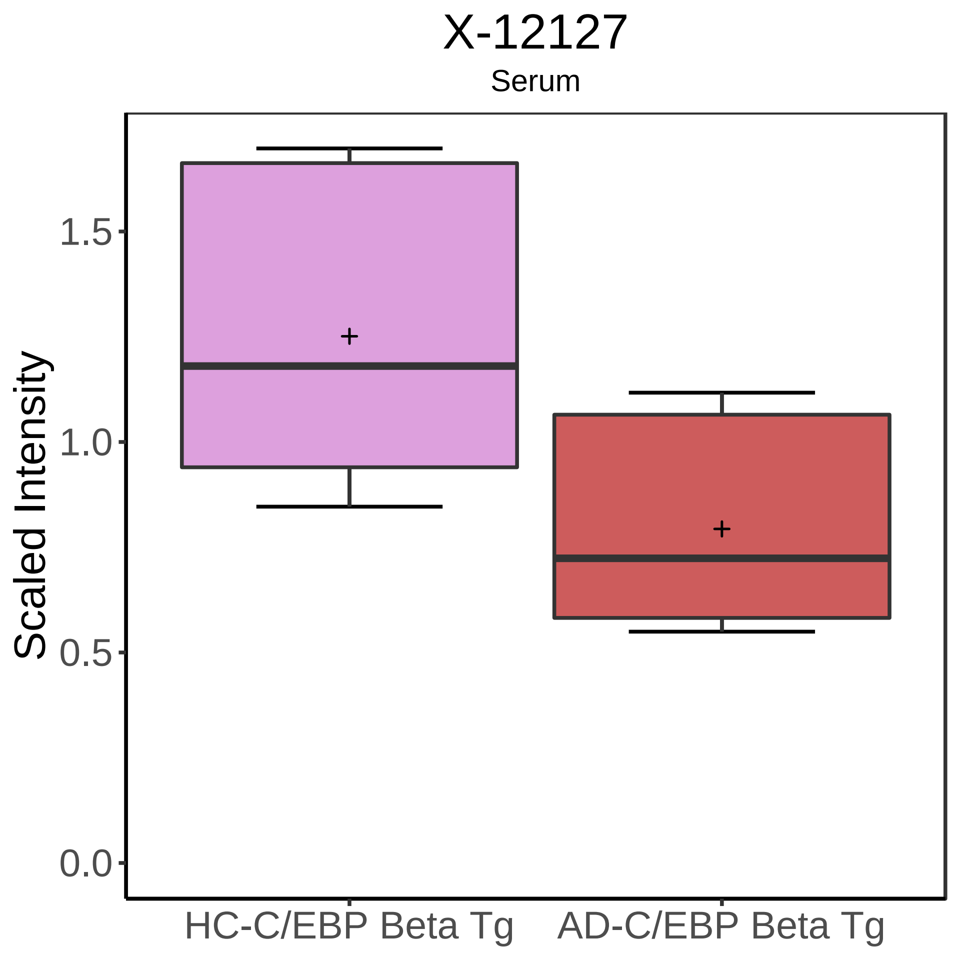
X-12127
Serum
Scaled Intensity
1.5
1.0
0.5
0.0
HC-C/EBP Beta Tg
AD-C/EBP Beta Tg

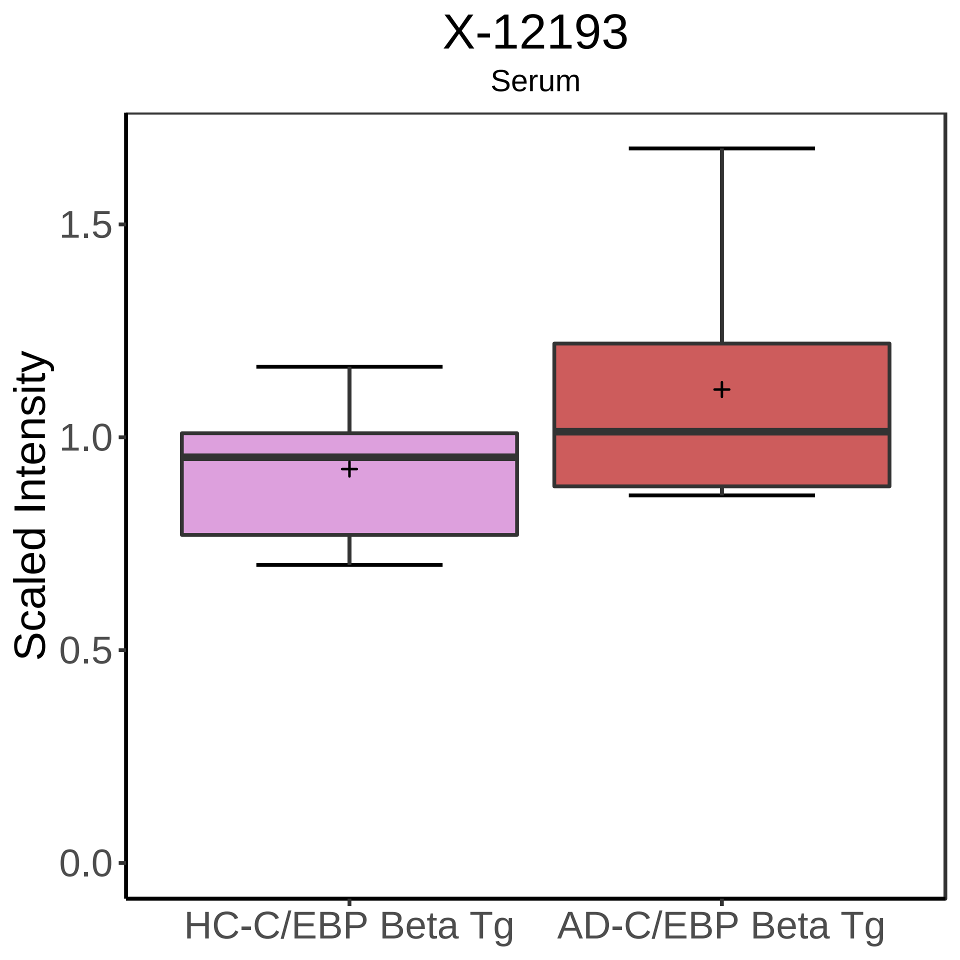
X-12193
Serum
Scaled Intensity
1.5
1.0
0.5
0.0
HC-C/EBP Beta Tg
AD-C/EBP Beta Tg

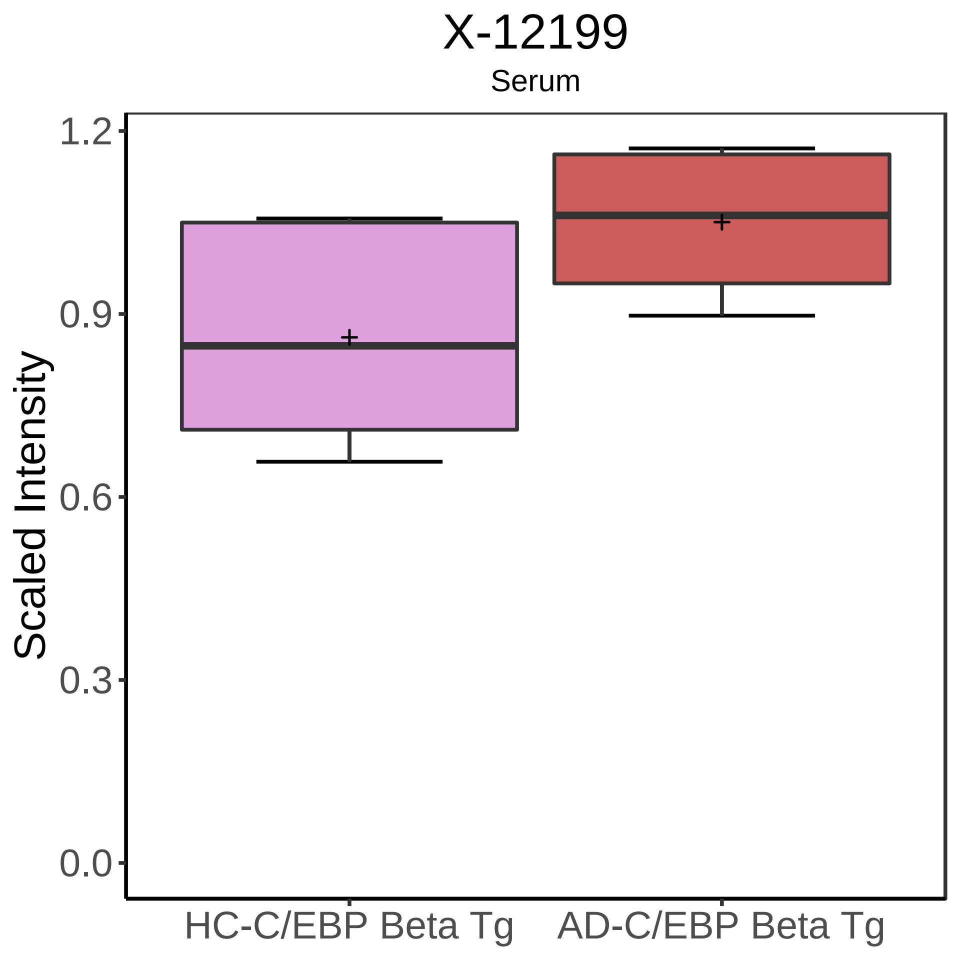
X-12199
Serum
Scaled Intensity
1.2
0.9
0.6
0.3
0.0
HC-C/EBP Beta Tg
AD-C/EBP Beta Tg

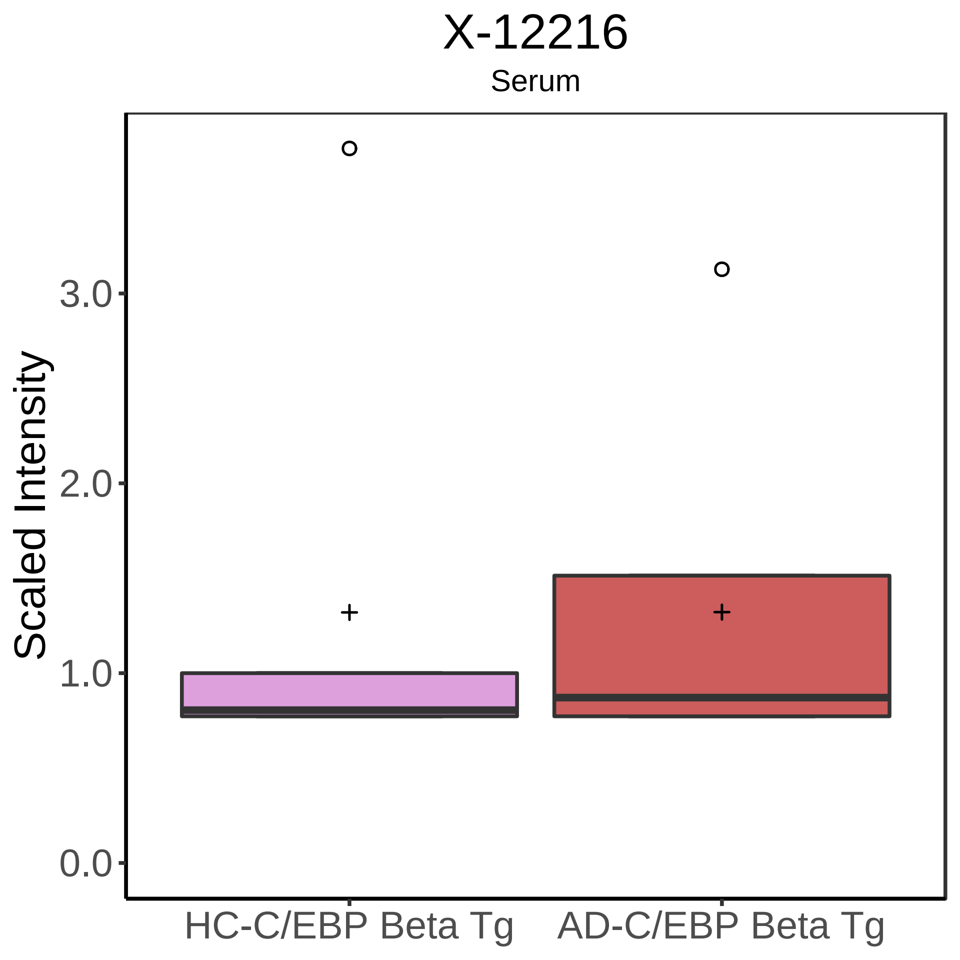

X-12216
Serum
Scaled Intensity
0.0
1.0
2.0
3.0
HC-C/EBP Beta Tg
AD-C/EBP Beta Tg

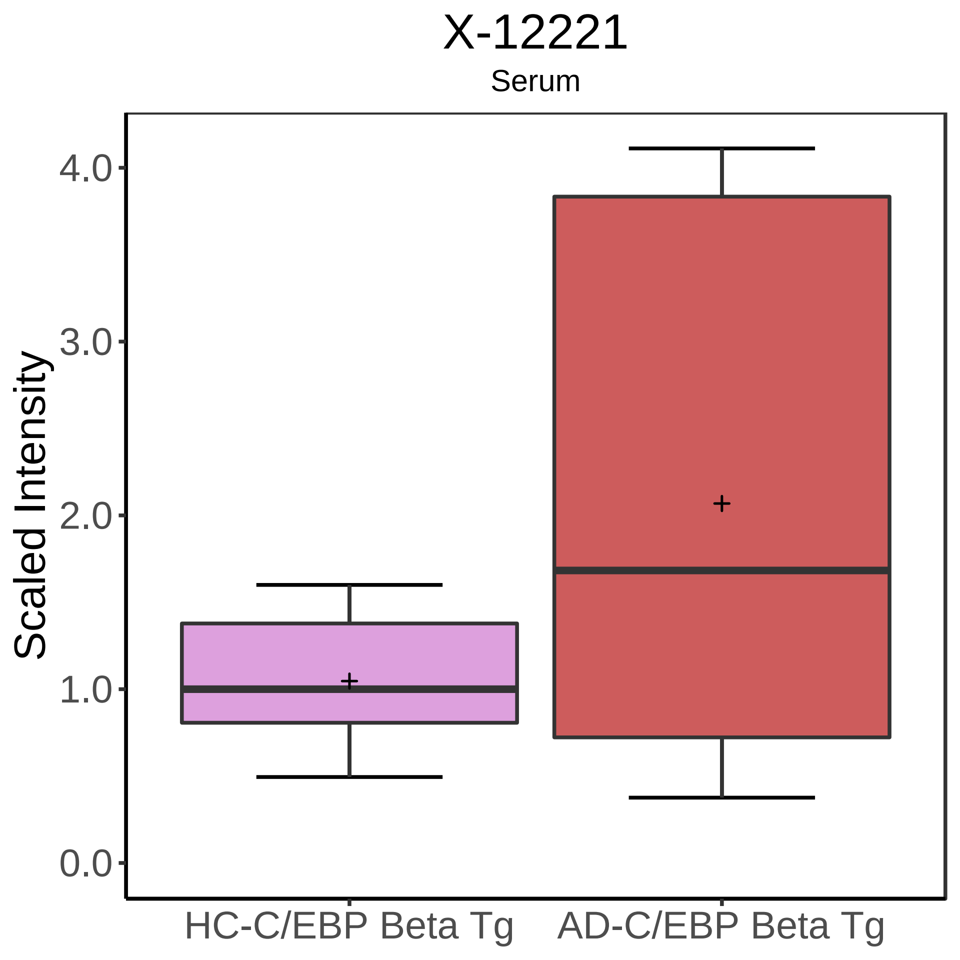

X-12221
Serum
Scaled Intensity
4.0
3.0
2.0
1.0
0.0
HC-C/EBP Beta Tg
AD-C/EBP Beta Tg

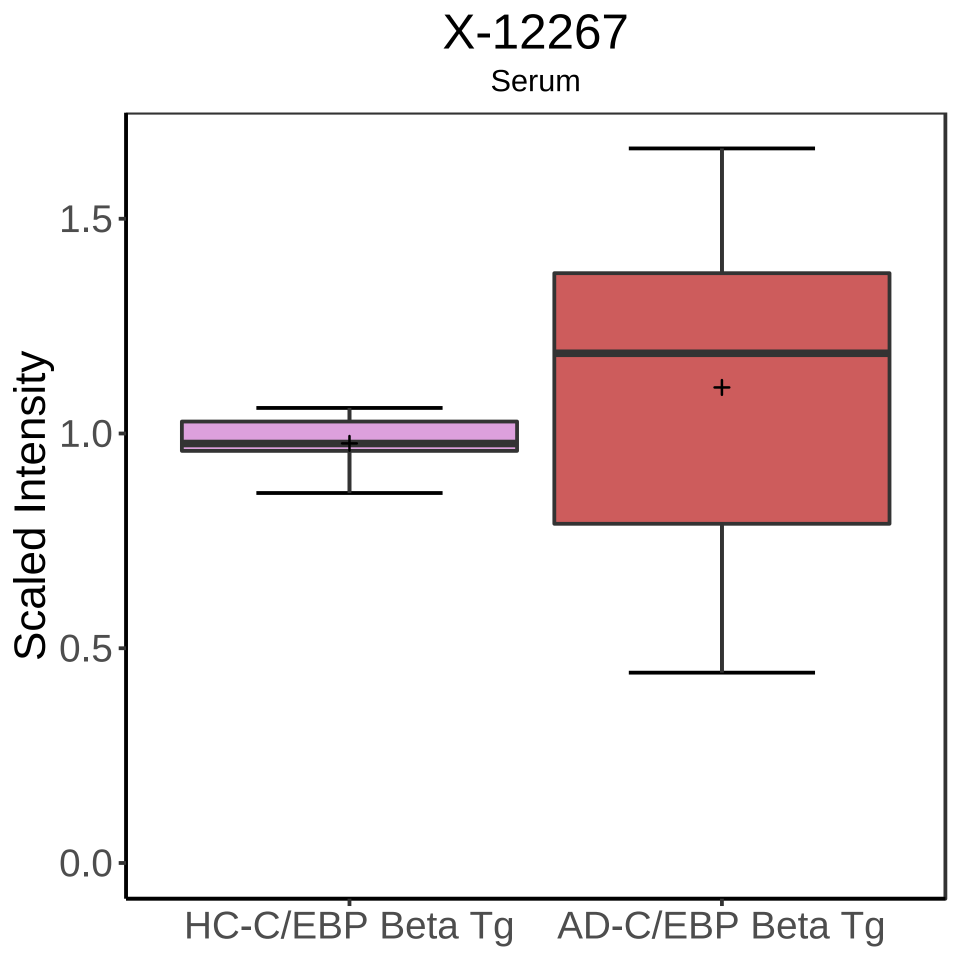
X-12267
Serum
Scaled Intensity
1.5
1.0
0.5
0.0
HC-C/EBP Beta Tg
AD-C/EBP Beta Tg

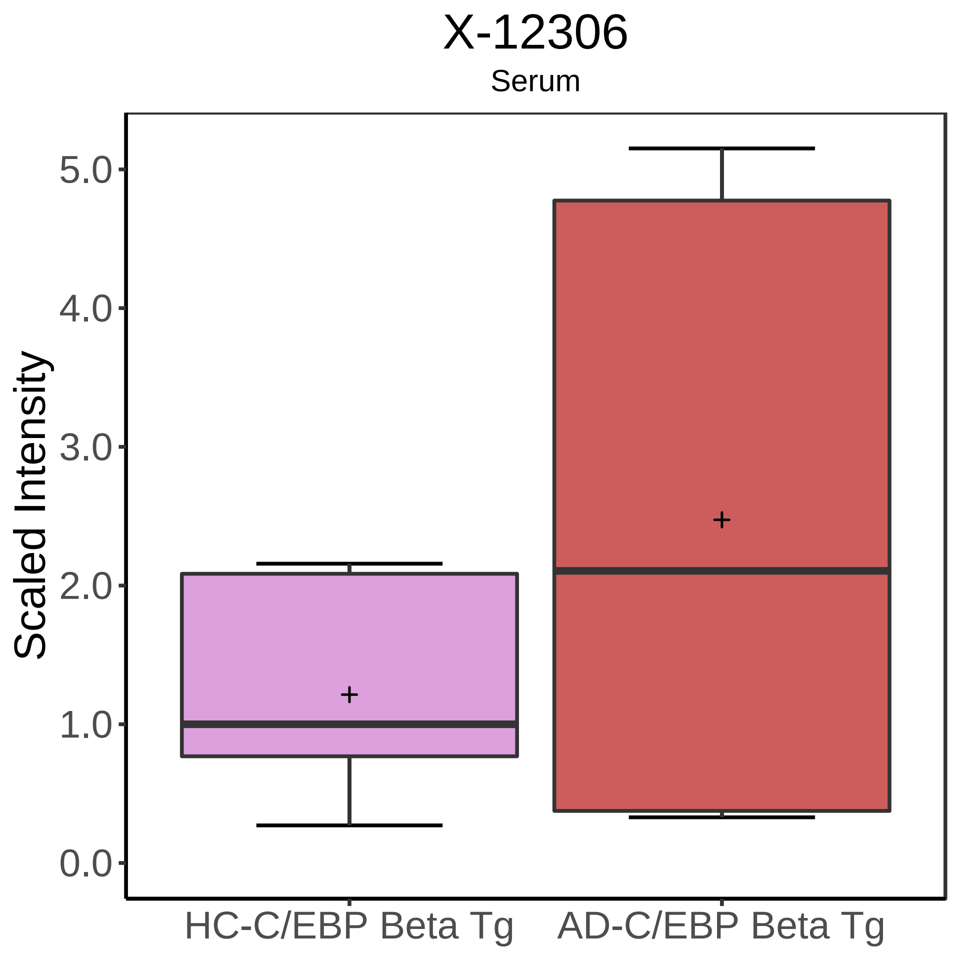
X-12306
Serum
Scaled Intensity
5.0
4.0
3.0
2.0
1.0
0.0
HC-C/EBP Beta Tg
AD-C/EBP Beta Tg

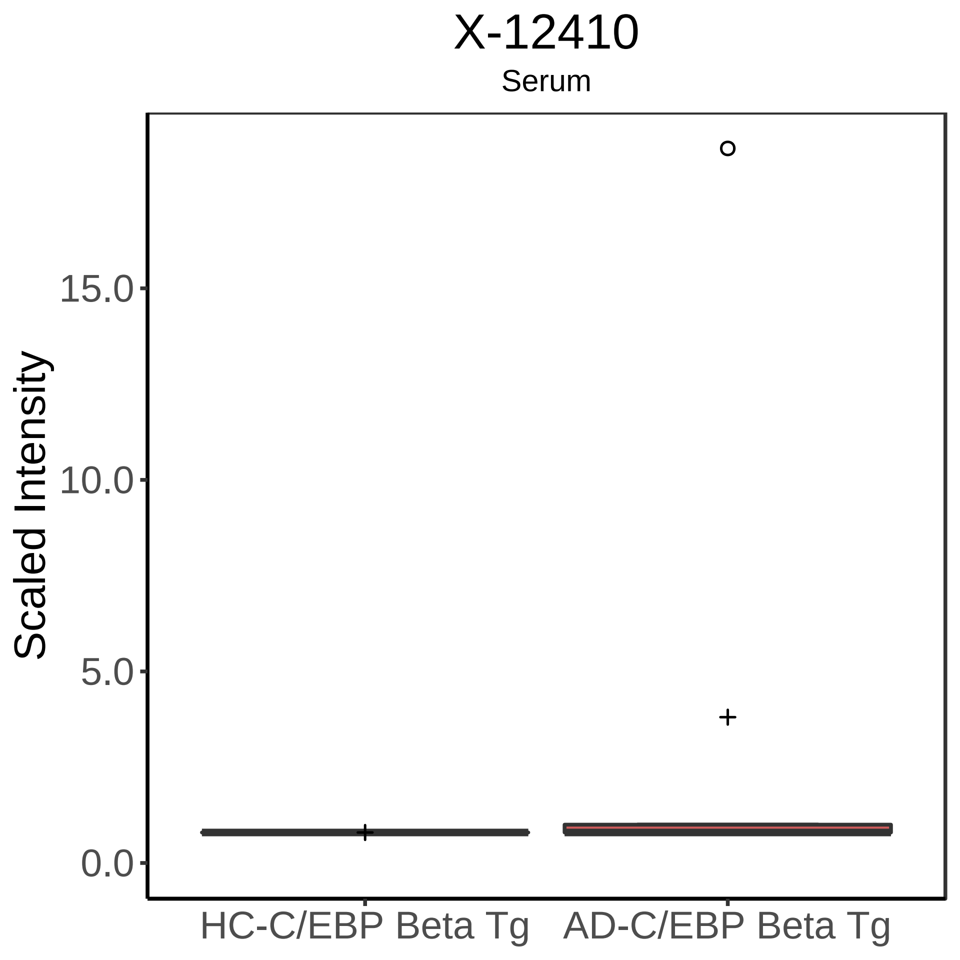
X-12410
Serum
Scaled Intensity
15.0
10.0
5.0
0.0
HC-C/EBP Beta Tg
AD-C/EBP Beta Tg

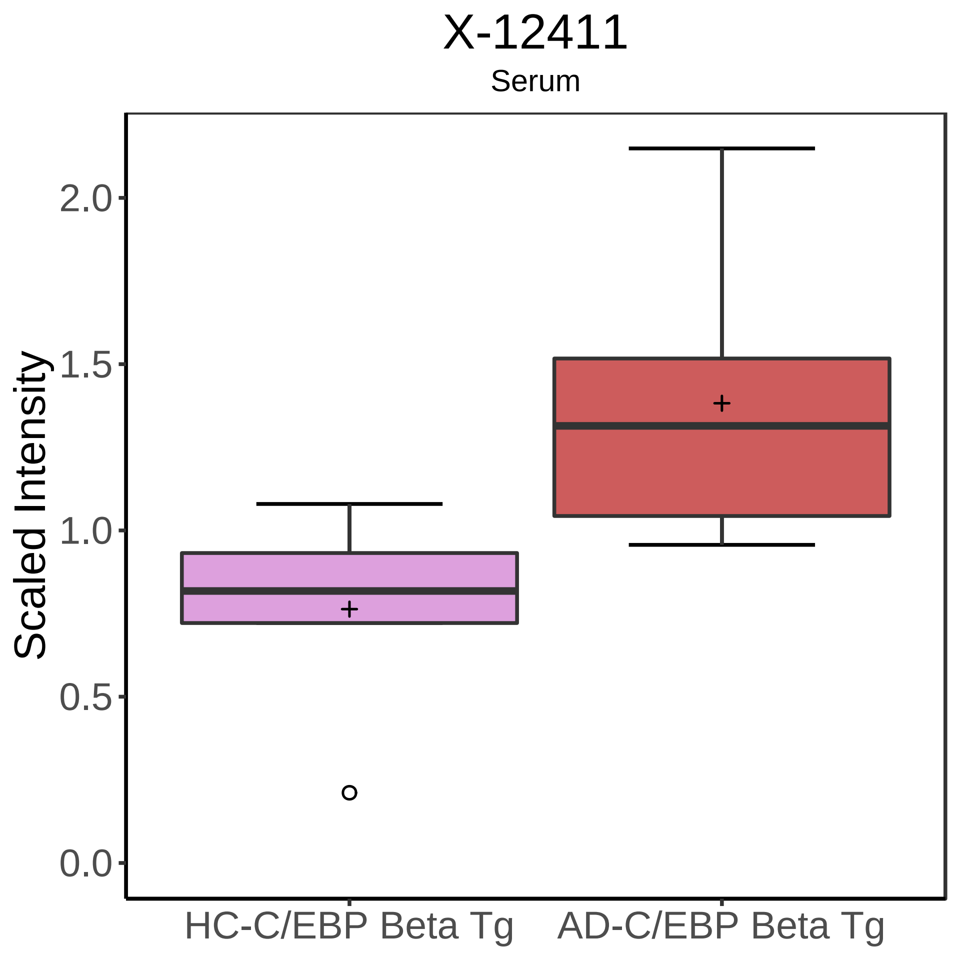
X-12411
Serum
Scaled Intensity
2.0
1.5
1.0
0.5
0.0
HC-C/EBP Beta Tg
AD-C/EBP Beta Tg

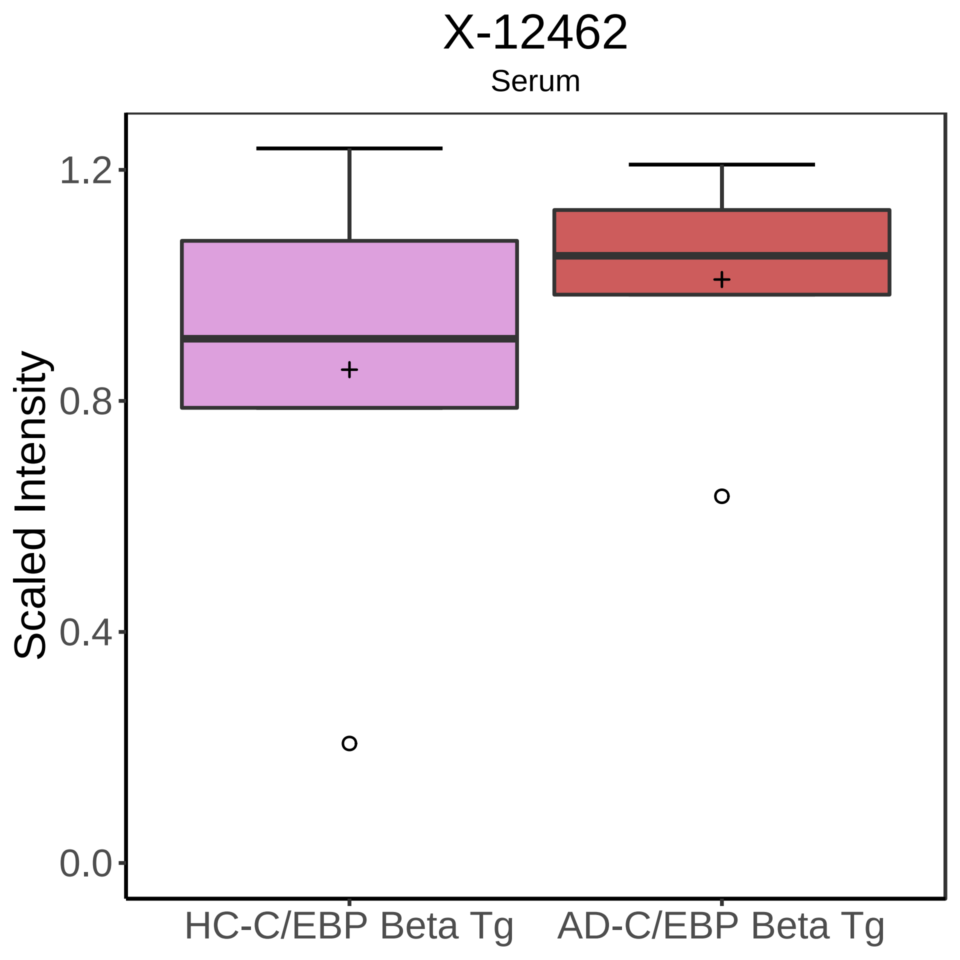
X-12462
Serum
Scaled Intensity
1.2
0.8
0.4
0.0
HC-C/EBP Beta Tg
AD-C/EBP Beta Tg

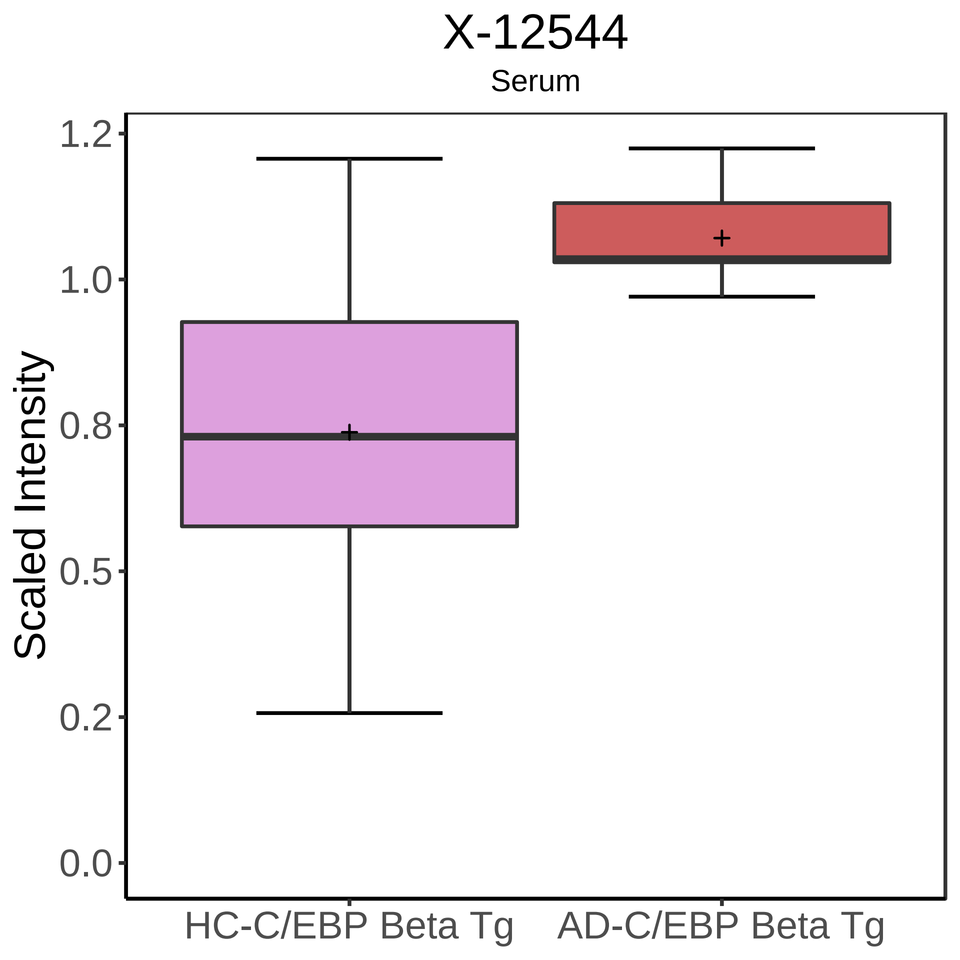
X-12544
Serum
Scaled Intensity
1.2
1.0
0.8
0.5
0.2
0.0
HC-C/EBP Beta Tg
AD-C/EBP Beta Tg

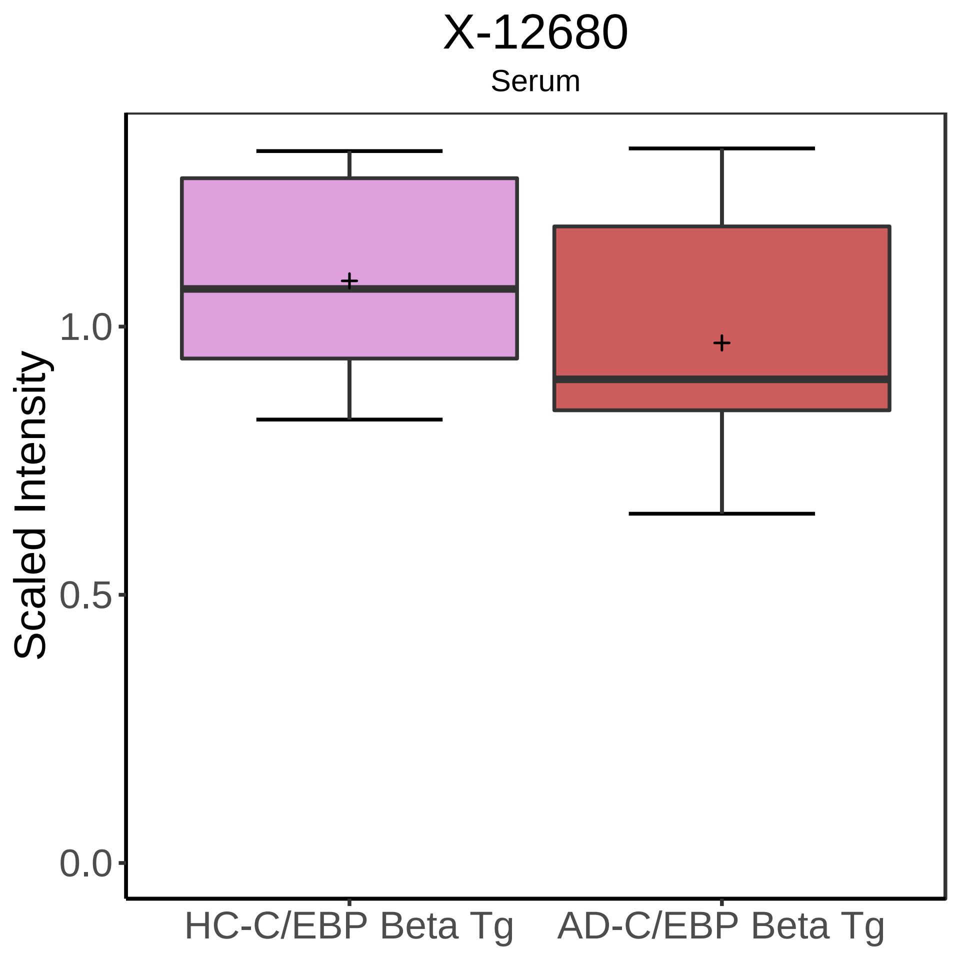
X-12680
Serum
Scaled Intensity
1.0
0.5
0.0
HC-C/EBP Beta Tg
AD-C/EBP Beta Tg

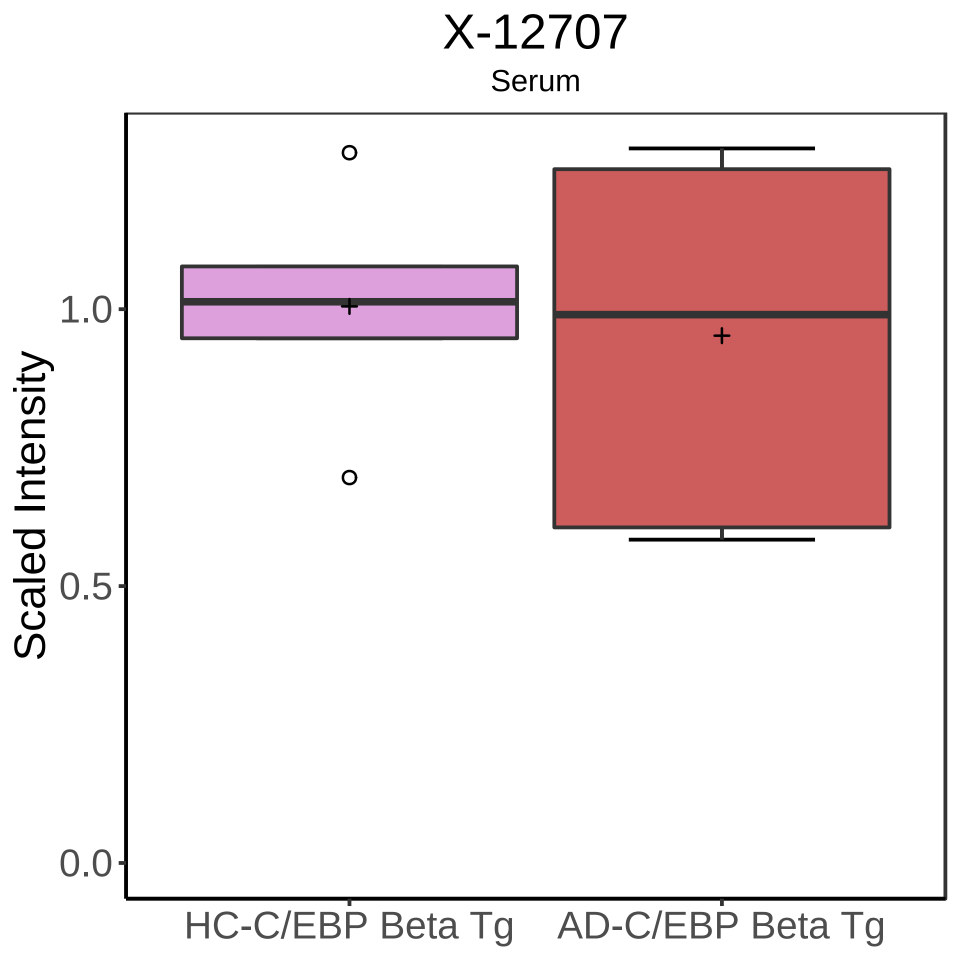
X-12707
Serum
Scaled Intensity
1.0
0.5
0.0
HC-C/EBP Beta Tg
AD-C/EBP Beta Tg

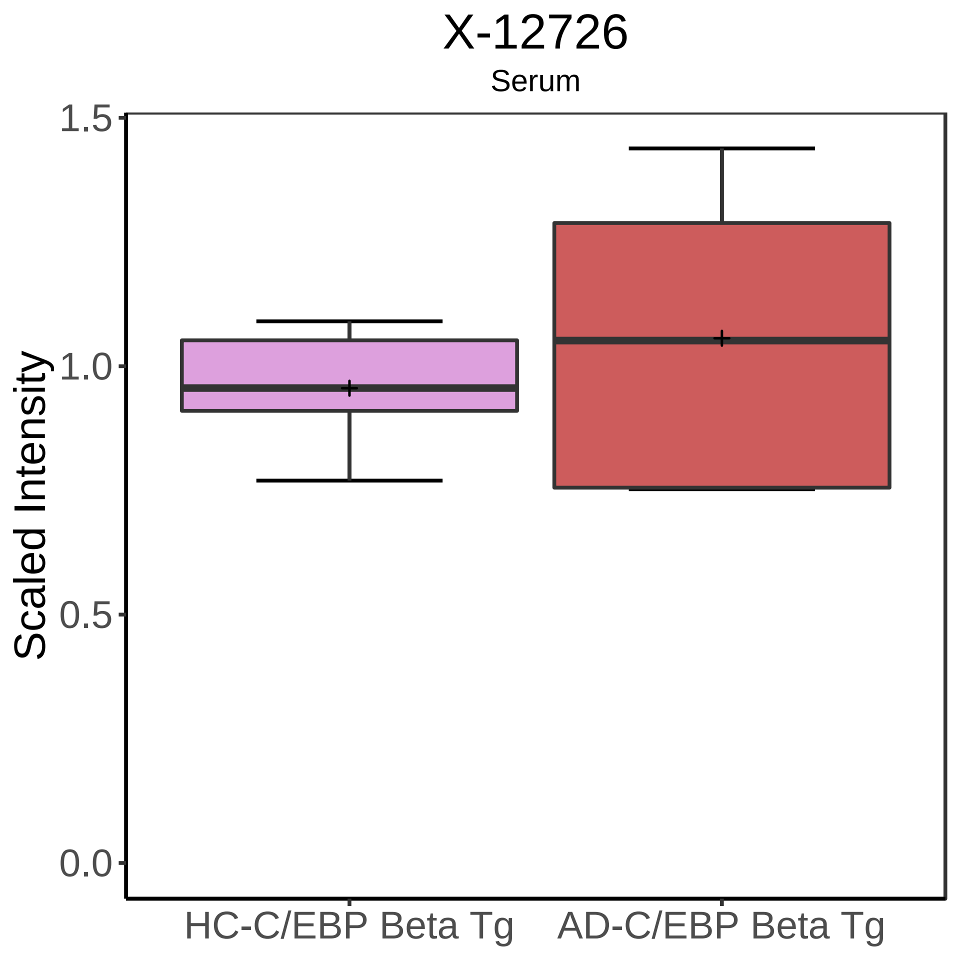
X-12726
Serum
Scaled Intensity
1.5
1.0
0.5
0.0
HC-C/EBP Beta Tg
AD-C/EBP Beta Tg

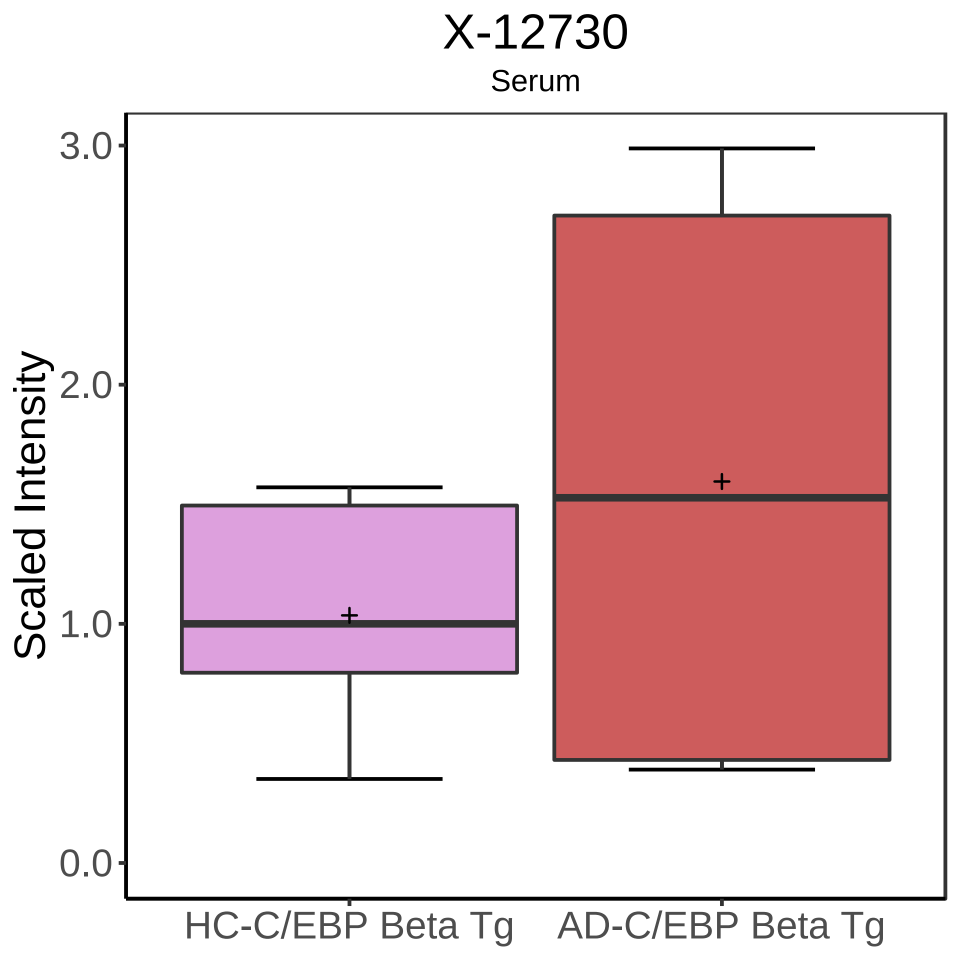

X-12730
Serum
Scaled Intensity
3.0
2.0
1.0
0.0
HC-C/EBP Beta Tg
AD-C/EBP Beta Tg

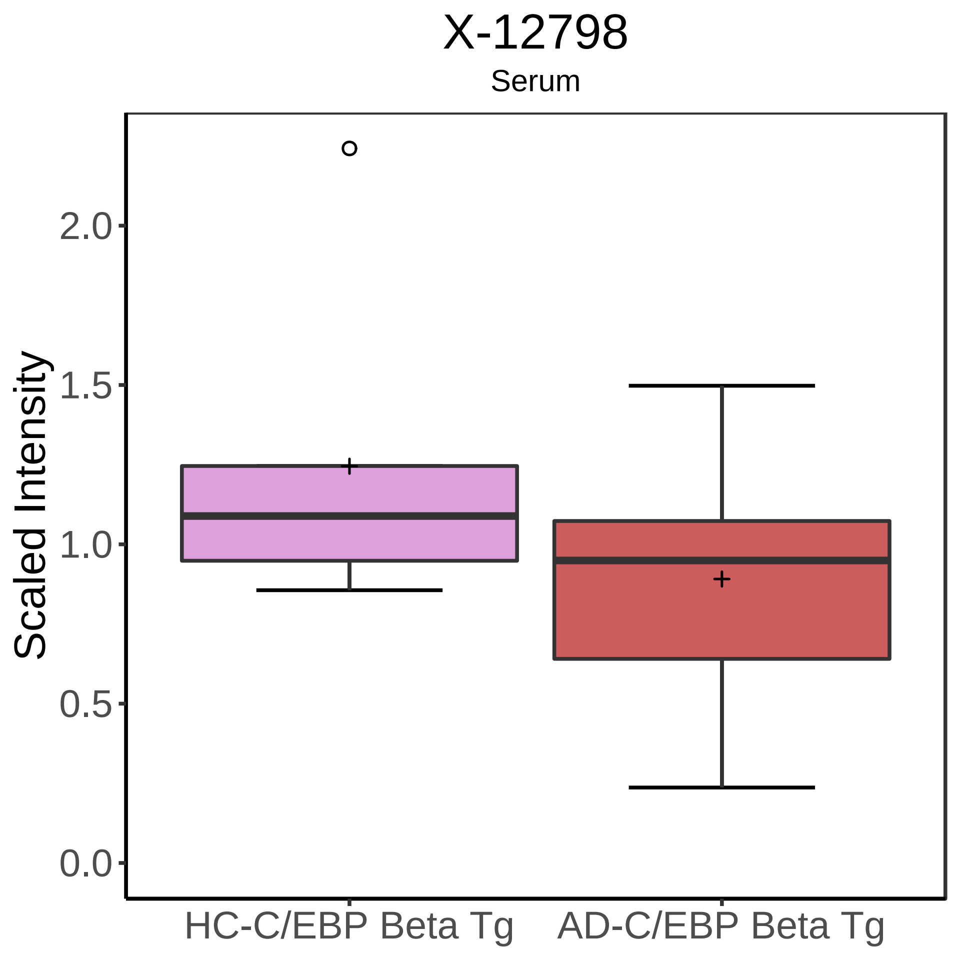
X-12798
Serum
Scaled Intensity
2.0
1.5
1.0
0.5
0.0
HC-C/EBP Beta Tg
AD-C/EBP Beta Tg

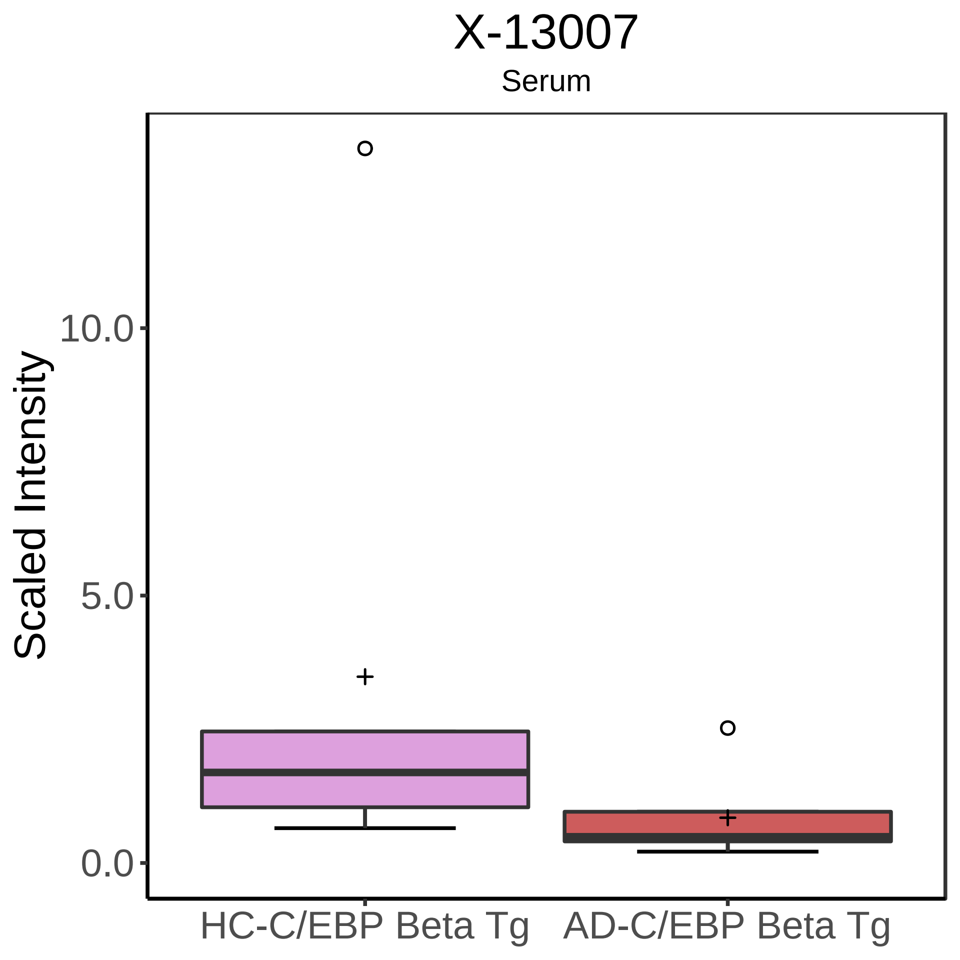
X-13007
Serum
Scaled Intensity
10.0
5.0
0.0
HC-C/EBP Beta Tg
AD-C/EBP Beta Tg

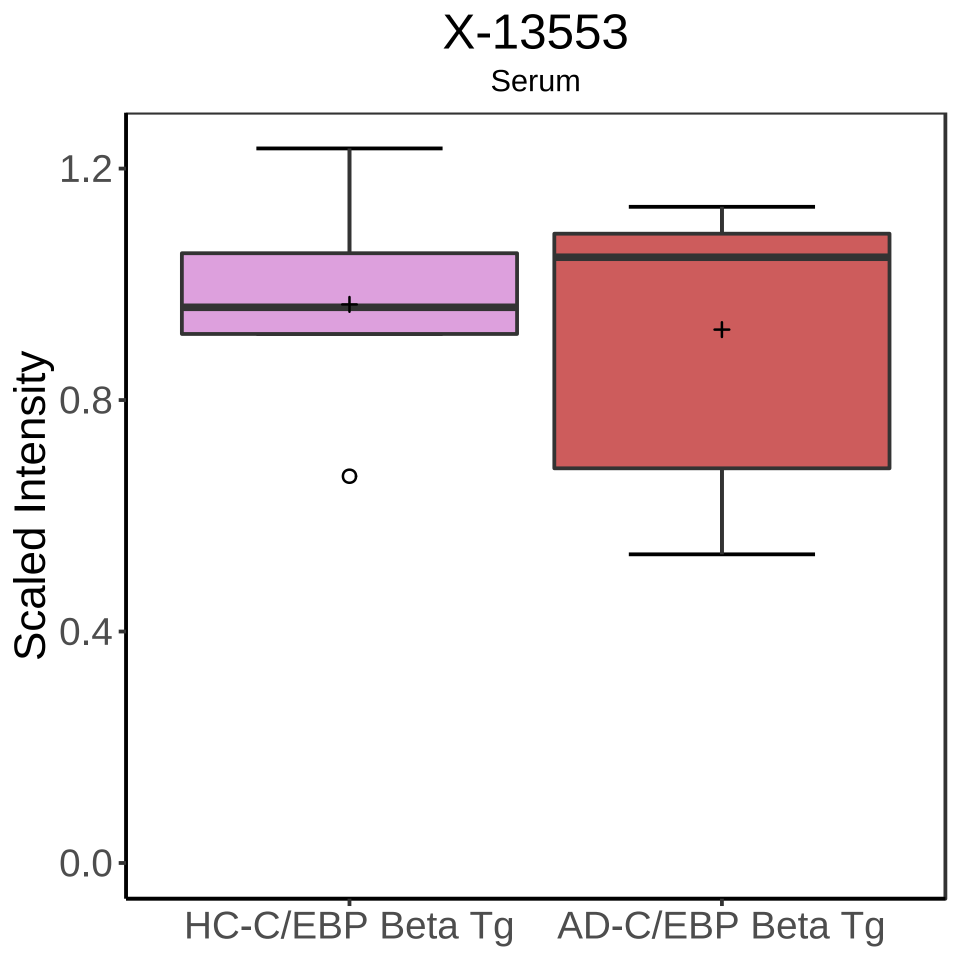
X-13553
Serum
Scaled Intensity
1.2
0.8
0.4
0.0
HC-C/EBP Beta Tg
AD-C/EBP Beta Tg

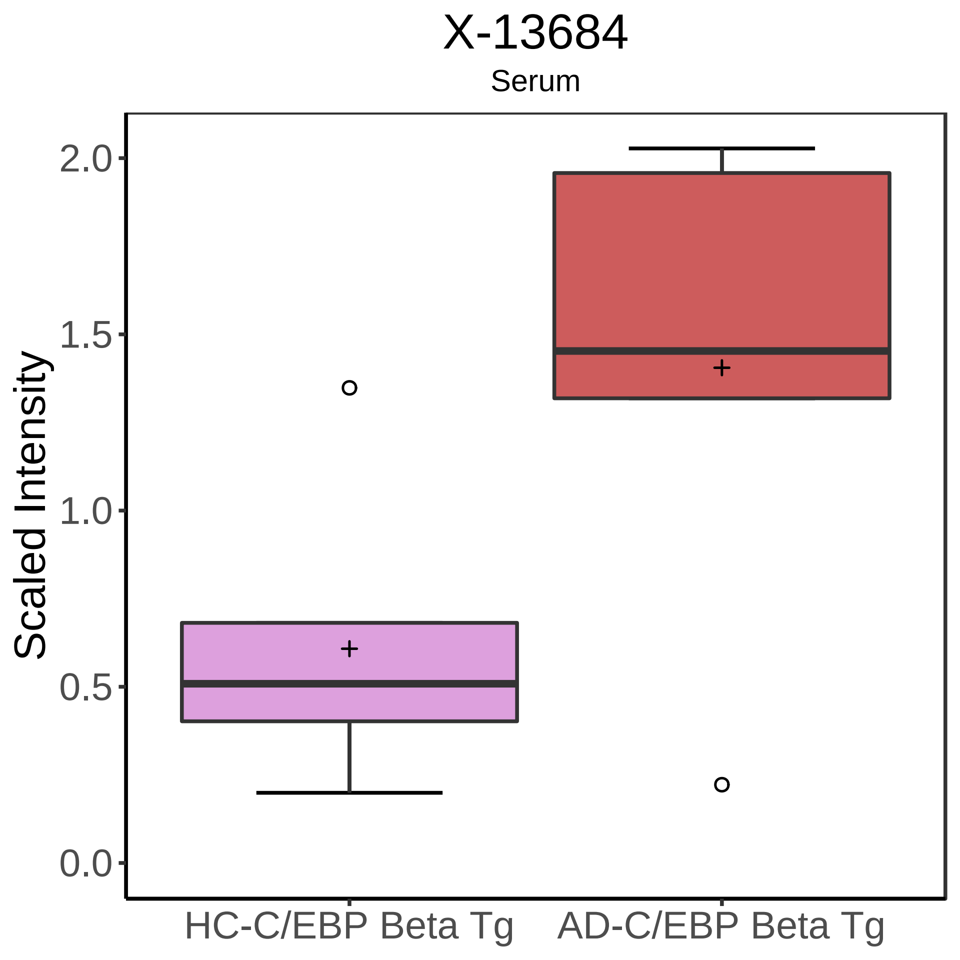

X-13684
Serum
Scaled Intensity
2.0
1.5
1.0
0.5
0.0
HC-C/EBP Beta Tg
AD-C/EBP Beta Tg

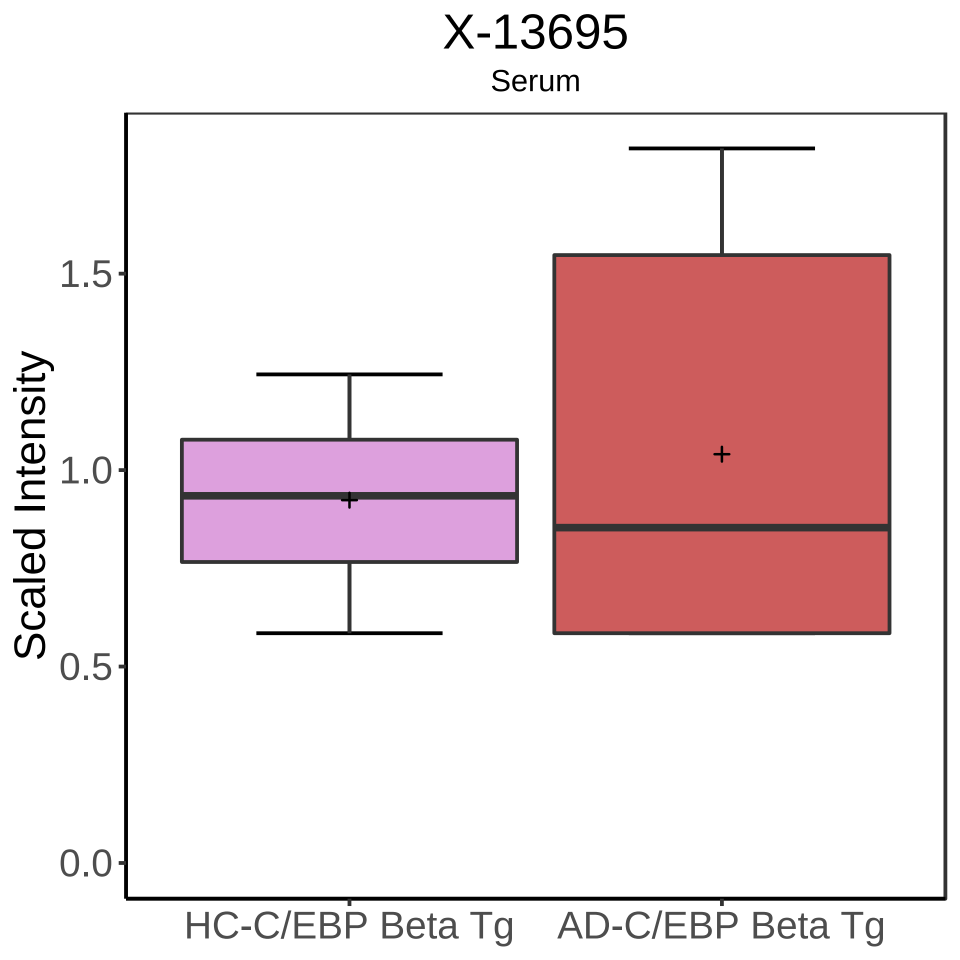
X-13695
Serum
Scaled Intensity
1.5
1.0
0.5
0.0
HC-C/EBP Beta Tg
AD-C/EBP Beta Tg

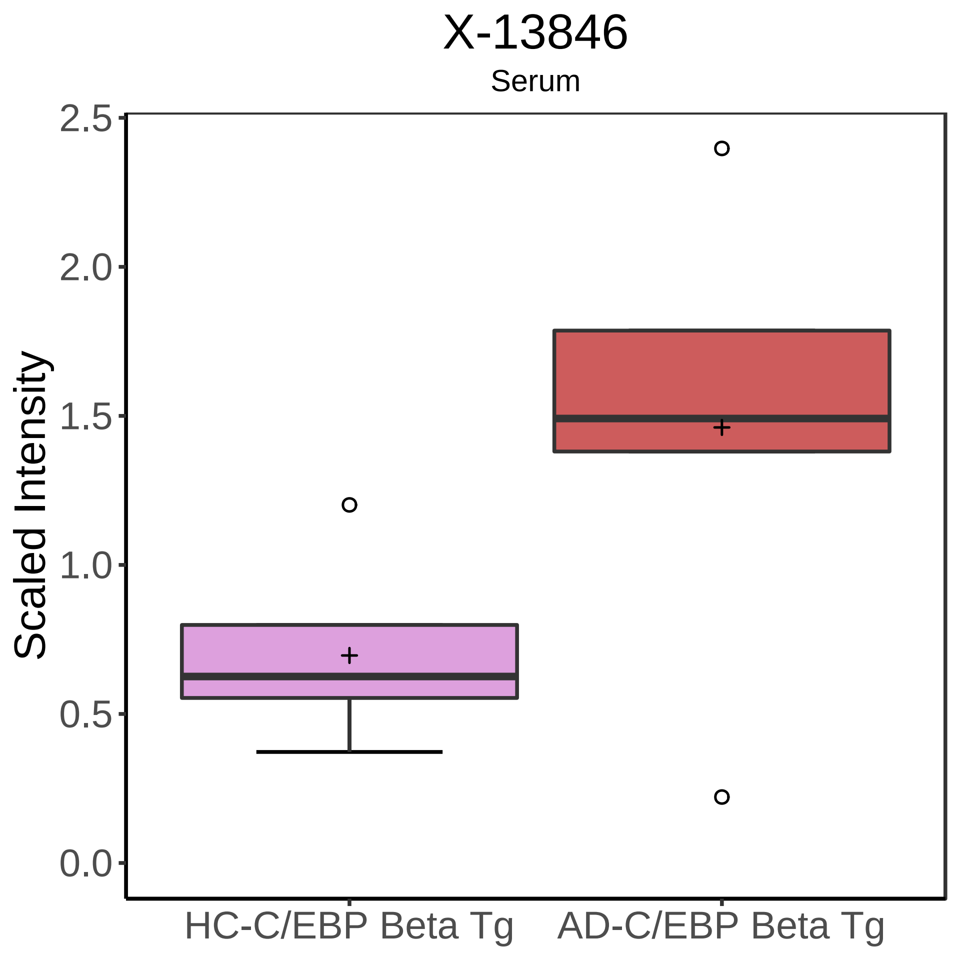
X-13846
Serum
Scaled Intensity
2.5
2.0
1.5
1.0
0.5
0.0
HC-C/EBP Beta Tg
AD-C/EBP Beta Tg

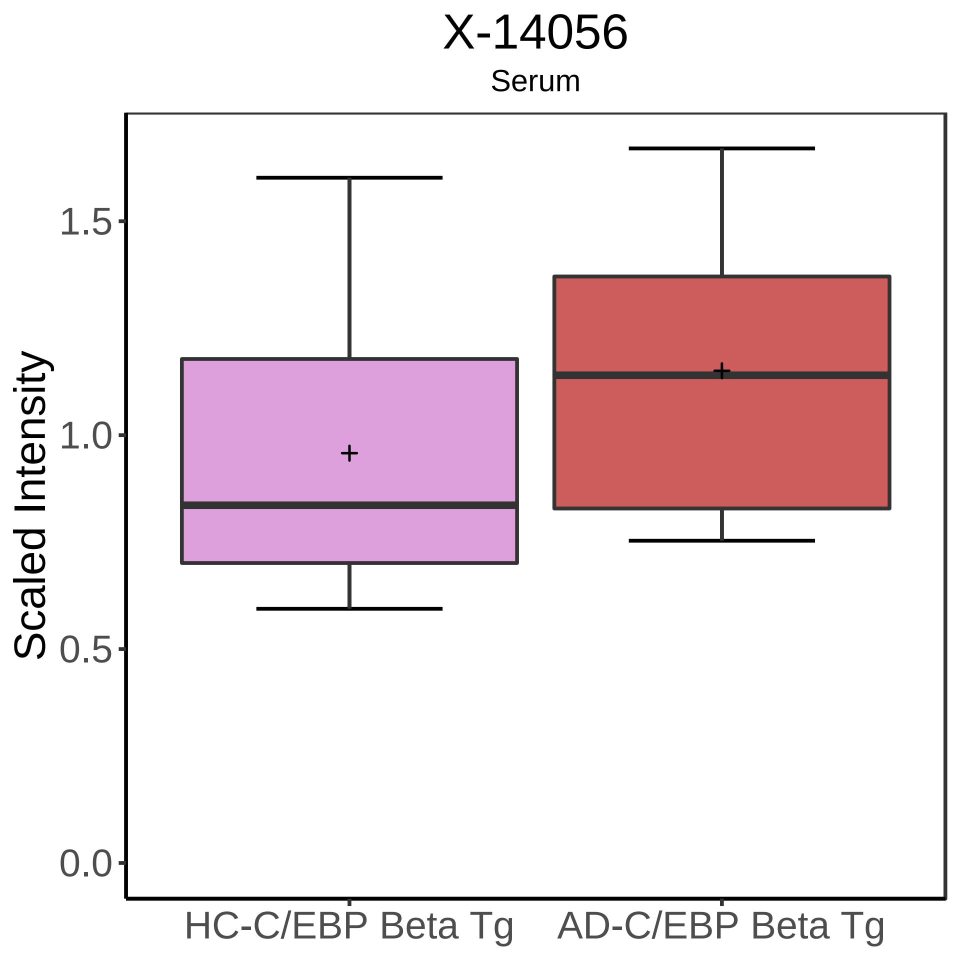
X-14056
Serum
Scaled Intensity
1.5
1.0
0.5
0.0
HC-C/EBP Beta Tg
AD-C/EBP Beta Tg

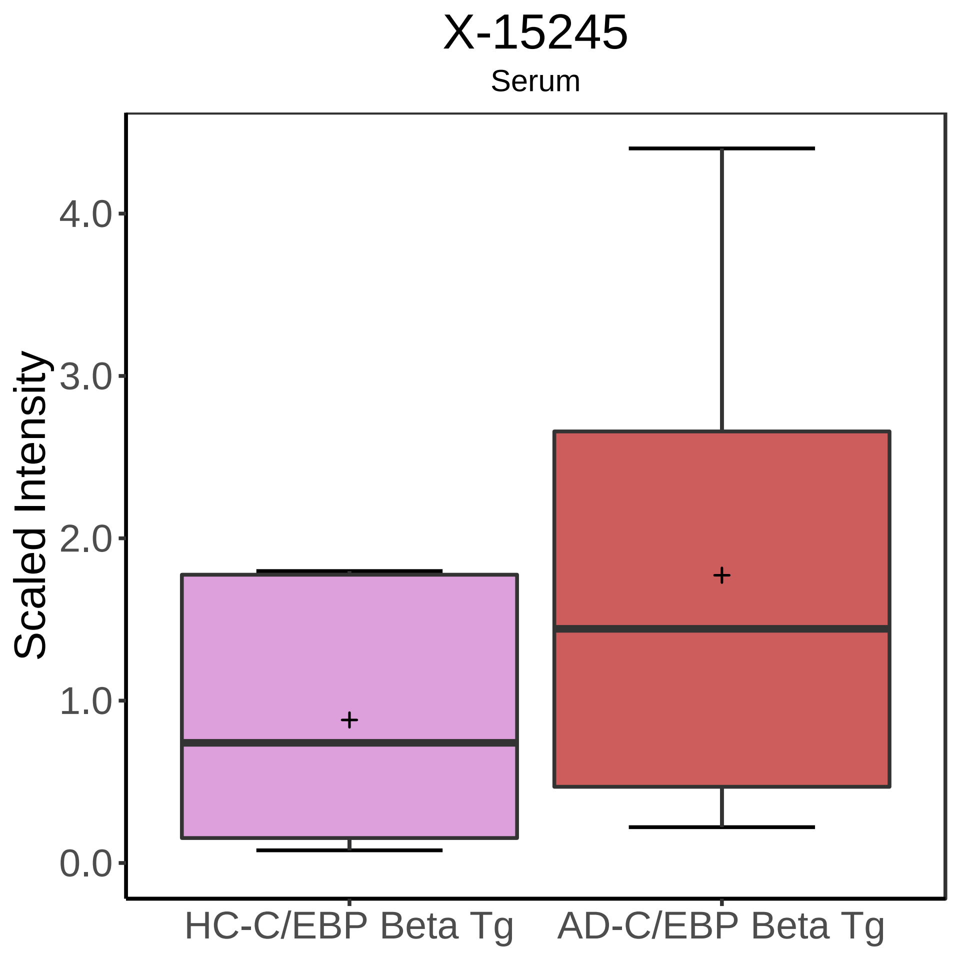
X-15245
Serum
Scaled Intensity
4.0
3.0
2.0
1.0
0.0
HC-C/EBP Beta Tg
AD-C/EBP Beta Tg

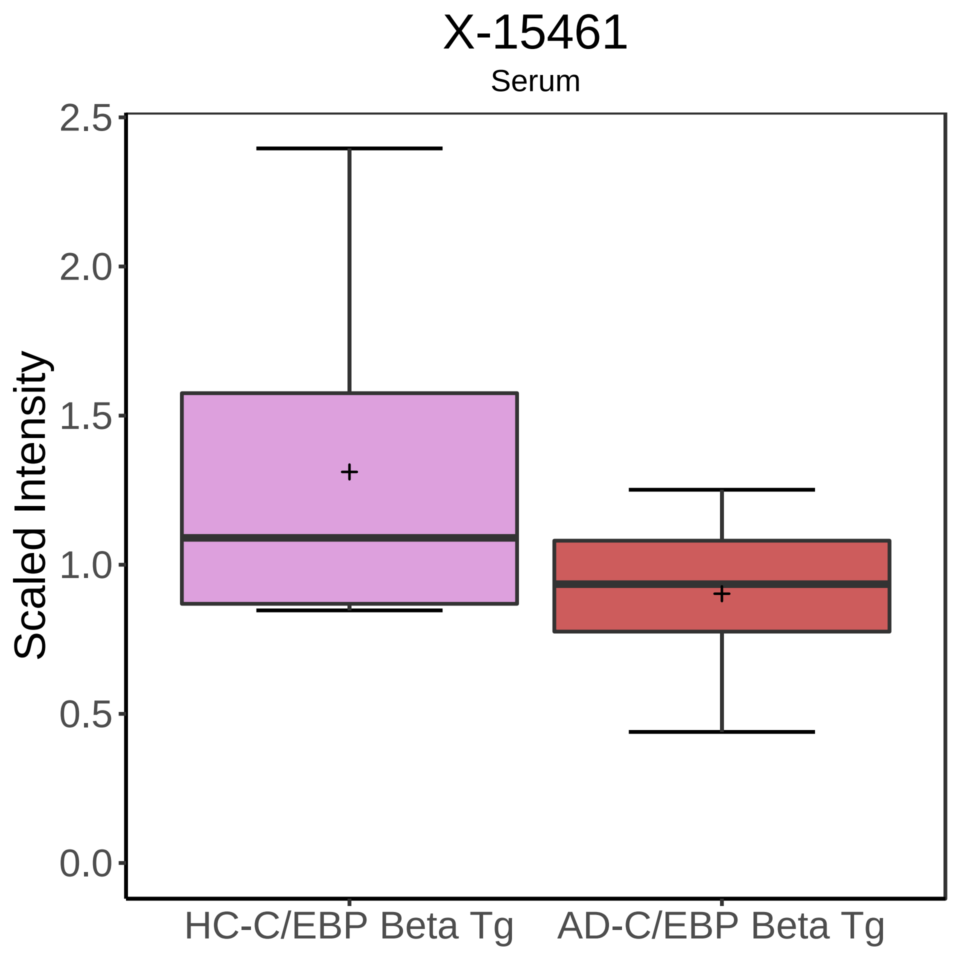
X-15461
Serum
Scaled Intensity
2.5
2.0
1.5
1.0
0.5
0.0
HC-C/EBP Beta Tg
AD-C/EBP Beta Tg

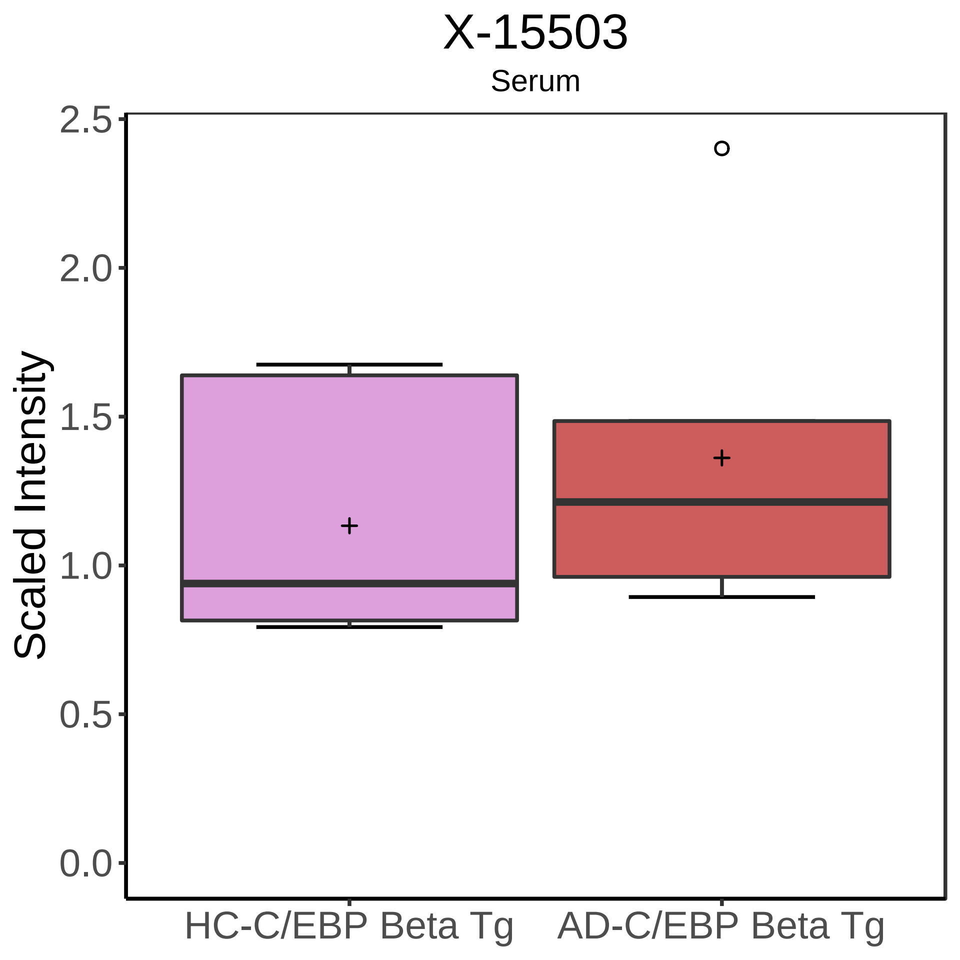

X-15503
Serum
Scaled Intensity
2.5
2.0
1.5
1.0
0.5
0.0
HC-C/EBP Beta Tg
AD-C/EBP Beta Tg

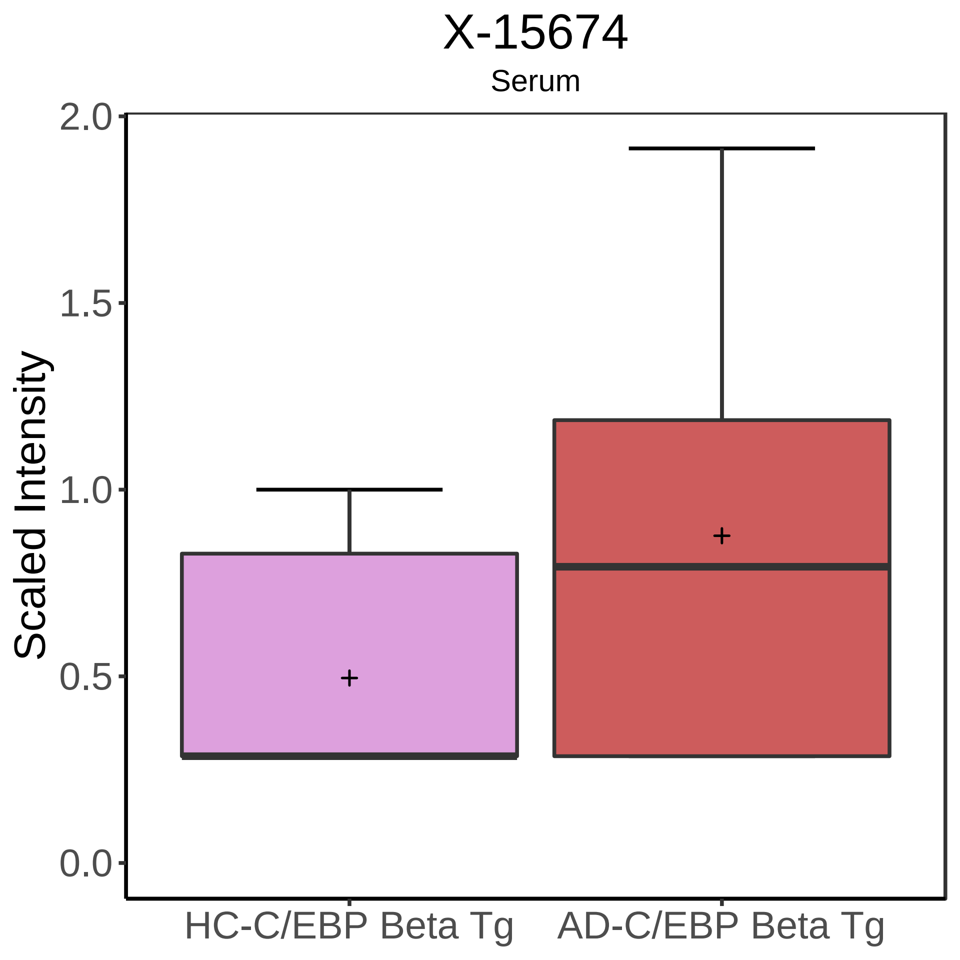
X-15674
Serum
Scaled Intensity
2.0
1.5
1.0
0.5
0.0
HC-C/EBP Beta Tg
AD-C/EBP Beta Tg

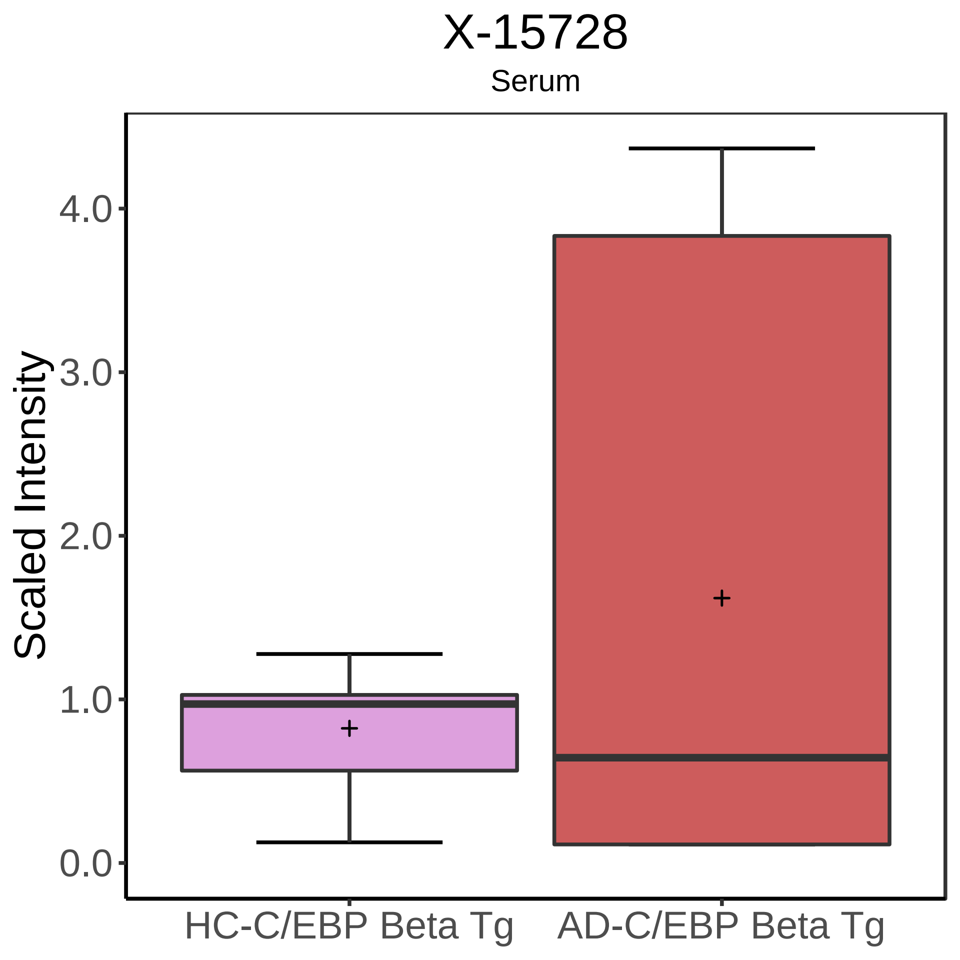
X-15728
Serum
Scaled Intensity
4.0
3.0
2.0
1.0
0.0
HC-C/EBP Beta Tg
AD-C/EBP Beta Tg

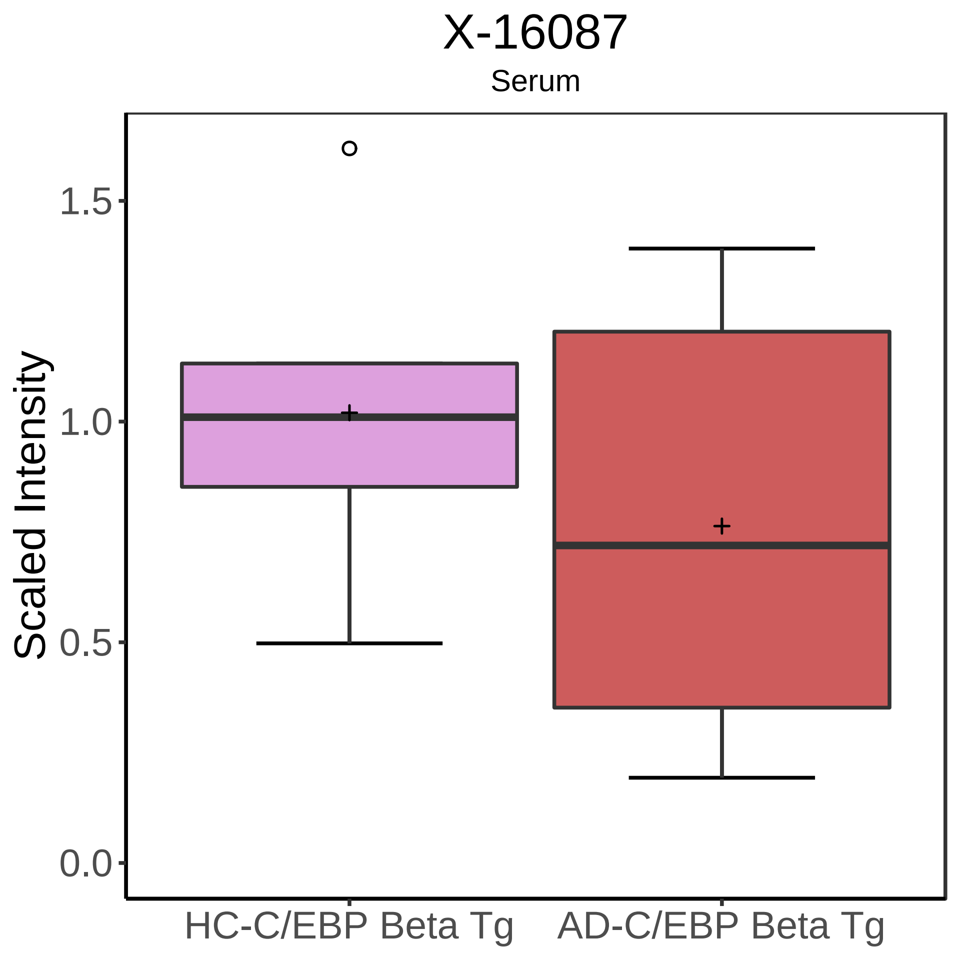
X-16087
Serum
Scaled Intensity
1.5
1.0
0.5
0.0
HC-C/EBP Beta Tg
AD-C/EBP Beta Tg

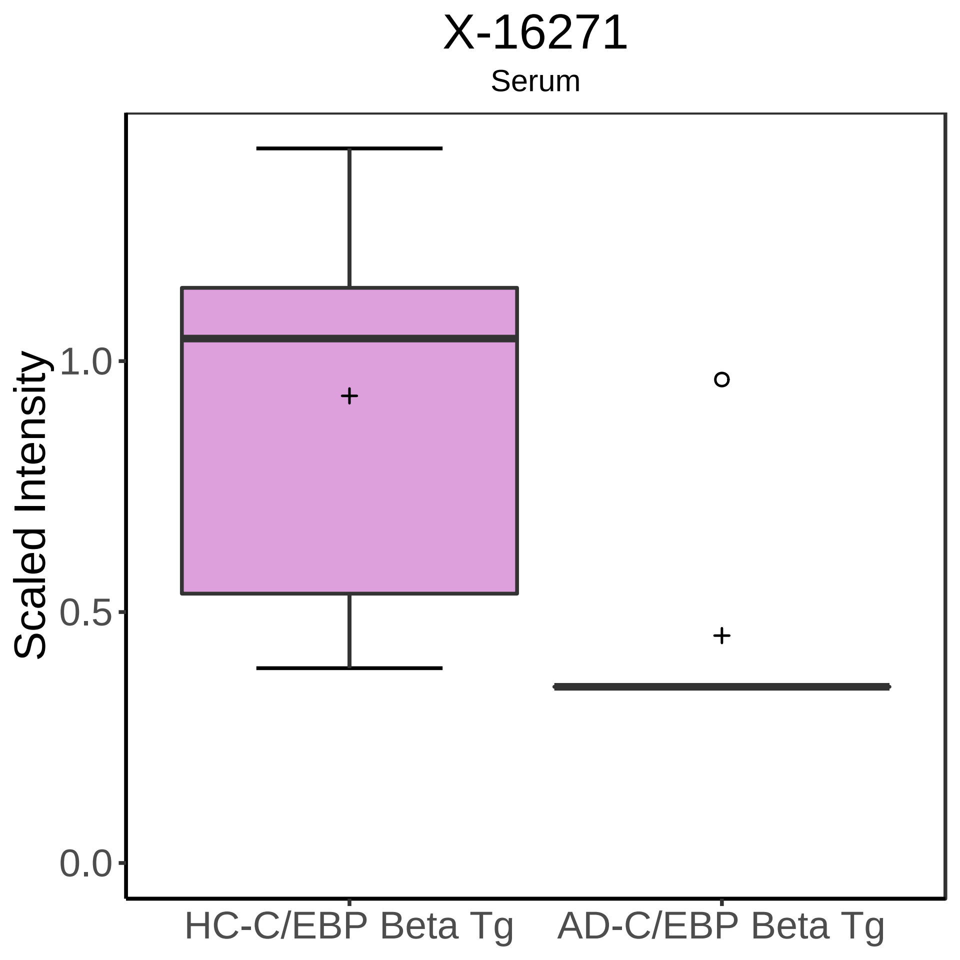
X-16271
Serum
Scaled Intensity
1.0
0.5
0.0
HC-C/EBP Beta Tg
AD-C/EBP Beta Tg

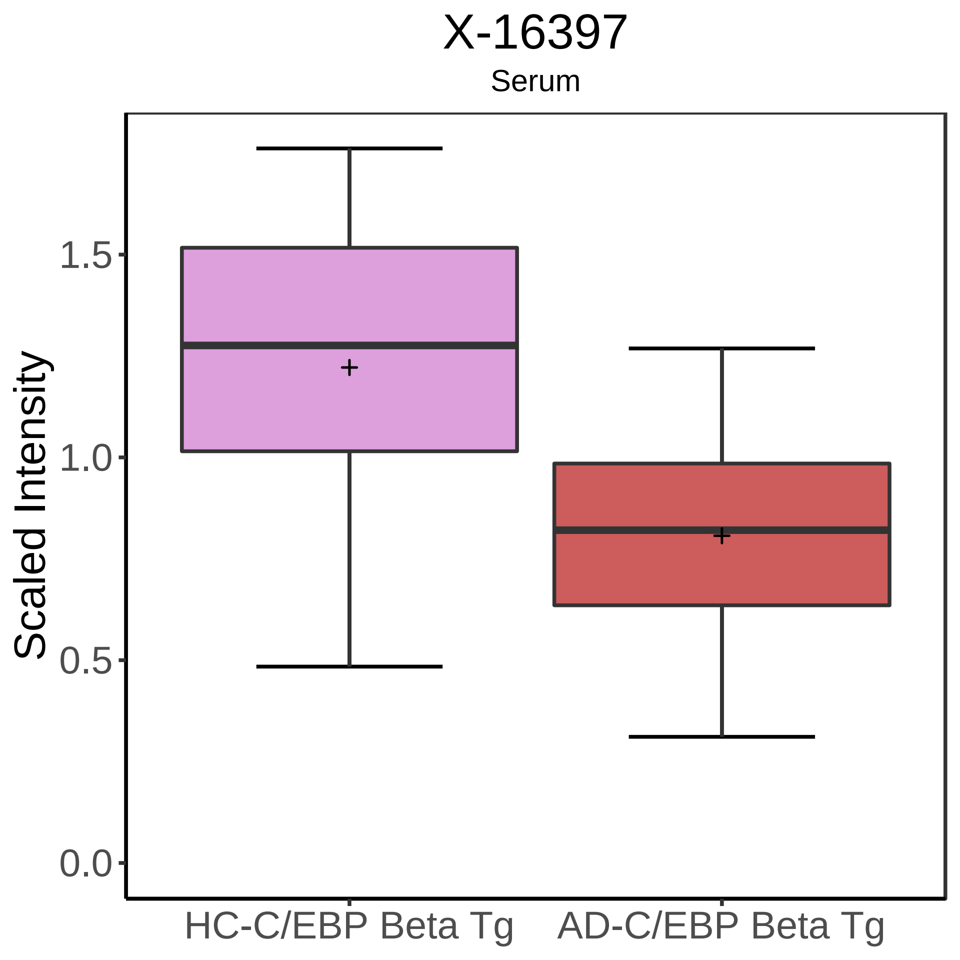

X-16397
Serum
Scaled Intensity
0.0
0.5
1.0
1.5
HC-C/EBP Beta Tg
AD-C/EBP Beta Tg

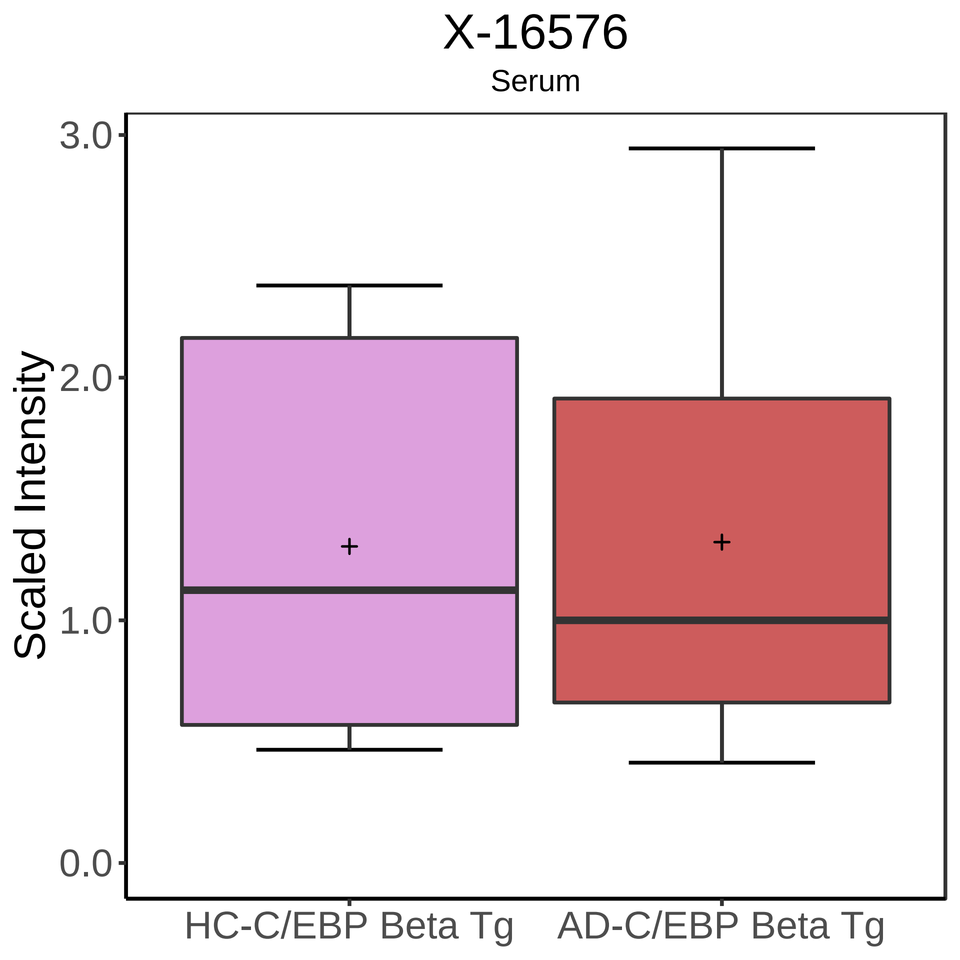

X-16576
Serum
Scaled Intensity
3.0
2.0
1.0
0.0
HC-C/EBP Beta Tg
AD-C/EBP Beta Tg

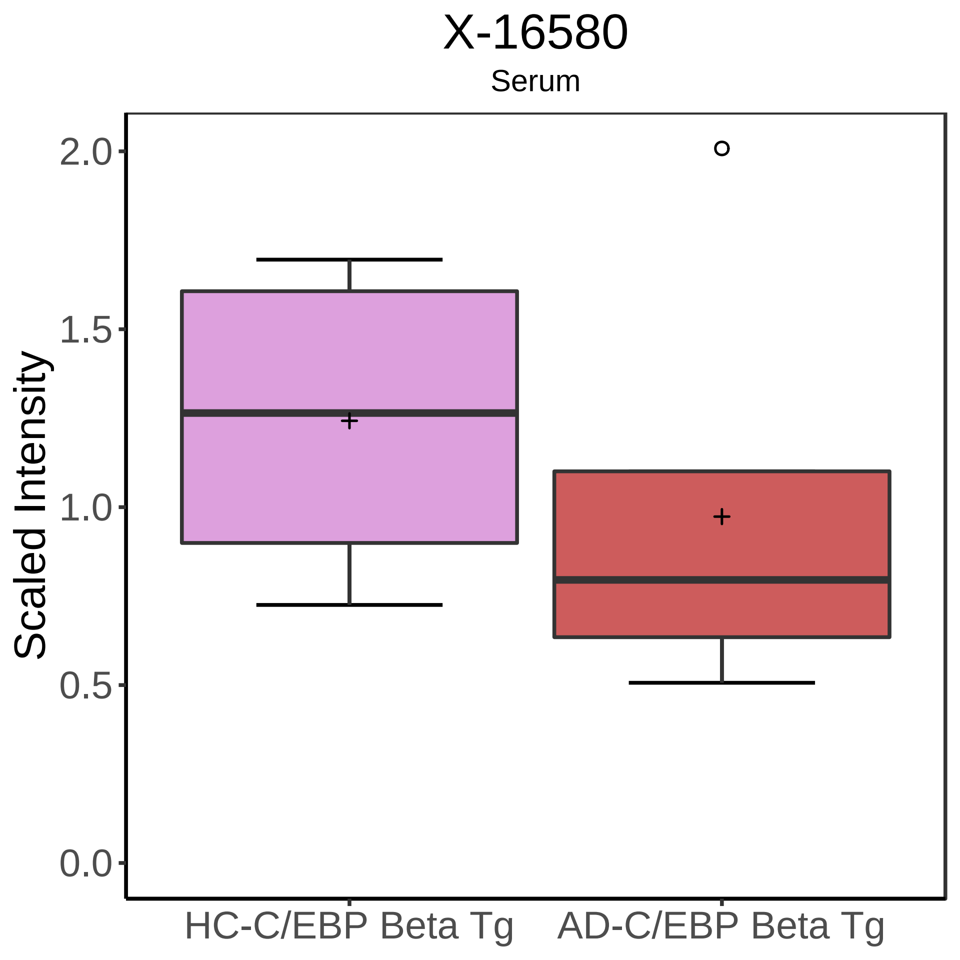
X-16580
Serum
Scaled Intensity
2.0
1.5
1.0
0.5
0.0
HC-C/EBP Beta Tg
AD-C/EBP Beta Tg

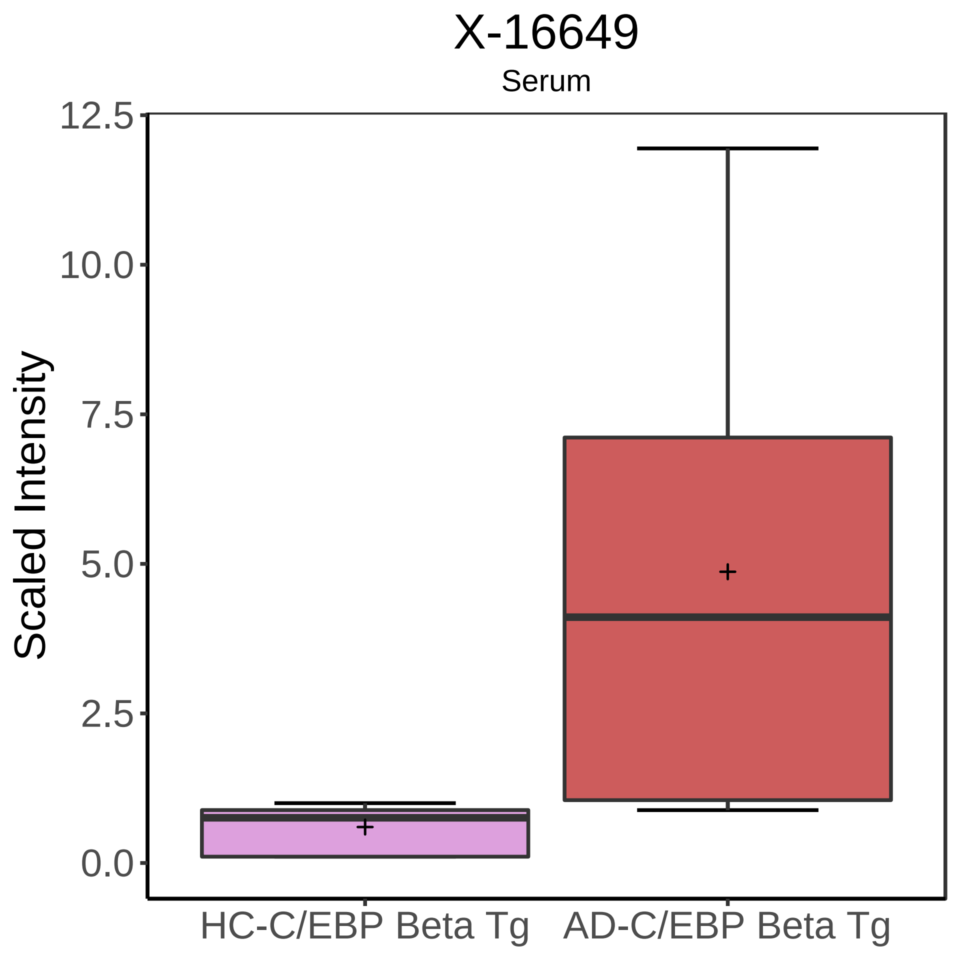
X-16649
Serum
Scaled Intensity
12.5
10.0
7.5
5.0
2.5
0.0
HC-C/EBP Beta Tg
AD-C/EBP Beta Tg

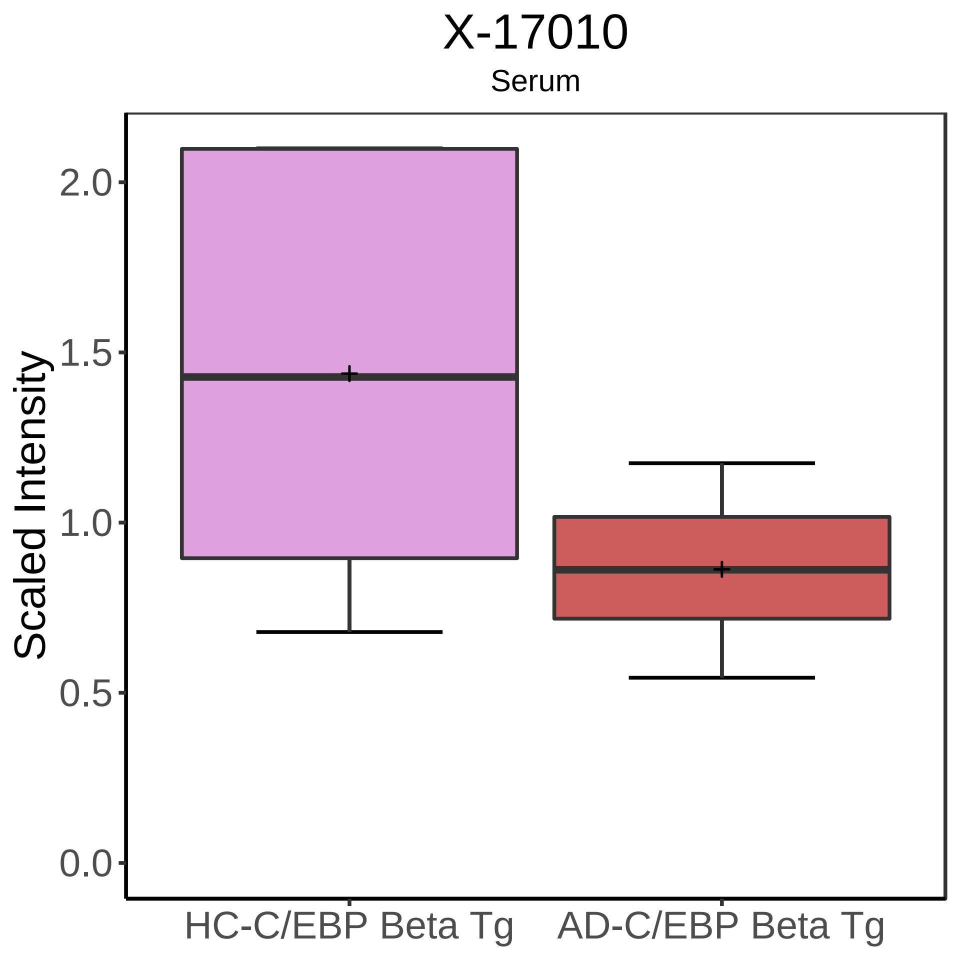
X-17010
Serum
Scaled Intensity
2.0
1.5
1.0
0.5
0.0
HC-C/EBP Beta Tg
AD-C/EBP Beta Tg

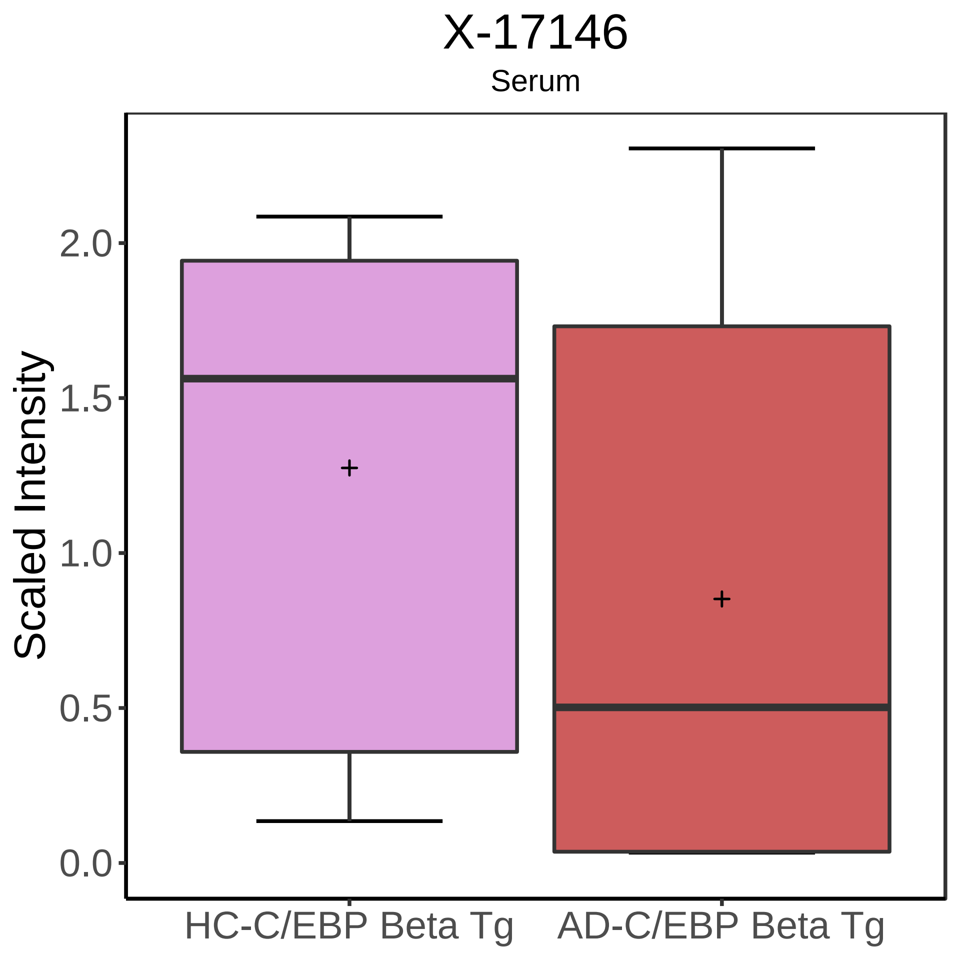

X-17146
Serum
Scaled Intensity
2.0
1.5
1.0
0.5
0.0
HC-C/EBP Beta Tg
AD-C/EBP Beta Tg

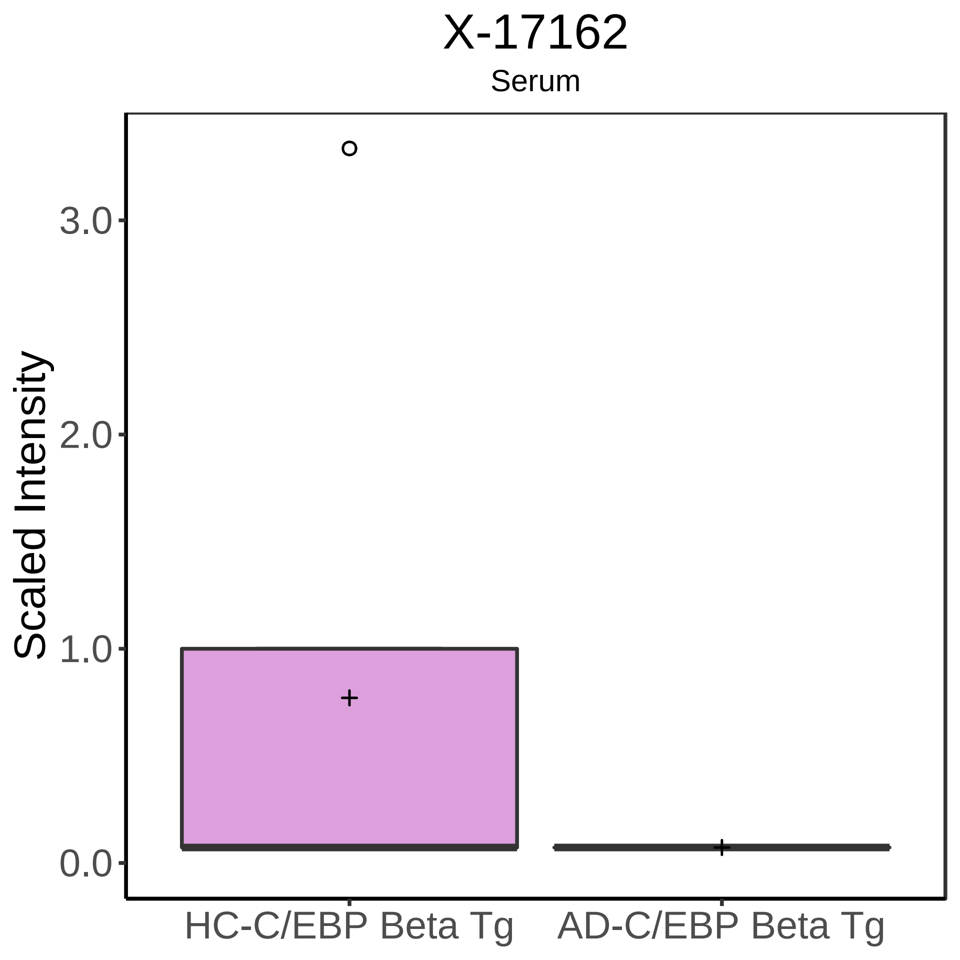
X-17162
Serum
Scaled Intensity
3.0
2.0
1.0
0.0
HC-C/EBP Beta Tg
AD-C/EBP Beta Tg

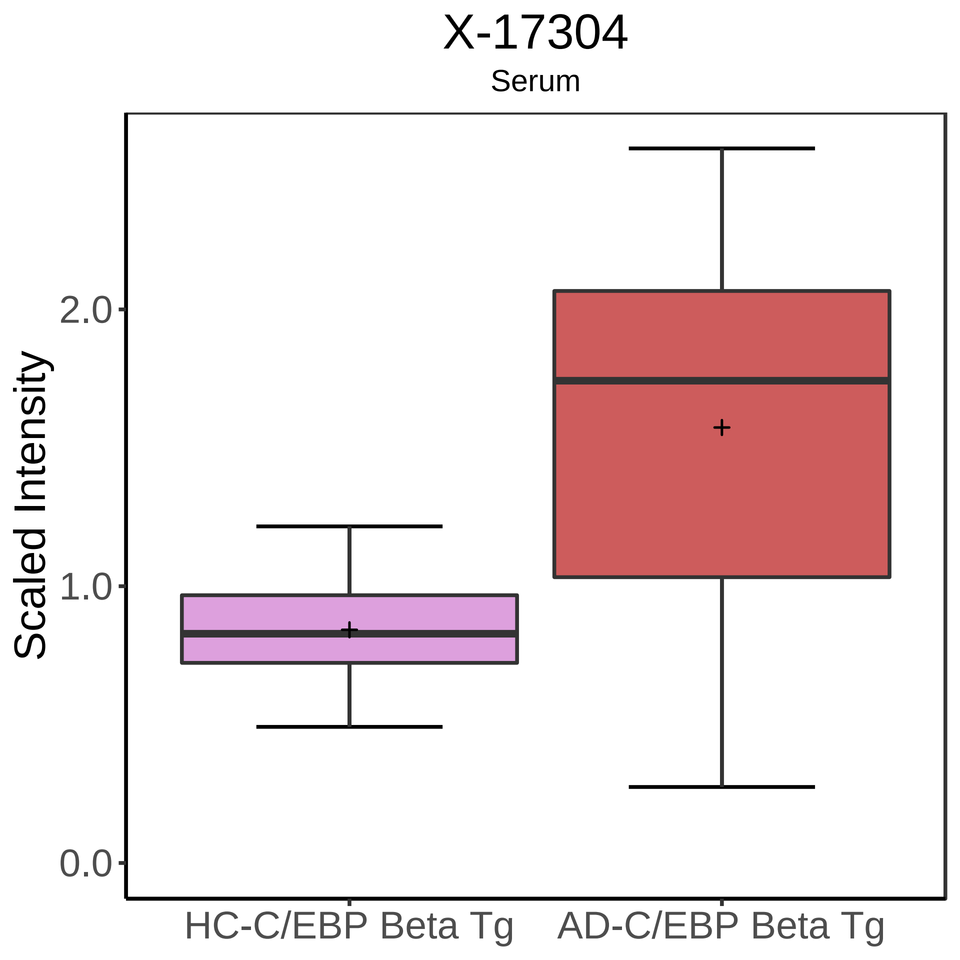
X-17304
Serum
Scaled Intensity
2.0
1.0
0.0
HC-C/EBP Beta Tg
AD-C/EBP Beta Tg

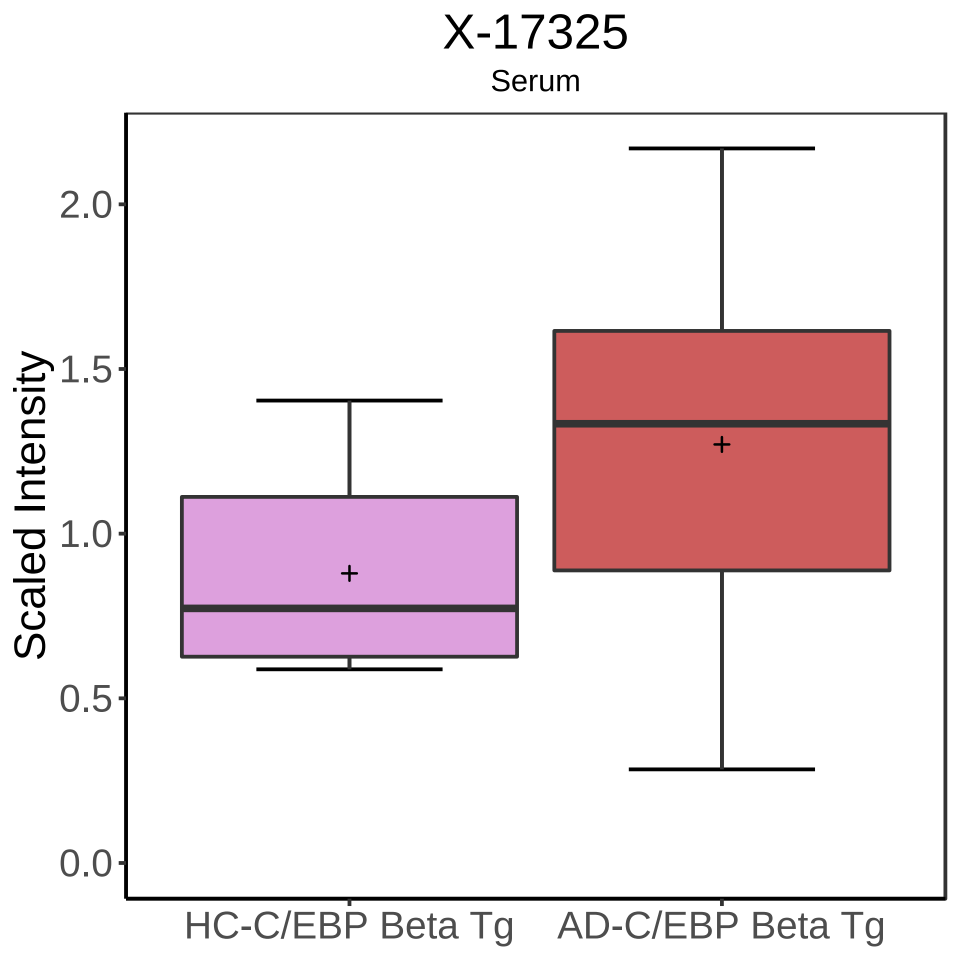
X-17325
Serum
Scaled Intensity
2.0
1.5
1.0
0.5
0.0
HC-C/EBP Beta Tg
AD-C/EBP Beta Tg

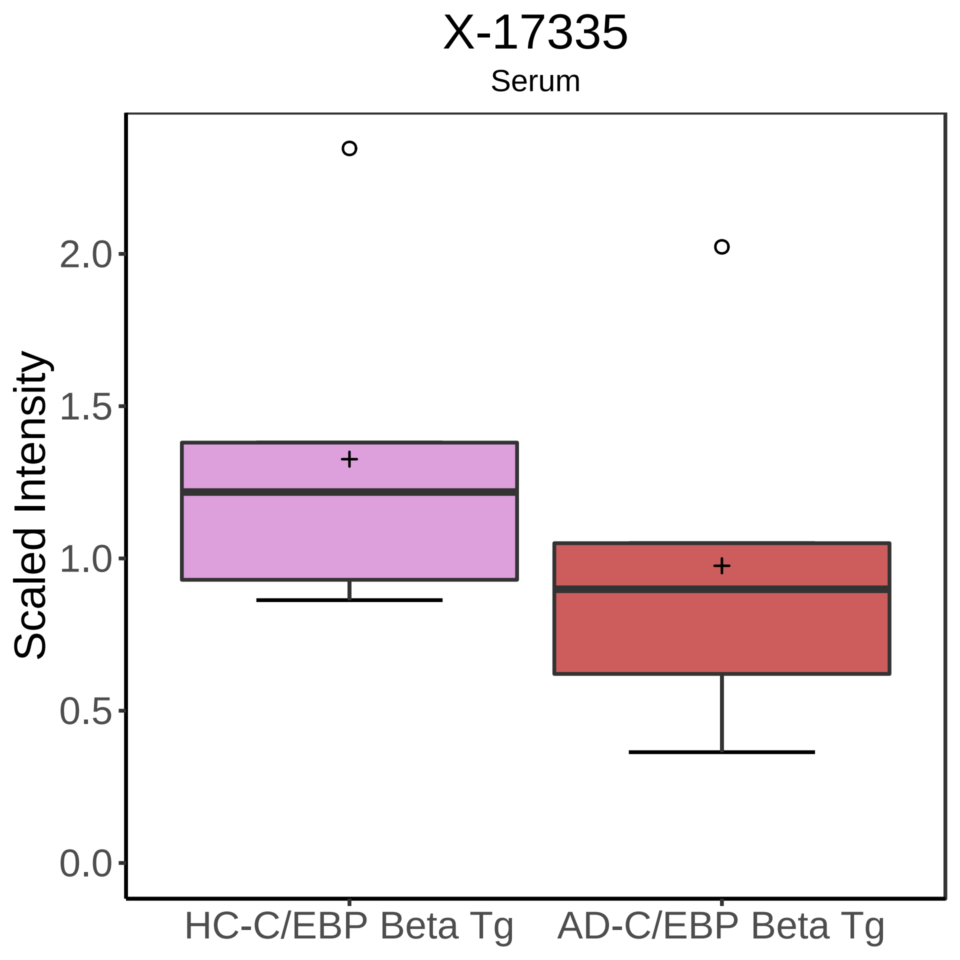
X-17335
Serum
Scaled Intensity
2.0
1.5
1.0
0.5
0.0
HC-C/EBP Beta Tg
AD-C/EBP Beta Tg

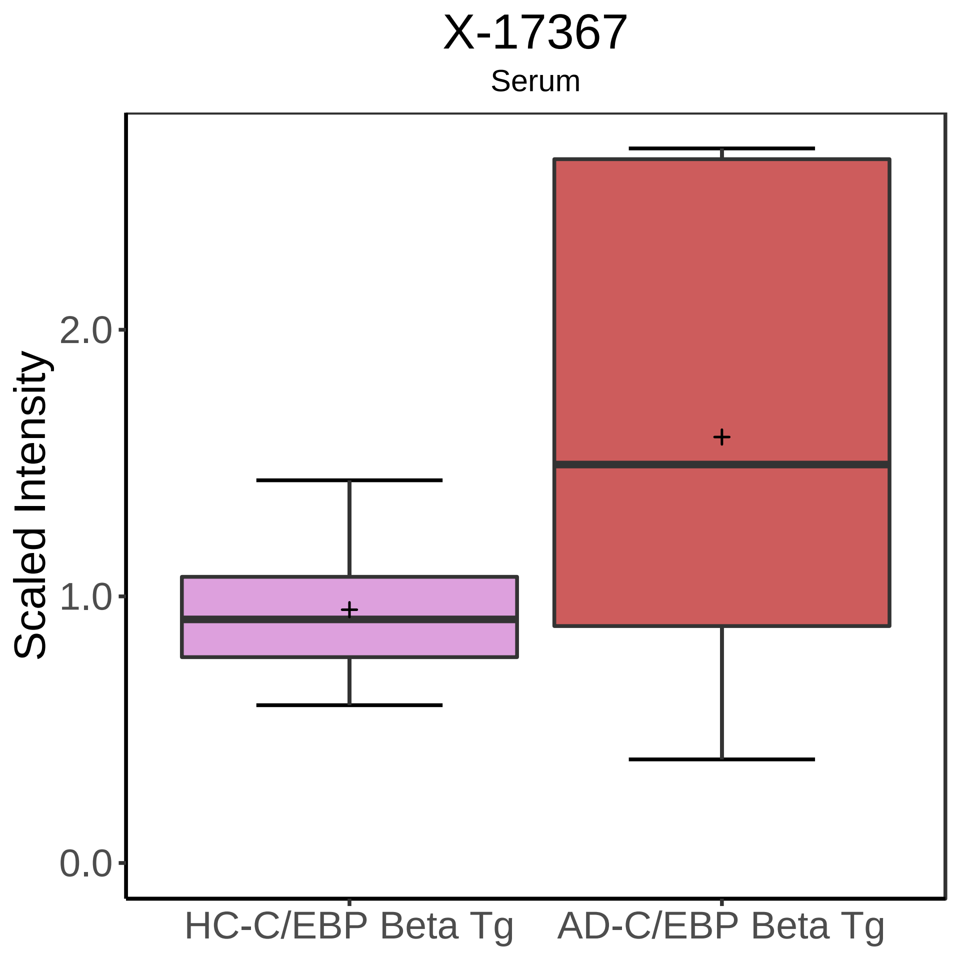

X-17367
Serum
Scaled Intensity
2.0
1.0
0.0
HC-C/EBP Beta Tg
AD-C/EBP Beta Tg

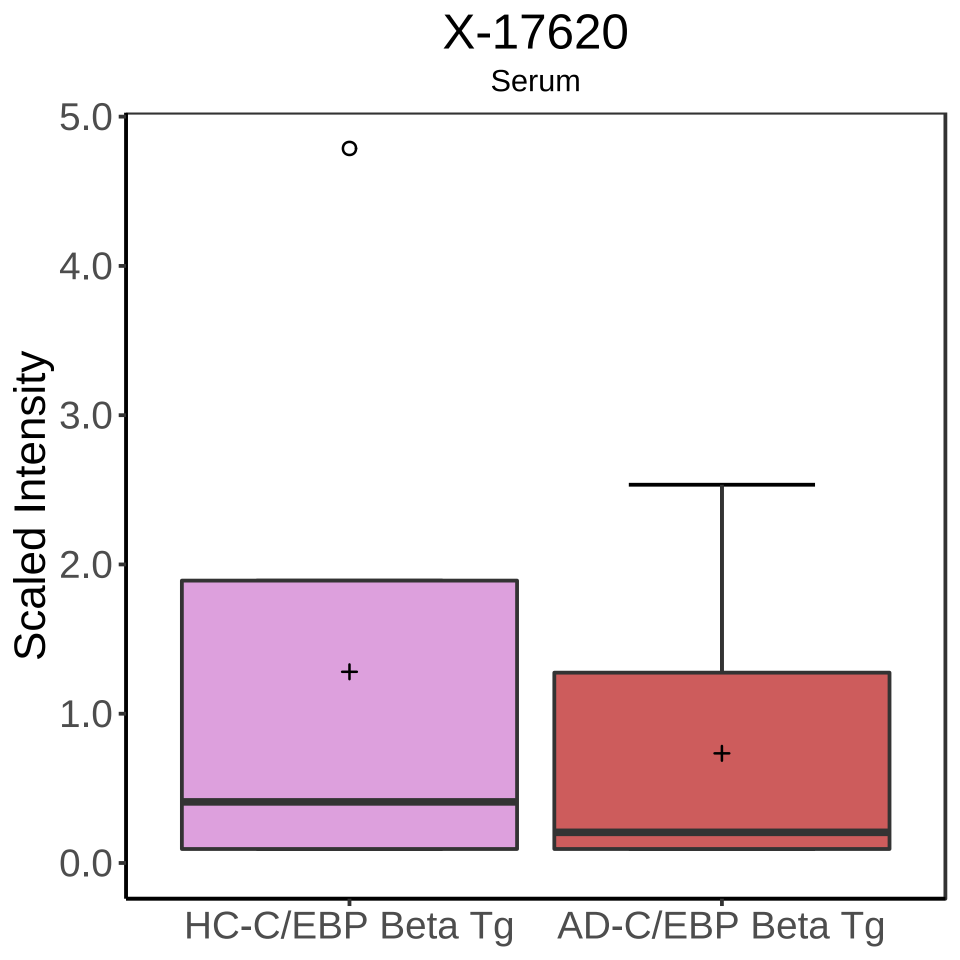
X-17620
Serum
Scaled Intensity
5.0
4.0
3.0
2.0
1.0
0.0
HC-C/EBP Beta Tg
AD-C/EBP Beta Tg

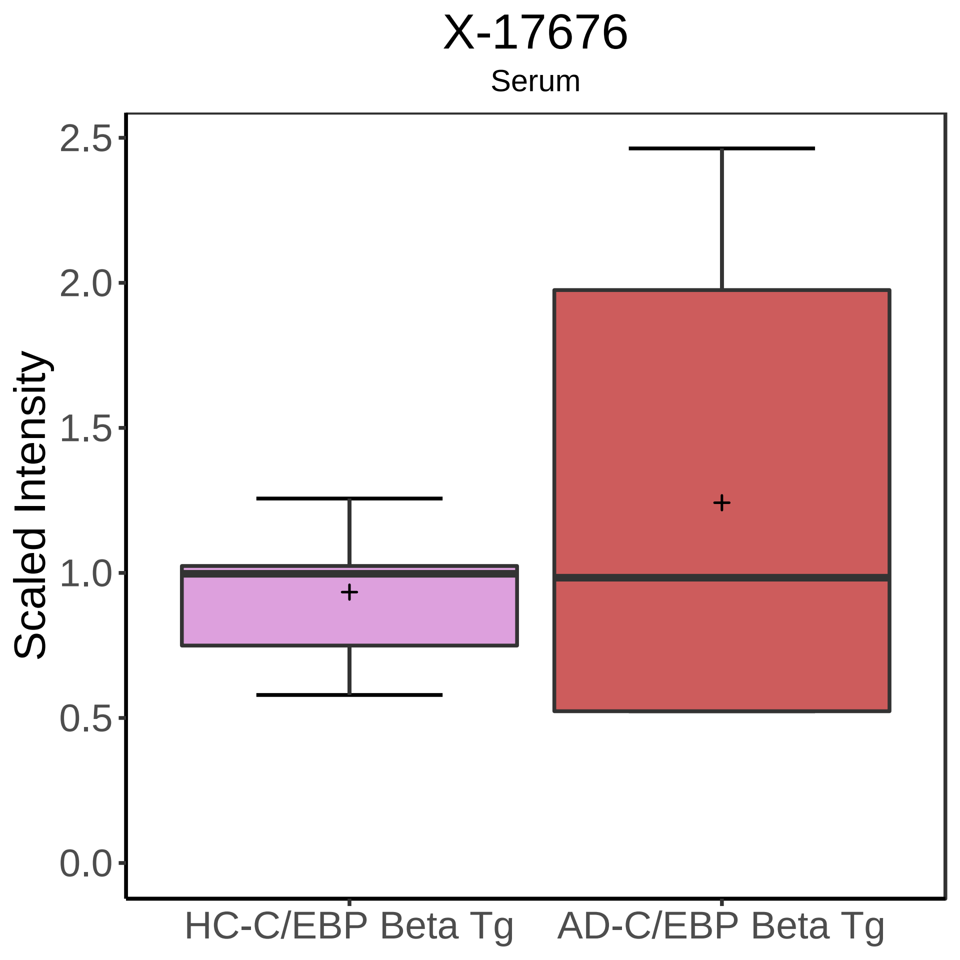
X-17676
Serum
Scaled Intensity
2.5
2.0
1.5
1.0
0.5
0.0
HC-C/EBP Beta Tg
AD-C/EBP Beta Tg

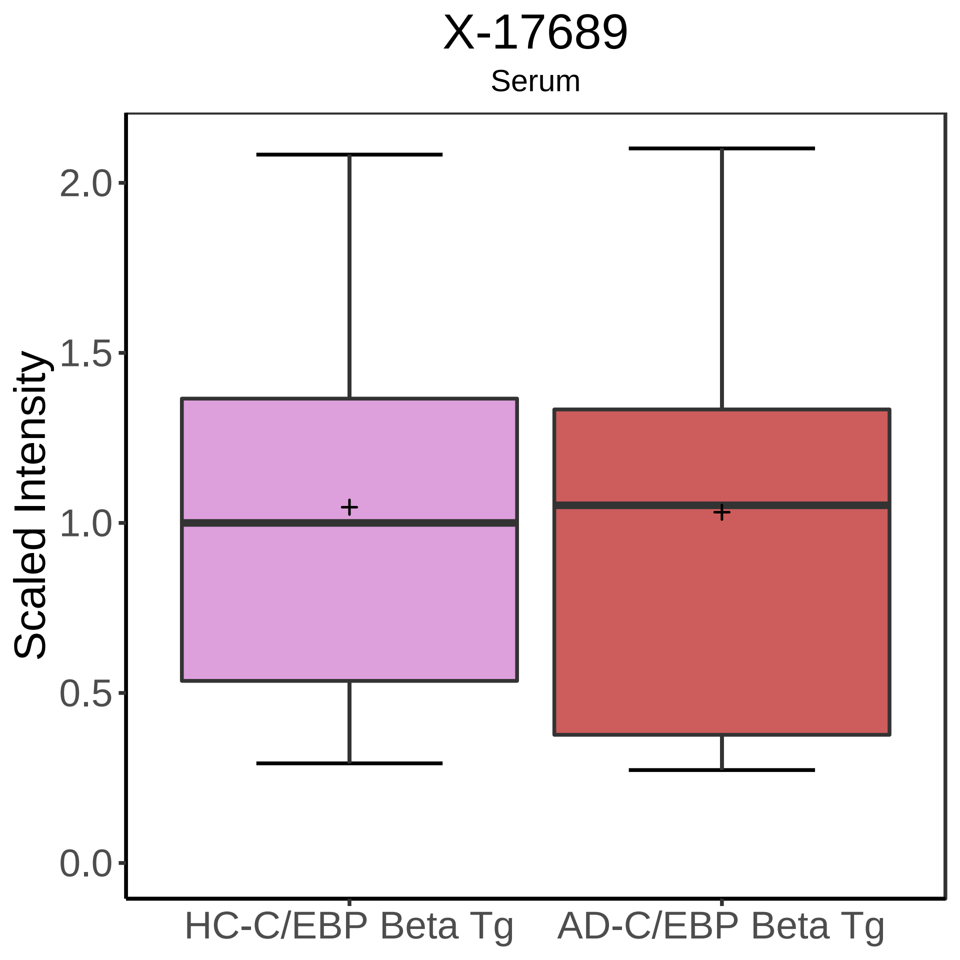
X-17689
Serum
Scaled Intensity
2.0
1.5
1.0
0.5
0.0
HC-C/EBP Beta Tg
AD-C/EBP Beta Tg

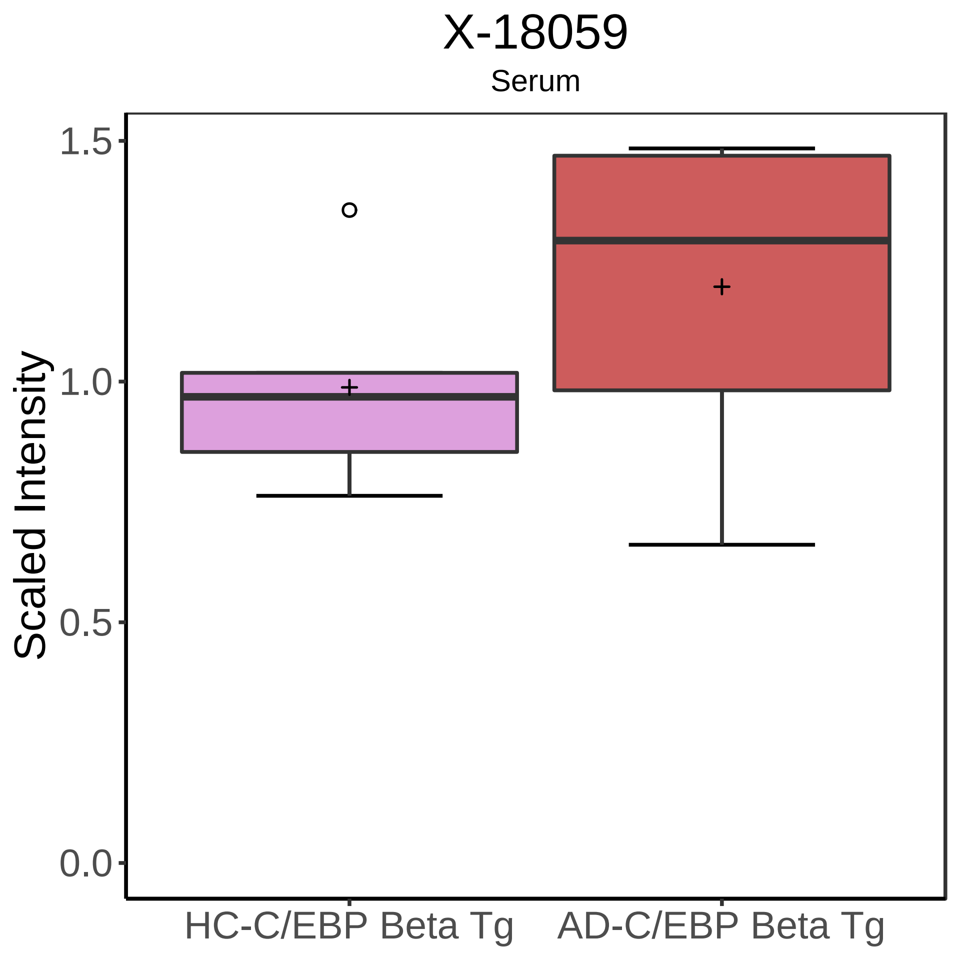
X-18059
Serum
Scaled Intensity
1.5
1.0
0.5
0.0
HC-C/EBP Beta Tg
AD-C/EBP Beta Tg

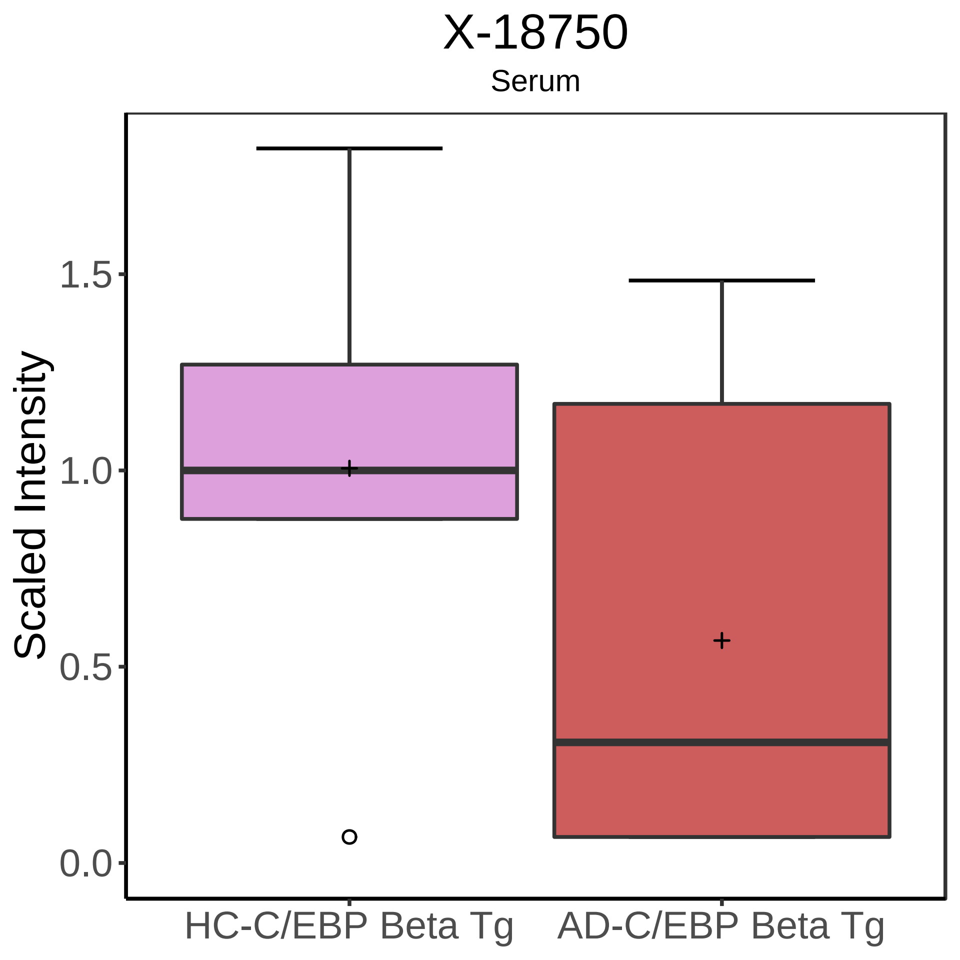
X-18750
Serum
Scaled Intensity
1.5
1.0
0.5
0.0
HC-C/EBP Beta Tg
AD-C/EBP Beta Tg

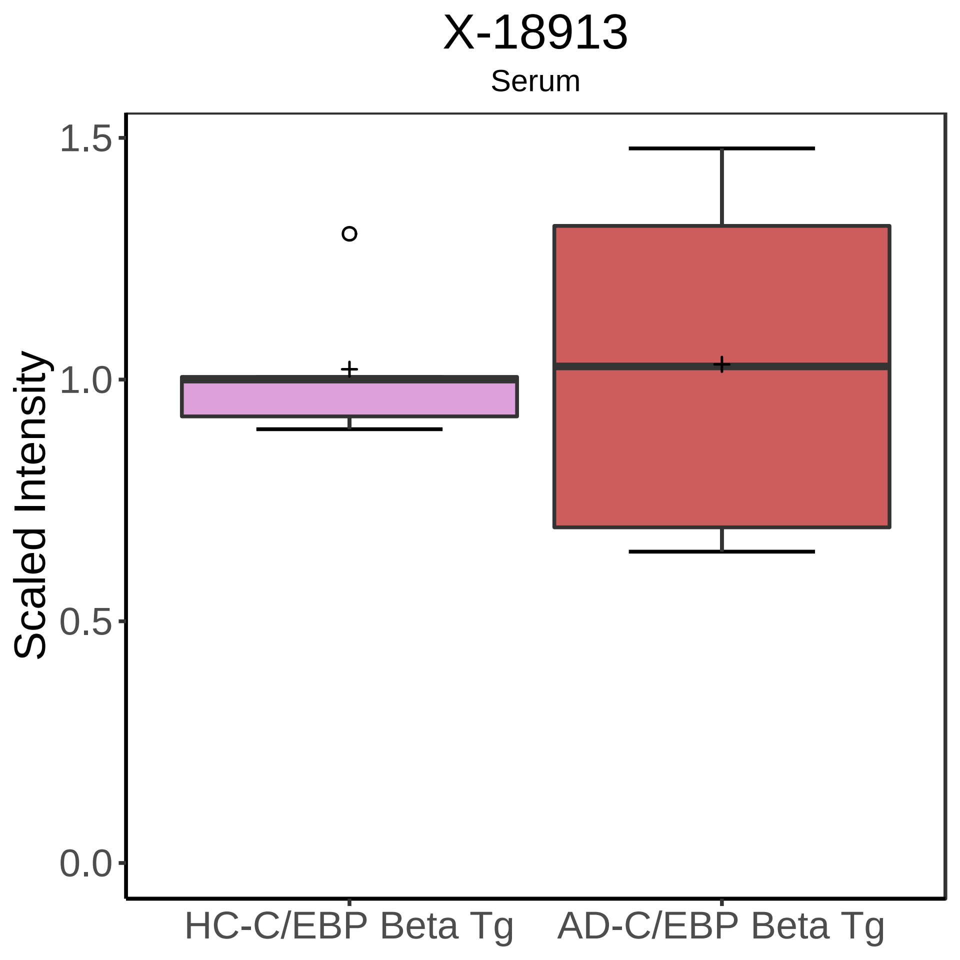
X-18913
Serum
Scaled Intensity
1.5
1.0
0.5
0.0
HC-C/EBP Beta Tg
AD-C/EBP Beta Tg

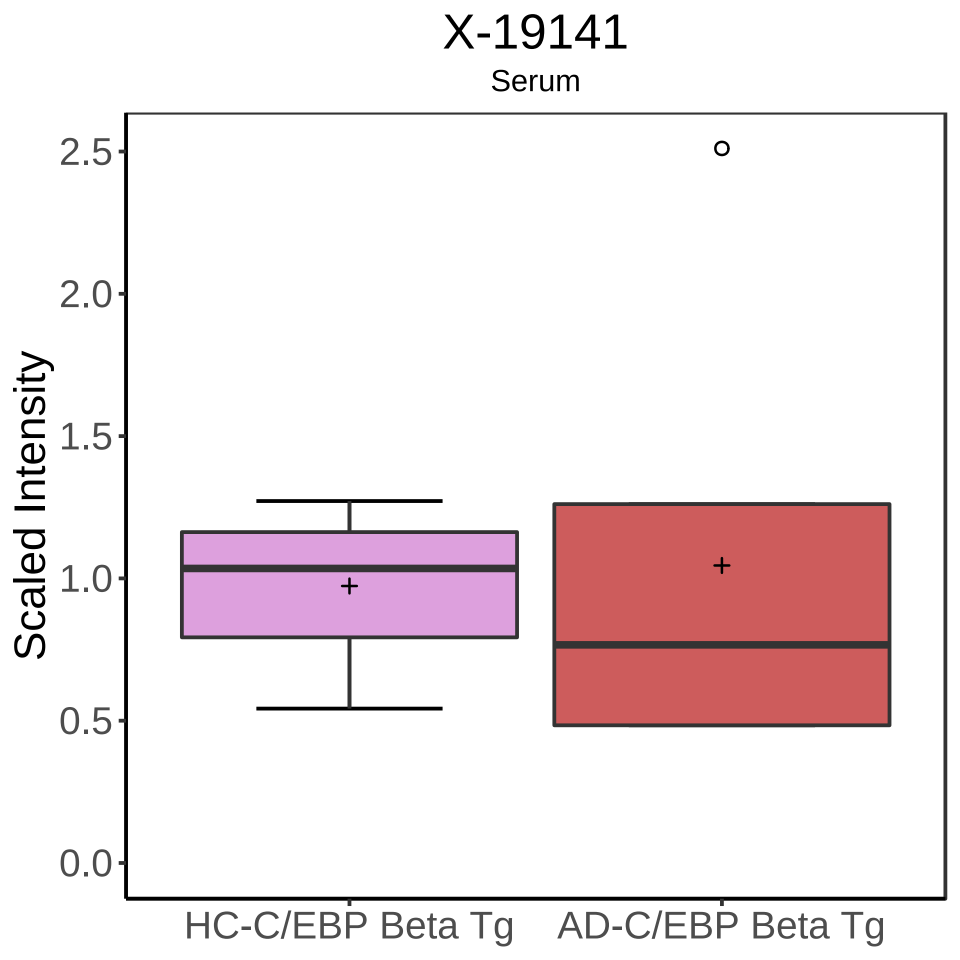
X-19141
Serum
Scaled Intensity
2.5
2.0
1.5
1.0
0.5
0.0
HC-C/EBP Beta Tg
AD-C/EBP Beta Tg

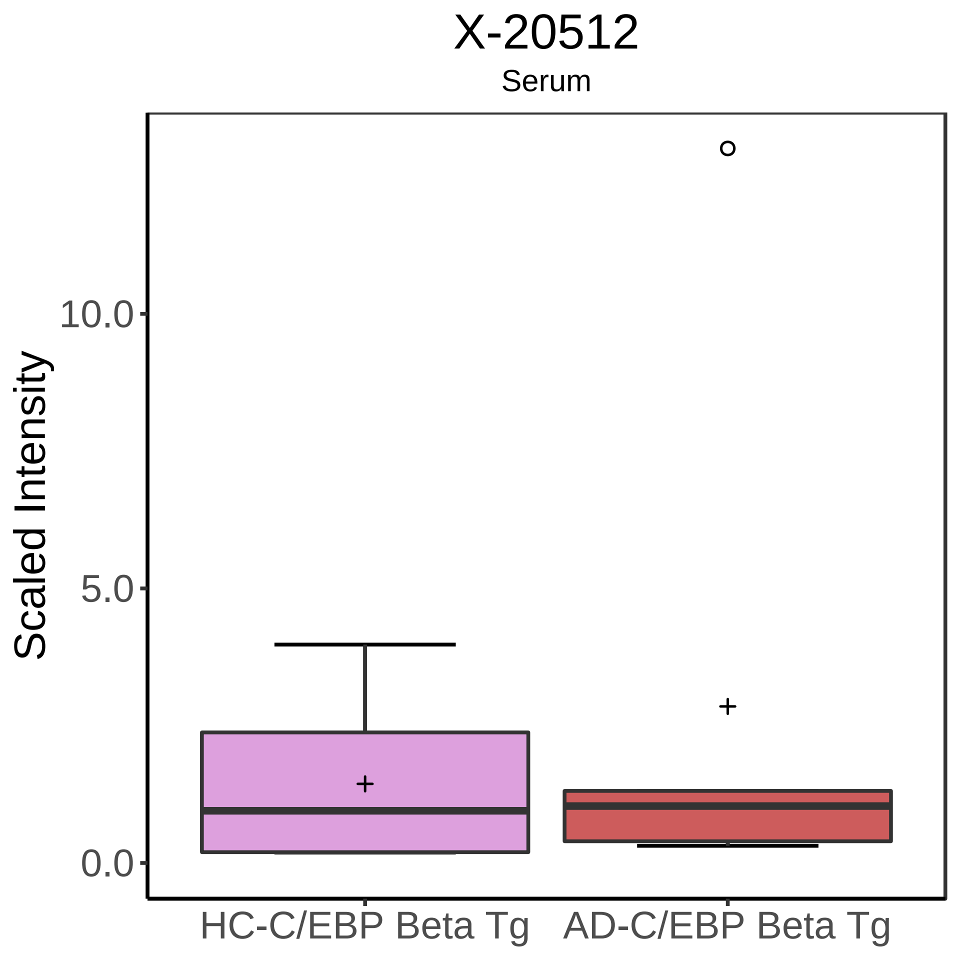
X-20512
Serum
Scaled Intensity
10.0
5.0
0.0
HC-C/EBP Beta Tg
AD-C/EBP Beta Tg

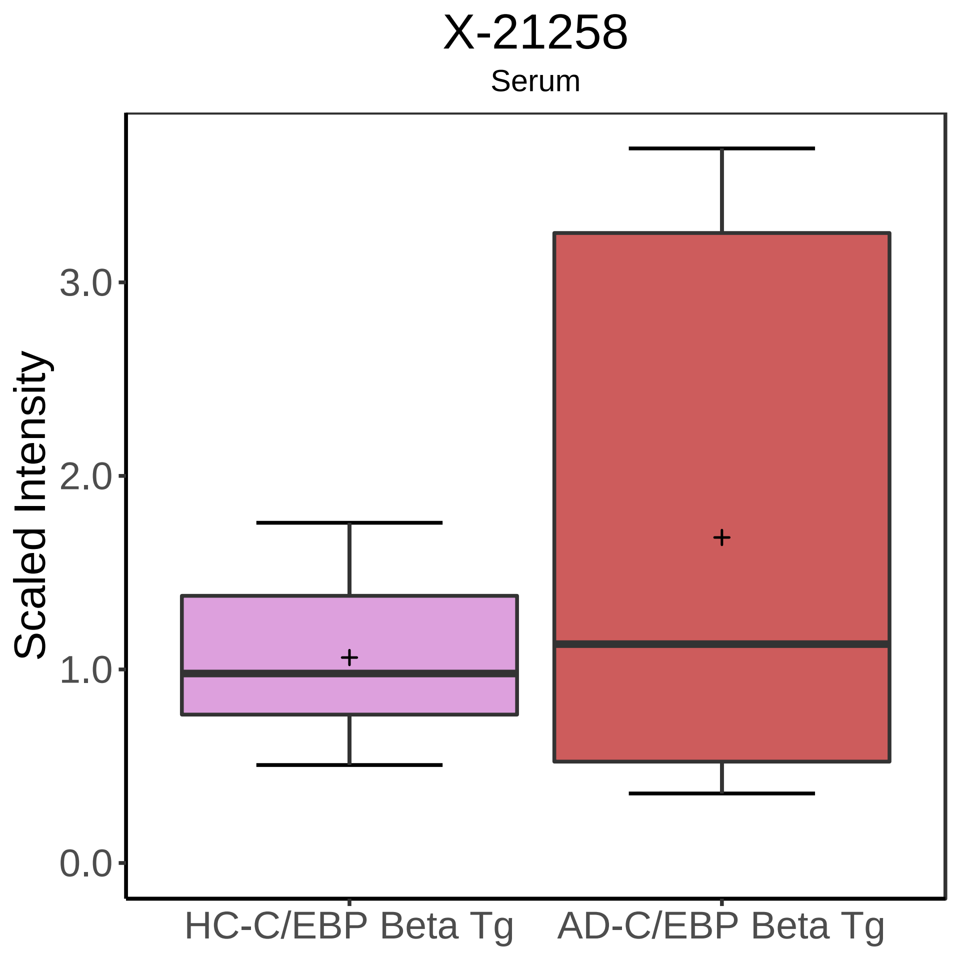
X-21258
Serum
Scaled Intensity
3.0
2.0
1.0
0.0
HC-C/EBP Beta Tg
AD-C/EBP Beta Tg

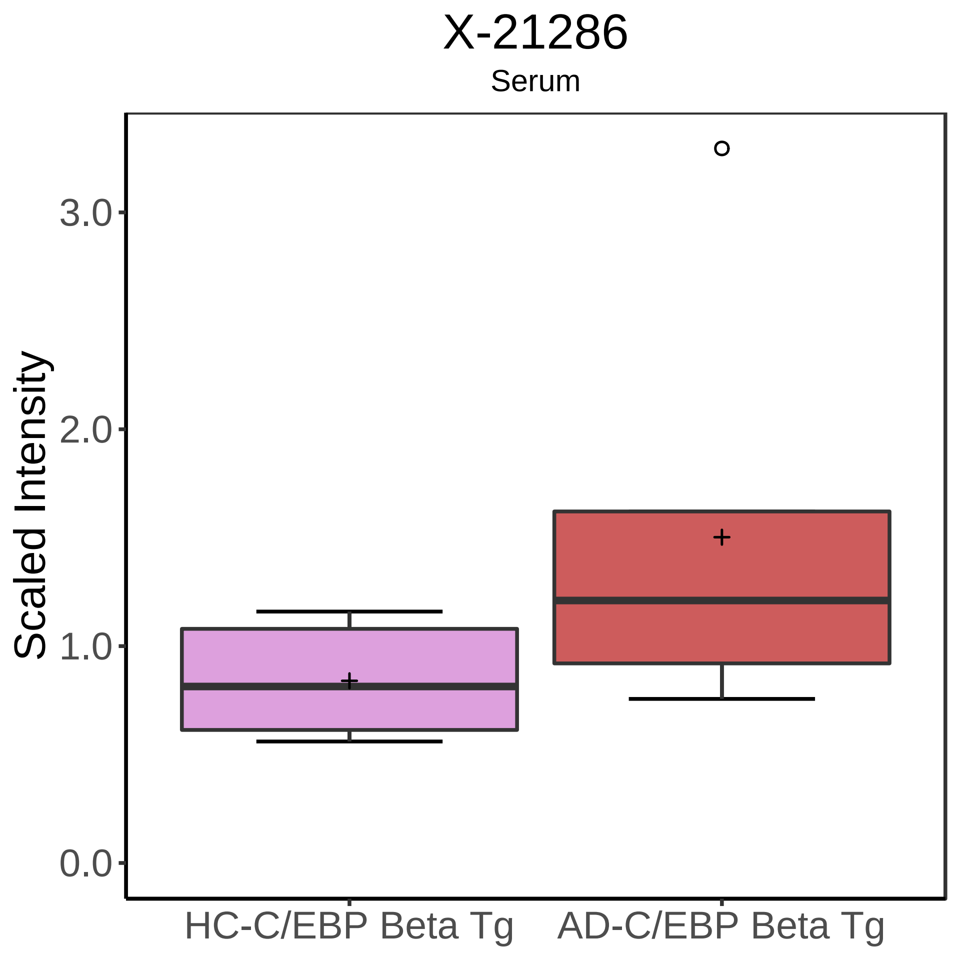
X-21286
Serum
Scaled Intensity
3.0
2.0
1.0
0.0
HC-C/EBP Beta Tg
AD-C/EBP Beta Tg

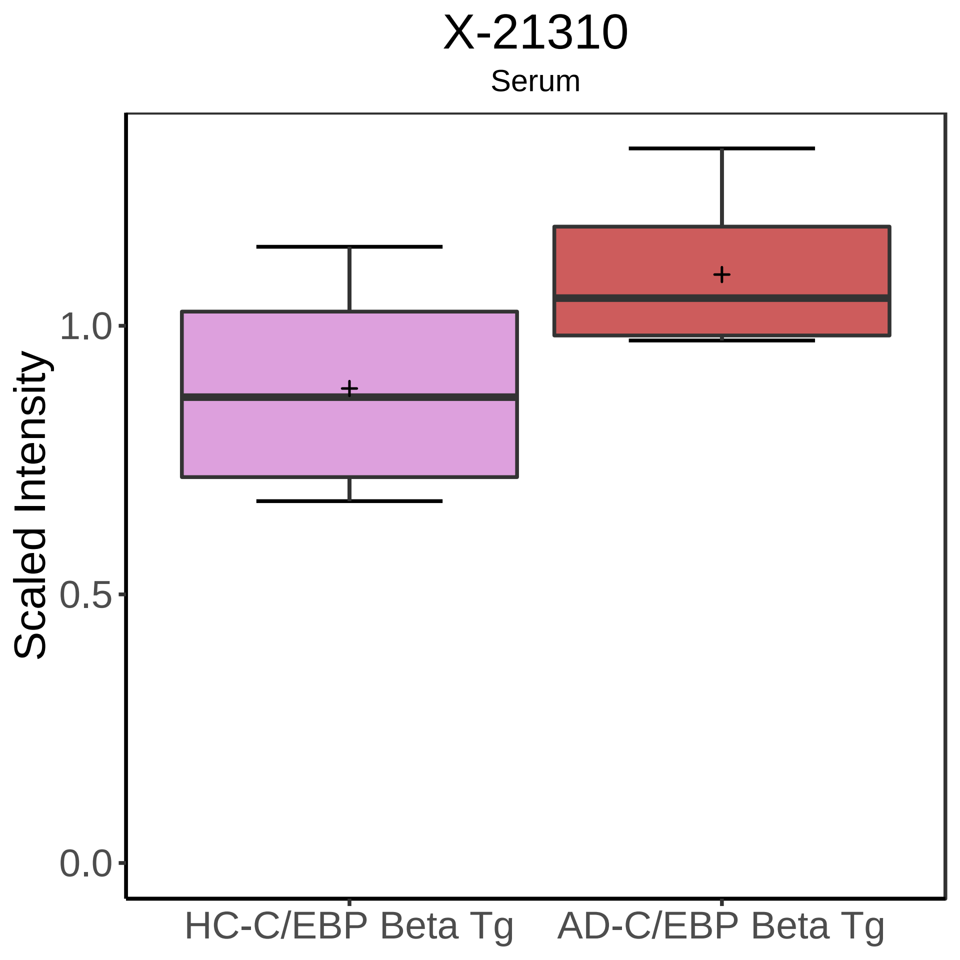
X-21310
Serum
Scaled Intensity
1.0
0.5
0.0
HC-C/EBP Beta Tg
AD-C/EBP Beta Tg

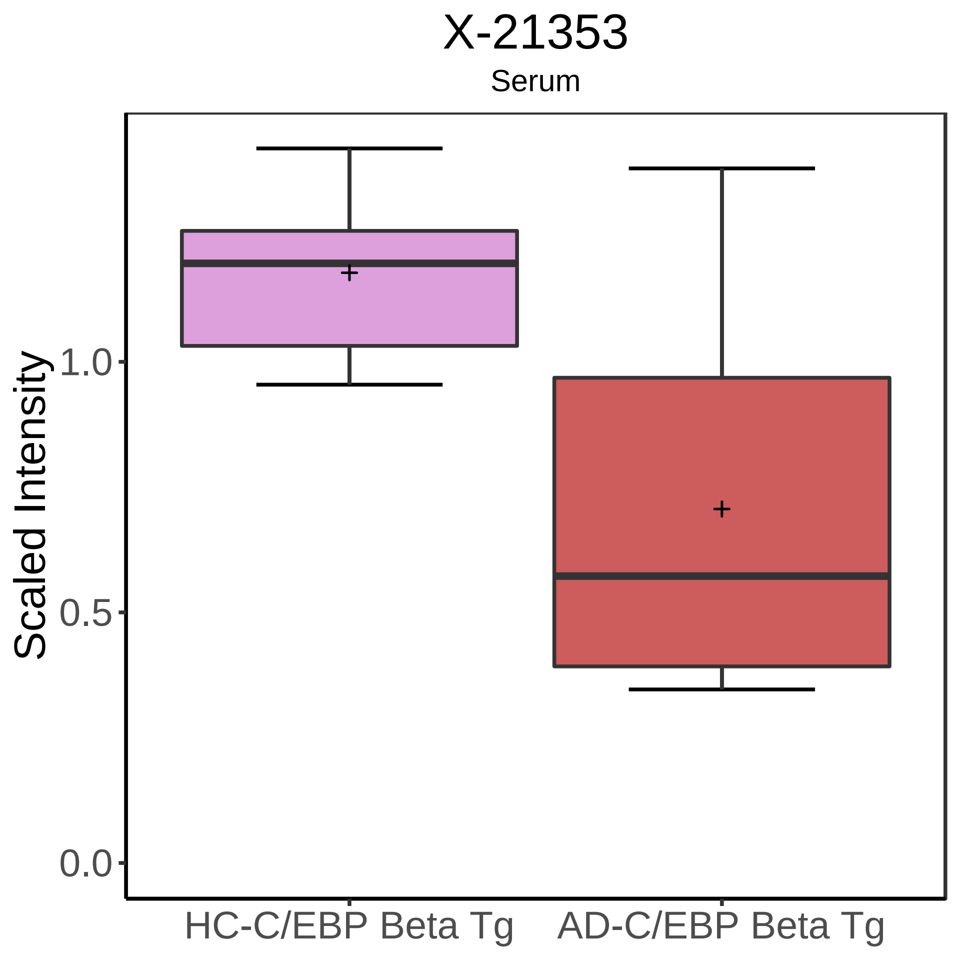

X-21353
Serum
Scaled Intensity
1.0
0.5
0.0
HC-C/EBP Beta Tg
AD-C/EBP Beta Tg

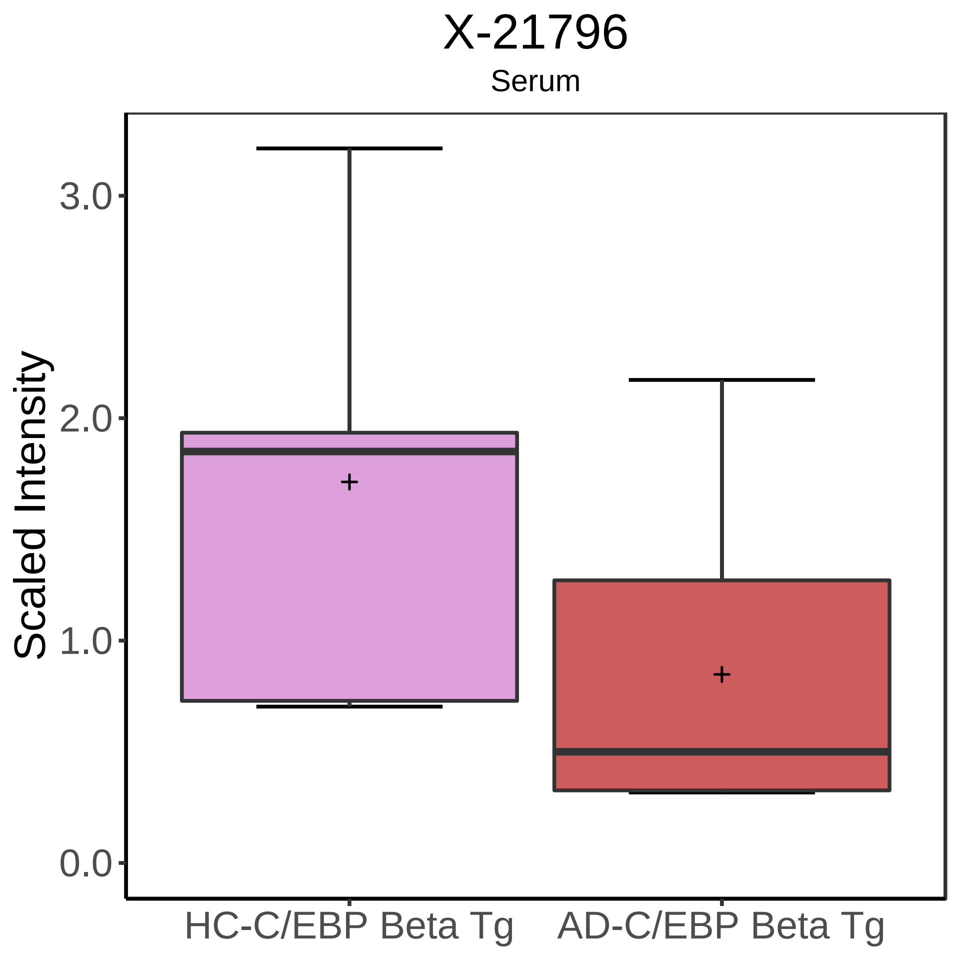
X-21796
Serum
Scaled Intensity
3.0
2.0
1.0
0.0
HC-C/EBP Beta Tg
AD-C/EBP Beta Tg

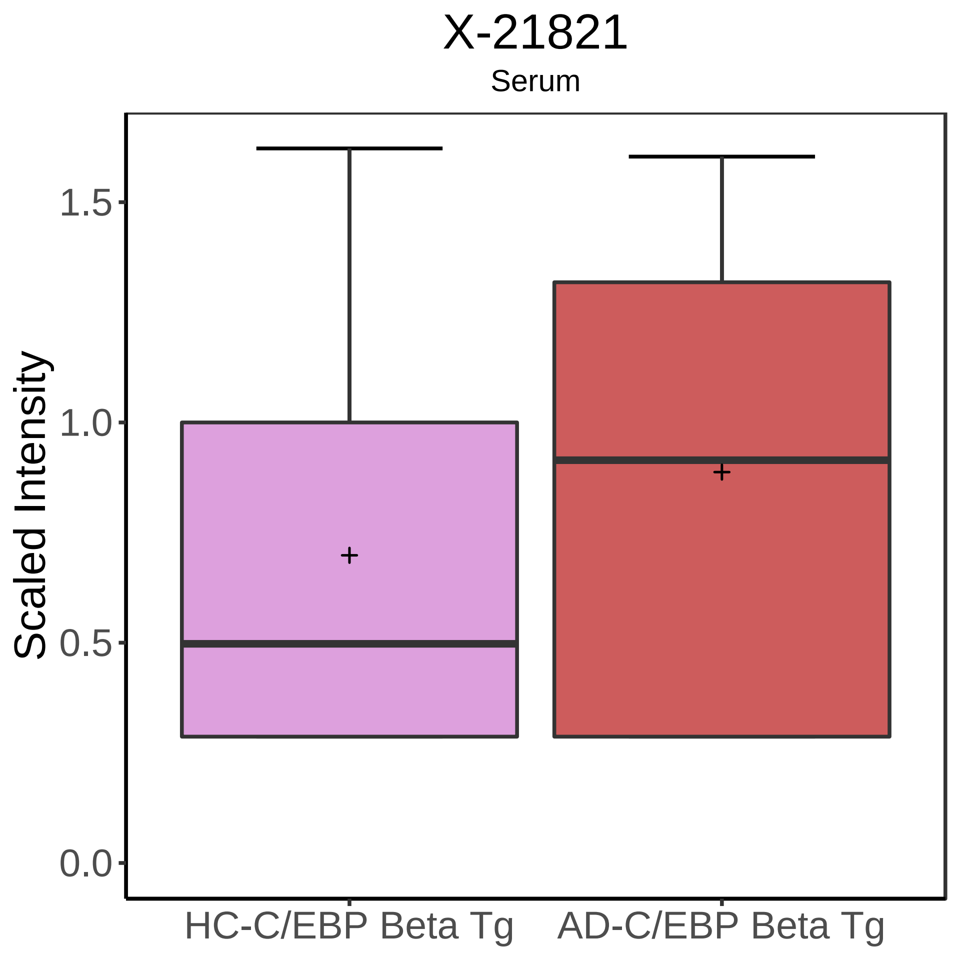
X-21821
Serum
Scaled Intensity
1.5
1.0
0.5
0.0
HC-C/EBP Beta Tg
AD-C/EBP Beta Tg

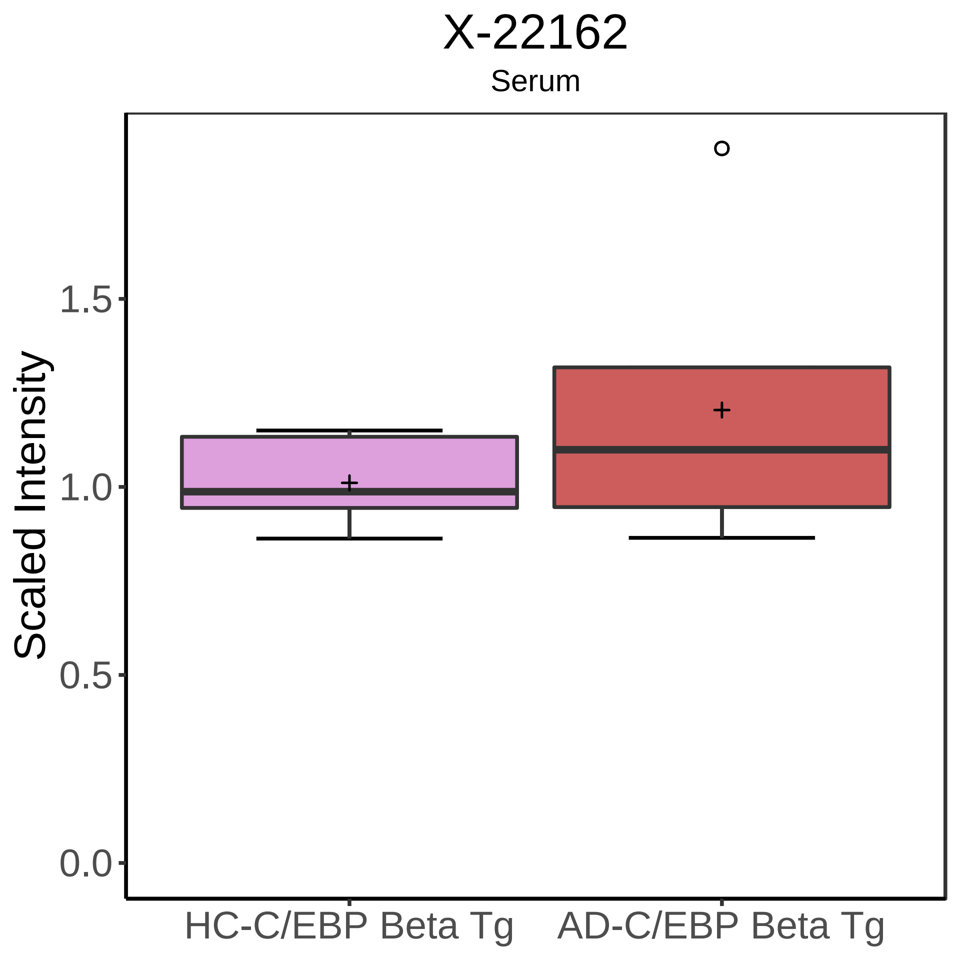
X-22162
Serum
Scaled Intensity
0.0
0.5
1.0
1.5
HC-C/EBP Beta Tg
AD-C/EBP Beta Tg

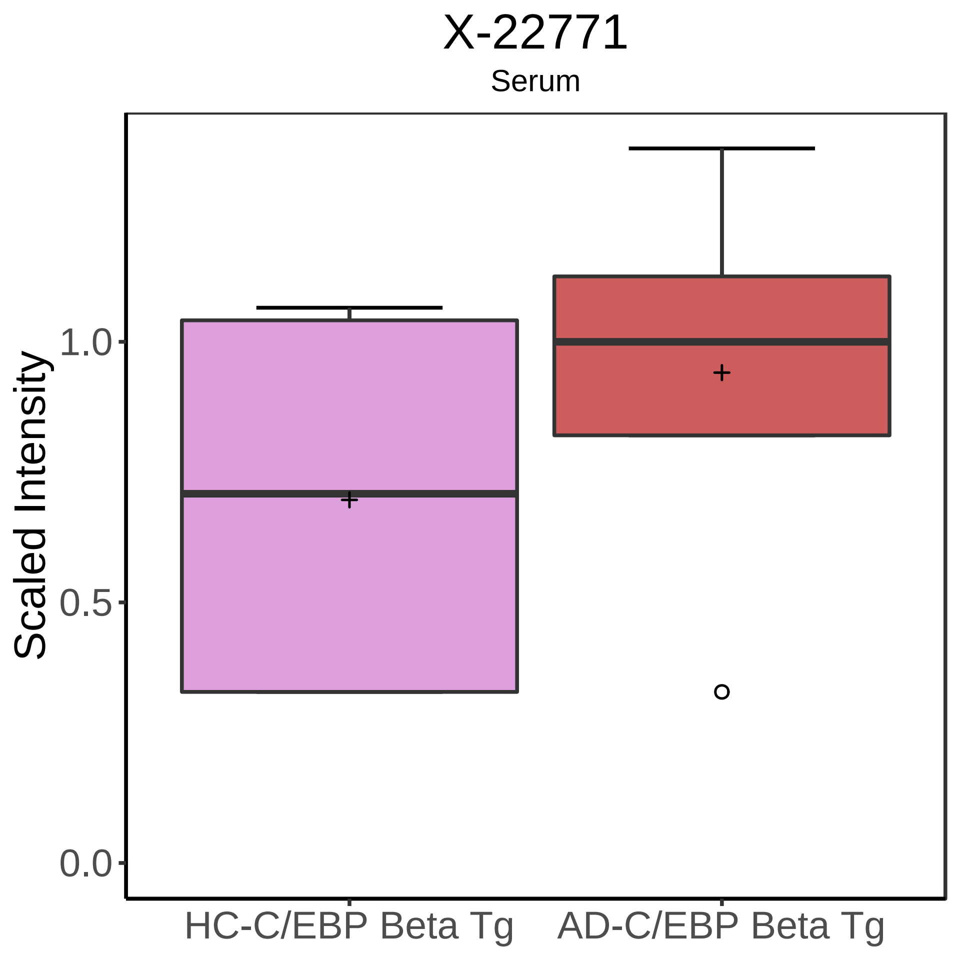
X-22771
Serum
Scaled Intensity
1.0
0.5
0.0
HC-C/EBP Beta Tg
AD-C/EBP Beta Tg

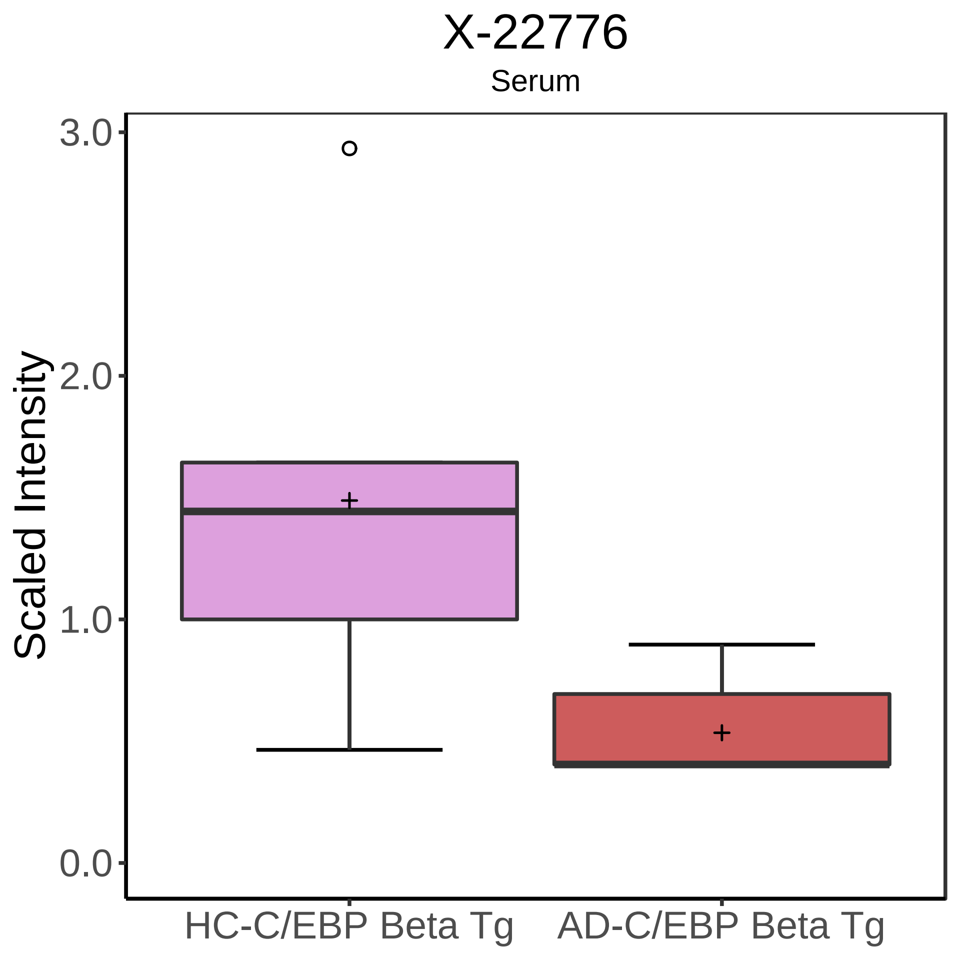

X-22776
Serum
Scaled Intensity
3.0
2.0
1.0
0.0
HC-C/EBP Beta Tg
AD-C/EBP Beta Tg

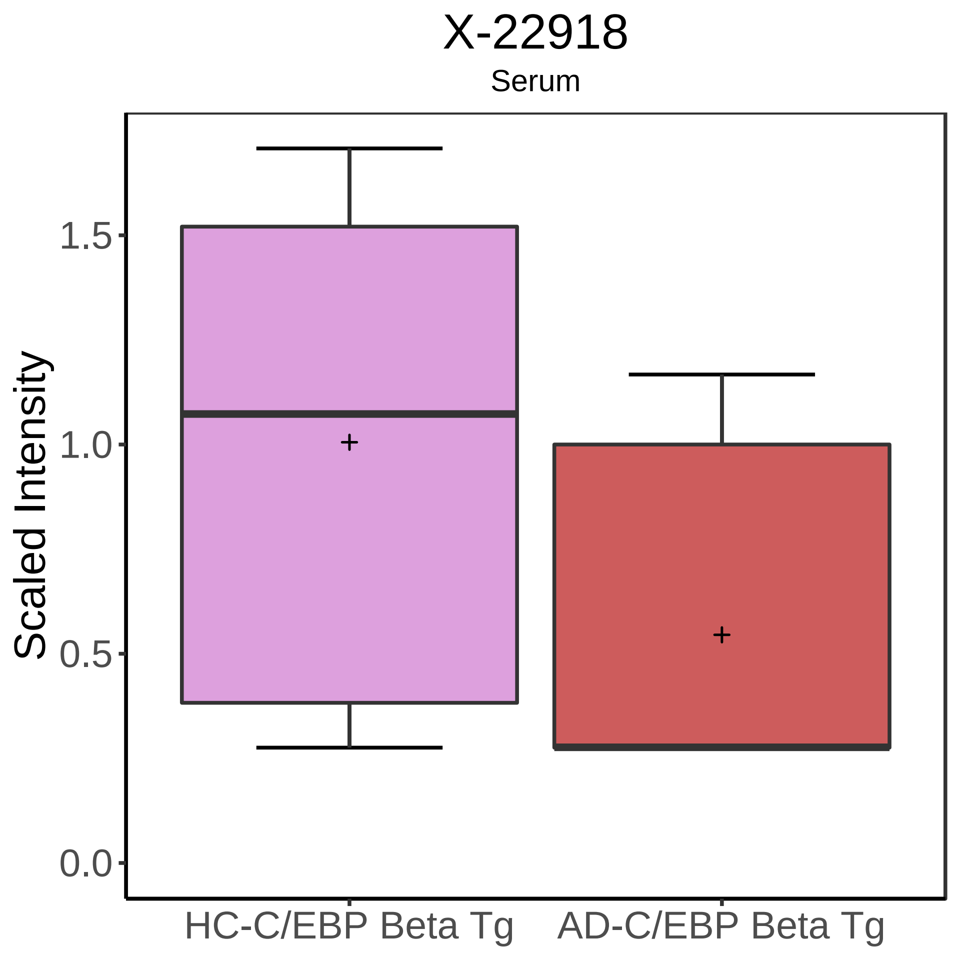
X-22918
Serum
Scaled Intensity
0.0
0.5
1.0
1.5
HC-C/EBP Beta Tg
AD-C/EBP Beta Tg

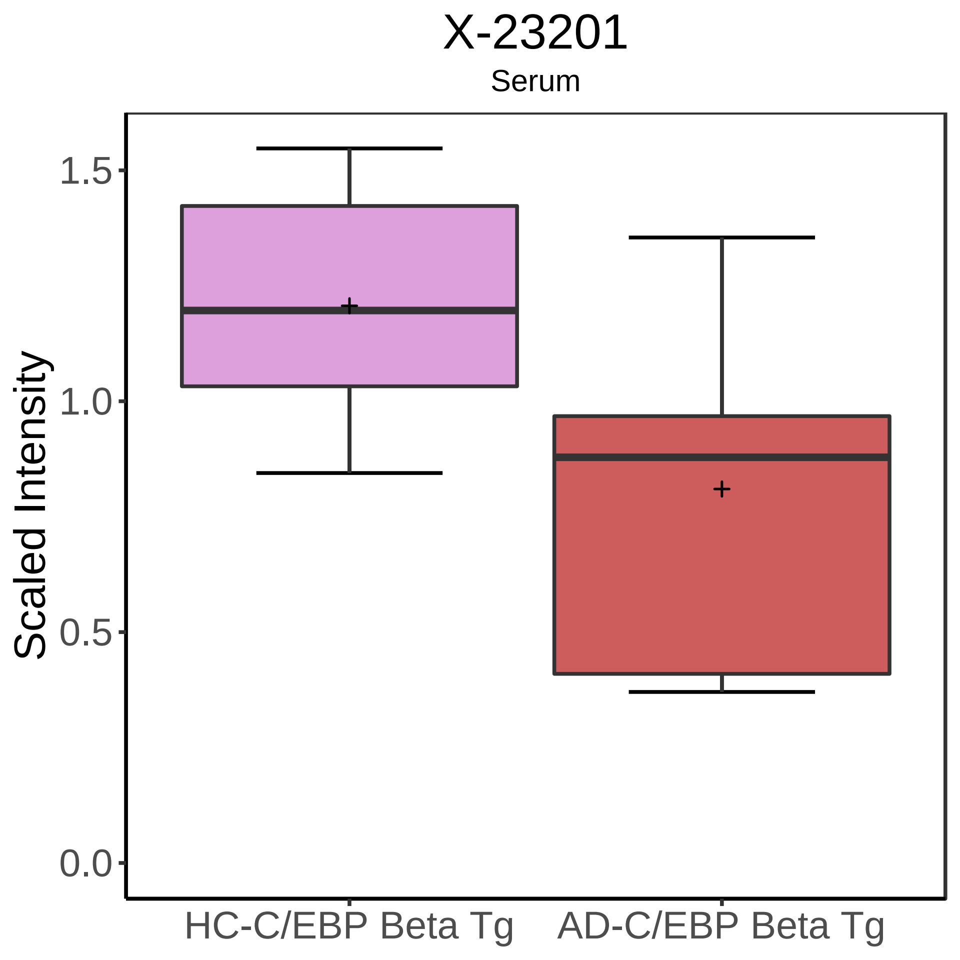
X-23201
Serum
Scaled Intensity
1.5
1.0
0.5
0.0
HC-C/EBP Beta Tg
AD-C/EBP Beta Tg

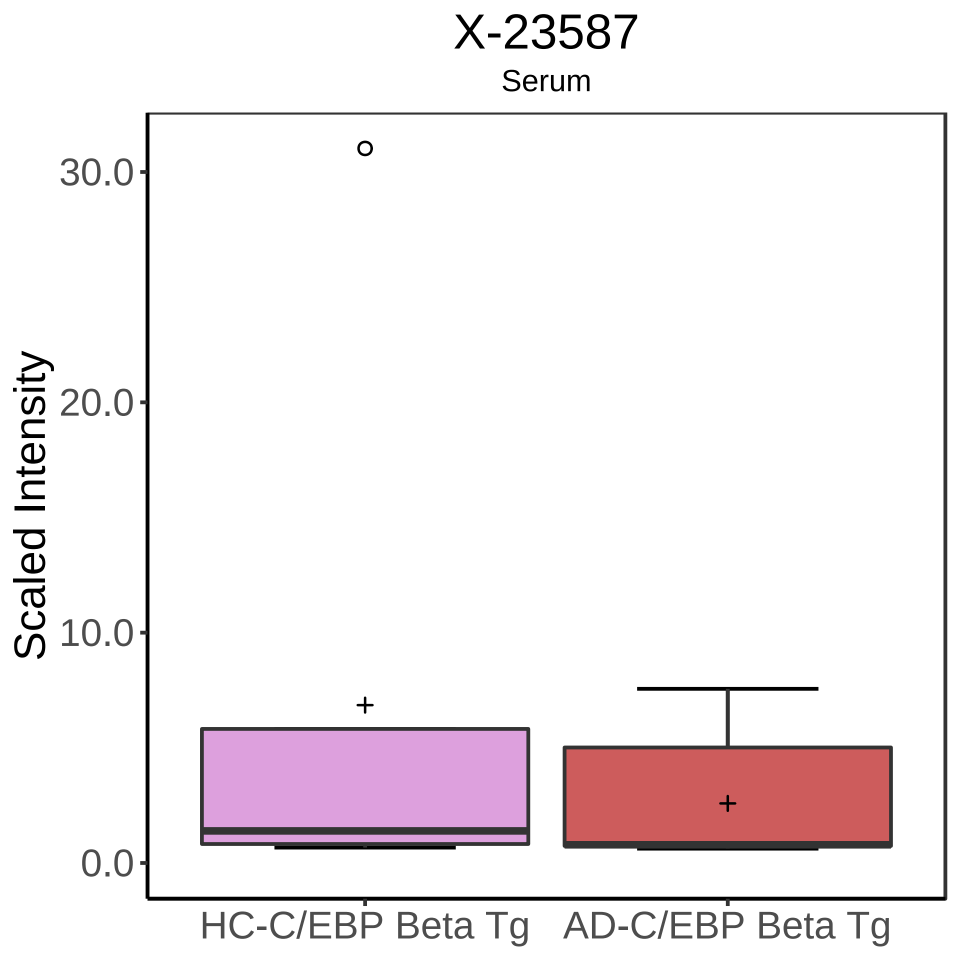
X-23587
Serum
Scaled Intensity
30.0
20.0
10.0
0.0
HC-C/EBP Beta Tg
AD-C/EBP Beta Tg

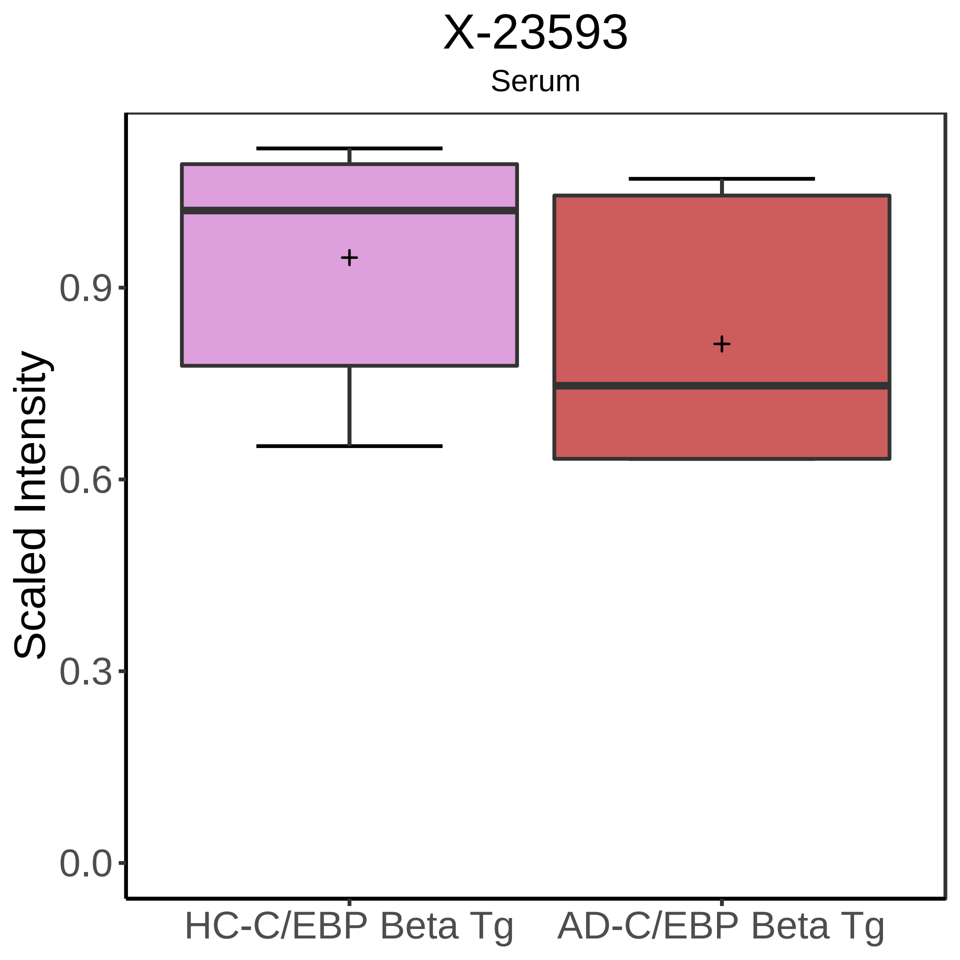
X-23593
Serum
Scaled Intensity
0.9
0.6
0.3
0.0
HC-C/EBP Beta Tg
AD-C/EBP Beta Tg

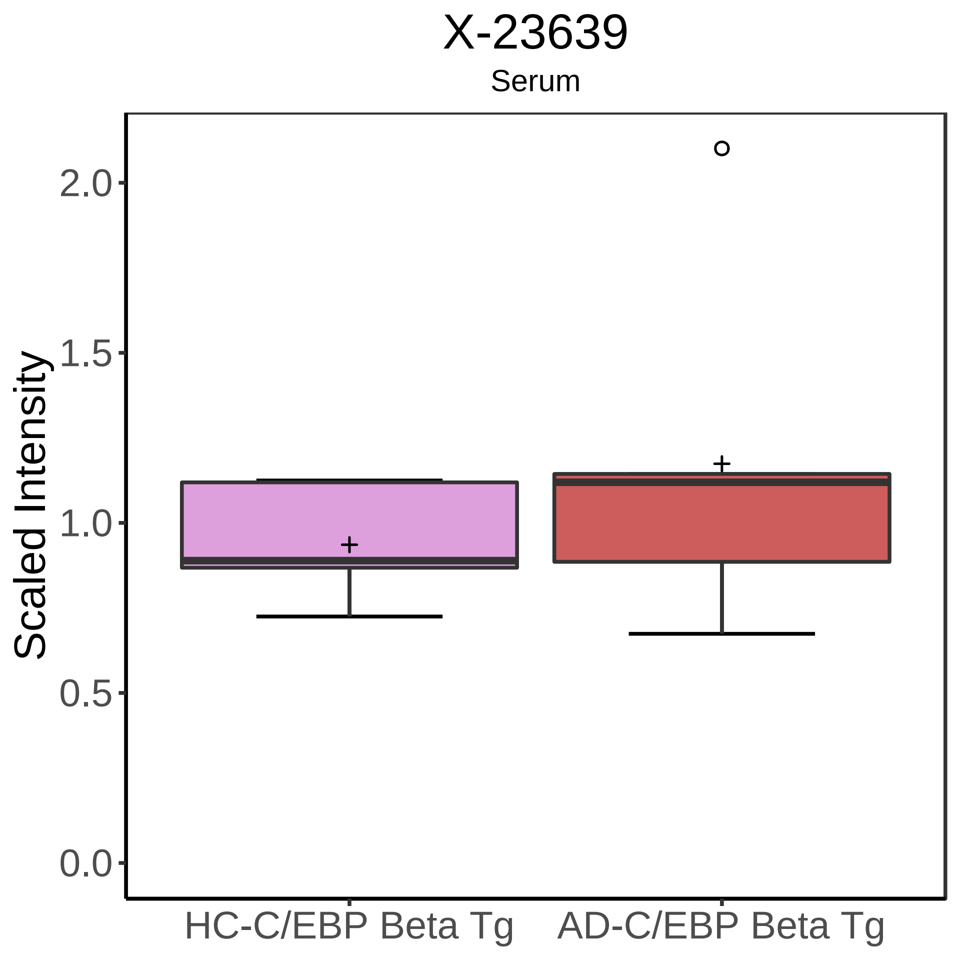

X-23639
Serum
Scaled Intensity
2.0
1.5
1.0
0.5
0.0
HC-C/EBP Beta Tg
AD-C/EBP Beta Tg

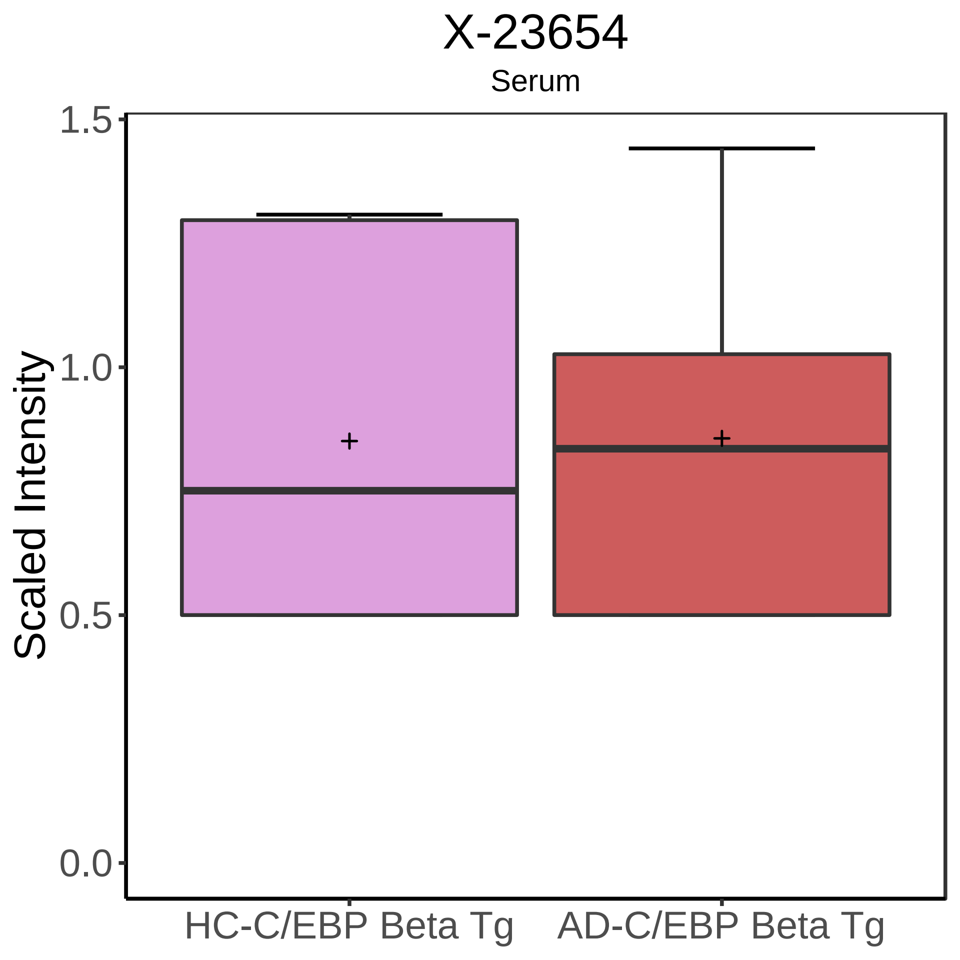
X-23654
Serum
Scaled Intensity
1.5
1.0
0.5
0.0
HC-C/EBP Beta Tg
AD-C/EBP Beta Tg

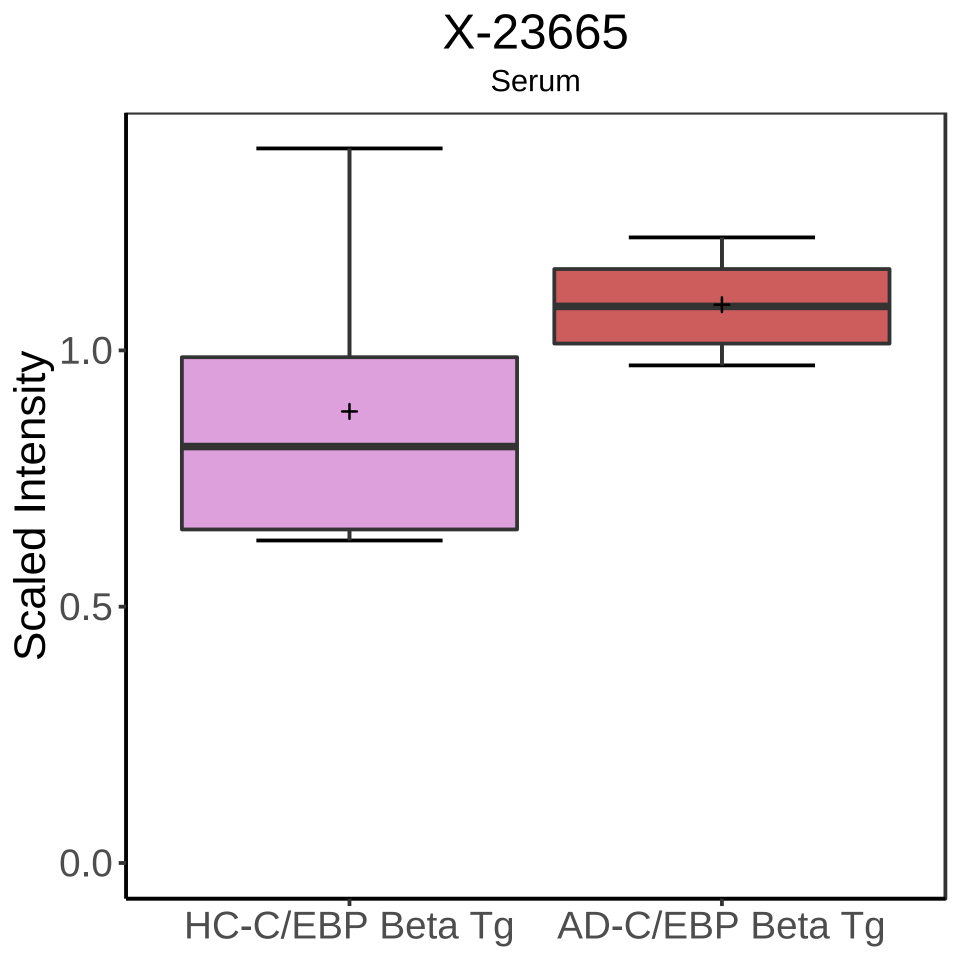
X-23665
Serum
Scaled Intensity
1.0
0.5
0.0
HC-C/EBP Beta Tg
AD-C/EBP Beta Tg

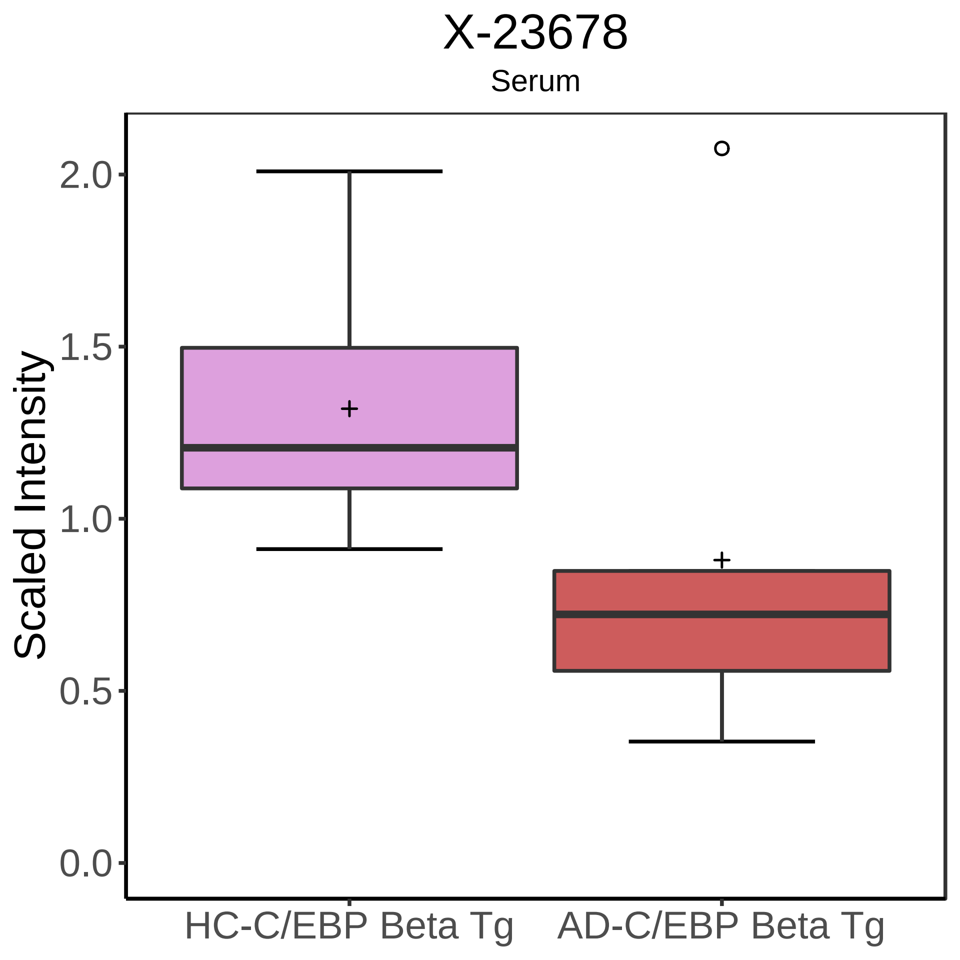
X-23678
Serum
Scaled Intensity
2.0
1.5
1.0
0.5
0.0
HC-C/EBP Beta Tg
AD-C/EBP Beta Tg

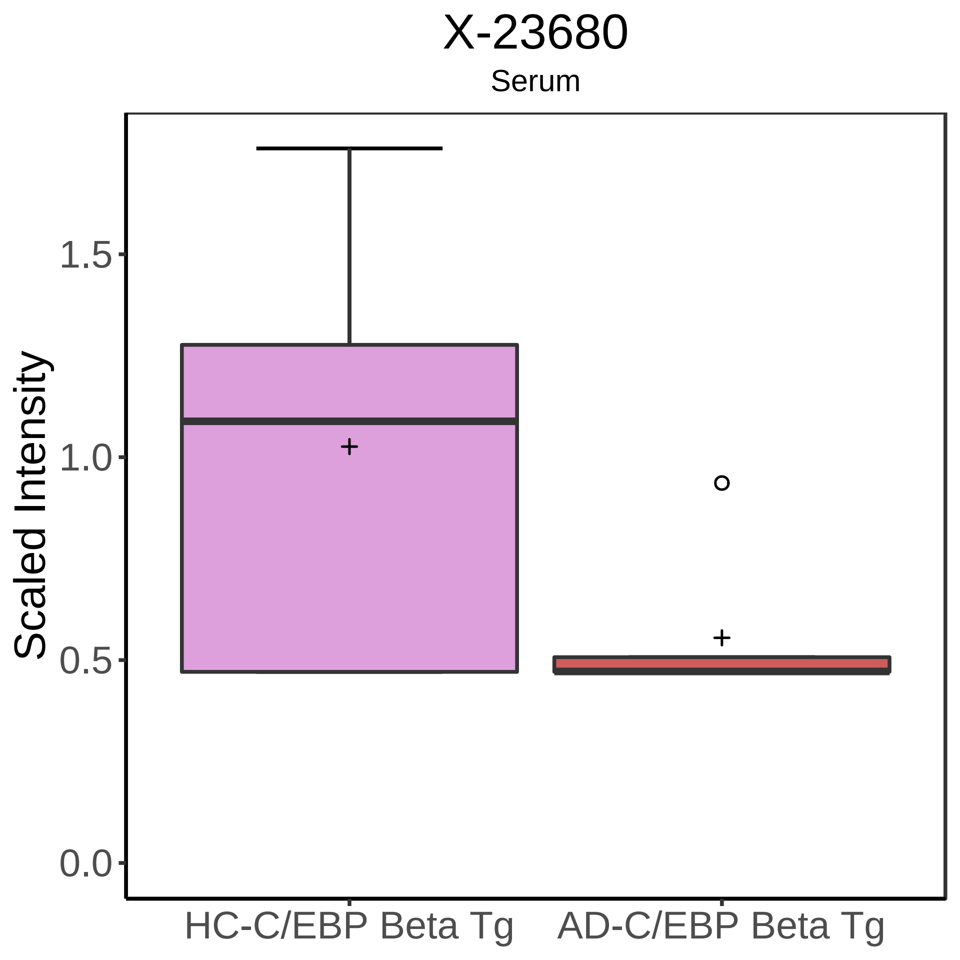

X-23680
Serum
Scaled Intensity
1.5
1.0
0.5
0.0
HC-C/EBP Beta Tg
AD-C/EBP Beta Tg

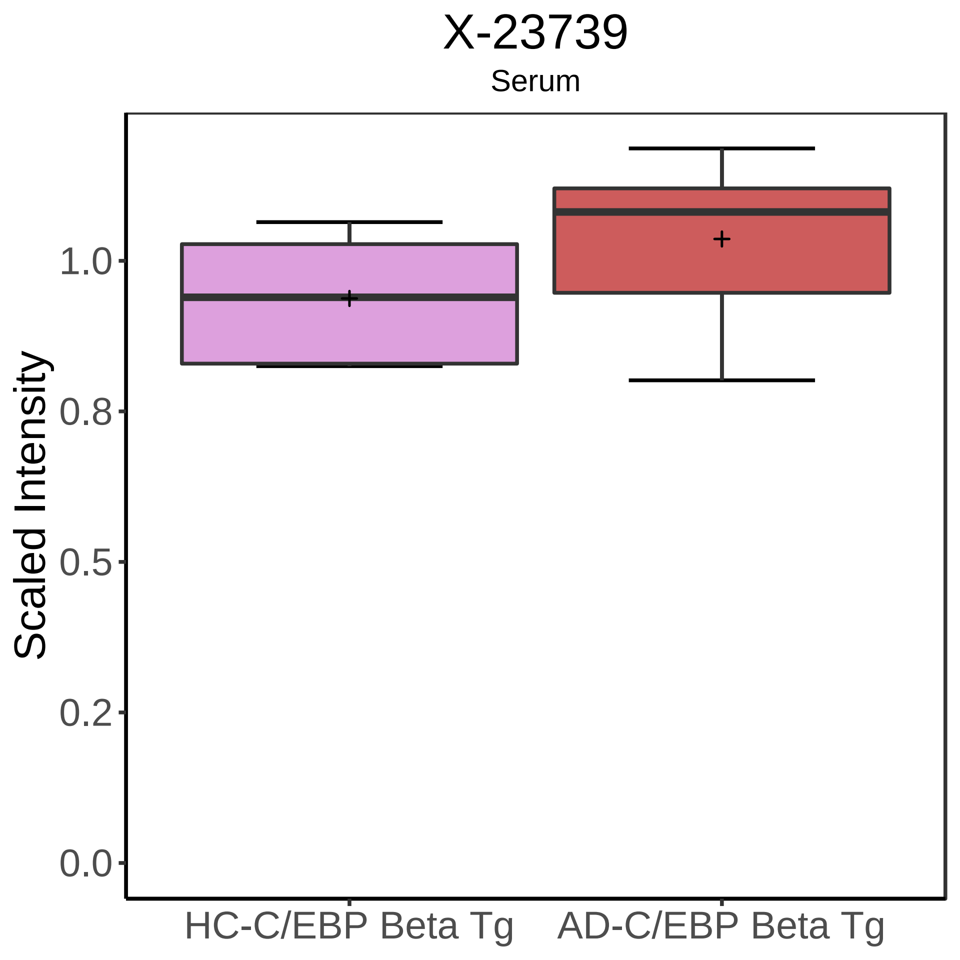
X-23739
Serum
Scaled Intensity
1.0
0.8
0.5
0.2
0.0
HC-C/EBP Beta Tg
AD-C/EBP Beta Tg

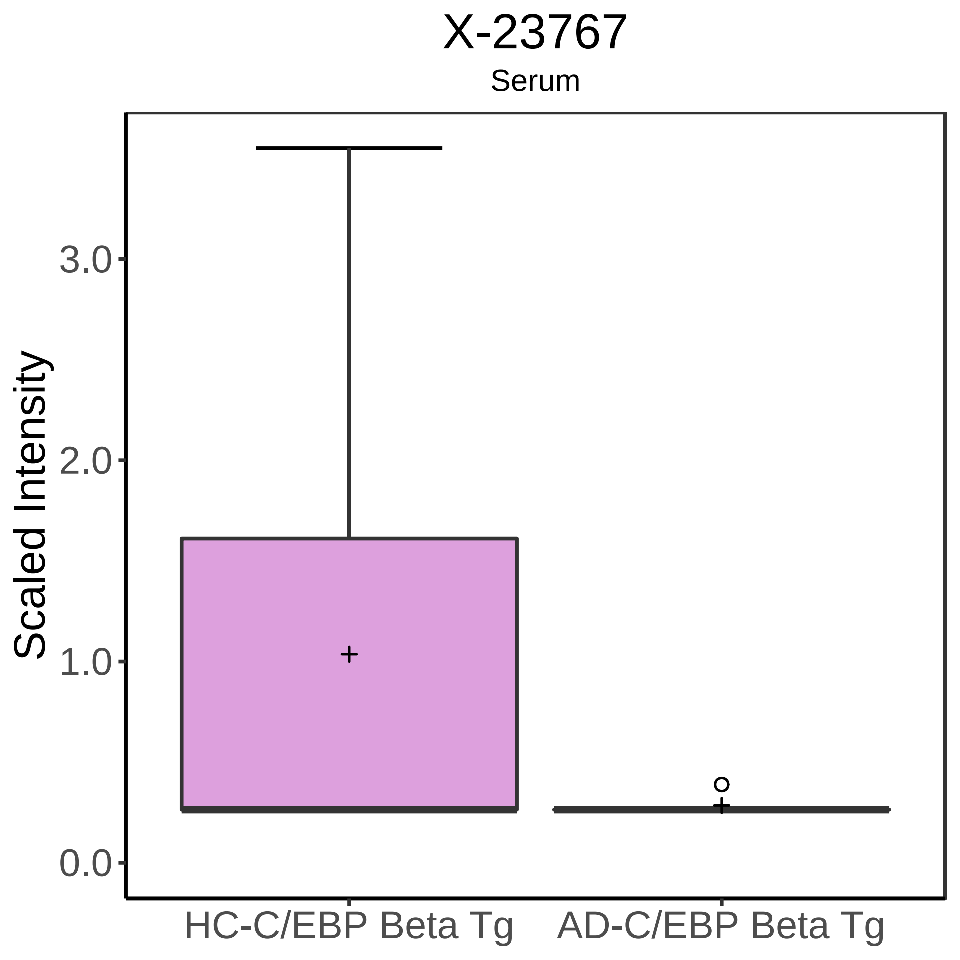
X-23767
Serum
Scaled Intensity
3.0
2.0
1.0
0.0
HC-C/EBP Beta Tg
AD-C/EBP Beta Tg

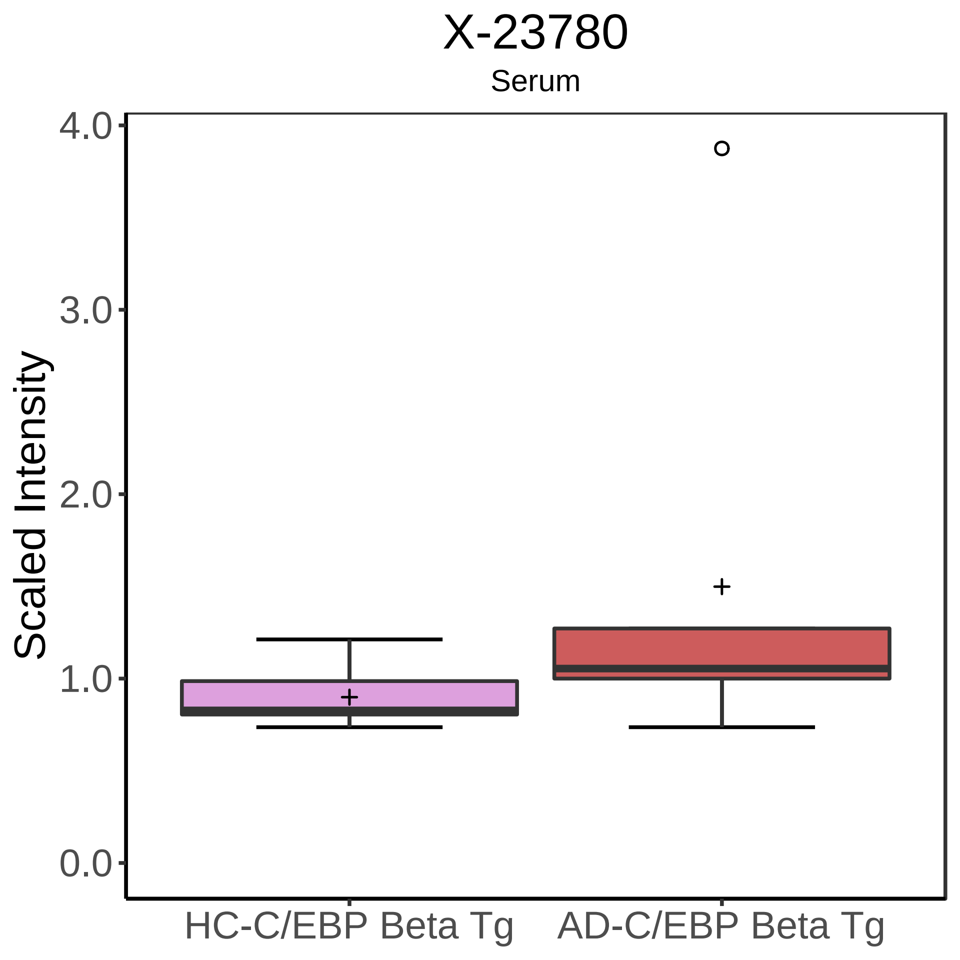

X-23780
Serum
Scaled Intensity
4.0
3.0
2.0
1.0
0.0
HC-C/EBP Beta Tg
AD-C/EBP Beta Tg

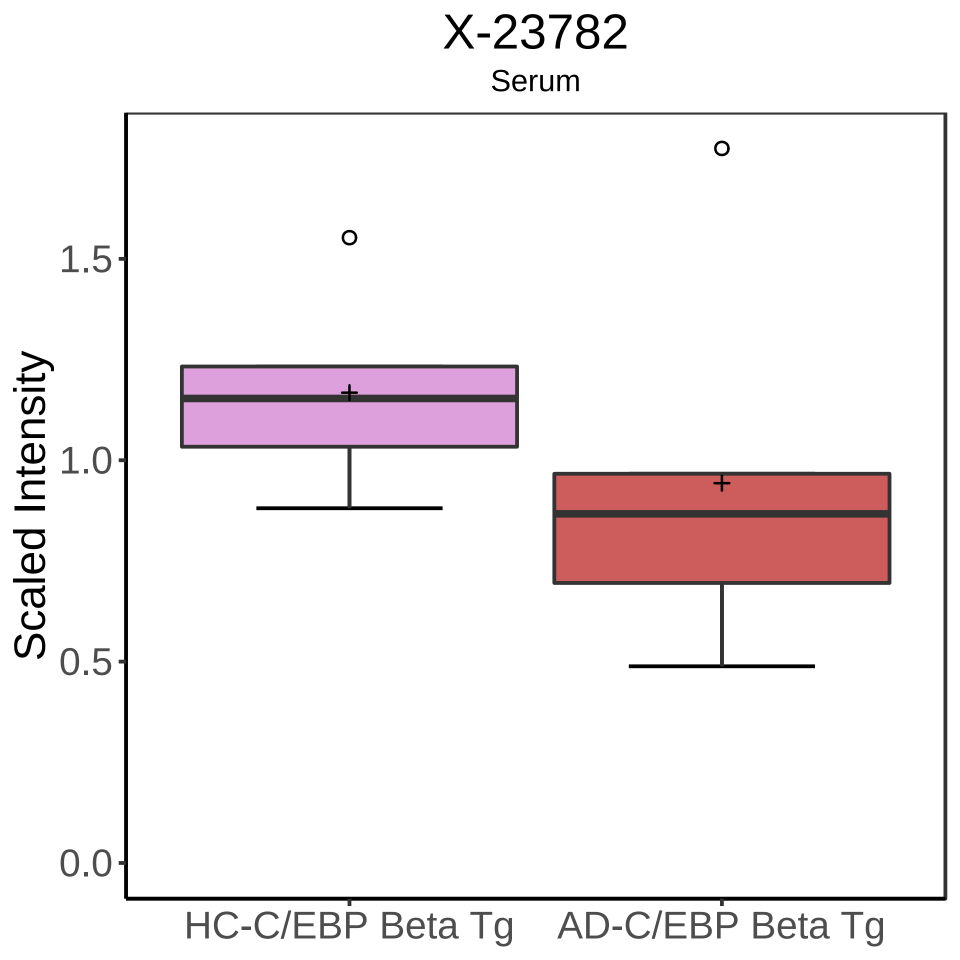
X-23782
Serum
Scaled Intensity
1.5
1.0
0.5
0.0
HC-C/EBP Beta Tg
AD-C/EBP Beta Tg

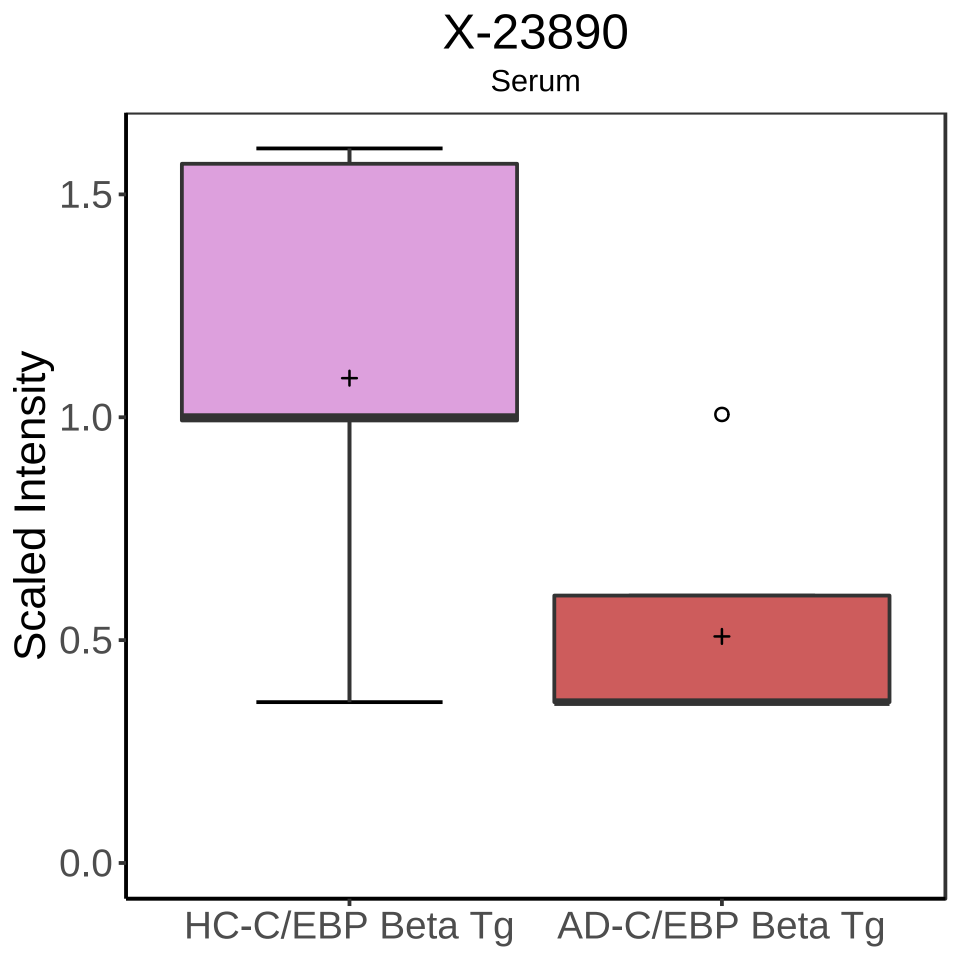
X-23890
Serum
Scaled Intensity
1.5
1.0
0.5
0.0
HC-C/EBP Beta Tg
AD-C/EBP Beta Tg

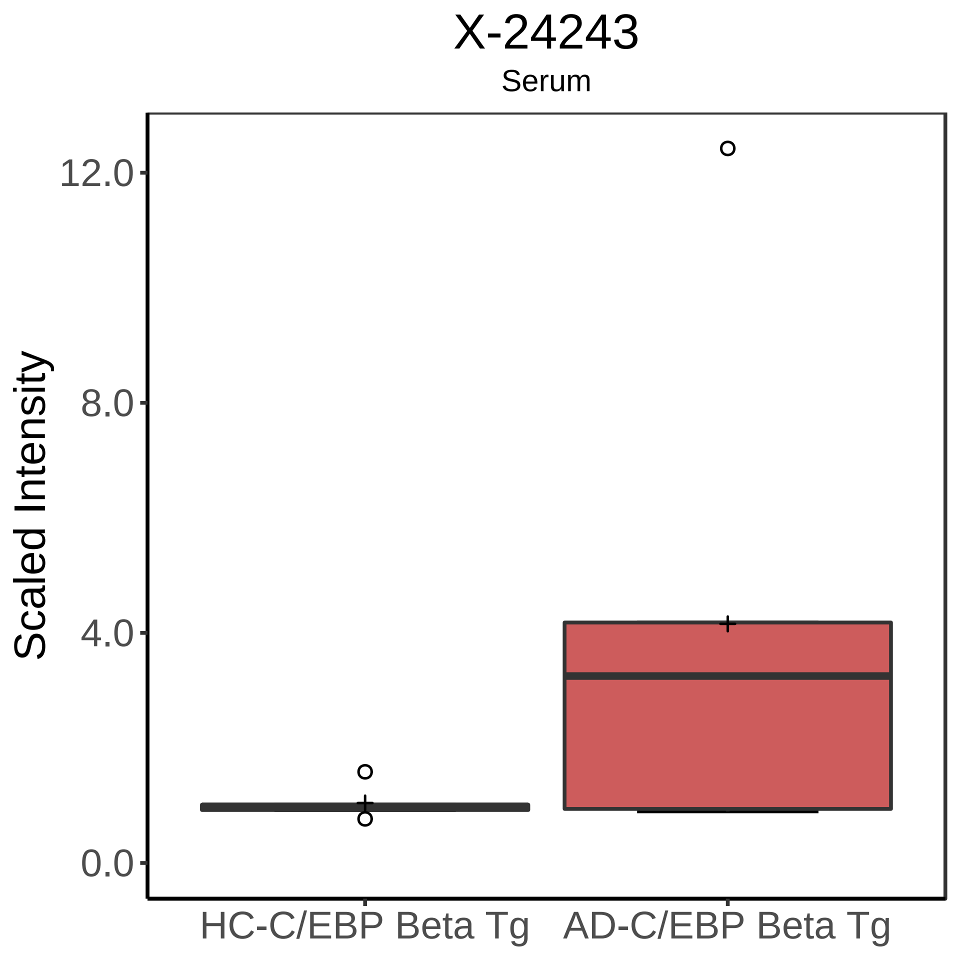
X-24243
Serum
Scaled Intensity
12.0
8.0
4.0
0.0
HC-C/EBP Beta Tg
AD-C/EBP Beta Tg

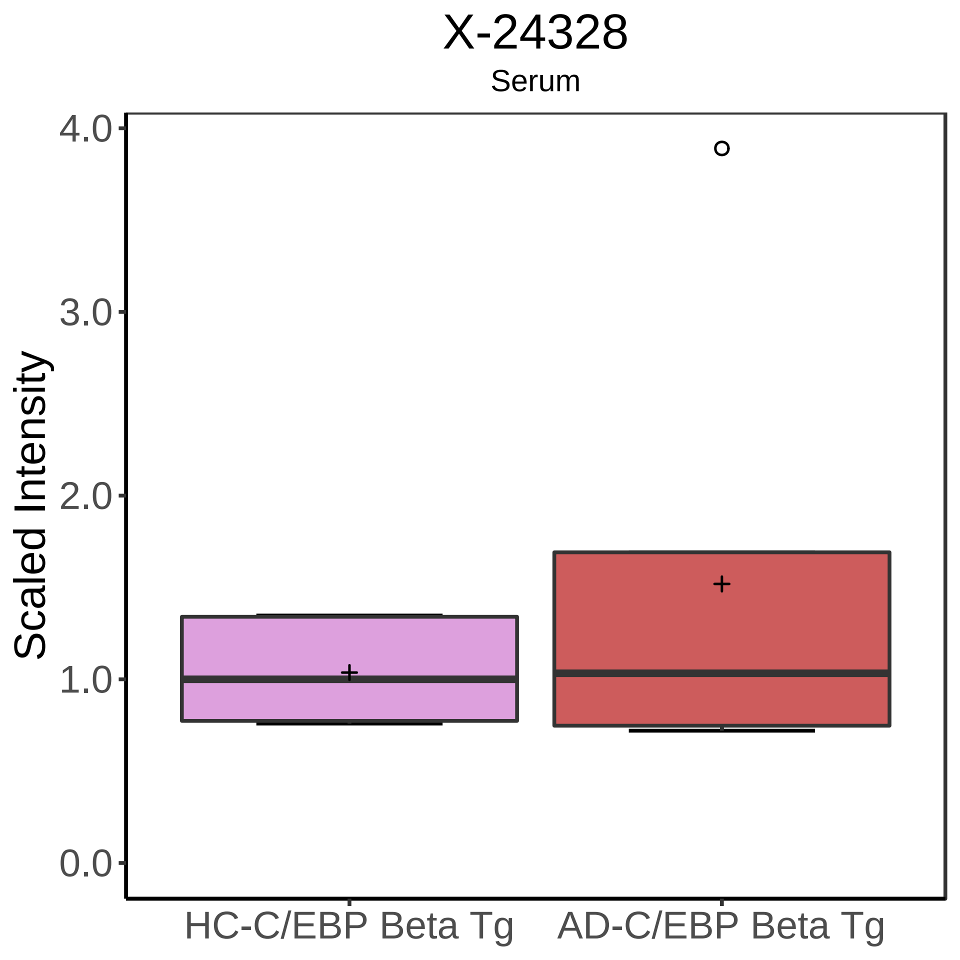
X-24328
Serum
Scaled Intensity
4.0
3.0
2.0
1.0
0.0
HC-C/EBP Beta Tg
AD-C/EBP Beta Tg

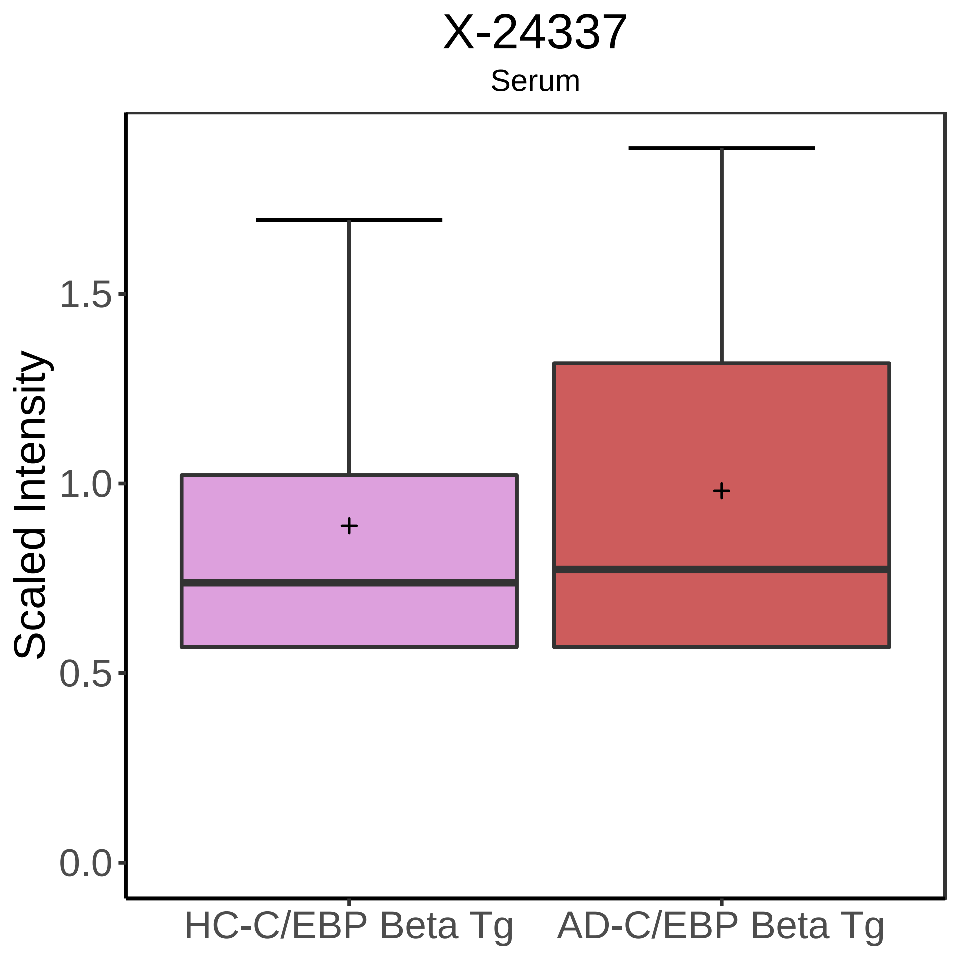

X-24337
Serum
Scaled Intensity
0.0
0.5
1.0
1.5
HC-C/EBP Beta Tg
AD-C/EBP Beta Tg

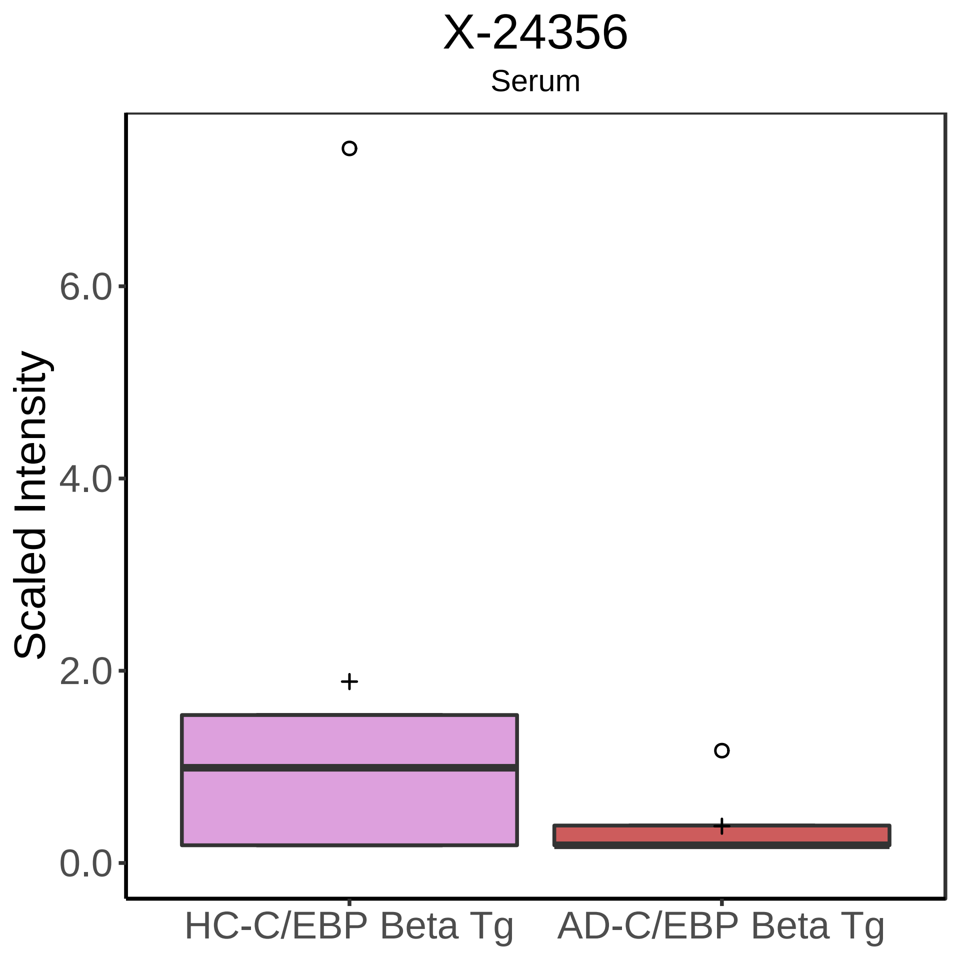

X-24356
Serum
Scaled Intensity
6.0
4.0
2.0
0.0
HC-C/EBP Beta Tg
AD-C/EBP Beta Tg

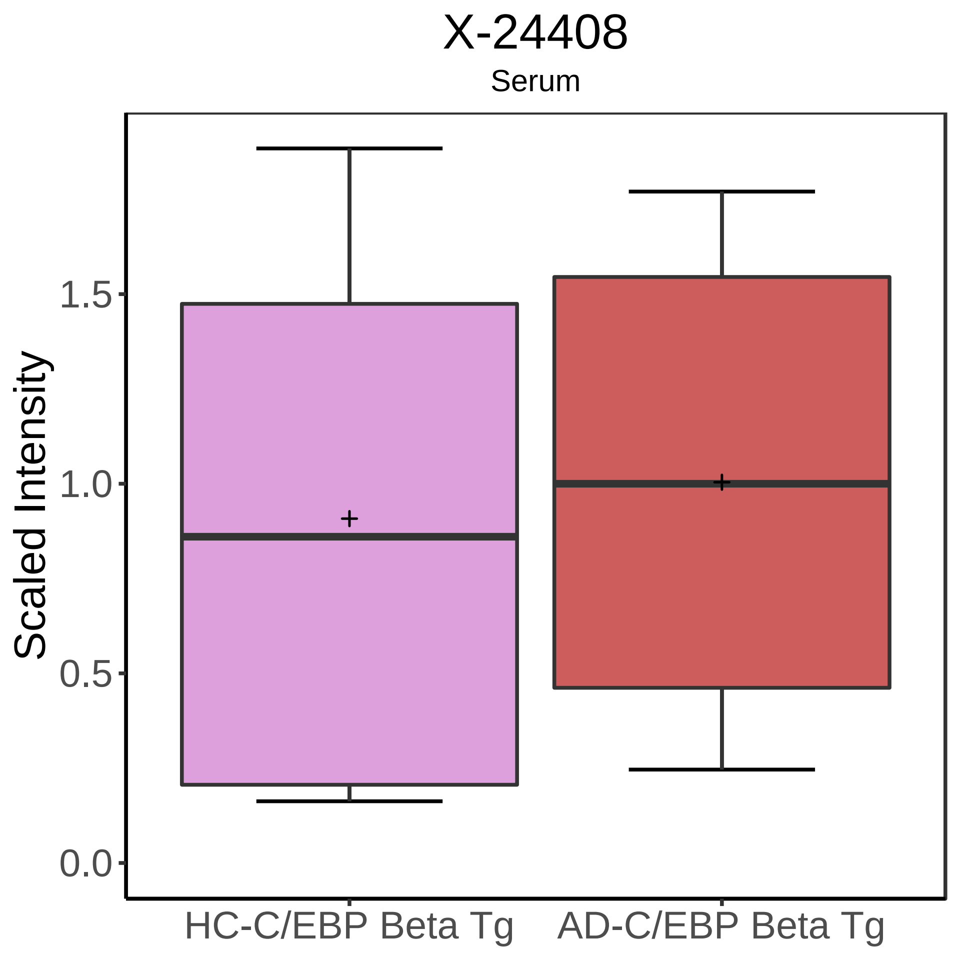
X-24408
Serum
Scaled Intensity
1.5
1.0
0.5
0.0
HC-C/EBP Beta Tg
AD-C/EBP Beta Tg

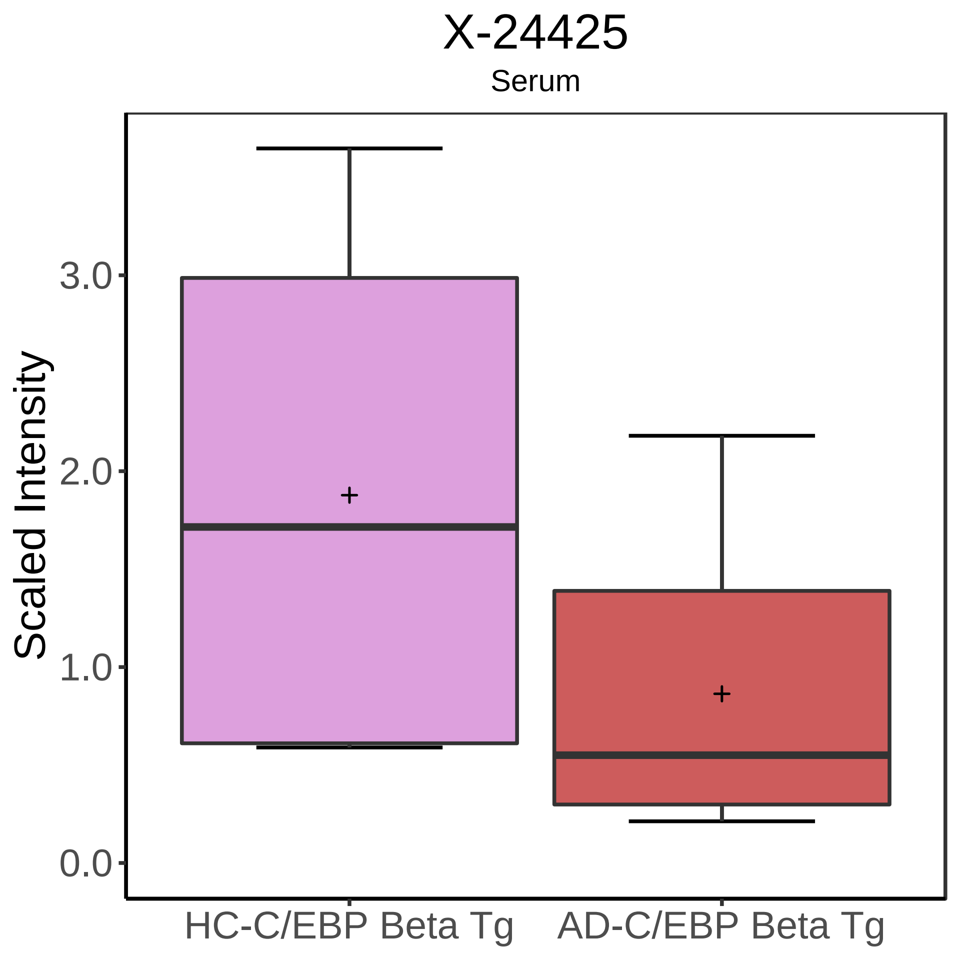
X-24425
Serum
Scaled Intensity
3.0
2.0
1.0
0.0
HC-C/EBP Beta Tg
AD-C/EBP Beta Tg

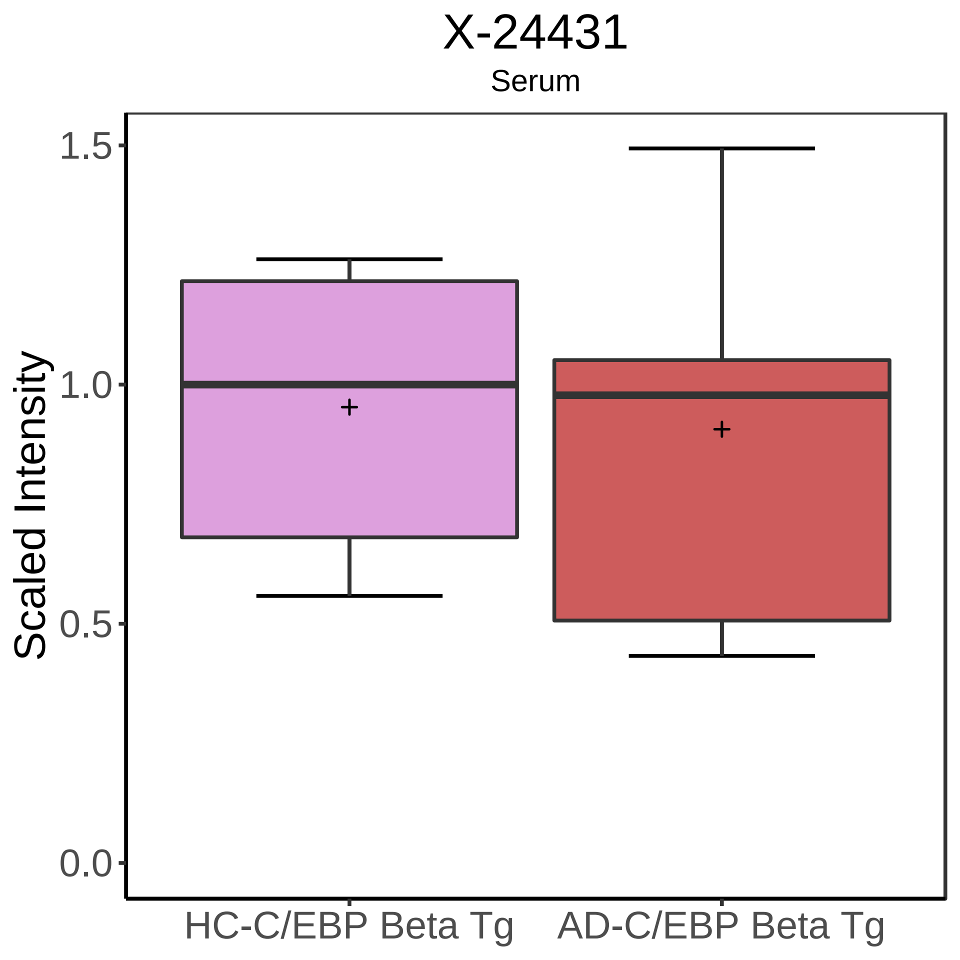

X-24431
Serum
Scaled Intensity
1.5
1.0
0.5
0.0
HC-C/EBP Beta Tg
AD-C/EBP Beta Tg

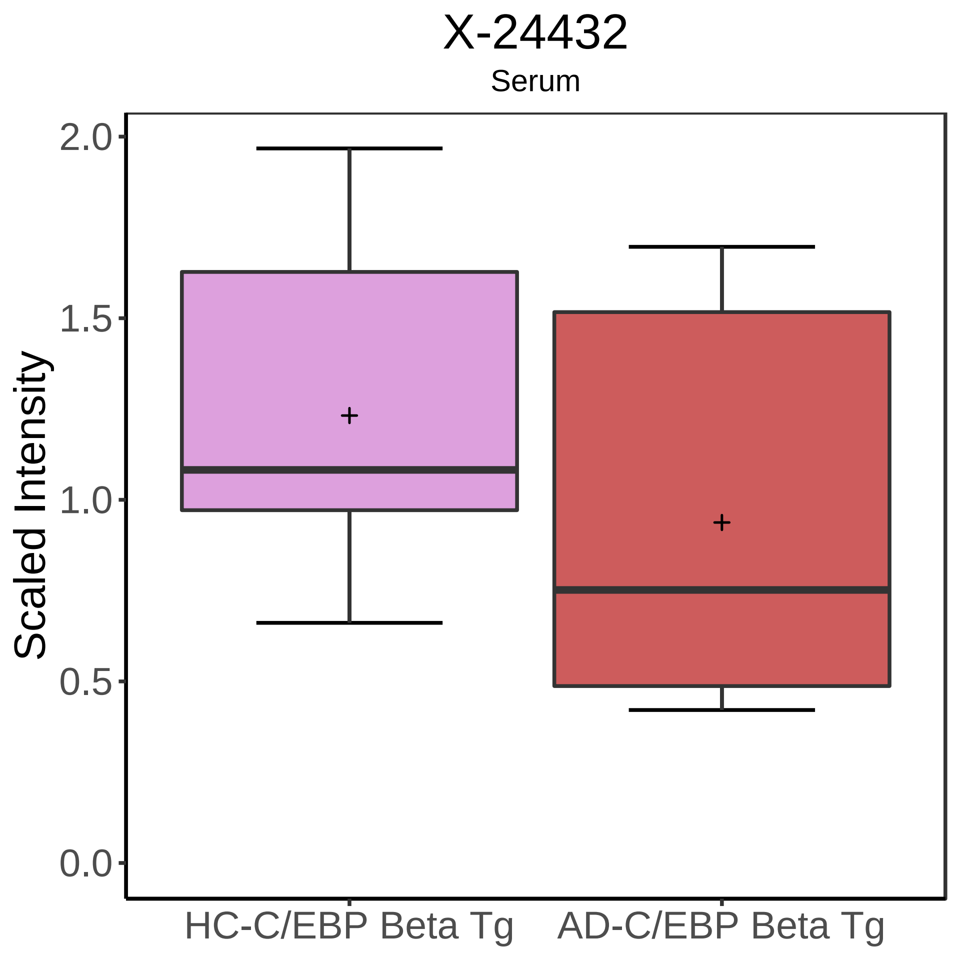
X-24432
Serum
Scaled Intensity
2.0
1.5
1.0
0.5
0.0
HC-C/EBP Beta Tg
AD-C/EBP Beta Tg

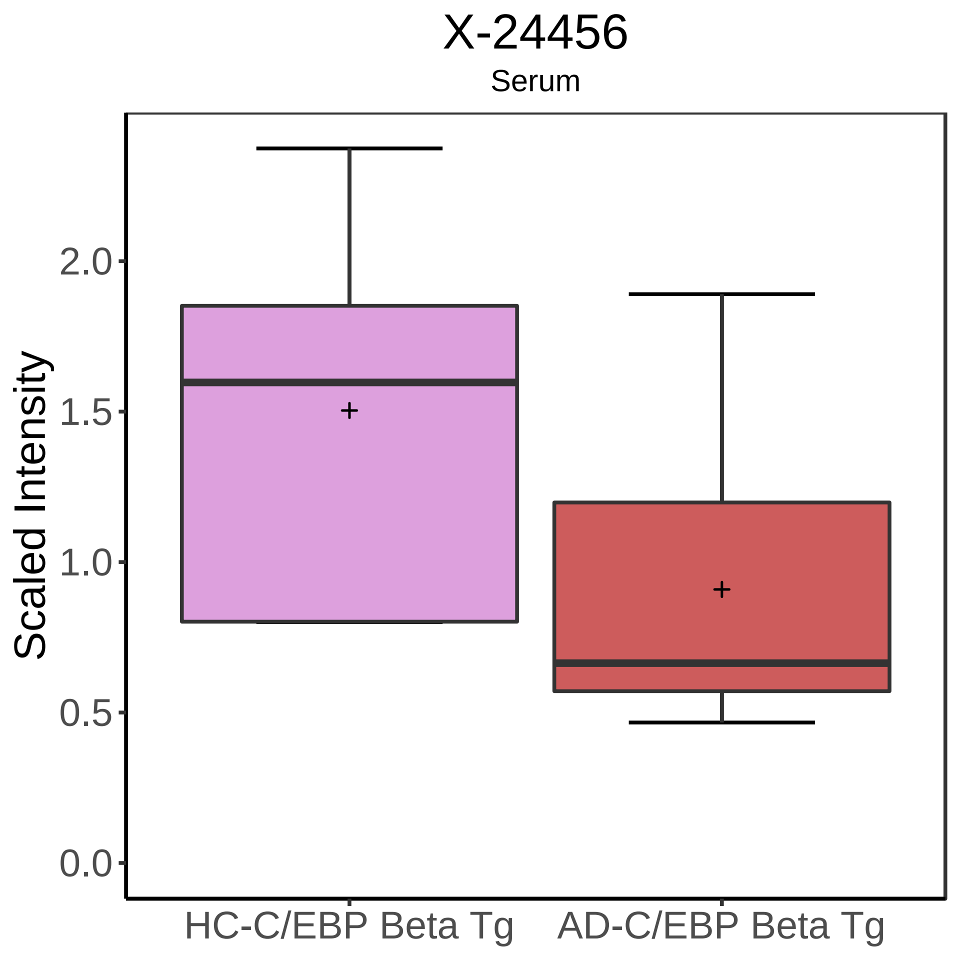
X-24456
Serum
Scaled Intensity
2.0
1.5
1.0
0.5
0.0
HC-C/EBP Beta Tg
AD-C/EBP Beta Tg

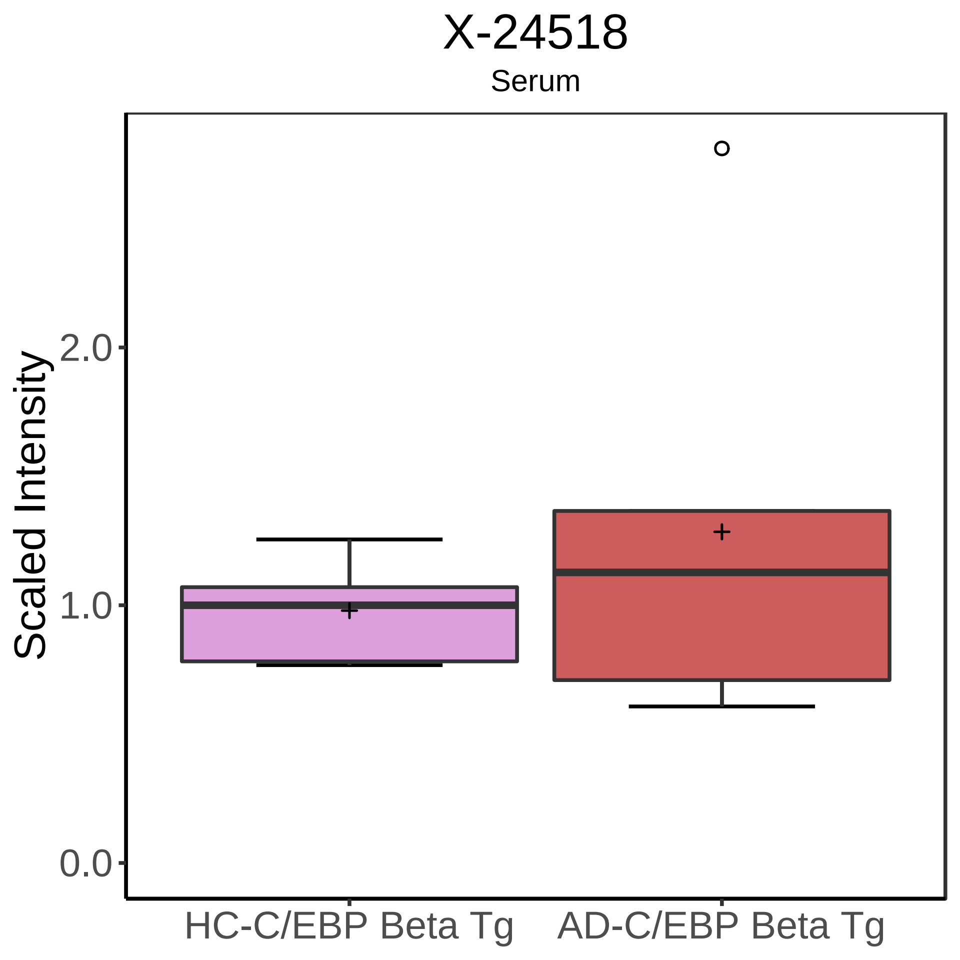

X-24518
Serum
Scaled Intensity
2.0
1.0
0.0
HC-C/EBP Beta Tg
AD-C/EBP Beta Tg

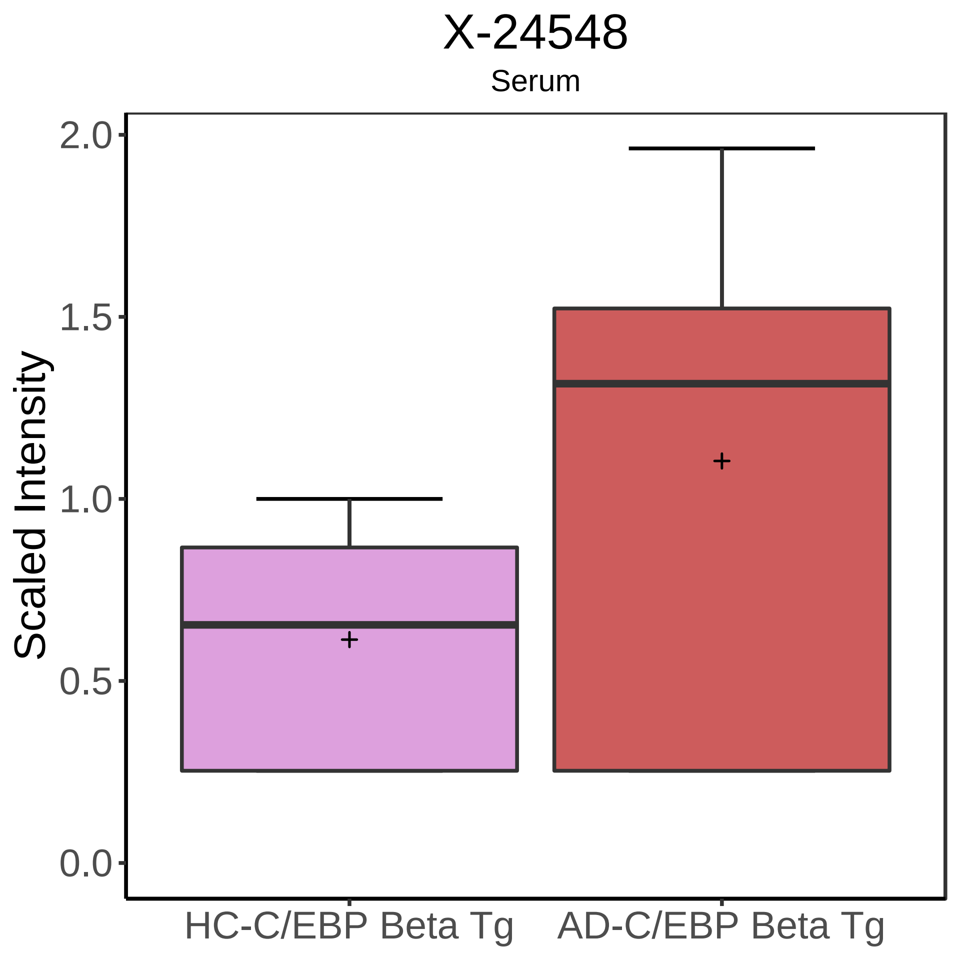
X-24548
Serum
Scaled Intensity
2.0
1.5
1.0
0.5
0.0
HC-C/EBP Beta Tg
AD-C/EBP Beta Tg

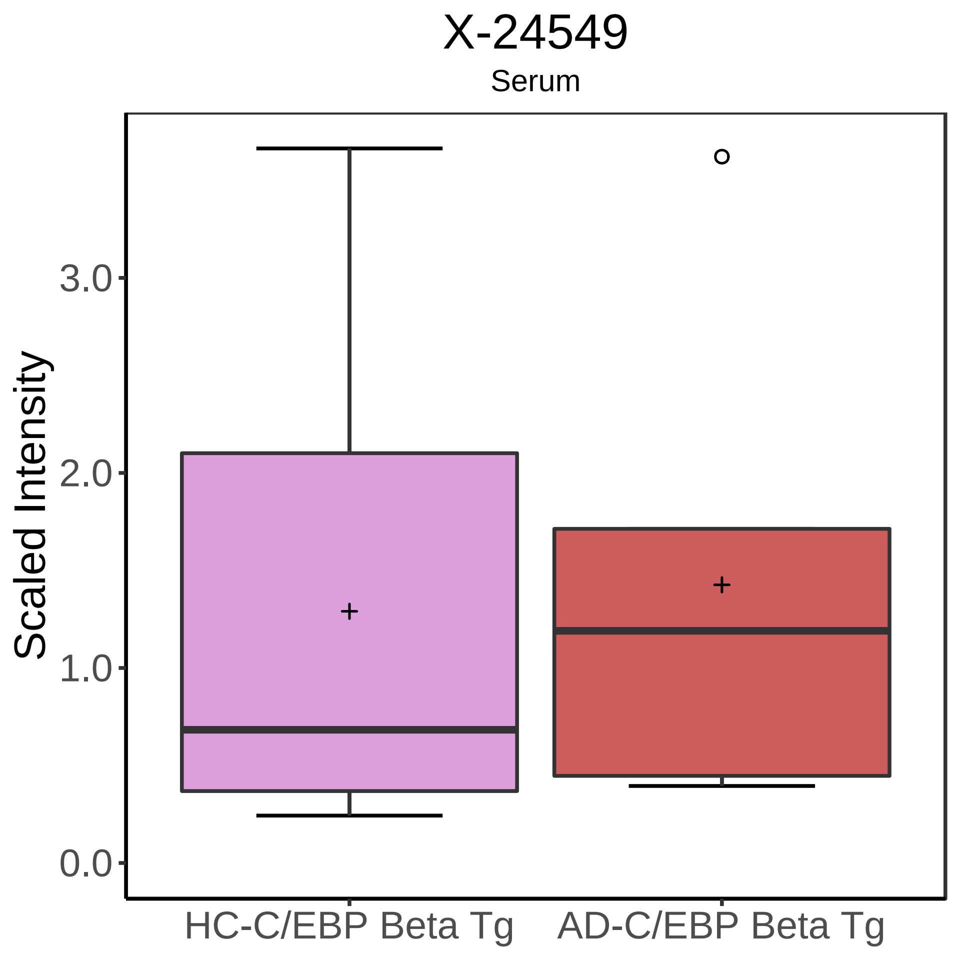
X-24549
Serum
Scaled Intensity
3.0
2.0
1.0
0.0
HC-C/EBP Beta Tg
AD-C/EBP Beta Tg

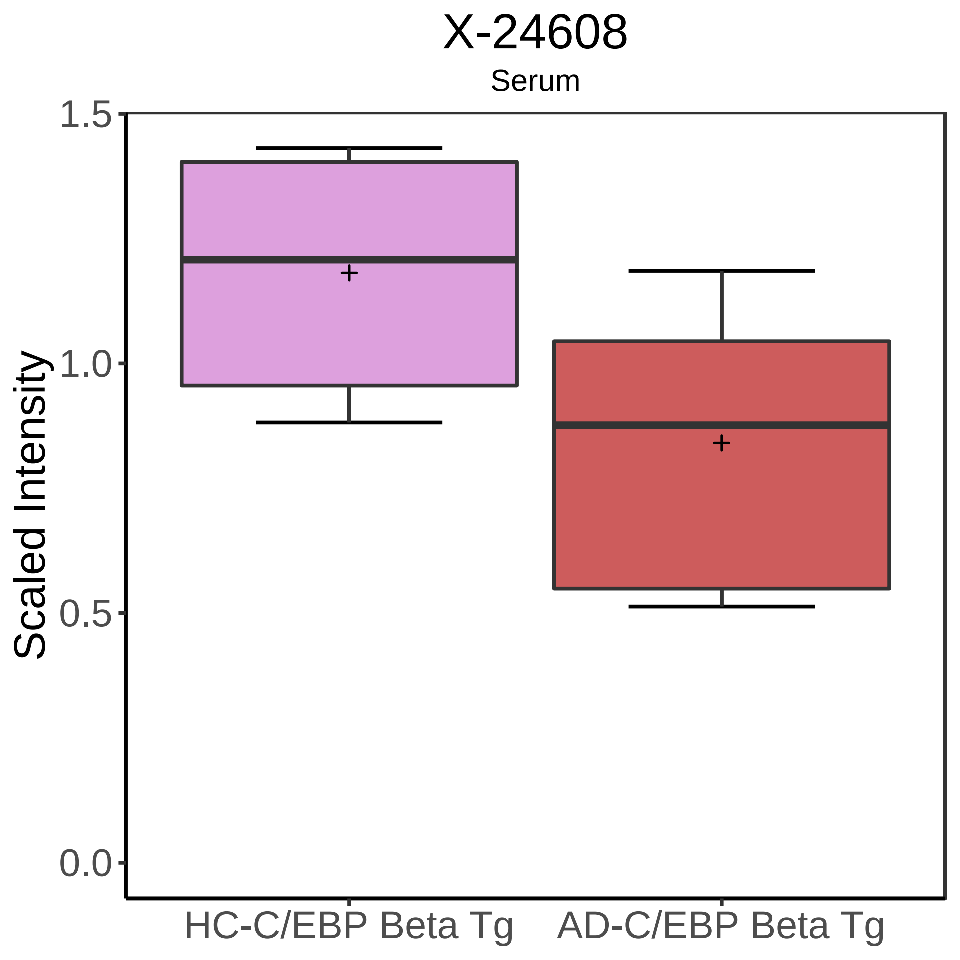

X-24608
Serum
Scaled Intensity
1.5
1.0
0.5
0.0
HC-C/EBP Beta Tg
AD-C/EBP Beta Tg

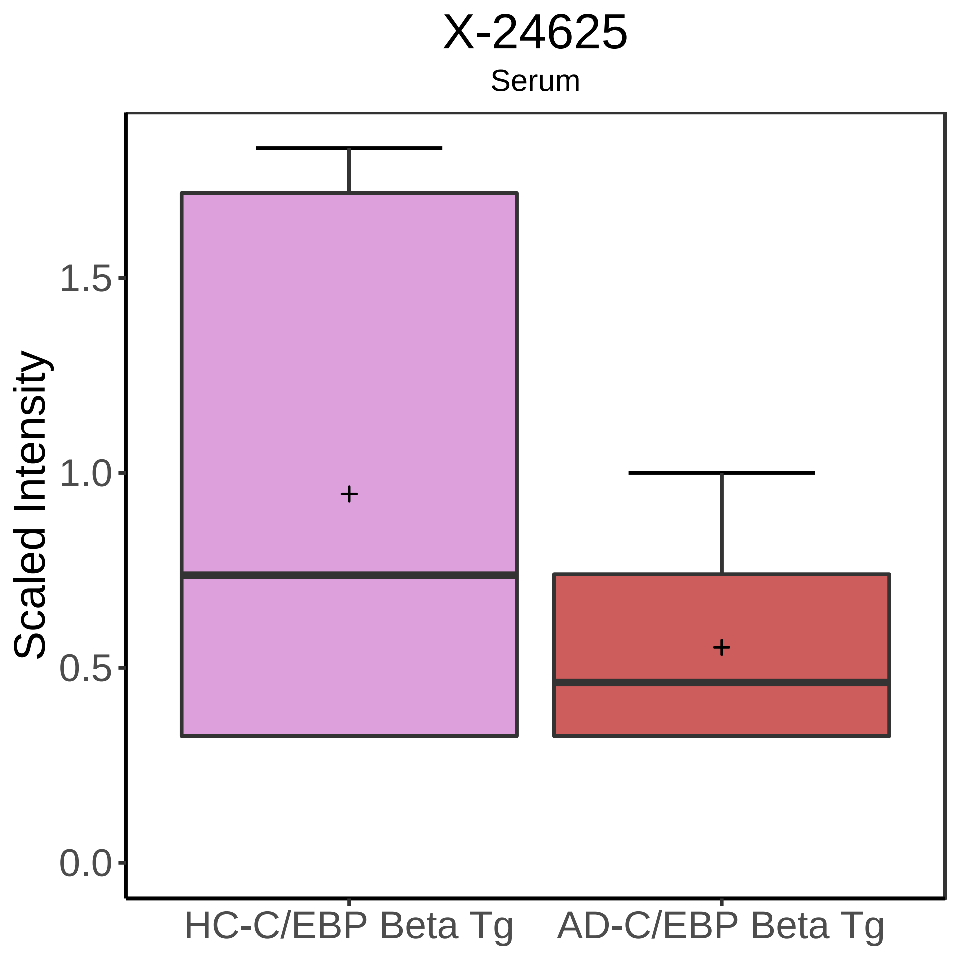
X-24625
Serum
Scaled Intensity
0.0
0.5
1.0
1.5
HC-C/EBP Beta Tg
AD-C/EBP Beta Tg

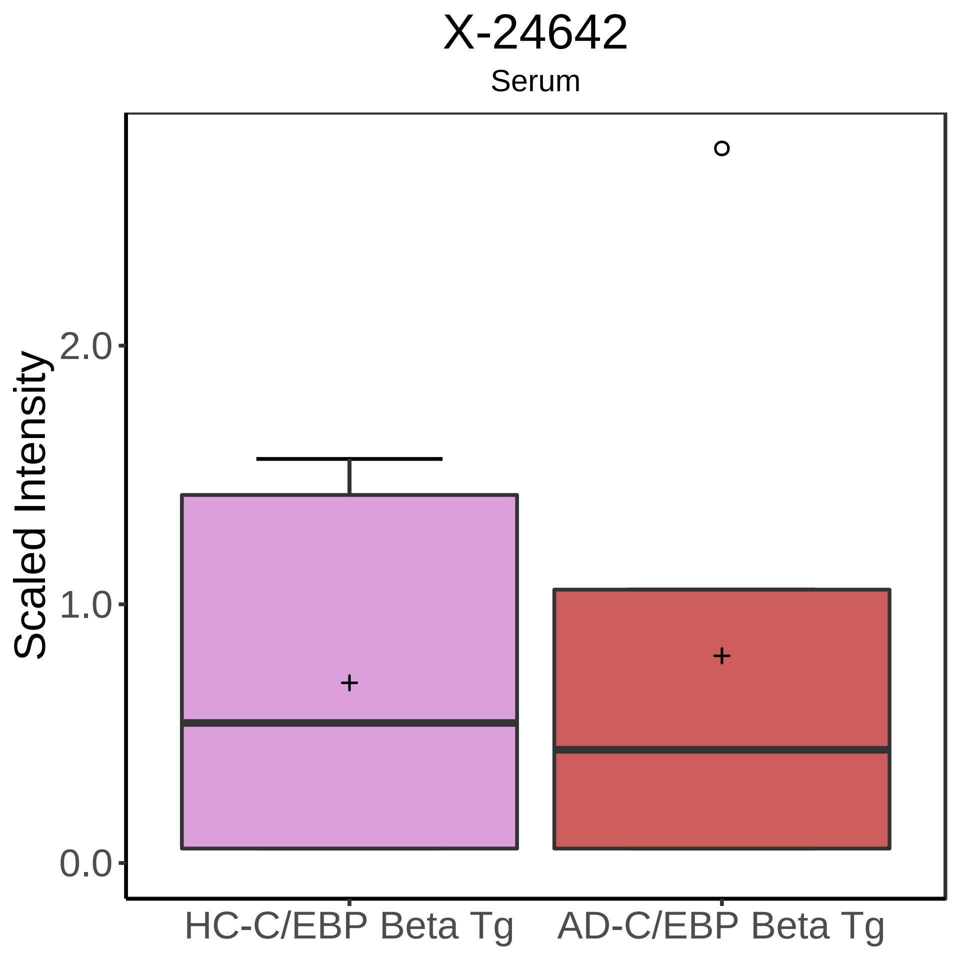

X-24642
Serum
Scaled Intensity
2.0
1.0
0.0
HC-C/EBP Beta Tg
AD-C/EBP Beta Tg

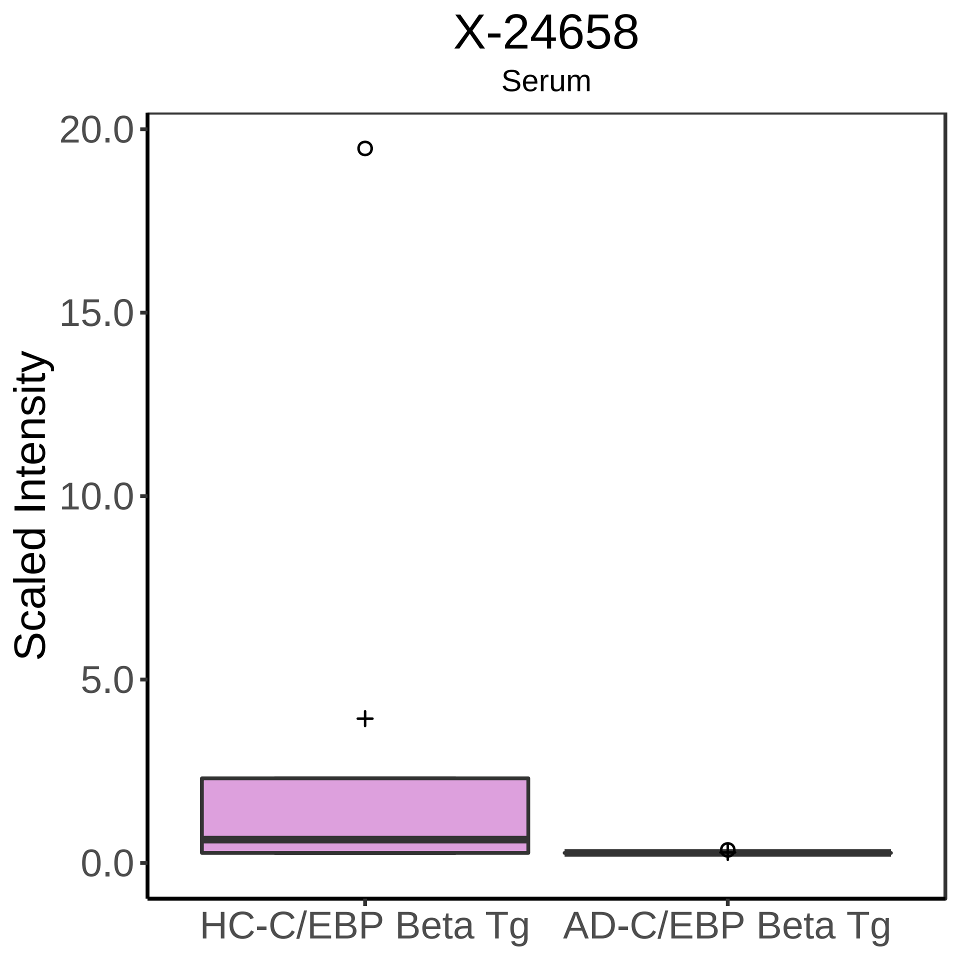

X-24658
Serum
Scaled Intensity
20.0
15.0
10.0
5.0
0.0
HC-C/EBP Beta Tg
AD-C/EBP Beta Tg

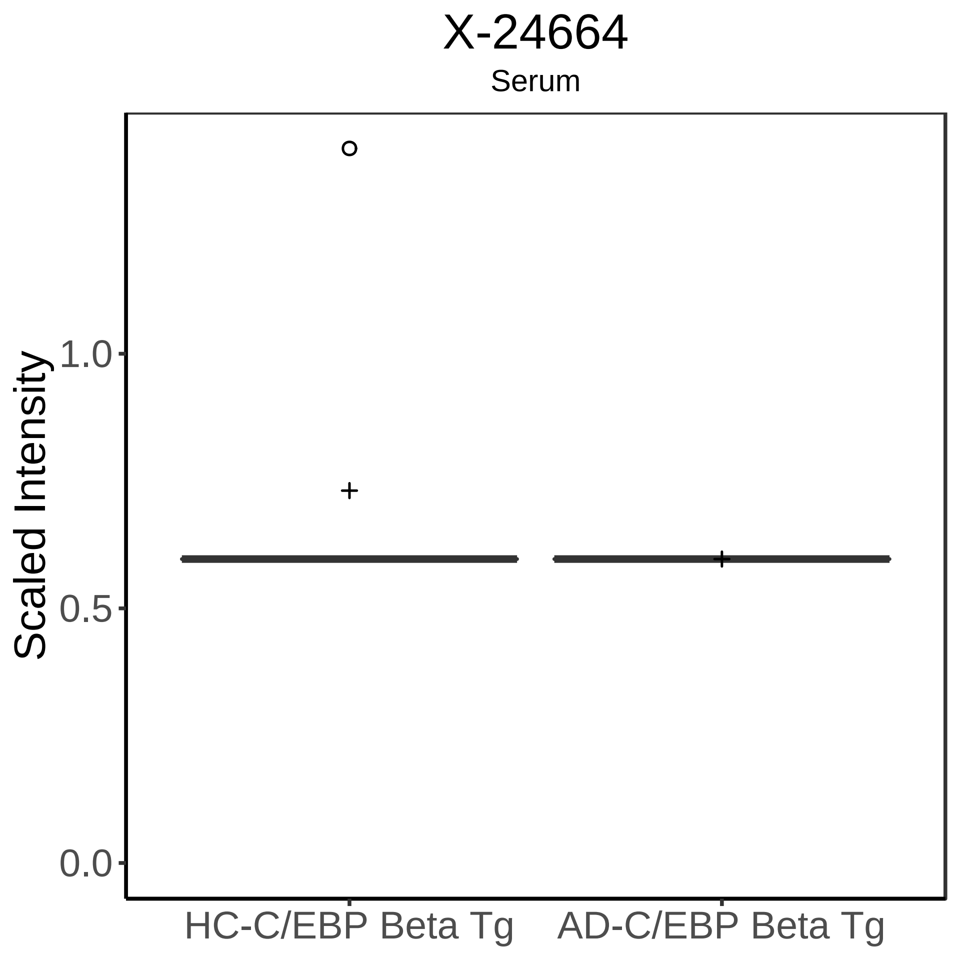
X-24664
Serum
Scaled Intensity
1.0
0.5
0.0
HC-C/EBP Beta Tg
AD-C/EBP Beta Tg

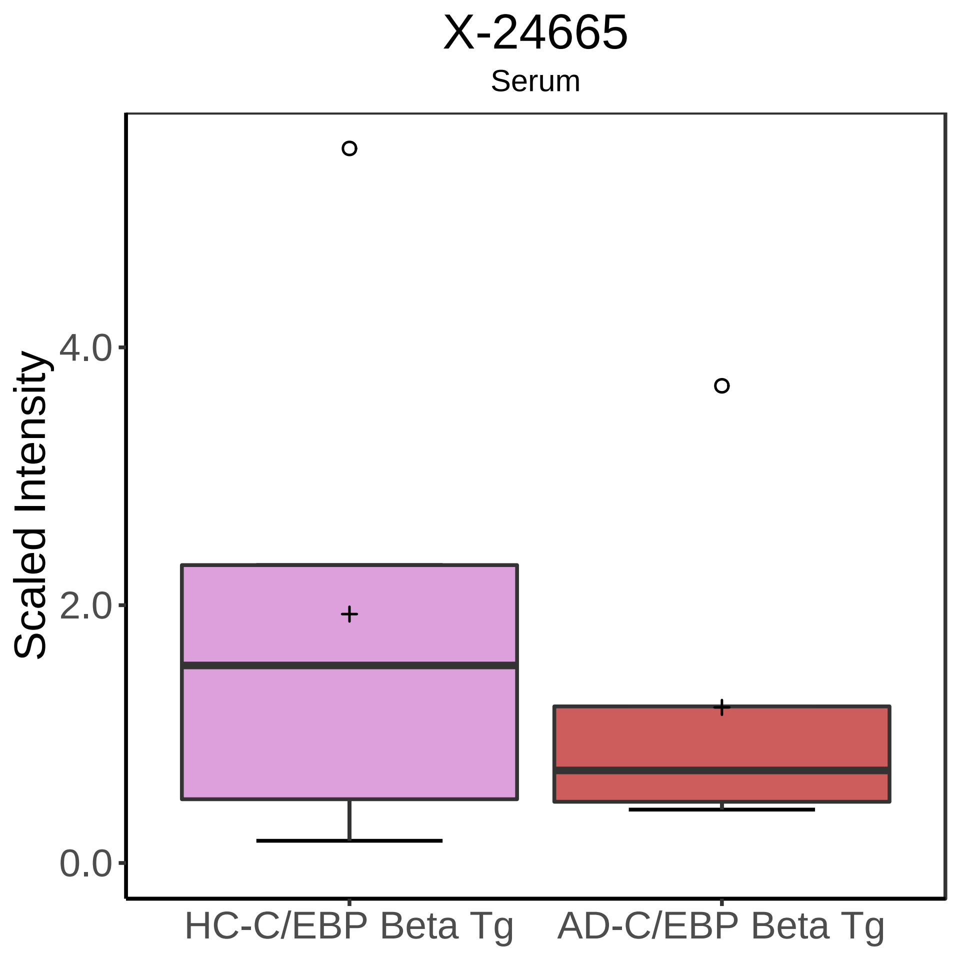
X-24665
Serum
Scaled Intensity
4.0
2.0
0.0
HC-C/EBP Beta Tg
AD-C/EBP Beta Tg

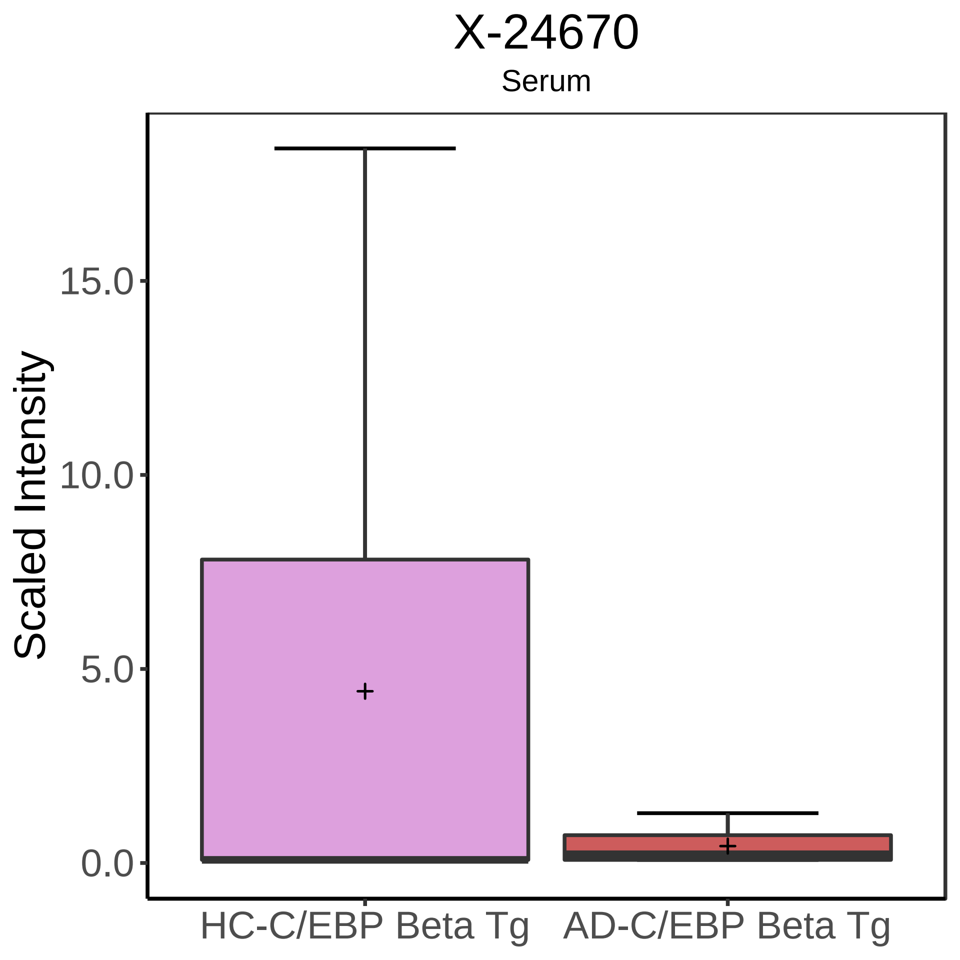

X-24670
Serum
Scaled Intensity
15.0
10.0
5.0
0.0
HC-C/EBP Beta Tg
AD-C/EBP Beta Tg

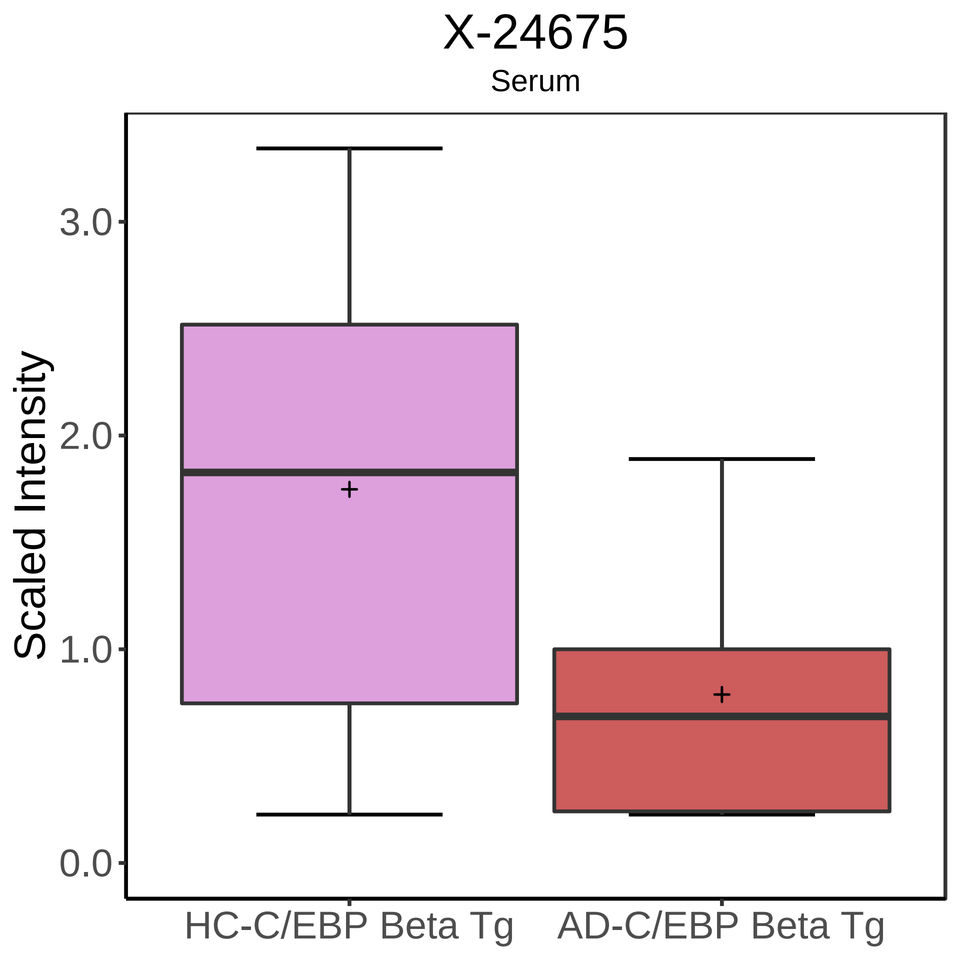

X-24675
Serum
Scaled Intensity
3.0
2.0
1.0
0.0
HC-C/EBP Beta Tg
AD-C/EBP Beta Tg

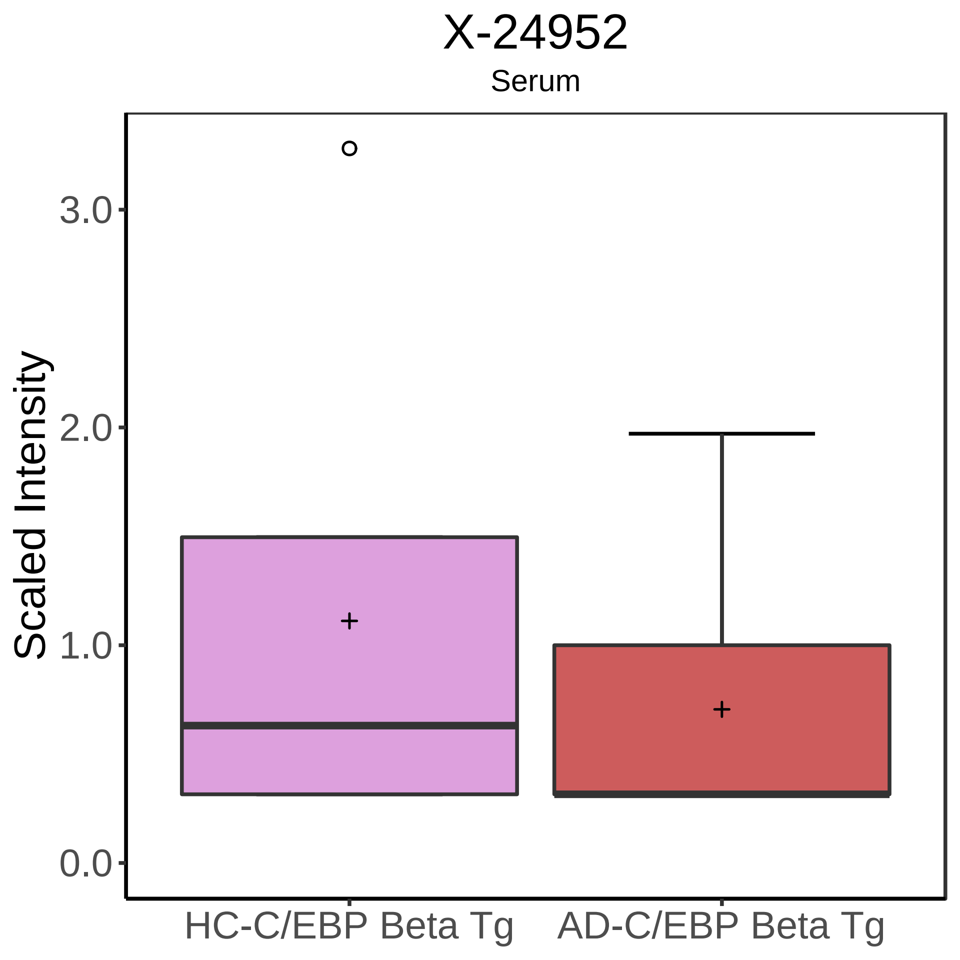
X-24952
Serum
Scaled Intensity
3.0
2.0
1.0
0.0
HC-C/EBP Beta Tg
AD-C/EBP Beta Tg

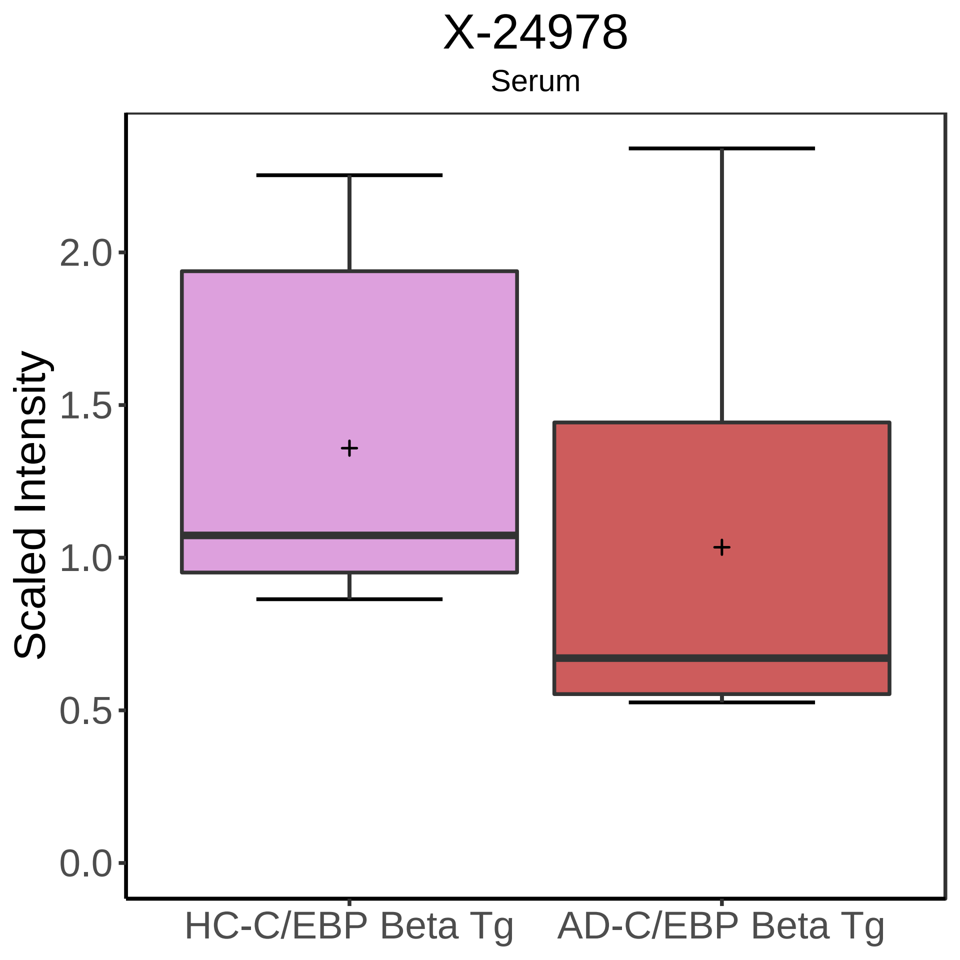
X-24978
Serum
Scaled Intensity
2.0
1.5
1.0
0.5
0.0
HC-C/EBP Beta Tg
AD-C/EBP Beta Tg

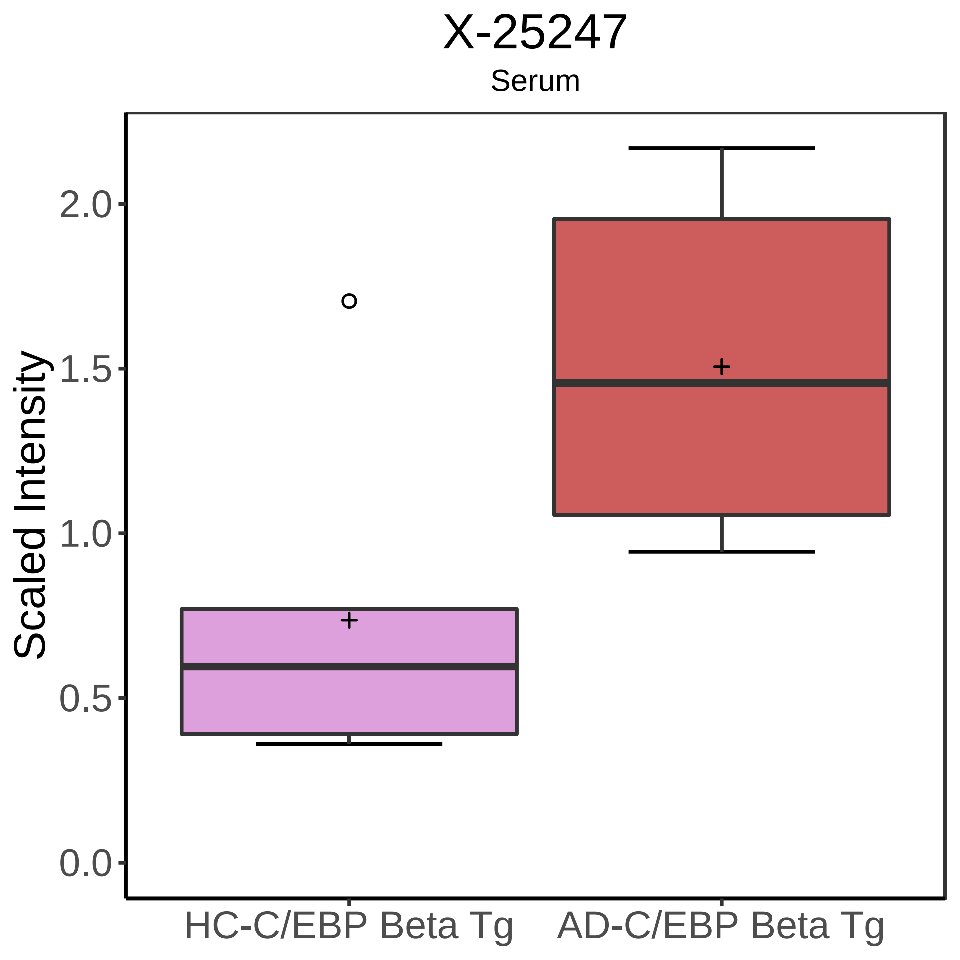

X-25247
Serum
Scaled Intensity
2.0
1.5
1.0
0.5
0.0
HC-C/EBP Beta Tg
AD-C/EBP Beta Tg

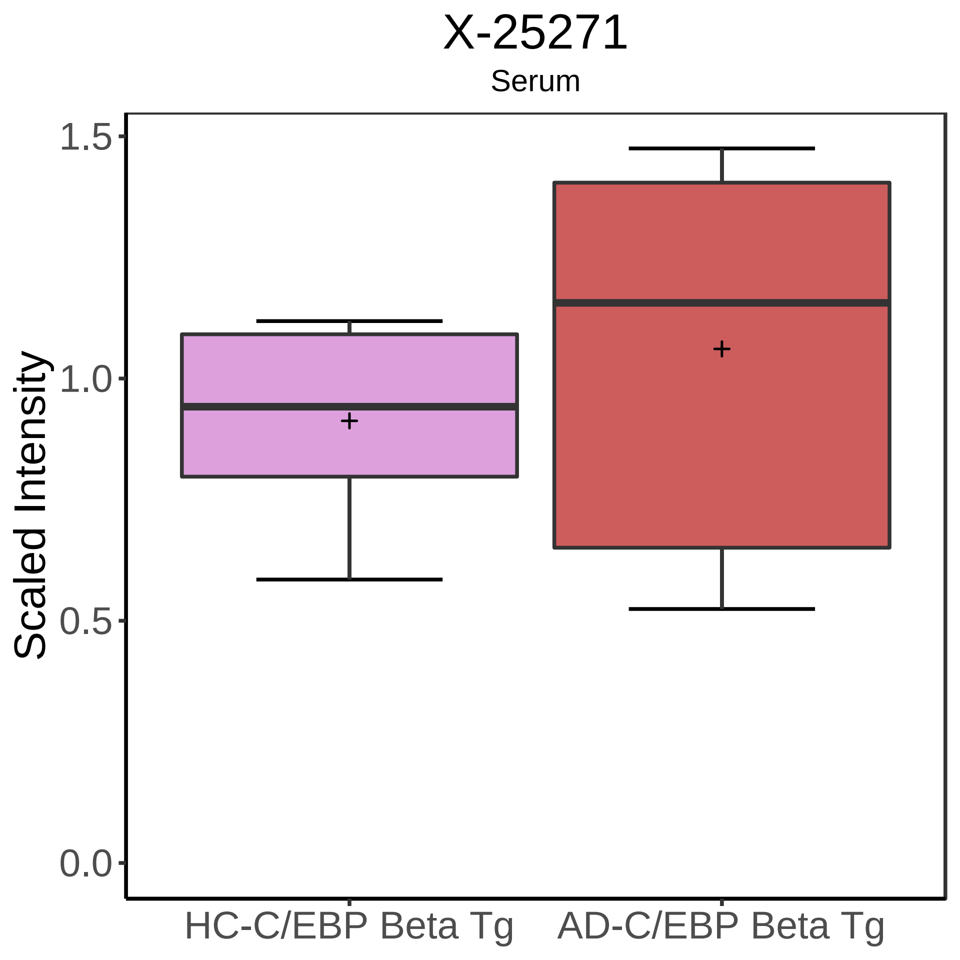
X-25271
Serum
Scaled Intensity
1.5
1.0
0.5
0.0
HC-C/EBP Beta Tg
AD-C/EBP Beta Tg

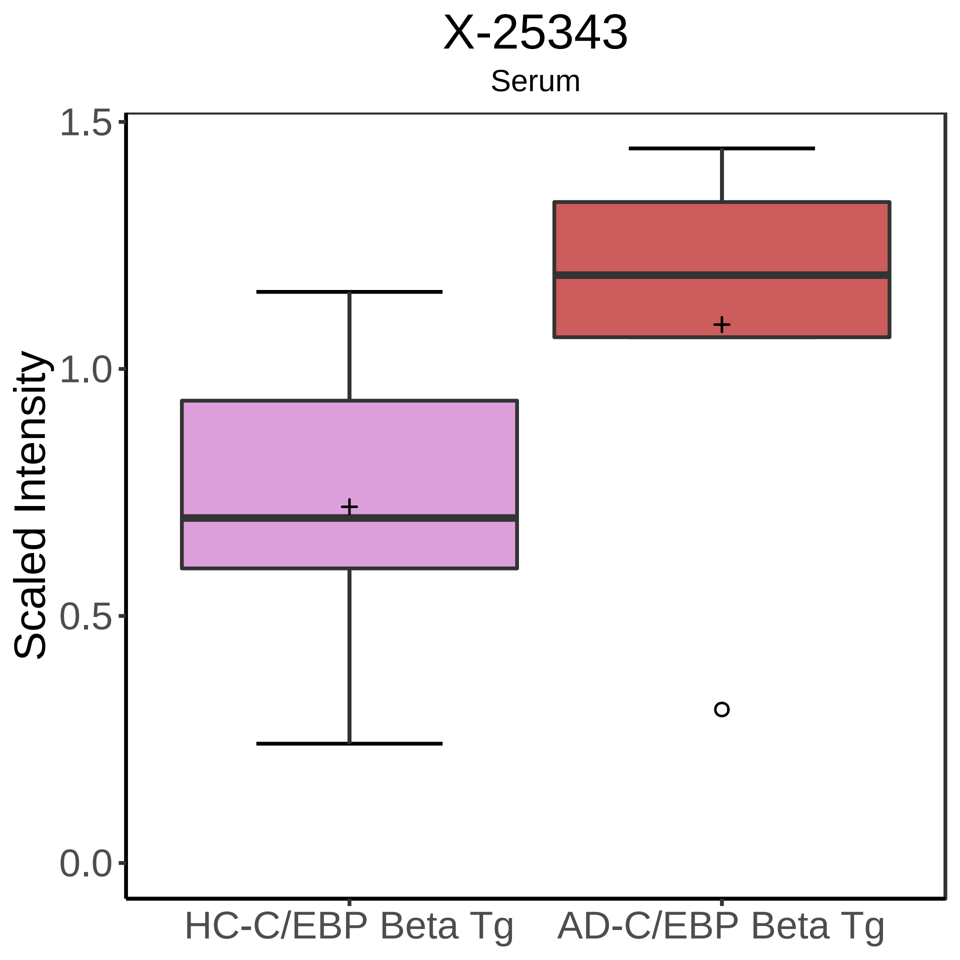
X-25343
Serum
Scaled Intensity
1.5
1.0
0.5
0.0
HC-C/EBP Beta Tg
AD-C/EBP Beta Tg

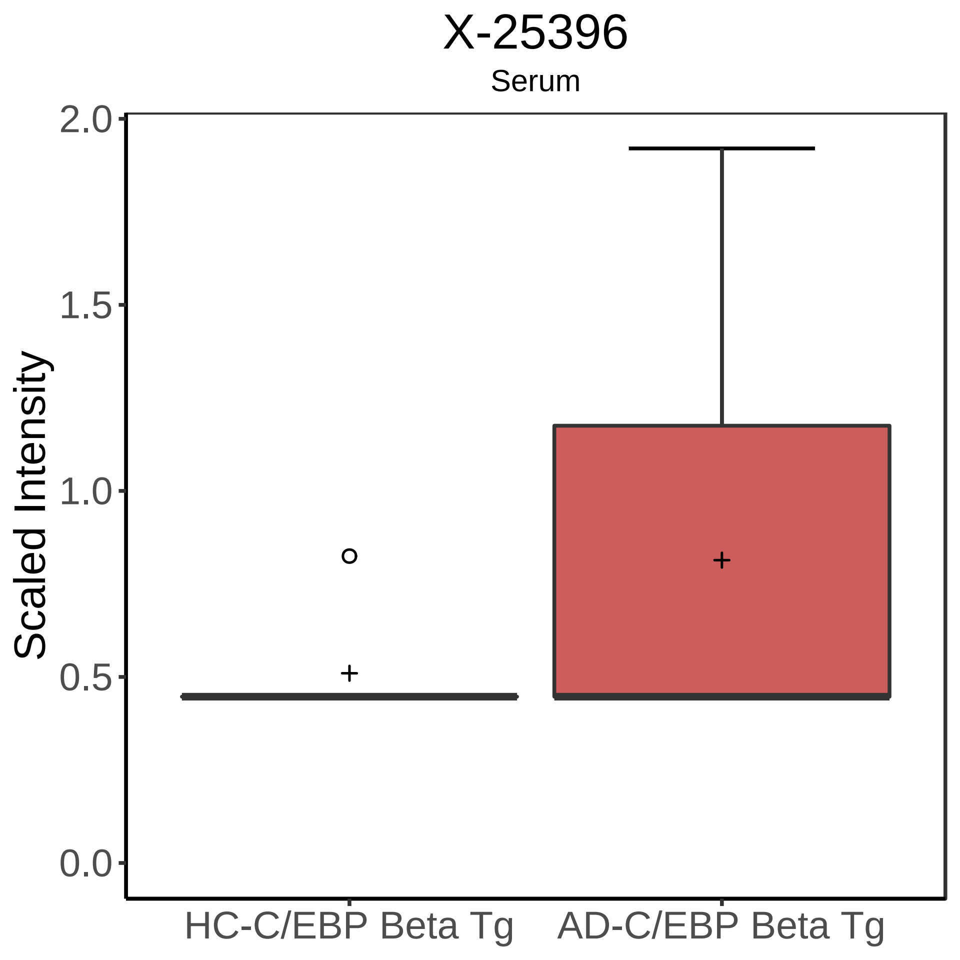
X-25396
Serum
Scaled Intensity
2.0
1.5
1.0
0.5
0.0
HC-C/EBP Beta Tg
AD-C/EBP Beta Tg

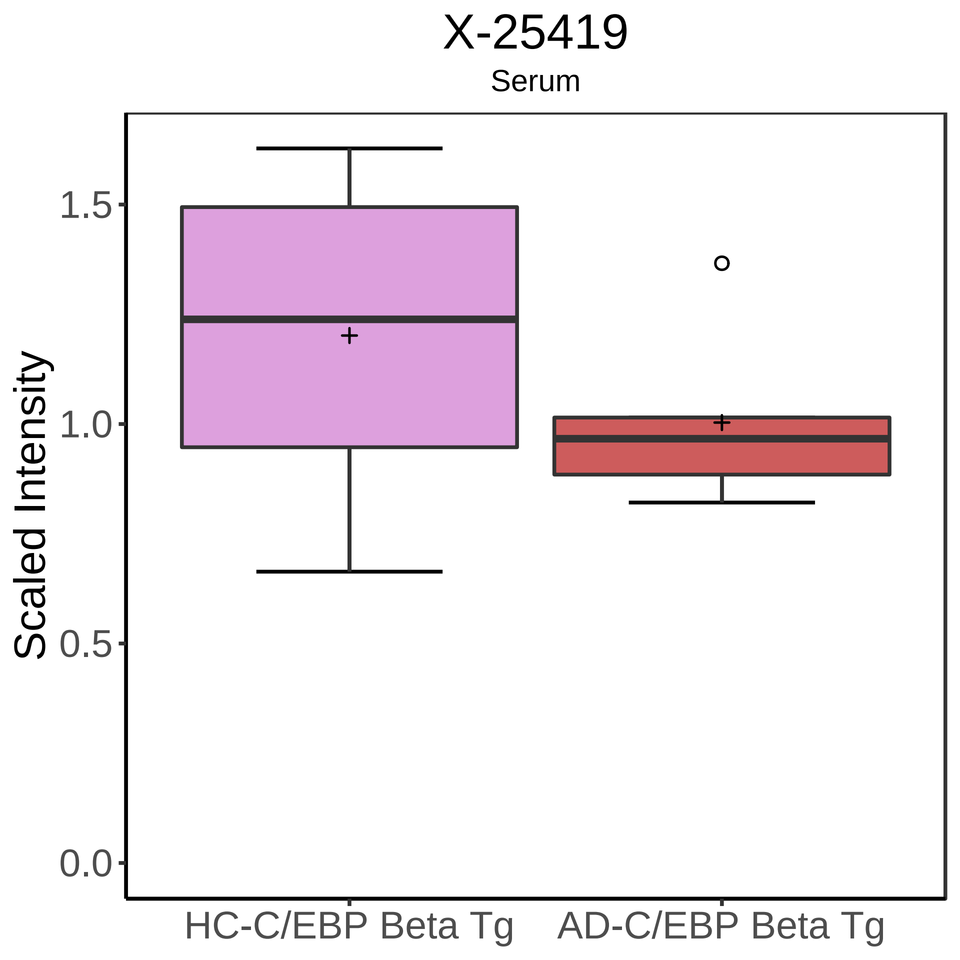
X-25419
Serum
Scaled Intensity
1.5
1.0
0.5
0.0
HC-C/EBP Beta Tg
AD-C/EBP Beta Tg

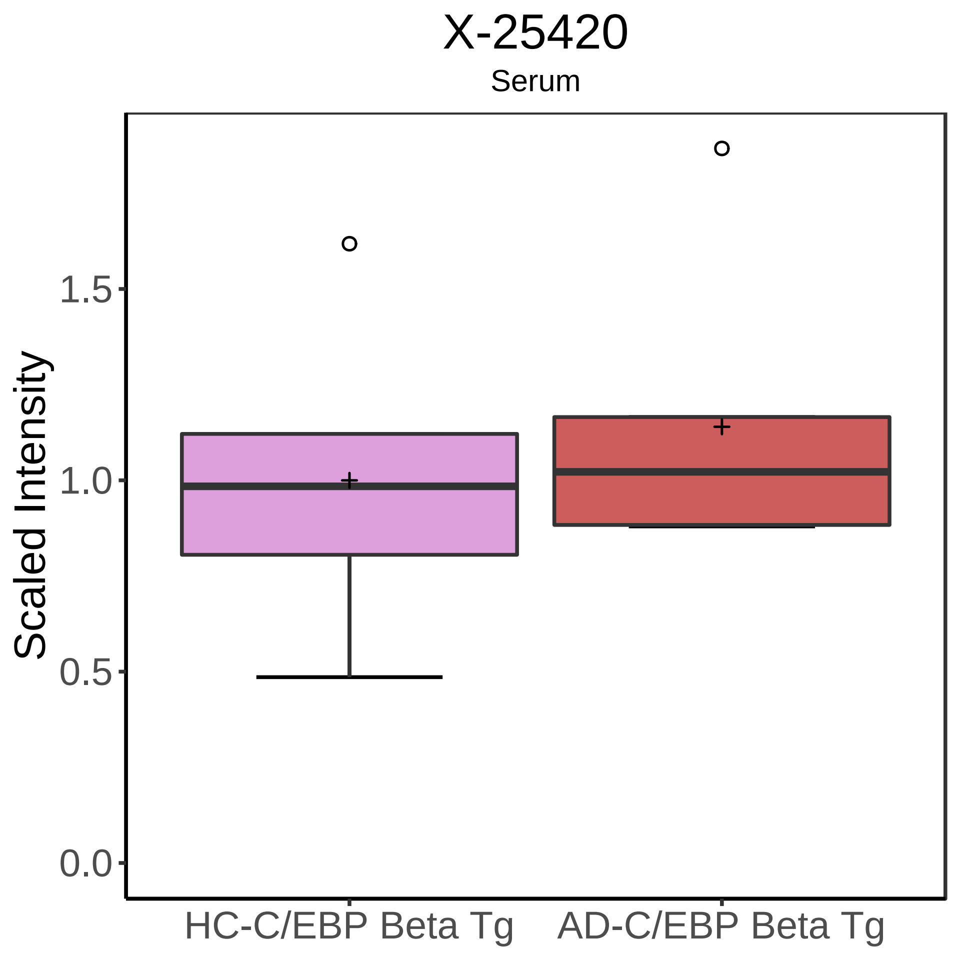
X-25420
Serum
Scaled Intensity
1.5
1.0
0.5
0.0
HC-C/EBP Beta Tg
AD-C/EBP Beta Tg

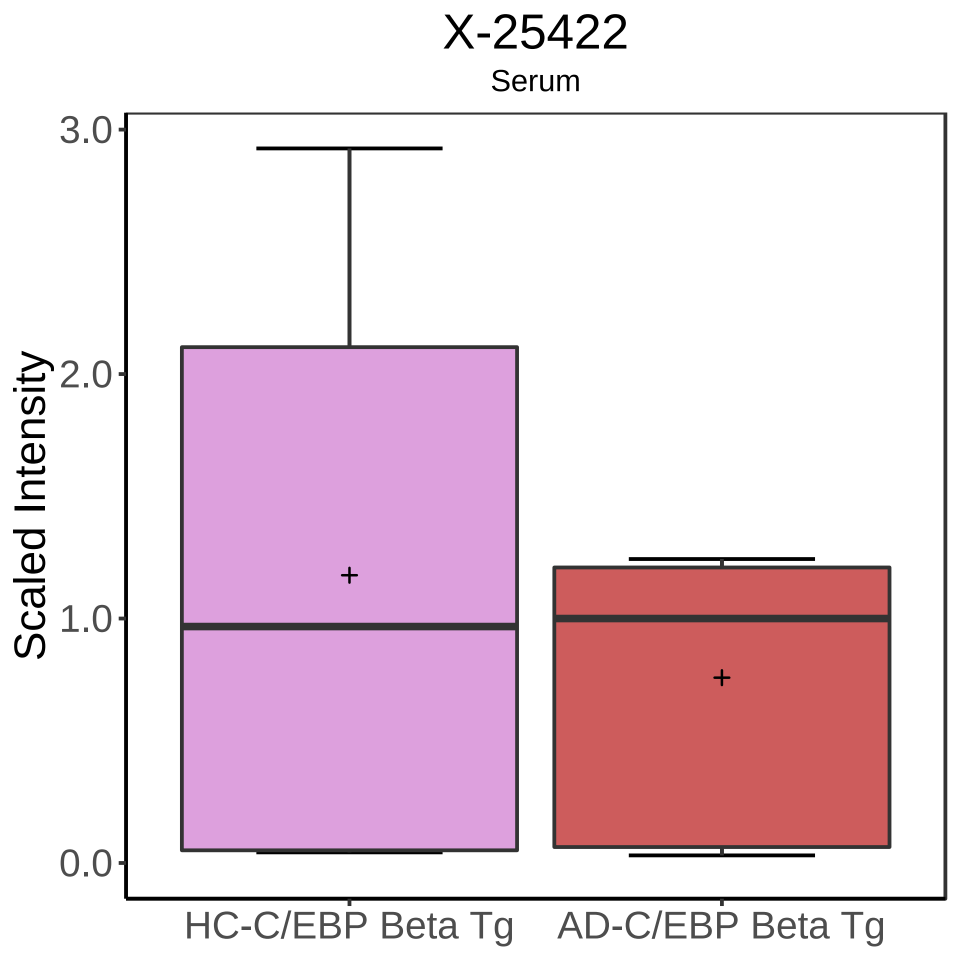
X-25422
Serum
Scaled Intensity
3.0
2.0
1.0
0.0
HC-C/EBP Beta Tg
AD-C/EBP Beta Tg

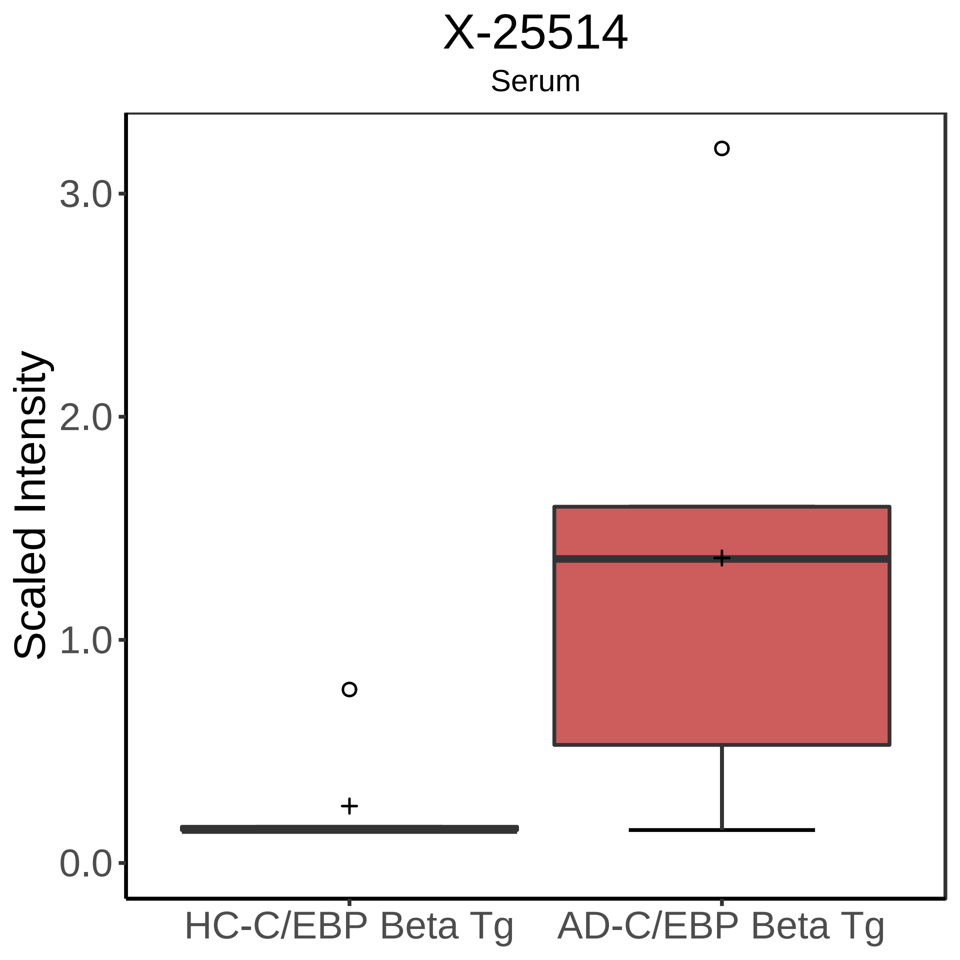
X-25514
Serum
Scaled Intensity
3.0
2.0
1.0
0.0
HC-C/EBP Beta Tg
AD-C/EBP Beta Tg

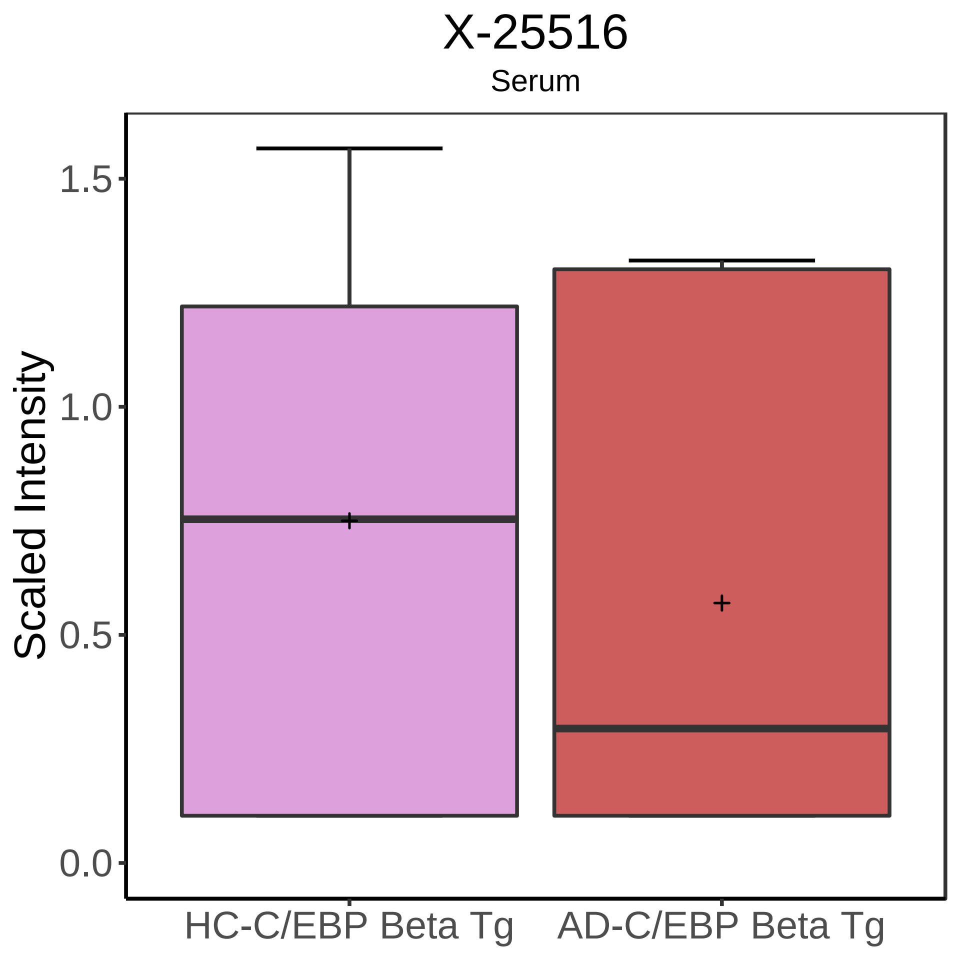

X-25516
Serum
Scaled Intensity
1.5
1.0
0.5
0.0
HC-C/EBP Beta Tg
AD-C/EBP Beta Tg

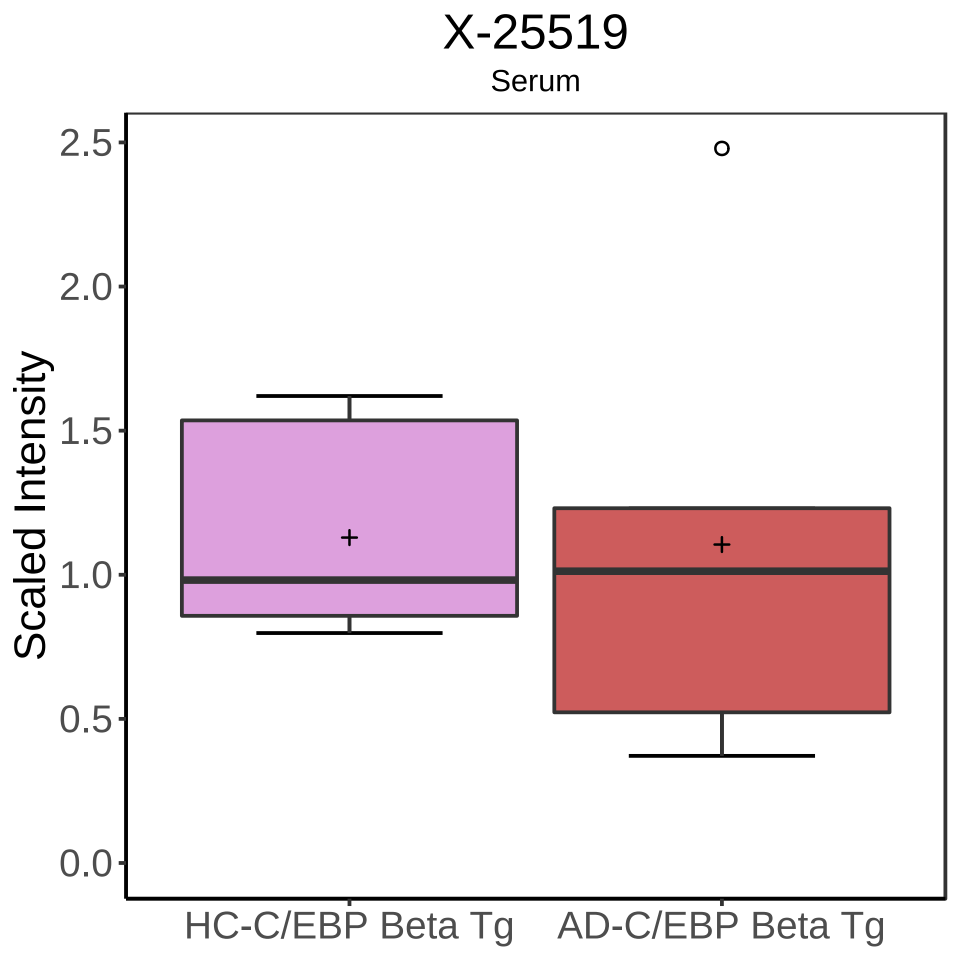
X-25519
Serum
Scaled Intensity
2.5
2.0
1.5
1.0
0.5
0.0
HC-C/EBP Beta Tg
AD-C/EBP Beta Tg

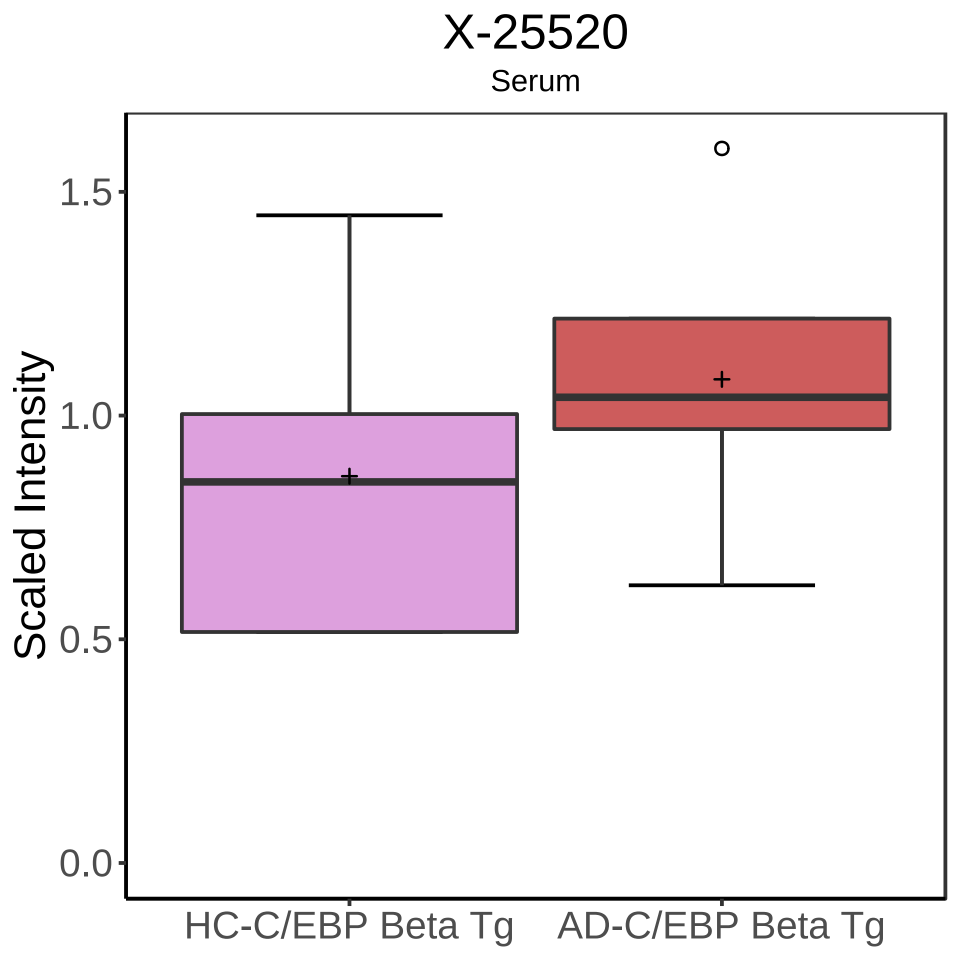
X-25520
Serum
Scaled Intensity
1.5
1.0
0.5
0.0
HC-C/EBP Beta Tg
AD-C/EBP Beta Tg

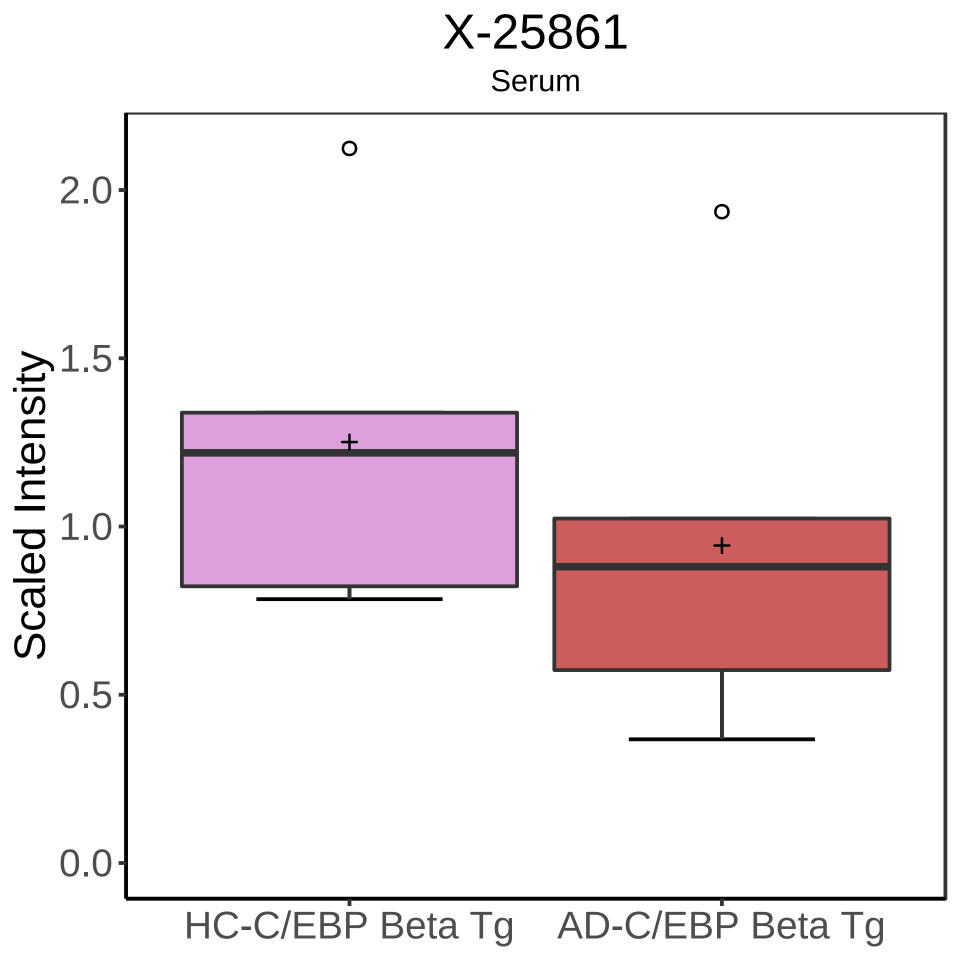
X-25861
Serum
Scaled Intensity
2.0
1.5
1.0
0.5
0.0
HC-C/EBP Beta Tg
AD-C/EBP Beta Tg

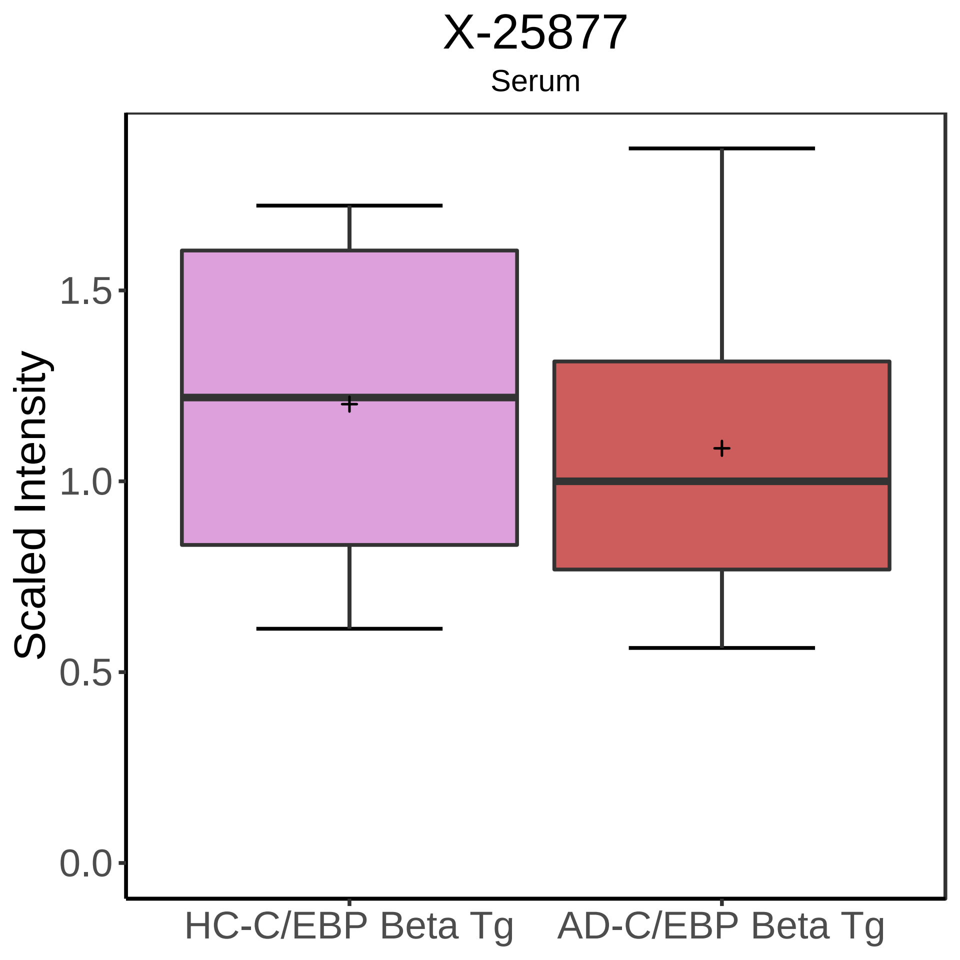

X-25877
Serum
Scaled Intensity
1.5
1.0
0.5
0.0
HC-C/EBP Beta Tg
AD-C/EBP Beta Tg

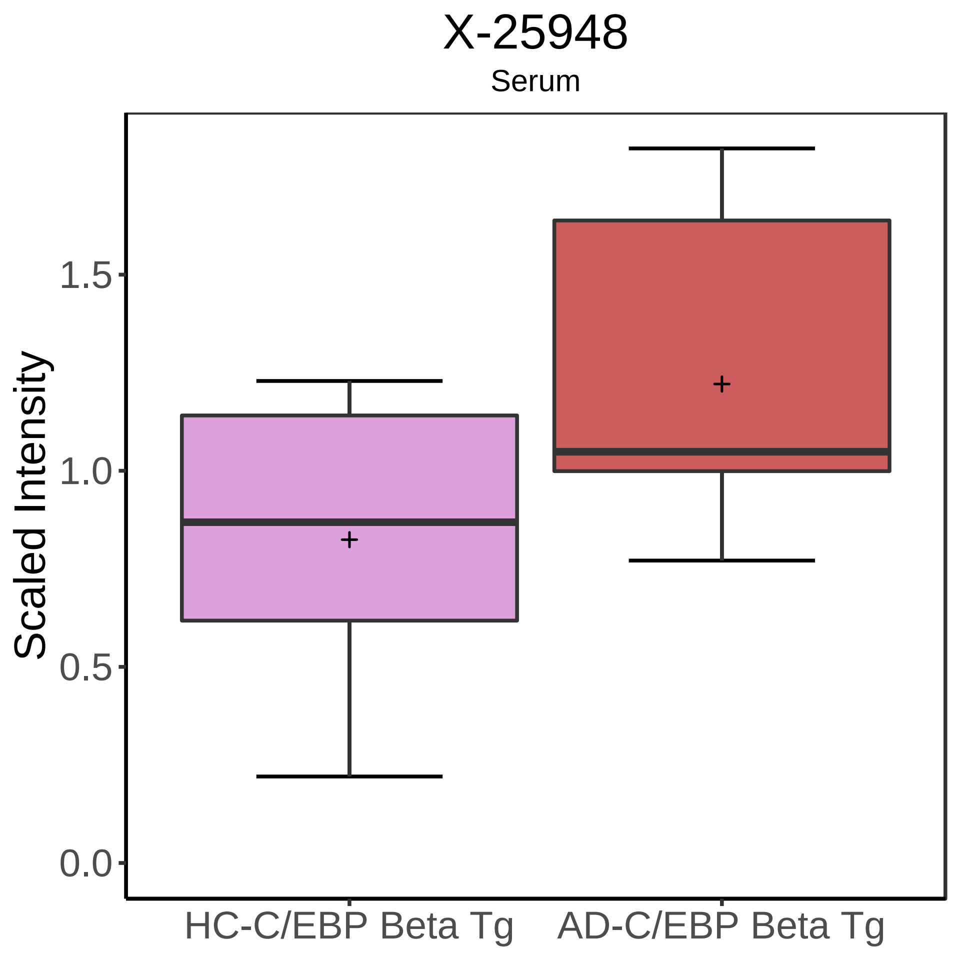

X-25948
Serum
Scaled Intensity
0.0
0.5
1.0
1.5
HC-C/EBP Beta Tg
AD-C/EBP Beta Tg

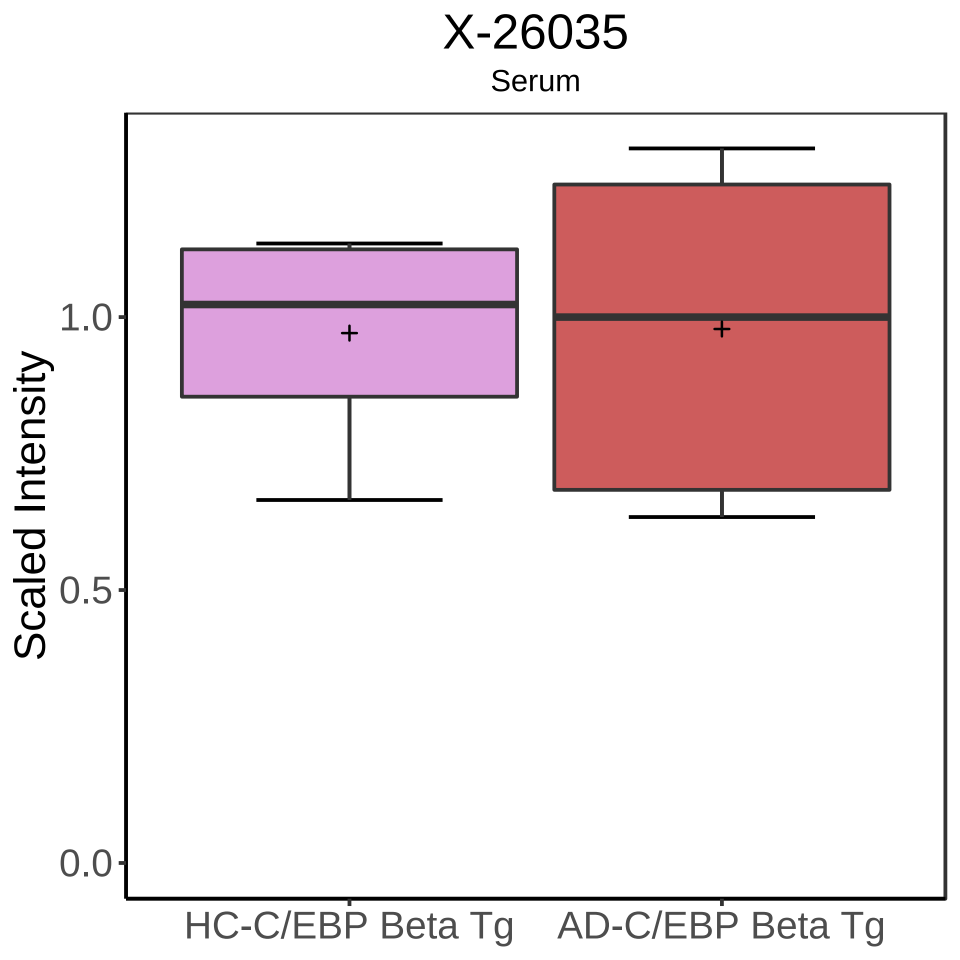
X-26035
Serum
Scaled Intensity
1.0
0.5
0.0
HC-C/EBP Beta Tg
AD-C/EBP Beta Tg

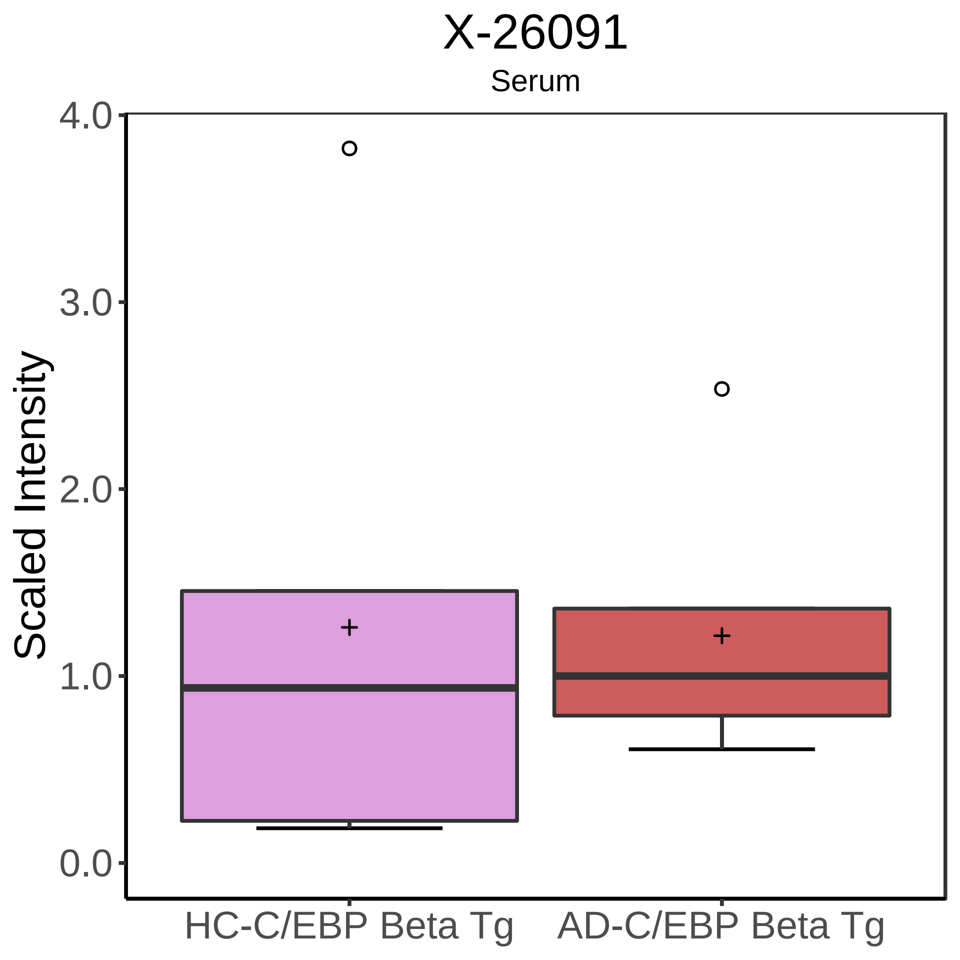
X-26091
Serum
Scaled Intensity
4.0
3.0
2.0
1.0
0.0
HC-C/EBP Beta Tg
AD-C/EBP Beta Tg

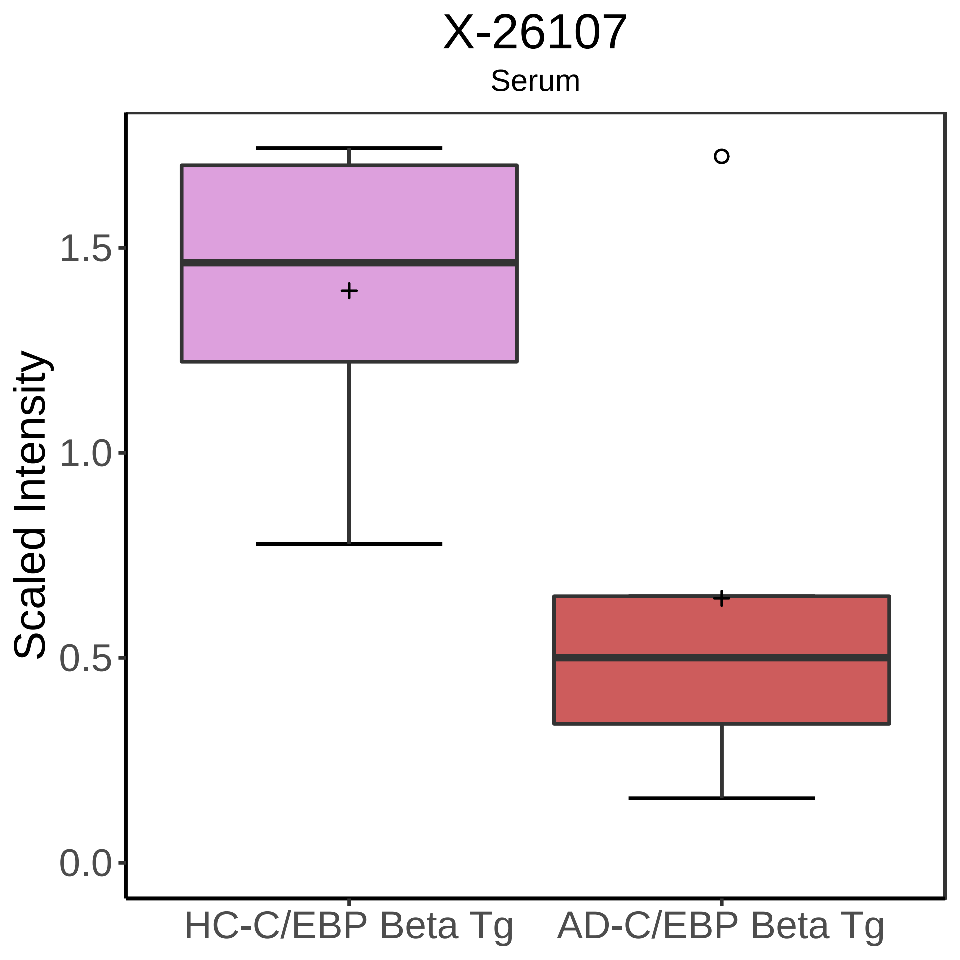

X-26107
Serum
Scaled Intensity
1.5
1.0
0.5
0.0
HC-C/EBP Beta Tg
AD-C/EBP Beta Tg

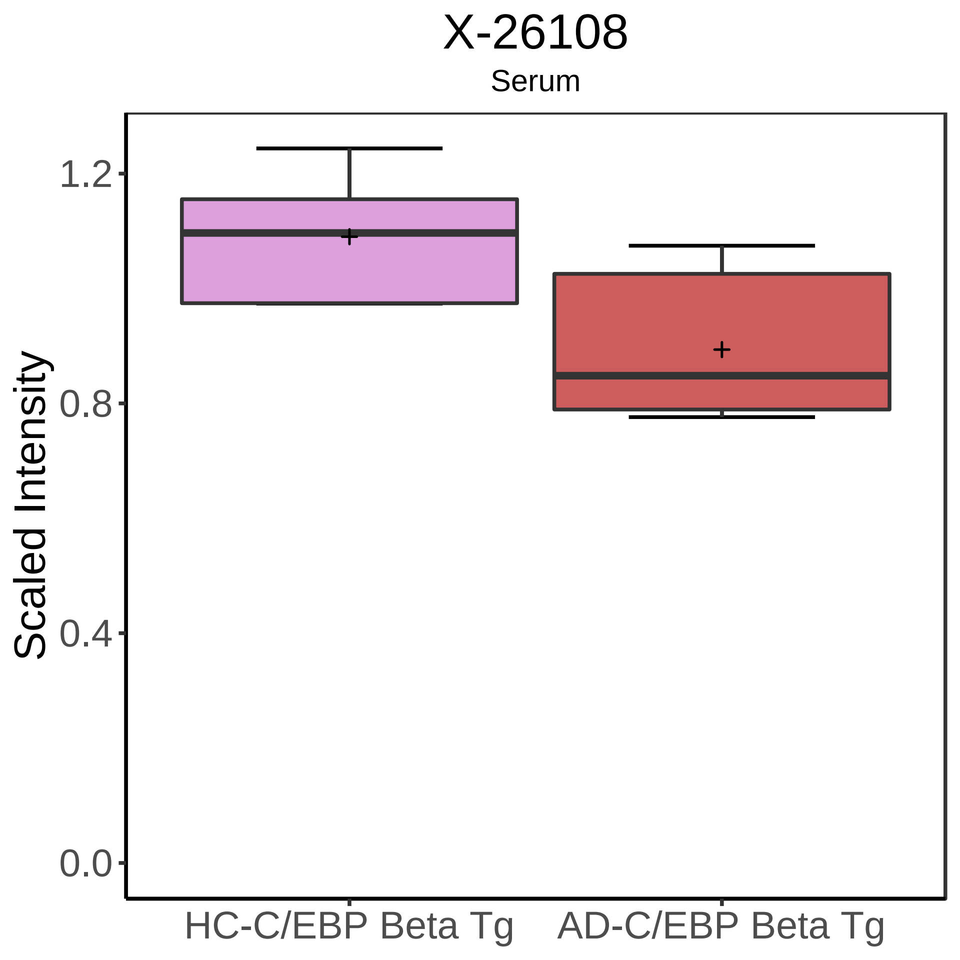

X-26108
Serum
Scaled Intensity
1.2
0.8
0.4
0.0
HC-C/EBP Beta Tg
AD-C/EBP Beta Tg
