# Supplementary material for: Bacteroides Fragilis in the gut microbiomes of Alzheimer’s disease activates microglia and triggers pathogenesis in neuronal C/EBPβ transgenic mice
Source: Nat Commun. 2023 Sep 6;14:5471. doi: 10.1038/s41467-023-41283-w (PMC10482867; doi:10.1038/s41467-023-41283-w)
Supplement: Supplementary file 5 — Reporting Summary [file 41467_2023_41283_MOESM5_ESM.pdf]

nature portfolio

Corresponding author(s): Keqiang Ye, Xiaochuan Wang, Xiji Shu

Last updated by author(s): Aug 18, 2023

Reporting Summary

Nature Portfolio wishes to improve the reproducibility of the work that we publish. This form provides structure for consistency and transparency in reporting. For further information on Nature Portfolio policies, see our Editorial Policies and the Editorial Policy Checklist.

Statistics

For all statistical analyses, confirm that the following items are present in the figure legend, table legend, main text, or Methods section.

n/a	Confirmed	
☐	☒	The exact sample size (n) for each experimental group/condition, given as a discrete number and unit of measurement
☐	☒	A statement on whether measurements were taken from distinct samples or whether the same sample was measured repeatedly
☐	☒	The statistical test(s) used AND whether they are one- or two-sided<br>*Only common tests should be described solely by name; describe more complex techniques in the Methods section.*
☒	☐	A description of all covariates tested
☐	☒	A description of any assumptions or corrections, such as tests of normality and adjustment for multiple comparisons
☐	☒	A full description of the statistical parameters including central tendency (e.g. means) or other basic estimates (e.g. regression coefficient) AND variation (e.g. standard deviation) or associated estimates of uncertainty (e.g. confidence intervals)
☐	☒	For null hypothesis testing, the test statistic (e.g. F, t, r) with confidence intervals, effect sizes, degrees of freedom and P value noted<br>*Give P values as exact values whenever suitable.*
☒	☐	For Bayesian analysis, information on the choice of priors and Markov chain Monte Carlo settings
☒	☐	For hierarchical and complex designs, identification of the appropriate level for tests and full reporting of outcomes
☒	☐	Estimates of effect sizes (e.g. Cohen's d, Pearson's r), indicating how they were calculated

Our web collection on statistics for biologists contains articles on many of the points above.

Software and code

Policy information about availability of computer code

Data collection	Noted in Methods section.
Data analysis	Sample size was determined by Power and Precision V2 (Biostat). Image J 1.51J8, SPSS 12.0, Prism-GraphPad 7.04, QIIME2 r2023.2, PyNAST 1.0, and Illumina's bcl2fastq were used for analysis.

For manuscripts utilizing custom algorithms or software that are central to the research but not yet described in published literature, software must be made available to editors and reviewers. We strongly encourage code deposition in a community repository (e.g. GitHub). See the Nature Portfolio guidelines for submitting code & software for further information.

Data

Policy information about availability of data

All manuscripts must include a data availability statement. This statement should provide the following information, where applicable:

- Accession codes, unique identifiers, or web links for publicly available datasets
- A description of any restrictions on data availability
- For clinical datasets or third party data, please ensure that the statement adheres to our policy

Source data are provided with this paper. Source Data file contains Source Data.xlsx and EMOR-0201-20VW.zip. Source Data.xlsx includes the relevant raw data from each graphic. EMOR-0201-20VW.zip file contains the metabolomic data of brain, serum, and fecal samples from the Alzheimer's disease mouse model. Raw RNA sequencing data used in this study are available in the Bioproject database under accession codes PRJNA1001130 (https://www.ncbi.nlm.nih.gov/bioproject/1001130)

Research involving human participants, their data, or biological material

Policy information about studies with human participants or human data. See also policy information about sex, gender (identity/presentation), and sexual orientation and race, ethnicity and racism.

Reporting on sex and gender

Sample ID	Age	Gender
AD_1	85	F
AD_2	85	F
AD_3	94	F
HC_1	91	F
HC_2	94	F
HC_3	93	F

Reporting on race, ethnicity, or other socially relevant groupings: Please contact Dr. John P. Haran and Dr. Beth A. McCormick for details.

Population characteristics: Nursing home elders who are 65 years of age and lived in one of four nursing home facilities located in central Massachusetts.

Recruitment: Within the preceding 4 weeks. No elders suffered from dysphagia or had a feeding tube. Any elders with antimicrobial exposure or a diarrheal illness during the conduct of the study were excluded from this analysis.

Ethics oversight: This study was approved by the UMass Chan Medical School Institutional Review Board (IRB) at the University of Massachusetts Medical School (docket H00010892).

Note that full information on the approval of the study protocol must also be provided in the manuscript.

Field-specific reporting

Please select the one below that is the best fit for your research. If you are not sure, read the appropriate sections before making your selection.

[x] Life sciences   [ ] Behavioural & social sciences   [ ] Ecological, evolutionary & environmental sciences

For a reference copy of the document with all sections, see nature.com/documents/nr-reporting-summary-flat.pdf

Life sciences study design

All studies must disclose on these points even when the disclosure is negative.

Sample size: Sample size was determined by Power and Precision V2(Biostat).

Data exclusions: No data were excluded.

Replication: Noted in figure legends.

Randomization: For all experiments, samples/mice were randomly allocated into experimental groups.

Blinding: Investigators were blind to the group allocation of samples and mice, during data collection and analysis, For other experiments, each experiment was repeated by different investigators and reached similar results.

Reporting for specific materials, systems and methods

We require information from authors about some types of materials, experimental systems and methods used in many studies. Here, indicate whether each material, system or method listed is relevant to your study. If you are not sure if a list item applies to your research, read the appropriate section before selecting a response.

Materials & experimental systems

n/a	Involved in the study
☐	☒ Antibodies
☒	☐ Eukaryotic cell lines
☒	☐ Palaeontology and archaeology
☐	☒ Animals and other organisms
☐	☒ Clinical data
☒	☐ Dual use research of concern
☒	☐ Plants

Methods

n/a	Involved in the study
☒	☐ ChIP-seq
☒	☐ Flow cytometry
☒	☐ MRI-based neuroimaging

Antibodies

Antibodies used	See details from Methods section: Key Resources Table.
Validation	Anti-AEP(11b7) PMID:34166772, 32782380 ; Anti-Tau N368 PMID: 37257680, 33895869; Anti-APP N585 PMID:26549211, 31778772; Other commercially available antibodies are used in published literature.

Animals and other research organisms

Policy information about studies involving animals; ARRIVE guidelines recommended for reporting animal research, and Sex and Gender in Research

Laboratory animals	Thyl-human C/EBP transgenic mouse of C57BL/6J background were generated as method section reported. The C57BL/6J mice were purchased from Jax lab (Cat#: 000664). These mice were free access to sterile water and food with 5 mice/ cage. They were kept in a specific pathogen-free (SPF) condition with 20-25°C temperature and 45-55% humidity on a regular 12-hour light/dark cycle.
Wild animals	Didn't involve.
Reporting on sex	All sex of animal were used for experiments.
Field-collected samples	Didn't involve.
Ethics oversight	The experimental protocol was approved by the Emory University Institutional Animal Care and Ethical Committee (DAR3000226ELMNTS-N).

Note that full information on the approval of the study protocol must also be provided in the manuscript.

Clinical data

Policy information about clinical studies

All manuscripts should comply with the ICMJEguidelines for publication of clinical research and a completedCONSORT checklist must be included with all submissions.

Clinical trial registration	This was not applicable to the current study.
Study protocol	This was not applicable to the current study.
Data collection	This was not applicable to the current study.
Outcomes	This was not applicable to the current study.
